# Simultaneous Analysis of Proteome, Phospho- and Glycoproteome of Rat Kidney Tissue with Electrostatic Repulsion Hydrophilic Interaction Chromatography

**DOI:** 10.1371/journal.pone.0016884

**Published:** 2011-02-23

**Authors:** Piliang Hao, Tiannan Guo, Siu Kwan Sze

**Affiliations:** School of Biological Sciences, Nanyang Technological University, Singapore, Singapore; National University of Singapore, Singapore

## Abstract

Protein post-translational modifications (PTMs) are regulated separately from protein expression levels. Thus, simultaneous characterization of the proteome and its PTMs is pivotal to an understanding of protein regulation, function and activity. However, concurrent analysis of the proteome and its PTMs by mass spectrometry is a challenging task because the peptides bearing PTMs are present in sub-stoichiometric amounts and their ionization is often suppressed by unmodified peptides of high abundance. We describe here a method for concurrent analysis of phosphopeptides, glycopeptides and unmodified peptides in a tryptic digest of rat kidney tissue with a sequence of ERLIC and RP-LC-MS/MS in a single experimental run, thereby avoiding inter-experimental variation. Optimization of loading solvents and elution gradients permitted ERLIC to be performed with totally volatile solvents. Two SCX and four ERLIC gradients were compared in details, and one ERLIC gradient was found to perform the best, which identified 2929 proteins, 583 phosphorylation sites in 338 phosphoproteins and 722 N-glycosylation sites in 387 glycoproteins from rat kidney tissue. Two hundred low-abundance proteins with important functions were identified only from the glyco- or phospho-subproteomes, reflecting the importance of the enrichment and separation of modified peptides by ERLIC. In addition, this strategy enables identification of unmodified and corresponding modified peptides (partial phosphorylation and N-glycosylation) from the same protein. Interestingly, partially modified proteins tend to occur on proteins involved in transport. Moreover, some membrane or extracellular proteins, such as versican core protein and fibronectin, were found to have both phosphorylation and N-glycosylation, which may permit an assessment of the potential for cross talk between these two vital PTMs and their roles in regulation.

## Introduction

As proteins and their modifications are directly involved in nearly all biological processes, the identification and quantification of as many proteins and their post-translational modifications (PTMs) as possible from the same sample are the prerequisites for biological discovery. As peptides are more compatible with liquid chromatography (LC) separation and mass spectrometry (MS) detection, protein extracts are usually digested with a protease to yield a complex mixture of peptides in shotgun proteomics. To thoroughly characterize the proteome, multidimensional protein identification technology (MudPIT) [1] is commonly employed in which multidimensional liquid chromatography (MDLC) is used to reduce sample complexity and increase dynamic range of protein identification, and separate experiments with different types of enrichment methods are used for concentration of peptides of low abundance with PTMs for mass spectrometric characterization.

In recent years, considerable attention has been paid to the study of information-rich subsets of the proteome, such as the phosphoproteome and glycoproteome, in order to improve the dynamic range of identified proteins [2]. It has been estimated that about 50% of all proteins are glycosylated [3] and over one third of all proteins are phosphorylated in mammals [4]. Phosphorylation is a dynamic and reversible modification involved in the regulation of many biological processes including metabolism, cell division, signal transduction and enzymatic activity [5]–[7]. Glycosylation also plays important roles in many biological processes including embryonic development, cell-to-cell interactions, cell division, and protein regulation and interaction [8]. Because of the frequently low stoichiometry of PTMs and the ion suppression effect from unmodified peptides of high abundance, phosphopeptides and glycopeptides have to be enriched before MS analysis to minimize such suppression [9]. Immunoprecipitation, immobilized metal affinity chromatography (IMAC), strong-cation exchange (SCX) and titanium dioxide (TiO2) chromatography have become popular for phosphopeptide enrichment [10]–[13], and lectin-based affinity enrichment, hydrophilic interaction liquid chromatography (HILIC), SCX and hydrazide covalent chromatography have been extensively used in the enrichment of glycoproteins or glycopeptides [14]–[18].

Regulatory protein phosphorylation is a dynamic modification of low occupancy; many sites are only partially phosphorylated at a given time. The phosphorylation of a protein has been found not necessarily to be consistent with its level of expression [19], [20]. Similarly, it has been reported that protein glycosylation changes significantly during inflammation, sepsis and cancers [21], [22]. The determination of the stoichiometry of phosphorylation and glycosylation at certain sites is helpful to understand the mechanism of some regulatory pathways [23], [24]. Furthermore, downstream gene expression regulated by phosphorylation may also include some unmodified proteins. Accordingly, appreciable efforts have been made toward the analysis of the proteome and phospho- and glycoproteomes. A method that permitted this to be done simultaneously would be convenient and would provide information on both protein expression and modification. Furthermore, the inter-experimental variation now resulted from separate analysis of modified and unmodified peptides would be avoided. Such an analysis has not been possible to date. Analysis of phosphopeptides and glycopeptides requires their specific enrichment from unmodified peptides as well as their fractionation. The analysis of the proteome overall requires comprehensive fractionation to reduce sample complexity, but unmodified peptides tend to elute in the flow-through in methods that selectively enrich the concentration of modified peptides. It is very difficult to achieve both types of separations in one analysis. In this study we propose to do so using electrostatic repulsion-hydrophilic interaction chromatography (ERLIC).

ERLIC was first introduced by Alpert for separation of biomolecules and phosphopeptide enrichment [25]. It has since been extended to the enrichment of phosphopeptides from cell extracts and fractionation of N-linked glycopeptides from complex samples [26]–[28]. Recently, the simultaneous characterization of the glycoproteome and phosphoproteome of mouse brain membrane has been achieved with ERLIC [29]. By performing gradient elution with unbuffered acids, ERLIC was optimized for whole proteome fractionation [30]. In the present study the conditions for ERLIC have been optimized further so that for the first time, the simultaneous analysis of proteome, phosphoproteome and glycoproteome has been achieved in one run. Since SCX and ERLIC have both been used for fractionation of both modified and unmodified peptides to some extent, they were compared here in detail for the analysis of tryptic peptides of rat kidney tissue.

## Materials and Methods

### Ethics Statement

The use of rat kidneys for proteomics research was approved by the Institutional Animal Care and Use Committee of Nanyang Technological University (NTU-IACUC) with the reference number of ARF SBS/NIE-A 0083.

### Sample Preparation and Digestion

Male Sprague Dawley rats (230–250g) were kept in a temperature-controlled environment (24°C) on a 12-h light:12-h dark cycle with free access to food and water. Animals were handled in accordance with the guidelines of NTU Institutional Animal Care and Use Committee (NTU-IACUC), NTU, Singapore. The animals were sacrificed under deep anesthesia and transcardially perfused with 300 ml of ice-cold PBS to flush out the blood from the circulatory system. The kidney was subsequently collected, snap-frozen in liquid nitrogen and kept at −80°C until use. The tissue was cut into small pieces and ground into fine powders in liquid nitrogen with a pestle. The powders were then suspended in lysis buffer (8 M urea, 50 mM Tris-HCl, pH 8.0) with protease inhibitor cocktail (P8340, Sigma) and phosphatase inhibitors (04906837001, Roche) added according to the manufacturers' instructions. The suspension was sonicated for 10 s thrice on ice and centrifuged at 20 000 g at 4°C for 30 min. The protein concentration of the supernatant was then determined by the bicinchoninic acid (BCA) assay. About 20 mg of sample lysate was reduced with 20 mM DTT at 37°C for 3–4 h and alkylated with 80 mM iodoacetamide for 45 min in the dark. After the concentration of urea was diluted to 1 M with 50 mM NH_4_HCO_3_, trypsin was added at a ratio of 1∶100 (trypsin/sample). It was then incubated at 37°C for 4 h. For complete digestion, incubation was continued at 37°C for about 12 h after a second addition of the same amount of trypsin. The obtained tryptic peptides were desalted using a Sep-Pak C18 artridge (Waters, Milford, MA) and dried in a SpeedVac Thermo Electron, Waltham, MA) [30].

### ERLIC Separation

Peptides from 2 mg proteins were fractionated using a PolyWAX LP weak anion-exchange column (4.6×200 mm, 5 µm, 300 Å, PolyLC, Columbia, MD) on a Shimadzu Prominence UFLC system. Forty six fractions were collected with a 140 min gradient of 100% buffer A for 10 min, 0%–8% buffer B for 20 min, 8%–27% buffer B for 30 min, 27%–45% buffer B for 10 min, 45%–81% buffer B for 20 min and 81%–100% buffer B for 20 min followed by 30 min at 100% buffer B at a flow rate of 0.5 mL/min. To optimize the ERLIC condition for simultaneous characterization of the overall proteome and peptides with the two specific PTMs, four different combinations of Buffer A and Buffer B were used in the ERLIC separation: ERLIC01, 10 mM ammonium formate in 90% ACN, pH 2.6 and 25% ACN, 2% formic acid (FA); ERLIC02, 10 mM ammonium formate in 90% ACN, pH 3.9 and 25% ACN, 2% FA; ERLIC03, 85% ACN, 0.05% FA and 25% ACN, 2% FA; ERLIC04, 80% ACN, 0.1% FA and 10% ACN, 2% FA. The collected fractions were then dried with a vacuum centrifuge.

### SCX Separation

This was performed with peptides from 2 mg protein in replicate as previously described except that the pH of the mobile phases was 2.7, not 3.0 [30].

### PNGase F Treatment

The SCX- and ERLIC-fractionated peptides were redissolved in 25 mM NH_4_HCO_3_. To the solution, about 15 units of PNGase F (P0705L, New England Biolabs Inc.) were added and incubated at 37°C for 5 h for complete deglycosylation. After the glycopeptides are digested with PNGase F, the NH_2_ group in asparagine changes into OH group at the glycosylation site (i.e. deamidation of asparagine) so that the mass of deglycosylated peptides increases 0.984 Dalton, which can be detected unambiguously by FTMS. However, the reaction can also happen spontaneously due to in vivo deamidation or sample preparation. Thus, an independent control was processed with the same procedure except without adding PNGase F to the sample for estimation of false positive identifications of glycosylation sites by measuring the number of pre-existing sites of deamidation. They were then dried with a vacuum centrifuge and redissolved in 100 µL 0.1% FA for LC-MS/MS analysis.

### LC-MS/MS

The deglycosylated peptides were separated and analyzed on a Shimadzu UFLC system coupled to a LTQ-FT Ultra (Thermo Electron, Bremen, Germany). One third of the peptides in each fraction were injected into a Zorbax peptide trap column (Agilent, CA, USA) via the auto-sampler of the Shimadzu UFLC for desalting. The peptides were separated in a capillary column (200 µm ×10 cm) packed with C18 AQ (5 µm, 300 Å, Michrom BioResources, Auburn, CA, USA) at a flow rate of 500 nl/min. Mobile phase A (0.1% FA in H2O) and mobile phase B (0.1% FA in acetonitrile) were used to establish the 60 min gradient comprised of 45 min of 8–35% B, 8 min of 35–50% B and 2 min of 80% B followed by re-equilibrating at 5% B for 5 min. The peptides were then analyzed on LTQ-FT with an ADVANCE™ CaptiveSpray™ Source (Michrom BioResources) at an electrospray potential of 1.5 kV. A gas flow of 2, ion transfer tube temperature of 180°C and collision gas pressure of 0.85 mTorr were used. The LTQ-FT was set to perform data acquisition in the positive ion mode as previously described except that the m/z range of 350–1600 was used in the full MS scan [27].

### Data Analysis

The raw data were first converted into the dta format using the extract_msn (version 4.0) in Bioworks Browser (version 3.3, Thermo Fisher Scientific Inc), and then the dta files were converted into Mascot generic file format using an in-house program as described [31]. Intensity values and fragment ion m/z ratios were not manipulated. The IPI rat protein database (version 3.40, 40381 sequences, 20547209 residues) and its reversed complement were combined and used for database searches. The database search was performed using an in-house Mascot server (version 2.2.04, Matrix Science, Boston, MA, USA) with MS tolerance of 5 ppm and MS/MS tolerance of 0.5 Da. Two missed cleavage sites of trypsin were allowed. Carbamidomethylation (C) was set as a fixed modification, and oxidation (M), phosphorylation (S, T and Y) and deamidation (N) were set as variable modifications. The obtained peptide/protein list for each fraction was either exported to Microsoft Excel or processed using an in-house script for further analysis. The dta files of peptides of which the Mascot score was over 20 in each fraction were combined and converted into Mascot generic file format using an in-house program. It was then searched again using Mascot to generate the protein list for false discovery rates (FDR) evaluation. FDRs were evaluated according to the target-decoy strategy as previously described [32] and were set to 0.01. Peptides identified with a consensus N-X-S/T (with X not proline) and a modification of deamidation at asparagine were regarded as N-linked glycopeptides. For high confidence peptide identification, peptide matches were filtered with an expectation value of less than 0.05 in the Mascot search. After filtering, the FDRs of glycopeptide and phosphopeptide identification were estimated to be less than 1% in each analysis.

### Protein Classification and Functional Annotation

Proteins, phosphoproteins and glycoproteins identified in this study were categorized according to their respective subcellular locations and biological processes using the RGD database with AmiGo Go Slimmer online annotation tools [33], [34].

## Results and Discussion

### The mechanism of concurrent analysis of proteome, phosphoproteome and glycoproteome in SCX and ERLIC fractionations

**Figure 1:**
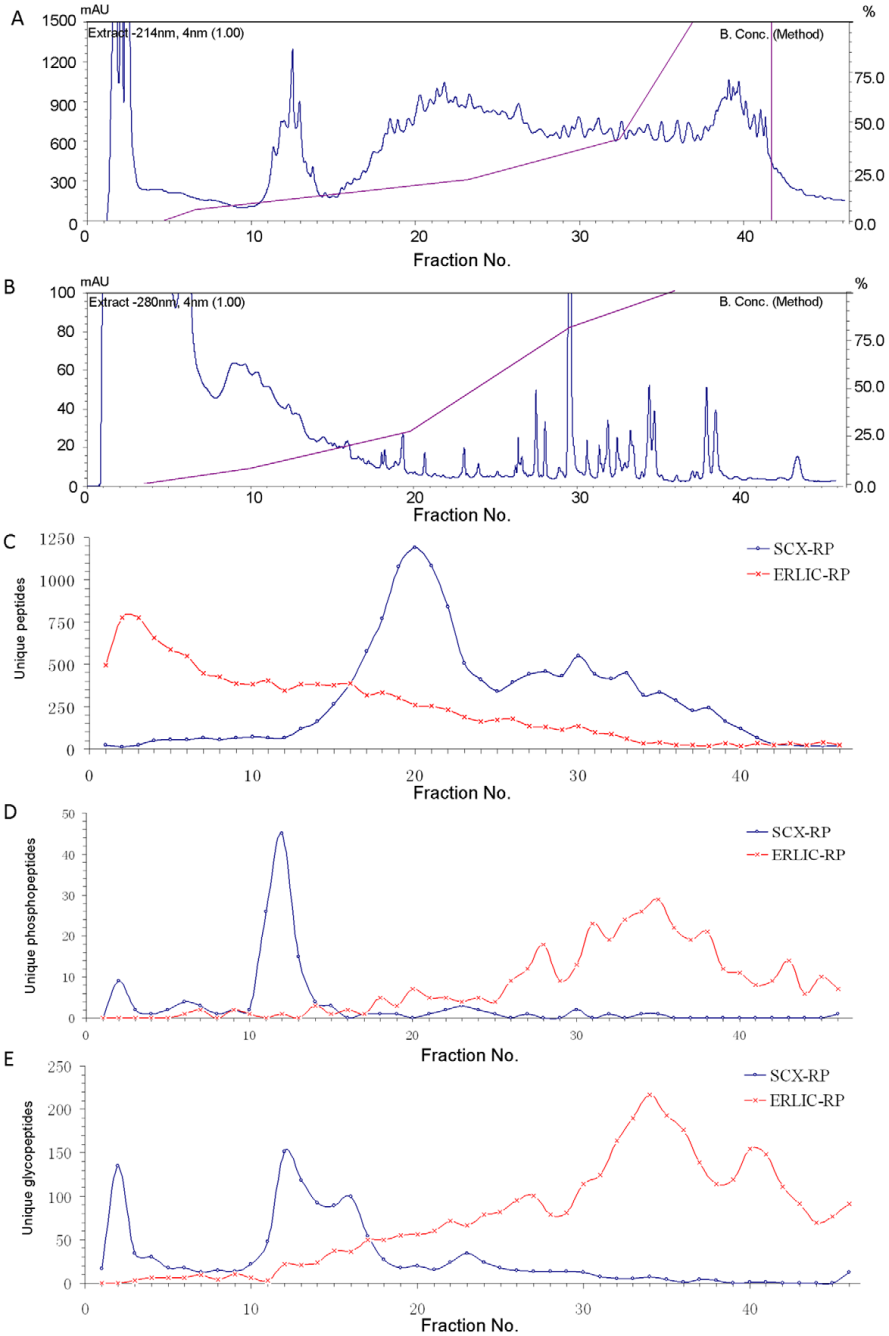
Chromatograms and peptides distribution. SCX (A) and ERLIC (B) chromatograms of rat kidney tryptic peptides and distribution of total peptides (C), phosphopeptides (D), and glycopeptides (E). The scales of the Y-axes are different because 214 nm was monitored in (A) and 280 nm in (B).

As shown in Figure 1A and 1B, SCX and ERLIC fractionations generate completely different chromatograms due to their different separation principles. When SCX fractionation is conducted at pH 2.7, most of the tryptic peptides carry a net charge of +2 due to the positive charge at the C-terminal arginine/lysine and at their N-terminus [35]. Because of the negative charge from a phosphate group or sialic acid, most mono-phosphorylated peptides and mono-sialylated glycopeptides have a net charge of +1 and so are less well-retained by SCX materials and elute before most unmodified peptides [17]. Most multi-phosphorylated peptides and multi-sialylated glycopeptides are neutral or negatively charged and elute even earlier, frequently in the flow-through. Thus, unmodified peptides are separated from phosphopeptides and sialylated glycopeptides to a significant extent, and concurrent analysis of proteome, phosphoproteome and glycoproteome is potentially achieved in one run (Figure 1C-1E). Practically speaking, this approach is not completely successful. Peptides eluted in the flow-through are difficult to identify without further fractionation. Also, only about 30% of the phosphopeptides in a complex digest have a net charge of +1 or less at pH 2.7. The rest are distributed throughout the SCX gradient and so a second enrichment step such as titania or IMAC affinity chromatography is necessary to achieve good phosphopeptide identification.

ERLIC is a mixed-mode chromatography method that separates peptides based on both charge and polarity. It can be manipulated more effectively than SCX to achieve phosphopeptide and glycopeptide enrichment with simultaneous fractionation of these two classes of modified peptides. They were characterized simultaneously from a digest of mouse brain membrane but under conditions where the unmodified peptides eluted in the flow-through [29]. Also, the use of non-volatile salts in the gradient buffers in that study required subsequent desalting of each fraction with reverse phase C18 cartridges. That may have led to the loss of some hydrophilic phosphopeptides [36]. In this study, no salts or only volatile salts were used in the loading and elution solvents so that desalting was not necessary for the ERLIC fractions, and the pH and concentration of organic reagents in the buffers were also optimized so that unmodified and modified peptides were all retained and fractionated simultaneously. In ERLIC fractionation, unmodified peptides with net charge of +2 in the pH range 2.6–3.9 are repelled electrostatically by the weak anion-exchange (WAX) material but are still retained on the column with high organic mobile phase through hydrophilic interaction. They can then be distributed into multiple fractions during elution through the simultaneous effect of electrostatic repulsion and a decrease in hydrophilic interaction. Most of the unmodified peptides are eluted from the column in the range of 80–65% ACN in the gradient (Figure 1B and 1C). Phosphopeptides and glycopeptides tend to elute after unmodified peptides due to their high hydrophilicity plus the electrostatic attraction to the column by the negatively charged phosphate or sialyl- group (Figure 1C–1E). In addition to this enrichment process, another obvious advantage of ERLIC over SCX is that few, if any, phosphopeptides and glycopeptides elute in the flow-through, facilitating their separations and subsequent MS analysis. As false positive discovery is always a concern for high throughput analysis, we performed a control experiment here with the sample preparation procedure unchanged except without adding PNGase F to the sample in order to estimate the extent of non-specific deamidation. We found that 72 NXS/T deamidated sites were identified both in ERLIC04 and in the control samples. The false positive glycosylation sites corresponded to 10% of the total in the final result, which is due to the non-specific deamidaton that happens spontaneously either in vivo or during the sample preparation.

### Comparison of the performance of SCX and ERLIC in the concurrent analysis of proteome, phosphoproteome and glycoproteome in rat kidney tissue

For SCX fractionation, a shallow gradient was used to optimize the separation of phosphopeptides and sialylated glycopeptides from unmodified peptides. As expected, most of the phosphopeptides and glycopeptides identified were eluted before unmodified peptides, some of them in the flow-through. In ERLIC fractionation, solvent A was also used as the sample solvent, and different combinations of solvent A and solvent B were used to produce different elution gradients for the optimized fractionation of phosphopeptides and glycopeptides without significantly affecting the separation of unmodified peptides. The retention and fractionation of unmodified peptides were successfully achieved with the solvents used here.

**Figure 2:**
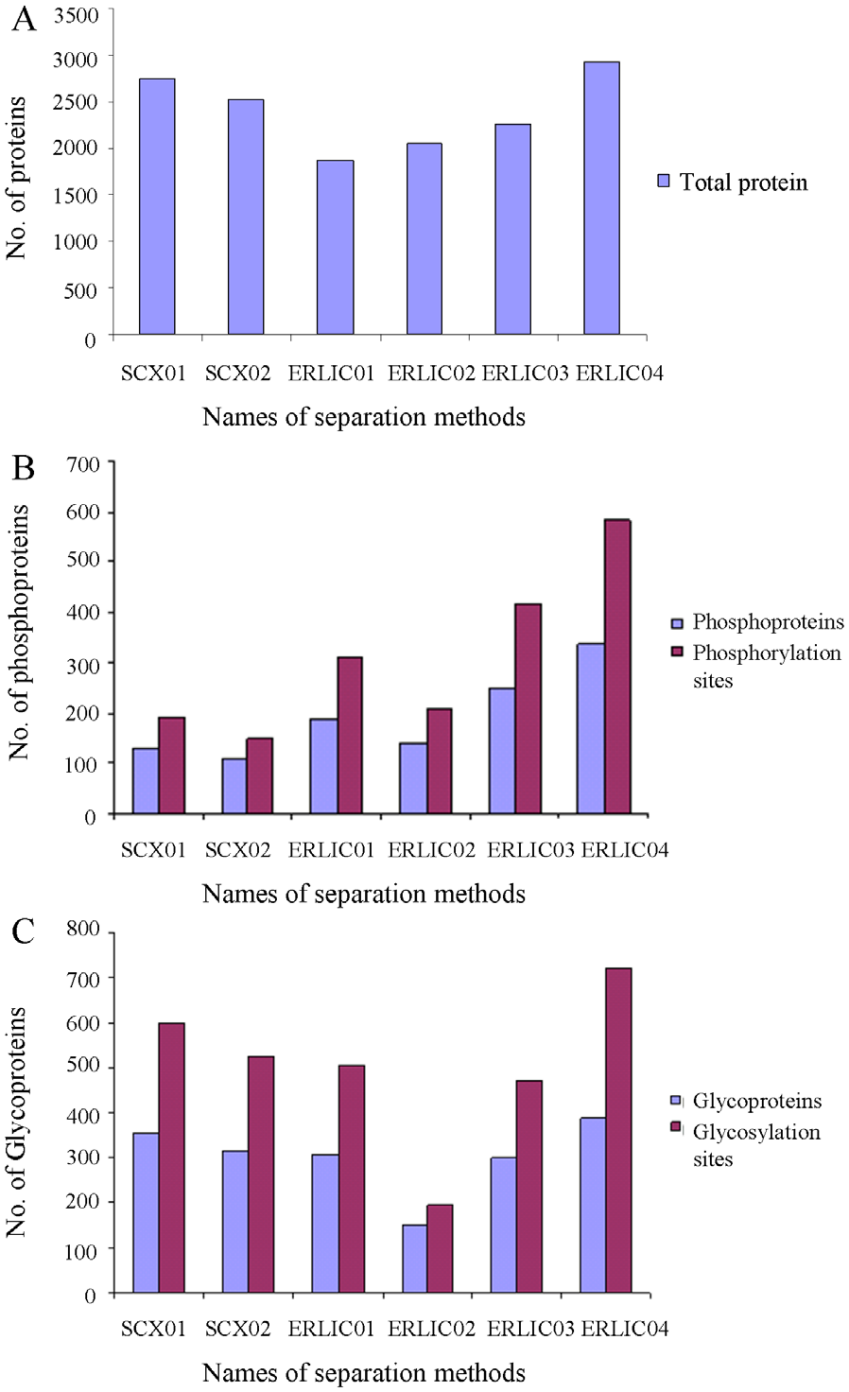
Comparison of the efficiency of SCX and ERLIC methods. Comparison of total protein (A), phosphoprotein (B) and glycoprotein (C) identification in SCX and ERLIC methods using different gradients. For the gradients used in each separation method, see Materials and Methods.

As shown in Figure 2A, the number of proteins identified in ERLIC04 was the highest in all six fractionation methods, i.e. 2929, better than with the SCX method that is so widely used for the fractionation of unmodified peptides. As illustrated in Figure 2B, 338 phosphoproteins and 583 phosphorylation sites (The MS/MS spectra are shown in Data S1) were identified in ERLIC04, the highest of the six fractionation methods (158 and 204 percent higher, respectively, than with SCX01 in which 131 phosphoproteins and 192 phosphorylation sites were identified). The identification of significantly less phosphoproteins in SCX may be attributed to 1) the loss of some hydrophilic phosphopeptides during the desalting step and 2) the distribution of most phosphopeptides amongst coeluting unmodified peptides. The better separation and enrichment efficiency of ERLIC can be another important factor. The number of glycoproteins (387) and glycosylation sites (722) (The MS/MS spectra are shown in Data S2) identified in ERLIC04 was also the highest compared with the other fractionation methods (Figure 2C). The MS/MS spectra for the identification of phosphopeptides and glycopeptides were shown in Data S3–S4. For SCX01, 598 glycosylation sites in 353 glycoproteins were identified. In conclusion, ERLIC04 using 80% ACN, 0.1% FA as buffer A and 10% ACN, 2% FA as buffer B performed the best in the concurrent fractionation of unmodified peptides, phosphopeptides and glycopeptides. The complete list of proteins, phosphoproteins and glycoproteins identified in this study is supplied in Table S1–S3. Use of salt-containing mobile phases (*e.g.*, ERLIC01 and ERLIC02) led to a better separation of modified from unmodified peptides but not to the identification of more modified peptides. A possible explanation is that the distribution of phosphopeptides or glycopeptides into more fractions in the salt-free ERLIC gradient facilitates their MS analysis.

### Annotation of identified phosphorylation and glycosylation sites with SWISS-PROT database

**Figure 3:**
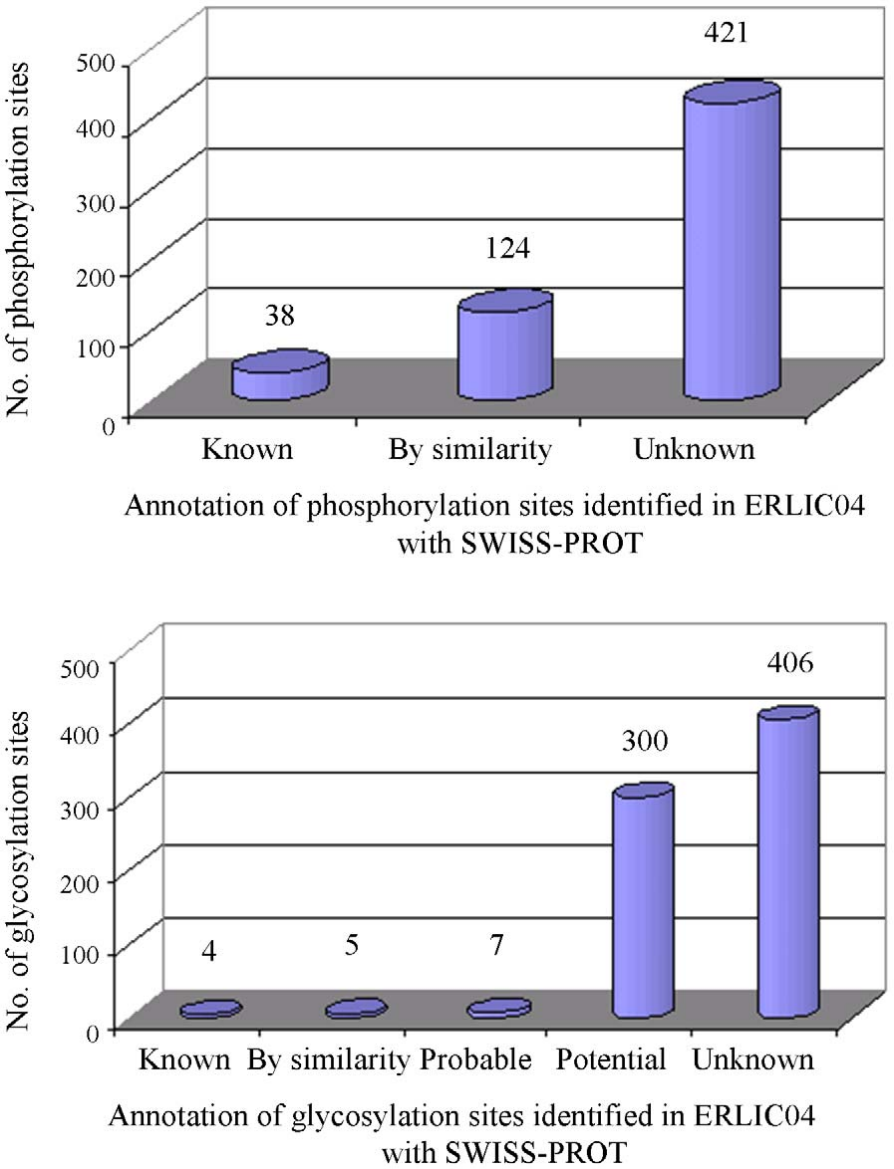
Annotation of identified phosphorylation and glycosylation sites. Matches of identified phosphorylation sites (A) and glycosylation sites (B) were with the SWISS-PROT knowledge database of *Rattus*.

A total of 583 putative phosphorylation sites and 722 putative glycosylation sites were identified in the ERLIC04 method via LC-MS/MS in this study (Figure 2B and 2C). They were annotated by matching them with the SWISS-PROT database. Among the identified phosphorylation sites, only 38 were annotated as known phosphorylation sites, 124 (21.3%) of them were annotated “by similarity”, and 421 (72.2%) were unknown either because the sites were not annotated in the database or because the corresponding proteins did not have a SWISS-PROT entry (Figure 3A). Similarly, for the glycosylation sites, only 4 were annotated as known glycosylation sites, 312 (43.2%) of them were annotated “probable”, “potential” or “by similarity”, and 406 (56.2%) of them were not documented as glycosylation sites or the corresponding proteins did not have a SWISS-PROT entry (Figure 3B). These results indicate that phosphorylation and glycosylation sites are underestimated in the current database. The strategy presented not only helps to validate known and potential modification sites but also identifies many new ones. Information for each identified phosphoprotein and glycoprotein including protein accession number (IPI), protein description, identified phosphopeptides or glycopeptides, unique phosphorylation or glycosylation sites, and their annotation in SWISS-PROT are listed in Table S2–S3.

The number of phosphorylation and glycosylation sites identified in this study using one-step enrichment methods was comparable to those from studies aimed only at either phosphoproteome or glycoproteome analysis in tissue samples. For example, Han et al identified 274 phosphorylation sites corresponding to 168 proteins from human liver tissue [37]; Feng et al reported 163 phosphorylation sites from 133 proteins in mouse liver lysate [38]; Zhang et al identified 445 glycosylation sites from prostate cancer tissue and 176 glycosylation sites from bladder cancer tissue [39]. Of course, the combination of additional enrichment steps for specific subproteomes would presumably have resulted in the identification of many more modification sites, but the incorporation of an additional enrichment step would have made difficult the simultaneous analysis of unmodified peptides.

### Functional analysis of identified proteins, phosphoproteins and glycoproteins

In total, 2929 proteins, 338 phosphoproteins and 387 glycoproteins were identified in the ERLIC04 method followed by LC-MS/MS (Figure 2A–2C). Each was categorized according to their subcellular locations and biological processes using online Gene Ontology tools in order to determine whether peptides from particular categories of proteins were significantly enriched. As shown in Table 1, the subcellular locations of the proteins identified in whole proteome and subproteome groups were quite different. Compared with the cellular components of the whole proteome, some cellular components were better represented in the phosphoproteome, such as cytoskeleton (7% to 11%) and nucleus (12% to 19%), while proteins in the extracellular region (5% to 2%) and mitochondrion (11% to 4%) decreased significantly. This confirms that peptides from phosphoproteins are selectively enriched in the ERLIC04 method, and suggests that phosphorylation mainly happens inside the cells in order to achieve its regulatory roles in some pathways. As with the phosphoproteome, some cellular components in the glycoproteome also increased significantly relative to the whole proteome: extracellular region (5% to 13%), proteinaceous extracellular matrix, (1% to 4%), plasma membrane (10% to 17%), and other cytoplasmic organelle (5% to 7%). It is noteworthy that less N-glycoproteins come from cytosol, cytoskeleton, mitochondrion and nucleus (Table 1), indicating that N-glycosylation mainly occurs on membrane proteins and extracellular matrix proteins located outside the cells.

**Table 1:** Gene ontology classification of identified proteins, phosphoproteins and glycoproteins in ERLIC04 according to their respective subcelluar locations using AmiGo Go Slimmer online annotation tools.

Subcellular location	Proteins	Phosphoproteins	Glycoproteins
Cytoskeleton	7%	11%	2%
Cytosol	10%	10%	4%
ER/Golgi	8%	7%	8%
extracellular region	5%	2%	13%
mitochondrion	11%	4%	6%
Nucleus	12%	19%	4%
other cell component	6%	6%	5%
other cytoplasmic organelle	5%	5%	7%
other membranes	24%	23%	30%
plasma membrane	10%	12%	17%
proteinaceous extracellular matrix	1%	1%	4%

As shown in Table 2, proteins identified from rat kidney tissue in the ERLIC04 method are involved in various biological processes such as protein metabolism, RNA metabolism, cellular component organization, transport and developmental processes. Here again, the distribution of the phosphoproteins and glycoproteins identified was found to be different in various biological processes than that of proteins of the whole proteome. Compared with the whole proteome, phosphoproteins were more significantly represented in the biological processes of RNA metabolism (7% to 10%), DNA metabolism (1% to 2%) and cellular component organization (9% to 13%), while N-glycoproteins were more likely to take part in various processes such as cell adhesion (2% to 7%), cell-cell signaling (1% to 2%), developmental processes (8% to 12%), and stress response (6% to 8%). This suggests again that protein phosphorylation plays important roles in intracellular processes while protein N-glycosylation is mainly involved in extracellular processes.

**Table 2:** Gene ontology classification of identified proteins, phosphoproteins and glycoproteins in ERLIC04 according to their respective biological processes using AmiGo Go Slimmer online annotation tools.

Biological process	Proteins	Phosphoproteins	Glycoproteins
biological_process	5%	5%	4%
cell adhesion	2%	2%	7%
cell cycle or proliferation	5%	6%	5%
cell-cell signaling	1%	1%	2%
cellular component organization	9%	13%	9%
cell death	4%	5%	3%
developmental processes	8%	9%	12%
DNA metabolism	1%	2%	1%
other metabolic processes	24%	20%	21%
protein metabolism	10%	9%	11%
RNA metabolism	7%	10%	3%
signal transduction	5%	6%	4%
stress response	6%	5%	8%
transport	11%	9%	11%

### The improvement of protein identification in subproteome enrichment

Since subproteome enrichment reduces the complexity of samples and facilitates MS analysis, it has been predicted that it will improve the sensitivity of protein identification significantly [9]. With the concurrent analysis of proteome, phosphoproteome and glycoproteome, it is convenient to determine whether subproteome enrichment improves the sensitivity of protein identification significantly in a single analysis and reduces the inter-experimental variation between proteome and subproteome analysis. In this study, 200 phosphoproteins or glycoproteins were identified solely from phosphopeptides and/or glycopeptides but not identified from unmodified peptides in ERLIC04 (Table S4), suggesting that the subproteome enrichment led to the identification of some low-abundance proteins. Based on the functional analysis using AmiGO Go Slimmer, 9% (27) of these proteins were involved in signal transduction, a significantly higher percentage than that in whole proteome (5%) and in the phosphoproteome (6%) and glycoproteome (4%) overall (Table 2). Since most proteins involved in signal transduction are of relatively low abundance, the results indicate that subproteome enrichment improves the sensitivity of identification of low-abundance proteins. Checking the listings of these 27 proteins in the SWISS-PROT database: 23 of them (85.2%) were annotated “evidence at transcript level”; 1 had no SWISS-PROT entry; only 3 were annotated “evidence at protein level”. This sketchy record seems to confirm that most of them are of low abundance.

In addition, subproteome enrichment leads to the identification of many low-abundance proteins with known important functions that cannot be identified from whole proteome analysis. For example, integrin alpha-5/beta-6 and angiopoietin-like 3 are identified only in the PTM-enriched fraction. Integrin alpha-5/beta-6 is a receptor for fibronectin and cytotactin, and their binding inhibits tumor growth, angiogenesis and metastasis [40]. Angiopoietin-like 3, present at extremely low levels in kidney, can bind integrin alpha-5/beta-3, which induces cell adhesion and migration and regulates angiogenesis [41]. The expression of many other cancer related genes, such as CD63, CD36, Cd164, Gpld1, Ace and Braf, was also detected only from the set of enriched modified peptides.

### The occurrence of partial phosphorylation on some phosphorylation sites

**Figure 4:**
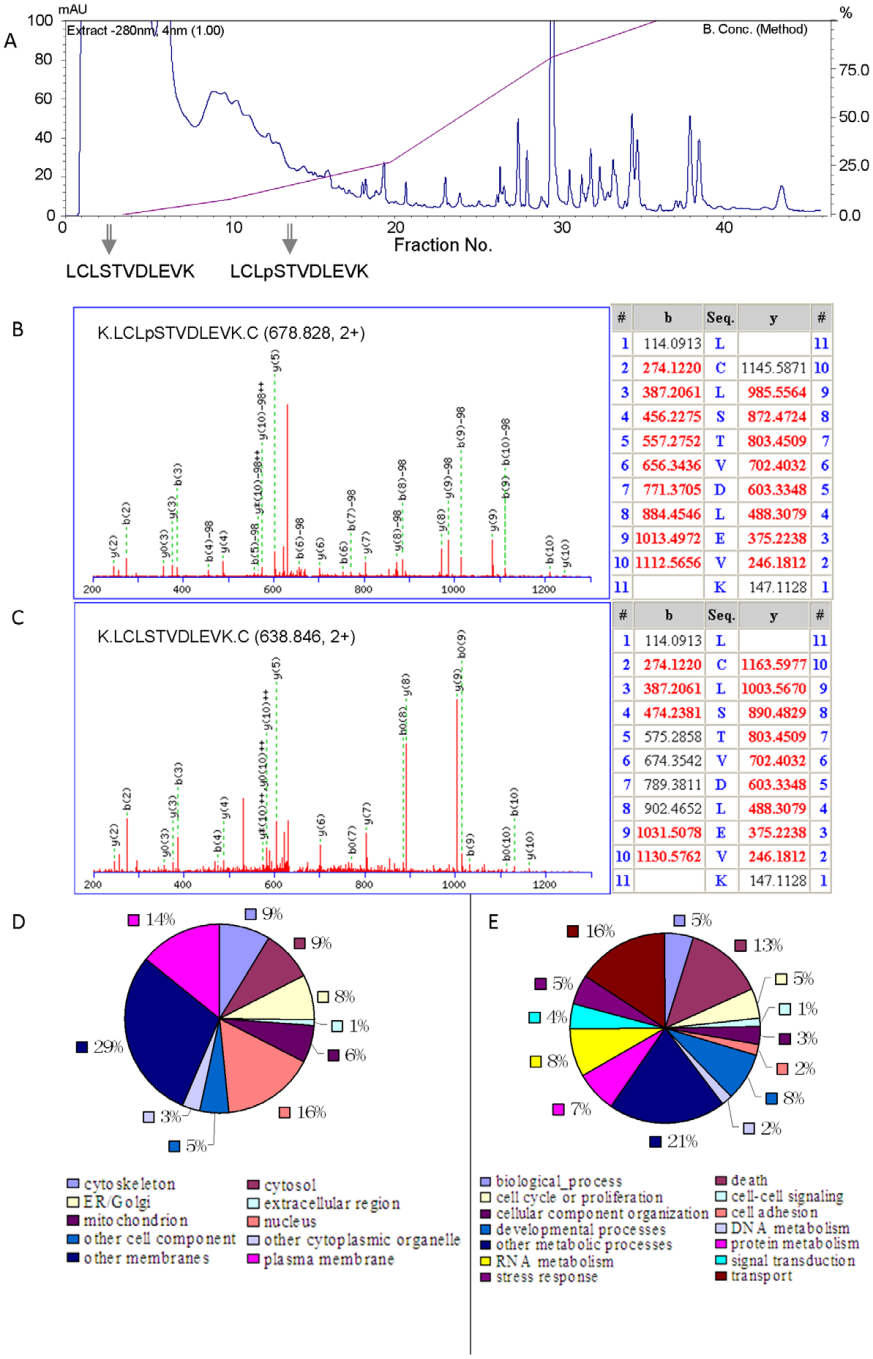
The occurrence of partial phosphorylation of a representative peptide. The ERLIC chromatogram shows the fractions in which the phosphopeptide and its unmodified form were eluted (A); Representative MS/MS spectra for identification of the phosphopeptide (B) and its unmodified form (C); Gene ontology analysis of partially phosphorylated proteins according to their subcellular locations (D) and biological processes (E) using AmiGo Go Slimmer.

Protein phosphorylation can vary quickly in response to intracellular or extracellular stimuli independent of protein expression, and a change in phosphorylation level of some proteins may be an indicator of physiological state [19]. Sometimes a change in protein phosphorylation also accompanies a protein's expression [42]. Thus, it is necessary to distinguish between the two. The presented method (ERLIC04) for concurrent analysis of proteome and subproteomes is a promising means of doing so. By comparing phosphopeptides with unmodified peptides identified here, the corresponding unmodified peptides were found for 96 unique phosphopeptides in 84 phosphoproteins (Table S5). As shown in Figure 4A, the phosphopeptide of LCLpSTVDLEVK was eluted 11 fractions later than its unmodified form, due of course to the phosphate group that prolongs its retention in the ERLIC mode. The phosphorylation level of the protein at this site was estimated to be about 51% using the peak intensities of the extracted ion chromatogram (XIC) of the phosphopeptide and its unmodified form. This estimate presumes that they ionize with equal efficiency, which may or may not be the case.

The MS/MS spectra of the peptide of LCLpSTVDLEVK and its unmodified form are shown in Figure 4B and 4C, respectively. In the MS/MS spectrum, its phosphorylation site is sandwiched by fragments still bearing the modification as well as the corresponding neutral loss (-98Da) fragments, i.e. y4, y5, y6, y7, y8-98, y8, y9-98, y9, y10, which validates the phosphorylation site assignment.

Phosphoproteins with substoichiometrically phosphorylated sites were categorized according to their subcellular locations and biological processes using Amigo Go Slimmer. As shown in Figure 4D and 4E, partial phosphorylation can occur on proteins in all biological processes and subcellular locations except for proteinaceous extracellular matrix. In comparison with that of total phosphoproteins in Table 1, partial phosphorylation is more likely to happen in mitochondria (6% versus 4%) and other membranes (29% versus 23%), and it also tends to occur on proteins involved in the biological process of transport (16% versus 9%). Of the 8 partially phosphorylated mitochondrion proteins identified in this study, 5 of them are involved in transport. For example, monocarboxylate transporter 1 is a proton-linked monocarboxylate transporter that catalyzes the rapid transport across the plasma membrane of many monocarboxylates; mitochondrial import receptor subunit TOM70 is a receptor to accelerate the import of all mitochondrial precursor proteins; 14-3-3 protein zeta/delta and 14-3-3 protein epsilon are adapter proteins involved in many general and specialized signaling pathway, and their interactions generally affects the activity of the binding partners. However, it is still elusive how partial phosphorylation affects the function of these proteins. Partial phosphorylation has been reported in the regulation of transport process. For example, the phosphorylation degree of tau protein regulates its axonal transport by controlling its binding to kinesin [43]; dysregulation of tau phosphorylation is observed in Alzheimer's disease [44]; cyclin-dependent kinase 5 can increase the phosphorylation degree of perikaryal neurofilament and inhibits neurofilament axonal transport in response to oxidative stress [45]; and the phosphorylation degree of the nuclear transport machinery has been reported to negatively regulate entire nuclear transport pathways for the global control of cellular activities [46]. The systematic study of partial phosphorylation will deepen the understanding of its roles in other biological processes.

### The occurrence of partial N-glycosylation on some glycosylation sites

**Figure 5:**
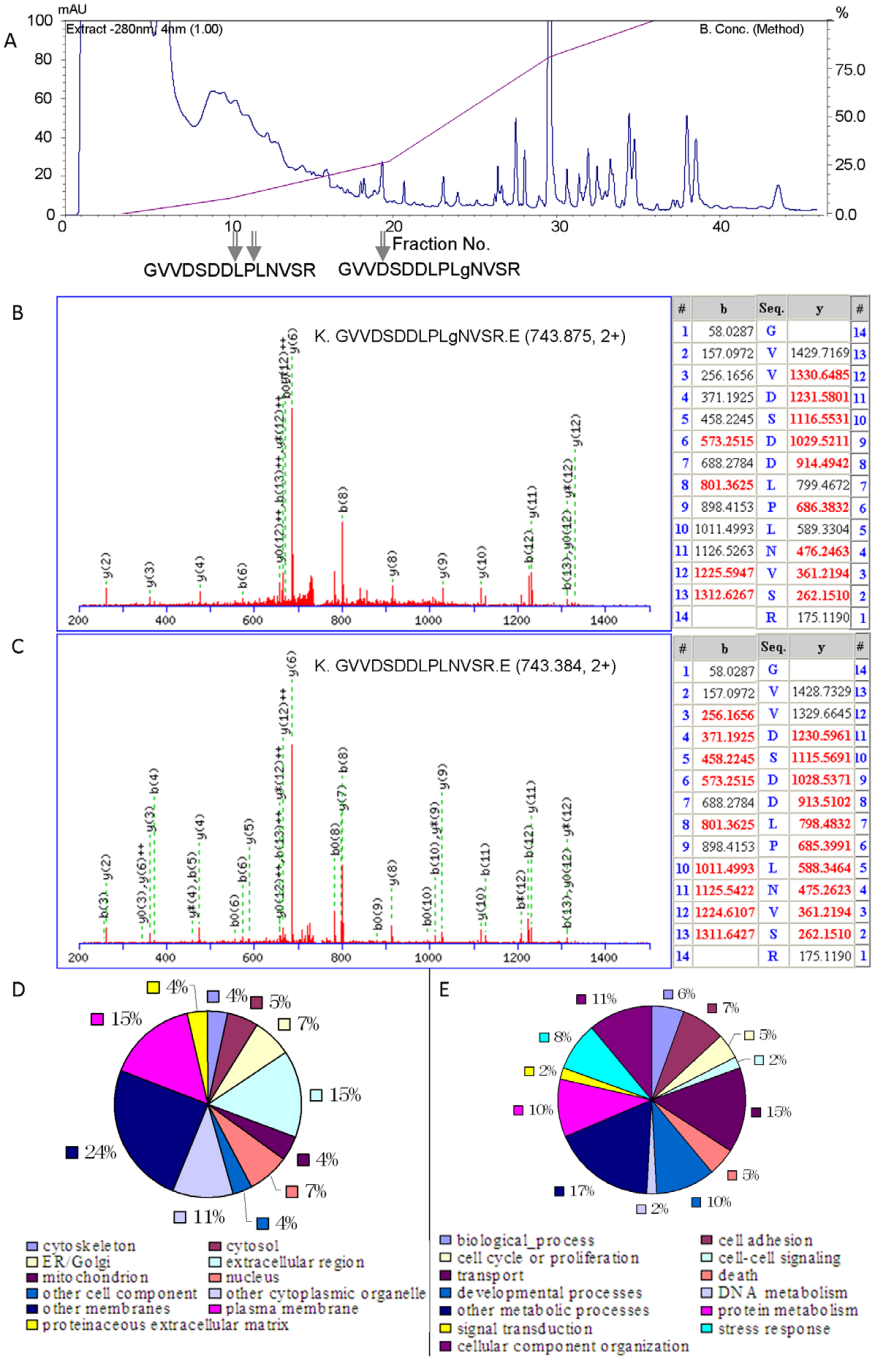
The occurrence of partial N-glycosylation of a representative peptide. The ERLIC chromatogram shows the fractions in which the glycopeptide and its unmodified form were eluted (A); Representative MS/MS spectra for identification of the glycopeptide (B) and its unmodified form (C); Gene ontology analysis of partially glycosylated proteins according to their subcellular locations (D) and biological processes (E) using AmiGo Go.

The role of partial N-glycosylation in glycoproteins has seldom been studied because most current glycoprotein analysis methods are incapable of distinguishing partially from fully glycosylated sites. In this study, by comparing glycopeptides with unmodified peptides identified by the ERLIC04 method, the corresponding unmodified peptides were identified for 60 unique glycopeptides in 53 glycoproteins (Table S6). As shown in Figure 5A, the glycopeptide of GVVDSDDLPLgNVSR eluted 8 or 9 fractions later than its unmodified form, a significant shift for so acidic a peptide. This is evidence of the degree to which the negative charge from sialic acid residues (and possibly the hydrophilic interaction with the rest of the glycan) prolongs retention in ERLIC. The glycosylation level at this site *in vivo* was estimated to be about 1.2% using the peak intensity of the XICs of the glycopeptide and its unmodified form, indicating that our strategy is very sensitive in detecting partial glycosylation. The MS/MS spectra of the peptide of GVVDSDDLPLgNVSR and its unmodified form are shown in Figure 5B and 5C, respectively. In the MS/MS spectrum, its glycosylation site is sandwiched between fragments with the deamidated asparagine (aspartic acid), i.e. b12, b13, y6 and y8-y12, the mass of which was about 0.98 Da higher than that of the corresponding b or y ions detected in the MS/MS spectrum of the unmodified peptides. This further validates the assignment of the glycosylation site.

The glycoproteins with partially glycosylated sites were also categorized according to their subcellular locations and biological processes using Amigo Go Slimmer. As shown in Figure 5D and 5E, partial glycosylation can occur on proteins in all of the subcellular locations and biological processes except for RNA metabolism and transcription. However, when compared with the distribution of glycoproteins listed in Table 1, partial glycosylation tends to happen on cytoskeleton (4% versus 2%), nucleus (7% versus 4%) and other cytoplasmic organelles including endosome, lysosome and vacuole (11% versus 7%), and is also prone to occur on proteins involved in the biological process of cellular component organization (11% versus 9%) and transport (15% versus 11%). Altered N-glycosylation was reported to reduce but not completely eliminate intracellular transport of lactase-phlorizin hydrolase to the microvillus membrane in rat small intestine [47]. Another study, about human solute carrier PAT1, reported that N-glycans are not essential for transport function but are important for membrane targeting [48]. The degree of N-glycosylation may play important roles in controlling the rate of transport. To the best of our knowledge, there are still no reports about the role of partial N-glycosylation in cellular component organization. The list of partially glycosylated proteins discovered in this study will be helpful to an understanding of its biological significance.

### Analysis of proteins with both phosphorylation sites and glycosylation sites

In this study, the concurrent analysis of proteome, phosphoproteome and glycoproteome led to the identification of 12 proteins with both phosphorylation and glycosylation, of which 11 were membrane proteins or extracellular matrix proteins. The existence of the remaining one was only predicted from the DNA. Information for each of them including protein accession number and annotation in SWISS-PROT, protein description, functions, and unique phosphorylation or glycosylation sites are listed in Table S7. Some of the proteins were found to be involved in both intracellular and extracellular processes. For example, versican core protein may play a role in intercellular signaling and in connecting cells with the extracellular matrix, and it may also be involved in the regulation of cell motility, growth and differentiation. Its phosphorylation sites have been predicted to be present in the extracellular domain in SWISS-PROT. As an extracellular matrix protein, fibronectin plays a major role in cell adhesion, growth, migration and differentiation [49]. It induces fibril formation after binding to anastellin, and the fibronectin polymer enhances cell adhesion so that tumor growth, angiogenesis and metastasis can be inhibited. It is reported that altered fibronectin expression, degradation and organization is closely related to many diseases including cancer and fibrosis [40]. Fibronectin not only exhibits functions typical of N-glycoproteins, but also signals to intracellular spaces via adhesion receptors, i.e. the typical functions of phosphoproteins. The formation of fibronectin and integrin complex results in a range of downstream effects, including phosphorylation of FAK [50]. FAK has been found to be directly involved in sensing the extracellular matrix and responding efficiently to the changing microenvironment [51]. Thus, fibronectin successfully connects intracellular events with the changes of extracellular matrix, which are closely related to glycoproteins. However, other proteins in Table S7 have been less studied with few functional annotations in SWISS-PROT. In the past, the study of N-glycosylation and phosphorylation has been conducted independently, which prevented the elucidation of the relationship between phosphorylation and N-glycosylation in the same protein. The present strategy will facilitate such analysis by identifying proteins with both these major modifications.

### Comparison of the ERLIC approach with two previously published ERLIC based approaches

We have published two novel ERLIC based fractionation approaches for the simultaneous characterization of glyco- and phosphoproteomes of mouse brain membrane [29] and the comprehensive profiling of rat kidney proteome [30]. In the first one, both glycopeptides and phosphopeptides were selectively enriched due to their hydrophilic interaction and/or electrostatic interaction of the negative charged phosphoric and sialyl groups with the stationary phase of the ERLIC column, but most unmodified peptides were excluded as flow-through since they were repelled by the stationary phase at 70% ACN at pH 2. In the second one, 90% ACN/0.1% acetic acid was used as mobile phase A so that nearly all peptides will be retained on the ERLIC column through hydrophilic and/or electrostatic interactions, and they were evenly distributed into multiple fractions based on both p*I* and polarity when eluted using a shallow gradient of increasing water content and decreasing pH value. However, both glycopeptides and phosphopeptides cannot be characterized effectively due to their sub-stoichiometric amounts and the ionization suppression from unmodified peptides. In this study, we further optimized the ERLIC conditions so that as many as unmodified peptides were retained and fractionated by the column when phosphopeptides and glycopeptides were enriched. Its limitation is that both the analysis of modified peptides and unmodified peptides was compromised to a certain extent compared with the two previously published approaches. However, it provided a global analysis of both unmodified peptides and modified peptides in one run, which could not be achieved with the two previously published ERLIC approaches. It was also capable of detecting partial phosphorylation and N-glycosylation with potential biological significance regarding the control of some biological processes, such as cellular component organization and transport. At the same time, it identified some proteins having both these modifications, which would facilitate the future evaluation of cross-talk between these two vital PTMs.

In the future, when the present method is employed together with some quantitative methods, such as SILAC, iTRAQ or label-free quantification methods, it will be capable of assessing the changes in protein expression and these two PTMs in one analysis, which reduces the inter-experimental variations in the quantitation. Better understanding of substoichiometric modifications may be helpful in elucidation of how some biological processes are controlled inside the cell.

## Supporting Information

Data S1 **Tandem MS spectra of_glycopeptides from ERLIC enrichment.**
(PDF)

Data S2 **Tandem MS spectra_of phosphopeptides from ERLIC enrichment.**
(PDF)

Data S3 **Tandem MS spectra_of glycopeptides from SCX enrichment.**
(PDF)

Data S4 **Tandem MS spectra_of phosphopeptides from SCX enrichment.**
(PDF)

Table S1 **Summary of the protein/peptide identified in all analyses.**
(XLS)

Table S2 **Summary of the phosphoproteins and their phosphorylation sites identified in all analyses.**
(XLS)

Table S3 **Summary of the glycoproteins and their glycosylation sites identified in all analyses.**
(XLS)

Table S4 **Summary of the proteins identified from modified peptides but not unmodified peptides in ERLIC04.**
(XLS)

Table S5 **Partially phosphorylated proteins identified in ERLIC04.**
(XLS)

Table S6 **Partially glycosylated proteins identified in ERLIC04.**
(XLS)

Table S7 **Information about proteins with both phosphorylation and N-glycosylation identified in ERLIC04.**
(DOC)
