# Supplementary material for: Simultaneous Analysis of Proteome, Phospho- and Glycoproteome of Rat Kidney Tissue with Electrostatic Repulsion Hydrophilic Interaction Chromatography
Source: PLoS One. 2011 Feb 23;6(2):e16884. doi: 10.1371/journal.pone.0016884 (PMC3044146; doi:10.1371/journal.pone.0016884)
Supplement: Data S4 — Tandem MS spectra_of phosphopeptides from SCX enrichment. (PDF) [file pone.0016884.s004.pdf]

{MATRIX SCIENCE} Mascot Search Results

Results Generated by a Script Modified from Mascot Pepitde View
by Newman Sze, School of Biological Sciences, Nanyang Technological University

---

Spectrum No: 1; Query: 653; Rank: 1

Peptide View

MS/MS Fragmentation of **SSGSPYGGGYGSGGGSGGYGSR**
Found in **IPI00382376**, Tax_Id=10116 Gene_Symbol=Hnrpa3 Isoform 1 of Heterogeneous nuclear ribonucleoprotein A3

Match to Query 653: 1989.753808 from(995.884180,2+)
Title: 091127RatKid_SCX01_13.924.924.2.dta
Data file K:\NewmanPaper\Piliang\3SubProteomes\Piliang3SP\mgf5ppm\SCX_3SubProteomes5ppm.mgf

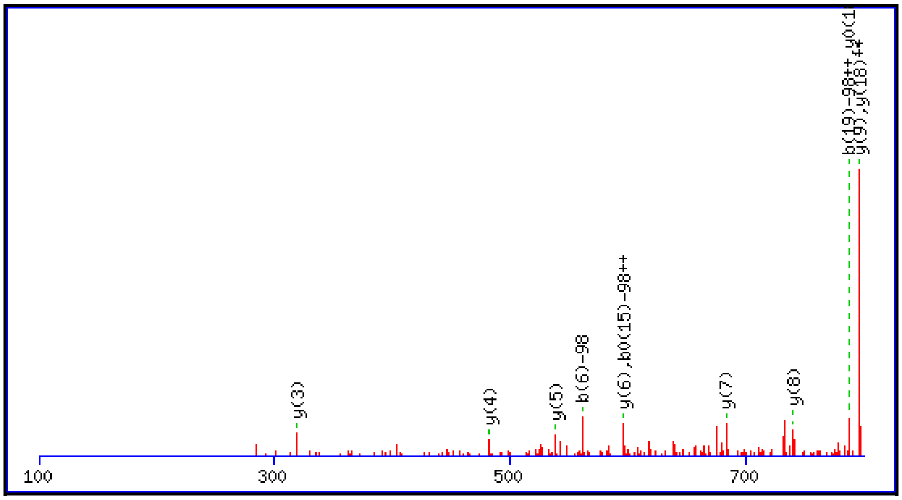

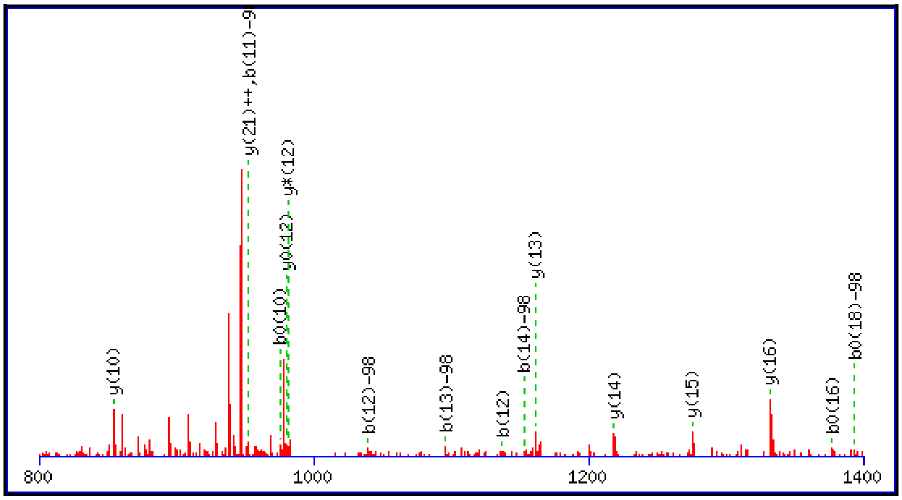

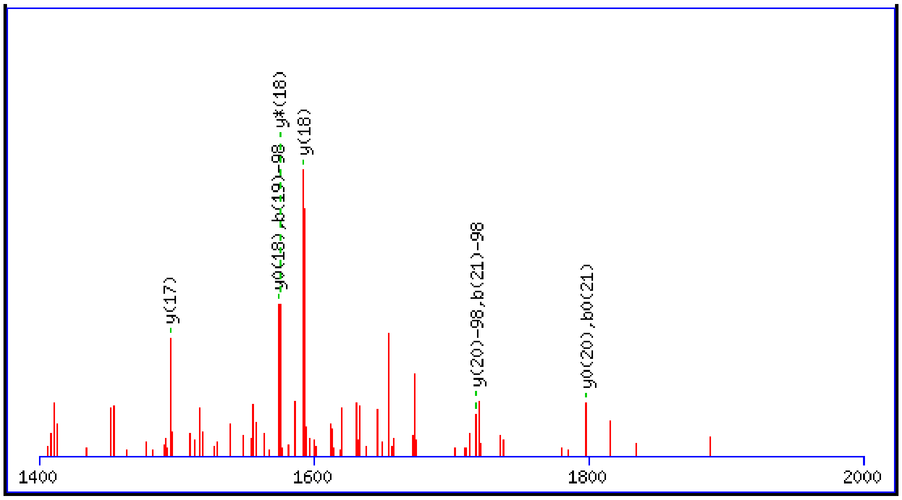

Monoisotopic mass of neutral peptide Mr(calc): 1989.7491
Fixed modifications: Carbamidomethyl (C)
Variable modifications:
S4 : Phospho (ST), with neutral losses 97.9769(shown in table), 0.0000
Ions Score: 97 **Expect:** 2.8e-008
Matches (Bold Red): 38/298 fragment ions using 66 most intense peaks

#	b	b^{++}	b^0	b^{0++}	Seq.	y	y^{++}	y*	y^{*++}	y^0	y^{0++}	#
1	88.0393	44.5233	70.0287	35.5180	S							22
2	175.0713	88.0393	157.0608	79.0340	S	1805.7474	903.3773	1788.7208	894.8641	1787.7368	894.3720	21
3	232.0928	116.5500	214.0822	107.5448	G	**1718.7153**	859.8613	1701.6888	851.3480	1700.7048	850.8560	20
4	301.1143	151.0608	283.1037	142.0555	S	1661.6939	831.3506	1644.6673	822.8373	1643.6833	822.3453	19
5	398.1670	199.5871	380.1565	190.5819	P	**1592.6724**	**796.8399**	**1575.6459**	**788.3266**	**1574.6619**	**787.8346**	18
6	**561.2303**	281.1188	543.2198	272.1135	Y	**1495.6197**	748.3135	1478.5931	739.8002	1477.6091	739.3082	17
7	618.2518	309.6295	600.2412	300.6243	G	**1332.5563**	666.7818	1315.5298	658.2685	1314.5458	657.7765	16
8	675.2733	338.1403	657.2627	329.1350	G	**1275.5349**	638.2711	1258.5083	629.7578	1257.5243	629.2658	15
9	732.2947	366.6510	714.2842	357.6457	G	**1218.5134**	609.7603	1201.4869	601.2471	1200.5028	600.7551	14
10	895.3581	448.1827	877.3475	439.1774	Y	**1161.4919**	581.2496	1144.4654	572.7363	1143.4814	572.2443	13
11	**952.3795**	476.6934	934.3690	467.6881	G	998.4286	499.7179	**981.4021**	491.2047	**980.4180**	490.7127	12
12	**1039.4116**	520.2094	1021.4010	511.2041	S	941.4071	471.2072	924.3806	462.6939	923.3966	462.2019	11
13	**1096.4330**	548.7201	1078.4225	539.7149	G	**854.3751**	427.6912	837.3486	419.1779	836.3646	418.6859	10
14	**1153.4545**	577.2309	1135.4439	568.2256	G	**797.3537**	399.1805	780.3271	390.6672	779.3431	390.1752	9
15	1210.4760	605.7416	1192.4654	**596.7363**	G	**740.3322**	370.6697	723.3056	362.1565	722.3216	361.6645	8
16	1297.5080	649.2576	1279.4974	640.2523	S	**683.3107**	342.1590	666.2842	333.6457	665.3002	333.1537	7
17	1354.5294	677.7684	1336.5189	668.7631	G	**596.2787**	298.6430	579.2522	290.1297	578.2681	289.6377	6
18	1411.5509	706.2791	**1393.5403**	697.2738	G	**539.2572**	270.1323	522.2307	261.6190	521.2467	261.1270	5
19	**1574.6142**	**787.8108**	1556.6037	778.8055	Y	**482.2358**	241.6215	465.2092	233.1083	464.2252	232.6162	4
20	1631.6357	816.3215	1613.6251	807.3162	G	**319.1724**	160.0899	302.1459	151.5766	301.1619	151.0846	3
21	**1718.6677**	859.8375	1700.6572	850.8322	S	262.1510	131.5791	245.1244	123.0659	244.1404	122.5738	2
22					R	175.1190	88.0631	158.0924	79.5498			1

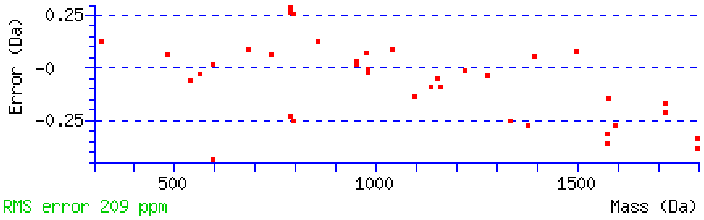

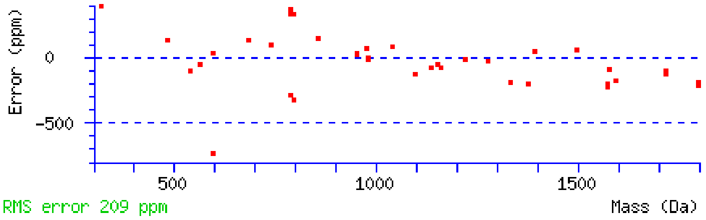

All matches to this query

Score	Mr(calc):	Delta	Sequence
97.0	1989.7491	0.0048	SSGSPYGGGYGSGGGSGGYGSR
88.0	1989.7491	0.0048	SSGSPYGGGYGSGGGSGGYGSR
88.0	1989.7491	0.0048	SSGSPYGGGYGSGGGSGGYGSR
70.1	1989.7491	0.0048	SSGSPYGGGYGSGGGSGGYGSR
44.6	1989.7491	0.0048	SSGSPYGGGYGSGGGSGGYGSR
26.5	1989.7491	0.0048	SSGSPYGGGYGSGGGSGGYGSR
11.8	1989.7491	0.0048	SSGSPYGGGYGSGGGSGGYGSR
6.0	1989.7659	-0.0121	LKFGNNWSQEYGSSGR
5.1	1989.7659	-0.0121	LKFGNNWSQEYGSSGR
4.7	1989.7659	-0.0121	LKFGNNWSQEYGSSGR

Spectrum No: 2; Query: 554; Rank: 1

Peptide View

MS/MS Fragmentation of **SSSVGSSSSYPISSAVPR**
Found in **IPI00209000**, Tax_Id=10116 Gene_Symbol=Plec1 Plectin 6

Match to Query 554: 1833.815948 from(917.915250,2+)
Title: 091127RatKid_SCX01_12.2077.2077.2.dta
Data file K:\NewmanPaper\Piliang\3SubProteomes\Piliang3SP\mgf5ppm\SCX_3SubProteomes5ppm.mgf

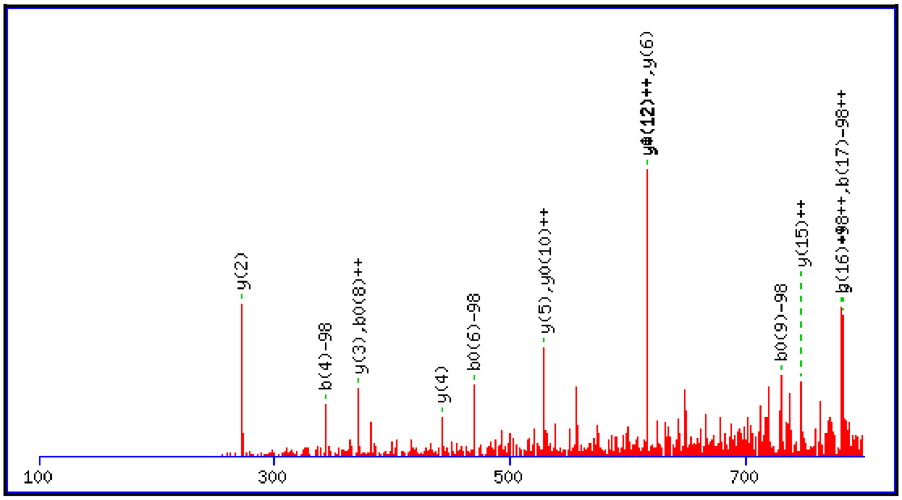

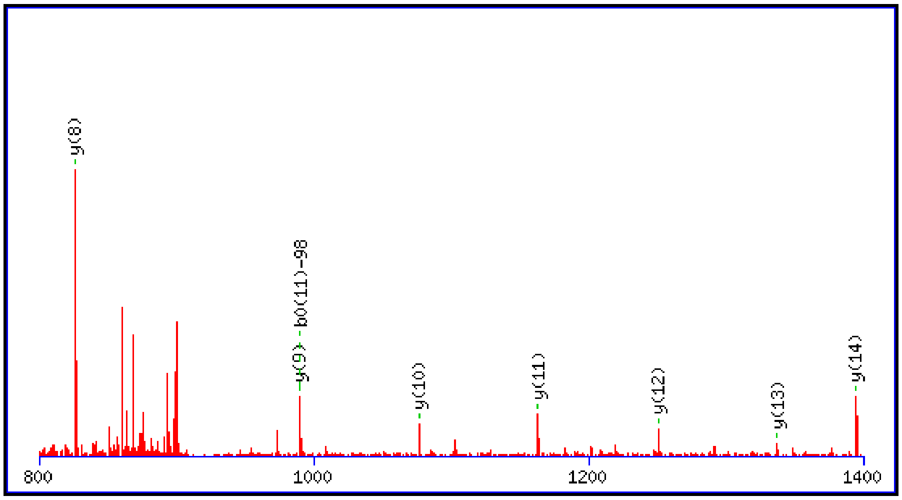

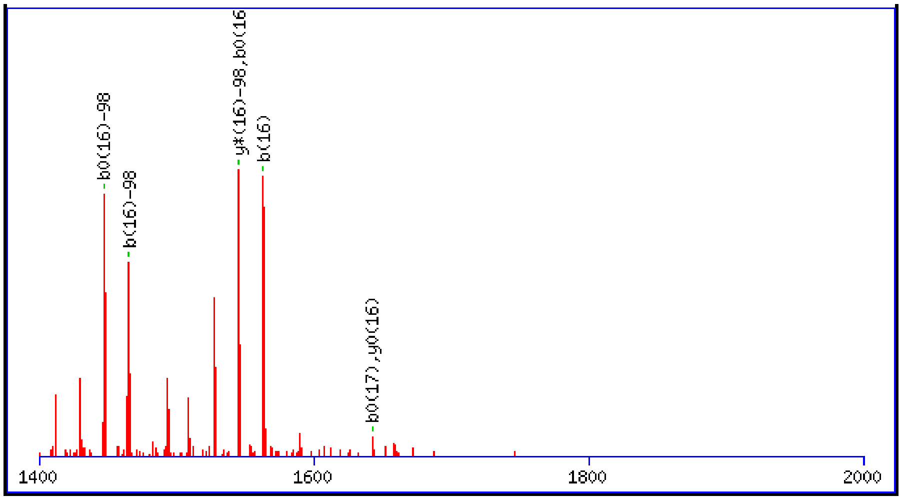

Monoisotopic mass of neutral peptide Mr(calc): 1833.8146
Fixed modifications: Carbamidomethyl (C)
Variable modifications:
S3 : Phospho (ST), with neutral losses 97.9769(shown in table), 0.0000
Ions Score: 95 **Expect:** 6.3e-008
Matches (Bold Red): 31/234 fragment ions using 37 most intense peaks

#	b	b^{++}	b^0	b^{0++}	Seq.	y	y^{++}	y*	y^{*++}	y^0	y^{0++}	#
1	88.0393	44.5233	70.0287	35.5180	S							18
2	175.0713	88.0393	157.0608	79.0340	S	1649.8129	825.4101	1632.7864	816.8968	1631.8024	816.4048	17
3	244.0928	122.5500	226.0822	113.5447	S	1562.7809	**781.8941**	**1545.7544**	773.3808	1544.7703	772.8888	16
4	**343.1612**	172.0842	325.1506	163.0790	V	1493.7594	**747.3834**	1476.7329	738.8701	1475.7489	738.3781	15
5	400.1827	200.5950	382.1721	191.5897	G	**1394.6910**	697.8492	1377.6645	689.3359	1376.6805	688.8439	14
6	487.2147	244.1110	**469.2041**	235.1057	S	**1337.6696**	669.3384	1320.6430	660.8251	1319.6590	660.3331	13
7	574.2467	287.6270	556.2362	278.6217	S	**1250.6375**	625.8224	1233.6110	**617.3091**	1232.6270	**616.8171**	12
8	661.2787	331.1430	643.2682	322.1377	S	**1163.6055**	582.3064	1146.5790	573.7931	1145.5949	573.3011	11
9	748.3108	374.6590	**730.3002**	365.6537	S	**1076.5735**	538.7904	1059.5469	530.2771	1058.5629	**529.7851**	10
10	911.3741	456.1907	893.3635	447.1854	Y	**989.5415**	495.2744	972.5149	486.7611	971.5309	486.2691	9
11	1008.4269	504.7171	**990.4163**	495.7118	P	**826.4781**	413.7427	809.4516	405.2294	808.4676	404.7374	8
12	1121.5109	561.2591	1103.5004	552.2538	I	729.4254	365.2163	712.3988	356.7030	711.4148	356.2110	7
13	1208.5430	604.7751	1190.5324	595.7698	S	**616.3413**	308.6743	599.3148	300.1610	598.3307	299.6690	6
14	1295.5750	648.2911	1277.5644	639.2859	S	**529.3093**	265.1583	512.2827	256.6450	511.2987	256.1530	5
15	1366.6121	683.8097	1348.6015	674.8044	A	**442.2772**	221.6423	425.2507	213.1290			4
16	**1465.6805**	733.3439	**1447.6700**	724.3386	V	**371.2401**	186.1237	354.2136	177.6104			3
17	1562.7333	**781.8703**	1544.7227	772.8650	P	**272.1717**	136.5895	255.1452	128.0762			2
18					R	175.1190	88.0631	158.0924	79.5498			1

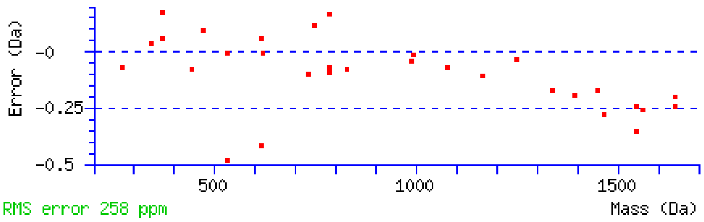

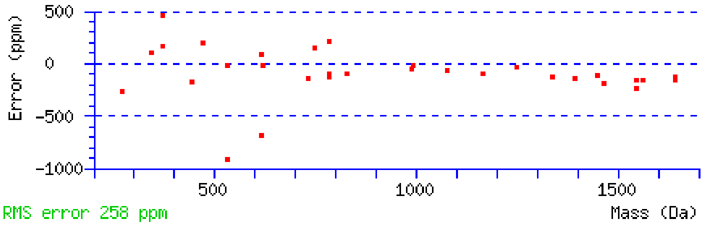

All matches to this query

Score	Mr(calc):	Delta	Sequence
95.2	1833.8146	0.0014	SSSVGSSSSYPISSAVPR
92.2	1833.8146	0.0014	SSSVGSSSSYPISSAVPR
92.2	1833.8146	0.0014	SSSVGSSSSYPISSAVPR

58.1	1833.8146	0.0014	SSSVGSSSSYPISSAVPR
58.1	1833.8146	0.0014	SSSVGSSSSYPISSAVPR
44.2	1833.8146	0.0014	SSSVGSSSSYPISSAVPR
32.0	1833.8146	0.0014	SSSVGSSSSYPISSAVPR
14.2	1833.8146	0.0014	SSSVGSSSSYPISSAVPR
11.3	1833.8006	0.0153	NRAGSPNPQSSSGELPR
11.3	1833.8006	0.0153	NRAGSPNPQSSSGELPR

Spectrum No: 3; Query: 565; Rank: 1

Peptide View

MS/MS Fragmentation of **SASSDTSEELNAQDSPK**
Found in **IPI00200898**, Tax_Id=10116 Gene_Symbol=Slc9a3r1 Ezrin-radixin-moesin-binding phosphoprotein 50

Match to Query 565: 1844.733368 from(923.373960,2+)
Title: 091127RatKid_SCX01_05.603.603.2.dta
Data file K:\NewmanPaper\Piliang\3SubProteomes\Piliang3SP\mgf5ppm\SCX_3SubProteomes5ppm.mgf

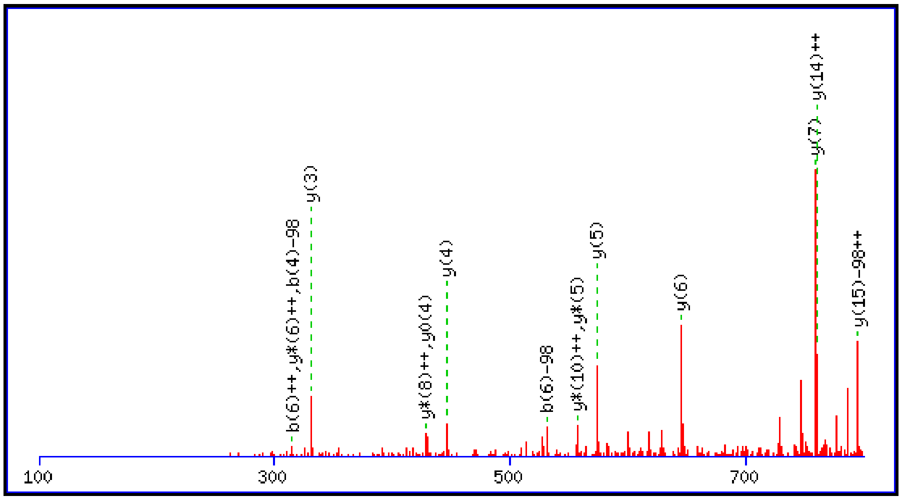

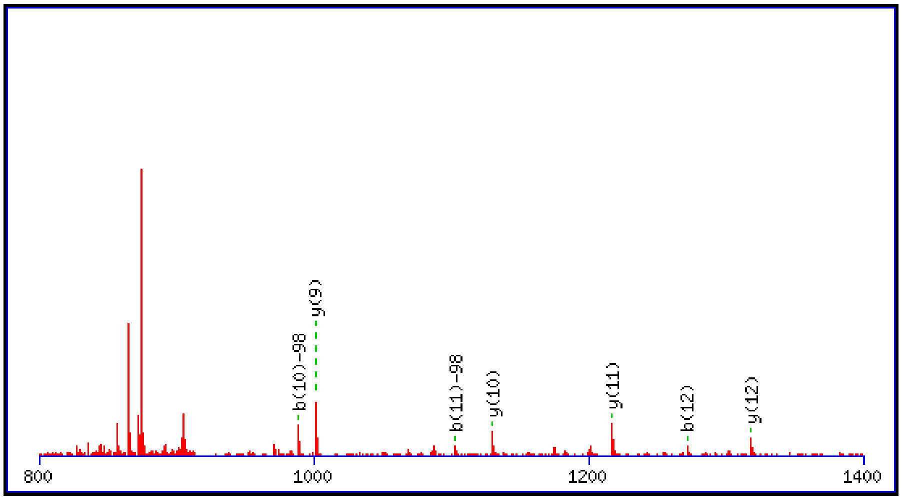

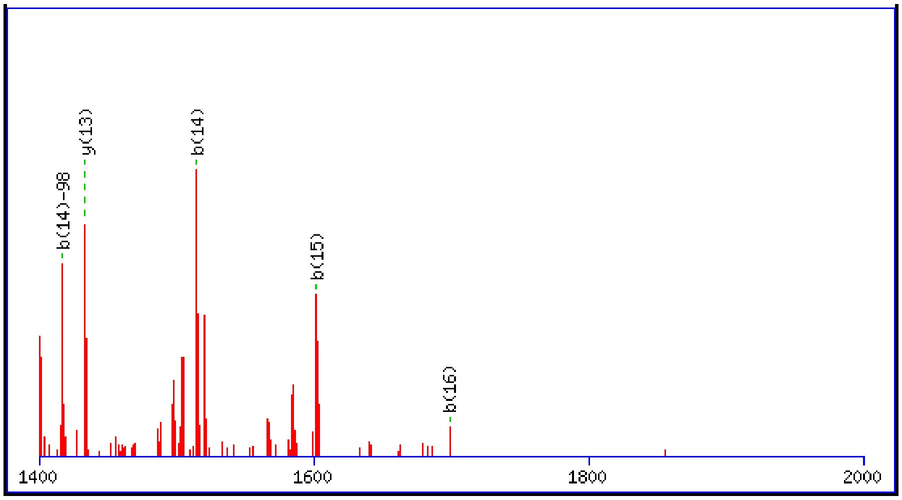

Monoisotopic mass of neutral peptide Mr(calc): 1844.7313
Fixed modifications: Carbamidomethyl (C)
Variable modifications:
S3 : Phospho (ST), with neutral losses 97.9769(shown in table), 0.0000
Ions Score: 89 **Expect:** 2.1e-007
Matches (Bold Red): 27/248 fragment ions using 34 most intense peaks

#	b	b^{++}	b*	b^{*++}	b^{0}	b^{0++}	Seq.	y	y^{++}	y*	y^{*++}	y^{0}	y^{0++}	#
1	88.0393	44.5233			70.0287	35.5180	S							17
2	159.0764	80.0418			141.0659	71.0366	A	1660.7297	830.8685	1643.7031	822.3552	1642.7191	821.8632	16
3	228.0979	114.5526			210.0873	105.5473	S	1589.6925	**795.3499**	1572.6660	786.8366	1571.6820	786.3446	15
4	**315.1299**	158.0686			297.1193	149.0633	S	1520.6711	**760.8392**	1503.6445	752.3259	1502.6605	751.8339	14
5	430.1568	215.5821			412.1463	206.5768	D	**1433.6391**	717.3232	1416.6125	708.8099	1415.6285	708.3179	13
6	**531.2045**	266.1059			513.1940	257.1006	T	**1318.6121**	659.8097	1301.5856	651.2964	1300.6016	650.8044	12
7	618.2366	309.6219			600.2260	300.6166	S	**1217.5644**	609.2859	1200.5379	600.7726	1199.5539	600.2806	11
8	747.2791	374.1432			729.2686	365.1379	E	**1130.5324**	565.7698	1113.5059	**557.2566**	1112.5218	556.7646	10
9	876.3217	438.6645			858.3112	429.6592	E	**1001.4898**	501.2485	984.4633	492.7353	983.4793	492.2433	9
10	**989.4058**	495.2065			971.3952	486.2013	L	872.4472	436.7272	855.4207	**428.2140**	854.4367	427.7220	8
11	**1103.4487**	552.2280	1086.4222	543.7147	1085.4382	543.2227	N	**759.3632**	380.1852	742.3366	371.6719	741.3526	371.1799	7
12	1174.4858	587.7466	1157.4593	579.2333	1156.4753	578.7413	A	**645.3202**	323.1638	628.2937	**314.6505**	627.3097	314.1585	6
13	1302.5444	651.7758	1285.5179	643.2626	1284.5339	642.7706	Q	**574.2831**	287.6452	**557.2566**	279.1319	556.2726	278.6399	5
14	**1417.5714**	709.2893	1400.5448	700.7760	1399.5608	700.2840	D	**446.2245**	223.6159	429.1980	215.1026	**428.2140**	214.6106	4
15	1504.6034	752.8053	1487.5768	744.2921	1486.5928	743.8001	S	**331.1976**	166.1024	314.1710	157.5892	313.1870	157.0972	3
16	1601.6562	801.3317	1584.6296	792.8184	1583.6456	792.3264	P	244.1656	122.5864	227.1390	114.0731			2
17							K	147.1128	74.0600	130.0863	65.5468			1

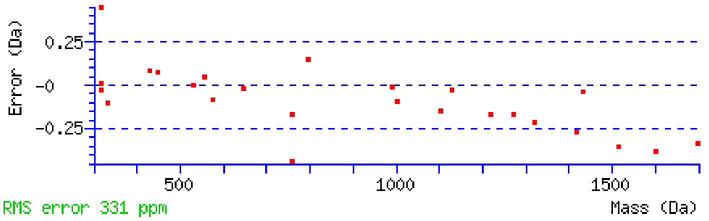

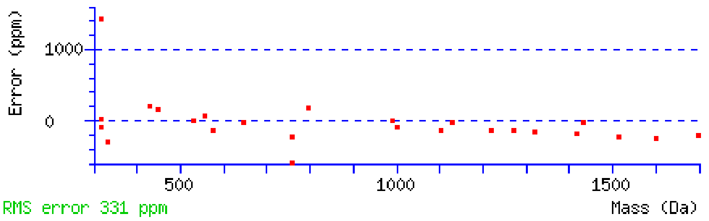

All matches to this query

Score	Mr(calc):	Delta	Sequence
88.7	1844.7313	0.0021	SASSDTSEELNAQDSPK
82.8	1844.7313	0.0021	SASSDTSEELNAQDSPK
80.0	1844.7313	0.0021	SASSDTSEELNAQDSPK
53.2	1844.7313	0.0021	SASSDTSEELNAQDSPK

41.5	1844.7313	0.0021	SASSDTSEELNAQDSPK
14.9	1842.7421	1.9912	ASGDFGSGLEESSNLHR
12.5	1843.7261	1.0072	SYDEGPQNASSGEPGLR
12.5	1843.7261	1.0072	SYDEGPQNASSGEPGLR
8.4	1842.7438	1.9896	FTLSSELEEERTSR
8.4	1842.7438	1.9896	FTLSSELEEERTSR

Spectrum No: 4; Query: 1178; Rank: 1

Peptide View

MS/MS Fragmentation of **YGPVSVADTTGSGAADAKDDDDIDLFGSDDEEESEDAKR**
Found in **IPI00476899**, Tax_Id=10116 Gene_Symbol=Eef1b2_predicted eukaryotic translation elongation factor 1 beta 2

Match to Query 1178: 4142.698542 from(1381.906790,3+)
Title: 091127RatKid_SCX01_23.3400.3400.3.dta
Data file K:\NewmanPaper\Piliang\3SubProteomes\Piliang3SP\mgf5ppm\SCX_3SubProteomes5ppm.mgf

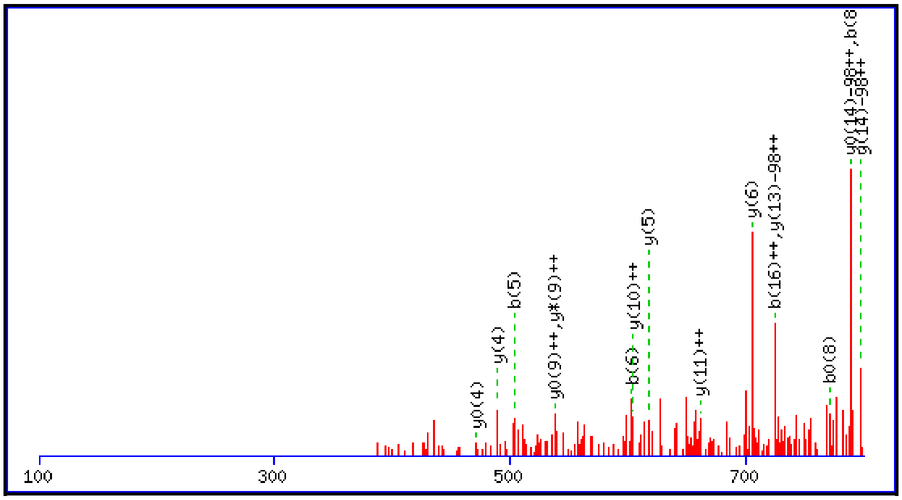

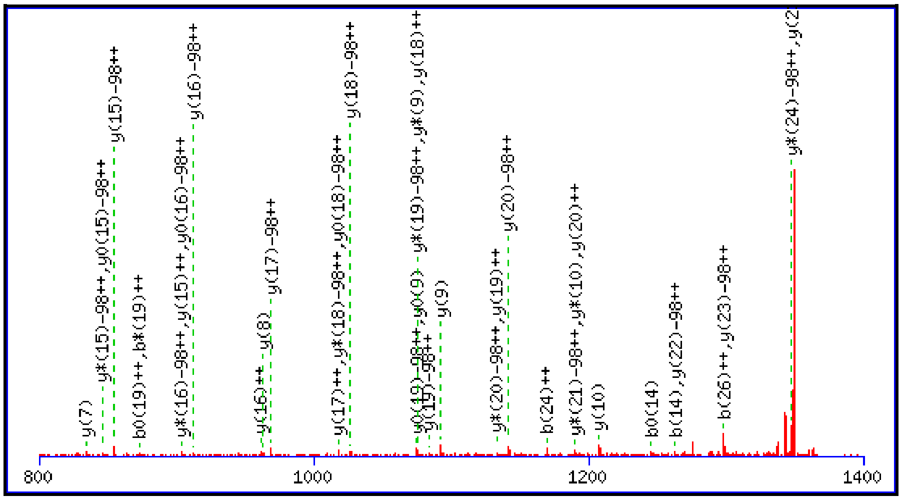

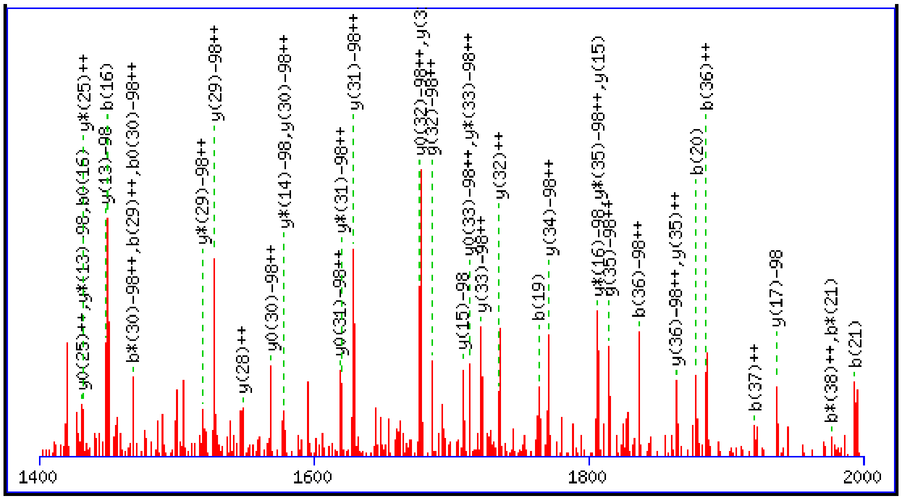

Monoisotopic mass of neutral peptide Mr(calc): 4141.6921
Fixed modifications: Carbamidomethyl (C)
Variable modifications:
S28 : Phospho (ST), with neutral losses 97.9769(shown in table), 0.0000
Ions Score: 86 **Expect:** 1.3e-006
Matches (Bold Red): 99/636 fragment ions using 135 most intense peaks

#	b	b^{++}	b*	b^{*++}	b^{0}	b^{0++}	Seq.	y	y^{++}	y*	y^{*++}	y^{0}	y^{0++}	#
1	164.0706	82.5389					Y							39
2	221.0921	111.0497					G	3881.6591	1941.3332	3864.6326	1932.8199	3863.6486	1932.3279	38
3	318.1448	159.5761					P	3824.6377	1912.8225	3807.6111	1904.3092	3806.6271	1903.8172	37
4	417.2132	209.1103					V	3727.5849	**1864.2961**	3710.5584	1855.7828	3709.5743	1855.2908	36
5	**504.2453**	252.6263			486.2347	243.6210	S	3628.5165	**1814.7619**	3611.4899	**1806.2486**	3610.5059	1805.7566	35
6	**603.3137**	302.1605			585.3031	293.1552	V	3541.4845	**1771.2459**	3524.4579	1762.7326	3523.4739	1762.2406	34
7	674.3508	337.6790			656.3402	328.6738	A	3442.4161	**1721.7117**	3425.3895	**1713.1984**	3424.4055	**1712.7064**	33
8	**789.3777**	395.1925			**771.3672**	386.1872	D	3371.3789	**1686.1931**	3354.3524	**1677.6798**	3353.3684	**1677.1878**	32
9	890.4254	445.7164			872.4149	436.7111	T	3256.3520	**1628.6796**	3239.3254	**1620.1664**	3238.3414	**1619.6744**	31
10	991.4731	496.2402			973.4625	487.2349	T	3155.3043	**1578.1558**	3138.2778	1569.6425	3137.2938	**1569.1505**	30
11	1048.4946	524.7509			1030.4840	515.7456	G	3054.2566	**1527.6320**	3037.2301	**1519.1187**	3036.2461	1518.6267	29
12	1135.5266	568.2669			1117.5160	559.2617	S	2997.2352	1499.1212	2980.2086	1490.6080	2979.2246	1490.1159	28
13	1192.5481	596.7777			1174.5375	587.7724	G	2910.2031	1455.6052	2893.1766	1447.0919	2892.1926	1446.5999	27
14	**1263.5852**	632.2962			**1245.5746**	623.2909	A	2853.1817	1427.0945	2836.1551	1418.5812	2835.1711	1418.0892	26
15	1334.6223	667.8148			1316.6117	658.8095	A	2782.1446	1391.5759	2765.1180	1383.0626	2764.1340	1382.5706	25
16	**1449.6492**	**725.3283**			**1431.6387**	716.3230	D	2711.1075	1356.0574	2694.0809	**1347.5441**	2693.0969	1347.0521	24
17	1520.6863	760.8468			1502.6758	751.8415	A	2596.0805	**1298.5439**	2579.0540	1290.0306	2578.0699	1289.5386	23
18	1648.7813	824.8943	1631.7548	816.3810	1630.7707	815.8890	K	2525.0434	**1263.0253**	2508.0168	1254.5121	2507.0328	1254.0201	22
19	**1763.8083**	882.4078	1746.7817	**873.8945**	1745.7977	**873.4025**	D	2396.9484	1198.9779	2379.9219	**1190.4646**	2378.9379	1189.9726	21
20	**1878.8352**	939.9212	1861.8086	931.4080	1860.8246	930.9160	D	2281.9215	**1141.4644**	2264.8949	**1132.9511**	2263.9109	1132.4591	20
21	**1993.8621**	997.4347	**1976.8356**	988.9214	1975.8516	988.4294	D	2166.8945	**1083.9509**	2149.8680	**1075.4376**	2148.8840	**1074.9456**	19
22	2108.8891	1054.9482	2091.8625	1046.4349	2090.8785	1045.9429	D	2051.8676	**1026.4374**	2034.8411	**1017.9242**	2033.8570	**1017.4322**	18
23	2221.9731	1111.4902	2204.9466	1102.9769	2203.9626	1102.4849	I	**1936.8407**	**968.9240**	1919.8141	960.4107	1918.8301	959.9187	17
24	2337.0001	**1169.0037**	2319.9735	1160.4904	2318.9895	1159.9984	D	1823.7566	**912.3819**	**1806.7301**	**903.8687**	1805.7460	**903.3767**	16
25	2450.0842	1225.5457	2433.0576	1217.0324	2432.0736	1216.5404	L	**1708.7297**	**854.8685**	1691.7031	**846.3552**	1690.7191	**845.8632**	15
26	2597.1526	**1299.0799**	2580.1260	1290.5666	2579.1420	1290.0746	F	1595.6456	**798.3264**	**1578.6190**	**789.8132**	1577.6350	**789.3212**	14
27	2654.1740	1327.5907	2637.1475	1319.0774	2636.1635	1318.5854	G	**1448.5772**	**724.7922**	**1431.5506**	716.2790	1430.5666	715.7869	13
28	2723.1955	1362.1014	2706.1689	1353.5881	2705.1849	1353.0961	S	1391.5557	696.2815	1374.5292	687.7682	1373.5451	687.2762	12
29	2838.2224	1419.6149	2821.1959	1411.1016	2820.2119	1410.6096	D	1322.5343	**661.7708**	1305.5077	653.2575	1304.5237	652.7655	11
30	2953.2494	1477.1283	2936.2228	**1468.6150**	2935.2388	**1468.1230**	D	**1207.5073**	**604.2573**	**1190.4808**	595.7440	1189.4968	595.2520	10
31	3082.2920	1541.6496	3065.2654	1533.1363	3064.2814	1532.6443	E	**1092.4804**	546.7438	**1075.4538**	**538.2305**	**1074.4698**	**537.7385**	9
32	3211.3346	1606.1709	3194.3080	1597.6576	3193.3240	1597.1656	E	**963.4378**	482.2225	946.4112	473.7093	945.4272	473.2172	8

**33**	3340.3771	1670.6922	3323.3506	1662.1789	3322.3666	1661.6869	**E**	**834.3952**	417.7012	817.3686	409.1880	816.3846	408.6959	**7**
**34**	3427.4092	1714.2082	3410.3826	1705.6950	3409.3986	1705.2029	**S**	**705.3526**	353.1799	688.3260	344.6667	687.3420	344.1747	**6**
**35**	3556.4518	1778.7295	3539.4252	1770.2162	3538.4412	1769.7242	**E**	**618.3206**	309.6639	601.2940	301.1506	600.3100	300.6586	**5**
**36**	3671.4787	**1836.2430**	3654.4522	1827.7297	3653.4681	1827.2377	**D**	**489.2780**	245.1426	472.2514	236.6293	**471.2674**	236.1373	**4**
**37**	3742.5158	1871.7616	3725.4893	1863.2483	3724.5053	1862.7563	**A**	374.2510	187.6292	357.2245	179.1159			**3**
**38**	3870.6108	1935.8090	3853.5842	1927.2958	3852.6002	1926.8038	**K**	303.2139	152.1106	286.1874	143.5973			**2**
**39**							**R**	175.1190	88.0631	158.0924	79.5498			**1**

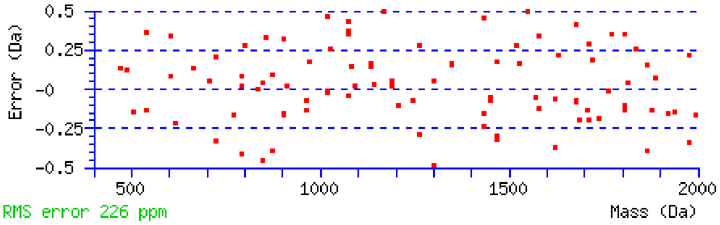

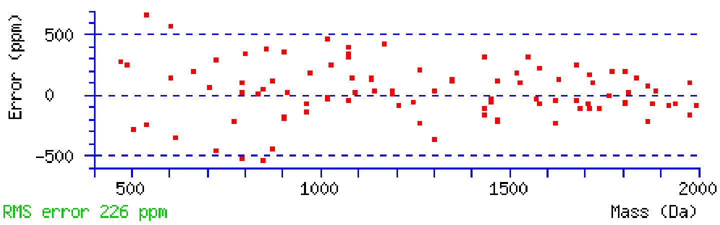

All matches to this query

Score	Mr(calc):	Delta	Sequence
85.8	4141.6921	1.0064	YGPVSVADTTGSGAADAKDDDDIDLFGSDDEEESEDAKR
70.8	4141.6921	1.0064	YGPVSVADTTGSGAADAKDDDDIDLFGSDDEEESEDAKR
38.9	4141.6921	1.0064	YGPVSVADTTGSGAADAKDDDDIDLFGSDDEEESEDAKR
34.4	4141.6921	1.0064	YGPVSVADTTGSGAADAKDDDDIDLFGSDDEEESEDAKR
30.2	4141.6921	1.0064	YGPVSVADTTGSGAADAKDDDDIDLFGSDDEEESEDAKR
12.9	4141.6921	1.0064	YGPVSVADTTGSGAADAKDDDDIDLFGSDDEEESEDAKR
9.7	4141.7253	0.9732	ASSWACSSASIETRPLNFFISYDAPPLRTRTIK
9.4	4141.6555	1.0431	VEELQYMGDESSANSSSDPESNPTSPMAGRRHVASNK
8.6	4140.6619	2.0367	SSLGPAERTTENNYMEIVNVSCVSGAIPNNSTQGSSK
8.0	4141.6921	1.0064	YGPVSVADTTGSGAADAKDDDDIDLFGSDDEEESEDAKR

Spectrum No: 5; Query: 701; Rank: 1

Peptide View

MS/MS Fragmentation of **KETESEAEDDNLDDLER**
Found in **IPI00779470**, Tax_Id=10116 Gene_Symbol=Srrm1_predicted 106 kDa protein

Match to Query 701: 2086.825308 from(1044.419930,2+)
Title: 091127RatKid_SCX01_23.1639.1639.2.dta
Data file K:\NewmanPaper\Piliang\3SubProteomes\Piliang3SP\mgf5ppm\SCX_3SubProteomes5ppm.mgf

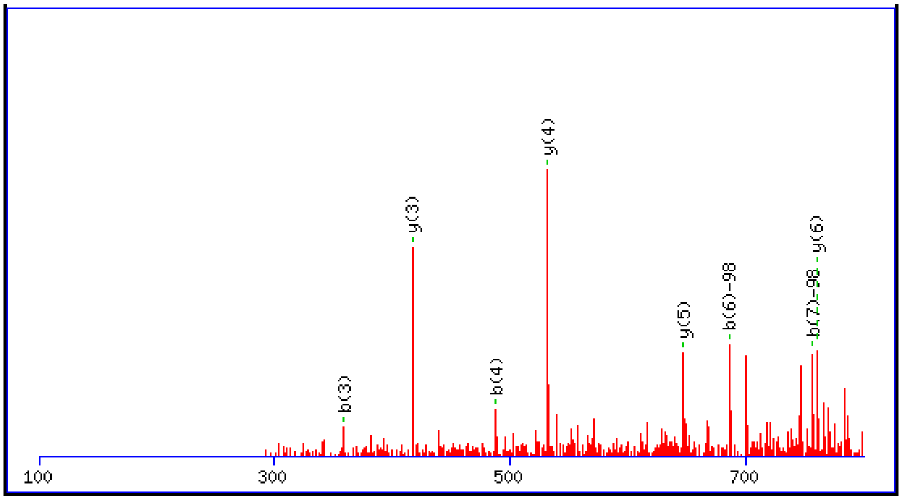

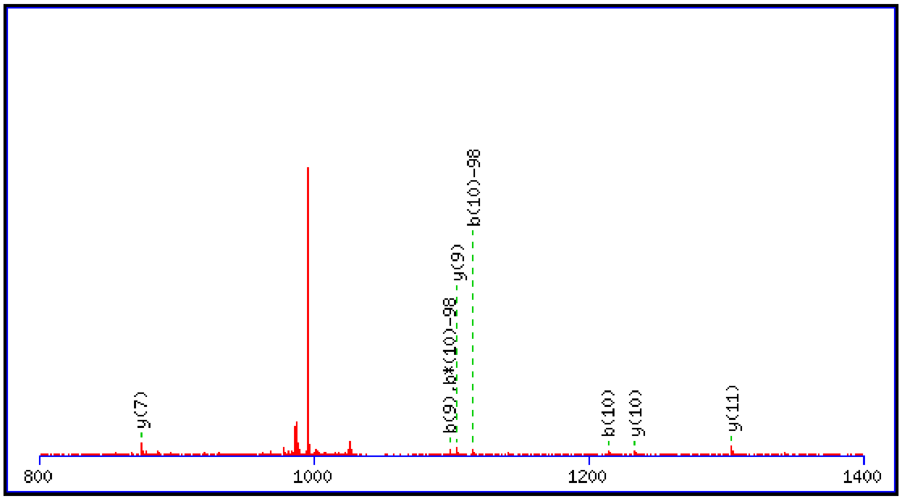

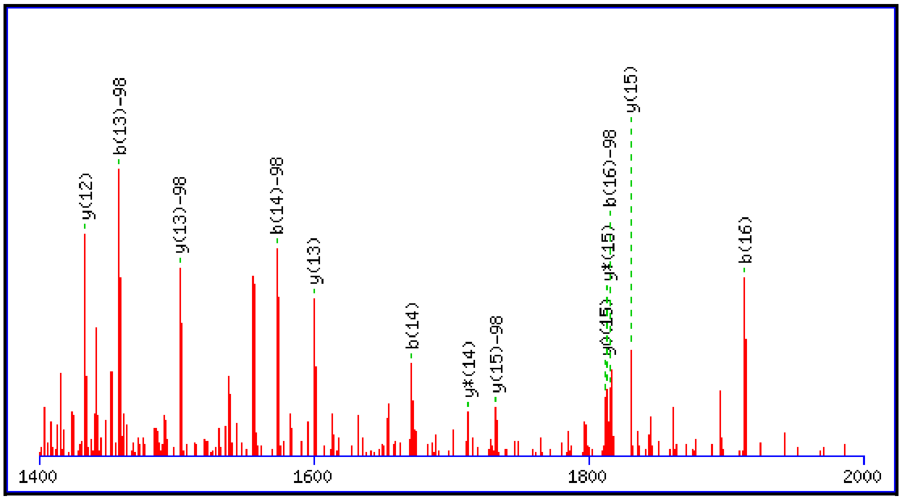

Monoisotopic mass of neutral peptide Mr(calc): 2086.8215
Fixed modifications: Carbamidomethyl (C)
Variable modifications:
S5 : Phospho (ST), with neutral losses 97.9769(shown in table), 0.0000
Ions Score: 85 **Expect:** 6e-007
Matches (Bold Red): 29/284 fragment ions using 41 most intense peaks

#	b	b^{++}	b*	b^{*++}	b^{0}	b^{0++}	Seq.	y	y^{++}	y*	y^{*++}	y^{0}	y^{0++}	#
1	129.1022	65.0548	112.0757	56.5415			K							17
2	258.1448	129.5761	241.1183	121.0628	240.1343	120.5708	E	1861.7570	931.3821	1844.7304	922.8689	1843.7464	922.3769	16
3	**359.1925**	180.0999	342.1660	171.5866	341.1819	171.0946	T	**1732.7144**	866.8608	1715.6879	858.3476	1714.7038	857.8556	15
4	**488.2351**	244.6212	471.2086	236.1079	470.2245	235.6159	E	1631.6667	816.3370	1614.6402	807.8237	1613.6562	807.3317	14
5	557.2566	279.1319	540.2300	270.6186	539.2460	270.1266	S	**1502.6241**	751.8157	1485.5976	743.3024	1484.6136	742.8104	13

**6**	**686.2992**	343.6532	669.2726	335.1399	668.2886	334.6479	**E**	**1433.6027**	717.3050	1416.5761	708.7917	1415.5921	708.2997	**12**
**7**	**757.3363**	379.1718	740.3097	370.6585	739.3257	370.1665	**A**	**1304.5601**	652.7837	1287.5335	644.2704	1286.5495	643.7784	**11**
**8**	886.3789	443.6931	869.3523	435.1798	868.3683	434.6878	**E**	**1233.5230**	617.2651	1216.4964	608.7518	1215.5124	608.2598	**10**
**9**	1001.4058	501.2065	984.3793	492.6933	983.3952	492.2013	**D**	**1104.4804**	552.7438	1087.4538	544.2305	1086.4698	543.7385	**9**
**10**	**1116.4327**	558.7200	**1099.4062**	550.2067	1098.4222	549.7147	**D**	989.4534	495.2304	972.4269	486.7171	971.4429	486.2251	**8**
**11**	1230.4757	615.7415	1213.4491	607.2282	1212.4651	606.7362	**N**	**874.4265**	437.7169	857.3999	429.2036	856.4159	428.7116	**7**
**12**	1343.5597	672.2835	1326.5332	663.7702	1325.5492	663.2782	**L**	**760.3836**	380.6954	743.3570	372.1821	742.3730	371.6901	**6**
**13**	**1458.5867**	729.7970	1441.5601	721.2837	1440.5761	720.7917	**D**	**647.2995**	324.1534	630.2729	315.6401	629.2889	315.1481	**5**
**14**	**1573.6136**	787.3104	1556.5871	778.7972	1555.6031	778.3052	**D**	**532.2726**	266.6399	515.2460	258.1266	514.2620	257.6346	**4**
**15**	1686.6977	843.8525	1669.6711	835.3392	1668.6871	834.8472	**L**	**417.2456**	209.1264	400.2191	200.6132	399.2350	200.1212	**3**
**16**	**1815.7403**	908.3738	1798.7137	899.8605	1797.7297	899.3685	**E**	304.1615	152.5844	287.1350	144.0711	286.1510	143.5791	**2**
**17**							**R**	175.1190	88.0631	158.0924	79.5498			**1**

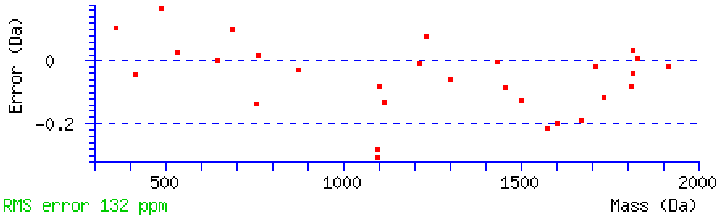

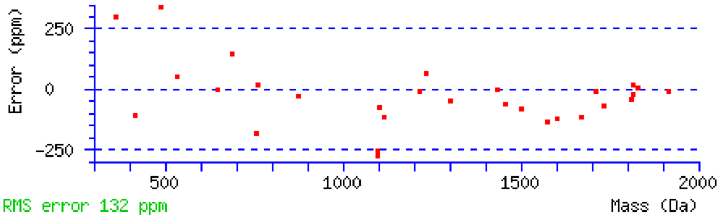

All matches to this query

Score	Mr(calc):	Delta	Sequence
84.9	2086.8215	0.0038	KETESEAEDDNLDDLER
68.6	2086.8215	0.0038	KETESEAEDDNLDDLER
9.0	2086.8047	0.0206	DSGTGMMMSVNFSTQELR
6.3	2086.8438	-0.0185	QFISWLQDVDDKYDR
5.9	2086.8047	0.0206	DSGTGMMMSVNFSTQELR
5.9	2086.8410	-0.0157	NNMTASMFDLSMKDKTR
3.8	2086.8449	-0.0196	ASEESRDMDSKCHQTVK
3.7	2086.8254	-0.0001	NYDVTIQGCKIQNMSR
3.4	2086.8220	0.0033	SMKNRPNPYYEDLER
3.4	2086.8220	0.0033	SMKNRPNPYYEDLER

Spectrum No: 6; Query: 513; Rank: 1

Peptide View

MS/MS Fragmentation of **SASPAPADVAPAQEDLR**
Found in **IPI00193648**, Tax_Id=10116 Gene_Symbol=G3bp similar to Ras-GTPase-activating protein binding protein 1

Match to Query 513: 1773.790608 from(887.902580,2+)
Title: 091129RatKid_SCX02_12.1156.1156.2.dta
Data file K:\NewmanPaper\Piliang\3SubProteomes\Piliang3SP\mgf5ppm\SCX_3SubProteomes5ppm.mgf

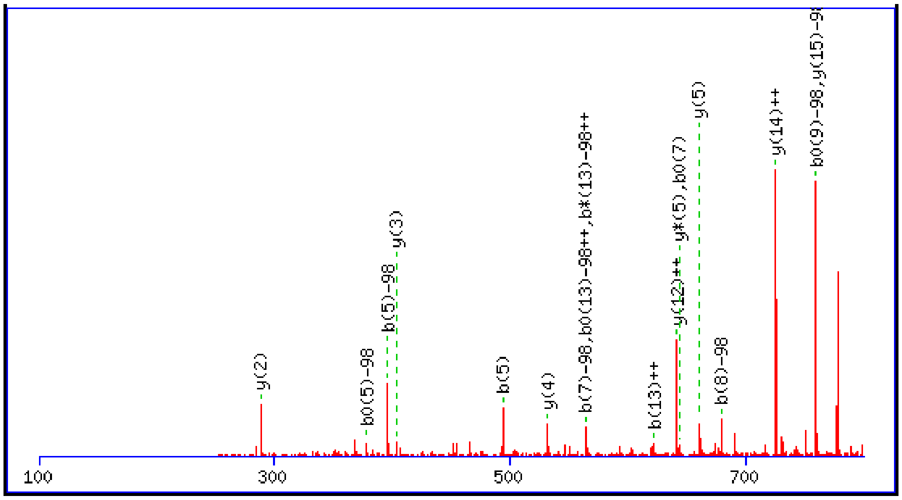

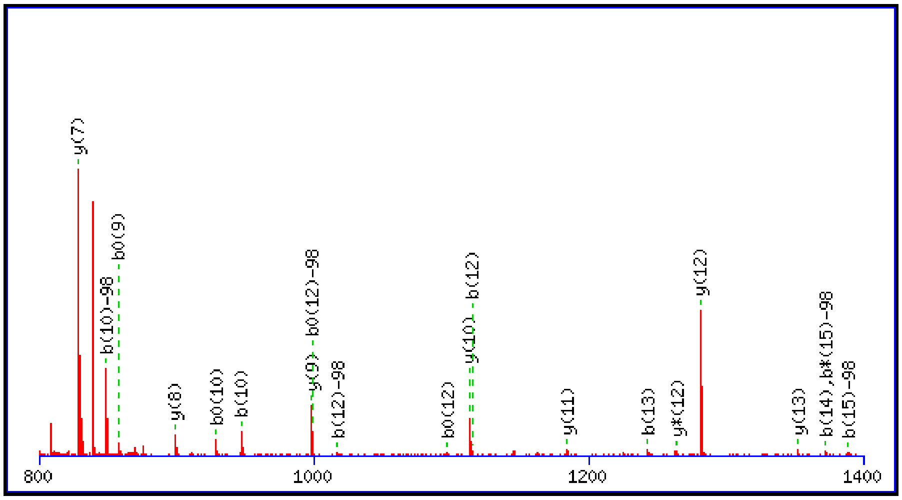

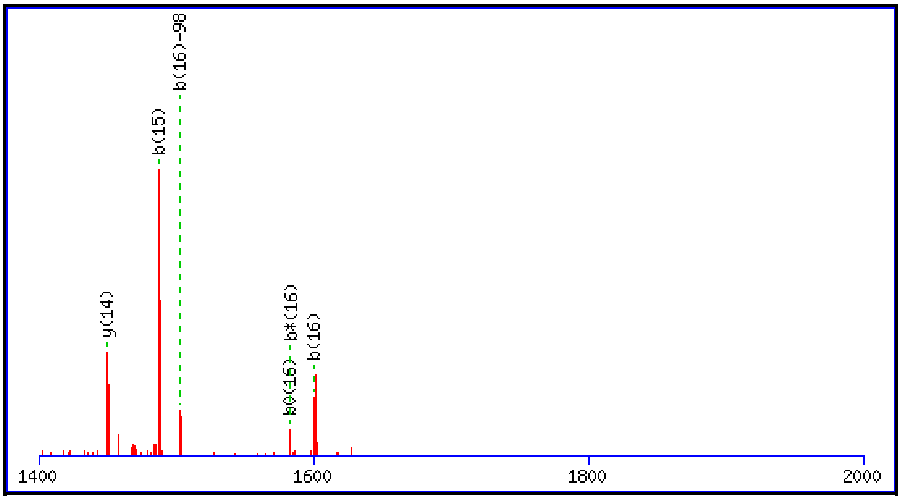

Monoisotopic mass of neutral peptide Mr(calc): 1773.7934
Fixed modifications: Carbamidomethyl (C)
Variable modifications:
S3 : Phospho (ST), with neutral losses 97.9769(shown in table), 0.0000
Ions Score: 83 **Expect:** 1.1e-006
Matches (Bold Red): 44/240 fragment ions using 59 most intense peaks

#	b	b^{++}	b*	b^{*++}	b^{0}	b^{0++}	Seq.	y	y^{++}	y*	y^{*++}	y^{0}	y^{0++}	#
1	88.0393	44.5233			70.0287	35.5180	S							17
2	159.0764	80.0418			141.0659	71.0366	A	1589.7918	795.3995	1572.7653	786.8863	1571.7812	786.3943	16
3	228.0979	114.5526			210.0873	105.5473	S	1518.7547	**759.8810**	1501.7281	751.3677	1500.7441	750.8757	15
4	325.1506	163.0790			307.1401	154.0737	P	**1449.7332**	**725.3703**	1432.7067	716.8570	1431.7227	716.3650	14
5	**396.1878**	198.5975			**378.1772**	189.5922	A	**1352.6805**	676.8439	1335.6539	668.3306	1334.6699	667.8386	13

6	493.2405	247.1239			475.2300	238.1186	P	1281.6434	641.3253	1264.6168	632.8120	1263.6328	632.3200	12
7	564.2776	282.6425			546.2671	273.6372	A	1184.5906	592.7989	1167.5640	584.2857	1166.5800	583.7937	11
8	679.3046	340.1559			661.2940	331.1506	D	1113.5535	557.2804	1096.5269	548.7671	1095.5429	548.2751	10
9	778.3730	389.6901			760.3624	380.6848	V	998.5265	499.7669	981.5000	491.2536	980.5160	490.7616	9
10	849.4101	425.2087			831.3995	416.2034	A	899.4581	450.2327	882.4316	441.7194	881.4476	441.2274	8
11	946.4629	473.7351			928.4523	464.7298	P	828.4210	414.7141	811.3945	406.2009	810.4104	405.7089	7
12	1017.5000	509.2536			999.4894	500.2483	A	731.3682	366.1878	714.3417	357.6745	713.3577	357.1825	6
13	1145.5586	573.2829	1128.5320	564.7696	1127.5480	564.2776	Q	660.3311	330.6692	643.3046	322.1559	642.3206	321.6639	5
14	1274.6012	637.8042	1257.5746	629.2909	1256.5906	628.7989	E	532.2726	266.6399	515.2460	258.1266	514.2620	257.6346	4
15	1389.6281	695.3177	1372.6015	686.8044	1371.6175	686.3124	D	403.2300	202.1186	386.2034	193.6053	385.2194	193.1133	3
16	1502.7122	751.8597	1485.6856	743.3464	1484.7016	742.8544	L	288.2030	144.6051	271.1765	136.0919			2
17							R	175.1190	88.0631	158.0924	79.5498			1

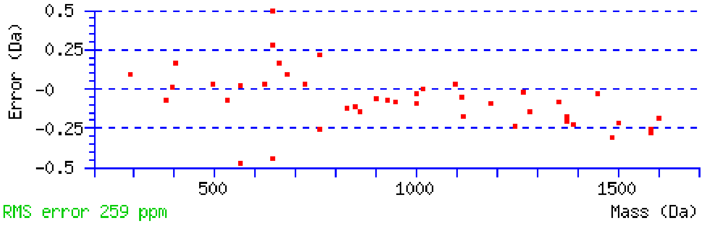

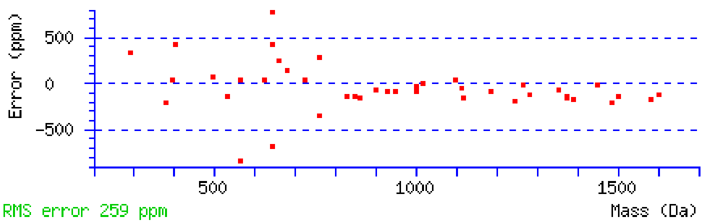

All matches to this query

Score	Mr(calc):	Delta	Sequence
82.9	1773.7934	-0.0028	SASPAPADVAPAQEDLR
81.4	1773.7934	-0.0028	SASPAPADVAPAQEDLR
13.4	1771.7699	2.0207	TCVLDANDGKGIVDSK
8.0	1773.7757	0.0149	VQVSSQNPPPSPMPGR
5.4	1773.8008	-0.0102	LDPTGKFEKDMIER
3.2	1773.7869	0.0037	MFGSQKAVEREQER
3.1	1773.8047	-0.0140	VKEHQESLDKDNPR
2.2	1771.7778	2.0128	FQKASVSGPNSPSETR
2.2	1771.7778	2.0128	FQKASVSGPNSPSETR
1.7	1771.7760	2.0146	RSPAAQHMCPKHTR

Spectrum No: 7; Query: 504; Rank: 1

Peptide View

MS/MS Fragmentation of **GSPSGGSTAEVSDTASIR**
Found in **IPI00197098**, Tax_Id=10116 Gene_Symbol=RGD1561149_predicted hypothetical protein

Match to Query 504: 1757.748748 from(879.881650,2+)
Title: 091127RatKid_SCX01_12.1294.1294.2.dta
Data file K:\NewmanPaper\Piliang\3SubProteomes\Piliang3SP\mgf5ppm\SCX_3SubProteomes5ppm.mgf

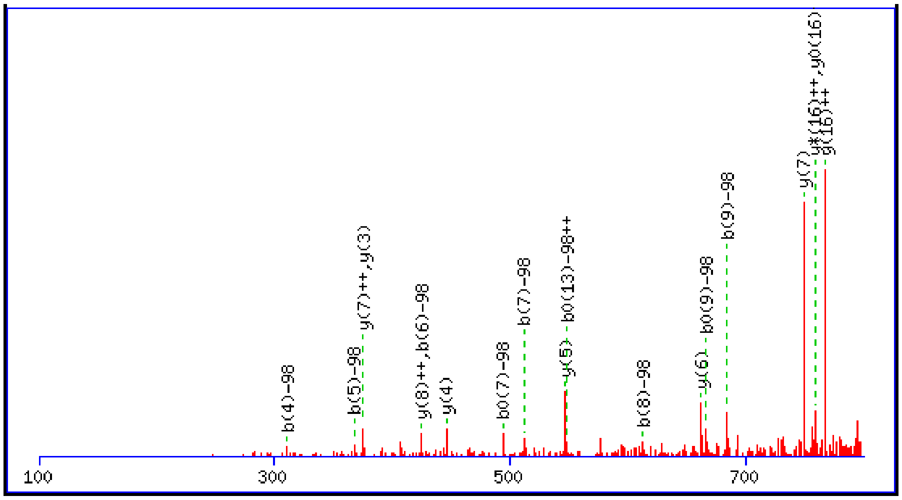

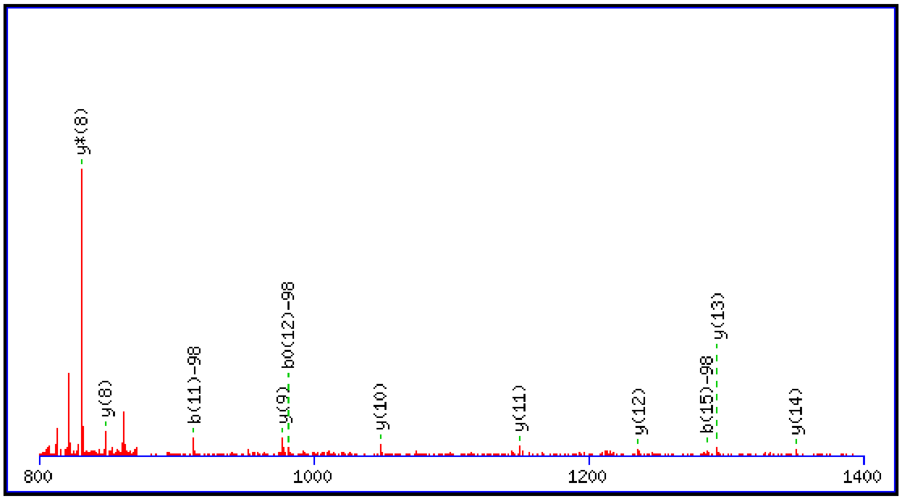

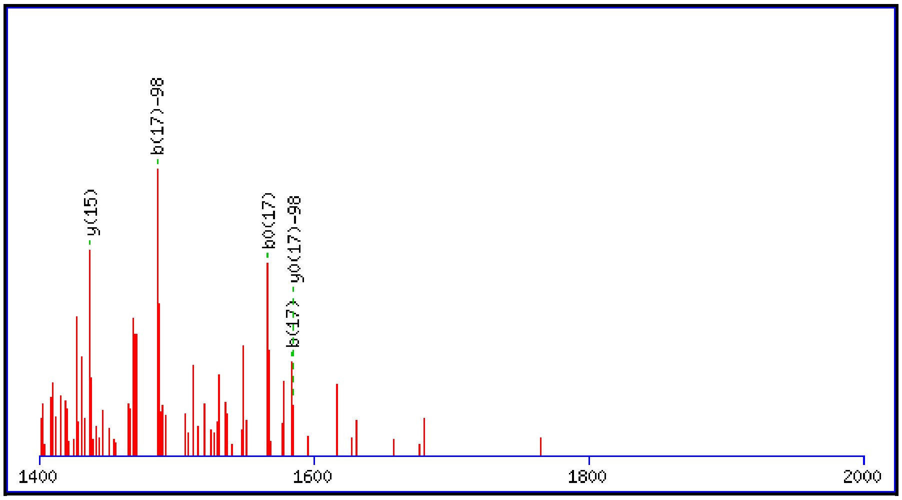

Monoisotopic mass of neutral peptide Mr(calc): 1757.7469
Fixed modifications: Carbamidomethyl (C)
Variable modifications:
S2 : Phospho (ST), with neutral losses 97.9769(shown in table), 0.0000
Ions Score: 82 **Expect:** 1.3e-006
Matches (Bold Red): 35/234 fragment ions using 61 most intense peaks

#	b	b++	b0	b0++	Seq.	y	y++	y*	y*++	y0	y0++	#
1	58.0287	29.5180			G							18
2	127.0502	64.0287	109.0396	55.0235	S	1603.7558	802.3815	1586.7293	793.8683	**1585.7452**	793.3763	17
3	224.1030	112.5551	206.0924	103.5498	P	1534.7344	**767.8708**	1517.7078	**759.3575**	1516.7238	**758.8655**	16
4	**311.1350**	156.0711	293.1244	147.0658	S	**1437.6816**	719.3444	1420.6550	710.8312	1419.6710	710.3392	15
5	**368.1565**	184.5819	350.1459	175.5766	G	**1350.6496**	675.8284	1333.6230	667.3151	1332.6390	666.8231	14

6	**425.1779**	213.0926	407.1674	204.0873	G	**1293.6281**	647.3177	1276.6016	638.8044	1275.6175	638.3124	13
7	**512.2099**	256.6086	**494.1994**	247.6033	S	**1236.6066**	618.8070	1219.5801	610.2937	1218.5961	609.8017	12
8	**613.2576**	307.1324	595.2471	298.1272	T	**1149.5746**	575.2909	1132.5481	566.7777	1131.5640	566.2857	11
9	**684.2947**	342.6510	**666.2842**	333.6457	A	**1048.5269**	524.7671	1031.5004	516.2538	1030.5164	515.7618	10
10	813.3373	407.1723	795.3268	398.1670	E	**977.4898**	489.2485	960.4633	480.7353	959.4792	480.2433	9
11	**912.4057**	456.7065	894.3952	447.7012	V	**848.4472**	**424.7272**	**831.4207**	416.2140	830.4367	415.7220	8
12	999.4378	500.2225	**981.4272**	491.2172	S	**749.3788**	**375.1930**	732.3523	366.6798	731.3682	366.1878	7
13	1114.4647	557.7360	1096.4542	**548.7307**	D	**662.3468**	331.6770	645.3202	323.1638	644.3362	322.6717	6
14	1215.5124	608.2598	1197.5018	599.2546	T	**547.3198**	274.1636	530.2933	265.6503	529.3093	265.1583	5
15	**1286.5495**	643.7784	1268.5389	634.7731	A	**446.2722**	223.6397	429.2456	215.1264	428.2616	214.6344	4
16	1373.5815	687.2944	1355.5710	678.2891	S	**375.2350**	188.1212	358.2085	179.6079	357.2245	179.1159	3
17	**1486.6656**	743.8364	1468.6550	734.8312	I	288.2030	144.6051	271.1765	136.0919			2
18					R	175.1190	88.0631	158.0924	79.5498			1

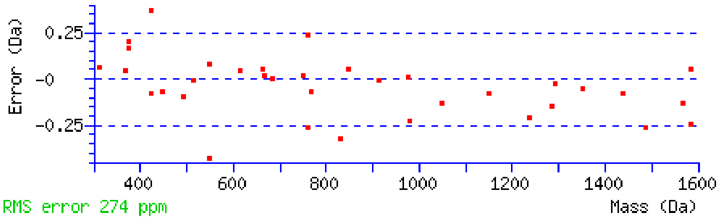

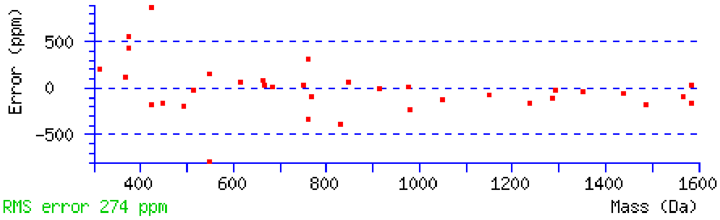

All matches to this query

Score	Mr(calc):	Delta	Sequence
81.6	1757.7469	0.0018	GSPSGGSTAEVSDTASIR
78.9	1757.7469	0.0018	GSPSGGSTAEVSDTASIR
47.7	1757.7469	0.0018	GSPSGGSTAEVSDTASIR
38.6	1757.7469	0.0018	GSPSGGSTAEVSDTASIR
18.3	1757.7469	0.0018	GSPSGGSTAEVSDTASIR
9.6	1757.7469	0.0018	GSPSGGSTAEVSDTASIR
6.6	1757.7491	-0.0004	VNCICVDWRRGSR
2.4	1756.7508	0.9980	KASINFSGKSPVMSK
2.3	1757.7543	-0.0055	MGILNTDTLGNSLNGR
1.9	1756.7388	1.0100	NMKCSVKXGPFSYK

Spectrum No: 8; Query: 228; Rank: 1

Peptide View

MS/MS Fragmentation of **TASGSSVTSLEGTR**
Found in **IPI00421389**, Tax_Id=10116 Gene_Symbol=Ndrg1 Protein NDRG1

Match to Query 228: 1431.625828 from(716.820190,2+)
Title: 091129RatKid_SCX02_12.925.925.2.dta
Data file K:\NewmanPaper\Piliang\3SubProteomes\Piliang3SP\mgf5ppm\SCX_3SubProteomes5ppm.mgf

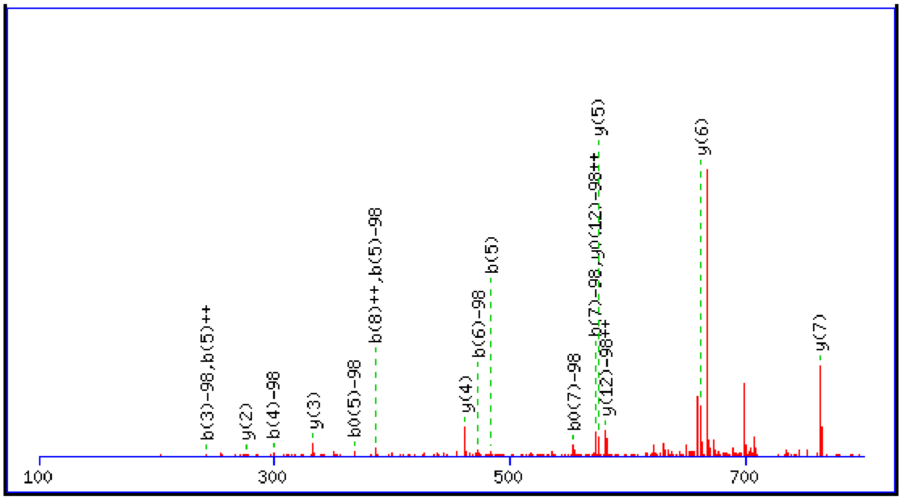

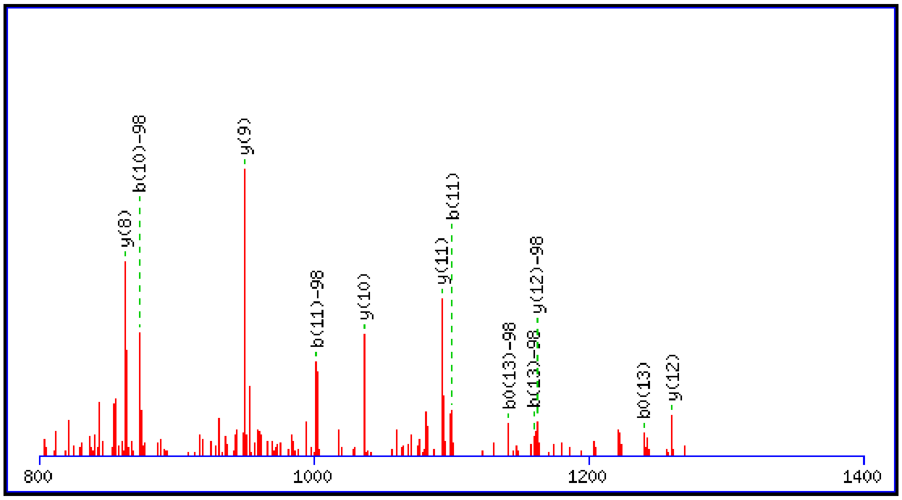

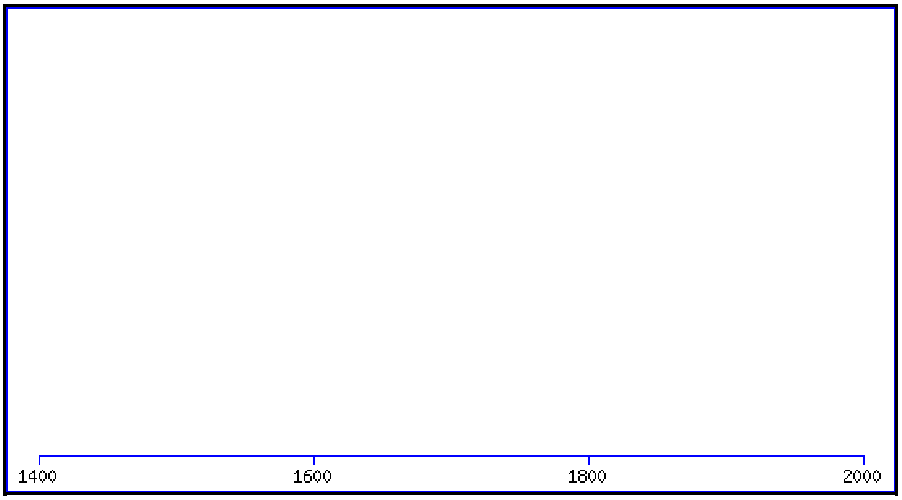

Monoisotopic mass of neutral peptide Mr(calc): 1431.6243
Fixed modifications: Carbamidomethyl (C)
Variable modifications:
S3 : Phospho (ST), with neutral losses 97.9769(shown in table), 0.0000
Ions Score: 78 **Expect:** 2.7e-006
Matches (Bold Red): 30/184 fragment ions using 49 most intense peaks

#	b	b^{++}	b^0	b^{0++}	Seq.	y	y^{++}	y*	y^{*++}	y^0	y^{0++}	#
1	102.0550	51.5311	84.0444	42.5258	T							14
2	173.0921	87.0497	155.0815	78.0444	A	1233.6070	617.3071	1216.5804	608.7938	1215.5964	608.3018	13
3	**242.1135**	121.5604	224.1030	112.5551	S	**1162.5698**	**581.7886**	1145.5433	573.2753	1144.5593	**572.7833**	12
4	**299.1350**	150.0711	281.1244	141.0658	G	**1093.5484**	547.2778	1076.5218	538.7646	1075.5378	538.2726	11
5	**386.1670**	193.5871	**368.1565**	184.5819	S	**1036.5269**	518.7671	1019.5004	510.2538	1018.5164	509.7618	10

**6**	**473.1990**	237.1032	455.1885	228.0979	**S**	**949.4949**	475.2511	932.4684	466.7378	931.4843	466.2458	**9**
**7**	**572.2675**	286.6374	**554.2569**	277.6321	**V**	**862.4629**	431.7351	845.4363	423.2218	844.4523	422.7298	**8**
**8**	673.3151	337.1612	655.3046	328.1559	**T**	**763.3945**	382.2009	746.3679	373.6876	745.3839	373.1956	**7**
**9**	760.3472	380.6772	742.3366	371.6719	**S**	**662.3468**	331.6770	645.3202	323.1638	644.3362	322.6717	**6**
**10**	**873.4312**	437.2193	855.4207	428.2140	**L**	**575.3148**	288.1610	558.2882	279.6477	557.3042	279.1557	**5**
**11**	**1002.4738**	501.7405	984.4633	492.7353	**E**	**462.2307**	231.6190	445.2041	223.1057	444.2201	222.6137	**4**
**12**	1059.4953	530.2513	1041.4847	521.2460	**G**	**333.1881**	167.0977	316.1615	158.5844	315.1775	158.0924	**3**
**13**	**1160.5430**	580.7751	**1142.5324**	571.7698	**T**	**276.1666**	138.5870	259.1401	130.0737	258.1561	129.5817	**2**
**14**					**R**	175.1190	88.0631	158.0924	79.5498			**1**

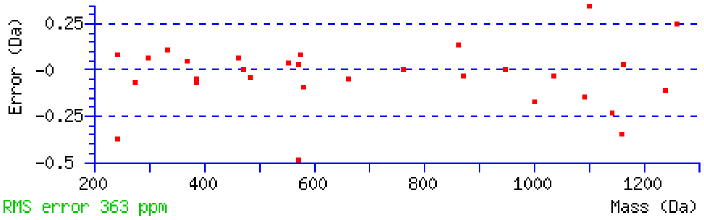

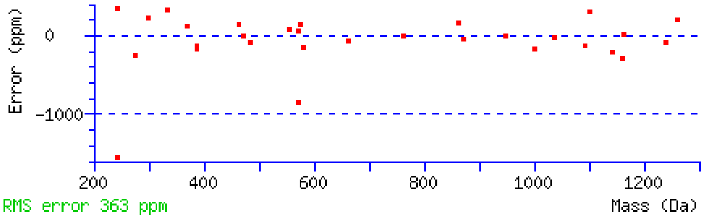

All matches to this query

Score	Mr(calc):	Delta	Sequence
78.1	1431.6243	0.0016	TASGSSVTSLEGTR
74.3	1431.6243	0.0016	TASGSSVTSLEGTR
52.9	1431.6243	0.0016	TASGSSVTSLEGTR
42.2	1431.6243	0.0016	TASGSSVTSLEGTR
31.5	1431.6243	0.0016	TASGSSVTSLEGTR
21.8	1431.6243	0.0016	TASGSSVTSLEGTR
6.3	1430.6191	1.0067	GADAASPPPATGSPR
6.3	1430.6191	1.0067	GADAASPPPATGSPR
5.0	1429.6312	1.9946	NKSAWMGLSDLK
3.5	1431.6330	-0.0072	QIGMGFRPSSTR

Spectrum No: 9; Query: 812; Rank: 1

Peptide View

MS/MS Fragmentation of **TPEELDDSDFETEDFDVR**
Found in **IPI00358406**, Tax_Id=10116 Gene_Symbol=Catna1 Catenin (Cadherin-associated protein), alpha 1

Match to Query 812: 2239.862928 from(1120.938740,2+)
Title: 091127RatKid_SCX01_12.3377.3377.2.dta
Data file K:\NewmanPaper\Piliang\3SubProteomes\Piliang3SP\mgf5ppm\SCX_3SubProteomes5ppm.mgf

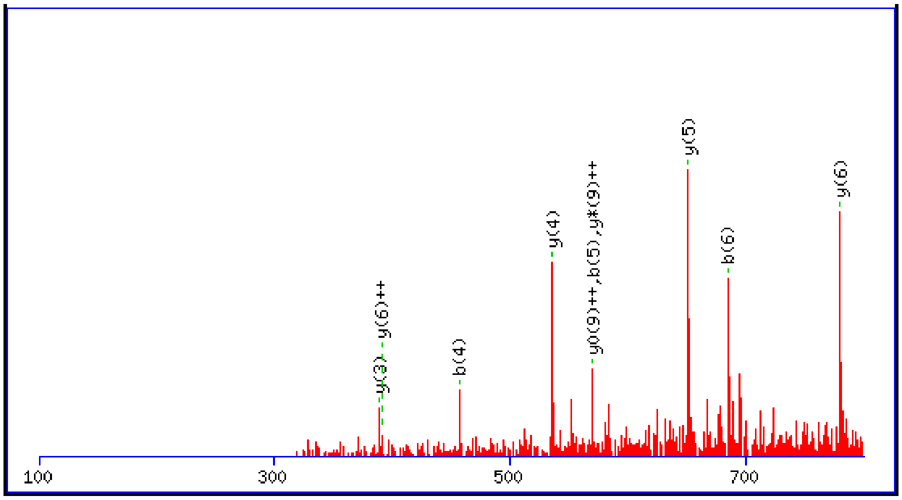

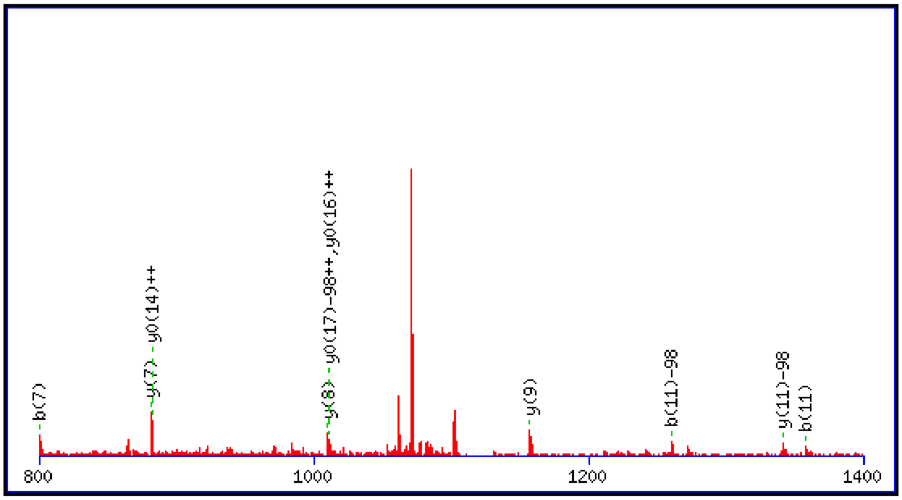

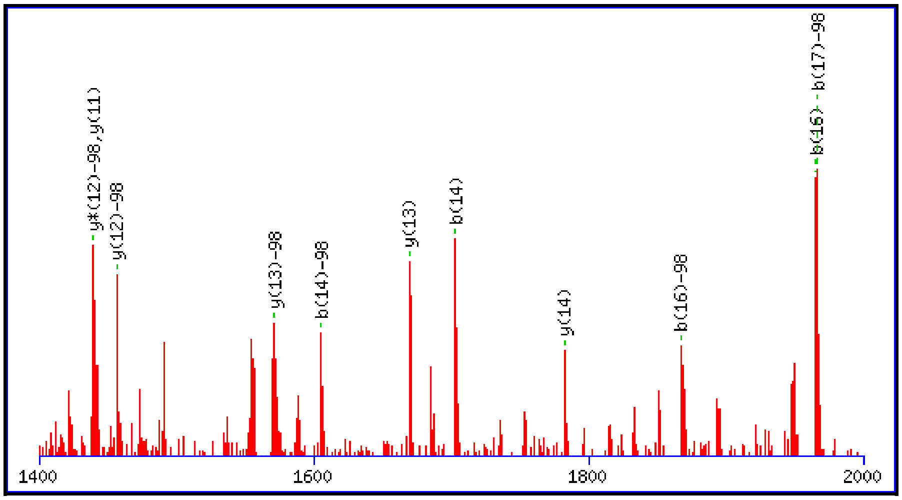

Monoisotopic mass of neutral peptide Mr(calc): 2237.8526
Fixed modifications: Carbamidomethyl (C)
Variable modifications:
S8 : Phospho (ST), with neutral losses 97.9769(shown in table), 0.0000
Ions Score: 77 **Expect:** 3.2e-006
Matches (Bold Red): 31/248 fragment ions using 41 most intense peaks

#	b	b^{++}	b^{0}	b^{0++}	Seq.	y	y^{++}	y*	y^{*++}	y^{0}	y^{0++}	#
1	102.0550	51.5311	84.0444	42.5258	T							18
2	199.1077	100.0575	181.0972	91.0522	P	2039.8352	1020.4213	2022.8087	1011.9080	2021.8247	**1011.4160**	17
3	328.1503	164.5788	310.1397	155.5735	E	1942.7825	971.8949	1925.7559	963.3816	1924.7719	962.8896	16
4	**457.1929**	229.1001	439.1823	220.0948	E	1813.7399	907.3736	1796.7133	898.8603	1795.7293	898.3683	15
5	**570.2770**	285.6421	552.2664	276.6368	L	1684.6973	842.8523	1667.6707	834.3390	1666.6867	833.8470	14

**6**	**685.3039**	343.1556	667.2933	334.1503	**D**	**1571.6132**	786.3103	1554.5867	777.7970	1553.6027	777.3050	**13**
**7**	**800.3309**	400.6691	782.3203	391.6638	**D**	**1456.5863**	728.7968	**1439.5597**	720.2835	1438.5757	719.7915	**12**
**8**	869.3523	435.1798	851.3417	426.1745	**S**	**1341.5593**	671.2833	1324.5328	662.7700	1323.5488	662.2780	**11**
**9**	984.3793	492.6933	966.3687	483.6880	**D**	1272.5379	636.7726	1255.5113	628.2593	1254.5273	627.7673	**10**
**10**	1131.4477	566.2275	1113.4371	557.2222	**F**	**1157.5109**	579.2591	1140.4844	**570.7458**	1139.5004	**570.2538**	**9**
**11**	**1260.4903**	630.7488	1242.4797	621.7435	**E**	**1010.4425**	505.7249	993.4160	497.2116	992.4320	496.7196	**8**
**12**	1361.5379	681.2726	1343.5274	672.2673	**T**	**881.3999**	441.2036	864.3734	432.6903	863.3894	432.1983	**7**
**13**	1490.5805	745.7939	1472.5700	736.7886	**E**	**780.3523**	**390.6798**	763.3257	382.1665	762.3417	381.6745	**6**
**14**	**1605.6075**	803.3074	1587.5969	794.3021	**D**	**651.3097**	326.1585	634.2831	317.6452	633.2991	317.1532	**5**
**15**	1752.6759	876.8416	1734.6653	867.8363	**F**	**536.2827**	268.6450	519.2562	260.1317	518.2722	259.6397	**4**
**16**	**1867.7028**	934.3551	1849.6923	925.3498	**D**	**389.2143**	195.1108	372.1878	186.5975	371.2037	186.1055	**3**
**17**	**1966.7712**	983.8893	1948.7607	974.8840	**V**	274.1874	137.5973	257.1608	129.0840			**2**
**18**					**R**	175.1190	88.0631	158.0924	79.5498			**1**

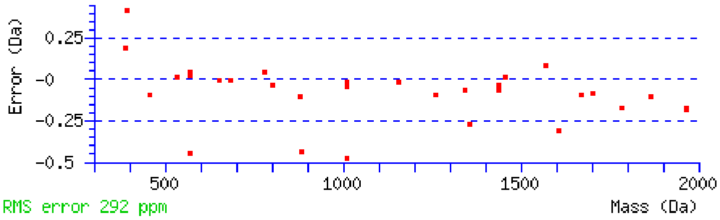

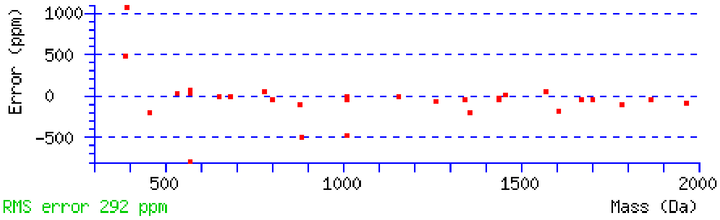

All matches to this query

Score	Mr(calc):	Delta	Sequence
77.2	2237.8526	2.0104	TPEELDDSDFETEDFDVR
47.5	2237.8526	2.0104	TPEELDDSDFETEDFDVR
35.4	2237.8526	2.0104	TPEELDDSDFETEDFDVR
2.3	2237.8661	1.9968	DSSSGADACARPTEALQAGSK
2.3	2237.8661	1.9968	DSSSGADACARPTEALQAGSK
2.2	2237.8451	2.0178	GIAAQPLFTGYCNYENKM
1.6	2237.8658	1.9972	GGSLRVGGGSFGGGSLYGGGGSR
1.4	2237.8661	1.9968	DSSSGADACARPTEALQAGSK
0.5	2237.8378	2.0252	SYATGNWTYGDVLCISNR

Spectrum No: 10; Query: 712; Rank: 1

Peptide View

MS/MS Fragmentation of **SLDSDESEDEDDDYQQK**
Found in **IPI00208277**, Tax_Id=10116 Gene_Symbol=Pdap1 28 kDa heat- and acid-stable phosphoprotein

Match to Query 712: 2096.726488 from(1049.370520,2+)
Title: 091127RatKid_SCX01_12.1124.1124.2.dta
Data file K:\NewmanPaper\Piliang\3SubProteomes\Piliang3SP\mgf5ppm\SCX_3SubProteomes5ppm.mgf

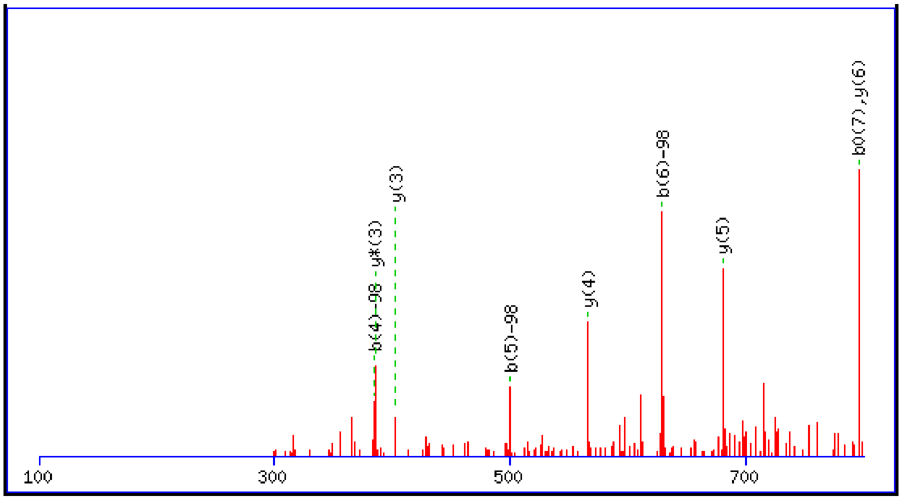

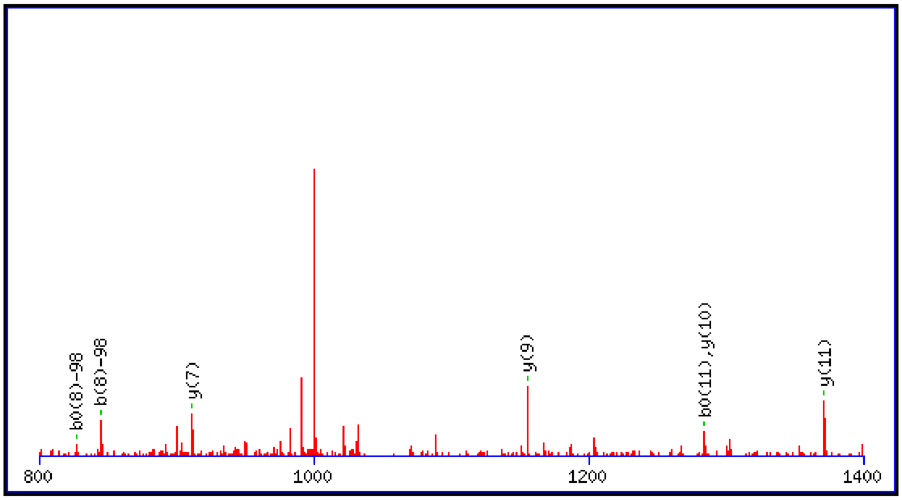

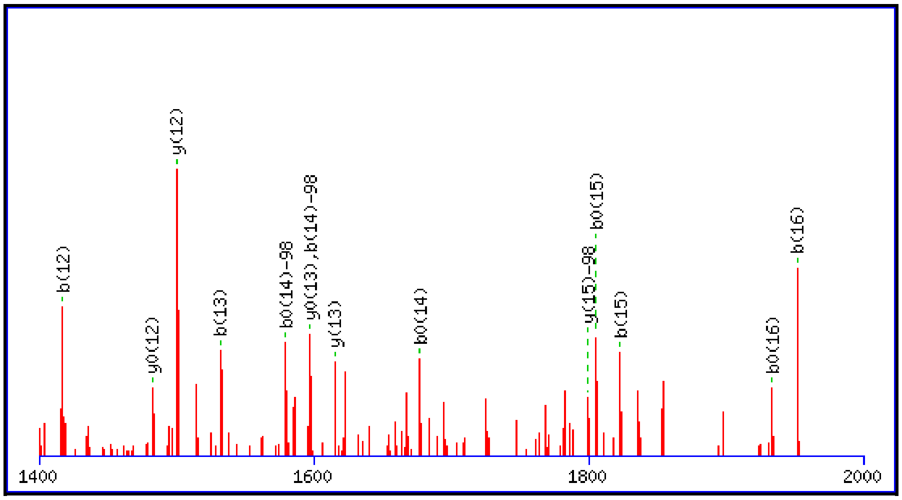

Monoisotopic mass of neutral peptide Mr(calc): 2096.7219
Fixed modifications: Carbamidomethyl (C)
Variable modifications:
S4 : Phospho (ST), with neutral losses 97.9769(shown in table), 0.0000
Ions Score: 76 **Expect:** 1.7e-006
Matches (Bold Red): 30/230 fragment ions using 38 most intense peaks

#	b	b^{++}	b*	b^{*++}	b^0	b^{0++}	Seq.	y	y^{++}	y*	y^{*++}	y^0	y^{0++}	#
1	88.0393	44.5233			70.0287	35.5180	S							17
2	201.1234	101.0653			183.1128	92.0600	L	1912.7203	956.8638	1895.6937	948.3505	1894.7097	947.8585	16
3	316.1503	158.5788			298.1397	149.5735	D	**1799.6362**	900.3217	1782.6097	891.8085	1781.6256	891.3165	15
4	**385.1718**	193.0895			367.1612	184.0842	S	1684.6093	842.8083	1667.5827	834.2950	1666.5987	833.8030	14
5	**500.1987**	250.6030			482.1881	241.5977	D	**1615.5878**	808.2975	1598.5613	799.7843	**1597.5772**	799.2923	13

**6**	**629.2413**	315.1243			611.2307	306.1190	**E**	**1500.5609**	750.7841	1483.5343	742.2708	**1482.5503**	741.7788	**12**
**7**	716.2733	358.6403			698.2628	349.6350	**S**	**1371.5183**	686.2628	1354.4917	677.7495	1353.5077	677.2575	**11**
**8**	**845.3159**	423.1616			**827.3054**	414.1563	**E**	**1284.4862**	642.7468	1267.4597	634.2335	1266.4757	633.7415	**10**
**9**	960.3429	480.6751			942.3323	471.6698	**D**	**1155.4437**	578.2255	1138.4171	569.7122	1137.4331	569.2202	**9**
**10**	1089.3855	545.1964			1071.3749	536.1911	**E**	1040.4167	520.7120	1023.3902	512.1987	1022.4061	511.7067	**8**
**11**	1204.4124	602.7098			1186.4018	593.7046	**D**	**911.3741**	456.1907	894.3476	447.6774	893.3636	447.1854	**7**
**12**	1319.4393	660.2233			1301.4288	651.2180	**D**	**796.3472**	398.6772	779.3206	390.1640	778.3366	389.6719	**6**
**13**	1434.4663	717.7368			1416.4557	708.7315	**D**	**681.3202**	341.1638	664.2937	332.6505	663.3097	332.1585	**5**
**14**	**1597.5296**	799.2684			**1579.5191**	790.2632	**Y**	**566.2933**	283.6503	549.2667	275.1370			**4**
**15**	1725.5882	863.2977	1708.5616	854.7845	1707.5776	854.2925	**Q**	**403.2300**	202.1186	**386.2034**	193.6053			**3**
**16**	1853.6468	927.3270	1836.6202	918.8138	1835.6362	918.3217	**Q**	275.1714	138.0893	258.1448	129.5761			**2**
**17**							**K**	147.1128	74.0600	130.0863	65.5468			**1**

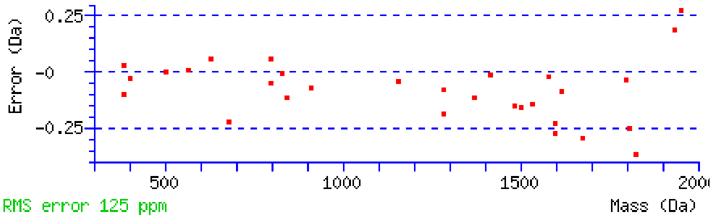

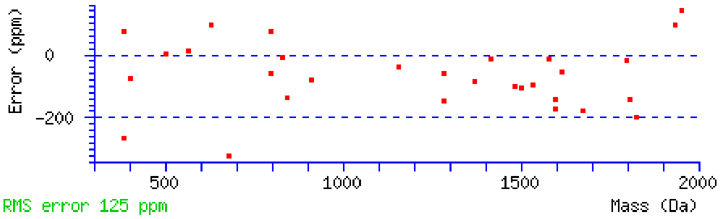

All matches to this query

Score	Mr(calc):	Delta	Sequence
76.4	2096.7219	0.0046	SLDSDESEDEDDDYQQK
65.7	2096.7219	0.0046	SLDSDESEDEDDDYQQK
50.3	2096.7219	0.0046	SLDSDESEDEDDDYQQK

Spectrum No: 11; Query: 594; Rank: 1

Peptide View

MS/MS Fragmentation of **SQSLPNSLDYAQTSER**
Found in **IPI00471759**, Tax_Id=10116 Gene_Symbol=RGD1308697 Protein FAM82C

Match to Query 594: 1874.813188 from(938.413870,2+)
Title: 091129RatKid_SCX02_12.1646.1646.2.dta
Data file K:\NewmanPaper\Piliang\3SubProteomes\Piliang3SP\mgf5ppm\SCX_3SubProteomes5ppm.mgf

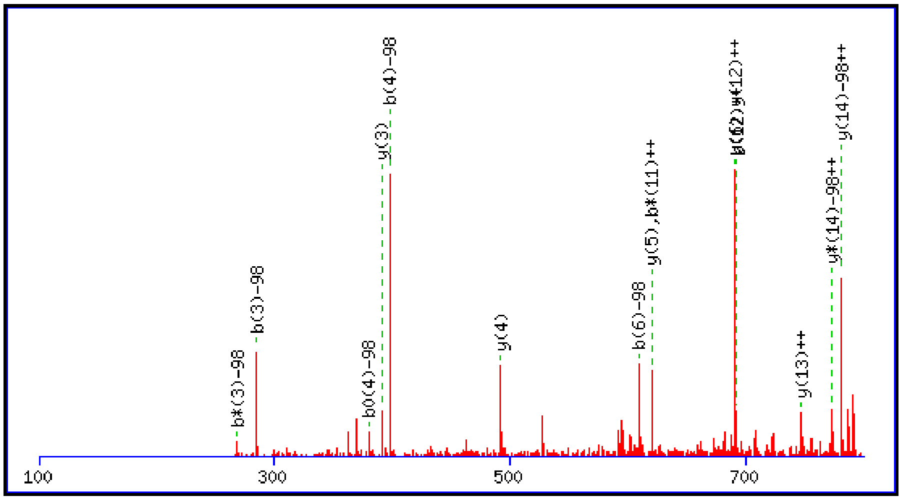

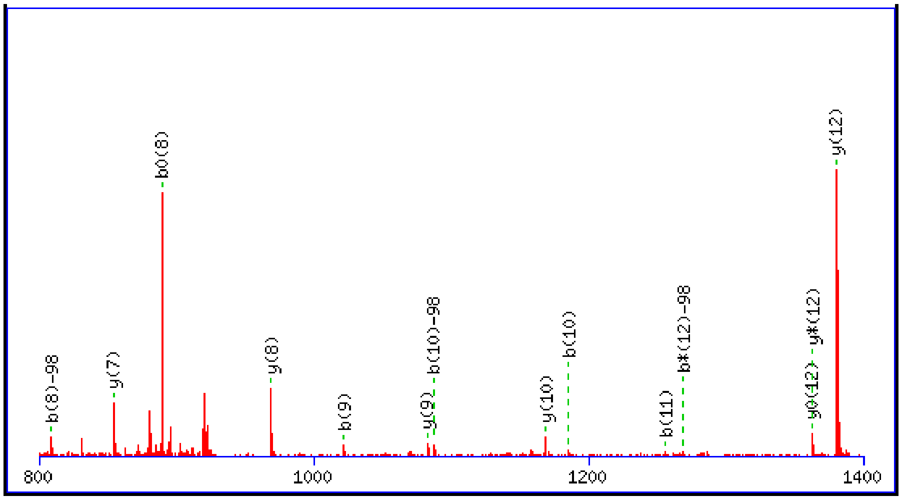

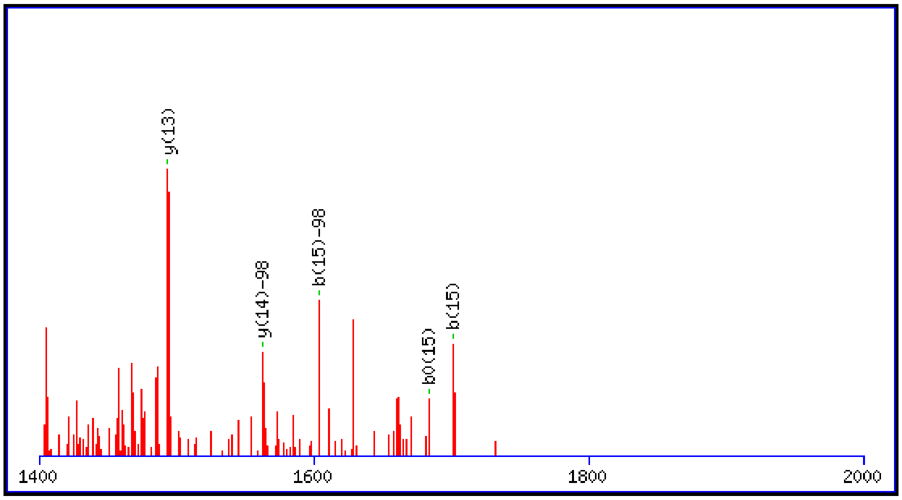

Monoisotopic mass of neutral peptide Mr(calc): 1874.8047
Fixed modifications: Carbamidomethyl (C)
Variable modifications:
S3 : Phospho (ST), with neutral losses 97.9769(shown in table), 0.0000
Ions Score: 76 **Expect:** 5.1e-006
Matches (Bold Red): 34/266 fragment ions using 49 most intense peaks

#	b	b^{++}	b*	b^{*++}	b^{0}	b^{0++}	Seq.	y	y^{++}	y*	y^{*++}	y^{0}	y^{0++}	#
1	88.0393	44.5233			70.0287	35.5180	S							16
2	216.0979	108.5526	199.0713	100.0393	198.0873	99.5473	Q	1690.8031	845.9052	1673.7765	837.3919	1672.7925	836.8999	15
3	**285.1193**	143.0633	**268.0928**	134.5500	267.1088	134.0580	S	**1562.7445**	**781.8759**	1545.7180	**773.3626**	1544.7340	772.8706	14
4	**398.2034**	199.6053	381.1769	191.0921	**380.1928**	190.6001	L	**1493.7231**	**747.3652**	1476.6965	738.8519	1475.7125	738.3599	13
5	495.2562	248.1317	478.2296	239.6184	477.2456	239.1264	P	**1380.6390**	**690.8231**	**1363.6125**	682.3099	**1362.6284**	681.8179	12
6	**609.2991**	305.1532	592.2725	296.6399	591.2885	296.1479	N	1283.5862	642.2968	1266.5597	633.7835	1265.5757	633.2915	11
7	696.3311	348.6692	679.3046	340.1559	678.3206	339.6639	S	**1169.5433**	585.2753	1152.5168	576.7620	1151.5327	576.2700	10
8	**809.4152**	405.2112	792.3886	396.6980	791.4046	396.2059	L	**1082.5113**	541.7593	1065.4847	533.2460	1064.5007	532.7540	9
9	924.4421	462.7247	907.4156	454.2114	906.4316	453.7194	D	**969.4272**	485.2172	952.4007	476.7040	951.4167	476.2120	8
10	**1087.5055**	544.2564	1070.4789	535.7431	1069.4949	535.2511	Y	**854.4003**	427.7038	837.3737	419.1905	836.3897	418.6985	7
11	1158.5426	579.7749	1141.5160	571.2616	1140.5320	570.7696	A	**691.3369**	346.1721	674.3104	337.6588	673.3264	337.1668	6
12	1286.6011	643.8042	**1269.5746**	635.2909	1268.5906	634.7989	Q	**620.2998**	310.6536	603.2733	302.1403	602.2893	301.6483	5
13	1387.6488	694.3281	1370.6223	685.8148	1369.6383	685.3228	T	**492.2413**	246.6243	475.2147	238.1110	474.2307	237.6190	4
14	1474.6809	737.8441	1457.6543	729.3308	1456.6703	728.8388	S	**391.1936**	196.1004	374.1670	187.5871	373.1830	187.0951	3
15	**1603.7234**	802.3654	1586.6969	793.8521	1585.7129	793.3601	E	304.1615	152.5844	287.1350	144.0711	286.1510	143.5791	2
16							R	175.1190	88.0631	158.0924	79.5498			1

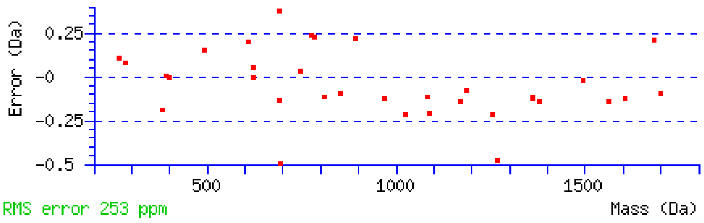

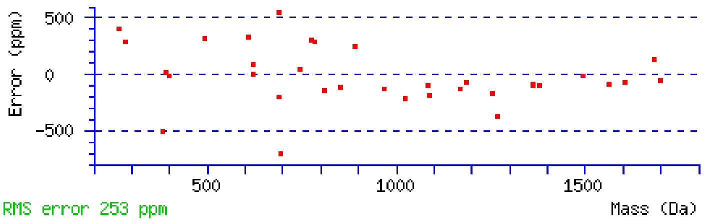

All matches to this query

Score	Mr(calc):	Delta	Sequence
76.2	1874.8047	0.0085	SQSLPNSLDYAQTSER
72.2	1874.8047	0.0085	SQSLPNSLDYAQTSER
46.7	1874.8047	0.0085	SQSLPNSLDYAQTSER
9.8	1874.8077	0.0055	QRLGSAALDSIHEYR
9.7	1874.8047	0.0085	SQSLPNSLDYAQTSER
8.7	1874.8274	-0.0142	FHLYEEMIELATGSR
8.7	1874.8274	-0.0142	FHLYEEMIELATGSR
8.7	1874.8077	0.0055	QRLGSAALDSIHEYR
5.2	1872.7965	2.0167	SKFEDNEHTVVMETK
4.0	1874.8064	0.0068	LETTSNQDNLAPITAK

Spectrum No: 12; Query: 603; Rank: 1

Peptide View

MS/MS Fragmentation of **SVPTIDSGNEDDDSSFK**
Found in **IPI00366370**, Tax_Id=10116 Gene_Symbol=Eif5b eukaryotic translation initiation factor 5B

Match to Query 603: 1891.740588 from(946.877570,2+)
Title: 091127RatKid_SCX01_12.1795.1795.2.dta
Data file K:\NewmanPaper\Piliang\3SubProteomes\Piliang3SP\mgf5ppm\SCX_3SubProteomes5ppm.mgf

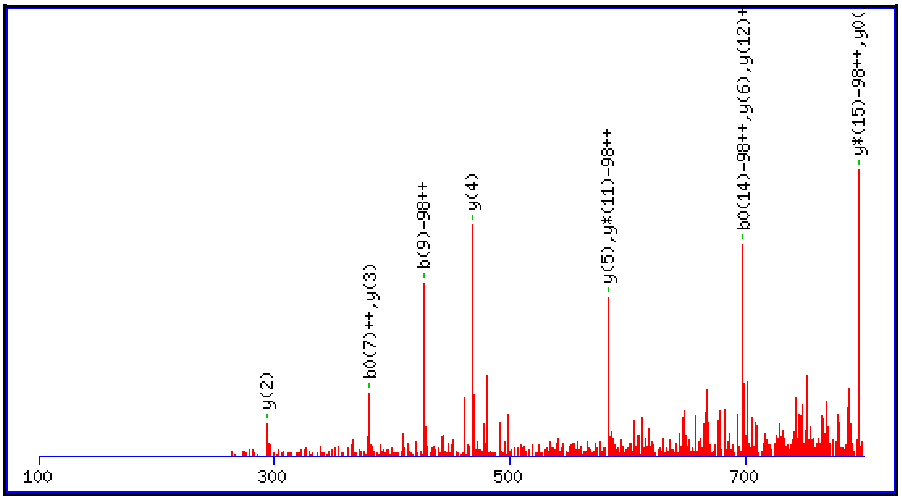

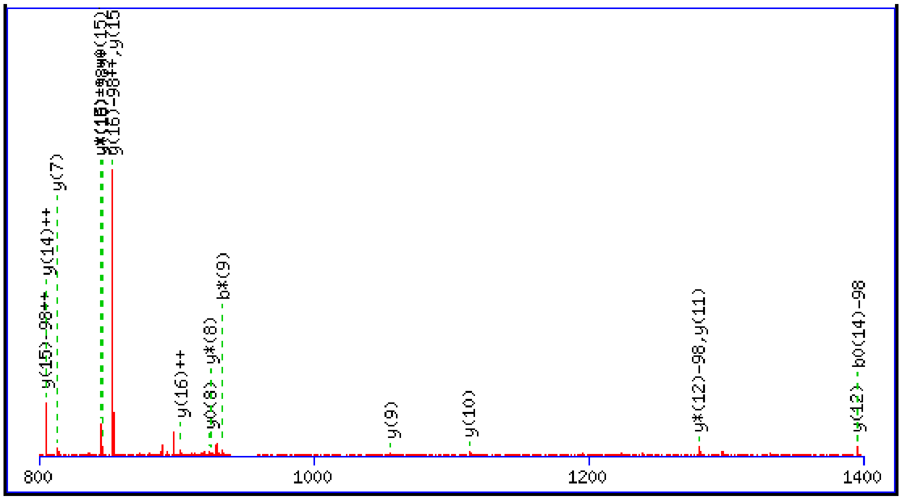

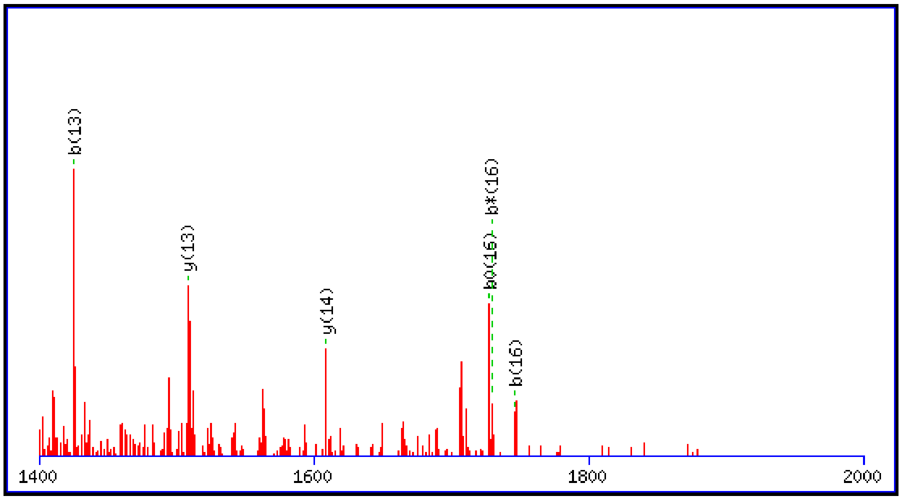

Monoisotopic mass of neutral peptide Mr(calc): 1891.7361
Fixed modifications: Carbamidomethyl (C)
Variable modifications:
S7 : Phospho (ST), with neutral losses 0.0000(shown in table), 97.9769
Ions Score: 76 **Expect:** 5.9e-006
Matches (Bold Red): 39/264 fragment ions using 44 most intense peaks

#	b	b^{++}	b*	b^{*++}	b^{0}	b^{0++}	Seq.	y	y^{++}	y*	y^{*++}	y^{0}	y^{0++}	#
1	88.0393	44.5233			70.0287	35.5180	S							17
2	187.1077	94.0575			169.0972	85.0522	V	1805.7113	**903.3593**	1788.6848	894.8460	1787.7007	894.3540	16
3	284.1605	142.5839			266.1499	133.5786	P	1706.6429	**853.8251**	1689.6163	**845.3118**	1688.6323	**844.8198**	15
4	385.2082	193.1077			367.1976	184.1024	T	**1609.5901**	**805.2987**	1592.5636	796.7854	1591.5796	**796.2934**	14
5	498.2922	249.6498			480.2817	240.6445	I	**1508.5424**	754.7749	1491.5159	746.2616	1490.5319	745.7696	13
6	613.3192	307.1632			595.3086	298.1579	D	**1395.4584**	**698.2328**	1378.4318	689.7196	1377.4478	689.2275	12
7	780.3175	390.6624			762.3070	**381.6571**	S	**1280.4314**	640.7194	1263.4049	632.2061	1262.4209	631.7141	11
8	837.3390	419.1731			819.3284	410.1678	G	**1113.4331**	557.2202	1096.4065	548.7069	1095.4225	548.2149	10
9	951.3819	476.1946	**934.3554**	467.6813	933.3713	467.1893	N	**1056.4116**	528.7094	1039.3851	520.1962	1038.4011	519.7042	9
10	1080.4245	540.7159	1063.3980	532.2026	1062.4139	531.7106	E	942.3687	471.6880	**925.3421**	463.1747	**924.3581**	462.6827	8
11	1195.4514	598.2294	1178.4249	589.7161	1177.4409	589.2241	D	**813.3261**	407.1667	**796.2996**	398.6534	795.3155	398.1614	7
12	1310.4784	655.7428	1293.4518	647.2296	1292.4678	646.7376	D	**698.2992**	349.6532	681.2726	341.1399	680.2886	340.6479	6
13	**1425.5053**	713.2563	1408.4788	704.7430	1407.4948	704.2510	D	**583.2722**	292.1397	566.2457	283.6265	565.2617	283.1345	5
14	1512.5374	756.7723	1495.5108	748.2590	1494.5268	747.7670	S	**468.2453**	234.6263	451.2187	226.1130	450.2347	225.6210	4
15	1599.5694	800.2883	1582.5428	791.7751	1581.5588	791.2831	S	**381.2132**	191.1103	364.1867	182.5970	363.2027	182.1050	3
16	**1746.6378**	873.8225	**1729.6113**	865.3093	**1728.6272**	864.8173	F	**294.1812**	147.5942	277.1547	139.0810			2
17							K	147.1128	74.0600	130.0863	65.5468			1

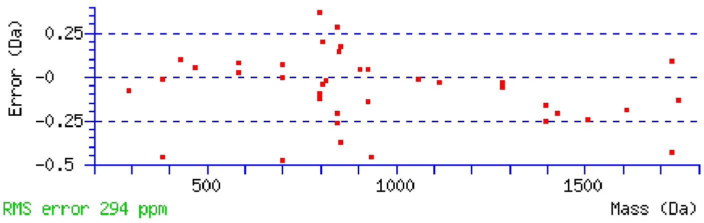

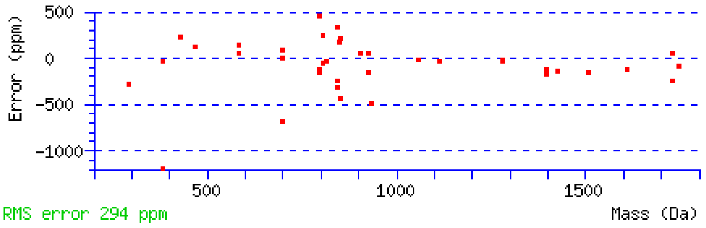

All matches to this query

Score	Mr(calc):	Delta	Sequence
75.7	1891.7361	0.0045	SVPTIDSGNEDDDSSFK
40.8	1891.7361	0.0045	SVPTIDSGNEDDDSSFK
28.1	1891.7361	0.0045	SVPTIDSGNEDDDSSFK
19.1	1891.7361	0.0045	SVPTIDSGNEDDDSSFK
19.1	1891.7361	0.0045	SVPTIDSGNEDDDSSFK
7.2	1890.7455	0.9951	LASIFSGTGTDPAVSTK
4.1	1891.7390	0.0016	WNDDPEARELSKSGK
3.4	1889.7306	2.0100	SNSXGEGARSERSPGNK
3.1	1890.7455	0.9951	LASIFSGTGTDPAVSTK
3.0	1889.7519	1.9887	MISEVTGGVSDTISYR

Spectrum No: 13; Query: 991; Rank: 1

Peptide View

MS/MS Fragmentation of **SLAALDALNTDDENDEEEYEAWK**
Found in **IPI00209527**, Tax_Id=10116 Gene_Symbol=RGD1562232_predicted similar to microfibrillar-associated protein 1

Match to Query 991: 2721.116268 from(1361.565410,2+)
Title: 091129RatKid_SCX02_12.3638.3638.2.dta
Data file K:\NewmanPaper\Piliang\3SubProteomes\Piliang3SP\mgf5ppm\SCX_3SubProteomes5ppm.mgf

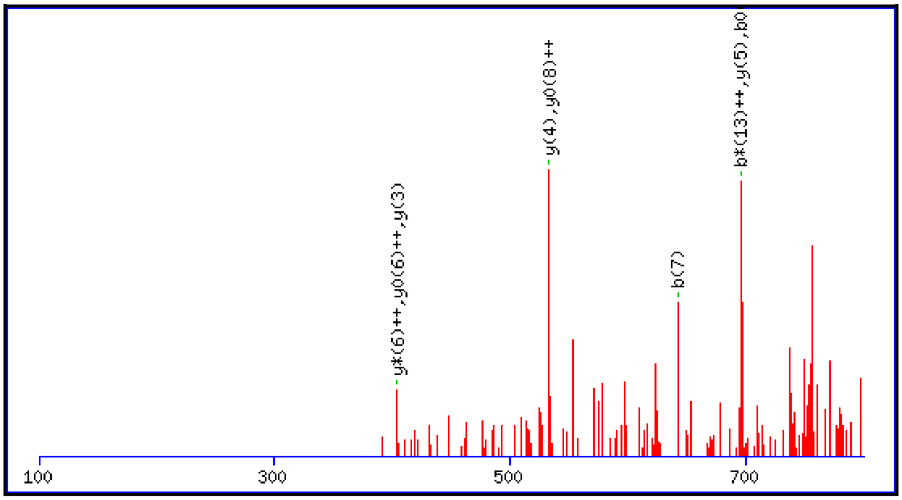

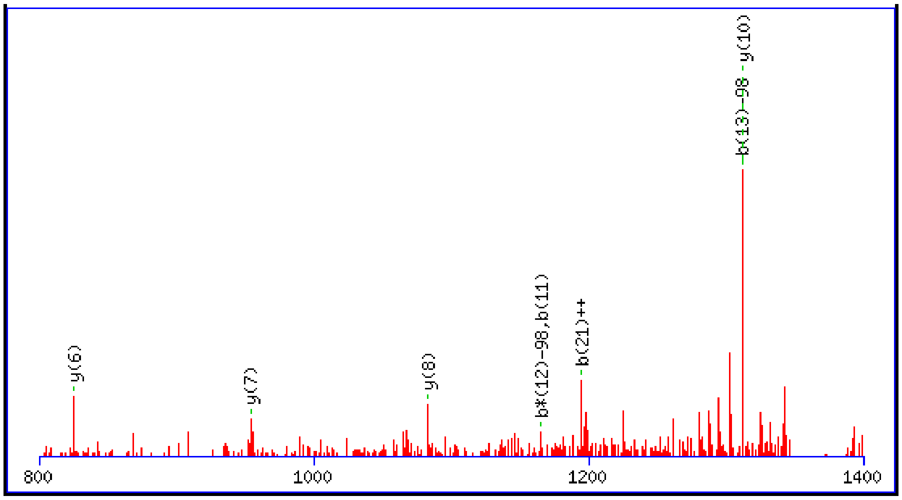

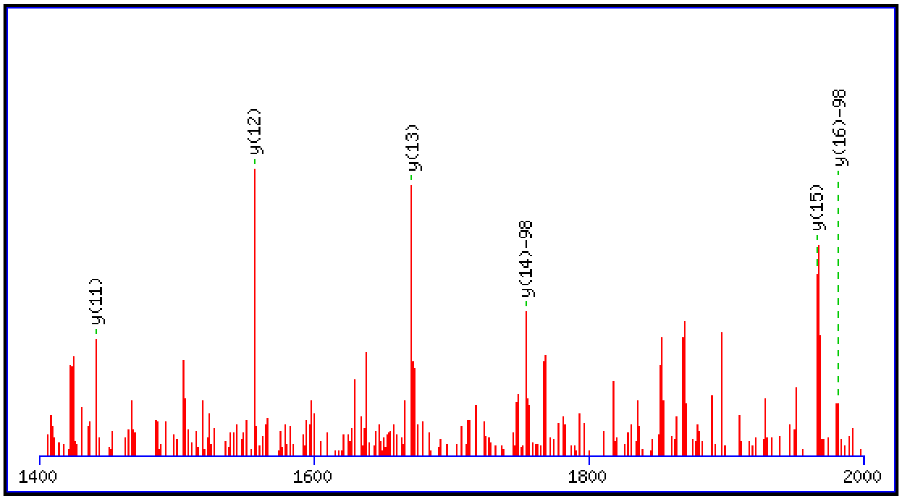

Monoisotopic mass of neutral peptide Mr(calc): 2720.1014
Fixed modifications: Carbamidomethyl (C)
Variable modifications:
T10 : Phospho (ST), with neutral losses 97.9769(shown in table), 0.0000
Ions Score: 75 **Expect:** 9.1e-006
Matches (Bold Red): 23/374 fragment ions using 26 most intense peaks

#	b	b^{++}	b*	b*$^{++}$	b^{0}	b^{0++}	Seq.	y	y^{++}	y*	y*$^{++}$	y^{0}	y^{0++}	#
1	88.0393	44.5233			70.0287	35.5180	S							23
2	201.1234	101.0653			183.1128	92.0600	L	2536.0998	1268.5535	2519.0732	1260.0403	2518.0892	1259.5483	22
3	272.1605	136.5839			254.1499	127.5786	A	2423.0157	1212.0115	2405.9892	1203.4982	2405.0052	1203.0062	21
4	343.1976	172.1024			325.1870	163.0972	A	2351.9786	1176.4929	2334.9521	1167.9797	2333.9680	1167.4877	20
5	456.2817	228.6445			438.2711	219.6392	L	2280.9415	1140.9744	2263.9150	1132.4611	2262.9309	1131.9691	19
6	571.3086	286.1579			553.2980	277.1527	D	2167.8574	1084.4324	2150.8309	1075.9191	2149.8469	1075.4271	18
7	**642.3457**	321.6765			624.3352	312.6712	A	2052.8305	1026.9189	2035.8039	1018.4056	2034.8199	1017.9136	17
8	755.4298	378.2185			737.4192	369.2132	L	**1981.7934**	991.4003	1964.7668	982.8871	1963.7828	982.3950	16
9	869.4727	435.2400	852.4462	426.7267	851.4621	426.2347	N	1868.7093	934.8583	1851.6828	926.3450	1850.6988	925.8530	15
10	952.5098	476.7585	935.4833	468.2453	934.4992	467.7533	T	**1754.6664**	877.8368	1737.6398	869.3236	1736.6558	868.8315	14
11	1067.5368	534.2720	1050.5102	525.7587	1049.5262	525.2667	D	**1671.6293**	836.3183	1654.6027	827.8050	1653.6187	827.3130	13
12	1182.5637	591.7855	**1165.5372**	583.2722	1164.5531	582.7802	D	**1556.6023**	778.8048	1539.5758	770.2915	1538.5918	769.7995	12
13	**1311.6063**	656.3068	1294.5797	647.7935	1293.5957	647.3015	E	**1441.5754**	721.2913	1424.5488	712.7781	1423.5648	712.2861	11
14	1425.6492	713.3282	1408.6227	704.8150	1407.6387	704.3230	N	**1312.5328**	656.7700	1295.5063	648.2568	1294.5222	647.7648	10
15	1540.6762	770.8417	1523.6496	762.3284	1522.6656	761.8364	D	1198.4899	599.7486	1181.4633	591.2353	1180.4793	590.7433	9
16	1669.7188	835.3630	1652.6922	826.8497	1651.7082	826.3577	E	**1083.4629**	542.2351	1066.4364	533.7218	1065.4524	**533.2298**	8
17	1798.7614	899.8843	1781.7348	891.3710	1780.7508	890.8790	E	**954.4203**	477.7138	937.3938	469.2005	936.4098	468.7085	7
18	1927.8039	964.4056	1910.7774	955.8923	1909.7934	955.4003	E	**825.3777**	413.1925	808.3512	**404.6792**	807.3672	**404.1872**	6

**19**	2090.8673	1045.9373	2073.8407	1037.4240	2072.8567	1036.9320	**Y**	**696.3352**	348.6712	679.3086	340.1579	678.3246	339.6659	**5**
**20**	2219.9099	1110.4586	2202.8833	1101.9453	2201.8993	1101.4533	**E**	**533.2718**	267.1396	516.2453	258.6263	515.2613	258.1343	**4**
**21**	2290.9470	1145.9771	2273.9204	1137.4639	2272.9364	1136.9718	**A**	**404.2292**	202.6183	387.2027	194.1050			**3**
**22**	2477.0263	1239.0168	2459.9997	1230.5035	2459.0157	1230.0115	**W**	333.1921	167.0997	316.1656	158.5864			**2**
**23**							**K**	147.1128	74.0600	130.0863	65.5468			**1**

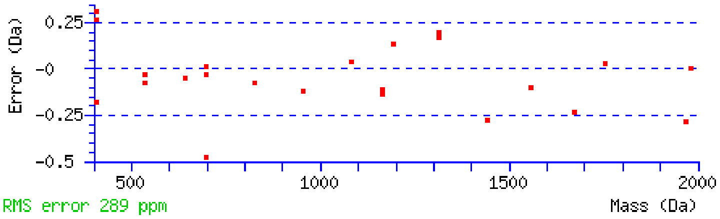

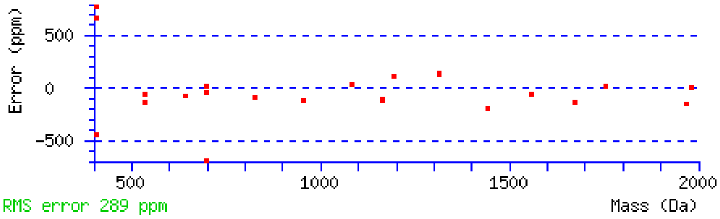

All matches to this query

Score	Mr(calc):	Delta	Sequence
75.1	2720.1014	1.0149	SLAALDALNTDDENDEEEYEAWK
68.6	2720.1014	1.0149	SLAALDALNTDDENDEEEYEAWK
5.7	2720.1345	0.9817	LMASTQPFKDIINNTSLAELEK
5.7	2720.1345	0.9817	LMASTQPFKDIINNTSLAELEK
4.2	2721.1347	-0.0184	NEKGDITTESEEIQNIISSYYK
4.2	2721.1347	-0.0184	NEKGDITTESEEIQNIISSYYK
4.2	2721.1347	-0.0184	NEKGDITTESEEIQNIISSYYK
4.2	2721.1347	-0.0184	NEKGDITTESEEIQNIISSYYK
4.1	2721.1225	-0.0062	SDLRQKENDPQIFNDSGMDSNSK
2.5	2720.1376	0.9786	SSLMQHLMAHAQEQIPTPNPEGK

Spectrum No: 14; Query: 1053; Rank: 1

Peptide View

MS/MS Fragmentation of **YEVAAQNEADEADGSAQGDGAGPAAEQVK**
Found in **IPI00470251**, Tax_Id=10116 Gene_Symbol=Slc7a7 Y+L amino acid transporter 1

Match to Query 1053: 2927.214072 from(976.745300,3+)
Title: 091127RatKid_SCX01_11.1091.1091.3.dta
Data file K:\NewmanPaper\Piliang\3SubProteomes\Piliang3SP\mgf5ppm\SCX_3SubProteomes5ppm.mgf

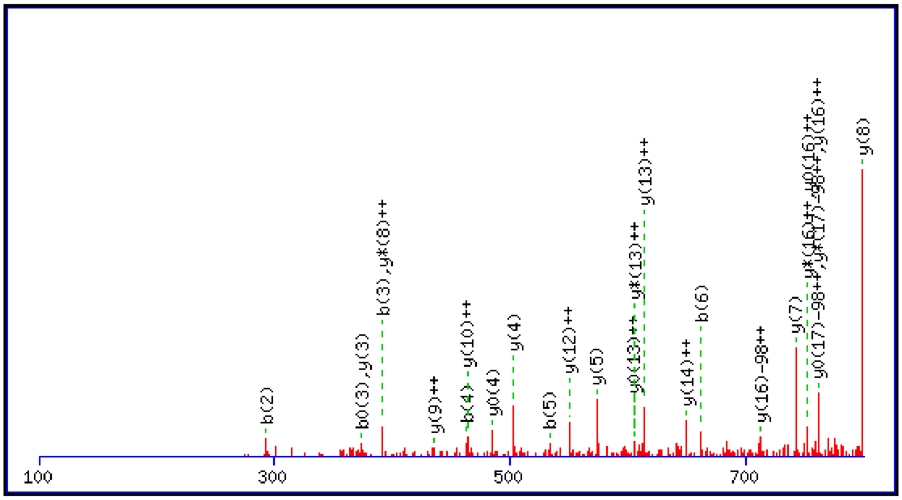

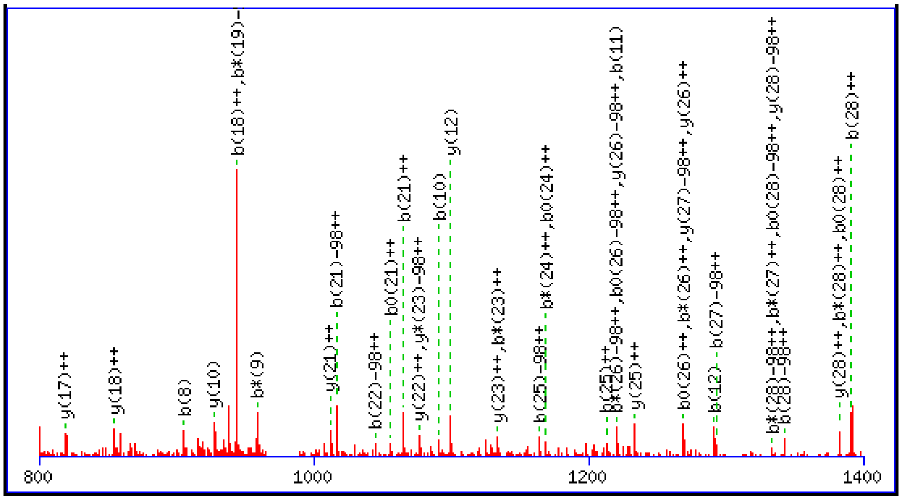

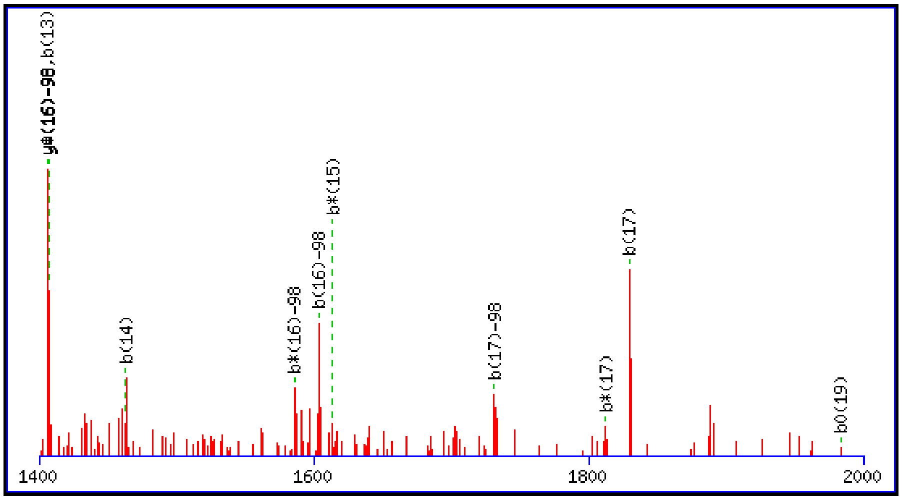

Monoisotopic mass of neutral peptide Mr(calc): 2927.2094
Fixed modifications: Carbamidomethyl (C)
Variable modifications:
S15 : Phospho (ST), with neutral losses 0.0000(shown in table), 97.9769
Ions Score: 73 **Expect:** 1.6e-005
Matches (Bold Red): 79/486 fragment ions using 100 most intense peaks

#	b	b^{++}	b*	b^{*++}	b^0	b^{0++}	Seq.	y	y^{++}	y*	y^{*++}	y^0	y^{0++}	#
1	164.0706	82.5389					Y							29
2	**293.1132**	147.0602			275.1026	138.0550	E	2765.1534	**1383.0803**	2748.1268	1374.5671	2747.1428	1374.0750	28
3	**392.1816**	196.5944			**374.1710**	187.5892	V	2636.1108	1318.5590	2619.0842	1310.0458	2618.1002	1309.5537	27
4	**463.2187**	232.1130			445.2082	223.1077	A	2537.0424	**1269.0248**	2520.0158	1260.5115	2519.0318	1260.0195	26
5	**534.2558**	267.6316			516.2453	258.6263	A	2466.0053	**1233.5063**	2448.9787	1224.9930	2447.9947	1224.5010	25
6	**662.3144**	331.6608	645.2879	323.1476	644.3039	322.6556	Q	2394.9681	1197.9877	2377.9416	1189.4744	2376.9576	1188.9824	24
7	776.3573	388.6823	759.3308	380.1690	758.3468	379.6770	N	2266.9096	**1133.9584**	2249.8830	1125.4451	2248.8990	1124.9531	23
8	**905.3999**	453.2036	888.3734	444.6903	887.3894	444.1983	E	2152.8666	**1076.9370**	2135.8401	1068.4237	2134.8561	1067.9317	22
9	976.4371	488.7222	**959.4105**	480.2089	958.4265	479.7169	A	2023.8240	**1012.4157**	2006.7975	1003.9024	2005.8135	1003.4104	21
10	**1091.4640**	546.2356	1074.4374	537.7224	1073.4534	537.2304	D	1952.7869	976.8971	1935.7604	968.3838	1934.7764	967.8918	20
11	**1220.5066**	610.7569	1203.4800	602.2437	1202.4960	601.7516	E	1837.7600	919.3836	1820.7334	910.8704	1819.7494	910.3783	19
12	**1291.5437**	646.2755	1274.5172	637.7622	1273.5331	637.2702	A	1708.7174	**854.8623**	1691.6908	846.3491	1690.7068	845.8571	18
13	**1406.5706**	703.7890	1389.5441	695.2757	1388.5601	694.7837	D	1637.6803	**819.3438**	1620.6537	810.8305	1619.6697	810.3385	17
14	**1463.5921**	732.2997	1446.5656	723.7864	1445.5815	723.2944	G	1522.6533	**761.8303**	1505.6268	**753.3170**	1504.6428	**752.8250**	16
15	1630.5905	815.7989	**1613.5639**	807.2856	1612.5799	806.7936	S	1465.6319	733.3196	1448.6053	724.8063	1447.6213	724.3143	15
16	1701.6276	851.3174	1684.6010	842.8042	1683.6170	842.3121	A	1298.6335	**649.8204**	1281.6070	641.3071	1280.6230	640.8151	14
17	**1829.6862**	915.3467	**1812.6596**	906.8334	1811.6756	906.3414	Q	1227.5964	**614.3018**	1210.5699	**605.7886**	1209.5858	**605.2966**	13
18	1886.7076	**943.8574**	1869.6811	935.3442	1868.6971	934.8522	G	**1099.5378**	**550.2726**	1082.5113	541.7593	1081.5273	541.2673	12

**19**	2001.7346	1001.3709	1984.7080	992.8576	**1983.7240**	992.3656	**D**	1042.5164	521.7618	1025.4898	513.2485	1024.5058	512.7565	**11**
**20**	2058.7560	1029.8816	2041.7295	1021.3684	2040.7455	1020.8764	**G**	**927.4894**	**464.2483**	910.4629	455.7351	909.4789	455.2431	**10**
**21**	2129.7931	**1065.4002**	2112.7666	1056.8869	2111.7826	**1056.3949**	**A**	870.4680	**435.7376**	853.4414	427.2243	852.4574	426.7323	**9**
**22**	2186.8146	1093.9109	2169.7881	1085.3977	2168.8040	1084.9057	**G**	**799.4308**	400.2191	782.4043	**391.7058**	781.4203	391.2138	**8**
**23**	2283.8674	1142.4373	2266.8408	**1133.9240**	2265.8568	1133.4320	**P**	**742.4094**	371.7083	725.3828	363.1951	724.3988	362.7030	**7**
**24**	2354.9045	1177.9559	2337.8779	**1169.4426**	2336.8939	**1168.9506**	**A**	645.3566	323.1819	628.3301	314.6687	627.3461	314.1767	**6**
**25**	2425.9416	**1213.4744**	2408.9150	1204.9612	2407.9310	1204.4692	**A**	**574.3195**	287.6634	557.2930	279.1501	556.3089	278.6581	**5**
**26**	2554.9842	1277.9957	2537.9576	**1269.4825**	2536.9736	**1268.9904**	**E**	**503.2824**	252.1448	486.2558	243.6316	**485.2718**	243.1396	**4**
**27**	2683.0428	1342.0250	2666.0162	**1333.5117**	2665.0322	1333.0197	**Q**	**374.2398**	187.6235	357.2132	179.1103			**3**
**28**	2782.1112	**1391.5592**	2765.0846	**1383.0460**	2764.1006	**1382.5539**	**V**	246.1812	123.5942	229.1547	115.0810			**2**
**29**							**K**	147.1128	74.0600	130.0863	65.5468			**1**

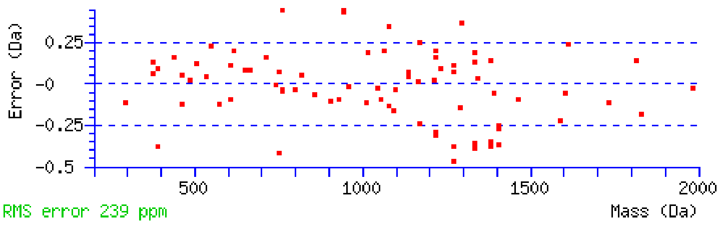

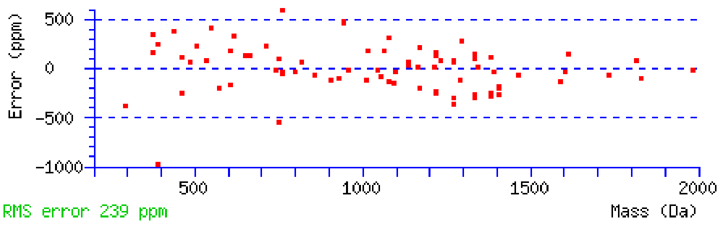

All matches to this query

Score	Mr(calc):	Delta	Sequence
73.1	2927.2094	0.0047	YEVAAQNEADEADGSAQGDGAGPAAEQVK
19.3	2927.2094	0.0047	YEVAAQNEADEADGSAQGDGAGPAAEQVK
5.4	2926.1994	1.0147	MAEAEATQLKEEGNQHFQRQDYK
5.2	2926.2384	0.9756	DLCLVENSISFYDHLANLEGSLEK
4.3	2926.2050	1.0091	SEACGYPVIIQKYKPYSNGAANQK
4.3	2926.2050	1.0091	SEACGYPVIIQKYKPYSNGAANQK
4.3	2926.2050	1.0091	SEACGYPVIIQKYKPYSNGAANQK
3.0	2927.2224	-0.0083	NTSMENYYNLVYRVSIINNSLEK
3.0	2927.2224	-0.0083	NTSMENYYNLVYRVSIINNSLEK
2.1	2925.2130	2.0011	RCAGPDSSSSSPASAAPRGAELVQSLK

Spectrum No: 15; Query: 1150; Rank: 1

Peptide View

MS/MS Fragmentation of **EGQVEAAQPEQAAEAPAESSAQPNQLETGASSPER**
Found in **IPI00768543**, Tax_Id=10116 Gene_Symbol=Zbtb20_predicted zinc finger and BTB domain containing 20

Match to Query 1150: 3643.597242 from(1215.539690,3+)
Title: 091127RatKid_SCX01_03.1874.1874.3.dta
Data file K:\NewmanPaper\Piliang\3SubProteomes\Piliang3SP\mgf5ppm\SCX_3SubProteomes5ppm.mgf

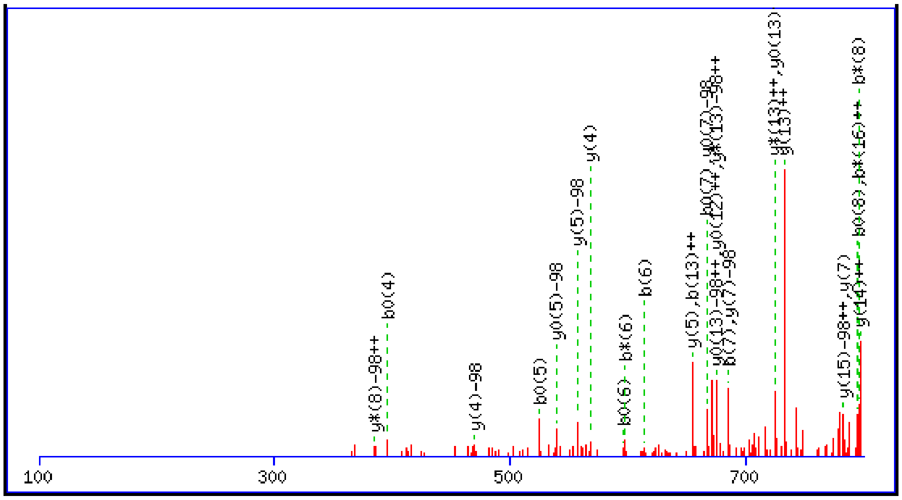

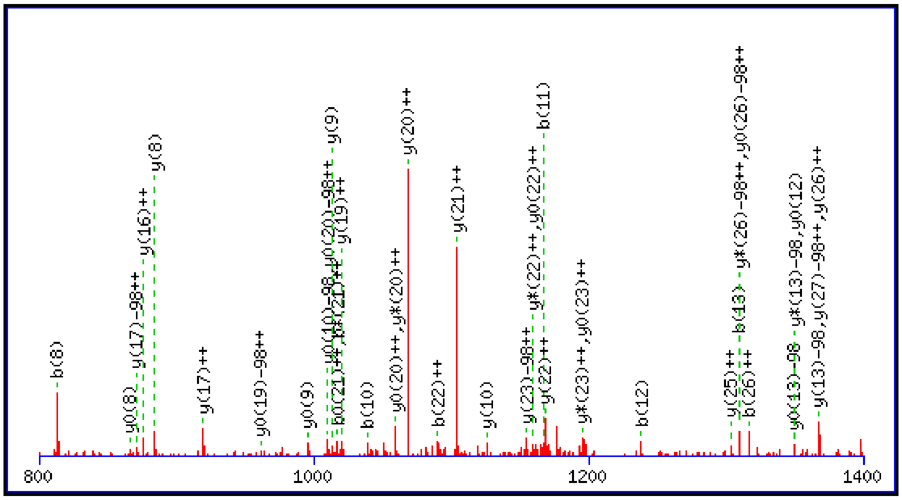

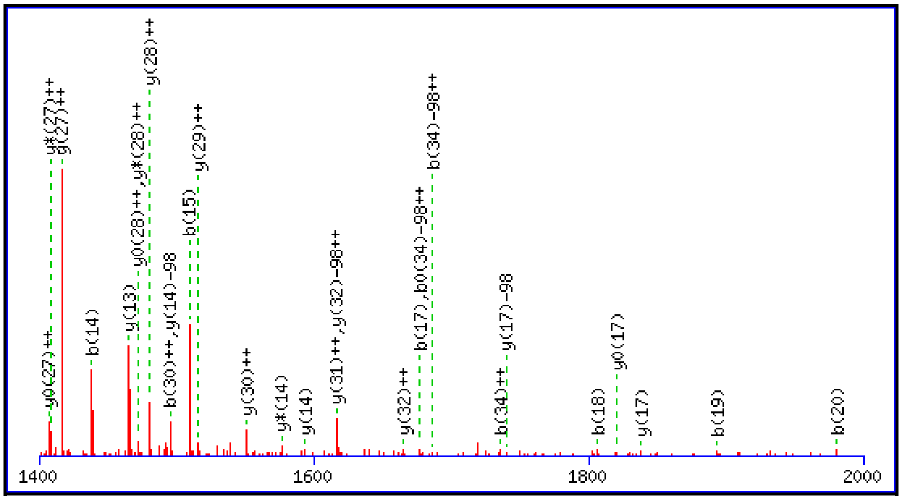

Monoisotopic mass of neutral peptide Mr(calc): 3643.5911
Fixed modifications: Carbamidomethyl (C)
Variable modifications:
S32 : Phospho (ST), with neutral losses 0.0000(shown in table), 97.9769
Ions Score: 73 **Expect:** 2.5e-005
Matches (Bold Red): 96/606 fragment ions using 132 most intense peaks

#	b	b^{++}	b*	b^{*++}	b^0	b^{0++}	Seq.	y	y^{++}	y*	y^{*++}	y^0	y^{0++}	#
1	130.0499	65.5286			112.0393	56.5233	E							35
2	187.0713	94.0393			169.0608	85.0340	G	3515.5558	1758.2815	3498.5293	1749.7683	3497.5453	1749.2763	34
3	315.1299	158.0686	298.1034	149.5553	297.1193	149.0633	Q	3458.5344	1729.7708	3441.5078	1721.2575	3440.5238	1720.7655	33
4	414.1983	207.6028	397.1718	199.0895	**396.1878**	198.5975	V	3330.4758	**1665.7415**	3313.4492	1657.2283	3312.4652	1656.7362	32
5	543.2409	272.1241	526.2144	263.6108	**525.2304**	263.1188	E	3231.4074	**1616.2073**	3214.3808	1607.6940	3213.3968	1607.2020	31

6	**614.2780**	307.6427	**597.2515**	299.1294	**596.2675**	298.6374	A	3102.3648	**1551.6860**	3085.3382	1543.1727	3084.3542	1542.6807	30
7	**685.3151**	343.1612	668.2886	334.6479	**667.3046**	334.1559	A	3031.3277	**1516.1675**	3014.3011	1507.6542	3013.3171	1507.1622	29
8	**813.3737**	407.1905	**796.3472**	398.6772	**795.3632**	398.1852	Q	2960.2905	**1480.6489**	2943.2640	**1472.1356**	2942.2800	**1471.6436**	28
9	910.4265	455.7169	893.3999	447.2036	892.4159	446.7116	P	2832.2320	**1416.6196**	2815.2054	**1408.1063**	2814.2214	**1407.6143**	27
10	**1039.4691**	520.2382	1022.4425	511.7249	1021.4585	511.2329	E	2735.1792	**1368.0932**	2718.1526	1359.5800	2717.1686	1359.0880	26
11	**1167.5277**	584.2675	1150.5011	575.7542	1149.5171	575.2622	Q	2606.1366	**1303.5719**	2589.1101	1295.0587	2588.1260	1294.5667	25
12	**1238.5648**	619.7860	1221.5382	611.2728	1220.5542	610.7807	A	2478.0780	1239.5427	2461.0515	1231.0294	2460.0675	1230.5374	24
13	**1309.6019**	**655.3046**	1292.5753	646.7913	1291.5913	646.2993	A	2407.0409	1204.0241	2390.0144	**1195.5108**	2389.0303	**1195.0188**	23
14	**1438.6445**	719.8259	1421.6179	711.3126	1420.6339	710.8206	E	2336.0038	**1168.5055**	2318.9772	**1159.9923**	2317.9932	**1159.5003**	22
15	**1509.6816**	755.3444	1492.6550	746.8312	1491.6710	746.3392	A	2206.9612	**1103.9842**	2189.9347	1095.4710	2188.9506	1094.9790	21
16	1606.7344	803.8708	1589.7078	**795.3575**	1588.7238	794.8655	P	2135.9241	**1068.4657**	2118.8975	**1059.9524**	2117.9135	**1059.4604**	20
17	**1677.7715**	839.3894	1660.7449	830.8761	1659.7609	830.3841	A	2038.8713	**1019.9393**	2021.8448	1011.4260	2020.8608	1010.9340	19
18	**1806.8141**	903.9107	1789.7875	895.3974	1788.8035	894.9054	E	1967.8342	984.4207	1950.8077	975.9075	1949.8236	975.4155	18
19	**1893.8461**	947.4267	1876.8195	938.9134	1875.8355	938.4214	S	**1838.7916**	**919.8994**	1821.7651	911.3862	**1820.7811**	910.8942	17
20	**1980.8781**	990.9427	1963.8516	982.4294	1962.8676	981.9374	S	1751.7596	**876.3834**	1734.7330	867.8702	1733.7490	867.3782	16
21	2051.9152	1026.4613	2034.8887	**1017.9480**	2033.9047	**1017.4560**	A	1664.7276	832.8674	1647.7010	824.3541	1646.7170	823.8621	15
22	2179.9738	**1090.4905**	2162.9473	1081.9773	2161.9632	1081.4853	Q	**1593.6905**	**797.3489**	**1576.6639**	788.8356	1575.6799	788.3436	14
23	2277.0266	1139.0169	2260.0000	1130.5037	2259.0160	1130.0116	P	**1465.6319**	**733.3196**	1448.6053	**724.8063**	1447.6213	**724.3143**	13
24	2391.0695	1196.0384	2374.0430	1187.5251	2373.0589	1187.0331	N	1368.5791	684.7932	1351.5526	676.2799	**1350.5685**	**675.7879**	12
25	2519.1281	1260.0677	2502.1015	1251.5544	2501.1175	1251.0624	Q	1254.5362	627.7717	1237.5096	619.2585	1236.5256	618.7664	11
26	2632.2121	**1316.6097**	2615.1856	1308.0964	2614.2016	1307.6044	L	**1126.4776**	563.7424	1109.4511	555.2292	1108.4670	554.7372	10
27	2761.2547	1381.1310	2744.2282	1372.6177	2743.2442	1372.1257	E	**1013.3935**	507.2004	996.3670	498.6871	**995.3830**	498.1951	9
28	2862.3024	1431.6548	2845.2759	1423.1416	2844.2919	1422.6496	T	**884.3509**	442.6791	867.3244	434.1658	**866.3404**	433.6738	8
29	2919.3239	1460.1656	2902.2973	1451.6523	2901.3133	1451.1603	G	**783.3033**	392.1553	766.2767	383.6420	765.2927	383.1500	7
30	2990.3610	**1495.6841**	2973.3344	1487.1709	2972.3504	1486.6789	A	726.2818	363.6445	709.2553	355.1313	708.2712	354.6393	6
31	3077.3930	1539.2002	3060.3665	1530.6869	3059.3825	1530.1949	S	**655.2447**	328.1260	638.2181	319.6127	637.2341	319.1207	5
32	3244.3914	1622.6993	3227.3648	1614.1861	3226.3808	1613.6940	S	**568.2127**	284.6100	551.1861	276.0967	550.2021	275.6047	4
33	3341.4441	1671.2257	3324.4176	1662.7124	3323.4336	1662.2204	P	401.2143	201.1108	384.1878	192.5975	383.2037	192.1055	3
34	3470.4867	**1735.7470**	3453.4602	1727.2337	3452.4762	1726.7417	E	304.1615	152.5844	287.1350	144.0711	286.1510	143.5791	2
35							R	175.1190	88.0631	158.0924	79.5498			1

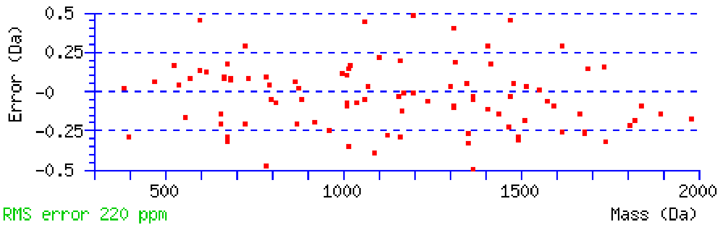

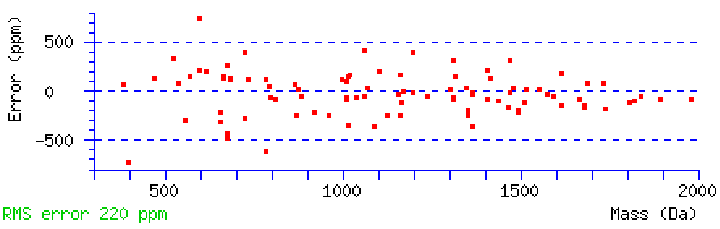

All matches to this query

Score	Mr(calc):	Delta	Sequence
73.0	3643.5911	0.0062	EGQVEAAQPEQAAEAPAESSAQPNQLETGASSPER
70.7	3643.5911	0.0062	EGQVEAAQPEQAAEAPAESSAQPNQLETGASSPER
69.5	3643.5911	0.0062	EGQVEAAQPEQAAEAPAESSAQPNQLETGASSPER
32.8	3643.5911	0.0062	EGQVEAAQPEQAAEAPAESSAQPNQLETGASSPER
26.1	3643.5911	0.0062	EGQVEAAQPEQAAEAPAESSAQPNQLETGASSPER
6.0	3642.5924	1.0049	EDRIIGMTVIQLQNIAEKGSYGAWYPLLK
5.5	3642.5699	1.0274	QEASTPERVDFPSSMEIASLQHTLDSQAGHSK
4.7	3643.6119	-0.0147	DDPAAPGPSGSPANDNGNGNGNGNGNGGKGKPAVPKGR
4.6	3642.6202	0.9770	HNPCTEFTIQSLVATVTDVFVAGSETTSTTLR
4.5	3643.6089	-0.0117	VSEQTFPCNVCGGVAGRQETLPAVLVSPPEER

Spectrum No: 16; Query: 641; Rank: 1

Peptide View

MS/MS Fragmentation of **AADPPAENSSAPEAEQGGAE**
Found in **IPI00551815**, Tax_Id=10116 Gene_Symbol=Ybx1-ps3 Ybx1 protein

Match to Query 641: 1976.770568 from(989.392560,2+)
Title: 091127RatKid_SCX01_02.701.701.2.dta
Data file K:\NewmanPaper\Piliang\3SubProteomes\Piliang3SP\mgf5ppm\SCX_3SubProteomes5ppm.mgf

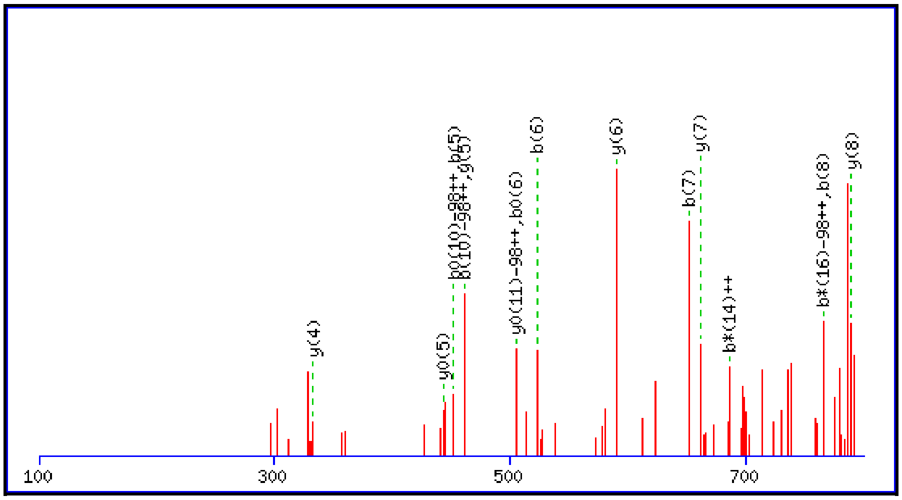

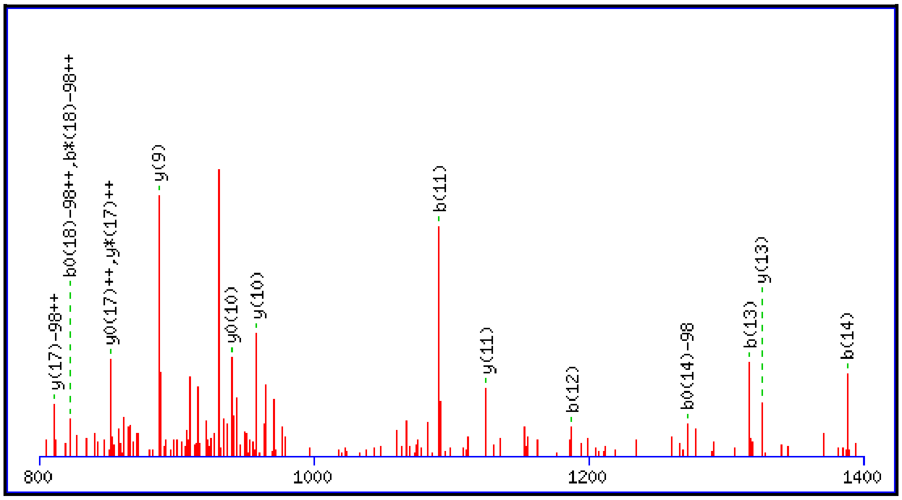

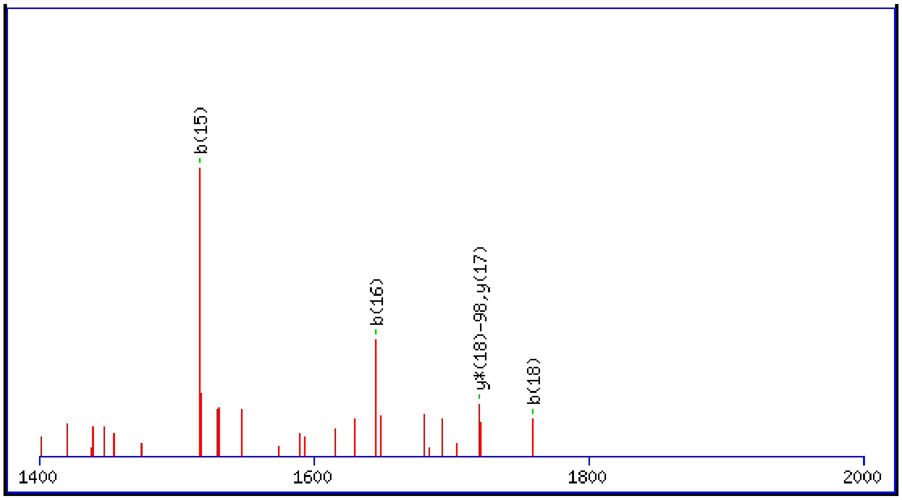

Monoisotopic mass of neutral peptide Mr(calc): 1976.7636
Fixed modifications: Carbamidomethyl (C)
Variable modifications:
S10 : Phospho (ST), with neutral losses 0.0000(shown in table), 97.9769
Ions Score: 73 **Expect:** 8.3e-006
Matches (Bold Red): 37/316 fragment ions using 51 most intense peaks

#	b	b^{++}	b*	b^{*++}	b^0	b^{0++}	Seq.	y	y^{++}	y*	y^{*++}	y^0	y^{0++}	#
1	72.0444	36.5258					A							20
2	143.0815	72.0444					A	1906.7338	953.8706	1889.7073	945.3573	1888.7233	944.8653	19
3	258.1084	129.5579			240.0979	120.5526	D	1835.6967	918.3520	1818.6702	909.8387	1817.6862	909.3467	18
4	355.1612	178.0842			337.1506	169.0790	P	**1720.6698**	860.8385	1703.6432	**852.3253**	1702.6592	**851.8332**	17
5	**452.2140**	226.6106			434.2034	217.6053	P	1623.6170	812.3121	1606.5905	803.7989	1605.6064	803.3069	16
6	**523.2511**	262.1292			**505.2405**	253.1239	A	1526.5642	763.7858	1509.5377	755.2725	1508.5537	754.7805	15
7	**652.2937**	326.6505			634.2831	317.6452	E	1455.5271	728.2672	1438.5006	719.7539	1437.5166	719.2619	14
8	**766.3366**	383.6719	749.3101	375.1587	748.3260	374.6667	N	**1326.4845**	663.7459	1309.4580	655.2326	1308.4740	654.7406	13
9	853.3686	427.1880	836.3421	418.6747	835.3581	418.1827	S	1212.4416	606.7244	1195.4151	598.2112	1194.4310	597.7192	12
10	1020.3670	510.6871	1003.3404	502.1739	1002.3564	501.6819	S	**1125.4096**	563.2084	1108.3830	554.6952	1107.3990	554.2031	11
11	**1091.4041**	546.2057	1074.3776	537.6924	1073.3935	537.2004	A	**958.4112**	479.7093	941.3847	471.1960	**940.4007**	470.7040	10
12	**1188.4569**	594.7321	1171.4303	586.2188	1170.4463	585.7268	P	**887.3741**	444.1907	870.3476	435.6774	869.3636	435.1854	9
13	**1317.4995**	659.2534	1300.4729	650.7401	1299.4889	650.2481	E	**790.3214**	395.6643	773.2948	387.1510	772.3108	386.6590	8
14	**1388.5366**	694.7719	1371.5100	**686.2587**	1370.5260	685.7666	A	**661.2788**	331.1430	644.2522	322.6297	643.2682	322.1377	7
15	**1517.5792**	759.2932	1500.5526	750.7799	1499.5686	750.2879	E	**590.2416**	295.6245	573.2151	287.1112	572.2311	286.6192	6
16	**1645.6377**	823.3225	1628.6112	814.8092	1627.6272	814.3172	Q	**461.1991**	231.1032	444.1725	222.5899	**443.1885**	222.0979	5
17	1702.6592	**851.8332**	1685.6327	843.3200	1684.6486	842.8280	G	**333.1405**	167.0739			315.1299	158.0686	4
18	**1759.6807**	880.3440	1742.6541	871.8307	1741.6701	871.3387	G	276.1190	138.5631			258.1084	129.5579	3
19	1830.7178	915.8625	1813.6912	907.3493	1812.7072	906.8572	A	219.0975	110.0524			201.0870	101.0471	2
20							E	148.0604	74.5339			130.0499	65.5286	1

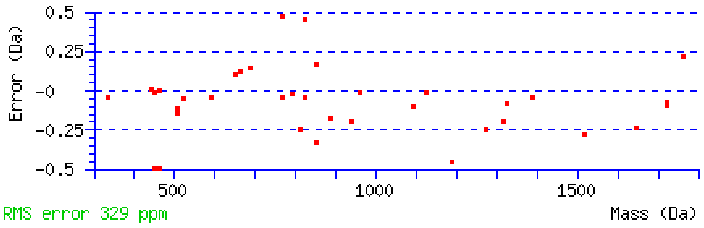

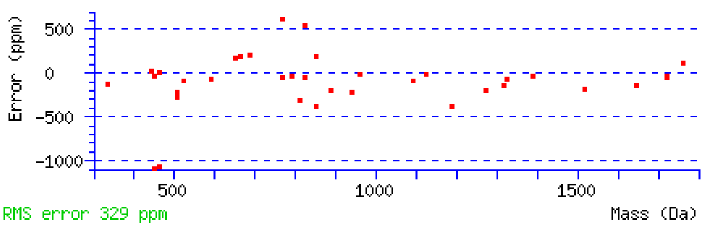

All matches to this query

Score	Mr(calc):	Delta	Sequence
72.5	1976.7636	0.0069	AADPPAENSSAPEAEQGGAE

61.6	1976.7636	0.0069	AADPPAENSSAPEAEQGGAE
0.7	1975.7693	1.0013	SAYDSTMETMNYAQIR
0.7	1975.7693	1.0013	SAYDSTMETMNYAQIR

Spectrum No: 17; Query: 283; Rank: 1

Peptide View

MS/MS Fragmentation of **ASAAEGSEASPPSLR**
Found in **IPI00358407**, Tax_Id=10116 Gene_Symbol=RGD1563576_predicted hypothetical protein LOC306617

Match to Query 283: 1508.650708 from(755.332630,2+)
Title: 091127RatKid_SCX01_13.951.951.2.dta
Data file K:\NewmanPaper\Piliang\3SubProteomes\Piliang3SP\mgf5ppm\SCX_3SubProteomes5ppm.mgf

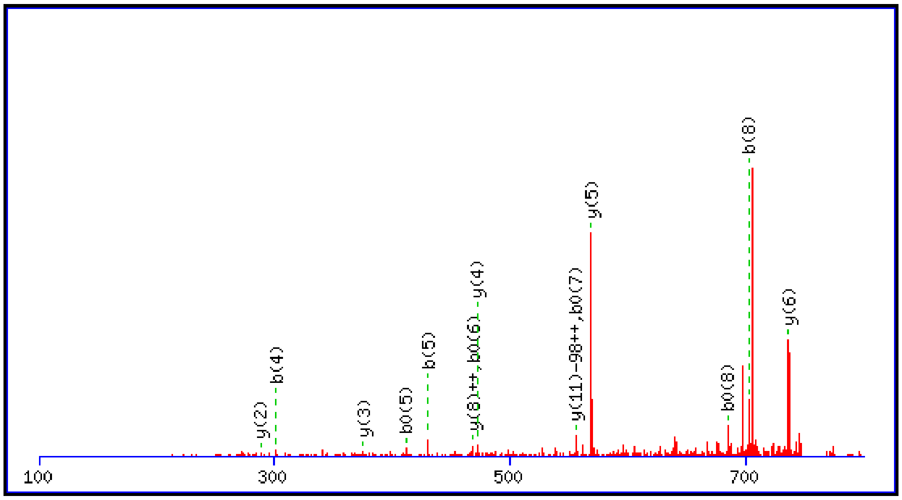

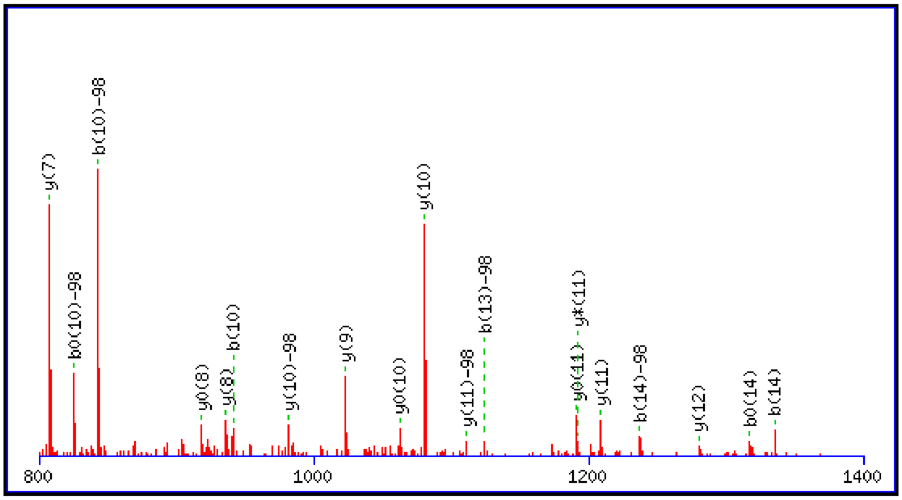

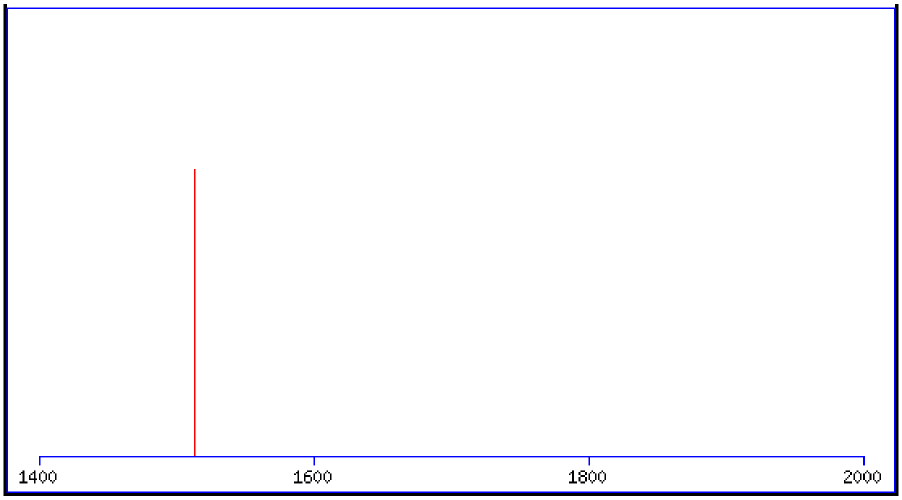

Monoisotopic mass of neutral peptide Mr(calc): 1508.6508
Fixed modifications: Carbamidomethyl (C)
Variable modifications:
S10 : Phospho (ST), with neutral losses 0.0000(shown in table), 97.9769
Ions Score: 72 **Expect:** 8.1e-006
Matches (Bold Red): 33/208 fragment ions using 52 most intense peaks

#	b	b^{++}	b^0	b^{0++}	Seq.	y	y^{++}	y*	y^{*++}	y^0	y^{0++}	#
1	72.0444	36.5258			A							15
2	159.0764	80.0418	141.0659	71.0366	S	1438.6210	719.8141	1421.5944	711.3008	1420.6104	710.8088	14
3	230.1135	115.5604	212.1030	106.5551	A	1351.5889	676.2981	1334.5624	667.7848	1333.5784	667.2928	13
4	**301.1506**	151.0790	283.1401	142.0737	A	**1280.5518**	640.7796	1263.5253	632.2663	1262.5413	631.7743	12
5	**430.1932**	215.6003	**412.1827**	206.5950	E	**1209.5147**	605.2610	**1192.4882**	596.7477	**1191.5042**	596.2557	11
6	487.2147	244.1110	**469.2041**	235.1057	G	**1080.4721**	540.7397	1063.4456	532.2264	**1062.4616**	531.7344	10
7	574.2467	287.6270	**556.2362**	278.6217	S	**1023.4507**	512.2290	1006.4241	503.7157	1005.4401	503.2237	9
8	**703.2893**	352.1483	**685.2788**	343.1430	E	**936.4186**	**468.7130**	919.3921	460.1997	**918.4081**	459.7077	8
9	774.3264	387.6669	756.3159	378.6616	A	**807.3760**	404.1917	790.3495	395.6784	789.3655	395.1864	7
10	**941.3248**	471.1660	923.3142	462.1608	S	**736.3389**	368.6731	719.3124	360.1598	718.3284	359.6678	6
11	1038.3776	519.6924	1020.3670	510.6871	P	**569.3406**	285.1739	552.3140	276.6606	551.3300	276.1686	5
12	1135.4303	568.2188	1117.4198	559.2135	P	**472.2878**	236.6475	455.2613	228.1343	454.2772	227.6423	4
13	1222.4623	611.7348	1204.4518	602.7295	S	**375.2350**	188.1212	358.2085	179.6079	357.2245	179.1159	3
14	**1335.5464**	668.2768	**1317.5358**	659.2716	L	**288.2030**	144.6051	271.1765	136.0919			2
15					R	175.1190	88.0631	158.0924	79.5498			1

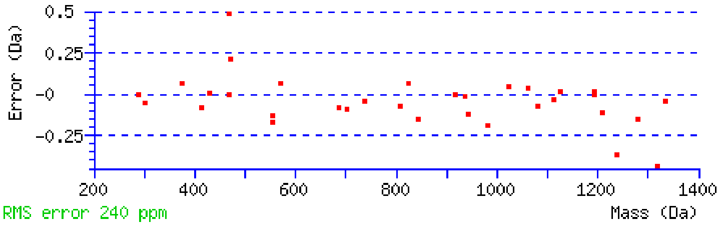

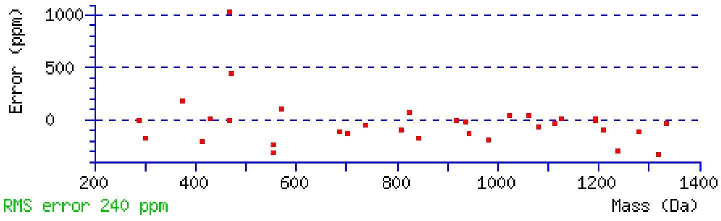

All matches to this query

Score	Mr(calc):	Delta	Sequence
72.2	1508.6508	-0.0001	ASAAEGSEASPPSLR
45.2	1508.6508	-0.0001	ASAAEGSEASPPSLR
36.3	1508.6508	-0.0001	ASAAEGSEASPPSLR
22.8	1508.6508	-0.0001	ASAAEGSEASPPSLR
9.0	1508.6426	0.0081	YVEDVTNVVRR
5.8	1508.6384	0.0123	TFQAYLPHCHR

3.4	1508.6466	0.0041	VGIFPSNYVTPR
2.7	1508.6409	0.0098	NRTEYLPAHGDR
2.0	1508.6517	-0.0010	ICRQDTSFMKK
1.7	1508.6473	0.0034	ANSCPHHQCSRR

Spectrum No: 18; Query: 414; Rank: 1

Peptide View

MS/MS Fragmentation of **TLSPTPSAEGFQDGR**
Found in **IPI00198339**, Tax_Id=10116 Gene_Symbol=Ablim1_predicted 78 kDa protein

Match to Query 414: 1641.700428 from(821.857490,2+)
Title: 091129RatKid_SCX02_12.1247.1247.2.dta
Data file K:\NewmanPaper\Piliang\3SubProteomes\Piliang3SP\mgf5ppm\SCX_3SubProteomes5ppm.mgf

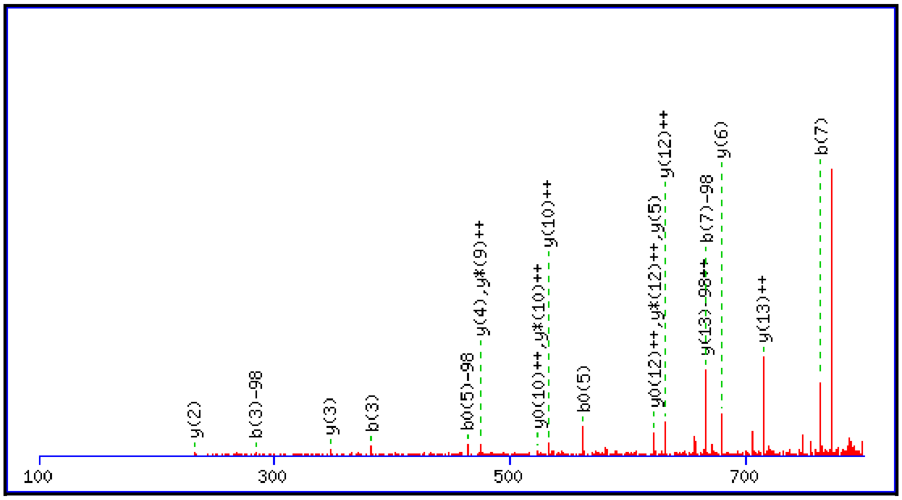

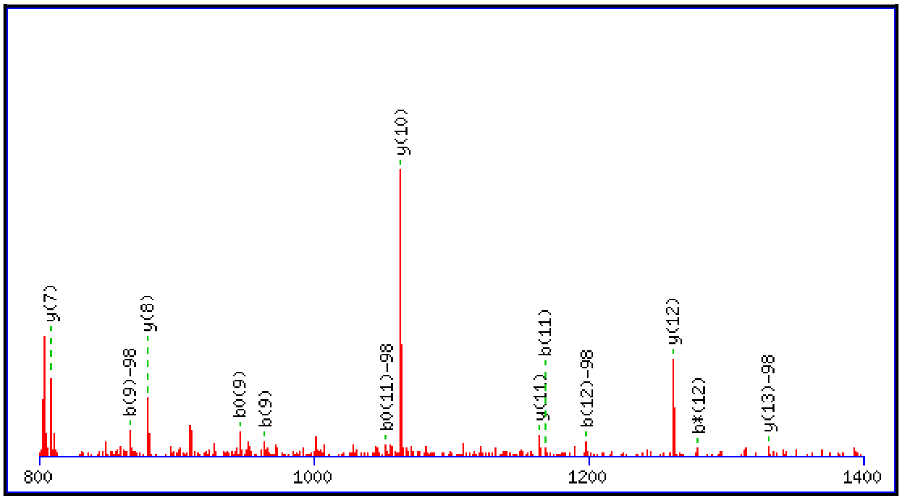

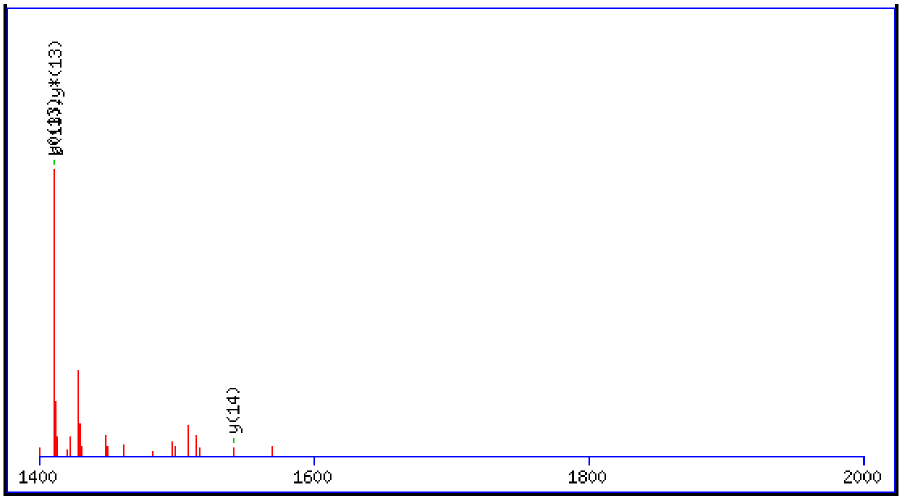

Monoisotopic mass of neutral peptide Mr(calc): 1641.7036
Fixed modifications: Carbamidomethyl (C)
Variable modifications:
S3 : Phospho (ST), with neutral losses 97.9769(shown in table), 0.0000
Ions Score: 71 **Expect:** 1.3e-005
Matches (Bold Red): 37/208 fragment ions using 57 most intense peaks

#	b	b^{++}	b*	b^{*++}	b^{0}	b^{0++}	Seq.	y	y^{++}	y*	y^{*++}	y^{0}	y^{0++}	#
1	102.0550	51.5311			84.0444	42.5258	T							15
2	215.1390	108.0731			197.1285	99.0679	L	1443.6863	722.3468	1426.6597	713.8335	1425.6757	713.3415	14
3	**284.1605**	142.5839			266.1499	133.5786	S	**1330.6022**	**665.8047**	1313.5757	657.2915	1312.5916	656.7995	13
4	381.2132	191.1103			363.2027	182.1050	P	**1261.5808**	**631.2940**	1244.5542	**622.7807**	1243.5702	**622.2887**	12
5	482.2609	241.6341			**464.2504**	232.6288	T	**1164.5280**	582.7676	1147.5014	574.2544	1146.5174	573.7624	11
6	579.3137	290.1605			561.3031	281.1552	P	**1063.4803**	**532.2438**	1046.4538	**523.7305**	1045.4697	**523.2385**	10
7	**666.3457**	333.6765			648.3351	324.6712	S	966.4276	483.7174	949.4010	**475.2041**	948.4170	474.7121	9
8	737.3828	369.1951			719.3723	360.1898	A	**879.3955**	440.2014	862.3690	431.6881	861.3850	431.1961	8
9	**866.4254**	433.7163			848.4149	424.7111	E	**808.3584**	404.6828	791.3319	396.1696	790.3478	395.6776	7
10	923.4469	462.2271			905.4363	453.2218	G	**679.3158**	340.1615	662.2893	331.6483	661.3053	331.1563	6
11	1070.5153	535.7613			**1052.5047**	526.7560	F	**622.2944**	311.6508	605.2678	303.1375	604.2838	302.6455	5
12	**1198.5739**	599.7906	1181.5473	591.2773	1180.5633	590.7853	Q	**475.2259**	238.1166	458.1994	229.6033	457.2154	229.1113	4
13	1313.6008	657.3040	1296.5743	648.7908	1295.5903	648.2988	D	**347.1674**	174.0873	330.1408	165.5740	329.1568	165.0820	3
14	1370.6223	685.8148	1353.5957	677.3015	1352.6117	676.8095	G	**232.1404**	116.5738	215.1139	108.0606			2
15							R	175.1190	88.0631	158.0924	79.5498			1

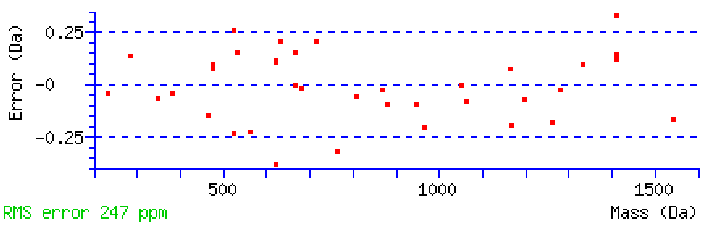

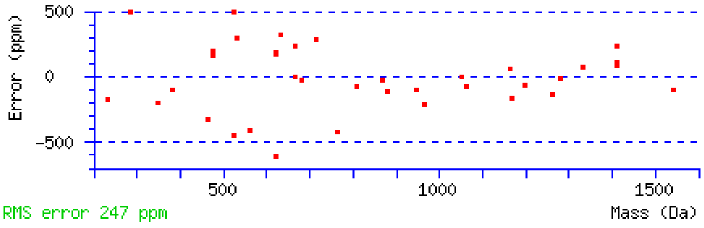

All matches to this query

Score	Mr(calc):	Delta	Sequence
71.1	1641.7036	-0.0032	TLSPTPSAEGFQDGR
57.5	1641.7036	-0.0032	TLSPTPSAEGFQDGR
46.6	1641.7036	-0.0032	TLSPTPSAEGFQDGR
40.8	1641.7036	-0.0032	TLSPTPSAEGFQDGR
6.9	1641.6892	0.0112	DVPSTQKCPLMDR
5.9	1641.7126	-0.0121	NSVAILMKNLYSK

1.0	1641.6892	0.0112	DVPSTQKCPLMDR

Spectrum No: 19; Query: 494; Rank: 1

Peptide View

MS/MS Fragmentation of **ATWGDGGDSSPSNVVSK**
Found in **IPI00197605**, Tax_Id=10116 Gene_Symbol=Snap23 Synaptosomal-associated protein 23

Match to Query 494: 1742.718108 from(872.366330,2+)
Title: 091127RatKid_SCX01_12.1559.1559.2.dta
Data file K:\NewmanPaper\Piliang\3SubProteomes\Piliang3SP\mgf5ppm\SCX_3SubProteomes5ppm.mgf

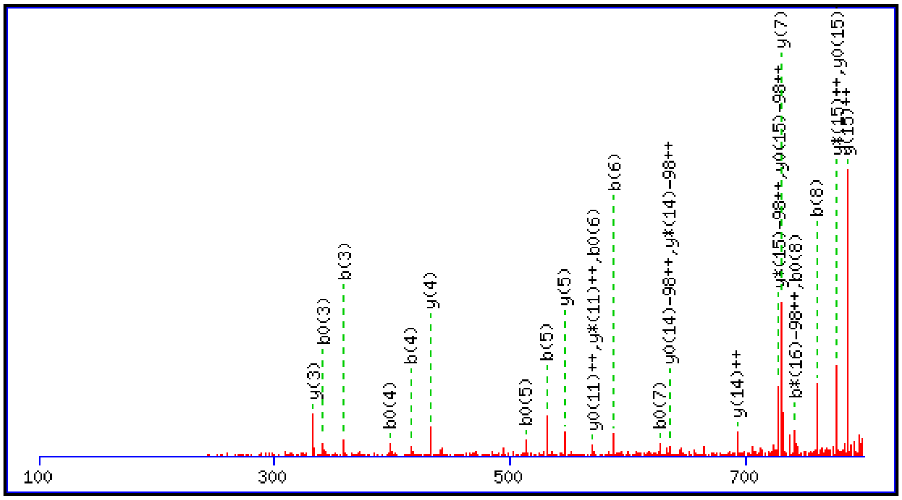

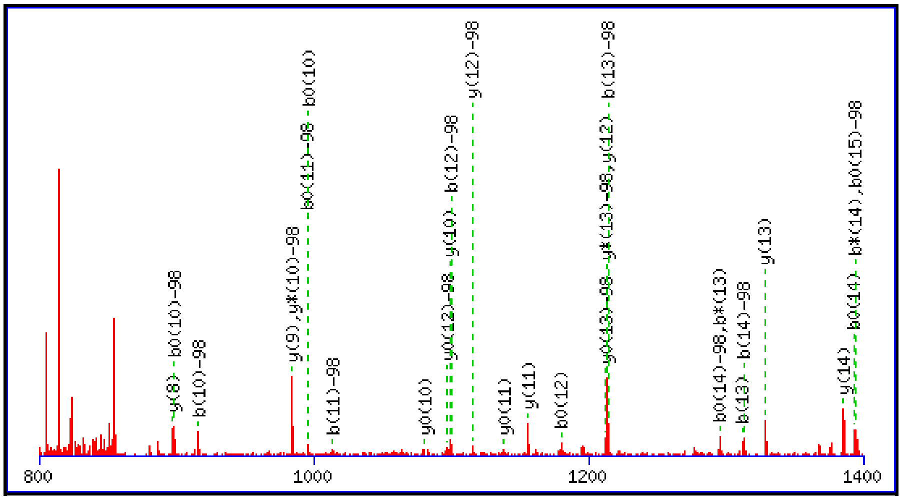

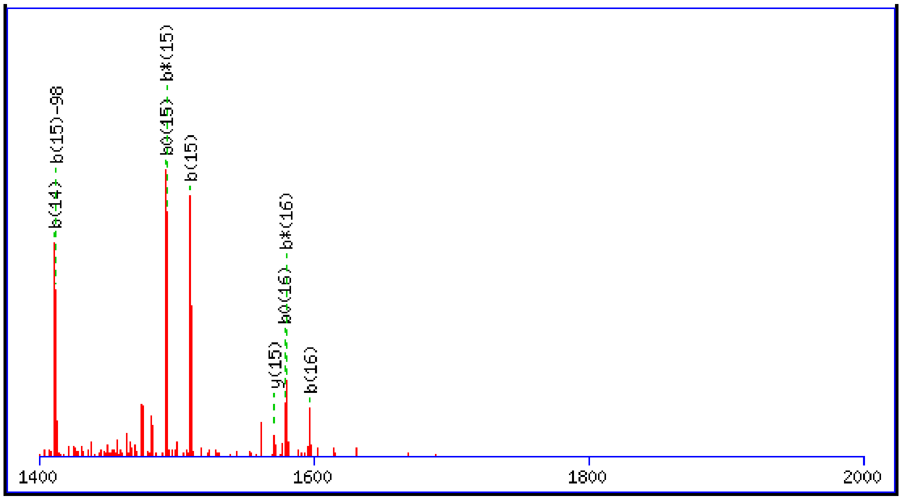

Monoisotopic mass of neutral peptide Mr(calc): 1742.7149
Fixed modifications: Carbamidomethyl (C)
Variable modifications:
S10 : Phospho (ST), with neutral losses 0.0000(shown in table), 97.9769
Ions Score: 71 **Expect:** 1.2e-005
Matches (Bold Red): 64/254 fragment ions using 81 most intense peaks

#	b	b^{++}	b*	b^{*++}	b^{0}	b^{0++}	Seq.	y	y^{++}	y*	y^{*++}	y^{0}	y^{0++}	#
1	72.0444	36.5258					A							17
2	173.0921	87.0497			155.0815	78.0444	T	1672.6850	836.8462	1655.6585	828.3329	1654.6745	827.8409	16
3	**359.1714**	180.0893			**341.1608**	171.0840	W	**1571.6374**	**786.3223**	1554.6108	**777.8090**	1553.6268	**777.3170**	15
4	**416.1928**	208.6001			**398.1823**	199.5948	G	**1385.5580**	**693.2827**	1368.5315	684.7694	1367.5475	684.2774	14
5	**531.2198**	266.1135			**513.2092**	257.1083	D	**1328.5366**	664.7719	1311.5100	656.2587	1310.5260	655.7666	13
6	**588.2413**	294.6243			**570.2307**	285.6190	G	**1213.5096**	607.2585	1196.4831	598.7452	1195.4991	598.2532	12
7	645.2627	323.1350			**627.2522**	314.1297	G	**1156.4882**	578.7477	1139.4616	**570.2344**	**1138.4776**	**569.7424**	11
8	**760.2897**	380.6485			**742.2791**	371.6432	D	**1099.4667**	550.2370	1082.4402	541.7237	**1081.4561**	541.2317	10
9	847.3217	424.1645			829.3111	415.1592	S	**984.4398**	492.7235	967.4132	484.2102	966.4292	483.7182	9
10	1014.3200	507.6637			**996.3095**	498.6584	S	**897.4077**	449.2075	880.3812	440.6942	879.3972	440.2022	8
11	1111.3728	556.1900			1093.3622	547.1848	P	**730.4094**	365.7083	713.3828	357.1951	712.3988	356.7030	7
12	1198.4048	599.7061			**1180.3943**	590.7008	S	633.3566	317.1819	616.3301	308.6687	615.3461	308.1767	6
13	**1312.4478**	656.7275	**1295.4212**	648.2142	1294.4372	647.7222	N	**546.3246**	273.6659	529.2980	265.1527	528.3140	264.6606	5
14	**1411.5162**	706.2617	**1394.4896**	697.7485	**1393.5056**	697.2564	V	**432.2817**	216.6445	415.2551	208.1312	414.2711	207.6392	4
15	**1510.5846**	755.7959	**1493.5580**	747.2827	**1492.5740**	746.7906	V	**333.2132**	167.1103	316.1867	158.5970	315.2027	158.1050	3
16	**1597.6166**	799.3119	**1580.5901**	790.7987	**1579.6061**	790.3067	S	234.1448	117.5761	217.1183	109.0628	216.1343	108.5708	2
17							K	147.1128	74.0600	130.0863	65.5468			1

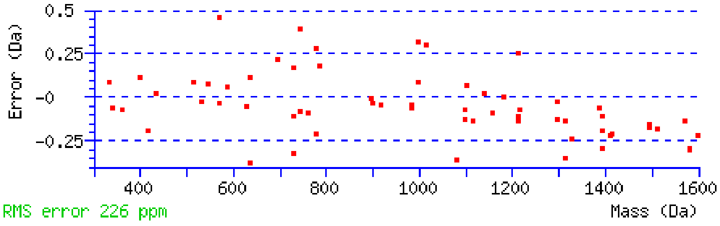

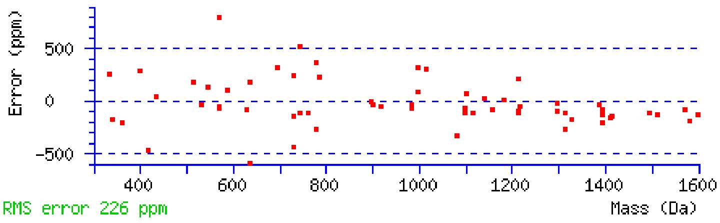

All matches to this query

Score	Mr(calc):	Delta	Sequence
70.9	1742.7149	0.0032	ATWGDGGDSSPSNVVSK
63.6	1742.7149	0.0032	ATWGDGGDSSPSNVVSK
60.4	1742.7149	0.0032	ATWGDGGDSSPSNVVSK
52.5	1742.7149	0.0032	ATWGDGGDSSPSNVVSK

27.1	1742.7149	0.0032	ATWGDGGDSSPSNVVSK
6.7	1740.7233	1.9948	YSVKVRADGGTNSAR
5.1	1742.7229	-0.0048	MSTNNMSDPRRPNK
4.6	1742.7317	-0.0136	SGKYLATEWNTVSK
4.1	1742.7317	-0.0136	SGKYLATEWNTVSK
3.9	1740.7233	1.9948	YSVKVRADGGTNSAR

Spectrum No: 20; Query: 1112; Rank: 1

Peptide View

MS/MS Fragmentation of **GATPAEDDEDNDIDLFGSDEEEEDKEAAR**
Found in **IPI00197900**, Tax_Id=10116 Gene_Symbol=Eef1d Translation elongation factor 1-delta subunit

Match to Query 1112: 3261.267972 from(1088.096600,3+)
Title: 091127RatKid_SCX01_18.2713.2713.3.dta
Data file K:\NewmanPaper\Piliang\3SubProteomes\Piliang3SP\mgf5ppm\SCX_3SubProteomes5ppm.mgf

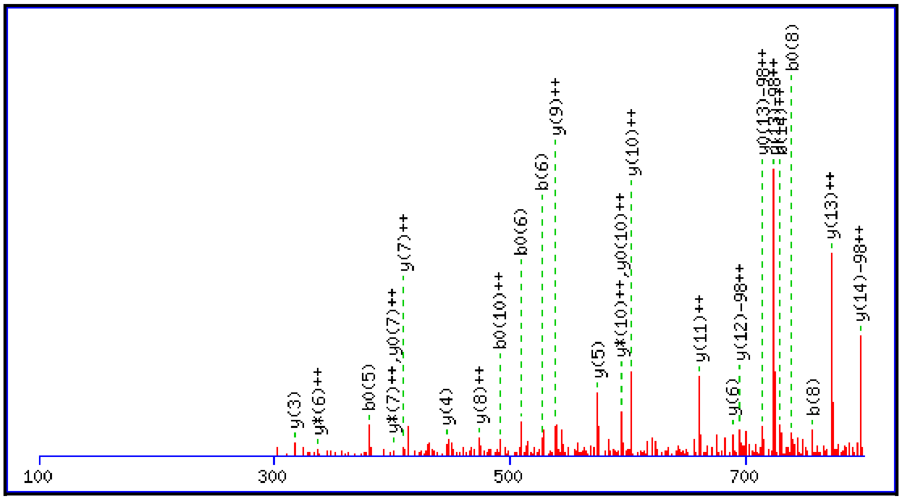

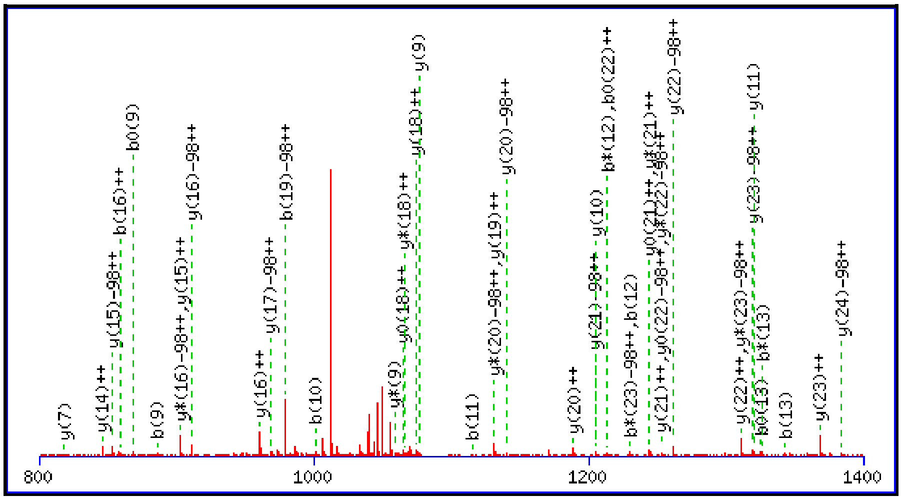

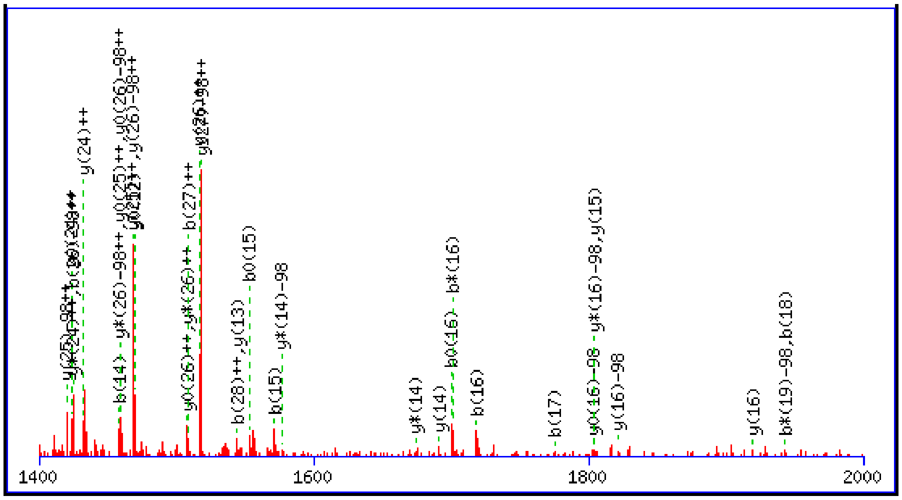

Monoisotopic mass of neutral peptide Mr(calc): 3261.2630
Fixed modifications: Carbamidomethyl (C)
Variable modifications:
S18 : Phospho (ST), with neutral losses 97.9769(shown in table), 0.0000
Ions Score: 68 **Expect:** 4.6e-005
Matches (Bold Red): 105/474 fragment ions using 188 most intense peaks

#	b	b++	b*	b*++	b0	b0++	Seq.	y	y++	y*	y*++	y0	y0++	#
1	58.0287	29.5180					G							29
2	129.0659	65.0366					A	3107.2720	1554.1396	3090.2454	1545.6263	3089.2614	1545.1343	28
3	230.1135	115.5604			212.1030	106.5551	T	3036.2348	**1518.6211**	3019.2083	1510.1078	3018.2243	1509.6158	27
4	327.1663	164.0868			309.1557	155.0815	P	2935.1872	**1468.0972**	2918.1606	**1459.5839**	2917.1766	**1459.0919**	26
5	398.2034	199.6053			**380.1928**	190.6001	A	2838.1344	**1419.5708**	2821.1078	1411.0576	2820.1238	1410.5656	25
6	**527.2460**	264.1266			**509.2354**	255.1214	E	2767.0973	**1384.0523**	2750.0707	1375.5390	2749.0867	1375.0470	24
7	642.2729	321.6401			624.2624	312.6348	D	2638.0547	**1319.5310**	2621.0281	**1311.0177**	2620.0441	1310.5257	23
8	**757.2999**	379.1536			**739.2893**	370.1483	D	2523.0277	**1262.0175**	2506.0012	**1253.5042**	2505.0172	**1253.0122**	22
9	**886.3425**	443.6749			**868.3319**	434.6696	E	2408.0008	**1204.5040**	2390.9743	1195.9908	2389.9902	1195.4988	21
10	**1001.3694**	501.1884			983.3589	**492.1831**	D	2278.9582	**1139.9827**	2261.9317	**1131.4695**	2260.9476	1130.9775	20
11	**1115.4124**	558.2098	1098.3858	549.6965	1097.4018	549.2045	N	2163.9313	1082.4693	2146.9047	1073.9560	2145.9207	1073.4640	19
12	**1230.4393**	615.7233	**1213.4127**	607.2100	1212.4287	606.7180	D	2049.8883	1025.4478	2032.8618	1016.9345	2031.8778	1016.4425	18
13	**1343.5234**	672.2653	**1326.4968**	663.7520	**1325.5128**	663.2600	I	1934.8614	**967.9343**	1917.8349	959.4211	1916.8508	958.9291	17
14	**1458.5503**	**729.7788**	1441.5238	721.2655	1440.5397	720.7735	D	**1821.7773**	**911.3923**	**1804.7508**	**902.8790**	**1803.7668**	902.3870	16
15	**1571.6344**	786.3208	1554.6078	777.8075	**1553.6238**	777.3155	L	1706.7504	**853.8788**	1689.7238	845.3656	1688.7398	844.8736	15
16	**1718.7028**	**859.8550**	**1701.6762**	851.3418	**1700.6922**	850.8497	F	1593.6663	**797.3368**	**1576.6398**	788.8235	1575.6558	788.3315	14
17	**1775.7242**	888.3658	1758.6977	879.8525	1757.7137	879.3605	G	1446.5979	**723.8026**	1429.5714	715.2893	1428.5873	**714.7973**	13
18	1844.7457	922.8765	1827.7192	914.3632	1826.7351	913.8712	S	1389.5765	**695.2919**	1372.5499	686.7786	1371.5659	686.2866	12
19	1959.7726	**980.3900**	**1942.7461**	971.8767	1941.7621	971.3847	D	**1320.5550**	**660.7811**	1303.5284	652.2679	1302.5444	651.7759	11
20	2088.8152	1044.9113	2071.7887	1036.3980	2070.8047	1035.9060	E	**1205.5281**	**603.2677**	1188.5015	**594.7544**	1187.5175	**594.2624**	10
21	2217.8578	1109.4326	2200.8313	1100.9193	2199.8473	1100.4273	E	**1076.4855**	**538.7464**	**1059.4589**	530.2331	1058.4749	529.7411	9
22	2346.9004	1173.9538	2329.8739	1165.4406	2328.8899	1164.9486	E	947.4429	**474.2251**	930.4163	465.7118	929.4323	465.2198	8
23	2475.9430	1238.4751	2458.9165	**1229.9619**	2457.9325	1229.4699	E	**818.4003**	**409.7038**	801.3737	**401.1905**	800.3897	**400.6985**	7
24	2590.9700	1295.9886	2573.9434	1287.4753	2572.9594	1286.9833	D	**689.3577**	345.1825	672.3311	**336.6692**	671.3471	336.1772	6
25	2719.0649	1360.0361	2702.0384	1351.5228	2701.0544	1351.0308	K	**574.3307**	287.6690	557.3042	279.1557	556.3202	278.6637	5
26	2848.1075	**1424.5574**	2831.0810	1416.0441	2830.0970	1415.5521	E	**446.2358**	223.6215	429.2092	215.1083	428.2252	214.6162	4
27	2919.1446	1460.0760	2902.1181	1451.5627	2901.1341	1451.0707	A	**317.1932**	159.1002	300.1666	150.5870			3
28	2990.1817	1495.5945	2973.1552	1487.0812	2972.1712	1486.5892	A	246.1561	123.5817	229.1295	115.0684			2
29							R	175.1190	88.0631	158.0924	79.5498			1

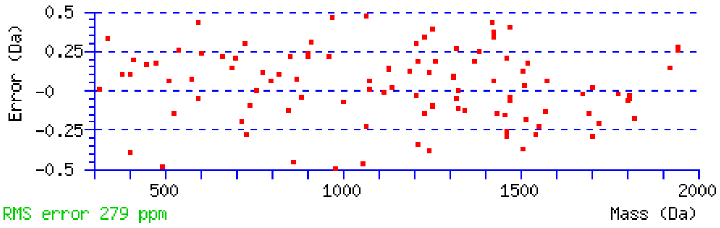

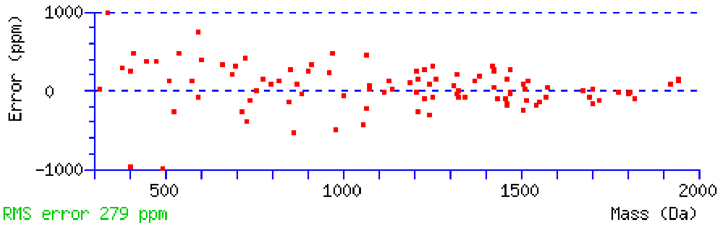

All matches to this query

Score	Mr(calc):	Delta	Sequence
68.5	3261.2630	0.0050	GATPAEDDEDNDIDLFGSDEEEEDKEAAR
7.0	3261.2630	0.0050	GATPAEDDEDNDIDLFGSDEEEEDKEAAR
5.4	3259.2535	2.0145	EEWELNPLYCDTVKQIYPYNSSNR
5.4	3259.2535	2.0145	EEWELNPLYCDTVKQIYPYNSSNR
2.2	3259.2914	1.9766	ESQGSLNSSASLDLGFLAFVSSKSESHR
2.1	3261.2810	-0.0130	AEMVSTNIRHSPPAERSFGFTSVLTK
2.0	3259.2456	2.0224	LISSYDNEYGYSNRVVDLMAYMASKE
1.8	3259.2456	2.0224	LISSYDNEYGYSNRVVDLMAYMASKE
1.8	3259.2456	2.0224	LISSYDNEYGYSNRVVDLMAYMASKE
1.8	3259.2456	2.0224	LISSYDNEYGYSNRVVDLMAYMASKE

Spectrum No: 21; Query: 667; Rank: 1

Peptide View

MS/MS Fragmentation of **TQPDGTSVPGEPASPISQR**
Found in **IPI00368473**, Tax_Id=10116 Gene_Symbol=Numa1 similar to nuclear mitotic apparatus protein 1

Match to Query 667: 2002.904588 from(1002.459570,2+)
Title: 091127RatKid_SCX01_12.1478.1478.2.dta
Data file K:\NewmanPaper\Piliang\3SubProteomes\Piliang3SP\mgf5ppm\SCX_3SubProteomes5ppm.mgf

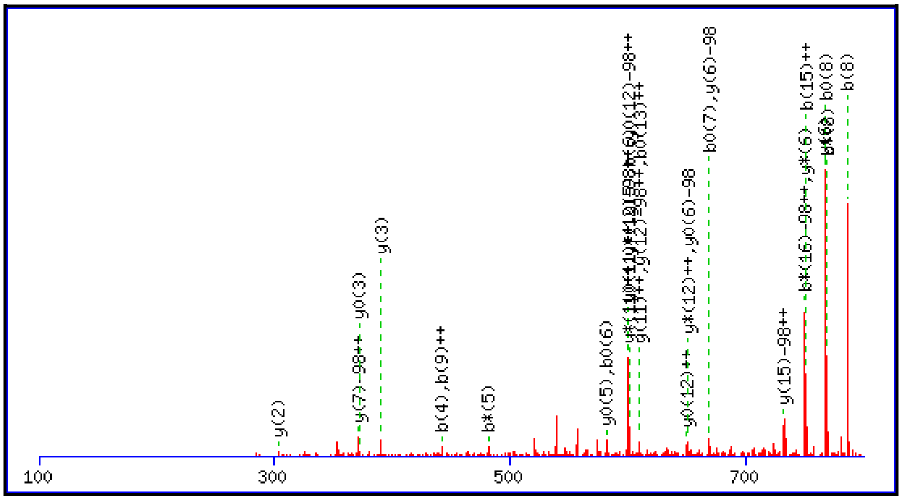

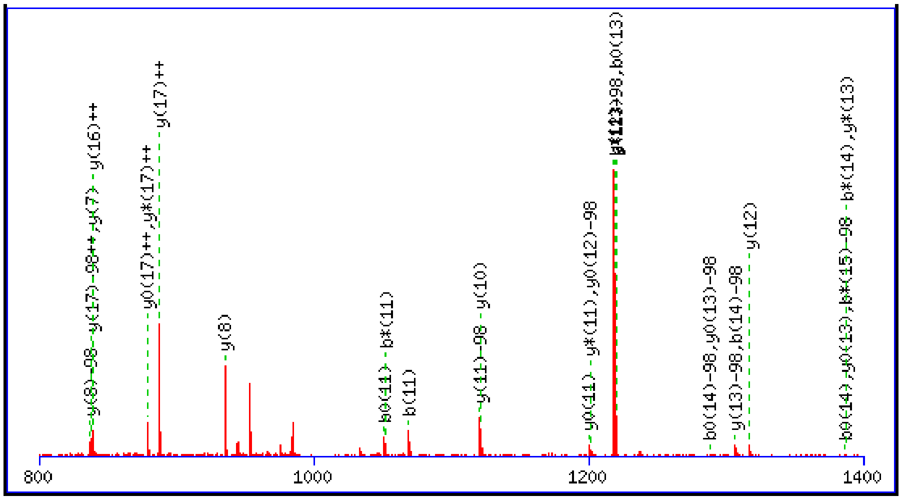

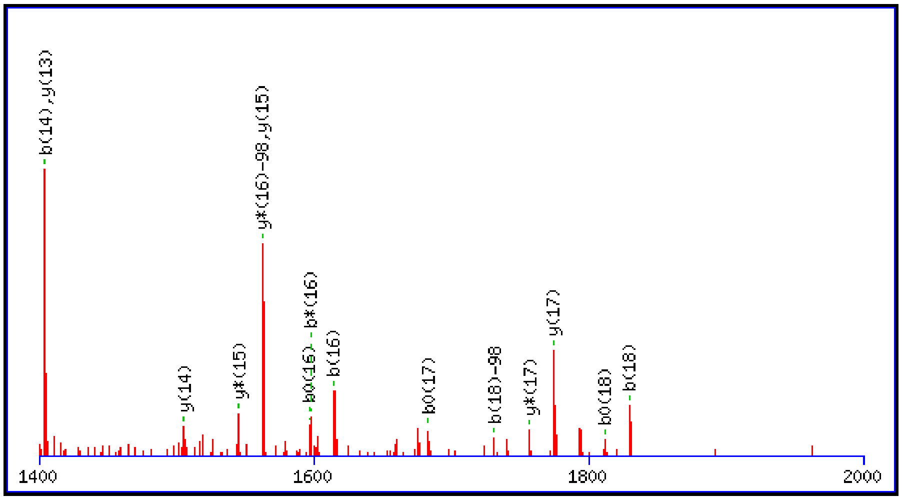

Monoisotopic mass of neutral peptide Mr(calc): 2002.8997
Fixed modifications: Carbamidomethyl (C)
Variable modifications:
S14 : Phospho (ST), with neutral losses 0.0000(shown in table), 97.9769
Ions Score: 67 **Expect:** 4.3e-005
Matches (Bold Red): 76/318 fragment ions using 100 most intense peaks

#	b	b^{++}	b*	b^{*++}	b^{0}	b^{0++}	Seq.	y	y^{++}	y*	y^{*++}	y^{0}	y^{0++}	#
1	102.0550	51.5311			84.0444	42.5258	T							19
2	230.1135	115.5604	213.0870	107.0471	212.1030	106.5551	Q	1902.8593	951.9333	1885.8328	943.4200	1884.8487	942.9280	18
3	327.1663	164.0868	310.1397	155.5735	309.1557	155.0815	P	**1774.8007**	**887.9040**	**1757.7742**	**879.3907**	1756.7902	**878.8987**	17
4	**442.1932**	221.6003	425.1667	213.0870	424.1827	212.5950	D	1677.7480	**839.3776**	1660.7214	830.8643	1659.7374	830.3723	16
5	499.2147	250.1110	**482.1882**	241.5977	481.2041	241.1057	G	**1562.7210**	781.8641	**1545.6945**	773.3509	1544.7105	772.8589	15
6	**600.2624**	300.6348	583.2358	292.1216	**582.2518**	291.6295	T	**1505.6996**	753.3534	1488.6730	744.8401	1487.6890	744.3481	14
7	687.2944	344.1508	670.2679	335.6376	**669.2838**	335.1456	S	**1404.6519**	702.8296	**1387.6253**	694.3163	**1386.6413**	693.8243	13
8	**786.3628**	393.6851	**769.3363**	385.1718	**768.3523**	384.6798	V	**1317.6199**	659.3136	1300.5933	**650.8003**	1299.6093	**650.3083**	12
9	883.4156	**442.2114**	866.3890	433.6982	865.4050	433.2062	P	**1218.5514**	**609.7794**	**1201.5249**	**601.2661**	**1200.5409**	**600.7741**	11
10	940.4371	470.7222	923.4105	462.2089	922.4265	461.7169	G	**1121.4987**	561.2530	1104.4721	552.7397	1103.4881	552.2477	10
11	**1069.4796**	535.2435	**1052.4531**	526.7302	**1051.4691**	526.2382	E	1064.4772	532.7422	1047.4507	524.2290	1046.4666	523.7370	9
12	1166.5324	583.7698	1149.5059	575.2566	1148.5218	574.7646	P	**935.4346**	468.2209	918.4081	459.7077	917.4241	459.2157	8
13	1237.5695	619.2884	**1220.5430**	610.7751	**1219.5590**	**610.2831**	A	**838.3819**	419.6946	821.3553	411.1813	820.3713	410.6893	7
14	**1404.5679**	702.7876	**1387.5413**	694.2743	**1386.5573**	693.7823	S	**767.3447**	384.1760	**750.3182**	375.6627	749.3342	375.1707	6
15	1501.6206	**751.3140**	1484.5941	742.8007	1483.6101	742.3087	P	**600.3464**	300.6768	583.3198	292.1636	**582.3358**	291.6715	5
16	**1614.7047**	807.8560	**1597.6782**	799.3427	**1596.6941**	798.8507	I	503.2936	252.1504	486.2671	243.6372	485.2831	243.1452	4
17	1701.7367	851.3720	1684.7102	842.8587	**1683.7262**	842.3667	S	**390.2096**	195.6084	373.1830	187.0951	**372.1990**	186.6031	3
18	**1829.7953**	915.4013	1812.7688	906.8880	**1811.7847**	906.3960	Q	**303.1775**	152.0924	286.1510	143.5791			2

**19**							**R**	175.1190	88.0631	158.0924	79.5498			**1**

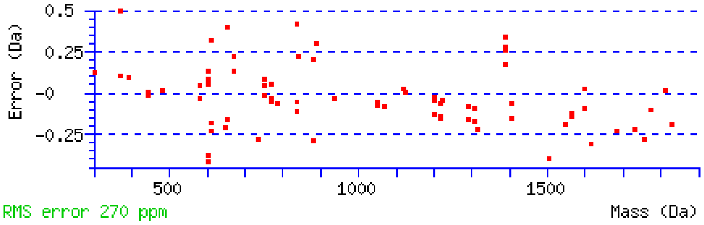

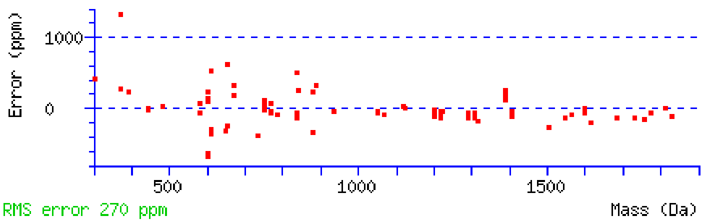

All matches to this query

Score	Mr(calc):	Delta	Sequence
67.4	2002.8997	0.0049	TQPDGTSVPGEPASPISQR
46.5	2002.8997	0.0049	TQPDGTSVPGEPASPISQR
28.4	2002.8997	0.0049	TQPDGTSVPGEPASPISQR
28.1	2002.8997	0.0049	TQPDGTSVPGEPASPISQR
25.6	2002.8997	0.0049	TQPDGTSVPGEPASPISQR
11.7	2002.9205	-0.0159	DGGARGANSSFQNDGQPLGR
8.4	2002.9082	-0.0036	SRSPIQRQNGTGHNSQR
3.2	2002.9158	-0.0112	TLTGLNWVNKYCPMAR
3.1	2002.8901	0.0145	DILINQSPASLTVSAGEK
3.1	2002.9238	-0.0192	SAVNGTVSSPAALLSRASR

Spectrum No: 22; Query: 622; Rank: 1

Peptide View

MS/MS Fragmentation of **SASSDTSEELNAQDSPK**
Found in **IPI00200898**, Tax_Id=10116 Gene_Symbol=Slc9a3r1 Ezrin-radixin-moesin-binding phosphoprotein 50

Match to Query 622: 1924.699548 from(963.357050,2+)
Title: 091127RatKid_SCX01_02.881.881.2.dta
Data file K:\NewmanPaper\Piliang\3SubProteomes\Piliang3SP\mgf5ppm\SCX_3SubProteomes5ppm.mgf

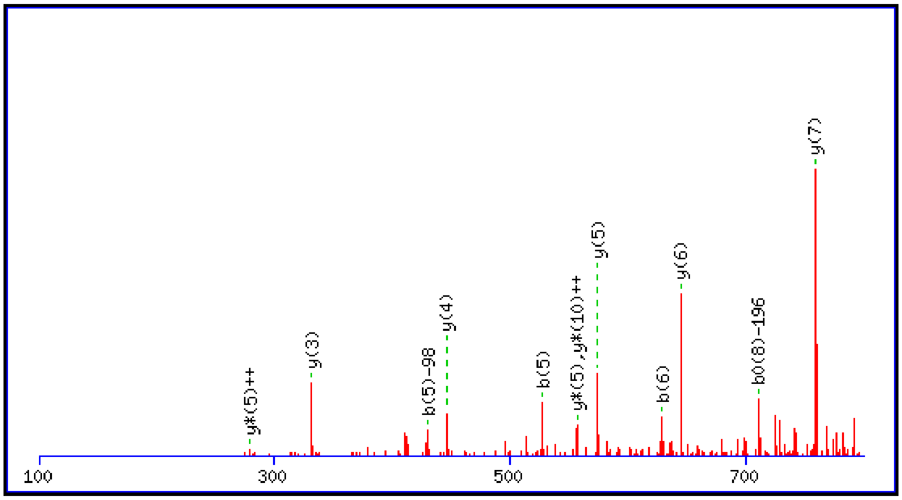

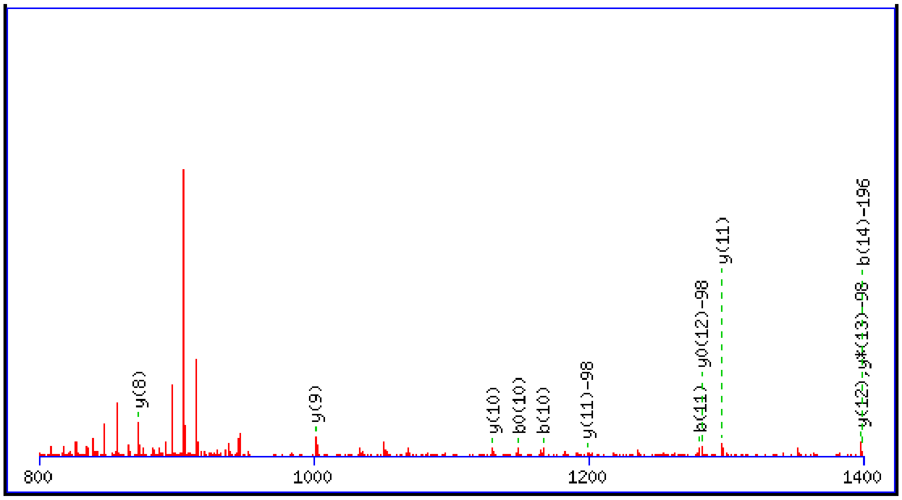

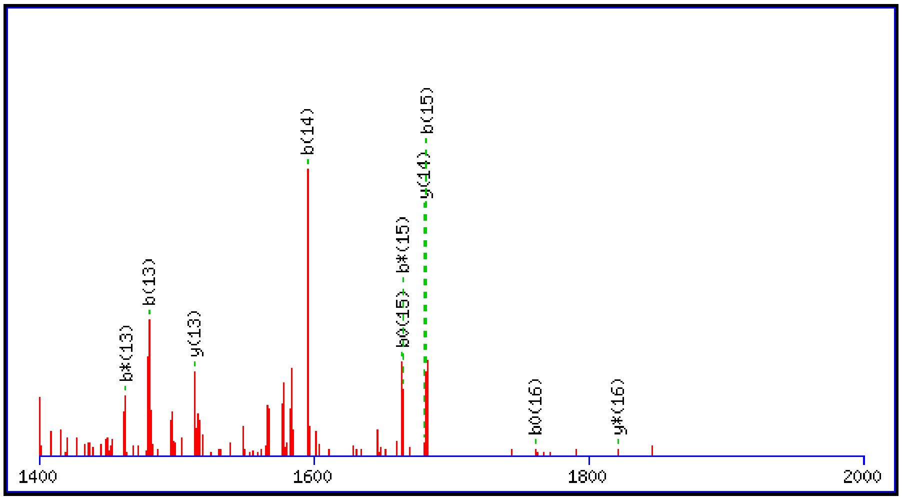

Monoisotopic mass of neutral peptide Mr(calc): 1924.6976
Fixed modifications: Carbamidomethyl (C)
Variable modifications:
S4 : Phospho (ST), with neutral losses 0.0000(shown in table), 97.9769
S7 : Phospho (ST), with neutral losses 0.0000(shown in table), 97.9769
Ions Score: 67 **Expect:** 2e-005
Matches (Bold Red): 34/268 fragment ions using 57 most intense peaks

#	b	b^{++}	b*	b^{*++}	b^0	b^{0++}	Seq.	y	y^{++}	y*	y^{*++}	y^0	y^{0++}	#
1	88.0393	44.5233			70.0287	35.5180	S							17
2	159.0764	80.0418			141.0659	71.0366	A	1838.6729	919.8401	**1821.6463**	911.3268	1820.6623	910.8348	16
3	246.1084	123.5579			228.0979	114.5526	S	1767.6358	884.3215	1750.6092	875.8082	1749.6252	875.3162	15
4	413.1068	207.0570			395.0962	198.0518	S	**1680.6037**	840.8055	1663.5772	832.2922	1662.5932	831.8002	14
5	**528.1337**	264.5705			510.1232	255.5652	D	**1513.6054**	757.3063	1496.5788	748.7931	1495.5948	748.3010	13
6	**629.1814**	315.0943			611.1709	306.0891	T	**1398.5784**	699.7929	1381.5519	691.2796	1380.5679	690.7876	12
7	796.1798	398.5935			778.1692	389.5882	S	**1297.5308**	649.2690	1280.5042	640.7557	1279.5202	640.2637	11
8	925.2224	463.1148			907.2118	454.1095	E	**1130.5324**	565.7698	1113.5059	**557.2566**	1112.5218	556.7646	10
9	1054.2650	527.6361			1036.2544	518.6308	E	**1001.4898**	501.2485	984.4633	492.7353	983.4793	492.2433	9
10	**1167.3490**	584.1781			**1149.3385**	575.1729	L	**872.4472**	436.7272	855.4207	428.2140	854.4367	427.7220	8
11	**1281.3919**	641.1996	1264.3654	632.6863	1263.3814	632.1943	N	**759.3632**	380.1852	742.3366	371.6719	741.3526	371.1799	7
12	1352.4291	676.7182	1335.4025	668.2049	1334.4185	667.7129	A	**645.3202**	323.1638	628.2937	314.6505	627.3097	314.1585	6
13	**1480.4876**	740.7475	**1463.4611**	732.2342	1462.4771	731.7422	Q	**574.2831**	287.6452	**557.2566**	**279.1319**	556.2726	278.6399	5
14	**1595.5146**	798.2609	1578.4880	789.7477	1577.5040	789.2556	D	**446.2245**	223.6159	429.1980	215.1026	428.2140	214.6106	4
15	**1682.5466**	841.7769	**1665.5201**	833.2637	**1664.5360**	832.7717	S	**331.1976**	166.1024	314.1710	157.5892	313.1870	157.0972	3
16	1779.5994	890.3033	1762.5728	881.7901	**1761.5888**	881.2980	P	244.1656	122.5864	227.1390	114.0731			2
17							K	147.1128	74.0600	130.0863	65.5468			1

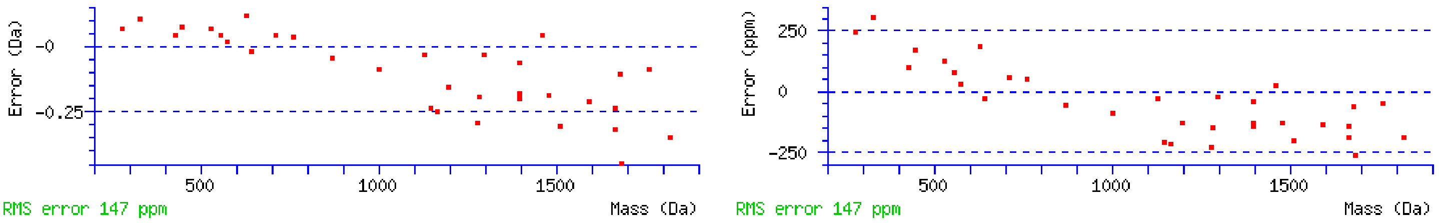

All matches to this query

Score	Mr(calc):	Delta	Sequence
66.9	1924.6976	0.0019	SASSDTSEELNAQDSPK
61.9	1924.6976	0.0019	SASSDTSEELNAQDSPK
61.9	1924.6976	0.0019	SASSDTSEELNAQDSPK
55.0	1924.6976	0.0019	SASSDTSEELNAQDSPK
47.5	1924.6976	0.0019	SASSDTSEELNAQDSPK
47.5	1924.6976	0.0019	SASSDTSEELNAQDSPK
47.5	1924.6976	0.0019	SASSDTSEELNAQDSPK
34.5	1924.6976	0.0019	SASSDTSEELNAQDSPK
34.5	1924.6976	0.0019	SASSDTSEELNAQDSPK
34.5	1924.6976	0.0019	SASSDTSEELNAQDSPK

Spectrum No: 23; Query: 562; Rank: 1

Peptide View

MS/MS Fragmentation of **SASSDTSEELNAQDSPK**
Found in **IPI00200898**, Tax_Id=10116 Gene_Symbol=Slc9a3r1 Ezrin-radixin-moesin-binding phosphoprotein 50

Match to Query 562: 1844.728108 from(923.371330,2+)
Title: 091129RatKid_SCX02_11.371.371.2.dta
Data file K:\NewmanPaper\Piliang\3SubProteomes\Piliang3SP\mgf5ppm\SCX_3SubProteomes5ppm.mgf

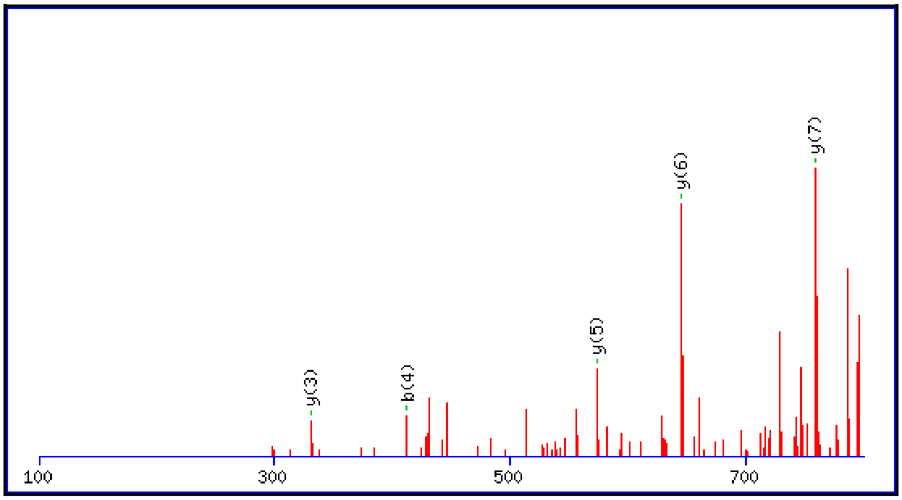

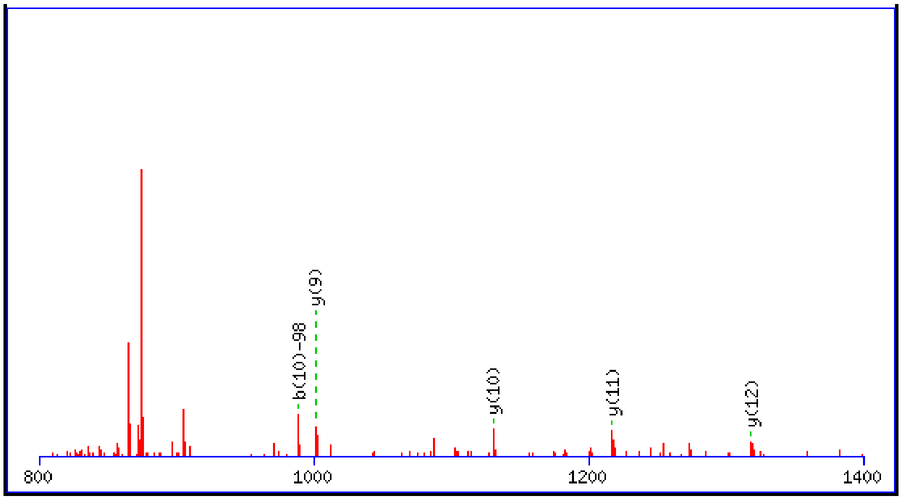

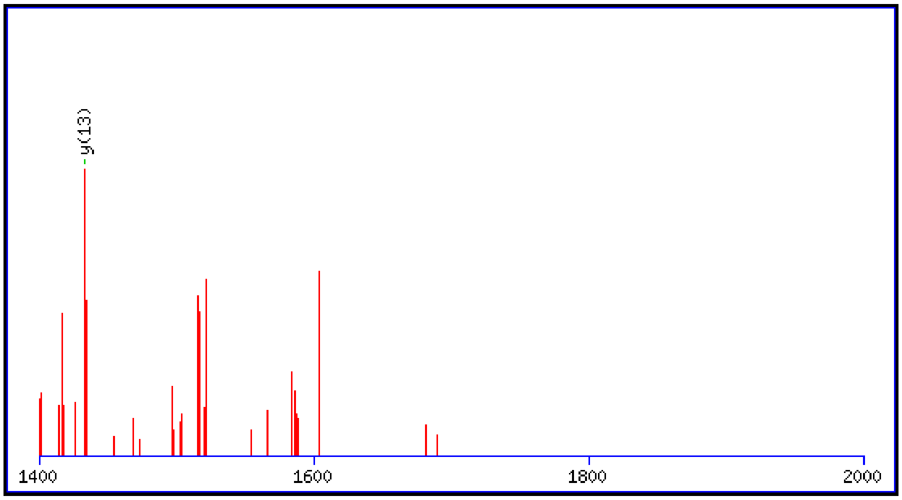

Monoisotopic mass of neutral peptide Mr(calc): 1844.7313
Fixed modifications: Carbamidomethyl (C)
Variable modifications:
S4 : Phospho (ST), with neutral losses 97.9769(shown in table), 0.0000
Ions Score: 67 **Expect:** 3.2e-005
Matches (Bold Red): 11/250 fragment ions using 18 most intense peaks

#	b	b^{++}	b*	b^{*++}	b^{0}	b^{0++}	Seq.	y	y^{++}	y*	y^{*++}	y^{0}	y^{0++}	#
1	88.0393	44.5233			70.0287	35.5180	S							17
2	159.0764	80.0418			141.0659	71.0366	A	1660.7297	830.8685	1643.7031	822.3552	1642.7191	821.8632	16
3	246.1084	123.5579			228.0979	114.5526	S	1589.6925	795.3499	1572.6660	786.8366	1571.6820	786.3446	15
4	315.1299	158.0686			297.1193	149.0633	S	1502.6605	751.8339	1485.6340	743.3206	1484.6499	742.8286	14
5	430.1568	215.5821			412.1463	206.5768	D	**1433.6391**	717.3232	1416.6125	708.8099	1415.6285	708.3179	13
6	531.2045	266.1059			513.1940	257.1006	T	**1318.6121**	659.8097	1301.5856	651.2964	1300.6016	650.8044	12
7	618.2366	309.6219			600.2260	300.6166	S	**1217.5644**	609.2859	1200.5379	600.7726	1199.5539	600.2806	11
8	747.2791	374.1432			729.2686	365.1379	E	**1130.5324**	565.7698	1113.5059	557.2566	1112.5218	556.7646	10
9	876.3217	438.6645			858.3112	429.6592	E	**1001.4898**	501.2485	984.4633	492.7353	983.4793	492.2433	9
10	**989.4058**	495.2065			971.3952	486.2013	L	872.4472	436.7272	855.4207	428.2140	854.4367	427.7220	8
11	1103.4487	552.2280	1086.4222	543.7147	1085.4382	543.2227	N	**759.3632**	380.1852	742.3366	371.6719	741.3526	371.1799	7
12	1174.4858	587.7466	1157.4593	579.2333	1156.4753	578.7413	A	**645.3202**	323.1638	628.2937	314.6505	627.3097	314.1585	6
13	1302.5444	651.7758	1285.5179	643.2626	1284.5339	642.7706	Q	**574.2831**	287.6452	557.2566	279.1319	556.2726	278.6399	5
14	1417.5714	709.2893	1400.5448	700.7760	1399.5608	700.2840	D	446.2245	223.6159	429.1980	215.1026	428.2140	214.6106	4
15	1504.6034	752.8053	1487.5768	744.2921	1486.5928	743.8001	S	**331.1976**	166.1024	314.1710	157.5892	313.1870	157.0972	3
16	1601.6562	801.3317	1584.6296	792.8184	1583.6456	792.3264	P	244.1656	122.5864	227.1390	114.0731			2
17							K	147.1128	74.0600	130.0863	65.5468			1

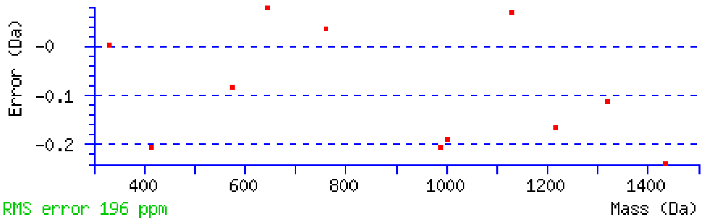

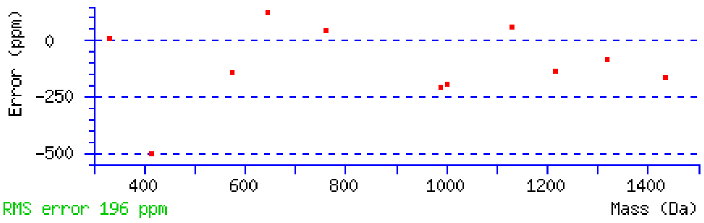

All matches to this query

Score	Mr(calc):	Delta	Sequence
66.5	1844.7313	-0.0032	SASSDTSEELNAQDSPK
66.5	1844.7313	-0.0032	SASSDTSEELNAQDSPK
66.5	1844.7313	-0.0032	SASSDTSEELNAQDSPK
38.9	1844.7313	-0.0032	SASSDTSEELNAQDSPK
27.3	1844.7313	-0.0032	SASSDTSEELNAQDSPK
6.1	1843.7139	1.0143	ESNPNVSQNSTNHKK
5.3	1843.7139	1.0143	ESNPNVSQNSTNHKK
2.4	1844.7434	-0.0153	GDGDLSCINGDMEVRK
1.6	1844.7118	0.0163	EEQKKYDSEESVSK
0.5	1844.7321	-0.0040	EHMETTMDIENQKK

Spectrum No: 24; Query: 634; Rank: 1

Peptide View

MS/MS Fragmentation of **DWEDDSDEDMSNFDR**
Found in **IPI00365935**, Tax_Id=10116 Gene_Symbol=Ptges3_predicted Prostaglandin E synthase 3

Match to Query 634: 1954.624968 from(978.319760,2+)
Title: 091127RatKid_SCX01_12.2836.2836.2.dta
Data file K:\NewmanPaper\Piliang\3SubProteomes\Piliang3SP\mgf5ppm\SCX_3SubProteomes5ppm.mgf

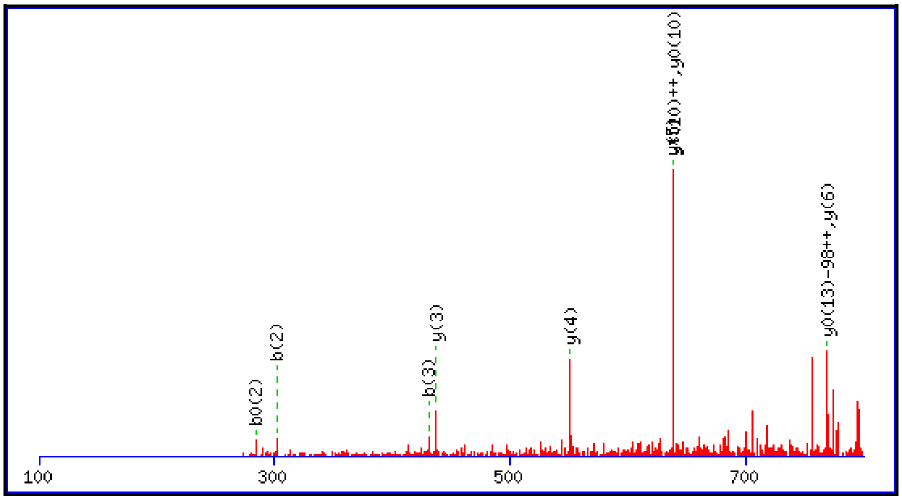

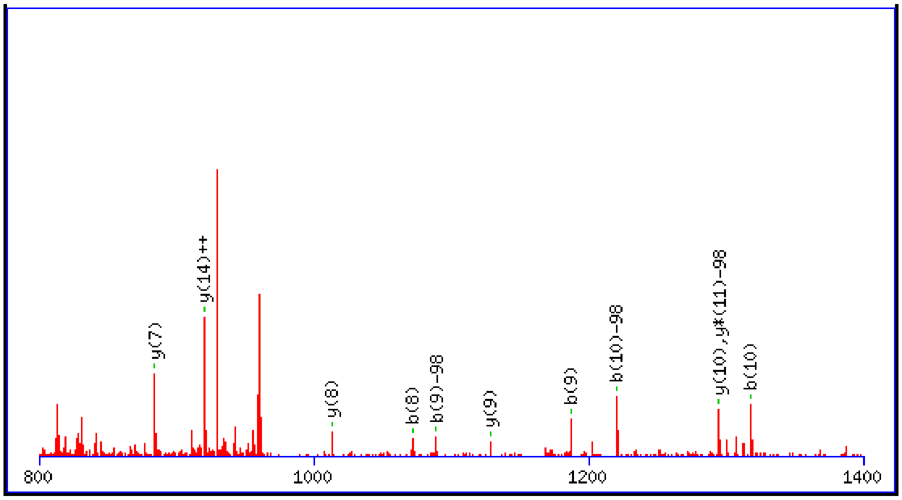

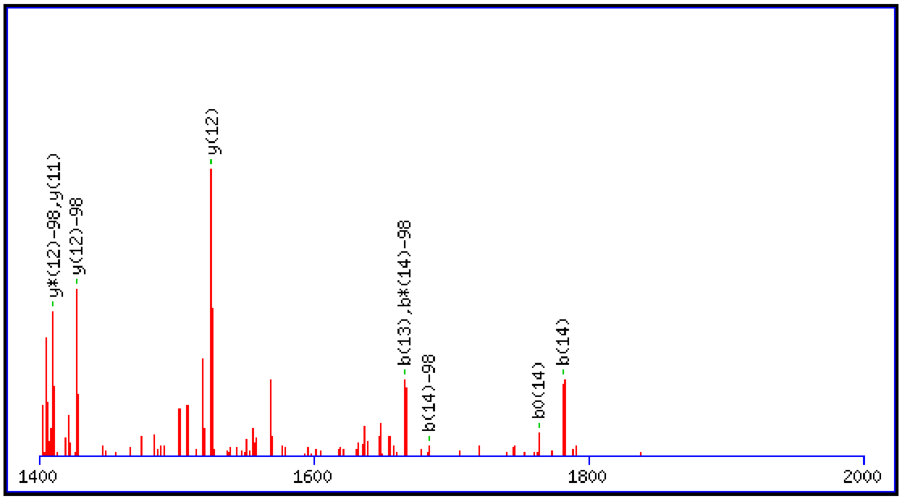

Monoisotopic mass of neutral peptide Mr(calc): 1954.6200
Fixed modifications: Carbamidomethyl (C)
Variable modifications:
S6 : Phospho (ST), with neutral losses 0.0000(shown in table), 97.9769
Ions Score: 66 **Expect:** 8e-006
Matches (Bold Red): 30/216 fragment ions using 35 most intense peaks

#	b	b^{++}	b*	b^{*++}	b^0	b^{0++}	Seq.	y	y^{++}	y*	y^{*++}	y^0	y^{0++}	#
1	116.0342	58.5207			98.0237	49.5155	D							15
2	**302.1135**	151.5604			**284.1030**	142.5551	W	1840.6004	**920.8038**	1823.5738	912.2905	1822.5898	911.7985	14
3	**431.1561**	216.0817			413.1456	207.0764	E	1654.5211	827.7642	1637.4945	819.2509	1636.5105	818.7589	13
4	546.1831	273.5952			528.1725	264.5899	D	**1525.4785**	763.2429	1508.4519	754.7296	1507.4679	754.2376	12
5	661.2100	331.1086			643.1994	322.1034	D	**1410.4515**	705.7294	1393.4250	697.2161	1392.4410	696.7241	11
6	828.2084	414.6078			810.1978	405.6025	S	**1295.4246**	648.2159	1278.3980	**639.7027**	1277.4140	**639.2106**	10
7	943.2353	472.1213			925.2247	463.1160	D	**1128.4262**	564.7168	1111.3997	556.2035	1110.4157	555.7115	9
8	**1072.2779**	536.6426			1054.2673	527.6373	E	**1013.3993**	507.2033	996.3727	498.6900	995.3887	498.1980	8
9	**1187.3048**	594.1561			1169.2943	585.1508	D	**884.3567**	442.6820	867.3301	434.1687	866.3461	433.6767	7
10	**1318.3453**	659.6763			1300.3348	650.6710	M	**769.3297**	385.1685	752.3032	376.6552	751.3192	376.1632	6
11	1405.3774	703.1923			1387.3668	694.1870	S	**638.2893**	319.6483	621.2627	311.1350	620.2787	310.6430	5
12	1519.4203	760.2138	1502.3937	751.7005	1501.4097	751.2085	N	**551.2572**	276.1323	534.2307	267.6190	533.2467	267.1270	4
13	**1666.4887**	833.7480	1649.4621	825.2347	1648.4781	824.7427	F	**437.2143**	219.1108	420.1878	210.5975	419.2037	210.1055	3
14	**1781.5156**	891.2615	1764.4891	882.7482	**1763.5051**	882.2562	D	290.1459	145.5766	273.1193	137.0633	272.1353	136.5713	2
15							R	175.1190	88.0631	158.0924	79.5498			1

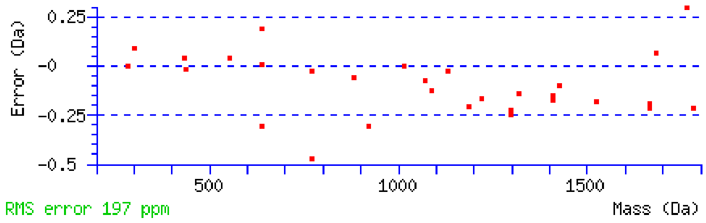

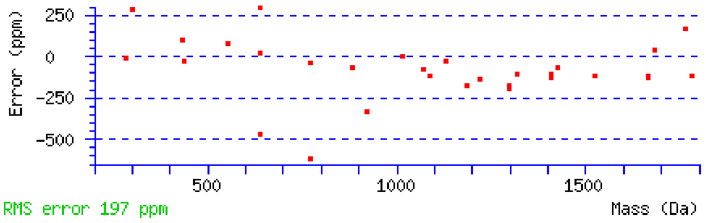

All matches to this query

Score	Mr(calc):	Delta	Sequence
66.4	1954.6200	0.0049	DWEDDSDEDMSNFDR
23.0	1954.6200	0.0049	DWEDDSDEDMSNFDR
1.1	1954.6175	0.0075	SYFGNMGPQYVTTYA

Spectrum No: 25; Query: 521; Rank: 1

Peptide View

MS/MS Fragmentation of **TLSNAEDYLDDEDSD**
Found in **IPI00202703**, Tax_Id=10116 Gene_Symbol=Ostf1 Osteoclast-stimulating factor 1

Match to Query 521: 1780.623608 from(891.319080,2+)
Title: 091127RatKid_SCX01_02.2868.2868.2.dta
Data file K:\NewmanPaper\Piliang\3SubProteomes\Piliang3SP\mgf5ppm\SCX_3SubProteomes5ppm.mgf

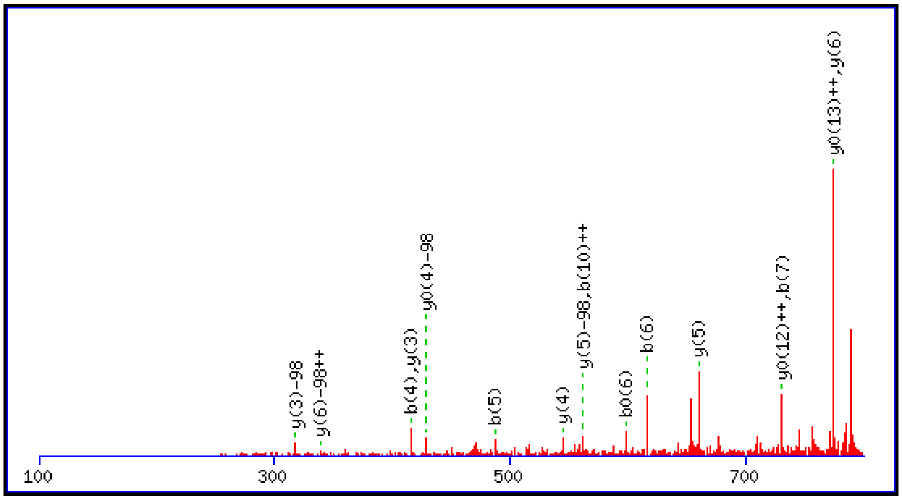

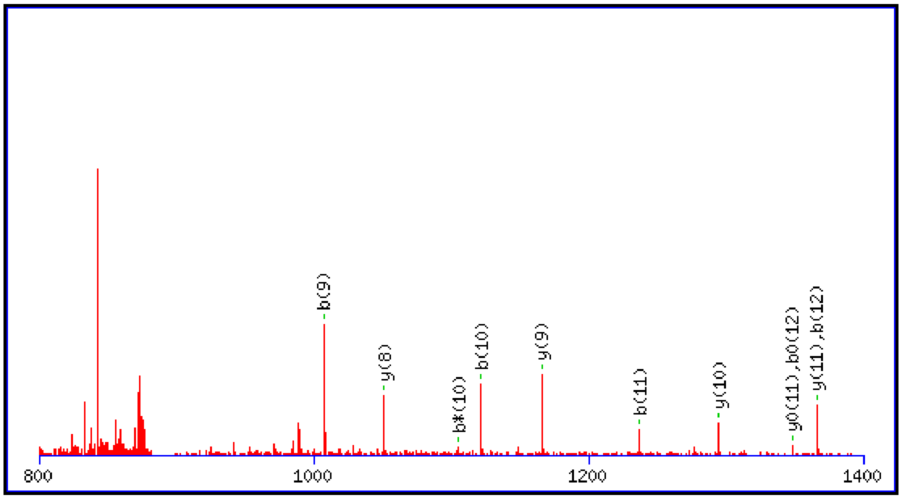

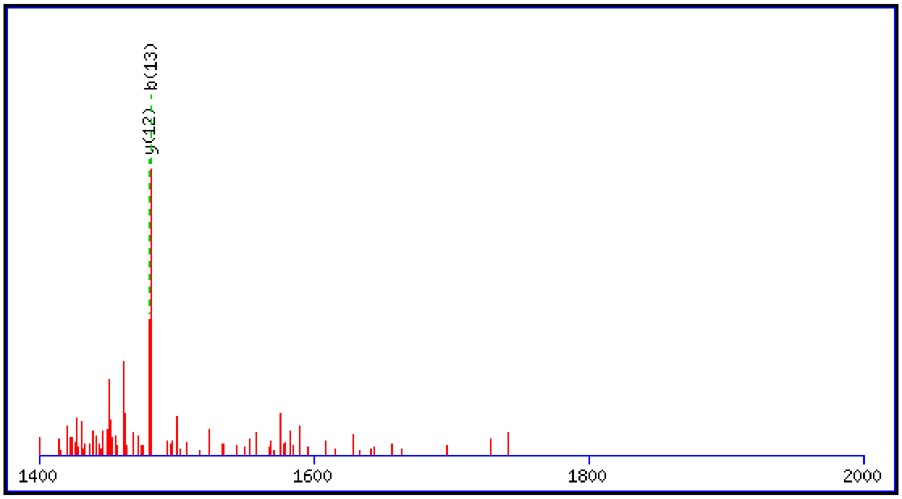

Monoisotopic mass of neutral peptide Mr(calc): 1780.6200
Fixed modifications: Carbamidomethyl (C)
Variable modifications:
S14 : Phospho (ST), with neutral losses 0.0000(shown in table), 97.9769
Ions Score: 66 **Expect:** 1.8e-005
Matches (Bold Red): 29/204 fragment ions using 34 most intense peaks

#	b	b++	b*	b*++	b0	b0++	Seq.	y	y++	y*	y*++	y0	y0++	#
1	102.0550	51.5311			84.0444	42.5258	T							15
2	215.1390	108.0731			197.1285	99.0679	L	1680.5796	840.7934	1663.5531	832.2802	1662.5691	831.7882	14
3	302.1710	151.5892			284.1605	142.5839	S	1567.4956	784.2514	1550.4690	775.7381	1549.4850	**775.2461**	13
4	**416.2140**	208.6106	399.1874	200.0974	398.2034	199.6053	N	**1480.4635**	740.7354	1463.4370	732.2221	1462.4530	**731.7301**	12
5	**487.2511**	244.1292	470.2245	235.6159	469.2405	235.1239	A	**1366.4206**	683.7139			**1348.4100**	674.7087	11
6	**616.2937**	308.6505	599.2671	300.1372	**598.2831**	299.6452	E	**1295.3835**	648.1954			1277.3729	639.1901	10
7	**731.3206**	366.1640	714.2941	357.6507	713.3101	357.1587	D	**1166.3409**	583.6741			1148.3303	574.6688	9
8	894.3840	447.6956	877.3574	439.1823	876.3734	438.6903	Y	**1051.3140**	526.1606			1033.3034	517.1553	8
9	**1007.4680**	504.2376	990.4415	495.7244	989.4575	495.2324	L	888.2506	444.6289			870.2401	435.6237	7
10	**1122.4950**	**561.7511**	**1105.4684**	553.2378	1104.4844	552.7458	D	**775.1666**	388.0869			757.1560	379.0816	6
11	**1237.5219**	619.2646	1220.4954	610.7513	1219.5113	610.2593	D	**660.1396**	330.5734			642.1291	321.5682	5
12	**1366.5645**	683.7859	1349.5379	675.2726	**1348.5539**	674.7806	E	**545.1127**	273.0600			527.1021	264.0547	4
13	**1481.5914**	741.2994	1464.5649	732.7861	1463.5809	732.2941	D	**416.0701**	208.5387			398.0595	199.5334	3
14	1648.5898	824.7985	1631.5632	816.2853	1630.5792	815.7933	S	301.0431	151.0252			283.0326	142.0199	2
15							D	134.0448	67.5260			116.0342	58.5207	1

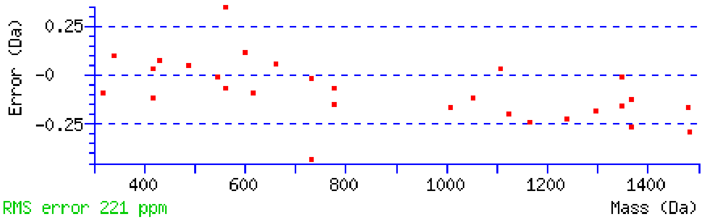

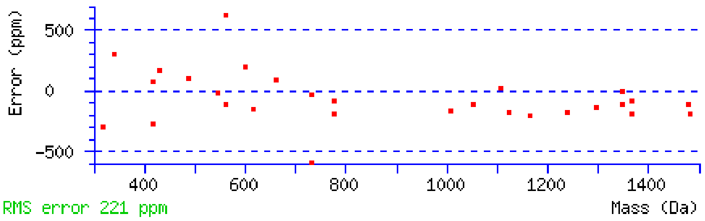

All matches to this query

Score	Mr(calc):	Delta	Sequence
65.9	1780.6200	0.0036	TLSNAEDYLDDEDSD
16.1	1780.6200	0.0036	TLSNAEDYLDDEDSD
5.5	1780.6200	0.0036	TLSNAEDYLDDEDSD
5.5	1780.6200	0.0036	TLSNAEDYLDDEDSD

Spectrum No: 26; Query: 924; Rank: 1

Peptide View

MS/MS Fragmentation of **EGEEPTVYSDDEEPKDEAARK**
Found in **IPI00480820**, Tax_Id=10116 Gene_Symbol=Pgrmc1 Membrane-associated progesterone receptor component 1

Match to Query 924: 2473.023792 from(825.348540,3+)
Title: 091129RatKid_SCX02_30.865.865.3.dta
Data file K:\NewmanPaper\Piliang\3SubProteomes\Piliang3SP\mgf5ppm\SCX_3SubProteomes5ppm.mgf

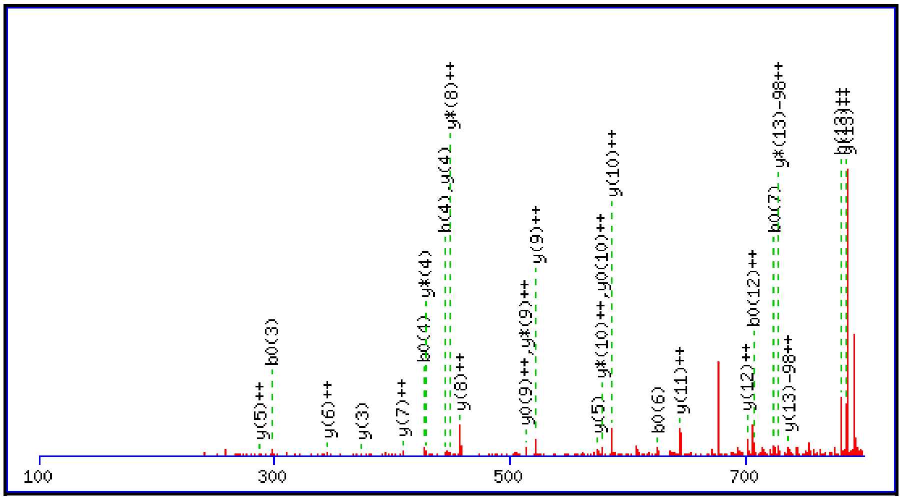

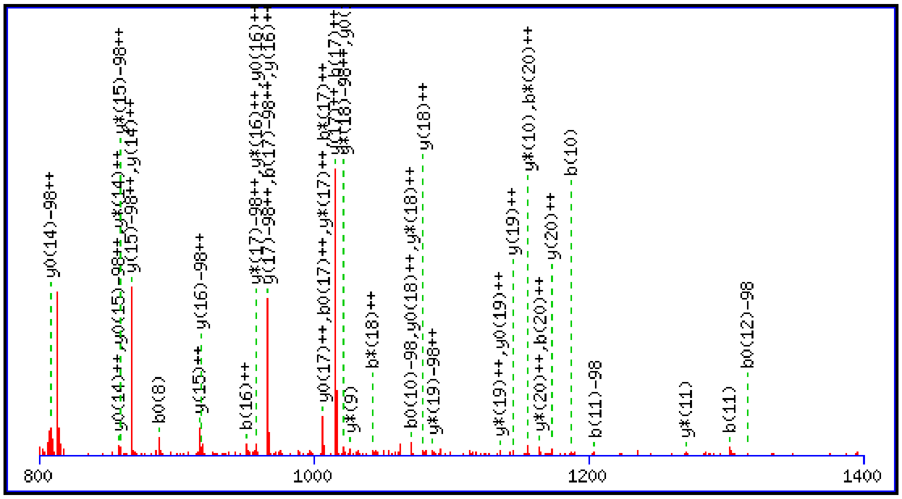

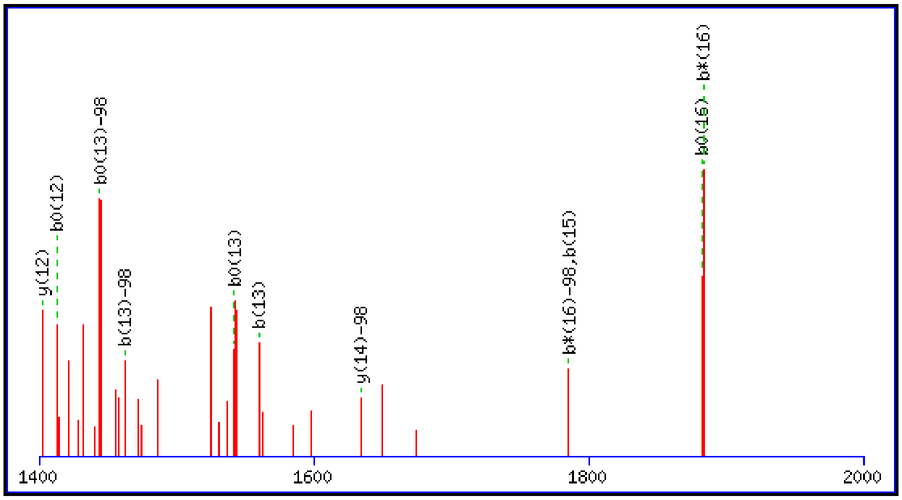

Monoisotopic mass of neutral peptide Mr(calc): 2473.0169
Fixed modifications: Carbamidomethyl (C)

```
Variable modifications:
S9      : Phospho (ST), with neutral losses 0.0000(shown in table), 97.9769
Ions Score: 66  Expect: 7e-005
Matches (Bold Red): 84/312 fragment ions using 137 most intense peaks
```

#	b	b^{++}	b*	b^{*++}	b^{0}	b^{0++}	Seq.	y	y^{++}	y*	y^{*++}	y^{0}	y^{0++}	#
1	130.0499	65.5286			112.0393	56.5233	E							21
2	187.0713	94.0393			169.0608	85.0340	G	2344.9817	**1172.9945**	2327.9551	**1164.4812**	2326.9711	1163.9892	20
3	316.1139	158.5606			**298.1034**	149.5553	E	2287.9602	**1144.4837**	2270.9337	**1135.9705**	2269.9496	**1135.4785**	19
4	**445.1565**	223.0819			**427.1460**	214.0766	E	2158.9176	**1079.9624**	2141.8911	**1071.4492**	2140.9070	**1070.9572**	18
5	542.2093	271.6083			524.1987	262.6030	P	2029.8750	**1015.4411**	2012.8485	**1006.9279**	2011.8645	**1006.4359**	17
6	643.2570	322.1321			**625.2464**	313.1268	T	1932.8223	**966.9148**	1915.7957	**958.4015**	1914.8117	**957.9095**	16
7	742.3254	371.6663			**724.3148**	362.6610	V	1831.7746	**916.3909**	1814.7480	907.8777	1813.7640	907.3856	15
8	905.3887	453.1980			**887.3781**	444.1927	Y	1732.7062	**866.8567**	1715.6796	**858.3434**	1714.6956	**857.8514**	14
9	1072.3871	536.6972			1054.3765	527.6919	S	1569.6428	**785.3251**	1552.6163	776.8118	1551.6323	776.3198	13
10	**1187.4140**	594.2106			1169.4034	585.2054	D	**1402.6445**	**701.8259**	1385.6179	693.3126	1384.6339	692.8206	12
11	**1302.4409**	651.7241			1284.4304	642.7188	D	1287.6175	**644.3124**	**1270.5910**	635.7991	1269.6070	635.3071	11
12	1431.4835	716.2454			**1413.4730**	**707.2401**	E	1172.5906	**586.7989**	**1155.5640**	**578.2857**	1154.5800	**577.7937**	10
13	**1560.5261**	**780.7667**			**1542.5156**	771.7614	E	1043.5480	**522.2776**	**1026.5215**	**513.7644**	1025.5374	**513.2724**	9
14	1657.5789	829.2931			1639.5683	820.2878	P	914.5054	**457.7563**	897.4789	**449.2431**	896.4948	448.7511	8
15	**1785.6739**	893.3406	1768.6473	884.8273	1767.6633	884.3353	K	817.4526	**409.2300**	800.4261	400.7167	799.4421	400.2247	7
16	1900.7008	**950.8540**	**1883.6743**	942.3408	**1882.6902**	941.8488	D	689.3577	**345.1825**	672.3311	336.6692	671.3471	336.1772	6
17	2029.7434	**1015.3753**	2012.7168	**1006.8621**	2011.7328	**1006.3701**	E	**574.3307**	**287.6690**	557.3042	279.1557	556.3202	278.6637	5
18	2100.7805	1050.8939	2083.7540	**1042.3806**	2082.7699	1041.8886	A	**445.2881**	223.1477	**428.2616**	214.6344			4
19	2171.8176	1086.4124	2154.7911	1077.8992	2153.8071	1077.4072	A	**374.2510**	187.6292	357.2245	179.1159			3
20	2327.9187	**1164.4630**	2310.8922	**1155.9497**	2309.9082	1155.4577	R	303.2139	152.1106	286.1874	143.5973			2
21							K	147.1128	74.0600	130.0863	65.5468			1

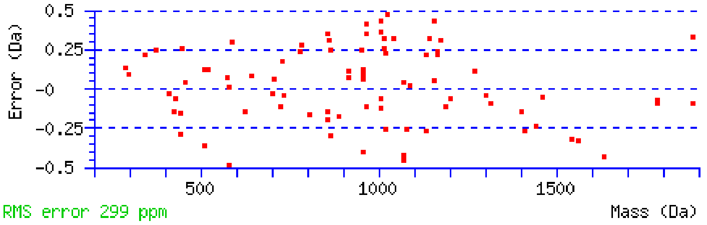

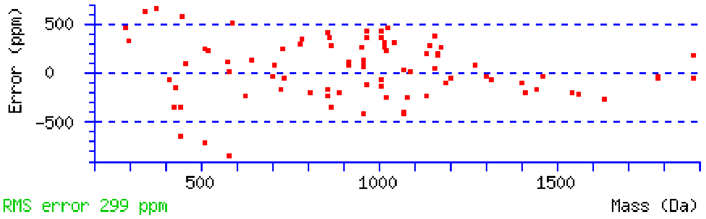

All matches to this query

Score	Mr(calc):	Delta	Sequence
65.9	2473.0169	0.0069	EGEEPTVYSDDEEPKDEAARK
58.3	2473.0169	0.0069	EGEEPTVYSDDEEPKDEAARK
41.9	2473.0169	0.0069	EGEEPTVYSDDEEPKDEAARK
2.8	2472.0177	1.0061	ESQDPNSKSDITKGESQDPNSK
2.6	2471.0337	1.9901	ESQDPNSKSDITKGESQDPNSK
2.5	2471.0187	2.0051	FYRWISERYPCLSEVVK
2.0	2471.0262	1.9976	NMVYVLTITTPLKTSDAKR
1.5	2472.0182	1.0056	TSQDSNYGGKIPIVCFAQGGGR
1.5	2472.0182	1.0056	TSQDSNYGGKIPIVCFAQGGGR
1.5	2472.0182	1.0056	TSQDSNYGGKIPIVCFAQGGGR

Spectrum No: 27; Query: 567; Rank: 1

Peptide View

MS/MS Fragmentation of **EEASDDDMEGDEAVVR**
Found in **IPI00373118**, Tax_Id=10116 Gene_Symbol=Ascc3l1 79 kDa protein

Match to Query 567: 1845.665988 from(923.840270,2+)
Title: 091127RatKid_SCX01_12.1513.1513.2.dta
Data file K:\NewmanPaper\Piliang\3SubProteomes\Piliang3SP\mgf5ppm\SCX_3SubProteomes5ppm.mgf

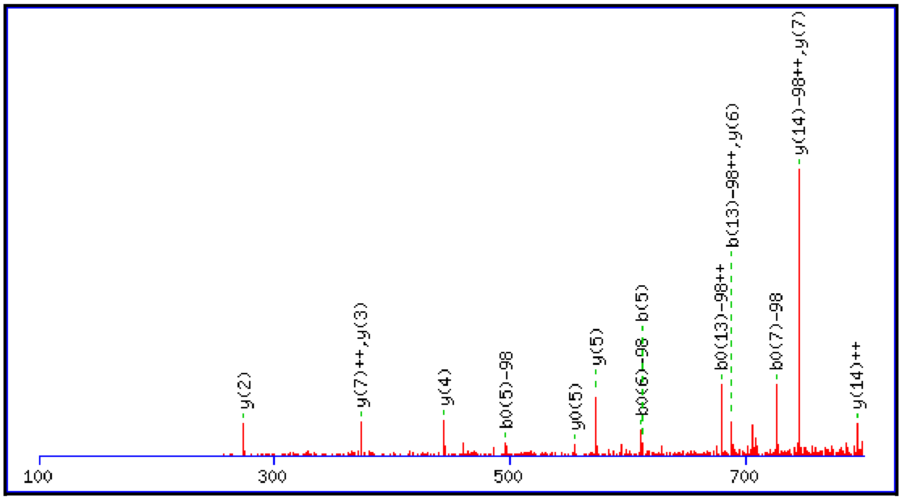

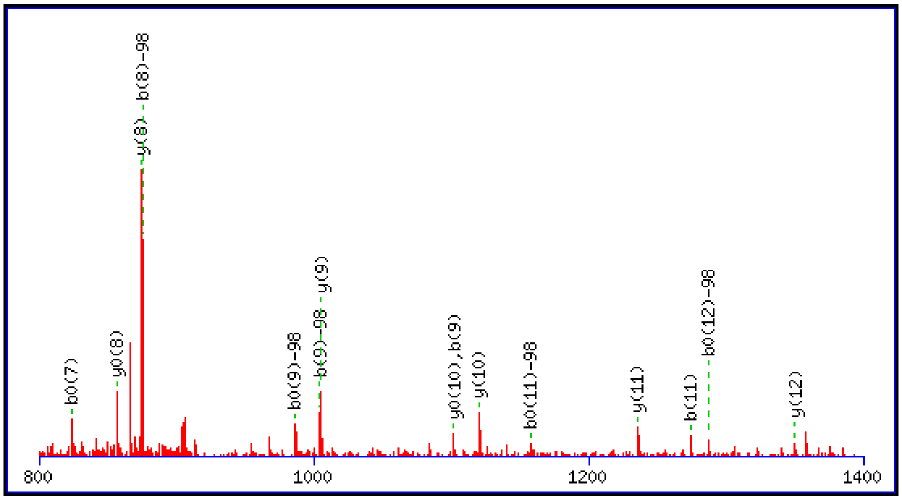

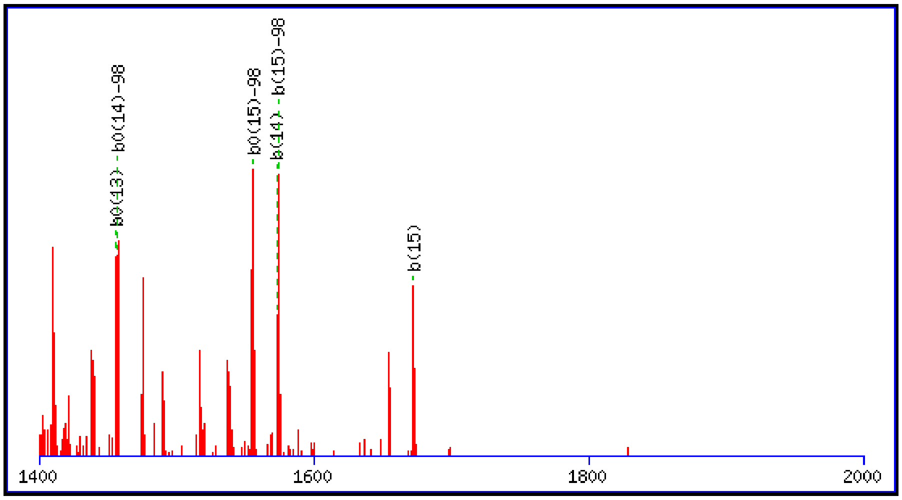

Monoisotopic mass of neutral peptide Mr(calc): 1845.6612
Fixed modifications: Carbamidomethyl (C)
Variable modifications:
S4 : Phospho (ST), with neutral losses 97.9769(shown in table), 0.0000
Ions Score: 66 **Expect:** 2.2e-005
Matches (Bold Red): 37/208 fragment ions using 53 most intense peaks

#	b	b++	b0	b0++	Seq.	y	y++	y*	y*++	y0	y0++	#
1	130.0499	65.5286	112.0393	56.5233	E							16
2	259.0925	130.0499	241.0819	121.0446	E	1619.6490	810.3281	1602.6224	801.8148	1601.6384	801.3228	15
3	330.1296	165.5684	312.1190	156.5631	A	1490.6064	**745.8068**	1473.5798	737.2935	1472.5958	736.8015	14
4	399.1510	200.0792	381.1405	191.0739	S	1419.5693	710.2883	1402.5427	701.7750	1401.5587	701.2830	13
5	514.1780	257.5926	**496.1674**	248.5873	D	**1350.5478**	675.7775	1333.5213	667.2643	1332.5372	666.7723	12
6	629.2049	315.1061	**611.1944**	306.1008	D	**1235.5209**	618.2641	1218.4943	609.7508	1217.5103	609.2588	11
7	744.2319	372.6196	**726.2213**	363.6143	D	**1120.4939**	560.7506	1103.4674	552.2373	**1102.4833**	551.7453	10
8	**875.2723**	438.1398	857.2618	429.1345	M	**1005.4670**	503.2371	988.4404	494.7238	987.4564	494.2318	9
9	**1004.3149**	502.6611	**986.3044**	493.6558	E	**874.4265**	437.7169	857.3999	429.2036	**856.4159**	428.7116	8
10	1061.3364	531.1718	1043.3258	522.1666	G	**745.3839**	**373.1956**	728.3573	364.6823	727.3733	364.1903	7
11	1176.3633	588.6853	**1158.3528**	579.6800	D	**688.3624**	344.6849	671.3359	336.1716	670.3519	335.6796	6
12	1305.4059	653.2066	**1287.3954**	644.2013	E	**573.3355**	287.1714	556.3089	278.6581	**555.3249**	278.1661	5
13	1376.4431	**688.7252**	1358.4325	**679.7199**	A	**444.2929**	222.6501	427.2663	214.1368			4
14	1475.5115	738.2594	**1457.5009**	729.2541	V	**373.2558**	187.1315	356.2292	178.6183			3
15	**1574.5799**	787.7936	**1556.5693**	778.7883	V	**274.1874**	137.5973	257.1608	129.0840			2
16					R	175.1190	88.0631	158.0924	79.5498			1

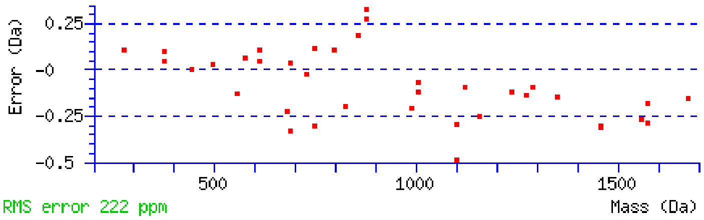

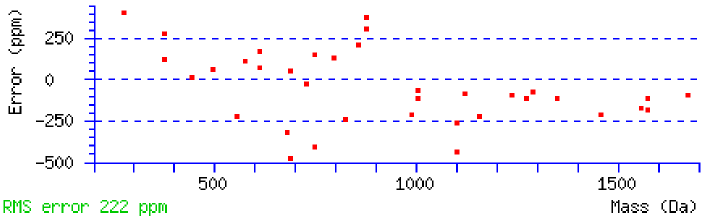

All matches to this query

Score	Mr(calc):	Delta	Sequence
65.5	1845.6612	0.0048	EEASDDDMEGDEAVVR
7.4	1843.6650	2.0010	ESGQEGSTDNDDSCSEK
0.3	1845.6789	-0.0129	EEEDDEKETSTNPSR
0.3	1845.6789	-0.0129	EEEDDEKETSTNPSR
0.3	1845.6789	-0.0129	EEEDDEKETSTNPSR
0.3	1845.6789	-0.0129	EEEDDEKETSTNPSR

Spectrum No: 28; Query: 378; Rank: 1

Peptide View

MS/MS Fragmentation of **DAVAVAPPPSPSLPAK**
Found in **IPI00205325**, Tax_Id=10116 Gene_Symbol=Lrp2 Low-density lipoprotein receptor-related protein 2 precursor

Match to Query 378: 1595.797428 from(798.905990,2+)
Title: 091129RatKid_SCX02_12.1464.1464.2.dta
Data file K:\NewmanPaper\Piliang\3SubProteomes\Piliang3SP\mgf5ppm\SCX_3SubProteomes5ppm.mgf

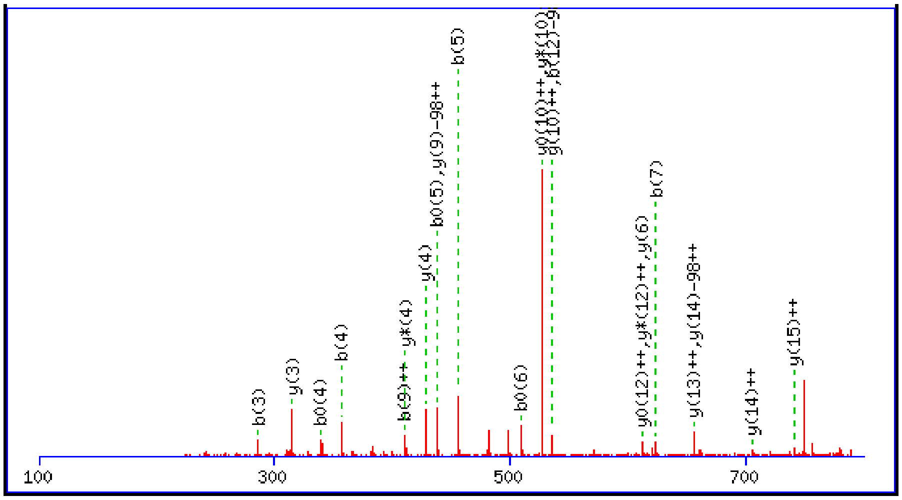

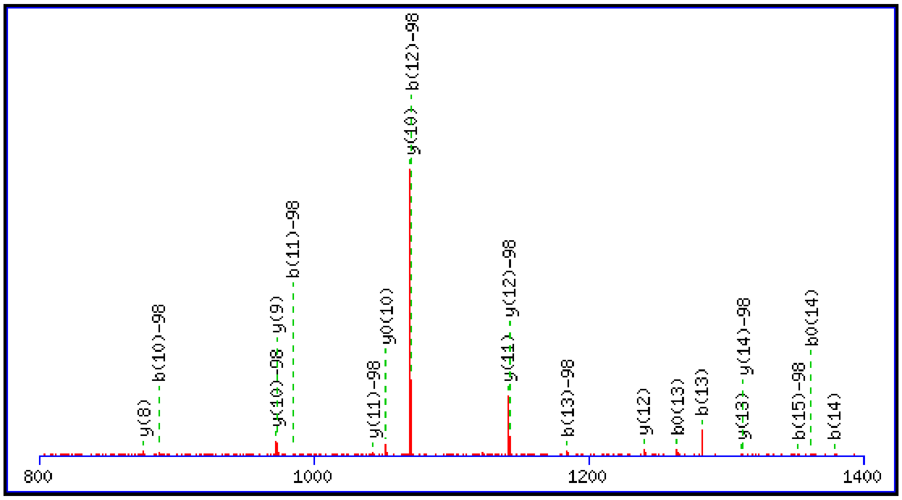

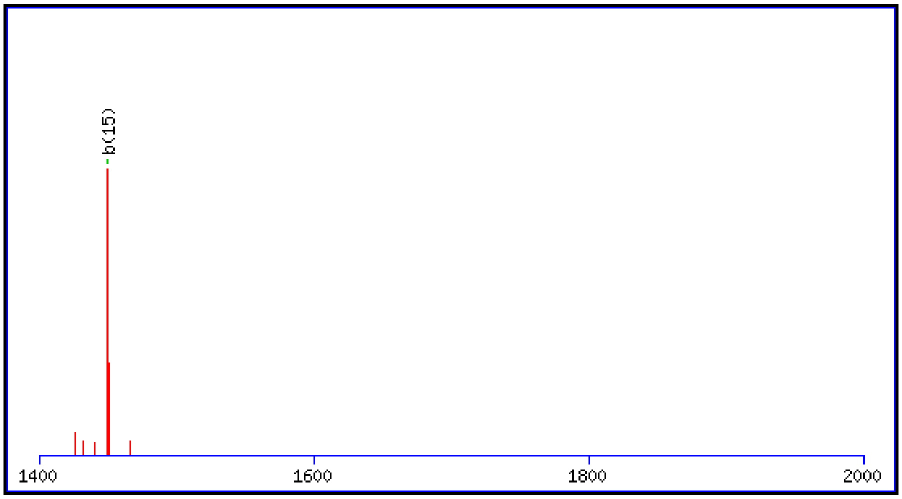

Monoisotopic mass of neutral peptide Mr(calc): 1595.7960
Fixed modifications: Carbamidomethyl (C)
Variable modifications:
S10 : Phospho (ST), with neutral losses 97.9769(shown in table), 0.0000
Ions Score: 65 **Expect:** 5.2e-005
Matches (Bold Red): 46/220 fragment ions using 56 most intense peaks

#	b	b^{++}	b^{0}	b^{0++}	Seq.	y	y^{++}	y^{*}	y^{*++}	y^{0}	y^{0++}	#
1	116.0342	58.5207	98.0237	49.5155	D							16
2	187.0713	94.0393	169.0608	85.0340	A	1383.7995	692.4034	1366.7729	683.8901	1365.7889	683.3981	15
3	**286.1397**	143.5735	268.1292	134.5682	V	**1312.7623**	**656.8848**	1295.7358	648.3715	1294.7518	647.8795	14
4	**357.1769**	179.0921	**339.1663**	170.0868	A	1213.6939	607.3506	1196.6674	598.8373	1195.6834	598.3453	13
5	**456.2453**	228.6263	**438.2347**	219.6210	V	**1142.6568**	571.8320	1125.6303	563.3188	1124.6462	562.8268	12

6	**527.2824**	264.1448	**509.2718**	255.1396	A	**1043.5884**	522.2978	1026.5619	513.7846	1025.5778	513.2926	11
7	**624.3352**	312.6712	606.3246	303.6659	P	**972.5513**	486.7793	955.5247	478.2660	954.5407	477.7740	10
8	721.3879	361.1976	703.3774	352.1923	P	875.4985	**438.2529**	858.4720	429.7396	857.4880	429.2476	9
9	818.4407	**409.7240**	800.4301	400.7187	P	778.4458	389.7265	761.4192	381.2132	760.4352	380.7212	8
10	**887.4621**	444.2347	869.4516	435.2294	S	681.3930	341.2001	664.3664	332.6869	663.3824	332.1949	7
11	**984.5149**	492.7611	966.5043	483.7558	P	**612.3715**	306.6894	595.3450	298.1761	594.3610	297.6841	6
12	**1071.5469**	**536.2771**	1053.5364	**527.2718**	S	515.3188	258.1630	498.2922	249.6498	497.3082	249.1577	5
13	**1184.6310**	592.8191	1166.6204	583.8139	L	**428.2867**	214.6470	**411.2602**	206.1337			4
14	1281.6838	641.3455	1263.6732	632.3402	P	**315.2027**	158.1050	298.1761	149.5917			3
15	**1352.7209**	676.8641	1334.7103	667.8588	A	218.1499	109.5786	201.1234	101.0653			2
16					K	147.1128	74.0600	130.0863	65.5468			1

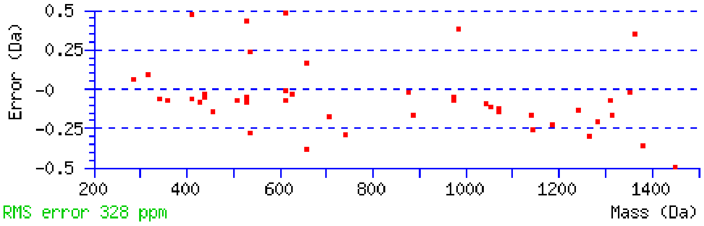

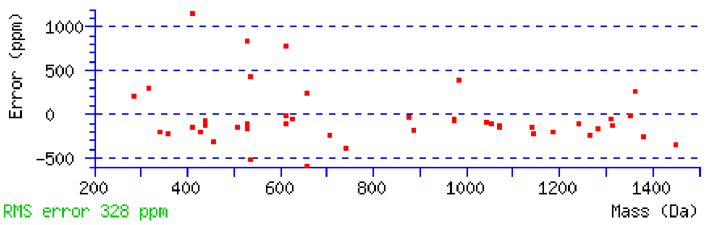

All matches to this query

Score	Mr(calc):	Delta	Sequence
64.6	1595.7960	0.0014	DAVAVAPPPSPSLPAK
52.0	1595.7960	0.0014	DAVAVAPPPSPSLPAK
16.1	1595.7848	0.0127	IEQEFLTEALPVK
10.7	1595.7823	0.0152	SFLVFINLYCIK
6.5	1595.8072	-0.0098	IKNPEGGLYVAVTR
5.4	1595.7928	0.0046	ERLMKLLPCSAAK
5.4	1595.8128	-0.0154	SAPEAAPGSTRPGRSR
5.2	1595.7960	0.0014	WLEEKAVLTTQAK
5.0	1595.7928	0.0046	LVLSLPVNMRCSK
4.5	1595.7977	-0.0003	ELMSPPDATQAAPLR

Spectrum No: 29; Query: 429; Rank: 1

Peptide View

MS/MS Fragmentation of **TNGSVDLGEEEEAAR**
Found in **IPI00464886**, Tax_Id=10116 Gene_Symbol=LOC652956 p55 protein

Match to Query 429: 1655.669988 from(828.842270,2+)
Title: 091127RatKid_SCX01_12.1528.1528.2.dta
Data file K:\NewmanPaper\Piliang\3SubProteomes\Piliang3SP\mgf5ppm\SCX_3SubProteomes5ppm.mgf

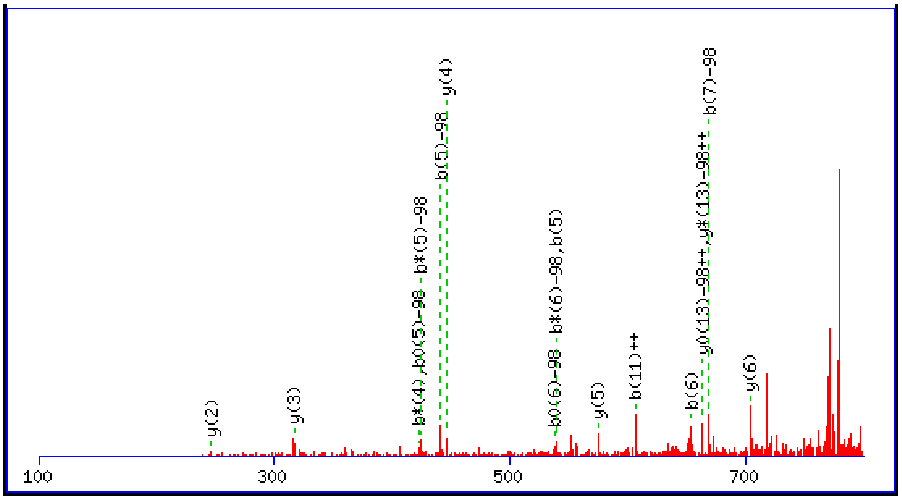

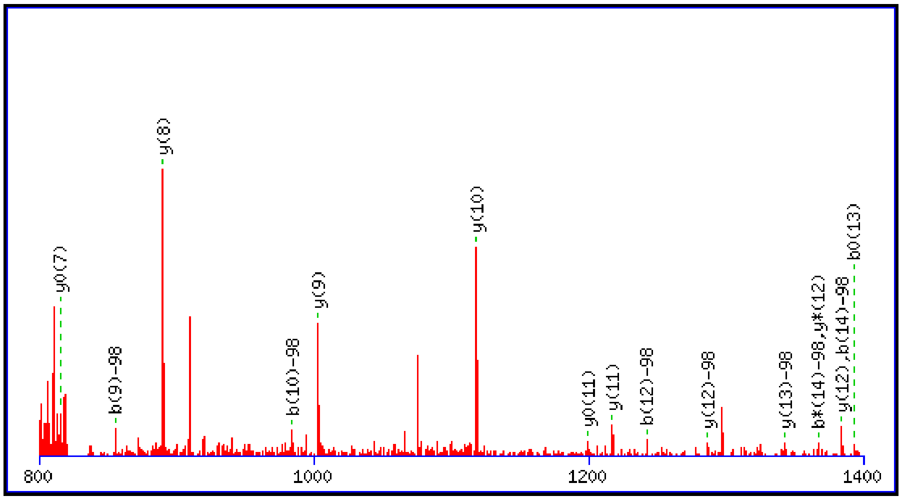

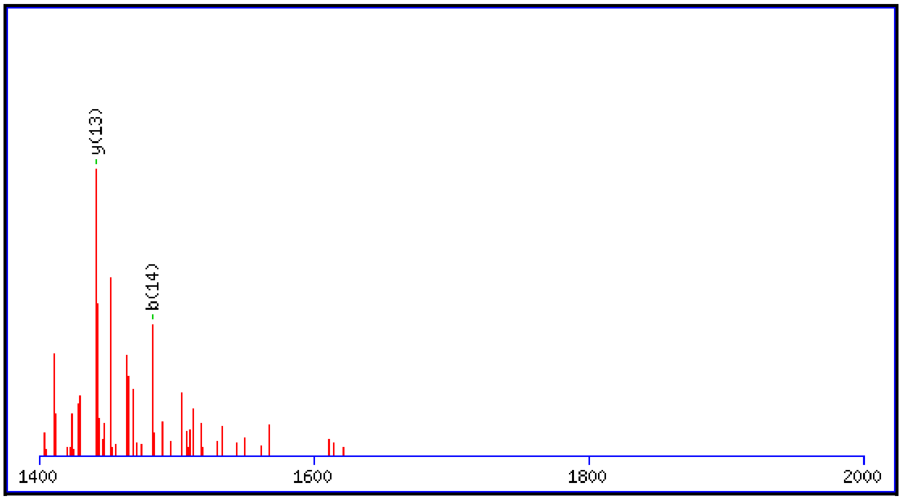

Monoisotopic mass of neutral peptide Mr(calc): 1655.6676
Fixed modifications: Carbamidomethyl (C)
Variable modifications:
S4 : Phospho (ST), with neutral losses 97.9769(shown in table), 0.0000
Ions Score: 64 **Expect:** 4.7e-005
Matches (Bold Red): 35/244 fragment ions using 70 most intense peaks

#	b	b^{++}	b*	b^{*++}	b^0	b^{0++}	Seq.	y	y^{++}	y*	y^{*++}	y^0	y^{0++}	#
1	102.0550	51.5311			84.0444	42.5258	T							15
2	216.0979	108.5526	199.0713	100.0393	198.0873	99.5473	N	1457.6503	729.3288	1440.6237	720.8155	1439.6397	720.3235	14
3	273.1193	137.0633	256.0928	128.5500	255.1088	128.0580	G	**1343.6074**	672.3073	1326.5808	**663.7940**	1325.5968	**663.3020**	13
4	342.1408	171.5740	325.1143	163.0608	324.1302	162.5688	S	**1286.5859**	643.7966	1269.5593	635.2833	1268.5753	634.7913	12
5	**441.2092**	221.1082	**424.1827**	212.5950	**423.1987**	212.1030	V	**1217.5644**	609.2859	1200.5379	600.7726	**1199.5539**	600.2806	11

6	556.2362	278.6217	**539.2096**	270.1084	**538.2256**	269.6164	D	**1118.4960**	559.7516	1101.4695	551.2384	1100.4855	550.7464	10
7	**669.3202**	335.1637	652.2937	326.6505	651.3097	326.1585	L	**1003.4691**	502.2382	986.4425	493.7249	985.4585	493.2329	9
8	726.3417	363.6745	709.3151	355.1612	708.3311	354.6692	G	**890.3850**	445.6961	873.3585	437.1829	872.3745	436.6909	8
9	**855.3843**	428.1958	838.3577	419.6825	837.3737	419.1905	E	833.3636	417.1854	816.3370	408.6721	**815.3530**	408.1801	7
10	**984.4269**	492.7171	967.4003	484.2038	966.4163	483.7118	E	**704.3210**	352.6641	687.2944	344.1508	686.3104	343.6588	6
11	1113.4695	557.2384	1096.4429	548.7251	1095.4589	548.2331	E	**575.2784**	288.1428	558.2518	279.6295	557.2678	279.1375	5
12	**1242.5121**	621.7597	1225.4855	613.2464	1224.5015	612.7544	E	**446.2358**	223.6215	429.2092	215.1083	428.2252	214.6162	4
13	1313.5492	657.2782	1296.5226	648.7650	1295.5386	648.2729	A	**317.1932**	159.1002	300.1666	150.5870			3
14	**1384.5863**	692.7968	**1367.5597**	684.2835	1366.5757	683.7915	A	**246.1561**	123.5817	229.1295	115.0684			2
15							R	175.1190	88.0631	158.0924	79.5498			1

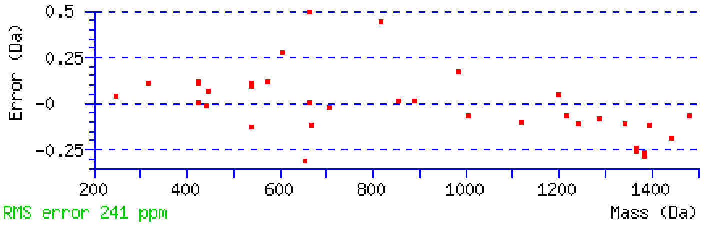

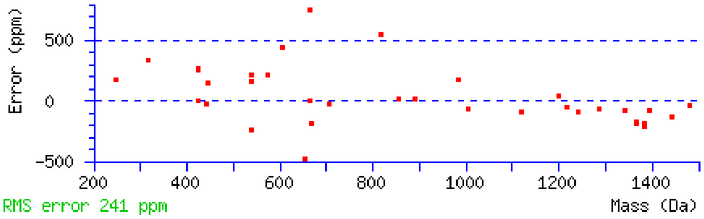

All matches to this query

Score	Mr(calc):	Delta	Sequence
64.4	1655.6676	0.0024	TNGSVDLGEEEEAAR
42.4	1655.6676	0.0024	TNGSVDLGEEEEAAR
0.8	1655.6788	-0.0088	GSAQDSLAPGTNTDSR
0.8	1655.6534	0.0165	SYQRFTAFYASR

Spectrum No: 30; Query: 685; Rank: 1

Peptide View

MS/MS Fragmentation of **ELGSLEEDANPDEEGVQK**
Found in **IPI00371736**, Tax_Id=10116 Gene_Symbol=LOC685174 similar to trinucleotide repeat containing 5

Match to Query 685: 2037.846968 from(1019.930760,2+)
Title: 091127RatKid_SCX01_11.1696.1696.2.dta
Data file K:\NewmanPaper\Piliang\3SubProteomes\Piliang3SP\mgf5ppm\SCX_3SubProteomes5ppm.mgf

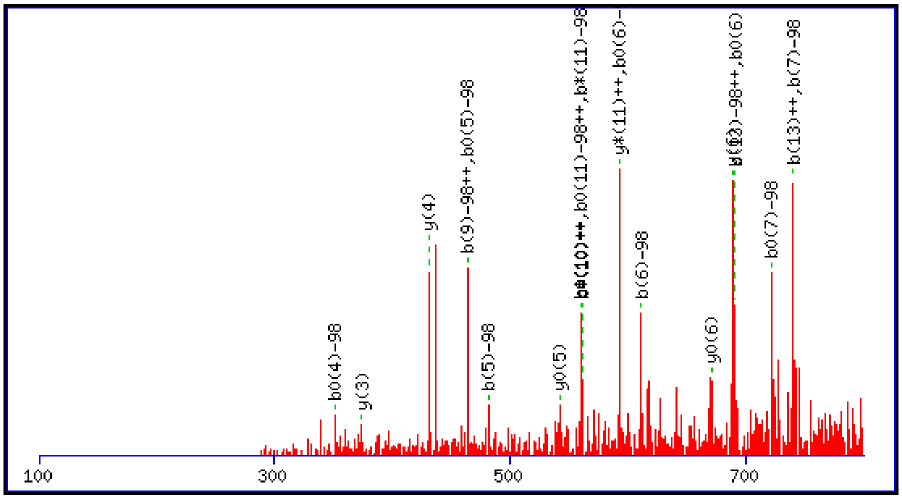

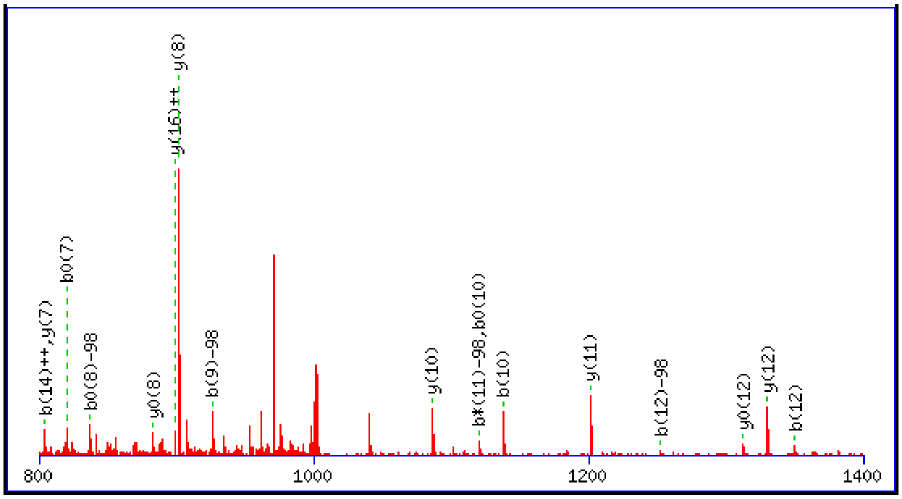

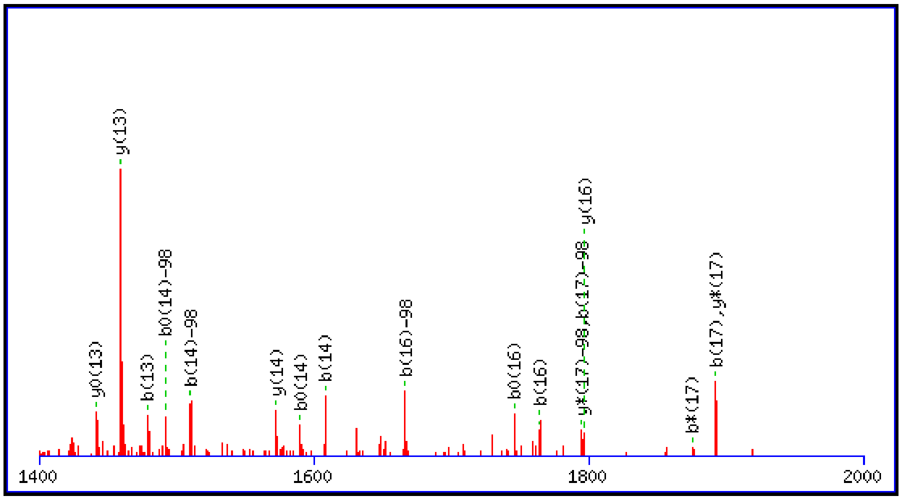

Monoisotopic mass of neutral peptide Mr(calc): 2037.8416
Fixed modifications: Carbamidomethyl (C)
Variable modifications:
S4 : Phospho (ST), with neutral losses 0.0000(shown in table), 97.9769
Ions Score: 63 **Expect:** 9.1e-005
Matches (Bold Red): 56/268 fragment ions using 69 most intense peaks

#	b	b^{++}	b*	b^{*++}	b^0	b^{0++}	Seq.	y	y^{++}	y*	y^{*++}	y^0	y^{0++}	#
1	130.0499	65.5286			112.0393	56.5233	E							18
2	243.1339	122.0706			225.1234	113.0653	L	1909.8063	955.4068	**1892.7797**	946.8935	1891.7957	946.4015	17
3	300.1554	150.5813			282.1448	141.5761	G	**1796.7222**	**898.8647**	1779.6957	890.3515	1778.7116	889.8595	16
4	467.1537	234.0805			449.1432	225.0752	S	1739.7007	870.3540	1722.6742	861.8407	1721.6902	861.3487	15
5	580.2378	290.6225			562.2272	281.6173	L	**1572.7024**	786.8548	1555.6758	778.3416	1554.6918	777.8495	14
6	709.2804	355.1438			**691.2698**	346.1386	E	**1459.6183**	730.3128	1442.5918	721.7995	**1441.6078**	721.3075	13
7	838.3230	419.6651			**820.3124**	410.6599	E	**1330.5757**	665.7915	1313.5492	657.2782	**1312.5652**	656.7862	12
8	953.3499	477.1786			935.3394	468.1733	D	**1201.5331**	601.2702	1184.5066	**592.7569**	1183.5226	592.2649	11
9	1024.3871	512.6972			1006.3765	503.6919	A	**1086.5062**	543.7567	1069.4796	535.2435	1068.4956	534.7515	10
10	**1138.4300**	569.7186	1121.4034	**561.2054**	**1120.4194**	**560.7133**	N	1015.4691	508.2382	998.4425	499.7249	997.4585	499.2329	9
11	1235.4827	618.2450	1218.4562	609.7317	1217.4722	609.2397	P	**901.4262**	451.2167	884.3996	442.7034	**883.4156**	442.2114	8
12	**1350.5097**	675.7585	1333.4831	667.2452	1332.4991	666.7532	D	**804.3734**	402.6903	787.3468	394.1771	786.3628	393.6851	7
13	**1479.5523**	**740.2798**	1462.5257	731.7665	1461.5417	731.2745	E	**689.3464**	345.1769	672.3199	336.6636	**671.3359**	336.1716	6
14	**1608.5949**	**804.8011**	1591.5683	796.2878	**1590.5843**	795.7958	E	**560.3039**	280.6556	543.2773	272.1423	**542.2933**	271.6503	5
15	1665.6163	833.3118	1648.5898	824.7985	1647.6058	824.3065	G	**431.2613**	216.1343	414.2347	207.6210			4
16	**1764.6848**	882.8460	1747.6582	874.3327	**1746.6742**	873.8407	V	**374.2398**	187.6235	357.2132	179.1103			3
17	**1892.7433**	946.8753	**1875.7168**	938.3620	1874.7328	937.8700	Q	275.1714	138.0893	258.1448	129.5761			2
18							K	147.1128	74.0600	130.0863	65.5468			1

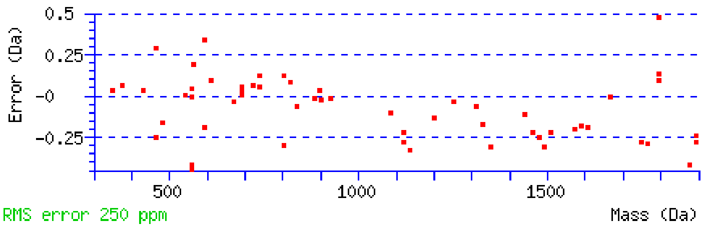

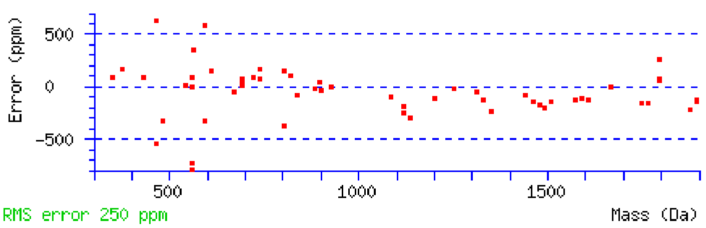

All matches to this query

Score	Mr(calc):	Delta	Sequence
63.5	2037.8416	0.0054	ELGSLEEDANPDEEGVQK
3.8	2037.8631	-0.0161	IVSSILKVPSSQGIYK
1.7	2037.8446	0.0024	GSKVSGLQYAGPDTENQK
1.3	2037.8631	-0.0161	IVSSILKVPSSQGIYK
0.2	2036.8394	1.0076	ASSQREGNQTAFXGLWK
0.1	2037.8631	-0.0161	IVSSILKVPSSQGIYK

Spectrum No: 31; Query: 692; Rank: 1

Peptide View

MS/MS Fragmentation of **VLGPSSSENQEGTLTDSMK**
Found in **IPI00199060**, Tax_Id=10116 Gene_Symbol=Tgoln2 Trans golgi network (TGN) specific integral membrane protein TGN38 precursor

Match to Query 692: 2058.890028 from(1030.452290,2+)
Title: 091127RatKid_SCX01_12.2030.2030.2.dta
Data file K:\NewmanPaper\Piliang\3SubProteomes\Piliang3SP\mgf5ppm\SCX_3SubProteomes5ppm.mgf

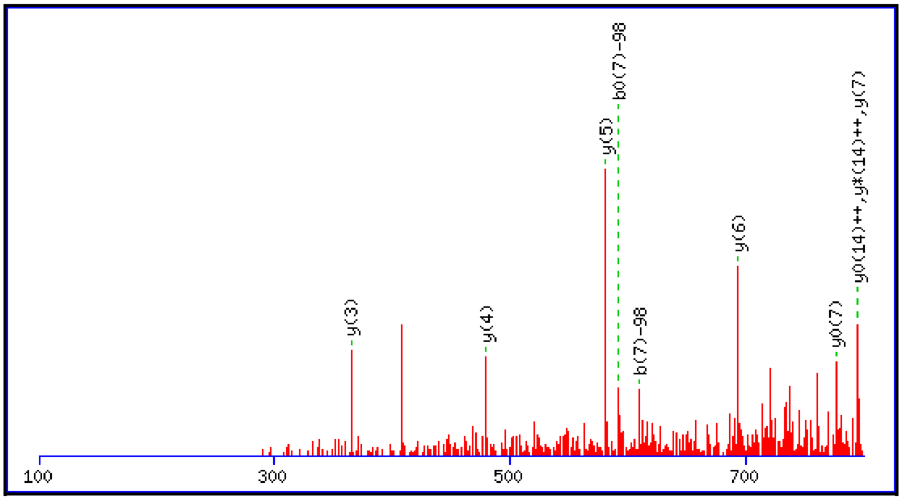

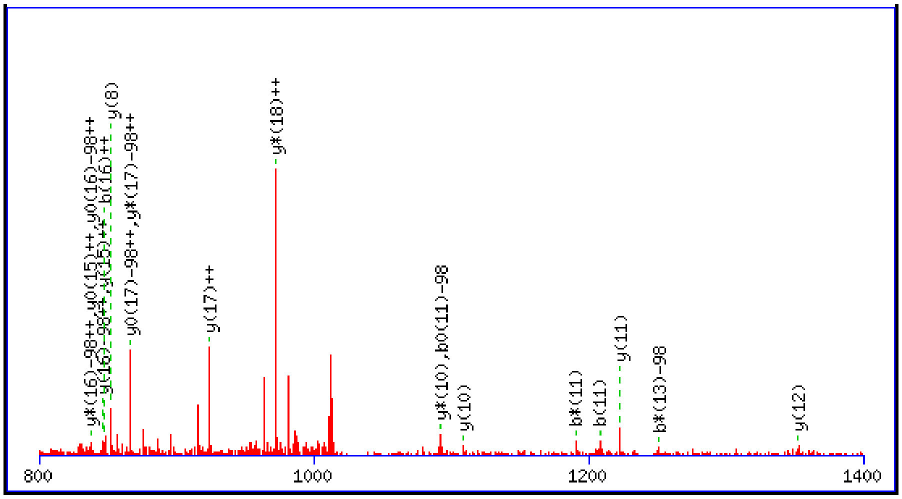

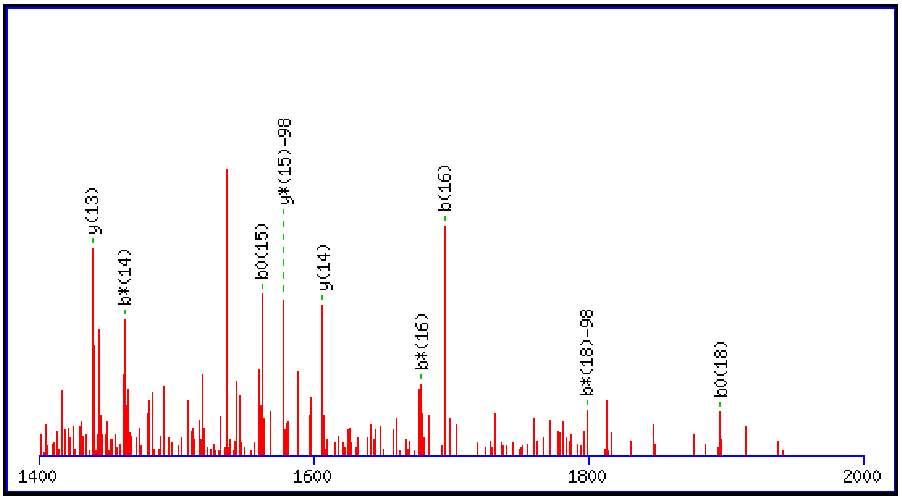

Monoisotopic mass of neutral peptide Mr(calc): 2058.8817
Fixed modifications: Carbamidomethyl (C)
Variable modifications:
S6 : Phospho (ST), with neutral losses 0.0000(shown in table), 97.9769
Ions Score: 63 **Expect:** 0.00011
Matches (Bold Red): 38/290 fragment ions using 46 most intense peaks

#	b	b^{++}	b*	b^{*++}	b^0	b^{0++}	Seq.	y	y^{++}	y*	y^{*++}	y^0	y^{0++}	#
1	100.0757	50.5415					V							19
2	213.1598	107.0835					L	1960.8205	980.9139	1943.7940	**972.4006**	1942.8100	971.9086	18
3	270.1812	135.5942					G	1847.7365	**924.3719**	1830.7099	915.8586	1829.7259	915.3666	17
4	367.2340	184.1206					P	1790.7150	895.8611	1773.6885	887.3479	1772.7044	886.8559	16
5	454.2660	227.6366			436.2554	218.6314	S	1693.6622	**847.3348**	1676.6357	838.8215	1675.6517	**838.3295**	15
6	621.2644	311.1358			603.2538	302.1305	S	**1606.6302**	803.8187	1589.6037	**795.3055**	1588.6197	**794.8135**	14
7	708.2964	354.6518			690.2858	345.6466	S	**1439.6319**	720.3196	1422.6053	711.8063	1421.6213	711.3143	13
8	837.3390	419.1731			819.3284	410.1678	E	**1352.5998**	676.8036	1335.5733	668.2903	1334.5893	667.7983	12
9	951.3819	476.1946	934.3554	467.6813	933.3713	467.1893	N	**1223.5572**	612.2823	1206.5307	603.7690	1205.5467	603.2770	11
10	1079.4405	540.2239	1062.4139	531.7106	1061.4299	531.2186	Q	**1109.5143**	555.2608	**1092.4878**	546.7475	1091.5038	546.2555	10
11	**1208.4831**	604.7452	**1191.4565**	596.2319	1190.4725	595.7399	E	981.4557	491.2315	964.4292	482.7182	963.4452	482.2262	9
12	1265.5045	633.2559	1248.4780	624.7426	1247.4940	624.2506	G	**852.4131**	426.7102	835.3866	418.1969	834.4026	417.7049	8
13	1366.5522	683.7798	1349.5257	675.2665	1348.5417	674.7745	T	**795.3917**	398.1995	778.3651	389.6862	**777.3811**	389.1942	7
14	1479.6363	740.3218	**1462.6097**	731.8085	1461.6257	731.3165	L	**694.3440**	347.6756	677.3175	339.1624	676.3334	338.6704	6
15	1580.6840	790.8456	1563.6574	782.3323	**1562.6734**	781.8403	T	**581.2599**	291.1336	564.2334	282.6203	563.2494	282.1283	5
16	**1695.7109**	**848.3591**	**1678.6844**	839.8458	1677.7003	839.3538	D	**480.2123**	240.6098	463.1857	232.0965	462.2017	231.6045	4
17	1782.7429	891.8751	1765.7164	883.3618	1764.7324	882.8698	S	**365.1853**	183.0963	348.1588	174.5830	347.1748	174.0910	3
18	1913.7834	957.3953	1896.7569	948.8821	**1895.7729**	948.3901	M	278.1533	139.5803	261.1267	131.0670			2

19							K	147.1128	74.0600	130.0863	65.5468			1

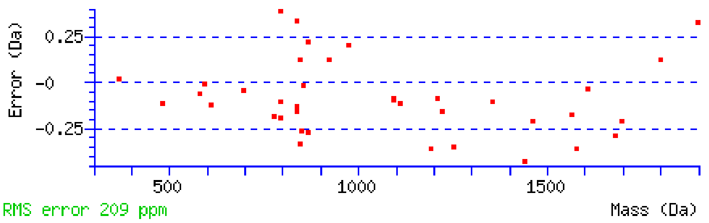

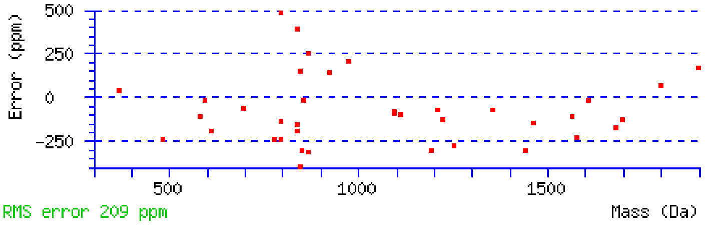

All matches to this query

Score	Mr(calc):	Delta	Sequence
63.1	2058.8817	0.0084	VLGPSSSENQEGTLTDSMK
51.3	2058.8817	0.0084	VLGPSSSENQEGTLTDSMK
51.3	2058.8817	0.0084	VLGPSSSENQEGTLTDSMK
12.2	2058.8817	0.0084	VLGPSSSENQEGTLTDSMK
7.3	2058.8817	0.0084	VLGPSSSENQEGTLTDSMK
0.2	2056.8795	2.0105	IDPANGNTIYAEKFKSK
0.0	2057.9070	0.9830	DNPEIWCHYLRLFSK

Spectrum No: 32; Query: 1151; Rank: 1

Peptide View

MS/MS Fragmentation of **EGQVEAAQPEQAAEAPAESSAQPNQLETGASSPER**
Found in **IPI00768543**, Tax_Id=10116 Gene_Symbol=Zbtb20_predicted zinc finger and BTB domain containing 20

Match to Query 1151: 3643.599042 from(1215.540290,3+)
Title: 091129RatKid_SCX02_46.1366.1366.3.dta
Data file K:\NewmanPaper\Piliang\3SubProteomes\Piliang3SP\mgf5ppm\SCX_3SubProteomes5ppm.mgf

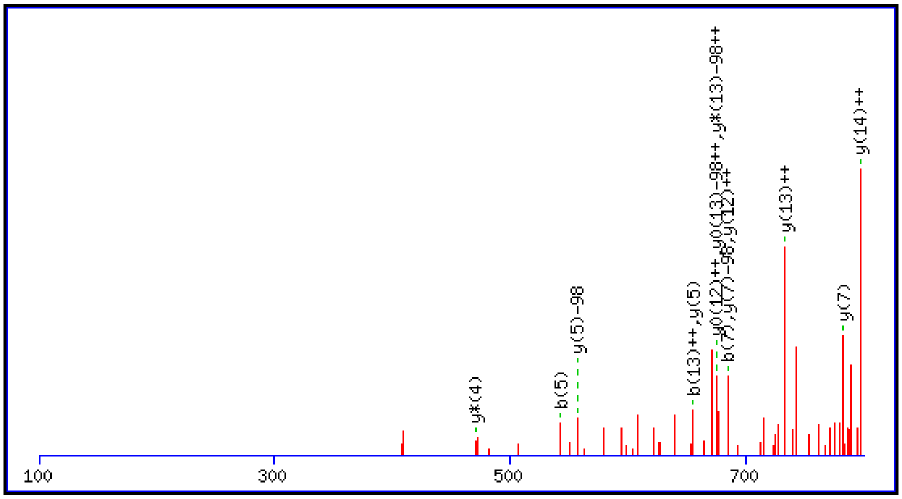

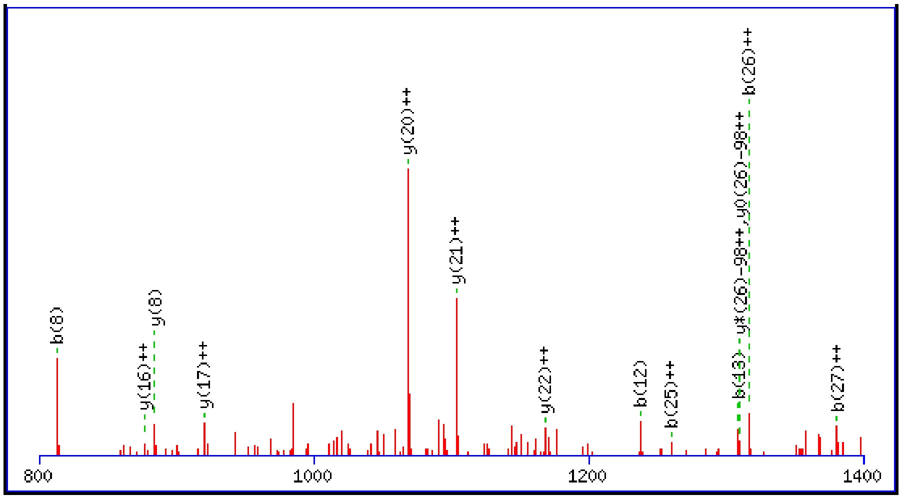

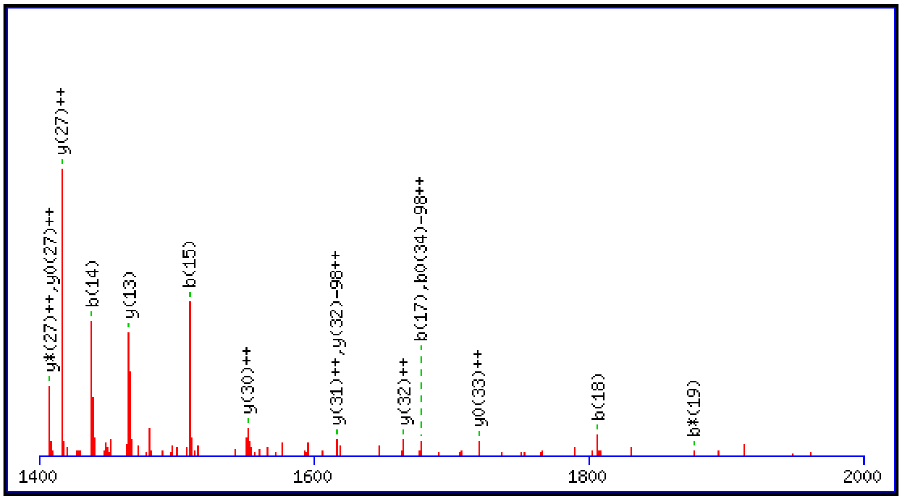

Monoisotopic mass of neutral peptide Mr(calc): 3643.5911
Fixed modifications: Carbamidomethyl (C)
Variable modifications:
S31 : Phospho (ST), with neutral losses 0.0000(shown in table), 97.9769
Ions Score: 62 **Expect:** 0.0003
Matches (Bold Red): 43/606 fragment ions using 52 most intense peaks

#	b	b^{++}	b*	b^{*++}	b^0	b^{0++}	Seq.	y	y^{++}	y*	y^{*++}	y^0	y^{0++}	#
1	130.0499	65.5286			112.0393	56.5233	E							35
2	187.0713	94.0393			169.0608	85.0340	G	3515.5558	1758.2815	3498.5293	1749.7683	3497.5453	1749.2763	34
3	315.1299	158.0686	298.1034	149.5553	297.1193	149.0633	Q	3458.5344	1729.7708	3441.5078	1721.2575	3440.5238	**1720.7655**	33
4	414.1983	207.6028	397.1718	199.0895	396.1878	198.5975	V	3330.4758	**1665.7415**	3313.4492	1657.2283	3312.4652	1656.7362	32
5	**543.2409**	272.1241	526.2144	263.6108	525.2304	263.1188	E	3231.4074	**1616.2073**	3214.3808	1607.6940	3213.3968	1607.2020	31
6	614.2780	307.6427	597.2515	299.1294	596.2675	298.6374	A	3102.3648	**1551.6860**	3085.3382	1543.1727	3084.3542	1542.6807	30
7	**685.3151**	343.1612	668.2886	334.6479	667.3046	334.1559	A	3031.3277	1516.1675	3014.3011	1507.6542	3013.3171	1507.1622	29
8	**813.3737**	407.1905	796.3472	398.6772	795.3632	398.1852	Q	2960.2905	1480.6489	2943.2640	1472.1356	2942.2800	1471.6436	28
9	910.4265	455.7169	893.3999	447.2036	892.4159	446.7116	P	2832.2320	**1416.6196**	2815.2054	**1408.1063**	2814.2214	**1407.6143**	27
10	1039.4691	520.2382	1022.4425	511.7249	1021.4585	511.2329	E	2735.1792	1368.0932	2718.1526	1359.5800	2717.1686	1359.0880	26
11	1167.5277	584.2675	1150.5011	575.7542	1149.5171	575.2622	Q	2606.1366	1303.5719	2589.1101	1295.0587	2588.1260	1294.5667	25
12	**1238.5648**	619.7860	1221.5382	611.2728	1220.5542	610.7807	A	2478.0780	1239.5427	2461.0515	1231.0294	2460.0675	1230.5374	24
13	**1309.6019**	**655.3046**	1292.5753	646.7913	1291.5913	646.2993	A	2407.0409	1204.0241	2390.0144	1195.5108	2389.0303	1195.0188	23
14	**1438.6445**	719.8259	1421.6179	711.3126	1420.6339	710.8206	E	2336.0038	**1168.5055**	2318.9772	1159.9923	2317.9932	1159.5003	22
15	**1509.6816**	755.3444	1492.6550	746.8312	1491.6710	746.3392	A	2206.9612	**1103.9842**	2189.9347	1095.4710	2188.9506	1094.9790	21
16	1606.7344	803.8708	1589.7078	795.3575	1588.7238	794.8655	P	2135.9241	**1068.4657**	2118.8975	1059.9524	2117.9135	1059.4604	20
17	**1677.7715**	839.3894	1660.7449	830.8761	1659.7609	830.3841	A	2038.8713	1019.9393	2021.8448	1011.4260	2020.8608	1010.9340	19
18	**1806.8141**	903.9107	1789.7875	895.3974	1788.8035	894.9054	E	1967.8342	984.4207	1950.8077	975.9075	1949.8236	975.4155	18

19	1893.8461	947.4267	**1876.8195**	938.9134	1875.8355	938.4214	S	1838.7916	**919.8994**	1821.7651	911.3862	1820.7811	910.8942	17
20	1980.8781	990.9427	1963.8516	982.4294	1962.8676	981.9374	S	1751.7596	**876.3834**	1734.7330	867.8702	1733.7490	867.3782	16
21	2051.9152	1026.4613	2034.8887	1017.9480	2033.9047	1017.4560	A	1664.7276	832.8674	1647.7010	824.3541	1646.7170	823.8621	15
22	2179.9738	1090.4905	2162.9473	1081.9773	2161.9632	1081.4853	Q	1593.6905	**797.3489**	1576.6639	788.8356	1575.6799	788.3436	14
23	2277.0266	1139.0169	2260.0000	1130.5037	2259.0160	1130.0116	P	**1465.6319**	**733.3196**	1448.6053	724.8063	1447.6213	724.3143	13
24	2391.0695	1196.0384	2374.0430	1187.5251	2373.0589	1187.0331	N	1368.5791	**684.7932**	1351.5526	676.2799	1350.5685	**675.7879**	12
25	2519.1281	**1260.0677**	2502.1015	1251.5544	2501.1175	1251.0624	Q	1254.5362	627.7717	1237.5096	619.2585	1236.5256	618.7664	11
26	2632.2121	**1316.6097**	2615.1856	1308.0964	2614.2016	1307.6044	L	1126.4776	563.7424	1109.4511	555.2292	1108.4670	554.7372	10
27	2761.2547	**1381.1310**	2744.2282	1372.6177	2743.2442	1372.1257	E	1013.3935	507.2004	996.3670	498.6871	995.3830	498.1951	9
28	2862.3024	1431.6548	2845.2759	1423.1416	2844.2919	1422.6496	T	**884.3509**	442.6791	867.3244	434.1658	866.3404	433.6738	8
29	2919.3239	1460.1656	2902.2973	1451.6523	2901.3133	1451.1603	G	**783.3033**	392.1553	766.2767	383.6420	765.2927	383.1500	7
30	2990.3610	1495.6841	2973.3344	1487.1709	2972.3504	1486.6789	A	726.2818	363.6445	709.2553	355.1313	708.2712	354.6393	6
31	3157.3593	1579.1833	3140.3328	1570.6700	3139.3488	1570.1780	S	**655.2447**	328.1260	638.2181	319.6127	637.2341	319.1207	5
32	3244.3914	1622.6993	3227.3648	1614.1861	3226.3808	1613.6940	S	488.2463	244.6268	**471.2198**	236.1135	470.2358	235.6215	4
33	3341.4441	1671.2257	3324.4176	1662.7124	3323.4336	1662.2204	P	401.2143	201.1108	384.1878	192.5975	383.2037	192.1055	3
34	3470.4867	1735.7470	3453.4602	1727.2337	3452.4762	1726.7417	E	304.1615	152.5844	287.1350	144.0711	286.1510	143.5791	2
35							R	175.1190	88.0631	158.0924	79.5498			1

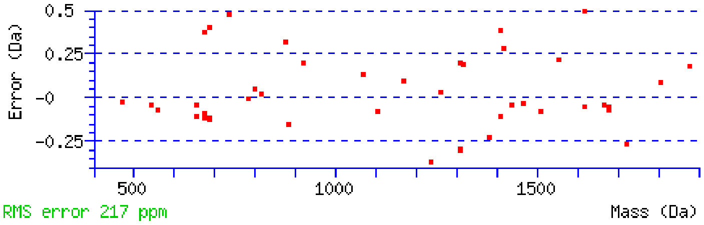

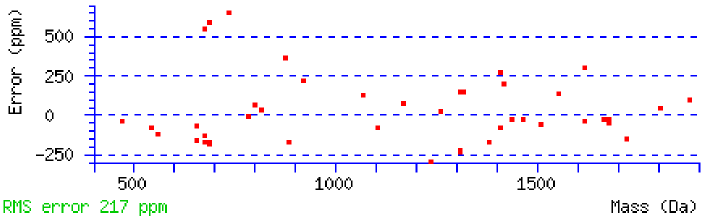

All matches to this query

Score	Mr(calc):	Delta	Sequence
62.2	3643.5911	0.0080	EGQVEAAQPEQAAEAPAESSAQPNQLETGASSPER
60.5	3643.5911	0.0080	EGQVEAAQPEQAAEAPAESSAQPNQLETGASSPER
50.8	3643.5911	0.0080	EGQVEAAQPEQAAEAPAESSAQPNQLETGASSPER
30.1	3643.5911	0.0080	EGQVEAAQPEQAAEAPAESSAQPNQLETGASSPER
24.0	3643.5911	0.0080	EGQVEAAQPEQAAEAPAESSAQPNQLETGASSPER
4.5	3641.5809	2.0181	LKDFKETVSNMIHSRPSLASQTNAASPCVGR
4.2	3643.6247	-0.0257	KGYGLCHGAAGNAYAFLALYNLTQDAKYLYR
4.0	3641.5614	2.0377	RGQPNGTGWNRSPVGEDSNLPGSQADYSLFFK
3.9	3643.6038	-0.0047	EIAEAYLGHPVTNAVITVPAYFNDSQRQATK
3.5	3642.5990	1.0000	YSPLHNVKLPEADDIQYPSMLLLTADHDDR

Spectrum No: 33; Query: 978; Rank: 1

Peptide View

MS/MS Fragmentation of **TENQPAVLEDAPDNTETGSVCTKV**
Found in **IPI00203446**, Tax_Id=10116 Gene_Symbol=Slc23a1 Solute carrier family 23 member 1

Match to Query 978: 2654.147548 from(1328.081050,2+)
Title: 091129RatKid_SCX02_11.1670.1670.2.dta
Data file K:\NewmanPaper\Piliang\3SubProteomes\Piliang3SP\mgf5ppm\SCX_3SubProteomes5ppm.mgf

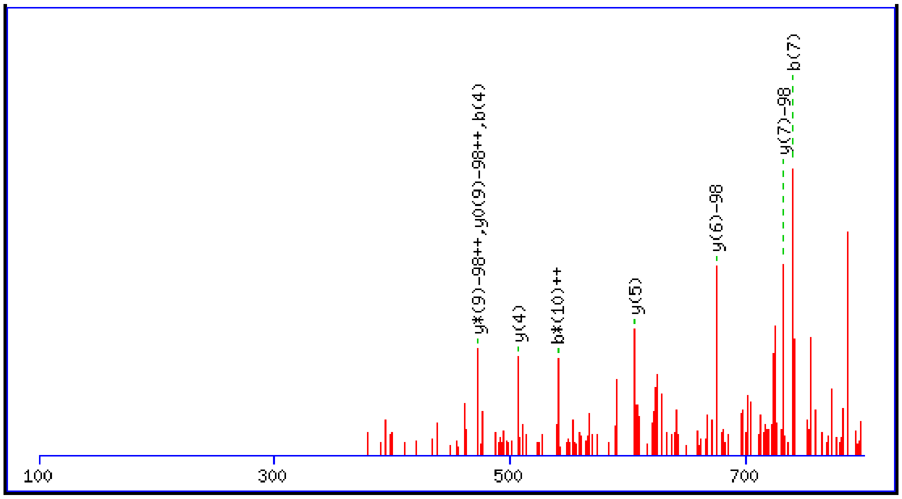

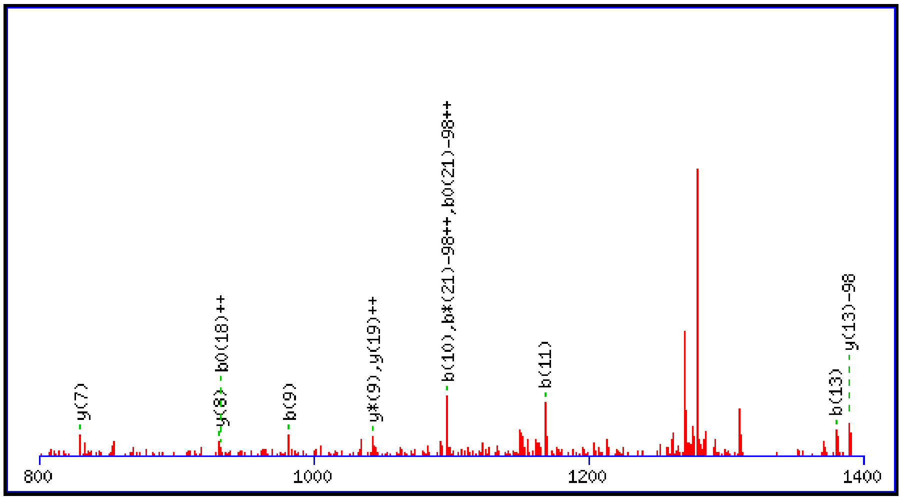

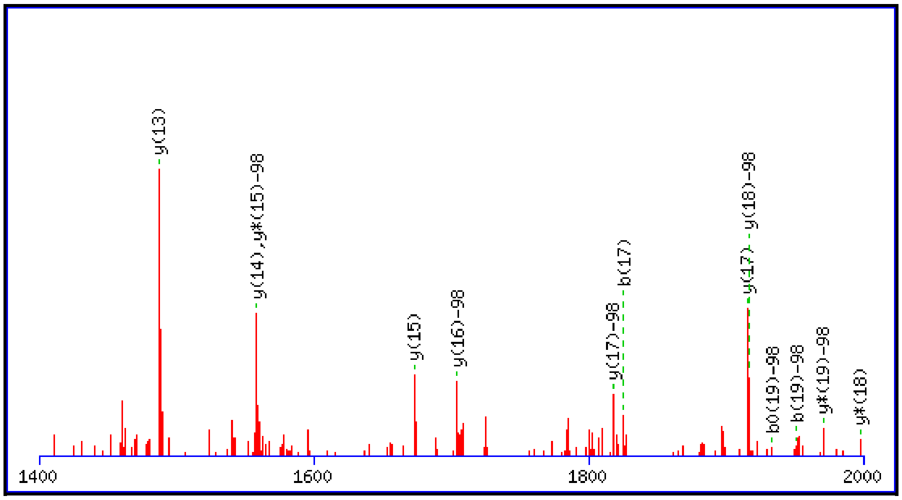

Monoisotopic mass of neutral peptide Mr(calc): 2654.1419
Fixed modifications: Carbamidomethyl (C)
Variable modifications:
S19 : Phospho (ST), with neutral losses 97.9769(shown in table), 0.0000
Ions Score: 60 **Expect:** 0.00032
Matches (Bold Red): 34/404 fragment ions using 45 most intense peaks

#	b	b^{++}	b*	b^{*++}	b^0	b^{0++}	Seq.	y	y^{++}	y*	y^{*++}	y^0	y^{0++}	#
1	102.0550	51.5311			84.0444	42.5258	T							24
2	231.0975	116.0524			213.0870	107.0471	E	2456.1246	1228.5659	2439.0980	1220.0526	2438.1140	1219.5606	23
3	345.1405	173.0739	328.1139	164.5606	327.1299	164.0686	N	2327.0820	1164.0446	2310.0554	1155.5314	2309.0714	1155.0393	22
4	**473.1991**	237.1032	456.1725	228.5899	455.1885	228.0979	Q	2213.0390	1107.0232	2196.0125	1098.5099	2195.0285	1098.0179	21
5	570.2518	285.6295	553.2253	277.1163	552.2413	276.6243	P	2084.9805	1042.9939	2067.9539	1034.4806	2066.9699	1033.9886	20

**6**	641.2889	321.1481	624.2624	312.6348	623.2784	312.1428	**A**	1987.9277	994.4675	**1970.9012**	985.9542	1969.9171	985.4622	**19**
**7**	**740.3573**	370.6823	723.3308	362.1690	722.3468	361.6770	**V**	**1916.8906**	958.9489	1899.8640	950.4357	1898.8800	949.9437	**18**
**8**	853.4414	427.2243	836.4149	418.7111	835.4308	418.2191	**L**	**1817.8222**	909.4147	1800.7956	900.9015	1799.8116	900.4094	**17**
**9**	**982.4840**	491.7456	965.4575	483.2324	964.4734	482.7404	**E**	**1704.7381**	852.8727	1687.7116	844.3594	1686.7275	843.8674	**16**
**10**	**1097.5109**	549.2591	1080.4844	**540.7458**	1079.5004	540.2538	**D**	1575.6955	788.3514	**1558.6690**	779.8381	1557.6850	779.3461	**15**
**11**	**1168.5481**	584.7777	1151.5215	576.2644	1150.5375	575.7724	**A**	1460.6686	730.8379	1443.6420	722.3247	1442.6580	721.8326	**14**
**12**	1265.6008	633.3040	1248.5743	624.7908	1247.5903	624.2988	**P**	**1389.6315**	695.3194	1372.6049	686.8061	1371.6209	686.3141	**13**
**13**	**1380.6278**	690.8175	1363.6012	682.3042	1362.6172	681.8122	**D**	1292.5787	646.7930	1275.5522	638.2797	1274.5681	637.7877	**12**
**14**	1494.6707	747.8390	1477.6441	739.3257	1476.6601	738.8337	**N**	1177.5518	589.2795	1160.5252	580.7662	1159.5412	580.2742	**11**
**15**	1595.7184	798.3628	1578.6918	789.8495	1577.7078	789.3575	**T**	1063.5088	532.2581	1046.4823	523.7448	1045.4983	523.2528	**10**
**16**	1724.7610	862.8841	1707.7344	854.3708	1706.7504	853.8788	**E**	962.4612	481.7342	945.4346	**473.2209**	944.4506	**472.7289**	**9**
**17**	**1825.8086**	913.4080	1808.7821	904.8947	1807.7981	904.4027	**T**	833.4186	417.2129	816.3920	408.6996	815.4080	408.2076	**8**
**18**	1882.8301	941.9187	1865.8036	933.4054	1864.8195	**932.9134**	**G**	**732.3709**	366.6891	715.3443	358.1758	714.3603	357.6838	**7**
**19**	**1951.8516**	976.4294	1934.8250	967.9161	**1933.8410**	967.4241	**S**	**675.3494**	338.1783	658.3229	329.6651	657.3389	329.1731	**6**
**20**	2050.9200	1025.9636	2033.8934	1017.4504	2032.9094	1016.9583	**V**	**606.3280**	303.6676	589.3014	295.1543	588.3174	294.6623	**5**
**21**	2210.9506	1105.9790	2193.9241	**1097.4657**	2192.9401	**1096.9737**	**C**	**507.2595**	254.1334	490.2330	245.6201	489.2490	245.1281	**4**
**22**	2311.9983	1156.5028	2294.9718	1147.9895	2293.9877	1147.4975	**T**	347.2289	174.1181	330.2023	165.6048	329.2183	165.1128	**3**
**23**	2440.0933	1220.5503	2423.0667	1212.0370	2422.0827	1211.5450	**K**	246.1812	123.5942	229.1547	115.0810			**2**
**24**							**V**	118.0863	59.5468					**1**

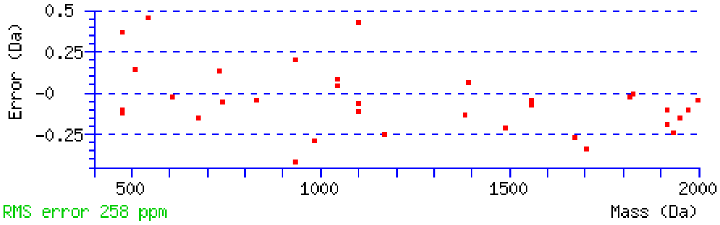

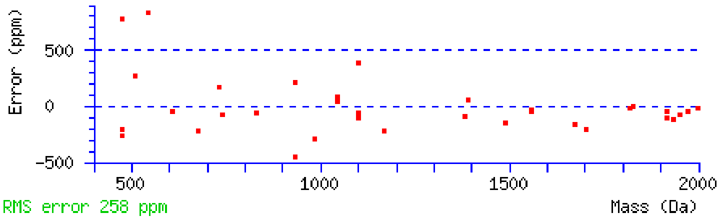

All matches to this query

Score	Mr(calc):	Delta	Sequence
60.4	2654.1419	0.0057	TENQPAVLEDAPDNTETGSVCTKV
43.4	2654.1419	0.0057	TENQPAVLEDAPDNTETGSVCTKV
38.8	2654.1419	0.0057	TENQPAVLEDAPDNTETGSVCTKV
37.2	2654.1419	0.0057	TENQPAVLEDAPDNTETGSVCTKV
4.5	2653.1454	1.0022	AWYREVPNTVHLMQLDITVK
3.8	2654.1271	0.0205	GVAASSTQKTPSRLENHYMICK
3.5	2652.1451	2.0025	THAAYNLLFFASGGGKFNYQGTK
3.5	2653.1291	1.0184	THAAYNLLFFASGGGKFNYQGTK
3.5	2654.1419	0.0057	TENQPAVLEDAPDNTETGSVCTKV
1.6	2654.1626	-0.0150	EVNPPNEGKNPTEASSKSQQKPK

Spectrum No: 34; Query: 766; Rank: 1

Peptide View

MS/MS Fragmentation of **ATSNVFAMFDQSQIQEFK**
Found in **IPI00421625**, Tax_Id=10116 Gene_Symbol=Mrlcb;RGD1565978_predicted Myosin regulatory light chain 2-B, smooth muscle isoform

Match to Query 766: 2169.948228 from(1085.981390,2+)
Title: 091129RatKid_SCX02_11.4364.4364.2.dta
Data file K:\NewmanPaper\Piliang\3SubProteomes\Piliang3SP\mgf5ppm\SCX_3SubProteomes5ppm.mgf

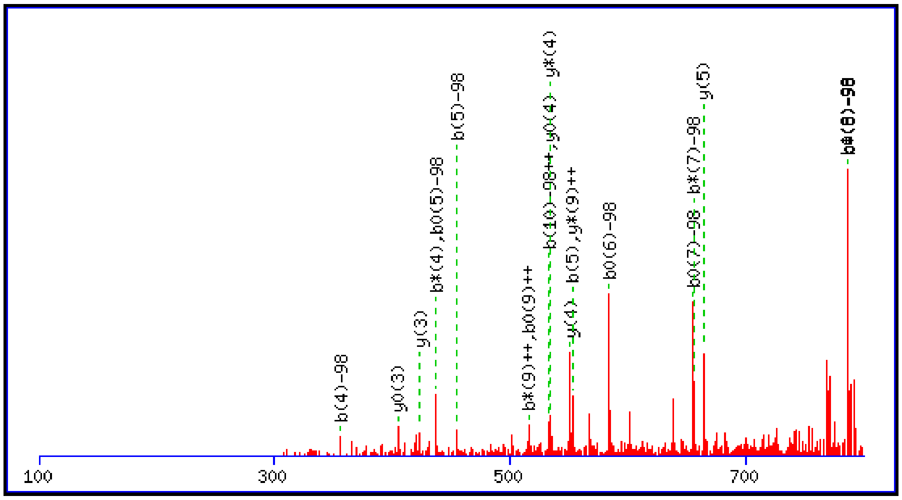

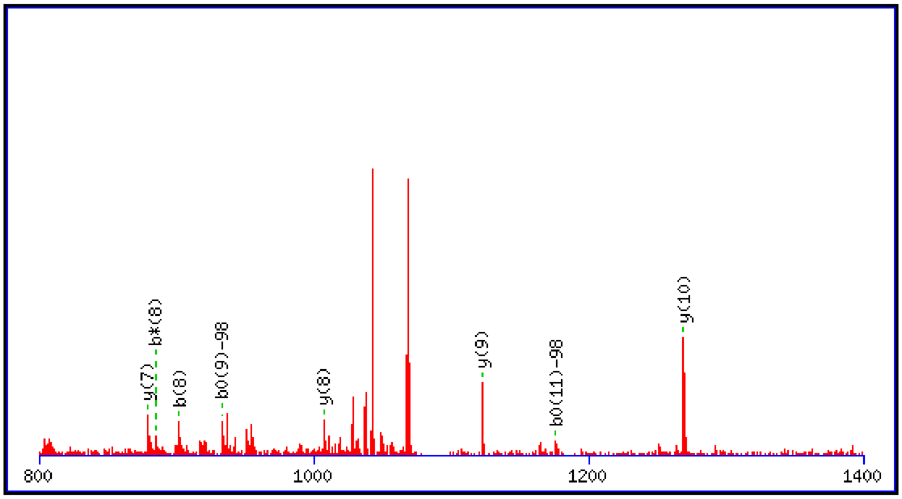

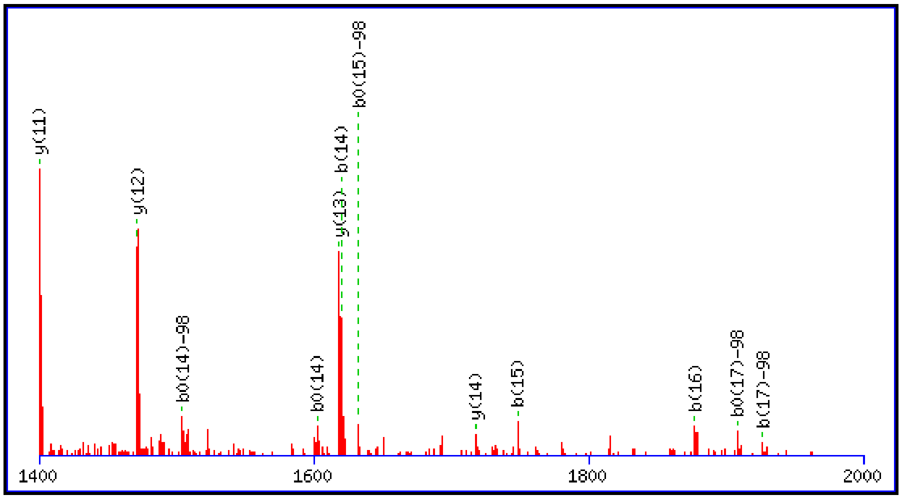

Monoisotopic mass of neutral peptide Mr(calc): 2169.9442
Fixed modifications: Carbamidomethyl (C)
Variable modifications:
S3 : Phospho (ST), with neutral losses 97.9769(shown in table), 0.0000
Ions Score: 60 **Expect:** 0.00024
Matches (Bold Red): 40/292 fragment ions using 61 most intense peaks

#	b	b^{++}	b*	b^{*++}	b^0	b^{0++}	Seq.	y	y^{++}	y*	y^{*++}	y^0	y^{0++}	#
1	72.0444	36.5258					A							18
2	173.0921	87.0497			155.0815	78.0444	T	2001.9375	1001.4724	1984.9109	992.9591	1983.9269	992.4671	17
3	242.1135	121.5604			224.1030	112.5551	S	1900.8898	950.9485	1883.8633	942.4353	1882.8792	941.9433	16
4	**356.1565**	178.5819	339.1299	170.0686	338.1459	169.5766	N	1831.8683	916.4378	1814.8418	907.9245	1813.8578	907.4325	15
5	**455.2249**	228.1161	438.1983	219.6028	**437.2143**	219.1108	V	**1717.8254**	859.4163	1700.7989	850.9031	1699.8149	850.4111	14

**6**	602.2933	301.6503	585.2667	293.1370	**584.2827**	292.6450	**F**	**1618.7570**	809.8821	1601.7305	801.3689	1600.7464	800.8769	**13**
**7**	673.3304	337.1688	**656.3038**	328.6556	**655.3198**	328.1636	**A**	**1471.6886**	736.3479	1454.6620	727.8347	1453.6780	727.3427	**12**
**8**	804.3709	402.6891	**787.3443**	394.1758	**786.3603**	393.6838	**M**	**1400.6515**	700.8294	1383.6249	692.3161	1382.6409	691.8241	**11**
**9**	951.4393	476.2233	934.4127	467.7100	**933.4287**	467.2180	**F**	**1269.6110**	635.3091	1252.5844	626.7959	1251.6004	626.3039	**10**
**10**	1066.4662	**533.7368**	1049.4397	525.2235	1048.4557	524.7315	**D**	**1122.5426**	561.7749	1105.5160	**553.2617**	1104.5320	552.7696	**9**
**11**	1194.5248	597.7660	1177.4983	589.2528	**1176.5142**	588.7608	**Q**	**1007.5156**	504.2615	990.4891	495.7482	989.5051	495.2562	**8**
**12**	1281.5568	641.2821	1264.5303	632.7688	1263.5463	632.2768	**S**	**879.4571**	440.2322	862.4305	431.7189	861.4465	431.2269	**7**
**13**	1409.6154	705.3113	1392.5889	696.7981	1391.6049	696.3061	**Q**	792.4250	396.7162	775.3985	388.2029	774.4145	387.7109	**6**
**14**	1522.6995	761.8534	1505.6729	753.3401	**1504.6889**	752.8481	**I**	**664.3665**	332.6869	647.3399	324.1736	646.3559	323.6816	**5**
**15**	1650.7581	825.8827	1633.7315	817.3694	**1632.7475**	816.8774	**Q**	**551.2824**	276.1448	**534.2558**	267.6316	**533.2718**	267.1396	**4**
**16**	1779.8007	890.4040	1762.7741	881.8907	1761.7901	881.3987	**E**	**423.2238**	212.1155	406.1973	203.6023	**405.2132**	203.1103	**3**
**17**	**1926.8691**	963.9382	1909.8425	955.4249	**1908.8585**	954.9329	**F**	294.1812	147.5942	277.1547	139.0810			**2**
**18**							**K**	147.1128	74.0600	130.0863	65.5468			**1**

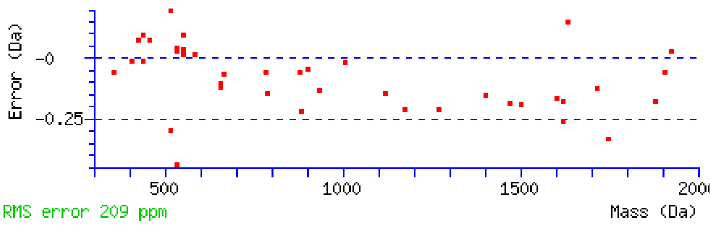

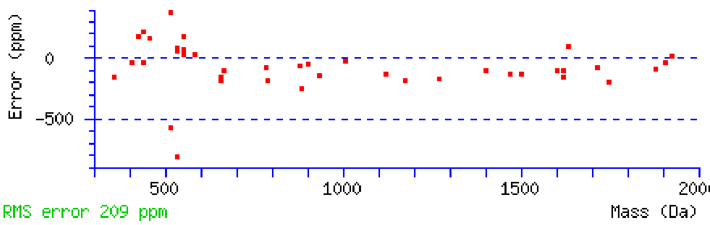

All matches to this query

Score	Mr(calc):	Delta	Sequence
60.2	2169.9442	0.0040	ATSNVFAMFDQSQIQEFK
60.2	2169.9442	0.0040	ATSNVFAMFDQSQIQEFK
5.8	2169.9613	-0.0131	QTVPASDQEMNSVLAELSR
1.5	2168.9612	0.9870	TLQKMMASGLTERVVSDK
0.9	2169.9497	-0.0014	WGSRPSSSKSDTALEKFK
0.7	2169.9497	-0.0014	WGSRPSSSKSDTALEKFK

Spectrum No: 35; Query: 454; Rank: 1

Peptide View

MS/MS Fragmentation of **KYVISDEEEEEDD**
Found in **IPI00471640**, Tax_Id=10116 Gene_Symbol=Leo1 RNA polymerase-associated protein LEO1

Match to Query 454: 1678.616968 from(840.315760,2+)
Title: 091127RatKid_SCX01_14.1699.1699.2.dta
Data file K:\NewmanPaper\Piliang\3SubProteomes\Piliang3SP\mgf5ppm\SCX_3SubProteomes5ppm.mgf

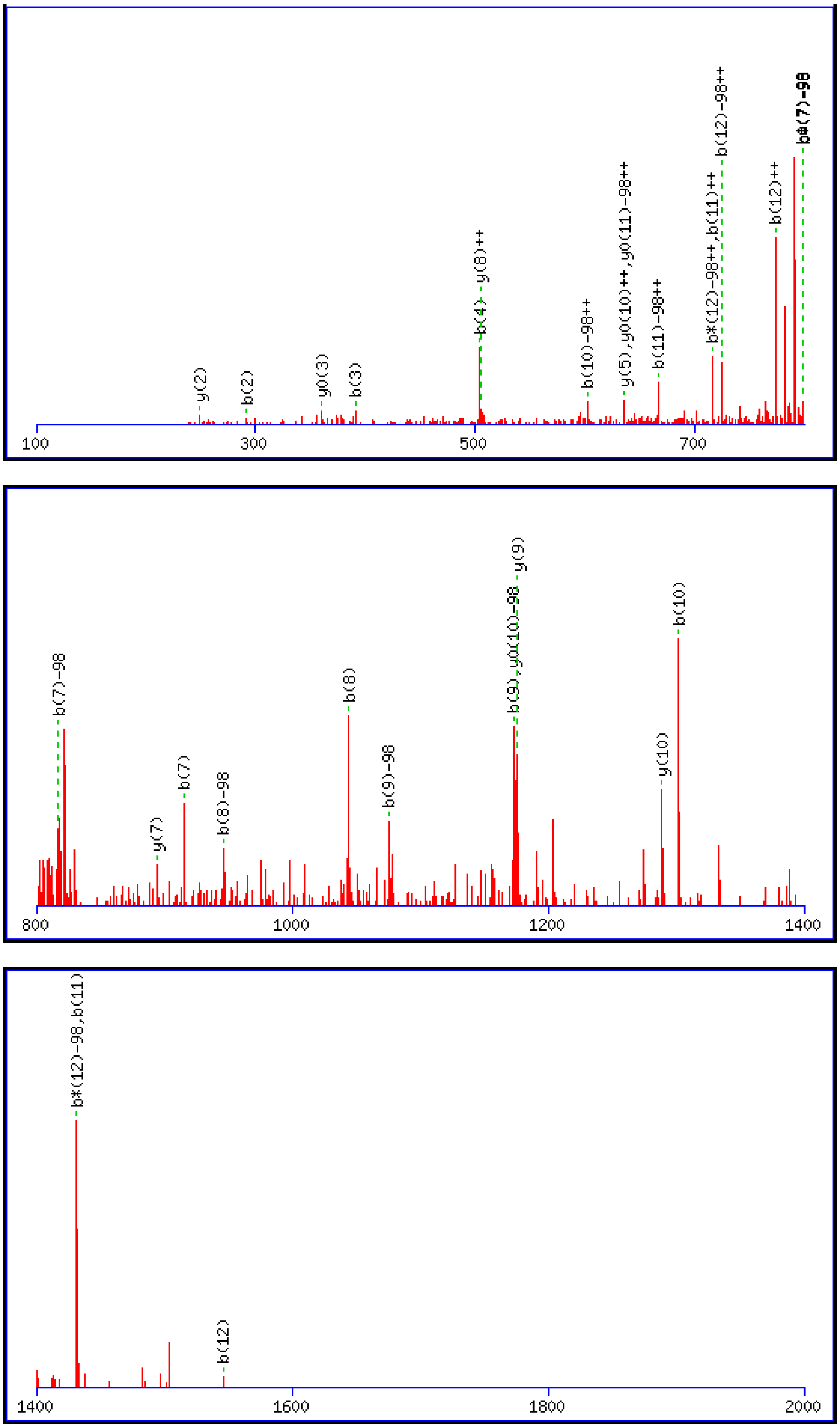

Monoisotopic mass of neutral peptide Mr(calc): 1678.6134
Fixed modifications: Carbamidomethyl (C)
Variable modifications:
S5 : Phospho (ST), with neutral losses 0.0000(shown in table), 97.9769
Ions Score: 60 **Expect:** 9.4e-005
Matches (Bold Red): 31/176 fragment ions using 49 most intense peaks

#	b	b^{++}	b*	b^{*++}	b^0	b^{0++}	Seq.	y	y^{++}	y^0	y^{0++}	#
1	129.1022	65.0548	112.0757	56.5415			K					13
2	**292.1656**	146.5864	275.1390	138.0731			Y	1551.5258	776.2665	1533.5152	767.2613	12
3	**391.2340**	196.1206	374.2074	187.6074			V	1388.4625	694.7349	1370.4519	685.7296	11
4	**504.3180**	252.6627	487.2915	244.1494			I	**1289.3941**	645.2007	1271.3835	**636.1954**	10
5	671.3164	336.1618	654.2898	327.6486	653.3058	327.1566	S	**1176.3100**	588.6586	1158.2994	579.6533	9

6	786.3433	393.6753	769.3168	385.1620	768.3328	384.6700	D	1009.3116	**505.1595**	991.3011	496.1542	8
7	**915.3859**	458.1966	898.3594	449.6833	897.3754	449.1913	E	**894.2847**	447.6460	876.2741	438.6407	7
8	**1044.4285**	522.7179	1027.4020	514.2046	1026.4180	513.7126	E	765.2421	383.1247	747.2315	374.1194	6
9	**1173.4711**	587.2392	1156.4446	578.7259	1155.4606	578.2339	E	**636.1995**	318.6034	618.1889	309.5981	5
10	**1302.5137**	651.7605	1285.4872	643.2472	1284.5031	642.7552	E	507.1569	254.0821	489.1463	245.0768	4
11	**1431.5563**	**716.2818**	1414.5298	707.7685	1413.5457	707.2765	E	378.1143	189.5608	**360.1038**	180.5555	3
12	**1546.5832**	**773.7953**	1529.5567	765.2820	1528.5727	764.7900	D	**249.0717**	125.0395	231.0612	116.0342	2
13							D	134.0448	67.5260	116.0342	58.5207	1

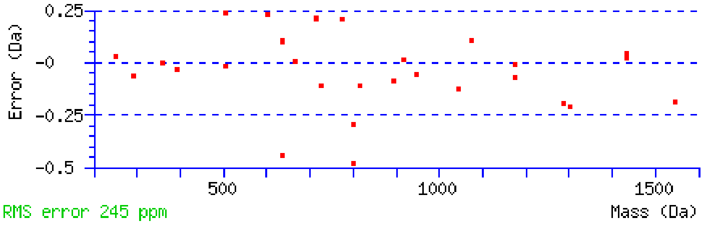

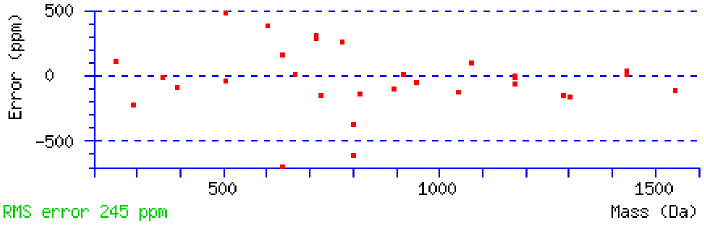

All matches to this query

Score	Mr(calc):	Delta	Sequence
59.5	1678.6134	0.0035	KYVISDEEEEEDD
33.1	1678.6134	0.0035	KYVISDEEEEEDD

Spectrum No: 36; Query: 1099; Rank: 1

Peptide View

MS/MS Fragmentation of **VDIITEEMPENALPSDEDDKDPNDPYR**
Found in **IPI00198486**, Tax_Id=10116 Gene_Symbol=Ap3d1 similar to adaptor-related protein complex 3, delta 1 subunit

Match to Query 1099: 3197.357202 from(1066.793010,3+)
Title: 091127RatKid_SCX01_17.3295.3295.3.dta
Data file K:\NewmanPaper\Piliang\3SubProteomes\Piliang3SP\mgf5ppm\SCX_3SubProteomes5ppm.mgf

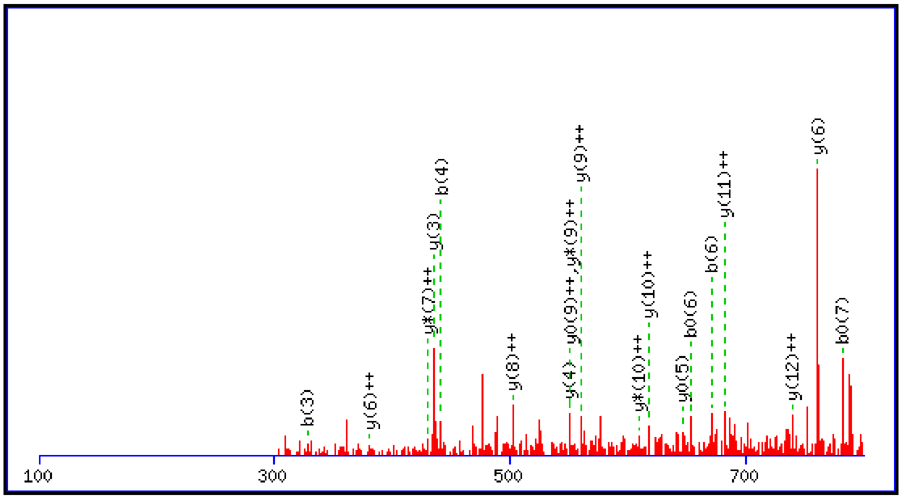

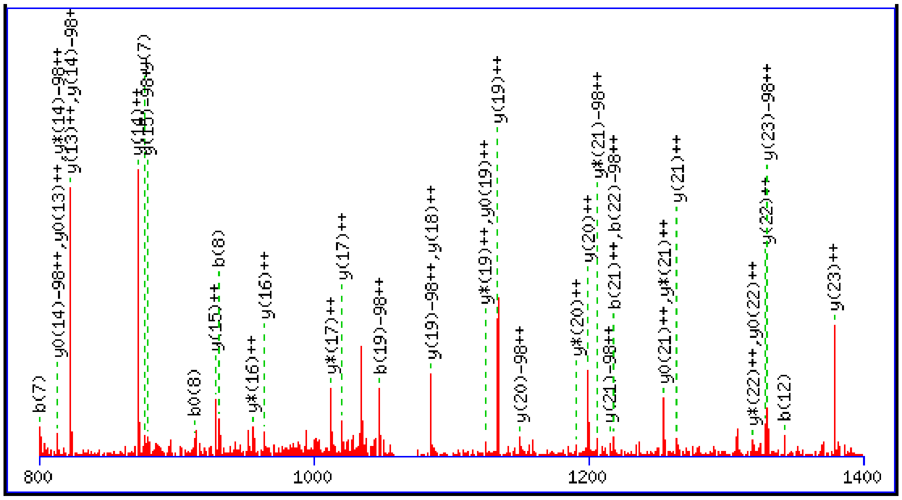

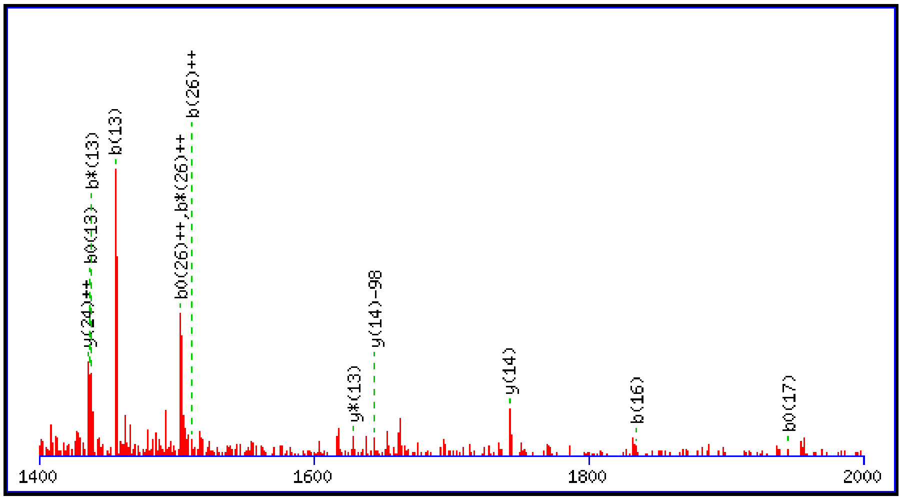

Monoisotopic mass of neutral peptide Mr(calc): 3196.3431
Fixed modifications: Carbamidomethyl (C)
Variable modifications:
S15 : Phospho (ST), with neutral losses 0.0000(shown in table), 97.9769
Ions Score: 59 **Expect:** 0.00056
Matches (Bold Red): 69/440 fragment ions using 158 most intense peaks

#	b	b^{++}	b*	b^{*++}	b^0	b^{0++}	Seq.	y	y^{++}	y*	y^{*++}	y^0	y^{0++}	#
1	100.0757	50.5415					V							27
2	215.1026	108.0550			197.0921	99.0497	D	3098.2820	1549.6446	3081.2555	1541.1314	3080.2714	1540.6394	26
3	**328.1867**	164.5970			310.1761	155.5917	I	2983.2551	1492.1312	2966.2285	1483.6179	2965.2445	1483.1259	25
4	**441.2708**	221.1390			423.2602	212.1337	I	2870.1710	**1435.5891**	2853.1444	1427.0759	2852.1604	1426.5839	24
5	542.3184	271.6629			524.3079	262.6576	T	2757.0869	**1379.0471**	2740.0604	1370.5338	2739.0764	1370.0418	23
6	**671.3610**	336.1842			**653.3505**	327.1789	E	2656.0393	**1328.5233**	2639.0127	**1320.0100**	2638.0287	**1319.5180**	22
7	**800.4036**	400.7055			**782.3931**	391.7002	E	2526.9967	**1264.0020**	2509.9701	**1255.4887**	2508.9861	**1254.9967**	21
8	**931.4441**	466.2257			**913.4335**	457.2204	M	2397.9541	**1199.4807**	2380.9275	**1190.9674**	2379.9435	1190.4754	20
9	1028.4969	514.7521			1010.4863	505.7468	P	2266.9136	**1133.9604**	2249.8870	**1125.4472**	2248.9030	**1124.9551**	19
10	1157.5395	579.2734			1139.5289	570.2681	E	2169.8608	**1085.4340**	2152.8343	1076.9208	2151.8503	1076.4288	18
11	1271.5824	636.2948	1254.5558	627.7816	1253.5718	627.2896	N	2040.8182	**1020.9128**	2023.7917	**1012.3995**	2022.8077	1011.9075	17
12	**1342.6195**	671.8134	1325.5930	663.3001	1324.6089	662.8081	A	1926.7753	**963.8913**	1909.7488	**955.3780**	1908.7647	954.8860	16
13	**1455.7036**	728.3554	**1438.6770**	719.8422	**1437.6930**	719.3501	L	1855.7382	**928.3727**	1838.7116	919.8595	1837.7276	919.3674	15
14	1552.7563	776.8818	1535.7298	768.3685	1534.7458	767.8765	P	**1742.6541**	**871.8307**	1725.6276	863.3174	1724.6436	862.8254	14
15	1719.7547	860.3810	1702.7281	851.8677	1701.7441	851.3757	S	1645.6014	**823.3043**	**1628.5748**	814.7910	1627.5908	**814.2990**	13
16	**1834.7816**	917.8945	1817.7551	909.3812	1816.7711	908.8892	D	1478.6030	**739.8051**	1461.5765	731.2919	1460.5924	730.7999	12
17	1963.8242	982.4158	1946.7977	973.9025	**1945.8137**	973.4105	E	1363.5761	**682.2917**	1346.5495	673.7784	1345.5655	673.2864	11
18	2078.8512	1039.9292	2061.8246	1031.4159	2060.8406	1030.9239	D	1234.5335	**617.7704**	1217.5069	**609.2571**	1216.5229	608.7651	10

19	2193.8781	1097.4427	2176.8516	1088.9294	2175.8675	1088.4374	D	1119.5065	560.2569	1102.4800	551.7436	1101.4960	551.2516	9
20	2321.9731	1161.4902	2304.9465	1152.9769	2303.9625	1152.4849	K	1004.4796	502.7434	987.4530	494.2302	986.4690	493.7381	8
21	2437.0000	1219.0036	2419.9735	1210.4904	2418.9895	1209.9984	D	876.3846	438.6959	859.3581	430.1827	858.3741	429.6907	7
22	2534.0528	1267.5300	2517.0262	1259.0168	2516.0422	1258.5247	P	761.3577	381.1825	744.3311	372.6692	743.3471	372.1772	6
23	2648.0957	1324.5515	2631.0692	1316.0382	2630.0851	1315.5462	N	664.3049	332.6561	647.2784	324.1428	646.2943	323.6508	5
24	2763.1227	1382.0650	2746.0961	1373.5517	2745.1121	1373.0597	D	550.2620	275.6346	533.2354	267.1214	532.2514	266.6293	4
25	2860.1754	1430.5913	2843.1489	1422.0781	2842.1648	1421.5861	P	435.2350	218.1212	418.2085	209.6079			3
26	3023.2387	1512.1230	3006.2122	1503.6097	3005.2282	1503.1177	Y	338.1823	169.5948	321.1557	161.0815			2
27							R	175.1190	88.0631	158.0924	79.5498			1

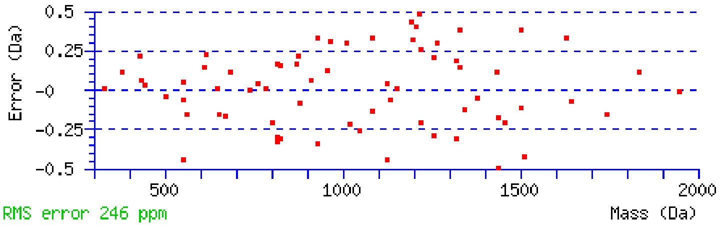

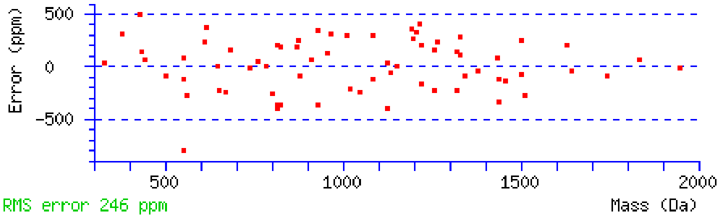

All matches to this query

Score	Mr(calc):	Delta	Sequence
58.8	3196.3431	1.0141	VDIITEEMPENALPSDEDDKDPNDPYR
48.4	3197.3271	0.0301	VDIITEEMPENALPSDEDDKDPNDPYR
35.7	3197.3271	0.0301	VDIITEEMPENALPSDEDDKDPNDPYR
20.9	3196.3431	1.0141	VDIITEEMPENALPSDEDDKDPNDPYR
16.9	3197.3271	0.0301	VDIITEEMPENALPSDEDDKDPNDPYR
16.6	3197.3271	0.0301	VDIITEEMPENALPSDEDDKDPNDPYR
7.0	3196.3291	1.0281	NAGKSTITVIAEDISGNNGYVELSFQAR
5.1	3196.3291	1.0281	NAGKSTITVIAEDISGNNGYVELSFQAR
3.8	3196.3431	1.0141	VDIITEEMPENALPSDEDDKDPNDPYR
3.7	3197.3271	0.0301	VDIITEEMPENALPSDEDDKDPNDPYR

Spectrum No: 37; Query: 1115; Rank: 1

Peptide View

MS/MS Fragmentation of **GENEETLGRPAQPPSAGETPHSPGVEDAPIAK**
Found in **IPI00373011**, Tax_Id=10116 Gene_Symbol=eplin similar to Epithelial protein lost in neoplasm

Match to Query 1115: 3317.524572 from(1106.848800,3+)
Title: 091129RatKid_SCX02_24.1567.1567.3.dta
Data file K:\NewmanPaper\Piliang\3SubProteomes\Piliang3SP\mgf5ppm\SCX_3SubProteomes5ppm.mgf

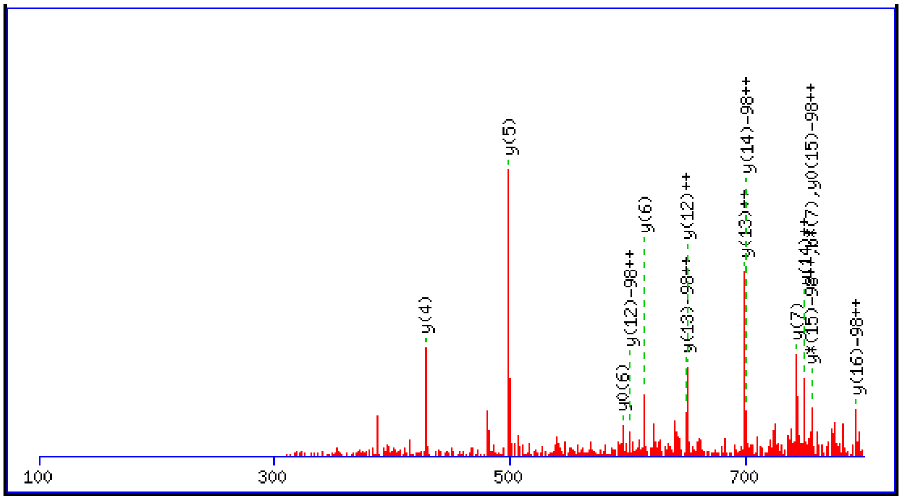

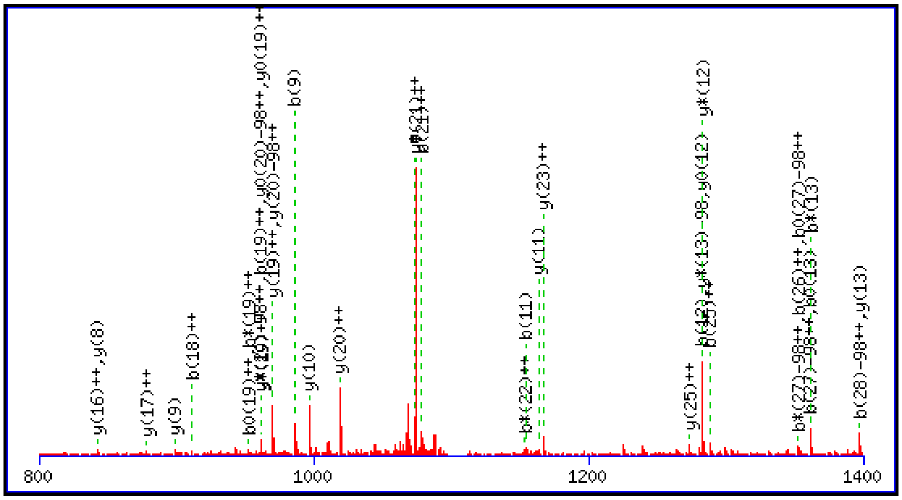

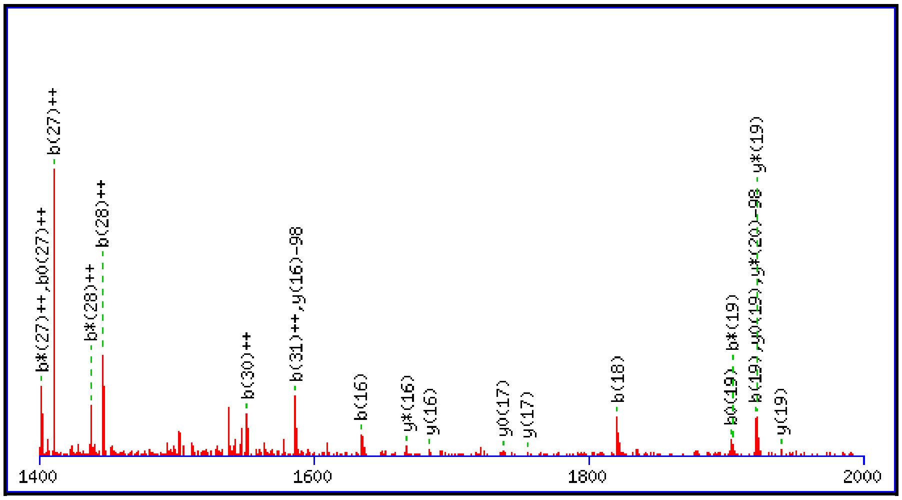

Monoisotopic mass of neutral peptide Mr(calc): 3317.5201
Fixed modifications: Carbamidomethyl (C)
Variable modifications:
S22 : Phospho (ST), with neutral losses 0.0000(shown in table), 97.9769
Ions Score: 59 **Expect:** 0.00061
Matches (Bold Red): 74/542 fragment ions using 110 most intense peaks

#	b	b++	b*	b*++	b0	b0++	Seq.	y	y++	y*	y*++	y0	y0++	#
1	58.0287	29.5180					G							32
2	187.0713	94.0393			169.0608	85.0340	E	3261.5060	1631.2566	3244.4794	1622.7433	3243.4954	1622.2513	31
3	301.1143	151.0608	284.0877	142.5475	283.1037	142.0555	N	3132.4634	1566.7353	3115.4368	1558.2220	3114.4528	1557.7300	30
4	430.1569	215.5821	413.1303	207.0688	412.1463	206.5768	E	3018.4204	1509.7139	3001.3939	1501.2006	3000.4099	1500.7086	29
5	559.1994	280.1034	542.1729	271.5901	541.1889	271.0981	E	2889.3778	1445.1926	2872.3513	1436.6793	2871.3673	1436.1873	28

6	660.2471	330.6272	643.2206	322.1139	642.2366	321.6219	T	2760.3352	1380.6713	2743.3087	1372.1580	2742.3247	1371.6660	27
7	773.3312	387.1692	**756.3046**	378.6560	755.3206	378.1640	L	2659.2876	1330.1474	2642.2610	1321.6341	2641.2770	1321.1421	26
8	830.3527	415.6800	813.3261	407.1667	812.3421	406.6747	G	2546.2035	**1273.6054**	2529.1770	1265.0921	2528.1929	1264.6001	25
9	**986.4538**	493.7305	969.4272	485.2172	968.4432	484.7252	R	2489.1820	1245.0947	2472.1555	1236.5814	2471.1715	1236.0894	24
10	1083.5065	542.2569	1066.4800	533.7436	1065.4960	533.2516	P	2333.0809	**1167.0441**	2316.0544	1158.5308	2315.0704	1158.0388	23
11	**1154.5436**	577.7755	1137.5171	569.2622	1136.5331	568.7702	A	2236.0282	1118.5177	2219.0016	1110.0044	2218.0176	1109.5124	22
12	**1282.6022**	641.8047	1265.5757	633.2915	1264.5917	632.7995	Q	2164.9911	1082.9992	2147.9645	**1074.4859**	2146.9805	**1073.9939**	21
13	1379.6550	690.3311	**1362.6284**	681.8179	**1361.6444**	681.3258	P	2036.9325	**1018.9699**	2019.9059	1010.4566	2018.9219	1009.9646	20
14	1476.7077	738.8575	1459.6812	730.3442	1458.6972	729.8522	P	**1939.8797**	**970.4435**	**1922.8532**	**961.9302**	**1921.8691**	**961.4382**	19
15	1563.7398	782.3735	1546.7132	773.8603	1545.7292	773.3682	S	1842.8269	921.9171	1825.8004	913.4038	1824.8164	912.9118	18
16	**1634.7769**	817.8921	1617.7503	809.3788	1616.7663	808.8868	A	**1755.7949**	**878.4011**	1738.7684	869.8878	**1737.7844**	869.3958	17
17	1691.7984	846.4028	1674.7718	837.8895	1673.7878	837.3975	G	**1684.7578**	**842.8825**	**1667.7313**	834.3693	1666.7472	833.8773	16
18	**1820.8409**	**910.9241**	1803.8144	902.4108	1802.8304	901.9188	E	1627.7363	814.3718	1610.7098	805.8585	1609.7258	805.3665	15
19	**1921.8886**	**961.4480**	**1904.8621**	**952.9347**	**1903.8781**	**952.4427**	T	1498.6937	**749.8505**	1481.6672	741.3372	1480.6832	740.8452	14
20	2018.9414	1009.9743	2001.9148	1001.4611	2000.9308	1000.9691	P	**1397.6461**	**699.3267**	1380.6195	690.8134	1379.6355	690.3214	13
21	2156.0003	**1078.5038**	2138.9738	1069.9905	2137.9897	1069.4985	H	1300.5933	**650.8003**	**1283.5668**	642.2870	**1282.5827**	641.7950	12
22	2322.9987	1162.0030	2305.9721	**1153.4897**	2304.9881	1152.9977	S	**1163.5344**	582.2708	1146.5078	573.7576	1145.5238	573.2656	11
23	2420.0514	1210.5293	2403.0249	1202.0161	2402.0409	1201.5241	P	**996.5360**	498.7717	979.5095	490.2584	978.5255	489.7664	10
24	2477.0729	1239.0401	2460.0463	1230.5268	2459.0623	1230.0348	G	**899.4833**	450.2453	882.4567	441.7320	881.4727	441.2400	9
25	2576.1413	**1288.5743**	2559.1147	1280.0610	2558.1307	1279.5690	V	**842.4618**	421.7345	825.4353	413.2213	824.4512	412.7293	8
26	2705.1839	**1353.0956**	2688.1573	1344.5823	2687.1733	1344.0903	E	**743.3934**	372.2003	726.3668	363.6871	725.3828	363.1951	7
27	2820.2108	**1410.6091**	2803.1843	**1402.0958**	2802.2003	**1401.6038**	D	**614.3508**	307.6790	597.3243	299.1658	**596.3402**	298.6738	6
28	2891.2479	**1446.1276**	2874.2214	**1437.6143**	2873.2374	1437.1223	A	**499.3239**	250.1656	482.2973	241.6523			5
29	2988.3007	1494.6540	2971.2742	1486.1407	2970.2901	1485.6487	P	**428.2867**	214.6470	411.2602	206.1337			4
30	3101.3848	**1551.1960**	3084.3582	1542.6828	3083.3742	1542.1907	I	331.2340	166.1206	314.2074	157.6074			3
31	3172.4219	**1586.7146**	3155.3953	1578.2013	3154.4113	1577.7093	A	218.1499	109.5786	201.1234	101.0653			2
32							K	147.1128	74.0600	130.0863	65.5468			1

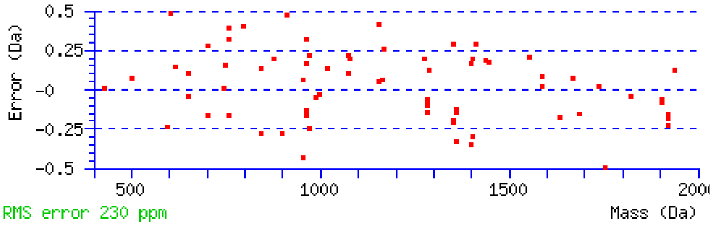

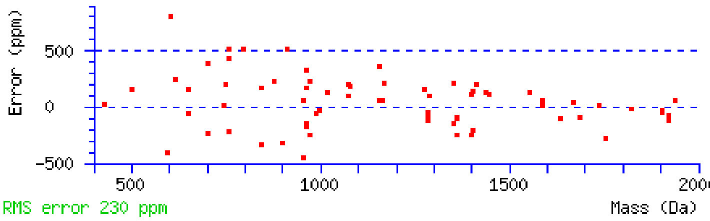

All matches to this query

Score	Mr(calc):	Delta	Sequence
58.6	3317.5201	0.0045	GENEETLGRPAQPPSAGETPHSPGVEDAPIAK
37.2	3317.5201	0.0045	GENEETLGRPAQPPSAGETPHSPGVEDAPIAK
27.7	3317.5201	0.0045	GENEETLGRPAQPPSAGETPHSPGVEDAPIAK
12.1	3317.5201	0.0045	GENEETLGRPAQPPSAGETPHSPGVEDAPIAK
4.3	3315.4941	2.0305	EPYRETSMGVKLNIAYQIGASEQCQGYK
4.1	3317.5410	-0.0164	VQNHYITEALSEEEARFPLAFTLTIHK
4.0	3317.5518	-0.0272	DVAAAAMYTVVTPMLNPFIYSLRNKDIK
3.3	3317.5560	-0.0314	LDNGALLCQLAATVQEKFKESMDTNKPAK
2.8	3316.5029	1.0217	REDPEAGWLLYLKTGQMYPVPANHLDK
2.8	3316.5101	1.0145	WLNTPNTYLRVNVADEVQRNMGSPRPK

Spectrum No: 38; Query: 495; Rank: 1

Peptide View

MS/MS Fragmentation of **GVENPAFVPSSPDTPR**
Found in **IPI00393407**, Tax_Id=10116 Gene_Symbol=Slco4c1 Kidney-specific organic anion transporter

Match to Query 495: 1748.783668 from(875.399110,2+)
Title: 091127RatKid_SCX01_12.1966.1966.2.dta
Data file K:\NewmanPaper\Piliang\3SubProteomes\Piliang3SP\mgf5ppm\SCX_3SubProteomes5ppm.mgf

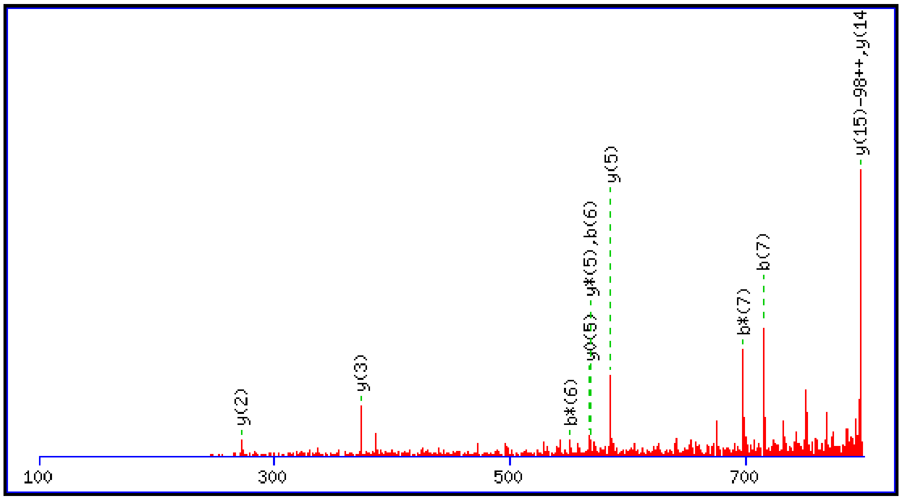

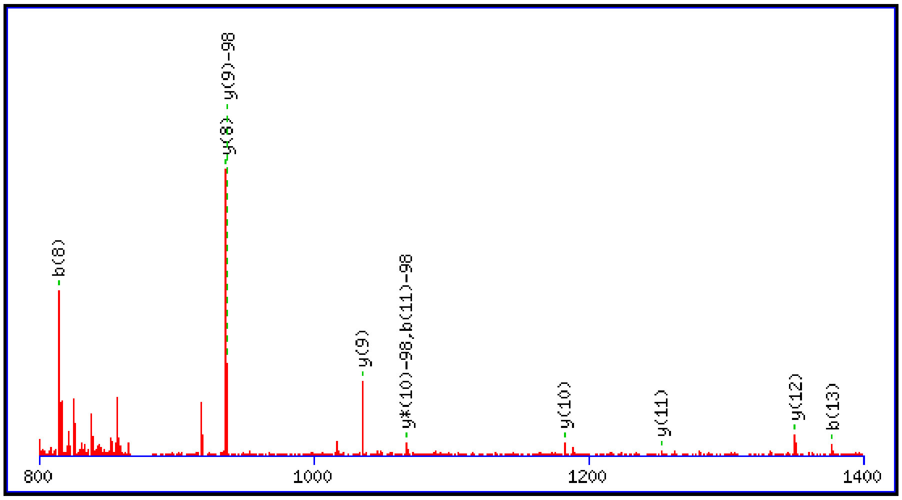

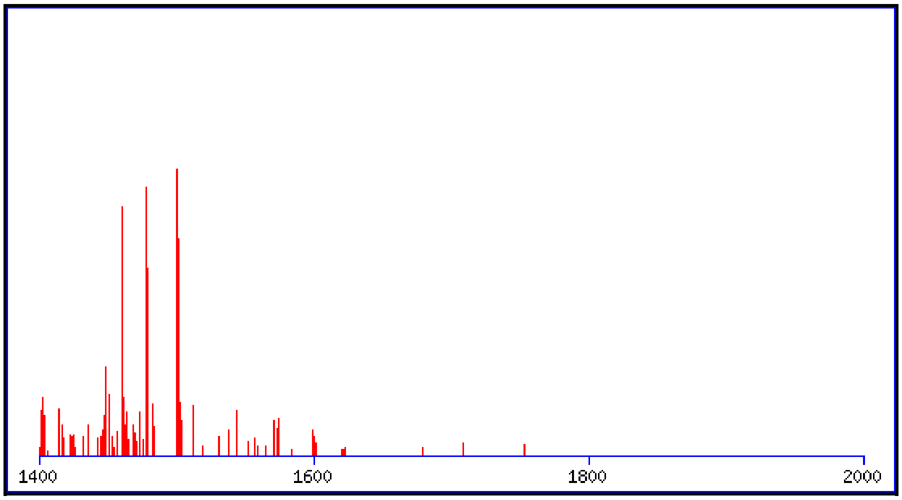

Monoisotopic mass of neutral peptide Mr(calc): 1748.7771
Fixed modifications: Carbamidomethyl (C)
Variable modifications:

S11 : Phospho (ST), with neutral losses 0.0000(shown in table), 97.9769
Ions Score: 58 **Expect:** 0.00035
Matches (Bold Red): 22/256 fragment ions using 34 most intense peaks

#	b	b^{++}	b*	b^{*++}	b^{0}	b^{0++}	Seq.	y	y^{++}	y*	y^{*++}	y^{0}	y^{0++}	#
1	58.0287	29.5180					G							16
2	157.0972	79.0522					V	1692.7629	846.8851	1675.7363	838.3718	1674.7523	837.8798	15
3	286.1397	143.5735			268.1292	134.5682	E	1593.6945	**797.3509**	1576.6679	788.8376	1575.6839	788.3456	14
4	400.1827	200.5950	383.1561	192.0817	382.1721	191.5897	N	1464.6519	732.8296	1447.6253	724.3163	1446.6413	723.8243	13
5	497.2354	249.1214	480.2089	240.6081	479.2249	240.1161	P	**1350.6090**	675.8081	1333.5824	667.2948	1332.5984	666.8028	12
6	**568.2726**	284.6399	**551.2460**	276.1266	550.2620	275.6346	A	**1253.5562**	627.2817	1236.5296	618.7685	1235.5456	618.2764	11
7	**715.3410**	358.1741	**698.3144**	349.6608	697.3304	349.1688	F	**1182.5191**	591.7632	1165.4925	583.2499	1164.5085	582.7579	10
8	**814.4094**	407.7083	**797.3828**	399.1951	796.3988	398.7030	V	**1035.4507**	518.2290	1018.4241	509.7157	1017.4401	509.2237	9
9	911.4621	456.2347	894.4356	447.7214	893.4516	447.2294	P	**936.3822**	468.6948	919.3557	460.1815	918.3717	459.6895	8
10	998.4942	499.7507	981.4676	491.2374	980.4836	490.7454	S	839.3295	420.1684	822.3029	411.6551	821.3189	411.1631	7
11	1165.4925	583.2499	1148.4660	574.7366	1147.4820	574.2446	S	752.2975	376.6524	735.2709	368.1391	734.2869	367.6471	6
12	1262.5453	631.7763	1245.5187	623.2630	1244.5347	622.7710	P	**585.2991**	293.1532	**568.2726**	284.6399	**567.2885**	284.1479	5
13	**1377.5722**	689.2898	1360.5457	680.7765	1359.5617	680.2845	D	488.2463	244.6268	471.2198	236.1135	470.2358	235.6215	4
14	1478.6199	739.8136	1461.5934	731.3003	1460.6093	730.8083	T	**373.2194**	187.1133	356.1928	178.6001	355.2088	178.1081	3
15	1575.6727	788.3400	1558.6461	779.8267	1557.6621	779.3347	P	**272.1717**	136.5895	255.1452	128.0762			2
16							R	175.1190	88.0631	158.0924	79.5498			1

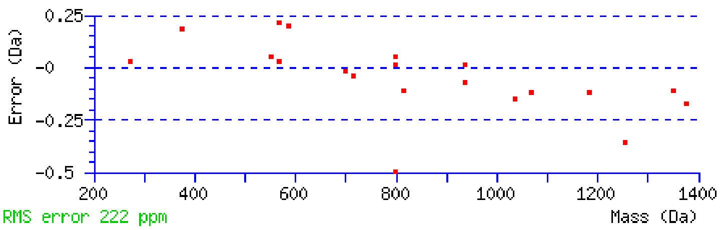

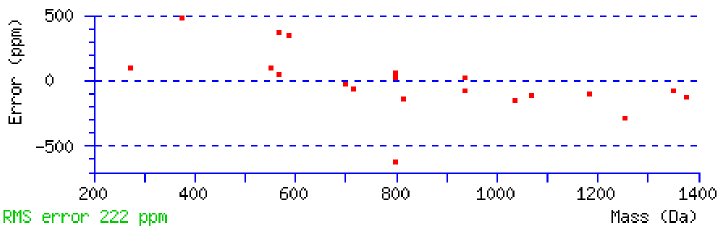

All matches to this query

Score	Mr(calc):	Delta	Sequence
57.8	1748.7771	0.0066	GVENPAFVPSSPDTPR
57.8	1748.7771	0.0066	GVENPAFVPSSPDTPR
34.6	1748.7771	0.0066	GVENPAFVPSSPDTPR
7.5	1747.7818	1.0018	SAGVATFVIQEEFDR
7.5	1747.7818	1.0018	SAGVATFVLQEEFDR
6.6	1748.7982	-0.0145	ANDPSLQEVNLNNIK
3.9	1748.7899	-0.0063	SSPDPAVNPVPKPKR
3.7	1747.7818	1.0018	SAGVATFVIQEEFDR
3.7	1747.7818	1.0018	SAGVATFVLQEEFDR
1.1	1747.7818	1.0019	TQGYNEIFLDSPKR

Spectrum No: 39; Query: 5; Rank: 1

Peptide View

MS/MS Fragmentation of **LGVSVSPSR**
Found in **IPI00400666**, Tax_Id=10116 Gene_Symbol=Nhn1 Isoform A of Zinc finger CCCH domain-containing protein 18

Match to Query 5: 980.468888 from(491.241720,2+)
Title: 091129RatKid_SCX02_12.628.628.2.dta
Data file K:\NewmanPaper\Piliang\3SubProteomes\Piliang3SP\mgf5ppm\SCX_3SubProteomes5ppm.mgf

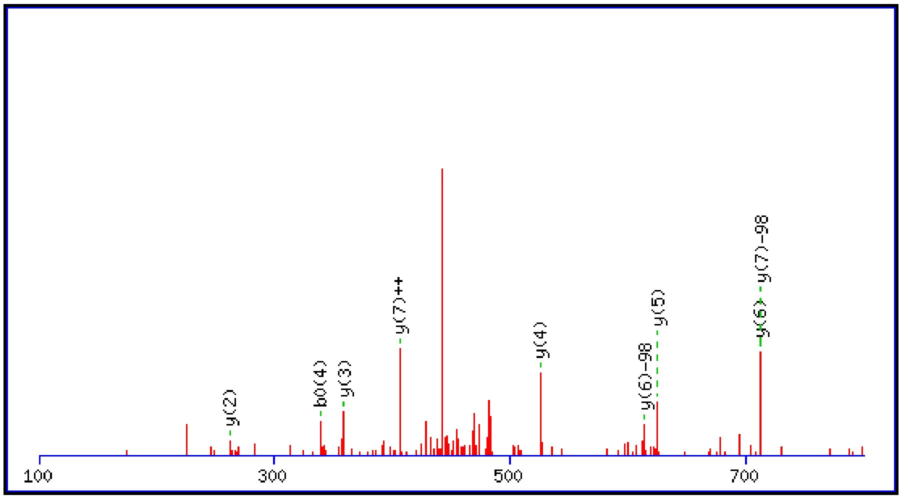

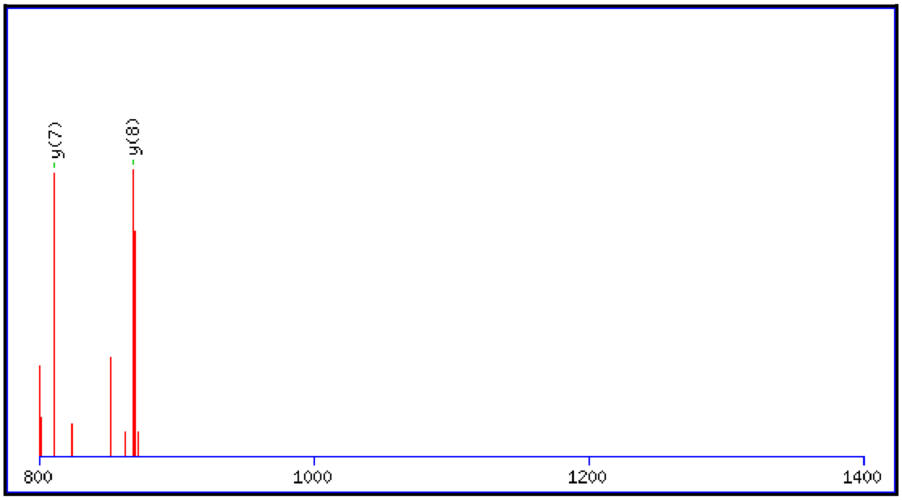

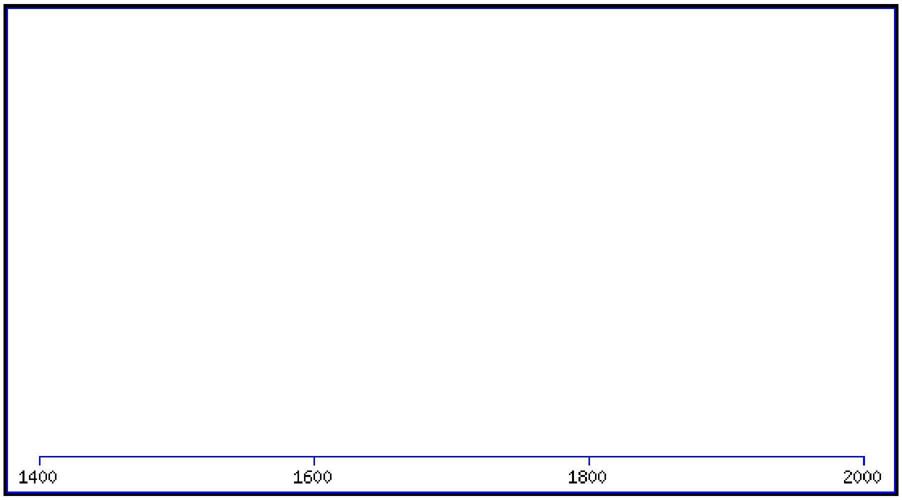

Monoisotopic mass of neutral peptide Mr(calc): 980.4692
Fixed modifications: Carbamidomethyl (C)
Variable modifications:
S6 : Phospho (ST), with neutral losses 0.0000(shown in table), 97.9769
Ions Score: 58 **Expect:** 0.00015
Matches (Bold Red): 11/114 fragment ions using 15 most intense peaks

#	b	b^{++}	b^{0}	b^{0++}	Seq.	y	y^{++}	y*	y^{*++}	y^{0}	y^{0++}	#
1	114.0913	57.5493			L							9
2	171.1128	86.0600			G	**868.3924**	434.6998	851.3659	426.1866	850.3819	425.6946	8
3	270.1812	135.5942			V	**811.3710**	**406.1891**	794.3444	397.6758	793.3604	397.1838	7
4	357.2132	179.1103	**339.2027**	170.1050	S	**712.3025**	356.6549	695.2760	348.1416	694.2920	347.6496	6

5	456.2817	228.6445	438.2711	219.6392	V	625.2705	313.1389	608.2440	304.6256	607.2599	304.1336	5
6	623.2800	312.1436	605.2694	303.1384	S	526.2021	263.6047	509.1755	255.0914	508.1915	254.5994	4
7	720.3328	360.6700	702.3222	351.6647	P	359.2037	180.1055	342.1772	171.5922	341.1932	171.1002	3
8	807.3648	404.1860	789.3542	395.1808	S	262.1510	131.5791	245.1244	123.0659	244.1404	122.5738	2
9					R	175.1190	88.0631	158.0924	79.5498			1

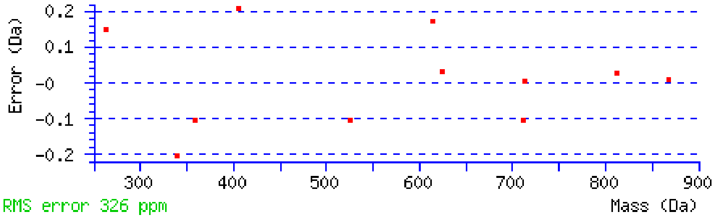

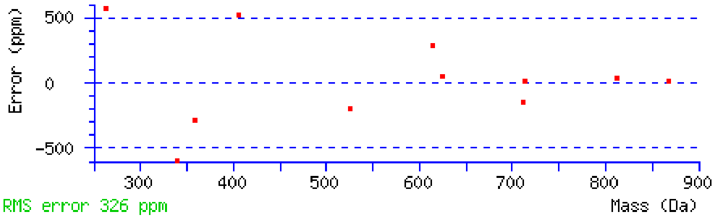

All matches to this query

Score	Mr(calc):	Delta	Sequence
57.7	980.4692	-0.0003	LGVSVSPSR
32.6	980.4692	-0.0003	LGVSVSPSR
27.5	980.4692	-0.0003	LGVSVSPSR
12.0	980.4593	0.0096	LWRSPSR
8.5	979.4739	0.9950	NIKVSDPK
5.7	980.4593	0.0096	RLWPSSR
4.0	980.4692	-0.0003	GIVIATGDR
3.7	978.4536	2.0153	TTPTGGLPR
3.7	980.4692	-0.0003	AESTIKPR
3.3	979.4627	1.0062	DILLSPDK

Spectrum No: 40; Query: 576; Rank: 1

Peptide View

MS/MS Fragmentation of **GSDTSPEAEASSGGGGVALK**
Found in **IPI00205295**, Tax_Id=10116 Gene_Symbol=Slc7a8 Large neutral amino acids transporter small subunit 2

Match to Query 576: 1855.786808 from(928.900680,2+)
Title: 091127RatKid_SCX01_12.1283.1283.2.dta
Data file K:\NewmanPaper\Piliang\3SubProteomes\Piliang3SP\mgf5ppm\SCX_3SubProteomes5ppm.mgf

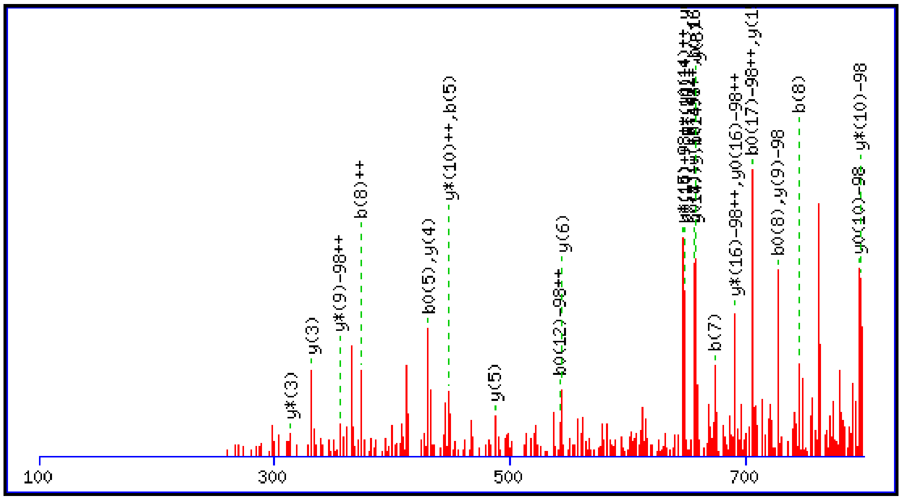

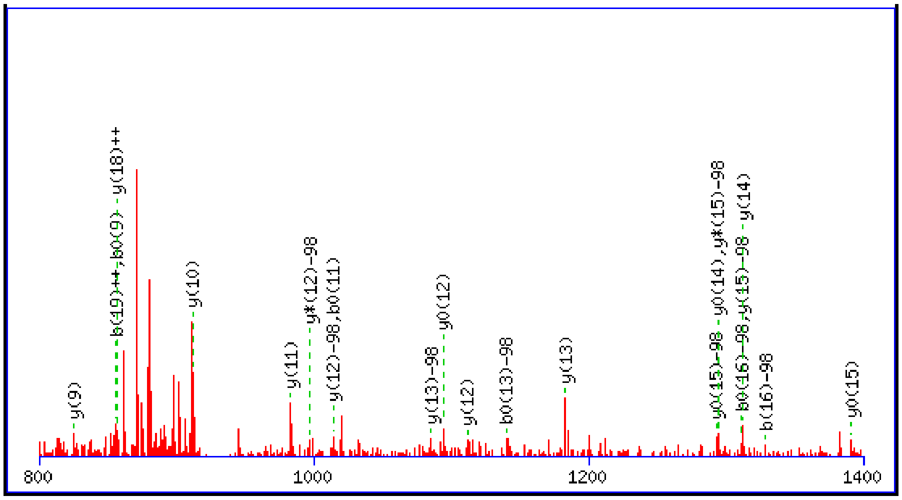

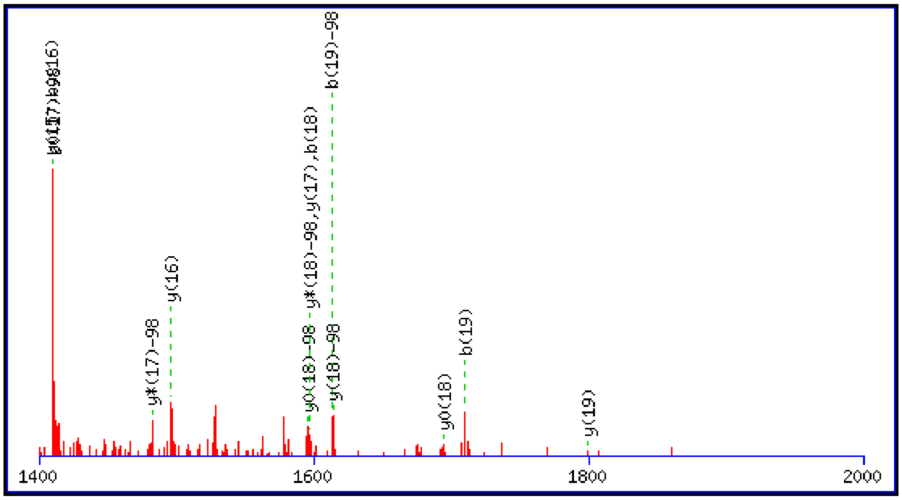

Monoisotopic mass of neutral peptide Mr(calc): 1855.7837
Fixed modifications: Carbamidomethyl (C)
Variable modifications:
S12 : Phospho (ST), with neutral losses 0.0000(shown in table), 97.9769
Ions Score: 58 **Expect:** 0.00035
Matches (Bold Red): 69/270 fragment ions using 98 most intense peaks

#	b	b^{++}	b^0	b^{0++}	Seq.	y	y^{++}	y^*	y^{*++}	y^0	y^{0++}	#
1	58.0287	29.5180			G							20
2	145.0608	73.0340	127.0502	64.0287	S	**1799.7695**	900.3884	1782.7429	891.8751	1781.7589	891.3831	19
3	260.0877	130.5475	242.0771	121.5422	D	1712.7375	**856.8724**	1695.7109	848.3591	**1694.7269**	847.8671	18
4	361.1354	181.0713	343.1248	172.0661	T	**1597.7105**	799.3589	1580.6840	790.8456	1579.7000	790.3536	17
5	**448.1674**	224.5873	**430.1569**	215.5821	S	**1496.6628**	748.8351	1479.6363	740.3218	1478.6523	739.8298	16
6	545.2202	273.1137	527.2096	264.1084	P	**1409.6308**	**705.3190**	1392.6043	696.8058	**1391.6202**	696.3138	15
7	**674.2628**	337.6350	**656.2522**	328.6297	E	**1312.5780**	**656.7927**	1295.5515	**648.2794**	**1294.5675**	**647.7874**	14
8	**745.2999**	**373.1536**	**727.2893**	364.1483	A	**1183.5355**	592.2714	1166.5089	583.7581	1165.5249	583.2661	13
9	874.3425	437.6749	**856.3319**	428.6696	E	**1112.4983**	556.7528	1095.4718	548.2395	**1094.4878**	547.7475	12
10	945.3796	473.1934	927.3690	464.1882	A	**983.4557**	492.2315	966.4292	483.7182	965.4452	483.2262	11
11	1032.4116	516.7095	**1014.4011**	507.7042	S	**912.4186**	456.7130	895.3921	**448.1997**	894.4081	447.7077	10
12	1199.4100	600.2086	1181.3994	591.2033	S	**825.3866**	413.1969	808.3601	404.6837	807.3760	404.1917	9
13	1256.4314	628.7194	1238.4209	619.7141	G	**658.3883**	329.6978	641.3617	321.1845			8
14	1313.4529	**657.2301**	1295.4423	**648.2248**	G	601.3668	301.1870	584.3402	292.6738			7
15	1370.4744	685.7408	1352.4638	676.7355	G	**544.3453**	272.6763	527.3188	264.1630			6
16	1427.4958	714.2516	**1409.4853**	**705.2463**	G	**487.3239**	244.1656	470.2973	235.6523			5
17	1526.5642	763.7858	1508.5537	754.7805	V	**430.3024**	215.6548	413.2758	207.1416			4
18	**1597.6014**	799.3043	1579.5908	790.2990	A	**331.2340**	166.1206	**314.2074**	157.6074			3

**19**	**1710.6854**	**855.8463**	1692.6749	846.8411	**L**	260.1969	130.6021	243.1703	122.0888			**2**
**20**					**K**	147.1128	74.0600	130.0863	65.5468			**1**

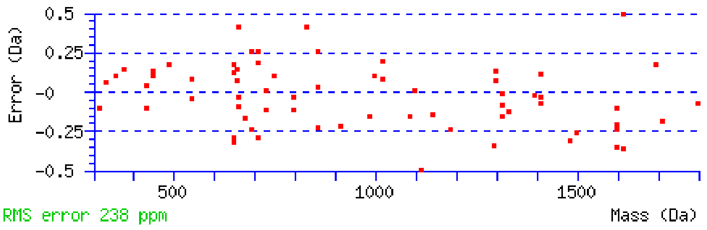

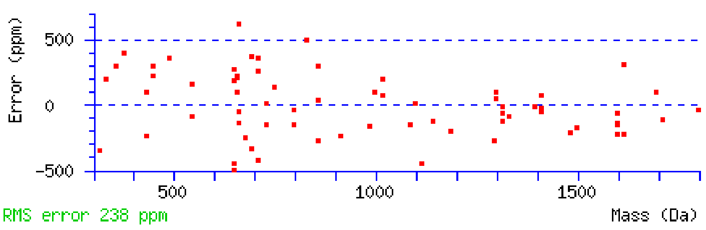

All matches to this query

Score	Mr(calc):	Delta	Sequence
57.6	1855.7837	0.0031	GSDTSPEAEASSGGGGVALK
55.1	1855.7837	0.0031	GSDTSPEAEASSGGGGVALK
31.6	1855.7837	0.0031	GSDTSPEAEASSGGGGVALK
29.9	1855.7837	0.0031	GSDTSPEAEASSGGGGVALK
22.9	1855.7837	0.0031	GSDTSPEAEASSGGGGVALK
6.9	1853.7979	1.9890	NKSTESQQTNMQHLK
4.7	1854.7819	1.0049	NKSTESQQTNMQHLK
3.4	1854.7689	1.0179	ELANQVSKDFSDITK
3.1	1854.7743	1.0125	HLPESLYPHTYNPK
2.6	1853.7727	2.0141	DNEKGSCHESSQRIK

Spectrum No: 41; Query: 959; Rank: 1

Peptide View

MS/MS Fragmentation of **KVEEEQEADEEDVSEEETENR**
Found in **IPI00365626**, Tax_Id=10116 Gene_Symbol=Txndc1 Thioredoxin domain containing 1

Match to Query 959: 2602.006668 from(1302.010610,2+)
Title: 091129RatKid_SCX02_24.791.791.2.dta
Data file K:\NewmanPaper\Piliang\3SubProteomes\Piliang3SP\mgf5ppm\SCX_3SubProteomes5ppm.mgf

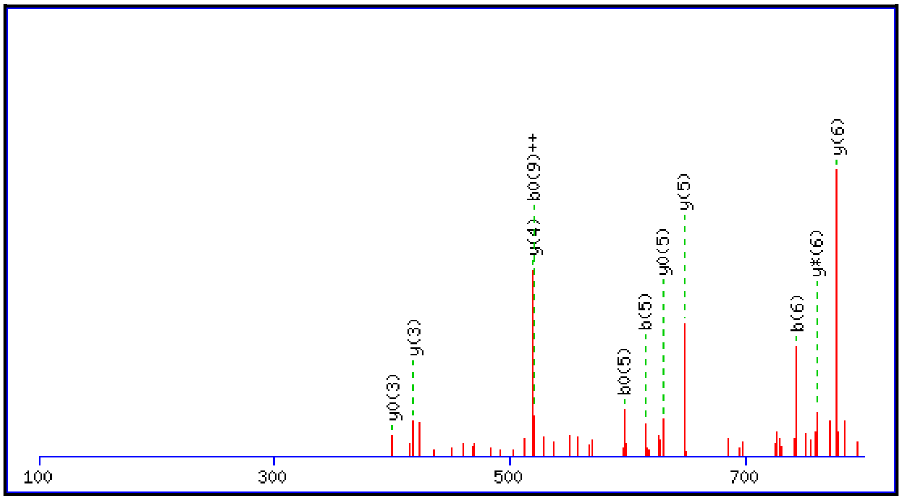

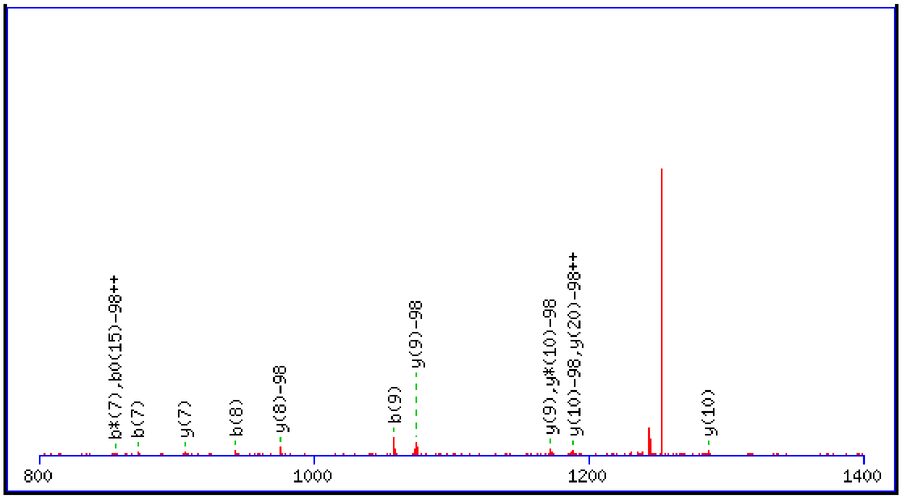

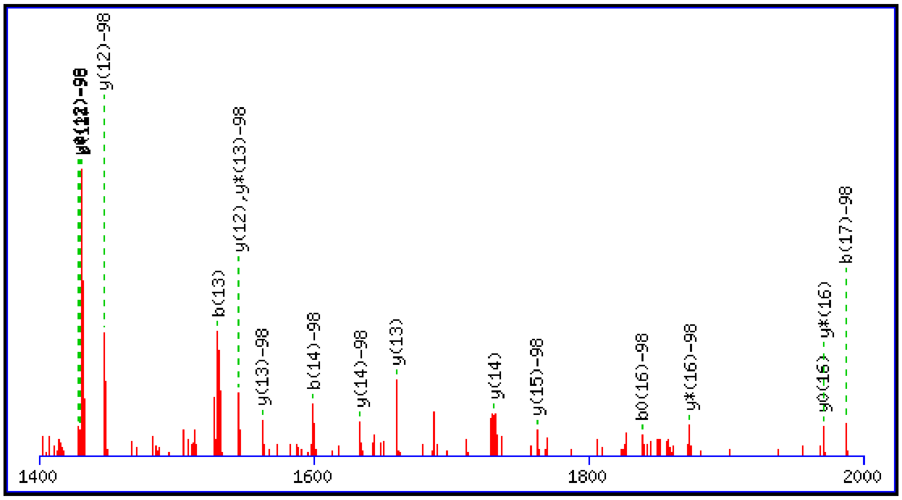

Monoisotopic mass of neutral peptide Mr(calc): 2602.0079
Fixed modifications: Carbamidomethyl (C)
Variable modifications:
S14 : Phospho (ST), with neutral losses 97.9769(shown in table), 0.0000
Ions Score: 58 **Expect:** 0.00035
Matches (Bold Red): 42/352 fragment ions using 56 most intense peaks

#	b	b++	b*	b*++	b0	b0++	Seq.	y	y++	y*	y*++	y0	y0++	#
1	129.1022	65.0548	112.0757	56.5415			K							21
2	228.1707	114.5890	211.1441	106.0757			V	2376.9434	**1188.9753**	2359.9168	1180.4620	2358.9328	1179.9700	20
3	357.2132	179.1103	340.1867	170.5970	339.2027	170.1050	E	2277.8749	1139.4411	2260.8484	1130.9278	2259.8644	1130.4358	19
4	486.2558	243.6316	469.2293	235.1183	468.2453	234.6263	E	2148.8323	1074.9198	2131.8058	1066.4065	2130.8218	1065.9145	18
5	**615.2984**	308.1529	598.2719	299.6396	**597.2879**	299.1476	E	2019.7898	1010.3985	2002.7632	1001.8852	2001.7792	1001.3932	17
6	**743.3570**	372.1821	726.3305	363.6689	725.3464	363.1769	Q	1890.7472	945.8772	**1873.7206**	937.3639	1872.7366	936.8719	16
7	**872.3996**	436.7034	**855.3731**	428.1902	854.3890	427.6982	E	**1762.6886**	881.8479	1745.6620	873.3347	1744.6780	872.8426	15
8	**943.4367**	472.2220	926.4102	463.7087	925.4262	463.2167	A	**1633.6460**	817.3266	1616.6194	808.8134	1615.6354	808.3213	14
9	**1058.4637**	529.7355	1041.4371	521.2222	1040.4531	**520.7302**	D	**1562.6089**	781.8081	**1545.5823**	773.2948	1544.5983	772.8028	13
10	1187.5063	594.2568	1170.4797	585.7435	1169.4957	585.2515	E	**1447.5819**	724.2946	**1430.5554**	715.7813	**1429.5714**	715.2893	12
11	1316.5488	658.7781	1299.5223	650.2648	1298.5383	649.7728	E	1318.5393	659.7733	1301.5128	651.2600	1300.5288	650.7680	11
12	**1431.5758**	716.2915	1414.5492	707.7783	1413.5652	707.2863	D	**1189.4967**	595.2520	**1172.4702**	586.7387	1171.4862	586.2467	10
13	**1530.6442**	765.8257	1513.6177	757.3125	1512.6336	756.8205	V	**1074.4698**	537.7385	1057.4433	529.2253	1056.4592	528.7333	9
14	**1599.6657**	800.3365	1582.6391	791.8232	1581.6551	791.3312	S	**975.4014**	488.2043	958.3748	479.6911	957.3908	479.1990	8
15	1728.7083	864.8578	1711.6817	856.3445	1710.6977	**855.8525**	E	**906.3799**	453.6936	889.3534	445.1803	888.3694	444.6883	7
16	1857.7508	929.3791	1840.7243	920.8658	**1839.7403**	920.3738	E	**777.3373**	389.1723	**760.3108**	380.6590	759.3268	380.1670	6
17	**1986.7934**	993.9004	1969.7669	985.3871	1968.7829	984.8951	E	**648.2947**	324.6510	631.2682	316.1377	**630.2842**	315.6457	5
18	2087.8411	1044.4242	2070.8146	1035.9109	2069.8306	1035.4189	T	**519.2522**	260.1297	502.2256	251.6164	501.2416	251.1244	4

**19**	2216.8837	1108.9455	2199.8572	1100.4322	2198.8731	1099.9402	**E**	**418.2045**	209.6059	401.1779	201.0926	**400.1939**	200.6006	**3**
**20**	2330.9266	1165.9670	2313.9001	1157.4537	2312.9161	1156.9617	**N**	289.1619	145.0846	272.1353	136.5713			**2**
**21**							**R**	175.1190	88.0631	158.0924	79.5498			**1**

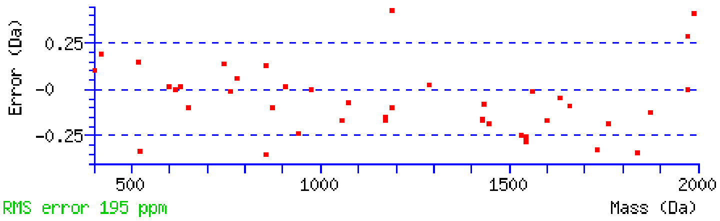

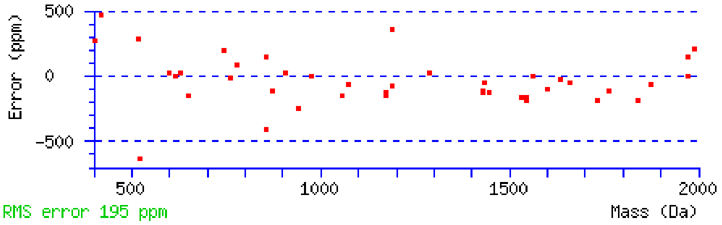

All matches to this query

Score	Mr(calc):	Delta	Sequence
57.7	2602.0079	-0.0012	KVEEEQEADEEDVSEEETENR
37.8	2602.0079	-0.0012	KVEEEQEADEEDVSEEETENR
7.1	2601.0206	0.9861	DAASPSVDMSKTVGMTAEKWWE
4.3	2601.9872	0.0195	YEAQRYDGFLKTYETASLK
3.9	2602.0132	-0.0066	XFDDDEGEEEDEAAAAAAAAIGYR
3.8	2601.9914	0.0153	NRDLLGSSYTETYSAANGSEVK
3.7	2600.9937	1.0130	GSYPMIENVTKGFGIAFSNGNR
3.7	2600.9937	1.0130	GSYPMIENVTKGFGIAFSNGNR
3.7	2602.0295	-0.0229	YGKNMAEATSGEAPENGVTTGSVR
3.3	2602.0295	-0.0229	YGKNMAEATSGEAPENGVTTGSVR

Spectrum No: 42; Query: 327; Rank: 1

Peptide View

MS/MS Fragmentation of **DEILPTTPISEQK**
Found in **IPI00195999**, Tax_Id=10116 Gene_Symbol=RGD1560831_predicted similar to 40S ribosomal protein S3

Match to Query 327: 1549.731648 from(775.873100,2+)
Title: 091127RatKid_SCX01_12.2182.2182.2.dta
Data file K:\NewmanPaper\Piliang\3SubProteomes\Piliang3SP\mgf5ppm\SCX_3SubProteomes5ppm.mgf

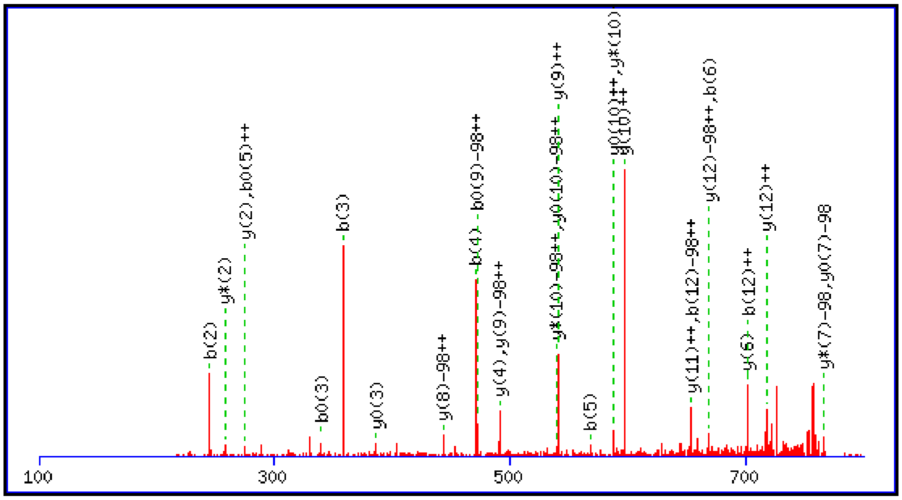

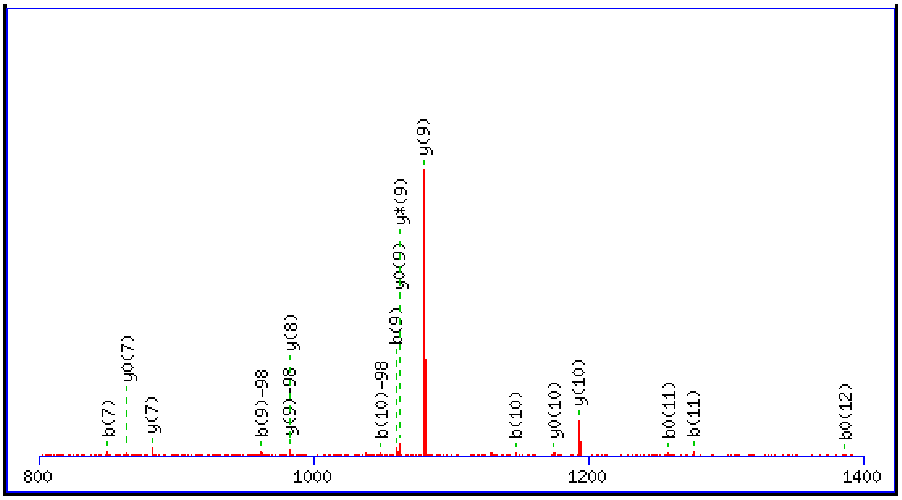

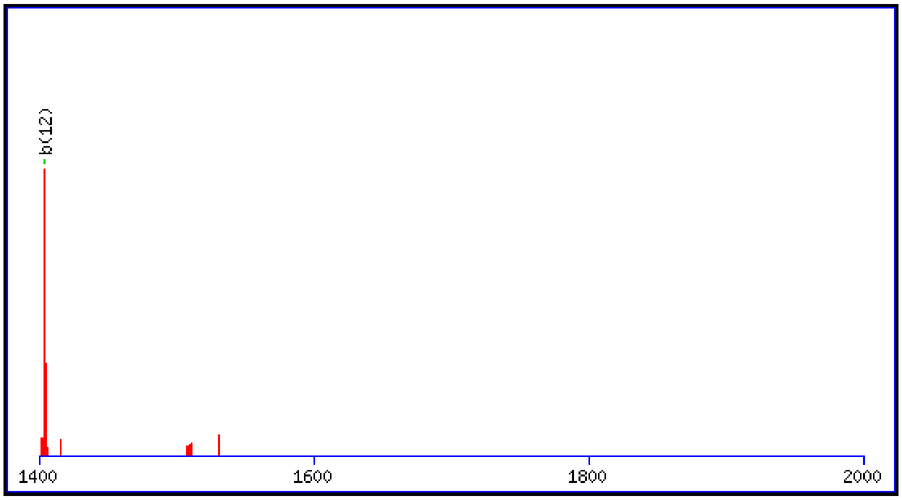

Monoisotopic mass of neutral peptide Mr(calc): 1549.7276
Fixed modifications: Carbamidomethyl (C)
Variable modifications:
T7 : Phospho (ST), with neutral losses 0.0000(shown in table), 97.9769
Ions Score: 57 **Expect:** 0.00033
Matches (Bold Red): 46/180 fragment ions using 73 most intense peaks

#	b	b^{++}	b*	b^{*++}	b^0	b^{0++}	Seq.	y	y^{++}	y*	y^{*++}	y^0	y^{0++}	#
1	116.0342	58.5207			98.0237	49.5155	D							13
2	**245.0768**	123.0420			227.0662	114.0368	E	1435.7080	**718.3576**	1418.6814	709.8444	1417.6974	709.3524	12
3	**358.1609**	179.5841			**340.1503**	170.5788	I	1306.6654	**653.8363**	1289.6389	645.3231	1288.6548	644.8311	11
4	**471.2449**	236.1261			453.2344	227.1208	L	**1193.5813**	**597.2943**	1176.5548	**588.7810**	**1175.5708**	**588.2890**	10
5	**568.2977**	284.6525			550.2871	**275.6472**	P	**1080.4973**	**540.7523**	**1063.4707**	532.2390	**1062.4867**	531.7470	9
6	**669.3454**	335.1763			651.3348	326.1710	T	**983.4445**	492.2259	966.4180	483.7126	965.4339	483.2206	8
7	**850.3594**	425.6833			832.3488	416.6780	T	**882.3968**	441.7021	865.3703	433.1888	**864.3863**	432.6968	7
8	947.4122	474.2097			929.4016	465.2044	P	**701.3828**	351.1951	684.3563	342.6818	683.3723	342.1898	6
9	**1060.4962**	530.7517			1042.4856	521.7465	I	604.3301	302.6687	587.3035	294.1554	586.3195	293.6634	5
10	**1147.5282**	574.2678			1129.5177	565.2625	S	**491.2460**	246.1266	474.2195	237.6134	473.2354	237.1214	4
11	**1276.5708**	638.7891			**1258.5603**	629.7838	E	404.2140	202.6106	387.1874	194.0974	**386.2034**	193.6053	3
12	**1404.6294**	**702.8183**	1387.6029	694.3051	**1386.6188**	693.8131	Q	**275.1714**	138.0893	**258.1448**	129.5761			2
13							K	147.1128	74.0600	130.0863	65.5468			1

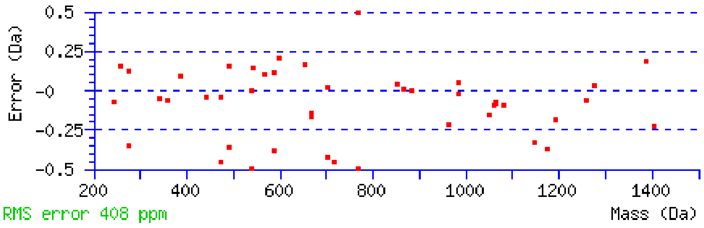

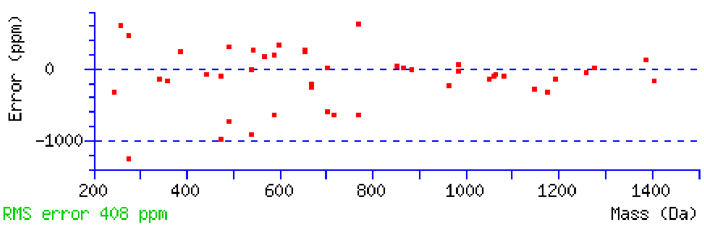

All matches to this query

Score	Mr(calc):	Delta	Sequence
57.1	1549.7276	0.0040	DEILPTTPISEQK
52.3	1549.7276	0.0040	DEILPTTPISEQK
35.9	1549.7276	0.0040	DEILPTTPISEQK
12.9	1549.7406	-0.0090	GAQTLSDLRSEMDK
11.5	1549.7177	0.0139	NLEPVSWSSLNPK
11.5	1549.7177	0.0139	NLEPVSWSSLNPK
11.5	1549.7177	0.0139	NLEPVSWSSLNPK
10.4	1549.7372	-0.0056	ENNLELQGYQTNK
9.1	1549.7372	-0.0056	DLQNYQSNQLAQK
8.3	1547.7241	2.0076	KLIISRNQMFMS

Spectrum No: 43; Query: 446; Rank: 1

Peptide View

MS/MS Fragmentation of **TNPSPTNPFSSDAQK**
Found in **IPI00202443**, Tax_Id=10116 Gene_Symbol=Numb Numb isoform o/i

Match to Query 446: 1669.701628 from(835.858090,2+)
Title: 091127RatKid_SCX01_12.1628.1628.2.dta
Data file K:\NewmanPaper\Piliang\3SubProteomes\Piliang3SP\mgf5ppm\SCX_3SubProteomes5ppm.mgf

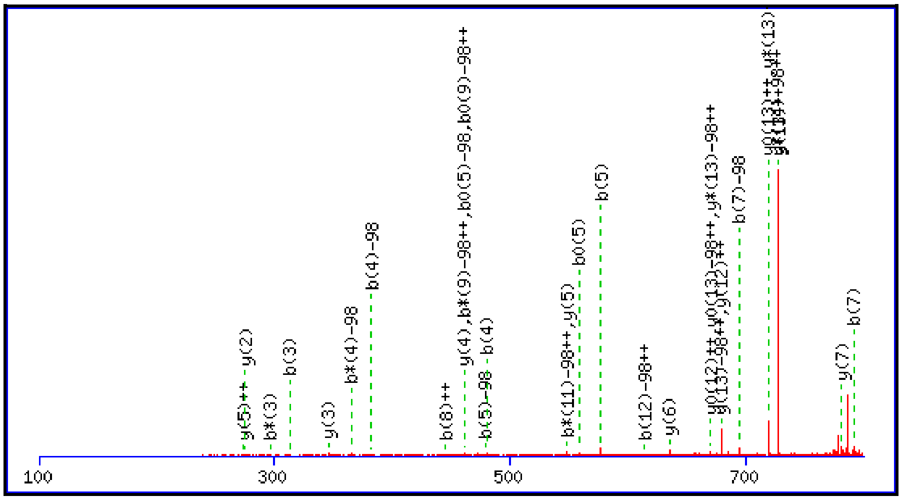

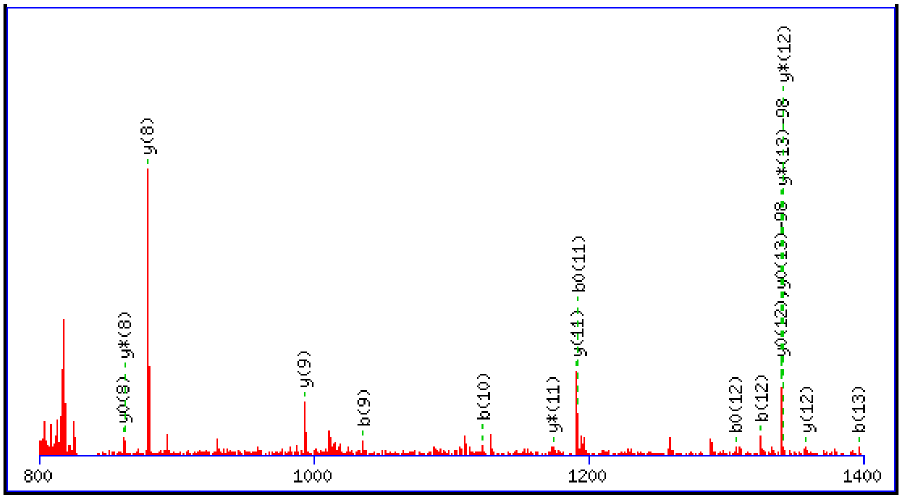

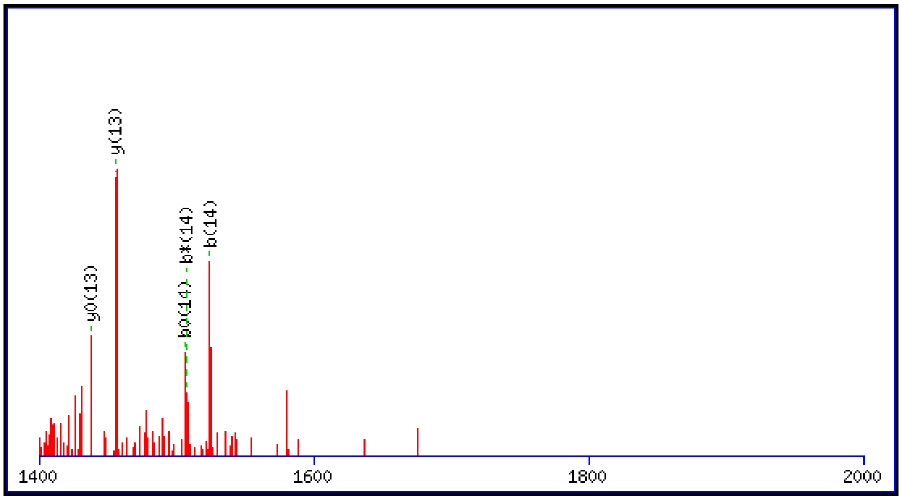

Monoisotopic mass of neutral peptide Mr(calc): 1669.6985
Fixed modifications: Carbamidomethyl (C)
Variable modifications:
S4 : Phospho (ST), with neutral losses 0.0000(shown in table), 97.9769
Ions Score: 57 **Expect:** 0.00036
Matches (Bold Red): 54/244 fragment ions using 92 most intense peaks

#	b	b^{++}	b*	b^{*++}	b^{0}	b^{0++}	Seq.	y	y^{++}	y*	y^{*++}	y^{0}	y^{0++}	#
1	102.0550	51.5311			84.0444	42.5258	T							15
2	216.0979	108.5526	199.0713	100.0393	198.0873	99.5473	N	1569.6581	785.3327	1552.6315	776.8194	1551.6475	776.3274	14
3	**313.1506**	157.0790	**296.1241**	148.5657	295.1401	148.0737	P	**1455.6152**	**728.3112**	1438.5886	**719.7979**	**1437.6046**	**719.3059**	13
4	**480.1490**	240.5781	463.1224	232.0649	462.1384	231.5729	S	**1358.5624**	**679.7848**	**1341.5358**	671.2716	**1340.5518**	**670.7796**	12
5	**577.2018**	289.1045	560.1752	280.5912	**559.1912**	280.0992	P	**1191.5640**	596.2857	**1174.5375**	587.7724	1173.5535	587.2804	11
6	678.2494	339.6284	661.2229	331.1151	660.2389	330.6231	T	1094.5113	547.7593	1077.4847	539.2460	1076.5007	538.7540	10
7	**792.2924**	396.6498	775.2658	388.1365	774.2818	387.6445	N	**993.4636**	497.2354	976.4371	488.7222	975.4530	488.2302	9
8	889.3451	**445.1762**	872.3186	436.6629	871.3346	436.1709	P	**879.4207**	440.2140	**862.3941**	431.7007	**861.4101**	431.2087	8
9	**1036.4135**	518.7104	1019.3870	510.1971	1018.4030	509.7051	F	**782.3679**	391.6876	765.3414	383.1743	764.3573	382.6823	7
10	**1123.4456**	562.2264	1106.4190	553.7132	1105.4350	553.2211	S	**635.2995**	318.1534	618.2729	309.6401	617.2889	309.1481	6
11	1210.4776	605.7424	1193.4511	597.2292	**1192.4670**	596.7372	S	**548.2675**	**274.6374**	531.2409	266.1241	530.2569	265.6321	5
12	**1325.5045**	663.2559	1308.4780	654.7426	**1307.4940**	654.2506	D	**461.2354**	231.1214	444.2089	222.6081	443.2249	222.1161	4
13	**1396.5417**	698.7745	1379.5151	690.2612	1378.5311	689.7692	A	**346.2085**	173.6079	329.1819	165.0946			3
14	**1524.6002**	762.8038	**1507.5737**	754.2905	**1506.5897**	753.7985	Q	**275.1714**	138.0893	258.1448	129.5761			2
15							K	147.1128	74.0600	130.0863	65.5468			1

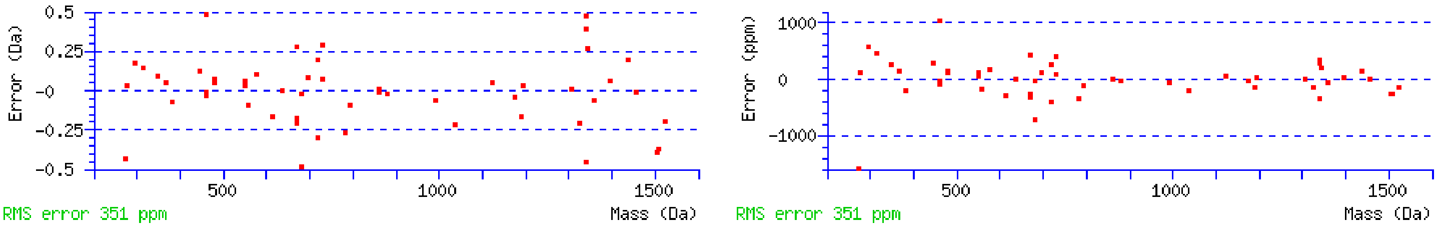

All matches to this query

Score	Mr(calc):	Delta	Sequence
56.5	1669.6985	0.0031	TNPSPTNPFSSDAQK
56.4	1669.6985	0.0031	TNPSPTNPFSSDAQK
36.1	1669.6985	0.0031	TNPSPTNPFSSDAQK
30.2	1669.6985	0.0031	TNPSPTNPFSSDAQK
22.4	1669.6985	0.0031	TNPSPTNPFSSDAQK
6.3	1669.7137	-0.0121	KSDWTWIDNNPSK
4.1	1668.7073	0.9944	LTFDSFFSPNTGEK
3.5	1668.6992	1.0024	DGGQLASIETADEQR
2.4	1668.6821	1.0196	DNYWIGSSYNNKK
2.4	1668.6821	1.0196	DNYWIGSSYNNKK

Spectrum No: 44; Query: 363; Rank: 1

Peptide View

MS/MS Fragmentation of **EIDQEAAVEVSQLR**
Found in **IPI00362949**, Tax_Id=10116 Gene_Symbol=Uqcrfs1 Cytochrome b-c1 complex subunit Rieske, mitochondrial precursor

Match to Query 363: 1585.796532 from(529.606120,3+)
Title: 091127RatKid_SCX01_19.2751.2751.3.dta
Data file K:\NewmanPaper\Piliang\3SubProteomes\Piliang3SP\mgf5ppm\SCX_3SubProteomes5ppm.mgf

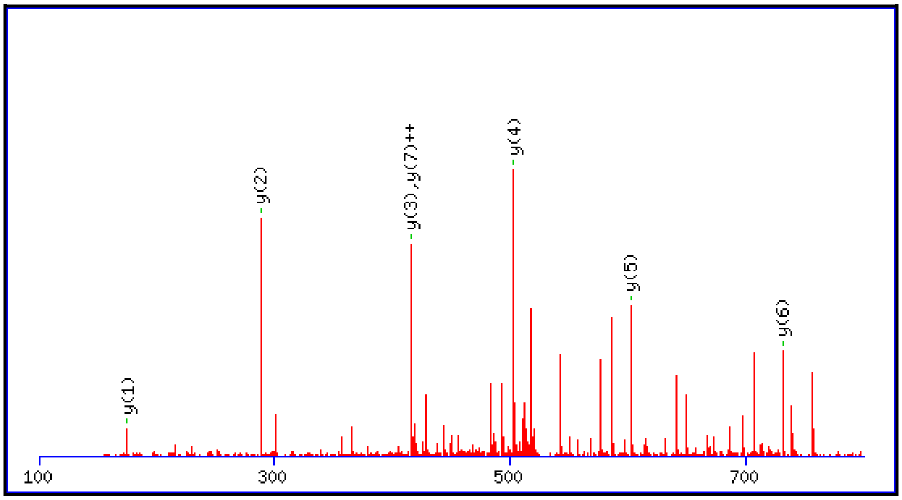

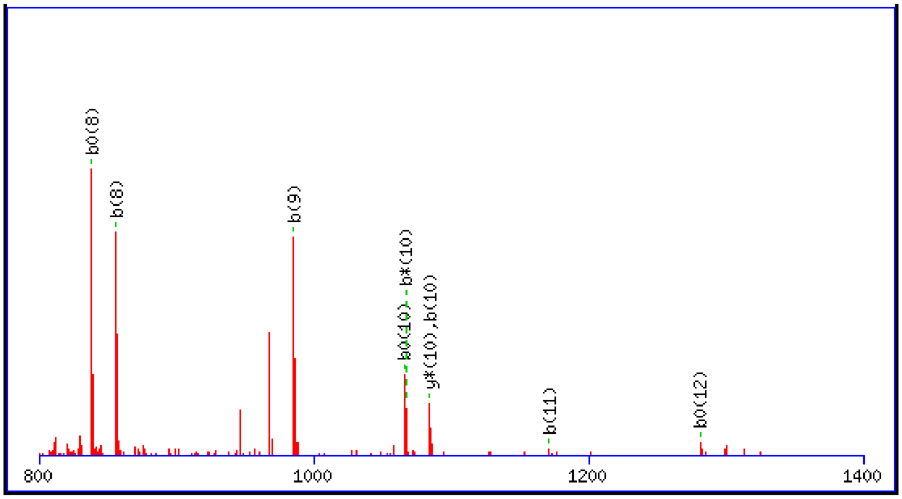

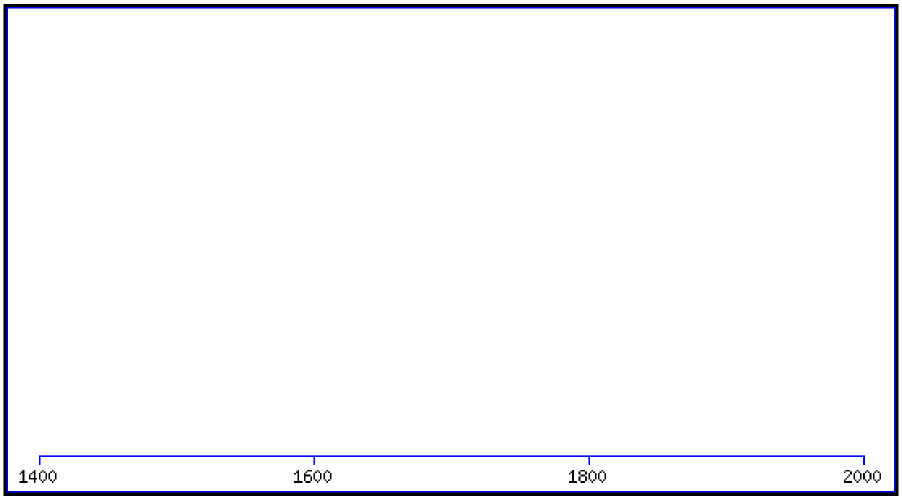

Monoisotopic mass of neutral peptide Mr(calc): 1585.7947
Fixed modifications: Carbamidomethyl (C)
Ions Score: 56 **Expect:** 0.00038
Matches (Bold Red): 16/144 fragment ions using 17 most intense peaks

#	b	b^{++}	b*	b*$^{++}$	b^{0}	b^{0++}	Seq.	y	y^{++}	y*	y*$^{++}$	y^{0}	y^{0++}	#
1	130.0499	65.5286			112.0393	56.5233	E							14
2	243.1339	122.0706			225.1234	113.0653	I	1457.7594	729.3834	1440.7329	720.8701	1439.7489	720.3781	13
3	358.1609	179.5841			340.1503	170.5788	D	1344.6754	672.8413	1327.6488	664.3281	1326.6648	663.8360	12
4	486.2195	243.6134	469.1929	235.1001	468.2089	234.6081	Q	1229.6484	615.3279	1212.6219	606.8146	1211.6379	606.3226	11
5	615.2620	308.1347	598.2355	299.6214	597.2515	299.1294	E	1101.5899	551.2986	**1084.5633**	542.7853	1083.5793	542.2933	10
6	686.2992	343.6532	669.2726	335.1399	668.2886	334.6479	A	972.5473	486.7773	955.5207	478.2640	954.5367	477.7720	9
7	757.3363	379.1718	740.3097	370.6585	739.3257	370.1665	A	901.5102	451.2587	884.4836	442.7454	883.4996	442.2534	8
8	**856.4047**	428.7060	839.3781	420.1927	**838.3941**	419.7007	V	830.4730	**415.7402**	813.4465	407.2269	812.4625	406.7349	7
9	**985.4473**	493.2273	968.4207	484.7140	967.4367	484.2220	E	**731.4046**	366.2060	714.3781	357.6927	713.3941	357.2007	6
10	**1084.5157**	542.7615	**1067.4891**	534.2482	**1066.5051**	533.7562	V	**602.3620**	301.6847	585.3355	293.1714	584.3515	292.6794	5
11	**1171.5477**	586.2775	1154.5212	577.7642	1153.5372	577.2722	S	**503.2936**	252.1504	486.2671	243.6372	485.2831	243.1452	4
12	1299.6063	650.3068	1282.5798	641.7935	**1281.5957**	641.3015	Q	**416.2616**	208.6344	399.2350	200.1212			3
13	1412.6904	706.8488	1395.6638	698.3355	1394.6798	697.8435	L	**288.2030**	144.6051	271.1765	136.0919			2
14							R	**175.1190**	88.0631	158.0924	79.5498			1

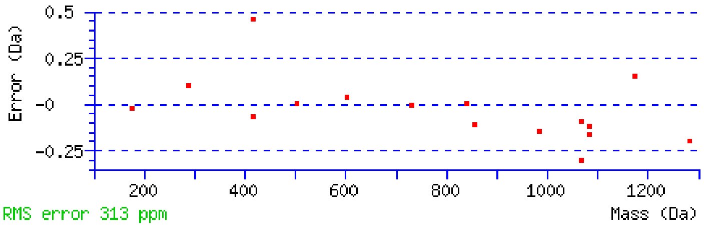

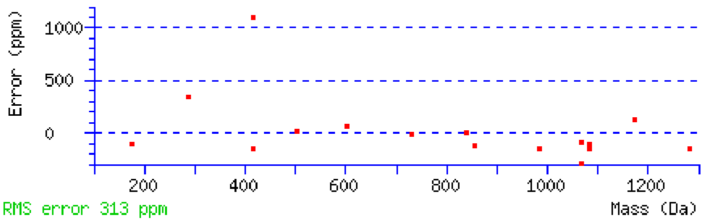

All matches to this query

Score	Mr(calc):	Delta	Sequence
55.8	1585.7947	0.0018	EIDQEAAVEVSQLR
55.8	1585.7977	-0.0012	YQAGSLREVSKIR
55.8	1585.7977	-0.0012	YQAGSLREVSKIR
19.0	1585.8059	-0.0094	EEQALREENKALR
13.9	1584.8009	0.9957	WDDAGGQVVRDVIR
13.6	1585.8011	-0.0046	SLKSSLRVNLMSR
12.9	1583.7950	2.0016	ALPVKTGRVSQLR
12.6	1584.8025	0.9940	TSRSKVFPTVNIR
11.1	1584.8065	0.9900	GSFFPLKEPSLKR
9.6	1583.7920	2.0046	ATSSATTLASTPKLR

Spectrum No: 45; Query: 618; Rank: 1

Peptide View

MS/MS Fragmentation of **SATPPPTEPASLPQEPPK**
Found in **IPI00560565**, Tax_Id=10116 Gene_Symbol=RGD1309571 54 kDa protein

Match to Query 618: 1922.911302 from(641.977710,3+)
Title: 091127RatKid_SCX01_11.1151.1151.3.dta
Data file K:\NewmanPaper\Piliang\3SubProteomes\Piliang3SP\mgf5ppm\SCX_3SubProteomes5ppm.mgf

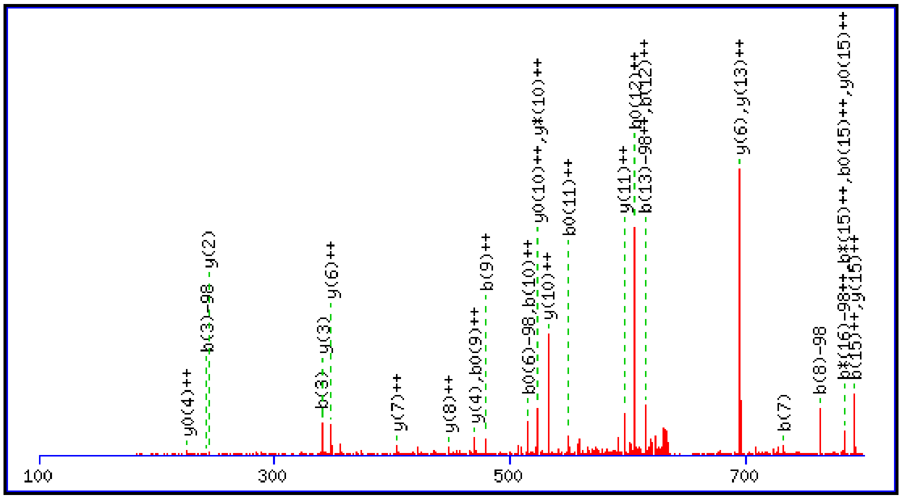

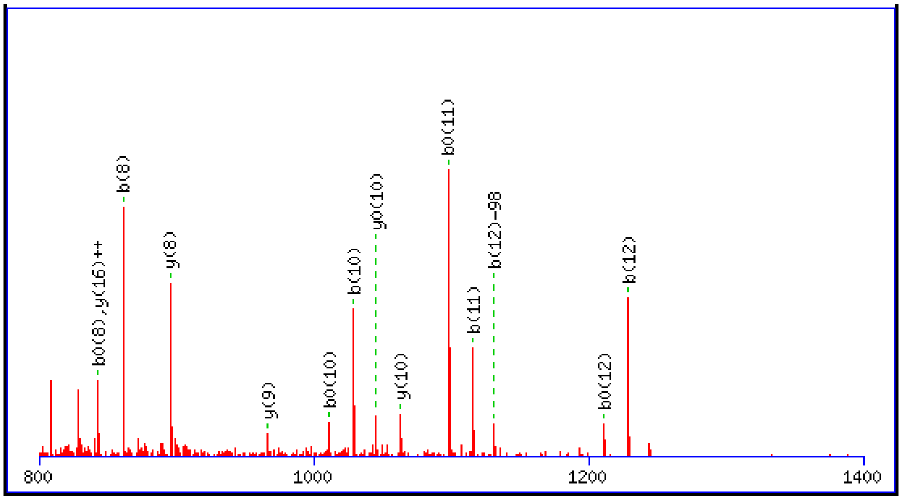

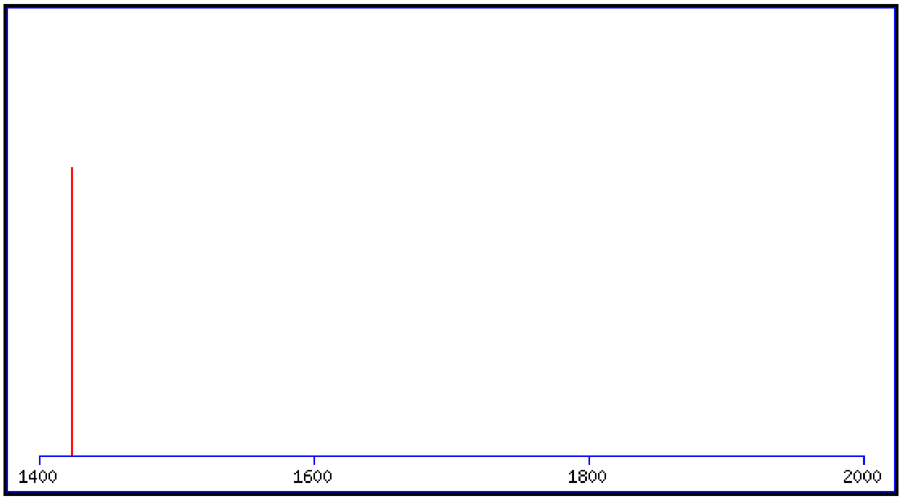

Monoisotopic mass of neutral peptide Mr(calc): 1922.9026
Fixed modifications: Carbamidomethyl (C)
Variable modifications:
S1 : Phospho (ST), with neutral losses 97.9769(shown in table), 0.0000
Ions Score: 56 **Expect:** 0.00067
Matches (Bold Red): 45/248 fragment ions using 53 most intense peaks

#	b	b^{++}	b*	b^{*++}	b^0	b^{0++}	Seq.	y	y^{++}	y*	y^{*++}	y^0	y^{0++}	#
1	70.0287	35.5180			52.0182	26.5127	S							18
2	141.0658	71.0366			123.0553	62.0313	A	1756.9116	878.9594	1739.8850	870.4462	1738.9010	869.9542	17
3	**242.1135**	121.5604			224.1030	112.5551	T	1685.8745	**843.4409**	1668.8479	834.9276	1667.8639	834.4356	16
4	339.1663	170.0868			321.1557	161.0815	P	1584.8268	**792.9170**	1567.8003	784.4038	1566.8162	**783.9118**	15
5	436.2191	218.6132			418.2085	209.6079	P	1487.7740	744.3907	1470.7475	735.8774	1469.7635	735.3854	14
6	533.2718	267.1395			**515.2613**	258.1343	P	1390.7213	**695.8643**	1373.6947	687.3510	1372.7107	686.8590	13
7	634.3195	317.6634			616.3089	308.6581	T	1293.6685	647.3379	1276.6420	638.8246	1275.6579	638.3326	12
8	**763.3621**	382.1847			745.3515	373.1794	E	1192.6208	**596.8141**	1175.5943	588.3008	1174.6103	587.8088	11
9	860.4149	430.7111			842.4043	421.7058	P	**1063.5782**	**532.2928**	1046.5517	**523.7795**	**1045.5677**	**523.2875**	10
10	931.4520	466.2296			913.4414	457.2243	A	**966.5255**	483.7664	949.4989	475.2531	948.5149	474.7611	9
11	1018.4840	509.7456			1000.4734	500.7404	S	**895.4884**	**448.2478**	878.4618	439.7345	877.4778	439.2425	8
12	**1131.5681**	566.2877			1113.5575	557.2824	L	808.4563	**404.7318**	791.4298	396.2185	790.4458	395.7265	7
13	1228.6208	**614.8140**			1210.6103	605.8088	P	**695.3723**	**348.1898**	678.3457	339.6765	677.3617	339.1845	6
14	1356.6794	678.8433	1339.6529	670.3301	1338.6688	669.8381	Q	598.3195	299.6634	581.2930	291.1501	580.3089	290.6581	5
15	1485.7220	743.3646	1468.6954	734.8514	1467.7114	734.3594	E	**470.2609**	235.6341	453.2344	227.1208	452.2504	**226.6288**	4
16	1582.7748	791.8910	1565.7482	**783.3777**	1564.7642	782.8857	P	**341.2183**	171.1128	324.1918	162.5995			3
17	1679.8275	840.4174	1662.8010	831.9041	1661.8170	831.4121	P	**244.1656**	122.5864	227.1390	114.0731			2
18							K	147.1128	74.0600	130.0863	65.5468			1

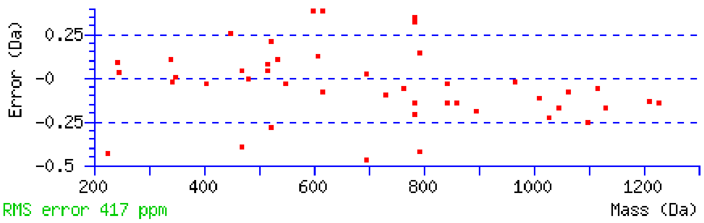

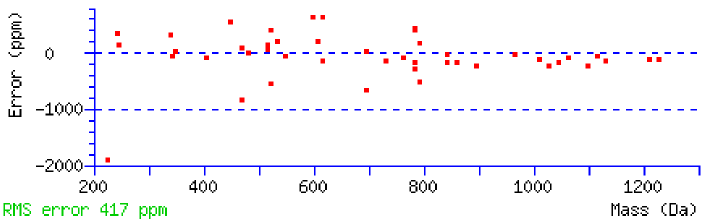

All matches to this query

Score	Mr(calc):	Delta	Sequence
55.6	1922.9026	0.0087	SATPPPTEPASLPQEPPK
52.5	1922.9026	0.0087	SATPPPTEPASLPQEPPK
41.5	1922.9026	0.0087	SATPPPTEPASLPQEPPK
16.2	1922.9026	0.0087	SATPPPTEPASLPQEPPK
7.5	1922.9155	-0.0042	QVKKTQEAVSELIYK
7.2	1922.9163	-0.0050	SPNSPIVEEFQFPYNR
5.8	1921.9216	0.9897	NRVKLAANFSFAPVTK
5.8	1922.9056	0.0057	NRVKLAANFSFAPVTK
5.2	1921.9047	1.0066	LEHGEELQVSNRRXK
3.6	1922.8945	0.0168	GVWKEMNPPTHPDASGGK

Spectrum No: 46; Query: 617; Rank: 1

Peptide View

MS/MS Fragmentation of **SATPPPTEPASLPQEPPK**
Found in **IPI00560565**, Tax_Id=10116 Gene_Symbol=RGD1309571 54 kDa protein

Match to Query 617: 1922.907208 from(962.460880,2+)
Title: 091127RatKid_SCX01_11.1262.1262.2.dta
Data file K:\NewmanPaper\Piliang\3SubProteomes\Piliang3SP\mgf5ppm\SCX_3SubProteomes5ppm.mgf

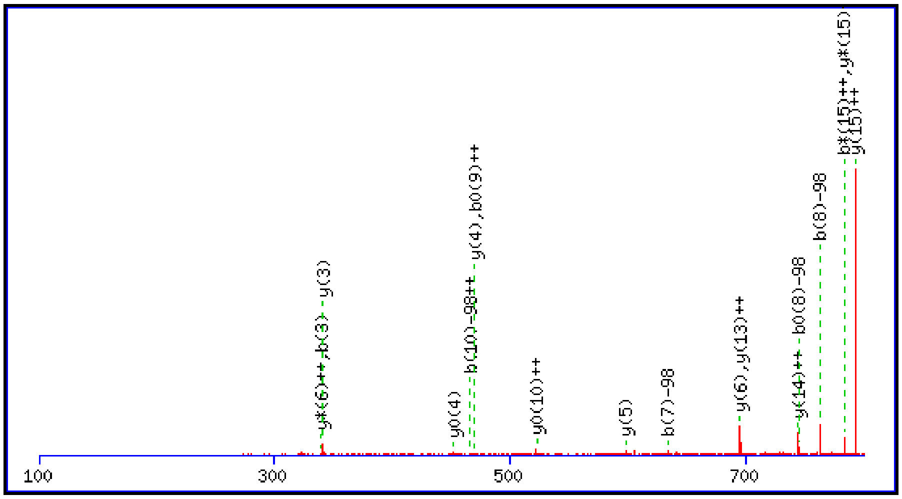

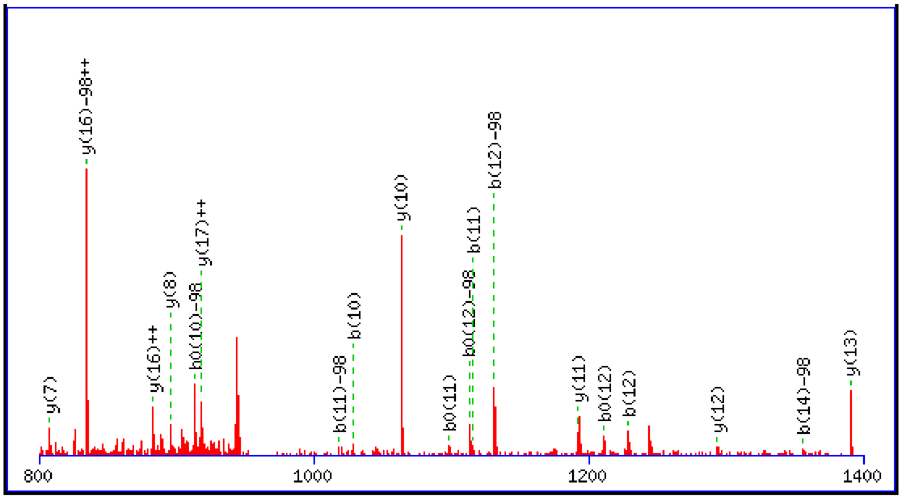

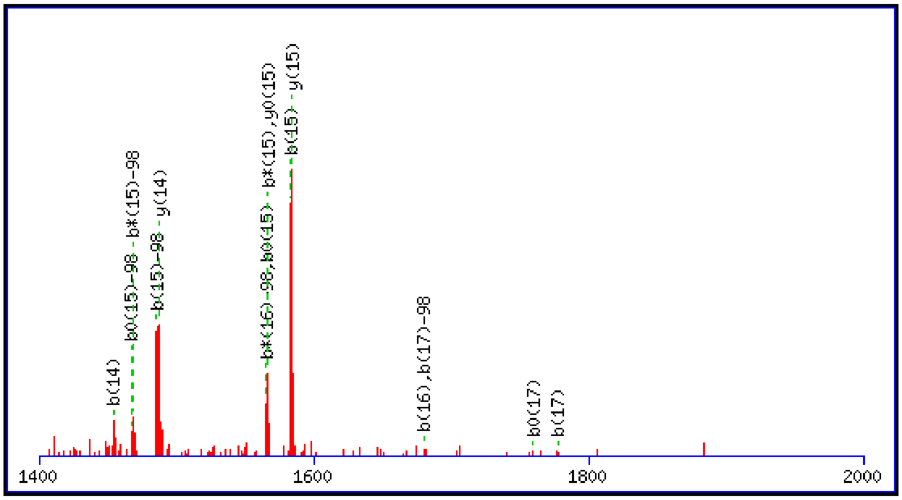

Monoisotopic mass of neutral peptide Mr(calc): 1922.9026
Fixed modifications: Carbamidomethyl (C)
Variable modifications:
T3 : Phospho (ST), with neutral losses 97.9769(shown in table), 0.0000
Ions Score: 55 **Expect:** 0.00068
Matches (Bold Red): 53/252 fragment ions using 99 most intense peaks

#	b	b^{++}	b*	b^{*++}	b^0	b^{0++}	Seq.	y	y^{++}	y*	y^{*++}	y^0	y^{0++}	#
1	88.0393	44.5233			70.0287	35.5180	S							18
2	159.0764	80.0418			141.0659	71.0366	A	1738.9010	869.9541	1721.8745	861.4409	1720.8905	860.9489	17
3	242.1135	121.5604			224.1030	112.5551	T	1667.8639	**834.4356**	1650.8374	825.9223	1649.8533	825.4303	16
4	339.1663	170.0868			321.1557	161.0815	P	**1584.8268**	**792.9170**	1567.8003	**784.4038**	**1566.8162**	**783.9118**	15
5	436.2191	218.6132			418.2085	209.6079	P	**1487.7740**	**744.3907**	1470.7475	735.8774	1469.7635	735.3854	14
6	533.2718	267.1395			515.2613	258.1343	P	**1390.7213**	**695.8643**	1373.6947	687.3510	1372.7107	686.8590	13
7	**634.3195**	317.6634			616.3089	308.6581	T	**1293.6685**	647.3379	1276.6420	638.8246	1275.6579	638.3326	12
8	**763.3621**	382.1847			**745.3515**	373.1794	E	**1192.6208**	596.8141	1175.5943	588.3008	1174.6103	587.8088	11
9	860.4149	430.7111			842.4043	421.7058	P	**1063.5782**	532.2928	1046.5517	523.7795	1045.5677	**523.2875**	10
10	931.4520	**466.2296**			**913.4414**	457.2243	A	966.5255	483.7664	949.4989	475.2531	948.5149	474.7611	9
11	**1018.4840**	509.7456			1000.4734	500.7404	S	**895.4884**	448.2478	878.4618	439.7345	877.4778	439.2425	8
12	**1131.5681**	566.2877			**1113.5575**	557.2824	L	**808.4563**	404.7318	791.4298	396.2185	790.4458	395.7265	7
13	1228.6208	614.8140			1210.6103	605.8088	P	**695.3723**	348.1898	678.3457	**339.6765**	677.3617	339.1845	6
14	**1356.6794**	678.8433	1339.6529	670.3301	1338.6688	669.8381	Q	**598.3195**	299.6634	581.2930	291.1501	580.3089	290.6581	5
15	**1485.7220**	743.3646	**1468.6954**	734.8514	**1467.7114**	734.3594	E	**470.2609**	235.6341	453.2344	227.1208	**452.2504**	226.6288	4
16	1582.7748	791.8910	**1565.7482**	783.3777	1564.7642	782.8857	P	**341.2183**	171.1128	324.1918	162.5995			3
17	**1679.8275**	840.4174	1662.8010	831.9041	1661.8170	831.4121	P	244.1656	122.5864	227.1390	114.0731			2
18							K	147.1128	74.0600	130.0863	65.5468			1

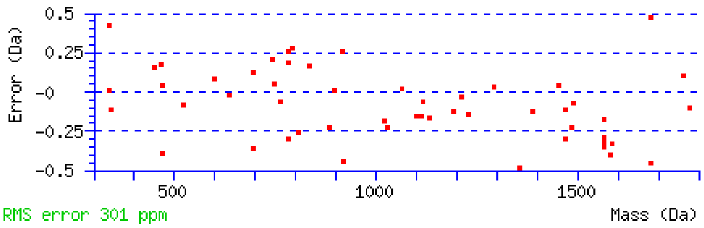

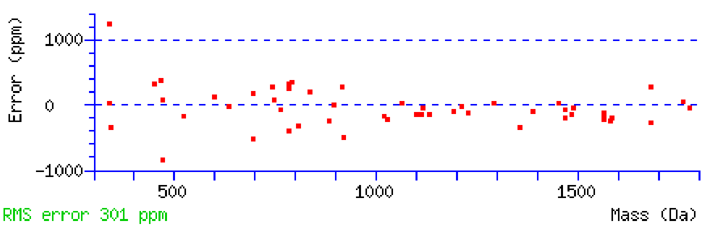

All matches to this query

Score	Mr(calc):	Delta	Sequence
55.3	1922.9026	0.0046	SATPPPTEPASLPQEPPK
54.3	1922.9026	0.0046	SATPPPTEPASLPQEPPK
29.1	1922.9026	0.0046	SATPPPTEPASLPQEPPK
14.7	1922.9026	0.0046	SATPPPTEPASLPQEPPK
2.5	1920.8940	2.0132	GLSLYPWIPTSPKFR
2.4	1920.9146	1.9926	NGMVIMRSGQPLTGTNGR
1.8	1922.9172	-0.0100	VGTTLLLLALLPGTSSK
1.8	1922.9172	-0.0100	VGTTLLLLALLPGTSSK
1.8	1922.9172	-0.0100	VGTTLLLLALLPGTSSK
1.8	1920.9056	2.0016	SGLPLYCLEKYTEIR

Spectrum No: 47; Query: 183; Rank: 1

Peptide View

MS/MS Fragmentation of **GEPNVSYICSR**
Found in **IPI00189904**, Tax_Id=10116 Gene_Symbol=Gsk3a Glycogen synthase kinase-3 alpha

Match to Query 183: 1360.550628 from(681.282590,2+)
Title: 091127RatKid_SCX01_13.1556.1556.2.dta
Data file K:\NewmanPaper\Piliang\3SubProteomes\Piliang3SP\mgf5ppm\SCX_3SubProteomes5ppm.mgf

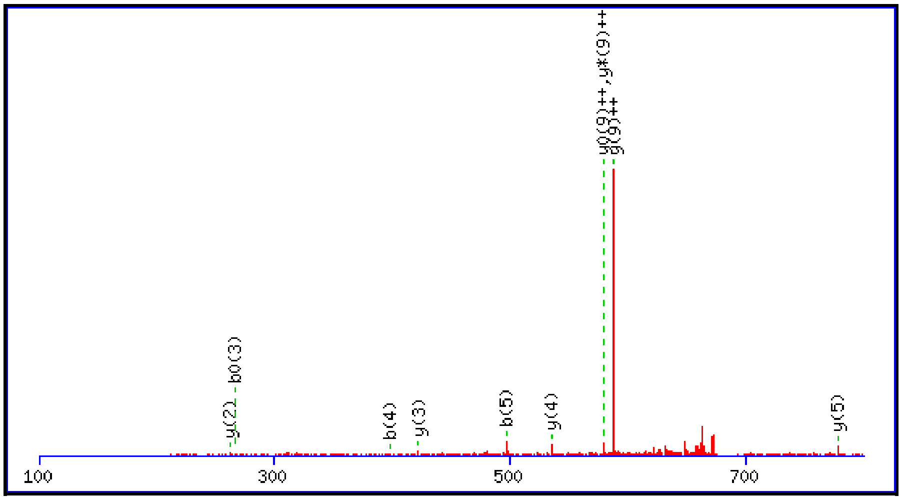

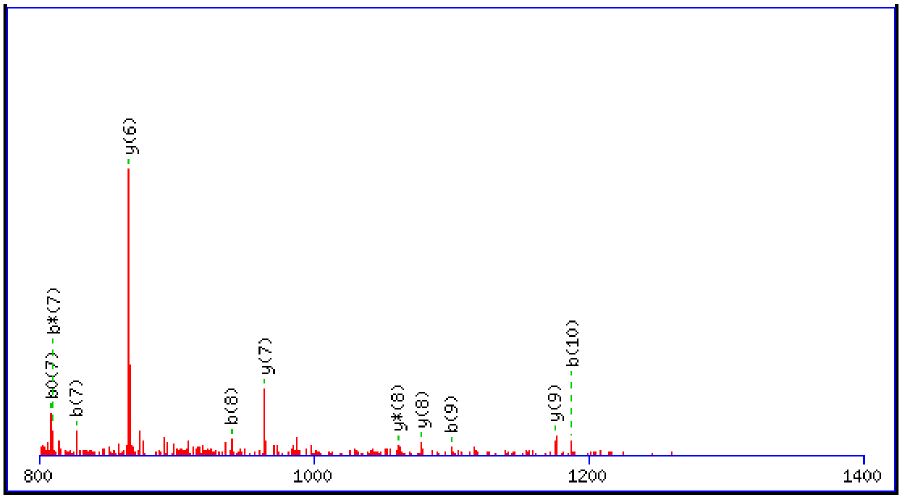

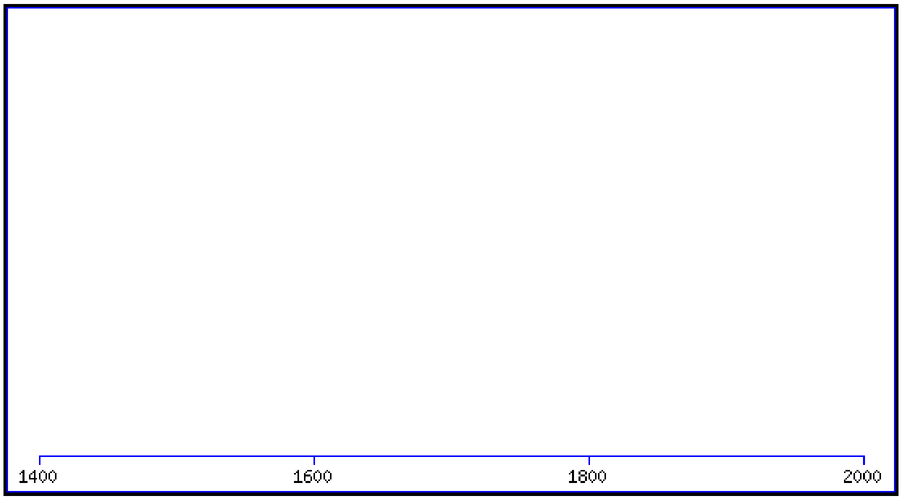

Monoisotopic mass of neutral peptide Mr(calc): 1360.5482
Fixed modifications: Carbamidomethyl (C)
Variable modifications:
Y7 : Phospho (Y)
Ions Score: 55 **Expect:** 0.00031
Matches (Bold Red): 21/110 fragment ions using 40 most intense peaks

#	b	b^{++}	b*	b^{*++}	b^{0}	b^{0++}	Seq.	y	y^{++}	y*	y^{*++}	y^{0}	y^{0++}	#
1	58.0287	29.5180					G							11
2	187.0713	94.0393			169.0608	85.0340	E	1304.5341	652.7707	1287.5075	644.2574	1286.5235	643.7654	10
3	284.1241	142.5657			**266.1135**	133.5604	P	**1175.4915**	**588.2494**	1158.4649	**579.7361**	1157.4809	**579.2441**	9
4	**398.1670**	199.5872	381.1405	191.0739	380.1565	190.5819	N	**1078.4387**	539.7230	**1061.4122**	531.2097	1060.4282	530.7177	8
5	**497.2354**	249.1214	480.2089	240.6081	479.2249	240.1161	V	**964.3958**	482.7015	947.3692	474.1883	946.3852	473.6962	7
6	584.2675	292.6374	567.2409	284.1241	566.2569	283.6321	S	**865.3274**	433.1673	848.3008	424.6541	847.3168	424.1620	6
7	**827.2971**	414.1522	**810.2706**	405.6389	**809.2866**	405.1469	Y	**778.2953**	389.6513	761.2688	381.1380	760.2848	380.6460	5
8	**940.3812**	470.6942	923.3546	462.1810	922.3706	461.6889	I	**535.2657**	268.1365	518.2391	259.6232	517.2551	259.1312	4
9	**1100.4118**	550.7096	1083.3853	542.1963	1082.4013	541.7043	C	**422.1816**	211.5945	405.1551	203.0812	404.1711	202.5892	3
10	**1187.4439**	594.2256	1170.4173	585.7123	1169.4333	585.2203	S	**262.1510**	131.5791	245.1244	123.0659	244.1404	122.5738	2
11							R	175.1190	88.0631	158.0924	79.5498			1

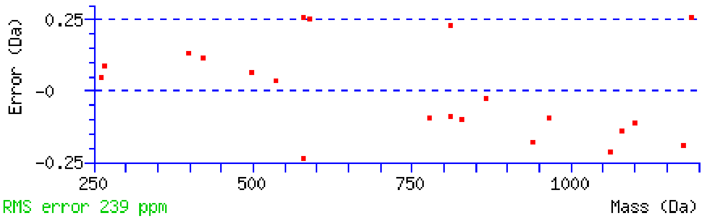

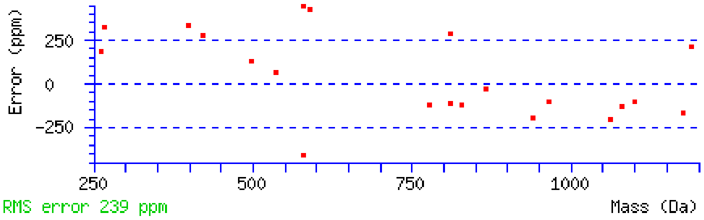

All matches to this query

Score	Mr(calc):	Delta	Sequence
55.2	1360.5482	0.0024	GEPNVSYICSR
45.8	1360.5482	0.0024	GEPNVSYICSR
21.6	1360.5482	0.0024	GEPNVSYICSR
10.5	1359.5391	1.0115	CVPQDGQSPHR
9.8	1360.5516	-0.0010	IDTSCVCALSR
9.1	1360.5516	-0.0010	IDTSCVCALSR
8.2	1360.5483	0.0024	WTSRDVGMADK
7.9	1360.5561	-0.0055	WFSNPSRSGSR
6.3	1360.5561	-0.0055	WFSNPSRSGSR
5.8	1360.5377	0.0129	SXCKARQCSR

Spectrum No: 48; Query: 608; Rank: 1

Peptide View

MS/MS Fragmentation of **KPASVSPTTPPSPTEGEAS**
Found in **IPI00213552**, Tax_Id=10116 Gene_Symbol=Dync1li1 Cytoplasmic dynein 1 light intermediate chain 1

Match to Query 608: 1918.858668 from(960.436610,2+)
Title: 091129RatKid_SCX02_14.749.749.2.dta
Data file K:\NewmanPaper\Piliang\3SubProteomes\Piliang3SP\mgf5ppm\SCX_3SubProteomes5ppm.mgf

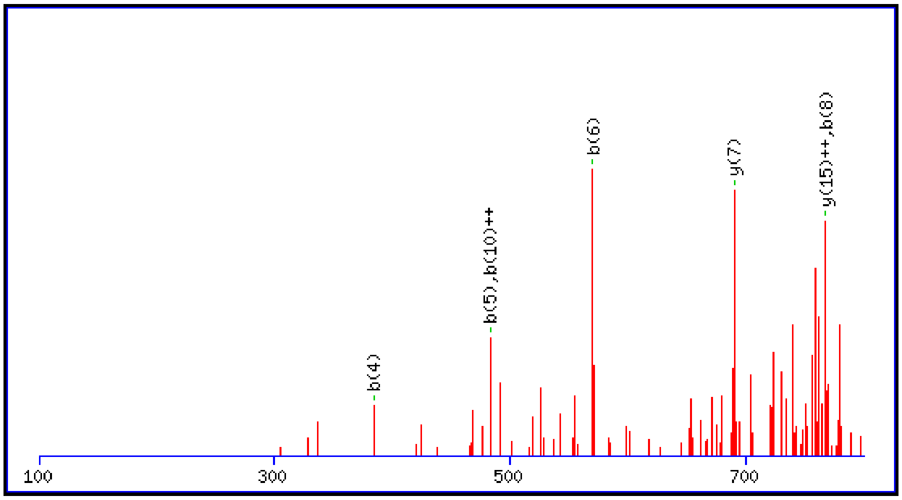

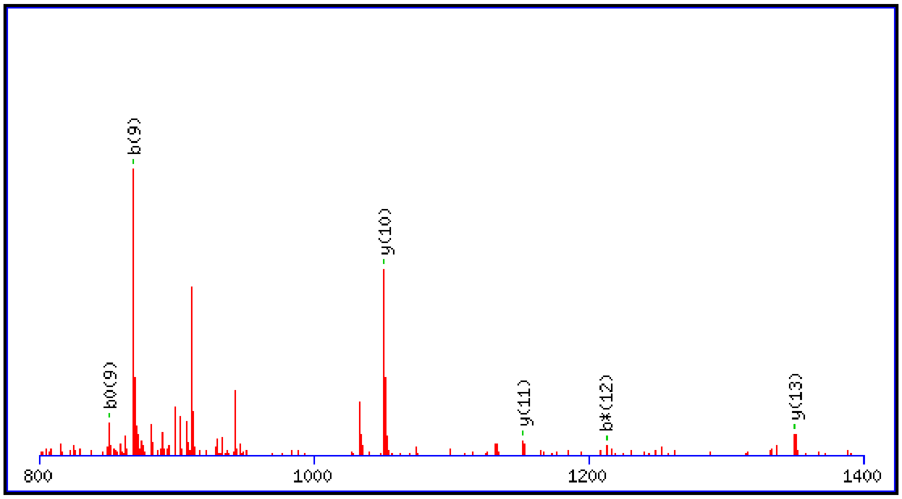

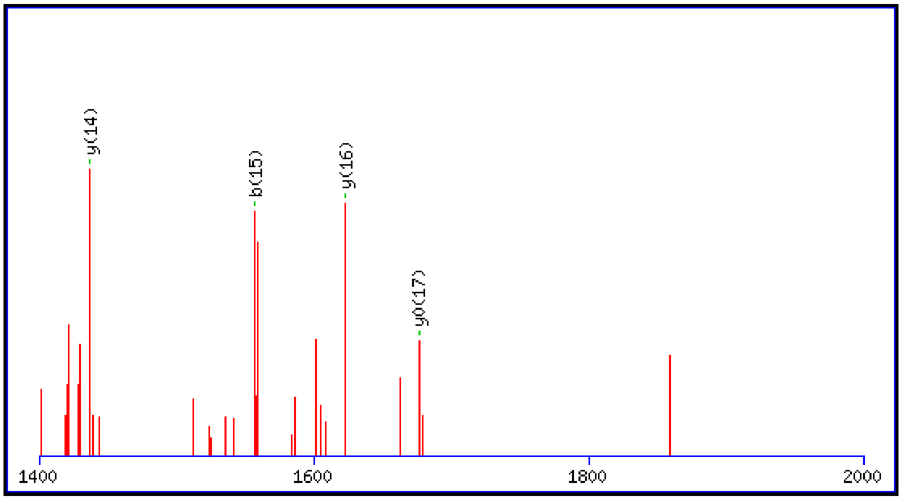

Monoisotopic mass of neutral peptide Mr(calc): 1918.8561
Fixed modifications: Carbamidomethyl (C)
Variable modifications:
S12 : Phospho (ST), with neutral losses 0.0000(shown in table), 97.9769
Ions Score: 55 **Expect:** 0.00073
Matches (Bold Red): 17/260 fragment ions using 22 most intense peaks

#	b	b^{++}	b*	b^{*++}	b^0	b^{0++}	Seq.	y	y^{++}	y^0	y^{0++}	#
1	129.1022	65.0548	112.0757	56.5415			K					19
2	226.1550	113.5811	209.1285	105.0679			P	1791.7684	896.3879	1773.7579	887.3826	18
3	297.1921	149.0997	280.1656	140.5864			A	1694.7157	847.8615	**1676.7051**	838.8562	17
4	**384.2241**	192.6157	367.1976	184.1024	366.2136	183.6104	S	**1623.6785**	812.3429	1605.6680	803.3376	16
5	**483.2926**	242.1499	466.2660	233.6366	465.2820	233.1446	V	1536.6465	**768.8269**	1518.6360	759.8216	15
6	**570.3246**	285.6659	553.2980	277.1527	552.3140	276.6606	S	**1437.5781**	719.2927	1419.5675	710.2874	14
7	667.3774	334.1923	650.3508	325.6790	649.3668	325.1870	P	**1350.5461**	675.7767	1332.5355	666.7714	13
8	**768.4250**	384.7162	751.3985	376.2029	750.4145	375.7109	T	1253.4933	627.2503	1235.4827	618.2450	12
9	**869.4727**	435.2400	852.4462	426.7267	**851.4621**	426.2347	T	**1152.4456**	576.7265	1134.4351	567.7212	11
10	966.5255	**483.7664**	949.4989	475.2531	948.5149	474.7611	P	**1051.3980**	526.2026	1033.3874	517.1973	10
11	1063.5782	532.2928	1046.5517	523.7795	1045.5677	523.2875	P	954.3452	477.6762	936.3346	468.6710	9
12	1230.5766	615.7919	**1213.5500**	607.2787	1212.5660	606.7867	S	857.2924	429.1499	839.2819	420.1446	8
13	1327.6294	664.3183	1310.6028	655.8050	1309.6188	655.3130	P	**690.2941**	345.6507	672.2835	336.6454	7
14	1428.6770	714.8422	1411.6505	706.3289	1410.6665	705.8369	T	593.2413	297.1243	575.2307	288.1190	6
15	**1557.7196**	779.3635	1540.6931	770.8502	1539.7091	770.3582	E	492.1936	246.6005	474.1831	237.5952	5
16	1614.7411	807.8742	1597.7145	799.3609	1596.7305	798.8689	G	363.1510	182.0792	345.1405	173.0739	4
17	1743.7837	872.3955	1726.7571	863.8822	1725.7731	863.3902	E	306.1296	153.5684	288.1190	144.5631	3
18	1814.8208	907.9140	1797.7942	899.4008	1796.8102	898.9088	A	177.0870	89.0471	159.0764	80.0418	2
19							S	106.0499	53.5286	88.0393	44.5233	1

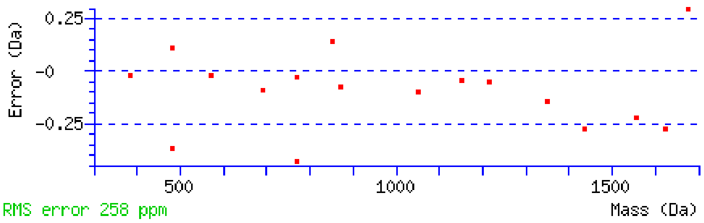

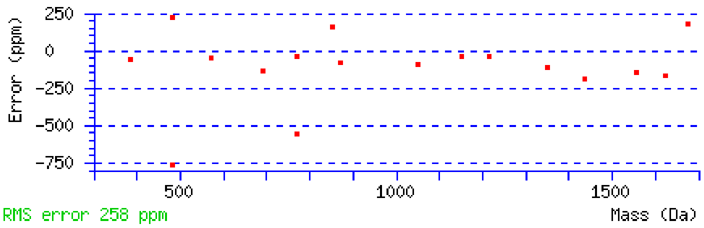

All matches to this query

Score	Mr(calc):	Delta	Sequence
55.1	1918.8561	0.0026	KPASVSPTTPPSPTEGEAS
40.9	1918.8561	0.0026	KPASVSPTTPPSPTEGEAS

33.1	1918.8561	0.0026	KPASVSPTTPPSPTEGEAS
31.8	1918.8561	0.0026	KPASVSPTTPPSPTEGEAS
23.0	1918.8561	0.0026	KPASVSPTTPPSPTEGEAS
6.7	1918.8561	0.0026	KPASVSPTTPPSPTEGEAS
0.3	1918.8574	0.0012	TEIGAPSNRGGHPGPYTK

Spectrum No: 49; Query: 776; Rank: 1

Peptide View

MS/MS Fragmentation of **GAEEEEEEEEDDDSEEEIK**
Found in **IPI00778976**, Tax_Id=10116 Gene_Symbol=Epb4.9_predicted 45 kDa protein

Match to Query 776: 2189.757348 from(1095.885950,2+)
Title: 091127RatKid_SCX01_12.1022.1022.2.dta
Data file K:\NewmanPaper\Piliang\3SubProteomes\Piliang3SP\mgf5ppm\SCX_3SubProteomes5ppm.mgf

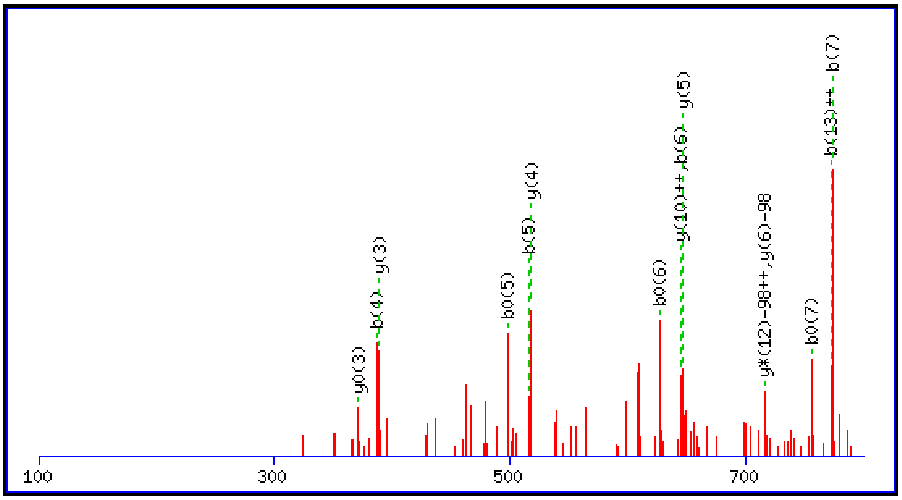

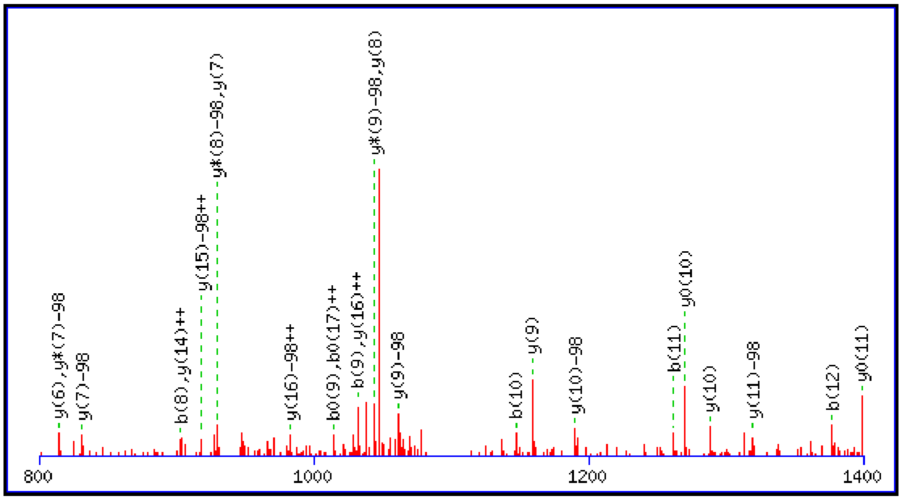

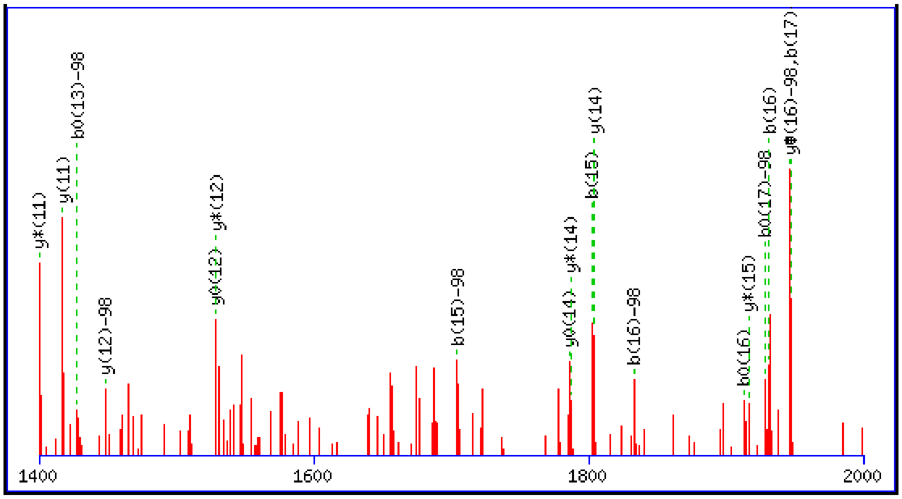

Monoisotopic mass of neutral peptide Mr(calc): 2189.7532
Fixed modifications: Carbamidomethyl (C)
Variable modifications:
S13 : Phospho (ST), with neutral losses 97.9769(shown in table), 0.0000
Ions Score: 55 **Expect:** 0.00026
Matches (Bold Red): 59/254 fragment ions using 90 most intense peaks

#	b	b^{++}	b^{0}	b^{0++}	Seq.	y	y^{++}	y*	y^{*++}	y^{0}	y^{0++}	#
1	58.0287	29.5180			G							18
2	129.0659	65.0366			A	2035.7622	1018.3847	2018.7356	1009.8715	2017.7516	1009.3795	17
3	258.1084	129.5579	240.0979	120.5526	E	1964.7251	**982.8662**	**1947.6985**	974.3529	**1946.7145**	973.8609	16
4	**387.1510**	194.0792	369.1405	185.0739	E	1835.6825	**918.3449**	1818.6559	909.8316	1817.6719	909.3396	15
5	**516.1936**	258.6005	**498.1831**	249.5952	E	1706.6399	853.8236	1689.6133	845.3103	1688.6293	844.8183	14
6	**645.2362**	323.1218	**627.2257**	314.1165	E	1577.5973	789.3023	1560.5708	780.7890	1559.5867	780.2970	13
7	**774.2788**	387.6430	**756.2683**	378.6378	E	**1448.5547**	724.7810	1431.5282	**716.2677**	1430.5441	715.7757	12
8	**903.3214**	452.1643	885.3108	443.1591	E	**1319.5121**	660.2597	1302.4856	651.7464	1301.5016	651.2544	11
9	**1032.3640**	516.6856	**1014.3534**	507.6804	E	**1190.4695**	595.7384	1173.4430	587.2251	1172.4590	586.7331	10
10	**1147.3909**	574.1991	1129.3804	565.1938	D	**1061.4269**	531.2171	**1044.4004**	522.7038	1043.4164	522.2118	9
11	**1262.4179**	631.7126	1244.4073	622.7073	D	946.4000	473.7036	**929.3734**	465.1904	928.3894	464.6984	8
12	**1377.4448**	689.2261	1359.4343	680.2208	D	**831.3730**	416.1902	**814.3465**	407.6769	813.3625	407.1849	7
13	1446.4663	723.7368	**1428.4557**	714.7315	S	**716.3461**	358.6767	699.3196	350.1634	698.3355	349.6714	6
14	1575.5089	788.2581	1557.4983	779.2528	E	**647.3246**	324.1660	630.2981	315.6527	629.3141	315.1607	5
15	**1704.5515**	852.7794	1686.5409	843.7741	E	**518.2821**	259.6447	501.2555	251.1314	500.2715	250.6394	4
16	**1833.5941**	917.3007	1815.5835	908.2954	E	**389.2395**	195.1234	372.2129	186.6101	**371.2289**	186.1181	3
17	**1946.6781**	973.8427	**1928.6676**	964.8374	I	260.1969	130.6021	243.1703	122.0888			2
18					K	147.1128	74.0600	130.0863	65.5468			1

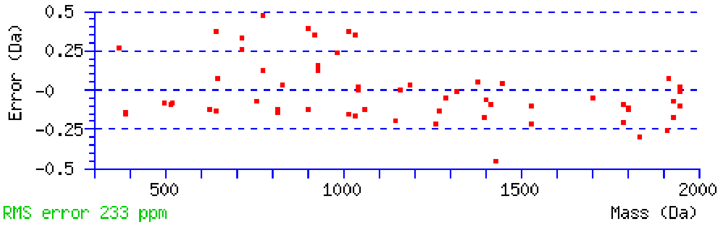

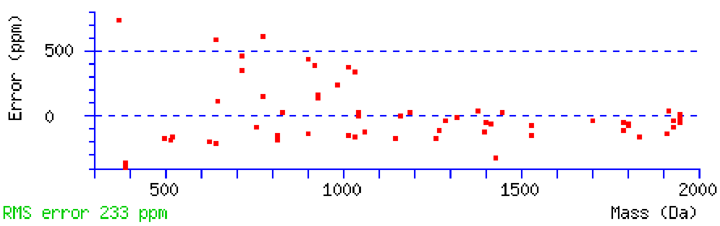

All matches to this query

Score	Mr(calc):	Delta	Sequence
54.9	2189.7532	0.0041	GAEEEEEEEDDDSEEEIK

Spectrum No: 50; Query: 55; Rank: 1

Peptide View

MS/MS Fragmentation of **QLSSGVSEIR**
Found in **IPI00201586**, Tax_Id=10116 Gene_Symbol=Hspb1 Heat shock protein beta-1

Match to Query 55: 1154.532588 from(578.273570,2+)
Title: 091127RatKid_SCX01_13.1279.1279.2.dta
Data file K:\NewmanPaper\Piliang\3SubProteomes\Piliang3SP\mgf5ppm\SCX_3SubProteomes5ppm.mgf

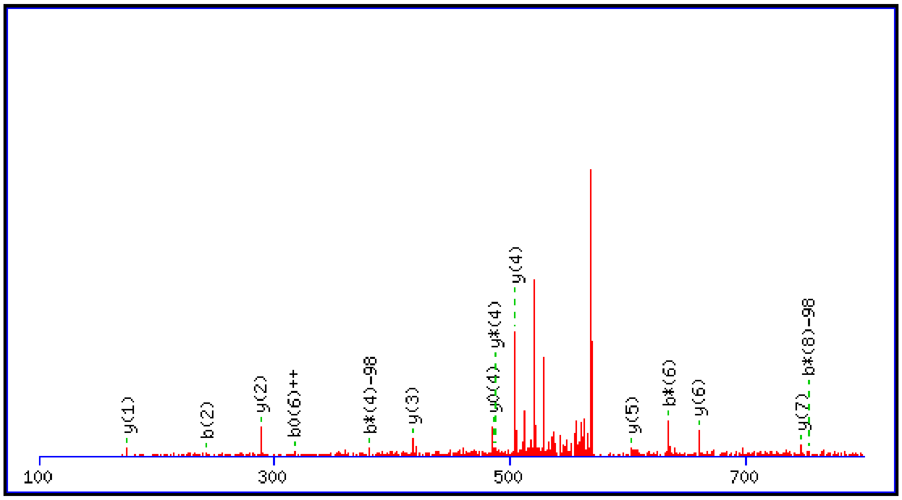

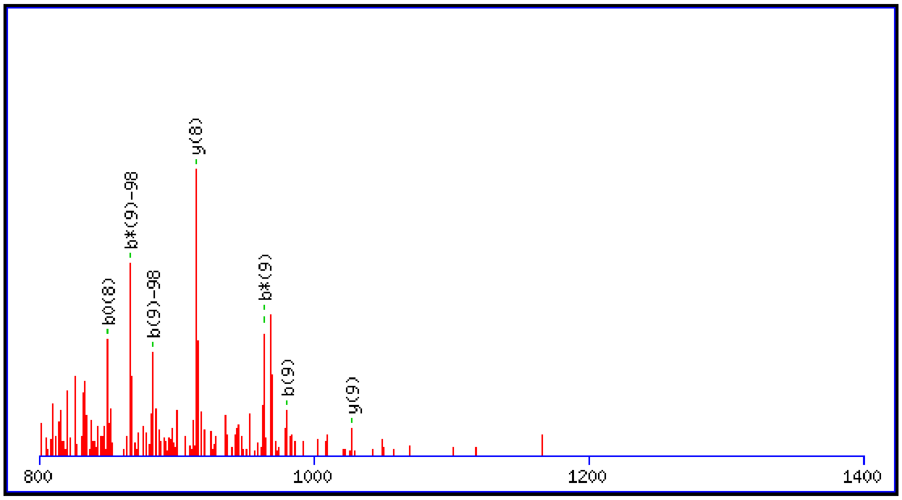

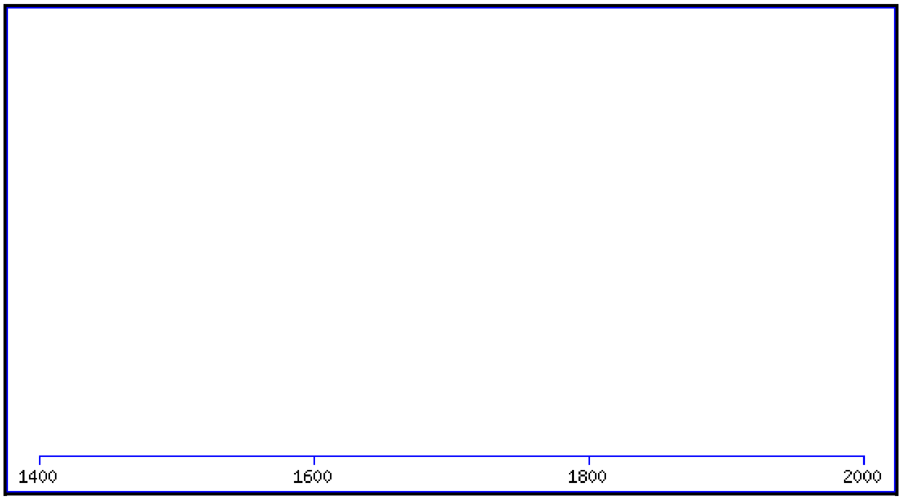

Monoisotopic mass of neutral peptide Mr(calc): 1154.5332
Fixed modifications: Carbamidomethyl (C)
Variable modifications:

```
S3     : Phospho (ST), with neutral losses 0.0000(shown in table), 97.9769
Ions Score: 55  Expect: 0.00042
Matches (Bold Red): 21/154 fragment ions using 48 most intense peaks
```

#	b	b^{++}	b*	b^{*++}	b^0	b^{0++}	Seq.	y	y^{++}	y*	y^{*++}	y^0	y^{0++}	#
1	129.0659	65.0366	112.0393	56.5233			Q							10
2	**242.1499**	121.5786	225.1234	113.0653			L	**1027.4820**	514.2446	1010.4554	505.7313	1009.4714	505.2393	9
3	409.1483	205.0778	392.1217	196.5645	391.1377	196.0725	S	**914.3979**	457.7026	897.3713	449.1893	896.3873	448.6973	8
4	496.1803	248.5938	479.1537	240.0805	478.1697	239.5885	S	**747.3995**	374.2034	730.3730	365.6901	729.3890	365.1981	7
5	553.2018	277.1045	536.1752	268.5912	535.1912	268.0992	G	**660.3675**	330.6874	643.3410	322.1741	642.3570	321.6821	6
6	652.2702	326.6387	**635.2436**	318.1255	634.2596	**317.6334**	V	**603.3461**	302.1767	586.3195	293.6634	585.3355	293.1714	5
7	739.3022	370.1547	722.2757	361.6415	721.2916	361.1495	S	**504.2776**	252.6425	**487.2511**	244.1292	**486.2671**	243.6372	4
8	868.3448	434.6760	851.3182	426.1628	**850.3342**	425.6708	E	**417.2456**	209.1264	400.2191	200.6132	399.2350	200.1212	3
9	**981.4289**	491.2181	**964.4023**	482.7048	963.4183	482.2128	I	**288.2030**	144.6051	271.1765	136.0919			2
10							R	**175.1190**	88.0631	158.0924	79.5498			1

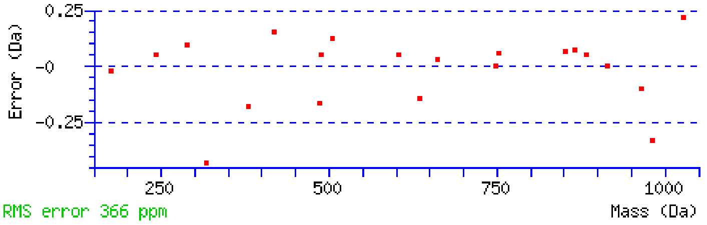

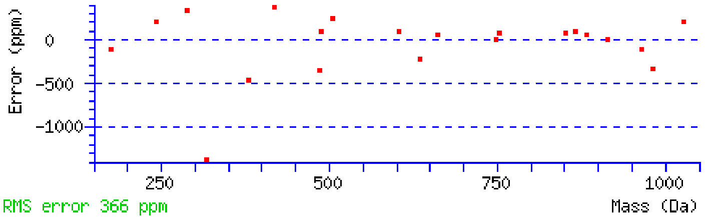

All matches to this query

Score	Mr(calc):	Delta	Sequence
54.9	1154.5332	-0.0007	QLSSGVSEIR
42.7	1154.5332	-0.0007	QLSSGVSEIR
18.7	1154.5332	-0.0007	QLSSGVSEIR
14.0	1154.5332	-0.0007	SVSELSLQGR
11.2	1154.5332	-0.0007	SVSELSLQGR
9.8	1152.5288	2.0038	PRTTENSLR
9.0	1152.5328	1.9998	KHYEVEIR
9.0	1152.5176	2.0150	LSQQAEELR
9.0	1152.5176	2.0150	SEAQLQEIR
9.0	1152.5288	2.0038	VTRENGELR

Spectrum No: 51; Query: 687; Rank: 1

Peptide View

MS/MS Fragmentation of **GSSQPNLSTSYSEQEYGK**
Found in **IPI00209348**, Tax_Id=10116 Gene_Symbol=Epn2 Epsin-2

Match to Query 687: 2040.837548 from(1021.426050,2+)
Title: 091127RatKid_SCX01_11.1196.1196.2.dta
Data file K:\NewmanPaper\Piliang\3SubProteomes\Piliang3SP\mgf5ppm\SCX_3SubProteomes5ppm.mgf

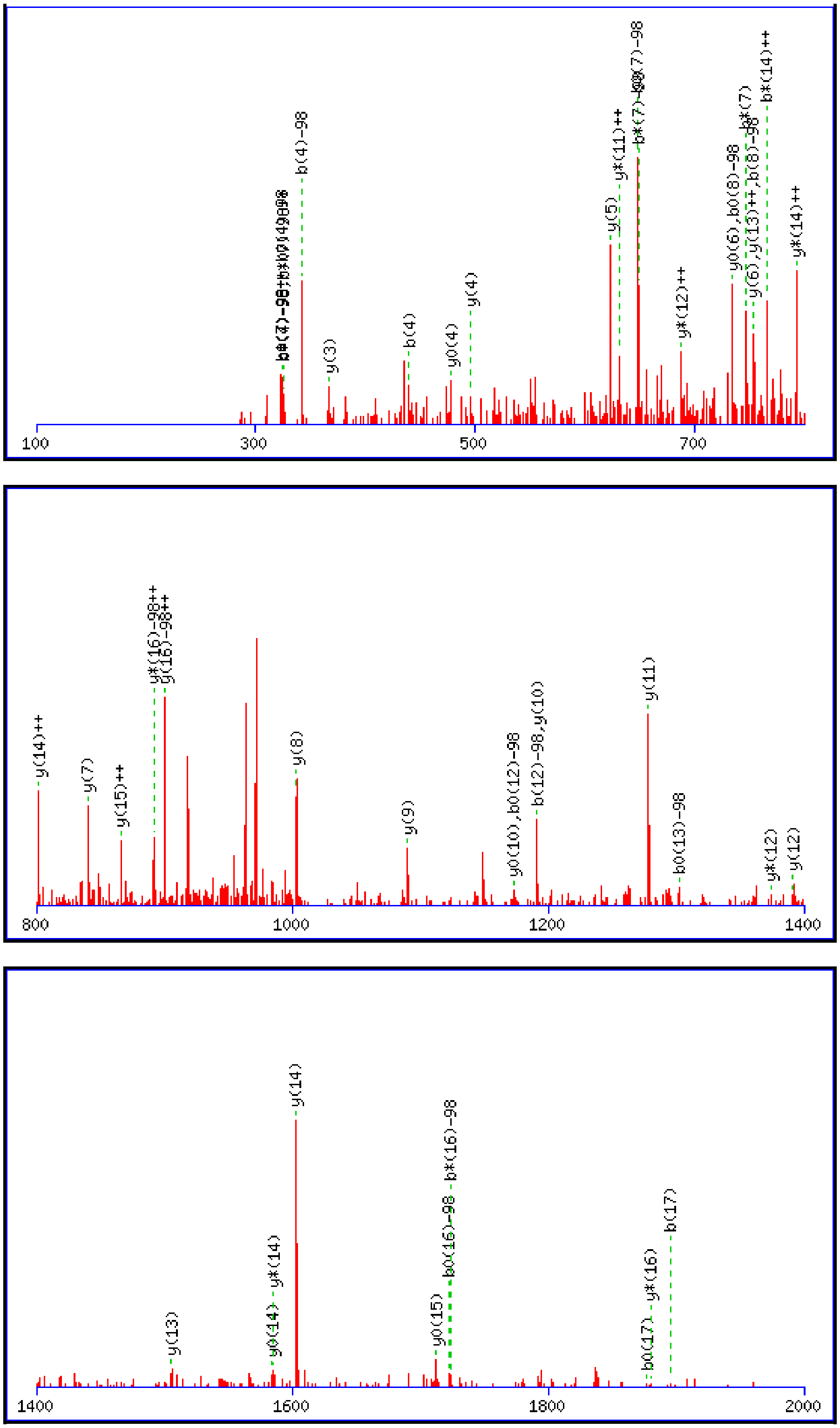

Monoisotopic mass of neutral peptide Mr(calc): 2040.8313
Fixed modifications: Carbamidomethyl (C)
Variable modifications:
S3 : Phospho (ST), with neutral losses 97.9769(shown in table), 0.0000
Ions Score: 55 **Expect:** 0.00071
Matches (Bold Red): 47/290 fragment ions using 93 most intense peaks

#	b	b^{++}	b*	b^{*++}	b^0	b^{0++}	Seq.	y	y^{++}	y*	y^{*++}	y^0	y^{0++}	#
1	58.0287	29.5180					G							18
2	145.0608	73.0340			127.0502	64.0287	S	1886.8403	943.9238	1869.8137	935.4105	1868.8297	934.9185	17
3	214.0822	107.5448			196.0717	98.5395	S	1799.8082	**900.4078**	1782.7817	**891.8945**	1781.7977	891.4025	16
4	**342.1408**	171.5740	**325.1143**	163.0608	**324.1302**	162.5688	Q	1730.7868	**865.8970**	1713.7602	857.3838	**1712.7762**	856.8917	15
5	439.1936	220.1004	422.1670	211.5871	421.1830	211.0951	P	**1602.7282**	**801.8677**	**1585.7017**	**793.3545**	**1584.7176**	792.8625	14

**6**	553.2365	277.1219	536.2099	268.6086	535.2259	268.1166	**N**	**1505.6754**	**753.3414**	1488.6489	744.8281	1487.6649	744.3361	**13**
**7**	666.3206	333.6639	**649.2940**	**325.1506**	**648.3100**	**324.6586**	**L**	**1391.6325**	696.3199	**1374.6060**	**687.8066**	1373.6220	687.3146	**12**
**8**	**753.3526**	377.1799	736.3260	368.6667	**735.3420**	368.1746	**S**	**1278.5485**	639.7779	1261.5219	**631.2646**	1260.5379	630.7726	**11**
**9**	854.4003	427.7038	837.3737	419.1905	836.3897	418.6985	**T**	**1191.5164**	596.2619	1174.4899	587.7486	**1173.5059**	587.2566	**10**
**10**	941.4323	471.2198	924.4057	462.7065	923.4217	462.2145	**S**	**1090.4687**	545.7380	1073.4422	537.2247	1072.4582	536.7327	**9**
**11**	1104.4956	552.7514	1087.4691	544.2382	1086.4851	543.7462	**Y**	**1003.4367**	502.2220	986.4102	493.7087	985.4262	493.2167	**8**
**12**	**1191.5276**	596.2675	1174.5011	587.7542	**1173.5171**	587.2622	**S**	**840.3734**	420.6903	823.3468	412.1771	822.3628	411.6851	**7**
**13**	1320.5702	660.7888	1303.5437	652.2755	**1302.5597**	651.7835	**E**	**753.3414**	377.1743	736.3148	368.6610	**735.3308**	368.1690	**6**
**14**	1448.6288	724.8180	1431.6023	716.3048	1430.6183	715.8128	**Q**	**624.2988**	312.6530	607.2722	304.1397	606.2882	303.6477	**5**
**15**	1577.6714	789.3393	1560.6449	780.8261	1559.6608	780.3341	**E**	**496.2402**	248.6237	479.2136	240.1105	**478.2296**	239.6185	**4**
**16**	1740.7347	870.8710	**1723.7082**	862.3577	**1722.7242**	861.8657	**Y**	**367.1976**	184.1024	350.1710	175.5892			**3**
**17**	1797.7562	899.3817	1780.7297	890.8685	1779.7456	890.3765	**G**	204.1343	102.5708	187.1077	94.0575			**2**
**18**							**K**	147.1128	74.0600	130.0863	65.5468			**1**

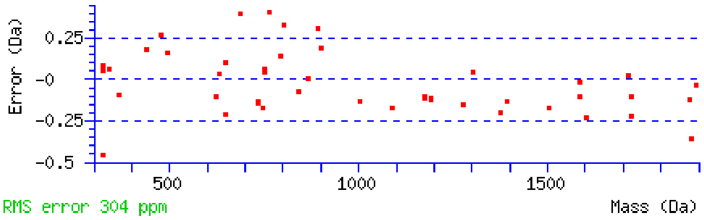

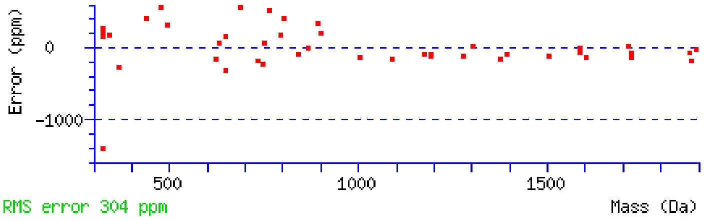

All matches to this query

Score	Mr(calc):	Delta	Sequence
54.6	2040.8313	0.0062	GSSQPNLSTSYSEQEYGK
54.6	2040.8313	0.0062	GSSQPNLSTSYSEQEYGK
20.7	2040.8313	0.0062	GSSQPNLSTSYSEQEYGK
19.9	2040.8313	0.0062	GSSQPNLSTSYSEQEYGK
15.6	2040.8313	0.0062	GSSQPNLSTSYSEQEYGK
7.4	2040.8313	0.0062	GSSQPNLSTSYSEQEYGK

Spectrum No: 52; Query: 966; Rank: 1

Peptide View

MS/MS Fragmentation of **GIPLPTGDTSPEPELLPGDPLPPPK**
Found in **IPI00365487**, Tax_Id=10116 Gene_Symbol=Eif3s4 Eukaryotic translation initiation factor 3, subunit 4

Match to Query 966: 2613.294128 from(1307.654340,2+)
Title: 091129RatKid_SCX02_10.3388.3388.2.dta
Data file K:\NewmanPaper\Piliang\3SubProteomes\Piliang3SP\mgf5ppm\SCX_3SubProteomes5ppm.mgf

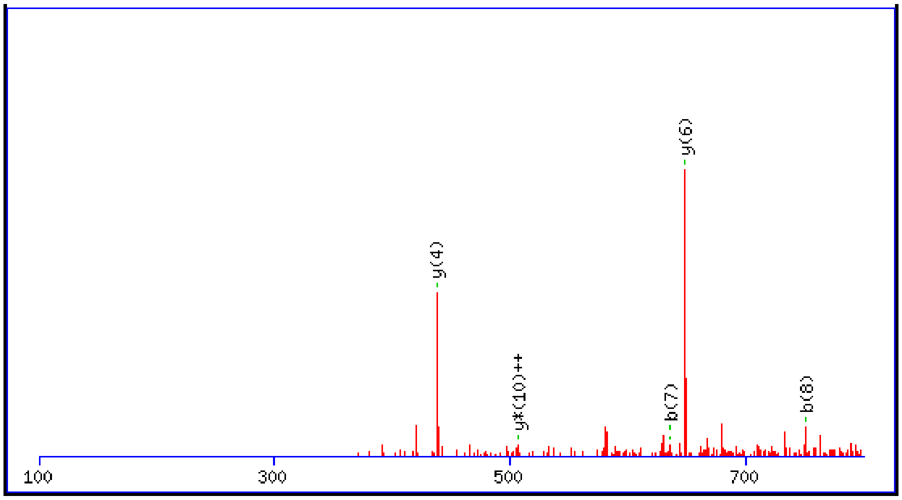

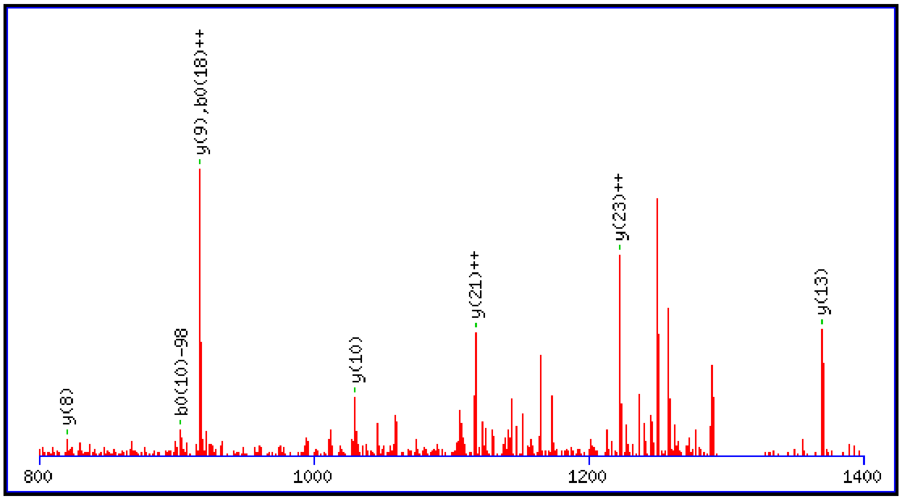

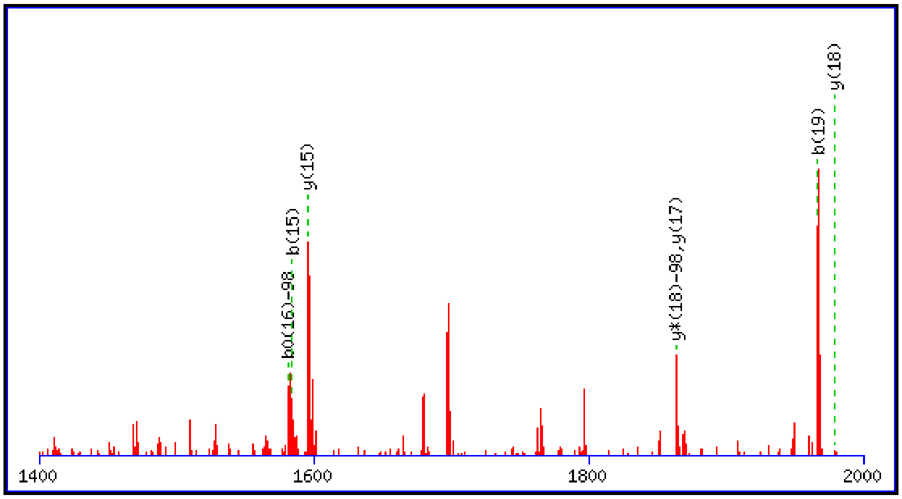

Monoisotopic mass of neutral peptide Mr(calc): 2613.2979
Fixed modifications: Carbamidomethyl (C)
Variable modifications:
S10 : Phospho (ST), with neutral losses 0.0000(shown in table), 97.9769
Ions Score: 55 **Expect:** 0.00086
Matches (Bold Red): 20/332 fragment ions using 41 most intense peaks

#	b	b^{++}	b^{0}	b^{0++}	Seq.	y	y^{++}	y*	y^{*++}	y^{0}	y^{0++}	#
1	58.0287	29.5180			G							25
2	171.1128	86.0600			I	2557.2837	1279.1455	2540.2572	1270.6322	2539.2731	1270.1402	24
3	268.1656	134.5864			P	2444.1996	**1222.6035**	2427.1731	1214.0902	2426.1891	1213.5982	23
4	381.2496	191.1285			L	2347.1469	1174.0771	2330.1203	1165.5638	2329.1363	1165.0718	22
5	478.3024	239.6548			P	2234.0628	**1117.5350**	2217.0363	1109.0218	2216.0523	1108.5298	21

**6**	579.3501	290.1787	561.3395	281.1734	**T**	2137.0101	1069.0087	2119.9835	1060.4954	2118.9995	1060.0034	**20**
**7**	**636.3715**	318.6894	618.3610	309.6841	**G**	2035.9624	1018.4848	2018.9358	1009.9716	2017.9518	1009.4795	**19**
**8**	**751.3985**	376.2029	733.3879	367.1976	**D**	**1978.9409**	989.9741	1961.9144	981.4608	1960.9303	980.9688	**18**
**9**	852.4462	426.7267	834.4356	417.7214	**T**	**1863.9140**	932.4606	1846.8874	923.9473	1845.9034	923.4553	**17**
**10**	1019.4445	510.2259	1001.4339	501.2206	**S**	1762.8663	881.9368	1745.8397	873.4235	1744.8557	872.9315	**16**
**11**	1116.4973	558.7523	1098.4867	549.7470	**P**	**1595.8679**	798.4376	1578.8414	789.9243	1577.8574	789.4323	**15**
**12**	1245.5399	623.2736	1227.5293	614.2683	**E**	1498.8152	749.9112	1481.7886	741.3980	1480.8046	740.9059	**14**
**13**	1342.5926	671.8000	1324.5821	662.7947	**P**	**1369.7726**	685.3899	1352.7460	676.8767	1351.7620	676.3846	**13**
**14**	1471.6352	736.3213	1453.6247	727.3160	**E**	1272.7198	636.8635	1255.6933	628.3503	1254.7093	627.8583	**12**
**15**	**1584.7193**	792.8633	1566.7087	783.8580	**L**	1143.6772	572.3422	1126.6507	563.8290	1125.6667	563.3370	**11**
**16**	1697.8034	849.4053	1679.7928	840.4000	**L**	**1030.5932**	515.8002	1013.5666	**507.2869**	1012.5826	506.7949	**10**
**17**	1794.8561	897.9317	1776.8456	888.9264	**P**	**917.5091**	459.2582	900.4825	450.7449	899.4985	450.2529	**9**
**18**	1851.8776	926.4424	1833.8670	**917.4371**	**G**	**820.4563**	410.7318	803.4298	402.2185	802.4458	401.7265	**8**
**19**	**1966.9045**	983.9559	1948.8940	974.9506	**D**	763.4349	382.2211	746.4083	373.7078	745.4243	373.2158	**7**
**20**	2063.9573	1032.4823	2045.9467	1023.4770	**P**	**648.4079**	324.7076	631.3814	316.1943			**6**
**21**	2177.0414	1089.0243	2159.0308	1080.0190	**L**	551.3552	276.1812	534.3286	267.6679			**5**
**22**	2274.0941	1137.5507	2256.0836	1128.5454	**P**	**438.2711**	219.6392	421.2445	211.1259			**4**
**23**	2371.1469	1186.0771	2353.1363	1177.0718	**P**	341.2183	171.1128	324.1918	162.5995			**3**
**24**	2468.1996	1234.6035	2450.1891	1225.5982	**P**	244.1656	122.5864	227.1390	114.0731			**2**
**25**					**K**	147.1128	74.0600	130.0863	65.5468			**1**

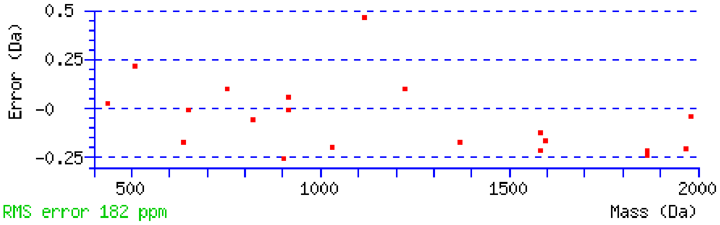

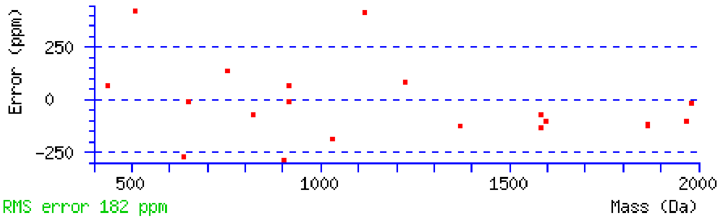

All matches to this query

Score	Mr(calc):	Delta	Sequence
54.5	2613.2979	-0.0038	GIPLPTGDTSPEPELLPGDPLPPPK
54.5	2613.2979	-0.0038	GIPLPTGDTSPEPELLPGDPLPPPK
34.5	2613.2979	-0.0038	GIPLPTGDTSPEPELLPGDPLPPPK
3.2	2611.2666	2.0275	NKNYNYATYYAESLEGRVTISR
1.8	2612.2652	1.0289	SPSLAPTQRLSPGEALPSVVYVGK

Spectrum No: 53; Query: 846; Rank: 1

Peptide View

MS/MS Fragmentation of **VVDYSQFQESDDADEDYGR**
Found in **IPI00189138**, Tax_Id=10116 Gene_Symbol=Nucks Nuclear ubiquitous casein and cyclin-dependent kinases substrate

Match to Query 846: 2316.872928 from(1159.443740,2+)
Title: 091129RatKid_SCX02_12.1833.1833.2.dta
Data file K:\NewmanPaper\Piliang\3SubProteomes\Piliang3SP\mgf5ppm\SCX_3SubProteomes5ppm.mgf

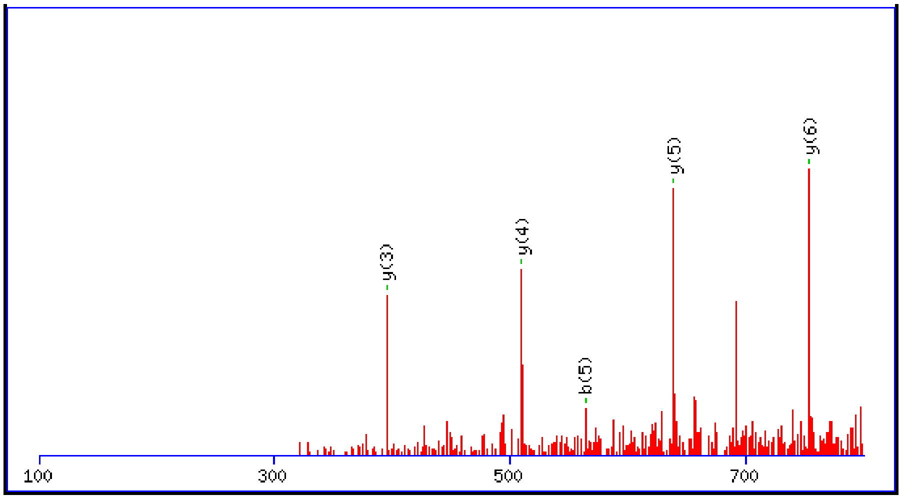

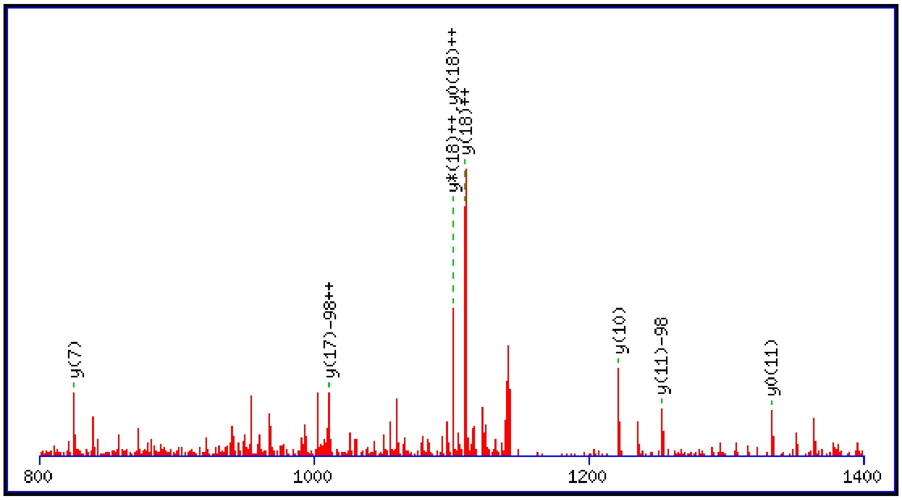

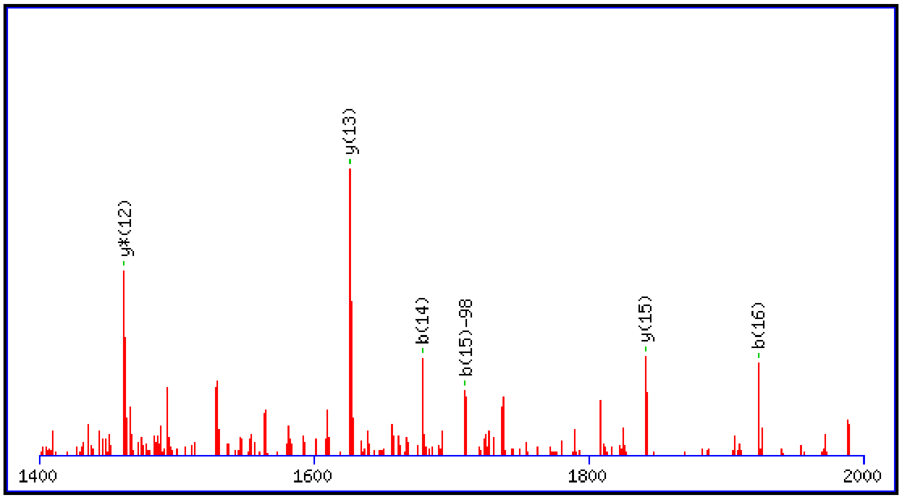

Monoisotopic mass of neutral peptide Mr(calc): 2316.8696
Fixed modifications: Carbamidomethyl (C)
Variable modifications:
S10 : Phospho (ST), with neutral losses 0.0000(shown in table), 97.9769
Ions Score: 54 **Expect:** 0.00063
Matches (Bold Red): 19/304 fragment ions using 27 most intense peaks

#	b	b^{++}	b*	b^{*++}	b^0	b^{0++}	Seq.	y	y^{++}	y*	y^{*++}	y^0	y^{0++}	#
1	100.0757	50.5415					V							19
2	199.1441	100.0757					V	2218.8085	**1109.9079**	2201.7819	**1101.3946**	2200.7979	**1100.9026**	18
3	314.1710	157.5892			296.1605	148.5839	D	2119.7400	1060.3737	2102.7135	1051.8604	2101.7295	1051.3684	17
4	477.2344	239.1208			459.2238	230.1155	Y	2004.7131	1002.8602	1987.6865	994.3469	1986.7025	993.8549	16
5	**564.2664**	282.6368			546.2558	273.6316	S	**1841.6498**	921.3285	1824.6232	912.8152	1823.6392	912.3232	15

**6**	692.3250	346.6661	675.2984	338.1529	674.3144	337.6608	**Q**	1754.6177	877.8125	1737.5912	869.2992	1736.6072	868.8072	**14**
**7**	839.3934	420.2003	822.3668	411.6871	821.3828	411.1951	**F**	**1626.5592**	813.7832	1609.5326	805.2699	1608.5486	804.7779	**13**
**8**	967.4520	484.2296	950.4254	475.7164	949.4414	475.2243	**Q**	1479.4907	740.2490	**1462.4642**	731.7357	1461.4802	731.2437	**12**
**9**	1096.4946	548.7509	1079.4680	540.2376	1078.4840	539.7456	**E**	1351.4322	676.2197	1334.4056	667.7064	**1333.4216**	667.2144	**11**
**10**	1263.4929	632.2501	1246.4664	623.7368	1245.4824	623.2448	**S**	**1222.3896**	611.6984	1205.3630	603.1852	1204.3790	602.6931	**10**
**11**	1378.5199	689.7636	1361.4933	681.2503	1360.5093	680.7583	**D**	1055.3912	528.1993	1038.3647	519.6860	1037.3807	519.1940	**9**
**12**	1493.5468	747.2770	1476.5203	738.7638	1475.5362	738.2718	**D**	940.3643	470.6858	923.3377	462.1725	922.3537	461.6805	**8**
**13**	1564.5839	782.7956	1547.5574	774.2823	1546.5734	773.7903	**A**	**825.3373**	413.1723	808.3108	404.6590	807.3268	404.1670	**7**
**14**	**1679.6109**	840.3091	1662.5843	831.7958	1661.6003	831.3038	**D**	**754.3002**	377.6538	737.2737	369.1405	736.2897	368.6485	**6**
**15**	1808.6535	904.8304	1791.6269	896.3171	1790.6429	895.8251	**E**	**639.2733**	320.1403	622.2467	311.6270	621.2627	311.1350	**5**
**16**	**1923.6804**	962.3438	1906.6538	953.8306	1905.6698	953.3386	**D**	**510.2307**	255.6190	493.2041	247.1057	492.2201	246.6137	**4**
**17**	2086.7437	1043.8755	2069.7172	1035.3622	2068.7332	1034.8702	**Y**	**395.2037**	198.1055	378.1772	189.5922			**3**
**18**	2143.7652	1072.3862	2126.7386	1063.8730	2125.7546	1063.3810	**G**	232.1404	116.5738	215.1139	108.0606			**2**
**19**							**R**	175.1190	88.0631	158.0924	79.5498			**1**

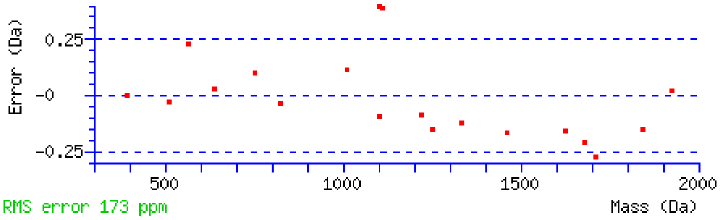

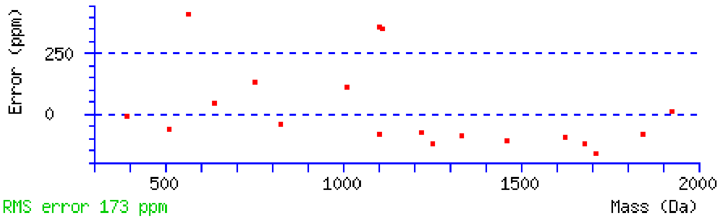

All matches to this query

Score	Mr(calc):	Delta	Sequence
53.9	2316.8696	0.0033	VVDYSQFQESDDADEDYGR
20.6	2316.8696	0.0033	VVDYSQFQESDDADEDYGR
16.4	2316.8696	0.0033	VVDYSQFQESDDADEDYGR
10.9	2316.8696	0.0033	VVDYSQFQESDDADEDYGR

Spectrum No: 54; Query: 466; Rank: 1

Peptide View

MS/MS Fragmentation of **MQLASESADDDDEDS**
Found in **IPI00391633**, Tax_Id=10116 Gene_Symbol=Hdgfrp2 hepatoma-derived growth factor-related protein 2

Match to Query 466: 1706.555028 from(854.284790,2+)
Title: 091127RatKid_SCX01_02.1294.1294.2.dta
Data file K:\NewmanPaper\Piliang\3SubProteomes\Piliang3SP\mgf5ppm\SCX_3SubProteomes5ppm.mgf

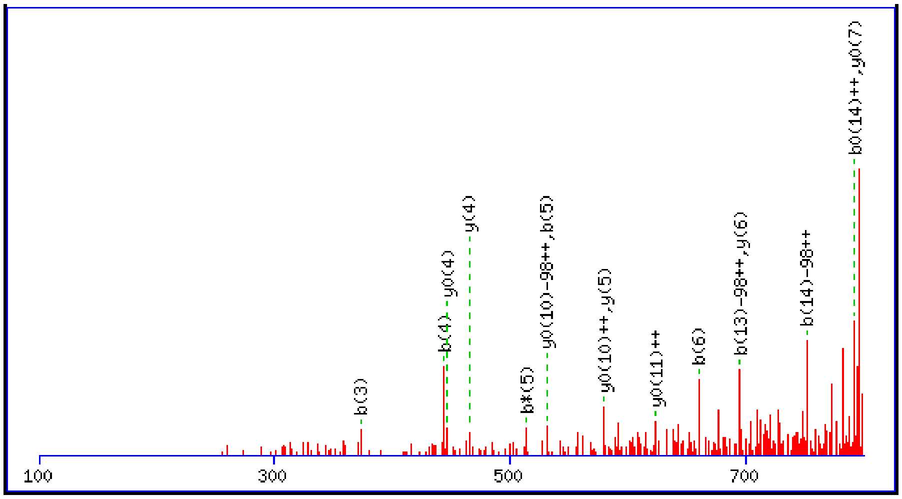

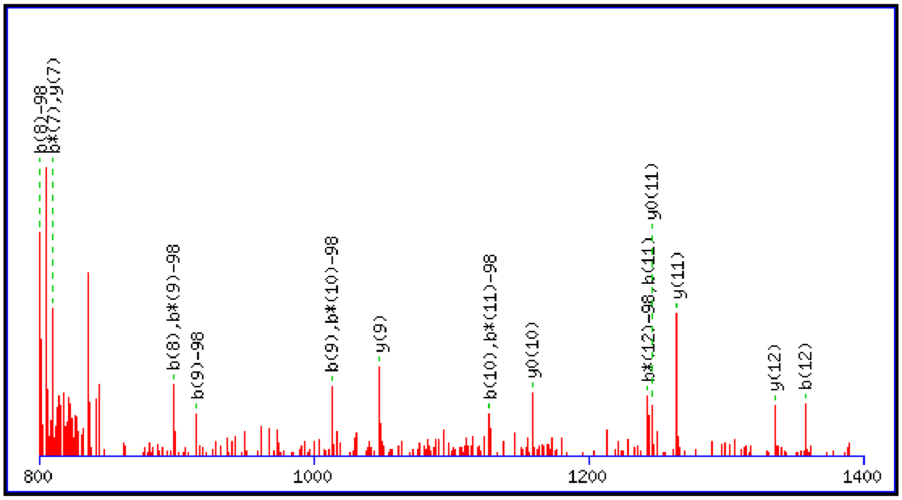

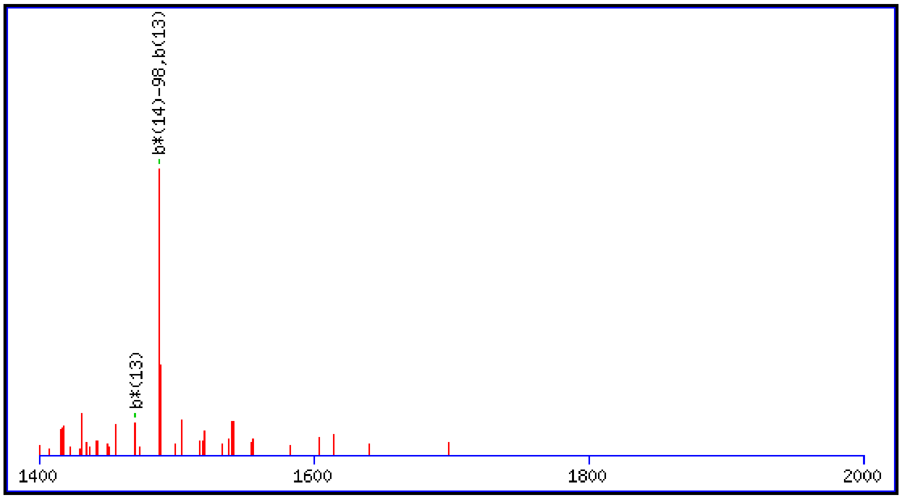

Monoisotopic mass of neutral peptide Mr(calc): 1706.5502
Fixed modifications: Carbamidomethyl (C)
Variable modifications:
S7 : Phospho (ST), with neutral losses 0.0000(shown in table), 97.9769
Ions Score: 53 **Expect:** 0.00017
Matches (Bold Red): 37/206 fragment ions using 54 most intense peaks

#	b	b^{++}	b*	b^{*++}	b^0	b^{0++}	Seq.	y	y^{++}	y*	y^{*++}	y^0	y^{0++}	#
1	132.0478	66.5275					M							15
2	260.1063	130.5568	243.0798	122.0435			Q	1576.5170	788.7621	1559.4905	780.2489	1558.5065	779.7569	14
3	**373.1904**	187.0988	356.1639	178.5856			L	1448.4584	724.7329			1430.4479	715.7276	13
4	**444.2275**	222.6174	427.2010	214.1041			A	**1335.3744**	668.1908			1317.3638	659.1855	12
5	**531.2595**	266.1334	**514.2330**	257.6201	513.2490	257.1281	S	**1264.3373**	632.6723			**1246.3267**	**623.6670**	11

6	660.3021	330.6547	643.2756	322.1414	642.2916	321.6494	E	1177.3052	589.1563			1159.2947	580.1510	10
7	827.3005	414.1539	810.2739	405.6406	809.2899	405.1486	S	1048.2626	524.6350			1030.2521	515.6297	9
8	898.3376	449.6724	881.3111	441.1592	880.3270	440.6672	A	881.2643	441.1358			863.2537	432.1305	8
9	1013.3645	507.1859	996.3380	498.6726	995.3540	498.1806	D	810.2272	405.6172			792.2166	396.6119	7
10	1128.3915	564.6994	1111.3649	556.1861	1110.3809	555.6941	D	695.2002	348.1038			677.1897	339.0985	6
11	1243.4184	622.2129	1226.3919	613.6996	1225.4079	613.2076	D	580.1733	290.5903			562.1627	281.5850	5
12	1358.4454	679.7263	1341.4188	671.2131	1340.4348	670.7210	D	465.1463	233.0768			447.1358	224.0715	4
13	1487.4880	744.2476	1470.4614	735.7343	1469.4774	735.2423	E	350.1194	175.5633			332.1088	166.5581	3
14	1602.5149	801.7611	1585.4884	793.2478	1584.5043	792.7558	D	221.0768	111.0420			203.0662	102.0368	2
15							S	106.0499	53.5286			88.0393	44.5233	1

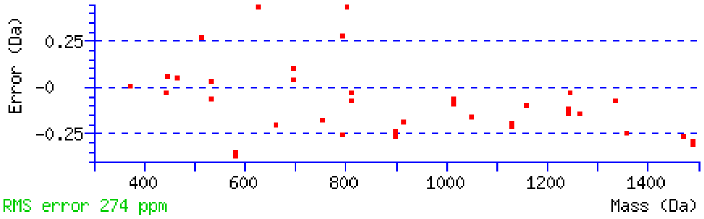

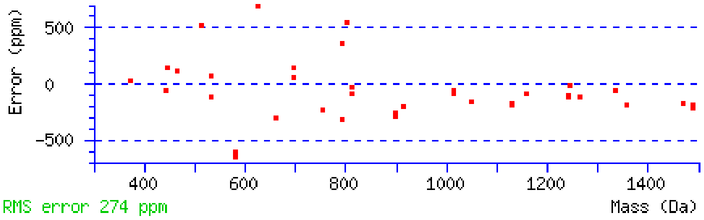

All matches to this query

Score	Mr(calc):	Delta	Sequence
53.3	1706.5502	0.0048	MQLASESADDDDEDS
27.9	1706.5502	0.0048	MQLASESADDDDEDS
9.9	1706.5502	0.0048	MQLASESADDDDEDS

Spectrum No: 55; Query: 211; Rank: 1

Peptide View

MS/MS Fragmentation of **KVMDSDEDDDY**
Found in **IPI00193547**, Tax_Id=10116 Gene_Symbol=Pdcd5_predicted programmed cell death 5

Match to Query 211: 1410.455368 from(706.234960,2+)
Title: 091127RatKid_SCX01_14.1114.1114.2.dta
Data file K:\NewmanPaper\Piliang\3SubProteomes\Piliang3SP\mgf5ppm\SCX_3SubProteomes5ppm.mgf

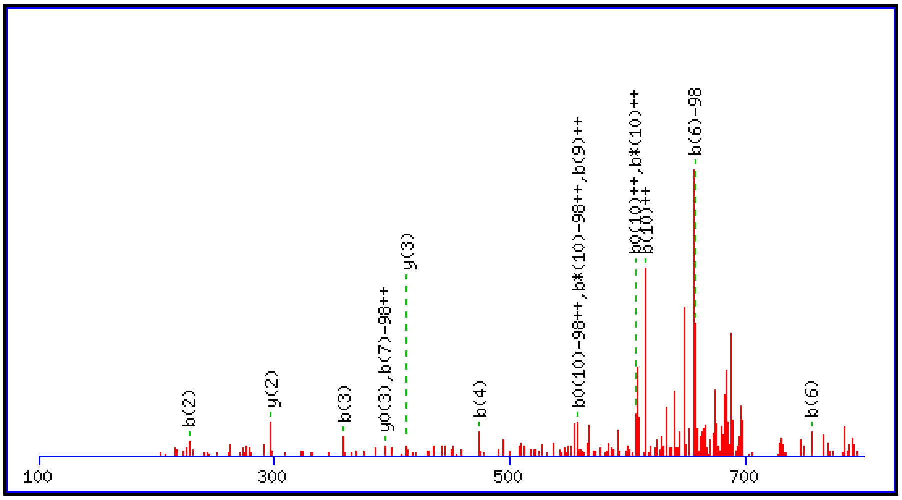

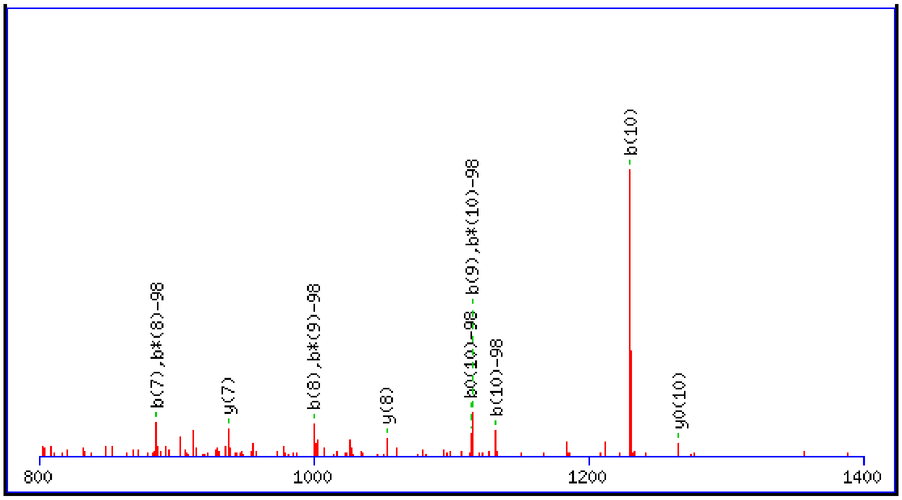

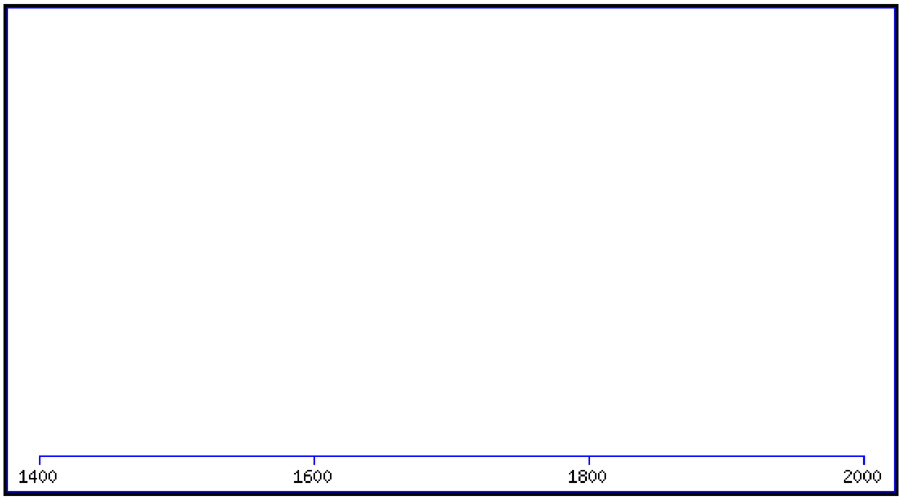

Monoisotopic mass of neutral peptide Mr(calc): 1410.4534
Fixed modifications: Carbamidomethyl (C)
Variable modifications:
S5 : Phospho (ST), with neutral losses 0.0000(shown in table), 97.9769
Ions Score: 52 **Expect:** 0.00016
Matches (Bold Red): 27/144 fragment ions using 49 most intense peaks

#	b	b^{++}	b*	b^{*++}	b^0	b^{0++}	Seq.	y	y^{++}	y^0	y^{0++}	#
1	129.1022	65.0548	112.0757	56.5415			K					11
2	**228.1707**	114.5890	211.1441	106.0757			V	1283.3657	642.1865	**1265.3552**	633.1812	10
3	**359.2111**	180.1092	342.1846	171.5959			M	1184.2973	592.6523	1166.2868	583.6470	9
4	**474.2381**	237.6227	457.2115	229.1094	456.2275	228.6174	D	**1053.2568**	527.1321	1035.2463	518.1268	8
5	641.2364	321.1219	624.2099	312.6086	623.2259	312.1166	S	**938.2299**	469.6186	920.2193	460.6133	7
6	**756.2634**	378.6353	739.2368	370.1221	738.2528	369.6300	D	771.2315	386.1194	753.2210	377.1141	6
7	**885.3060**	443.1566	868.2794	434.6433	867.2954	434.1513	E	656.2046	328.6059	638.1940	319.6007	5
8	**1000.3329**	500.6701	983.3064	492.1568	982.3223	491.6648	D	527.1620	264.0846	509.1514	255.0794	4
9	**1115.3599**	**558.1836**	1098.3333	549.6703	1097.3493	549.1783	D	**412.1351**	206.5712	**394.1245**	197.5659	3
10	**1230.3868**	**615.6970**	1213.3602	**607.1838**	1212.3762	**606.6918**	D	**297.1081**	149.0577	279.0975	140.0524	2
11							Y	182.0812	91.5442			1

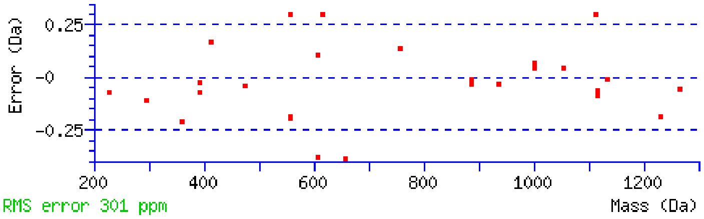

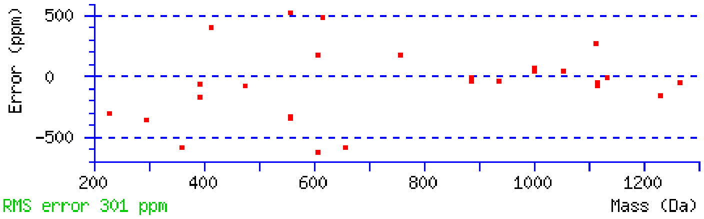

All matches to this query

Score	Mr(calc):	Delta	Sequence
52.2	1410.4534	0.0020	KVMDSDEDDDY
3.3	1410.4534	0.0020	KVMDSDEDDDY

Spectrum No: 56; Query: 381; Rank: 1

Peptide View

MS/MS Fragmentation of **DAVAVAPPPSPSLPAK**
Found in **IPI00205325**, Tax_Id=10116 Gene_Symbol=Lrp2 Low-density lipoprotein receptor-related protein 2 precursor

Match to Query 381: 1595.800128 from(798.907340,2+)
Title: 091129RatKid_SCX02_11.1178.1178.2.dta
Data file K:\NewmanPaper\Piliang\3SubProteomes\Piliang3SP\mgf5ppm\SCX_3SubProteomes5ppm.mgf

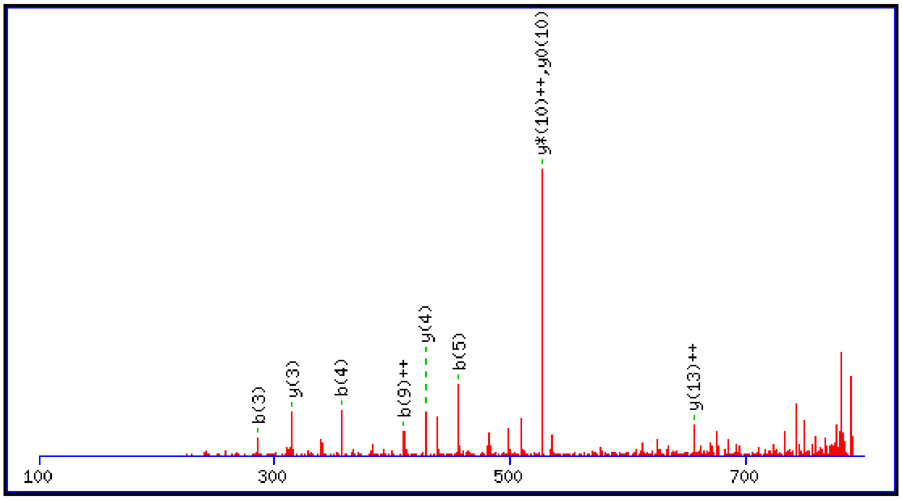

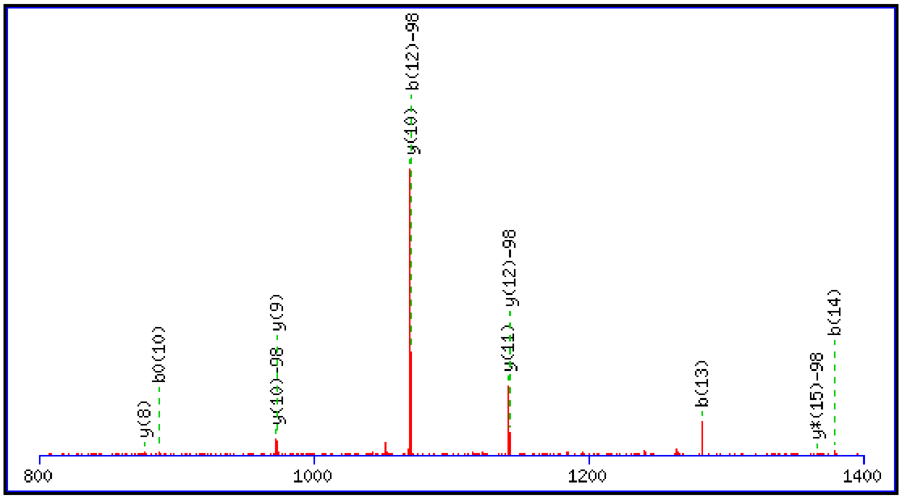

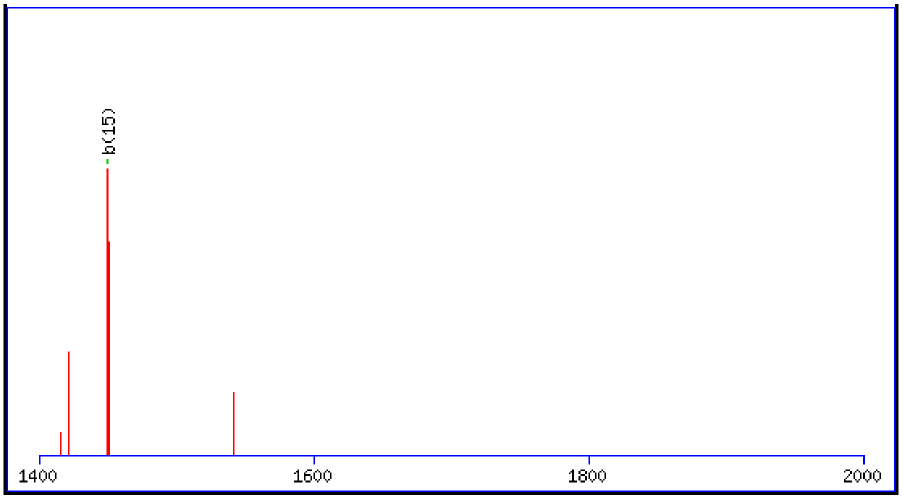

Monoisotopic mass of neutral peptide Mr(calc): 1595.7960
Fixed modifications: Carbamidomethyl (C)
Variable modifications:
S12 : Phospho (ST), with neutral losses 0.0000(shown in table), 97.9769
Ions Score: 52 **Expect:** 0.0009
Matches (Bold Red): 23/224 fragment ions using 34 most intense peaks

#	b	b++	b0	b0++	Seq.	y	y++	y*	y*++	y0	y0++	#
1	116.0342	58.5207	98.0237	49.5155	D							16
2	187.0713	94.0393	169.0608	85.0340	A	1481.7764	741.3918	1464.7498	732.8785	1463.7658	732.3865	15
3	**286.1397**	143.5735	268.1292	134.5682	V	1410.7392	705.8733	1393.7127	697.3600	1392.7287	696.8680	14
4	**357.1769**	179.0921	339.1663	170.0868	A	1311.6708	**656.3391**	1294.6443	647.8258	1293.6603	647.3338	13
5	**456.2453**	228.6263	438.2347	219.6210	V	1240.6337	620.8205	1223.6072	612.3072	1222.6231	611.8152	12
6	**527.2824**	264.1448	509.2718	255.1396	A	**1141.5653**	571.2863	1124.5387	562.7730	1123.5547	562.2810	11
7	624.3352	312.6712	606.3246	303.6659	P	**1070.5282**	535.7677	1053.5016	**527.2545**	1052.5176	**526.7624**	10
8	721.3879	361.1976	703.3774	352.1923	P	**973.4754**	487.2413	956.4489	478.7281	955.4649	478.2361	9
9	818.4407	**409.7240**	800.4301	400.7187	P	**876.4227**	438.7150	859.3961	430.2017	858.4121	429.7097	8
10	905.4727	453.2400	**887.4621**	444.2347	S	779.3699	390.1886	762.3433	381.6753	761.3593	381.1833	7
11	1002.5255	501.7664	984.5149	492.7611	P	692.3379	346.6726	675.3113	338.1593	674.3273	337.6673	6
12	1169.5238	585.2656	1151.5133	576.2603	S	595.2851	298.1462	578.2585	289.6329	577.2745	289.1409	5
13	**1282.6079**	641.8076	1264.5973	632.8023	L	**428.2867**	214.6470	411.2602	206.1337			4
14	**1379.6607**	690.3340	1361.6501	681.3287	P	**315.2027**	158.1050	298.1761	149.5917			3
15	**1450.6978**	725.8525	1432.6872	716.8472	A	218.1499	109.5786	201.1234	101.0653			2
16					K	147.1128	74.0600	130.0863	65.5468			1

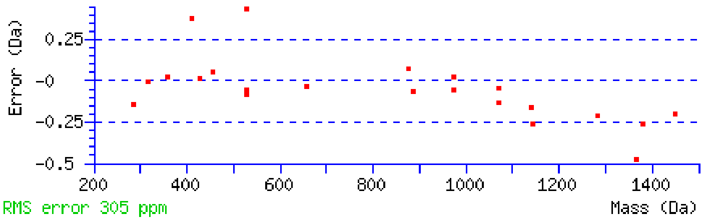

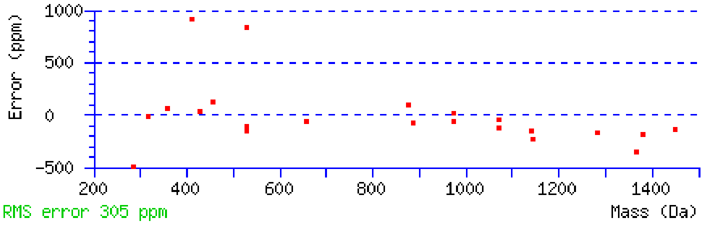

All matches to this query

Score	Mr(calc):	Delta	Sequence
52.1	1595.7960	0.0041	DAVAVAPPPSPSLPAK
50.0	1595.7960	0.0041	DAVAVAPPPSPSLPAK
15.7	1595.7848	0.0154	IEQEFLTEALPVK
10.9	1595.8072	-0.0071	SAPVTALEPGRKYK
10.4	1595.7928	0.0073	LVLSLPVNMRCSK

6.9	1595.7928	0.0073	ERLMKLLPCSAAK
5.3	1594.8120	0.9881	QLNLGAPFLSSLVR
4.9	1595.8072	-0.0071	IKNPEGGLYVAVTR
4.7	1595.7960	0.0041	WLEEKAVLTTQAK
4.7	1593.7780	2.0222	KSITTSAGKSLTLK

Spectrum No: 57; Query: 826; Rank: 1

Peptide View

MS/MS Fragmentation of **TWTLCGTPEYLAPEIILSK**
Found in **IPI00200013**, Tax_Id=10116 Gene_Symbol=Prkaca Isoform 1 of cAMP-dependent protein kinase, alpha-catalytic subunit

Match to Query 826: 2272.102408 from(1137.058480,2+)
Title: 091127RatKid_SCX01_11.5210.5210.2.dta
Data file K:\NewmanPaper\Piliang\3SubProteomes\Piliang3SP\mgf5ppm\SCX_3SubProteomes5ppm.mgf

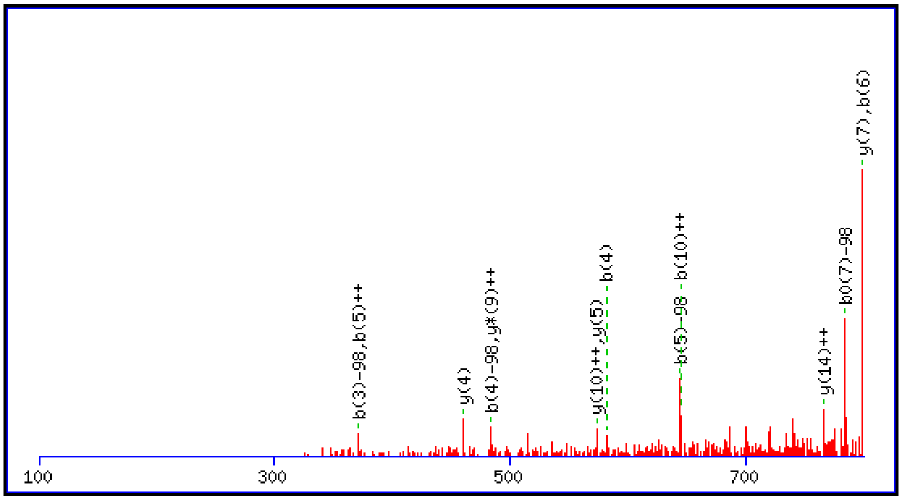

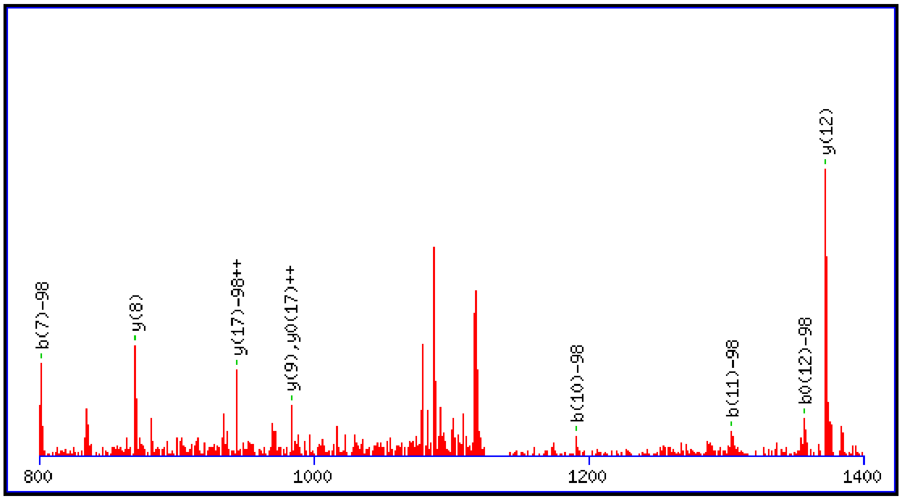

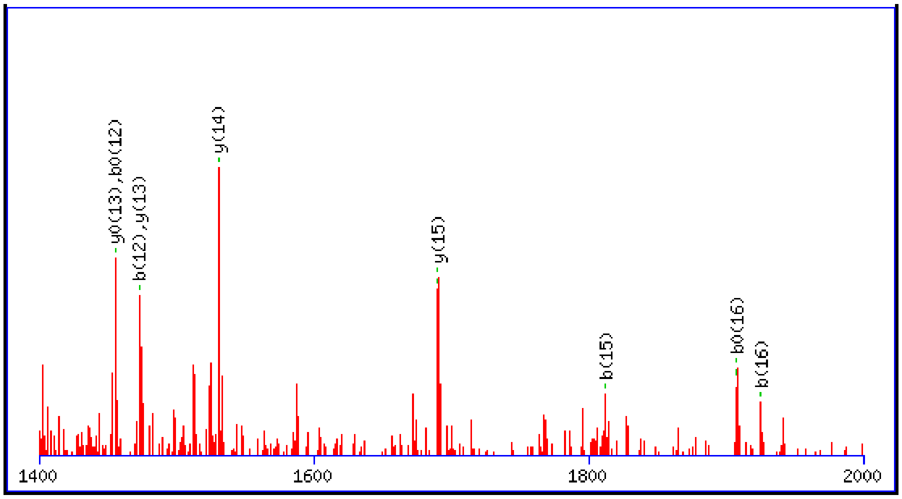

Monoisotopic mass of neutral peptide Mr(calc): 2271.0898
Fixed modifications: Carbamidomethyl (C)
Variable modifications:
T3 : Phospho (ST), with neutral losses 97.9769(shown in table), 0.0000
Ions Score: 52 **Expect:** 0.0015
Matches (Bold Red): 32/254 fragment ions using 46 most intense peaks

#	b	b^{++}	b^{0}	b^{0++}	Seq.	y	y^{++}	y*	y^{*++}	y^{0}	y^{0++}	#
1	102.0550	51.5311	84.0444	42.5258	T							19
2	288.1343	144.5708	270.1237	135.5655	W	2073.0725	1037.0399	2056.0460	1028.5266	2055.0620	1028.0346	18
3	**371.1714**	186.0893	353.1608	177.0840	T	1886.9932	**944.0002**	1869.9667	935.4870	1868.9826	934.9950	17
4	**484.2554**	242.6314	466.2449	233.6261	L	1803.9561	902.4817	1786.9296	893.9684	1785.9455	893.4764	16
5	**644.2861**	322.6467	626.2755	313.6414	C	**1690.8720**	845.9397	1673.8455	837.4264	1672.8615	836.9344	15
6	701.3076	351.1574	683.2970	342.1521	G	**1530.8414**	**765.9243**	1513.8148	757.4111	1512.8308	756.9190	14
7	**802.3552**	401.6813	**784.3447**	392.6760	T	**1473.8199**	737.4136	1456.7934	728.9003	**1455.8094**	728.4083	13
8	899.4080	450.2076	881.3974	441.2024	P	**1372.7722**	686.8898	1355.7457	678.3765	1354.7617	677.8845	12
9	1028.4506	514.7289	1010.4400	505.7236	E	1275.7195	638.3634	1258.6929	629.8501	1257.7089	629.3581	11
10	**1191.5139**	596.2606	1173.5034	587.2553	Y	1146.6769	**573.8421**	1129.6503	565.3288	1128.6663	564.8368	10
11	**1304.5980**	652.8026	1286.5874	643.7973	L	**983.6136**	492.3104	966.5870	**483.7971**	965.6030	483.3051	9
12	1375.6351	688.3212	**1357.6245**	679.3159	A	**870.5295**	435.7684	853.5029	427.2551	852.5189	426.7631	8
13	1472.6879	736.8476	1454.6773	727.8423	P	**799.4924**	400.2498	782.4658	391.7366	781.4818	391.2445	7
14	1601.7305	801.3689	1583.7199	792.3636	E	702.4396	351.7234	685.4131	343.2102	684.4291	342.7182	6
15	1714.8145	857.9109	1696.8040	848.9056	I	**573.3970**	287.2021	556.3705	278.6889	555.3865	278.1969	5
16	1827.8986	914.4529	1809.8880	905.4476	I	**460.3130**	230.6601	443.2864	222.1468	442.3024	221.6548	4
17	1940.9826	970.9950	1922.9721	961.9897	L	347.2289	174.1181	330.2023	165.6048	329.2183	165.1128	3
18	2028.0147	1014.5110	2010.0041	1005.5057	S	234.1448	117.5761	217.1183	109.0628	216.1343	108.5708	2
19					K	147.1128	74.0600	130.0863	65.5468			1

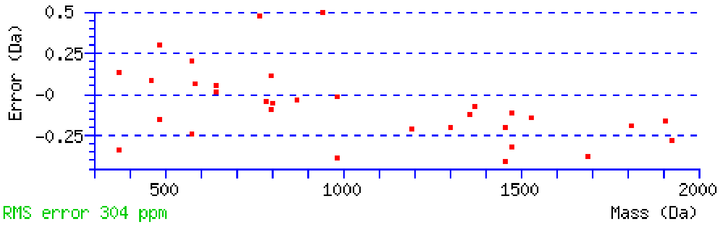

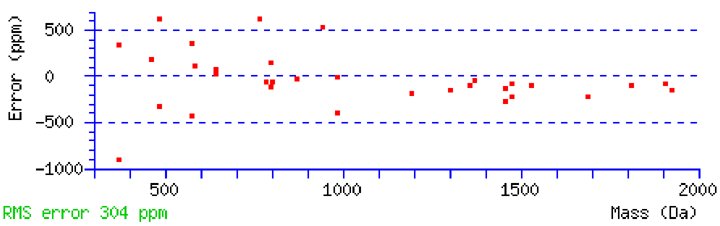

All matches to this query

Score	Mr(calc):	Delta	Sequence
51.8	2271.0898	1.0126	TWTLCGTPEYLAPEIILSK
50.0	2271.0898	1.0126	TWTLCGTPEYLAPEIILSK

38.1	2271.0898	1.0126	TWTLCGTPEYLAPEIILSK
10.2	2271.0898	1.0126	TWTLCGTPEYLAPEIILSK
3.0	2271.1066	0.9958	MDGRPSLLPVDFNTPDGRER
0.1	2271.1057	0.9967	MRADMGGTNIFSPLKWVLR

Spectrum No: 58; Query: 216; Rank: 1

Peptide View

MS/MS Fragmentation of **TASLTSAASIDGSR**
Found in **IPI00326606**, Tax_Id=10116 Gene_Symbol=Ndrg2 Isoform 2 of Protein NDRG2

Match to Query 216: 1415.627008 from(708.820780,2+)
Title: 091129RatKid_SCX02_12.1114.1114.2.dta
Data file K:\NewmanPaper\Piliang\3SubProteomes\Piliang3SP\mgf5ppm\SCX_3SubProteomes5ppm.mgf

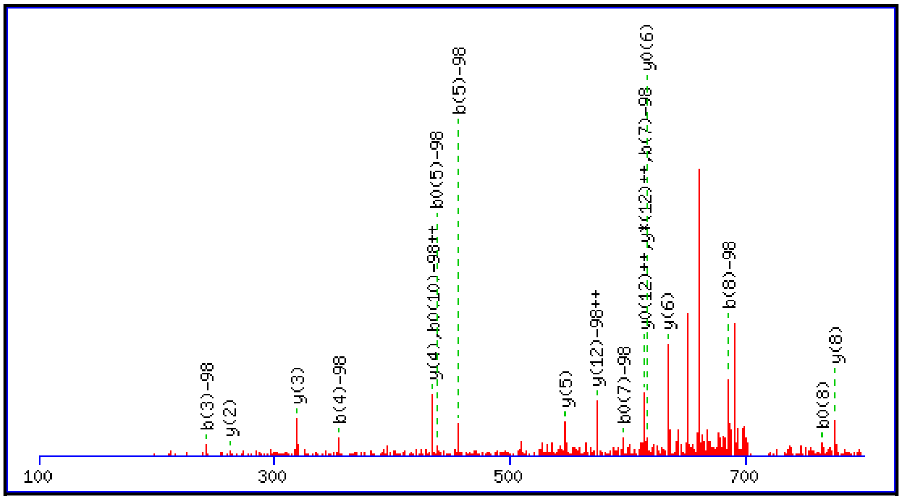

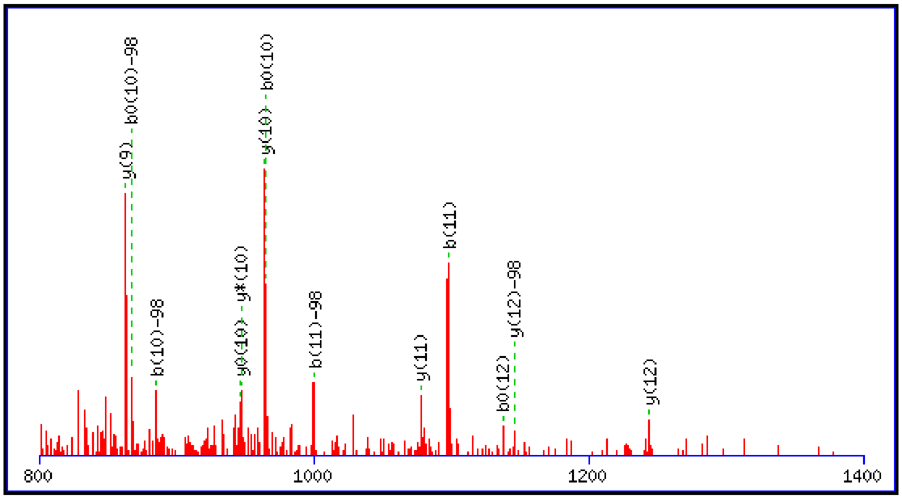

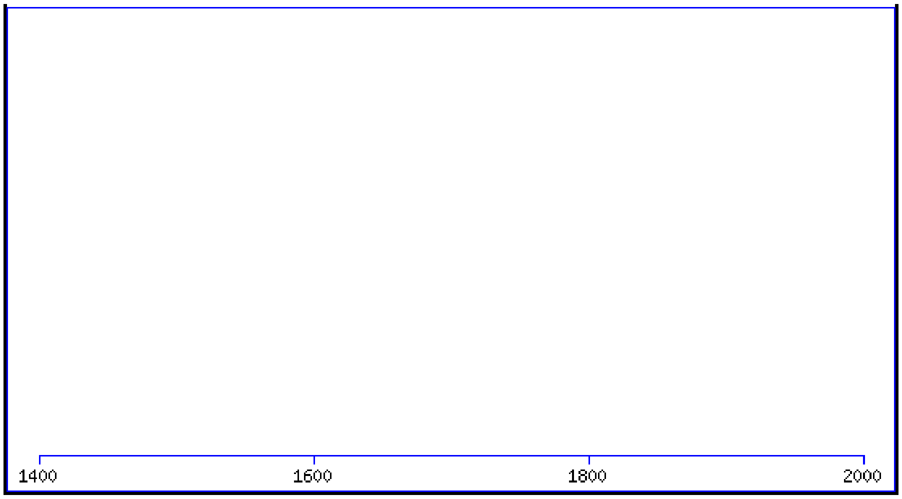

Monoisotopic mass of neutral peptide Mr(calc): 1415.6293
Fixed modifications: Carbamidomethyl (C)
Variable modifications:
S3 : Phospho (ST), with neutral losses 97.9769(shown in table), 0.0000
Ions Score: 51 **Expect:** 0.0011
Matches (Bold Red): 32/184 fragment ions using 63 most intense peaks

#	b	b++	b0	b0++	Seq.	y	y++	y*	y*++	y0	y0++	#
1	102.0550	51.5311	84.0444	42.5258	T							14
2	173.0921	87.0497	155.0815	78.0444	A	1217.6120	609.3097	1200.5855	600.7964	1199.6015	600.3044	13
3	**242.1135**	121.5604	224.1030	112.5551	S	**1146.5749**	**573.7911**	1129.5484	565.2778	1128.5644	564.7858	12
4	**355.1976**	178.1024	337.1870	169.0971	L	**1077.5535**	539.2804	1060.5269	530.7671	1059.5429	530.2751	11
5	**456.2453**	228.6263	**438.2347**	219.6210	T	**964.4694**	482.7383	**947.4429**	474.2251	**946.4588**	473.7331	10
6	543.2773	272.1423	525.2667	263.1370	S	**863.4217**	432.2145	846.3952	423.7012	845.4112	423.2092	9
7	**614.3144**	307.6608	**596.3038**	298.6556	A	**776.3897**	388.6985	759.3632	380.1852	758.3791	379.6932	8
8	**685.3515**	343.1794	667.3410	334.1741	A	705.3526	353.1799	688.3260	344.6667	687.3420	344.1747	7
9	772.3836	386.6954	754.3730	377.6901	S	**634.3155**	317.6614	617.2889	309.1481	**616.3049**	308.6561	6
10	**885.4676**	443.2374	**867.4571**	**434.2322**	I	**547.2835**	274.1454	530.2569	265.6321	529.2729	265.1401	5
11	**1000.4946**	500.7509	982.4840	491.7456	D	**434.1994**	217.6033	417.1728	209.0901	416.1888	208.5980	4
12	1057.5160	529.2616	1039.5055	520.2564	G	**319.1724**	160.0899	302.1459	151.5766	301.1619	151.0846	3
13	1144.5481	572.7777	1126.5375	563.7724	S	**262.1510**	131.5791	245.1244	123.0659	244.1404	122.5738	2
14					R	175.1190	88.0631	158.0924	79.5498			1

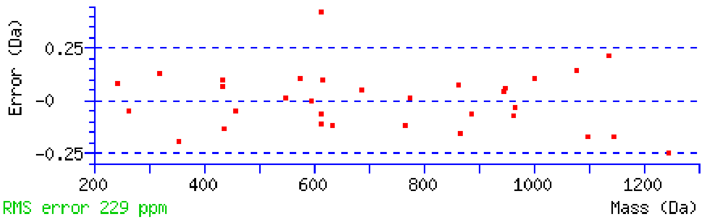

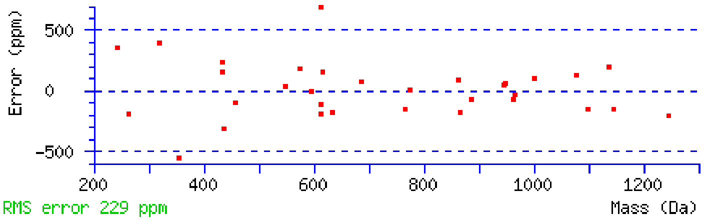

All matches to this query

Score	Mr(calc):	Delta	Sequence
51.1	1415.6293	-0.0023	TASLTSAASIDGSR
42.0	1415.6293	-0.0023	TASLTSAASIDGSR
38.3	1415.6293	-0.0023	TASLTSAASIDGSR
30.4	1415.6293	-0.0023	TASLTSAASIDGSR
6.6	1415.6293	-0.0023	TASLTSAASIDGSR
1.4	1414.6371	0.9899	VSPSSLPGRLSR

Spectrum No: 59; Query: 628; Rank: 1

Peptide View

MS/MS Fragmentation of **FSTVAGESGSADTVRDPR**
Found in **IPI00231742**, Tax_Id=10116 Gene_Symbol=Cat Catalase

Match to Query 628: 1930.834268 from(966.424410,2+)
Title: 091127RatKid_SCX01_22.1068.1068.2.dta
Data file K:\NewmanPaper\Piliang\3SubProteomes\Piliang3SP\mgf5ppm\SCX_3SubProteomes5ppm.mgf

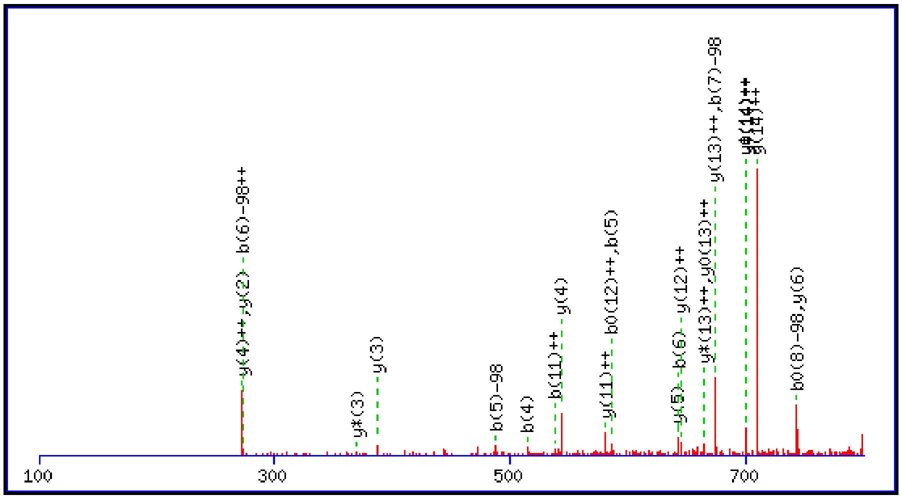

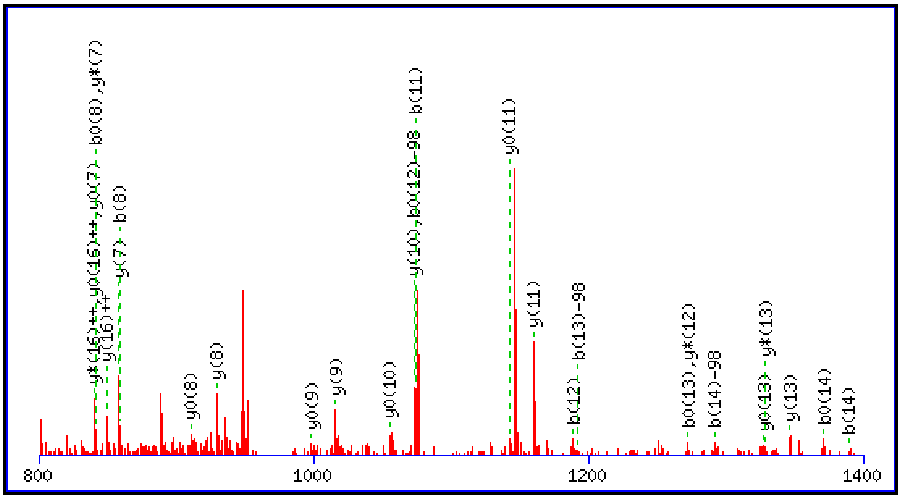

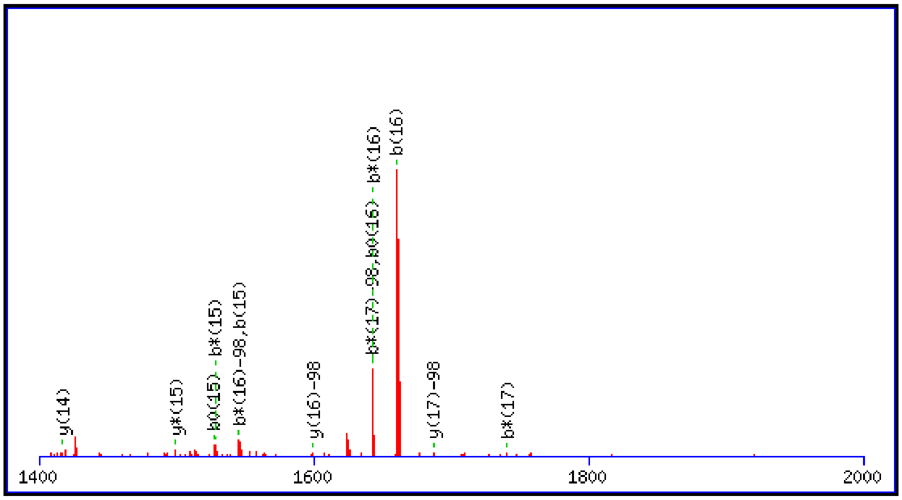

Monoisotopic mass of neutral peptide Mr(calc): 1930.8422

Fixed modifications: Carbamidomethyl (C)
Variable modifications:
T3 : Phospho (ST), with neutral losses 97.9769(shown in table), 0.0000
Ions Score: 51 **Expect:** 0.0017
Matches (Bold Red): 65/248 fragment ions using 154 most intense peaks

#	b	b^{++}	b*	b^{*++}	b^{0}	b^{0++}	Seq.	y	y^{++}	y*	y^{*++}	y^{0}	y^{0++}	#
1	148.0757	74.5415					F							18
2	235.1077	118.0575			217.0972	109.0522	S	**1686.8042**	843.9057	1669.7776	835.3924	1668.7936	834.9004	17
3	318.1448	159.5761			300.1343	150.5708	T	**1599.7721**	800.3897	1582.7456	791.8764	1581.7616	791.3844	16
4	417.2132	209.1103			399.2027	200.1050	V	1516.7350	758.8712	**1499.7085**	750.3579	1498.7245	749.8659	15
5	**488.2504**	244.6288			470.2398	235.6235	A	**1417.6666**	**709.3369**	1400.6401	**700.8237**	1399.6560	**700.3317**	14
6	545.2718	**273.1395**			527.2613	264.1343	G	**1346.6295**	**673.8184**	**1329.6029**	**665.3051**	**1328.6189**	**664.8131**	13
7	**674.3144**	337.6608			656.3038	328.6556	E	1289.6080	**645.3077**	**1272.5815**	636.7944	1271.5975	636.3024	12
8	761.3464	381.1769			**743.3359**	372.1716	S	**1160.5654**	**580.7864**	1143.5389	572.2731	**1142.5549**	571.7811	11
9	818.3679	409.6876			800.3573	400.6823	G	**1073.5334**	537.2703	1056.5069	528.7571	**1055.5228**	528.2651	10
10	905.3999	453.2036			887.3894	444.1983	S	**1016.5119**	508.7596	999.4854	500.2463	**998.5014**	499.7543	9
11	976.4370	488.7222			958.4265	479.7169	A	**929.4799**	465.2436	912.4534	456.7303	**911.4694**	456.2383	8
12	1091.4640	546.2356			**1073.4534**	537.2303	D	**858.4428**	429.7250	**841.4163**	421.2118	**840.4322**	420.7198	7
13	**1192.5117**	596.7595			1174.5011	587.7542	T	**743.4159**	372.2116	726.3893	363.6983	725.4053	363.2063	6
14	**1291.5801**	646.2937			1273.5695	637.2884	V	**642.3682**	321.6877	625.3416	313.1745	624.3576	312.6824	5
15	1447.6812	724.3442	1430.6546	715.8310	1429.6706	715.3390	R	**543.2998**	**272.1535**	526.2732	263.6402	525.2892	263.1482	4
16	1562.7081	781.8577	**1545.6816**	773.3444	1544.6976	772.8524	D	**387.1987**	194.1030	**370.1721**	185.5897	369.1881	185.0977	3
17	1659.7609	830.3841	**1642.7343**	821.8708	1641.7503	821.3788	P	**272.1717**	136.5895	255.1452	128.0762			2
18							R	175.1190	88.0631	158.0924	79.5498			1

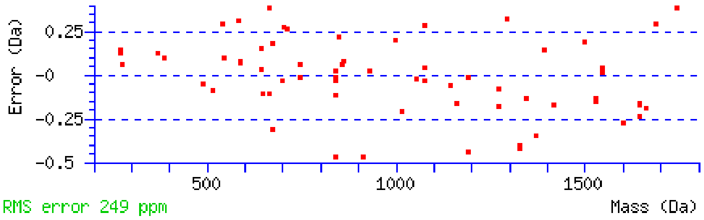

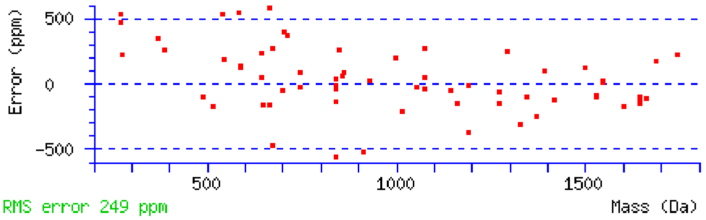

All matches to this query

Score	Mr(calc):	Delta	Sequence
51.1	1930.8422	-0.0079	FSTVAGESGSADTVRDPR
43.2	1930.8422	-0.0079	FSTVAGESGSADTVRDPR
38.2	1930.8422	-0.0079	FSTVAGESGSADTVRDPR
27.9	1930.8422	-0.0079	FSTVAGESGSADTVRDPR
9.4	1930.8422	-0.0079	FSTVAGESGSADTVRDPR
2.5	1930.8284	0.0059	MERPNGLYPNYLNPR
2.4	1929.8251	1.0091	SDCTLPDSASSGNKGRAK
2.3	1930.8244	0.0098	LDANGFSGAXGCGGTAGLAR
1.9	1928.8265	2.0078	VDKGASNNPEFEETRR
1.8	1928.8217	2.0126	LRSVNCPGTGDPQLVR

Spectrum No: 60; Query: 1189; Rank: 1

Peptide View

MS/MS Fragmentation of **TSVTVYPGDVFCLHTPGGGGYGDPEDPAPPPGSPPLFPAFPER**

Found in **IPI00326436**, Tax_Id=10116 Gene_Symbol=Oplah 5-oxoprolinase

Match to Query 1189: 4533.079662 from(1512.033830,3+)
Title: 091129RatKid_SCX02_14.4857.4857.3.dta
Data file K:\NewmanPaper\Piliang\3SubProteomes\Piliang3SP\mgf5ppm\SCX_3SubProteomes5ppm.mgf

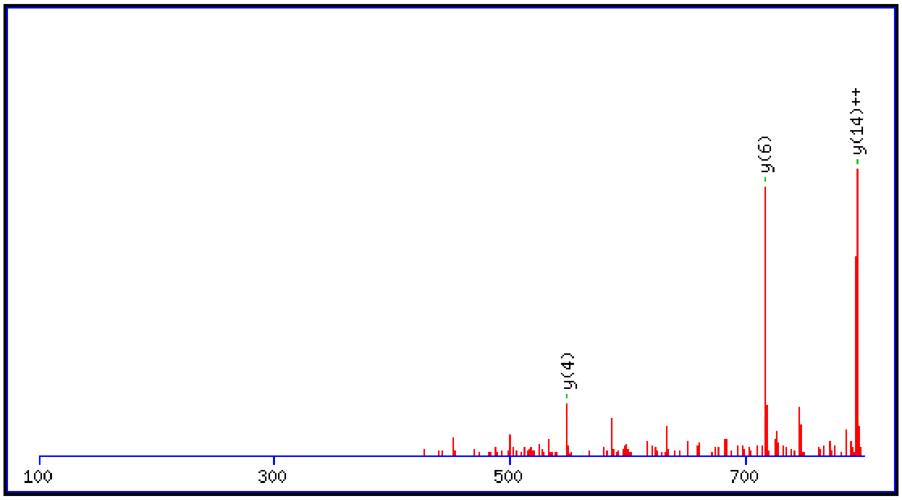

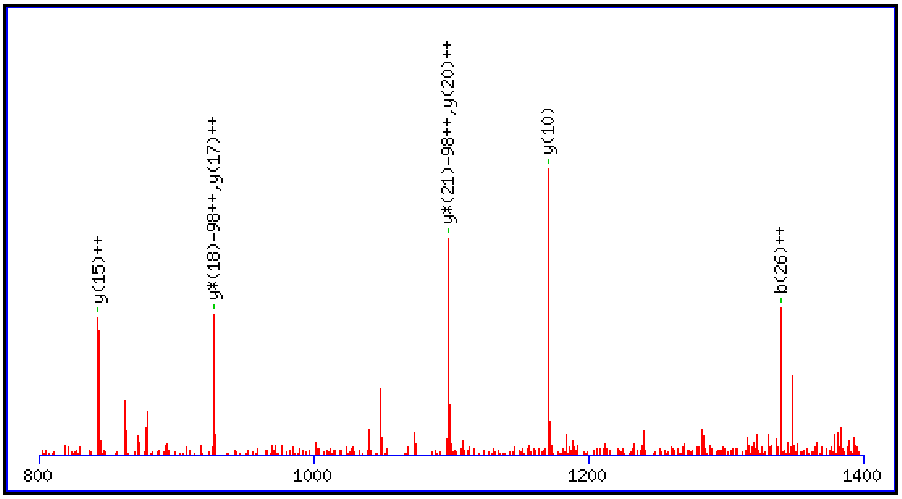

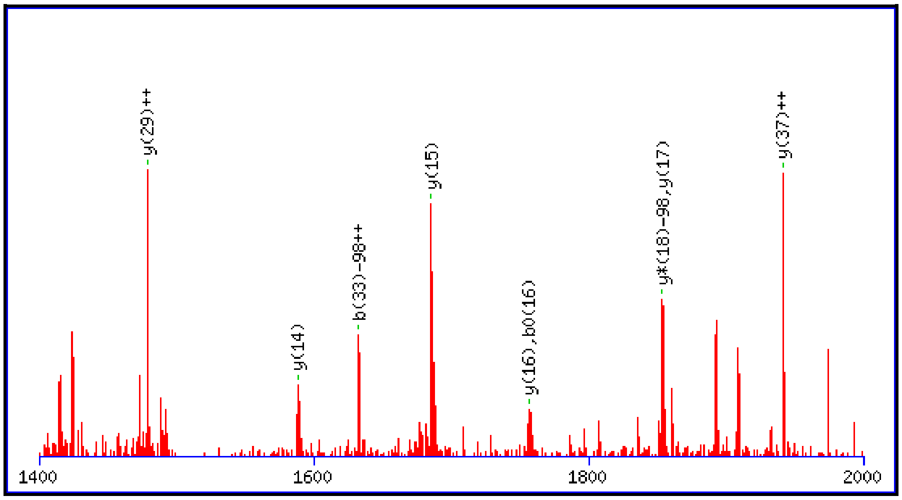

Monoisotopic mass of neutral peptide Mr(calc): 4531.0509
Fixed modifications: Carbamidomethyl (C)
Variable modifications:
S33 : Phospho (ST), with neutral losses 0.0000(shown in table), 97.9769
Ions Score: 50 **Expect:** 0.0046
Matches (Bold Red): 19/650 fragment ions using 24 most intense peaks

#	b	b^{++}	b^0	b^{0++}	Seq.	y	y^{++}	y*	y^{*++}	y^0	y^{0++}	#
1	102.0550	51.5311	84.0444	42.5258	T							43

2	189.0870	95.0471	171.0764	86.0418	S	4431.0104	2216.0088	4413.9839	2207.4956	4412.9999	2207.0036	42
3	288.1554	144.5813	270.1448	135.5761	V	4343.9784	2172.4928	4326.9518	2163.9796	4325.9678	2163.4875	41
4	389.2031	195.1052	371.1925	186.0999	T	4244.9100	2122.9586	4227.8834	2114.4454	4226.8994	2113.9533	40
5	488.2715	244.6394	470.2609	235.6341	V	4143.8623	2072.4348	4126.8357	2063.9215	4125.8517	2063.4295	39
6	651.3348	326.1710	633.3243	317.1658	Y	4044.7939	2022.9006	4027.7673	2014.3873	4026.7833	2013.8953	38
7	748.3876	374.6974	730.3770	365.6921	P	3881.7306	**1941.3689**	3864.7040	1932.8556	3863.7200	1932.3636	37
8	805.4090	403.2082	787.3985	394.2029	G	3784.6778	1892.8425	3767.6512	1884.3293	3766.6672	1883.8372	36
9	920.4360	460.7216	902.4254	451.7164	D	3727.6563	1864.3318	3710.6298	1855.8185	3709.6458	1855.3265	35
10	1019.5044	510.2558	1001.4938	501.2506	V	3612.6294	1806.8183	3595.6028	1798.3051	3594.6188	1797.8130	34
11	1166.5728	583.7900	1148.5623	574.7848	F	3513.5610	1757.2841	3496.5344	1748.7708	3495.5504	1748.2788	33
12	1326.6035	663.8054	1308.5929	654.8001	C	3366.4926	1683.7499	3349.4660	1675.2366	3348.4820	1674.7446	32
13	1439.6875	720.3474	1421.6770	711.3421	L	3206.4619	1603.7346	3189.4354	1595.2213	3188.4513	1594.7293	31
14	1576.7464	788.8769	1558.7359	779.8716	H	3093.3778	1547.1926	3076.3513	1538.6793	3075.3673	1538.1873	30
15	1677.7941	839.4007	1659.7836	830.3954	T	2956.3189	**1478.6631**	2939.2924	1470.1498	2938.3084	1469.6578	29
16	1774.8469	887.9271	**1756.8363**	878.9218	P	2855.2712	1428.1393	2838.2447	1419.6260	2837.2607	1419.1340	28
17	1831.8684	916.4378	1813.8578	907.4325	G	2758.2185	1379.6129	2741.1919	1371.0996	2740.2079	1370.6076	27
18	1888.8898	944.9485	1870.8792	935.9433	G	2701.1970	1351.1021	2684.1705	1342.5889	2683.1865	1342.0969	26
19	1945.9113	973.4593	1927.9007	964.4540	G	2644.1756	1322.5914	2627.1490	1314.0781	2626.1650	1313.5861	25
20	2002.9327	1001.9700	1984.9222	992.9647	G	2587.1541	1294.0807	2570.1275	1285.5674	2569.1435	1285.0754	24
21	2165.9961	1083.5017	2147.9855	1074.4964	Y	2530.1326	1265.5700	2513.1061	1257.0567	2512.1221	1256.5647	23
22	2223.0175	1112.0124	2205.0070	1103.0071	G	2367.0693	1184.0383	2350.0428	1175.5250	2349.0587	1175.0330	22
23	2338.0445	1169.5259	2320.0339	1160.5206	D	2310.0478	1155.5276	2293.0213	1147.0143	2292.0373	1146.5223	21
24	2435.0972	1218.0523	2417.0867	1209.0470	P	2195.0209	**1098.0141**	2177.9943	1089.5008	2177.0103	1089.0088	20
25	2564.1398	1282.5736	2546.1293	1273.5683	E	2097.9681	1049.4877	2080.9416	1040.9744	2079.9576	1040.4824	19
26	2679.1668	**1340.0870**	2661.1562	1331.0817	D	1968.9255	984.9664	1951.8990	976.4531	1950.9150	975.9611	18
27	2776.2195	1388.6134	2758.2090	1379.6081	P	**1853.8986**	**927.4529**	1836.8720	918.9397	1835.8880	918.4477	17
28	2847.2567	1424.1320	2829.2461	1415.1267	A	**1756.8458**	878.9266	1739.8193	870.4133	1738.8353	869.9213	16
29	2944.3094	1472.6583	2926.2989	1463.6531	P	**1685.8087**	**843.4080**	1668.7822	834.8947	1667.7982	834.4027	15
30	3041.3622	1521.1847	3023.3516	1512.1794	P	**1588.7560**	**794.8816**	1571.7294	786.3683	1570.7454	785.8763	14
31	3138.4149	1569.7111	3120.4044	1560.7058	P	1491.7032	746.3552	1474.6766	737.8420	1473.6926	737.3499	13
32	3195.4364	1598.2218	3177.4258	1589.2166	G	1394.6504	697.8288	1377.6239	689.3156	1376.6399	688.8236	12
33	3362.4348	1681.7210	3344.4242	1672.7157	S	1337.6290	669.3181	1320.6024	660.8048	1319.6184	660.3128	11
34	3459.4875	1730.2474	3441.4770	1721.2421	P	**1170.6306**	585.8189	1153.6041	577.3057	1152.6200	576.8137	10
35	3556.5403	1778.7738	3538.5297	1769.7685	P	1073.5778	537.2926	1056.5513	528.7793	1055.5673	528.2873	9
36	3669.6244	1835.3158	3651.6138	1826.3105	L	976.5251	488.7662	959.4985	480.2529	958.5145	479.7609	8
37	3816.6928	1908.8500	3798.6822	1899.8447	F	863.4410	432.2241	846.4145	423.7109	845.4305	423.2189	7
38	3913.7455	1957.3764	3895.7350	1948.3711	P	**716.3726**	358.6899	699.3461	350.1767	698.3620	349.6847	6
39	3984.7826	1992.8950	3966.7721	1983.8897	A	619.3198	310.1636	602.2933	301.6503	601.3093	301.1583	5
40	4131.8511	2066.4292	4113.8405	2057.4239	F	**548.2827**	274.6450	531.2562	266.1317	530.2722	265.6397	4
41	4228.9038	2114.9556	4210.8933	2105.9503	P	401.2143	201.1108	384.1878	192.5975	383.2037	192.1055	3
42	4357.9464	2179.4768	4339.9359	2170.4716	E	304.1615	152.5844	287.1350	144.0711	286.1510	143.5791	2
43					R	175.1190	88.0631	158.0924	79.5498			1

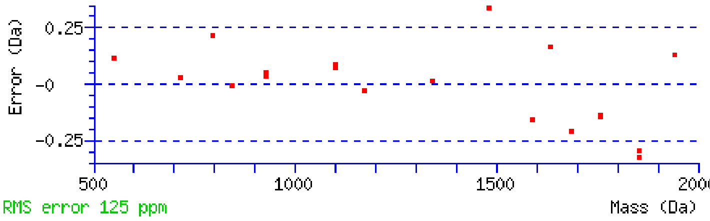

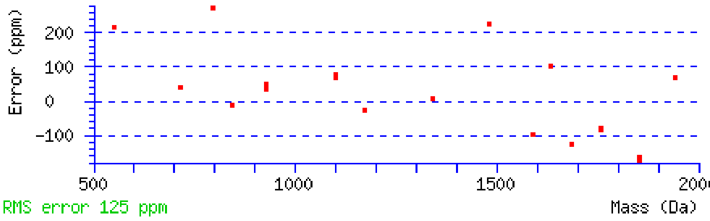

All matches to this query

Score	Mr(calc):	Delta	Sequence
50.5	4531.0509	2.0288	TSVTVYPGDVFCLHTPGGGGYGDPEDPAPPPGSPPLFPAFPER
12.0	4531.0509	2.0288	TSVTVYPGDVFCLHTPGGGGYGDPEDPAPPPGSPPLFPAFPER
8.4	4531.0509	2.0288	TSVTVYPGDVFCLHTPGGGGYGDPEDPAPPPGSPPLFPAFPER
7.3	4531.0509	2.0288	TSVTVYPGDVFCLHTPGGGGYGDPEDPAPPPGSPPLFPAFPER
7.3	4531.0509	2.0288	TSVTVYPGDVFCLHTPGGGGYGDPEDPAPPPGSPPLFPAFPER
7.3	4531.0509	2.0288	TSVTVYPGDVFCLHTPGGGGYGDPEDPAPPPGSPPLFPAFPER
5.2	4531.0638	2.0159	NIAPVQLVNFAYRDLPLAAVDLSTGGSQLLSNLDEEYQR
5.2	4531.0638	2.0159	NIAPVQLVNFAYRDLPLAAVDLSTGGSQLLSNLDEEYQR
5.2	4533.0575	0.0221	LDIINESWLQDYIGITMNHFFSLMQAEVNRFQDTKR
5.0	4531.0509	2.0288	TSVTVYPGDVFCLHTPGGGGYGDPEDPAPPPGSPPLFPAFPER

Spectrum No: 61; Query: 686; Rank: 1

Peptide View

MS/MS Fragmentation of **GSSQPNLSTSYSEQEYGK**
Found in **IPI00209348**, Tax_Id=10116 Gene_Symbol=Epn2 Epsin-2

Match to Query 686: 2040.834688 from(1021.424620,2+)
Title: 091127RatKid_SCX01_11.1186.1186.2.dta
Data file K:\NewmanPaper\Piliang\3SubProteomes\Piliang3SP\mgf5ppm\SCX_3SubProteomes5ppm.mgf

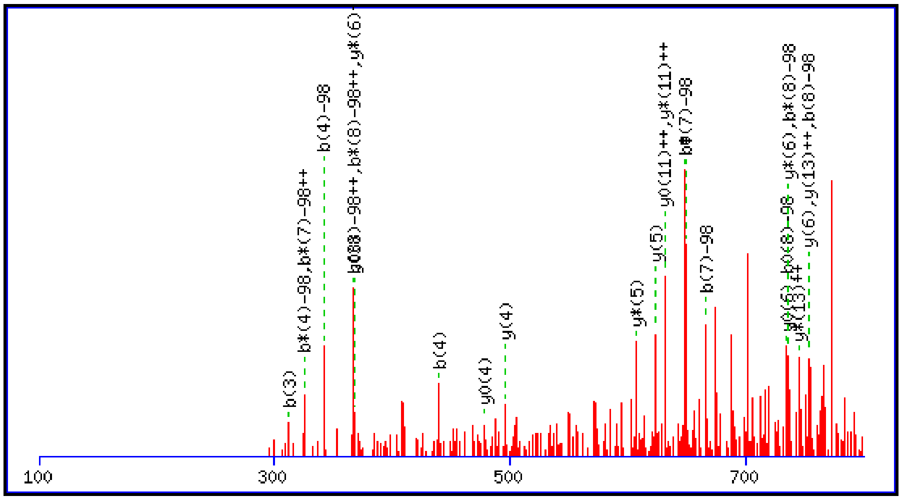

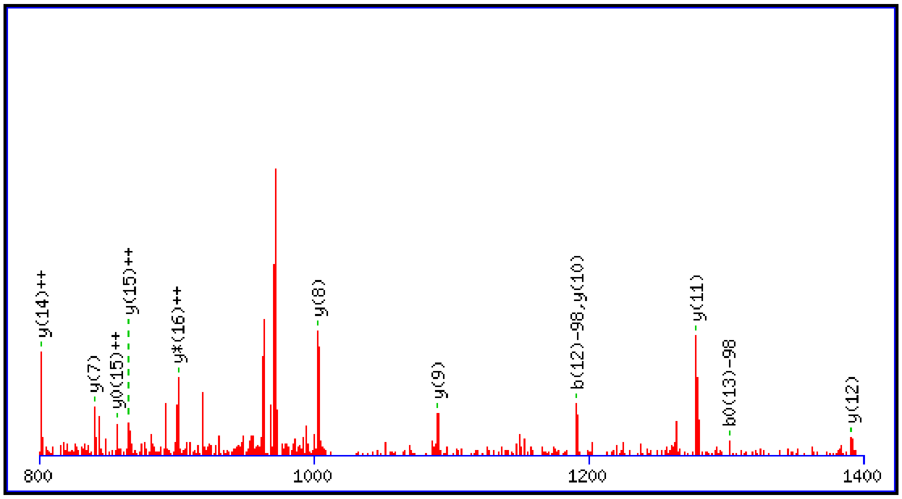

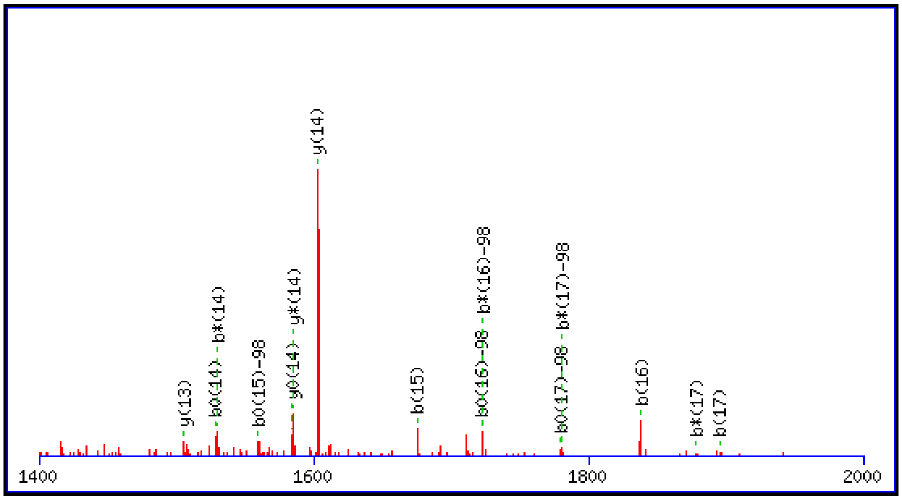

Monoisotopic mass of neutral peptide Mr(calc): 2040.8313
Fixed modifications: Carbamidomethyl (C)
Variable modifications:
S2 : Phospho (ST), with neutral losses 97.9769(shown in table), 0.0000
Ions Score: 50 **Expect:** 0.0019
Matches (Bold Red): 54/288 fragment ions using 105 most intense peaks

#	b	b^{++}	b*	b^{*++}	b^{0}	b^{0++}	Seq.	y	y^{++}	y*	y^{*++}	y^{0}	y^{0++}	#
1	58.0287	29.5180					G							18
2	127.0502	64.0287			109.0396	55.0235	S	1886.8403	943.9238	1869.8137	935.4105	1868.8297	934.9185	17
3	214.0822	107.5447			196.0717	98.5395	S	1817.8188	909.4130	1800.7923	**900.8998**	1799.8082	900.4078	16
4	**342.1408**	171.5740	**325.1143**	163.0608	324.1302	162.5688	Q	1730.7868	**865.8970**	1713.7602	857.3838	1712.7762	**856.8917**	15
5	439.1936	220.1004	422.1670	211.5871	421.1830	211.0951	P	**1602.7282**	**801.8677**	**1585.7017**	793.3545	**1584.7176**	792.8625	14
6	553.2365	277.1219	536.2099	268.6086	535.2259	268.1166	N	**1505.6754**	**753.3414**	1488.6489	**744.8281**	1487.6649	744.3361	13
7	**666.3206**	333.6639	**649.2940**	**325.1506**	**648.3100**	324.6586	L	**1391.6325**	696.3199	1374.6060	687.8066	1373.6220	687.3146	12
8	**753.3526**	377.1799	**736.3260**	**368.6667**	**735.3420**	**368.1746**	S	**1278.5485**	639.7779	1261.5219	**631.2646**	1260.5379	**630.7726**	11
9	854.4003	427.7038	837.3737	419.1905	836.3897	418.6985	T	**1191.5164**	596.2619	1174.4899	587.7486	1173.5059	587.2566	10
10	941.4323	471.2198	924.4057	462.7065	923.4217	462.2145	S	**1090.4687**	545.7380	1073.4422	537.2247	1072.4582	536.7327	9
11	1104.4956	552.7514	1087.4691	544.2382	1086.4851	543.7462	Y	**1003.4367**	502.2220	986.4102	493.7087	985.4262	493.2167	8
12	**1191.5276**	596.2675	1174.5011	587.7542	1173.5171	587.2622	S	**840.3734**	420.6903	823.3468	412.1771	822.3628	411.6851	7
13	1320.5702	660.7888	1303.5437	652.2755	**1302.5597**	651.7835	E	**753.3414**	377.1743	**736.3148**	**368.6610**	**735.3308**	**368.1690**	6
14	1448.6288	724.8180	1431.6023	716.3048	1430.6183	715.8128	Q	**624.2988**	312.6530	**607.2722**	304.1397	606.2882	303.6477	5
15	1577.6714	789.3393	1560.6449	780.8261	**1559.6608**	780.3341	E	**496.2402**	248.6237	479.2136	240.1105	**478.2296**	239.6185	4
16	1740.7347	870.8710	**1723.7082**	862.3577	**1722.7242**	861.8657	Y	**367.1976**	184.1024	350.1710	175.5892			3
17	1797.7562	899.3817	**1780.7297**	890.8685	**1779.7456**	890.3765	G	204.1343	102.5708	187.1077	94.0575			2
18							K	147.1128	74.0600	130.0863	65.5468			1

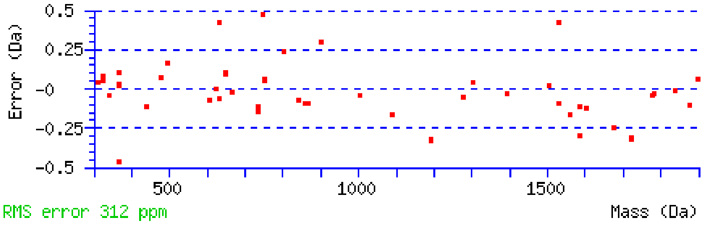

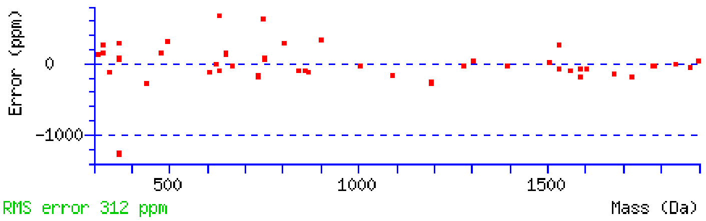

All matches to this query

Score	Mr(calc):	Delta	Sequence
50.3	2040.8313	0.0034	GSSQPNLSTSYSEQEYGK
50.2	2040.8313	0.0034	GSSQPNLSTSYSEQEYGK
18.5	2040.8313	0.0034	GSSQPNLSTSYSEQEYGK

7.5	2040.8313	0.0034	GSSQPNLSTSYSEQEYGK
4.9	2040.8313	0.0034	GSSQPNLSTSYSEQEYGK
1.4	2040.8313	0.0034	GSSQPNLSTSYSEQEYGK

Spectrum No: 62; Query: 767; Rank: 1

Peptide View

MS/MS Fragmentation of **ATSNVFAMFDQSQIQEFK**
Found in **IPI00421625**, Tax_Id=10116 Gene_Symbol=Mrlcb;RGD1565978_predicted Myosin regulatory light chain 2-B, smooth muscle isoform

Match to Query 767: 2169.952508 from(1085.983530,2+)
Title: 091127RatKid_SCX01_11.4868.4868.2.dta
Data file K:\NewmanPaper\Piliang\3SubProteomes\Piliang3SP\mgf5ppm\SCX_3SubProteomes5ppm.mgf

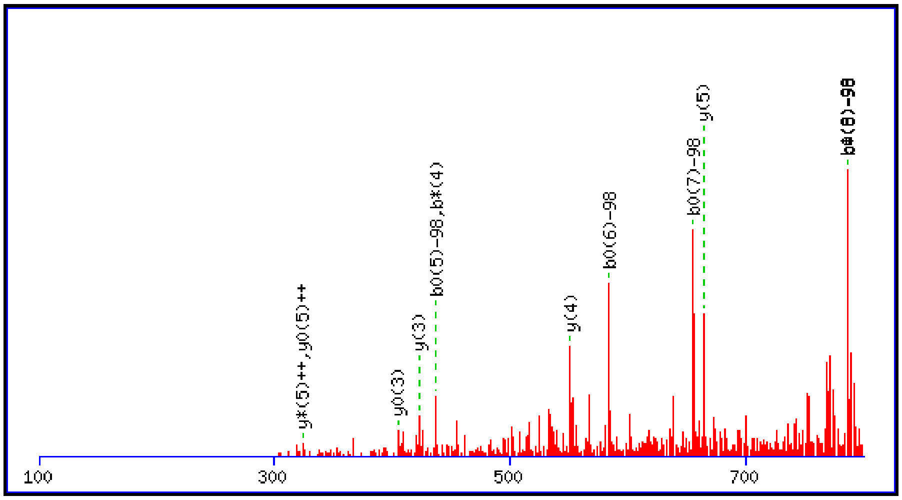

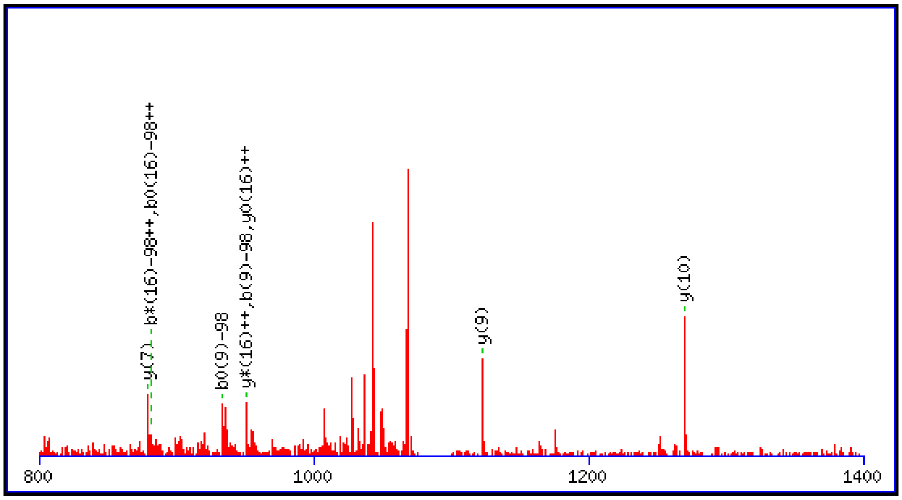

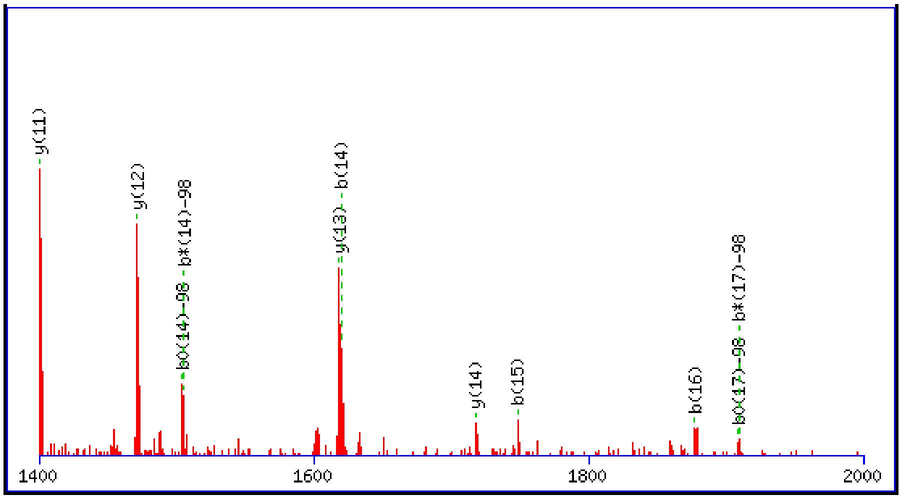

Monoisotopic mass of neutral peptide Mr(calc): 2169.9442
Fixed modifications: Carbamidomethyl (C)
Variable modifications:
T2 : Phospho (ST), with neutral losses 0.0000(shown in table), 97.9769
Ions Score: 50 **Expect:** 0.0026
Matches (Bold Red): 32/290 fragment ions using 44 most intense peaks

#	b	b^{++}	b*	b^{*++}	b^0	b^{0++}	Seq.	y	y^{++}	y*	y^{*++}	y^0	y^{0++}	#
1	72.0444	36.5258					A							18
2	253.0584	127.0328			235.0478	118.0276	T	2099.9144	1050.4608	2082.8878	1041.9476	2081.9038	1041.4555	17
3	340.0904	170.5488			322.0799	161.5436	S	1918.9004	959.9538	1901.8738	**951.4406**	1900.8898	**950.9485**	16
4	454.1333	227.5703	**437.1068**	219.0570	436.1228	218.5650	N	1831.8683	916.4378	1814.8418	907.9245	1813.8578	907.4325	15
5	553.2018	277.1045	536.1752	268.5912	535.1912	268.0992	V	**1717.8254**	859.4163	1700.7989	850.9031	1699.8149	850.4111	14
6	700.2702	350.6387	683.2436	342.1255	682.2596	341.6334	F	**1618.7570**	809.8821	1601.7305	801.3689	1600.7464	800.8769	13
7	771.3073	386.1573	754.2807	377.6440	753.2967	377.1520	A	**1471.6886**	736.3479	1454.6620	727.8347	1453.6780	727.3427	12
8	902.3478	451.6775	885.3212	443.1643	884.3372	442.6722	M	**1400.6515**	700.8294	1383.6249	692.3161	1382.6409	691.8241	11
9	1049.4162	525.2117	1032.3896	516.6985	1031.4056	516.2065	F	**1269.6110**	635.3091	1252.5844	626.7959	1251.6004	626.3039	10
10	1164.4431	582.7252	1147.4166	574.2119	1146.4326	573.7199	D	**1122.5426**	561.7749	1105.5160	553.2617	1104.5320	552.7696	9
11	1292.5017	646.7545	1275.4752	638.2412	1274.4911	637.7492	Q	1007.5156	504.2615	990.4891	495.7482	989.5051	495.2562	8
12	1379.5337	690.2705	1362.5072	681.7572	1361.5232	681.2652	S	**879.4571**	440.2322	862.4305	431.7189	861.4465	431.2269	7
13	1507.5923	754.2998	1490.5658	745.7865	1489.5818	745.2945	Q	792.4250	396.7162	775.3985	388.2029	774.4145	387.7109	6
14	**1620.6764**	810.8418	1603.6498	802.3286	1602.6658	801.8365	I	**664.3665**	332.6869	647.3399	**324.1736**	646.3559	**323.6816**	5
15	**1748.7350**	874.8711	1731.7084	866.3578	1730.7244	865.8658	Q	**551.2824**	276.1448	534.2558	267.6316	533.2718	267.1396	4
16	**1877.7776**	939.3924	1860.7510	930.8791	1859.7670	930.3871	E	**423.2238**	212.1155	406.1973	203.6023	**405.2132**	203.1103	3
17	2024.8460	1012.9266	2007.8194	1004.4133	2006.8354	1003.9213	F	294.1812	147.5942	277.1547	139.0810			2
18							K	147.1128	74.0600	130.0863	65.5468			1

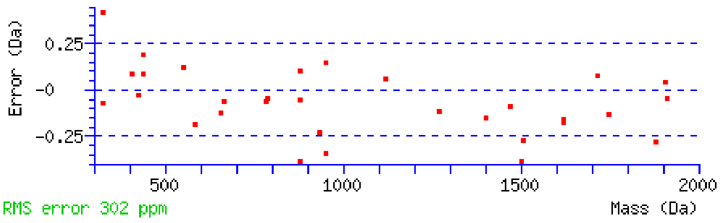

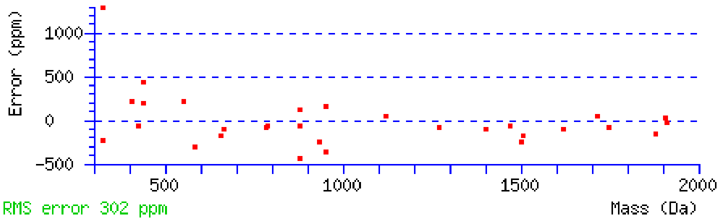

All matches to this query

Score	Mr(calc):	Delta	Sequence
49.9	2169.9442	0.0083	ATSNVFAMFDQSQIQEFK
49.7	2169.9442	0.0083	ATSNVFAMFDQSQIQEFK
3.4	2169.9705	-0.0180	LKGREADYQNLEHSHHR

Spectrum No: 63; Query: 1057; Rank: 1

Peptide View

MS/MS Fragmentation of **IQQQAGAVPPSQEEDSQEEEEEASSR**
Found in **IPI00197321**, Tax_Id=10116 Gene_Symbol=RGD1565452_predicted hypothetical protein LOC500578

Match to Query 1057: 2937.220212 from(980.080680,3+)
Title: 091127RatKid_SCX01_11.724.724.3.dta
Data file K:\NewmanPaper\Piliang\3SubProteomes\Piliang3SP\mgf5ppm\SCX_3SubProteomes5ppm.mgf

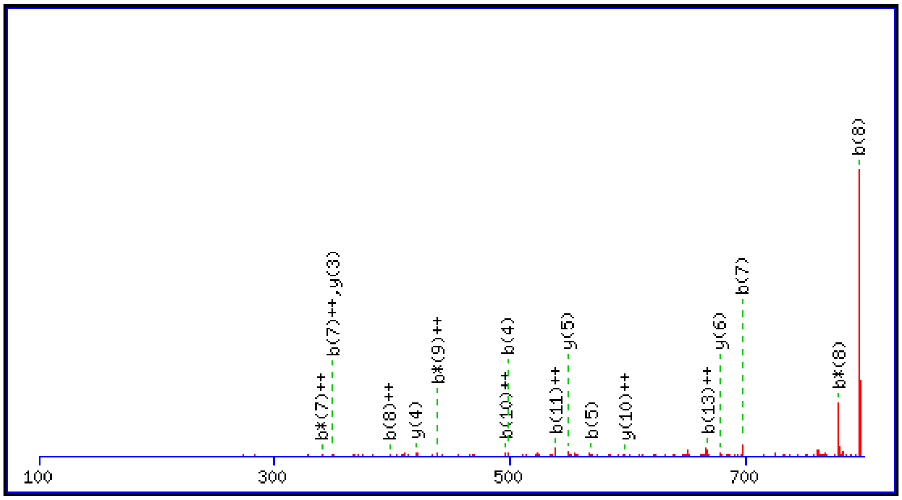

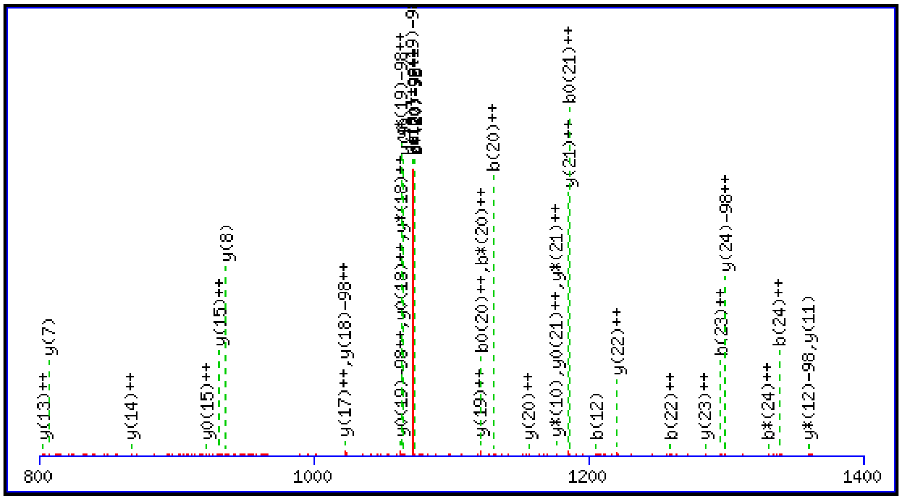

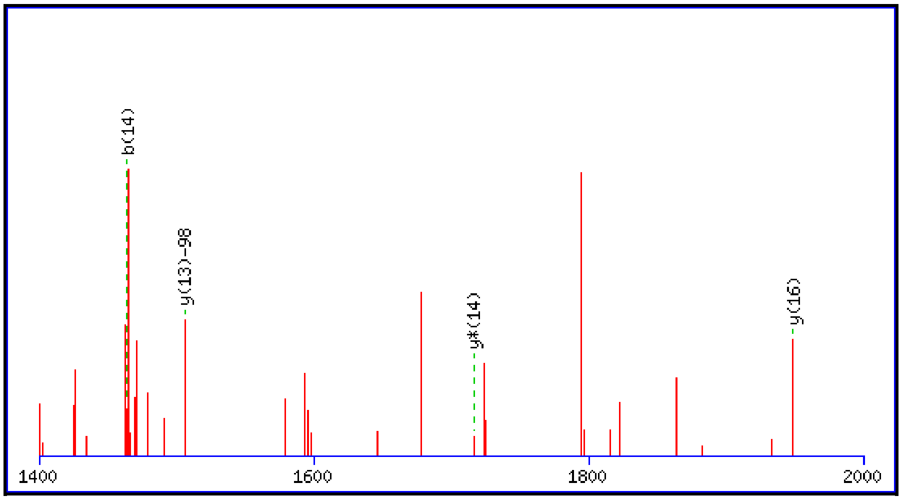

Monoisotopic mass of neutral peptide Mr(calc): 2937.2149
Fixed modifications: Carbamidomethyl (C)
Variable modifications:
S16 : Phospho (ST), with neutral losses 0.0000(shown in table), 97.9769
Ions Score: 50 **Expect:** 0.004
Matches (Bold Red): 58/426 fragment ions using 132 most intense peaks

#	b	b++	b*	b*++	b0	b0++	Seq.	y	y++	y*	y*++	y0	y0++	#
1	114.0913	57.5493					I							26
2	242.1499	121.5786	225.1234	113.0653			Q	2825.1381	1413.0727	2808.1116	1404.5594	2807.1276	1404.0674	25
3	370.2085	185.6079	353.1819	177.0946			Q	2697.0795	1349.0434	2680.0530	1340.5301	2679.0690	1340.0381	24
4	**498.2671**	249.6372	481.2405	241.1239			Q	2569.0210	**1285.0141**	2551.9944	1276.5008	2551.0104	1276.0088	23
5	**569.3042**	285.1557	552.2776	276.6425			A	2440.9624	**1220.9848**	2423.9358	1212.4716	2422.9518	1211.9795	22
6	626.3257	313.6665	609.2991	305.1532			G	2369.9253	**1185.4663**	2352.8987	**1176.9530**	2351.9147	**1176.4610**	21
7	**697.3628**	**349.1850**	680.3362	**340.6717**			A	2312.9038	**1156.9555**	2295.8773	1148.4423	2294.8932	1147.9503	20
8	**796.4312**	**398.7192**	**779.4046**	390.2060			V	2241.8667	**1121.4370**	2224.8401	1112.9237	2223.8561	1112.4317	19
9	893.4839	447.2456	876.4574	**438.7323**			P	2142.7983	**1071.9028**	2125.7717	**1063.3895**	2124.7877	**1062.8975**	18
10	990.5367	**495.7720**	973.5102	487.2587			P	2045.7455	**1023.3764**	2028.7190	1014.8631	2027.7350	1014.3711	17
11	1077.5687	**539.2880**	1060.5422	530.7747	1059.5582	530.2827	S	**1948.6928**	974.8500	1931.6662	966.3367	1930.6822	965.8447	16
12	**1205.6273**	603.3173	1188.6008	594.8040	1187.6167	594.3120	Q	1861.6607	**931.3340**	1844.6342	922.8207	1843.6502	**922.3287**	15
13	1334.6699	**667.8386**	1317.6434	659.3253	1316.6593	658.8333	E	1733.6021	**867.3047**	**1716.5756**	858.7914	1715.5916	858.2994	14
14	**1463.7125**	732.3599	1446.6860	723.8466	1445.7019	723.3546	E	1604.5596	**802.7834**	1587.5330	794.2701	1586.5490	793.7781	13
15	1578.7394	789.8734	1561.7129	781.3601	1560.7289	780.8681	D	1475.5170	738.2621	1458.4904	729.7488	1457.5064	729.2568	12
16	1745.7378	873.3725	1728.7112	864.8593	1727.7272	864.3673	S	**1360.4900**	680.7486	1343.4635	672.2354	1342.4795	671.7434	11
17	1873.7964	937.4018	1856.7698	928.8886	1855.7858	928.3965	Q	1193.4917	**597.2495**	**1176.4651**	588.7362	1175.4811	588.2442	10
18	2002.8390	1001.9231	1985.8124	993.4098	1984.8284	992.9178	E	**1065.4331**	533.2202	1048.4065	524.7069	1047.4225	524.2149	9
19	2131.8816	1066.4444	2114.8550	1057.9311	2113.8710	1057.4391	E	**936.3905**	468.6989	919.3639	460.1856	918.3799	459.6936	8
20	2260.9242	**1130.9657**	2243.8976	**1122.4524**	2242.9136	**1121.9604**	E	**807.3479**	404.1776	790.3214	395.6643	789.3373	395.1723	7
21	2389.9667	1195.4870	2372.9402	1186.9737	2371.9562	**1186.4817**	E	**678.3053**	339.6563	661.2788	331.1430	660.2947	330.6510	6
22	2519.0093	**1260.0083**	2501.9828	1251.4950	2500.9988	1251.0030	E	**549.2627**	275.1350	532.2362	266.6217	531.2522	266.1297	5
23	2590.0465	**1295.5269**	2573.0199	1287.0136	2572.0359	1286.5216	A	**420.2201**	210.6137	403.1936	202.1004	402.2096	201.6084	4
24	2677.0785	**1339.0429**	2660.0519	**1330.5296**	2659.0679	1330.0376	S	**349.1830**	175.0951	332.1565	166.5819	331.1724	166.0899	3
25	2764.1105	1382.5589	2747.0840	1374.0456	2746.0999	1373.5536	S	262.1510	131.5791	245.1244	123.0659	244.1404	122.5738	2
26							R	175.1190	88.0631	158.0924	79.5498			1

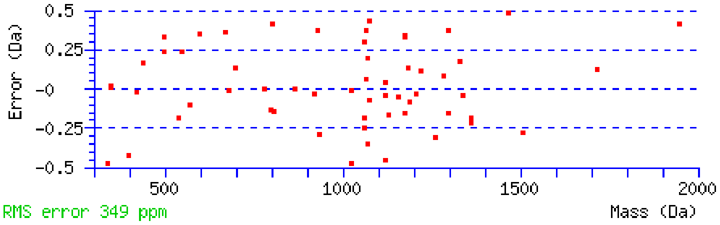

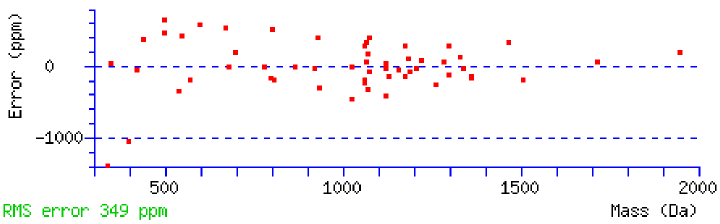

All matches to this query

Score	Mr(calc):	Delta	Sequence
49.5	2937.2149	0.0054	IQQQAGAVPPSQEEDSQEEEEEASSR
35.6	2937.2149	0.0054	IQQQAGAVPPSQEEDSQEEEEEASSR
17.2	2937.2149	0.0054	IQQQAGAVPPSQEEDSQEEEEEASSR
13.8	2937.2149	0.0054	IQQQAGAVPPSQEEDSQEEEEEASSR
9.5	2937.2108	0.0094	GSKMMPTLAPVVTKLGNSGVPSSSSGK
8.4	2937.2063	0.0139	YPEDIYLHVLWHSFFNCSFFR
7.1	2937.1993	0.0209	GYSTQVLLQEVYQSPCTMGQRPR
7.1	2937.1993	0.0209	GYSTQVLLQEVYQSPCTMGQRPR

7.1	2937.1993	0.0209	GYSTQVLLQEVYQSPCTMGQRPR
7.1	2937.1993	0.0209	GYSTQVLLQEVYQSPCTMGQRPR

Spectrum No: 64; Query: 853; Rank: 1

Peptide View

MS/MS Fragmentation of **ASVDSETESSPGVNETAAASGQR**
Found in **IPI00211999**, Tax_Id=10116 Gene_Symbol=Rnmt 46 kDa protein

Match to Query 853: 2328.974202 from(777.332010,3+)
Title: 091127RatKid_SCX01_12.1109.1109.3.dta
Data file K:\NewmanPaper\Piliang\3SubProteomes\Piliang3SP\mgf5ppm\SCX_3SubProteomes5ppm.mgf

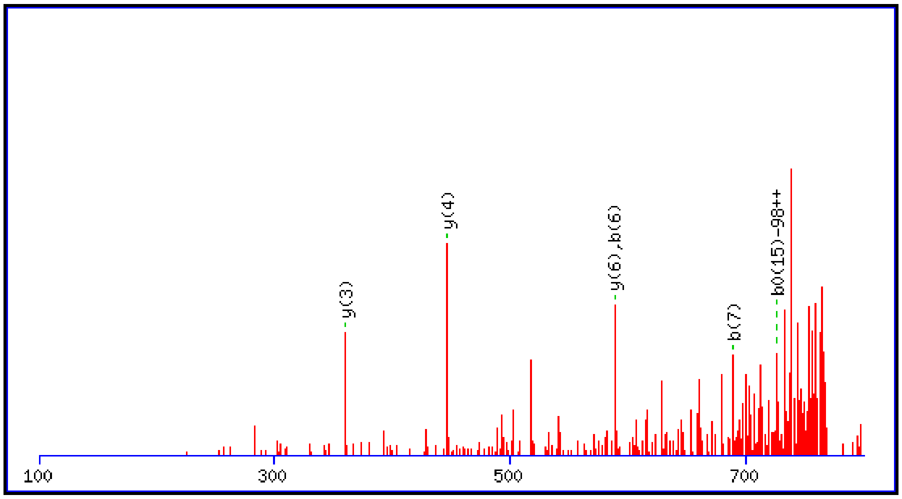

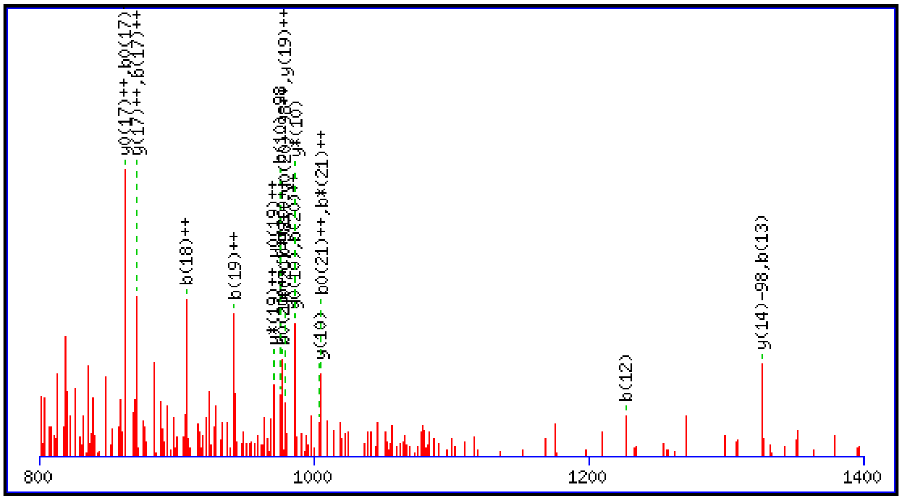

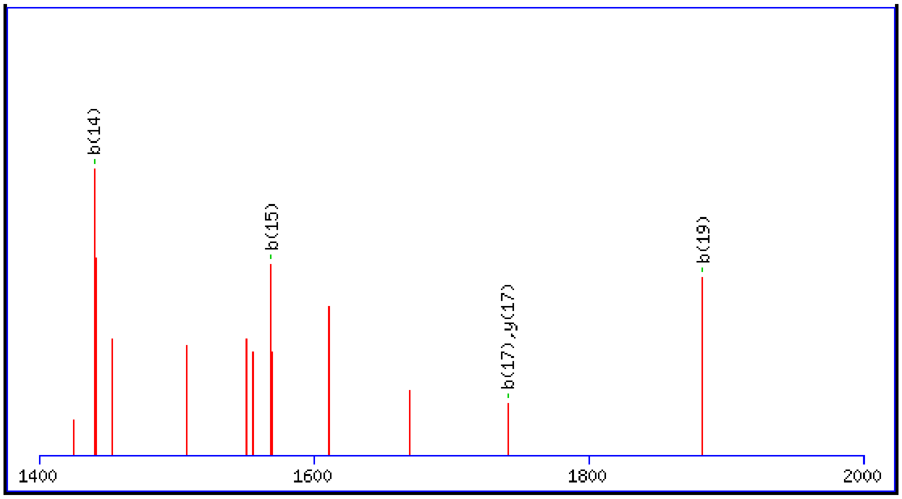

Monoisotopic mass of neutral peptide Mr(calc): 2328.9707
Fixed modifications: Carbamidomethyl (C)
Variable modifications:
S10 : Phospho (ST), with neutral losses 0.0000(shown in table), 97.9769
Ions Score: 49 **Expect:** 0.0035
Matches (Bold Red): 36/354 fragment ions using 39 most intense peaks

#	b	b^{++}	b*	b^{*++}	b^0	b^{0++}	Seq.	y	y^{++}	y*	y^{*++}	y^0	y^{0++}	#
1	72.0444	36.5258					A							23
2	159.0764	80.0418			141.0659	71.0366	S	2258.9409	1129.9741	2241.9143	1121.4608	2240.9303	1120.9688	22
3	258.1448	129.5761			240.1343	120.5708	V	2171.9088	1086.4581	2154.8823	1077.9448	2153.8983	1077.4528	21
4	373.1718	187.0895			355.1612	178.0842	D	2072.8404	1036.9238	2055.8139	1028.4106	2054.8299	1027.9186	20
5	460.2038	230.6055			442.1932	221.6003	S	1957.8135	**979.4104**	1940.7869	**970.8971**	1939.8029	**970.4051**	19
6	**589.2464**	295.1268			571.2358	286.1216	E	1870.7815	935.8944	1853.7549	927.3811	1852.7709	926.8891	18
7	**690.2941**	345.6507			672.2835	336.6454	T	**1741.7389**	**871.3731**	1724.7123	**862.8598**	1723.7283	**862.3678**	17
8	819.3367	410.1720			801.3261	401.1667	E	1640.6912	820.8492	1623.6646	812.3360	1622.6806	811.8439	16
9	906.3687	453.6880			888.3581	444.6827	S	1511.6486	756.3279	1494.6220	747.8147	1493.6380	747.3226	15
10	1073.3670	537.1872			1055.3565	528.1819	S	1424.6166	712.8119	1407.5900	704.2986	1406.6060	703.8066	14
11	1170.4198	585.7135			1152.4092	576.7083	P	1257.6182	629.3127	1240.5917	620.7995	1239.6076	620.3075	13
12	**1227.4413**	614.2243			1209.4307	605.2190	G	1160.5654	580.7864	1143.5389	572.2731	1142.5549	571.7811	12
13	**1326.5097**	663.7585			1308.4991	654.7532	V	1103.5440	552.2756	1086.5174	543.7624	1085.5334	543.2703	11
14	**1440.5526**	720.7799	1423.5261	712.2667	1422.5421	711.7747	N	**1004.4756**	502.7414	**987.4490**	494.2281	**986.4650**	493.7361	10
15	**1569.5952**	785.3012	1552.5687	776.7880	1551.5846	776.2960	E	890.4326	445.7200	873.4061	437.2067	872.4221	436.7147	9
16	1670.6429	835.8251	1653.6163	827.3118	1652.6323	826.8198	T	761.3900	381.1987	744.3635	372.6854	743.3795	372.1934	8
17	**1741.6800**	**871.3436**	1724.6535	**862.8304**	1723.6694	**862.3384**	A	660.3424	330.6748	643.3158	322.1615	642.3318	321.6695	7
18	1812.7171	**906.8622**	1795.6906	898.3489	1794.7066	897.8569	A	**589.3053**	295.1563	572.2787	286.6430	571.2947	286.1510	6
19	**1883.7542**	**942.3808**	1866.7277	933.8675	1865.7437	933.3755	A	518.2681	259.6377	501.2416	251.1244	500.2576	250.6324	5
20	1970.7863	**985.8968**	1953.7597	**977.3835**	1952.7757	**976.8915**	S	**447.2310**	224.1191	430.2045	215.6059	429.2205	215.1139	4
21	2027.8077	1014.4075	2010.7812	**1005.8942**	2009.7972	**1005.4022**	G	**360.1990**	180.6031	343.1724	172.0899			3
22	2155.8663	1078.4368	2138.8398	1069.9235	2137.8557	1069.4315	Q	303.1775	152.0924	286.1510	143.5791			2
23							R	175.1190	88.0631	158.0924	79.5498			1

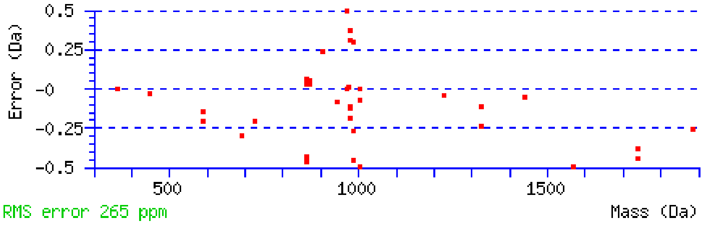

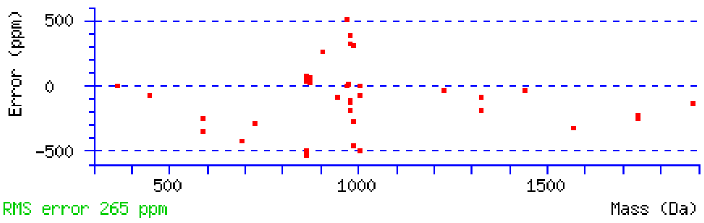

All matches to this query

Score	Mr(calc):	Delta	Sequence
49.3	2328.9707	0.0035	ASVDSETESSPGVNETAAASGQR
49.3	2328.9707	0.0035	ASVDSETESSPGVNETAAASGQR
36.7	2328.9707	0.0035	ASVDSETESSPGVNETAAASGQR
28.3	2328.9707	0.0035	ASVDSETESSPGVNETAAASGQR
28.2	2328.9707	0.0035	ASVDSETESSPGVNETAAASGQR
14.6	2328.9707	0.0035	ASVDSETESSPGVNETAAASGQR
2.5	2328.9531	0.0211	ASIMFNQNINSVVEFCTEK
1.2	2327.9858	0.9884	SPSSNGVSPGPGAGMLKTPSPSR

Spectrum No: 65; Query: 771; Rank: 1

Peptide View

MS/MS Fragmentation of **SLDSDESEDEDDDYQQK**
Found in **IPI00208277**, Tax_Id=10116 Gene_Symbol=Pdap1 28 kDa heat- and acid-stable phosphoprotein

Match to Query 771: 2176.695568 from(1089.355060,2+)
Title: 091127RatKid_SCX01_02.1064.1064.2.dta
Data file K:\NewmanPaper\Piliang\3SubProteomes\Piliang3SP\mgf5ppm\SCX_3SubProteomes5ppm.mgf

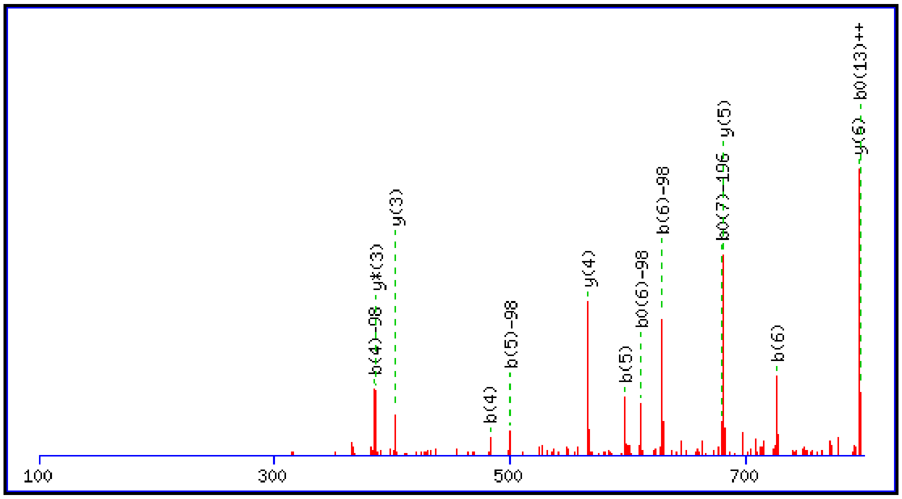

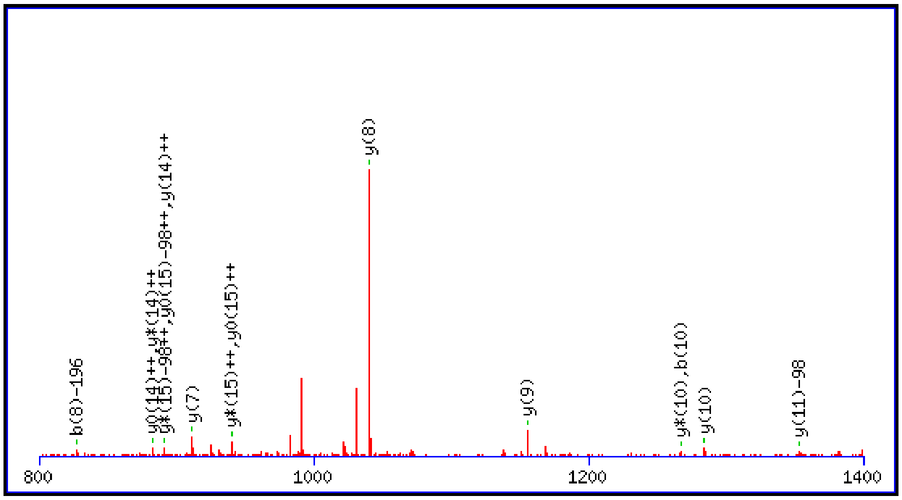

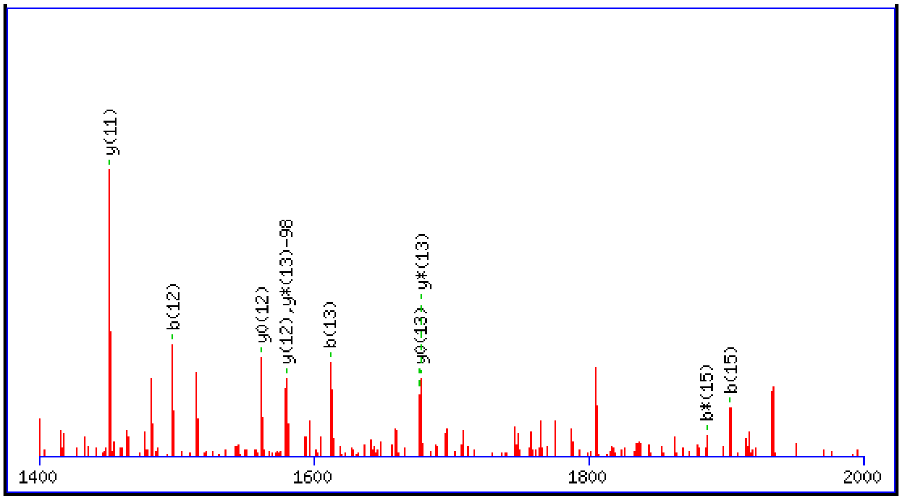

Monoisotopic mass of neutral peptide Mr(calc): 2176.6882
Fixed modifications: Carbamidomethyl (C)
Variable modifications:
S1 : Phospho (ST), with neutral losses 0.0000(shown in table), 97.9769
S7 : Phospho (ST), with neutral losses 0.0000(shown in table), 97.9769
Ions Score: 49 **Expect:** 0.00053
Matches (Bold Red): 39/260 fragment ions using 53 most intense peaks

#	b	b^{++}	b*	b^{*++}	b^{0}	b^{0++}	Seq.	y	y^{++}	y*	y^{*++}	y^{0}	y^{0++}	#
1	168.0056	84.5065			149.9951	75.5012	S							17
2	281.0897	141.0485			263.0791	132.0432	L	2010.6972	1005.8522	1993.6706	997.3389	1992.6866	996.8469	16
3	396.1166	198.5620			378.1061	189.5567	D	1897.6131	949.3102	1880.5866	**940.7969**	1879.6025	**940.3049**	15
4	**483.1487**	242.0780			465.1381	233.0727	S	1782.5862	**891.7967**	1765.5596	**883.2834**	1764.5756	**882.7914**	14
5	**598.1756**	299.5914			580.1650	290.5862	D	1695.5541	848.2807	**1678.5276**	839.7674	**1677.5436**	839.2754	13
6	**727.2182**	364.1127			709.2076	355.1075	E	**1580.5272**	790.7672	1563.5006	782.2540	**1562.5166**	781.7620	12
7	894.2166	447.6119			876.2060	438.6066	S	**1451.4846**	726.2459	1434.4581	717.7327	1433.4740	717.2407	11
8	1023.2591	512.1332			1005.2486	503.1279	E	**1284.4862**	642.7468	**1267.4597**	634.2335	1266.4757	633.7415	10
9	1138.2861	569.6467			1120.2755	560.6414	D	**1155.4437**	578.2255	1138.4171	569.7122	1137.4331	569.2202	9
10	**1267.3287**	634.1680			1249.3181	625.1627	E	**1040.4167**	520.7120	1023.3902	512.1987	1022.4061	511.7067	8
11	1382.3556	691.6814			1364.3451	682.6762	D	**911.3741**	456.1907	894.3476	447.6774	893.3636	447.1854	7
12	**1497.3826**	749.1949			1479.3720	740.1896	D	**796.3472**	398.6772	779.3206	390.1640	778.3366	389.6719	6
13	**1612.4095**	806.7084			1594.3989	**797.7031**	D	**681.3202**	341.1638	664.2937	332.6505	663.3097	332.1585	5
14	1775.4728	888.2401			1757.4623	879.2348	Y	**566.2933**	283.6503	549.2667	275.1370			4
15	**1903.5314**	952.2693	**1886.5049**	943.7561	1885.5209	943.2641	Q	**403.2300**	202.1186	**386.2034**	193.6053			3
16	2031.5900	1016.2986	2014.5634	1007.7854	2013.5794	1007.2934	Q	275.1714	138.0893	258.1448	129.5761			2
17							K	147.1128	74.0600	130.0863	65.5468			1

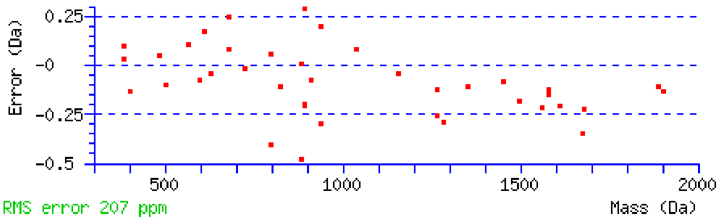

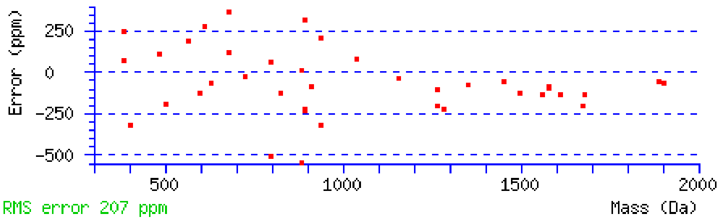

All matches to this query

Score	Mr(calc):	Delta	Sequence
49.2	2176.6882	0.0073	SLDSDESEDEDDDYQQK
49.1	2176.6882	0.0073	SLDSDESEDEDDDYQQK
32.8	2176.6882	0.0073	SLDSDESEDEDDDYQQK
21.9	2176.6882	0.0073	SLDSDESEDEDDDYQQK

21.9	2176.6882	0.0073	SLDSDESEDEDDDYQQK
7.3	2176.6882	0.0073	SLDSDESEDEDDDYQQK
2.0	2175.6949	1.0007	LPSNQHSNDSANGNGSKK
1.9	2174.6756	2.0200	YFLQGMGYMPSASMTR
1.9	2174.6756	2.0200	YFLQGMGYMPSASMTR
1.7	2176.7050	-0.0094	ECPHLSSSVCIAPDSAK

Spectrum No: 66; Query: 256; Rank: 1

Peptide View

MS/MS Fragmentation of **FFGNSWSETYR**
Found in **IPI00325765**, Tax_Id=10116 Gene_Symbol=Akr7a2 Aflatoxin B1 aldehyde reductase member 2

Match to Query 256: 1472.577588 from(737.296070,2+)
Title: 091127RatKid_SCX01_13.3461.3461.2.dta
Data file K:\NewmanPaper\Piliang\3SubProteomes\Piliang3SP\mgf5ppm\SCX_3SubProteomes5ppm.mgf

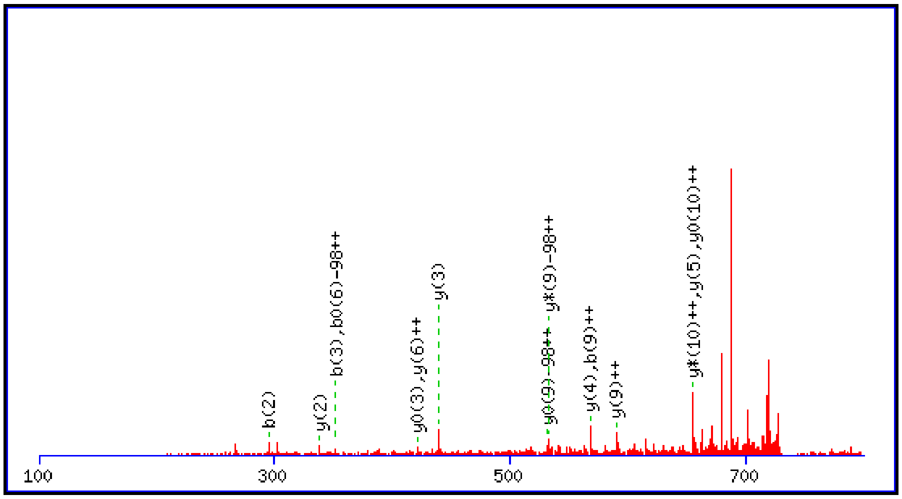

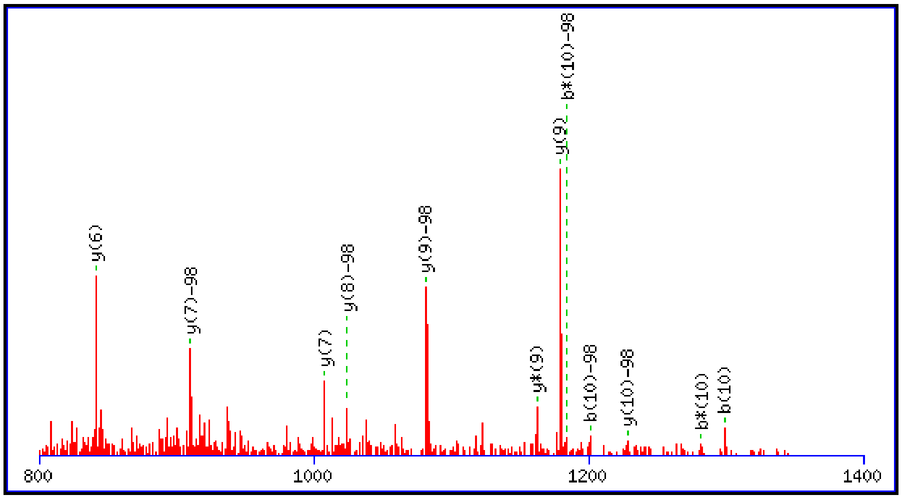

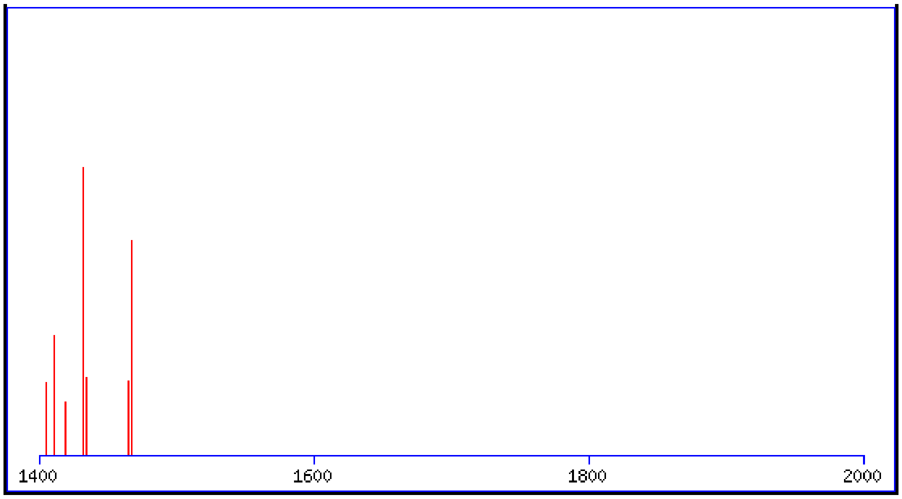

Monoisotopic mass of neutral peptide Mr(calc): 1472.5762
Fixed modifications: Carbamidomethyl (C)
Variable modifications:
S5 : Phospho (ST), with neutral losses 97.9769(shown in table), 0.0000
Ions Score: 49 **Expect:** 0.0013
Matches (Bold Red): 27/162 fragment ions using 43 most intense peaks

#	b	b^{++}	b*	b*$^{++}$	b^{0}	b^{0++}	Seq.	y	y^{++}	y*	y*$^{++}$	y^{0}	y^{0++}	#
1	148.0757	74.5415					F							11
2	**295.1441**	148.0757					F	**1228.5382**	614.7727	1211.5116	606.2594	1210.5276	605.7674	10
3	**352.1656**	176.5864					G	**1081.4697**	541.2385	1064.4432	**532.7252**	1063.4592	**532.2332**	9
4	466.2085	233.6079	449.1819	225.0946			N	**1024.4483**	512.7278	1007.4217	504.2145	1006.4377	503.7225	8
5	535.2300	268.1186	518.2034	259.6053	517.2194	259.1133	S	**910.4054**	455.7063	893.3788	447.1930	892.3948	446.7010	7
6	721.3093	361.1583	704.2827	352.6450	703.2987	**352.1530**	W	**841.3839**	**421.1956**	824.3573	412.6823	823.3733	412.1903	6
7	808.3413	404.6743	791.3147	396.1610	790.3307	395.6690	S	**655.3046**	328.1559	638.2780	319.6427	637.2940	319.1506	5
8	937.3839	469.1956	920.3573	460.6823	919.3733	460.1903	E	**568.2726**	284.6399	551.2460	276.1266	550.2620	275.6346	4
9	1038.4316	519.7194	1021.4050	511.2061	1020.4210	510.7141	T	**439.2300**	220.1186	422.2034	211.6053	**421.2194**	211.1133	3
10	**1201.4949**	601.2511	**1184.4683**	592.7378	1183.4843	592.2458	Y	**338.1823**	169.5948	321.1557	161.0815			2
11							R	175.1190	88.0631	158.0924	79.5498			1

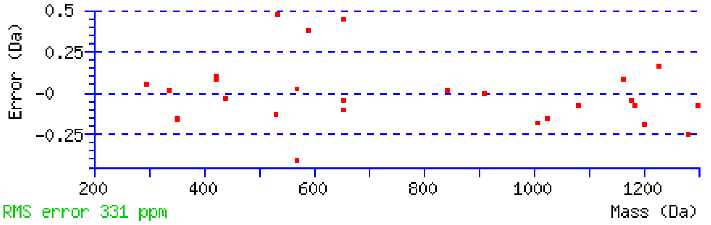

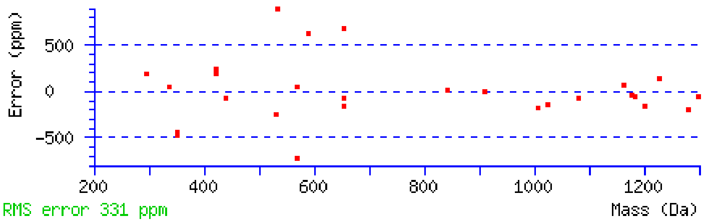

All matches to this query

Score	Mr(calc):	Delta	Sequence
49.2	1472.5762	0.0014	FFGNSWSETYR
28.3	1472.5762	0.0014	FFGNSWSETYR
20.9	1472.5762	0.0014	FFGNSWSETYR
6.1	1470.5615	2.0160	SYSTVTTKCHK
3.9	1472.5738	0.0038	LTQPSSFYSQR
3.9	1472.5738	0.0038	LTQPSSFYSQR
0.8	1471.5625	1.0151	CFSRGESVYCK
0.7	1472.5869	-0.0093	MFPKEMPYYR
0.2	1472.5751	0.0025	MFMATQITSGMK
0.2	1472.5751	0.0025	MFMATQITSGMK

Spectrum No: 67; Query: 563; Rank: 1

Peptide View

MS/MS Fragmentation of **SASSDTSEELNAQDSPK**
Found in **IPI00200898**, Tax_Id=10116 Gene_Symbol=Slc9a3r1 Ezrin-radixin-moesin-binding phosphoprotein 50

Match to Query 563: 1844.728248 from(923.371400,2+)
Title: 091129RatKid_SCX02_06.418.418.2.dta
Data file K:\NewmanPaper\Piliang\3SubProteomes\Piliang3SP\mgf5ppm\SCX_3SubProteomes5ppm.mgf

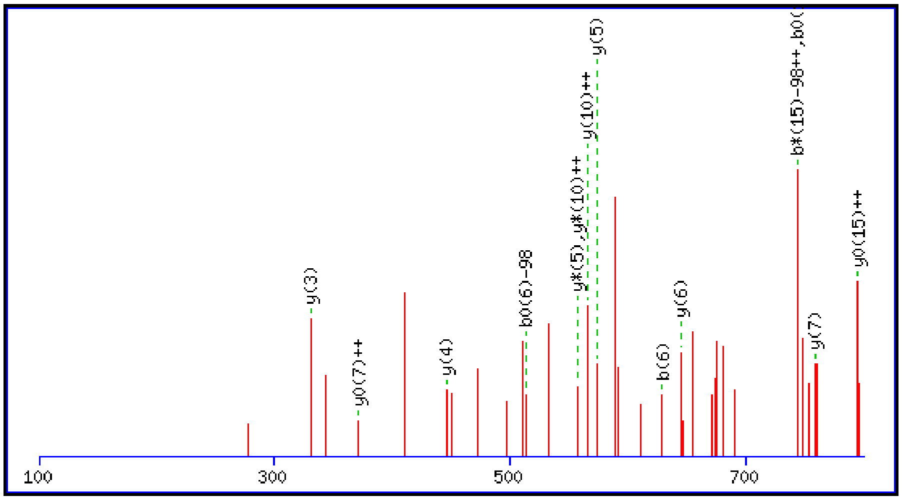

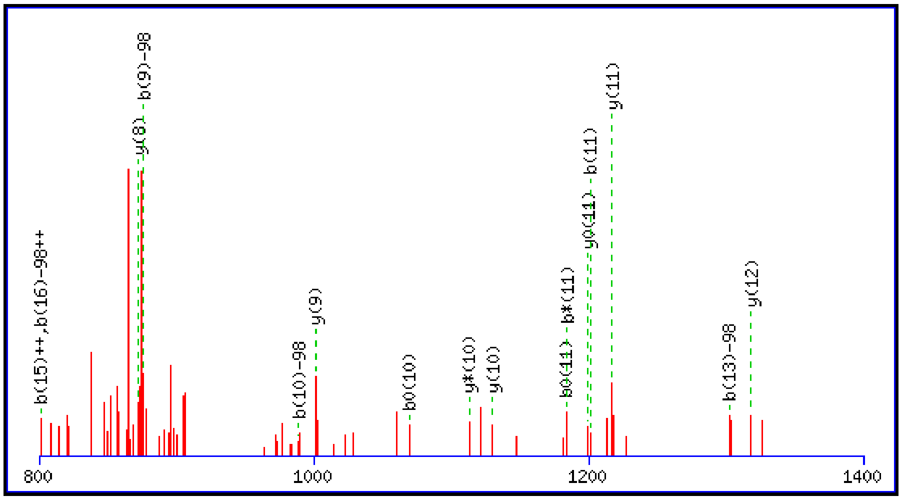

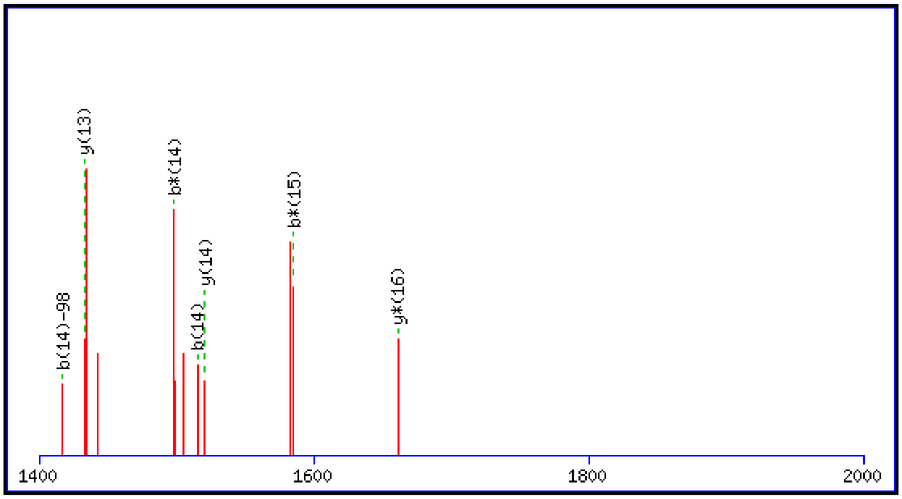

Monoisotopic mass of neutral peptide Mr(calc): 1844.7313
Fixed modifications: Carbamidomethyl (C)
Variable modifications:
S1 : Phospho (ST), with neutral losses 97.9769(shown in table), 0.0000
Ions Score: 49 **Expect:** 0.0018
Matches (Bold Red): 37/244 fragment ions using 88 most intense peaks

#	b	b^{++}	b*	b^{*++}	b^0	b^{0++}	Seq.	y	y^{++}	y*	y^{*++}	y^0	y^{0++}	#
1	70.0287	35.5180			52.0182	26.5127	S							17
2	141.0658	71.0366			123.0553	62.0313	A	1678.7402	839.8738	**1661.7137**	831.3605	1660.7297	830.8685	16
3	228.0979	114.5526			210.0873	105.5473	S	1607.7031	804.3552	1590.6766	795.8419	1589.6925	**795.3499**	15
4	315.1299	158.0686			297.1193	149.0633	S	**1520.6711**	760.8392	1503.6445	752.3259	1502.6605	751.8339	14
5	430.1568	215.5821			412.1463	206.5768	D	**1433.6391**	717.3232	1416.6125	708.8099	1415.6285	708.3179	13
6	531.2045	266.1059			**513.1940**	257.1006	T	**1318.6121**	659.8097	1301.5856	651.2964	1300.6016	650.8044	12
7	618.2366	309.6219			600.2260	300.6166	S	**1217.5644**	609.2859	1200.5379	600.7726	**1199.5539**	600.2806	11
8	747.2791	374.1432			729.2686	365.1379	E	**1130.5324**	**565.7698**	**1113.5059**	**557.2566**	1112.5218	556.7646	10
9	**876.3217**	438.6645			858.3112	429.6592	E	**1001.4898**	501.2485	984.4633	492.7353	983.4793	492.2433	9
10	**989.4058**	495.2065			971.3952	486.2013	L	**872.4472**	436.7272	855.4207	428.2140	854.4367	427.7220	8
11	1103.4487	552.2280	1086.4222	543.7147	1085.4382	543.2227	N	**759.3632**	380.1852	742.3366	371.6719	741.3526	**371.1799**	7
12	1174.4858	587.7466	1157.4593	579.2333	1156.4753	578.7413	A	**645.3202**	323.1638	628.2937	314.6505	627.3097	314.1585	6
13	**1302.5444**	651.7758	1285.5179	643.2626	1284.5339	642.7706	Q	**574.2831**	287.6452	**557.2566**	279.1319	556.2726	278.6399	5
14	**1417.5714**	709.2893	1400.5448	700.7760	1399.5608	700.2840	D	**446.2245**	223.6159	429.1980	215.1026	428.2140	214.6106	4
15	1504.6034	752.8053	1487.5768	**744.2921**	1486.5928	**743.8001**	S	**331.1976**	166.1024	314.1710	157.5892	313.1870	157.0972	3
16	1601.6562	**801.3317**	1584.6296	792.8184	1583.6456	792.3264	P	244.1656	122.5864	227.1390	114.0731			2
17							K	147.1128	74.0600	130.0863	65.5468			1

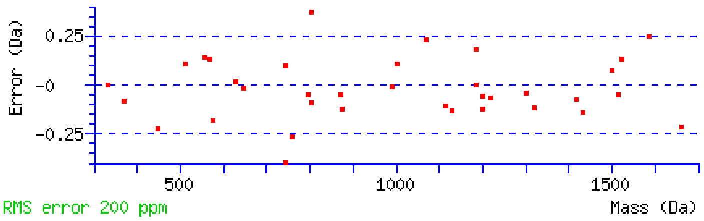

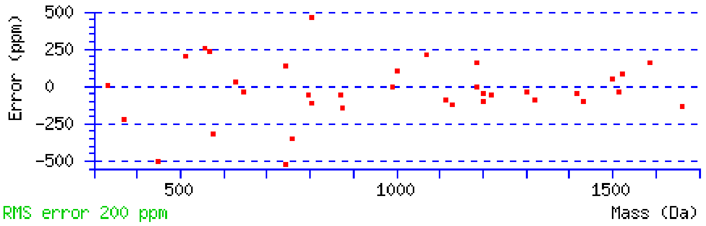

All matches to this query

Score	Mr(calc):	Delta	Sequence
49.1	1844.7313	-0.0030	SASSDTSEELNAQDSPK
47.9	1844.7313	-0.0030	SASSDTSEELNAQDSPK
38.8	1844.7313	-0.0030	SASSDTSEELNAQDSPK
23.4	1844.7313	-0.0030	SASSDTSEELNAQDSPK
21.3	1844.7313	-0.0030	SASSDTSEELNAQDSPK

Spectrum No: 68; Query: 500; Rank: 1

Peptide View

MS/MS Fragmentation of **SRTASGSSVTSLEGTR**
Found in **IPI00421389**, Tax_Id=10116 Gene_Symbol=Ndrg1 Protein NDRG1

Match to Query 500: 1754.725602 from(585.915810,3+)
Title: 091127RatKid_SCX01_15.1304.1304.3.dta
Data file K:\NewmanPaper\Piliang\3SubProteomes\Piliang3SP\mgf5ppm\SCX_3SubProteomes5ppm.mgf

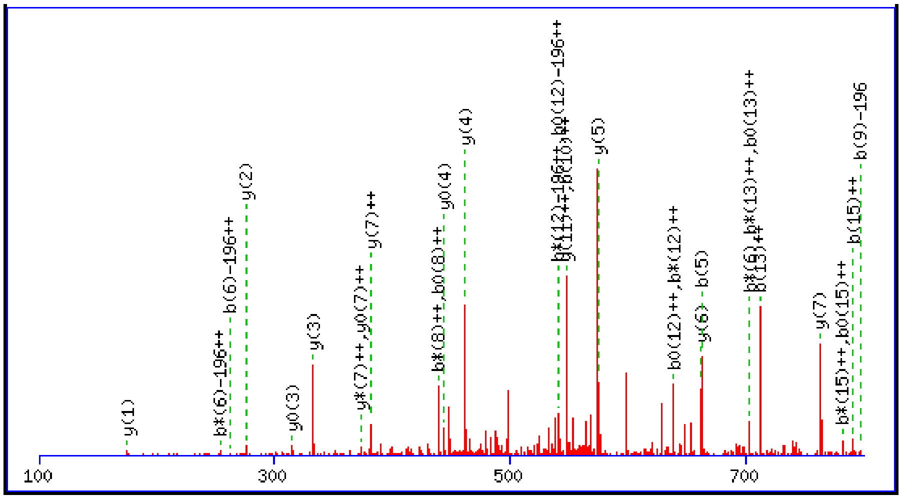

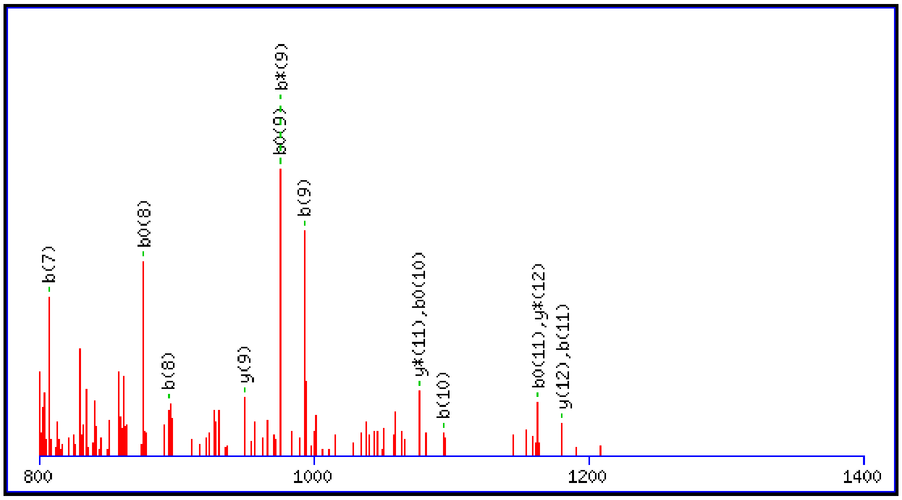

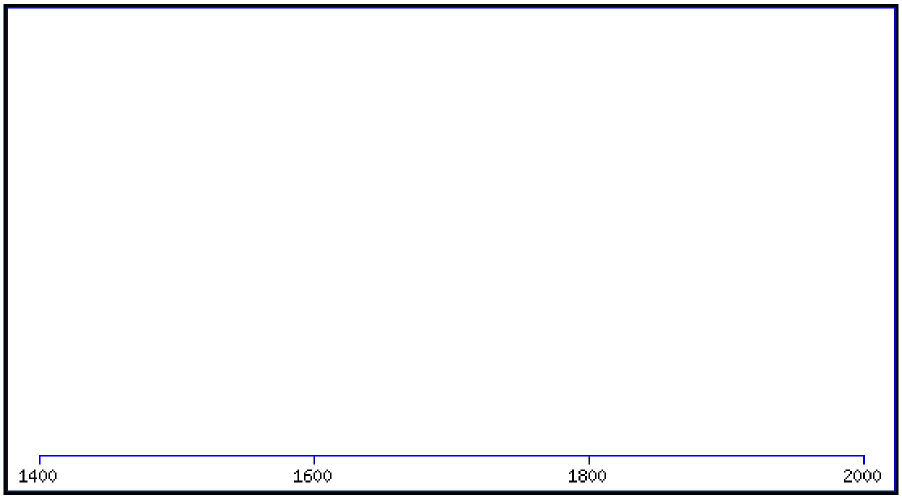

Monoisotopic mass of neutral peptide Mr(calc): 1754.7237
Fixed modifications: Carbamidomethyl (C)
Variable modifications:
S1 : Phospho (ST), with neutral losses 0.0000(shown in table), 97.9769
T3 : Phospho (ST), with neutral losses 0.0000(shown in table), 97.9769
Ions Score: 49 **Expect:** 0.002
Matches (Bold Red): 45/276 fragment ions using 54 most intense peaks

#	b	b^{++}	b*	b^{*++}	b^0	b^{0++}	Seq.	y	y^{++}	y*	y^{*++}	y^0	y^{0++}	#
1	168.0056	84.5065			149.9951	75.5012	S							16
2	324.1067	162.5570	307.0802	154.0437	306.0962	153.5517	R	1588.7326	794.8700	1571.7061	786.3567	1570.7221	785.8647	15
3	505.1207	253.0640	488.0942	244.5507	487.1102	244.0587	T	1432.6315	716.8194	1415.6050	708.3061	1414.6210	707.8141	14
4	576.1579	288.5826	559.1313	280.0693	558.1473	279.5773	A	1251.6175	626.3124	1234.5910	617.7991	1233.6070	617.3071	13

5	**663.1899**	332.0986	646.1633	323.5853	645.1793	323.0933	S	**1180.5804**	590.7938	**1163.5539**	582.2806	1162.5699	581.7886	12
6	720.2113	360.6093	**703.1848**	352.0960	702.2008	351.6040	G	1093.5484	**547.2778**	**1076.5218**	538.7646	1075.5378	538.2726	11
7	**807.2434**	404.1253	790.2168	395.6121	789.2328	395.1200	S	1036.5269	518.7671	1019.5004	510.2538	1018.5164	509.7618	10
8	**894.2754**	447.6413	877.2489	**439.1281**	**876.2648**	**438.6361**	S	**949.4949**	475.2511	932.4684	466.7378	931.4843	466.2458	9
9	**993.3438**	497.1755	**976.3173**	488.6623	**975.3333**	488.1703	V	862.4629	431.7351	845.4363	423.2218	844.4523	422.7298	8
10	**1094.3915**	**547.6994**	1077.3649	539.1861	**1076.3809**	538.6941	T	**763.3945**	**382.2009**	746.3679	**373.6876**	745.3839	**373.1956**	7
11	**1181.4235**	591.2154	1164.3970	582.7021	**1163.4130**	582.2101	S	**662.3468**	331.6770	645.3202	323.1638	644.3362	322.6717	6
12	1294.5076	647.7574	1277.4810	**639.2442**	1276.4970	**638.7521**	L	**575.3148**	288.1610	558.2882	279.6477	557.3042	279.1557	5
13	1423.5502	**712.2787**	1406.5236	**703.7655**	1405.5396	**703.2734**	E	**462.2307**	231.6190	445.2041	223.1057	**444.2201**	222.6137	4
14	1480.5716	740.7895	1463.5451	732.2762	1462.5611	731.7842	G	**333.1881**	167.0977	316.1615	158.5844	**315.1775**	158.0924	3
15	1581.6193	**791.3133**	1564.5928	**782.8000**	1563.6088	**782.3080**	T	**276.1666**	138.5870	259.1401	130.0737	258.1561	129.5817	2
16							R	**175.1190**	88.0631	158.0924	79.5498			1

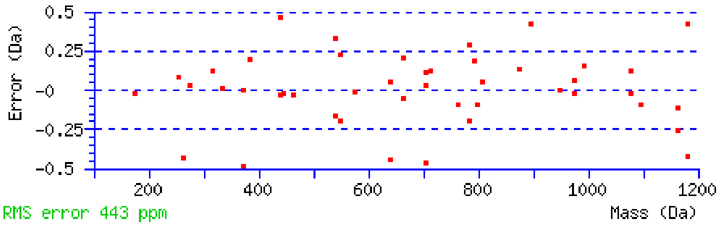

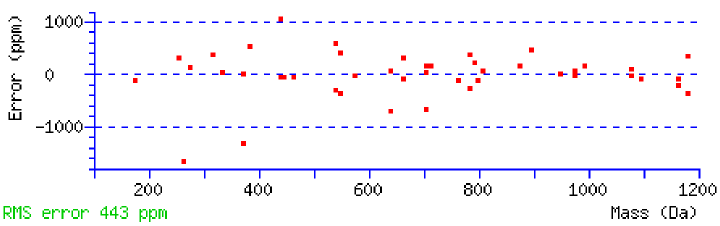

All matches to this query

Score	Mr(calc):	Delta	Sequence
49.1	1754.7237	0.0019	SRTASGSSVTSLEGTR
41.9	1754.7237	0.0019	SRTASGSSVTSLEGTR
38.7	1754.7237	0.0019	SRTASGSSVTSLEGTR
29.9	1754.7237	0.0019	SRTASGSSVTSLEGTR
13.3	1754.7237	0.0019	SRTASGSSVTSLEGTR
11.3	1754.7352	-0.0096	DVSCSLTNTHFCASR
8.3	1754.7252	0.0004	IPMNASWKTAFGTR
8.1	1753.7266	0.9990	SINPWERKSYWK
8.1	1753.7266	0.9990	SINPWERKSYWK
8.0	1754.7259	-0.0003	GKYFPFYVNFSAR

Spectrum No: 69; Query: 489; Rank: 1

Peptide View

MS/MS Fragmentation of **AELGMNDSPSQSPPVK**
Found in **IPI00869599**, Tax_Id=10116 Gene_Symbol=Acss2_predicted acyl-CoA synthetase short-chain family member 2

Match to Query 489: 1735.754268 from(868.884410,2+)
Title: 091127RatKid_SCX01_12.1546.1546.2.dta
Data file K:\NewmanPaper\Piliang\3SubProteomes\Piliang3SP\mgf5ppm\SCX_3SubProteomes5ppm.mgf

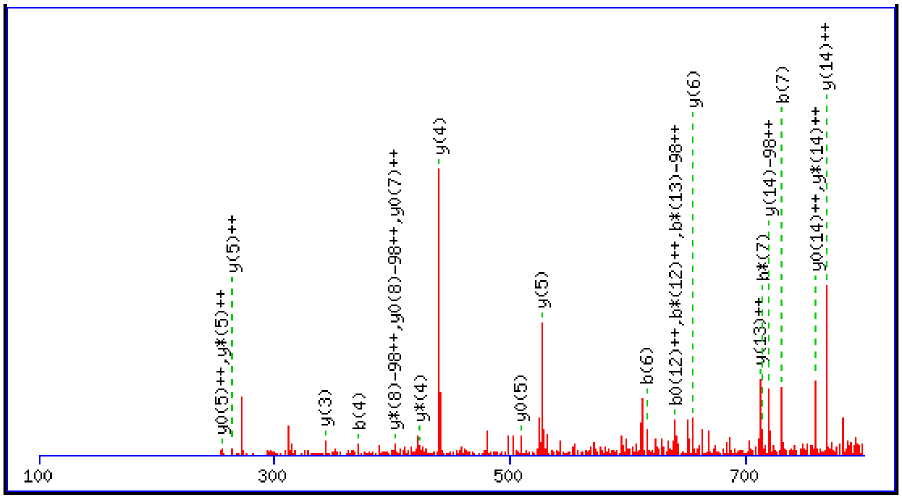

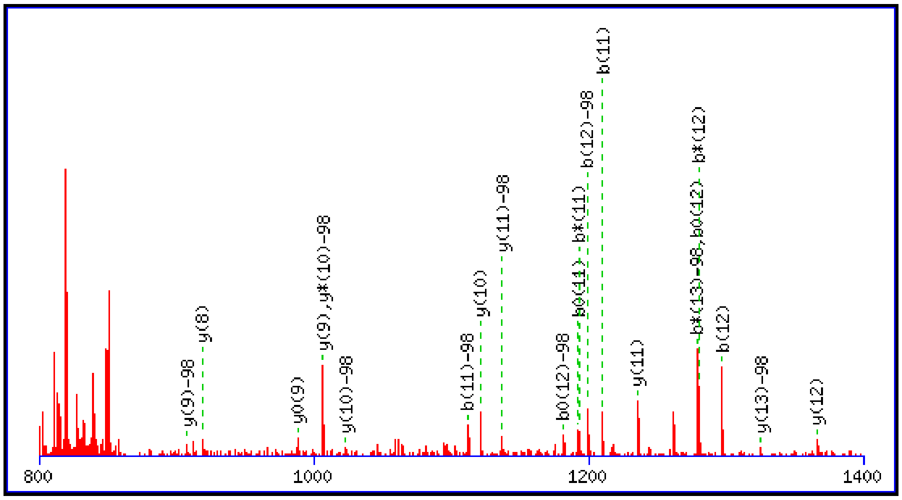

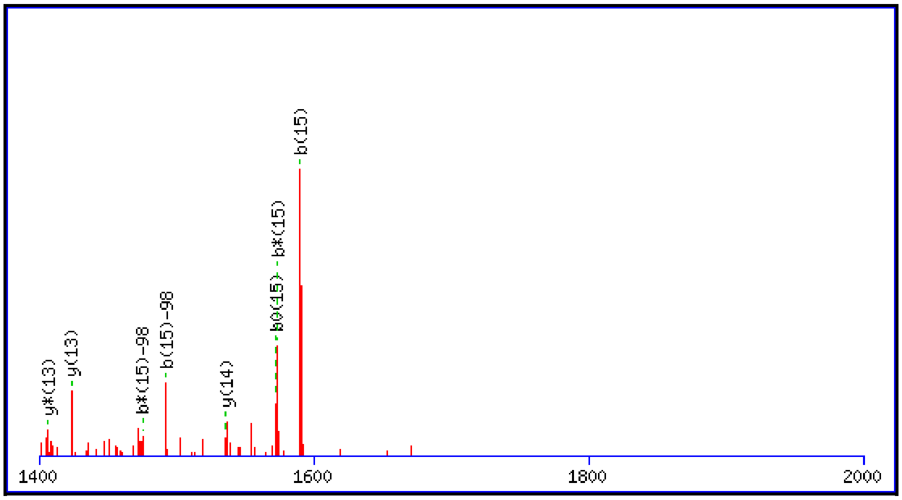

Monoisotopic mass of neutral peptide Mr(calc): 1735.7488
Fixed modifications: Carbamidomethyl (C)
Variable modifications:
S10 : Phospho (ST), with neutral losses 0.0000(shown in table), 97.9769
Ions Score: 49 **Expect:** 0.0026
Matches (Bold Red): 53/250 fragment ions using 91 most intense peaks

#	b	b^{++}	b*	b^{*++}	b^{0}	b^{0++}	Seq.	y	y^{++}	y*	y^{*++}	y^{0}	y^{0++}	#
1	72.0444	36.5258					A							16
2	201.0870	101.0471			183.0764	92.0418	E	1665.7190	833.3631	1648.6924	824.8498	1647.7084	824.3578	15
3	314.1710	157.5892			296.1605	148.5839	L	**1536.6764**	**768.8418**	1519.6498	**760.3286**	1518.6658	**759.8365**	14
4	**371.1925**	186.0999			353.1819	177.0946	G	**1423.5923**	**712.2998**	**1406.5658**	703.7865	1405.5818	703.2945	13
5	502.2330	251.6201			484.2224	242.6149	M	**1366.5709**	683.7891	1349.5443	675.2758	1348.5603	674.7838	12

**6**	**616.2759**	308.6416	599.2494	300.1283	598.2654	299.6363	**N**	**1235.5304**	618.2688	1218.5038	609.7555	1217.5198	609.2635	**11**
**7**	**731.3029**	366.1551	**714.2763**	357.6418	713.2923	357.1498	**D**	**1121.4874**	561.2474	1104.4609	552.7341	1103.4769	552.2421	**10**
**8**	818.3349	409.6711	801.3083	401.1578	800.3243	400.6658	**S**	**1006.4605**	503.7339	989.4339	495.2206	**988.4499**	494.7286	**9**
**9**	915.3877	458.1975	898.3611	449.6842	897.3771	449.1922	**P**	**919.4285**	460.2179	902.4019	451.7046	901.4179	451.2126	**8**
**10**	1082.3860	541.6966	1065.3595	533.1834	1064.3754	532.6914	**S**	822.3757	411.6915	805.3492	403.1782	804.3651	**402.6862**	**7**
**11**	**1210.4446**	605.7259	**1193.4180**	597.2127	**1192.4340**	596.7206	**Q**	**655.3774**	328.1923	638.3508	319.6790	637.3668	319.1870	**6**
**12**	**1297.4766**	649.2419	**1280.4501**	**640.7287**	**1279.4661**	**640.2367**	**S**	**527.3188**	**264.1630**	510.2922	**255.6498**	**509.3082**	**255.1577**	**5**
**13**	1394.5294	697.7683	1377.5028	689.2551	1376.5188	688.7630	**P**	**440.2867**	220.6470	**423.2602**	212.1337			**4**
**14**	1491.5821	746.2947	1474.5556	737.7814	1473.5716	737.2894	**P**	**343.2340**	172.1206	326.2074	163.6074			**3**
**15**	**1590.6506**	795.8289	**1573.6240**	787.3156	**1572.6400**	786.8236	**V**	246.1812	123.5942	229.1547	115.0810			**2**
**16**							**K**	147.1128	74.0600	130.0863	65.5468			**1**

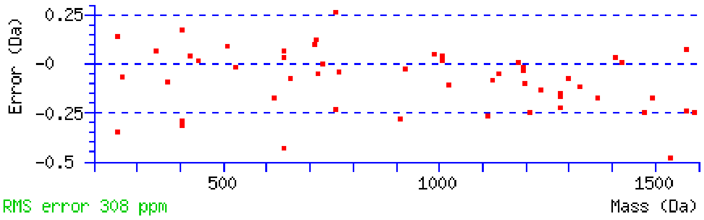

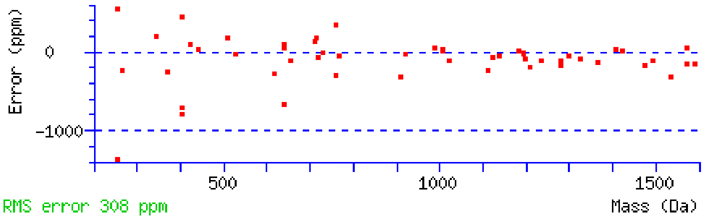

All matches to this query

Score	Mr(calc):	Delta	Sequence
48.6	1735.7488	0.0055	AELGMNDSPSQSPPVK
40.0	1735.7488	0.0055	AELGMNDSPSQSPPVK
32.4	1735.7488	0.0055	AELGMNDSPSQSPPVK
13.1	1735.7430	0.0112	VTSAVEALLSADSASR
13.1	1735.7430	0.0112	VTSAVEALLSADSASR
7.7	1735.7488	0.0055	CPSQTYXSSTKQEK
7.7	1735.7488	0.0055	CPSQTYXSSTKQEK
7.7	1735.7488	0.0055	CPSQTYXSSTKQEK
7.7	1735.7488	0.0055	CPSQTYXSSTKQEK
7.7	1735.7488	0.0055	CPSQTYXSSTKQEK

Spectrum No: 70; Query: 683; Rank: 1

Peptide View

MS/MS Fragmentation of **KEESEESDDDMGFGLFD**
Found in **IPI00188804**, Tax_Id=10116 Gene_Symbol=Rplp2 60S acidic ribosomal protein P2

Match to Query 683: 2028.718908 from(1015.366730,2+)
Title: 091129RatKid_SCX02_12.3800.3800.2.dta
Data file K:\NewmanPaper\Piliang\3SubProteomes\Piliang3SP\mgf5ppm\SCX_3SubProteomes5ppm.mgf

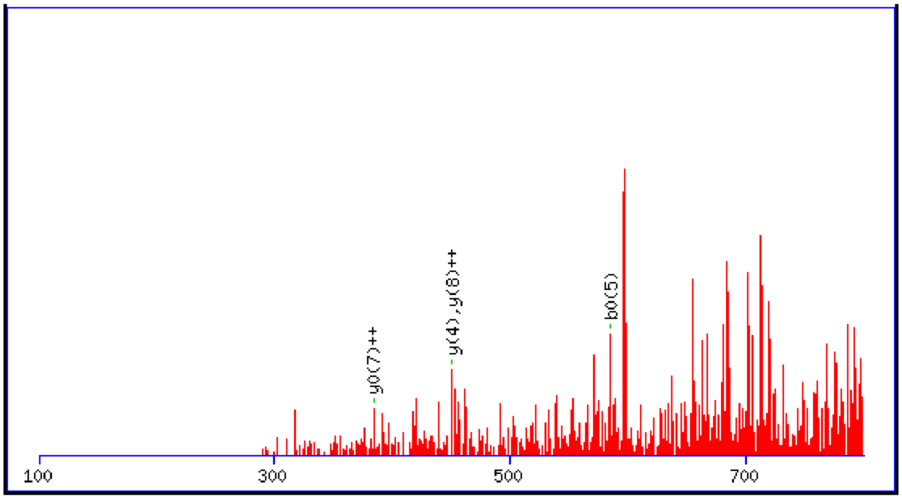

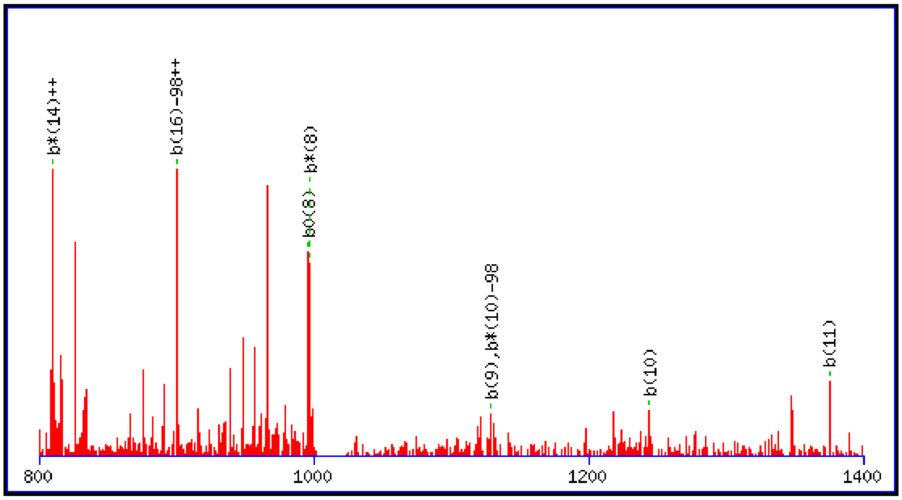

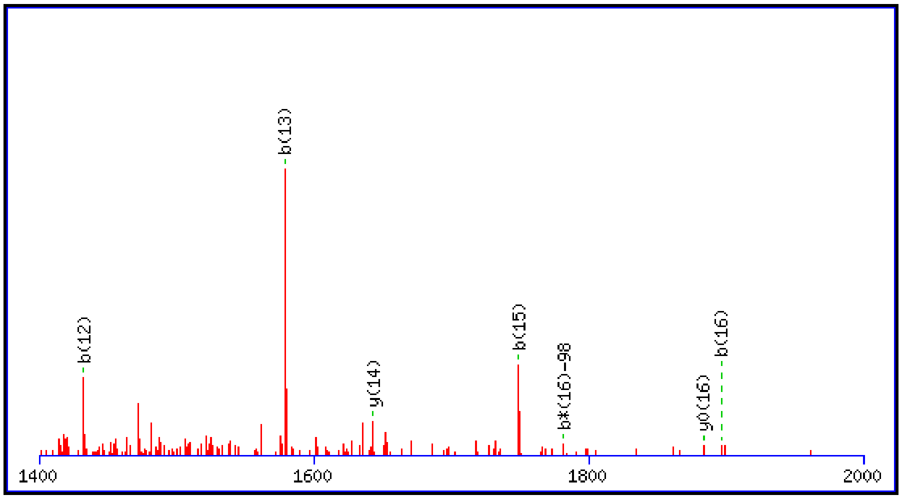

Monoisotopic mass of neutral peptide Mr(calc): 2028.7184
Fixed modifications: Carbamidomethyl (C)
Variable modifications:
S7 : Phospho (ST), with neutral losses 0.0000(shown in table), 97.9769
Ions Score: 48 **Expect:** 0.0014
Matches (Bold Red): 19/242 fragment ions using 32 most intense peaks

#	b	b^{++}	b*	b^{*++}	b^0	b^{0++}	Seq.	y	y^{++}	y^0	y^{0++}	#
1	129.1022	65.0548	112.0757	56.5415			K					17
2	258.1448	129.5761	241.1183	121.0628	240.1343	120.5708	E	1901.6307	951.3190	**1883.6201**	942.3137	16
3	387.1874	194.0974	370.1609	185.5841	369.1769	185.0921	E	1772.5881	886.7977	1754.5775	877.7924	15
4	474.2195	237.6134	457.1929	229.1001	456.2089	228.6081	S	**1643.5455**	822.2764	1625.5349	813.2711	14
5	603.2620	302.1347	586.2355	293.6214	**585.2515**	293.1294	E	1556.5135	778.7604	1538.5029	769.7551	13

6	732.3046	366.6560	715.2781	358.1427	714.2941	357.6507	E	1427.4709	714.2391	1409.4603	705.2338	12
7	899.3030	450.1551	882.2764	441.6419	881.2924	441.1499	S	1298.4283	649.7178	1280.4177	640.7125	11
8	1014.3299	507.6686	**997.3034**	499.1553	**996.3194**	498.6633	D	1131.4299	566.2186	1113.4194	557.2133	10
9	**1129.3569**	565.1821	1112.3303	556.6688	1111.3463	556.1768	D	1016.4030	508.7051	998.3924	499.6998	9
10	**1244.3838**	622.6955	1227.3573	614.1823	1226.3733	613.6903	D	901.3760	**451.1917**	883.3655	442.1864	8
11	**1375.4243**	688.2158	1358.3978	679.7025	1357.4137	679.2105	M	786.3491	393.6782	768.3385	**384.6729**	7
12	**1432.4458**	716.7265	1415.4192	708.2132	1414.4352	707.7212	G	655.3086	328.1579	637.2980	319.1527	6
13	**1579.5142**	790.2607	1562.4876	781.7475	1561.5036	781.2554	F	598.2871	299.6472	580.2766	290.6419	5
14	1636.5356	818.7715	1619.5091	**810.2582**	1618.5251	809.7662	G	**451.2187**	226.1130	433.2082	217.1077	4
15	**1749.6197**	875.3135	1732.5932	866.8002	1731.6091	866.3082	L	394.1973	197.6023	376.1867	188.5970	3
16	**1896.6881**	948.8477	1879.6616	940.3344	1878.6776	939.8424	F	281.1132	141.0602	263.1026	132.0550	2
17							D	134.0448	67.5260	116.0342	58.5207	1

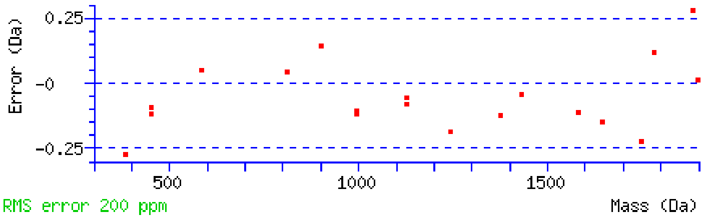

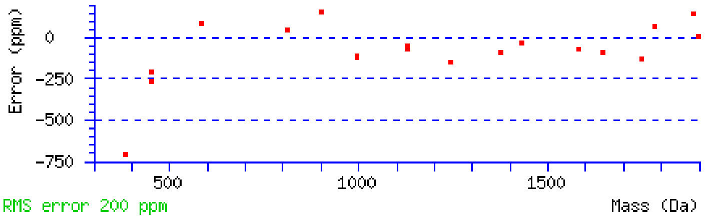

All matches to this query

Score	Mr(calc):	Delta	Sequence
48.2	2028.7184	0.0006	KEESEEESDDDMGFGLFD
45.0	2028.7184	0.0006	KEESEEESDDDMGFGLFD

Spectrum No: 71; Query: 1141; Rank: 1

Peptide View

MS/MS Fragmentation of **DLGHPVEEEDESGDQEDDDDELDDGDRDQDI**
Found in **IPI00324618**, Tax_Id=10116 Gene_Symbol=Vdp General vesicular transport factor p115

Match to Query 1141: 3596.304852 from(1199.775560,3+)
Title: 091129RatKid_SCX02_18.1913.1913.3.dta
Data file K:\NewmanPaper\Piliang\3SubProteomes\Piliang3SP\mgf5ppm\SCX_3SubProteomes5ppm.mgf

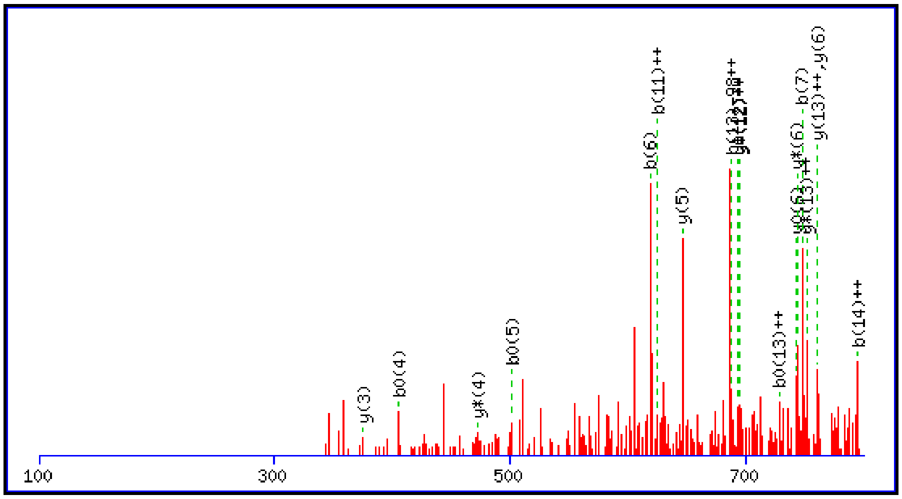

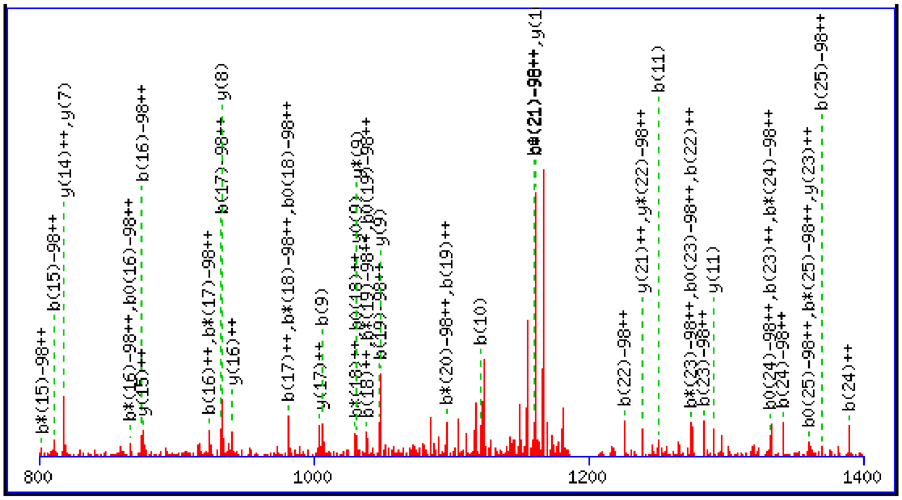

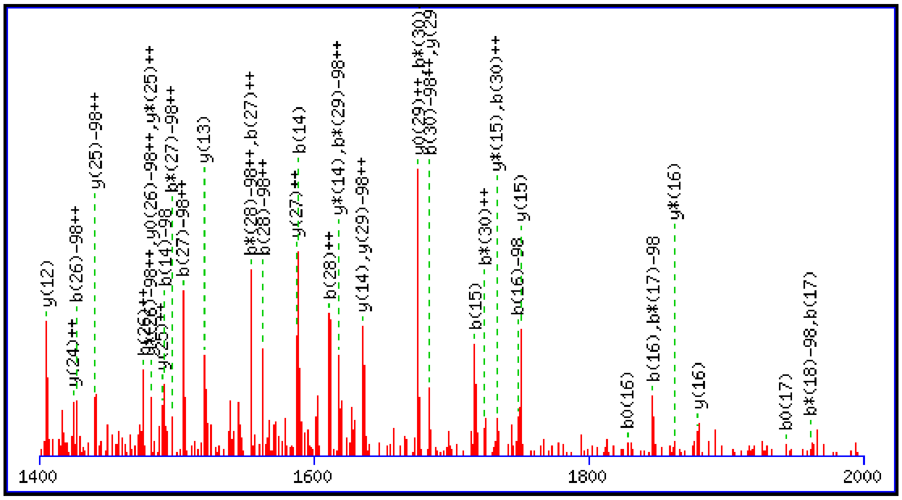

Monoisotopic mass of neutral peptide Mr(calc): 3595.3027
Fixed modifications: Carbamidomethyl (C)
Variable modifications:
S12 : Phospho (ST), with neutral losses 97.9769(shown in table), 0.0000
Ions Score: 48 **Expect:** 0.0033
Matches (Bold Red): 112/500 fragment ions using 178 most intense peaks

#	b	b^{++}	b*	b^{*++}	b^0	b^{0++}	Seq.	y	y^{++}	y*	y^{*++}	y^0	y^{0++}	#
1	116.0342	58.5207			98.0237	49.5155	D							31
2	229.1183	115.0628			211.1077	106.0575	L	3383.3062	1692.1567	3366.2796	1683.6434	3365.2956	1683.1514	30
3	286.1397	143.5735			268.1292	134.5682	G	3270.2221	**1635.6147**	3253.1956	1627.1014	3252.2115	1626.6094	29
4	423.1987	212.1030			**405.1881**	203.0977	H	3213.2006	1607.1040	3196.1741	1598.5907	3195.1901	1598.0987	28
5	520.2514	260.6293			**502.2409**	251.6241	P	3076.1417	1538.5745	3059.1152	1530.0612	3058.1312	1529.5692	27
6	**619.3198**	310.1636			601.3093	301.1583	V	2979.0890	1490.0481	2962.0624	**1481.5348**	2961.0784	**1481.0428**	26
7	**748.3624**	374.6849			730.3519	365.6796	E	2880.0206	**1440.5139**	2862.9940	1432.0006	2862.0100	1431.5086	25
8	877.4050	439.2061			859.3945	430.2009	E	2750.9780	1375.9926	2733.9514	1367.4793	2732.9674	1366.9873	24
9	**1006.4476**	503.7274			988.4371	494.7222	E	2621.9354	1311.4713	2604.9088	1302.9580	2603.9248	1302.4660	23
10	**1121.4746**	561.2409			1103.4640	552.2356	D	2492.8928	1246.9500	2475.8662	**1238.4367**	2474.8822	1237.9447	22
11	**1250.5172**	**625.7622**			1232.5066	616.7569	E	2377.8658	1189.4366	2360.8393	1180.9233	2359.8553	1180.4313	21
12	1319.5386	660.2729			1301.5280	651.2677	S	2248.8232	1124.9153	2231.7967	1116.4020	2230.8127	1115.9100	20
13	1376.5601	**688.7837**			1358.5495	679.7784	G	2179.8018	1090.4045	2162.7752	1081.8913	2161.7912	1081.3992	19
14	**1491.5870**	746.2971			1473.5765	737.2919	D	2122.7803	1061.8938	2105.7538	1053.3805	2104.7698	1052.8885	18
15	1619.6456	**810.3264**	1602.6190	**801.8132**	1601.6350	801.3212	Q	2007.7534	**1004.3803**	1990.7268	995.8670	1989.7428	995.3750	17
16	**1748.6882**	**874.8477**	1731.6616	**866.3345**	1730.6776	**865.8424**	E	**1879.6948**	**940.3510**	**1862.6682**	931.8378	1861.6842	931.3458	16
17	1863.7151	**932.3612**	**1846.6886**	**923.8479**	1845.7046	923.3559	D	**1750.6522**	**875.8297**	**1733.6257**	867.3165	1732.6416	866.8245	15
18	1978.7421	989.8747	**1961.7155**	**981.3614**	1960.7315	**980.8694**	D	**1635.6253**	**818.3163**	**1618.5987**	809.8030	1617.6147	809.3110	14

19	2093.7690	1047.3881	2076.7425	1038.8749	2075.7584	1038.3829	D	1520.5983	760.8028	1503.5718	752.2895	1502.5878	751.7975	13
20	2208.7960	1104.9016	2191.7694	1096.3883	2190.7854	1095.8963	D	1405.5714	703.2893	1388.5448	694.7760	1387.5608	694.2840	12
21	2337.8386	1169.4229	2320.8120	1160.9096	2319.8280	1160.4176	E	1290.5444	645.7759	1273.5179	637.2626	1272.5339	636.7706	11
22	2450.9226	1225.9649	2433.8961	1217.4517	2432.9120	1216.9597	L	1161.5018	581.2546	1144.4753	572.7413	1143.4913	572.2493	10
23	2565.9496	1283.4784	2548.9230	1274.9651	2547.9390	1274.4731	D	1048.4178	524.7125	1031.3912	516.1992	1030.4072	515.7072	9
24	2680.9765	1340.9919	2663.9500	1332.4786	2662.9659	1331.9866	D	933.3908	467.1991	916.3643	458.6858	915.3803	458.1938	8
25	2737.9980	1369.5026	2720.9714	1360.9893	2719.9874	1360.4973	G	818.3639	409.6856	801.3373	401.1723	800.3533	400.6803	7
26	2853.0249	1427.0161	2835.9984	1418.5028	2835.0143	1418.0108	D	761.3424	381.1748	744.3159	372.6616	743.3319	372.1696	6
27	3009.1260	1505.0666	2992.0995	1496.5534	2991.1155	1496.0614	R	646.3155	323.6614	629.2889	315.1481	628.3049	314.6561	5
28	3124.1530	1562.5801	3107.1264	1554.0668	3106.1424	1553.5748	D	490.2144	245.6108	473.1878	237.0975	472.2038	236.6055	4
29	3252.2115	1626.6094	3235.1850	1618.0961	3234.2010	1617.6041	Q	375.1874	188.0974	358.1609	179.5841	357.1769	179.0921	3
30	3367.2385	1684.1229	3350.2119	1675.6096	3349.2279	1675.1176	D	247.1288	124.0681			229.1183	115.0628	2
31							I	132.1019	66.5546					1

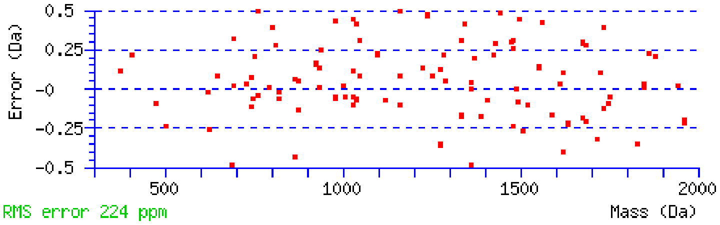

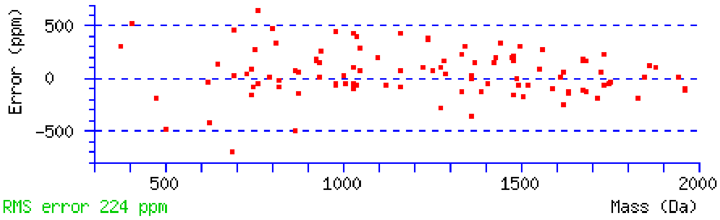

All matches to this query

Score	Mr(calc):	Delta	Sequence
47.8	3595.3027	1.0021	DLGHPVEEEDESGDQEDDDDELDDGDRDQDI
2.7	3595.3242	0.9807	CFTFMDRGFVFKMVNNYVSMFSSGEFK
2.0	3595.3242	0.9807	CFTFMDRGFVFKMVNNYVSMFSSGEFK
1.6	3595.3242	0.9807	CFTFMDRGFVFKMVNNYVSMFSSGEFK
0.9	3595.3347	0.9701	SGFSIQFSTSIASTCNDPGMPQNGTRYGDSR
0.4	3595.3274	0.9774	YELVLSKNSTLFWNTESSELPDSFCTK

Spectrum No: 72; Query: 805; Rank: 1

Peptide View

MS/MS Fragmentation of **IQQFDDGGSDEEDIWEEK**
Found in **IPI00766645**, Tax_Id=10116 Gene_Symbol=Saps3_predicted similar to SAPS domain family, member 3

Match to Query 805: 2218.862308 from(1110.438430,2+)
Title: 091127RatKid_SCX01_12.3101.3101.2.dta
Data file K:\NewmanPaper\Piliang\3SubProteomes\Piliang3SP\mgf5ppm\SCX_3SubProteomes5ppm.mgf

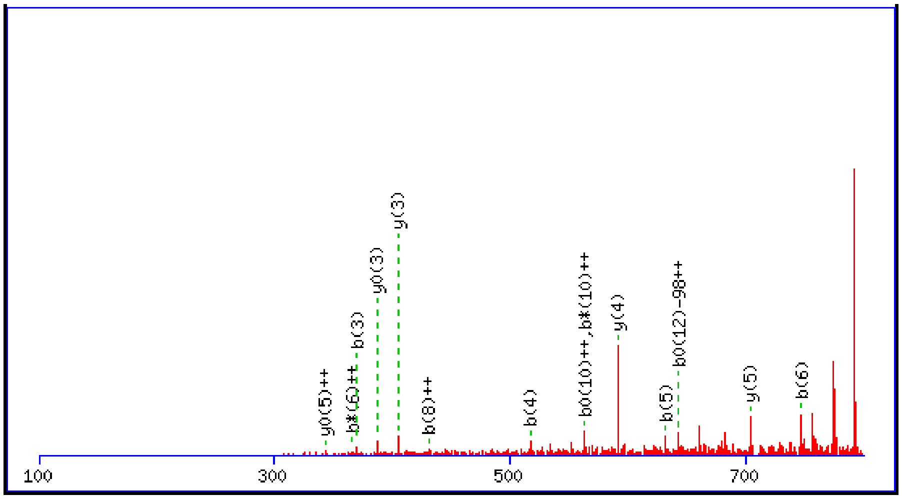

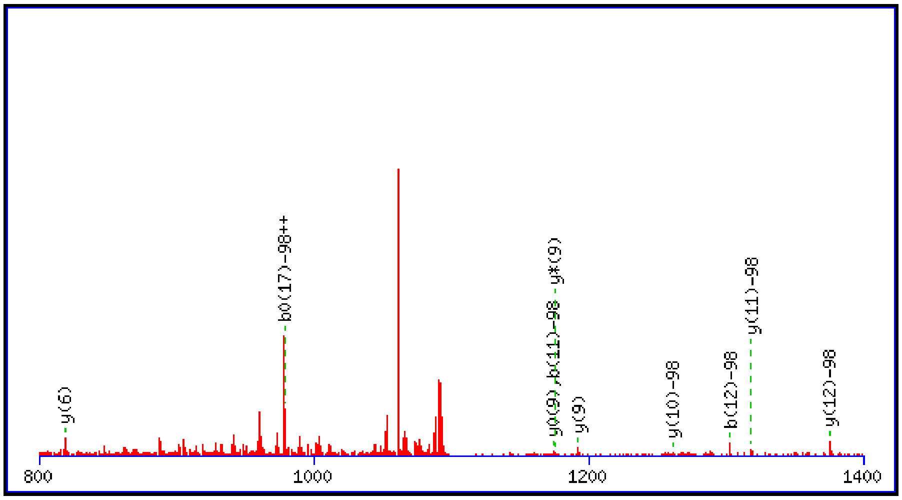

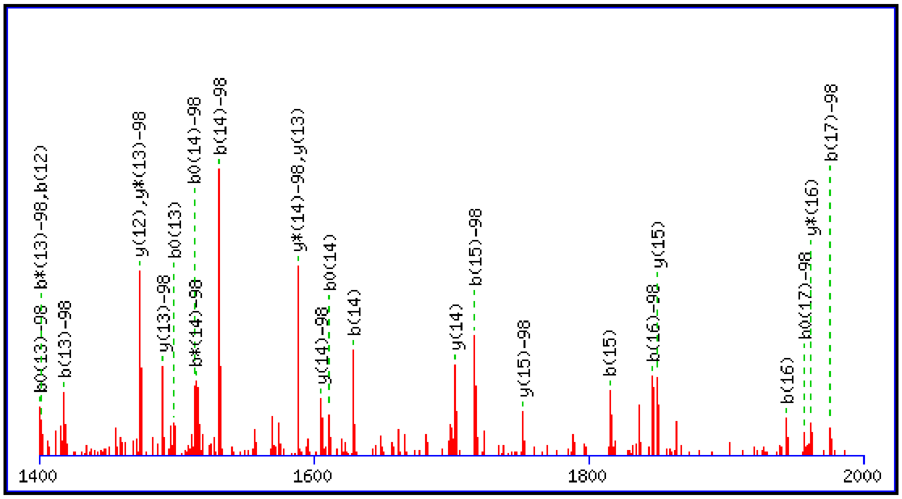

Monoisotopic mass of neutral peptide Mr(calc): 2218.8580
Fixed modifications: Carbamidomethyl (C)
Variable modifications:
S9 : Phospho (ST), with neutral losses 97.9769(shown in table), 0.0000
Ions Score: 48 **Expect:** 0.0028
Matches (Bold Red): 50/294 fragment ions using 92 most intense peaks

#	b	b^{++}	b*	b^{*++}	b^{0}	b^{0++}	Seq.	y	y^{++}	y*	y^{*++}	y^{0}	y^{0++}	#
1	114.0913	57.5493					I							18
2	242.1499	121.5786	225.1234	113.0653			Q	2008.8043	1004.9058	1991.7777	996.3925	1990.7937	995.9005	17
3	**370.2085**	185.6079	353.1819	177.0946			Q	1880.7457	940.8765	1863.7192	932.3632	1862.7351	931.8712	16
4	**517.2769**	259.1421	500.2504	250.6288			F	**1752.6871**	876.8472	1735.6606	868.3339	1734.6766	867.8419	15
5	**632.3039**	316.6556	615.2773	308.1423	614.2933	307.6503	D	**1605.6187**	803.3130	**1588.5922**	794.7997	1587.6081	794.3077	14

**6**	**747.3308**	374.1690	730.3042	**365.6558**	729.3202	365.1638	**D**	**1490.5918**	745.7995	**1473.5652**	737.2862	1472.5812	736.7942	**13**
**7**	804.3523	402.6798	787.3257	394.1665	786.3417	393.6745	**G**	**1375.5648**	688.2860	1358.5383	679.7728	1357.5543	679.2808	**12**
**8**	861.3737	**431.1905**	844.3472	422.6772	843.3632	422.1852	**G**	**1318.5434**	659.7753	1301.5168	651.2620	1300.5328	650.7700	**11**
**9**	930.3952	465.7012	913.3686	457.1880	912.3846	456.6959	**S**	**1261.5219**	631.2646	1244.4953	622.7513	1243.5113	622.2593	**10**
**10**	1045.4221	523.2147	1028.3956	514.7014	1027.4116	514.2094	**D**	**1192.5004**	596.7539	**1175.4739**	588.2406	**1174.4899**	587.7486	**9**
**11**	**1174.4647**	587.7360	1157.4382	579.2227	1156.4542	578.7307	**E**	1077.4735	539.2404	1060.4469	530.7271	1059.4629	530.2351	**8**
**12**	**1303.5073**	652.2573	1286.4808	643.7440	1285.4967	**643.2520**	**E**	948.4309	474.7191	931.4044	466.2058	930.4203	465.7138	**7**
**13**	**1418.5343**	709.7708	**1401.5077**	701.2575	**1400.5237**	700.7655	**D**	**819.3883**	410.1978	802.3618	401.6845	801.3777	401.1925	**6**
**14**	**1531.6183**	766.3128	**1514.5918**	757.7995	**1513.6078**	757.3075	**I**	**704.3614**	352.6843	687.3348	344.1710	686.3508	**343.6790**	**5**
**15**	**1717.6976**	859.3525	1700.6711	850.8392	1699.6871	850.3472	**W**	**591.2773**	296.1423	574.2508	287.6290	573.2667	287.1370	**4**
**16**	**1846.7402**	923.8737	1829.7137	915.3605	1828.7297	914.8685	**E**	**405.1980**	203.1026	388.1714	194.5894	**387.1874**	194.0974	**3**
**17**	**1975.7828**	988.3950	1958.7563	979.8818	**1957.7723**	**979.3898**	**E**	276.1554	138.5813	259.1288	130.0681	258.1448	129.5761	**2**
**18**							**K**	147.1128	74.0600	130.0863	65.5468			**1**

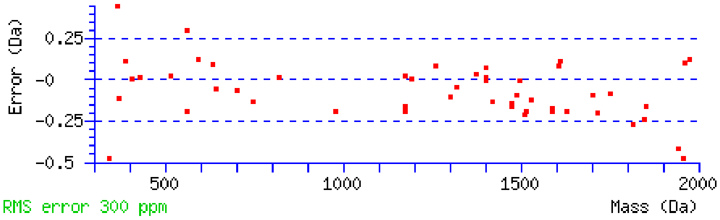

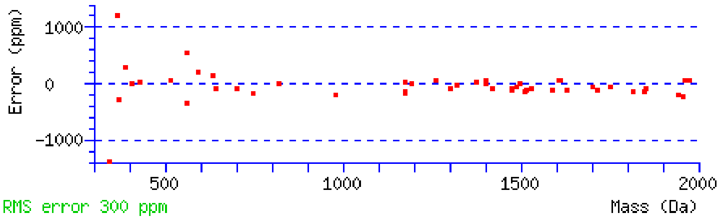

All matches to this query

Score	Mr(calc):	Delta	Sequence
47.8	2218.8580	0.0044	IQQFDDGGSDEEDIWEEK
1.3	2218.8697	-0.0074	IWTAHYDPNHCFIETR
0.2	2216.8721	1.9902	TVENFVTLATGEKGYGYK

Spectrum No: 73; Query: 635; Rank: 1

Peptide View

MS/MS Fragmentation of **DWEDDSDEDMSNFDR**
Found in **IPI00365935**, Tax_Id=10116 Gene_Symbol=Ptges3_predicted Prostaglandin E synthase 3

Match to Query 635: 1970.618548 from(986.316550,2+)
Title: 091129RatKid_SCX02_12.1584.1584.2.dta
Data file K:\NewmanPaper\Piliang\3SubProteomes\Piliang3SP\mgf5ppm\SCX_3SubProteomes5ppm.mgf

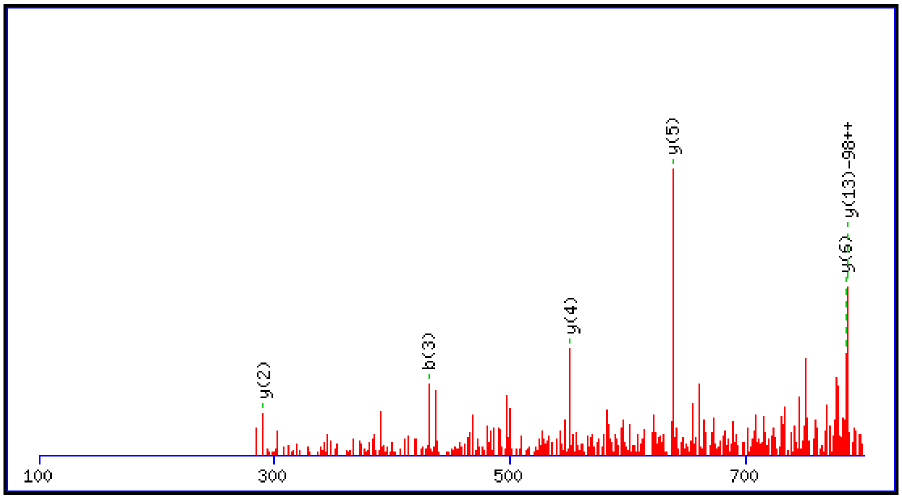

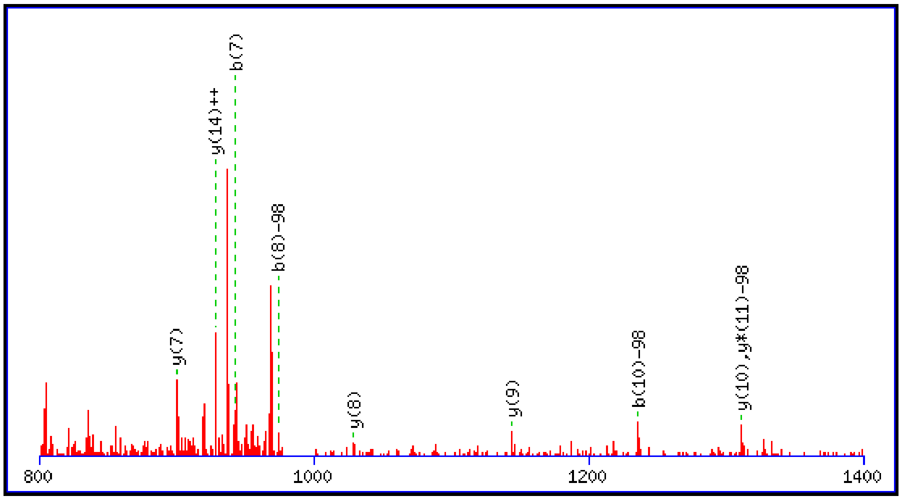

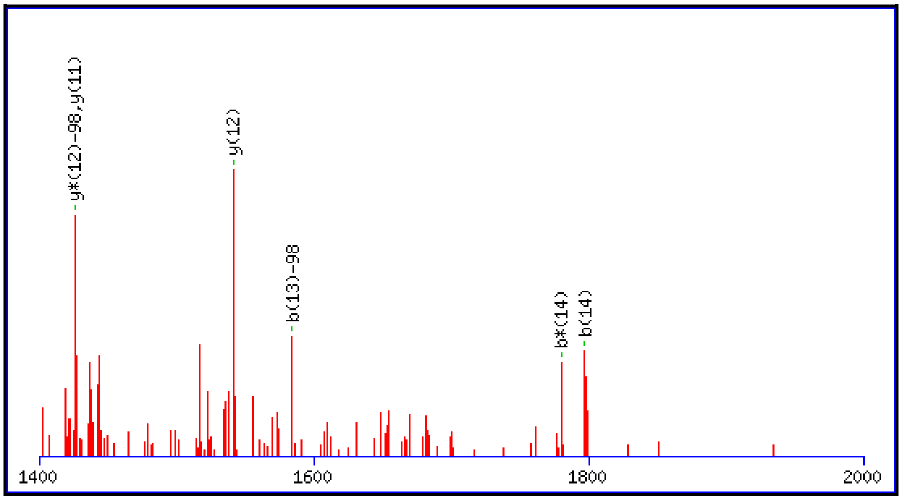

Monoisotopic mass of neutral peptide Mr(calc): 1970.6150
Fixed modifications: Carbamidomethyl (C)
Variable modifications:
S6 : Phospho (ST), with neutral losses 0.0000(shown in table), 97.9769
M10 : Oxidation (M)
Ions Score: 48 **Expect:** 0.00053
Matches (Bold Red): 21/216 fragment ions using 45 most intense peaks

#	b	b^{++}	b*	$b*^{++}$	b^0	b^{0++}	Seq.	y	y^{++}	y*	$y*^{++}$	y^0	y^{0++}	#
1	116.0342	58.5207			98.0237	49.5155	D							15
2	302.1135	151.5604			284.1030	142.5551	W	1856.5953	**928.8013**	1839.5687	920.2880	1838.5847	919.7960	14
3	**431.1561**	216.0817			413.1456	207.0764	E	1670.5160	835.7616	1653.4894	827.2484	1652.5054	826.7563	13
4	546.1831	273.5952			528.1725	264.5899	D	**1541.4734**	771.2403	1524.4468	762.7271	1523.4628	762.2350	12
5	661.2100	331.1086			643.1994	322.1034	D	**1426.4464**	713.7269	1409.4199	705.2136	1408.4359	704.7216	11
6	828.2084	414.6078			810.1978	405.6025	S	**1311.4195**	656.2134	1294.3930	647.7001	1293.4089	647.2081	10
7	**943.2353**	472.1213			925.2247	463.1160	D	**1144.4211**	572.7142	1127.3946	564.2009	1126.4106	563.7089	9
8	1072.2779	536.6426			1054.2673	527.6373	E	**1029.3942**	515.2007	1012.3677	506.6875	1011.3836	506.1955	8
9	1187.3048	594.1561			1169.2943	585.1508	D	**900.3516**	450.6794	883.3251	442.1662	882.3410	441.6742	7
10	1334.3402	667.6738			1316.3297	658.6685	M	**785.3247**	393.1660	768.2981	384.6527	767.3141	384.1607	6
11	1421.3723	711.1898			1403.3617	702.1845	S	**638.2893**	319.6483	621.2627	311.1350	620.2787	310.6430	5
12	1535.4152	768.2112	1518.3887	759.6980	1517.4046	759.2060	N	**551.2572**	276.1323	534.2307	267.6190	533.2467	267.1270	4
13	1682.4836	841.7454	1665.4571	833.2322	1664.4731	832.7402	F	437.2143	219.1108	420.1878	210.5975	419.2037	210.1055	3
14	**1797.5106**	899.2589	**1780.4840**	890.7456	1779.5000	890.2536	D	**290.1459**	145.5766	273.1193	137.0633	272.1353	136.5713	2
15							R	175.1190	88.0631	158.0924	79.5498			1

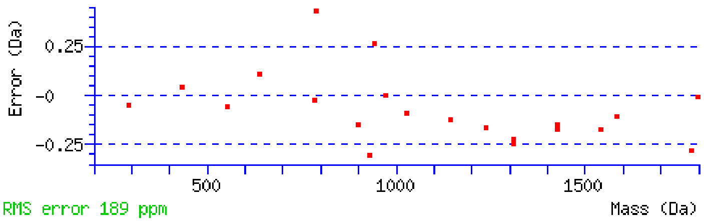

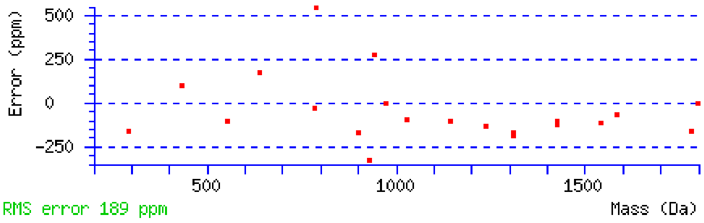

All matches to this query

Score	Mr(calc):	Delta	Sequence
47.8	1970.6150	0.0036	DWEDDSDEDMSNFDR
16.4	1970.6150	0.0036	DWEDDSDEDMSNFDR

Spectrum No: 74; Query: 664; Rank: 1

Peptide View

MS/MS Fragmentation of **ADSEGDLSENDDGAGDLR**
Found in **IPI00204776**, Tax_Id=10116 Gene_Symbol=Akap8 A-kinase anchor protein 8

Match to Query 664: 1994.669348 from(998.341950,2+)
Title: 091129RatKid_SCX02_02.1039.1039.2.dta
Data file K:\NewmanPaper\Piliang\3SubProteomes\Piliang3SP\mgf5ppm\SCX_3SubProteomes5ppm.mgf

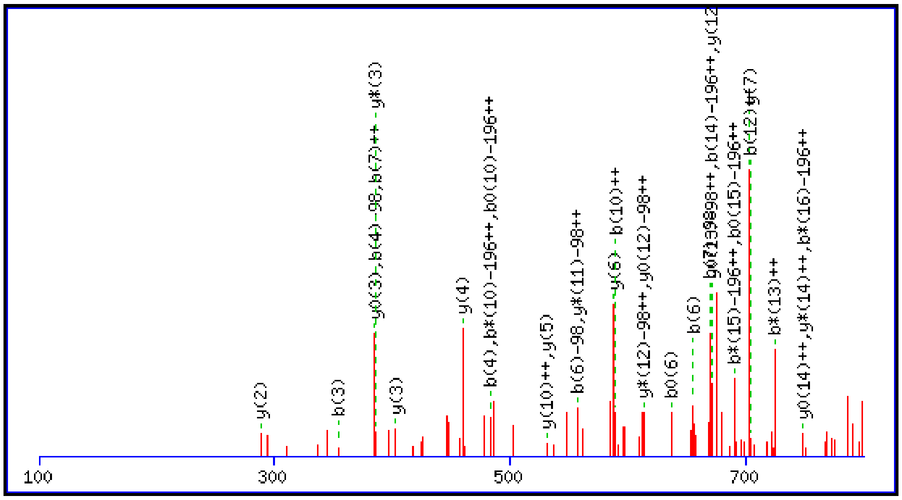

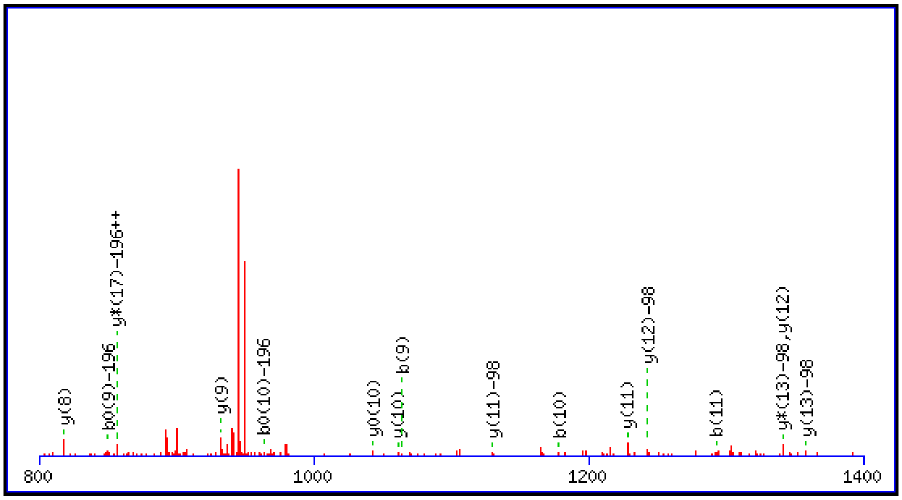

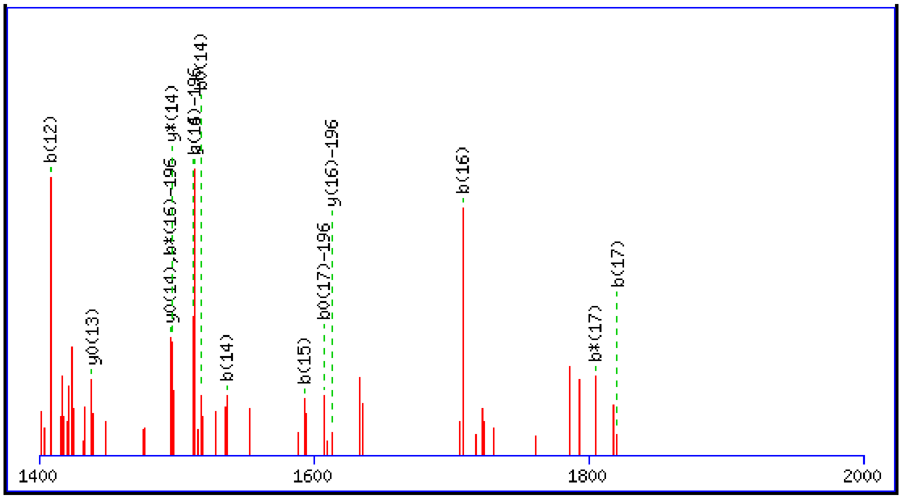

Monoisotopic mass of neutral peptide Mr(calc): 1994.6779
Fixed modifications: Carbamidomethyl (C)
Variable modifications:
S3 : Phospho (ST), with neutral losses 97.9769(shown in table), 0.0000
S8 : Phospho (ST), with neutral losses 97.9769(shown in table), 0.0000
Ions Score: 48 **Expect:** 0.00093
Matches (Bold Red): 65/298 fragment ions using 141 most intense peaks

#	b	b^{++}	b*	b^{*++}	b^0	b^{0++}	Seq.	y	y^{++}	y*	y^{*++}	y^0	y^{0++}	#
1	72.0444	36.5258					A							18
2	187.0713	94.0393			169.0608	85.0340	D	1728.6943	864.8508	1711.6678	**856.3375**	1710.6838	855.8455	17
3	256.0928	128.5500			238.0822	119.5448	S	**1613.6674**	807.3373	1596.6408	798.8241	1595.6568	798.3320	16
4	**385.1354**	193.0713			367.1248	184.0660	E	1544.6459	772.8266	1527.6194	764.3133	1526.6354	763.8213	15
5	442.1568	221.5821			424.1463	212.5768	G	1415.6033	708.3053	1398.5768	699.7920	1397.5928	699.3000	14
6	**557.1838**	279.0955			539.1732	270.0903	D	**1358.5819**	679.7946	**1341.5553**	**671.2813**	1340.5713	**670.7893**	13
7	**670.2679**	335.6376			652.2573	326.6323	L	**1243.5549**	622.2811	1226.5284	**613.7678**	1225.5444	**613.2758**	12
8	739.2893	370.1483			721.2787	361.1430	S	**1130.4709**	565.7391	1113.4443	**557.2258**	1112.4603	556.7338	11
9	868.3319	434.6696			**850.3213**	425.6643	E	**1061.4494**	**531.2283**	1044.4229	522.7151	**1043.4388**	522.2231	10
10	982.3748	491.6911	965.3483	**483.1778**	**964.3643**	**482.6858**	N	**932.4068**	466.7070	915.3803	458.1938	914.3962	457.7018	9
11	1097.4018	549.2045	1080.3752	540.6912	1079.3912	540.1992	D	**818.3639**	409.6856	801.3373	401.1723	800.3533	400.6803	8
12	1212.4287	606.7180	1195.4022	598.2047	1194.4182	597.7127	D	**703.3369**	352.1721	686.3104	343.6588	685.3264	343.1668	7
13	1269.4502	635.2287	1252.4236	626.7155	1251.4396	626.2234	G	**588.3100**	294.6586	571.2835	286.1454	570.2994	285.6534	6
14	1340.4873	**670.7473**	1323.4607	662.2340	1322.4767	661.7420	A	**531.2885**	266.1479	514.2620	257.6346	513.2780	257.1426	5
15	1397.5088	699.2580	1380.4822	**690.7447**	1379.4982	**690.2527**	G	**460.2514**	230.6293	443.2249	222.1161	442.2409	221.6241	4
16	**1512.5357**	756.7715	**1495.5092**	**748.2582**	1494.5251	747.7662	D	**403.2300**	202.1186	**386.2034**	193.6053	**385.2194**	193.1133	3
17	1625.6198	813.3135	1608.5932	804.8002	**1607.6092**	804.3082	L	**288.2030**	144.6051	271.1765	136.0919			2
18							R	175.1190	88.0631	158.0924	79.5498			1

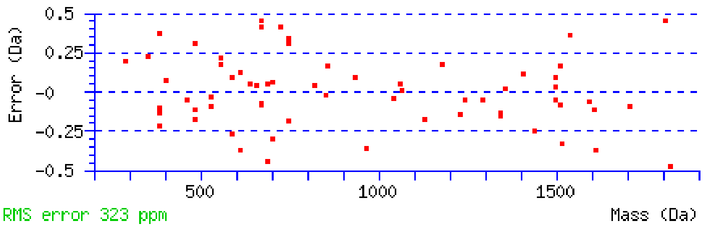

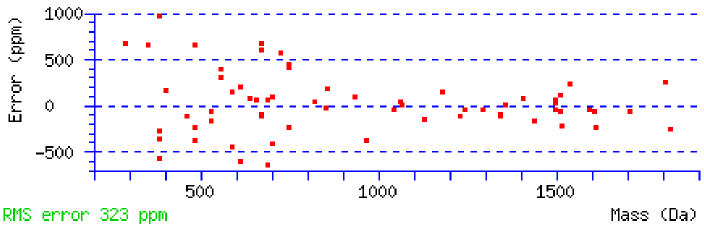

All matches to this query

Score	Mr(calc):	Delta	Sequence
47.6	1994.6779	-0.0086	ADSEGDLSENDDGAGDLR

Spectrum No: 75; Query: 848; Rank: 1

Peptide View

MS/MS Fragmentation of **KGAEEEEEEEEDDDSEEEIK**
Found in **IPI00778976**, Tax_Id=10116 Gene_Symbol=Epb4.9_predicted 45 kDa protein

Match to Query 848: 2317.850868 from(1159.932710,2+)
Title: 091129RatKid_SCX02_25.548.548.2.dta
Data file K:\NewmanPaper\Piliang\3SubProteomes\Piliang3SP\mgf5ppm\SCX_3SubProteomes5ppm.mgf

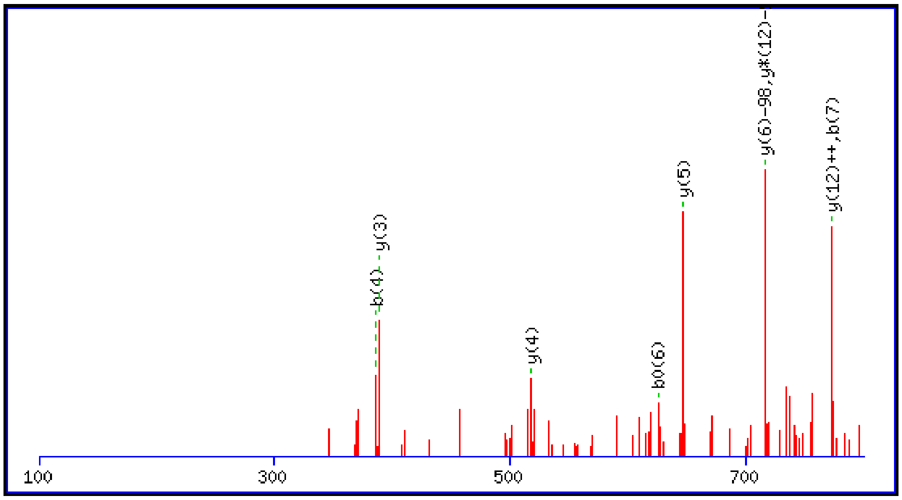

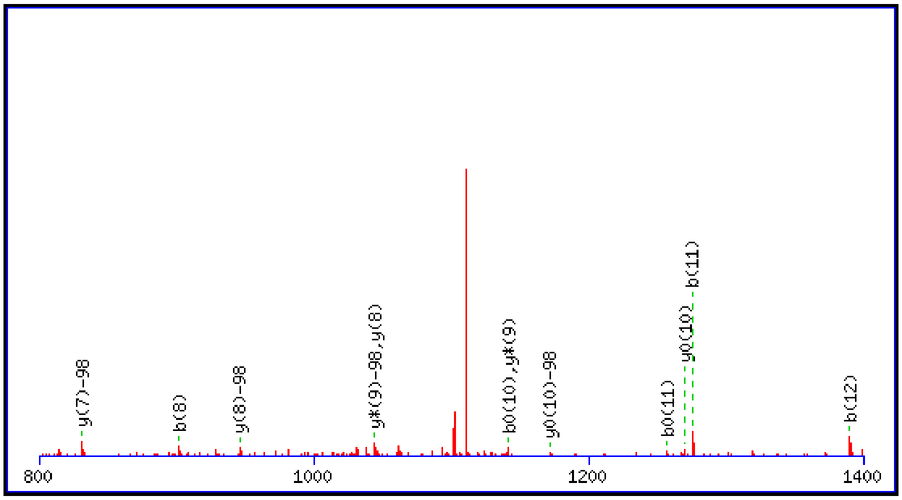

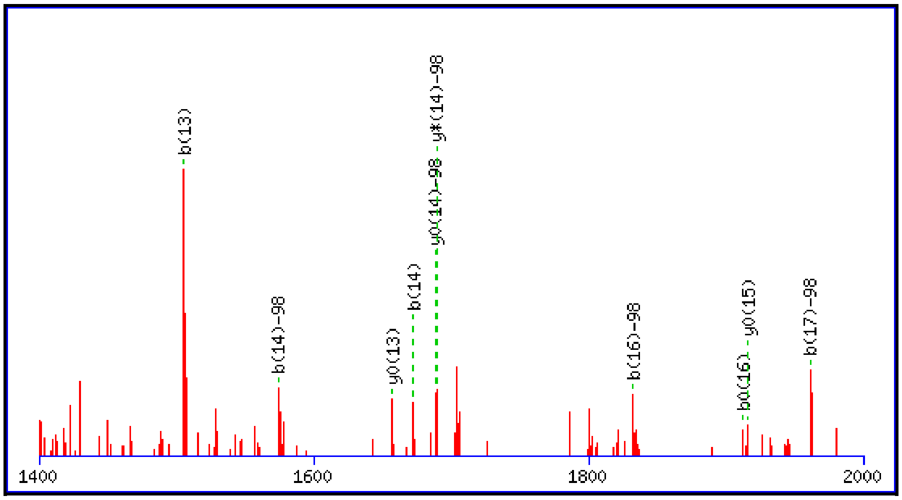

Monoisotopic mass of neutral peptide Mr(calc): 2317.8482
Fixed modifications: Carbamidomethyl (C)

Variable modifications:
S14 : Phospho (ST), with neutral losses 97.9769(shown in table), 0.0000
Ions Score: 48 **Expect:** 0.0024
Matches (Bold Red): 31/314 fragment ions using 48 most intense peaks

#	b	b++	b*	b*++	b0	b0++	Seq.	y	y++	y*	y*++	y0	y0++	#
1	129.1022	65.0548	112.0757	56.5415			K							19
2	186.1237	93.5655	169.0972	85.0522			G	2092.7837	1046.8955	2075.7571	1038.3822	2074.7731	1037.8902	18
3	257.1608	129.0840	240.1343	120.5708			A	2035.7622	1018.3847	2018.7356	1009.8715	2017.7516	1009.3795	17
4	**386.2034**	193.6053	369.1769	185.0921	368.1928	184.6001	E	1964.7251	982.8662	1947.6985	974.3529	1946.7145	973.8609	16
5	515.2460	258.1266	498.2195	249.6134	497.2354	249.1214	E	1835.6825	918.3449	1818.6559	909.8316	1817.6719	909.3396	15
6	644.2886	322.6479	627.2620	314.1347	**626.2780**	313.6427	E	1706.6399	853.8236	**1689.6133**	845.3103	**1688.6293**	844.8183	14
7	**773.3312**	387.1692	756.3046	378.6560	755.3206	378.1640	E	1577.5973	789.3023	1560.5708	780.7890	1559.5867	780.2970	13
8	**902.3738**	451.6905	885.3472	443.1773	884.3632	442.6852	E	1448.5547	724.7810	1431.5282	**716.2677**	1430.5441	715.7757	12
9	1031.4164	516.2118	1014.3898	507.6986	1013.4058	507.2065	E	1319.5121	660.2597	1302.4856	651.7464	1301.5016	651.2544	11
10	1160.4590	580.7331	1143.4324	572.2198	**1142.4484**	571.7278	E	1190.4695	595.7384	1173.4430	587.2251	**1172.4590**	586.7331	10
11	**1275.4859**	638.2466	1258.4594	629.7333	**1257.4753**	629.2413	D	1061.4269	531.2171	**1044.4004**	522.7038	1043.4164	522.2118	9
12	**1390.5129**	695.7601	1373.4863	687.2468	1372.5023	686.7548	D	**946.4000**	473.7036	929.3734	465.1904	928.3894	464.6984	8
13	**1505.5398**	753.2735	1488.5132	744.7603	1487.5292	744.2683	D	**831.3730**	416.1902	814.3465	407.6769	813.3625	407.1849	7
14	**1574.5613**	787.7843	1557.5347	779.2710	1556.5507	778.7790	S	**716.3461**	358.6767	699.3196	350.1634	698.3355	349.6714	6
15	1703.6038	852.3056	1686.5773	843.7923	1685.5933	843.3003	E	**647.3246**	324.1660	630.2981	315.6527	629.3141	315.1607	5
16	**1832.6464**	916.8269	1815.6199	908.3136	1814.6359	907.8216	E	**518.2821**	259.6447	501.2555	251.1314	500.2715	250.6394	4
17	**1961.6890**	981.3482	1944.6625	972.8349	1943.6785	972.3429	E	**389.2395**	195.1234	372.2129	186.6101	371.2289	186.1181	3
18	2074.7731	1037.8902	2057.7465	1029.3769	2056.7625	1028.8849	I	260.1969	130.6021	243.1703	122.0888			2
19							K	147.1128	74.0600	130.0863	65.5468			1

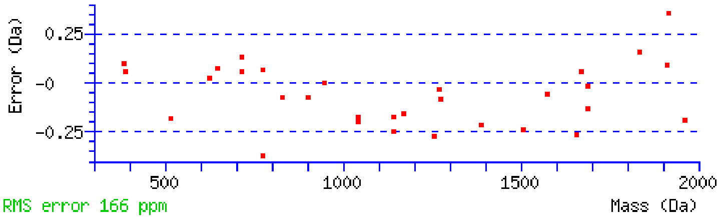

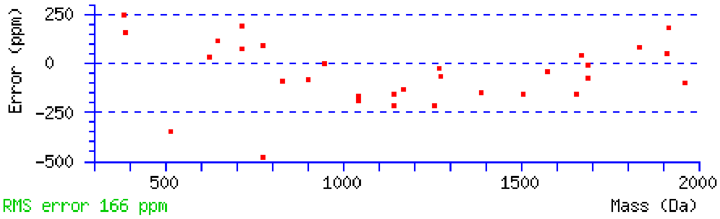

All matches to this query

Score	Mr(calc):	Delta	Sequence
47.5	2317.8482	0.0027	KGAEEEEEEEDDDSEEEIK
4.7	2317.8447	0.0062	NPSNRNQDDMAPSGPNSPIK
3.0	2316.8606	0.9902	NPSNRNQDDMAPSGPNSPIK
2.8	2316.8606	0.9902	NPSNRNQDDMAPSGPNSPIK
2.5	2316.8606	0.9902	NPSNRNQDDMAPSGPNSPIK
2.0	2317.8734	-0.0226	HELHNLKESWETTYYK
1.9	2317.8595	-0.0086	NNPCTNYSMLPETMIDLK
1.7	2317.8595	-0.0086	NNPCTNYSMLPETMIDLK
1.6	2317.8595	-0.0086	NNPCTNYSMLPETMIDLK
1.6	2315.8612	1.9897	VHSPWNTETVALNFMGEK

Spectrum No: 76; Query: 648; Rank: 1

Peptide View

MS/MS Fragmentation of **GSLDSDNDDSDCPYSEK**

Found in **IPI00199985**, Tax_Id=10116 Gene_Symbol=Slc4a4 Isoform 1 of Electrogenic sodium bicarbonate cotransporter 1

Match to Query 648: 1982.664508 from(992.339530,2+)
Title: 091129RatKid_SCX02_12.708.708.2.dta
Data file K:\NewmanPaper\Piliang\3SubProteomes\Piliang3SP\mgf5ppm\SCX_3SubProteomes5ppm.mgf

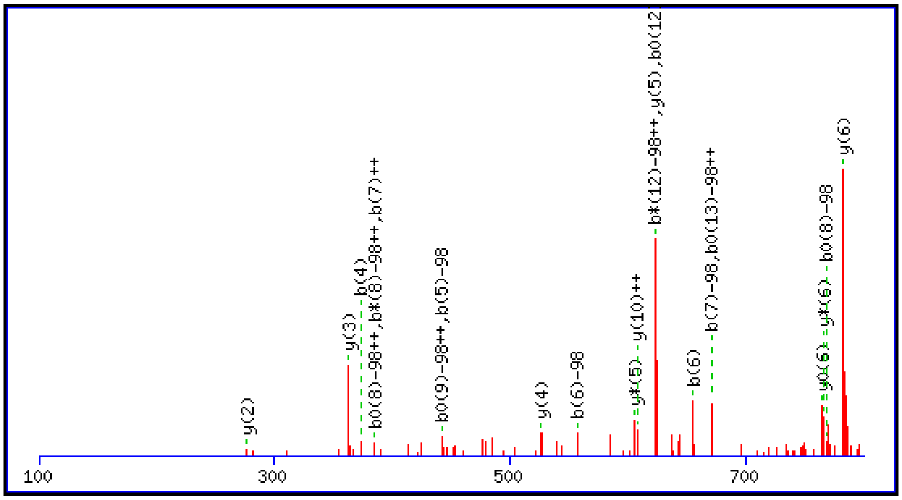

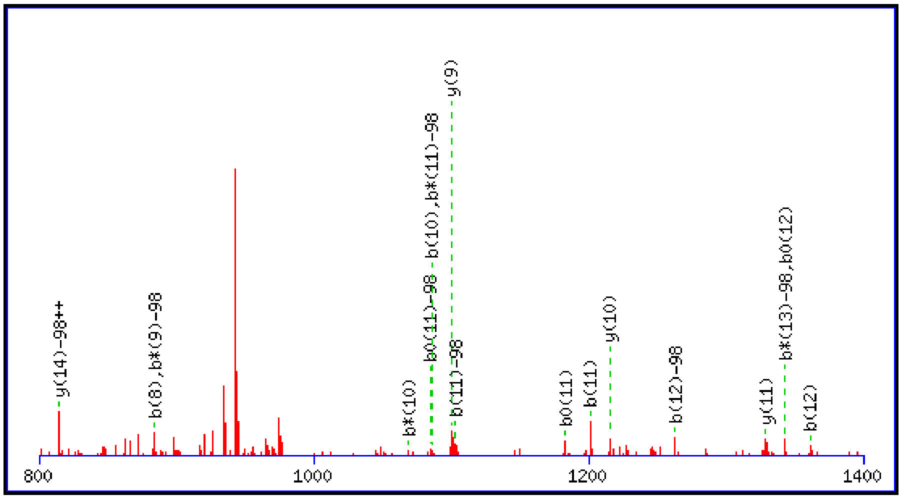

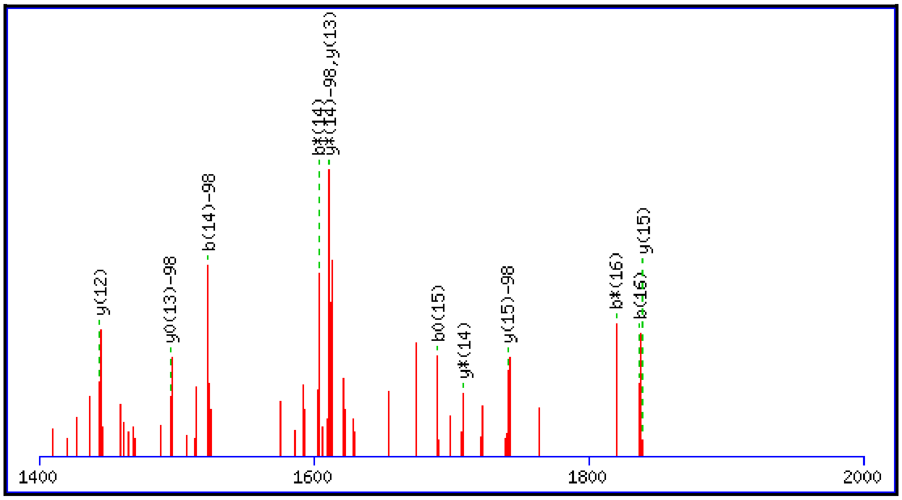

Monoisotopic mass of neutral peptide Mr(calc): 1982.6725
Fixed modifications: Carbamidomethyl (C)
Variable modifications:
S5 : Phospho (ST), with neutral losses 0.0000(shown in table), 97.9769
Ions Score: 47 **Expect:** 0.0011
Matches (Bold Red): 51/268 fragment ions using 75 most intense peaks

#	b	b^{++}	b*	b^{*++}	b^0	b^{0++}	Seq.	y	y^{++}	y*	y^{*++}	y^0	y^{0++}	#
1	58.0287	29.5180					G							17

2	145.0608	73.0340			127.0502	64.0287	S	1926.6583	963.8328	1909.6317	955.3195	1908.6477	954.8275	16
3	258.1448	129.5761			240.1343	120.5708	L	**1839.6263**	920.3168	1822.5997	911.8035	1821.6157	911.3115	15
4	**373.1718**	187.0895			355.1612	178.0842	D	1726.5422	863.7747	**1709.5156**	855.2615	1708.5316	854.7695	14
5	540.1701	270.5887			522.1596	261.5834	S	**1611.5152**	806.2613	1594.4887	797.7480	1593.5047	797.2560	13
6	**655.1971**	328.1022			637.1865	319.0969	D	**1444.5169**	722.7621	1427.4903	714.2488	1426.5063	713.7568	12
7	769.2400	**385.1236**	752.2134	376.6104	751.2294	376.1184	N	**1329.4900**	665.2486	1312.4634	656.7353	1311.4794	656.2433	11
8	**884.2669**	442.6371	867.2404	434.1238	866.2564	433.6318	D	**1215.4470**	**608.2272**	1198.4205	599.7139	1197.4365	599.2219	10
9	999.2939	500.1506	982.2673	491.6373	981.2833	491.1453	D	**1100.4201**	550.7137	1083.3935	542.2004	1082.4095	541.7084	9
10	**1086.3259**	543.6666	**1069.2994**	535.1533	1068.3153	534.6613	S	985.3931	493.2002	968.3666	484.6869	967.3826	484.1949	8
11	**1201.3529**	601.1801	1184.3263	592.6668	**1183.3423**	592.1748	D	898.3611	449.6842	881.3346	441.1709	880.3505	440.6789	7
12	**1361.3835**	681.1954	1344.3570	672.6821	**1343.3729**	672.1901	C	**783.3342**	392.1707	**766.3076**	383.6574	**765.3236**	383.1654	6
13	1458.4363	729.7218	1441.4097	721.2085	1440.4257	720.7165	P	**623.3035**	312.1554	**606.2770**	303.6421	605.2930	303.1501	5
14	1621.4996	811.2534	**1604.4730**	802.7402	1603.4890	802.2482	Y	**526.2508**	263.6290	509.2242	255.1157	508.2402	254.6237	4
15	1708.5316	854.7695	1691.5051	846.2562	**1690.5211**	845.7642	S	**363.1874**	182.0974	346.1609	173.5841	345.1769	173.0921	3
16	**1837.5742**	919.2907	**1820.5477**	910.7775	1819.5637	910.2855	E	**276.1554**	138.5813	259.1288	130.0681	258.1448	129.5761	2
17							K	147.1128	74.0600	130.0863	65.5468			1

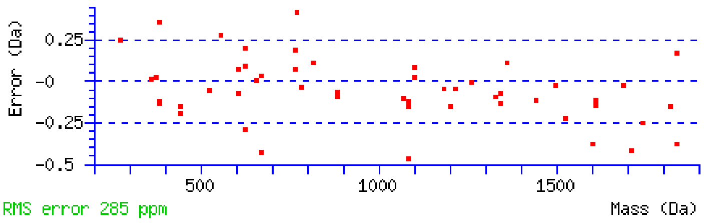

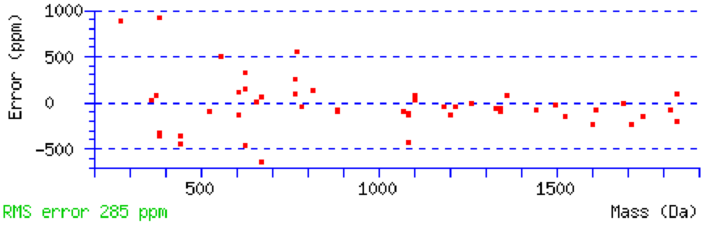

All matches to this query

Score	Mr(calc):	Delta	Sequence
47.4	1982.6725	-0.0080	GSLDSDNDDSDCPYSEK
37.4	1982.6725	-0.0080	GSLDSDNDDSDCPYSEK
22.2	1982.6725	-0.0080	GSLDSDNDDSDCPYSEK
7.5	1982.6725	-0.0080	GSLDSDNDDSDCPYSEK
3.2	1982.6725	-0.0080	GSLDSDNDDSDCPYSEK
0.4	1982.6686	-0.0041	TSRRSDEERPSTSR

Spectrum No: 77; Query: 962; Rank: 1

Peptide View

MS/MS Fragmentation of **SKEPSSPGTDDVFTPGPSDSPGSQR**
Found in **IPI00193369**, Tax_Id=10116 Gene_Symbol=Slc9a1 Sodium/hydrogen exchanger 1

Match to Query 962: 2611.095702 from(871.372510,3+)
Title: 091129RatKid_SCX02_22.1526.1526.3.dta
Data file K:\NewmanPaper\Piliang\3SubProteomes\Piliang3SP\mgf5ppm\SCX_3SubProteomes5ppm.mgf

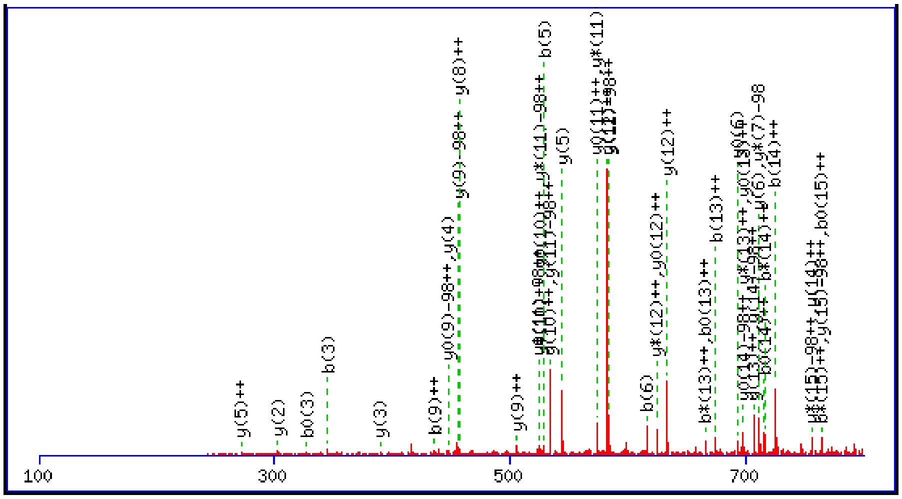

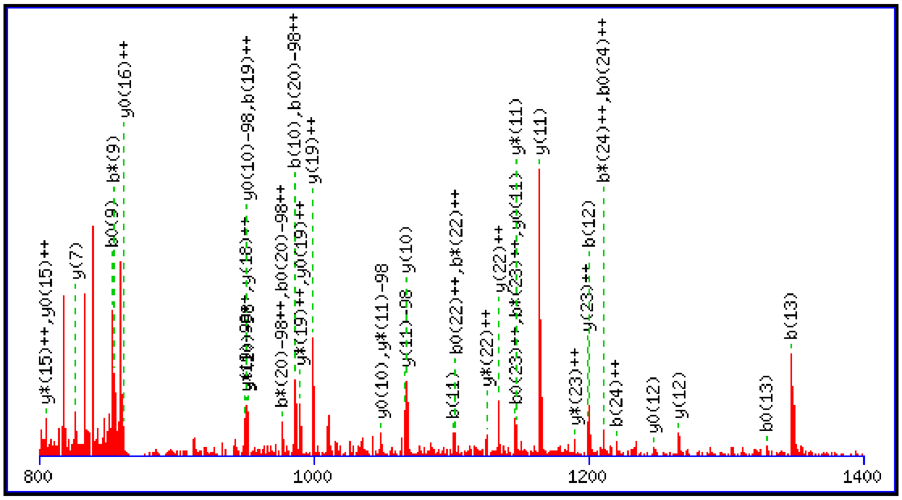

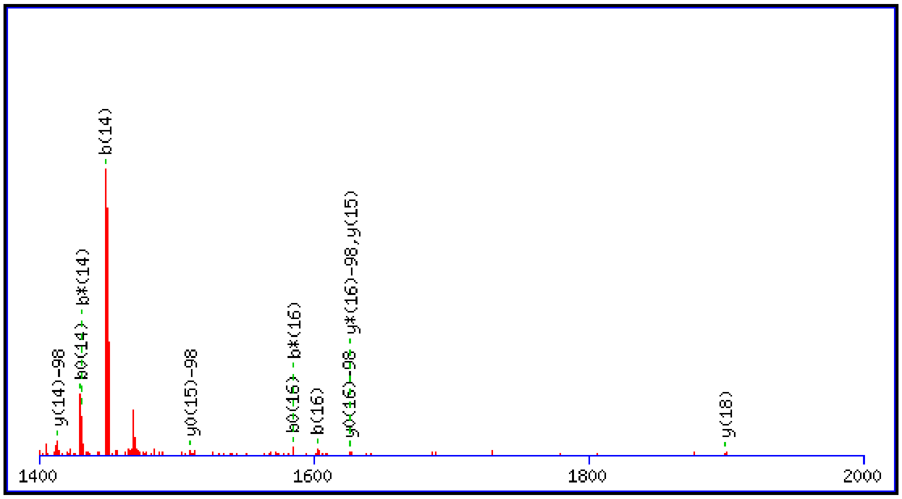

Monoisotopic mass of neutral peptide Mr(calc): 2611.1076
Fixed modifications: Carbamidomethyl (C)
Variable modifications:
S20 : Phospho (ST), with neutral losses 0.0000(shown in table), 97.9769
Ions Score: 47 **Expect:** 0.006
Matches (Bold Red): 100/426 fragment ions using 156 most intense peaks

#	b	b++	b*	b*++	b0	b0++	Seq.	y	y++	y*	y*++	y0	y0++	#
1	88.0393	44.5233			70.0287	35.5180	S							25
2	216.1343	108.5708	199.1077	100.0575	198.1237	99.5655	K	2525.0828	1263.0450	2508.0562	1254.5318	2507.0722	1254.0397	24
3	**345.1769**	173.0921	328.1503	164.5788	**327.1663**	164.0868	E	2396.9878	**1198.9975**	2379.9613	**1190.4843**	2378.9772	1189.9923	23
4	442.2296	221.6185	425.2031	213.1052	424.2191	212.6132	P	2267.9452	**1134.4762**	2250.9187	**1125.9630**	2249.9347	1125.4710	22
5	**529.2617**	265.1345	512.2351	256.6212	511.2511	256.1292	S	2170.8925	1085.9499	2153.8659	1077.4366	2152.8819	1076.9446	21

6	616.2937	308.6505	599.2671	300.1372	598.2831	299.6452	S	2083.8604	1042.4339	2066.8339	1033.9206	2065.8499	1033.4286	20
7	713.3464	357.1769	696.3199	348.6636	695.3359	348.1716	P	1996.8284	998.9178	1979.8019	990.4046	1978.8178	989.9126	19
8	770.3679	385.6876	753.3414	377.1743	752.3573	376.6823	G	1899.7756	950.3915	1882.7491	941.8782	1881.7651	941.3862	18
9	871.4156	436.2114	854.3890	427.6982	853.4050	427.2061	T	1842.7542	921.8807	1825.7276	913.3675	1824.7436	912.8754	17
10	986.4425	493.7249	969.4160	485.2116	968.4320	484.7196	D	1741.7065	871.3569	1724.6799	862.8436	1723.6959	862.3516	16
11	1101.4695	551.2384	1084.4429	542.7251	1083.4589	542.2331	D	1626.6796	813.8434	1609.6530	805.3301	1608.6690	804.8381	15
12	1200.5379	600.7726	1183.5113	592.2593	1182.5273	591.7673	V	1511.6526	756.3299	1494.6261	747.8167	1493.6420	747.3247	14
13	1347.6063	674.3068	1330.5798	665.7935	1329.5957	665.3015	F	1412.5842	706.7957	1395.5576	698.2825	1394.5736	697.7905	13
14	1448.6540	724.8306	1431.6274	716.3174	1430.6434	715.8253	T	1265.5158	633.2615	1248.4892	624.7483	1247.5052	624.2562	12
15	1545.7067	773.3570	1528.6802	764.8437	1527.6962	764.3517	P	1164.4681	582.7377	1147.4416	574.2244	1146.4575	573.7324	11
16	1602.7282	801.8677	1585.7017	793.3545	1584.7176	792.8625	G	1067.4153	534.2113	1050.3888	525.6980	1049.4048	525.2060	10
17	1699.7810	850.3941	1682.7544	841.8808	1681.7704	841.3888	P	1010.3939	505.7006	993.3673	497.1873	992.3833	496.6953	9
18	1786.8130	893.9101	1769.7865	885.3969	1768.8024	884.9049	S	913.3411	457.1742	896.3146	448.6609	895.3305	448.1689	8
19	1901.8399	951.4236	1884.8134	942.9103	1883.8294	942.4183	D	826.3091	413.6582	809.2825	405.1449	808.2985	404.6529	7
20	2068.8383	1034.9228	2051.8117	1026.4095	2050.8277	1025.9175	S	711.2821	356.1447	694.2556	347.6314	693.2716	347.1394	6
21	2165.8911	1083.4492	2148.8645	1074.9359	2147.8805	1074.4439	P	544.2838	272.6455	527.2572	264.1323	526.2732	263.6402	5
22	2222.9125	1111.9599	2205.8860	1103.4466	2204.9020	1102.9546	G	447.2310	224.1191	430.2045	215.6059	429.2205	215.1139	4
23	2309.9446	1155.4759	2292.9180	1146.9626	2291.9340	1146.4706	S	390.2096	195.6084	373.1830	187.0951	372.1990	186.6031	3
24	2438.0031	1219.5052	2420.9766	1210.9919	2419.9926	1210.4999	Q	303.1775	152.0924	286.1510	143.5791			2
25							R	175.1190	88.0631	158.0924	79.5498			1

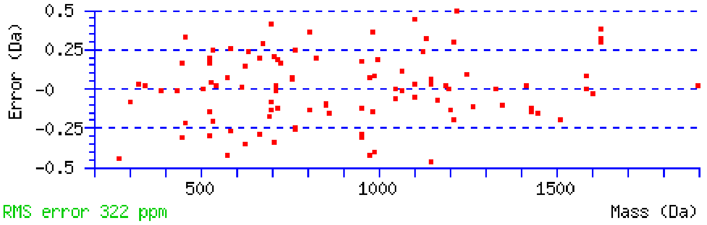

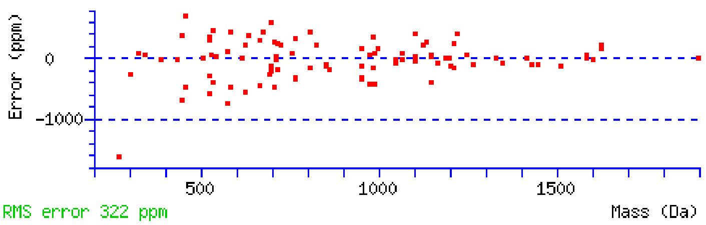

All matches to this query

Score	Mr(calc):	Delta	Sequence
47.4	2611.1076	-0.0119	SKEPSSPGTDDVFTPGPSDSPGSQR
40.5	2611.1076	-0.0119	SKEPSSPGTDDVFTPGPSDSPGSQR
36.3	2611.1076	-0.0119	SKEPSSPGTDDVFTPGPSDSPGSQR
22.8	2611.1076	-0.0119	SKEPSSPGTDDVFTPGPSDSPGSQR
19.8	2611.1076	-0.0119	SKEPSSPGTDDVFTPGPSDSPGSQR
15.4	2611.1076	-0.0119	SKEPSSPGTDDVFTPGPSDSPGSQR
13.9	2611.0783	0.0174	AYKMHQNISLTGGDMPSCLRR
11.9	2611.1076	-0.0119	SKEPSSPGTDDVFTPGPSDSPGSQR
10.9	2611.1076	-0.0119	SKEPSSPGTDDVFTPGPSDSPGSQR
7.0	2611.0783	0.0174	AYKMHQNISLTGGDMPSCLRR

Spectrum No: 78; Query: 292; Rank: 1

Peptide View

MS/MS Fragmentation of **TASGSSVTSLEGTR**
Found in **IPI00421389**, Tax_Id=10116 Gene_Symbol=Ndrg1 Protein NDRG1

Match to Query 292: 1511.593448 from(756.804000,2+)
Title: 091129RatKid_SCX02_02.979.979.2.dta

Data file K:\NewmanPaper\Piliang\3SubProteomes\Piliang3SP\mgf5ppm\SCX_3SubProteomes5ppm.mgf

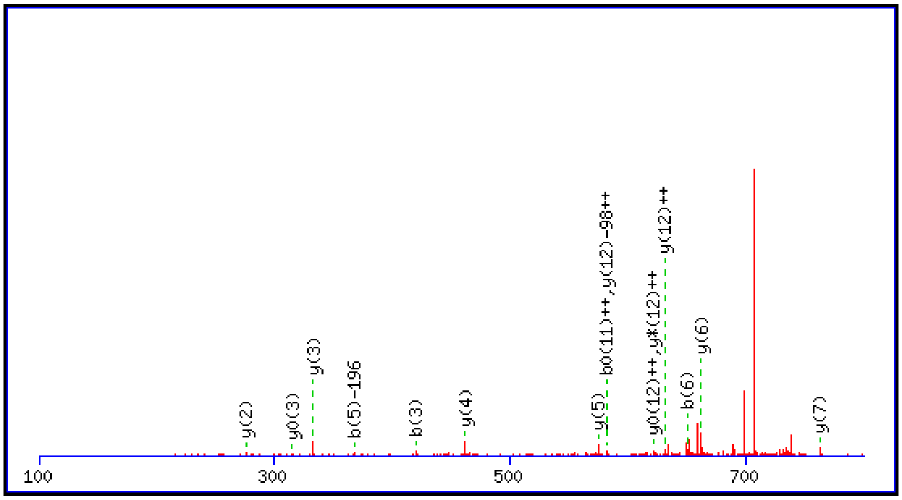

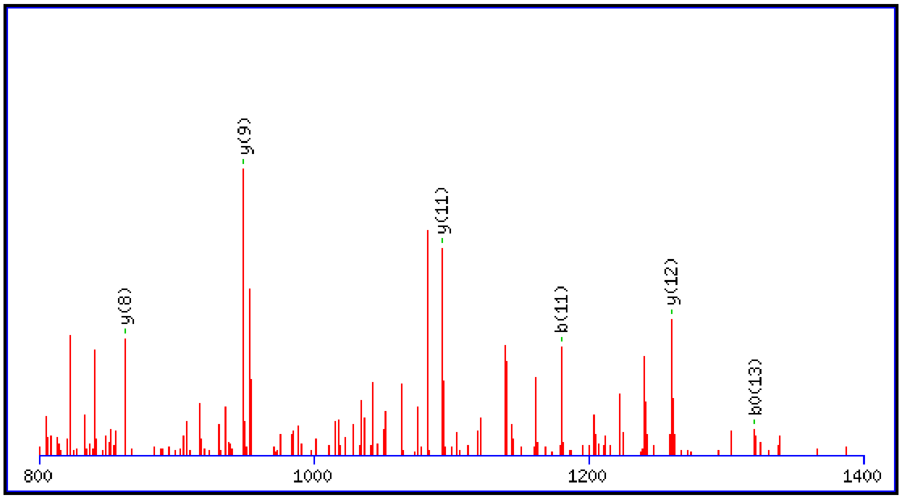

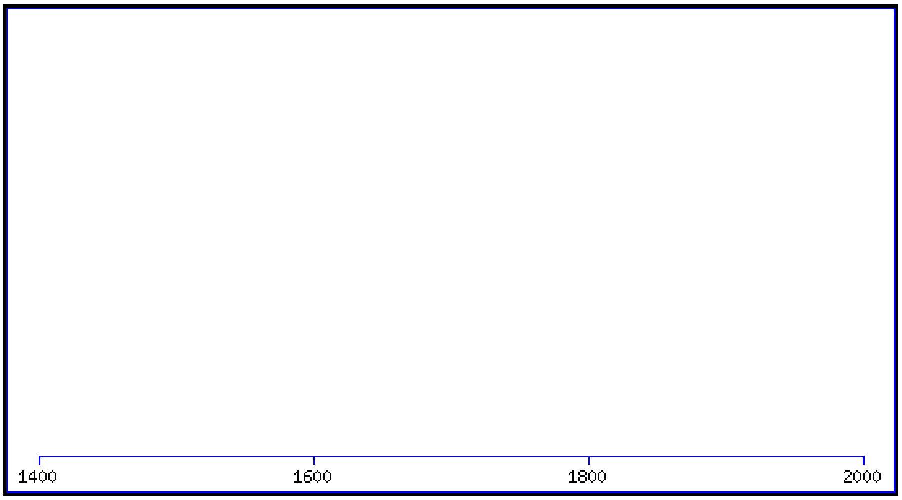

Monoisotopic mass of neutral peptide Mr(calc): 1511.5906
Fixed modifications: Carbamidomethyl (C)
Variable modifications:
T1 : Phospho (ST), with neutral losses 0.0000(shown in table), 97.9769
S3 : Phospho (ST), with neutral losses 0.0000(shown in table), 97.9769
Ions Score: 47 **Expect:** 0.0028
Matches (Bold Red): 21/192 fragment ions using 39 most intense peaks

#	b	b^{++}	b^{0}	b^{0++}	Seq.	y	y^{++}	y*	y^{*++}	y^{0}	y^{0++}	#
1	182.0213	91.5143	164.0107	82.5090	T							14
2	253.0584	127.0328	235.0478	118.0276	A	1331.5839	666.2956	1314.5573	657.7823	1313.5733	657.2903	13
3	**420.0567**	210.5320	402.0462	201.5267	S	**1260.5467**	**630.7770**	1243.5202	**622.2637**	1242.5362	**621.7717**	12

**4**	477.0782	239.0427	459.0676	230.0375	**G**	**1093.5484**	547.2778	1076.5218	538.7646	1075.5378	538.2726	**11**
**5**	564.1102	282.5588	546.0997	273.5535	**S**	1036.5269	518.7671	1019.5004	510.2538	1018.5164	509.7618	**10**
**6**	**651.1423**	326.0748	633.1317	317.0695	**S**	**949.4949**	475.2511	932.4684	466.7378	931.4843	466.2458	**9**
**7**	750.2107	375.6090	732.2001	366.6037	**V**	**862.4629**	431.7351	845.4363	423.2218	844.4523	422.7298	**8**
**8**	851.2584	426.1328	833.2478	417.1275	**T**	**763.3945**	382.2009	746.3679	373.6876	745.3839	373.1956	**7**
**9**	938.2904	469.6488	920.2798	460.6435	**S**	**662.3468**	331.6770	645.3202	323.1638	644.3362	322.6717	**6**
**10**	1051.3744	526.1909	1033.3639	517.1856	**L**	**575.3148**	288.1610	558.2882	279.6477	557.3042	279.1557	**5**
**11**	**1180.4170**	590.7122	1162.4065	**581.7069**	**E**	**462.2307**	231.6190	445.2041	223.1057	444.2201	222.6137	**4**
**12**	1237.4385	619.2229	1219.4279	610.2176	**G**	**333.1881**	167.0977	316.1615	158.5844	**315.1775**	158.0924	**3**
**13**	1338.4862	669.7467	**1320.4756**	660.7414	**T**	**276.1666**	138.5870	259.1401	130.0737	258.1561	129.5817	**2**
**14**					**R**	175.1190	88.0631	158.0924	79.5498			**1**

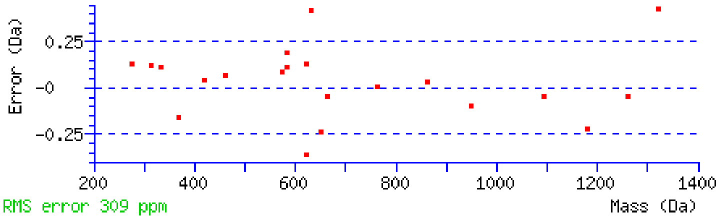

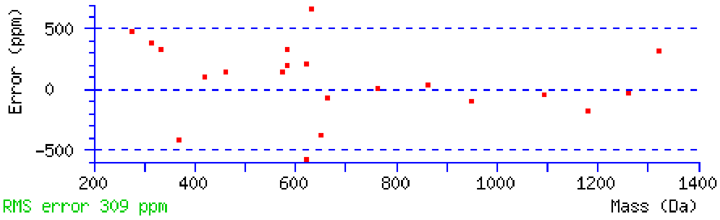

All matches to this query

Score	Mr(calc):	Delta	Sequence
47.0	1511.5906	0.0029	TASGSSVTSLEGTR
24.1	1511.5906	0.0029	TASGSSVTSLEGTR
9.6	1511.5938	-0.0004	NFEAFASLCMAR
9.3	1511.5889	0.0045	INSNHINSNSTGSS
9.3	1511.5889	0.0045	INSNHINSNSTGSS
7.5	1511.5889	0.0045	INSNHINSNSTGSS
6.7	1511.5824	0.0111	MSSYSSDRDRGR
6.5	1511.5889	0.0045	INSNHINSNSTGSS
6.5	1511.5889	0.0045	INSNHINSNSTGSS
6.4	1511.5889	0.0045	INSNHINSNSTGSS

Spectrum No: 79; Query: 501; Rank: 1

Peptide View

MS/MS Fragmentation of **SRTASGSSVTSLEGTR**
Found in **IPI00421389**, Tax_Id=10116 Gene_Symbol=Ndrg1 Protein NDRG1

Match to Query 501: 1754.727668 from(878.371110,2+)
Title: 091127RatKid_SCX01_14.1354.1354.2.dta
Data file K:\NewmanPaper\Piliang\3SubProteomes\Piliang3SP\mgf5ppm\SCX_3SubProteomes5ppm.mgf

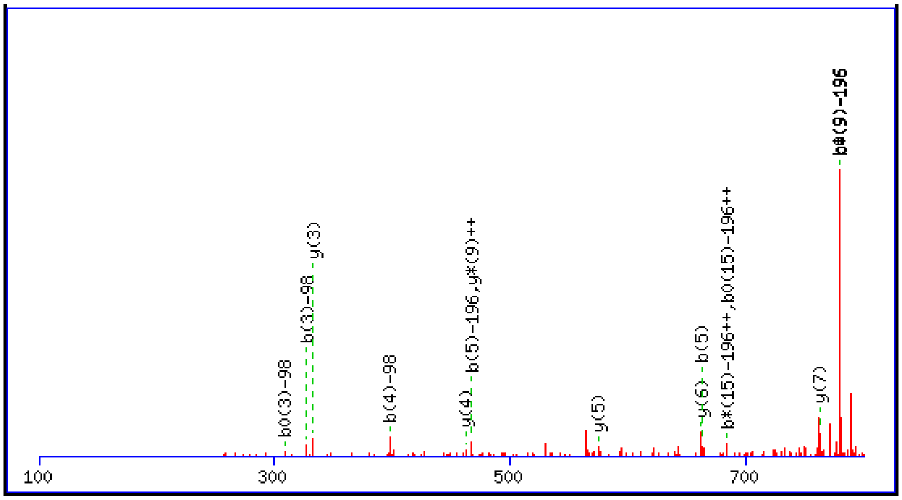

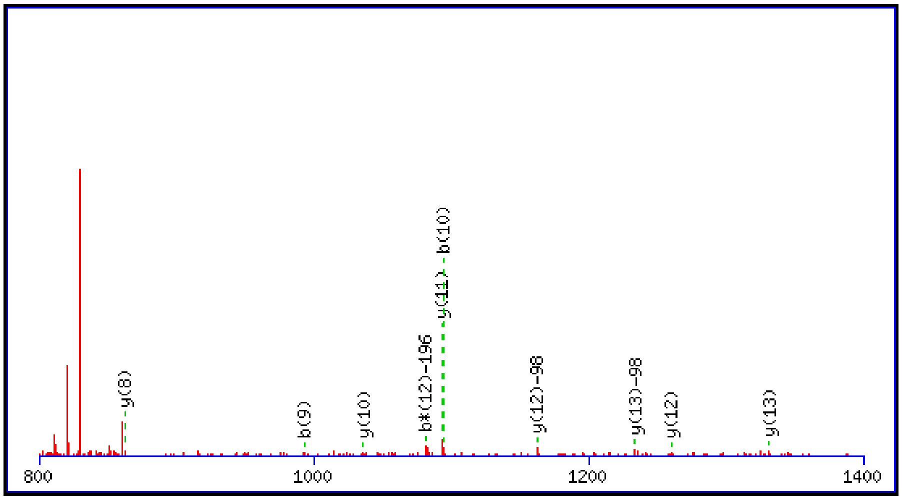

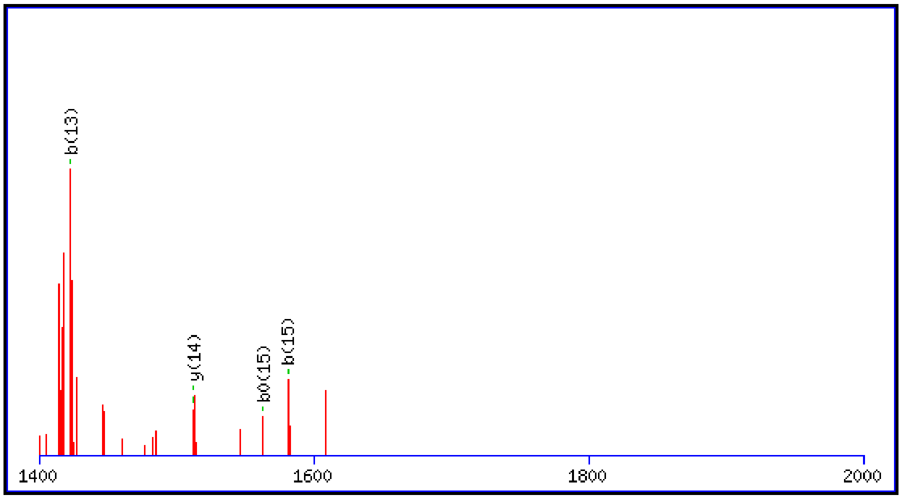

Monoisotopic mass of neutral peptide Mr(calc): 1754.7237
Fixed modifications: Carbamidomethyl (C)
Variable modifications:
T3 : Phospho (ST), with neutral losses 97.9769(shown in table), 0.0000
S5 : Phospho (ST), with neutral losses 97.9769(shown in table), 0.0000
Ions Score: 47 **Expect:** 0.0033
Matches (Bold Red): 29/278 fragment ions using 55 most intense peaks

#	b	b^{++}	b*	b^{*++}	b^0	b^{0++}	Seq.	y	y^{++}	y*	y^{*++}	y^0	y^{0++}	#
1	88.0393	44.5233			70.0287	35.5180	S							16
2	244.1404	122.5738	227.1139	114.0606	226.1298	113.5686	R	1472.7452	736.8762	1455.7186	728.3630	1454.7346	727.8709	15
3	**327.1775**	164.0924	310.1510	155.5791	**309.1670**	155.0871	T	1316.6441	658.8257	1299.6175	650.3124	1298.6335	649.8204	14
4	**398.2146**	199.6110	381.1881	191.0977	380.2041	190.6057	A	**1233.6070**	617.3071	1216.5804	608.7938	1215.5964	608.3018	13

**5**	**467.2361**	234.1217	450.2095	225.6084	449.2255	225.1164	**S**	**1162.5698**	581.7886	1145.5433	573.2753	1144.5593	572.7833	**12**
**6**	524.2576	262.6324	507.2310	254.1191	506.2470	253.6271	**G**	**1093.5484**	547.2778	1076.5218	538.7646	1075.5378	538.2726	**11**
**7**	611.2896	306.1484	594.2630	297.6352	593.2790	297.1431	**S**	**1036.5269**	518.7671	1019.5004	510.2538	1018.5164	509.7618	**10**
**8**	698.3216	349.6644	681.2951	341.1512	680.3110	340.6592	**S**	949.4949	475.2511	932.4684	**466.7378**	931.4843	466.2458	**9**
**9**	797.3900	399.1987	**780.3635**	390.6854	**779.3795**	390.1934	**V**	**862.4629**	431.7351	845.4363	423.2218	844.4523	422.7298	**8**
**10**	898.4377	449.7225	881.4112	441.2092	880.4271	440.7172	**T**	**763.3945**	382.2009	746.3679	373.6876	745.3839	373.1956	**7**
**11**	985.4697	493.2385	968.4432	484.7252	967.4592	484.2332	**S**	**662.3468**	331.6770	645.3202	323.1638	644.3362	322.6717	**6**
**12**	1098.5538	549.7805	**1081.5272**	541.2673	1080.5432	540.7753	**L**	**575.3148**	288.1610	558.2882	279.6477	557.3042	279.1557	**5**
**13**	1227.5964	614.3018	1210.5698	605.7886	1209.5858	605.2966	**E**	**462.2307**	231.6190	445.2041	223.1057	444.2201	222.6137	**4**
**14**	1284.6179	642.8126	1267.5913	634.2993	1266.6073	633.8073	**G**	**333.1881**	167.0977	316.1615	158.5844	315.1775	158.0924	**3**
**15**	1385.6655	693.3364	1368.6390	**684.8231**	1367.6550	**684.3311**	**T**	276.1666	138.5870	259.1401	130.0737	258.1561	129.5817	**2**
**16**							**R**	175.1190	88.0631	158.0924	79.5498			**1**

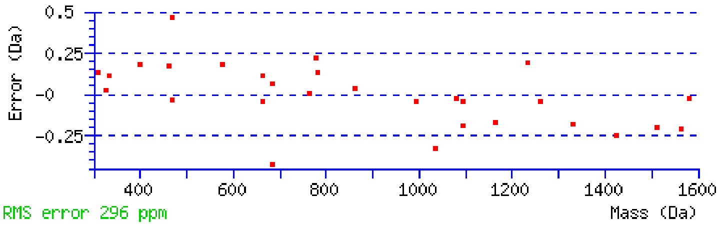

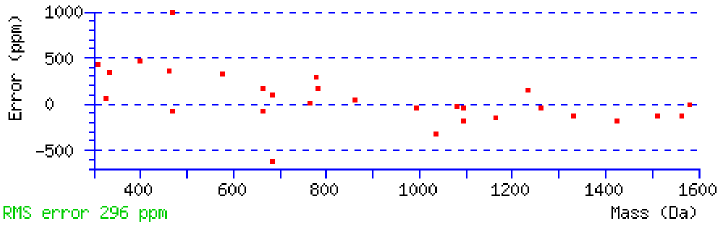

All matches to this query

Score	Mr(calc):	Delta	Sequence
46.9	1754.7237	0.0039	SRTASGSSVTSLEGTR
23.2	1754.7237	0.0039	SRTASGSSVTSLEGTR
15.2	1754.7237	0.0039	SRTASGSSVTSLEGTR
13.4	1754.7237	0.0039	SRTASGSSVTSLEGTR
7.5	1754.7237	0.0039	SRTASGSSVTSLEGTR
5.6	1754.7307	-0.0030	XRKSLGLQTSSWR
5.6	1754.7307	-0.0030	XRKSLGLQTSSWR
5.6	1754.7307	-0.0030	XRKSLGLQTSSWR
3.8	1752.7089	2.0187	GMCLSPSSLVSGRAR
3.8	1752.7089	2.0187	GMCLSPSSLVSGRAR

Spectrum No: 80; Query: 918; Rank: 1

Peptide View

MS/MS Fragmentation of **RVSVCAETFNPDEEEDNDPR**
Found in **IPI00196684**, Tax_Id=10116 Gene_Symbol=Prkar2a cAMP-dependent protein kinase type II-alpha regulatory subunit

Match to Query 918: 2457.967508 from(1229.991030,2+)
Title: 091127RatKid_SCX01_21.2044.2044.2.dta
Data file K:\NewmanPaper\Piliang\3SubProteomes\Piliang3SP\mgf5ppm\SCX_3SubProteomes5ppm.mgf

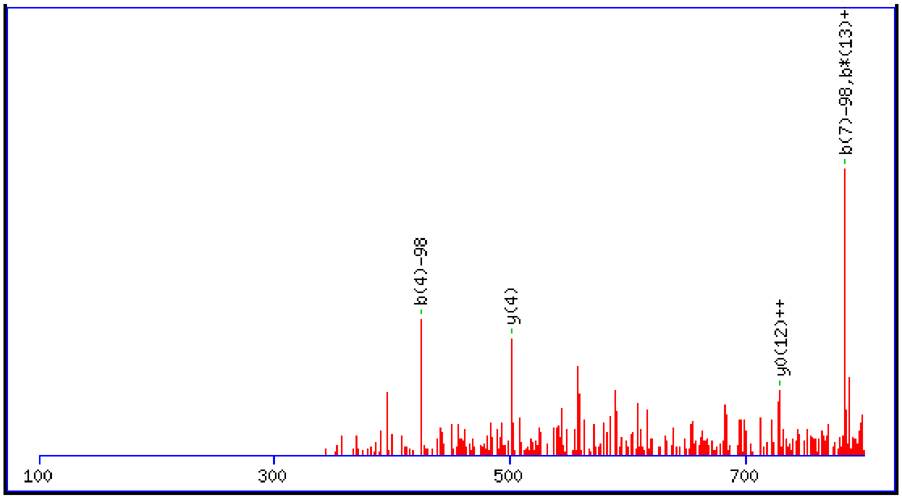

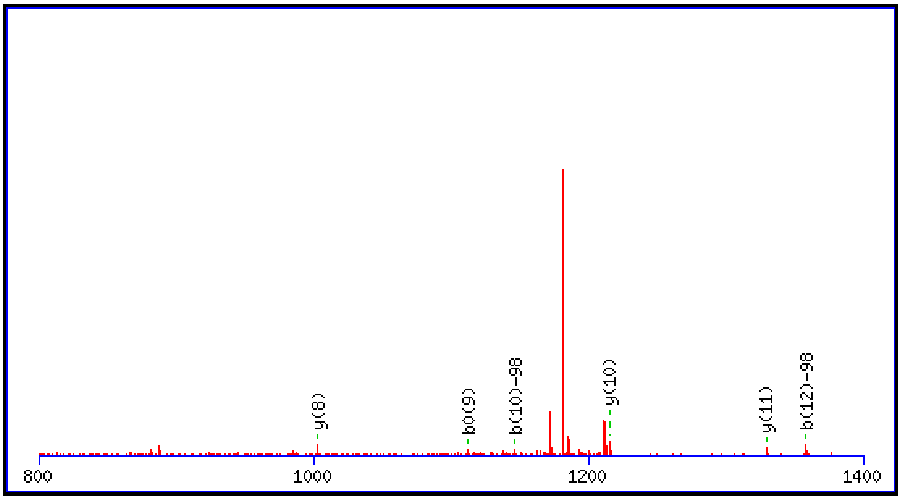

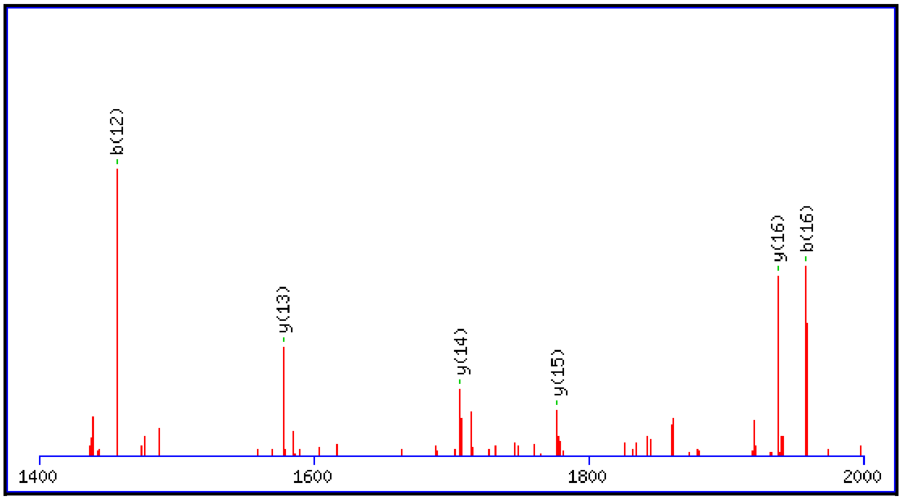

Monoisotopic mass of neutral peptide Mr(calc): 2457.9744
Fixed modifications: Carbamidomethyl (C)
Variable modifications:
S3 : Phospho (ST), with neutral losses 97.9769(shown in table), 0.0000
Ions Score: 47 **Expect:** 0.0042
Matches (Bold Red): 18/334 fragment ions using 29 most intense peaks

#	b	b^{++}	b*	b^{*++}	b^0	b^{0++}	Seq.	y	y^{++}	y*	y^{*++}	y^0	y^{0++}	#
1	157.1084	79.0578	140.0818	70.5446			R							20
2	256.1768	128.5920	239.1503	120.0788			V	2204.9037	1102.9555	2187.8771	1094.4422	2186.8931	1093.9502	19
3	325.1983	163.1028	308.1717	154.5895	307.1877	154.0975	S	2105.8353	1053.4213	2088.8087	1044.9080	2087.8247	1044.4160	18
4	**424.2667**	212.6370	407.2401	204.1237	406.2561	203.6317	V	2036.8138	1018.9105	2019.7873	1010.3973	2018.8032	1009.9053	17
5	584.2973	292.6523	567.2708	284.1390	566.2868	283.6470	C	**1937.7454**	969.3763	1920.7188	960.8631	1919.7348	960.3711	16

6	655.3344	328.1709	638.3079	319.6576	637.3239	319.1656	A	1777.7147	889.3610	1760.6882	880.8477	1759.7042	880.3557	15
7	784.3770	392.6922	767.3505	384.1789	766.3665	383.6869	E	1706.6776	853.8425	1689.6511	845.3292	1688.6671	844.8372	14
8	885.4247	443.2160	868.3982	434.7027	867.4141	434.2107	T	1577.6350	789.3212	1560.6085	780.8079	1559.6245	780.3159	13
9	1032.4931	516.7502	1015.4666	508.2369	1014.4826	507.7449	F	1476.5874	738.7973	1459.5608	730.2840	1458.5768	729.7920	12
10	1146.5360	573.7717	1129.5095	565.2584	1128.5255	564.7664	N	1329.5189	665.2631	1312.4924	656.7498	1311.5084	656.2578	11
11	1243.5888	622.2980	1226.5623	613.7848	1225.5782	613.2928	P	1215.4760	608.2416	1198.4495	599.7284	1197.4655	599.2364	10
12	1358.6158	679.8115	1341.5892	671.2982	1340.6052	670.8062	D	1118.4233	559.7153	1101.3967	551.2020	1100.4127	550.7100	9
13	1487.6583	744.3328	1470.6318	735.8195	1469.6478	735.3275	E	1003.3963	502.2018	986.3698	493.6885	985.3857	493.1965	8
14	1616.7009	808.8541	1599.6744	800.3408	1598.6904	799.8488	E	874.3537	437.6805	857.3272	429.1672	856.3432	428.6752	7
15	1745.7435	873.3754	1728.7170	864.8621	1727.7330	864.3701	E	745.3111	373.1592	728.2846	364.6459	727.3006	364.1539	6
16	1860.7705	930.8889	1843.7439	922.3756	1842.7599	921.8836	D	616.2685	308.6379	599.2420	300.1246	598.2580	299.6326	5
17	1974.8134	987.9103	1957.7869	979.3971	1956.8028	978.9051	N	501.2416	251.1244	484.2150	242.6112	483.2310	242.1191	4
18	2089.8403	1045.4238	2072.8138	1036.9105	2071.8298	1036.4185	D	387.1987	194.1030	370.1721	185.5897	369.1881	185.0977	3
19	2186.8931	1093.9502	2169.8666	1085.4369	2168.8825	1084.9449	P	272.1717	136.5895	255.1452	128.0762			2
20							R	175.1190	88.0631	158.0924	79.5498			1

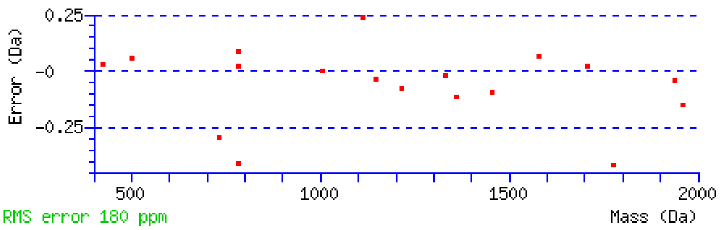

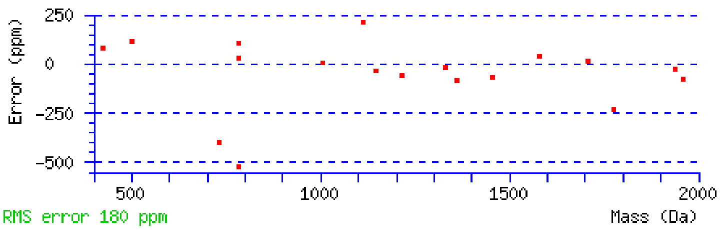

All matches to this query

Score	Mr(calc):	Delta	Sequence
46.7	2457.9744	-0.0069	RVSVCAETFNPDEEEDNDPR
21.4	2457.9744	-0.0069	RVSVCAETFNPDEEEDNDPR
7.6	2457.9437	0.0238	EQYSEPDMEGVSYGVPQDLR
4.6	2457.9784	-0.0109	FYSKEETGPVQSSHDMNSYR
3.0	2457.9437	0.0238	EQYSEPDMEGVSYGVPQDLR
2.8	2457.9443	0.0232	ESLSRLMATLSNTNPSFVR
2.8	2457.9443	0.0232	ESLSRLMATLSNTNPSFVR
2.8	2457.9443	0.0232	ESLSRLMATLSNTNPSFVR
2.8	2457.9443	0.0232	ESLSRLMATLSNTNPSFVR
2.8	2457.9510	0.0165	ARYSMEKIMPEEEYSEFK

Spectrum No: 81; Query: 1087; Rank: 1

Peptide View

MS/MS Fragmentation of **KVEEEQEADEEDVSEEETENREGESK**
Found in **IPI00365626**, Tax_Id=10116 Gene_Symbol=Txndc1 Thioredoxin domain containing 1

Match to Query 1087: 3132.239052 from(1045.086960,3+)
Title: 091129RatKid_SCX02_32.1002.1002.3.dta
Data file K:\NewmanPaper\Piliang\3SubProteomes\Piliang3SP\mgf5ppm\SCX_3SubProteomes5ppm.mgf

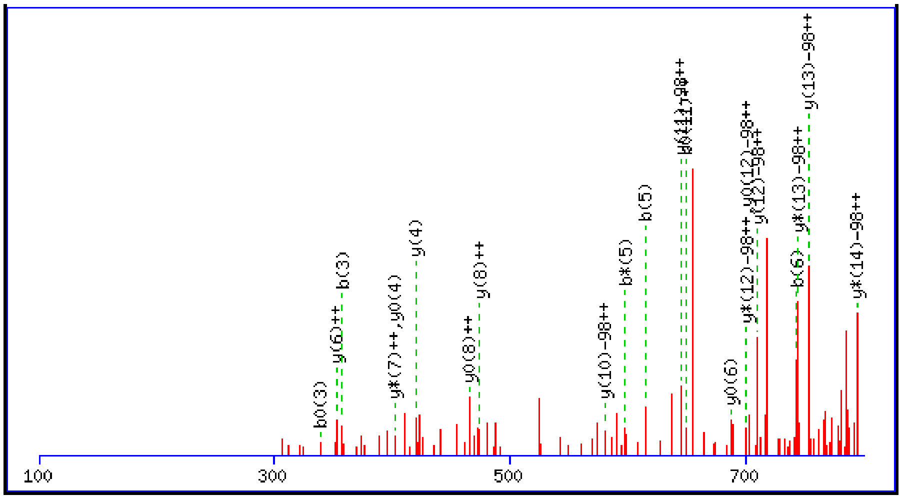

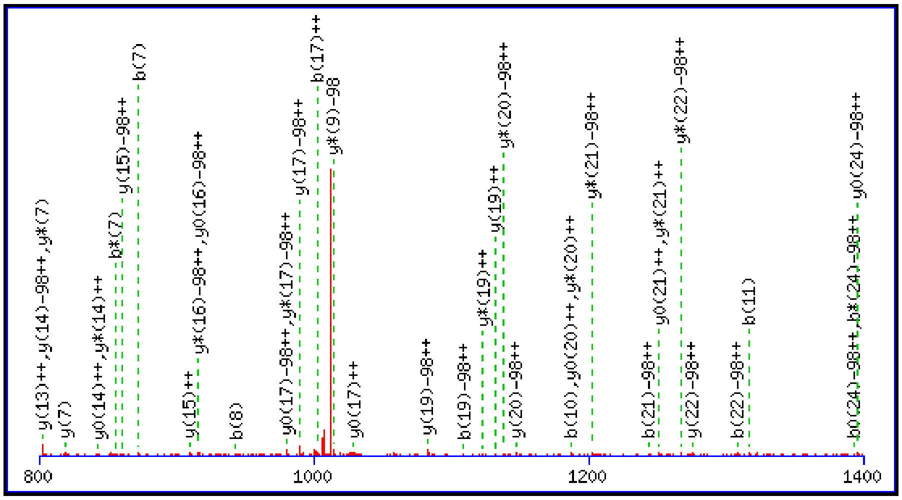

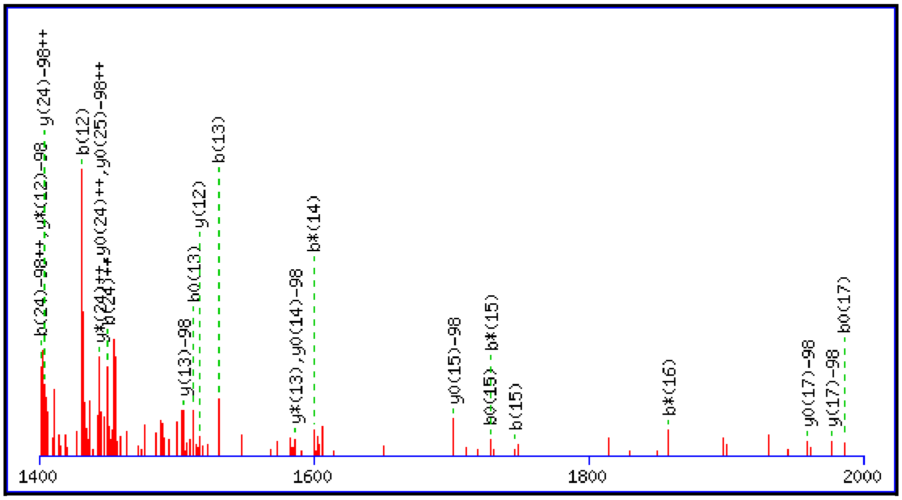

Monoisotopic mass of neutral peptide Mr(calc): 3132.2415
Fixed modifications: Carbamidomethyl (C)
Variable modifications:
T18 : Phospho (ST), with neutral losses 97.9769(shown in table), 0.0000
Ions Score: 46 **Expect:** 0.0069
Matches (Bold Red): 84/444 fragment ions using 155 most intense peaks

#	b	b^{++}	b*	b^{*++}	b^0	b^{0++}	Seq.	y	y^{++}	y*	y^{*++}	y^0	y^{0++}	#
1	129.1022	65.0548	112.0757	56.5415			K							26
2	228.1707	114.5890	211.1441	106.0757			V	2907.1770	1454.0921	2890.1504	1445.5789	2889.1664	**1445.0869**	25
3	**357.2132**	179.1103	340.1867	170.5970	**339.2027**	170.1050	E	2808.1086	**1404.5579**	2791.0820	1396.0447	2790.0980	**1395.5526**	24
4	486.2558	243.6316	469.2293	235.1183	468.2453	234.6263	E	2679.0660	1340.0366	2662.0394	1331.5234	2661.0554	1331.0313	23
5	**615.2984**	308.1529	**598.2719**	299.6396	597.2879	299.1476	E	2550.0234	**1275.5153**	2532.9968	**1267.0021**	2532.0128	1266.5101	22

6	**743.3570**	372.1821	726.3305	363.6689	725.3464	363.1769	Q	2420.9808	1210.9940	2403.9542	**1202.4808**	2402.9702	1201.9888	21
7	**872.3996**	436.7034	**855.3731**	428.1902	854.3890	427.6982	E	2292.9222	**1146.9647**	2275.8957	**1138.4515**	2274.9117	1137.9595	20
8	**943.4367**	472.2220	926.4102	463.7087	925.4262	463.2167	A	2163.8796	**1082.4435**	2146.8531	1073.9302	2145.8691	1073.4382	19
9	1058.4637	529.7355	1041.4371	521.2222	1040.4531	520.7302	D	2092.8425	1046.9249	2075.8160	1038.4116	2074.8319	1037.9196	18
10	**1187.5063**	594.2568	1170.4797	585.7435	1169.4957	585.2515	E	**1977.8156**	**989.4114**	1960.7890	**980.8981**	**1959.8050**	**980.4061**	17
11	**1316.5488**	658.7781	1299.5223	650.2648	1298.5383	**649.7728**	E	1848.7730	924.8901	1831.7464	**916.3769**	1830.7624	**915.8848**	16
12	**1431.5758**	716.2915	1414.5492	707.7783	1413.5652	707.2863	D	1719.7304	**860.3688**	1702.7038	851.8556	**1701.7198**	851.3635	15
13	**1530.6442**	765.8257	1513.6177	757.3125	**1512.6336**	756.8205	V	1604.7034	**802.8554**	1587.6769	**794.3421**	**1586.6929**	793.8501	14
14	1617.6762	809.3418	**1600.6497**	800.8285	1599.6657	800.3365	S	**1505.6350**	**753.3212**	1488.6085	**744.8079**	1487.6245	744.3159	13
15	**1746.7188**	873.8631	**1729.6923**	865.3498	**1728.7083**	864.8578	E	1418.6030	**709.8051**	**1401.5765**	**701.2919**	1400.5924	**700.7999**	12
16	1875.7614	938.3843	**1858.7349**	929.8711	1857.7509	929.3791	E	1289.5604	**645.2838**	1272.5339	636.7706	1271.5498	636.2786	11
17	2004.8040	**1002.9056**	1987.7775	994.3924	**1986.7934**	993.9004	E	1160.5178	**580.7625**	1143.4913	572.2493	1142.5072	571.7573	10
18	2087.8411	1044.4242	2070.8146	1035.9109	2069.8306	1035.4189	T	1031.4752	516.2412	**1014.4487**	507.7280	1013.4647	507.2360	9
19	2216.8837	**1108.9455**	2199.8572	1100.4322	2198.8731	1099.9402	E	948.4381	**474.7227**	931.4116	466.2094	930.4275	**465.7174**	8
20	2330.9266	1165.9670	2313.9001	1157.4537	2312.9161	1156.9617	N	**819.3955**	410.2014	**802.3690**	**401.6881**	801.3850	401.1961	7
21	2487.0277	**1244.0175**	2470.0012	1235.5042	2469.0172	1235.0122	R	705.3526	**353.1799**	688.3260	344.6667	**687.3420**	344.1747	6
22	2616.0703	**1308.5388**	2599.0438	1300.0255	2598.0598	1299.5335	E	549.2515	275.1294	532.2249	266.6161	531.2409	266.1241	5
23	2673.0918	1337.0495	2656.0653	1328.5363	2655.0812	1328.0443	G	**420.2089**	210.6081	403.1823	202.0948	**402.1983**	201.6028	4
24	2802.1344	**1401.5708**	2785.1078	**1393.0576**	2784.1238	**1392.5656**	E	363.1874	182.0974	346.1609	173.5841	345.1769	173.0921	3
25	2889.1664	**1445.0869**	2872.1399	1436.5736	2871.1559	1436.0816	S	234.1448	117.5761	217.1183	109.0628	216.1343	108.5708	2
26							K	147.1128	74.0600	130.0863	65.5468			1

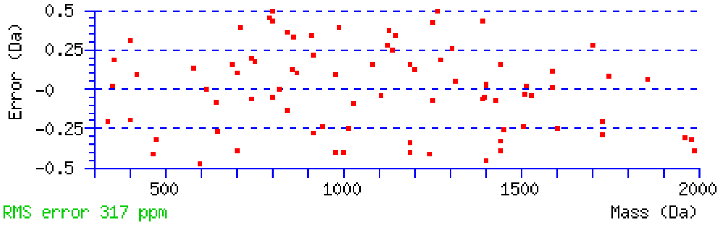

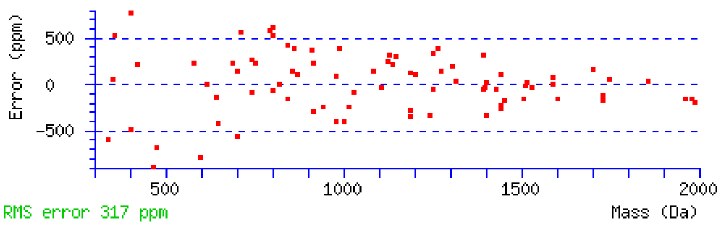

All matches to this query

Score	Mr(calc):	Delta	Sequence
46.5	3132.2415	-0.0024	KVEEEQEADEEDVSEEETENREGESK
42.7	3132.2415	-0.0024	KVEEEQEADEEDVSEEETENREGESK
30.6	3132.2415	-0.0024	KVEEEQEADEEDVSEEETENREGESK
5.0	3131.2089	1.0302	NRNSSPLVASSLESMDMNPECLVSPR
5.0	3131.2089	1.0302	NRNSSPLVASSLESMDMNPECLVSPR
5.0	3131.2089	1.0302	NRNSSPLVASSLESMDMNPECLVSPR
4.9	3131.2515	0.9876	SCKYQSGHHKEAINPADAAGMSADDPSK
4.7	3131.2480	0.9910	QLKRQLAFFSSELSENSTFGSGHEL
4.7	3131.2480	0.9910	QLKRQLAFFSSELSENSTFGSGHEL
4.3	3131.2480	0.9910	QLKRQLAFFSSELSENSTFGSGHEL

Spectrum No: 82; Query: 845; Rank: 1

Peptide View

MS/MS Fragmentation of **KLEKEEEEGISQESSEEEQ**
Found in **IPI00203725**, Tax_Id=10116 Gene_Symbol=Hmga1 Isoform HMG-I of High mobility group protein HMG-I/HMG-Y

Match to Query 845: 2315.954232 from(772.992020,3+)

Title: 091129RatKid_SCX02_28.338.338.3.dta
Data file K:\NewmanPaper\Piliang\3SubProteomes\Piliang3SP\mgf5ppm\SCX_3SubProteomes5ppm.mgf

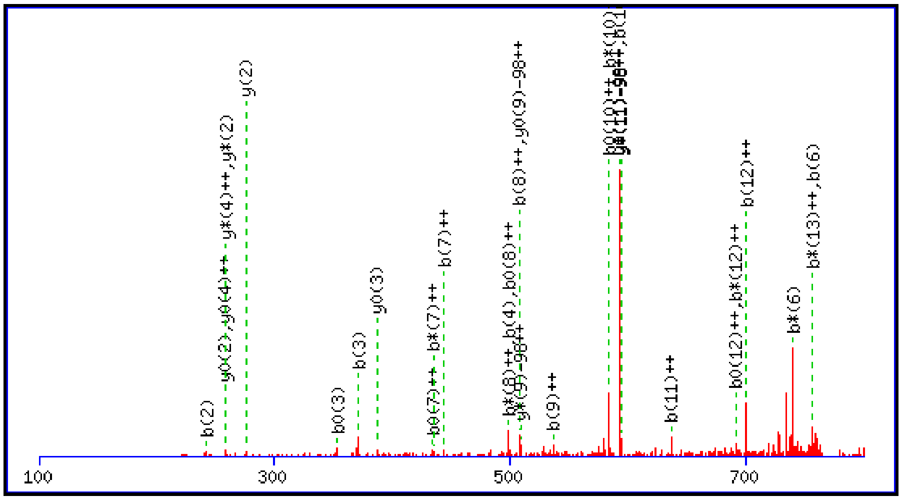

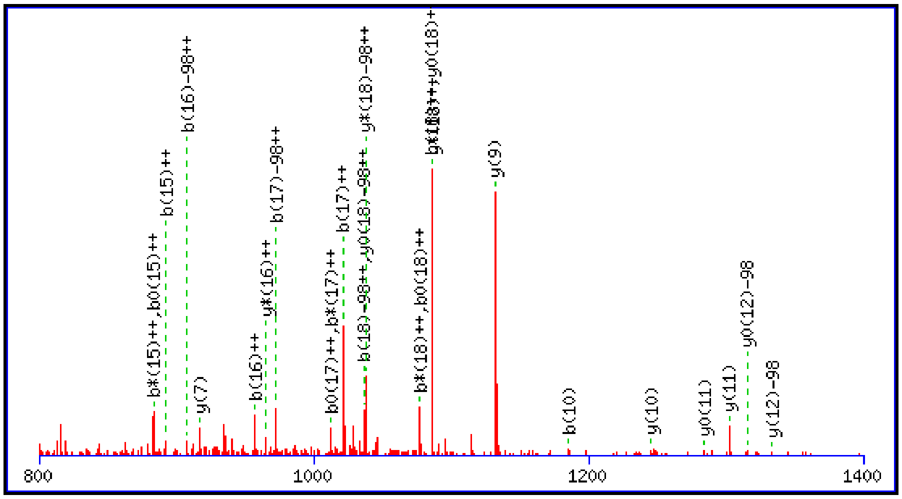

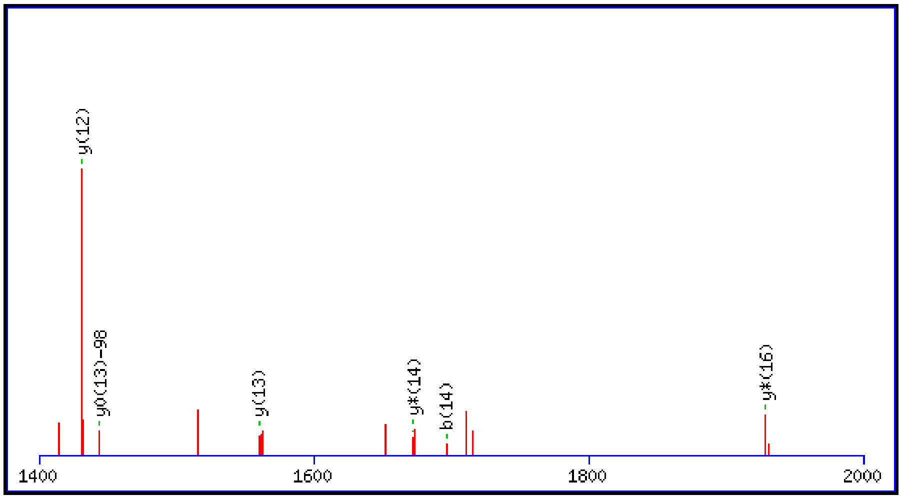

Monoisotopic mass of neutral peptide Mr(calc): 2315.9529
Fixed modifications: Carbamidomethyl (C)
Variable modifications:
S14 : Phospho (ST), with neutral losses 0.0000(shown in table), 97.9769
Ions Score: 46 **Expect:** 0.0062
Matches (Bold Red): 63/318 fragment ions using 93 most intense peaks

#	b	b++	b*	b*++	b0	b0++	Seq.	y	y++	y*	y*++	y0	y0++	#
1	129.1022	65.0548	112.0757	56.5415			K							19
2	**242.1863**	121.5968	225.1598	113.0835			L	2188.8653	1094.9363	2171.8387	**1086.4230**	2170.8547	**1085.9310**	18
3	**371.2289**	186.1181	354.2023	177.6048	**353.2183**	177.1128	E	2075.7812	1038.3943	2058.7547	1029.8810	2057.7707	1029.3890	17

4	**499.3239**	250.1656	482.2973	241.6523	481.3133	241.1603	K	1946.7386	973.8730	**1929.7121**	**965.3597**	1928.7281	964.8677	16
5	628.3665	314.6869	611.3399	306.1736	610.3559	305.6816	E	1818.6437	909.8255	1801.6171	901.3122	1800.6331	900.8202	15
6	**757.4090**	379.2082	**740.3825**	370.6949	739.3985	370.2029	E	1689.6011	845.3042	**1672.5745**	836.7909	1671.5905	836.2989	14
7	886.4516	**443.7295**	869.4251	**435.2162**	868.4411	**434.7242**	E	**1560.5585**	780.7829	1543.5319	772.2696	1542.5479	771.7776	13
8	1015.4942	**508.2508**	998.4677	**499.7375**	997.4837	**499.2455**	E	**1431.5159**	716.2616	1414.4893	707.7483	1413.5053	707.2563	12
9	1072.5157	**536.7615**	1055.4891	528.2482	1054.5051	527.7562	G	**1302.4733**	651.7403	1285.4468	643.2270	**1284.4627**	642.7350	11
10	**1185.5998**	**593.3035**	1168.5732	**584.7902**	1167.5892	**584.2982**	I	**1245.4518**	623.2296	1228.4253	614.7163	1227.4413	614.2243	10
11	1272.6318	**636.8195**	1255.6052	628.3063	1254.6212	627.8142	S	**1132.3678**	566.6875	1115.3412	558.1743	1114.3572	557.6822	9
12	1400.6904	**700.8488**	1383.6638	**692.3355**	1382.6798	**691.8435**	Q	1045.3357	523.1715	1028.3092	514.6582	1027.3252	514.1662	8
13	1529.7330	765.3701	1512.7064	**756.8568**	1511.7224	756.3648	E	**917.2772**	459.1422	900.2506	450.6289	899.2666	450.1369	7
14	**1696.7313**	848.8693	1679.7048	840.3560	1678.7207	839.8640	S	788.2346	394.6209	771.2080	386.1077	770.2240	385.6156	6
15	1783.7633	**892.3853**	1766.7368	**883.8720**	1765.7528	**883.3800**	S	621.2362	311.1218	604.2097	302.6085	603.2257	302.1165	5
16	1912.8059	**956.9066**	1895.7794	948.3933	1894.7954	947.9013	E	534.2042	267.6057	517.1776	**259.0925**	516.1936	**258.6005**	4
17	2041.8485	**1021.4279**	2024.8220	**1012.9146**	2023.8380	**1012.4226**	E	405.1616	203.0844	388.1351	194.5712	**387.1510**	194.0792	3
18	2170.8911	**1085.9492**	2153.8646	**1077.4359**	2152.8806	**1076.9439**	E	**276.1190**	138.5631	**259.0925**	130.0499	**258.1084**	129.5579	2
19							Q	147.0764	74.0418	130.0499	65.5286			1

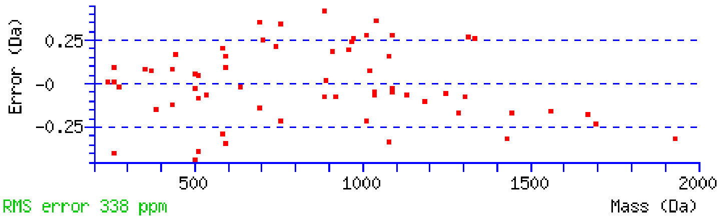

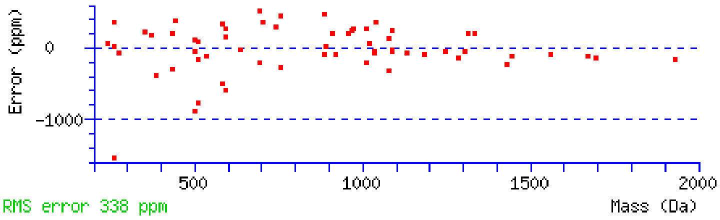

All matches to this query

Score	Mr(calc):	Delta	Sequence
46.5	2315.9529	0.0013	KLEKEEEEGISQESSEEEQ
40.5	2315.9529	0.0013	KLEKEEEEGISQESSEEEQ
37.3	2315.9529	0.0013	KLEKEEEEGISQESSEEEQ
12.1	2315.9388	0.0154	EKYPYHSFIGEESVASGEK
11.5	2315.9388	0.0154	EKYPYHSFIGEESVASGEK
11.5	2315.9388	0.0154	EKYPYHSFIGEESVASGEK
11.5	2315.9388	0.0154	EKYPYHSFIGEESVASGEK
10.9	2315.9388	0.0154	EKYPYHSFIGEESVASGEK
10.8	2315.9388	0.0154	EKYPYHSFIGEESVASGEK
7.6	2314.9694	0.9848	SYCAEIAPNVSFENGKTIR

Spectrum No: 83; Query: 512; Rank: 1

Peptide View

MS/MS Fragmentation of **VIENTDGSEEEMDAR**
Found in **IPI00464464**, Tax_Id=10116 Gene_Symbol=Myh11 Isoform 1 of Myosin-11 (Fragment)

Match to Query 512: 1773.678328 from(887.846440,2+)
Title: 091129RatKid_SCX02_12.623.623.2.dta
Data file K:\NewmanPaper\Piliang\3SubProteomes\Piliang3SP\mgf5ppm\SCX_3SubProteomes5ppm.mgf

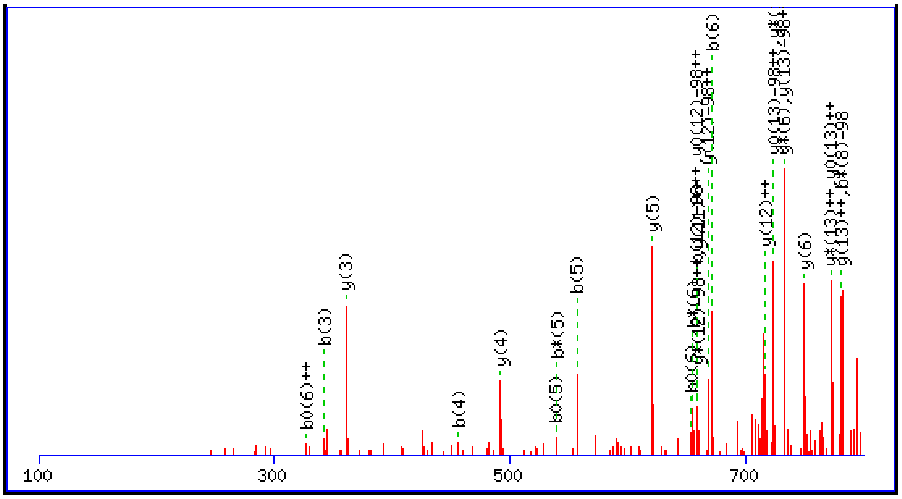

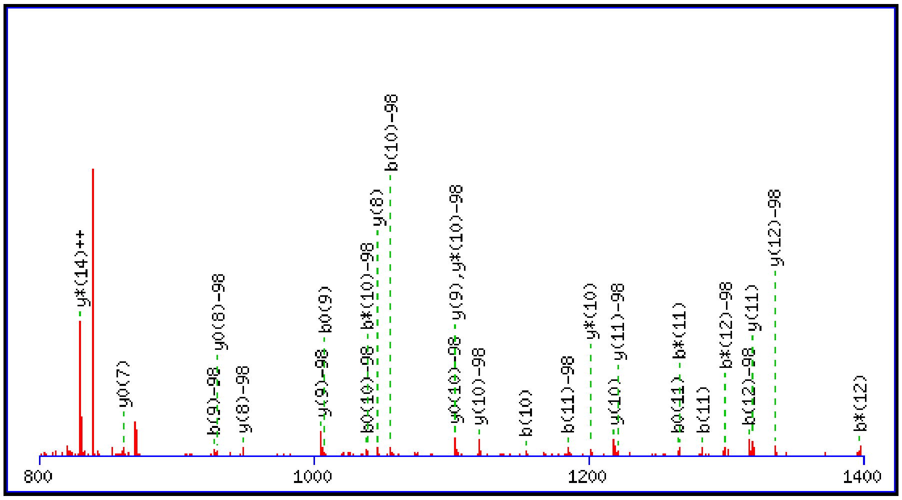

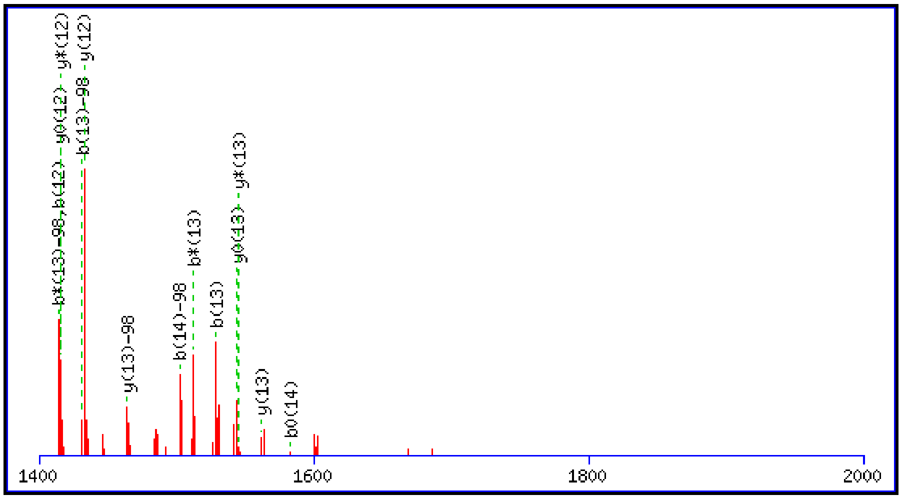

Monoisotopic mass of neutral peptide Mr(calc): 1773.6764
Fixed modifications: Carbamidomethyl (C)
Variable modifications:
S8 : Phospho (ST), with neutral losses 97.9769(shown in table), 0.0000
Ions Score: 46 **Expect:** 0.0029
Matches (Bold Red): 69/238 fragment ions using 109 most intense peaks

#	b	b^{++}	b*	b^{*++}	b^{0}	b^{0++}	Seq.	y	y^{++}	y*	y^{*++}	y^{0}	y^{0++}	#
1	100.0757	50.5415					V							15
2	213.1598	107.0835					I	1577.6384	789.3228	1560.6119	780.8096	1559.6278	780.3176	14
3	**342.2023**	171.6048			324.1918	162.5995	E	**1464.5543**	**732.7808**	1447.5278	**724.2675**	1446.5438	**723.7755**	13
4	**456.2453**	228.6263	439.2187	220.1130	438.2347	219.6210	N	**1335.5117**	**668.2595**	1318.4852	**659.7462**	1317.5012	**659.2542**	12
5	**557.2930**	279.1501	**540.2664**	270.6368	**539.2824**	270.1448	T	**1221.4688**	611.2380	1204.4423	602.7248	1203.4583	602.2328	11

**6**	**672.3199**	336.6636	**655.2933**	328.1503	**654.3093**	**327.6583**	**D**	**1120.4211**	560.7142	**1103.3946**	552.2009	**1102.4106**	551.7089	**10**
**7**	729.3414	365.1743	712.3148	356.6610	711.3308	356.1690	**G**	**1005.3942**	503.2007	988.3676	494.6875	987.3836	494.1955	**9**
**8**	798.3628	399.6850	**781.3363**	391.1718	780.3523	390.6798	**S**	**948.3727**	474.6900	931.3462	466.1767	**930.3622**	465.6847	**8**
**9**	**927.4054**	464.2063	910.3789	455.6931	909.3948	455.2011	**E**	879.3513	440.1793	862.3247	431.6660	**861.3407**	431.1740	**7**
**10**	**1056.4480**	528.7276	**1039.4215**	520.2144	**1038.4374**	519.7224	**E**	**750.3087**	375.6580	**733.2821**	367.1447	732.2981	366.6527	**6**
**11**	**1185.4906**	593.2489	1168.4640	584.7357	1167.4800	584.2437	**E**	**621.2661**	311.1367	604.2395	302.6234	603.2555	302.1314	**5**
**12**	**1316.5311**	**658.7692**	**1299.5045**	650.2559	1298.5205	649.7639	**M**	**492.2235**	246.6154	475.1969	238.1021	474.2129	237.6101	**4**
**13**	**1431.5580**	716.2826	**1414.5315**	707.7694	1413.5475	707.2774	**D**	**361.1830**	181.0951	344.1565	172.5819	343.1724	172.0899	**3**
**14**	**1502.5951**	751.8012	1485.5686	743.2879	1484.5846	742.7959	**A**	246.1561	123.5817	229.1295	115.0684			**2**
**15**							**R**	175.1190	88.0631	158.0924	79.5498			**1**

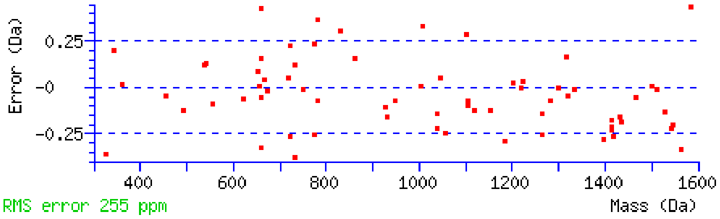

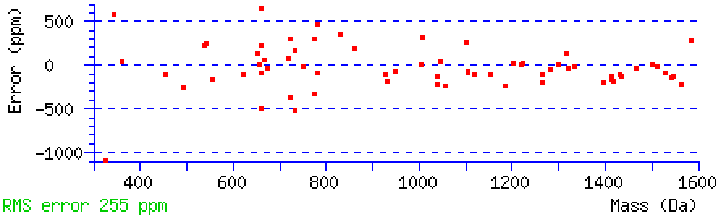

All matches to this query

Score	Mr(calc):	Delta	Sequence
46.4	1773.6764	0.0019	VIENTDGSEEEMDAR
30.9	1773.6764	0.0019	VIENTDGSEEEMDAR
7.5	1772.6672	1.0112	DTEIDDLKTQLSR
1.1	1772.6825	0.9958	NCPTNGDKNFESGPR
0.6	1772.6620	1.0163	CPGLXPSRRGQSR
0.4	1773.6760	0.0023	IENVSYQDRGNYR
0.3	1772.6601	1.0183	DMFLDSNFTDTESR
0.3	1772.6831	0.9952	TCRSGTLSPGVRSR
0.3	1772.6601	1.0183	DMFLDSNFTDTESR
0.3	1773.6739	0.0044	YTDTCRQNTPMYK

Spectrum No: 84; Query: 455; Rank: 1

Peptide View

MS/MS Fragmentation of **DTEDTGSVGAPVSSPGK**
Found in **IPI00364728**, Tax_Id=10116 Gene_Symbol=RGD1304644 Similar to RIKEN cDNA 2310046K01

Match to Query 455: 1682.702348 from(842.358450,2+)
Title: 091129RatKid_SCX02_12.441.441.2.dta
Data file K:\NewmanPaper\Piliang\3SubProteomes\Piliang3SP\mgf5ppm\SCX_3SubProteomes5ppm.mgf

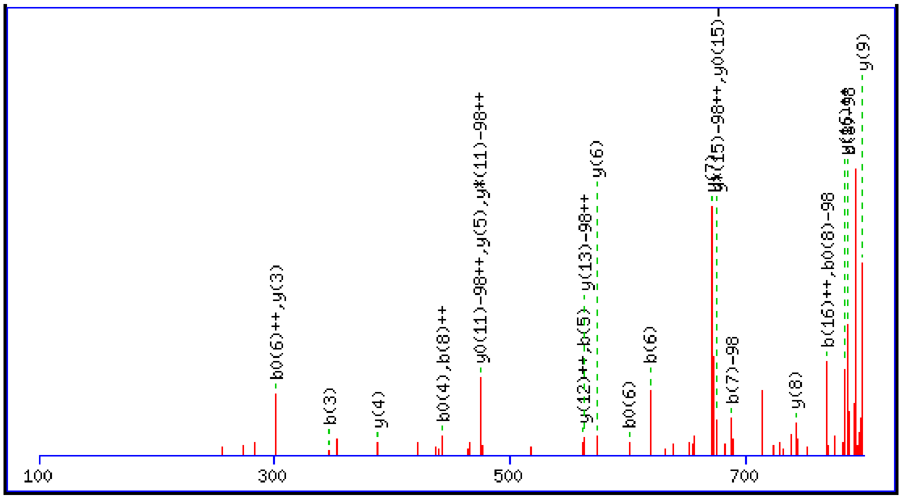

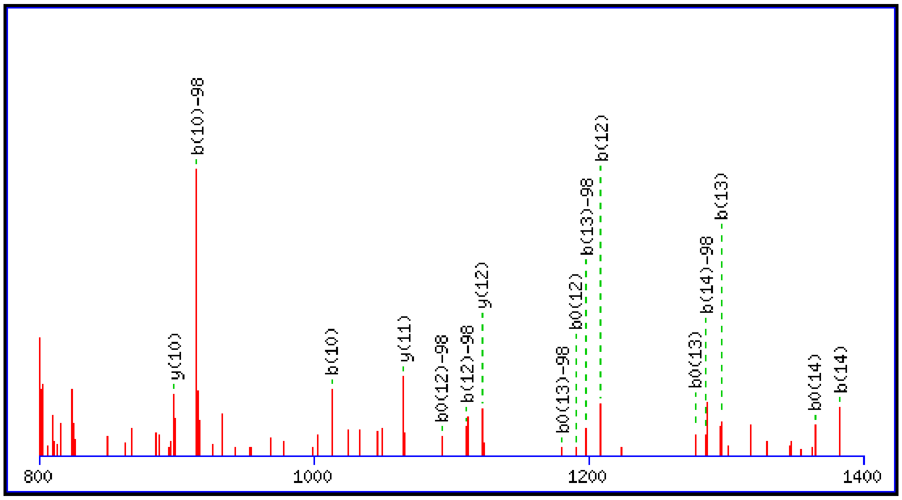

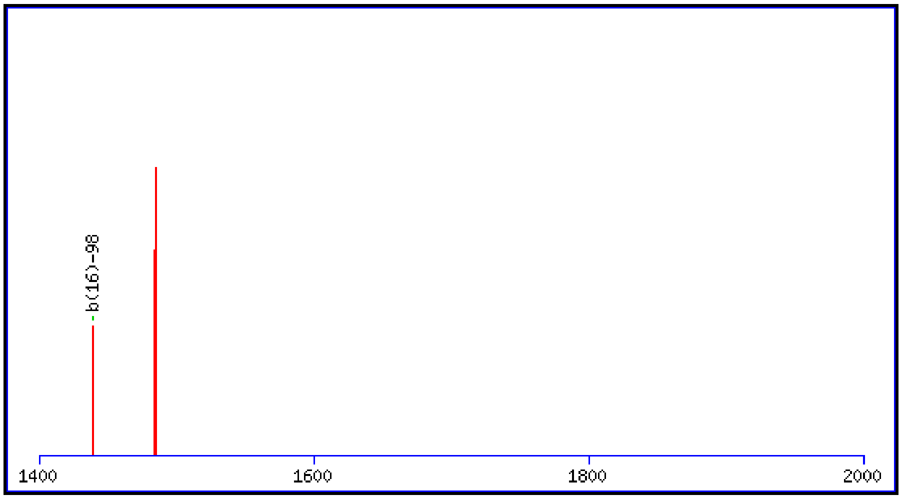

Monoisotopic mass of neutral peptide Mr(calc): 1682.7037
Fixed modifications: Carbamidomethyl (C)
Variable modifications:
S7 : Phospho (ST), with neutral losses 97.9769(shown in table), 0.0000
Ions Score: 46 **Expect:** 0.0045
Matches (Bold Red): 42/230 fragment ions using 78 most intense peaks

#	b	b^{++}	b^{0}	b^{0++}	Seq.	y	y^{++}	y*	y^{*++}	y^{0}	y^{0++}	#
1	116.0342	58.5207	98.0237	49.5155	D							17
2	217.0819	109.0446	199.0713	100.0393	T	1470.7071	735.8572	1453.6805	727.3439	1452.6965	726.8519	16
3	**346.1245**	173.5659	328.1139	164.5606	E	1369.6594	685.3333	1352.6328	**676.8201**	1351.6488	**676.3281**	15
4	461.1514	231.0794	**443.1409**	222.0741	D	1240.6168	620.8120	1223.5903	612.2988	1222.6062	611.8068	14
5	**562.1991**	281.6032	544.1885	272.5979	T	1125.5899	**563.2986**	1108.5633	554.7853	1107.5793	554.2933	13

**6**	**619.2206**	310.1139	**601.2100**	**301.1086**	**G**	1024.5422	512.7747	1007.5156	504.2615	1006.5316	503.7694	**12**
**7**	**688.2420**	344.6247	670.2315	335.6194	**S**	967.5207	484.2640	950.4942	**475.7507**	949.5101	**475.2587**	**11**
**8**	**787.3104**	394.1589	**769.2999**	385.1536	**V**	**898.4993**	449.7533	881.4727	441.2400	880.4887	440.7480	**10**
**9**	844.3319	422.6696	826.3213	413.6643	**G**	**799.4308**	400.2191	782.4043	391.7058	781.4203	391.2138	**9**
**10**	**915.3690**	458.1882	897.3585	449.1829	**A**	**742.4094**	371.7083	725.3828	363.1951	724.3988	362.7030	**8**
**11**	1012.4218	506.7145	994.4112	497.7092	**P**	**671.3723**	336.1898	654.3457	327.6765	653.3617	327.1845	**7**
**12**	**1111.4902**	556.2487	**1093.4796**	547.2435	**V**	**574.3195**	287.6634	557.2930	279.1501	556.3089	278.6581	**6**
**13**	**1198.5222**	599.7648	**1180.5117**	590.7595	**S**	**475.2511**	238.1292	458.2245	229.6159	457.2405	229.1239	**5**
**14**	**1285.5543**	643.2808	1267.5437	634.2755	**S**	**388.2191**	194.6132	371.1925	186.0999	370.2085	185.6079	**4**
**15**	1382.6070	691.8071	1364.5965	682.8019	**P**	**301.1870**	151.0972	284.1605	142.5839			**3**
**16**	**1439.6285**	720.3179	1421.6179	711.3126	**G**	204.1343	102.5708	187.1077	94.0575			**2**
**17**					**K**	147.1128	74.0600	130.0863	65.5468			**1**

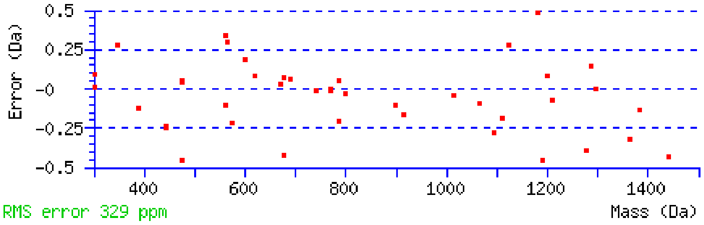

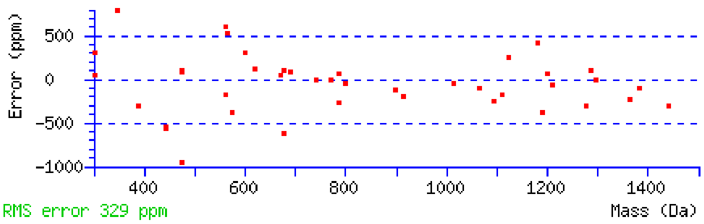

All matches to this query

Score	Mr(calc):	Delta	Sequence
45.9	1682.7037	-0.0013	DTEDTGSVGAPVSSPGK
35.9	1682.7037	-0.0013	DTEDTGSVGAPVSSPGK
33.9	1682.7037	-0.0013	DTEDTGSVGAPVSSPGK
12.9	1682.7037	-0.0013	DTEDTGSVGAPVSSPGK
6.8	1682.7037	-0.0013	DTEDTGSVGAPVSSPGK
6.5	1682.7124	-0.0100	DNSFLQVHNCIQK
4.3	1682.6937	0.0086	GDSHSWGSDLSSLQK
3.5	1682.6937	0.0086	GDSHSWGSDLSSLQK
1.6	1682.7178	-0.0155	TTSQDRQRQLYK
1.2	1682.7123	-0.0100	NMTWRENQPNAIK

Spectrum No: 85; Query: 1129; Rank: 1

Peptide View

MS/MS Fragmentation of **AENDVDNELLDYEDDEVETAAGADGTEAPAKK**
Found in **IPI00215291**, Tax_Id=10116 Gene_Symbol=Bat1a;Atp6v1g2 Spliceosome RNA helicase Bat1

Match to Query 1129: 3473.455456 from(869.371140,4+)
Title: 091127RatKid_SCX01_15.3634.3634.4.dta
Data file K:\NewmanPaper\Piliang\3SubProteomes\Piliang3SP\mgf5ppm\SCX_3SubProteomes5ppm.mgf

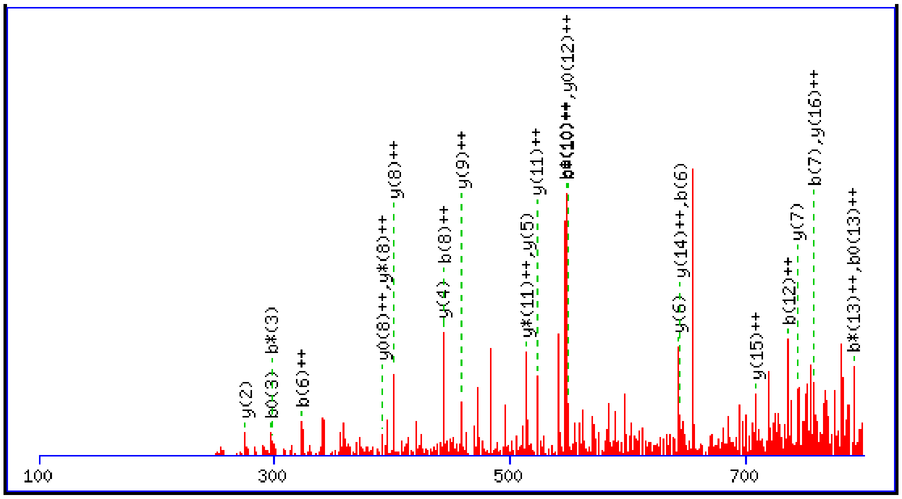

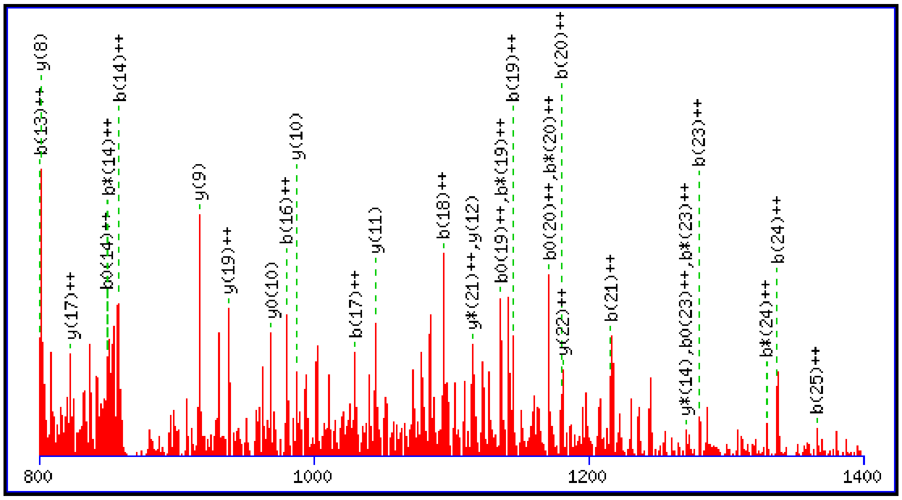

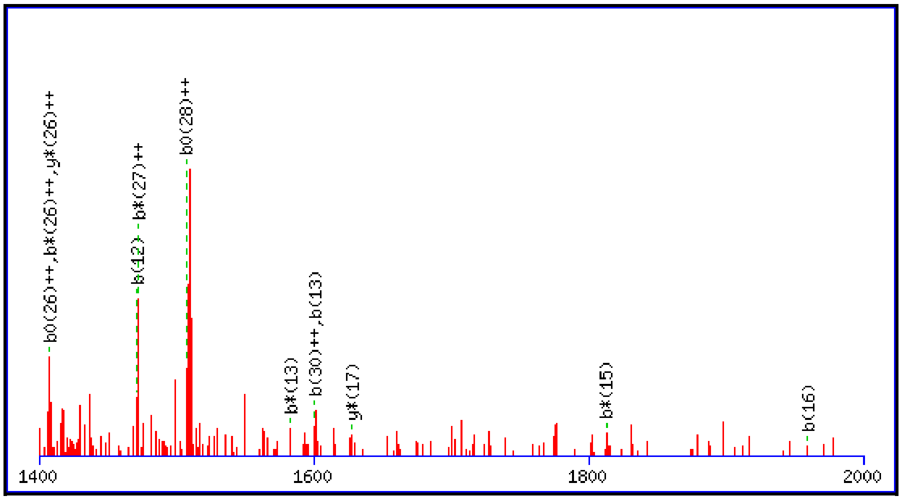

Monoisotopic mass of neutral peptide Mr(calc): 3473.4519
Fixed modifications: Carbamidomethyl (C)
Variable modifications:
Y12 : Phospho (Y)
Ions Score: 45 **Expect:** 0.013
Matches (Bold Red): 69/356 fragment ions using 147 most intense peaks

#	b	b++	b*	b*++	b0	b0++	Seq.	y	y++	y*	y*++	y0	y0++	#
1	72.0444	36.5258					A							32
2	201.0870	101.0471			183.0764	92.0418	E	3403.4221	1702.2147	3386.3955	1693.7014	3385.4115	1693.2094	31
3	315.1299	158.0686	**298.1034**	149.5553	**297.1193**	149.0633	N	3274.3795	1637.6934	3257.3529	1629.1801	3256.3689	1628.6881	30
4	430.1569	215.5821	413.1303	207.0688	412.1463	206.5768	D	3160.3365	1580.6719	3143.3100	1572.1586	3142.3260	1571.6666	29
5	529.2253	265.1163	512.1987	256.6030	511.2147	256.1110	V	3045.3096	1523.1584	3028.2831	1514.6452	3027.2990	1514.1532	28

6	644.2522	322.6297	627.2257	314.1165	626.2416	313.6245	D	2946.2412	1473.6242	2929.2146	1465.1110	2928.2306	1464.6190	27
7	758.2951	379.6512	741.2686	371.1379	740.2846	370.6459	N	2831.2142	1416.1108	2814.1877	1407.5975	2813.2037	1407.1055	26
8	887.3377	444.1725	870.3112	435.6592	869.3272	435.1672	E	2717.1713	1359.0893	2700.1448	1350.5760	2699.1608	1350.0840	25
9	1000.4218	500.7145	983.3952	492.2013	982.4112	491.7093	L	2588.1287	1294.5680	2571.1022	1286.0547	2570.1182	1285.5627	24
10	1113.5059	557.2566	1096.4793	548.7433	1095.4953	548.2513	L	2475.0447	1238.0260	2458.0181	1229.5127	2457.0341	1229.0207	23
11	1228.5328	614.7700	1211.5063	606.2568	1210.5222	605.7648	D	2361.9606	1181.4839	2344.9340	1172.9707	2343.9500	1172.4787	22
12	1471.5625	736.2849	1454.5359	727.7716	1453.5519	727.2796	Y	2246.9337	1123.9705	2229.9071	1115.4572	2228.9231	1114.9652	21
13	1600.6050	800.8062	1583.5785	792.2929	1582.5945	791.8009	E	2003.9040	1002.4556	1986.8775	993.9424	1985.8934	993.4504	20
14	1715.6320	858.3196	1698.6054	849.8064	1697.6214	849.3144	D	1874.8614	937.9343	1857.8349	929.4211	1856.8508	928.9291	19
15	1830.6589	915.8331	1813.6324	907.3198	1812.6484	906.8278	D	1759.8345	880.4209	1742.8079	871.9076	1741.8239	871.4156	18
16	1959.7015	980.3544	1942.6750	971.8411	1941.6910	971.3491	E	1644.8075	822.9074	1627.7810	814.3941	1626.7970	813.9021	17
17	2058.7699	1029.8886	2041.7434	1021.3753	2040.7594	1020.8833	V	1515.7649	758.3861	1498.7384	749.8728	1497.7544	749.3808	16
18	2187.8125	1094.4099	2170.7860	1085.8966	2169.8020	1085.4046	E	1416.6965	708.8519	1399.6700	700.3386	1398.6860	699.8466	15
19	2288.8602	1144.9337	2271.8337	1136.4205	2270.8496	1135.9285	T	1287.6539	644.3306	1270.6274	635.8173	1269.6434	635.3253	14
20	2359.8973	1180.4523	2342.8708	1171.9390	2341.8868	1171.4470	A	1186.6062	593.8068	1169.5797	585.2935	1168.5957	584.8015	13
21	2430.9344	1215.9709	2413.9079	1207.4576	2412.9239	1206.9656	A	1115.5691	558.2882	1098.5426	549.7749	1097.5586	549.2829	12
22	2487.9559	1244.4816	2470.9294	1235.9683	2469.9453	1235.4763	G	1044.5320	522.7696	1027.5055	514.2564	1026.5215	513.7644	11
23	2558.9930	1280.0001	2541.9665	1271.4869	2540.9825	1270.9949	A	987.5106	494.2589	970.4840	485.7456	969.5000	485.2536	10
24	2674.0200	1337.5136	2656.9934	1329.0003	2656.0094	1328.5083	D	916.4734	458.7404	899.4469	450.2271	898.4629	449.7351	9
25	2731.0414	1366.0244	2714.0149	1357.5111	2713.0309	1357.0191	G	801.4465	401.2269	784.4199	392.7136	783.4359	392.2216	8
26	2832.0891	1416.5482	2815.0626	1408.0349	2814.0785	1407.5429	T	744.4250	372.7162	727.3985	364.2029	726.4145	363.7109	7
27	2961.1317	1481.0695	2944.1051	1472.5562	2943.1211	1472.0642	E	643.3774	322.1923	626.3508	313.6790	625.3668	313.1870	6
28	3032.1688	1516.5880	3015.1423	1508.0748	3014.1582	1507.5828	A	514.3348	257.6710	497.3082	249.1577			5
29	3129.2216	1565.1144	3112.1950	1556.6012	3111.2110	1556.1091	P	443.2976	222.1525	426.2711	213.6392			4
30	3200.2587	1600.6330	3183.2321	1592.1197	3182.2481	1591.6277	A	346.2449	173.6261	329.2183	165.1128			3
31	3328.3537	1664.6805	3311.3271	1656.1672	3310.3431	1655.6752	K	275.2078	138.1075	258.1812	129.5942			2
32							K	147.1128	74.0600	130.0863	65.5468			1

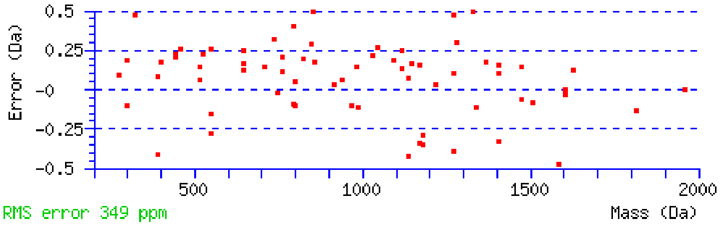

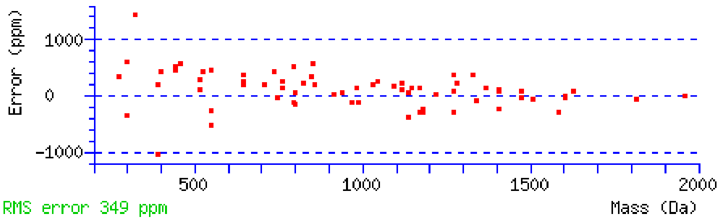

All matches to this query

Score	Mr(calc):	Delta	Sequence
45.3	3473.4519	0.0036	AENDVDNELLDYEDDEVETAAGADGTEAPAKK
9.8	3473.4519	0.0036	AENDVDNELLDYEDDEVETAAGADGTEAPAKK
6.3	3471.4496	2.0059	EDAGWYTVSAKNEAGIVSCTARLDVYISR
1.7	3473.4421	0.0133	IHDSLNNTLSVGLMPTNSTNTIMDQKNLK
0.7	3471.4790	1.9765	VAGYQSNTASDVLETITNIQPKESGGGMGETR
0.5	3473.4595	-0.0041	TKVENGEGTIPVESSDIVPTWDGIRLGER
0.3	3472.4471	1.0083	GEMMYGNETELKMSSFSDMAYPSKSTVVPK

Spectrum No: 86; Query: 488; Rank: 1

Peptide View

MS/MS Fragmentation of **AELGMNDSPSQSPPVK**
Found in **IPI00869599**, Tax_Id=10116 Gene_Symbol=Acss2_predicted acyl-CoA synthetase short-chain family member 2

Match to Query 488: 1735.752428 from(868.883490,2+)
Title: 091127RatKid_SCX01_12.1632.1632.2.dta
Data file K:\NewmanPaper\Piliang\3SubProteomes\Piliang3SP\mgf5ppm\SCX_3SubProteomes5ppm.mgf

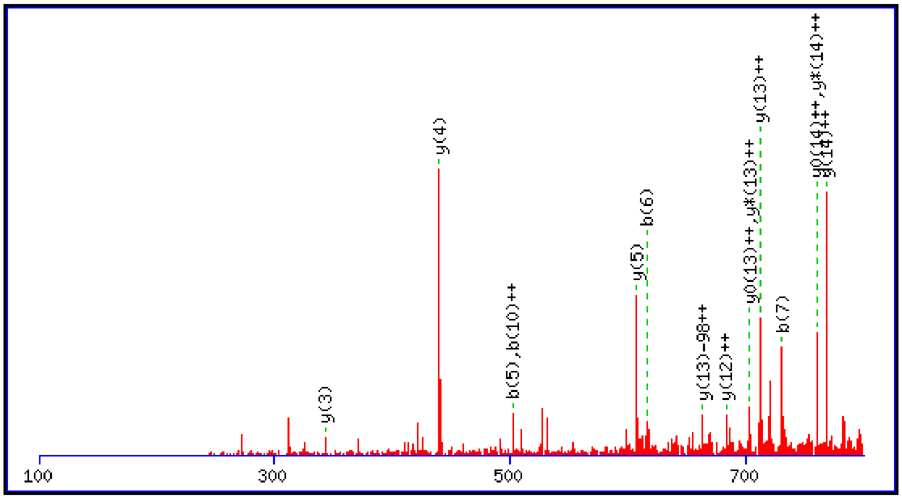

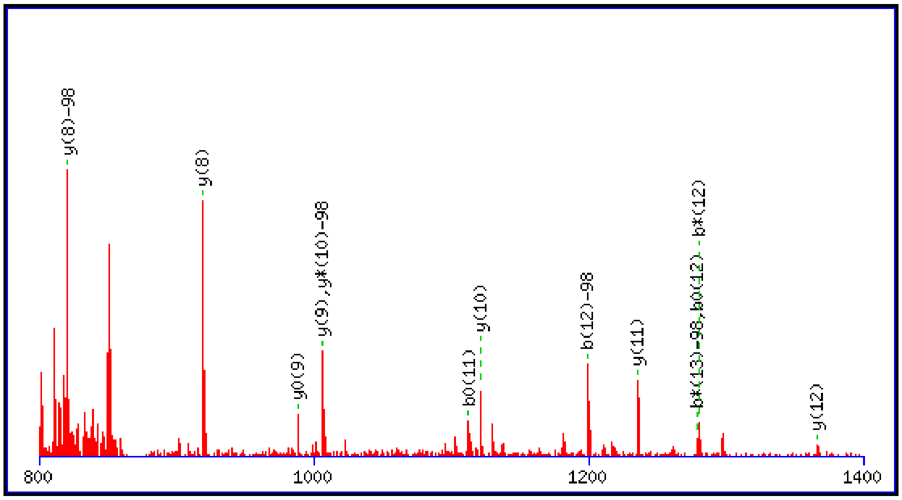

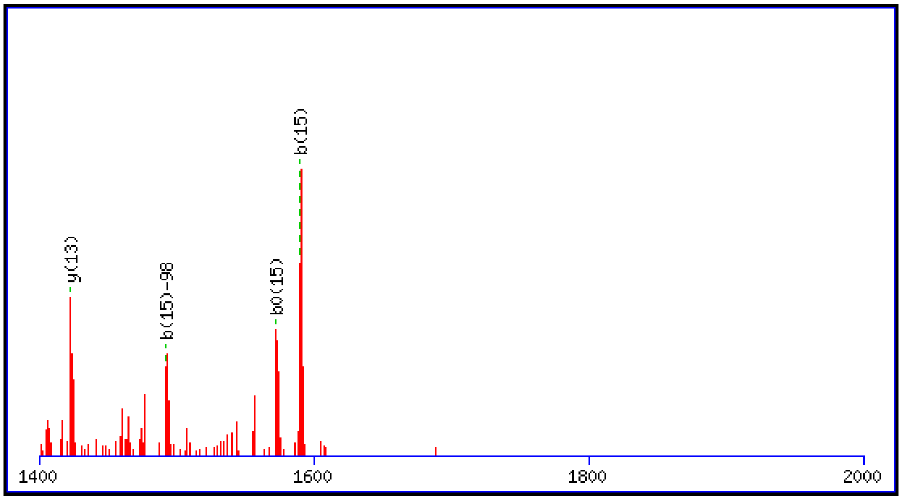

Monoisotopic mass of neutral peptide Mr(calc): 1735.7488
Fixed modifications: Carbamidomethyl (C)
Variable modifications:
S12 : Phospho (ST), with neutral losses 0.0000(shown in table), 97.9769
Ions Score: 45 **Expect:** 0.0057
Matches (Bold Red): 32/250 fragment ions using 55 most intense peaks

#	b	b^{++}	b*	b^{*++}	b^{0}	b^{0++}	Seq.	y	y^{++}	y*	y^{*++}	y^{0}	y^{0++}	#

1	72.0444	36.5258					A							16
2	201.0870	101.0471			183.0764	92.0418	E	1665.7190	833.3631	1648.6924	824.8498	1647.7084	824.3578	15
3	314.1710	157.5892			296.1605	148.5839	L	1536.6764	**768.8418**	1519.6498	**760.3286**	1518.6658	**759.8365**	14
4	371.1925	186.0999			353.1819	177.0946	G	**1423.5923**	**712.2998**	1406.5658	**703.7865**	1405.5818	**703.2945**	13
5	**502.2330**	251.6201			484.2224	242.6149	M	**1366.5709**	**683.7891**	1349.5443	675.2758	1348.5603	674.7838	12
6	**616.2759**	308.6416	599.2494	300.1283	598.2654	299.6363	N	**1235.5304**	618.2688	1218.5038	609.7555	1217.5198	609.2635	11
7	**731.3029**	366.1551	714.2763	357.6418	713.2923	357.1498	D	**1121.4874**	561.2474	1104.4609	552.7341	1103.4769	552.2421	10
8	818.3349	409.6711	801.3083	401.1578	800.3243	400.6658	S	**1006.4605**	503.7339	989.4339	495.2206	**988.4499**	494.7286	9
9	915.3877	458.1975	898.3611	449.6842	897.3771	449.1922	P	**919.4285**	460.2179	902.4019	451.7046	901.4179	451.2126	8
10	1002.4197	**501.7135**	985.3931	493.2002	984.4091	492.7082	S	822.3757	411.6915	805.3492	403.1782	804.3651	402.6862	7
11	1130.4783	565.7428	1113.4517	557.2295	**1112.4677**	556.7375	Q	735.3437	368.1755	718.3171	359.6622	717.3331	359.1702	6
12	1297.4766	649.2419	**1280.4501**	640.7287	**1279.4661**	640.2367	S	**607.2851**	304.1462	590.2585	295.6329	589.2745	295.1409	5
13	1394.5294	697.7683	1377.5028	689.2551	1376.5188	688.7630	P	**440.2867**	220.6470	423.2602	212.1337			4
14	1491.5821	746.2947	1474.5556	737.7814	1473.5716	737.2894	P	**343.2340**	172.1206	326.2074	163.6074			3
15	**1590.6506**	795.8289	1573.6240	787.3156	**1572.6400**	786.8236	V	246.1812	123.5942	229.1547	115.0810			2
16							K	147.1128	74.0600	130.0863	65.5468			1

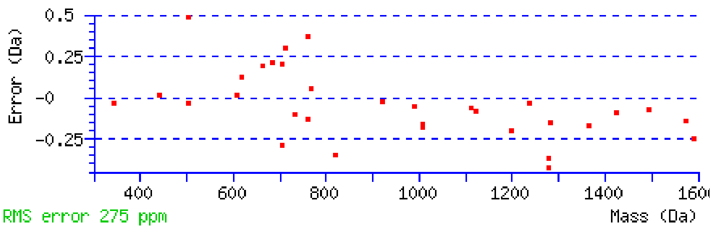

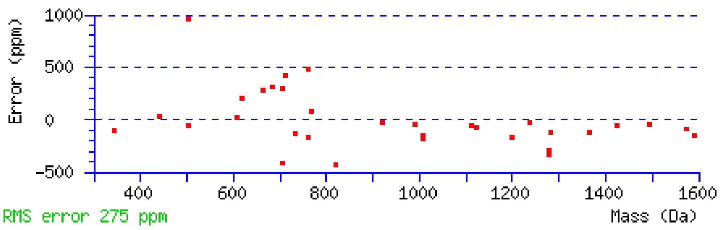

All matches to this query

Score	Mr(calc):	Delta	Sequence
45.2	1735.7488	0.0036	AELGMNDSPSQSPPVK
43.5	1735.7488	0.0036	AELGMNDSPSQSPPVK
36.0	1735.7488	0.0036	AELGMNDSPSQSPPVK
3.5	1735.7639	-0.0114	RNSIPQXSGSSSHRK
3.5	1735.7638	-0.0114	RNSIPQXSGSSSHRK
3.4	1735.7639	-0.0114	RNSIPQXSGSSSHRK
3.4	1735.7638	-0.0114	RNSIPQXSGSSSHRK
3.3	1735.7657	-0.0133	MSFISPQPGSAITLK
3.3	1735.7657	-0.0133	MSFISPQPGSAITLK
3.0	1735.7639	-0.0114	RNSIPQXSGSSSHRK

Spectrum No: 87; Query: 227; Rank: 1

Peptide View

MS/MS Fragmentation of **TASGSSVTSLEGTR**
Found in **IPI00421389**, Tax_Id=10116 Gene_Symbol=Ndrg1 Protein NDRG1

Match to Query 227: 1431.625508 from(716.820030,2+)
Title: 091129RatKid_SCX02_13.877.877.2.dta
Data file K:\NewmanPaper\Piliang\3SubProteomes\Piliang3SP\mgf5ppm\SCX_3SubProteomes5ppm.mgf

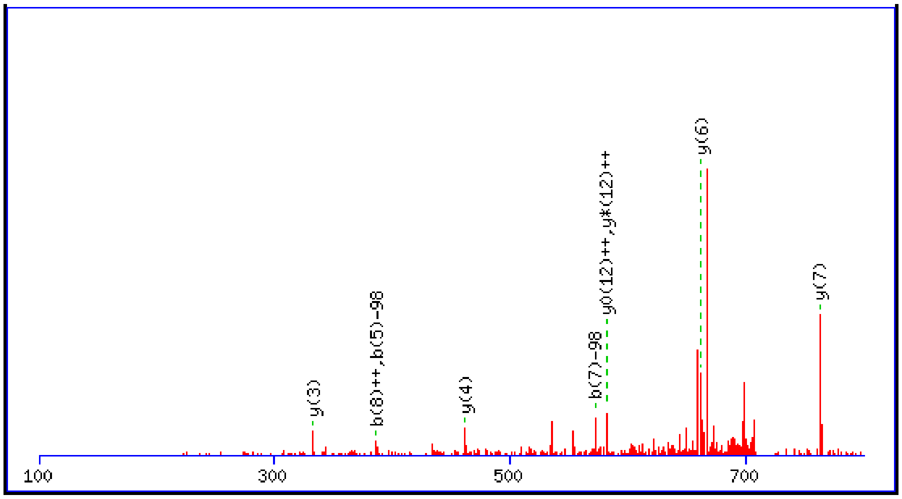

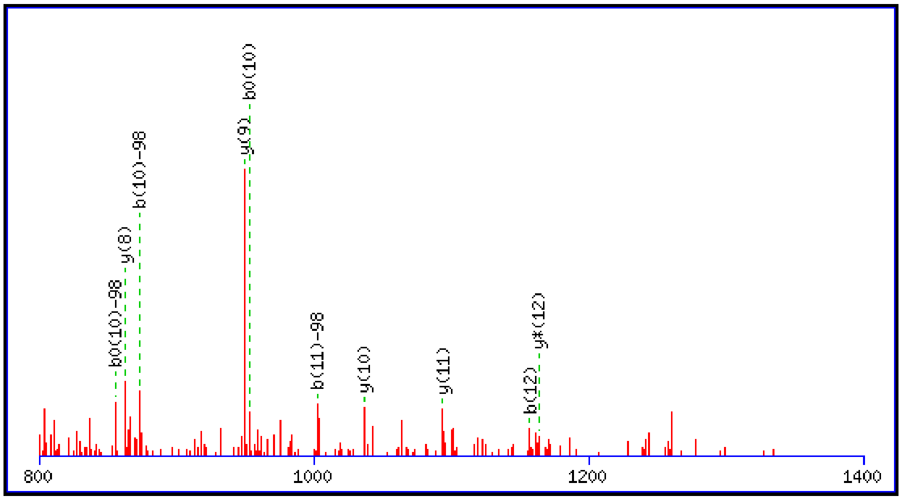

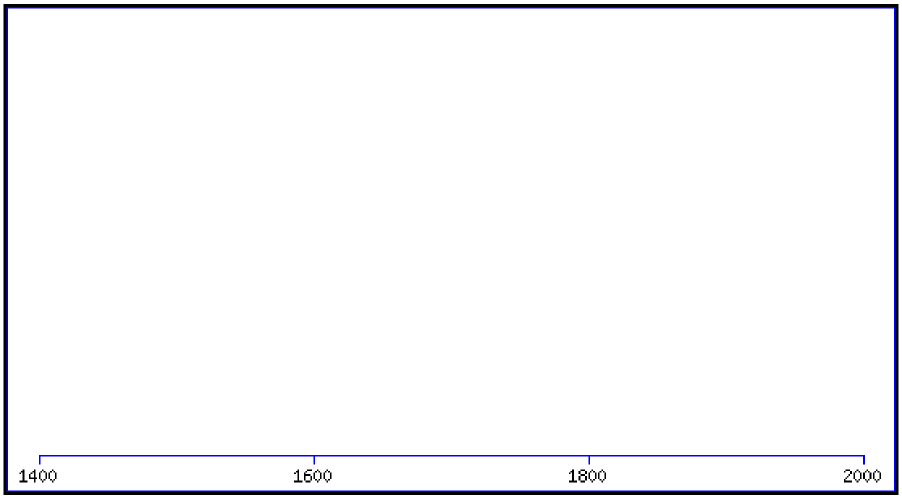

Monoisotopic mass of neutral peptide Mr(calc): 1431.6243
Fixed modifications: Carbamidomethyl (C)
Variable modifications:
T1 : Phospho (ST), with neutral losses 97.9769(shown in table), 0.0000
Ions Score: 45 **Expect:** 0.0056
Matches (Bold Red): 19/180 fragment ions using 38 most intense peaks

#	b	b^{++}	b^{0}	b^{0++}	Seq.	y	y^{++}	y^{*}	y^{*++}	y^{0}	y^{0++}	#
1	84.0444	42.5258	66.0338	33.5205	T							14
2	155.0815	78.0444	137.0709	69.0391	A	1251.6175	626.3124	1234.5910	617.7991	1233.6070	617.3071	13
3	242.1135	121.5604	224.1030	112.5551	S	1180.5804	590.7938	**1163.5539**	**582.2806**	1162.5699	**581.7886**	12
4	299.1350	150.0711	281.1244	141.0658	G	**1093.5484**	547.2778	1076.5218	538.7646	1075.5378	538.2726	11
5	**386.1670**	193.5871	368.1565	184.5819	S	**1036.5269**	518.7671	1019.5004	510.2538	1018.5164	509.7618	10

**6**	473.1990	237.1032	455.1885	228.0979	**S**	**949.4949**	475.2511	932.4684	466.7378	931.4843	466.2458	**9**
**7**	**572.2675**	286.6374	554.2569	277.6321	**V**	**862.4629**	431.7351	845.4363	423.2218	844.4523	422.7298	**8**
**8**	673.3151	337.1612	655.3046	328.1559	**T**	**763.3945**	382.2009	746.3679	373.6876	745.3839	373.1956	**7**
**9**	760.3472	380.6772	742.3366	371.6719	**S**	**662.3468**	331.6770	645.3202	323.1638	644.3362	322.6717	**6**
**10**	**873.4312**	437.2193	**855.4207**	428.2140	**L**	575.3148	288.1610	558.2882	279.6477	557.3042	279.1557	**5**
**11**	**1002.4738**	501.7405	984.4633	492.7353	**E**	**462.2307**	231.6190	445.2041	223.1057	444.2201	222.6137	**4**
**12**	1059.4953	530.2513	1041.4847	521.2460	**G**	**333.1881**	167.0977	316.1615	158.5844	315.1775	158.0924	**3**
**13**	1160.5430	580.7751	1142.5324	571.7698	**T**	276.1666	138.5870	259.1401	130.0737	258.1561	129.5817	**2**
**14**					**R**	175.1190	88.0631	158.0924	79.5498			**1**

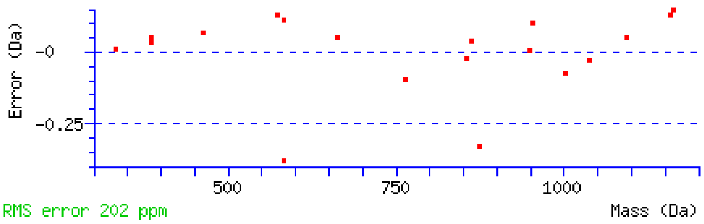

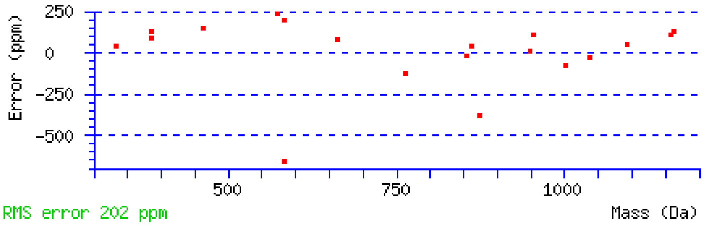

All matches to this query

Score	Mr(calc):	Delta	Sequence
45.0	1431.6243	0.0012	TASGSSVTSLEGTR
44.6	1431.6243	0.0012	TASGSSVTSLEGTR
31.2	1431.6243	0.0012	TASGSSVTSLEGTR
18.7	1431.6243	0.0012	TASGSSVTSLEGTR
7.7	1429.6312	1.9943	NKSAWMGLSDLK
6.3	1431.6330	-0.0075	RSCFPASLTASR
0.9	1431.6243	0.0012	TASGSSVTSLEGTR
0.9	1431.6243	0.0012	TASGSSVTSLEGTR
0.8	1431.6282	-0.0027	QNYNKLADSVAK

Spectrum No: 88; Query: 927; Rank: 1

Peptide View

MS/MS Fragmentation of **VEEEQEADEEDVSEEETENR**
Found in **IPI00365626**, Tax_Id=10116 Gene_Symbol=Txndc1 Thioredoxin domain containing 1

Match to Query 927: 2473.915782 from(825.645870,3+)
Title: 091127RatKid_SCX01_11.739.739.3.dta
Data file K:\NewmanPaper\Piliang\3SubProteomes\Piliang3SP\mgf5ppm\SCX_3SubProteomes5ppm.mgf

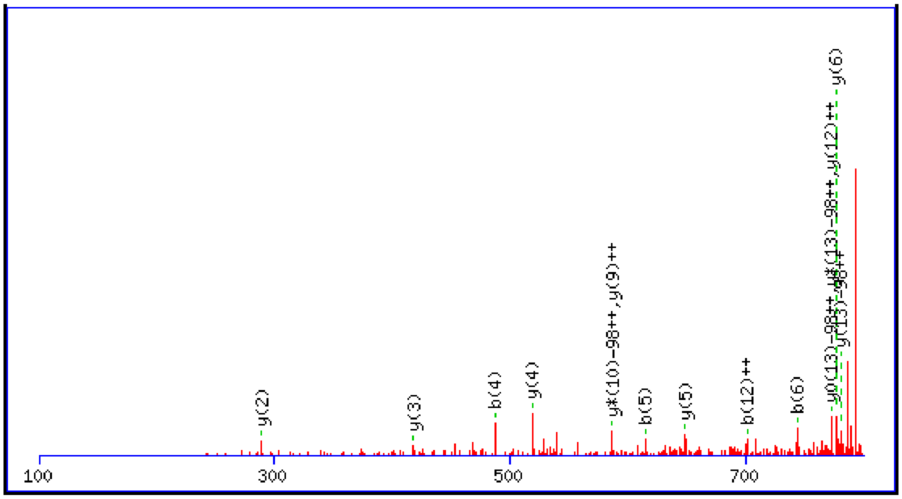

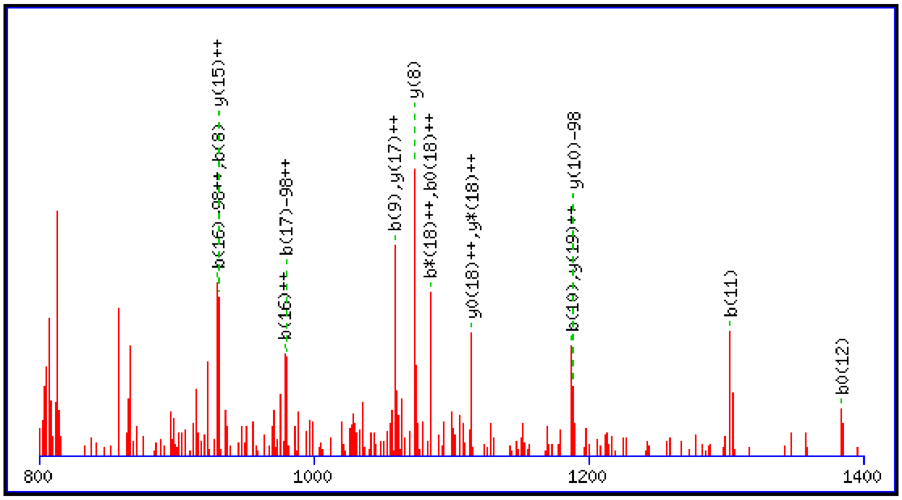

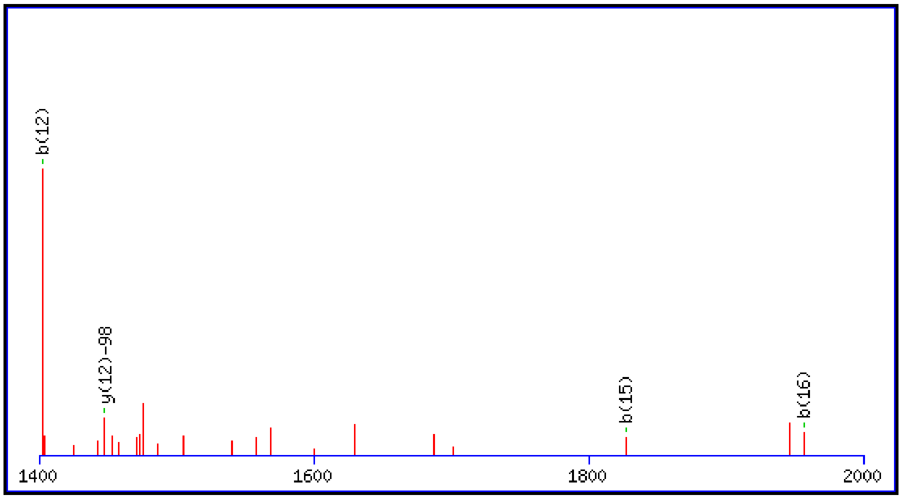

Monoisotopic mass of neutral peptide Mr(calc): 2473.9129
Fixed modifications: Carbamidomethyl (C)
Variable modifications:
S13 : Phospho (ST), with neutral losses 97.9769(shown in table), 0.0000
Ions Score: 45 **Expect:** 0.0048
Matches (Bold Red): 36/328 fragment ions using 46 most intense peaks

#	b	b^{++}	b*	b^{*++}	b^{0}	b^{0++}	Seq.	y	y^{++}	y*	y^{*++}	y^{0}	y^{0++}	#
1	100.0757	50.5415					V							20
2	229.1183	115.0628			211.1077	106.0575	E	2277.8749	1139.4411	2260.8484	1130.9278	2259.8644	1130.4358	19
3	358.1609	179.5841			340.1503	170.5788	E	2148.8323	1074.9198	2131.8058	1066.4065	2130.8218	1065.9145	18
4	**487.2035**	244.1054			469.1929	235.1001	E	2019.7898	1010.3985	2002.7632	1001.8852	2001.7792	1001.3932	17
5	**615.2620**	308.1347	598.2355	299.6214	597.2515	299.1294	Q	1890.7472	945.8772	1873.7206	937.3639	1872.7366	936.8719	16

6	**744.3046**	372.6560	727.2781	364.1427	726.2941	363.6507	E	1762.6886	881.8479	1745.6620	873.3347	1744.6780	872.8426	15
7	815.3418	408.1745	798.3152	399.6612	797.3312	399.1692	A	1633.6460	817.3266	1616.6194	808.8134	1615.6354	808.3213	14
8	**930.3687**	465.6880	913.3421	457.1747	912.3581	456.6827	D	1562.6089	**781.8081**	1545.5823	**773.2948**	1544.5983	**772.8028**	13
9	**1059.4113**	530.2093	1042.3847	521.6960	1041.4007	521.2040	E	**1447.5819**	724.2946	1430.5554	715.7813	1429.5714	715.2893	12
10	**1188.4539**	594.7306	1171.4273	586.2173	1170.4433	585.7253	E	1318.5393	659.7733	1301.5128	651.2600	1300.5288	650.7680	11
11	**1303.4808**	652.2441	1286.4543	643.7308	1285.4703	643.2388	D	**1189.4967**	595.2520	1172.4702	**586.7387**	1171.4862	586.2467	10
12	**1402.5492**	**701.7783**	1385.5227	693.2650	**1384.5387**	692.7730	V	1074.4698	537.7385	1057.4433	529.2253	1056.4592	528.7333	9
13	1471.5707	736.2890	1454.5441	727.7757	1453.5601	727.2837	S	975.4014	488.2043	958.3748	479.6911	957.3908	479.1990	8
14	1600.6133	800.8103	1583.5867	792.2970	1582.6027	791.8050	E	906.3799	453.6936	889.3534	445.1803	888.3694	444.6883	7
15	1729.6559	865.3316	1712.6293	856.8183	1711.6453	856.3263	E	**777.3373**	389.1723	760.3108	380.6590	759.3268	380.1670	6
16	1858.6985	**929.8529**	1841.6719	921.3396	1840.6879	920.8476	E	**648.2947**	324.6510	631.2682	316.1377	630.2842	315.6457	5
17	1959.7462	**980.3767**	1942.7196	971.8634	1941.7356	971.3714	T	**519.2522**	260.1297	502.2256	251.6164	501.2416	251.1244	4
18	2088.7887	1044.8980	2071.7622	1036.3847	2070.7782	1035.8927	E	**418.2045**	209.6059	401.1779	201.0926	400.1939	200.6006	3
19	2202.8317	1101.9195	2185.8051	1093.4062	2184.8211	1092.9142	N	**289.1619**	145.0846	272.1353	136.5713			2
20							R	175.1190	88.0631	158.0924	79.5498			1

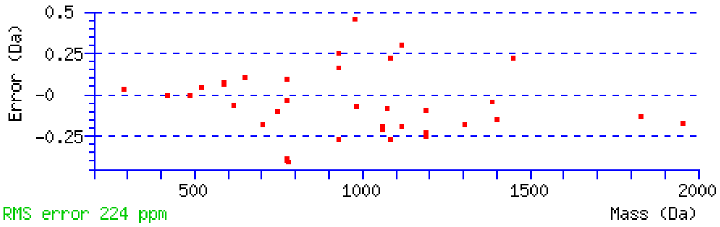

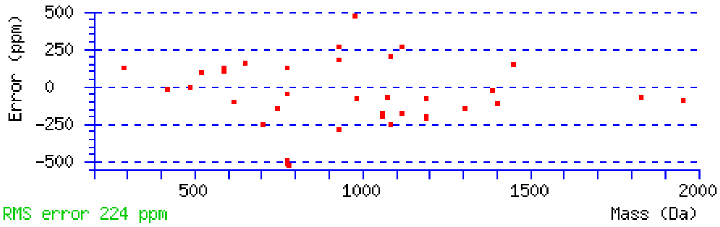

All matches to this query

Score	Mr(calc):	Delta	Sequence
45.0	2473.9129	0.0029	VEEEQEADEEDVSEEETENR
25.3	2473.9129	0.0029	VEEEQEADEEDVSEEETENR
2.5	2471.9043	2.0115	SSENYEDLSSSDESWPVPQR
1.3	2471.9149	2.0009	ENSVCSDTSESSAADVDIDRR
1.3	2471.9149	2.0009	ENSVCSDTSESSAADVDIDRR
0.3	2473.9345	-0.0187	SLTDLPAAVGDSESVTLPTGPQ
0.3	2473.9345	-0.0187	SLTDLPAAVGDSESVTLPTGPQ
0.1	2473.9335	-0.0177	LTRENTQAENKNLCSQGNR

Spectrum No: 89; Query: 347; Rank: 1

Peptide View

MS/MS Fragmentation of **GSGTASDDEFENLR**
Found in **IPI00201213**, Tax_Id=10116 Gene_Symbol=LOC501546 LOC501546 protein

Match to Query 347: 1576.609128 from(789.311840,2+)
Title: 091129RatKid_SCX02_12.1514.1514.2.dta
Data file K:\NewmanPaper\Piliang\3SubProteomes\Piliang3SP\mgf5ppm\SCX_3SubProteomes5ppm.mgf

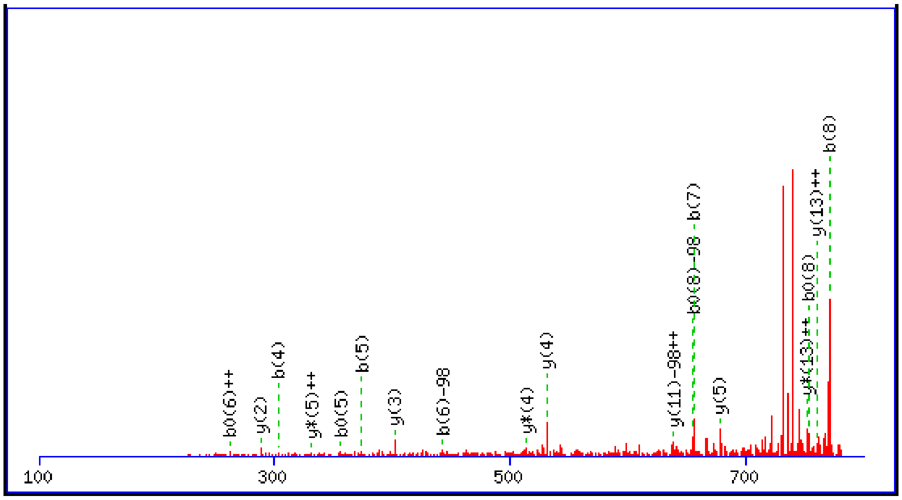

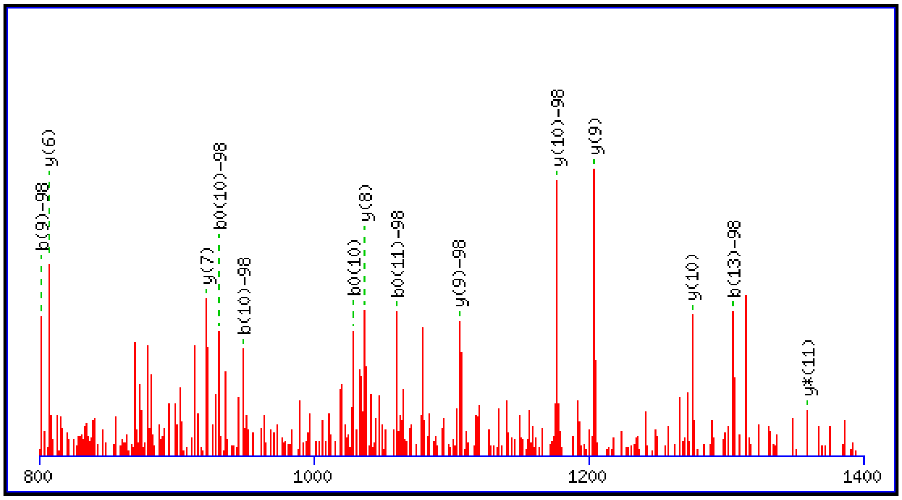

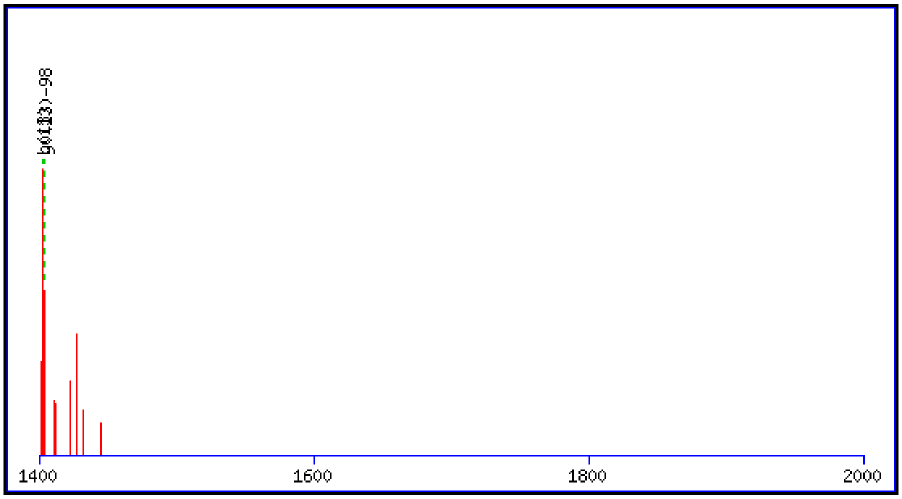

Monoisotopic mass of neutral peptide Mr(calc): 1576.6042
Fixed modifications: Carbamidomethyl (C)
Variable modifications:
S6 : Phospho (ST), with neutral losses 97.9769(shown in table), 0.0000
Ions Score: 45 **Expect:** 0.0033
Matches (Bold Red): 34/192 fragment ions using 68 most intense peaks

#	b	b^{++}	b*	b^{*++}	b^0	b^{0++}	Seq.	y	y^{++}	y*	y^{*++}	y^0	y^{0++}	#
1	58.0287	29.5180					G							14
2	145.0608	73.0340			127.0502	64.0287	S	1422.6132	711.8102	1405.5866	703.2969	**1404.6026**	702.8049	13
3	202.0822	101.5448			184.0717	92.5395	G	1335.5811	668.2942	1318.5546	659.7809	1317.5706	659.2889	12
4	**303.1299**	152.0686			285.1193	143.0633	T	1278.5597	**639.7835**	1261.5331	631.2702	1260.5491	630.7782	11
5	**374.1670**	187.5872			**356.1565**	178.5819	A	**1177.5120**	589.2596	1160.4854	580.7464	1159.5014	580.2544	10

**6**	**443.1885**	222.0979			425.1779	213.0926	**S**	**1106.4749**	553.7411	1089.4483	545.2278	1088.4643	544.7358	**9**
**7**	558.2154	279.6114			540.2049	270.6061	**D**	**1037.4534**	519.2304	1020.4269	510.7171	1019.4429	510.2251	**8**
**8**	673.2424	337.1248			**655.2318**	328.1195	**D**	**922.4265**	461.7169	905.3999	453.2036	904.4159	452.7116	**7**
**9**	**802.2850**	401.6461			784.2744	392.6408	**E**	**807.3995**	404.2034	790.3730	395.6901	789.3890	395.1981	**6**
**10**	**949.3534**	475.1803			**931.3428**	466.1750	**F**	**678.3569**	339.6821	661.3304	**331.1688**	660.3464	330.6768	**5**
**11**	1078.3960	539.7016			**1060.3854**	530.6963	**E**	**531.2885**	266.1479	**514.2620**	257.6346	513.2780	257.1426	**4**
**12**	1192.4389	596.7231	1175.4123	588.2098	1174.4283	587.7178	**N**	**402.2459**	201.6266	385.2194	193.1133			**3**
**13**	**1305.5230**	653.2651	1288.4964	644.7518	1287.5124	644.2598	**L**	**288.2030**	144.6051	271.1765	136.0919			**2**
**14**							**R**	175.1190	88.0631	158.0924	79.5498			**1**

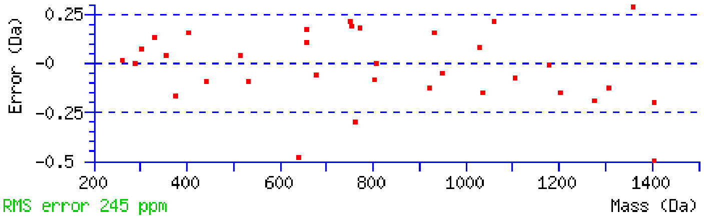

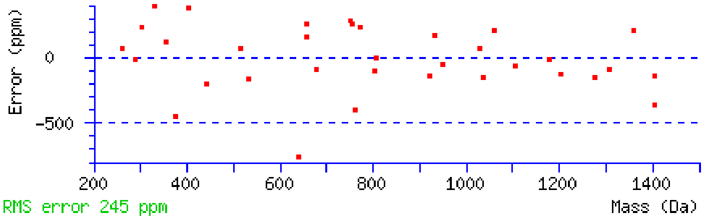

All matches to this query

Score	Mr(calc):	Delta	Sequence
44.9	1576.6042	0.0049	GSGTASDDEFENLR
25.7	1576.6042	0.0049	GSGTASDDEFENLR
25.7	1576.6042	0.0049	GSGTASDDEFENLR
6.5	1576.6150	-0.0059	NEESMEGTMVTLR
2.9	1576.6076	0.0015	MAGTEQSLNSASER
2.9	1576.6076	0.0015	MAGTEQSLNSASER
2.1	1576.6146	-0.0055	ANYNSAKMALYR
1.0	1575.6120	0.9972	SAFKEPSRDHNK
1.0	1576.6116	-0.0025	GGFEDEMEDSLLR
1.0	1576.6051	0.0040	SATMWXTPSPSTR

Spectrum No: 90; Query: 939; Rank: 1

Peptide View

MS/MS Fragmentation of **ESDDKPEIEDVGSDEEEEEKK**
Found in **IPI00210566**, Tax_Id=10116 Gene_Symbol=Hspca Heat shock protein HSP 90-alpha

Match to Query 939: 2515.004322 from(839.342050,3+)
Title: 091127RatKid_SCX01_30.938.938.3.dta
Data file K:\NewmanPaper\Piliang\3SubProteomes\Piliang3SP\mgf5ppm\SCX_3SubProteomes5ppm.mgf

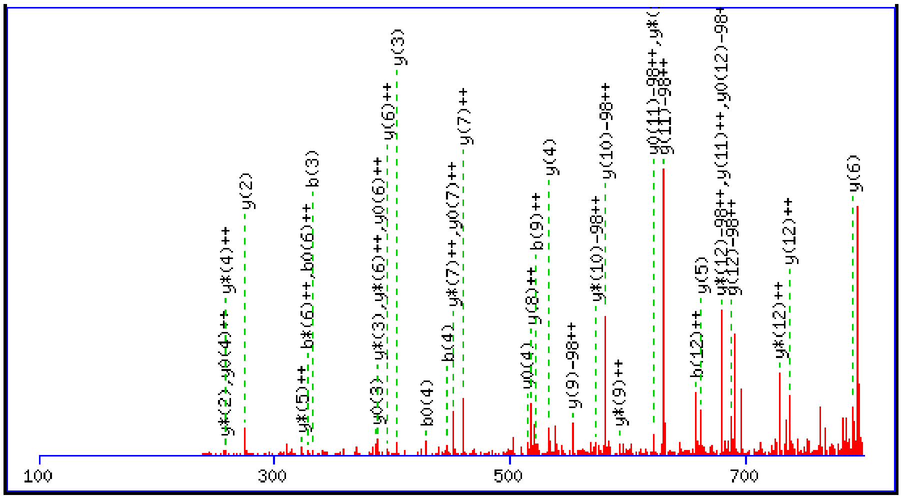

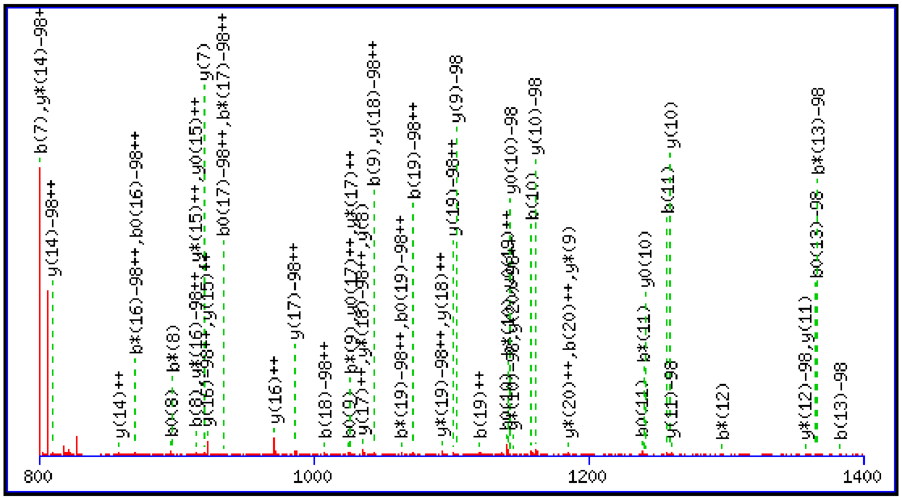

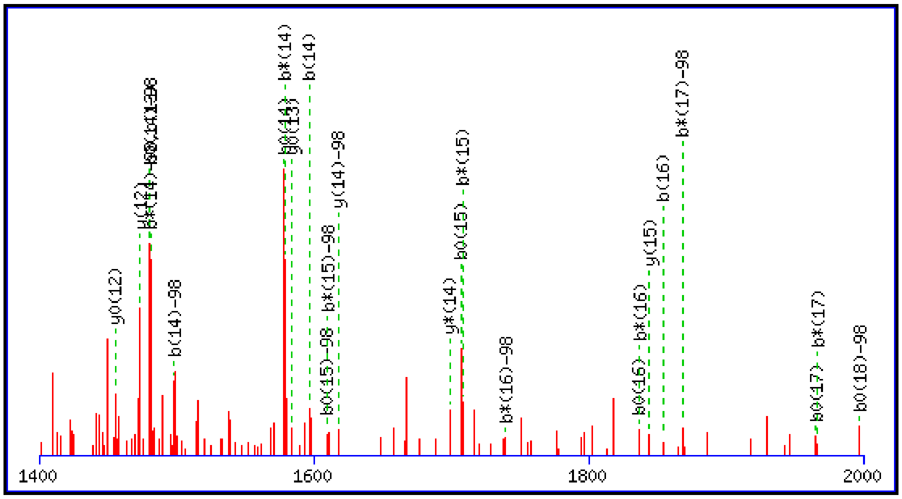

Monoisotopic mass of neutral peptide Mr(calc): 2515.0010
Fixed modifications: Carbamidomethyl (C)
Variable modifications:
S13 : Phospho (ST), with neutral losses 97.9769(shown in table), 0.0000
Ions Score: 45 **Expect:** 0.0084
Matches (Bold Red): 125/348 fragment ions using 182 most intense peaks

#	b	b^{++}	b*	b^{*++}	b^0	b^{0++}	Seq.	y	y^{++}	y*	y^{*++}	y^0	y^{0++}	#
1	130.0499	65.5286			112.0393	56.5233	E							21
2	217.0819	109.0446			199.0713	100.0393	S	2288.9888	**1144.9981**	2271.9623	1136.4848	2270.9783	1135.9928	20
3	**332.1088**	166.5581			314.0983	157.5528	D	2201.9568	**1101.4820**	2184.9303	**1092.9688**	2183.9463	1092.4768	19
4	**447.1358**	224.0715			**429.1252**	215.0662	D	2086.9299	**1043.9686**	2069.9033	**1035.4553**	2068.9193	1034.9633	18
5	575.2307	288.1190	558.2042	279.6057	557.2202	279.1137	K	1971.9029	**986.4551**	1954.8764	977.9418	1953.8924	977.4498	17

6	672.2835	336.6454	655.2570	328.1321	654.2729	327.6401	P	1843.8080	922.4076	1826.7814	913.8943	1825.7974	913.4023	16
7	801.3261	401.1667	784.2996	392.6534	783.3155	392.1614	E	1746.7552	873.8812	1729.7287	865.3680	1728.7446	864.8760	15
8	914.4102	457.7087	897.3836	449.1954	896.3996	448.7034	I	1617.7126	809.3599	1600.6861	800.8467	1599.7020	800.3547	14
9	1043.4528	522.2300	1026.4262	513.7167	1025.4422	513.2247	E	1504.6285	752.8179	1487.6020	744.3046	1486.6180	743.8126	13
10	1158.4797	579.7435	1141.4532	571.2302	1140.4691	570.7382	D	1375.5860	688.2966	1358.5594	679.7833	1357.5754	679.2913	12
11	1257.5481	629.2777	1240.5216	620.7644	1239.5376	620.2724	V	1260.5590	630.7831	1243.5325	622.2699	1242.5484	621.7779	11
12	1314.5696	657.7884	1297.5430	649.2752	1296.5590	648.7831	G	1161.4906	581.2489	1144.4640	572.7357	1143.4800	572.2437	10
13	1383.5910	692.2992	1366.5645	683.7859	1365.5805	683.2939	S	1104.4691	552.7382	1087.4426	544.2249	1086.4586	543.7329	9
14	1498.6180	749.8126	1481.5914	741.2994	1480.6074	740.8073	D	1035.4477	518.2275	1018.4211	509.7142	1017.4371	509.2222	8
15	1627.6606	814.3339	1610.6340	805.8207	1609.6500	805.3286	E	920.4207	460.7140	903.3942	452.2007	902.4102	451.7087	7
16	1756.7032	878.8552	1739.6766	870.3419	1738.6926	869.8499	E	791.3781	396.1927	774.3516	387.6794	773.3676	387.1874	6
17	1885.7458	943.3765	1868.7192	934.8632	1867.7352	934.3712	E	662.3355	331.6714	645.3090	323.1581	644.3250	322.6661	5
18	2014.7884	1007.8978	1997.7618	999.3845	1996.7778	998.8925	E	533.2930	267.1501	516.2664	258.6368	515.2824	258.1448	4
19	2143.8309	1072.4191	2126.8044	1063.9058	2125.8204	1063.4138	E	404.2504	202.6288	387.2238	194.1155	386.2398	193.6235	3
20	2271.9259	1136.4666	2254.8994	1127.9533	2253.9153	1127.4613	K	275.2078	138.1075	258.1812	129.5942			2
21							K	147.1128	74.0600	130.0863	65.5468			1

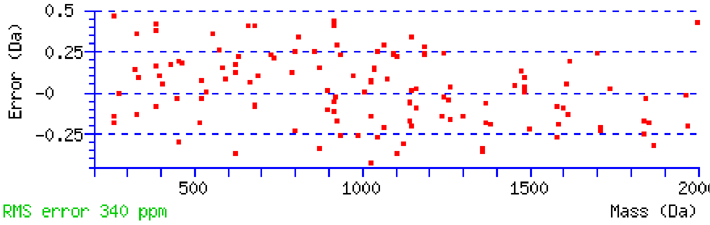

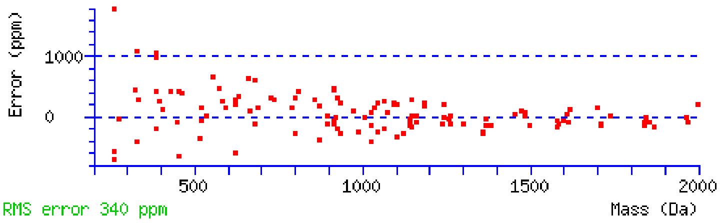

All matches to this query

Score	Mr(calc):	Delta	Sequence
44.8	2515.0010	0.0033	ESDDKPEIEDVGSDEEEEEKK
20.0	2515.0010	0.0033	ESDDKPEIEDVGSDEEEEEKK
6.2	2512.9770	2.0273	KMVNSSPEEISTCAQVTHQK
5.8	2512.9788	2.0256	LEPIPEELEEDTSATDEKTK
3.4	2512.9875	2.0168	WDTGAEYVIESTSICTSVKK
2.7	2514.9926	0.0117	NGTVMAPDLPEMLDLAGTRSR
2.5	2512.9847	2.0196	LAHTKSSVALAPALVETYSR
2.5	2512.9847	2.0196	LAHTKSSVALAPALVETYSR
2.4	2512.9930	2.0113	SRTPLDKDLINTGIYESSGK
2.3	2513.9919	1.0124	LPDTLEDSKSSDSHSDSDDEQK

Spectrum No: 91; Query: 870; Rank: 1

Peptide View

MS/MS Fragmentation of **SAEDLTEGSYDAILSAEQLEK**
Found in **IPI00372341**, Tax_Id=10116 Gene_Symbol=RGD1305915 armadillo repeat containing 10

Match to Query 870: 2348.040548 from(1175.027550,2+)
Title: 091129RatKid_SCX02_03.4048.4048.2.dta
Data file K:\NewmanPaper\Piliang\3SubProteomes\Piliang3SP\mgf5ppm\SCX_3SubProteomes5ppm.mgf

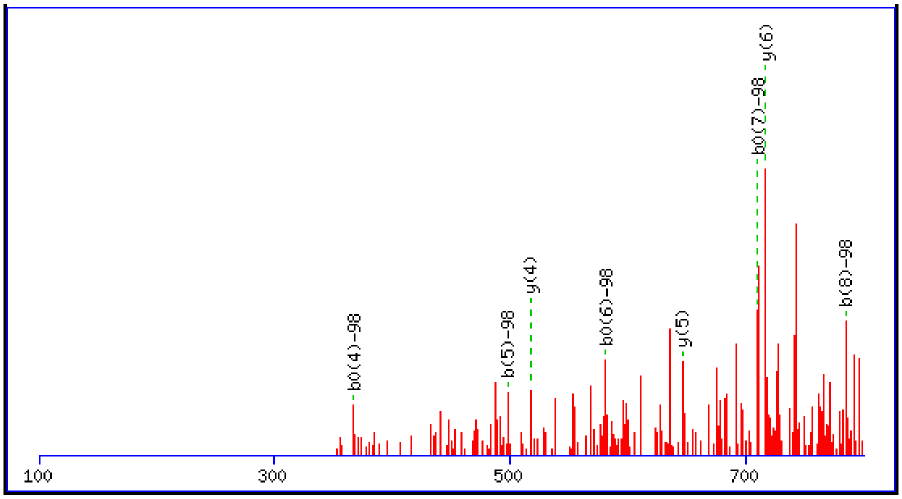

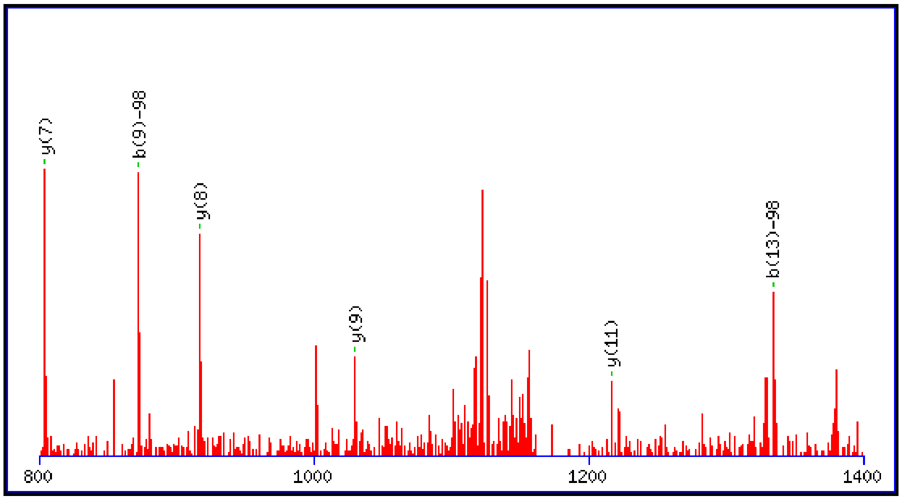

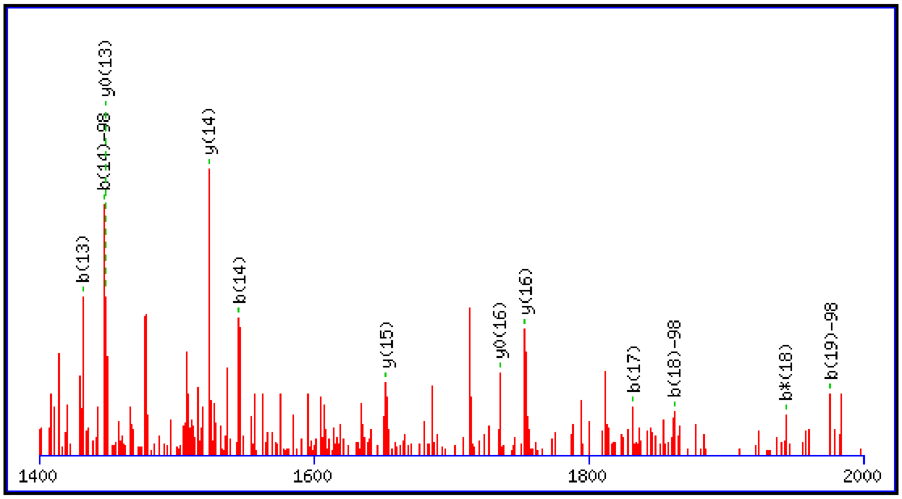

Monoisotopic mass of neutral peptide Mr(calc): 2348.0308
Fixed modifications: Carbamidomethyl (C)
Variable modifications:
S1 : Phospho (ST), with neutral losses 97.9769(shown in table), 0.0000
Ions Score: 45 **Expect:** 0.011
Matches (Bold Red): 26/290 fragment ions using 61 most intense peaks

#	b	b^{++}	b*	b^{*++}	b^{0}	b^{0++}	Seq.	y	y^{++}	y*	y^{*++}	y^{0}	y^{0++}	#
1	70.0287	35.5180			52.0182	26.5127	S							21
2	141.0658	71.0366			123.0553	62.0313	A	2182.0398	1091.5235	2165.0132	1083.0102	2164.0292	1082.5182	20
3	270.1084	135.5579			252.0979	126.5526	E	2111.0027	1056.0050	2093.9761	1047.4917	2092.9921	1046.9997	19
4	385.1354	193.0713			**367.1248**	184.0660	D	1981.9601	991.4837	1964.9335	982.9704	1963.9495	982.4784	18
5	**498.2194**	249.6134			480.2089	240.6081	L	1866.9331	933.9702	1849.9066	925.4569	1848.9226	924.9649	17

6	599.2671	300.1372			**581.2566**	291.1319	T	**1753.8491**	877.4282	1736.8225	868.9149	**1735.8385**	868.4229	16
7	728.3097	364.6585			**710.2992**	355.6532	E	**1652.8014**	826.9043	1635.7748	818.3911	1634.7908	817.8990	15
8	**785.3312**	393.1692			767.3206	384.1639	G	**1523.7588**	762.3830	1506.7322	753.8698	1505.7482	753.3777	14
9	**872.3632**	436.6852			854.3526	427.6800	S	1466.7373	733.8723	1449.7108	725.3590	**1448.7268**	724.8670	13
10	1035.4265	518.2169			1017.4160	509.2116	Y	1379.7053	690.3563	1362.6787	681.8430	1361.6947	681.3510	12
11	1150.4535	575.7304			1132.4429	566.7251	D	**1216.6420**	608.8246	1199.6154	600.3113	1198.6314	599.8193	11
12	1221.4906	611.2489			1203.4800	602.2437	A	1101.6150	551.3111	1084.5885	542.7979	1083.6045	542.3059	10
13	**1334.5747**	667.7910			1316.5641	658.7857	I	**1030.5779**	515.7926	1013.5514	507.2793	1012.5673	506.7873	9
14	**1447.6587**	724.3330			1429.6482	715.3277	L	**917.4938**	459.2506	900.4673	450.7373	899.4833	450.2453	8
15	1534.6908	767.8490			1516.6802	758.8437	S	**804.4098**	402.7085	787.3832	394.1953	786.3992	393.7032	7
16	1605.7279	803.3676			1587.7173	794.3623	A	**717.3777**	359.1925	700.3512	350.6792	699.3672	350.1872	6
17	1734.7705	867.8889			1716.7599	858.8836	E	**646.3406**	323.6740	629.3141	315.1607	628.3301	314.6687	5
18	**1862.8290**	931.9182	1845.8025	923.4049	1844.8185	922.9129	Q	**517.2980**	259.1527	500.2715	250.6394	499.2875	250.1474	4
19	**1975.9131**	988.4602	1958.8866	979.9469	1957.9025	979.4549	L	389.2395	195.1234	372.2129	186.6101	371.2289	186.1181	3
20	2104.9557	1052.9815	2087.9291	1044.4682	2086.9451	1043.9762	E	276.1554	138.5813	259.1288	130.0681	258.1448	129.5761	2
21							K	147.1128	74.0600	130.0863	65.5468			1

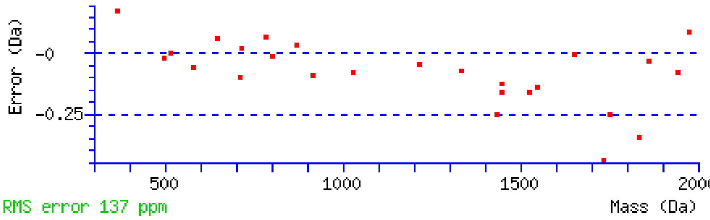

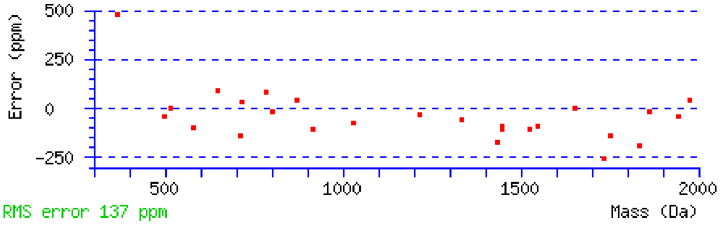

All matches to this query

Score	Mr(calc):	Delta	Sequence
44.7	2348.0308	0.0098	SAEDLTEGSYDAILSAEQLEK
39.8	2348.0308	0.0098	SAEDLTEGSYDAILSAEQLEK
24.0	2348.0308	0.0098	SAEDLTEGSYDAILSAEQLEK
7.8	2348.0308	0.0098	SAEDLTEGSYDAILSAEQLEK
6.5	2348.0178	0.0228	TLPDNSGWMCATGNKIKTTR
2.7	2348.0178	0.0228	TLPDNSGWMCATGNKIKTTR
1.4	2347.0320	1.0085	ISFSGLTNLMVKYNNDKSR
0.0	2348.0368	0.0038	IELSVLSYHSSFSIRKSR

Spectrum No: 92; Query: 818; Rank: 1

Peptide View

MS/MS Fragmentation of **GETPQGSSEECDLSGSCTER**
Found in **IPI00203529**, Tax_Id=10116 Gene_Symbol=Slc34a3 Sodium-dependent phosphate transport protein 2C

Match to Query 818: 2264.824728 from(1133.419640,2+)
Title: 091127RatKid_SCX01_12.1115.1115.2.dta
Data file K:\NewmanPaper\Piliang\3SubProteomes\Piliang3SP\mgf5ppm\SCX_3SubProteomes5ppm.mgf

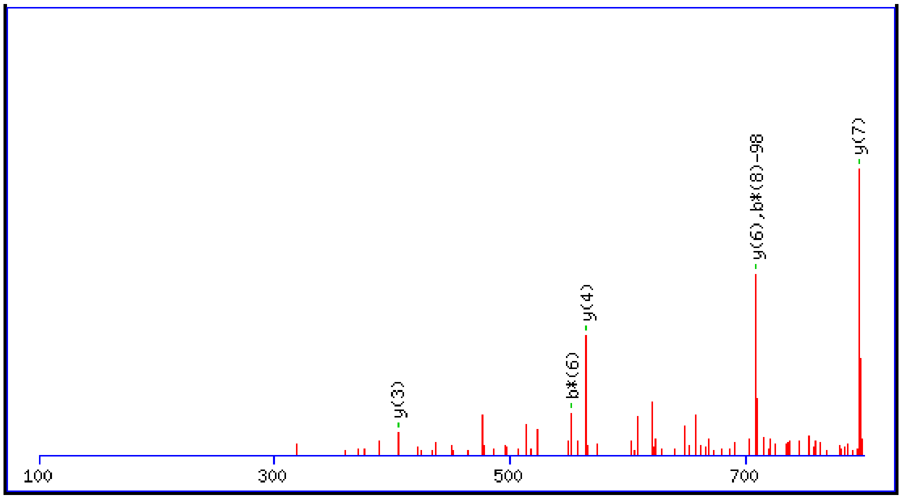

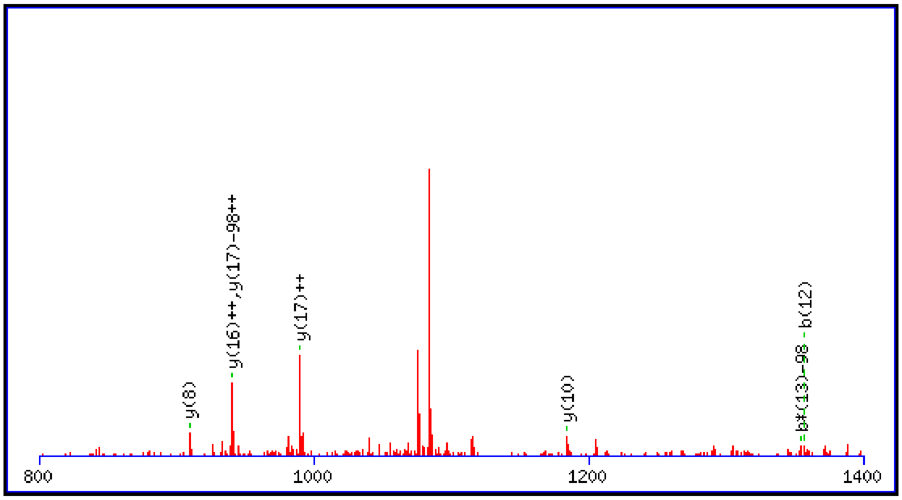

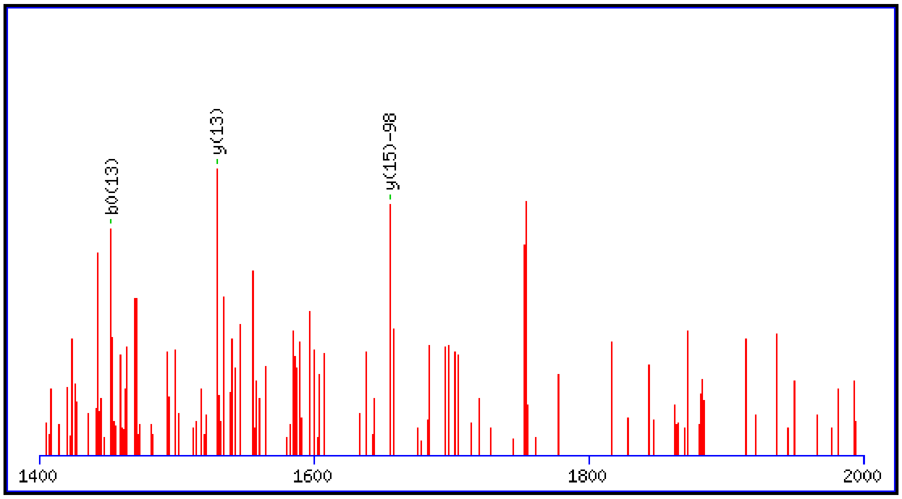

Monoisotopic mass of neutral peptide Mr(calc): 2264.8199
Fixed modifications: Carbamidomethyl (C)
Variable modifications:
S7 : Phospho (ST), with neutral losses 97.9769(shown in table), 0.0000
Ions Score: 45 **Expect:** 0.0041
Matches (Bold Red): 16/330 fragment ions using 27 most intense peaks

#	b	b^{++}	b*	b^{*++}	b^{0}	b^{0++}	Seq.	y	y^{++}	y*	y^{*++}	y^{0}	y^{0++}	#
1	58.0287	29.5180					G							20
2	187.0713	94.0393			169.0608	85.0340	E	2110.8288	1055.9180	2093.8022	1047.4048	2092.8182	1046.9128	19
3	288.1190	144.5631			270.1084	135.5579	T	1981.7862	991.3967	1964.7597	982.8835	1963.7756	982.3915	18
4	385.1718	193.0895			367.1612	184.0842	P	1880.7385	**940.8729**	1863.7120	932.3596	1862.7280	931.8676	17
5	513.2304	257.1188	496.2038	248.6055	495.2198	248.1135	Q	1783.6858	892.3465	1766.6592	883.8332	1765.6752	883.3412	16

6	570.2518	285.6295	553.2253	277.1163	552.2413	276.6243	G	1655.6272	828.3172	1638.6006	819.8040	1637.6166	819.3119	15
7	639.2733	320.1403	622.2467	311.6270	621.2627	311.1350	S	1598.6057	799.8065	1581.5792	791.2932	1580.5952	790.8012	14
8	726.3053	363.6563	709.2788	355.1430	708.2947	354.6510	S	1529.5843	765.2958	1512.5577	756.7825	1511.5737	756.2905	13
9	855.3479	428.1776	838.3213	419.6643	837.3373	419.1723	E	1442.5522	721.7798	1425.5257	713.2665	1424.5417	712.7745	12
10	984.3905	492.6989	967.3639	484.1856	966.3799	483.6936	E	1313.5096	657.2585	1296.4831	648.7452	1295.4991	648.2532	11
11	1144.4211	572.7142	1127.3946	564.2009	1126.4106	563.7089	C	1184.4670	592.7372	1167.4405	584.2239	1166.4565	583.7319	10
12	1259.4481	630.2277	1242.4215	621.7144	1241.4375	621.2224	D	1024.4364	512.7218	1007.4099	504.2086	1006.4258	503.7166	9
13	1372.5321	686.7697	1355.5056	678.2564	1354.5216	677.7644	L	909.4095	455.2084	892.3829	446.6951	891.3989	446.2031	8
14	1459.5642	730.2857	1442.5376	721.7724	1441.5536	721.2804	S	796.3254	398.6663	779.2988	390.1531	778.3148	389.6611	7
15	1516.5856	758.7965	1499.5591	750.2832	1498.5751	749.7912	G	709.2934	355.1503	692.2668	346.6370	691.2828	346.1450	6
16	1603.6177	802.3125	1586.5911	793.7992	1585.6071	793.3072	S	652.2719	326.6396	635.2454	318.1263	634.2613	317.6343	5
17	1763.6483	882.3278	1746.6218	873.8145	1745.6377	873.3225	C	565.2399	283.1236	548.2133	274.6103	547.2293	274.1183	4
18	1864.6960	932.8516	1847.6694	924.3384	1846.6854	923.8464	T	405.2092	203.1082	388.1827	194.5950	387.1987	194.1030	3
19	1993.7386	997.3729	1976.7120	988.8597	1975.7280	988.3676	E	304.1615	152.5844	287.1350	144.0711	286.1510	143.5791	2
20							R	175.1190	88.0631	158.0924	79.5498			1

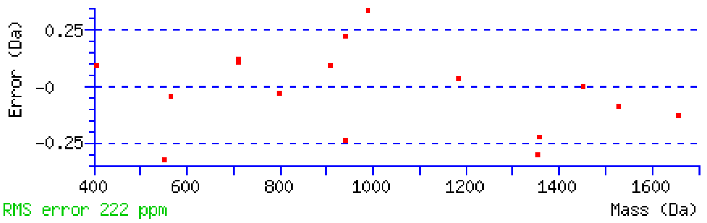

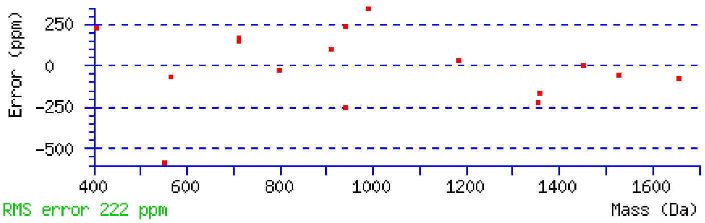

All matches to this query

Score	Mr(calc):	Delta	Sequence
44.5	2264.8199	0.0048	GETPQGSSEECDLSGSCTER
33.3	2264.8199	0.0048	GETPQGSSEECDLSGSCTER
32.4	2264.8199	0.0048	GETPQGSSEECDLSGSCTER
5.5	2264.8199	0.0048	GETPQGSSEECDLSGSCTER
1.5	2264.8199	0.0048	GETPQGSSEECDLSGSCTER

Spectrum No: 93; Query: 761; Rank: 1

Peptide View

MS/MS Fragmentation of **DYEEVGADSAEGDDEGEEY**
Found in **IPI00364046**, Tax_Id=10116 Gene_Symbol=Tuba1c Tubulin alpha-1C chain

Match to Query 761: 2157.712388 from(1079.863470,2+)
Title: 091127RatKid_SCX01_02.2242.2242.2.dta
Data file K:\NewmanPaper\Piliang\3SubProteomes\Piliang3SP\mgf5ppm\SCX_3SubProteomes5ppm.mgf

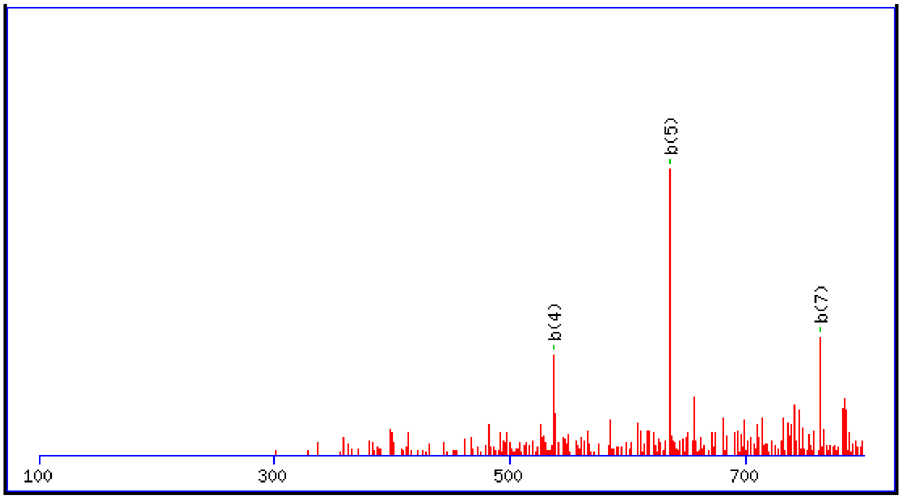

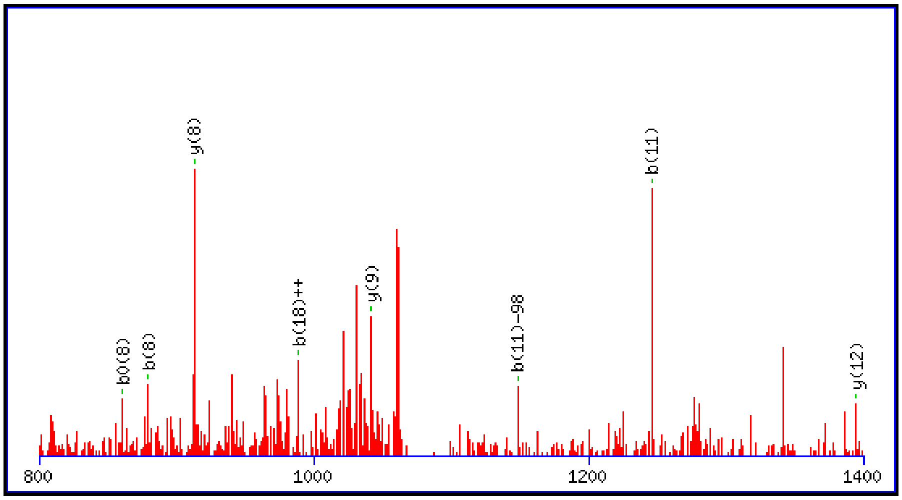

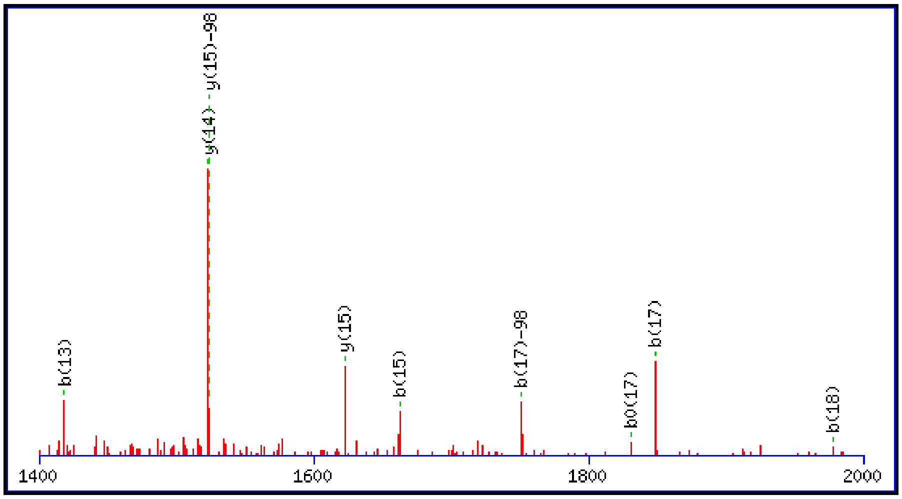

Monoisotopic mass of neutral peptide Mr(calc): 2157.7059
Fixed modifications: Carbamidomethyl (C)
Variable modifications:
S9 : Phospho (ST), with neutral losses 0.0000(shown in table), 97.9769
Ions Score: 44 **Expect:** 0.0021
Matches (Bold Red): 20/214 fragment ions using 41 most intense peaks

#	b	b^{++}	b^0	b^{0++}	Seq.	y	y^{++}	y^0	y^{0++}	#
1	116.0342	58.5207	98.0237	49.5155	D					19
2	279.0975	140.0524	261.0870	131.0471	Y	2043.6863	1022.3468	2025.6757	1013.3415	18
3	408.1401	204.5737	390.1296	195.5684	E	1880.6229	940.8151	1862.6124	931.8098	17
4	**537.1827**	269.0950	519.1722	260.0897	E	1751.5804	876.2938	1733.5698	867.2885	16
5	**636.2511**	318.6292	618.2406	309.6239	V	**1622.5378**	811.7725	1604.5272	802.7672	15

**6**	693.2726	347.1399	675.2620	338.1347	**G**	**1523.4693**	762.2383	1505.4588	753.2330	**14**
**7**	**764.3097**	382.6585	746.2992	373.6532	**A**	1466.4479	733.7276	1448.4373	724.7223	**13**
**8**	**879.3367**	440.1720	**861.3261**	431.1667	**D**	**1395.4108**	698.2090	1377.4002	689.2037	**12**
**9**	1046.3350	523.6711	1028.3245	514.6659	**S**	1280.3838	640.6955	1262.3733	631.6903	**11**
**10**	1117.3721	559.1897	1099.3616	550.1844	**A**	1113.3855	557.1964	1095.3749	548.1911	**10**
**11**	**1246.4147**	623.7110	1228.4042	614.7057	**E**	**1042.3484**	521.6778	1024.3378	512.6725	**9**
**12**	1303.4362	652.2217	1285.4256	643.2165	**G**	**913.3058**	457.1565	895.2952	448.1512	**8**
**13**	**1418.4631**	709.7352	1400.4526	700.7299	**D**	856.2843	428.6458	838.2737	419.6405	**7**
**14**	1533.4901	767.2487	1515.4795	758.2434	**D**	741.2574	371.1323	723.2468	362.1270	**6**
**15**	**1662.5327**	831.7700	1644.5221	822.7647	**E**	626.2304	313.6188	608.2198	304.6136	**5**
**16**	1719.5541	860.2807	1701.5436	851.2754	**G**	497.1878	249.0975	479.1773	240.0923	**4**
**17**	**1848.5967**	924.8020	**1830.5862**	915.7967	**E**	440.1664	220.5868	422.1558	211.5815	**3**
**18**	**1977.6393**	**989.3233**	1959.6288	980.3180	**E**	311.1238	156.0655	293.1132	147.0602	**2**
**19**					**Y**	182.0812	91.5442			**1**

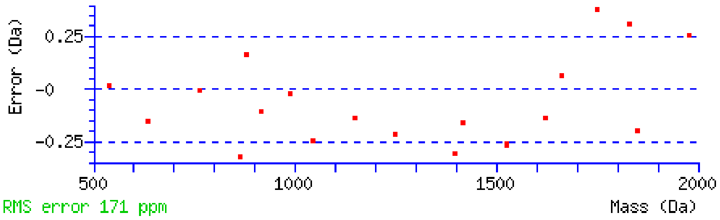

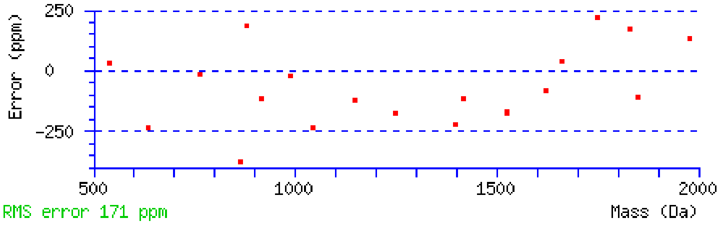

All matches to this query

Score	Mr(calc):	Delta	Sequence
44.2	2157.7059	0.0065	DYEEVGADSAEGDDEGEEY
10.8	2157.7059	0.0065	DYEEVGADSAEGDDEGEEY
9.9	2157.7059	0.0065	DYEEVGADSAEGDDEGEEY
4.9	2157.7290	-0.0166	GSRPSGNQNGEGDQINASK

Spectrum No: 94; Query: 394; Rank: 1

Peptide View

MS/MS Fragmentation of **SFSLASSGNSPISQR**
Found in **IPI00362151**, Tax_Id=10116 Gene_Symbol=Osbpl11_predicted oxysterol binding protein-like 11

Match to Query 394: 1616.720908 from(809.367730,2+)
Title: 091127RatKid_SCX01_13.1967.1967.2.dta
Data file K:\NewmanPaper\Piliang\3SubProteomes\Piliang3SP\mgf5ppm\SCX_3SubProteomes5ppm.mgf

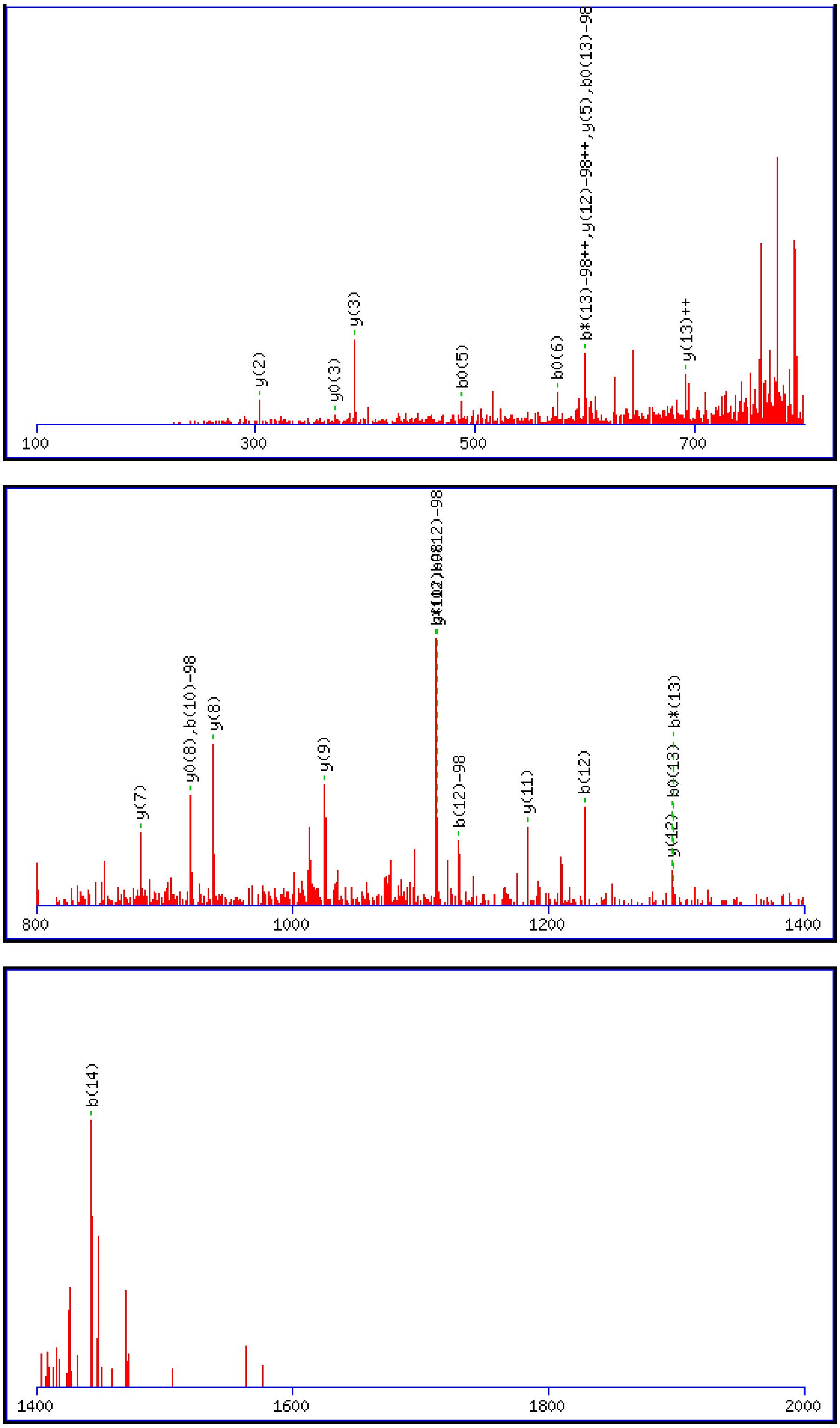

Monoisotopic mass of neutral peptide Mr(calc): 1616.7195
Fixed modifications: Carbamidomethyl (C)
Variable modifications:
S10 : Phospho (ST), with neutral losses 0.0000(shown in table), 97.9769
Ions Score: 44 **Expect:** 0.007
Matches (Bold Red): 25/232 fragment ions using 50 most intense peaks

#	b	b^{++}	b*	b^{*++}	b^{0}	b^{0++}	Seq.	y	y^{++}	y*	y^{*++}	y^{0}	y^{0++}	#
1	88.0393	44.5233			70.0287	35.5180	S							15
2	235.1077	118.0575			217.0972	109.0522	F	1530.6948	765.8510	1513.6683	757.3378	1512.6842	756.8458	14
3	322.1397	161.5735			304.1292	152.5682	S	1383.6264	**692.3168**	1366.5998	683.8036	1365.6158	683.3116	13
4	435.2238	218.1155			417.2132	209.1103	L	**1296.5944**	648.8008	1279.5678	640.2875	1278.5838	639.7955	12
5	506.2609	253.6341			**488.2504**	244.6288	A	**1183.5103**	592.2588	1166.4837	583.7455	1165.4997	583.2535	11

**6**	593.2930	297.1501			**575.2824**	288.1448	**S**	**1112.4732**	556.7402	1095.4466	548.2270	1094.4626	547.7349	**10**
**7**	680.3250	340.6661			662.3144	331.6608	**S**	**1025.4412**	513.2242	1008.4146	504.7109	1007.4306	504.2189	**9**
**8**	737.3464	369.1769			719.3359	360.1716	**G**	**938.4091**	469.7082	921.3826	461.1949	**920.3986**	460.7029	**8**
**9**	851.3894	426.1983	834.3628	417.6850	833.3788	417.1930	**N**	**881.3877**	441.1975	864.3611	432.6842	863.3771	432.1922	**7**
**10**	1018.3877	509.6975	1001.3612	501.1842	1000.3772	500.6922	**S**	767.3447	384.1760	750.3182	375.6627	749.3342	375.1707	**6**
**11**	1115.4405	558.2239	1098.4139	549.7106	1097.4299	549.2186	**P**	**600.3464**	300.6768	583.3198	292.1636	582.3358	291.6715	**5**
**12**	**1228.5246**	614.7659	1211.4980	606.2526	1210.5140	605.7606	**I**	503.2936	252.1504	486.2671	243.6372	485.2831	243.1452	**4**
**13**	1315.5566	658.2819	**1298.5300**	649.7687	**1297.5460**	649.2766	**S**	**390.2096**	195.6084	373.1830	187.0951	**372.1990**	186.6031	**3**
**14**	**1443.6152**	722.3112	1426.5886	713.7979	1425.6046	713.3059	**Q**	**303.1775**	152.0924	286.1510	143.5791			**2**
**15**							**R**	175.1190	88.0631	158.0924	79.5498			**1**

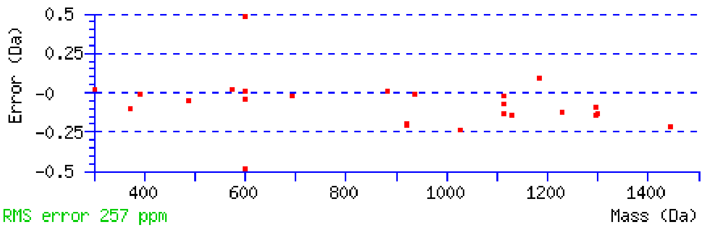

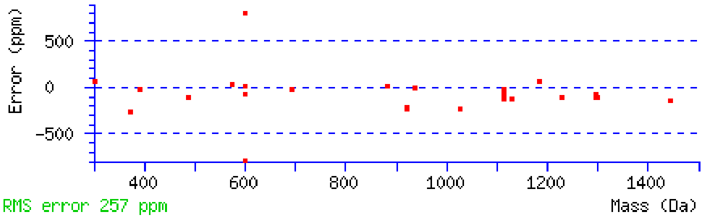

All matches to this query

Score	Mr(calc):	Delta	Sequence
44.2	1616.7195	0.0014	SFSLASSGNSPISQR
25.1	1616.7195	0.0014	SFSLASSGNSPISQR
21.4	1616.7195	0.0014	SFSLASSGNSPISQR
18.9	1616.7195	0.0014	SFSLASSGNSPISQR
9.7	1616.7195	0.0014	SFSLASSGNSPISQR
8.4	1616.7195	0.0014	SFSLASSGNSPISQR
6.6	1615.7218	0.9991	EALPFWMNSTGKR
6.6	1615.7218	0.9991	EALPFWMNSTGKR
4.0	1616.7096	0.0113	NYNNTWTRLAER
3.1	1616.7117	0.0093	MSDKSDLKAELER

Spectrum No: 95; Query: 510; Rank: 1

Peptide View

MS/MS Fragmentation of **SSSPILSEEEAEGSLR**
Found in **IPI00764230**, Tax_Id=10116 Gene_Symbol=LOC682920 similar to phosphatase and actin regulator 4

Match to Query 510: 1769.774508 from(885.894530,2+)
Title: 091129RatKid_SCX02_12.1862.1862.2.dta
Data file K:\NewmanPaper\Piliang\3SubProteomes\Piliang3SP\mgf5ppm\SCX_3SubProteomes5ppm.mgf

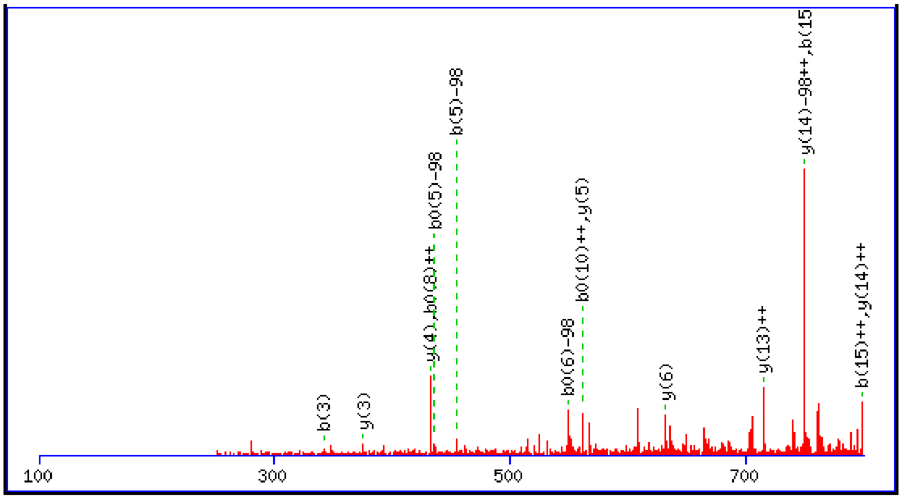

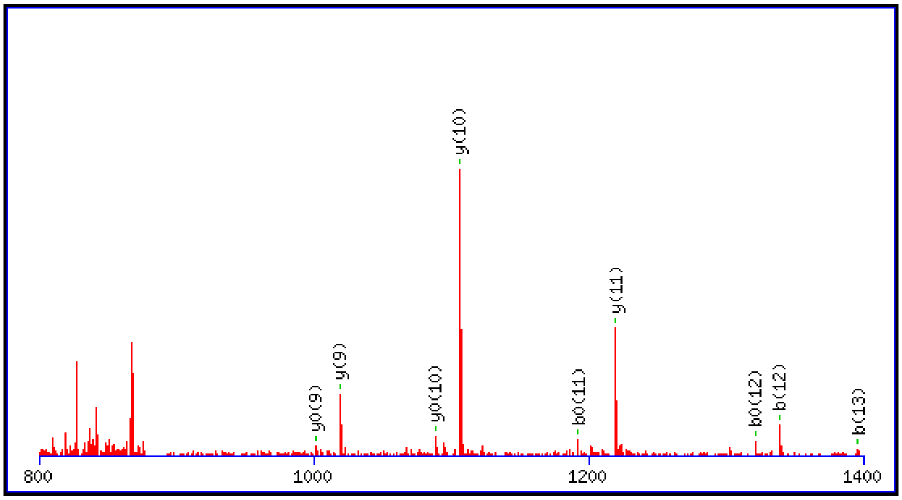

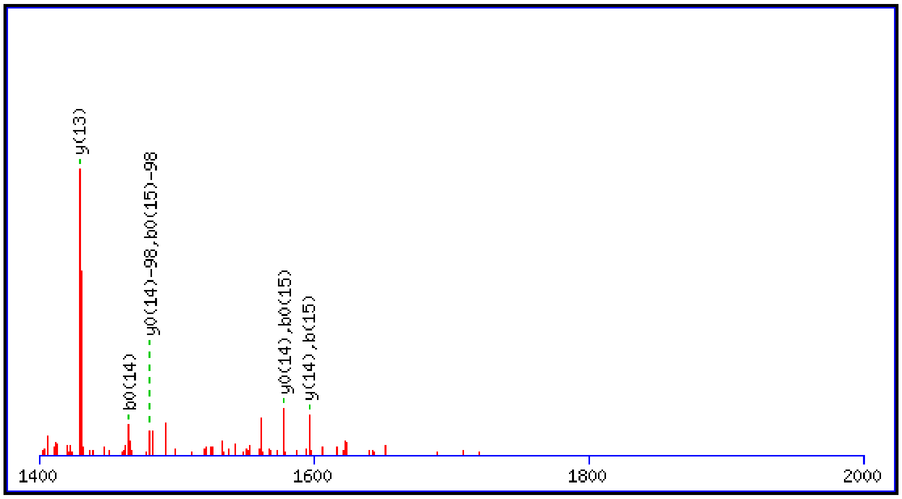

Monoisotopic mass of neutral peptide Mr(calc): 1769.7720
Fixed modifications: Carbamidomethyl (C)
Variable modifications:
S3 : Phospho (ST), with neutral losses 0.0000(shown in table), 97.9769
Ions Score: 44 **Expect:** 0.0089
Matches (Bold Red): 32/210 fragment ions using 60 most intense peaks

#	b	b^{++}	b^0	b^{0++}	Seq.	y	y^{++}	y*	y^{*++}	y^0	y^{0++}	#
1	88.0393	44.5233	70.0287	35.5180	S							16
2	175.0713	88.0393	157.0608	79.0340	S	1683.7473	842.3773	1666.7207	833.8640	1665.7367	833.3720	15
3	**342.0697**	171.5385	324.0591	162.5332	S	**1596.7153**	**798.8613**	1579.6887	790.3480	**1578.7047**	789.8560	14
4	439.1224	220.0649	421.1119	211.0596	P	**1429.7169**	**715.3621**	1412.6904	706.8488	1411.7063	706.3568	13
5	552.2065	276.6069	534.1959	267.6016	I	1332.6641	666.8357	1315.6376	658.3224	1314.6536	657.8304	12

**6**	665.2906	333.1489	647.2800	324.1436	**L**	**1219.5801**	610.2937	1202.5535	601.7804	1201.5695	601.2884	**11**
**7**	752.3226	376.6649	734.3120	367.6597	**S**	**1106.4960**	553.7516	1089.4695	545.2384	**1088.4855**	544.7464	**10**
**8**	881.3652	441.1862	863.3546	**432.1810**	**E**	**1019.4640**	510.2356	1002.4374	501.7224	**1001.4534**	501.2304	**9**
**9**	1010.4078	505.7075	992.3972	496.7023	**E**	890.4214	445.7143	873.3949	437.2011	872.4108	436.7091	**8**
**10**	1139.4504	570.2288	1121.4398	**561.2235**	**E**	761.3788	381.1930	744.3523	372.6798	743.3682	372.1878	**7**
**11**	1210.4875	605.7474	**1192.4769**	596.7421	**A**	**632.3362**	316.6717	615.3097	308.1585	614.3256	307.6665	**6**
**12**	**1339.5301**	670.2687	**1321.5195**	661.2634	**E**	**561.2991**	281.1532	544.2726	272.6399	543.2885	272.1479	**5**
**13**	**1396.5516**	698.7794	1378.5410	689.7741	**G**	**432.2565**	216.6319	415.2300	208.1186	414.2459	207.6266	**4**
**14**	1483.5836	742.2954	**1465.5730**	733.2901	**S**	**375.2350**	188.1212	358.2085	179.6079	357.2245	179.1159	**3**
**15**	**1596.6676**	**798.8375**	**1578.6571**	789.8322	**L**	288.2030	144.6051	271.1765	136.0919			**2**
**16**					**R**	175.1190	88.0631	158.0924	79.5498			**1**

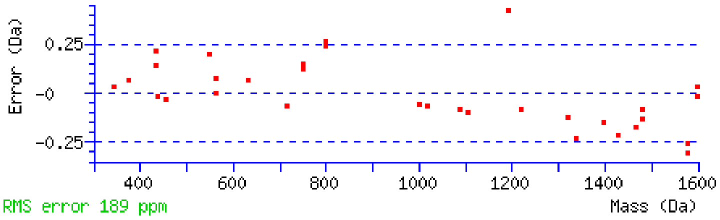

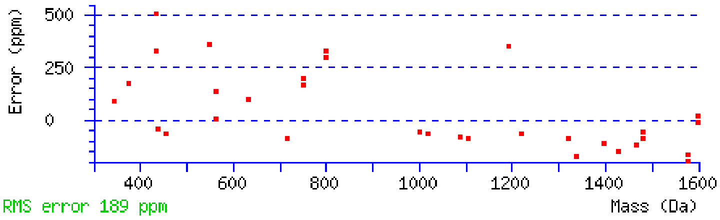

All matches to this query

Score	Mr(calc):	Delta	Sequence
43.6	1769.7720	0.0025	SSSPILSEEEAEGSLR
40.7	1769.7720	0.0025	SSSPILSEEEAEGSLR
39.8	1769.7720	0.0025	SSSPILSEEEAEGSLR
19.4	1769.7720	0.0025	SSSPILSEEEAEGSLR
6.8	1769.7790	-0.0045	LELGHNPLTYTVPR
4.3	1769.7862	-0.0117	ASAAKYSGSRSLGLSR
2.5	1767.7651	2.0094	TTHDNLAWSLNCKK
2.5	1767.7651	2.0094	TTHDNLAWSLNCKK
2.3	1769.7794	-0.0049	EPGTSLSLPADEVTMK
2.3	1769.7794	-0.0049	EPGTSLSLPADEVTMK

Spectrum No: 96; Query: 382; Rank: 1

Peptide View

MS/MS Fragmentation of **EDEISPPPPNPVVK**
Found in **IPI00231770**, Tax_Id=10116 Gene_Symbol=Prkar1a cAMP-dependent protein kinase type I-alpha regulatory subunit

Match to Query 382: 1596.747988 from(799.381270,2+)
Title: 091129RatKid_SCX02_12.1258.1258.2.dta
Data file K:\NewmanPaper\Piliang\3SubProteomes\Piliang3SP\mgf5ppm\SCX_3SubProteomes5ppm.mgf

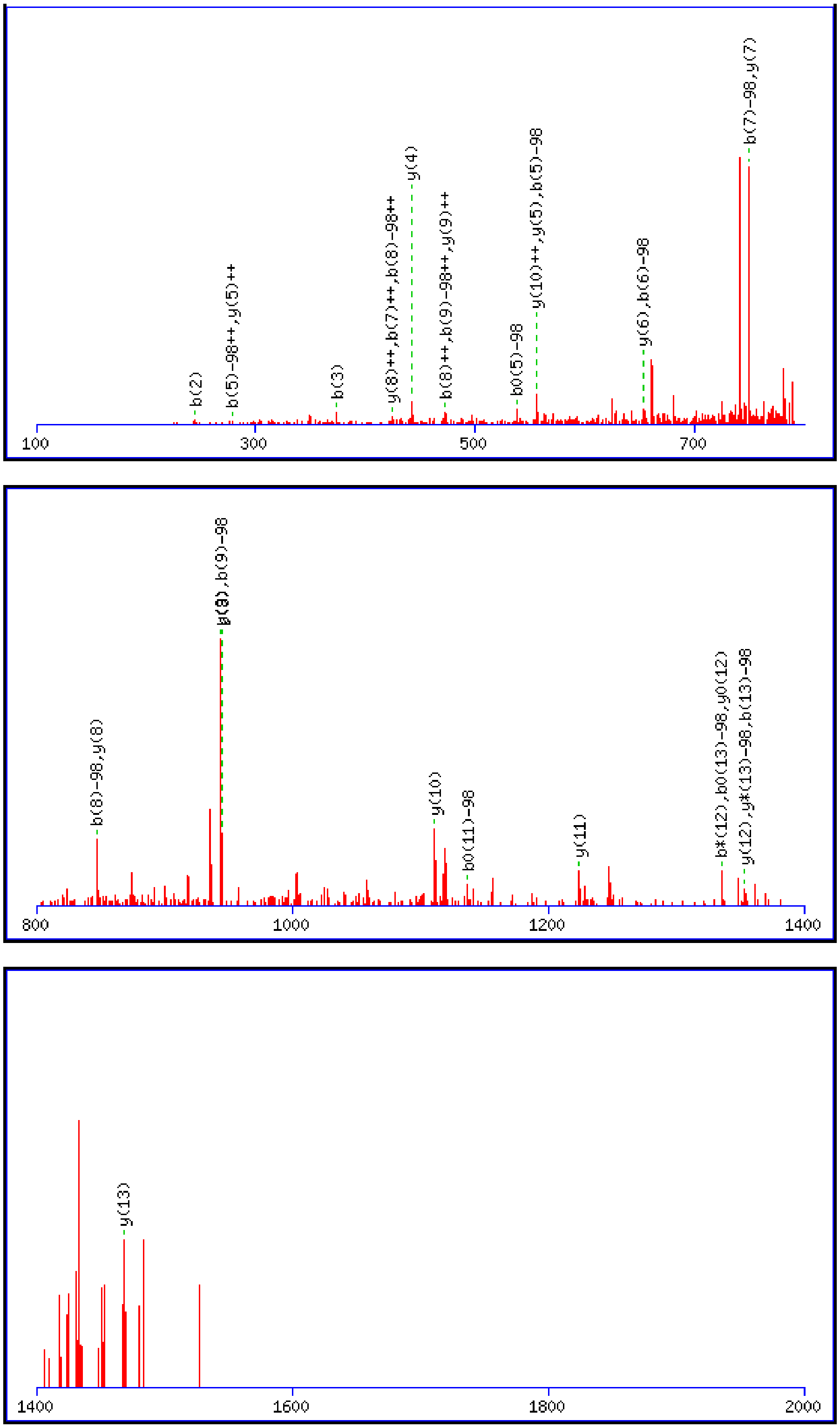

Monoisotopic mass of neutral peptide Mr(calc): 1596.7436
Fixed modifications: Carbamidomethyl (C)
Variable modifications:
S5 : Phospho (ST), with neutral losses 0.0000(shown in table), 97.9769
Ions Score: 44 **Expect:** 0.0093
Matches (Bold Red): 34/188 fragment ions using 55 most intense peaks

#	b	b^{++}	b*	b^{*++}	b^{0}	b^{0++}	Seq.	y	y^{++}	y*	y^{*++}	y^{0}	y^{0++}	#
1	130.0499	65.5286			112.0393	56.5233	E							14
2	**245.0768**	123.0420			227.0662	114.0368	D	**1468.7083**	734.8578	1451.6818	726.3445	1450.6978	725.8525	13
3	**374.1194**	187.5633			356.1088	178.5581	E	**1353.6814**	677.3443	1336.6548	668.8311	**1335.6708**	668.3390	12
4	487.2035	244.1054			469.1929	235.1001	I	**1224.6388**	612.8230	1207.6122	604.3098	1206.6282	603.8178	11
5	654.2018	327.6045			636.1913	318.5993	S	**1111.5547**	**556.2810**	1094.5282	547.7677	1093.5442	547.2757	10

**6**	751.2546	376.1309			733.2440	367.1256	**P**	**944.5564**	**472.7818**	927.5298	464.2686			**9**
**7**	848.3073	**424.6573**			830.2968	415.6520	**P**	**847.5036**	**424.2554**	830.4771	415.7422			**8**
**8**	**945.3601**	**473.1837**			927.3495	464.1784	**P**	**750.4509**	375.7291	733.4243	367.2158			**7**
**9**	1042.4129	521.7101			1024.4023	512.7048	**P**	**653.3981**	327.2027	636.3715	318.6894			**6**
**10**	1156.4558	578.7315	1139.4293	570.2183	1138.4452	569.7263	**N**	**556.3453**	**278.6763**	539.3188	270.1630			**5**
**11**	1253.5086	627.2579	1236.4820	618.7446	1235.4980	618.2526	**P**	**442.3024**	221.6548	425.2758	213.1416			**4**
**12**	1352.5770	676.7921	**1335.5504**	668.2789	1334.5664	667.7868	**V**	345.2496	173.1285	328.2231	164.6152			**3**
**13**	1451.6454	726.3263	1434.6188	717.8131	1433.6348	717.3211	**V**	246.1812	123.5942	229.1547	115.0810			**2**
**14**							**K**	147.1128	74.0600	130.0863	65.5468			**1**

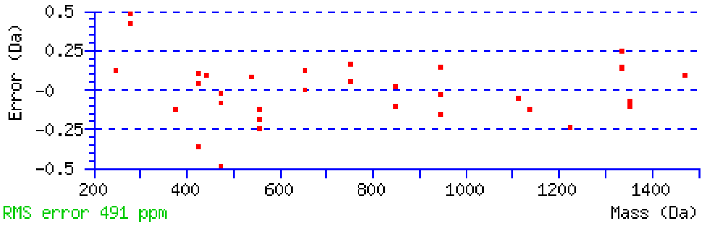

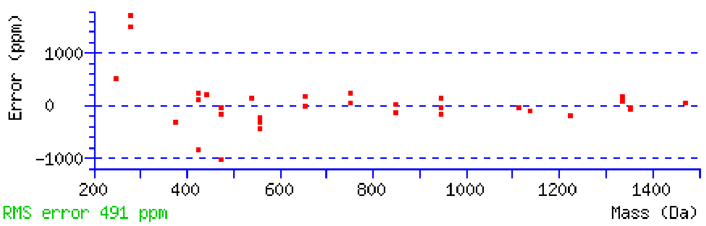

All matches to this query

Score	Mr(calc):	Delta	Sequence
43.5	1596.7436	0.0044	EDEISPPPPNPVVK
11.9	1596.7548	-0.0068	NYEPLQTPSARIK
7.5	1596.7623	-0.0143	MQYILLSPPSPQK
5.3	1596.7506	-0.0027	IKPFSWISKKGF
4.3	1595.7345	1.0135	GGVWRNTEDEILK
4.1	1596.7396	0.0084	TDDQNVNASLLLXK
3.5	1596.7331	0.0149	KEMGTPDVRIDTR
2.9	1596.7532	-0.0052	EEIDDLHQKWER
2.3	1596.7548	-0.0068	NYEPLQTPSARIK
0.7	1595.7444	1.0036	NPVSSSSSTPLLAQK

Spectrum No: 97; Query: 178; Rank: 1

Peptide View

MS/MS Fragmentation of **LCLSTVDLEVK**
Found in **IPI00369678**, Tax_Id=10116 Gene_Symbol=Slc43a2_predicted solute carrier family 43, member 2

Match to Query 178: 1355.643368 from(678.828960,2+)
Title: 091127RatKid_SCX01_12.3207.3207.2.dta
Data file K:\NewmanPaper\Piliang\3SubProteomes\Piliang3SP\mgf5ppm\SCX_3SubProteomes5ppm.mgf

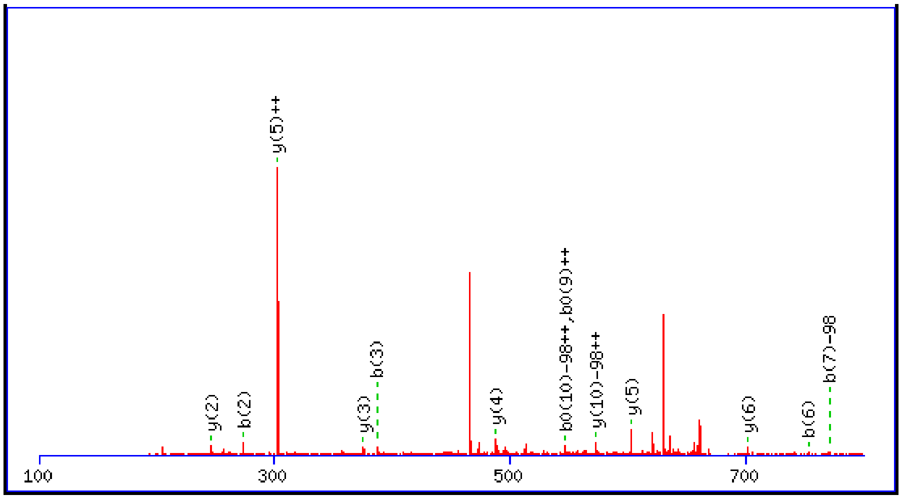

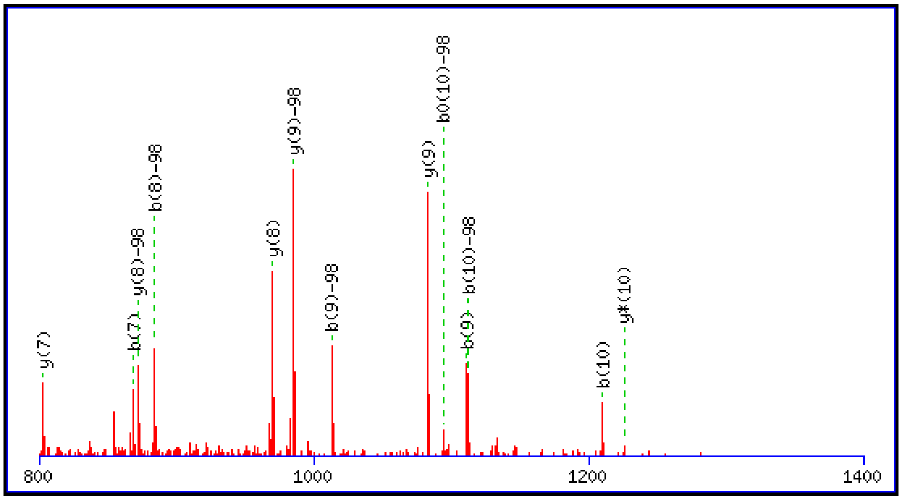

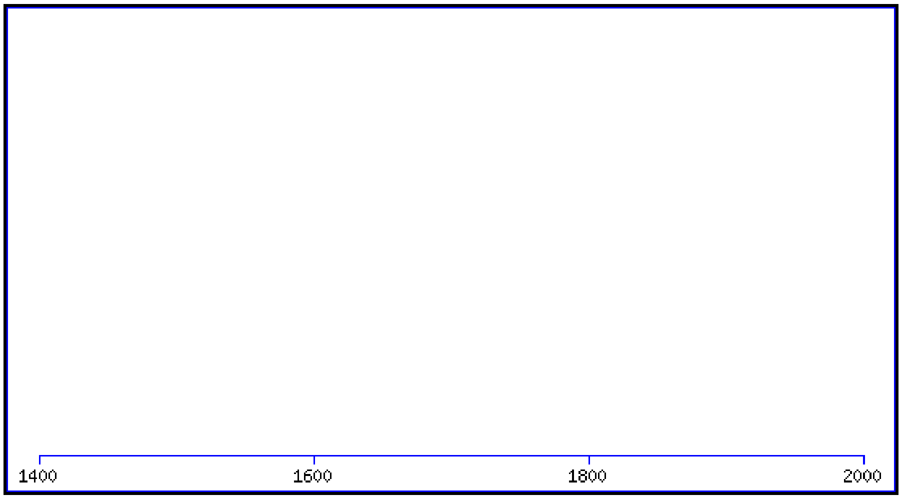

Monoisotopic mass of neutral peptide Mr(calc): 1355.6408
Fixed modifications: Carbamidomethyl (C)
Variable modifications:
S4 : Phospho (ST), with neutral losses 97.9769(shown in table), 0.0000
Ions Score: 44 **Expect:** 0.0067
Matches (Bold Red): 26/136 fragment ions using 45 most intense peaks

#	b	b++	b0	b0++	Seq.	y	y++	y*	y*++	y0	y0++	#
1	114.0913	57.5493			L							11
2	**274.1220**	137.5646			C	1145.5871	**573.2972**	1128.5605	564.7839	1127.5765	564.2919	10
3	**387.2061**	194.1067			L	**985.5564**	493.2819	968.5299	484.7686	967.5459	484.2766	9
4	456.2275	228.6174	438.2169	219.6121	S	**872.4724**	436.7398	855.4458	428.2265	854.4618	427.7345	8
5	557.2752	279.1412	539.2646	270.1359	T	**803.4509**	402.2291	786.4244	393.7158	785.4403	393.2238	7

**6**	656.3436	328.6754	638.3330	319.6702	**V**	**702.4032**	351.7053	685.3767	343.1920	684.3927	342.7000	**6**
**7**	**771.3705**	386.1889	753.3600	377.1836	**D**	**603.3348**	**302.1710**	586.3083	293.6578	585.3243	293.1658	**5**
**8**	**884.4546**	442.7309	866.4440	433.7257	**L**	**488.3079**	244.6576	471.2813	236.1443	470.2973	235.6523	**4**
**9**	**1013.4972**	507.2522	995.4866	498.2470	**E**	**375.2238**	188.1155	358.1973	179.6023	357.2132	179.1103	**3**
**10**	**1112.5656**	556.7864	**1094.5551**	**547.7812**	**V**	**246.1812**	123.5942	229.1547	115.0810			**2**
**11**					**K**	147.1128	74.0600	130.0863	65.5468			**1**

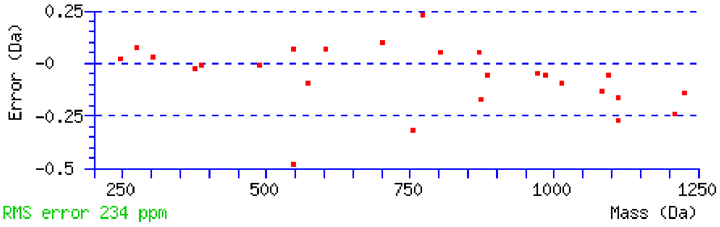

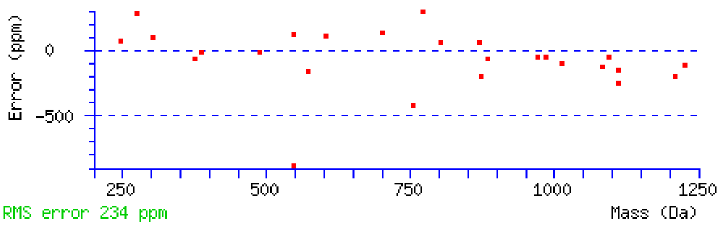

All matches to this query

Score	Mr(calc):	Delta	Sequence
43.5	1355.6408	0.0026	LCLSTVDLEVK
36.5	1355.6408	0.0026	LCLSTVDLEVK
10.2	1355.6421	0.0013	MNIPFRIGNSK
6.5	1355.6380	0.0053	MELRNNTRVK
6.1	1355.6374	0.0060	ESITENFIPVK
5.7	1354.6428	1.0006	AKCSRLSSAAPK
5.5	1355.6374	0.0060	ESITENFIPVK
4.9	1354.6435	0.9999	FTFSYKKAQR
4.9	1354.6435	0.9999	FTFSYKKAQR
4.5	1355.6374	0.0060	FPGANVTILTDK

Spectrum No: 98; Query: 1039; Rank: 1

Peptide View

MS/MS Fragmentation of **VLEIPDRDSEEELEHVIEQIAYR**
Found in **IPI00231379**, Tax_Id=10116 Gene_Symbol=Slc4a1 solute carrier family 4, member 1

Match to Query 1039: 2861.362182 from(954.794670,3+)
Title: 091129RatKid_SCX02_25.6003.6003.3.dta
Data file K:\NewmanPaper\Piliang\3SubProteomes\Piliang3SP\mgf5ppm\SCX_3SubProteomes5ppm.mgf

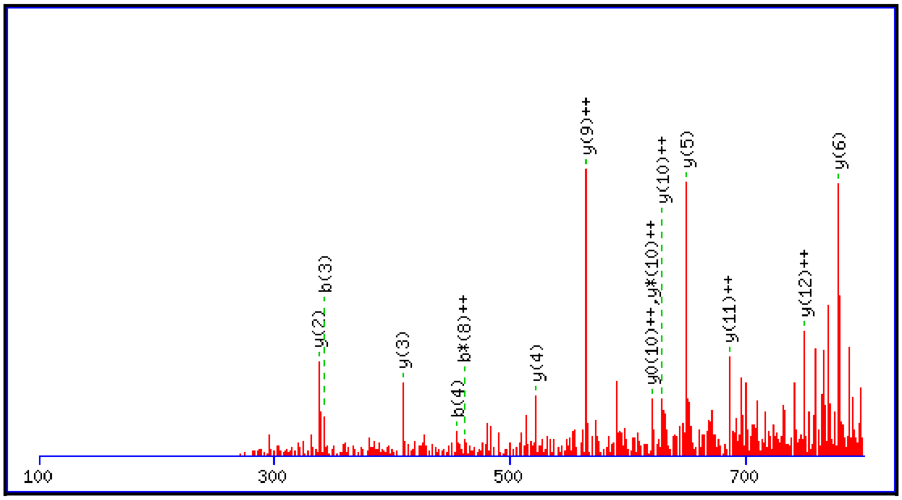

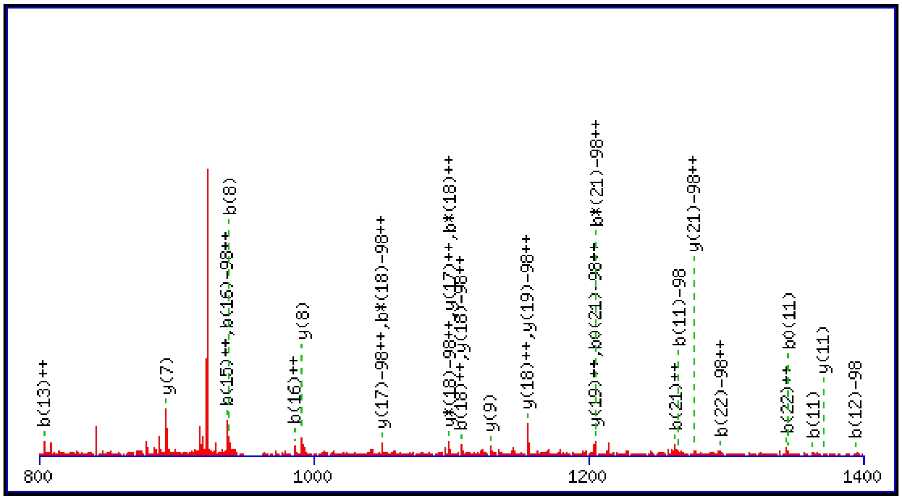

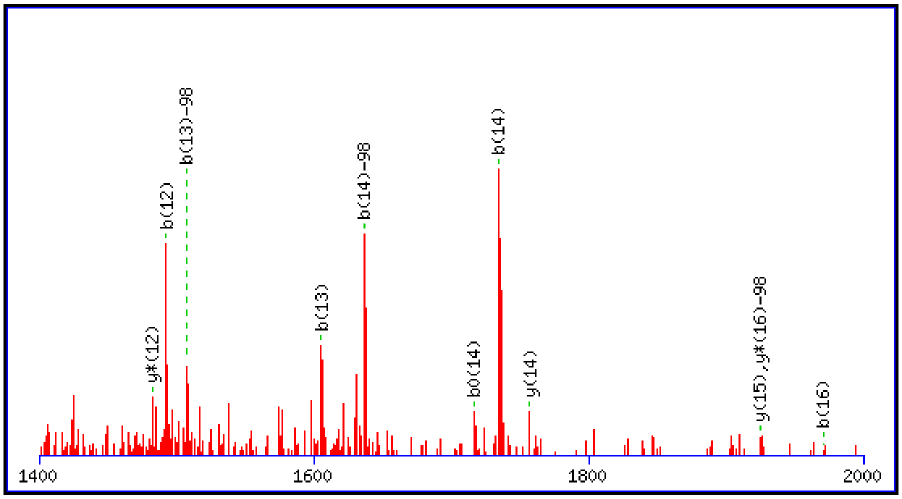

Monoisotopic mass of neutral peptide Mr(calc): 2861.3484
Fixed modifications: Carbamidomethyl (C)
Variable modifications:
S9 : Phospho (ST), with neutral losses 97.9769(shown in table), 0.0000
Ions Score: 43 **Expect:** 0.016
Matches (Bold Red): 54/370 fragment ions using 105 most intense peaks

#	b	b^{++}	b*	b^{*++}	b^{0}	b^{0++}	Seq.	y	y^{++}	y*	y^{*++}	y^{0}	y^{0++}	#
1	100.0757	50.5415					V							23
2	213.1598	107.0835					L	2665.3104	1333.1588	2648.2838	1324.6456	2647.2998	1324.1536	22
3	**342.2023**	171.6048			324.1918	162.5995	E	2552.2263	**1276.6168**	2535.1998	1268.1035	2534.2158	1267.6115	21
4	**455.2864**	228.1468			437.2758	219.1416	I	2423.1837	1212.0955	2406.1572	1203.5822	2405.1732	1203.0902	20
5	552.3392	276.6732			534.3286	267.6679	P	2310.0997	**1155.5535**	2293.0731	1147.0402	2292.0891	1146.5482	19
6	667.3661	334.1867			649.3556	325.1814	D	2213.0469	**1107.0271**	2196.0204	**1098.5138**	2195.0363	1098.0218	18
7	823.4672	412.2373	806.4407	403.7240	805.4567	403.2320	R	2098.0200	**1049.5136**	2080.9934	1041.0003	2080.0094	1040.5083	17
8	**938.4942**	469.7507	921.4676	**461.2374**	920.4836	460.7454	D	1941.9189	971.4631	**1924.8923**	962.9498	1923.9083	962.4578	16
9	1007.5156	504.2615	990.4891	495.7482	989.5051	495.2562	S	1826.8919	913.9496	1809.8654	905.4363	1808.8813	904.9443	15
10	1136.5582	568.7827	1119.5317	560.2695	1118.5477	559.7775	E	**1757.8705**	879.4389	1740.8439	870.9256	1739.8599	870.4336	14
11	**1265.6008**	633.3040	1248.5743	624.7908	1247.5902	624.2988	E	1628.8279	814.9176	1611.8013	806.4043	1610.8173	805.9123	13
12	**1394.6434**	697.8253	1377.6169	689.3121	1376.6328	688.8201	E	1499.7853	**750.3963**	**1482.7587**	741.8830	1481.7747	741.3910	12
13	**1507.7275**	754.3674	1490.7009	745.8541	1489.7169	745.3621	L	**1370.7427**	**685.8750**	1353.7161	677.3617	1352.7321	676.8697	11
14	**1636.7701**	818.8887	1619.7435	810.3754	1618.7595	809.8834	E	1257.6586	**629.3329**	1240.6321	**620.8197**	1239.6480	**620.3277**	10
15	1773.8290	887.4181	1756.8024	878.9049	1755.8184	878.4128	H	**1128.6160**	**564.8116**	1111.5895	556.2984	1110.6055	555.8064	9
16	1872.8974	**936.9523**	1855.8708	928.4391	1854.8868	927.9471	V	**991.5571**	496.2822	974.5306	487.7689	973.5465	487.2769	8
17	1985.9815	993.4944	1968.9549	984.9811	1967.9709	984.4891	I	**892.4887**	446.7480	875.4621	438.2347	874.4781	437.7427	7
18	2115.0240	1058.0157	2097.9975	**1049.5024**	2097.0135	1049.0104	E	**779.4046**	390.2060	762.3781	381.6927	761.3941	381.2007	6

**19**	2243.0826	1122.0450	2226.0561	1113.5317	2225.0721	1113.0397	**Q**	**650.3620**	325.6847	633.3355	317.1714			**5**
**20**	2356.1667	1178.5870	2339.1401	1170.0737	2338.1561	1169.5817	**I**	**522.3035**	261.6554	505.2769	253.1421			**4**
**21**	2427.2038	1214.1055	2410.1773	**1205.5923**	2409.1932	**1205.1003**	**A**	**409.2194**	205.1133	392.1928	196.6001			**3**
**22**	2590.2671	**1295.6372**	2573.2406	1287.1239	2572.2566	1286.6319	**Y**	**338.1823**	169.5948	321.1557	161.0815			**2**
**23**							**R**	175.1190	88.0631	158.0924	79.5498			**1**

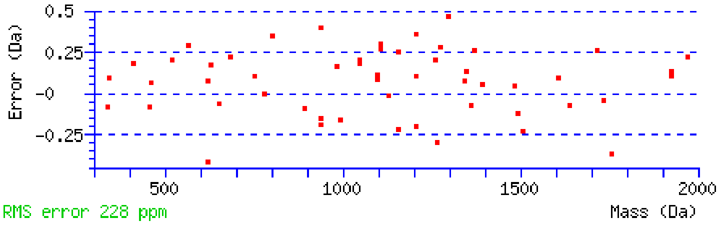

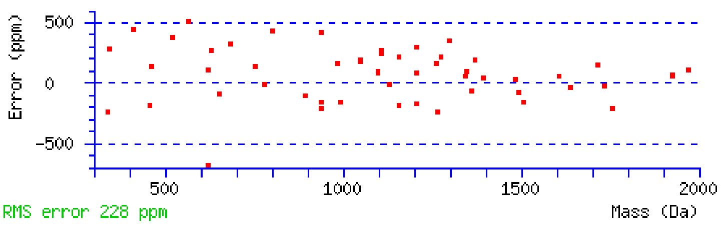

All matches to this query

Score	Mr(calc):	Delta	Sequence
43.3	2861.3484	0.0138	VLEIPDRDSEEELEHVIEQIAYR
4.6	2860.3597	1.0025	DKIMSVFYTVVTPMLNPIIYSLR
3.3	2860.3813	0.9809	LPPRSSVLSLLTSSYSGTEKYLFR
2.9	2860.3813	0.9809	LPPRSSVLSLLTSSYSGTEKYLFR
1.5	2860.3602	1.0020	QFPSHDLPSVLAKFSLPVSLSEFR
1.4	2860.3602	1.0020	QFPSHDLPSVLAKFSLPVSLSEFR
1.4	2860.3465	1.0157	FELYWPAHCLTVTPQHGYIMPER
1.4	2861.3719	-0.0098	MPPPVYRPVVSPEFAPPECLQLMK
1.3	2861.3513	0.0108	QEPAPKPNNKTPAILYTYSGLRSR
1.2	2861.3743	-0.0121	SLNPALDGLTCGLTSHDKRISDLGNK

Spectrum No: 99; Query: 1043; Rank: 1

Peptide View

MS/MS Fragmentation of **TAAVQEECGPDPRPATPPGGREETLR**
Found in **IPI00191563**, Tax_Id=10116 Gene_Symbol=LOC690489 hypothetical protein LOC690489

Match to Query 1043: 2870.310912 from(957.777580,3+)
Title: 091129RatKid_SCX02_27.1229.1229.3.dta
Data file K:\NewmanPaper\Piliang\3SubProteomes\Piliang3SP\mgf5ppm\SCX_3SubProteomes5ppm.mgf

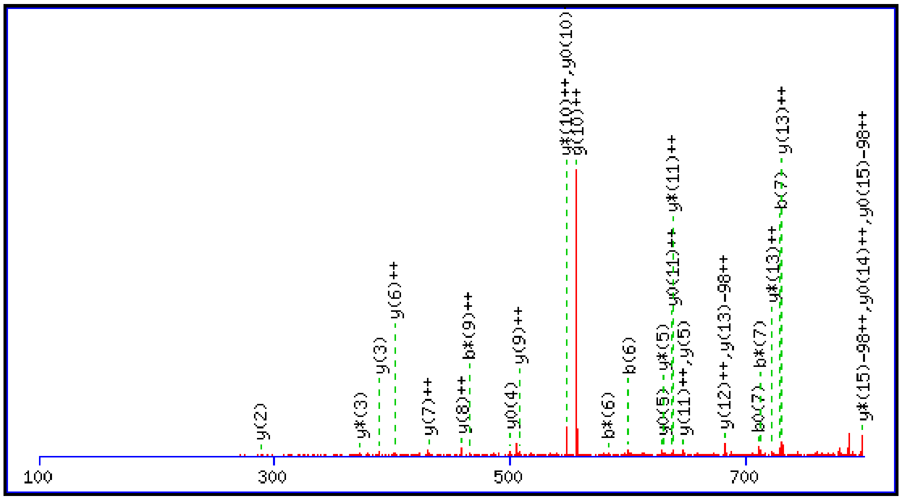

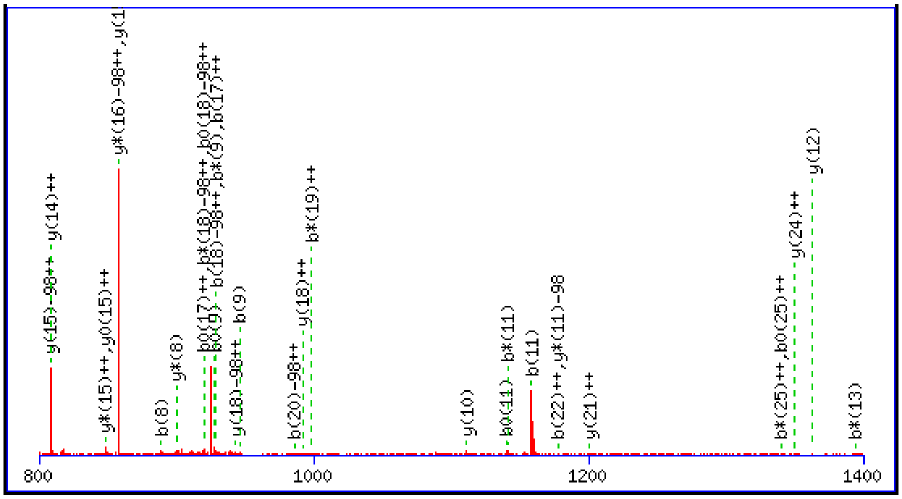

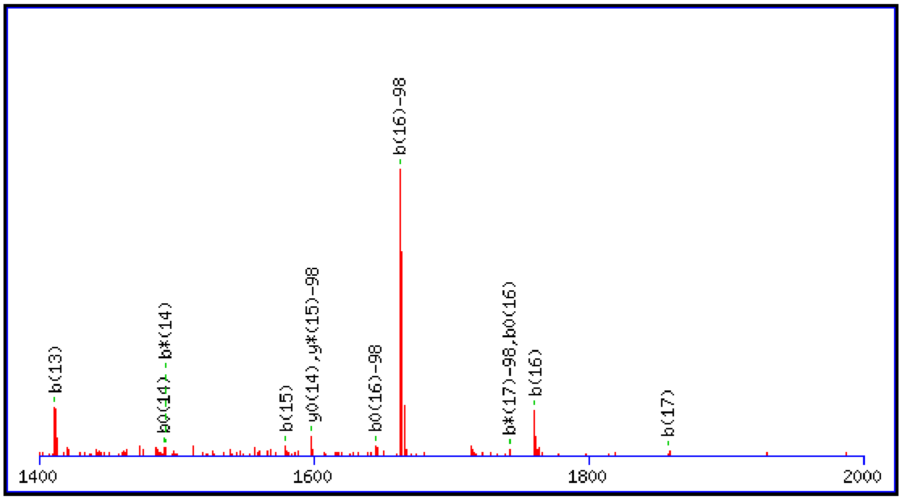

Monoisotopic mass of neutral peptide Mr(calc): 2870.3018
Fixed modifications: Carbamidomethyl (C)
Variable modifications:
T16 : Phospho (ST), with neutral losses 0.0000(shown in table), 97.9769
Ions Score: 43 **Expect:** 0.017
Matches (Bold Red): 74/438 fragment ions using 141 most intense peaks

#	b	b^{++}	b*	b^{*++}	b^0	b^{0++}	Seq.	y	y^{++}	y*	y^{*++}	y^0	y^{0++}	#
1	102.0550	51.5311			84.0444	42.5258	T							26
2	173.0921	87.0497			155.0815	78.0444	A	2770.2614	1385.6344	2753.2349	1377.1211	2752.2509	1376.6291	25
3	244.1292	122.5682			226.1186	113.5629	A	2699.2243	**1350.1158**	2682.1978	1341.6025	2681.2138	1341.1105	24
4	343.1976	172.1024			325.1870	163.0972	V	2628.1872	1314.5972	2611.1607	1306.0840	2610.1766	1305.5920	23
5	471.2562	236.1317	454.2296	227.6185	453.2456	227.1264	Q	2529.1188	1265.0630	2512.0922	1256.5498	2511.1082	1256.0577	22
6	**600.2988**	300.6530	**583.2722**	292.1397	582.2882	291.6477	E	2401.0602	**1201.0337**	2384.0337	1192.5205	2383.0496	1192.0285	21
7	**729.3414**	365.1743	**712.3148**	356.6610	**711.3308**	356.1690	E	2272.0176	1136.5124	2254.9911	1127.9992	2254.0071	1127.5072	20
8	**889.3720**	445.1896	872.3455	436.6764	871.3614	436.1844	C	2142.9750	1071.9911	2125.9485	1063.4779	2124.9645	1062.9859	19
9	**946.3935**	473.7004	**929.3669**	**465.1871**	**928.3829**	464.6951	G	1982.9444	**991.9758**	1965.9178	983.4626	1964.9338	982.9705	18
10	1043.4462	522.2268	1026.4197	513.7135	1025.4357	513.2215	P	1925.9229	963.4651	1908.8964	954.9518	1907.9123	954.4598	17
11	**1158.4732**	579.7402	**1141.4466**	571.2270	**1140.4626**	570.7349	D	1828.8701	914.9387	1811.8436	906.4254	1810.8596	905.9334	16
12	1255.5259	628.2666	1238.4994	619.7533	1237.5154	619.2613	P	1713.8432	**857.4252**	1696.8167	**848.9120**	1695.8326	**848.4200**	15
13	**1411.6271**	706.3172	**1394.6005**	697.8039	1393.6165	697.3119	R	1616.7904	**808.8989**	1599.7639	800.3856	**1598.7799**	**799.8936**	14
14	1508.6798	754.8435	**1491.6533**	746.3303	**1490.6693**	745.8383	P	1460.6893	**730.8483**	1443.6628	**722.3350**	1442.6788	721.8430	13
15	**1579.7169**	790.3621	1562.6904	781.8488	1561.7064	781.3568	A	**1363.6366**	**682.3219**	1346.6100	673.8086	1345.6260	673.3166	12
16	**1760.7309**	880.8691	1743.7044	872.3558	**1742.7204**	871.8638	T	1292.5995	**646.8034**	1275.5729	**638.2901**	1274.5889	**637.7981**	11
17	**1857.7837**	**929.3955**	1840.7572	920.8822	1839.7731	**920.3902**	P	**1111.5854**	**556.2964**	1094.5589	**547.7831**	1093.5749	**547.2911**	10
18	1954.8365	977.9219	1937.8099	969.4086	1936.8259	968.9166	P	1014.5327	**507.7700**	997.5061	499.2567	996.5221	498.7647	9

**19**	2011.8579	1006.4326	1994.8314	**997.9193**	1993.8474	997.4273	**G**	917.4799	**459.2436**	**900.4534**	450.7303	899.4694	450.2383	**8**
**20**	2068.8794	1034.9433	2051.8528	1026.4301	2050.8688	1025.9381	**G**	860.4585	**430.7329**	843.4319	422.2196	842.4479	421.7276	**7**
**21**	2224.9805	1112.9939	2207.9540	1104.4806	2206.9699	1103.9886	**R**	803.4370	**402.2221**	786.4104	393.7089	785.4264	393.2169	**6**
**22**	2354.0231	**1177.5152**	2336.9965	1169.0019	2336.0125	1168.5099	**E**	**647.3359**	324.1716	**630.3093**	315.6583	**629.3253**	315.1663	**5**
**23**	2483.0657	1242.0365	2466.0391	1233.5232	2465.0551	1233.0312	**E**	518.2933	259.6503	501.2667	251.1370	**500.2827**	250.6450	**4**
**24**	2584.1134	1292.5603	2567.0868	1284.0470	2566.1028	1283.5550	**T**	**389.2507**	195.1290	**372.2241**	186.6157	371.2401	186.1237	**3**
**25**	2697.1974	1349.1024	2680.1709	**1340.5891**	2679.1869	**1340.0971**	**L**	**288.2030**	144.6051	271.1765	136.0919			**2**
**26**							**R**	175.1190	88.0631	158.0924	79.5498			**1**

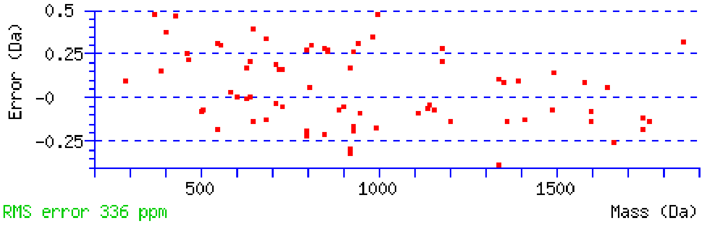

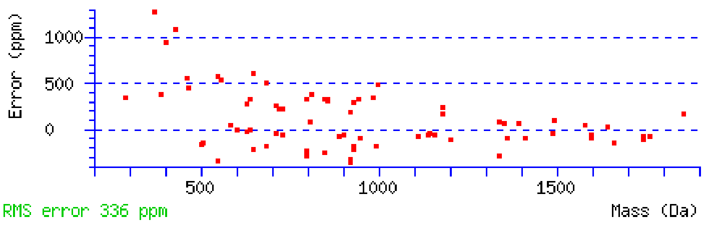

All matches to this query

Score	Mr(calc):	Delta	Sequence
43.3	2870.3018	0.0091	TAAVQEECGPDPRPATPPGGREETLR
19.3	2870.3018	0.0091	TAAVQEECGPDPRPATPPGGREETLR
12.1	2870.3018	0.0091	TAAVQEECGPDPRPATPPGGREETLR
4.1	2869.2911	1.0198	QWLQDVLRENEAGSCFIFLVGTK
3.0	2870.2995	0.0114	SFRFYMTTKMPNPHYLPEVCIK
3.0	2870.2995	0.0114	SFRFYMTTKMPNPHYLPEVCIK
2.9	2870.2952	0.0157	MQTLPVASALSSHRTGPPPISPSKR
2.9	2870.2995	0.0114	SFRFYMTTKMPNPHYLPEVCIK
2.9	2870.2995	0.0114	SFRFYMTTKMPNPHYLPEVCIK
2.8	2870.3295	-0.0186	SFRGLSLCTTCMLSVLQAITLSPR

Spectrum No: 100; Query: 615; Rank: 1

Peptide View

MS/MS Fragmentation of **SATPPPTEPASLPQEPPK**
Found in **IPI00560565**, Tax_Id=10116 Gene_Symbol=RGD1309571 54 kDa protein

Match to Query 615: 1922.904822 from(641.975550,3+)
Title: 091127RatKid_SCX01_11.1123.1123.3.dta
Data file K:\NewmanPaper\Piliang\3SubProteomes\Piliang3SP\mgf5ppm\SCX_3SubProteomes5ppm.mgf

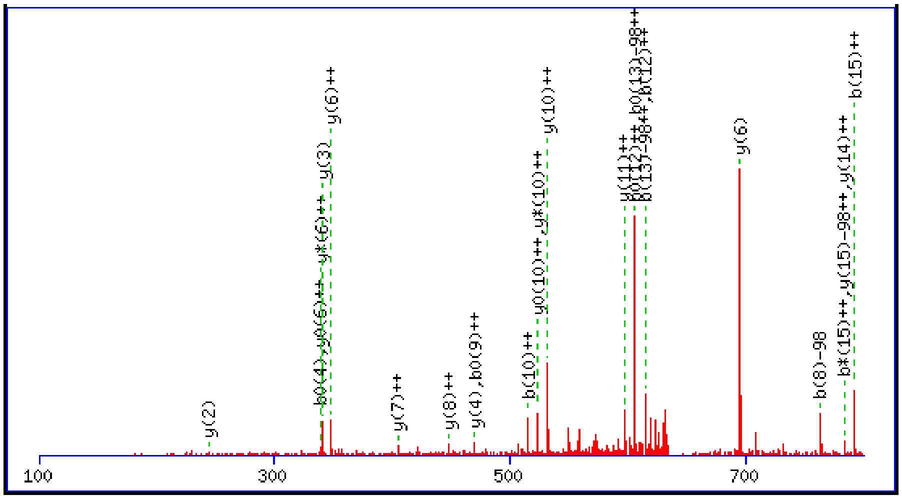

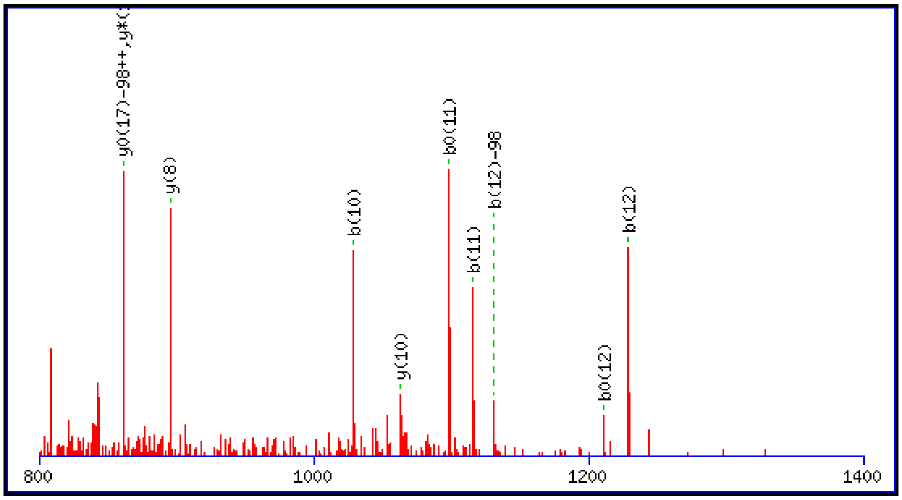

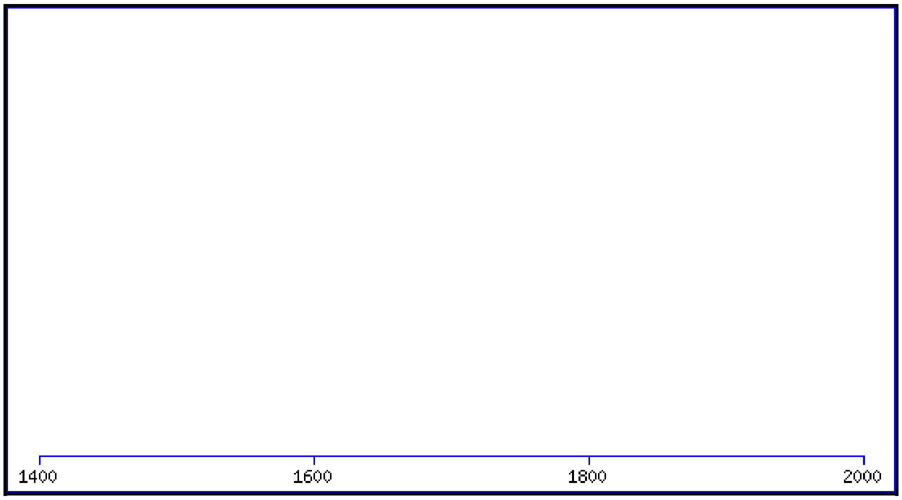

Monoisotopic mass of neutral peptide Mr(calc): 1922.9026
Fixed modifications: Carbamidomethyl (C)
Variable modifications:
T7 : Phospho (ST), with neutral losses 0.0000(shown in table), 97.9769
Ions Score: 43 **Expect:** 0.011
Matches (Bold Red): 36/260 fragment ions using 40 most intense peaks

#	b	b^{++}	b*	b^{*++}	b^0	b^{0++}	Seq.	y	y^{++}	y*	y^{*++}	y^0	y^{0++}	#
1	88.0393	44.5233			70.0287	35.5180	S							18
2	159.0764	80.0418			141.0659	71.0366	A	1836.8779	918.9426	1819.8514	910.4293	1818.8674	909.9373	17
3	260.1241	130.5657			242.1135	121.5604	T	1765.8408	883.4240	1748.8143	874.9108	1747.8302	874.4188	16
4	357.1769	179.0921			**339.1663**	170.0868	P	1664.7931	832.9002	1647.7666	824.3869	1646.7826	823.8949	15
5	454.2296	227.6185			436.2191	218.6132	P	1567.7404	**784.3738**	1550.7138	775.8605	1549.7298	775.3685	14

**6**	551.2824	276.1448			533.2718	267.1396	**P**	1470.6876	735.8474	1453.6610	727.3342	1452.6770	726.8422	**13**
**7**	732.2964	366.6518			714.2858	357.6466	**T**	1373.6348	687.3211	1356.6083	678.8078	1355.6243	678.3158	**12**
**8**	**861.3390**	431.1731			843.3284	422.1678	**E**	1192.6208	**596.8141**	1175.5943	588.3008	1174.6103	587.8088	**11**
**9**	958.3917	479.6995			940.3812	**470.6942**	**P**	**1063.5782**	**532.2928**	1046.5517	**523.7795**	1045.5677	**523.2875**	**10**
**10**	**1029.4289**	**515.2181**			1011.4183	506.2128	**A**	966.5255	483.7664	949.4989	475.2531	948.5149	474.7611	**9**
**11**	**1116.4609**	558.7341			**1098.4503**	549.7288	**S**	**895.4884**	**448.2478**	878.4618	439.7345	877.4778	439.2425	**8**
**12**	**1229.5450**	**615.2761**			**1211.5344**	**606.2708**	**L**	808.4563	**404.7318**	791.4298	396.2185	790.4458	395.7265	**7**
**13**	1326.5977	663.8025			1308.5872	654.7972	**P**	**695.3723**	**348.1898**	678.3457	**339.6765**	677.3617	**339.1845**	**6**
**14**	1454.6563	727.8318	1437.6297	719.3185	1436.6457	718.8265	**Q**	598.3195	299.6634	581.2930	291.1501	580.3089	290.6581	**5**
**15**	1583.6989	**792.3531**	1566.6723	**783.8398**	1565.6883	783.3478	**E**	**470.2609**	235.6341	453.2344	227.1208	452.2504	226.6288	**4**
**16**	1680.7517	840.8795	1663.7251	832.3662	1662.7411	831.8742	**P**	**341.2183**	171.1128	324.1918	162.5995			**3**
**17**	1777.8044	889.4058	1760.7779	880.8926	1759.7939	880.4006	**P**	**244.1656**	122.5864	227.1390	114.0731			**2**
**18**							**K**	147.1128	74.0600	130.0863	65.5468			**1**

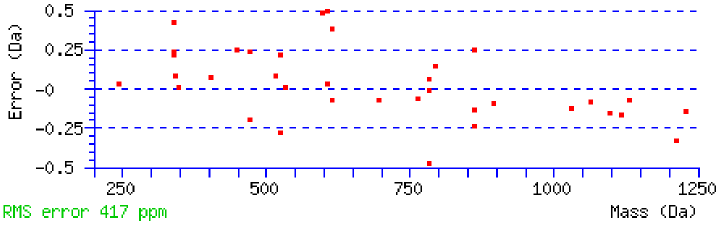

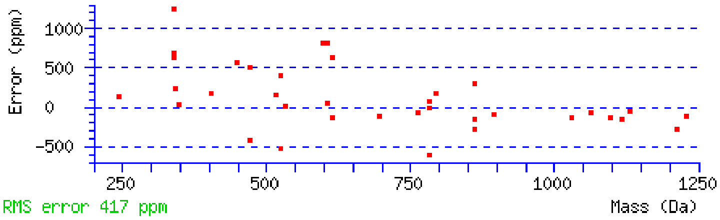

All matches to this query

Score	Mr(calc):	Delta	Sequence
43.2	1922.9026	0.0022	SATPPPTEPASLPQEPPK
39.7	1922.9026	0.0022	SATPPPTEPASLPQEPPK
39.7	1922.9026	0.0022	SATPPPTEPASLPQEPPK
20.0	1922.9026	0.0022	SATPPPTEPASLPQEPPK
6.7	1922.9056	-0.0008	NRVKLAANFSFAPVTK
4.9	1922.9155	-0.0107	RALIITLNSFGTELSK
4.7	1920.9111	1.9937	LSPVTYKLQPGSKTSR
4.4	1922.9155	-0.0107	RALIITLNSFGTELSK
4.3	1922.8995	0.0053	TVXNLLGMAGMTPNVNSK
4.0	1920.9090	1.9959	ILENCMPNASLELLPK

Spectrum No: 101; Query: 1110; Rank: 1

Peptide View

MS/MS Fragmentation of **AKPAAQSEEETAASPAASPTPQSAQEPSAPGK**
Found in **IPI00363771**, Tax_Id=10116 Gene_Symbol=Eif3s9 108 kDa protein

Match to Query 1110: 3250.397772 from(1084.473200,3+)
Title: 091129RatKid_SCX02_15.458.458.3.dta
Data file K:\NewmanPaper\Piliang\3SubProteomes\Piliang3SP\mgf5ppm\SCX_3SubProteomes5ppm.mgf

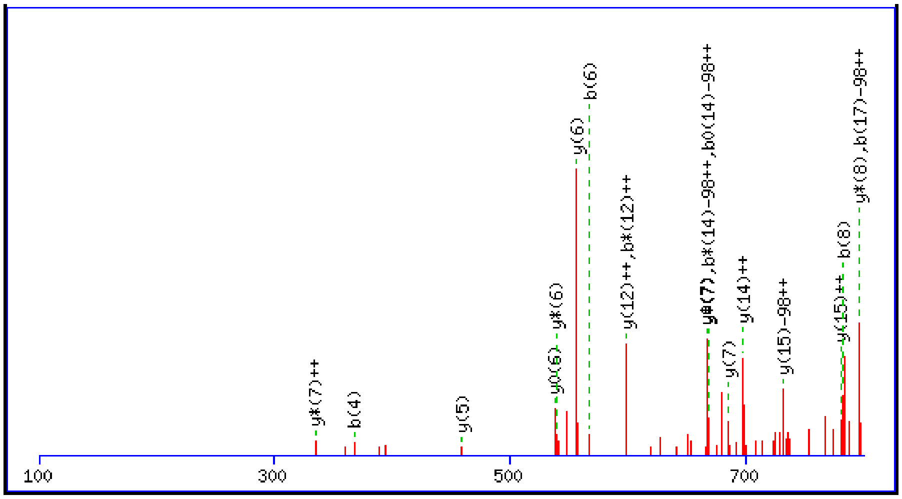

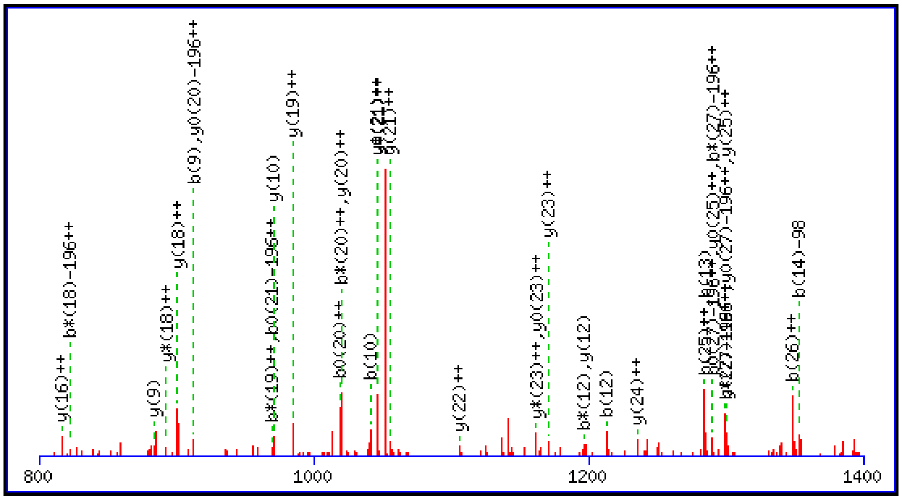

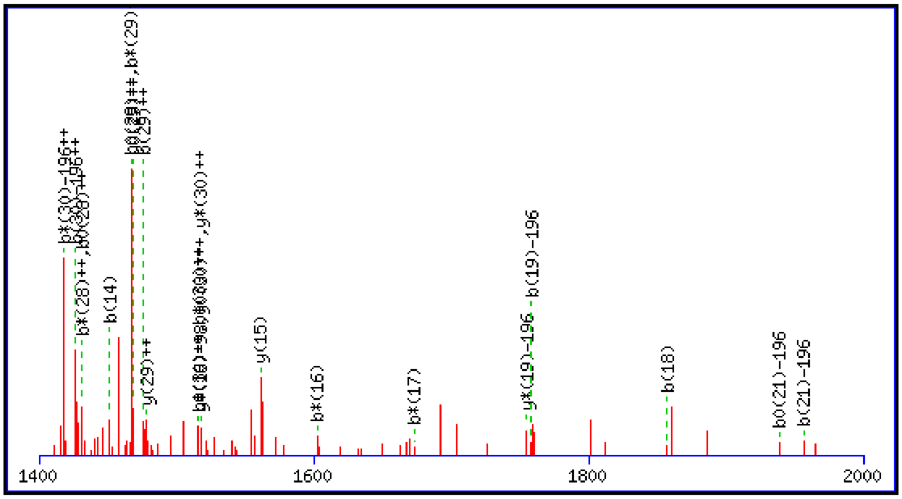

Monoisotopic mass of neutral peptide Mr(calc): 3250.4068
Fixed modifications: Carbamidomethyl (C)
Variable modifications:
S14 : Phospho (ST), with neutral losses 0.0000(shown in table), 97.9769
S18 : Phospho (ST), with neutral losses 0.0000(shown in table), 97.9769
Ions Score: 43 **Expect:** 0.021
Matches (Bold Red): 80/560 fragment ions using 120 most intense peaks

#	b	b++	b*	b*++	b0	b0++	Seq.	y	y++	y*	y*++	y0	y0++	#
1	72.0444	36.5258					A							32
2	200.1394	100.5733	183.1128	92.0600			K	3180.3770	1590.6921	3163.3504	1582.1789	3162.3664	1581.6868	31
3	297.1921	149.0997	280.1656	140.5864			P	3052.2820	1526.6446	3035.2555	**1518.1314**	3034.2715	**1517.6394**	30
4	**368.2292**	184.6183	351.2027	176.1050			A	2955.2293	**1478.1183**	2938.2027	1469.6050	2937.2187	**1469.1130**	29

5	439.2663	220.1368	422.2398	211.6235			A	2884.1921	1442.5997	2867.1656	1434.0864	2866.1816	1433.5944	28
6	567.3249	284.1661	550.2984	275.6528			Q	2813.1550	1407.0812	2796.1285	1398.5679	2795.1445	1398.0759	27
7	654.3570	327.6821	637.3304	319.1688	636.3464	318.6768	S	2685.0964	1343.0519	2668.0699	1334.5386	2667.0859	1334.0466	26
8	783.3995	392.2034	766.3730	383.6901	765.3890	383.1981	E	2598.0644	1299.5358	2581.0379	1291.0226	2580.0539	1290.5306	25
9	912.4421	456.7247	895.4156	448.2114	894.4316	447.7194	E	2469.0218	1235.0146	2451.9953	1226.5013	2451.0113	1226.0093	24
10	1041.4847	521.2460	1024.4582	512.7327	1023.4742	512.2407	E	2339.9792	1170.4933	2322.9527	1161.9800	2321.9687	1161.4880	23
11	1142.5324	571.7698	1125.5059	563.2566	1124.5218	562.7646	T	2210.9366	1105.9720	2193.9101	1097.4587	2192.9261	1096.9667	22
12	1213.5695	607.2884	1196.5430	598.7751	1195.5590	598.2831	A	2109.8890	1055.4481	2092.8624	1046.9348	2091.8784	1046.4428	21
13	1284.6066	642.8070	1267.5801	634.2937	1266.5961	633.8017	A	2038.8518	1019.9296	2021.8253	1011.4163	2020.8413	1010.9243	20
14	1451.6050	726.3061	1434.5784	717.7929	1433.5944	717.3008	S	1967.8147	984.4110	1950.7882	975.8977	1949.8042	975.4057	19
15	1548.6578	774.8325	1531.6312	766.3192	1530.6472	765.8272	P	1800.8164	900.9118	1783.7898	892.3986	1782.8058	891.9065	18
16	1619.6949	810.3511	1602.6683	801.8378	1601.6843	801.3458	A	1703.7636	852.3854	1686.7371	843.8722	1685.7531	843.3802	17
17	1690.7320	845.8696	1673.7054	837.3564	1672.7214	836.8643	A	1632.7265	816.8669	1615.7000	808.3536	1614.7159	807.8616	16
18	1857.7303	929.3688	1840.7038	920.8555	1839.7198	920.3635	S	1561.6894	781.3483	1544.6628	772.8351	1543.6788	772.3430	15
19	1954.7831	977.8952	1937.7565	969.3819	1936.7725	968.8899	P	1394.6910	697.8492	1377.6645	689.3359	1376.6805	688.8439	14
20	2055.8308	1028.4190	2038.8042	1019.9058	2037.8202	1019.4137	T	1297.6383	649.3228	1280.6117	640.8095	1279.6277	640.3175	13
21	2152.8835	1076.9454	2135.8570	1068.4321	2134.8730	1067.9401	P	1196.5906	598.7989	1179.5640	590.2857	1178.5800	589.7937	12
22	2280.9421	1140.9747	2263.9156	1132.4614	2262.9316	1131.9694	Q	1099.5378	550.2726	1082.5113	541.7593	1081.5273	541.2673	11
23	2367.9741	1184.4907	2350.9476	1175.9774	2349.9636	1175.4854	S	971.4793	486.2433	954.4527	477.7300	953.4687	477.2380	10
24	2439.0113	1220.0093	2421.9847	1211.4960	2421.0007	1211.0040	A	884.4472	442.7272	867.4207	434.2140	866.4367	433.7220	9
25	2567.0698	1284.0386	2550.0433	1275.5253	2549.0593	1275.0333	Q	813.4101	407.2087	796.3836	398.6954	795.3995	398.2034	8
26	2696.1124	1348.5599	2679.0859	1340.0466	2678.1019	1339.5546	E	685.3515	343.1794	668.3250	334.6661	667.3410	334.1741	7
27	2793.1652	1397.0862	2776.1386	1388.5730	2775.1546	1388.0810	P	556.3089	278.6581	539.2824	270.1448	538.2984	269.6528	6
28	2880.1972	1440.6022	2863.1707	1432.0890	2862.1867	1431.5970	S	459.2562	230.1317	442.2296	221.6185	441.2456	221.1264	5
29	2951.2343	1476.1208	2934.2078	1467.6075	2933.2238	1467.1155	A	372.2241	186.6157	355.1976	178.1024			4
30	3048.2871	1524.6472	3031.2606	1516.1339	3030.2765	1515.6419	P	301.1870	151.0972	284.1605	142.5839			3
31	3105.3086	1553.1579	3088.2820	1544.6446	3087.2980	1544.1526	G	204.1343	102.5708	187.1077	94.0575			2
32							K	147.1128	74.0600	130.0863	65.5468			1

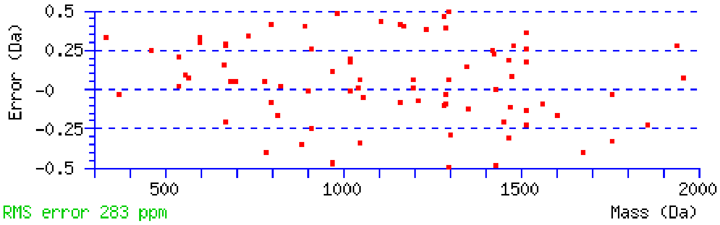

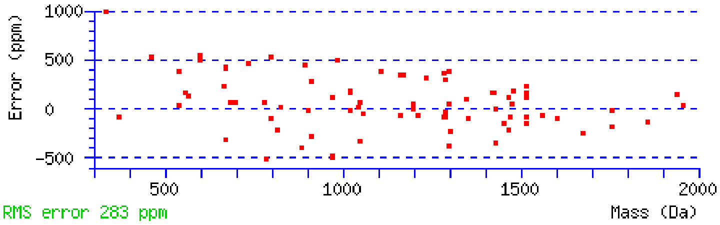

All matches to this query

Score	Mr(calc):	Delta	Sequence
42.8	3250.4068	-0.0090	AKPAAQSEEETAASPAASPTPQSAQEPSAPGK
28.4	3250.4068	-0.0090	AKPAAQSEEETAASPAASPTPQSAQEPSAPGK
24.8	3250.4068	-0.0090	AKPAAQSEEETAASPAASPTPQSAQEPSAPGK
17.8	3250.4068	-0.0090	AKPAAQSEEETAASPAASPTPQSAQEPSAPGK
12.1	3250.4068	-0.0090	AKPAAQSEEETAASPAASPTPQSAQEPSAPGK
3.5	3248.4111	1.9867	RNSQWVPTLPNSSHHLDAVPCSTTINR
3.5	3249.3951	1.0026	RNSQWVPTLPNSSHHLDAVPCSTTINR
1.5	3249.3902	1.0076	ETAFLYAVSAAALTHALARACSAGRMER
0.7	3249.3920	1.0058	NVKETSLQVDNLPLSLREASEEAYFR
0.7	3248.4080	1.9898	NVKETSLQVDNLPLSLREASEEAYFR

Spectrum No: 102; Query: 892; Rank: 1

Peptide View

MS/MS Fragmentation of **KLEKEEEEGISQESSEEEQ**
Found in **IPI00203725**, Tax_Id=10116 Gene_Symbol=Hmga1 Isoform HMG-I of High mobility group protein HMG-I/HMG-Y

Match to Query 892: 2395.913592 from(799.645140,3+)
Title: 091129RatKid_SCX02_22.724.724.3.dta
Data file K:\NewmanPaper\Piliang\3SubProteomes\Piliang3SP\mgf5ppm\SCX_3SubProteomes5ppm.mgf

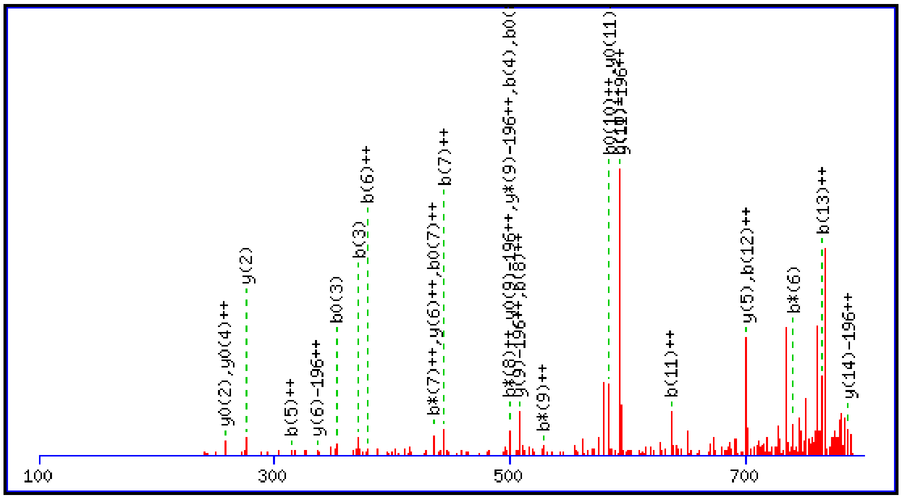

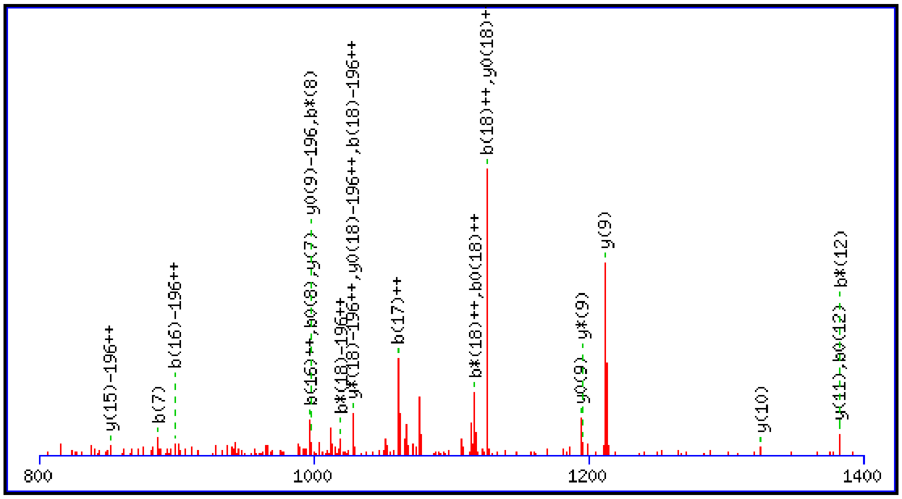

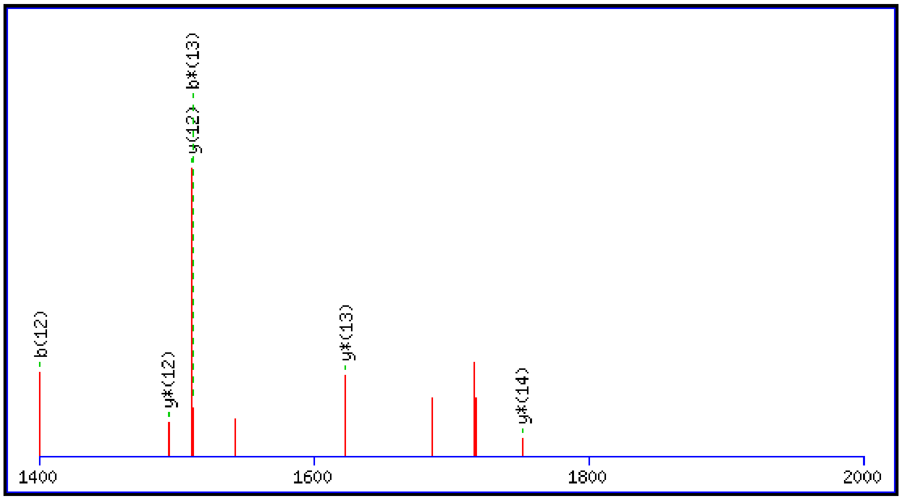

Monoisotopic mass of neutral peptide Mr(calc): 2395.9192
Fixed modifications: Carbamidomethyl (C)

Variable modifications:
S14 : Phospho (ST), with neutral losses 0.0000(shown in table), 97.9769
S15 : Phospho (ST), with neutral losses 0.0000(shown in table), 97.9769
Ions Score: 42 **Expect:** 0.011
Matches (Bold Red): 62/324 fragment ions using 88 most intense peaks

#	b	b^{++}	b^{*}	b^{*++}	b^{0}	b^{0++}	Seq.	y	y^{++}	y^{*}	y^{*++}	y^{0}	y^{0++}	#
1	129.1022	65.0548	112.0757	56.5415			K							19
2	242.1863	121.5968	225.1598	113.0835			L	2268.8316	1134.9194	2251.8051	**1126.4062**	2250.8211	**1125.9142**	18
3	**371.2289**	186.1181	354.2023	177.6048	**353.2183**	177.1128	E	2155.7476	1078.3774	2138.7210	1069.8641	2137.7370	1069.3721	17
4	**499.3239**	250.1656	482.2973	241.6523	481.3133	241.1603	K	2026.7050	1013.8561	2009.6784	1005.3428	2008.6944	1004.8508	16
5	628.3665	**314.6869**	611.3399	306.1736	610.3559	305.6816	E	1898.6100	949.8086	1881.5835	941.2954	1880.5994	940.8034	15
6	757.4090	**379.2082**	**740.3825**	370.6949	739.3985	370.2029	E	1769.5674	885.2873	**1752.5409**	876.7741	1751.5568	876.2821	14
7	**886.4516**	**443.7295**	869.4251	**435.2162**	868.4411	**434.7242**	E	1640.5248	820.7660	**1623.4983**	812.2528	1622.5143	811.7608	13
8	1015.4942	**508.2508**	**998.4677**	**499.7375**	**997.4837**	**499.2455**	E	**1511.4822**	756.2447	**1494.4557**	747.7315	1493.4717	747.2395	12
9	1072.5157	536.7615	1055.4891	**528.2482**	1054.5051	527.7562	G	**1382.4396**	691.7235	1365.4131	683.2102	1364.4291	682.7182	11
10	1185.5998	**593.3035**	1168.5732	**584.7902**	1167.5892	**584.2982**	I	**1325.4182**	663.2127	1308.3916	654.6994	1307.4076	654.2074	10
11	1272.6318	**636.8195**	1255.6052	628.3063	1254.6212	627.8142	S	**1212.3341**	606.6707	**1195.3076**	598.1574	**1194.3235**	597.6654	9
12	**1400.6904**	**700.8488**	**1383.6638**	692.3355	**1382.6798**	691.8435	Q	1125.3021	563.1547	1108.2755	554.6414	1107.2915	554.1494	8
13	1529.7330	**765.3701**	**1512.7064**	756.8568	1511.7224	756.3648	E	**997.2435**	499.1254	980.2169	490.6121	979.2329	490.1201	7
14	1696.7313	848.8693	1679.7048	840.3560	1678.7207	839.8640	S	868.2009	**434.6041**	851.1744	426.0908	850.1903	425.5988	6
15	1863.7297	932.3685	1846.7031	923.8552	1845.7191	923.3632	S	**701.2026**	351.1049	684.1760	342.5916	683.1920	342.0996	5
16	1992.7723	**996.8898**	1975.7457	988.3765	1974.7617	987.8845	E	534.2042	267.6057	517.1776	259.0925	516.1936	**258.6005**	4
17	2121.8148	**1061.4111**	2104.7883	1052.8978	2103.8043	1052.4058	E	405.1616	203.0844	388.1351	194.5712	387.1510	194.0792	3
18	2250.8574	**1125.9324**	2233.8309	**1117.4191**	2232.8469	**1116.9271**	E	**276.1190**	138.5631	259.0925	130.0499	**258.1084**	129.5579	2
19							Q	147.0764	74.0418	130.0499	65.5286			1

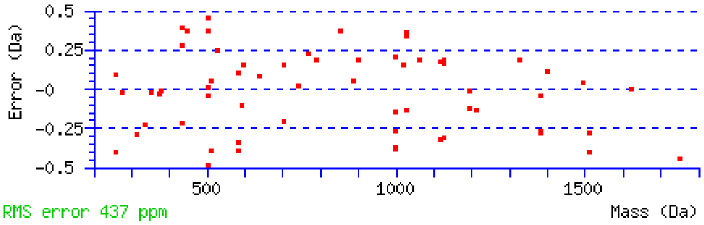

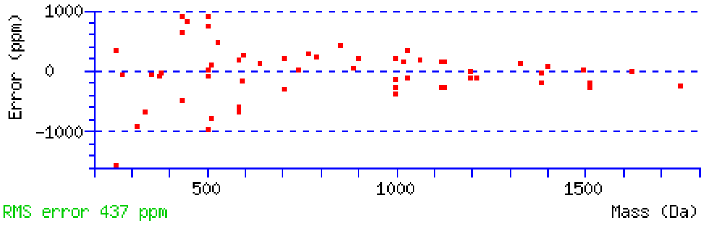

All matches to this query

Score	Mr(calc):	Delta	Sequence
42.5	2395.9192	-0.0056	KLEKEEEEGISQESSEEEQ
34.4	2395.9192	-0.0056	KLEKEEEEGISQESSEEEQ
28.3	2395.9192	-0.0056	KLEKEEEEGISQESSEEEQ
21.6	2395.9052	0.0084	EKYPYHSFIGEESVASGEK
21.6	2395.9052	0.0084	EKYPYHSFIGEESVASGEK
21.6	2395.9052	0.0084	EKYPYHSFIGEESVASGEK
21.6	2395.9052	0.0084	EKYPYHSFIGEESVASGEK
15.3	2395.9052	0.0084	EKYPYHSFIGEESVASGEK
15.3	2395.9052	0.0084	EKYPYHSFIGEESVASGEK
4.2	2395.9154	-0.0018	RLGFAHWQSTTSSVTRDK

Spectrum No: 103; Query: 478; Rank: 1

Peptide View

MS/MS Fragmentation of **GSYGSDPEEEEEYR**
Found in **IPI00190553**, Tax_Id=10116 Gene_Symbol=Tjp2 Zonula occludens 2 protein (Fragment)

Match to Query 478: 1725.607828 from(863.811190,2+)
Title: 091127RatKid_SCX01_12.1328.1328.2.dta
Data file K:\NewmanPaper\Piliang\3SubProteomes\Piliang3SP\mgf5ppm\SCX_3SubProteomes5ppm.mgf

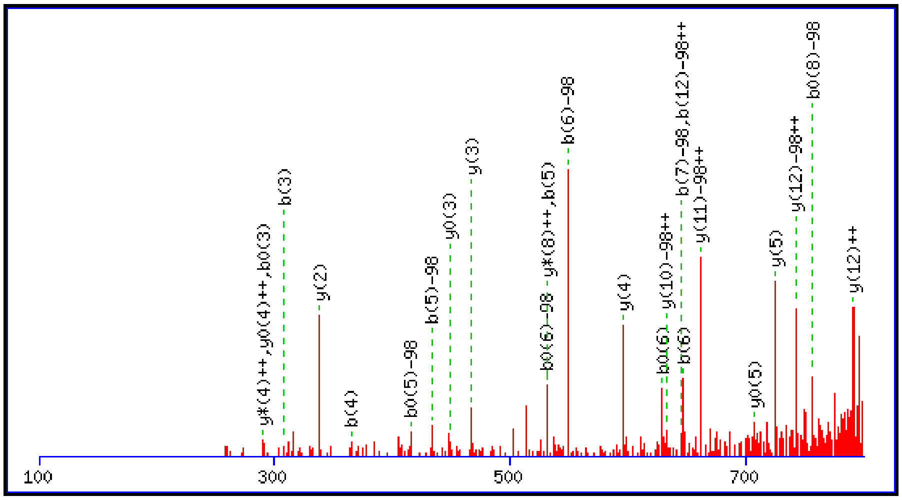

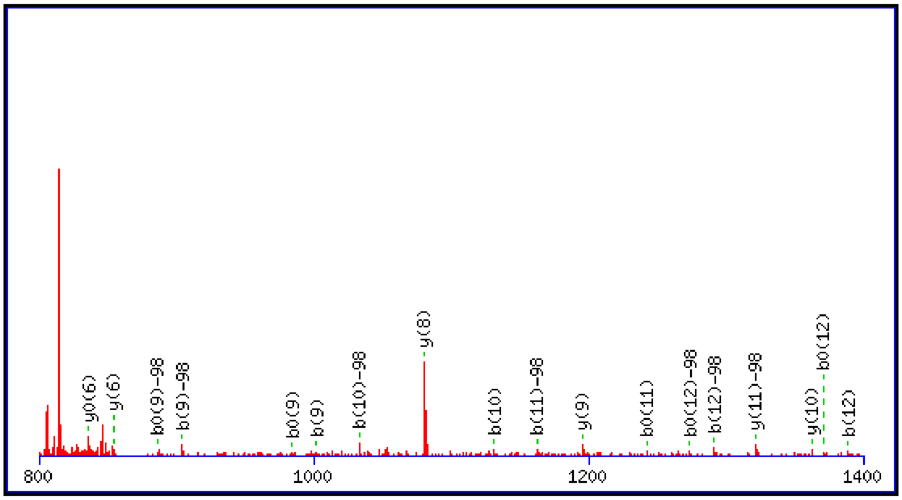

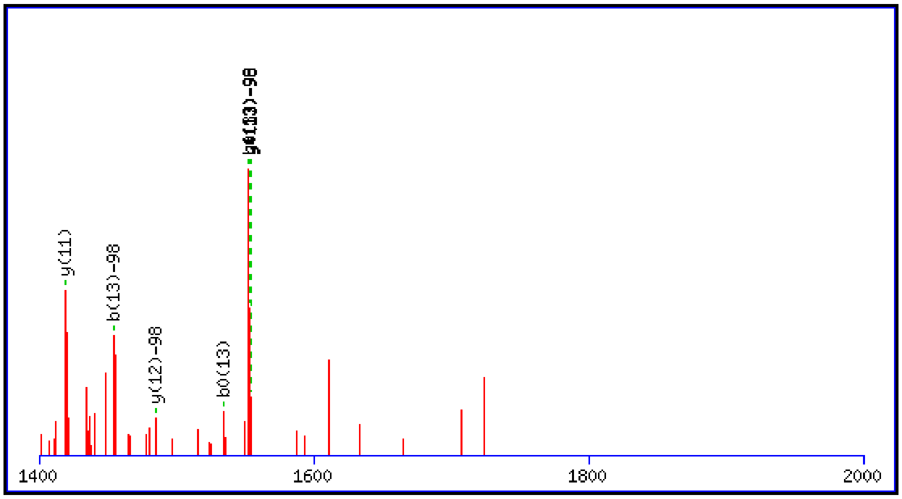

Monoisotopic mass of neutral peptide Mr(calc): 1725.6043
Fixed modifications: Carbamidomethyl (C)
Variable modifications:
S5 : Phospho (ST), with neutral losses 97.9769(shown in table), 0.0000
Ions Score: 42 **Expect:** 0.0038
Matches (Bold Red): 51/184 fragment ions using 110 most intense peaks

#	b	b^{++}	b^0	b^{0++}	Seq.	y	y^{++}	y*	y^{*++}	y^0	y^{0++}	#

1	58.0287	29.5180			G							14
2	145.0608	73.0340	127.0502	64.0287	S	1571.6132	786.3103	**1554.5867**	777.7970	**1553.6027**	777.3050	13
3	**308.1241**	154.5657	**290.1135**	145.5604	Y	**1484.5812**	**742.7942**	1467.5547	734.2810	1466.5706	733.7890	12
4	**365.1456**	183.0764	347.1350	174.0711	G	**1321.5179**	**661.2626**	1304.4913	652.7493	1303.5073	652.2573	11
5	**434.1670**	217.5871	**416.1565**	208.5819	S	1264.4964	**632.7518**	1247.4699	624.2386	1246.4858	623.7466	10
6	**549.1940**	275.1006	**531.1834**	266.0953	D	**1195.4750**	598.2411	1178.4484	589.7278	1177.4644	589.2358	9
7	**646.2467**	323.6270	628.2362	314.6217	P	**1080.4480**	540.7276	1063.4215	**532.2144**	1062.4374	531.7224	8
8	775.2893	388.1483	**757.2788**	379.1430	E	983.3952	492.2013	966.3687	483.6880	965.3847	483.1960	7
9	**904.3319**	452.6696	**886.3213**	443.6643	E	**854.3527**	427.6800	837.3261	419.1667	**836.3421**	418.6747	6
10	**1033.3745**	517.1909	1015.3639	508.1856	E	**725.3101**	363.1587	708.2835	354.6454	**707.2995**	354.1534	5
11	**1162.4171**	581.7122	1144.4065	572.7069	E	**596.2675**	298.6374	579.2409	**290.1241**	578.2569	**289.6321**	4
12	**1291.4597**	**646.2335**	**1273.4491**	637.2282	E	**467.2249**	234.1161	450.1983	225.6028	**449.2143**	225.1108	3
13	**1454.5230**	727.7651	1436.5125	718.7599	Y	**338.1823**	169.5948	321.1557	161.0815			2
14					R	175.1190	88.0631	158.0924	79.5498			1

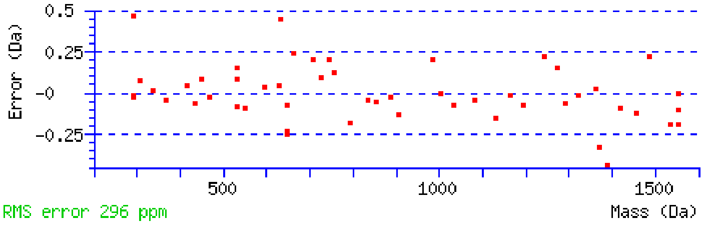

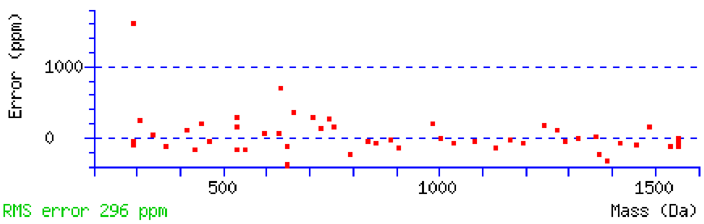

All matches to this query

Score	Mr(calc):	Delta	Sequence
42.4	1725.6043	0.0035	GSYGSDPEEEEEYR
37.8	1725.6043	0.0035	GSYGSDPEEEEEYR
9.5	1725.6043	0.0035	GSYGSDPEEEEEYR
2.3	1724.6093	0.9986	MSMNTNGAEDDGKMK
2.3	1724.6093	0.9986	MSMNTNGAEDDGKMK
2.3	1724.6093	0.9986	MSMNTNGAEDDGKMK
2.3	1724.6093	0.9986	MSMNTNGAEDDGKMK

Spectrum No: 104; Query: 114; Rank: 1

Peptide View

MS/MS Fragmentation of **SASADNLILPR**
Found in **IPI00364913**, Tax_Id=10116 Gene_Symbol=RGD1565969_predicted similar to cyclin fold protein 1

Match to Query 114: 1235.593708 from(618.804130,2+)
Title: 091127RatKid_SCX01_12.2283.2283.2.dta
Data file K:\NewmanPaper\Piliang\3SubProteomes\Piliang3SP\mgf5ppm\SCX_3SubProteomes5ppm.mgf

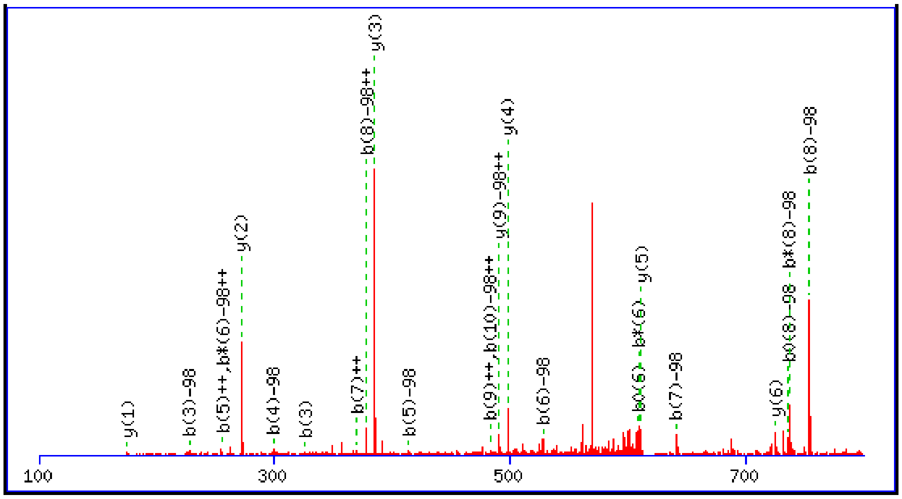

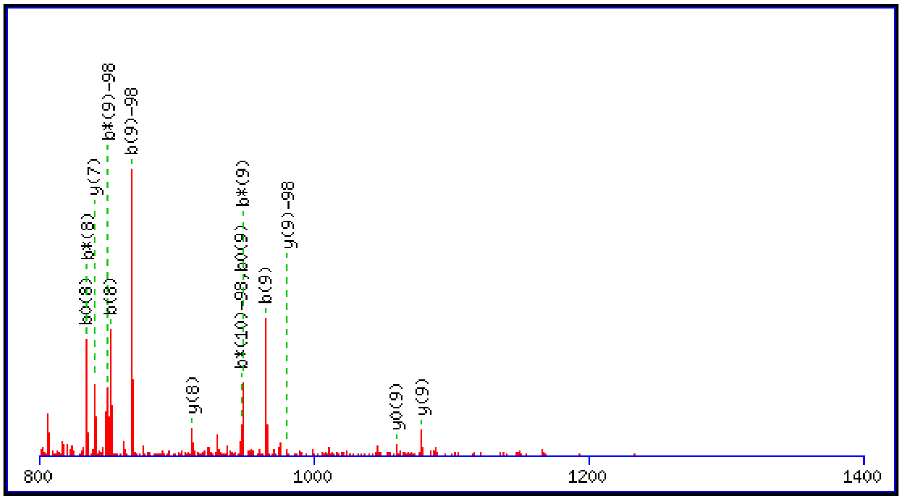

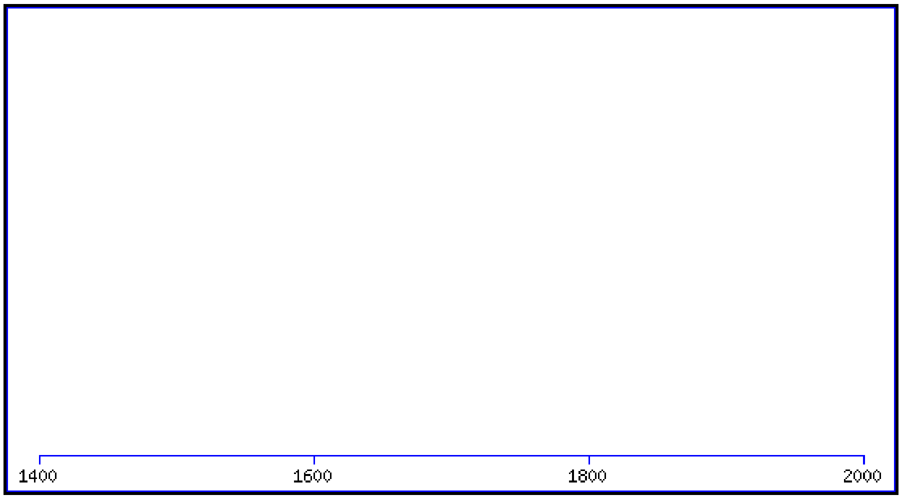

Monoisotopic mass of neutral peptide Mr(calc): 1235.5911
Fixed modifications: Carbamidomethyl (C)
Variable modifications:
S3 : Phospho (ST), with neutral losses 97.9769(shown in table), 0.0000
Ions Score: 42 **Expect:** 0.0074
Matches (Bold Red): 38/152 fragment ions using 79 most intense peaks

#	b	b^{++}	b*	b^{*++}	b^0	b^{0++}	Seq.	y	y^{++}	y*	y^{*++}	y^0	y^{0++}	#
1	88.0393	44.5233			70.0287	35.5180	S							11
2	159.0764	80.0418			141.0659	71.0366	A	1051.5895	526.2984	1034.5629	517.7851	1033.5789	517.2931	10
3	**228.0979**	114.5526			210.0873	105.5473	S	**980.5523**	**490.7798**	963.5258	482.2665	962.5418	481.7745	9
4	**299.1350**	150.0711			281.1244	141.0658	A	**911.5309**	456.2691	894.5043	447.7558	893.5203	447.2638	8
5	**414.1619**	207.5846			396.1514	198.5793	D	**840.4938**	420.7505	823.4672	412.2373	822.4832	411.7452	7

**6**	**528.2049**	264.6061	511.1783	**256.0928**	510.1943	255.6008	**N**	**725.4668**	363.2371	708.4403	354.7238			**6**
**7**	**641.2889**	321.1481	624.2624	312.6348	623.2784	312.1428	**L**	**611.4239**	306.2156	594.3974	297.7023			**5**
**8**	**754.3730**	**377.6901**	**737.3464**	369.1769	**736.3624**	368.6848	**I**	**498.3398**	249.6736	481.3133	241.1603			**4**
**9**	**867.4571**	434.2322	**850.4305**	425.7189	849.4465	425.2269	**L**	**385.2558**	193.1315	368.2292	184.6183			**3**
**10**	964.5098	**482.7585**	**947.4833**	474.2453	946.4992	473.7533	**P**	**272.1717**	136.5895	255.1452	128.0762			**2**
**11**							**R**	**175.1190**	88.0631	158.0924	79.5498			**1**

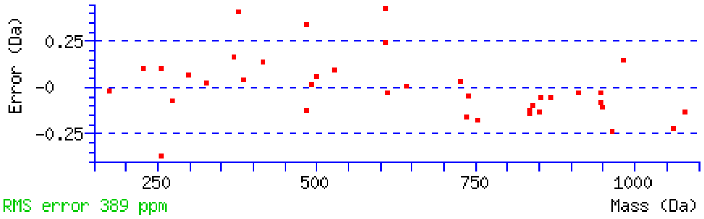

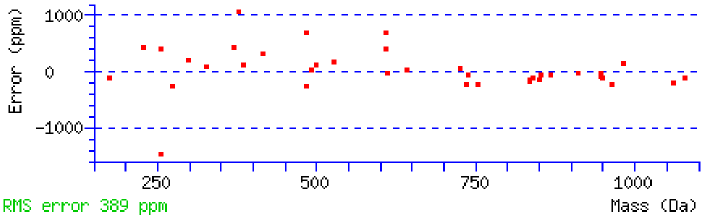

All matches to this query

Score	Mr(calc):	Delta	Sequence
42.3	1235.5911	0.0026	SASADNLILPR
33.7	1235.5911	0.0026	SASADNLILPR
5.2	1233.5850	2.0087	NRNPQGSAFSR
4.7	1235.5968	-0.0031	RDIPDYLCGK
4.2	1233.5979	1.9958	ARDRSPSPLR
2.4	1233.5979	1.9958	TGQLHTRVSR
1.9	1235.6023	-0.0086	RLNPDGKSIR
1.4	1233.5979	1.9958	VRGSNLQQPR
0.7	1235.5911	0.0026	DTSQIPLLNR
0.7	1235.5911	0.0026	DTSQIPLLNR

Spectrum No: 105; Query: 469; Rank: 1

Peptide View

MS/MS Fragmentation of **SSTPLPTVSSSAENTR**
Found in **IPI00208114**, Tax_Id=10116 Gene_Symbol=Tmpo Lamina-associated polypeptide 2 isoform beta

Match to Query 469: 1712.765248 from(857.389900,2+)
Title: 091127RatKid_SCX01_12.1309.1309.2.dta
Data file K:\NewmanPaper\Piliang\3SubProteomes\Piliang3SP\mgf5ppm\SCX_3SubProteomes5ppm.mgf

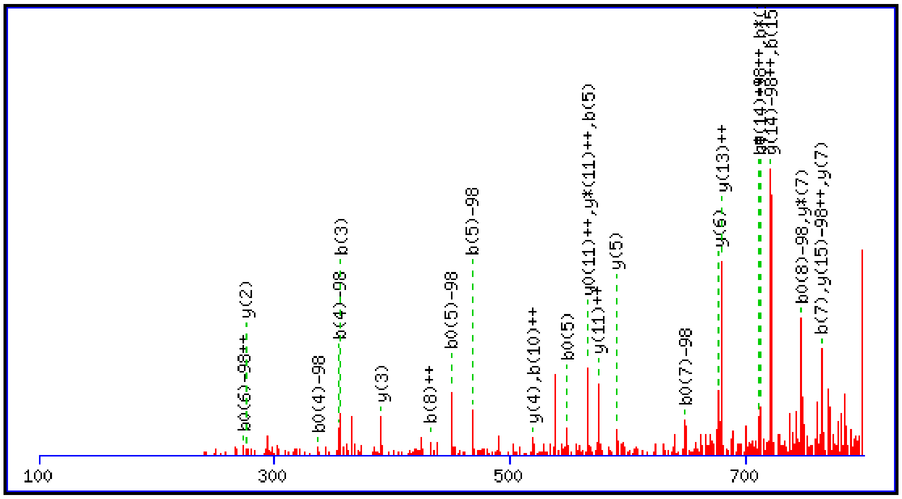

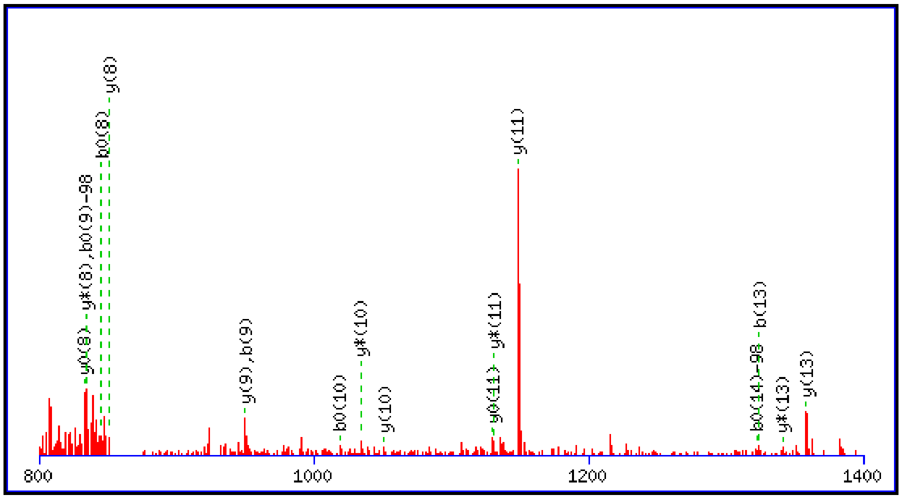

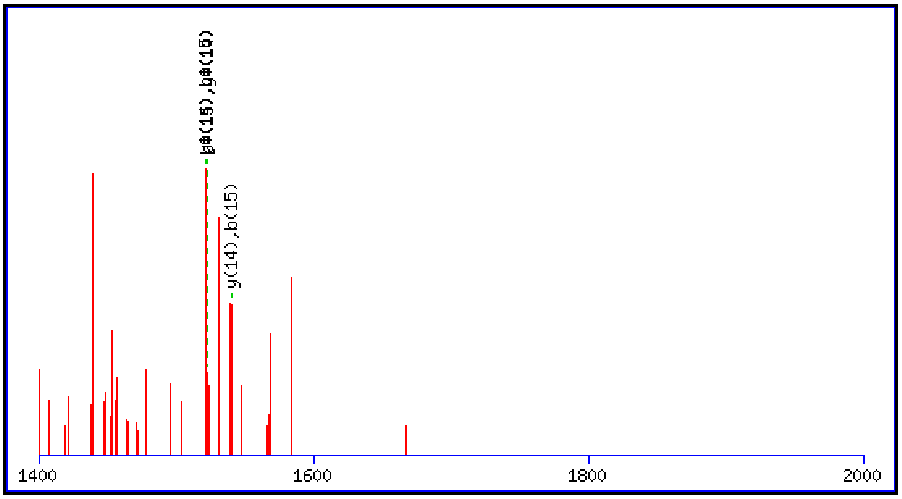

Monoisotopic mass of neutral peptide Mr(calc): 1712.7618
Fixed modifications: Carbamidomethyl (C)
Variable modifications:
T3 : Phospho (ST), with neutral losses 0.0000(shown in table), 97.9769
Ions Score: 42 **Expect:** 0.014
Matches (Bold Red): 55/220 fragment ions using 114 most intense peaks

#	b	b^{++}	b*	b^{*++}	b^0	b^{0++}	Seq.	y	y^{++}	y*	y^{*++}	y^0	y^{0++}	#
1	88.0393	44.5233			70.0287	35.5180	S							16
2	175.0713	88.0393			157.0608	79.0340	S	1626.7371	813.8722	1609.7105	805.3589	1608.7265	804.8669	15
3	**356.0853**	178.5463			338.0748	169.5410	T	**1539.7050**	770.3562	**1522.6785**	761.8429	**1521.6945**	761.3509	14
4	453.1381	227.0727			435.1275	218.0674	P	**1358.6910**	**679.8492**	**1341.6645**	671.3359	1340.6805	670.8439	13
5	**566.2222**	283.6147			**548.2116**	274.6094	L	1261.6383	631.3228	1244.6117	622.8095	1243.6277	622.3175	12
6	663.2749	332.1411			645.2644	323.1358	P	**1148.5542**	**574.7807**	**1131.5277**	**566.2675**	**1130.5436**	**565.7755**	11
7	**764.3226**	382.6649			746.3120	373.6597	T	**1051.5014**	526.2544	**1034.4749**	517.7411	1033.4909	517.2491	10
8	863.3910	**432.1991**			**845.3805**	423.1939	V	**950.4538**	475.7305	933.4272	467.2172	932.4432	466.7252	9
9	**950.4230**	475.7152			932.4125	466.7099	S	**851.3853**	426.1963	**834.3588**	417.6830	**833.3748**	417.1910	8
10	1037.4551	**519.2312**			**1019.4445**	510.2259	S	**764.3533**	382.6803	**747.3268**	374.1670	746.3428	373.6750	7
11	1124.4871	562.7472			1106.4765	553.7419	S	**677.3213**	339.1643	660.2947	330.6510	659.3107	330.1590	6
12	1195.5242	598.2657			1177.5137	589.2605	A	**590.2893**	295.6483	573.2627	287.1350	572.2787	286.6430	5
13	**1324.5668**	662.7870			1306.5562	653.7818	E	**519.2522**	260.1297	502.2256	251.6164	501.2416	251.1244	4
14	1438.6097	719.8085	1421.5832	**711.2952**	1420.5992	710.8032	N	**390.2096**	195.6084	373.1830	187.0951	372.1990	186.6031	3
15	**1539.6574**	770.3323	**1522.6309**	761.8191	**1521.6469**	761.3271	T	**276.1666**	138.5870	259.1401	130.0737	258.1561	129.5817	2
16							R	175.1190	88.0631	158.0924	79.5498			1

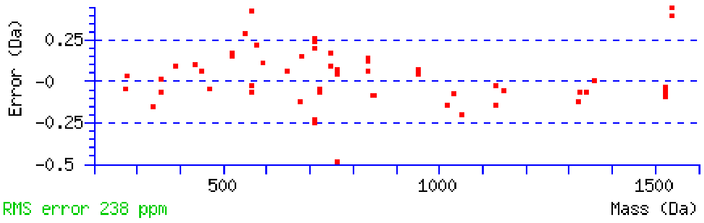

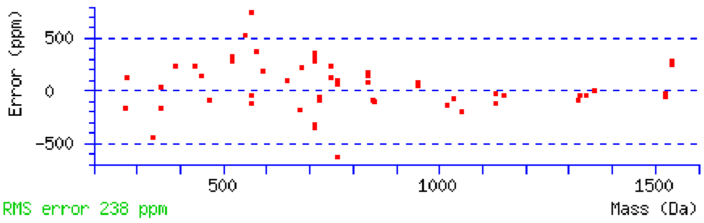

All matches to this query

Score	Mr(calc):	Delta	Sequence
42.2	1712.7618	0.0034	SSTPLPTVSSSAENTR
37.3	1712.7618	0.0034	SSTPLPTVSSSAENTR
37.3	1712.7618	0.0034	SSTPLPTVSSSAENTR
27.9	1712.7618	0.0034	SSTPLPTVSSSAENTR
11.7	1712.7618	0.0034	SSTPLPTVSSSAENTR
11.4	1712.7618	0.0034	SSTPLPTVSSSAENTR
6.6	1710.7620	2.0032	LRQSTSAKTKPVR
6.0	1712.7531	0.0121	TTIAVGLMSXTLTMK
5.4	1712.7618	0.0034	SSTPLPTVSSSAENTR
2.3	1712.7531	0.0121	TTIAVGLMSXTLTMK

Spectrum No: 106; Query: 31; Rank: 1

Peptide View

MS/MS Fragmentation of **GWSPPPEVR**
Found in **IPI00869599**, Tax_Id=10116 Gene_Symbol=Acss2_predicted acyl-CoA synthetase short-chain family member 2

Match to Query 31: 1103.480188 from(552.747370,2+)
Title: 091127RatKid_SCX01_13.2193.2193.2.dta
Data file K:\NewmanPaper\Piliang\3SubProteomes\Piliang3SP\mgf5ppm\SCX_3SubProteomes5ppm.mgf

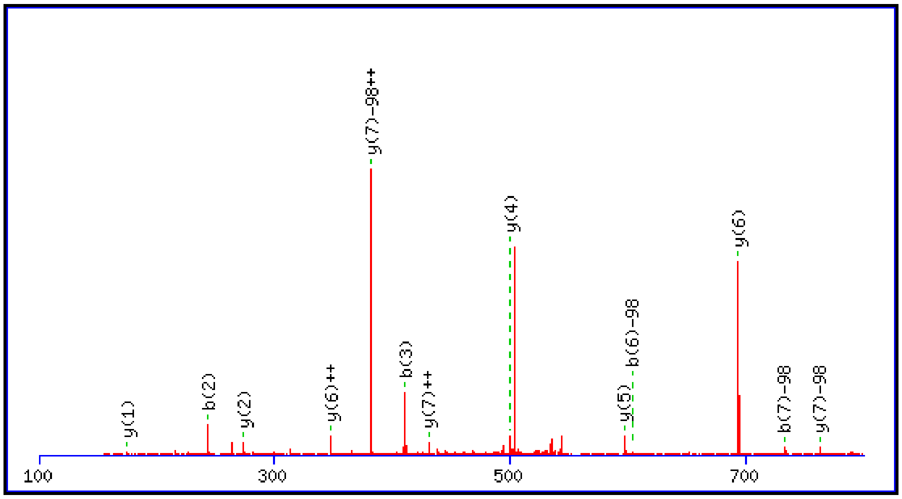

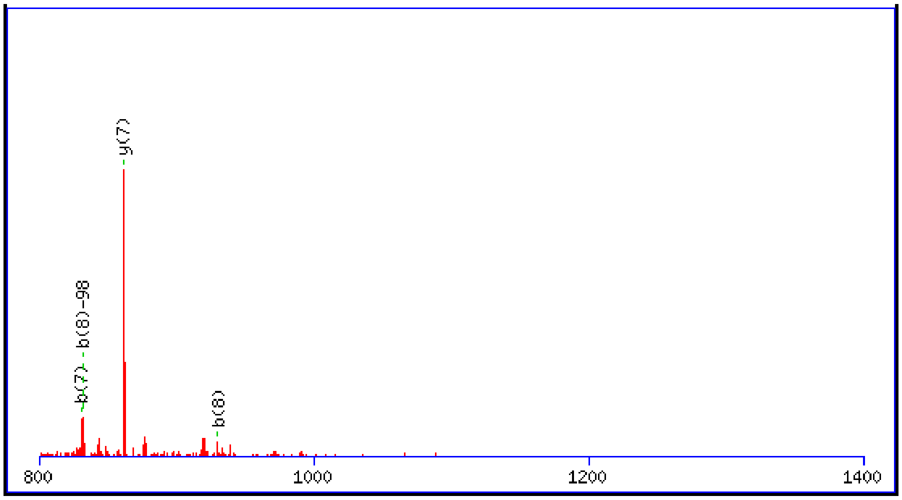

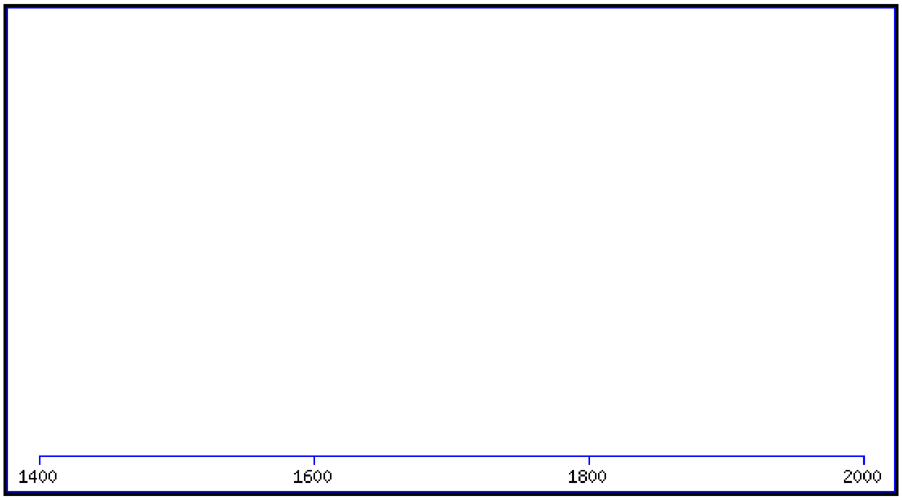

Monoisotopic mass of neutral peptide Mr(calc): 1103.4801
Fixed modifications: Carbamidomethyl (C)
Variable modifications:
S3 : Phospho (ST), with neutral losses 97.9769(shown in table), 0.0000
Ions Score: 42 **Expect:** 0.0061
Matches (Bold Red): 17/108 fragment ions using 41 most intense peaks

#	b	b^{++}	b^{0}	b^{0++}	Seq.	y	y^{++}	y*	y^{*++}	y^{0}	y^{0++}	#
1	58.0287	29.5180			G							9
2	**244.1081**	122.5577			W	949.4890	475.2481	932.4625	466.7349	931.4785	466.2429	8
3	313.1295	157.0684	295.1189	148.0631	S	**763.4097**	**382.2085**	746.3832	373.6952	745.3991	373.2032	7
4	410.1823	205.5948	392.1717	196.5895	P	**694.3883**	**347.6978**	677.3617	339.1845	676.3777	338.6925	6
5	507.2350	254.1212	489.2245	245.1159	P	**597.3355**	299.1714	580.3089	290.6581	579.3249	290.1661	5
6	**604.2878**	302.6475	586.2772	293.6423	P	**500.2827**	250.6450	483.2562	242.1317	482.2722	241.6397	4
7	**733.3304**	367.1688	715.3198	358.1636	E	403.2300	202.1186	386.2034	193.6053	385.2194	193.1133	3
8	**832.3988**	416.7030	814.3882	407.6978	V	**274.1874**	137.5973	257.1608	129.0840			2
9					R	**175.1190**	88.0631	158.0924	79.5498			1

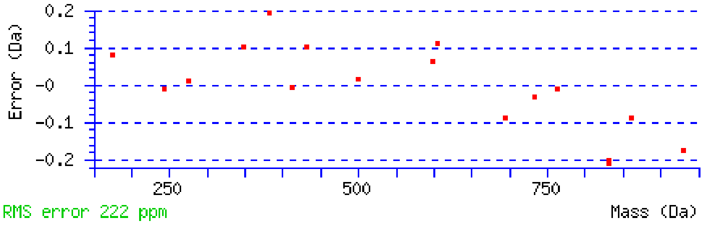

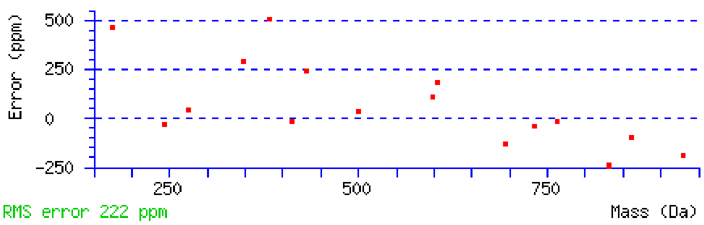

All matches to this query

Score	Mr(calc):	Delta	Sequence
41.8	1103.4801	0.0001	GWSPPPEVR
15.9	1101.4743	2.0059	IQSSESFPK
15.0	1103.4834	-0.0033	MAASAALFSR
11.7	1103.4761	0.0041	SRFKEDSR
11.1	1103.4835	-0.0033	RSSMKPGFV
9.6	1103.4900	-0.0098	NVSNVPAPPK
9.5	1103.4760	0.0041	SRENYLSR
8.3	1103.4900	-0.0098	SYKTVDSPK
7.7	1102.4696	1.0106	ELSEYVQR
7.5	1103.4794	0.0008	SGMSKKTNR

Spectrum No: 107; Query: 625; Rank: 1

Peptide View

MS/MS Fragmentation of **SGSLCASVPSNSVSQLQK**
Found in **IPI00373579**, Tax_Id=10116 Gene_Symbol=Vill_predicted similar to Villin-like protein

Match to Query 625: 1927.872608 from(964.943580,2+)
Title: 091127RatKid_SCX01_11.1556.1556.2.dta
Data file K:\NewmanPaper\Piliang\3SubProteomes\Piliang3SP\mgf5ppm\SCX_3SubProteomes5ppm.mgf

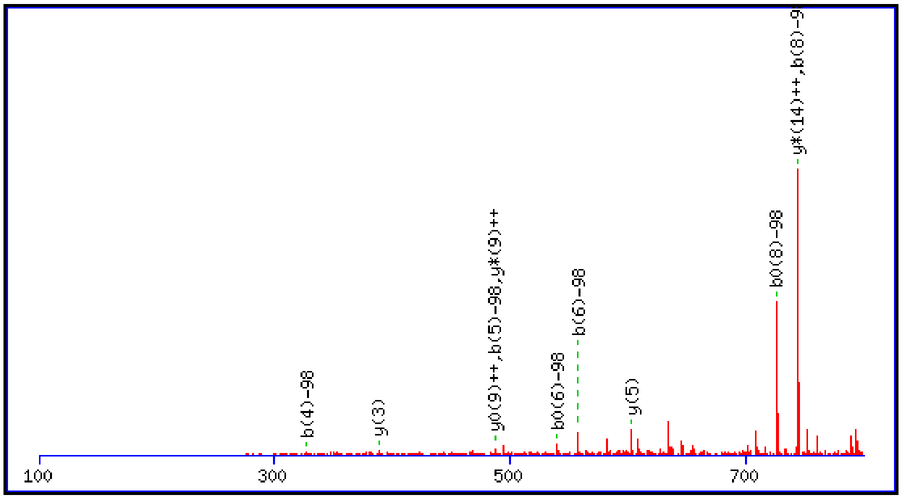

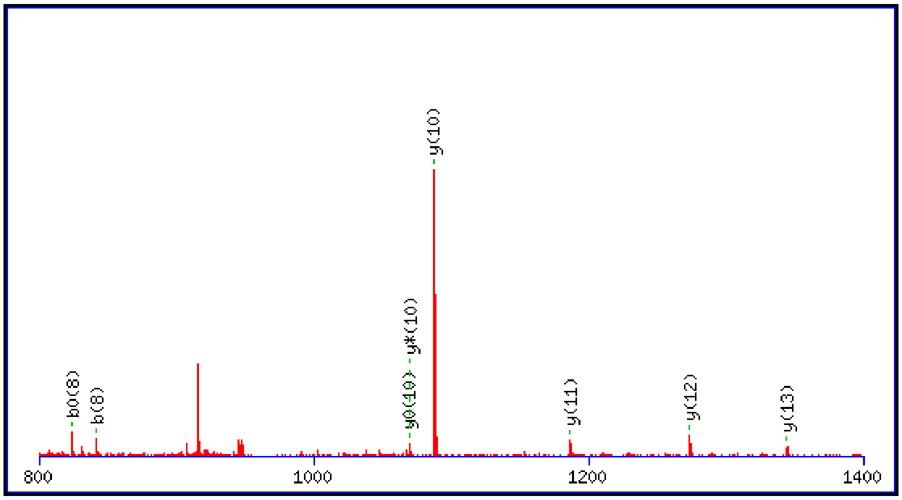

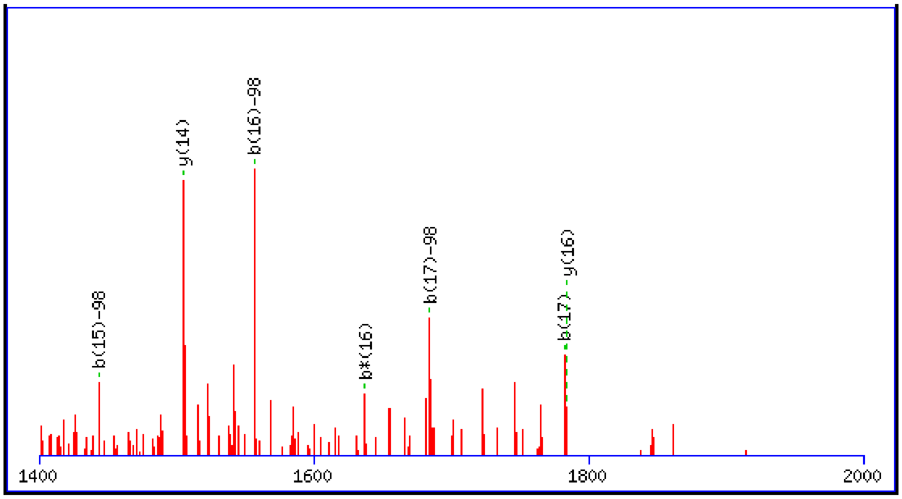

Monoisotopic mass of neutral peptide Mr(calc): 1927.8710
Fixed modifications: Carbamidomethyl (C)
Variable modifications:
S3 : Phospho (ST), with neutral losses 97.9769(shown in table), 0.0000
Ions Score: 42 **Expect:** 0.018
Matches (Bold Red): 27/262 fragment ions using 50 most intense peaks

#	b	b^{++}	b*	b^{*++}	b^0	b^{0++}	Seq.	y	y^{++}	y*	y^{*++}	y^0	y^{0++}	#
1	88.0393	44.5233			70.0287	35.5180	S							18
2	145.0608	73.0340			127.0502	64.0287	G	1743.8694	872.4383	1726.8429	863.9251	1725.8588	863.4331	17
3	214.0822	107.5448			196.0717	98.5395	S	1686.8479	843.9276	1669.8214	835.4143	1668.8374	834.9223	16
4	**327.1663**	164.0868			309.1557	155.0815	L	1617.8265	809.4169	1600.7999	800.9036	1599.8159	800.4116	15
5	**487.1969**	244.1021			469.1864	235.0968	C	**1504.7424**	752.8748	1487.7159	**744.3616**	1486.7319	**743.8696**	14
6	**558.2341**	279.6207			**540.2235**	270.6154	A	**1344.7118**	672.8595	1327.6852	664.3462	1326.7012	663.8542	13
7	645.2661	323.1367			627.2555	314.1314	S	**1273.6747**	637.3410	1256.6481	628.8277	1255.6641	628.3357	12
8	**744.3345**	372.6709			**726.3239**	363.6656	V	**1186.6426**	593.8250	1169.6161	585.3117	1168.6321	584.8197	11
9	841.3873	421.1973			823.3767	412.1920	P	**1087.5742**	544.2907	**1070.5477**	535.7775	**1069.5636**	535.2855	10
10	928.4193	464.7133			910.4087	455.7080	S	990.5214	495.7644	973.4949	**487.2511**	972.5109	**486.7591**	9
11	1042.4622	521.7347	1025.4357	513.2215	1024.4516	512.7295	N	903.4894	452.2483	886.4629	443.7351	885.4789	443.2431	8
12	1129.4942	565.2508	1112.4677	556.7375	1111.4837	556.2455	S	789.4465	395.2269	772.4199	386.7136	771.4359	386.2216	7
13	1228.5627	614.7850	1211.5361	606.2717	1210.5521	605.7797	V	702.4145	351.7109	685.3879	343.1976	684.4039	342.7056	6
14	1315.5947	658.3010	1298.5681	649.7877	1297.5841	649.2957	S	**603.3461**	302.1767	586.3195	293.6634	585.3355	293.1714	5
15	**1443.6533**	722.3303	1426.6267	713.8170	1425.6427	713.3250	Q	516.3140	258.6607	499.2875	250.1474			4
16	**1556.7373**	778.8723	1539.7108	770.3590	1538.7268	769.8670	L	**388.2554**	194.6314	371.2289	186.1181			3
17	**1684.7959**	842.9016	1667.7694	834.3883	1666.7853	833.8963	Q	275.1714	138.0893	258.1448	129.5761			2
18							K	147.1128	74.0600	130.0863	65.5468			1

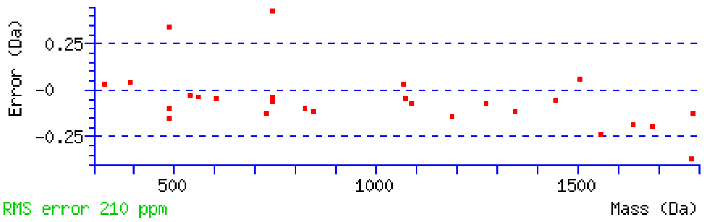

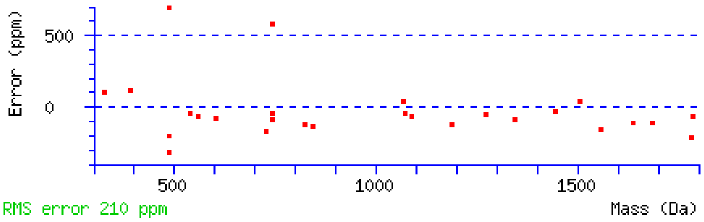

All matches to this query

Score	Mr(calc):	Delta	Sequence
41.8	1927.8710	0.0016	SGSLCASVPSNSVSQLQK
40.3	1927.8710	0.0016	SGSLCASVPSNSVSQLQK
16.5	1927.8710	0.0016	SGSLCASVPSNSVSQLQK

4.0	1927.8710	0.0016	SGSLCASVPSNSVSQLQK
4.0	1927.8710	0.0016	SGSLCASVPSNSVSQLQK
3.8	1927.8888	-0.0162	SSAQNKQVDENSLISTK
3.8	1927.8888	-0.0162	SSAQNKQVDENSLISTK
3.8	1927.8888	-0.0162	SSAQNKQVDENSLISTK
3.4	1927.8539	0.0187	TENMYHSKSFIGYKK
1.1	1927.8645	0.0082	LRASNAMMNNNDLVRK

Spectrum No: 108; Query: 784; Rank: 1

Peptide View

MS/MS Fragmentation of **GPLEAPQDGEAEEGTTSDGEK**
Found in **IPI00198250**, Tax_Id=10116 Gene_Symbol=Akap12 Isoform 1 of A-kinase anchor protein 12

Match to Query 784: 2195.876188 from(1098.945370,2+)
Title: 091127RatKid_SCX01_11.793.793.2.dta
Data file K:\NewmanPaper\Piliang\3SubProteomes\Piliang3SP\mgf5ppm\SCX_3SubProteomes5ppm.mgf

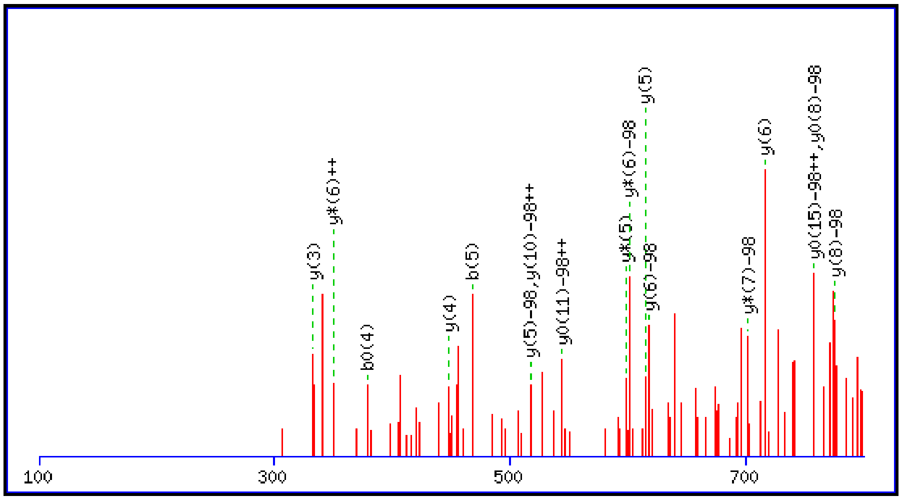

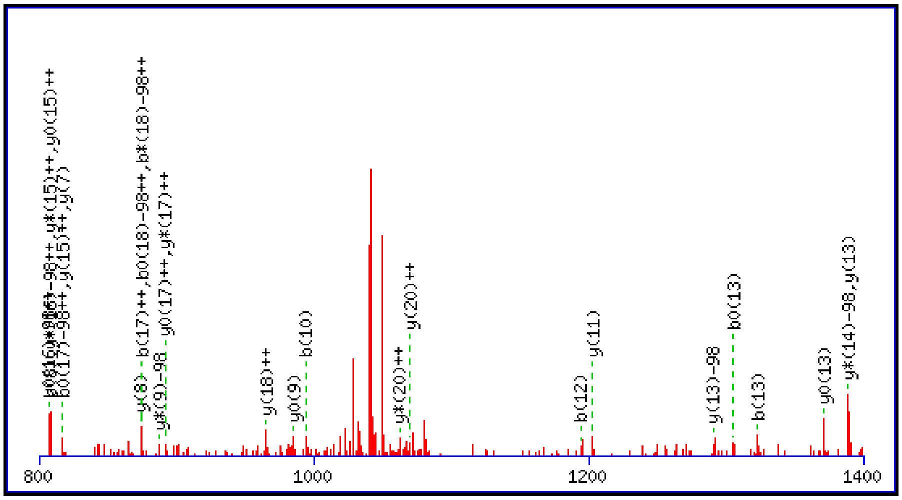

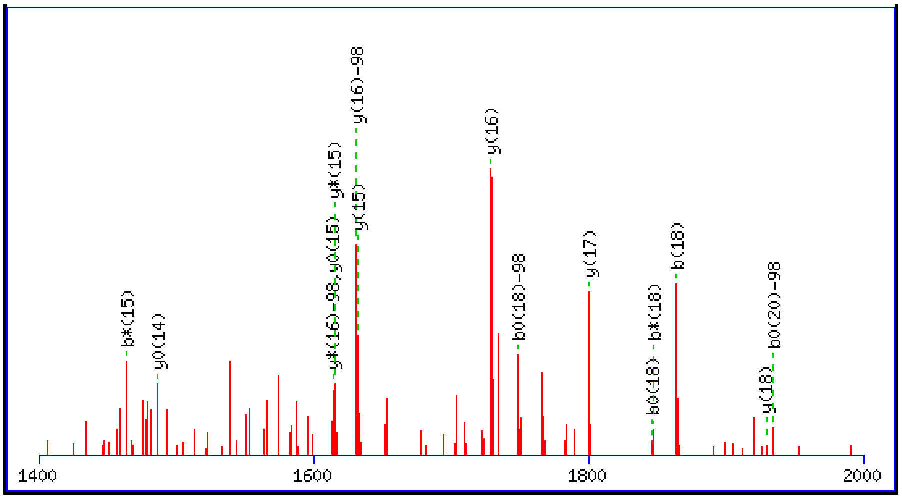

Monoisotopic mass of neutral peptide Mr(calc): 2195.8743
Fixed modifications: Carbamidomethyl (C)
Variable modifications:
S17 : Phospho (ST), with neutral losses 0.0000(shown in table), 97.9769
Ions Score: 42 **Expect:** 0.013
Matches (Bold Red): 60/340 fragment ions using 102 most intense peaks

#	b	b^{++}	b^{*}	b^{*++}	b^{0}	b^{0++}	Seq.	y	y^{++}	y^{*}	y^{*++}	y^{0}	y^{0++}	#
1	58.0287	29.5180					G							21
2	155.0815	78.0444					P	2139.8602	**1070.4337**	2122.8336	**1061.9204**	2121.8496	1061.4284	20
3	268.1656	134.5864					L	2042.8074	1021.9073	2025.7808	1013.3941	2024.7968	1012.9021	19
4	397.2082	199.1077			**379.1976**	190.1024	E	**1929.7233**	**965.3653**	1912.6968	956.8520	1911.7128	956.3600	18
5	**468.2453**	234.6263			450.2347	225.6210	A	**1800.6807**	900.8440	1783.6542	**892.3307**	1782.6702	**891.8387**	17
6	565.2980	283.1527			547.2875	274.1474	P	**1729.6436**	865.3254	1712.6171	856.8122	1711.6331	856.3202	16
7	693.3566	347.1819	676.3301	338.6687	675.3461	338.1767	Q	**1632.5909**	**816.7991**	**1615.5643**	**808.2858**	**1614.5803**	**807.7938**	15
8	**808.3836**	404.6954	791.3570	396.1821	790.3730	395.6901	D	1504.5323	752.7698	1487.5057	744.2565	**1486.5217**	743.7645	14
9	865.4050	433.2062	848.3785	424.6929	847.3945	424.2009	G	**1389.5053**	695.2563	1372.4788	686.7430	**1371.4948**	686.2510	13
10	**994.4476**	497.7274	977.4211	489.2142	976.4371	488.7222	E	1332.4839	666.7456	1315.4573	658.2323	1314.4733	657.7403	12
11	1065.4847	533.2460	1048.4582	524.7327	1047.4742	524.2407	A	**1203.4413**	602.2243	1186.4147	593.7110	1185.4307	593.2190	11
12	**1194.5273**	597.7673	1177.5008	589.2540	1176.5168	588.7620	E	1132.4042	566.7057	1115.3776	558.1924	1114.3936	557.7004	10
13	**1323.5699**	662.2886	1306.5434	653.7753	**1305.5594**	653.2833	E	1003.3616	502.1844	986.3350	493.6711	**985.3510**	493.1791	9
14	1380.5914	690.7993	1363.5648	682.2861	1362.5808	681.7940	G	**874.3190**	437.6631	857.2924	429.1499	856.3084	428.6578	8
15	1481.6391	741.3232	**1464.6125**	732.8099	1463.6285	732.3179	T	**817.2975**	409.1524	800.2710	400.6391	799.2869	400.1471	7
16	1582.6867	791.8470	1565.6602	783.3337	1564.6762	782.8417	T	**716.2498**	358.6286	699.2233	**350.1153**	698.2393	349.6233	6
17	1749.6851	**875.3462**	1732.6585	866.8329	1731.6745	866.3409	S	**615.2022**	308.1047	**598.1756**	299.5914	597.1916	299.0994	5
18	**1864.7120**	932.8597	**1847.6855**	924.3464	**1846.7015**	923.8544	D	**448.2038**	224.6055	431.1773	216.0923	430.1932	215.6003	4
19	1921.7335	961.3704	1904.7069	952.8571	1903.7229	952.3651	G	**333.1769**	167.0921	316.1503	158.5788	315.1663	158.0868	3
20	2050.7761	1025.8917	2033.7495	1017.3784	2032.7655	1016.8864	E	276.1554	138.5813	259.1288	130.0681	258.1448	129.5761	2
21							K	147.1128	74.0600	130.0863	65.5468			1

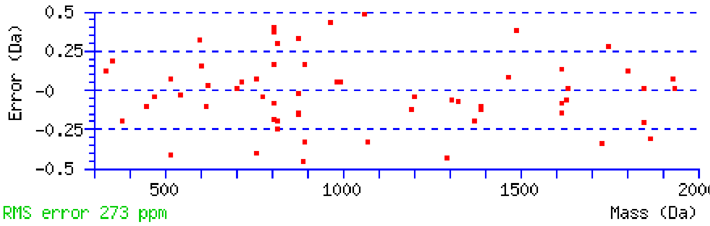

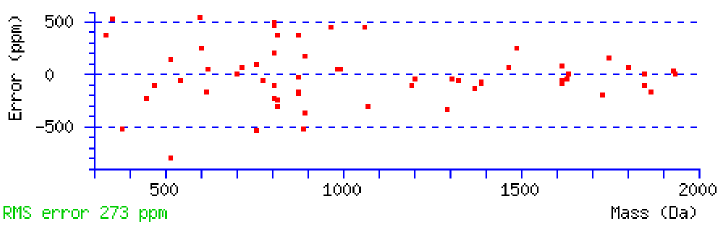

All matches to this query

Score	Mr(calc):	Delta	Sequence

41.7	2195.8743	0.0019	GPLEAPQDGEAEEGTTSDGEK
33.5	2195.8743	0.0019	GPLEAPQDGEAEEGTTSDGEK
25.0	2195.8743	0.0019	GPLEAPQDGEAEEGTTSDGEK

Spectrum No: 109; Query: 110; Rank: 1

Peptide View

MS/MS Fragmentation of **SLSPGGAALGYR**
Found in **IPI00763238**, Tax_Id=10116 Gene_Symbol=LOC684233 similar to Putative RNA-binding protein 15

Match to Query 110: 1227.566388 from(614.790470,2+)
Title: 091127RatKid_SCX01_12.1883.1883.2.dta
Data file K:\NewmanPaper\Piliang\3SubProteomes\Piliang3SP\mgf5ppm\SCX_3SubProteomes5ppm.mgf

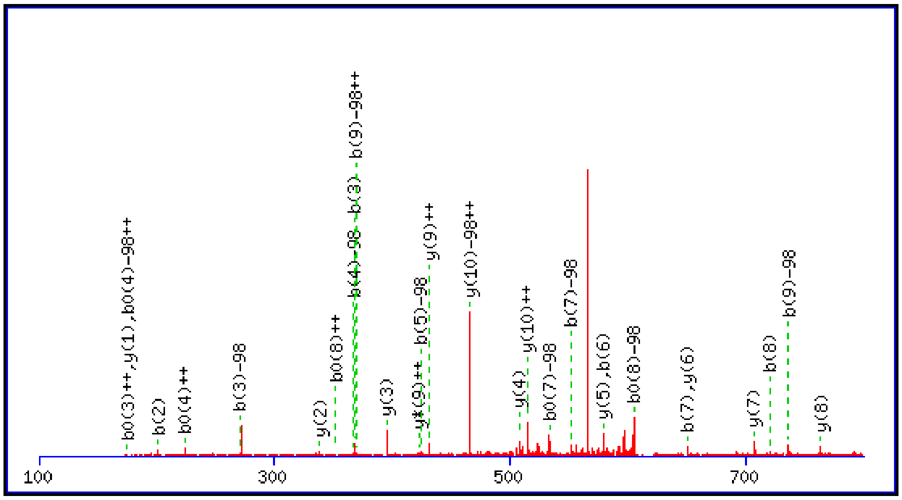

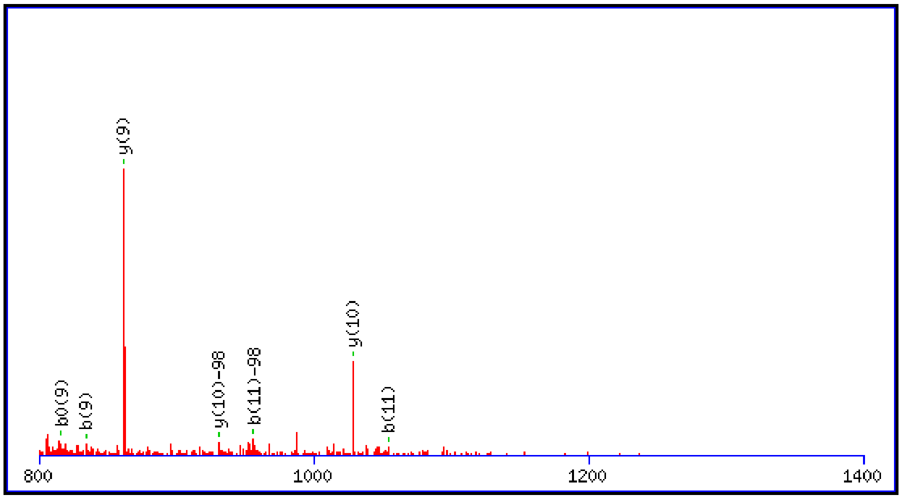

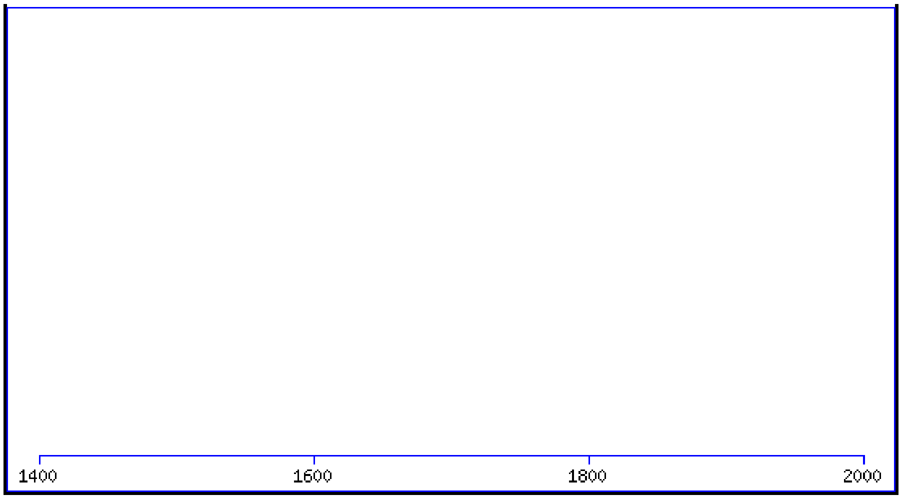

Monoisotopic mass of neutral peptide Mr(calc): 1227.5649
Fixed modifications: Carbamidomethyl (C)
Variable modifications:
S3 : Phospho (ST), with neutral losses 97.9769(shown in table), 0.0000
Ions Score: 41 **Expect:** 0.011
Matches (Bold Red): 36/140 fragment ions using 98 most intense peaks

#	b	b^{++}	b^{0}	b^{0++}	Seq.	y	y^{++}	y*	y^{*++}	y^{0}	y^{0++}	#
1	88.0393	44.5233	70.0287	35.5180	S							12
2	**201.1234**	101.0653	183.1128	92.0600	L	1043.5632	522.2853	1026.5367	513.7720	1025.5527	513.2800	11
3	**270.1448**	135.5760	252.1343	126.5708	S	**930.4792**	**465.7432**	913.4526	457.2300	912.4686	456.7379	10
4	**367.1976**	184.1024	349.1870	**175.0971**	P	**861.4577**	**431.2325**	844.4312	**422.7192**			9
5	**424.2191**	212.6132	406.2085	203.6079	G	**764.4050**	382.7061	747.3784	374.1928			8
6	481.2405	241.1239	463.2300	232.1186	G	**707.3835**	354.1954	690.3570	345.6821			7
7	**552.2776**	276.6425	**534.2671**	267.6372	A	**650.3620**	325.6847	633.3355	317.1714			6
8	623.3147	312.1610	**605.3042**	303.1557	A	**579.3249**	290.1661	562.2984	281.6528			5
9	**736.3988**	**368.7030**	718.3882	359.6978	L	**508.2878**	254.6475	491.2613	246.1343			4
10	793.4203	397.2138	775.4097	388.2085	G	**395.2037**	198.1055	378.1772	189.5922			3
11	**956.4836**	478.7454	938.4730	469.7402	Y	**338.1823**	169.5948	321.1557	161.0815			2
12					R	**175.1190**	88.0631	158.0924	79.5498			1

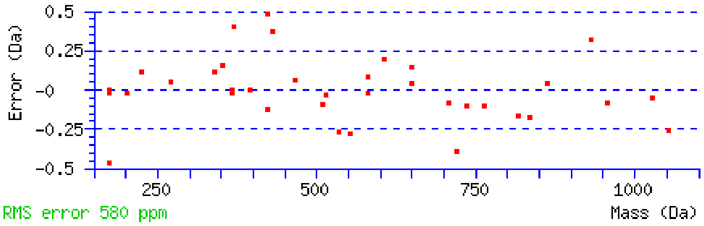

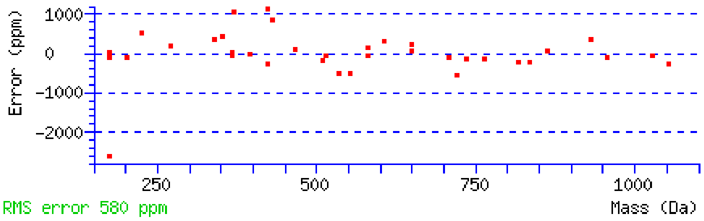

All matches to this query

Score	Mr(calc):	Delta	Sequence
41.1	1227.5649	0.0015	SLSPGGAALGYR
32.0	1227.5649	0.0015	SLSPGGAALGYR
8.4	1227.5748	-0.0084	TVSVEQKETK
2.7	1227.5649	0.0015	FNAGELITQR
2.6	1227.5761	-0.0097	GSLQPRTSFR
2.6	1227.5609	0.0055	KTRDDLSASR
2.6	1227.5649	0.0015	TIGNEKAPYR
2.4	1225.5509	2.0154	MTTANSPHNPR
2.3	1227.5570	0.0094	AEAMEILLSR

2.1	1226.5696	0.9967	VSQATPEFLR

Spectrum No: 110; Query: 745; Rank: 1

Peptide View

MS/MS Fragmentation of **ANSFVGTAQYVSPELLTEK**
Found in **IPI00195987**, Tax_Id=10116 Gene_Symbol=Pdpk1 3-phosphoinositide-dependent protein kinase 1

Match to Query 745: 2133.009128 from(1067.511840,2+)
Title: 091129RatKid_SCX02_10.2955.2955.2.dta
Data file K:\NewmanPaper\Piliang\3SubProteomes\Piliang3SP\mgf5ppm\SCX_3SubProteomes5ppm.mgf

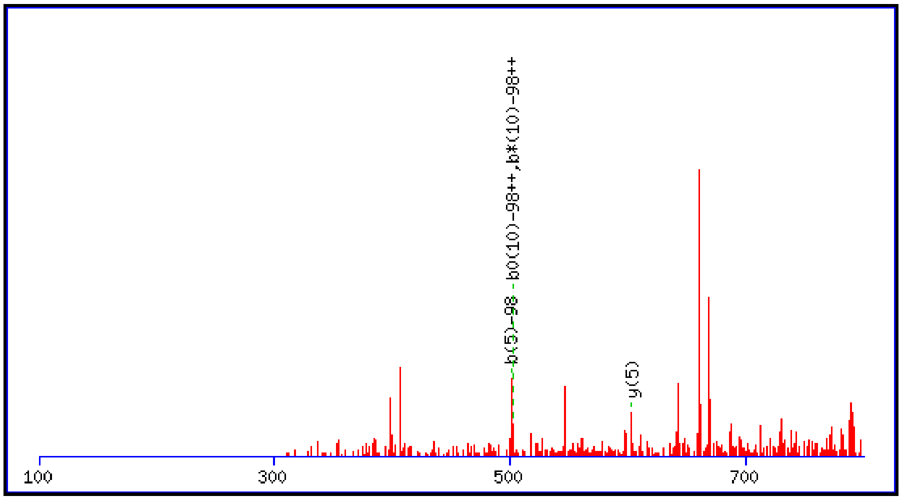

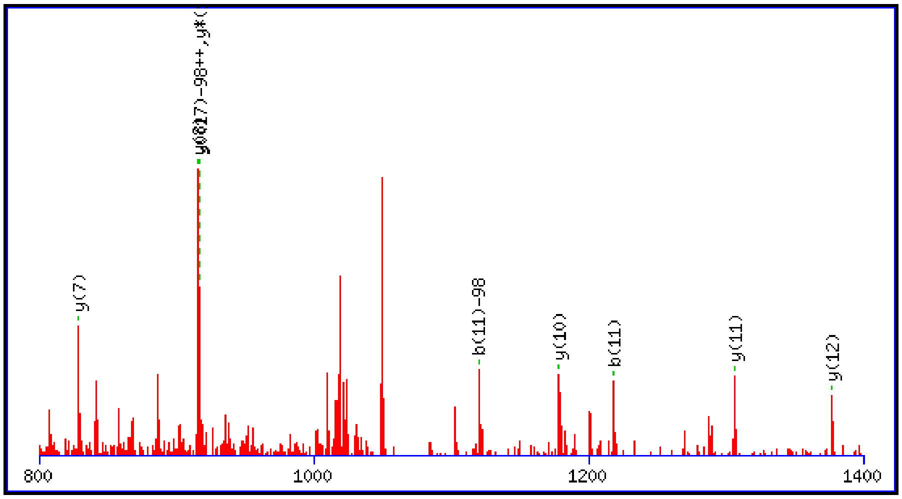

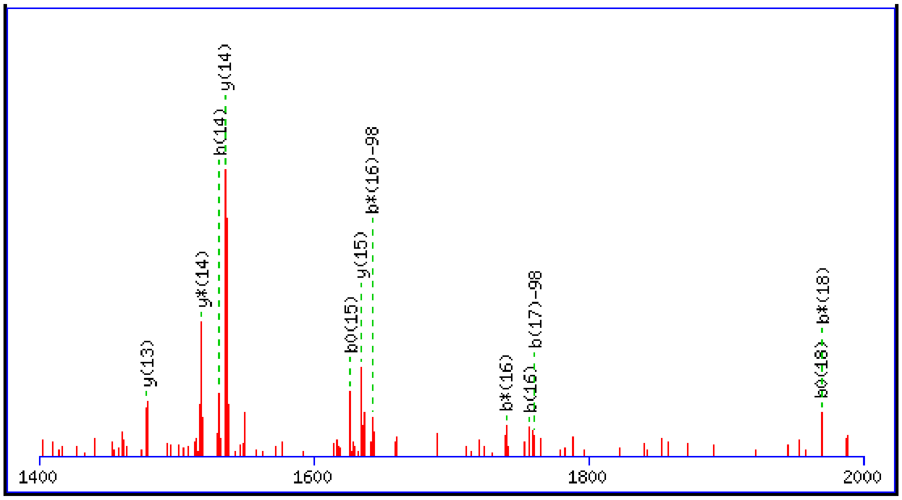

Monoisotopic mass of neutral peptide Mr(calc): 2133.0031
Fixed modifications: Carbamidomethyl (C)
Variable modifications:
S3 : Phospho (ST), with neutral losses 0.0000(shown in table), 97.9769
Ions Score: 41 **Expect:** 0.021
Matches (Bold Red): 25/316 fragment ions using 46 most intense peaks

#	b	b^{++}	b*	b^{*++}	b^0	b^{0++}	Seq.	y	y^{++}	y*	y^{*++}	y^0	y^{0++}	#
1	72.0444	36.5258					A							19
2	186.0873	93.5473	169.0608	85.0340			N	2062.9733	1031.9903	2045.9467	1023.4770	2044.9627	1022.9850	18
3	353.0857	177.0465	336.0591	168.5332	335.0751	168.0412	S	1948.9303	974.9688	1931.9038	966.4555	1930.9198	965.9635	17
4	500.1541	250.5807	483.1275	242.0674	482.1435	241.5754	F	1781.9320	891.4696	1764.9054	882.9564	1763.9214	882.4644	16
5	599.2225	300.1149	582.1959	291.6016	581.2119	291.1096	V	**1634.8636**	817.9354	1617.8370	809.4222	1616.8530	808.9301	15
6	656.2440	328.6256	639.2174	320.1123	638.2334	319.6203	G	**1535.7952**	768.4012	**1518.7686**	759.8879	1517.7846	759.3959	14
7	757.2916	379.1495	740.2651	370.6362	739.2811	370.1442	T	**1478.7737**	739.8905	1461.7472	731.3772	1460.7631	730.8852	13
8	828.3288	414.6680	811.3022	406.1547	810.3182	405.6627	A	**1377.7260**	689.3666	1360.6995	680.8534	1359.7155	680.3614	12
9	956.3873	478.6973	939.3608	470.1840	938.3768	469.6920	Q	**1306.6889**	653.8481	1289.6624	645.3348	1288.6783	644.8428	11
10	1119.4507	560.2290	1102.4241	551.7157	1101.4401	551.2237	Y	**1178.6303**	589.8188	1161.6038	581.3055	1160.6198	580.8135	10
11	**1218.5191**	609.7632	1201.4925	601.2499	1200.5085	600.7579	V	1015.5670	508.2871	998.5405	499.7739	997.5564	499.2819	9
12	1305.5511	653.2792	1288.5246	644.7659	1287.5405	644.2739	S	**916.4986**	458.7529	899.4720	450.2397	898.4880	449.7476	8
13	1402.6039	701.8056	1385.5773	693.2923	1384.5933	692.8003	P	**829.4666**	415.2369	812.4400	406.7236	811.4560	406.2316	7
14	**1531.6465**	766.3269	1514.6199	757.8136	1513.6359	757.3216	E	732.4138	366.7105	715.3872	358.1973	714.4032	357.7053	6
15	1644.7305	822.8689	1627.7040	814.3556	**1626.7200**	813.8636	L	**603.3712**	302.1892	586.3447	293.6760	585.3606	293.1840	5
16	**1757.8146**	879.4109	**1740.7880**	870.8977	1739.8040	870.4056	L	490.2871	245.6472	473.2606	237.1339	472.2766	236.6419	4
17	1858.8623	929.9348	1841.8357	921.4215	1840.8517	920.9295	T	377.2031	189.1052	360.1765	180.5919	359.1925	180.0999	3
18	1987.9049	994.4561	**1970.8783**	985.9428	**1969.8943**	985.4508	E	276.1554	138.5813	259.1288	130.0681	258.1448	129.5761	2
19							K	147.1128	74.0600	130.0863	65.5468			1

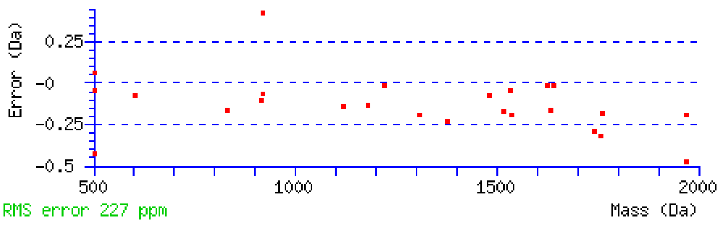

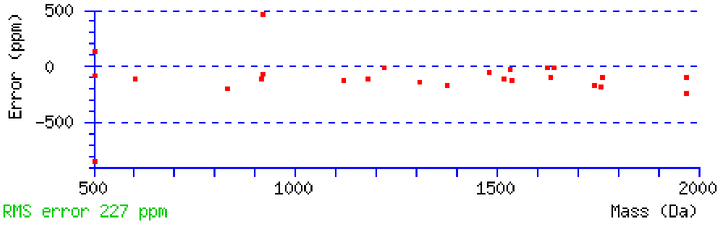

All matches to this query

Score	Mr(calc):	Delta	Sequence
40.8	2133.0031	0.0060	ANSFVGTAQYVSPELLTEK
25.1	2133.0031	0.0060	ANSFVGTAQYVSPELLTEK

Spectrum No: 111; Query: 1025; Rank: 1

Peptide View

MS/MS Fragmentation of **DSSSSSSSSSDSDSDGEEHGSDIGPR**
Found in **IPI00197986**, Tax_Id=10116 Gene_Symbol=Ndufv3l;LOC685005 NADH dehydrogenase (ubiquinone) flavoprotein 3-like isoform 1

Match to Query 1025: 2816.933772 from(939.985200,3+)
Title: 091129RatKid_SCX02_12.290.290.3.dta
Data file K:\NewmanPaper\Piliang\3SubProteomes\Piliang3SP\mgf5ppm\SCX_3SubProteomes5ppm.mgf

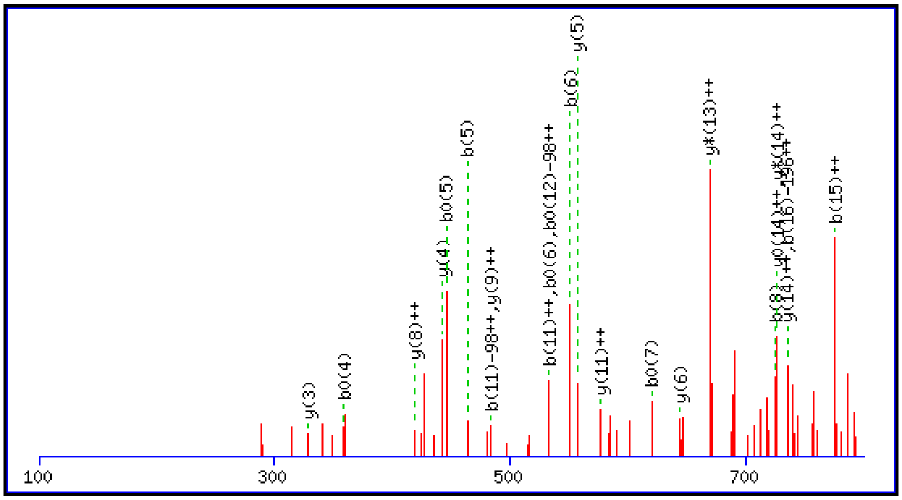

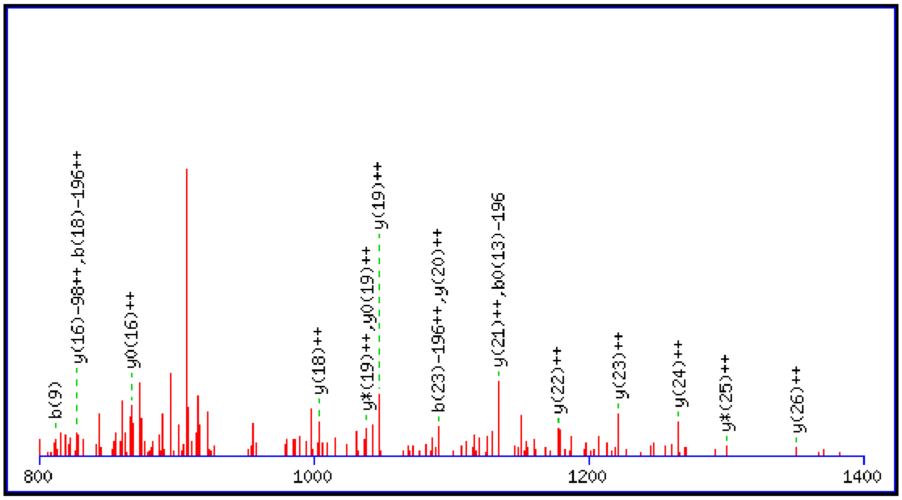

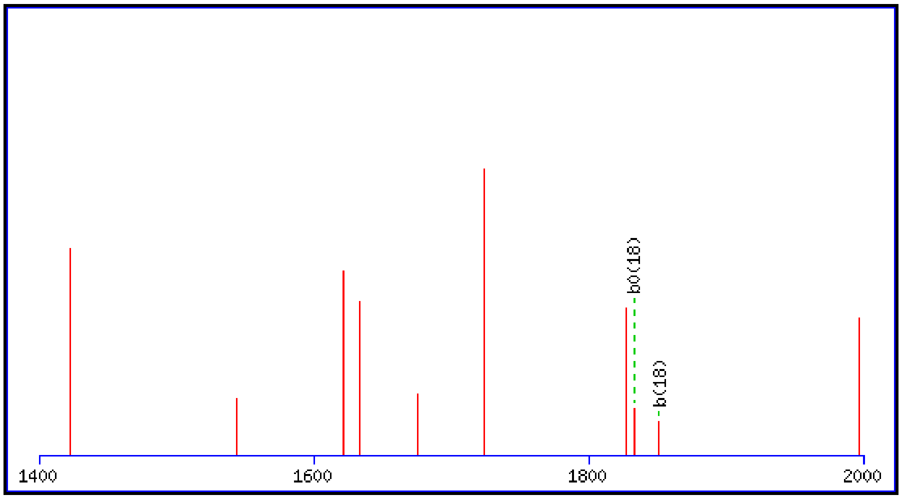

Monoisotopic mass of neutral peptide Mr(calc): 2816.9407
Fixed modifications: Carbamidomethyl (C)
Variable modifications:
S11 : Phospho (ST), with neutral losses 0.0000(shown in table), 97.9769
S13 : Phospho (ST), with neutral losses 0.0000(shown in table), 97.9769
Ions Score: 40 **Expect:** 0.007
Matches (Bold Red): 42/388 fragment ions using 87 most intense peaks

#	b	b^{++}	b^{0}	b^{0++}	Seq.	y	y^{++}	y*	y^{*++}	y^{0}	y^{0++}	#
1	116.0342	58.5207	98.0237	49.5155	D							27
2	203.0662	102.0368	185.0557	93.0315	S	2702.9211	**1351.9642**	2685.8945	1343.4509	2684.9105	1342.9589	26
3	290.0983	145.5528	272.0877	136.5475	S	2615.8890	1308.4482	2598.8625	**1299.9349**	2597.8785	1299.4429	25
4	377.1303	189.0688	**359.1197**	180.0635	S	2528.8570	**1264.9321**	2511.8304	1256.4189	2510.8464	1255.9269	24
5	**464.1623**	232.5848	**446.1518**	223.5795	S	2441.8250	**1221.4161**	2424.7984	1212.9028	2423.8144	1212.4108	23
6	**551.1944**	276.1008	**533.1838**	267.0955	S	2354.7929	**1177.9001**	2337.7664	1169.3868	2336.7824	1168.8948	22
7	638.2264	319.6168	**620.2158**	310.6115	S	2267.7609	**1134.3841**	2250.7344	1125.8708	2249.7503	1125.3788	21
8	**725.2584**	363.1328	707.2478	354.1276	S	2180.7289	**1090.8681**	2163.7023	1082.3548	2162.7183	1081.8628	20
9	**812.2904**	406.6489	794.2799	397.6436	S	2093.6969	**1047.3521**	2076.6703	**1038.8388**	2075.6863	**1038.3468**	19
10	899.3225	450.1649	881.3119	441.1596	S	2006.6648	**1003.8361**	1989.6383	995.3228	1988.6543	994.8308	18
11	1066.3208	**533.6640**	1048.3103	524.6588	S	1919.6328	960.3200	1902.6063	951.8068	1901.6222	951.3148	17
12	1181.3478	591.1775	1163.3372	582.1722	D	1752.6345	876.8209	1735.6079	868.3076	1734.6239	**867.8156**	16
13	1348.3461	674.6767	1330.3356	665.6714	S	1637.6075	819.3074	1620.5810	810.7941	1619.5969	810.3021	15
14	1463.3731	732.1902	1445.3625	723.1849	D	1470.6092	**735.8082**	1453.5826	**727.2949**	1452.5986	**726.8029**	14
15	1550.4051	**775.7062**	1532.3945	766.7009	S	1355.5822	678.2947	1338.5557	**669.7815**	1337.5716	669.2895	13
16	1665.4320	833.2197	1647.4215	824.2144	D	1268.5502	634.7787	1251.5236	626.2655	1250.5396	625.7734	12
17	1722.4535	861.7304	1704.4429	852.7251	G	1153.5232	**577.2653**	1136.4967	568.7520	1135.5127	568.2600	11
18	**1851.4961**	926.2517	**1833.4855**	917.2464	E	1096.5018	548.7545	1079.4752	540.2413	1078.4912	539.7492	10
19	1980.5387	990.7730	1962.5281	981.7677	E	967.4592	**484.2332**	950.4326	475.7200	949.4486	475.2279	9
20	2117.5976	1059.3024	2099.5870	1050.2972	H	838.4166	**419.7119**	821.3900	411.1987	820.4060	410.7067	8
21	2174.6191	1087.8132	2156.6085	1078.8079	G	701.3577	351.1825	684.3311	342.6692	683.3471	342.1772	7
22	2261.6511	1131.3292	2243.6405	1122.3239	S	**644.3362**	322.6717	627.3097	314.1585	626.3257	313.6665	6
23	2376.6780	1188.8427	2358.6675	1179.8374	D	**557.3042**	279.1557	540.2776	270.6425	539.2936	270.1504	5
24	2489.7621	1245.3847	2471.7515	1236.3794	I	**442.2772**	221.6423	425.2507	213.1290			4
25	2546.7836	1273.8954	2528.7730	1264.8901	G	**329.1932**	165.1002	312.1666	156.5870			3
26	2643.8363	1322.4218	2625.8258	1313.4165	P	272.1717	136.5895	255.1452	128.0762			2
27					R	175.1190	88.0631	158.0924	79.5498			1

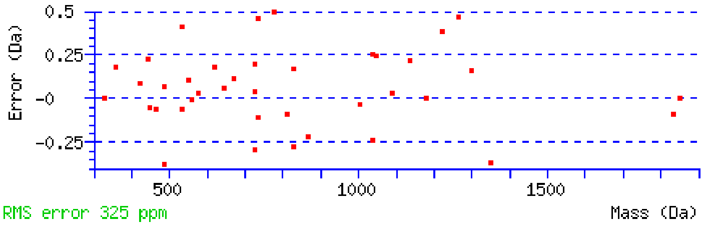

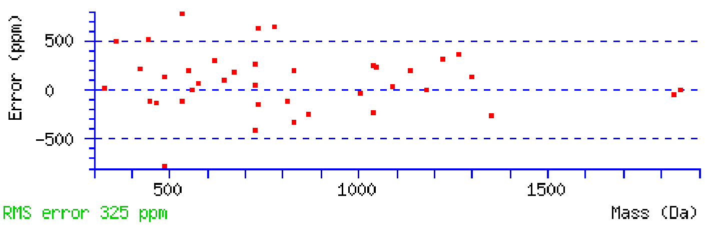

All matches to this query

Score	Mr(calc):	Delta	Sequence
40.3	2816.9407	-0.0070	DSSSSSSSSSSDSDSDGEEHGSDIGPR
39.3	2816.9407	-0.0070	DSSSSSSSSSSDSDSDGEEHGSDIGPR
38.9	2816.9407	-0.0070	DSSSSSSSSSSDSDSDGEEHGSDIGPR
31.9	2816.9407	-0.0070	DSSSSSSSSSSDSDSDGEEHGSDIGPR
25.9	2816.9407	-0.0070	DSSSSSSSSSSDSDSDGEEHGSDIGPR
20.0	2816.9407	-0.0070	DSSSSSSSSSSDSDSDGEEHGSDIGPR
19.8	2816.9407	-0.0070	DSSSSSSSSSSDSDSDGEEHGSDIGPR

11.5	2816.9407	-0.0070	DSSSSSSSSSDSDSDGEEHGSDIGPR
9.6	2816.9407	-0.0070	DSSSSSSSSSDSDSDGEEHGSDIGPR
7.9	2816.9407	-0.0070	DSSSSSSSSSDSDSDGEEHGSDIGPR

Spectrum No: 112; Query: 42; Rank: 1

Peptide View

MS/MS Fragmentation of **IDISPSTFR**
Found in **IPI00359323**, Tax_Id=10116 Gene_Symbol=Thrap3 Thyroid hormone receptor-associated protein 3

Match to Query 42: 1114.508008 from(558.261280,2+)
Title: 091129RatKid_SCX02_13.2157.2157.2.dta
Data file K:\NewmanPaper\Piliang\3SubProteomes\Piliang3SP\mgf5ppm\SCX_3SubProteomes5ppm.mgf

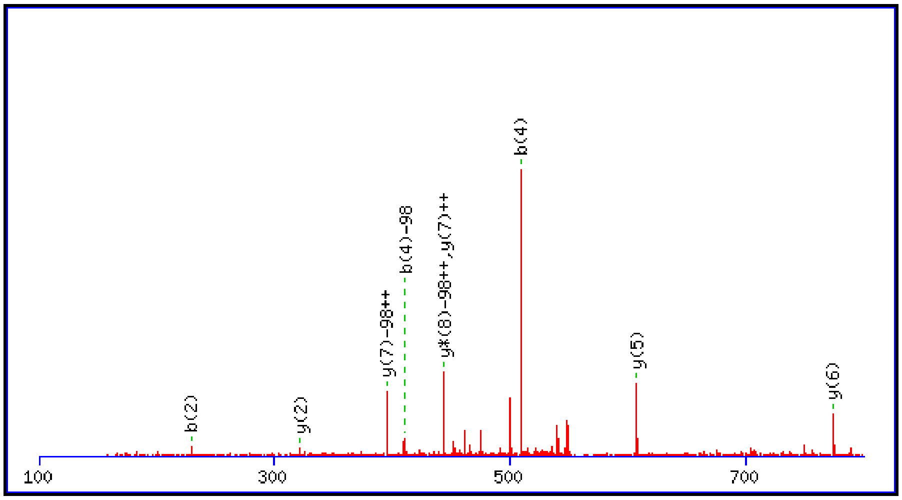

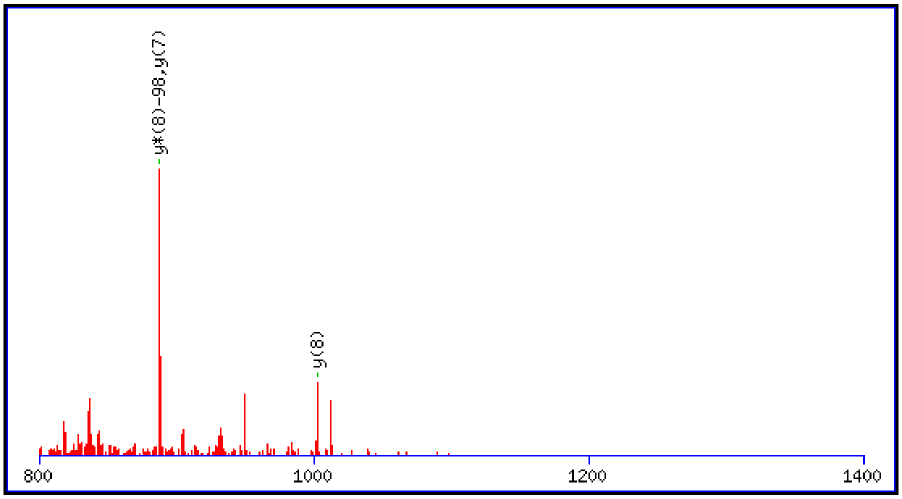

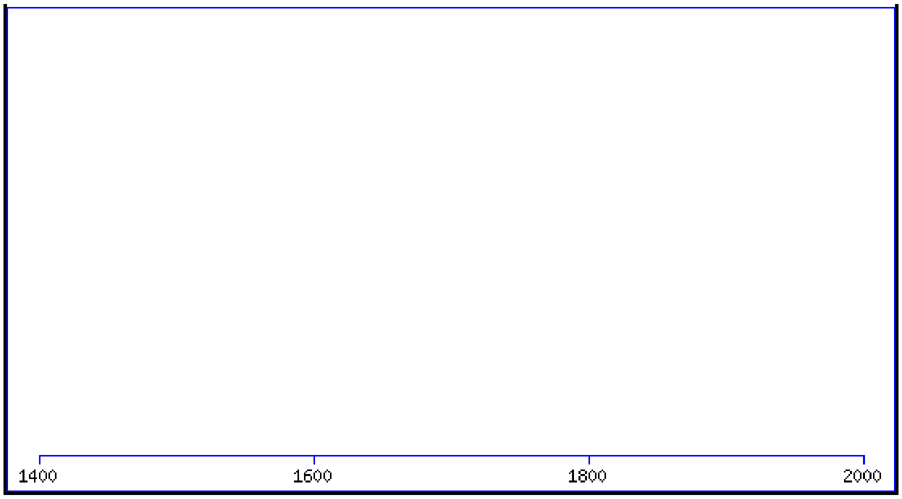

Monoisotopic mass of neutral peptide Mr(calc): 1114.5060
Fixed modifications: Carbamidomethyl (C)
Variable modifications:
S4 : Phospho (ST), with neutral losses 0.0000(shown in table), 97.9769
Ions Score: 40 **Expect:** 0.0097
Matches (Bold Red): 12/112 fragment ions using 22 most intense peaks

#	b	b^{++}	b^{0}	b^{0++}	Seq.	y	y^{++}	y*	y^{*++}	y^{0}	y^{0++}	#
1	114.0913	57.5493			I							9
2	**229.1183**	115.0628	211.1077	106.0575	D	**1002.4292**	501.7182	985.4026	493.2050	984.4186	492.7130	8
3	342.2023	171.6048	324.1918	162.5995	I	**887.4023**	**444.2048**	870.3757	435.6915	869.3917	435.1995	7
4	**509.2007**	255.1040	491.1901	246.0987	S	**774.3182**	387.6627	757.2916	379.1495	756.3076	378.6574	6
5	606.2535	303.6304	588.2429	294.6251	P	**607.3198**	304.1636	590.2933	295.6503	589.3093	295.1583	5
6	693.2855	347.1464	675.2749	338.1411	S	510.2671	255.6372	493.2405	247.1239	492.2565	246.6319	4
7	794.3332	397.6702	776.3226	388.6649	T	423.2350	212.1212	406.2085	203.6079	405.2245	203.1159	3
8	941.4016	471.2044	923.3910	462.1991	F	**322.1874**	161.5973	305.1608	153.0840			2
9					R	175.1190	88.0631	158.0924	79.5498			1

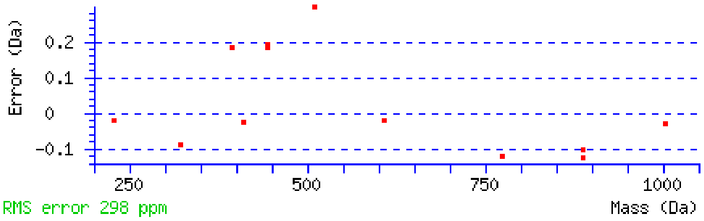

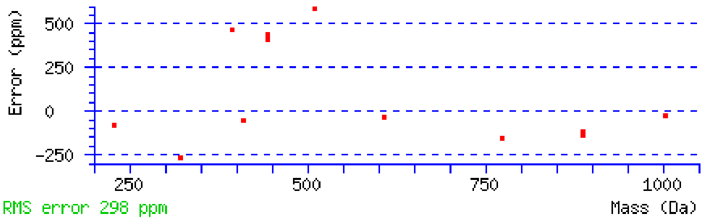

All matches to this query

Score	Mr(calc):	Delta	Sequence
40.2	1114.5060	0.0020	IDISPSTFR
16.8	1114.5060	0.0020	IDISPSTFR
16.8	1114.5060	0.0020	IDISPSTFR
14.6	1114.5151	-0.0071	LDIMAMSFR
12.6	1114.5020	0.0061	NILTSSTGSR
11.7	1113.5080	1.0000	ELQHSKHR
5.5	1114.5020	0.0061	NILTSSTGSR
5.3	1114.5172	-0.0092	IDIYAERR
3.6	1114.5060	0.0020	EAGIEITFR
1.3	1113.5107	0.9973	PETVAVGFSK

Spectrum No: 113; Query: 624; Rank: 1

Peptide View

MS/MS Fragmentation of **LRSEDGVEGDLGETQSR**
Found in **IPI00769072**, Tax_Id=10116 Gene_Symbol=Ahnak similar to AHNAK nucleoprotein isoform 1 isoform 2

Match to Query 624: 1926.832992 from(643.284940,3+)
Title: 091129RatKid_SCX02_23.874.874.3.dta
Data file K:\NewmanPaper\Piliang\3SubProteomes\Piliang3SP\mgf5ppm\SCX_3SubProteomes5ppm.mgf

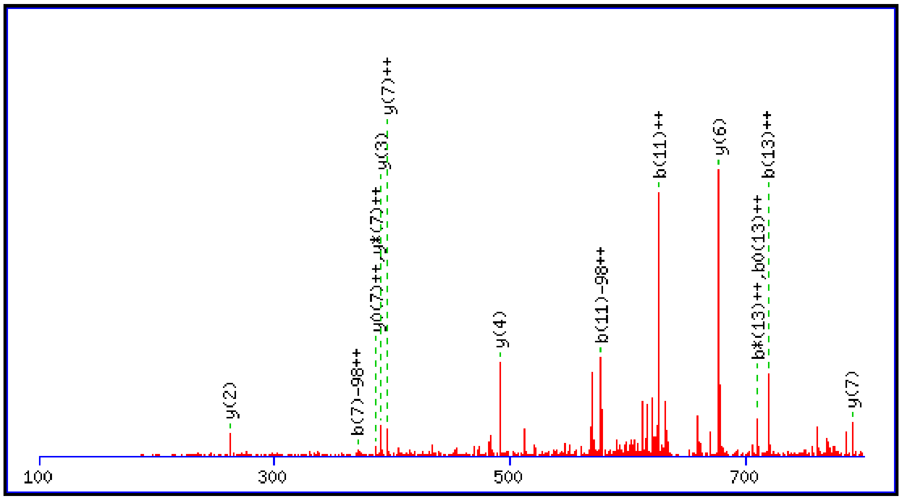

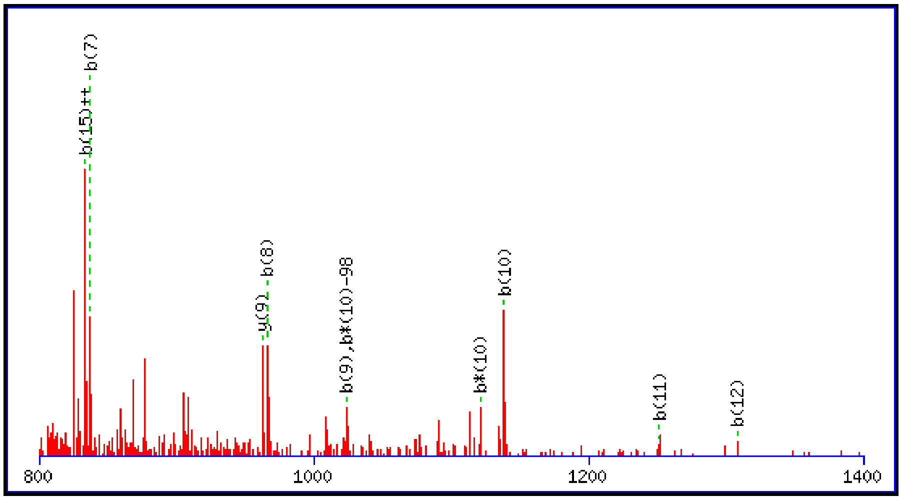

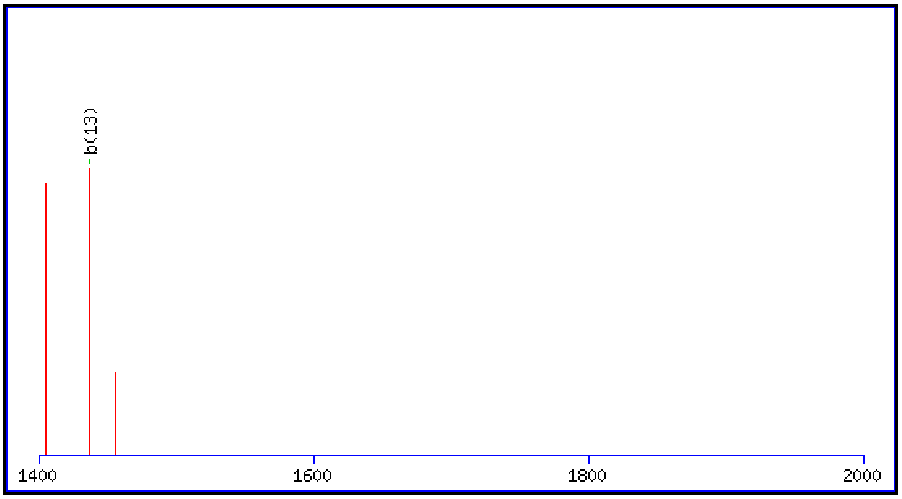

Monoisotopic mass of neutral peptide Mr(calc): 1926.8320
Fixed modifications: Carbamidomethyl (C)
Variable modifications:

S3 : Phospho (ST), with neutral losses 0.0000(shown in table), 97.9769
Ions Score: 40 **Expect:** 0.021
Matches (Bold Red): 25/280 fragment ions using 30 most intense peaks

#	b	b++	b*	b*++	b0	b0++	Seq.	y	y++	y*	y*++	y0	y0++	#
1	114.0913	57.5493					L							17
2	270.1925	135.5999	253.1659	127.0866			R	1814.7552	907.8813	1797.7287	899.3680	1796.7447	898.8760	16
3	437.1908	219.0990	420.1643	210.5858	419.1802	210.0938	S	1658.6541	829.8307	1641.6276	821.3174	1640.6436	820.8254	15
4	566.2334	283.6203	549.2068	275.1071	548.2228	274.6151	E	1491.6558	746.3315	1474.6292	737.8182	1473.6452	737.3262	14
5	681.2603	341.1338	664.2338	332.6205	663.2498	332.1285	D	1362.6132	681.8102	1345.5866	673.2970	1344.6026	672.8049	13
6	738.2818	369.6445	721.2553	361.1313	720.2712	360.6393	G	1247.5862	624.2968	1230.5597	615.7835	1229.5757	615.2915	12
7	**837.3502**	419.1787	820.3237	410.6655	819.3397	410.1735	V	1190.5648	595.7860	1173.5382	587.2727	1172.5542	586.7807	11
8	**966.3928**	483.7000	949.3663	475.1868	948.3822	474.6948	E	1091.4964	546.2518	1074.4698	537.7385	1073.4858	537.2465	10
9	**1023.4143**	512.2108	1006.3877	503.6975	1005.4037	503.2055	G	**962.4538**	481.7305	945.4272	473.2172	944.4432	472.7252	9
10	**1138.4412**	569.7242	**1121.4147**	561.2110	1120.4307	560.7190	D	905.4323	453.2198	888.4058	444.7065	887.4217	444.2145	8
11	**1251.5253**	**626.2663**	1234.4987	617.7530	1233.5147	617.2610	L	**790.4054**	**395.7063**	773.3788	**387.1930**	772.3948	**386.7010**	7
12	**1308.5467**	654.7770	1291.5202	646.2637	1290.5362	645.7717	G	**677.3213**	339.1643	660.2947	330.6510	659.3107	330.1590	6
13	**1437.5893**	**719.2983**	1420.5628	**710.7850**	1419.5788	**710.2930**	E	620.2998	310.6536	603.2733	302.1403	602.2893	301.6483	5
14	1538.6370	769.8221	1521.6105	761.3089	1520.6265	760.8169	T	**491.2572**	246.1323	474.2307	237.6190	473.2467	237.1270	4
15	1666.6956	**833.8514**	1649.6690	825.3382	1648.6850	824.8462	Q	**390.2096**	195.6084	373.1830	187.0951	372.1990	186.6031	3
16	1753.7276	877.3674	1736.7011	868.8542	1735.7171	868.3622	S	**262.1510**	131.5791	245.1244	123.0659	244.1404	122.5738	2
17							R	175.1190	88.0631	158.0924	79.5498			1

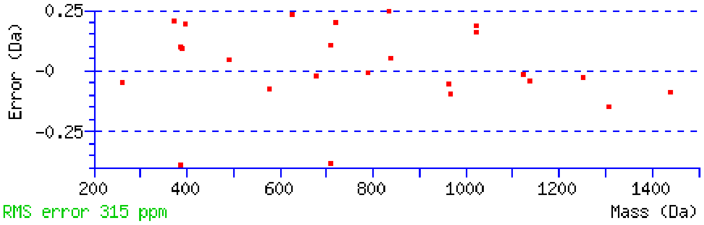

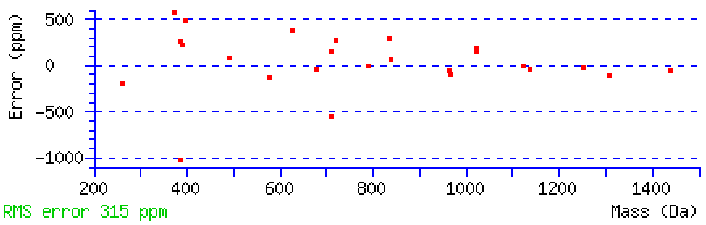

All matches to this query

Score	Mr(calc):	Delta	Sequence
40.2	1926.8320	0.0010	LRSEDGVEGDLGETQSR
8.1	1926.8183	0.0147	HMTKEETQGLWEQSK
8.0	1926.8183	0.0147	HMTKEETQGLWEQSK
6.8	1924.8356	1.9974	ALYQAEAFTADFQQSR
5.8	1926.8401	-0.0071	LVSAVDYYFIQDDGSR
5.8	1926.8401	-0.0071	LVSAVDYYFIQDDGSR
5.8	1926.8401	-0.0071	LVSAVDYYFIQDDGSR
5.4	1925.8246	1.0083	NPLGSLQAMYTNISLK
5.0	1926.8143	0.0187	AVGTPGGNSGGAGPGISTMSR
5.0	1926.8143	0.0187	AVGTPGGNSGGAGPGISTMSR

Spectrum No: 114; Query: 166; Rank: 1

Peptide View

MS/MS Fragmentation of **VQIPNSPSNFR**
Found in **IPI00193371**, Tax_Id=10116 Gene_Symbol=Slc9a3 Sodium/hydrogen exchanger 3

Match to Query 166: 1337.616708 from(669.815630,2+)

Title: 091129RatKid_SCX02_13.1557.1557.2.dta
Data file K:\NewmanPaper\Piliang\3SubProteomes\Piliang3SP\mgf5ppm\SCX_3SubProteomes5ppm.mgf

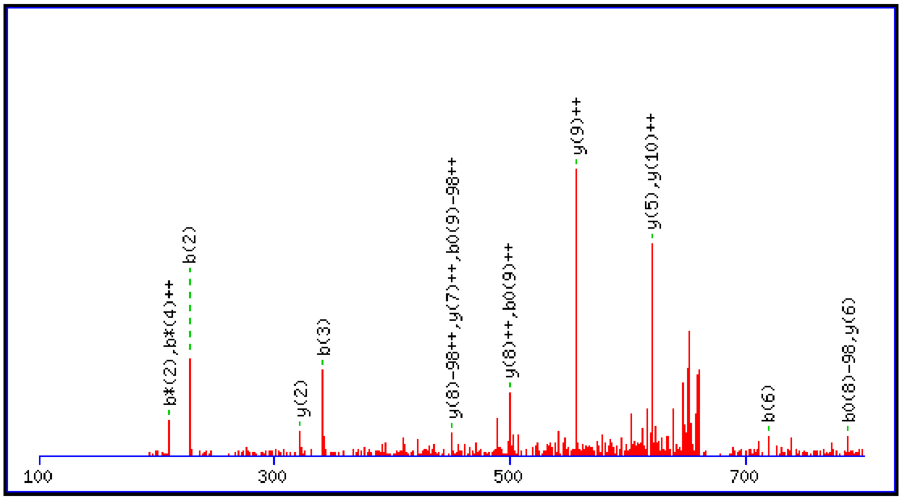

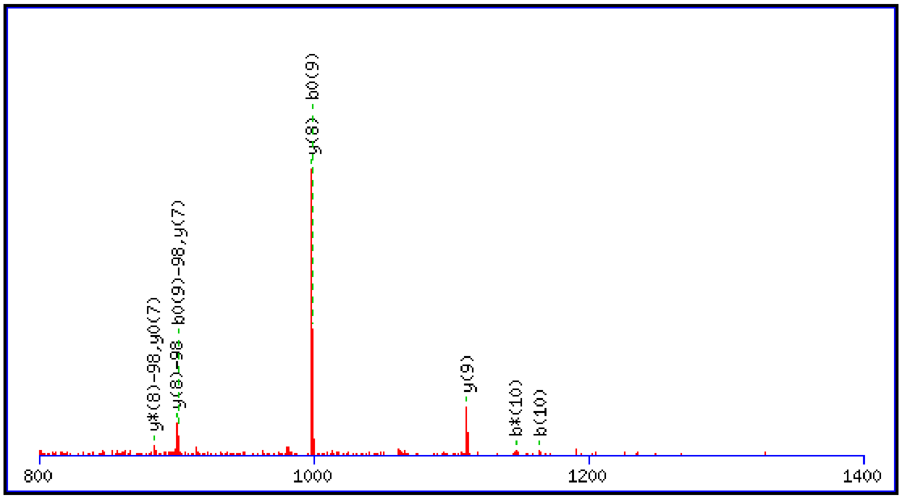

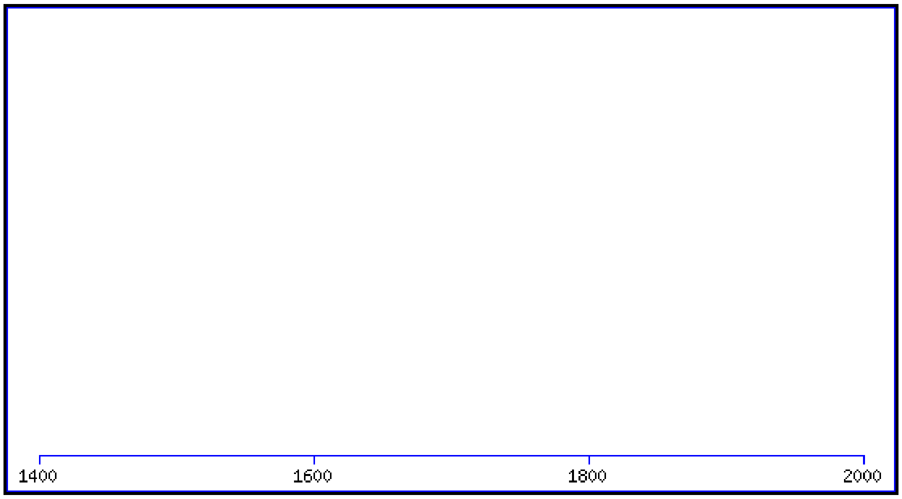

Monoisotopic mass of neutral peptide Mr(calc): 1337.6129
Fixed modifications: Carbamidomethyl (C)
Variable modifications:
S6 : Phospho (ST), with neutral losses 0.0000(shown in table), 97.9769
Ions Score: 40 **Expect:** 0.013
Matches (Bold Red): 26/162 fragment ions using 35 most intense peaks

#	b	b^{++}	b*	b^{*++}	b^0	b^{0++}	Seq.	y	y^{++}	y*	y^{*++}	y^0	y^{0++}	#
1	100.0757	50.5415					V							11
2	**228.1343**	114.5708	**211.1077**	106.0575			Q	1239.5518	**620.2795**	1222.5252	611.7662	1221.5412	611.2742	10
3	**341.2183**	171.1128	324.1918	162.5995			I	**1111.4932**	**556.2502**	1094.4666	547.7370	1093.4826	547.2450	9

**4**	438.2711	219.6392	421.2445	**211.1259**			**P**	**998.4091**	**499.7082**	981.3826	491.1949	980.3986	490.7029	**8**
**5**	552.3140	276.6606	535.2875	268.1474			**N**	**901.3564**	**451.1818**	884.3298	442.6685	**883.3458**	442.1765	**7**
**6**	**719.3124**	360.1598	702.2858	351.6466	701.3018	351.1545	**S**	**787.3134**	394.1604	770.2869	385.6471	769.3029	385.1551	**6**
**7**	816.3651	408.6862	799.3386	400.1729	798.3546	399.6809	**P**	**620.3151**	310.6612	603.2885	302.1479	602.3045	301.6559	**5**
**8**	903.3972	452.2022	886.3706	443.6889	885.3866	443.1969	**S**	523.2623	262.1348	506.2358	253.6215	505.2518	253.1295	**4**
**9**	1017.4401	509.2237	1000.4135	500.7104	**999.4295**	**500.2184**	**N**	436.2303	218.6188	419.2037	210.1055			**3**
**10**	**1164.5085**	582.7579	**1147.4820**	574.2446	1146.4979	573.7526	**F**	**322.1874**	161.5973	305.1608	153.0840			**2**
**11**							**R**	175.1190	88.0631	158.0924	79.5498			**1**

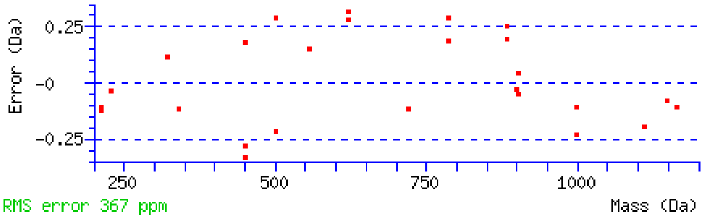

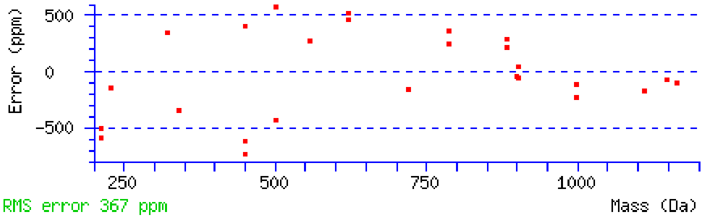

All matches to this query

Score	Mr(calc):	Delta	Sequence
40.2	1337.6129	0.0038	VQIPNSPSNFR
32.2	1337.6129	0.0038	VQIPNSPSNFR
16.9	1337.6091	0.0076	VKIMLPWDPSG
12.7	1337.6067	0.0100	LNLSQNMMSQR
12.7	1337.6067	0.0100	LNLSQNMMSQR
10.9	1337.6241	-0.0074	INSRTQFAGHK
10.9	1337.6241	-0.0074	INSRTQFAGHK
10.3	1337.6146	0.0021	KVITSGGITFR
9.2	1337.6146	0.0021	KVITSGGITFR
8.7	1337.6115	0.0052	ELPETDADLKK

Spectrum No: 115; Query: 751; Rank: 1

Peptide View

MS/MS Fragmentation of **LDGPIENVSEDEAQSSSQR**
Found in **IPI00471864**, Tax_Id=10116 Gene_Symbol=RGD1307509 Similar to RIKEN cDNA 1700108L22

Match to Query 751: 2139.895988 from(1070.955270,2+)
Title: 091127RatKid_SCX01_12.1707.1707.2.dta
Data file K:\NewmanPaper\Piliang\3SubProteomes\Piliang3SP\mgf5ppm\SCX_3SubProteomes5ppm.mgf

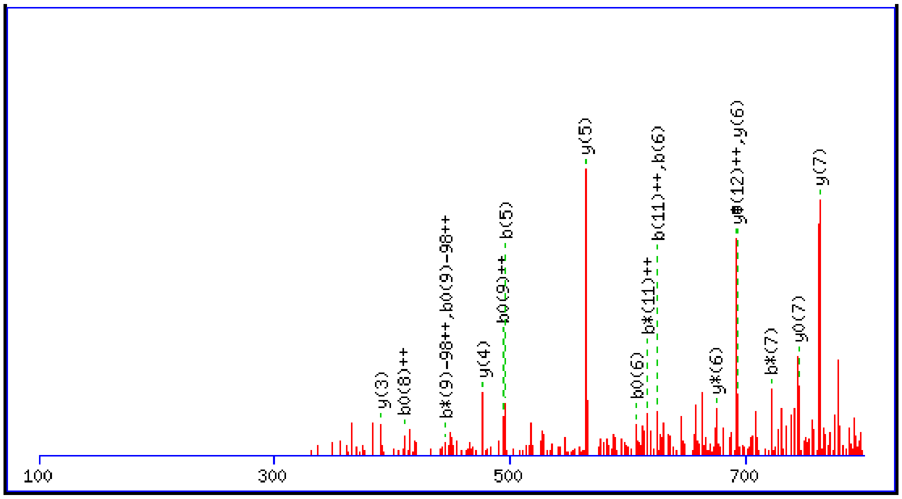

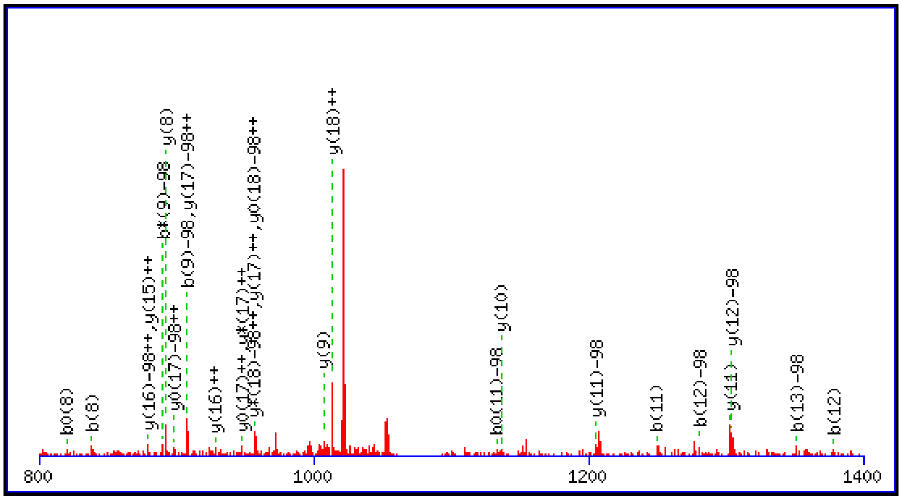

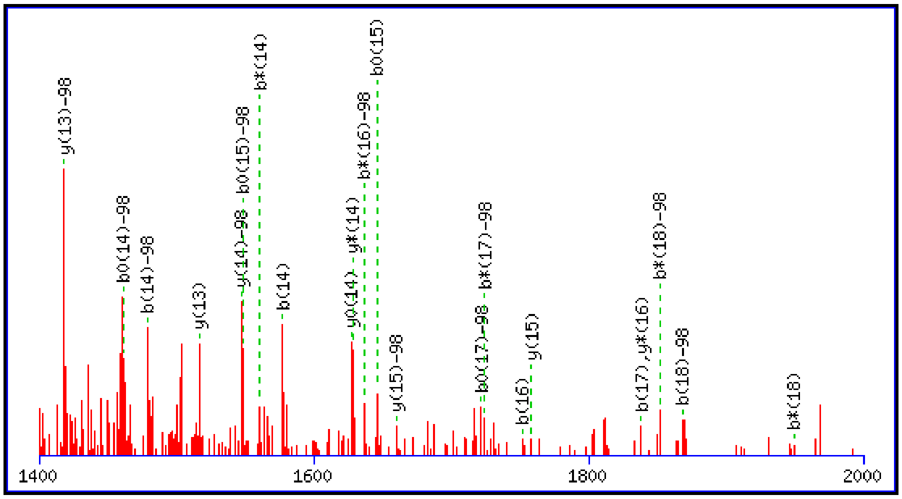

Monoisotopic mass of neutral peptide Mr(calc): 2139.8957
Fixed modifications: Carbamidomethyl (C)
Variable modifications:
S9 : Phospho (ST), with neutral losses 97.9769(shown in table), 0.0000
Ions Score: 40 **Expect:** 0.022
Matches (Bold Red): 67/306 fragment ions using 155 most intense peaks

#	b	b^{++}	b*	b^{*++}	b^0	b^{0++}	Seq.	y	y^{++}	y*	y^{*++}	y^0	y^{0++}	#
1	114.0913	57.5493					L							19
2	229.1183	115.0628			211.1077	106.0575	D	1929.8421	965.4247	1912.8155	**956.9114**	1911.8315	**956.4194**	18
3	286.1397	143.5735			268.1292	134.5682	G	1814.8151	**907.9112**	1797.7886	899.3979	1796.8046	**898.9059**	17
4	383.1925	192.0999			365.1819	183.0946	P	1757.7937	**879.4005**	1740.7671	870.8872	1739.7831	870.3952	16
5	**496.2766**	248.6419			478.2660	239.6366	I	**1660.7409**	830.8741	1643.7143	822.3608	1642.7303	821.8688	15

**6**	**625.3192**	313.1632			**607.3086**	304.1579	**E**	**1547.6568**	774.3321	1530.6303	765.8188	1529.6463	765.3268	**14**
**7**	739.3621	370.1847	**722.3355**	361.6714	721.3515	361.1794	**N**	**1418.6142**	709.8108	1401.5877	701.2975	1400.6037	700.8055	**13**
**8**	**838.4305**	419.7189	821.4040	411.2056	**820.4199**	**410.7136**	**V**	**1304.5713**	652.7893	1287.5448	644.2760	1286.5607	643.7840	**12**
**9**	**907.4520**	454.2296	**890.4254**	**445.7163**	889.4414	**445.2243**	**S**	**1205.5029**	603.2551	1188.4763	594.7418	1187.4923	594.2498	**11**
**10**	1036.4946	518.7509	1019.4680	510.2376	1018.4840	509.7456	**E**	**1136.4814**	568.7444	1119.4549	560.2311	1118.4709	559.7391	**10**
**11**	1151.5215	576.2644	1134.4950	567.7511	**1133.5109**	567.2591	**D**	**1007.4388**	504.2231	990.4123	495.7098	989.4283	495.2178	**9**
**12**	**1280.5641**	640.7857	1263.5375	632.2724	1262.5535	631.7804	**E**	**892.4119**	446.7096	875.3854	438.1963	874.4013	437.7043	**8**
**13**	**1351.6012**	676.3042	1334.5747	667.7910	1333.5906	667.2990	**A**	**763.3693**	382.1883	746.3428	373.6750	**745.3587**	373.1830	**7**
**14**	**1479.6598**	740.3335	1462.6332	731.8203	**1461.6492**	731.3282	**Q**	**692.3322**	346.6697	**675.3056**	338.1565	674.3216	337.6645	**6**
**15**	1566.6918	783.8495	1549.6653	775.3363	**1548.6812**	774.8443	**S**	**564.2736**	282.6404	547.2471	274.1272	546.2630	273.6352	**5**
**16**	1653.7238	827.3656	**1636.6973**	818.8523	1635.7133	818.3603	**S**	**477.2416**	239.1244	460.2150	230.6112	459.2310	230.1191	**4**
**17**	1740.7559	870.8816	**1723.7293**	862.3683	**1722.7453**	861.8763	**S**	**390.2096**	195.6084	373.1830	187.0951	372.1990	186.6031	**3**
**18**	**1868.8144**	934.9109	**1851.7879**	926.3976	1850.8039	925.9056	**Q**	303.1775	152.0924	286.1510	143.5791			**2**
**19**							**R**	175.1190	88.0631	158.0924	79.5498			**1**

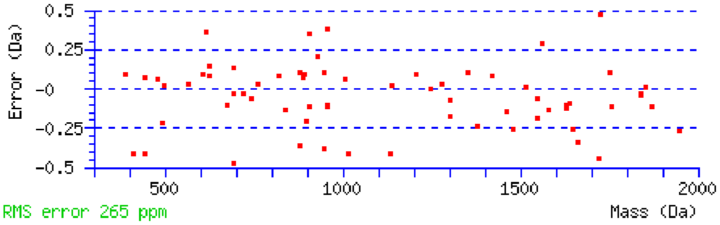

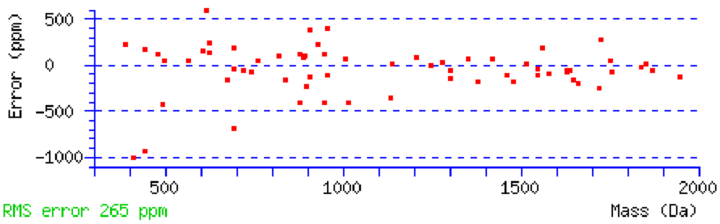

All matches to this query

Score	Mr(calc):	Delta	Sequence
40.0	2139.8957	0.0003	LDGPIENVSEDEAQSSSQR
13.3	2139.8957	0.0003	LDGPIENVSEDEAQSSSQR
9.6	2139.8957	0.0003	LDGPIENVSEDEAQSSSQR
6.5	2139.8957	0.0003	LDGPIENVSEDEAQSSSQR
0.5	2137.8793	2.0167	MSSAPTTPPSVDKVDGFSR
0.5	2137.8793	2.0167	MSSAPTTPPSVDKVDGFSR
0.5	2137.8793	2.0167	MSSAPTTPPSVDKVDGFSR
0.5	2137.8793	2.0167	MSSAPTTPPSVDKVDGFSR
0.5	2137.8793	2.0167	MSSAPTTPPSVDKVDGFSR
0.5	2137.8793	2.0167	MSSAPTTPPSVDKVDGFSR

Spectrum No: 116; Query: 575; Rank: 1

Peptide View

MS/MS Fragmentation of **GSDTSPEAEASSGGGGVALK**
Found in **IPI00205295**, Tax_Id=10116 Gene_Symbol=Slc7a8 Large neutral amino acids transporter small subunit 2

Match to Query 575: 1855.782388 from(928.898470,2+)
Title: 091129RatKid_SCX02_12.840.840.2.dta
Data file K:\NewmanPaper\Piliang\3SubProteomes\Piliang3SP\mgf5ppm\SCX_3SubProteomes5ppm.mgf

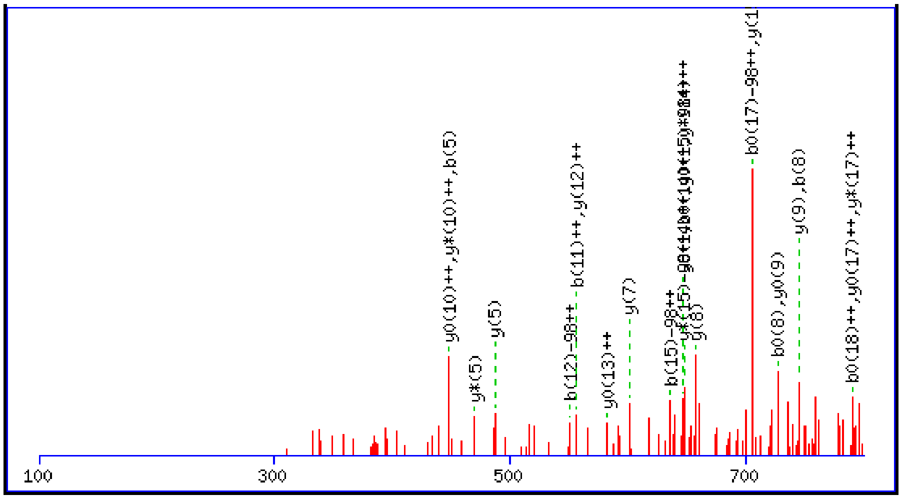

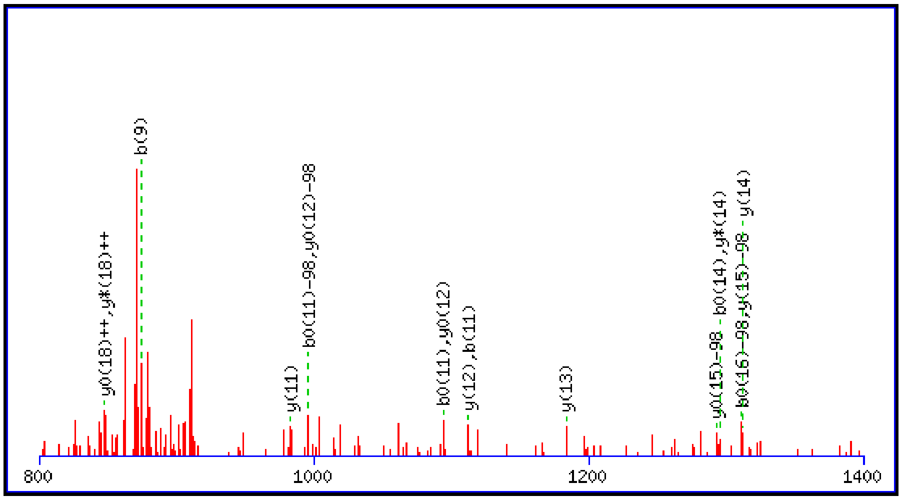

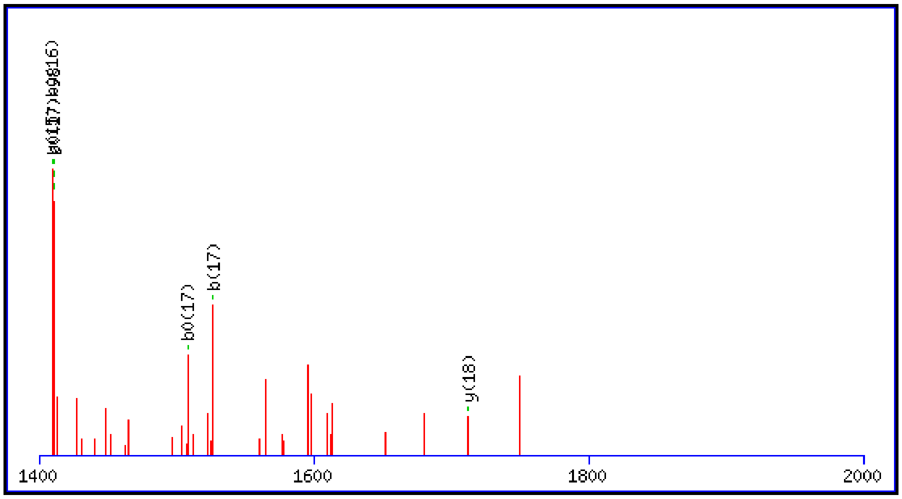

Monoisotopic mass of neutral peptide Mr(calc): 1855.7837
Fixed modifications: Carbamidomethyl (C)
Variable modifications:
S11 : Phospho (ST), with neutral losses 0.0000(shown in table), 97.9769
Ions Score: 39 **Expect:** 0.022
Matches (Bold Red): 50/268 fragment ions using 66 most intense peaks

#	b	b^{++}	b^{0}	b^{0++}	Seq.	y	y^{++}	y*	y^{*++}	y^{0}	y^{0++}	#
1	58.0287	29.5180			G							20
2	145.0608	73.0340	127.0502	64.0287	S	1799.7695	900.3884	1782.7429	891.8751	1781.7589	891.3831	19
3	260.0877	130.5475	242.0771	121.5422	D	**1712.7375**	856.8724	1695.7109	**848.3591**	1694.7269	**847.8671**	18
4	361.1354	181.0713	343.1248	172.0661	T	1597.7105	799.3589	1580.6840	**790.8456**	1579.7000	**790.3536**	17
5	**448.1674**	224.5873	430.1569	215.5821	S	1496.6628	748.8351	1479.6363	740.3218	1478.6523	739.8298	16

6	545.2202	273.1137	527.2096	264.1084	P	1409.6308	705.3190	1392.6043	696.8058	1391.6202	696.3138	15
7	674.2628	337.6350	656.2522	328.6297	E	1312.5780	656.7927	1295.5515	648.2794	1294.5675	647.7874	14
8	745.2999	373.1536	727.2893	364.1483	A	1183.5355	592.2714	1166.5089	583.7581	1165.5249	583.2661	13
9	874.3425	437.6749	856.3319	428.6696	E	1112.4983	556.7528	1095.4718	548.2395	1094.4878	547.7475	12
10	945.3796	473.1934	927.3690	464.1882	A	983.4557	492.2315	966.4292	483.7182	965.4452	483.2262	11
11	1112.3779	556.6926	1094.3674	547.6873	S	912.4186	456.7130	895.3921	448.1997	894.4081	447.7077	10
12	1199.4100	600.2086	1181.3994	591.2033	S	745.4203	373.2138	728.3937	364.7005	727.4097	364.2085	9
13	1256.4314	628.7194	1238.4209	619.7141	G	658.3883	329.6978	641.3617	321.1845			8
14	1313.4529	657.2301	1295.4423	648.2248	G	601.3668	301.1870	584.3402	292.6738			7
15	1370.4744	685.7408	1352.4638	676.7355	G	544.3453	272.6763	527.3188	264.1630			6
16	1427.4958	714.2516	1409.4853	705.2463	G	487.3239	244.1656	470.2973	235.6523			5
17	1526.5642	763.7858	1508.5537	754.7805	V	430.3024	215.6548	413.2758	207.1416			4
18	1597.6014	799.3043	1579.5908	790.2990	A	331.2340	166.1206	314.2074	157.6074			3
19	1710.6854	855.8463	1692.6749	846.8411	L	260.1969	130.6021	243.1703	122.0888			2
20					K	147.1128	74.0600	130.0863	65.5468			1

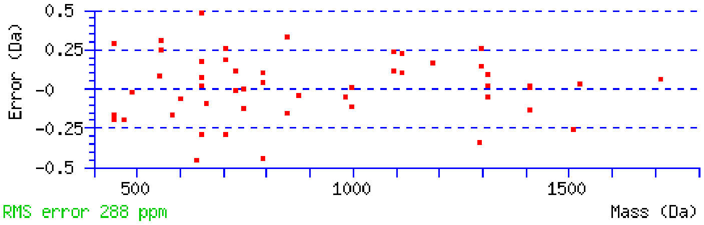

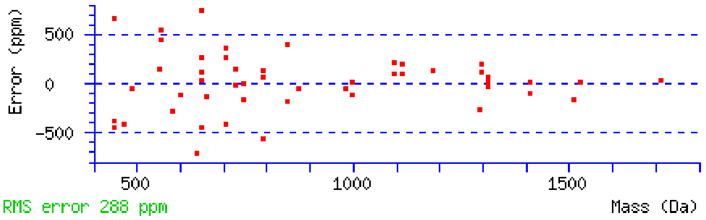

All matches to this query

Score	Mr(calc):	Delta	Sequence
39.5	1855.7837	-0.0013	GSDTSPEAEASSGGGVALK
34.2	1855.7837	-0.0013	GSDTSPEAEASSGGGVALK
13.5	1855.7837	-0.0013	GSDTSPEAEASSGGGVALK
13.5	1855.7837	-0.0013	GSDTSPEAEASSGGGVALK
7.1	1855.7837	-0.0013	GSDTSPEAEASSGGGVALK
2.7	1854.7706	1.0117	MEADKTSEGPPATQDAK
2.4	1853.7940	1.9884	ASLADSGEYMCKVISK
2.4	1853.7940	1.9884	ASLADSGEYMCKVISK
2.4	1853.7940	1.9884	ASLADSXEYMCKVISK
2.4	1853.7940	1.9884	ASLADSXEYMCKVISK

Spectrum No: 117; Query: 979; Rank: 1

Peptide View

MS/MS Fragmentation of **TENQPAVLEDAPDNTETGSVCTKV**
Found in **IPI00203446**, Tax_Id=10116 Gene_Symbol=Slc23a1 Solute carrier family 23 member 1

Match to Query 979: 2654.148072 from(885.723300,3+)
Title: 091127RatKid_SCX01_11.1943.1943.3.dta
Data file K:\NewmanPaper\Piliang\3SubProteomes\Piliang3SP\mgf5ppm\SCX_3SubProteomes5ppm.mgf

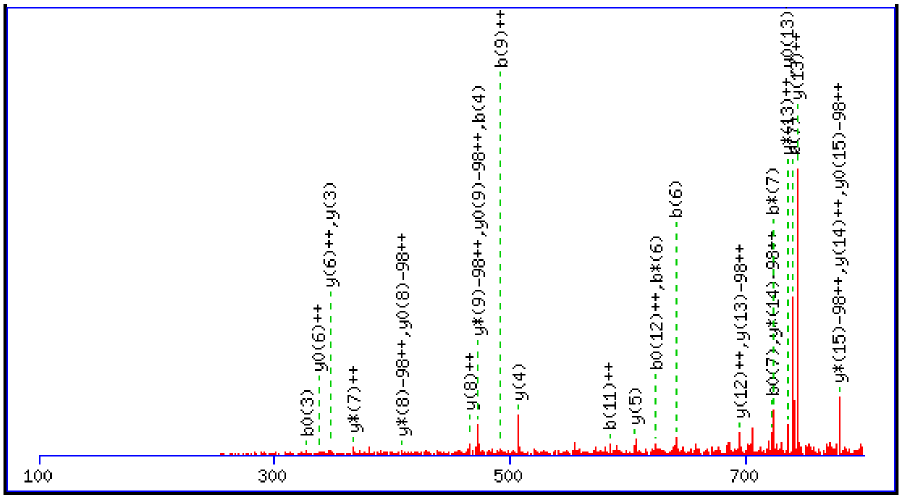

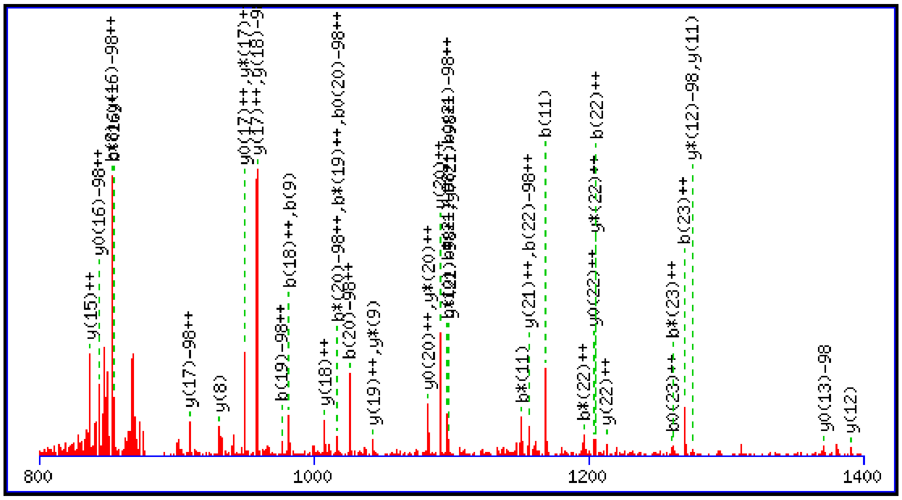

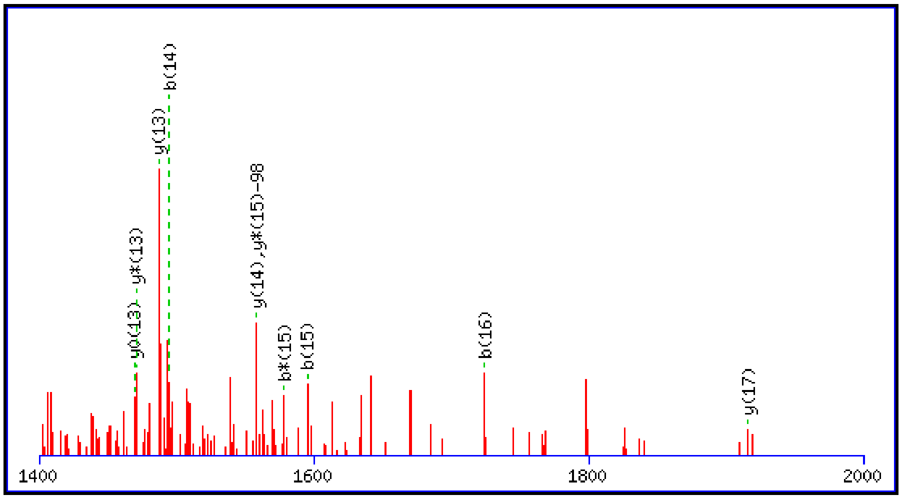

Monoisotopic mass of neutral peptide Mr(calc): 2654.1419
Fixed modifications: Carbamidomethyl (C)
Variable modifications:
T17 : Phospho (ST), with neutral losses 0.0000(shown in table), 97.9769
Ions Score: 39 **Expect:** 0.041
Matches (Bold Red): 86/404 fragment ions using 133 most intense peaks

#	b	b^{++}	b*	b^{*++}	b^0	b^{0++}	Seq.	y	y^{++}	y*	y^{*++}	y^0	y^{0++}	#
1	102.0550	51.5311			84.0444	42.5258	T							24
2	231.0975	116.0524			213.0870	107.0471	E	2554.1015	1277.5544	2537.0749	1269.0411	2536.0909	1268.5491	23
3	345.1405	173.0739	328.1139	164.5606	**327.1299**	164.0686	N	2425.0589	**1213.0331**	2408.0323	**1204.5198**	2407.0483	**1204.0278**	22
4	**473.1991**	237.1032	456.1725	228.5899	455.1885	228.0979	Q	2311.0159	**1156.0116**	2293.9894	1147.4983	2293.0054	1147.0063	21
5	570.2518	285.6295	553.2253	277.1163	552.2413	276.6243	P	2182.9574	**1091.9823**	2165.9308	**1083.4690**	2164.9468	**1082.9770**	20

**6**	**641.2889**	321.1481	**624.2624**	312.6348	623.2784	312.1428	**A**	2085.9046	**1043.4559**	2068.8781	1034.9427	2067.8940	1034.4507	**19**
**7**	**740.3573**	370.6823	**723.3308**	362.1690	**722.3468**	361.6770	**V**	2014.8675	**1007.9374**	1997.8409	999.4241	1996.8569	998.9321	**18**
**8**	**853.4414**	427.2243	836.4149	418.7111	835.4308	418.2191	**L**	**1915.7991**	**958.4032**	1898.7725	**949.8899**	1897.7885	**949.3979**	**17**
**9**	**982.4840**	**491.7456**	965.4575	483.2324	964.4734	482.7404	**E**	1802.7150	901.8611	1785.6885	893.3479	1784.7044	892.8559	**16**
**10**	**1097.5109**	549.2591	1080.4844	540.7458	1079.5004	540.2538	**D**	1673.6724	**837.3398**	1656.6459	828.8266	1655.6619	828.3346	**15**
**11**	**1168.5481**	**584.7777**	**1151.5215**	576.2644	1150.5375	575.7724	**A**	**1558.6455**	**779.8264**	1541.6189	771.3131	1540.6349	770.8211	**14**
**12**	1265.6008	633.3040	1248.5743	624.7908	1247.5903	**624.2988**	**P**	**1487.6084**	**744.3078**	**1470.5818**	**735.7945**	**1469.5978**	**735.3025**	**13**
**13**	1380.6278	690.8175	1363.6012	682.3042	1362.6172	681.8122	**D**	**1390.5556**	**695.7814**	1373.5290	687.2682	1372.5450	686.7762	**12**
**14**	**1494.6707**	747.8390	1477.6441	739.3257	1476.6601	738.8337	**N**	**1275.5287**	638.2680	1258.5021	629.7547	1257.5181	629.2627	**11**
**15**	**1595.7184**	798.3628	**1578.6918**	789.8495	1577.7078	789.3575	**T**	1161.4857	581.2465	1144.4592	572.7332	1143.4752	572.2412	**10**
**16**	**1724.7610**	862.8841	1707.7344	**854.3708**	1706.7504	853.8788	**E**	1060.4380	530.7227	**1043.4115**	522.2094	1042.4275	521.7174	**9**
**17**	1905.7750	953.3911	1888.7484	944.8778	1887.7644	944.3858	**T**	**931.3955**	**466.2014**	914.3689	457.6881	913.3849	457.1961	**8**
**18**	1962.7964	**981.9019**	1945.7699	973.3886	1944.7859	972.8966	**G**	750.3815	375.6944	733.3549	**367.1811**	732.3709	366.6891	**7**
**19**	2049.8285	1025.4179	2032.8019	**1016.9046**	2031.8179	1016.4126	**S**	693.3600	**347.1836**	676.3334	338.6704	675.3494	**338.1783**	**6**
**20**	2148.8969	1074.9521	2131.8703	1066.4388	2130.8863	1065.9468	**V**	**606.3280**	303.6676	589.3014	295.1543	588.3174	294.6623	**5**
**21**	2308.9275	1154.9674	2291.9010	1146.4541	2290.9170	1145.9621	**C**	**507.2595**	254.1334	490.2330	245.6201	489.2490	245.1281	**4**
**22**	2409.9752	**1205.4912**	2392.9487	**1196.9780**	2391.9646	1196.4860	**T**	**347.2289**	174.1181	330.2023	165.6048	329.2183	165.1128	**3**
**23**	2538.0702	**1269.5387**	2521.0436	**1261.0254**	2520.0596	**1260.5334**	**K**	246.1812	123.5942	229.1547	115.0810			**2**
**24**							**V**	118.0863	59.5468					**1**

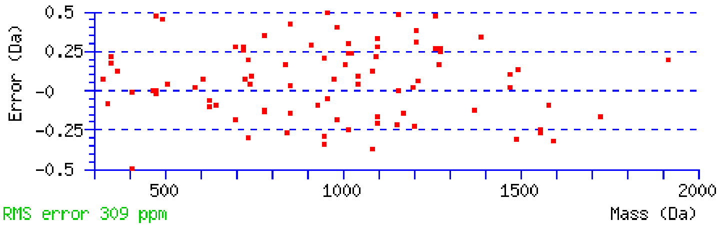

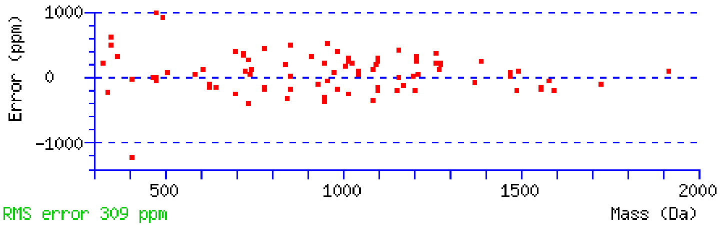

All matches to this query

Score	Mr(calc):	Delta	Sequence
39.3	2654.1419	0.0062	TENQPAVLEDAPDNTETGSVCTKV
38.4	2654.1419	0.0062	TENQPAVLEDAPDNTETGSVCTKV
32.3	2654.1419	0.0062	TENQPAVLEDAPDNTETGSVCTKV
30.9	2654.1419	0.0062	TENQPAVLEDAPDNTETGSVCTKV
6.2	2654.1656	-0.0175	SNMLRGRNSATSADEQPHIGNYR
4.7	2654.1367	0.0114	SQHAHVIEDLHCNLCDVDVSAR
4.0	2654.1700	-0.0220	AMDKMYLSNNPNSHTDNSAKSSDK
3.9	2654.1656	-0.0175	SNMLRGRNSATSADEQPHIGNYR
1.6	2653.1203	1.0277	STSAKTGEIVKTIQATLGASSQR
0.8	2653.1690	0.9791	NALATTAPKETLATSTVTSPSPQK

Spectrum No: 118; Query: 118; Rank: 1

Peptide View

MS/MS Fragmentation of **LGGPAVSPLPVR**
Found in **IPI00193397**, Tax_Id=10116 Gene_Symbol=Slc34a1 Sodium-dependent phosphate transport protein 2A

Match to Query 118: 1241.655288 from(621.834920,2+)
Title: 091127RatKid_SCX01_12.2051.2051.2.dta
Data file K:\NewmanPaper\Piliang\3SubProteomes\Piliang3SP\mgf5ppm\SCX_3SubProteomes5ppm.mgf

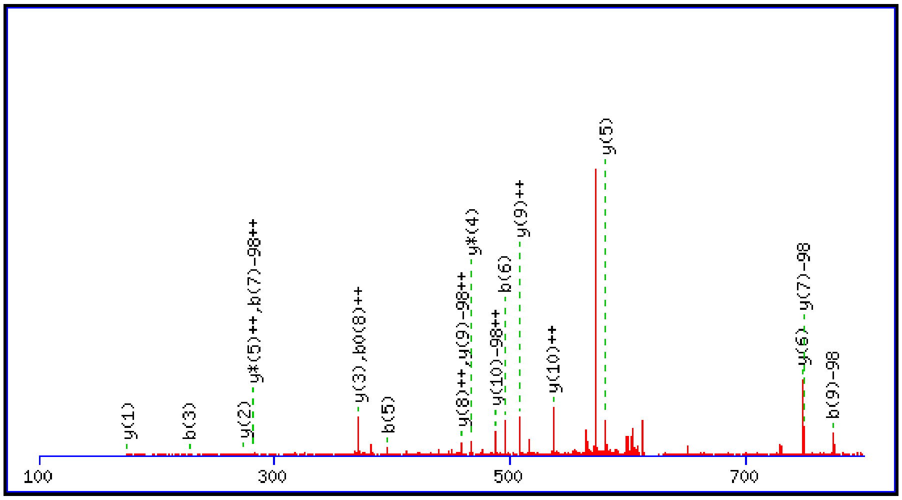

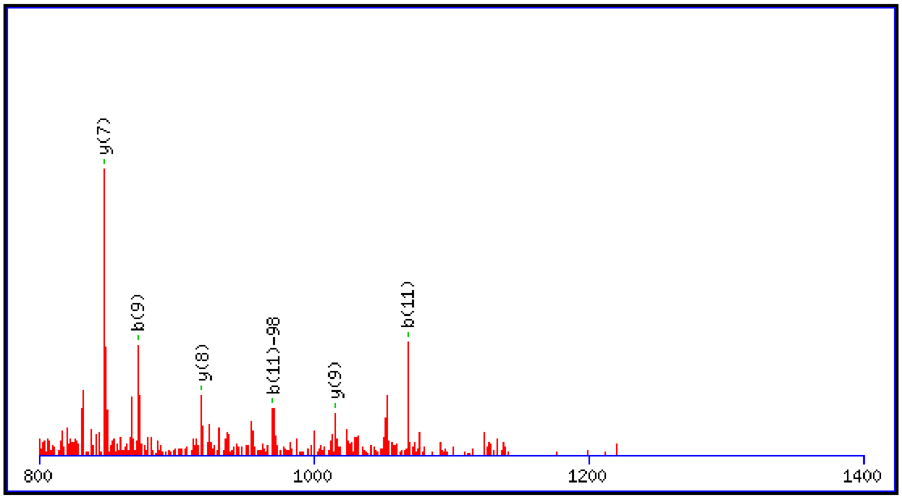

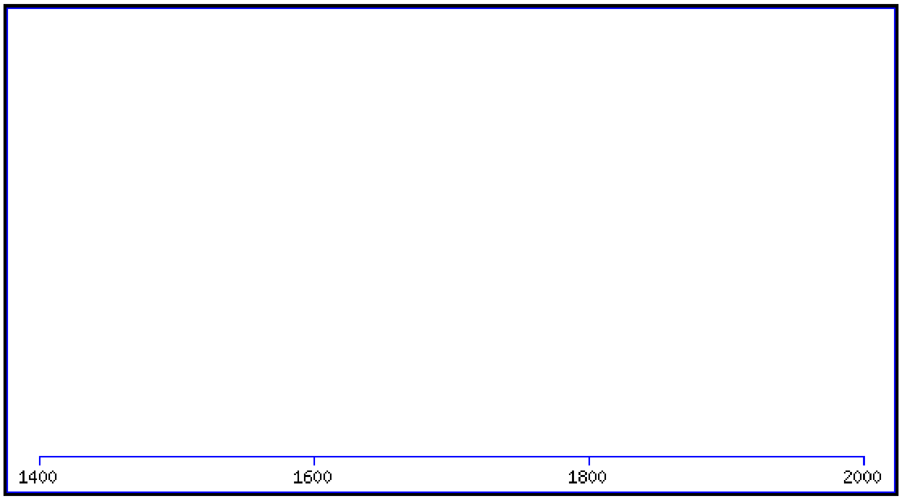

Monoisotopic mass of neutral peptide Mr(calc): 1241.6533
Fixed modifications: Carbamidomethyl (C)
Variable modifications:
S7 : Phospho (ST), with neutral losses 0.0000(shown in table), 97.9769
Ions Score: 39 **Expect:** 0.0099
Matches (Bold Red): 25/144 fragment ions using 50 most intense peaks

#	b	b^{++}	b^0	b^{0++}	Seq.	y	y^{++}	y*	y^{*++}	y^0	y^{0++}	#
1	114.0913	57.5493			L							12
2	171.1128	86.0600			G	1129.5765	565.2919	1112.5500	556.7786	1111.5660	556.2866	11
3	**228.1343**	114.5708			G	1072.5551	**536.7812**	1055.5285	528.2679	1054.5445	527.7759	10
4	325.1870	163.0972			P	**1015.5336**	**508.2704**	998.5071	499.7572	997.5230	499.2652	9
5	**396.2241**	198.6157			A	**918.4808**	**459.7441**	901.4543	451.2308	900.4703	450.7388	8

6	**495.2926**	248.1499			V	**847.4437**	424.2255	830.4172	415.7122	829.4332	415.2202	7
7	662.2909	331.6491	644.2803	322.6438	S	**748.3753**	374.6913	731.3488	366.1780	730.3647	365.6860	6
8	759.3437	380.1755	741.3331	**371.1702**	P	**581.3770**	291.1921	564.3504	**282.6788**			5
9	**872.4277**	436.7175	854.4172	427.7122	L	484.3242	242.6657	**467.2976**	234.1525			4
10	969.4805	485.2439	951.4699	476.2386	P	**371.2401**	186.1237	354.2136	177.6104			3
11	**1068.5489**	534.7781	1050.5384	525.7728	V	**274.1874**	137.5973	257.1608	129.0840			2
12					R	**175.1190**	88.0631	158.0924	79.5498			1

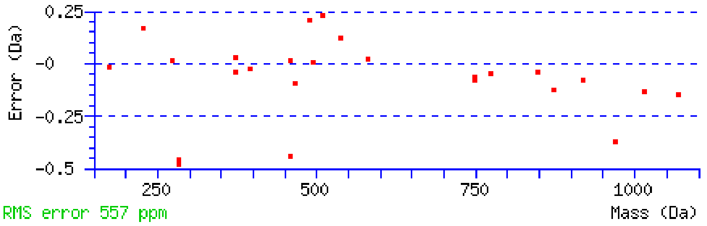

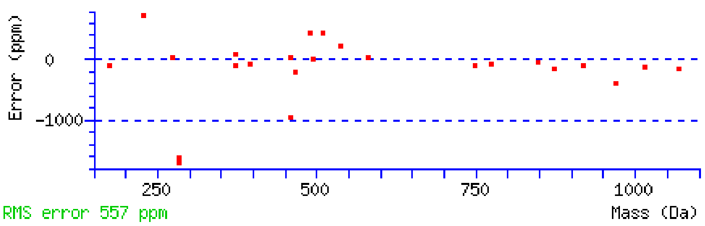

All matches to this query

Score	Mr(calc):	Delta	Sequence
39.2	1241.6533	0.0020	LGGPAVSPLPVR
7.2	1241.6589	-0.0036	DGVELRQRNR
6.7	1240.6524	1.0029	VSTQPRNSQPK
3.9	1240.6540	1.0013	ISTRLTTITR
1.7	1240.6540	1.0013	VGSLKSSKLSR
1.1	1241.6557	-0.0004	WWISGILDPR
0.7	1239.6571	1.9982	RVLADINSSHK
0.6	1239.6546	2.0007	MFFLENKRR
0.6	1240.6537	1.0016	QDRRLNHFR
0.3	1240.6411	1.0142	KELNSKQEHK

Spectrum No: 119; Query: 1142; Rank: 1

Peptide View

MS/MS Fragmentation of **SLSPVLPAAHGSAPAPDPCFPAPSPVPAATAPEAFK**
Found in **IPI00763200**, Tax_Id=10116 Gene_Symbol=Arhgef18_predicted rho/rac guanine nucleotide exchange factor (GEF) 18

Match to Query 1142: 3602.746362 from(1201.922730,3+)
Title: 091127RatKid_SCX01_13.4095.4095.3.dta
Data file K:\NewmanPaper\Piliang\3SubProteomes\Piliang3SP\mgf5ppm\SCX_3SubProteomes5ppm.mgf

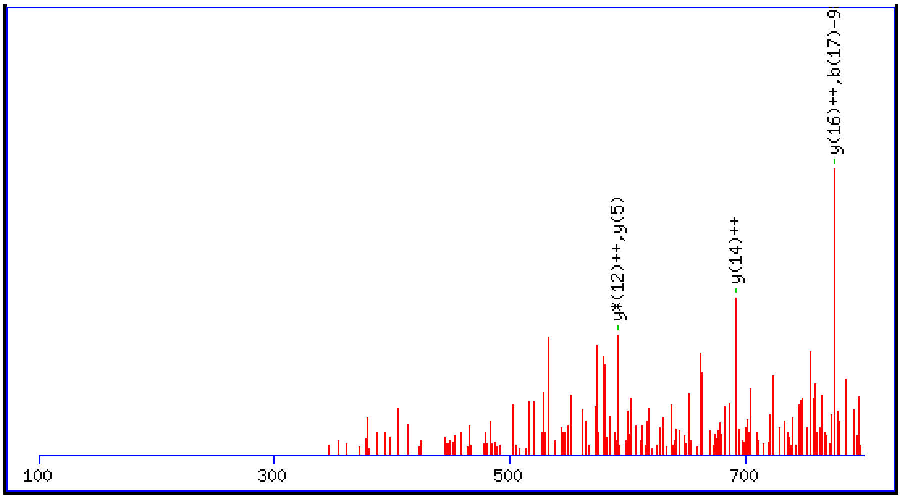

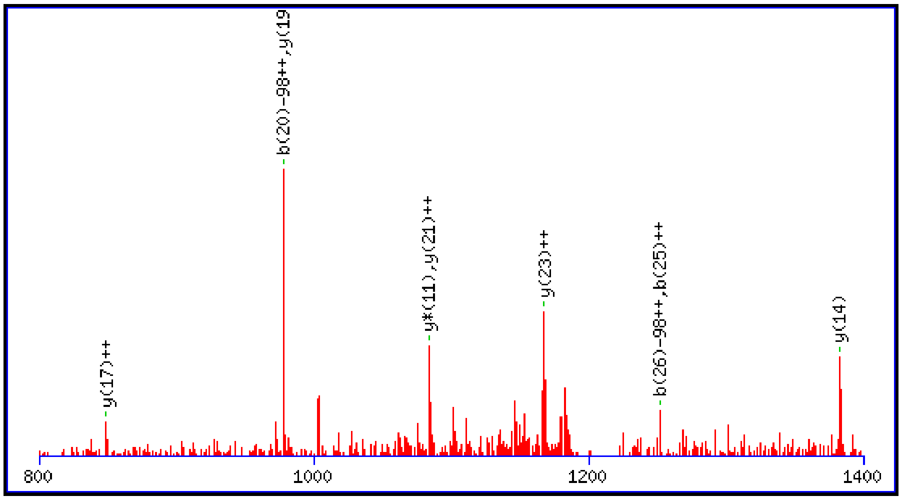

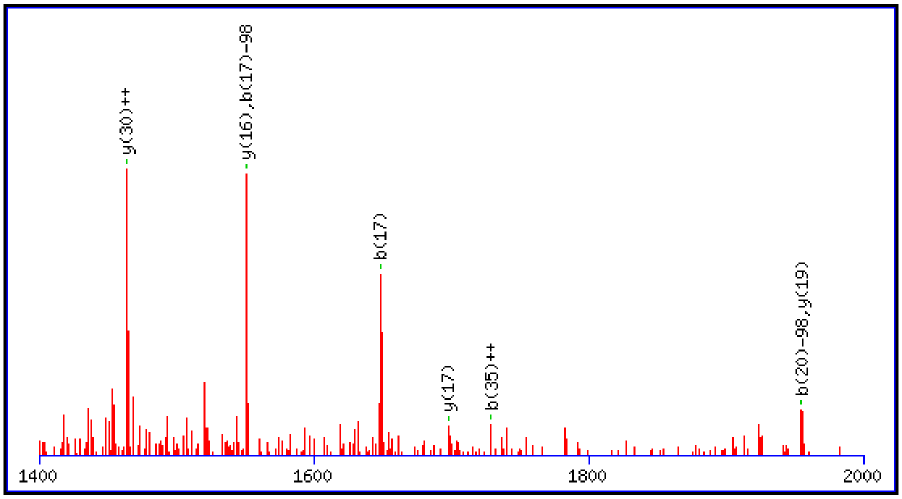

Monoisotopic mass of neutral peptide Mr(calc): 3601.7316
Fixed modifications: Carbamidomethyl (C)
Variable modifications:
S3 : Phospho (ST), with neutral losses 97.9769(shown in table), 0.0000
Ions Score: 39 **Expect:** 0.038
Matches (Bold Red): 22/488 fragment ions using 29 most intense peaks

#	b	b^{++}	b^{0}	b^{0++}	Seq.	y	y^{++}	y*	y^{*++}	y^{0}	y^{0++}	#
1	88.0393	44.5233	70.0287	35.5180	S							36
2	201.1234	101.0653	183.1128	92.0600	L	3417.7300	1709.3686	3400.7035	1700.8554	3399.7194	1700.3634	35
3	270.1448	135.5760	252.1343	126.5708	S	3304.6459	1652.8266	3287.6194	1644.3133	3286.6354	1643.8213	34
4	367.1976	184.1024	349.1870	175.0971	P	3235.6245	1618.3159	3218.5979	1609.8026	3217.6139	1609.3106	33
5	466.2660	233.6366	448.2554	224.6314	V	3138.5717	1569.7895	3121.5452	1561.2762	3120.5612	1560.7842	32

6	579.3501	290.1787	561.3395	281.1734	L	3039.5033	1520.2553	3022.4768	1511.7420	3021.4927	1511.2500	31
7	676.4028	338.7051	658.3923	329.6998	P	2926.4192	1463.7133	2909.3927	1455.2000	2908.4087	1454.7080	30
8	747.4399	374.2236	729.4294	365.2183	A	2829.3665	1415.1869	2812.3399	1406.6736	2811.3559	1406.1816	29
9	818.4771	409.7422	800.4665	400.7369	A	2758.3294	1379.6683	2741.3028	1371.1550	2740.3188	1370.6630	28
10	955.5360	478.2716	937.5254	469.2663	H	2687.2923	1344.1498	2670.2657	1335.6365	2669.2817	1335.1445	27
11	1012.5574	506.7824	994.5469	497.7771	G	2550.2333	1275.6203	2533.2068	1267.1070	2532.2228	1266.6150	26
12	1099.5895	550.2984	1081.5789	541.2931	S	2493.2119	1247.1096	2476.1853	1238.5963	2475.2013	1238.1043	25
13	1170.6266	585.8169	1152.6160	576.8116	A	2406.1798	1203.5936	2389.1533	1195.0803	2388.1693	1194.5883	24
14	1267.6793	634.3433	1249.6688	625.3380	P	2335.1427	1168.0750	2318.1162	1159.5617	2317.1322	1159.0697	23
15	1338.7165	669.8619	1320.7059	660.8566	A	2238.0900	1119.5486	2221.0634	1111.0353	2220.0794	1110.5433	22
16	1435.7692	718.3882	1417.7587	709.3830	P	2167.0529	1084.0301	2150.0263	1075.5168	2149.0423	1075.0248	21
17	1550.7962	775.9017	1532.7856	766.8964	D	2070.0001	1035.5037	2052.9735	1026.9904	2051.9895	1026.4984	20
18	1647.8489	824.4281	1629.8384	815.4228	P	1954.9731	977.9902	1937.9466	969.4769	1936.9626	968.9849	19
19	1807.8796	904.4434	1789.8690	895.4381	C	1857.9204	929.4638	1840.8938	920.9506	1839.9098	920.4585	18
20	1954.9480	977.9776	1936.9374	968.9723	F	1697.8897	849.4485	1680.8632	840.9352	1679.8792	840.4432	17
21	2052.0008	1026.5040	2033.9902	1017.4987	P	1550.8213	775.9143	1533.7948	767.4010	1532.8108	766.9090	16
22	2123.0379	1062.0226	2105.0273	1053.0173	A	1453.7686	727.3879	1436.7420	718.8746	1435.7580	718.3826	15
23	2220.0906	1110.5490	2202.0801	1101.5437	P	1382.7314	691.8694	1365.7049	683.3561	1364.7209	682.8641	14
24	2307.1227	1154.0650	2289.1121	1145.0597	S	1285.6787	643.3430	1268.6521	634.8297	1267.6681	634.3377	13
25	2404.1754	1202.5913	2386.1649	1193.5861	P	1198.6467	599.8270	1181.6201	591.3137	1180.6361	590.8217	12
26	2503.2438	1252.1256	2485.2333	1243.1203	V	1101.5939	551.3006	1084.5673	542.7873	1083.5833	542.2953	11
27	2600.2966	1300.6519	2582.2860	1291.6467	P	1002.5255	501.7664	985.4989	493.2531	984.5149	492.7611	10
28	2671.3337	1336.1705	2653.3231	1327.1652	A	905.4727	453.2400	888.4462	444.7267	887.4621	444.2347	9
29	2742.3708	1371.6891	2724.3603	1362.6838	A	834.4356	417.7214	817.4090	409.2082	816.4250	408.7162	8
30	2843.4185	1422.2129	2825.4079	1413.2076	T	763.3985	382.2029	746.3719	373.6896	745.3879	373.1976	7
31	2914.4556	1457.7314	2896.4451	1448.7262	A	662.3508	331.6790	645.3243	323.1658	644.3402	322.6738	6
32	3011.5084	1506.2578	2993.4978	1497.2525	P	591.3137	296.1605	574.2871	287.6472	573.3031	287.1552	5
33	3140.5510	1570.7791	3122.5404	1561.7738	E	494.2609	247.6341	477.2344	239.1208	476.2504	238.6288	4
34	3211.5881	1606.2977	3193.5775	1597.2924	A	365.2183	183.1128	348.1918	174.5995			3
35	3358.6565	1679.8319	3340.6459	1670.8266	F	294.1812	147.5942	277.1547	139.0810			2
36					K	147.1128	74.0600	130.0863	65.5468			1

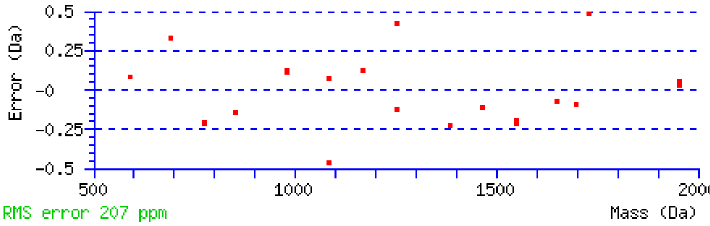

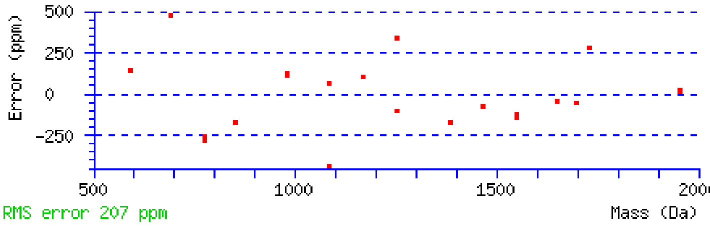

All matches to this query

Score	Mr(calc):	Delta	Sequence
38.9	3601.7316	1.0147	SLSPVLPAAHGSAPAPDPCFPAPSPVPAATAPEAFK
38.9	3601.7316	1.0147	SLSPVLPAAHGSAPAPDPCFPAPSPVPAATAPEAFK
28.0	3601.7316	1.0147	SLSPVLPAAHGSAPAPDPCFPAPSPVPAATAPEAFK
7.7	3601.7464	1.0000	QMKPPAPAAISDSVAAPASLLSTGKASTTSSVKTR
7.7	3601.7464	1.0000	QMKPPAPAAISDSVAAPASLLSTGKASTTSSVKTR
6.8	3600.7431	2.0033	QLEILNHVTNAFSSLLNGVNTLQSQDEEKAER
6.5	3601.7464	1.0000	QMKPPAPAAISDSVAAPASLLSTGKASTTSSVKTR
5.8	3600.7216	2.0248	YFLSCPVVPGHAQATVKWMSKLWNAIIAPR
5.6	3602.7231	0.0232	VFNFSEAVTQLSILVSSLERMAKELDASTLK

3.7	3600.7216	2.0248	YFLSCPVVPGHAQATVKWMSKLWNAIIAPR

Spectrum No: 120; Query: 115; Rank: 1

Peptide View

MS/MS Fragmentation of **GSEAEGQLLKK**
Found in **IPI00215165**, Tax_Id=10116 Gene_Symbol=Chrnb1 Acetylcholine receptor subunit beta precursor

Match to Query 115: 1238.590828 from(620.302690,2+)
Title: 091129RatKid_SCX02_23.2816.2816.2.dta
Data file K:\NewmanPaper\Piliang\3SubProteomes\Piliang3SP\mgf5ppm\SCX_3SubProteomes5ppm.mgf

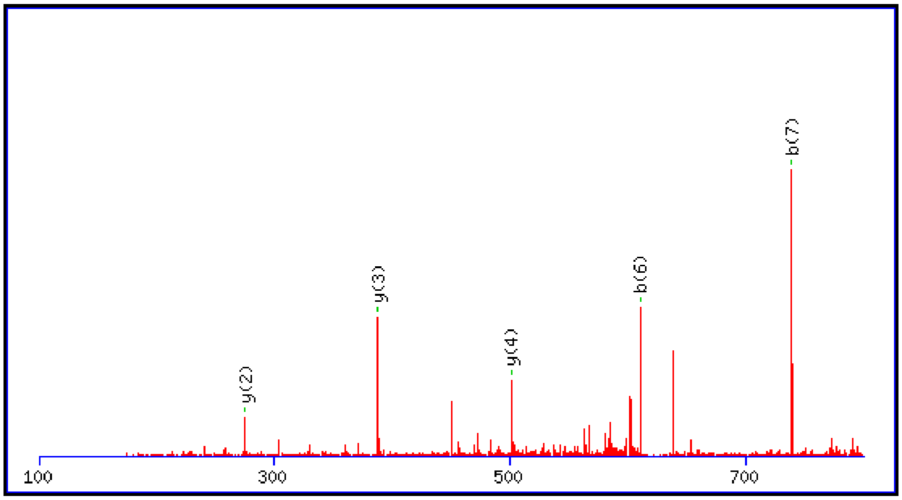

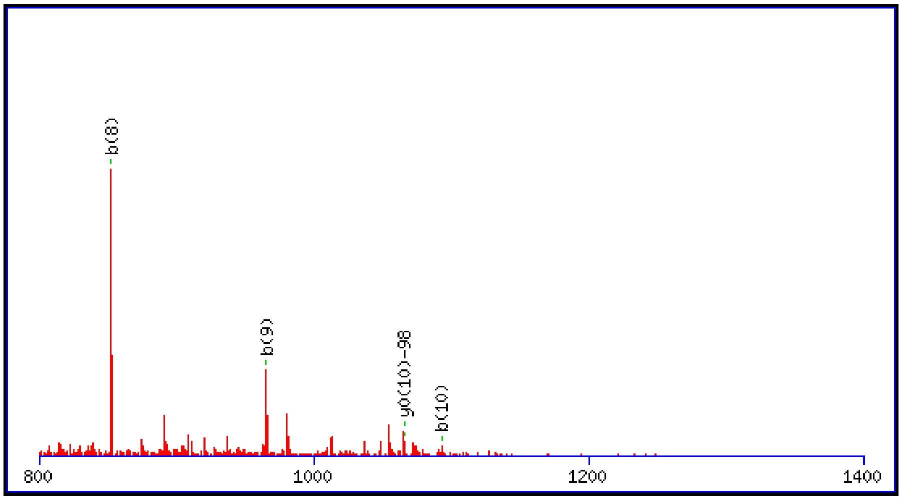

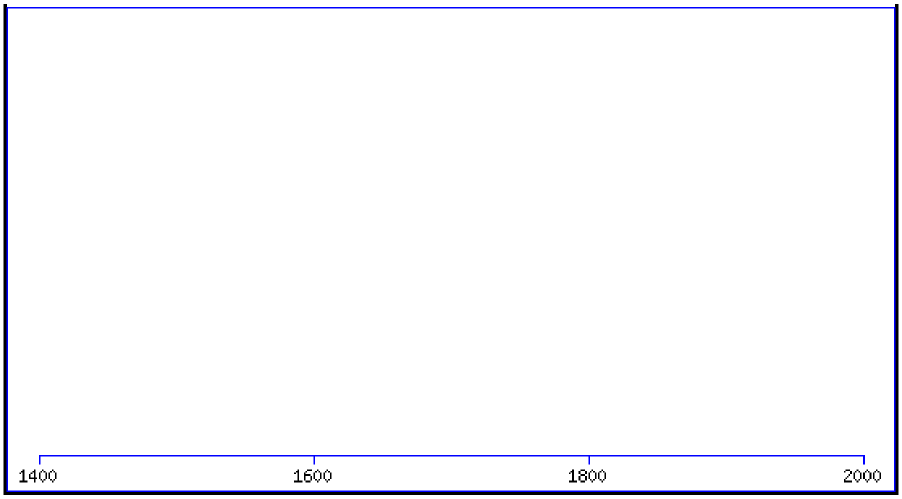

Monoisotopic mass of neutral peptide Mr(calc): 1238.5907
Fixed modifications: Carbamidomethyl (C)
Variable modifications:
S2 : Phospho (ST), with neutral losses 0.0000(shown in table), 97.9769
Ions Score: 39 **Expect:** 0.017
Matches (Bold Red): 9/144 fragment ions using 35 most intense peaks

#	b	b++	b*	b*++	b0	b0++	Seq.	y	y++	y*	y*++	y0	y0++	#
1	58.0287	29.5180					G							11
2	225.0271	113.0172			207.0165	104.0119	S	1182.5766	591.7919	1165.5500	583.2787	1164.5660	582.7867	10
3	354.0697	177.5385			336.0591	168.5332	E	1015.5782	508.2928	998.5517	499.7795	997.5677	499.2875	9
4	425.1068	213.0570			407.0962	204.0518	A	886.5356	443.7715	869.5091	435.2582	868.5251	434.7662	8
5	554.1494	277.5783			536.1388	268.5731	E	815.4985	408.2529	798.4720	399.7396	797.4880	399.2476	7
6	**611.1709**	306.0891			593.1603	297.0838	G	686.4559	343.7316	669.4294	335.2183			6
7	**739.2294**	370.1184	722.2029	361.6051	721.2189	361.1131	Q	629.4345	315.2209	612.4079	306.7076			5
8	**852.3135**	426.6604	835.2869	418.1471	834.3029	417.6551	L	**501.3759**	251.1916	484.3493	242.6783			4
9	**965.3976**	483.2024	948.3710	474.6891	947.3870	474.1971	L	**388.2918**	194.6496	371.2653	186.1363			3
10	**1093.4925**	547.2499	1076.4660	538.7366	1075.4820	538.2446	K	**275.2078**	138.1075	258.1812	129.5942			2
11							K	147.1128	74.0600	130.0863	65.5468			1

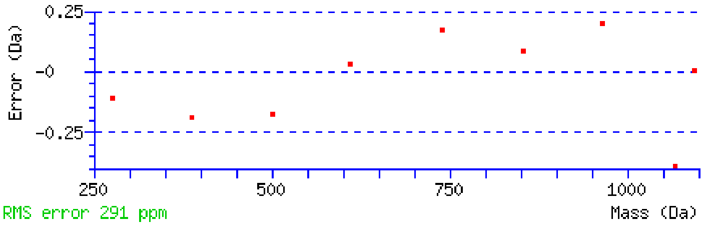

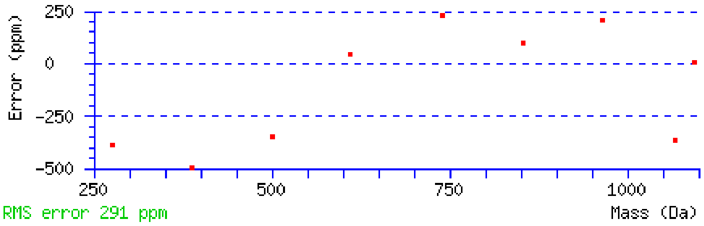

All matches to this query

Score	Mr(calc):	Delta	Sequence
38.7	1238.5907	0.0001	GSEAEGQLLKK
13.2	1238.5907	0.0001	QELKSDALQK
12.2	1238.6030	-0.0122	EYSTLEELQK
12.2	1238.5788	0.0121	SMMNTWTLQK
8.5	1238.6020	-0.0111	ANTVGGRNKLK
8.5	1238.5842	0.0066	CSKPNGQKLK
8.1	1237.5760	1.0148	YITEALIKK
7.3	1238.5907	0.0001	IISNTENLVR
6.6	1238.5886	0.0022	VLMLNSNMVGK
6.2	1238.5907	0.0001	IISNTENLVR

Spectrum No: 121; Query: 472; Rank: 1

Peptide View

MS/MS Fragmentation of **GGSSGEELEDEEPVK**
Found in **IPI00391633**, Tax_Id=10116 Gene_Symbol=Hdgfrp2 hepatoma-derived growth factor-related protein 2

Match to Query 472: 1720.618408 from(861.316480,2+)
Title: 091127RatKid_SCX01_02.1759.1759.2.dta
Data file K:\NewmanPaper\Piliang\3SubProteomes\Piliang3SP\mgf5ppm\SCX_3SubProteomes5ppm.mgf

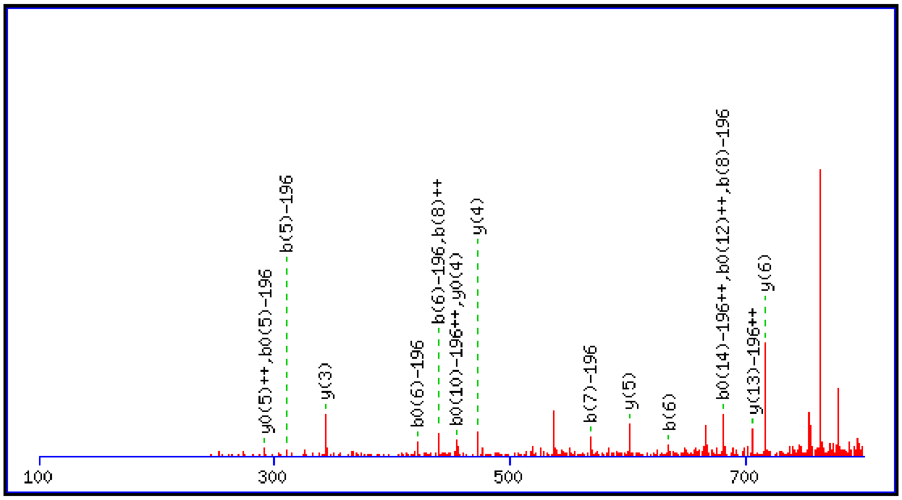

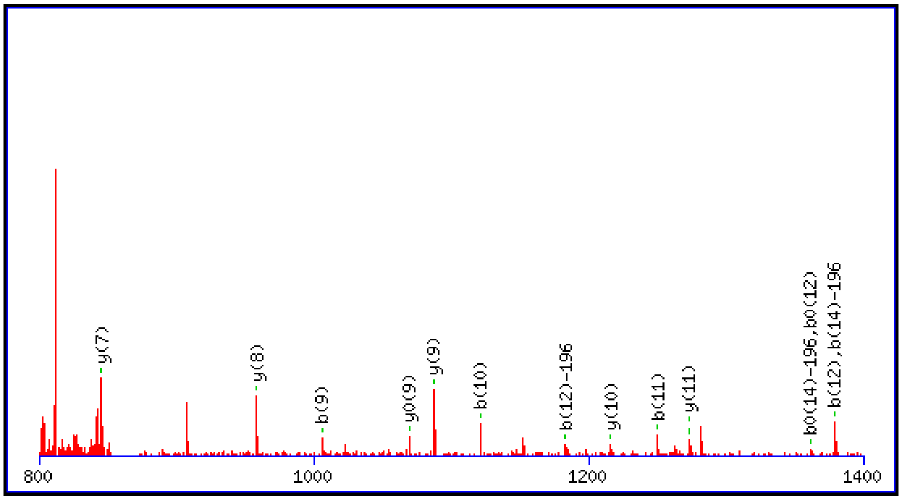

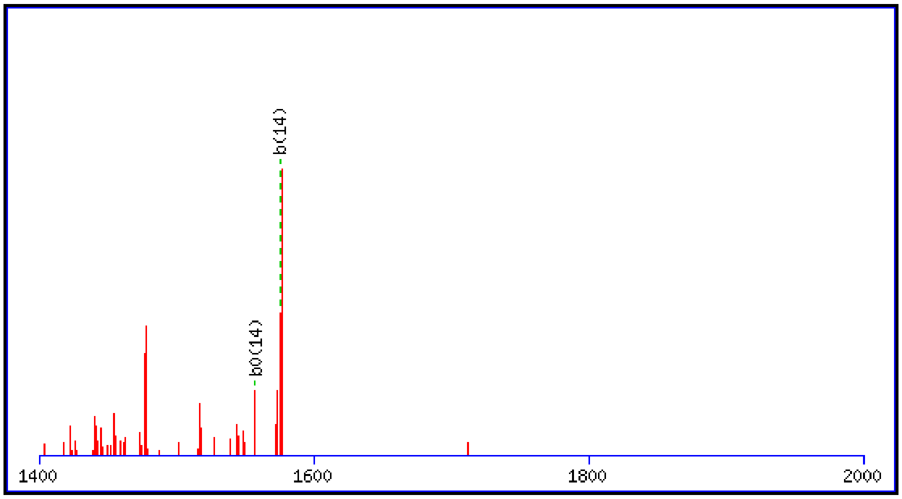

Monoisotopic mass of neutral peptide Mr(calc): 1720.6118
Fixed modifications: Carbamidomethyl (C)
Variable modifications:
S3 : Phospho (ST), with neutral losses 97.9769(shown in table), 0.0000
S4 : Phospho (ST), with neutral losses 97.9769(shown in table), 0.0000
Ions Score: 39 **Expect:** 0.0096
Matches (Bold Red): 34/196 fragment ions using 57 most intense peaks

#	b	b^{++}	b^{0}	b^{0++}	Seq.	y	y^{++}	y*	y^{*++}	y^{0}	y^{0++}	#
1	58.0287	29.5180			G							15
2	115.0502	58.0287			G	1468.6438	734.8255	1451.6172	726.3123	1450.6332	725.8203	14
3	184.0717	92.5395	166.0611	83.5342	S	1411.6223	**706.3148**	1394.5958	697.8015	1393.6118	697.3095	13
4	253.0931	127.0502	235.0826	118.0449	S	1342.6009	671.8041	1325.5743	663.2908	1324.5903	662.7988	12
5	**310.1146**	155.5609	**292.1040**	146.5556	G	**1273.5794**	637.2933	1256.5529	628.7801	1255.5689	628.2881	11
6	**439.1572**	220.0822	**421.1466**	211.0769	E	**1216.5580**	608.7826	1199.5314	600.2693	1198.5474	599.7773	10
7	**568.1998**	284.6035	550.1892	275.5982	E	**1087.5154**	544.2613	1070.4888	535.7480	**1069.5048**	535.2560	9
8	**681.2838**	341.1456	663.2733	332.1403	L	**958.4728**	479.7400	941.4462	471.2267	940.4622	470.7347	8
9	810.3264	405.6668	792.3159	396.6616	E	**845.3887**	423.1980	828.3622	414.6847	827.3781	414.1927	7
10	925.3534	463.1803	907.3428	**454.1750**	D	**716.3461**	358.6767	699.3196	350.1634	698.3355	349.6714	6
11	1054.3960	527.7016	1036.3854	518.6963	E	**601.3192**	301.1632	584.2926	292.6499	583.3086	**292.1579**	5
12	**1183.4386**	592.2229	1165.4280	583.2176	E	**472.2766**	236.6419	455.2500	228.1287	**454.2660**	227.6366	4
13	1280.4913	640.7493	1262.4808	631.7440	P	**343.2340**	172.1206	326.2074	163.6074			3
14	**1379.5597**	690.2835	**1361.5492**	**681.2782**	V	246.1812	123.5942	229.1547	115.0810			2
15					K	147.1128	74.0600	130.0863	65.5468			1

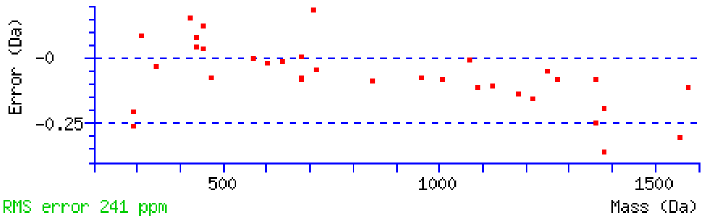

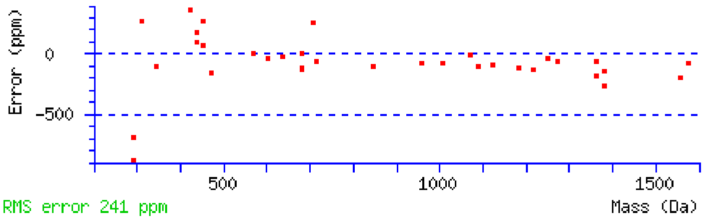

All matches to this query

Score	Mr(calc):	Delta	Sequence
38.7	1720.6118	0.0067	GGSSGEELEDEEPVK
8.5	1720.6147	0.0037	QLQSKYQSQDEK
6.6	1718.6089	2.0095	AASEELVEKNSSSK
6.6	1718.6089	2.0095	AASEELVEKNSSSK
6.6	1718.6089	2.0095	AASEELVEKNSSSK
5.3	1720.6187	-0.0003	KTLNPNQDPYYK
3.5	1719.6100	1.0084	SGDEMTSLSEYVSR
2.7	1719.6240	0.9944	ECETDQECETYEK
0.1	1720.6277	-0.0093	SCSGKGHQTEDLSR

Spectrum No: 122; Query: 195; Rank: 1

Peptide View

MS/MS Fragmentation of **DIISLAPGSPNPK**
Found in **IPI00214373**, Tax_Id=10116 Gene_Symbol=Aadat Kynurenine/alpha-aminoadipate aminotransferase mitochondrial precursor

Match to Query 195: 1387.679308 from(694.846930,2+)
Title: 091127RatKid_SCX01_12.2406.2406.2.dta

Data file K:\NewmanPaper\Piliang\3SubProteomes\Piliang3SP\mgf5ppm\SCX_3SubProteomes5ppm.mgf

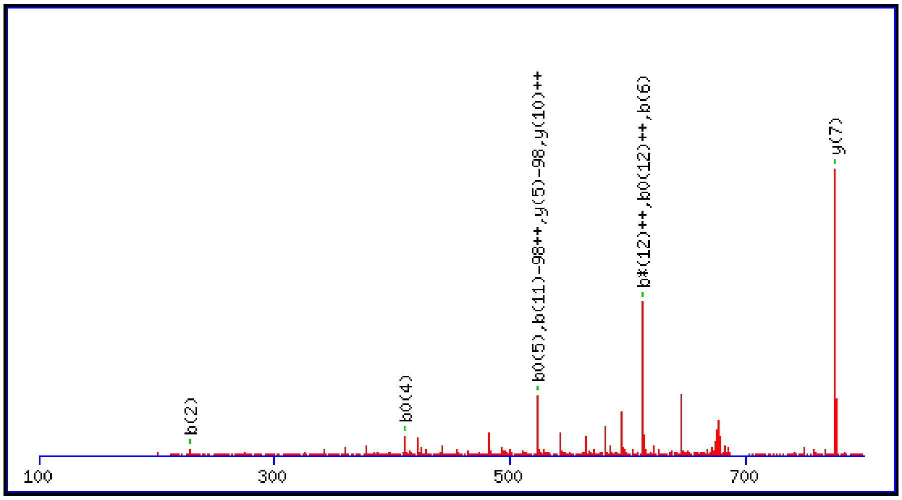

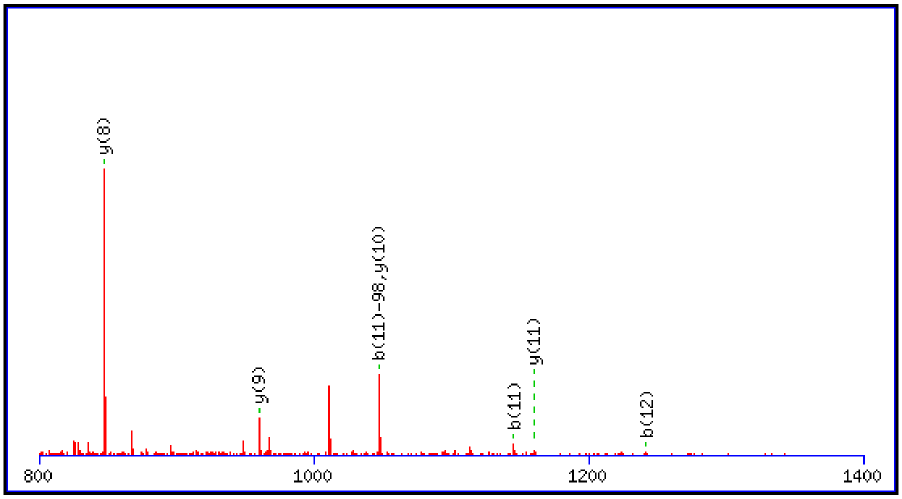

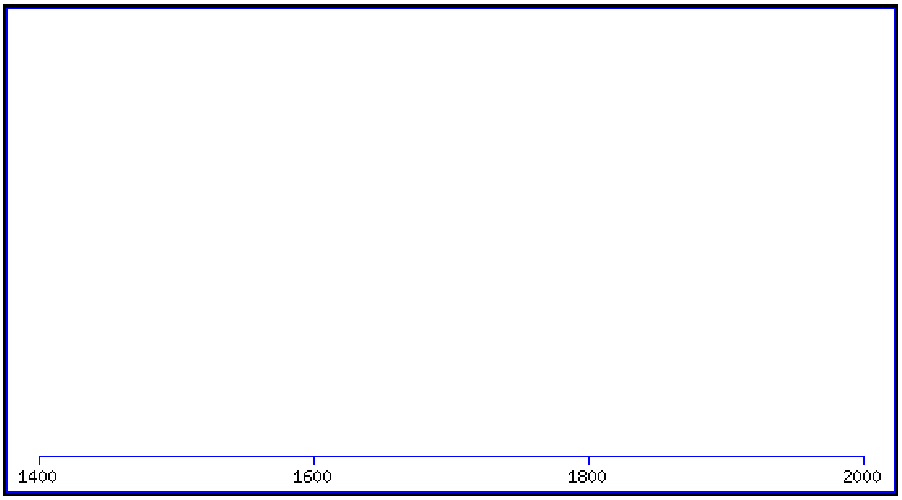

Monoisotopic mass of neutral peptide Mr(calc): 1387.6748
Fixed modifications: Carbamidomethyl (C)
Variable modifications:
S9 : Phospho (ST), with neutral losses 0.0000(shown in table), 97.9769
Ions Score: 39 **Expect:** 0.019
Matches (Bold Red): 17/184 fragment ions using 22 most intense peaks

#	b	b^{++}	b*	b^{*++}	b^0	b^{0++}	Seq.	y	y^{++}	y*	y^{*++}	y^0	y^{0++}	#
1	116.0342	58.5207			98.0237	49.5155	D							13
2	**229.1183**	115.0628			211.1077	106.0575	I	1273.6552	637.3312	1256.6286	628.8180	1255.6446	628.3259	12
3	342.2023	171.6048			324.1918	162.5995	I	**1160.5711**	580.7892	1143.5446	572.2759	1142.5605	571.7839	11
4	429.2344	215.1208			**411.2238**	206.1155	S	**1047.4870**	**524.2472**	1030.4605	515.7339	1029.4765	515.2419	10

**5**	542.3184	271.6629			**524.3079**	262.6576	**L**	**960.4550**	480.7311	943.4285	472.2179	942.4445	471.7259	**9**
**6**	**613.3556**	307.1814			595.3450	298.1761	**A**	**847.3710**	424.1891	830.3444	415.6758	829.3604	415.1838	**8**
**7**	710.4083	355.7078			692.3978	346.7025	**P**	**776.3338**	388.6706	759.3073	380.1573	758.3233	379.6653	**7**
**8**	767.4298	384.2185			749.4192	375.2132	**G**	679.2811	340.1442	662.2545	331.6309	661.2705	331.1389	**6**
**9**	934.4281	467.7177			916.4176	458.7124	**S**	622.2596	311.6334	605.2331	303.1202	604.2490	302.6282	**5**
**10**	1031.4809	516.2441			1013.4703	507.2388	**P**	455.2613	228.1343	438.2347	219.6210			**4**
**11**	**1145.5238**	573.2655	1128.4973	564.7523	1127.5133	564.2603	**N**	358.2085	179.6079	341.1819	171.0946			**3**
**12**	**1242.5766**	621.7919	1225.5500	**613.2787**	1224.5660	**612.7866**	**P**	244.1656	122.5864	227.1390	114.0731			**2**
**13**							**K**	147.1128	74.0600	130.0863	65.5468			**1**

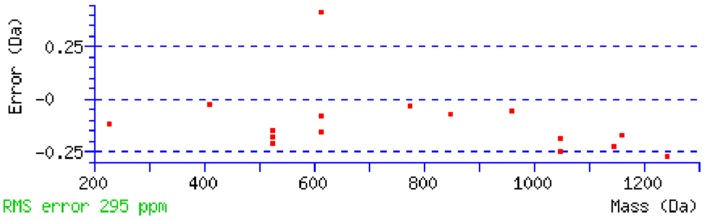

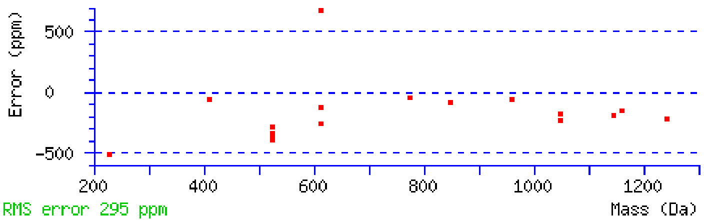

All matches to this query

Score	Mr(calc):	Delta	Sequence
38.6	1387.6748	0.0045	DIISLAPGSPNPK
23.4	1387.6748	0.0045	DIISLAPGSPNPK
12.7	1387.6765	0.0029	ILNSLTLPTKK
10.8	1387.6918	-0.0125	NEWKMAANIPAK
7.9	1385.6592	2.0201	GALLSSIQNFQK
7.1	1387.6748	0.0045	TAIQKAISEGYK
7.1	1387.6748	0.0045	TAIQKAISEGYK
5.8	1387.6683	0.0110	NNSKKALFQMK
5.4	1385.6625	2.0168	KISLIKNCDSK
5.3	1387.6740	0.0053	WCHEIQIMKK

Spectrum No: 123; Query: 119; Rank: 1

Peptide View

MS/MS Fragmentation of **SFLSEPSSPGR**
Found in **IPI00778008**, Tax_Id=10116 Gene_Symbol=RGD1559904_predicted 70 kDa protein

Match to Query 119: 1242.530608 from(622.272580,2+)
Title: 091129RatKid_SCX02_13.1234.1234.2.dta
Data file K:\NewmanPaper\Piliang\3SubProteomes\Piliang3SP\mgf5ppm\SCX_3SubProteomes5ppm.mgf

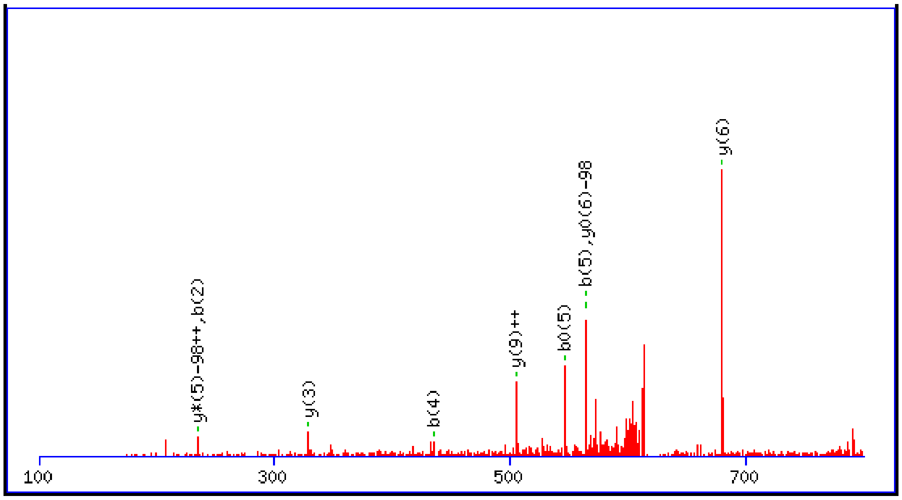

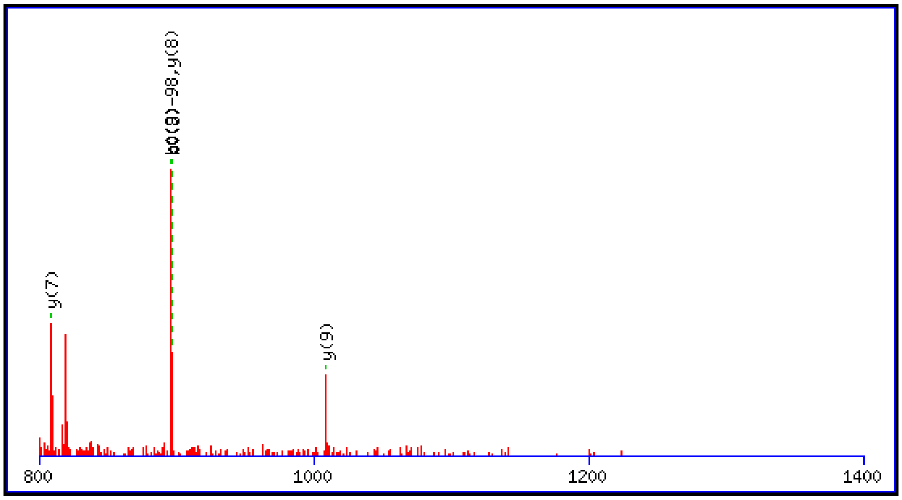

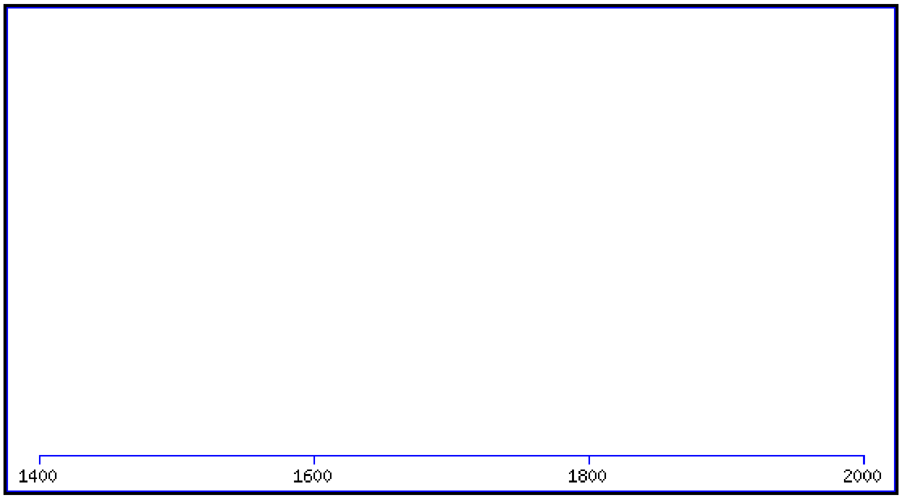

Monoisotopic mass of neutral peptide Mr(calc): 1242.5282
Fixed modifications: Carbamidomethyl (C)
Variable modifications:
S8 : Phospho (ST), with neutral losses 0.0000(shown in table), 97.9769
Ions Score: 39 **Expect:** 0.015
Matches (Bold Red): 14/148 fragment ions using 20 most intense peaks

#	b	b^{++}	b^{0}	b^{0++}	Seq.	y	y^{++}	y*	y^{*++}	y^{0}	y^{0++}	#
1	88.0393	44.5233	70.0287	35.5180	S							11
2	**235.1077**	118.0575	217.0972	109.0522	F	1156.5034	578.7553	1139.4769	570.2421	1138.4929	569.7501	10
3	348.1918	174.5995	330.1812	165.5942	L	**1009.4350**	**505.2211**	992.4085	496.7079	991.4244	496.2159	9
4	**435.2238**	218.1155	417.2132	209.1103	S	**896.3509**	448.6791	879.3244	440.1658	878.3404	439.6738	8
5	**564.2664**	282.6368	**546.2558**	273.6316	E	**809.3189**	405.1631	792.2924	396.6498	791.3084	396.1578	7

**6**	661.3192	331.1632	643.3086	322.1579	**P**	**680.2763**	340.6418	663.2498	332.1285	662.2658	331.6365	**6**
**7**	748.3512	374.6792	730.3406	365.6740	**S**	583.2236	292.1154	566.1970	283.6021	565.2130	283.1101	**5**
**8**	915.3495	458.1784	**897.3390**	449.1731	**S**	496.1915	248.5994	479.1650	240.0861	478.1810	239.5941	**4**
**9**	1012.4023	506.7048	994.3917	497.6995	**P**	**329.1932**	165.1002	312.1666	156.5870			**3**
**10**	1069.4238	535.2155	1051.4132	526.2102	**G**	232.1404	116.5738	215.1139	108.0606			**2**
**11**					**R**	175.1190	88.0631	158.0924	79.5498			**1**

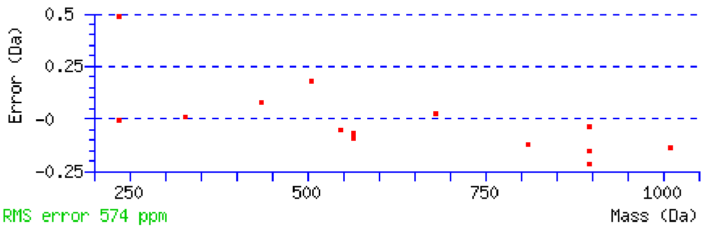

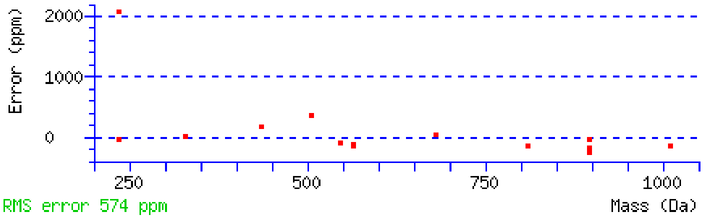

All matches to this query

Score	Mr(calc):	Delta	Sequence
38.5	1242.5282	0.0024	SFLSEPSSPGR
38.5	1242.5282	0.0024	SFLSEPSSPGR
6.0	1242.5187	0.0119	NSLGFCDTNSK
5.4	1242.5282	0.0024	SFLSEPSSPGR
3.5	1241.5177	1.0130	AVPSESPSSSSK

Spectrum No: 124; Query: 702; Rank: 1

Peptide View

MS/MS Fragmentation of **GWLRDPNASPGDAGEQAIR**
Found in **IPI00365286**, Tax_Id=10116 Gene_Symbol=Vcl_predicted vinculin

Match to Query 702: 2088.943182 from(697.321670,3+)
Title: 091129RatKid_SCX02_23.1643.1643.3.dta
Data file K:\NewmanPaper\Piliang\3SubProteomes\Piliang3SP\mgf5ppm\SCX_3SubProteomes5ppm.mgf

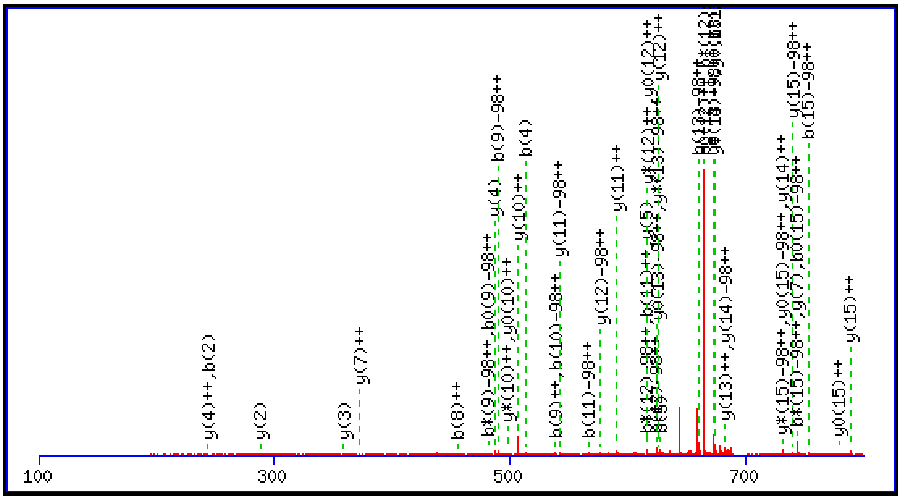

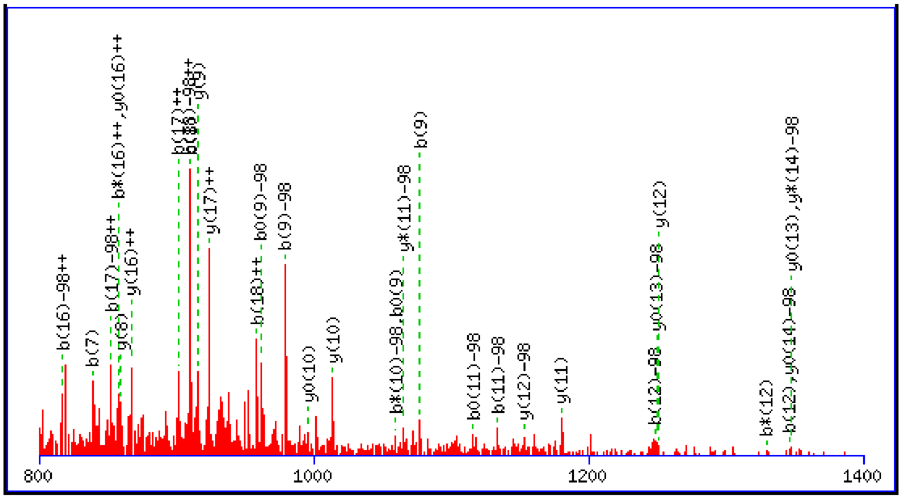

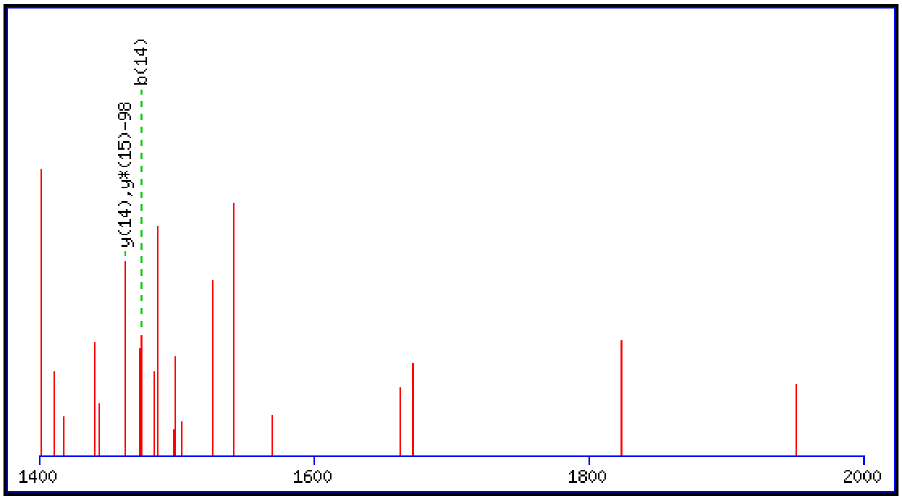

Monoisotopic mass of neutral peptide Mr(calc): 2088.9378
Fixed modifications: Carbamidomethyl (C)
Variable modifications:
S9 : Phospho (ST), with neutral losses 0.0000(shown in table), 97.9769
Ions Score: 38 **Expect:** 0.037
Matches (Bold Red): 87/302 fragment ions using 145 most intense peaks

#	b	b^{++}	b*	b^{*++}	b^{0}	b^{0++}	Seq.	y	y^{++}	y*	y^{*++}	y^{0}	y^{0++}	#
1	58.0287	29.5180					G							19
2	**244.1081**	122.5577					W	2032.9236	1016.9655	2015.8971	1008.4522	2014.9131	1007.9602	18
3	357.1921	179.0997					L	1846.8443	**923.9258**	1829.8178	915.4125	1828.8338	914.9205	17
4	**513.2932**	257.1503	496.2667	248.6370			R	1733.7603	**867.3838**	1716.7337	858.8705	1715.7497	**858.3785**	16
5	**628.3202**	314.6637	611.2936	306.1504	610.3096	305.6584	D	1577.6592	**789.3332**	1560.6326	780.8199	1559.6486	**780.3279**	15
6	725.3729	363.1901	708.3464	354.6768	707.3624	354.1848	P	**1462.6322**	**731.8197**	1445.6057	723.3065	1444.6216	722.8145	14
7	**839.4159**	420.2116	822.3893	411.6983	821.4053	411.2063	N	1365.5794	**683.2934**	1348.5529	**674.7801**	**1347.5689**	**674.2881**	13
8	**910.4530**	**455.7301**	893.4264	447.2169	892.4424	446.7248	A	**1251.5365**	**626.2719**	1234.5100	**617.7586**	1233.5260	**617.2666**	12
9	**1077.4513**	**539.2293**	1060.4248	530.7160	**1059.4408**	530.2240	S	**1180.4994**	**590.7533**	1163.4729	582.2401	1162.4888	581.7481	11
10	1174.5041	587.7557	1157.4775	579.2424	1156.4935	578.7504	P	**1013.5011**	**507.2542**	996.4745	**498.7409**	**995.4905**	**498.2489**	10
11	1231.5256	**616.2664**	1214.4990	607.7531	1213.5150	607.2611	G	**916.4483**	458.7278	899.4217	450.2145	898.4377	449.7225	9
12	**1346.5525**	**673.7799**	**1329.5260**	**665.2666**	1328.5419	**664.7746**	D	**859.4268**	430.2170	842.4003	421.7038	841.4163	421.2118	8
13	1417.5896	709.2984	1400.5631	700.7852	1399.5790	700.2932	A	**744.3999**	**372.7036**	727.3733	364.1903	726.3893	363.6983	7
14	**1474.6111**	737.8092	1457.5845	729.2959	1456.6005	728.8039	G	**673.3628**	337.1850	656.3362	328.6717	655.3522	328.1797	6
15	1603.6537	802.3305	1586.6271	793.8172	1585.6431	793.3252	E	**616.3413**	308.6743	599.3148	300.1610	598.3307	299.6690	5
16	1731.7122	866.3598	1714.6857	**857.8465**	1713.7017	857.3545	Q	**487.2987**	**244.1530**	470.2722	235.6397			4
17	1802.7494	**901.8783**	1785.7228	893.3650	1784.7388	892.8730	A	**359.2401**	180.1237	342.2136	171.6104			3
18	1915.8334	**958.4204**	1898.8069	949.9071	1897.8229	949.4151	I	**288.2030**	144.6051	271.1765	136.0919			2

19							R	175.1190	88.0631	158.0924	79.5498			1

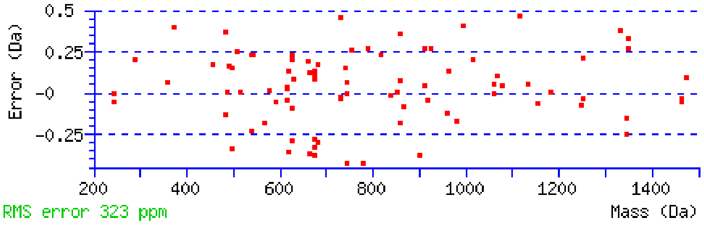

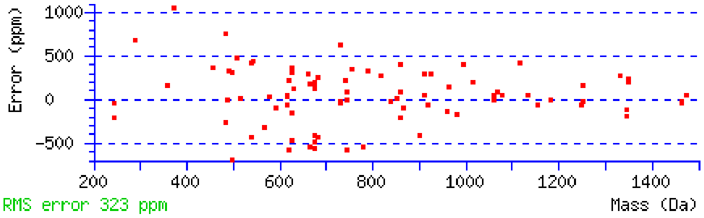

All matches to this query

Score	Mr(calc):	Delta	Sequence
38.3	2088.9378	0.0054	GWLRDPNASPGDAGEQAIR
7.4	2088.9252	0.0179	EEITESLTEVTDIGFGNR
7.4	2088.9252	0.0179	EEITESLTEVTDIGFGNR
7.4	2088.9252	0.0179	EEITESLTEVTDIGFGNR
4.7	2087.9360	1.0072	SFRRNWIQASDCGAALR
4.2	2087.9347	1.0085	SGSAMGTGWGLSGLSPTALSR
3.7	2088.9323	0.0109	EAAFVYAISSAGVAFAVTR
3.7	2086.9520	1.9912	SFRRNWIQASDCGAALR
3.6	2088.9354	0.0078	TAANAARSSEQNLQVTLR
3.6	2088.9323	0.0109	EAAFVYAISSAGVAFAVTR

Spectrum No: 125; Query: 734; Rank: 1

Peptide View

MS/MS Fragmentation of **KEESEESDDDMGFGLFD**
Found in **IPI00188804**, Tax_Id=10116 Gene_Symbol=Rplp2 60S acidic ribosomal protein P2

Match to Query 734: 2124.682388 from(1063.348470,2+)
Title: 091127RatKid_SCX01_02.4104.4104.2.dta
Data file K:\NewmanPaper\Piliang\3SubProteomes\Piliang3SP\mgf5ppm\SCX_3SubProteomes5ppm.mgf

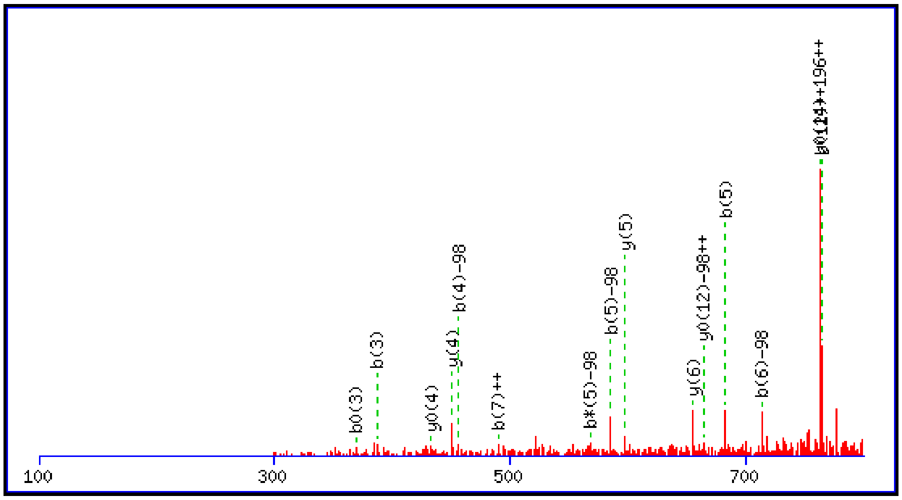

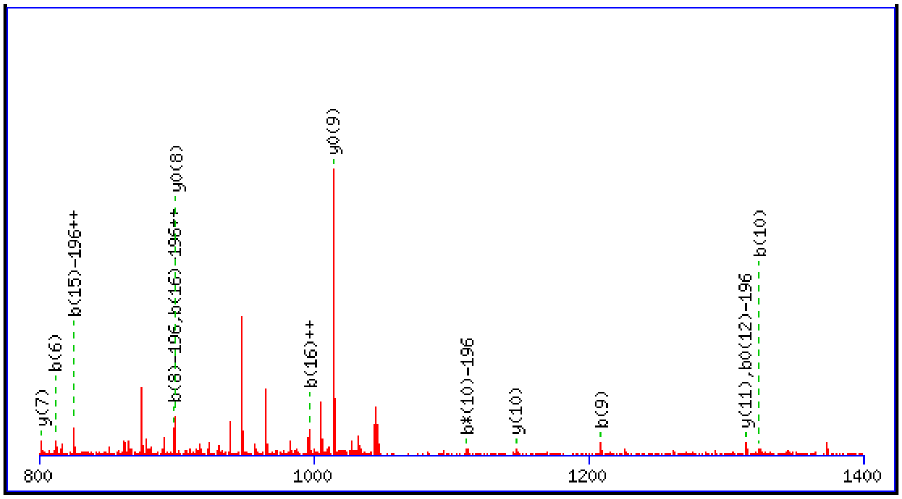

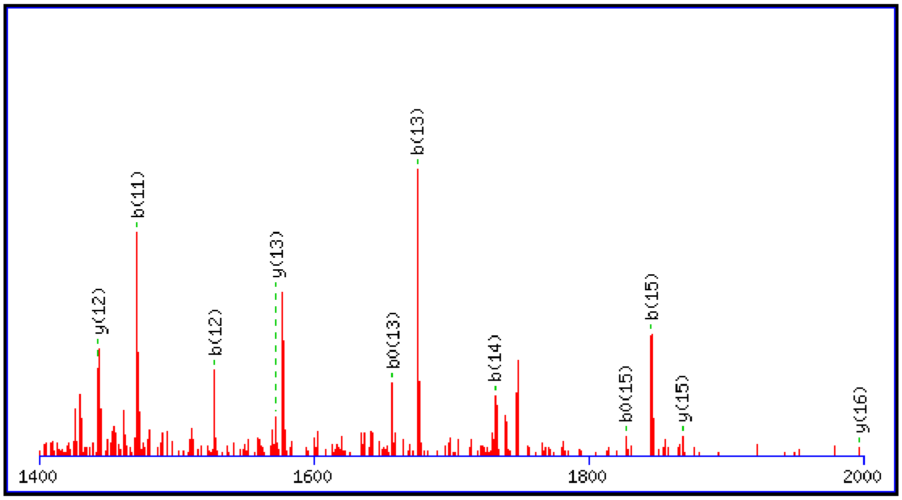

Monoisotopic mass of neutral peptide Mr(calc): 2124.6796
Fixed modifications: Carbamidomethyl (C)
Variable modifications:
S4 : Phospho (ST), with neutral losses 0.0000(shown in table), 97.9769
S7 : Phospho (ST), with neutral losses 0.0000(shown in table), 97.9769
M11 : Oxidation (M)
Ions Score: 38 **Expect:** 0.0067
Matches (Bold Red): 40/260 fragment ions using 78 most intense peaks

#	b	b^{++}	b*	b^{*++}	b^0	b^{0++}	Seq.	y	y^{++}	y^0	y^{0++}	#
1	129.1022	65.0548	112.0757	56.5415			K					17
2	258.1448	129.5761	241.1183	121.0628	240.1343	120.5708	E	**1997.5919**	999.2996	1979.5813	990.2943	16
3	**387.1874**	194.0974	370.1609	185.5841	**369.1769**	185.0921	E	**1868.5493**	934.7783	1850.5388	925.7730	15
4	554.1858	277.5965	537.1592	269.0833	536.1752	268.5912	S	1739.5067	870.2570	1721.4962	861.2517	14
5	**683.2284**	342.1178	666.2018	333.6045	665.2178	333.1125	E	**1572.5084**	786.7578	1554.4978	777.7525	13
6	**812.2710**	406.6391	795.2444	398.1258	794.2604	397.6338	E	**1443.4658**	722.2365	1425.4552	713.2312	12
7	979.2693	**490.1383**	962.2428	481.6250	961.2587	481.1330	S	**1314.4232**	657.7152	1296.4126	648.7099	11
8	1094.2963	547.6518	1077.2697	539.1385	1076.2857	538.6465	D	**1147.4248**	574.2161	1129.4143	565.2108	10
9	**1209.3232**	605.1652	1192.2967	596.6520	1191.3126	596.1600	D	1032.3979	516.7026	**1014.3873**	507.6973	9
10	**1324.3501**	662.6787	1307.3236	654.1654	1306.3396	653.6734	D	917.3710	459.1891	**899.3604**	450.1838	8
11	**1471.3855**	736.1964	1454.3590	727.6831	1453.3750	727.1911	M	**802.3440**	401.6756	784.3334	392.6704	7
12	**1528.4070**	**764.7071**	1511.3805	756.1939	1510.3964	755.7019	G	**655.3086**	328.1579	637.2980	319.1527	6
13	**1675.4754**	838.2414	1658.4489	829.7281	**1657.4649**	829.2361	F	**598.2871**	299.6472	580.2766	290.6419	5
14	**1732.4969**	866.7521	1715.4703	858.2388	1714.4863	857.7468	G	**451.2187**	226.1130	**433.2082**	217.1077	4
15	**1845.5810**	923.2941	1828.5544	914.7808	**1827.5704**	914.2888	L	394.1973	197.6023	376.1867	188.5970	3
16	1992.6494	**996.8283**	1975.6228	988.3150	1974.6388	987.8230	F	281.1132	141.0602	263.1026	132.0550	2
17							D	134.0448	67.5260	116.0342	58.5207	1

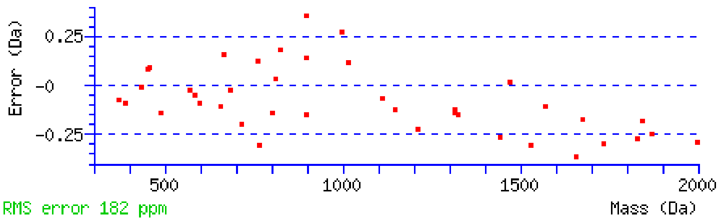

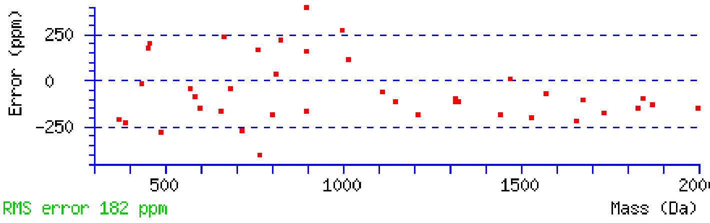

All matches to this query

Score	Mr(calc):	Delta	Sequence
38.3	2124.6796	0.0028	KEESEESDDDMGFGLFD
0.3	2124.6663	0.0161	MATSGANGPGSATASASNPR

Spectrum No: 126; Query: 556; Rank: 1

Peptide View

MS/MS Fragmentation of **SSSVGSSSSYPISSAVPR**
Found in **IPI00209000**, Tax_Id=10116 Gene_Symbol=Plec1 Plectin 6

Match to Query 556: 1834.812968 from(918.413760,2+)
Title: 091129RatKid_SCX02_12.1684.1684.2.dta
Data file K:\NewmanPaper\Piliang\3SubProteomes\Piliang3SP\mgf5ppm\SCX_3SubProteomes5ppm.mgf

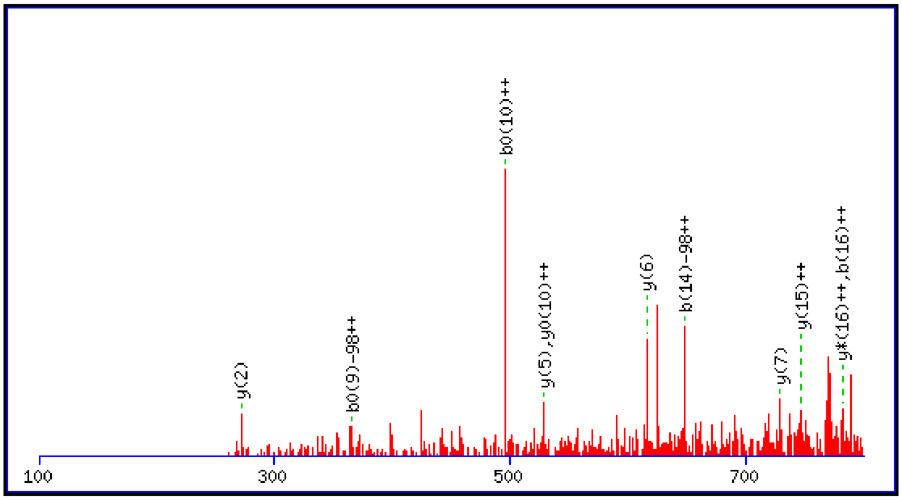

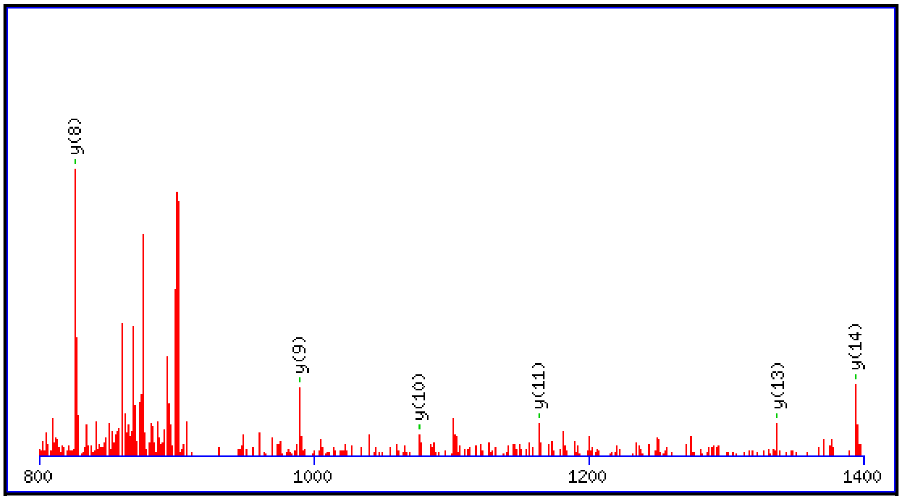

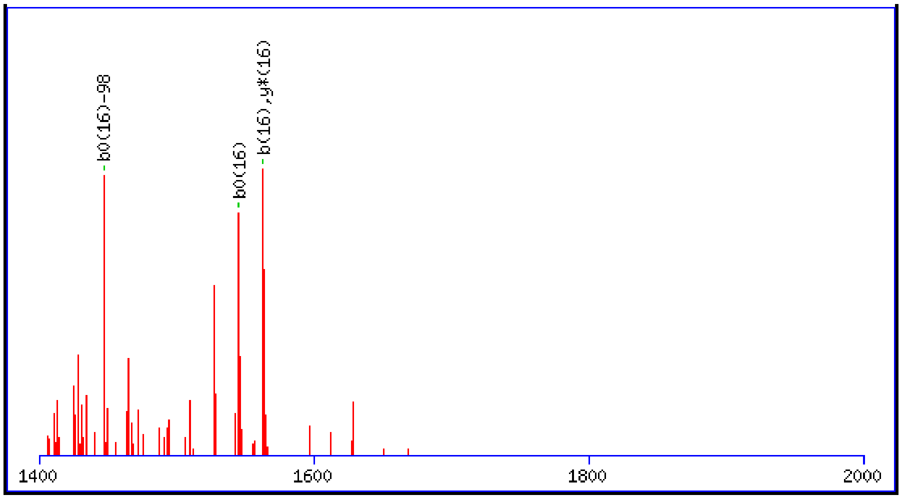

Monoisotopic mass of neutral peptide Mr(calc): 1833.8146
Fixed modifications: Carbamidomethyl (C)
Variable modifications:
S2 : Phospho (ST), with neutral losses 97.9769(shown in table), 0.0000
Ions Score: 38 **Expect:** 0.031
Matches (Bold Red): 21/232 fragment ions using 49 most intense peaks

#	b	b^{++}	b^{0}	b^{0++}	Seq.	y	y^{++}	y*	y^{*++}	y^{0}	y^{0++}	#
1	88.0393	44.5233	70.0287	35.5180	S							18
2	157.0608	79.0340	139.0502	70.0287	S	1649.8129	825.4101	1632.7864	816.8968	1631.8024	816.4048	17
3	244.0928	122.5500	226.0822	113.5447	S	1580.7915	790.8994	**1563.7649**	**782.3861**	1562.7809	781.8941	16
4	343.1612	172.0842	325.1506	163.0790	V	1493.7594	**747.3834**	1476.7329	738.8701	1475.7489	738.3781	15
5	400.1827	200.5950	382.1721	191.5897	G	**1394.6910**	697.8492	1377.6645	689.3359	1376.6805	688.8439	14
6	487.2147	244.1110	469.2041	235.1057	S	**1337.6696**	669.3384	1320.6430	660.8251	1319.6590	660.3331	13
7	574.2467	287.6270	556.2362	278.6217	S	1250.6375	625.8224	1233.6110	617.3091	1232.6270	616.8171	12
8	661.2787	331.1430	643.2682	322.1377	S	**1163.6055**	582.3064	1146.5790	573.7931	1145.5949	573.3011	11
9	748.3108	374.6590	730.3002	**365.6537**	S	**1076.5735**	538.7904	1059.5469	530.2771	1058.5629	**529.7851**	10
10	911.3741	456.1907	893.3635	447.1854	Y	**989.5415**	495.2744	972.5149	486.7611	971.5309	486.2691	9
11	1008.4269	504.7171	990.4163	495.7118	P	**826.4781**	413.7427	809.4516	405.2294	808.4676	404.7374	8
12	1121.5109	561.2591	1103.5004	552.2538	I	**729.4254**	365.2163	712.3988	356.7030	711.4148	356.2110	7
13	1208.5430	604.7751	1190.5324	595.7698	S	**616.3413**	308.6743	599.3148	300.1610	598.3307	299.6690	6
14	1295.5750	**648.2911**	1277.5644	639.2859	S	**529.3093**	265.1583	512.2827	256.6450	511.2987	256.1530	5
15	1366.6121	683.8097	1348.6015	674.8044	A	442.2772	221.6423	425.2507	213.1290			4
16	1465.6805	733.3439	**1447.6700**	724.3386	V	371.2401	186.1237	354.2136	177.6104			3
17	1562.7333	781.8703	1544.7227	772.8650	P	**272.1717**	136.5895	255.1452	128.0762			2
18					R	175.1190	88.0631	158.0924	79.5498			1

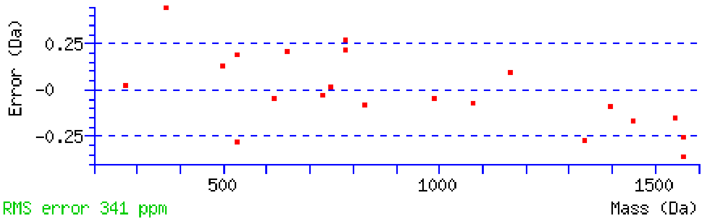

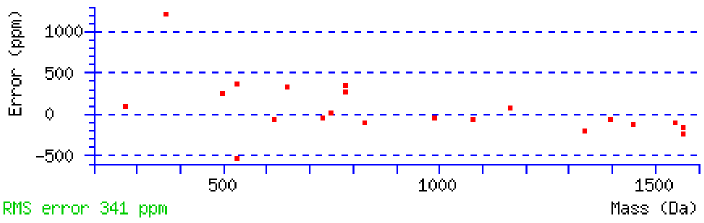

All matches to this query

Score	Mr(calc):	Delta	Sequence
38.3	1833.8146	0.9984	SSSVGSSSSYPISSAVPR
38.3	1833.8146	0.9984	SSSVGSSSSYPISSAVPR
38.2	1833.8146	0.9984	SSSVGSSSSYPISSAVPR

20.9	1833.8146	0.9984	SSSVGSSSSYPISSAVPR
19.0	1833.8146	0.9984	SSSVGSSSSYPISSAVPR
11.6	1833.8146	0.9984	SSSVGSSSSYPISSAVPR
6.9	1833.8146	0.9984	SSSVGSSSSYPISSAVPR
6.4	1833.7977	1.0153	RDSDSDADEATPTTTPR
4.3	1834.8098	0.0032	SLSATSDYSSSLERPR
4.3	1834.8098	0.0032	SLSATSDYSSSLERPR

Spectrum No: 127; Query: 325; Rank: 1

Peptide View

MS/MS Fragmentation of **VIEPPAASCPSSPR**
Found in **IPI00362539**, Tax_Id=10116 Gene_Symbol=Rgl3_predicted 78 kDa protein

Match to Query 325: 1546.691448 from(774.353000,2+)
Title: 091129RatKid_SCX02_12.721.721.2.dta
Data file K:\NewmanPaper\Piliang\3SubProteomes\Piliang3SP\mgf5ppm\SCX_3SubProteomes5ppm.mgf

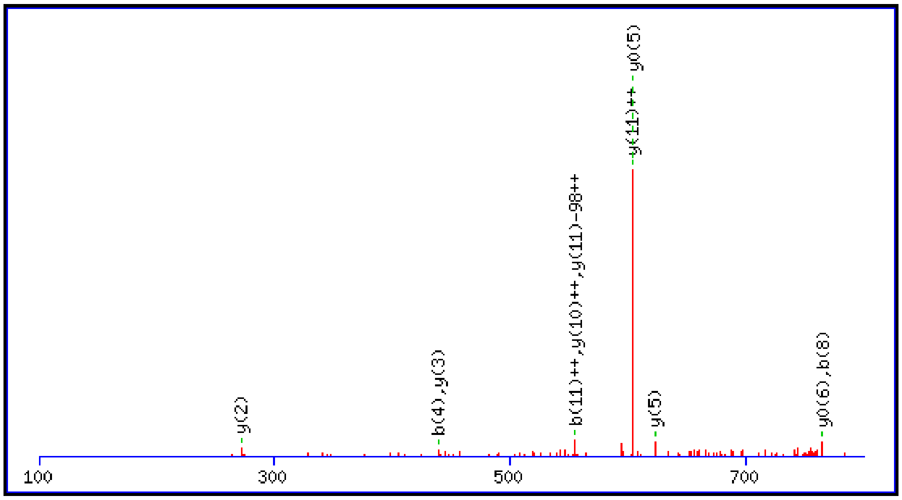

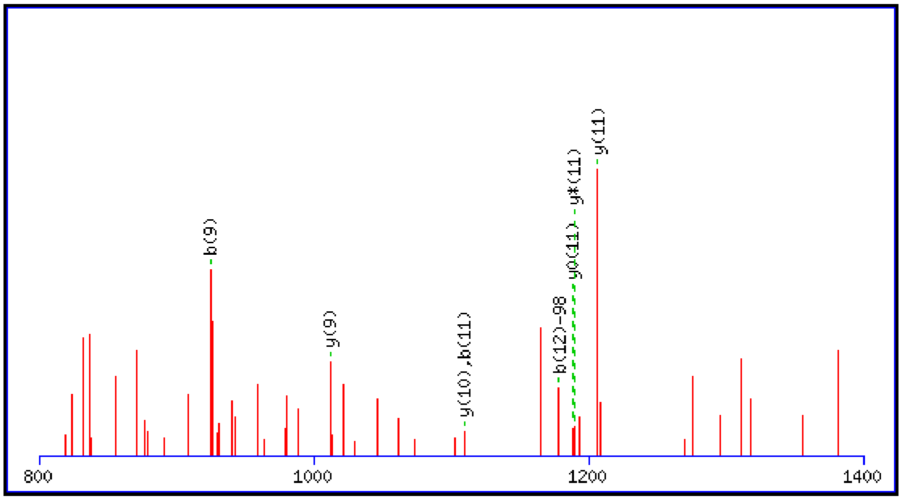

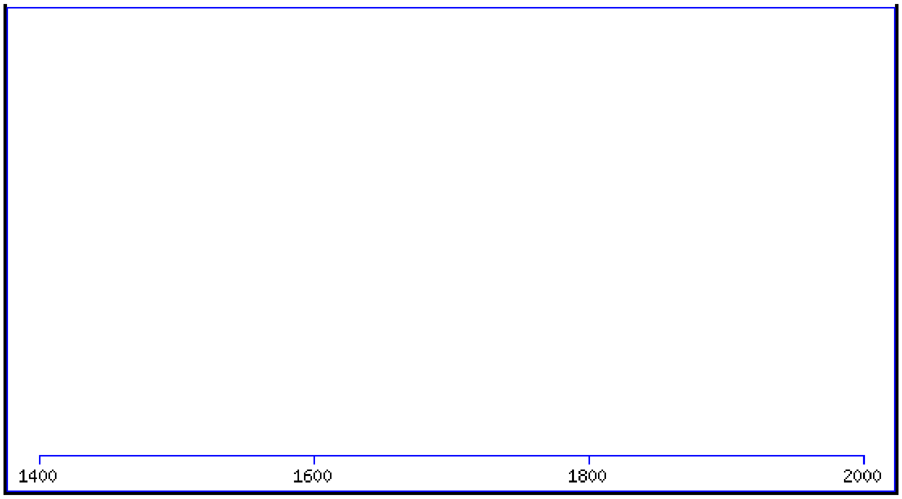

Monoisotopic mass of neutral peptide Mr(calc): 1546.6851
Fixed modifications: Carbamidomethyl (C)
Variable modifications:
S12 : Phospho (ST), with neutral losses 0.0000(shown in table), 97.9769
Ions Score: 38 **Expect:** 0.024
Matches (Bold Red): 19/196 fragment ions using 23 most intense peaks

#	b	b++	b0	b0++	Seq.	y	y++	y*	y*++	y0	y0++	#
1	100.0757	50.5415			V							14
2	213.1598	107.0835			I	1448.6240	724.8156	1431.5974	716.3023	1430.6134	715.8103	13
3	342.2023	171.6048	324.1918	162.5995	E	1335.5399	668.2736	1318.5133	659.7603	1317.5293	659.2683	12
4	**439.2551**	220.1312	421.2445	211.1259	P	**1206.4973**	**603.7523**	**1189.4707**	595.2390	**1188.4867**	594.7470	11
5	536.3079	268.6576	518.2973	259.6523	P	**1109.4445**	**555.2259**	1092.4180	546.7126	1091.4340	546.2206	10
6	607.3450	304.1761	589.3344	295.1709	A	**1012.3918**	506.6995	995.3652	498.1862	994.3812	497.6942	9
7	678.3821	339.6947	660.3715	330.6894	A	941.3547	471.1810	924.3281	462.6677	923.3441	462.1757	8
8	**765.4141**	383.2107	747.4036	374.2054	S	870.3175	435.6624	853.2910	427.1491	852.3070	426.6571	7
9	**925.4448**	463.2260	907.4342	454.2207	C	783.2855	392.1464	766.2590	383.6331	**765.2749**	383.1411	6
10	1022.4975	511.7524	1004.4870	502.7471	P	**623.2549**	312.1311	606.2283	303.6178	**605.2443**	303.1258	5
11	**1109.5296**	**555.2684**	1091.5190	546.2631	S	526.2021	263.6047	509.1755	255.0914	508.1915	254.5994	4
12	1276.5279	638.7676	1258.5174	629.7623	S	**439.1701**	220.0887	422.1435	211.5754	421.1595	211.0834	3
13	1373.5807	687.2940	1355.5701	678.2887	P	**272.1717**	136.5895	255.1452	128.0762			2
14					R	175.1190	88.0631	158.0924	79.5498			1

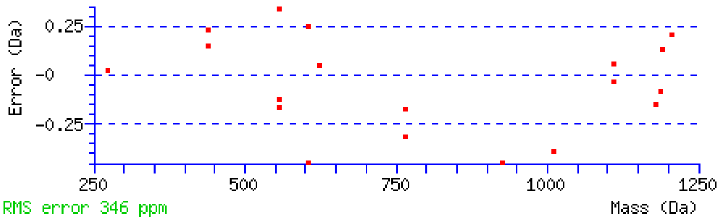

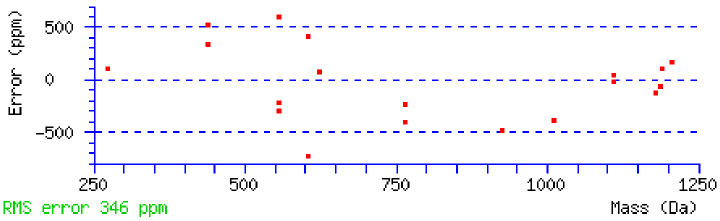

All matches to this query

Score	Mr(calc):	Delta	Sequence
38.3	1546.6851	0.0064	VIEPPAASCPSSPR
28.0	1546.6851	0.0064	VIEPPAASCPSSPR
21.4	1546.6851	0.0064	VIEPPAASCPSSPR
10.9	1544.6807	2.0108	LLIDPNCSGHSPR
5.3	1544.6749	2.0165	GPSPDSKVVTRSR
5.2	1544.6984	1.9930	KLQGGGPQEPPNSR
5.1	1546.6929	-0.0015	GQRPNLSTFFSGR

5.1	1546.6929	-0.0015	GQRPNLSTFFSGR
5.1	1546.6929	-0.0015	GQRPNLSTFFSGR
3.7	1546.7062	-0.0147	KNSLESMGVRTTK

Spectrum No: 128; Query: 676; Rank: 1

Peptide View

MS/MS Fragmentation of **EGPEPPEEVPAPTTPPAPK**
Found in **IPI00417730**, Tax_Id=10116 Gene_Symbol=Bat2 Large proline-rich protein BAT2

Match to Query 676: 2018.926968 from(1010.470760,2+)
Title: 091127RatKid_SCX01_11.1350.1350.2.dta
Data file K:\NewmanPaper\Piliang\3SubProteomes\Piliang3SP\mgf5ppm\SCX_3SubProteomes5ppm.mgf

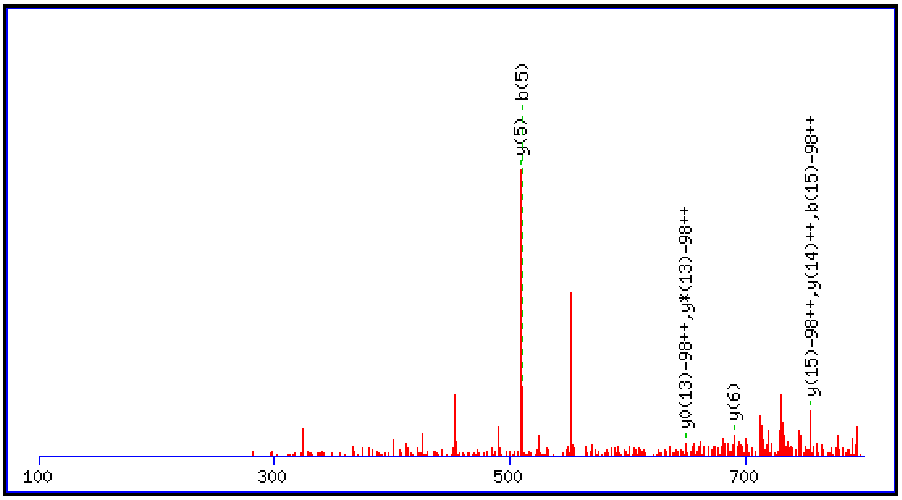

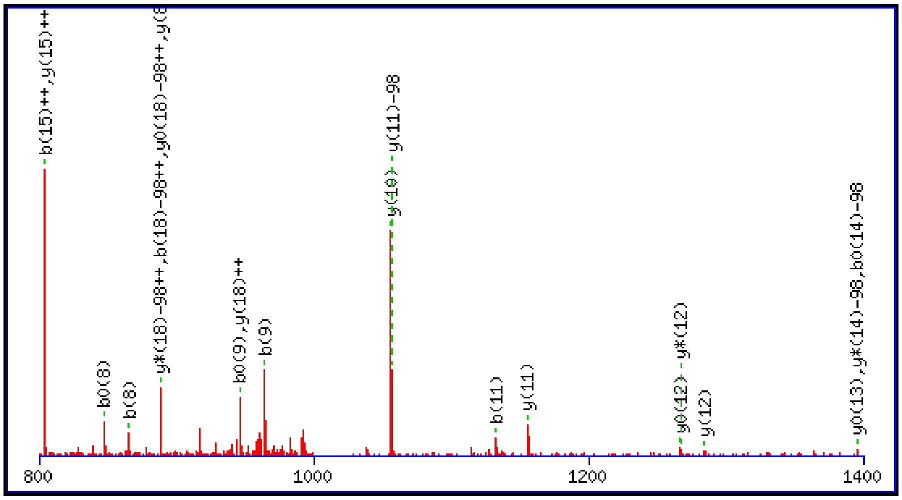

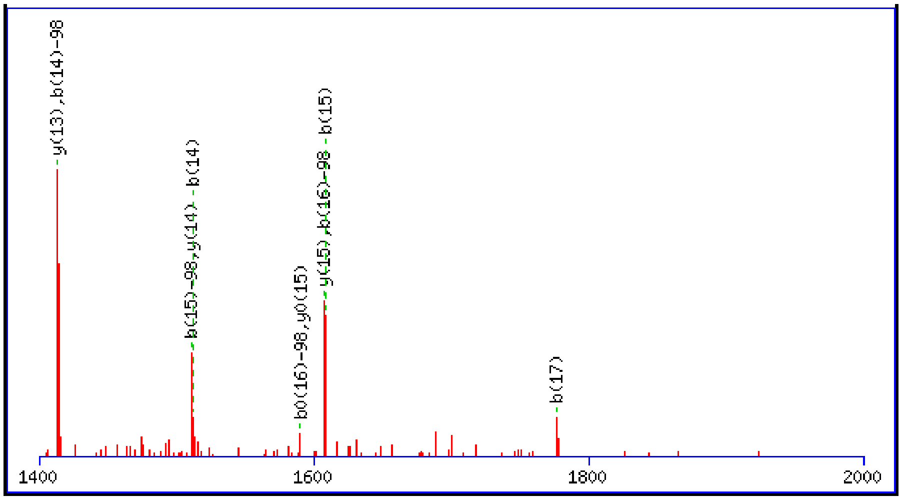

Monoisotopic mass of neutral peptide Mr(calc): 2018.9238
Fixed modifications: Carbamidomethyl (C)
Variable modifications:
T14 : Phospho (ST), with neutral losses 0.0000(shown in table), 97.9769
Ions Score: 38 **Expect:** 0.037
Matches (Bold Red): 41/268 fragment ions using 54 most intense peaks

#	b	b^{++}	b^{0}	b^{0++}	Seq.	y	y^{++}	y*	y^{*++}	y^{0}	y^{0++}	#
1	130.0499	65.5286	112.0393	56.5233	E							19
2	187.0713	94.0393	169.0608	85.0340	G	1890.8885	**945.9479**	1873.8619	937.4346	1872.8779	936.9426	18
3	284.1241	142.5657	266.1135	133.5604	P	1833.8670	917.4371	1816.8405	908.9239	1815.8565	908.4319	17
4	413.1667	207.0870	395.1561	198.0817	E	1736.8143	868.9108	1719.7877	860.3975	1718.8037	859.9055	16
5	**510.2195**	255.6134	492.2089	246.6081	P	**1607.7717**	**804.3895**	1590.7451	795.8762	**1589.7611**	795.3842	15
6	607.2722	304.1397	589.2617	295.1345	P	**1510.7189**	**755.8631**	1493.6923	747.3498	1492.7083	746.8578	14
7	736.3148	368.6610	718.3042	359.6558	E	**1413.6661**	707.3367	1396.6396	698.8234	**1395.6556**	698.3314	13
8	**865.3574**	433.1823	**847.3468**	424.1771	E	**1284.6235**	642.8154	**1267.5970**	634.3021	**1266.6130**	633.8101	12
9	**964.4258**	482.7165	**946.4153**	473.7113	V	**1155.5809**	578.2941	1138.5544	569.7808	1137.5704	569.2888	11
10	1061.4786	531.2429	1043.4680	522.2376	P	**1056.5125**	528.7599	1039.4860	520.2466	1038.5020	519.7546	10
11	**1132.5157**	566.7615	1114.5051	557.7562	A	959.4598	480.2335	942.4332	471.7202	941.4492	471.2282	9
12	1229.5685	615.2879	1211.5579	606.2826	P	**888.4227**	444.7150	871.3961	436.2017	870.4121	435.7097	8
13	1330.6161	665.8117	1312.6056	656.8064	T	791.3699	396.1886	774.3433	387.6753	773.3593	387.1833	7
14	**1511.6301**	756.3187	1493.6196	747.3134	T	**690.3222**	345.6647	673.2957	337.1515	672.3116	336.6595	6
15	**1608.6829**	**804.8451**	1590.6723	795.8398	P	**509.3082**	255.1577	492.2817	246.6445			5
16	1705.7357	853.3715	1687.7251	844.3662	P	412.2554	206.6314	395.2289	198.1181			4
17	**1776.7728**	888.8900	1758.7622	879.8847	A	315.2027	158.1050	298.1761	149.5917			3
18	1873.8255	937.4164	1855.8150	928.4111	P	244.1656	122.5864	227.1390	114.0731			2
19					K	147.1128	74.0600	130.0863	65.5468			1

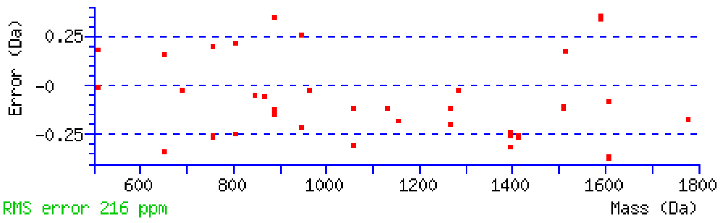

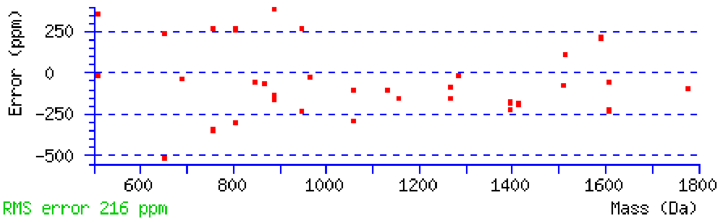

All matches to this query

Score	Mr(calc):	Delta	Sequence
38.3	2018.9238	0.0032	EGPEPPEEVPAPTTPPAPK
38.3	2018.9238	0.0032	EGPEPPEEVPAPTTPPAPK

1.5	2017.9048	1.0222	GTGPVQIPPGEWHSSFXR
1.5	2017.9048	1.0222	GTGPVQIPPGEWHSSFXR
1.5	2017.9048	1.0222	GTGPVQIPPGEWHSSFXR
1.0	2016.9234	2.0036	ELSPPLPDKFFDYVDR
0.7	2018.9075	0.0195	SRELTGGVDGIASIESIR
0.3	2018.9132	0.0137	ALQGDYRDVVNMKESSK
0.2	2017.9431	0.9838	SVEELTDFLTEVLCKR

Spectrum No: 129; Query: 940; Rank: 1

Peptide View

MS/MS Fragmentation of **IYHLPDAESDEDEDFKEQTR**
Found in **IPI00208304**, Tax_Id=10116 Gene_Symbol=Sept2 Septin-2

Match to Query 940: 2516.039292 from(839.687040,3+)
Title: 091129RatKid_SCX02_30.2139.2139.3.dta
Data file K:\NewmanPaper\Piliang\3SubProteomes\Piliang3SP\mgf5ppm\SCX_3SubProteomes5ppm.mgf

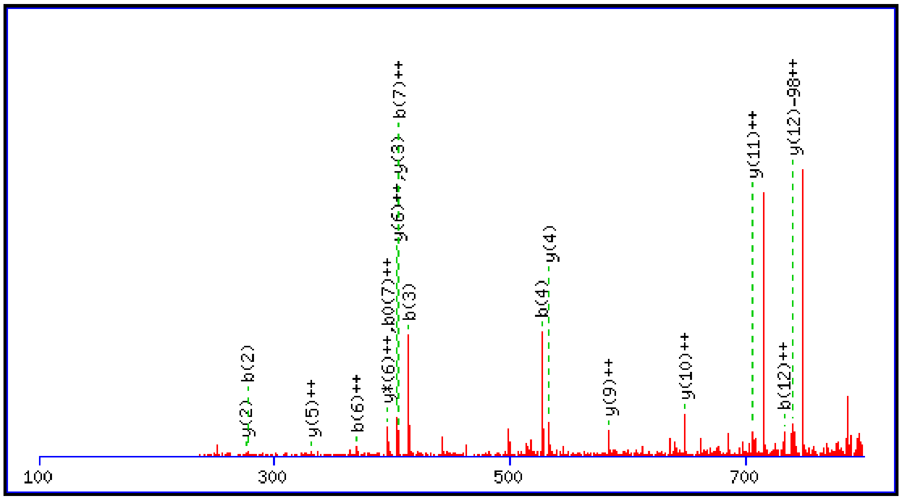

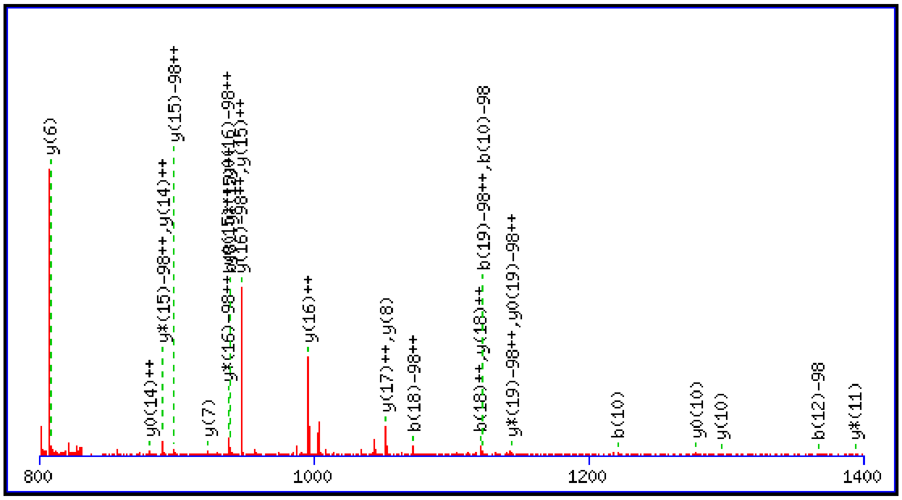

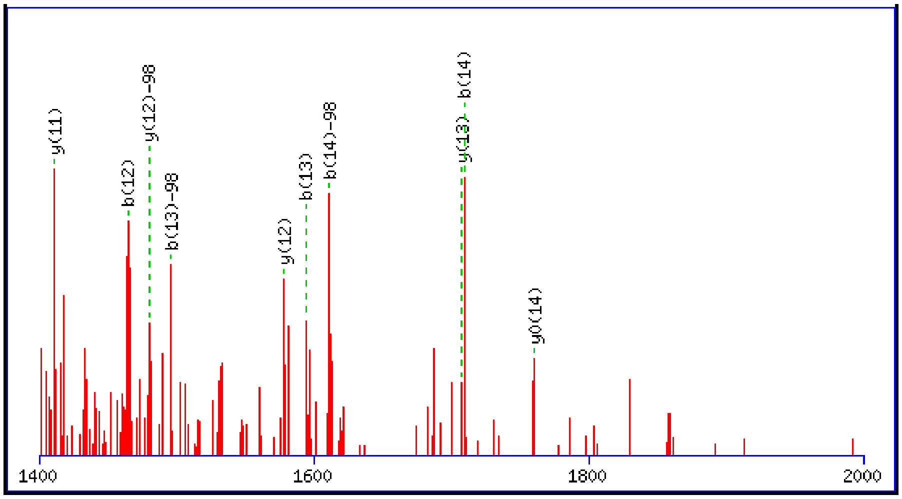

Monoisotopic mass of neutral peptide Mr(calc): 2516.0380
Fixed modifications: Carbamidomethyl (C)
Variable modifications:
S9 : Phospho (ST), with neutral losses 0.0000(shown in table), 97.9769
Ions Score: 38 **Expect:** 0.048
Matches (Bold Red): 55/286 fragment ions using 101 most intense peaks

#	b	b^{++}	b*	b^{*++}	b^{0}	b^{0++}	Seq.	y	y^{++}	y*	y^{*++}	y^{0}	y^{0++}	#
1	114.0913	57.5493					I							20
2	**277.1547**	139.0810					Y	2403.9613	1202.4843	2386.9347	1193.9710	2385.9507	1193.4790	19
3	**414.2136**	207.6104					H	2240.8979	**1120.9526**	2223.8714	1112.4393	2222.8874	1111.9473	18
4	**527.2976**	264.1525					L	2103.8390	**1052.4231**	2086.8125	1043.9099	2085.8285	1043.4179	17
5	624.3504	312.6788					P	1990.7550	**995.8811**	1973.7284	987.3678	1972.7444	986.8758	16
6	739.3774	**370.1923**			721.3668	361.1870	D	1893.7022	**947.3547**	1876.6756	**938.8415**	1875.6916	**938.3495**	15
7	810.4145	**405.7109**			792.4039	**396.7056**	A	1778.6753	**889.8413**	1761.6487	881.3280	**1760.6647**	**880.8360**	14
8	**939.4571**	470.2322			921.4465	461.2269	E	**1707.6381**	854.3227	1690.6116	845.8094	1689.6276	845.3174	13
9	1106.4554	553.7313			1088.4448	544.7261	S	**1578.5955**	789.8014	1561.5690	781.2881	1560.5850	780.7961	12
10	**1221.4824**	611.2448			1203.4718	602.2395	D	**1411.5972**	**706.3022**	**1394.5706**	697.7890	1393.5866	697.2970	11
11	1350.5249	675.7661			1332.5144	666.7608	E	**1296.5703**	**648.7888**	1279.5437	640.2755	**1278.5597**	639.7835	10
12	**1465.5519**	**733.2796**			1447.5413	724.2743	D	1167.5277	**584.2675**	1150.5011	575.7542	1149.5171	575.2622	9
13	**1594.5945**	797.8009			1576.5839	788.7956	E	**1052.5007**	526.7540	1035.4742	518.2407	1034.4902	517.7487	8
14	**1709.6214**	855.3144			1691.6109	846.3091	D	**923.4581**	462.2327	906.4316	453.7194	905.4476	453.2274	7
15	1856.6898	928.8486			1838.6793	919.8433	F	**808.4312**	**404.7192**	791.4046	**396.2060**	790.4206	395.7139	6
16	1984.7848	992.8960	1967.7583	984.3828	1966.7742	983.8908	K	661.3628	**331.1850**	644.3362	322.6717	643.3522	322.1797	5
17	2113.8274	1057.4173	2096.8008	1048.9041	2095.8168	1048.4121	E	**533.2678**	267.1375	516.2413	258.6243	515.2572	258.1323	4
18	2241.8860	**1121.4466**	2224.8594	1112.9334	2223.8754	1112.4413	Q	**404.2252**	202.6162	387.1987	194.1030	386.2146	193.6110	3
19	2342.9337	1171.9705	2325.9071	1163.4572	2324.9231	1162.9652	T	**276.1666**	138.5870	259.1401	130.0737	258.1561	129.5817	2
20							R	175.1190	88.0631	158.0924	79.5498			1

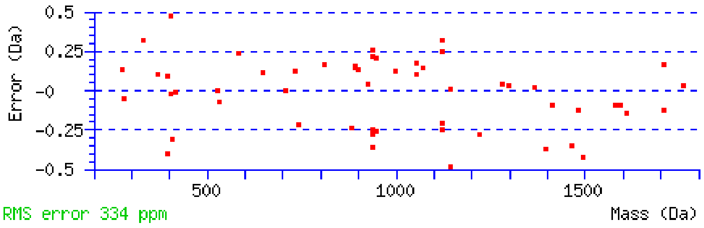

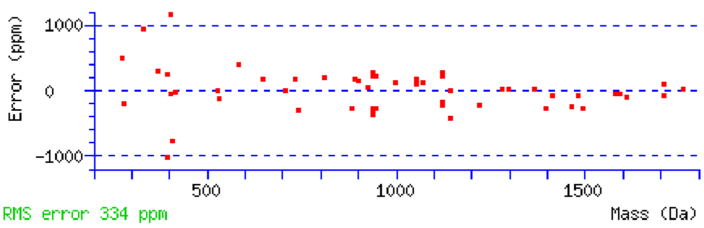

All matches to this query

Score	Mr(calc):	Delta	Sequence
38.1	2516.0380	0.0013	IYHLPDAESDEDEDFKEQTR

18.6	2516.0380	0.0013	IYHLPDAESDEDEDFKEQTR
6.2	2514.0539	1.9854	ASATSSYENPWTIPNLLSMTR
6.2	2514.0539	1.9854	ASATSSYENPWTIPNLLSMTR
5.8	2516.0175	0.0218	VSLGGAYGAGGYGSRSLYNVGGSK
5.5	2516.0175	0.0218	VSLGGAYGAGGYGSRSLYNVGGSK
4.3	2514.0117	2.0275	VDQGAATALRKESTSNTFYSK
2.9	2514.0134	2.0259	NLTPKPPVEDSPRISISNTK
2.6	2516.0175	0.0218	QGAASSTXSIPEEAKHGTFVGR
2.5	2516.0322	0.0071	SVMVHRVFSQGVASQEGTVSR

Spectrum No: 130; Query: 605; Rank: 1

Peptide View

MS/MS Fragmentation of **SPPDQSAVPNTPPSTPVK**
Found in **IPI00231810**, Tax_Id=10116 Gene_Symbol=Add1 Isoform 2 of Alpha-adducin

Match to Query 605: 1897.887128 from(949.950840,2+)
Title: 091127RatKid_SCX01_12.1473.1473.2.dta
Data file K:\NewmanPaper\Piliang\3SubProteomes\Piliang3SP\mgf5ppm\SCX_3SubProteomes5ppm.mgf

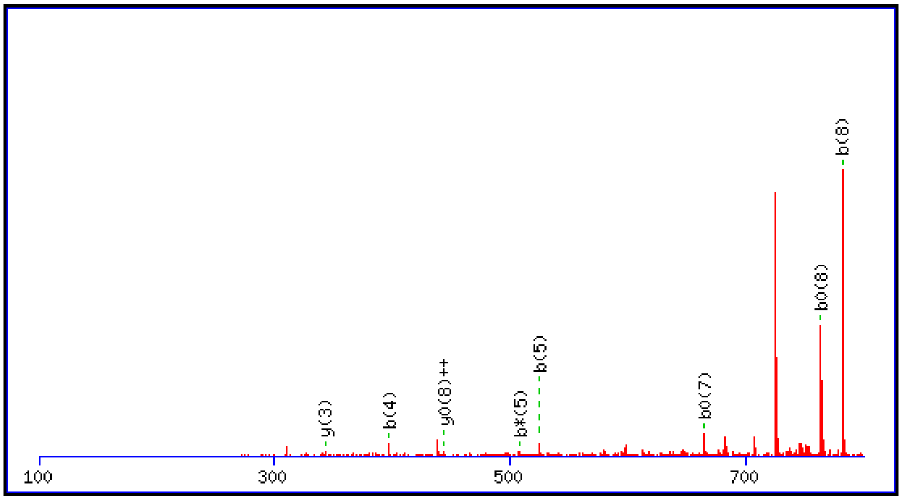

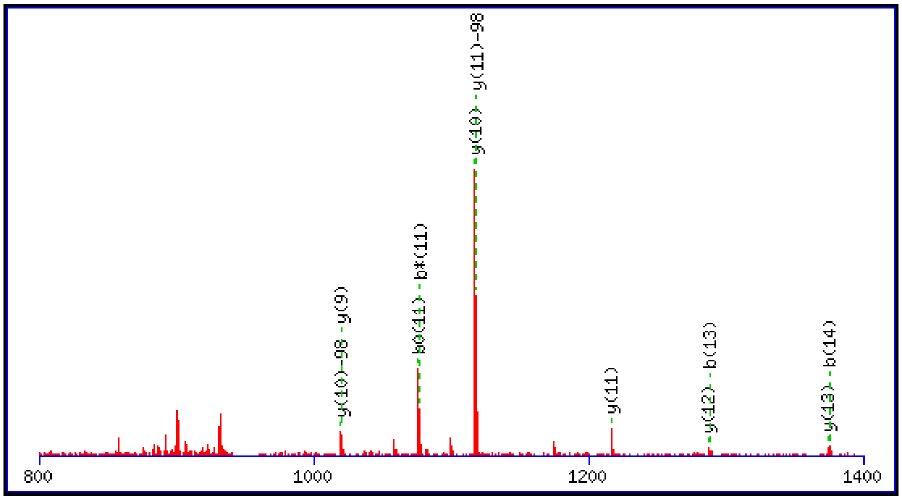

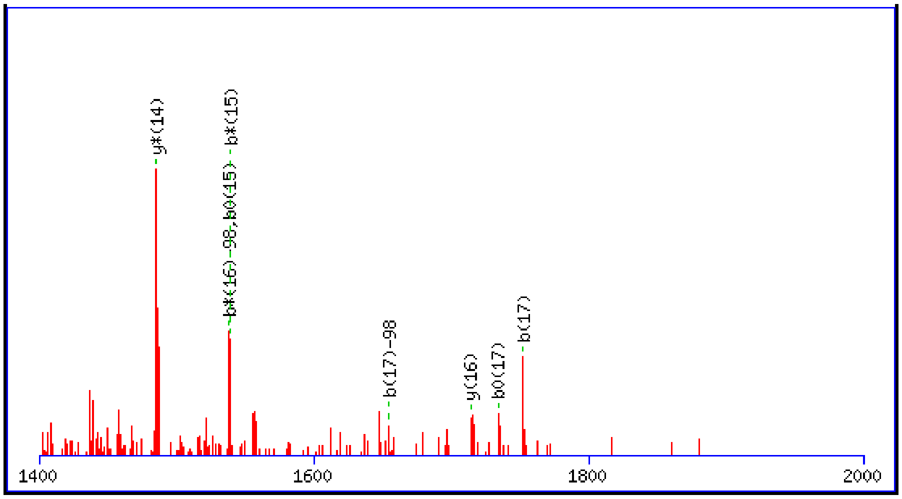

Monoisotopic mass of neutral peptide Mr(calc): 1897.8823
Fixed modifications: Carbamidomethyl (C)
Variable modifications:
T15 : Phospho (ST), with neutral losses 0.0000(shown in table), 97.9769
Ions Score: 38 **Expect:** 0.039
Matches (Bold Red): 27/292 fragment ions using 45 most intense peaks

#	b	b^{++}	b*	b^{*++}	b^0	b^{0++}	Seq.	y	y^{++}	y*	y^{*++}	y^0	y^{0++}	#
1	88.0393	44.5233			70.0287	35.5180	S							18
2	185.0921	93.0497			167.0815	84.0444	P	1811.8575	906.4324	1794.8310	897.9191	1793.8470	897.4271	17
3	282.1448	141.5761			264.1343	132.5708	P	**1714.8048**	857.9060	1697.7782	849.3927	1696.7942	848.9007	16
4	**397.1718**	199.0895			379.1612	190.0842	D	1617.7520	809.3796	1600.7254	800.8664	1599.7414	800.3743	15
5	**525.2304**	263.1188	**508.2038**	254.6055	507.2198	254.1135	Q	1502.7250	751.8662	**1485.6985**	743.3529	1484.7145	742.8609	14
6	612.2624	306.6348	595.2358	298.1216	594.2518	297.6295	S	**1374.6665**	687.8369	1357.6399	679.3236	1356.6559	678.8316	13
7	683.2995	342.1534	666.2729	333.6401	**665.2889**	333.1481	A	**1287.6344**	644.3209	1270.6079	635.8076	1269.6239	635.3156	12
8	**782.3679**	391.6876	765.3414	383.1743	**764.3573**	382.6823	V	**1216.5973**	608.8023	1199.5708	600.2890	1198.5868	599.7970	11
9	879.4207	440.2140	862.3941	431.7007	861.4101	431.2087	P	**1117.5289**	559.2681	1100.5024	550.7548	1099.5183	550.2628	10
10	993.4636	497.2354	976.4371	488.7222	975.4530	488.2302	N	**1020.4761**	510.7417	1003.4496	502.2284	1002.4656	501.7364	9
11	1094.5113	547.7593	**1077.4847**	539.2460	**1076.5007**	538.7540	T	906.4332	453.7202	889.4067	445.2070	888.4227	**444.7150**	8
12	1191.5640	596.2857	1174.5375	587.7724	1173.5535	587.2804	P	805.3855	403.1964	788.3590	394.6831	787.3750	394.1911	7
13	**1288.6168**	644.8120	1271.5903	636.2988	1270.6062	635.8068	P	708.3328	354.6700	691.3062	346.1568	690.3222	345.6647	6
14	**1375.6488**	688.3281	1358.6223	679.8148	1357.6383	679.3228	S	611.2800	306.1436	594.2535	297.6304	593.2694	297.1384	5
15	1556.6628	778.8351	**1539.6363**	770.3218	**1538.6523**	769.8298	T	524.2480	262.6276	507.2214	254.1144	506.2374	253.6223	4
16	1653.7156	827.3614	1636.6891	818.8482	1635.7050	818.3562	P	**343.2340**	172.1206	326.2074	163.6074			3
17	**1752.7840**	876.8956	1735.7575	868.3824	**1734.7735**	867.8904	V	246.1812	123.5942	229.1547	115.0810			2
18							K	147.1128	74.0600	130.0863	65.5468			1

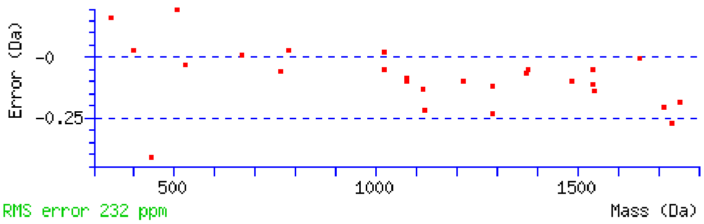

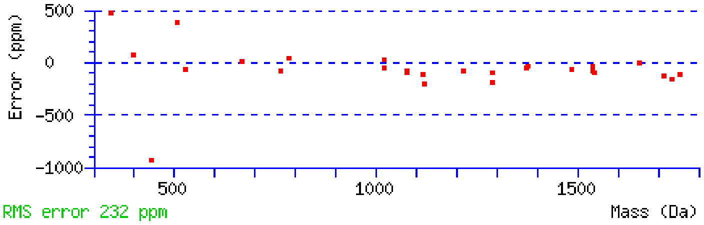

All matches to this query

Score	Mr(calc):	Delta	Sequence
38.0	1897.8823	0.0049	SPPDQSAVPNTPPSTPVK
37.4	1897.8823	0.0049	SPPDQSAVPNTPPSTPVK
35.9	1897.8823	0.0049	SPPDQSAVPNTPPSTPVK

13.4	1897.8823	0.0049	SPPDQSAVPNTPPSTPVK
12.2	1897.8740	0.0131	WKLPSPVTQVNAPSSK
10.9	1897.8740	0.0131	WKLPSPVTQVNAPSSK
9.5	1897.8700	0.0171	RTVSEPSLSGLHLNTK
9.5	1897.8700	0.0171	RTVSEPSLSGLHLNTK
9.5	1897.8700	0.0171	RTVSEPSLSGLHLNTK
9.5	1897.8700	0.0171	RTVSEPSLSGLHLNTK

Spectrum No: 131; Query: 343; Rank: 1

Peptide View

MS/MS Fragmentation of **QGDNISDDEDEVR**
Found in **IPI00204739**, Tax_Id=10116 Gene_Symbol=Stx4a Syntaxin-4

Match to Query 343: 1570.583108 from(786.298830,2+)
Title: 091129RatKid_SCX02_12.717.717.2.dta
Data file K:\NewmanPaper\Piliang\3SubProteomes\Piliang3SP\mgf5ppm\SCX_3SubProteomes5ppm.mgf

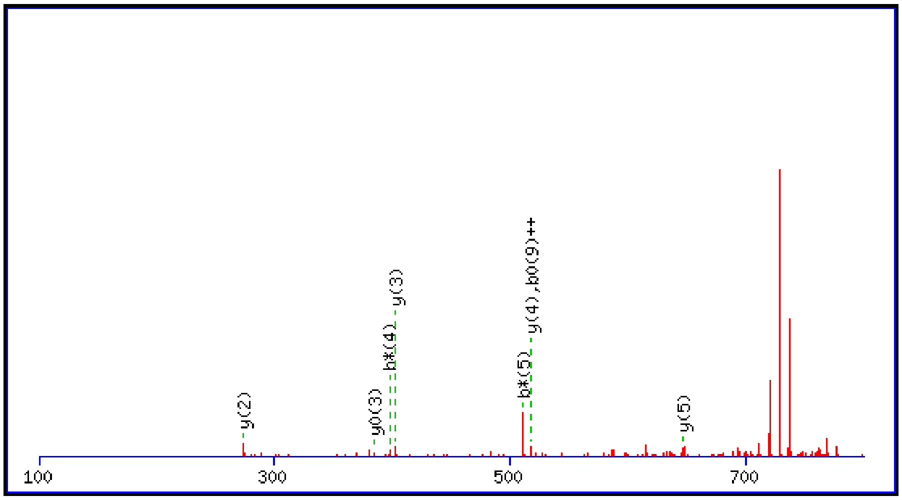

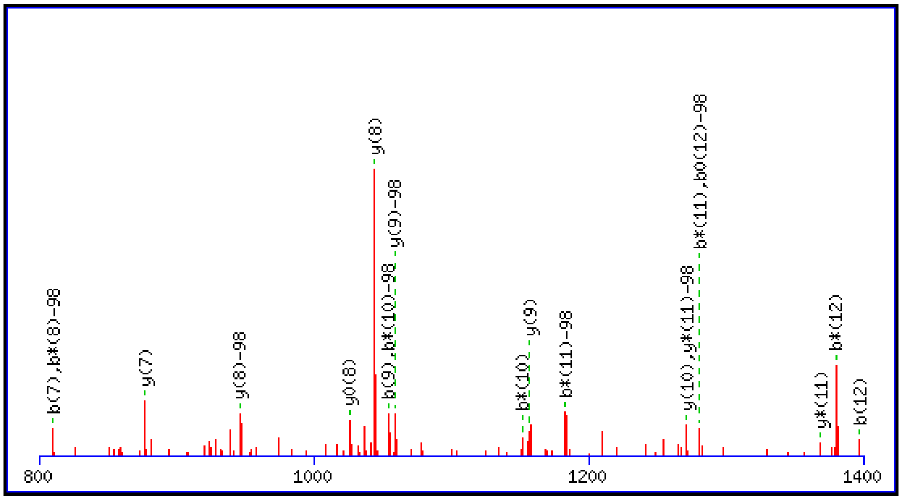

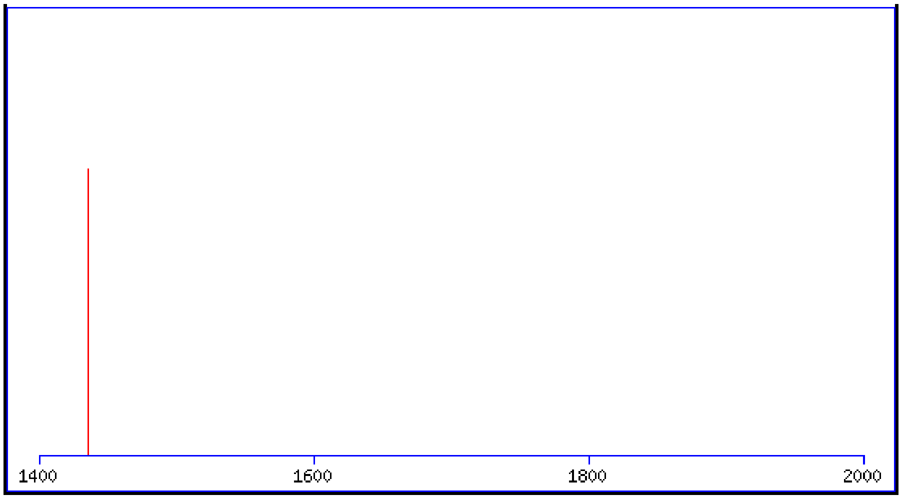

Monoisotopic mass of neutral peptide Mr(calc): 1570.5784
Fixed modifications: Carbamidomethyl (C)
Variable modifications:
S6 : Phospho (ST), with neutral losses 0.0000(shown in table), 97.9769
Ions Score: 38 **Expect:** 0.014
Matches (Bold Red): 27/208 fragment ions using 46 most intense peaks

#	b	b^{++}	b*	b^{*++}	b^{0}	b^{0++}	Seq.	y	y^{++}	y*	y^{*++}	y^{0}	y^{0++}	#
1	129.0659	65.0366	112.0393	56.5233			Q							13
2	186.0873	93.5473	169.0608	85.0340			G	1443.5271	722.2672	1426.5006	713.7539	1425.5166	713.2619	12
3	301.1143	151.0608	284.0877	142.5475	283.1037	142.0555	D	1386.5057	693.7565	**1369.4791**	685.2432	1368.4951	684.7512	11
4	415.1572	208.0822	**398.1306**	199.5690	397.1466	199.0769	N	**1271.4787**	636.2430	1254.4522	627.7297	1253.4682	627.2377	10
5	528.2413	264.6243	**511.2147**	256.1110	510.2307	255.6190	I	**1157.4358**	579.2215	1140.4092	570.7083	1139.4252	570.2163	9
6	695.2396	348.1234	678.2131	339.6102	677.2290	339.1182	S	**1044.3517**	522.6795	1027.3252	514.1662	**1026.3412**	513.6742	8
7	**810.2665**	405.6369	793.2400	397.1236	792.2560	396.6316	D	**877.3534**	439.1803	860.3268	430.6671	859.3428	430.1750	7
8	925.2935	463.1504	908.2669	454.6371	907.2829	454.1451	D	762.3264	381.6669	745.2999	373.1536	744.3159	372.6616	6
9	**1054.3361**	527.6717	1037.3095	519.1584	1036.3255	**518.6664**	E	**647.2995**	324.1534	630.2729	315.6401	629.2889	315.1481	5
10	1169.3630	585.1852	**1152.3365**	576.6719	1151.3525	576.1799	D	**518.2569**	259.6321	501.2304	251.1188	500.2463	250.6268	4
11	1298.4056	649.7064	**1281.3791**	641.1932	1280.3951	640.7012	E	**403.2300**	202.1186	386.2034	193.6053	**385.2194**	193.1133	3
12	**1397.4740**	699.2407	**1380.4475**	690.7274	1379.4635	690.2354	V	**274.1874**	137.5973	257.1608	129.0840			2
13							R	175.1190	88.0631	158.0924	79.5498			1

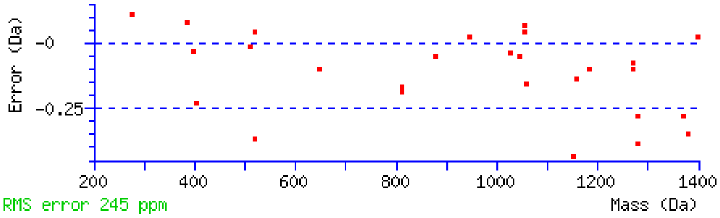

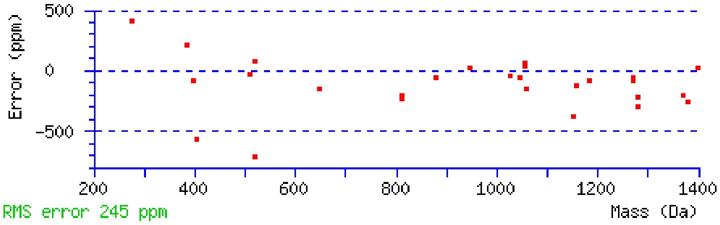

All matches to this query

Score	Mr(calc):	Delta	Sequence
37.9	1570.5784	0.0047	QGDNISDDEDEVR
1.0	1568.5644	2.0187	ITNSESGEKNGGSK
0.6	1570.5697	0.0134	SILTLSPMDSSTC
0.5	1570.5697	0.0134	SILTLSPMDSSTC
0.5	1568.5871	1.9961	EMESNWVSLVSK
0.4	1569.5935	0.9896	NPLSGKSYCLDR
0.3	1570.5697	0.0134	SILTLSPMDSSTC

Spectrum No: 132; Query: 597; Rank: 1

Peptide View

MS/MS Fragmentation of **TTATLDPSSPAPGEGPSGR**
Found in **IPI00204663**, Tax_Id=10116 Gene_Symbol=Tfpt TCF3 fusion partner homolog

Match to Query 597: 1876.827688 from(939.421120,2+)
Title: 091127RatKid_SCX01_12.1204.1204.2.dta
Data file K:\NewmanPaper\Piliang\3SubProteomes\Piliang3SP\mgf5ppm\SCX_3SubProteomes5ppm.mgf

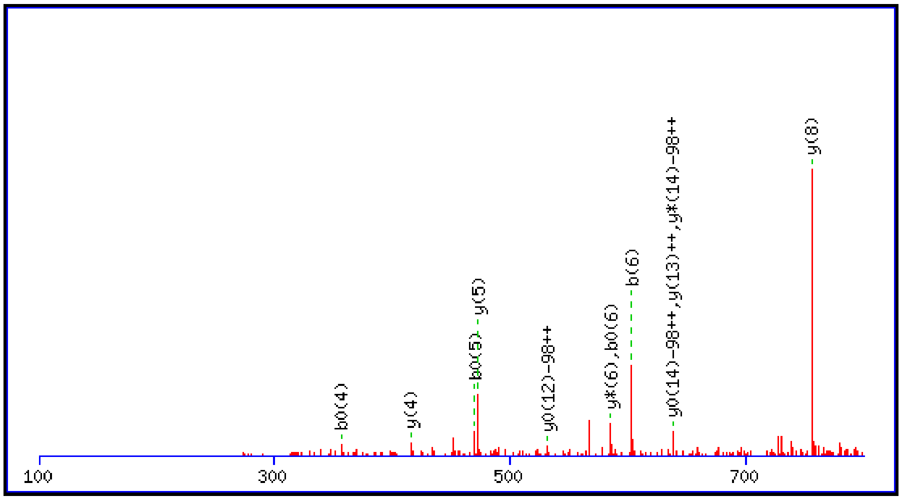

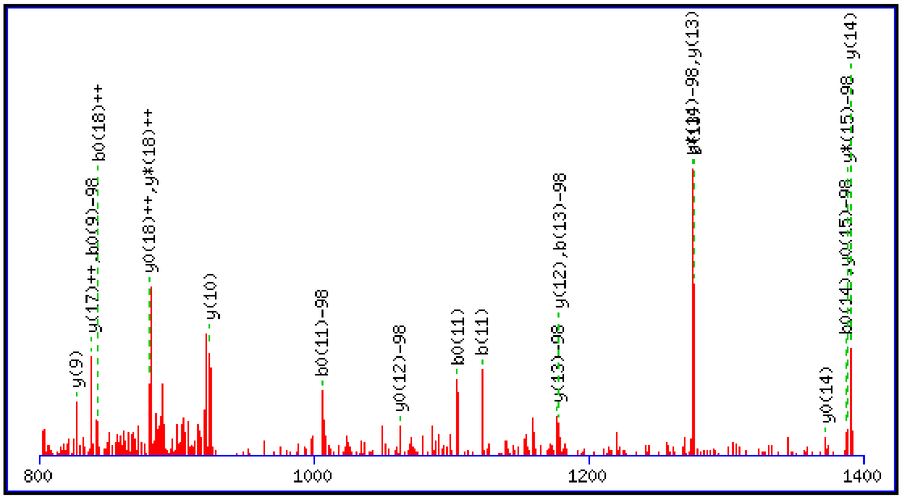

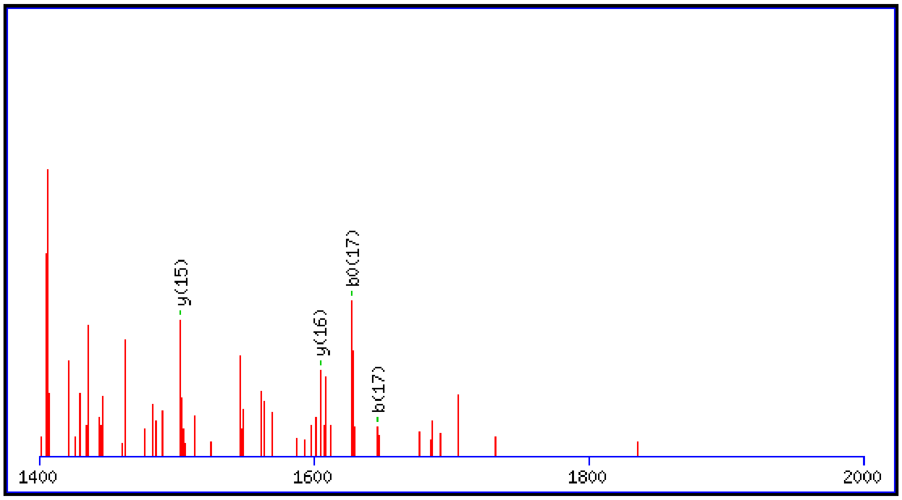

Monoisotopic mass of neutral peptide Mr(calc): 1876.8204

Fixed modifications: Carbamidomethyl (C)
Variable modifications:
S9 : Phospho (ST), with neutral losses 0.0000(shown in table), 97.9769
Ions Score: 38 **Expect:** 0.037
Matches (Bold Red): 38/264 fragment ions using 68 most intense peaks

#	b	b^{++}	b^{0}	b^{0++}	Seq.	y	y^{++}	y*	y^{*++}	y^{0}	y^{0++}	#
1	102.0550	51.5311	84.0444	42.5258	T							19
2	203.1026	102.0550	185.0921	93.0497	T	1776.7800	888.8936	1759.7534	**880.3804**	1758.7694	**879.8884**	18
3	274.1397	137.5735	256.1292	128.5682	A	1675.7323	**838.3698**	1658.7058	829.8565	1657.7217	829.3645	17
4	375.1874	188.0974	**357.1769**	179.0921	T	**1604.6952**	802.8512	1587.6687	794.3380	1586.6846	793.8460	16
5	488.2715	244.6394	**470.2609**	235.6341	L	**1503.6475**	752.3274	1486.6210	743.8141	1485.6370	743.3221	15
6	**603.2984**	302.1529	**585.2879**	293.1476	D	**1390.5635**	695.7854	1373.5369	687.2721	**1372.5529**	686.7801	14
7	700.3512	350.6792	682.3406	341.6740	P	**1275.5365**	**638.2719**	1258.5100	629.7586	1257.5260	629.2666	13
8	787.3832	394.1953	769.3727	385.1900	S	**1178.4838**	589.7455	1161.4572	581.2322	1160.4732	580.7402	12
9	954.3816	477.6944	936.3710	468.6891	S	1091.4517	546.2295	1074.4252	537.7162	1073.4412	537.2242	11
10	1051.4343	526.2208	1033.4238	517.2155	P	**924.4534**	462.7303	907.4268	454.2170	906.4428	453.7250	10
11	**1122.4715**	561.7394	**1104.4609**	552.7341	A	**827.4006**	414.2039	810.3741	405.6907	809.3900	405.1987	9
12	1219.5242	610.2657	1201.5137	601.2605	P	**756.3635**	378.6854	739.3369	370.1721	738.3529	369.6801	8
13	**1276.5457**	638.7765	1258.5351	629.7712	G	659.3107	330.1590	642.2842	321.6457	641.3002	321.1537	7
14	1405.5883	703.2978	**1387.5777**	694.2925	E	602.2893	301.6483	**585.2627**	293.1350	584.2787	292.6430	6
15	1462.6097	731.8085	1444.5992	722.8032	G	**473.2467**	237.1270	456.2201	228.6137	455.2361	228.1217	5
16	1559.6625	780.3349	1541.6519	771.3296	P	**416.2252**	208.6162	399.1987	200.1030	398.2146	199.6110	4
17	**1646.6945**	823.8509	**1628.6840**	814.8456	S	319.1724	160.0899	302.1459	151.5766	301.1619	151.0846	3
18	1703.7160	852.3616	1685.7054	**843.3564**	G	232.1404	116.5738	215.1139	108.0606			2
19					R	175.1190	88.0631	158.0924	79.5498			1

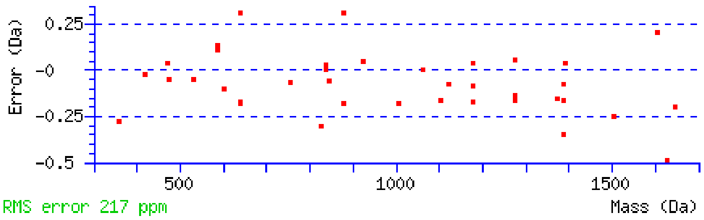

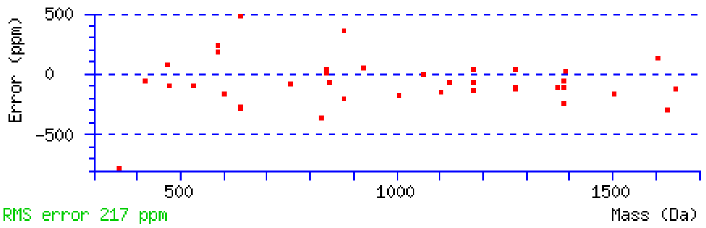

All matches to this query

Score	Mr(calc):	Delta	Sequence
37.8	1876.8204	0.0073	TTATLDPSSPAPGEGPSGR
37.8	1876.8204	0.0073	TTATLDPSSPAPGEGPSGR
20.9	1876.8204	0.0073	TTATLDPSSPAPGEGPSGR
15.6	1876.8204	0.0073	TTATLDPSSPAPGEGPSGR
15.6	1876.8204	0.0073	TTATLDPSSPAPGEGPSGR
1.9	1876.8445	-0.0168	NASSQLRLTRNTDLK

Spectrum No: 133; Query: 487; Rank: 1

Peptide View

MS/MS Fragmentation of **ESLKEEDESDDDNM**
Found in **IPI00476178**, Tax_Id=10116 Gene_Symbol=Psma3;Psma3l Proteasome subunit alpha type-3

Match to Query 487: 1734.584348 from(868.299450,2+)
Title: 091129RatKid_SCX02_14.605.605.2.dta

Data file K:\NewmanPaper\Piliang\3SubProteomes\Piliang3SP\mgf5ppm\SCX_3SubProteomes5ppm.mgf

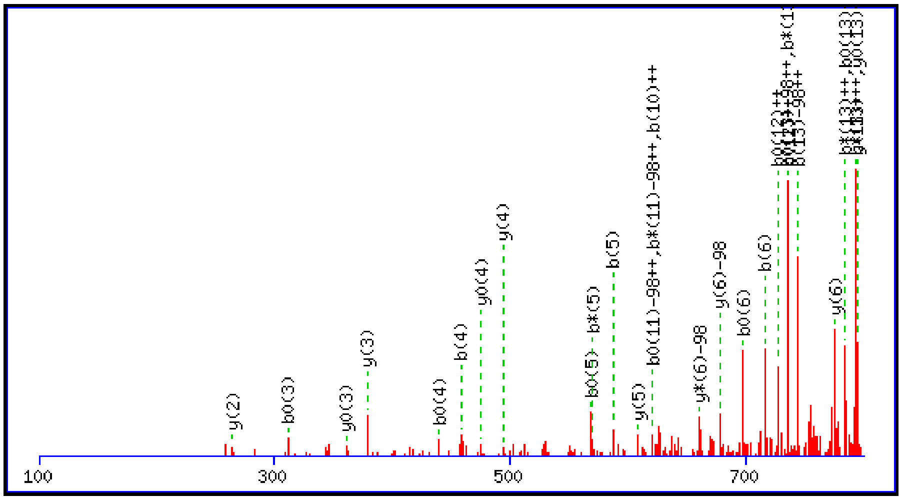

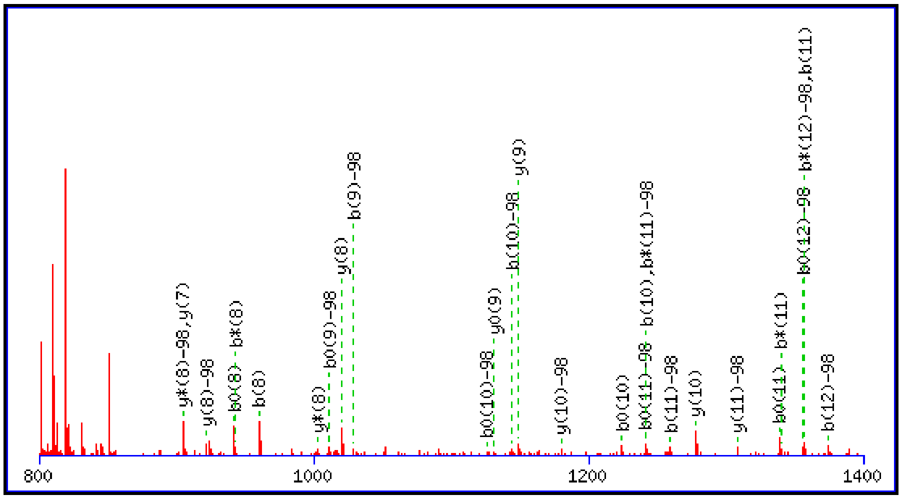

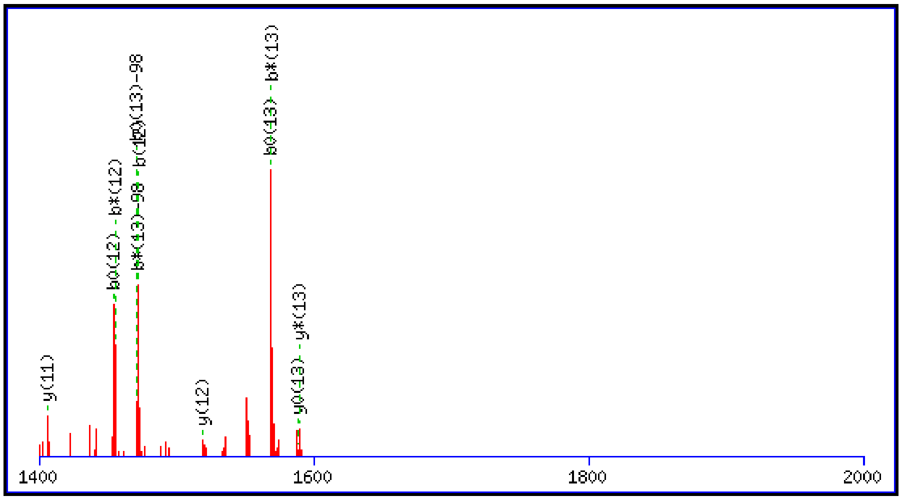

Monoisotopic mass of neutral peptide Mr(calc): 1734.5815
Fixed modifications: Carbamidomethyl (C)
Variable modifications:
S9 : Phospho (ST), with neutral losses 0.0000(shown in table), 97.9769
Ions Score: 38 **Expect:** 0.0086
Matches (Bold Red): 69/222 fragment ions using 126 most intense peaks

#	b	b++	b*	b*++	b0	b0++	Seq.	y	y++	y*	y*++	y0	y0++	#
1	130.0499	65.5286			112.0393	56.5233	E							14
2	217.0819	109.0446			199.0713	100.0393	S	1606.5462	803.7767	**1589.5197**	**795.2635**	**1588.5356**	**794.7715**	13
3	330.1660	165.5866			**312.1554**	156.5813	L	**1519.5142**	760.2607	1502.4876	751.7475	1501.5036	751.2554	12
4	**458.2609**	229.6341	441.2344	221.1208	**440.2504**	220.6288	K	**1406.4301**	703.7187	1389.4036	695.2054	1388.4196	694.7134	11

5	**587.3035**	294.1554	**570.2770**	285.6421	**569.2930**	285.1501	E	**1278.3352**	639.6712	1261.3086	631.1579	1260.3246	630.6659	10
6	**716.3461**	358.6767	699.3196	350.1634	**698.3355**	349.6714	E	**1149.2926**	575.1499	1132.2660	566.6366	**1131.2820**	566.1446	9
7	831.3731	416.1902	814.3465	407.6769	813.3625	407.1849	D	**1020.2500**	510.6286	**1003.2234**	502.1153	1002.2394	501.6233	8
8	**960.4156**	480.7115	**943.3891**	472.1982	**942.4051**	471.7062	E	**905.2230**	453.1152	888.1965	444.6019	887.2125	444.1099	7
9	1127.4140	564.2106	1110.3874	555.6974	1109.4034	555.2054	S	**776.1804**	388.5939	759.1539	380.0806	758.1699	379.5886	6
10	**1242.4409**	**621.7241**	1225.4144	613.2108	**1224.4304**	612.7188	D	**609.1821**	305.0947	592.1555	296.5814	591.1715	296.0894	5
11	**1357.4679**	679.2376	**1340.4413**	670.7243	**1339.4573**	670.2323	D	**494.1551**	247.5812	477.1286	239.0679	**476.1446**	238.5759	4
12	**1472.4948**	**736.7511**	**1455.4683**	728.2378	**1454.4843**	**727.7458**	D	**379.1282**	190.0677	362.1016	181.5545	**361.1176**	181.0625	3
13	1586.5378	**793.7725**	**1569.5112**	**785.2592**	**1568.5272**	**784.7672**	N	**264.1013**	132.5543	247.0747	124.0410			2
14							M	150.0583	75.5328					1

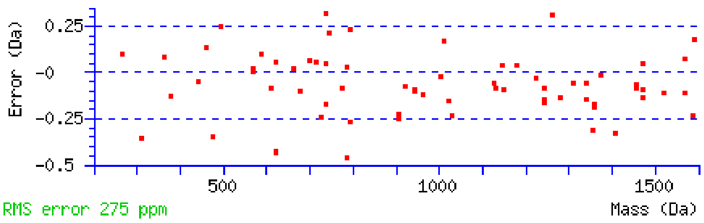

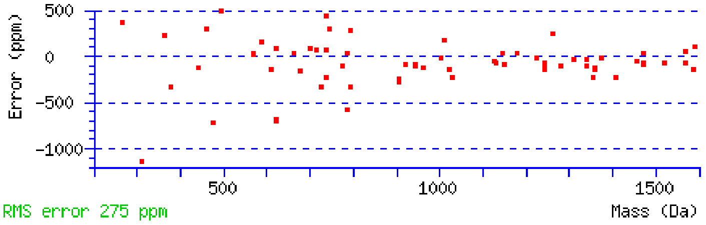

All matches to this query

Score	Mr(calc):	Delta	Sequence
37.7	1734.5815	0.0029	ESLKEEDESDDDNM
26.4	1734.5815	0.0029	ESLKEEDESDDDNM
3.0	1734.5940	-0.0096	SNTISHLTYNTSR
0.7	1734.5940	-0.0096	SNTISHLTYNTSR
0.7	1734.5940	-0.0096	SNTISHLTYNTSR
0.7	1734.5940	-0.0096	SNTISHLTYNTSR

Spectrum No: 134; Query: 585; Rank: 1

Peptide View

MS/MS Fragmentation of **GLLSQGSPLSWEETQR**
Found in **IPI00231862**, Tax_Id=10116 Gene_Symbol=Gclc Glutamate--cysteine ligase catalytic subunit

Match to Query 585: 1866.851508 from(934.433030,2+)
Title: 091127RatKid_SCX01_12.3281.3281.2.dta
Data file K:\NewmanPaper\Piliang\3SubProteomes\Piliang3SP\mgf5ppm\SCX_3SubProteomes5ppm.mgf

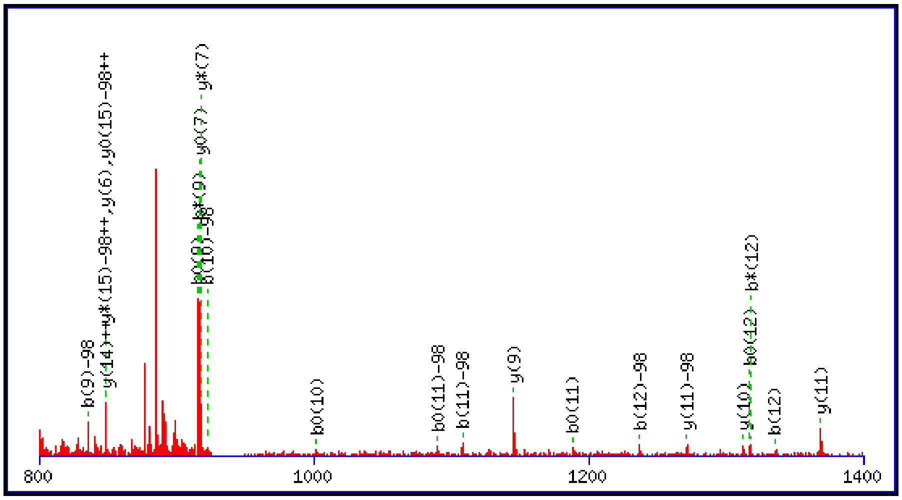

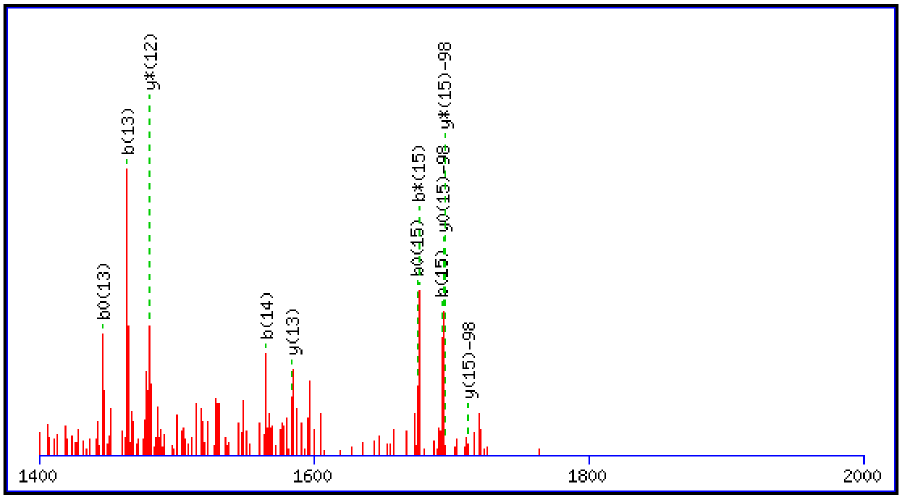

Monoisotopic mass of neutral peptide Mr(calc): 1866.8513
Fixed modifications: Carbamidomethyl (C)
Variable modifications:
S7 : Phospho (ST), with neutral losses 0.0000(shown in table), 97.9769
Ions Score: 38 **Expect:** 0.039
Matches (Bold Red): 42/252 fragment ions using 82 most intense peaks

#	b	b^{++}	b*	b^{*++}	b^0	b^{0++}	Seq.	y	y^{++}	y*	y^{*++}	y^0	y^{0++}	#
1	58.0287	29.5180					G							16
2	171.1128	86.0600					L	1810.8371	905.9222	1793.8106	897.4089	1792.8265	896.9169	15
3	284.1969	142.6021					L	1697.7531	**849.3802**	1680.7265	840.8669	1679.7425	840.3749	14
4	371.2289	186.1181			353.2183	177.1128	S	**1584.6690**	**792.8381**	1567.6424	784.3249	1566.6584	783.8328	13
5	**499.2875**	250.1474	482.2609	241.6341	481.2769	241.1421	Q	1497.6370	749.3221	**1480.6104**	740.8088	1479.6264	740.3168	12

**6**	556.3089	278.6581	539.2824	270.1448	538.2984	269.6528	**G**	**1369.5784**	685.2928	1352.5518	676.7796	1351.5678	676.2875	**11**
**7**	723.3073	362.1573	706.2807	353.6440	705.2967	353.1520	**S**	**1312.5569**	**656.7821**	1295.5304	648.2688	1294.5464	647.7768	**10**
**8**	820.3601	410.6837	803.3335	402.1704	802.3495	401.6784	**P**	**1145.5586**	573.2829	1128.5320	564.7696	1127.5480	564.2776	**9**
**9**	933.4441	467.2257	**916.4176**	458.7124	**915.4336**	458.2204	**L**	1048.5058	524.7565	1031.4793	516.2433	1030.4952	515.7513	**8**
**10**	1020.4761	510.7417	1003.4496	502.2284	**1002.4656**	501.7364	**S**	935.4217	468.2145	**918.3952**	459.7012	**917.4112**	459.2092	**7**
**11**	1206.5555	603.7814	1189.5289	595.2681	**1188.5449**	594.7761	**W**	**848.3897**	424.6985	831.3632	416.1852	830.3791	415.6932	**6**
**12**	**1335.5981**	668.3027	**1318.5715**	659.7894	**1317.5875**	659.2974	**E**	**662.3104**	331.6588	645.2838	323.1456	644.2998	322.6536	**5**
**13**	**1464.6406**	732.8240	1447.6141	724.3107	**1446.6301**	723.8187	**E**	**533.2678**	267.1375	516.2413	258.6243	515.2572	258.1323	**4**
**14**	**1565.6883**	783.3478	1548.6618	774.8345	1547.6778	774.3425	**T**	**404.2252**	202.6162	**387.1987**	194.1030	386.2146	193.6110	**3**
**15**	**1693.7469**	847.3771	**1676.7204**	838.8638	**1675.7363**	838.3718	**Q**	**303.1775**	152.0924	286.1510	143.5791			**2**
**16**							**R**	175.1190	88.0631	158.0924	79.5498			**1**

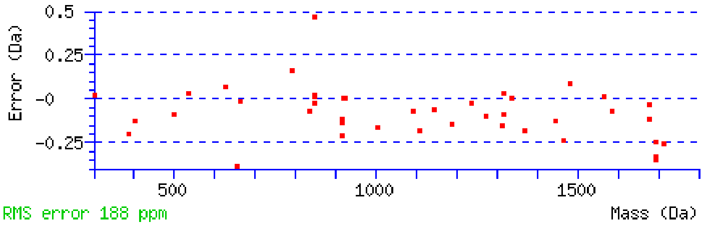

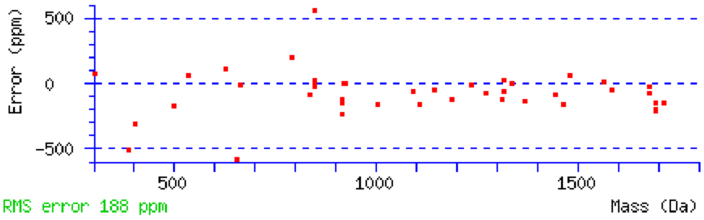

All matches to this query

Score	Mr(calc):	Delta	Sequence
37.6	1866.8513	0.0002	GLLSQGSPLSWEETQR
29.4	1866.8513	0.0002	GLLSQGSPLSWEETQR
22.7	1866.8513	0.0002	GLLSQGSPLSWEETQR
3.0	1866.8513	0.0002	GLLSQGSPLSWEETQR
1.7	1866.8401	0.0114	GVENVDATVSVPESWAK
1.7	1866.8401	0.0114	GVENVDATVSVPESWAK
1.6	1866.8430	0.0085	DKLLTQGFTNWTKR
0.8	1866.8530	-0.0015	SSKASFNGVSNEMAHKR
0.3	1866.8401	0.0114	GVENVDATVSVPESWAK

Spectrum No: 135; Query: 665; Rank: 1

Peptide View

MS/MS Fragmentation of **VLHAQCHSTPDSAEDVR**
Found in **IPI00327469**, Tax_Id=10116 Gene_Symbol=Ahsg Alpha-2-HS-glycoprotein precursor

Match to Query 665: 2000.840742 from(667.954190,3+)
Title: 091127RatKid_SCX01_35.595.595.3.dta
Data file K:\NewmanPaper\Piliang\3SubProteomes\Piliang3SP\mgf5ppm\SCX_3SubProteomes5ppm.mgf

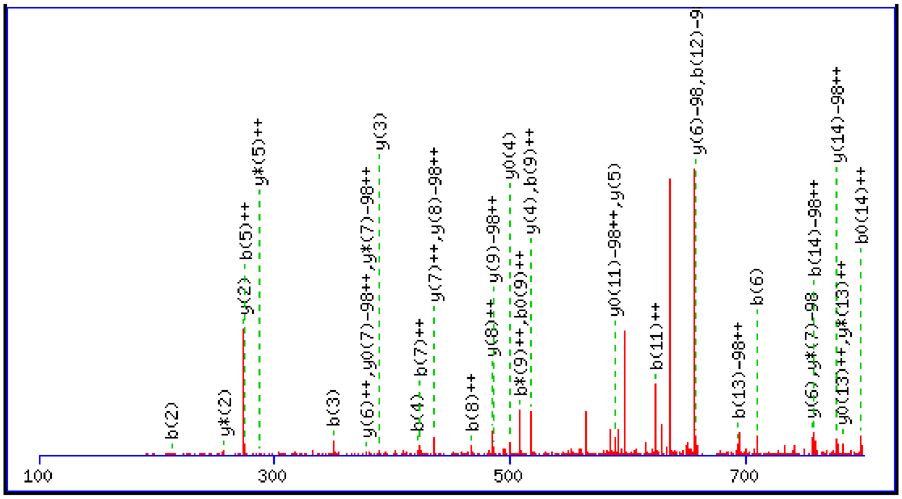

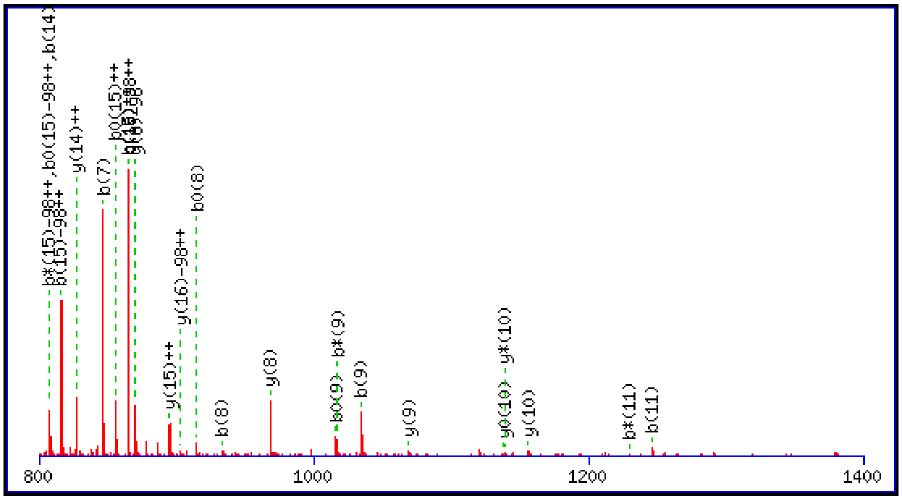

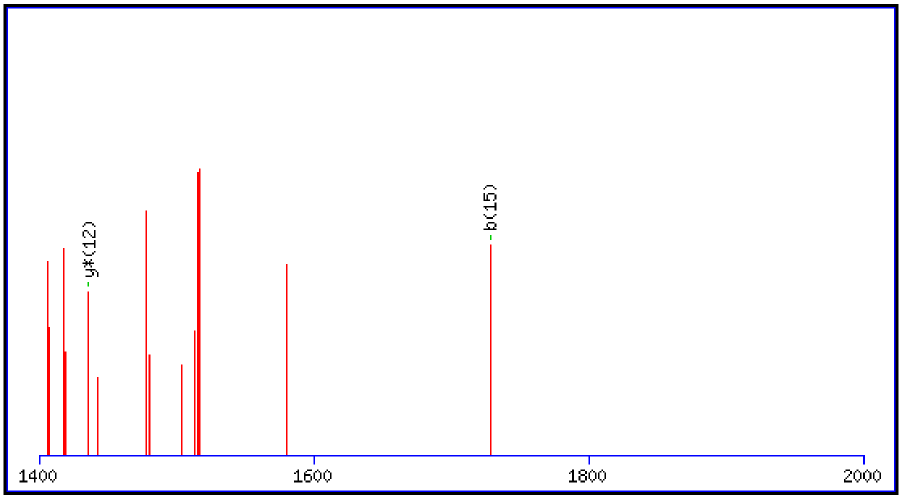

Monoisotopic mass of neutral peptide Mr(calc): 2000.8412
Fixed modifications: Carbamidomethyl (C)
Variable modifications:
S12 : Phospho (ST), with neutral losses 97.9769(shown in table), 0.0000
Ions Score: 37 **Expect:** 0.039
Matches (Bold Red): 62/262 fragment ions using 123 most intense peaks

#	b	b^{++}	b*	b^{*++}	b^0	b^{0++}	Seq.	y	y^{++}	y*	y^{*++}	y^0	y^{0++}	#
1	100.0757	50.5415					V							17
2	**213.1598**	107.0835					L	1804.8031	**902.9052**	1787.7766	894.3919	1786.7926	893.8999	16
3	**350.2187**	175.6130					H	1691.7191	846.3632	1674.6925	837.8499	1673.7085	837.3579	15
4	**421.2558**	211.1315					A	1554.6601	**777.8337**	1537.6336	769.3204	1536.6496	768.8284	14
5	549.3144	**275.1608**	532.2878	266.6475			Q	1483.6230	742.3152	1466.5965	733.8019	1465.6125	733.3099	13

6	709.3450	355.1761	692.3185	346.6629			C	1355.5644	678.2859	1338.5379	669.7726	1337.5539	669.2806	12
7	846.4039	423.7056	829.3774	415.1923			H	1195.5338	598.2705	1178.5072	589.7573	1177.5232	589.2653	11
8	933.4359	467.2216	916.4094	458.7083	915.4254	458.2163	S	1058.4749	529.7411	1041.4483	521.2278	1040.4643	520.7358	10
9	1034.4836	517.7455	1017.4571	509.2322	1016.4731	508.7402	T	971.4429	486.2251	954.4163	477.7118	953.4323	477.2198	9
10	1131.5364	566.2718	1114.5098	557.7586	1113.5258	557.2666	P	870.3952	435.7012	853.3686	427.1880	852.3846	426.6959	8
11	1246.5633	623.7853	1229.5368	615.2720	1228.5528	614.7800	D	773.3424	387.1748	756.3159	378.6616	755.3319	378.1696	7
12	1315.5848	658.2960	1298.5582	649.7828	1297.5742	649.2908	S	658.3155	329.6614	641.2889	321.1481	640.3049	320.6561	6
13	1386.6219	693.8146	1369.5954	685.3013	1368.6113	684.8093	A	589.2940	295.1506	572.2675	286.6374	571.2835	286.1454	5
14	1515.6645	758.3359	1498.6379	749.8226	1497.6539	749.3306	E	518.2569	259.6321	501.2304	251.1188	500.2463	250.6268	4
15	1630.6914	815.8494	1613.6649	807.3361	1612.6809	806.8441	D	389.2143	195.1108	372.1878	186.5975	371.2037	186.1055	3
16	1729.7599	865.3836	1712.7333	856.8703	1711.7493	856.3783	V	274.1874	137.5973	257.1608	129.0840			2
17							R	175.1190	88.0631	158.0924	79.5498			1

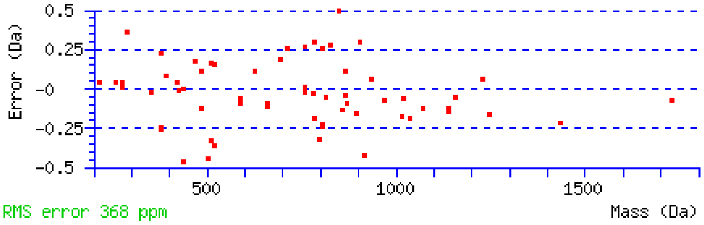

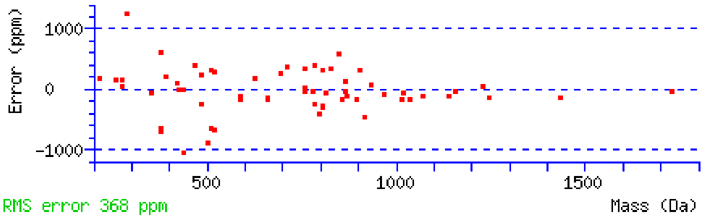

All matches to this query

Score	Mr(calc):	Delta	Sequence
37.4	2000.8412	-0.0004	VLHAQCHSTPDSAEDVR
27.6	2000.8412	-0.0004	VLHAQCHSTPDSAEDVR
26.7	2000.8412	-0.0004	VLHAQCHSTPDSAEDVR
3.8	1999.8409	0.9998	VWRTSCXVCWWPMR
3.8	1999.8409	0.9998	VWRTSCXVCWWPMR
3.5	1999.8498	0.9909	LSSFLFSYLSVQNKK
3.0	1999.8475	0.9932	GSESHKMIDLSGNPVLR
1.2	1999.8492	0.9916	ETLPTVLKSTAAQMVR
0.9	1999.8492	0.9916	ETLPTVLKSTAAQMVR
0.6	1999.8363	1.0045	ATNVVMNYSEIESKVR

Spectrum No: 136; Query: 387; Rank: 1

Peptide View

MS/MS Fragmentation of **VDSTTCLFPVEEK**
Found in **IPI00190908**, Tax_Id=10116 Gene_Symbol=Gfpt1 79 kDa protein

Match to Query 387: 1603.688968 from(802.851760,2+)
Title: 091127RatKid_SCX01_11.2418.2418.2.dta
Data file K:\NewmanPaper\Piliang\3SubProteomes\Piliang3SP\mgf5ppm\SCX_3SubProteomes5ppm.mgf

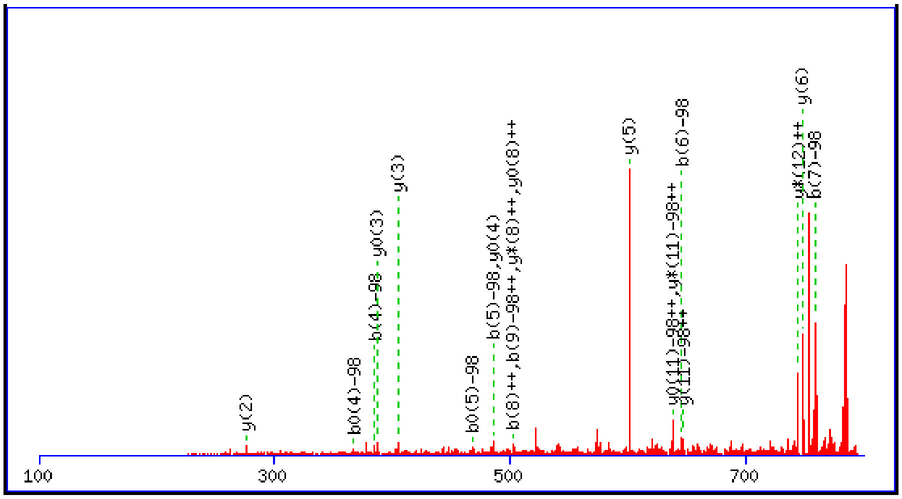

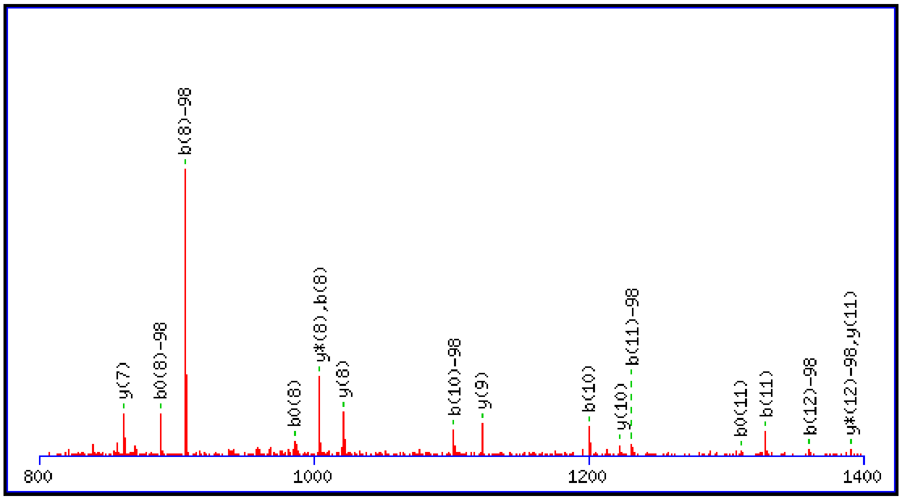

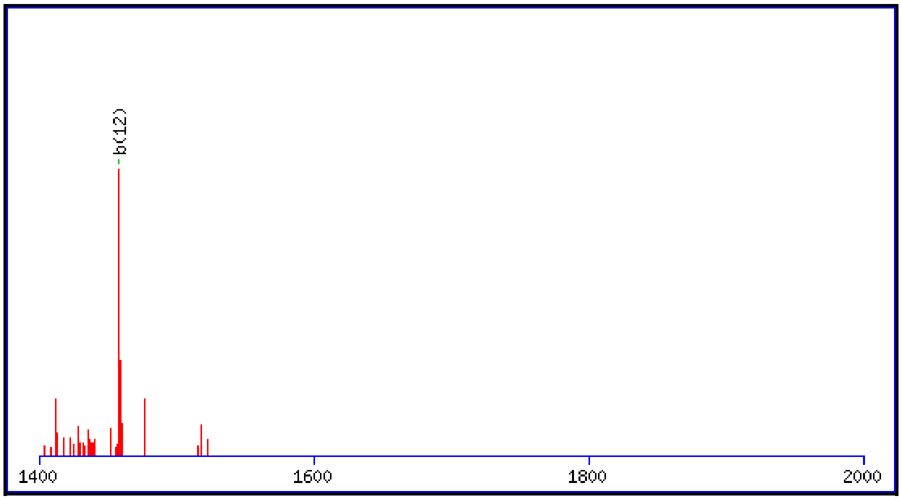

Monoisotopic mass of neutral peptide Mr(calc): 1603.6841
Fixed modifications: Carbamidomethyl (C)
Variable modifications:
S3 : Phospho (ST), with neutral losses 97.9769(shown in table), 0.0000
Ions Score: 37 **Expect:** 0.031
Matches (Bold Red): 38/168 fragment ions using 58 most intense peaks

#	b	b++	b0	b0++	Seq.	y	y++	y*	y*++	y0	y0++	#
1	100.0757	50.5415			V							13
2	215.1026	108.0550	197.0921	99.0497	D	1407.6461	704.3267	**1390.6195**	695.8134	1389.6355	695.3214	12
3	284.1241	142.5657	266.1135	133.5604	S	1292.6191	**646.8132**	1275.5926	**638.2999**	1274.6085	**637.8079**	11
4	**385.1718**	193.0895	**367.1612**	184.0842	T	**1223.5977**	612.3025	1206.5711	603.7892	1205.5871	603.2972	10
5	**486.2194**	243.6134	**468.2089**	234.6081	T	**1122.5500**	561.7786	1105.5234	553.2654	1104.5394	552.7733	9

**6**	**646.2501**	323.6287	628.2395	314.6234	**C**	**1021.5023**	511.2548	**1004.4757**	**502.7415**	1003.4917	**502.2495**	**8**
**7**	**759.3342**	380.1707	741.3236	371.1654	**L**	**861.4716**	431.2395	844.4451	422.7262	843.4611	422.2342	**7**
**8**	**906.4026**	453.7049	**888.3920**	444.6996	**F**	**748.3876**	374.6974	731.3610	366.1842	730.3770	365.6921	**6**
**9**	1003.4553	**502.2313**	985.4448	493.2260	**P**	**601.3192**	301.1632	584.2926	292.6499	583.3086	292.1579	**5**
**10**	**1102.5238**	551.7655	1084.5132	542.7602	**V**	504.2664	252.6368	487.2399	244.1236	**486.2558**	243.6316	**4**
**11**	**1231.5663**	616.2868	1213.5558	607.2815	**E**	**405.1980**	203.1026	388.1714	194.5894	**387.1874**	194.0974	**3**
**12**	**1360.6089**	680.8081	1342.5984	671.8028	**E**	**276.1554**	138.5813	259.1288	130.0681	258.1448	129.5761	**2**
**13**					**K**	147.1128	74.0600	130.0863	65.5468			**1**

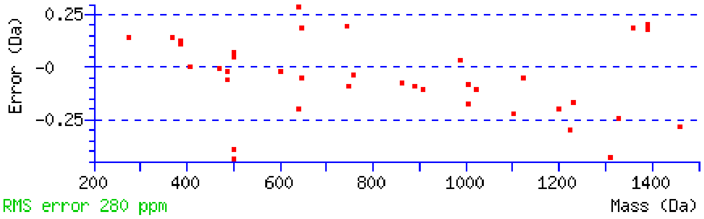

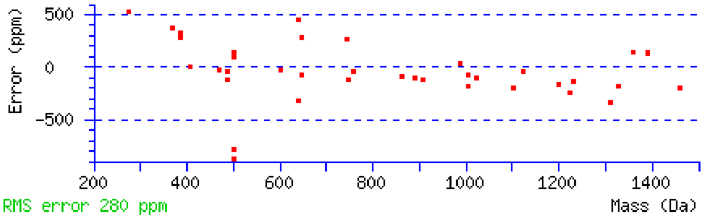

All matches to this query

Score	Mr(calc):	Delta	Sequence
37.4	1603.6841	0.0049	VDSTTCLFPVEEK
36.4	1603.6841	0.0049	VDSTTCLFPVEEK
30.1	1603.6841	0.0049	VDSTTCLFPVEEK
11.9	1603.6888	0.0002	NCFSHLMKSEKK
11.6	1603.6953	-0.0063	CQKSLNEFLEEK
9.4	1603.6987	-0.0097	DAKMAETCVVKEK
7.5	1603.6991	-0.0102	THSPVRDQAEGAEK
7.5	1603.6991	-0.0102	THSPVRDQAEGAEK
6.6	1603.7008	-0.0118	RASSPNVISKQEK
6.6	1603.7008	-0.0118	RASSPNVISKQEK

Spectrum No: 137; Query: 733; Rank: 1

Peptide View

MS/MS Fragmentation of **KEESEESEDDMGFGLFD**
Found in **IPI00200145**, Tax_Id=10116 Gene_Symbol=Rplp1 60S acidic ribosomal protein P1

Match to Query 733: 2122.707108 from(1062.360830,2+)
Title: 091129RatKid_SCX02_02.4095.4095.2.dta
Data file K:\NewmanPaper\Piliang\3SubProteomes\Piliang3SP\mgf5ppm\SCX_3SubProteomes5ppm.mgf

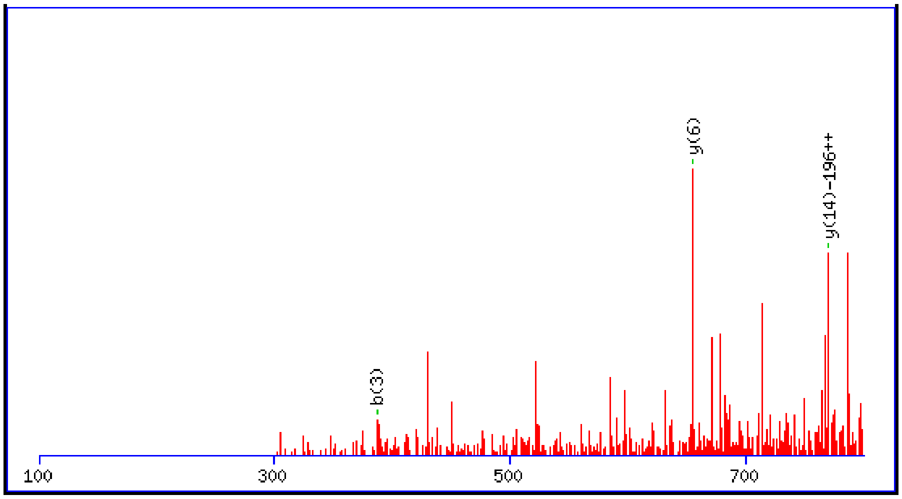

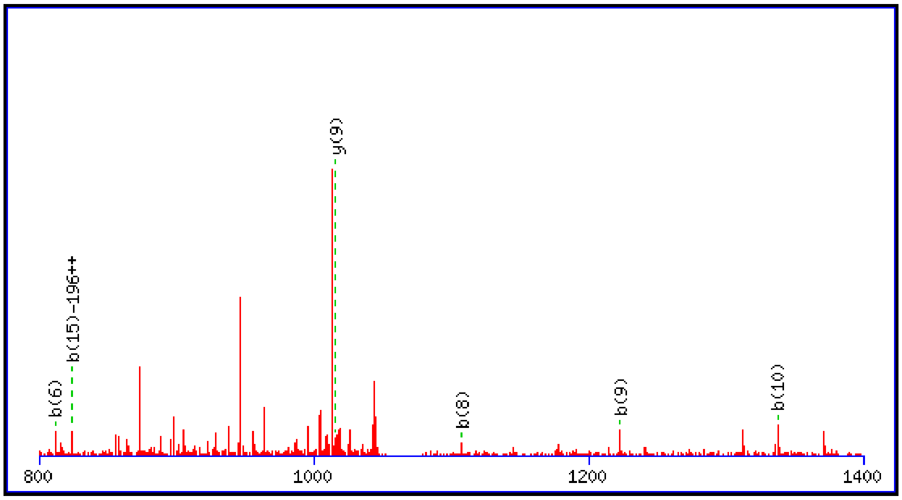

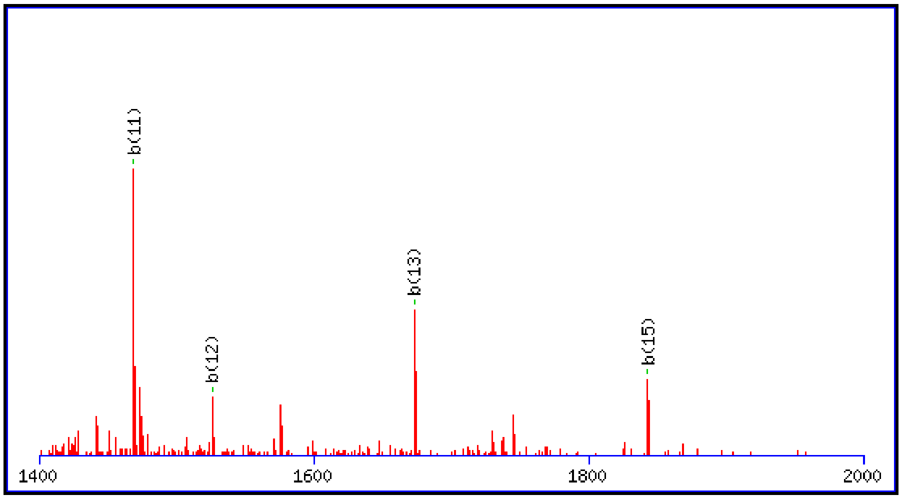

Monoisotopic mass of neutral peptide Mr(calc): 2122.7003
Fixed modifications: Carbamidomethyl (C)
Variable modifications:
S4 : Phospho (ST), with neutral losses 0.0000(shown in table), 97.9769
S7 : Phospho (ST), with neutral losses 0.0000(shown in table), 97.9769
Ions Score: 37 **Expect:** 0.012
Matches (Bold Red): 13/260 fragment ions using 36 most intense peaks

#	b	b^{++}	b*	b^{*++}	b^{0}	b^{0++}	Seq.	y	y^{++}	y^{0}	y^{0++}	#
1	129.1022	65.0548	112.0757	56.5415			K					17
2	258.1448	129.5761	241.1183	121.0628	240.1343	120.5708	E	1995.6126	998.3100	1977.6021	989.3047	16
3	**387.1874**	194.0974	370.1609	185.5841	369.1769	185.0921	E	1866.5701	933.7887	1848.5595	924.7834	15
4	554.1858	277.5965	537.1592	269.0833	536.1752	268.5912	S	1737.5275	869.2674	1719.5169	860.2621	14

5	683.2284	342.1178	666.2018	333.6045	665.2178	333.1125	E	1570.5291	785.7682	1552.5185	776.7629	13
6	**812.2710**	406.6391	795.2444	398.1258	794.2604	397.6338	E	1441.4865	721.2469	1423.4759	712.2416	12
7	979.2693	490.1383	962.2428	481.6250	961.2587	481.1330	S	1312.4439	656.7256	1294.4334	647.7203	11
8	**1108.3119**	554.6596	1091.2854	546.1463	1090.3013	545.6543	E	1145.4456	573.2264	1127.4350	564.2211	10
9	**1223.3389**	612.1731	1206.3123	603.6598	1205.3283	603.1678	D	**1016.4030**	508.7051	998.3924	499.6998	9
10	**1338.3658**	669.6865	1321.3392	661.1733	1320.3552	660.6813	D	901.3760	451.1917	883.3655	442.1864	8
11	**1469.4063**	735.2068	1452.3797	726.6935	1451.3957	726.2015	M	786.3491	393.6782	768.3385	384.6729	7
12	**1526.4277**	763.7175	1509.4012	755.2042	1508.4172	754.7122	G	**655.3086**	328.1579	637.2980	319.1527	6
13	**1673.4962**	837.2517	1656.4696	828.7384	1655.4856	828.2464	F	598.2871	299.6472	580.2766	290.6419	5
14	1730.5176	865.7624	1713.4911	857.2492	1712.5071	856.7572	G	451.2187	226.1130	433.2082	217.1077	4
15	**1843.6017**	922.3045	1826.5751	913.7912	1825.5911	913.2992	L	394.1973	197.6023	376.1867	188.5970	3
16	1990.6701	995.8387	1973.6436	987.3254	1972.6595	986.8334	F	281.1132	141.0602	263.1026	132.0550	2
17							D	134.0448	67.5260	116.0342	58.5207	1

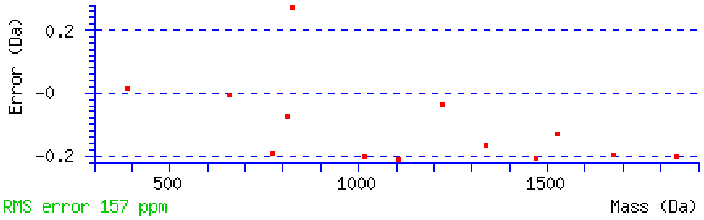

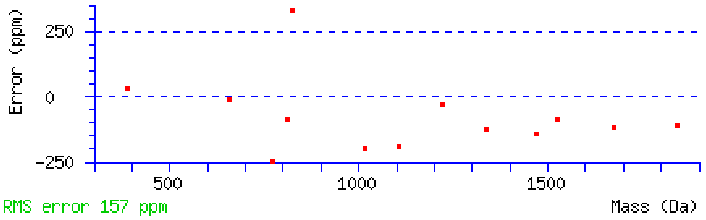

All matches to this query

Score	Mr(calc):	Delta	Sequence
37.4	2122.7003	0.0068	KEESEESEDDMGFGLFD
26.4	2122.7003	0.0068	KEESEESDEDMGFGLFD
24.1	2122.7181	-0.0110	KEESEESEDDTSFGLFD
4.3	2122.7181	-0.0110	KEESEESEDDTSFGLFD
3.3	2122.7181	-0.0110	KEESEESEDDTSFGLFD
2.1	2122.7042	0.0029	SLMYWMTVQYDSMGR
2.1	2122.7042	0.0029	SLMYWMTVQYDSMGR
1.3	2122.7042	0.0029	SLMYWMTVQYDSMGR

Spectrum No: 138; Query: 497; Rank: 1

Peptide View

MS/MS Fragmentation of **GQSQTWPDTSSPEVR**
Found in **IPI00368692**, Tax_Id=10116 Gene_Symbol=Srrm2_predicted similar to serine/arginine repetitive matrix 2

Match to Query 497: 1753.733368 from(877.873960,2+)
Title: 091127RatKid_SCX01_13.1581.1581.2.dta
Data file K:\NewmanPaper\Piliang\3SubProteomes\Piliang3SP\mgf5ppm\SCX_3SubProteomes5ppm.mgf

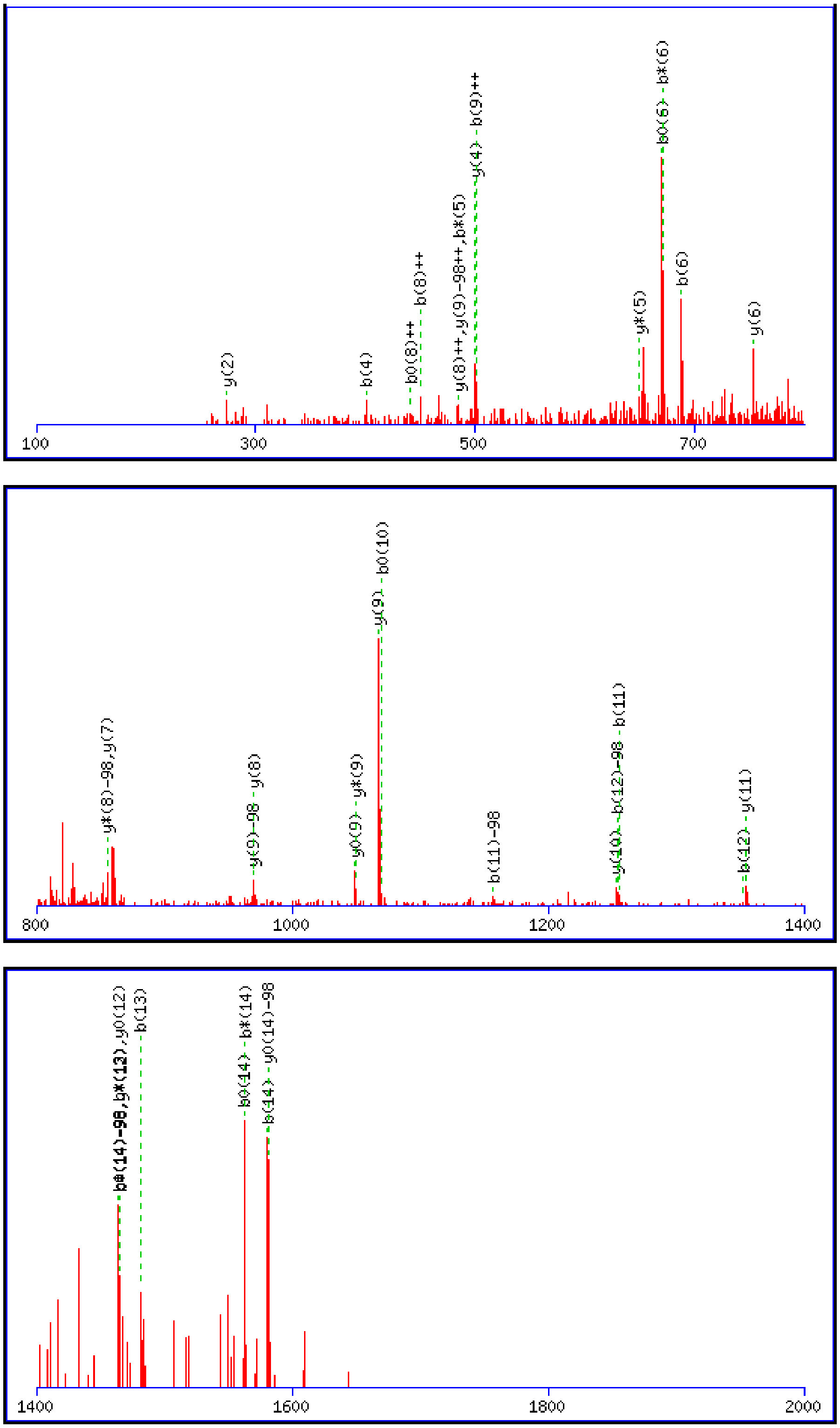

Monoisotopic mass of neutral peptide Mr(calc): 1753.7309
Fixed modifications: Carbamidomethyl (C)
Variable modifications:
S11 : Phospho (ST), with neutral losses 0.0000(shown in table), 97.9769
Ions Score: 37 **Expect:** 0.033
Matches (Bold Red): 38/242 fragment ions using 57 most intense peaks

#	b	b^{++}	b*	b^{*++}	b^0	b^{0++}	Seq.	y	y^{++}	y*	y^{*++}	y^0	y^{0++}	#
1	58.0287	29.5180					G							15
2	186.0873	93.5473	169.0608	85.0340			Q	1697.7167	849.3620	1680.6901	840.8487	1679.7061	840.3567	14
3	273.1193	137.0633	256.0928	128.5500	255.1088	128.0580	S	1569.6581	785.3327	1552.6315	776.8194	1551.6475	776.3274	13
4	**401.1779**	201.0926	384.1514	192.5793	383.1674	192.0873	Q	1482.6261	741.8167	**1465.5995**	733.3034	**1464.6155**	732.8114	12
5	502.2256	251.6164	**485.1991**	243.1032	484.2150	242.6112	T	**1354.5675**	677.7874	1337.5409	669.2741	1336.5569	668.7821	11

6	**688.3049**	344.6561	**671.2784**	336.1428	**670.2944**	335.6508	W	**1253.5198**	627.2635	1236.4933	618.7503	1235.5092	618.2583	10
7	785.3577	393.1825	768.3311	384.6692	767.3471	384.1772	P	**1067.4405**	534.2239	**1050.4139**	525.7106	**1049.4299**	525.2186	9
8	900.3846	**450.6959**	883.3581	442.1827	882.3741	**441.6907**	D	**970.3877**	**485.6975**	953.3612	477.1842	952.3772	476.6922	8
9	1001.4323	**501.2198**	984.4058	492.7065	983.4217	492.2145	T	**855.3608**	428.1840	838.3342	419.6708	837.3502	419.1787	7
10	1088.4643	544.7358	1071.4378	536.2225	**1070.4538**	535.7305	S	**754.3131**	377.6602	737.2866	369.1469	736.3025	368.6549	6
11	**1255.4627**	628.2350	1238.4361	619.7217	1237.4521	619.2297	S	667.2811	334.1442	**650.2545**	325.6309	649.2705	325.1389	5
12	**1352.5154**	676.7614	1335.4889	668.2481	1334.5049	667.7561	P	**500.2827**	250.6450	483.2562	242.1317	482.2722	241.6397	4
13	**1481.5580**	741.2827	**1464.5315**	732.7694	1463.5475	732.2774	E	403.2300	202.1186	386.2034	193.6053	385.2194	193.1133	3
14	**1580.6265**	790.8169	**1563.5999**	782.3036	**1562.6159**	781.8116	V	**274.1874**	137.5973	257.1608	129.0840			2
15							R	175.1190	88.0631	158.0924	79.5498			1

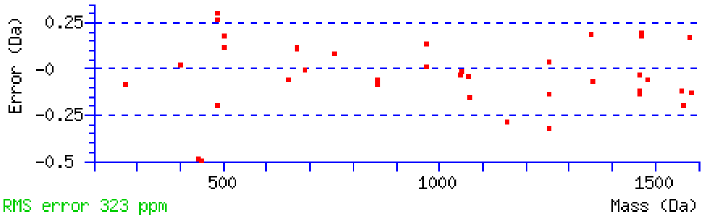

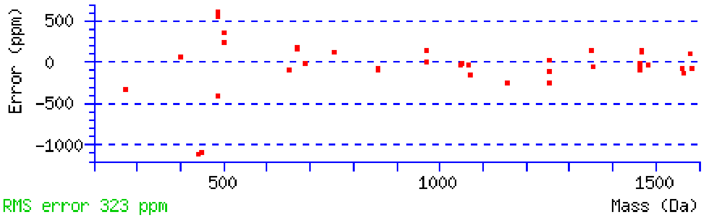

All matches to this query

Score	Mr(calc):	Delta	Sequence
36.9	1753.7309	0.0025	GQSQTWPDTSSPEVR
34.9	1753.7309	0.0025	GQSQTWPDTSSPEVR
28.3	1753.7309	0.0025	GQSQTWPDTSSPEVR
10.3	1751.7103	2.0230	TTTWQRPTMESVR
8.9	1753.7342	-0.0009	SNAAGVPCDLVTGEER
6.6	1753.7309	0.0025	GQSQTWPDTSSPEVR
5.8	1753.7325	0.0009	DAKLYEIGGGTSEVR
5.8	1753.7325	0.0009	DAKLYEIGGGTSEVR
5.4	1752.7428	0.9906	DASQREPAETEERR
4.0	1753.7461	-0.0127	DSAFWDHTAQNLIR

Spectrum No: 139; Query: 428; Rank: 1

Peptide View

MS/MS Fragmentation of **VLHGAQTSDEEKDF**
Found in **IPI00365864**, Tax_Id=10116 Gene_Symbol=LOC688717 hypothetical protein LOC688717

Match to Query 428: 1654.686188 from(828.350370,2+)
Title: 091129RatKid_SCX02_24.1003.1003.2.dta
Data file K:\NewmanPaper\Piliang\3SubProteomes\Piliang3SP\mgf5ppm\SCX_3SubProteomes5ppm.mgf

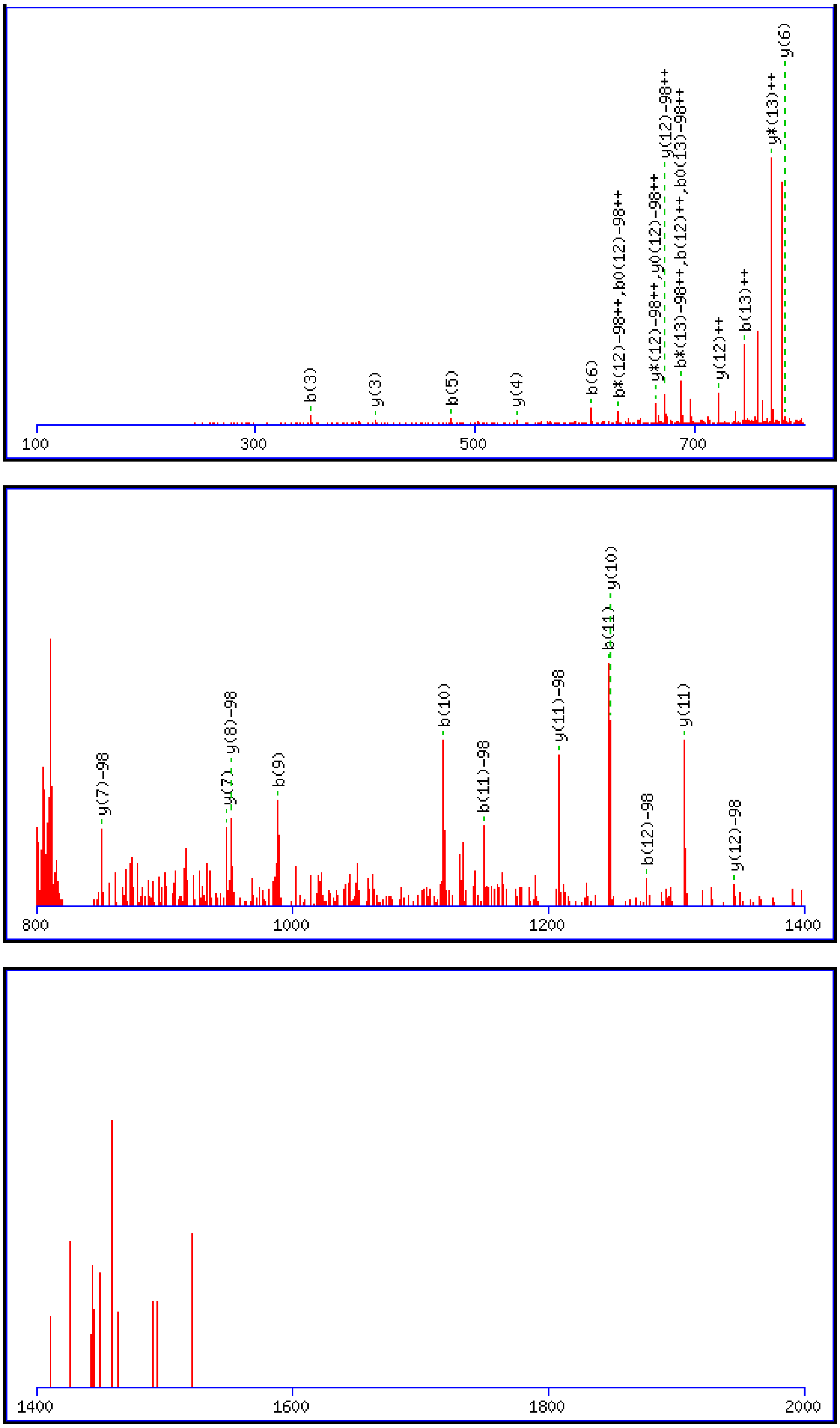

Monoisotopic mass of neutral peptide Mr(calc): 1654.6876
Fixed modifications: Carbamidomethyl (C)
Variable modifications:
S8 : Phospho (ST), with neutral losses 97.9769(shown in table), 0.0000
Ions Score: 37 **Expect:** 0.032
Matches (Bold Red): 29/206 fragment ions using 49 most intense peaks

#	b	b^{++}	b*	b^{*++}	b^{0}	b^{0++}	Seq.	y	y^{++}	y*	y^{*++}	y^{0}	y^{0++}	#
1	100.0757	50.5415					V							14
2	213.1598	107.0835					L	1458.6496	729.8284	1441.6230	721.3151	1440.6390	720.8231	13
3	**350.2187**	175.6130					H	**1345.5655**	**673.2864**	1328.5389	**664.7731**	1327.5549	**664.2811**	12
4	407.2401	204.1237					G	**1208.5066**	604.7569	1191.4800	596.2437	1190.4960	595.7516	11
5	**478.2772**	239.6423					A	1151.4851	576.2462	1134.4586	567.7329	1133.4746	567.2409	10

**6**	**606.3358**	303.6715	589.3093	295.1583			**Q**	1080.4480	540.7276	1063.4215	532.2144	1062.4374	531.7224	**9**
**7**	707.3835	354.1954	690.3570	345.6821	689.3729	345.1901	**T**	**952.3894**	476.6984	935.3629	468.1851	934.3789	467.6931	**8**
**8**	776.4050	388.7061	759.3784	380.1928	758.3944	379.7008	**S**	**851.3417**	426.1745	834.3152	417.6612	833.3312	417.1692	**7**
**9**	891.4319	446.2196	874.4054	437.7063	873.4213	437.2143	**D**	**782.3203**	391.6638	765.2937	383.1505	764.3097	382.6585	**6**
**10**	1020.4745	510.7409	1003.4479	502.2276	1002.4639	501.7356	**E**	667.2933	334.1503	650.2668	325.6370	649.2828	325.1450	**5**
**11**	**1149.5171**	575.2622	1132.4905	566.7489	1131.5065	566.2569	**E**	**538.2508**	269.6290	521.2242	261.1157	520.2402	260.6237	**4**
**12**	**1277.6120**	639.3097	1260.5855	**630.7964**	1259.6015	**630.3044**	**K**	**409.2082**	205.1077	392.1816	196.5944	391.1976	196.1024	**3**
**13**	1392.6390	696.8231	1375.6124	**688.3099**	1374.6284	**687.8179**	**D**	281.1132	141.0602			263.1026	132.0550	**2**
**14**							**F**	166.0863	83.5468					**1**

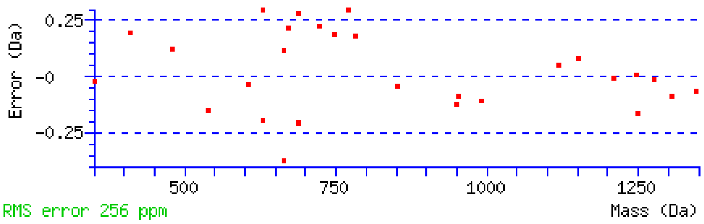

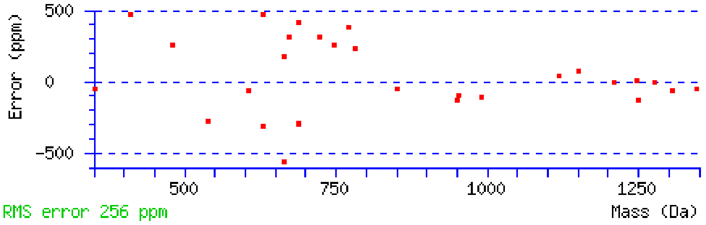

All matches to this query

Score	Mr(calc):	Delta	Sequence
36.8	1654.6876	-0.0014	VLHGAQTSDEEKDF
36.5	1654.6876	-0.0014	VLHGAQTSDEEKDF
4.2	1654.6865	-0.0003	LSKSNSQNVEPHR
2.0	1653.6968	0.9894	NNQSRRAQNNQSR
2.0	1654.6808	0.0054	NNQSRRAQNNQSR
2.0	1654.6745	0.0116	CDLCGKAFQHPSR
2.0	1654.6892	-0.0030	LLTIYKENGSNNK
1.6	1654.6947	-0.0086	NNSRNNSSGRAPSVI
1.5	1654.6892	-0.0030	LLTIYKENGSNNK
1.4	1654.6950	-0.0088	SQVYQLESTFDMK

Spectrum No: 140; Query: 87; Rank: 1

Peptide View

MS/MS Fragmentation of **NSKFHRVIK**
Found in **IPI00213033**, Tax_Id=10116 Gene_Symbol=Ppib 15 kDa protein

Match to Query 87: 1207.623748 from(604.819150,2+)
Title: 091129RatKid_SCX02_06.2945.2945.2.dta
Data file K:\NewmanPaper\Piliang\3SubProteomes\Piliang3SP\mgf5ppm\SCX_3SubProteomes5ppm.mgf

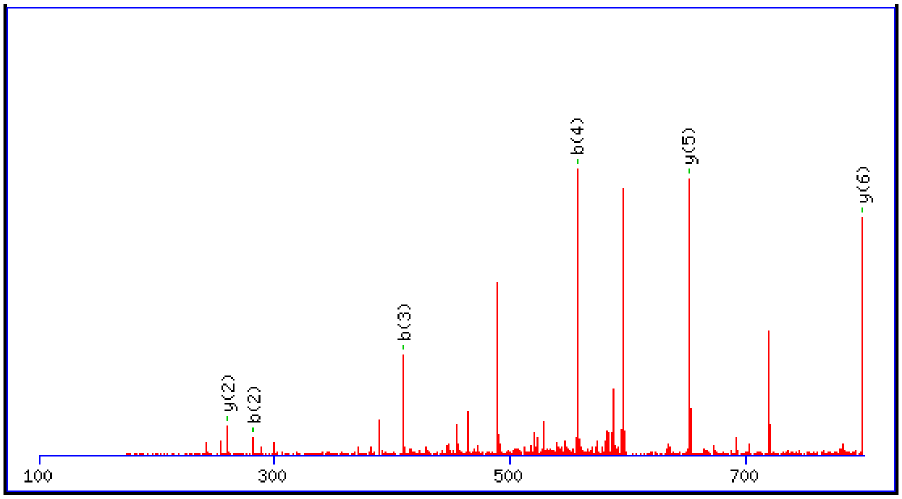

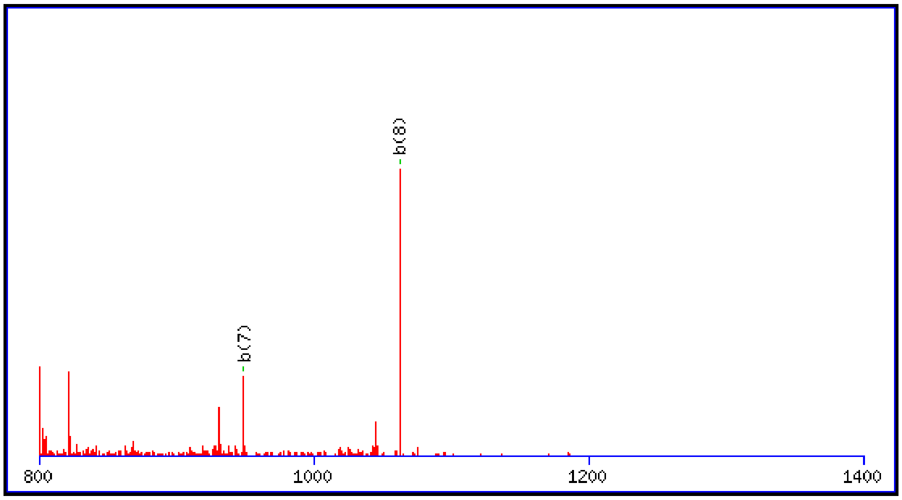

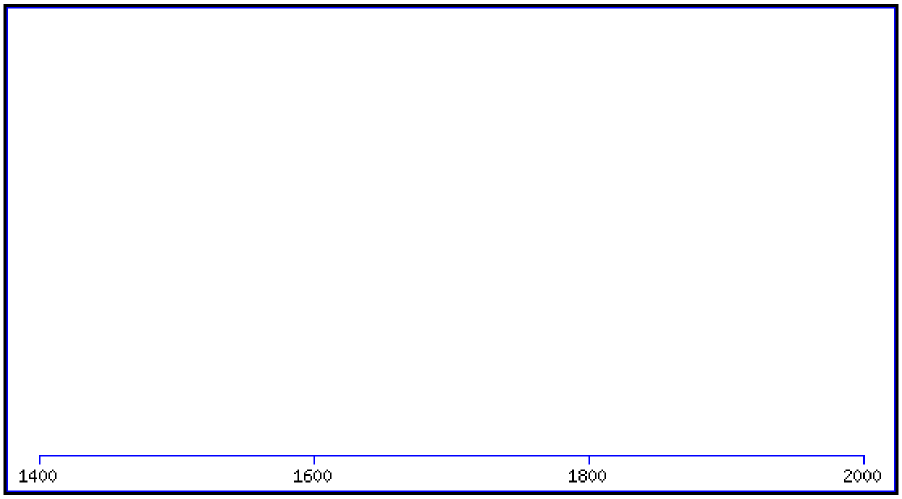

Monoisotopic mass of neutral peptide Mr(calc): 1207.6227
Fixed modifications: Carbamidomethyl (C)
Variable modifications:
S2 : Phospho (ST), with neutral losses 0.0000(shown in table), 97.9769
Ions Score: 37 **Expect:** 0.025
Matches (Bold Red): 8/128 fragment ions using 17 most intense peaks

#	b	b^{++}	b*	b^{*++}	b^0	b^{0++}	Seq.	y	y^{++}	y*	y^{*++}	y^0	y^{0++}	#
1	115.0502	58.0287	98.0237	49.5155			N							9
2	**282.0486**	141.5279	265.0220	133.0146	264.0380	132.5226	S	1094.5870	547.7972	1077.5605	539.2839	1076.5765	538.7919	8
3	**410.1435**	205.5754	393.1170	197.0621	392.1330	196.5701	K	927.5887	464.2980	910.5621	455.7847			7
4	**557.2119**	279.1096	540.1854	270.5963	539.2014	270.1043	F	**799.4937**	400.2505	782.4672	391.7372			6
5	694.2708	347.6391	677.2443	339.1258	676.2603	338.6338	H	**652.4253**	326.7163	635.3988	318.2030			5

6	850.3720	425.6896	833.3454	417.1763	832.3614	416.6843	R	515.3664	258.1868	498.3398	249.6736			4
7	**949.4404**	475.2238	932.4138	466.7105	931.4298	466.2185	V	359.2653	180.1363	342.2387	171.6230			3
8	**1062.5244**	531.7659	1045.4979	523.2526	1044.5139	522.7606	I	**260.1969**	130.6021	243.1703	122.0888			2
9							K	147.1128	74.0600	130.0863	65.5468			1

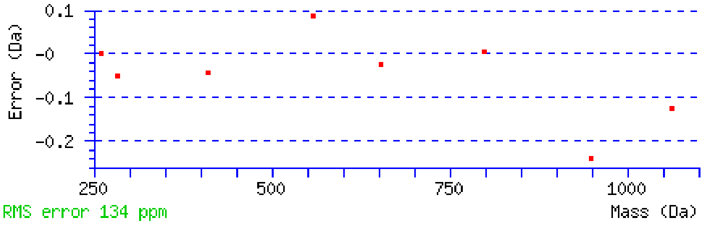

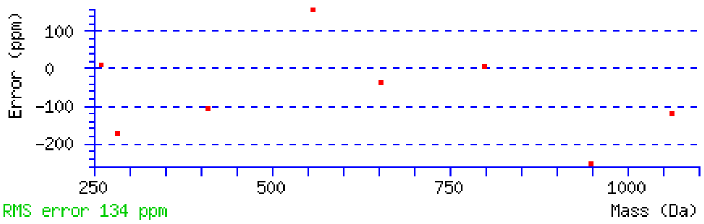

All matches to this query

Score	Mr(calc):	Delta	Sequence
36.6	1207.6227	0.0011	NSKFHRVIK
10.7	1207.6325	-0.0088	ILSLGRNNIK
10.5	1207.6326	-0.0088	NTNQIVAIKK
9.2	1207.6325	-0.0088	ILSLGRNNIK
9.1	1207.6237	0.0001	QIWNDYKLK
8.9	1207.6118	0.0119	DGSMNVSLKLK
5.2	1207.6213	0.0024	LPENLSSKIK
5.2	1207.6213	0.0024	LPENLSSKIK
5.1	1207.6349	-0.0112	IYEISNRWK
3.4	1207.6325	-0.0088	LNKALQSNLK

Spectrum No: 141; Query: 652; Rank: 1

Peptide View

MS/MS Fragmentation of **SSGSPYGGGYGSGGGSGGYGSR**
Found in **IPI00382376**, Tax_Id=10116 Gene_Symbol=Hnrpa3 Isoform 1 of Heterogeneous nuclear ribonucleoprotein A3

Match to Query 652: 1989.743068 from(995.878810,2+)
Title: 091129RatKid_SCX02_12.709.709.2.dta
Data file K:\NewmanPaper\Piliang\3SubProteomes\Piliang3SP\mgf5ppm\SCX_3SubProteomes5ppm.mgf

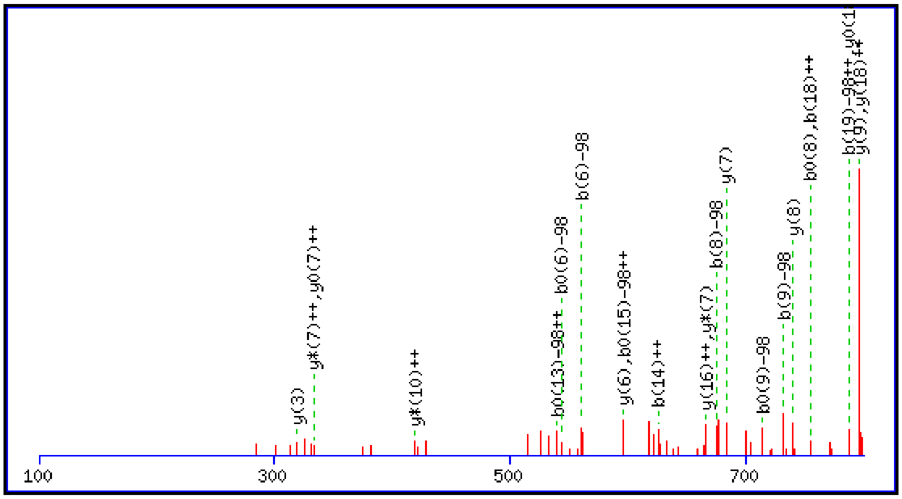

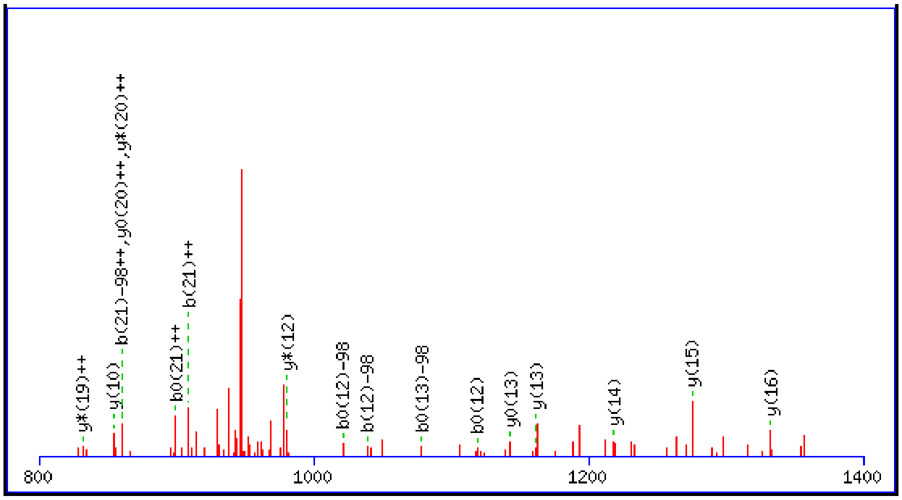

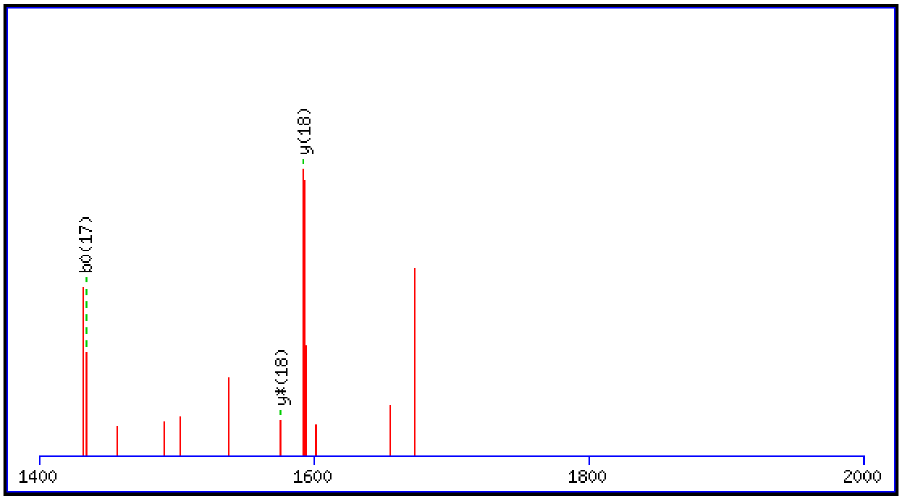

Monoisotopic mass of neutral peptide Mr(calc): 1989.7491
Fixed modifications: Carbamidomethyl (C)
Variable modifications:
S2 : Phospho (ST), with neutral losses 97.9769(shown in table), 0.0000
Ions Score: 36 **Expect:** 0.027
Matches (Bold Red): 44/294 fragment ions using 85 most intense peaks

#	b	b^{++}	b^{0}	b^{0++}	Seq.	y	y^{++}	y*	y^{*++}	y^{0}	y^{0++}	#
1	88.0393	44.5233	70.0287	35.5180	S							22
2	157.0608	79.0340	139.0502	70.0287	S	1805.7474	903.3773	1788.7208	894.8641	1787.7368	894.3720	21
3	214.0822	107.5447	196.0717	98.5395	G	1736.7259	868.8666	1719.6994	**860.3533**	1718.7154	**859.8613**	20
4	301.1143	151.0608	283.1037	142.0555	S	1679.7045	840.3559	1662.6779	**831.8426**	1661.6939	831.3506	19
5	398.1670	199.5871	380.1565	190.5819	P	**1592.6724**	**796.8399**	**1575.6459**	**788.3266**	1574.6619	**787.8346**	18
6	**561.2303**	281.1188	**543.2198**	272.1135	Y	1495.6197	748.3135	1478.5931	739.8002	1477.6091	739.3082	17
7	618.2518	309.6295	600.2412	300.6243	G	**1332.5563**	**666.7818**	1315.5298	658.2685	1314.5458	657.7765	16
8	**675.2733**	338.1403	657.2627	329.1350	G	**1275.5349**	638.2711	1258.5083	629.7578	1257.5243	629.2658	15
9	**732.2947**	366.6510	**714.2842**	357.6457	G	**1218.5134**	609.7603	1201.4869	601.2471	1200.5028	600.7551	14
10	895.3581	448.1827	877.3475	439.1774	Y	**1161.4919**	581.2496	1144.4654	572.7363	**1143.4814**	572.2443	13
11	952.3795	476.6934	934.3690	467.6881	G	998.4286	499.7179	**981.4021**	491.2047	980.4180	490.7127	12
12	**1039.4116**	520.2094	**1021.4010**	511.2041	S	941.4071	471.2072	924.3806	462.6939	923.3966	462.2019	11
13	1096.4330	548.7201	**1078.4225**	**539.7149**	G	**854.3751**	427.6912	837.3486	**419.1779**	836.3646	418.6859	10
14	1153.4545	577.2309	1135.4439	568.2256	G	**797.3537**	399.1805	780.3271	390.6672	779.3431	390.1752	9
15	1210.4760	605.7416	1192.4654	**596.7363**	G	**740.3322**	370.6697	723.3056	362.1565	722.3216	361.6645	8
16	1297.5080	649.2576	1279.4974	640.2523	S	**683.3107**	342.1590	**666.2842**	**333.6457**	665.3002	**333.1537**	7
17	1354.5294	677.7684	1336.5189	668.7631	G	**596.2787**	298.6430	579.2522	290.1297	578.2681	289.6377	6
18	1411.5509	706.2791	1393.5403	697.2738	G	539.2572	270.1323	522.2307	261.6190	521.2467	261.1270	5

**19**	1574.6142	**787.8108**	1556.6037	778.8055	**Y**	482.2358	241.6215	465.2092	233.1083	464.2252	232.6162	**4**
**20**	1631.6357	816.3215	1613.6251	807.3162	**G**	**319.1724**	160.0899	302.1459	151.5766	301.1619	151.0846	**3**
**21**	1718.6677	**859.8375**	1700.6572	850.8322	**S**	262.1510	131.5791	245.1244	123.0659	244.1404	122.5738	**2**
**22**					**R**	175.1190	88.0631	158.0924	79.5498			**1**

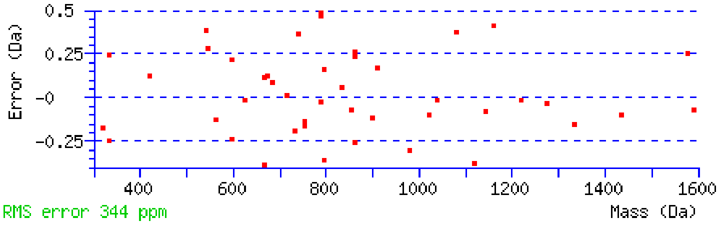

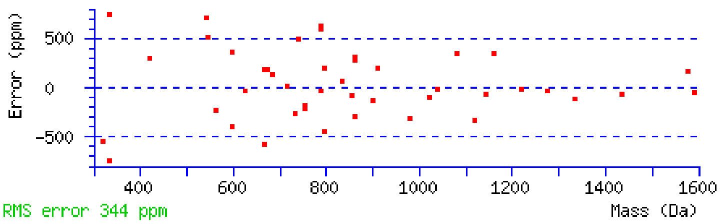

All matches to this query

Score	Mr(calc):	Delta	Sequence
36.5	1989.7491	-0.0060	SSGSPYGGGYGSGGGSGGYGSR
36.5	1989.7491	-0.0060	SSGSPYGGGYGSGGGSGGYGSR
36.4	1989.7491	-0.0060	SSGSPYGGGYGSGGGSGGYGSR
28.3	1989.7491	-0.0060	SSGSPYGGGYGSGGGSGGYGSR
23.1	1989.7491	-0.0060	SSGSPYGGGYGSGGGSGGYGSR
12.6	1989.7491	-0.0060	SSGSPYGGGYGSGGGSGGYGSR
11.8	1989.7491	-0.0060	SSGSPYGGGYGSGGGSGGYGSR
11.4	1989.7491	-0.0060	SSGSPYGGGYGSGGGSGGYGSR
5.1	1989.7515	-0.0085	NWLKTGSCLYGNTCR
2.6	1989.7491	-0.0060	SSGSPYGGGYGSGGGSGGYGSR

Spectrum No: 142; Query: 316; Rank: 1

Peptide View

MS/MS Fragmentation of **SMTYNSSTAILLR**
Found in **IPI00393555**, Tax_Id=10116 Gene_Symbol=Eml5 similar to echinoderm microtubule associated protein like 5

Match to Query 316: 1535.698208 from(768.856380,2+)
Title: 091127RatKid_SCX01_07.3477.3477.2.dta
Data file K:\NewmanPaper\Piliang\3SubProteomes\Piliang3SP\mgf5ppm\SCX_3SubProteomes5ppm.mgf

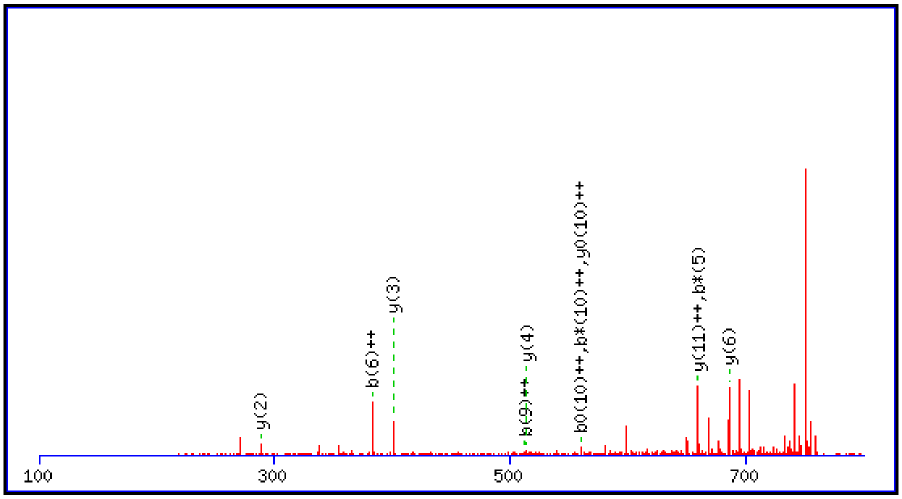

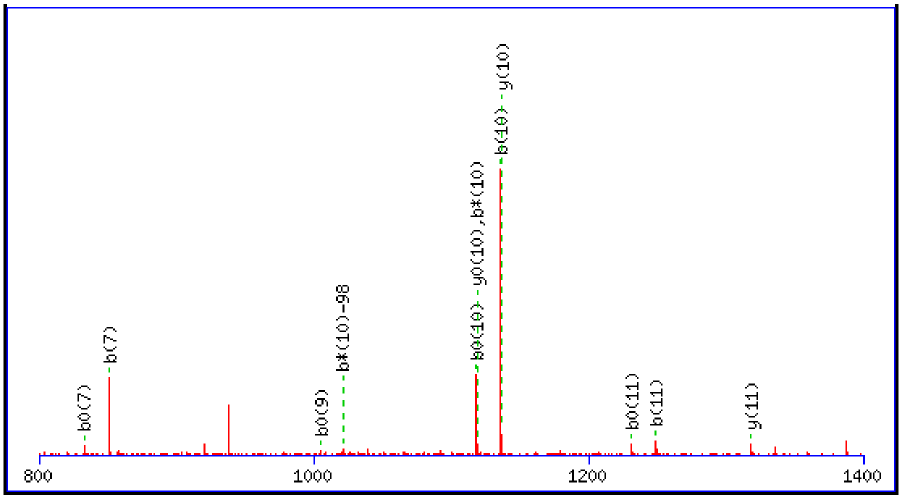

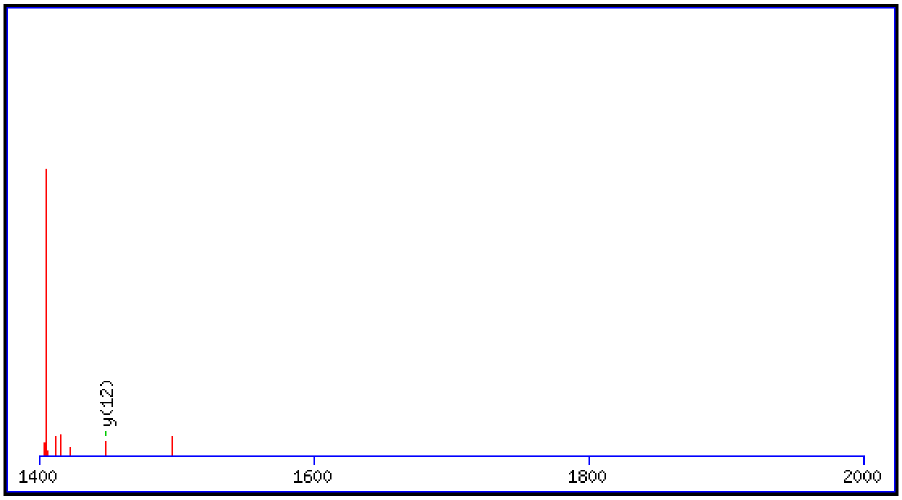

Monoisotopic mass of neutral peptide Mr(calc): 1535.7055
Fixed modifications: Carbamidomethyl (C)
Variable modifications:
T3 : Phospho (ST), with neutral losses 0.0000(shown in table), 97.9769
Ions Score: 36 **Expect:** 0.037
Matches (Bold Red): 24/194 fragment ions using 45 most intense peaks

#	b	b^{++}	b*	b^{*++}	b^0	b^{0++}	Seq.	y	y^{++}	y*	y^{*++}	y^0	y^{0++}	#
1	88.0393	44.5233			70.0287	35.5180	S							13
2	219.0798	110.0435			201.0692	101.0382	M	**1449.6807**	725.3440	1432.6542	716.8307	1431.6702	716.3387	12
3	400.0938	200.5505			382.0832	191.5453	T	**1318.6403**	**659.8238**	1301.6137	651.3105	1300.6297	650.8185	11
4	563.1571	282.0822			545.1466	273.0769	Y	**1137.6262**	569.3168	1120.5997	560.8035	**1119.6157**	**560.3115**	10
5	677.2000	339.1037	**660.1735**	330.5904	659.1895	330.0984	N	974.5629	487.7851	957.5364	479.2718	956.5524	478.7798	9
6	764.2321	**382.6197**	747.2055	374.1064	746.2215	373.6144	S	860.5200	430.7636	843.4934	422.2504	842.5094	421.7584	8
7	**851.2641**	426.1357	834.2376	417.6224	**833.2535**	417.1304	S	773.4880	387.2476	756.4614	378.7343	755.4774	378.2423	7
8	952.3118	476.6595	935.2852	468.1463	934.3012	467.6542	T	**686.4559**	343.7316	669.4294	335.2183	668.4454	334.7263	6
9	1023.3489	**512.1781**	1006.3223	503.6648	**1005.3383**	503.1728	A	585.4083	293.2078	568.3817	284.6945			5
10	**1136.4330**	568.7201	**1119.4064**	**560.2068**	**1118.4224**	**559.7148**	I	**514.3711**	257.6892	497.3446	249.1759			4
11	**1249.5170**	625.2622	1232.4905	616.7489	**1231.5065**	616.2569	L	**401.2871**	201.1472	384.2605	192.6339			3
12	1362.6011	681.8042	1345.5745	673.2909	1344.5905	672.7989	L	**288.2030**	144.6051	271.1765	136.0919			2
13							R	175.1190	88.0631	158.0924	79.5498			1

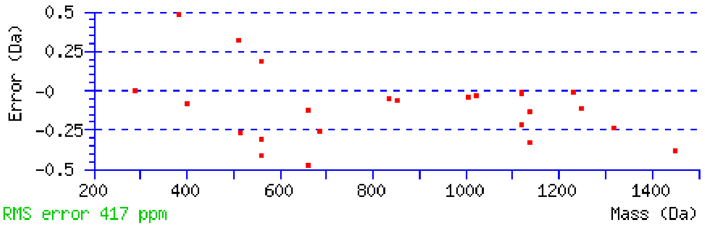

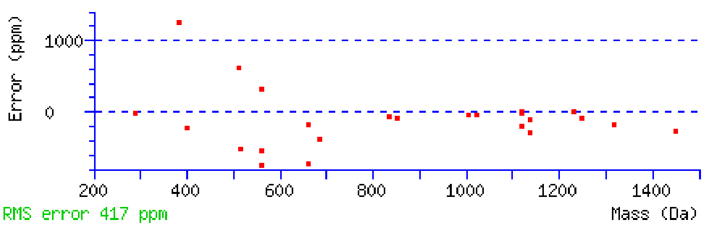

All matches to this query

Score	Mr(calc):	Delta	Sequence
36.4	1535.7055	-0.0073	SXTYNSSTAILLR
27.0	1535.7055	-0.0073	SXTYNSSTAILLR
27.0	1535.7055	-0.0073	SXTYNSSTAILLR
25.8	1535.7055	-0.0073	SXTYNSSTAILLR
18.9	1535.7055	-0.0073	SXTYNSSTAILLR
18.7	1535.7055	-0.0073	SXTYNSSTAILLR
9.1	1535.6915	0.0067	IIRDHGDMTNRK
8.5	1535.7072	-0.0090	EEARAMGMEDILR
8.0	1533.6854	2.0128	EASLLSRWRTR
7.3	1535.6885	0.0097	DTAIESSVVTKVK

Spectrum No: 143; Query: 765; Rank: 1

Peptide View

MS/MS Fragmentation of **KETESEAEDDNLDDLER**
Found in **IPI00779470**, Tax_Id=10116 Gene_Symbol=Srrm1_predicted 106 kDa protein

Match to Query 765: 2166.790908 from(1084.402730,2+)
Title: 091127RatKid_SCX01_13.1900.1900.2.dta
Data file K:\NewmanPaper\Piliang\3SubProteomes\Piliang3SP\mgf5ppm\SCX_3SubProteomes5ppm.mgf

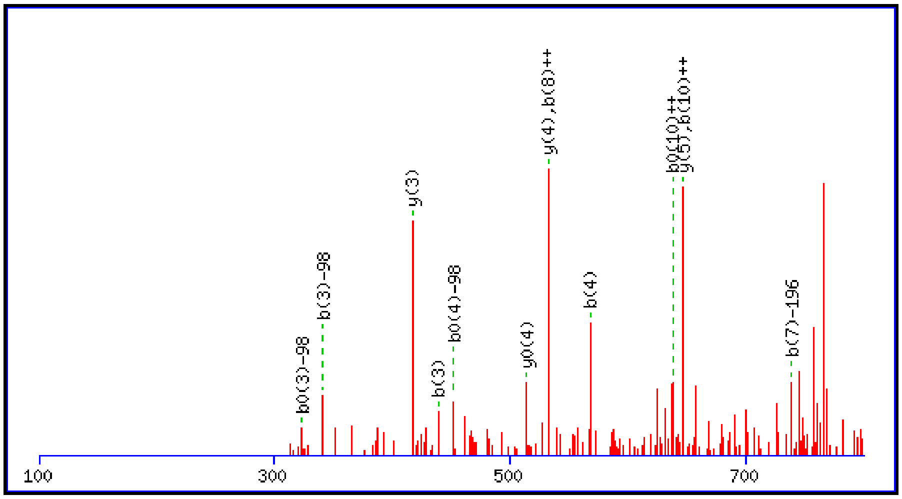

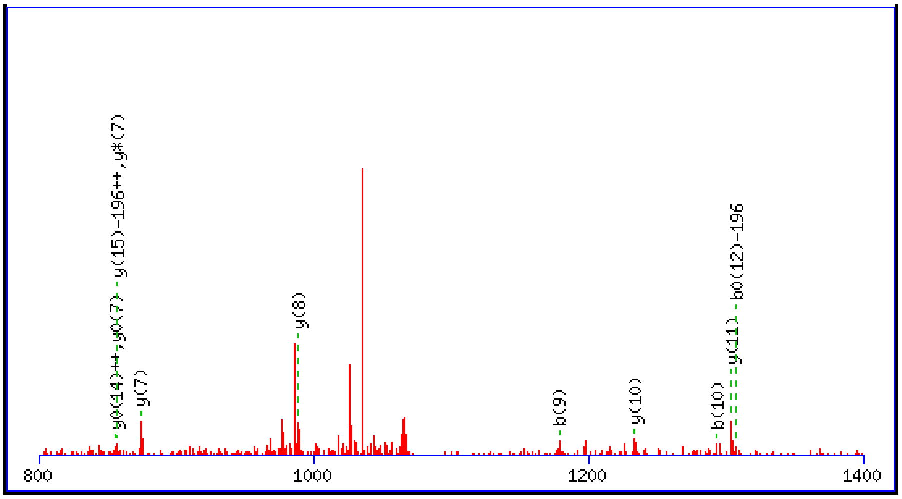

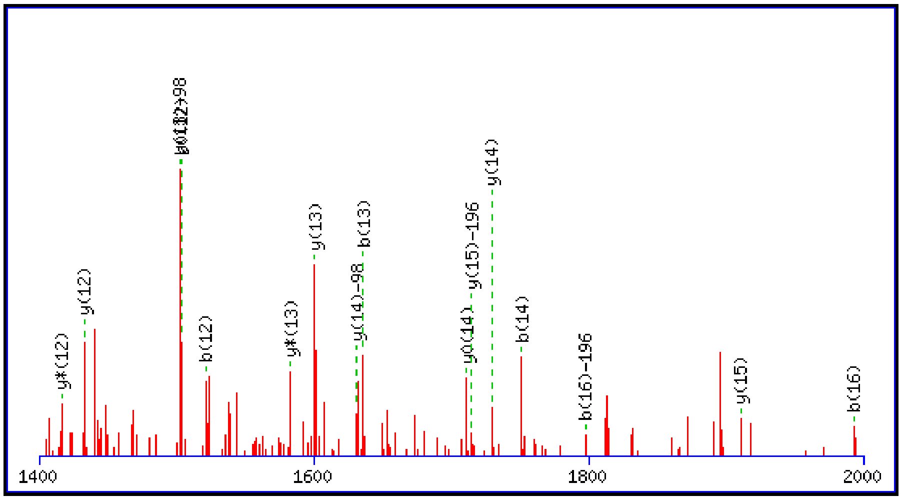

Monoisotopic mass of neutral peptide Mr(calc): 2166.7879
Fixed modifications: Carbamidomethyl (C)
Variable modifications:
T3 : Phospho (ST), with neutral losses 97.9769(shown in table), 0.0000
S5 : Phospho (ST), with neutral losses 97.9769(shown in table), 0.0000
Ions Score: 36 **Expect:** 0.029
Matches (Bold Red): 40/296 fragment ions using 91 most intense peaks

#	b	b^{++}	b*	b^{*++}	b^0	b^{0++}	Seq.	y	y^{++}	y*	y^{*++}	y^0	y^{0++}	#
1	129.1022	65.0548	112.0757	56.5415			K							17
2	258.1448	129.5761	241.1183	121.0628	240.1343	120.5708	E	1843.7464	922.3768	1826.7199	913.8636	1825.7359	913.3716	16
3	**341.1819**	171.0946	324.1554	162.5813	**323.1714**	162.0893	T	**1714.7038**	**857.8556**	1697.6773	849.3423	1696.6933	848.8503	15
4	470.2245	235.6159	453.1980	227.1026	**452.2140**	226.6106	E	**1631.6667**	816.3370	1614.6402	807.8237	1613.6562	807.3317	14
5	539.2460	270.1266	522.2194	261.6134	521.2354	261.1213	S	**1502.6241**	751.8157	1485.5976	743.3024	1484.6136	742.8104	13
6	668.2886	334.6479	651.2620	326.1347	650.2780	325.6426	E	**1433.6027**	717.3050	**1416.5761**	708.7917	1415.5921	708.2997	12
7	**739.3257**	370.1665	722.2991	361.6532	721.3151	361.1612	A	**1304.5601**	652.7837	1287.5335	644.2704	1286.5495	643.7784	11
8	868.3683	434.6878	851.3417	426.1745	850.3577	425.6825	E	**1233.5230**	617.2651	1216.4964	608.7518	1215.5124	608.2598	10
9	983.3952	492.2013	966.3687	483.6880	965.3847	483.1960	D	1104.4804	552.7438	1087.4538	544.2305	1086.4698	543.7385	9
10	1098.4222	549.7147	1081.3956	541.2015	1080.4116	540.7094	D	**989.4534**	495.2304	972.4269	486.7171	971.4429	486.2251	8
11	1212.4651	606.7362	1195.4386	598.2229	1194.4545	597.7309	N	**874.4265**	437.7169	**857.3999**	429.2036	**856.4159**	428.7116	7
12	1325.5492	663.2782	1308.5226	654.7649	**1307.5386**	654.2729	L	760.3836	380.6954	743.3570	372.1821	742.3730	371.6901	6
13	1440.5761	720.7917	1423.5496	712.2784	1422.5655	711.7864	D	**647.2995**	324.1534	630.2729	315.6401	629.2889	315.1481	5
14	1555.6031	778.3052	1538.5765	769.7919	1537.5925	769.2999	D	**532.2726**	266.6399	515.2460	258.1266	**514.2620**	257.6346	4
15	1668.6871	834.8472	1651.6606	826.3339	1650.6766	825.8419	L	**417.2456**	209.1264	400.2191	200.6132	399.2350	200.1212	3
16	**1797.7297**	899.3685	1780.7032	890.8552	1779.7191	890.3632	E	304.1615	152.5844	287.1350	144.0711	286.1510	143.5791	2
17							R	175.1190	88.0631	158.0924	79.5498			1

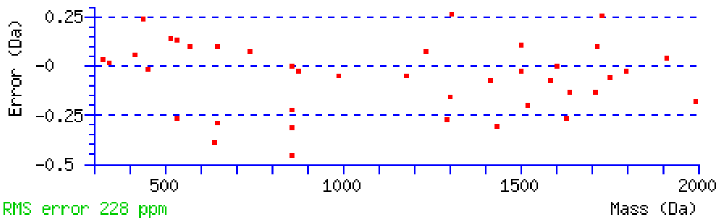

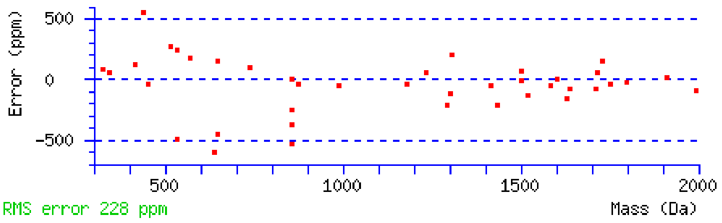

All matches to this query

Score	Mr(calc):	Delta	Sequence
36.3	2166.7879	0.0031	KETESEAEDDNLDDLER

Spectrum No: 144; Query: 317; Rank: 1

Peptide View

MS/MS Fragmentation of **ALDIDSDEEPEPK**
Found in **IPI00367996**, Tax_Id=10116 Gene_Symbol=RGD1562559_predicted similar to Src homology 3 domain-containing guanine nucleotide exchange factor

Match to Query 317: 1536.624928 from(769.319740,2+)
Title: 091127RatKid_SCX01_12.1623.1623.2.dta
Data file K:\NewmanPaper\Piliang\3SubProteomes\Piliang3SP\mgf5ppm\SCX_3SubProteomes5ppm.mgf

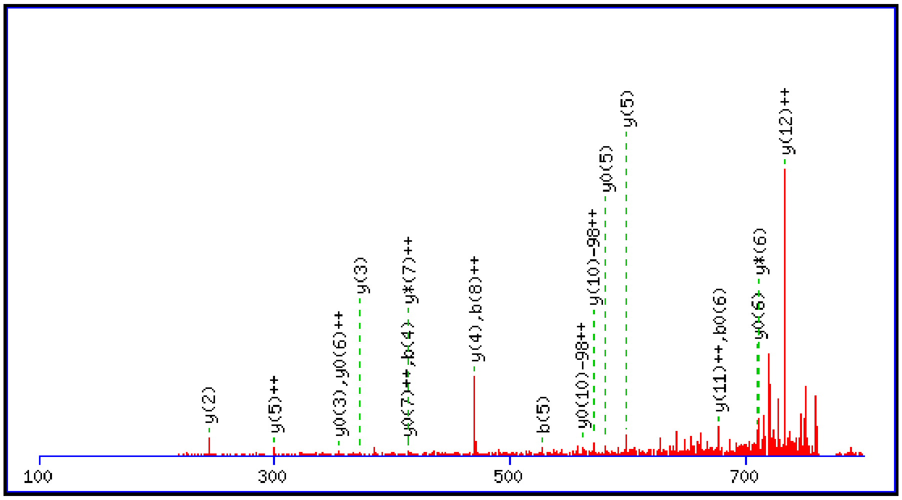

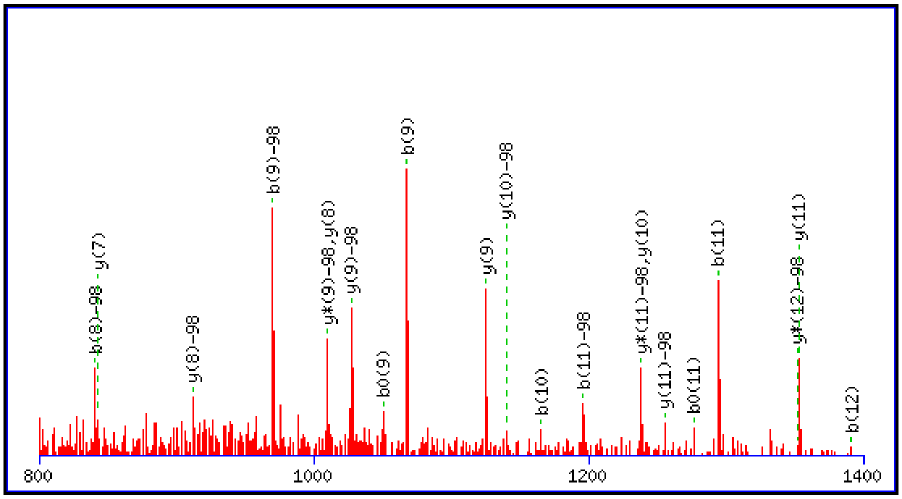

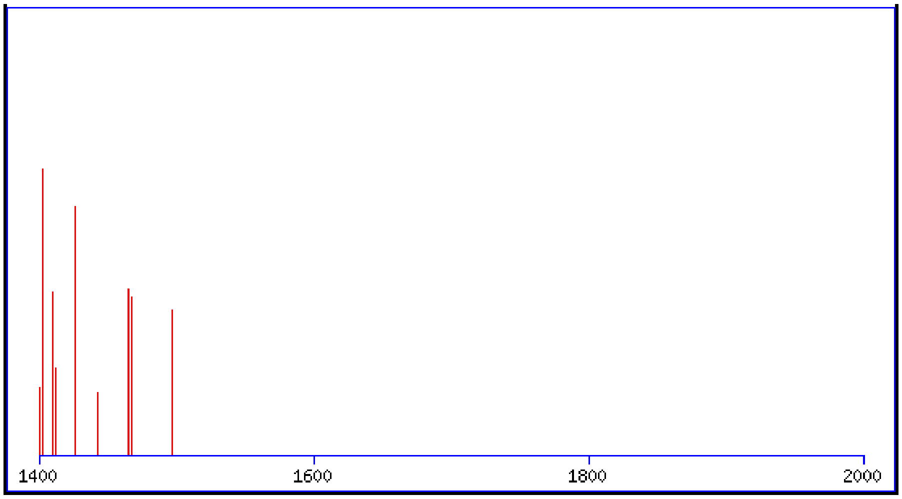

Monoisotopic mass of neutral peptide Mr(calc): 1536.6232
Fixed modifications: Carbamidomethyl (C)
Variable modifications:
S6 : Phospho (ST), with neutral losses 97.9769(shown in table), 0.0000
Ions Score: 36 **Expect:** 0.031
Matches (Bold Red): 42/170 fragment ions using 90 most intense peaks

#	b	b^{++}	b^{0}	b^{0++}	Seq.	y	y^{++}	y^{*}	y^{*++}	y^{0}	y^{0++}	#
1	72.0444	36.5258			A							13
2	185.1285	93.0679			L	1368.6165	684.8119	**1351.5900**	676.2986	1350.6060	675.8066	12
3	**300.1554**	150.5813	282.1448	141.5761	D	**1255.5325**	628.2699	**1238.5059**	619.7566	1237.5219	619.2646	11
4	**413.2395**	207.1234	395.2289	198.1181	I	**1140.5055**	**570.7564**	1123.4790	562.2431	1122.4950	**561.7511**	10
5	**528.2664**	264.6368	510.2558	255.6316	D	**1027.4215**	514.2144	**1010.3949**	505.7011	1009.4109	505.2091	9
6	597.2879	299.1476	579.2773	290.1423	S	**912.3945**	456.7009	895.3680	448.1876	894.3839	447.6956	8
7	712.3148	356.6610	694.3042	347.6558	D	**843.3731**	422.1902	826.3465	**413.6769**	825.3625	**413.1849**	7
8	**841.3574**	421.1823	823.3468	412.1771	E	728.3461	364.6767	**711.3196**	356.1634	**710.3355**	**355.6714**	6
9	**970.4000**	485.7036	952.3894	476.6983	E	**599.3035**	**300.1554**	582.2770	291.6421	**581.2930**	291.1501	5
10	1067.4528	534.2300	1049.4422	525.2247	P	**470.2609**	235.6341	453.2344	227.1208	452.2504	226.6288	4
11	**1196.4953**	598.7513	1178.4848	589.7460	E	**373.2082**	187.1077	356.1816	178.5944	**355.1976**	178.1024	3
12	1293.5481	647.2777	1275.5375	638.2724	P	**244.1656**	122.5864	227.1390	114.0731			2
13					K	147.1128	74.0600	130.0863	65.5468			1

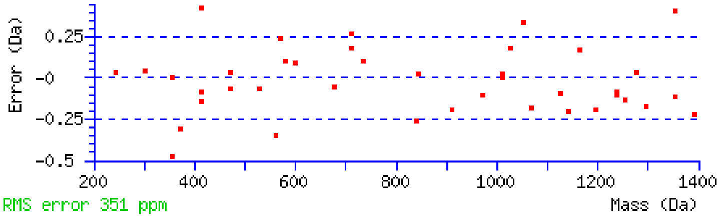

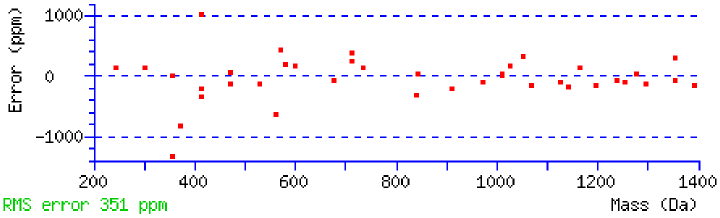

All matches to this query

Score	Mr(calc):	Delta	Sequence
36.2	1536.6232	0.0017	ALDIDSDEEPEPK
7.4	1536.6262	-0.0013	NKPLLSASDFER
3.5	1536.6123	0.0126	QNGLTYDRAPSR

Spectrum No: 145; Query: 523; Rank: 1

Peptide View

MS/MS Fragmentation of **FQEQECPPSPEPTR**
Found in **IPI00190290**, Tax_Id=10116 Gene_Symbol=Rras2 Related RAS viral (R-ras) oncogene homolog 2

Match to Query 523: 1780.718168 from(891.366360,2+)
Title: 091127RatKid_SCX01_12.1055.1055.2.dta
Data file K:\NewmanPaper\Piliang\3SubProteomes\Piliang3SP\mgf5ppm\SCX_3SubProteomes5ppm.mgf

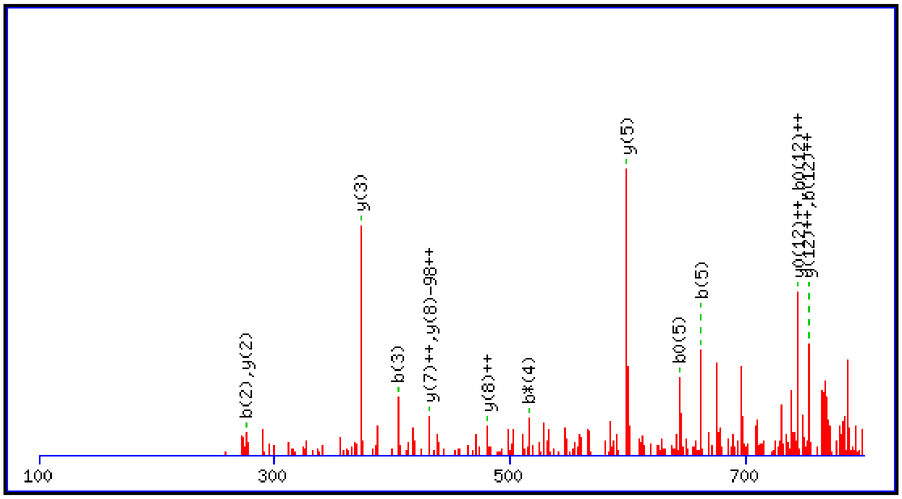

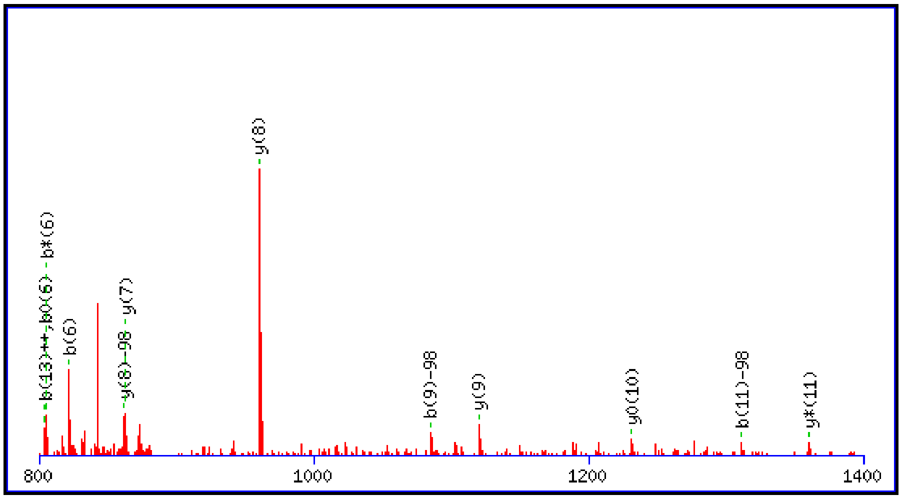

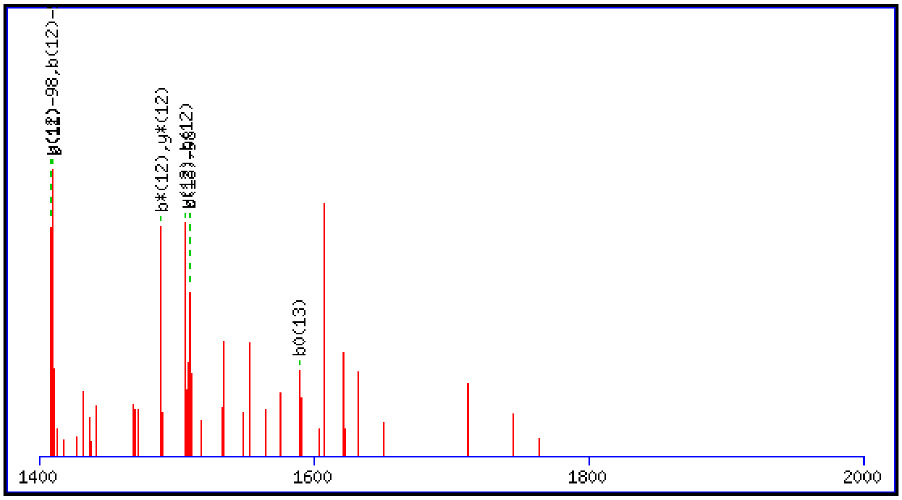

Monoisotopic mass of neutral peptide Mr(calc): 1780.7128
Fixed modifications: Carbamidomethyl (C)
Variable modifications:
S9 : Phospho (ST), with neutral losses 97.9769(shown in table), 0.0000
Ions Score: 36 **Expect:** 0.04
Matches (Bold Red): 36/226 fragment ions using 52 most intense peaks

#	b	++	b*	++	0	0++	Seq.	y	++	y*	++	0	0++	#

		b		b*	b	b			y		y*	y	y	
1	148.0757	74.5415					F							14
2	**276.1343**	138.5708	259.1077	130.0575			Q	1536.6747	768.8410	1519.6482	760.3277	1518.6642	759.8357	13
3	**405.1769**	203.0921	388.1503	194.5788	387.1663	194.0868	E	**1408.6161**	704.8117	1391.5896	696.2984	1390.6056	695.8064	12
4	533.2354	267.1214	**516.2089**	258.6081	515.2249	258.1161	Q	1279.5736	640.2904	1262.5470	631.7771	1261.5630	631.2851	11
5	**662.2780**	331.6427	645.2515	323.1294	**644.2675**	322.6374	E	1151.5150	576.2611	1134.4884	567.7479	1133.5044	567.2558	10
6	**822.3087**	411.6580	**805.2821**	403.1447	**804.2981**	402.6527	C	1022.4724	511.7398	1005.4458	503.2266	1004.4618	502.7345	9
7	919.3614	460.1844	902.3349	451.6711	901.3509	451.1791	P	**862.4417**	**431.7245**	845.4152	423.2112	844.4312	422.7192	8
8	1016.4142	508.7107	999.3877	500.1975	998.4036	499.7055	P	765.3890	383.1981	748.3624	374.6848	747.3784	374.1928	7
9	**1085.4357**	543.2215	1068.4091	534.7082	1067.4251	534.2162	S	668.3362	334.6717	651.3097	326.1585	650.3256	325.6665	6
10	1182.4884	591.7479	1165.4619	583.2346	1164.4779	582.7426	P	**599.3148**	300.1610	582.2882	291.6477	581.3042	291.1557	5
11	**1311.5310**	656.2691	1294.5045	647.7559	1293.5205	647.2639	E	502.2620	251.6346	485.2354	243.1214	484.2514	242.6293	4
12	**1408.5838**	704.7955	1391.5572	696.2823	1390.5732	695.7902	P	**373.2194**	187.1133	356.1928	178.6001	355.2088	178.1081	3
13	**1509.6315**	755.3194	1492.6049	746.8061	1491.6209	746.3141	T	**276.1666**	138.5870	259.1401	130.0737	258.1561	129.5817	2
14							R	175.1190	88.0631	158.0924	79.5498			1

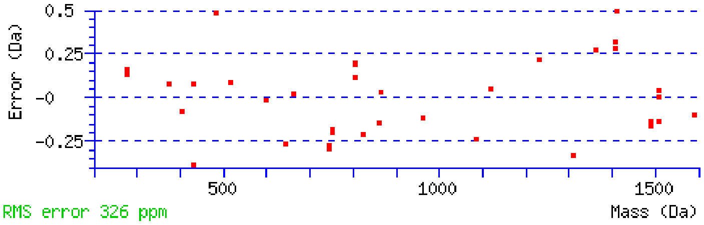

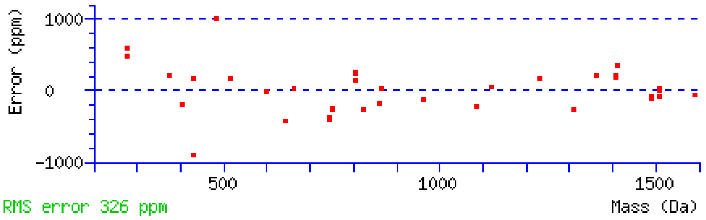

All matches to this query

Score	Mr(calc):	Delta	Sequence
35.9	1780.7128	0.0054	FQEQECPPSPEPTR
20.6	1780.7128	0.0054	FQEQECPPSPEPTR
12.9	1780.7096	0.0086	ILCCNCVSDFRTR
7.9	1780.7096	0.0086	ILCCNCVSDFRTR
1.9	1780.7263	-0.0081	FLQSAEFFNYTVR
1.8	1780.7005	0.0177	FQHVMSITTRNNR
0.8	1779.7012	1.0169	KSRGDVCGQVSQGDK
0.6	1780.7057	0.0125	MQNNSAENETAEGEEK
0.6	1779.7135	1.0047	AAEPPPCDVSTGQESR
0.1	1780.7057	0.0125	MQNNSAENETAEGEEK

Spectrum No: 146; Query: 791; Rank: 1

Peptide View

MS/MS Fragmentation of **KGTGDCSDEEVDGKADGADAK**
Found in **IPI00209113**, Tax_Id=10116 Gene_Symbol=Myh9 Myosin-9

Match to Query 791: 2203.858092 from(735.626640,3+)
Title: 091127RatKid_SCX01_32.224.224.3.dta
Data file K:\NewmanPaper\Piliang\3SubProteomes\Piliang3SP\mgf5ppm\SCX_3SubProteomes5ppm.mgf

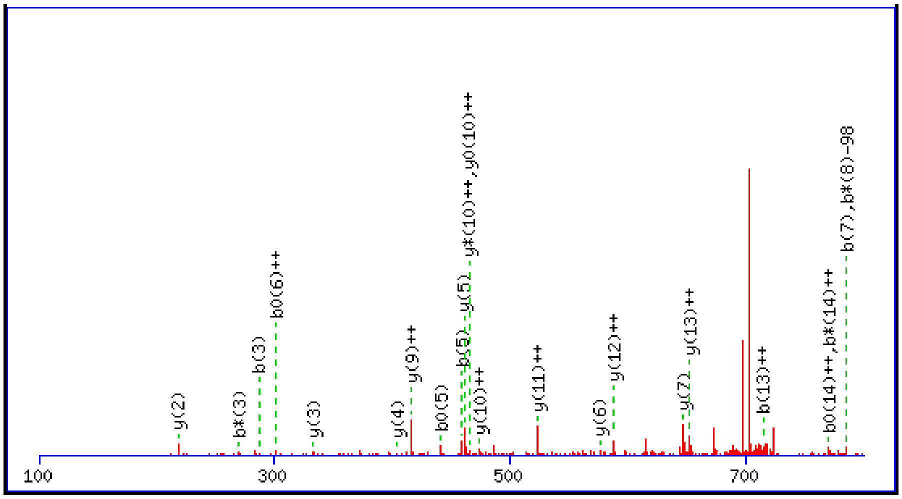

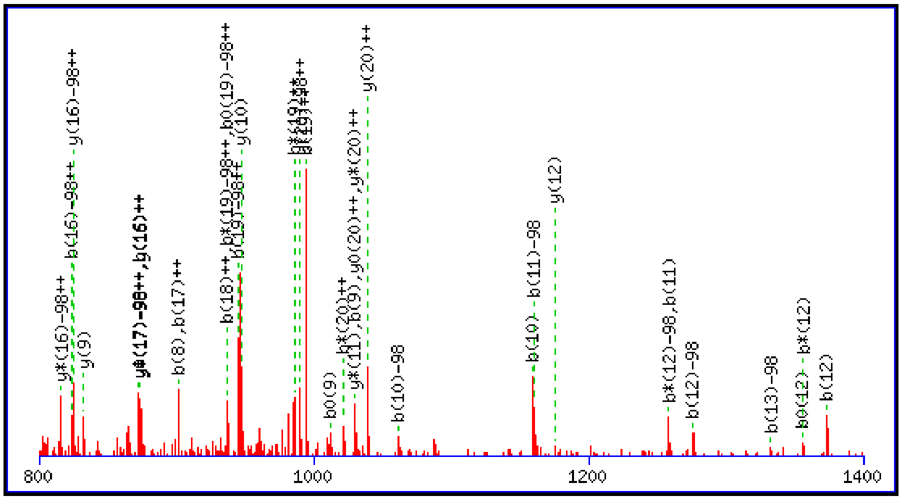

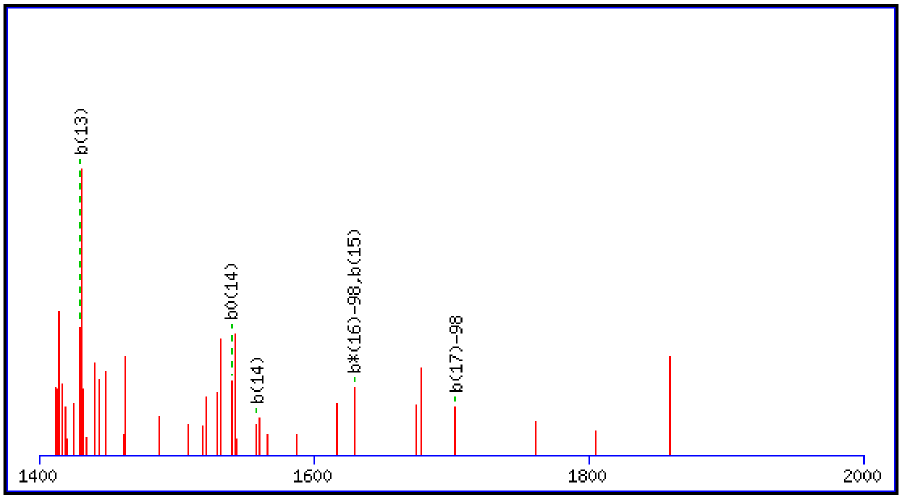

Monoisotopic mass of neutral peptide Mr(calc): 2203.8577
Fixed modifications: Carbamidomethyl (C)
Variable modifications:
S7 : Phospho (ST), with neutral losses 0.0000(shown in table), 97.9769
Ions Score: 36 **Expect:** 0.048
Matches (Bold Red): 66/352 fragment ions using 127 most intense peaks

#	b	b^{++}	b*	b^{*++}	b^0	b^{0++}	Seq.	y	y^{++}	y*	y^{*++}	y^0	y^{0++}	#
1	129.1022	65.0548	112.0757	56.5415			K							21
2	186.1237	93.5655	169.0972	85.0522			G	2076.7700	**1038.8886**	2059.7434	**1030.3753**	2058.7594	**1029.8833**	20
3	**287.1714**	144.0893	**270.1448**	135.5761	269.1608	135.0840	T	2019.7485	1010.3779	2002.7219	1001.8646	2001.7379	1001.3726	19
4	344.1928	172.6001	327.1663	164.0868	326.1823	163.5948	G	1918.7008	959.8540	1901.6743	951.3408	1900.6903	950.8488	18
5	**459.2198**	230.1135	442.1932	221.6003	**441.2092**	221.1083	D	1861.6794	931.3433	1844.6528	922.8300	1843.6688	922.3380	17

6	619.2504	310.1289	602.2239	301.6156	601.2399	301.1236	C	1746.6524	873.8298	1729.6259	865.3166	1728.6418	864.8246	16
7	786.2488	393.6280	769.2222	385.1148	768.2382	384.6228	S	1586.6218	793.8145	1569.5952	785.3012	1568.6112	784.8092	15
8	901.2757	451.1415	884.2492	442.6282	883.2652	442.1362	D	1419.6234	710.3153	1402.5969	701.8021	1401.6128	701.3101	14
9	1030.3183	515.6628	1013.2918	507.1495	1012.3078	506.6575	E	1304.5965	652.8019	1287.5699	644.2886	1286.5859	643.7966	13
10	1159.3609	580.1841	1142.3344	571.6708	1141.3504	571.1788	E	1175.5539	588.2806	1158.5273	579.7673	1157.5433	579.2753	12
11	1258.4293	629.7183	1241.4028	621.2050	1240.4188	620.7130	V	1046.5113	523.7593	1029.4847	515.2460	1028.5007	514.7540	11
12	1373.4563	687.2318	1356.4297	678.7185	1355.4457	678.2265	D	947.4429	474.2251	930.4163	465.7118	929.4323	465.2198	10
13	1430.4777	715.7425	1413.4512	707.2292	1412.4672	706.7372	G	832.4159	416.7116	815.3894	408.1983	814.4054	407.7063	9
14	1558.5727	779.7900	1541.5462	771.2767	1540.5621	770.7847	K	775.3945	388.2009	758.3679	379.6876	757.3839	379.1956	8
15	1629.6098	815.3085	1612.5833	806.7953	1611.5993	806.3033	A	647.2995	324.1534	630.2729	315.6401	629.2889	315.1481	7
16	1744.6368	872.8220	1727.6102	864.3087	1726.6262	863.8167	D	576.2624	288.6348	559.2358	280.1216	558.2518	279.6295	6
17	1801.6582	901.3328	1784.6317	892.8195	1783.6477	892.3275	G	461.2354	231.1214	444.2089	222.6081	443.2249	222.1161	5
18	1872.6953	936.8513	1855.6688	928.3380	1854.6848	927.8460	A	404.2140	202.6106	387.1874	194.0974	386.2034	193.6053	4
19	1987.7223	994.3648	1970.6957	985.8515	1969.7117	985.3595	D	333.1769	167.0921	316.1503	158.5788	315.1663	158.0868	3
20	2058.7594	1029.8833	2041.7328	1021.3701	2040.7488	1020.8781	A	218.1499	109.5786	201.1234	101.0653			2
21							K	147.1128	74.0600	130.0863	65.5468			1

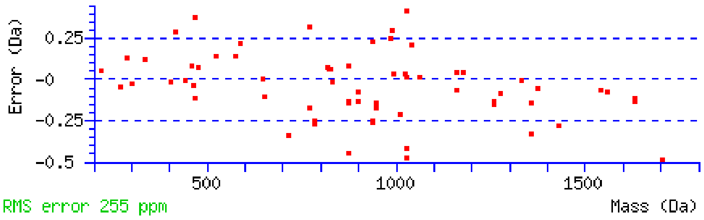

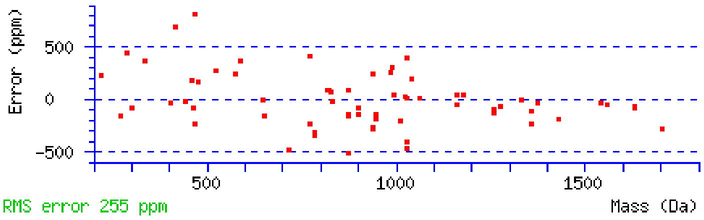

All matches to this query

Score	Mr(calc):	Delta	Sequence
35.8	2203.8577	0.0004	KGTGDCSDEEVDGKADGADAK
25.0	2203.8577	0.0004	KGTGDCSDEEVDGKADGADAK
10.5	2203.8680	-0.0099	DRKMVGDMNGAQTYASTAK
9.5	2203.8680	-0.0099	DRKMVGDMNGAQTYASTAK
7.7	2201.8564	2.0017	FGQMLGSNMTEFHSQISK
7.3	2203.8680	-0.0099	DRKMVGDMNGAQTYASTAK
6.6	2203.8680	-0.0099	DRKMVGDMNGAQTYASTAK
5.8	2203.8680	-0.0099	DRKMVGDMNGAQTYASTAK
4.4	2201.8564	2.0017	FGQMLGSNMTEFHSQISK
4.2	2203.8680	-0.0099	DRKMVGDMNGAQTYASTAK

Spectrum No: 147; Query: 328; Rank: 1

Peptide View

MS/MS Fragmentation of **YVISDEEEEEDD**
Found in **IPI00471640**, Tax_Id=10116 Gene_Symbol=Leo1 RNA polymerase-associated protein LEO1

Match to Query 328: 1550.520928 from(776.267740,2+)
Title: 091127RatKid_SCX01_02.1962.1962.2.dta
Data file K:\NewmanPaper\Piliang\3SubProteomes\Piliang3SP\mgf5ppm\SCX_3SubProteomes5ppm.mgf

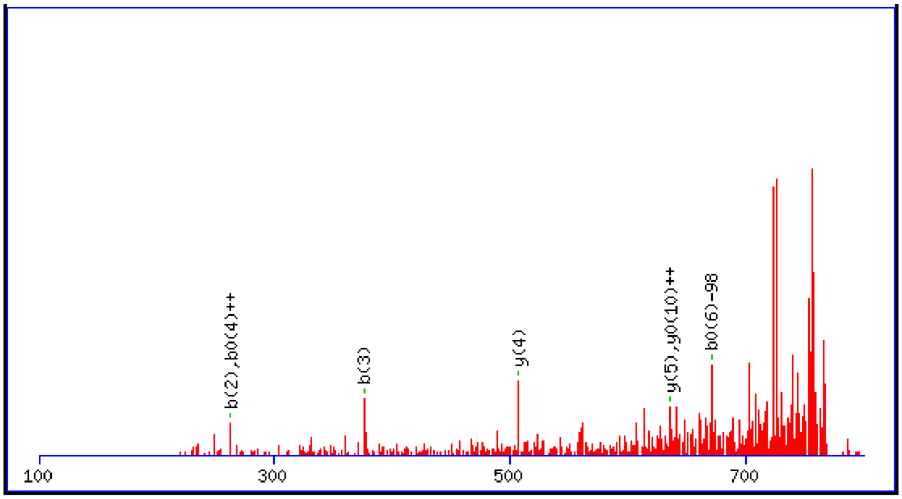

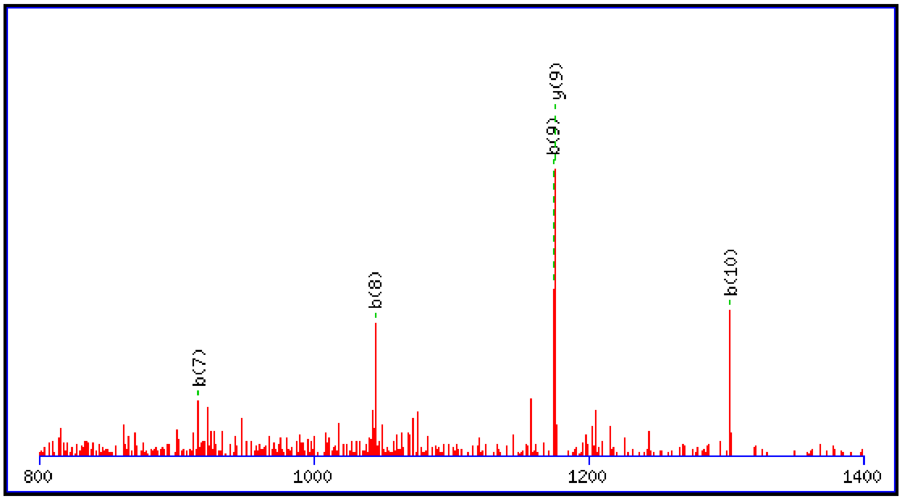

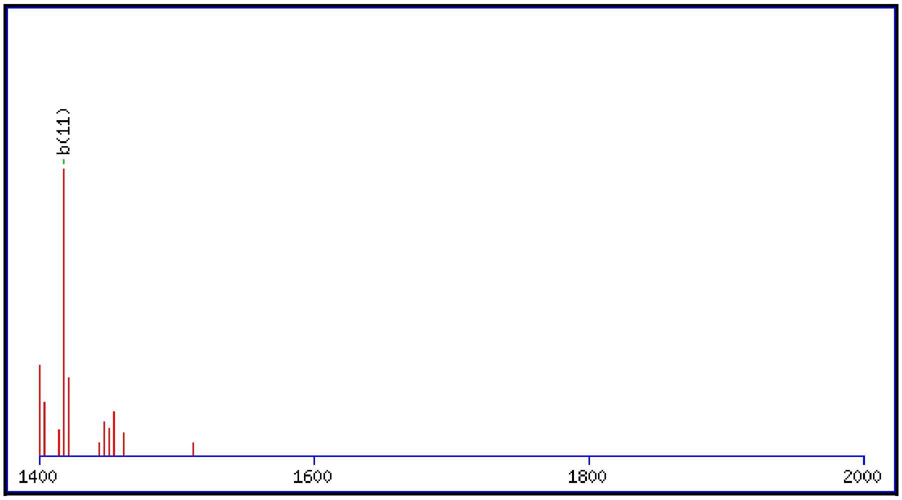

Monoisotopic mass of neutral peptide Mr(calc): 1550.5185
Fixed modifications: Carbamidomethyl (C)
Variable modifications:
S4 : Phospho (ST), with neutral losses 0.0000(shown in table), 97.9769
Ions Score: 34 **Expect:** 0.017
Matches (Bold Red): 13/126 fragment ions using 27 most intense peaks

#	b	b^{++}	b^0	b^{0++}	Seq.	y	y^{++}	y^0	y^{0++}	#
1	164.0706	82.5389			Y					12
2	**263.1390**	132.0731			V	1388.4625	694.7349	1370.4519	685.7296	11
3	**376.2231**	188.6152			I	1289.3941	645.2007	1271.3835	**636.1954**	10
4	543.2214	272.1144	525.2109	**263.1091**	S	**1176.3100**	588.6586	1158.2994	579.6533	9
5	658.2484	329.6278	640.2378	320.6225	D	1009.3116	505.1595	991.3011	496.1542	8

**6**	787.2910	394.1491	769.2804	385.1438	**E**	894.2847	447.6460	876.2741	438.6407	**7**
**7**	**916.3336**	458.6704	898.3230	449.6651	**E**	765.2421	383.1247	747.2315	374.1194	**6**
**8**	**1045.3762**	523.1917	1027.3656	514.1864	**E**	**636.1995**	318.6034	618.1889	309.5981	**5**
**9**	**1174.4188**	587.7130	1156.4082	578.7077	**E**	**507.1569**	254.0821	489.1463	245.0768	**4**
**10**	**1303.4613**	652.2343	1285.4508	643.2290	**E**	378.1143	189.5608	360.1038	180.5555	**3**
**11**	**1418.4883**	709.7478	1400.4777	700.7425	**D**	249.0717	125.0395	231.0612	116.0342	**2**
**12**					**D**	134.0448	67.5260	116.0342	58.5207	**1**

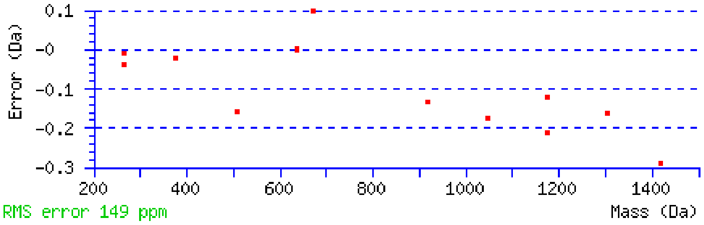

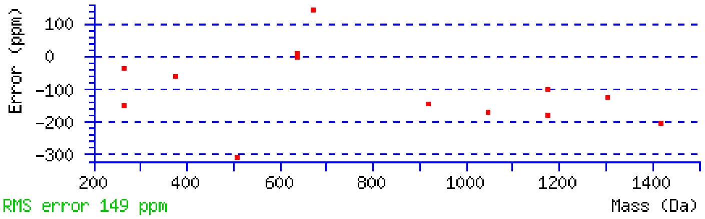

All matches to this query

Score	Mr(calc):	Delta	Sequence
34.4	1550.5185	0.0024	YVISDEEEEEDD
13.3	1550.5185	0.0024	YVISDEEEEEDD

Spectrum No: 148; Query: 7; Rank: 1

Peptide View

MS/MS Fragmentation of **MELGTPLR**
Found in **IPI00368692**, Tax_Id=10116 Gene_Symbol=Srrm2_predicted similar to serine/arginine repetitive matrix 2

Match to Query 7: 995.450768 from(498.732660,2+)
Title: 091127RatKid_SCX01_13.1799.1799.2.dta
Data file K:\NewmanPaper\Piliang\3SubProteomes\Piliang3SP\mgf5ppm\SCX_3SubProteomes5ppm.mgf

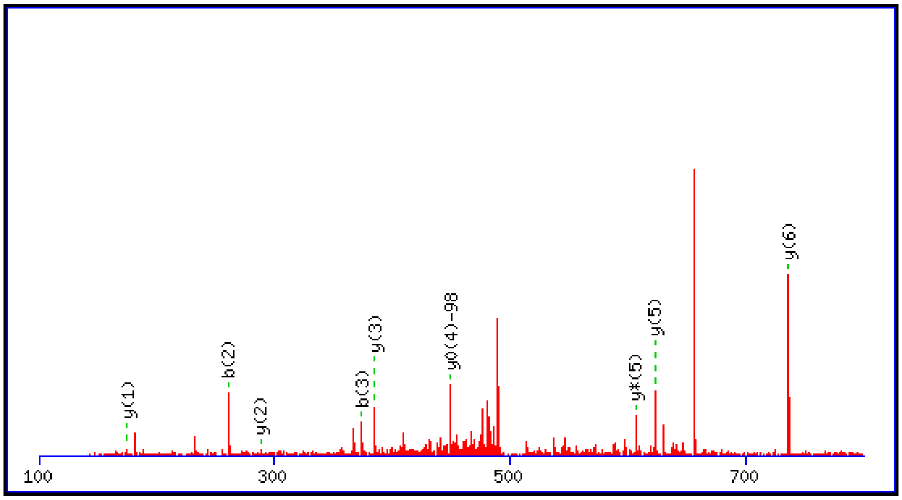

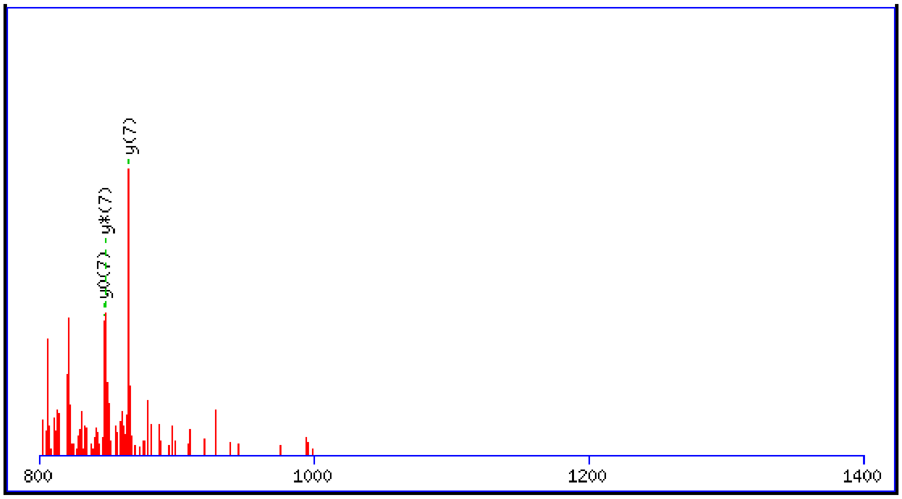

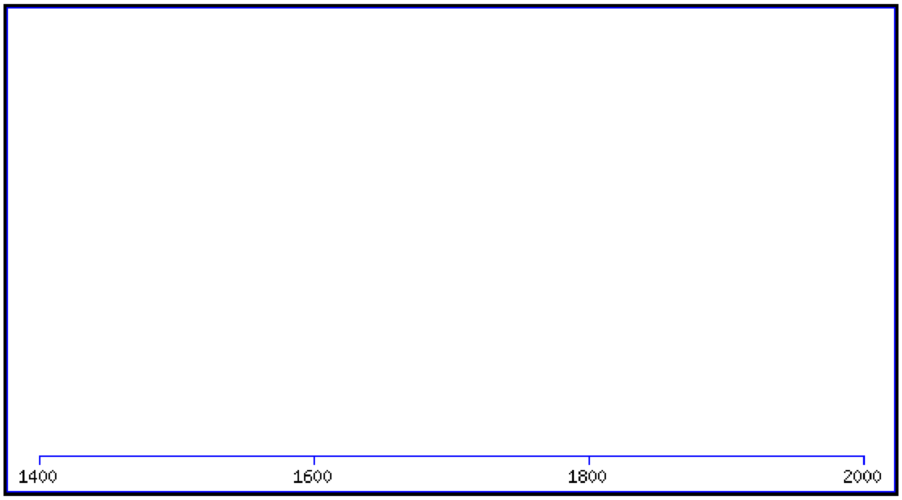

Monoisotopic mass of neutral peptide Mr(calc): 995.4511
Fixed modifications: Carbamidomethyl (C)
Variable modifications:
T5 : Phospho (ST), with neutral losses 0.0000(shown in table), 97.9769
Ions Score: 33 **Expect:** 0.036
Matches (Bold Red): 12/98 fragment ions using 42 most intense peaks

#	b	b++	b0	b0++	Seq.	y	y++	y*	y*++	y0	y0++	#
1	132.0478	66.5275			M							8
2	**261.0904**	131.0488	243.0798	122.0435	E	**865.4179**	433.2126	**848.3914**	424.6993	**847.4073**	424.2073	7
3	**374.1744**	187.5908	356.1639	178.5856	L	**736.3753**	368.6913	719.3488	360.1780	718.3647	359.6860	6
4	431.1959	216.1016	413.1853	207.0963	G	**623.2912**	312.1493	**606.2647**	303.6360	605.2807	303.1440	5
5	612.2099	306.6086	594.1993	297.6033	T	566.2698	283.6385	549.2432	275.1253	548.2592	274.6332	4
6	709.2626	355.1350	691.2521	346.1297	P	**385.2558**	193.1315	368.2292	184.6183			3
7	822.3467	411.6770	804.3361	402.6717	L	**288.2030**	144.6051	271.1765	136.0919			2
8					R	**175.1190**	88.0631	158.0924	79.5498			1

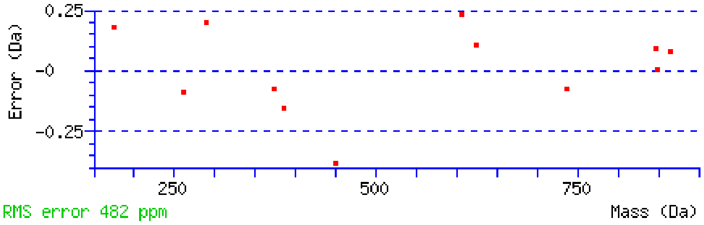

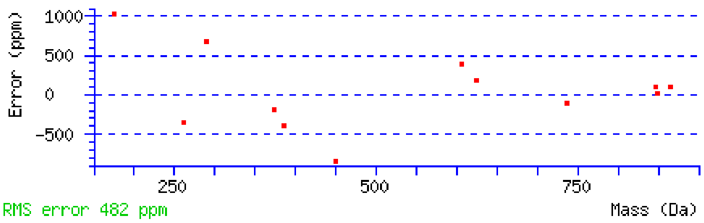

All matches to this query

Score	Mr(calc):	Delta	Sequence

33.3	995.4511	-0.0003	MELGTPLR
9.7	995.4528	-0.0020	MEASKQMR
4.9	994.4430	1.0078	MNLDPFNK
4.0	994.4485	1.0023	EIPNSVTR
4.0	993.4437	2.0071	KEMTNDEK
3.5	993.4437	2.0071	KDCTLDNK
3.3	995.4590	-0.0082	FISSVHAR
3.3	995.4590	-0.0082	FISSVHAR
3.2	994.4463	1.0044	NMMIDNLK
2.6	994.4509	0.9999	PFQFDDAR

Mascot: http://www.matrixscience.com/
