# Supplementary material for: Simultaneous Analysis of Proteome, Phospho- and Glycoproteome of Rat Kidney Tissue with Electrostatic Repulsion Hydrophilic Interaction Chromatography
Source: PLoS One. 2011 Feb 23;6(2):e16884. doi: 10.1371/journal.pone.0016884 (PMC3044146; doi:10.1371/journal.pone.0016884)
Supplement: Table S7 — Information about proteins with both phosphorylation and N-glycosylation identified in ERLIC04. (DOC) [file pone.0016884.s011.doc]

Table S7. Information about proteins with both phosphorylation and N-glycosylation identified in ERLIC04

| SWISSPROT ID. | Name | Phos_Sites | Glyco_site | Function annotations in SWISS-PROT |
| --- | --- | --- | --- | --- |
| P98158  4660 A | Low-density lipoprotein receptor-related protein 2 | s4624 (new), s4626 (new), s4658 (new), | 159, 178, 259, 299, 340, 462, 657, 865, 1187, 1497, 1551, 1733, 2396, 2488, 2782, 2989, 3127, 3213, 3317, 3566, 3682, 3840, 3980, 4329 (all potential) | Cell Membrane. Acts together with cubilin to mediate HDL endocytosis By similarity. Receptor-mediated uptake of polybasic drugs such as aprotinin, aminoglycosides and polymyxin B. |
| Q66H67  830 A | Cadherin 16 | S504 (new), S823 (new) | 519 (new), 604 (new) | Membrane protein. |
| P50123  945 A | Glutamyl aminopeptidase | s246 (new) | 236, 316, 584, 601, 640, 754, 766, 792 (all potential) | Membrane protein. Appears to have a role in the catabolic pathway of the renin-angiotensin system. Isoform 1 has aminopeptidase activity while isoform 2 does not. |
| P24090  352 A | Alpha-2-HS-glycoprotein | s138 (known) | 99, 156, 176 (all potential) | Extracellular matrix. Could inhibit both insulin-receptor tyrosine kinase activity and insulin-stimulated receptor autophosphorylation and, concomitantly, antagonize the mitogenic effect of the hormone in cultured rat hepatoma cells. |
| P04937  2477 A | Fibronectin | s2475 (by similarity) | 528 (potential), 1006 (potential) | Extracellular matrix. Fibronectins bind cell surfaces and various compounds including collagen, fibrin, heparin, DNA, and actin. Fibronectins are involved in cell adhesion, cell motility, opsonization, wound healing, and maintenance of cell shape. Anastellin binds fibronectin and induces fibril formation. This fibronectin polymer, named superfibronectin, exhibits enhanced adhesive properties. Both anastellin and superfibronectin inhibit tumor growth, angiogenesis and metastasis. |
| Q9WUH8 | Fibrillin-1 | s2703 (New) | 449, 1068, 1582 (all new) | Extracellular matrix. Kidney development (inferred) |
| O70594 | Solute carrier family 22 member 5 | s548 (new) | 57 (potential), 91 (potential) | Membrane protein. Sodium-ion dependent, high affinity carnitine transporter. Involved in the active cellular uptake of carnitine. Transports one sodium ion with one molecule of carnitine. Also transports organic cations such as tetraethylammonium (TEA) without the involvement of sodium. Also Relative uptake activity ratio of carnitine to TEA is 11.3. |
| Q9ERB4  2738 A | Versican core protein | T794 (new) | 216 (new) | Extracellular matrix. May play a role in intercellular signaling and in connecting cells with the extracellular matrix. May take part in the regulation of cell motility, growth and differentiation. Binds hyaluronic acid. Phosphorylation sites are present in the extracelllular medium. |
| Q6AYP2  409 AA | Microfibrillar-associated protein 3-like | s298 (new), s307 (new) | 67 (potential), 111 (potential) | Cell membrane. |
| Q8R520  598 A | Low affinity Na-dependent glucose transporter SGLT2 delta e trans | s547 (new) | 248 (new) | Membrane. |
| Q71MB6  724 A | Solute carrier organic anion transporter family member 4C1 | s16 (new) | 547 (new) | Plasma Membrane. Organic anion transporter, capable of transporting pharmacological substances such as digoxin, ouabain, thyroxine, methotrexate and cAMP. May participate in the regulation of membrane transport of ouabain. Involved in the uptake of the dipeptidyl peptidase-4 inhibitor sitagliptin and hence may play a role in its transport into and out of renal proximal tubule cells. May be involved in the first step of the transport pathway of digoxin and various compounds into the urine in the kidney. May be involved in sperm maturation by enabling directed movement of organic anions and compounds within or between cells. This ion-transporting process is important to maintain the strict epididymal homeostasis necessary for sperm maturation. May have a role in secretory functions since seminal vesicle epithelial cells are assumed to secrete proteins involved in decapacitation by modifying surface proteins to facilitate the acquisition of the ability to fertilize the egg. |
| IPI00371736 | No record in Swissprot | S 335 (new) | 237 (new) | Predicted existence. |
