# Supplementary material for: Simultaneous Analysis of Proteome, Phospho- and Glycoproteome of Rat Kidney Tissue with Electrostatic Repulsion Hydrophilic Interaction Chromatography
Source: PLoS One. 2011 Feb 23;6(2):e16884. doi: 10.1371/journal.pone.0016884 (PMC3044146; doi:10.1371/journal.pone.0016884)
Supplement: Data S1 — Tandem MS spectra of_glycopeptides from ERLIC enrichment. (PDF) [file pone.0016884.s001.pdf]

# Mascot Search Results

Results Generated by a Script Modified from Mascot Pepitde View
by Newman Sze, School of Biological Sciences, Nanyang Technological University

---

Spectrum No: 1; Query: 1525; Rank: 1

---

## Peptide View

MS/MS Fragmentation of **SNSTAGELDALQAEAGSLDK**
Found in **IPI00365542**, Tax_Id=10116 Gene_Symbol=Lamb1_predicted laminin, beta 1

Match to Query 1525: 1976.923288 from(989.468920,2+)
Title: 100101RatKid_NS_deglyco_19.3877.3877.2.dta
Data file K:\NewmanPaper\Piliang\3SubProteomes\Piliang3SP\mgf5ppm\ERLIC_3SubProteomes5ppm.mgf

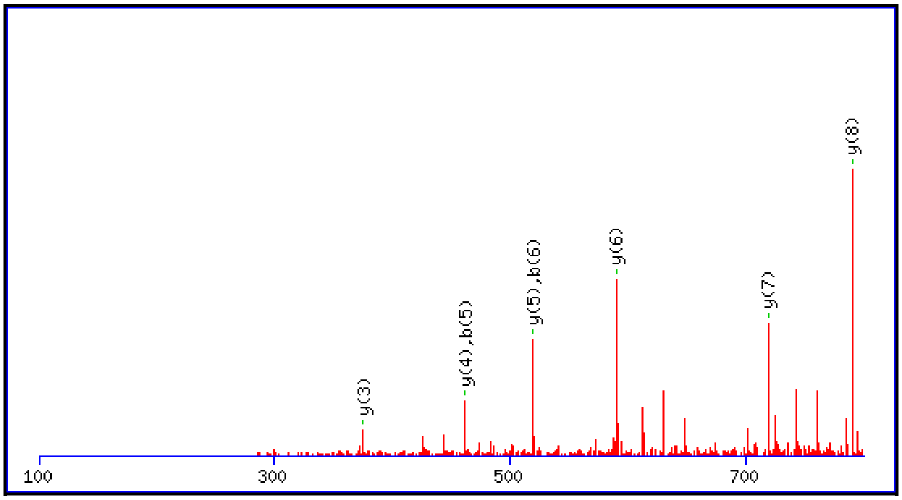


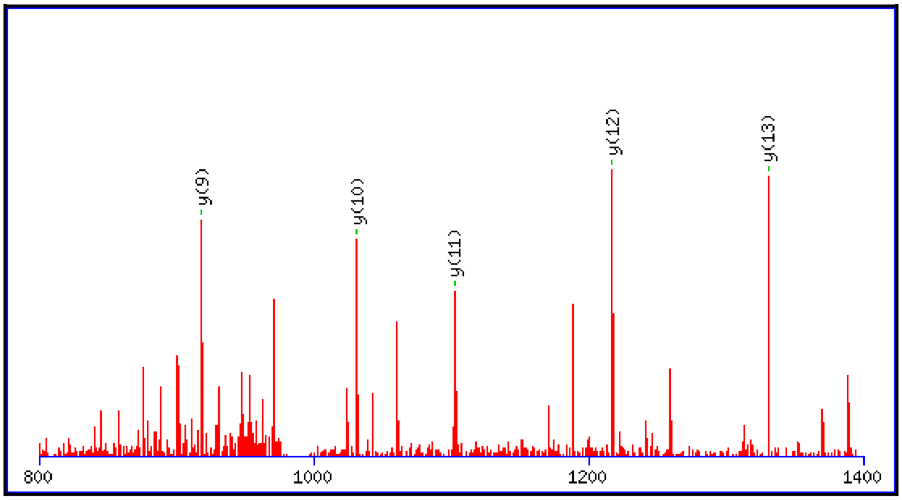

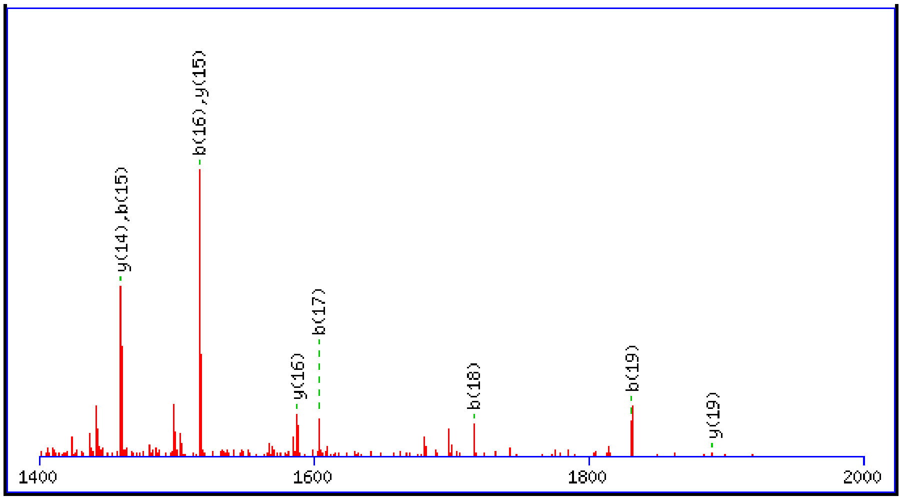


**Monoisotopic mass of neutral peptide Mr(calc):** 1976.9174
**Fixed modifications:** Carbamidomethyl (C)
**Variable modifications:**
**N2** : Deamidated_N (N)
**Ions Score:** 161 **Expect:** 1.7e-014
**Matches (Bold Red):** 22/224 fragment ions using 20 most intense peaks

| # | b | $b^{++}$ | b* | $b^{*++}$ | $b^{0}$ | $b^{0++}$ | Seq. | y | $y^{++}$ | y* | $y^{*++}$ | $y^{0}$ | $y^{0++}$ | # |
|---|---|---|---|---|---|---|---|---|---|---|---|---|---|---|
| 1 | 88.0393 | 44.5233 | | | 70.0287 | 35.5180 | S | | | | | | | 20 |
| 2 | 203.0662 | 102.0368 | 186.0397 | 93.5235 | 185.0557 | 93.0315 | N | **1890.8927** | 945.9500 | 1873.8662 | 937.4367 | 1872.8821 | 936.9447 | 19 |
| 3 | 290.0983 | 145.5528 | 273.0717 | 137.0395 | 272.0877 | 136.5475 | S | 1775.8658 | 888.4365 | 1758.8392 | 879.9232 | 1757.8552 | 879.4312 | 18 |
| 4 | 391.1459 | 196.0766 | 374.1194 | 187.5633 | 373.1354 | 187.0713 | T | 1688.8337 | 844.9205 | 1671.8072 | 836.4072 | 1670.8232 | 835.9152 | 17 |
| 5 | **462.1831** | 231.5952 | 445.1565 | 223.0819 | 444.1725 | 222.5899 | A | **1587.7861** | 794.3967 | 1570.7595 | 785.8834 | 1569.7755 | 785.3914 | 16 |
| 6 | **519.2045** | 260.1059 | 502.1780 | 251.5926 | 501.1940 | 251.1006 | G | **1516.7489** | 758.8781 | 1499.7224 | 750.3648 | 1498.7384 | 749.8728 | 15 |
| 7 | 648.2471 | 324.6272 | 631.2206 | 316.1139 | 630.2366 | 315.6219 | E | **1459.7275** | 730.3674 | 1442.7009 | 721.8541 | 1441.7169 | 721.3621 | 14 |
| 8 | 761.3312 | 381.1692 | 744.3046 | 372.6560 | 743.3206 | 372.1639 | L | **1330.6849** | 665.8461 | 1313.6583 | 657.3328 | 1312.6743 | 656.8408 | 13 |
| 9 | 876.3581 | 438.6827 | 859.3316 | 430.1694 | 858.3476 | 429.6774 | D | **1217.6008** | 609.3040 | 1200.5743 | 600.7908 | 1199.5903 | 600.2988 | 12 |
| 10 | 947.3952 | 474.2013 | 930.3687 | 465.6880 | 929.3847 | 465.1960 | A | **1102.5739** | 551.7906 | 1085.5473 | 543.2773 | 1084.5633 | 542.7853 | 11 |
| 11 | 1060.4793 | 530.7433 | 1043.4528 | 522.2300 | 1042.4687 | 521.7380 | L | **1031.5368** | 516.2720 | 1014.5102 | 507.7587 | 1013.5262 | 507.2667 | 10 |
| 12 | 1188.5379 | 594.7726 | 1171.5113 | 586.2593 | 1170.5273 | 585.7673 | Q | **918.4527** | 459.7300 | 901.4262 | 451.2167 | 900.4421 | 450.7247 | 9 |
| 13 | 1259.5750 | 630.2911 | 1242.5484 | 621.7779 | 1241.5644 | 621.2859 | A | **790.3941** | 395.7007 | 773.3676 | 387.1874 | 772.3836 | 386.6954 | 8 |
| 14 | 1388.6176 | 694.8124 | 1371.5910 | 686.2992 | 1370.6070 | 685.8071 | E | **719.3570** | 360.1821 | 702.3305 | 351.6689 | 701.3464 | 351.1769 | 7 |
| 15 | **1459.6547** | 730.3310 | 1442.6282 | 721.8177 | 1441.6441 | 721.3257 | A | **590.3144** | 295.6608 | 573.2879 | 287.1476 | 572.3039 | 286.6556 | 6 |
| 16 | **1516.6762** | 758.8417 | 1499.6496 | 750.3284 | 1498.6656 | 749.8364 | G | **519.2773** | 260.1423 | 502.2508 | 251.6290 | 501.2667 | 251.1370 | 5 |
| 17 | **1603.7082** | 802.3577 | 1586.6816 | 793.8445 | 1585.6976 | 793.3525 | S | **462.2558** | 231.6316 | 445.2293 | 223.1183 | 444.2453 | 222.6263 | 4 |
| 18 | **1716.7923** | 858.8998 | 1699.7657 | 850.3865 | 1698.7817 | 849.8945 | L | **375.2238** | 188.1155 | 358.1973 | 179.6023 | 357.2132 | 179.1103 | 3 |
| 19 | **1831.8192** | 916.4132 | 1814.7927 | 907.9000 | 1813.8086 | 907.4080 | D | 262.1397 | 131.5735 | 245.1132 | 123.0602 | 244.1292 | 122.5682 | 2 |
| 20 | | | | | | | K | 147.1128 | 74.0600 | 130.0863 | 65.5468 | | | 1 |

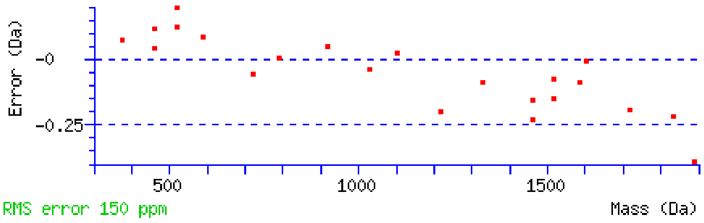


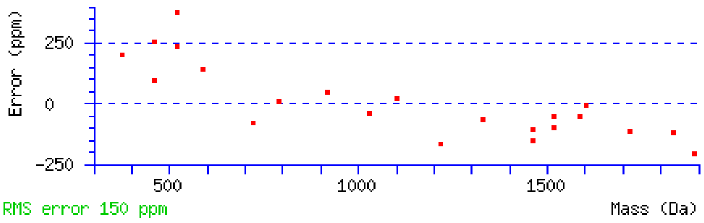


### All matches to this query

| Score | Mr(calc): | Delta | Sequence |
|---|---|---|---|
| 161.4 | 1976.9174 | 0.0059 | SNSTAGELDALQAEAGSLDK |
| | | | |

| | | | |
|---|---|---|---|
| 134.8 | 1975.9334 | 0.9899 | SNSTAGELDALQAEAGSLDK |
| 6.4 | 1976.9327 | -0.0094 | QSLDGFLDETNKENLPR |
| 5.3 | 1976.9035 | 0.0198 | YMRQQCIKQELICK |
| 4.8 | 1976.9206 | 0.0027 | YMVLVPSQLYTETPEK |
| 4.8 | 1976.9206 | 0.0027 | YMVLVPSQLYTETPEK |
| 4.1 | 1976.9196 | 0.0037 | ILMHHIPEQTTQLLK |
| 3.9 | 1976.9206 | 0.0027 | YMVLVPSQLYTETPEK |
| 2.4 | 1974.9201 | 2.0032 | SDWIHDTPVSLGKADVR |
| 2.0 | 1976.9258 | -0.0025 | NPPRWXTAGLAGSLTWR |

---

Spectrum No: 2; Query: 1414; Rank: 1

---

## Peptide View

MS/MS Fragmentation of **IYAGMVSLLDEAVGNVTK**
Found in **IPI00198405**, Tax_Id=10116 Gene_Symbol=Arsb Arylsulfatase B

Match to Query 1414: 1895.951128 from(948.982840,2+)
Title: 091008RatKidney_NH4Format01_23.3772.3772.2.dta
Data file K:\NewmanPaper\Piliang\3SubProteomes\Piliang3SP\mgf5ppm\ERLIC_3SubProteomes5ppm.mgf

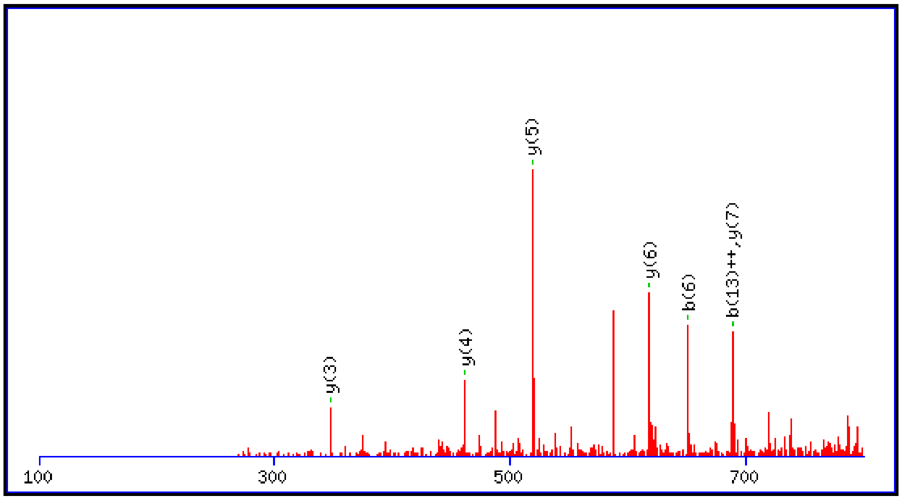


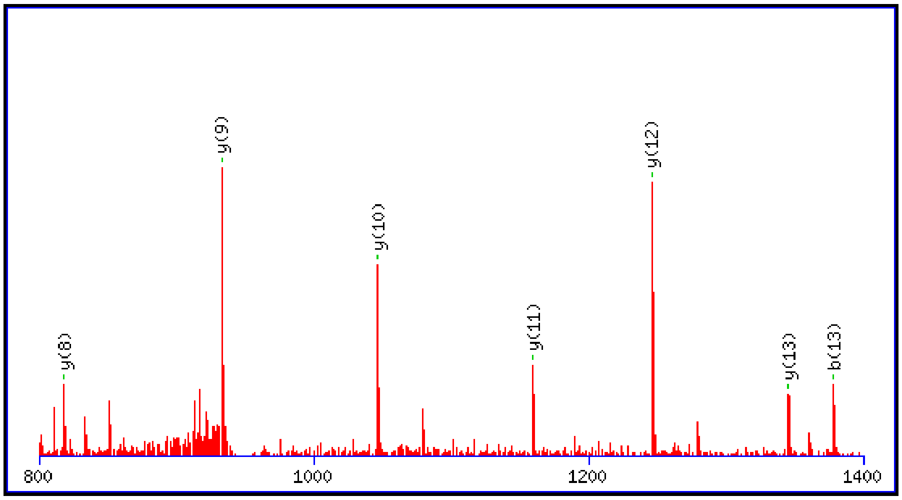

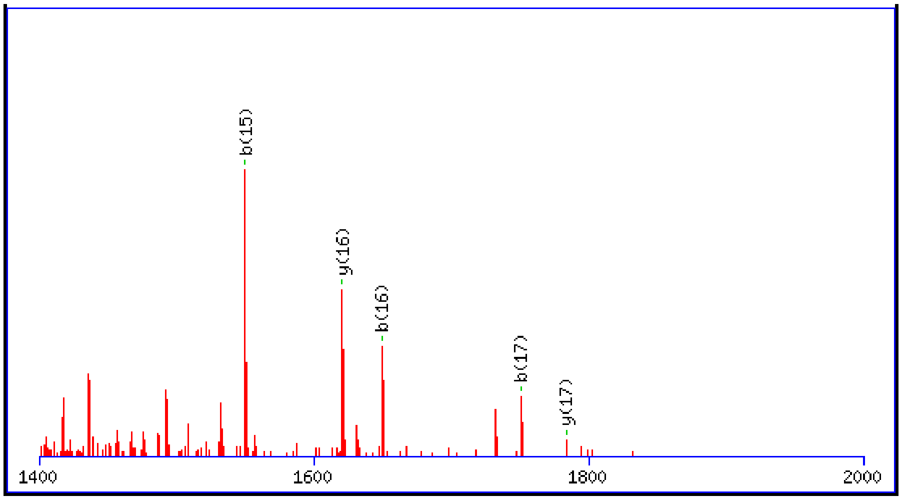


**Monoisotopic mass of neutral peptide Mr(calc):** 1895.9550
**Fixed modifications:** Carbamidomethyl (C)
**Variable modifications:**
**M5** : Oxidation (M)
**N15** : Deamidated_N (N)
**Ions Score:** 145 **Expect:** 6.2e-013
**Matches (Bold Red):** 19/162 fragment ions using 23 most intense peaks

| # | b | $b^{++}$ | $b^{*}$ | $b^{*++}$ | $b^{0}$ | $b^{0++}$ | Seq. | y | $y^{++}$ | $y^{*}$ | $y^{*++}$ | $y^{0}$ | $y^{0++}$ | # |
|---|---|---|---|---|---|---|---|---|---|---|---|---|---|---|
| 1 | 114.0913 | 57.5493 | | | | | I | | | | | | | 18 |
| 2 | 277.1547 | 139.0810 | | | | | Y | **1783.8782** | 892.4428 | 1766.8517 | 883.9295 | 1765.8677 | 883.4375 | 17 |
| 3 | 348.1918 | 174.5995 | | | | | A | **1620.8149** | 810.9111 | 1603.7884 | 802.3978 | 1602.8043 | 801.9058 | 16 |
| 4 | 405.2132 | 203.1103 | | | | | G | 1549.7778 | 775.3925 | 1532.7513 | 766.8793 | 1531.7672 | 766.3873 | 15 |
| 5 | 552.2487 | 276.6280 | | | | | M | 1492.7563 | 746.8818 | 1475.7298 | 738.3685 | 1474.7458 | 737.8765 | 14 |
| 6 | **651.3171** | 326.1622 | | | | | V | **1345.7209** | 673.3641 | 1328.6944 | 664.8508 | 1327.7104 | 664.3588 | 13 |
| 7 | 738.3491 | 369.6782 | | | 720.3385 | 360.6729 | S | **1246.6525** | 623.8299 | 1229.6260 | 615.3166 | 1228.6420 | 614.8246 | 12 |
| 8 | 851.4332 | 426.2202 | | | 833.4226 | 417.2149 | L | **1159.6205** | 580.3139 | 1142.5939 | 571.8006 | 1141.6099 | 571.3086 | 11 |
| 9 | 964.5172 | 482.7622 | | | 946.5067 | 473.7570 | L | **1046.5364** | 523.7719 | 1029.5099 | 515.2586 | 1028.5259 | 514.7666 | 10 |
| 10 | 1079.5442 | 540.2757 | | | 1061.5336 | 531.2704 | D | **933.4524** | 467.2298 | 916.4258 | 458.7165 | 915.4418 | 458.2245 | 9 |
| 11 | 1208.5868 | 604.7970 | | | 1190.5762 | 595.7917 | E | **818.4254** | 409.7163 | 801.3989 | 401.2031 | 800.4149 | 400.7111 | 8 |
| 12 | 1279.6239 | 640.3156 | | | 1261.6133 | 631.3103 | A | **689.3828** | 345.1951 | 672.3563 | 336.6818 | 671.3723 | 336.1898 | 7 |
| 13 | **1378.6923** | **689.8498** | | | 1360.6817 | 680.8445 | V | **618.3457** | 309.6765 | 601.3192 | 301.1632 | 600.3351 | 300.6712 | 6 |
| 14 | 1435.7137 | 718.3605 | | | 1417.7032 | 709.3552 | G | **519.2773** | 260.1423 | 502.2507 | 251.6290 | 501.2667 | 251.1370 | 5 |
| 15 | **1550.7407** | 775.8740 | 1533.7141 | 767.3607 | 1532.7301 | 766.8687 | N | **462.2558** | 231.6316 | 445.2293 | 223.1183 | 444.2453 | 222.6263 | 4 |
| 16 | **1649.8091** | 825.4082 | 1632.7826 | 816.8949 | 1631.7985 | 816.4029 | V | **347.2289** | 174.1181 | 330.2023 | 165.6048 | 329.2183 | 165.1128 | 3 |
| 17 | **1750.8568** | 875.9320 | 1733.8302 | 867.4188 | 1732.8462 | 866.9267 | T | 248.1605 | 124.5839 | 231.1339 | 116.0706 | 230.1499 | 115.5786 | 2 |
| 18 | | | | | | | K | 147.1128 | 74.0600 | 130.0863 | 65.5468 | | | 1 |

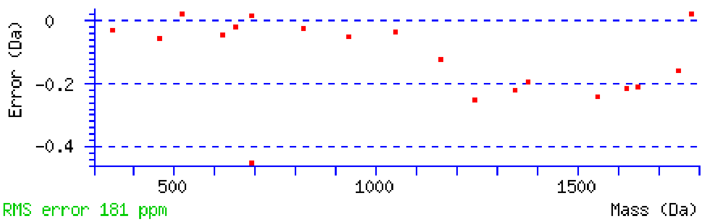


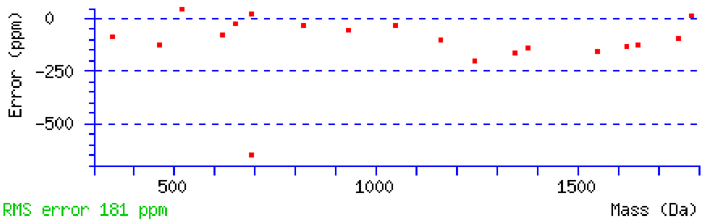


### All matches to this query

| Score | Mr(calc): | Delta | Sequence |
|---|---|---|---|
| 145.0 | 1895.9550 | -0.0039 | IYAGMVSLLDEAVGNVTK |
| 0.4 | 1895.9332 | 0.0179 | CTLSANLVASGELMSSKK |

Spectrum No: 3; Query: 2284; Rank: 1

# Peptide View

MS/MS Fragmentation of **LVQDVANNTNEEAGDGTTTATVLAR**
Found in **IPI00339148**, Tax_Id=10116 Gene_Symbol=Hspd1 60 kDa heat shock protein, mitochondrial precursor

Match to Query 2284: 2560.230988 from(1281.122770,2+)
Title: 091008RatKidney_NoSalt_21.2179.2179.2.dta
Data file K:\NewmanPaper\Piliang\3SubProteomes\Piliang3SP\mgf5ppm\ERLIC_3SubProteomes5ppm.mgf

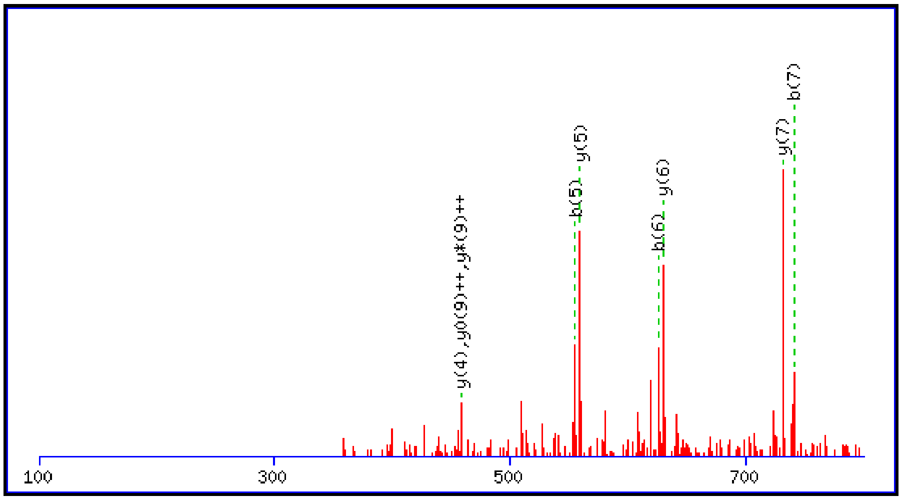


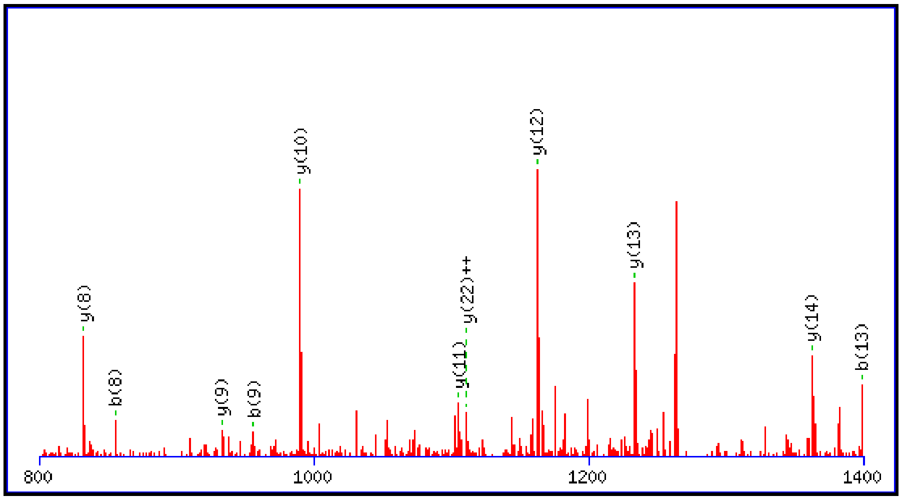


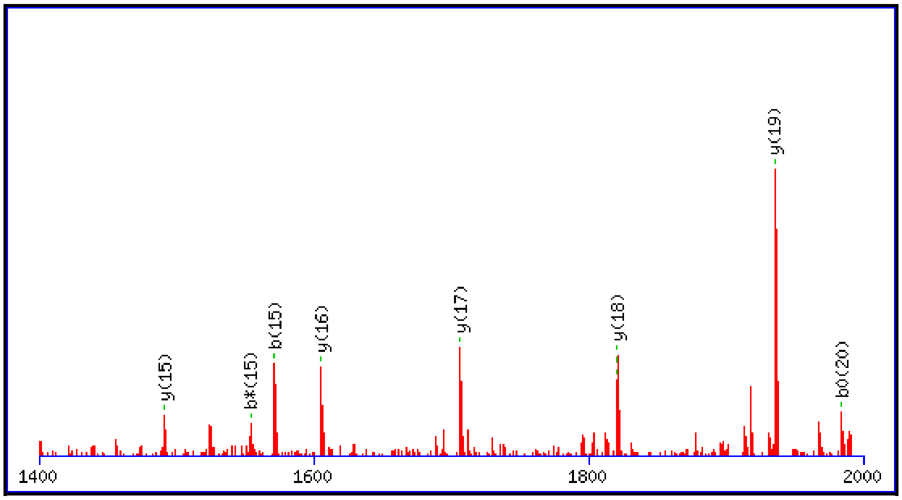

**Monoisotopic mass of neutral peptide Mr(calc):** 2560.2253
**Fixed modifications:** Carbamidomethyl (C)
**Variable modifications:**
**N7** : Deamidated_N (N)
**Ions Score:** 139 **Expect:** 3.6e-012
**Matches (Bold Red):** 28/270 fragment ions using 37 most intense peaks

| # | b | $b^{++}$ | b* | $b^{*++}$ | $b^{0}$ | $b^{0++}$ | Seq. | y | $y^{++}$ | y* | $y^{*++}$ | $y^{0}$ | $y^{0++}$ | # |
|---|---|---|---|---|---|---|---|---|---|---|---|---|---|---|
| 1 | 114.0913 | 57.5493 | | | | | L | | | | | | | 25 |
| 2 | 213.1598 | 107.0835 | | | | | V | 2448.1485 | 1224.5779 | 2431.1219 | 1216.0646 | 2430.1379 | 1215.5726 | 24 |
| 3 | 341.2183 | 171.1128 | 324.1918 | 162.5995 | | | Q | 2349.0801 | 1175.0437 | 2332.0535 | 1166.5304 | 2331.0695 | 1166.0384 | 23 |
| 4 | 456.2453 | 228.6263 | 439.2187 | 220.1130 | 438.2347 | 219.6210 | D | 2221.0215 | **1111.0144** | 2203.9949 | 1102.5011 | 2203.0109 | 1102.0091 | 22 |
| 5 | **555.3137** | 278.1605 | 538.2871 | 269.6472 | 537.3031 | 269.1552 | V | 2105.9945 | 1053.5009 | 2088.9680 | 1044.9876 | 2087.9840 | 1044.4956 | 21 |
| 6 | **626.3508** | 313.6790 | 609.3243 | 305.1658 | 608.3402 | 304.6738 | A | 2006.9261 | 1003.9667 | 1989.8996 | 995.4534 | 1988.9156 | 994.9614 | 20 |
| 7 | **741.3777** | 371.1925 | 724.3512 | 362.6792 | 723.3672 | 362.1872 | N | **1935.8890** | 968.4481 | 1918.8625 | 959.9349 | 1917.8784 | 959.4429 | 19 |
| 8 | **855.4207** | 428.2140 | 838.3941 | 419.7007 | 837.4101 | 419.2087 | N | **1820.8621** | 910.9347 | 1803.8355 | 902.4214 | 1802.8515 | 901.9294 | 18 |
| 9 | **956.4683** | 478.7378 | 939.4418 | 470.2245 | 938.4578 | 469.7325 | T | **1706.8192** | 853.9132 | 1689.7926 | 845.3999 | 1688.8086 | 844.9079 | 17 |
| 10 | 1070.5113 | 535.7593 | 1053.4847 | 527.2460 | 1052.5007 | 526.7540 | N | **1605.7715** | 803.3894 | 1588.7449 | 794.8761 | 1587.7609 | 794.3841 | 16 |
| 11 | 1199.5539 | 600.2806 | 1182.5273 | 591.7673 | 1181.5433 | 591.2753 | E | **1491.7285** | 746.3679 | 1474.7020 | 737.8546 | 1473.7180 | 737.3626 | 15 |
| 12 | 1328.5965 | 664.8019 | 1311.5699 | 656.2886 | 1310.5859 | 655.7966 | E | **1362.6860** | 681.8466 | 1345.6594 | 673.3333 | 1344.6754 | 672.8413 | 14 |
| 13 | **1399.6336** | 700.3204 | 1382.6070 | 691.8071 | 1381.6230 | 691.3151 | A | **1233.6434** | 617.3253 | 1216.6168 | 608.8120 | 1215.6328 | 608.3200 | 13 |
| 14 | 1456.6550 | 728.8312 | 1439.6285 | 720.3179 | 1438.6445 | 719.8259 | G | **1162.6062** | 581.8068 | 1145.5797 | 573.2935 | 1144.5957 | 572.8015 | 12 |
| 15 | **1571.6820** | 786.3446 | **1554.6554** | 777.8314 | 1553.6714 | 777.3393 | D | **1105.5848** | 553.2960 | 1088.5582 | 544.7828 | 1087.5742 | 544.2907 | 11 |
| 16 | 1628.7034 | 814.8554 | 1611.6769 | 806.3421 | 1610.6929 | 805.8501 | G | **990.5578** | 495.7826 | 973.5313 | 487.2693 | 972.5473 | 486.7773 | 10 |
| 17 | 1729.7511 | 865.3792 | 1712.7246 | 856.8659 | 1711.7406 | 856.3739 | T | **933.5364** | 467.2718 | 916.5098 | **458.7586** | 915.5258 | **458.2665** | 9 |
| 18 | 1830.7988 | 915.9030 | 1813.7723 | 907.3898 | 1812.7882 | 906.8978 | T | **832.4887** | 416.7480 | 815.4621 | 408.2347 | 814.4781 | 407.7427 | 8 |
| 19 | 1931.8465 | 966.4269 | 1914.8199 | 957.9136 | 1913.8359 | 957.4216 | T | **731.4410** | 366.2241 | 714.4145 | 357.7109 | 713.4305 | 357.2189 | 7 |
| 20 | 2002.8836 | 1001.9454 | 1985.8570 | 993.4322 | **1984.8730** | 992.9402 | A | **630.3933** | 315.7003 | 613.3668 | 307.1870 | 612.3828 | 306.6950 | 6 |
| 21 | 2103.9313 | 1052.4693 | 2086.9047 | 1043.9560 | 2085.9207 | 1043.4640 | T | **559.3562** | 280.1817 | 542.3297 | 271.6685 | 541.3457 | 271.1765 | 5 |
| 22 | 2202.9997 | 1102.0035 | 2185.9731 | 1093.4902 | 2184.9891 | 1092.9982 | V | **458.3085** | 229.6579 | 441.2820 | 221.1446 | | | 4 |
| 23 | 2316.0838 | 1158.5455 | 2299.0572 | 1150.0322 | 2298.0732 | 1149.5402 | L | 359.2401 | 180.1237 | 342.2136 | 171.6104 | | | 3 |
| 24 | 2387.1209 | 1194.0641 | 2370.0943 | 1185.5508 | 2369.1103 | 1185.0588 | A | 246.1561 | 123.5817 | 229.1295 | 115.0684 | | | 2 |
| 25 | | | | | | | R | 175.1190 | 88.0631 | 158.0924 | 79.5498 | | | 1 |

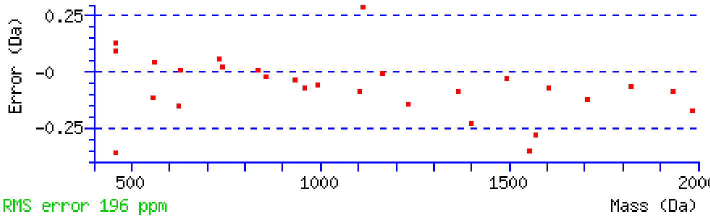


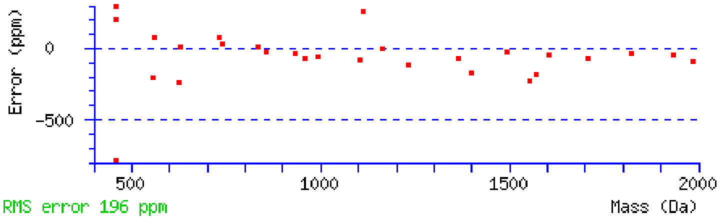


### All matches to this query

| Score | Mr(calc): | Delta | Sequence |
|---|---|---|---|
| 138.9 | 2560.2253 | 0.0057 | LVQDVANNTNEEAGDGTTTATVLAR |
| 135.4 | 2560.2253 | 0.0057 | LVQDVANNTNEEAGDGTTTATVLAR |
| 113.3 | 2560.2253 | 0.0057 | LVQDVANNTNEEAGDGTTTATVLAR |
| 103.0 | 2559.2413 | 0.9897 | LVQDVANNTNEEAGDGTTTATVLAR |
| 1.9 | 2559.2080 | 1.0230 | QYLFVSNKTGTLHEACEQLLK |

Spectrum No: 4; Query: 1901; Rank: 1

# Peptide View

MS/MS Fragmentation of **DGGSTTAGNSSQVSDGAAAVLLAR**
Found in **IPI00370596**, Tax_Id=10116 Gene_Symbol=RGD1562373_predicted 3-ketoacyl-CoA thiolase B, peroxisomal precursor

Match to Query 1901: 2205.050968 from(1103.532760,2+)
Title: 091008RatKidney_NoSalt_22.2961.2961.2.dta
Data file K:\NewmanPaper\Piliang\3SubProteomes\Piliang3SP\mgf5ppm\ERLIC_3SubProteomes5ppm.mgf

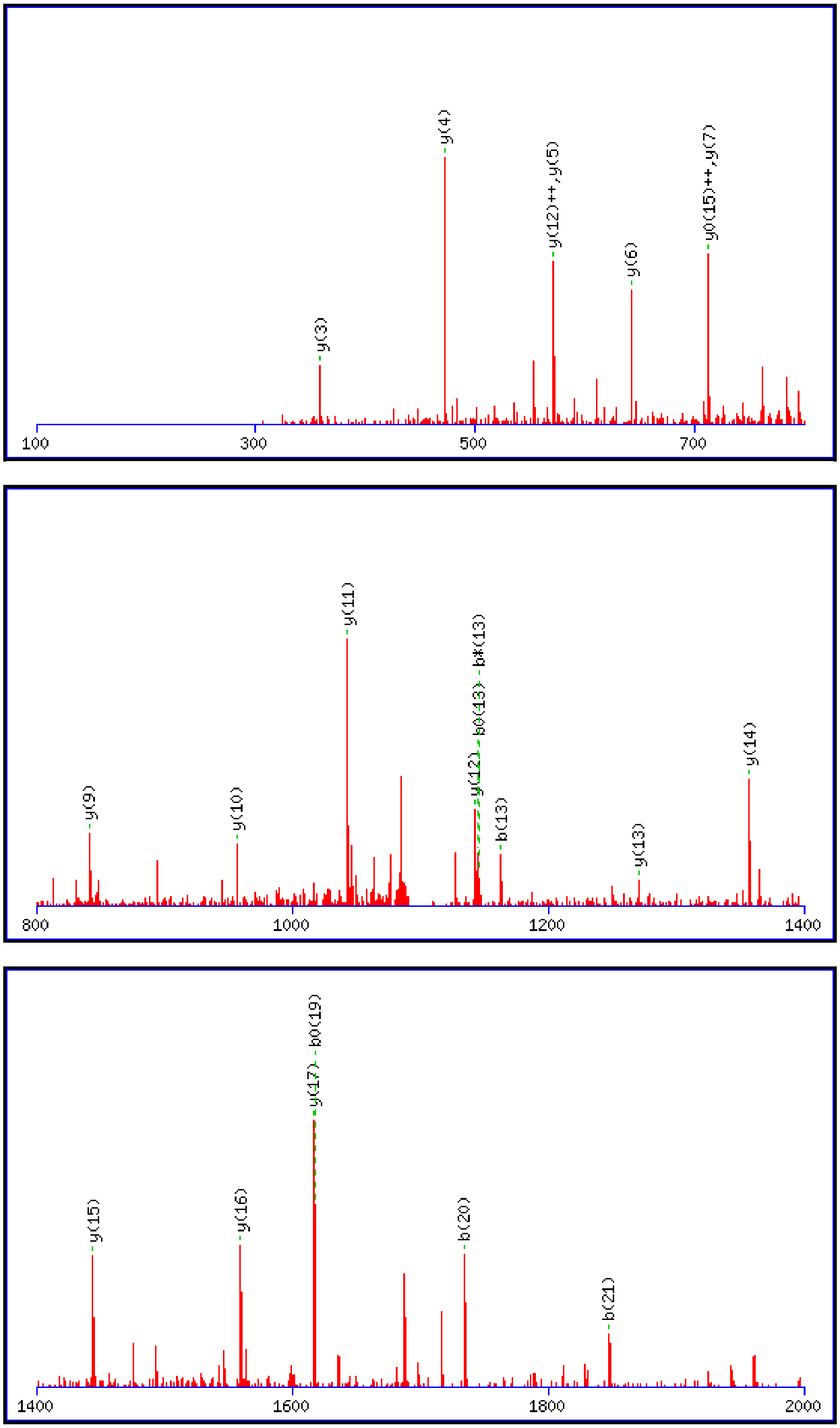


**Monoisotopic mass of neutral peptide Mr(calc):** 2205.0509
**Fixed modifications:** Carbamidomethyl (C)
**Variable modifications:**
**N9** : Deamidated_N (N)
**Ions Score:** 139 **Expect:** 3.4e-012
**Matches (Bold Red):** 22/242 fragment ions using 25 most intense peaks

| # | b | b++ | b* | b*++ | b0 | b0++ | Seq. | y | y++ | y* | y*++ | y0 | y0++ | # |
|---|---|---|---|---|---|---|---|---|---|---|---|---|---|---|
| 1 | 116.0342 | 58.5207 | | | 98.0237 | 49.5155 | D | | | | | | | 24 |
| 2 | 173.0557 | 87.0315 | | | 155.0451 | 78.0262 | G | 2091.0313 | 1046.0193 | 2074.0047 | 1037.5060 | 2073.0207 | 1037.0140 | 23 |
| 3 | 230.0771 | 115.5422 | | | 212.0666 | 106.5369 | G | 2034.0098 | 1017.5085 | 2016.9832 | 1008.9953 | 2015.9992 | 1008.5033 | 22 |
| 4 | 317.1092 | 159.0582 | | | 299.0986 | 150.0529 | S | 1976.9883 | 988.9978 | 1959.9618 | 980.4845 | 1958.9778 | 979.9925 | 21 |
| 5 | 418.1569 | 209.5821 | | | 400.1463 | 200.5768 | T | 1889.9563 | 945.4818 | 1872.9298 | 936.9685 | 1871.9457 | 936.4765 | 20 |
| 6 | 519.2045 | 260.1059 | | | 501.1940 | 251.1006 | T | 1788.9086 | 894.9580 | 1771.8821 | 886.4447 | 1770.8981 | 885.9527 | 19 |
| 7 | 590.2416 | 295.6245 | | | 572.2311 | 286.6192 | A | 1687.8609 | 844.4341 | 1670.8344 | 835.9208 | 1669.8504 | 835.4288 | 18 |
| 8 | 647.2631 | 324.1352 | | | 629.2525 | 315.1299 | G | **1616.8238** | 808.9156 | 1599.7973 | 800.4023 | 1598.8133 | 799.9103 | 17 |
| 9 | 762.2900 | 381.6487 | 745.2635 | 373.1354 | 744.2795 | 372.6434 | N | **1559.8024** | 780.4048 | 1542.7758 | 771.8915 | 1541.7918 | 771.3995 | 16 |
| 10 | 849.3221 | 425.1647 | 832.2955 | 416.6514 | 831.3115 | 416.1594 | S | **1444.7754** | 722.8914 | 1427.7489 | 714.3781 | 1426.7649 | **713.8861** | 15 |
| 11 | 936.3541 | 468.6807 | 919.3276 | 460.1674 | 918.3435 | 459.6754 | S | **1357.7434** | 679.3753 | 1340.7169 | 670.8621 | 1339.7328 | 670.3701 | 14 |
| 12 | 1064.4127 | 532.7100 | 1047.3861 | 524.1967 | 1046.4021 | 523.7047 | Q | **1270.7114** | 635.8593 | 1253.6848 | 627.3461 | 1252.7008 | 626.8540 | 13 |
| 13 | **1163.4811** | 582.2442 | **1146.4545** | 573.7309 | **1145.4705** | 573.2389 | V | **1142.6528** | **571.8300** | 1125.6263 | 563.3168 | 1124.6422 | 562.8248 | 12 |
| 14 | 1250.5131 | 625.7602 | 1233.4866 | 617.2469 | 1232.5026 | 616.7549 | S | **1043.5844** | 522.2958 | 1026.5578 | 513.7826 | 1025.5738 | 513.2905 | 11 |
| 15 | 1365.5401 | 683.2737 | 1348.5135 | 674.7604 | 1347.5295 | 674.2684 | D | **956.5524** | 478.7798 | 939.5258 | 470.2665 | 938.5418 | 469.7745 | 10 |
| 16 | 1422.5615 | 711.7844 | 1405.5350 | 703.2711 | 1404.5510 | 702.7791 | G | **841.5254** | 421.2663 | 824.4989 | 412.7531 | | | 9 |
| 17 | 1493.5986 | 747.3030 | 1476.5721 | 738.7897 | 1475.5881 | 738.2977 | A | 784.5040 | 392.7556 | 767.4774 | 384.2423 | | | 8 |
| 18 | 1564.6358 | 782.8215 | 1547.6092 | 774.3082 | 1546.6252 | 773.8162 | A | **713.4668** | 357.2371 | 696.4403 | 348.7238 | | | 7 |
| 19 | 1635.6729 | 818.3401 | 1618.6463 | 809.8268 | **1617.6623** | 809.3348 | A | **642.4297** | 321.7185 | 625.4032 | 313.2052 | | | 6 |
| 20 | **1734.7413** | 867.8743 | 1717.7147 | 859.3610 | 1716.7307 | 858.8690 | V | **571.3926** | 286.1999 | 554.3661 | 277.6867 | | | 5 |
| 21 | **1847.8254** | 924.4163 | 1830.7988 | 915.9030 | 1829.8148 | 915.4110 | L | **472.3242** | 236.6657 | 455.2976 | 228.1525 | | | 4 |
| 22 | 1960.9094 | 980.9583 | 1943.8829 | 972.4451 | 1942.8988 | 971.9531 | L | **359.2401** | 180.1237 | 342.2136 | 171.6104 | | | 3 |
| 23 | 2031.9465 | 1016.4769 | 2014.9200 | 1007.9636 | 2013.9360 | 1007.4716 | A | 246.1561 | 123.5817 | 229.1295 | 115.0684 | | | 2 |
| 24 | | | | | | | R | 175.1190 | 88.0631 | 158.0924 | 79.5498 | | | 1 |

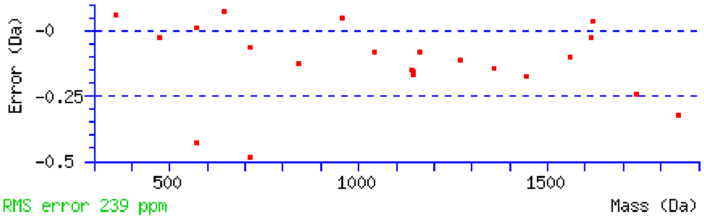


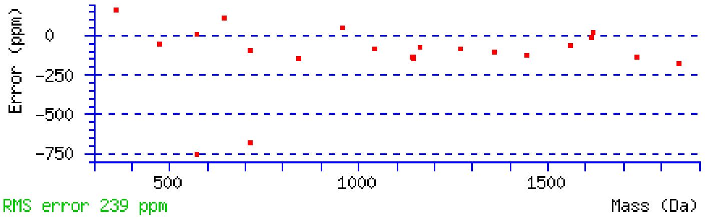


### All matches to this query

| Score | Mr(calc): | Delta | Sequence |
|---|---|---|---|
| 138.8 | 2205.0509 | 0.0000 | DGGSTTAGNSSQVSDGAAAVLLAR |
| 91.7 | 2204.0669 | 0.9840 | DGGSTTAGNSSQVSDGAAAVLLAR |
| 12.8 | 2203.0438 | 2.0072 | GFSWWPAMVVSWKATSKR |
| 12.2 | 2203.0438 | 2.0072 | GFSWWPAMVVSWKATSKR |
| 3.7 | 2205.0428 | 0.0082 | IMASFGNFANDNEIKVLLK |
| 2.5 | 2204.0588 | 0.9922 | IMASFGNFANDNEIKVLLK |
| 2.5 | 2204.0588 | 0.9922 | IMASFGNFANDNEIKVLLK |
| 2.0 | 2205.0496 | 0.0014 | ESPKMQMMAVSITSILALK |
| 1.6 | 2203.0270 | 2.0239 | NFLLQGSSTNRPTASTSNTK |
| 1.6 | 2203.0270 | 2.0239 | NFLLQGSSTNRPTASTSNTK |

Spectrum No: 5; Query: 2792; Rank: 1

# Peptide View

MS/MS Fragmentation of **VSGILFNDEMDDFSSPNFTNQFGVAPSPANFIKPGK**
Found in **IPI00206254**, Tax_Id=10116 Gene_Symbol=Ggt1 Gamma-glutamyltranspeptidase 1 precursor

Match to Query 2792: 3887.852742 from(1296.958190,3+)
Title: 100101RatKid_NS_deglyco_14.6003.6003.3.dta
Data file K:\NewmanPaper\Piliang\3SubProteomes\Piliang3SP\mgf5ppm\ERLIC_3SubProteomes5ppm.mgf

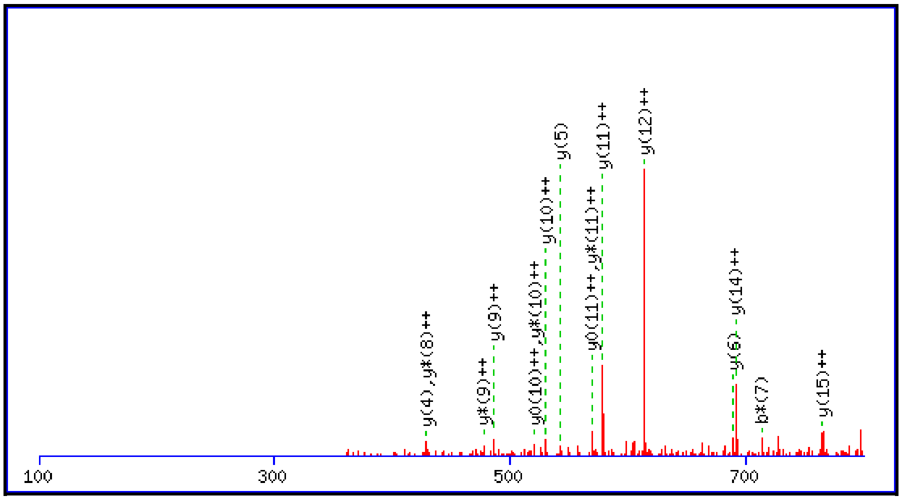


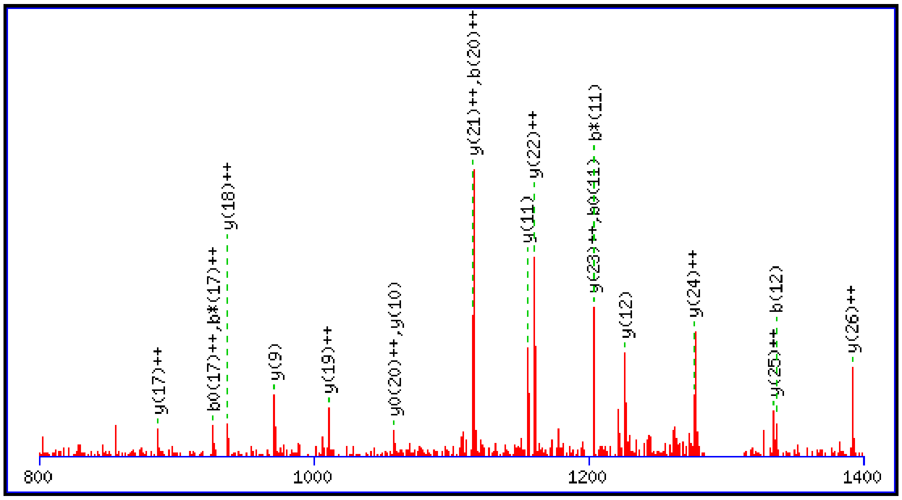


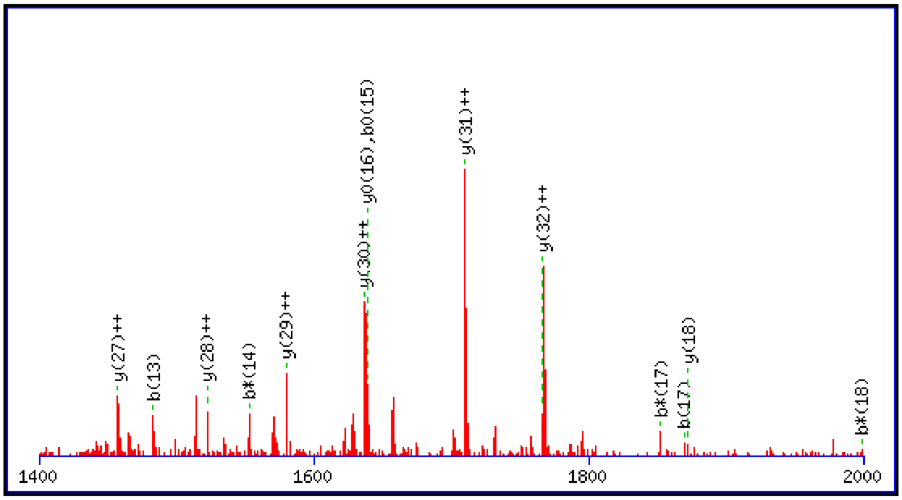


**Monoisotopic mass of neutral peptide Mr(calc):** 3887.8353
**Fixed modifications:** Carbamidomethyl (C)
**Variable modifications:**
**N17** : Deamidated_N (N)
**Ions Score:** 136 **Expect:** 1e-011
**Matches (Bold Red):** 50/388 fragment ions using 71 most intense peaks

| # | b | b++ | b* | b*++ | b0 | b0++ | Seq. | y | y++ | y* | y*++ | y0 | y0++ | # |
|---|---|---|---|---|---|---|---|---|---|---|---|---|---|---|
| 1 | 100.0757 | 50.5415 | | | | | V | | | | | | | 36 |
| 2 | 187.1077 | 94.0575 | | | 169.0972 | 85.0522 | S | 3789.7741 | 1895.3907 | 3772.7476 | 1886.8774 | 3771.7635 | 1886.3854 | 35 |
| 3 | 244.1292 | 122.5682 | | | 226.1186 | 113.5629 | G | 3702.7421 | 1851.8747 | 3685.7155 | 1843.3614 | 3684.7315 | 1842.8694 | 34 |
| 4 | 357.2132 | 179.1103 | | | 339.2027 | 170.1050 | I | 3645.7206 | 1823.3639 | 3628.6941 | 1814.8507 | 3627.7101 | 1814.3587 | 33 |
| 5 | 470.2973 | 235.6523 | | | 452.2867 | 226.6470 | L | 3532.6366 | **1766.8219** | 3515.6100 | 1758.3086 | 3514.6260 | 1757.8166 | 32 |
| 6 | 617.3657 | 309.1865 | | | 599.3552 | 300.1812 | F | 3419.5525 | **1710.2799** | 3402.5259 | 1701.7666 | 3401.5419 | 1701.2746 | 31 |
| 7 | 731.4087 | 366.2080 | **714.3821** | 357.6947 | 713.3981 | 357.2027 | N | 3272.4841 | **1636.7457** | 3255.4575 | 1628.2324 | 3254.4735 | 1627.7404 | 30 |
| 8 | 846.4356 | 423.7214 | 829.4090 | 415.2082 | 828.4250 | 414.7162 | D | 3158.4412 | **1579.7242** | 3141.4146 | 1571.2109 | 3140.4306 | 1570.7189 | 29 |
| 9 | 975.4782 | 488.2427 | 958.4516 | 479.7295 | 957.4676 | 479.2374 | E | 3043.4142 | **1522.2107** | 3026.3877 | 1513.6975 | 3025.4036 | 1513.2055 | 28 |
| 10 | 1106.5187 | 553.7630 | 1089.4921 | 545.2497 | 1088.5081 | 544.7577 | M | 2914.3716 | **1457.6894** | 2897.3451 | 1449.1762 | 2896.3611 | 1448.6842 | 27 |
| 11 | 1221.5456 | 611.2764 | **1204.5191** | 602.7632 | **1203.5351** | 602.2712 | D | 2783.3311 | **1392.1692** | 2766.3046 | 1383.6559 | 2765.3206 | 1383.1639 | 26 |
| 12 | **1336.5726** | 668.7899 | 1319.5460 | 660.2766 | 1318.5620 | 659.7846 | D | 2668.3042 | **1334.6557** | 2651.2776 | 1326.1425 | 2650.2936 | 1325.6504 | 25 |
| 13 | **1483.6410** | 742.3241 | 1466.6144 | 733.8108 | 1465.6304 | 733.3188 | F | 2553.2772 | **1277.1423** | 2536.2507 | 1268.6290 | 2535.2667 | 1268.1370 | 24 |
| 14 | 1570.6730 | 785.8401 | **1553.6465** | 777.3269 | 1552.6624 | 776.8349 | S | 2406.2088 | **1203.6081** | 2389.1823 | 1195.0948 | 2388.1983 | 1194.6028 | 23 |
| 15 | 1657.7050 | 829.3562 | 1640.6785 | 820.8429 | **1639.6945** | 820.3509 | S | 2319.1768 | **1160.0920** | 2302.1503 | 1151.5788 | 2301.1662 | 1151.0868 | 22 |
| 16 | 1754.7578 | 877.8825 | 1737.7312 | 869.3693 | 1736.7472 | 868.8773 | P | 2232.1448 | **1116.5760** | 2215.1182 | 1108.0628 | 2214.1342 | 1107.5707 | 21 |
| 17 | **1869.7847** | 935.3960 | **1852.7582** | **926.8827** | 1851.7742 | **926.3907** | N | 2135.0920 | 1068.0496 | 2118.0655 | 1059.5364 | 2117.0814 | **1059.0444** | 20 |
| 18 | 2016.8531 | 1008.9302 | **1999.8266** | 1000.4169 | 1998.8426 | 999.9249 | F | 2020.0651 | **1010.5362** | 2003.0385 | 1002.0229 | 2002.0545 | 1001.5309 | 19 |
| 19 | 2117.9008 | 1059.4540 | 2100.8743 | 1050.9408 | 2099.8903 | 1050.4488 | T | **1872.9967** | **937.0020** | 1855.9701 | 928.4887 | 1854.9861 | 927.9967 | 18 |
| 20 | 2231.9437 | **1116.4755** | 2214.9172 | 1107.9622 | 2213.9332 | 1107.4702 | N | 1771.9490 | **886.4781** | 1754.9224 | 877.9649 | 1753.9384 | 877.4728 | 17 |
| 21 | 2360.0023 | 1180.5048 | 2342.9758 | 1171.9915 | 2341.9918 | 1171.4995 | Q | 1657.9061 | 829.4567 | 1640.8795 | 820.9434 | **1639.8955** | 820.4514 | 16 |
| 22 | 2507.0707 | 1254.0390 | 2490.0442 | 1245.5257 | 2489.0602 | 1245.0337 | F | 1529.8475 | **765.4274** | 1512.8209 | 756.9141 | 1511.8369 | 756.4221 | 15 |
| 23 | 2564.0922 | 1282.5497 | 2547.0657 | 1274.0365 | 2546.0816 | 1273.5445 | G | 1382.7791 | **691.8932** | 1365.7525 | 683.3799 | 1364.7685 | 682.8879 | 14 |
| 24 | 2663.1606 | 1332.0839 | 2646.1341 | 1323.5707 | 2645.1501 | 1323.0787 | V | 1325.7576 | 663.3824 | 1308.7310 | 654.8692 | 1307.7470 | 654.3772 | 13 |
| 25 | 2734.1977 | 1367.6025 | 2717.1712 | 1359.0892 | 2716.1872 | 1358.5972 | A | **1226.6892** | **613.8482** | 1209.6626 | 605.3350 | 1208.6786 | 604.8429 | 12 |
| 26 | 2831.2505 | 1416.1289 | 2814.2239 | 1407.6156 | 2813.2399 | 1407.1236 | P | **1155.6521** | **578.3297** | 1138.6255 | **569.8164** | 1137.6415 | **569.3244** | 11 |
| 27 | 2918.2825 | 1459.6449 | 2901.2560 | 1451.1316 | 2900.2720 | 1450.6396 | S | **1058.5993** | **529.8033** | 1041.5728 | **521.2900** | 1040.5887 | **520.7980** | 10 |
| 28 | 3015.3353 | 1508.1713 | 2998.3087 | 1499.6580 | 2997.3247 | 1499.1660 | P | **971.5673** | **486.2873** | 954.5407 | **477.7740** | | | 9 |
| 29 | 3086.3724 | 1543.6898 | 3069.3459 | 1535.1766 | 3068.3618 | 1534.6846 | A | 874.5145 | 437.7609 | 857.4880 | **429.2476** | | | 8 |
| 30 | 3200.4153 | 1600.7113 | 3183.3888 | 1592.1980 | 3182.4048 | 1591.7060 | N | 803.4774 | 402.2423 | 786.4509 | 393.7291 | | | 7 |
| 31 | 3347.4837 | 1674.2455 | 3330.4572 | 1665.7322 | 3329.4732 | 1665.2402 | F | **689.4345** | 345.2209 | 672.4079 | 336.7076 | | | 6 |
| 32 | 3460.5678 | 1730.7875 | 3443.5413 | 1722.2743 | 3442.5572 | 1721.7823 | I | **542.3661** | 271.6867 | 525.3395 | 263.1734 | | | 5 |
| 33 | 3588.6628 | 1794.8350 | 3571.6362 | 1786.3217 | 3570.6522 | 1785.8297 | K | **429.2820** | 215.1446 | 412.2554 | 206.6314 | | | 4 |
| 34 | 3685.7155 | 1843.3614 | 3668.6890 | 1834.8481 | 3667.7050 | 1834.3561 | P | 301.1870 | 151.0972 | 284.1605 | 142.5839 | | | 3 |
| 35 | 3742.7370 | 1871.8721 | 3725.7104 | 1863.3589 | 3724.7264 | 1862.8669 | G | 204.1343 | 102.5708 | 187.1077 | 94.0575 | | | 2 |
| 36 | | | | | | | K | 147.1128 | 74.0600 | 130.0863 | 65.5468 | | | 1 |

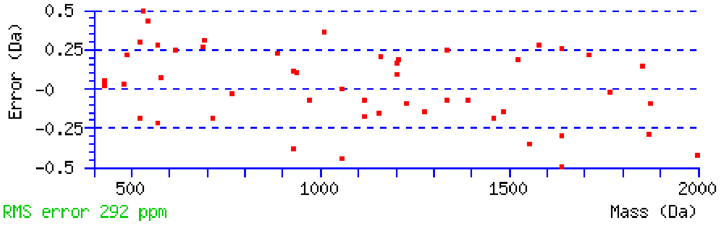


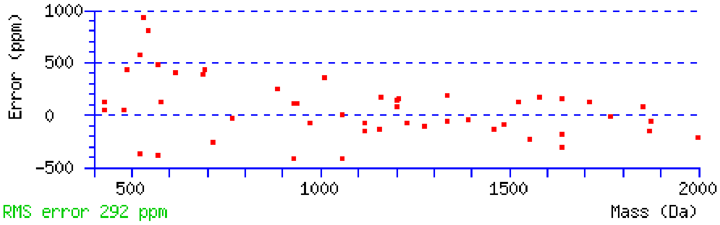


### All matches to this query

| Score | Mr(calc): | Delta | Sequence |
|---|---|---|---|
| 135.6 | 3887.8353 | 0.0175 | VSGILFNDEMDDFSSPNFTNQFGVAPSPANFIKPGK |
| 123.7 | 3887.8353 | 0.0175 | VSGILFNDEMDDFSSPNFTNQFGVAPSPANFIKPGK |
| | | | |

| | | | |
|---|---|---|---|
| 98.7 | 3887.8353 | 0.0175 | VSGILFNDEMDDFSSPNFTNQFGVAPSPANFIKPGK |
| 69.5 | 3887.8353 | 0.0175 | VSGILFNDEMDDFSSPNFTNQFGVAPSPANFIKPGK |
| 55.8 | 3886.8513 | 1.0015 | VSGILFNDEMDDFSSPNFTNQFGVAPSPANFIKPGK |
| 3.8 | 3885.8155 | 2.0373 | NCRSLMNQVQNIAELATLSLERLTGILGHSGNAK |
| 3.6 | 3885.8099 | 2.0428 | EEKPMPTASLLSPVVLAPVPSRRNPSGGMSSLILN |
| 2.5 | 3887.8436 | 0.0091 | NVSIPALNDSKYIGMSVYNVGIMCIIGAAVSFLTR |
| 2.1 | 3885.8747 | 1.9780 | LAALKDEPQTVPDVPSFGDSPPLSPIDMDTQERIK |
| 1.9 | 3886.8790 | 0.9737 | TGPMKEINLSPDSTSVIVSGLMVATKYEVSVYALK |

Spectrum No: 6; Query: 1942; Rank: 1

## Peptide View

MS/MS Fragmentation of **YGYENDTEIANLYDEMVAK**
Found in **IPI00191437**, Tax_Id=10116 Gene_Symbol=- 64 kDa protein

Match to Query 1942: 2237.971288 from(1119.992920,2+)
Title: 091008RatKidney_NoSalt_21.5392.5392.2.dta
Data file K:\NewmanPaper\Piliang\3SubProteomes\Piliang3SP\mgf5ppm\ERLIC_3SubProteomes5ppm.mgf

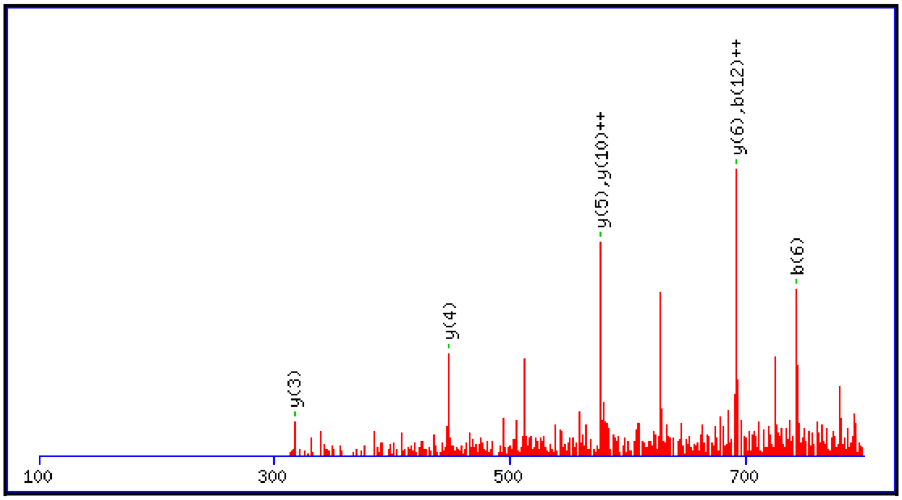


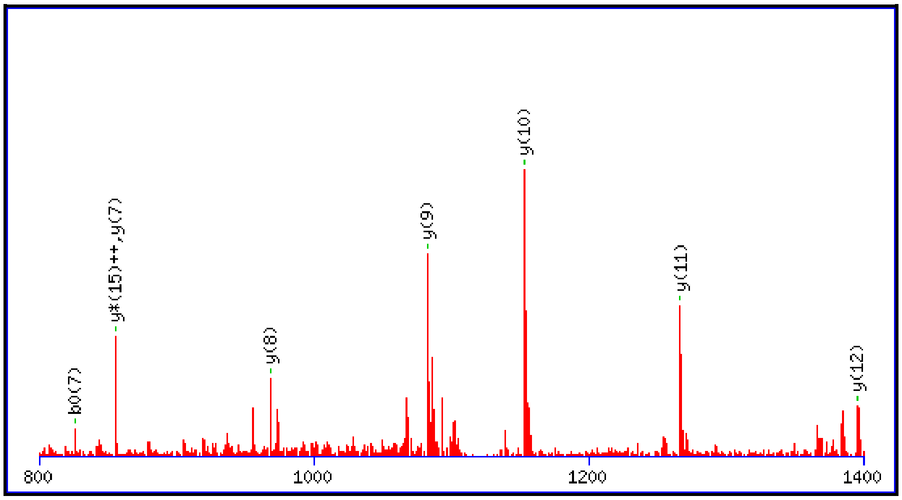

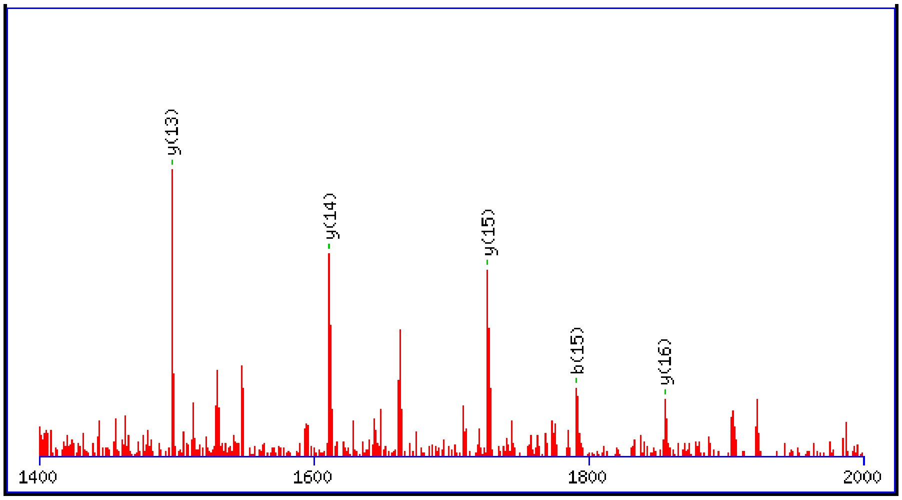


**Monoisotopic mass of neutral peptide Mr(calc):** 2237.9674
**Fixed modifications:** Carbamidomethyl (C)
**Variable modifications:**
**N5** : Deamidated_N (N)
**Ions Score:** 132 **Expect:** 1.8e-011
**Matches (Bold Red):** 20/194 fragment ions using 25 most intense peaks

| # | b | $b^{++}$ | b* | $b^{*++}$ | $b^0$ | $b^{0++}$ | Seq. | y | $y^{++}$ | y* | $y^{*++}$ | $y^0$ | $y^{0++}$ | # |
|---|---|---|---|---|---|---|---|---|---|---|---|---|---|---|
| 1 | 164.0706 | 82.5389 | | | | | Y | | | | | | | 19 |
| 2 | 221.0921 | 111.0497 | | | | | G | 2075.9114 | 1038.4593 | 2058.8848 | 1029.9461 | 2057.9008 | 1029.4540 | 18 |
| 3 | 384.1554 | 192.5813 | | | | | Y | 2018.8899 | 1009.9486 | 2001.8634 | 1001.4353 | 2000.8794 | 1000.9433 | 17 |
| 4 | 513.1980 | 257.1026 | | | 495.1874 | 248.0974 | E | **1855.8266** | 928.4169 | 1838.8000 | 919.9037 | 1837.8160 | 919.4117 | 16 |
| 5 | 628.2249 | 314.6161 | 611.1984 | 306.1028 | 610.2144 | 305.6108 | N | **1726.7840** | 863.8956 | 1709.7575 | **855.3824** | 1708.7734 | 854.8904 | 15 |
| 6 | **743.2519** | 372.1296 | 726.2253 | 363.6163 | 725.2413 | 363.1243 | D | **1611.7571** | 806.3822 | 1594.7305 | 797.8689 | 1593.7465 | 797.3769 | 14 |
| 7 | 844.2995 | 422.6534 | 827.2730 | 414.1401 | **826.2890** | 413.6481 | T | **1496.7301** | 748.8687 | 1479.7036 | 740.3554 | 1478.7196 | 739.8634 | 13 |
| 8 | 973.3421 | 487.1747 | 956.3156 | 478.6614 | 955.3316 | 478.1694 | E | **1395.6824** | 698.3449 | 1378.6559 | 689.8316 | 1377.6719 | 689.3396 | 12 |
| 9 | 1086.4262 | 543.7167 | 1069.3997 | 535.2035 | 1068.4156 | 534.7115 | I | **1266.6399** | 633.8236 | 1249.6133 | 625.3103 | 1248.6293 | 624.8183 | 11 |
| 10 | 1157.4633 | 579.2353 | 1140.4368 | 570.7220 | 1139.4528 | 570.2300 | A | **1153.5558** | **577.2815** | 1136.5292 | 568.7683 | 1135.5452 | 568.2762 | 10 |
| 11 | 1271.5062 | 636.2568 | 1254.4797 | 627.7435 | 1253.4957 | 627.2515 | N | **1082.5187** | 541.7630 | 1065.4921 | 533.2497 | 1064.5081 | 532.7577 | 9 |
| 12 | 1384.5903 | **692.7988** | 1367.5638 | 684.2855 | 1366.5797 | 683.7935 | L | **968.4757** | 484.7415 | 951.4492 | 476.2282 | 950.4652 | 475.7362 | 8 |
| 13 | 1547.6536 | 774.3305 | 1530.6271 | 765.8172 | 1529.6431 | 765.3252 | Y | **855.3917** | 428.1995 | 838.3651 | 419.6862 | 837.3811 | 419.1942 | 7 |
| 14 | 1662.6806 | 831.8439 | 1645.6540 | 823.3307 | 1644.6700 | 822.8386 | D | **692.3284** | 346.6678 | 675.3018 | 338.1545 | 674.3178 | 337.6625 | 6 |
| 15 | **1791.7232** | 896.3652 | 1774.6966 | 887.8520 | 1773.7126 | 887.3599 | E | **577.3014** | 289.1543 | 560.2749 | 280.6411 | 559.2908 | 280.1491 | 5 |
| 16 | 1922.7637 | 961.8855 | 1905.7371 | 953.3722 | 1904.7531 | 952.8802 | M | **448.2588** | 224.6330 | 431.2323 | 216.1198 | | | 4 |
| 17 | 2021.8321 | 1011.4197 | 2004.8055 | 1002.9064 | 2003.8215 | 1002.4144 | V | **317.2183** | 159.1128 | 300.1918 | 150.5995 | | | 3 |
| 18 | 2092.8692 | 1046.9382 | 2075.8426 | 1038.4250 | 2074.8586 | 1037.9329 | A | 218.1499 | 109.5786 | 201.1234 | 101.0653 | | | 2 |
| 19 | | | | | | | K | 147.1128 | 74.0600 | 130.0863 | 65.5468 | | | 1 |

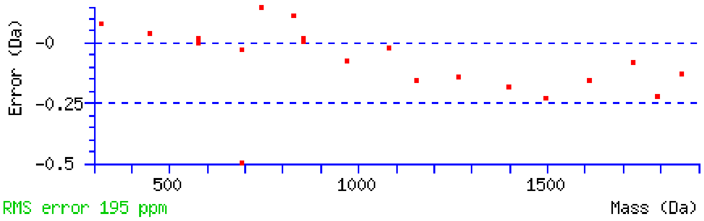


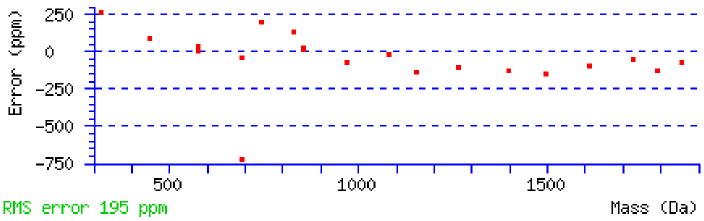


### All matches to this query

| Score | Mr(calc): | Delta | Sequence |
|---|---|---|---|
| 132.0 | 2237.9674 | 0.0039 | YGYENDTEIANLYDEMVAK |
| 98.7 | 2237.9674 | 0.0039 | YGYENDTEIANLYDEMVAK |
| | | | |

| | | | |
|---|---|---|---|
| 96.6 | 2236.9834 | 0.9879 | YGYENDTEIANLYDEMVAK |
| 2.4 | 2235.9618 | 2.0095 | KSASGDSSQRSSHSDTHVQR |
| 2.0 | 2237.9858 | -0.0145 | EQLEYTIEEEVAKQTIR |
| 1.2 | 2235.9844 | 1.9869 | KATGAVSSNVLQWGSHXKK |
| 0.7 | 2235.9792 | 1.9920 | SMLGNGNYDVNVIMAALQTK |
| 0.4 | 2236.9729 | 0.9984 | TCTNPAPLNGGAFCEGQSVQK |

---

Spectrum No: 7; Query: 1942; Rank: 1

---

## Peptide View

MS/MS Fragmentation of **YGYENDTEIANLYDEMVAK**
Found in **IPI00191437**, Tax_Id=10116 Gene_Symbol=- 64 kDa protein

Match to Query 1942: 2237.971288 from(1119.992920,2+)
Title: 091008RatKidney_NoSalt_21.5392.5392.2.dta
Data file K:\NewmanPaper\Piliang\3SubProteomes\Piliang3SP\mgf5ppm\ERLIC_3SubProteomes5ppm.mgf

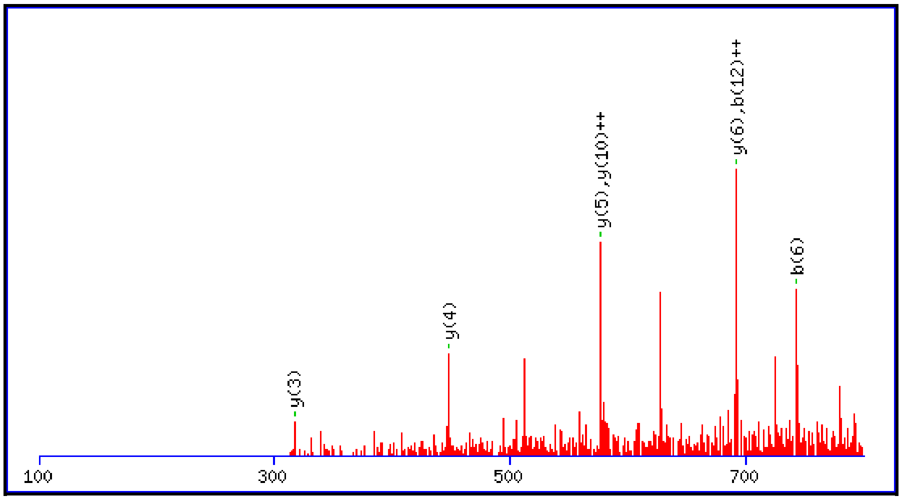


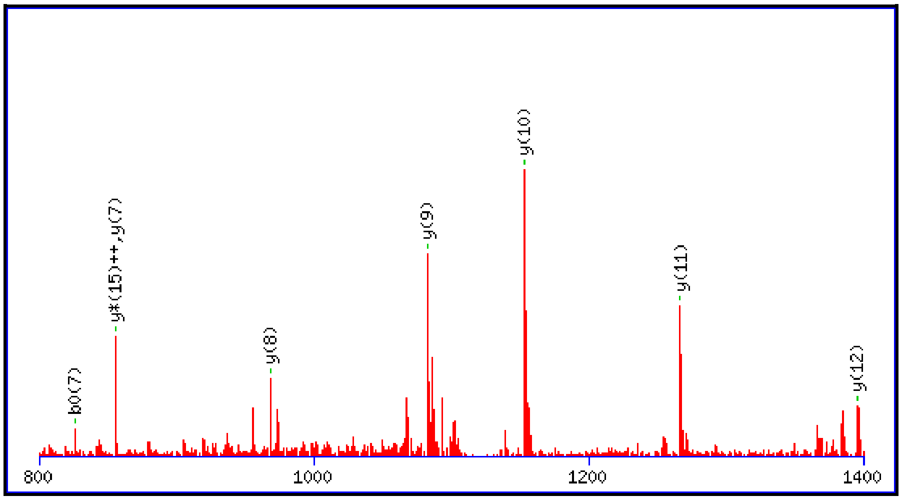

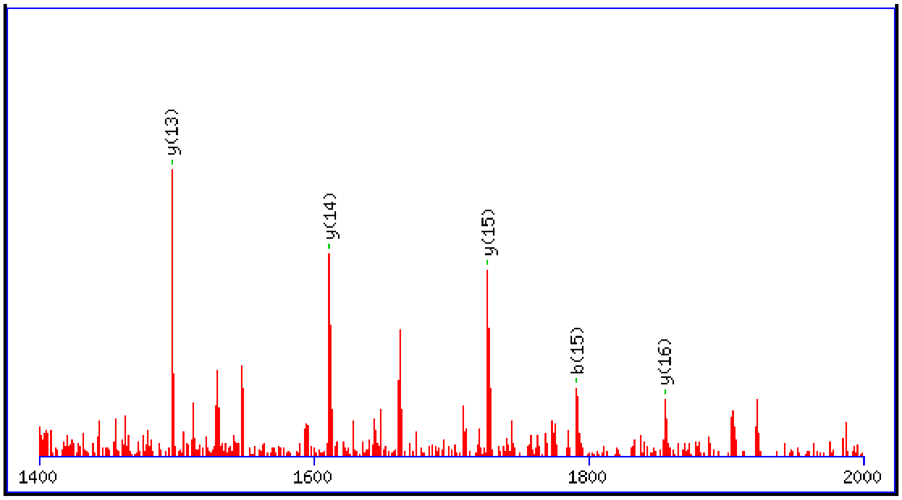


**Monoisotopic mass of neutral peptide Mr(calc):** 2237.9674
**Fixed modifications:** Carbamidomethyl (C)
**Variable modifications:**
**N5** : Deamidated_N (N)
**Ions Score:** 132 **Expect:** 1.8e-011
**Matches (Bold Red):** 20/194 fragment ions using 25 most intense peaks

| # | b | b++ | b* | b*++ | b0 | b0++ | Seq. | y | y++ | y* | y*++ | y0 | y0++ | # |
|---|---|---|---|---|---|---|---|---|---|---|---|---|---|---|
| 1 | 164.0706 | 82.5389 | | | | | Y | | | | | | | 19 |
| 2 | 221.0921 | 111.0497 | | | | | G | 2075.9114 | 1038.4593 | 2058.8848 | 1029.9461 | 2057.9008 | 1029.4540 | 18 |
| 3 | 384.1554 | 192.5813 | | | | | Y | 2018.8899 | 1009.9486 | 2001.8634 | 1001.4353 | 2000.8794 | 1000.9433 | 17 |
| 4 | 513.1980 | 257.1026 | | | 495.1874 | 248.0974 | E | **1855.8266** | 928.4169 | 1838.8000 | 919.9037 | 1837.8160 | 919.4117 | 16 |
| 5 | 628.2249 | 314.6161 | 611.1984 | 306.1028 | 610.2144 | 305.6108 | N | **1726.7840** | 863.8956 | 1709.7575 | **855.3824** | 1708.7734 | 854.8904 | 15 |
| 6 | **743.2519** | 372.1296 | 726.2253 | 363.6163 | 725.2413 | 363.1243 | D | **1611.7571** | 806.3822 | 1594.7305 | 797.8689 | 1593.7465 | 797.3769 | 14 |
| 7 | 844.2995 | 422.6534 | 827.2730 | 414.1401 | **826.2890** | 413.6481 | T | **1496.7301** | 748.8687 | 1479.7036 | 740.3554 | 1478.7196 | 739.8634 | 13 |
| 8 | 973.3421 | 487.1747 | 956.3156 | 478.6614 | 955.3316 | 478.1694 | E | **1395.6824** | 698.3449 | 1378.6559 | 689.8316 | 1377.6719 | 689.3396 | 12 |
| 9 | 1086.4262 | 543.7167 | 1069.3997 | 535.2035 | 1068.4156 | 534.7115 | I | **1266.6399** | 633.8236 | 1249.6133 | 625.3103 | 1248.6293 | 624.8183 | 11 |
| 10 | 1157.4633 | 579.2353 | 1140.4368 | 570.7220 | 1139.4528 | 570.2300 | A | **1153.5558** | **577.2815** | 1136.5292 | 568.7683 | 1135.5452 | 568.2762 | 10 |
| 11 | 1271.5062 | 636.2568 | 1254.4797 | 627.7435 | 1253.4957 | 627.2515 | N | **1082.5187** | 541.7630 | 1065.4921 | 533.2497 | 1064.5081 | 532.7577 | 9 |
| 12 | 1384.5903 | **692.7988** | 1367.5638 | 684.2855 | 1366.5797 | 683.7935 | L | **968.4757** | 484.7415 | 951.4492 | 476.2282 | 950.4652 | 475.7362 | 8 |
| 13 | 1547.6536 | 774.3305 | 1530.6271 | 765.8172 | 1529.6431 | 765.3252 | Y | **855.3917** | 428.1995 | 838.3651 | 419.6862 | 837.3811 | 419.1942 | 7 |
| 14 | 1662.6806 | 831.8439 | 1645.6540 | 823.3307 | 1644.6700 | 822.8386 | D | **692.3284** | 346.6678 | 675.3018 | 338.1545 | 674.3178 | 337.6625 | 6 |
| 15 | **1791.7232** | 896.3652 | 1774.6966 | 887.8520 | 1773.7126 | 887.3599 | E | **577.3014** | 289.1543 | 560.2749 | 280.6411 | 559.2908 | 280.1491 | 5 |
| 16 | 1922.7637 | 961.8855 | 1905.7371 | 953.3722 | 1904.7531 | 952.8802 | M | **448.2588** | 224.6330 | 431.2323 | 216.1198 | | | 4 |
| 17 | 2021.8321 | 1011.4197 | 2004.8055 | 1002.9064 | 2003.8215 | 1002.4144 | V | **317.2183** | 159.1128 | 300.1918 | 150.5995 | | | 3 |
| 18 | 2092.8692 | 1046.9382 | 2075.8426 | 1038.4250 | 2074.8586 | 1037.9329 | A | 218.1499 | 109.5786 | 201.1234 | 101.0653 | | | 2 |
| 19 | | | | | | | K | 147.1128 | 74.0600 | 130.0863 | 65.5468 | | | 1 |

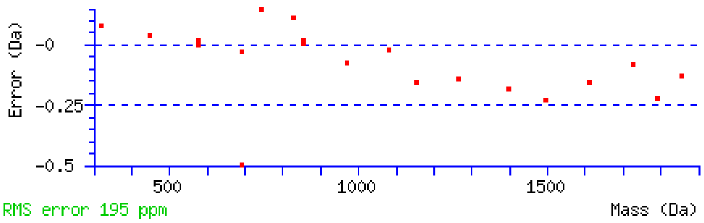


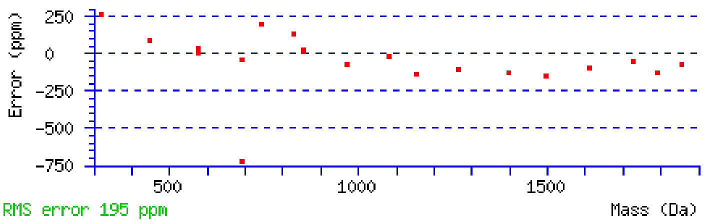


## All matches to this query

| Score | Mr(calc): | Delta | Sequence |
|---|---|---|---|
| 132.0 | 2237.9674 | 0.0039 | YGYENDTEIANLYDEMVAK |
| 98.7 | 2237.9674 | 0.0039 | YGYENDTEIANLYDEMVAK |
| | | | |

| | | | |
|---|---|---|---|
| 96.6 | 2236.9834 | 0.9879 | YGYENDTEIANLYDEMVAK |
| 2.4 | 2235.9618 | 2.0095 | KSASGDSSQRSSHSDTHVQR |
| 2.0 | 2237.9858 | -0.0145 | EQLEYTIEEEVAKQTIR |
| 1.2 | 2235.9844 | 1.9869 | KATGAVSSNVLQWGSHXKK |
| 0.7 | 2235.9792 | 1.9920 | SMLGNGNYDVNVIMAALQTK |
| 0.4 | 2236.9729 | 0.9984 | TCTNPAPLNGGAFCEGQSVQK |

---

Spectrum No: 8; Query: 1425; Rank: 1

---

## Peptide View

MS/MS Fragmentation of **TLAGENQTALEIEELNR**
Found in **IPI00363849**, Tax_Id=10116 Gene_Symbol=Lamc1 similar to Laminin gamma-1 chain precursor

Match to Query 1425: 1900.944208 from(951.479380,2+)
Title: 100101RatKid_NS_deglyco_25.3189.3189.2.dta
Data file K:\NewmanPaper\Piliang\3SubProteomes\Piliang3SP\mgf5ppm\ERLIC_3SubProteomes5ppm.mgf

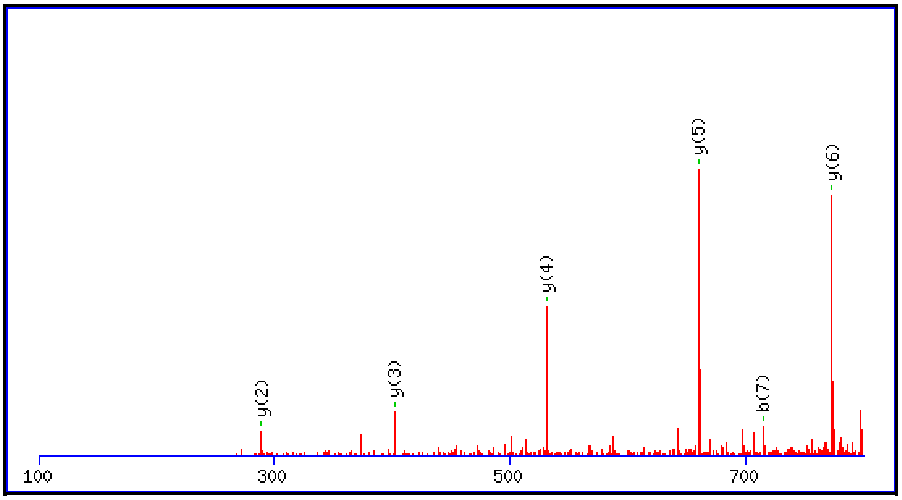


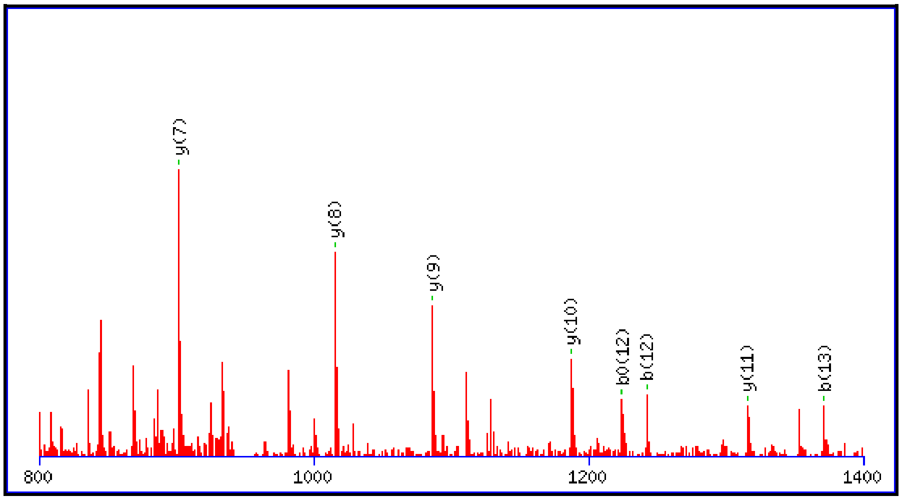

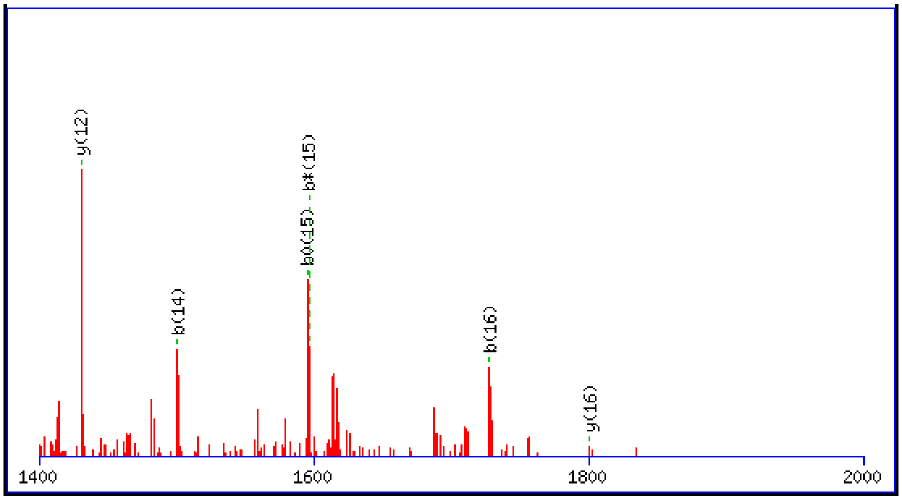


**Monoisotopic mass of neutral peptide Mr(calc):** 1900.9377
**Fixed modifications:** Carbamidomethyl (C)
**Variable modifications:**
**N6** : Deamidated_N (N)
**Ions Score:** 130 **Expect:** 2.1e-011
**Matches (Bold Red):** 20/176 fragment ions using 27 most intense peaks

| # | b | $b^{++}$ | b* | $b^{*++}$ | $b^{0}$ | $b^{0++}$ | Seq. | y | $y^{++}$ | y* | $y^{*++}$ | $y^{0}$ | $y^{0++}$ | # |
|---|---|---|---|---|---|---|---|---|---|---|---|---|---|---|
| 1 | 102.0550 | 51.5311 | | | 84.0444 | 42.5258 | T | | | | | | | 17 |
| 2 | 215.1390 | 108.0731 | | | 197.1285 | 99.0679 | L | **1800.8974** | 900.9523 | 1783.8708 | 892.4391 | 1782.8868 | 891.9471 | 16 |
| 3 | 286.1761 | 143.5917 | | | 268.1656 | 134.5864 | A | 1687.8133 | 844.4103 | 1670.7868 | 835.8970 | 1669.8028 | 835.4050 | 15 |
| 4 | 343.1976 | 172.1024 | | | 325.1870 | 163.0972 | G | 1616.7762 | 808.8917 | 1599.7497 | 800.3785 | 1598.7656 | 799.8865 | 14 |
| 5 | 472.2402 | 236.6237 | | | 454.2296 | 227.6185 | E | 1559.7548 | 780.3810 | 1542.7282 | 771.8677 | 1541.7442 | 771.3757 | 13 |
| 6 | 587.2671 | 294.1372 | 570.2406 | 285.6239 | 569.2566 | 285.1319 | N | **1430.7122** | 715.8597 | 1413.6856 | 707.3464 | 1412.7016 | 706.8544 | 12 |
| 7 | **715.3257** | 358.1665 | 698.2992 | 349.6532 | 697.3151 | 349.1612 | Q | **1315.6852** | 658.3462 | 1298.6587 | 649.8330 | 1297.6747 | 649.3410 | 11 |
| 8 | 816.3734 | 408.6903 | 799.3468 | 400.1771 | 798.3628 | 399.6850 | T | **1187.6266** | 594.3170 | 1170.6001 | 585.8037 | 1169.6161 | 585.3117 | 10 |
| 9 | 887.4105 | 444.2089 | 870.3839 | 435.6956 | 869.3999 | 435.2036 | A | **1086.5790** | 543.7931 | 1069.5524 | 535.2798 | 1068.5684 | 534.7878 | 9 |
| 10 | 1000.4946 | 500.7509 | 983.4680 | 492.2376 | 982.4840 | 491.7456 | L | **1015.5419** | 508.2746 | 998.5153 | 499.7613 | 997.5313 | 499.2693 | 8 |
| 11 | 1129.5372 | 565.2722 | 1112.5106 | 556.7589 | 1111.5266 | 556.2669 | E | **902.4578** | 451.7325 | 885.4312 | 443.2193 | 884.4472 | 442.7272 | 7 |
| 12 | **1242.6212** | 621.8142 | 1225.5947 | 613.3010 | **1224.6107** | 612.8090 | I | **773.4152** | 387.2112 | 756.3886 | 378.6980 | 755.4046 | 378.2060 | 6 |
| 13 | **1371.6638** | 686.3355 | 1354.6373 | 677.8223 | 1353.6532 | 677.3303 | E | **660.3311** | 330.6692 | 643.3046 | 322.1559 | 642.3206 | 321.6639 | 5 |
| 14 | **1500.7064** | 750.8568 | 1483.6799 | 742.3436 | 1482.6958 | 741.8516 | E | **531.2885** | 266.1479 | 514.2620 | 257.6346 | 513.2780 | 257.1426 | 4 |
| 15 | 1613.7905 | 807.3989 | **1596.7639** | 798.8856 | **1595.7799** | 798.3936 | L | **402.2459** | 201.6266 | 385.2194 | 193.1133 | | | 3 |
| 16 | **1727.8334** | 864.4203 | 1710.8068 | 855.9071 | 1709.8228 | 855.4151 | N | **289.1619** | 145.0846 | 272.1353 | 136.5713 | | | 2 |
| 17 | | | | | | | R | 175.1190 | 88.0631 | 158.0924 | 79.5498 | | | 1 |

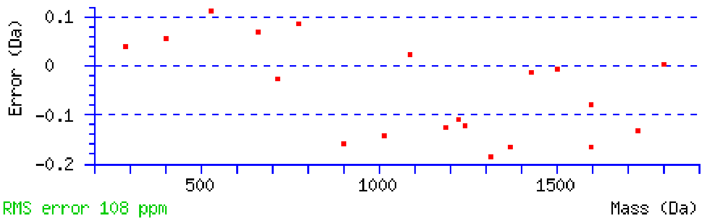


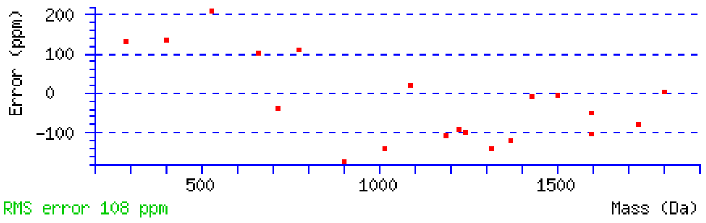


## All matches to this query

| Score | Mr(calc): | Delta | Sequence |
|---|---|---|---|
| 129.8 | 1900.9377 | 0.0065 | TLAGENQTALEIEELNR |
| 73.4 | 1899.9537 | 0.9905 | TLAGENQTALEIEELNR |
| 58.1 | 1900.9377 | 0.0065 | TLAGENQTALEIEELNR |
| 9.8 | 1899.9482 | 0.9960 | TELDSFLIEITANILK |
| | | | |

| | | | |
|---|---|---|---|
| 9.8 | 1899.9482 | 0.9960 | TELDSFLIEITANILK |
| 8.2 | 1900.9274 | 0.0168 | SVSTMLYLMALQELNR |
| 5.4 | 1900.9457 | -0.0015 | GFLMTKIFIGCPPGKR |
| 3.5 | 1900.9376 | 0.0066 | EDVFWLPLLEKAYAK |
| 2.3 | 1900.9560 | -0.0118 | VYTLTPFLANNREKR |
| 2.3 | 1900.9560 | -0.0118 | VYTLTPFLANNREKR |

Spectrum No: 9; Query: 2109; Rank: 1

## Peptide View

MS/MS Fragmentation of **FKLDWLGNCSGLNDESYGYK**
Found in **IPI00339124**, Tax_Id=10116 Gene_Symbol=Atp1b1 Sodium/potassium-transporting ATPase subunit beta-1

Match to Query 2109: 2366.063148 from(1184.038850,2+)
Title: 100101RatKid_NS_deglyco_14.4549.4549.2.dta
Data file K:\NewmanPaper\Piliang\3SubProteomes\Piliang3SP\mgf5ppm\ERLIC_3SubProteomes5ppm.mgf

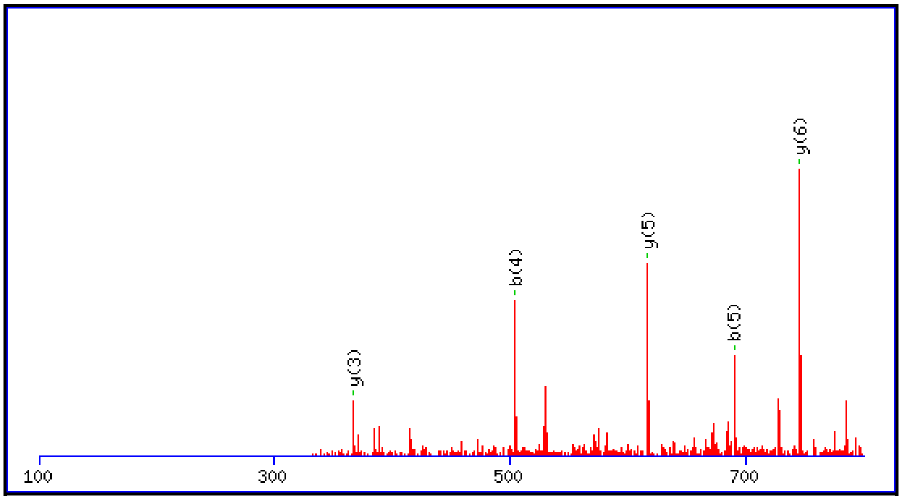


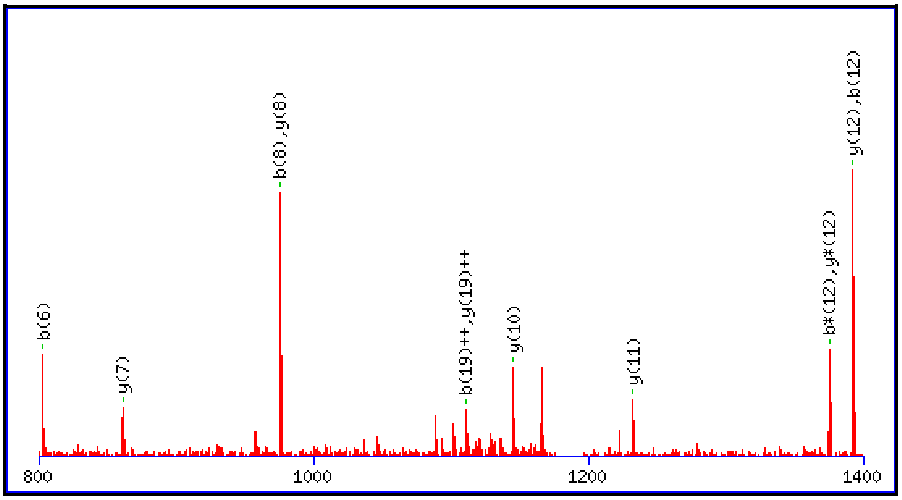

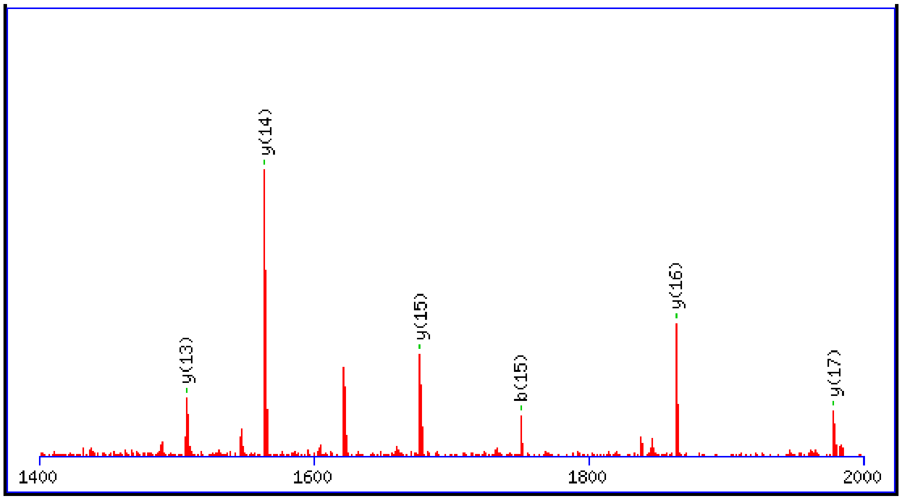


**Monoisotopic mass of neutral peptide Mr(calc):** 2366.0525
**Fixed modifications:** Carbamidomethyl (C)
**Variable modifications:**
**N8** : Deamidated_N (N)
**Ions Score:** 129 **Expect:** 3.7e-011
**Matches (Bold Red):** 23/212 fragment ions using 26 most intense peaks

| # | b | $b^{++}$ | b* | $b^{*++}$ | $b^0$ | $b^{0++}$ | Seq. | y | $y^{++}$ | y* | $y^{*++}$ | $y^0$ | $y^{0++}$ | # |
|---|---|---|---|---|---|---|---|---|---|---|---|---|---|---|
| 1 | 148.0757 | 74.5415 | | | | | F | | | | | | | 20 |
| 2 | 276.1707 | 138.5890 | 259.1441 | 130.0757 | | | K | 2219.9914 | **1110.4993** | 2202.9648 | 1101.9860 | 2201.9808 | 1101.4940 | 19 |
| 3 | 389.2547 | 195.1310 | 372.2282 | 186.6177 | | | L | 2091.8964 | 1046.4518 | 2074.8699 | 1037.9386 | 2073.8858 | 1037.4466 | 18 |
| 4 | **504.2817** | 252.6445 | 487.2551 | 244.1312 | 486.2711 | 243.6392 | D | **1978.8123** | 989.9098 | 1961.7858 | 981.3965 | 1960.8018 | 980.9045 | 17 |
| 5 | **690.3610** | 345.6841 | 673.3344 | 337.1709 | 672.3504 | 336.6788 | W | **1863.7854** | 932.3963 | 1846.7589 | 923.8831 | 1845.7748 | 923.3911 | 16 |
| 6 | **803.4450** | 402.2262 | 786.4185 | 393.7129 | 785.4345 | 393.2209 | L | **1677.7061** | 839.3567 | 1660.6795 | 830.8434 | 1659.6955 | 830.3514 | 15 |
| 7 | 860.4665 | 430.7369 | 843.4400 | 422.2236 | 842.4559 | 421.7316 | G | **1564.6220** | 782.8146 | 1547.5955 | 774.3014 | 1546.6115 | 773.8094 | 14 |
| 8 | **975.4934** | 488.2504 | 958.4669 | 479.7371 | 957.4829 | 479.2451 | N | **1507.6006** | 754.3039 | 1490.5740 | 745.7906 | 1489.5900 | 745.2986 | 13 |
| 9 | 1135.5241 | 568.2657 | 1118.4975 | 559.7524 | 1117.5135 | 559.2604 | C | **1392.5736** | 696.7904 | **1375.5471** | 688.2772 | 1374.5631 | 687.7852 | 12 |
| 10 | 1222.5561 | 611.7817 | 1205.5296 | 603.2684 | 1204.5455 | 602.7764 | S | **1232.5430** | 616.7751 | 1215.5164 | 608.2619 | 1214.5324 | 607.7698 | 11 |
| 11 | 1279.5776 | 640.2924 | 1262.5510 | 631.7792 | 1261.5670 | 631.2871 | G | **1145.5109** | 573.2591 | 1128.4844 | 564.7458 | 1127.5004 | 564.2538 | 10 |
| 12 | **1392.6616** | 696.8345 | **1375.6351** | 688.3212 | 1374.6511 | 687.8292 | L | 1088.4895 | 544.7484 | 1071.4629 | 536.2351 | 1070.4789 | 535.7431 | 9 |
| 13 | 1506.7046 | 753.8559 | 1489.6780 | 745.3426 | 1488.6940 | 744.8506 | N | **975.4054** | 488.2063 | 958.3789 | 479.6931 | 957.3949 | 479.2011 | 8 |
| 14 | 1621.7315 | 811.3694 | 1604.7050 | 802.8561 | 1603.7209 | 802.3641 | D | **861.3625** | 431.1849 | 844.3359 | 422.6716 | 843.3519 | 422.1796 | 7 |
| 15 | **1750.7741** | 875.8907 | 1733.7476 | 867.3774 | 1732.7635 | 866.8854 | E | **746.3355** | 373.6714 | 729.3090 | 365.1581 | 728.3250 | 364.6661 | 6 |
| 16 | 1837.8061 | 919.4067 | 1820.7796 | 910.8934 | 1819.7956 | 910.4014 | S | **617.2930** | 309.1501 | 600.2664 | 300.6368 | 599.2824 | 300.1448 | 5 |
| 17 | 2000.8695 | 1000.9384 | 1983.8429 | 992.4251 | 1982.8589 | 991.9331 | Y | 530.2609 | 265.6341 | 513.2344 | 257.1208 | | | 4 |
| 18 | 2057.8909 | 1029.4491 | 2040.8644 | 1020.9358 | 2039.8804 | 1020.4438 | G | **367.1976** | 184.1024 | 350.1710 | 175.5892 | | | 3 |
| 19 | 2220.9543 | **1110.9808** | 2203.9277 | 1102.4675 | 2202.9437 | 1101.9755 | Y | 310.1761 | 155.5917 | 293.1496 | 147.0784 | | | 2 |
| 20 | | | | | | | K | 147.1128 | 74.0600 | 130.0863 | 65.5468 | | | 1 |

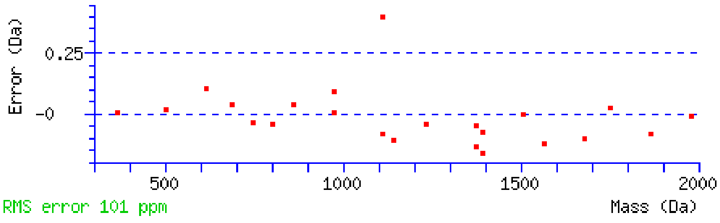


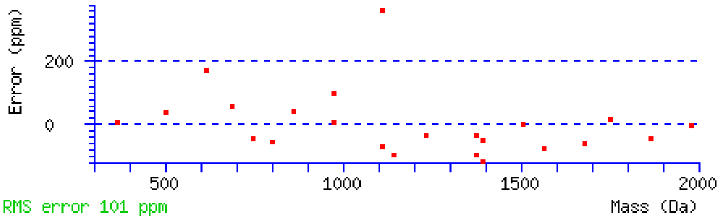


### All matches to this query

| Score | Mr(calc): | Delta | Sequence |
|---|---|---|---|
| 129.2 | 2366.0525 | 0.0107 | FKLDWLGNCSGLNDESYGYK |
| | | | |

| | | | |
|---|---|---|---|
| 92.3 | 2366.0525 | 0.0107 | FKLDWLGNCSGLNDESYGYK |
| 55.0 | 2365.0685 | 0.9947 | FKLDWLGNCSGLNDESYGYK |
| 9.2 | 2366.0630 | 0.0001 | LQGGKDFNMPLTISSLKDGGK |
| 9.2 | 2366.0630 | 0.0001 | LQGGKDFNMPLTISSLKDGGK |
| 9.2 | 2366.0630 | 0.0001 | LQGGKDFNMPLTISSLKDGGK |
| 5.3 | 2366.0759 | -0.0127 | SFSFSVNWNEIINFPLEIK |
| 5.3 | 2366.0759 | -0.0127 | SFSFSVNWNEIINFPLEIK |
| 5.3 | 2366.0759 | -0.0127 | SFSFSVNWNEIINFPLEIK |
| 4.6 | 2366.0776 | -0.0145 | MGANNLERMGLERMGANSLER |

Spectrum No: 10; Query: 2162; Rank: 1

## Peptide View

MS/MS Fragmentation of **IYYSDFSNQTINSMAEDGSNR**
Found in **IPI00205325**, Tax_Id=10116 Gene_Symbol=Lrp2 Low-density lipoprotein receptor-related protein 2 precursor

Match to Query 2162: 2412.025568 from(1207.020060,2+)
Title: 100101RatKid_NS_deglyco_18.3791.3791.2.dta
Data file K:\NewmanPaper\Piliang\3SubProteomes\Piliang3SP\mgf5ppm\ERLIC_3SubProteomes5ppm.mgf

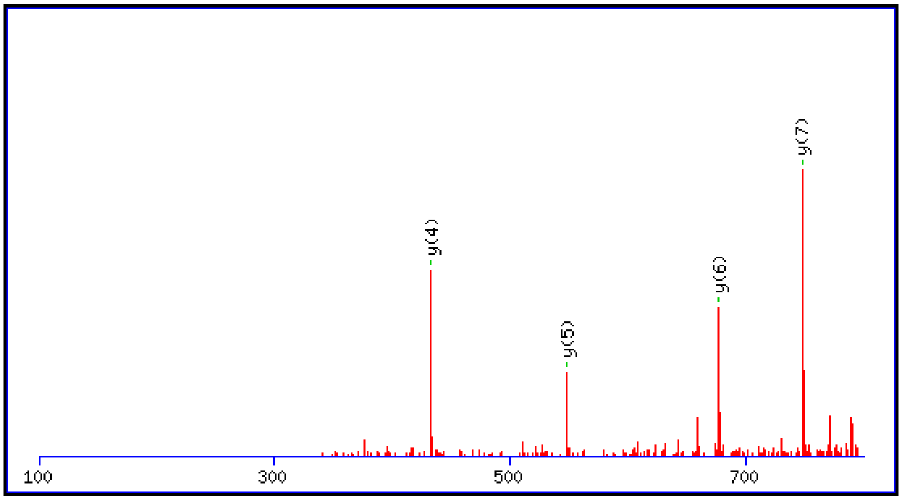


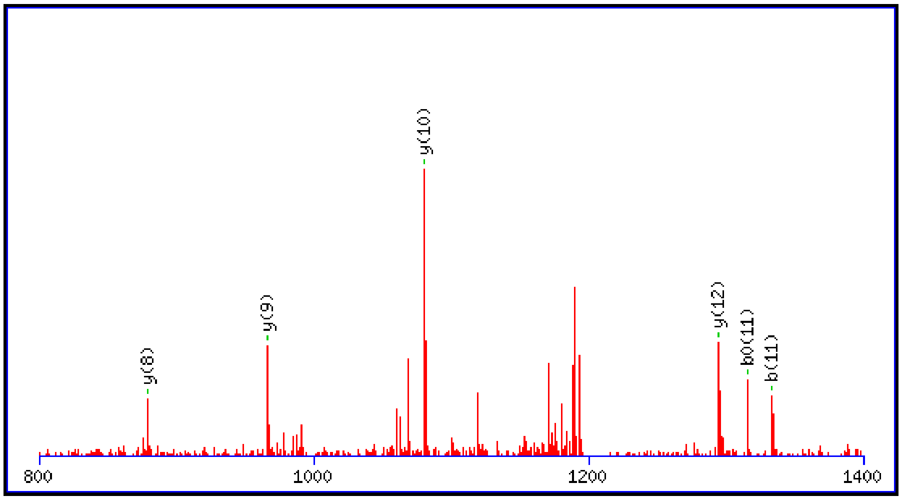

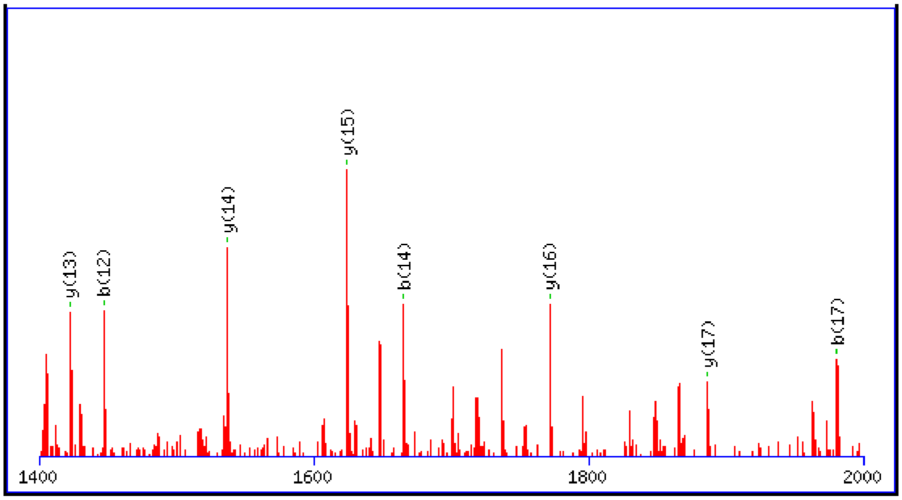


**Monoisotopic mass of neutral peptide Mr(calc):** 2412.0175
**Fixed modifications:** Carbamidomethyl (C)
**Variable modifications:**
**N8** : Deamidated_N (N)
**Ions Score:** 129 **Expect:** 4e-011
**Matches (Bold Red):** 18/216 fragment ions using 23 most intense peaks

| # | b | $b^{++}$ | b* | $b^{*++}$ | $b^{0}$ | $b^{0++}$ | Seq. | y | $y^{++}$ | y* | $y^{*++}$ | $y^{0}$ | $y^{0++}$ | # |
|---|---|---|---|---|---|---|---|---|---|---|---|---|---|---|
| 1 | 114.0913 | 57.5493 | | | | | I | | | | | | | 21 |
| 2 | 277.1547 | 139.0810 | | | | | Y | 2299.9408 | 1150.4740 | 2282.9142 | 1141.9608 | 2281.9302 | 1141.4687 | 20 |
| 3 | 440.2180 | 220.6126 | | | | | Y | 2136.8775 | 1068.9424 | 2119.8509 | 1060.4291 | 2118.8669 | 1059.9371 | 19 |
| 4 | 527.2500 | 264.1287 | | | 509.2395 | 255.1234 | S | 1973.8141 | 987.4107 | 1956.7876 | 978.8974 | 1955.8036 | 978.4054 | 18 |
| 5 | 642.2770 | 321.6421 | | | 624.2664 | 312.6368 | D | **1886.7821** | 943.8947 | 1869.7556 | 935.3814 | 1868.7715 | 934.8894 | 17 |
| 6 | 789.3454 | 395.1763 | | | 771.3348 | 386.1710 | F | **1771.7552** | 886.3812 | 1754.7286 | 877.8679 | 1753.7446 | 877.3759 | 16 |
| 7 | 876.3774 | 438.6923 | | | 858.3668 | 429.6871 | S | **1624.6867** | 812.8470 | 1607.6602 | 804.3337 | 1606.6762 | 803.8417 | 15 |
| 8 | 991.4043 | 496.2058 | 974.3778 | 487.6925 | 973.3938 | 487.2005 | N | **1537.6547** | 769.3310 | 1520.6282 | 760.8177 | 1519.6442 | 760.3257 | 14 |
| 9 | 1119.4629 | 560.2351 | 1102.4364 | 551.7218 | 1101.4524 | 551.2298 | Q | **1422.6278** | 711.8175 | 1405.6012 | 703.3043 | 1404.6172 | 702.8122 | 13 |
| 10 | 1220.5106 | 610.7589 | 1203.4841 | 602.2457 | 1202.5000 | 601.7537 | T | **1294.5692** | 647.7882 | 1277.5427 | 639.2750 | 1276.5586 | 638.7830 | 12 |
| 11 | **1333.5947** | 667.3010 | 1316.5681 | 658.7877 | **1315.5841** | 658.2957 | I | 1193.5215 | 597.2644 | 1176.4950 | 588.7511 | 1175.5110 | 588.2591 | 11 |
| 12 | **1447.6376** | 724.3224 | 1430.6110 | 715.8092 | 1429.6270 | 715.3172 | N | **1080.4375** | 540.7224 | 1063.4109 | 532.2091 | 1062.4269 | 531.7171 | 10 |
| 13 | 1534.6696 | 767.8384 | 1517.6431 | 759.3252 | 1516.6591 | 758.8332 | S | **966.3945** | 483.7009 | 949.3680 | 475.1876 | 948.3840 | 474.6956 | 9 |
| 14 | **1665.7101** | 833.3587 | 1648.6836 | 824.8454 | 1647.6995 | 824.3534 | M | **879.3625** | 440.1849 | 862.3360 | 431.6716 | 861.3519 | 431.1796 | 8 |
| 15 | 1736.7472 | 868.8772 | 1719.7207 | 860.3640 | 1718.7367 | 859.8720 | A | **748.3220** | 374.6646 | 731.2955 | 366.1514 | 730.3115 | 365.6594 | 7 |
| 16 | 1865.7898 | 933.3985 | 1848.7633 | 924.8853 | 1847.7792 | 924.3933 | E | **677.2849** | 339.1461 | 660.2584 | 330.6328 | 659.2743 | 330.1408 | 6 |
| 17 | **1980.8168** | 990.9120 | 1963.7902 | 982.3987 | 1962.8062 | 981.9067 | D | **548.2423** | 274.6248 | 531.2158 | 266.1115 | 530.2317 | 265.6195 | 5 |
| 18 | 2037.8382 | 1019.4227 | 2020.8117 | 1010.9095 | 2019.8277 | 1010.4175 | G | **433.2154** | 217.1113 | 416.1888 | 208.5980 | 415.2048 | 208.1060 | 4 |
| 19 | 2124.8702 | 1062.9388 | 2107.8437 | 1054.4255 | 2106.8597 | 1053.9335 | S | 376.1939 | 188.6006 | 359.1674 | 180.0873 | 358.1833 | 179.5953 | 3 |
| 20 | 2238.9132 | 1119.9602 | 2221.8866 | 1111.4470 | 2220.9026 | 1110.9549 | N | 289.1619 | 145.0846 | 272.1353 | 136.5713 | | | 2 |
| 21 | | | | | | | R | 175.1190 | 88.0631 | 158.0924 | 79.5498 | | | 1 |

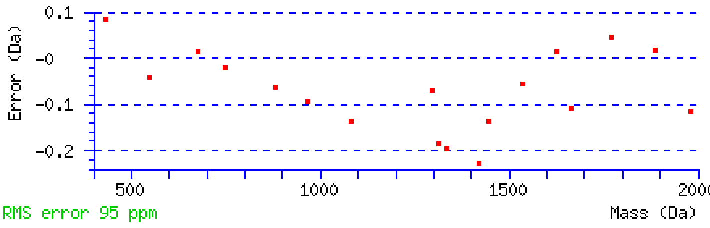


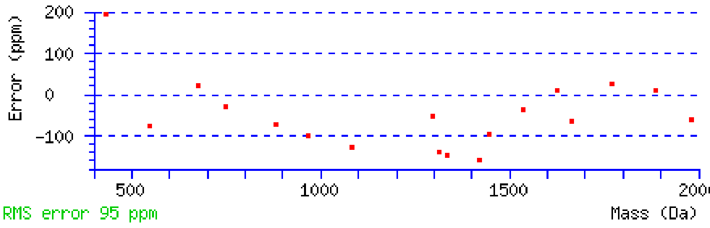


### All matches to this query

| Score | Mr(calc): | Delta | Sequence |
|---|---|---|---|
| | | | |

| | | | |
|---|---|---|---|
| 128.8 | 2412.0175 | 0.0080 | IYYSDFSNQTINSMAEDGSNR |
| 88.6 | 2412.0175 | 0.0080 | IYYSDFSNQTINSMAEDGSNR |
| 56.9 | 2412.0175 | 0.0080 | IYYSDFSNQTINSMAEDGSNR |
| 50.9 | 2411.0335 | 0.9920 | IYYSDFSNQTINSMAEDGSNR |
| 3.3 | 2410.0299 | 1.9957 | DSRAVSEQTAPNSNSNGSWAPR |
| 2.3 | 2412.0403 | -0.0147 | EGICGSCAMNINGGNTLACTRR |
| 1.9 | 2411.0287 | 0.9969 | QSEWHKMTVSKNCPDQDLK |
| 0.8 | 2412.0279 | -0.0024 | TNYLLSSTFPEXMALCWNR |
| 0.8 | 2412.0279 | -0.0024 | TNYLLSSTFPEXMALCWNR |
| 0.8 | 2412.0279 | -0.0024 | TNYLLSSTFPEXMALCWNR |

---

Spectrum No: 11; Query: 1176; Rank: 1

---

## Peptide View

MS/MS Fragmentation of **MESDLANTTAGFTAALK**
Found in **IPI00392894**, Tax_Id=10116 Gene_Symbol=Mgam_predicted 89 kDa protein

Match to Query 1176: 1756.816828 from(879.415690,2+)
Title: 091008RatKidney_NoSalt_30.2788.2788.2.dta
Data file K:\NewmanPaper\Piliang\3SubProteomes\Piliang3SP\mgf5ppm\ERLIC_3SubProteomes5ppm.mgf

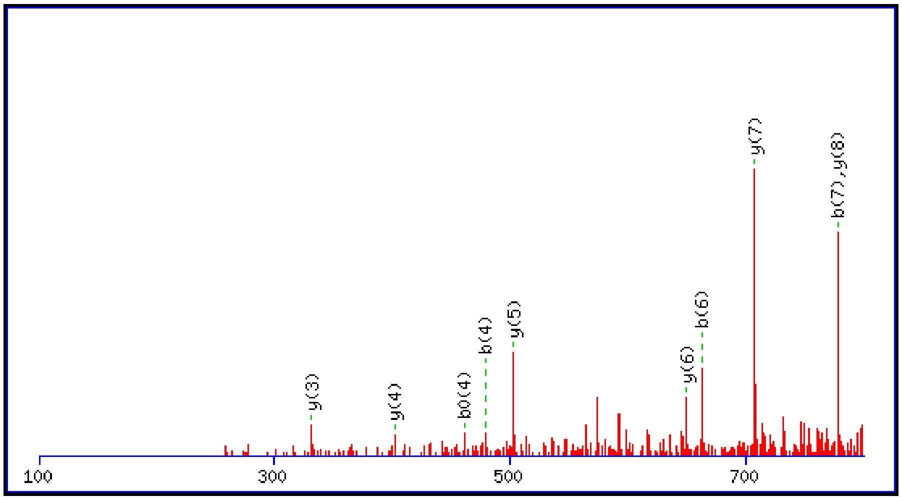


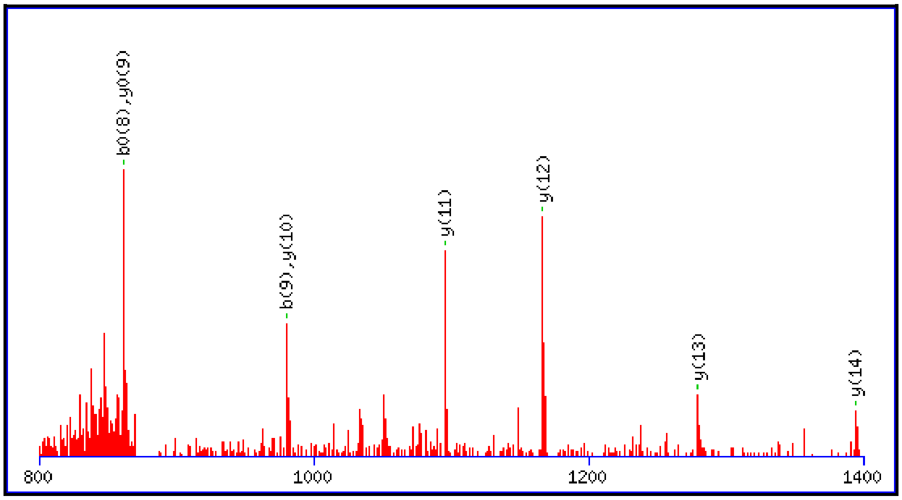

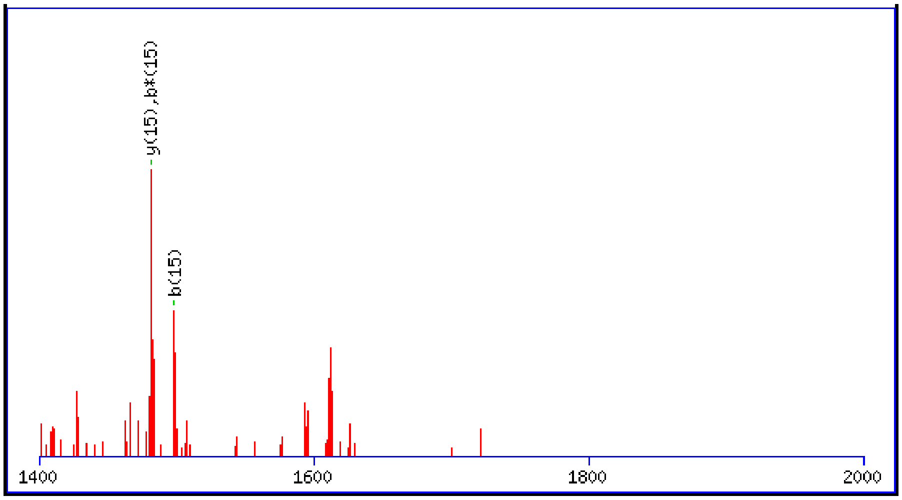


**Monoisotopic mass of neutral peptide Mr(calc):** 1756.8189
**Fixed modifications:** Carbamidomethyl (C)
**Variable modifications:**
**M1** : Oxidation (M)
**N7** : Deamidated_N (N)
**Ions Score:** 125 **Expect:** 7e-011
**Matches (Bold Red):** 21/170 fragment ions using 23 most intense peaks

| # | b | $b^{++}$ | b* | $b^{*++}$ | $b^{0}$ | $b^{0++}$ | Seq. | y | $y^{++}$ | y* | $y^{*++}$ | $y^{0}$ | $y^{0++}$ | # |
|---|---|---|---|---|---|---|---|---|---|---|---|---|---|---|
| 1 | 148.0427 | 74.5250 | | | | | M | | | | | | | 17 |
| 2 | 277.0853 | 139.0463 | | | 259.0747 | 130.0410 | E | 1610.7908 | 805.8990 | 1593.7643 | 797.3858 | 1592.7802 | 796.8938 | 16 |
| 3 | 364.1173 | 182.5623 | | | 346.1067 | 173.5570 | S | **1481.7482** | 741.3777 | 1464.7217 | 732.8645 | 1463.7376 | 732.3725 | 15 |
| 4 | **479.1442** | 240.0758 | | | **461.1337** | 231.0705 | D | **1394.7162** | 697.8617 | 1377.6896 | 689.3485 | 1376.7056 | 688.8564 | 14 |
| 5 | 592.2283 | 296.6178 | | | 574.2177 | 287.6125 | L | **1279.6892** | 640.3483 | 1262.6627 | 631.8350 | 1261.6787 | 631.3430 | 13 |
| 6 | **663.2654** | 332.1363 | | | 645.2549 | 323.1311 | A | **1166.6052** | 583.8062 | 1149.5786 | 575.2930 | 1148.5946 | 574.8009 | 12 |
| 7 | **778.2924** | 389.6498 | 761.2658 | 381.1365 | 760.2818 | 380.6445 | N | **1095.5681** | 548.2877 | 1078.5415 | 539.7744 | 1077.5575 | 539.2824 | 11 |
| 8 | 879.3400 | 440.1737 | 862.3135 | 431.6604 | **861.3295** | 431.1684 | T | **980.5411** | 490.7742 | 963.5146 | 482.2609 | 962.5306 | 481.7689 | 10 |
| 9 | **980.3877** | 490.6975 | 963.3612 | 482.1842 | 962.3772 | 481.6922 | T | 879.4934 | 440.2504 | 862.4669 | 431.7371 | **861.4829** | 431.2451 | 9 |
| 10 | 1051.4248 | 526.2161 | 1034.3983 | 517.7028 | 1033.4143 | 517.2108 | A | **778.4458** | 389.7265 | 761.4192 | 381.2132 | 760.4352 | 380.7212 | 8 |
| 11 | 1108.4463 | 554.7268 | 1091.4197 | 546.2135 | 1090.4357 | 545.7215 | G | **707.4087** | 354.2080 | 690.3821 | 345.6947 | 689.3981 | 345.2027 | 7 |
| 12 | 1255.5147 | 628.2610 | 1238.4882 | 619.7477 | 1237.5041 | 619.2557 | F | **650.3872** | 325.6972 | 633.3606 | 317.1840 | 632.3766 | 316.6920 | 6 |
| 13 | 1356.5624 | 678.7848 | 1339.5358 | 670.2716 | 1338.5518 | 669.7795 | T | **503.3188** | 252.1630 | 486.2922 | 243.6498 | 485.3082 | 243.1577 | 5 |
| 14 | 1427.5995 | 714.3034 | 1410.5730 | 705.7901 | 1409.5889 | 705.2981 | A | **402.2711** | 201.6392 | 385.2445 | 193.1259 | | | 4 |
| 15 | **1498.6366** | 749.8219 | **1481.6101** | 741.3087 | 1480.6260 | 740.8167 | A | **331.2340** | 166.1206 | 314.2074 | 157.6074 | | | 3 |
| 16 | 1611.7207 | 806.3640 | 1594.6941 | 797.8507 | 1593.7101 | 797.3587 | L | 260.1969 | 130.6021 | 243.1703 | 122.0888 | | | 2 |
| 17 | | | | | | | K | 147.1128 | 74.0600 | 130.0863 | 65.5468 | | | 1 |

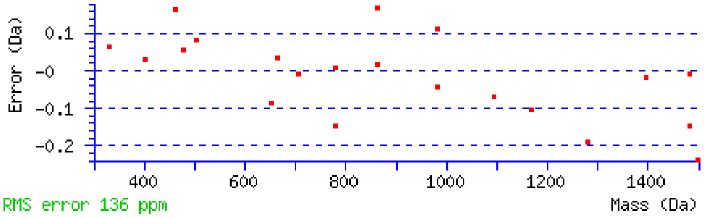


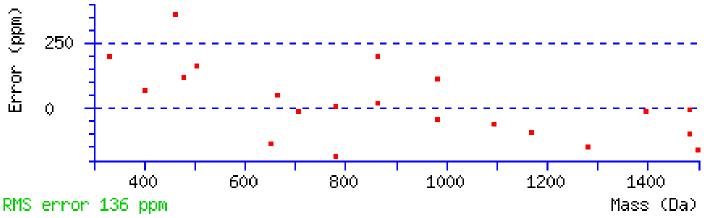


## All matches to this query

| Score | Mr(calc): | Delta | Sequence |
|---|---|---|---|
| 124.8 | 1756.8189 | -0.0021 | MESDLANTTAGFTAALK |
| 5.9 | 1756.8045 | 0.0123 | TLLNTSCDNMLMAIK |
| 5.4 | 1754.7997 | 2.0171 | MMSFGKEHRPPSKK |
| 5.4 | 1754.7997 | 2.0171 | MMSFGKEHRPPSKK |

| | | | |
|---|---|---|---|
| 3.5 | 1756.8318 | -0.0149 | TSILINALGSNTCLAK |
| 3.2 | 1756.8219 | -0.0051 | LKVTHQAESNLYMK |
| 3.1 | 1756.8050 | 0.0118 | GSSARPSDDVPPMNQAK |
| 2.8 | 1756.8318 | -0.0149 | TSILINALGSNTCLAK |
| 2.8 | 1756.8318 | -0.0149 | TSILINALGSNTCLAK |
| 1.5 | 1755.7975 | 1.0193 | APQAAKACXQTSVSTR |

---

Spectrum No: 12; Query: 714; Rank: 1

---

## Peptide View

MS/MS Fragmentation of **VFGANQSLSAAQLGR**
Found in **IPI00392474**, Tax_Id=10116 Gene_Symbol=Slc39a8 Zinc transporter ZIP8

Match to Query 714: 1518.780108 from(760.397330,2+)
Title: 100101RatKid_NS_deglyco_25.2513.2513.2.dta
Data file K:\NewmanPaper\Piliang\3SubProteomes\Piliang3SP\mgf5ppm\ERLIC_3SubProteomes5ppm.mgf

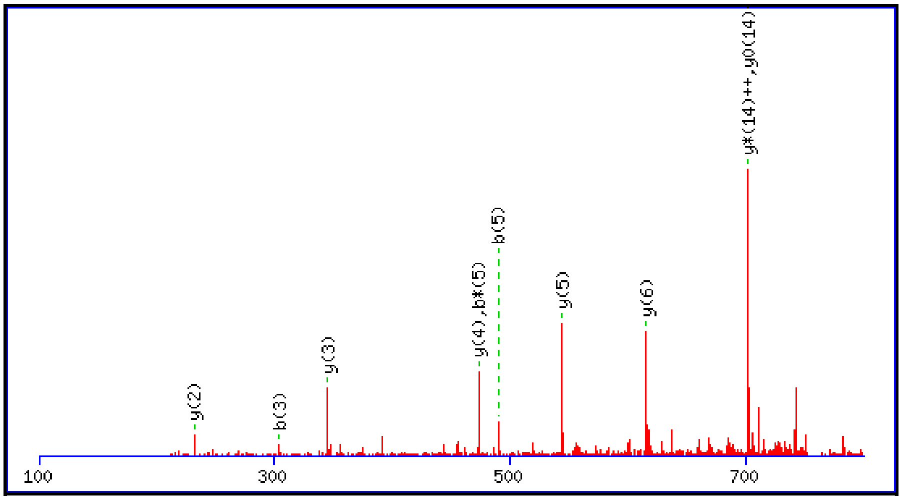


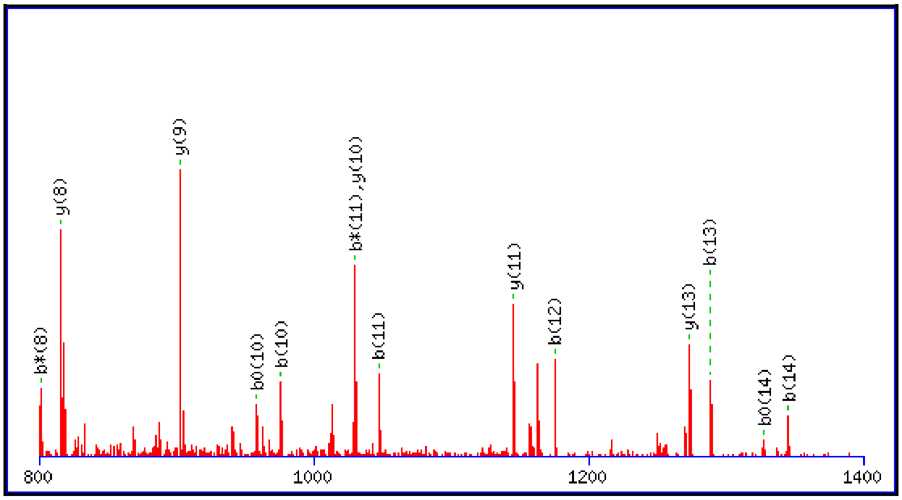

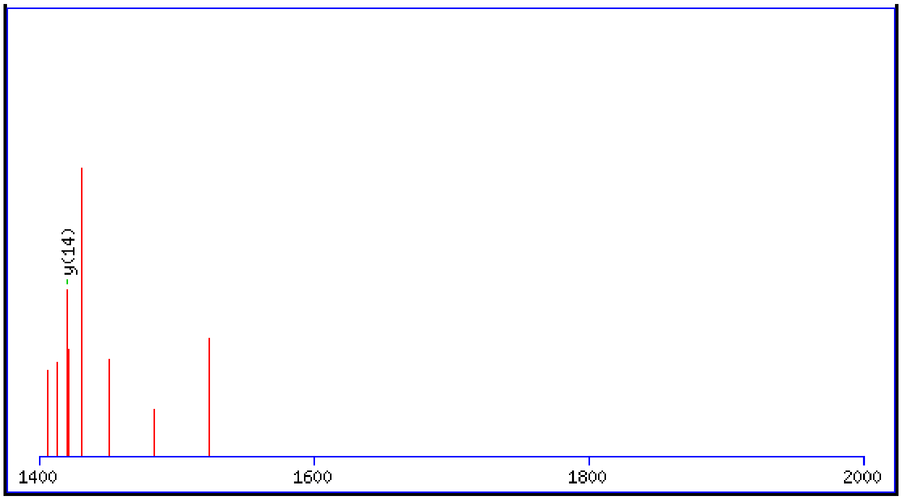


**Monoisotopic mass of neutral peptide Mr(calc):** 1518.7790
**Fixed modifications:** Carbamidomethyl (C)
**Variable modifications:**
**N5** : Deamidated_N (N)
**Ions Score:** 123 **Expect:** 6.4e-011
**Matches (Bold Red):** 26/136 fragment ions using 32 most intense peaks

| # | b | $b^{++}$ | b* | $b^{*++}$ | $b^{0}$ | $b^{0++}$ | Seq. | y | $y^{++}$ | y* | $y^{*++}$ | $y^{0}$ | $y^{0++}$ | # |
|---|---|---|---|---|---|---|---|---|---|---|---|---|---|---|
| 1 | 100.0757 | 50.5415 | | | | | V | | | | | | | 15 |
| 2 | 247.1441 | 124.0757 | | | | | F | **1420.7179** | 710.8626 | 1403.6914 | **702.3493** | 1402.7073 | **701.8573** | 14 |
| 3 | **304.1656** | 152.5864 | | | | | G | **1273.6495** | 637.3284 | 1256.6229 | 628.8151 | 1255.6389 | 628.3231 | 13 |
| 4 | 375.2027 | 188.1050 | | | | | A | 1216.6280 | 608.8177 | 1199.6015 | 600.3044 | 1198.6175 | 599.8124 | 12 |
| 5 | **490.2296** | 245.6184 | **473.2031** | 237.1052 | | | N | **1145.5909** | 573.2991 | 1128.5644 | 564.7858 | 1127.5804 | 564.2938 | 11 |
| 6 | 618.2882 | 309.6477 | 601.2616 | 301.1345 | | | Q | **1030.5640** | 515.7856 | 1013.5374 | 507.2724 | 1012.5534 | 506.7803 | 10 |
| 7 | 705.3202 | 353.1637 | 688.2937 | 344.6505 | 687.3097 | 344.1585 | S | **902.5054** | 451.7563 | 885.4789 | 443.2431 | 884.4948 | 442.7511 | 9 |
| 8 | 818.4043 | 409.7058 | **801.3777** | 401.1925 | 800.3937 | 400.7005 | L | **815.4734** | 408.2403 | 798.4468 | 399.7271 | 797.4628 | 399.2350 | 8 |
| 9 | 905.4363 | 453.2218 | 888.4098 | 444.7085 | 887.4258 | 444.2165 | S | **702.3893** | 351.6983 | 685.3628 | 343.1850 | 684.3787 | 342.6930 | 7 |
| 10 | **976.4734** | 488.7404 | 959.4469 | 480.2271 | **958.4629** | 479.7351 | A | **615.3573** | 308.1823 | 598.3307 | 299.6690 | | | 6 |
| 11 | **1047.5105** | 524.2589 | **1030.4840** | 515.7456 | 1029.5000 | 515.2536 | A | **544.3202** | 272.6637 | 527.2936 | 264.1504 | | | 5 |
| 12 | **1175.5691** | 588.2882 | 1158.5426 | 579.7749 | 1157.5586 | 579.2829 | Q | **473.2831** | 237.1452 | 456.2565 | 228.6319 | | | 4 |
| 13 | **1288.6532** | 644.8302 | 1271.6266 | 636.3170 | 1270.6426 | 635.8249 | L | **345.2245** | 173.1159 | 328.1979 | 164.6026 | | | 3 |
| 14 | **1345.6746** | 673.3410 | 1328.6481 | 664.8277 | **1327.6641** | 664.3357 | G | **232.1404** | 116.5738 | 215.1139 | 108.0606 | | | 2 |
| 15 | | | | | | | R | 175.1190 | 88.0631 | 158.0924 | 79.5498 | | | 1 |

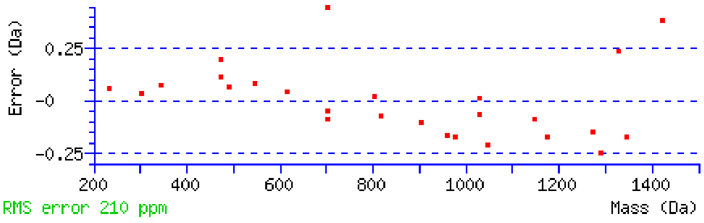


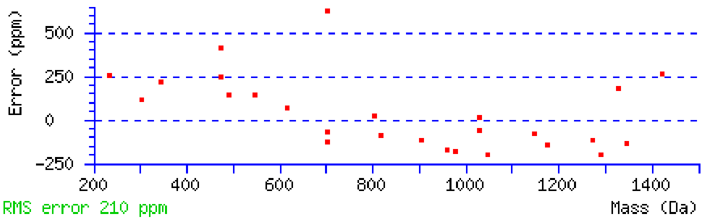


### All matches to this query

| Score | Mr(calc): | Delta | Sequence |
|---|---|---|---|
| 123.5 | 1518.7790 | 0.0011 | VFGANQSLSAAQLGR |
| 11.4 | 1518.7903 | -0.0102 | VEFEGRVLSRNGR |
| 10.4 | 1517.7755 | 1.0046 | NLFSRYGKVVGAK |
| 7.1 | 1518.7748 | 0.0053 | FASIWYAKKLGR |
| 7.1 | 1518.7748 | 0.0053 | FASIWYAKKLGR |
| 6.5 | 1518.7807 | -0.0006 | VKASNPLEQQVVK |
| | | | |

| | | | |
|---|---|---|---|
| 5.8 | 1517.7616 | 1.0185 | INFSHPKAAARAR |
| 5.5 | 1517.7732 | 1.0069 | SKAGAGAASRLMNER |
| 5.1 | 1518.7711 | 0.0090 | NKTAGEEEMIKIR |
| 4.9 | 1517.7912 | 0.9889 | VMLTNVLWTDLGR |

---

Spectrum No: 13; Query: 1841; Rank: 1

---

## Peptide View

MS/MS Fragmentation of **ISASGAELEALEAQVLNLSLK**
Found in **IPI00188541**, Tax_Id=10116 Gene_Symbol=Itih4 inter-alpha-inhibitor H4 heavy chain

Match to Query 1841: 2156.161228 from(1079.087890,2+)
Title: 091008RatKidney_NoSalt_26.5458.5458.2.dta
Data file K:\NewmanPaper\Piliang\3SubProteomes\Piliang3SP\mgf5ppm\ERLIC_3SubProteomes5ppm.mgf

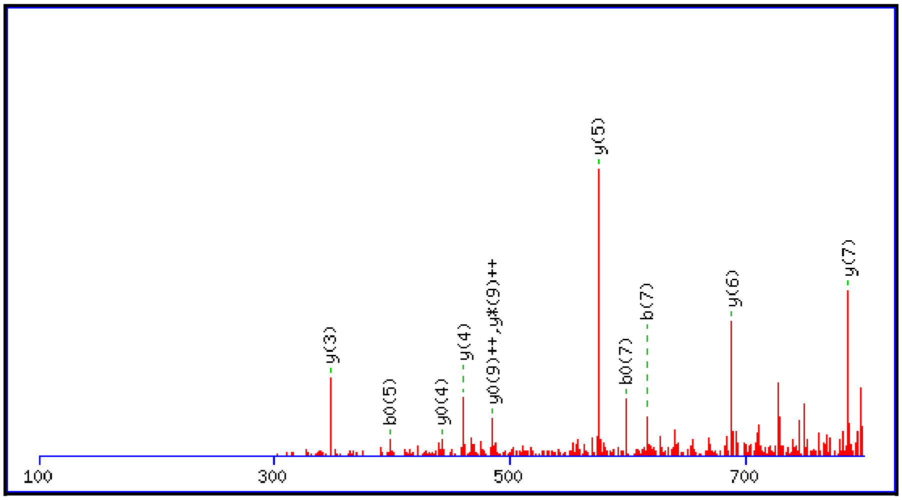


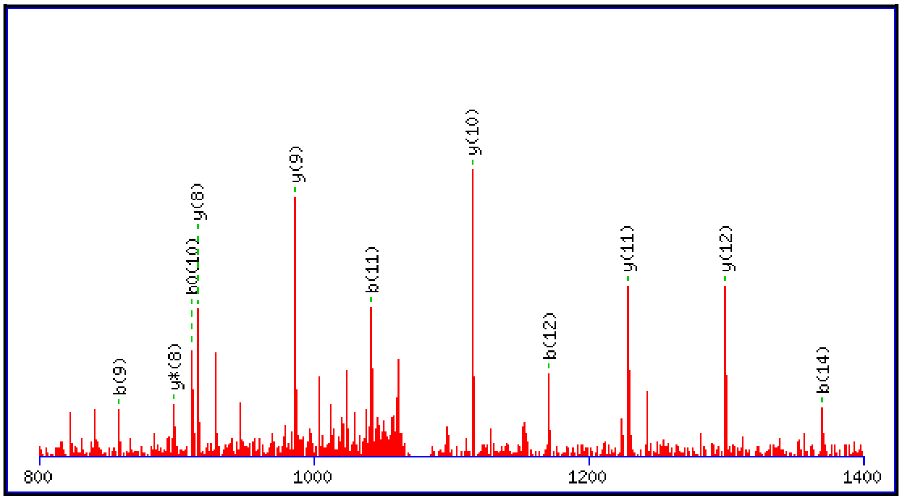

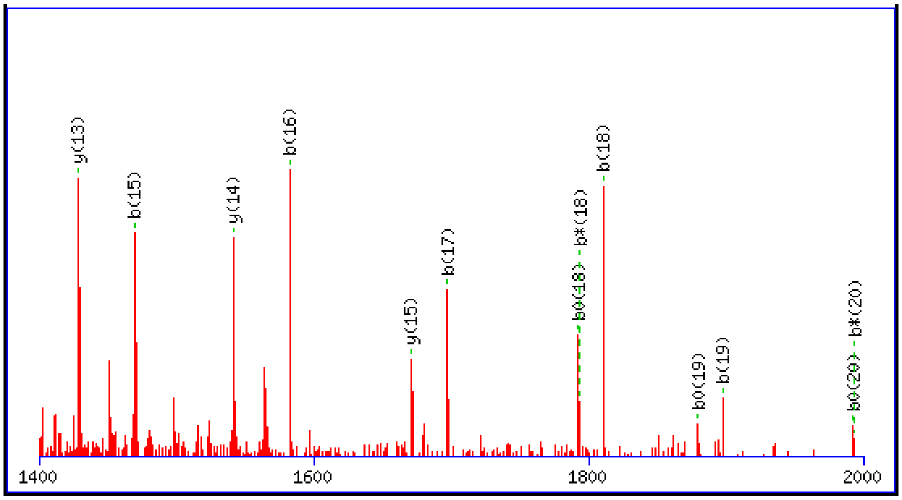


**Monoisotopic mass of neutral peptide Mr(calc):** 2156.1576
**Fixed modifications:** Carbamidomethyl (C)
**Variable modifications:**
**N17** : Deamidated_N (N)
**Ions Score:** 123 **Expect:** 3.3e-011
**Matches (Bold Red):** 35/208 fragment ions using 42 most intense peaks

| # | b | b++ | b* | b*++ | b0 | b0++ | Seq. | y | y++ | y* | y*++ | y0 | y0++ | # |
|---|---|---|---|---|---|---|---|---|---|---|---|---|---|---|
| 1 | 114.0913 | 57.5493 | | | | | I | | | | | | | 21 |
| 2 | 201.1234 | 101.0653 | | | 183.1128 | 92.0600 | S | 2044.0808 | 1022.5441 | 2027.0543 | 1014.0308 | 2026.0703 | 1013.5388 | 20 |
| 3 | 272.1605 | 136.5839 | | | 254.1499 | 127.5786 | A | 1957.0488 | 979.0280 | 1940.0223 | 970.5148 | 1939.0382 | 970.0228 | 19 |
| 4 | 359.1925 | 180.0999 | | | 341.1819 | 171.0946 | S | 1886.0117 | 943.5095 | 1868.9851 | 934.9962 | 1868.0011 | 934.5042 | 18 |
| 5 | 416.2140 | 208.6106 | | | **398.2034** | 199.6053 | G | 1798.9797 | 899.9935 | 1781.9531 | 891.4802 | 1780.9691 | 890.9882 | 17 |
| 6 | 487.2511 | 244.1292 | | | 469.2405 | 235.1239 | A | 1741.9582 | 871.4827 | 1724.9317 | 862.9695 | 1723.9476 | 862.4775 | 16 |
| 7 | **616.2937** | 308.6505 | | | **598.2831** | 299.6452 | E | **1670.9211** | 835.9642 | 1653.8945 | 827.4509 | 1652.9105 | 826.9589 | 15 |
| 8 | 729.3777 | 365.1925 | | | 711.3672 | 356.1872 | L | **1541.8785** | 771.4429 | 1524.8519 | 762.9296 | 1523.8679 | 762.4376 | 14 |
| 9 | **858.4203** | 429.7138 | | | 840.4098 | 420.7085 | E | **1428.7944** | 714.9009 | 1411.7679 | 706.3876 | 1410.7839 | 705.8956 | 13 |
| 10 | 929.4575 | 465.2324 | | | **911.4469** | 456.2271 | A | **1299.7518** | 650.3796 | 1282.7253 | 641.8663 | 1281.7413 | 641.3743 | 12 |
| 11 | **1042.5415** | 521.7744 | | | 1024.5310 | 512.7691 | L | **1228.7147** | 614.8610 | 1211.6882 | 606.3477 | 1210.7042 | 605.8557 | 11 |
| 12 | **1171.5841** | 586.2957 | | | 1153.5735 | 577.2904 | E | **1115.6307** | 558.3190 | 1098.6041 | 549.8057 | 1097.6201 | 549.3137 | 10 |
| 13 | 1242.6212 | 621.8142 | | | 1224.6107 | 612.8090 | A | **986.5881** | 493.7977 | 969.5615 | **485.2844** | 968.5775 | **484.7924** | 9 |
| 14 | **1370.6798** | 685.8435 | 1353.6533 | 677.3303 | 1352.6692 | 676.8383 | Q | **915.5510** | 458.2791 | **898.5244** | 449.7658 | 897.5404 | 449.2738 | 8 |
| 15 | **1469.7482** | 735.3777 | 1452.7217 | 726.8645 | 1451.7377 | 726.3725 | V | **787.4924** | 394.2498 | 770.4658 | 385.7366 | 769.4818 | 385.2445 | 7 |
| 16 | **1582.8323** | 791.9198 | 1565.8057 | 783.4065 | 1564.8217 | 782.9145 | L | **688.4240** | 344.7156 | 671.3974 | 336.2023 | 670.4134 | 335.7103 | 6 |
| 17 | **1697.8592** | 849.4332 | 1680.8327 | 840.9200 | 1679.8486 | 840.4280 | N | **575.3399** | 288.1736 | 558.3133 | 279.6603 | 557.3293 | 279.1683 | 5 |
| 18 | **1810.9433** | 905.9753 | **1793.9167** | 897.4620 | **1792.9327** | 896.9700 | L | **460.3130** | 230.6601 | 443.2864 | 222.1468 | **442.3024** | 221.6548 | 4 |
| 19 | **1897.9753** | 949.4913 | 1880.9488 | 940.9780 | **1879.9647** | 940.4860 | S | **347.2289** | 174.1181 | 330.2023 | 165.6048 | 329.2183 | 165.1128 | 3 |
| 20 | 2011.0594 | 1006.0333 | **1994.0328** | 997.5200 | **1993.0488** | 997.0280 | L | 260.1969 | 130.6021 | 243.1703 | 122.0888 | | | 2 |
| 21 | | | | | | | K | 147.1128 | 74.0600 | 130.0863 | 65.5468 | | | 1 |

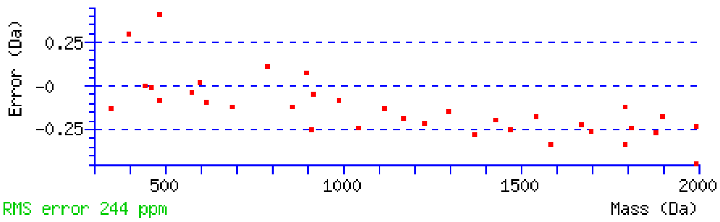


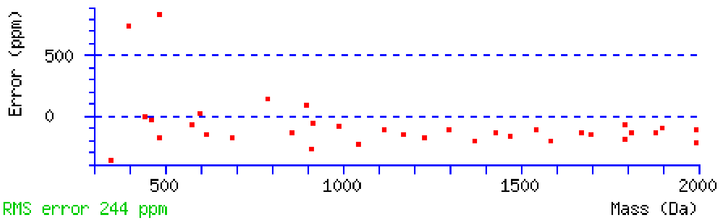


## All matches to this query

| Score | Mr(calc): | Delta | Sequence |
|---|---|---|---|
| | | | |

| | | | |
|---|---|---|---|
| 123.4 | 2156.1576 | 0.0037 | ISASGAELEALEAQVLNLSLK |
| 26.8 | 2155.1736 | 0.9877 | ISASGAELEALEAQVLNLSLK |
| 7.2 | 2156.1801 | -0.0188 | TVQIEASTVEIEERGVKLR |
| 5.7 | 2156.1412 | 0.0201 | WCGDPALLNSLKQKSALVR |
| 1.8 | 2156.1549 | 0.0063 | TINLTVDGNALGREAGGTRLK |
| 0.6 | 2156.1814 | -0.0202 | NAEIFLALQNQRGGRISLR |

---

Spectrum No: 14; Query: 458; Rank: 1

---

## Peptide View

MS/MS Fragmentation of **VSLTNVSISDEGR**
Found in **IPI00464765**, Tax_Id=10116 Gene_Symbol=Igsf4a Immunoglobulin superfamily, member 4A

Match to Query 458: 1376.680908 from(689.347730,2+)
Title: 100101RatKid_NS_deglyco_27.2032.2032.2.dta
Data file K:\NewmanPaper\Piliang\3SubProteomes\Piliang3SP\mgf5ppm\ERLIC_3SubProteomes5ppm.mgf

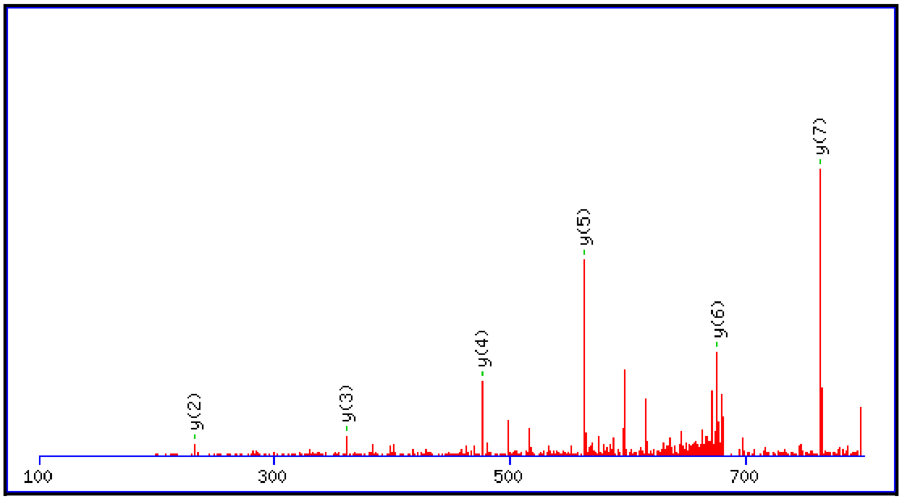


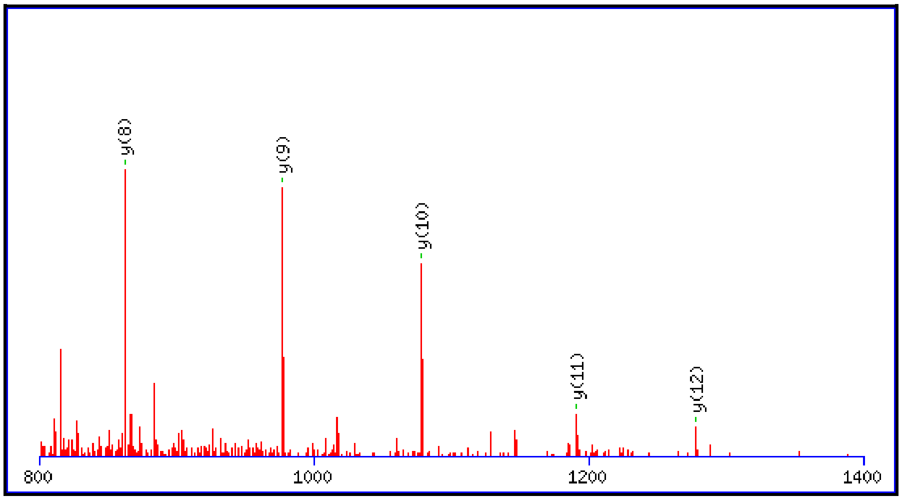

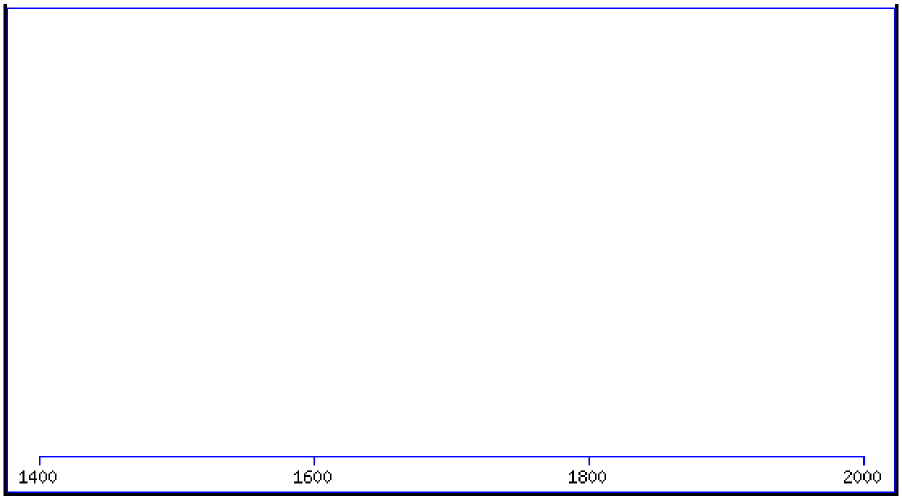


**Monoisotopic mass of neutral peptide Mr(calc):** 1376.6783
**Fixed modifications:** Carbamidomethyl (C)
**Variable modifications:**
**N5** : Deamidated_N (N)
**Ions Score:** 122 **Expect:** 8.7e-011
**Matches (Bold Red):** 11/130 fragment ions using 15 most intense peaks

| # | b | $b^{++}$ | b* | $b^{*++}$ | $b^{0}$ | $b^{0++}$ | Seq. | y | $y^{++}$ | y* | $y^{*++}$ | $y^{0}$ | $y^{0++}$ | # |
|---|---|---|---|---|---|---|---|---|---|---|---|---|---|---|
| 1 | 100.0757 | 50.5415 | | | | | V | | | | | | | 13 |
| 2 | 187.1077 | 94.0575 | | | 169.0972 | 85.0522 | S | **1278.6172** | 639.8122 | 1261.5906 | 631.2990 | 1260.6066 | 630.8070 | 12 |
| 3 | 300.1918 | 150.5995 | | | 282.1812 | 141.5942 | L | **1191.5852** | 596.2962 | 1174.5586 | 587.7829 | 1173.5746 | 587.2909 | 11 |
| 4 | 401.2395 | 201.1234 | | | 383.2289 | 192.1181 | T | **1078.5011** | 539.7542 | 1061.4746 | 531.2409 | 1060.4905 | 530.7489 | 10 |
| 5 | 516.2664 | 258.6368 | 499.2398 | 250.1236 | 498.2558 | 249.6316 | N | **977.4534** | 489.2303 | 960.4269 | 480.7171 | 959.4429 | 480.2251 | 9 |
| 6 | 615.3348 | 308.1710 | 598.3083 | 299.6578 | 597.3242 | 299.1658 | V | **862.4265** | 431.7169 | 845.3999 | 423.2036 | 844.4159 | 422.7116 | 8 |
| 7 | 702.3668 | 351.6871 | 685.3403 | 343.1738 | 684.3563 | 342.6818 | S | **763.3581** | 382.1827 | 746.3315 | 373.6694 | 745.3475 | 373.1774 | 7 |
| 8 | 815.4509 | 408.2291 | 798.4244 | 399.7158 | 797.4403 | 399.2238 | I | **676.3260** | 338.6667 | 659.2995 | 330.1534 | 658.3155 | 329.6614 | 6 |
| 9 | 902.4829 | 451.7451 | 885.4564 | 443.2318 | 884.4724 | 442.7398 | S | **563.2420** | 282.1246 | 546.2154 | 273.6114 | 545.2314 | 273.1193 | 5 |
| 10 | 1017.5099 | 509.2586 | 1000.4833 | 500.7453 | 999.4993 | 500.2533 | D | **476.2100** | 238.6086 | 459.1834 | 230.0953 | 458.1994 | 229.6033 | 4 |
| 11 | 1146.5525 | 573.7799 | 1129.5259 | 565.2666 | 1128.5419 | 564.7746 | E | **361.1830** | 181.0951 | 344.1565 | 172.5819 | 343.1724 | 172.0899 | 3 |
| 12 | 1203.5739 | 602.2906 | 1186.5474 | 593.7773 | 1185.5634 | 593.2853 | G | **232.1404** | 116.5738 | 215.1139 | 108.0606 | | | 2 |
| 13 | | | | | | | R | 175.1190 | 88.0631 | 158.0924 | 79.5498 | | | 1 |

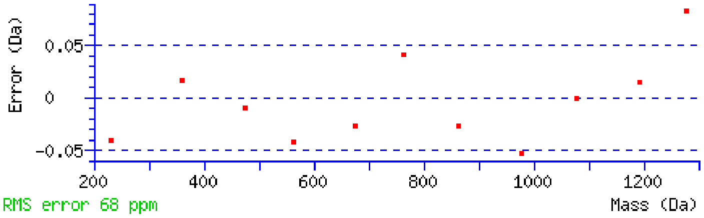


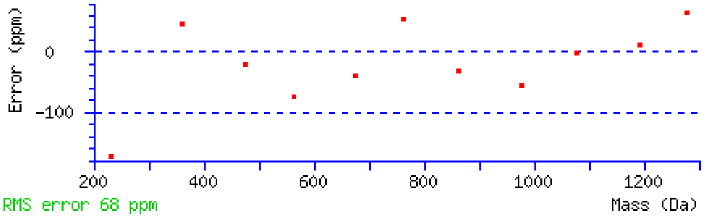


### All matches to this query

| Score | Mr(calc): | Delta | Sequence |
|---|---|---|---|
| 122.0 | 1376.6783 | 0.0026 | VSLTNVSISDEGR |
| 3.6 | 1376.6871 | -0.0061 | IFKSSDHIQMR |
| 3.2 | 1376.6837 | -0.0028 | DAPFFRESPVGR |
| 2.1 | 1376.6718 | 0.0091 | KNIEEKSGMEGR |
| 2.0 | 1376.6693 | 0.0116 | MHIASLQYMAGR |
| 1.8 | 1374.6853 | 1.9956 | YLELMIVNDHK |
| 1.8 | 1376.6909 | -0.0100 | RGHPIDSQSPQR |
| 1.1 | 1375.6732 | 1.0077 | SHFNKVSTNVDK |
| | | | |

| | | | |
|---|---|---|---|
| 0.3 | 1375.6766 | 1.0043 | TSEPKAMVVGEGR |

Spectrum No: 15; Query: 2222; Rank: 1

## Peptide View

MS/MS Fragmentation of **LVSDDLDSSLANLVGNLGIGNGTTK**
Found in **IPI00194958**, Tax_Id=10116 Gene_Symbol=Picalm Isoform 2 of Phosphatidylinositol-binding clathrin assembly protein

Match to Query 2222: 2473.261408 from(1237.637980,2+)
Title: 091008RatKidney_NoSalt_17.6783.6783.2.dta
Data file K:\NewmanPaper\Piliang\3SubProteomes\Piliang3SP\mgf5ppm\ERLIC_3SubProteomes5ppm.mgf

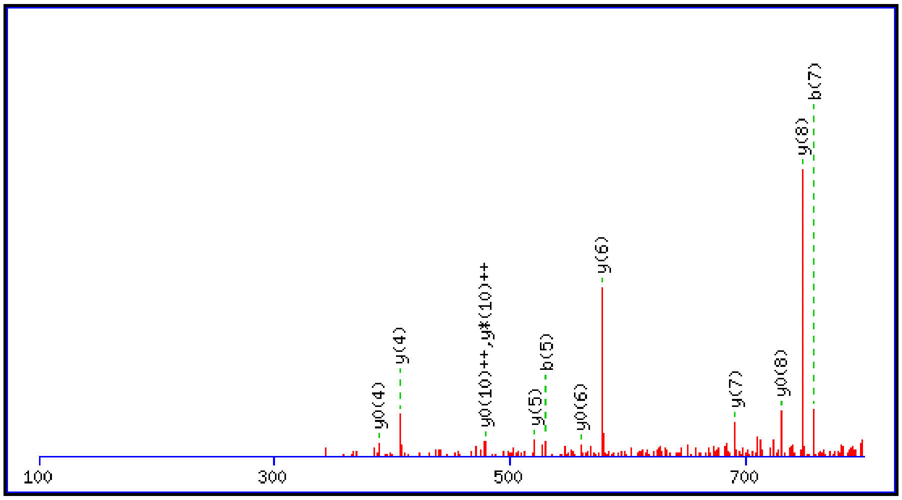


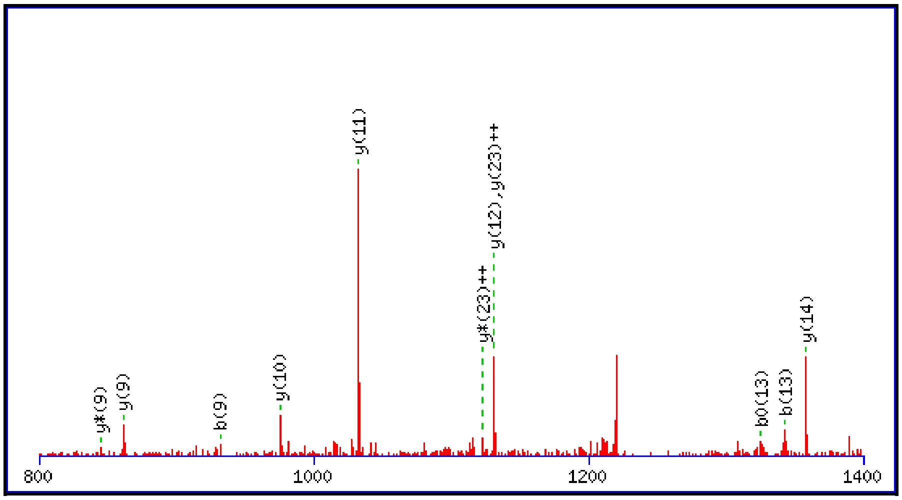

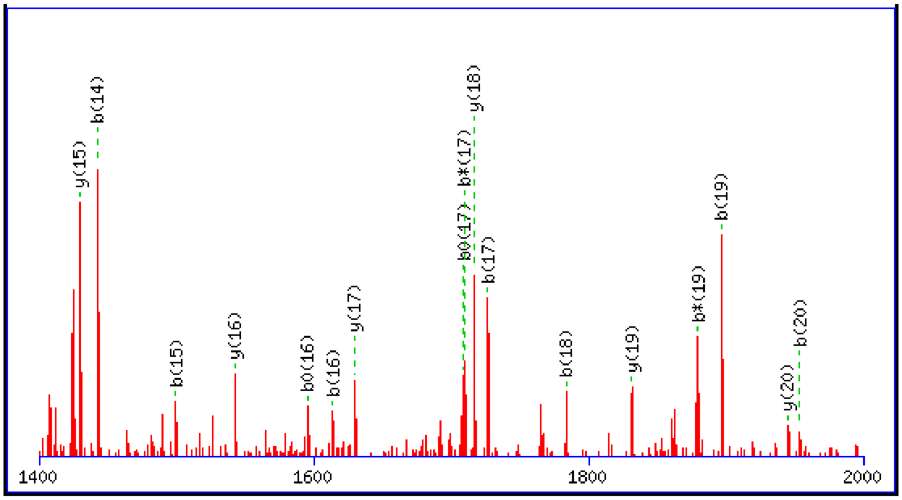


**Monoisotopic mass of neutral peptide Mr(calc):** 2473.2548
**Fixed modifications:** Carbamidomethyl (C)
**Variable modifications:**
**N21** : Deamidated_N (N)
**Ions Score:** 122 **Expect:** 1.2e-010
**Matches (Bold Red):** 40/260 fragment ions using 58 most intense peaks

| # | b | $b^{++}$ | b* | $b^{*++}$ | $b^0$ | $b^{0++}$ | Seq. | y | $y^{++}$ | y* | $y^{*++}$ | $y^0$ | $y^{0++}$ | # |
|---|---|---|---|---|---|---|---|---|---|---|---|---|---|---|
| 1 | 114.0913 | 57.5493 | | | | | L | | | | | | | 25 |
| 2 | 213.1598 | 107.0835 | | | | | V | 2361.1780 | 1181.0926 | 2344.1514 | 1172.5794 | 2343.1674 | 1172.0873 | 24 |
| 3 | 300.1918 | 150.5995 | | | 282.1812 | 141.5942 | S | 2262.1096 | **1131.5584** | 2245.0830 | **1123.0451** | 2244.0990 | 1122.5531 | 23 |
| 4 | 415.2187 | 208.1130 | | | 397.2082 | 199.1077 | D | 2175.0775 | 1088.0424 | 2158.0510 | 1079.5291 | 2157.0670 | 1079.0371 | 22 |
| 5 | **530.2457** | 265.6265 | | | 512.2351 | 256.6212 | D | 2060.0506 | 1030.5289 | 2043.0240 | 1022.0157 | 2042.0400 | 1021.5237 | 21 |
| 6 | 643.3297 | 322.1685 | | | 625.3192 | 313.1632 | L | **1945.0237** | 973.0155 | 1927.9971 | 964.5022 | 1927.0131 | 964.0102 | 20 |
| 7 | **758.3567** | 379.6820 | | | 740.3461 | 370.6767 | D | **1831.9396** | 916.4734 | 1814.9130 | 907.9602 | 1813.9290 | 907.4682 | 19 |
| 8 | 845.3887 | 423.1980 | | | 827.3781 | 414.1927 | S | **1716.9126** | 858.9600 | 1699.8861 | 850.4467 | 1698.9021 | 849.9547 | 18 |
| 9 | **932.4207** | 466.7140 | | | 914.4102 | 457.7087 | S | **1629.8806** | 815.4439 | 1612.8541 | 806.9307 | 1611.8701 | 806.4387 | 17 |
| 10 | 1045.5048 | 523.2560 | | | 1027.4942 | 514.2508 | L | **1542.8486** | 771.9279 | 1525.8220 | 763.4147 | 1524.8380 | 762.9227 | 16 |
| 11 | 1116.5419 | 558.7746 | | | 1098.5313 | 549.7693 | A | **1429.7645** | 715.3859 | 1412.7380 | 706.8726 | 1411.7540 | 706.3806 | 15 |
| 12 | 1230.5848 | 615.7961 | 1213.5583 | 607.2828 | 1212.5743 | 606.7908 | N | **1358.7274** | 679.8673 | 1341.7009 | 671.3541 | 1340.7168 | 670.8621 | 14 |
| 13 | **1343.6689** | 672.3381 | 1326.6424 | 663.8248 | **1325.6583** | 663.3328 | L | 1244.6845 | 622.8459 | 1227.6579 | 614.3326 | 1226.6739 | 613.8406 | 13 |
| 14 | **1442.7373** | 721.8723 | 1425.7108 | 713.3590 | 1424.7267 | 712.8670 | V | **1131.6004** | 566.3038 | 1114.5739 | 557.7906 | 1113.5899 | 557.2986 | 12 |
| 15 | **1499.7588** | 750.3830 | 1482.7322 | 741.8698 | 1481.7482 | 741.3777 | G | **1032.5320** | 516.7696 | 1015.5055 | 508.2564 | 1014.5214 | 507.7644 | 11 |
| 16 | **1613.8017** | 807.4045 | 1596.7752 | 798.8912 | **1595.7911** | 798.3992 | N | **975.5105** | 488.2589 | 958.4840 | **479.7456** | 957.5000 | **479.2536** | 10 |
| 17 | **1726.8858** | 863.9465 | **1709.8592** | 855.4332 | **1708.8752** | 854.9412 | L | **861.4676** | 431.2374 | **844.4411** | 422.7242 | 843.4571 | 422.2322 | 9 |
| 18 | **1783.9072** | 892.4573 | 1766.8807 | 883.9440 | 1765.8967 | 883.4520 | G | **748.3836** | 374.6954 | 731.3570 | 366.1821 | **730.3730** | 365.6901 | 8 |
| 19 | **1896.9913** | 948.9993 | **1879.9647** | 940.4860 | 1878.9807 | 939.9940 | I | **691.3621** | 346.1847 | 674.3355 | 337.6714 | 673.3515 | 337.1794 | 7 |
| 20 | **1954.0128** | 977.5100 | 1936.9862 | 968.9967 | 1936.0022 | 968.5047 | G | **578.2780** | 289.6427 | 561.2515 | 281.1294 | **560.2675** | 280.6374 | 6 |
| 21 | 2069.0397 | 1035.0235 | 2052.0131 | 1026.5102 | 2051.0291 | 1026.0182 | N | **521.2566** | 261.1319 | 504.2300 | 252.6186 | 503.2460 | 252.1266 | 5 |
| 22 | 2126.0612 | 1063.5342 | 2109.0346 | 1055.0209 | 2108.0506 | 1054.5289 | G | **406.2296** | 203.6185 | 389.2031 | 195.1052 | **388.2191** | 194.6132 | 4 |
| 23 | 2227.1088 | 1114.0581 | 2210.0823 | 1105.5448 | 2209.0983 | 1105.0528 | T | 349.2082 | 175.1077 | 332.1816 | 166.5944 | 331.1976 | 166.1024 | 3 |
| 24 | 2328.1565 | 1164.5819 | 2311.1300 | 1156.0686 | 2310.1460 | 1155.5766 | T | 248.1605 | 124.5839 | 231.1339 | 116.0706 | 230.1499 | 115.5786 | 2 |
| 25 | | | | | | | K | 147.1128 | 74.0600 | 130.0863 | 65.5468 | | | 1 |

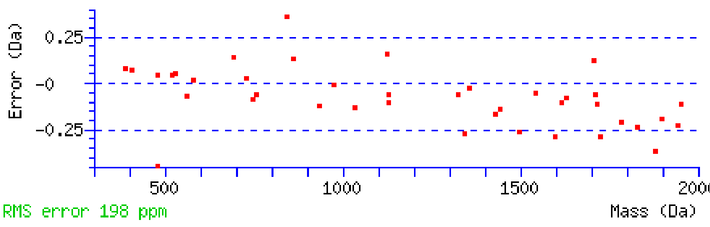


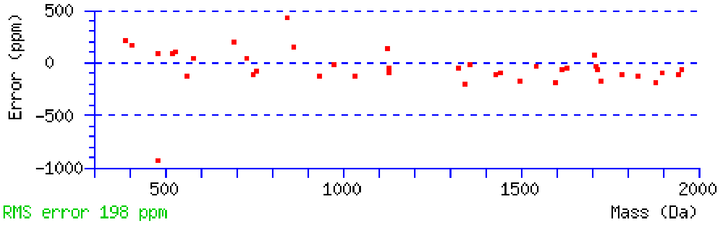

### All matches to this query

| Score | Mr(calc): | Delta | Sequence |
|---|---|---|---|
| 121.5 | 2473.2548 | 0.0066 | LVSDDLDSSLANLVGNLGIGNGTTK |
| 63.7 | 2473.2548 | 0.0066 | LVSDDLDSSLANLVGNLGIGNGTTK |
| 44.7 | 2473.2548 | 0.0066 | LVSDDLDSSLANLVGNLGIGNGTTK |
| 38.5 | 2472.2708 | 0.9906 | LVSDDLDSSLANLVGNLGIGNGTTK |
| 0.8 | 2473.2617 | -0.0003 | SSADPPITPNPERILNVFAVKK |
| 0.8 | 2473.2617 | -0.0003 | SSADPPITPNPERILNVFAVKK |
| 0.5 | 2473.2804 | -0.0190 | RNLLLGTACAIYLGFLVSQVGK |
| 0.1 | 2472.2348 | 1.0266 | CLEGTSDLRLVYTHHPLLLR |

---

Spectrum No: 16; Query: 1889; Rank: 1

---

## Peptide View

MS/MS Fragmentation of **DQCIVDDITYNVNDTFHK**
Found in **IPI00200757**, Tax_Id=10116 Gene_Symbol=Fn1 Isoform 1 of Fibronectin precursor

Match to Query 1889: 2196.970848 from(1099.492700,2+)
Title: 100101RatKid_NS_deglyco_08.4368.4368.2.dta
Data file K:\NewmanPaper\Piliang\3SubProteomes\Piliang3SP\mgf5ppm\ERLIC_3SubProteomes5ppm.mgf

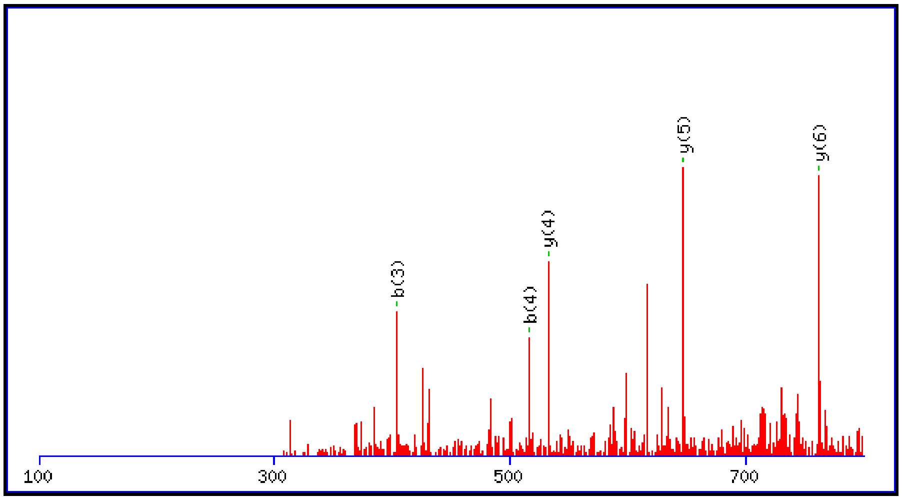


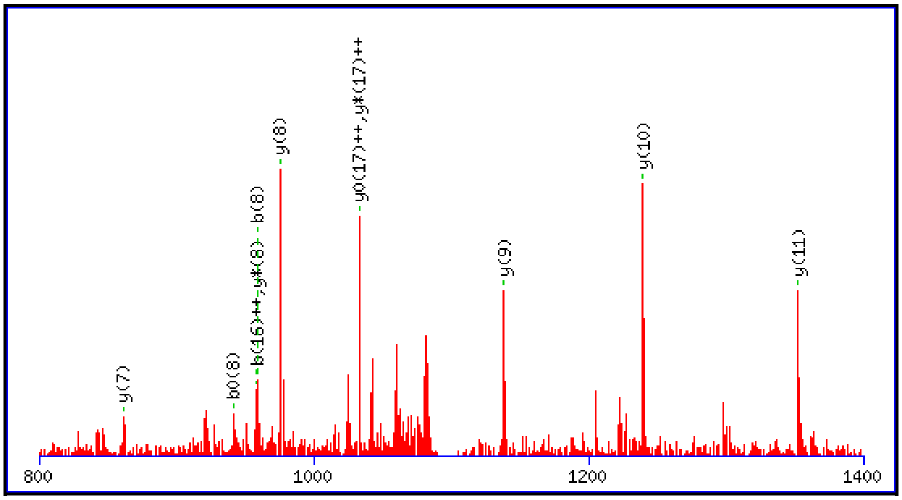

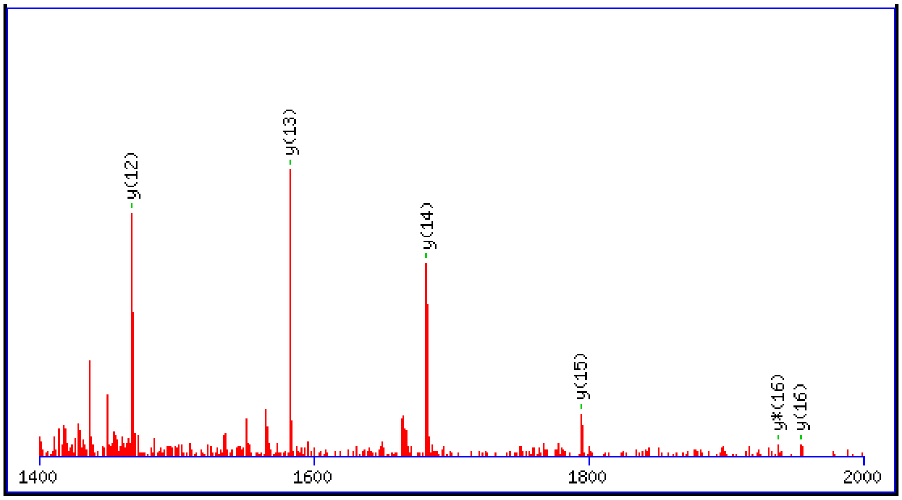


**Monoisotopic mass of neutral peptide Mr(calc):** 2196.9634
**Fixed modifications:** Carbamidomethyl (C)
**Variable modifications:**
**N13** : Deamidated_N (N)
**Ions Score:** 121 **Expect:** 2.3e-010
**Matches (Bold Red):** 22/196 fragment ions using 26 most intense peaks

| # | b | $b^{++}$ | b* | $b^{*++}$ | $b^{0}$ | $b^{0++}$ | Seq. | y | $y^{++}$ | y* | $y^{*++}$ | $y^{0}$ | $y^{0++}$ | # |
|---|---|---|---|---|---|---|---|---|---|---|---|---|---|---|
| 1 | 116.0342 | 58.5207 | | | 98.0237 | 49.5155 | D | | | | | | | 18 |
| 2 | 244.0928 | 122.5500 | 227.0662 | 114.0368 | 226.0822 | 113.5448 | Q | 2082.9437 | 1041.9755 | 2065.9171 | **1033.4622** | 2064.9331 | **1032.9702** | 17 |
| 3 | **404.1234** | 202.5654 | 387.0969 | 194.0521 | 386.1129 | 193.5601 | C | **1954.8851** | 977.9462 | **1937.8586** | 969.4329 | 1936.8745 | 968.9409 | 16 |
| 4 | **517.2075** | 259.1074 | 500.1810 | 250.5941 | 499.1969 | 250.1021 | I | **1794.8545** | 897.9309 | 1777.8279 | 889.4176 | 1776.8439 | 888.9256 | 15 |
| 5 | 616.2759 | 308.6416 | 599.2494 | 300.1283 | 598.2654 | 299.6363 | V | **1681.7704** | 841.3888 | 1664.7439 | 832.8756 | 1663.7598 | 832.3836 | 14 |
| 6 | 731.3029 | 366.1551 | 714.2763 | 357.6418 | 713.2923 | 357.1498 | D | **1582.7020** | 791.8546 | 1565.6754 | 783.3414 | 1564.6914 | 782.8493 | 13 |
| 7 | 846.3298 | 423.6685 | 829.3033 | 415.1553 | 828.3192 | 414.6633 | D | **1467.6750** | 734.3412 | 1450.6485 | 725.8279 | 1449.6645 | 725.3359 | 12 |
| 8 | **959.4139** | 480.2106 | 942.3873 | 471.6973 | **941.4033** | 471.2053 | I | **1352.6481** | 676.8277 | 1335.6216 | 668.3144 | 1334.6375 | 667.8224 | 11 |
| 9 | 1060.4616 | 530.7344 | 1043.4350 | 522.2211 | 1042.4510 | 521.7291 | T | **1239.5640** | 620.2857 | 1222.5375 | 611.7724 | 1221.5535 | 611.2804 | 10 |
| 10 | 1223.5249 | 612.2661 | 1206.4983 | 603.7528 | 1205.5143 | 603.2608 | Y | **1138.5164** | 569.7618 | 1121.4898 | 561.2485 | 1120.5058 | 560.7565 | 9 |
| 11 | 1337.5678 | 669.2875 | 1320.5413 | 660.7743 | 1319.5572 | 660.2823 | N | **975.4530** | 488.2302 | **958.4265** | 479.7169 | 957.4425 | 479.2249 | 8 |
| 12 | 1436.6362 | 718.8217 | 1419.6097 | 710.3085 | 1418.6257 | 709.8165 | V | **861.4101** | 431.2087 | 844.3836 | 422.6954 | 843.3995 | 422.2034 | 7 |
| 13 | 1551.6632 | 776.3352 | 1534.6366 | 767.8219 | 1533.6526 | 767.3299 | N | **762.3417** | 381.6745 | 745.3151 | 373.1612 | 744.3311 | 372.6692 | 6 |
| 14 | 1666.6901 | 833.8487 | 1649.6636 | 825.3354 | 1648.6795 | 824.8434 | D | **647.3148** | 324.1610 | 630.2882 | 315.6477 | 629.3042 | 315.1557 | 5 |
| 15 | 1767.7378 | 884.3725 | 1750.7112 | 875.8593 | 1749.7272 | 875.3672 | T | **532.2878** | 266.6475 | 515.2613 | 258.1343 | 514.2772 | 257.6423 | 4 |
| 16 | 1914.8062 | **957.9067** | 1897.7796 | 949.3935 | 1896.7956 | 948.9015 | F | 431.2401 | 216.1237 | 414.2136 | 207.6104 | | | 3 |
| 17 | 2051.8651 | 1026.4362 | 2034.8386 | 1017.9229 | 2033.8545 | 1017.4309 | H | 284.1717 | 142.5895 | 267.1452 | 134.0762 | | | 2 |
| 18 | | | | | | | K | 147.1128 | 74.0600 | 130.0863 | 65.5468 | | | 1 |

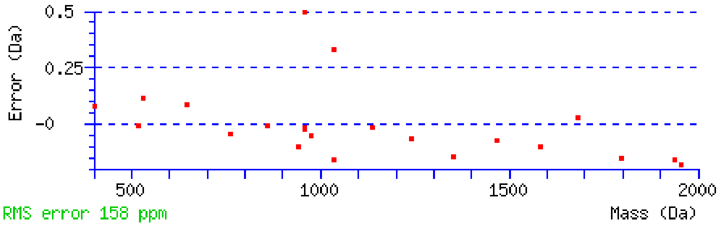


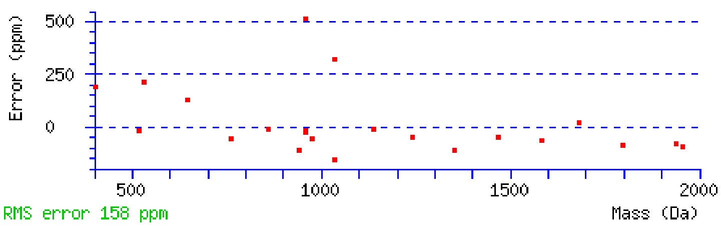


## All matches to this query

| Score | Mr(calc): | Delta | Sequence |
|---|---|---|---|
| 120.9 | 2196.9634 | 0.0075 | DQCIVDDITYNVNDTFHK |
| 86.5 | 2196.9634 | 0.0075 | DQCIVDDITYNVNDTFHK |
| 21.0 | 2195.9794 | 0.9915 | DQCIVDDITYNVNDTFHK |
| | | | |

| | | | |
|---|---|---|---|
| 12.9 | 2196.9752 | -0.0044 | HGSLXLSVTPTLRGSMHNK |
| 10.4 | 2196.9583 | 0.0126 | DDQGKNQSNRNVSSAICPK |
| 8.6 | 2196.9752 | -0.0044 | HGSLXLSVTPTLRGSMHNK |
| 6.7 | 2196.9752 | -0.0044 | HGSLXLSVTPTLRGSMHNK |
| 6.1 | 2196.9857 | -0.0149 | VIHLSNLPHSGYSDSAVLK |
| 4.4 | 2196.9752 | -0.0044 | HGSLXLSVTPTLRGSMHNK |
| 4.3 | 2196.9752 | -0.0044 | HGSLXLSVTPTLRGSMHNK |

Spectrum No: 17; Query: 1778; Rank: 1

## Peptide View

MS/MS Fragmentation of **MYVTNDTEVAENNYQALK**
Found in **IPI00464785**, Tax_Id=10116 Gene_Symbol=Ppgb Protective protein for beta-galactosidase

Match to Query 1778: 2118.945948 from(1060.480250,2+)
Title: 091008RatKidney_NoSalt_21.2270.2270.2.dta
Data file K:\NewmanPaper\Piliang\3SubProteomes\Piliang3SP\mgf5ppm\ERLIC_3SubProteomes5ppm.mgf

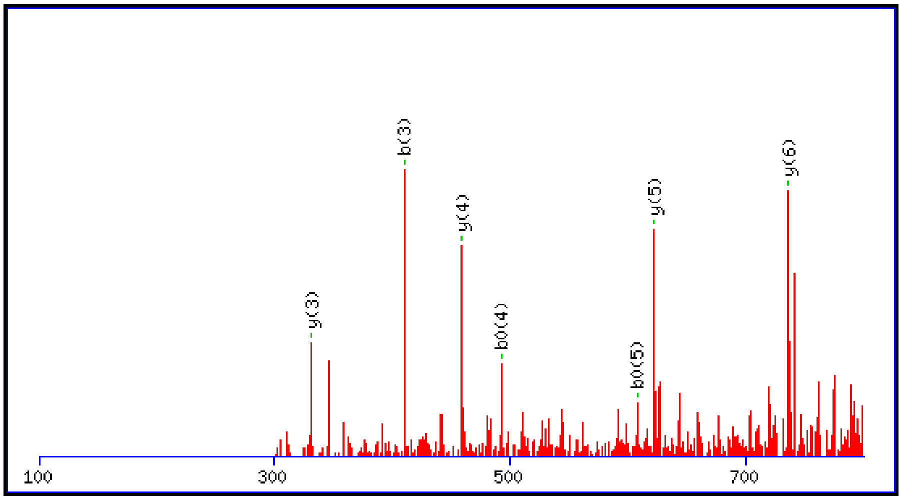


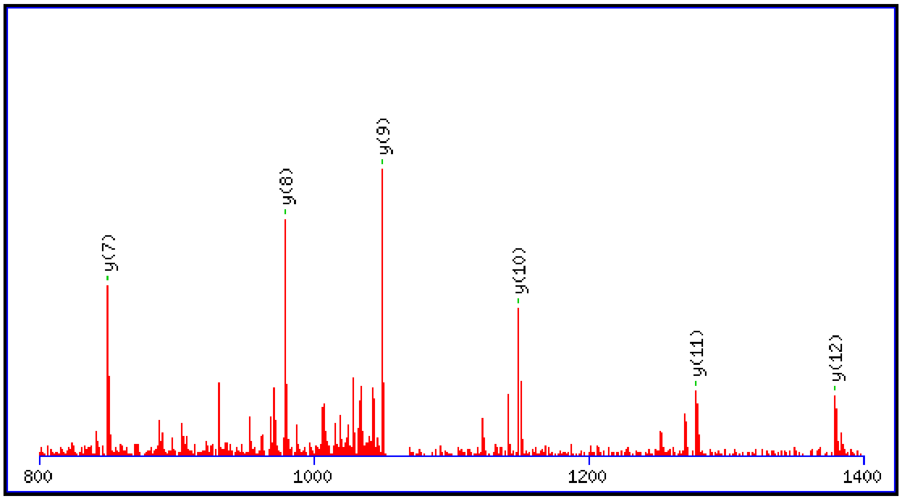

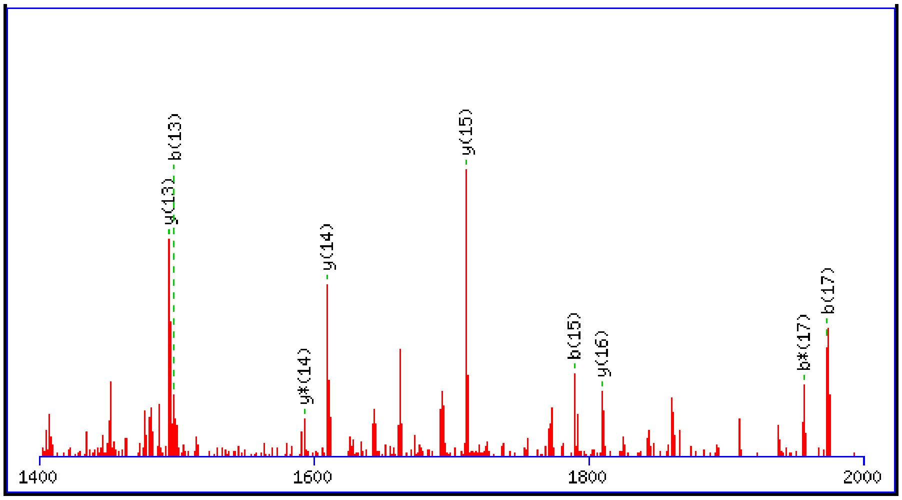


**Monoisotopic mass of neutral peptide Mr(calc):** 2118.9415
**Fixed modifications:** Carbamidomethyl (C)
**Variable modifications:**
**M1** : Oxidation (M)
**N5** : Deamidated_N (N)
**Ions Score:** 121 **Expect:** 2.2e-010
**Matches (Bold Red):** 22/176 fragment ions using 30 most intense peaks

| # | b | $b^{++}$ | b* | $b^{*++}$ | $b^0$ | $b^{0++}$ | Seq. | y | $y^{++}$ | y* | $y^{*++}$ | $y^0$ | $y^{0++}$ | # |
|---|---|---|---|---|---|---|---|---|---|---|---|---|---|---|
| 1 | 148.0427 | 74.5250 | | | | | M | | | | | | | 18 |
| 2 | 311.1060 | 156.0566 | | | | | Y | 1972.9134 | 986.9604 | 1955.8869 | 978.4471 | 1954.9029 | 977.9551 | 17 |
| 3 | **410.1744** | 205.5908 | | | | | V | **1809.8501** | 905.4287 | 1792.8236 | 896.9154 | 1791.8395 | 896.4234 | 16 |
| 4 | 511.2221 | 256.1147 | | | **493.2115** | 247.1094 | T | **1710.7817** | 855.8945 | 1693.7551 | 847.3812 | 1692.7711 | 846.8892 | 15 |
| 5 | 626.2490 | 313.6282 | 609.2225 | 305.1149 | **608.2385** | 304.6229 | N | **1609.7340** | 805.3706 | **1592.7075** | 796.8574 | 1591.7235 | 796.3654 | 14 |
| 6 | 741.2760 | 371.1416 | 724.2494 | 362.6284 | 723.2654 | 362.1363 | D | **1494.7071** | 747.8572 | 1477.6805 | 739.3439 | 1476.6965 | 738.8519 | 13 |
| 7 | 842.3237 | 421.6655 | 825.2971 | 413.1522 | 824.3131 | 412.6602 | T | **1379.6801** | 690.3437 | 1362.6536 | 681.8304 | 1361.6696 | 681.3384 | 12 |
| 8 | 971.3663 | 486.1868 | 954.3397 | 477.6735 | 953.3557 | 477.1815 | E | **1278.6325** | 639.8199 | 1261.6059 | 631.3066 | 1260.6219 | 630.8146 | 11 |
| 9 | 1070.4347 | 535.7210 | 1053.4081 | 527.2077 | 1052.4241 | 526.7157 | V | **1149.5899** | 575.2986 | 1132.5633 | 566.7853 | 1131.5793 | 566.2933 | 10 |
| 10 | 1141.4718 | 571.2395 | 1124.4452 | 562.7263 | 1123.4612 | 562.2342 | A | **1050.5215** | 525.7644 | 1033.4949 | 517.2511 | 1032.5109 | 516.7591 | 9 |
| 11 | 1270.5144 | 635.7608 | 1253.4878 | 627.2476 | 1252.5038 | 626.7555 | E | **979.4843** | 490.2458 | 962.4578 | 481.7325 | 961.4738 | 481.2405 | 8 |
| 12 | 1384.5573 | 692.7823 | 1367.5308 | 684.2690 | 1366.5467 | 683.7770 | N | **850.4417** | 425.7245 | 833.4152 | 417.2112 | | | 7 |
| 13 | **1498.6002** | 749.8038 | 1481.5737 | 741.2905 | 1480.5897 | 740.7985 | N | **736.3988** | 368.7030 | 719.3723 | 360.1898 | | | 6 |
| 14 | 1661.6636 | 831.3354 | 1644.6370 | 822.8221 | 1643.6530 | 822.3301 | Y | **622.3559** | 311.6816 | 605.3293 | 303.1683 | | | 5 |
| 15 | **1789.7221** | 895.3647 | 1772.6956 | 886.8514 | 1771.7116 | 886.3594 | Q | **459.2926** | 230.1499 | 442.2660 | 221.6366 | | | 4 |
| 16 | 1860.7592 | 930.8833 | 1843.7327 | 922.3700 | 1842.7487 | 921.8780 | A | **331.2340** | 166.1206 | 314.2074 | 157.6074 | | | 3 |
| 17 | **1973.8433** | 987.4253 | **1956.8168** | 978.9120 | 1955.8327 | 978.4200 | L | 260.1969 | 130.6021 | 243.1703 | 122.0888 | | | 2 |
| 18 | | | | | | | K | 147.1128 | 74.0600 | 130.0863 | 65.5468 | | | 1 |

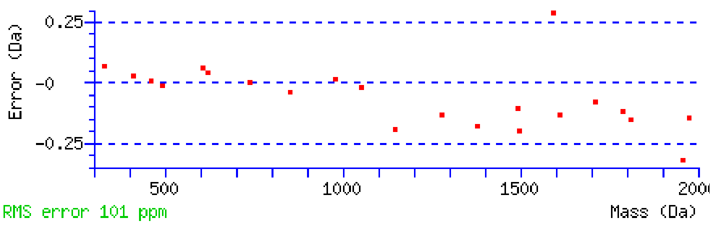


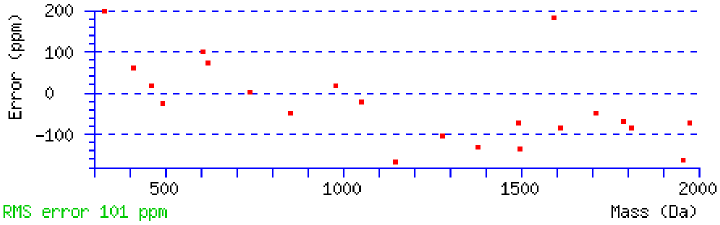


### All matches to this query

| Score | Mr(calc): | Delta | Sequence |
|---|---|---|---|
| 120.8 | 2118.9415 | 0.0044 | MYVTNDTEVAENNYQALK |
| 86.9 | 2118.9415 | 0.0044 | MYVTNDTEVAENNYQALK |
| 72.3 | 2118.9415 | 0.0044 | MYVTNDTEVAENNYQALK |

| | | | |
|---|---|---|---|
| 67.3 | 2117.9575 | 0.9884 | MYVTNDTEVAENNYQALK |
| 3.7 | 2117.9493 | 0.9967 | AGSKPFNPVLGETYECIR |
| 0.1 | 2117.9560 | 0.9899 | KTVXNLLGMAGMTPNVNSK |

---

Spectrum No: 18; Query: 2181; Rank: 1

---

## Peptide View

MS/MS Fragmentation of **IYYSDFSNQTINSMAEDGSNR**
Found in **IPI00205325**, Tax_Id=10116 Gene_Symbol=Lrp2 Low-density lipoprotein receptor-related protein 2 precursor

Match to Query 2181: 2428.017308 from(1215.015930,2+)
Title: 100101RatKid_NS_deglyco_19.2718.2718.2.dta
Data file K:\NewmanPaper\Piliang\3SubProteomes\Piliang3SP\mgf5ppm\ERLIC_3SubProteomes5ppm.mgf

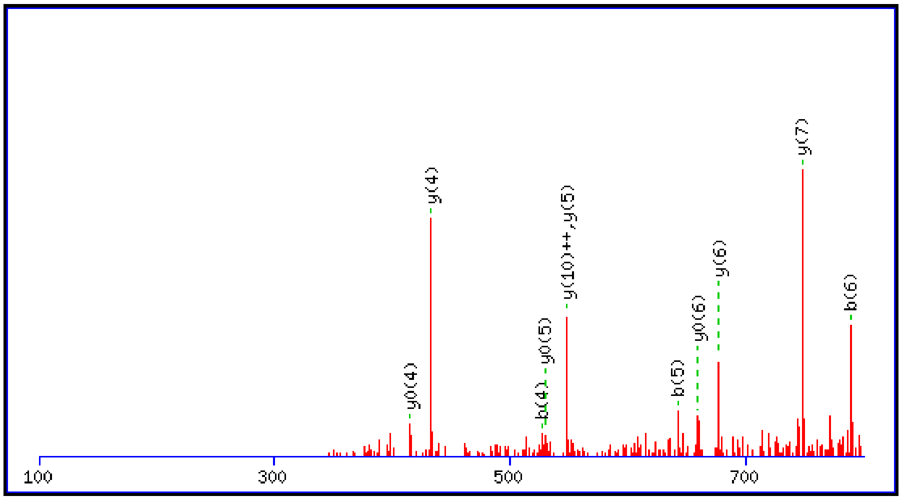


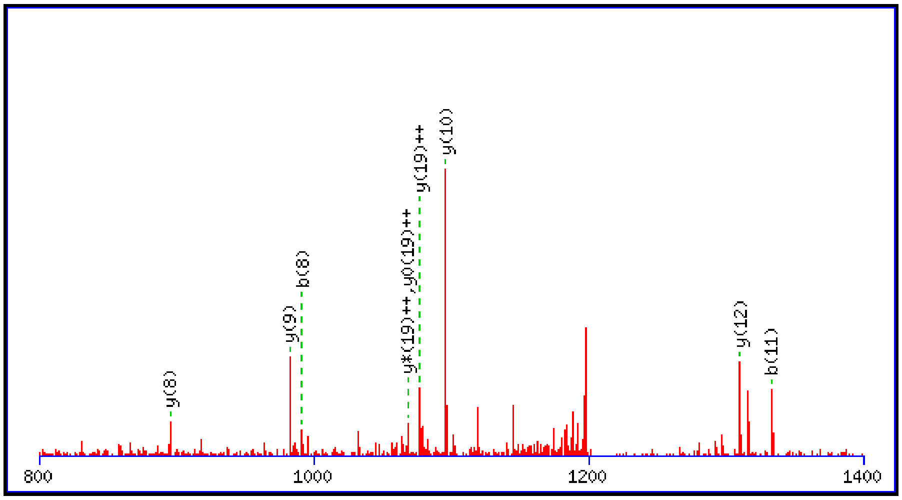

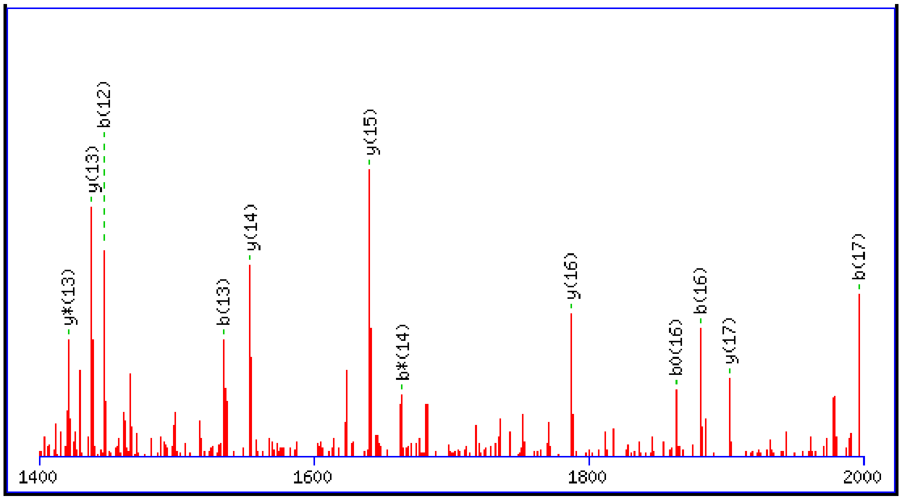


**Monoisotopic mass of neutral peptide Mr(calc):** 2428.0125
**Fixed modifications:** Carbamidomethyl (C)
**Variable modifications:**
**N8** : Deamidated_N (N)
**M14** : Oxidation (M)
**Ions Score:** 118 **Expect:** 5.5e-010
**Matches (Bold Red):** 32/216 fragment ions using 38 most intense peaks

| # | b | $b^{++}$ | b* | $b^{*++}$ | $b^0$ | $b^{0++}$ | Seq. | y | $y^{++}$ | y* | $y^{*++}$ | $y^0$ | $y^{0++}$ | # |
|---|---|---|---|---|---|---|---|---|---|---|---|---|---|---|
| 1 | 114.0913 | 57.5493 | | | | | I | | | | | | | 21 |
| 2 | 277.1547 | 139.0810 | | | | | Y | 2315.9357 | 1158.4715 | 2298.9092 | 1149.9582 | 2297.9251 | 1149.4662 | 20 |
| 3 | 440.2180 | 220.6126 | | | | | Y | 2152.8724 | **1076.9398** | 2135.8458 | **1068.4266** | 2134.8618 | **1067.9345** | 19 |
| 4 | **527.2500** | 264.1287 | | | 509.2395 | 255.1234 | S | 1989.8091 | 995.4082 | 1972.7825 | 986.8949 | 1971.7985 | 986.4029 | 18 |
| 5 | **642.2770** | 321.6421 | | | 624.2664 | 312.6368 | D | **1902.7770** | 951.8921 | 1885.7505 | 943.3789 | 1884.7665 | 942.8869 | 17 |
| 6 | **789.3454** | 395.1763 | | | 771.3348 | 386.1710 | F | **1787.7501** | 894.3787 | 1770.7235 | 885.8654 | 1769.7395 | 885.3734 | 16 |
| 7 | 876.3774 | 438.6923 | | | 858.3668 | 429.6871 | S | **1640.6817** | 820.8445 | 1623.6551 | 812.3312 | 1622.6711 | 811.8392 | 15 |
| 8 | **991.4043** | 496.2058 | 974.3778 | 487.6925 | 973.3938 | 487.2005 | N | **1553.6496** | 777.3285 | 1536.6231 | 768.8152 | 1535.6391 | 768.3232 | 14 |
| 9 | 1119.4629 | 560.2351 | 1102.4364 | 551.7218 | 1101.4524 | 551.2298 | Q | **1438.6227** | 719.8150 | **1421.5962** | 711.3017 | 1420.6121 | 710.8097 | 13 |
| 10 | 1220.5106 | 610.7589 | 1203.4841 | 602.2457 | 1202.5000 | 601.7537 | T | **1310.5641** | 655.7857 | 1293.5376 | 647.2724 | 1292.5536 | 646.7804 | 12 |
| 11 | **1333.5947** | 667.3010 | 1316.5681 | 658.7877 | 1315.5841 | 658.2957 | I | 1209.5164 | 605.2619 | 1192.4899 | 596.7486 | 1191.5059 | 596.2566 | 11 |
| 12 | **1447.6376** | 724.3224 | 1430.6110 | 715.8092 | 1429.6270 | 715.3172 | N | **1096.4324** | **548.7198** | 1079.4058 | 540.2066 | 1078.4218 | 539.7145 | 10 |
| 13 | **1534.6696** | 767.8384 | 1517.6431 | 759.3252 | 1516.6591 | 758.8332 | S | **982.3895** | 491.6984 | 965.3629 | 483.1851 | 964.3789 | 482.6931 | 9 |
| 14 | 1681.7050 | 841.3562 | **1664.6785** | 832.8429 | 1663.6945 | 832.3509 | M | **895.3574** | 448.1824 | 878.3309 | 439.6691 | 877.3469 | 439.1771 | 8 |
| 15 | 1752.7421 | 876.8747 | 1735.7156 | 868.3614 | 1734.7316 | 867.8694 | A | **748.3220** | 374.6646 | 731.2955 | 366.1514 | 730.3115 | 365.6594 | 7 |
| 16 | **1881.7847** | 941.3960 | 1864.7582 | 932.8827 | **1863.7742** | 932.3907 | E | **677.2849** | 339.1461 | 660.2584 | 330.6328 | **659.2743** | 330.1408 | 6 |
| 17 | **1996.8117** | 998.9095 | 1979.7851 | 990.3962 | 1978.8011 | 989.9042 | D | **548.2423** | 274.6248 | 531.2158 | 266.1115 | **530.2317** | 265.6195 | 5 |
| 18 | 2053.8331 | 1027.4202 | 2036.8066 | 1018.9069 | 2035.8226 | 1018.4149 | G | **433.2154** | 217.1113 | 416.1888 | 208.5980 | **415.2048** | 208.1060 | 4 |
| 19 | 2140.8652 | 1070.9362 | 2123.8386 | 1062.4229 | 2122.8546 | 1061.9309 | S | 376.1939 | 188.6006 | 359.1674 | 180.0873 | 358.1833 | 179.5953 | 3 |
| 20 | 2254.9081 | 1127.9577 | 2237.8815 | 1119.4444 | 2236.8975 | 1118.9524 | N | 289.1619 | 145.0846 | 272.1353 | 136.5713 | | | 2 |
| 21 | | | | | | | R | 175.1190 | 88.0631 | 158.0924 | 79.5498 | | | 1 |

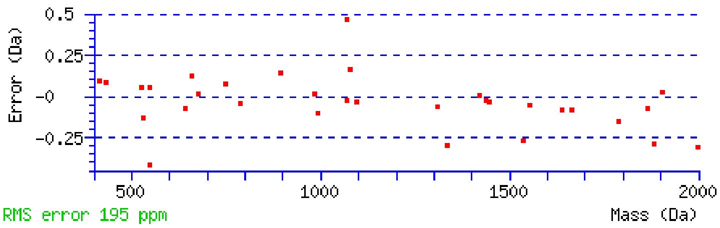


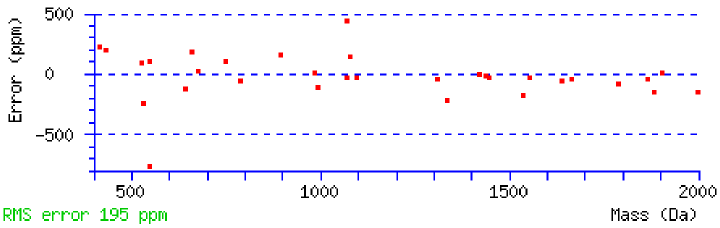


### All matches to this query

| Score | Mr(calc): | Delta | Sequence |
|---|---|---|---|

| | | | |
|---|---|---|---|
| 117.8 | 2428.0125 | 0.0048 | IYYSDFSNQTINSMAEDGSNR |
| 64.9 | 2428.0125 | 0.0048 | IYYSDFSNQTINSMAEDGSNR |
| 51.3 | 2427.0285 | 0.9889 | IYYSDFSNQTINSMAEDGSNR |
| 31.3 | 2428.0125 | 0.0048 | IYYSDFSNQTINSMAEDGSNR |
| 6.3 | 2428.0352 | -0.0179 | EGICGSCAMNINGGNTLACTRR |
| 4.4 | 2428.0302 | -0.0129 | KAYSFAMGCWPKNGLLDMNK |
| 3.0 | 2428.0397 | -0.0224 | MSYREVKYFSFPGELLMR |
| 2.4 | 2428.0325 | -0.0151 | KLSKATQDSLAEATQLAEER |
| 1.5 | 2428.0391 | -0.0218 | ENGINGELTSADRETAEEVSAR |
| 1.5 | 2428.0391 | -0.0218 | ENGINGELTSADRETAEEVSAR |

Spectrum No: 19; Query: 1299; Rank: 1

## Peptide View

MS/MS Fragmentation of **GVGIISEGNETVEDIAAR**
Found in **IPI00205693**, Tax_Id=10116 Gene_Symbol=Atp1a2 Sodium/potassium-transporting ATPase subunit alpha-2 precursor

Match to Query 1299: 1829.903548 from(915.959050,2+)
Title: 091008RatKidney_NoSalt_18.2651.2651.2.dta
Data file K:\NewmanPaper\Piliang\3SubProteomes\Piliang3SP\mgf5ppm\ERLIC_3SubProteomes5ppm.mgf

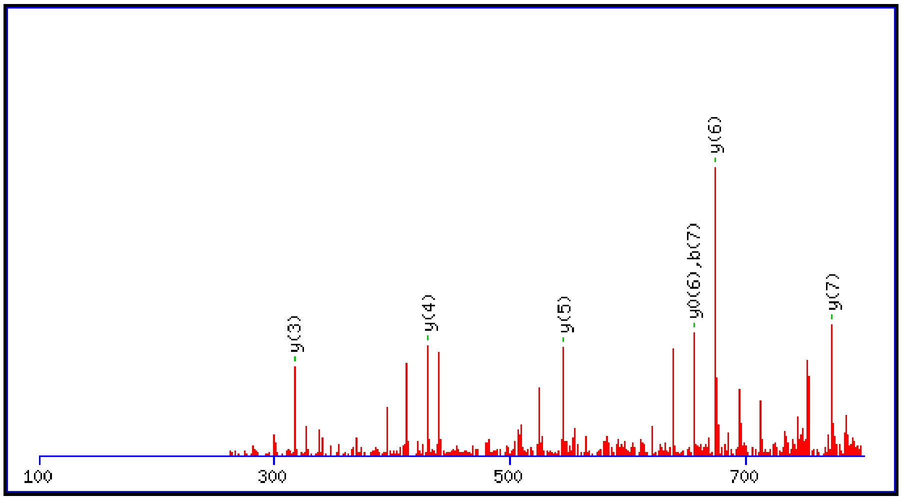


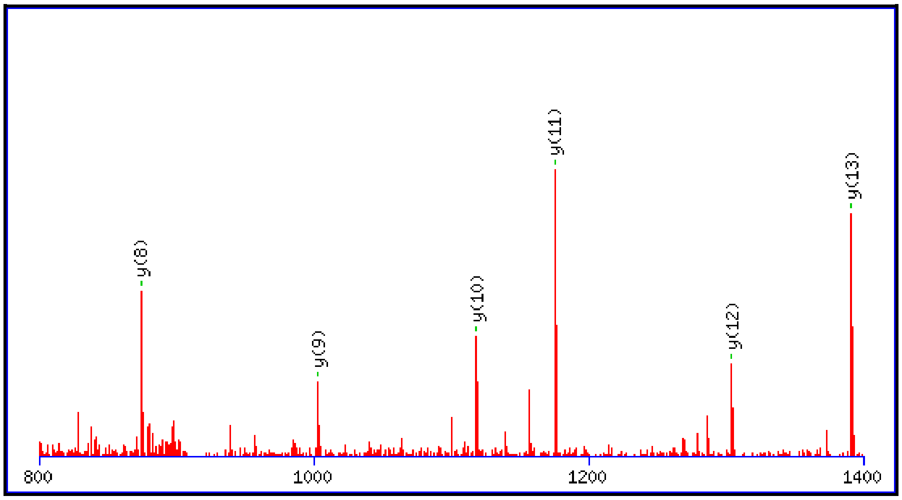

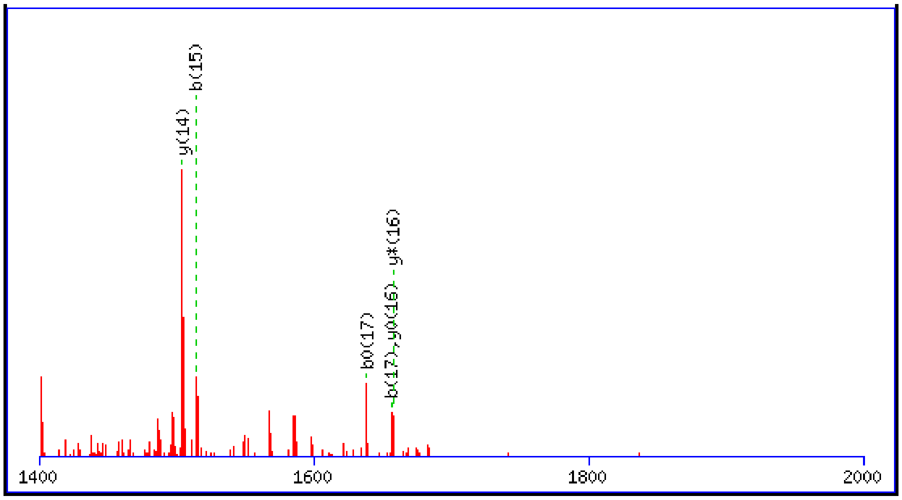


**Monoisotopic mass of neutral peptide Mr(calc):** 1829.9007
**Fixed modifications:** Carbamidomethyl (C)
**Variable modifications:**
**N9** : Deamidated_N (N)
**Ions Score:** 117 **Expect:** 3.4e-010
**Matches (Bold Red):** 19/170 fragment ions using 23 most intense peaks

| # | b | $b^{++}$ | b* | $b^{*++}$ | $b^0$ | $b^{0++}$ | Seq. | y | $y^{++}$ | y* | $y^{*++}$ | $y^0$ | $y^{0++}$ | # |
|---|---|---|---|---|---|---|---|---|---|---|---|---|---|---|
| 1 | 58.0287 | 29.5180 | | | | | G | | | | | | | 18 |
| 2 | 157.0972 | 79.0522 | | | | | V | 1773.8865 | 887.4469 | 1756.8599 | 878.9336 | 1755.8759 | 878.4416 | 17 |
| 3 | 214.1186 | 107.5629 | | | | | G | 1674.8181 | 837.9127 | **1657.7915** | 829.3994 | **1656.8075** | 828.9074 | 16 |
| 4 | 327.2027 | 164.1050 | | | | | I | 1617.7966 | 809.4019 | 1600.7701 | 800.8887 | 1599.7861 | 800.3967 | 15 |
| 5 | 440.2867 | 220.6470 | | | | | I | **1504.7126** | 752.8599 | 1487.6860 | 744.3466 | 1486.7020 | 743.8546 | 14 |
| 6 | 527.3188 | 264.1630 | | | 509.3082 | 255.1577 | S | **1391.6285** | 696.3179 | 1374.6019 | 687.8046 | 1373.6179 | 687.3126 | 13 |
| 7 | **656.3614** | 328.6843 | | | 638.3508 | 319.6790 | E | **1304.5965** | 652.8019 | 1287.5699 | 644.2886 | 1286.5859 | 643.7966 | 12 |
| 8 | 713.3828 | 357.1951 | | | 695.3723 | 348.1898 | G | **1175.5539** | 588.2806 | 1158.5273 | 579.7673 | 1157.5433 | 579.2753 | 11 |
| 9 | 828.4098 | 414.7085 | 811.3832 | 406.1952 | 810.3992 | 405.7032 | N | **1118.5324** | 559.7698 | 1101.5059 | 551.2566 | 1100.5218 | 550.7646 | 10 |
| 10 | 957.4524 | 479.2298 | 940.4258 | 470.7165 | 939.4418 | 470.2245 | E | **1003.5055** | 502.2564 | 986.4789 | 493.7431 | 985.4949 | 493.2511 | 9 |
| 11 | 1058.5000 | 529.7537 | 1041.4735 | 521.2404 | 1040.4895 | 520.7484 | T | **874.4629** | 437.7351 | 857.4363 | 429.2218 | 856.4523 | 428.7298 | 8 |
| 12 | 1157.5685 | 579.2879 | 1140.5419 | 570.7746 | 1139.5579 | 570.2826 | V | **773.4152** | 387.2112 | 756.3886 | 378.6980 | 755.4046 | 378.2060 | 7 |
| 13 | 1286.6110 | 643.8092 | 1269.5845 | 635.2959 | 1268.6005 | 634.8039 | E | **674.3468** | 337.6770 | 657.3202 | 329.1638 | **656.3362** | 328.6717 | 6 |
| 14 | 1401.6380 | 701.3226 | 1384.6114 | 692.8094 | 1383.6274 | 692.3174 | D | **545.3042** | 273.1557 | 528.2776 | 264.6425 | 527.2936 | 264.1504 | 5 |
| 15 | **1514.7221** | 757.8647 | 1497.6955 | 749.3514 | 1496.7115 | 748.8594 | I | **430.2772** | 215.6423 | 413.2507 | 207.1290 | | | 4 |
| 16 | 1585.7592 | 793.3832 | 1568.7326 | 784.8699 | 1567.7486 | 784.3779 | A | **317.1932** | 159.1002 | 300.1666 | 150.5870 | | | 3 |
| 17 | **1656.7963** | 828.9018 | 1639.7697 | 820.3885 | **1638.7857** | 819.8965 | A | 246.1561 | 123.5817 | 229.1295 | 115.0684 | | | 2 |
| 18 | | | | | | | R | 175.1190 | 88.0631 | 158.0924 | 79.5498 | | | 1 |

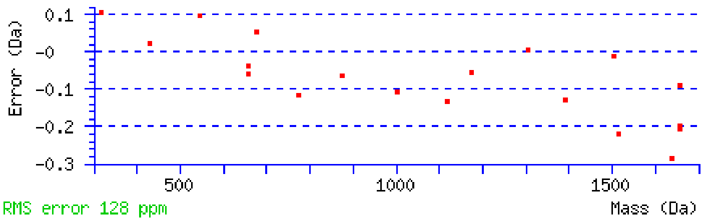


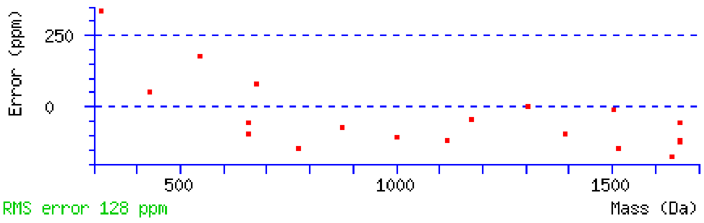


### All matches to this query

| Score | Mr(calc): | Delta | Sequence |
|---|---|---|---|
| 117.4 | 1829.9007 | 0.0029 | GVGIISEGNETVEDIAAR |
| 36.0 | 1828.9167 | 0.9869 | GVGIISEGNETVEDIAAR |
| 2.1 | 1829.9119 | -0.0083 | ARSQVNGNNGTLIVEEK |
| | | | |

| | | | |
|---|---|---|---|
| 1.6 | 1829.9149 | -0.0113 | RTISALEVELQAQHR |
| 0.8 | 1827.9053 | 1.9983 | SDLKVILMSATLNAEK |
| 0.8 | 1827.8871 | 2.0164 | KSMIIGFFLRTSLR |
| 0.8 | 1827.8871 | 2.0164 | KSMIIGFFLRTSLR |
| 0.3 | 1827.8963 | 2.0073 | TSQIASAGVPAPGGSQSADK |

---

Spectrum No: 20; Query: 1071; Rank: 1

---

## Peptide View

MS/MS Fragmentation of **AITCGGIYNESSGILR**
Found in **IPI00196620**, Tax_Id=10116 Gene_Symbol=Cubn Cubilin precursor

Match to Query 1071: 1710.825448 from(856.420000,2+)
Title: 091008RatKidney_NoSalt_25.2890.2890.2.dta
Data file K:\NewmanPaper\Piliang\3SubProteomes\Piliang3SP\mgf5ppm\ERLIC_3SubProteomes5ppm.mgf

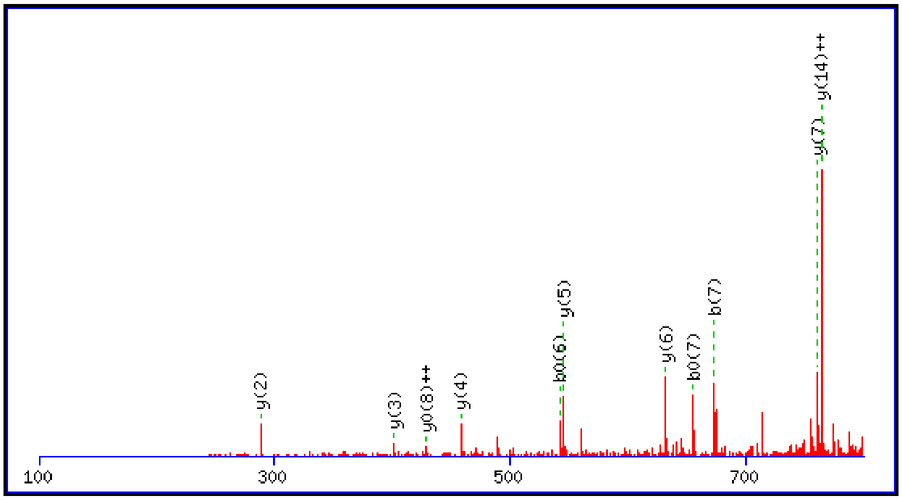


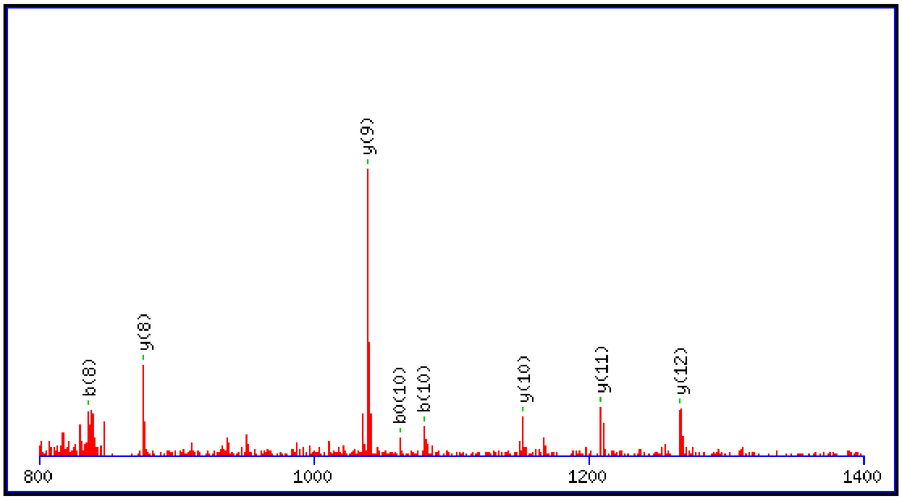

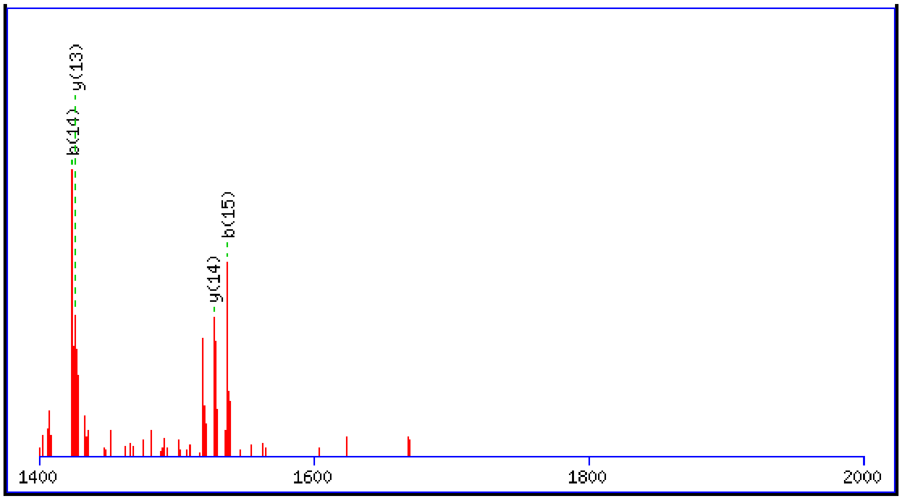


**Monoisotopic mass of neutral peptide Mr(calc):** 1710.8247
**Fixed modifications:** Carbamidomethyl (C)
**Variable modifications:**
**N9** : Deamidated_N (N)
**Ions Score:** 117 **Expect:** 3.7e-010
**Matches (Bold Red):** 23/152 fragment ions using 36 most intense peaks

| # | b | $b^{++}$ | b* | $b^{*++}$ | $b^0$ | $b^{0++}$ | Seq. | y | $y^{++}$ | y* | $y^{*++}$ | $y^0$ | $y^{0++}$ | # |
|---|---|---|---|---|---|---|---|---|---|---|---|---|---|---|
| 1 | 72.0444 | 36.5258 | | | | | A | | | | | | | 16 |
| 2 | 185.1285 | 93.0679 | | | | | I | 1640.7948 | 820.9011 | 1623.7683 | 812.3878 | 1622.7843 | 811.8958 | 15 |
| 3 | 286.1761 | 143.5917 | | | 268.1656 | 134.5864 | T | **1527.7108** | **764.3590** | 1510.6842 | 755.8458 | 1509.7002 | 755.3537 | 14 |
| 4 | 446.2068 | 223.6070 | | | 428.1962 | 214.6017 | C | **1426.6631** | 713.8352 | 1409.6365 | 705.3219 | 1408.6525 | 704.8299 | 13 |
| 5 | 503.2282 | 252.1178 | | | 485.2177 | 243.1125 | G | **1266.6324** | 633.8199 | 1249.6059 | 625.3066 | 1248.6219 | 624.8146 | 12 |
| 6 | 560.2497 | 280.6285 | | | **542.2391** | 271.6232 | G | **1209.6110** | 605.3091 | 1192.5844 | 596.7959 | 1191.6004 | 596.3038 | 11 |
| 7 | **673.3338** | 337.1705 | | | **655.3232** | 328.1652 | I | **1152.5895** | 576.7984 | 1135.5630 | 568.2851 | 1134.5790 | 567.7931 | 10 |
| 8 | **836.3971** | 418.7022 | | | 818.3865 | 409.6969 | Y | **1039.5055** | 520.2564 | 1022.4789 | 511.7431 | 1021.4949 | 511.2511 | 9 |
| 9 | 951.4240 | 476.2157 | 934.3975 | 467.7024 | 933.4135 | 467.2104 | N | **876.4421** | 438.7247 | 859.4156 | 430.2114 | 858.4316 | **429.7194** | 8 |
| 10 | **1080.4666** | 540.7370 | 1063.4401 | 532.2237 | **1062.4561** | 531.7317 | E | **761.4152** | 381.2112 | 744.3886 | 372.6980 | 743.4046 | 372.2060 | 7 |
| 11 | 1167.4987 | 584.2530 | 1150.4721 | 575.7397 | 1149.4881 | 575.2477 | S | **632.3726** | 316.6899 | 615.3461 | 308.1767 | 614.3620 | 307.6847 | 6 |
| 12 | 1254.5307 | 627.7690 | 1237.5041 | 619.2557 | 1236.5201 | 618.7637 | S | **545.3406** | 273.1739 | 528.3140 | 264.6606 | 527.3300 | 264.1686 | 5 |
| 13 | 1311.5522 | 656.2797 | 1294.5256 | 647.7664 | 1293.5416 | 647.2744 | G | **458.3085** | 229.6579 | 441.2820 | 221.1446 | | | 4 |
| 14 | **1424.6362** | 712.8217 | 1407.6097 | 704.3085 | 1406.6257 | 703.8165 | I | **401.2871** | 201.1472 | 384.2605 | 192.6339 | | | 3 |
| 15 | **1537.7203** | 769.3638 | 1520.6937 | 760.8505 | 1519.7097 | 760.3585 | L | **288.2030** | 144.6051 | 271.1765 | 136.0919 | | | 2 |
| 16 | | | | | | | R | 175.1190 | 88.0631 | 158.0924 | 79.5498 | | | 1 |

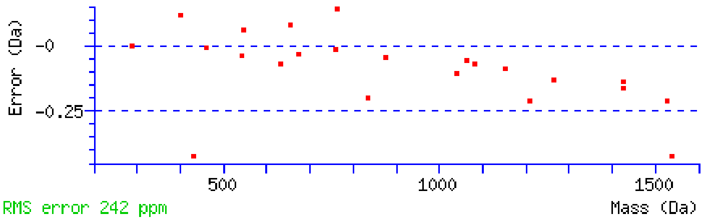


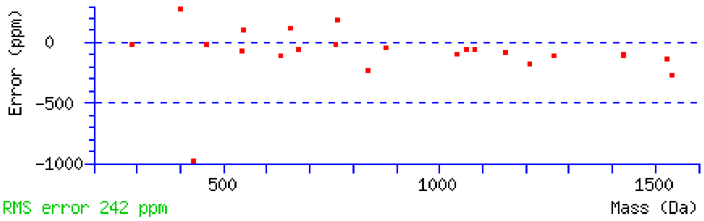


### All matches to this query

| Score | Mr(calc): | Delta | Sequence |
|---|---|---|---|
| 117.4 | 1710.8247 | 0.0008 | AITCGGIYNESSGILR |
| 8.7 | 1710.8375 | -0.0121 | KSSEQLLGSMPVSLR |
| 6.2 | 1710.8103 | 0.0152 | LMELNMEIRDMIR |
| 6.0 | 1710.8375 | -0.0121 | KSSEQLLGSMPVSLR |
| 5.0 | 1710.8237 | 0.0018 | VVMLLTAGSGGGAGGGRR |
| | | | |

| | | | |
|---|---|---|---|
| 3.6 | 1710.8124 | 0.0131 | EVVVNMLNSLSRNR |
| 3.6 | 1710.8124 | 0.0131 | EVVVNMLNSLSRNR |
| 2.9 | 1709.8250 | 1.0005 | RQGASTAXAATPIPAR |
| 2.7 | 1710.8189 | 0.0066 | QELILSNSEDKSIR |
| 2.6 | 1708.8172 | 2.0083 | GETLETLVLLNPSDK |

---

Spectrum No: 21; Query: 1956; Rank: 1

---

## Peptide View

MS/MS Fragmentation of **YGYENDTEIANLYDEMVAK**
Found in **IPI00191437**, Tax_Id=10116 Gene_Symbol=- 64 kDa protein

Match to Query 1956: 2253.969528 from(1127.992040,2+)
Title: 100101RatKid_NS_deglyco_25.3594.3594.2.dta
Data file K:\NewmanPaper\Piliang\3SubProteomes\Piliang3SP\mgf5ppm\ERLIC_3SubProteomes5ppm.mgf

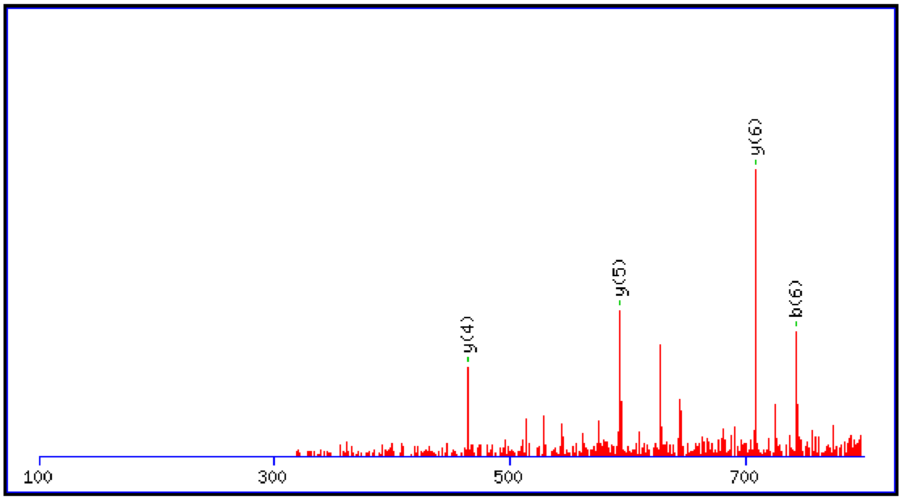


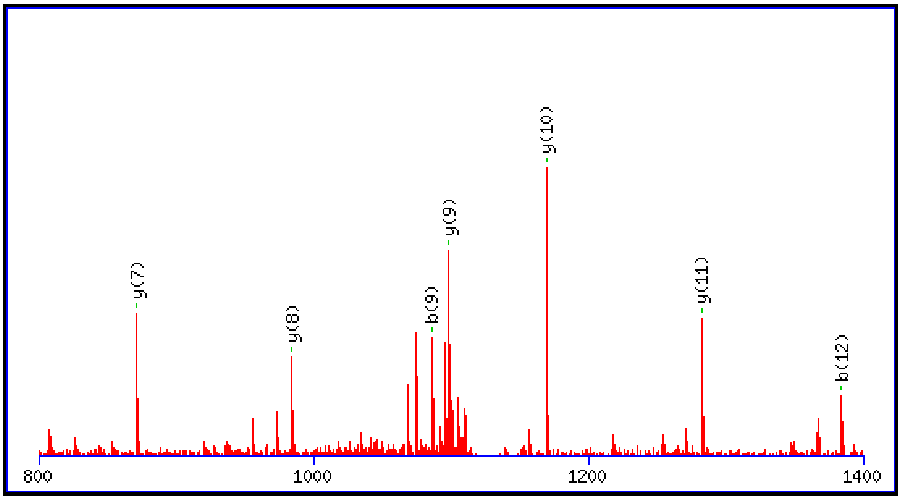

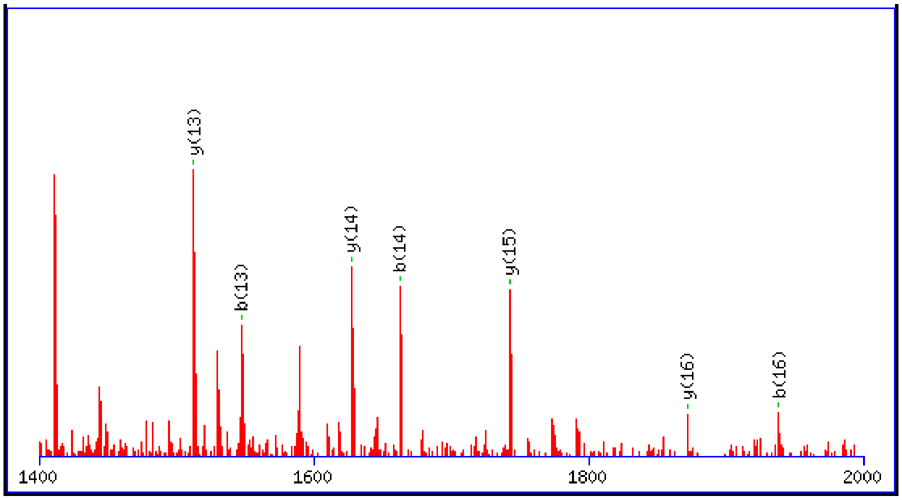


**Monoisotopic mass of neutral peptide Mr(calc):** 2253.9623
**Fixed modifications:** Carbamidomethyl (C)
**Variable modifications:**
**N5** : Deamidated_N (N)
**M16** : Oxidation (M)
**Ions Score:** 117 **Expect:** 5.5e-010
**Matches (Bold Red):** 18/194 fragment ions using 25 most intense peaks

| # | b | $b^{++}$ | b* | $b^{*++}$ | $b^0$ | $b^{0++}$ | Seq. | y | $y^{++}$ | y* | $y^{*++}$ | $y^0$ | $y^{0++}$ | # |
|---|---|---|---|---|---|---|---|---|---|---|---|---|---|---|
| 1 | 164.0706 | 82.5389 | | | | | Y | | | | | | | 19 |
| 2 | 221.0921 | 111.0497 | | | | | G | 2091.9063 | 1046.4568 | 2074.8798 | 1037.9435 | 2073.8957 | 1037.4515 | 18 |
| 3 | 384.1554 | 192.5813 | | | | | Y | 2034.8848 | 1017.9461 | 2017.8583 | 1009.4328 | 2016.8743 | 1008.9408 | 17 |
| 4 | 513.1980 | 257.1026 | | | 495.1874 | 248.0974 | E | **1871.8215** | 936.4144 | 1854.7950 | 927.9011 | 1853.8109 | 927.4091 | 16 |
| 5 | 628.2249 | 314.6161 | 611.1984 | 306.1028 | 610.2144 | 305.6108 | N | **1742.7789** | 871.8931 | 1725.7524 | 863.3798 | 1724.7684 | 862.8878 | 15 |
| 6 | **743.2519** | 372.1296 | 726.2253 | 363.6163 | 725.2413 | 363.1243 | D | **1627.7520** | 814.3796 | 1610.7254 | 805.8664 | 1609.7414 | 805.3743 | 14 |
| 7 | 844.2995 | 422.6534 | 827.2730 | 414.1401 | 826.2890 | 413.6481 | T | **1512.7250** | 756.8662 | 1495.6985 | 748.3529 | 1494.7145 | 747.8609 | 13 |
| 8 | 973.3421 | 487.1747 | 956.3156 | 478.6614 | 955.3316 | 478.1694 | E | 1411.6774 | 706.3423 | 1394.6508 | 697.8290 | 1393.6668 | 697.3370 | 12 |
| 9 | **1086.4262** | 543.7167 | 1069.3997 | 535.2035 | 1068.4156 | 534.7115 | I | **1282.6348** | 641.8210 | 1265.6082 | 633.3077 | 1264.6242 | 632.8157 | 11 |
| 10 | 1157.4633 | 579.2353 | 1140.4368 | 570.7220 | 1139.4528 | 570.2300 | A | **1169.5507** | 585.2790 | 1152.5242 | 576.7657 | 1151.5401 | 576.2737 | 10 |
| 11 | 1271.5062 | 636.2568 | 1254.4797 | 627.7435 | 1253.4957 | 627.2515 | N | **1098.5136** | 549.7604 | 1081.4870 | 541.2472 | 1080.5030 | 540.7552 | 9 |
| 12 | **1384.5903** | 692.7988 | 1367.5638 | 684.2855 | 1366.5797 | 683.7935 | L | **984.4707** | 492.7390 | 967.4441 | 484.2257 | 966.4601 | 483.7337 | 8 |
| 13 | **1547.6536** | 774.3305 | 1530.6271 | 765.8172 | 1529.6431 | 765.3252 | Y | **871.3866** | 436.1969 | 854.3601 | 427.6837 | 853.3760 | 427.1917 | 7 |
| 14 | **1662.6806** | 831.8439 | 1645.6540 | 823.3307 | 1644.6700 | 822.8386 | D | **708.3233** | 354.6653 | 691.2967 | 346.1520 | 690.3127 | 345.6600 | 6 |
| 15 | 1791.7232 | 896.3652 | 1774.6966 | 887.8520 | 1773.7126 | 887.3599 | E | **593.2963** | 297.1518 | 576.2698 | 288.6385 | 575.2858 | 288.1465 | 5 |
| 16 | **1938.7586** | 969.8829 | 1921.7320 | 961.3697 | 1920.7480 | 960.8776 | M | **464.2537** | 232.6305 | 447.2272 | 224.1172 | | | 4 |
| 17 | 2037.8270 | 1019.4171 | 2020.8004 | 1010.9039 | 2019.8164 | 1010.4119 | V | 317.2183 | 159.1128 | 300.1918 | 150.5995 | | | 3 |
| 18 | 2108.8641 | 1054.9357 | 2091.8376 | 1046.4224 | 2090.8535 | 1045.9304 | A | 218.1499 | 109.5786 | 201.1234 | 101.0653 | | | 2 |
| 19 | | | | | | | K | 147.1128 | 74.0600 | 130.0863 | 65.5468 | | | 1 |

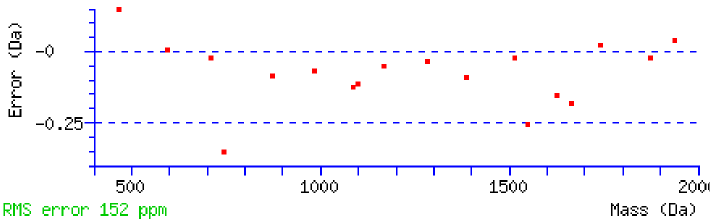


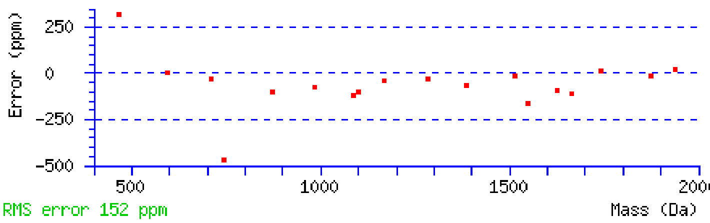


### All matches to this query

| Score | Mr(calc): | Delta | Sequence |
|---|---|---|---|
| 117.0 | 2253.9623 | 0.0072 | YGYENDTEIANLYDEMVAK |
| 66.0 | 2252.9783 | 0.9912 | YGYENDTEIANLYDEMVAK |

| | | | |
|---|---|---|---|
| 54.6 | 2253.9623 | 0.0072 | YGYENDTEIANLYDEMVAK |
| 8.4 | 2251.9482 | 2.0214 | GNQSDEGSDIDSEPDLPLKR |
| 6.3 | 2251.9482 | 2.0214 | GNQSDEGSDIDSEPDLPLKR |
| 4.8 | 2253.9596 | 0.0099 | LDSTAASLISNGPGGSVSFWK |
| 3.9 | 2251.9763 | 1.9932 | GKKDDNGIGTAIDFVLSNAR |
| 3.8 | 2252.9464 | 1.0231 | HPSNPKVRSSPSGNTQSSPK |
| 3.1 | 2252.9500 | 1.0195 | GKFYSCNDLSKMTEEECR |
| 2.7 | 2251.9829 | 1.9866 | YSMYNSVSQKLMAKMGFR |

---

Spectrum No: 22; Query: 1956; Rank: 1

---

## Peptide View

MS/MS Fragmentation of **YGYENDTEIANLYDEMVAK**
Found in **IPI00191437**, Tax_Id=10116 Gene_Symbol=- 64 kDa protein

Match to Query 1956: 2253.969528 from(1127.992040,2+)
Title: 100101RatKid_NS_deglyco_25.3594.3594.2.dta
Data file K:\NewmanPaper\Piliang\3SubProteomes\Piliang3SP\mgf5ppm\ERLIC_3SubProteomes5ppm.mgf

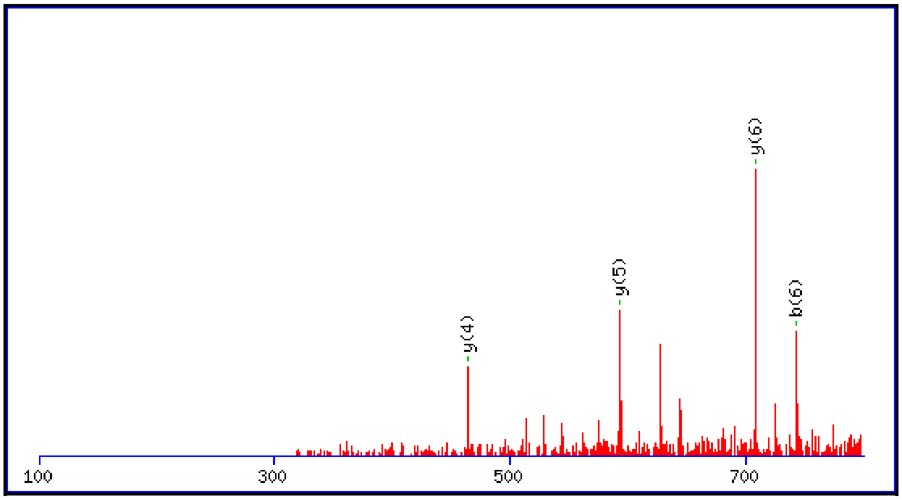


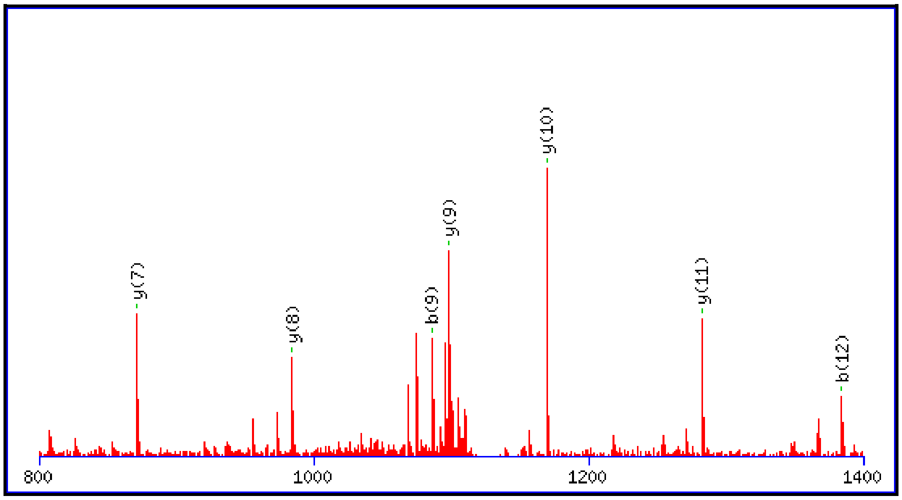

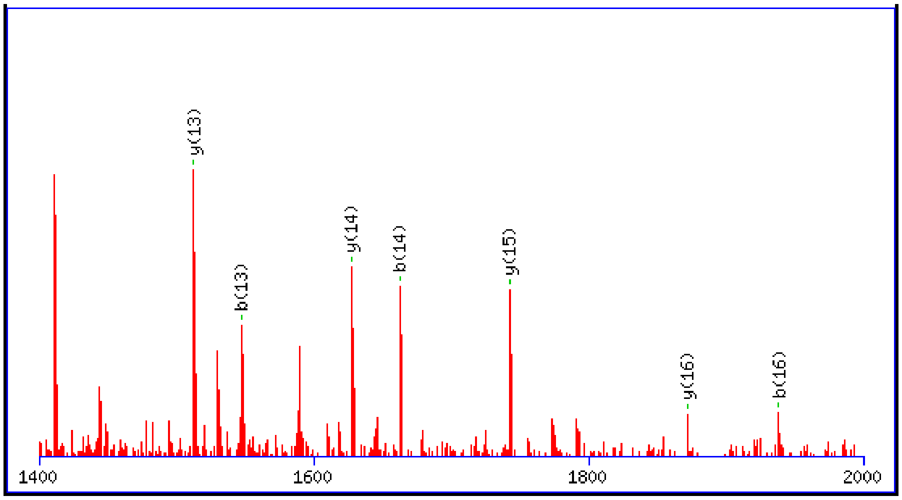


**Monoisotopic mass of neutral peptide Mr(calc):** 2253.9623
**Fixed modifications:** Carbamidomethyl (C)
**Variable modifications:**
**N5** : Deamidated_N (N)
**M16** : Oxidation (M)
**Ions Score:** 117 **Expect:** 5.5e-010
**Matches (Bold Red):** 18/194 fragment ions using 25 most intense peaks

| # | b | b$^{++}$ | b* | b*$^{++}$ | b$^{0}$ | b$^{0++}$ | Seq. | y | y$^{++}$ | y* | y*$^{++}$ | y$^{0}$ | y$^{0++}$ | # |
|---|---|---|---|---|---|---|---|---|---|---|---|---|---|---|
| 1 | 164.0706 | 82.5389 | | | | | Y | | | | | | | 19 |
| 2 | 221.0921 | 111.0497 | | | | | G | 2091.9063 | 1046.4568 | 2074.8798 | 1037.9435 | 2073.8957 | 1037.4515 | 18 |
| 3 | 384.1554 | 192.5813 | | | | | Y | 2034.8848 | 1017.9461 | 2017.8583 | 1009.4328 | 2016.8743 | 1008.9408 | 17 |
| 4 | 513.1980 | 257.1026 | | | 495.1874 | 248.0974 | E | **1871.8215** | 936.4144 | 1854.7950 | 927.9011 | 1853.8109 | 927.4091 | 16 |
| 5 | 628.2249 | 314.6161 | 611.1984 | 306.1028 | 610.2144 | 305.6108 | N | **1742.7789** | 871.8931 | 1725.7524 | 863.3798 | 1724.7684 | 862.8878 | 15 |
| 6 | **743.2519** | 372.1296 | 726.2253 | 363.6163 | 725.2413 | 363.1243 | D | **1627.7520** | 814.3796 | 1610.7254 | 805.8664 | 1609.7414 | 805.3743 | 14 |
| 7 | 844.2995 | 422.6534 | 827.2730 | 414.1401 | 826.2890 | 413.6481 | T | **1512.7250** | 756.8662 | 1495.6985 | 748.3529 | 1494.7145 | 747.8609 | 13 |
| 8 | 973.3421 | 487.1747 | 956.3156 | 478.6614 | 955.3316 | 478.1694 | E | 1411.6774 | 706.3423 | 1394.6508 | 697.8290 | 1393.6668 | 697.3370 | 12 |
| 9 | **1086.4262** | 543.7167 | 1069.3997 | 535.2035 | 1068.4156 | 534.7115 | I | **1282.6348** | 641.8210 | 1265.6082 | 633.3077 | 1264.6242 | 632.8157 | 11 |
| 10 | 1157.4633 | 579.2353 | 1140.4368 | 570.7220 | 1139.4528 | 570.2300 | A | **1169.5507** | 585.2790 | 1152.5242 | 576.7657 | 1151.5401 | 576.2737 | 10 |
| 11 | 1271.5062 | 636.2568 | 1254.4797 | 627.7435 | 1253.4957 | 627.2515 | N | **1098.5136** | 549.7604 | 1081.4870 | 541.2472 | 1080.5030 | 540.7552 | 9 |
| 12 | **1384.5903** | 692.7988 | 1367.5638 | 684.2855 | 1366.5797 | 683.7935 | L | **984.4707** | 492.7390 | 967.4441 | 484.2257 | 966.4601 | 483.7337 | 8 |
| 13 | **1547.6536** | 774.3305 | 1530.6271 | 765.8172 | 1529.6431 | 765.3252 | Y | **871.3866** | 436.1969 | 854.3601 | 427.6837 | 853.3760 | 427.1917 | 7 |
| 14 | **1662.6806** | 831.8439 | 1645.6540 | 823.3307 | 1644.6700 | 822.8386 | D | **708.3233** | 354.6653 | 691.2967 | 346.1520 | 690.3127 | 345.6600 | 6 |
| 15 | 1791.7232 | 896.3652 | 1774.6966 | 887.8520 | 1773.7126 | 887.3599 | E | **593.2963** | 297.1518 | 576.2698 | 288.6385 | 575.2858 | 288.1465 | 5 |
| 16 | **1938.7586** | 969.8829 | 1921.7320 | 961.3697 | 1920.7480 | 960.8776 | M | **464.2537** | 232.6305 | 447.2272 | 224.1172 | | | 4 |
| 17 | 2037.8270 | 1019.4171 | 2020.8004 | 1010.9039 | 2019.8164 | 1010.4119 | V | 317.2183 | 159.1128 | 300.1918 | 150.5995 | | | 3 |
| 18 | 2108.8641 | 1054.9357 | 2091.8376 | 1046.4224 | 2090.8535 | 1045.9304 | A | 218.1499 | 109.5786 | 201.1234 | 101.0653 | | | 2 |
| 19 | | | | | | | K | 147.1128 | 74.0600 | 130.0863 | 65.5468 | | | 1 |

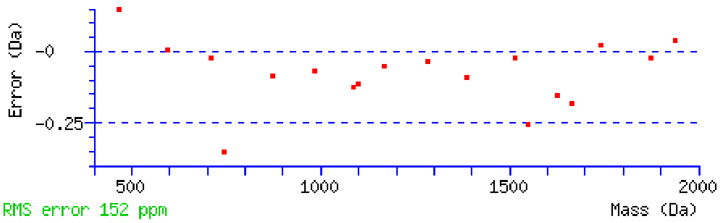


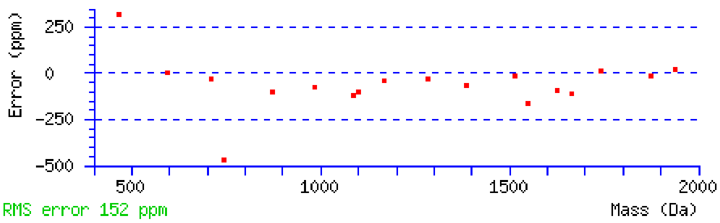


## All matches to this query

| Score | Mr(calc): | Delta | Sequence |
|---|---|---|---|
| 117.0 | 2253.9623 | 0.0072 | YGYENDTEIANLYDEMVAK |
| 66.0 | 2252.9783 | 0.9912 | YGYENDTEIANLYDEMVAK |

| | | | |
|---|---|---|---|
| 54.6 | 2253.9623 | 0.0072 | YGYENDTEIANLYDEMVAK |
| 8.4 | 2251.9482 | 2.0214 | GNQSDEGSDIDSEPDLPLKR |
| 6.3 | 2251.9482 | 2.0214 | GNQSDEGSDIDSEPDLPLKR |
| 4.8 | 2253.9596 | 0.0099 | LDSTAASLISNGPGGSVSFWK |
| 3.9 | 2251.9763 | 1.9932 | GKKDDNGIGTAIDFVLSNAR |
| 3.8 | 2252.9464 | 1.0231 | HPSNPKVRSSPSGNTQSSPK |
| 3.1 | 2252.9500 | 1.0195 | GKFYSCNDLSKMTEEECR |
| 2.7 | 2251.9829 | 1.9866 | YSMYNSVSQKLMAKMGFR |

Spectrum No: 23; Query: 1912; Rank: 1

## Peptide View

MS/MS Fragmentation of **IGCFALSEPGNGSDAGAASTTAR**
Found in **IPI00231359**, Tax_Id=10116 Gene_Symbol=Acads Acetyl-Coenzyme A dehydrogenase, short chain

Match to Query 1912: 2209.988948 from(1106.001750,2+)
Title: 091008RatKidney_NH4Format02_24.2775.2775.2.dta
Data file K:\NewmanPaper\Piliang\3SubProteomes\Piliang3SP\mgf5ppm\ERLIC_3SubProteomes5ppm.mgf

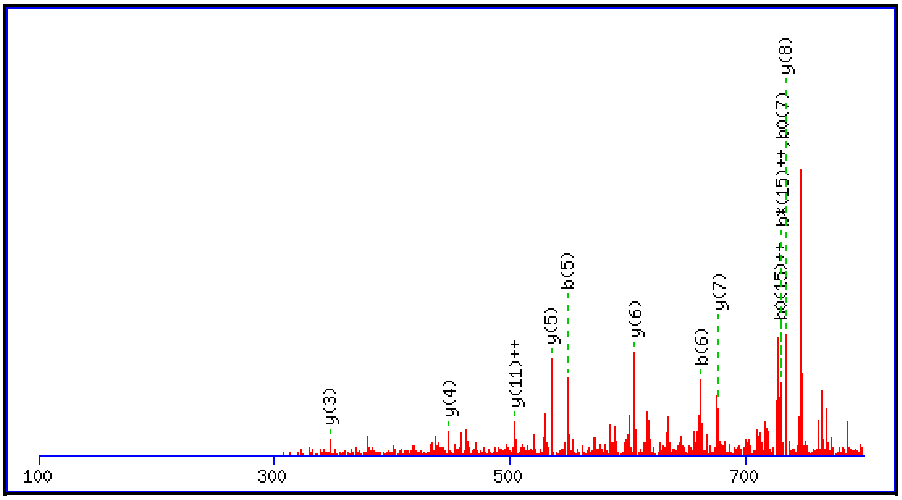


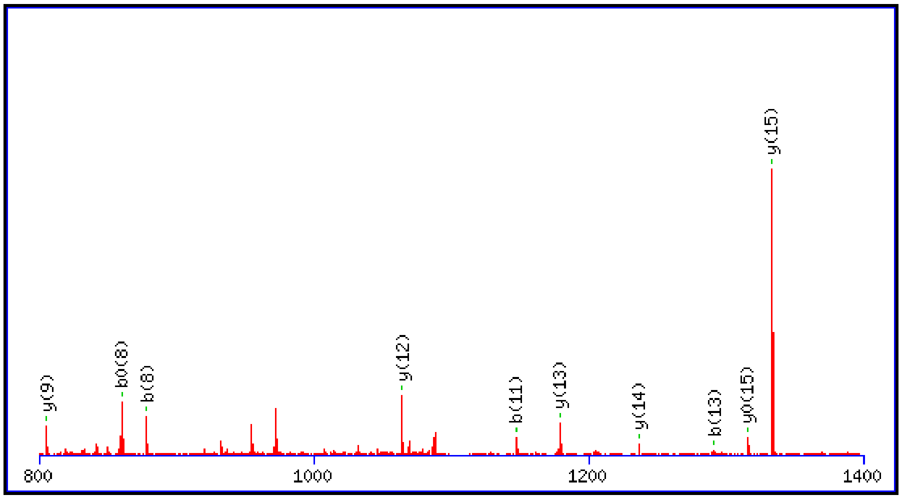

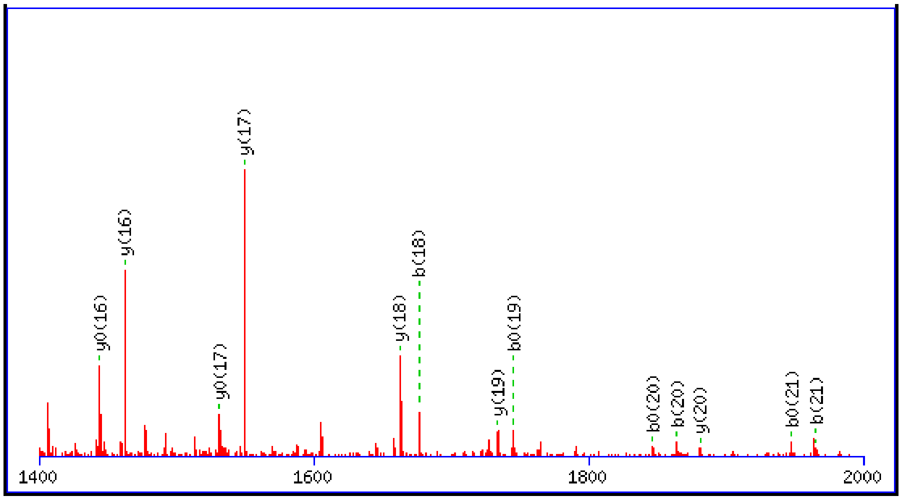


**Monoisotopic mass of neutral peptide Mr(calc):** 2209.9910
**Fixed modifications:** Carbamidomethyl (C)
**Variable modifications:**
**N11** : Deamidated_N (N)
**Ions Score:** 116 **Expect:** 6.5e-010
**Matches (Bold Red):** 35/228 fragment ions using 63 most intense peaks

| # | b | $b^{++}$ | b* | $b^{*++}$ | $b^0$ | $b^{0++}$ | Seq. | y | $y^{++}$ | y* | $y^{*++}$ | $y^0$ | $y^{0++}$ | # |
|---|---|---|---|---|---|---|---|---|---|---|---|---|---|---|
| 1 | 114.0913 | 57.5493 | | | | | I | | | | | | | 23 |
| 2 | 171.1128 | 86.0600 | | | | | G | 2097.9142 | 1049.4607 | 2080.8876 | 1040.9475 | 2079.9036 | 1040.4554 | 22 |
| 3 | 331.1435 | 166.0754 | | | | | C | 2040.8927 | 1020.9500 | 2023.8662 | 1012.4367 | 2022.8822 | 1011.9447 | 21 |
| 4 | 478.2119 | 239.6096 | | | | | F | **1880.8621** | 940.9347 | 1863.8355 | 932.4214 | 1862.8515 | 931.9294 | 20 |
| 5 | **549.2490** | 275.1281 | | | | | A | **1733.7937** | 867.4005 | 1716.7671 | 858.8872 | 1715.7831 | 858.3952 | 19 |
| 6 | **662.3330** | 331.6702 | | | | | L | **1662.7565** | 831.8819 | 1645.7300 | 823.3686 | 1644.7460 | 822.8766 | 18 |
| 7 | 749.3651 | 375.1862 | | | **731.3545** | 366.1809 | S | **1549.6725** | 775.3399 | 1532.6459 | 766.8266 | **1531.6619** | 766.3346 | 17 |
| 8 | **878.4077** | 439.7075 | | | **860.3971** | 430.7022 | E | **1462.6405** | 731.8239 | 1445.6139 | 723.3106 | **1444.6299** | 722.8186 | 16 |
| 9 | 975.4604 | 488.2339 | | | 957.4499 | 479.2286 | P | **1333.5979** | 667.3026 | 1316.5713 | 658.7893 | **1315.5873** | 658.2973 | 15 |
| 10 | 1032.4819 | 516.7446 | | | 1014.4713 | 507.7393 | G | **1236.5451** | 618.7762 | 1219.5185 | 610.2629 | 1218.5345 | 609.7709 | 14 |
| 11 | **1147.5088** | 574.2581 | 1130.4823 | 565.7448 | 1129.4983 | 565.2528 | N | **1179.5236** | 590.2655 | 1162.4971 | 581.7522 | 1161.5131 | 581.2602 | 13 |
| 12 | 1204.5303 | 602.7688 | 1187.5037 | 594.2555 | 1186.5197 | 593.7635 | G | **1064.4967** | 532.7520 | 1047.4701 | 524.2387 | 1046.4861 | 523.7467 | 12 |
| 13 | **1291.5623** | 646.2848 | 1274.5358 | 637.7715 | 1273.5518 | 637.2795 | S | 1007.4752 | **504.2413** | 990.4487 | 495.7280 | 989.4647 | 495.2360 | 11 |
| 14 | 1406.5893 | 703.7983 | 1389.5627 | 695.2850 | 1388.5787 | 694.7930 | D | 920.4432 | 460.7252 | 903.4167 | 452.2120 | 902.4326 | 451.7200 | 10 |
| 15 | 1477.6264 | 739.3168 | 1460.5998 | **730.8036** | 1459.6158 | **730.3115** | A | **805.4163** | 403.2118 | 788.3897 | 394.6985 | 787.4057 | 394.2065 | 9 |
| 16 | 1534.6478 | 767.8276 | 1517.6213 | 759.3143 | 1516.6373 | 758.8223 | G | **734.3791** | 367.6932 | 717.3526 | 359.1799 | 716.3686 | 358.6879 | 8 |
| 17 | 1605.6850 | 803.3461 | 1588.6584 | 794.8328 | 1587.6744 | 794.3408 | A | **677.3577** | 339.1825 | 660.3311 | 330.6692 | 659.3471 | 330.1772 | 7 |
| 18 | **1676.7221** | 838.8647 | 1659.6955 | 830.3514 | 1658.7115 | 829.8594 | A | **606.3206** | 303.6639 | 589.2940 | 295.1506 | 588.3100 | 294.6586 | 6 |
| 19 | 1763.7541 | 882.3807 | 1746.7276 | 873.8674 | **1745.7435** | 873.3754 | S | **535.2835** | 268.1454 | 518.2569 | 259.6321 | 517.2729 | 259.1401 | 5 |
| 20 | **1864.8018** | 932.9045 | 1847.7752 | 924.3913 | **1846.7912** | 923.8992 | T | **448.2514** | 224.6294 | 431.2249 | 216.1161 | 430.2409 | 215.6241 | 4 |
| 21 | **1965.8495** | 983.4284 | 1948.8229 | 974.9151 | **1947.8389** | 974.4231 | T | **347.2037** | 174.1055 | 330.1772 | 165.5922 | 329.1932 | 165.1002 | 3 |
| 22 | 2036.8866 | 1018.9469 | 2019.8600 | 1010.4336 | 2018.8760 | 1009.9416 | A | 246.1561 | 123.5817 | 229.1295 | 115.0684 | | | 2 |
| 23 | | | | | | | R | 175.1190 | 88.0631 | 158.0924 | 79.5498 | | | 1 |

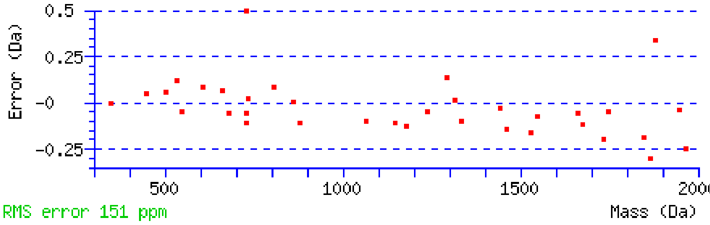


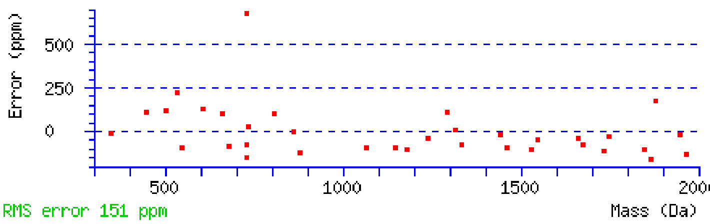


**All matches to this query**

| Score | Mr(calc): | Delta | Sequence |
|---|---|---|---|
| 116.2 | 2209.9910 | -0.0020 | IGCFALSEPGNGSDAGAASTTAR |
| 26.3 | 2209.0070 | 0.9820 | IGCFALSEPGNGSDAGAASTTAR |
| 11.2 | 2208.9673 | 1.0216 | LNATTDVMRGLLSNVLCR |
| 2.3 | 2207.9613 | 2.0277 | EHLSLSIEENVQPRSPGR |
| 1.7 | 2209.0007 | 0.9882 | SLAEMLMSKNTRTSDTLSK |
| 1.0 | 2209.9674 | 0.0215 | EPRKSAEEALAAAGDMDNAGK |
| 0.7 | 2209.9711 | 0.0178 | KGTGPVQIPPGEWHSSFXR |
| 0.4 | 2207.9683 | 2.0206 | SPWRPGSSKVWTPISKR |
| 0.3 | 2208.9704 | 1.0185 | NQINIRNSETDTIIFIR |
| 0.1 | 2209.9787 | 0.0103 | TRPRAESDDPENEAKMIR |

---

Spectrum No: 24; Query: 2727; Rank: 1

---

## Peptide View

MS/MS Fragmentation of **QAEVADQQTTDLPAENGETENQSPASEEEKEAK**
Found in **IPI00373052**, Tax_Id=10116 Gene_Symbol=Hmgn1 High mobility group nucleosomal binding domain 1

Match to Query 2727: 3573.575502 from(1192.199110,3+)
Title: 091008RatKidney_NoSalt_21.1314.1314.3.dta
Data file K:\NewmanPaper\Piliang\3SubProteomes\Piliang3SP\mgf5ppm\ERLIC_3SubProteomes5ppm.mgf

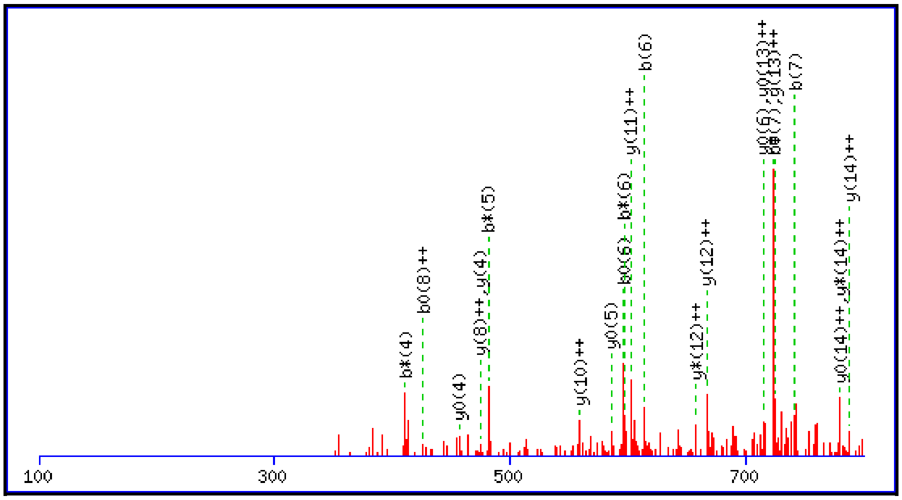


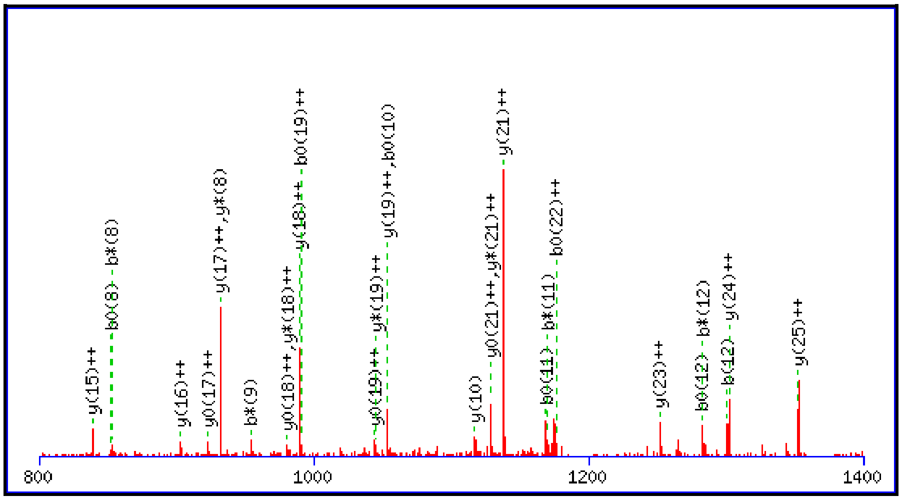

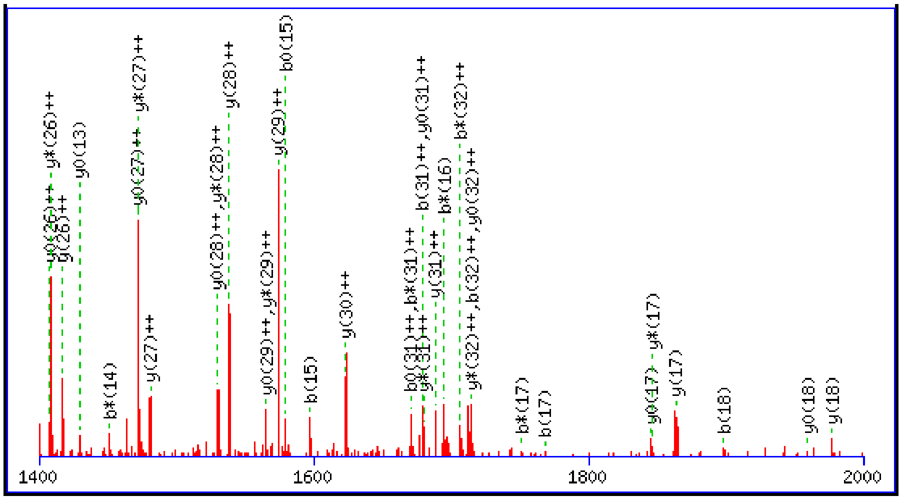


**Monoisotopic mass of neutral peptide Mr(calc):** 3573.5714
**Fixed modifications:** Carbamidomethyl (C)
**Variable modifications:**
**N21** : Deamidated_N (N)
**Ions Score:** 115 **Expect:** 1.4e-009
**Matches (Bold Red):** 88/376 fragment ions using 142 most intense peaks

| # | b | b$^{++}$ | b* | b*$^{++}$ | b$^{0}$ | b$^{0++}$ | Seq. | y | y$^{++}$ | y* | y*$^{++}$ | y$^{0}$ | y$^{0++}$ | # |
|---|---|---|---|---|---|---|---|---|---|---|---|---|---|---|
| 1 | 129.0659 | 65.0366 | 112.0393 | 56.5233 | | | Q | | | | | | | 33 |
| 2 | 200.1030 | 100.5551 | 183.0764 | 92.0418 | | | A | 3446.5201 | 1723.7637 | 3429.4936 | **1715.2504** | 3428.5096 | **1714.7584** | 32 |
| 3 | 329.1456 | 165.0764 | 312.1190 | 156.5631 | 311.1350 | 156.0711 | E | 3375.4830 | **1688.2451** | 3358.4565 | **1679.7319** | 3357.4724 | **1679.2399** | 31 |
| 4 | 428.2140 | 214.6106 | **411.1874** | 206.0974 | 410.2034 | 205.6053 | V | 3246.4404 | **1623.7238** | 3229.4139 | 1615.2106 | 3228.4299 | 1614.7186 | 30 |
| 5 | 499.2511 | 250.1292 | **482.2245** | 241.6159 | 481.2405 | 241.1239 | A | 3147.3720 | **1574.1896** | 3130.3455 | **1565.6764** | 3129.3614 | **1565.1844** | 29 |
| 6 | **614.2780** | 307.6427 | **597.2515** | 299.1294 | **596.2675** | 298.6374 | D | 3076.3349 | **1538.6711** | 3059.3083 | **1530.1578** | 3058.3243 | **1529.6658** | 28 |
| 7 | **742.3366** | 371.6719 | **725.3101** | 363.1587 | **724.3260** | 362.6667 | Q | 2961.3079 | **1481.1576** | 2944.2814 | **1472.6443** | 2943.2974 | **1472.1523** | 27 |
| 8 | 870.3952 | 435.7012 | **853.3686** | 427.1880 | **852.3846** | **426.6959** | Q | 2833.2494 | **1417.1283** | 2816.2228 | **1408.6150** | 2815.2388 | **1408.1230** | 26 |
| 9 | 971.4429 | 486.2251 | **954.4163** | 477.7118 | 953.4323 | 477.2198 | T | 2705.1908 | **1353.0990** | 2688.1642 | 1344.5858 | 2687.1802 | 1344.0938 | 25 |
| 10 | 1072.4905 | 536.7489 | 1055.4640 | 528.2356 | **1054.4800** | 527.7436 | T | 2604.1431 | **1302.5752** | 2587.1166 | 1294.0619 | 2586.1325 | 1293.5699 | 24 |
| 11 | 1187.5175 | 594.2624 | **1170.4909** | 585.7491 | **1169.5069** | 585.2571 | D | 2503.0954 | **1252.0514** | 2486.0689 | 1243.5381 | 2485.0849 | 1243.0461 | 23 |
| 12 | **1300.6016** | 650.8044 | **1283.5750** | 642.2911 | **1282.5910** | 641.7991 | L | 2388.0685 | 1194.5379 | 2371.0419 | 1186.0246 | 2370.0579 | 1185.5326 | 22 |
| 13 | 1397.6543 | 699.3308 | 1380.6278 | 690.8175 | 1379.6438 | 690.3255 | P | 2274.9844 | **1137.9959** | 2257.9579 | **1129.4826** | 2256.9739 | **1128.9906** | 21 |
| 14 | 1468.6914 | 734.8494 | **1451.6649** | 726.3361 | 1450.6809 | 725.8441 | A | 2177.9317 | 1089.4695 | 2160.9051 | 1080.9562 | 2159.9211 | 1080.4642 | 20 |
| 15 | **1597.7340** | 799.3707 | 1580.7075 | 790.8574 | **1579.7235** | 790.3654 | E | 2106.8946 | **1053.9509** | 2089.8680 | **1045.4376** | 2088.8840 | **1044.9456** | 19 |
| 16 | 1711.7770 | 856.3921 | **1694.7504** | 847.8788 | 1693.7664 | 847.3868 | N | **1977.8520** | **989.4296** | 1960.8254 | **980.9163** | **1959.8414** | **980.4243** | 18 |
| 17 | **1768.7984** | 884.9028 | **1751.7719** | 876.3896 | 1750.7878 | 875.8976 | G | **1863.8090** | **932.4082** | **1846.7825** | 923.8949 | **1845.7985** | **923.4029** | 17 |
| 18 | **1897.8410** | 949.4241 | 1880.8145 | 940.9109 | 1879.8304 | 940.4189 | E | 1806.7876 | **903.8974** | 1789.7610 | 895.3841 | 1788.7770 | 894.8921 | 16 |
| 19 | 1998.8887 | 999.9480 | 1981.8621 | 991.4347 | 1980.8781 | **990.9427** | T | 1677.7450 | **839.3761** | 1660.7184 | 830.8629 | 1659.7344 | 830.3708 | 15 |
| 20 | 2127.9313 | 1064.4693 | 2110.9047 | 1055.9560 | 2109.9207 | 1055.4640 | E | 1576.6973 | **788.8523** | 1559.6707 | **780.3390** | 1558.6867 | **779.8470** | 14 |
| 21 | 2242.9582 | 1121.9827 | 2225.9317 | 1113.4695 | 2224.9477 | 1112.9775 | N | 1447.6547 | **724.3310** | 1430.6282 | 715.8177 | **1429.6441** | **715.3257** | 13 |
| 22 | 2371.0168 | 1186.0120 | 2353.9902 | 1177.4988 | 2353.0062 | **1177.0068** | Q | 1332.6278 | **666.8175** | 1315.6012 | **658.3042** | 1314.6172 | 657.8122 | 12 |
| 23 | 2458.0488 | 1229.5280 | 2441.0223 | 1221.0148 | 2440.0383 | 1220.5228 | S | 1204.5692 | **602.7882** | 1187.5426 | 594.2750 | 1186.5586 | 593.7829 | 11 |
| 24 | 2555.1016 | 1278.0544 | 2538.0750 | 1269.5412 | 2537.0910 | 1269.0491 | P | **1117.5372** | **559.2722** | 1100.5106 | 550.7589 | 1099.5266 | 550.2669 | 10 |
| 25 | 2626.1387 | 1313.5730 | 2609.1122 | 1305.0597 | 2608.1281 | 1304.5677 | A | 1020.4844 | 510.7458 | 1003.4578 | 502.2326 | 1002.4738 | 501.7406 | 9 |
| 26 | 2713.1707 | 1357.0890 | 2696.1442 | 1348.5757 | 2695.1602 | 1348.0837 | S | 949.4473 | **475.2273** | **932.4207** | 466.7140 | 931.4367 | 466.2220 | 8 |
| 27 | 2842.2133 | 1421.6103 | 2825.1868 | 1413.0970 | 2824.2028 | 1412.6050 | E | 862.4153 | 431.7113 | 845.3887 | 423.1980 | 844.4047 | 422.7060 | 7 |
| 28 | 2971.2559 | 1486.1316 | 2954.2294 | 1477.6183 | 2953.2453 | 1477.1263 | E | 733.3727 | 367.1900 | 716.3461 | 358.6767 | **715.3621** | 358.1847 | 6 |
| 29 | 3100.2985 | 1550.6529 | 3083.2720 | 1542.1396 | 3082.2879 | 1541.6476 | E | 604.3301 | 302.6687 | 587.3035 | 294.1554 | **586.3195** | 293.6634 | 5 |
| 30 | 3228.3935 | 1614.7004 | 3211.3669 | 1606.1871 | 3210.3829 | 1605.6951 | K | **475.2875** | 238.1474 | 458.2609 | 229.6341 | **457.2769** | 229.1421 | 4 |
| 31 | 3357.4361 | **1679.2217** | 3340.4095 | **1670.7084** | 3339.4255 | **1670.2164** | E | 347.1925 | 174.0999 | 330.1660 | 165.5866 | 329.1819 | 165.0946 | 3 |
| 32 | 3428.4732 | **1714.7402** | 3411.4466 | **1706.2270** | 3410.4626 | 1705.7349 | A | 218.1499 | 109.5786 | 201.1234 | 101.0653 | | | 2 |

| | | | | | | | | | | | | | |
|---|---|---|---|---|---|---|---|---|---|---|---|---|---|
| 33 | | | | | | | K | 147.1128 | 74.0600 | 130.0863 | 65.5468 | | 1 |

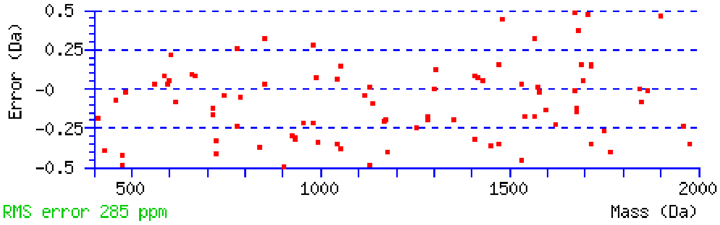


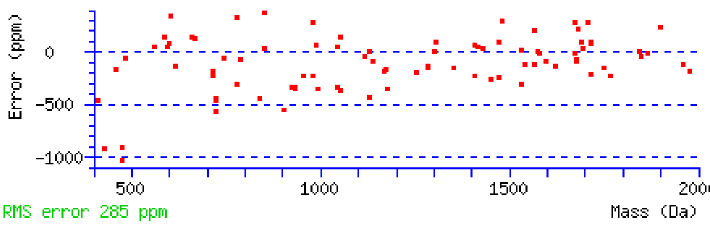


**All matches to this query**

| Score | Mr(calc): | Delta | Sequence |
|---|---|---|---|
| 115.4 | 3573.5714 | 0.0041 | QAEVADQQTTDLPAENGETENQSPASEEEKEAK |
| 112.1 | 3573.5714 | 0.0041 | QAEVADQQTTDLPAENGETENQSPASEEEKEAK |
| 54.8 | 3572.5874 | 0.9881 | QAEVADQQTTDLPAENGETENQSPASEEEKEAK |
| 12.2 | 3573.5809 | -0.0054 | EETFALSSTKTLEDAVNNIITFLGMQPCER |
| 12.2 | 3573.5809 | -0.0054 | EETFALSSTKTLEDAVNNIITFLGMQPCER |
| 12.2 | 3573.5809 | -0.0054 | EETFALSSTKTLEDAVNNIITFLGMQPCER |
| 12.2 | 3573.5809 | -0.0054 | EETFALSSTKTLEDAVNNIITFLGMQPCER |
| 8.9 | 3573.5684 | 0.0071 | CXHSQNTSLACQTLAPSVWQLESGWSARAR |
| 8.9 | 3573.5684 | 0.0071 | CXHSQNTSLACQTLAPSVWQLESGWSARAR |
| 8.4 | 3573.5677 | 0.0078 | MALFPAFAGVSEASGDGASRKELDWLSNPSFR |

---

Spectrum No: 25; Query: 1309; Rank: 1

---

## Peptide View

MS/MS Fragmentation of **GTFTDCALANMTQQIR**
Found in **IPI00372839**, Tax_Id=10116 Gene_Symbol=Col6a2 similar to procollagen, type VI, alpha 2

Match to Query 1309: 1842.819008 from(922.416780,2+)
Title: 091008RatKidney_NH4Format01_23.2093.2093.2.dta
Data file K:\NewmanPaper\Piliang\3SubProteomes\Piliang3SP\mgf5ppm\ERLIC_3SubProteomes5ppm.mgf

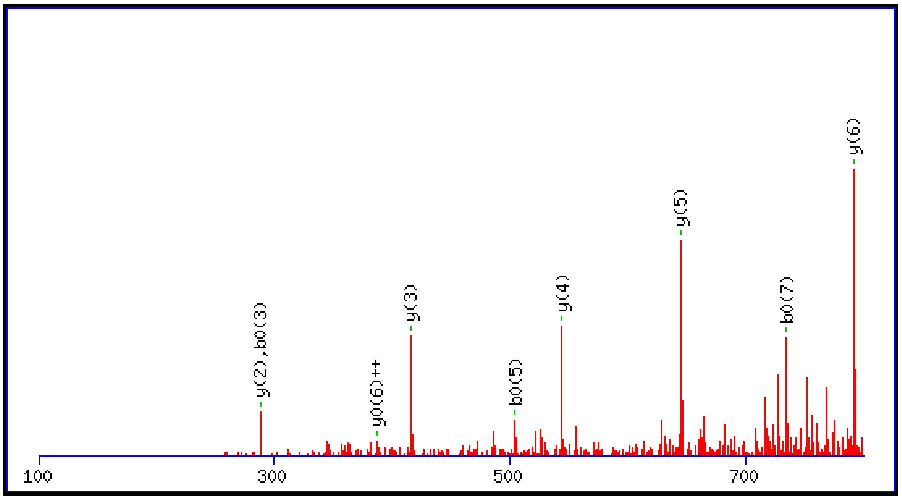

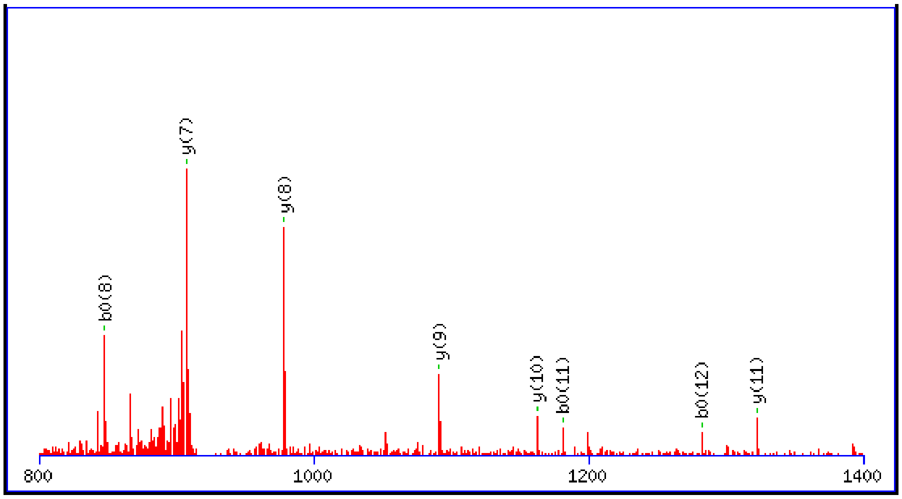


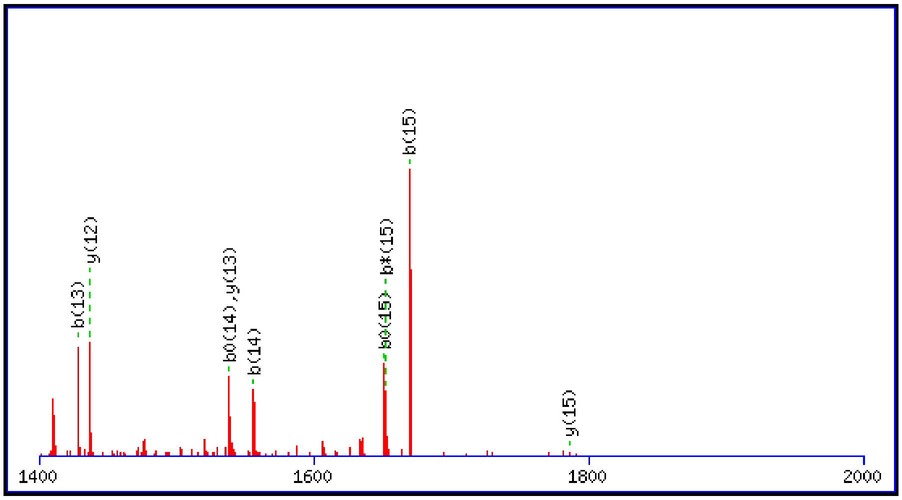


**Monoisotopic mass of neutral peptide Mr(calc):** 1842.8240
**Fixed modifications:** Carbamidomethyl (C)
**Variable modifications:**
**N10** : Deamidated_N (N)
**M11** : Oxidation (M)
**Ions Score:** 115 **Expect:** 6.2e-010
**Matches (Bold Red):** 26/152 fragment ions using 36 most intense peaks

| # | b | $b^{++}$ | b* | $b^{*++}$ | $b^0$ | $b^{0++}$ | Seq. | y | $y^{++}$ | y* | $y^{*++}$ | $y^0$ | $y^{0++}$ | # |
|---|---|---|---|---|---|---|---|---|---|---|---|---|---|---|
| 1 | 58.0287 | 29.5180 | | | | | G | | | | | | | 16 |
| 2 | 159.0764 | 80.0418 | | | 141.0659 | 71.0366 | T | **1786.8098** | 893.9086 | 1769.7833 | 885.3953 | 1768.7993 | 884.9033 | 15 |
| 3 | 306.1448 | 153.5761 | | | **288.1343** | 144.5708 | F | 1685.7622 | 843.3847 | 1668.7356 | 834.8714 | 1667.7516 | 834.3794 | 14 |
| 4 | 407.1925 | 204.0999 | | | 389.1819 | 195.0946 | T | **1538.6938** | 769.8505 | 1521.6672 | 761.3372 | 1520.6832 | 760.8452 | 13 |
| 5 | 522.2195 | 261.6134 | | | **504.2089** | 252.6081 | D | **1437.6461** | 719.3267 | 1420.6195 | 710.8134 | 1419.6355 | 710.3214 | 12 |
| 6 | 682.2501 | 341.6287 | | | 664.2395 | 332.6234 | C | **1322.6191** | 661.8132 | 1305.5926 | 653.2999 | 1304.6086 | 652.8079 | 11 |
| 7 | 753.2872 | 377.1472 | | | **735.2767** | 368.1420 | A | **1162.5885** | 581.7979 | 1145.5619 | 573.2846 | 1144.5779 | 572.7926 | 10 |
| 8 | 866.3713 | 433.6893 | | | **848.3607** | 424.6840 | L | **1091.5514** | 546.2793 | 1074.5248 | 537.7660 | 1073.5408 | 537.2740 | 9 |
| 9 | 937.4084 | 469.2078 | | | 919.3978 | 460.2026 | A | **978.4673** | 489.7373 | 961.4408 | 481.2240 | 960.4567 | 480.7320 | 8 |
| 10 | 1052.4353 | 526.7213 | 1035.4088 | 518.2080 | 1034.4248 | 517.7160 | N | **907.4302** | 454.2187 | 890.4036 | 445.7055 | 889.4196 | 445.2135 | 7 |
| 11 | 1199.4707 | 600.2390 | 1182.4442 | 591.7257 | **1181.4602** | 591.2337 | M | **792.4033** | 396.7053 | 775.3767 | 388.1920 | 774.3927 | **387.7000** | 6 |
| 12 | 1300.5184 | 650.7628 | 1283.4919 | 642.2496 | **1282.5079** | 641.7576 | T | **645.3679** | 323.1876 | 628.3413 | 314.6743 | 627.3573 | 314.1823 | 5 |
| 13 | **1428.5770** | 714.7921 | 1411.5504 | 706.2789 | 1410.5664 | 705.7869 | Q | **544.3202** | 272.6637 | 527.2936 | 264.1504 | | | 4 |
| 14 | **1556.6356** | 778.8214 | 1539.6090 | 770.3081 | **1538.6250** | 769.8161 | Q | **416.2616** | 208.6344 | 399.2350 | 200.1212 | | | 3 |
| 15 | **1669.7196** | 835.3635 | **1652.6931** | 826.8502 | **1651.7091** | 826.3582 | I | **288.2030** | 144.6051 | 271.1765 | 136.0919 | | | 2 |
| 16 | | | | | | | R | 175.1190 | 88.0631 | 158.0924 | 79.5498 | | | 1 |

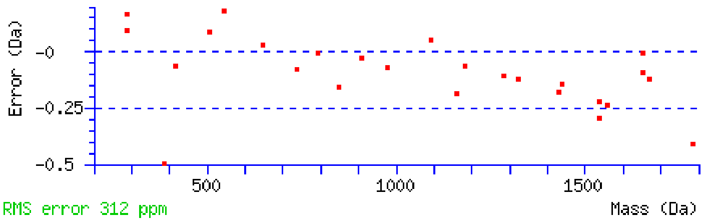


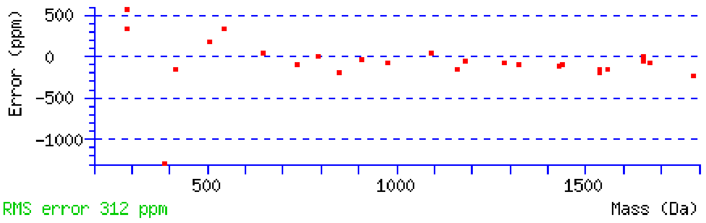


### All matches to this query

| Score | Mr(calc): | Delta | Sequence |
|---|---|---|---|
| 115.1 | 1842.8240 | -0.0050 | GTFTDCALANMTQQIR |
| 9.5 | 1842.8278 | -0.0088 | SSLQDTNVRSPIPIR |
| 8.6 | 1842.8278 | -0.0088 | SSLQDTNVRSPIPIR |
| 8.6 | 1842.8278 | -0.0088 | SSLQDTNVRSPIPIR |
| 6.8 | 1842.8374 | -0.0184 | TSRAVHPTQPSSNPAGR |
| 5.8 | 1842.8067 | 0.0124 | FQLSNGHLLSPLGSGR |
| 5.6 | 1842.8067 | 0.0124 | FQLSNGHLLSPLGSGR |
| 4.7 | 1842.8070 | 0.0120 | LMQVNDTLTSEDAGLR |
| 4.4 | 1840.8025 | 2.0165 | AVLINNLGTIAKSGTK |
| 4.2 | 1842.8118 | 0.0073 | GATLCVICQDRNSLR |

---

Spectrum No: 26; Query: 516; Rank: 1

---

## Peptide View

MS/MS Fragmentation of **AFYNGSLTAQIVK**
Found in **IPI00206254**, Tax_Id=10116 Gene_Symbol=Ggt1 Gamma-glutamyltranspeptidase 1 precursor

Match to Query 516: 1411.741148 from(706.877850,2+)
Title: 091008RatKidney_NoSalt_24.3462.3462.2.dta
Data file K:\NewmanPaper\Piliang\3SubProteomes\Piliang3SP\mgf5ppm\ERLIC_3SubProteomes5ppm.mgf

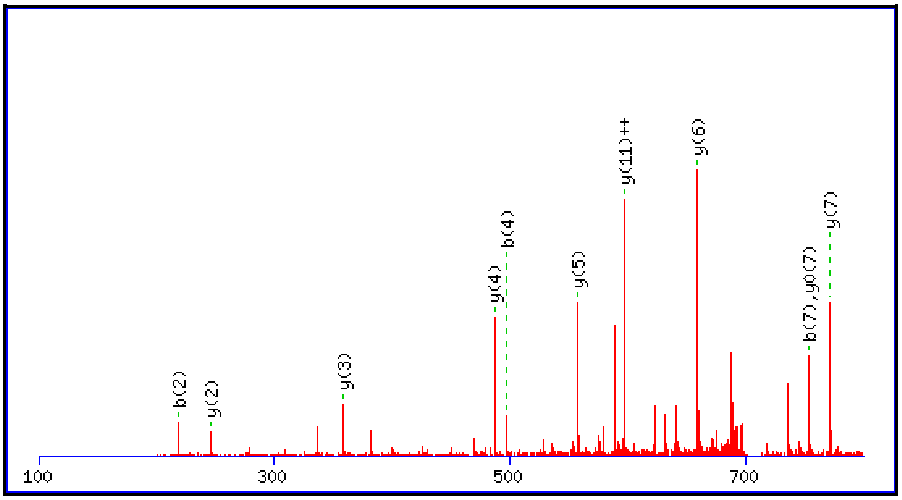

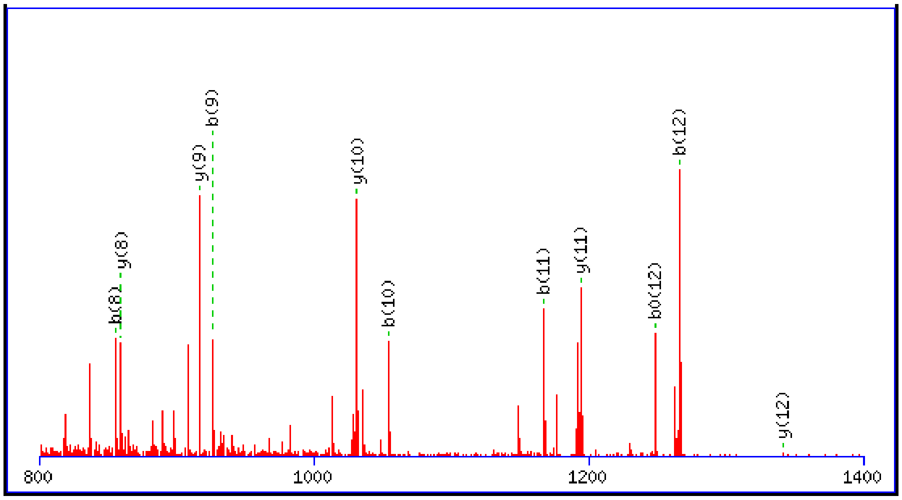


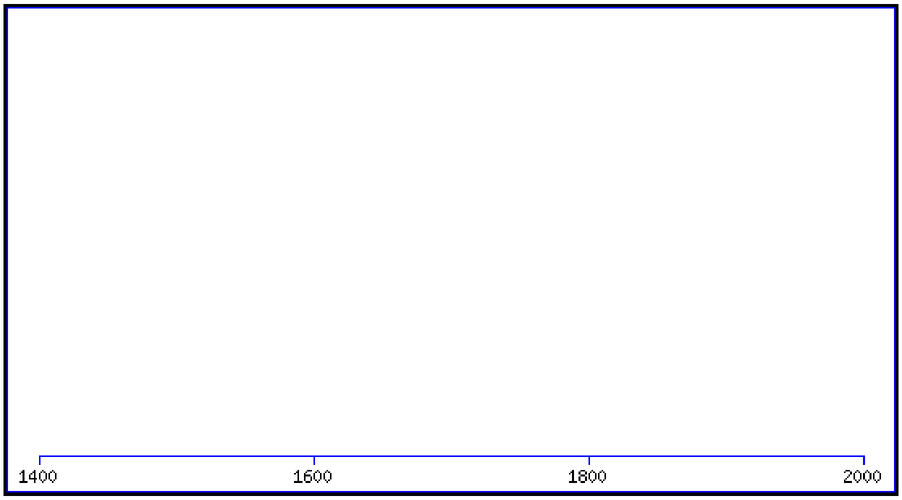


**Monoisotopic mass of neutral peptide Mr(calc):** 1411.7347
**Fixed modifications:** Carbamidomethyl (C)
**Variable modifications:**
**N4** : Deamidated_N (N)
**Ions Score:** 115 **Expect:** 3.1e-010
**Matches (Bold Red):** 22/118 fragment ions using 25 most intense peaks

| # | b | $b^{++}$ | b* | $b^{*++}$ | $b^{0}$ | $b^{0++}$ | Seq. | y | $y^{++}$ | y* | $y^{*++}$ | $y^{0}$ | $y^{0++}$ | # |
|---|---|---|---|---|---|---|---|---|---|---|---|---|---|---|
| 1 | 72.0444 | 36.5258 | | | | | A | | | | | | | 13 |
| 2 | **219.1128** | 110.0600 | | | | | F | **1341.7049** | 671.3561 | 1324.6783 | 662.8428 | 1323.6943 | 662.3508 | 12 |
| 3 | 382.1761 | 191.5917 | | | | | Y | **1194.6365** | **597.8219** | 1177.6099 | 589.3086 | 1176.6259 | 588.8166 | 11 |
| 4 | **497.2031** | 249.1052 | 480.1765 | 240.5919 | | | N | **1031.5731** | 516.2902 | 1014.5466 | 507.7769 | 1013.5626 | 507.2849 | 10 |
| 5 | 554.2245 | 277.6159 | 537.1980 | 269.1026 | | | G | **916.5462** | 458.7767 | 899.5197 | 450.2635 | 898.5356 | 449.7715 | 9 |
| 6 | 641.2566 | 321.1319 | 624.2300 | 312.6186 | 623.2460 | 312.1266 | S | **859.5247** | 430.2660 | 842.4982 | 421.7527 | 841.5142 | 421.2607 | 8 |
| 7 | **754.3406** | 377.6740 | 737.3141 | 369.1607 | 736.3301 | 368.6687 | L | **772.4927** | 386.7500 | 755.4662 | 378.2367 | **754.4822** | 377.7447 | 7 |
| 8 | **855.3883** | 428.1978 | 838.3618 | 419.6845 | 837.3777 | 419.1925 | T | **659.4087** | 330.2080 | 642.3821 | 321.6947 | 641.3981 | 321.2027 | 6 |
| 9 | **926.4254** | 463.7163 | 909.3989 | 455.2031 | 908.4149 | 454.7111 | A | **558.3610** | 279.6841 | 541.3344 | 271.1709 | | | 5 |
| 10 | **1054.4840** | 527.7456 | 1037.4574 | 519.2324 | 1036.4734 | 518.7404 | Q | **487.3239** | 244.1656 | 470.2973 | 235.6523 | | | 4 |
| 11 | **1167.5681** | 584.2877 | 1150.5415 | 575.7744 | 1149.5575 | 575.2824 | I | **359.2653** | 180.1363 | 342.2387 | 171.6230 | | | 3 |
| 12 | **1266.6365** | 633.8219 | 1249.6099 | 625.3086 | **1248.6259** | 624.8166 | V | **246.1812** | 123.5942 | 229.1547 | 115.0810 | | | 2 |
| 13 | | | | | | | K | 147.1128 | 74.0600 | 130.0863 | 65.5468 | | | 1 |

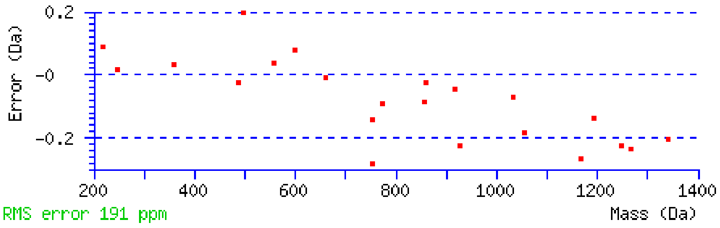


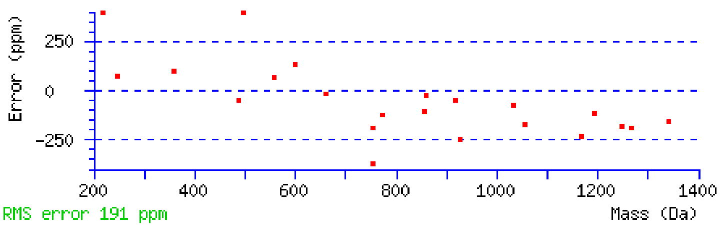


### All matches to this query

| Score | Mr(calc): | Delta | Sequence |
|---|---|---|---|
| 114.9 | 1411.7347 | 0.0064 | AFYNGSLTAQIVK |
| 45.8 | 1410.7507 | 0.9904 | AFYNGSLTAQIVK |
| 9.7 | 1411.7476 | -0.0065 | LTGQIFLGGSIVK |
| 8.9 | 1411.7320 | 0.0091 | AFSDHSSLRKHK |
| 4.8 | 1411.7364 | 0.0048 | FLISDLTLSPVK |
| 4.6 | 1411.7298 | 0.0113 | FMTDPIRILVK |
| 3.1 | 1409.7375 | 2.0037 | KDLQHLSREER |
| 2.5 | 1411.7476 | -0.0065 | LTGQIFLGGSIVK |
| 1.5 | 1411.7363 | 0.0048 | NLDLYLEILVK |
| 1.2 | 1409.7263 | 2.0149 | LHNAIEGTAQLSR |

---

Spectrum No: 27; Query: 356; Rank: 1

---

## Peptide View

MS/MS Fragmentation of **LNLSGGEVSATEK**
Found in **IPI00326462**, Tax_Id=10116 Gene_Symbol=Enpp3 Ectonucleotide pyrophosphatase/phosphodiesterase family member 3

Match to Query 356: 1304.643548 from(653.329050,2+)
Title: 091008RatKidney_NH4Format01_25.1340.1340.2.dta
Data file K:\NewmanPaper\Piliang\3SubProteomes\Piliang3SP\mgf5ppm\ERLIC_3SubProteomes5ppm.mgf

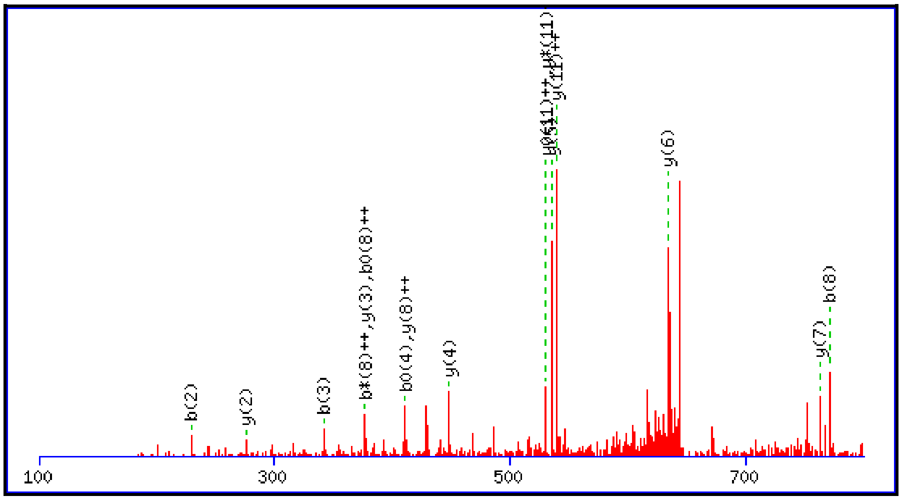

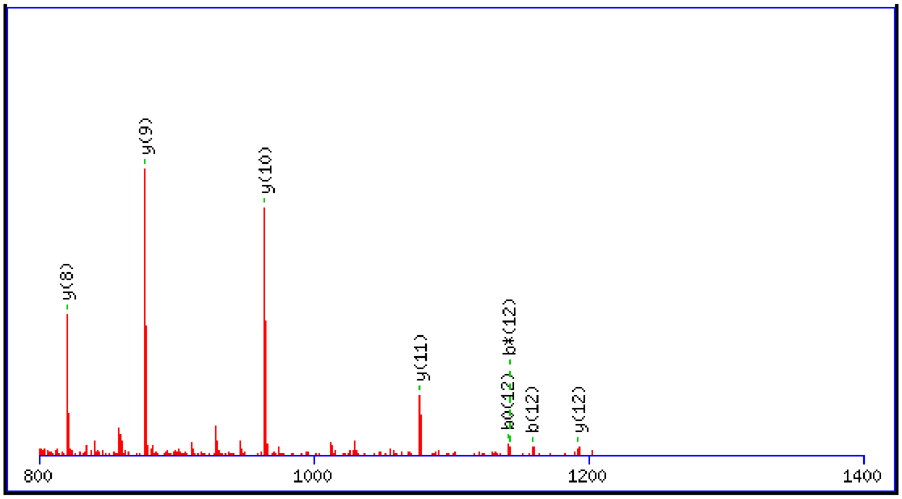


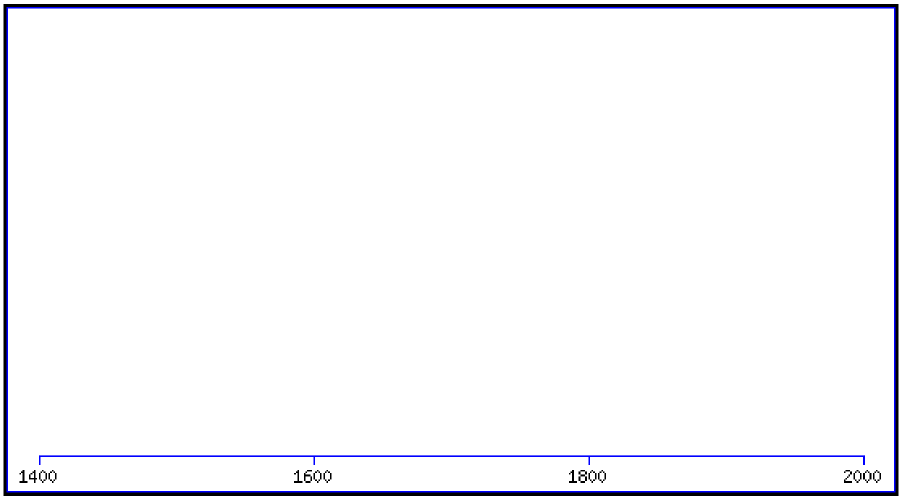


**Monoisotopic mass of neutral peptide Mr(calc):** 1304.6460
**Fixed modifications:** Carbamidomethyl (C)
**Variable modifications:**
**N2** : Deamidated_N (N)
**Ions Score:** 114 **Expect:** 4.3e-010
**Matches (Bold Red):** 24/134 fragment ions using 25 most intense peaks

| # | b | $b^{++}$ | b* | $b^{*++}$ | $b^0$ | $b^{0++}$ | Seq. | y | $y^{++}$ | y* | $y^{*++}$ | $y^0$ | $y^{0++}$ | # |
|---|---|---|---|---|---|---|---|---|---|---|---|---|---|---|
| 1 | 114.0913 | 57.5493 | | | | | L | | | | | | | 13 |
| 2 | **229.1183** | 115.0628 | 212.0917 | 106.5495 | | | N | **1192.5692** | 596.7882 | 1175.5426 | 588.2750 | 1174.5586 | 587.7829 | 12 |
| 3 | **342.2023** | 171.6048 | 325.1758 | 163.0915 | | | L | **1077.5422** | **539.2748** | 1060.5157 | **530.7615** | 1059.5317 | **530.2695** | 11 |
| 4 | 429.2344 | 215.1208 | 412.2078 | 206.6075 | **411.2238** | 206.1155 | S | **964.4582** | 482.7327 | 947.4316 | 474.2195 | 946.4476 | 473.7274 | 10 |
| 5 | 486.2558 | 243.6316 | 469.2293 | 235.1183 | 468.2453 | 234.6263 | G | **877.4262** | 439.2167 | 860.3996 | 430.7034 | 859.4156 | 430.2114 | 9 |
| 6 | 543.2773 | 272.1423 | 526.2507 | 263.6290 | 525.2667 | 263.1370 | G | **820.4047** | **410.7060** | 803.3781 | 402.1927 | 802.3941 | 401.7007 | 8 |
| 7 | 672.3199 | 336.6636 | 655.2933 | 328.1503 | 654.3093 | 327.6583 | E | **763.3832** | 382.1953 | 746.3567 | 373.6820 | 745.3727 | 373.1900 | 7 |
| 8 | **771.3883** | 386.1978 | 754.3618 | **377.6845** | 753.3777 | **377.1925** | V | **634.3406** | 317.6740 | 617.3141 | 309.1607 | 616.3301 | 308.6687 | 6 |
| 9 | 858.4203 | 429.7138 | 841.3938 | 421.2005 | 840.4098 | 420.7085 | S | **535.2722** | 268.1397 | 518.2457 | 259.6265 | 517.2617 | 259.1345 | 5 |
| 10 | 929.4574 | 465.2324 | 912.4309 | 456.7191 | 911.4469 | 456.2271 | A | **448.2402** | 224.6237 | 431.2136 | 216.1105 | 430.2296 | 215.6185 | 4 |
| 11 | 1030.5051 | 515.7562 | 1013.4786 | 507.2429 | 1012.4946 | 506.7509 | T | **377.2031** | 189.1052 | 360.1765 | 180.5919 | 359.1925 | 180.0999 | 3 |
| 12 | **1159.5477** | 580.2775 | **1142.5212** | 571.7642 | **1141.5372** | 571.2722 | E | **276.1554** | 138.5813 | 259.1288 | 130.0681 | 258.1448 | 129.5761 | 2 |
| 13 | | | | | | | K | 147.1128 | 74.0600 | 130.0863 | 65.5468 | | | 1 |

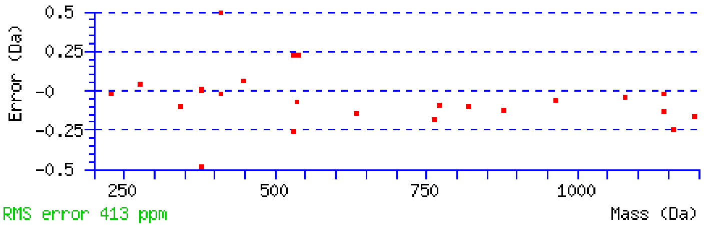


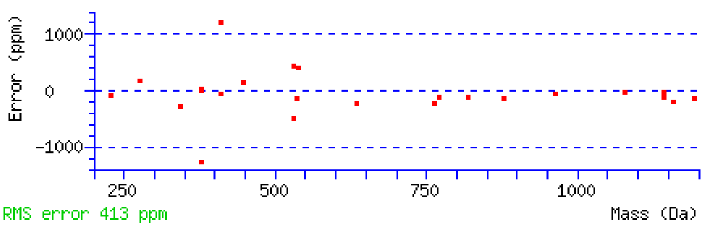


### All matches to this query

| Score | Mr(calc): | Delta | Sequence |
|---|---|---|---|
| 114.4 | 1304.6460 | -0.0024 | LNLSGGEVSATEK |
| 7.2 | 1304.6401 | 0.0034 | DVTGLPNWFEK |
| 4.9 | 1304.6394 | 0.0041 | DLEALSKQCNK |
| 4.8 | 1304.6312 | 0.0124 | ILAMIQGSHQK |
| 4.8 | 1304.6394 | 0.0041 | IVQNCGTEKEK |
| 4.6 | 1304.6312 | 0.0124 | LTHTHTIKMK |
| 4.5 | 1304.6473 | -0.0037 | NVQSLAAAHHEK |
| 3.9 | 1304.6312 | 0.0124 | LTHTHTIKMK |
| 3.9 | 1304.6489 | -0.0054 | TVINNKAHLSK |
| 3.7 | 1304.6460 | -0.0024 | LSVELEASDSQK |

---

Spectrum No: 28; Query: 813; Rank: 1

---

## Peptide View

MS/MS Fragmentation of **TNGSVDLGEEEEAAR**
Found in **IPI00464886**, Tax_Id=10116 Gene_Symbol=LOC652956 p55 protein

Match to Query 813: 1576.679608 from(789.347080,2+)
Title: 091008RatKidney_NoSalt_23.1182.1182.2.dta
Data file K:\NewmanPaper\Piliang\3SubProteomes\Piliang3SP\mgf5ppm\ERLIC_3SubProteomes5ppm.mgf

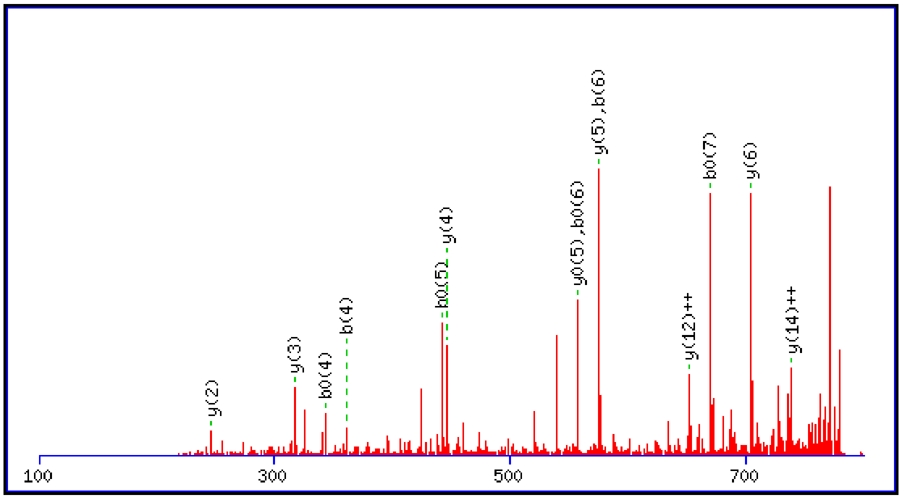

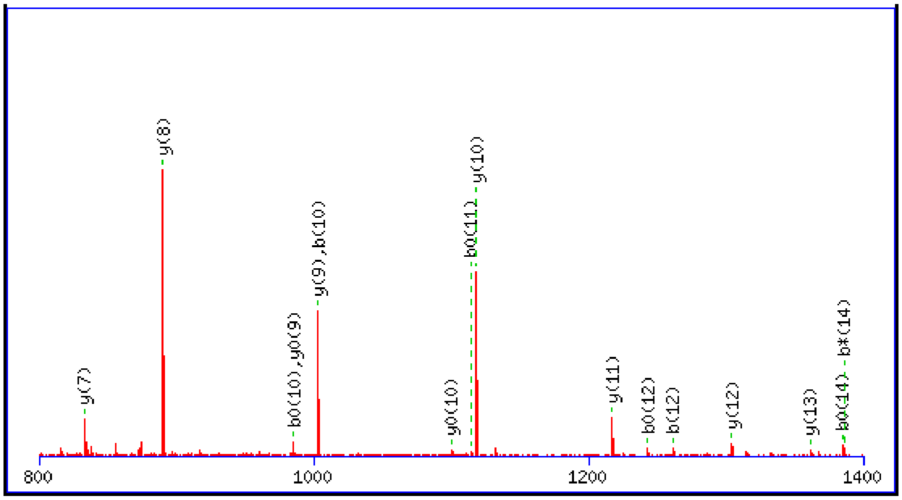


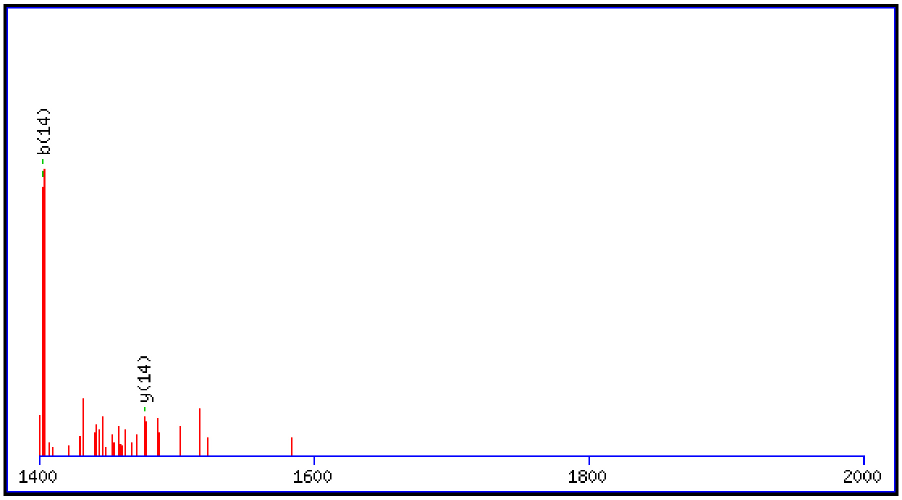


**Monoisotopic mass of neutral peptide Mr(calc):** 1576.6852
**Fixed modifications:** Carbamidomethyl (C)
**Variable modifications:**
**N2** : Deamidated_N (N)
**Ions Score:** 114 **Expect:** 6.2e-010
**Matches (Bold Red):** 32/160 fragment ions using 43 most intense peaks

| # | b | b$^{++}$ | b* | b*$^{++}$ | b$^{0}$ | b$^{0++}$ | Seq. | y | y$^{++}$ | y* | y*$^{++}$ | y$^{0}$ | y$^{0++}$ | # |
|---|---|---|---|---|---|---|---|---|---|---|---|---|---|---|
| 1 | 102.0550 | 51.5311 | | | 84.0444 | 42.5258 | T | | | | | | | 15 |
| 2 | 217.0819 | 109.0446 | 200.0553 | 100.5313 | 199.0713 | 100.0393 | N | **1476.6449** | **738.8261** | 1459.6183 | 730.3128 | 1458.6343 | 729.8208 | 14 |
| 3 | 274.1034 | 137.5553 | 257.0768 | 129.0420 | 256.0928 | 128.5500 | G | **1361.6179** | 681.3126 | 1344.5914 | 672.7993 | 1343.6074 | 672.3073 | 13 |
| 4 | **361.1354** | 181.0713 | 344.1088 | 172.5581 | **343.1248** | 172.0660 | S | **1304.5965** | **652.8019** | 1287.5699 | 644.2886 | 1286.5859 | 643.7966 | 12 |
| 5 | 460.2038 | 230.6055 | 443.1772 | 222.0923 | **442.1932** | 221.6003 | V | **1217.5644** | 609.2859 | 1200.5379 | 600.7726 | 1199.5539 | 600.2806 | 11 |
| 6 | **575.2307** | 288.1190 | 558.2042 | 279.6057 | **557.2202** | 279.1137 | D | **1118.4960** | 559.7516 | 1101.4695 | 551.2384 | **1100.4855** | 550.7464 | 10 |
| 7 | 688.3148 | 344.6610 | 671.2883 | 336.1478 | **670.3042** | 335.6558 | L | **1003.4691** | 502.2382 | 986.4425 | 493.7249 | **985.4585** | 493.2329 | 9 |
| 8 | 745.3363 | 373.1718 | 728.3097 | 364.6585 | 727.3257 | 364.1665 | G | **890.3850** | 445.6961 | 873.3585 | 437.1829 | 872.3745 | 436.6909 | 8 |
| 9 | 874.3789 | 437.6931 | 857.3523 | 429.1798 | 856.3683 | 428.6878 | E | **833.3636** | 417.1854 | 816.3370 | 408.6721 | 815.3530 | 408.1801 | 7 |
| 10 | **1003.4215** | 502.2144 | 986.3949 | 493.7011 | **985.4109** | 493.2091 | E | **704.3210** | 352.6641 | 687.2944 | 344.1508 | 686.3104 | 343.6588 | 6 |
| 11 | 1132.4640 | 566.7357 | 1115.4375 | 558.2224 | **1114.4535** | 557.7304 | E | **575.2784** | 288.1428 | 558.2518 | 279.6295 | **557.2678** | 279.1375 | 5 |
| 12 | **1261.5066** | 631.2570 | 1244.4801 | 622.7437 | **1243.4961** | 622.2517 | E | **446.2358** | 223.6215 | 429.2092 | 215.1083 | 428.2252 | 214.6162 | 4 |
| 13 | 1332.5438 | 666.7755 | 1315.5172 | 658.2622 | 1314.5332 | 657.7702 | A | **317.1932** | 159.1002 | 300.1666 | 150.5870 | | | 3 |
| 14 | **1403.5809** | 702.2941 | **1386.5543** | 693.7808 | **1385.5703** | 693.2888 | A | **246.1561** | 123.5817 | 229.1295 | 115.0684 | | | 2 |
| 15 | | | | | | | R | 175.1190 | 88.0631 | 158.0924 | 79.5498 | | | 1 |

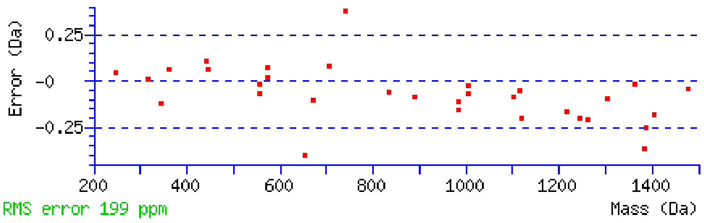


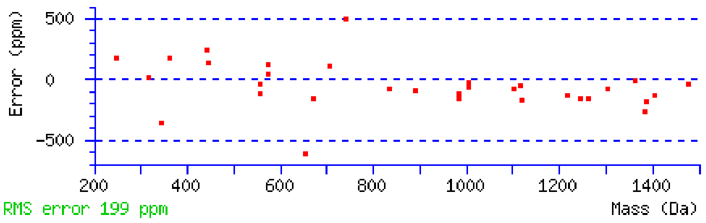


### All matches to this query

| Score | Mr(calc): | Delta | Sequence |
|---|---|---|---|
| 114.0 | 1576.6852 | -0.0056 | TNGSVDLGEEEEAAR |
| 9.6 | 1576.6658 | 0.0139 | STQSVYQENLAEK |
| 9.6 | 1576.6658 | 0.0139 | STQSVYQENLAEK |
| 4.7 | 1576.6844 | -0.0047 | MKAEDGENYAIKK |
| 4.5 | 1576.6658 | 0.0139 | STQSVYQENLAEK |
| 4.5 | 1576.6658 | 0.0139 | STQSVYQENLAEK |
| 2.4 | 1576.6891 | -0.0095 | CSFIVRDCVSVR |
| 2.0 | 1576.6770 | 0.0026 | SPEQIESHEVTNK |
| 1.3 | 1576.6779 | 0.0017 | NTPFMGIMDKTAR |
| 1.3 | 1576.6779 | 0.0017 | NTPFMGIMDKTAR |

---

Spectrum No: 29; Query: 1377; Rank: 1

---

## Peptide View

MS/MS Fragmentation of **FTSDTSVAGTGFNASFHK**
Found in **IPI00196620**, Tax_Id=10116 Gene_Symbol=Cubn Cubilin precursor

Match to Query 1377: 1873.852428 from(937.933490,2+)
Title: 100101RatKid_NS_deglyco_13.2504.2504.2.dta
Data file K:\NewmanPaper\Piliang\3SubProteomes\Piliang3SP\mgf5ppm\ERLIC_3SubProteomes5ppm.mgf

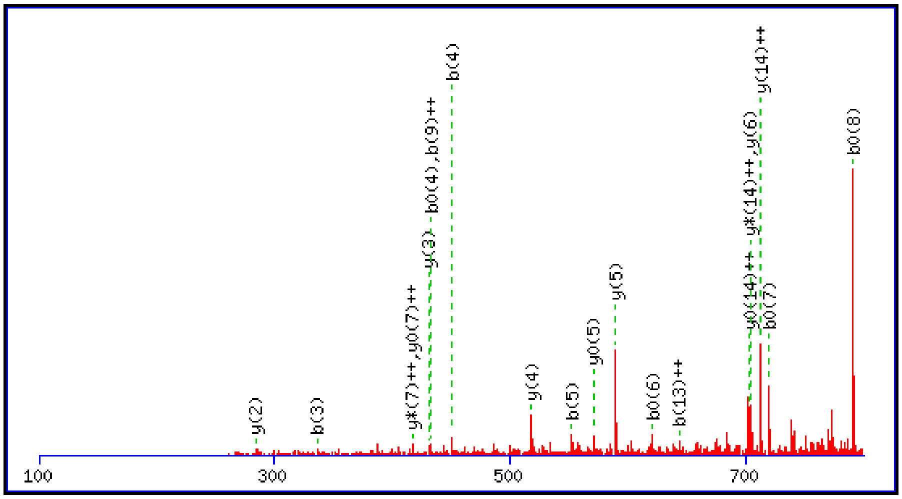

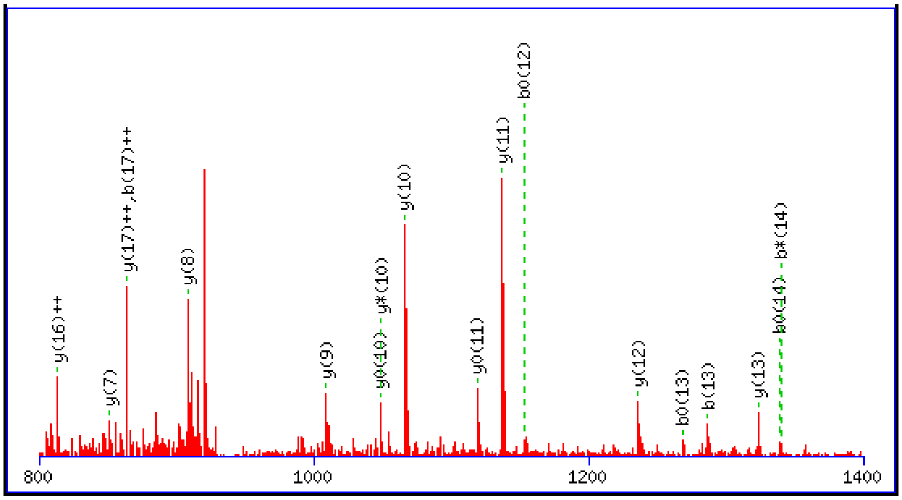


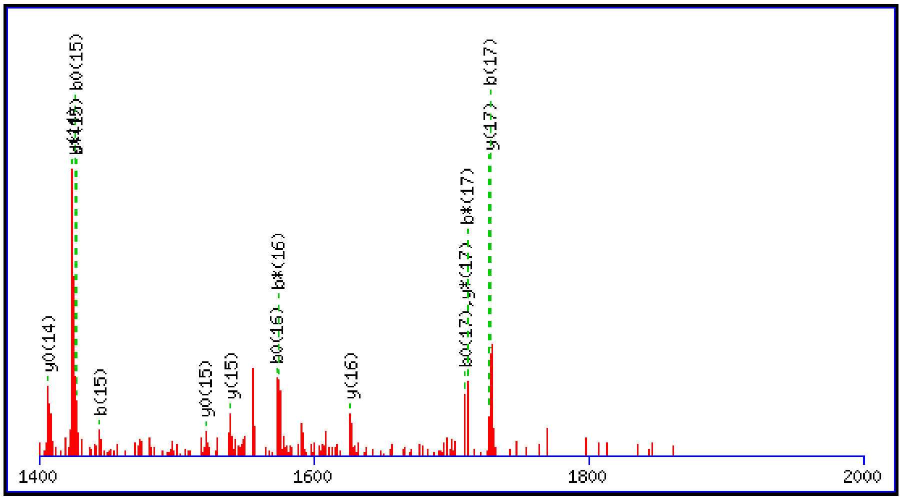


**Monoisotopic mass of neutral peptide Mr(calc):** 1873.8483
**Fixed modifications:** Carbamidomethyl (C)
**Variable modifications:**
**N13** : Deamidated_N (N)
**Ions Score:** 114 **Expect:** 8.9e-010
**Matches (Bold Red):** 53/172 fragment ions using 95 most intense peaks

| # | b | $b^{++}$ | b* | $b^{*++}$ | $b^{0}$ | $b^{0++}$ | Seq. | y | $y^{++}$ | y* | $y^{*++}$ | $y^{0}$ | $y^{0++}$ | # |
|---|---|---|---|---|---|---|---|---|---|---|---|---|---|---|
| 1 | 148.0757 | 74.5415 | | | | | F | | | | | | | 18 |
| 2 | 249.1234 | 125.0653 | | | 231.1128 | 116.0600 | T | **1727.7871** | **864.3972** | **1710.7606** | 855.8839 | 1709.7765 | 855.3919 | 17 |
| 3 | **336.1554** | 168.5813 | | | 318.1448 | 159.5761 | S | **1626.7394** | **813.8734** | 1609.7129 | 805.3601 | 1608.7289 | 804.8681 | 16 |
| 4 | **451.1823** | 226.0948 | | | **433.1718** | 217.0895 | D | **1539.7074** | 770.3573 | 1522.6809 | 761.8441 | **1521.6968** | 761.3521 | 15 |
| 5 | **552.2300** | 276.6186 | | | 534.2195 | 267.6134 | T | **1424.6805** | **712.8439** | 1407.6539 | **704.3306** | **1406.6699** | **703.8386** | 14 |
| 6 | 639.2620 | 320.1347 | | | **621.2515** | 311.1294 | S | **1323.6328** | 662.3200 | 1306.6062 | 653.8068 | 1305.6222 | 653.3147 | 13 |
| 7 | 738.3305 | 369.6689 | | | **720.3199** | 360.6636 | V | **1236.6008** | 618.8040 | 1219.5742 | 610.2907 | 1218.5902 | 609.7987 | 12 |
| 8 | 809.3676 | 405.1874 | | | **791.3570** | 396.1821 | A | **1137.5323** | 569.2698 | 1120.5058 | 560.7565 | **1119.5218** | 560.2645 | 11 |
| 9 | 866.3890 | **433.6982** | | | 848.3785 | 424.6929 | G | **1066.4952** | 533.7513 | **1049.4687** | 525.2380 | **1048.4847** | 524.7460 | 10 |
| 10 | 967.4367 | 484.2220 | | | 949.4262 | 475.2167 | T | **1009.4738** | 505.2405 | 992.4472 | 496.7272 | 991.4632 | 496.2352 | 9 |
| 11 | 1024.4582 | 512.7327 | | | 1006.4476 | 503.7274 | G | **908.4261** | 454.7167 | 891.3995 | 446.2034 | 890.4155 | 445.7114 | 8 |
| 12 | 1171.5266 | 586.2669 | | | **1153.5160** | 577.2617 | F | **851.4046** | 426.2059 | 834.3781 | **417.6927** | 833.3941 | **417.2007** | 7 |
| 13 | **1286.5535** | **643.7804** | 1269.5270 | 635.2671 | **1268.5430** | 634.7751 | N | **704.3362** | 352.6717 | 687.3097 | 344.1585 | 686.3256 | 343.6665 | 6 |
| 14 | 1357.5906 | 679.2990 | **1340.5641** | 670.7857 | **1339.5801** | 670.2937 | A | **589.3093** | 295.1583 | 572.2827 | 286.6450 | **571.2987** | 286.1530 | 5 |
| 15 | **1444.6227** | 722.8150 | **1427.5961** | 714.3017 | **1426.6121** | 713.8097 | S | **518.2722** | 259.6397 | 501.2456 | 251.1264 | 500.2616 | 250.6344 | 4 |
| 16 | 1591.6911 | 796.3492 | **1574.6645** | 787.8359 | **1573.6805** | 787.3439 | F | **431.2401** | 216.1237 | 414.2136 | 207.6104 | | | 3 |
| 17 | **1728.7500** | **864.8786** | **1711.7235** | 856.3654 | **1710.7394** | 855.8734 | H | **284.1717** | 142.5895 | 267.1452 | 134.0762 | | | 2 |
| 18 | | | | | | | K | 147.1128 | 74.0600 | 130.0863 | 65.5468 | | | 1 |

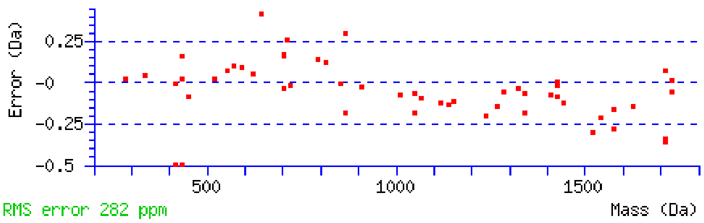


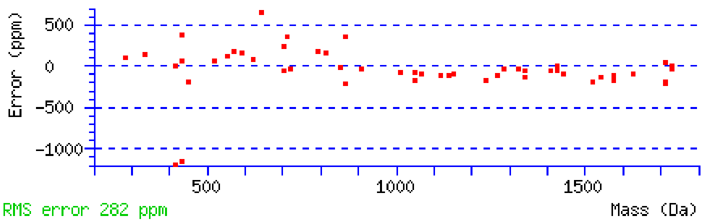


### All matches to this query

| Score | Mr(calc): | Delta | Sequence |
|---|---|---|---|
| 114.0 | 1873.8483 | 0.0041 | FTSDTSVAGTGFNASFHK |
| 14.3 | 1872.8643 | 0.9882 | FTSDTSVAGTGFNASFHK |
| 7.3 | 1872.8492 | 1.0032 | MERTSVMERTSVMER |
| 4.3 | 1873.8628 | -0.0104 | EFYRGRCVLNEDSTK |
| 2.8 | 1873.8628 | -0.0104 | APSTPVPPSPAPTPGLTK |
| 2.1 | 1871.8349 | 2.0175 | MSHPSPAGKPSNSKNPR |
| 1.2 | 1872.8492 | 1.0032 | MERTSVMERTSVMER |

---

Spectrum No: 30; Query: 2355; Rank: 1

---

## Peptide View

MS/MS Fragmentation of **YEVDWNQSTLDCVDPLSSLAADR**
Found in **IPI00188881**, Tax_Id=10116 Gene_Symbol=Slc22a2 Organic cation transporter OCT2

Match to Query 2355: 2654.186168 from(1328.100360,2+)
Title: 100101RatKid_NS_deglyco_25.4833.4833.2.dta
Data file K:\NewmanPaper\Piliang\3SubProteomes\Piliang3SP\mgf5ppm\ERLIC_3SubProteomes5ppm.mgf

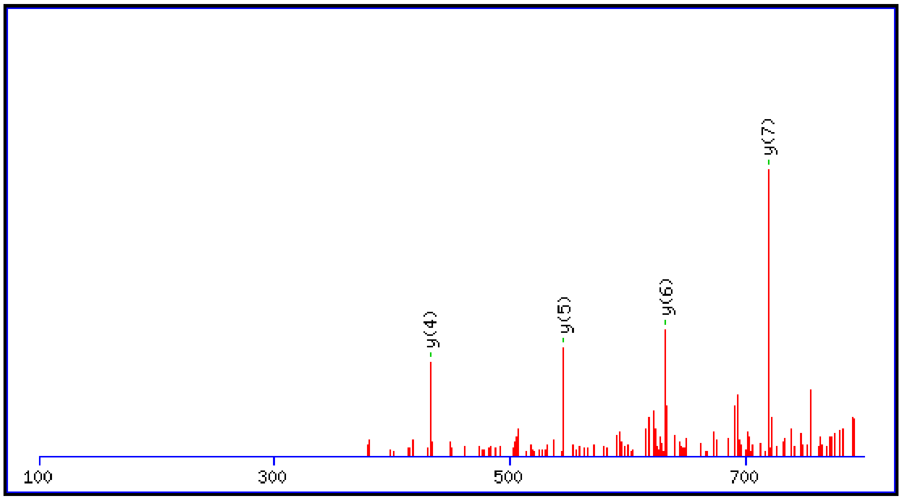

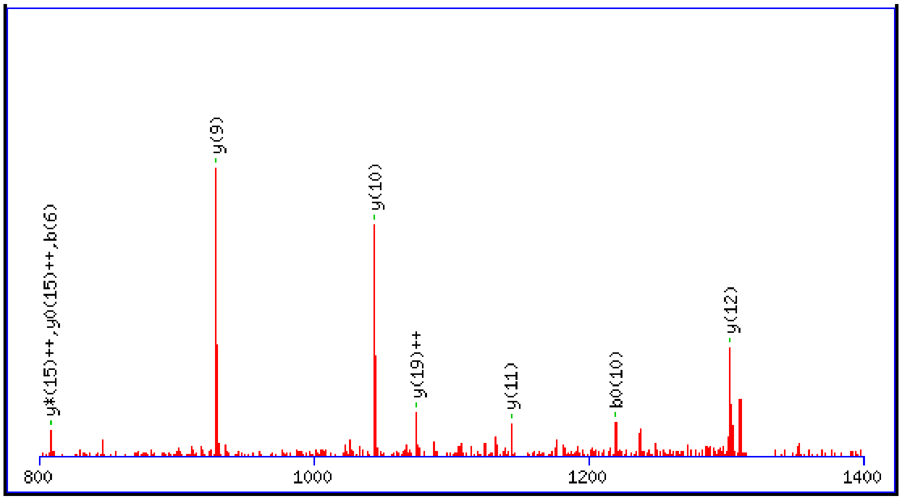


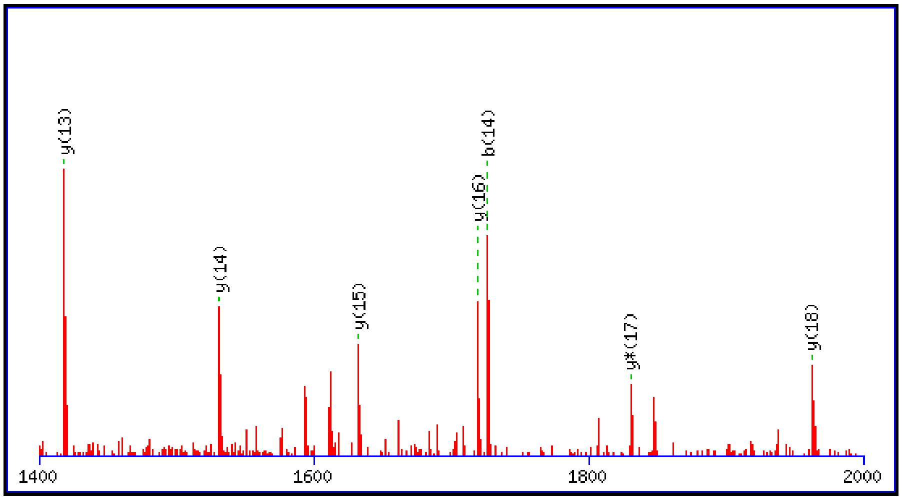


**Monoisotopic mass of neutral peptide Mr(calc):** 2654.1806
**Fixed modifications:** Carbamidomethyl (C)
**Variable modifications:**
**N6** : Deamidated_N (N)
**Ions Score:** 113 **Expect:** 1.6e-009
**Matches (Bold Red):** 20/250 fragment ions using 24 most intense peaks

| # | b | $b^{++}$ | b* | $b^{*++}$ | $b^0$ | $b^{0++}$ | Seq. | y | $y^{++}$ | y* | $y^{*++}$ | $y^0$ | $y^{0++}$ | # |
|---|---|---|---|---|---|---|---|---|---|---|---|---|---|---|
| 1 | 164.0706 | 82.5389 | | | | | Y | | | | | | | 23 |
| 2 | 293.1132 | 147.0602 | | | 275.1026 | 138.0550 | E | 2492.1246 | 1246.5659 | 2475.0980 | 1238.0526 | 2474.1140 | 1237.5606 | 22 |
| 3 | 392.1816 | 196.5944 | | | 374.1710 | 187.5892 | V | 2363.0820 | 1182.0446 | 2346.0554 | 1173.5314 | 2345.0714 | 1173.0393 | 21 |
| 4 | 507.2086 | 254.1079 | | | 489.1980 | 245.1026 | D | 2264.0136 | 1132.5104 | 2246.9870 | 1123.9971 | 2246.0030 | 1123.5051 | 20 |
| 5 | 693.2879 | 347.1476 | | | 675.2773 | 338.1423 | W | 2148.9866 | **1074.9969** | 2131.9601 | 1066.4837 | 2130.9761 | 1065.9917 | 19 |
| 6 | **808.3148** | 404.6610 | 791.2883 | 396.1478 | 790.3042 | 395.6558 | N | **1962.9073** | 981.9573 | 1945.8808 | 973.4440 | 1944.8967 | 972.9520 | 18 |
| 7 | 936.3734 | 468.6903 | 919.3468 | 460.1771 | 918.3628 | 459.6850 | Q | 1847.8804 | 924.4438 | **1830.8538** | 915.9305 | 1829.8698 | 915.4385 | 17 |
| 8 | 1023.4054 | 512.2063 | 1006.3789 | 503.6931 | 1005.3948 | 503.2011 | S | **1719.8218** | 860.4145 | 1702.7952 | 851.9013 | 1701.8112 | 851.4093 | 16 |
| 9 | 1124.4531 | 562.7302 | 1107.4265 | 554.2169 | 1106.4425 | 553.7249 | T | **1632.7898** | 816.8985 | 1615.7632 | **808.3852** | 1614.7792 | **807.8932** | 15 |
| 10 | 1237.5372 | 619.2722 | 1220.5106 | 610.7589 | **1219.5266** | 610.2669 | L | **1531.7421** | 766.3747 | 1514.7155 | 757.8614 | 1513.7315 | 757.3694 | 14 |
| 11 | 1352.5641 | 676.7857 | 1335.5375 | 668.2724 | 1334.5535 | 667.7804 | D | **1418.6580** | 709.8326 | 1401.6315 | 701.3194 | 1400.6475 | 700.8274 | 13 |
| 12 | 1512.5947 | 756.8010 | 1495.5682 | 748.2877 | 1494.5842 | 747.7957 | C | **1303.6311** | 652.3192 | 1286.6045 | 643.8059 | 1285.6205 | 643.3139 | 12 |
| 13 | 1611.6632 | 806.3352 | 1594.6366 | 797.8219 | 1593.6526 | 797.3299 | V | **1143.6004** | 572.3039 | 1126.5739 | 563.7906 | 1125.5899 | 563.2986 | 11 |
| 14 | **1726.6901** | 863.8487 | 1709.6636 | 855.3354 | 1708.6795 | 854.8434 | D | **1044.5320** | 522.7696 | 1027.5055 | 514.2564 | 1026.5214 | 513.7644 | 10 |
| 15 | 1823.7429 | 912.3751 | 1806.7163 | 903.8618 | 1805.7323 | 903.3698 | P | **929.5051** | 465.2562 | 912.4785 | 456.7429 | 911.4945 | 456.2509 | 9 |
| 16 | 1936.8269 | 968.9171 | 1919.8004 | 960.4038 | 1918.8164 | 959.9118 | L | 832.4523 | 416.7298 | 815.4258 | 408.2165 | 814.4417 | 407.7245 | 8 |
| 17 | 2023.8590 | 1012.4331 | 2006.8324 | 1003.9198 | 2005.8484 | 1003.4278 | S | **719.3682** | 360.1878 | 702.3417 | 351.6745 | 701.3577 | 351.1825 | 7 |
| 18 | 2110.8910 | 1055.9491 | 2093.8644 | 1047.4359 | 2092.8804 | 1046.9438 | S | **632.3362** | 316.6717 | 615.3097 | 308.1585 | 614.3257 | 307.6665 | 6 |

| | | | | | | | | | | | | | | |
|---|---|---|---|---|---|---|---|---|---|---|---|---|---|---|
| **19** | 2223.9751 | 1112.4912 | 2206.9485 | 1103.9779 | 2205.9645 | 1103.4859 | **L** | **545.3042** | 273.1557 | 528.2776 | 264.6425 | 527.2936 | 264.1504 | **5** |
| **20** | 2295.0122 | 1148.0097 | 2277.9856 | 1139.4964 | 2277.0016 | 1139.0044 | **A** | **432.2201** | 216.6137 | 415.1936 | 208.1004 | 414.2096 | 207.6084 | **4** |
| **21** | 2366.0493 | 1183.5283 | 2349.0227 | 1175.0150 | 2348.0387 | 1174.5230 | **A** | 361.1830 | 181.0951 | 344.1565 | 172.5819 | 343.1724 | 172.0899 | **3** |
| **22** | 2481.0762 | 1241.0417 | 2464.0497 | 1232.5285 | 2463.0657 | 1232.0365 | **D** | 290.1459 | 145.5766 | 273.1193 | 137.0633 | 272.1353 | 136.5713 | **2** |
| **23** | | | | | | | **R** | 175.1190 | 88.0631 | 158.0924 | 79.5498 | | | **1** |

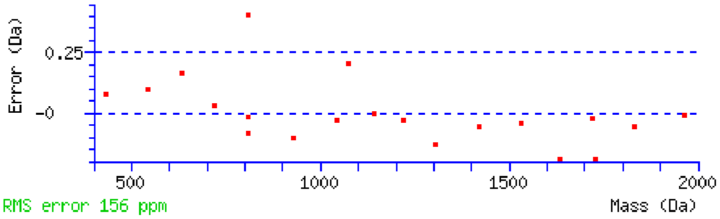


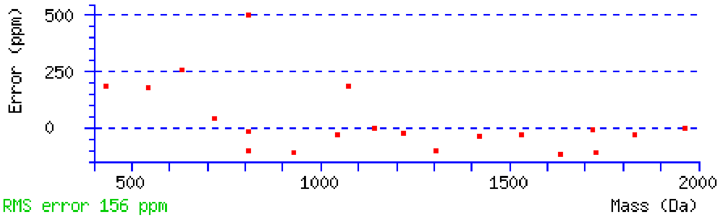


### All matches to this query

| Score | Mr(calc): | Delta | Sequence |
|---|---|---|---|
| 113.4 | 2654.1806 | 0.0056 | YEVDWNQSTLDCVDPLSSLAADR |
| 85.8 | 2653.1966 | 0.9896 | YEVDWNQSTLDCVDPLSSLAADR |
| 3.4 | 2653.2014 | 0.9847 | VISLFYTVVTPMFNPMVYSLR |
| 2.8 | 2652.1737 | 2.0125 | SISIAALLDVNKEEPNKELNSK |
| 2.8 | 2652.1737 | 2.0125 | SISIAALLDVNKEEPNKELNSK |
| 2.8 | 2653.1577 | 1.0285 | SISIAALLDVNKEEPNKELNSK |
| 2.8 | 2652.1737 | 2.0125 | SISIAALLDVNKEEPNKELNSK |
| 2.8 | 2653.1577 | 1.0285 | SISIAALLDVNKEEPNKELNSK |
| 2.8 | 2653.1577 | 1.0285 | SISIAALLDVNKEEPNKELNSK |
| 0.8 | 2653.2056 | 0.9806 | TMSKSDMLNALEEVINNPFYKK |

---

Spectrum No: 31; Query: 1004; Rank: 1

---

## Peptide View

MS/MS Fragmentation of **TANETSAEAYNLLLR**
Found in **IPI00363849**, Tax_Id=10116 Gene_Symbol=Lamc1 similar to Laminin gamma-1 chain precursor

Match to Query 1004: 1665.820168 from(833.917360,2+)
Title: 091008RatKidney_NH4Format01_23.2929.2929.2.dta
Data file K:\NewmanPaper\Piliang\3SubProteomes\Piliang3SP\mgf5ppm\ERLIC_3SubProteomes5ppm.mgf

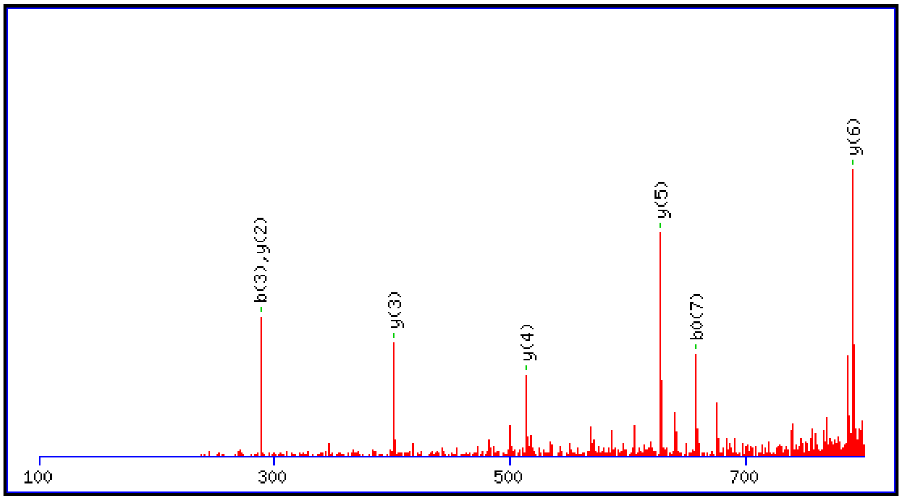

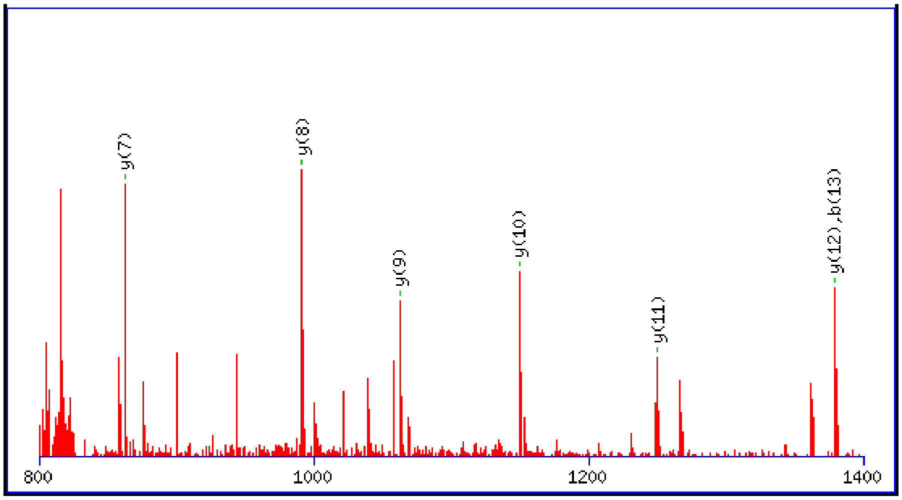


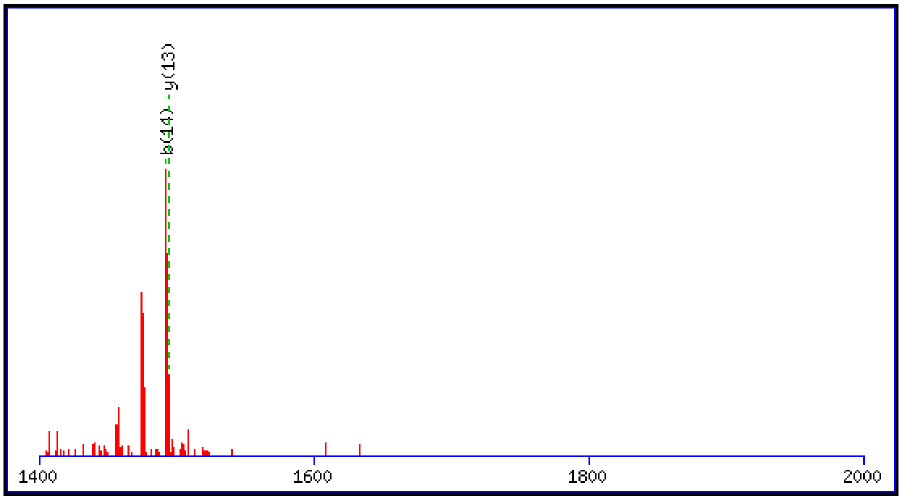


**Monoisotopic mass of neutral peptide Mr(calc):** 1665.8209
**Fixed modifications:** Carbamidomethyl (C)
**Variable modifications:**
**N3** : Deamidated_N (N)
**Ions Score:** 113 **Expect:** 8.3e-010
**Matches (Bold Red):** 16/150 fragment ions using 19 most intense peaks

| # | b | $b^{++}$ | b* | $b^{*++}$ | $b^0$ | $b^{0++}$ | Seq. | y | $y^{++}$ | y* | $y^{*++}$ | $y^0$ | $y^{0++}$ | # |
|---|---|---|---|---|---|---|---|---|---|---|---|---|---|---|
| 1 | 102.0550 | 51.5311 | | | 84.0444 | 42.5258 | T | | | | | | | 15 |
| 2 | 173.0921 | 87.0497 | | | 155.0815 | 78.0444 | A | 1565.7806 | 783.3939 | 1548.7540 | 774.8806 | 1547.7700 | 774.3886 | 14 |
| 3 | **288.1190** | 144.5631 | 271.0925 | 136.0499 | 270.1084 | 135.5579 | N | **1494.7435** | 747.8754 | 1477.7169 | 739.3621 | 1476.7329 | 738.8701 | 13 |
| 4 | 417.1616 | 209.0844 | 400.1350 | 200.5712 | 399.1510 | 200.0792 | E | **1379.7165** | 690.3619 | 1362.6900 | 681.8486 | 1361.7060 | 681.3566 | 12 |
| 5 | 518.2093 | 259.6083 | 501.1827 | 251.0950 | 500.1987 | 250.6030 | T | **1250.6739** | 625.8406 | 1233.6474 | 617.3273 | 1232.6634 | 616.8353 | 11 |
| 6 | 605.2413 | 303.1243 | 588.2148 | 294.6110 | 587.2307 | 294.1190 | S | **1149.6262** | 575.3168 | 1132.5997 | 566.8035 | 1131.6157 | 566.3115 | 10 |
| 7 | 676.2784 | 338.6428 | 659.2519 | 330.1296 | **658.2679** | 329.6376 | A | **1062.5942** | 531.8007 | 1045.5677 | 523.2875 | 1044.5837 | 522.7955 | 9 |
| 8 | 805.3210 | 403.1641 | 788.2945 | 394.6509 | 787.3104 | 394.1589 | E | **991.5571** | 496.2822 | 974.5306 | 487.7689 | 973.5465 | 487.2769 | 8 |
| 9 | 876.3581 | 438.6827 | 859.3316 | 430.1694 | 858.3476 | 429.6774 | A | **862.5145** | 431.7609 | 845.4880 | 423.2476 | | | 7 |
| 10 | 1039.4215 | 520.2144 | 1022.3949 | 511.7011 | 1021.4109 | 511.2091 | Y | **791.4774** | 396.2423 | 774.4509 | 387.7291 | | | 6 |
| 11 | 1153.4644 | 577.2358 | 1136.4378 | 568.7226 | 1135.4538 | 568.2305 | N | **628.4141** | 314.7107 | 611.3875 | 306.1974 | | | 5 |
| 12 | 1266.5484 | 633.7779 | 1249.5219 | 625.2646 | 1248.5379 | 624.7726 | L | **514.3711** | 257.6892 | 497.3446 | 249.1759 | | | 4 |
| 13 | **1379.6325** | 690.3199 | 1362.6060 | 681.8066 | 1361.6219 | 681.3146 | L | **401.2871** | 201.1472 | 384.2605 | 192.6339 | | | 3 |
| 14 | **1492.7166** | 746.8619 | 1475.6900 | 738.3487 | 1474.7060 | 737.8566 | L | **288.2030** | 144.6051 | 271.1765 | 136.0919 | | | 2 |
| 15 | | | | | | | R | 175.1190 | 88.0631 | 158.0924 | 79.5498 | | | 1 |

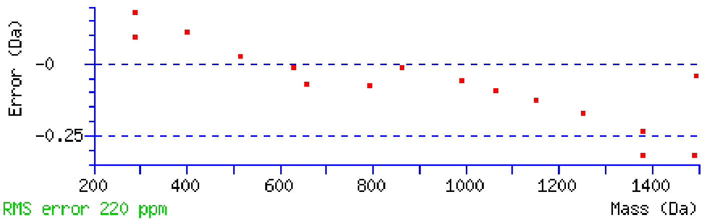


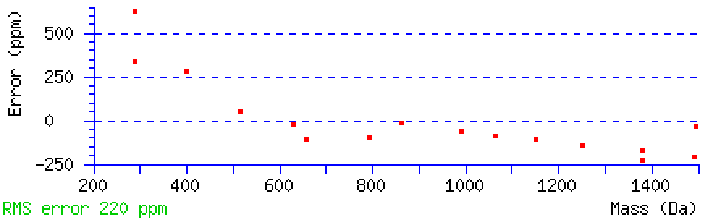


### All matches to this query

| Score | Mr(calc): | Delta | Sequence |
|---|---|---|---|
| 113.2 | 1665.8209 | -0.0008 | TANETSAEAYNLLLR |
| 66.1 | 1665.8209 | -0.0008 | TANETSAEAYNLLLR |
| 19.9 | 1665.8087 | 0.0115 | NNSTPLNISETKLR |
| 12.0 | 1665.8199 | 0.0002 | DGKQQTNQTKGLLR |
| 8.8 | 1664.8022 | 1.0180 | GDVENTILDILGGLR |
| 7.7 | 1665.8087 | 0.0115 | EIEQKDSEIARLR |
| 7.5 | 1665.8352 | -0.0150 | SVSRSLWRTPELR |
| 7.1 | 1663.8182 | 2.0020 | GDVENTILDILGGLR |
| 5.9 | 1665.8239 | -0.0037 | NPDYRSNKPIILR |
| 5.3 | 1665.8239 | -0.0037 | NPDYRSNKPIILR |

Spectrum No: 32; Query: 1653; Rank: 1

## Peptide View

MS/MS Fragmentation of **ADNLYSQVVGLSAAQANLSK**
Found in **IPI00204504**, Tax_Id=10116 Gene_Symbol=Plvap Plasmalemma vesicle-associated protein

Match to Query 1653: 2049.044068 from(1025.529310,2+)
Title: 100101RatKid_NS_deglyco_08.4414.4414.2.dta
Data file K:\NewmanPaper\Piliang\3SubProteomes\Piliang3SP\mgf5ppm\ERLIC_3SubProteomes5ppm.mgf

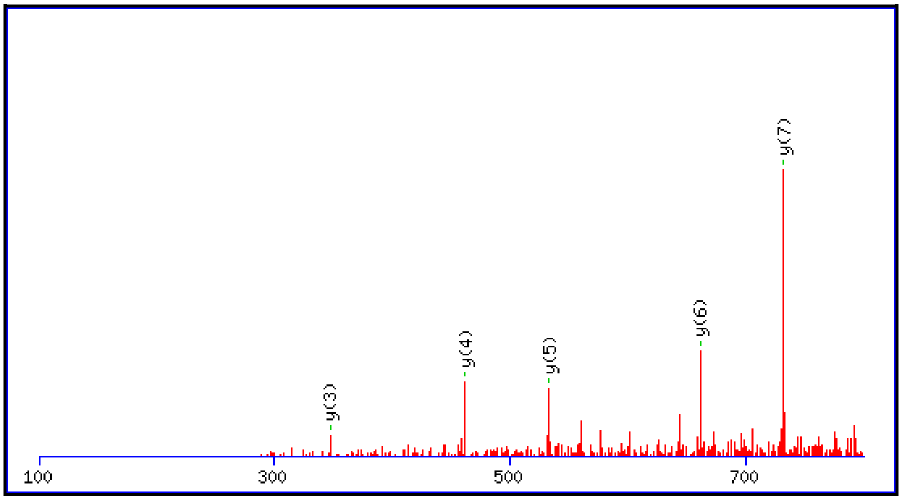

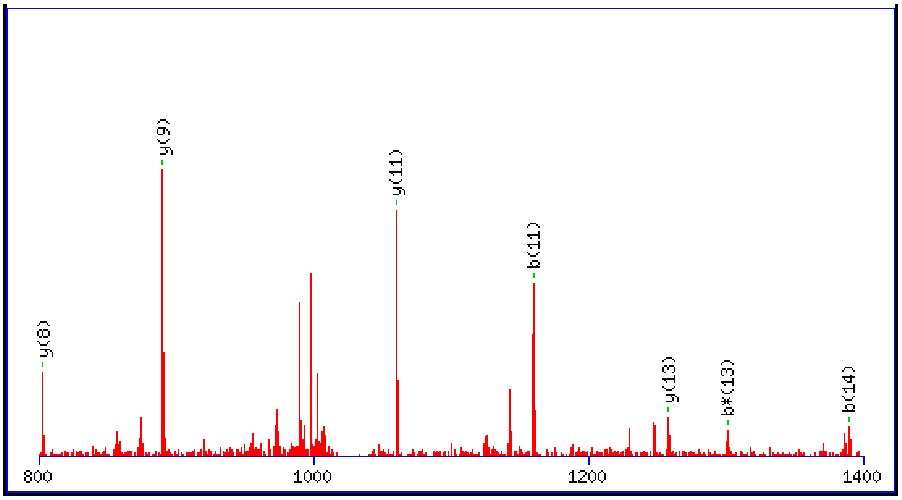


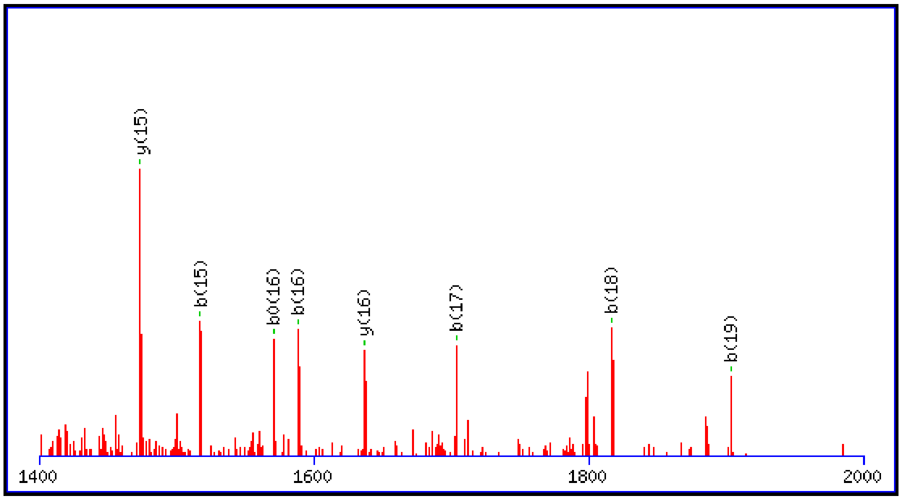


**Monoisotopic mass of neutral peptide Mr(calc):** 2049.0378
**Fixed modifications:** Carbamidomethyl (C)
**Variable modifications:**
**N17** : Deamidated_N (N)
**Ions Score:** 113 **Expect:** 9.2e-010
**Matches (Bold Red):** 20/220 fragment ions using 23 most intense peaks

| # | b | $b^{++}$ | b* | $b^{*++}$ | $b^0$ | $b^{0++}$ | Seq. | y | $y^{++}$ | y* | $y^{*++}$ | $y^0$ | $y^{0++}$ | # |
|---|---|---|---|---|---|---|---|---|---|---|---|---|---|---|
| 1 | 72.0444 | 36.5258 | | | | | A | | | | | | | 20 |
| 2 | 187.0713 | 94.0393 | | | 169.0608 | 85.0340 | D | 1979.0080 | 990.0076 | 1961.9815 | 981.4944 | 1960.9974 | 981.0024 | 19 |
| 3 | 301.1143 | 151.0608 | 284.0877 | 142.5475 | 283.1037 | 142.0555 | N | 1863.9811 | 932.4942 | 1846.9545 | 923.9809 | 1845.9705 | 923.4889 | 18 |
| 4 | 414.1983 | 207.6028 | 397.1718 | 199.0895 | 396.1878 | 198.5975 | L | 1749.9381 | 875.4727 | 1732.9116 | 866.9594 | 1731.9276 | 866.4674 | 17 |
| 5 | 577.2617 | 289.1345 | 560.2351 | 280.6212 | 559.2511 | 280.1292 | Y | **1636.8541** | 818.9307 | 1619.8275 | 810.4174 | 1618.8435 | 809.9254 | 16 |
| 6 | 664.2937 | 332.6505 | 647.2671 | 324.1372 | 646.2831 | 323.6452 | S | **1473.7907** | 737.3990 | 1456.7642 | 728.8857 | 1455.7802 | 728.3937 | 15 |
| 7 | 792.3523 | 396.6798 | 775.3257 | 388.1665 | 774.3417 | 387.6745 | Q | 1386.7587 | 693.8830 | 1369.7322 | 685.3697 | 1368.7481 | 684.8777 | 14 |
| 8 | 891.4207 | 446.2140 | 874.3941 | 437.7007 | 873.4101 | 437.2087 | V | **1258.7001** | 629.8537 | 1241.6736 | 621.3404 | 1240.6896 | 620.8484 | 13 |
| 9 | 990.4891 | 495.7482 | 973.4625 | 487.2349 | 972.4785 | 486.7429 | V | 1159.6317 | 580.3195 | 1142.6052 | 571.8062 | 1141.6212 | 571.3142 | 12 |
| 10 | 1047.5106 | 524.2589 | 1030.4840 | 515.7456 | 1029.5000 | 515.2536 | G | **1060.5633** | 530.7853 | 1043.5368 | 522.2720 | 1042.5527 | 521.7800 | 11 |
| 11 | **1160.5946** | 580.8009 | 1143.5681 | 572.2877 | 1142.5840 | 571.7957 | L | 1003.5418 | 502.2746 | 986.5153 | 493.7613 | 985.5313 | 493.2693 | 10 |
| 12 | 1247.6266 | 624.3170 | 1230.6001 | 615.8037 | 1229.6161 | 615.3117 | S | **890.4578** | 445.7325 | 873.4312 | 437.2193 | 872.4472 | 436.7272 | 9 |
| 13 | 1318.6638 | 659.8355 | **1301.6372** | 651.3222 | 1300.6532 | 650.8302 | A | **803.4258** | 402.2165 | 786.3992 | 393.7032 | 785.4152 | 393.2112 | 8 |
| 14 | **1389.7009** | 695.3541 | 1372.6743 | 686.8408 | 1371.6903 | 686.3488 | A | **732.3886** | 366.6980 | 715.3621 | 358.1847 | 714.3781 | 357.6927 | 7 |
| 15 | **1517.7594** | 759.3834 | 1500.7329 | 750.8701 | 1499.7489 | 750.3781 | Q | **661.3515** | 331.1794 | 644.3250 | 322.6661 | 643.3410 | 322.1741 | 6 |
| 16 | **1588.7966** | 794.9019 | 1571.7700 | 786.3886 | **1570.7860** | 785.8966 | A | **533.2929** | 267.1501 | 516.2664 | 258.6368 | 515.2824 | 258.1448 | 5 |
| 17 | **1703.8235** | 852.4154 | 1686.7969 | 843.9021 | 1685.8129 | 843.4101 | N | **462.2558** | 231.6316 | 445.2293 | 223.1183 | 444.2453 | 222.6263 | 4 |
| 18 | **1816.9076** | 908.9574 | 1799.8810 | 900.4441 | 1798.8970 | 899.9521 | L | **347.2289** | 174.1181 | 330.2023 | 165.6048 | 329.2183 | 165.1128 | 3 |

| | | | | | | | | | | | | | | |
|---|---|---|---|---|---|---|---|---|---|---|---|---|---|---|
| **19** | **1903.9396** | 952.4734 | 1886.9130 | 943.9602 | 1885.9290 | 943.4682 | **S** | 234.1448 | 117.5761 | 217.1183 | 109.0628 | 216.1343 | 108.5708 | **2** |
| **20** | | | | | | | **K** | 147.1128 | 74.0600 | 130.0863 | 65.5468 | | | **1** |

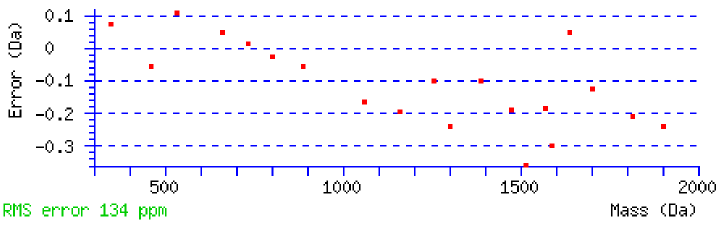


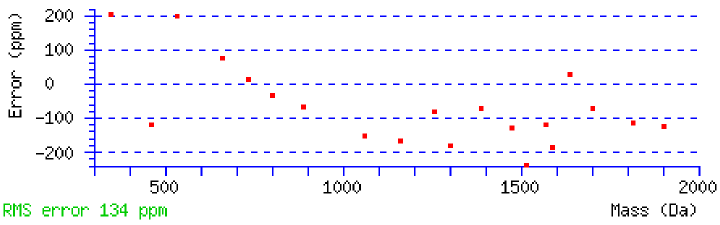


### All matches to this query

| Score | Mr(calc): | Delta | Sequence |
|---|---|---|---|
| 112.6 | 2049.0378 | 0.0063 | ADNLYSQVVGLSAAQANLSK |
| 28.8 | 2048.0538 | 0.9903 | ADNLYSQVVGLSAAQANLSK |
| 19.1 | 2049.0378 | 0.0063 | ADNLYSQVVGLSAAQANLSK |
| 8.7 | 2047.0474 | 1.9967 | FTATAGIQVVGDDLTVTNPK |
| 3.3 | 2048.0249 | 1.0192 | FTATAGIQVVGDDLTMANPK |
| 1.6 | 2049.0565 | -0.0124 | ELRLYNTGLTEVPSGICK |

---

Spectrum No: 33; Query: 590; Rank: 1

---

## Peptide View

MS/MS Fragmentation of **SLGNVNFSVSAEAR**
Found in **IPI00201262**, Tax_Id=10116 Gene_Symbol=LOC297568;Mug2;Mug1 Alpha-1-inhibitor 3 precursor

Match to Query 590: 1450.708868 from(726.361710,2+)
Title: 100101RatKid_NS_deglyco_21.3904.3904.2.dta
Data file K:\NewmanPaper\Piliang\3SubProteomes\Piliang3SP\mgf5ppm\ERLIC_3SubProteomes5ppm.mgf

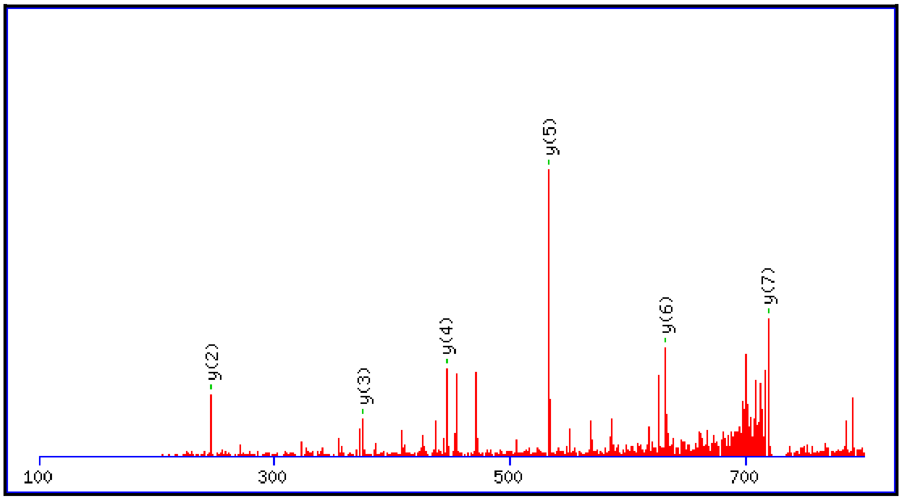

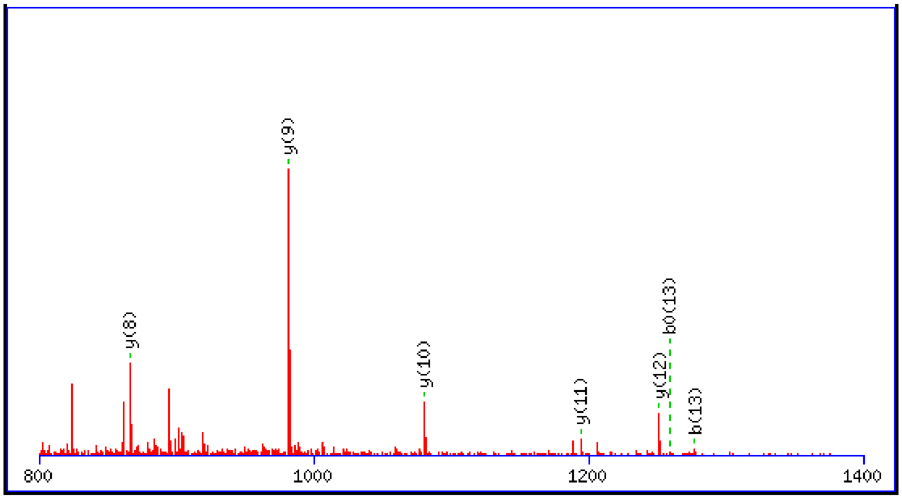


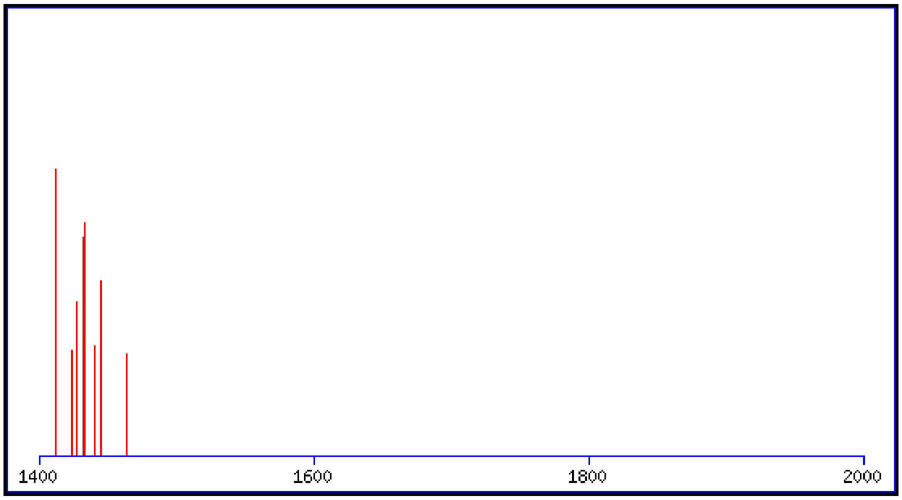


**Monoisotopic mass of neutral peptide Mr(calc):** 1450.7052
**Fixed modifications:** Carbamidomethyl (C)
**Variable modifications:**
**N6** : Deamidated_N (N)
**Ions Score:** 112 **Expect:** 8.8e-010
**Matches (Bold Red):** 13/146 fragment ions using 22 most intense peaks

| # | b | b++ | b* | b*++ | b0 | b0++ | Seq. | y | y++ | y* | y*++ | y0 | y0++ | # |
|---|---|---|---|---|---|---|---|---|---|---|---|---|---|---|
| 1 | 88.0393 | 44.5233 | | | 70.0287 | 35.5180 | S | | | | | | | 14 |
| 2 | 201.1234 | 101.0653 | | | 183.1128 | 92.0600 | L | 1364.6805 | 682.8439 | 1347.6539 | 674.3306 | 1346.6699 | 673.8386 | 13 |
| 3 | 258.1448 | 129.5761 | | | 240.1343 | 120.5708 | G | **1251.5964** | 626.3018 | 1234.5698 | 617.7886 | 1233.5858 | 617.2966 | 12 |
| 4 | 372.1878 | 186.5975 | 355.1612 | 178.0842 | 354.1772 | 177.5922 | N | **1194.5749** | 597.7911 | 1177.5484 | 589.2778 | 1176.5644 | 588.7858 | 11 |
| 5 | 471.2562 | 236.1317 | 454.2296 | 227.6185 | 453.2456 | 227.1264 | V | **1080.5320** | 540.7696 | 1063.5055 | 532.2564 | 1062.5214 | 531.7644 | 10 |
| 6 | 586.2831 | 293.6452 | 569.2566 | 285.1319 | 568.2725 | 284.6399 | N | **981.4636** | 491.2354 | 964.4370 | 482.7222 | 963.4530 | 482.2302 | 9 |
| 7 | 733.3515 | 367.1794 | 716.3250 | 358.6661 | 715.3410 | 358.1741 | F | **866.4367** | 433.7220 | 849.4101 | 425.2087 | 848.4261 | 424.7167 | 8 |
| 8 | 820.3836 | 410.6954 | 803.3570 | 402.1821 | 802.3730 | 401.6901 | S | **719.3682** | 360.1878 | 702.3417 | 351.6745 | 701.3577 | 351.1825 | 7 |
| 9 | 919.4520 | 460.2296 | 902.4254 | 451.7163 | 901.4414 | 451.2243 | V | **632.3362** | 316.6717 | 615.3097 | 308.1585 | 614.3256 | 307.6665 | 6 |
| 10 | 1006.4840 | 503.7456 | 989.4574 | 495.2324 | 988.4734 | 494.7404 | S | **533.2678** | 267.1375 | 516.2413 | 258.6243 | 515.2572 | 258.1323 | 5 |
| 11 | 1077.5211 | 539.2642 | 1060.4946 | 530.7509 | 1059.5105 | 530.2589 | A | **446.2358** | 223.6215 | 429.2092 | 215.1082 | 428.2252 | 214.6162 | 4 |
| 12 | 1206.5637 | 603.7855 | 1189.5372 | 595.2722 | 1188.5531 | 594.7802 | E | **375.1987** | 188.1030 | 358.1721 | 179.5897 | 357.1881 | 179.0977 | 3 |
| 13 | **1277.6008** | 639.3040 | 1260.5743 | 630.7908 | **1259.5902** | 630.2988 | A | **246.1561** | 123.5817 | 229.1295 | 115.0684 | | | 2 |
| 14 | | | | | | | R | 175.1190 | 88.0631 | 158.0924 | 79.5498 | | | 1 |

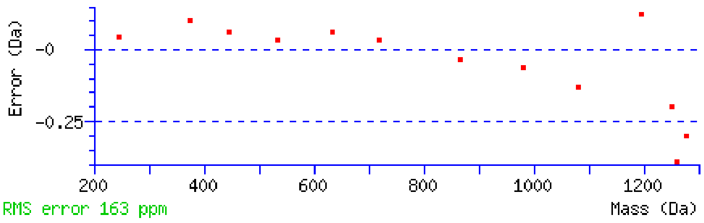


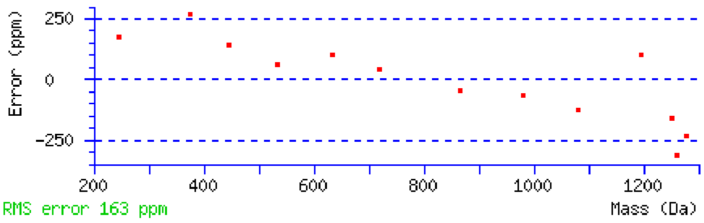


### All matches to this query

| Score | Mr(calc): | Delta | Sequence |
|---|---|---|---|
| 112.2 | 1450.7052 | 0.0037 | SLGNVNFSVSAEAR |
| 73.0 | 1450.7052 | 0.0037 | SLGNVNFSVSAEAR |
| 0.1 | 1450.7181 | -0.0092 | TSTKARGPTPVEK |

---

Spectrum No: 34; Query: 1729; Rank: 1

---

## Peptide View

MS/MS Fragmentation of **AEYAVANDTGFVDIPQQEK**
Found in **IPI00326070**, Tax_Id=10116 Gene_Symbol=Ctsl Cathepsin L1 precursor

Match to Query 1729: 2094.973008 from(1048.493780,2+)
Title: 091008RatKidney_NH4Format01_24.2755.2755.2.dta
Data file K:\NewmanPaper\Piliang\3SubProteomes\Piliang3SP\mgf5ppm\ERLIC_3SubProteomes5ppm.mgf

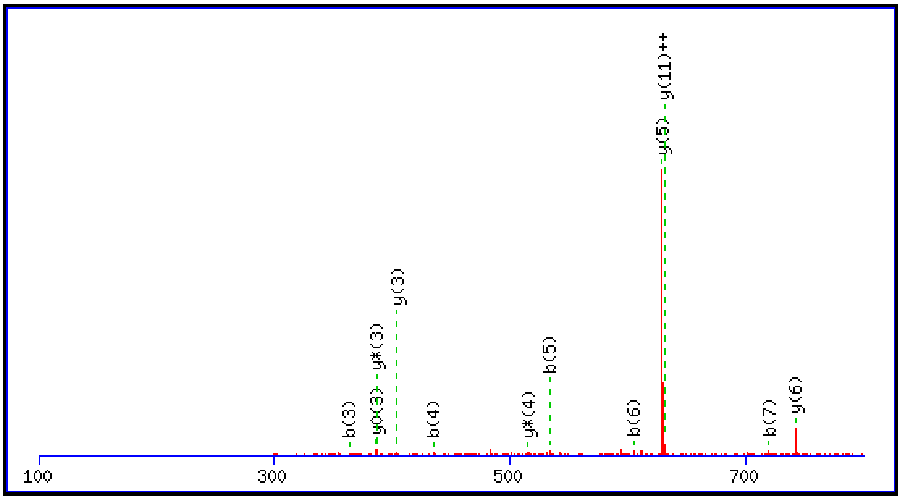


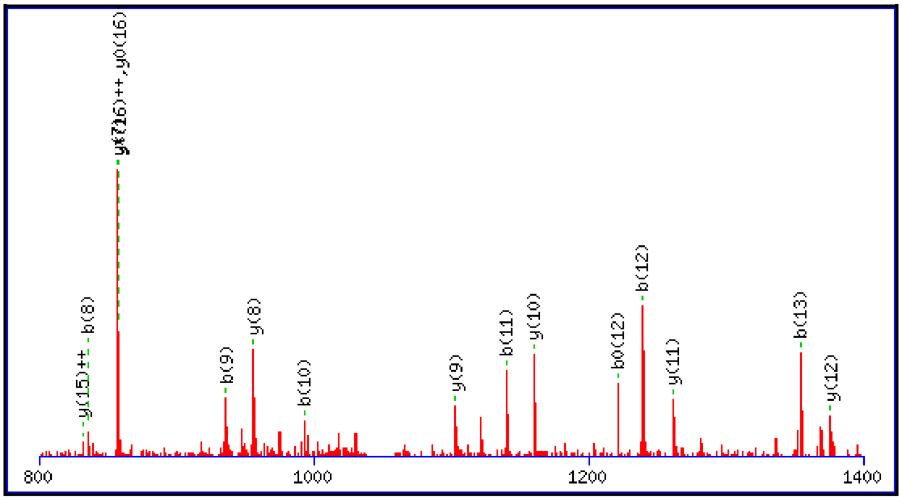

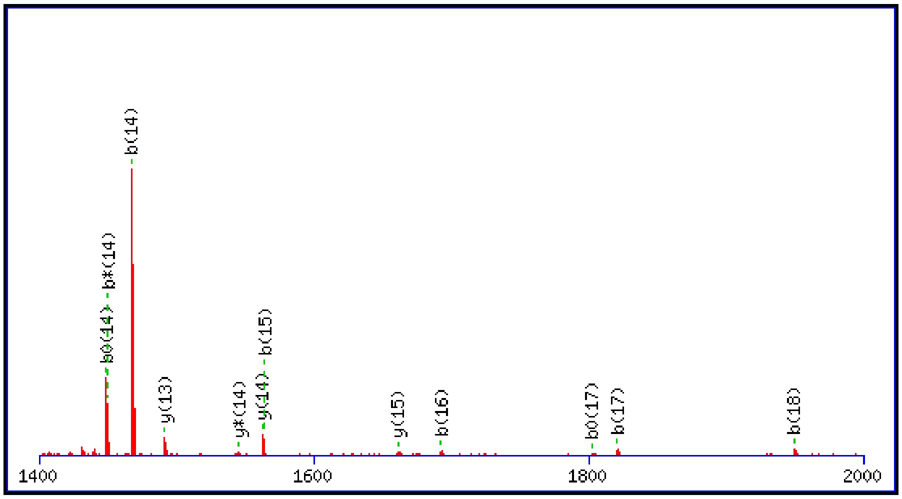


**Monoisotopic mass of neutral peptide Mr(calc):** 2094.9746
**Fixed modifications:** Carbamidomethyl (C)
**Variable modifications:**
**N7** : Deamidated_N (N)
**Ions Score:** 112 **Expect:** 1.6e-009
**Matches (Bold Red):** 40/200 fragment ions using 78 most intense peaks

| # | b | $b^{++}$ | b* | $b^{*++}$ | $b^0$ | $b^{0++}$ | Seq. | y | $y^{++}$ | y* | $y^{*++}$ | $y^0$ | $y^{0++}$ | # |
|---|---|---|---|---|---|---|---|---|---|---|---|---|---|---|
| 1 | 72.0444 | 36.5258 | | | | | A | | | | | | | 19 |
| 2 | 201.0870 | 101.0471 | | | 183.0764 | 92.0418 | E | 2024.9447 | 1012.9760 | 2007.9182 | 1004.4627 | 2006.9342 | 1003.9707 | 18 |
| 3 | **364.1503** | 182.5788 | | | 346.1397 | 173.5735 | Y | 1895.9021 | 948.4547 | 1878.8756 | 939.9414 | 1877.8916 | 939.4494 | 17 |
| 4 | **435.1874** | 218.0974 | | | 417.1769 | 209.0921 | A | 1732.8388 | 866.9230 | 1715.8123 | **858.4098** | 1714.8283 | **857.9178** | 16 |
| 5 | **534.2558** | 267.6316 | | | 516.2453 | 258.6263 | V | **1661.8017** | **831.4045** | 1644.7752 | 822.8912 | 1643.7911 | 822.3992 | 15 |
| 6 | **605.2930** | 303.1501 | | | 587.2824 | 294.1448 | A | **1562.7333** | 781.8703 | **1545.7067** | 773.3570 | 1544.7227 | 772.8650 | 14 |
| 7 | **720.3199** | 360.6636 | 703.2933 | 352.1503 | 702.3093 | 351.6583 | N | **1491.6962** | 746.3517 | 1474.6696 | 737.8385 | 1473.6856 | 737.3464 | 13 |
| 8 | **835.3468** | 418.1771 | 818.3203 | 409.6638 | 817.3363 | 409.1718 | D | **1376.6692** | 688.8383 | 1359.6427 | 680.3250 | 1358.6587 | 679.8330 | 12 |
| 9 | **936.3945** | 468.7009 | 919.3680 | 460.1876 | 918.3839 | 459.6956 | T | **1261.6423** | **631.3248** | 1244.6157 | 622.8115 | 1243.6317 | 622.3195 | 11 |
| 10 | **993.4160** | 497.2116 | 976.3894 | 488.6984 | 975.4054 | 488.2063 | G | **1160.5946** | 580.8009 | 1143.5681 | 572.2877 | 1142.5841 | 571.7957 | 10 |
| 11 | **1140.4844** | 570.7458 | 1123.4578 | 562.2326 | 1122.4738 | 561.7406 | F | **1103.5732** | 552.2902 | 1086.5466 | 543.7769 | 1085.5626 | 543.2849 | 9 |
| 12 | **1239.5528** | 620.2800 | 1222.5263 | 611.7668 | **1221.5422** | 611.2748 | V | **956.5047** | 478.7560 | 939.4782 | 470.2427 | 938.4942 | 469.7507 | 8 |
| 13 | **1354.5797** | 677.7935 | 1337.5532 | 669.2802 | 1336.5692 | 668.7882 | D | **857.4363** | 429.2218 | 840.4098 | 420.7085 | 839.4258 | 420.2165 | 7 |
| 14 | **1467.6638** | 734.3355 | **1450.6373** | 725.8223 | **1449.6532** | 725.3303 | I | **742.4094** | 371.7083 | 725.3828 | 363.1951 | 724.3988 | 362.7030 | 6 |
| 15 | **1564.7166** | 782.8619 | 1547.6900 | 774.3487 | 1546.7060 | 773.8566 | P | **629.3253** | 315.1663 | 612.2988 | 306.6530 | 611.3148 | 306.1610 | 5 |
| 16 | **1692.7752** | 846.8912 | 1675.7486 | 838.3779 | 1674.7646 | 837.8859 | Q | 532.2726 | 266.6399 | **515.2460** | 258.1266 | 514.2620 | 257.6346 | 4 |
| 17 | **1820.8337** | 910.9205 | 1803.8072 | 902.4072 | **1802.8232** | 901.9152 | Q | **404.2140** | 202.6106 | **387.1874** | 194.0974 | **386.2034** | 193.6053 | 3 |
| 18 | **1949.8763** | 975.4418 | 1932.8498 | 966.9285 | 1931.8658 | 966.4365 | E | 276.1554 | 138.5813 | 259.1288 | 130.0681 | 258.1448 | 129.5761 | 2 |
| 19 | | | | | | | K | 147.1128 | 74.0600 | 130.0863 | 65.5468 | | | 1 |

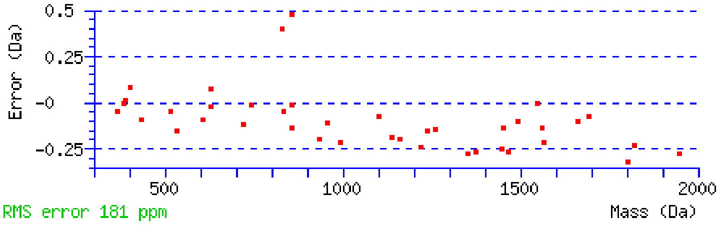


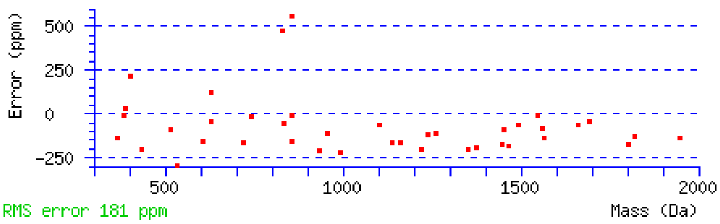


### All matches to this query

| Score | Mr(calc): | Delta | Sequence |
|---|---|---|---|
| 112.1 | 2094.9746 | -0.0016 | AEYAVANDTGFVDIPQQEK |
| 34.5 | 2093.9906 | 0.9825 | AEYAVANDTGFVDIPQQEK |
| | | | |

| | | | |
|---|---|---|---|
| 13.7 | 2094.9760 | -0.0030 | FKGPMTVLRLNNLMASK |
| 12.0 | 2092.9764 | 1.9966 | TISSYTAFSRLILILR |
| 11.1 | 2094.9834 | -0.0104 | KEIEELQSQAQALSQEGK |
| 5.3 | 2094.9623 | 0.0107 | VTGNQGANIAYPPGSTPTIR |
| 5.3 | 2094.9623 | 0.0107 | VTGNQGANIAYPPGSTPTIR |
| 5.3 | 2094.9623 | 0.0107 | VTGNQGANIAYPPGSTPTIR |
| 5.2 | 2094.9623 | 0.0107 | VTGNQGANIAYPPGSTPTIR |
| 5.0 | 2094.9834 | -0.0104 | KEIEELQSQAQALSQEGK |

---

Spectrum No: 35; Query: 1507; Rank: 1

---

## Peptide View

MS/MS Fragmentation of **ALASETNGTDSNGSNSSNIQ**
Found in **IPI00201410**, Tax_Id=10116 Gene_Symbol=Ppp3ca Isoform 1 of Serine/threonine-protein phosphatase 2B catalytic subunit alpha isoform

Match to Query 1507: 1967.810968 from(984.912760,2+)
Title: 091008RatKidney_NoSalt_29.797.797.2.dta
Data file K:\NewmanPaper\Piliang\3SubProteomes\Piliang3SP\mgf5ppm\ERLIC_3SubProteomes5ppm.mgf

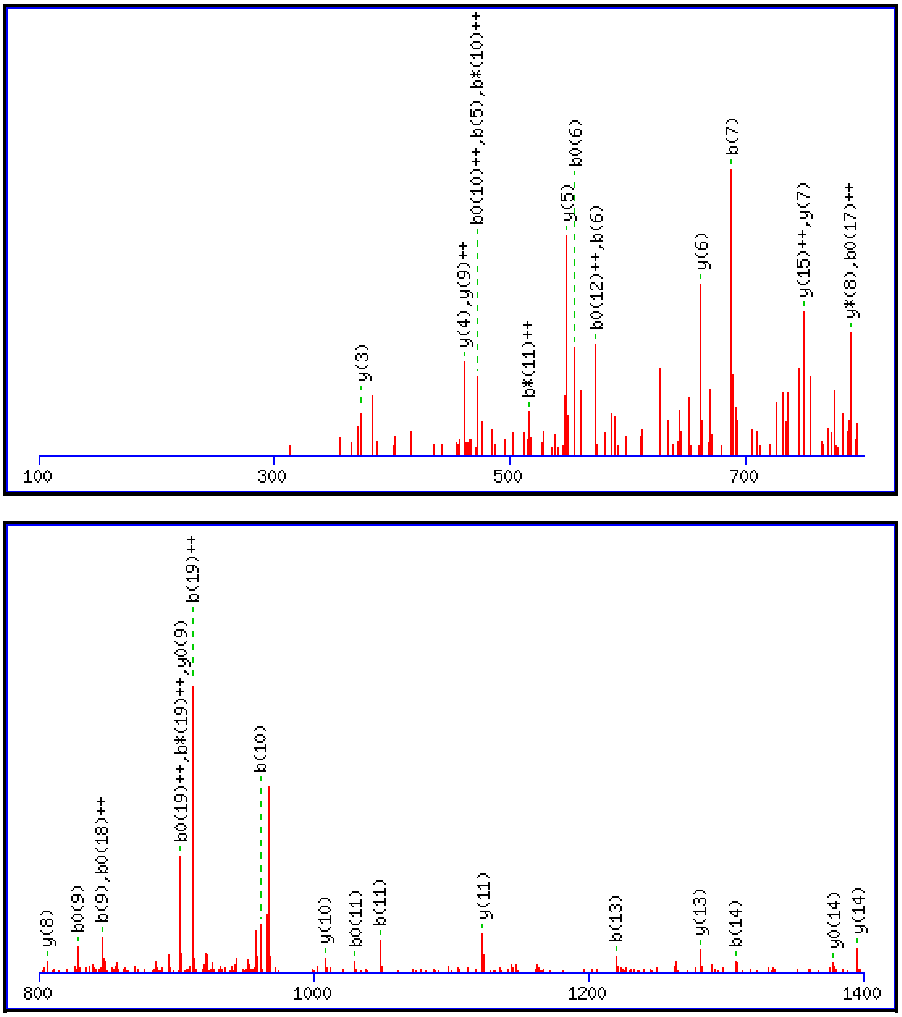

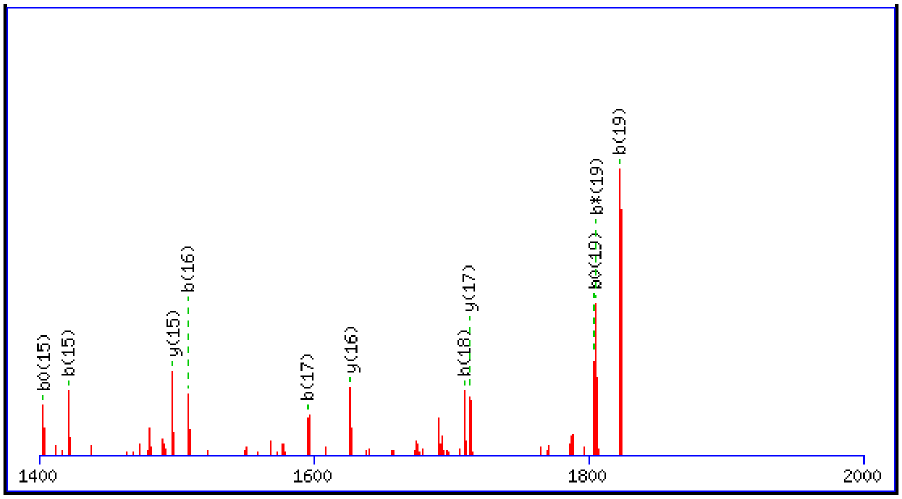


**Monoisotopic mass of neutral peptide Mr(calc):** 1967.8192
**Fixed modifications:** Carbamidomethyl (C)
**Variable modifications:**
**N7** : Deamidated_N (N)
**N12** : Deamidated_N (N)
**Ions Score:** 112 **Expect:** 1.2e-009
**Matches (Bold Red):** 46/204 fragment ions using 54 most intense peaks

| # | b | $b^{++}$ | b* | $b^{*++}$ | $b^{0}$ | $b^{0++}$ | Seq. | y | $y^{++}$ | y* | $y^{*++}$ | $y^{0}$ | $y^{0++}$ | # |
|---|---|---|---|---|---|---|---|---|---|---|---|---|---|---|
| 1 | 72.0444 | 36.5258 | | | | | A | | | | | | | 20 |
| 2 | 185.1285 | 93.0679 | | | | | L | 1897.7893 | 949.3983 | 1880.7628 | 940.8850 | 1879.7788 | 940.3930 | 19 |
| 3 | 256.1656 | 128.5864 | | | | | A | 1784.7053 | 892.8563 | 1767.6787 | 884.3430 | 1766.6947 | 883.8510 | 18 |
| 4 | 343.1976 | 172.1024 | | | 325.1870 | 163.0972 | S | **1713.6682** | 857.3377 | 1696.6416 | 848.8244 | 1695.6576 | 848.3324 | 17 |
| 5 | **472.2402** | 236.6237 | | | 454.2296 | 227.6185 | E | **1626.6361** | 813.8217 | 1609.6096 | 805.3084 | 1608.6256 | 804.8164 | 16 |
| 6 | **573.2879** | 287.1476 | | | **555.2773** | 278.1423 | T | **1497.5936** | **749.3004** | 1480.5670 | 740.7871 | 1479.5830 | 740.2951 | 15 |
| 7 | **688.3148** | 344.6610 | 671.2883 | 336.1478 | 670.3042 | 335.6558 | N | **1396.5459** | 698.7766 | 1379.5193 | 690.2633 | **1378.5353** | 689.7713 | 14 |
| 8 | 745.3363 | 373.1718 | 728.3097 | 364.6585 | 727.3257 | 364.1665 | G | **1281.5189** | 641.2631 | 1264.4924 | 632.7498 | 1263.5084 | 632.2578 | 13 |
| 9 | **846.3839** | 423.6956 | 829.3574 | 415.1823 | **828.3734** | 414.6903 | T | 1224.4975 | 612.7524 | 1207.4709 | 604.2391 | 1206.4869 | 603.7471 | 12 |
| 10 | **961.4109** | 481.2091 | 944.3843 | **472.6958** | 943.4003 | **472.2038** | D | **1123.4498** | 562.2285 | 1106.4232 | 553.7153 | 1105.4392 | 553.2233 | 11 |
| 11 | **1048.4429** | 524.7251 | 1031.4164 | **516.2118** | **1030.4324** | 515.7198 | S | **1008.4228** | 504.7151 | 991.3963 | 496.2018 | 990.4123 | 495.7098 | 10 |
| 12 | 1163.4699 | 582.2386 | 1146.4433 | 573.7253 | 1145.4593 | **573.2333** | N | 921.3908 | **461.1990** | 904.3643 | 452.6858 | **903.3803** | 452.1938 | 9 |
| 13 | **1220.4913** | 610.7493 | 1203.4648 | 602.2360 | 1202.4808 | 601.7440 | G | **806.3639** | 403.6856 | **789.3373** | 395.1723 | 788.3533 | 394.6803 | 8 |
| 14 | **1307.5233** | 654.2653 | 1290.4968 | 645.7520 | 1289.5128 | 645.2600 | S | **749.3424** | 375.1748 | 732.3159 | 366.6616 | 731.3319 | 366.1696 | 7 |
| 15 | **1421.5663** | 711.2868 | 1404.5397 | 702.7735 | **1403.5557** | 702.2815 | N | **662.3104** | 331.6588 | 645.2838 | 323.1456 | 644.2998 | 322.6536 | 6 |
| 16 | **1508.5983** | 754.8028 | 1491.5718 | 746.2895 | 1490.5877 | 745.7975 | S | **548.2675** | 274.6374 | 531.2409 | 266.1241 | 530.2569 | 265.6321 | 5 |
| 17 | **1595.6303** | 798.3188 | 1578.6038 | 789.8055 | 1577.6198 | **789.3135** | S | **461.2354** | 231.1214 | 444.2089 | 222.6081 | 443.2249 | 222.1161 | 4 |
| 18 | **1709.6733** | 855.3403 | 1692.6467 | 846.8270 | 1691.6627 | **846.3350** | N | **374.2034** | 187.6053 | 357.1769 | 179.0921 | | | 3 |
| 19 | **1822.7573** | **911.8823** | **1805.7308** | **903.3690** | **1804.7468** | **902.8770** | I | 260.1605 | 130.5839 | 243.1339 | 122.0706 | | | 2 |
| 20 | | | | | | | Q | 147.0764 | 74.0418 | 130.0499 | 65.5286 | | | 1 |

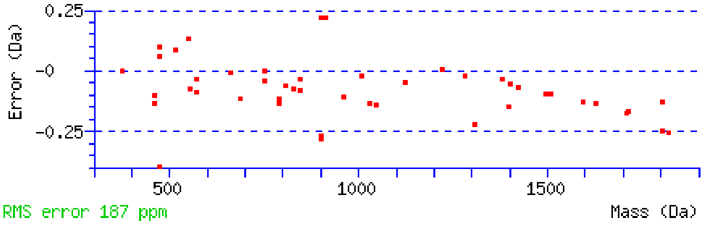


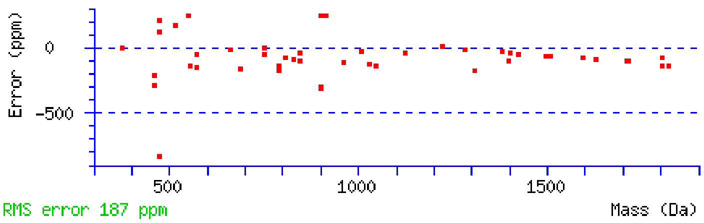


### All matches to this query

| Score | Mr(calc): | Delta | Sequence |
|---|---|---|---|
| 111.8 | 1967.8192 | -0.0082 | ALASETNGTDSNGSNSSNIQ |

| | | | |
|---|---|---|---|
| 72.9 | 1967.8192 | -0.0082 | ALASETNGTDSNGSNSSNIQ |
| 41.5 | 1967.8192 | -0.0082 | ALASETNGTDSNGSNSSNIQ |
| 37.7 | 1967.8192 | -0.0082 | ALASETNGTDSNGSNSSNIQ |
| 16.5 | 1967.8192 | -0.0082 | ALASETNGTDSNGSNSSNIQ |
| 15.3 | 1967.8192 | -0.0082 | ALASETNGTDSNGSNSSNIQ |
| 10.6 | 1967.8006 | 0.0104 | ENEFSTTGTRMDLQMK |
| 8.3 | 1967.7923 | 0.0187 | MEALSMAHYSSPAILR |
| 8.3 | 1967.7923 | 0.0187 | MEALSMAHYSSPAILR |
| 8.1 | 1967.8093 | 0.0017 | CNICNKSFTWCASLK |

---

Spectrum No: 36; Query: 1522; Rank: 1

---

## Peptide View

MS/MS Fragmentation of **AQEAGYTNATTTDQHGLAK**
Found in **IPI00212666**, Tax_Id=10116 Gene_Symbol=Mug1 Isoform 1 of Murinoglobulin-1 precursor

Match to Query 1522: 1976.911128 from(989.462840,2+)
Title: 100101RatKid_NS_deglyco_24.855.855.2.dta
Data file K:\NewmanPaper\Piliang\3SubProteomes\Piliang3SP\mgf5ppm\ERLIC_3SubProteomes5ppm.mgf

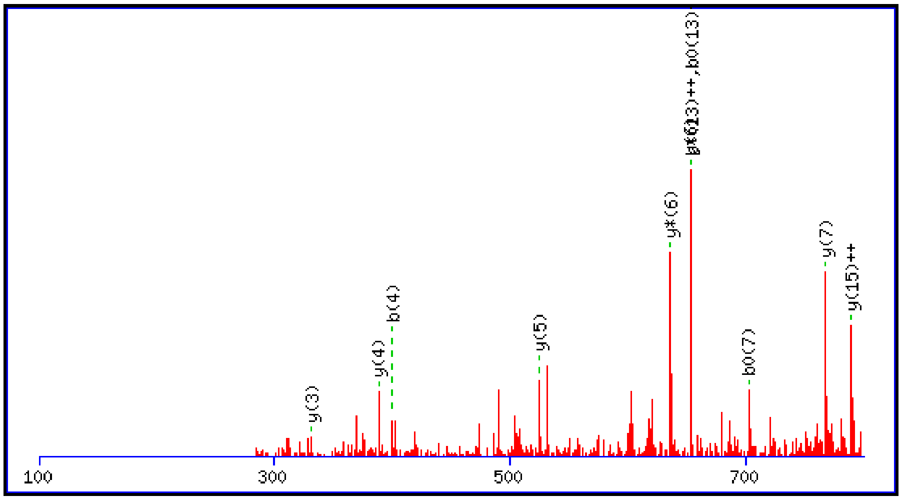


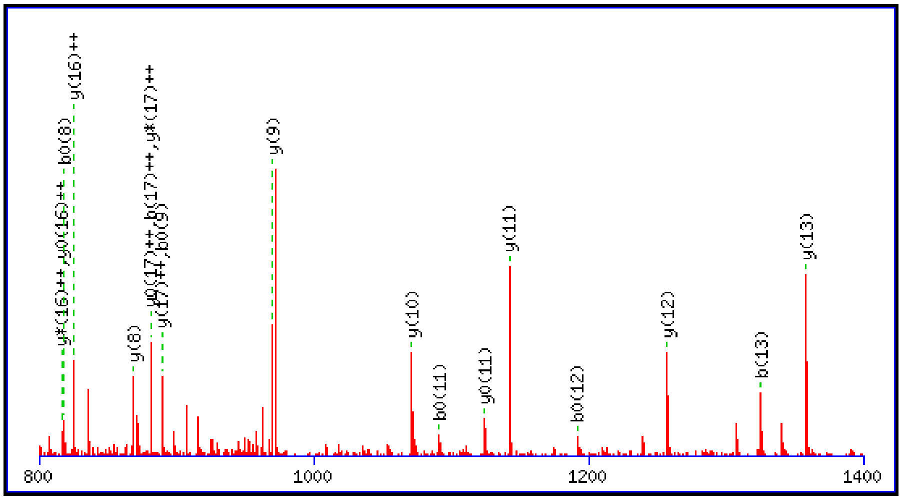

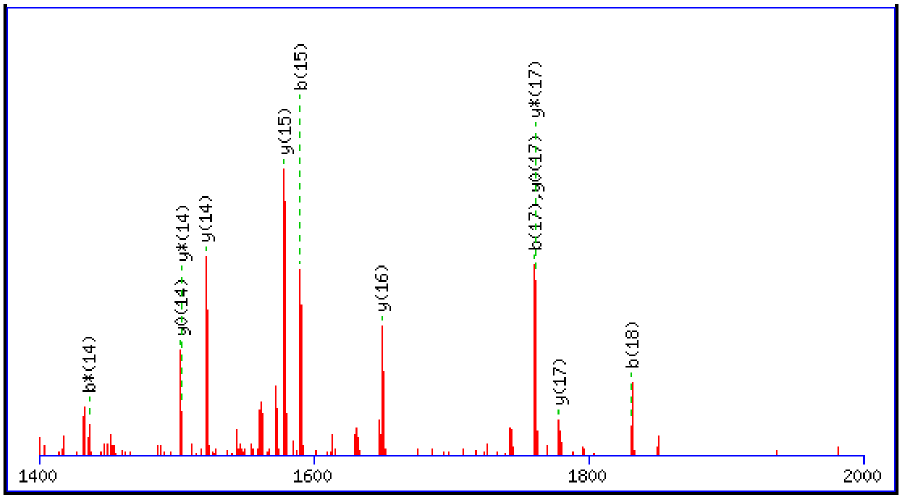


**Monoisotopic mass of neutral peptide Mr(calc):** 1976.9075
**Fixed modifications:** Carbamidomethyl (C)
**Variable modifications:**
**N8** : Deamidated_N (N)
**Ions Score:** 112 **Expect:** 1.6e-009
**Matches (Bold Red):** 42/198 fragment ions using 59 most intense peaks

| # | b | $b^{++}$ | b* | $b^{*++}$ | $b^0$ | $b^{0++}$ | Seq. | y | $y^{++}$ | y* | $y^{*++}$ | $y^0$ | $y^{0++}$ | # |
|---|---|---|---|---|---|---|---|---|---|---|---|---|---|---|
| 1 | 72.0444 | 36.5258 | | | | | A | | | | | | | 19 |
| 2 | 200.1030 | 100.5551 | 183.0764 | 92.0418 | | | Q | 1906.8777 | 953.9425 | 1889.8512 | 945.4292 | 1888.8672 | 944.9372 | 18 |
| 3 | 329.1456 | 165.0764 | 312.1190 | 156.5631 | 311.1350 | 156.0711 | E | **1778.8191** | **889.9132** | **1761.7926** | **881.3999** | **1760.8086** | **880.9079** | 17 |
| 4 | **400.1827** | 200.5950 | 383.1561 | 192.0817 | 382.1721 | 191.5897 | A | **1649.7766** | **825.3919** | 1632.7500 | **816.8786** | 1631.7660 | **816.3866** | 16 |
| 5 | 457.2041 | 229.1057 | 440.1776 | 220.5924 | 439.1936 | 220.1004 | G | **1578.7394** | **789.8734** | 1561.7129 | 781.3601 | 1560.7289 | 780.8681 | 15 |
| 6 | 620.2675 | 310.6374 | 603.2409 | 302.1241 | 602.2569 | 301.6321 | Y | **1521.7180** | 761.3626 | **1504.6914** | 752.8494 | **1503.7074** | 752.3573 | 14 |
| 7 | 721.3151 | 361.1612 | 704.2886 | 352.6479 | **703.3046** | 352.1559 | T | **1358.6546** | 679.8310 | 1341.6281 | 671.3177 | 1340.6441 | 670.8257 | 13 |
| 8 | 836.3421 | 418.6747 | 819.3155 | 410.1614 | **818.3315** | 409.6694 | N | **1257.6070** | 629.3071 | 1240.5804 | 620.7938 | 1239.5964 | 620.3018 | 12 |
| 9 | 907.3792 | 454.1932 | 890.3526 | 445.6800 | **889.3686** | 445.1880 | A | **1142.5800** | 571.7937 | 1125.5535 | 563.2804 | **1124.5695** | 562.7884 | 11 |
| 10 | 1008.4269 | 504.7171 | 991.4003 | 496.2038 | 990.4163 | 495.7118 | T | **1071.5429** | 536.2751 | 1054.5164 | 527.7618 | 1053.5324 | 527.2698 | 10 |
| 11 | 1109.4746 | 555.2409 | 1092.4480 | 546.7276 | **1091.4640** | 546.2356 | T | **970.4952** | 485.7513 | 953.4687 | 477.2380 | 952.4847 | 476.7460 | 9 |
| 12 | 1210.5222 | 605.7648 | 1193.4957 | 597.2515 | **1192.5117** | 596.7595 | T | **869.4476** | 435.2274 | 852.4210 | 426.7141 | 851.4370 | 426.2221 | 8 |
| 13 | **1325.5492** | 663.2782 | 1308.5226 | **654.7650** | 1307.5386 | **654.2729** | D | **768.3999** | 384.7036 | 751.3733 | 376.1903 | 750.3893 | 375.6983 | 7 |
| 14 | 1453.6078 | 727.3075 | **1436.5812** | 718.7942 | 1435.5972 | 718.3022 | Q | **653.3729** | 327.1901 | **636.3464** | 318.6768 | | | 6 |
| 15 | **1590.6667** | 795.8370 | 1573.6401 | 787.3237 | 1572.6561 | 786.8317 | H | **525.3144** | 263.1608 | 508.2878 | 254.6475 | | | 5 |
| 16 | 1647.6881 | 824.3477 | 1630.6616 | 815.8344 | 1629.6776 | 815.3424 | G | **388.2554** | 194.6314 | 371.2289 | 186.1181 | | | 4 |
| 17 | **1760.7722** | **880.8897** | 1743.7456 | 872.3765 | 1742.7616 | 871.8845 | L | **331.2340** | 166.1206 | 314.2074 | 157.6074 | | | 3 |
| 18 | **1831.8093** | 916.4083 | 1814.7828 | 907.8950 | 1813.7987 | 907.4030 | A | 218.1499 | 109.5786 | 201.1234 | 101.0653 | | | 2 |
| 19 | | | | | | | K | 147.1128 | 74.0600 | 130.0863 | 65.5468 | | | 1 |

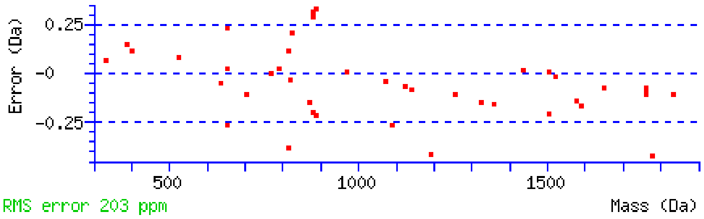


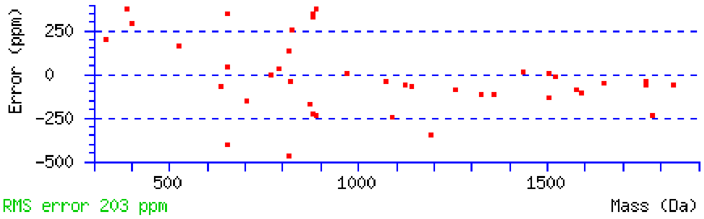


## All matches to this query

| Score | Mr(calc): | Delta | Sequence |
|---|---|---|---|
| 111.6 | 1976.9075 | 0.0036 | AQEAGYTNATTTDQHGLAK |
| 30.5 | 1975.9235 | 0.9876 | AQEAGYTNATTTDQHGLAK |
| | | | |

| | | | |
|---|---|---|---|
| 5.8 | 1976.9050 | 0.0061 | TFGESHPFTKLVVDLK |
| 2.7 | 1976.9027 | 0.0085 | STLDSHPMNISGIPERK |
| 1.3 | 1976.8993 | 0.0118 | AQEALDFYGEVRTRDK |

---

Spectrum No: 37; Query: 2170; Rank: 1

---

## Peptide View

MS/MS Fragmentation of **VILILDPAISGNETEPYPAFTR**
Found in **IPI00191437**, Tax_Id=10116 Gene_Symbol=- 64 kDa protein

Match to Query 2170: 2416.261828 from(1209.138190,2+)
Title: 091008RatKidney_NoSalt_26.4795.4795.2.dta
Data file K:\NewmanPaper\Piliang\3SubProteomes\Piliang3SP\mgf5ppm\ERLIC_3SubProteomes5ppm.mgf

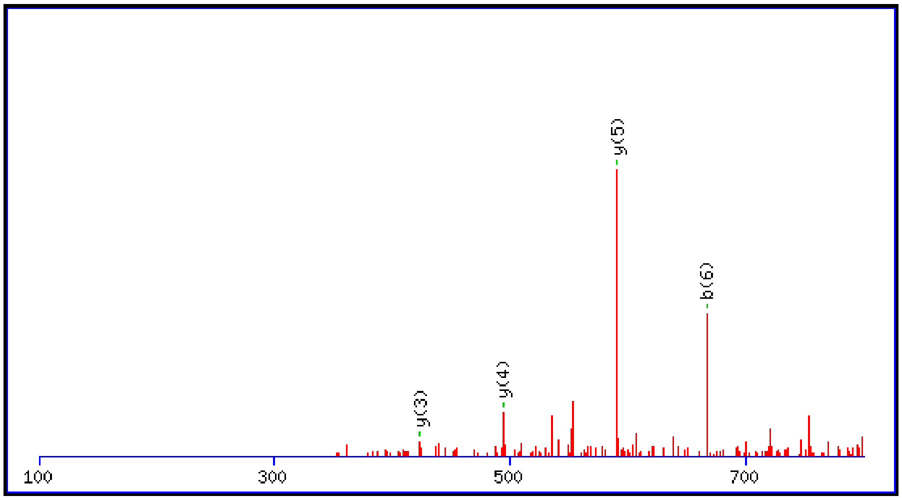


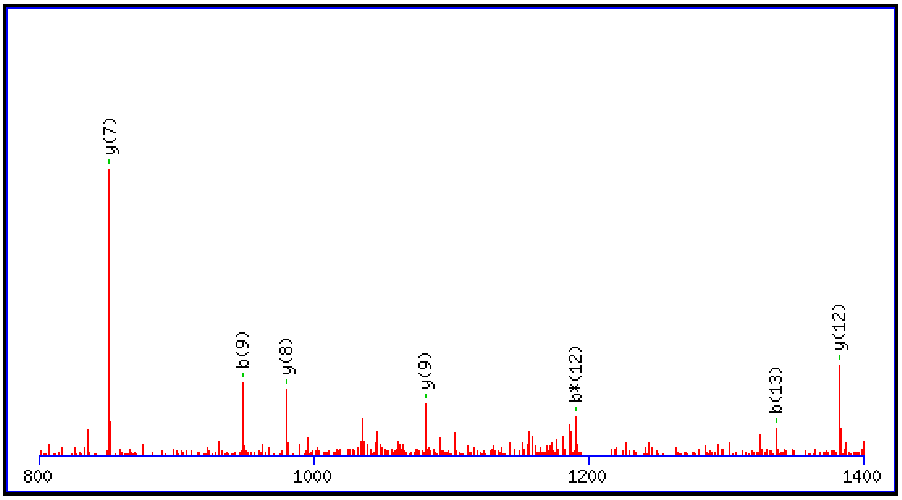

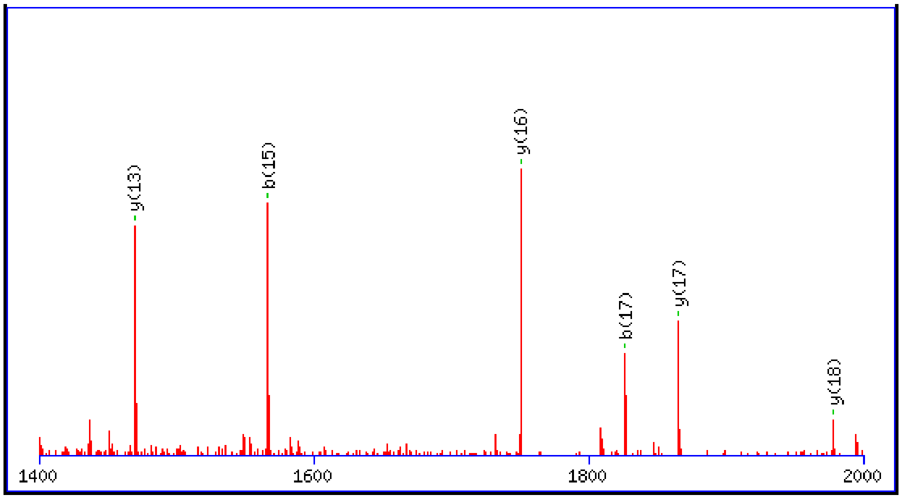


**Monoisotopic mass of neutral peptide Mr(calc):** 2416.2526
**Fixed modifications:** Carbamidomethyl (C)
**Variable modifications:**
**N12** : Deamidated_N (N)
**Ions Score:** 111 **Expect:** 8.1e-010
**Matches (Bold Red):** 17/218 fragment ions using 18 most intense peaks

| # | b | $b^{++}$ | $b^{*}$ | $b^{*++}$ | $b^{0}$ | $b^{0++}$ | Seq. | y | $y^{++}$ | $y^{*}$ | $y^{*++}$ | $y^{0}$ | $y^{0++}$ | # |
|---|---|---|---|---|---|---|---|---|---|---|---|---|---|---|
| 1 | 100.0757 | 50.5415 | | | | | V | | | | | | | 22 |
| 2 | 213.1598 | 107.0835 | | | | | I | 2318.1914 | 1159.5994 | 2301.1649 | 1151.0861 | 2300.1809 | 1150.5941 | 21 |
| 3 | 326.2438 | 163.6255 | | | | | L | 2205.1074 | 1103.0573 | 2188.0808 | 1094.5441 | 2187.0968 | 1094.0520 | 20 |
| 4 | 439.3279 | 220.1676 | | | | | I | 2092.0233 | 1046.5153 | 2074.9968 | 1038.0020 | 2074.0128 | 1037.5100 | 19 |
| 5 | 552.4119 | 276.7096 | | | | | L | **1978.9393** | 989.9733 | 1961.9127 | 981.4600 | 1960.9287 | 980.9680 | 18 |
| 6 | **667.4389** | 334.2231 | | | 649.4283 | 325.2178 | D | **1865.8552** | 933.4312 | 1848.8286 | 924.9180 | 1847.8446 | 924.4260 | 17 |
| 7 | 764.4917 | 382.7495 | | | 746.4811 | 373.7442 | P | **1750.8283** | 875.9178 | 1733.8017 | 867.4045 | 1732.8177 | 866.9125 | 16 |
| 8 | 835.5288 | 418.2680 | | | 817.5182 | 409.2627 | A | 1653.7755 | 827.3914 | 1636.7489 | 818.8781 | 1635.7649 | 818.3861 | 15 |
| 9 | **948.6128** | 474.8101 | | | 930.6023 | 465.8048 | I | 1582.7384 | 791.8728 | 1565.7118 | 783.3596 | 1564.7278 | 782.8675 | 14 |
| 10 | 1035.6449 | 518.3261 | | | 1017.6343 | 509.3208 | S | **1469.6543** | 735.3308 | 1452.6278 | 726.8175 | 1451.6437 | 726.3255 | 13 |
| 11 | 1092.6663 | 546.8368 | | | 1074.6558 | 537.8315 | G | **1382.6223** | 691.8148 | 1365.5957 | 683.3015 | 1364.6117 | 682.8095 | 12 |
| 12 | 1207.6933 | 604.3503 | **1190.6667** | 595.8370 | 1189.6827 | 595.3450 | N | 1325.6008 | 663.3040 | 1308.5743 | 654.7908 | 1307.5903 | 654.2988 | 11 |
| 13 | **1336.7359** | 668.8716 | 1319.7093 | 660.3583 | 1318.7253 | 659.8663 | E | 1210.5739 | 605.7906 | 1193.5473 | 597.2773 | 1192.5633 | 596.7853 | 10 |
| 14 | 1437.7835 | 719.3954 | 1420.7570 | 710.8821 | 1419.7730 | 710.3901 | T | **1081.5313** | 541.2693 | 1064.5047 | 532.7560 | 1063.5207 | 532.2640 | 9 |
| 15 | **1566.8261** | 783.9167 | 1549.7996 | 775.4034 | 1548.8156 | 774.9114 | E | **980.4836** | 490.7454 | 963.4571 | 482.2322 | 962.4730 | 481.7402 | 8 |
| 16 | 1663.8789 | 832.4431 | 1646.8523 | 823.9298 | 1645.8683 | 823.4378 | P | **851.4410** | 426.2241 | 834.4145 | 417.7109 | 833.4305 | 417.2189 | 7 |
| 17 | **1826.9422** | 913.9747 | 1809.9157 | 905.4615 | 1808.9317 | 904.9695 | Y | 754.3883 | 377.6978 | 737.3617 | 369.1845 | 736.3777 | 368.6925 | 6 |
| 18 | 1923.9950 | 962.5011 | 1906.9684 | 953.9879 | 1905.9844 | 953.4958 | P | **591.3249** | 296.1661 | 574.2984 | 287.6528 | 573.3144 | 287.1608 | 5 |
| 19 | 1995.0321 | 998.0197 | 1978.0055 | 989.5064 | 1977.0215 | 989.0144 | A | **494.2722** | 247.6397 | 477.2456 | 239.1264 | 476.2616 | 238.6344 | 4 |
| 20 | 2142.1005 | 1071.5539 | 2125.0740 | 1063.0406 | 2124.0899 | 1062.5486 | F | **423.2350** | 212.1212 | 406.2085 | 203.6079 | 405.2245 | 203.1159 | 3 |
| 21 | 2243.1482 | 1122.0777 | 2226.1216 | 1113.5645 | 2225.1376 | 1113.0724 | T | 276.1666 | 138.5870 | 259.1401 | 130.0737 | 258.1561 | 129.5817 | 2 |
| 22 | | | | | | | R | 175.1190 | 88.0631 | 158.0924 | 79.5498 | | | 1 |

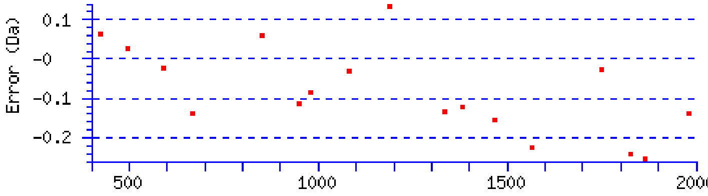


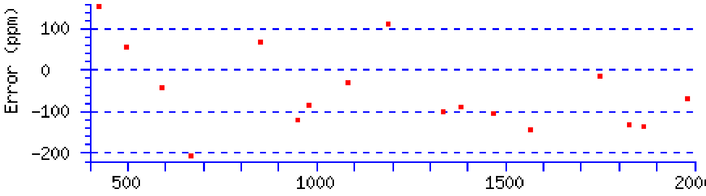


## All matches to this query

| | | | |
|---|---|---|---|

| Score | Mr(calc): | Delta | Sequence |
|---|---|---|---|
| 111.4 | 2416.2526 | 0.0093 | VILILDPAISGNETEPYPAFTR |
| 30.0 | 2415.2686 | 0.9933 | VILILDPAISGNETEPYPAFTR |
| 5.8 | 2416.2515 | 0.0103 | EIQRLLPSPLKDAVEVSTWR |
| 5.1 | 2416.2646 | -0.0028 | FAFQAEVNRMMKLIINSLYK |
| 0.2 | 2414.2570 | 2.0048 | GLNLTSLSITHTNITAVPAAALR |
| 0.2 | 2414.2570 | 2.0048 | GLNLTSLSITHTNITAVPAAALR |

---

Spectrum No: 38; Query: 1722; Rank: 1

---

## Peptide View

MS/MS Fragmentation of **LDWLGNCSGLNDESYGYK**
Found in **IPI00339124**, Tax_Id=10116 Gene_Symbol=Atp1b1 Sodium/potassium-transporting ATPase subunit beta-1

Match to Query 1722: 2090.894568 from(1046.454560,2+)
Title: 100101RatKid_NS_deglyco_27.3469.3469.2.dta
Data file K:\NewmanPaper\Piliang\3SubProteomes\Piliang3SP\mgf5ppm\ERLIC_3SubProteomes5ppm.mgf

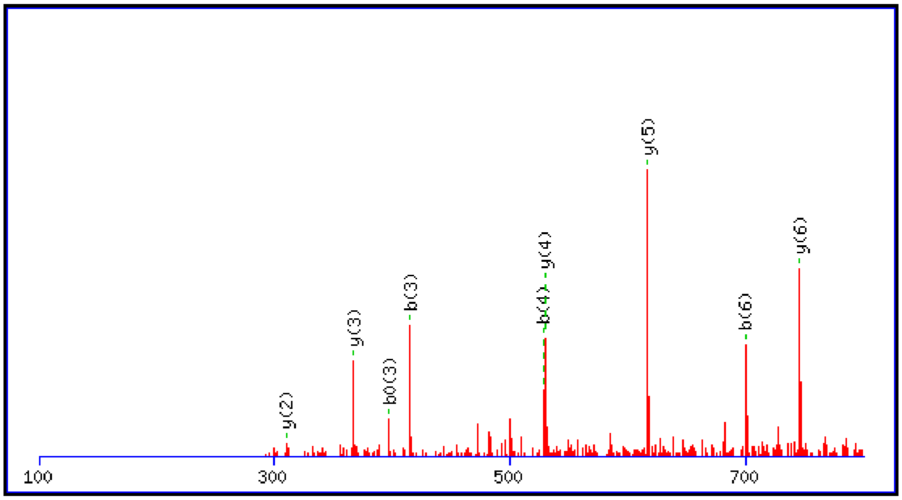


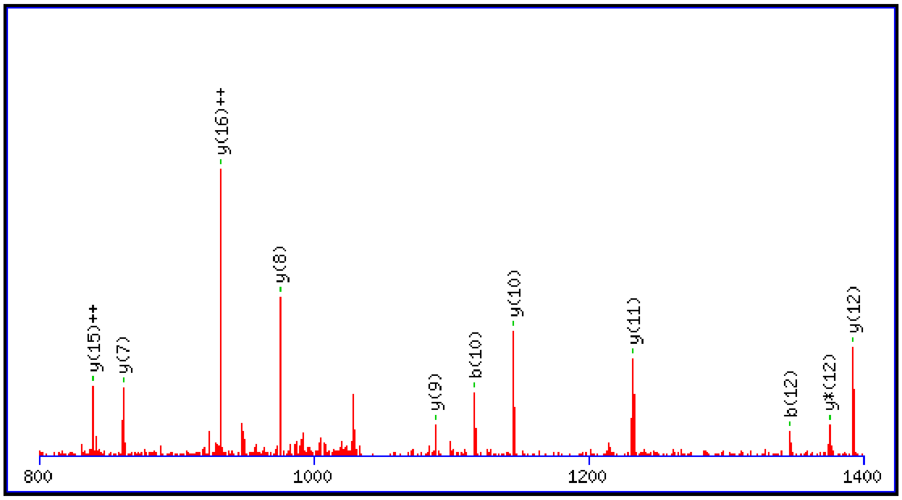

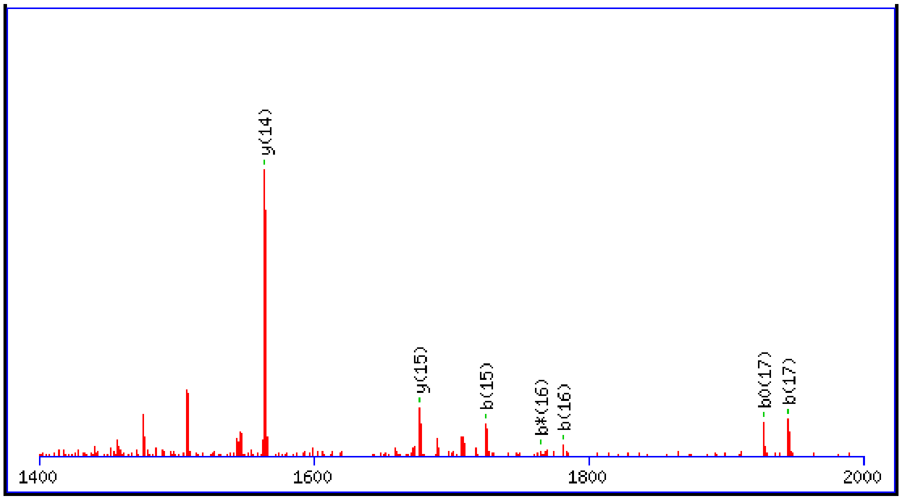


**Monoisotopic mass of neutral peptide Mr(calc):** 2090.8891
**Fixed modifications:** Carbamidomethyl (C)
**Variable modifications:**
**N6** : Deamidated_N (N)
**Ions Score:** 111 **Expect:** 2.5e-009
**Matches (Bold Red):** 27/184 fragment ions using 43 most intense peaks

| # | b | b++ | b* | b*++ | b0 | b0++ | Seq. | y | y++ | y* | y*++ | y0 | y0++ | # |
|---|---|---|---|---|---|---|---|---|---|---|---|---|---|---|
| 1 | 114.0913 | 57.5493 | | | | | L | | | | | | | 18 |
| 2 | 229.1183 | 115.0628 | | | 211.1077 | 106.0575 | D | 1978.8123 | 989.9098 | 1961.7858 | 981.3965 | 1960.8018 | 980.9045 | 17 |
| 3 | **415.1976** | 208.1024 | | | **397.1870** | 199.0972 | W | 1863.7854 | **932.3963** | 1846.7589 | 923.8831 | 1845.7748 | 923.3911 | 16 |
| 4 | **528.2817** | 264.6445 | | | 510.2711 | 255.6392 | L | **1677.7061** | **839.3567** | 1660.6795 | 830.8434 | 1659.6955 | 830.3514 | 15 |
| 5 | 585.3031 | 293.1552 | | | 567.2926 | 284.1499 | G | **1564.6220** | 782.8146 | 1547.5955 | 774.3014 | 1546.6115 | 773.8094 | 14 |
| 6 | **700.3301** | 350.6687 | 683.3035 | 342.1554 | 682.3195 | 341.6634 | N | 1507.6006 | 754.3039 | 1490.5740 | 745.7906 | 1489.5900 | 745.2986 | 13 |
| 7 | 860.3607 | 430.6840 | 843.3342 | 422.1707 | 842.3501 | 421.6787 | C | **1392.5736** | 696.7904 | **1375.5471** | 688.2772 | 1374.5631 | 687.7852 | 12 |
| 8 | 947.3927 | 474.2000 | 930.3662 | 465.6867 | 929.3822 | 465.1947 | S | **1232.5430** | 616.7751 | 1215.5164 | 608.2619 | 1214.5324 | 607.7698 | 11 |
| 9 | 1004.4142 | 502.7107 | 987.3877 | 494.1975 | 986.4036 | 493.7055 | G | **1145.5109** | 573.2591 | 1128.4844 | 564.7458 | 1127.5004 | 564.2538 | 10 |
| 10 | **1117.4983** | 559.2528 | 1100.4717 | 550.7395 | 1099.4877 | 550.2475 | L | **1088.4895** | 544.7484 | 1071.4629 | 536.2351 | 1070.4789 | 535.7431 | 9 |
| 11 | 1231.5412 | 616.2742 | 1214.5146 | 607.7610 | 1213.5306 | 607.2690 | N | **975.4054** | 488.2063 | 958.3789 | 479.6931 | 957.3949 | 479.2011 | 8 |
| 12 | **1346.5681** | 673.7877 | 1329.5416 | 665.2744 | 1328.5576 | 664.7824 | D | **861.3625** | 431.1849 | 844.3359 | 422.6716 | 843.3519 | 422.1796 | 7 |
| 13 | 1475.6107 | 738.3090 | 1458.5842 | 729.7957 | 1457.6002 | 729.3037 | E | **746.3355** | 373.6714 | 729.3090 | 365.1581 | 728.3250 | 364.6661 | 6 |
| 14 | 1562.6428 | 781.8250 | 1545.6162 | 773.3117 | 1544.6322 | 772.8197 | S | **617.2930** | 309.1501 | 600.2664 | 300.6368 | 599.2824 | 300.1448 | 5 |
| 15 | **1725.7061** | 863.3567 | 1708.6795 | 854.8434 | 1707.6955 | 854.3514 | Y | **530.2609** | 265.6341 | 513.2344 | 257.1208 | | | 4 |
| 16 | **1782.7275** | 891.8674 | **1765.7010** | 883.3541 | 1764.7170 | 882.8621 | G | **367.1976** | 184.1024 | 350.1710 | 175.5892 | | | 3 |
| 17 | **1945.7909** | 973.3991 | 1928.7643 | 964.8858 | **1927.7803** | 964.3938 | Y | **310.1761** | 155.5917 | 293.1496 | 147.0784 | | | 2 |
| 18 | | | | | | | K | 147.1128 | 74.0600 | 130.0863 | 65.5468 | | | 1 |

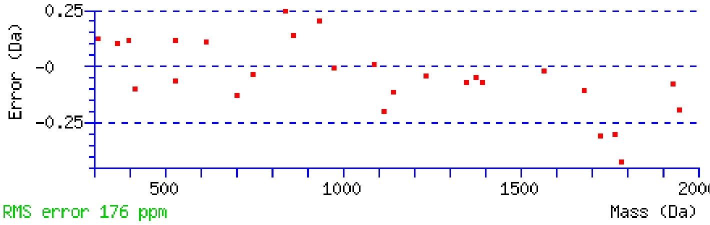


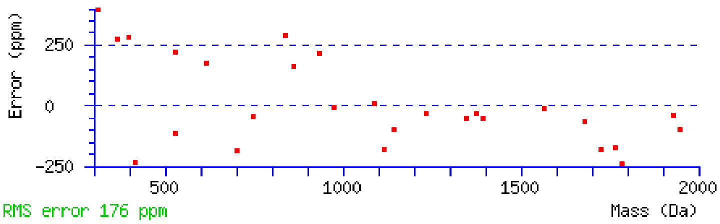


## All matches to this query

| Score | Mr(calc): | Delta | Sequence |
|---|---|---|---|
| 110.8 | 2090.8891 | 0.0055 | LDWLGNCSGLNDESYGYK |
| 82.5 | 2090.8891 | 0.0055 | LDWLGNCSGLNDESYGYK |
| 63.0 | 2089.9051 | 0.9895 | LDWLGNCSGLNDESYGYK |
| | | | |

| | | | |
|---|---|---|---|
| 5.7 | 2090.8979 | -0.0034 | EREEMQRQYEEDILK |
| 3.6 | 2090.9132 | -0.0186 | IFHRYEALNSQTMNTGK |
| 3.0 | 2090.8768 | 0.0177 | NIGGNKDWCKENFVNSK |
| 1.4 | 2089.9065 | 0.9880 | ESSESTNNRFAANQGKLR |
| 1.4 | 2089.9065 | 0.9880 | ESSESTNNRFAANQGKLR |
| 1.4 | 2089.9065 | 0.9880 | ESSESTNNRFAANQGKLR |
| 1.4 | 2089.9065 | 0.9880 | ESSESTNNRFAANQGKLR |

---

Spectrum No: 39; Query: 1347; Rank: 1

---

## Peptide View

MS/MS Fragmentation of **VIDLWDLAQSANFTEK**
Found in **IPI00364124**, Tax_Id=10116 Gene_Symbol=Lrpap1 Alpha-2-macroglobulin receptor-associated protein precursor

Match to Query 1347: 1849.912148 from(925.963350,2+)
Title: 091008RatKidney_NoSalt_20.5946.5946.2.dta
Data file K:\NewmanPaper\Piliang\3SubProteomes\Piliang3SP\mgf5ppm\ERLIC_3SubProteomes5ppm.mgf

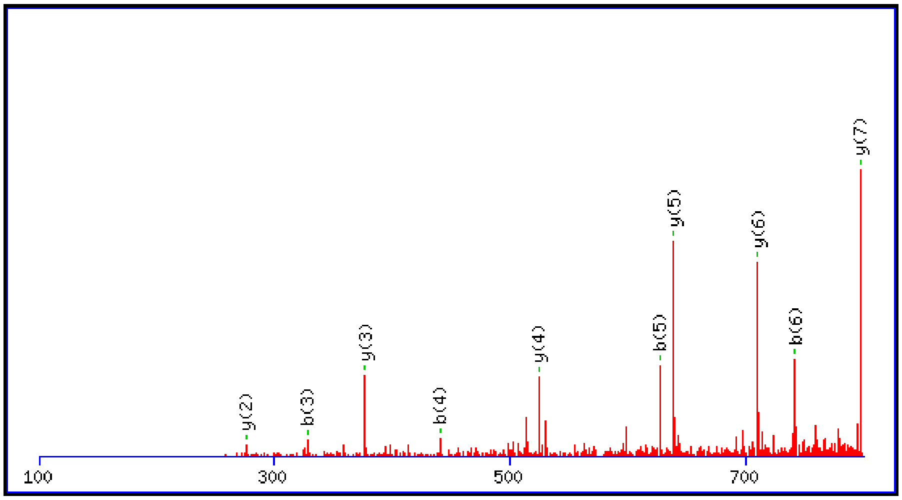


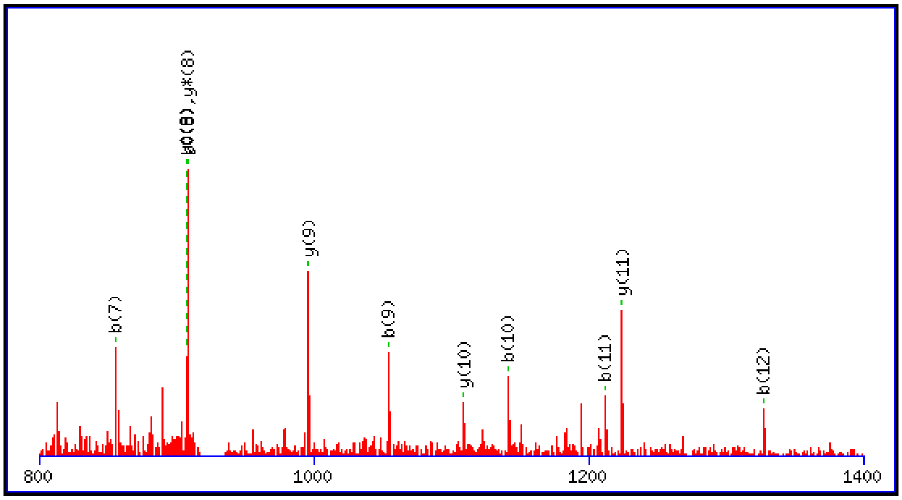

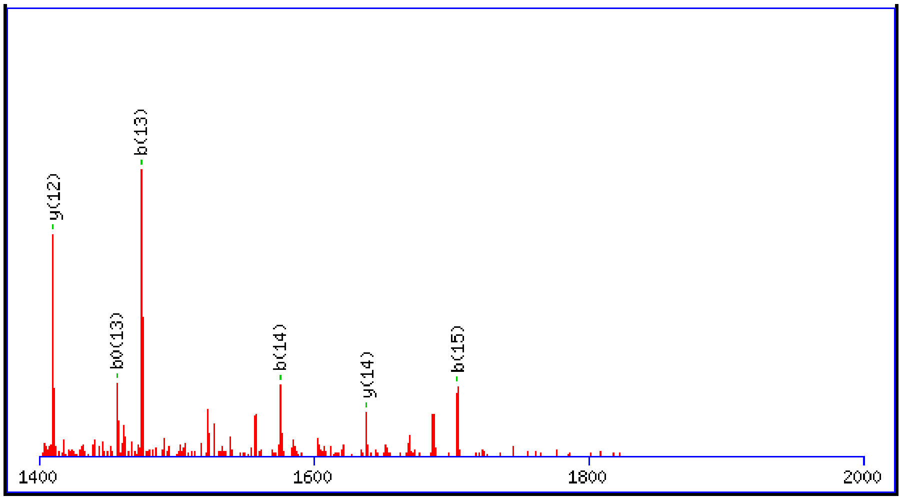


**Monoisotopic mass of neutral peptide Mr(calc):** 1849.9098
**Fixed modifications:** Carbamidomethyl (C)
**Variable modifications:**
**N12** : Deamidated_N (N)
**Ions Score:** 111 **Expect:** 2e-009
**Matches (Bold Red):** 27/158 fragment ions using 36 most intense peaks

| # | b | $b^{++}$ | b* | $b^{*++}$ | $b^{0}$ | $b^{0++}$ | Seq. | y | $y^{++}$ | y* | $y^{*++}$ | $y^{0}$ | $y^{0++}$ | # |
|---|---|---|---|---|---|---|---|---|---|---|---|---|---|---|
| 1 | 100.0757 | 50.5415 | | | | | V | | | | | | | 16 |
| 2 | 213.1598 | 107.0835 | | | | | I | 1751.8487 | 876.4280 | 1734.8221 | 867.9147 | 1733.8381 | 867.4227 | 15 |
| 3 | **328.1867** | 164.5970 | | | 310.1761 | 155.5917 | D | **1638.7646** | 819.8859 | 1621.7380 | 811.3727 | 1620.7540 | 810.8806 | 14 |
| 4 | **441.2708** | 221.1390 | | | 423.2602 | 212.1337 | L | 1523.7376 | 762.3725 | 1506.7111 | 753.8592 | 1505.7271 | 753.3672 | 13 |
| 5 | **627.3501** | 314.1787 | | | 609.3395 | 305.1734 | W | **1410.6536** | 705.8304 | 1393.6270 | 697.3172 | 1392.6430 | 696.8251 | 12 |
| 6 | **742.3770** | 371.6921 | | | 724.3665 | 362.6869 | D | **1224.5743** | 612.7908 | 1207.5477 | 604.2775 | 1206.5637 | 603.7855 | 11 |
| 7 | **855.4611** | 428.2342 | | | 837.4505 | 419.2289 | L | **1109.5473** | 555.2773 | 1092.5208 | 546.7640 | 1091.5368 | 546.2720 | 10 |
| 8 | 926.4982 | 463.7527 | | | **908.4876** | 454.7475 | A | **996.4633** | 498.7353 | 979.4367 | 490.2220 | 978.4527 | 489.7300 | 9 |
| 9 | **1054.5568** | 527.7820 | 1037.5302 | 519.2688 | 1036.5462 | 518.7767 | Q | 925.4261 | 463.2167 | **908.3996** | 454.7034 | **907.4156** | 454.2114 | 8 |
| 10 | **1141.5888** | 571.2980 | 1124.5623 | 562.7848 | 1123.5782 | 562.2928 | S | **797.3676** | 399.1874 | 780.3410 | 390.6741 | 779.3570 | 390.1821 | 7 |
| 11 | **1212.6259** | 606.8166 | 1195.5994 | 598.3033 | 1194.6154 | 597.8113 | A | **710.3355** | 355.6714 | 693.3090 | 347.1581 | 692.3250 | 346.6661 | 6 |
| 12 | **1327.6529** | 664.3301 | 1310.6263 | 655.8168 | 1309.6423 | 655.3248 | N | **639.2984** | 320.1529 | 622.2719 | 311.6396 | 621.2879 | 311.1476 | 5 |
| 13 | **1474.7213** | 737.8643 | 1457.6947 | 729.3510 | **1456.7107** | 728.8590 | F | **524.2715** | 262.6394 | 507.2449 | 254.1261 | 506.2609 | 253.6341 | 4 |
| 14 | **1575.7689** | 788.3881 | 1558.7424 | 779.8748 | 1557.7584 | 779.3828 | T | **377.2031** | 189.1052 | 360.1765 | 180.5919 | 359.1925 | 180.0999 | 3 |
| 15 | **1704.8115** | 852.9094 | 1687.7850 | 844.3961 | 1686.8010 | 843.9041 | E | **276.1554** | 138.5813 | 259.1288 | 130.0681 | 258.1448 | 129.5761 | 2 |
| 16 | | | | | | | K | 147.1128 | 74.0600 | 130.0863 | 65.5468 | | | 1 |

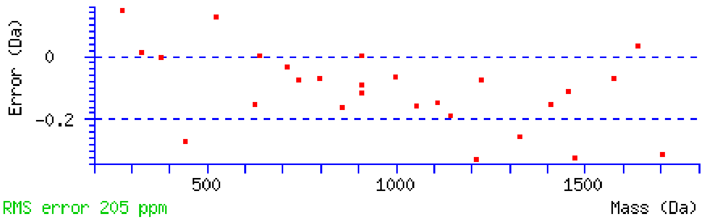


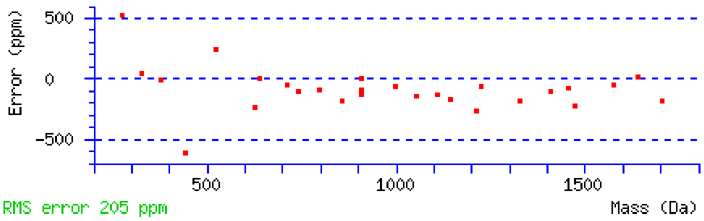


### All matches to this query

| Score | Mr(calc): | Delta | Sequence |
|---|---|---|---|
| 110.6 | 1849.9098 | 0.0024 | VIDLWDLAQSANFTEK |
| 34.5 | 1848.9258 | 0.9864 | VIDLWDLAQSANFTEK |
| 5.4 | 1849.9244 | -0.0122 | NLVHYLTTVERMETK |
| 4.0 | 1849.9267 | -0.0145 | VLYESLKYPSFPLSK |
| 1.5 | 1849.9298 | -0.0177 | KDLRNDLNTIQELAK |
| | | | |

| | | | |
|---|---|---|---|
| 0.6 | 1849.8959 | 0.0163 | DPYLAGPGSRPLSYSDR |

---

Spectrum No: 40; Query: 2776; Rank: 1

---

## Peptide View

MS/MS Fragmentation of **EGNCSVQSGLTWQDCDFKDAEEAATGECTTTLGK**
Found in **IPI00327182**, Tax_Id=10116 Gene_Symbol=Kng1 T-kininogen 1 precursor

Match to Query 2776: 3765.583392 from(1256.201740,3+)
Title: 100101RatKid_NS_deglyco_24.4023.4023.3.dta
Data file K:\NewmanPaper\Piliang\3SubProteomes\Piliang3SP\mgf5ppm\ERLIC_3SubProteomes5ppm.mgf

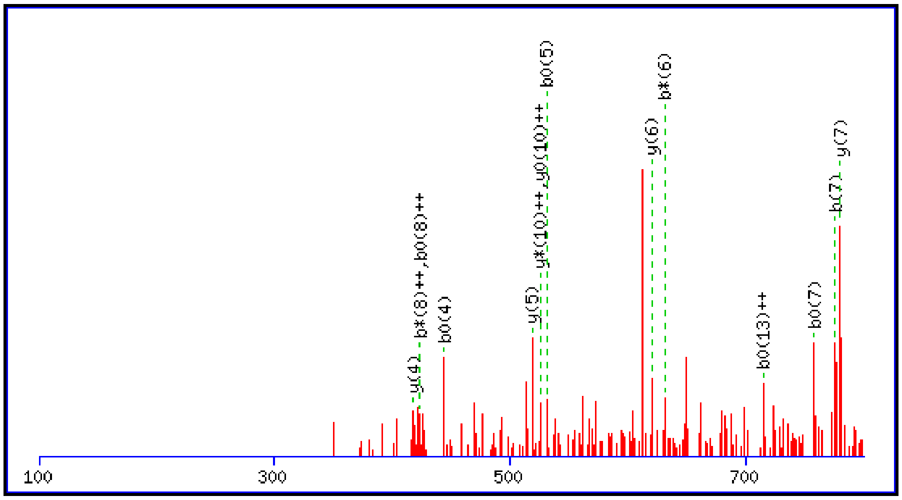


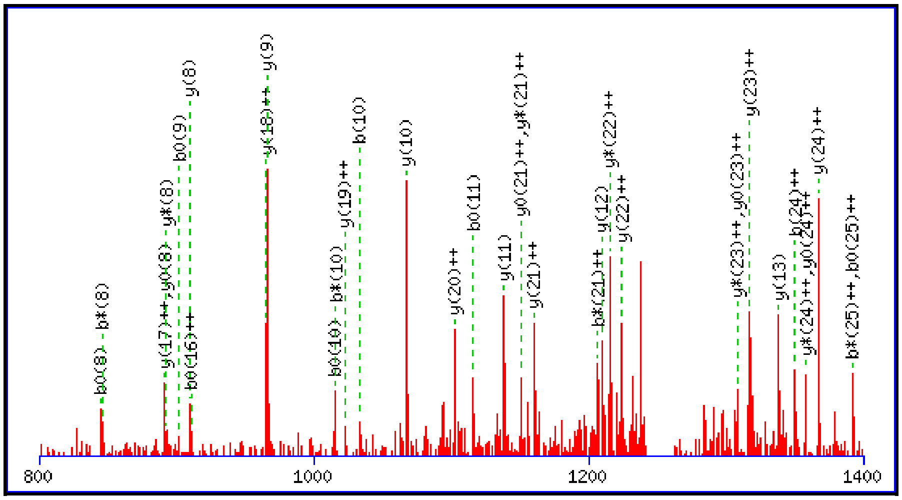

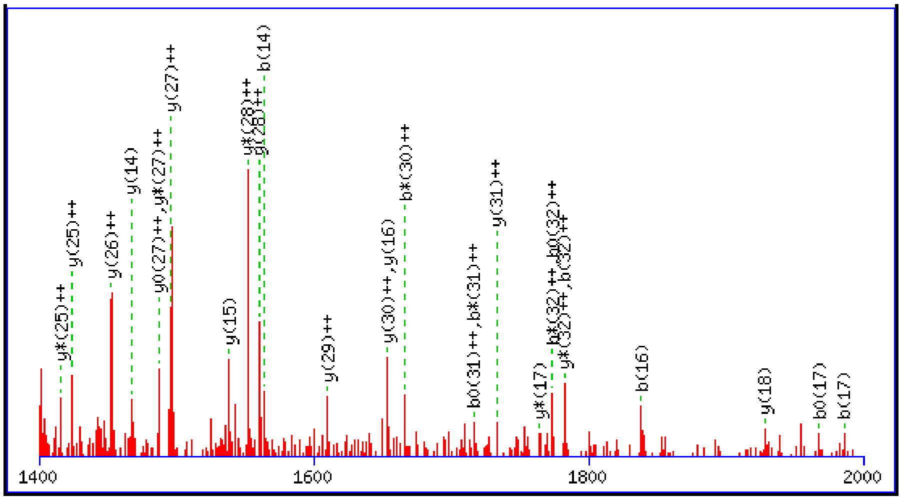


**Monoisotopic mass of neutral peptide Mr(calc):** 3765.5717
**Fixed modifications:** Carbamidomethyl (C)
**Variable modifications:**
**N3** : Deamidated_N (N)
**Ions Score:** 110 **Expect:** 4.9e-009
**Matches (Bold Red):** 76/386 fragment ions using 121 most intense peaks

| # | b | $b^{++}$ | b* | $b^{*++}$ | $b^0$ | $b^{0++}$ | Seq. | y | $y^{++}$ | y* | $y^{*++}$ | $y^0$ | $y^{0++}$ | # |
|---|---|---|---|---|---|---|---|---|---|---|---|---|---|---|
| 1 | 130.0499 | 65.5286 | | | 112.0393 | 56.5233 | E | | | | | | | 34 |
| 2 | 187.0713 | 94.0393 | | | 169.0608 | 85.0340 | G | 3637.5363 | 1819.2718 | 3620.5098 | 1810.7585 | 3619.5258 | 1810.2665 | 33 |
| 3 | 302.0983 | 151.5528 | 285.0717 | 143.0395 | 284.0877 | 142.5475 | N | 3580.5149 | 1790.7611 | 3563.4883 | **1782.2478** | 3562.5043 | 1781.7558 | 32 |
| 4 | 462.1289 | 231.5681 | 445.1024 | 223.0548 | **444.1184** | 222.5628 | C | 3465.4879 | **1733.2476** | 3448.4614 | 1724.7343 | 3447.4774 | 1724.2423 | 31 |
| 5 | 549.1609 | 275.0841 | 532.1344 | 266.5708 | **531.1504** | 266.0788 | S | 3305.4573 | **1653.2323** | 3288.4307 | 1644.7190 | 3287.4467 | 1644.2270 | 30 |
| 6 | 648.2294 | 324.6183 | **631.2028** | 316.1050 | 630.2188 | 315.6130 | V | 3218.4253 | **1609.7163** | 3201.3987 | 1601.2030 | 3200.4147 | 1600.7110 | 29 |
| 7 | **776.2879** | 388.6476 | 759.2614 | 380.1343 | **758.2774** | 379.6423 | Q | 3119.3568 | **1560.1821** | 3102.3303 | **1551.6688** | 3101.3463 | 1551.1768 | 28 |
| 8 | 863.3200 | 432.1636 | **846.2934** | **423.6503** | **845.3094** | **423.1583** | S | 2991.2983 | **1496.1528** | 2974.2717 | **1487.6395** | 2973.2877 | **1487.1475** | 27 |
| 9 | 920.3414 | 460.6744 | 903.3149 | 452.1611 | **902.3309** | 451.6691 | G | 2904.2662 | **1452.6368** | 2887.2397 | 1444.1235 | 2886.2557 | 1443.6315 | 26 |
| 10 | **1033.4255** | 517.2164 | **1016.3989** | 508.7031 | **1015.4149** | 508.2111 | L | 2847.2448 | **1424.1260** | 2830.2182 | **1415.6127** | 2829.2342 | 1415.1207 | 25 |
| 11 | 1134.4732 | 567.7402 | 1117.4466 | 559.2270 | **1116.4626** | 558.7349 | T | 2734.1607 | **1367.5840** | 2717.1342 | **1359.0707** | 2716.1501 | **1358.5787** | 24 |
| 12 | 1320.5525 | 660.7799 | 1303.5259 | 652.2666 | 1302.5419 | 651.7746 | W | 2633.1130 | **1317.0602** | 2616.0865 | **1308.5469** | 2615.1025 | **1308.0549** | 23 |
| 13 | 1448.6111 | 724.8092 | 1431.5845 | 716.2959 | 1430.6005 | **715.8039** | Q | 2447.0337 | **1224.0205** | 2430.0072 | **1215.5072** | 2429.0231 | 1215.0152 | 22 |
| 14 | **1563.6380** | 782.3226 | 1546.6115 | 773.8094 | 1545.6274 | 773.3174 | D | 2318.9751 | **1159.9912** | 2301.9486 | **1151.4779** | 2300.9646 | **1150.9859** | 21 |
| 15 | 1723.6687 | 862.3380 | 1706.6421 | 853.8247 | 1705.6581 | 853.3327 | C | 2203.9482 | **1102.4777** | 2186.9216 | 1093.9645 | 2185.9376 | 1093.4725 | 20 |
| 16 | **1838.6956** | 919.8514 | 1821.6691 | 911.3382 | 1820.6850 | **910.8462** | D | 2043.9175 | **1022.4624** | 2026.8910 | 1013.9491 | 2025.9070 | 1013.4571 | 19 |
| 17 | **1985.7640** | 993.3856 | 1968.7375 | 984.8724 | **1967.7534** | 984.3804 | F | **1928.8906** | **964.9489** | 1911.8641 | 956.4357 | 1910.8800 | 955.9437 | 18 |
| 18 | 2113.8590 | 1057.4331 | 2096.8324 | 1048.9199 | 2095.8484 | 1048.4278 | K | 1781.8222 | **891.4147** | **1764.7956** | 882.9015 | 1763.8116 | 882.4094 | 17 |
| 19 | 2228.8859 | 1114.9466 | 2211.8594 | 1106.4333 | 2210.8754 | 1105.9413 | D | **1653.7272** | 827.3673 | 1636.7007 | 818.8540 | 1635.7167 | 818.3620 | 16 |
| 20 | 2299.9230 | 1150.4652 | 2282.8965 | 1141.9519 | 2281.9125 | 1141.4599 | A | **1538.7003** | 769.8538 | 1521.6737 | 761.3405 | 1520.6897 | 760.8485 | 15 |
| 21 | 2428.9656 | 1214.9865 | 2411.9391 | **1206.4732** | 2410.9551 | 1205.9812 | E | **1467.6632** | 734.3352 | 1450.6366 | 725.8219 | 1449.6526 | 725.3299 | 14 |
| 22 | 2558.0082 | 1279.5077 | 2540.9817 | 1270.9945 | 2539.9977 | 1270.5025 | E | **1338.6206** | 669.8139 | 1321.5940 | 661.3007 | 1320.6100 | 660.8086 | 13 |
| 23 | 2629.0453 | 1315.0263 | 2612.0188 | 1306.5130 | 2611.0348 | 1306.0210 | A | **1209.5780** | 605.2926 | 1192.5514 | 596.7794 | 1191.5674 | 596.2873 | 12 |
| 24 | 2700.0824 | **1350.5449** | 2683.0559 | 1342.0316 | 2682.0719 | 1341.5396 | A | **1138.5409** | 569.7741 | 1121.5143 | 561.2608 | 1120.5303 | 560.7688 | 11 |
| 25 | 2801.1301 | 1401.0687 | 2784.1036 | **1392.5554** | 2783.1196 | **1392.0634** | T | **1067.5038** | 534.2555 | 1050.4772 | **525.7422** | 1049.4932 | **525.2502** | 10 |
| 26 | 2858.1516 | 1429.5794 | 2841.1250 | 1421.0662 | 2840.1410 | 1420.5742 | G | **966.4561** | 483.7317 | 949.4295 | 475.2184 | 948.4455 | 474.7264 | 9 |
| 27 | 2987.1942 | 1494.1007 | 2970.1676 | 1485.5875 | 2969.1836 | 1485.0954 | E | **909.4346** | 455.2209 | **892.4081** | 446.7077 | **891.4240** | 446.2157 | 8 |
| 28 | 3147.2248 | 1574.1161 | 3130.1983 | 1565.6028 | 3129.2143 | 1565.1108 | C | **780.3920** | 390.6996 | 763.3655 | 382.1864 | 762.3815 | 381.6944 | 7 |
| 29 | 3248.2725 | 1624.6399 | 3231.2460 | 1616.1266 | 3230.2619 | 1615.6346 | T | **620.3614** | 310.6843 | 603.3348 | 302.1710 | 602.3508 | 301.6790 | 6 |
| 30 | 3349.3202 | 1675.1637 | 3332.2936 | **1666.6505** | 3331.3096 | 1666.1585 | T | **519.3137** | 260.1605 | 502.2871 | 251.6472 | 501.3031 | 251.1552 | 5 |
| 31 | 3450.3679 | 1725.6876 | 3433.3413 | **1717.1743** | 3432.3573 | **1716.6823** | T | **418.2660** | 209.6366 | 401.2395 | 201.1234 | 400.2554 | 200.6314 | 4 |
| 32 | 3563.4519 | **1782.2296** | 3546.4254 | **1773.7163** | 3545.4414 | **1773.2243** | L | 317.2183 | 159.1128 | 300.1918 | 150.5995 | | | 3 |

| | | | | | | | | | | | | | | |
|---|---|---|---|---|---|---|---|---|---|---|---|---|---|---|
| 33 | 3620.4734 | 1810.7403 | 3603.4468 | 1802.2271 | 3602.4628 | 1801.7351 | G | 204.1343 | 102.5708 | 187.1077 | 94.0575 | | | 2 |
| 34 | | | | | | | K | 147.1128 | 74.0600 | 130.0863 | 65.5468 | | | 1 |

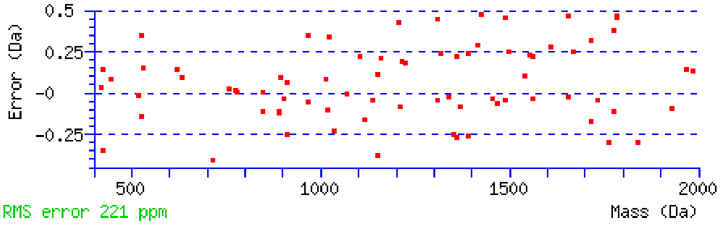


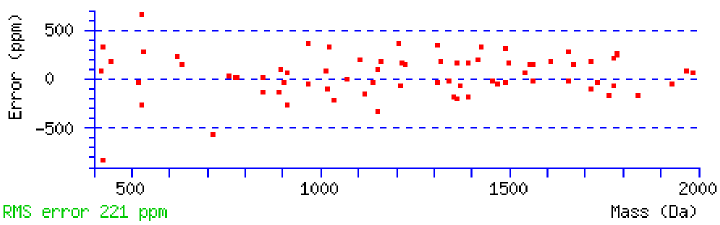


### All matches to this query

| Score | Mr(calc): | Delta | Sequence |
|---|---|---|---|
| 110.3 | 3765.5717 | 0.0117 | EGNCSVQSGLTWQDCDFKDAEEAATGECTTTLGK |
| 79.5 | 3764.5877 | 0.9957 | EGNCSVQSGLTWQDCDFKDAEEAATGECTTTLGK |
| 5.5 | 3763.6105 | 1.9728 | DFHGSLLGFYENGELCDVTLKVGSKLISCHK |
| 2.2 | 3763.5614 | 2.0220 | DSATGETEVSTPPKASMKLVNFQQSEPTSANEK |
| 1.5 | 3764.5454 | 1.0380 | DSATGETEVSTPPKASMKLVNFQQSEPTSANEK |
| 1.4 | 3763.6105 | 1.9728 | DFHGSLLGFYENGELCDVTLKVGSKLISCHK |
| 1.2 | 3765.5914 | -0.0080 | IPDLESETWMSPLAWLEKGVNTSVMLQSLR |
| 1.2 | 3764.6062 | 0.9772 | AGSMTVTVGETCSLECKVAGTPELSVEWYKDGK |
| 1.1 | 3765.6107 | -0.0273 | NGSSNPLNWNSSKNGDWHLEANTSTLYYLVSGR |
| 0.3 | 3763.5924 | 1.9910 | AFSTCSSHMVVVSITYESCMFMYMKTSAKER |

---

Spectrum No: 41; Query: 1568; Rank: 1

---

## Peptide View

MS/MS Fragmentation of **MGAGEALGNASQLFEAWLK**
Found in **IPI00327398**, Tax_Id=10116 Gene_Symbol=Enpep Isoform 1 of Glutamyl aminopeptidase

Match to Query 1568: 1992.966768 from(997.490660,2+)
Title: 100101RatKid_NS_deglyco_19.6506.6506.2.dta
Data file K:\NewmanPaper\Piliang\3SubProteomes\Piliang3SP\mgf5ppm\ERLIC_3SubProteomes5ppm.mgf

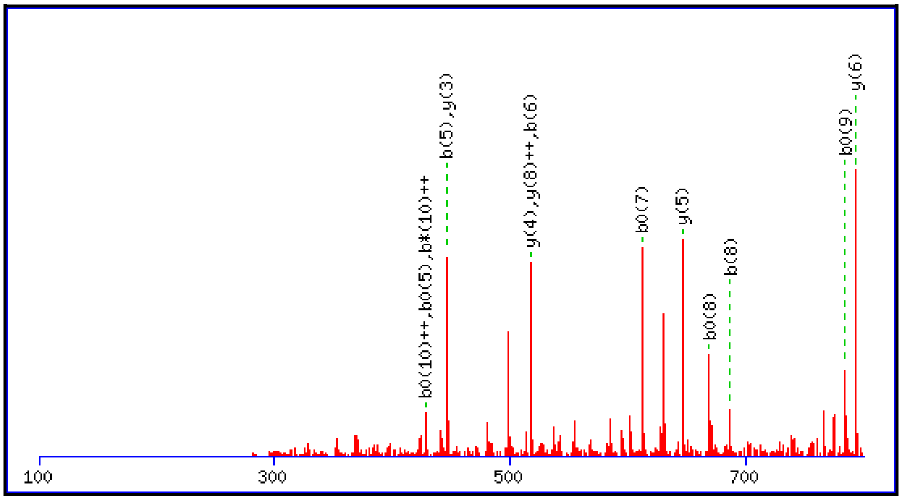

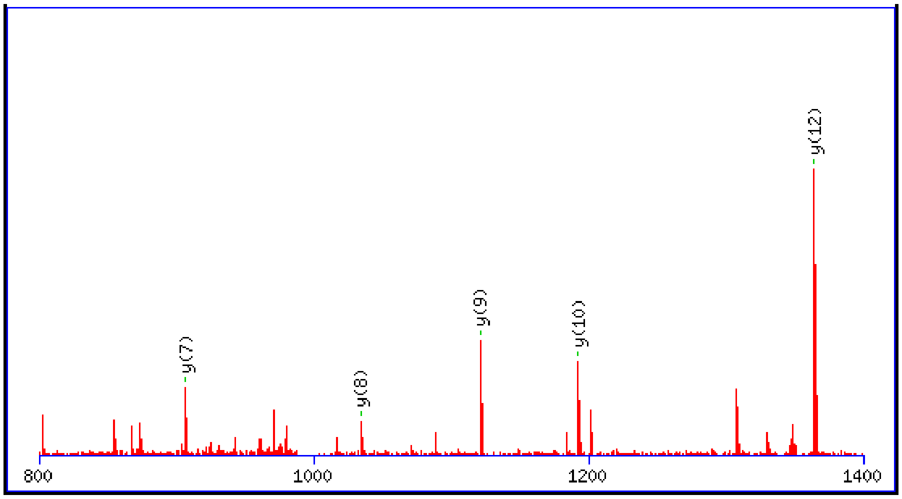


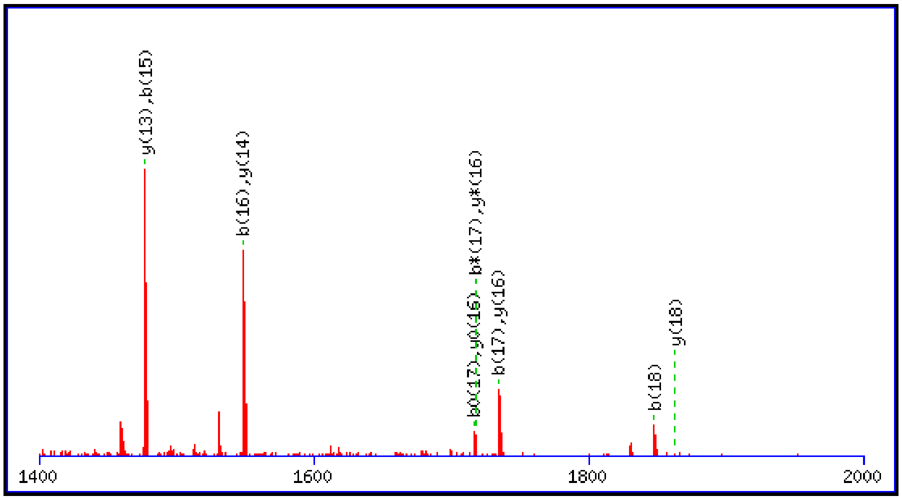


**Monoisotopic mass of neutral peptide Mr(calc):** 1992.9615
**Fixed modifications:** Carbamidomethyl (C)
**Variable modifications:**
**N9** : Deamidated_N (N)
**Ions Score:** 110 **Expect:** 2.1e-009
**Matches (Bold Red):** 31/184 fragment ions using 39 most intense peaks

| # | b | b++ | b* | b*++ | b0 | b0++ | Seq. | y | y++ | y* | y*++ | y0 | y0++ | # |
|---|---|---|---|---|---|---|---|---|---|---|---|---|---|---|
| 1 | 132.0478 | 66.5275 | | | | | M | | | | | | | 19 |
| 2 | 189.0692 | 95.0383 | | | | | G | **1862.9283** | 931.9678 | 1845.9018 | 923.4545 | 1844.9177 | 922.9625 | 18 |
| 3 | 260.1063 | 130.5568 | | | | | A | 1805.9068 | 903.4571 | 1788.8803 | 894.9438 | 1787.8963 | 894.4518 | 17 |
| 4 | 317.1278 | 159.0675 | | | | | G | **1734.8697** | 867.9385 | **1717.8432** | 859.4252 | **1716.8592** | 858.9332 | 16 |
| 5 | **446.1704** | 223.5888 | | | **428.1598** | 214.5836 | E | 1677.8483 | 839.4278 | 1660.8217 | 830.9145 | 1659.8377 | 830.4225 | 15 |
| 6 | **517.2075** | 259.1074 | | | 499.1969 | 250.1021 | A | **1548.8057** | 774.9065 | 1531.7791 | 766.3932 | 1530.7951 | 765.9012 | 14 |
| 7 | 630.2916 | 315.6494 | | | **612.2810** | 306.6441 | L | **1477.7686** | 739.3879 | 1460.7420 | 730.8746 | 1459.7580 | 730.3826 | 13 |
| 8 | **687.3130** | 344.1602 | | | **669.3025** | 335.1549 | G | **1364.6845** | 682.8459 | 1347.6579 | 674.3326 | 1346.6739 | 673.8406 | 12 |
| 9 | 802.3400 | 401.6736 | 785.3134 | 393.1604 | **784.3294** | 392.6683 | N | 1307.6630 | 654.3351 | 1290.6365 | 645.8219 | 1289.6525 | 645.3299 | 11 |
| 10 | 873.3771 | 437.1922 | 856.3505 | **428.6789** | 855.3665 | **428.1869** | A | **1192.6361** | 596.8217 | 1175.6095 | 588.3084 | 1174.6255 | 587.8164 | 10 |
| 11 | 960.4091 | 480.7082 | 943.3826 | 472.1949 | 942.3986 | 471.7029 | S | **1121.5990** | 561.3031 | 1104.5724 | 552.7898 | 1103.5884 | 552.2978 | 9 |
| 12 | 1088.4677 | 544.7375 | 1071.4411 | 536.2242 | 1070.4571 | 535.7322 | Q | **1034.5669** | **517.7871** | 1017.5404 | 509.2738 | 1016.5564 | 508.7818 | 8 |
| 13 | 1201.5518 | 601.2795 | 1184.5252 | 592.7662 | 1183.5412 | 592.2742 | L | **906.5084** | 453.7578 | 889.4818 | 445.2445 | 888.4978 | 444.7525 | 7 |
| 14 | 1348.6202 | 674.8137 | 1331.5936 | 666.3004 | 1330.6096 | 665.8084 | F | **793.4243** | 397.2158 | 776.3978 | 388.7025 | 775.4137 | 388.2105 | 6 |
| 15 | **1477.6628** | 739.3350 | 1460.6362 | 730.8217 | 1459.6522 | 730.3297 | E | **646.3559** | 323.6816 | 629.3293 | 315.1683 | 628.3453 | 314.6763 | 5 |
| 16 | **1548.6999** | 774.8536 | 1531.6733 | 766.3403 | 1530.6893 | 765.8483 | A | **517.3133** | 259.1603 | 500.2867 | 250.6470 | | | 4 |
| 17 | **1734.7792** | 867.8932 | **1717.7526** | 859.3800 | **1716.7686** | 858.8880 | W | **446.2762** | 223.6417 | 429.2496 | 215.1285 | | | 3 |
| 18 | **1847.8633** | 924.4353 | 1830.8367 | 915.9220 | 1829.8527 | 915.4300 | L | 260.1969 | 130.6021 | 243.1703 | 122.0888 | | | 2 |

| | | | | | | | | | | | | | | |
|---|---|---|---|---|---|---|---|---|---|---|---|---|---|---|
| 19 | | | | | | | K | 147.1128 | 74.0600 | 130.0863 | 65.5468 | | | 1 |

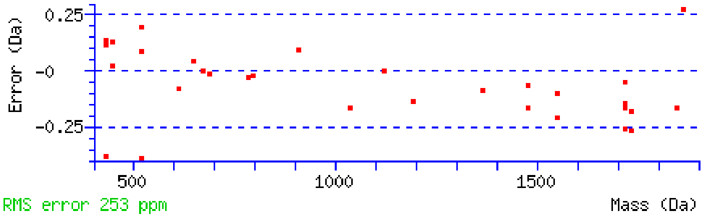


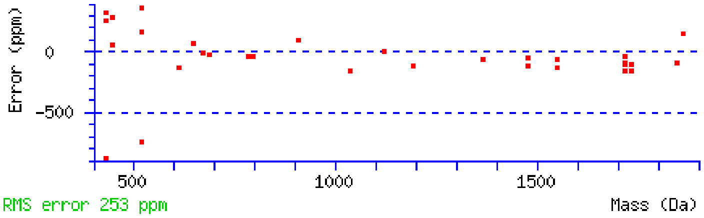


### All matches to this query

| Score | Mr(calc): | Delta | Sequence |
|---|---|---|---|
| 110.1 | 1992.9615 | 0.0053 | MGAGEALGNASQLFEAWLK |
| 43.2 | 1991.9775 | 0.9893 | MGAGEALGNASQLFEAWLK |
| 12.7 | 1992.9629 | 0.0038 | DLNTLREAQENINKQK |
| 12.0 | 1992.9686 | -0.0019 | KVTVTTYNTSITIHKK |
| 10.3 | 1992.9686 | -0.0019 | KVTVTTYNTSITIHKK |
| 7.1 | 1992.9856 | -0.0188 | VCKEIANIPSKNIWNK |
| 4.3 | 1992.9526 | 0.0142 | SATICLAYLMRTNRVK |
| 4.0 | 1992.9686 | -0.0019 | KVTVTTYNTSITIHKK |
| 3.3 | 1992.9686 | -0.0019 | KVTVTTYNTSITIHKK |
| 2.7 | 1991.9679 | 0.9988 | GSILQFMPFTTVSELVK |

---

Spectrum No: 42; Query: 1670; Rank: 1

---

## Peptide View

MS/MS Fragmentation of **YCGNSTPSSVDTSSNVASVK**
Found in **IPI00196620**, Tax_Id=10116 Gene_Symbol=Cubn Cubilin precursor

Match to Query 1670: 2059.892388 from(1030.953470,2+)
Title: 091008RatKidney_NoSalt_19.1023.1023.2.dta
Data file K:\NewmanPaper\Piliang\3SubProteomes\Piliang3SP\mgf5ppm\ERLIC_3SubProteomes5ppm.mgf

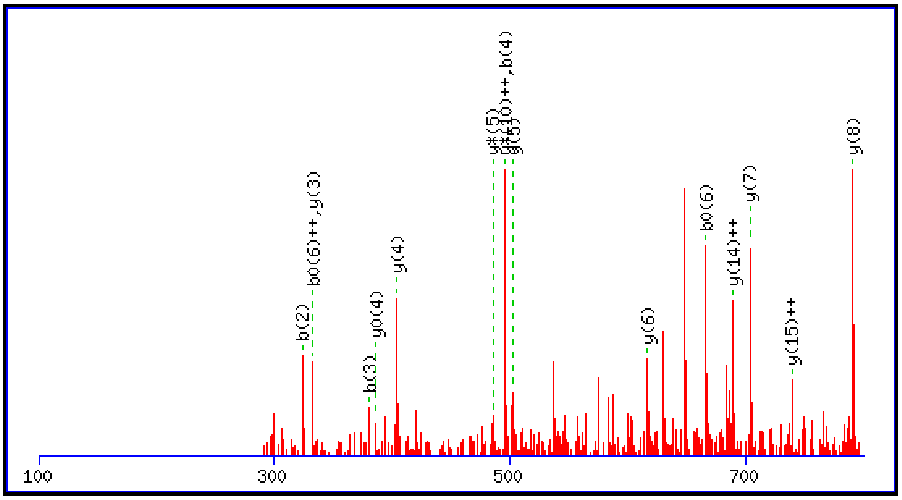

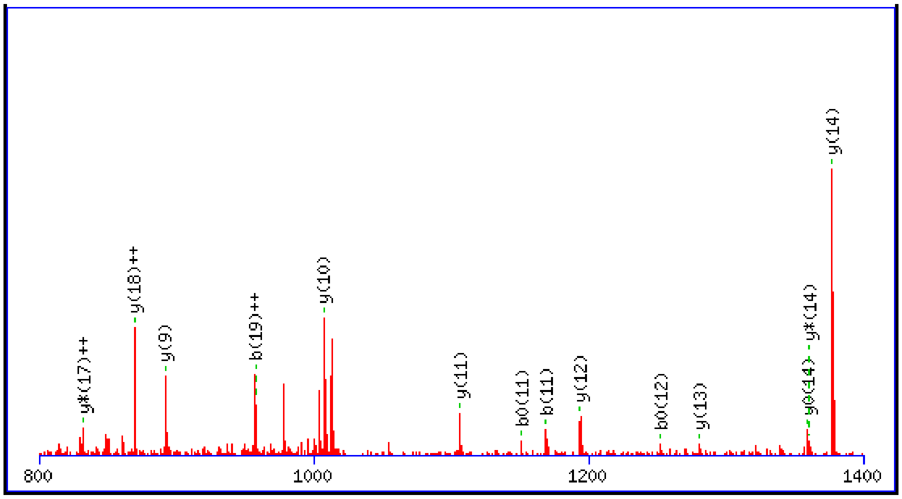


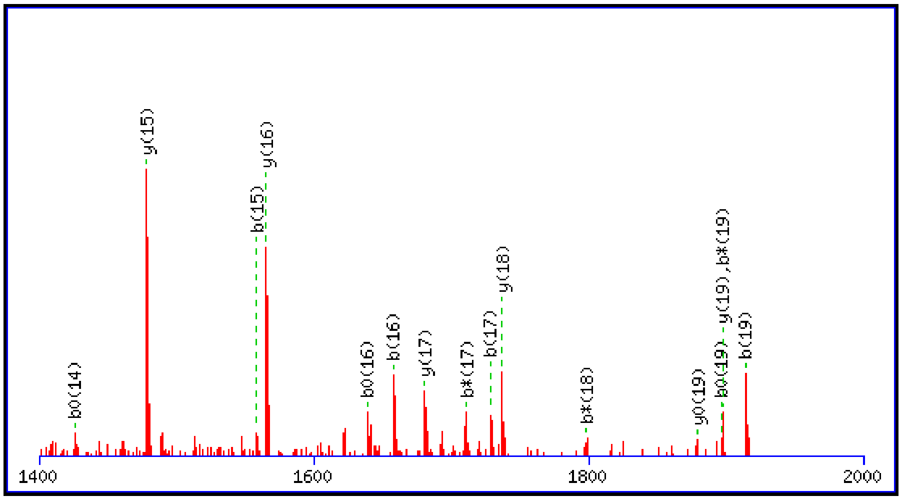


**Monoisotopic mass of neutral peptide Mr(calc):** 2059.9004
**Fixed modifications:** Carbamidomethyl (C)
**Variable modifications:**
**N4** : Deamidated_N (N)
**Ions Score:** 110 **Expect:** 2.6e-009
**Matches (Bold Red):** 46/210 fragment ions using 87 most intense peaks

| # | b | $b^{++}$ | b* | $b^{*++}$ | $b^{0}$ | $b^{0++}$ | Seq. | y | $y^{++}$ | y* | $y^{*++}$ | $y^{0}$ | $y^{0++}$ | # |
|---|---|---|---|---|---|---|---|---|---|---|---|---|---|---|
| 1 | 164.0706 | 82.5389 | | | | | Y | | | | | | | 20 |
| 2 | **324.1013** | 162.5543 | | | | | C | **1897.8444** | 949.4258 | 1880.8178 | 940.9125 | **1879.8338** | 940.4205 | 19 |
| 3 | **381.1227** | 191.0650 | | | | | G | **1737.8137** | **869.4105** | 1720.7872 | 860.8972 | 1719.8032 | 860.4052 | 18 |
| 4 | **496.1497** | 248.5785 | 479.1231 | 240.0652 | | | N | **1680.7923** | 840.8998 | 1663.7657 | **832.3865** | 1662.7817 | 831.8945 | 17 |
| 5 | 583.1817 | 292.0945 | 566.1551 | 283.5812 | 565.1711 | 283.0892 | S | **1565.7653** | 783.3863 | 1548.7388 | 774.8730 | 1547.7548 | 774.3810 | 16 |
| 6 | 684.2294 | 342.6183 | 667.2028 | 334.1050 | **666.2188** | **333.6130** | T | **1478.7333** | **739.8703** | 1461.7067 | 731.3570 | 1460.7227 | 730.8650 | 15 |
| 7 | 781.2821 | 391.1447 | 764.2556 | 382.6314 | 763.2716 | 382.1394 | P | **1377.6856** | **689.3464** | **1360.6591** | 680.8332 | **1359.6750** | 680.3412 | 14 |
| 8 | 868.3142 | 434.6607 | 851.2876 | 426.1474 | 850.3036 | 425.6554 | S | **1280.6328** | 640.8201 | 1263.6063 | 632.3068 | 1262.6223 | 631.8148 | 13 |
| 9 | 955.3462 | 478.1767 | 938.3196 | 469.6635 | 937.3356 | 469.1714 | S | **1193.6008** | 597.3040 | 1176.5743 | 588.7908 | 1175.5903 | 588.2988 | 12 |
| 10 | 1054.4146 | 527.7109 | 1037.3880 | 519.1977 | 1036.4040 | 518.7057 | V | **1106.5688** | 553.7880 | 1089.5422 | 545.2748 | 1088.5582 | 544.7828 | 11 |
| 11 | **1169.4415** | 585.2244 | 1152.4150 | 576.7111 | **1151.4310** | 576.2191 | D | **1007.5004** | 504.2538 | 990.4738 | **495.7406** | 989.4898 | 495.2485 | 10 |
| 12 | 1270.4892 | 635.7482 | 1253.4627 | 627.2350 | **1252.4787** | 626.7430 | T | **892.4734** | 446.7404 | 875.4469 | 438.2271 | 874.4629 | 437.7351 | 9 |
| 13 | 1357.5212 | 679.2643 | 1340.4947 | 670.7510 | 1339.5107 | 670.2590 | S | **791.4258** | 396.2165 | 774.3992 | 387.7032 | 773.4152 | 387.2112 | 8 |
| 14 | 1444.5533 | 722.7803 | 1427.5267 | 714.2670 | **1426.5427** | 713.7750 | S | **704.3937** | 352.7005 | 687.3672 | 344.1872 | 686.3832 | 343.6952 | 7 |
| 15 | **1558.5962** | 779.8017 | 1541.5697 | 771.2885 | 1540.5856 | 770.7965 | N | **617.3617** | 309.1845 | 600.3352 | 300.6712 | 599.3511 | 300.1792 | 6 |
| 16 | **1657.6646** | 829.3359 | 1640.6381 | 820.8227 | **1639.6540** | 820.3307 | V | **503.3188** | 252.1630 | **486.2922** | 243.6498 | 485.3082 | 243.1577 | 5 |
| 17 | **1728.7017** | 864.8545 | **1711.6752** | 856.3412 | 1710.6912 | 855.8492 | A | **404.2504** | 202.6288 | 387.2238 | 194.1155 | **386.2398** | 193.6235 | 4 |
| 18 | 1815.7338 | 908.3705 | **1798.7072** | 899.8572 | 1797.7232 | 899.3652 | S | **333.2132** | 167.1103 | 316.1867 | 158.5970 | 315.2027 | 158.1050 | 3 |
| | | | | | | | | | | | | | | |

| | | | | | | | | | | | | | |
|---|---|---|---|---|---|---|---|---|---|---|---|---|---|
| 19 | 1914.8022 | 957.9047 | 1897.7756 | 949.3914 | 1896.7916 | 948.8994 | V | 246.1812 | 123.5942 | 229.1547 | 115.0810 | | | 2 |
| 20 | | | | | | | K | 147.1128 | 74.0600 | 130.0863 | 65.5468 | | | 1 |

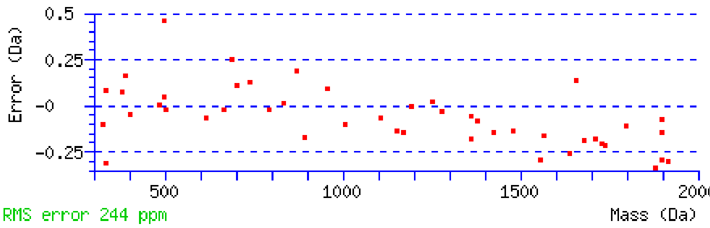


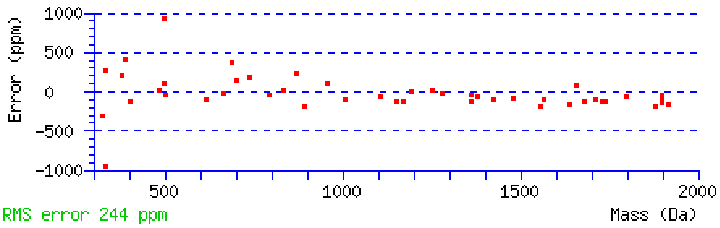


### All matches to this query

| Score | Mr(calc): | Delta | Sequence |
|---|---|---|---|
| 109.8 | 2059.9004 | -0.0080 | YCGNSTPSSVDTSSNVASVK |
| 72.0 | 2059.9004 | -0.0080 | YCGNSTPSSVDTSSNVASVK |
| 4.9 | 2059.8985 | -0.0061 | RSGNMGIPGTMNQISPLK |
| 4.1 | 2059.8985 | -0.0061 | RSGNMGIPGTMNQISPLK |
| 1.0 | 2059.8750 | 0.0174 | MTSSSQLLLXKPKRR |
| 0.4 | 2059.8951 | -0.0027 | LPLRSVSMNRGNGPSWK |

---

Spectrum No: 43; Query: 2450; Rank: 1

---

## Peptide View

MS/MS Fragmentation of **MCQLYPETFECVTNSSDILQAFR**
Found in **IPI00327697**, Tax_Id=10116 Gene_Symbol=Dpep1 Dipeptidase 1 precursor

Match to Query 2450: 2810.247608 from(1406.131080,2+)
Title: 100101RatKid_NS_deglyco_21.5660.5660.2.dta
Data file K:\NewmanPaper\Piliang\3SubProteomes\Piliang3SP\mgf5ppm\ERLIC_3SubProteomes5ppm.mgf

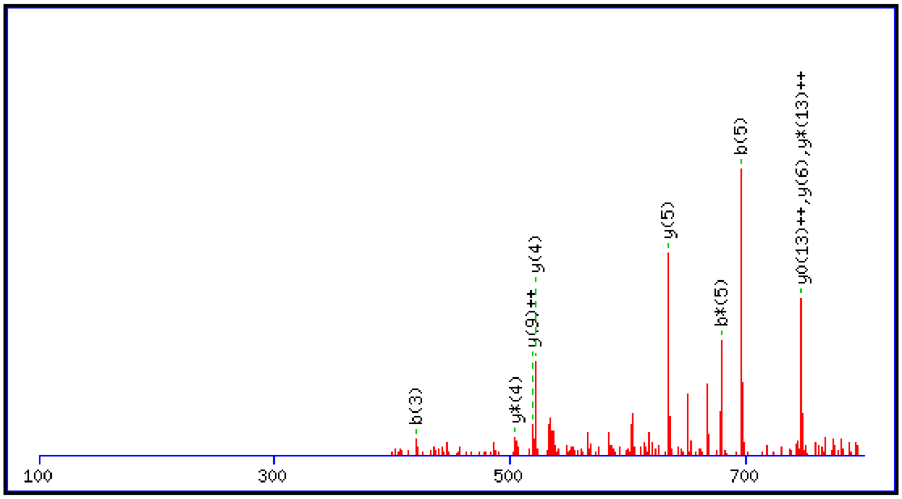

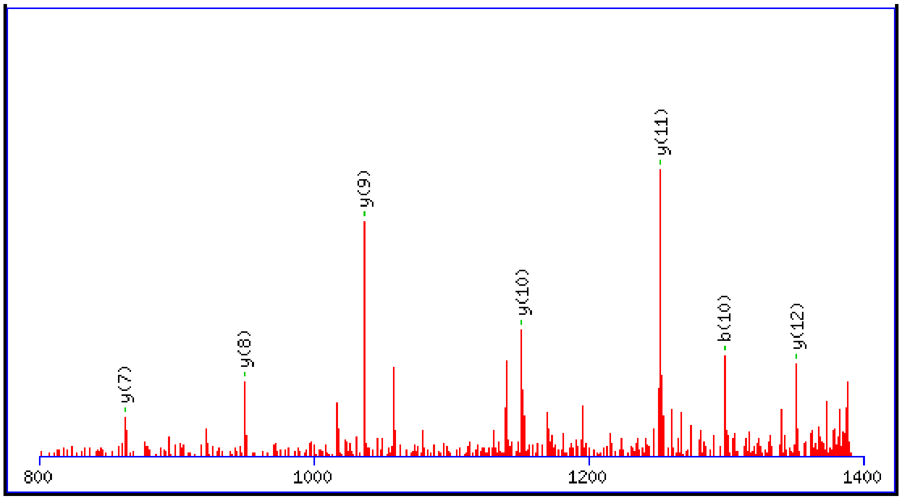


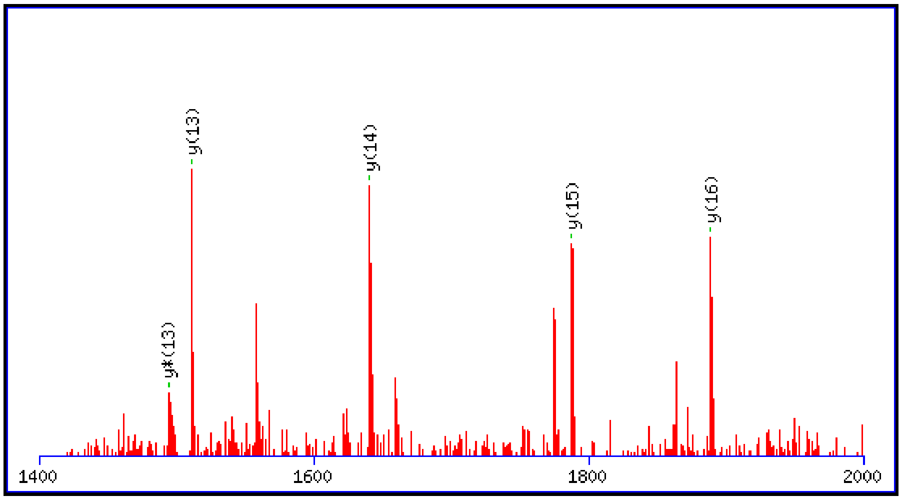


**Monoisotopic mass of neutral peptide Mr(calc):** 2809.2397
**Fixed modifications:** Carbamidomethyl (C)
**Variable modifications:**
**N14** : Deamidated_N (N)
**Ions Score:** 110 **Expect:** 3.8e-009
**Matches (Bold Red):** 22/236 fragment ions using 27 most intense peaks

| # | b | $b^{++}$ | b* | $b^{*++}$ | $b^{0}$ | $b^{0++}$ | Seq. | y | $y^{++}$ | y* | $y^{*++}$ | $y^{0}$ | $y^{0++}$ | # |
|---|---|---|---|---|---|---|---|---|---|---|---|---|---|---|
| 1 | 132.0478 | 66.5275 | | | | | M | | | | | | | 23 |
| 2 | 292.0784 | 146.5428 | | | | | C | 2679.2065 | 1340.1069 | 2662.1800 | 1331.5936 | 2661.1960 | 1331.1016 | 22 |
| 3 | **420.1370** | 210.5721 | 403.1104 | 202.0589 | | | Q | 2519.1759 | 1260.0916 | 2502.1493 | 1251.5783 | 2501.1653 | 1251.0863 | 21 |
| 4 | 533.2211 | 267.1142 | 516.1945 | 258.6009 | | | L | 2391.1173 | 1196.0623 | 2374.0907 | 1187.5490 | 2373.1067 | 1187.0570 | 20 |
| 5 | **696.2844** | 348.6458 | **679.2578** | 340.1326 | | | Y | 2278.0332 | 1139.5203 | 2261.0067 | 1131.0070 | 2260.0227 | 1130.5150 | 19 |
| 6 | 793.3371 | 397.1722 | 776.3106 | 388.6589 | | | P | 2114.9699 | 1057.9886 | 2097.9434 | 1049.4753 | 2096.9593 | 1048.9833 | 18 |
| 7 | 922.3797 | 461.6935 | 905.3532 | 453.1802 | 904.3692 | 452.6882 | E | 2017.9171 | 1009.4622 | 2000.8906 | 1000.9489 | 1999.9066 | 1000.4569 | 17 |
| 8 | 1023.4274 | 512.2173 | 1006.4009 | 503.7041 | 1005.4169 | 503.2121 | T | **1888.8745** | 944.9409 | 1871.8480 | 936.4276 | 1870.8640 | 935.9356 | 16 |
| 9 | 1170.4958 | 585.7516 | 1153.4693 | 577.2383 | 1152.4853 | 576.7463 | F | **1787.8269** | 894.4171 | 1770.8003 | 885.9038 | 1769.8163 | 885.4118 | 15 |
| 10 | **1299.5384** | 650.2729 | 1282.5119 | 641.7596 | 1281.5279 | 641.2676 | E | **1640.7585** | 820.8829 | 1623.7319 | 812.3696 | 1622.7479 | 811.8776 | 14 |
| 11 | 1459.5691 | 730.2882 | 1442.5425 | 721.7749 | 1441.5585 | 721.2829 | C | **1511.7159** | 756.3616 | **1494.6893** | **747.8483** | 1493.7053 | **747.3563** | 13 |
| 12 | 1558.6375 | 779.8224 | 1541.6109 | 771.3091 | 1540.6269 | 770.8171 | V | **1351.6852** | 676.3462 | 1334.6587 | 667.8330 | 1333.6746 | 667.3410 | 12 |
| 13 | 1659.6852 | 830.3462 | 1642.6586 | 821.8329 | 1641.6746 | 821.3409 | T | **1252.6168** | 626.8120 | 1235.5903 | 618.2988 | 1234.6062 | 617.8068 | 11 |
| 14 | 1774.7121 | 887.8597 | 1757.6856 | 879.3464 | 1756.7015 | 878.8544 | N | **1151.5691** | 576.2882 | 1134.5426 | 567.7749 | 1133.5586 | 567.2829 | 10 |
| 15 | 1861.7441 | 931.3757 | 1844.7176 | 922.8624 | 1843.7336 | 922.3704 | S | **1036.5422** | **518.7747** | 1019.5156 | 510.2615 | 1018.5316 | 509.7694 | 9 |
| 16 | 1948.7762 | 974.8917 | 1931.7496 | 966.3784 | 1930.7656 | 965.8864 | S | **949.5102** | 475.2587 | 932.4836 | 466.7454 | 931.4996 | 466.2534 | 8 |
| 17 | 2063.8031 | 1032.4052 | 2046.7766 | 1023.8919 | 2045.7925 | 1023.3999 | D | **862.4781** | 431.7427 | 845.4516 | 423.2294 | 844.4676 | 422.7374 | 7 |
| 18 | 2176.8872 | 1088.9472 | 2159.8606 | 1080.4339 | 2158.8766 | 1079.9419 | I | **747.4512** | 374.2292 | 730.4246 | 365.7160 | | | 6 |

| | | | | | | | | | | | | | | |
|---|---|---|---|---|---|---|---|---|---|---|---|---|---|---|
| **19** | 2289.9712 | 1145.4893 | 2272.9447 | 1136.9760 | 2271.9607 | 1136.4840 | **L** | **634.3671** | 317.6872 | 617.3406 | 309.1739 | | | **5** |
| **20** | 2418.0298 | 1209.5185 | 2401.0033 | 1201.0053 | 2400.0192 | 1200.5133 | **Q** | **521.2831** | 261.1452 | **504.2565** | 252.6319 | | | **4** |
| **21** | 2489.0669 | 1245.0371 | 2472.0404 | 1236.5238 | 2471.0564 | 1236.0318 | **A** | 393.2245 | 197.1159 | 376.1979 | 188.6026 | | | **3** |
| **22** | 2636.1353 | 1318.5713 | 2619.1088 | 1310.0580 | 2618.1248 | 1309.5660 | **F** | 322.1874 | 161.5973 | 305.1608 | 153.0840 | | | **2** |
| **23** | | | | | | | **R** | 175.1190 | 88.0631 | 158.0924 | 79.5498 | | | **1** |

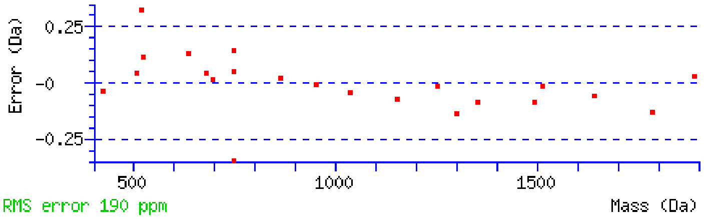


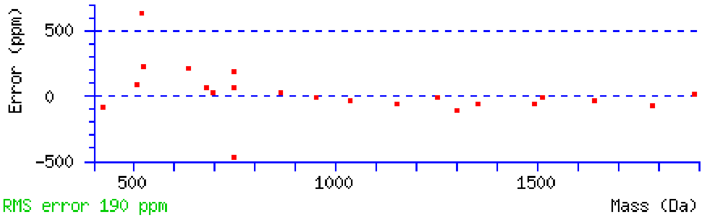


### All matches to this query

| Score | Mr(calc): | Delta | Sequence |
|---|---|---|---|
| 109.7 | 2809.2397 | 1.0079 | MCQLYPETFECVTNSSDILQAFR |
| 23.4 | 2808.2557 | 1.9919 | MCQLYPETFECVTNSSDILQAFR |
| 2.3 | 2809.2541 | 0.9935 | FRDIPNTSSMENPAPNKNPWDTFK |
| 2.0 | 2809.2477 | 0.9999 | QMAPVERLSQPDGEGVSSELTEENK |
| 0.1 | 2810.2533 | -0.0057 | HTTDVIYAAKMISESGSRMDVLAR |
| 0.1 | 2810.2533 | -0.0057 | HTTDVIYAAKMISESGSRMDVLAR |
| 0.1 | 2810.2533 | -0.0057 | HTTDVIYAAKMISESGSRMDVLAR |
| 0.1 | 2810.2533 | -0.0057 | HTTDVIYAAKMISESGSRMDVLAR |

---

Spectrum No: 44; Query: 2062; Rank: 1

---

## Peptide View

MS/MS Fragmentation of **NNLSFIETSALDSTNVEEAFK**
Found in **IPI00210381**, Tax_Id=10116 Gene_Symbol=Rab11b Ras-related protein Rab-11B

Match to Query 2062: 2329.107068 from(1165.560810,2+)
Title: 100101RatKid_NS_deglyco_13.6301.6301.2.dta
Data file K:\NewmanPaper\Piliang\3SubProteomes\Piliang3SP\mgf5ppm\ERLIC_3SubProteomes5ppm.mgf

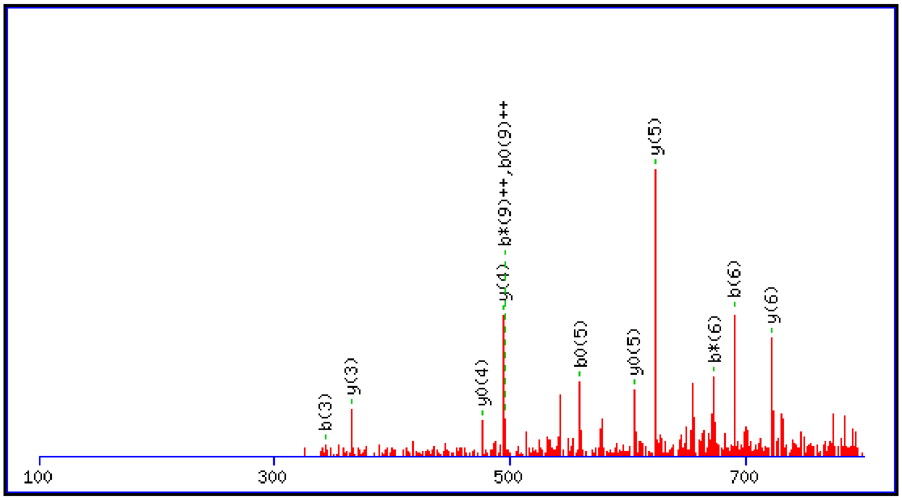

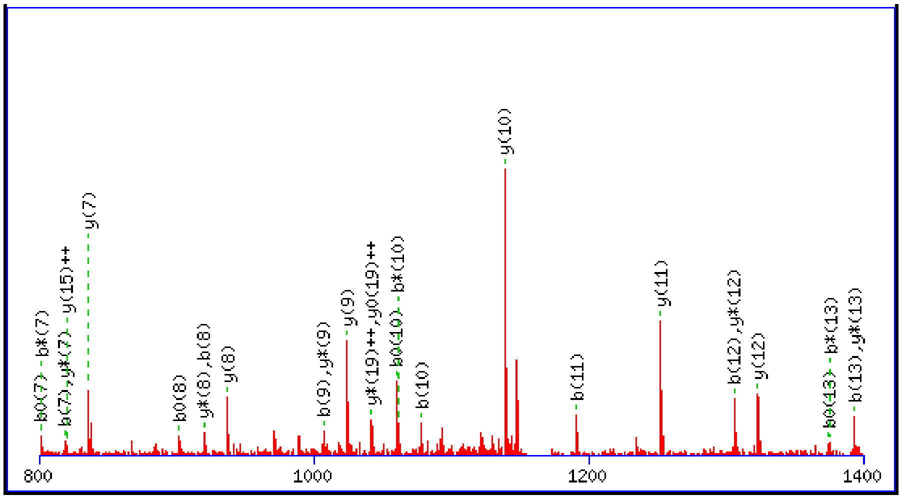


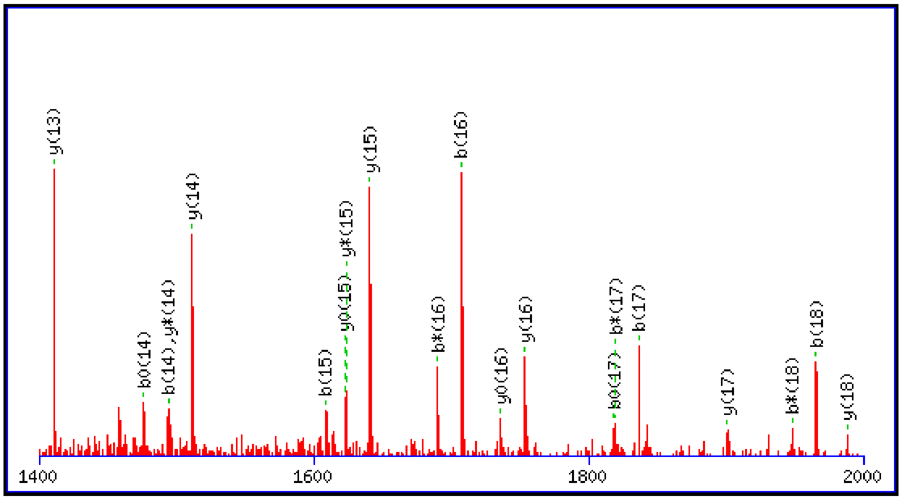


**Monoisotopic mass of neutral peptide Mr(calc):** 2329.0961
**Fixed modifications:** Carbamidomethyl (C)
**Variable modifications:**
**N2** : Deamidated_N (N)
**Ions Score:** 110 **Expect:** 3.1e-009
**Matches (Bold Red):** 60/228 fragment ions using 86 most intense peaks

| # | b | $b^{++}$ | b* | $b^{*++}$ | $b^0$ | $b^{0++}$ | Seq. | y | $y^{++}$ | y* | $y^{*++}$ | $y^0$ | $y^{0++}$ | # |
|---|---|---|---|---|---|---|---|---|---|---|---|---|---|---|
| 1 | 115.0502 | 58.0287 | 98.0237 | 49.5155 | | | N | | | | | | | 21 |
| 2 | 230.0771 | 115.5422 | 213.0506 | 107.0289 | | | N | 2216.0605 | 1108.5339 | 2199.0339 | 1100.0206 | 2198.0499 | 1099.5286 | 20 |
| 3 | **343.1612** | 172.0842 | 326.1347 | 163.5710 | | | L | 2101.0336 | 1051.0204 | 2084.0070 | **1042.5071** | 2083.0230 | **1042.0151** | 19 |
| 4 | 430.1932 | 215.6003 | 413.1667 | 207.0870 | 412.1827 | 206.5950 | S | **1987.9495** | 994.4784 | 1970.9229 | 985.9651 | 1969.9389 | 985.4731 | 18 |
| 5 | 577.2616 | 289.1345 | 560.2351 | 280.6212 | **559.2511** | 280.1292 | F | **1900.9175** | 950.9624 | 1883.8909 | 942.4491 | 1882.9069 | 941.9571 | 17 |
| 6 | **690.3457** | 345.6765 | **673.3192** | 337.1632 | 672.3351 | 336.6712 | I | **1753.8491** | 877.4282 | 1736.8225 | 868.9149 | **1735.8385** | 868.4229 | 16 |
| 7 | **819.3883** | 410.1978 | **802.3618** | 401.6845 | **801.3777** | 401.1925 | E | **1640.7650** | **820.8861** | **1623.7384** | 812.3729 | **1622.7544** | 811.8808 | 15 |
| 8 | **920.4360** | 460.7216 | 903.4094 | 452.2084 | **902.4254** | 451.7163 | T | **1511.7224** | 756.3648 | **1494.6958** | 747.8516 | 1493.7118 | 747.3596 | 14 |
| 9 | **1007.4680** | 504.2376 | 990.4415 | **495.7244** | 989.4574 | **495.2324** | S | **1410.6747** | 705.8410 | **1393.6482** | 697.3277 | 1392.6642 | 696.8357 | 13 |
| 10 | **1078.5051** | 539.7562 | **1061.4786** | 531.2429 | **1060.4946** | 530.7509 | A | **1323.6427** | 662.3250 | **1306.6161** | 653.8117 | 1305.6321 | 653.3197 | 12 |
| 11 | **1191.5892** | 596.2982 | 1174.5626 | 587.7850 | 1173.5786 | 587.2929 | L | **1252.6056** | 626.8064 | 1235.5790 | 618.2932 | 1234.5950 | 617.8011 | 11 |
| 12 | **1306.6161** | 653.8117 | 1289.5896 | 645.2984 | 1288.6056 | 644.8064 | D | **1139.5215** | 570.2644 | 1122.4950 | 561.7511 | 1121.5109 | 561.2591 | 10 |
| 13 | **1393.6482** | 697.3277 | **1376.6216** | 688.8144 | **1375.6376** | 688.3224 | S | **1024.4946** | 512.7509 | **1007.4680** | 504.2376 | 1006.4840 | 503.7456 | 9 |
| 14 | **1494.6958** | 747.8516 | 1477.6693 | 739.3383 | **1476.6853** | 738.8463 | T | **937.4625** | 469.2349 | **920.4360** | 460.7216 | 919.4520 | 460.2296 | 8 |
| 15 | **1608.7388** | 804.8730 | 1591.7122 | 796.3597 | 1590.7282 | 795.8677 | N | **836.4149** | 418.7111 | **819.3883** | 410.1978 | 818.4043 | 409.7058 | 7 |
| 16 | **1707.8072** | 854.4072 | **1690.7806** | 845.8940 | 1689.7966 | 845.4019 | V | **722.3719** | 361.6896 | 705.3454 | 353.1763 | 704.3614 | 352.6843 | 6 |
| 17 | **1836.8498** | 918.9285 | **1819.8232** | 910.4152 | **1818.8392** | 909.9232 | E | **623.3035** | 312.1554 | 606.2770 | 303.6421 | **605.2930** | 303.1501 | 5 |
| 18 | **1965.8924** | 983.4498 | **1948.8658** | 974.9365 | 1947.8818 | 974.4445 | E | **494.2609** | 247.6341 | 477.2344 | 239.1208 | **476.2504** | 238.6288 | 4 |

| | | | | | | | | | | | | | |
|---|---|---|---|---|---|---|---|---|---|---|---|---|---|
| **19** | 2036.9295 | 1018.9684 | 2019.9029 | 1010.4551 | 2018.9189 | 1009.9631 | **A** | **365.2183** | 183.1128 | 348.1918 | 174.5995 | | | **3** |
| **20** | 2183.9979 | 1092.5026 | 2166.9713 | 1083.9893 | 2165.9873 | 1083.4973 | **F** | 294.1812 | 147.5942 | 277.1547 | 139.0810 | | | **2** |
| **21** | | | | | | | **K** | 147.1128 | 74.0600 | 130.0863 | 65.5468 | | | **1** |

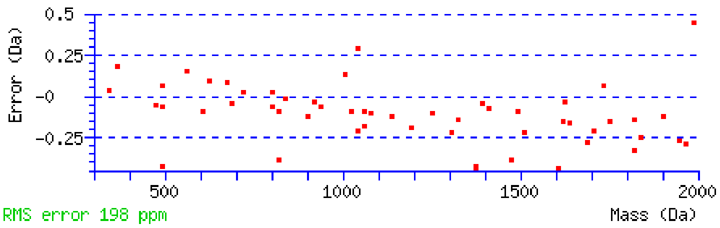


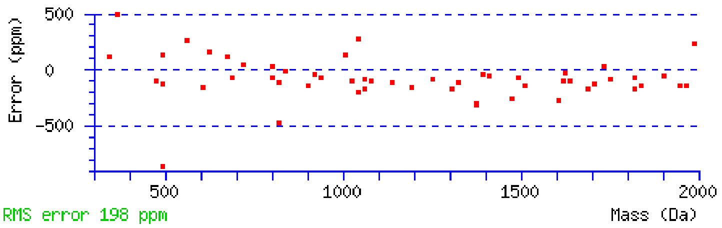


**All matches to this query**

| Score | Mr(calc): | Delta | Sequence |
|---|---|---|---|
| 109.6 | 2329.0961 | 0.0110 | NNLSFIETSALDSTNVEEAFK |
| 109.6 | 2329.0961 | 0.0110 | NNLSFIETSALDSTNVEEAFK |
| 80.5 | 2328.1121 | 0.9950 | NNLSFIETSALDSTNVEEAFK |
| 76.8 | 2329.0961 | 0.0110 | NNLSFIETSALDSTNVEEAFK |
| 4.2 | 2329.1082 | -0.0012 | DIGNSAFITFICKGNCEILR |
| 0.1 | 2327.1059 | 2.0012 | IRNIFHQNDIIYSLSSNGR |
| 0.1 | 2328.0899 | 1.0171 | IRNIFHQNDIIYSLSSNGR |
| 0.1 | 2327.1059 | 2.0012 | IRNIFHQNDIIYSLSSNGR |
| 0.1 | 2328.0899 | 1.0171 | IRNIFHQNDIIYSLSSNGR |
| 0.1 | 2327.1059 | 2.0012 | IRNIFHQNDIIYSLSSNGR |

---

Spectrum No: 45; Query: 1741; Rank: 1

---

## Peptide View

MS/MS Fragmentation of **TTLVDNNTWNNSHIALVGK**
Found in **IPI00382320**, Tax_Id=10116 Gene_Symbol=RGD1311563 93 kDa protein

Match to Query 1741: 2097.058928 from(1049.536740,2+)
Title: 100101RatKid_NS_deglyco_12.3324.3324.2.dta
Data file K:\NewmanPaper\Piliang\3SubProteomes\Piliang3SP\mgf5ppm\ERLIC_3SubProteomes5ppm.mgf

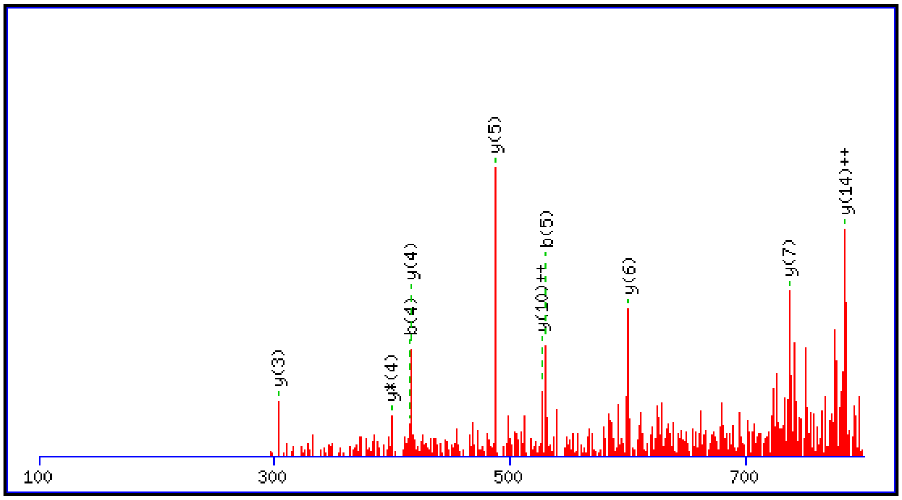

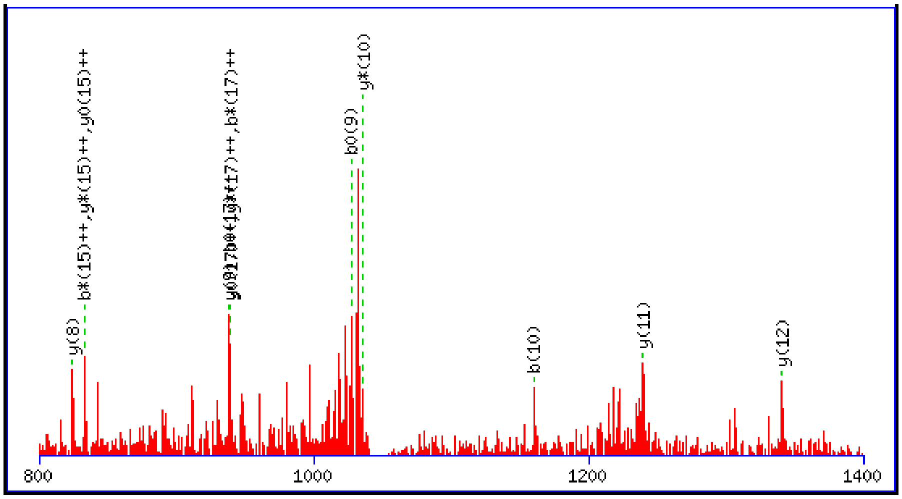


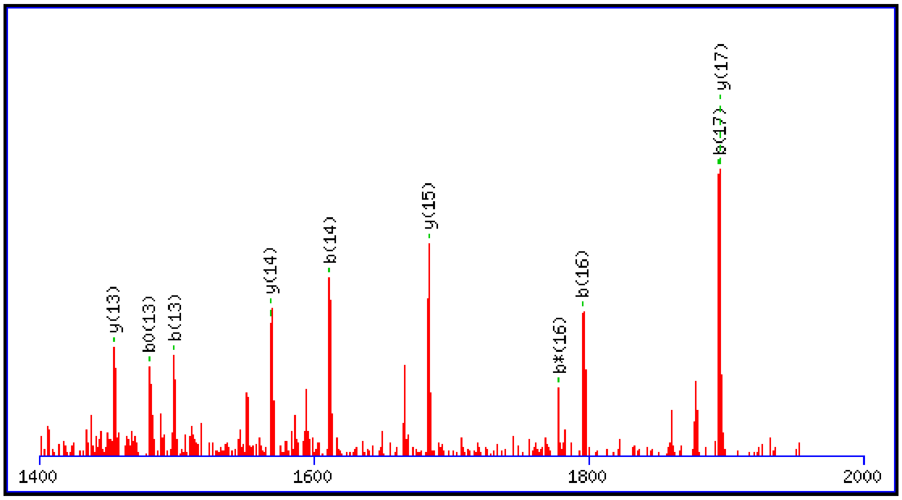


**Monoisotopic mass of neutral peptide Mr(calc):** 2097.0491
**Fixed modifications:** Carbamidomethyl (C)
**Variable modifications:**
**N10** : Deamidated_N (N)
**Ions Score:** 109 **Expect:** 2.2e-009
**Matches (Bold Red):** 34/192 fragment ions using 46 most intense peaks

| # | b | $b^{++}$ | b* | $b^{*++}$ | $b^0$ | $b^{0++}$ | Seq. | y | $y^{++}$ | y* | $y^{*++}$ | $y^0$ | $y^{0++}$ | # |
|---|---|---|---|---|---|---|---|---|---|---|---|---|---|---|
| 1 | 102.0550 | 51.5311 | | | 84.0444 | 42.5258 | T | | | | | | | 19 |
| 2 | 203.1026 | 102.0550 | | | 185.0921 | 93.0497 | T | 1997.0087 | 999.0080 | 1979.9821 | 990.4947 | 1978.9981 | 990.0027 | 18 |
| 3 | 316.1867 | 158.5970 | | | 298.1761 | 149.5917 | L | **1895.9610** | 948.4841 | 1878.9344 | **939.9709** | 1877.9504 | **939.4789** | 17 |
| 4 | **415.2551** | 208.1312 | | | 397.2445 | 199.1259 | V | 1782.8769 | 891.9421 | 1765.8504 | 883.4288 | 1764.8664 | 882.9368 | 16 |
| 5 | **530.2821** | 265.6447 | | | 512.2715 | 256.6394 | D | **1683.8085** | 842.4079 | 1666.7820 | **833.8946** | 1665.7980 | **833.4026** | 15 |
| 6 | 644.3250 | 322.6661 | 627.2984 | 314.1529 | 626.3144 | 313.6608 | N | **1568.7816** | **784.8944** | 1551.7550 | 776.3812 | 1550.7710 | 775.8891 | 14 |
| 7 | 758.3679 | 379.6876 | 741.3414 | 371.1743 | 740.3573 | 370.6823 | N | **1454.7386** | 727.8730 | 1437.7121 | 719.3597 | 1436.7281 | 718.8677 | 13 |
| 8 | 859.4156 | 430.2114 | 842.3890 | 421.6982 | 841.4050 | 421.2061 | T | **1340.6957** | 670.8515 | 1323.6692 | 662.3382 | 1322.6852 | 661.8462 | 12 |
| 9 | 1045.4949 | 523.2511 | 1028.4684 | 514.7378 | **1027.4843** | 514.2458 | W | **1239.6480** | 620.3277 | 1222.6215 | 611.8144 | 1221.6375 | 611.3224 | 11 |
| 10 | **1160.5218** | 580.7646 | 1143.4953 | 572.2513 | 1142.5113 | 571.7593 | N | 1053.5687 | **527.2880** | **1036.5422** | 518.7747 | 1035.5582 | 518.2827 | 10 |
| 11 | 1274.5648 | 637.7860 | 1257.5382 | 629.2727 | 1256.5542 | 628.7807 | N | **938.5418** | 469.7745 | 921.5152 | 461.2613 | 920.5312 | 460.7693 | 9 |
| 12 | 1361.5968 | 681.3020 | 1344.5702 | 672.7888 | 1343.5862 | 672.2968 | S | **824.4989** | 412.7531 | 807.4723 | 404.2398 | 806.4883 | 403.7478 | 8 |
| 13 | **1498.6557** | 749.8315 | 1481.6292 | 741.3182 | **1480.6451** | 740.8262 | H | **737.4668** | 369.2371 | 720.4403 | 360.7238 | | | 7 |
| 14 | **1611.7398** | 806.3735 | 1594.7132 | 797.8602 | 1593.7292 | 797.3682 | I | **600.4079** | 300.7076 | 583.3814 | 292.1943 | | | 6 |
| 15 | 1682.7769 | 841.8921 | 1665.7503 | **833.3788** | 1664.7663 | 832.8868 | A | **487.3239** | 244.1656 | 470.2973 | 235.6523 | | | 5 |
| 16 | **1795.8609** | 898.4341 | **1778.8344** | 889.9208 | 1777.8504 | 889.4288 | L | **416.2867** | 208.6470 | **399.2602** | 200.1337 | | | 4 |
| 17 | **1894.9294** | 947.9683 | 1877.9028 | **939.4550** | 1876.9188 | **938.9630** | V | **303.2027** | 152.1050 | 286.1761 | 143.5917 | | | 3 |
| 18 | 1951.9508 | 976.4790 | 1934.9243 | 967.9658 | 1933.9403 | 967.4738 | G | 204.1343 | 102.5708 | 187.1077 | 94.0575 | | | 2 |

| | | | | | | | | | | | | | | |
|---|---|---|---|---|---|---|---|---|---|---|---|---|---|---|
| 19 | | | | | | | K | 147.1128 | 74.0600 | 130.0863 | 65.5468 | | | 1 |

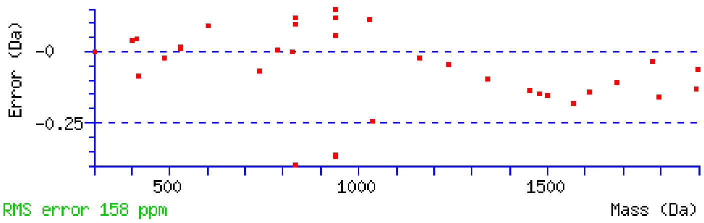


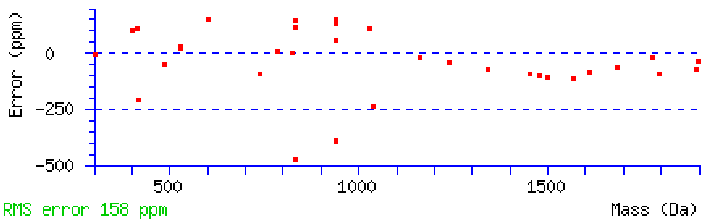


### All matches to this query

| Score | Mr(calc): | Delta | Sequence |
|---|---|---|---|
| 108.9 | 2097.0491 | 0.0099 | TTLVDNNTWNNSHIALVGK |
| 105.8 | 2097.0491 | 0.0099 | TTLVDNNTWNNSHIALVGK |
| 74.4 | 2097.0491 | 0.0099 | TTLVDNNTWNNSHIALVGK |
| 61.6 | 2097.0491 | 0.0099 | TTLVDNNTWNNSHIALVGK |
| 21.2 | 2096.0651 | 0.9939 | TTLVDNNTWNNSHIALVGK |
| 10.9 | 2097.0701 | -0.0112 | QLETQNNNLQAQILALQR |
| 5.1 | 2097.0531 | 0.0058 | WDNASGDWDLILLLVPDR |
| 3.1 | 2097.0660 | -0.0071 | FNQPLDLSLFSLVGSPRK |
| 2.6 | 2097.0660 | -0.0071 | FNQPLDLSLFSLVGSPRK |

---

Spectrum No: 46; Query: 2190; Rank: 1

---

## Peptide View

MS/MS Fragmentation of **SLGEVNFTATAEALQSPELCGNK**
Found in **IPI00326140**, Tax_Id=10116 Gene_Symbol=Pzp Alpha-1-macroglobulin precursor

Match to Query 2190: 2436.156668 from(1219.085610,2+)
Title: 100101RatKid_NS_deglyco_21.4347.4347.2.dta
Data file K:\NewmanPaper\Piliang\3SubProteomes\Piliang3SP\mgf5ppm\ERLIC_3SubProteomes5ppm.mgf

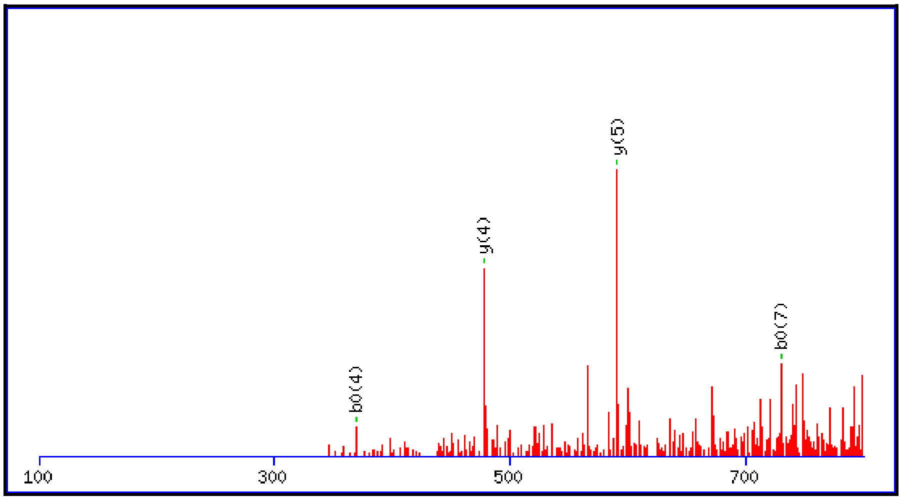

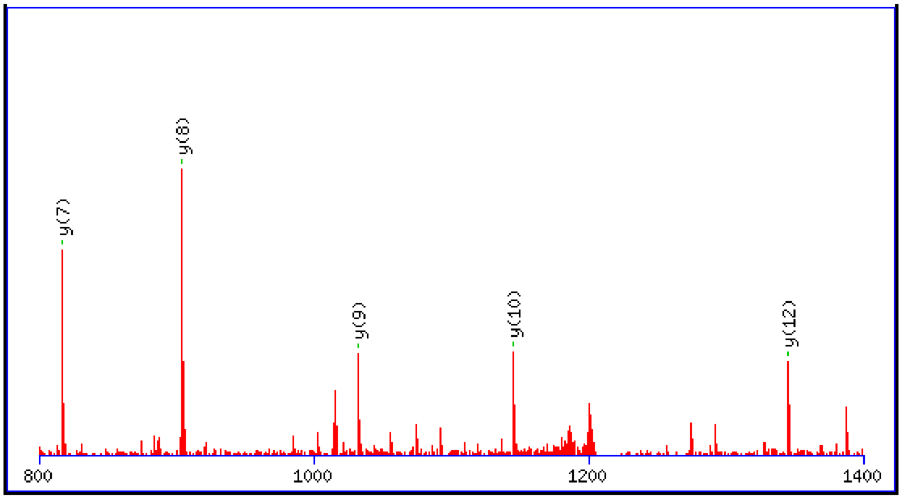


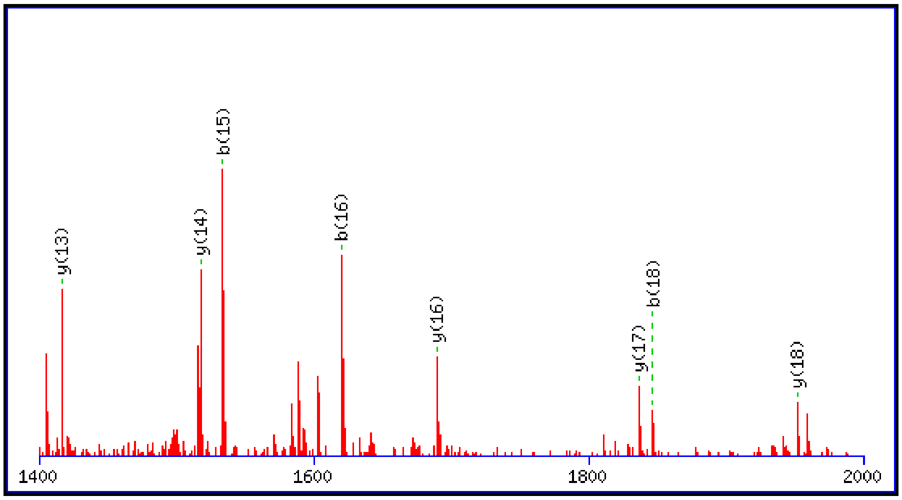


**Monoisotopic mass of neutral peptide Mr(calc):** 2436.1478
**Fixed modifications:** Carbamidomethyl (C)
**Variable modifications:**
**N6** : Deamidated_N (N)
**Ions Score:** 109 **Expect:** 3.8e-009
**Matches (Bold Red):** 17/244 fragment ions using 18 most intense peaks

| # | b | $b^{++}$ | b* | $b^{*++}$ | $b^0$ | $b^{0++}$ | Seq. | y | $y^{++}$ | y* | $y^{*++}$ | $y^0$ | $y^{0++}$ | # |
|---|---|---|---|---|---|---|---|---|---|---|---|---|---|---|
| 1 | 88.0393 | 44.5233 | | | 70.0287 | 35.5180 | S | | | | | | | 23 |
| 2 | 201.1234 | 101.0653 | | | 183.1128 | 92.0600 | L | 2350.1231 | 1175.5652 | 2333.0966 | 1167.0519 | 2332.1125 | 1166.5599 | 22 |
| 3 | 258.1448 | 129.5761 | | | 240.1343 | 120.5708 | G | 2237.0390 | 1119.0232 | 2220.0125 | 1110.5099 | 2219.0285 | 1110.0179 | 21 |
| 4 | 387.1874 | 194.0974 | | | **369.1769** | 185.0921 | E | 2180.0176 | 1090.5124 | 2162.9910 | 1081.9992 | 2162.0070 | 1081.5071 | 20 |
| 5 | 486.2558 | 243.6316 | | | 468.2453 | 234.6263 | V | 2050.9750 | 1025.9911 | 2033.9484 | 1017.4779 | 2032.9644 | 1016.9859 | 19 |
| 6 | 601.2828 | 301.1450 | 584.2562 | 292.6318 | 583.2722 | 292.1397 | N | **1951.9066** | 976.4569 | 1934.8800 | 967.9437 | 1933.8960 | 967.4516 | 18 |
| 7 | 748.3512 | 374.6792 | 731.3246 | 366.1660 | **730.3406** | 365.6739 | F | **1836.8796** | 918.9435 | 1819.8531 | 910.4302 | 1818.8691 | 909.9382 | 17 |
| 8 | 849.3989 | 425.2031 | 832.3723 | 416.6898 | 831.3883 | 416.1978 | T | **1689.8112** | 845.4093 | 1672.7847 | 836.8960 | 1671.8007 | 836.4040 | 16 |
| 9 | 920.4360 | 460.7216 | 903.4094 | 452.2084 | 902.4254 | 451.7163 | A | 1588.7635 | 794.8854 | 1571.7370 | 786.3721 | 1570.7530 | 785.8801 | 15 |
| 10 | 1021.4837 | 511.2455 | 1004.4571 | 502.7322 | 1003.4731 | 502.2402 | T | **1517.7264** | 759.3669 | 1500.6999 | 750.8536 | 1499.7159 | 750.3616 | 14 |
| 11 | 1092.5208 | 546.7640 | 1075.4942 | 538.2508 | 1074.5102 | 537.7587 | A | **1416.6788** | 708.8430 | 1399.6522 | 700.3297 | 1398.6682 | 699.8377 | 13 |
| 12 | 1221.5634 | 611.2853 | 1204.5368 | 602.7720 | 1203.5528 | 602.2800 | E | **1345.6416** | 673.3245 | 1328.6151 | 664.8112 | 1327.6311 | 664.3192 | 12 |
| 13 | 1292.6005 | 646.8039 | 1275.5739 | 638.2906 | 1274.5899 | 637.7986 | A | 1216.5990 | 608.8032 | 1199.5725 | 600.2899 | 1198.5885 | 599.7979 | 11 |
| 14 | 1405.6845 | 703.3459 | 1388.6580 | 694.8326 | 1387.6740 | 694.3406 | L | **1145.5619** | 573.2846 | 1128.5354 | 564.7713 | 1127.5514 | 564.2793 | 10 |
| 15 | **1533.7431** | 767.3752 | 1516.7166 | 758.8619 | 1515.7326 | 758.3699 | Q | **1032.4779** | 516.7426 | 1015.4513 | 508.2293 | 1014.4673 | 507.7373 | 9 |
| 16 | **1620.7752** | 810.8912 | 1603.7486 | 802.3779 | 1602.7646 | 801.8859 | S | **904.4193** | 452.7133 | 887.3927 | 444.2000 | 886.4087 | 443.7080 | 8 |
| 17 | 1717.8279 | 859.4176 | 1700.8014 | 850.9043 | 1699.8174 | 850.4123 | P | **817.3873** | 409.1973 | 800.3607 | 400.6840 | 799.3767 | 400.1920 | 7 |
| 18 | **1846.8705** | 923.9389 | 1829.8440 | 915.4256 | 1828.8599 | 914.9336 | E | 720.3345 | 360.6709 | 703.3080 | 352.1576 | 702.3239 | 351.6656 | 6 |

| | | | | | | | | | | | | | |
|---|---|---|---|---|---|---|---|---|---|---|---|---|---|
| **19** | 1959.9546 | 980.4809 | 1942.9280 | 971.9677 | 1941.9440 | 971.4756 | **L** | **591.2919** | 296.1496 | 574.2654 | 287.6363 | | | **5** |
| **20** | 2119.9852 | 1060.4962 | 2102.9587 | 1051.9830 | 2101.9747 | 1051.4910 | **C** | **478.2078** | 239.6076 | 461.1813 | 231.0943 | | | **4** |
| **21** | 2177.0067 | 1089.0070 | 2159.9801 | 1080.4937 | 2158.9961 | 1080.0017 | **G** | 318.1772 | 159.5922 | 301.1506 | 151.0790 | | | **3** |
| **22** | 2291.0496 | 1146.0284 | 2274.0231 | 1137.5152 | 2273.0390 | 1137.0232 | **N** | 261.1557 | 131.0815 | 244.1292 | 122.5682 | | | **2** |
| **23** | | | | | | | **K** | 147.1128 | 74.0600 | 130.0863 | 65.5468 | | | **1** |

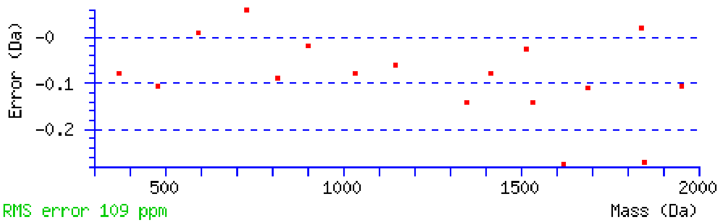


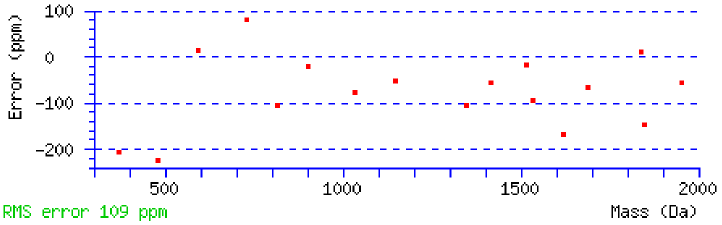


### All matches to this query

| Score | Mr(calc): | Delta | Sequence |
|---|---|---|---|
| 108.7 | 2436.1478 | 0.0088 | SLGEVNFTATAEALQSPELCGNK |
| 64.0 | 2435.1638 | 0.9928 | SLGEVNFTATAEALQSPELCGNK |
| 29.8 | 2436.1478 | 0.0088 | SLGEVNFTATAEALQSPELCGNK |
| 10.7 | 2436.1394 | 0.0173 | VTSATQLNGRQAQPSSKPASGNR |
| 10.7 | 2436.1394 | 0.0173 | VTSATQLNGRQAQPSSKPASGNR |
| 10.7 | 2436.1394 | 0.0173 | VTSATQLNGRQAQPSSKPASGNR |
| 3.9 | 2436.1378 | 0.0188 | LSSGTKWIIEQNLSQLPYAK |
| 3.9 | 2436.1378 | 0.0188 | LSSGTKWIIEQNLSQLPYAK |
| 3.9 | 2436.1378 | 0.0188 | LSSGTKWIIEQNLSQLPYAK |
| 3.7 | 2434.1273 | 2.0293 | EMQRNSVCLAFVSIIISDEK |

---

Spectrum No: 47; Query: 2436; Rank: 1

---

## Peptide View

MS/MS Fragmentation of **DLVVGLYSQWMNNSDNNPIHPNLR**
Found in **IPI00230862**, Tax_Id=10116 Gene_Symbol=Anpep Aminopeptidase N

Match to Query 2436: 2796.332802 from(933.118210,3+)
Title: 100101RatKid_NS_deglyco_19.5135.5135.3.dta
Data file K:\NewmanPaper\Piliang\3SubProteomes\Piliang3SP\mgf5ppm\ERLIC_3SubProteomes5ppm.mgf

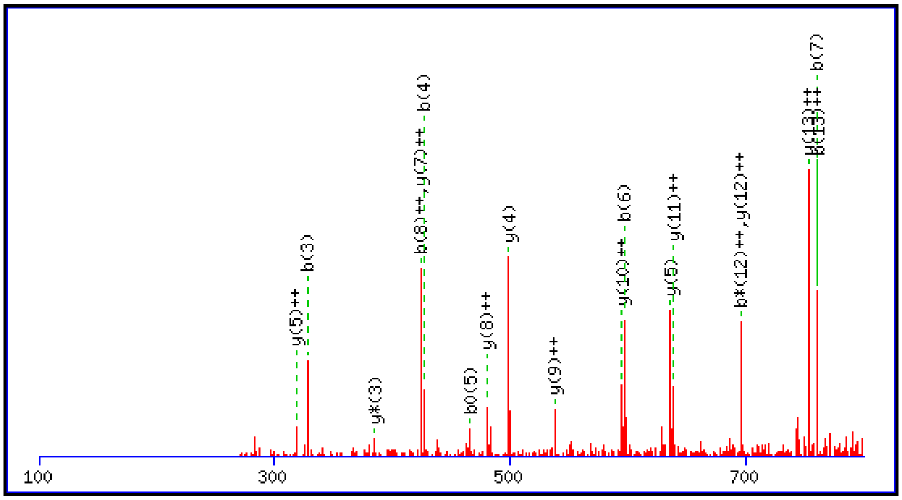

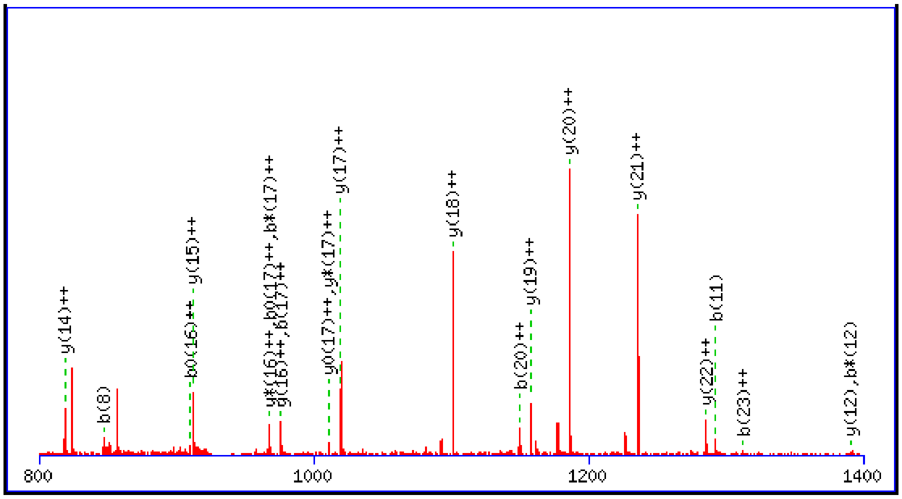


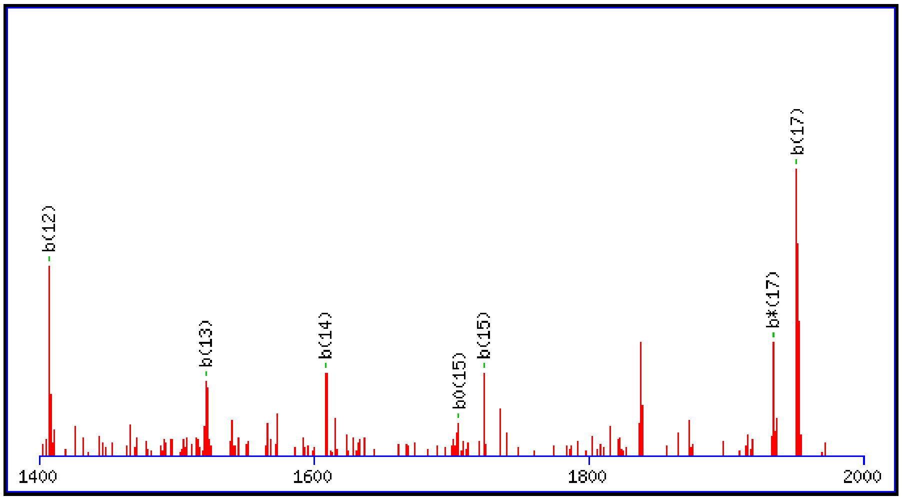


**Monoisotopic mass of neutral peptide Mr(calc):** 2796.3289
**Fixed modifications:** Carbamidomethyl (C)
**Variable modifications:**
**N12** : Deamidated_N (N)
**Ions Score:** 108 **Expect:** 4.5e-009
**Matches (Bold Red):** 48/242 fragment ions using 74 most intense peaks

| # | b | b++ | b* | b*++ | b0 | b0++ | Seq. | y | y++ | y* | y*++ | y0 | y0++ | # |
|---|---|---|---|---|---|---|---|---|---|---|---|---|---|---|
| 1 | 116.0342 | 58.5207 | | | 98.0237 | 49.5155 | D | | | | | | | 24 |
| 2 | 229.1183 | 115.0628 | | | 211.1077 | 106.0575 | L | 2682.3093 | 1341.6583 | 2665.2827 | 1333.1450 | 2664.2987 | 1332.6530 | 23 |
| 3 | **328.1867** | 164.5970 | | | 310.1761 | 155.5917 | V | 2569.2252 | **1285.1162** | 2552.1987 | 1276.6030 | 2551.2147 | 1276.1110 | 22 |
| 4 | **427.2551** | 214.1312 | | | 409.2445 | 205.1259 | V | 2470.1568 | **1235.5820** | 2453.1303 | 1227.0688 | 2452.1462 | 1226.5768 | 21 |
| 5 | 484.2766 | 242.6419 | | | **466.2660** | 233.6366 | G | 2371.0884 | **1186.0478** | 2354.0618 | 1177.5346 | 2353.0778 | 1177.0426 | 20 |
| 6 | **597.3606** | 299.1840 | | | 579.3501 | 290.1787 | L | 2314.0669 | **1157.5371** | 2297.0404 | 1149.0238 | 2296.0564 | 1148.5318 | 19 |
| 7 | **760.4240** | 380.7156 | | | 742.4134 | 371.7103 | Y | 2200.9829 | **1100.9951** | 2183.9563 | 1092.4818 | 2182.9723 | 1091.9898 | 18 |
| 8 | **847.4560** | **424.2316** | | | 829.4454 | 415.2264 | S | 2037.9195 | **1019.4634** | 2020.8930 | **1010.9501** | 2019.9090 | **1010.4581** | 17 |
| 9 | 975.5146 | 488.2609 | 958.4880 | 479.7477 | 957.5040 | 479.2556 | Q | 1950.8875 | **975.9474** | 1933.8610 | **967.4341** | 1932.8769 | 966.9421 | 16 |
| 10 | 1161.5939 | 581.3006 | 1144.5673 | 572.7873 | 1143.5833 | 572.2953 | W | 1822.8289 | **911.9181** | 1805.8024 | 903.4048 | 1804.8184 | 902.9128 | 15 |
| 11 | **1292.6344** | 646.8208 | 1275.6078 | 638.3075 | 1274.6238 | 637.8155 | M | 1636.7496 | **818.8784** | 1619.7231 | 810.3652 | 1618.7391 | 809.8732 | 14 |
| 12 | **1407.6613** | 704.3343 | **1390.6348** | **695.8210** | 1389.6507 | 695.3290 | N | 1505.7091 | **753.3582** | 1488.6826 | 744.8449 | 1487.6986 | 744.3529 | 13 |
| 13 | **1521.7042** | **761.3558** | 1504.6777 | 752.8425 | 1503.6937 | 752.3505 | N | **1390.6822** | **695.8447** | 1373.6557 | 687.3315 | 1372.6716 | 686.8395 | 12 |
| 14 | **1608.7363** | 804.8718 | 1591.7097 | 796.3585 | 1590.7257 | 795.8665 | S | 1276.6393 | **638.8233** | 1259.6127 | 630.3100 | 1258.6287 | 629.8180 | 11 |
| 15 | **1723.7632** | 862.3852 | 1706.7367 | 853.8720 | **1705.7526** | 853.3800 | D | 1189.6072 | **595.3073** | 1172.5807 | 586.7940 | 1171.5967 | 586.3020 | 10 |
| 16 | 1837.8061 | 919.4067 | 1820.7796 | 910.8934 | 1819.7956 | **910.4014** | N | 1074.5803 | **537.7938** | 1057.5538 | 529.2805 | | | 9 |
| 17 | **1951.8491** | **976.4282** | **1934.8225** | **967.9149** | 1933.8385 | **967.4229** | N | 960.5374 | **480.7723** | 943.5108 | 472.2591 | | | 8 |
| 18 | 2048.9018 | 1024.9545 | 2031.8753 | 1016.4413 | 2030.8913 | 1015.9493 | P | 846.4944 | **423.7509** | 829.4679 | 415.2376 | | | 7 |

| | | | | | | | | | | | | | | |
|---|---|---|---|---|---|---|---|---|---|---|---|---|---|---|
| **19** | 2161.9859 | 1081.4966 | 2144.9593 | 1072.9833 | 2143.9753 | 1072.4913 | **I** | 749.4417 | 375.2245 | 732.4151 | 366.7112 | | | **6** |
| **20** | 2299.0448 | **1150.0260** | 2282.0183 | 1141.5128 | 2281.0342 | 1141.0208 | **H** | **636.3576** | **318.6824** | 619.3311 | 310.1692 | | | **5** |
| **21** | 2396.0976 | 1198.5524 | 2379.0710 | 1190.0391 | 2378.0870 | 1189.5471 | **P** | **499.2987** | 250.1530 | 482.2722 | 241.6397 | | | **4** |
| **22** | 2510.1405 | 1255.5739 | 2493.1139 | 1247.0606 | 2492.1299 | 1246.5686 | **N** | 402.2459 | 201.6266 | **385.2194** | 193.1133 | | | **3** |
| **23** | 2623.2246 | **1312.1159** | 2606.1980 | 1303.6026 | 2605.2140 | 1303.1106 | **L** | 288.2030 | 144.6051 | 271.1765 | 136.0919 | | | **2** |
| **24** | | | | | | | **R** | 175.1190 | 88.0631 | 158.0924 | 79.5498 | | | **1** |

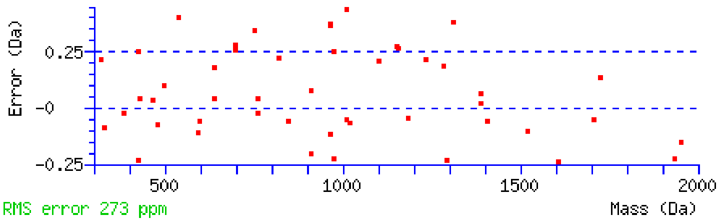


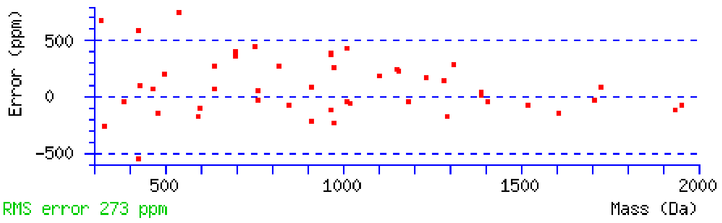


**All matches to this query**

| Score | Mr(calc): | Delta | Sequence |
|---|---|---|---|
| 108.3 | 2796.3289 | 0.0039 | DLVVGLYSQWMNNSDNNPIHPNLR |
| 108.3 | 2796.3289 | 0.0039 | DLVVGLYSQWMNNSDNNPIHPNLR |
| 91.1 | 2796.3289 | 0.0039 | DLVVGLYSQWMNNSDNNPIHPNLR |
| 89.5 | 2796.3289 | 0.0039 | DLVVGLYSQWMNNSDNNPIHPNLR |
| 75.7 | 2796.3289 | 0.0039 | DLVVGLYSQWMNNSDNNPIHPNLR |
| 29.9 | 2795.3449 | 0.9879 | DLVVGLYSQWMNNSDNNPIHPNLR |
| 3.9 | 2794.3228 | 2.0100 | ALNSAANNVYQYGREWITHKLHK |
| 3.9 | 2794.3228 | 2.0100 | ALNSAANNVYQYGREWITHKLHK |
| 3.9 | 2794.3228 | 2.0100 | ALNSAANNVYQYGREWITHKLHK |
| 2.6 | 2795.3493 | 0.9835 | NIPGIPLTGLVGFPAGFATMPTGEDVK |

---

Spectrum No: 48; Query: 1113; Rank: 1

---

## Peptide View

MS/MS Fragmentation of **TALAAFNAQNNGTYFK**
Found in **IPI00327469**, Tax_Id=10116 Gene_Symbol=Ahsg Alpha-2-HS-glycoprotein precursor

Match to Query 1113: 1730.824108 from(866.419330,2+)
Title: 091008RatKidney_NoSalt_34.2213.2213.2.dta
Data file K:\NewmanPaper\Piliang\3SubProteomes\Piliang3SP\mgf5ppm\ERLIC_3SubProteomes5ppm.mgf

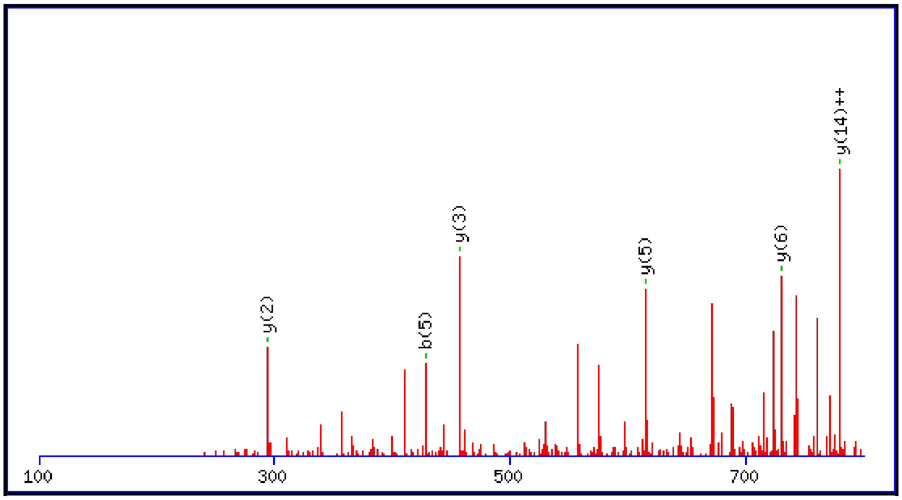

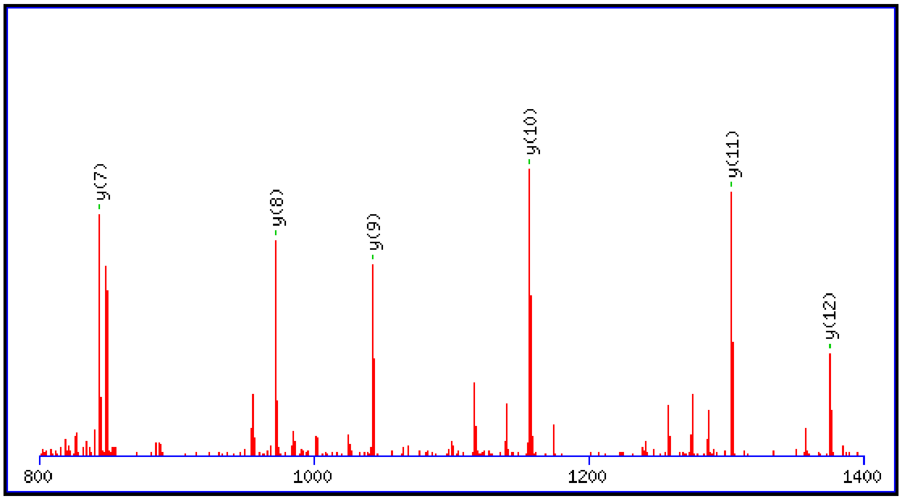


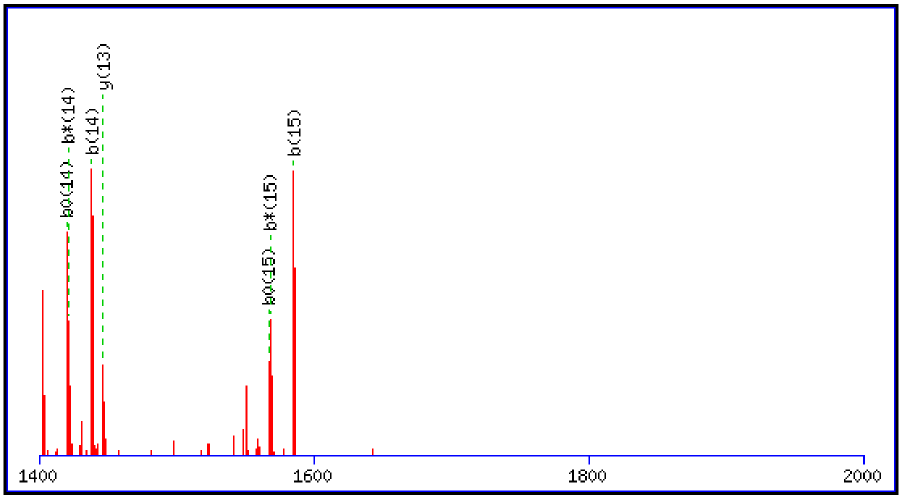


**Monoisotopic mass of neutral peptide Mr(calc):** 1730.8264
**Fixed modifications:** Carbamidomethyl (C)
**Variable modifications:**
**N11** : Deamidated_N (N)
**Ions Score:** 108 **Expect:** 4.1e-009
**Matches (Bold Red):** 20/162 fragment ions using 25 most intense peaks

| # | b | $b^{++}$ | b* | $b^{*++}$ | $b^0$ | $b^{0++}$ | Seq. | y | $y^{++}$ | y* | $y^{*++}$ | $y^0$ | $y^{0++}$ | # |
|---|---|---|---|---|---|---|---|---|---|---|---|---|---|---|
| 1 | 102.0550 | 51.5311 | | | 84.0444 | 42.5258 | T | | | | | | | 16 |
| 2 | 173.0921 | 87.0497 | | | 155.0815 | 78.0444 | A | 1630.7860 | 815.8966 | 1613.7594 | 807.3834 | 1612.7754 | 806.8914 | 15 |
| 3 | 286.1761 | 143.5917 | | | 268.1656 | 134.5864 | L | 1559.7489 | **780.3781** | 1542.7223 | 771.8648 | 1541.7383 | 771.3728 | 14 |
| 4 | 357.2132 | 179.1103 | | | 339.2027 | 170.1050 | A | **1446.6648** | 723.8360 | 1429.6383 | 715.3228 | 1428.6542 | 714.8308 | 13 |
| 5 | **428.2504** | 214.6288 | | | 410.2398 | 205.6235 | A | **1375.6277** | 688.3175 | 1358.6012 | 679.8042 | 1357.6171 | 679.3122 | 12 |
| 6 | 575.3188 | 288.1630 | | | 557.3082 | 279.1577 | F | **1304.5906** | 652.7989 | 1287.5640 | 644.2857 | 1286.5800 | 643.7936 | 11 |
| 7 | 689.3617 | 345.1845 | 672.3352 | 336.6712 | 671.3511 | 336.1792 | N | **1157.5222** | 579.2647 | 1140.4956 | 570.7514 | 1139.5116 | 570.2594 | 10 |
| 8 | 760.3988 | 380.7030 | 743.3723 | 372.1898 | 742.3883 | 371.6978 | A | **1043.4792** | 522.2433 | 1026.4527 | 513.7300 | 1025.4687 | 513.2380 | 9 |
| 9 | 888.4574 | 444.7323 | 871.4308 | 436.2191 | 870.4468 | 435.7271 | Q | **972.4421** | 486.7247 | 955.4156 | 478.2114 | 954.4316 | 477.7194 | 8 |
| 10 | 1002.5003 | 501.7538 | 985.4738 | 493.2405 | 984.4898 | 492.7485 | N | **844.3836** | 422.6954 | 827.3570 | 414.1821 | 826.3730 | 413.6901 | 7 |
| 11 | 1117.5273 | 559.2673 | 1100.5007 | 550.7540 | 1099.5167 | 550.2620 | N | **730.3406** | 365.6740 | 713.3141 | 357.1607 | 712.3301 | 356.6687 | 6 |
| 12 | 1174.5487 | 587.7780 | **1157.5222** | 579.2647 | 1156.5382 | 578.7727 | G | **615.3137** | 308.1605 | 598.2871 | 299.6472 | 597.3031 | 299.1552 | 5 |
| 13 | 1275.5964 | 638.3018 | 1258.5699 | 629.7886 | 1257.5858 | 629.2966 | T | 558.2922 | 279.6498 | 541.2657 | 271.1365 | 540.2817 | 270.6445 | 4 |
| 14 | **1438.6597** | 719.8335 | **1421.6332** | 711.3202 | **1420.6492** | 710.8282 | Y | **457.2445** | 229.1259 | 440.2180 | 220.6126 | | | 3 |
| 15 | **1585.7281** | 793.3677 | **1568.7016** | 784.8544 | **1567.7176** | 784.3624 | F | **294.1812** | 147.5942 | 277.1547 | 139.0810 | | | 2 |
| 16 | | | | | | | K | 147.1128 | 74.0600 | 130.0863 | 65.5468 | | | 1 |

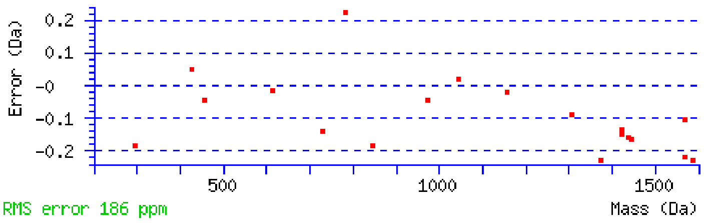


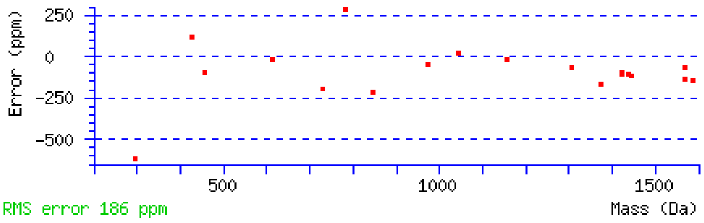


### All matches to this query

| Score | Mr(calc): | Delta | Sequence |
|---|---|---|---|
| 107.7 | 1730.8264 | -0.0023 | TALAAFNAQNNGTYFK |
| 89.2 | 1730.8264 | -0.0023 | TALAAFNAQNNGTYFK |
| 46.5 | 1730.8264 | -0.0023 | TALAAFNAQNNGTYFK |
| 7.4 | 1730.8253 | -0.0012 | TQTRAGSHSLRYFK |
| 7.4 | 1730.8253 | -0.0012 | TQTRAGSHSLRYFK |
| 5.7 | 1729.8036 | 1.0205 | DAAAEKPGAPGSPLNVR |
| 3.1 | 1730.8322 | -0.0081 | KISQGTELQNPDNGTK |
| 3.1 | 1730.8069 | 0.0172 | YHFKALDPEFGTVK |
| 3.0 | 1730.8103 | 0.0139 | LFSSKSSAQFKMYK |
| 2.9 | 1730.8158 | 0.0083 | MNINGQWEGEVNGRK |

---

Spectrum No: 49; Query: 1118; Rank: 1

---

## Peptide View

MS/MS Fragmentation of **LDAPTNLQFVNETDR**
Found in **IPI00200757**, Tax_Id=10116 Gene_Symbol=Fn1 Isoform 1 of Fibronectin precursor

Match to Query 1118: 1732.823828 from(867.419190,2+)
Title: 091008RatKidney_NoSalt_31.2396.2396.2.dta
Data file K:\NewmanPaper\Piliang\3SubProteomes\Piliang3SP\mgf5ppm\ERLIC_3SubProteomes5ppm.mgf

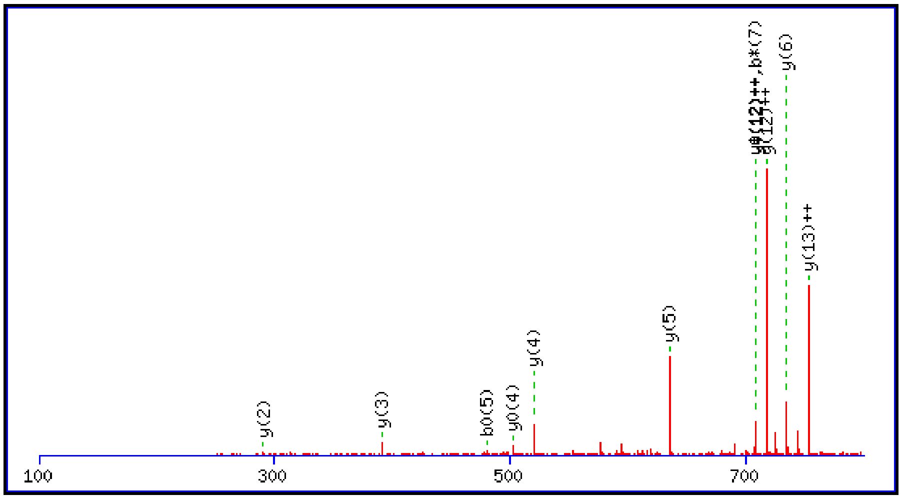

Mascot Search Results: Peptide View

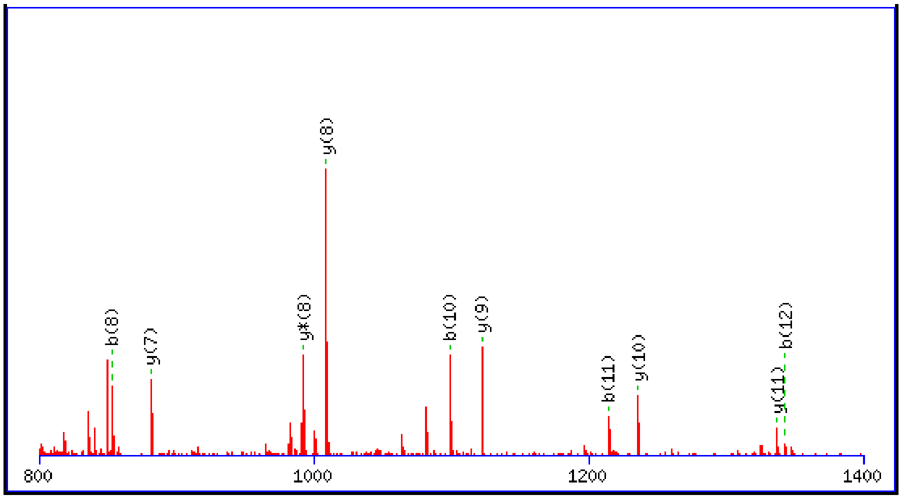


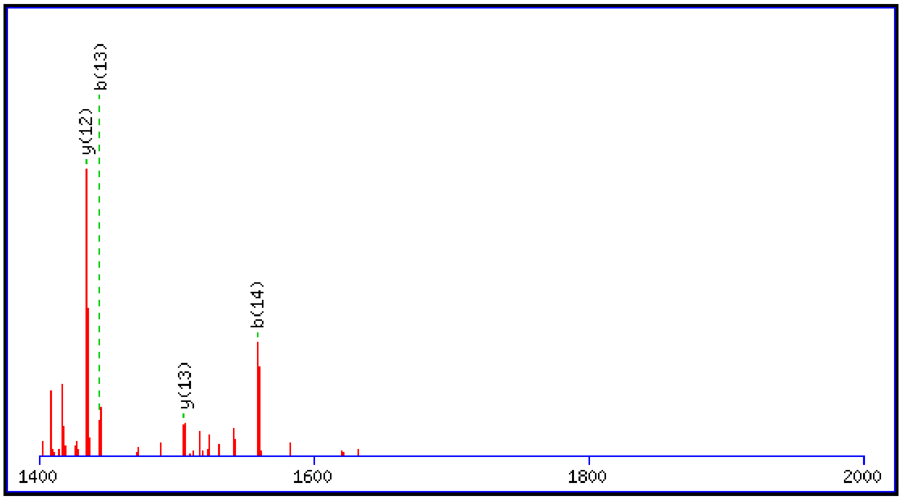


**Monoisotopic mass of neutral peptide Mr(calc):** 1732.8268
**Fixed modifications:** Carbamidomethyl (C)
**Variable modifications:**
**N11** : Deamidated_N (N)
**Ions Score:** 107 **Expect:** 4.5e-009
**Matches (Bold Red):** 26/154 fragment ions using 37 most intense peaks

| # | b | $b^{++}$ | b* | $b^{*++}$ | $b^0$ | $b^{0++}$ | Seq. | y | $y^{++}$ | y* | $y^{*++}$ | $y^0$ | $y^{0++}$ | # |
|---|---|---|---|---|---|---|---|---|---|---|---|---|---|---|
| 1 | 114.0913 | 57.5493 | | | | | L | | | | | | | 15 |
| 2 | 229.1183 | 115.0628 | | | 211.1077 | 106.0575 | D | 1620.7500 | 810.8786 | 1603.7235 | 802.3654 | 1602.7394 | 801.8734 | 14 |
| 3 | 300.1554 | 150.5813 | | | 282.1448 | 141.5761 | A | **1505.7231** | **753.3652** | 1488.6965 | 744.8519 | 1487.7125 | 744.3599 | 13 |
| 4 | 397.2082 | 199.1077 | | | 379.1976 | 190.1024 | P | **1434.6859** | **717.8466** | 1417.6594 | **709.3333** | 1416.6754 | **708.8413** | 12 |
| 5 | 498.2558 | 249.6316 | | | **480.2453** | 240.6263 | T | **1337.6332** | 669.3202 | 1320.6066 | 660.8070 | 1319.6226 | 660.3149 | 11 |
| 6 | 612.2988 | 306.6530 | 595.2722 | 298.1397 | 594.2882 | 297.6477 | N | **1236.5855** | 618.7964 | 1219.5590 | 610.2831 | 1218.5749 | 609.7911 | 10 |
| 7 | 725.3828 | 363.1951 | **708.3563** | 354.6818 | 707.3723 | 354.1898 | L | **1122.5426** | 561.7749 | 1105.5160 | 553.2617 | 1104.5320 | 552.7696 | 9 |
| 8 | **853.4414** | 427.2243 | 836.4149 | 418.7111 | 835.4308 | 418.2191 | Q | **1009.4585** | 505.2329 | **992.4320** | 496.7196 | 991.4479 | 496.2276 | 8 |
| 9 | 1000.5098 | 500.7585 | 983.4833 | 492.2453 | 982.4993 | 491.7533 | F | **881.3999** | 441.2036 | 864.3734 | 432.6903 | 863.3894 | 432.1983 | 7 |
| 10 | **1099.5782** | 550.2928 | 1082.5517 | 541.7795 | 1081.5677 | 541.2875 | V | **734.3315** | 367.6694 | 717.3050 | 359.1561 | 716.3210 | 358.6641 | 6 |
| 11 | **1214.6052** | 607.8062 | 1197.5786 | 599.2930 | 1196.5946 | 598.8009 | N | **635.2631** | 318.1352 | 618.2366 | 309.6219 | 617.2525 | 309.1299 | 5 |
| 12 | **1343.6478** | 672.3275 | 1326.6212 | 663.8142 | 1325.6372 | 663.3222 | E | **520.2362** | 260.6217 | 503.2096 | 252.1084 | **502.2256** | 251.6164 | 4 |
| 13 | **1444.6954** | 722.8514 | 1427.6689 | 714.3381 | 1426.6849 | 713.8461 | T | **391.1936** | 196.1004 | 374.1670 | 187.5872 | 373.1830 | 187.0951 | 3 |
| 14 | **1559.7224** | 780.3648 | 1542.6958 | 771.8516 | 1541.7118 | 771.3595 | D | **290.1459** | 145.5766 | 273.1193 | 137.0633 | 272.1353 | 136.5713 | 2 |
| 15 | | | | | | | R | 175.1190 | 88.0631 | 158.0924 | 79.5498 | | | 1 |

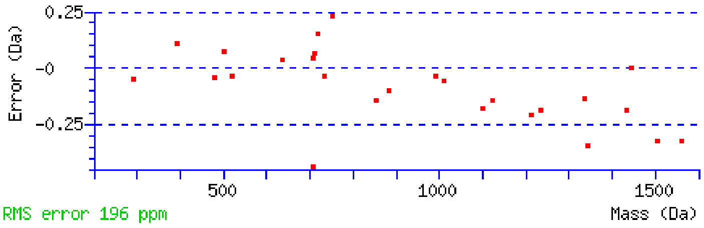


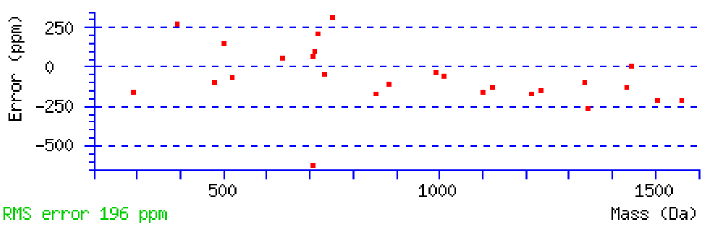


### All matches to this query

| Score | Mr(calc): | Delta | Sequence |
|---|---|---|---|
| 107.2 | 1732.8268 | -0.0030 | LDAPTNLQFVNETDR |
| 37.5 | 1732.8268 | -0.0030 | LDAPTNLQFVNETDR |
| 18.0 | 1731.8080 | 1.0158 | VTSSGVLLLDNYTDR |
| 18.0 | 1731.8080 | 1.0158 | VTSSGVLLLDNYTDR |
| 13.3 | 1731.8080 | 1.0158 | VTSSGVLLLDNYTDR |
| 7.9 | 1731.8120 | 1.0118 | EPNGLIVLYEVSYR |
| 6.9 | 1732.8331 | -0.0093 | LLEAAVQQHSTMIGR |
| 5.9 | 1732.8096 | 0.0142 | AVTVEGMLKISRNR |
| 3.2 | 1732.8331 | -0.0093 | LLEAAVQQHSTMIGR |
| 2.2 | 1732.8331 | -0.0093 | EASLLVSCRTSKFR |

---

Spectrum No: 50; Query: 1830; Rank: 1

---

## Peptide View

MS/MS Fragmentation of **SVCADAPYVNVTAEDLDGPR**
Found in **IPI00358687**, Tax_Id=10116 Gene_Symbol=Dsg2_predicted similar to desmoglein 2

Match to Query 1830: 2148.966048 from(1075.490300,2+)
Title: 100101RatKid_NS_deglyco_25.3198.3198.2.dta
Data file K:\NewmanPaper\Piliang\3SubProteomes\Piliang3SP\mgf5ppm\ERLIC_3SubProteomes5ppm.mgf

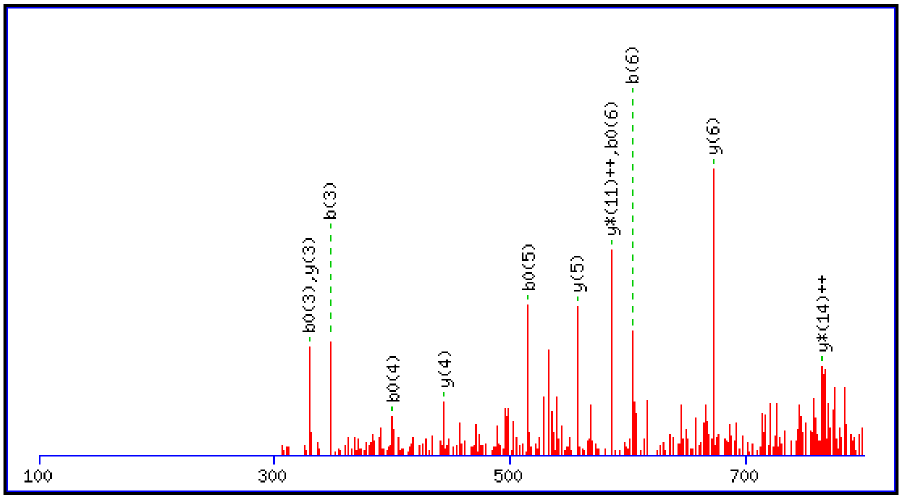

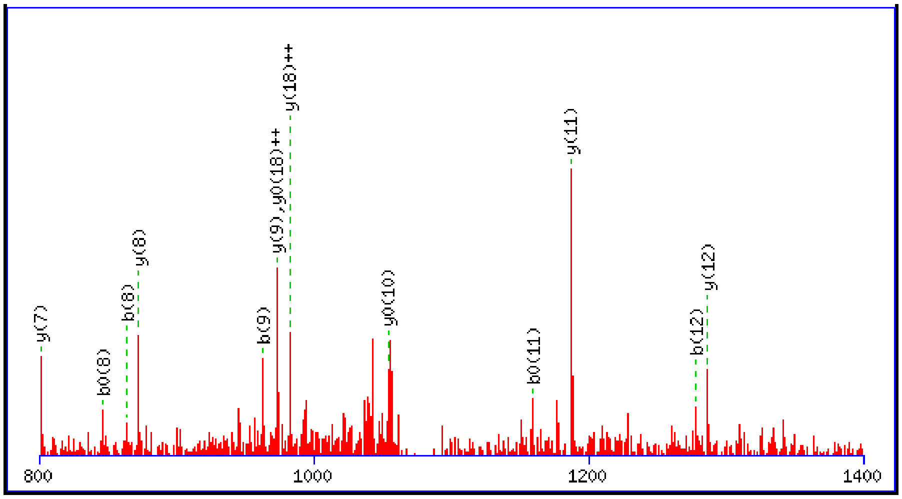


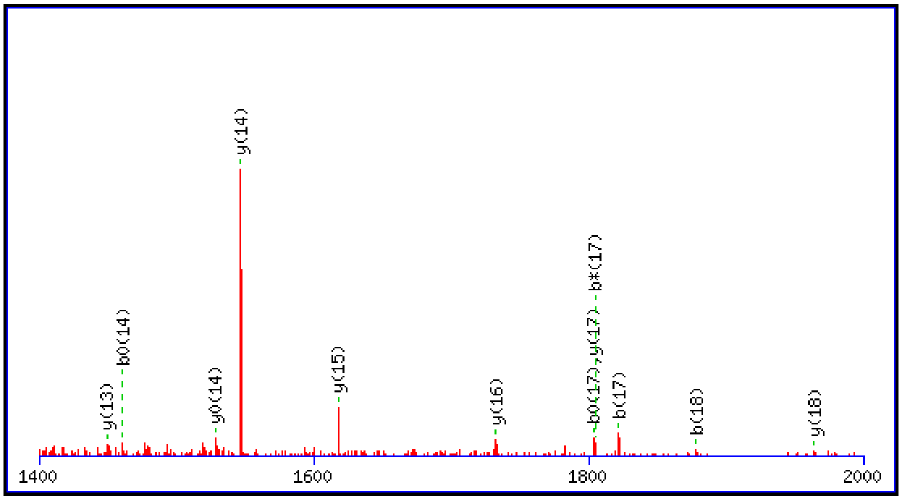


**Monoisotopic mass of neutral peptide Mr(calc):** 2148.9634
**Fixed modifications:** Carbamidomethyl (C)
**Variable modifications:**
**N10** : Deamidated_N (N)
**Ions Score:** 107 **Expect:** 5.2e-009
**Matches (Bold Red):** 37/204 fragment ions using 59 most intense peaks

| # | b | $b^{++}$ | b* | $b^{*++}$ | $b^0$ | $b^{0++}$ | Seq. | y | $y^{++}$ | y* | $y^{*++}$ | $y^0$ | $y^{0++}$ | # |
|---|---|---|---|---|---|---|---|---|---|---|---|---|---|---|
| 1 | 88.0393 | 44.5233 | | | 70.0287 | 35.5180 | S | | | | | | | 20 |
| 2 | 187.1077 | 94.0575 | | | 169.0972 | 85.0522 | V | 2062.9386 | 1031.9729 | 2045.9121 | 1023.4597 | 2044.9280 | 1022.9677 | 19 |
| 3 | **347.1384** | 174.0728 | | | **329.1278** | 165.0675 | C | **1963.8702** | **982.4387** | 1946.8436 | 973.9255 | 1945.8596 | **973.4335** | 18 |
| 4 | 418.1755 | 209.5914 | | | **400.1649** | 200.5861 | A | **1803.8395** | 902.4234 | 1786.8130 | 893.9101 | 1785.8290 | 893.4181 | 17 |
| 5 | 533.2024 | 267.1049 | | | **515.1919** | 258.0996 | D | **1732.8024** | 866.9049 | 1715.7759 | 858.3916 | 1714.7919 | 857.8996 | 16 |
| 6 | **604.2395** | 302.6234 | | | **586.2290** | 293.6181 | A | **1617.7755** | 809.3914 | 1600.7489 | 800.8781 | 1599.7649 | 800.3861 | 15 |
| 7 | 701.2923 | 351.1498 | | | 683.2817 | 342.1445 | P | **1546.7384** | 773.8728 | 1529.7118 | **765.3596** | **1528.7278** | 764.8675 | 14 |
| 8 | **864.3556** | 432.6815 | | | **846.3451** | 423.6762 | Y | **1449.6856** | 725.3464 | 1432.6591 | 716.8332 | 1431.6750 | 716.3412 | 13 |
| 9 | **963.4240** | 482.2157 | | | 945.4135 | 473.2104 | V | **1286.6223** | 643.8148 | 1269.5957 | 635.3015 | 1268.6117 | 634.8095 | 12 |
| 10 | 1078.4510 | 539.7291 | 1061.4244 | 531.2159 | 1060.4404 | 530.7238 | N | **1187.5539** | 594.2806 | 1170.5273 | **585.7673** | 1169.5433 | 585.2753 | 11 |
| 11 | 1177.5194 | 589.2633 | 1160.4928 | 580.7501 | **1159.5088** | 580.2581 | V | 1072.5269 | 536.7671 | 1055.5004 | 528.2538 | **1054.5164** | 527.7618 | 10 |
| 12 | **1278.5671** | 639.7872 | 1261.5405 | 631.2739 | 1260.5565 | 630.7819 | T | **973.4585** | 487.2329 | 956.4320 | 478.7196 | 955.4480 | 478.2276 | 9 |
| 13 | 1349.6042 | 675.3057 | 1332.5776 | 666.7925 | 1331.5936 | 666.3004 | A | **872.4108** | 436.7091 | 855.3843 | 428.1958 | 854.4003 | 427.7038 | 8 |
| 14 | 1478.6468 | 739.8270 | 1461.6202 | 731.3138 | **1460.6362** | 730.8217 | E | **801.3737** | 401.1905 | 784.3472 | 392.6772 | 783.3632 | 392.1852 | 7 |
| 15 | 1593.6737 | 797.3405 | 1576.6472 | 788.8272 | 1575.6632 | 788.3352 | D | **672.3311** | 336.6692 | 655.3046 | 328.1559 | 654.3206 | 327.6639 | 6 |
| 16 | 1706.7578 | 853.8825 | 1689.7312 | 845.3693 | 1688.7472 | 844.8772 | L | **557.3042** | 279.1557 | 540.2776 | 270.6425 | 539.2936 | 270.1504 | 5 |
| 17 | **1821.7847** | 911.3960 | **1804.7582** | 902.8827 | **1803.7742** | 902.3907 | D | **444.2201** | 222.6137 | 427.1936 | 214.1004 | 426.2096 | 213.6084 | 4 |
| 18 | **1878.8062** | 939.9067 | 1861.7796 | 931.3935 | 1860.7956 | 930.9015 | G | **329.1932** | 165.1002 | 312.1666 | 156.5870 | | | 3 |

| | | | | | | | | | | | | | | |
|---|---|---|---|---|---|---|---|---|---|---|---|---|---|---|
| **19** | 1975.8590 | 988.4331 | 1958.8324 | 979.9198 | 1957.8484 | 979.4278 | **P** | 272.1717 | 136.5895 | 255.1452 | 128.0762 | | | **2** |
| **20** | | | | | | | **R** | 175.1190 | 88.0631 | 158.0924 | 79.5498 | | | **1** |

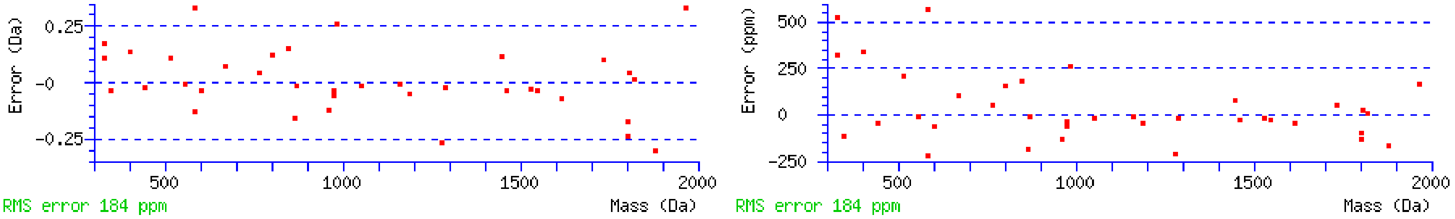


### All matches to this query

| Score | Mr(calc): | Delta | Sequence |
|---|---|---|---|
| 106.8 | 2148.9634 | 0.0027 | SVCADAPYVNVTAEDLDGPR |
| 31.1 | 2147.9794 | 0.9867 | SVCADAPYVNVTAEDLDGPR |
| 3.0 | 2147.9671 | 0.9990 | QESVSALSASMHPQLSPQR |
| 2.8 | 2148.9558 | 0.0102 | AVCGGAGFGSRSLCGVGSSKR |
| 2.3 | 2147.9792 | 0.9868 | TDTLLYSVLPPSVANELR |
| 2.3 | 2147.9792 | 0.9868 | TDTLLYSVLPPSVANELR |
| 2.3 | 2147.9792 | 0.9868 | TDTLLYSVLPPSVANELR |
| 2.3 | 2147.9792 | 0.9868 | TDTLLYSVLPPSVANELR |
| 0.6 | 2147.9840 | 0.9821 | TINFQIECTPVKTGPRK |
| 0.4 | 2148.9558 | 0.0102 | AVCGGAGFGSRSLCGVGSSKR |

---

Spectrum No: 51; Query: 2508; Rank: 1

---

## Peptide View

MS/MS Fragmentation of **LLEFDSTNVSEGTQPPGKPYPPYSLAK**
Found in **IPI00196226**, Tax_Id=10116 Gene_Symbol=RGD1303130 Uncharacterized protein C1orf85 homolog precursor

Match to Query 2508: 2935.453032 from(979.491620,3+)
Title: 100101RatKid_NS_deglyco_18.3782.3782.3.dta
Data file K:\NewmanPaper\Piliang\3SubProteomes\Piliang3SP\mgf5ppm\ERLIC_3SubProteomes5ppm.mgf

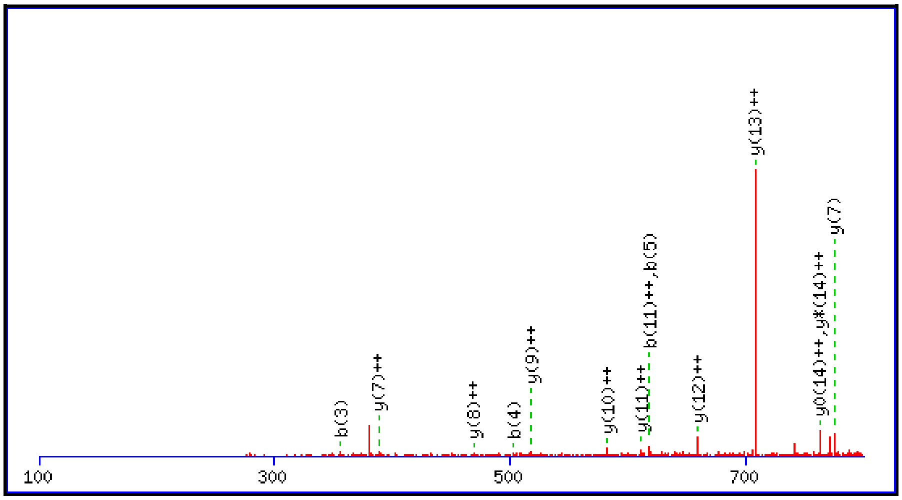

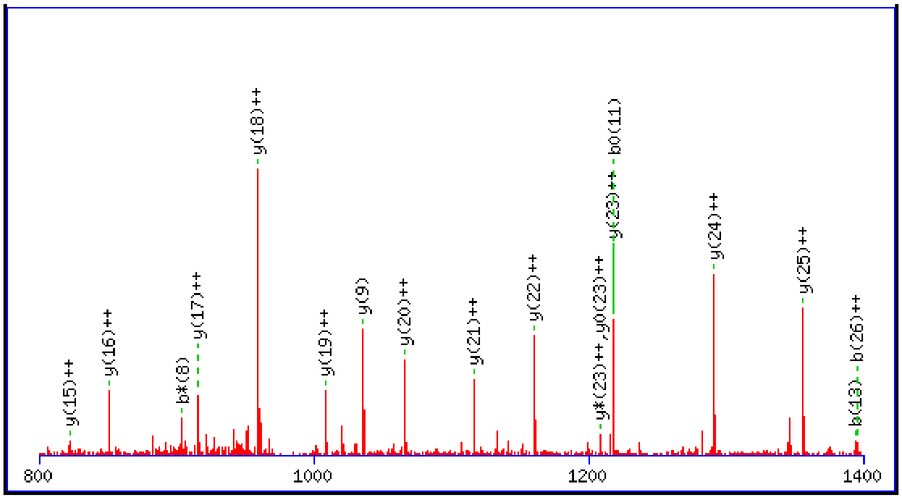


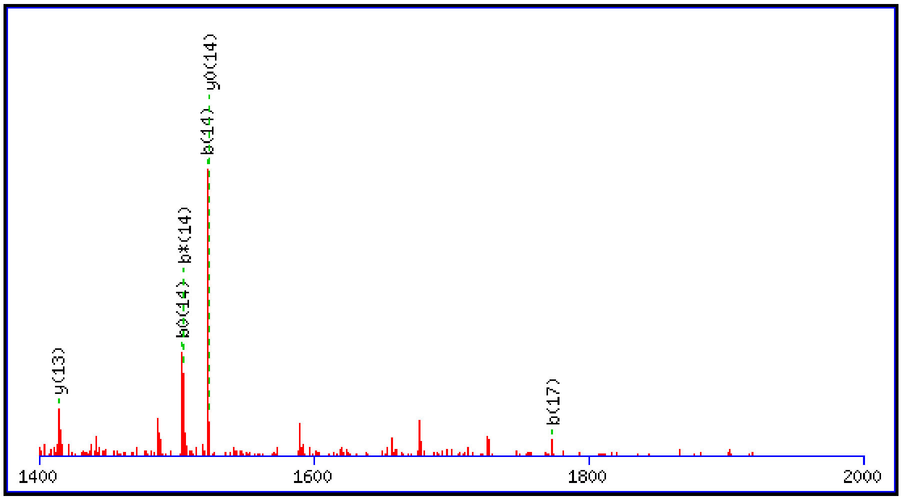


**Monoisotopic mass of neutral peptide Mr(calc):** 2935.4491
**Fixed modifications:** Carbamidomethyl (C)
**Variable modifications:**
**N8** : Deamidated_N (N)
**Ions Score:** 106 **Expect:** 5.5e-009
**Matches (Bold Red):** 38/288 fragment ions using 70 most intense peaks

| # | b | $b^{++}$ | b* | $b^{*++}$ | $b^{0}$ | $b^{0++}$ | Seq. | y | $y^{++}$ | y* | $y^{*++}$ | $y^{0}$ | $y^{0++}$ | # |
|---|---|---|---|---|---|---|---|---|---|---|---|---|---|---|
| 1 | 114.0913 | 57.5493 | | | | | L | | | | | | | 27 |
| 2 | 227.1754 | 114.0913 | | | | | L | 2823.3723 | 1412.1898 | 2806.3458 | 1403.6765 | 2805.3618 | 1403.1845 | 26 |
| 3 | **356.2180** | 178.6126 | | | 338.2074 | 169.6074 | E | 2710.2883 | **1355.6478** | 2693.2617 | 1347.1345 | 2692.2777 | 1346.6425 | 25 |
| 4 | **503.2864** | 252.1468 | | | 485.2758 | 243.1416 | F | 2581.2457 | **1291.1265** | 2564.2191 | 1282.6132 | 2563.2351 | 1282.1212 | 24 |
| 5 | **618.3134** | 309.6603 | | | 600.3028 | 300.6550 | D | 2434.1773 | **1217.5923** | 2417.1507 | **1209.0790** | 2416.1667 | **1208.5870** | 23 |
| 6 | 705.3454 | 353.1763 | | | 687.3348 | 344.1710 | S | 2319.1503 | **1160.0788** | 2302.1238 | 1151.5655 | 2301.1397 | 1151.0735 | 22 |
| 7 | 806.3931 | 403.7002 | | | 788.3825 | 394.6949 | T | 2232.1183 | **1116.5628** | 2215.0917 | 1108.0495 | 2214.1077 | 1107.5575 | 21 |
| 8 | 921.4200 | 461.2136 | **904.3934** | 452.7004 | 903.4094 | 452.2084 | N | 2131.0706 | **1066.0389** | 2114.0441 | 1057.5257 | 2113.0600 | 1057.0337 | 20 |
| 9 | 1020.4884 | 510.7478 | 1003.4619 | 502.2346 | 1002.4778 | 501.7426 | V | 2016.0437 | **1008.5255** | 1999.0171 | 1000.0122 | 1998.0331 | 999.5202 | 19 |
| 10 | 1107.5204 | 554.2639 | 1090.4939 | 545.7506 | 1089.5099 | 545.2586 | S | 1916.9753 | **958.9913** | 1899.9487 | 950.4780 | 1898.9647 | 949.9860 | 18 |
| 11 | 1236.5630 | **618.7852** | 1219.5365 | 610.2719 | **1218.5525** | 609.7799 | E | 1829.9432 | **915.4753** | 1812.9167 | 906.9620 | 1811.9327 | 906.4700 | 17 |
| 12 | 1293.5845 | 647.2959 | 1276.5579 | 638.7826 | 1275.5739 | 638.2906 | G | 1700.9006 | **850.9540** | 1683.8741 | 842.4407 | 1682.8901 | 841.9487 | 16 |
| 13 | **1394.6322** | 697.8197 | 1377.6056 | 689.3065 | 1376.6216 | 688.8144 | T | 1643.8792 | **822.4432** | 1626.8526 | 813.9299 | 1625.8686 | 813.4379 | 15 |
| 14 | **1522.6908** | 761.8490 | **1505.6642** | 753.3357 | **1504.6802** | 752.8437 | Q | 1542.8315 | 771.9194 | 1525.8049 | **763.4061** | **1524.8209** | **762.9141** | 14 |
| 15 | 1619.7435 | 810.3754 | 1602.7170 | 801.8621 | 1601.7330 | 801.3701 | P | **1414.7729** | **707.8901** | 1397.7464 | 699.3768 | 1396.7624 | 698.8848 | 13 |
| 16 | 1716.7963 | 858.9018 | 1699.7697 | 850.3885 | 1698.7857 | 849.8965 | P | 1317.7202 | **659.3637** | 1300.6936 | 650.8504 | 1299.7096 | 650.3584 | 12 |
| 17 | **1773.8177** | 887.4125 | 1756.7912 | 878.8992 | 1755.8072 | 878.4072 | G | 1220.6674 | **610.8373** | 1203.6408 | 602.3241 | 1202.6568 | 601.8320 | 11 |
| 18 | 1901.9127 | 951.4600 | 1884.8862 | 942.9467 | 1883.9021 | 942.4547 | K | 1163.6459 | **582.3266** | 1146.6194 | 573.8133 | 1145.6354 | 573.3213 | 10 |

| | | | | | | | | | | | | | | |
|---|---|---|---|---|---|---|---|---|---|---|---|---|---|---|
| **19** | 1998.9655 | 999.9864 | 1981.9389 | 991.4731 | 1980.9549 | 990.9811 | **P** | **1035.5510** | **518.2791** | 1018.5244 | 509.7658 | 1017.5404 | 509.2738 | **9** |
| **20** | 2162.0288 | 1081.5180 | 2145.0023 | 1073.0048 | 2144.0182 | 1072.5128 | **Y** | 938.4982 | **469.7527** | 921.4716 | 461.2395 | 920.4876 | 460.7475 | **8** |
| **21** | 2259.0816 | 1130.0444 | 2242.0550 | 1121.5311 | 2241.0710 | 1121.0391 | **P** | **775.4349** | **388.2211** | 758.4083 | 379.7078 | 757.4243 | 379.2158 | **7** |
| **22** | 2356.1343 | 1178.5708 | 2339.1078 | 1170.0575 | 2338.1238 | 1169.5655 | **P** | 678.3821 | 339.6947 | 661.3556 | 331.1814 | 660.3715 | 330.6894 | **6** |
| **23** | 2519.1977 | 1260.1025 | 2502.1711 | 1251.5892 | 2501.1871 | 1251.0972 | **Y** | 581.3293 | 291.1683 | 564.3028 | 282.6550 | 563.3188 | 282.1630 | **5** |
| **24** | 2606.2297 | 1303.6185 | 2589.2031 | 1295.1052 | 2588.2191 | 1294.6132 | **S** | 418.2660 | 209.6366 | 401.2395 | 201.1234 | 400.2554 | 200.6314 | **4** |
| **25** | 2719.3137 | 1360.1605 | 2702.2872 | 1351.6472 | 2701.3032 | 1351.1552 | **L** | 331.2340 | 166.1206 | 314.2074 | 157.6074 | | | **3** |
| **26** | 2790.3509 | **1395.6791** | 2773.3243 | 1387.1658 | 2772.3403 | 1386.6738 | **A** | 218.1499 | 109.5786 | 201.1234 | 101.0653 | | | **2** |
| **27** | | | | | | | **K** | 147.1128 | 74.0600 | 130.0863 | 65.5468 | | | **1** |

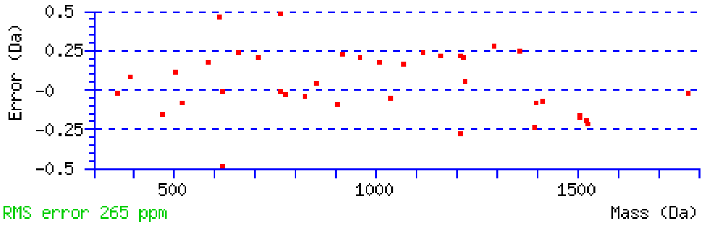


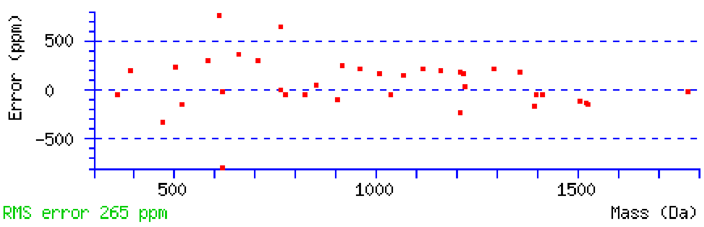


### All matches to this query

| Score | Mr(calc): | Delta | Sequence |
|---|---|---|---|
| 106.4 | 2935.4491 | 0.0039 | LLEFDSTNVSEGTQPPGKPYPPYSLAK |
| 40.6 | 2934.4651 | 0.9879 | LLEFDSTNVSEGTQPPGKPYPPYSLAK |
| 9.5 | 2935.4613 | -0.0083 | EVILSGGAINSPQLLMLSGVGNADDLKK |
| 5.5 | 2935.4375 | 0.0155 | NSAMLVQNPYITVRSDGSLHIERVR |
| 3.7 | 2935.4553 | -0.0022 | EEAEAAPTPGLGGGPGPRQRTPLDILTR |
| 1.9 | 2935.4613 | -0.0083 | EVILSGGAINSPQLLMLSGVGNADDLKK |
| 1.5 | 2935.4493 | 0.0037 | LLQGGTLMYEQVPMVEIDGMNLVQTR |
| 1.4 | 2935.4613 | -0.0083 | EVILSGGAINSPQLLMLSGVGNADDLKK |
| 1.3 | 2935.4643 | -0.0113 | LLEPLIIQVTTLVNCPQNPSNRKK |
| 1.3 | 2935.4643 | -0.0113 | LLEPLIIQVTTLVNCPQNPSNRKK |

---

Spectrum No: 52; Query: 1603; Rank: 1

---

## Peptide View

MS/MS Fragmentation of **TQLTCFLNSSGIDIVGHR**
Found in **IPI00193425**, Tax_Id=10116 Gene_Symbol=Bsg Isoform 2 of Basigin precursor

Match to Query 1603: 2017.994488 from(1010.004520,2+)
Title: 100101RatKid_NS_deglyco_26.4122.4122.2.dta
Data file K:\NewmanPaper\Piliang\3SubProteomes\Piliang3SP\mgf5ppm\ERLIC_3SubProteomes5ppm.mgf

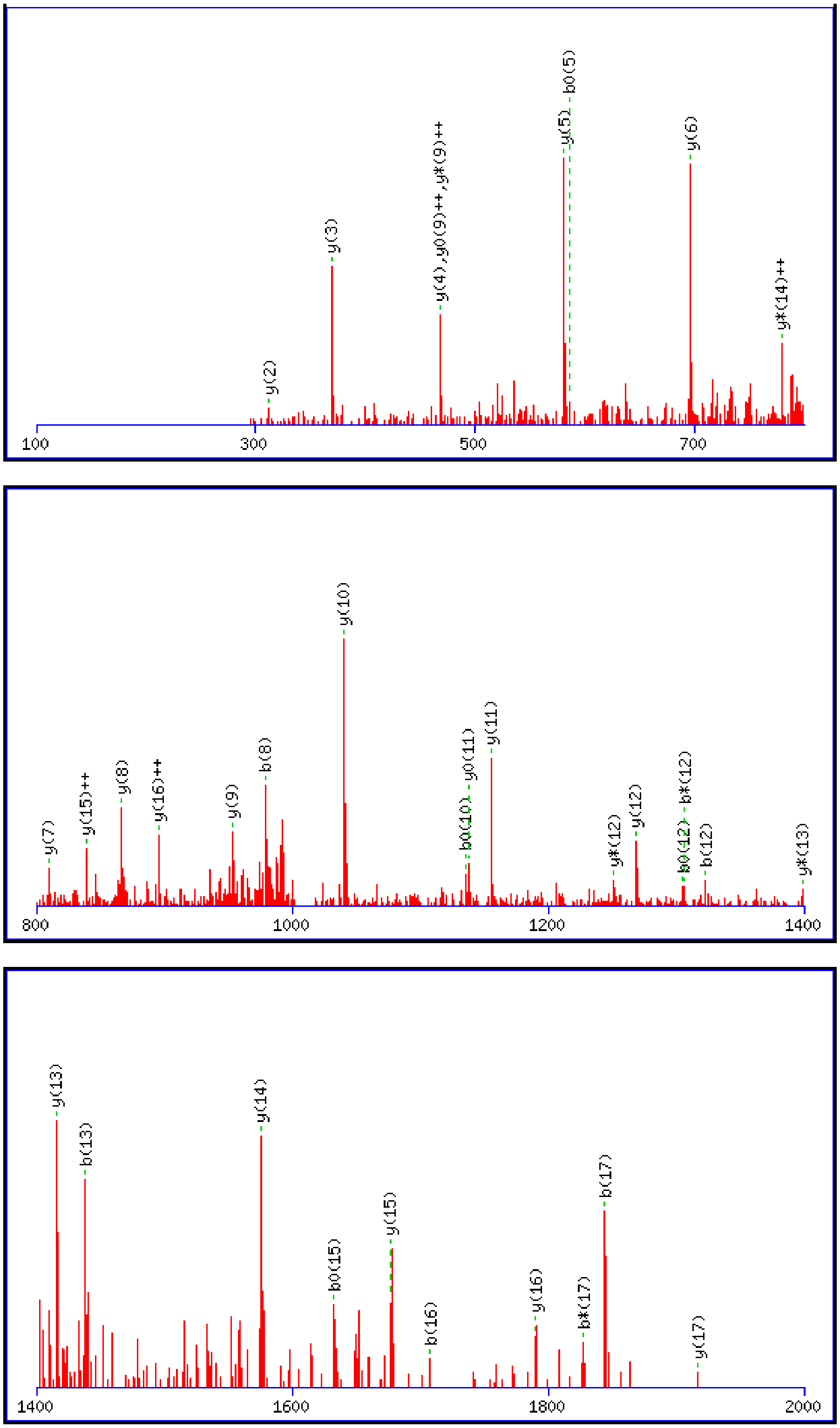


**Monoisotopic mass of neutral peptide Mr(calc):** 2017.9891
**Fixed modifications:** Carbamidomethyl (C)
**Variable modifications:**
**N8** : Deamidated_N (N)
**Ions Score:** 106 **Expect:** 5.5e-009
**Matches (Bold Red):** 35/192 fragment ions using 72 most intense peaks

| # | b | $b^{++}$ | b* | $b^{*++}$ | $b^0$ | $b^{0++}$ | Seq. | y | $y^{++}$ | y* | $y^{*++}$ | $y^0$ | $y^{0++}$ | # |
|---|---|---|---|---|---|---|---|---|---|---|---|---|---|---|
| 1 | 102.0550 | 51.5311 | | | 84.0444 | 42.5258 | T | | | | | | | 18 |
| 2 | 230.1135 | 115.5604 | 213.0870 | 107.0471 | 212.1030 | 106.5551 | Q | **1917.9487** | 959.4780 | 1900.9222 | 950.9647 | 1899.9382 | 950.4727 | 17 |
| 3 | 343.1976 | 172.1024 | 326.1710 | 163.5892 | 325.1870 | 163.0972 | L | **1789.8901** | **895.4487** | 1772.8636 | 886.9354 | 1771.8796 | 886.4434 | 16 |
| 4 | 444.2453 | 222.6263 | 427.2187 | 214.1130 | 426.2347 | 213.6210 | T | **1676.8061** | **838.9067** | 1659.7795 | 830.3934 | 1658.7955 | 829.9014 | 15 |
| 5 | 604.2759 | 302.6416 | 587.2494 | 294.1283 | **586.2654** | 293.6363 | C | **1575.7584** | 788.3828 | 1558.7318 | **779.8696** | 1557.7478 | 779.3776 | 14 |

| | | | | | | | | | | | | | | |
|---|---|---|---|---|---|---|---|---|---|---|---|---|---|---|
| **6** | 751.3443 | 376.1758 | 734.3178 | 367.6625 | 733.3338 | 367.1705 | **F** | **1415.7277** | 708.3675 | **1398.7012** | 699.8542 | 1397.7172 | 699.3622 | **13** |
| **7** | 864.4284 | 432.7178 | 847.4019 | 424.2046 | 846.4178 | 423.7126 | **L** | **1268.6593** | 634.8333 | **1251.6328** | 626.3200 | 1250.6488 | 625.8280 | **12** |
| **8** | **979.4553** | 490.2313 | 962.4288 | 481.7180 | 961.4448 | 481.2260 | **N** | **1155.5753** | 578.2913 | 1138.5487 | 569.7780 | **1137.5647** | 569.2860 | **11** |
| **9** | 1066.4874 | 533.7473 | 1049.4608 | 525.2340 | 1048.4768 | 524.7420 | **S** | **1040.5483** | 520.7778 | 1023.5218 | 512.2645 | 1022.5378 | 511.7725 | **10** |
| **10** | 1153.5194 | 577.2633 | 1136.4928 | 568.7501 | **1135.5088** | 568.2581 | **S** | **953.5163** | 477.2618 | 936.4898 | **468.7485** | 935.5057 | **468.2565** | **9** |
| **11** | 1210.5409 | 605.7741 | 1193.5143 | 597.2608 | 1192.5303 | 596.7688 | **G** | **866.4843** | 433.7458 | 849.4577 | 425.2325 | 848.4737 | 424.7405 | **8** |
| **12** | **1323.6249** | 662.3161 | **1306.5984** | 653.8028 | **1305.6144** | 653.3108 | **I** | **809.4628** | 405.2350 | 792.4363 | 396.7218 | 791.4522 | 396.2298 | **7** |
| **13** | **1438.6519** | 719.8296 | 1421.6253 | 711.3163 | 1420.6413 | 710.8243 | **D** | **696.3787** | 348.6930 | 679.3522 | 340.1797 | 678.3682 | 339.6877 | **6** |
| **14** | 1551.7359 | 776.3716 | 1534.7094 | 767.8583 | 1533.7254 | 767.3663 | **I** | **581.3518** | 291.1795 | 564.3253 | 282.6663 | | | **5** |
| **15** | 1650.8043 | 825.9058 | 1633.7778 | 817.3925 | **1632.7938** | 816.9005 | **V** | **468.2677** | 234.6375 | 451.2412 | 226.1242 | | | **4** |
| **16** | **1707.8258** | 854.4165 | 1690.7993 | 845.9033 | 1689.8152 | 845.4113 | **G** | **369.1993** | 185.1033 | 352.1728 | 176.5900 | | | **3** |
| **17** | **1844.8847** | 922.9460 | **1827.8582** | 914.4327 | 1826.8742 | 913.9407 | **H** | **312.1779** | 156.5926 | 295.1513 | 148.0793 | | | **2** |
| **18** | | | | | | | **R** | 175.1190 | 88.0631 | 158.0924 | 79.5498 | | | **1** |

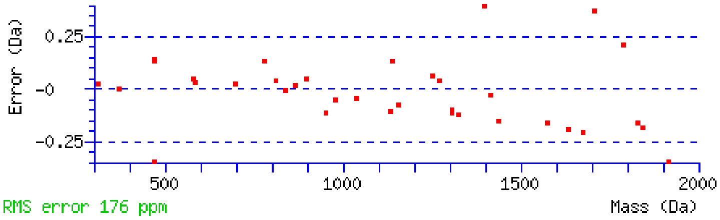


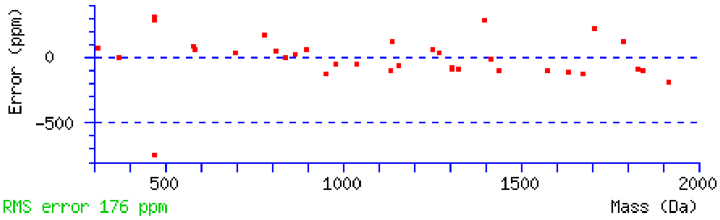


### All matches to this query

| Score | Mr(calc): | Delta | Sequence |
|---|---|---|---|
| 106.0 | 2017.9891 | 0.0054 | TQLTCFLNSSGIDIVGHR |
| 19.6 | 2017.0051 | 0.9894 | TQLTCFLNSSGIDIVGHR |
| 4.8 | 2017.9850 | 0.0095 | NANVSITIASSALRTLVK |
| 4.8 | 2017.9850 | 0.0095 | NANVSITIASSALRTLVK |
| 1.9 | 2017.0010 | 0.9935 | NANVSITIASSALRTLVK |

---

Spectrum No: 53; Query: 2405; Rank: 1

---

## Peptide View

MS/MS Fragmentation of **HIDCASVYGNETEIGEALKESVGAGK**
Found in **IPI00230859**, Tax_Id=10116 Gene_Symbol=Akr1a1 Alcohol dehydrogenase

Match to Query 2405: 2734.286862 from(912.436230,3+)
Title: 091008RatKidney_NH4Format01_14.3949.3949.3.dta
Data file K:\NewmanPaper\Piliang\3SubProteomes\Piliang3SP\mgf5ppm\ERLIC_3SubProteomes5ppm.mgf

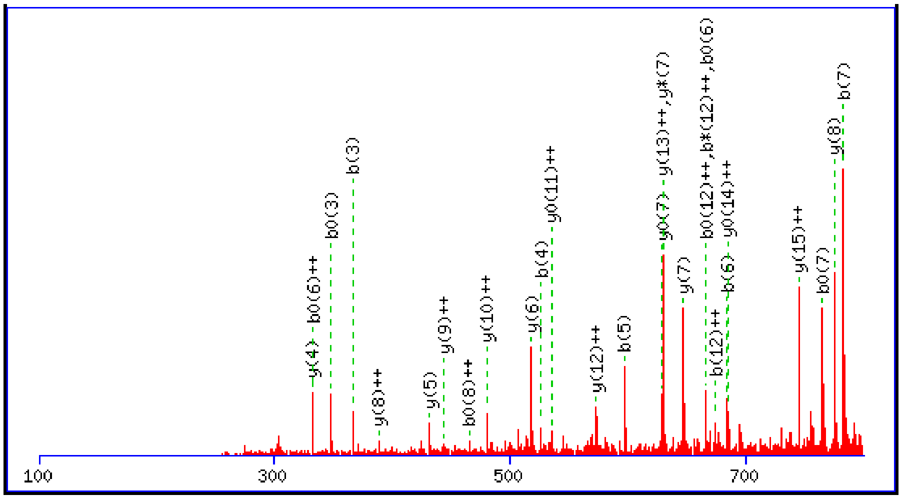


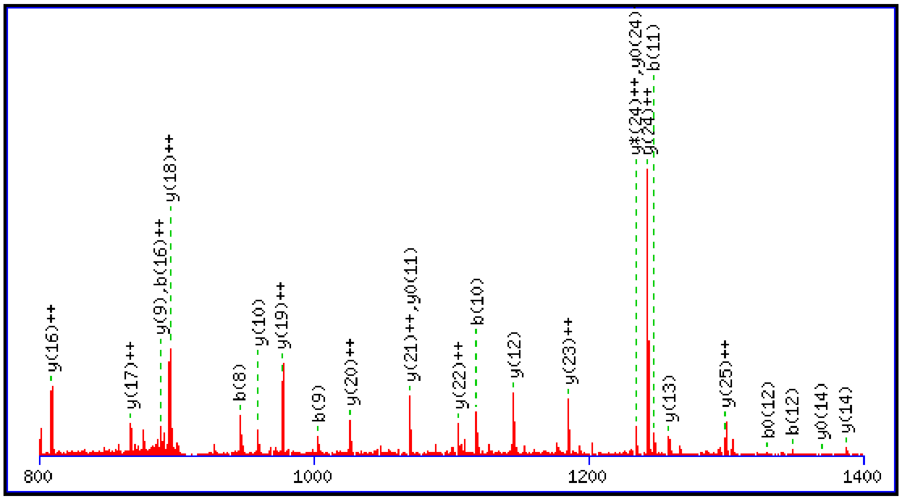


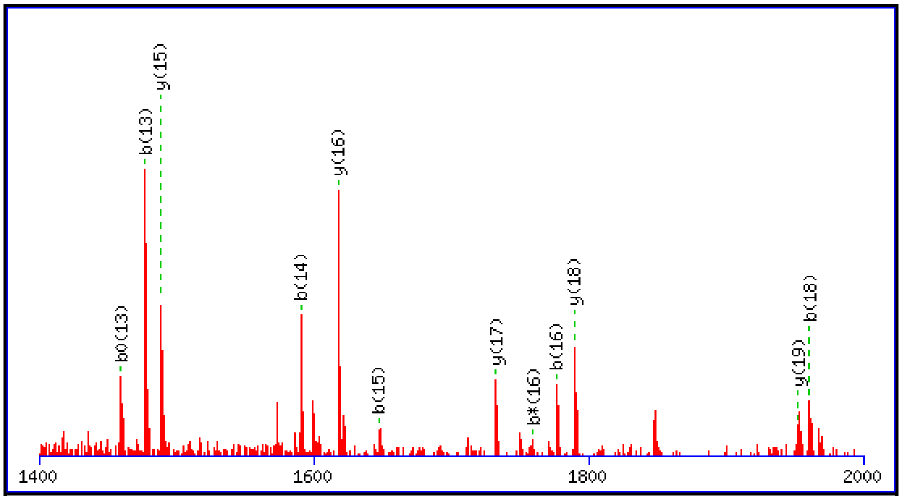


**Monoisotopic mass of neutral peptide Mr(calc):** 2734.2756
**Fixed modifications:** Carbamidomethyl (C)
**Variable modifications:**
**N10** : Deamidated_N (N)
**Ions Score:** 106 **Expect:** 8.4e-009
**Matches (Bold Red):** 66/268 fragment ions using 110 most intense peaks

| # | b | $b^{++}$ | b* | $b^{*++}$ | $b^{0}$ | $b^{0++}$ | Seq. | y | $y^{++}$ | y* | $y^{*++}$ | $y^{0}$ | $y^{0++}$ | # |
|---|---|---|---|---|---|---|---|---|---|---|---|---|---|---|
| 1 | 138.0662 | 69.5367 | | | | | H | | | | | | | 26 |
| 2 | 251.1503 | 126.0788 | | | | | I | 2598.2239 | **1299.6156** | 2581.1974 | 1291.1023 | 2580.2134 | 1290.6103 | 25 |
| 3 | **366.1772** | 183.5922 | | | **348.1666** | 174.5870 | D | 2485.1399 | **1243.0736** | 2468.1133 | **1234.5603** | 2467.1293 | **1234.0683** | 24 |
| 4 | **526.2078** | 263.6076 | | | 508.1973 | 254.6023 | C | 2370.1129 | **1185.5601** | 2353.0864 | 1177.0468 | 2352.1024 | 1176.5548 | 23 |
| 5 | **597.2450** | 299.1261 | | | 579.2344 | 290.1208 | A | 2210.0823 | **1105.5448** | 2193.0557 | 1097.0315 | 2192.0717 | 1096.5395 | 22 |

| | | | | | | | | | | | | | | |
|---|---|---|---|---|---|---|---|---|---|---|---|---|---|---|
| **6** | **684.2770** | 342.6421 | | | **666.2664** | **333.6368** | **S** | 2139.0452 | **1070.0262** | 2122.0186 | 1061.5130 | 2121.0346 | 1061.0209 | **21** |
| **7** | **783.3454** | 392.1763 | | | **765.3348** | 383.1711 | **V** | 2052.0132 | **1026.5102** | 2034.9866 | 1017.9969 | 2034.0026 | 1017.5049 | **20** |
| **8** | **946.4087** | 473.7080 | | | 928.3982 | **464.7027** | **Y** | **1952.9447** | **976.9760** | 1935.9182 | 968.4627 | 1934.9342 | 967.9707 | **19** |
| **9** | **1003.4302** | 502.2187 | | | 985.4196 | 493.2135 | **G** | **1789.8814** | **895.4443** | 1772.8549 | 886.9311 | 1771.8708 | 886.4391 | **18** |
| **10** | **1118.4571** | 559.7322 | 1101.4306 | 551.2189 | 1100.4466 | 550.7269 | **N** | **1732.8599** | **866.9336** | 1715.8334 | 858.4203 | 1714.8494 | 857.9283 | **17** |
| **11** | **1247.4997** | 624.2535 | 1230.4732 | 615.7402 | 1229.4892 | 615.2482 | **E** | **1617.8330** | **809.4201** | 1600.8065 | 800.9069 | 1599.8224 | 800.4149 | **16** |
| **12** | **1348.5474** | **674.7773** | 1331.5209 | **666.2641** | **1330.5368** | **665.7721** | **T** | **1488.7904** | **744.8988** | 1471.7639 | 736.3856 | 1470.7798 | 735.8936 | **15** |
| **13** | **1477.5900** | 739.2986 | 1460.5634 | 730.7854 | **1459.5794** | 730.2934 | **E** | **1387.7427** | 694.3750 | 1370.7162 | 685.8617 | **1369.7322** | **685.3697** | **14** |
| **14** | **1590.6741** | 795.8407 | 1573.6475 | 787.3274 | 1572.6635 | 786.8354 | **I** | **1258.7001** | **629.8537** | 1241.6736 | 621.3404 | 1240.6896 | 620.8484 | **13** |
| **15** | **1647.6955** | 824.3514 | 1630.6690 | 815.8381 | 1629.6850 | 815.3461 | **G** | **1145.6161** | **573.3117** | 1128.5895 | 564.7984 | 1127.6055 | 564.3064 | **12** |
| **16** | **1776.7381** | **888.8727** | **1759.7116** | 880.3594 | 1758.7275 | 879.8674 | **E** | 1088.5946 | 544.8009 | 1071.5681 | 536.2877 | **1070.5840** | **535.7957** | **11** |
| **17** | 1847.7752 | 924.3913 | 1830.7487 | 915.8780 | 1829.7647 | 915.3860 | **A** | **959.5520** | **480.2796** | 942.5255 | 471.7664 | 941.5415 | 471.2744 | **10** |
| **18** | **1960.8593** | 980.9333 | 1943.8327 | 972.4200 | 1942.8487 | 971.9280 | **L** | **888.5149** | **444.7611** | 871.4884 | 436.2478 | 870.5043 | 435.7558 | **9** |
| **19** | 2088.9543 | 1044.9808 | 2071.9277 | 1036.4675 | 2070.9437 | 1035.9755 | **K** | **775.4308** | **388.2191** | 758.4043 | 379.7058 | 757.4203 | 379.2138 | **8** |
| **20** | 2217.9968 | 1109.5021 | 2200.9703 | 1100.9888 | 2199.9863 | 1100.4968 | **E** | **647.3359** | 324.1716 | **630.3093** | 315.6583 | **629.3253** | 315.1663 | **7** |
| **21** | 2305.0289 | 1153.0181 | 2288.0023 | 1144.5048 | 2287.0183 | 1144.0128 | **S** | **518.2933** | 259.6503 | 501.2667 | 251.1370 | 500.2827 | 250.6450 | **6** |
| **22** | 2404.0973 | 1202.5523 | 2387.0707 | 1194.0390 | 2386.0867 | 1193.5470 | **V** | **431.2613** | 216.1343 | 414.2347 | 207.6210 | | | **5** |
| **23** | 2461.1188 | 1231.0630 | 2444.0922 | 1222.5497 | 2443.1082 | 1222.0577 | **G** | **332.1928** | 166.6001 | 315.1663 | 158.0868 | | | **4** |
| **24** | 2532.1559 | 1266.5816 | 2515.1293 | 1258.0683 | 2514.1453 | 1257.5763 | **A** | 275.1714 | 138.0893 | 258.1448 | 129.5761 | | | **3** |
| **25** | 2589.1773 | 1295.0923 | 2572.1508 | 1286.5790 | 2571.1668 | 1286.0870 | **G** | 204.1343 | 102.5708 | 187.1077 | 94.0575 | | | **2** |
| **26** | | | | | | | **K** | 147.1128 | 74.0600 | 130.0863 | 65.5468 | | | **1** |

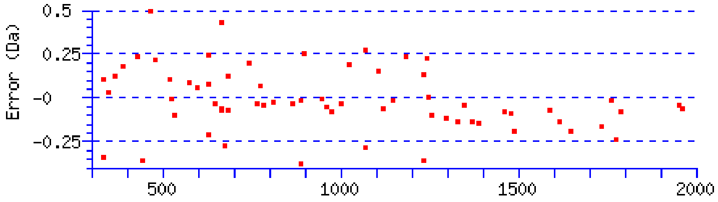

RMS error 292 ppm

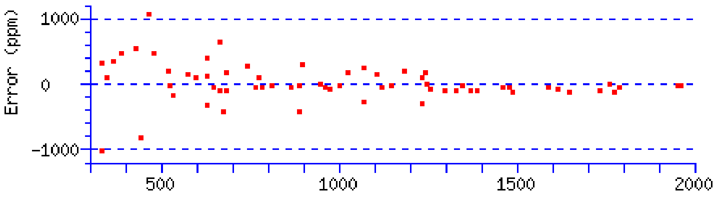

RMS error 292 ppm

**All matches to this query**

| Score | Mr(calc): | Delta | Sequence |
|---|---|---|---|
| 105.8 | 2734.2756 | 0.0113 | HIDCASVYGNETEIGEALKESVGAGK |
| 51.7 | 2733.2915 | 0.9953 | HIDCASVYGNETEIGEALKESVGAGK |
| 4.9 | 2732.2550 | 2.0318 | GPDLPNAIAGVSACVCALNPRLEEK |
| 0.7 | 2734.2906 | -0.0038 | HLRGEASGSSSSSSSSDNELAPFARAK |

---

Spectrum No: 54; Query: 2325; Rank: 1

---

## Peptide View

MS/MS Fragmentation of **LVIILDPAISNNSLSSNPYGPYDR**
Found in **IPI00193894**, Tax_Id=10116 Gene_Symbol=Mgam_predicted 198 kDa protein

Match to Query 2325: 2618.331428 from(1310.172990,2+)
Title: 100101RatKid_NS_deglyco_21.4862.4862.2.dta
Data file K:\NewmanPaper\Piliang\3SubProteomes\Piliang3SP\mgf5ppm\ERLIC_3SubProteomes5ppm.mgf

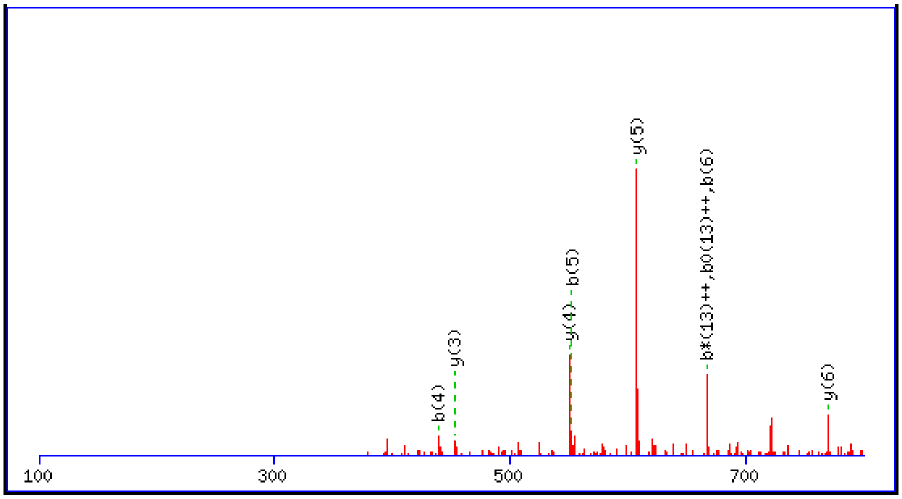


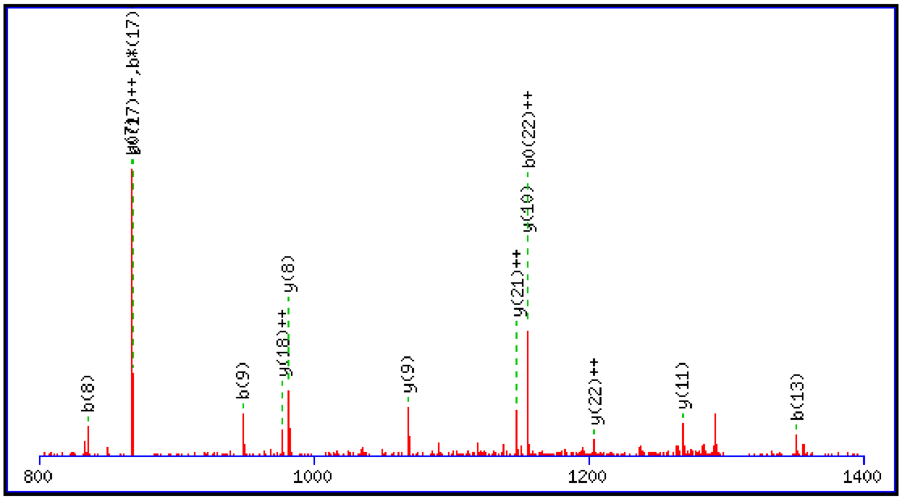


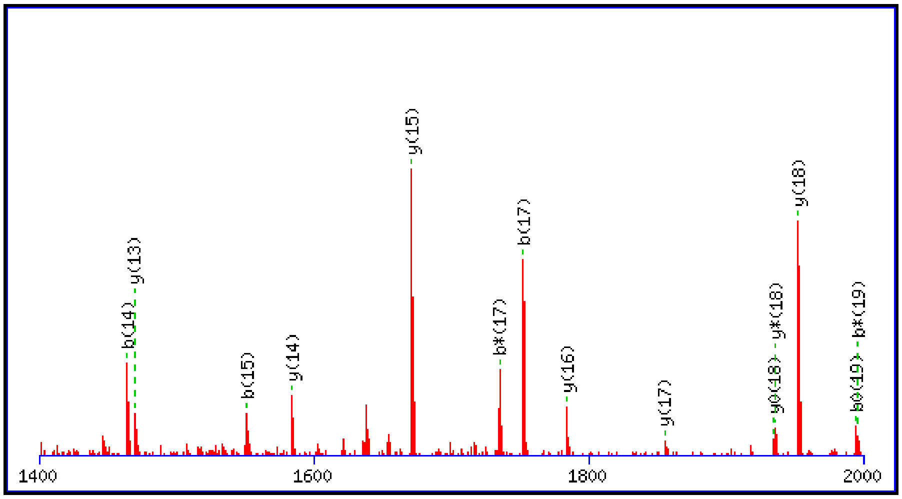


**Monoisotopic mass of neutral peptide Mr(calc):** 2618.3228
**Fixed modifications:** Carbamidomethyl (C)
**Variable modifications:**
**N11** : Deamidated_N (N)
**Ions Score:** 106 **Expect:** 4.8e-009
**Matches (Bold Red):** 37/244 fragment ions using 55 most intense peaks

| # | b | b++ | b* | b*++ | b0 | b0++ | Seq. | y | y++ | y* | y*++ | y0 | y0++ | # |
|---|---|---|---|---|---|---|---|---|---|---|---|---|---|---|
| 1 | 114.0913 | 57.5493 | | | | | L | | | | | | | 24 |
| 2 | 213.1598 | 107.0835 | | | | | V | 2506.2460 | 1253.6266 | 2489.2195 | 1245.1134 | 2488.2354 | 1244.6214 | 23 |
| 3 | 326.2438 | 163.6255 | | | | | I | 2407.1776 | **1204.0924** | 2390.1510 | 1195.5792 | 2389.1670 | 1195.0871 | 22 |
| 4 | **439.3279** | 220.1676 | | | | | I | 2294.0935 | **1147.5504** | 2277.0670 | 1139.0371 | 2276.0830 | 1138.5451 | 21 |
| 5 | **552.4119** | 276.7096 | | | | | L | 2181.0095 | 1091.0084 | 2163.9829 | 1082.4951 | 2162.9989 | 1082.0031 | 20 |

| | | | | | | | | | | | | | | |
|---|---|---|---|---|---|---|---|---|---|---|---|---|---|---|
| 6 | 667.4389 | 334.2231 | | | 649.4283 | 325.2178 | D | 2067.9254 | 1034.4663 | 2050.8988 | 1025.9531 | 2049.9148 | 1025.4611 | 19 |
| 7 | 764.4917 | 382.7495 | | | 746.4811 | 373.7442 | P | 1952.8985 | 976.9529 | 1935.8719 | 968.4396 | 1934.8879 | 967.9476 | 18 |
| 8 | 835.5288 | 418.2680 | | | 817.5182 | 409.2627 | A | 1855.8457 | 928.4265 | 1838.8191 | 919.9132 | 1837.8351 | 919.4212 | 17 |
| 9 | 948.6128 | 474.8101 | | | 930.6023 | 465.8048 | I | 1784.8086 | 892.9079 | 1767.7820 | 884.3947 | 1766.7980 | 883.9026 | 16 |
| 10 | 1035.6449 | 518.3261 | | | 1017.6343 | 509.3208 | S | 1671.7245 | 836.3659 | 1654.6980 | 827.8526 | 1653.7139 | 827.3606 | 15 |
| 11 | 1150.6718 | 575.8395 | 1133.6452 | 567.3263 | 1132.6612 | 566.8343 | N | 1584.6925 | 792.8499 | 1567.6659 | 784.3366 | 1566.6819 | 783.8446 | 14 |
| 12 | 1264.7147 | 632.8610 | 1247.6882 | 624.3477 | 1246.7042 | 623.8557 | N | 1469.6655 | 735.3364 | 1452.6390 | 726.8231 | 1451.6550 | 726.3311 | 13 |
| 13 | 1351.7468 | 676.3770 | 1334.7202 | 667.8637 | 1333.7362 | 667.3717 | S | 1355.6226 | 678.3149 | 1338.5961 | 669.8017 | 1337.6121 | 669.3097 | 12 |
| 14 | 1464.8308 | 732.9190 | 1447.8043 | 724.4058 | 1446.8202 | 723.9138 | L | 1268.5906 | 634.7989 | 1251.5640 | 626.2857 | 1250.5800 | 625.7937 | 11 |
| 15 | 1551.8628 | 776.4351 | 1534.8363 | 767.9218 | 1533.8523 | 767.4298 | S | 1155.5065 | 578.2569 | 1138.4800 | 569.7436 | 1137.4960 | 569.2516 | 10 |
| 16 | 1638.8949 | 819.9511 | 1621.8683 | 811.4378 | 1620.8843 | 810.9458 | S | 1068.4745 | 534.7409 | 1051.4480 | 526.2276 | 1050.4639 | 525.7356 | 9 |
| 17 | 1752.9378 | 876.9725 | 1735.9112 | 868.4593 | 1734.9272 | 867.9673 | N | 981.4425 | 491.2249 | 964.4159 | 482.7116 | 963.4319 | 482.2196 | 8 |
| 18 | 1849.9906 | 925.4989 | 1832.9640 | 916.9856 | 1831.9800 | 916.4936 | P | 867.3995 | 434.2034 | 850.3730 | 425.6901 | 849.3890 | 425.1981 | 7 |
| 19 | 2013.0539 | 1007.0306 | 1996.0273 | 998.5173 | 1995.0433 | 998.0253 | Y | 770.3468 | 385.6770 | 753.3202 | 377.1638 | 752.3362 | 376.6717 | 6 |
| 20 | 2070.0754 | 1035.5413 | 2053.0488 | 1027.0280 | 2052.0648 | 1026.5360 | G | 607.2835 | 304.1454 | 590.2569 | 295.6321 | 589.2729 | 295.1401 | 5 |
| 21 | 2167.1281 | 1084.0677 | 2150.1016 | 1075.5544 | 2149.1176 | 1075.0624 | P | 550.2620 | 275.6346 | 533.2354 | 267.1214 | 532.2514 | 266.6293 | 4 |
| 22 | 2330.1914 | 1165.5994 | 2313.1649 | 1157.0861 | 2312.1809 | 1156.5941 | Y | 453.2092 | 227.1082 | 436.1827 | 218.5950 | 435.1987 | 218.1030 | 3 |
| 23 | 2445.2184 | 1223.1128 | 2428.1918 | 1214.5996 | 2427.2078 | 1214.1076 | D | 290.1459 | 145.5766 | 273.1193 | 137.0633 | 272.1353 | 136.5713 | 2 |
| 24 | | | | | | | R | 175.1190 | 88.0631 | 158.0924 | 79.5498 | | | 1 |

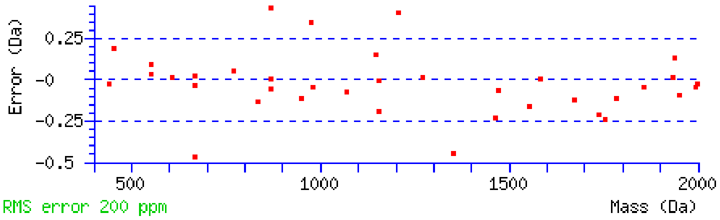


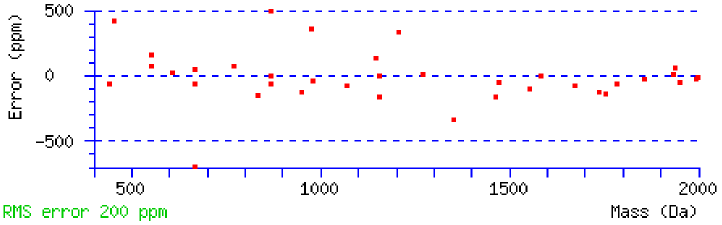


### All matches to this query

| Score | Mr(calc): | Delta | Sequence |
|---|---|---|---|
| 105.6 | 2618.3228 | 0.0087 | LVIILDPAISNNSLSSNPYGPYDR |
| 95.8 | 2618.3228 | 0.0087 | LVIILDPAISNNSLSSNPYGPYDR |
| 81.3 | 2618.3228 | 0.0087 | LVIILDPAISNNSLSSNPYGPYDR |
| 40.1 | 2617.3387 | 0.9927 | LVIILDPAISNNSLSSNPYGPYDR |
| 7.8 | 2618.3551 | -0.0237 | QNLSQQLDVKLVPSSYSEAVASVR |
| 3.2 | 2617.3460 | 0.9855 | EETVLATVQALHTASHLSQQADLR |
| 0.9 | 2616.3080 | 2.0235 | SRAAYNVTLLNFMDPQKMPYLK |

---

Spectrum No: 55; Query: 1924; Rank: 1

---

## Peptide View

MS/MS Fragmentation of **SCATSQEDFLIYSLNNSLR**
Found in **IPI00205325**, Tax_Id=10116 Gene_Symbol=Lrp2 Low-density lipoprotein receptor-related protein 2 precursor

Match to Query 1924: 2219.018988 from(1110.516770,2+)
Title: 091008RatKidney_NH4Format01_24.4275.4275.2.dta
Data file K:\NewmanPaper\Piliang\3SubProteomes\Piliang3SP\mgf5ppm\ERLIC_3SubProteomes5ppm.mgf

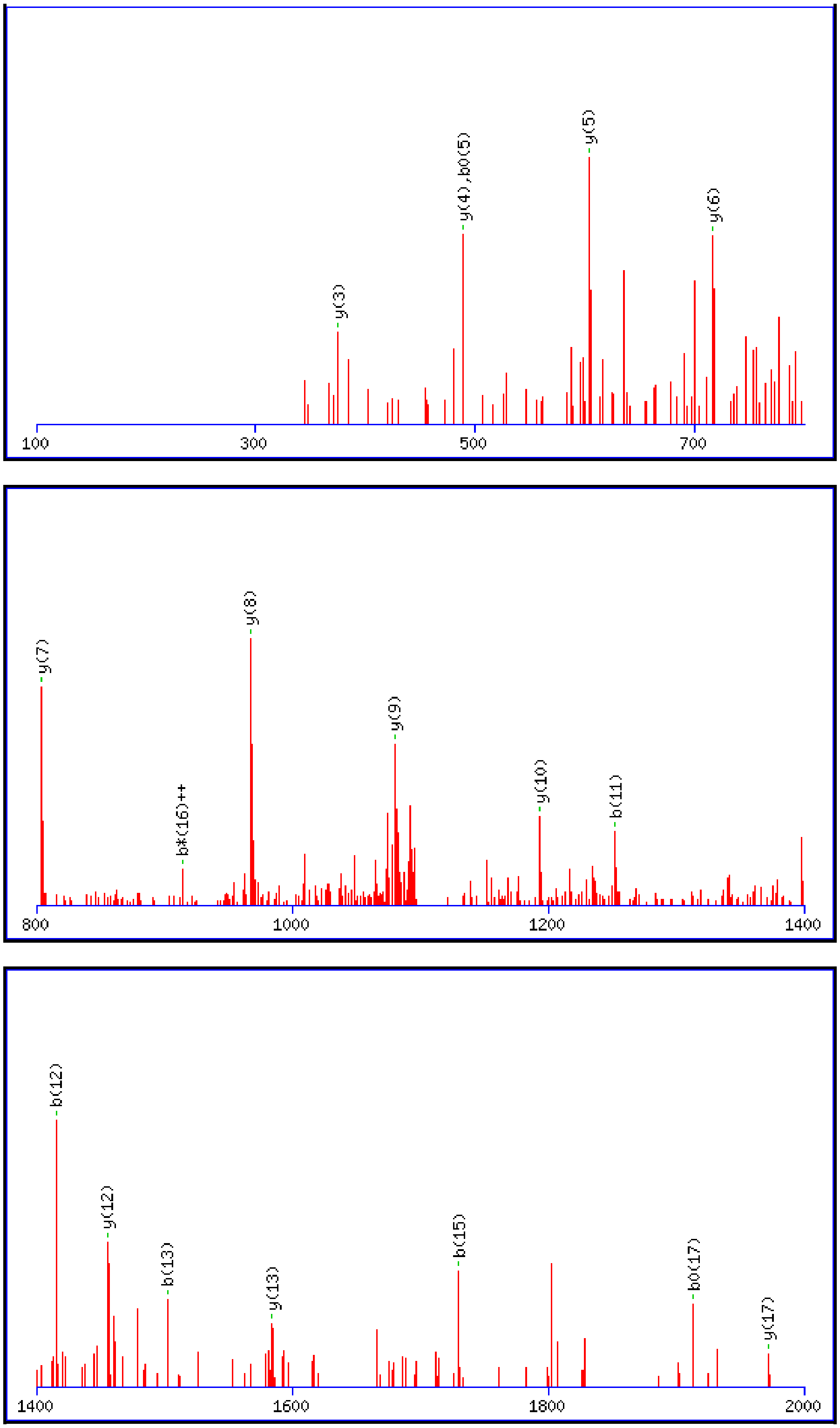


**Monoisotopic mass of neutral peptide Mr(calc):** 2218.0212
**Fixed modifications:** Carbamidomethyl (C)
**Variable modifications:**
**N15** : Deamidated_N (N)
**Ions Score:** 105 **Expect:** 8.7e-009
**Matches (Bold Red):** 18/202 fragment ions using 25 most intense peaks

| # | b | b++ | b* | b*++ | b0 | b0++ | Seq. | y | y++ | y* | y*++ | y0 | y0++ | # |
|---|---|---|---|---|---|---|---|---|---|---|---|---|---|---|
| 1 | 88.0393 | 44.5233 | | | 70.0287 | 35.5180 | S | | | | | | | 19 |
| 2 | 248.0700 | 124.5386 | | | 230.0594 | 115.5333 | C | 2131.9965 | 1066.5019 | 2114.9699 | 1057.9886 | 2113.9859 | 1057.4966 | 18 |
| 3 | 319.1071 | 160.0572 | | | 301.0965 | 151.0519 | A | **1971.9658** | 986.4865 | 1954.9393 | 977.9733 | 1953.9552 | 977.4813 | 17 |
| 4 | 420.1547 | 210.5810 | | | 402.1442 | 201.5757 | T | 1900.9287 | 950.9680 | 1883.9021 | 942.4547 | 1882.9181 | 941.9627 | 16 |
| 5 | 507.1868 | 254.0970 | | | **489.1762** | 245.0917 | S | 1799.8810 | 900.4441 | 1782.8545 | 891.9309 | 1781.8704 | 891.4389 | 15 |

| | | | | | | | | | | | | | | |
|---|---|---|---|---|---|---|---|---|---|---|---|---|---|---|
| **6** | 635.2454 | 318.1263 | 618.2188 | 309.6130 | 617.2348 | 309.1210 | **Q** | 1712.8490 | 856.9281 | 1695.8224 | 848.4149 | 1694.8384 | 847.9228 | **14** |
| **7** | 764.2879 | 382.6476 | 747.2614 | 374.1343 | 746.2774 | 373.6423 | **E** | **1584.7904** | 792.8988 | 1567.7639 | 784.3856 | 1566.7798 | 783.8936 | **13** |
| **8** | 879.3149 | 440.1611 | 862.2883 | 431.6478 | 861.3043 | 431.1558 | **D** | **1455.7478** | 728.3775 | 1438.7213 | 719.8643 | 1437.7372 | 719.3723 | **12** |
| **9** | 1026.3833 | 513.6953 | 1009.3568 | 505.1820 | 1008.3727 | 504.6900 | **F** | 1340.7209 | 670.8641 | 1323.6943 | 662.3508 | 1322.7103 | 661.8588 | **11** |
| **10** | 1139.4674 | 570.2373 | 1122.4408 | 561.7240 | 1121.4568 | 561.2320 | **L** | **1193.6525** | 597.3299 | 1176.6259 | 588.8166 | 1175.6419 | 588.3246 | **10** |
| **11** | **1252.5514** | 626.7794 | 1235.5249 | 618.2661 | 1234.5409 | 617.7741 | **I** | **1080.5684** | 540.7878 | 1063.5418 | 532.2746 | 1062.5578 | 531.7826 | **9** |
| **12** | **1415.6148** | 708.3110 | 1398.5882 | 699.7977 | 1397.6042 | 699.3057 | **Y** | **967.4843** | 484.2458 | 950.4578 | 475.7325 | 949.4738 | 475.2405 | **8** |
| **13** | **1502.6468** | 751.8270 | 1485.6202 | 743.3138 | 1484.6362 | 742.8217 | **S** | **804.4210** | 402.7141 | 787.3945 | 394.2009 | 786.4104 | 393.7089 | **7** |
| **14** | 1615.7309 | 808.3691 | 1598.7043 | 799.8558 | 1597.7203 | 799.3638 | **L** | **717.3890** | 359.1981 | 700.3624 | 350.6848 | 699.3784 | 350.1928 | **6** |
| **15** | **1730.7578** | 865.8825 | 1713.7312 | 857.3693 | 1712.7472 | 856.8772 | **N** | **604.3049** | 302.6561 | 587.2784 | 294.1428 | 586.2943 | 293.6508 | **5** |
| **16** | 1844.8007 | 922.9040 | 1827.7742 | **914.3907** | 1826.7901 | 913.8987 | **N** | **489.2780** | 245.1426 | 472.2514 | 236.6293 | 471.2674 | 236.1373 | **4** |
| **17** | 1931.8327 | 966.4200 | 1914.8062 | 957.9067 | **1913.8222** | 957.4147 | **S** | **375.2350** | 188.1212 | 358.2085 | 179.6079 | 357.2245 | 179.1159 | **3** |
| **18** | 2044.9168 | 1022.9620 | 2027.8903 | 1014.4488 | 2026.9062 | 1013.9568 | **L** | 288.2030 | 144.6051 | 271.1765 | 136.0919 | | | **2** |
| **19** | | | | | | | **R** | 175.1190 | 88.0631 | 158.0924 | 79.5498 | | | **1** |

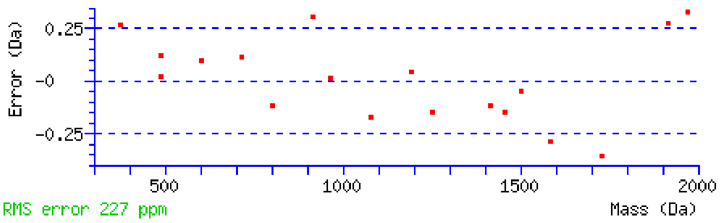


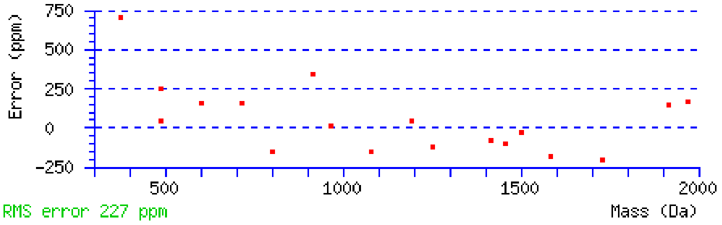


### All matches to this query

| Score | Mr(calc): | Delta | Sequence |
|---|---|---|---|
| 105.3 | 2218.0212 | 0.9978 | SCATSQEDFLIYSLNNSLR |
| 87.3 | 2218.0212 | 0.9978 | SCATSQEDFLIYSLNNSLR |
| 56.6 | 2219.0052 | 0.0138 | SCATSQEDFLIYSLNNSLR |
| 6.0 | 2217.0191 | 1.9999 | TKGPSRRPSLISSDSNNLK |
| 6.0 | 2217.0191 | 1.9999 | TKGPSRRPSLISSDSNNLK |
| 5.0 | 2218.0031 | 1.0158 | TKGPSRRPSLISSDSNNLK |
| 5.0 | 2218.0031 | 1.0158 | TKGPSRRPSLISSDSNNLK |
| 5.0 | 2218.0031 | 1.0158 | TKGPSRRPSLISSDSNNLK |
| 4.8 | 2219.0161 | 0.0029 | LPGASLSVHTWQGNGYSHTK |
| 4.1 | 2219.0161 | 0.0029 | LPGASLSVHTWQGNGYSHTK |

---

Spectrum No: 56; Query: 1915; Rank: 1

---

## Peptide View

MS/MS Fragmentation of **LDNNGCSTQEVNITEFQLK**
Found in **IPI00201262**, Tax_Id=10116 Gene_Symbol=LOC297568;Mug2;Mug1 Alpha-1-inhibitor 3 precursor

Match to Query 1915: 2210.025308 from(1106.019930,2+)
Title: 100101RatKid_NS_deglyco_26.3319.3319.2.dta
Data file K:\NewmanPaper\Piliang\3SubProteomes\Piliang3SP\mgf5ppm\ERLIC_3SubProteomes5ppm.mgf

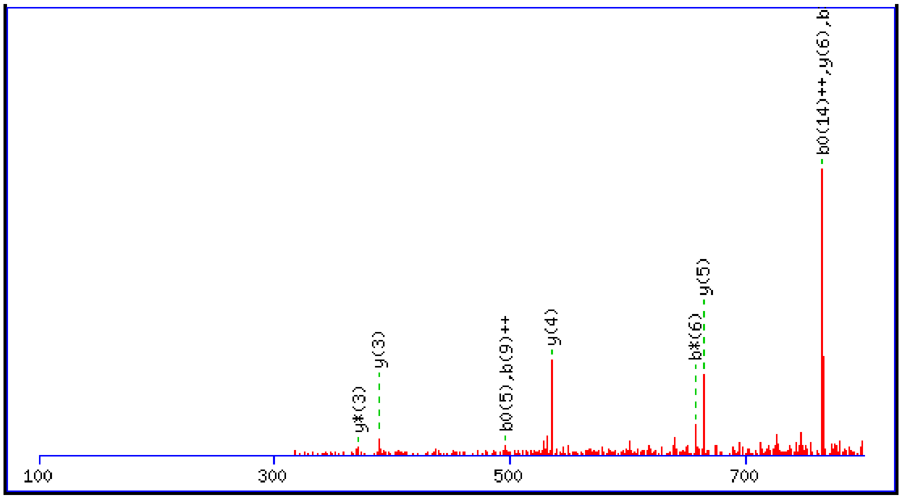


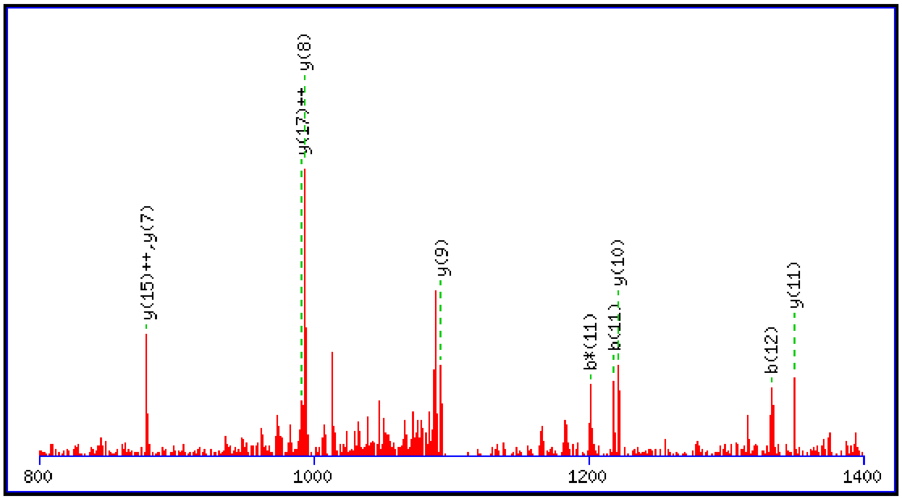


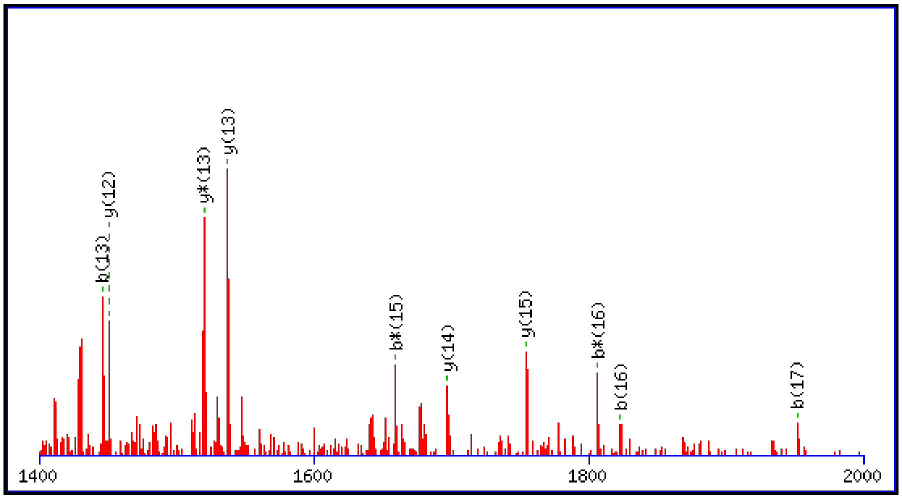


**Monoisotopic mass of neutral peptide Mr(calc):** 2210.0161
**Fixed modifications:** Carbamidomethyl (C)
**Variable modifications:**
**N12** : Deamidated_N (N)
**Ions Score:** 105 **Expect:** 8.8e-009
**Matches (Bold Red):** 30/202 fragment ions using 44 most intense peaks

| # | b | $b^{++}$ | b* | $b^{*++}$ | $b^0$ | $b^{0++}$ | Seq. | y | $y^{++}$ | y* | $y^{*++}$ | $y^0$ | $y^{0++}$ | # |
|---|---|---|---|---|---|---|---|---|---|---|---|---|---|---|
| 1 | 114.0913 | 57.5493 | | | | | L | | | | | | | 19 |
| 2 | 229.1183 | 115.0628 | | | 211.1077 | 106.0575 | D | 2097.9393 | 1049.4733 | 2080.9128 | 1040.9600 | 2079.9288 | 1040.4680 | 18 |
| 3 | 343.1612 | 172.0842 | 326.1347 | 163.5710 | 325.1506 | 163.0790 | N | 1982.9124 | **991.9598** | 1965.8858 | 983.4466 | 1964.9018 | 982.9546 | 17 |
| 4 | 457.2041 | 229.1057 | 440.1776 | 220.5924 | 439.1936 | 220.1004 | N | 1868.8695 | 934.9384 | 1851.8429 | 926.4251 | 1850.8589 | 925.9331 | 16 |
| 5 | 514.2256 | 257.6164 | 497.1991 | 249.1032 | **496.2150** | 248.6112 | G | **1754.8265** | **877.9169** | 1737.8000 | 869.4036 | 1736.8160 | 868.9116 | 15 |

| | | | | | | | | | | | | | | |
|---|---|---|---|---|---|---|---|---|---|---|---|---|---|---|
| 6 | 674.2563 | 337.6318 | **657.2297** | 329.1185 | 656.2457 | 328.6265 | C | **1697.8051** | 849.4062 | 1680.7785 | 840.8929 | 1679.7945 | 840.4009 | 14 |
| 7 | 761.2883 | 381.1478 | 744.2617 | 372.6345 | 743.2777 | 372.1425 | S | **1537.7744** | 769.3909 | **1520.7479** | 760.8776 | 1519.7639 | 760.3856 | 13 |
| 8 | 862.3360 | 431.6716 | 845.3094 | 423.1583 | 844.3254 | 422.6663 | T | **1450.7424** | 725.8748 | 1433.7158 | 717.3616 | 1432.7318 | 716.8696 | 12 |
| 9 | 990.3945 | **495.7009** | 973.3680 | 487.1876 | 972.3840 | 486.6956 | Q | **1349.6947** | 675.3510 | 1332.6682 | 666.8377 | 1331.6842 | 666.3457 | 11 |
| 10 | 1119.4371 | 560.2222 | 1102.4106 | 551.7089 | 1101.4266 | 551.2169 | E | **1221.6361** | 611.3217 | 1204.6096 | 602.8084 | 1203.6256 | 602.3164 | 10 |
| 11 | **1218.5055** | 609.7564 | **1201.4790** | 601.2431 | 1200.4950 | 600.7511 | V | **1092.5935** | 546.8004 | 1075.5670 | 538.2871 | 1074.5830 | 537.7951 | 9 |
| 12 | **1333.5325** | 667.2699 | 1316.5059 | 658.7566 | 1315.5219 | 658.2646 | N | **993.5251** | 497.2662 | 976.4986 | 488.7529 | 975.5146 | 488.2609 | 8 |
| 13 | **1446.6165** | 723.8119 | 1429.5900 | 715.2986 | 1428.6060 | 714.8066 | I | **878.4982** | 439.7527 | 861.4716 | 431.2395 | 860.4876 | 430.7475 | 7 |
| 14 | 1547.6642 | 774.3357 | 1530.6377 | **765.8225** | 1529.6537 | **765.3305** | T | **765.4141** | 383.2107 | 748.3876 | 374.6974 | 747.4036 | 374.2054 | 6 |
| 15 | 1676.7068 | 838.8570 | **1659.6803** | 830.3438 | 1658.6962 | 829.8518 | E | **664.3665** | 332.6869 | 647.3399 | 324.1736 | 646.3559 | 323.6816 | 5 |
| 16 | **1823.7752** | 912.3913 | **1806.7487** | 903.8780 | 1805.7647 | 903.3860 | F | **535.3239** | 268.1656 | 518.2973 | 259.6523 | | | 4 |
| 17 | **1951.8338** | 976.4205 | 1934.8073 | 967.9073 | 1933.8232 | 967.4153 | Q | **388.2554** | 194.6314 | **371.2289** | 186.1181 | | | 3 |
| 18 | 2064.9179 | 1032.9626 | 2047.8913 | 1024.4493 | 2046.9073 | 1023.9573 | L | 260.1969 | 130.6021 | 243.1703 | 122.0888 | | | 2 |
| 19 | | | | | | | K | 147.1128 | 74.0600 | 130.0863 | 65.5468 | | | 1 |

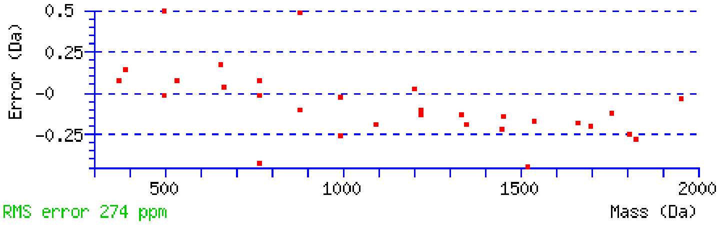


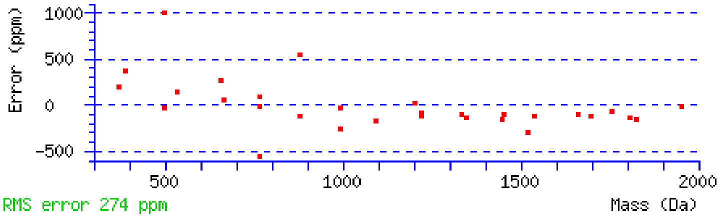


### All matches to this query

| Score | Mr(calc): | Delta | Sequence |
|---|---|---|---|
| 104.8 | 2210.0161 | 0.0092 | LDNNGCSTQEVNITEFQLK |
| 19.9 | 2210.0161 | 0.0092 | LDNNGCSTQEVNITEFQLK |
| 19.9 | 2210.0161 | 0.0092 | LDNNGCSTQEVNITEFQLK |
| 10.0 | 2209.0321 | 0.9932 | LDNNGCSTQEVNITEFQLK |
| 6.3 | 2210.0248 | 0.0006 | LKSHPXTVMNLKNYFLK |
| 2.6 | 2210.0402 | -0.0149 | QDLGGLINVNPETNKSMKR |
| 2.5 | 2210.0157 | 0.0096 | EQLDTIWSDLSHHPLSPR |
| 2.3 | 2208.0059 | 2.0194 | NRVTLTLDNDAASPAPETSR |
| 2.1 | 2209.0126 | 1.0127 | DCNLVYLIPHAVGSSEDLK |
| 1.7 | 2209.0126 | 1.0127 | DCNLVYLIPHAVGSSEDLK |

---

Spectrum No: 57; Query: 1915; Rank: 1

---

## Peptide View

MS/MS Fragmentation of **LDNNGCSTQEVNITEFQLK**
Found in **IPI00201262**, Tax_Id=10116 Gene_Symbol=LOC297568;Mug2;Mug1 Alpha-1-inhibitor 3 precursor

Match to Query 1915: 2210.025308 from(1106.019930,2+)
Title: 100101RatKid_NS_deglyco_26.3319.3319.2.dta
Data file K:\NewmanPaper\Piliang\3SubProteomes\Piliang3SP\mgf5ppm\ERLIC_3SubProteomes5ppm.mgf

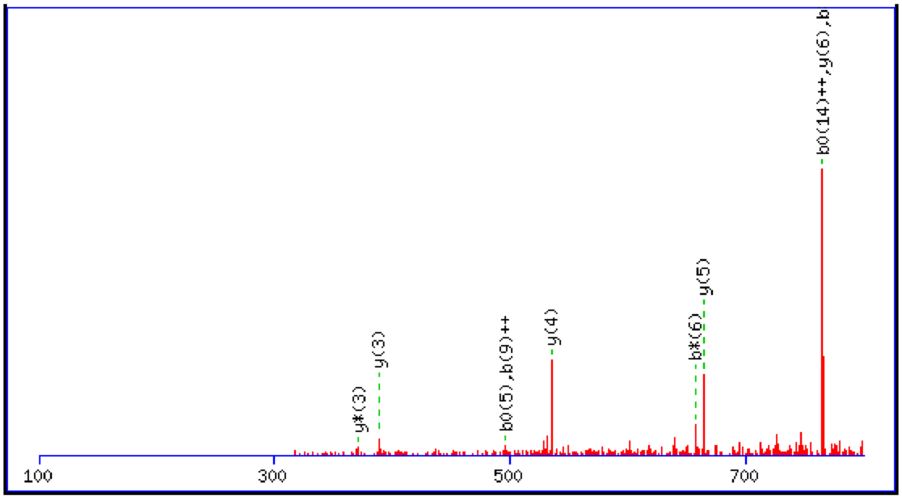


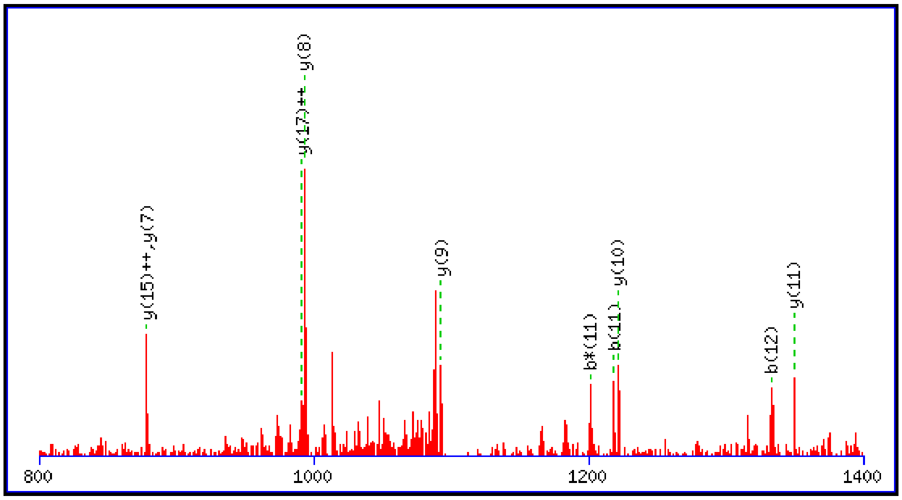


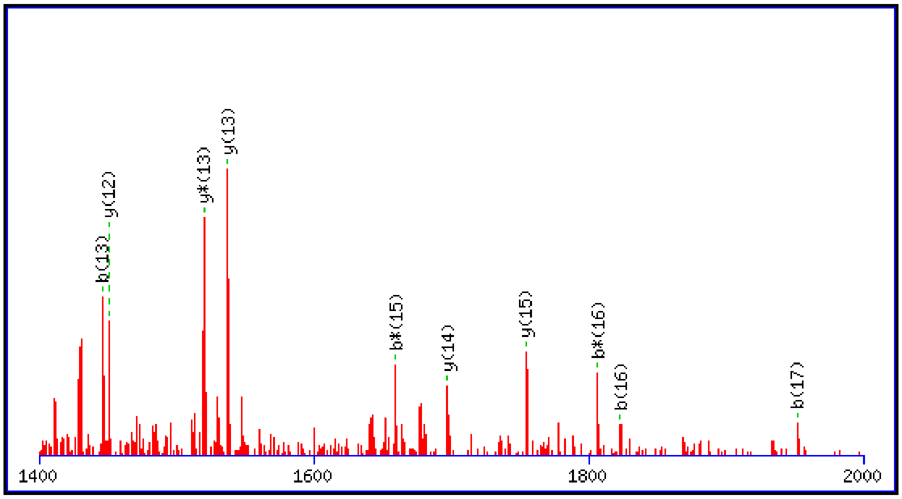


**Monoisotopic mass of neutral peptide Mr(calc):** 2210.0161
**Fixed modifications:** Carbamidomethyl (C)
**Variable modifications:**
**N12** : Deamidated_N (N)
**Ions Score:** 105 **Expect:** 8.8e-009
**Matches (Bold Red):** 30/202 fragment ions using 44 most intense peaks

| # | b | b++ | b* | b*++ | b0 | b0++ | Seq. | y | y++ | y* | y*++ | y0 | y0++ | # |
|---|---|---|---|---|---|---|---|---|---|---|---|---|---|---|
| 1 | 114.0913 | 57.5493 | | | | | L | | | | | | | 19 |
| 2 | 229.1183 | 115.0628 | | | 211.1077 | 106.0575 | D | 2097.9393 | 1049.4733 | 2080.9128 | 1040.9600 | 2079.9288 | 1040.4680 | 18 |
| 3 | 343.1612 | 172.0842 | 326.1347 | 163.5710 | 325.1506 | 163.0790 | N | 1982.9124 | **991.9598** | 1965.8858 | 983.4466 | 1964.9018 | 982.9546 | 17 |
| 4 | 457.2041 | 229.1057 | 440.1776 | 220.5924 | 439.1936 | 220.1004 | N | 1868.8695 | 934.9384 | 1851.8429 | 926.4251 | 1850.8589 | 925.9331 | 16 |
| 5 | 514.2256 | 257.6164 | 497.1991 | 249.1032 | **496.2150** | 248.6112 | G | **1754.8265** | **877.9169** | 1737.8000 | 869.4036 | 1736.8160 | 868.9116 | 15 |

| | | | | | | | | | | | | | | |
|---|---|---|---|---|---|---|---|---|---|---|---|---|---|---|
| 6 | 674.2563 | 337.6318 | **657.2297** | 329.1185 | 656.2457 | 328.6265 | C | **1697.8051** | 849.4062 | 1680.7785 | 840.8929 | 1679.7945 | 840.4009 | 14 |
| 7 | 761.2883 | 381.1478 | 744.2617 | 372.6345 | 743.2777 | 372.1425 | S | **1537.7744** | 769.3909 | **1520.7479** | 760.8776 | 1519.7639 | 760.3856 | 13 |
| 8 | 862.3360 | 431.6716 | 845.3094 | 423.1583 | 844.3254 | 422.6663 | T | **1450.7424** | 725.8748 | 1433.7158 | 717.3616 | 1432.7318 | 716.8696 | 12 |
| 9 | 990.3945 | **495.7009** | 973.3680 | 487.1876 | 972.3840 | 486.6956 | Q | **1349.6947** | 675.3510 | 1332.6682 | 666.8377 | 1331.6842 | 666.3457 | 11 |
| 10 | 1119.4371 | 560.2222 | 1102.4106 | 551.7089 | 1101.4266 | 551.2169 | E | **1221.6361** | 611.3217 | 1204.6096 | 602.8084 | 1203.6256 | 602.3164 | 10 |
| 11 | **1218.5055** | 609.7564 | **1201.4790** | 601.2431 | 1200.4950 | 600.7511 | V | **1092.5935** | 546.8004 | 1075.5670 | 538.2871 | 1074.5830 | 537.7951 | 9 |
| 12 | **1333.5325** | 667.2699 | 1316.5059 | 658.7566 | 1315.5219 | 658.2646 | N | **993.5251** | 497.2662 | 976.4986 | 488.7529 | 975.5146 | 488.2609 | 8 |
| 13 | **1446.6165** | 723.8119 | 1429.5900 | 715.2986 | 1428.6060 | 714.8066 | I | **878.4982** | 439.7527 | 861.4716 | 431.2395 | 860.4876 | 430.7475 | 7 |
| 14 | 1547.6642 | 774.3357 | 1530.6377 | **765.8225** | 1529.6537 | **765.3305** | T | **765.4141** | 383.2107 | 748.3876 | 374.6974 | 747.4036 | 374.2054 | 6 |
| 15 | 1676.7068 | 838.8570 | **1659.6803** | 830.3438 | 1658.6962 | 829.8518 | E | **664.3665** | 332.6869 | 647.3399 | 324.1736 | 646.3559 | 323.6816 | 5 |
| 16 | **1823.7752** | 912.3913 | **1806.7487** | 903.8780 | 1805.7647 | 903.3860 | F | **535.3239** | 268.1656 | 518.2973 | 259.6523 | | | 4 |
| 17 | **1951.8338** | 976.4205 | 1934.8073 | 967.9073 | 1933.8232 | 967.4153 | Q | **388.2554** | 194.6314 | **371.2289** | 186.1181 | | | 3 |
| 18 | 2064.9179 | 1032.9626 | 2047.8913 | 1024.4493 | 2046.9073 | 1023.9573 | L | 260.1969 | 130.6021 | 243.1703 | 122.0888 | | | 2 |
| 19 | | | | | | | K | 147.1128 | 74.0600 | 130.0863 | 65.5468 | | | 1 |

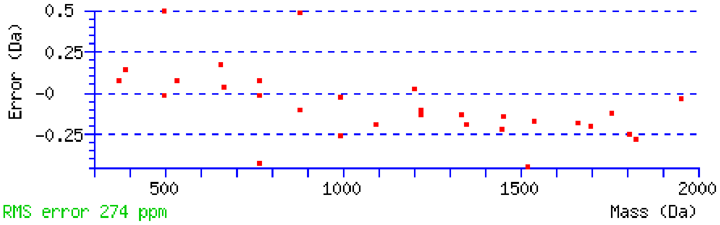


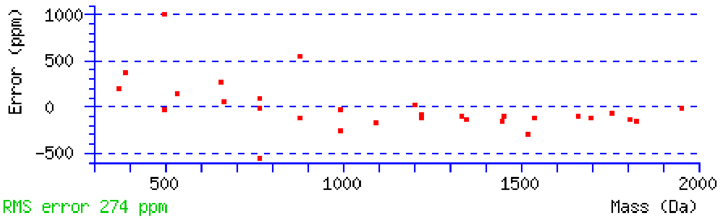


### All matches to this query

| Score | Mr(calc): | Delta | Sequence |
|---|---|---|---|
| 104.8 | 2210.0161 | 0.0092 | LDNNGCSTQEVNITEFQLK |
| 19.9 | 2210.0161 | 0.0092 | LDNNGCSTQEVNITEFQLK |
| 19.9 | 2210.0161 | 0.0092 | LDNNGCSTQEVNITEFQLK |
| 10.0 | 2209.0321 | 0.9932 | LDNNGCSTQEVNITEFQLK |
| 6.3 | 2210.0248 | 0.0006 | LKSHPXTVMNLKNYFLK |
| 2.6 | 2210.0402 | -0.0149 | QDLGGLINVNPETNKSMKR |
| 2.5 | 2210.0157 | 0.0096 | EQLDTIWSDLSHHPLSPR |
| 2.3 | 2208.0059 | 2.0194 | NRVTLTLDNDAASPAPETSR |
| 2.1 | 2209.0126 | 1.0127 | DCNLVYLIPHAVGSSEDLK |
| 1.7 | 2209.0126 | 1.0127 | DCNLVYLIPHAVGSSEDLK |

---

Spectrum No: 58; Query: 1206; Rank: 1

---

## Peptide View

MS/MS Fragmentation of **TSVGNSSFYQDTVVFK**
Found in **IPI00195241**, Tax_Id=10116 Gene_Symbol=Apoh Beta-2-glycoprotein 1 precursor

Match to Query 1206: 1778.837308 from(890.425930,2+)
Title: 100101RatKid_NS_deglyco_27.3109.3109.2.dta
Data file K:\NewmanPaper\Piliang\3SubProteomes\Piliang3SP\mgf5ppm\ERLIC_3SubProteomes5ppm.mgf

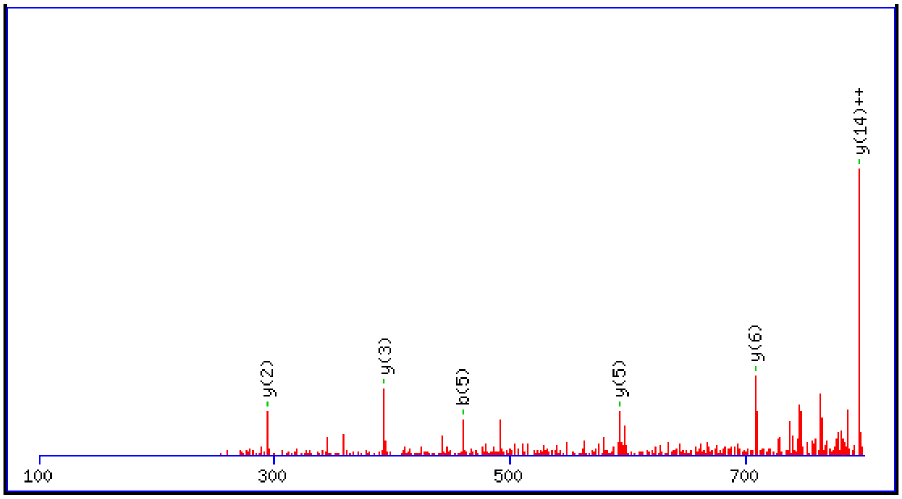


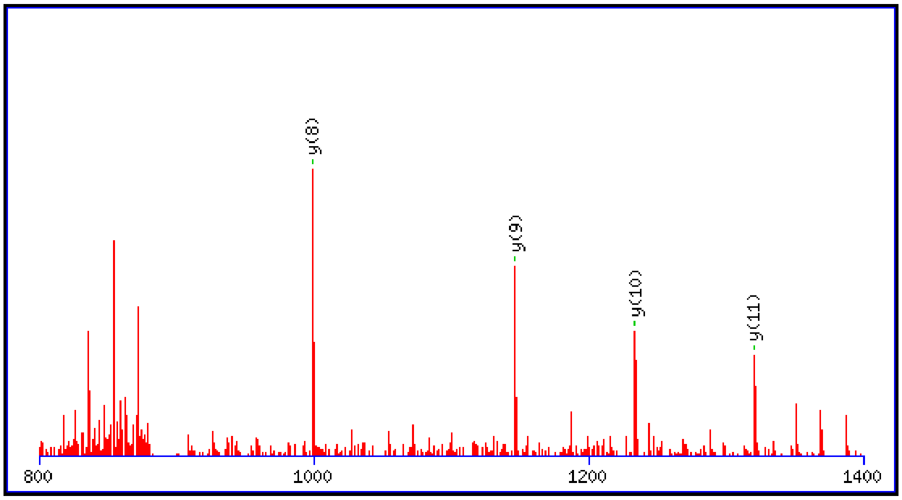


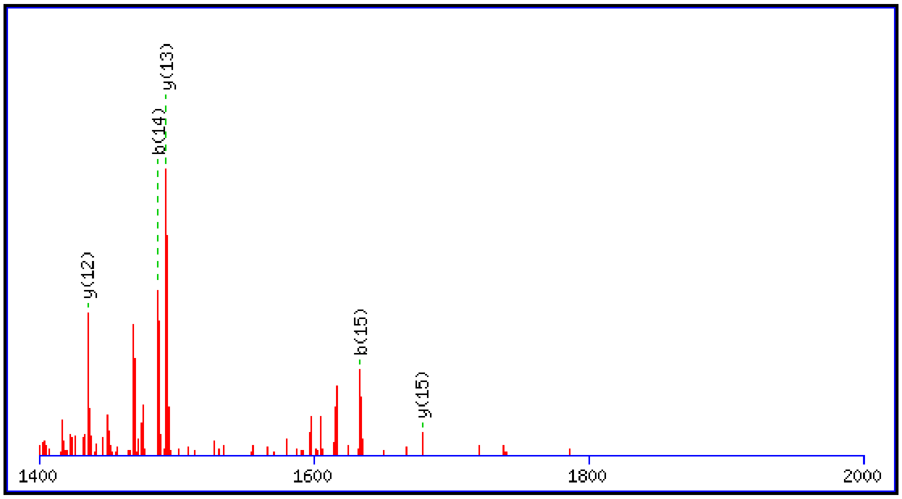


**Monoisotopic mass of neutral peptide Mr(calc):** 1778.8363
**Fixed modifications:** Carbamidomethyl (C)
**Variable modifications:**
**N5** : Deamidated_N (N)
**Ions Score:** 105 **Expect:** 7e-009
**Matches (Bold Red):** 15/164 fragment ions using 20 most intense peaks

| # | b | $b^{++}$ | b* | $b^{*++}$ | $b^{0}$ | $b^{0++}$ | Seq. | y | $y^{++}$ | y* | $y^{*++}$ | $y^{0}$ | $y^{0++}$ | # |
|---|---|---|---|---|---|---|---|---|---|---|---|---|---|---|
| 1 | 102.0550 | 51.5311 | | | 84.0444 | 42.5258 | T | | | | | | | 16 |
| 2 | 189.0870 | 95.0471 | | | 171.0764 | 86.0418 | S | **1678.7959** | 839.9016 | 1661.7693 | 831.3883 | 1660.7853 | 830.8963 | 15 |
| 3 | 288.1554 | 144.5813 | | | 270.1448 | 135.5761 | V | 1591.7639 | **796.3856** | 1574.7373 | 787.8723 | 1573.7533 | 787.3803 | 14 |
| 4 | 345.1769 | 173.0921 | | | 327.1663 | 164.0868 | G | **1492.6954** | 746.8514 | 1475.6689 | 738.3381 | 1474.6849 | 737.8461 | 13 |
| 5 | **460.2038** | 230.6055 | 443.1772 | 222.0923 | 442.1932 | 221.6003 | N | **1435.6740** | 718.3406 | 1418.6474 | 709.8274 | 1417.6634 | 709.3353 | 12 |

| | | | | | | | | | | | | | | |
|---|---|---|---|---|---|---|---|---|---|---|---|---|---|---|
| **6** | 547.2358 | 274.1216 | 530.2093 | 265.6083 | 529.2253 | 265.1163 | **S** | **1320.6470** | 660.8272 | 1303.6205 | 652.3139 | 1302.6365 | 651.8219 | **11** |
| **7** | 634.2679 | 317.6376 | 617.2413 | 309.1243 | 616.2573 | 308.6323 | **S** | **1233.6150** | 617.3111 | 1216.5885 | 608.7979 | 1215.6045 | 608.3059 | **10** |
| **8** | 781.3363 | 391.1718 | 764.3097 | 382.6585 | 763.3257 | 382.1665 | **F** | **1146.5830** | 573.7951 | 1129.5564 | 565.2819 | 1128.5724 | 564.7899 | **9** |
| **9** | 944.3996 | 472.7034 | 927.3730 | 464.1902 | 926.3890 | 463.6982 | **Y** | **999.5146** | 500.2609 | 982.4880 | 491.7477 | 981.5040 | 491.2556 | **8** |
| **10** | 1072.4582 | 536.7327 | 1055.4316 | 528.2195 | 1054.4476 | 527.7274 | **Q** | 836.4512 | 418.7293 | 819.4247 | 410.2160 | 818.4407 | 409.7240 | **7** |
| **11** | 1187.4851 | 594.2462 | 1170.4586 | 585.7329 | 1169.4746 | 585.2409 | **D** | **708.3927** | 354.7000 | 691.3661 | 346.1867 | 690.3821 | 345.6947 | **6** |
| **12** | 1288.5328 | 644.7700 | 1271.5062 | 636.2568 | 1270.5222 | 635.7648 | **T** | **593.3657** | 297.1865 | 576.3392 | 288.6732 | 575.3552 | 288.1812 | **5** |
| **13** | 1387.6012 | 694.3042 | 1370.5747 | 685.7910 | 1369.5906 | 685.2990 | **V** | 492.3180 | 246.6627 | 475.2915 | 238.1494 | | | **4** |
| **14** | **1486.6696** | 743.8385 | 1469.6431 | 735.3252 | 1468.6591 | 734.8332 | **V** | **393.2496** | 197.1285 | 376.2231 | 188.6152 | | | **3** |
| **15** | **1633.7380** | 817.3727 | 1616.7115 | 808.8594 | 1615.7275 | 808.3674 | **F** | **294.1812** | 147.5942 | 277.1547 | 139.0810 | | | **2** |
| **16** | | | | | | | **K** | 147.1128 | 74.0600 | 130.0863 | 65.5468 | | | **1** |

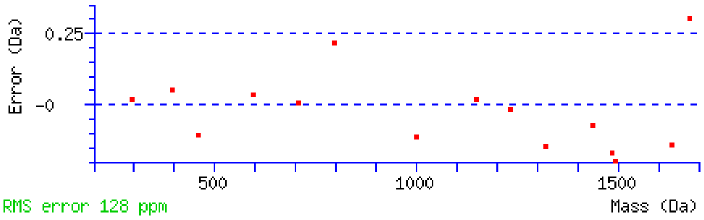


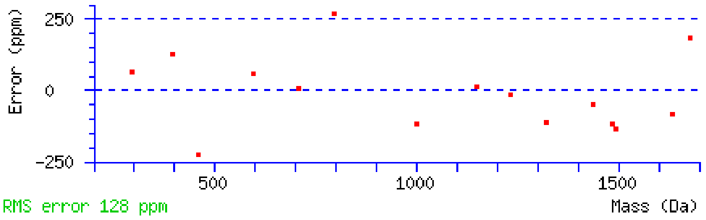


**All matches to this query**

| Score | Mr(calc): | Delta | Sequence |
|---|---|---|---|
| 104.7 | 1778.8363 | 0.0010 | TSVGNSSFYQDTVVFK |
| 9.3 | 1777.8264 | 1.0109 | AGTKTSSASLSSLALPK |
| 6.1 | 1778.8491 | -0.0118 | TLFINGISKYAESEK |
| 5.9 | 1778.8543 | -0.0170 | TASSXGPALATGNVVVMK |
| 5.6 | 1776.8369 | 2.0004 | SGSLSLTQFADMISLK |
| 3.1 | 1778.8304 | 0.0069 | EDQRAFHRMMTGLR |
| 1.4 | 1778.8509 | -0.0136 | TVNCSLWQENSLTVK |
| 1.4 | 1778.8509 | -0.0136 | TVNCSLWQENSLTVK |
| 1.4 | 1778.8491 | -0.0118 | TLFINGISKYAESEK |
| 0.2 | 1778.8465 | -0.0092 | SVLHGVNDNRSQVFK |

---

Spectrum No: 59; Query: 871; Rank: 1

---

## Peptide View

MS/MS Fragmentation of **MIENGSLSFLPTLR**
Found in **IPI00191090**, Tax_Id=10116 Gene_Symbol=Bgn Biglycan precursor

Match to Query 871: 1593.807468 from(797.911010,2+)
Title: 091008RatKidney_NH4Format01_26.3451.3451.2.dta
Data file K:\NewmanPaper\Piliang\3SubProteomes\Piliang3SP\mgf5ppm\ERLIC_3SubProteomes5ppm.mgf

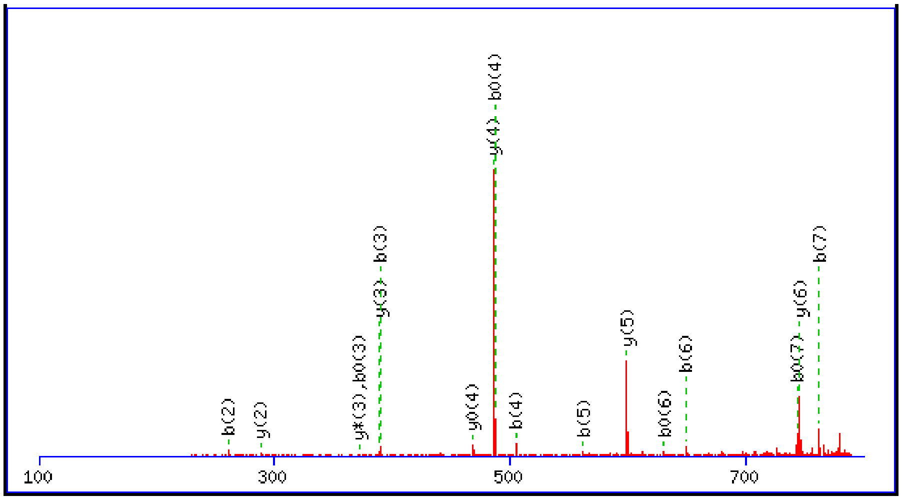


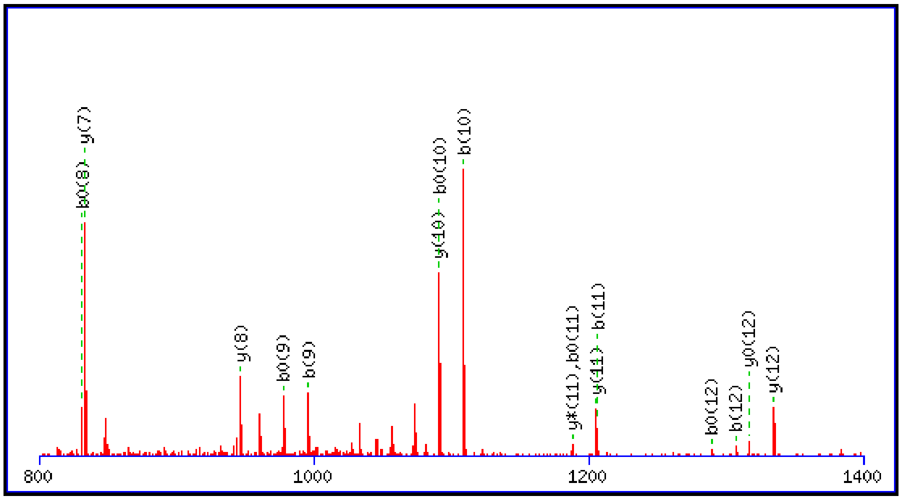


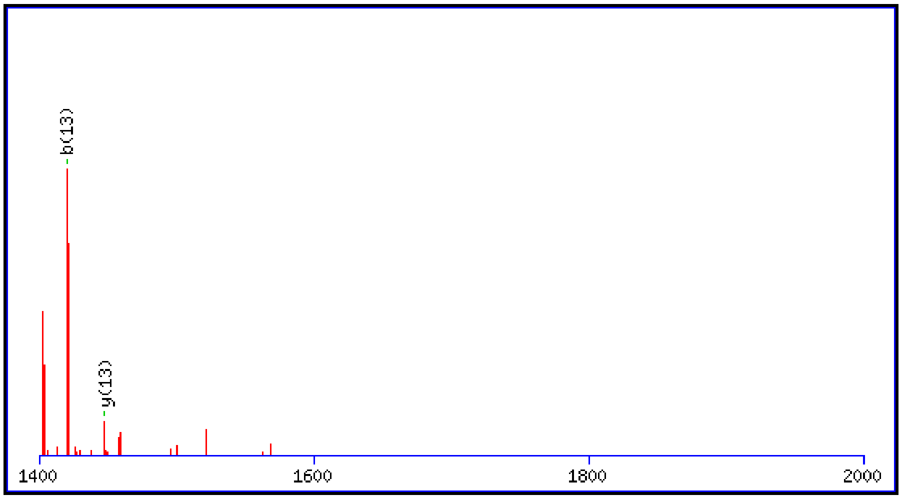


**Monoisotopic mass of neutral peptide Mr(calc):** 1593.8072
**Fixed modifications:** Carbamidomethyl (C)
**Variable modifications:**
**M1** : Oxidation (M)
**N4** : Deamidated_N (N)
**Ions Score:** 105 **Expect:** 5e-009
**Matches (Bold Red):** 35/142 fragment ions using 46 most intense peaks

| # | b | $b^{++}$ | b* | $b^{*++}$ | $b^{0}$ | $b^{0++}$ | Seq. | y | $y^{++}$ | y* | $y^{*++}$ | $y^{0}$ | $y^{0++}$ | # |
|---|---|---|---|---|---|---|---|---|---|---|---|---|---|---|
| 1 | 148.0427 | 74.5250 | | | | | M | | | | | | | 14 |
| 2 | **261.1267** | 131.0670 | | | | | I | **1447.7791** | 724.3932 | 1430.7526 | 715.8799 | 1429.7685 | 715.3879 | 13 |
| 3 | **390.1693** | 195.5883 | | | **372.1588** | 186.5830 | E | **1334.6951** | 667.8512 | 1317.6685 | 659.3379 | **1316.6845** | 658.8459 | 12 |
| 4 | **505.1963** | 253.1018 | 488.1697 | 244.5885 | **487.1857** | 244.0965 | N | **1205.6525** | 603.3299 | **1188.6259** | 594.8166 | 1187.6419 | 594.3246 | 11 |
| | | | | | | | | | | | | | | |

| | | | | | | | | | | | | | | |
|---|---|---|---|---|---|---|---|---|---|---|---|---|---|---|
| 5 | **562.2177** | 281.6125 | 545.1912 | 273.0992 | 544.2072 | 272.6072 | G | **1090.6255** | 545.8164 | 1073.5990 | 537.3031 | 1072.6150 | 536.8111 | 10 |
| 6 | **649.2498** | 325.1285 | 632.2232 | 316.6152 | **631.2392** | 316.1232 | S | 1033.6041 | 517.3057 | 1016.5775 | 508.7924 | 1015.5935 | 508.3004 | 9 |
| 7 | **762.3338** | 381.6706 | 745.3073 | 373.1573 | **744.3233** | 372.6653 | L | **946.5720** | 473.7897 | 929.5455 | 465.2764 | 928.5615 | 464.7844 | 8 |
| 8 | 849.3659 | 425.1866 | 832.3393 | 416.6733 | **831.3553** | 416.1813 | S | **833.4880** | 417.2476 | 816.4614 | 408.7343 | 815.4774 | 408.2423 | 7 |
| 9 | **996.4343** | 498.7208 | 979.4077 | 490.2075 | **978.4237** | 489.7155 | F | **746.4559** | 373.7316 | 729.4294 | 365.2183 | 728.4454 | 364.7263 | 6 |
| 10 | **1109.5183** | 555.2628 | 1092.4918 | 546.7495 | **1091.5078** | 546.2575 | L | **599.3875** | 300.1974 | 582.3610 | 291.6841 | 581.3770 | 291.1921 | 5 |
| 11 | **1206.5711** | 603.7892 | 1189.5446 | 595.2759 | **1188.5605** | 594.7839 | P | **486.3035** | 243.6554 | 469.2769 | 235.1421 | **468.2929** | 234.6501 | 4 |
| 12 | **1307.6188** | 654.3130 | 1290.5922 | 645.7998 | **1289.6082** | 645.3077 | T | **389.2507** | 195.1290 | **372.2241** | 186.6157 | 371.2401 | 186.1237 | 3 |
| 13 | **1420.7028** | 710.8551 | 1403.6763 | 702.3418 | 1402.6923 | 701.8498 | L | **288.2030** | 144.6051 | 271.1765 | 136.0919 | | | 2 |
| 14 | | | | | | | R | 175.1190 | 88.0631 | 158.0924 | 79.5498 | | | 1 |

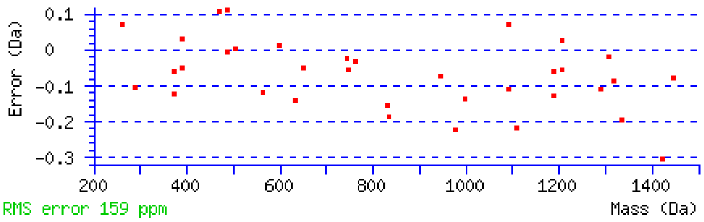


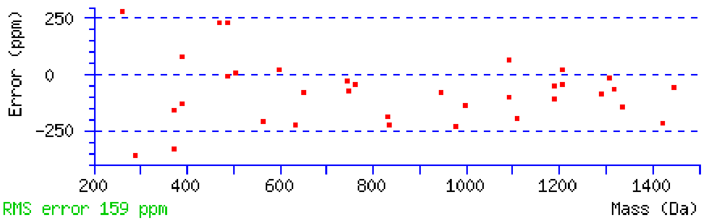


### All matches to this query

| Score | Mr(calc): | Delta | Sequence |
|---|---|---|---|
| 104.6 | 1593.8072 | 0.0002 | MIENGSLSFLPTLR |
| 16.6 | 1593.8223 | -0.0148 | SLRELHLQDNNVR |
| 14.3 | 1593.8223 | -0.0148 | SLRELHLQDNNVR |
| 12.2 | 1593.8055 | 0.0020 | FKEVIPISDPELK |
| 10.3 | 1593.8106 | -0.0031 | LMQDKEEMIGKLK |
| 9.2 | 1593.8223 | -0.0148 | RISELEHENAQLR |
| 8.5 | 1592.8079 | 0.9995 | MTEEQQSLKNKIK |
| 7.9 | 1593.8144 | -0.0070 | MLKAESSGERTTLR |
| 7.6 | 1593.8184 | -0.0110 | FLEGNSMKLASLER |
| 5.9 | 1593.8068 | 0.0006 | FYFIEAAKRTIR |

---

Spectrum No: 60; Query: 992; Rank: 1

---

## Peptide View

MS/MS Fragmentation of **DLNISLDTDAPLSVGK**
Found in **IPI00326990**, Tax_Id=10116 Gene_Symbol=Slc15a2 Oligopeptide transporter, kidney isoform

Match to Query 992: 1657.840788 from(829.927670,2+)
Title: 091008RatKidney_NH4Format01_27.2868.2868.2.dta
Data file K:\NewmanPaper\Piliang\3SubProteomes\Piliang3SP\mgf5ppm\ERLIC_3SubProteomes5ppm.mgf

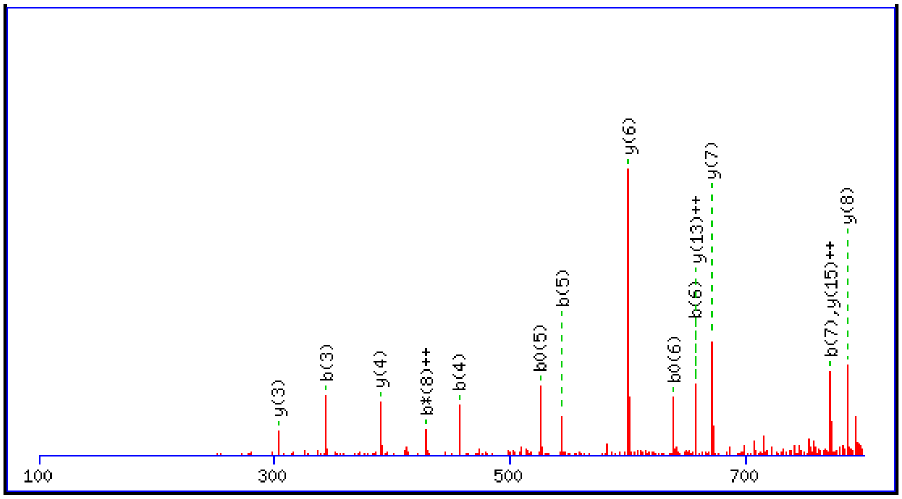


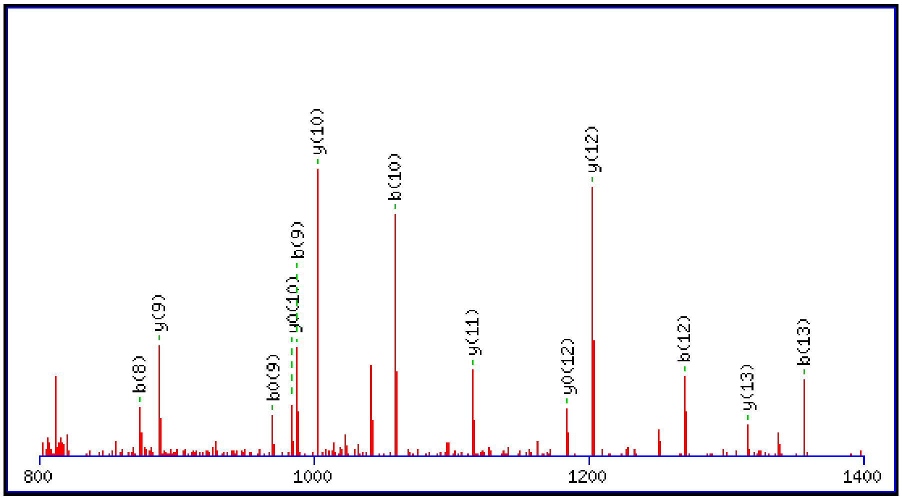


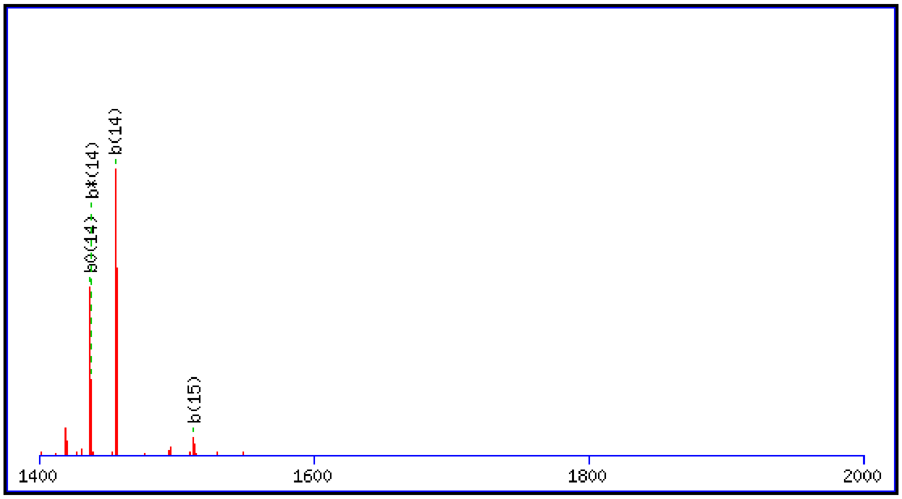


**Monoisotopic mass of neutral peptide Mr(calc):** 1657.8410
**Fixed modifications:** Carbamidomethyl (C)
**Variable modifications:**
**N3** : Deamidated_N (N)
**Ions Score:** 105 **Expect:** 5.2e-009
**Matches (Bold Red):** 32/170 fragment ions using 41 most intense peaks

| # | b | $b^{++}$ | b* | $b^{*++}$ | $b^0$ | $b^{0++}$ | Seq. | y | $y^{++}$ | y* | $y^{*++}$ | $y^0$ | $y^{0++}$ | # |
|---|---|---|---|---|---|---|---|---|---|---|---|---|---|---|
| 1 | 116.0342 | 58.5207 | | | 98.0237 | 49.5155 | D | | | | | | | 16 |
| 2 | 229.1183 | 115.0628 | | | 211.1077 | 106.0575 | L | 1543.8214 | **772.4143** | 1526.7948 | 763.9011 | 1525.8108 | 763.4090 | 15 |
| 3 | **344.1452** | 172.5762 | 327.1187 | 164.0630 | 326.1347 | 163.5710 | N | 1430.7373 | 715.8723 | 1413.7108 | 707.3590 | 1412.7267 | 706.8670 | 14 |
| 4 | **457.2293** | 229.1183 | 440.2027 | 220.6050 | 439.2187 | 220.1130 | I | **1315.7104** | **658.3588** | 1298.6838 | 649.8456 | 1297.6998 | 649.3535 | 13 |
| 5 | **544.2613** | 272.6343 | 527.2348 | 264.1210 | **526.2507** | 263.6290 | S | **1202.6263** | 601.8168 | 1185.5998 | 593.3035 | **1184.6157** | 592.8115 | 12 |

| | | | | | | | | | | | | | | |
|---|---|---|---|---|---|---|---|---|---|---|---|---|---|---|
| **6** | **657.3454** | 329.1763 | 640.3188 | 320.6631 | **639.3348** | 320.1710 | **L** | **1115.5943** | 558.3008 | 1098.5677 | 549.7875 | 1097.5837 | 549.2955 | **11** |
| **7** | **772.3723** | 386.6898 | 755.3458 | 378.1765 | 754.3618 | 377.6845 | **D** | **1002.5102** | 501.7587 | 985.4837 | 493.2455 | **984.4997** | 492.7535 | **10** |
| **8** | **873.4200** | 437.2136 | 856.3934 | **428.7004** | 855.4094 | 428.2084 | **T** | **887.4833** | 444.2453 | 870.4567 | 435.7320 | 869.4727 | 435.2400 | **9** |
| **9** | **988.4469** | 494.7271 | 971.4204 | 486.2138 | **970.4364** | 485.7218 | **D** | **786.4356** | 393.7214 | 769.4090 | 385.2082 | 768.4250 | 384.7162 | **8** |
| **10** | **1059.4841** | 530.2457 | 1042.4575 | 521.7324 | 1041.4735 | 521.2404 | **A** | **671.4087** | 336.2080 | 654.3821 | 327.6947 | 653.3981 | 327.2027 | **7** |
| **11** | 1156.5368 | 578.7720 | 1139.5103 | 570.2588 | 1138.5263 | 569.7668 | **P** | **600.3715** | 300.6894 | 583.3450 | 292.1761 | 582.3610 | 291.6841 | **6** |
| **12** | **1269.6209** | 635.3141 | 1252.5943 | 626.8008 | 1251.6103 | 626.3088 | **L** | 503.3188 | 252.1630 | 486.2922 | 243.6498 | 485.3082 | 243.1577 | **5** |
| **13** | **1356.6529** | 678.8301 | 1339.6264 | 670.3168 | 1338.6423 | 669.8248 | **S** | **390.2347** | 195.6210 | 373.2082 | 187.1077 | 372.2241 | 186.6157 | **4** |
| **14** | **1455.7213** | 728.3643 | **1438.6948** | 719.8510 | **1437.7108** | 719.3590 | **V** | **303.2027** | 152.1050 | 286.1761 | 143.5917 | | | **3** |
| **15** | **1512.7428** | 756.8750 | 1495.7162 | 748.3618 | 1494.7322 | 747.8697 | **G** | 204.1343 | 102.5708 | 187.1077 | 94.0575 | | | **2** |
| **16** | | | | | | | **K** | 147.1128 | 74.0600 | 130.0863 | 65.5468 | | | **1** |

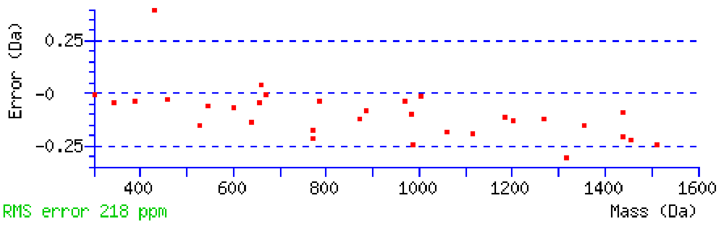


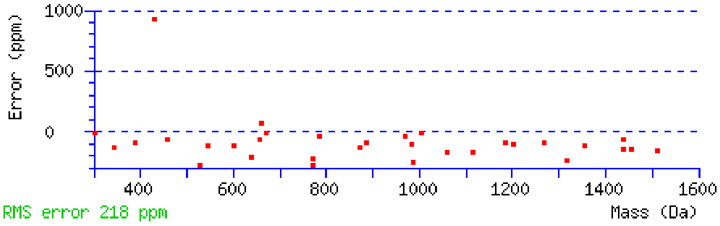


### All matches to this query

| Score | Mr(calc): | Delta | Sequence |
|---|---|---|---|
| 104.5 | 1657.8410 | -0.0002 | DLNISLDTDAPLSVGK |
| 14.8 | 1656.8488 | 0.9920 | GGGLFSVGETTAKILK |
| 10.7 | 1657.8383 | 0.0025 | KASESPGGAASPAGTSKR |
| 7.4 | 1657.8280 | 0.0128 | QHSSSNMVIMLIGNK |
| 6.5 | 1657.8385 | 0.0023 | NAPFLLNDPVLGGMAK |
| 3.9 | 1656.8220 | 1.0188 | GSPGNIGHPGSPGLPGEK |
| 3.5 | 1657.8440 | -0.0032 | VSKGILKAFGNSTEK |
| 3.4 | 1657.8497 | -0.0090 | MELSLRNIYGYVGK |
| 2.9 | 1657.8311 | 0.0096 | QFINPAGDNVVLDEK |
| 2.7 | 1655.8283 | 2.0124 | ALADFIRNNLSTIK |

---

Spectrum No: 61; Query: 1268; Rank: 1

---

## Peptide View

MS/MS Fragmentation of **NEIQVNTSQIPDTVTR**
Found in **IPI00211648**, Tax_Id=10116 Gene_Symbol=Slc3a1 Neutral and basic amino acid transport protein rBAT

Match to Query 1268: 1814.896088 from(908.455320,2+)
Title: 091008RatKidney_NoSalt_30.2060.2060.2.dta
Data file K:\NewmanPaper\Piliang\3SubProteomes\Piliang3SP\mgf5ppm\ERLIC_3SubProteomes5ppm.mgf

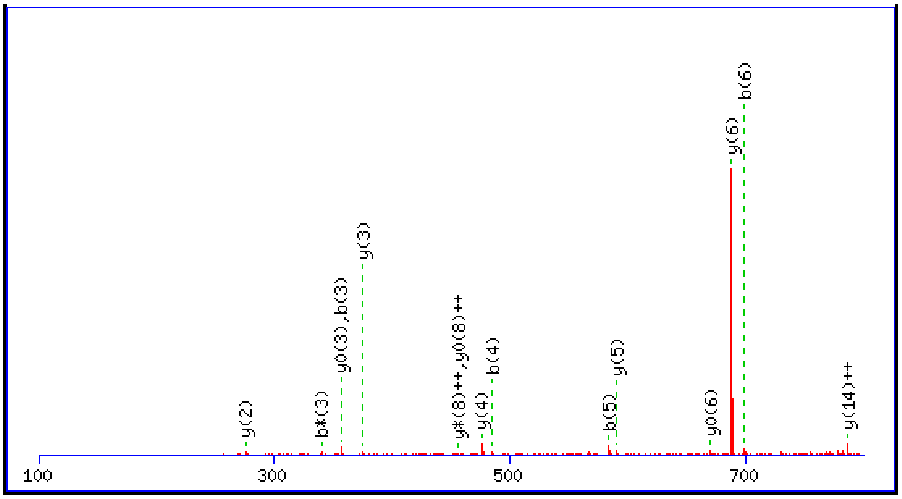


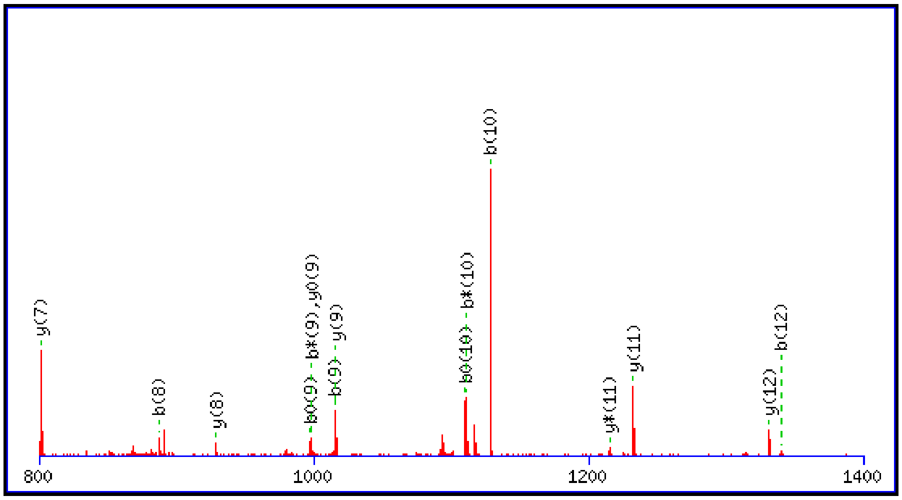


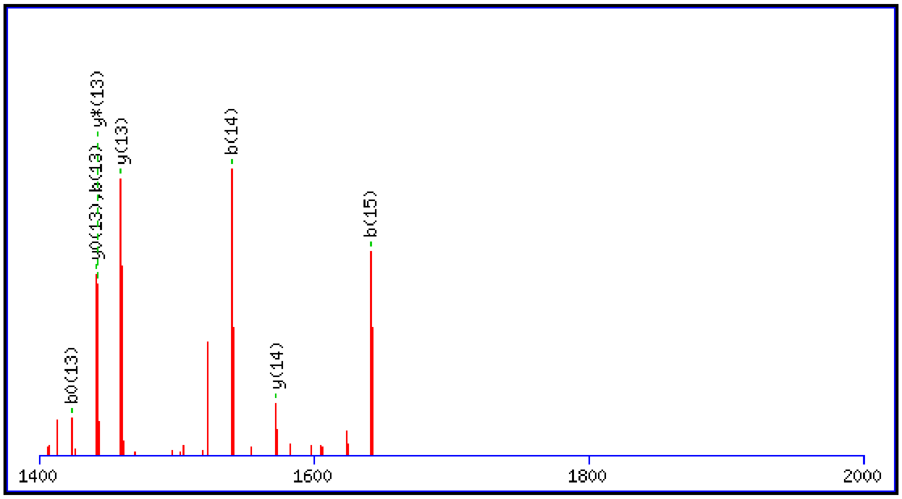


**Monoisotopic mass of neutral peptide Mr(calc):** 1814.9010
**Fixed modifications:** Carbamidomethyl (C)
**Variable modifications:**
**N6** : Deamidated_N (N)
**Ions Score:** 104 **Expect:** 6.9e-009
**Matches (Bold Red):** 38/176 fragment ions using 46 most intense peaks

| # | b | $b^{++}$ | b* | $b^{*++}$ | $b^0$ | $b^{0++}$ | Seq. | y | $y^{++}$ | y* | $y^{*++}$ | $y^0$ | $y^{0++}$ | # |
|---|---|---|---|---|---|---|---|---|---|---|---|---|---|---|
| 1 | 115.0502 | 58.0287 | 98.0237 | 49.5155 | | | N | | | | | | | 16 |
| 2 | 244.0928 | 122.5500 | 227.0662 | 114.0368 | 226.0822 | 113.5448 | E | 1701.8654 | 851.4363 | 1684.8388 | 842.9230 | 1683.8548 | 842.4310 | 15 |
| 3 | **357.1769** | 179.0921 | **340.1503** | 170.5788 | 339.1663 | 170.0868 | I | **1572.8228** | **786.9150** | 1555.7962 | 778.4017 | 1554.8122 | 777.9097 | 14 |
| 4 | **485.2354** | 243.1214 | 468.2089 | 234.6081 | 467.2249 | 234.1161 | Q | **1459.7387** | 730.3730 | **1442.7122** | 721.8597 | **1441.7281** | 721.3677 | 13 |
| 5 | **584.3039** | 292.6556 | 567.2773 | 284.1423 | 566.2933 | 283.6503 | V | **1331.6801** | 666.3437 | 1314.6536 | 657.8304 | 1313.6696 | 657.3384 | 12 |

| | | | | | | | | | | | | | | |
|---|---|---|---|---|---|---|---|---|---|---|---|---|---|---|
| 6 | **699.3308** | 350.1690 | 682.3042 | 341.6558 | 681.3202 | 341.1637 | N | **1232.6117** | 616.8095 | **1215.5852** | 608.2962 | 1214.6012 | 607.8042 | 11 |
| 7 | 800.3785 | 400.6929 | 783.3519 | 392.1796 | 782.3679 | 391.6876 | T | 1117.5848 | 559.2960 | 1100.5582 | 550.7828 | 1099.5742 | 550.2907 | 10 |
| 8 | **887.4105** | 444.2089 | 870.3839 | 435.6956 | 869.3999 | 435.2036 | S | **1016.5371** | 508.7722 | 999.5106 | 500.2589 | **998.5265** | 499.7669 | 9 |
| 9 | **1015.4691** | 508.2382 | **998.4425** | 499.7249 | **997.4585** | 499.2329 | Q | **929.5051** | 465.2562 | 912.4785 | **456.7429** | 911.4945 | **456.2509** | 8 |
| 10 | **1128.5531** | 564.7802 | **1111.5266** | 556.2669 | **1110.5426** | 555.7749 | I | **801.4465** | 401.2269 | 784.4199 | 392.7136 | 783.4359 | 392.2216 | 7 |
| 11 | 1225.6059 | 613.3066 | 1208.5794 | 604.7933 | 1207.5953 | 604.3013 | P | **688.3624** | 344.6849 | 671.3359 | 336.1716 | **670.3519** | 335.6796 | 6 |
| 12 | **1340.6328** | 670.8201 | 1323.6063 | 662.3068 | 1322.6223 | 661.8148 | D | **591.3097** | 296.1585 | 574.2831 | 287.6452 | 573.2991 | 287.1532 | 5 |
| 13 | **1441.6805** | 721.3439 | 1424.6540 | 712.8306 | **1423.6700** | 712.3386 | T | **476.2827** | 238.6450 | 459.2562 | 230.1317 | 458.2722 | 229.6397 | 4 |
| 14 | **1540.7489** | 770.8781 | 1523.7224 | 762.3648 | 1522.7384 | 761.8728 | V | **375.2350** | 188.1212 | 358.2085 | 179.6079 | **357.2245** | 179.1159 | 3 |
| 15 | **1641.7966** | 821.4019 | 1624.7701 | 812.8887 | 1623.7861 | 812.3967 | T | **276.1666** | 138.5870 | 259.1401 | 130.0737 | 258.1561 | 129.5817 | 2 |
| 16 | | | | | | | R | 175.1190 | 88.0631 | 158.0924 | 79.5498 | | | 1 |

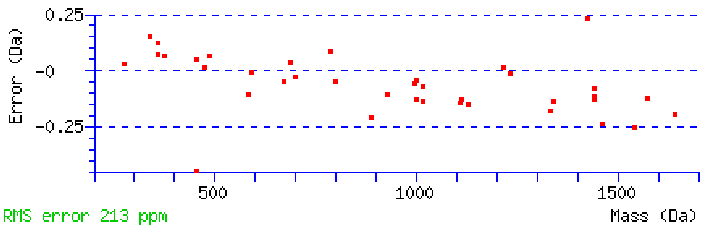


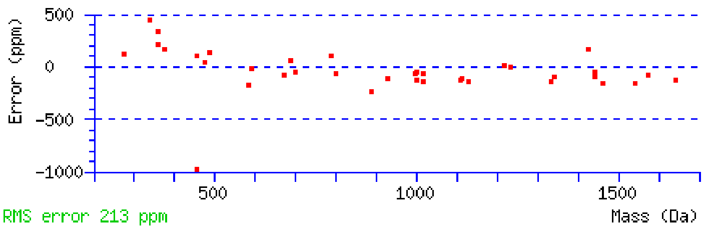


### All matches to this query

| Score | Mr(calc): | Delta | Sequence |
|---|---|---|---|
| 104.3 | 1814.9010 | -0.0049 | NEIQVNTSQIPDTVTR |
| 72.9 | 1814.9010 | -0.0049 | NEIQVNTSQIPDTVTR |
| 18.0 | 1814.9040 | -0.0079 | AQSALHEQKTLPGVTR |
| 16.2 | 1812.8870 | 2.0091 | EEKLSLTKSLDDVTR |
| 14.8 | 1814.8788 | 0.0173 | SDLRHLRAVPTDEAR |
| 10.1 | 1814.8985 | -0.0024 | LGPAQSGPCLFLSNPTR |
| 7.4 | 1812.8870 | 2.0091 | EEKLSLTKSLDDVTR |
| 6.5 | 1812.8870 | 2.0091 | EEKLSLTKSLDDVTR |
| 6.1 | 1814.9131 | -0.0170 | MAGGPTRMEGNLPAKLR |
| 4.6 | 1814.8981 | -0.0020 | AFRVWESATPLRFR |

---

Spectrum No: 62; Query: 2817; Rank: 1

---

## Peptide View

MS/MS Fragmentation of **CDVDNDCGDYSDEPIDECTTAAYNCDNHTEFSCK**
Found in **IPI00205325**, Tax_Id=10116 Gene_Symbol=Lrp2 Low-density lipoprotein receptor-related protein 2 precursor

Match to Query 2817: 4078.461192 from(1360.494340,3+)
Title: 100101RatKid_NS_deglyco_22.2718.2718.3.dta
Data file K:\NewmanPaper\Piliang\3SubProteomes\Piliang3SP\mgf5ppm\ERLIC_3SubProteomes5ppm.mgf

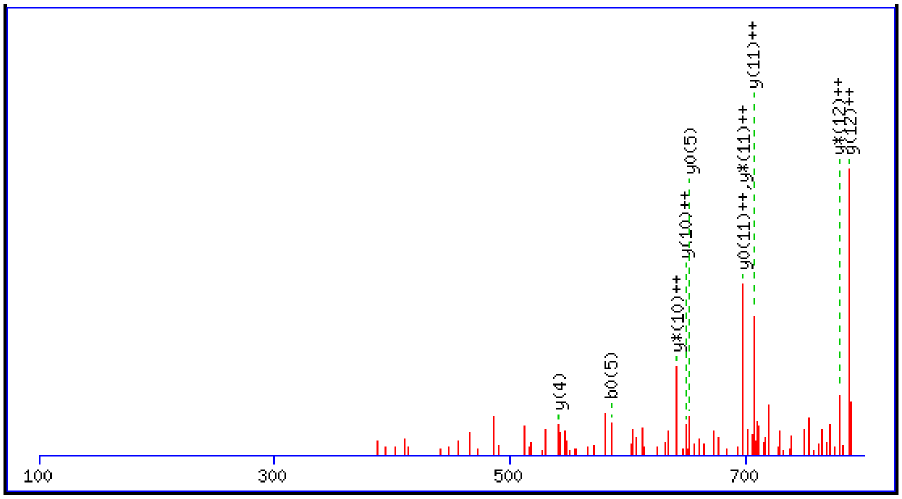


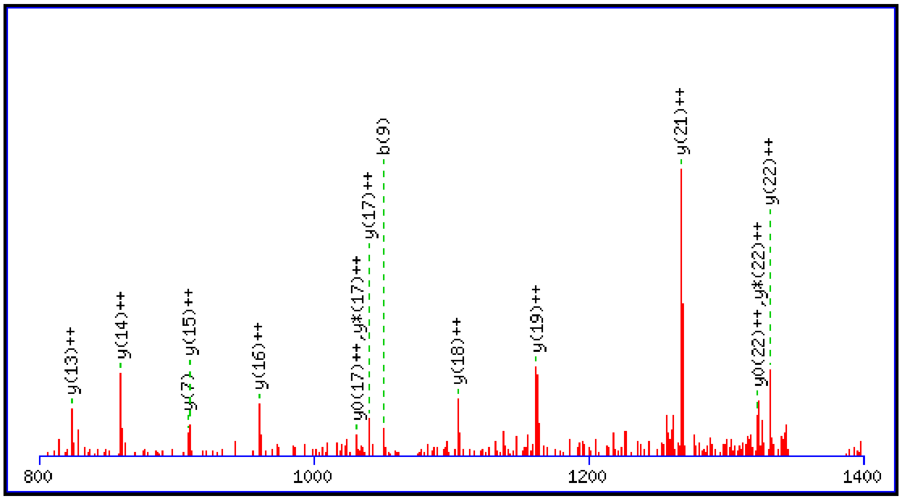


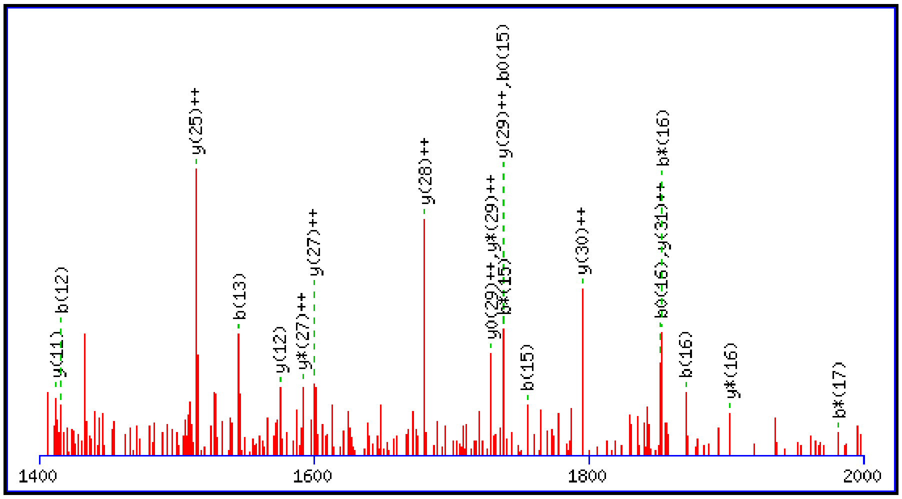


**Monoisotopic mass of neutral peptide Mr(calc):** 4077.4500
**Fixed modifications:** Carbamidomethyl (C)
**Variable modifications:**
**N27** : Deamidated_N (N)
**Ions Score:** 104 **Expect:** 8e-009
**Matches (Bold Red):** 46/382 fragment ions using 71 most intense peaks

| # | b | $b^{++}$ | b* | $b^{*++}$ | $b^0$ | $b^{0++}$ | Seq. | y | $y^{++}$ | y* | $y^{*++}$ | $y^0$ | $y^{0++}$ | # |
|---|---|---|---|---|---|---|---|---|---|---|---|---|---|---|
| 1 | 161.0379 | 81.0226 | | | | | C | | | | | | | 34 |
| 2 | 276.0649 | 138.5361 | | | 258.0543 | 129.5308 | D | 3918.4266 | 1959.7169 | 3901.4000 | 1951.2037 | 3900.4160 | 1950.7116 | 33 |
| 3 | 375.1333 | 188.0703 | | | 357.1227 | 179.0650 | V | 3803.3996 | 1902.2035 | 3786.3731 | 1893.6902 | 3785.3891 | 1893.1982 | 32 |
| 4 | 490.1602 | 245.5838 | | | 472.1497 | 236.5785 | D | 3704.3312 | **1852.6692** | 3687.3047 | 1844.1560 | 3686.3207 | 1843.6640 | 31 |
| 5 | 604.2032 | 302.6052 | 587.1766 | 294.0919 | **586.1926** | 293.5999 | N | 3589.3043 | **1795.1558** | 3572.2777 | 1786.6425 | 3571.2937 | 1786.1505 | 30 |

| | | | | | | | | | | | | | | |
|---|---|---|---|---|---|---|---|---|---|---|---|---|---|---|
| **6** | 719.2301 | 360.1187 | 702.2035 | 351.6054 | 701.2195 | 351.1134 | **D** | 3475.2614 | **1738.1343** | 3458.2348 | **1729.6210** | 3457.2508 | **1729.1290** | **29** |
| **7** | 879.2607 | 440.1340 | 862.2342 | 431.6207 | 861.2502 | 431.1287 | **C** | 3360.2344 | **1680.6208** | 3343.2079 | 1672.1076 | 3342.2238 | 1671.6156 | **28** |
| **8** | 936.2822 | 468.6447 | 919.2557 | 460.1315 | 918.2716 | 459.6395 | **G** | 3200.2038 | **1600.6055** | 3183.1772 | **1592.0922** | 3182.1932 | 1591.6002 | **27** |
| **9** | **1051.3092** | 526.1582 | 1034.2826 | 517.6449 | 1033.2986 | 517.1529 | **D** | 3143.1823 | 1572.0948 | 3126.1557 | 1563.5815 | 3125.1717 | 1563.0895 | **26** |
| **10** | 1214.3725 | 607.6899 | 1197.3459 | 599.1766 | 1196.3619 | 598.6846 | **Y** | 3028.1554 | **1514.5813** | 3011.1288 | 1506.0680 | 3010.1448 | 1505.5760 | **25** |
| **11** | 1301.4045 | 651.2059 | 1284.3780 | 642.6926 | 1283.3939 | 642.2006 | **S** | 2865.0920 | 1433.0497 | 2848.0655 | 1424.5364 | 2847.0815 | 1424.0444 | **24** |
| **12** | **1416.4315** | 708.7194 | 1399.4049 | 700.2061 | 1398.4209 | 699.7141 | **D** | 2778.0600 | 1389.5336 | 2761.0334 | 1381.0204 | 2760.0494 | 1380.5284 | **23** |
| **13** | **1545.4740** | 773.2407 | 1528.4475 | 764.7274 | 1527.4635 | 764.2354 | **E** | 2663.0331 | **1332.0202** | 2646.0065 | **1323.5069** | 2645.0225 | **1323.0149** | **22** |
| **14** | 1642.5268 | 821.7670 | 1625.5003 | 813.2538 | 1624.5162 | 812.7618 | **P** | 2533.9905 | **1267.4989** | 2516.9639 | 1258.9856 | 2515.9799 | 1258.4936 | **21** |
| **15** | **1755.6109** | 878.3091 | **1738.5843** | 869.7958 | **1737.6003** | 869.3038 | **I** | 2436.9377 | 1218.9725 | 2419.9111 | 1210.4592 | 2418.9271 | 1209.9672 | **20** |
| **16** | **1870.6378** | 935.8225 | **1853.6113** | 927.3093 | **1852.6273** | 926.8173 | **D** | 2323.8536 | **1162.4305** | 2306.8271 | 1153.9172 | 2305.8431 | 1153.4252 | **19** |
| **17** | 1999.6804 | 1000.3438 | **1982.6539** | 991.8306 | 1981.6698 | 991.3386 | **E** | 2208.8267 | **1104.9170** | 2191.8001 | 1096.4037 | 2190.8161 | 1095.9117 | **18** |
| **18** | 2159.7111 | 1080.3592 | 2142.6845 | 1071.8459 | 2141.7005 | 1071.3539 | **C** | 2079.7841 | **1040.3957** | 2062.7575 | **1031.8824** | 2061.7735 | **1031.3904** | **17** |
| **19** | 2260.7587 | 1130.8830 | 2243.7322 | 1122.3697 | 2242.7482 | 1121.8777 | **T** | 1919.7534 | **960.3804** | **1902.7269** | 951.8671 | 1901.7429 | 951.3751 | **16** |
| **20** | 2361.8064 | 1181.4068 | 2344.7799 | 1172.8936 | 2343.7958 | 1172.4016 | **T** | 1818.7058 | **909.8565** | 1801.6792 | 901.3432 | 1800.6952 | 900.8512 | **15** |
| **21** | 2432.8435 | 1216.9254 | 2415.8170 | 1208.4121 | 2414.8330 | 1207.9201 | **A** | 1717.6581 | **859.3327** | 1700.6315 | 850.8194 | 1699.6475 | 850.3274 | **14** |
| **22** | 2503.8806 | 1252.4440 | 2486.8541 | 1243.9307 | 2485.8701 | 1243.4387 | **A** | 1646.6210 | **823.8141** | 1629.5944 | 815.3009 | 1628.6104 | 814.8088 | **13** |
| **23** | 2666.9440 | 1333.9756 | 2649.9174 | 1325.4623 | 2648.9334 | 1324.9703 | **Y** | **1575.5839** | **788.2956** | 1558.5573 | **779.7823** | 1557.5733 | 779.2903 | **12** |
| **24** | 2780.9869 | 1390.9971 | 2763.9603 | 1382.4838 | 2762.9763 | 1381.9918 | **N** | **1412.5205** | **706.7639** | 1395.4940 | **698.2506** | 1394.5100 | **697.7586** | **11** |
| **25** | 2941.0175 | 1471.0124 | 2923.9910 | 1462.4991 | 2923.0070 | 1462.0071 | **C** | 1298.4776 | **649.7424** | 1281.4511 | **641.2292** | 1280.4670 | 640.7372 | **10** |
| **26** | 3056.0445 | 1528.5259 | 3039.0179 | 1520.0126 | 3038.0339 | 1519.5206 | **D** | 1138.4470 | 569.7271 | 1121.4204 | 561.2138 | 1120.4364 | 560.7218 | **9** |
| **27** | 3171.0714 | 1586.0394 | 3154.0449 | 1577.5261 | 3153.0609 | 1577.0341 | **N** | 1023.4200 | 512.2136 | 1006.3935 | 503.7004 | 1005.4094 | 503.2084 | **8** |
| **28** | 3308.1303 | 1654.5688 | 3291.1038 | 1646.0555 | 3290.1198 | 1645.5635 | **H** | **908.3931** | 454.7002 | 891.3665 | 446.1869 | 890.3825 | 445.6949 | **7** |
| **29** | 3409.1780 | 1705.0926 | 3392.1515 | 1696.5794 | 3391.1675 | 1696.0874 | **T** | 771.3342 | 386.1707 | 754.3076 | 377.6574 | 753.3236 | 377.1654 | **6** |
| **30** | 3538.2206 | 1769.6139 | 3521.1941 | 1761.1007 | 3520.2100 | 1760.6087 | **E** | 670.2865 | 335.6469 | 653.2599 | 327.1336 | **652.2759** | 326.6416 | **5** |
| **31** | 3685.2890 | 1843.1482 | 3668.2625 | 1834.6349 | 3667.2785 | 1834.1429 | **F** | **541.2439** | 271.1256 | 524.2173 | 262.6123 | 523.2333 | 262.1203 | **4** |
| **32** | 3772.3211 | 1886.6642 | 3755.2945 | 1878.1509 | 3754.3105 | 1877.6589 | **S** | 394.1755 | 197.5914 | 377.1489 | 189.0781 | 376.1649 | 188.5861 | **3** |
| **33** | 3932.3517 | 1966.6795 | 3915.3252 | 1958.1662 | 3914.3411 | 1957.6742 | **C** | 307.1435 | 154.0754 | 290.1169 | 145.5621 | | | **2** |
| **34** | | | | | | | **K** | 147.1128 | 74.0600 | 130.0863 | 65.5468 | | | **1** |

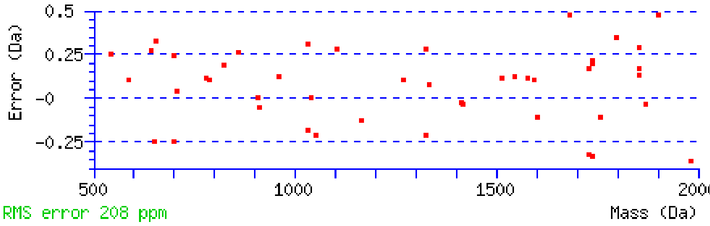


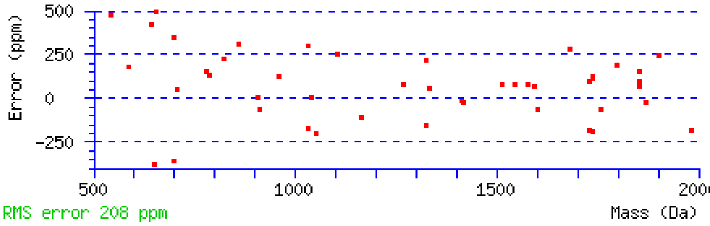


### All matches to this query

| Score | Mr(calc): | Delta | Sequence |
|---|---|---|---|
| 104.3 | 4077.4500 | 1.0112 | CDVDNDCGDYSDEPIDECTTAAYNCDNHTEFSCK |
| 102.6 | 4078.4340 | 0.0272 | CDVDNDCGDYSDEPIDECTTAAYNCDNHTEFSCK |
| 102.5 | 4077.4500 | 1.0112 | CDVDNDCGDYSDEPIDECTTAAYNCDNHTEFSCK |
| 89.5 | 4078.4340 | 0.0272 | CDVDNDCGDYSDEPIDECTTAAYNCDNHTEFSCK |
| 87.6 | 4078.4340 | 0.0272 | CDVDNDCGDYSDEPIDECTTAAYNCDNHTEFSCK |
| 9.7 | 4077.4218 | 1.0393 | NQDYMAPSEPNSPTKTNMEYPNTPGKQDLVSK |
| 9.3 | 4078.5013 | -0.0401 | NDFISVPSIQPLDPISDSDSENSFQDSKPENQK |
| 7.6 | 4078.5013 | -0.0401 | NDFISVPSIQPLDPISDSDSENSFQDSKPENQK |
| 6.6 | 4076.4660 | 1.9952 | CDVDNDCGDYSDEPIDECTTAAYNCDNHTEFSCK |
| 6.1 | 4078.4705 | -0.0093 | GTAGNALMEGASQLVGENRTMTIHNGMFFSTYDR |

Spectrum No: 63; Query: 2377; Rank: 1

## Peptide View

MS/MS Fragmentation of **ALSCLESSWENIEQGGNGSFVYTK**
Found in **IPI00201262**, Tax_Id=10116 Gene_Symbol=LOC297568;Mug2;Mug1 Alpha-1-inhibitor 3 precursor

Match to Query 2377: 2678.207088 from(1340.110820,2+)
Title: 100101RatKid_NS_deglyco_27.4299.4299.2.dta
Data file K:\NewmanPaper\Piliang\3SubProteomes\Piliang3SP\mgf5ppm\ERLIC_3SubProteomes5ppm.mgf

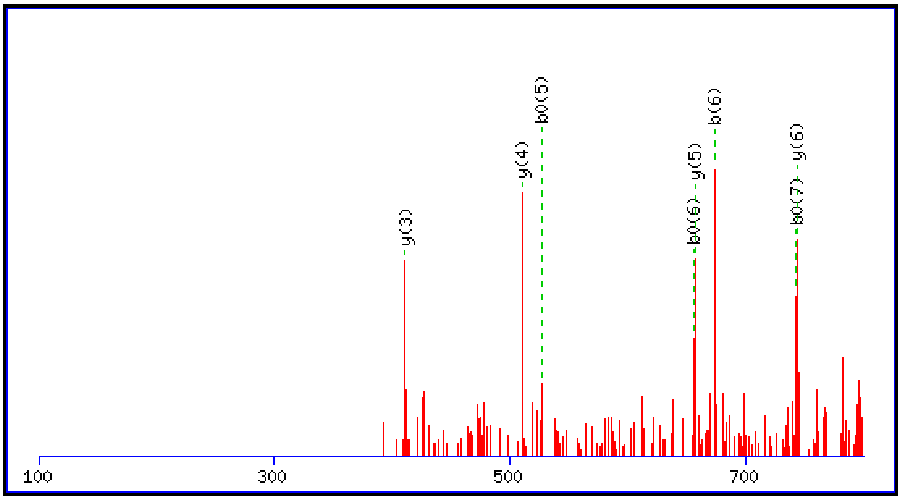


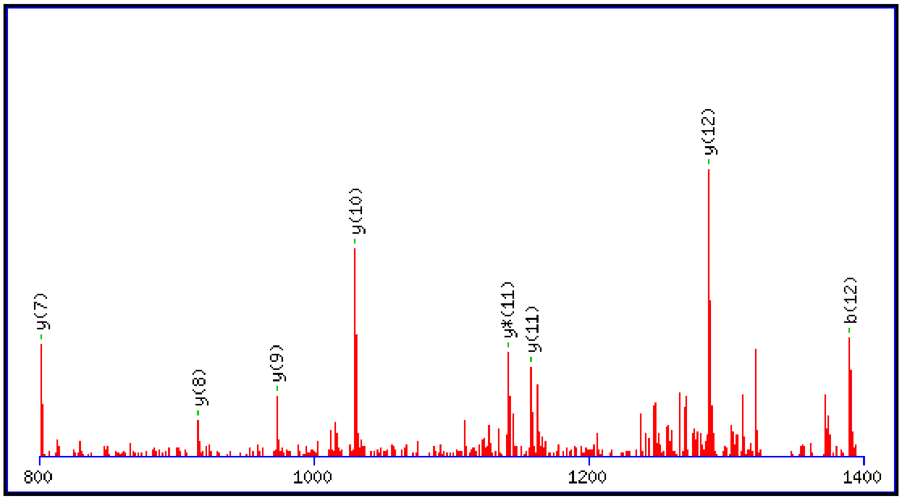


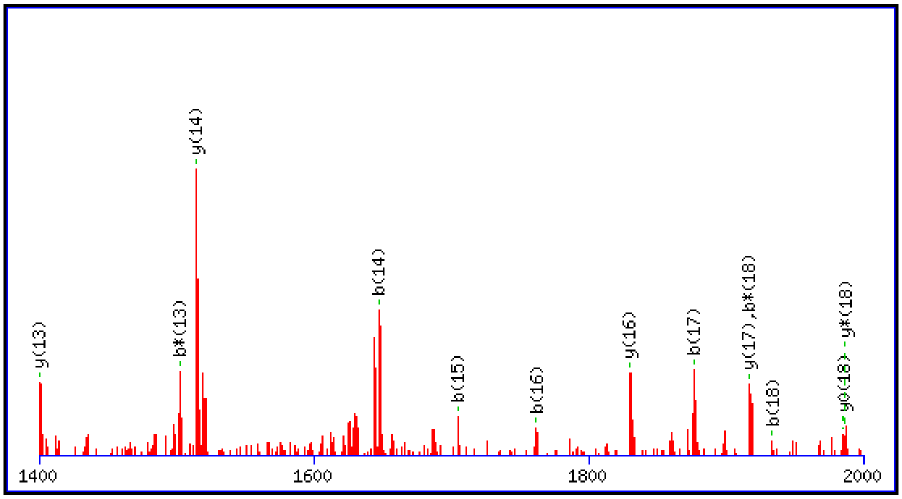

**Monoisotopic mass of neutral peptide Mr(calc):** 2676.2013
**Fixed modifications:** Carbamidomethyl (C)
**Variable modifications:**
**N17** : Deamidated_N (N)
**Ions Score:** 104 **Expect:** 1.3e-008
**Matches (Bold Red):** 29/250 fragment ions using 51 most intense peaks

| # | b | $b^{++}$ | b* | $b^{*++}$ | $b^0$ | $b^{0++}$ | Seq. | y | $y^{++}$ | y* | $y^{*++}$ | $y^0$ | $y^{0++}$ | # |
|---|---|---|---|---|---|---|---|---|---|---|---|---|---|---|
| 1 | 72.0444 | 36.5258 | | | | | A | | | | | | | 24 |
| 2 | 185.1285 | 93.0679 | | | | | L | 2606.1715 | 1303.5894 | 2589.1450 | 1295.0761 | 2588.1610 | 1294.5841 | 23 |
| 3 | 272.1605 | 136.5839 | | | 254.1499 | 127.5786 | S | 2493.0875 | 1247.0474 | 2476.0609 | 1238.5341 | 2475.0769 | 1238.0421 | 22 |
| 4 | 432.1911 | 216.5992 | | | 414.1806 | 207.5939 | C | 2406.0554 | 1203.5314 | 2389.0289 | 1195.0181 | 2388.0449 | 1194.5261 | 21 |
| 5 | 545.2752 | 273.1412 | | | **527.2646** | 264.1360 | L | 2246.0248 | 1123.5160 | 2228.9982 | 1115.0028 | 2228.0142 | 1114.5107 | 20 |
| 6 | **674.3178** | 337.6625 | | | **656.3072** | 328.6572 | E | 2132.9407 | 1066.9740 | 2115.9142 | 1058.4607 | 2114.9301 | 1057.9687 | 19 |
| 7 | 761.3498 | 381.1785 | | | **743.3393** | 372.1733 | S | 2003.8981 | 1002.4527 | **1986.8716** | 993.9394 | **1985.8876** | 993.4474 | 18 |
| 8 | 848.3818 | 424.6946 | | | 830.3713 | 415.6893 | S | **1916.8661** | 958.9367 | 1899.8395 | 950.4234 | 1898.8555 | 949.9314 | 17 |
| 9 | 1034.4612 | 517.7342 | | | 1016.4506 | 508.7289 | W | **1829.8341** | 915.4207 | 1812.8075 | 906.9074 | 1811.8235 | 906.4154 | 16 |
| 10 | 1163.5038 | 582.2555 | | | 1145.4932 | 573.2502 | E | 1643.7548 | 822.3810 | 1626.7282 | 813.8677 | 1625.7442 | 813.3757 | 15 |
| 11 | 1277.5467 | 639.2770 | 1260.5201 | 630.7637 | 1259.5361 | 630.2717 | N | **1514.7122** | 757.8597 | 1497.6856 | 749.3464 | 1496.7016 | 748.8544 | 14 |
| 12 | **1390.6307** | 695.8190 | 1373.6042 | 687.3057 | 1372.6202 | 686.8137 | I | **1400.6692** | 700.8383 | 1383.6427 | 692.3250 | 1382.6587 | 691.8330 | 13 |
| 13 | 1519.6733 | 760.3403 | **1502.6468** | 751.8270 | 1501.6628 | 751.3350 | E | **1287.5852** | 644.2962 | 1270.5586 | 635.7829 | 1269.5746 | 635.2909 | 12 |
| 14 | **1647.7319** | 824.3696 | 1630.7054 | 815.8563 | 1629.7213 | 815.3643 | Q | **1158.5426** | 579.7749 | **1141.5160** | 571.2617 | 1140.5320 | 570.7696 | 11 |
| 15 | **1704.7534** | 852.8803 | 1687.7268 | 844.3671 | 1686.7428 | 843.8750 | G | **1030.4840** | 515.7456 | 1013.4574 | 507.2324 | 1012.4734 | 506.7404 | 10 |
| 16 | **1761.7748** | 881.3911 | 1744.7483 | 872.8778 | 1743.7643 | 872.3858 | G | **973.4625** | 487.2349 | 956.4360 | 478.7216 | 955.4520 | 478.2296 | 9 |
| 17 | **1876.8018** | 938.9045 | 1859.7752 | 930.3913 | 1858.7912 | 929.8992 | N | **916.4411** | 458.7242 | 899.4145 | 450.2109 | 898.4305 | 449.7189 | 8 |
| 18 | **1933.8232** | 967.4153 | **1916.7967** | 958.9020 | 1915.8127 | 958.4100 | G | **801.4141** | 401.2107 | 784.3876 | 392.6974 | 783.4036 | 392.2054 | 7 |
| 19 | 2020.8553 | 1010.9313 | 2003.8287 | 1002.4180 | 2002.8447 | 1001.9260 | S | **744.3927** | 372.7000 | 727.3661 | 364.1867 | 726.3821 | 363.6947 | 6 |
| 20 | 2167.9237 | 1084.4655 | 2150.8971 | 1075.9522 | 2149.9131 | 1075.4602 | F | **657.3606** | 329.1840 | 640.3341 | 320.6707 | 639.3501 | 320.1787 | 5 |
| 21 | 2266.9921 | 1133.9997 | 2249.9655 | 1125.4864 | 2248.9815 | 1124.9944 | V | **510.2922** | 255.6498 | 493.2657 | 247.1365 | 492.2817 | 246.6445 | 4 |
| 22 | 2430.0554 | 1215.5314 | 2413.0289 | 1207.0181 | 2412.0449 | 1206.5261 | Y | **411.2238** | 206.1155 | 394.1973 | 197.6023 | 393.2132 | 197.1103 | 3 |
| 23 | 2531.1031 | 1266.0552 | 2514.0766 | 1257.5419 | 2513.0925 | 1257.0499 | T | 248.1605 | 124.5839 | 231.1339 | 116.0706 | 230.1499 | 115.5786 | 2 |
| 24 | | | | | | | K | 147.1128 | 74.0600 | 130.0863 | 65.5468 | | | 1 |

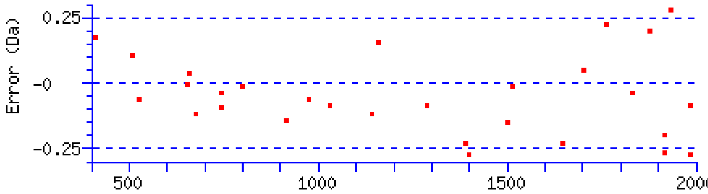


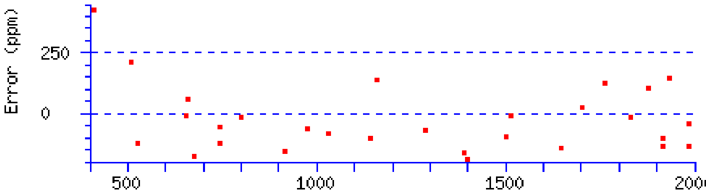


### All matches to this query

| Score | Mr(calc): | Delta | Sequence |
|---|---|---|---|
| 104.2 | 2676.2013 | 2.0057 | ALSCLESSWENIEQGGNGSFVYTK |
| 87.5 | 2677.1853 | 1.0217 | ALSCLESSWENIEQGGNGSFVYTK |
| 36.8 | 2676.2013 | 2.0057 | ALSCLESSWENIEQGGNGSFVYTK |
| 5.0 | 2677.2128 | 0.9943 | EKEVMSLLHNLEIKNTEHCQK |
| 4.6 | 2677.1966 | 1.0105 | VTYDMTEGDRGFFEIDQVNGEVR |
| 4.6 | 2677.1813 | 1.0258 | DNYVPEVSVLDQEITKVEPDTK |
| 4.4 | 2676.2126 | 1.9945 | VTYDMTEGDRGFFEIDQVNGEVR |
| 3.7 | 2677.2218 | 0.9853 | FKPEIMPDDSVDEFDETIHLER |
| 2.1 | 2676.1847 | 2.0224 | APYHGSRAGIATQFSTANGQVNLR |
| 2.1 | 2676.1847 | 2.0224 | APYHGSRAGIATQFSTANGQVNLR |

Spectrum No: 64; Query: 1570; Rank: 1

## Peptide View

MS/MS Fragmentation of **TNSTCSPNLDACLVAVSGK**
Found in **IPI00195173**, Tax_Id=10116 Gene_Symbol=Cd59 CD59 glycoprotein precursor

Match to Query 1570: 1993.913468 from(997.964010,2+)
Title: 100101RatKid_NS_deglyco_25.2956.2956.2.dta
Data file K:\NewmanPaper\Piliang\3SubProteomes\Piliang3SP\mgf5ppm\ERLIC_3SubProteomes5ppm.mgf

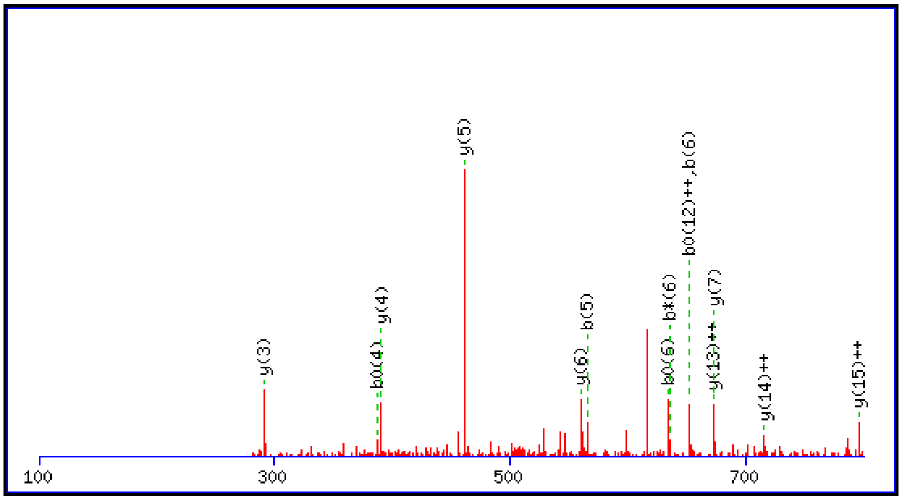


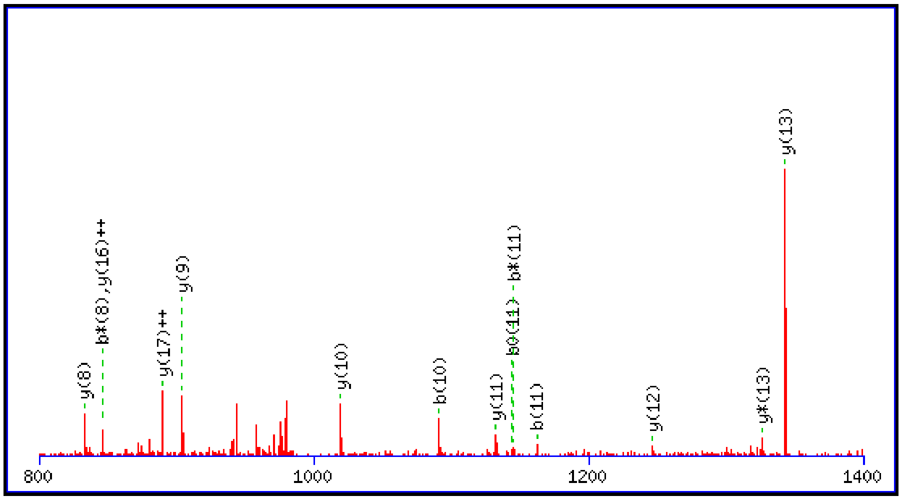


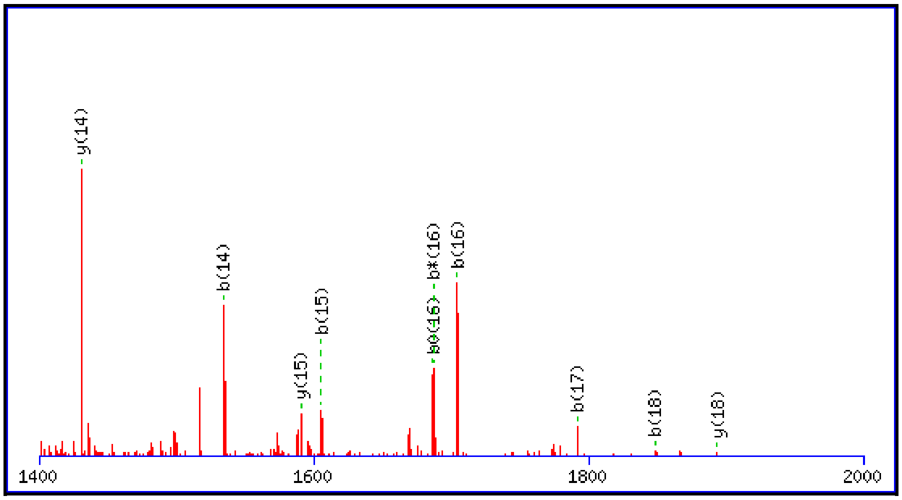

**Monoisotopic mass of neutral peptide Mr(calc):** 1993.9085
**Fixed modifications:** Carbamidomethyl (C)
**Variable modifications:**
**N2** : Deamidated_N (N)
**Ions Score:** 104 **Expect:** 1e-008
**Matches (Bold Red):** 38/210 fragment ions using 53 most intense peaks

| # | b | $b^{++}$ | b* | $b^{*++}$ | $b^0$ | $b^{0++}$ | Seq. | y | $y^{++}$ | y* | $y^{*++}$ | $y^0$ | $y^{0++}$ | # |
|---|---|---|---|---|---|---|---|---|---|---|---|---|---|---|
| 1 | 102.0550 | 51.5311 | | | 84.0444 | 42.5258 | T | | | | | | | 19 |
| 2 | 217.0819 | 109.0446 | 200.0553 | 100.5313 | 199.0713 | 100.0393 | N | **1893.8681** | 947.4377 | 1876.8415 | 938.9244 | 1875.8575 | 938.4324 | 18 |
| 3 | 304.1139 | 152.5606 | 287.0874 | 144.0473 | 286.1034 | 143.5553 | S | 1778.8411 | **889.9242** | 1761.8146 | 881.4109 | 1760.8306 | 880.9189 | 17 |
| 4 | 405.1616 | 203.0844 | 388.1350 | 194.5712 | **387.1510** | 194.0792 | T | 1691.8091 | **846.4082** | 1674.7826 | 837.8949 | 1673.7986 | 837.4029 | 16 |
| 5 | **565.1922** | 283.0998 | 548.1657 | 274.5865 | 547.1817 | 274.0945 | C | **1590.7614** | **795.8844** | 1573.7349 | 787.3711 | 1572.7509 | 786.8791 | 15 |
| 6 | **652.2243** | 326.6158 | **635.1977** | 318.1025 | **634.2137** | 317.6105 | S | **1430.7308** | **715.8690** | 1413.7042 | 707.3558 | 1412.7202 | 706.8638 | 14 |
| 7 | 749.2770 | 375.1422 | 732.2505 | 366.6289 | 731.2665 | 366.1369 | P | **1343.6988** | **672.3530** | **1326.6722** | 663.8397 | 1325.6882 | 663.3477 | 13 |
| 8 | 863.3200 | 432.1636 | **846.2934** | 423.6503 | 845.3094 | 423.1583 | N | **1246.6460** | 623.8266 | 1229.6195 | 615.3134 | 1228.6354 | 614.8214 | 12 |
| 9 | 976.4040 | 488.7057 | 959.3775 | 480.1924 | 958.3935 | 479.7004 | L | **1132.6031** | 566.8052 | 1115.5765 | 558.2919 | 1114.5925 | 557.7999 | 11 |
| 10 | **1091.4310** | 546.2191 | 1074.4044 | 537.7059 | 1073.4204 | 537.2138 | D | **1019.5190** | 510.2631 | 1002.4925 | 501.7499 | 1001.5084 | 501.2579 | 10 |
| 11 | **1162.4681** | 581.7377 | **1145.4415** | 573.2244 | **1144.4575** | 572.7324 | A | **904.4921** | 452.7497 | 887.4655 | 444.2364 | 886.4815 | 443.7444 | 9 |
| 12 | 1322.4987 | 661.7530 | 1305.4722 | 653.2397 | 1304.4882 | **652.7477** | C | **833.4550** | 417.2311 | 816.4284 | 408.7178 | 815.4444 | 408.2258 | 8 |
| 13 | 1435.5828 | 718.2950 | 1418.5563 | 709.7818 | 1417.5722 | 709.2898 | L | **673.4243** | 337.2158 | 656.3978 | 328.7025 | 655.4137 | 328.2105 | 7 |
| 14 | **1534.6512** | 767.8292 | 1517.6247 | 759.3160 | 1516.6406 | 758.8240 | V | **560.3402** | 280.6738 | 543.3137 | 272.1605 | 542.3297 | 271.6685 | 6 |
| 15 | **1605.6883** | 803.3478 | 1588.6618 | 794.8345 | 1587.6778 | 794.3425 | A | **461.2718** | 231.1396 | 444.2453 | 222.6263 | 443.2613 | 222.1343 | 5 |
| 16 | **1704.7567** | 852.8820 | **1687.7302** | 844.3687 | **1686.7462** | 843.8767 | V | **390.2347** | 195.6210 | 373.2082 | 187.1077 | 372.2241 | 186.6157 | 4 |
| 17 | **1791.7888** | 896.3980 | 1774.7622 | 887.8847 | 1773.7782 | 887.3927 | S | **291.1663** | 146.0868 | 274.1397 | 137.5735 | 273.1557 | 137.0815 | 3 |
| 18 | **1848.8102** | 924.9088 | 1831.7837 | 916.3955 | 1830.7997 | 915.9035 | G | 204.1343 | 102.5708 | 187.1077 | 94.0575 | | | 2 |
| 19 | | | | | | | K | 147.1128 | 74.0600 | 130.0863 | 65.5468 | | | 1 |

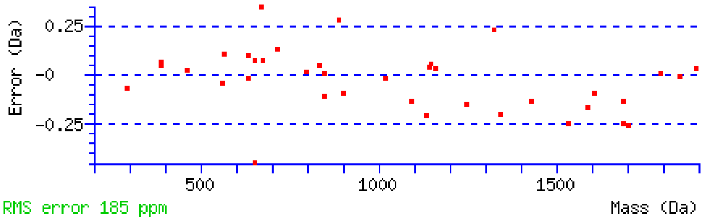


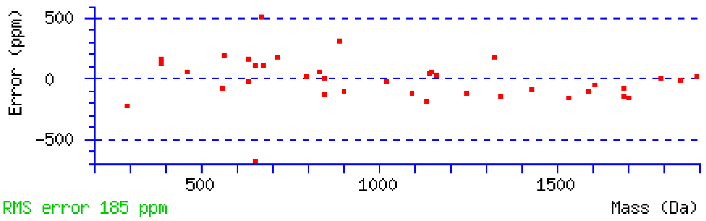


### All matches to this query

| Score | Mr(calc): | Delta | Sequence |
|---|---|---|---|
| 104.0 | 1993.9085 | 0.0050 | TNSTCSPNLDACLVAVSGK |
| 84.1 | 1993.9085 | 0.0050 | TNSTCSPNLDACLVAVSGK |
| 70.5 | 1992.9245 | 0.9890 | TNSTCSPNLDACLVAVSGK |
| 3.2 | 1991.8924 | 2.0211 | NLHDAICAVNSLHRYK |
| 0.8 | 1993.9285 | -0.0151 | EGDATPDNIPADIVFVLK |
| 0.1 | 1993.9267 | -0.0132 | RHLLEALWLSCGGAGMK |

Spectrum No: 65; Query: 1494; Rank: 1

## Peptide View

MS/MS Fragmentation of **DQDPASFGEDSLLLNSSR**
Found in **IPI00193894**, Tax_Id=10116 Gene_Symbol=Mgam_predicted 198 kDa protein

Match to Query 1494: 1950.886088 from(976.450320,2+)

Title: 100101RatKid_NS_deglyco_20.3676.3676.2.dta
Data file K:\NewmanPaper\Piliang\3SubProteomes\Piliang3SP\mgf5ppm\ERLIC_3SubProteomes5ppm.mgf

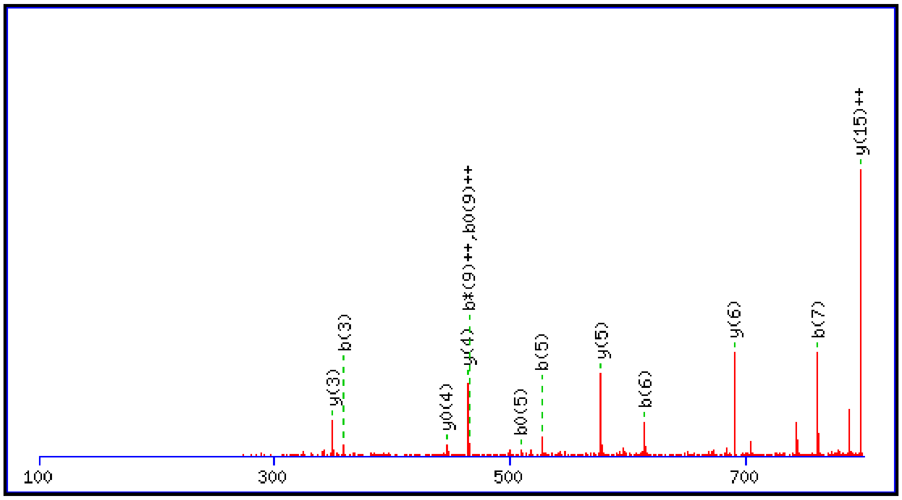


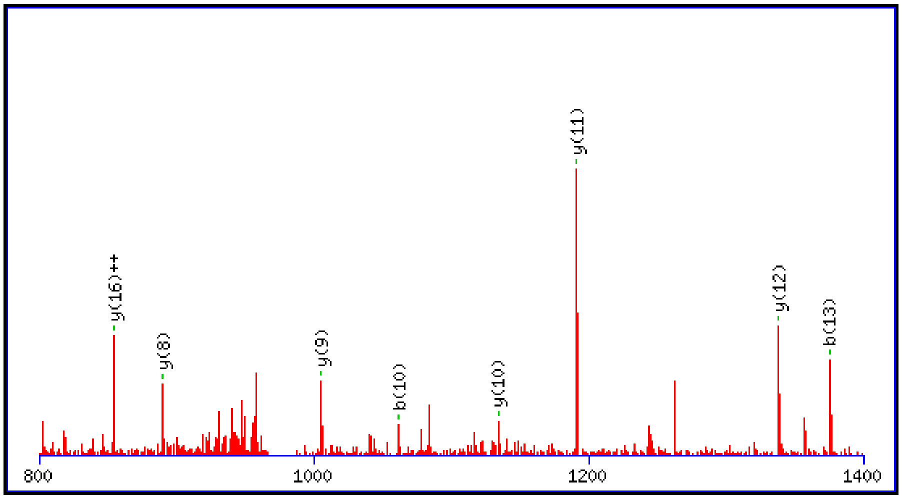


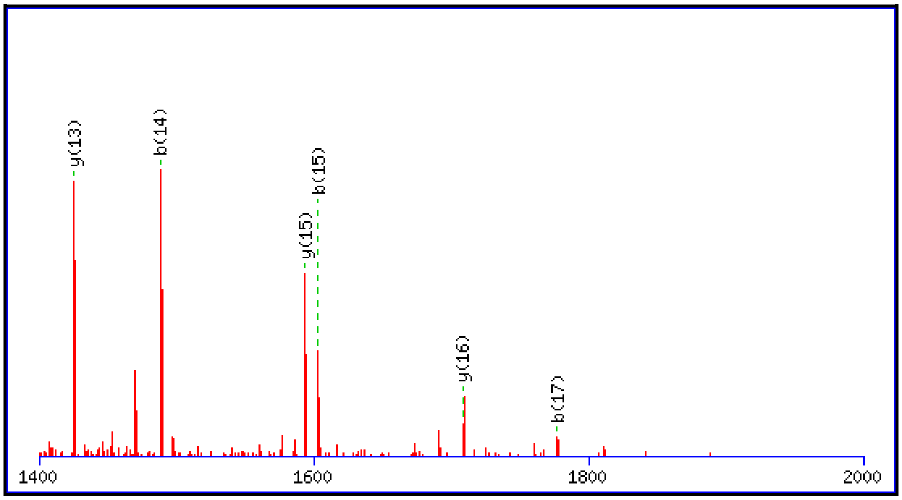


**Monoisotopic mass of neutral peptide Mr(calc):** 1950.8807
**Fixed modifications:** Carbamidomethyl (C)
**Variable modifications:**
**N15** : Deamidated_N (N)
**Ions Score:** 103 **Expect:** 1.3e-008
**Matches (Bold Red):** 27/200 fragment ions using 39 most intense peaks

| # | b | $b^{++}$ | b* | $b^{*++}$ | $b^0$ | $b^{0++}$ | Seq. | y | $y^{++}$ | y* | $y^{*++}$ | $y^0$ | $y^{0++}$ | # |
|---|---|---|---|---|---|---|---|---|---|---|---|---|---|---|
| 1 | 116.0342 | 58.5207 | | | 98.0237 | 49.5155 | D | | | | | | | 18 |
| 2 | 244.0928 | 122.5500 | 227.0662 | 114.0368 | 226.0822 | 113.5448 | Q | 1836.8610 | 918.9341 | 1819.8345 | 910.4209 | 1818.8504 | 909.9289 | 17 |
| 3 | **359.1197** | 180.0635 | 342.0932 | 171.5502 | 341.1092 | 171.0582 | D | **1708.8024** | **854.9049** | 1691.7759 | 846.3916 | 1690.7919 | 845.8996 | 16 |

| | | | | | | | | | | | | | | |
|---|---|---|---|---|---|---|---|---|---|---|---|---|---|---|
| 4 | 456.1725 | 228.5899 | 439.1460 | 220.0766 | 438.1619 | 219.5846 | P | **1593.7755** | **797.3914** | 1576.7489 | 788.8781 | 1575.7649 | 788.3861 | 15 |
| 5 | **527.2096** | 264.1084 | 510.1831 | 255.5952 | **509.1991** | 255.1032 | A | 1496.7227 | 748.8650 | 1479.6962 | 740.3517 | 1478.7122 | 739.8597 | 14 |
| 6 | **614.2416** | 307.6245 | 597.2151 | 299.1112 | 596.2311 | 298.6192 | S | **1425.6856** | 713.3464 | 1408.6591 | 704.8332 | 1407.6750 | 704.3412 | 13 |
| 7 | **761.3101** | 381.1587 | 744.2835 | 372.6454 | 743.2995 | 372.1534 | F | **1338.6536** | 669.8304 | 1321.6270 | 661.3172 | 1320.6430 | 660.8251 | 12 |
| 8 | 818.3315 | 409.6694 | 801.3050 | 401.1561 | 800.3210 | 400.6641 | G | **1191.5852** | 596.2962 | 1174.5586 | 587.7829 | 1173.5746 | 587.2909 | 11 |
| 9 | 947.3741 | 474.1907 | 930.3476 | **465.6774** | 929.3636 | **465.1854** | E | **1134.5637** | 567.7855 | 1117.5372 | 559.2722 | 1116.5531 | 558.7802 | 10 |
| 10 | **1062.4011** | 531.7042 | 1045.3745 | 523.1909 | 1044.3905 | 522.6989 | D | **1005.5211** | 503.2642 | 988.4946 | 494.7509 | 987.5105 | 494.2589 | 9 |
| 11 | 1149.4331 | 575.2202 | 1132.4065 | 566.7069 | 1131.4225 | 566.2149 | S | **890.4942** | 445.7507 | 873.4676 | 437.2374 | 872.4836 | 436.7454 | 8 |
| 12 | 1262.5172 | 631.7622 | 1245.4906 | 623.2489 | 1244.5066 | 622.7569 | L | 803.4621 | 402.2347 | 786.4356 | 393.7214 | 785.4516 | 393.2294 | 7 |
| 13 | **1375.6012** | 688.3042 | 1358.5747 | 679.7910 | 1357.5907 | 679.2990 | L | **690.3781** | 345.6927 | 673.3515 | 337.1794 | 672.3675 | 336.6874 | 6 |
| 14 | **1488.6853** | 744.8463 | 1471.6587 | 736.3330 | 1470.6747 | 735.8410 | L | **577.2940** | 289.1506 | 560.2675 | 280.6374 | 559.2834 | 280.1454 | 5 |
| 15 | **1603.7122** | 802.3597 | 1586.6857 | 793.8465 | 1585.7017 | 793.3545 | N | **464.2099** | 232.6086 | 447.1834 | 224.0953 | **446.1994** | 223.6033 | 4 |
| 16 | 1690.7442 | 845.8758 | 1673.7177 | 837.3625 | 1672.7337 | 836.8705 | S | **349.1830** | 175.0951 | 332.1565 | 166.5819 | 331.1724 | 166.0899 | 3 |
| 17 | **1777.7763** | 889.3918 | 1760.7497 | 880.8785 | 1759.7657 | 880.3865 | S | 262.1510 | 131.5791 | 245.1244 | 123.0659 | 244.1404 | 122.5738 | 2 |
| 18 | | | | | | | R | 175.1190 | 88.0631 | 158.0924 | 79.5498 | | | 1 |

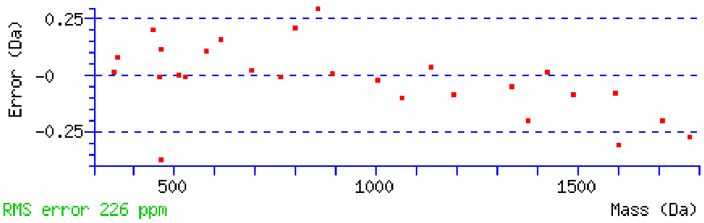


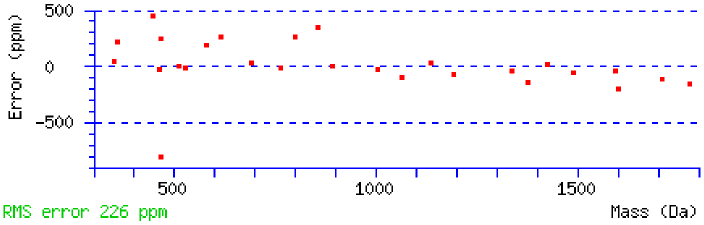


**All matches to this query**

| Score | Mr(calc): | Delta | Sequence |
|---|---|---|---|
| 103.1 | 1950.8807 | 0.0054 | DQDPASFGEDSLLLNSSR |
| 24.8 | 1949.8967 | 0.9894 | DQDPASFGEDSLLLNSSR |
| 7.9 | 1949.8983 | 0.9878 | QDAIVAVTGDGVNDSPALK |
| 7.3 | 1950.9040 | -0.0179 | SSSMALKGHGGSFIQECR |
| 4.5 | 1950.8740 | 0.0120 | SEYIIIPKSTLQEDR |
| 4.5 | 1950.8740 | 0.0120 | SEYIIIPKSTLQEDR |
| 3.5 | 1950.8838 | 0.0023 | EFIGSFLEMFGPEGALK |
| 3.5 | 1949.8983 | 0.9878 | QDAIVAVTGDGVNDSPALK |
| 3.0 | 1950.8714 | 0.0147 | TPGNPVNSARKPGSAGGPK |
| 2.6 | 1950.8737 | 0.0123 | RSLSPWGYSINHDPSR |

---

Spectrum No: 66; Query: 2745; Rank: 1

---

## Peptide View

MS/MS Fragmentation of **NSTPSEPDSGQGPPPEEEEGEEEAAKEEAEAQGVR**
Found in **IPI00326566**, Tax_Id=10116 Gene_Symbol=Hdgf Hepatoma-derived growth factor

Match to Query 2745: 3666.571992 from(1223.197940,3+)
Title: 100101RatKid_NS_deglyco_20.2758.2758.3.dta
Data file K:\NewmanPaper\Piliang\3SubProteomes\Piliang3SP\mgf5ppm\ERLIC_3SubProteomes5ppm.mgf

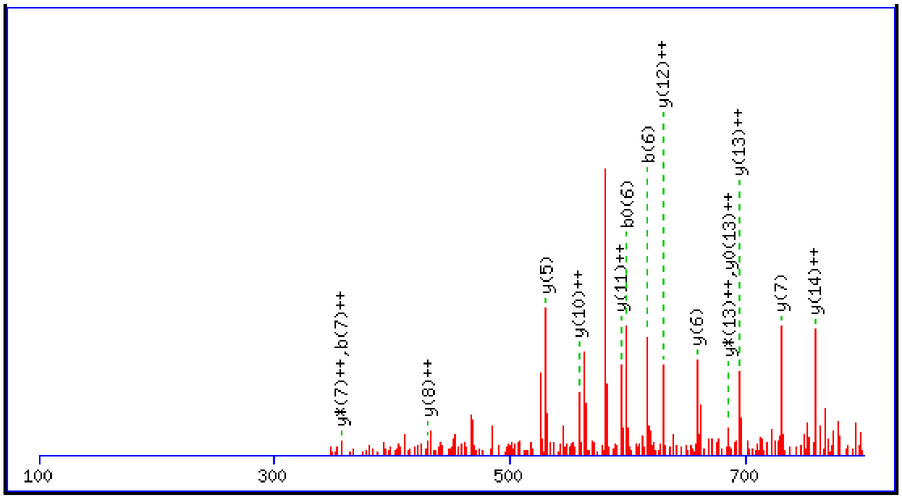


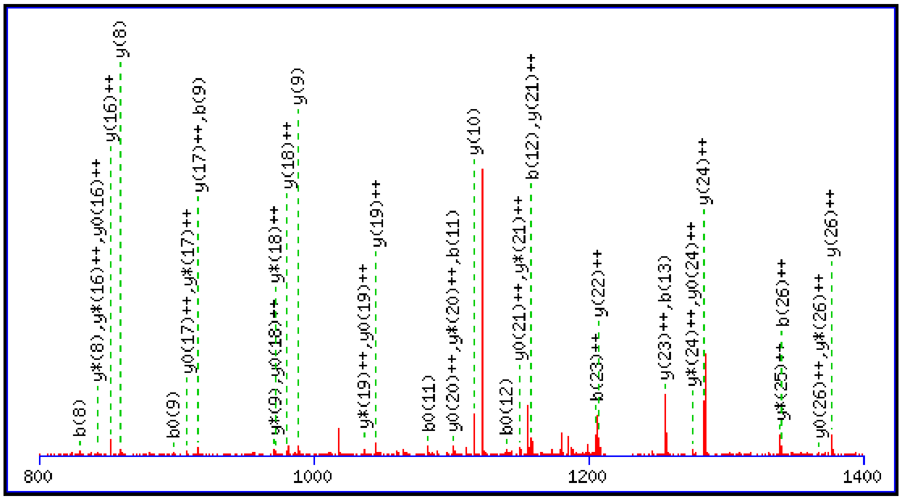


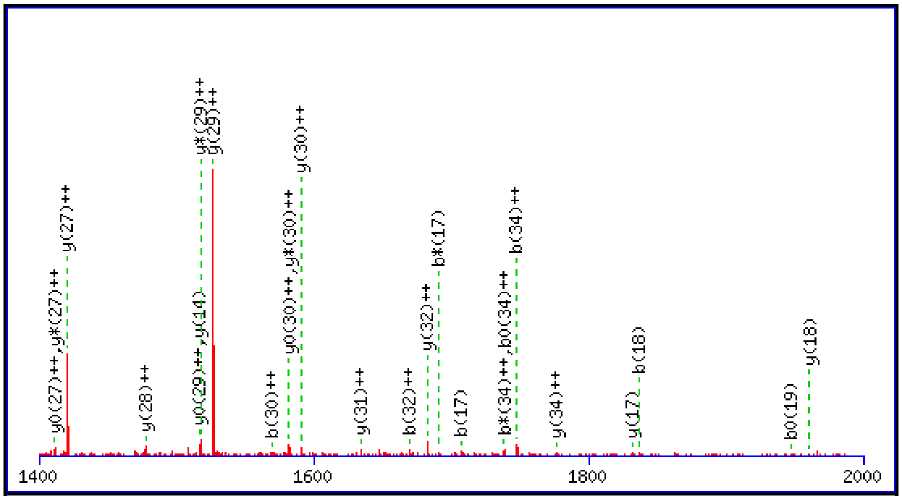


**Monoisotopic mass of neutral peptide Mr(calc):** 3666.5564
**Fixed modifications:** Carbamidomethyl (C)
**Variable modifications:**
**N1** : Deamidated_N (N)
**Ions Score:** 103 **Expect:** 2.7e-008
**Matches (Bold Red):** 81/396 fragment ions using 140 most intense peaks

| # | b | $b^{++}$ | b* | $b^{*++}$ | $b^0$ | $b^{0++}$ | Seq. | y | $y^{++}$ | y* | $y^{*++}$ | $y^0$ | $y^{0++}$ | # |
|---|---|---|---|---|---|---|---|---|---|---|---|---|---|---|
| 1 | 116.0342 | 58.5207 | 99.0077 | 50.0075 | | | N | | | | | | | 35 |
| 2 | 203.0662 | 102.0368 | 186.0397 | 93.5235 | 185.0557 | 93.0315 | S | 3552.5368 | **1776.7721** | 3535.5103 | 1768.2588 | 3534.5263 | 1767.7668 | 34 |
| 3 | 304.1139 | 152.5606 | 287.0874 | 144.0473 | 286.1034 | 143.5553 | T | 3465.5048 | 1733.2560 | 3448.4783 | 1724.7428 | 3447.4943 | 1724.2508 | 33 |
| 4 | 401.1667 | 201.0870 | 384.1401 | 192.5737 | 383.1561 | 192.0817 | P | 3364.4571 | **1682.7322** | 3347.4306 | 1674.2189 | 3346.4466 | 1673.7269 | 32 |
| 5 | 488.1987 | 244.6030 | 471.1722 | 236.0897 | 470.1881 | 235.5977 | S | 3267.4044 | **1634.2058** | 3250.3778 | 1625.6926 | 3249.3938 | 1625.2005 | 31 |

| | | | | | | | | | | | | | | |
|---|---|---|---|---|---|---|---|---|---|---|---|---|---|---|
| 6 | **617.2413** | 309.1243 | 600.2148 | 300.6110 | **599.2307** | 300.1190 | E | 3180.3723 | **1590.6898** | 3163.3458 | **1582.1765** | 3162.3618 | **1581.6845** | 30 |
| 7 | 714.2941 | **357.6507** | 697.2675 | 349.1374 | 696.2835 | 348.6454 | P | 3051.3298 | **1526.1685** | 3034.3032 | **1517.6552** | 3033.3192 | **1517.1632** | 29 |
| 8 | **829.3210** | 415.1641 | 812.2945 | 406.6509 | 811.3104 | 406.1589 | D | 2954.2770 | **1477.6421** | 2937.2504 | 1469.1289 | 2936.2664 | 1468.6369 | 28 |
| 9 | **916.3530** | 458.6802 | 899.3265 | 450.1669 | **898.3425** | 449.6749 | S | 2839.2500 | **1420.1287** | 2822.2235 | **1411.6154** | 2821.2395 | **1411.1234** | 27 |
| 10 | 973.3745 | 487.1909 | 956.3480 | 478.6776 | 955.3639 | 478.1856 | G | 2752.2180 | **1376.6126** | 2735.1915 | **1368.0994** | 2734.2075 | **1367.6074** | 26 |
| 11 | **1101.4331** | 551.2202 | 1084.4065 | 542.7069 | **1083.4225** | 542.2149 | Q | 2695.1966 | 1348.1019 | 2678.1700 | **1339.5886** | 2677.1860 | 1339.0966 | 25 |
| 12 | **1158.4545** | 579.7309 | 1141.4280 | 571.2176 | **1140.4440** | 570.7256 | G | 2567.1380 | **1284.0726** | 2550.1114 | **1275.5594** | 2549.1274 | **1275.0673** | 24 |
| 13 | **1255.5073** | 628.2573 | 1238.4808 | 619.7440 | 1237.4967 | 619.2520 | P | 2510.1165 | **1255.5619** | 2493.0900 | 1247.0486 | 2492.1059 | 1246.5566 | 23 |
| 14 | 1352.5601 | 676.7837 | 1335.5335 | 668.2704 | 1334.5495 | 667.7784 | P | 2413.0637 | **1207.0355** | 2396.0372 | 1198.5222 | 2395.0532 | 1198.0302 | 22 |
| 15 | 1449.6128 | 725.3101 | 1432.5863 | 716.7968 | 1431.6023 | 716.3048 | P | 2316.0110 | **1158.5091** | 2298.9844 | **1149.9959** | 2298.0004 | **1149.5038** | 21 |
| 16 | 1578.6554 | 789.8314 | 1561.6289 | 781.3181 | 1560.6449 | 780.8261 | E | 2218.9582 | 1109.9827 | 2201.9317 | **1101.4695** | 2200.9477 | **1100.9775** | 20 |
| 17 | **1707.6980** | 854.3526 | **1690.6715** | 845.8394 | 1689.6875 | 845.3474 | E | 2089.9156 | **1045.4615** | 2072.8891 | **1036.9482** | 2071.9051 | **1036.4562** | 19 |
| 18 | **1836.7406** | 918.8739 | 1819.7141 | 910.3607 | 1818.7301 | 909.8687 | E | **1960.8730** | **980.9402** | 1943.8465 | **972.4269** | 1942.8625 | **971.9349** | 18 |
| 19 | 1965.7832 | 983.3952 | 1948.7567 | 974.8820 | **1947.7726** | 974.3900 | E | **1831.8304** | **916.4189** | 1814.8039 | **907.9056** | 1813.8199 | **907.4136** | 17 |
| 20 | 2022.8047 | 1011.9060 | 2005.7781 | 1003.3927 | 2004.7941 | 1002.9007 | G | 1702.7878 | **851.8976** | 1685.7613 | **843.3843** | 1684.7773 | **842.8923** | 16 |
| 21 | 2151.8473 | 1076.4273 | 2134.8207 | 1067.9140 | 2133.8367 | 1067.4220 | E | 1645.7664 | 823.3868 | 1628.7398 | 814.8736 | 1627.7558 | 814.3815 | 15 |
| 22 | 2280.8899 | 1140.9486 | 2263.8633 | 1132.4353 | 2262.8793 | 1131.9433 | E | **1516.7238** | **758.8655** | 1499.6972 | 750.3523 | 1498.7132 | 749.8603 | 14 |
| 23 | 2409.9325 | **1205.4699** | 2392.9059 | 1196.9566 | 2391.9219 | 1196.4646 | E | 1387.6812 | **694.3442** | 1370.6547 | **685.8310** | 1369.6706 | **685.3390** | 13 |
| 24 | 2480.9696 | 1240.9884 | 2463.9430 | 1232.4751 | 2462.9590 | 1231.9831 | A | 1258.6386 | **629.8229** | 1241.6121 | 621.3097 | 1240.6280 | 620.8177 | 12 |
| 25 | 2552.0067 | 1276.5070 | 2534.9801 | 1267.9937 | 2533.9961 | 1267.5017 | A | 1187.6015 | **594.3044** | 1170.5749 | 585.7911 | 1169.5909 | 585.2991 | 11 |
| 26 | 2680.1016 | **1340.5545** | 2663.0751 | 1332.0412 | 2662.0911 | 1331.5492 | K | **1116.5644** | **558.7858** | 1099.5378 | 550.2726 | 1098.5538 | 549.7805 | 10 |
| 27 | 2809.1442 | 1405.0758 | 2792.1177 | 1396.5625 | 2791.1337 | 1396.0705 | E | **988.4694** | 494.7383 | **971.4429** | 486.2251 | 970.4589 | 485.7331 | 9 |
| 28 | 2938.1868 | 1469.5971 | 2921.1603 | 1461.0838 | 2920.1763 | 1460.5918 | E | **859.4268** | **430.2170** | **842.4003** | 421.7038 | 841.4163 | 421.2118 | 8 |
| 29 | 3009.2239 | 1505.1156 | 2992.1974 | 1496.6023 | 2991.2134 | 1496.1103 | A | **730.3842** | 365.6958 | 713.3577 | **357.1825** | 712.3737 | 356.6905 | 7 |
| 30 | 3138.2665 | **1569.6369** | 3121.2400 | 1561.1236 | 3120.2560 | 1560.6316 | E | **659.3471** | 330.1772 | 642.3206 | 321.6639 | 641.3366 | 321.1719 | 6 |
| 31 | 3209.3037 | 1605.1555 | 3192.2771 | 1596.6422 | 3191.2931 | 1596.1502 | A | **530.3045** | 265.6559 | 513.2780 | 257.1426 | | | 5 |
| 32 | 3337.3622 | **1669.1848** | 3320.3357 | 1660.6715 | 3319.3517 | 1660.1795 | Q | 459.2674 | 230.1373 | 442.2409 | 221.6241 | | | 4 |
| 33 | 3394.3837 | 1697.6955 | 3377.3571 | 1689.1822 | 3376.3731 | 1688.6902 | G | 331.2088 | 166.1081 | 314.1823 | 157.5948 | | | 3 |
| 34 | 3493.4521 | **1747.2297** | 3476.4256 | **1738.7164** | 3475.4415 | **1738.2244** | V | 274.1874 | 137.5973 | 257.1608 | 129.0840 | | | 2 |
| 35 | | | | | | | R | 175.1190 | 88.0631 | 158.0924 | 79.5498 | | | 1 |

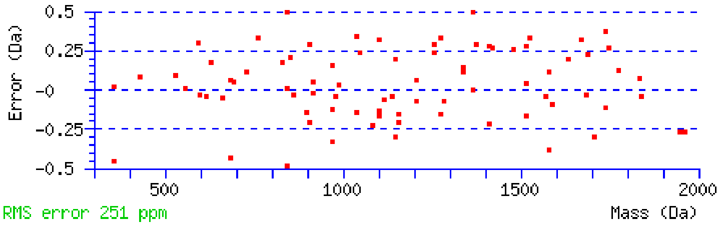


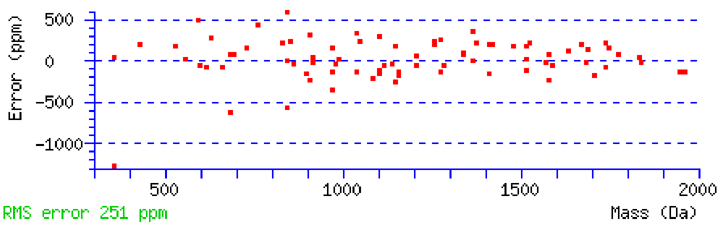


## All matches to this query

| Score | Mr(calc): | Delta | Sequence |
|---|---|---|---|
| 102.6 | 3666.5564 | 0.0156 | NSTPSEPDSGQGPPPEEEEGEEEAAKEEAEAQGVR |
| 86.6 | 3665.5724 | 0.9996 | NSTPSEPDSGQGPPPEEEEGEEEAAKEEAEAQGVR |
| 8.4 | 3665.5727 | 0.9993 | YFLTAVSRPGLGEPWFIIVGYVDDTEFVR |
| 8.4 | 3665.5727 | 0.9993 | YFLTAVSRPGLGEPWFIIVGYVDDTEFVR |
| 8.3 | 3666.5677 | 0.0043 | VYLIRGLSFETTDESLKTMENTSNFLVMR |
| 8.2 | 3665.5836 | 0.9883 | VYLIRGLSFETTDESLKTMENTSNFLVMR |
| 8.2 | 3666.5677 | 0.0043 | VYLIRGLSFETTDESLKTMENTSNFLVMR |
| 6.9 | 3666.5723 | -0.0004 | EVGGTNPQQHRGQKPESENVGDETSDIENLLK |
| 5.5 | 3666.5779 | -0.0059 | IYYIHTAEDEVKMDSFEFQVTDGRNPVFR |
| 5.4 | 3665.5940 | 0.9780 | YGDLLPADGVLIQGNDLKIDESSLTGESDHVR |

Spectrum No: 67; Query: 2088; Rank: 1

## Peptide View

MS/MS Fragmentation of **YLQPLLAVQFTNLTLDTEIR**
Found in **IPI00339124**, Tax_Id=10116 Gene_Symbol=Atp1b1 Sodium/potassium-transporting ATPase subunit beta-1

Match to Query 2088: 2348.267428 from(1175.140990,2+)
Title: 091008RatKidney_NH4Format01_25.4494.4494.2.dta
Data file K:\NewmanPaper\Piliang\3SubProteomes\Piliang3SP\mgf5ppm\ERLIC_3SubProteomes5ppm.mgf

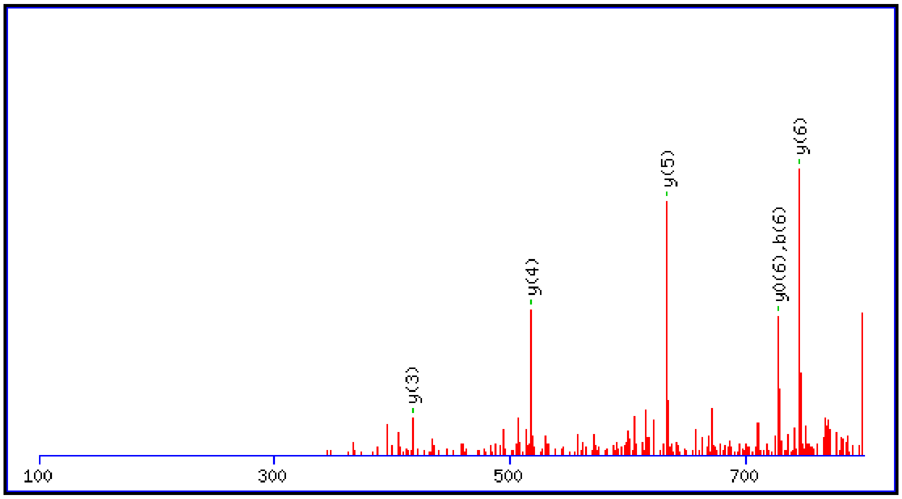


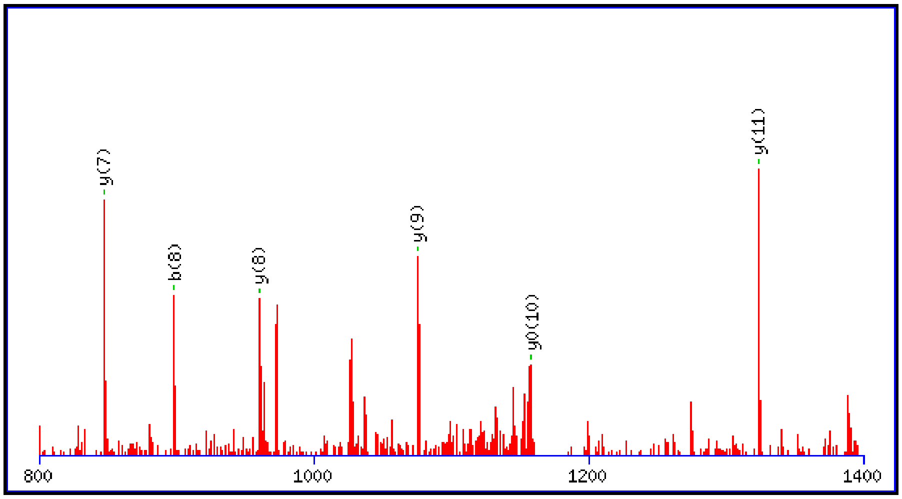

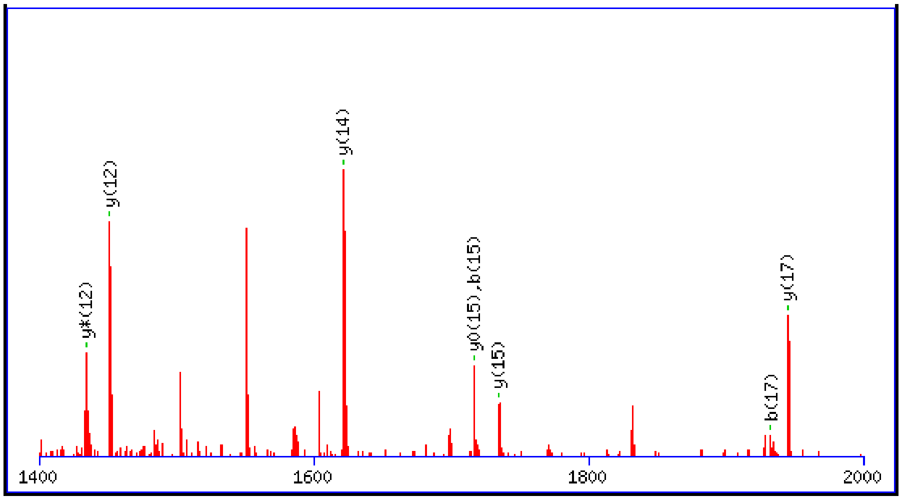


**Monoisotopic mass of neutral peptide Mr(calc):** 2348.2627
**Fixed modifications:** Carbamidomethyl (C)
**Variable modifications:**
**N12** : Deamidated_N (N)
**Ions Score:** 102 **Expect:** 3.6e-009
**Matches (Bold Red):** 20/200 fragment ions using 23 most intense peaks

| # | b | b++ | b* | b*++ | b0 | b0++ | Seq. | y | y++ | y* | y*++ | y0 | y0++ | # |
|---|---|---|---|---|---|---|---|---|---|---|---|---|---|---|
| 1 | 164.0706 | 82.5389 | | | | | Y | | | | | | | 20 |
| 2 | 277.1547 | 139.0810 | | | | | L | 2186.2067 | 1093.6070 | 2169.1802 | 1085.0937 | 2168.1961 | 1084.6017 | 19 |
| 3 | 405.2132 | 203.1103 | 388.1867 | 194.5970 | | | Q | 2073.1226 | 1037.0650 | 2056.0961 | 1028.5517 | 2055.1121 | 1028.0597 | 18 |
| 4 | 502.2660 | 251.6366 | 485.2395 | 243.1234 | | | P | **1945.0641** | 973.0357 | 1928.0375 | 964.5224 | 1927.0535 | 964.0304 | 17 |
| 5 | 615.3501 | 308.1787 | 598.3235 | 299.6654 | | | L | 1848.0113 | 924.5093 | 1830.9848 | 915.9960 | 1830.0007 | 915.5040 | 16 |
| 6 | **728.4341** | 364.7207 | 711.4076 | 356.2074 | | | L | **1734.9272** | 867.9673 | 1717.9007 | 859.4540 | **1716.9167** | 858.9620 | 15 |
| 7 | 799.4713 | 400.2393 | 782.4447 | 391.7260 | | | A | **1621.8432** | 811.4252 | 1604.8166 | 802.9120 | 1603.8326 | 802.4199 | 14 |
| 8 | **898.5397** | 449.7735 | 881.5131 | 441.2602 | | | V | 1550.8061 | 775.9067 | 1533.7795 | 767.3934 | 1532.7955 | 766.9014 | 13 |
| 9 | 1026.5982 | 513.8028 | 1009.5717 | 505.2895 | | | Q | **1451.7376** | 726.3725 | **1434.7111** | 717.8592 | 1433.7271 | 717.3672 | 12 |
| 10 | 1173.6667 | 587.3370 | 1156.6401 | 578.8237 | | | F | **1323.6791** | 662.3432 | 1306.6525 | 653.8299 | 1305.6685 | 653.3379 | 11 |
| 11 | 1274.7143 | 637.8608 | 1257.6878 | 629.3475 | 1256.7038 | 628.8555 | T | 1176.6107 | 588.8090 | 1159.5841 | 580.2957 | **1158.6001** | 579.8037 | 10 |
| 12 | 1389.7413 | 695.3743 | 1372.7147 | 686.8610 | 1371.7307 | 686.3690 | N | **1075.5630** | 538.2851 | 1058.5364 | 529.7719 | 1057.5524 | 529.2798 | 9 |
| 13 | 1502.8253 | 751.9163 | 1485.7988 | 743.4030 | 1484.8148 | 742.9110 | L | **960.5360** | 480.7717 | 943.5095 | 472.2584 | 942.5255 | 471.7664 | 8 |
| 14 | 1603.8730 | 802.4401 | 1586.8465 | 793.9269 | 1585.8625 | 793.4349 | T | **847.4520** | 424.2296 | 830.4254 | 415.7164 | 829.4414 | 415.2243 | 7 |
| 15 | **1716.9571** | 858.9822 | 1699.9305 | 850.4689 | 1698.9465 | 849.9769 | L | **746.4043** | 373.7058 | 729.3777 | 365.1925 | **728.3937** | 364.7005 | 6 |
| 16 | 1831.9840 | 916.4956 | 1814.9575 | 907.9824 | 1813.9735 | 907.4904 | D | **633.3202** | 317.1638 | 616.2937 | 308.6505 | 615.3097 | 308.1585 | 5 |
| 17 | **1933.0317** | 967.0195 | 1916.0052 | 958.5062 | 1915.0211 | 958.0142 | T | **518.2933** | 259.6503 | 501.2667 | 251.1370 | 500.2827 | 250.6450 | 4 |
| 18 | 2062.0743 | 1031.5408 | 2045.0477 | 1023.0275 | 2044.0637 | 1022.5355 | E | **417.2456** | 209.1264 | 400.2191 | 200.6132 | 399.2350 | 200.1212 | 3 |
| 19 | 2175.1584 | 1088.0828 | 2158.1318 | 1079.5695 | 2157.1478 | 1079.0775 | I | 288.2030 | 144.6051 | 271.1765 | 136.0919 | | | 2 |
| 20 | | | | | | | R | 175.1190 | 88.0631 | 158.0924 | 79.5498 | | | 1 |

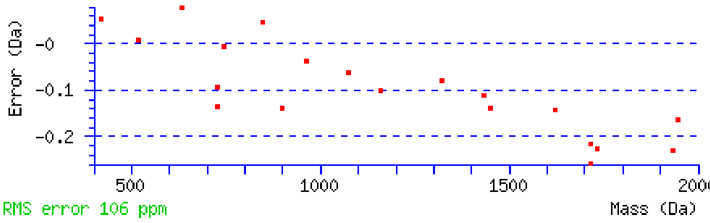


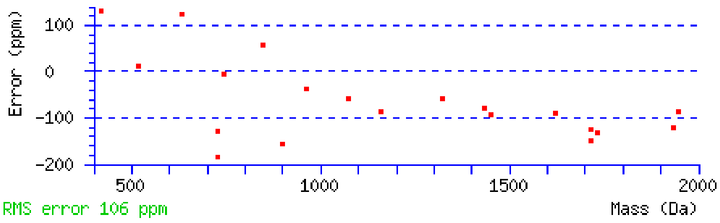


### All matches to this query

| Score | Mr(calc): | Delta | Sequence |
|---|---|---|---|
| 102.5 | 2348.2627 | 0.0047 | YLQPLLAVQFTNLTLDTEIR |
| | | | |

| | | | |
|---|---|---|---|
| 23.2 | 2347.2787 | 0.9887 | YLQPLLAVQFTNLTLDTEIR |
| 4.7 | 2348.2577 | 0.0098 | TLTSILVGLQGAARRESDLLR |

---

Spectrum No: 68; Query: 964; Rank: 1

---

## Peptide View

MS/MS Fragmentation of **NCNSTTEFTCSNGR**
Found in **IPI00205325**, Tax_Id=10116 Gene_Symbol=Lrp2 Low-density lipoprotein receptor-related protein 2 precursor

Match to Query 964: 1647.632248 from(824.823400,2+)
Title: 100101RatKid_NS_deglyco_24.766.766.2.dta
Data file K:\NewmanPaper\Piliang\3SubProteomes\Piliang3SP\mgf5ppm\ERLIC_3SubProteomes5ppm.mgf

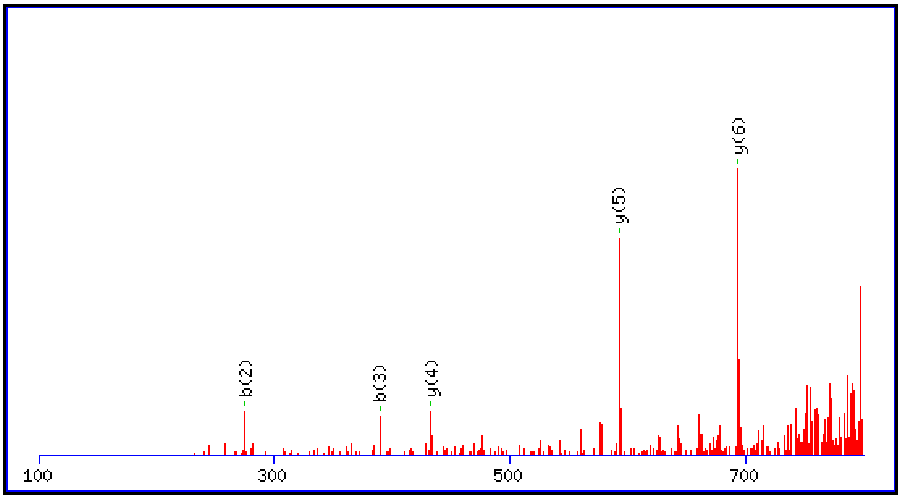


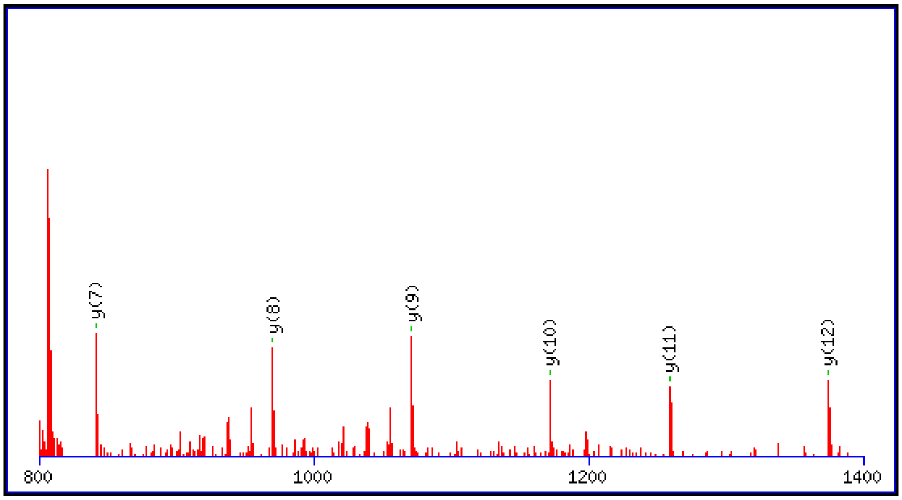

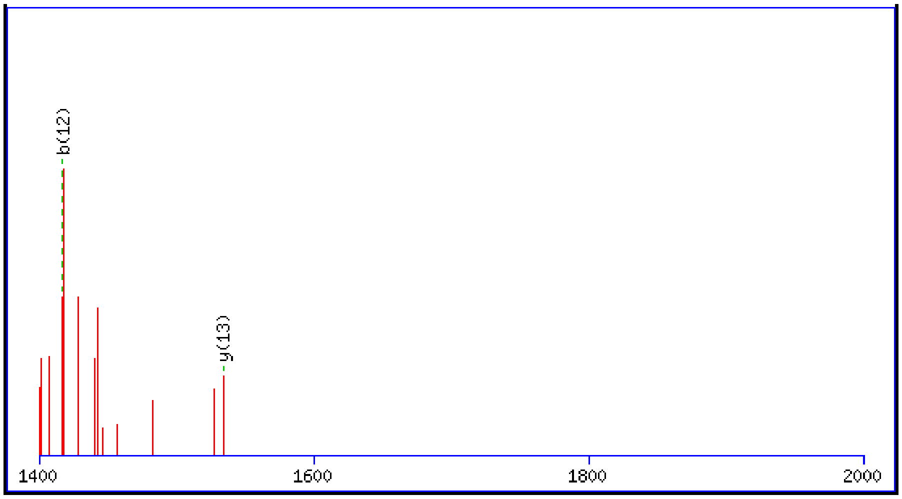


**Monoisotopic mass of neutral peptide Mr(calc):** 1647.6253
**Fixed modifications:** Carbamidomethyl (C)
**Variable modifications:**
**N3** : Deamidated_N (N)
**Ions Score:** 102 **Expect:** 7.4e-009
**Matches (Bold Red):** 13/144 fragment ions using 21 most intense peaks

| # | b | b++ | b* | b*++ | b0 | b0++ | Seq. | y | y++ | y* | y*++ | y0 | y0++ | # |
|---|---|---|---|---|---|---|---|---|---|---|---|---|---|---|
| 1 | 115.0502 | 58.0287 | 98.0237 | 49.5155 | | | N | | | | | | | 14 |
| 2 | **275.0809** | 138.0441 | 258.0543 | 129.5308 | | | C | **1534.5897** | 767.7985 | 1517.5631 | 759.2852 | 1516.5791 | 758.7932 | 13 |
| 3 | **390.1078** | 195.5575 | 373.0812 | 187.0443 | | | N | **1374.5590** | 687.7832 | 1357.5325 | 679.2699 | 1356.5485 | 678.7779 | 12 |
| 4 | 477.1398 | 239.0735 | 460.1133 | 230.5603 | 459.1293 | 230.0683 | S | **1259.5321** | 630.2697 | 1242.5055 | 621.7564 | 1241.5215 | 621.2644 | 11 |
| 5 | 578.1875 | 289.5974 | 561.1609 | 281.0841 | 560.1769 | 280.5921 | T | **1172.5001** | 586.7537 | 1155.4735 | 578.2404 | 1154.4895 | 577.7484 | 10 |
| 6 | 679.2352 | 340.1212 | 662.2086 | 331.6080 | 661.2246 | 331.1159 | T | **1071.4524** | 536.2298 | 1054.4258 | 527.7166 | 1053.4418 | 527.2245 | 9 |
| 7 | 808.2778 | 404.6425 | 791.2512 | 396.1292 | 790.2672 | 395.6372 | E | **970.4047** | 485.7060 | 953.3782 | 477.1927 | 952.3941 | 476.7007 | 8 |
| 8 | 955.3462 | 478.1767 | 938.3196 | 469.6635 | 937.3356 | 469.1714 | F | **841.3621** | 421.1847 | 824.3356 | 412.6714 | 823.3515 | 412.1794 | 7 |
| 9 | 1056.3939 | 528.7006 | 1039.3673 | 520.1873 | 1038.3833 | 519.6953 | T | **694.2937** | 347.6505 | 677.2672 | 339.1372 | 676.2831 | 338.6452 | 6 |
| 10 | 1216.4245 | 608.7159 | 1199.3980 | 600.2026 | 1198.4139 | 599.7106 | C | **593.2460** | 297.1266 | 576.2195 | 288.6134 | 575.2355 | 288.1214 | 5 |
| 11 | 1303.4565 | 652.2319 | 1286.4300 | 643.7186 | 1285.4460 | 643.2266 | S | **433.2154** | 217.1113 | 416.1888 | 208.5980 | 415.2048 | 208.1060 | 4 |
| 12 | **1417.4995** | 709.2534 | 1400.4729 | 700.7401 | 1399.4889 | 700.2481 | N | 346.1833 | 173.5953 | 329.1568 | 165.0820 | | | 3 |
| 13 | 1474.5209 | 737.7641 | 1457.4944 | 729.2508 | 1456.5104 | 728.7588 | G | 232.1404 | 116.5738 | 215.1139 | 108.0606 | | | 2 |
| 14 | | | | | | | R | 175.1190 | 88.0631 | 158.0924 | 79.5498 | | | 1 |

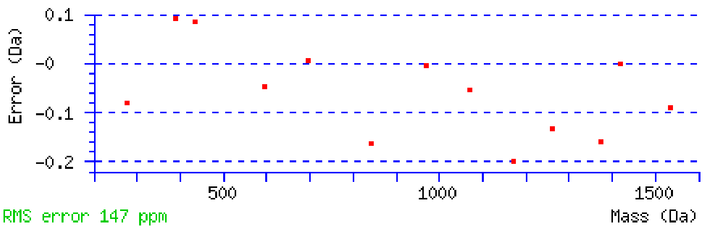


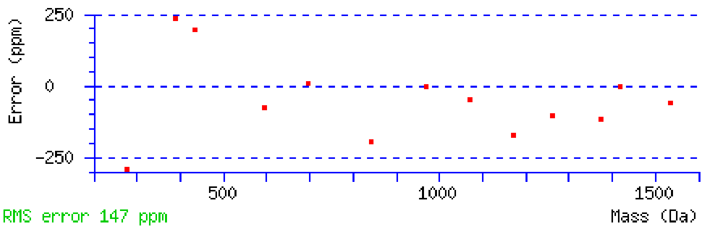


### All matches to this query

| Score | Mr(calc): | Delta | Sequence |
|---|---|---|---|
| 101.5 | 1647.6253 | 0.0069 | NCNSTTEFTCSNGR |
| 62.0 | 1646.6413 | 0.9909 | NCNSTTEFTCSNGR |
| 54.7 | 1647.6253 | 0.0069 | NCNSTTEFTCSNGR |
| 42.8 | 1647.6253 | 0.0069 | NCNSTTEFTCSNGR |
| 1.0 | 1647.6331 | -0.0008 | IFSGNSHQNLNQK |
| 1.0 | 1647.6331 | -0.0008 | IFSGNSHQNLNQK |
| 0.2 | 1647.6365 | -0.0042 | MSKQTARASDSYK |
| | | | |

| | | | |
|---|---|---|---|
| 0.2 | 1647.6365 | -0.0042 | MSKQTARASDSYK |

---

Spectrum No: 69; Query: 687; Rank: 1

---

## Peptide View

MS/MS Fragmentation of **TAFQNGTESTIITK**
Found in **IPI00204808**, Tax_Id=10116 Gene_Symbol=Mep1b Meprin A subunit beta precursor

Match to Query 687: 1510.749568 from(756.382060,2+)
Title: 091008RatKidney_NH4Format01_27.1460.1460.2.dta
Data file K:\NewmanPaper\Piliang\3SubProteomes\Piliang3SP\mgf5ppm\ERLIC_3SubProteomes5ppm.mgf

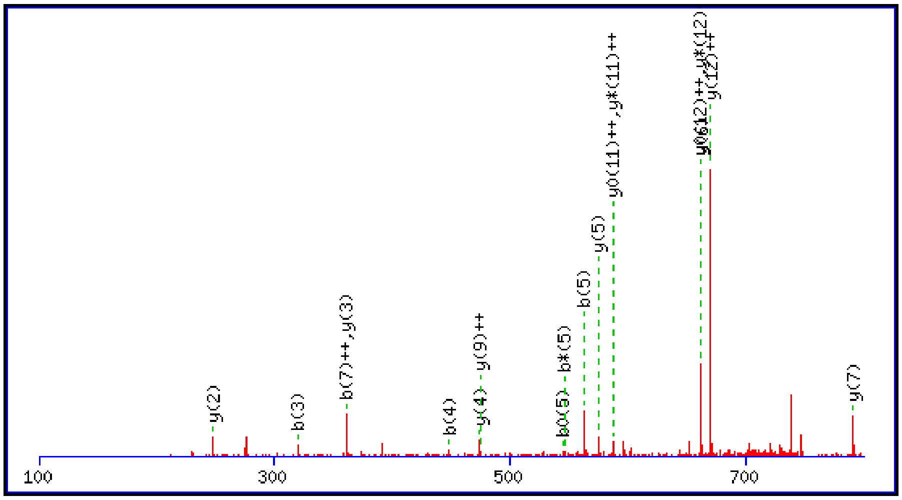


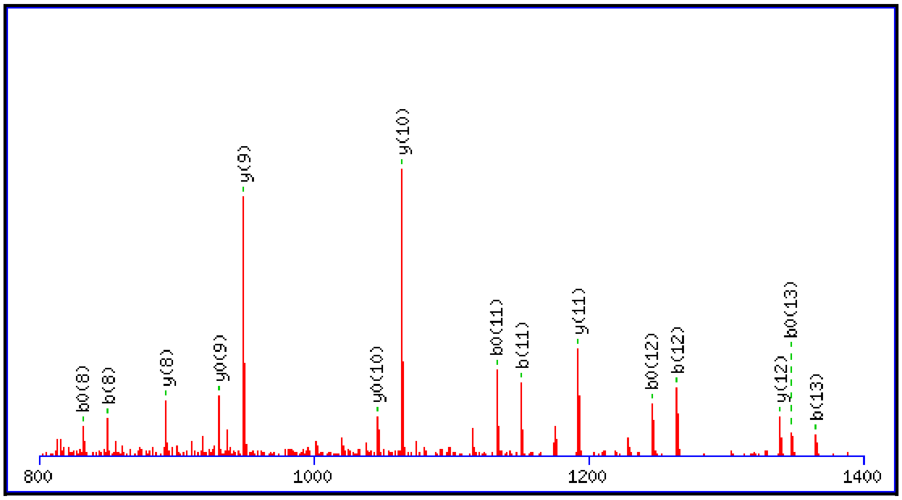

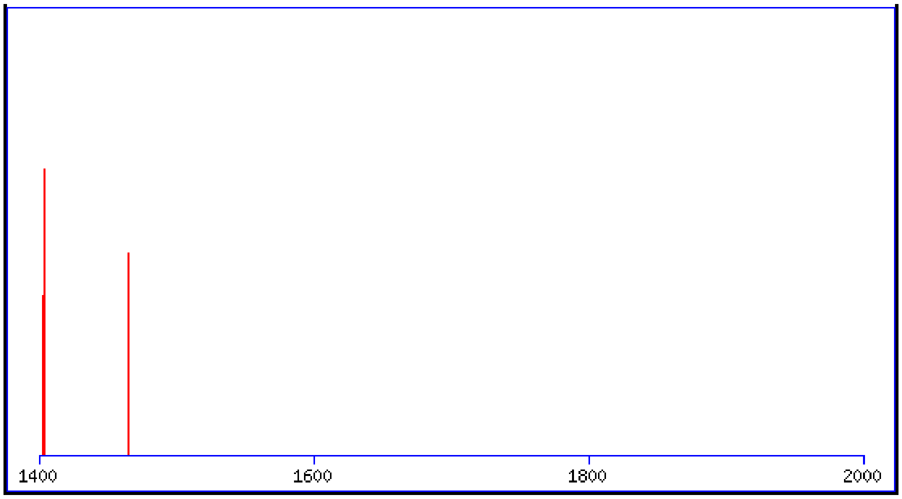


**Monoisotopic mass of neutral peptide Mr(calc):** 1510.7515
**Fixed modifications:** Carbamidomethyl (C)
**Variable modifications:**
**N5** : Deamidated_N (N)
**Ions Score:** 101 **Expect:** 1.1e-008
**Matches (Bold Red):** 33/148 fragment ions using 43 most intense peaks

| # | b | $b^{++}$ | b* | $b^{*++}$ | $b^0$ | $b^{0++}$ | Seq. | y | $y^{++}$ | y* | $y^{*++}$ | $y^0$ | $y^{0++}$ | # |
|---|---|---|---|---|---|---|---|---|---|---|---|---|---|---|
| 1 | 102.0550 | 51.5311 | | | 84.0444 | 42.5258 | T | | | | | | | 14 |
| 2 | 173.0921 | 87.0497 | | | 155.0815 | 78.0444 | A | 1410.7111 | 705.8592 | 1393.6845 | 697.3459 | 1392.7005 | 696.8539 | 13 |
| 3 | **320.1605** | 160.5839 | | | 302.1499 | 151.5786 | F | **1339.6740** | **670.3406** | 1322.6474 | **661.8274** | 1321.6634 | **661.3353** | 12 |
| 4 | **448.2191** | 224.6132 | 431.1925 | 216.0999 | 430.2085 | 215.6079 | Q | **1192.6056** | 596.8064 | 1175.5790 | **588.2931** | 1174.5950 | **587.8011** | 11 |
| 5 | **563.2460** | 282.1266 | **546.2194** | 273.6134 | **545.2354** | 273.1214 | N | **1064.5470** | 532.7771 | 1047.5204 | 524.2639 | **1046.5364** | 523.7719 | 10 |
| 6 | 620.2675 | 310.6374 | 603.2409 | 302.1241 | 602.2569 | 301.6321 | G | **949.5201** | **475.2637** | 932.4935 | 466.7504 | **931.5095** | 466.2584 | 9 |
| 7 | 721.3151 | **361.1612** | 704.2886 | 352.6479 | 703.3046 | 352.1559 | T | **892.4986** | 446.7529 | 875.4720 | 438.2397 | 874.4880 | 437.7477 | 8 |
| 8 | **850.3577** | 425.6825 | 833.3312 | 417.1692 | **832.3472** | 416.6772 | E | **791.4509** | 396.2291 | 774.4244 | 387.7158 | 773.4403 | 387.2238 | 7 |
| 9 | 937.3898 | 469.1985 | 920.3632 | 460.6852 | 919.3792 | 460.1932 | S | **662.4083** | 331.7078 | 645.3818 | 323.1945 | 644.3978 | 322.7025 | 6 |
| 10 | 1038.4374 | 519.7224 | 1021.4109 | 511.2091 | 1020.4269 | 510.7171 | T | **575.3763** | 288.1918 | 558.3497 | 279.6785 | 557.3657 | 279.1865 | 5 |
| 11 | **1151.5215** | 576.2644 | 1134.4950 | 567.7511 | **1133.5109** | 567.2591 | I | **474.3286** | 237.6679 | 457.3021 | 229.1547 | 456.3180 | 228.6627 | 4 |
| 12 | **1264.6056** | 632.8064 | 1247.5790 | 624.2931 | **1246.5950** | 623.8011 | I | **361.2445** | 181.1259 | 344.2180 | 172.6126 | 343.2340 | 172.1206 | 3 |
| 13 | **1365.6532** | 683.3303 | 1348.6267 | 674.8170 | **1347.6427** | 674.3250 | T | **248.1605** | 124.5839 | 231.1339 | 116.0706 | 230.1499 | 115.5786 | 2 |
| 14 | | | | | | | K | 147.1128 | 74.0600 | 130.0863 | 65.5468 | | | 1 |

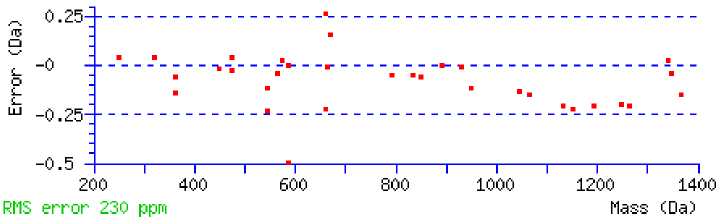


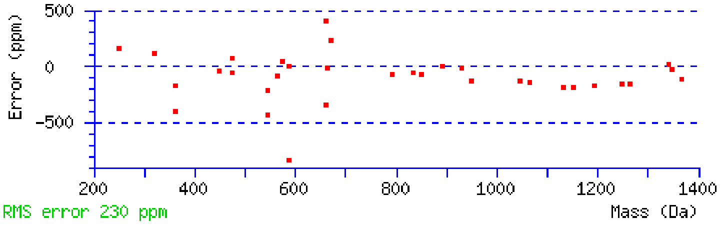


### All matches to this query

| Score | Mr(calc): | Delta | Sequence |
|---|---|---|---|
| 101.4 | 1510.7515 | -0.0019 | TAFQNGTESTIITK |
| 9.7 | 1508.7501 | 1.9995 | FQGPQPSSRAVKK |
| 5.8 | 1508.7501 | 1.9995 | FQGPQPSSRAVKK |
| 5.7 | 1510.7371 | 0.0125 | MQTSLDEVMKTTK |
| 5.3 | 1510.7456 | 0.0039 | TAFQEEFVHYLK |
| 4.2 | 1510.7502 | -0.0006 | TVPHQGTEGHHRR |
| 2.2 | 1510.7504 | -0.0009 | TELKQRLNSQSK |
| | | | |

| | | | |
|---|---|---|---|
| 1.9 | 1508.7545 | 1.9951 | GLFCDVTLIAGDTK |
| 1.8 | 1508.7505 | 1.9991 | SGNVTTVMETIGRK |

Spectrum No: 70; Query: 1748; Rank: 1

## Peptide View

MS/MS Fragmentation of **MYVTNDTEVAENNYQALK**
Found in **IPI00464785**, Tax_Id=10116 Gene_Symbol=Ppgb Protective protein for beta-galactosidase

Match to Query 1748: 2102.955528 from(1052.485040,2+)
Title: 100101RatKid_NS_deglyco_23.2834.2834.2.dta
Data file K:\NewmanPaper\Piliang\3SubProteomes\Piliang3SP\mgf5ppm\ERLIC_3SubProteomes5ppm.mgf

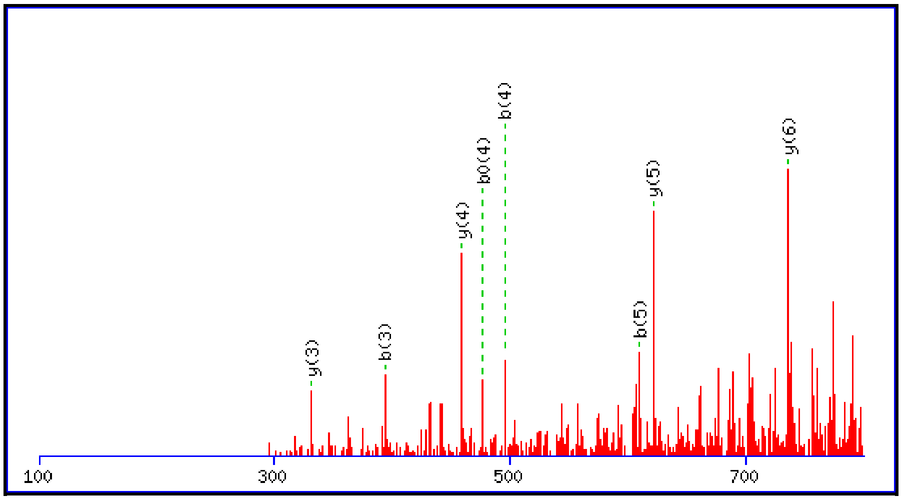


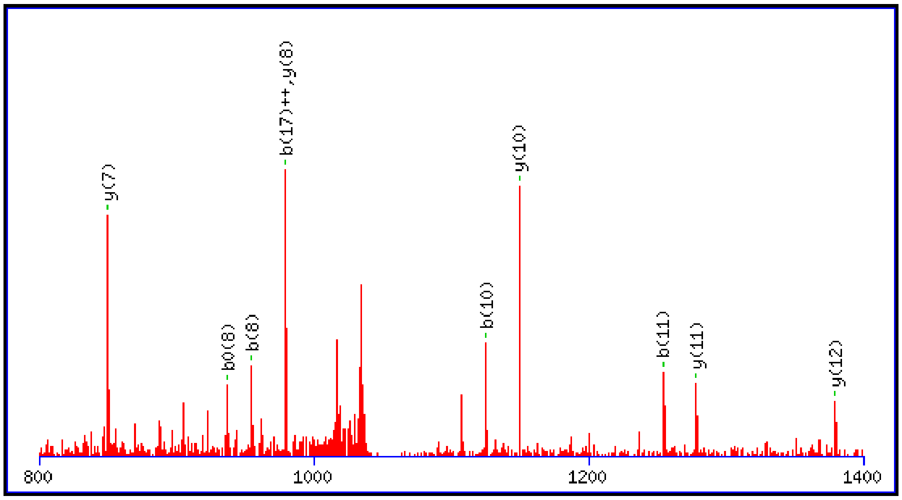

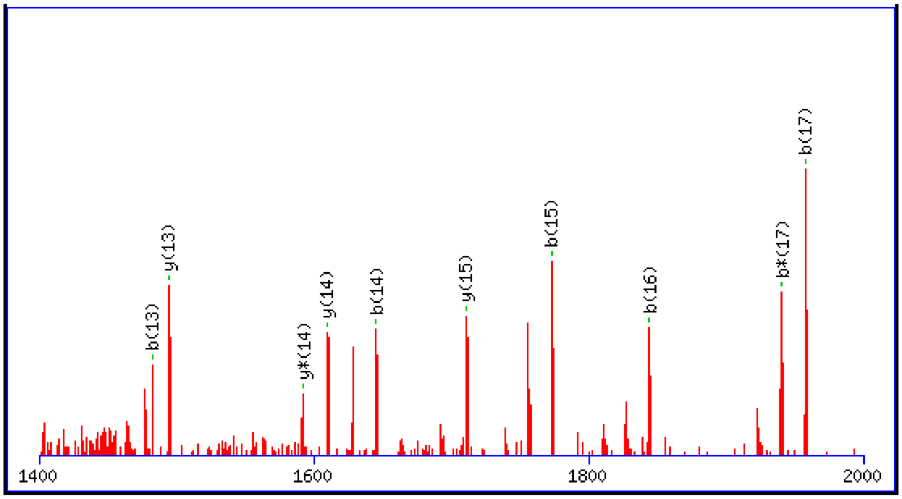


**Monoisotopic mass of neutral peptide Mr(calc):** 2102.9466
**Fixed modifications:** Carbamidomethyl (C)
**Variable modifications:**
**N5** : Deamidated_N (N)
**Ions Score:** 101 **Expect:** 1.9e-008
**Matches (Bold Red):** 28/176 fragment ions using 38 most intense peaks

| # | b | $b^{++}$ | b* | $b^{*++}$ | $b^{0}$ | $b^{0++}$ | Seq. | y | $y^{++}$ | y* | $y^{*++}$ | $y^{0}$ | $y^{0++}$ | # |
|---|---|---|---|---|---|---|---|---|---|---|---|---|---|---|
| 1 | 132.0478 | 66.5275 | | | | | M | | | | | | | 18 |
| 2 | 295.1111 | 148.0592 | | | | | Y | 1972.9134 | 986.9604 | 1955.8869 | 978.4471 | 1954.9029 | 977.9551 | 17 |
| 3 | **394.1795** | 197.5934 | | | | | V | 1809.8501 | 905.4287 | 1792.8236 | 896.9154 | 1791.8395 | 896.4234 | 16 |
| 4 | **495.2272** | 248.1172 | | | **477.2166** | 239.1119 | T | **1710.7817** | 855.8945 | 1693.7551 | 847.3812 | 1692.7711 | 846.8892 | 15 |
| 5 | **610.2541** | 305.6307 | 593.2276 | 297.1174 | 592.2436 | 296.6254 | N | **1609.7340** | 805.3706 | **1592.7075** | 796.8574 | 1591.7234 | 796.3654 | 14 |
| 6 | 725.2811 | 363.1442 | 708.2545 | 354.6309 | 707.2705 | 354.1389 | D | **1494.7071** | 747.8572 | 1477.6805 | 739.3439 | 1476.6965 | 738.8519 | 13 |
| 7 | 826.3287 | 413.6680 | 809.3022 | 405.1547 | 808.3182 | 404.6627 | T | **1379.6801** | 690.3437 | 1362.6536 | 681.8304 | 1361.6696 | 681.3384 | 12 |
| 8 | **955.3713** | 478.1893 | 938.3448 | 469.6760 | **937.3608** | 469.1840 | E | **1278.6325** | 639.8199 | 1261.6059 | 631.3066 | 1260.6219 | 630.8146 | 11 |
| 9 | 1054.4397 | 527.7235 | 1037.4132 | 519.2102 | 1036.4292 | 518.7182 | V | **1149.5899** | 575.2986 | 1132.5633 | 566.7853 | 1131.5793 | 566.2933 | 10 |
| 10 | **1125.4769** | 563.2421 | 1108.4503 | 554.7288 | 1107.4663 | 554.2368 | A | 1050.5215 | 525.7644 | 1033.4949 | 517.2511 | 1032.5109 | 516.7591 | 9 |
| 11 | **1254.5195** | 627.7634 | 1237.4929 | 619.2501 | 1236.5089 | 618.7581 | E | **979.4843** | 490.2458 | 962.4578 | 481.7325 | 961.4738 | 481.2405 | 8 |
| 12 | 1368.5624 | 684.7848 | 1351.5358 | 676.2716 | 1350.5518 | 675.7795 | N | **850.4417** | 425.7245 | 833.4152 | 417.2112 | | | 7 |
| 13 | **1482.6053** | 741.8063 | 1465.5788 | 733.2930 | 1464.5947 | 732.8010 | N | **736.3988** | 368.7030 | 719.3723 | 360.1898 | | | 6 |
| 14 | **1645.6686** | 823.3380 | 1628.6421 | 814.8247 | 1627.6581 | 814.3327 | Y | **622.3559** | 311.6816 | 605.3293 | 303.1683 | | | 5 |
| 15 | **1773.7272** | 887.3672 | 1756.7007 | 878.8540 | 1755.7167 | 878.3620 | Q | **459.2926** | 230.1499 | 442.2660 | 221.6366 | | | 4 |
| 16 | **1844.7643** | 922.8858 | 1827.7378 | 914.3725 | 1826.7538 | 913.8805 | A | **331.2340** | 166.1206 | 314.2074 | 157.6074 | | | 3 |
| 17 | **1957.8484** | **979.4278** | **1940.8218** | 970.9146 | 1939.8378 | 970.4226 | L | 260.1969 | 130.6021 | 243.1703 | 122.0888 | | | 2 |
| 18 | | | | | | | K | 147.1128 | 74.0600 | 130.0863 | 65.5468 | | | 1 |

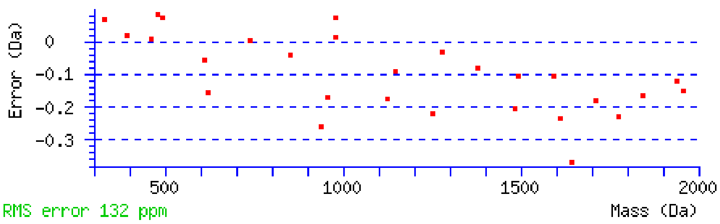


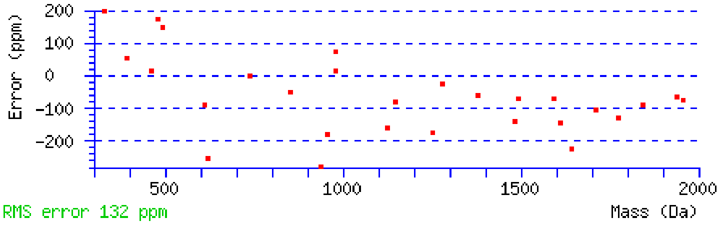


## All matches to this query

| Score | Mr(calc): | Delta | Sequence |
|---|---|---|---|
| 101.4 | 2102.9466 | 0.0089 | MYVTNDTEVAENNYQALK |
| 68.3 | 2102.9466 | 0.0089 | MYVTNDTEVAENNYQALK |
| 56.1 | 2102.9466 | 0.0089 | MYVTNDTEVAENNYQALK |
| | | | |

| | | | |
|---|---|---|---|
| 50.8 | 2101.9626 | 0.9929 | MYVTNDTEVAENNYQALK |
| 6.7 | 2102.9762 | -0.0207 | RPTSELGDARKTLDSVAK |
| 5.4 | 2102.9551 | 0.0004 | GAPSASASPTEAGPLRPHIK |
| 5.4 | 2102.9551 | 0.0004 | GAPSASASPTEAGPLRPHIK |
| 4.2 | 2102.9551 | 0.0004 | GAPSASASPTEAGPLRPHIK |
| 4.2 | 2102.9551 | 0.0004 | GAPSASASPTEAGPLRPHIK |
| 3.8 | 2102.9496 | 0.0060 | SAQYPFVASLTVVAVALK |

---

Spectrum No: 71; Query: 1265; Rank: 1

---

## Peptide View

MS/MS Fragmentation of **QGAIVAVTGDGVNDSPALK**
Found in **IPI00205693**, Tax_Id=10116 Gene_Symbol=Atp1a2 Sodium/potassium-transporting ATPase subunit alpha-2 precursor

Match to Query 1265: 1811.933048 from(906.973800,2+)
Title: 091008RatKidney_NoSalt_17.2326.2326.2.dta
Data file K:\NewmanPaper\Piliang\3SubProteomes\Piliang3SP\mgf5ppm\ERLIC_3SubProteomes5ppm.mgf

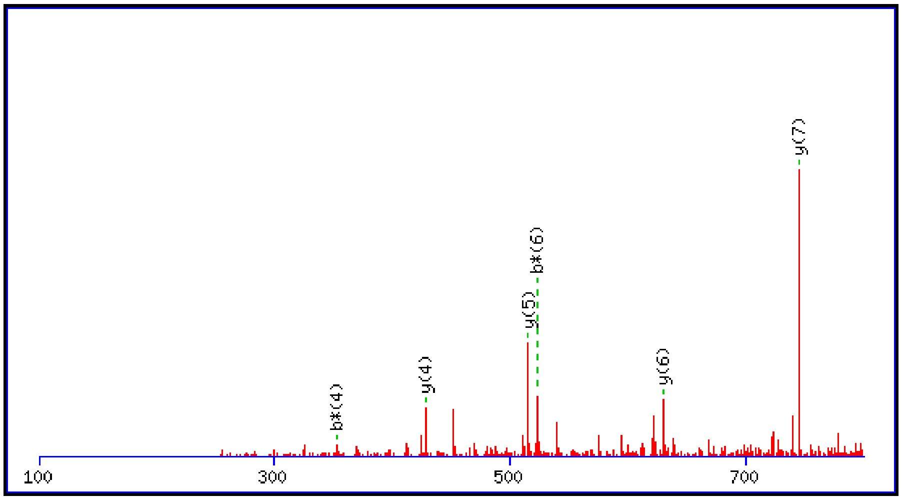


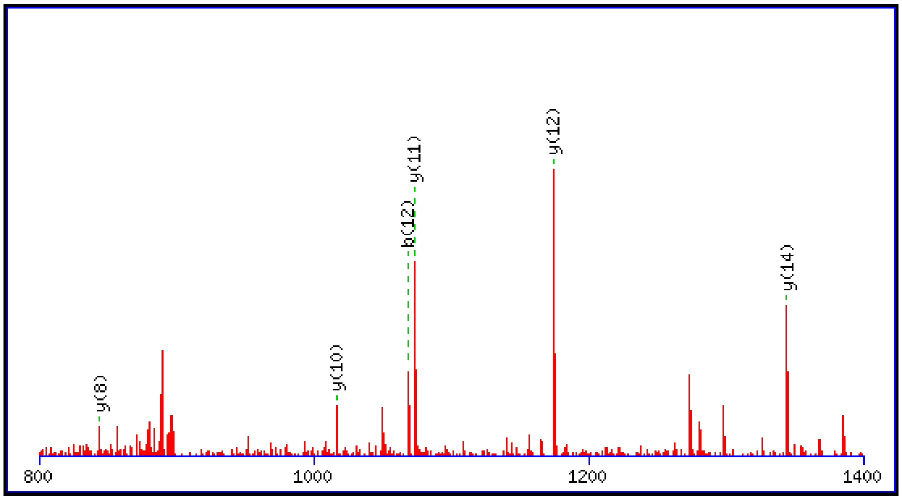

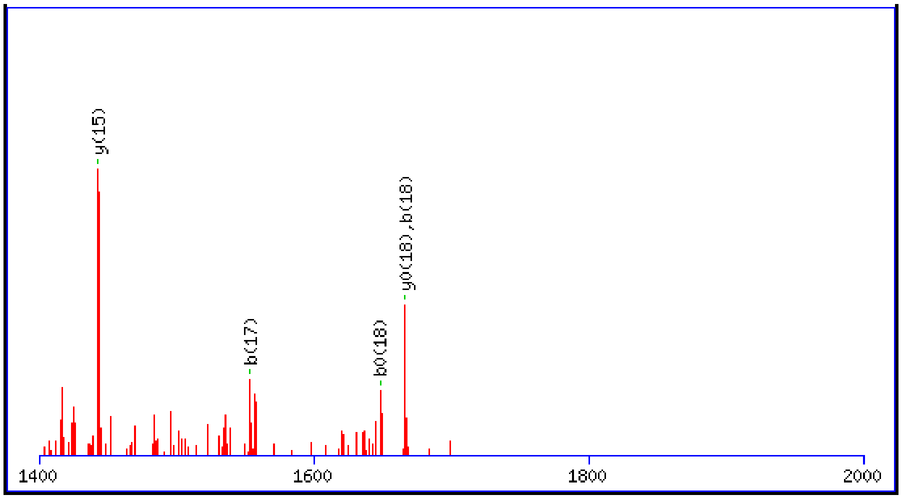


**Monoisotopic mass of neutral peptide Mr(calc):** 1811.9265
**Fixed modifications:** Carbamidomethyl (C)
**Variable modifications:**
**N13** : Deamidated_N (N)
**Ions Score:** 101 **Expect:** 1.1e-008
**Matches (Bold Red):** 17/194 fragment ions using 25 most intense peaks

| # | b | $b^{++}$ | b* | $b^{*++}$ | $b^{0}$ | $b^{0++}$ | Seq. | y | $y^{++}$ | y* | $y^{*++}$ | $y^{0}$ | $y^{0++}$ | # |
|---|---|---|---|---|---|---|---|---|---|---|---|---|---|---|
| 1 | 129.0659 | 65.0366 | 112.0393 | 56.5233 | | | Q | | | | | | | 19 |
| 2 | 186.0873 | 93.5473 | 169.0608 | 85.0340 | | | G | 1684.8752 | 842.9412 | 1667.8487 | 834.4280 | **1666.8646** | 833.9360 | 18 |
| 3 | 257.1244 | 129.0659 | 240.0979 | 120.5526 | | | A | 1627.8537 | 814.4305 | 1610.8272 | 805.9172 | 1609.8432 | 805.4252 | 17 |
| 4 | 370.2085 | 185.6079 | **353.1819** | 177.0946 | | | I | 1556.8166 | 778.9119 | 1539.7901 | 770.3987 | 1538.8061 | 769.9067 | 16 |
| 5 | 469.2769 | 235.1421 | 452.2504 | 226.6288 | | | V | **1443.7326** | 722.3699 | 1426.7060 | 713.8566 | 1425.7220 | 713.3646 | 15 |
| 6 | 540.3140 | 270.6607 | **523.2875** | 262.1474 | | | A | **1344.6641** | 672.8357 | 1327.6376 | 664.3224 | 1326.6536 | 663.8304 | 14 |
| 7 | 639.3824 | 320.1949 | 622.3559 | 311.6816 | | | V | 1273.6270 | 637.3172 | 1256.6005 | 628.8039 | 1255.6165 | 628.3119 | 13 |
| 8 | 740.4301 | 370.7187 | 723.4036 | 362.2054 | 722.4196 | 361.7134 | T | **1174.5586** | 587.7829 | 1157.5321 | 579.2697 | 1156.5481 | 578.7777 | 12 |
| 9 | 797.4516 | 399.2294 | 780.4250 | 390.7162 | 779.4410 | 390.2241 | G | **1073.5109** | 537.2591 | 1056.4844 | 528.7458 | 1055.5004 | 528.2538 | 11 |
| 10 | 912.4785 | 456.7429 | 895.4520 | 448.2296 | 894.4680 | 447.7376 | D | **1016.4895** | 508.7484 | 999.4629 | 500.2351 | 998.4789 | 499.7431 | 10 |
| 11 | 969.5000 | 485.2536 | 952.4734 | 476.7404 | 951.4894 | 476.2483 | G | 901.4625 | 451.2349 | 884.4360 | 442.7216 | 883.4520 | 442.2296 | 9 |
| 12 | **1068.5684** | 534.7878 | 1051.5419 | 526.2746 | 1050.5578 | 525.7826 | V | **844.4411** | 422.7242 | 827.4145 | 414.2109 | 826.4305 | 413.7189 | 8 |
| 13 | 1183.5953 | 592.3013 | 1166.5688 | 583.7880 | 1165.5848 | 583.2960 | N | **745.3727** | 373.1900 | 728.3461 | 364.6767 | 727.3621 | 364.1847 | 7 |
| 14 | 1298.6223 | 649.8148 | 1281.5957 | 641.3015 | 1280.6117 | 640.8095 | D | **630.3457** | 315.6765 | 613.3192 | 307.1632 | 612.3352 | 306.6712 | 6 |
| 15 | 1385.6543 | 693.3308 | 1368.6278 | 684.8175 | 1367.6437 | 684.3255 | S | **515.3188** | 258.1630 | 498.2922 | 249.6498 | 497.3082 | 249.1577 | 5 |
| 16 | 1482.7071 | 741.8572 | 1465.6805 | 733.3439 | 1464.6965 | 732.8519 | P | **428.2867** | 214.6470 | 411.2602 | 206.1337 | | | 4 |
| 17 | **1553.7442** | 777.3757 | 1536.7176 | 768.8625 | 1535.7336 | 768.3704 | A | 331.2340 | 166.1206 | 314.2074 | 157.6074 | | | 3 |
| 18 | **1666.8283** | 833.9178 | 1649.8017 | 825.4045 | **1648.8177** | 824.9125 | L | 260.1969 | 130.6021 | 243.1703 | 122.0888 | | | 2 |
| 19 | | | | | | | K | 147.1128 | 74.0600 | 130.0863 | 65.5468 | | | 1 |

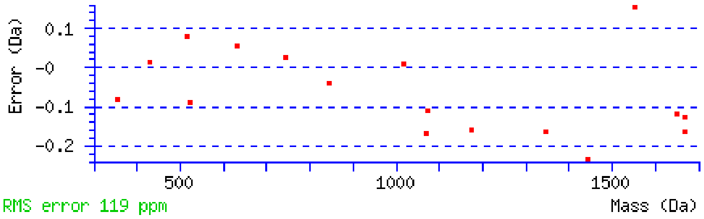


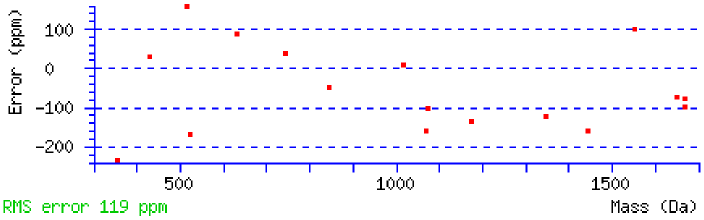


## All matches to this query

| Score | Mr(calc): | Delta | Sequence |
|---|---|---|---|
| 101.3 | 1811.9265 | 0.0065 | QGAIVAVTGDGVNDSPALK |
| 8.4 | 1810.9425 | 0.9905 | QGAIVAVTGDGVNDSPALK |
| | | | |

| | | | |
|---|---|---|---|
| 6.5 | 1811.9166 | 0.0164 | EKGLSGRGPDTGPDWLK |
| 3.5 | 1811.9264 | 0.0066 | APSPEKAEEESERLLK |
| 1.1 | 1810.9188 | 1.0142 | YFLQGMGYIAHLKDR |
| 0.3 | 1810.9230 | 1.0100 | ATTPSVTVKNSAAPIFK |
| 0.3 | 1810.9230 | 1.0100 | ATTPSVTVKNSAAPIFK |

---

Spectrum No: 72; Query: 1151; Rank: 1

---

## Peptide View

MS/MS Fragmentation of **DYYPINESLASLPLR**
Found in **IPI00193869**, Tax_Id=10116 Gene_Symbol=Clptm1_predicted cleft lip and palate associated transmembrane protein 1

Match to Query 1151: 1750.875988 from(876.445270,2+)
Title: 091008RatKidney_NH4Format01_23.3892.3892.2.dta
Data file K:\NewmanPaper\Piliang\3SubProteomes\Piliang3SP\mgf5ppm\ERLIC_3SubProteomes5ppm.mgf

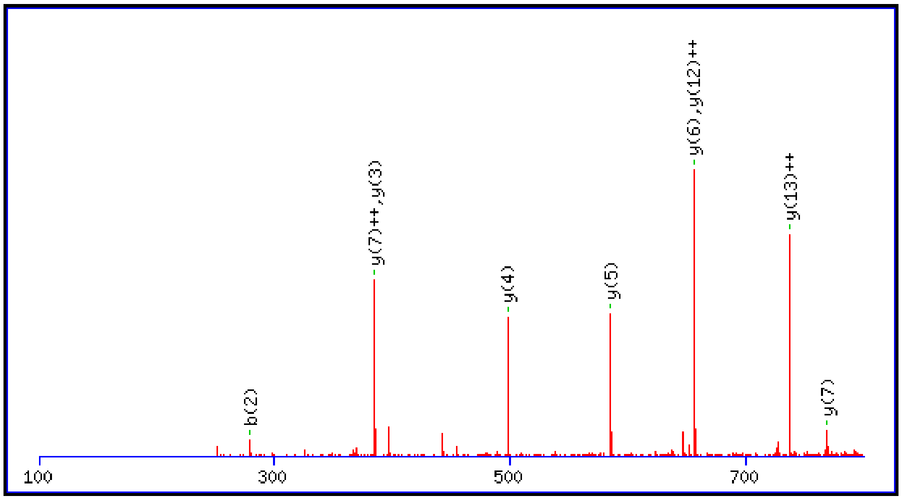


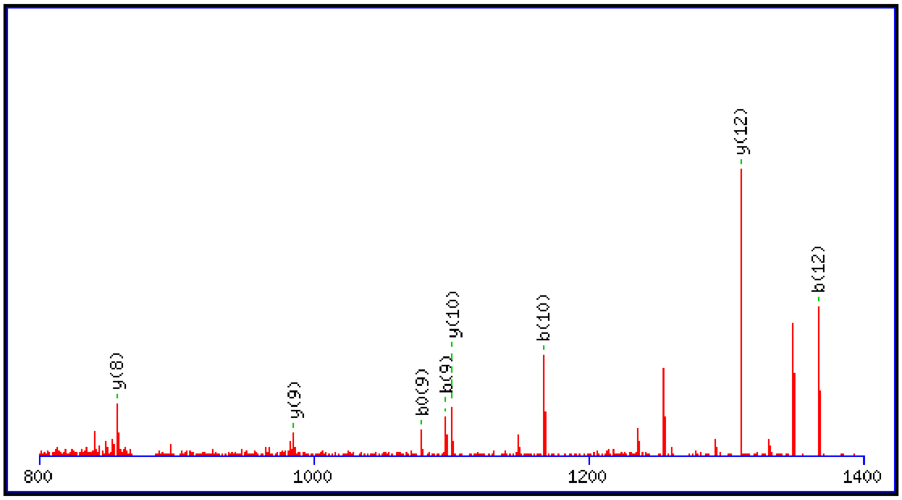

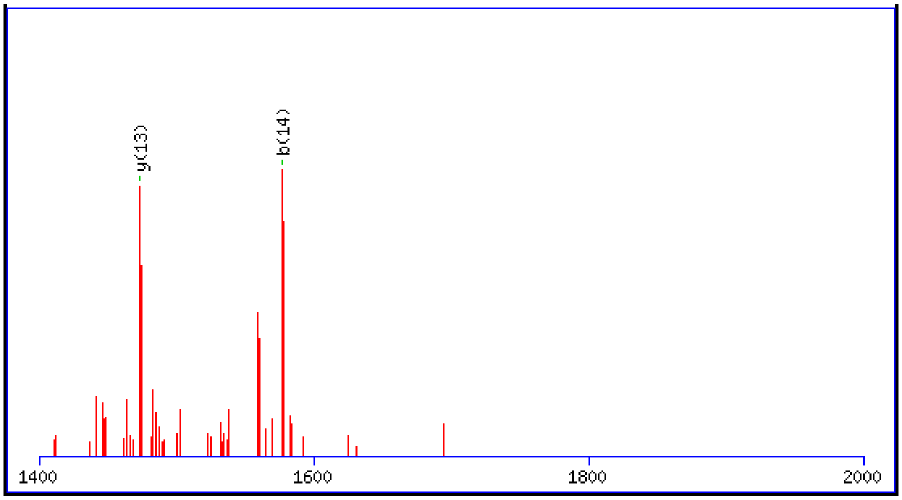


**Monoisotopic mass of neutral peptide Mr(calc):** 1750.8777
**Fixed modifications:** Carbamidomethyl (C)
**Variable modifications:**
**N6** : Deamidated_N (N)
**Ions Score:** 101 **Expect:** 1.4e-008
**Matches (Bold Red):** 19/150 fragment ions using 24 most intense peaks

| # | b | $b^{++}$ | b* | $b^{*++}$ | $b^{0}$ | $b^{0++}$ | Seq. | y | $y^{++}$ | y* | $y^{*++}$ | $y^{0}$ | $y^{0++}$ | # |
|---|---|---|---|---|---|---|---|---|---|---|---|---|---|---|
| 1 | 116.0342 | 58.5207 | | | 98.0237 | 49.5155 | D | | | | | | | 15 |
| 2 | **279.0975** | 140.0524 | | | 261.0870 | 131.0471 | Y | 1636.8581 | 818.9327 | 1619.8315 | 810.4194 | 1618.8475 | 809.9274 | 14 |
| 3 | 442.1609 | 221.5841 | | | 424.1503 | 212.5788 | Y | **1473.7948** | **737.4010** | 1456.7682 | 728.8877 | 1455.7842 | 728.3957 | 13 |
| 4 | 539.2136 | 270.1105 | | | 521.2031 | 261.1052 | P | **1310.7314** | **655.8694** | 1293.7049 | 647.3561 | 1292.7209 | 646.8641 | 12 |
| 5 | 652.2977 | 326.6525 | | | 634.2871 | 317.6472 | I | 1213.6787 | 607.3430 | 1196.6521 | 598.8297 | 1195.6681 | 598.3377 | 11 |
| 6 | 767.3246 | 384.1660 | 750.2981 | 375.6527 | 749.3141 | 375.1607 | N | **1100.5946** | 550.8009 | 1083.5681 | 542.2877 | 1082.5840 | 541.7957 | 10 |
| 7 | 896.3672 | 448.6873 | 879.3407 | 440.1740 | 878.3567 | 439.6820 | E | **985.5677** | 493.2875 | 968.5411 | 484.7742 | 967.5571 | 484.2822 | 9 |
| 8 | 983.3993 | 492.2033 | 966.3727 | 483.6900 | 965.3887 | 483.1980 | S | **856.5251** | 428.7662 | 839.4985 | 420.2529 | 838.5145 | 419.7609 | 8 |
| 9 | **1096.4833** | 548.7453 | 1079.4568 | 540.2320 | **1078.4728** | 539.7400 | L | **769.4930** | **385.2502** | 752.4665 | 376.7369 | 751.4825 | 376.2449 | 7 |
| 10 | **1167.5204** | 584.2639 | 1150.4939 | 575.7506 | 1149.5099 | 575.2586 | A | **656.4090** | 328.7081 | 639.3824 | 320.1949 | 638.3984 | 319.7028 | 6 |
| 11 | 1254.5525 | 627.7799 | 1237.5259 | 619.2666 | 1236.5419 | 618.7746 | S | **585.3719** | 293.1896 | 568.3453 | 284.6763 | 567.3613 | 284.1843 | 5 |
| 12 | **1367.6365** | 684.3219 | 1350.6100 | 675.8086 | 1349.6260 | 675.3166 | L | **498.3398** | 249.6736 | 481.3133 | 241.1603 | | | 4 |
| 13 | 1464.6893 | 732.8483 | 1447.6627 | 724.3350 | 1446.6787 | 723.8430 | P | **385.2558** | 193.1315 | 368.2292 | 184.6183 | | | 3 |
| 14 | **1577.7734** | 789.3903 | 1560.7468 | 780.8770 | 1559.7628 | 780.3850 | L | 288.2030 | 144.6051 | 271.1765 | 136.0919 | | | 2 |
| 15 | | | | | | | R | 175.1190 | 88.0631 | 158.0924 | 79.5498 | | | 1 |

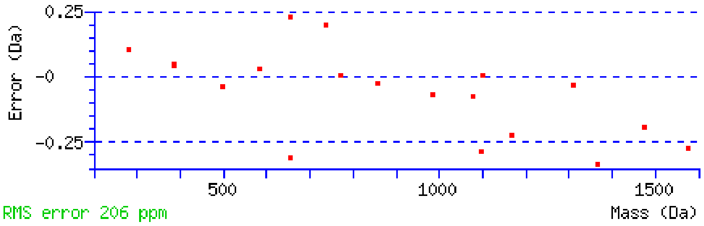


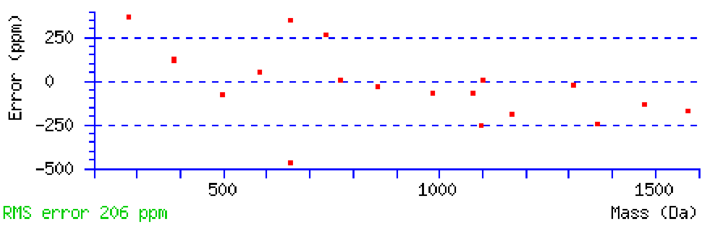


### All matches to this query

| Score | Mr(calc): | Delta | Sequence |
|---|---|---|---|
| 100.7 | 1750.8777 | -0.0017 | DYYPINESLASLPLR |
| 8.7 | 1750.8879 | -0.0120 | RQGYDLRVDAIPIR |
| 7.7 | 1750.8614 | 0.0146 | ELPASSAVSSALDRIR |
| 7.7 | 1750.8614 | 0.0146 | ELPASSAVSSALDRIR |
| 7.5 | 1750.8615 | 0.0145 | VDGLGLGVVTRQNSQK |
| 7.2 | 1749.8662 | 1.0098 | DLLVQTLENSGVLNR |
| | | | |

| | | | |
|---|---|---|---|
| 4.5 | 1750.8866 | -0.0106 | EASPLSSNKLILRDK |
| 4.2 | 1748.8822 | 1.9938 | NVAGEVVLITGAGSGLGR |
| 3.8 | 1749.8597 | 1.0163 | RATLPQAAMGVATVER |
| 3.5 | 1749.8662 | 1.0098 | NVAGEVVLITGAGSGLGR |

---

Spectrum No: 73; Query: 2137; Rank: 1

---

## Peptide View

MS/MS Fragmentation of **LDPPCTNTTAPSTYLNNPYVR**
Found in **IPI00464785**, Tax_Id=10116 Gene_Symbol=Ppgb Protective protein for beta-galactosidase

Match to Query 2137: 2394.113488 from(1198.064020,2+)
Title: 091008RatKidney_NH4Format01_24.2946.2946.2.dta
Data file K:\NewmanPaper\Piliang\3SubProteomes\Piliang3SP\mgf5ppm\ERLIC_3SubProteomes5ppm.mgf

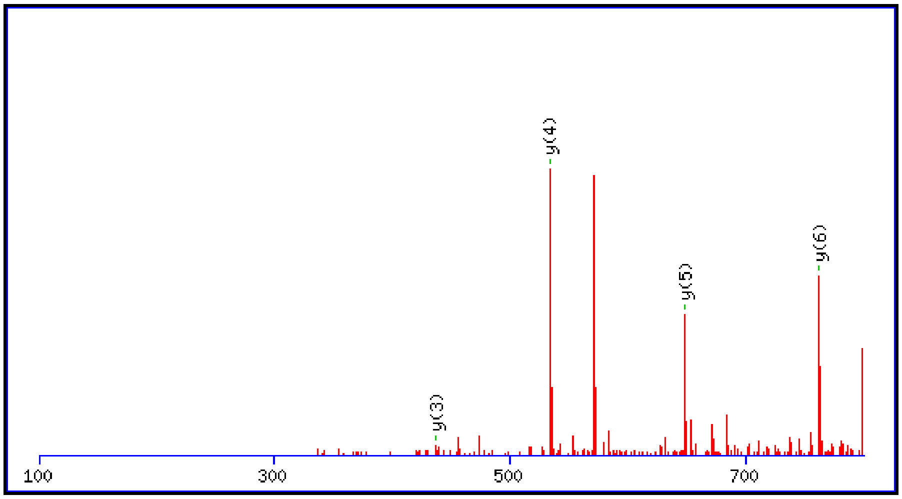


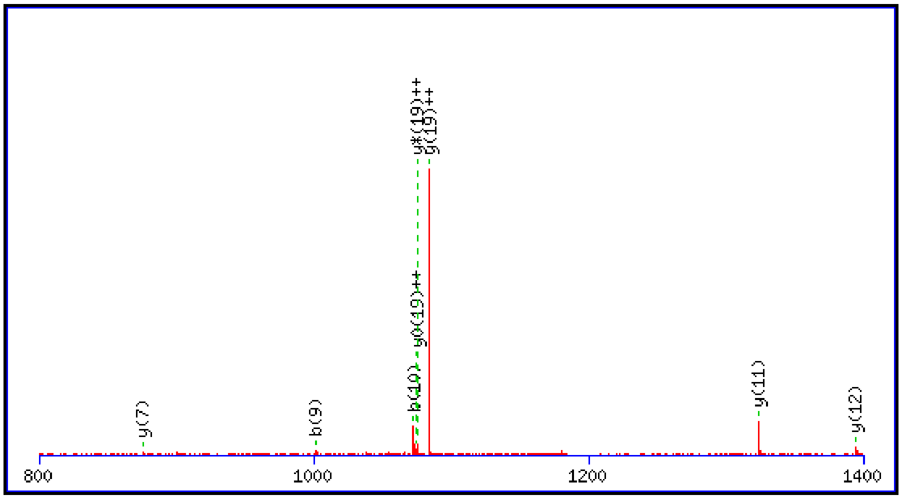

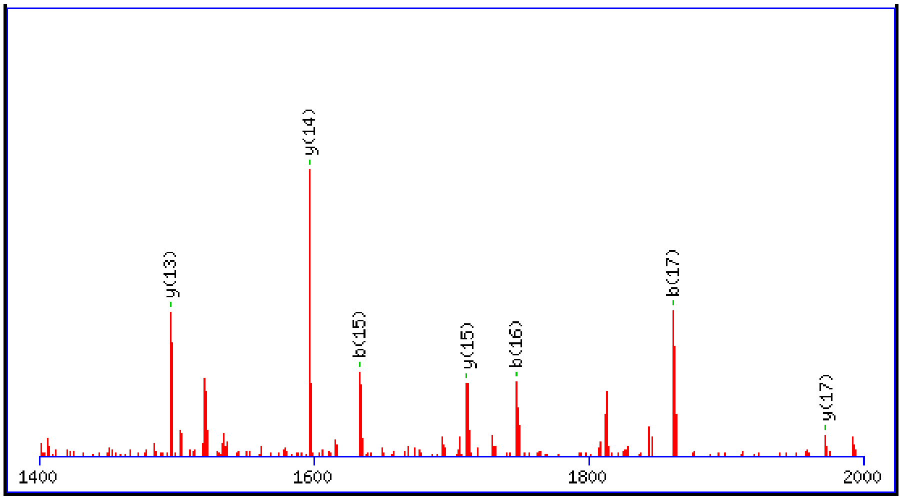


**Monoisotopic mass of neutral peptide Mr(calc):** 2394.1162
**Fixed modifications:** Carbamidomethyl (C)
**Variable modifications:**
**N7** : Deamidated_N (N)
**Ions Score:** 101 **Expect:** 2.5e-008
**Matches (Bold Red):** 19/210 fragment ions using 30 most intense peaks

| # | b | $b^{++}$ | b* | $b^{*++}$ | $b^0$ | $b^{0++}$ | Seq. | y | $y^{++}$ | y* | $y^{*++}$ | $y^0$ | $y^{0++}$ | # |
|---|---|---|---|---|---|---|---|---|---|---|---|---|---|---|
| 1 | 114.0913 | 57.5493 | | | | | L | | | | | | | 21 |
| 2 | 229.1183 | 115.0628 | | | 211.1077 | 106.0575 | D | 2282.0394 | 1141.5233 | 2265.0128 | 1133.0101 | 2264.0288 | 1132.5180 | 20 |
| 3 | 326.1710 | 163.5892 | | | 308.1605 | 154.5839 | P | 2167.0124 | **1084.0099** | 2149.9859 | **1075.4966** | 2149.0019 | **1075.0046** | 19 |
| 4 | 423.2238 | 212.1155 | | | 405.2132 | 203.1103 | P | 2069.9597 | 1035.4835 | 2052.9331 | 1026.9702 | 2051.9491 | 1026.4782 | 18 |
| 5 | 583.2545 | 292.1309 | | | 565.2439 | 283.1256 | C | **1972.9069** | 986.9571 | 1955.8804 | 978.4438 | 1954.8963 | 977.9518 | 17 |
| 6 | 684.3021 | 342.6547 | | | 666.2916 | 333.6494 | T | 1812.8763 | 906.9418 | 1795.8497 | 898.4285 | 1794.8657 | 897.9365 | 16 |
| 7 | 799.3291 | 400.1682 | 782.3025 | 391.6549 | 781.3185 | 391.1629 | N | **1711.8286** | 856.4179 | 1694.8020 | 847.9047 | 1693.8180 | 847.4126 | 15 |
| 8 | 900.3768 | 450.6920 | 883.3502 | 442.1787 | 882.3662 | 441.6867 | T | **1596.8016** | 798.9045 | 1579.7751 | 790.3912 | 1578.7911 | 789.8992 | 14 |
| 9 | **1001.4244** | 501.2159 | 984.3979 | 492.7026 | 983.4139 | 492.2106 | T | **1495.7540** | 748.3806 | 1478.7274 | 739.8673 | 1477.7434 | 739.3753 | 13 |
| 10 | **1072.4615** | 536.7344 | 1055.4350 | 528.2211 | 1054.4510 | 527.7291 | A | **1394.7063** | 697.8568 | 1377.6797 | 689.3435 | 1376.6957 | 688.8515 | 12 |
| 11 | 1169.5143 | 585.2608 | 1152.4878 | 576.7475 | 1151.5037 | 576.2555 | P | **1323.6692** | 662.3382 | 1306.6426 | 653.8250 | 1305.6586 | 653.3329 | 11 |
| 12 | 1256.5463 | 628.7768 | 1239.5198 | 620.2635 | 1238.5358 | 619.7715 | S | 1226.6164 | 613.8118 | 1209.5899 | 605.2986 | 1208.6058 | 604.8066 | 10 |
| 13 | 1357.5940 | 679.3006 | 1340.5675 | 670.7874 | 1339.5835 | 670.2954 | T | 1139.5844 | 570.2958 | 1122.5578 | 561.7826 | 1121.5738 | 561.2905 | 9 |
| 14 | 1520.6573 | 760.8323 | 1503.6308 | 752.3190 | 1502.6468 | 751.8270 | Y | 1038.5367 | 519.7720 | 1021.5102 | 511.2587 | | | 8 |
| 15 | **1633.7414** | 817.3743 | 1616.7149 | 808.8611 | 1615.7308 | 808.3691 | L | **875.4734** | 438.2403 | 858.4468 | 429.7271 | | | 7 |
| 16 | **1747.7843** | 874.3958 | 1730.7578 | 865.8825 | 1729.7738 | 865.3905 | N | **762.3893** | 381.6983 | 745.3628 | 373.1850 | | | 6 |
| 17 | **1861.8273** | 931.4173 | 1844.8007 | 922.9040 | 1843.8167 | 922.4120 | N | **648.3464** | 324.6768 | 631.3198 | 316.1636 | | | 5 |
| 18 | 1958.8800 | 979.9437 | 1941.8535 | 971.4304 | 1940.8695 | 970.9384 | P | **534.3035** | 267.6554 | 517.2769 | 259.1421 | | | 4 |
| 19 | 2121.9434 | 1061.4753 | 2104.9168 | 1052.9620 | 2103.9328 | 1052.4700 | Y | **437.2507** | 219.1290 | 420.2241 | 210.6157 | | | 3 |
| 20 | 2221.0118 | 1111.0095 | 2203.9852 | 1102.4962 | 2203.0012 | 1102.0042 | V | 274.1874 | 137.5973 | 257.1608 | 129.0840 | | | 2 |
| 21 | | | | | | | R | 175.1190 | 88.0631 | 158.0924 | 79.5498 | | | 1 |

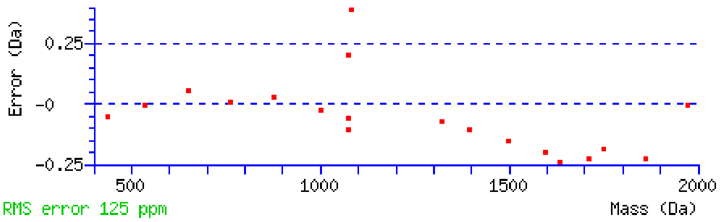


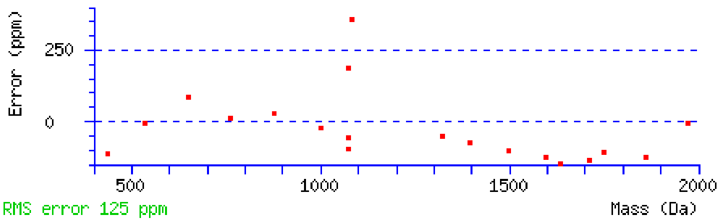


## All matches to this query

| Score | Mr(calc): | Delta | Sequence |
|---|---|---|---|
| | | | |

| | | | |
|---|---|---|---|
| 100.6 | 2394.1162 | -0.0027 | LDPPCTNTTAPSTYLNNPYVR |
| 55.5 | 2394.1162 | -0.0027 | LDPPCTNTTAPSTYLNNPYVR |
| 53.1 | 2394.1162 | -0.0027 | LDPPCTNTTAPSTYLNNPYVR |
| 48.0 | 2393.1322 | 0.9813 | LDPPCTNTTAPSTYLNNPYVR |
| 5.1 | 2394.1104 | 0.0031 | EFQENIIKQVEAHKEESQK |
| 5.0 | 2394.1121 | 0.0014 | AHETNLLIATSVVEEGVDIPK |
| 5.0 | 2394.1121 | 0.0014 | AHETNLLIATSVVEEGVDIPK |
| 5.0 | 2394.1121 | 0.0014 | AHETNLLIATSVVEEGVDIPK |
| 4.3 | 2393.1182 | 0.9953 | AGVSFALGITTIDRGNHTYLK |
| 4.3 | 2393.1182 | 0.9953 | AGVSFALGITTIDRGNHTYLK |

---

Spectrum No: 74; Query: 735; Rank: 1

---

## Peptide View

MS/MS Fragmentation of **IYGGILNLSEITNK**
Found in **IPI00203384**, Tax_Id=10116 Gene_Symbol=Klkb1 Plasma kallikrein precursor

Match to Query 735: 1534.822188 from(768.418370,2+)
Title: 091008RatKidney_NoSalt_29.3574.3574.2.dta
Data file K:\NewmanPaper\Piliang\3SubProteomes\Piliang3SP\mgf5ppm\ERLIC_3SubProteomes5ppm.mgf

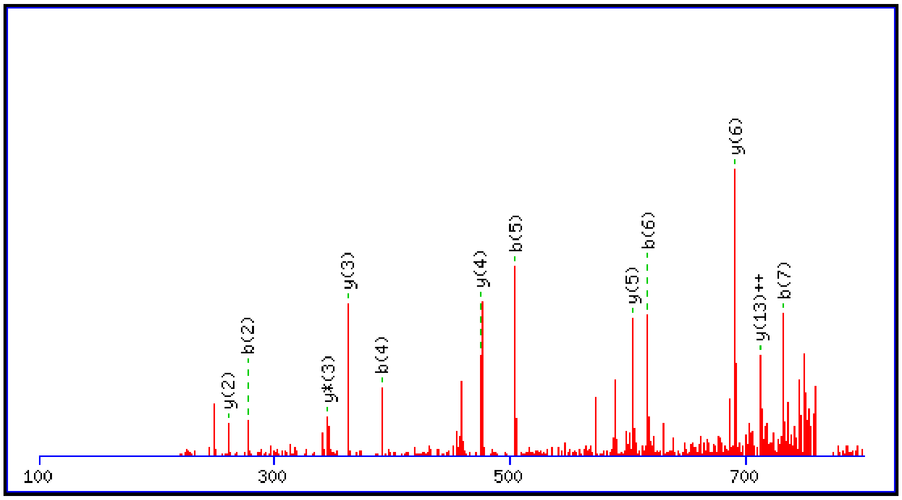


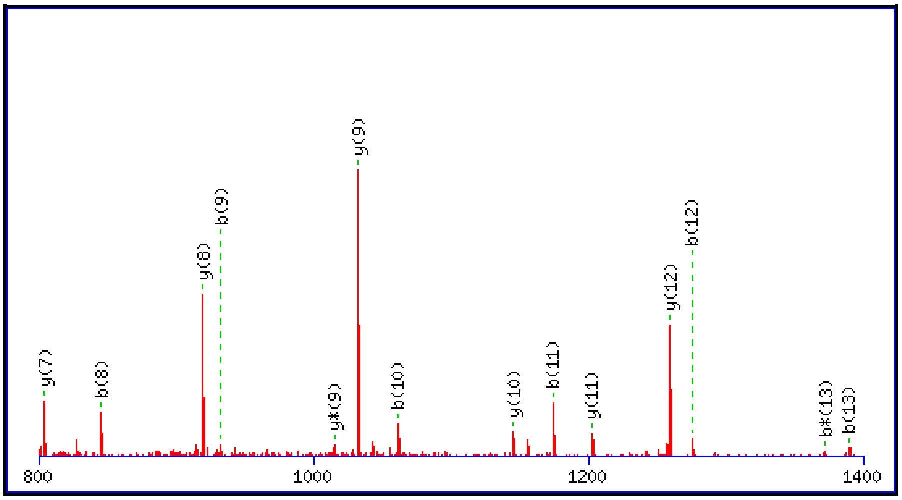

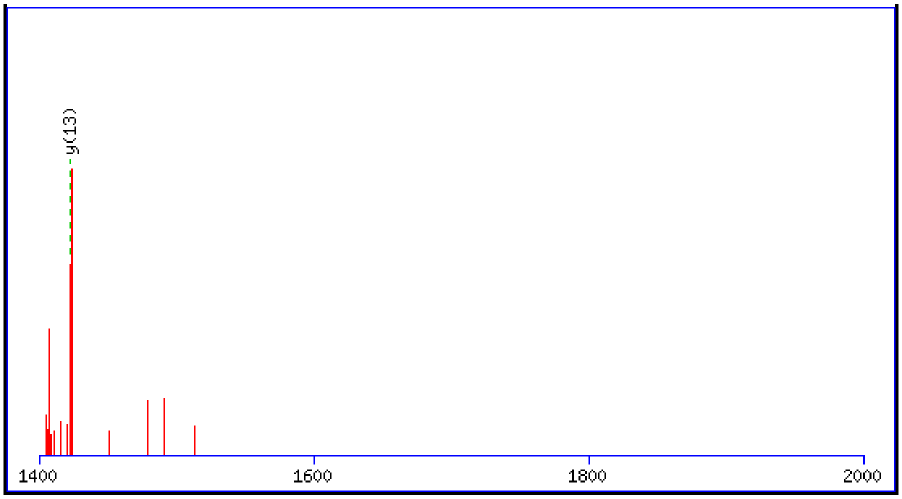


**Monoisotopic mass of neutral peptide Mr(calc):** 1534.8242
**Fixed modifications:** Carbamidomethyl (C)
**Variable modifications:**
**N7** : Deamidated_N (N)
**Ions Score:** 101 **Expect:** 8.3e-009
**Matches (Bold Red):** 27/124 fragment ions using 43 most intense peaks

| # | b | b++ | b* | b*++ | b0 | b0++ | Seq. | y | y++ | y* | y*++ | y0 | y0++ | # |
|---|---|---|---|---|---|---|---|---|---|---|---|---|---|---|
| 1 | 114.0913 | 57.5493 | | | | | I | | | | | | | 14 |
| 2 | **277.1547** | 139.0810 | | | | | Y | **1422.7475** | **711.8774** | 1405.7209 | 703.3641 | 1404.7369 | 702.8721 | 13 |
| 3 | 334.1761 | 167.5917 | | | | | G | **1259.6842** | 630.3457 | 1242.6576 | 621.8324 | 1241.6736 | 621.3404 | 12 |
| 4 | **391.1976** | 196.1024 | | | | | G | **1202.6627** | 601.8350 | 1185.6361 | 593.3217 | 1184.6521 | 592.8297 | 11 |
| 5 | **504.2817** | 252.6445 | | | | | I | **1145.6412** | 573.3242 | 1128.6147 | 564.8110 | 1127.6307 | 564.3190 | 10 |
| 6 | **617.3657** | 309.1865 | | | | | L | **1032.5572** | 516.7822 | **1015.5306** | 508.2689 | 1014.5466 | 507.7769 | 9 |
| 7 | **732.3927** | 366.7000 | 715.3661 | 358.1867 | | | N | **919.4731** | 460.2402 | 902.4465 | 451.7269 | 901.4625 | 451.2349 | 8 |
| 8 | **845.4767** | 423.2420 | 828.4502 | 414.7287 | | | L | **804.4462** | 402.7267 | 787.4196 | 394.2134 | 786.4356 | 393.7214 | 7 |
| 9 | **932.5088** | 466.7580 | 915.4822 | 458.2447 | 914.4982 | 457.7527 | S | **691.3621** | 346.1847 | 674.3355 | 337.6714 | 673.3515 | 337.1794 | 6 |
| 10 | **1061.5513** | 531.2793 | 1044.5248 | 522.7660 | 1043.5408 | 522.2740 | E | **604.3301** | 302.6687 | 587.3035 | 294.1554 | 586.3195 | 293.6634 | 5 |
| 11 | **1174.6354** | 587.8213 | 1157.6089 | 579.3081 | 1156.6248 | 578.8161 | I | **475.2875** | 238.1474 | 458.2609 | 229.6341 | 457.2769 | 229.1421 | 4 |
| 12 | **1275.6831** | 638.3452 | 1258.6565 | 629.8319 | 1257.6725 | 629.3399 | T | **362.2034** | 181.6053 | **345.1769** | 173.0921 | 344.1928 | 172.6001 | 3 |
| 13 | **1389.7260** | 695.3666 | **1372.6995** | 686.8534 | 1371.7155 | 686.3614 | N | **261.1557** | 131.0815 | 244.1292 | 122.5682 | | | 2 |
| 14 | | | | | | | K | 147.1128 | 74.0600 | 130.0863 | 65.5468 | | | 1 |

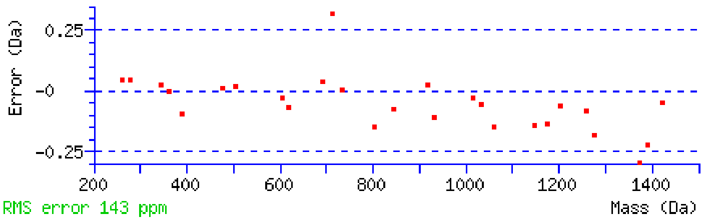


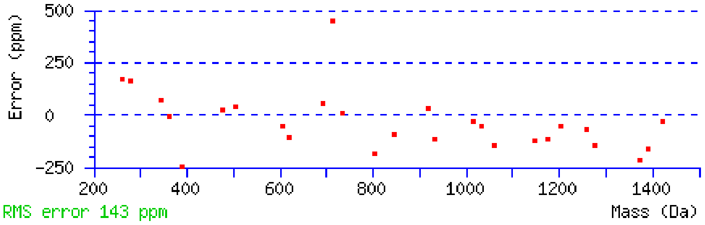


### All matches to this query

| Score | Mr(calc): | Delta | Sequence |
|---|---|---|---|
| 100.5 | 1534.8242 | -0.0020 | IYGGILNLSEITNK |
| 46.9 | 1534.8242 | -0.0020 | IYGGILNLSEITNK |
| 14.8 | 1534.8355 | -0.0133 | KFNLDATELSIRK |
| 10.6 | 1534.8242 | -0.0020 | LLNYLLDNLSGTAK |
| 9.8 | 1533.8072 | 1.0150 | IMNKNISLVDNKK |
| 7.4 | 1534.8251 | -0.0029 | YLMVPALQGALTMK |
| 7.2 | 1534.8355 | -0.0133 | DGYLELLKEATRK |
| | | | |

| | | | |
|---|---|---|---|
| 5.9 | 1534.8355 | -0.0133 | FLSQESGVAQTLKK |
| 5.2 | 1534.8218 | 0.0004 | NFTAFPSLPGLIMK |
| 3.6 | 1533.8072 | 1.0150 | IMNKNISLVDNKK |

Spectrum No: 75; Query: 917; Rank: 1

## Peptide View

MS/MS Fragmentation of **VDNSSLTGESEPQTR**
Found in **IPI00205693**, Tax_Id=10116 Gene_Symbol=Atp1a2 Sodium/potassium-transporting ATPase subunit alpha-2 precursor

Match to Query 917: 1619.724248 from(810.869400,2+)
Title: 091008RatKidney_NH4Format01_18.591.591.2.dta
Data file K:\NewmanPaper\Piliang\3SubProteomes\Piliang3SP\mgf5ppm\ERLIC_3SubProteomes5ppm.mgf

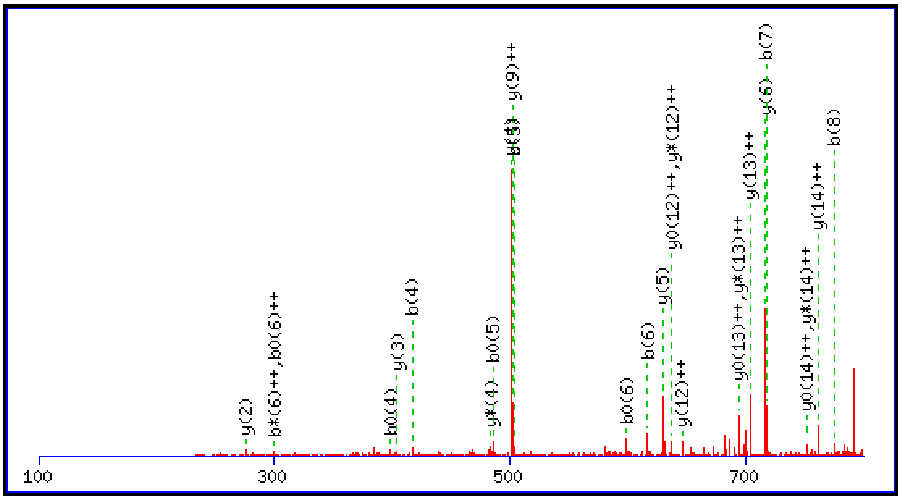


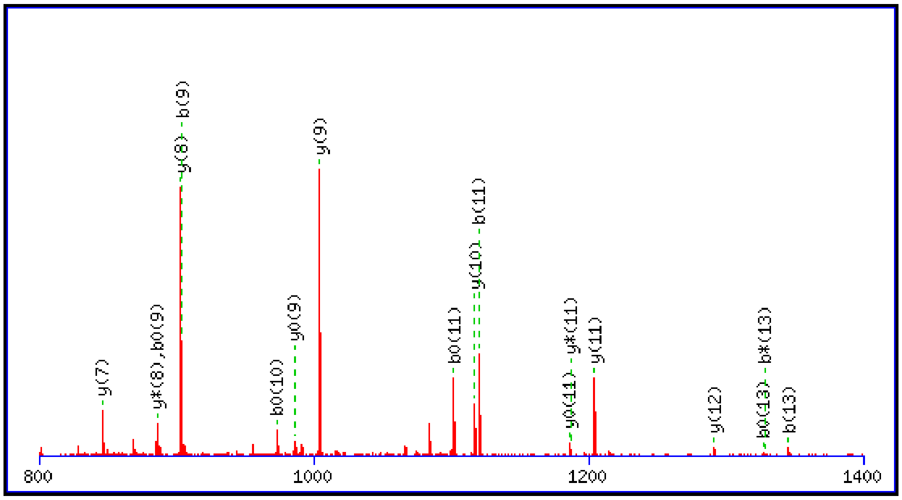

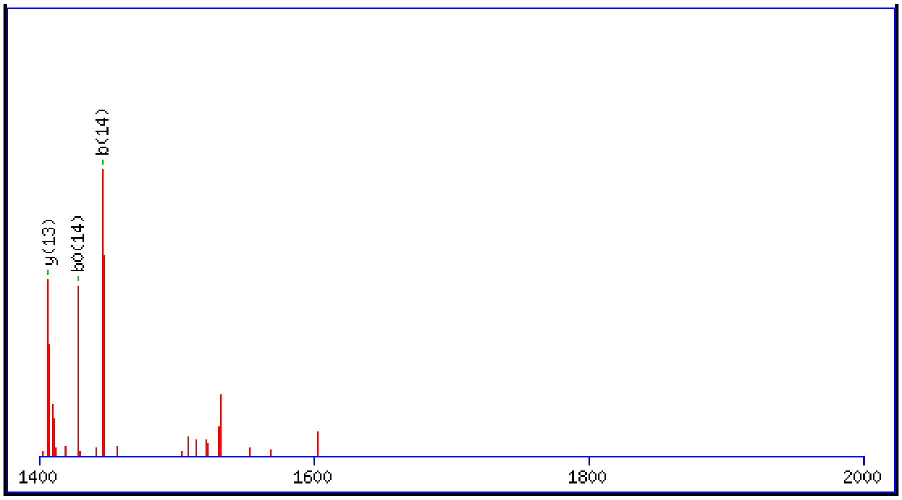


**Monoisotopic mass of neutral peptide Mr(calc):** 1619.7275
**Fixed modifications:** Carbamidomethyl (C)
**Variable modifications:**
**N3** : Deamidated_N (N)
**Ions Score:** 100 **Expect:** 1.8e-008
**Matches (Bold Red):** 47/160 fragment ions using 63 most intense peaks

| # | b | $b^{++}$ | b* | $b^{*++}$ | $b^{0}$ | $b^{0++}$ | Seq. | y | $y^{++}$ | y* | $y^{*++}$ | $y^{0}$ | $y^{0++}$ | # |
|---|---|---|---|---|---|---|---|---|---|---|---|---|---|---|
| 1 | 100.0757 | 50.5415 | | | | | V | | | | | | | 15 |
| 2 | 215.1026 | 108.0550 | | | 197.0921 | 99.0497 | D | 1521.6663 | **761.3368** | 1504.6398 | **752.8235** | 1503.6558 | **752.3315** | 14 |
| 3 | 330.1296 | 165.5684 | 313.1030 | 157.0551 | 312.1190 | 156.5631 | N | **1406.6394** | **703.8233** | 1389.6128 | **695.3101** | 1388.6288 | **694.8180** | 13 |
| 4 | **417.1616** | 209.0844 | 400.1350 | 200.5712 | **399.1510** | 200.0792 | S | **1291.6124** | **646.3099** | 1274.5859 | **637.7966** | 1273.6019 | **637.3046** | 12 |
| 5 | **504.1936** | 252.6005 | 487.1671 | 244.0872 | **486.1831** | 243.5952 | S | **1204.5804** | 602.7938 | **1187.5539** | 594.2806 | **1186.5699** | 593.7886 | 11 |
| 6 | **617.2777** | 309.1425 | 600.2511 | **300.6292** | **599.2671** | **300.1372** | L | **1117.5484** | 559.2778 | 1100.5218 | 550.7646 | 1099.5378 | 550.2726 | 10 |
| 7 | **718.3254** | 359.6663 | 701.2988 | 351.1530 | 700.3148 | 350.6610 | T | **1004.4643** | **502.7358** | 987.4378 | 494.2225 | **986.4538** | 493.7305 | 9 |
| 8 | **775.3468** | 388.1771 | 758.3203 | 379.6638 | 757.3363 | 379.1718 | G | **903.4167** | 452.2120 | **886.3901** | 443.6987 | 885.4061 | 443.2067 | 8 |
| 9 | **904.3894** | 452.6984 | 887.3629 | 444.1851 | **886.3789** | 443.6931 | E | **846.3952** | 423.7012 | 829.3686 | 415.1880 | 828.3846 | 414.6959 | 7 |
| 10 | 991.4215 | 496.2144 | 974.3949 | 487.7011 | **973.4109** | 487.2091 | S | **717.3526** | 359.1799 | 700.3260 | 350.6667 | 699.3420 | 350.1747 | 6 |
| 11 | **1120.4640** | 560.7357 | 1103.4375 | 552.2224 | **1102.4535** | 551.7304 | E | **630.3206** | 315.6639 | 613.2940 | 307.1506 | 612.3100 | 306.6586 | 5 |
| 12 | 1217.5168 | 609.2620 | 1200.4903 | 600.7488 | 1199.5062 | 600.2568 | P | **501.2780** | 251.1426 | **484.2514** | 242.6293 | 483.2674 | 242.1373 | 4 |
| 13 | **1345.5754** | 673.2913 | **1328.5488** | 664.7781 | **1327.5648** | 664.2860 | Q | **404.2252** | 202.6162 | 387.1987 | 194.1030 | 386.2146 | 193.6110 | 3 |
| 14 | **1446.6231** | 723.8152 | 1429.5965 | 715.3019 | **1428.6125** | 714.8099 | T | **276.1666** | 138.5870 | 259.1401 | 130.0737 | 258.1561 | 129.5817 | 2 |
| 15 | | | | | | | R | 175.1190 | 88.0631 | 158.0924 | 79.5498 | | | 1 |

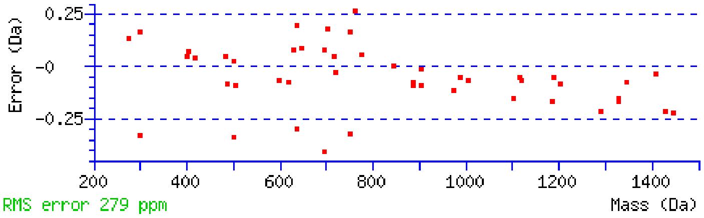


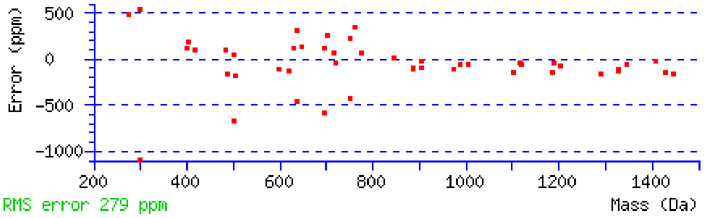


## All matches to this query

| Score | Mr(calc): | Delta | Sequence |
|---|---|---|---|
| 100.2 | 1619.7275 | -0.0032 | VDNSSLTGESEPQTR |
| 11.3 | 1619.7165 | 0.0077 | RNSIPQXSGSSSHR |
| 7.1 | 1619.7345 | -0.0102 | EKYSSAPNAVAFTR |
| 6.8 | 1618.7321 | 0.9922 | KMRVSGVMTQGASR |
| 4.1 | 1619.7279 | -0.0037 | FNHHSAMVLAAGLR |
| 2.7 | 1619.7226 | 0.0016 | ILDGTGTSGTMKSTR |
| | | | |

| | | | |
|---|---|---|---|
| 2.4 | 1619.7153 | 0.0089 | IEYECKLNTLEK |
| 2.1 | 1619.7127 | 0.0116 | GNGKNYMGNLSKTR |
| 2.1 | 1619.7127 | 0.0116 | GNGKNYMGNLSKTR |
| 1.9 | 1618.7313 | 0.9929 | IEYECKLNTLEK |

---

Spectrum No: 76; Query: 1132; Rank: 1

---

## Peptide View

MS/MS Fragmentation of **MESDLANTTAGFTAALK**
Found in **IPI00392894**, Tax_Id=10116 Gene_Symbol=Mgam_predicted 89 kDa protein

Match to Query 1132: 1740.824148 from(871.419350,2+)
Title: 091008RatKidney_NoSalt_26.3424.3424.2.dta
Data file K:\NewmanPaper\Piliang\3SubProteomes\Piliang3SP\mgf5ppm\ERLIC_3SubProteomes5ppm.mgf

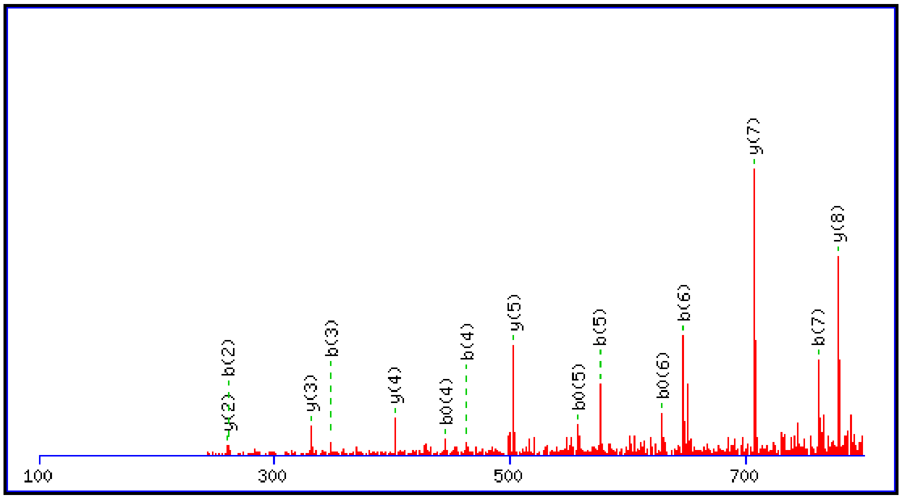


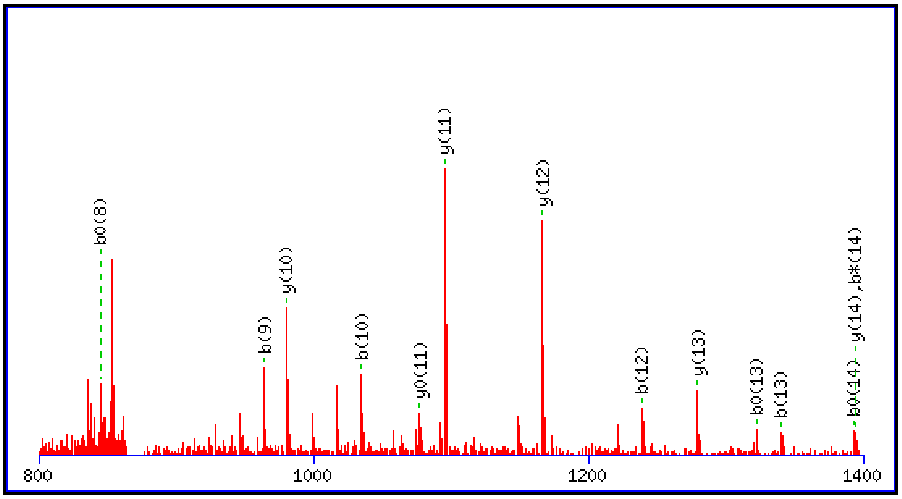

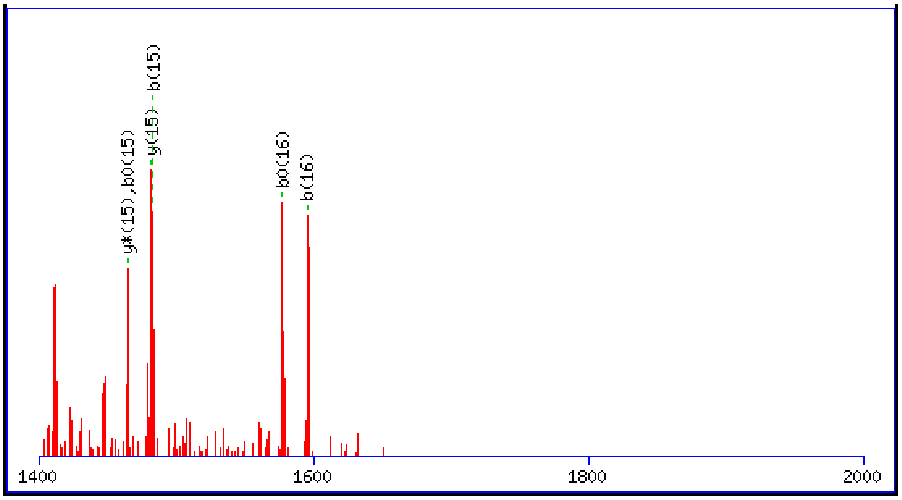


**Monoisotopic mass of neutral peptide Mr(calc):** 1740.8240
**Fixed modifications:** Carbamidomethyl (C)
**Variable modifications:**
**N7** : Deamidated_N (N)
**Ions Score:** 100 **Expect:** 1.8e-008
**Matches (Bold Red):** 35/170 fragment ions using 53 most intense peaks

| # | b | $b^{++}$ | b* | $b^{*++}$ | $b^0$ | $b^{0++}$ | Seq. | y | $y^{++}$ | y* | $y^{*++}$ | $y^0$ | $y^{0++}$ | # |
|---|---|---|---|---|---|---|---|---|---|---|---|---|---|---|
| 1 | 132.0478 | 66.5275 | | | | | M | | | | | | | 17 |
| 2 | **261.0904** | 131.0488 | | | 243.0798 | 122.0435 | E | 1610.7908 | 805.8990 | 1593.7643 | 797.3858 | 1592.7802 | 796.8938 | 16 |
| 3 | **348.1224** | 174.5648 | | | 330.1118 | 165.5595 | S | **1481.7482** | 741.3777 | **1464.7217** | 732.8645 | 1463.7376 | 732.3725 | 15 |
| 4 | **463.1493** | 232.0783 | | | **445.1388** | 223.0730 | D | **1394.7162** | 697.8617 | 1377.6896 | 689.3485 | 1376.7056 | 688.8564 | 14 |
| 5 | **576.2334** | 288.6203 | | | **558.2228** | 279.6151 | L | **1279.6892** | 640.3483 | 1262.6627 | 631.8350 | 1261.6787 | 631.3430 | 13 |
| 6 | **647.2705** | 324.1389 | | | **629.2599** | 315.1336 | A | **1166.6052** | 583.8062 | 1149.5786 | 575.2930 | 1148.5946 | 574.8009 | 12 |
| 7 | **762.2974** | 381.6524 | 745.2709 | 373.1391 | 744.2869 | 372.6471 | N | **1095.5681** | 548.2877 | 1078.5415 | 539.7744 | **1077.5575** | 539.2824 | 11 |
| 8 | 863.3451 | 432.1762 | 846.3186 | 423.6629 | **845.3346** | 423.1709 | T | **980.5411** | 490.7742 | 963.5146 | 482.2609 | 962.5306 | 481.7689 | 10 |
| 9 | **964.3928** | 482.7000 | 947.3662 | 474.1868 | 946.3822 | 473.6948 | T | 879.4934 | 440.2504 | 862.4669 | 431.7371 | 861.4829 | 431.2451 | 9 |
| 10 | **1035.4299** | 518.2186 | 1018.4034 | 509.7053 | 1017.4193 | 509.2133 | A | **778.4458** | 389.7265 | 761.4192 | 381.2132 | 760.4352 | 380.7212 | 8 |
| 11 | 1092.4514 | 546.7293 | 1075.4248 | 538.2161 | 1074.4408 | 537.7240 | G | **707.4087** | 354.2080 | 690.3821 | 345.6947 | 689.3981 | 345.2027 | 7 |
| 12 | **1239.5198** | 620.2635 | 1222.4932 | 611.7503 | 1221.5092 | 611.2582 | F | 650.3872 | 325.6972 | 633.3606 | 317.1840 | 632.3766 | 316.6920 | 6 |
| 13 | **1340.5675** | 670.7874 | 1323.5409 | 662.2741 | **1322.5569** | 661.7821 | T | **503.3188** | 252.1630 | 486.2922 | 243.6498 | 485.3082 | 243.1577 | 5 |
| 14 | 1411.6046 | 706.3059 | **1394.5780** | 697.7927 | **1393.5940** | 697.3006 | A | **402.2711** | 201.6392 | 385.2445 | 193.1259 | | | 4 |
| 15 | **1482.6417** | 741.8245 | 1465.6151 | 733.3112 | **1464.6311** | 732.8192 | A | **331.2340** | 166.1206 | 314.2074 | 157.6074 | | | 3 |
| 16 | **1595.7258** | 798.3665 | 1578.6992 | 789.8532 | **1577.7152** | 789.3612 | L | **260.1969** | 130.6021 | 243.1703 | 122.0888 | | | 2 |
| 17 | | | | | | | K | 147.1128 | 74.0600 | 130.0863 | 65.5468 | | | 1 |

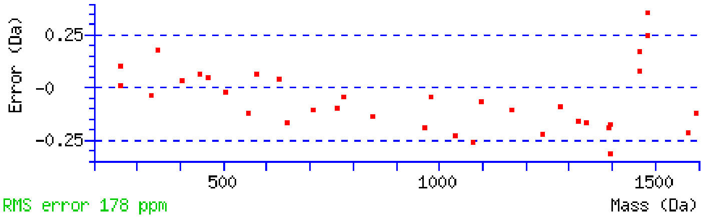


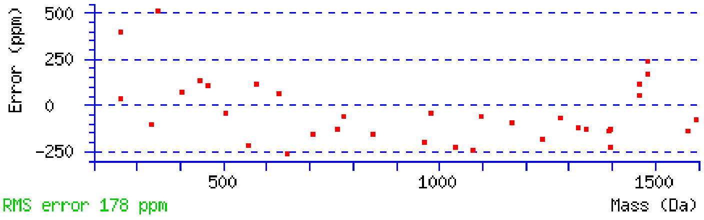


### All matches to this query

| Score | Mr(calc): | Delta | Sequence |
|---|---|---|---|
| 100.1 | 1740.8240 | 0.0002 | MESDLANTTAGFTAALK |
| 6.3 | 1739.8213 | 1.0028 | INSENSFSEASNLSLK |
| 5.5 | 1739.8213 | 1.0028 | INSENSFSEASNLSLK |
| 4.1 | 1740.8240 | 0.0002 | TSLMVDNQELGYLNK |
| | | | |

| | | | |
|---|---|---|---|
| 3.9 | 1740.8270 | -0.0028 | LKVTHQAESNLYMK |
| 3.5 | 1739.8188 | 1.0053 | EWMGYQGNKASVELK |
| 3.4 | 1740.8270 | -0.0028 | LKVTHQAESNLYMK |
| 3.4 | 1740.8196 | 0.0045 | GFVDDIIQPSSTRAR |
| 2.7 | 1740.8196 | 0.0045 | GFVDDIIQPSSTRAR |
| 2.0 | 1739.8213 | 1.0028 | INSENSFSEASNLSLK |

---

Spectrum No: 77; Query: 1044; Rank: 1

---

## Peptide View

MS/MS Fragmentation of **YTGNASALFILPDQGK**
Found in **IPI00200591**, Tax_Id=10116 Gene_Symbol=LOC299282 Serine protease inhibitor A3L precursor

Match to Query 1044: 1694.850428 from(848.432490,2+)
Title: 091008RatKidney_NoSalt_30.2971.2971.2.dta
Data file K:\NewmanPaper\Piliang\3SubProteomes\Piliang3SP\mgf5ppm\ERLIC_3SubProteomes5ppm.mgf

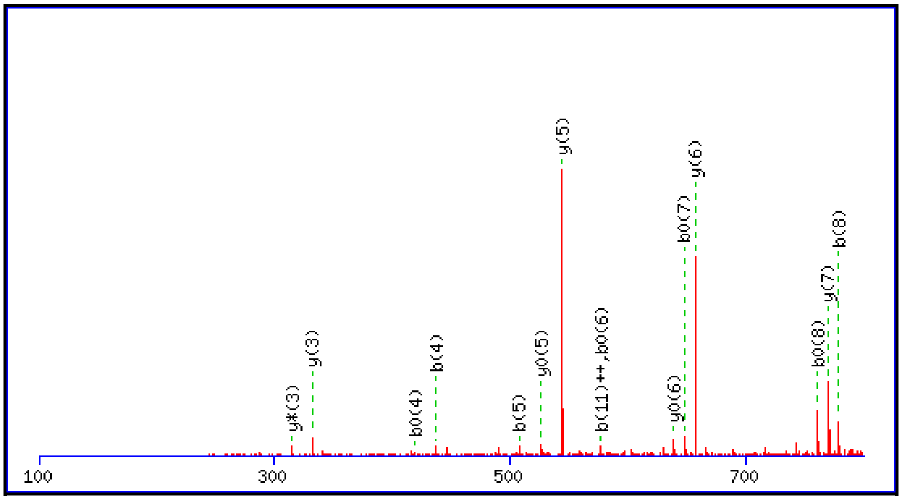


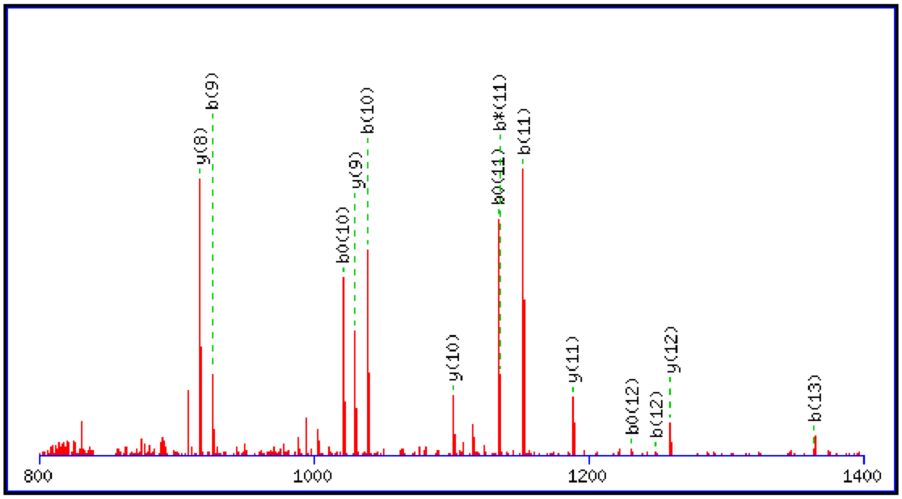

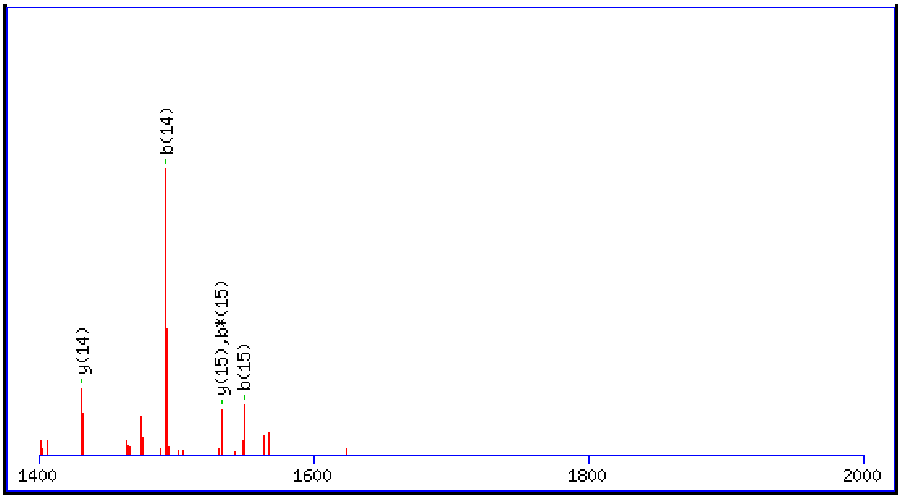


**Monoisotopic mass of neutral peptide Mr(calc):** 1694.8515
**Fixed modifications:** Carbamidomethyl (C)
**Variable modifications:**
**N4** : Deamidated_N (N)
**Ions Score:** 100 **Expect:** 1.7e-008
**Matches (Bold Red):** 34/166 fragment ions using 48 most intense peaks

| # | b | b$^{++}$ | b* | b*$^{++}$ | b$^{0}$ | b$^{0++}$ | Seq. | y | y$^{++}$ | y* | y*$^{++}$ | y$^{0}$ | y$^{0++}$ | # |
|---|---|---|---|---|---|---|---|---|---|---|---|---|---|---|
| 1 | 164.0706 | 82.5389 | | | | | Y | | | | | | | 16 |
| 2 | 265.1183 | 133.0628 | | | 247.1077 | 124.0575 | T | **1532.7955** | 766.9014 | 1515.7689 | 758.3881 | 1514.7849 | 757.8961 | 15 |
| 3 | 322.1397 | 161.5735 | | | 304.1292 | 152.5682 | G | **1431.7478** | 716.3775 | 1414.7213 | 707.8643 | 1413.7372 | 707.3723 | 14 |
| 4 | **437.1667** | 219.0870 | 420.1401 | 210.5737 | **419.1561** | 210.0817 | N | 1374.7264 | 687.8668 | 1357.6998 | 679.3535 | 1356.7158 | 678.8615 | 13 |
| 5 | **508.2038** | 254.6055 | 491.1772 | 246.0923 | 490.1932 | 245.6003 | A | **1259.6994** | 630.3533 | 1242.6729 | 621.8401 | 1241.6888 | 621.3481 | 12 |
| 6 | 595.2358 | 298.1216 | 578.2093 | 289.6083 | **577.2253** | 289.1163 | S | **1188.6623** | 594.8348 | 1171.6358 | 586.3215 | 1170.6517 | 585.8295 | 11 |
| 7 | 666.2729 | 333.6401 | 649.2464 | 325.1268 | **648.2624** | 324.6348 | A | **1101.6303** | 551.3188 | 1084.6037 | 542.8055 | 1083.6197 | 542.3135 | 10 |
| 8 | **779.3570** | 390.1821 | 762.3305 | 381.6689 | **761.3464** | 381.1769 | L | **1030.5932** | 515.8002 | 1013.5666 | 507.2869 | 1012.5826 | 506.7949 | 9 |
| 9 | **926.4254** | 463.7163 | 909.3989 | 455.2031 | 908.4149 | 454.7111 | F | **917.5091** | 459.2582 | 900.4825 | 450.7449 | 899.4985 | 450.2529 | 8 |
| 10 | **1039.5095** | 520.2584 | 1022.4829 | 511.7451 | **1021.4989** | 511.2531 | I | **770.4407** | 385.7240 | 753.4141 | 377.2107 | 752.4301 | 376.7187 | 7 |
| 11 | **1152.5935** | **576.8004** | **1135.5670** | 568.2871 | **1134.5830** | 567.7951 | L | **657.3566** | 329.1819 | 640.3301 | 320.6687 | **639.3461** | 320.1767 | 6 |
| 12 | **1249.6463** | 625.3268 | 1232.6198 | 616.8135 | **1231.6357** | 616.3215 | P | **544.2726** | 272.6399 | 527.2460 | 264.1266 | **526.2620** | 263.6346 | 5 |
| 13 | **1364.6733** | 682.8403 | 1347.6467 | 674.3270 | 1346.6627 | 673.8350 | D | 447.2198 | 224.1135 | 430.1932 | 215.6003 | 429.2092 | 215.1082 | 4 |
| 14 | **1492.7318** | 746.8696 | 1475.7053 | 738.3563 | 1474.7213 | 737.8643 | Q | **332.1928** | 166.6001 | **315.1663** | 158.0868 | | | 3 |
| 15 | **1549.7533** | 775.3803 | **1532.7267** | 766.8670 | 1531.7427 | 766.3750 | G | 204.1343 | 102.5708 | 187.1077 | 94.0575 | | | 2 |
| 16 | | | | | | | K | 147.1128 | 74.0600 | 130.0863 | 65.5468 | | | 1 |

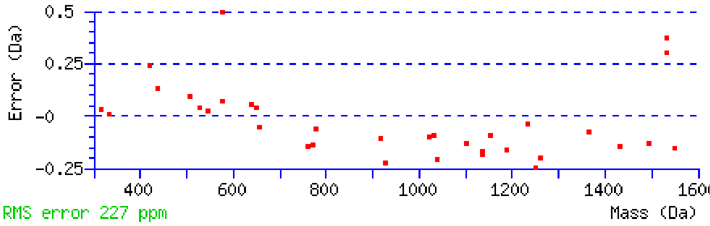


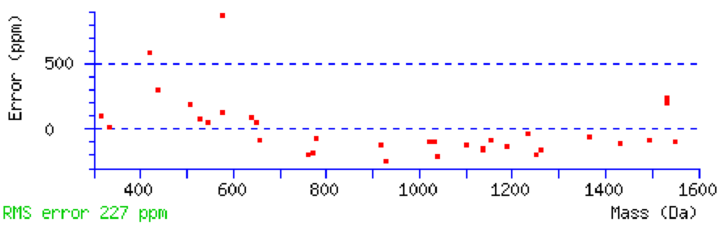


### All matches to this query

| Score | Mr(calc): | Delta | Sequence |
|---|---|---|---|
| 100.0 | 1694.8515 | -0.0011 | YTGNASALFILPDQGK |
| 10.9 | 1693.8440 | 1.0064 | FAENQGTPLLVIANK |
| 9.7 | 1694.8662 | -0.0157 | SLITFEGGSMIQIQR |
| 8.7 | 1694.8362 | 0.0142 | VLEPEAAEEALENGPK |
| 8.6 | 1694.8662 | -0.0157 | SQDNVIKVCASVTFK |
| | | | |

| | | | |
|---|---|---|---|
| 5.6 | 1694.8628 | -0.0124 | SDGGNLNFEFQIVKK |
| 5.5 | 1694.8426 | 0.0078 | VLPDTMRNLLSTQK |
| 4.0 | 1694.8379 | 0.0125 | LETEILNKEAVEVK |
| 3.6 | 1694.8426 | 0.0078 | VLPDTMRNLLSTQK |
| 2.8 | 1694.8406 | 0.0098 | IHHRIHTGEKPYK |

---

Spectrum No: 78; Query: 1084; Rank: 1

---

## Peptide View

MS/MS Fragmentation of **DSSDEANCTTLCSQK**
Found in **IPI00205325**, Tax_Id=10116 Gene_Symbol=Lrp2 Low-density lipoprotein receptor-related protein 2 precursor

Match to Query 1084: 1715.660968 from(858.837760,2+)
Title: 091008RatKidney_NoSalt_26.531.531.2.dta
Data file K:\NewmanPaper\Piliang\3SubProteomes\Piliang3SP\mgf5ppm\ERLIC_3SubProteomes5ppm.mgf

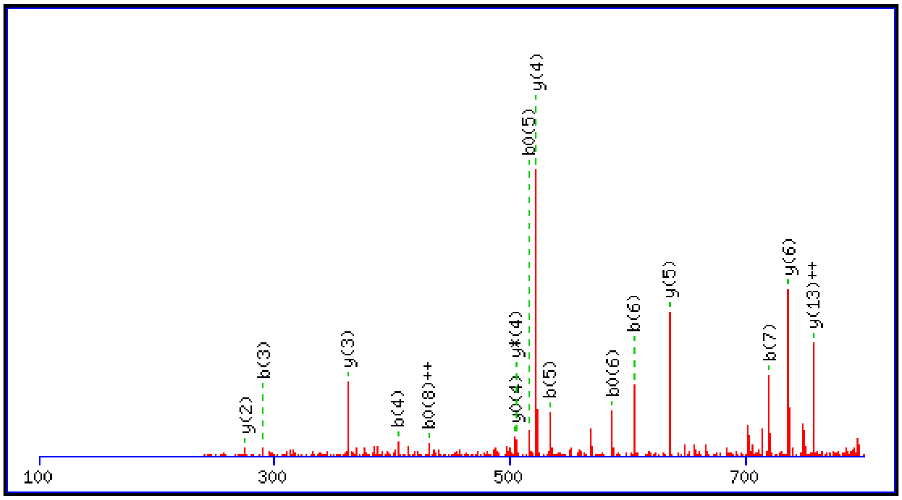


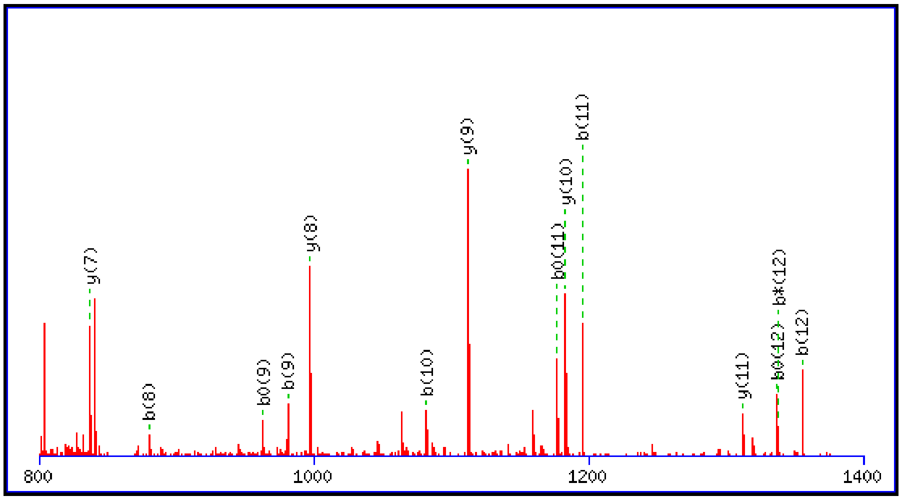

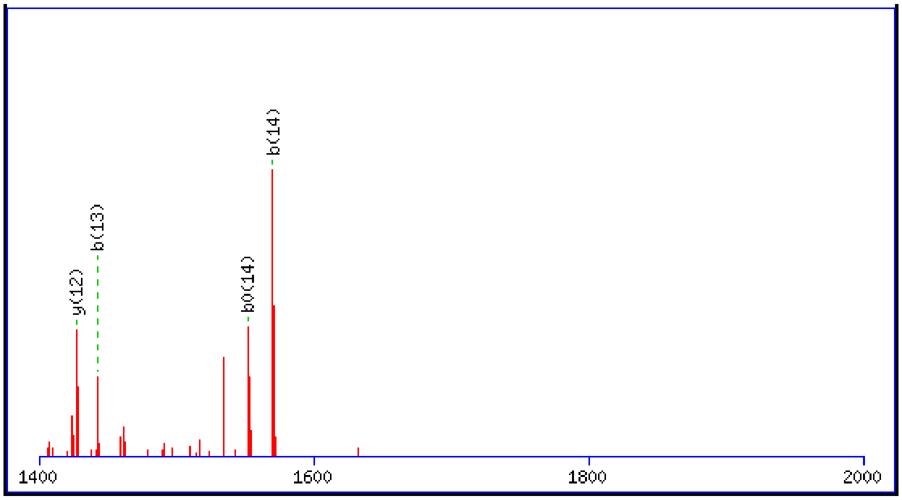


**Monoisotopic mass of neutral peptide Mr(calc):** 1715.6614
**Fixed modifications:** Carbamidomethyl (C)
**Variable modifications:**
**N7** : Deamidated_N (N)
**Ions Score:** 100 **Expect:** 1.3e-008
**Matches (Bold Red):** 34/152 fragment ions using 48 most intense peaks

| # | b | b$^{++}$ | b* | b*$^{++}$ | b$^{0}$ | b$^{0++}$ | Seq. | y | y$^{++}$ | y* | y*$^{++}$ | y$^{0}$ | y$^{0++}$ | # |
|---|---|---|---|---|---|---|---|---|---|---|---|---|---|---|
| 1 | 116.0342 | 58.5207 | | | 98.0237 | 49.5155 | D | | | | | | | 15 |
| 2 | 203.0662 | 102.0368 | | | 185.0557 | 93.0315 | S | 1601.6418 | 801.3245 | 1584.6152 | 792.8112 | 1583.6312 | 792.3192 | 14 |
| 3 | **290.0983** | 145.5528 | | | 272.0877 | 136.5475 | S | 1514.6097 | **757.8085** | 1497.5832 | 749.2952 | 1496.5992 | 748.8032 | 13 |
| 4 | **405.1252** | 203.0662 | | | 387.1147 | 194.0610 | D | **1427.5777** | 714.2925 | 1410.5512 | 705.7792 | 1409.5672 | 705.2872 | 12 |
| 5 | **534.1678** | 267.5875 | | | **516.1572** | 258.5823 | E | **1312.5508** | 656.7790 | 1295.5242 | 648.2658 | 1294.5402 | 647.7737 | 11 |
| 6 | **605.2049** | 303.1061 | | | **587.1944** | 294.1008 | A | **1183.5082** | 592.2577 | 1166.4816 | 583.7445 | 1165.4976 | 583.2524 | 10 |
| 7 | **720.2319** | 360.6196 | 703.2053 | 352.1063 | 702.2213 | 351.6143 | N | **1112.4711** | 556.7392 | 1095.4445 | 548.2259 | 1094.4605 | 547.7339 | 9 |
| 8 | **880.2625** | 440.6349 | 863.2360 | 432.1216 | 862.2519 | **431.6296** | C | **997.4441** | 499.2257 | 980.4176 | 490.7124 | 979.4336 | 490.2204 | 8 |
| 9 | **981.3102** | 491.1587 | 964.2836 | 482.6455 | **963.2996** | 482.1535 | T | **837.4135** | 419.2104 | 820.3869 | 410.6971 | 819.4029 | 410.2051 | 7 |
| 10 | **1082.3579** | 541.6826 | 1065.3313 | 533.1693 | 1064.3473 | 532.6773 | T | **736.3658** | 368.6865 | 719.3393 | 360.1733 | 718.3552 | 359.6813 | 6 |
| 11 | **1195.4419** | 598.2246 | 1178.4154 | 589.7113 | **1177.4314** | 589.2193 | L | **635.3181** | 318.1627 | 618.2916 | 309.6494 | 617.3076 | 309.1574 | 5 |
| 12 | **1355.4726** | 678.2399 | **1338.4460** | 669.7267 | **1337.4620** | 669.2346 | C | **522.2341** | 261.6207 | **505.2075** | 253.1074 | **504.2235** | 252.6154 | 4 |
| 13 | **1442.5046** | 721.7559 | 1425.4781 | 713.2427 | 1424.4940 | 712.7507 | S | **362.2034** | 181.6053 | 345.1769 | 173.0921 | 344.1928 | 172.6001 | 3 |
| 14 | **1570.5632** | 785.7852 | 1553.5366 | 777.2720 | **1552.5526** | 776.7799 | Q | **275.1714** | 138.0893 | 258.1448 | 129.5761 | | | 2 |
| 15 | | | | | | | K | 147.1128 | 74.0600 | 130.0863 | 65.5468 | | | 1 |

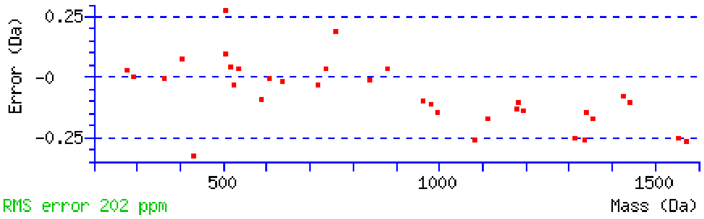


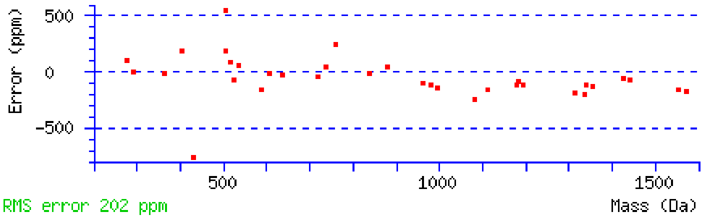


### All matches to this query

| Score | Mr(calc): | Delta | Sequence |
|---|---|---|---|
| 99.9 | 1715.6614 | -0.0005 | DSSDEANCTTLCSQK |
| 7.8 | 1715.6701 | -0.0091 | SADFTCSNLLAMVK |
| 7.3 | 1715.6548 | 0.0062 | MTVTNLLSRNMDK |
| 5.8 | 1715.6706 | -0.0096 | RFYRGDSPTDSQK |
| 4.6 | 1715.6481 | 0.0129 | YTHAANTVVYSSNK |
| 4.6 | 1715.6481 | 0.0129 | YTHAANTVVYSSNK |
| | | | |

| | | | |
|---|---|---|---|
| 3.8 | 1715.6593 | 0.0017 | NQNFNESFTISQK |
| 3.8 | 1715.6706 | -0.0096 | RFYRGDSPTDSQK |
| 3.3 | 1715.6706 | -0.0096 | RFYRGDSPTDSQK |
| 3.3 | 1715.6457 | 0.0152 | TDSLVTGTKPDSQK |

---

Spectrum No: 79; Query: 2372; Rank: 1

---

## Peptide View

MS/MS Fragmentation of **DGQQNISLLYTEPGASQTQTGASFR**
Found in **IPI00214859**, Tax_Id=10116 Gene_Symbol=Col18a1 similar to Collagen alpha-1(XVIII) chain precursor

Match to Query 2372: 2669.267428 from(1335.640990,2+)
Title: 100101RatKid_NS_deglyco_18.3864.3864.2.dta
Data file K:\NewmanPaper\Piliang\3SubProteomes\Piliang3SP\mgf5ppm\ERLIC_3SubProteomes5ppm.mgf

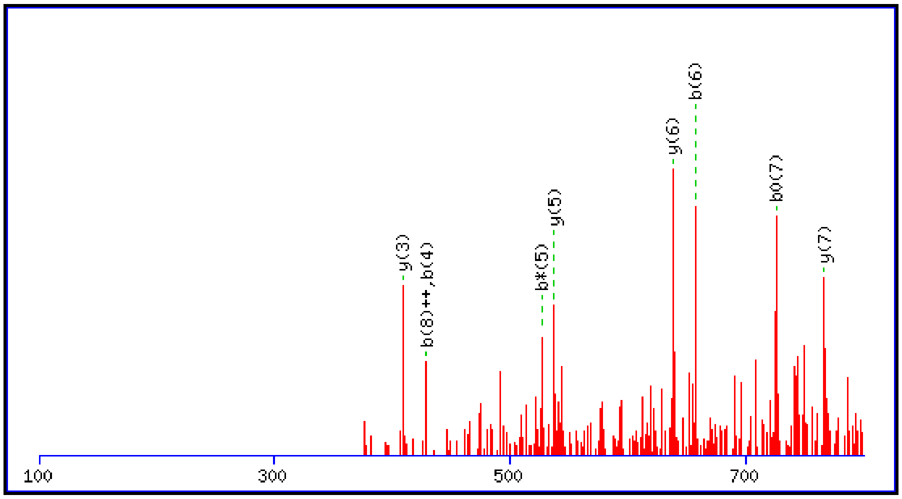


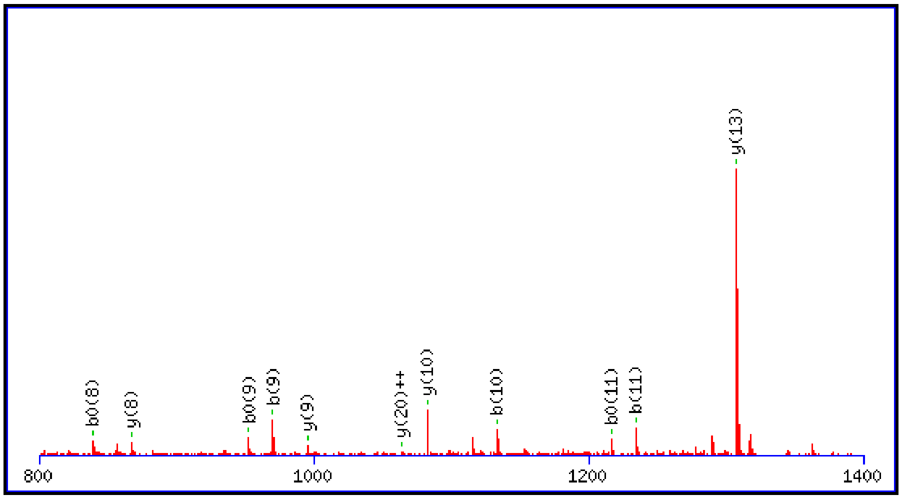

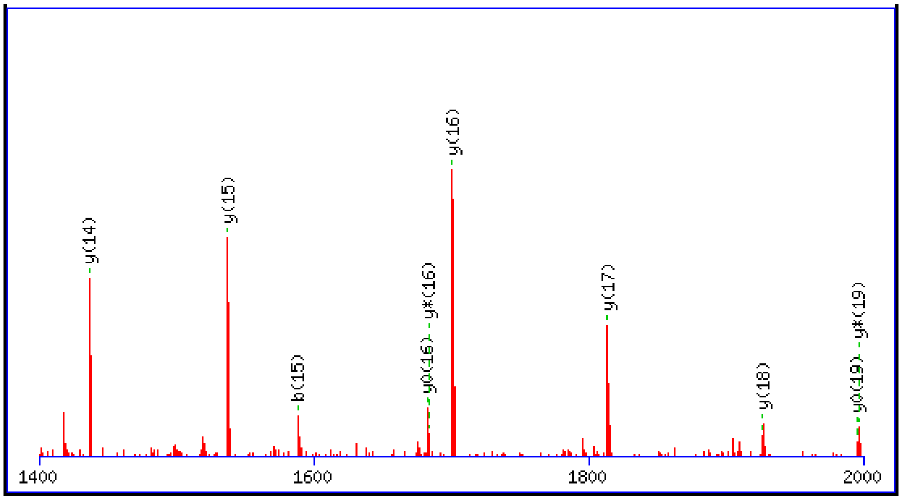


**Monoisotopic mass of neutral peptide Mr(calc):** 2669.2569
**Fixed modifications:** Carbamidomethyl (C)
**Variable modifications:**
**N5** : Deamidated_N (N)
**Ions Score:** 100 **Expect:** 3e-008
**Matches (Bold Red):** 30/280 fragment ions using 38 most intense peaks

| # | b | b++ | b* | b*++ | b0 | b0++ | Seq. | y | y++ | y* | y*++ | y0 | y0++ | # |
|---|---|---|---|---|---|---|---|---|---|---|---|---|---|---|
| 1 | 116.0342 | 58.5207 | | | 98.0237 | 49.5155 | D | | | | | | | 25 |
| 2 | 173.0557 | 87.0315 | | | 155.0451 | 78.0262 | G | 2555.2372 | 1278.1223 | 2538.2107 | 1269.6090 | 2537.2267 | 1269.1170 | 24 |
| 3 | 301.1143 | 151.0608 | 284.0877 | 142.5475 | 283.1037 | 142.0555 | Q | 2498.2158 | 1249.6115 | 2481.1892 | 1241.0982 | 2480.2052 | 1240.6062 | 23 |
| 4 | **429.1728** | 215.0901 | 412.1463 | 206.5768 | 411.1623 | 206.0848 | Q | 2370.1572 | 1185.5822 | 2353.1306 | 1177.0690 | 2352.1466 | 1176.5770 | 22 |
| 5 | 544.1998 | 272.6035 | **527.1732** | 264.0903 | 526.1892 | 263.5982 | N | 2242.0986 | 1121.5529 | 2225.0721 | 1113.0397 | 2224.0880 | 1112.5477 | 21 |
| 6 | **657.2838** | 329.1456 | 640.2573 | 320.6323 | 639.2733 | 320.1403 | I | 2127.0717 | **1064.0395** | 2110.0451 | 1055.5262 | 2109.0611 | 1055.0342 | 20 |
| 7 | 744.3159 | 372.6616 | 727.2893 | 364.1483 | **726.3053** | 363.6563 | S | 2013.9876 | 1007.4974 | **1996.9611** | 998.9842 | **1995.9770** | 998.4922 | 19 |
| 8 | 857.3999 | **429.2036** | 840.3734 | 420.6903 | **839.3894** | 420.1983 | L | **1926.9556** | 963.9814 | 1909.9290 | 955.4682 | 1908.9450 | 954.9761 | 18 |
| 9 | **970.4840** | 485.7456 | 953.4574 | 477.2324 | **952.4734** | 476.7404 | L | **1813.8715** | 907.4394 | 1796.8450 | 898.9261 | 1795.8610 | 898.4341 | 17 |
| 10 | **1133.5473** | 567.2773 | 1116.5208 | 558.7640 | 1115.5368 | 558.2720 | Y | **1700.7875** | 850.8974 | **1683.7609** | 842.3841 | **1682.7769** | 841.8921 | 16 |
| 11 | **1234.5950** | 617.8011 | 1217.5685 | 609.2879 | **1216.5844** | 608.7959 | T | **1537.7241** | 769.3657 | 1520.6976 | 760.8524 | 1519.7136 | 760.3604 | 15 |
| 12 | 1363.6376 | 682.3224 | 1346.6110 | 673.8092 | 1345.6270 | 673.3172 | E | **1436.6764** | 718.8419 | 1419.6499 | 710.3286 | 1418.6659 | 709.8366 | 14 |
| 13 | 1460.6904 | 730.8488 | 1443.6638 | 722.3355 | 1442.6798 | 721.8435 | P | **1307.6339** | 654.3206 | 1290.6073 | 645.8073 | 1289.6233 | 645.3153 | 13 |
| 14 | 1517.7118 | 759.3596 | 1500.6853 | 750.8463 | 1499.7013 | 750.3543 | G | 1210.5811 | 605.7942 | 1193.5545 | 597.2809 | 1192.5705 | 596.7889 | 12 |
| 15 | **1588.7489** | 794.8781 | 1571.7224 | 786.3648 | 1570.7384 | 785.8728 | A | 1153.5596 | 577.2835 | 1136.5331 | 568.7702 | 1135.5491 | 568.2782 | 11 |
| 16 | 1675.7810 | 838.3941 | 1658.7544 | 829.8808 | 1657.7704 | 829.3888 | S | **1082.5225** | 541.7649 | 1065.4960 | 533.2516 | 1064.5119 | 532.7596 | 10 |
| 17 | 1803.8395 | 902.4234 | 1786.8130 | 893.9101 | 1785.8290 | 893.4181 | Q | **995.4905** | 498.2489 | 978.4639 | 489.7356 | 977.4799 | 489.2436 | 9 |
| 18 | 1904.8872 | 952.9472 | 1887.8607 | 944.4340 | 1886.8767 | 943.9420 | T | **867.4319** | 434.2196 | 850.4054 | 425.7063 | 849.4213 | 425.2143 | 8 |
| 19 | 2032.9458 | 1016.9765 | 2015.9193 | 1008.4633 | 2014.9352 | 1007.9713 | Q | **766.3842** | 383.6958 | 749.3577 | 375.1825 | 748.3737 | 374.6905 | 7 |
| 20 | 2133.9935 | 1067.5004 | 2116.9669 | 1058.9871 | 2115.9829 | 1058.4951 | T | **638.3257** | 319.6665 | 621.2991 | 311.1532 | 620.3151 | 310.6612 | 6 |
| 21 | 2191.0149 | 1096.0111 | 2173.9884 | 1087.4978 | 2173.0044 | 1087.0058 | G | **537.2780** | 269.1426 | 520.2514 | 260.6293 | 519.2674 | 260.1373 | 5 |
| 22 | 2262.0521 | 1131.5297 | 2245.0255 | 1123.0164 | 2244.0415 | 1122.5244 | A | 480.2565 | 240.6319 | 463.2300 | 232.1186 | 462.2459 | 231.6266 | 4 |
| 23 | 2349.0841 | 1175.0457 | 2332.0575 | 1166.5324 | 2331.0735 | 1166.0404 | S | **409.2194** | 205.1133 | 392.1928 | 196.6001 | 391.2088 | 196.1081 | 3 |
| 24 | 2496.1525 | 1248.5799 | 2479.1260 | 1240.0666 | 2478.1419 | 1239.5746 | F | 322.1874 | 161.5973 | 305.1608 | 153.0840 | | | 2 |
| 25 | | | | | | | R | 175.1190 | 88.0631 | 158.0924 | 79.5498 | | | 1 |

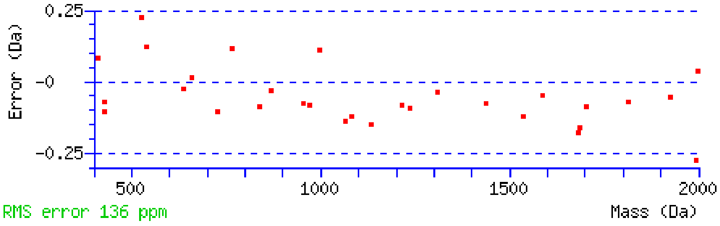


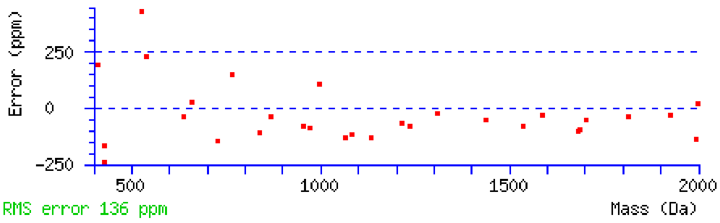

### All matches to this query

| Score | Mr(calc): | Delta | Sequence |
|---|---|---|---|
| 99.9 | 2669.2569 | 0.0105 | DGQQNISLLYTEPGASQTQTGASFR |
| 75.2 | 2668.2729 | 0.9945 | DGQQNISLLYTEPGASQTQTGASFR |
| 7.8 | 2667.2370 | 2.0304 | TVSVWSLINSNKDEFTNPFYAR |
| 7.3 | 2669.2808 | -0.0133 | YIAWYQQKPGKAPGLLIGYTSR |
| 7.3 | 2669.2808 | -0.0133 | YIAWYQQKPGKAPGLLIGYTSR |
| 7.3 | 2669.2808 | -0.0133 | YIAWYQQKPGKAPGLLIGYTSR |
| 4.5 | 2667.2458 | 2.0216 | FNFNSDLRPRITTAIETVREK |
| 4.5 | 2667.2458 | 2.0216 | FNFNSDLRPRITTAIETVREK |
| 3.5 | 2667.2458 | 2.0216 | FNFNSDLRPRITTAIETVREK |
| 3.4 | 2667.2370 | 2.0304 | TVSVWSLINSNKDEFTNPFYAR |

---

Spectrum No: 80; Query: 1822; Rank: 1

---

## Peptide View

MS/MS Fragmentation of **EDAQINTTIGSVAAQDPDAAR**
Found in **IPI00215357**, Tax_Id=10116 Gene_Symbol=Cdh6 Cadherin-6 precursor

Match to Query 1822: 2143.007088 from(1072.510820,2+)
Title: 100101RatKid_NS_deglyco_21.2614.2614.2.dta
Data file K:\NewmanPaper\Piliang\3SubProteomes\Piliang3SP\mgf5ppm\ERLIC_3SubProteomes5ppm.mgf

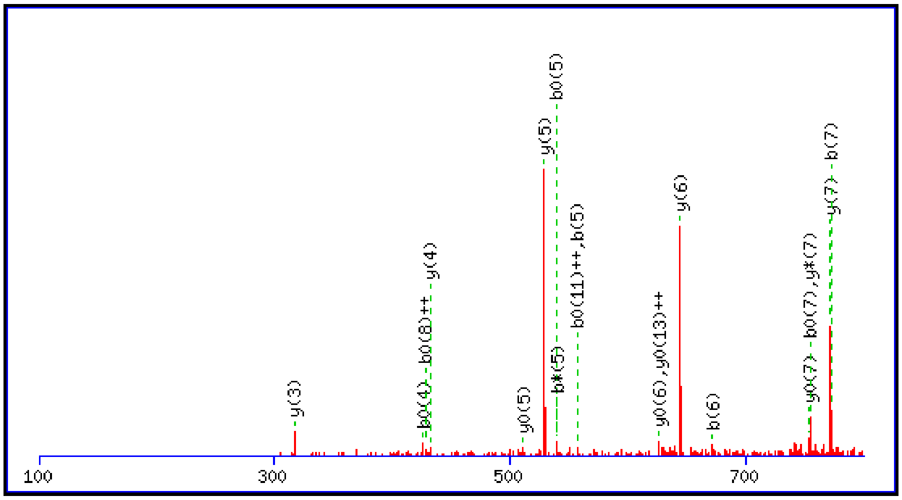

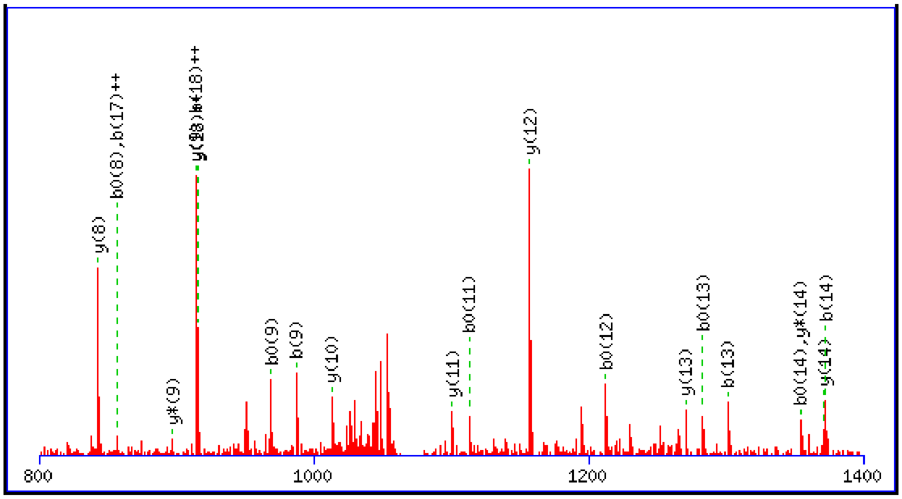


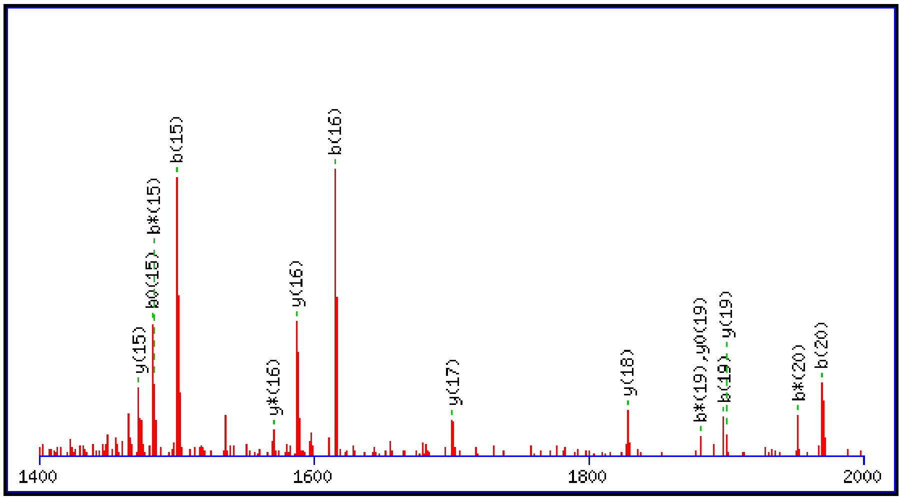


**Monoisotopic mass of neutral peptide Mr(calc):** 2143.0029
**Fixed modifications:** Carbamidomethyl (C)
**Variable modifications:**
**N6** : Deamidated_N (N)
**Ions Score:** 100 **Expect:** 2.7e-008
**Matches (Bold Red):** 55/228 fragment ions using 94 most intense peaks

| # | b | b$^{++}$ | b* | b*$^{++}$ | b$^{0}$ | b$^{0++}$ | Seq. | y | y$^{++}$ | y* | y*$^{++}$ | y$^{0}$ | y$^{0++}$ | # |
|---|---|---|---|---|---|---|---|---|---|---|---|---|---|---|
| 1 | 130.0499 | 65.5286 | | | 112.0393 | 56.5233 | E | | | | | | | 21 |
| 2 | 245.0768 | 123.0420 | | | 227.0662 | 114.0368 | D | 2014.9676 | 1007.9874 | 1997.9411 | 999.4742 | 1996.9570 | 998.9822 | 20 |
| 3 | 316.1139 | 158.5606 | | | 298.1034 | 149.5553 | A | **1899.9407** | 950.4740 | 1882.9141 | 941.9607 | **1881.9301** | 941.4687 | 19 |
| 4 | 444.1725 | 222.5899 | 427.1460 | 214.0766 | **426.1619** | 213.5846 | Q | **1828.9035** | **914.9554** | 1811.8770 | 906.4421 | 1810.8930 | 905.9501 | 18 |
| 5 | **557.2566** | 279.1319 | **540.2300** | 270.6186 | **539.2460** | 270.1266 | I | **1700.8450** | 850.9261 | 1683.8184 | 842.4128 | 1682.8344 | 841.9208 | 17 |
| 6 | **672.2835** | 336.6454 | 655.2570 | 328.1321 | 654.2729 | 327.6401 | N | **1587.7609** | 794.3841 | **1570.7344** | 785.8708 | 1569.7503 | 785.3788 | 16 |
| 7 | **773.3312** | 387.1692 | 756.3046 | 378.6560 | **755.3206** | 378.1639 | T | **1472.7340** | 736.8706 | 1455.7074 | 728.3573 | 1454.7234 | 727.8653 | 15 |
| 8 | 874.3789 | 437.6931 | 857.3523 | 429.1798 | **856.3683** | **428.6878** | T | **1371.6863** | 686.3468 | **1354.6597** | 677.8335 | 1353.6757 | 677.3415 | 14 |
| 9 | **987.4629** | 494.2351 | 970.4364 | 485.7218 | **969.4524** | 485.2298 | I | **1270.6386** | 635.8229 | 1253.6121 | 627.3097 | 1252.6280 | **626.8177** | 13 |
| 10 | 1044.4844 | 522.7458 | 1027.4578 | 514.2326 | 1026.4738 | 513.7406 | G | **1157.5545** | 579.2809 | 1140.5280 | 570.7676 | 1139.5440 | 570.2756 | 12 |
| 11 | 1131.5164 | 566.2618 | 1114.4899 | 557.7486 | **1113.5059** | **557.2566** | S | **1100.5331** | 550.7702 | 1083.5065 | 542.2569 | 1082.5225 | 541.7649 | 11 |
| 12 | 1230.5848 | 615.7961 | 1213.5583 | 607.2828 | **1212.5743** | 606.7908 | V | **1013.5011** | 507.2542 | 996.4745 | 498.7409 | 995.4905 | 498.2489 | 10 |
| 13 | **1301.6219** | 651.3146 | 1284.5954 | 642.8013 | **1283.6114** | 642.3093 | A | **914.4326** | 457.7200 | **897.4061** | 449.2067 | 896.4221 | 448.7147 | 9 |
| 14 | **1372.6591** | 686.8332 | 1355.6325 | 678.3199 | **1354.6485** | 677.8279 | A | **843.3955** | 422.2014 | 826.3690 | 413.6881 | 825.3850 | 413.1961 | 8 |
| 15 | **1500.7176** | 750.8625 | **1483.6911** | 742.3492 | **1482.7071** | 741.8572 | Q | **772.3584** | 386.6828 | **755.3319** | 378.1696 | **754.3478** | 377.6776 | 7 |
| 16 | **1615.7446** | 808.3759 | 1598.7180 | 799.8627 | 1597.7340 | 799.3706 | D | **644.2998** | 322.6536 | 627.2733 | 314.1403 | **626.2893** | 313.6483 | 6 |
| 17 | 1712.7973 | **856.9023** | 1695.7708 | 848.3890 | 1694.7868 | 847.8970 | P | **529.2729** | 265.1401 | 512.2463 | 256.6268 | **511.2623** | 256.1348 | 5 |
| 18 | 1827.8243 | **914.4158** | 1810.7977 | 905.9025 | 1809.8137 | 905.4105 | D | **432.2201** | 216.6137 | 415.1936 | 208.1004 | 414.2096 | 207.6084 | 4 |

| | | | | | | | | | | | | | | |
|---|---|---|---|---|---|---|---|---|---|---|---|---|---|---|
| **19** | **1898.8614** | 949.9343 | **1881.8349** | 941.4211 | 1880.8508 | 940.9291 | **A** | **317.1932** | 159.1002 | 300.1666 | 150.5870 | | | **3** |
| **20** | **1969.8985** | 985.4529 | **1952.8720** | 976.9396 | 1951.8880 | 976.4476 | **A** | 246.1561 | 123.5817 | 229.1295 | 115.0684 | | | **2** |
| **21** | | | | | | | **R** | 175.1190 | 88.0631 | 158.0924 | 79.5498 | | | **1** |

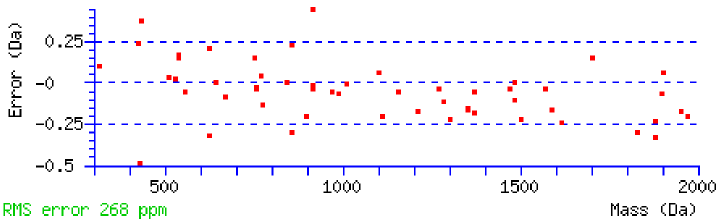


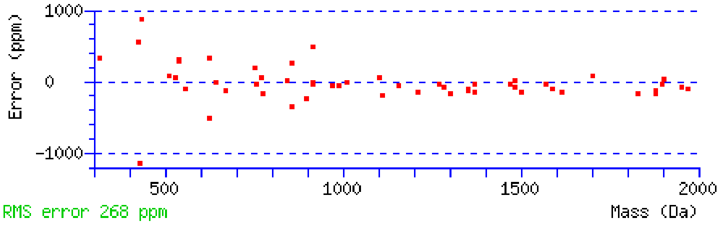


### All matches to this query

| Score | Mr(calc): | Delta | Sequence |
|---|---|---|---|
| 99.8 | 2143.0029 | 0.0042 | EDAQINTTIGSVAAQDPDAAR |
| 51.1 | 2142.0189 | 0.9882 | EDAQINTTIGSVAAQDPDAAR |
| 11.6 | 2143.0089 | -0.0018 | LRSSGLRGGGEAALGAWLGR |
| 9.3 | 2142.0106 | 0.9965 | KANGIFSTATLPENAERSR |
| 6.5 | 2140.9830 | 2.0241 | WLNTHFDTENLKSSIQK |
| 5.8 | 2142.9868 | 0.0203 | AAASLDTAALSATDMALALNR |
| 5.8 | 2142.9868 | 0.0203 | AAASLDTAALSATDMALALNR |
| 4.5 | 2142.9858 | 0.0213 | SLDVNQDSELKFNEYWR |
| 4.1 | 2142.9946 | 0.0125 | NIRDSLNDYEAKLQDLR |
| 4.1 | 2142.9946 | 0.0125 | NIRDSLNDYEAKLQDLR |

---

Spectrum No: 81; Query: 413; Rank: 1

---

## Peptide View

MS/MS Fragmentation of **KLDFIVLNETR**
Found in **IPI00208422**, Tax_Id=10116 Gene_Symbol=Dpp4 Dipeptidyl peptidase 4

Match to Query 413: 1347.736688 from(674.875620,2+)
Title: 091008RatKidney_NH4Format01_23.2662.2662.2.dta
Data file K:\NewmanPaper\Piliang\3SubProteomes\Piliang3SP\mgf5ppm\ERLIC_3SubProteomes5ppm.mgf

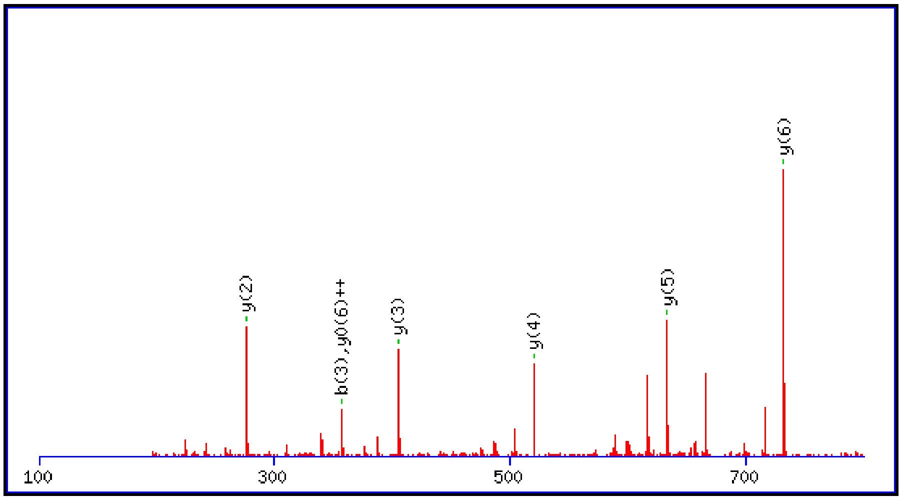

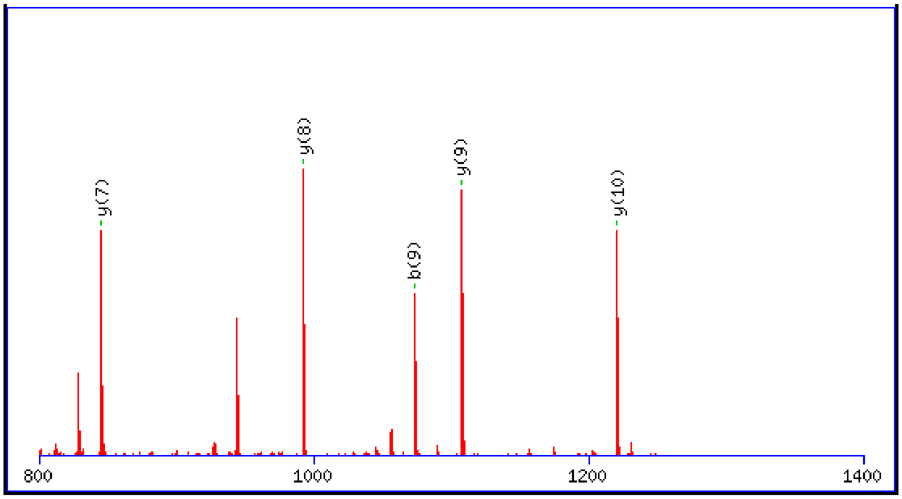


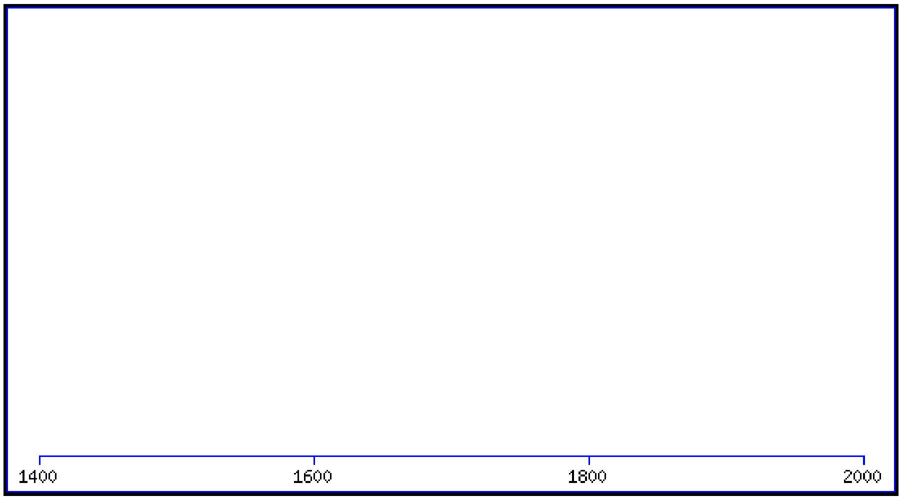


**Monoisotopic mass of neutral peptide Mr(calc):** 1347.7398
**Fixed modifications:** Carbamidomethyl (C)
**Variable modifications:**
**N8** : Deamidated_N (N)
**Ions Score:** 100 **Expect:** 8.9e-009
**Matches (Bold Red):** 12/114 fragment ions using 11 most intense peaks

| # | b | $b^{++}$ | b* | $b^{*++}$ | $b^0$ | $b^{0++}$ | Seq. | y | $y^{++}$ | y* | $y^{*++}$ | $y^0$ | $y^{0++}$ | # |
|---|---|---|---|---|---|---|---|---|---|---|---|---|---|---|
| 1 | 129.1022 | 65.0548 | 112.0757 | 56.5415 | | | K | | | | | | | 11 |
| 2 | 242.1863 | 121.5968 | 225.1598 | 113.0835 | | | L | **1220.6521** | 610.8297 | 1203.6256 | 602.3164 | 1202.6416 | 601.8244 | 10 |
| 3 | **357.2132** | 179.1103 | 340.1867 | 170.5970 | 339.2027 | 170.1050 | D | **1107.5681** | 554.2877 | 1090.5415 | 545.7744 | 1089.5575 | 545.2824 | 9 |
| 4 | 504.2817 | 252.6445 | 487.2551 | 244.1312 | 486.2711 | 243.6392 | F | **992.5411** | 496.7742 | 975.5146 | 488.2609 | 974.5306 | 487.7689 | 8 |
| 5 | 617.3657 | 309.1865 | 600.3392 | 300.6732 | 599.3552 | 300.1812 | I | **845.4727** | 423.2400 | 828.4462 | 414.7267 | 827.4621 | 414.2347 | 7 |
| 6 | 716.4341 | 358.7207 | 699.4076 | 350.2074 | 698.4236 | 349.7154 | V | **732.3886** | 366.6980 | 715.3621 | 358.1847 | 714.3781 | **357.6927** | 6 |
| 7 | 829.5182 | 415.2627 | 812.4917 | 406.7495 | 811.5076 | 406.2575 | L | **633.3202** | 317.1638 | 616.2937 | 308.6505 | 615.3097 | 308.1585 | 5 |
| 8 | 944.5451 | 472.7762 | 927.5186 | 464.2629 | 926.5346 | 463.7709 | N | **520.2362** | 260.6217 | 503.2096 | 252.1084 | 502.2256 | 251.6164 | 4 |
| 9 | **1073.5877** | 537.2975 | 1056.5612 | 528.7842 | 1055.5772 | 528.2922 | E | **405.2092** | 203.1082 | 388.1827 | 194.5950 | 387.1987 | 194.1030 | 3 |
| 10 | 1174.6354 | 587.8213 | 1157.6089 | 579.3081 | 1156.6248 | 578.8161 | T | **276.1666** | 138.5870 | 259.1401 | 130.0737 | 258.1561 | 129.5817 | 2 |
| 11 | | | | | | | R | 175.1190 | 88.0631 | 158.0924 | 79.5498 | | | 1 |

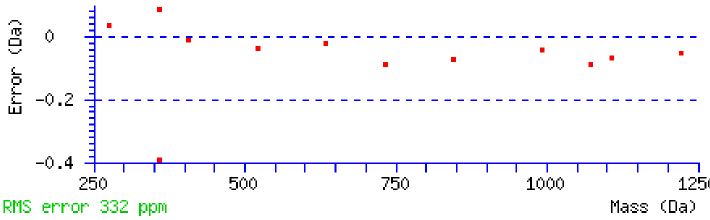


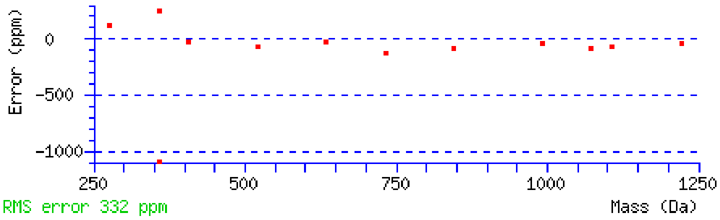

### All matches to this query

| Score | Mr(calc): | Delta | Sequence |
|---|---|---|---|
| 99.7 | 1347.7398 | -0.0031 | KLDFIVLNETR |
| 9.1 | 1347.7431 | -0.0065 | KITNLKITCEK |
| 8.3 | 1347.7428 | -0.0061 | ITQPLVKKWR |
| 7.7 | 1347.7431 | -0.0065 | QIVSLKEKMEK |
| 5.5 | 1347.7398 | -0.0031 | QNIEDVKKVFK |
| 5.4 | 1347.7387 | -0.0021 | QLLSIPRASKR |
| 5.1 | 1347.7431 | -0.0065 | KMKLVESQLEK |
| 3.5 | 1346.7228 | 1.0139 | QLDMELVSIKR |
| 3.0 | 1347.7398 | -0.0031 | QIYEKGLELQK |
| 2.6 | 1346.7405 | 0.9962 | VSSTKEVAAEAKK |

---

Spectrum No: 82; Query: 2815; Rank: 1

---

## Peptide View

MS/MS Fragmentation of **SSPSLSILAGGQCNESVGYFVEPCIIESKDPQEPIMK**
Found in **IPI00475676**, Tax_Id=10116 Gene_Symbol=Tas1r2 Delta-1-pyrroline-5-carboxylate dehydrogenase, mitochondrial precursor

Match to Query 2815: 4066.915662 from(1356.645830,3+)
Title: 100101RatKid_NS_deglyco_07.5803.5803.3.dta
Data file K:\NewmanPaper\Piliang\3SubProteomes\Piliang3SP\mgf5ppm\ERLIC_3SubProteomes5ppm.mgf

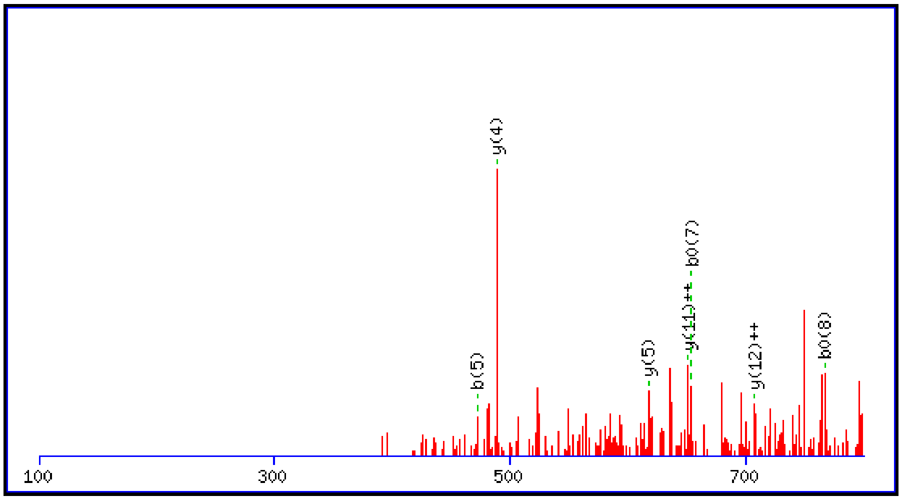


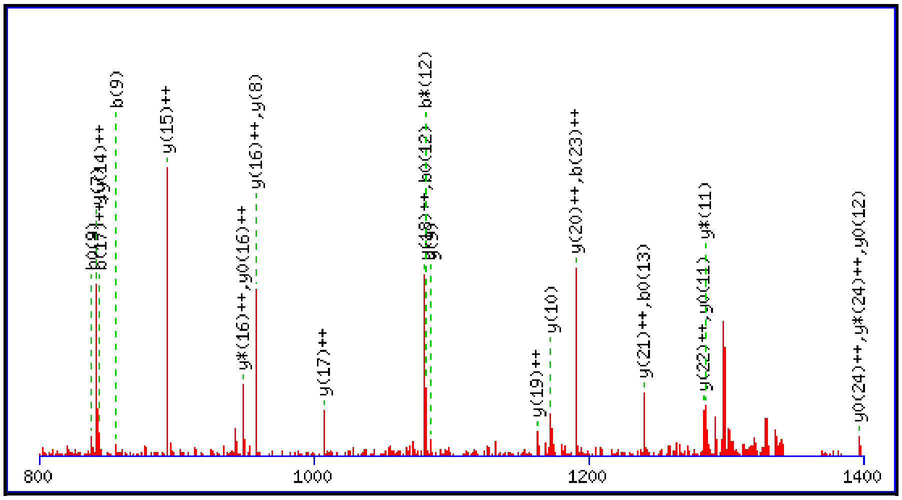

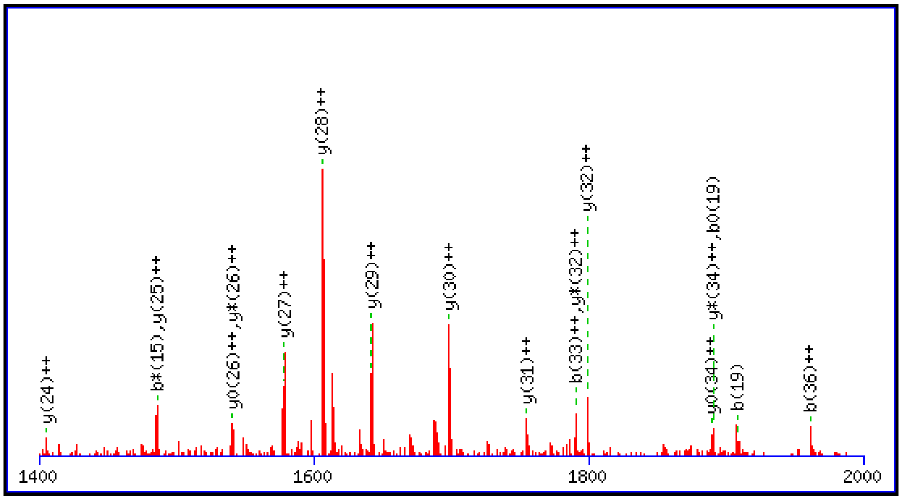


**Monoisotopic mass of neutral peptide Mr(calc):** 4066.9213
**Fixed modifications:** Carbamidomethyl (C)
**Variable modifications:**
**N14** : Deamidated_N (N)
**Ions Score:** 99 **Expect:** 4.8e-008
**Matches (Bold Red):** 52/402 fragment ions using 83 most intense peaks

| # | b | $b^{++}$ | b* | $b^{*++}$ | $b^0$ | $b^{0++}$ | Seq. | y | $y^{++}$ | y* | $y^{*++}$ | $y^0$ | $y^{0++}$ | # |
|---|---|---|---|---|---|---|---|---|---|---|---|---|---|---|
| 1 | 88.0393 | 44.5233 | | | 70.0287 | 35.5180 | S | | | | | | | 37 |
| 2 | 175.0713 | 88.0393 | | | 157.0608 | 79.0340 | S | 3980.8966 | 1990.9519 | 3963.8701 | 1982.4387 | 3962.8860 | 1981.9467 | 36 |
| 3 | 272.1241 | 136.5657 | | | 254.1135 | 127.5604 | P | 3893.8646 | 1947.4359 | 3876.8380 | 1938.9227 | 3875.8540 | 1938.4306 | 35 |
| 4 | 359.1561 | 180.0817 | | | 341.1456 | 171.0764 | S | 3796.8118 | 1898.9095 | 3779.7853 | **1890.3963** | 3778.8013 | **1889.9043** | 34 |
| 5 | **472.2402** | 236.6237 | | | 454.2296 | 227.6184 | L | 3709.7798 | 1855.3935 | 3692.7532 | 1846.8803 | 3691.7692 | 1846.3883 | 33 |
| 6 | 559.2722 | 280.1397 | | | 541.2617 | 271.1345 | S | 3596.6957 | **1798.8515** | 3579.6692 | **1790.3382** | 3578.6852 | 1789.8462 | 32 |
| 7 | 672.3563 | 336.6818 | | | **654.3457** | 327.6765 | I | 3509.6637 | **1755.3355** | 3492.6372 | 1746.8222 | 3491.6531 | 1746.3302 | 31 |
| 8 | 785.4403 | 393.2238 | | | **767.4298** | 384.2185 | L | 3396.5796 | **1698.7935** | 3379.5531 | 1690.2802 | 3378.5691 | 1689.7882 | 30 |
| 9 | **856.4775** | 428.7424 | | | **838.4669** | 419.7371 | A | 3283.4956 | **1642.2514** | 3266.4690 | 1633.7382 | 3265.4850 | 1633.2461 | 29 |
| 10 | 913.4989 | 457.2531 | | | 895.4884 | 448.2478 | G | 3212.4585 | **1606.7329** | 3195.4319 | 1598.2196 | 3194.4479 | 1597.7276 | 28 |
| 11 | 970.5204 | 485.7638 | | | 952.5098 | 476.7585 | G | 3155.4370 | **1578.2221** | 3138.4104 | 1569.7089 | 3137.4264 | 1569.2169 | 27 |
| 12 | 1098.5790 | 549.7931 | **1081.5524** | 541.2798 | **1080.5684** | 540.7878 | Q | 3098.4155 | 1549.7114 | 3081.3890 | **1541.1981** | 3080.4050 | **1540.7061** | 26 |
| 13 | 1258.6096 | 629.8084 | 1241.5831 | 621.2952 | **1240.5990** | 620.8032 | C | 2970.3570 | **1485.6821** | 2953.3304 | 1477.1688 | 2952.3464 | 1476.6768 | 25 |
| 14 | 1373.6365 | 687.3219 | 1356.6100 | 678.8086 | 1355.6260 | 678.3166 | N | 2810.3263 | **1405.6668** | 2793.2998 | **1397.1535** | 2792.3157 | **1396.6615** | 24 |
| 15 | 1502.6791 | 751.8432 | **1485.6526** | 743.3299 | 1484.6686 | 742.8379 | E | 2695.2994 | 1348.1533 | 2678.2728 | 1339.6400 | 2677.2888 | 1339.1480 | 23 |
| 16 | 1589.7112 | 795.3592 | 1572.6846 | 786.8459 | 1571.7006 | 786.3539 | S | 2566.2568 | **1283.6320** | 2549.2302 | 1275.1188 | 2548.2462 | 1274.6267 | 22 |
| 17 | 1688.7796 | **844.8934** | 1671.7530 | 836.3802 | 1670.7690 | 835.8881 | V | 2479.2247 | **1240.1160** | 2462.1982 | 1231.6027 | 2461.2142 | 1231.1107 | 21 |
| 18 | 1745.8010 | 873.4042 | 1728.7745 | 864.8909 | 1727.7905 | 864.3989 | G | 2380.1563 | **1190.5818** | 2363.1298 | 1182.0685 | 2362.1458 | 1181.5765 | 20 |
| 19 | **1908.8644** | 954.9358 | 1891.8378 | 946.4226 | **1890.8538** | 945.9305 | Y | 2323.1349 | **1162.0711** | 2306.1083 | 1153.5578 | 2305.1243 | 1153.0658 | 19 |
| 20 | 2055.9328 | 1028.4700 | 2038.9062 | 1019.9568 | 2037.9222 | 1019.4648 | F | 2160.0715 | **1080.5394** | 2143.0450 | 1072.0261 | 2142.0610 | 1071.5341 | 18 |
| 21 | 2155.0012 | 1078.0042 | 2137.9747 | 1069.4910 | 2136.9906 | 1068.9990 | V | 2013.0031 | **1007.0052** | 1995.9766 | 998.4919 | 1994.9926 | 997.9999 | 17 |
| 22 | 2284.0438 | 1142.5255 | 2267.0172 | 1134.0123 | 2266.0332 | 1133.5203 | E | 1913.9347 | **957.4710** | 1896.9082 | **948.9577** | 1895.9241 | **948.4657** | 16 |
| 23 | 2381.0966 | **1191.0519** | 2364.0700 | 1182.5386 | 2363.0860 | 1182.0466 | P | 1784.8921 | **892.9497** | 1767.8656 | 884.4364 | 1766.8816 | 883.9444 | 15 |
| 24 | 2541.1272 | 1271.0672 | 2524.1007 | 1262.5540 | 2523.1166 | 1262.0620 | C | 1687.8394 | **844.4233** | 1670.8128 | 835.9100 | 1669.8288 | 835.4180 | 14 |
| 25 | 2654.2113 | 1327.6093 | 2637.1847 | 1319.0960 | 2636.2007 | 1318.6040 | I | 1527.8087 | 764.4080 | 1510.7822 | 755.8947 | 1509.7981 | 755.4027 | 13 |
| 26 | 2767.2953 | 1384.1513 | 2750.2688 | 1375.6380 | 2749.2848 | 1375.1460 | I | 1414.7246 | **707.8660** | 1397.6981 | 699.3527 | **1396.7141** | 698.8607 | 12 |
| 27 | 2896.3379 | 1448.6726 | 2879.3114 | 1440.1593 | 2878.3274 | 1439.6673 | E | 1301.6406 | **651.3239** | **1284.6140** | 642.8107 | **1283.6300** | 642.3186 | 11 |
| 28 | 2983.3700 | 1492.1886 | 2966.3434 | 1483.6753 | 2965.3594 | 1483.1833 | S | **1172.5980** | 586.8026 | 1155.5714 | 578.2894 | 1154.5874 | 577.7973 | 10 |
| 29 | 3111.4649 | 1556.2361 | 3094.4384 | 1547.7228 | 3093.4544 | 1547.2308 | K | **1085.5660** | 543.2866 | 1068.5394 | 534.7733 | 1067.5554 | 534.2813 | 9 |
| 30 | 3226.4919 | 1613.7496 | 3209.4653 | 1605.2363 | 3208.4813 | 1604.7443 | D | **957.4710** | 479.2391 | 940.4444 | 470.7259 | 939.4604 | 470.2339 | 8 |
| 31 | 3323.5446 | 1662.2760 | 3306.5181 | 1653.7627 | 3305.5341 | 1653.2707 | P | **842.4441** | 421.7257 | 825.4175 | 413.2124 | 824.4335 | 412.7204 | 7 |

| | | | | | | | | | | | | | | |
|---|---|---|---|---|---|---|---|---|---|---|---|---|---|---|
| 32 | 3451.6032 | 1726.3052 | 3434.5767 | 1717.7920 | 3433.5926 | 1717.3000 | Q | 745.3913 | 373.1993 | 728.3647 | 364.6860 | 727.3807 | 364.1940 | 6 |
| 33 | 3580.6458 | **1790.8265** | 3563.6193 | 1782.3133 | 3562.6352 | 1781.8213 | E | **617.3327** | 309.1700 | 600.3062 | 300.6567 | 599.3221 | 300.1647 | 5 |
| 34 | 3677.6986 | 1839.3529 | 3660.6720 | 1830.8396 | 3659.6880 | 1830.3476 | P | **488.2901** | 244.6487 | 471.2636 | 236.1354 | | | 4 |
| 35 | 3790.7826 | 1895.8950 | 3773.7561 | 1887.3817 | 3772.7721 | 1886.8897 | I | 391.2374 | 196.1223 | 374.2108 | 187.6090 | | | 3 |
| 36 | 3921.8231 | **1961.4152** | 3904.7966 | 1952.9019 | 3903.8125 | 1952.4099 | M | 278.1533 | 139.5803 | 261.1267 | 131.0670 | | | 2 |
| 37 | | | | | | | K | 147.1128 | 74.0600 | 130.0863 | 65.5468 | | | 1 |

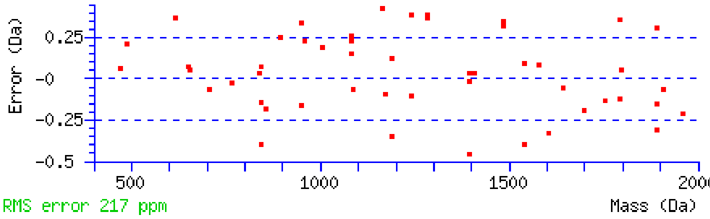


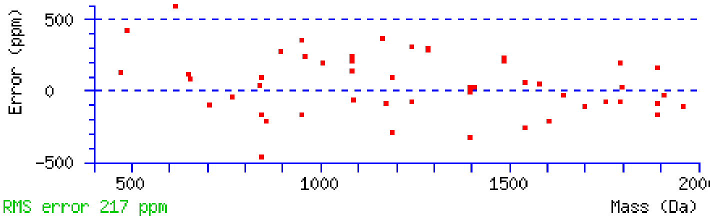


### All matches to this query

| Score | Mr(calc): | Delta | Sequence |
|---|---|---|---|
| 99.5 | 4066.9213 | -0.0057 | SSPSLSILAGGQCNESVGYFVEPCIIESKDPQEPIMK |
| 74.0 | 4065.9373 | 0.9783 | SSPSLSILAGGQCNESVGYFVEPCIIESKDPQEPIMK |
| 4.4 | 4065.9240 | 0.9916 | VFPVKVIALEDGDLEATVTVWVTGYCYNIEVHLR |
| 4.4 | 4066.8846 | 0.0311 | TYFAQEGCSFLYEKLPPSPSSPGNGDCTLLALAQLR |
| 4.0 | 4066.9055 | 0.0101 | RSENTPEPSSTQTPNPLSTLVPTPPSTETDNLMRHR |
| 4.0 | 4066.9055 | 0.0101 | RSENTPEPSSTQTPNPLSTLVPTPPSTETDNLMRHR |
| 4.0 | 4066.9055 | 0.0101 | RSENTPEPSSTQTPNPLSTLVPTPPSTETDNLMRHR |
| 3.5 | 4066.8846 | 0.0311 | TYFAQEGCSFLYEKLPPSPSSPGNGDCTLLALAQLR |
| 3.4 | 4066.9086 | 0.0070 | SIKATPMAVADLIPVDTVVNLTIAVGWYTAVHRPK |
| 3.4 | 4065.8829 | 1.0327 | EGAGDTGALETLGRMGDSMQLNQETAFQGVLLKLDIK |

---

Spectrum No: 83; Query: 966; Rank: 1

---

## Peptide View

MS/MS Fragmentation of **EGITLNANLSGDGFLK**
Found in **IPI00327398**, Tax_Id=10116 Gene_Symbol=Enpep Isoform 1 of Glutamyl aminopeptidase

Match to Query 966: 1648.832468 from(825.423510,2+)
Title: 091008RatKidney_NH4Format01_25.3412.3412.2.dta
Data file K:\NewmanPaper\Piliang\3SubProteomes\Piliang3SP\mgf5ppm\ERLIC_3SubProteomes5ppm.mgf

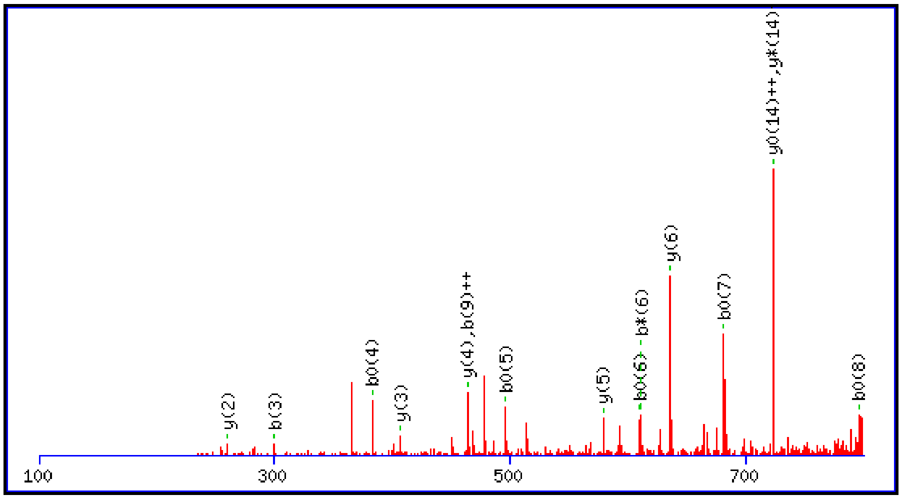

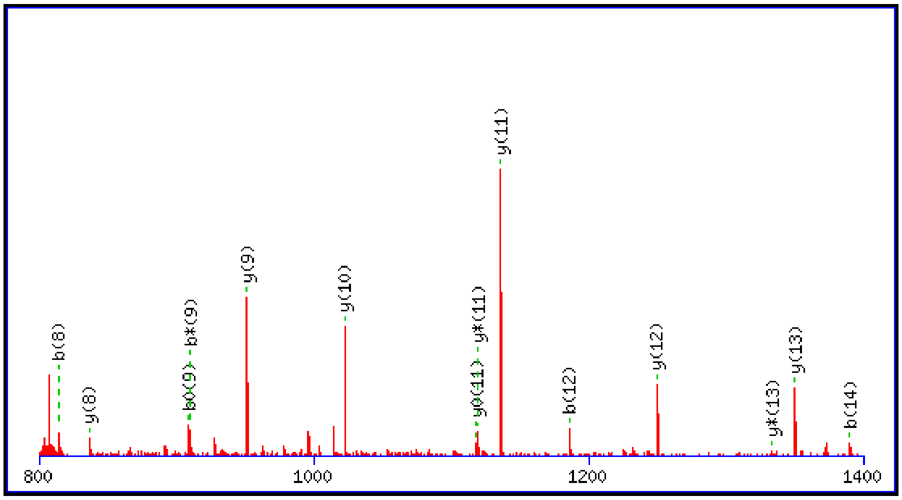


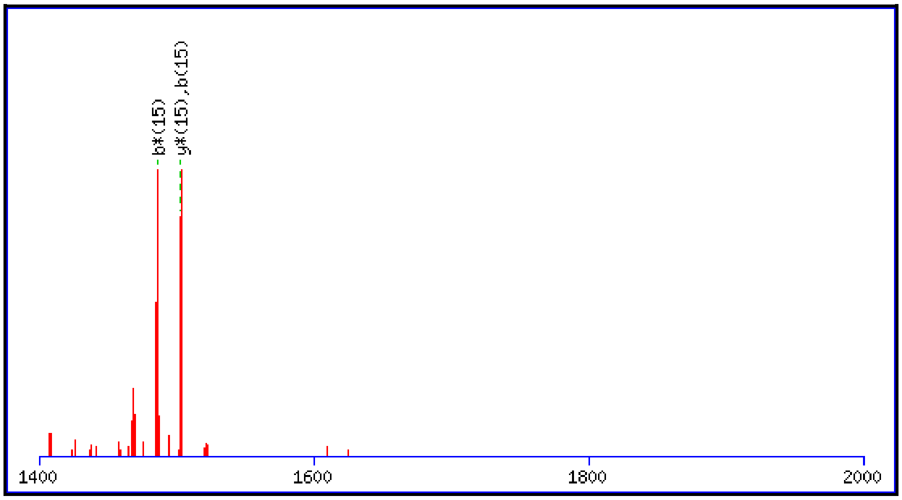


**Monoisotopic mass of neutral peptide Mr(calc):** 1648.8308
**Fixed modifications:** Carbamidomethyl (C)
**Variable modifications:**
**N8** : Deamidated_N (N)
**Ions Score:** 100 **Expect:** 1.9e-008
**Matches (Bold Red):** 33/162 fragment ions using 46 most intense peaks

| # | b | $b^{++}$ | b* | $b^{*++}$ | $b^{0}$ | $b^{0++}$ | Seq. | y | $y^{++}$ | y* | $y^{*++}$ | $y^{0}$ | $y^{0++}$ | # |
|---|---|---|---|---|---|---|---|---|---|---|---|---|---|---|
| 1 | 130.0499 | 65.5286 | | | 112.0393 | 56.5233 | E | | | | | | | 16 |
| 2 | 187.0713 | 94.0393 | | | 169.0608 | 85.0340 | G | 1520.7955 | 760.9014 | **1503.7689** | 752.3881 | 1502.7849 | 751.8961 | 15 |
| 3 | **300.1554** | 150.5813 | | | 282.1448 | 141.5761 | I | 1463.7740 | 732.3907 | 1446.7475 | **723.8774** | 1445.7635 | **723.3854** | 14 |
| 4 | 401.2031 | 201.1052 | | | **383.1925** | 192.0999 | T | **1350.6900** | 675.8486 | **1333.6634** | 667.3353 | 1332.6794 | 666.8433 | 13 |
| 5 | 514.2871 | 257.6472 | | | **496.2766** | 248.6419 | L | **1249.6423** | 625.3248 | 1232.6157 | 616.8115 | 1231.6317 | 616.3195 | 12 |
| 6 | 628.3301 | 314.6687 | **611.3035** | 306.1554 | **610.3195** | 305.6634 | N | **1136.5582** | 568.7827 | **1119.5317** | 560.2695 | **1118.5477** | 559.7775 | 11 |
| 7 | 699.3672 | 350.1872 | 682.3406 | 341.6740 | **681.3566** | 341.1819 | A | **1022.5153** | 511.7613 | 1005.4887 | 503.2480 | 1004.5047 | 502.7560 | 10 |
| 8 | **814.3941** | 407.7007 | 797.3676 | 399.1874 | **796.3836** | 398.6954 | N | **951.4782** | 476.2427 | 934.4516 | 467.7295 | 933.4676 | 467.2374 | 9 |
| 9 | 927.4782 | **464.2427** | **910.4516** | 455.7295 | **909.4676** | 455.2374 | L | **836.4512** | 418.7293 | 819.4247 | 410.2160 | 818.4407 | 409.7240 | 8 |
| 10 | 1014.5102 | 507.7587 | 997.4837 | 499.2455 | 996.4996 | 498.7535 | S | **723.3672** | 362.1872 | 706.3406 | 353.6740 | 705.3566 | 353.1819 | 7 |
| 11 | 1071.5317 | 536.2695 | 1054.5051 | 527.7562 | 1053.5211 | 527.2642 | G | **636.3352** | 318.6712 | 619.3086 | 310.1579 | 618.3246 | 309.6659 | 6 |
| 12 | **1186.5586** | 593.7829 | 1169.5321 | 585.2697 | 1168.5481 | 584.7777 | D | **579.3137** | 290.1605 | 562.2871 | 281.6472 | 561.3031 | 281.1552 | 5 |
| 13 | 1243.5801 | 622.2937 | 1226.5535 | 613.7804 | 1225.5695 | 613.2884 | G | **464.2867** | 232.6470 | 447.2602 | 224.1337 | | | 4 |
| 14 | **1390.6485** | 695.8279 | 1373.6219 | 687.3146 | 1372.6379 | 686.8226 | F | **407.2653** | 204.1363 | 390.2387 | 195.6230 | | | 3 |
| 15 | **1503.7326** | 752.3699 | **1486.7060** | 743.8566 | 1485.7220 | 743.3646 | L | **260.1969** | 130.6021 | 243.1703 | 122.0888 | | | 2 |
| 16 | | | | | | | K | 147.1128 | 74.0600 | 130.0863 | 65.5468 | | | 1 |

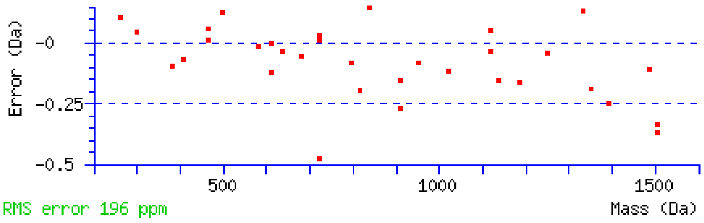


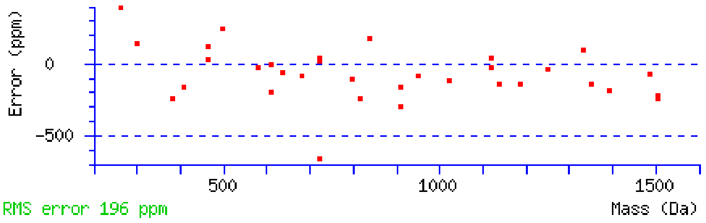


### All matches to this query

| Score | Mr(calc): | Delta | Sequence |
|---|---|---|---|
| 99.5 | 1648.8308 | 0.0017 | EGITLNANLSGDGFLK |
| 66.5 | 1648.8308 | 0.0017 | EGITLNANLSGDGFLK |
| 5.0 | 1648.8466 | -0.0142 | ALSYLIRAVSLKR |
| 0.2 | 1648.8243 | 0.0082 | MFNSPNNQALVSALK |
| 0.1 | 1647.8167 | 1.0157 | SMTLGSLPPKPRER |
| 0.0 | 1648.8308 | 0.0017 | EADENGALKKEGFIK |

---

Spectrum No: 84; Query: 868; Rank: 1

---

## Peptide View

MS/MS Fragmentation of **YLNETQQLTENIK**
Found in **IPI00564327**, Tax_Id=10116 Gene_Symbol=Mug2 Murinoglobulin-2 precursor

Match to Query 868: 1593.788508 from(797.901530,2+)
Title: 091008RatKidney_NoSalt_30.1986.1986.2.dta
Data file K:\NewmanPaper\Piliang\3SubProteomes\Piliang3SP\mgf5ppm\ERLIC_3SubProteomes5ppm.mgf

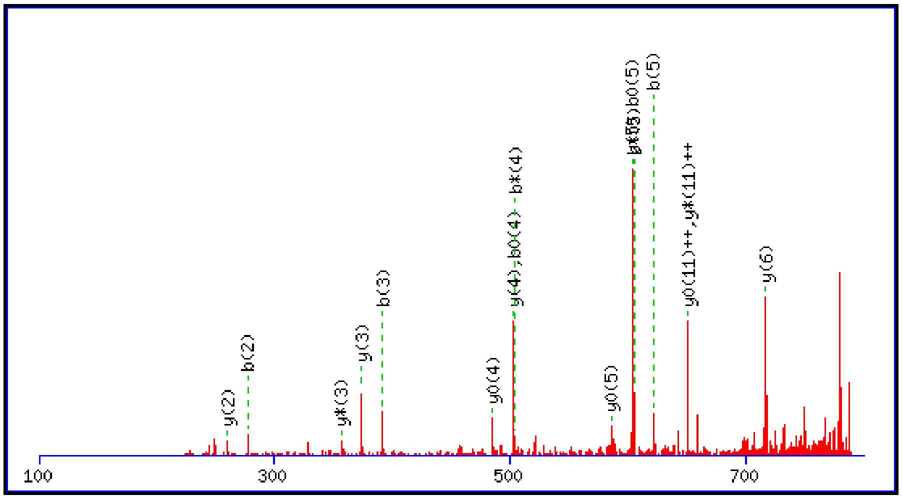

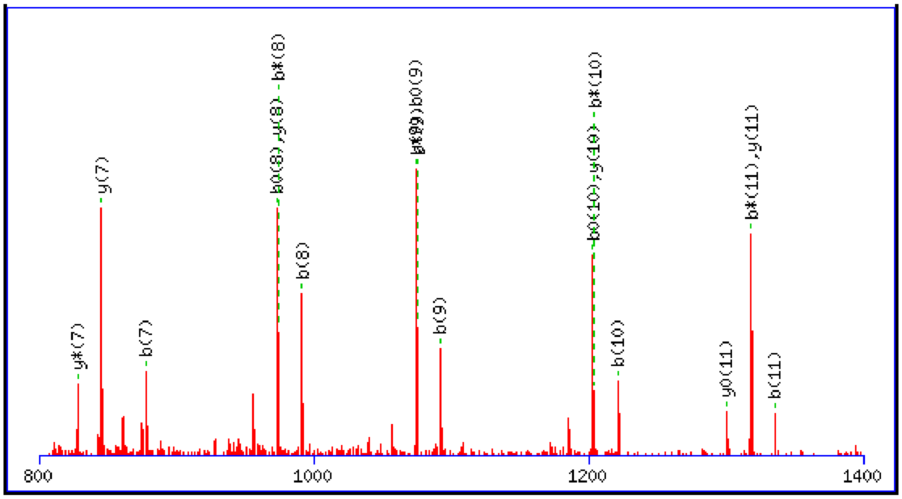


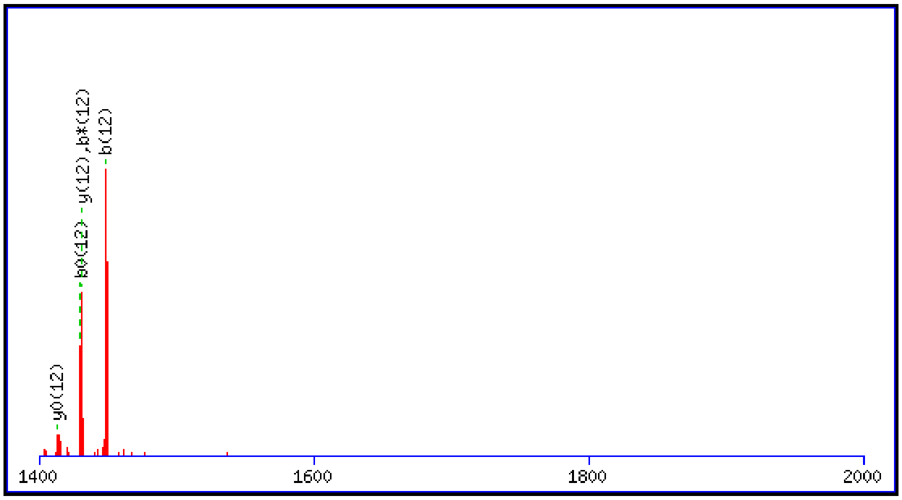


**Monoisotopic mass of neutral peptide Mr(calc):** 1593.7886
**Fixed modifications:** Carbamidomethyl (C)
**Variable modifications:**
**N3** : Deamidated_N (N)
**Ions Score:** 99 **Expect:** 2e-008
**Matches (Bold Red):** 41/128 fragment ions using 45 most intense peaks

| # | b | $b^{++}$ | b* | $b^{*++}$ | $b^{0}$ | $b^{0++}$ | Seq. | y | $y^{++}$ | y* | $y^{*++}$ | $y^{0}$ | $y^{0++}$ | # |
|---|---|---|---|---|---|---|---|---|---|---|---|---|---|---|
| 1 | 164.0706 | 82.5389 | | | | | Y | | | | | | | 13 |
| 2 | **277.1547** | 139.0810 | | | | | L | **1431.7326** | 716.3699 | 1414.7060 | 707.8566 | **1413.7220** | 707.3646 | 12 |
| 3 | **392.1816** | 196.5944 | 375.1551 | 188.0812 | | | N | **1318.6485** | 659.8279 | 1301.6219 | **651.3146** | **1300.6379** | **650.8226** | 11 |
| 4 | 521.2242 | 261.1157 | **504.1976** | 252.6025 | **503.2136** | 252.1105 | E | **1203.6216** | 602.3144 | 1186.5950 | 593.8011 | 1185.6110 | 593.3091 | 10 |
| 5 | **622.2719** | 311.6396 | **605.2453** | 303.1263 | **604.2613** | 302.6343 | T | **1074.5790** | 537.7931 | 1057.5524 | 529.2798 | 1056.5684 | 528.7878 | 9 |
| 6 | 750.3305 | 375.6689 | 733.3039 | 367.1556 | 732.3199 | 366.6636 | Q | **973.5313** | 487.2693 | 956.5047 | 478.7560 | 955.5207 | 478.2640 | 8 |
| 7 | **878.3890** | 439.6982 | 861.3625 | 431.1849 | 860.3785 | 430.6929 | Q | **845.4727** | 423.2400 | **828.4462** | 414.7267 | 827.4621 | 414.2347 | 7 |
| 8 | **991.4731** | 496.2402 | **974.4465** | 487.7269 | **973.4625** | 487.2349 | L | **717.4141** | 359.2107 | 700.3876 | 350.6974 | 699.4036 | 350.2054 | 6 |
| 9 | **1092.5208** | 546.7640 | **1075.4942** | 538.2508 | **1074.5102** | 537.7587 | T | **604.3301** | 302.6687 | 587.3035 | 294.1554 | **586.3195** | 293.6634 | 5 |
| 10 | **1221.5634** | 611.2853 | **1204.5368** | 602.7720 | **1203.5528** | 602.2800 | E | **503.2824** | 252.1448 | 486.2558 | 243.6316 | **485.2718** | 243.1395 | 4 |
| 11 | **1335.6063** | 668.3068 | **1318.5797** | 659.7935 | 1317.5957 | 659.3015 | N | **374.2398** | 187.6235 | **357.2132** | 179.1103 | | | 3 |
| 12 | **1448.6904** | 724.8488 | **1431.6638** | 716.3355 | **1430.6798** | 715.8435 | I | **260.1969** | 130.6021 | 243.1703 | 122.0888 | | | 2 |
| 13 | | | | | | | K | 147.1128 | 74.0600 | 130.0863 | 65.5468 | | | 1 |

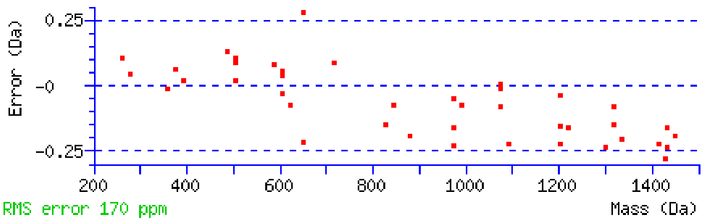


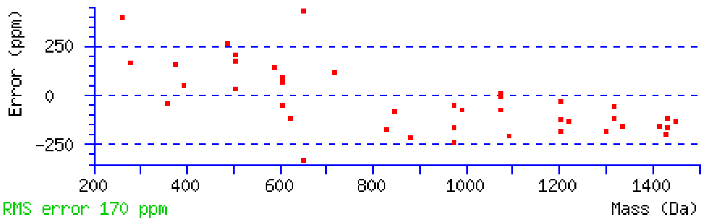


### All matches to this query

| Score | Mr(calc): | Delta | Sequence |
|---|---|---|---|
| 99.0 | 1593.7886 | -0.0001 | YLNETQQLTENIK |
| 66.3 | 1593.7886 | -0.0001 | YLNETQQLTENIK |
| 29.1 | 1593.7763 | 0.0122 | EQARISLLNDGGLK |
| 12.9 | 1593.7738 | 0.0147 | QNMQHQLYVILK |
| 9.8 | 1592.7923 | 0.9962 | EQARISLLNDGGLK |
| 9.6 | 1593.7928 | -0.0043 | IICTTQMLENMIK |
| 7.3 | 1592.7976 | 0.9909 | MKLELNTMQLPMK |
| 7.1 | 1592.7981 | 0.9905 | KMPETFSNLPRTR |
| 5.9 | 1593.7988 | -0.0103 | LTRGNRESVQINK |
| 5.8 | 1593.7933 | -0.0048 | GNDAVMTRGTFANIK |

---

Spectrum No: 85; Query: 859; Rank: 1

---

## Peptide View

MS/MS Fragmentation of **ESNSTSLTQAALLEK**
Found in **IPI00199867**, Tax_Id=10116 Gene_Symbol=Emilin1_predicted elastin microfibril interfacer 1

Match to Query 859: 1591.793788 from(796.904170,2+)
Title: 091008RatKidney_NH4Format01_24.2113.2113.2.dta
Data file K:\NewmanPaper\Piliang\3SubProteomes\Piliang3SP\mgf5ppm\ERLIC_3SubProteomes5ppm.mgf

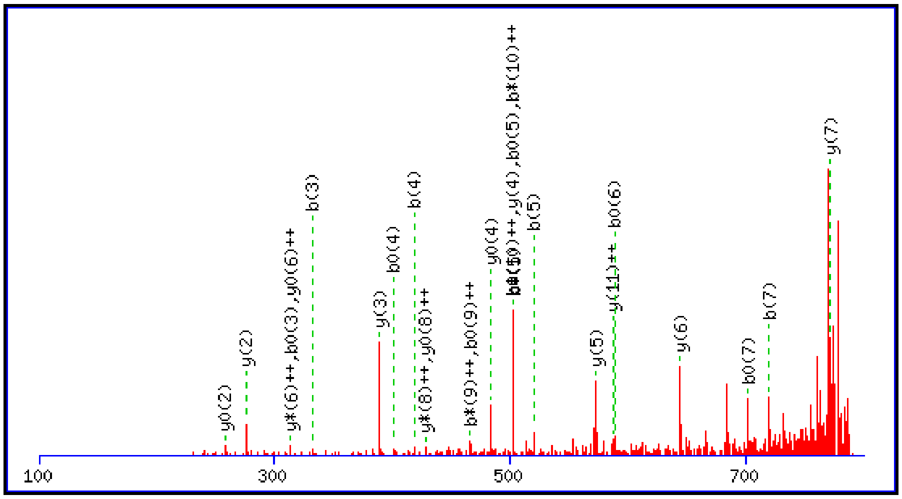

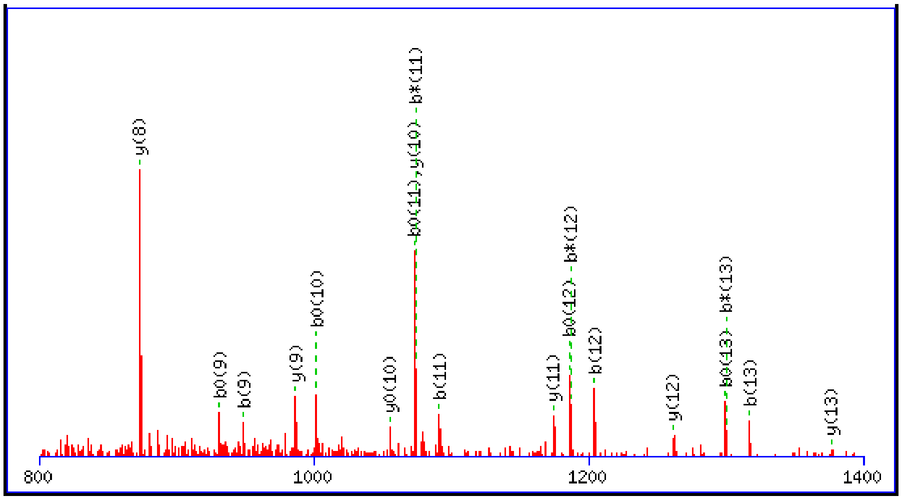


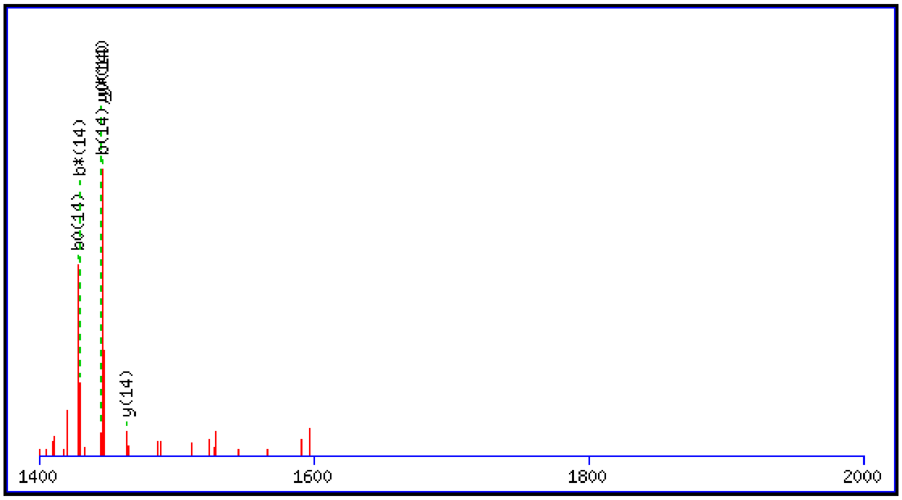


**Monoisotopic mass of neutral peptide Mr(calc):** 1591.7941
**Fixed modifications:** Carbamidomethyl (C)
**Variable modifications:**
**N3** : Deamidated_N (N)
**Ions Score:** 99 **Expect:** 2.1e-008
**Matches (Bold Red):** 52/162 fragment ions using 75 most intense peaks

| # | b | $b^{++}$ | b* | $b^{*++}$ | $b^0$ | $b^{0++}$ | Seq. | y | $y^{++}$ | y* | $y^{*++}$ | $y^0$ | $y^{0++}$ | # |
|---|---|---|---|---|---|---|---|---|---|---|---|---|---|---|
| 1 | 130.0499 | 65.5286 | | | 112.0393 | 56.5233 | E | | | | | | | 15 |
| 2 | 217.0819 | 109.0446 | | | 199.0713 | 100.0393 | S | **1463.7588** | 732.3830 | **1446.7322** | 723.8697 | **1445.7482** | 723.3777 | 14 |
| 3 | **332.1088** | 166.5581 | 315.0823 | 158.0448 | **314.0983** | 157.5528 | N | **1376.7267** | 688.8670 | 1359.7002 | 680.3537 | 1358.7162 | 679.8617 | 13 |
| 4 | **419.1409** | 210.0741 | 402.1143 | 201.5608 | **401.1303** | 201.0688 | S | **1261.6998** | 631.3535 | 1244.6733 | 622.8403 | 1243.6892 | 622.3483 | 12 |
| 5 | **520.1885** | 260.5979 | **503.1620** | 252.0846 | **502.1780** | 251.5926 | T | **1174.6678** | **587.8375** | 1157.6412 | 579.3243 | 1156.6572 | 578.8322 | 11 |
| 6 | 607.2206 | 304.1139 | 590.1940 | 295.6006 | **589.2100** | 295.1086 | S | **1073.6201** | 537.3137 | 1056.5936 | 528.8004 | **1055.6095** | 528.3084 | 10 |
| 7 | **720.3046** | 360.6560 | 703.2781 | 352.1427 | **702.2941** | 351.6507 | L | **986.5881** | 493.7977 | 969.5615 | 485.2844 | 968.5775 | 484.7924 | 9 |
| 8 | 821.3523 | 411.1798 | 804.3258 | 402.6665 | 803.3417 | 402.1745 | T | **873.5040** | 437.2556 | 856.4775 | **428.7424** | 855.4934 | **428.2504** | 8 |
| 9 | **949.4109** | 475.2091 | 932.3843 | **466.6958** | **931.4003** | **466.2038** | Q | **772.4563** | 386.7318 | 755.4298 | 378.2185 | 754.4458 | 377.7265 | 7 |
| 10 | 1020.4480 | 510.7276 | 1003.4215 | **502.2144** | **1002.4374** | **501.7224** | A | **644.3978** | 322.7025 | 627.3712 | **314.1892** | 626.3872 | **313.6972** | 6 |
| 11 | **1091.4851** | 546.2462 | **1074.4586** | 537.7329 | **1073.4746** | 537.2409 | A | **573.3606** | 287.1840 | 556.3341 | 278.6707 | 555.3501 | 278.1787 | 5 |
| 12 | **1204.5692** | 602.7882 | **1187.5426** | 594.2750 | **1186.5586** | 593.7829 | L | **502.3235** | 251.6654 | 485.2970 | 243.1521 | **484.3130** | 242.6601 | 4 |
| 13 | **1317.6532** | 659.3303 | **1300.6267** | 650.8170 | **1299.6427** | 650.3250 | L | **389.2395** | 195.1234 | 372.2129 | 186.6101 | 371.2289 | 186.1181 | 3 |
| 14 | **1446.6958** | 723.8516 | **1429.6693** | 715.3383 | **1428.6853** | 714.8463 | E | **276.1554** | 138.5813 | 259.1288 | 130.0681 | **258.1448** | 129.5761 | 2 |
| 15 | | | | | | | K | 147.1128 | 74.0600 | 130.0863 | 65.5468 | | | 1 |

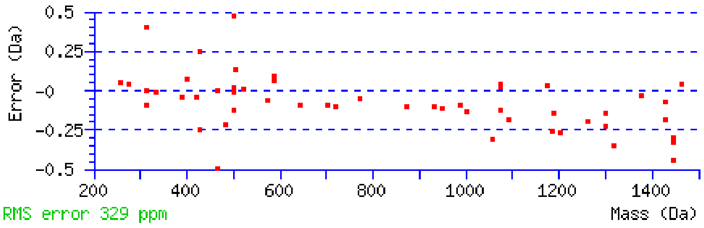


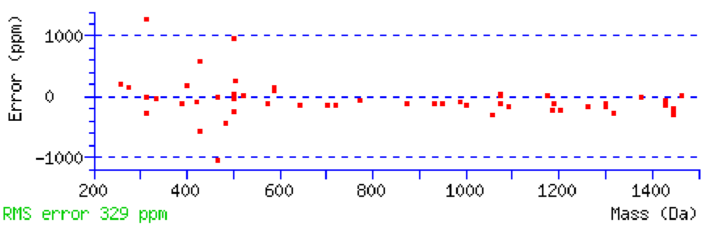


### All matches to this query

| Score | Mr(calc): | Delta | Sequence |
|---|---|---|---|
| 98.8 | 1591.7941 | -0.0003 | ESNSTSLTQAALLEK |
| 26.5 | 1591.7828 | 0.0110 | TSSLLILTENENEK |
| 18.7 | 1589.7970 | 1.9968 | GESMALNEKQILEK |
| 16.0 | 1589.7970 | 1.9968 | GEAMALNEKQILEK |
| 11.9 | 1591.8062 | -0.0124 | MKLMRSEGPDVSVK |
| 11.7 | 1591.7945 | -0.0008 | GNYRFMKLLLTR |
| 10.5 | 1591.8053 | -0.0115 | EKVAGEDVGTSSSAKK |
| 10.3 | 1590.7988 | 0.9950 | TSSLLILTENENEK |
| 7.5 | 1591.7810 | 0.0128 | CNKVKPMEGRTEK |
| 6.5 | 1590.8018 | 0.9920 | LALSLEPSQPSLTR |

---

Spectrum No: 86; Query: 820; Rank: 1

---

## Peptide View

MS/MS Fragmentation of **KNSTLCDLCIGPAK**
Found in **IPI00196656**, Tax_Id=10116 Gene_Symbol=Srprb Ba1-667

Match to Query 820: 1576.762428 from(789.388490,2+)
Title: 100101RatKid_NS_deglyco_24.2039.2039.2.dta
Data file K:\NewmanPaper\Piliang\3SubProteomes\Piliang3SP\mgf5ppm\ERLIC_3SubProteomes5ppm.mgf

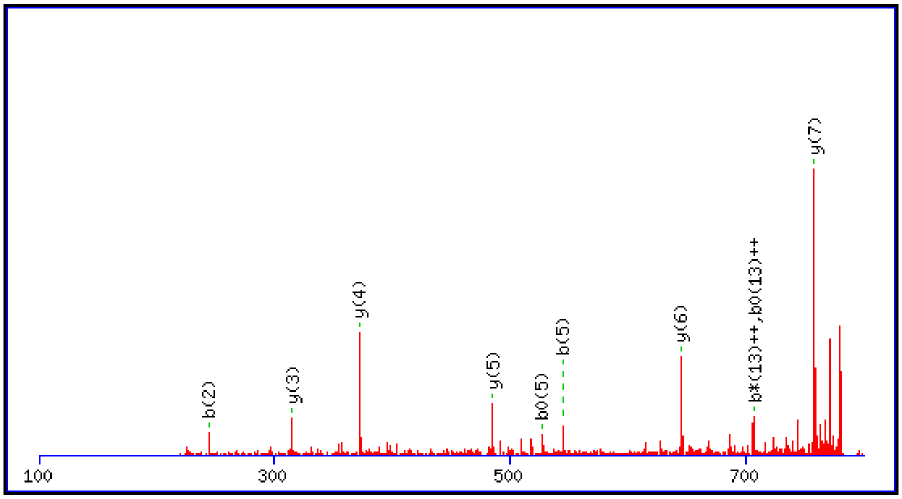

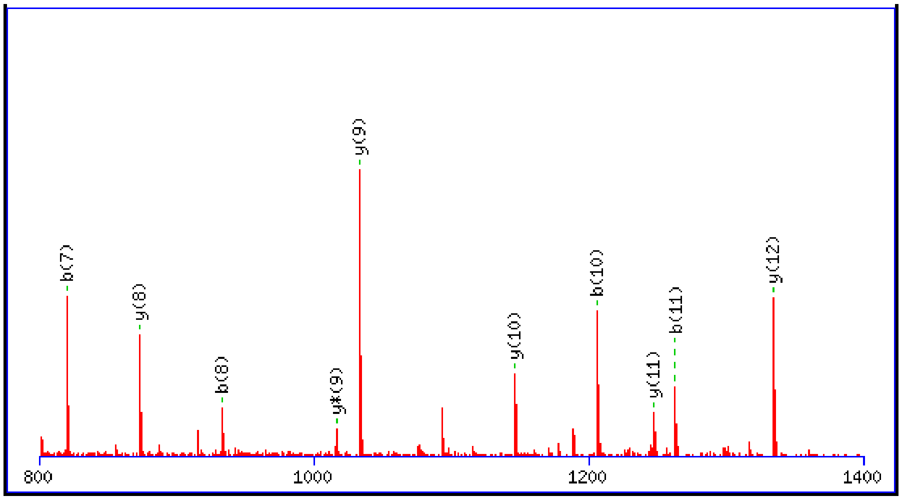


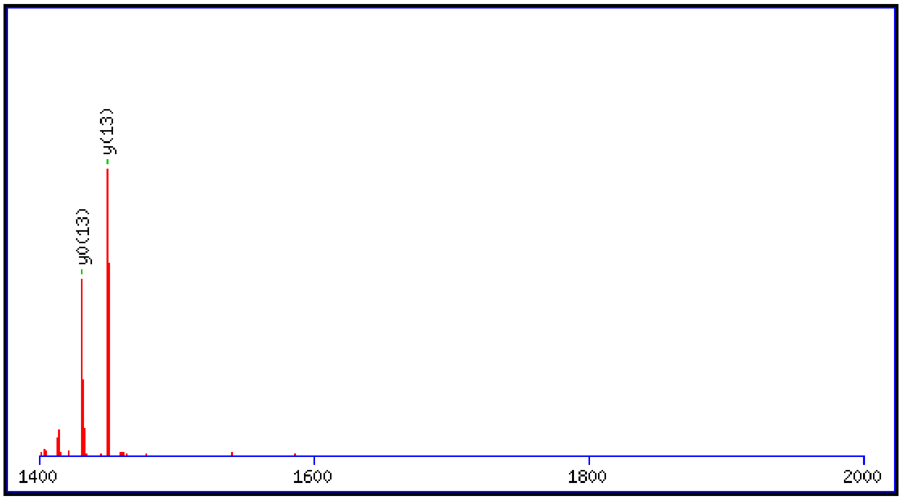


**Monoisotopic mass of neutral peptide Mr(calc):** 1576.7589
**Fixed modifications:** Carbamidomethyl (C)
**Variable modifications:**
**N2** : Deamidated_N (N)
**Ions Score:** 99 **Expect:** 2.4e-008
**Matches (Bold Red):** 23/138 fragment ions using 30 most intense peaks

| # | b | $b^{++}$ | b* | $b^{*++}$ | $b^{0}$ | $b^{0++}$ | Seq. | y | $y^{++}$ | y* | $y^{*++}$ | $y^{0}$ | $y^{0++}$ | # |
|---|---|---|---|---|---|---|---|---|---|---|---|---|---|---|
| 1 | 129.1022 | 65.0548 | 112.0757 | 56.5415 | | | K | | | | | | | 14 |
| 2 | **244.1292** | 122.5682 | 227.1026 | 114.0550 | | | N | **1449.6712** | 725.3392 | 1432.6447 | 716.8260 | **1431.6607** | 716.3340 | 13 |
| 3 | 331.1612 | 166.0842 | 314.1347 | 157.5710 | 313.1506 | 157.0790 | S | **1334.6443** | 667.8258 | 1317.6177 | 659.3125 | 1316.6337 | 658.8205 | 12 |
| 4 | 432.2089 | 216.6081 | 415.1823 | 208.0948 | 414.1983 | 207.6028 | T | **1247.6123** | 624.3098 | 1230.5857 | 615.7965 | 1229.6017 | 615.3045 | 11 |
| 5 | **545.2929** | 273.1501 | 528.2664 | 264.6368 | **527.2824** | 264.1448 | L | **1146.5646** | 573.7859 | 1129.5380 | 565.2727 | 1128.5540 | 564.7806 | 10 |
| 6 | 705.3236 | 353.1654 | 688.2970 | 344.6522 | 687.3130 | 344.1602 | C | **1033.4805** | 517.2439 | **1016.4540** | 508.7306 | 1015.4699 | 508.2386 | 9 |
| 7 | **820.3505** | 410.6789 | 803.3240 | 402.1656 | 802.3400 | 401.6736 | D | **873.4499** | 437.2286 | 856.4233 | 428.7153 | 855.4393 | 428.2233 | 8 |
| 8 | **933.4346** | 467.2209 | 916.4081 | 458.7077 | 915.4240 | 458.2157 | L | **758.4229** | 379.7151 | 741.3964 | 371.2018 | | | 7 |
| 9 | 1093.4653 | 547.2363 | 1076.4387 | 538.7230 | 1075.4547 | 538.2310 | C | **645.3389** | 323.1731 | 628.3123 | 314.6598 | | | 6 |
| 10 | **1206.5493** | 603.7783 | 1189.5228 | 595.2650 | 1188.5387 | 594.7730 | I | **485.3082** | 243.1577 | 468.2817 | 234.6445 | | | 5 |
| 11 | **1263.5708** | 632.2890 | 1246.5442 | 623.7758 | 1245.5602 | 623.2837 | G | **372.2241** | 186.6157 | 355.1976 | 178.1024 | | | 4 |
| 12 | 1360.6235 | 680.8154 | 1343.5970 | 672.3021 | 1342.6130 | 671.8101 | P | **315.2027** | 158.1050 | 298.1761 | 149.5917 | | | 3 |
| 13 | **1431.6607** | 716.3340 | 1414.6341 | **707.8207** | 1413.6501 | **707.3287** | A | 218.1499 | 109.5786 | 201.1234 | 101.0653 | | | 2 |
| 14 | | | | | | | K | 147.1128 | 74.0600 | 130.0863 | 65.5468 | | | 1 |

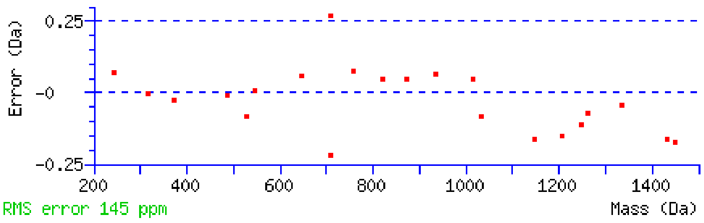


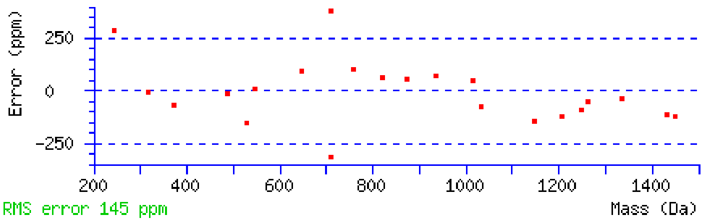


### All matches to this query

| Score | Mr(calc): | Delta | Sequence |
|---|---|---|---|
| 98.5 | 1576.7589 | 0.0035 | KNSTLCDLCIGPAK |
| 9.6 | 1576.7722 | -0.0098 | KDQSNLRQLNGPK |
| 8.3 | 1576.7498 | 0.0126 | QNSVADEVGLLPQK |
| 8.3 | 1576.7571 | 0.0053 | NSVAILMKNLYSK |
| 7.2 | 1576.7668 | -0.0043 | GGQELSWVSRPMSK |
| 6.4 | 1576.7780 | -0.0155 | KNEGVNWLRMASR |
| 5.2 | 1576.7498 | 0.0126 | FSGSSSGTTAILTIR |
| 5.2 | 1576.7538 | 0.0087 | QYEKSIVNYKPK |
| 4.7 | 1576.7538 | 0.0087 | QYEKSIVNYKPK |
| 4.5 | 1576.7555 | 0.0069 | ELEEEKMWAEKR |

---

Spectrum No: 87; Query: 1961; Rank: 1

---

## Peptide View

MS/MS Fragmentation of **EDSCQLNYSEGPCLGMQQK**
Found in **IPI00210900**, Tax_Id=10116 Gene_Symbol=Ambp AMBP protein precursor

Match to Query 1961: 2259.910208 from(1130.962380,2+)
Title: 091008RatKidney_NoSalt_31.1573.1573.2.dta
Data file K:\NewmanPaper\Piliang\3SubProteomes\Piliang3SP\mgf5ppm\ERLIC_3SubProteomes5ppm.mgf

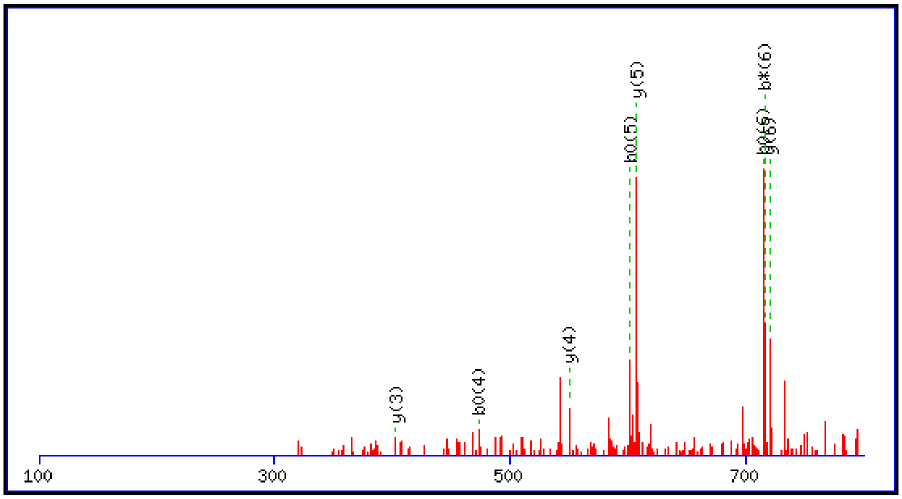

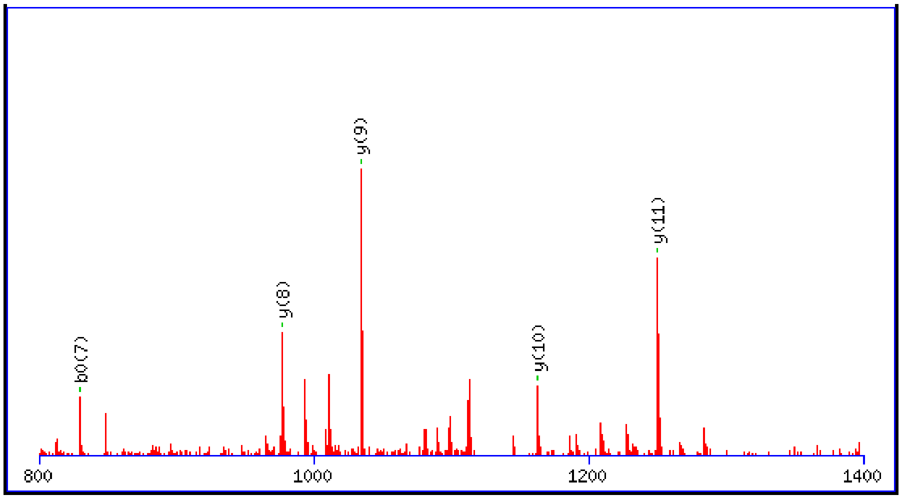


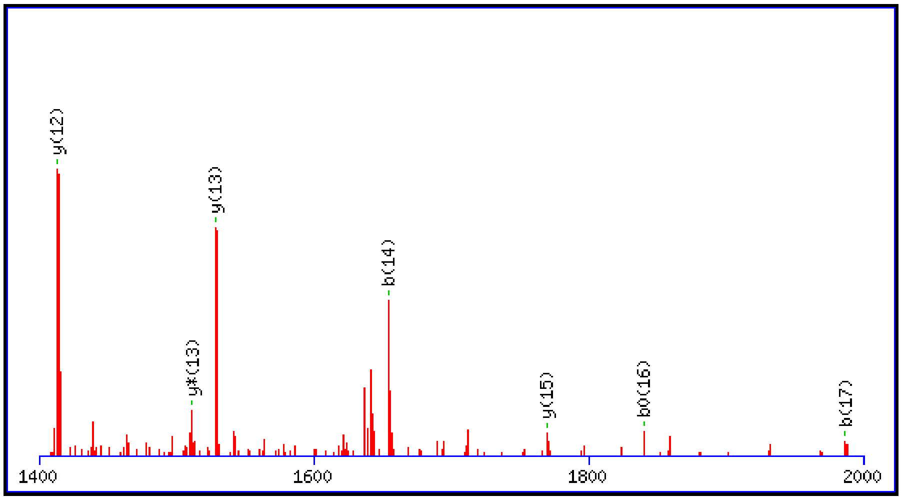


**Monoisotopic mass of neutral peptide Mr(calc):** 2259.9082
**Fixed modifications:** Carbamidomethyl (C)
**Variable modifications:**
**N7** : Deamidated_N (N)
**M16** : Oxidation (M)
**Ions Score:** 98 **Expect:** 3e-008
**Matches (Bold Red):** 20/190 fragment ions using 35 most intense peaks

| # | b | $b^{++}$ | b* | $b^{*++}$ | $b^0$ | $b^{0++}$ | Seq. | y | $y^{++}$ | y* | $y^{*++}$ | $y^0$ | $y^{0++}$ | # |
|---|---|---|---|---|---|---|---|---|---|---|---|---|---|---|
| 1 | 130.0499 | 65.5286 | | | 112.0393 | 56.5233 | E | | | | | | | 19 |
| 2 | 245.0768 | 123.0420 | | | 227.0662 | 114.0368 | D | 2131.8729 | 1066.4401 | 2114.8464 | 1057.9268 | 2113.8624 | 1057.4348 | 18 |
| 3 | 332.1088 | 166.5581 | | | 314.0983 | 157.5528 | S | 2016.8460 | 1008.9266 | 1999.8194 | 1000.4134 | 1998.8354 | 999.9213 | 17 |
| 4 | 492.1395 | 246.5734 | | | **474.1289** | 237.5681 | C | 1929.8139 | 965.4106 | 1912.7874 | 956.8973 | 1911.8034 | 956.4053 | 16 |
| 5 | 620.1981 | 310.6027 | 603.1715 | 302.0894 | **602.1875** | 301.5974 | Q | **1769.7833** | 885.3953 | 1752.7567 | 876.8820 | 1751.7727 | 876.3900 | 15 |
| 6 | 733.2821 | 367.1447 | **716.2556** | 358.6314 | **715.2716** | 358.1394 | L | 1641.7247 | 821.3660 | 1624.6982 | 812.8527 | 1623.7142 | 812.3607 | 14 |
| 7 | 848.3091 | 424.6582 | 831.2825 | 416.1449 | **830.2985** | 415.6529 | N | **1528.6407** | 764.8240 | **1511.6141** | 756.3107 | 1510.6301 | 755.8187 | 13 |
| 8 | 1011.3724 | 506.1898 | 994.3458 | 497.6766 | 993.3618 | 497.1846 | Y | **1413.6137** | 707.3105 | 1396.5872 | 698.7972 | 1395.6032 | 698.3052 | 12 |
| 9 | 1098.4044 | 549.7059 | 1081.3779 | 541.1926 | 1080.3939 | 540.7006 | S | **1250.5504** | 625.7788 | 1233.5238 | 617.2656 | 1232.5398 | 616.7736 | 11 |
| 10 | 1227.4470 | 614.2271 | 1210.4205 | 605.7139 | 1209.4365 | 605.2219 | E | **1163.5184** | 582.2628 | 1146.4918 | 573.7495 | 1145.5078 | 573.2575 | 10 |
| 11 | 1284.4685 | 642.7379 | 1267.4419 | 634.2246 | 1266.4579 | 633.7326 | G | **1034.4758** | 517.7415 | 1017.4492 | 509.2282 | | | 9 |
| 12 | 1381.5212 | 691.2643 | 1364.4947 | 682.7510 | 1363.5107 | 682.2590 | P | **977.4543** | 489.2308 | 960.4278 | 480.7175 | | | 8 |
| 13 | 1541.5519 | 771.2796 | 1524.5253 | 762.7663 | 1523.5413 | 762.2743 | C | 880.4015 | 440.7044 | 863.3750 | 432.1911 | | | 7 |
| 14 | **1654.6360** | 827.8216 | 1637.6094 | 819.3083 | 1636.6254 | 818.8163 | L | **720.3709** | 360.6891 | 703.3443 | 352.1758 | | | 6 |
| 15 | 1711.6574 | 856.3323 | 1694.6309 | 847.8191 | 1693.6469 | 847.3271 | G | **607.2868** | 304.1471 | 590.2603 | 295.6338 | | | 5 |
| 16 | 1858.6928 | 929.8501 | 1841.6663 | 921.3368 | **1840.6823** | 920.8448 | M | **550.2654** | 275.6363 | 533.2388 | 267.1230 | | | 4 |
| 17 | **1986.7514** | 993.8793 | 1969.7249 | 985.3661 | 1968.7408 | 984.8741 | Q | **403.2300** | 202.1186 | 386.2034 | 193.6053 | | | 3 |
| 18 | 2114.8100 | 1057.9086 | 2097.7834 | 1049.3954 | 2096.7994 | 1048.9033 | Q | 275.1714 | 138.0893 | 258.1448 | 129.5761 | | | 2 |

| | | | | | | | | | | | | | |
|---|---|---|---|---|---|---|---|---|---|---|---|---|---|
| 19 | | | | | | | K | 147.1128 | 74.0600 | 130.0863 | 65.5468 | | 1 |

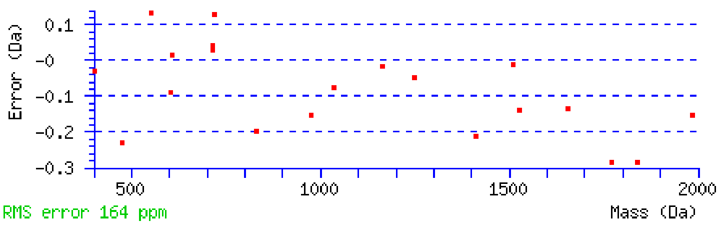


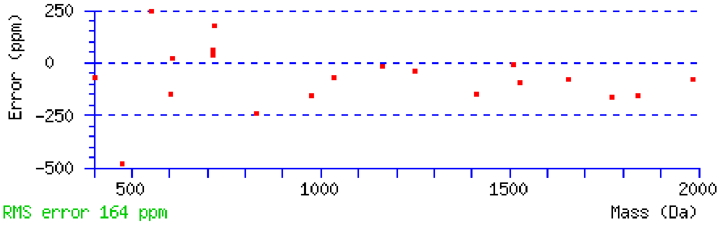


### All matches to this query

| Score | Mr(calc): | Delta | Sequence |
|---|---|---|---|
| 98.3 | 2259.9082 | 0.0020 | EDSCQLNYSEGPCLGMQQK |
| 40.3 | 2258.9242 | 0.9860 | EDSCQLNYSEGPCLGMQQK |
| 11.0 | 2259.9046 | 0.0056 | RPEEGTVDAQSSELSENPR |
| 7.1 | 2259.8951 | 0.0151 | MDSQNGDTEGEGRAEEGLIR |
| 4.5 | 2259.9281 | -0.0179 | ETPLPNNNHVASDAGETEER |
| 2.7 | 2259.8951 | 0.0151 | MDSQNGDTEGEGRAEEGLIR |
| 1.8 | 2257.9213 | 1.9889 | KPRPSCQGSAMALLKSCK |
| 1.0 | 2257.9157 | 1.9945 | QLSTEQAALQESLEKESK |
| 0.9 | 2258.9139 | 0.9963 | RPTSLPLNTKNSTKEPR |
| 0.7 | 2259.8997 | 0.0105 | STATVQICSGSVNLRGNVR |

---

Spectrum No: 88; Query: 2241; Rank: 1

---

## Peptide View

MS/MS Fragmentation of **YGMQNSGNEAAWNYTLEQYQK**
Found in **IPI00327398**, Tax_Id=10116 Gene_Symbol=Enpep Isoform 1 of Glutamyl aminopeptidase

Match to Query 2241: 2495.079988 from(1248.547270,2+)
Title: 100101RatKid_NS_deglyco_23.3857.3857.2.dta
Data file K:\NewmanPaper\Piliang\3SubProteomes\Piliang3SP\mgf5ppm\ERLIC_3SubProteomes5ppm.mgf

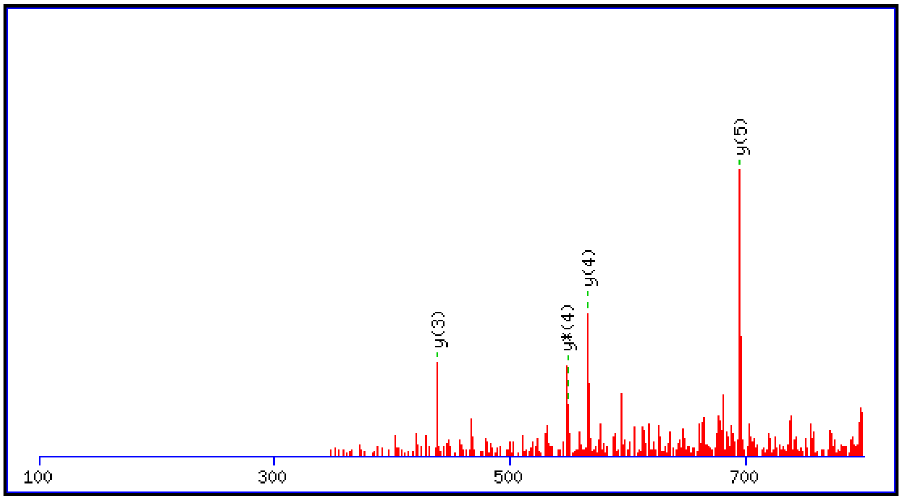

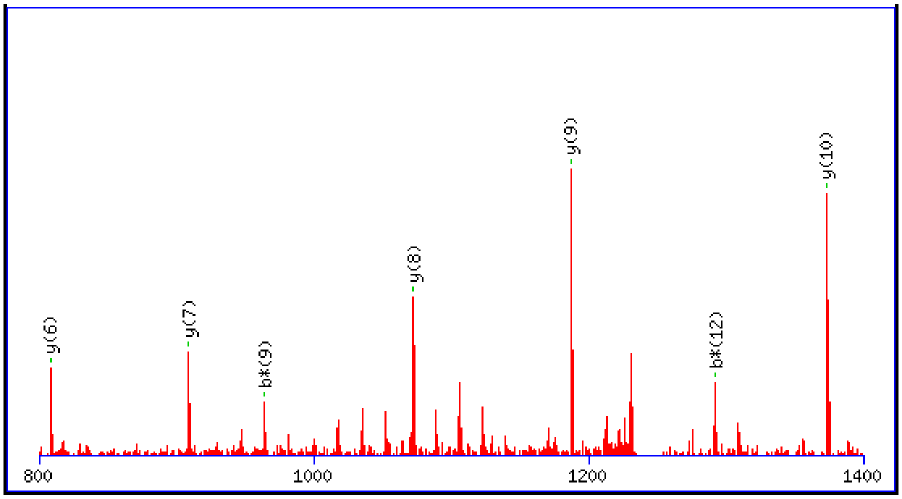


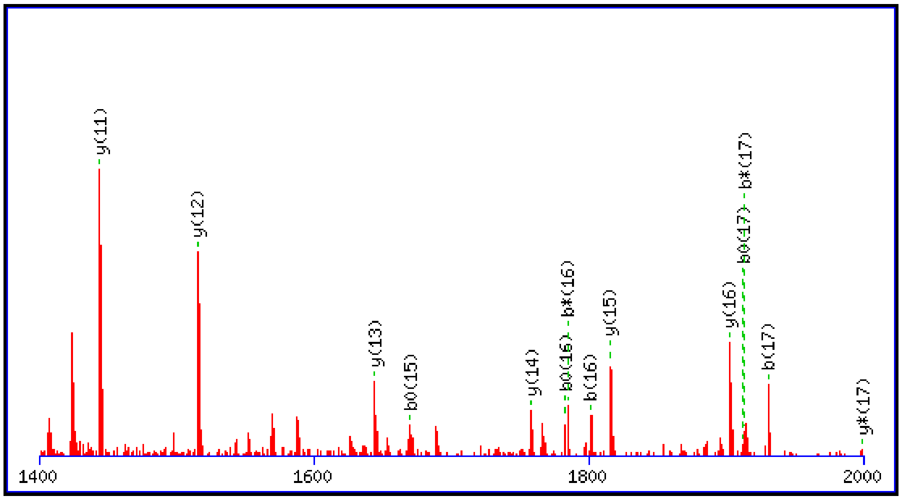


**Monoisotopic mass of neutral peptide Mr(calc):** 2495.0699
**Fixed modifications:** Carbamidomethyl (C)
**Variable modifications:**
**N13** : Deamidated_N (N)
**Ions Score:** 98 **Expect:** 4.5e-008
**Matches (Bold Red):** 25/216 fragment ions using 42 most intense peaks

| # | b | $b^{++}$ | b* | $b^{*++}$ | $b^0$ | $b^{0++}$ | Seq. | y | $y^{++}$ | y* | $y^{*++}$ | $y^0$ | $y^{0++}$ | # |
|---|---|---|---|---|---|---|---|---|---|---|---|---|---|---|
| 1 | 164.0706 | 82.5389 | | | | | Y | | | | | | | 21 |
| 2 | 221.0921 | 111.0497 | | | | | G | 2333.0139 | 1167.0106 | 2315.9873 | 1158.4973 | 2315.0033 | 1158.0053 | 20 |
| 3 | 352.1326 | 176.5699 | | | | | M | 2275.9924 | 1138.4999 | 2258.9659 | 1129.9866 | 2257.9819 | 1129.4946 | 19 |
| 4 | 480.1911 | 240.5992 | 463.1646 | 232.0859 | | | Q | 2144.9519 | 1072.9796 | 2127.9254 | 1064.4663 | 2126.9414 | 1063.9743 | 18 |
| 5 | 594.2341 | 297.6207 | 577.2075 | 289.1074 | | | N | 2016.8934 | 1008.9503 | **1999.8668** | 1000.4370 | 1998.8828 | 999.9450 | 17 |
| 6 | 681.2661 | 341.1367 | 664.2395 | 332.6234 | 663.2555 | 332.1314 | S | **1902.8504** | 951.9289 | 1885.8239 | 943.4156 | 1884.8399 | 942.9236 | 16 |
| 7 | 738.2876 | 369.6474 | 721.2610 | 361.1341 | 720.2770 | 360.6421 | G | **1815.8184** | 908.4128 | 1798.7919 | 899.8996 | 1797.8079 | 899.4076 | 15 |
| 8 | 852.3305 | 426.6689 | 835.3039 | 418.1556 | 834.3199 | 417.6636 | N | **1758.7970** | 879.9021 | 1741.7704 | 871.3888 | 1740.7864 | 870.8968 | 14 |
| 9 | 981.3731 | 491.1902 | **964.3465** | 482.6769 | 963.3625 | 482.1849 | E | **1644.7540** | 822.8807 | 1627.7275 | 814.3674 | 1626.7435 | 813.8754 | 13 |
| 10 | 1052.4102 | 526.7087 | 1035.3836 | 518.1955 | 1034.3996 | 517.7034 | A | **1515.7114** | 758.3594 | 1498.6849 | 749.8461 | 1497.7009 | 749.3541 | 12 |
| 11 | 1123.4473 | 562.2273 | 1106.4208 | 553.7140 | 1105.4367 | 553.2220 | A | **1444.6743** | 722.8408 | 1427.6478 | 714.3275 | 1426.6638 | 713.8355 | 11 |
| 12 | 1309.5266 | 655.2669 | **1292.5001** | 646.7537 | 1291.5160 | 646.2617 | W | **1373.6372** | 687.3222 | 1356.6107 | 678.8090 | 1355.6266 | 678.3170 | 10 |
| 13 | 1424.5535 | 712.7804 | 1407.5270 | 704.2671 | 1406.5430 | 703.7751 | N | **1187.5579** | 594.2826 | 1170.5313 | 585.7693 | 1169.5473 | 585.2773 | 9 |
| 14 | 1587.6169 | 794.3121 | 1570.5903 | 785.7988 | 1569.6063 | 785.3068 | Y | **1072.5310** | 536.7691 | 1055.5044 | 528.2558 | 1054.5204 | 527.7638 | 8 |
| 15 | 1688.6646 | 844.8359 | 1671.6380 | 836.3226 | **1670.6540** | 835.8306 | T | **909.4676** | 455.2375 | 892.4411 | 446.7242 | 891.4571 | 446.2322 | 7 |
| 16 | **1801.7486** | 901.3779 | **1784.7221** | 892.8647 | **1783.7381** | 892.3727 | L | **808.4199** | 404.7136 | 791.3934 | 396.2003 | 790.4094 | 395.7083 | 6 |
| 17 | **1930.7912** | 965.8992 | **1913.7647** | 957.3860 | **1912.7806** | 956.8940 | E | **695.3359** | 348.1716 | 678.3093 | 339.6583 | 677.3253 | 339.1663 | 5 |
| 18 | 2058.8498 | 1029.9285 | 2041.8232 | 1021.4153 | 2040.8392 | 1020.9233 | Q | **566.2933** | 283.6503 | **549.2667** | 275.1370 | | | 4 |
| | | | | | | | | | | | | | | |

| | | | | | | | | | | | | | |
|---|---|---|---|---|---|---|---|---|---|---|---|---|---|
| 19 | 2221.9131 | 1111.4602 | 2204.8866 | 1102.9469 | 2203.9026 | 1102.4549 | Y | 438.2347 | 219.6210 | 421.2082 | 211.1077 | | | 3 |
| 20 | 2349.9717 | 1175.4895 | 2332.9451 | 1166.9762 | 2331.9611 | 1166.4842 | Q | 275.1714 | 138.0893 | 258.1448 | 129.5761 | | | 2 |
| 21 | | | | | | | K | 147.1128 | 74.0600 | 130.0863 | 65.5468 | | | 1 |

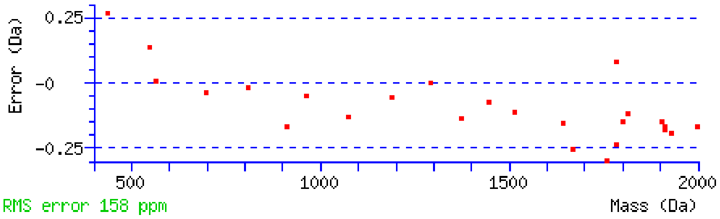


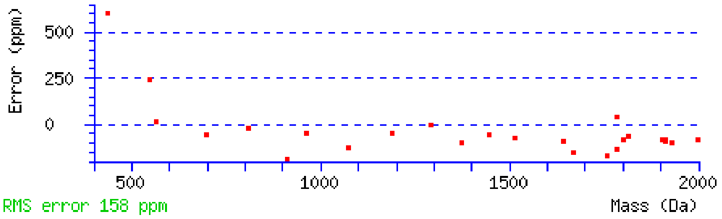


### All matches to this query

| Score | Mr(calc): | Delta | Sequence |
|---|---|---|---|
| 98.2 | 2495.0699 | 0.0101 | YGMQNSGNEAAWNYTLEQYQK |
| 39.4 | 2495.0699 | 0.0101 | YGMQNSGNEAAWNYTLEQYQK |
| 18.4 | 2495.0699 | 0.0101 | YGMQNSGNEAAWNYTLEQYQK |
| 16.4 | 2494.0859 | 0.9941 | YGMQNSGNEAAWNYTLEQYQK |
| 7.1 | 2495.1013 | -0.0213 | AFDMISQRSSGGHPGSGPQLGTGR |
| 0.8 | 2495.0553 | 0.0247 | GVHSGKMDENRFVAVTSTNAAK |
| 0.8 | 2495.0553 | 0.0247 | GVHSGKMDENRFVAVTSTNAAK |
| 0.8 | 2495.0553 | 0.0247 | GVHSGKMDENRFVAVTSTNAAK |

---

Spectrum No: 89; Query: 432; Rank: 1

---

## Peptide View

MS/MS Fragmentation of **AFINSSYTIDPK**
Found in **IPI00200067**, Tax_Id=10116 Gene_Symbol=F11r Junctional adhesion molecule A precursor

Match to Query 432: 1355.658988 from(678.836770,2+)
Title: 091008RatKidney_NoSalt_30.2083.2083.2.dta
Data file K:\NewmanPaper\Piliang\3SubProteomes\Piliang3SP\mgf5ppm\ERLIC_3SubProteomes5ppm.mgf

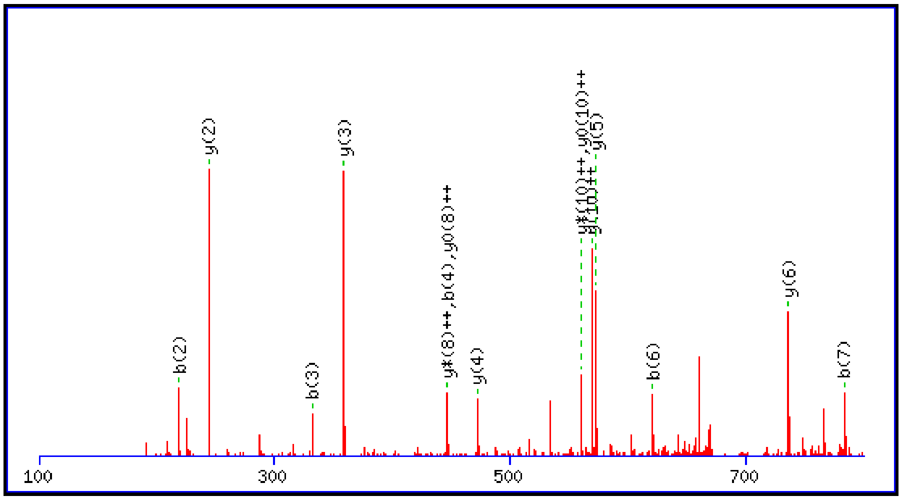

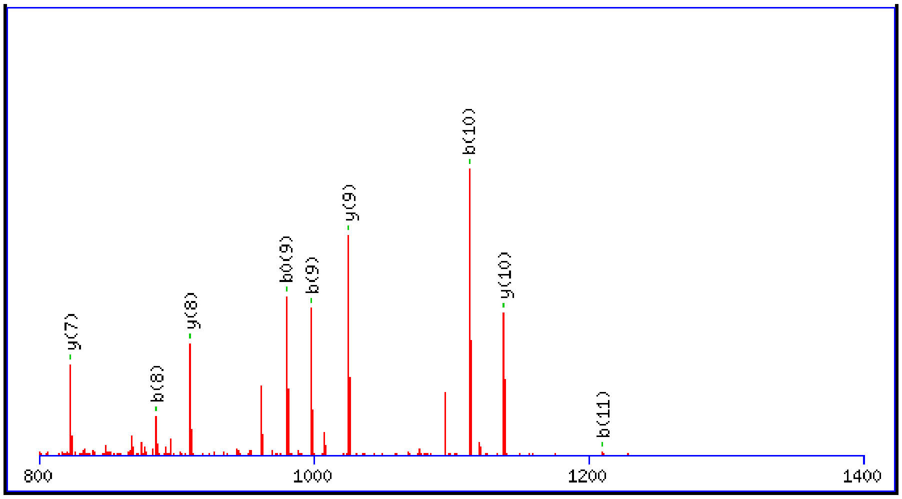


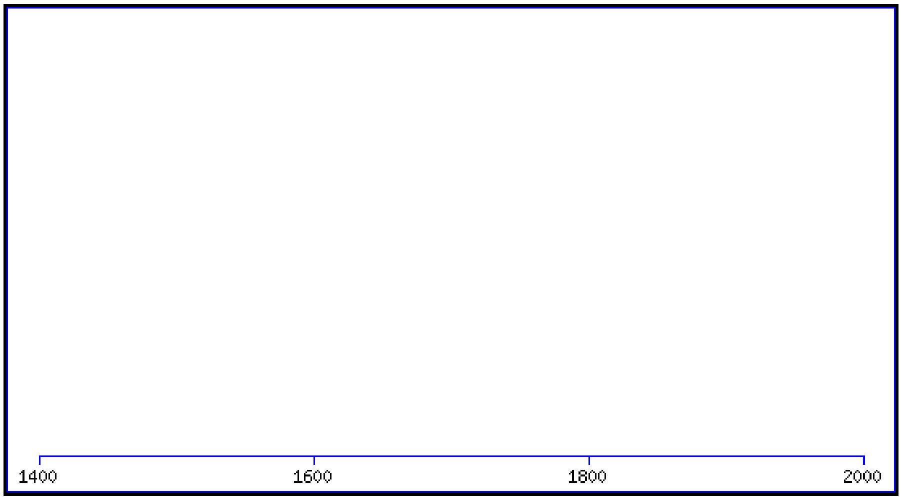


**Monoisotopic mass of neutral peptide Mr(calc):** 1355.6609
**Fixed modifications:** Carbamidomethyl (C)
**Variable modifications:**
**N4** : Deamidated_N (N)
**Ions Score:** 98 **Expect:** 2.2e-008
**Matches (Bold Red):** 24/114 fragment ions using 24 most intense peaks

| # | b | $b^{++}$ | b* | $b^{*++}$ | $b^{0}$ | $b^{0++}$ | Seq. | y | $y^{++}$ | y* | $y^{*++}$ | $y^{0}$ | $y^{0++}$ | # |
|---|---|---|---|---|---|---|---|---|---|---|---|---|---|---|
| 1 | 72.0444 | 36.5258 | | | | | A | | | | | | | 12 |
| 2 | **219.1128** | 110.0600 | | | | | F | 1285.6311 | 643.3192 | 1268.6045 | 634.8059 | 1267.6205 | 634.3139 | 11 |
| 3 | **332.1969** | 166.6021 | | | | | I | **1138.5626** | **569.7850** | 1121.5361 | **561.2717** | 1120.5521 | **560.7797** | 10 |
| 4 | **447.2238** | 224.1155 | 430.1973 | 215.6023 | | | N | **1025.4786** | 513.2429 | 1008.4520 | 504.7297 | 1007.4680 | 504.2376 | 9 |
| 5 | 534.2558 | 267.6316 | 517.2293 | 259.1183 | 516.2453 | 258.6263 | S | **910.4516** | 455.7295 | 893.4251 | **447.2162** | 892.4411 | **446.7242** | 8 |
| 6 | **621.2879** | 311.1476 | 604.2613 | 302.6343 | 603.2773 | 302.1423 | S | **823.4196** | 412.2134 | 806.3931 | 403.7002 | 805.4090 | 403.2082 | 7 |
| 7 | **784.3512** | 392.6792 | 767.3246 | 384.1660 | 766.3406 | 383.6739 | Y | **736.3876** | 368.6974 | 719.3610 | 360.1842 | 718.3770 | 359.6921 | 6 |
| 8 | **885.3989** | 443.2031 | 868.3723 | 434.6898 | 867.3883 | 434.1978 | T | **573.3243** | 287.1658 | 556.2977 | 278.6525 | 555.3137 | 278.1605 | 5 |
| 9 | **998.4829** | 499.7451 | 981.4564 | 491.2318 | **980.4724** | 490.7398 | I | **472.2766** | 236.6419 | 455.2500 | 228.1287 | 454.2660 | 227.6366 | 4 |
| 10 | **1113.5099** | 557.2586 | 1096.4833 | 548.7453 | 1095.4993 | 548.2533 | D | **359.1925** | 180.0999 | 342.1660 | 171.5866 | 341.1819 | 171.0946 | 3 |
| 11 | **1210.5626** | 605.7850 | 1193.5361 | 597.2717 | 1192.5521 | 596.7797 | P | **244.1656** | 122.5864 | 227.1390 | 114.0731 | | | 2 |
| 12 | | | | | | | K | 147.1128 | 74.0600 | 130.0863 | 65.5468 | | | 1 |

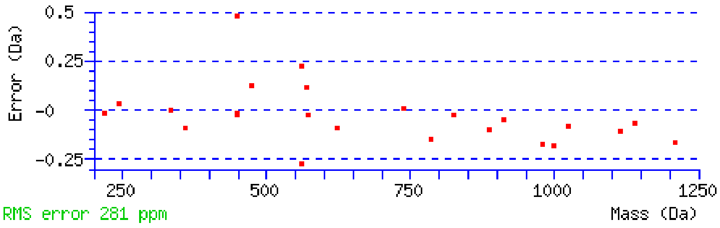


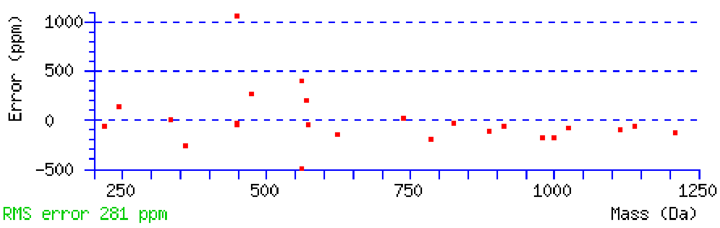


### All matches to this query

| Score | Mr(calc): | Delta | Sequence |
|---|---|---|---|
| 98.2 | 1355.6609 | -0.0019 | AFINSSYTIDPK |
| 12.7 | 1355.6520 | 0.0070 | KDMPTTKNTLK |
| 11.9 | 1355.6486 | 0.0104 | YDLSGRPLNIK |
| 11.4 | 1355.6520 | 0.0070 | KDMPTTKNTLK |
| 10.9 | 1355.6569 | 0.0021 | QNDVEIPSPTQK |
| 9.0 | 1355.6681 | -0.0091 | KNNLKDHEETK |
| 8.7 | 1355.6503 | 0.0087 | AKHTVSVCNNPK |
| 6.8 | 1355.6469 | 0.0120 | DHRXFLAENPK |
| 6.2 | 1355.6711 | -0.0121 | KTESHHKPKGK |
| 5.7 | 1355.6681 | -0.0091 | KNNKAEDEPSPK |

---

Spectrum No: 90; Query: 1234; Rank: 1

---

## Peptide View

MS/MS Fragmentation of **LDNTSMSTVIDGVVLPK**
Found in **IPI00195148**, Tax_Id=10116 Gene_Symbol=Es2 Liver carboxylesterase 1 precursor

Match to Query 1234: 1788.923948 from(895.469250,2+)
Title: 091008RatKidney_NoSalt_30.3470.3470.2.dta
Data file K:\NewmanPaper\Piliang\3SubProteomes\Piliang3SP\mgf5ppm\ERLIC_3SubProteomes5ppm.mgf

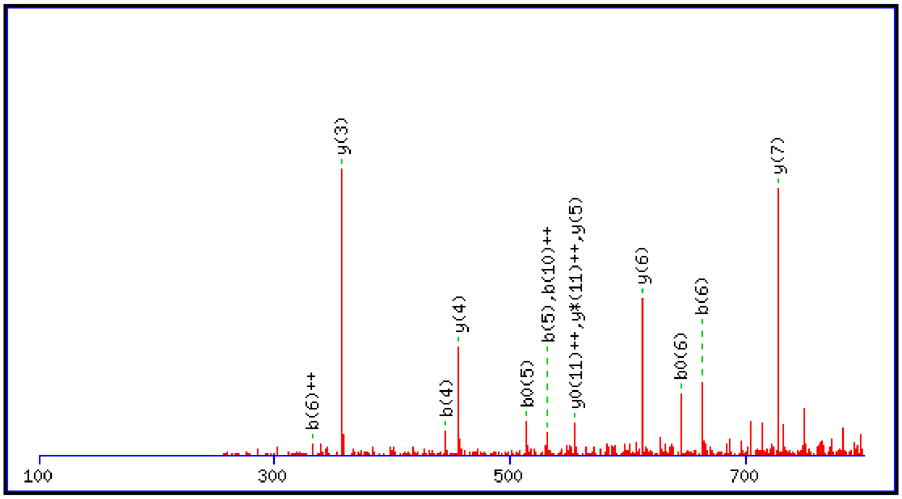

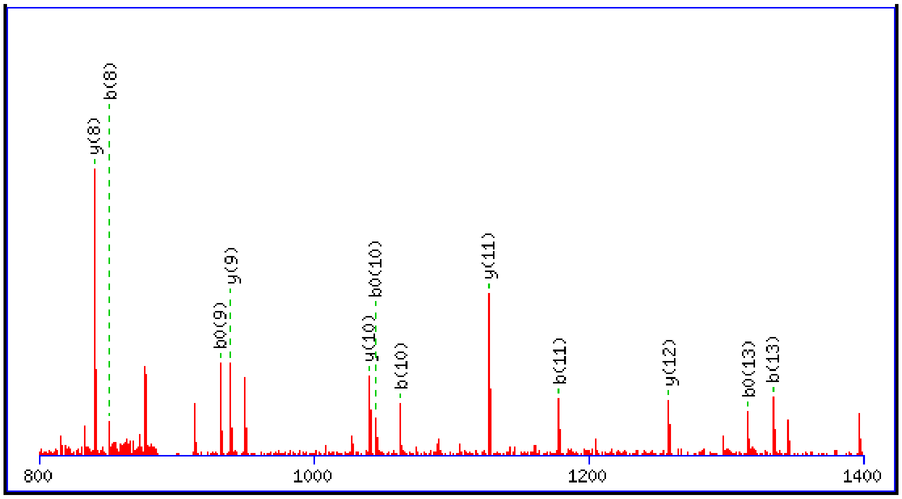


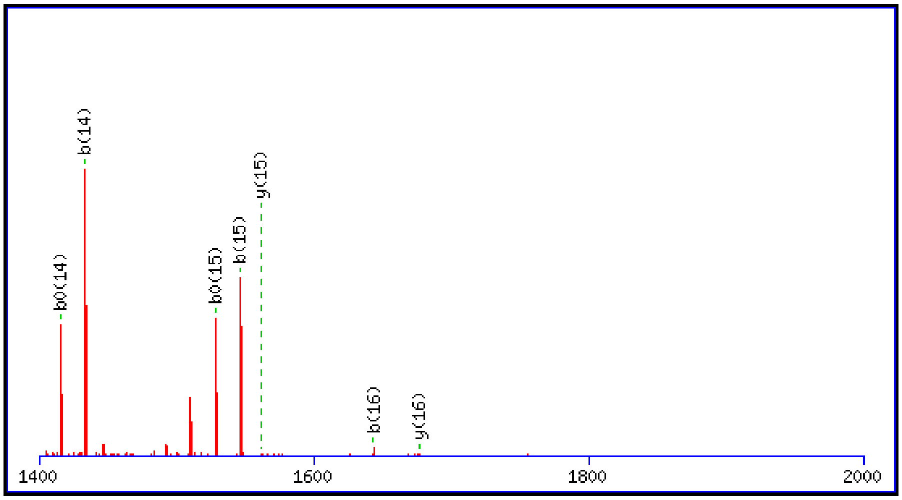


**Monoisotopic mass of neutral peptide Mr(calc):** 1788.9179
**Fixed modifications:** Carbamidomethyl (C)
**Variable modifications:**
**N3** : Deamidated_N (N)
**Ions Score:** 98 **Expect:** 2.3e-008
**Matches (Bold Red):** 33/174 fragment ions using 46 most intense peaks

| # | b | $b^{++}$ | b* | $b^{*++}$ | $b^0$ | $b^{0++}$ | Seq. | y | $y^{++}$ | y* | $y^{*++}$ | $y^0$ | $y^{0++}$ | # |
|---|---|---|---|---|---|---|---|---|---|---|---|---|---|---|
| 1 | 114.0913 | 57.5493 | | | | | L | | | | | | | 17 |
| 2 | 229.1183 | 115.0628 | | | 211.1077 | 106.0575 | D | **1676.8411** | 838.9242 | 1659.8146 | 830.4109 | 1658.8306 | 829.9189 | 16 |
| 3 | 344.1452 | 172.5762 | 327.1187 | 164.0630 | 326.1347 | 163.5710 | N | **1561.8142** | 781.4107 | 1544.7876 | 772.8975 | 1543.8036 | 772.4054 | 15 |
| 4 | **445.1929** | 223.1001 | 428.1663 | 214.5868 | 427.1823 | 214.0948 | T | 1446.7872 | 723.8973 | 1429.7607 | 715.3840 | 1428.7767 | 714.8920 | 14 |
| 5 | **532.2249** | 266.6161 | 515.1984 | 258.1028 | **514.2144** | 257.6108 | S | 1345.7396 | 673.3734 | 1328.7130 | 664.8601 | 1327.7290 | 664.3681 | 13 |
| 6 | **663.2654** | **332.1363** | 646.2389 | 323.6231 | **645.2548** | 323.1311 | M | **1258.7075** | 629.8574 | 1241.6810 | 621.3441 | 1240.6970 | 620.8521 | 12 |
| 7 | 750.2974 | 375.6524 | 733.2709 | 367.1391 | 732.2869 | 366.6471 | S | **1127.6671** | 564.3372 | 1110.6405 | **555.8239** | 1109.6565 | **555.3319** | 11 |
| 8 | **851.3451** | 426.1762 | 834.3186 | 417.6629 | 833.3346 | 417.1709 | T | **1040.6350** | 520.8212 | 1023.6085 | 512.3079 | 1022.6245 | 511.8159 | 10 |
| 9 | 950.4135 | 475.7104 | 933.3870 | 467.1971 | **932.4030** | 466.7051 | V | **939.5873** | 470.2973 | 922.5608 | 461.7840 | 921.5768 | 461.2920 | 9 |
| 10 | **1063.4976** | **532.2524** | 1046.4710 | 523.7392 | **1045.4870** | 523.2472 | I | **840.5189** | 420.7631 | 823.4924 | 412.2498 | 822.5084 | 411.7578 | 8 |
| 11 | **1178.5245** | 589.7659 | 1161.4980 | 581.2526 | 1160.5140 | 580.7606 | D | **727.4349** | 364.2211 | 710.4083 | 355.7078 | 709.4243 | 355.2158 | 7 |
| 12 | 1235.5460 | 618.2766 | 1218.5195 | 609.7634 | 1217.5354 | 609.2714 | G | **612.4079** | 306.7076 | 595.3814 | 298.1943 | | | 6 |
| 13 | **1334.6144** | 667.8108 | 1317.5879 | 659.2976 | **1316.6039** | 658.8056 | V | **555.3865** | 278.1969 | 538.3599 | 269.6836 | | | 5 |
| 14 | **1433.6828** | 717.3451 | 1416.6563 | 708.8318 | **1415.6723** | 708.3398 | V | **456.3180** | 228.6627 | 439.2915 | 220.1494 | | | 4 |
| 15 | **1546.7669** | 773.8871 | 1529.7403 | 765.3738 | **1528.7563** | 764.8818 | L | **357.2496** | 179.1285 | 340.2231 | 170.6152 | | | 3 |
| 16 | **1643.8197** | 822.4135 | 1626.7931 | 813.9002 | 1625.8091 | 813.4082 | P | 244.1656 | 122.5864 | 227.1390 | 114.0731 | | | 2 |
| 17 | | | | | | | K | 147.1128 | 74.0600 | 130.0863 | 65.5468 | | | 1 |

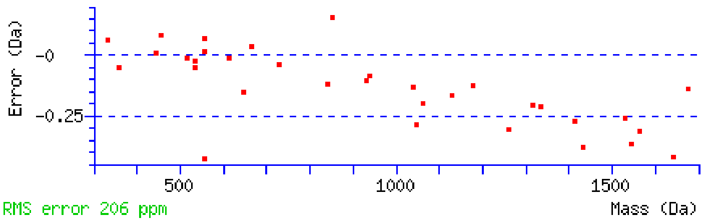


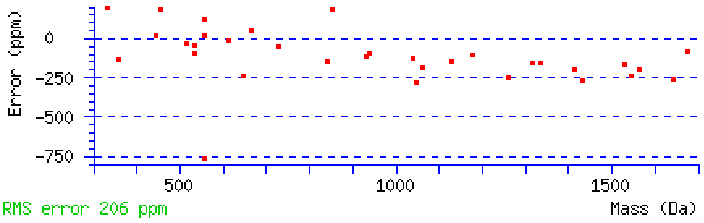


### All matches to this query

| Score | Mr(calc): | Delta | Sequence |
|---|---|---|---|
| 97.8 | 1788.9179 | 0.0060 | LDNTSMSTVIDGVVLPK |
| 45.7 | 1787.9339 | 0.9900 | LDNTSMSTVIDGVVLPK |
| 5.8 | 1788.9217 | 0.0023 | ILEQQNSSRTLEKNK |
| 1.5 | 1788.9291 | -0.0052 | DQLALAIGTLGENMSLK |

---

Spectrum No: 91; Query: 575; Rank: 1

---

## Peptide View

MS/MS Fragmentation of **VANLTSVVNSLDSK**
Found in **IPI00324102**, Tax_Id=10116 Gene_Symbol=Fgl2 fibrinogen-like 2

Match to Query 575: 1446.759988 from(724.387270,2+)
Title: 100101RatKid_NS_deglyco_23.3766.3766.2.dta
Data file K:\NewmanPaper\Piliang\3SubProteomes\Piliang3SP\mgf5ppm\ERLIC_3SubProteomes5ppm.mgf

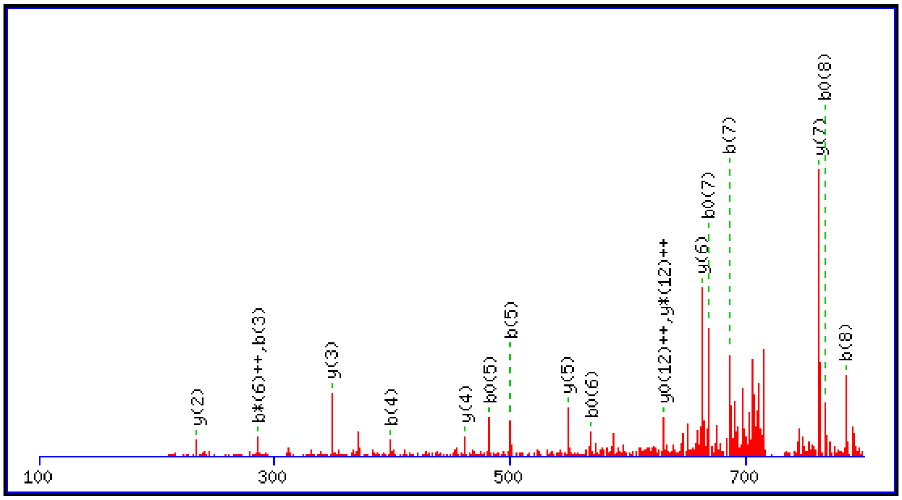

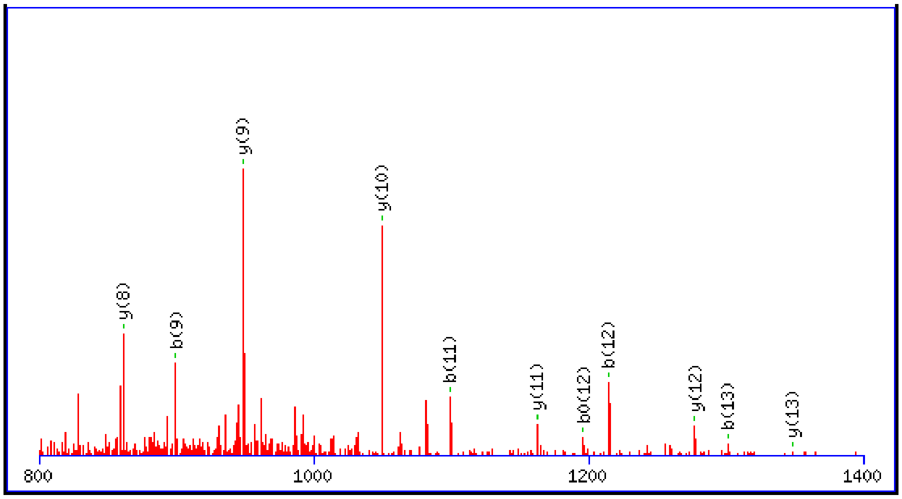


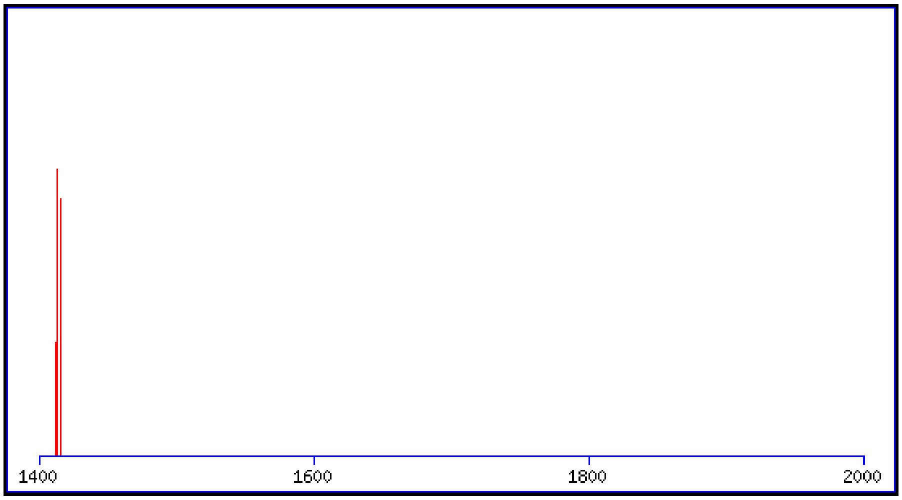


**Monoisotopic mass of neutral peptide Mr(calc):** 1446.7566
**Fixed modifications:** Carbamidomethyl (C)
**Variable modifications:**
**N3** : Deamidated_N (N)
**Ions Score:** 98 **Expect:** 2e-008
**Matches (Bold Red):** 29/142 fragment ions using 55 most intense peaks

| # | b | $b^{++}$ | b* | $b^{*++}$ | $b^{0}$ | $b^{0++}$ | Seq. | y | $y^{++}$ | y* | $y^{*++}$ | $y^{0}$ | $y^{0++}$ | # |
|---|---|---|---|---|---|---|---|---|---|---|---|---|---|---|
| 1 | 100.0757 | 50.5415 | | | | | V | | | | | | | 14 |
| 2 | 171.1128 | 86.0600 | | | | | A | **1348.6954** | 674.8514 | 1331.6689 | 666.3381 | 1330.6849 | 665.8461 | 13 |
| 3 | **286.1397** | 143.5735 | 269.1132 | 135.0602 | | | N | **1277.6583** | 639.3328 | 1260.6318 | **630.8195** | 1259.6478 | **630.3275** | 12 |
| 4 | **399.2238** | 200.1155 | 382.1973 | 191.6023 | | | L | **1162.6314** | 581.8193 | 1145.6048 | 573.3061 | 1144.6208 | 572.8141 | 11 |
| 5 | **500.2715** | 250.6394 | 483.2449 | 242.1261 | **482.2609** | 241.6341 | T | **1049.5473** | 525.2773 | 1032.5208 | 516.7640 | 1031.5368 | 516.2720 | 10 |
| 6 | 587.3035 | 294.1554 | 570.2770 | **285.6421** | **569.2929** | 285.1501 | S | **948.4997** | 474.7535 | 931.4731 | 466.2402 | 930.4891 | 465.7482 | 9 |
| 7 | **686.3719** | 343.6896 | 669.3454 | 335.1763 | **668.3614** | 334.6843 | V | **861.4676** | 431.2374 | 844.4411 | 422.7242 | 843.4571 | 422.2322 | 8 |
| 8 | **785.4403** | 393.2238 | 768.4138 | 384.7105 | **767.4298** | 384.2185 | V | **762.3992** | 381.7032 | 745.3727 | 373.1900 | 744.3886 | 372.6980 | 7 |
| 9 | **899.4833** | 450.2453 | 882.4567 | 441.7320 | 881.4727 | 441.2400 | N | **663.3308** | 332.1690 | 646.3042 | 323.6558 | 645.3202 | 323.1638 | 6 |
| 10 | 986.5153 | 493.7613 | 969.4887 | 485.2480 | 968.5047 | 484.7560 | S | **549.2879** | 275.1476 | 532.2613 | 266.6343 | 531.2773 | 266.1423 | 5 |
| 11 | **1099.5994** | 550.3033 | 1082.5728 | 541.7900 | 1081.5888 | 541.2980 | L | **462.2558** | 231.6316 | 445.2293 | 223.1183 | 444.2453 | 222.6263 | 4 |
| 12 | **1214.6263** | 607.8168 | 1197.5998 | 599.3035 | **1196.6157** | 598.8115 | D | **349.1718** | 175.0895 | 332.1452 | 166.5763 | 331.1612 | 166.0842 | 3 |
| 13 | **1301.6583** | 651.3328 | 1284.6318 | 642.8195 | 1283.6478 | 642.3275 | S | **234.1448** | 117.5761 | 217.1183 | 109.0628 | 216.1343 | 108.5708 | 2 |
| 14 | | | | | | | K | 147.1128 | 74.0600 | 130.0863 | 65.5468 | | | 1 |

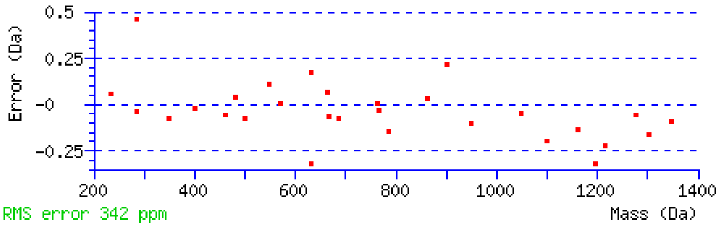


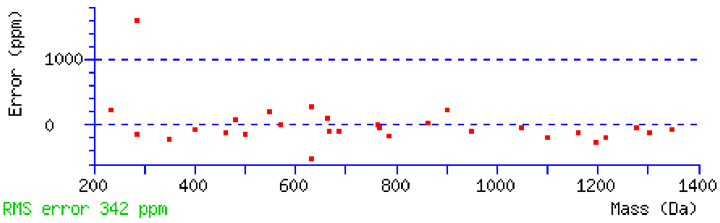


### All matches to this query

| Score | Mr(calc): | Delta | Sequence |
|---|---|---|---|
| 97.7 | 1446.7566 | 0.0034 | VANLTSVVNSLDSK |
| 58.4 | 1446.7566 | 0.0034 | VANLTSVVNSLDSK |
| 10.6 | 1446.7541 | 0.0059 | VALDSVTCVWGIK |
| 7.8 | 1444.7522 | 2.0078 | GLILTSNDSQQAAK |
| 3.5 | 1445.7436 | 1.0164 | MTDLSKGVIEEPK |
| 2.1 | 1446.7500 | 0.0100 | NISGKQSSLPAMSK |

---

Spectrum No: 92; Query: 1760; Rank: 1

---

## Peptide View

MS/MS Fragmentation of **HIDCASVYGNETEIGEALK**
Found in **IPI00230859**, Tax_Id=10116 Gene_Symbol=Akr1a1 Alcohol dehydrogenase

Match to Query 1760: 2105.963128 from(1053.988840,2+)
Title: 091008RatKidney_NH4Format02_22.2946.2946.2.dta
Data file K:\NewmanPaper\Piliang\3SubProteomes\Piliang3SP\mgf5ppm\ERLIC_3SubProteomes5ppm.mgf

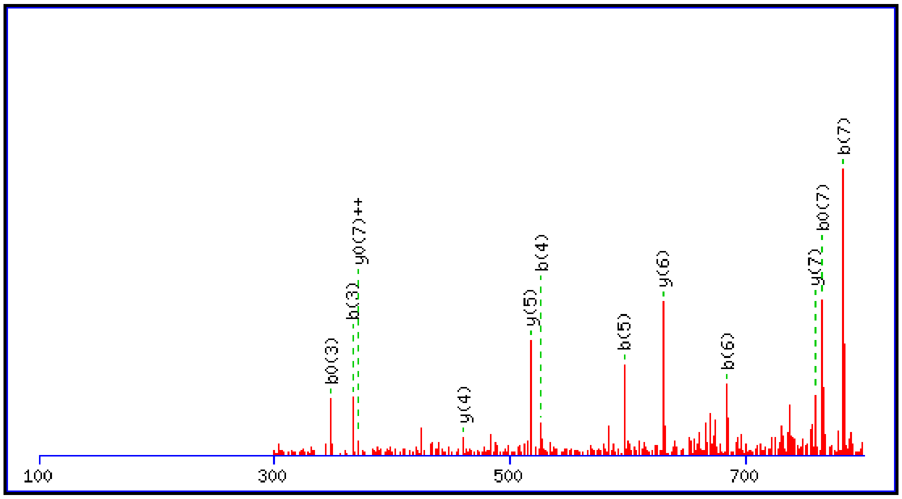

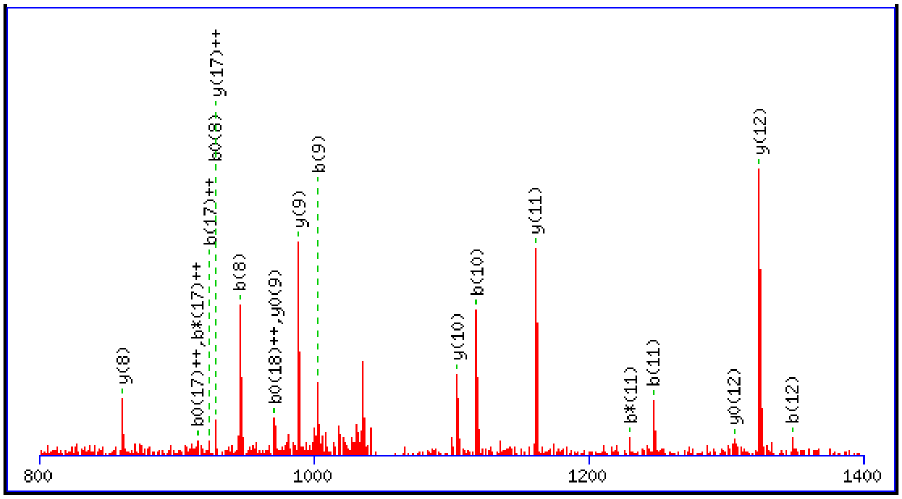


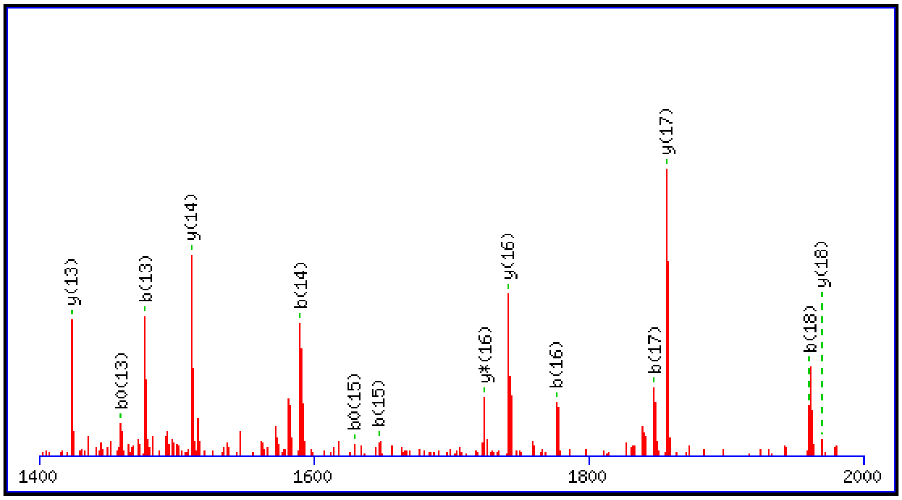


**Monoisotopic mass of neutral peptide Mr(calc):** 2105.9575
**Fixed modifications:** Carbamidomethyl (C)
**Variable modifications:**
**N10** : Deamidated_N (N)
**Ions Score:** 97 **Expect:** 5e-008
**Matches (Bold Red):** 45/188 fragment ions using 96 most intense peaks

| # | b | $b^{++}$ | b* | $b^{*++}$ | $b^{0}$ | $b^{0++}$ | Seq. | y | $y^{++}$ | y* | $y^{*++}$ | $y^{0}$ | $y^{0++}$ | # |
|---|---|---|---|---|---|---|---|---|---|---|---|---|---|---|
| 1 | 138.0662 | 69.5367 | | | | | H | | | | | | | 19 |
| 2 | 251.1503 | 126.0788 | | | | | I | **1969.9059** | 985.4566 | 1952.8794 | 976.9433 | 1951.8953 | 976.4513 | 18 |
| 3 | **366.1772** | 183.5922 | | | **348.1666** | 174.5870 | D | **1856.8218** | **928.9146** | 1839.7953 | 920.4013 | 1838.8113 | 919.9093 | 17 |
| 4 | **526.2078** | 263.6076 | | | 508.1973 | 254.6023 | C | **1741.7949** | 871.4011 | **1724.7684** | 862.8878 | 1723.7843 | 862.3958 | 16 |
| 5 | **597.2450** | 299.1261 | | | 579.2344 | 290.1208 | A | 1581.7643 | 791.3858 | 1564.7377 | 782.8725 | 1563.7537 | 782.3805 | 15 |
| 6 | **684.2770** | 342.6421 | | | 666.2664 | 333.6368 | S | **1510.7271** | 755.8672 | 1493.7006 | 747.3539 | 1492.7166 | 746.8619 | 14 |
| 7 | **783.3454** | 392.1763 | | | **765.3348** | 383.1711 | V | **1423.6951** | 712.3512 | 1406.6686 | 703.8379 | 1405.6845 | 703.3459 | 13 |
| 8 | **946.4087** | 473.7080 | | | **928.3982** | 464.7027 | Y | **1324.6267** | 662.8170 | 1307.6001 | 654.3037 | **1306.6161** | 653.8117 | 12 |
| 9 | **1003.4302** | 502.2187 | | | 985.4196 | 493.2135 | G | **1161.5634** | 581.2853 | 1144.5368 | 572.7720 | 1143.5528 | 572.2800 | 11 |
| 10 | **1118.4571** | 559.7322 | 1101.4306 | 551.2189 | 1100.4466 | 550.7269 | N | **1104.5419** | 552.7746 | 1087.5154 | 544.2613 | 1086.5313 | 543.7693 | 10 |
| 11 | **1247.4997** | 624.2535 | **1230.4732** | 615.7402 | 1229.4892 | 615.2482 | E | **989.5150** | 495.2611 | 972.4884 | 486.7478 | **971.5044** | 486.2558 | 9 |
| 12 | **1348.5474** | 674.7773 | 1331.5209 | 666.2641 | 1330.5368 | 665.7721 | T | **860.4724** | 430.7398 | 843.4458 | 422.2266 | 842.4618 | 421.7345 | 8 |
| 13 | **1477.5900** | 739.2986 | 1460.5634 | 730.7854 | **1459.5794** | 730.2934 | E | **759.4247** | 380.2160 | 742.3981 | 371.7027 | 741.4141 | **371.2107** | 7 |
| 14 | **1590.6741** | 795.8407 | 1573.6475 | 787.3274 | 1572.6635 | 786.8354 | I | **630.3821** | 315.6947 | 613.3556 | 307.1814 | 612.3715 | 306.6894 | 6 |
| 15 | **1647.6955** | 824.3514 | 1630.6690 | 815.8381 | **1629.6850** | 815.3461 | G | **517.2980** | 259.1527 | 500.2715 | 250.6394 | 499.2875 | 250.1474 | 5 |
| 16 | **1776.7381** | 888.8727 | 1759.7116 | 880.3594 | 1758.7275 | 879.8674 | E | **460.2766** | 230.6419 | 443.2500 | 222.1287 | 442.2660 | 221.6366 | 4 |
| 17 | **1847.7752** | **924.3913** | 1830.7487 | **915.8780** | 1829.7647 | **915.3860** | A | 331.2340 | 166.1206 | 314.2074 | 157.6074 | | | 3 |
| 18 | **1960.8593** | 980.9333 | 1943.8327 | 972.4200 | 1942.8487 | **971.9280** | L | 260.1969 | 130.6021 | 243.1703 | 122.0888 | | | 2 |

| | | | | | | | | | | | | | | |
|---|---|---|---|---|---|---|---|---|---|---|---|---|---|---|
| 19 | | | | | | | K | 147.1128 | 74.0600 | 130.0863 | 65.5468 | | | 1 |

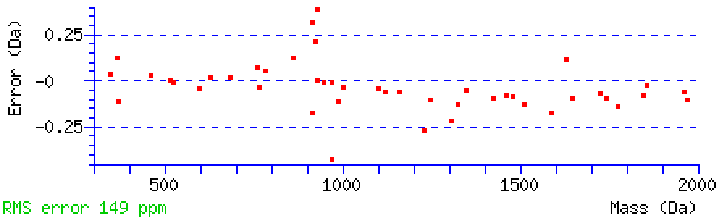


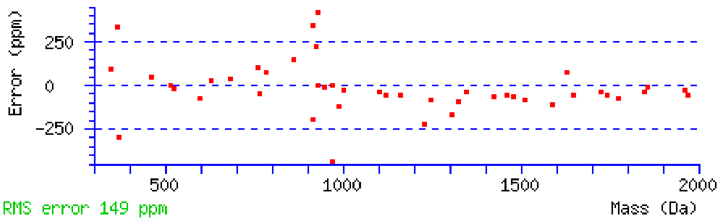


### All matches to this query

| Score | Mr(calc): | Delta | Sequence |
|---|---|---|---|
| 97.3 | 2105.9575 | 0.0056 | HIDCASVYGNETEIGEALK |
| 25.3 | 2104.9735 | 0.9896 | HIDCASVYGNETEIGEALK |

---

Spectrum No: 93; Query: 2020; Rank: 1

---

## Peptide View

MS/MS Fragmentation of **CGTLQDLYATVDFGTTGNITR**
Found in **IPI00371132**, Tax_Id=10116 Gene_Symbol=Glb1_predicted galactosidase, beta 1

Match to Query 2020: 2303.085108 from(1152.549830,2+)
Title: 100101RatKid_NS_deglyco_15.5409.5409.2.dta
Data file K:\NewmanPaper\Piliang\3SubProteomes\Piliang3SP\mgf5ppm\ERLIC_3SubProteomes5ppm.mgf

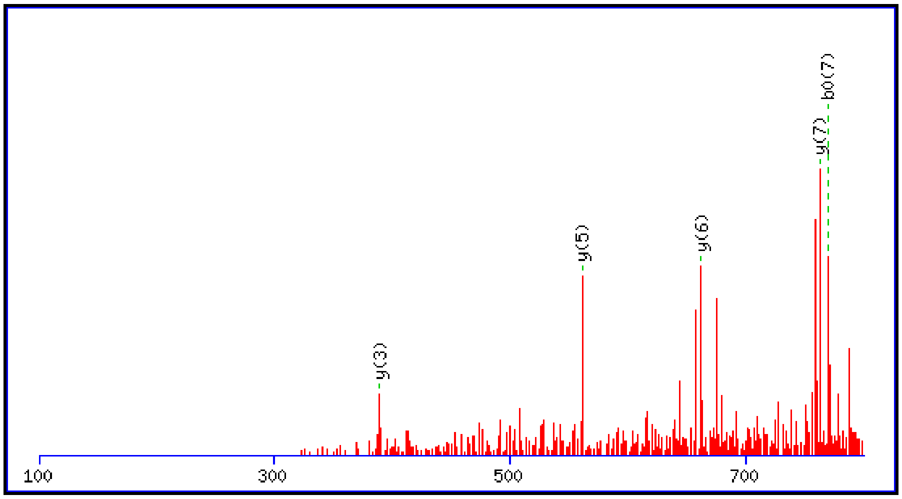

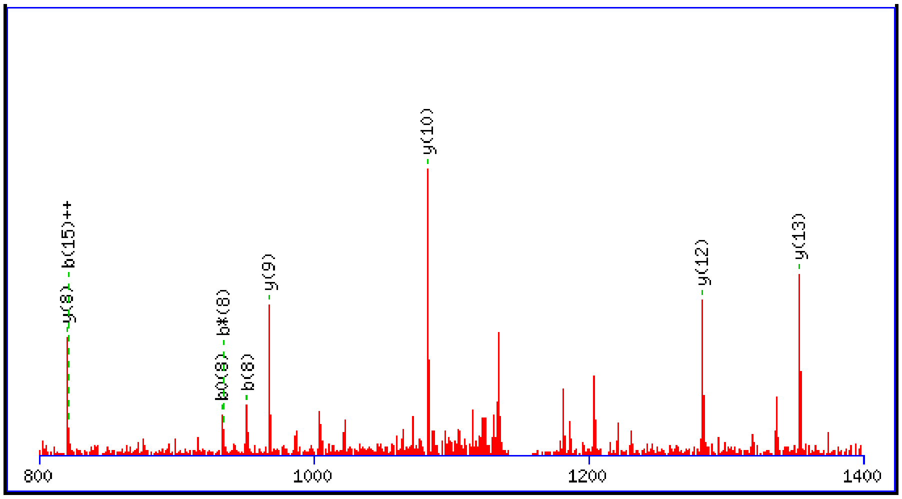


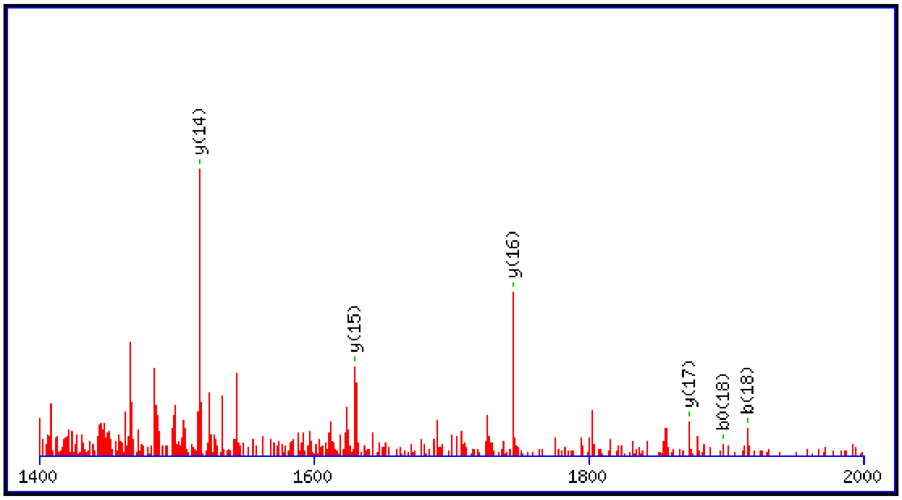


**Monoisotopic mass of neutral peptide Mr(calc):** 2303.0740
**Fixed modifications:** Carbamidomethyl (C)
**Variable modifications:**
**N18** : Deamidated_N (N)
**Ions Score:** 97 **Expect:** 5.7e-008
**Matches (Bold Red):** 20/226 fragment ions using 32 most intense peaks

| # | b | $b^{++}$ | b* | $b^{*++}$ | $b^{0}$ | $b^{0++}$ | Seq. | y | $y^{++}$ | y* | $y^{*++}$ | $y^{0}$ | $y^{0++}$ | # |
|---|---|---|---|---|---|---|---|---|---|---|---|---|---|---|
| 1 | 161.0379 | 81.0226 | | | | | C | | | | | | | 21 |
| 2 | 218.0594 | 109.5333 | | | | | G | 2144.0506 | 1072.5289 | 2127.0241 | 1064.0157 | 2126.0400 | 1063.5237 | 20 |
| 3 | 319.1071 | 160.0572 | | | 301.0965 | 151.0519 | T | 2087.0291 | 1044.0182 | 2070.0026 | 1035.5049 | 2069.0186 | 1035.0129 | 19 |
| 4 | 432.1911 | 216.5992 | | | 414.1806 | 207.5939 | L | 1985.9815 | 993.4944 | 1968.9549 | 984.9811 | 1967.9709 | 984.4891 | 18 |
| 5 | 560.2497 | 280.6285 | 543.2232 | 272.1152 | 542.2391 | 271.6232 | Q | **1872.8974** | 936.9523 | 1855.8708 | 928.4391 | 1854.8868 | 927.9471 | 17 |
| 6 | 675.2767 | 338.1420 | 658.2501 | 329.6287 | 657.2661 | 329.1367 | D | **1744.8388** | 872.9230 | 1727.8123 | 864.4098 | 1726.8283 | 863.9178 | 16 |
| 7 | 788.3607 | 394.6840 | 771.3342 | 386.1707 | **770.3502** | 385.6787 | L | **1629.8119** | 815.4096 | 1612.7853 | 806.8963 | 1611.8013 | 806.4043 | 15 |
| 8 | **951.4240** | 476.2157 | **934.3975** | 467.7024 | **933.4135** | 467.2104 | Y | **1516.7278** | 758.8675 | 1499.7013 | 750.3543 | 1498.7172 | 749.8623 | 14 |
| 9 | 1022.4612 | 511.7342 | 1005.4346 | 503.2209 | 1004.4506 | 502.7289 | A | **1353.6645** | 677.3359 | 1336.6379 | 668.8226 | 1335.6539 | 668.3306 | 13 |
| 10 | 1123.5088 | 562.2581 | 1106.4823 | 553.7448 | 1105.4983 | 553.2528 | T | **1282.6274** | 641.8173 | 1265.6008 | 633.3040 | 1264.6168 | 632.8120 | 12 |
| 11 | 1222.5773 | 611.7923 | 1205.5507 | 603.2790 | 1204.5667 | 602.7870 | V | 1181.5797 | 591.2935 | 1164.5531 | 582.7802 | 1163.5691 | 582.2882 | 11 |
| 12 | 1337.6042 | 669.3057 | 1320.5776 | 660.7925 | 1319.5936 | 660.3005 | D | **1082.5113** | 541.7593 | 1065.4847 | 533.2460 | 1064.5007 | 532.7540 | 10 |
| 13 | 1484.6726 | 742.8399 | 1467.6461 | 734.3267 | 1466.6620 | 733.8347 | F | **967.4843** | 484.2458 | 950.4578 | 475.7325 | 949.4738 | 475.2405 | 9 |
| 14 | 1541.6941 | 771.3507 | 1524.6675 | 762.8374 | 1523.6835 | 762.3454 | G | **820.4159** | 410.7116 | 803.3894 | 402.1983 | 802.4054 | 401.7063 | 8 |
| 15 | 1642.7418 | **821.8745** | 1625.7152 | 813.3612 | 1624.7312 | 812.8692 | T | **763.3945** | 382.2009 | 746.3679 | 373.6876 | 745.3839 | 373.1956 | 7 |
| 16 | 1743.7894 | 872.3984 | 1726.7629 | 863.8851 | 1725.7789 | 863.3931 | T | **662.3468** | 331.6770 | 645.3202 | 323.1638 | 644.3362 | 322.6717 | 6 |
| 17 | 1800.8109 | 900.9091 | 1783.7843 | 892.3958 | 1782.8003 | 891.9038 | G | **561.2991** | 281.1532 | 544.2725 | 272.6399 | 543.2885 | 272.1479 | 5 |
| 18 | **1915.8378** | 958.4226 | 1898.8113 | 949.9093 | **1897.8273** | 949.4173 | N | 504.2776 | 252.6425 | 487.2511 | 244.1292 | 486.2671 | 243.6372 | 4 |

| | | | | | | | | | | | | | |
|---|---|---|---|---|---|---|---|---|---|---|---|---|---|
| 19 | 2028.9219 | 1014.9646 | 2011.8953 | 1006.4513 | 2010.9113 | 1005.9593 | I | 389.2507 | 195.1290 | 372.2241 | 186.6157 | 371.2401 | 186.1237 | 3 |
| 20 | 2129.9696 | 1065.4884 | 2112.9430 | 1056.9752 | 2111.9590 | 1056.4831 | T | 276.1666 | 138.5870 | 259.1401 | 130.0737 | 258.1561 | 129.5817 | 2 |
| 21 | | | | | | | R | 175.1190 | 88.0631 | 158.0924 | 79.5498 | | | 1 |

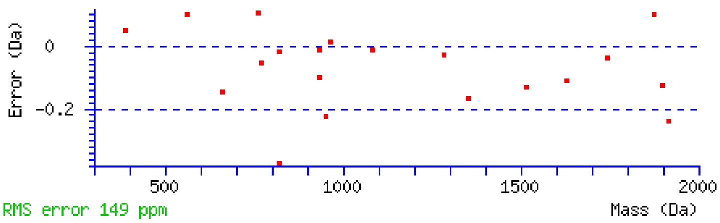


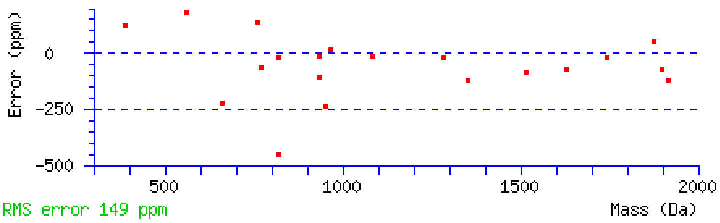


**All matches to this query**

| Score | Mr(calc): | Delta | Sequence |
|---|---|---|---|
| 97.1 | 2303.0740 | 0.0111 | CGTLQDLYATVDFGTTGNITR |
| 15.4 | 2302.0900 | 0.9951 | CGTLQDLYATVDFGTTGNITR |
| 2.5 | 2301.0589 | 2.0262 | DLANMTGLLHLSLSRNTIR |

---

Spectrum No: 94; Query: 1036; Rank: 1

---

## Peptide View

MS/MS Fragmentation of **TINISNLYIGGLPEGK**
Found in **IPI00363534**, Tax_Id=10116 Gene_Symbol=Lama1 laminin, alpha 1

Match to Query 1036: 1688.901988 from(845.458270,2+)
Title: 100101RatKid_NS_deglyco_13.4688.4688.2.dta
Data file K:\NewmanPaper\Piliang\3SubProteomes\Piliang3SP\mgf5ppm\ERLIC_3SubProteomes5ppm.mgf

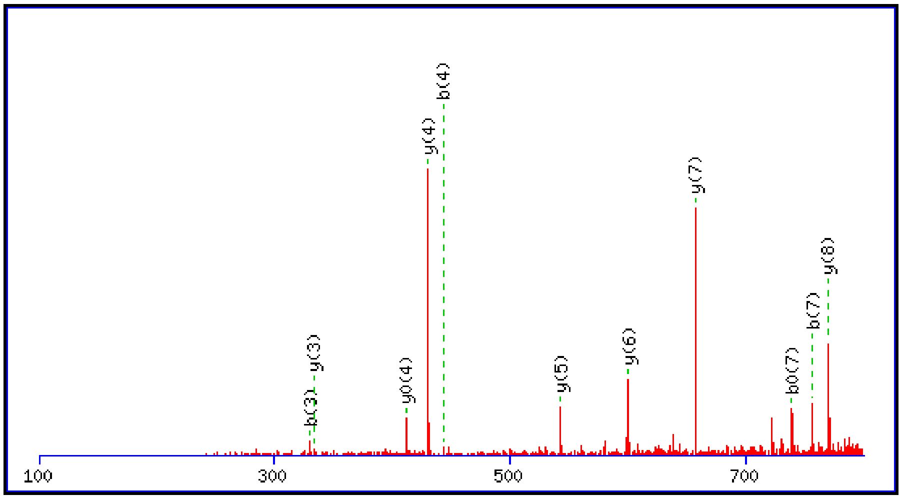

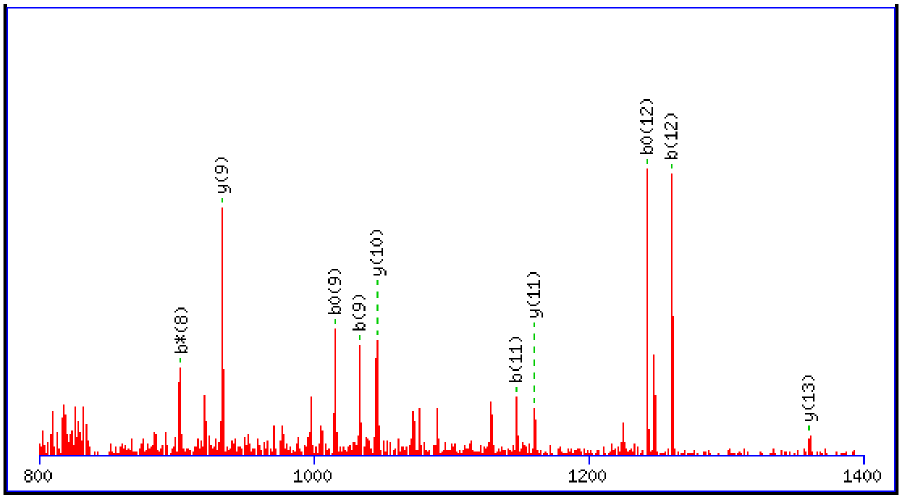


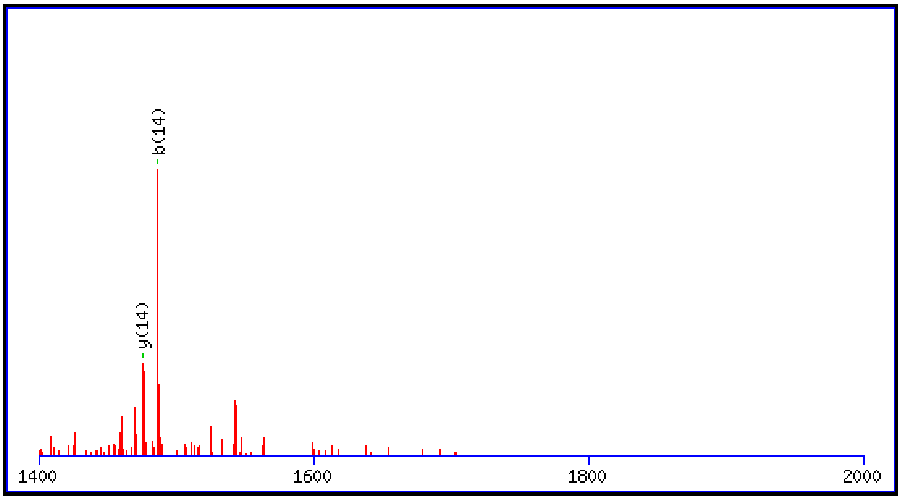


**Monoisotopic mass of neutral peptide Mr(calc):** 1688.8985
**Fixed modifications:** Carbamidomethyl (C)
**Variable modifications:**
**N3** : Deamidated_N (N)
**Ions Score:** 97 **Expect:** 2.2e-008
**Matches (Bold Red):** 23/172 fragment ions using 39 most intense peaks

| # | b | $b^{++}$ | $b^{*}$ | $b^{*++}$ | $b^{0}$ | $b^{0++}$ | Seq. | y | $y^{++}$ | $y^{*}$ | $y^{*++}$ | $y^{0}$ | $y^{0++}$ | # |
|---|---|---|---|---|---|---|---|---|---|---|---|---|---|---|
| 1 | 102.0550 | 51.5311 | | | 84.0444 | 42.5258 | T | | | | | | | 16 |
| 2 | 215.1390 | 108.0731 | | | 197.1285 | 99.0679 | I | 1588.8581 | 794.9327 | 1571.8315 | 786.4194 | 1570.8475 | 785.9274 | 15 |
| 3 | **330.1660** | 165.5866 | 313.1394 | 157.0733 | 312.1554 | 156.5813 | N | **1475.7740** | 738.3907 | 1458.7475 | 729.8774 | 1457.7635 | 729.3854 | 14 |
| 4 | **443.2500** | 222.1286 | 426.2235 | 213.6154 | 425.2395 | 213.1234 | I | **1360.7471** | 680.8772 | 1343.7205 | 672.3639 | 1342.7365 | 671.8719 | 13 |
| 5 | 530.2820 | 265.6447 | 513.2555 | 257.1314 | 512.2715 | 256.6394 | S | 1247.6630 | 624.3352 | 1230.6365 | 615.8219 | 1229.6525 | 615.3299 | 12 |
| 6 | 644.3250 | 322.6661 | 627.2984 | 314.1529 | 626.3144 | 313.6608 | N | **1160.6310** | 580.8191 | 1143.6045 | 572.3059 | 1142.6204 | 571.8139 | 11 |
| 7 | **757.4090** | 379.2082 | 740.3825 | 370.6949 | **739.3985** | 370.2029 | L | **1046.5881** | 523.7977 | 1029.5615 | 515.2844 | 1028.5775 | 514.7924 | 10 |
| 8 | 920.4724 | 460.7398 | **903.4458** | 452.2265 | 902.4618 | 451.7345 | Y | **933.5040** | 467.2556 | 916.4775 | 458.7424 | 915.4934 | 458.2504 | 9 |
| 9 | **1033.5564** | 517.2819 | 1016.5299 | 508.7686 | **1015.5459** | 508.2766 | I | **770.4407** | 385.7240 | 753.4141 | 377.2107 | 752.4301 | 376.7187 | 8 |
| 10 | 1090.5779 | 545.7926 | 1073.5513 | 537.2793 | 1072.5673 | 536.7873 | G | **657.3566** | 329.1819 | 640.3301 | 320.6687 | 639.3461 | 320.1767 | 7 |
| 11 | **1147.5994** | 574.3033 | 1130.5728 | 565.7900 | 1129.5888 | 565.2980 | G | **600.3352** | 300.6712 | 583.3086 | 292.1579 | 582.3246 | 291.6659 | 6 |
| 12 | **1260.6834** | 630.8453 | 1243.6569 | 622.3321 | **1242.6729** | 621.8401 | L | **543.3137** | 272.1605 | 526.2871 | 263.6472 | 525.3031 | 263.1552 | 5 |
| 13 | 1357.7362 | 679.3717 | 1340.7096 | 670.8585 | 1339.7256 | 670.3664 | P | **430.2296** | 215.6185 | 413.2031 | 207.1052 | **412.2191** | 206.6132 | 4 |
| 14 | **1486.7788** | 743.8930 | 1469.7522 | 735.3798 | 1468.7682 | 734.8877 | E | **333.1769** | 167.0921 | 316.1503 | 158.5788 | 315.1663 | 158.0868 | 3 |
| 15 | 1543.8002 | 772.4038 | 1526.7737 | 763.8905 | 1525.7897 | 763.3985 | G | 204.1343 | 102.5708 | 187.1077 | 94.0575 | | | 2 |
| 16 | | | | | | | K | 147.1128 | 74.0600 | 130.0863 | 65.5468 | | | 1 |

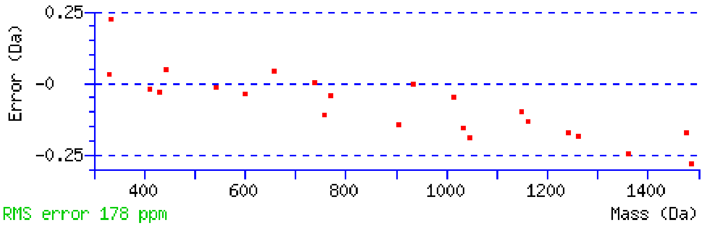


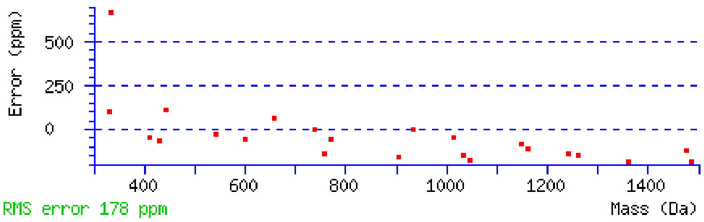


### All matches to this query

| Score | Mr(calc): | Delta | Sequence |
|---|---|---|---|
| 96.7 | 1688.8985 | 0.0035 | TINISNLYIGGLPEGK |
| 76.1 | 1688.8985 | 0.0035 | TINISNLYIGGLPEGK |
| 50.3 | 1687.9145 | 0.9875 | TINISNLYIGGLPEGK |
| 1.5 | 1686.9043 | 1.9977 | LRASGGPLGAAGLGVRR |
| 1.3 | 1686.8957 | 2.0063 | SKITILKTFNASGVK |

---

Spectrum No: 95; Query: 2093; Rank: 1

---

## Peptide View

MS/MS Fragmentation of **TEAGAFEYVADPTFENFTGGVK**
Found in **IPI00370450**, Tax_Id=10116 Gene_Symbol=Plxnb2 plexin B2

Match to Query 2093: 2350.075808 from(1176.045180,2+)
Title: 100101RatKid_NS_deglyco_16.5101.5101.2.dta
Data file K:\NewmanPaper\Piliang\3SubProteomes\Piliang3SP\mgf5ppm\ERLIC_3SubProteomes5ppm.mgf

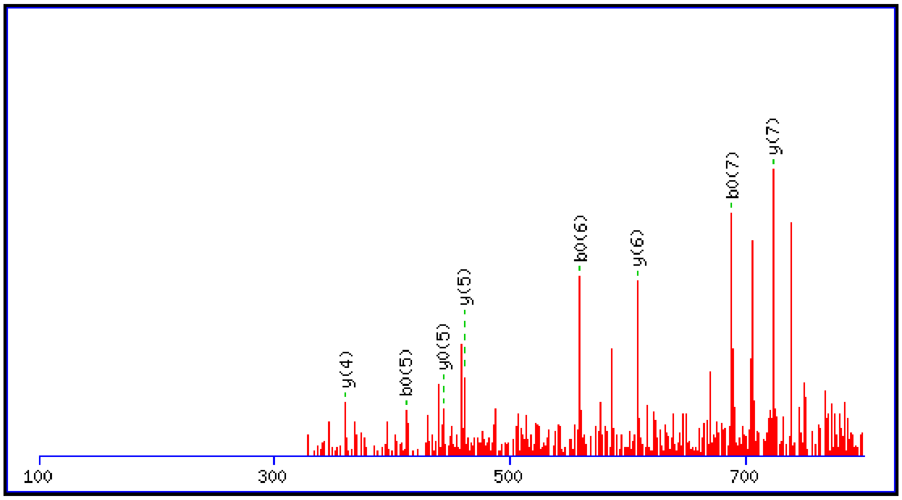

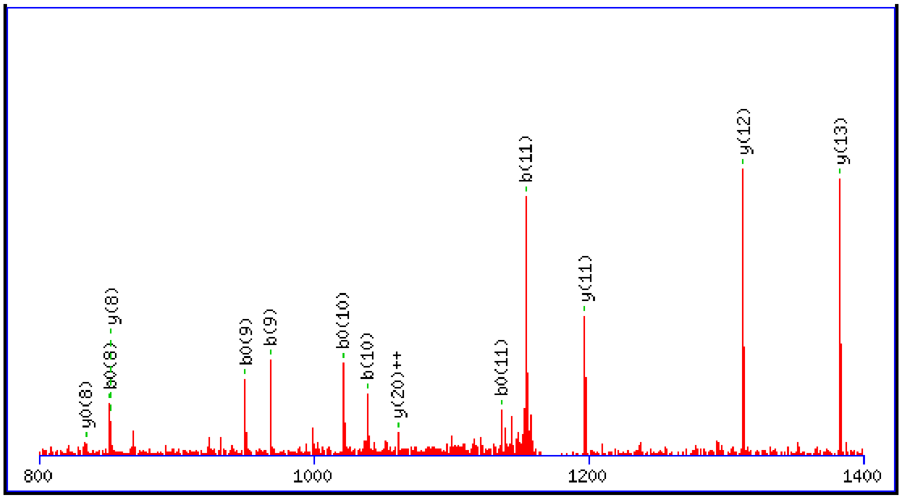


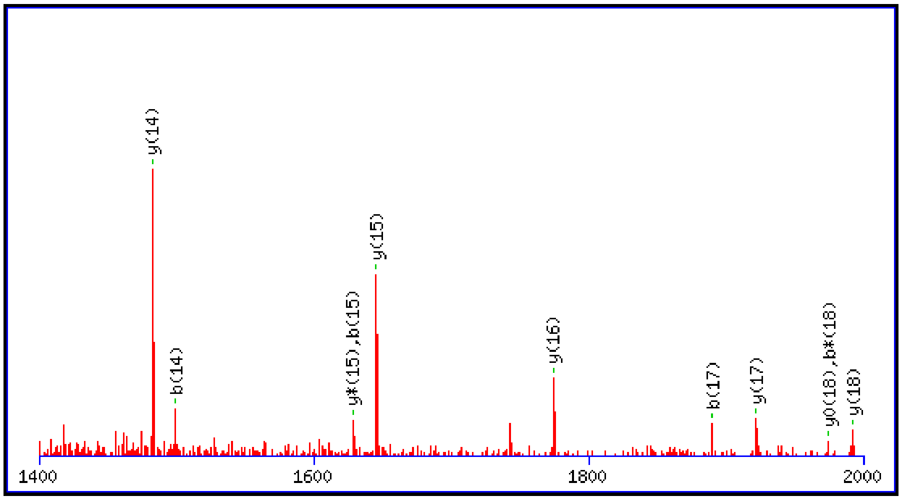


**Monoisotopic mass of neutral peptide Mr(calc):** 2350.0641
**Fixed modifications:** Carbamidomethyl (C)
**Variable modifications:**
**N16** : Deamidated_N (N)
**Ions Score:** 96 **Expect:** 6.9e-008
**Matches (Bold Red):** 32/214 fragment ions using 48 most intense peaks

| # | b | $b^{++}$ | b* | $b^{*++}$ | $b^0$ | $b^{0++}$ | Seq. | y | $y^{++}$ | y* | $y^{*++}$ | $y^0$ | $y^{0++}$ | # |
|---|---|---|---|---|---|---|---|---|---|---|---|---|---|---|
| 1 | 102.0550 | 51.5311 | | | 84.0444 | 42.5258 | T | | | | | | | 22 |
| 2 | 231.0975 | 116.0524 | | | 213.0870 | 107.0471 | E | 2250.0237 | 1125.5155 | 2232.9972 | 1117.0022 | 2232.0132 | 1116.5102 | 21 |
| 3 | 302.1347 | 151.5710 | | | 284.1241 | 142.5657 | A | 2120.9811 | **1060.9942** | 2103.9546 | 1052.4809 | 2102.9706 | 1051.9889 | 20 |
| 4 | 359.1561 | 180.0817 | | | 341.1456 | 171.0764 | G | 2049.9440 | 1025.4756 | 2032.9175 | 1016.9624 | 2031.9334 | 1016.4704 | 19 |
| 5 | 430.1932 | 215.6003 | | | **412.1827** | 206.5950 | A | **1992.9225** | 996.9649 | 1975.8960 | 988.4516 | **1974.9120** | 987.9596 | 18 |
| 6 | 577.2617 | 289.1345 | | | **559.2511** | 280.1292 | F | **1921.8854** | 961.4464 | 1904.8589 | 952.9331 | 1903.8749 | 952.4411 | 17 |
| 7 | 706.3042 | 353.6558 | | | **688.2937** | 344.6505 | E | **1774.8170** | 887.9121 | 1757.7905 | 879.3989 | 1756.8065 | 878.9069 | 16 |
| 8 | 869.3676 | 435.1874 | | | **851.3570** | 426.1821 | Y | **1645.7744** | 823.3909 | **1628.7479** | 814.8776 | 1627.7639 | 814.3856 | 15 |
| 9 | **968.4360** | 484.7216 | | | **950.4254** | 475.7164 | V | **1482.7111** | 741.8592 | 1465.6845 | 733.3459 | 1464.7005 | 732.8539 | 14 |
| 10 | **1039.4731** | 520.2402 | | | **1021.4625** | 511.2349 | A | **1383.6427** | 692.3250 | 1366.6161 | 683.8117 | 1365.6321 | 683.3197 | 13 |
| 11 | **1154.5000** | 577.7537 | | | **1136.4895** | 568.7484 | D | **1312.6056** | 656.8064 | 1295.5790 | 648.2931 | 1294.5950 | 647.8011 | 12 |
| 12 | 1251.5528 | 626.2800 | | | 1233.5422 | 617.2748 | P | **1197.5786** | 599.2930 | 1180.5521 | 590.7797 | 1179.5681 | 590.2877 | 11 |
| 13 | 1352.6005 | 676.8039 | | | 1334.5899 | 667.7986 | T | 1100.5259 | 550.7666 | 1083.4993 | 542.2533 | 1082.5153 | 541.7613 | 10 |
| 14 | **1499.6689** | 750.3381 | | | 1481.6583 | 741.3328 | F | 999.4782 | 500.2427 | 982.4516 | 491.7295 | 981.4676 | 491.2374 | 9 |
| 15 | **1628.7115** | 814.8594 | | | 1610.7009 | 805.8541 | E | **852.4098** | 426.7085 | 835.3832 | 418.1952 | **834.3992** | 417.7032 | 8 |
| 16 | 1743.7384 | 872.3729 | 1726.7119 | 863.8596 | 1725.7279 | 863.3676 | N | **723.3672** | 362.1872 | 706.3406 | 353.6740 | 705.3566 | 353.1819 | 7 |
| 17 | **1890.8068** | 945.9071 | 1873.7803 | 937.3938 | 1872.7963 | 936.9018 | F | **608.3402** | 304.6738 | 591.3137 | 296.1605 | 590.3297 | 295.6685 | 6 |
| 18 | 1991.8545 | 996.4309 | **1974.8280** | 987.9176 | 1973.8440 | 987.4256 | T | **461.2718** | 231.1396 | 444.2453 | 222.6263 | **443.2613** | 222.1343 | 5 |
| | | | | | | | | | | | | | | |

| | | | | | | | | | | | | | | |
|---|---|---|---|---|---|---|---|---|---|---|---|---|---|---|
| **19** | 2048.8760 | 1024.9416 | 2031.8494 | 1016.4284 | 2030.8654 | 1015.9364 | **G** | **360.2241** | 180.6157 | 343.1976 | 172.1024 | | | **4** |
| **20** | 2105.8975 | 1053.4524 | 2088.8709 | 1044.9391 | 2087.8869 | 1044.4471 | **G** | 303.2027 | 152.1050 | 286.1761 | 143.5917 | | | **3** |
| **21** | 2204.9659 | 1102.9866 | 2187.9393 | 1094.4733 | 2186.9553 | 1093.9813 | **V** | 246.1812 | 123.5942 | 229.1547 | 115.0810 | | | **2** |
| **22** | | | | | | | **K** | 147.1128 | 74.0600 | 130.0863 | 65.5468 | | | **1** |

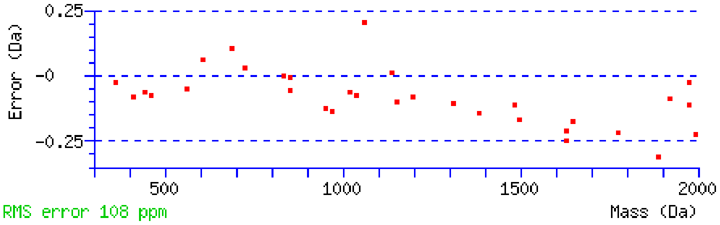


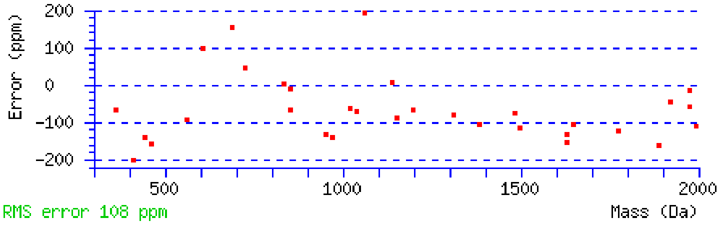


**All matches to this query**

| Score | Mr(calc): | Delta | Sequence |
|---|---|---|---|
| 96.3 | 2350.0641 | 0.0117 | TEAGAFEYVADPTFENFTGGVK |
| 25.2 | 2349.0801 | 0.9957 | TEAGAFEYVADPTFENFTGGVK |
| 2.9 | 2350.0755 | 0.0003 | NVFELGPPVMLDAATLKTMK |
| 2.6 | 2348.0787 | 1.9971 | SGRPPRRSVAAPADSSVPEPR |
| 2.6 | 2348.0787 | 1.9971 | SGRPPRRSVAAPADSSVPEPR |
| 2.1 | 2348.0474 | 2.0284 | EHAAVNQKGIYDDAISSFFR |
| 2.1 | 2348.0474 | 2.0284 | EHAAVNQKGIYDDAISSFFR |
| 1.3 | 2348.0686 | 2.0073 | RLSESPVFDAPPSPPDSLSDR |
| 0.2 | 2350.0621 | 0.0138 | GLRPQEGYFVRVTAVNDGGR |
| 0.1 | 2348.0703 | 2.0055 | LMDAWAGSARVPDEENTTGTAR |

---

Spectrum No: 96; Query: 2571; Rank: 1

---

## Peptide View

MS/MS Fragmentation of **ENGTDAVQEEEESPAEGSKDEPAEQGELK**
Found in **IPI00210975**, Tax_Id=10116 Gene_Symbol=Hyou1 Hypoxia up-regulated protein 1 precursor

Match to Query 2571: 3102.333702 from(1035.118510,3+)
Title: 100101RatKid_NS_deglyco_20.1634.1634.3.dta
Data file K:\NewmanPaper\Piliang\3SubProteomes\Piliang3SP\mgf5ppm\ERLIC_3SubProteomes5ppm.mgf

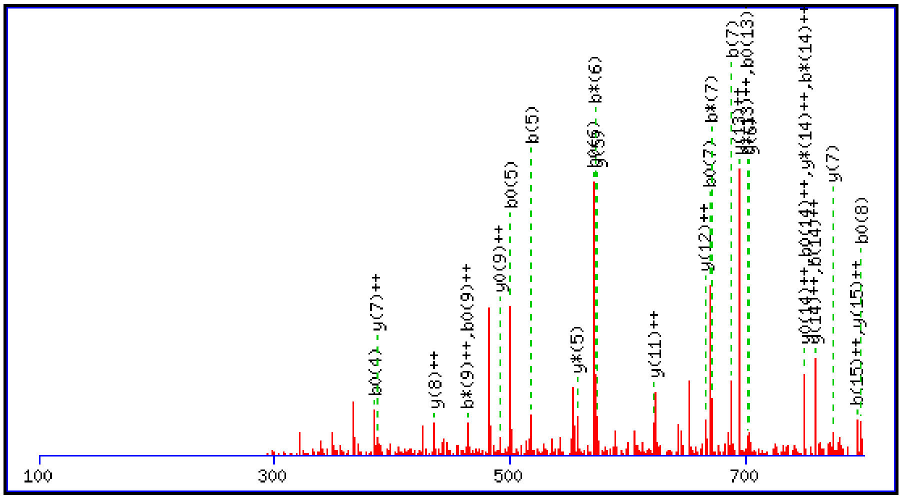

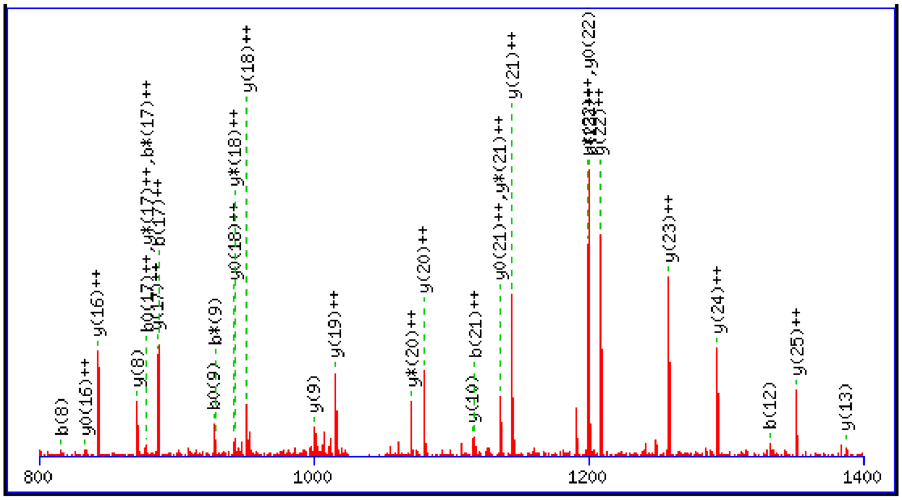


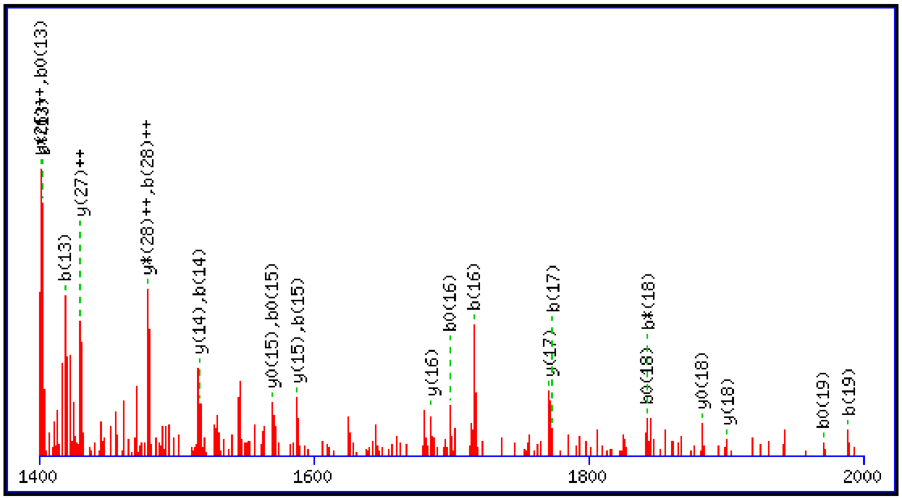


**Monoisotopic mass of neutral peptide Mr(calc):** 3102.3272
**Fixed modifications:** Carbamidomethyl (C)
**Variable modifications:**
**N2** : Deamidated_N (N)
**Ions Score:** 96 **Expect:** 1.1e-007
**Matches (Bold Red):** 87/330 fragment ions using 127 most intense peaks

| # | b | $b^{++}$ | b* | $b^{*++}$ | $b^0$ | $b^{0++}$ | Seq. | y | $y^{++}$ | y* | $y^{*++}$ | $y^0$ | $y^{0++}$ | # |
|---|---|---|---|---|---|---|---|---|---|---|---|---|---|---|
| 1 | 130.0499 | 65.5286 | | | 112.0393 | 56.5233 | E | | | | | | | 29 |
| 2 | 245.0768 | 123.0420 | 228.0503 | 114.5288 | 227.0662 | 114.0368 | N | 2974.2920 | 1487.6496 | 2957.2654 | **1479.1363** | 2956.2814 | 1478.6443 | 28 |
| 3 | 302.0983 | 151.5528 | 285.0717 | 143.0395 | 284.0877 | 142.5475 | G | 2859.2650 | **1430.1362** | 2842.2385 | 1421.6229 | 2841.2545 | 1421.1309 | 27 |
| 4 | 403.1459 | 202.0766 | 386.1194 | 193.5633 | **385.1354** | 193.0713 | T | 2802.2436 | **1401.6254** | 2785.2170 | 1393.1121 | 2784.2330 | 1392.6201 | 26 |
| 5 | **518.1729** | 259.5901 | 501.1463 | 251.0768 | **500.1623** | 250.5848 | D | 2701.1959 | **1351.1016** | 2684.1693 | 1342.5883 | 2683.1853 | 1342.0963 | 25 |
| 6 | 589.2100 | 295.1086 | **572.1835** | 286.5954 | **571.1994** | 286.1034 | A | 2586.1689 | **1293.5881** | 2569.1424 | 1285.0748 | 2568.1584 | 1284.5828 | 24 |
| 7 | **688.2784** | 344.6428 | **671.2519** | 336.1296 | **670.2679** | 335.6376 | V | 2515.1318 | **1258.0696** | 2498.1053 | 1249.5563 | 2497.1213 | 1249.0643 | 23 |
| 8 | **816.3370** | 408.6721 | 799.3104 | 400.1589 | **798.3264** | 399.6669 | Q | 2416.0634 | **1208.5353** | 2399.0369 | **1200.0221** | 2398.0528 | **1199.5301** | 22 |
| 9 | 945.3796 | 473.1934 | **928.3530** | **464.6802** | **927.3690** | **464.1882** | E | 2288.0048 | **1144.5061** | 2270.9783 | **1135.9928** | 2269.9943 | **1135.5008** | 21 |
| 10 | 1074.4222 | 537.7147 | 1057.3956 | 529.2015 | 1056.4116 | 528.7094 | E | 2158.9622 | **1079.9848** | 2141.9357 | **1071.4715** | 2140.9517 | 1070.9795 | 20 |
| 11 | 1203.4648 | 602.2360 | 1186.4382 | 593.7228 | 1185.4542 | 593.2307 | E | 2029.9197 | **1015.4635** | 2012.8931 | 1006.9502 | 2011.9091 | 1006.4582 | 19 |
| 12 | **1332.5074** | 666.7573 | 1315.4808 | 658.2440 | 1314.4968 | 657.7520 | E | **1900.8771** | **950.9422** | 1883.8505 | **942.4289** | **1882.8665** | **941.9369** | 18 |
| 13 | **1419.5394** | 710.2733 | **1402.5128** | **701.7601** | **1401.5288** | **701.2681** | S | **1771.8345** | **886.4209** | 1754.8079 | **877.9076** | 1753.8239 | 877.4156 | 17 |
| 14 | **1516.5922** | **758.7997** | 1499.5656 | **750.2864** | 1498.5816 | **749.7944** | P | **1684.8024** | **842.9049** | 1667.7759 | 834.3916 | 1666.7919 | **833.8996** | 16 |
| 15 | **1587.6293** | **794.3183** | 1570.6027 | 785.8050 | **1569.6187** | 785.3130 | A | **1587.7497** | **794.3785** | 1570.7231 | 785.8652 | **1569.7391** | 785.3732 | 15 |
| 16 | **1716.6719** | 858.8396 | 1699.6453 | 850.3263 | **1698.6613** | 849.8343 | E | **1516.7126** | **758.8599** | 1499.6860 | **750.3466** | 1498.7020 | **749.8546** | 14 |
| 17 | **1773.6933** | **887.3503** | 1756.6668 | **878.8370** | 1755.6828 | **878.3450** | G | **1387.6700** | **694.3386** | 1370.6434 | 685.8253 | 1369.6594 | 685.3333 | 13 |
| 18 | 1860.7254 | 930.8663 | **1843.6988** | 922.3530 | **1842.7148** | 921.8610 | S | 1330.6485 | **665.8279** | 1313.6220 | 657.3146 | 1312.6379 | 656.8226 | 12 |
| | | | | | | | | | | | | | | |

| | | | | | | | | | | | | | | |
|---|---|---|---|---|---|---|---|---|---|---|---|---|---|---|
| **19** | **1988.8203** | 994.9138 | 1971.7938 | 986.4005 | **1970.8098** | 985.9085 | **K** | 1243.6165 | **622.3119** | 1226.5899 | 613.7986 | 1225.6059 | 613.3066 | **11** |
| **20** | 2103.8473 | 1052.4273 | 2086.8207 | 1043.9140 | 2085.8367 | 1043.4220 | **D** | **1115.5215** | 558.2644 | 1098.4950 | 549.7511 | 1097.5109 | 549.2591 | **10** |
| **21** | 2232.8899 | **1116.9486** | 2215.8633 | 1108.4353 | 2214.8793 | 1107.9433 | **E** | **1000.4946** | 500.7509 | 983.4680 | 492.2376 | 982.4840 | **491.7456** | **9** |
| **22** | 2329.9426 | 1165.4749 | 2312.9161 | 1156.9617 | 2311.9321 | 1156.4697 | **P** | **871.4520** | **436.2296** | 854.4254 | 427.7164 | 853.4414 | 427.2243 | **8** |
| **23** | 2400.9797 | **1200.9935** | 2383.9532 | 1192.4802 | 2382.9692 | 1191.9882 | **A** | **774.3992** | **387.7032** | 757.3727 | 379.1900 | 756.3886 | 378.6980 | **7** |
| **24** | 2530.0223 | 1265.5148 | 2512.9958 | 1257.0015 | 2512.0118 | 1256.5095 | **E** | **703.3621** | 352.1847 | 686.3355 | 343.6714 | 685.3515 | 343.1794 | **6** |
| **25** | 2658.0809 | 1329.5441 | 2641.0544 | 1321.0308 | 2640.0703 | 1320.5388 | **Q** | **574.3195** | 287.6634 | **557.2930** | 279.1501 | 556.3089 | 278.6581 | **5** |
| **26** | 2715.1024 | 1358.0548 | 2698.0758 | 1349.5415 | 2697.0918 | 1349.0495 | **G** | 446.2609 | 223.6341 | 429.2344 | 215.1208 | 428.2504 | 214.6288 | **4** |
| **27** | 2844.1450 | 1422.5761 | 2827.1184 | 1414.0628 | 2826.1344 | 1413.5708 | **E** | 389.2395 | 195.1234 | 372.2129 | 186.6101 | 371.2289 | 186.1181 | **3** |
| **28** | 2957.2290 | **1479.1182** | 2940.2025 | 1470.6049 | 2939.2185 | 1470.1129 | **L** | 260.1969 | 130.6021 | 243.1703 | 122.0888 | | | **2** |
| **29** | | | | | | | **K** | 147.1128 | 74.0600 | 130.0863 | 65.5468 | | | **1** |

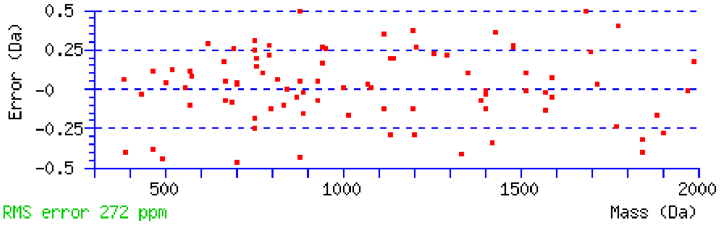


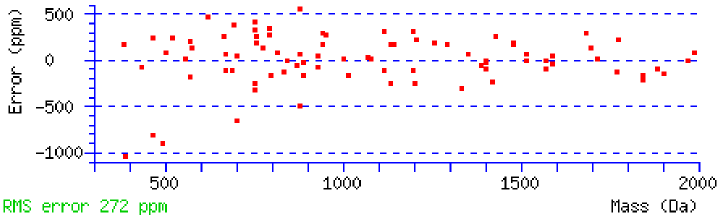


**All matches to this query**

| Score | Mr(calc): | Delta | Sequence |
|---|---|---|---|
| 96.2 | 3102.3272 | 0.0065 | ENGTDAVQEEEESPAEGSKDEPAEQGELK |
| 73.0 | 3101.3432 | 0.9905 | ENGTDAVQEEEESPAEGSKDEPAEQGELK |
| 7.4 | 3102.3522 | -0.0185 | NPSPGMSAKNAEKPNMQRNNTLGISTTK |
| 7.4 | 3102.3522 | -0.0185 | NPSPGMSAKNAEKPNMQRNNTLGISTTK |
| 7.4 | 3102.3522 | -0.0185 | NPSPGMSAKNAEKPNMQRNNTLGISTTK |
| 6.4 | 3101.3359 | 0.9978 | DLDFDMDSSMNGAGPLGPAEPSAKVALER |
| 5.2 | 3102.3196 | 0.0141 | TEQSGSELSSRLDTLNAEKEALSGAIR |
| 4.7 | 3102.3406 | -0.0069 | KEMVSQSPKYEEAVLPVHGEGQEESR |
| 4.4 | 3102.3199 | 0.0138 | DLDFDMDSSMNGAGPLGPAEPSAKVALER |
| 4.0 | 3102.3415 | -0.0078 | MNFLTNLELNFAECILDGGRATMGVR |

---

Spectrum No: 97; Query: 1879; Rank: 1

---

## Peptide View

MS/MS Fragmentation of **VPFNPNDTFESEFYLDEK**
Found in **IPI00200591**, Tax_Id=10116 Gene_Symbol=LOC299282 Serine protease inhibitor A3L precursor

Match to Query 1879: 2190.966228 from(1096.490390,2+)
Title: 091008RatKidney_NoSalt_28.3850.3850.2.dta
Data file K:\NewmanPaper\Piliang\3SubProteomes\Piliang3SP\mgf5ppm\ERLIC_3SubProteomes5ppm.mgf

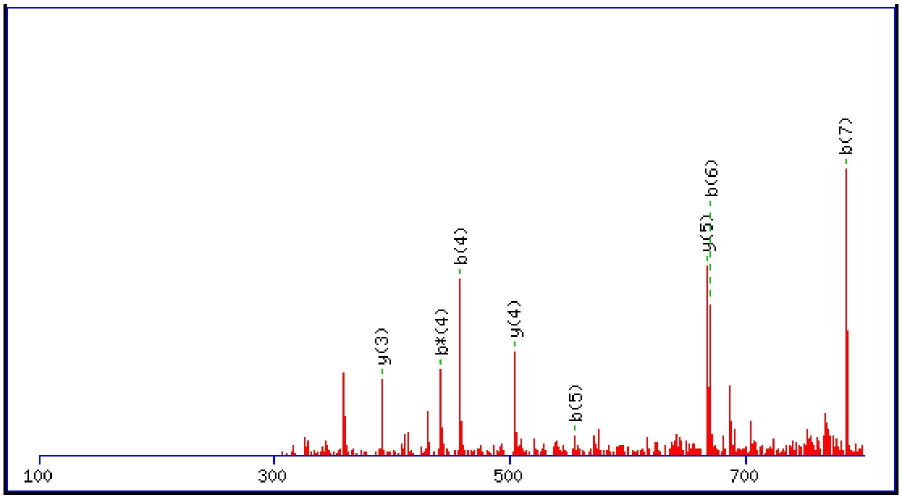


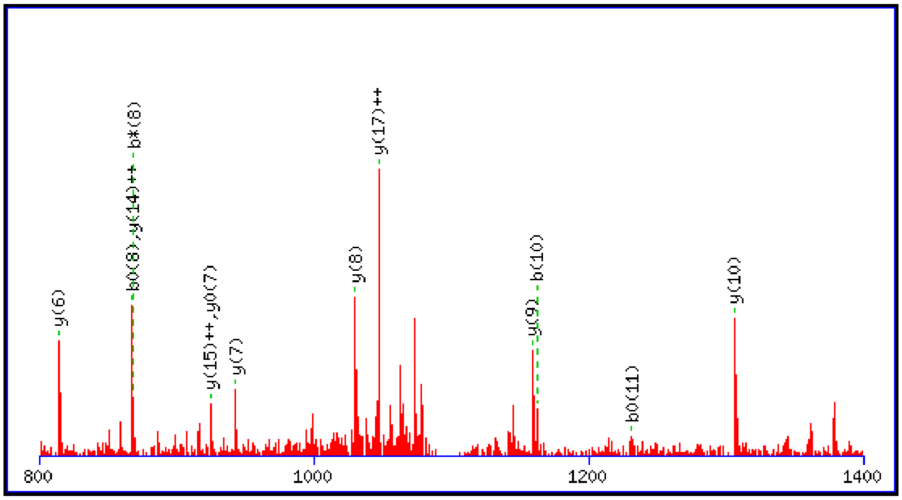


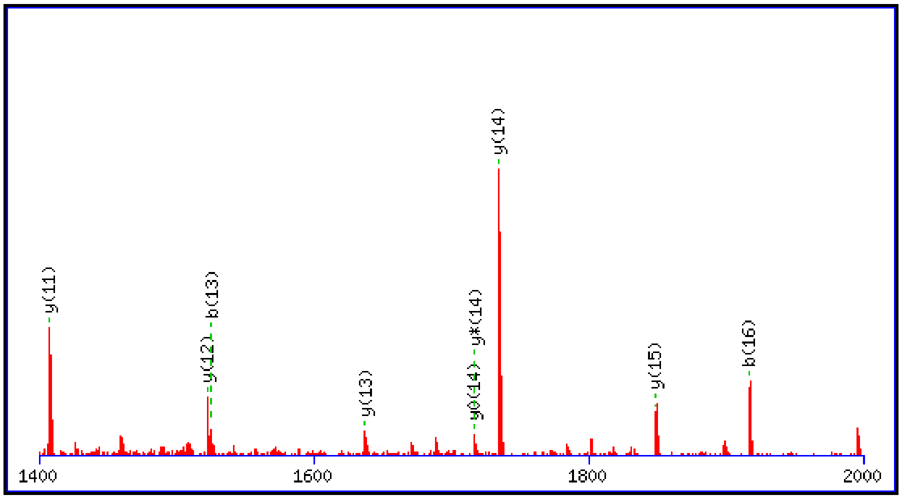


**Monoisotopic mass of neutral peptide Mr(calc):** 2190.9633
**Fixed modifications:** Carbamidomethyl (C)
**Variable modifications:**
**N6** : Deamidated_N (N)
**Ions Score:** 96 **Expect:** 7.3e-008
**Matches (Bold Red):** 30/184 fragment ions using 44 most intense peaks

| # | b | $b^{++}$ | b* | $b^{*++}$ | $b^0$ | $b^{0++}$ | Seq. | y | $y^{++}$ | y* | $y^{*++}$ | $y^0$ | $y^{0++}$ | # |
|---|---|---|---|---|---|---|---|---|---|---|---|---|---|---|
| 1 | 100.0757 | 50.5415 | | | | | V | | | | | | | 18 |
| 2 | 197.1285 | 99.0679 | | | | | P | 2092.9022 | **1046.9547** | 2075.8757 | 1038.4415 | 2074.8916 | 1037.9495 | 17 |
| 3 | 344.1969 | 172.6021 | | | | | F | 1995.8494 | 998.4284 | 1978.8229 | 989.9151 | 1977.8389 | 989.4231 | 16 |
| 4 | **458.2398** | 229.6235 | **441.2132** | 221.1103 | | | N | **1848.7810** | **924.8942** | 1831.7545 | 916.3809 | 1830.7705 | 915.8889 | 15 |
| 5 | **555.2926** | 278.1499 | 538.2660 | 269.6366 | | | P | **1734.7381** | **867.8727** | **1717.7115** | 859.3594 | **1716.7275** | 858.8674 | 14 |

| | | | | | | | | | | | | | | |
|---|---|---|---|---|---|---|---|---|---|---|---|---|---|---|
| **6** | **670.3195** | 335.6634 | 653.2929 | 327.1501 | | | **N** | **1637.6853** | 819.3463 | 1620.6588 | 810.8330 | 1619.6748 | 810.3410 | **13** |
| **7** | **785.3464** | 393.1769 | 768.3199 | 384.6636 | 767.3359 | 384.1716 | **D** | **1522.6584** | 761.8328 | 1505.6318 | 753.3196 | 1504.6478 | 752.8276 | **12** |
| **8** | 886.3941 | 443.7007 | **869.3676** | 435.1874 | **868.3836** | 434.6954 | **T** | **1407.6315** | 704.3194 | 1390.6049 | 695.8061 | 1389.6209 | 695.3141 | **11** |
| **9** | 1033.4625 | 517.2349 | 1016.4360 | 508.7216 | 1015.4520 | 508.2296 | **F** | **1306.5838** | 653.7955 | 1289.5572 | 645.2823 | 1288.5732 | 644.7902 | **10** |
| **10** | **1162.5051** | 581.7562 | 1145.4786 | 573.2429 | 1144.4946 | 572.7509 | **E** | **1159.5154** | 580.2613 | 1142.4888 | 571.7480 | 1141.5048 | 571.2560 | **9** |
| **11** | 1249.5372 | 625.2722 | 1232.5106 | 616.7589 | **1231.5266** | 616.2669 | **S** | **1030.4728** | 515.7400 | 1013.4462 | 507.2267 | 1012.4622 | 506.7347 | **8** |
| **12** | 1378.5797 | 689.7935 | 1361.5532 | 681.2802 | 1360.5692 | 680.7882 | **E** | **943.4407** | 472.2240 | 926.4142 | 463.7107 | **925.4302** | 463.2187 | **7** |
| **13** | **1525.6482** | 763.3277 | 1508.6216 | 754.8144 | 1507.6376 | 754.3224 | **F** | **814.3981** | 407.7027 | 797.3716 | 399.1894 | 796.3876 | 398.6974 | **6** |
| **14** | 1688.7115 | 844.8594 | 1671.6849 | 836.3461 | 1670.7009 | 835.8541 | **Y** | **667.3297** | 334.1685 | 650.3032 | 325.6552 | 649.3192 | 325.1632 | **5** |
| **15** | 1801.7956 | 901.4014 | 1784.7690 | 892.8881 | 1783.7850 | 892.3961 | **L** | **504.2664** | 252.6368 | 487.2399 | 244.1236 | 486.2558 | 243.6316 | **4** |
| **16** | **1916.8225** | 958.9149 | 1899.7959 | 950.4016 | 1898.8119 | 949.9096 | **D** | **391.1823** | 196.0948 | 374.1558 | 187.5815 | 373.1718 | 187.0895 | **3** |
| **17** | 2045.8651 | 1023.4362 | 2028.8385 | 1014.9229 | 2027.8545 | 1014.4309 | **E** | 276.1554 | 138.5813 | 259.1288 | 130.0681 | 258.1448 | 129.5761 | **2** |
| **18** | | | | | | | **K** | 147.1128 | 74.0600 | 130.0863 | 65.5468 | | | **1** |

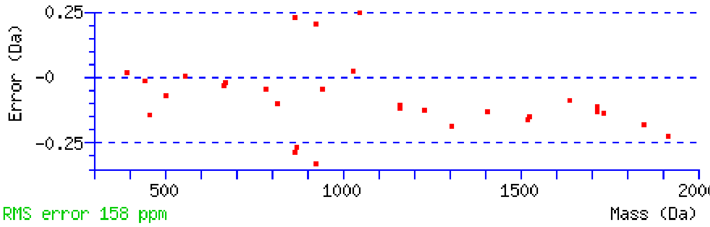


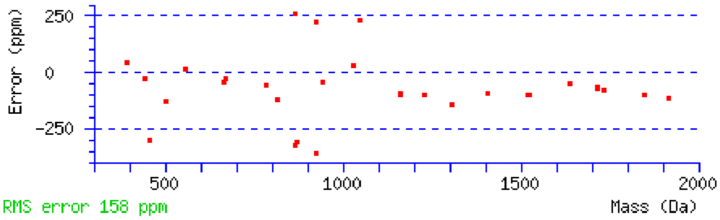


**All matches to this query**

| Score | Mr(calc): | Delta | Sequence |
|---|---|---|---|
| 96.2 | 2190.9633 | 0.0029 | VPFNPNDTFESEFYLDEK |
| 63.7 | 2190.9633 | 0.0029 | VPFNPNDTFESEFYLDEK |
| 46.5 | 2189.9793 | 0.9869 | VPFNPNDTFESEFYLDEK |
| 8.3 | 2190.9745 | -0.0083 | IEVMNTDGTGRRVLVEDK |
| 7.2 | 2188.9654 | 2.0008 | ESVATVALTVLDKNDNSPR |
| 7.2 | 2189.9494 | 1.0168 | ESVATVALTVLDKNDNSPR |
| 7.2 | 2188.9654 | 2.0008 | ESVATVALTVLDKNDNSPR |
| 7.2 | 2189.9494 | 1.0168 | ESVATVALTVLDKNDNSPR |
| 4.0 | 2190.9810 | -0.0148 | RSNLTIIGIEESEDSQLK |
| 3.3 | 2190.9731 | -0.0068 | EFNLMYDGTKEVPMNPVK |

---

Spectrum No: 98; Query: 1267; Rank: 1

---

## Peptide View

MS/MS Fragmentation of **LSPNCCNSSTVADAYR**
Found in **IPI00203176**, Tax_Id=10116 Gene_Symbol=Tpcn1 Two pore calcium channel protein 1

Match to Query 1267: 1814.761648 from(908.388100,2+)
Title: 100101RatKid_NS_deglyco_25.1508.1508.2.dta
Data file K:\NewmanPaper\Piliang\3SubProteomes\Piliang3SP\mgf5ppm\ERLIC_3SubProteomes5ppm.mgf

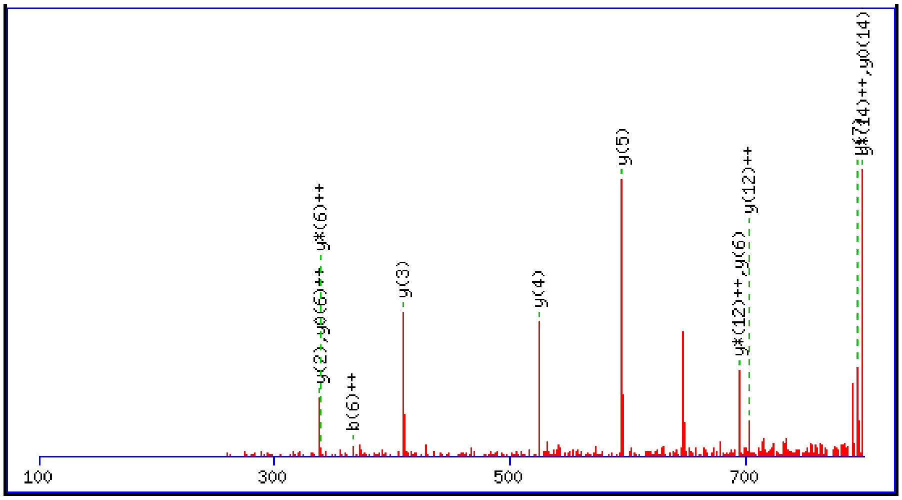


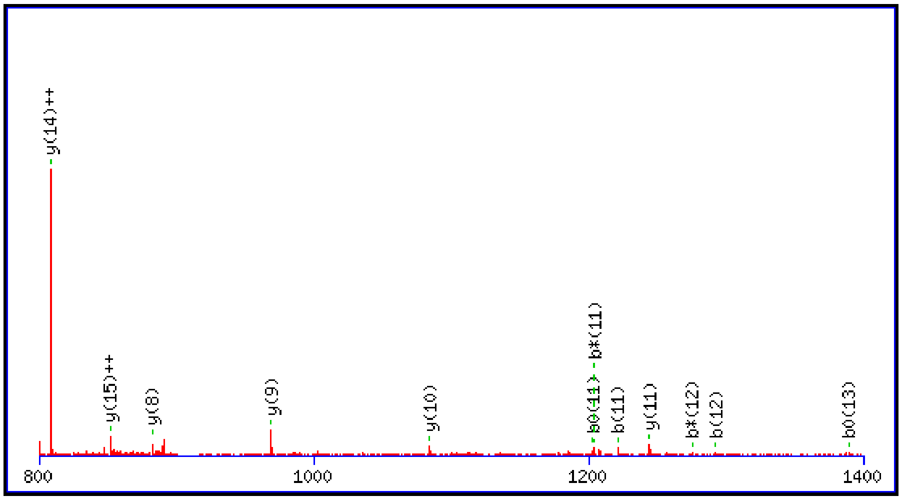


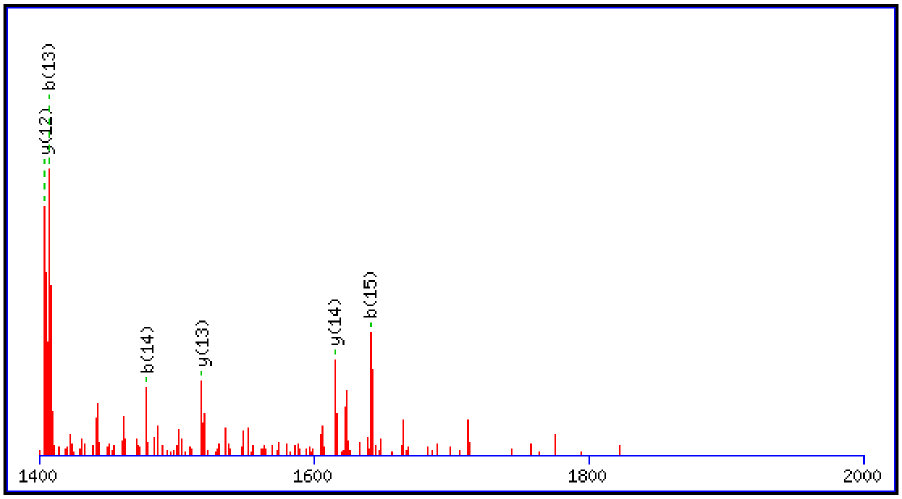


**Monoisotopic mass of neutral peptide Mr(calc):** 1814.7563
**Fixed modifications:** Carbamidomethyl (C)
**Variable modifications:**
**N7** : Deamidated_N (N)
**Ions Score:** 96 **Expect:** 4.7e-008
**Matches (Bold Red):** 31/166 fragment ions using 51 most intense peaks

| # | b | $b^{++}$ | b* | $b^{*++}$ | $b^0$ | $b^{0++}$ | Seq. | y | $y^{++}$ | y* | $y^{*++}$ | $y^0$ | $y^{0++}$ | # |
|---|---|---|---|---|---|---|---|---|---|---|---|---|---|---|
| 1 | 114.0913 | 57.5493 | | | | | L | | | | | | | 16 |
| 2 | 201.1234 | 101.0653 | | | 183.1128 | 92.0600 | S | 1702.6796 | **851.8434** | 1685.6530 | 843.3301 | 1684.6690 | 842.8381 | 15 |
| 3 | 298.1761 | 149.5917 | | | 280.1656 | 140.5864 | P | **1615.6475** | **808.3274** | 1598.6210 | **799.8141** | 1597.6370 | **799.3221** | 14 |
| 4 | 412.2191 | 206.6132 | 395.1925 | 198.0999 | 394.2085 | 197.6079 | N | **1518.5948** | 759.8010 | 1501.5682 | 751.2877 | 1500.5842 | 750.7957 | 13 |
| 5 | 572.2497 | 286.6285 | 555.2232 | 278.1152 | 554.2391 | 277.6232 | C | **1404.5518** | **702.7796** | 1387.5253 | **694.2663** | 1386.5413 | 693.7743 | 12 |

| | | | | | | | | | | | | | | |
|---|---|---|---|---|---|---|---|---|---|---|---|---|---|---|
| **6** | 732.2804 | **366.6438** | 715.2538 | 358.1305 | 714.2698 | 357.6385 | **C** | **1244.5212** | 622.7642 | 1227.4946 | 614.2510 | 1226.5106 | 613.7589 | **11** |
| **7** | 847.3073 | 424.1573 | 830.2807 | 415.6440 | 829.2967 | 415.1520 | **N** | **1084.4905** | 542.7489 | 1067.4640 | 534.2356 | 1066.4800 | 533.7436 | **10** |
| **8** | 934.3393 | 467.6733 | 917.3128 | 459.1600 | 916.3288 | 458.6680 | **S** | **969.4636** | 485.2354 | 952.4371 | 476.7222 | 951.4530 | 476.2302 | **9** |
| **9** | 1021.3713 | 511.1893 | 1004.3448 | 502.6760 | 1003.3608 | 502.1840 | **S** | **882.4316** | 441.7194 | 865.4050 | 433.2062 | 864.4210 | 432.7141 | **8** |
| **10** | 1122.4190 | 561.7132 | 1105.3925 | 553.1999 | 1104.4085 | 552.7079 | **T** | **795.3995** | 398.2034 | 778.3730 | 389.6901 | 777.3890 | 389.1981 | **7** |
| **11** | **1221.4874** | 611.2474 | **1204.4609** | 602.7341 | **1203.4769** | 602.2421 | **V** | **694.3519** | 347.6796 | 677.3253 | **339.1663** | 676.3413 | **338.6743** | **6** |
| **12** | **1292.5246** | 646.7659 | **1275.4980** | 638.2526 | 1274.5140 | 637.7606 | **A** | **595.2835** | 298.1454 | 578.2569 | 289.6321 | 577.2729 | 289.1401 | **5** |
| **13** | **1407.5515** | 704.2794 | 1390.5250 | 695.7661 | **1389.5409** | 695.2741 | **D** | **524.2463** | 262.6268 | 507.2198 | 254.1135 | 506.2358 | 253.6215 | **4** |
| **14** | **1478.5886** | 739.7979 | 1461.5621 | 731.2847 | 1460.5780 | 730.7927 | **A** | **409.2194** | 205.1133 | 392.1928 | 196.6001 | | | **3** |
| **15** | **1641.6519** | 821.3296 | 1624.6254 | 812.8163 | 1623.6414 | 812.3243 | **Y** | **338.1823** | 169.5948 | 321.1557 | 161.0815 | | | **2** |
| **16** | | | | | | | **R** | 175.1190 | 88.0631 | 158.0924 | 79.5498 | | | **1** |

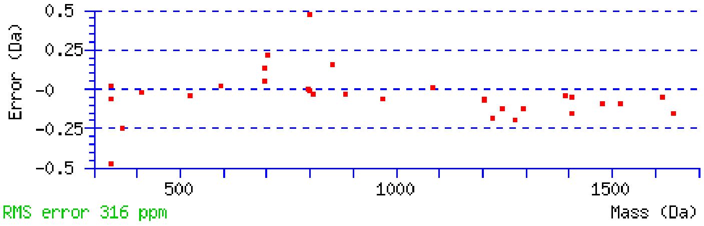


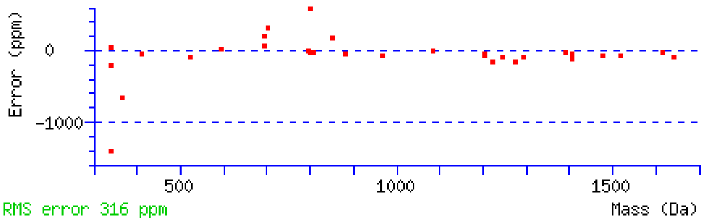


**All matches to this query**

| Score | Mr(calc): | Delta | Sequence |
|---|---|---|---|
| 96.0 | 1814.7563 | 0.0053 | LSPNCCNSSTVADAYR |
| 51.3 | 1814.7563 | 0.0053 | LSPNCCNSSTVADAYR |
| 25.2 | 1813.7723 | 0.9893 | LSPNCCNSSTVADAYR |
| 10.3 | 1813.7416 | 1.0201 | TELFCSINCLSAYR |
| 5.7 | 1814.7724 | -0.0107 | INTTSDEKDPTNPFR |
| 3.8 | 1814.7665 | -0.0048 | HYTPDNNFHSLYVK |
| 3.8 | 1814.7665 | -0.0048 | HYTPDNNFHSLYVK |
| 3.6 | 1812.7689 | 1.9928 | VWQCGGSVEVLPCSR |
| 3.1 | 1812.7567 | 2.0049 | TTYVNTNDEVVSHPR |
| 2.7 | 1812.7567 | 2.0049 | TTYVNTNDEVVSHPR |

---

Spectrum No: 99; Query: 2101; Rank: 1

---

## Peptide View

MS/MS Fragmentation of **GPNVPISTEVNATQFLALVQEK**
Found in **IPI00471666**, Tax_Id=10116 Gene_Symbol=MGC94145 Uncharacterized protein C1orf179 homolog

Match to Query 2101: 2355.243168 from(1178.628860,2+)
Title: 100101RatKid_NS_deglyco_19.5544.5544.2.dta
Data file K:\NewmanPaper\Piliang\3SubProteomes\Piliang3SP\mgf5ppm\ERLIC_3SubProteomes5ppm.mgf

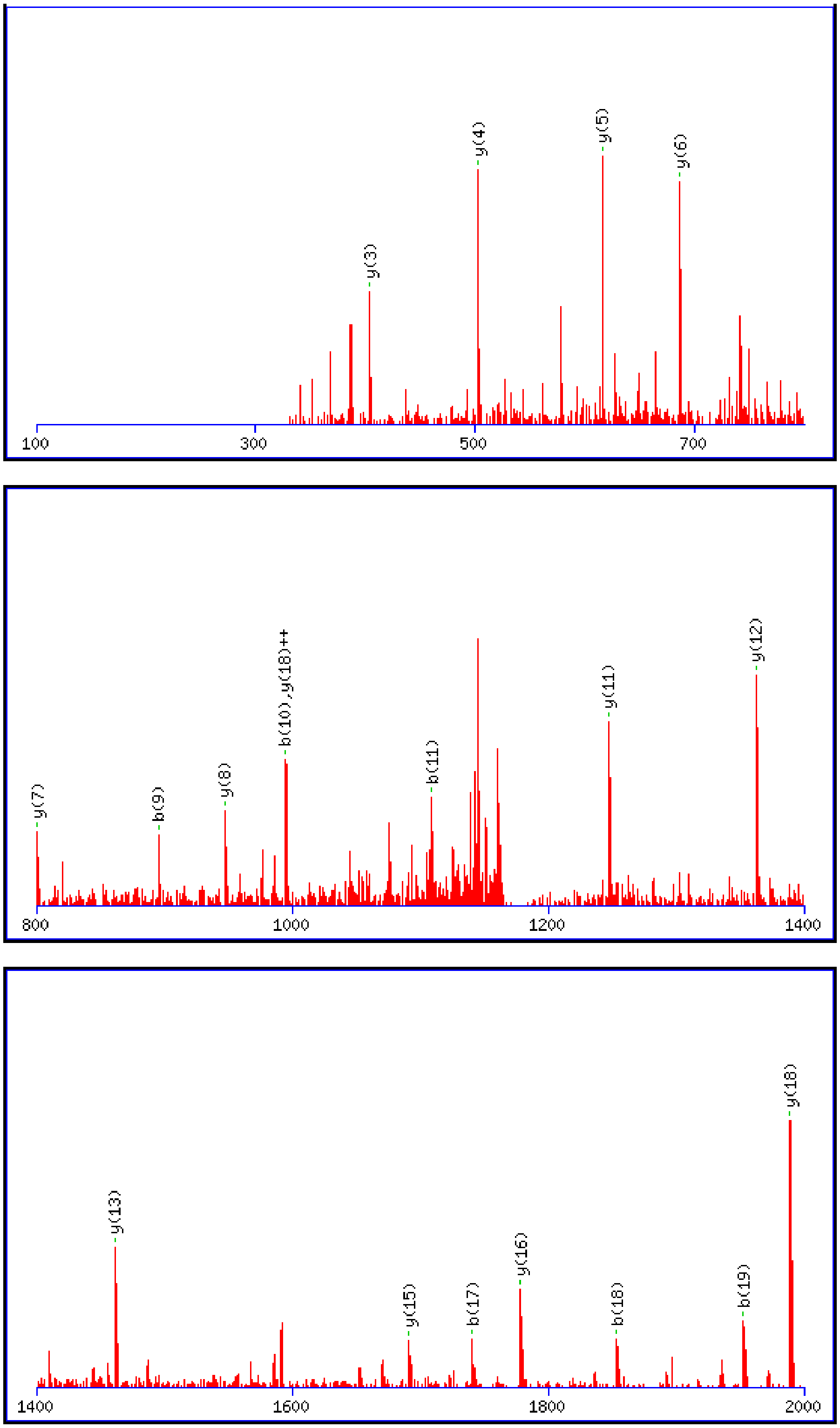


**Monoisotopic mass of neutral peptide Mr(calc):** 2355.2322
**Fixed modifications:** Carbamidomethyl (C)
**Variable modifications:**
**N11** : Deamidated_N (N)
**Ions Score:** 96 **Expect:** 2.5e-008
**Matches (Bold Red):** 19/234 fragment ions using 28 most intense peaks

| # | b | $b^{++}$ | b* | $b^{*++}$ | $b^0$ | $b^{0++}$ | Seq. | y | $y^{++}$ | y* | $y^{*++}$ | $y^0$ | $y^{0++}$ | # |
|---|---|---|---|---|---|---|---|---|---|---|---|---|---|---|
| 1 | 58.0287 | 29.5180 | | | | | G | | | | | | | 22 |
| 2 | 155.0815 | 78.0444 | | | | | P | 2299.2180 | 1150.1126 | 2282.1914 | 1141.5994 | 2281.2074 | 1141.1074 | 21 |
| 3 | 269.1244 | 135.0659 | 252.0979 | 126.5526 | | | N | 2202.1652 | 1101.5863 | 2185.1387 | 1093.0730 | 2184.1547 | 1092.5810 | 20 |
| 4 | 368.1928 | 184.6001 | 351.1663 | 176.0868 | | | V | 2088.1223 | 1044.5648 | 2071.0958 | 1036.0515 | 2070.1117 | 1035.5595 | 19 |
| 5 | 465.2456 | 233.1264 | 448.2191 | 224.6132 | | | P | **1989.0539** | **995.0306** | 1972.0273 | 986.5173 | 1971.0433 | 986.0253 | 18 |

| | | | | | | | | | | | | | | |
|---|---|---|---|---|---|---|---|---|---|---|---|---|---|---|
| **6** | 578.3297 | 289.6685 | 561.3031 | 281.1552 | | | **I** | 1892.0011 | 946.5042 | 1874.9746 | 937.9909 | 1873.9906 | 937.4989 | **17** |
| **7** | 665.3617 | 333.1845 | 648.3352 | 324.6712 | 647.3511 | 324.1792 | **S** | **1778.9171** | 889.9622 | 1761.8905 | 881.4489 | 1760.9065 | 880.9569 | **16** |
| **8** | 766.4094 | 383.7083 | 749.3828 | 375.1951 | 748.3988 | 374.7030 | **T** | **1691.8850** | 846.4462 | 1674.8585 | 837.9329 | 1673.8745 | 837.4409 | **15** |
| **9** | **895.4520** | 448.2296 | 878.4254 | 439.7164 | 877.4414 | 439.2243 | **E** | 1590.8374 | 795.9223 | 1573.8108 | 787.4090 | 1572.8268 | 786.9170 | **14** |
| **10** | **994.5204** | 497.7638 | 977.4938 | 489.2506 | 976.5098 | 488.7585 | **V** | **1461.7948** | 731.4010 | 1444.7682 | 722.8877 | 1443.7842 | 722.3957 | **13** |
| **11** | **1109.5473** | 555.2773 | 1092.5208 | 546.7640 | 1091.5368 | 546.2720 | **N** | **1362.7264** | 681.8668 | 1345.6998 | 673.3535 | 1344.7158 | 672.8615 | **12** |
| **12** | 1180.5844 | 590.7959 | 1163.5579 | 582.2826 | 1162.5739 | 581.7906 | **A** | **1247.6994** | 624.3533 | 1230.6729 | 615.8401 | 1229.6889 | 615.3481 | **11** |
| **13** | 1281.6321 | 641.3197 | 1264.6056 | 632.8064 | 1263.6216 | 632.3144 | **T** | 1176.6623 | 588.8348 | 1159.6358 | 580.3215 | 1158.6517 | 579.8295 | **10** |
| **14** | 1409.6907 | 705.3490 | 1392.6641 | 696.8357 | 1391.6801 | 696.3437 | **Q** | 1075.6146 | 538.3109 | 1058.5881 | 529.7977 | 1057.6041 | 529.3057 | **9** |
| **15** | 1556.7591 | 778.8832 | 1539.7326 | 770.3699 | 1538.7485 | 769.8779 | **F** | **947.5560** | 474.2817 | 930.5295 | 465.7684 | 929.5455 | 465.2764 | **8** |
| **16** | 1669.8432 | 835.4252 | 1652.8166 | 826.9119 | 1651.8326 | 826.4199 | **L** | **800.4876** | 400.7475 | 783.4611 | 392.2342 | 782.4771 | 391.7422 | **7** |
| **17** | **1740.8803** | 870.9438 | 1723.8537 | 862.4305 | 1722.8697 | 861.9385 | **A** | **687.4036** | 344.2054 | 670.3770 | 335.6921 | 669.3930 | 335.2001 | **6** |
| **18** | **1853.9644** | 927.4858 | 1836.9378 | 918.9725 | 1835.9538 | 918.4805 | **L** | **616.3665** | 308.6869 | 599.3399 | 300.1736 | 598.3559 | 299.6816 | **5** |
| **19** | **1953.0328** | 977.0200 | 1936.0062 | 968.5067 | 1935.0222 | 968.0147 | **V** | **503.2824** | 252.1448 | 486.2558 | 243.6316 | 485.2718 | 243.1396 | **4** |
| **20** | 2081.0913 | 1041.0493 | 2064.0648 | 1032.5360 | 2063.0808 | 1032.0440 | **Q** | **404.2140** | 202.6106 | 387.1874 | 194.0974 | 386.2034 | 193.6053 | **3** |
| **21** | 2210.1339 | 1105.5706 | 2193.1074 | 1097.0573 | 2192.1234 | 1096.5653 | **E** | 276.1554 | 138.5813 | 259.1288 | 130.0681 | 258.1448 | 129.5761 | **2** |
| **22** | | | | | | | **K** | 147.1128 | 74.0600 | 130.0863 | 65.5468 | | | **1** |

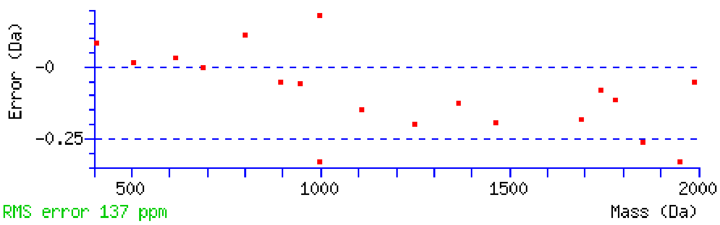


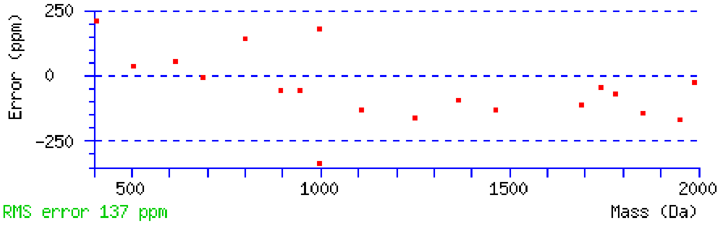


**All matches to this query**

| Score | Mr(calc): | Delta | Sequence |
|---|---|---|---|
| 96.0 | 2355.2322 | 0.0110 | GPNVPISTEVNATQFLALVQEK |
| 20.2 | 2355.2322 | 0.0110 | GPNVPISTEVNATQFLALVQEK |
| 19.6 | 2354.2482 | 0.9950 | GPNVPISTEVNATQFLALVQEK |

---

Spectrum No: 100; Query: 1396; Rank: 1

---

## Peptide View

MS/MS Fragmentation of **IYAGMVSLLDEAVGNVTK**
Found in **IPI00198405**, Tax_Id=10116 Gene_Symbol=Arsb Arylsulfatase B

Match to Query 1396: 1879.961068 from(940.987810,2+)
Title: 091008RatKidney_NH4Format02_25.5366.5366.2.dta
Data file K:\NewmanPaper\Piliang\3SubProteomes\Piliang3SP\mgf5ppm\ERLIC_3SubProteomes5ppm.mgf

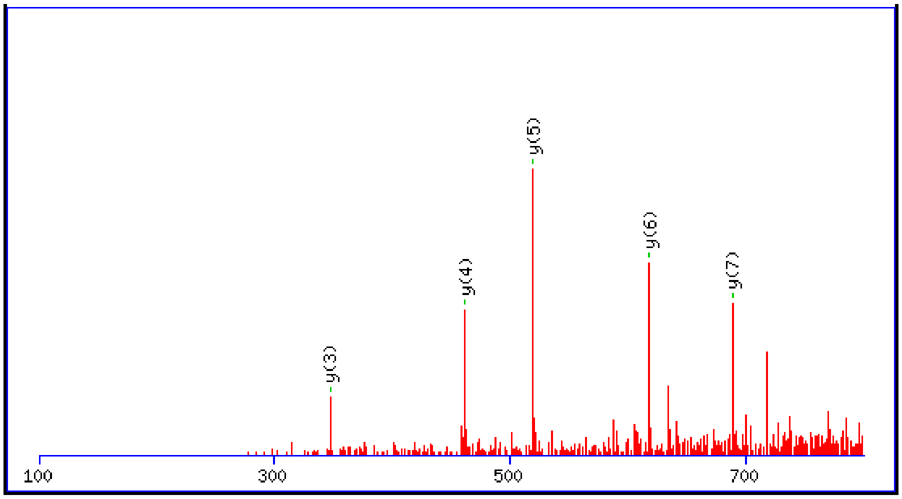


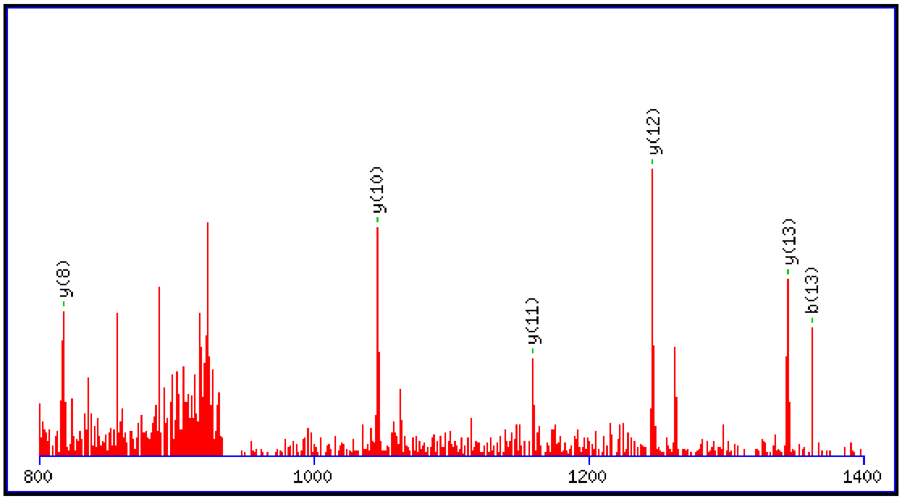


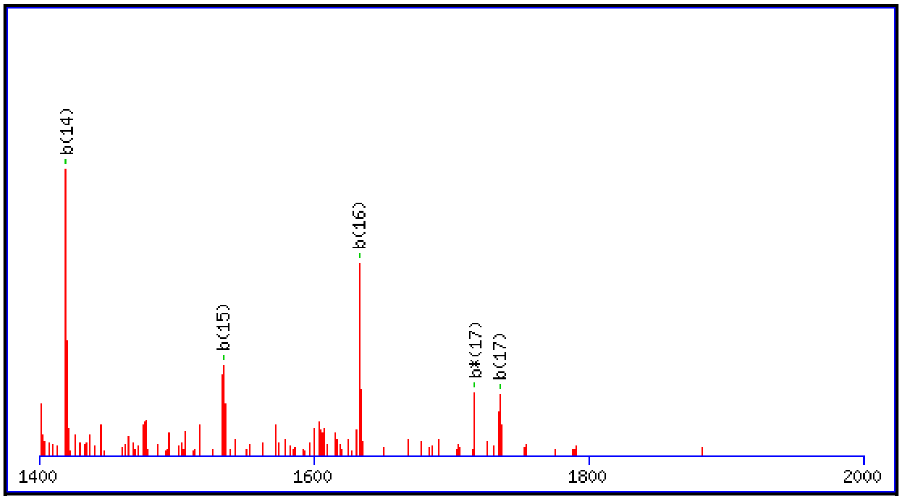


**Monoisotopic mass of neutral peptide Mr(calc):** 1879.9601
**Fixed modifications:** Carbamidomethyl (C)
**Variable modifications:**
**N15** : Deamidated_N (N)
**Ions Score:** 96 **Expect:** 4e-008
**Matches (Bold Red):** 16/162 fragment ions using 26 most intense peaks

| # | b | $b^{++}$ | b* | $b^{*++}$ | $b^{0}$ | $b^{0++}$ | Seq. | y | $y^{++}$ | y* | $y^{*++}$ | $y^{0}$ | $y^{0++}$ | # |
|---|---|---|---|---|---|---|---|---|---|---|---|---|---|---|
| 1 | 114.0913 | 57.5493 | | | | | I | | | | | | | 18 |
| 2 | 277.1547 | 139.0810 | | | | | Y | 1767.8833 | 884.4453 | 1750.8568 | 875.9320 | 1749.8728 | 875.4400 | 17 |
| 3 | 348.1918 | 174.5995 | | | | | A | 1604.8200 | 802.9136 | 1587.7934 | 794.4004 | 1586.8094 | 793.9084 | 16 |
| 4 | 405.2132 | 203.1103 | | | | | G | 1533.7829 | 767.3951 | 1516.7563 | 758.8818 | 1515.7723 | 758.3898 | 15 |
| 5 | 536.2537 | 268.6305 | | | | | M | 1476.7614 | 738.8843 | 1459.7349 | 730.3711 | 1458.7509 | 729.8791 | 14 |

| | | | | | | | | | | | | | | |
|---|---|---|---|---|---|---|---|---|---|---|---|---|---|---|
| **6** | 635.3221 | 318.1647 | | | | | **V** | 1345.7209 | 673.3641 | 1328.6944 | 664.8508 | 1327.7104 | 664.3588 | **13** |
| **7** | 722.3542 | 361.6807 | | | 704.3436 | 352.6754 | **S** | 1246.6525 | 623.8299 | 1229.6260 | 615.3166 | 1228.6420 | 614.8246 | **12** |
| **8** | 835.4382 | 418.2228 | | | 817.4277 | 409.2175 | **L** | 1159.6205 | 580.3139 | 1142.5939 | 571.8006 | 1141.6099 | 571.3086 | **11** |
| **9** | 948.5223 | 474.7648 | | | 930.5117 | 465.7595 | **L** | 1046.5364 | 523.7719 | 1029.5099 | 515.2586 | 1028.5259 | 514.7666 | **10** |
| **10** | 1063.5492 | 532.2783 | | | 1045.5387 | 523.2730 | **D** | 933.4524 | 467.2298 | 916.4258 | 458.7165 | 915.4418 | 458.2245 | **9** |
| **11** | 1192.5918 | 596.7996 | | | 1174.5813 | 587.7943 | **E** | 818.4254 | 409.7163 | 801.3989 | 401.2031 | 800.4149 | 400.7111 | **8** |
| **12** | 1263.6290 | 632.3181 | | | 1245.6184 | 623.3128 | **A** | 689.3828 | 345.1951 | 672.3563 | 336.6818 | 671.3723 | 336.1898 | **7** |
| **13** | 1362.6974 | 681.8523 | | | 1344.6868 | 672.8470 | **V** | 618.3457 | 309.6765 | 601.3192 | 301.1632 | 600.3351 | 300.6712 | **6** |
| **14** | 1419.7188 | 710.3631 | | | 1401.7083 | 701.3578 | **G** | 519.2773 | 260.1423 | 502.2507 | 251.6290 | 501.2667 | 251.1370 | **5** |
| **15** | 1534.7458 | 767.8765 | 1517.7192 | 759.3632 | 1516.7352 | 758.8712 | **N** | 462.2558 | 231.6316 | 445.2293 | 223.1183 | 444.2453 | 222.6263 | **4** |
| **16** | 1633.8142 | 817.4107 | 1616.7876 | 808.8975 | 1615.8036 | 808.4054 | **V** | 347.2289 | 174.1181 | 330.2023 | 165.6048 | 329.2183 | 165.1128 | **3** |
| **17** | 1734.8619 | 867.9346 | 1717.8353 | 859.4213 | 1716.8513 | 858.9293 | **T** | 248.1605 | 124.5839 | 231.1339 | 116.0706 | 230.1499 | 115.5786 | **2** |
| **18** | | | | | | | **K** | 147.1128 | 74.0600 | 130.0863 | 65.5468 | | | **1** |

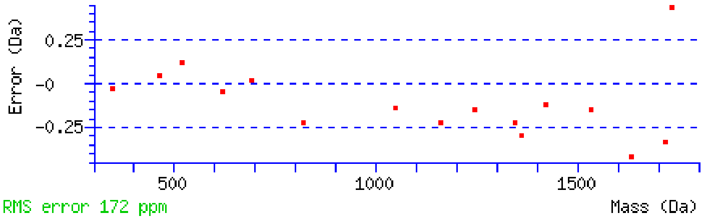


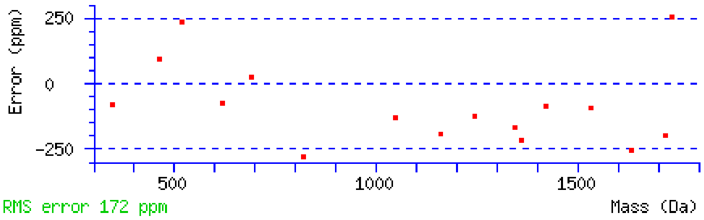


**All matches to this query**

| Score | Mr(calc): | Delta | Sequence |
|---|---|---|---|
| 95.7 | 1879.9601 | 0.0010 | IYAGMVSLLDEAVGNVTK |
| 24.8 | 1878.9761 | 0.9850 | IYAGMVSLLDEAVGNVTK |
| 8.9 | 1879.9536 | 0.0075 | SWVQCGISMKILNSLK |
| 5.1 | 1879.9502 | 0.0109 | AWEDMLIQFLRNSLK |
| 3.0 | 1878.9588 | 1.0022 | RTYVTAPGTGFLPGDTAR |
| 3.0 | 1877.9557 | 2.0054 | NFLQAVCTLLNSGGGVVK |
| 2.5 | 1879.9462 | 0.0149 | QFDKVREDMELSLVR |
| 1.6 | 1879.9639 | -0.0028 | FQSRTSEAAKLEAEVSK |
| 0.8 | 1877.9595 | 2.0015 | RSSGLVQNIDQLYSGGGK |
| 0.8 | 1878.9696 | 0.9915 | SWVQCGISMKILNSLK |

---

Spectrum No: 101; Query: 1885; Rank: 1

---

## Peptide View

MS/MS Fragmentation of **TGEANLTQLYLQEALDFIR**
Found in **IPI00359847**, Tax_Id=10116 Gene_Symbol=Galns N-acetylgalactosamine-6-sulfatase precursor

Match to Query 1885: 2195.118008 from(1098.566280,2+)
Title: 100101RatKid_NS_deglyco_11.7671.7671.2.dta
Data file K:\NewmanPaper\Piliang\3SubProteomes\Piliang3SP\mgf5ppm\ERLIC_3SubProteomes5ppm.mgf

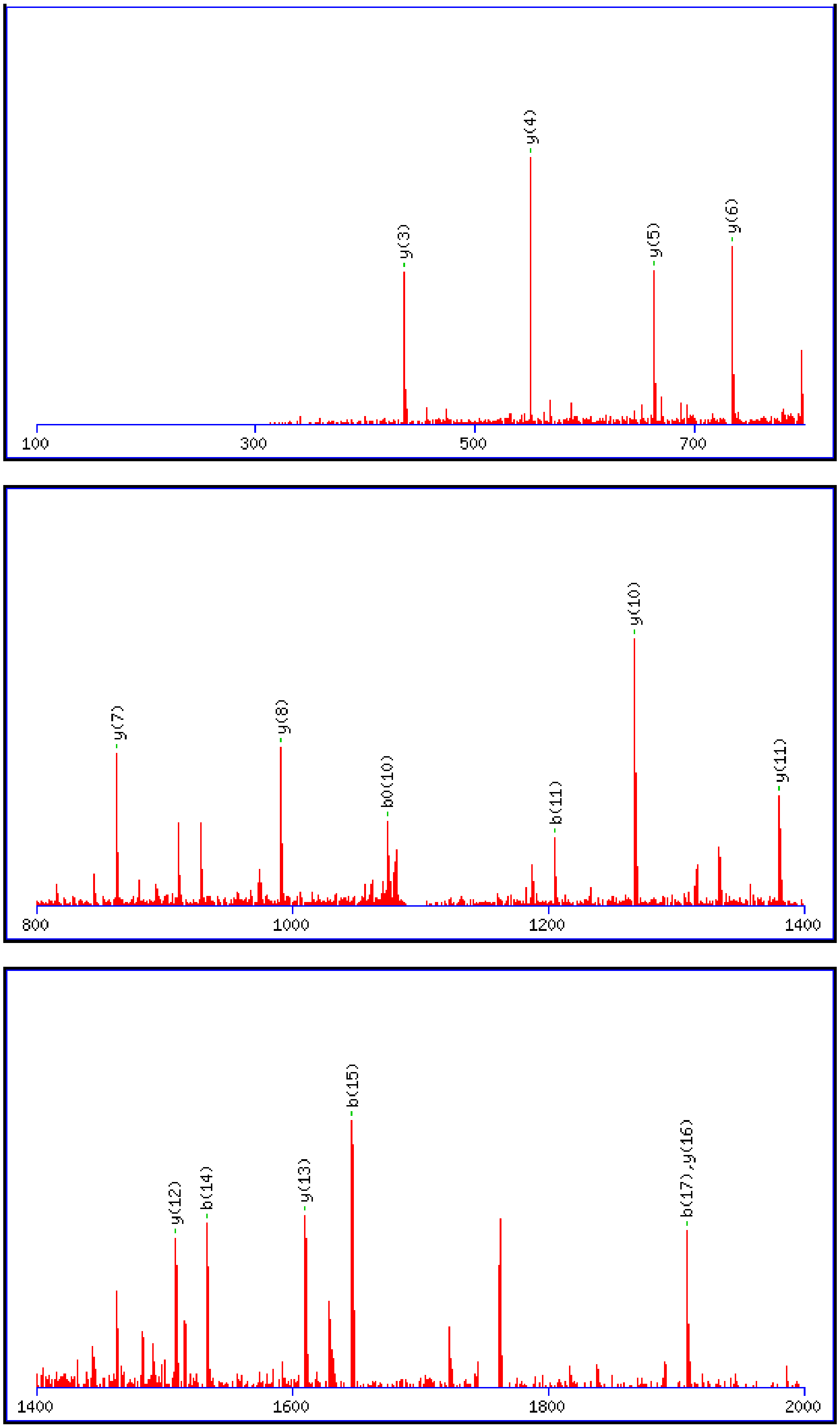


**Monoisotopic mass of neutral peptide Mr(calc):** 2195.1110
**Fixed modifications:** Carbamidomethyl (C)
**Variable modifications:**
**N5** : Deamidated_N (N)
**Ions Score:** 96 **Expect:** 4.5e-008
**Matches (Bold Red):** 16/202 fragment ions using 23 most intense peaks

| # | b | $b^{++}$ | b* | $b^{*++}$ | $b^0$ | $b^{0++}$ | Seq. | y | $y^{++}$ | y* | $y^{*++}$ | $y^0$ | $y^{0++}$ | # |
|---|---|---|---|---|---|---|---|---|---|---|---|---|---|---|
| 1 | 102.0550 | 51.5311 | | | 84.0444 | 42.5258 | T | | | | | | | 19 |
| 2 | 159.0764 | 80.0418 | | | 141.0659 | 71.0366 | G | 2095.0706 | 1048.0389 | 2078.0441 | 1039.5257 | 2077.0600 | 1039.0337 | 18 |
| 3 | 288.1190 | 144.5631 | | | 270.1084 | 135.5579 | E | 2038.0491 | 1019.5282 | 2021.0226 | 1011.0149 | 2020.0386 | 1010.5229 | 17 |
| 4 | 359.1561 | 180.0817 | | | 341.1456 | 171.0764 | A | **1909.0066** | 955.0069 | 1891.9800 | 946.4936 | 1890.9960 | 946.0016 | 16 |
| 5 | 474.1831 | 237.5952 | 457.1565 | 229.0819 | 456.1725 | 228.5899 | N | 1837.9694 | 919.4884 | 1820.9429 | 910.9751 | 1819.9589 | 910.4831 | 15 |

| | | | | | | | | | | | | | | |
|---|---|---|---|---|---|---|---|---|---|---|---|---|---|---|
| 6 | 587.2671 | 294.1372 | 570.2406 | 285.6239 | 569.2566 | 285.1319 | L | 1722.9425 | 861.9749 | 1705.9160 | 853.4616 | 1704.9319 | 852.9696 | 14 |
| 7 | 688.3148 | 344.6610 | 671.2883 | 336.1478 | 670.3042 | 335.6558 | T | **1609.8584** | 805.4329 | 1592.8319 | 796.9196 | 1591.8479 | 796.4276 | 13 |
| 8 | 816.3734 | 408.6903 | 799.3468 | 400.1771 | 798.3628 | 399.6850 | Q | **1508.8108** | 754.9090 | 1491.7842 | 746.3957 | 1490.8002 | 745.9037 | 12 |
| 9 | 929.4574 | 465.2324 | 912.4309 | 456.7191 | 911.4469 | 456.2271 | L | **1380.7522** | 690.8797 | 1363.7256 | 682.3665 | 1362.7416 | 681.8744 | 11 |
| 10 | 1092.5208 | 546.7640 | 1075.4942 | 538.2508 | **1074.5102** | 537.7587 | Y | **1267.6681** | 634.3377 | 1250.6416 | 625.8244 | 1249.6576 | 625.3324 | 10 |
| 11 | **1205.6048** | 603.3061 | 1188.5783 | 594.7928 | 1187.5943 | 594.3008 | L | 1104.6048 | 552.8060 | 1087.5782 | 544.2928 | 1086.5942 | 543.8007 | 9 |
| 12 | 1333.6634 | 667.3353 | 1316.6369 | 658.8221 | 1315.6529 | 658.3301 | Q | **991.5207** | 496.2640 | 974.4942 | 487.7507 | 973.5102 | 487.2587 | 8 |
| 13 | 1462.7060 | 731.8566 | 1445.6795 | 723.3434 | 1444.6954 | 722.8514 | E | **863.4621** | 432.2347 | 846.4356 | 423.7214 | 845.4516 | 423.2294 | 7 |
| 14 | **1533.7431** | 767.3752 | 1516.7166 | 758.8619 | 1515.7326 | 758.3699 | A | **734.4196** | 367.7134 | 717.3930 | 359.2001 | 716.4090 | 358.7081 | 6 |
| 15 | **1646.8272** | 823.9172 | 1629.8006 | 815.4040 | 1628.8166 | 814.9119 | L | **663.3824** | 332.1949 | 646.3559 | 323.6816 | 645.3719 | 323.1896 | 5 |
| 16 | 1761.8541 | 881.4307 | 1744.8276 | 872.9174 | 1743.8436 | 872.4254 | D | **550.2984** | 275.6528 | 533.2718 | 267.1396 | 532.2878 | 266.6475 | 4 |
| 17 | **1908.9225** | 954.9649 | 1891.8960 | 946.4516 | 1890.9120 | 945.9596 | F | **435.2714** | 218.1394 | 418.2449 | 209.6261 | | | 3 |
| 18 | 2022.0066 | 1011.5069 | 2004.9801 | 1002.9937 | 2003.9960 | 1002.5017 | I | 288.2030 | 144.6051 | 271.1765 | 136.0919 | | | 2 |
| 19 | | | | | | | R | 175.1190 | 88.0631 | 158.0924 | 79.5498 | | | 1 |

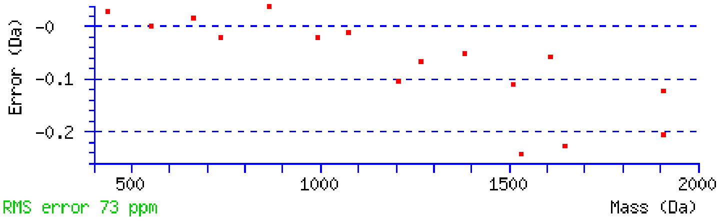


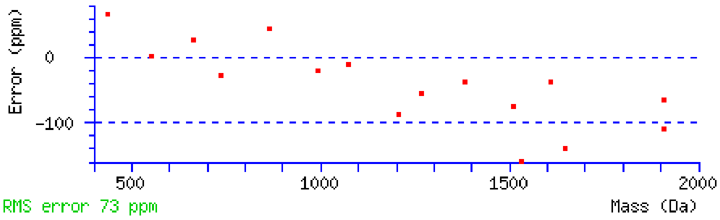


**All matches to this query**

| Score | Mr(calc): | Delta | Sequence |
|---|---|---|---|
| 95.7 | 2195.1110 | 0.0070 | TGEANLTQLYLQEALDFIR |
| 67.2 | 2194.1270 | 0.9910 | TGEANLTQLYLQEALDFIR |
| 2.9 | 2195.1395 | -0.0215 | TLAKVNTFMSETGNILNIVK |

---

Spectrum No: 102; Query: 952; Rank: 1

---

## Peptide View

MS/MS Fragmentation of **KFINDYVSNQTQGK**
Found in **IPI00200593**, Tax_Id=10116 Gene_Symbol=Serpina3k Serine protease inhibitor A3K precursor

Match to Query 952: 1641.799808 from(821.907180,2+)
Title: 091008RatKidney_NoSalt_31.1289.1289.2.dta
Data file K:\NewmanPaper\Piliang\3SubProteomes\Piliang3SP\mgf5ppm\ERLIC_3SubProteomes5ppm.mgf

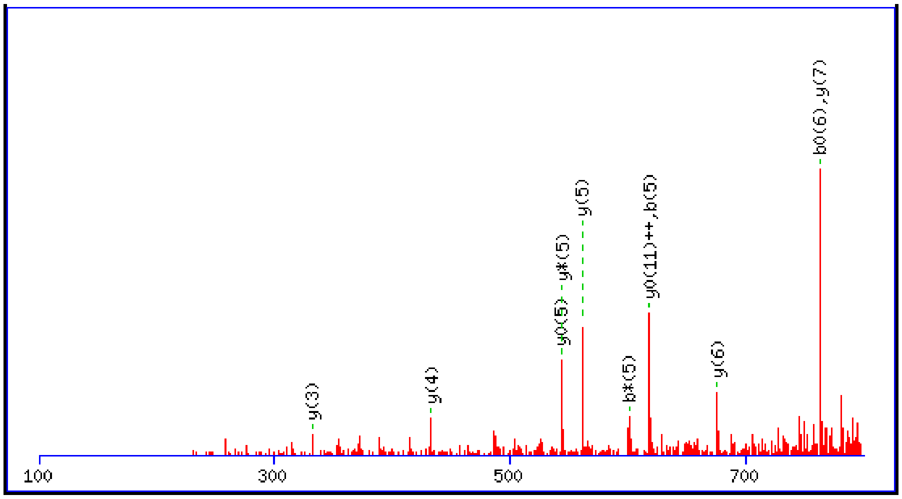


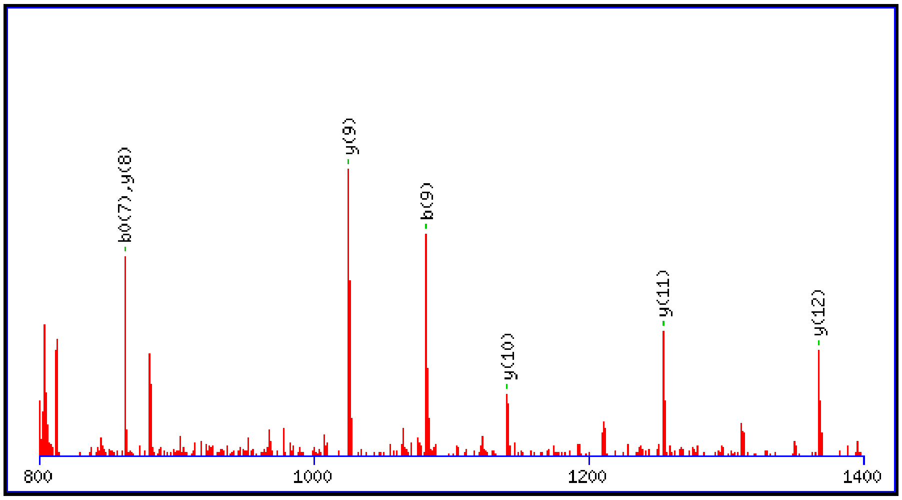


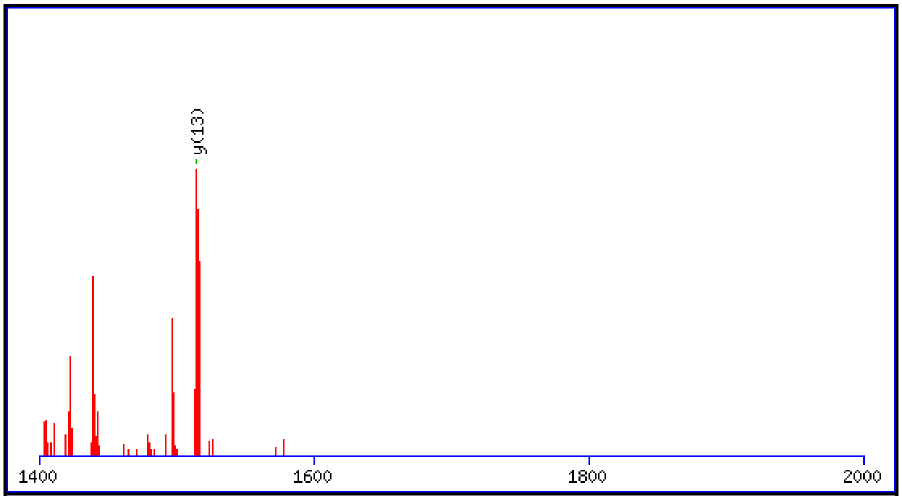


**Monoisotopic mass of neutral peptide Mr(calc):** 1641.7998
**Fixed modifications:** Carbamidomethyl (C)
**Variable modifications:**
**N9** : Deamidated_N (N)
**Ions Score:** 96 **Expect:** 5e-008
**Matches (Bold Red):** 19/142 fragment ions using 22 most intense peaks

| # | b | b++ | b* | b*++ | b0 | b0++ | Seq. | y | y++ | y* | y*++ | y0 | y0++ | # |
|---|---|---|---|---|---|---|---|---|---|---|---|---|---|---|
| 1 | 129.1022 | 65.0548 | 112.0757 | 56.5415 | | | K | | | | | | | 14 |
| 2 | 276.1707 | 138.5890 | 259.1441 | 130.0757 | | | F | **1514.7122** | 757.8597 | 1497.6856 | 749.3464 | 1496.7016 | 748.8544 | 13 |
| 3 | 389.2547 | 195.1310 | 372.2282 | 186.6177 | | | I | **1367.6437** | 684.3255 | 1350.6172 | 675.8122 | 1349.6332 | 675.3202 | 12 |
| 4 | 503.2976 | 252.1525 | 486.2711 | 243.6392 | | | N | **1254.5597** | 627.7835 | 1237.5331 | 619.2702 | 1236.5491 | **618.7782** | 11 |
| 5 | **618.3246** | 309.6659 | **601.2980** | 301.1527 | 600.3140 | 300.6606 | D | **1140.5168** | 570.7620 | 1123.4902 | 562.2487 | 1122.5062 | 561.7567 | 10 |

| | | | | | | | | | | | | | | |
|---|---|---|---|---|---|---|---|---|---|---|---|---|---|---|
| **6** | 781.3879 | 391.1976 | 764.3614 | 382.6843 | **763.3774** | 382.1923 | **Y** | **1025.4898** | 513.2485 | 1008.4633 | 504.7353 | 1007.4792 | 504.2433 | **9** |
| **7** | 880.4563 | 440.7318 | 863.4298 | 432.2185 | **862.4458** | 431.7265 | **V** | **862.4265** | 431.7169 | 845.3999 | 423.2036 | 844.4159 | 422.7116 | **8** |
| **8** | 967.4884 | 484.2478 | 950.4618 | 475.7345 | 949.4778 | 475.2425 | **S** | **763.3581** | 382.1827 | 746.3315 | 373.6694 | 745.3475 | 373.1774 | **7** |
| **9** | **1082.5153** | 541.7613 | 1065.4887 | 533.2480 | 1064.5047 | 532.7560 | **N** | **676.3260** | 338.6667 | 659.2995 | 330.1534 | 658.3155 | 329.6614 | **6** |
| **10** | 1210.5739 | 605.7906 | 1193.5473 | 597.2773 | 1192.5633 | 596.7853 | **Q** | **561.2991** | 281.1532 | **544.2726** | 272.6399 | **543.2885** | 272.1479 | **5** |
| **11** | 1311.6216 | 656.3144 | 1294.5950 | 647.8011 | 1293.6110 | 647.3091 | **T** | **433.2405** | 217.1239 | 416.2140 | 208.6106 | 415.2300 | 208.1186 | **4** |
| **12** | 1439.6801 | 720.3437 | 1422.6536 | 711.8304 | 1421.6696 | 711.3384 | **Q** | **332.1928** | 166.6001 | 315.1663 | 158.0868 | | | **3** |
| **13** | 1496.7016 | 748.8544 | 1479.6750 | 740.3412 | 1478.6910 | 739.8492 | **G** | 204.1343 | 102.5708 | 187.1077 | 94.0575 | | | **2** |
| **14** | | | | | | | **K** | 147.1128 | 74.0600 | 130.0863 | 65.5468 | | | **1** |

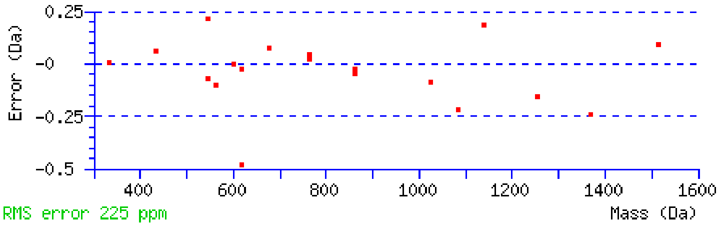


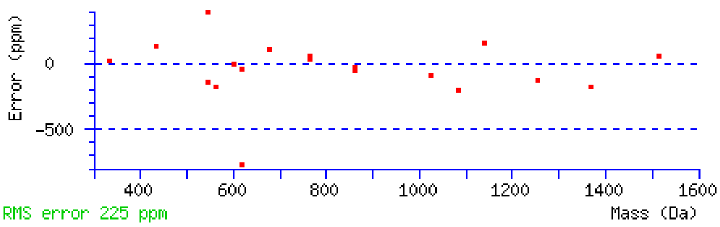


**All matches to this query**

| Score | Mr(calc): | Delta | Sequence |
|---|---|---|---|
| 95.5 | 1641.7998 | -0.0000 | KFINDYVSNQTQGK |
| 44.4 | 1641.7998 | -0.0000 | KFINDYVSNQTQGK |
| 17.5 | 1641.8032 | -0.0034 | MENIGAHLSNAVLEK |
| 7.0 | 1640.7957 | 1.0042 | QELDLNSSMRLKK |
| 6.5 | 1639.8093 | 1.9905 | VFLEEEDSFSALVR |
| 5.8 | 1641.8015 | -0.0017 | LTVGDPVTVEYITR |
| 5.5 | 1641.7988 | 0.0010 | SRKNRPAASGGFVSK |
| 4.9 | 1641.7875 | 0.0123 | WTTNXRSLNSNKK |
| 4.9 | 1641.7875 | 0.0123 | WTTNXRSLNSNKK |
| 4.8 | 1640.7866 | 1.0132 | RTGERQNLSPDDPR |

---

Spectrum No: 103; Query: 894; Rank: 1

---

## Peptide View

MS/MS Fragmentation of **VTINNWVANKTEGR**
Found in **IPI00372372**, Tax_Id=10116 Gene_Symbol=Serpinc1 Serine (Or cysteine) peptidase inhibitor, clade C (Antithrombin), member 1

Match to Query 894: 1601.819008 from(801.916780,2+)
Title: 091008RatKidney_NoSalt_27.1969.1969.2.dta
Data file K:\NewmanPaper\Piliang\3SubProteomes\Piliang3SP\mgf5ppm\ERLIC_3SubProteomes5ppm.mgf

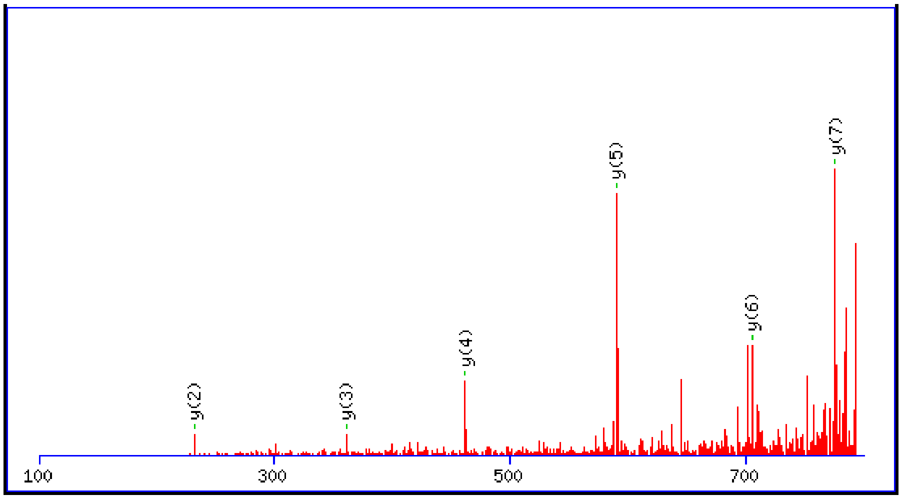


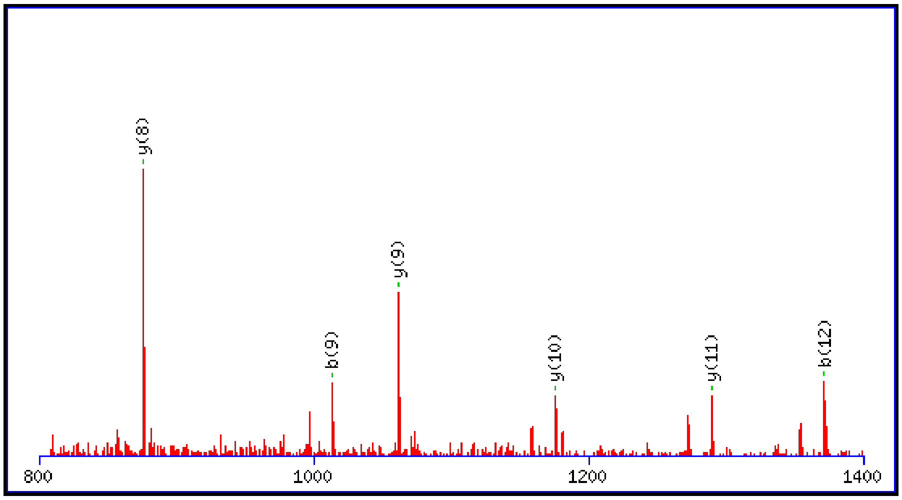


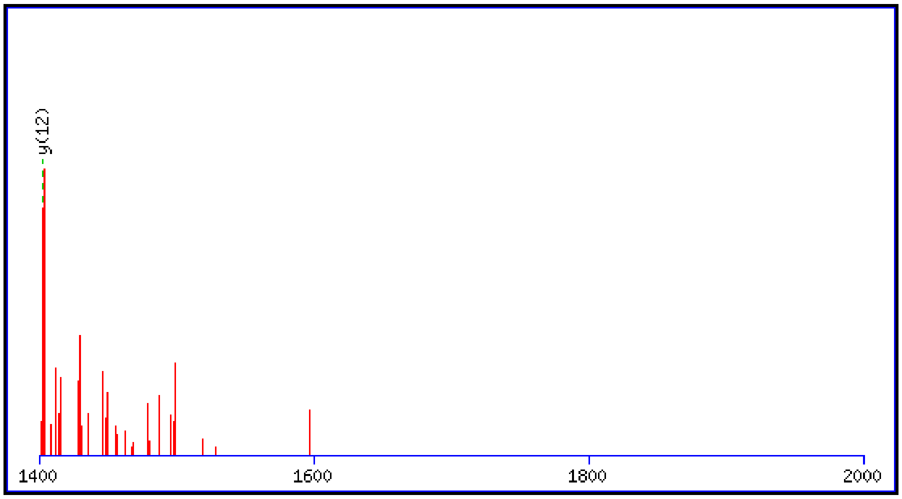


**Monoisotopic mass of neutral peptide Mr(calc):** 1601.8161
**Fixed modifications:** Carbamidomethyl (C)
**Variable modifications:**
**N9** : Deamidated_N (N)
**Ions Score:** 96 **Expect:** 3.7e-008
**Matches (Bold Red):** 13/144 fragment ions using 24 most intense peaks

| # | b | $b^{++}$ | b* | $b^{*++}$ | $b^{0}$ | $b^{0++}$ | Seq. | y | $y^{++}$ | y* | $y^{*++}$ | $y^{0}$ | $y^{0++}$ | # |
|---|---|---|---|---|---|---|---|---|---|---|---|---|---|---|
| 1 | 100.0757 | 50.5415 | | | | | V | | | | | | | 14 |
| 2 | 201.1234 | 101.0653 | | | 183.1128 | 92.0600 | T | 1503.7550 | 752.3812 | 1486.7285 | 743.8679 | 1485.7445 | 743.3759 | 13 |
| 3 | 314.2074 | 157.6074 | | | 296.1969 | 148.6021 | I | **1402.7073** | 701.8573 | 1385.6808 | 693.3440 | 1384.6968 | 692.8520 | 12 |
| 4 | 428.2504 | 214.6288 | 411.2238 | 206.1155 | 410.2398 | 205.6235 | N | **1289.6233** | 645.3153 | 1272.5967 | 636.8020 | 1271.6127 | 636.3100 | 11 |
| 5 | 542.2933 | 271.6503 | 525.2667 | 263.1370 | 524.2827 | 262.6450 | N | **1175.5804** | 588.2938 | 1158.5538 | 579.7805 | 1157.5698 | 579.2885 | 10 |

| | | | | | | | | | | | | | | |
|---|---|---|---|---|---|---|---|---|---|---|---|---|---|---|
| **6** | 728.3726 | 364.6899 | 711.3461 | 356.1767 | 710.3620 | 355.6847 | **W** | **1061.5374** | 531.2724 | 1044.5109 | 522.7591 | 1043.5269 | 522.2671 | **9** |
| **7** | 827.4410 | 414.2241 | 810.4145 | 405.7109 | 809.4304 | 405.2189 | **V** | **875.4581** | 438.2327 | 858.4316 | 429.7194 | 857.4475 | 429.2274 | **8** |
| **8** | 898.4781 | 449.7427 | 881.4516 | 441.2294 | 880.4676 | 440.7374 | **A** | **776.3897** | 388.6985 | 759.3632 | 380.1852 | 758.3791 | 379.6932 | **7** |
| **9** | **1013.5051** | 507.2562 | 996.4785 | 498.7429 | 995.4945 | 498.2509 | **N** | **705.3526** | 353.1799 | 688.3260 | 344.6667 | 687.3420 | 344.1746 | **6** |
| **10** | 1141.6000 | 571.3037 | 1124.5735 | 562.7904 | 1123.5895 | 562.2984 | **K** | **590.3257** | 295.6665 | 573.2991 | 287.1532 | 572.3151 | 286.6612 | **5** |
| **11** | 1242.6477 | 621.8275 | 1225.6212 | 613.3142 | 1224.6371 | 612.8222 | **T** | **462.2307** | 231.6190 | 445.2041 | 223.1057 | 444.2201 | 222.6137 | **4** |
| **12** | **1371.6903** | 686.3488 | 1354.6638 | 677.8355 | 1353.6797 | 677.3435 | **E** | **361.1830** | 181.0951 | 344.1565 | 172.5819 | 343.1724 | 172.0899 | **3** |
| **13** | 1428.7118 | 714.8595 | 1411.6852 | 706.3462 | 1410.7012 | 705.8542 | **G** | **232.1404** | 116.5738 | 215.1139 | 108.0606 | | | **2** |
| **14** | | | | | | | **R** | 175.1190 | 88.0631 | 158.0924 | 79.5498 | | | **1** |

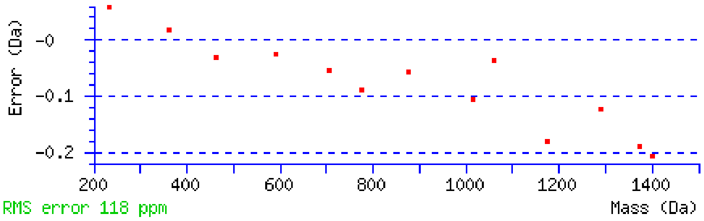


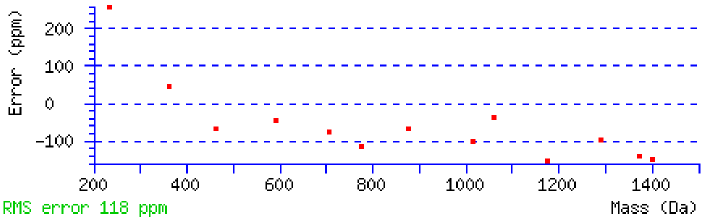


**All matches to this query**

| Score | Mr(calc): | Delta | Sequence |
|---|---|---|---|
| 95.5 | 1601.8161 | 0.0029 | VTINNWVANKTEGR |
| 31.0 | 1601.8161 | 0.0029 | VTINNWVANKTEGR |
| 19.6 | 1601.8161 | 0.0029 | VTINNWVANKTEGR |
| 2.6 | 1601.8168 | 0.0022 | GAAGGNLSSRCVRAAR |

Spectrum No: 104; Query: 1254; Rank: 1

## Peptide View

MS/MS Fragmentation of **LDNTSMSTVIDGVVLPK**
Found in **IPI00195148**, Tax_Id=10116 Gene_Symbol=Es2 Liver carboxylesterase 1 precursor

Match to Query 1254: 1804.911988 from(903.463270,2+)
Title: 091008RatKidney_NoSalt_30.2570.2570.2.dta
Data file K:\NewmanPaper\Piliang\3SubProteomes\Piliang3SP\mgf5ppm\ERLIC_3SubProteomes5ppm.mgf

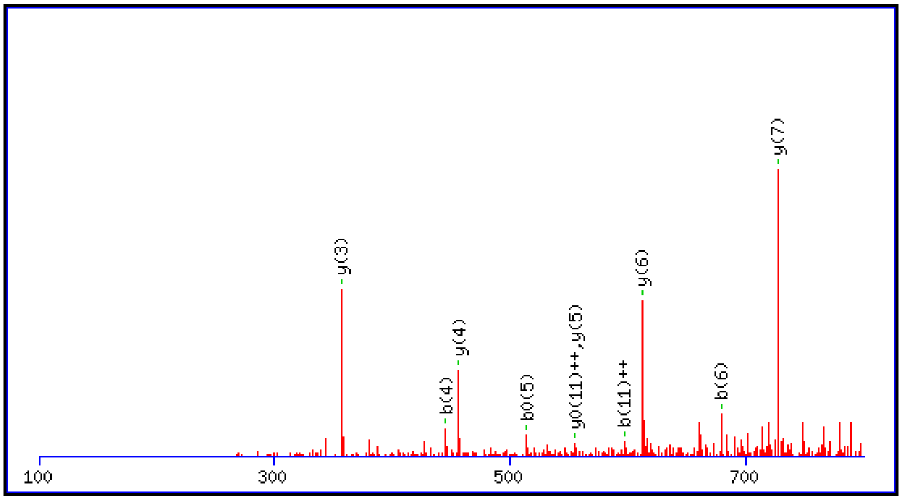

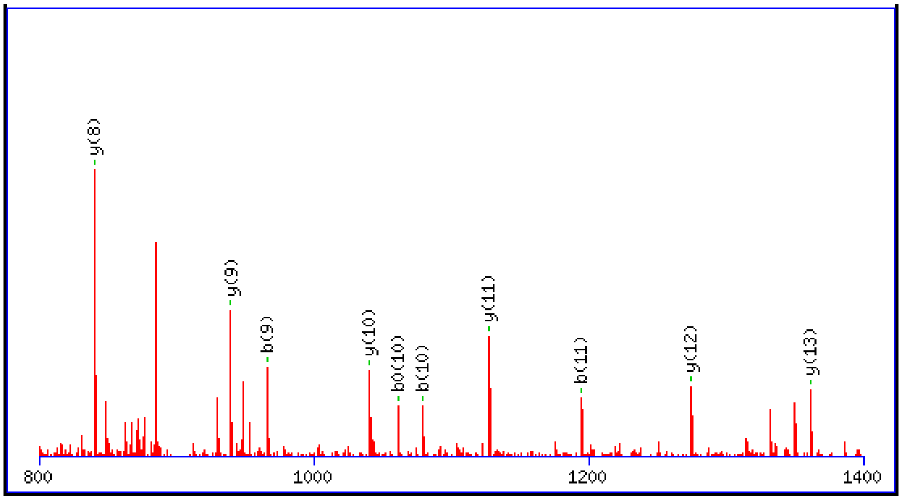


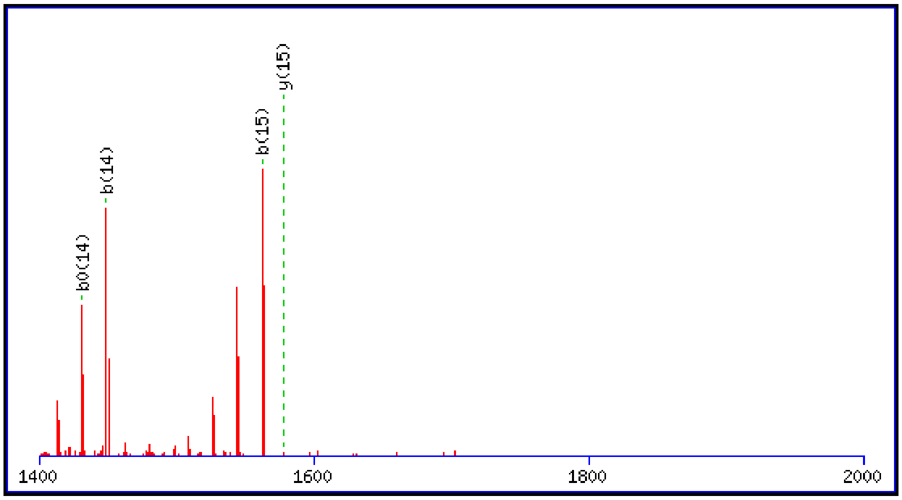


**Monoisotopic mass of neutral peptide Mr(calc):** 1804.9128
**Fixed modifications:** Carbamidomethyl (C)
**Variable modifications:**
**N3** : Deamidated_N (N)
**M6** : Oxidation (M)
**Ions Score:** 95 **Expect:** 4.8e-008
**Matches (Bold Red):** 24/174 fragment ions using 36 most intense peaks

| # | b | $b^{++}$ | b* | $b^{*++}$ | $b^{0}$ | $b^{0++}$ | Seq. | y | $y^{++}$ | y* | $y^{*++}$ | $y^{0}$ | $y^{0++}$ | # |
|---|---|---|---|---|---|---|---|---|---|---|---|---|---|---|
| 1 | 114.0913 | 57.5493 | | | | | L | | | | | | | 17 |
| 2 | 229.1183 | 115.0628 | | | 211.1077 | 106.0575 | D | 1692.8360 | 846.9217 | 1675.8095 | 838.4084 | 1674.8255 | 837.9164 | 16 |
| 3 | 344.1452 | 172.5762 | 327.1187 | 164.0630 | 326.1347 | 163.5710 | N | **1577.8091** | 789.4082 | 1560.7826 | 780.8949 | 1559.7985 | 780.4029 | 15 |
| 4 | **445.1929** | 223.1001 | 428.1663 | 214.5868 | 427.1823 | 214.0948 | T | 1462.7822 | 731.8947 | 1445.7556 | 723.3814 | 1444.7716 | 722.8894 | 14 |
| 5 | 532.2249 | 266.6161 | 515.1984 | 258.1028 | **514.2144** | 257.6108 | S | **1361.7345** | 681.3709 | 1344.7079 | 672.8576 | 1343.7239 | 672.3656 | 13 |
| 6 | **679.2603** | 340.1338 | 662.2338 | 331.6205 | 661.2498 | 331.1285 | M | **1274.7025** | 637.8549 | 1257.6759 | 629.3416 | 1256.6919 | 628.8496 | 12 |
| 7 | 766.2924 | 383.6498 | 749.2658 | 375.1365 | 748.2818 | 374.6445 | S | **1127.6671** | 564.3372 | 1110.6405 | 555.8239 | 1109.6565 | **555.3319** | 11 |
| 8 | 867.3400 | 434.1737 | 850.3135 | 425.6604 | 849.3295 | 425.1684 | T | **1040.6350** | 520.8212 | 1023.6085 | 512.3079 | 1022.6245 | 511.8159 | 10 |
| 9 | **966.4085** | 483.7079 | 949.3819 | 475.1946 | 948.3979 | 474.7026 | V | **939.5873** | 470.2973 | 922.5608 | 461.7840 | 921.5768 | 461.2920 | 9 |
| 10 | **1079.4925** | 540.2499 | 1062.4660 | 531.7366 | **1061.4819** | 531.2446 | I | **840.5189** | 420.7631 | 823.4924 | 412.2498 | 822.5084 | 411.7578 | 8 |
| 11 | **1194.5195** | **597.7634** | 1177.4929 | 589.2501 | 1176.5089 | 588.7581 | D | **727.4349** | 364.2211 | 710.4083 | 355.7078 | 709.4243 | 355.2158 | 7 |
| 12 | 1251.5409 | 626.2741 | 1234.5144 | 617.7608 | 1233.5304 | 617.2688 | G | **612.4079** | 306.7076 | 595.3814 | 298.1943 | | | 6 |
| 13 | 1350.6093 | 675.8083 | 1333.5828 | 667.2950 | 1332.5988 | 666.8030 | V | **555.3865** | 278.1969 | 538.3599 | 269.6836 | | | 5 |
| 14 | **1449.6778** | 725.3425 | 1432.6512 | 716.8292 | **1431.6672** | 716.3372 | V | **456.3180** | 228.6627 | 439.2915 | 220.1494 | | | 4 |
| 15 | **1562.7618** | 781.8845 | 1545.7353 | 773.3713 | 1544.7512 | 772.8793 | L | **357.2496** | 179.1285 | 340.2231 | 170.6152 | | | 3 |
| 16 | 1659.8146 | 830.4109 | 1642.7880 | 821.8977 | 1641.8040 | 821.4056 | P | 244.1656 | 122.5864 | 227.1390 | 114.0731 | | | 2 |
| 17 | | | | | | | K | 147.1128 | 74.0600 | 130.0863 | 65.5468 | | | 1 |

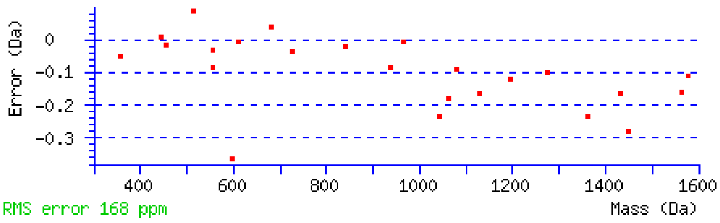


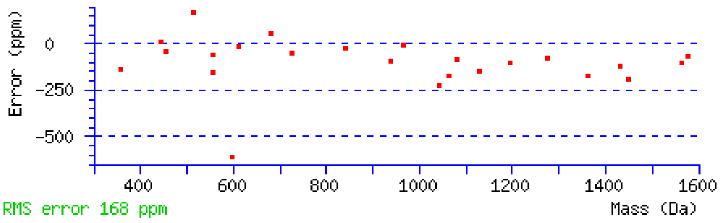


### All matches to this query

| Score | Mr(calc): | Delta | Sequence |
|---|---|---|---|
| 95.3 | 1804.9128 | -0.0009 | LDNTSMSTVIDGVVLPK |
| 8.1 | 1804.9100 | 0.0020 | LKKLSLSSLETASLR |
| 8.1 | 1804.9100 | 0.0020 | LKKLSLSSLETASLR |
| 4.5 | 1803.9132 | 0.9988 | DVLKGVDASNALVLPGR |
| 2.8 | 1803.9189 | 0.9931 | LECLHVPANGTTGPPIK |
| 2.2 | 1803.8992 | 1.0128 | RTRSPSPTLGESLAPR |
| 0.4 | 1803.8920 | 1.0200 | IIYKEISVTGPPHDR |
| 0.2 | 1804.9100 | 0.0020 | LKKLSLSSLETASLR |
| 0.2 | 1804.9100 | 0.0020 | LKKLSLSSLETASLR |

---

Spectrum No: 105; Query: 1311; Rank: 1

---

## Peptide View

MS/MS Fragmentation of **LENLSSTESGYTATLTR**
Found in **IPI00400579**, Tax_Id=10116 Gene_Symbol=Gaa Lysosomal alpha-glucosidase precursor

Match to Query 1311: 1842.890108 from(922.452330,2+)
Title: 100101RatKid_NS_deglyco_14.2819.2819.2.dta
Data file K:\NewmanPaper\Piliang\3SubProteomes\Piliang3SP\mgf5ppm\ERLIC_3SubProteomes5ppm.mgf

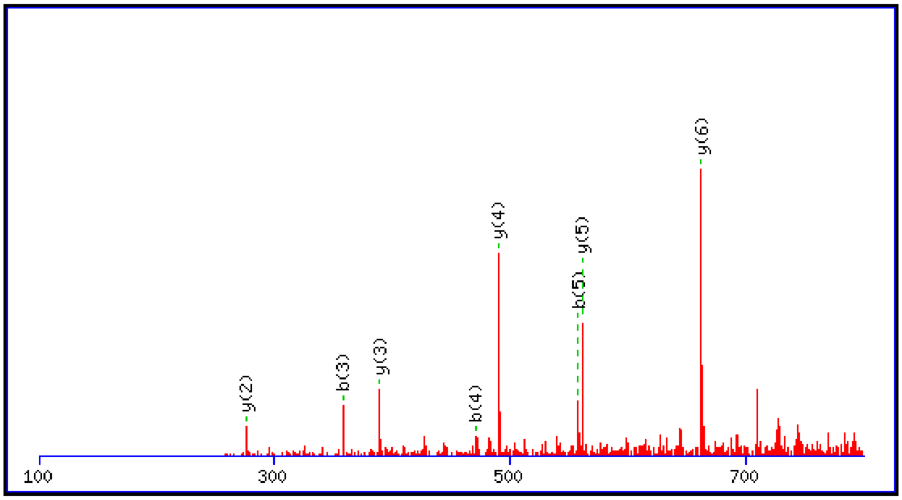

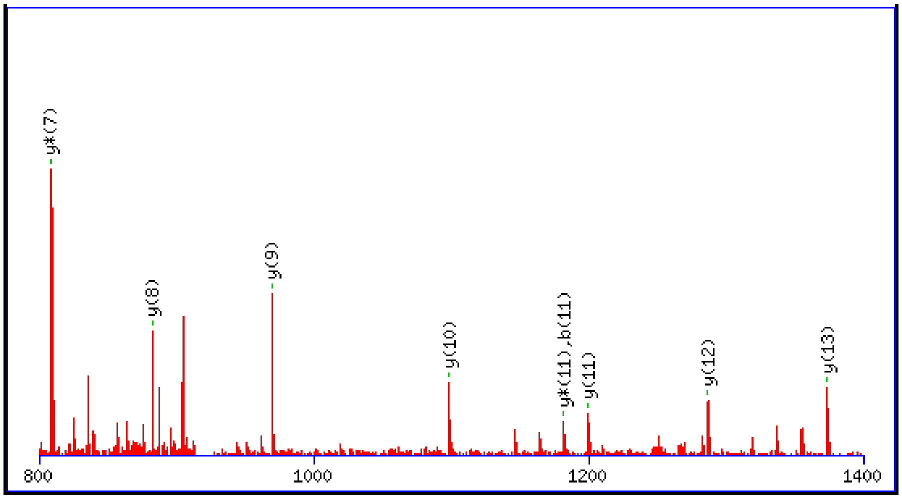


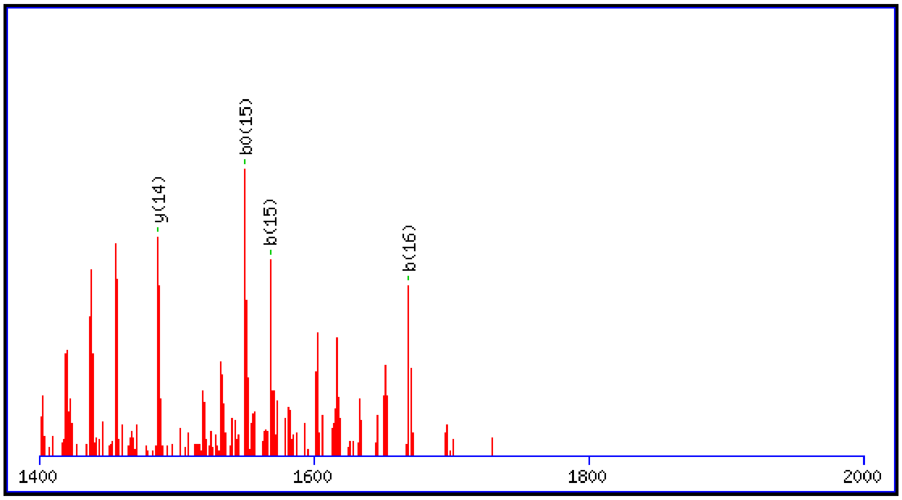


**Monoisotopic mass of neutral peptide Mr(calc):** 1842.8847
**Fixed modifications:** Carbamidomethyl (C)
**Variable modifications:**
**N3** : Deamidated_N (N)
**Ions Score:** 95 **Expect:** 6.7e-008
**Matches (Bold Red):** 21/184 fragment ions using 31 most intense peaks

| # | b | b++ | b* | b*++ | b0 | b0++ | Seq. | y | y++ | y* | y*++ | y0 | y0++ | # |
|---|---|---|---|---|---|---|---|---|---|---|---|---|---|---|
| 1 | 114.0913 | 57.5493 | | | | | L | | | | | | | 17 |
| 2 | 243.1339 | 122.0706 | | | 225.1234 | 113.0653 | E | 1730.8079 | 865.9076 | 1713.7814 | 857.3943 | 1712.7973 | 856.9023 | 16 |
| 3 | **358.1609** | 179.5841 | 341.1343 | 171.0708 | 340.1503 | 170.5788 | N | 1601.7653 | 801.3863 | 1584.7388 | 792.8730 | 1583.7548 | 792.3810 | 15 |
| 4 | **471.2449** | 236.1261 | 454.2184 | 227.6128 | 453.2344 | 227.1208 | L | **1486.7384** | 743.8728 | 1469.7118 | 735.3596 | 1468.7278 | 734.8675 | 14 |
| 5 | **558.2770** | 279.6421 | 541.2504 | 271.1288 | 540.2664 | 270.6368 | S | **1373.6543** | 687.3308 | 1356.6278 | 678.8175 | 1355.6438 | 678.3255 | 13 |
| 6 | 645.3090 | 323.1581 | 628.2824 | 314.6449 | 627.2984 | 314.1529 | S | **1286.6223** | 643.8148 | 1269.5957 | 635.3015 | 1268.6117 | 634.8095 | 12 |
| 7 | 746.3567 | 373.6820 | 729.3301 | 365.1687 | 728.3461 | 364.6767 | T | **1199.5903** | 600.2988 | **1182.5637** | 591.7855 | 1181.5797 | 591.2935 | 11 |
| 8 | 875.3993 | 438.2033 | 858.3727 | 429.6900 | 857.3887 | 429.1980 | E | **1098.5426** | 549.7749 | 1081.5160 | 541.2617 | 1080.5320 | 540.7696 | 10 |
| 9 | 962.4313 | 481.7193 | 945.4047 | 473.2060 | 944.4207 | 472.7140 | S | **969.5000** | 485.2536 | 952.4734 | 476.7404 | 951.4894 | 476.2483 | 9 |
| 10 | 1019.4528 | 510.2300 | 1002.4262 | 501.7167 | 1001.4422 | 501.2247 | G | **882.4680** | 441.7376 | 865.4414 | 433.2243 | 864.4574 | 432.7323 | 8 |
| 11 | **1182.5161** | 591.7617 | 1165.4895 | 583.2484 | 1164.5055 | 582.7564 | Y | 825.4465 | 413.2269 | **808.4199** | 404.7136 | 807.4359 | 404.2216 | 7 |
| 12 | 1283.5638 | 642.2855 | 1266.5372 | 633.7722 | 1265.5532 | 633.2802 | T | **662.3832** | 331.6952 | 645.3566 | 323.1819 | 644.3726 | 322.6899 | 6 |
| 13 | 1354.6009 | 677.8041 | 1337.5743 | 669.2908 | 1336.5903 | 668.7988 | A | **561.3355** | 281.1714 | 544.3089 | 272.6581 | 543.3249 | 272.1661 | 5 |
| 14 | 1455.6486 | 728.3279 | 1438.6220 | 719.8146 | 1437.6380 | 719.3226 | T | **490.2984** | 245.6528 | 473.2718 | 237.1396 | 472.2878 | 236.6475 | 4 |
| 15 | **1568.7326** | 784.8699 | 1551.7061 | 776.3567 | **1550.7221** | 775.8647 | L | **389.2507** | 195.1290 | 372.2241 | 186.6157 | 371.2401 | 186.1237 | 3 |
| 16 | **1669.7803** | 835.3938 | 1652.7537 | 826.8805 | 1651.7697 | 826.3885 | T | **276.1666** | 138.5870 | 259.1401 | 130.0737 | 258.1561 | 129.5817 | 2 |
| 17 | | | | | | | R | 175.1190 | 88.0631 | 158.0924 | 79.5498 | | | 1 |

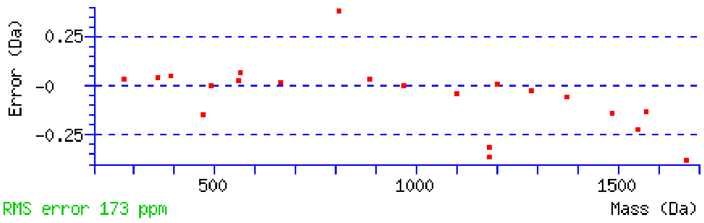


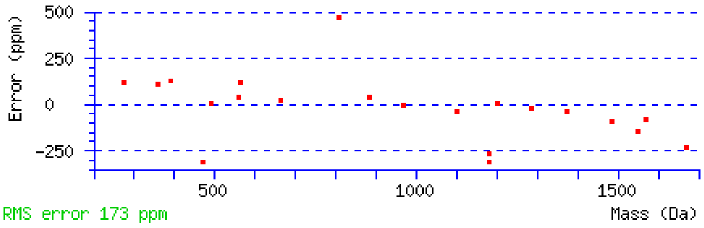


### All matches to this query

| Score | Mr(calc): | Delta | Sequence |
|---|---|---|---|
| 94.7 | 1842.8847 | 0.0054 | LENLSSTESGYTATLTR |
| 77.3 | 1841.9007 | 0.9894 | LENLSSTESGYTATLTR |
| 8.7 | 1842.8950 | -0.0049 | MKDYQLNALLPALTR |
| 5.2 | 1842.8877 | 0.0024 | FKSDQNLQTALELTR |
| 3.3 | 1842.8911 | -0.0010 | QTKVTEISLVVSGCSR |
| 2.9 | 1842.8794 | 0.0107 | VKNFLHSIVSGLDVR |
| 2.8 | 1842.8876 | 0.0025 | LVEERTQAYLEEKR |
| 2.6 | 1842.8887 | 0.0014 | LNEDYTKEYVQALEK |
| 2.3 | 1842.8798 | 0.0103 | DIESKMLSAILTGVNR |
| 1.5 | 1842.8959 | -0.0058 | LDEELQIQAQEDREK |

---

Spectrum No: 106; Query: 2827; Rank: 1

---

## Peptide View

MS/MS Fragmentation of **ALAGLVYNSSGMEPCFDIYQMYQSCADPTGCGTGSNAR**
Found in **IPI00230946**, Tax_Id=10116 Gene_Symbol=Dpp7 Dipeptidyl-peptidase 2 precursor

Match to Query 2827: 4149.743652 from(1384.255160,3+)
Title: 091008RatKidney_NoSalt_20.5990.5990.3.dta
Data file K:\NewmanPaper\Piliang\3SubProteomes\Piliang3SP\mgf5ppm\ERLIC_3SubProteomes5ppm.mgf

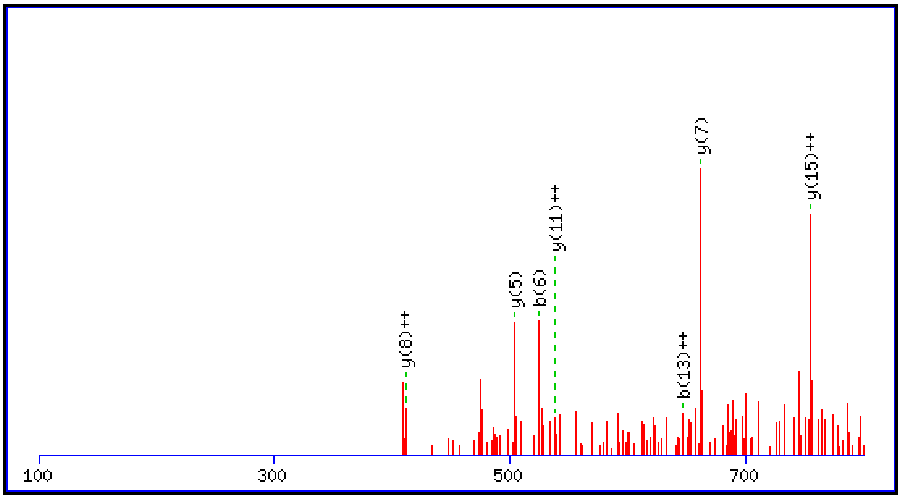

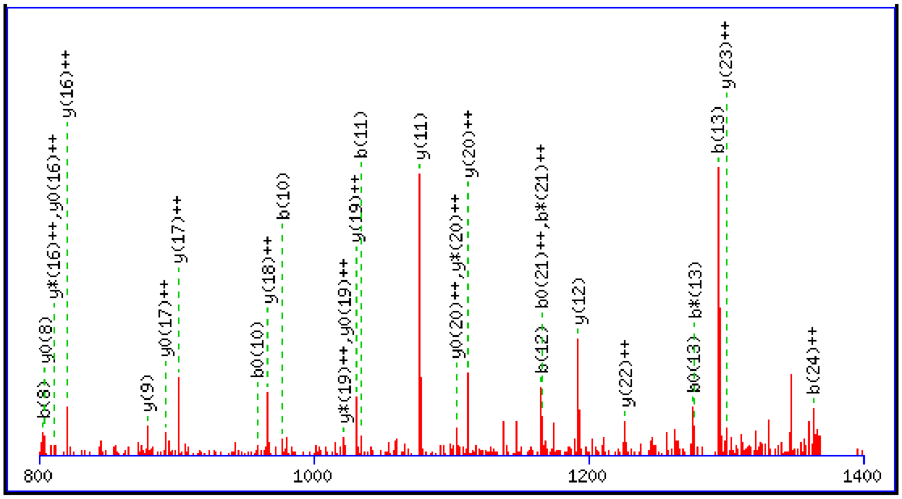


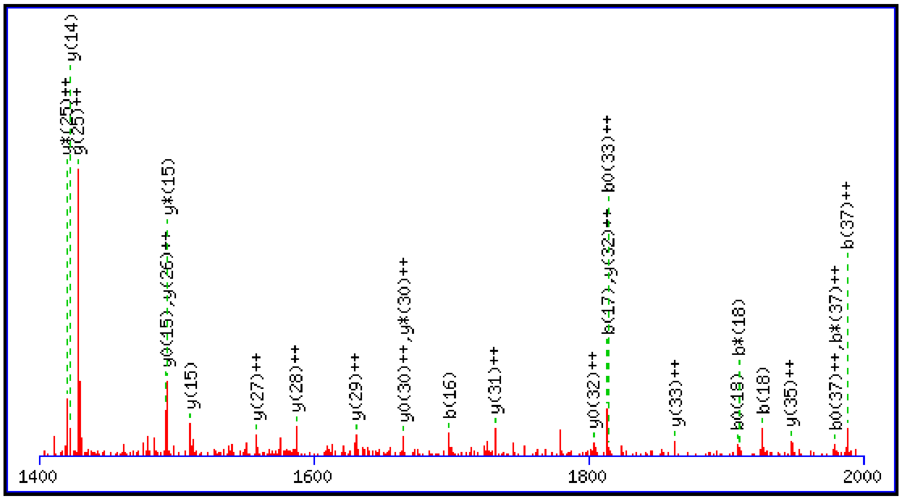


**Monoisotopic mass of neutral peptide Mr(calc):** 4148.7319
**Fixed modifications:** Carbamidomethyl (C)
**Variable modifications:**
**N8** : Deamidated_N (N)
**Ions Score:** 94 **Expect:** 1.7e-007
**Matches (Bold Red):** 62/408 fragment ions using 100 most intense peaks

| # | b | $b^{++}$ | b* | $b^{*++}$ | $b^{0}$ | $b^{0++}$ | Seq. | y | $y^{++}$ | y* | $y^{*++}$ | $y^{0}$ | $y^{0++}$ | # |
|---|---|---|---|---|---|---|---|---|---|---|---|---|---|---|
| 1 | 72.0444 | 36.5258 | | | | | A | | | | | | | 38 |
| 2 | 185.1285 | 93.0679 | | | | | L | 4078.7020 | 2039.8547 | 4061.6755 | 2031.3414 | 4060.6915 | 2030.8494 | 37 |
| 3 | 256.1656 | 128.5864 | | | | | A | 3965.6180 | 1983.3126 | 3948.5914 | 1974.7993 | 3947.6074 | 1974.3073 | 36 |
| 4 | 313.1870 | 157.0972 | | | | | G | 3894.5809 | **1947.7941** | 3877.5543 | 1939.2808 | 3876.5703 | 1938.7888 | 35 |
| 5 | 426.2711 | 213.6392 | | | | | L | 3837.5594 | 1919.2833 | 3820.5328 | 1910.7701 | 3819.5488 | 1910.2781 | 34 |
| 6 | **525.3395** | 263.1734 | | | | | V | 3724.4753 | **1862.7413** | 3707.4488 | 1854.2280 | 3706.4648 | 1853.7360 | 33 |
| 7 | 688.4028 | 344.7051 | | | | | Y | 3625.4069 | **1813.2071** | 3608.3804 | 1804.6938 | 3607.3963 | **1804.2018** | 32 |
| 8 | **803.4298** | 402.2185 | 786.4032 | 393.7053 | | | N | 3462.3436 | **1731.6754** | 3445.3170 | 1723.1622 | 3444.3330 | 1722.6701 | 31 |
| 9 | 890.4618 | 445.7345 | 873.4353 | 437.2213 | 872.4512 | 436.7293 | S | 3347.3166 | 1674.1620 | 3330.2901 | **1665.6487** | 3329.3061 | **1665.1567** | 30 |
| 10 | **977.4938** | 489.2506 | 960.4673 | 480.7373 | **959.4833** | 480.2453 | S | 3260.2846 | **1630.6459** | 3243.2581 | 1622.1327 | 3242.2741 | 1621.6407 | 29 |
| 11 | **1034.5153** | 517.7613 | 1017.4887 | 509.2480 | 1016.5047 | 508.7560 | G | 3173.2526 | **1587.1299** | 3156.2260 | 1578.6167 | 3155.2420 | 1578.1247 | 28 |
| 12 | **1165.5558** | 583.2815 | 1148.5292 | 574.7683 | 1147.5452 | 574.2762 | M | 3116.2311 | **1558.6192** | 3099.2046 | 1550.1059 | 3098.2206 | 1549.6139 | 27 |
| 13 | **1294.5984** | **647.8028** | **1277.5718** | 639.2896 | **1276.5878** | 638.7975 | E | 2985.1906 | **1493.0990** | 2968.1641 | 1484.5857 | 2967.1801 | 1484.0937 | 26 |
| 14 | 1391.6511 | 696.3292 | 1374.6246 | 687.8159 | 1373.6406 | 687.3239 | P | 2856.1481 | **1428.5777** | 2839.1215 | **1420.0644** | 2838.1375 | 1419.5724 | 25 |
| 15 | 1551.6818 | 776.3445 | 1534.6552 | 767.8313 | 1533.6712 | 767.3392 | C | 2759.0953 | 1380.0513 | 2742.0687 | 1371.5380 | 2741.0847 | 1371.0460 | 24 |
| 16 | **1698.7502** | 849.8787 | 1681.7237 | 841.3655 | 1680.7396 | 840.8735 | F | 2599.0646 | **1300.0360** | 2582.0381 | 1291.5227 | 2581.0541 | 1291.0307 | 23 |
| 17 | **1813.7771** | 907.3922 | 1796.7506 | 898.8789 | 1795.7666 | 898.3869 | D | 2451.9962 | **1226.5017** | 2434.9697 | 1217.9885 | 2433.9857 | 1217.4965 | 22 |
| 18 | **1926.8612** | 963.9342 | **1909.8347** | 955.4210 | **1908.8506** | 954.9290 | I | 2336.9693 | 1168.9883 | 2319.9427 | 1160.4750 | 2318.9587 | 1159.9830 | 21 |

| | | | | | | | | | | | | | | |
|---|---|---|---|---|---|---|---|---|---|---|---|---|---|---|
| 19 | 2089.9245 | 1045.4659 | 2072.8980 | 1036.9526 | 2071.9140 | 1036.4606 | Y | 2223.8852 | 1112.4462 | 2206.8587 | 1103.9330 | 2205.8747 | 1103.4410 | 20 |
| 20 | 2217.9831 | 1109.4952 | 2200.9566 | 1100.9819 | 2199.9725 | 1100.4899 | Q | 2060.8219 | 1030.9146 | 2043.7953 | 1022.4013 | 2042.8113 | 1021.9093 | 19 |
| 21 | 2349.0236 | 1175.0154 | 2331.9970 | 1166.5022 | 2331.0130 | 1166.0102 | M | 1932.7633 | 966.8853 | 1915.7368 | 958.3720 | 1914.7527 | 957.8800 | 18 |
| 22 | 2512.0869 | 1256.5471 | 2495.0604 | 1248.0338 | 2494.0764 | 1247.5418 | Y | 1801.7228 | 901.3651 | 1784.6963 | 892.8518 | 1783.7123 | 892.3598 | 17 |
| 23 | 2640.1455 | 1320.5764 | 2623.1190 | 1312.0631 | 2622.1349 | 1311.5711 | Q | 1638.6595 | 819.8334 | 1621.6329 | 811.3201 | 1620.6489 | 810.8281 | 16 |
| 24 | 2727.1775 | 1364.0924 | 2710.1510 | 1355.5791 | 2709.1670 | 1355.0871 | S | 1510.6009 | 755.8041 | 1493.5744 | 747.2908 | 1492.5904 | 746.7988 | 15 |
| 25 | 2887.2082 | 1444.1077 | 2870.1816 | 1435.5945 | 2869.1976 | 1435.1024 | C | 1423.5689 | 712.2881 | 1406.5423 | 703.7748 | 1405.5583 | 703.2828 | 14 |
| 26 | 2958.2453 | 1479.6263 | 2941.2187 | 1471.1130 | 2940.2347 | 1470.6210 | A | 1263.5382 | 632.2728 | 1246.5117 | 623.7595 | 1245.5277 | 623.2675 | 13 |
| 27 | 3073.2722 | 1537.1398 | 3056.2457 | 1528.6265 | 3055.2617 | 1528.1345 | D | 1192.5011 | 596.7542 | 1175.4746 | 588.2409 | 1174.4906 | 587.7489 | 12 |
| 28 | 3170.3250 | 1585.6661 | 3153.2985 | 1577.1529 | 3152.3144 | 1576.6609 | P | 1077.4742 | 539.2407 | 1060.4476 | 530.7275 | 1059.4636 | 530.2354 | 11 |
| 29 | 3271.3727 | 1636.1900 | 3254.3461 | 1627.6767 | 3253.3621 | 1627.1847 | T | 980.4214 | 490.7143 | 963.3949 | 482.2011 | 962.4109 | 481.7091 | 10 |
| 30 | 3328.3941 | 1664.7007 | 3311.3676 | 1656.1874 | 3310.3836 | 1655.6954 | G | 879.3737 | 440.1905 | 862.3472 | 431.6772 | 861.3632 | 431.1852 | 9 |
| 31 | 3488.4248 | 1744.7160 | 3471.3982 | 1736.2028 | 3470.4142 | 1735.7108 | C | 822.3523 | 411.6798 | 805.3257 | 403.1665 | 804.3417 | 402.6745 | 8 |
| 32 | 3545.4463 | 1773.2268 | 3528.4197 | 1764.7135 | 3527.4357 | 1764.2215 | G | 662.3216 | 331.6645 | 645.2951 | 323.1512 | 644.3111 | 322.6592 | 7 |
| 33 | 3646.4939 | 1823.7506 | 3629.4674 | 1815.2373 | 3628.4834 | 1814.7453 | T | 605.3002 | 303.1537 | 588.2736 | 294.6404 | 587.2896 | 294.1484 | 6 |
| 34 | 3703.5154 | 1852.2613 | 3686.4889 | 1843.7481 | 3685.5048 | 1843.2561 | G | 504.2525 | 252.6299 | 487.2259 | 244.1166 | 486.2419 | 243.6246 | 5 |
| 35 | 3790.5474 | 1895.7774 | 3773.5209 | 1887.2641 | 3772.5369 | 1886.7721 | S | 447.2310 | 224.1191 | 430.2045 | 215.6059 | 429.2205 | 215.1139 | 4 |
| 36 | 3904.5904 | 1952.7988 | 3887.5638 | 1944.2855 | 3886.5798 | 1943.7935 | N | 360.1990 | 180.6031 | 343.1724 | 172.0899 | | | 3 |
| 37 | 3975.6275 | 1988.3174 | 3958.6009 | 1979.8041 | 3957.6169 | 1979.3121 | A | 246.1561 | 123.5817 | 229.1295 | 115.0684 | | | 2 |
| 38 | | | | | | | R | 175.1190 | 88.0631 | 158.0924 | 79.5498 | | | 1 |

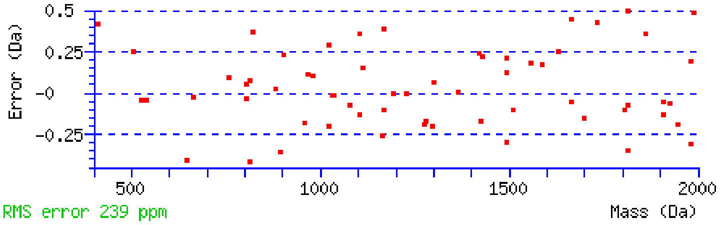


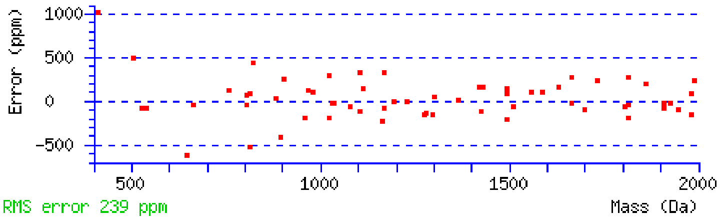


**All matches to this query**

| Score | Mr(calc): | Delta | Sequence |
|---|---|---|---|
| 94.4 | 4148.7319 | 1.0118 | ALAGLVYNSSGMEPCFDIYQMYQSCADPTGCGTGSNAR |
| 80.1 | 4149.7159 | 0.0278 | ALAGLVYNSSGMEPCFDIYQMYQSCADPTGCGTGSNAR |
| 63.9 | 4148.7319 | 1.0118 | ALAGLVYNSSGMEPCFDIYQMYQSCADPTGCGTGSNAR |
| 54.1 | 4147.7479 | 1.9958 | ALAGLVYNSSGMEPCFDIYQMYQSCADPTGCGTGSNAR |
| 7.1 | 4147.7044 | 2.0393 | TKSESMGSLLCDEGSKESDPMTWTSPYIPEVCGNSR |
| 7.1 | 4147.7044 | 2.0393 | TKSESMGSLLCDEGSKESDPMTWTSPYIPEVCGNSR |
| 7.1 | 4147.7044 | 2.0393 | TKSESMGSLLCDEGSKESDPMTWTSPYIPEVCGNSR |
| 5.9 | 4149.7494 | -0.0057 | ELQRYLFELNPNAEDEQEKTSNLQTQQNVVGTSS |
| 5.5 | 4149.7747 | -0.0311 | YTNGESFQPNCRYNCTCIDGTVGCTPLCLSPRPPR |
| 5.3 | 4148.7768 | 0.9668 | FPYLSESYFITTDAAGSIATATPDGGIVLISGTGSNCR |

---

Spectrum No: 107; Query: 1218; Rank: 1

---

## Peptide View

MS/MS Fragmentation of **ATEPCLDGWIYNSTR**
Found in **IPI00207702**, Tax_Id=10116 Gene_Symbol=Slc22a8 Solute carrier family 22 member 8

Match to Query 1218: 1782.787828 from(892.401190,2+)
Title: 091008RatKidney_NoSalt_26.3448.3448.2.dta

Data file K:\NewmanPaper\Piliang\3SubProteomes\Piliang3SP\mgf5ppm\ERLIC_3SubProteomes5ppm.mgf

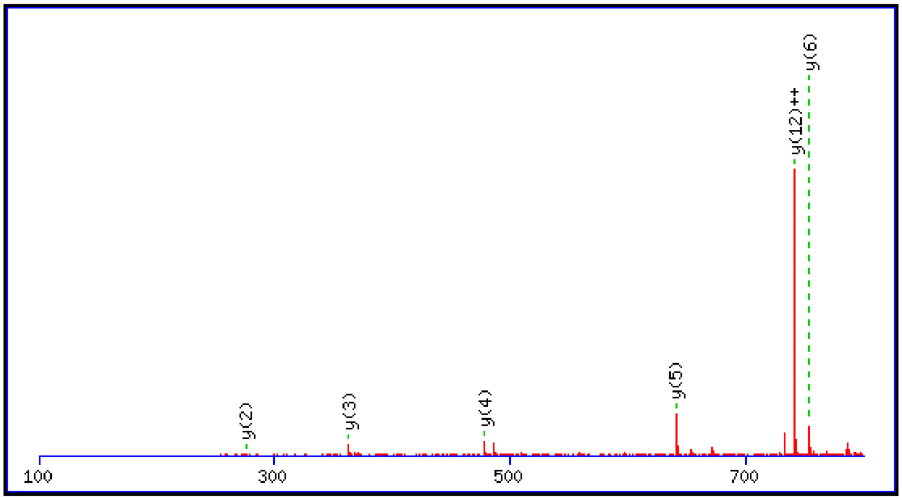


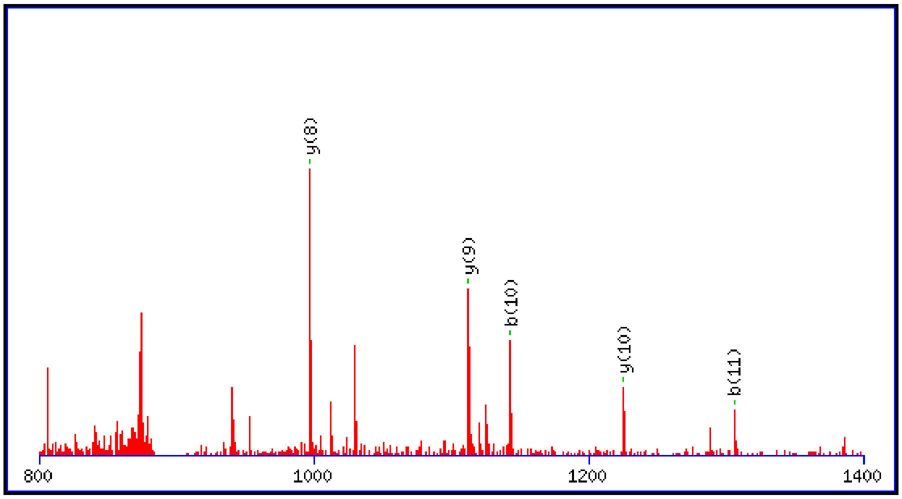


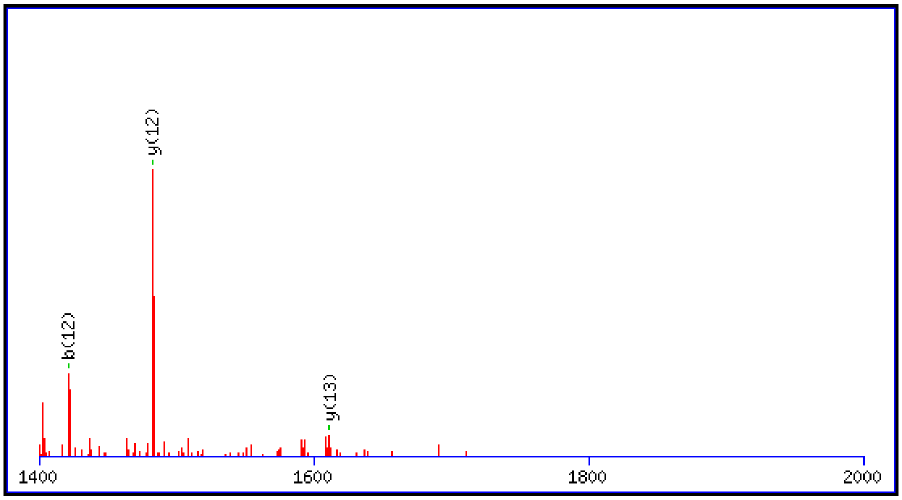


**Monoisotopic mass of neutral peptide Mr(calc):** 1782.7883
**Fixed modifications:** Carbamidomethyl (C)
**Variable modifications:**
**N12** : Deamidated_N (N)
**Ions Score:** 94 **Expect:** 7.5e-008
**Matches (Bold Red):** 14/142 fragment ions using 18 most intense peaks

| # | b | b++ | b* | b*++ | b0 | b0++ | Seq. | y | y++ | y* | y*++ | y0 | y0++ | # |
|---|---|---|---|---|---|---|---|---|---|---|---|---|---|---|
| 1 | 72.0444 | 36.5258 | | | | | A | | | | | | | 15 |
| 2 | 173.0921 | 87.0497 | | | 155.0815 | 78.0444 | T | 1712.7585 | 856.8829 | 1695.7319 | 848.3696 | 1694.7479 | 847.8776 | 14 |
| 3 | 302.1347 | 151.5710 | | | 284.1241 | 142.5657 | E | **1611.7108** | 806.3590 | 1594.6842 | 797.8458 | 1593.7002 | 797.3537 | 13 |
| 4 | 399.1874 | 200.0974 | | | 381.1769 | 191.0921 | P | **1482.6682** | **741.8377** | 1465.6416 | 733.3245 | 1464.6576 | 732.8324 | 12 |

| | | | | | | | | | | | | | | |
|---|---|---|---|---|---|---|---|---|---|---|---|---|---|---|
| 5 | 559.2181 | 280.1127 | | | 541.2075 | 271.1074 | C | 1385.6154 | 693.3113 | 1368.5889 | 684.7981 | 1367.6049 | 684.3061 | 11 |
| 6 | 672.3021 | 336.6547 | | | 654.2916 | 327.6494 | L | **1225.5848** | 613.2960 | 1208.5582 | 604.7827 | 1207.5742 | 604.2907 | 10 |
| 7 | 787.3291 | 394.1682 | | | 769.3185 | 385.1629 | D | **1112.5007** | 556.7540 | 1095.4742 | 548.2407 | 1094.4901 | 547.7487 | 9 |
| 8 | 844.3505 | 422.6789 | | | 826.3400 | 413.6736 | G | **997.4738** | 499.2405 | 980.4472 | 490.7272 | 979.4632 | 490.2352 | 8 |
| 9 | 1030.4299 | 515.7186 | | | 1012.4193 | 506.7133 | W | 940.4523 | 470.7298 | 923.4258 | 462.2165 | 922.4417 | 461.7245 | 7 |
| 10 | **1143.5139** | 572.2606 | | | 1125.5034 | 563.2553 | I | **754.3730** | 377.6901 | 737.3464 | 369.1769 | 736.3624 | 368.6848 | 6 |
| 11 | **1306.5773** | 653.7923 | | | 1288.5667 | 644.7870 | Y | **641.2889** | 321.1481 | 624.2624 | 312.6348 | 623.2784 | 312.1428 | 5 |
| 12 | **1421.6042** | 711.3057 | 1404.5776 | 702.7925 | 1403.5936 | 702.3004 | N | **478.2256** | 239.6164 | 461.1990 | 231.1032 | 460.2150 | 230.6112 | 4 |
| 13 | 1508.6362 | 754.8217 | 1491.6097 | 746.3085 | 1490.6257 | 745.8165 | S | **363.1987** | 182.1030 | 346.1721 | 173.5897 | 345.1881 | 173.0977 | 3 |
| 14 | 1609.6839 | 805.3456 | 1592.6573 | 796.8323 | 1591.6733 | 796.3403 | T | **276.1666** | 138.5870 | 259.1401 | 130.0737 | 258.1561 | 129.5817 | 2 |
| 15 | | | | | | | R | 175.1190 | 88.0631 | 158.0924 | 79.5498 | | | 1 |

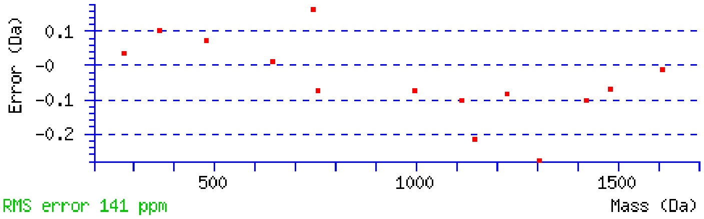


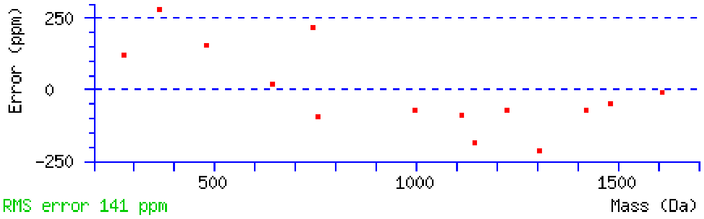


### All matches to this query

| Score | Mr(calc): | Delta | Sequence |
|---|---|---|---|
| 94.3 | 1782.7883 | -0.0005 | ATEPCLDGWIYNSTR |
| 7.2 | 1781.7695 | 1.0183 | GQLCTYFLNTDLTR |
| 7.2 | 1780.7637 | 2.0241 | MSGPNGDLGMPVDAGRK |
| 4.3 | 1781.7729 | 1.0149 | DVMNGEQTGLLMLHK |
| 4.0 | 1782.8036 | -0.0158 | EKGPNNTANSSEVKVK |
| 4.0 | 1782.8036 | -0.0158 | EKGPNNTANSSEVKVK |
| 3.7 | 1782.8050 | -0.0172 | SNTRYYTGTQSRIR |
| 2.2 | 1780.7920 | 1.9958 | VEAEFHLVTAEEAEK |
| 1.5 | 1782.8050 | -0.0172 | SNTRYYTGTQSRIR |
| 1.3 | 1782.8036 | -0.0158 | EKGPNNTANSSEVKVK |

---

Spectrum No: 108; Query: 1835; Rank: 1

---

## Peptide View

MS/MS Fragmentation of **YPVPFQNENVTAAFVELGR**
Found in **IPI00213569**, Tax_Id=10116 Gene_Symbol=Ugt1a1 UDP-glucuronosyltransferase 1-1 precursor

Match to Query 1835: 2151.070828 from(1076.542690,2+)
Title: 100101RatKid_NS_deglyco_12.5821.5821.2.dta
Data file K:\NewmanPaper\Piliang\3SubProteomes\Piliang3SP\mgf5ppm\ERLIC_3SubProteomes5ppm.mgf

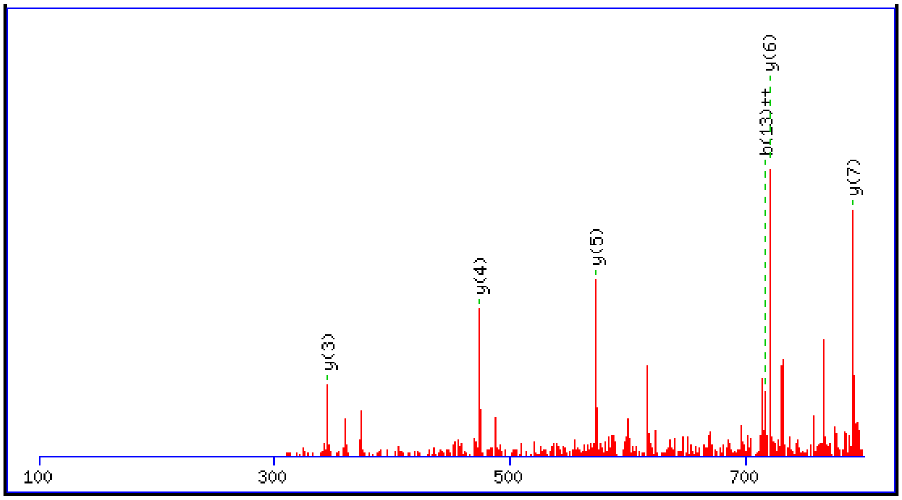


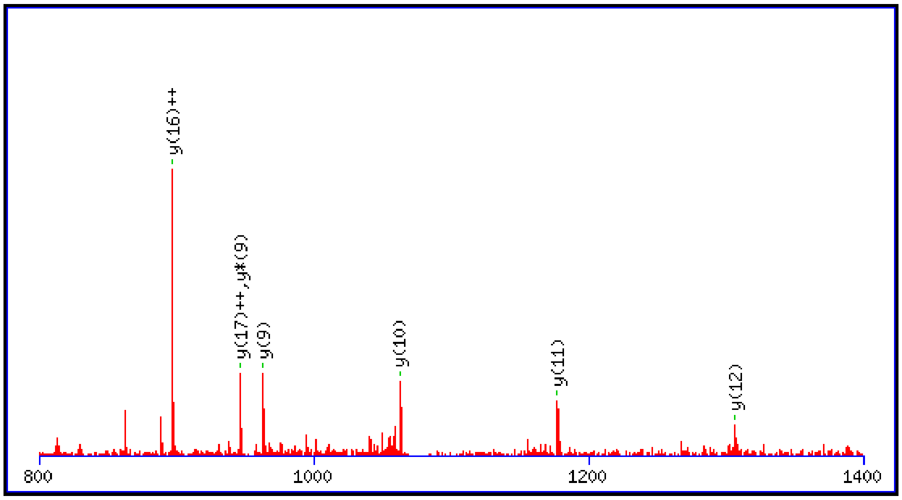


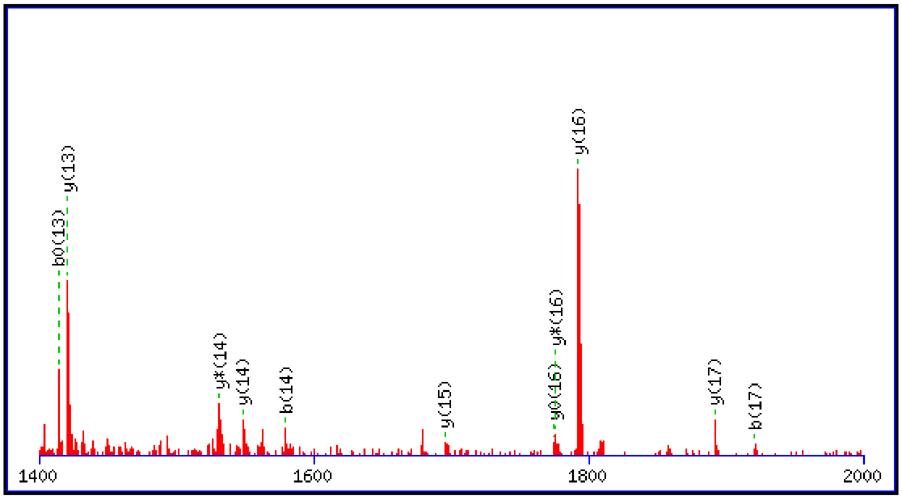


**Monoisotopic mass of neutral peptide Mr(calc):** 2151.0637
**Fixed modifications:** Carbamidomethyl (C)
**Variable modifications:**
**N9** : Deamidated_N (N)
**Ions Score:** 94 **Expect:** 8.2e-008
**Matches (Bold Red):** 24/186 fragment ions using 43 most intense peaks

| # | b | $b^{++}$ | b* | $b^{*++}$ | $b^0$ | $b^{0++}$ | Seq. | y | $y^{++}$ | y* | $y^{*++}$ | $y^0$ | $y^{0++}$ | # |
|---|---|---|---|---|---|---|---|---|---|---|---|---|---|---|
| 1 | 164.0706 | 82.5389 | | | | | Y | | | | | | | 19 |
| 2 | 261.1234 | 131.0653 | | | | | P | 1989.0076 | 995.0074 | 1971.9811 | 986.4942 | 1970.9970 | 986.0022 | 18 |
| 3 | 360.1918 | 180.5995 | | | | | V | **1891.9548** | **946.4811** | 1874.9283 | 937.9678 | 1873.9443 | 937.4758 | 17 |
| 4 | 457.2445 | 229.1259 | | | | | P | **1792.8864** | **896.9469** | **1775.8599** | 888.4336 | **1774.8759** | 887.9416 | 16 |
| 5 | 604.3130 | 302.6601 | | | | | F | **1695.8337** | 848.4205 | 1678.8071 | 839.9072 | 1677.8231 | 839.4152 | 15 |

| | | | | | | | | | | | | | | |
|---|---|---|---|---|---|---|---|---|---|---|---|---|---|---|
| **6** | 732.3715 | 366.6894 | 715.3450 | 358.1761 | | | **Q** | **1548.7653** | 774.8863 | **1531.7387** | 766.3730 | 1530.7547 | 765.8810 | **14** |
| **7** | 846.4145 | 423.7109 | 829.3879 | 415.1976 | | | **N** | **1420.7067** | 710.8570 | 1403.6801 | 702.3437 | 1402.6961 | 701.8517 | **13** |
| **8** | 975.4571 | 488.2322 | 958.4305 | 479.7189 | 957.4465 | 479.2269 | **E** | **1306.6638** | 653.8355 | 1289.6372 | 645.3222 | 1288.6532 | 644.8302 | **12** |
| **9** | 1090.4840 | 545.7456 | 1073.4574 | 537.2324 | 1072.4734 | 536.7404 | **N** | **1177.6212** | 589.3142 | 1160.5946 | 580.8009 | 1159.6106 | 580.3089 | **11** |
| **10** | 1189.5524 | 595.2798 | 1172.5259 | 586.7666 | 1171.5418 | 586.2746 | **V** | **1062.5942** | 531.8007 | 1045.5677 | 523.2875 | 1044.5837 | 522.7955 | **10** |
| **11** | 1290.6001 | 645.8037 | 1273.5735 | 637.2904 | 1272.5895 | 636.7984 | **T** | **963.5258** | 482.2665 | **946.4993** | 473.7533 | 945.5152 | 473.2613 | **9** |
| **12** | 1361.6372 | 681.3222 | 1344.6107 | 672.8090 | 1343.6266 | 672.3170 | **A** | 862.4781 | 431.7427 | 845.4516 | 423.2294 | 844.4676 | 422.7374 | **8** |
| **13** | 1432.6743 | **716.8408** | 1415.6478 | 708.3275 | **1414.6638** | 707.8355 | **A** | **791.4410** | 396.2241 | 774.4145 | 387.7109 | 773.4305 | 387.2189 | **7** |
| **14** | **1579.7427** | 790.3750 | 1562.7162 | 781.8617 | 1561.7322 | 781.3697 | **F** | **720.4039** | 360.7056 | 703.3774 | 352.1923 | 702.3933 | 351.7003 | **6** |
| **15** | 1678.8111 | 839.9092 | 1661.7846 | 831.3959 | 1660.8006 | 830.9039 | **V** | **573.3355** | 287.1714 | 556.3089 | 278.6581 | 555.3249 | 278.1661 | **5** |
| **16** | 1807.8537 | 904.4305 | 1790.8272 | 895.9172 | 1789.8432 | 895.4252 | **E** | **474.2671** | 237.6372 | 457.2405 | 229.1239 | 456.2565 | 228.6319 | **4** |
| **17** | **1920.9378** | 960.9725 | 1903.9113 | 952.4593 | 1902.9272 | 951.9673 | **L** | **345.2245** | 173.1159 | 328.1979 | 164.6026 | | | **3** |
| **18** | 1977.9593 | 989.4833 | 1960.9327 | 980.9700 | 1959.9487 | 980.4780 | **G** | 232.1404 | 116.5738 | 215.1139 | 108.0606 | | | **2** |
| **19** | | | | | | | **R** | 175.1190 | 88.0631 | 158.0924 | 79.5498 | | | **1** |

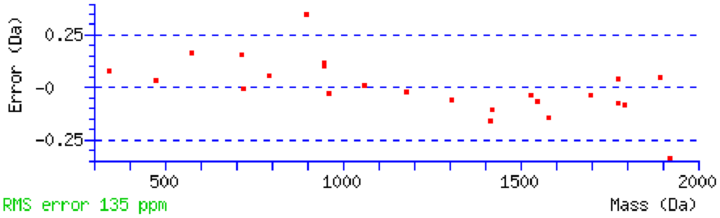


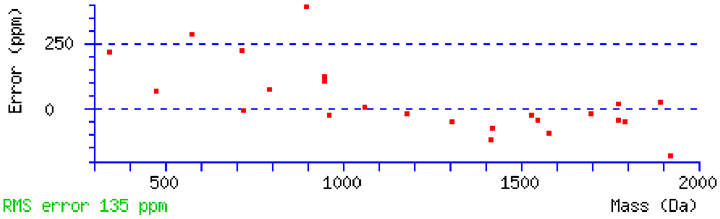


### All matches to this query

| Score | Mr(calc): | Delta | Sequence |
|---|---|---|---|
| 94.2 | 2151.0637 | 0.0072 | YPVPFQNENVTAAFVELGR |
| 67.4 | 2151.0637 | 0.0072 | YPVPFQNENVTAAFVELGR |
| 17.2 | 2150.0797 | 0.9912 | YPVPFQNENVTAAFVELGR |
| 3.3 | 2151.0583 | 0.0126 | DDEIIKKAVNEYDETLEK |
| 3.1 | 2150.0578 | 1.0131 | YDILPYSIRVLLEAAVR |
| 0.7 | 2149.0539 | 2.0169 | WINGSTLNSDVLSITGDTEK |
| 0.4 | 2151.0590 | 0.0118 | NSTGSTLAAMVGAGGSSLSVAASR |

---

Spectrum No: 109; Query: 1754; Rank: 1

---

## Peptide View

MS/MS Fragmentation of **LAGGTNFNNSIQADPQTVTR**
Found in **IPI00192495**, Tax_Id=10116 Gene_Symbol=Ncstn Isoform 1 of Nicastrin precursor

Match to Query 1754: 2104.023548 from(1053.019050,2+)
Title: 100101RatKid_NS_deglyco_25.2200.2200.2.dta
Data file K:\NewmanPaper\Piliang\3SubProteomes\Piliang3SP\mgf5ppm\ERLIC_3SubProteomes5ppm.mgf

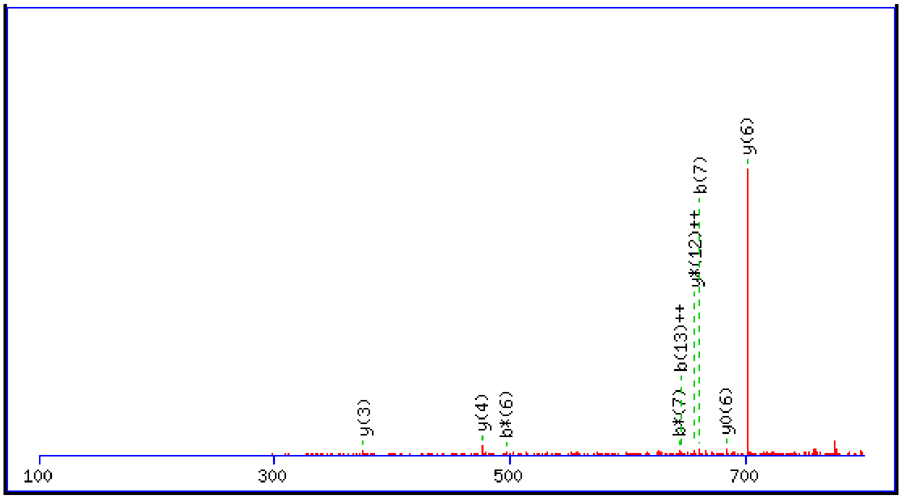


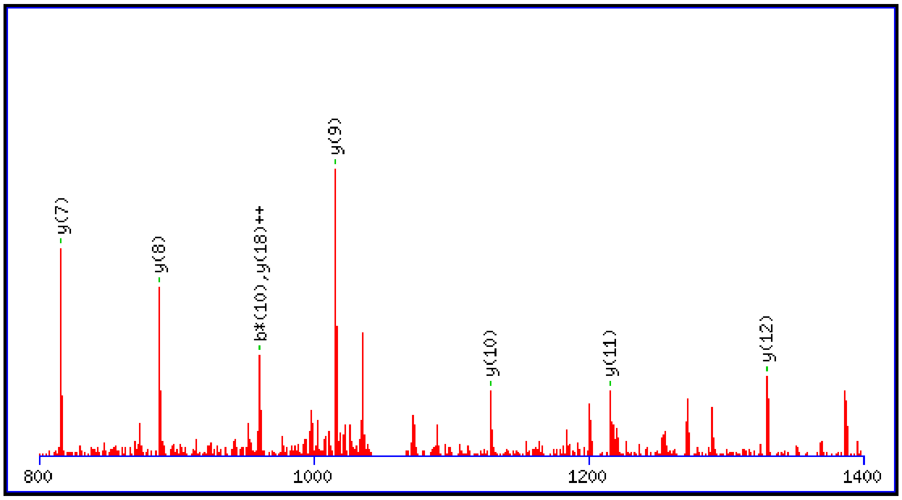


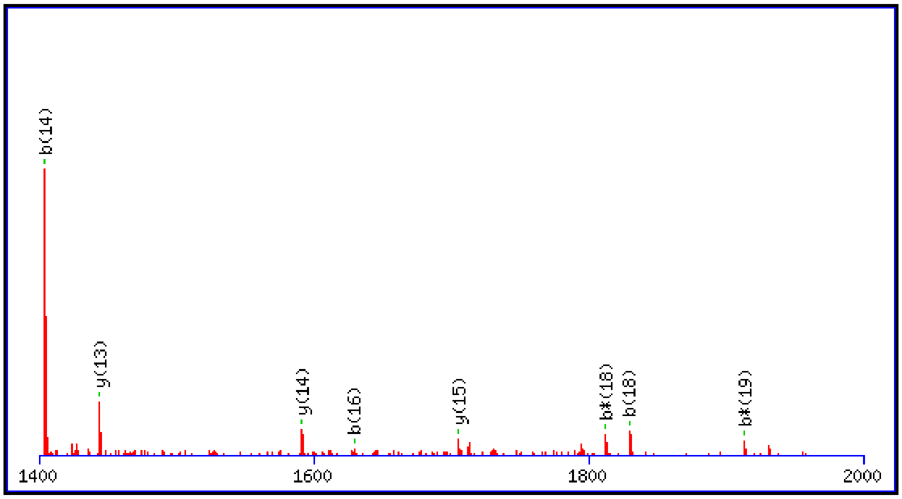


**Monoisotopic mass of neutral peptide Mr(calc):** 2104.0185
**Fixed modifications:** Carbamidomethyl (C)
**Variable modifications:**
**N8** : Deamidated_N (N)
**Ions Score:** 94 **Expect:** 9.2e-008
**Matches (Bold Red):** 25/208 fragment ions using 37 most intense peaks

| # | b | $b^{++}$ | b* | $b^{*++}$ | $b^{0}$ | $b^{0++}$ | Seq. | y | $y^{++}$ | y* | $y^{*++}$ | $y^{0}$ | $y^{0++}$ | # |
|---|---|---|---|---|---|---|---|---|---|---|---|---|---|---|
| 1 | 114.0913 | 57.5493 | | | | | L | | | | | | | 20 |
| 2 | 185.1285 | 93.0679 | | | | | A | 1991.9417 | 996.4745 | 1974.9152 | 987.9612 | 1973.9312 | 987.4692 | 19 |
| 3 | 242.1499 | 121.5786 | | | | | G | 1920.9046 | **960.9559** | 1903.8781 | 952.4427 | 1902.8940 | 951.9507 | 18 |
| 4 | 299.1714 | 150.0893 | | | | | G | 1863.8831 | 932.4452 | 1846.8566 | 923.9319 | 1845.8726 | 923.4399 | 17 |
| 5 | 400.2191 | 200.6132 | | | 382.2085 | 191.6079 | T | 1806.8617 | 903.9345 | 1789.8351 | 895.4212 | 1788.8511 | 894.9292 | 16 |

| | | | | | | | | | | | | | | |
|---|---|---|---|---|---|---|---|---|---|---|---|---|---|---|
| 6 | 514.2620 | 257.6346 | **497.2354** | 249.1214 | 496.2514 | 248.6293 | N | **1705.8140** | 853.4106 | 1688.7874 | 844.8974 | 1687.8034 | 844.4054 | 15 |
| 7 | **661.3304** | 331.1688 | **644.3039** | 322.6556 | 643.3198 | 322.1636 | F | **1591.7711** | 796.3892 | 1574.7445 | 787.8759 | 1573.7605 | 787.3839 | 14 |
| 8 | 776.3573 | 388.6823 | 759.3308 | 380.1690 | 758.3468 | 379.6770 | N | **1444.7027** | 722.8550 | 1427.6761 | 714.3417 | 1426.6921 | 713.8497 | 13 |
| 9 | 890.4003 | 445.7038 | 873.3737 | 437.1905 | 872.3897 | 436.6985 | N | **1329.6757** | 665.3415 | 1312.6492 | **656.8282** | 1311.6652 | 656.3362 | 12 |
| 10 | 977.4323 | 489.2198 | **960.4057** | 480.7065 | 959.4217 | 480.2145 | S | **1215.6328** | 608.3200 | 1198.6062 | 599.8068 | 1197.6222 | 599.3148 | 11 |
| 11 | 1090.5164 | 545.7618 | 1073.4898 | 537.2485 | 1072.5058 | 536.7565 | I | **1128.6008** | 564.8040 | 1111.5742 | 556.2907 | 1110.5902 | 555.7987 | 10 |
| 12 | 1218.5749 | 609.7911 | 1201.5484 | 601.2778 | 1200.5644 | 600.7858 | Q | **1015.5167** | 508.2620 | 998.4902 | 499.7487 | 997.5061 | 499.2567 | 9 |
| 13 | 1289.6120 | **645.3097** | 1272.5855 | 636.7964 | 1271.6015 | 636.3044 | A | **887.4581** | 444.2327 | 870.4316 | 435.7194 | 869.4476 | 435.2274 | 8 |
| 14 | **1404.6390** | 702.8231 | 1387.6124 | 694.3099 | 1386.6284 | 693.8179 | D | **816.4210** | 408.7141 | 799.3945 | 400.2009 | 798.4104 | 399.7089 | 7 |
| 15 | 1501.6918 | 751.3495 | 1484.6652 | 742.8362 | 1483.6812 | 742.3442 | P | **701.3941** | 351.2007 | 684.3675 | 342.6874 | **683.3835** | 342.1954 | 6 |
| 16 | **1629.7503** | 815.3788 | 1612.7238 | 806.8655 | 1611.7398 | 806.3735 | Q | 604.3413 | 302.6743 | 587.3148 | 294.1610 | 586.3307 | 293.6690 | 5 |
| 17 | 1730.7980 | 865.9026 | 1713.7715 | 857.3894 | 1712.7874 | 856.8974 | T | **476.2827** | 238.6450 | 459.2562 | 230.1317 | 458.2722 | 229.6397 | 4 |
| 18 | **1829.8664** | 915.4369 | **1812.8399** | 906.9236 | 1811.8559 | 906.4316 | V | **375.2350** | 188.1212 | 358.2085 | 179.6079 | 357.2245 | 179.1159 | 3 |
| 19 | 1930.9141 | 965.9607 | **1913.8876** | 957.4474 | 1912.9035 | 956.9554 | T | 276.1666 | 138.5870 | 259.1401 | 130.0737 | 258.1561 | 129.5817 | 2 |
| 20 | | | | | | | R | 175.1190 | 88.0631 | 158.0924 | 79.5498 | | | 1 |

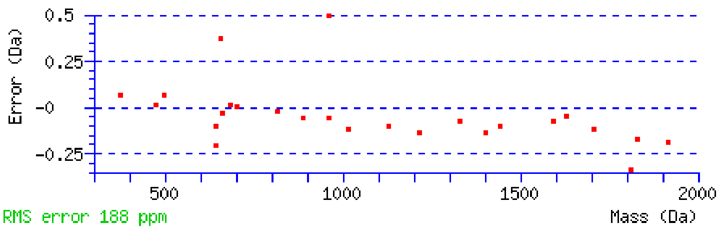


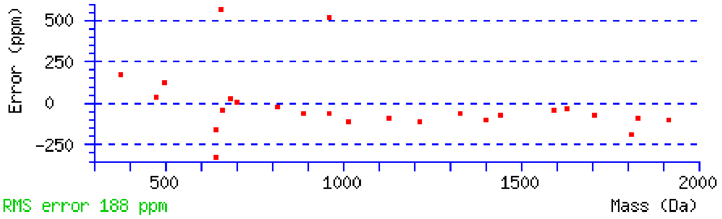


### All matches to this query

| Score | Mr(calc): | Delta | Sequence |
|---|---|---|---|
| 94.1 | 2104.0185 | 0.0050 | LAGGTNFNNSIQADPQTVTR |
| 77.0 | 2104.0185 | 0.0050 | LAGGTNFNNSIQADPQTVTR |
| 76.9 | 2104.0185 | 0.0050 | LAGGTNFNNSIQADPQTVTR |
| 45.2 | 2103.0345 | 0.9890 | LAGGTNFNNSIQADPQTVTR |
| 4.3 | 2104.0354 | -0.0118 | SLFQDKNRTLEAFSLVR |
| 2.1 | 2102.0092 | 2.0144 | CSQLLRENAKAGHTPALR |
| 1.6 | 2104.0201 | 0.0034 | DASSSSKLFSAKLEQLASR |
| 1.1 | 2103.0080 | 1.0156 | KKSSDTAGSGAPNSDSQLPEK |
| 0.6 | 2102.0296 | 1.9939 | ELHIALLKLSADLDTNXE |

---

Spectrum No: 110; Query: 2068; Rank: 1

---

## Peptide View

MS/MS Fragmentation of **FFNDSIVDPVDSEWFGFYR**
Found in **IPI00208382**, Tax_Id=10116 Gene_Symbol=Ppt1 Palmitoyl-protein thioesterase 1 precursor

Match to Query 2068: 2340.045288 from(1171.029920,2+)
Title: 091008RatKidney_NoSalt_18.6114.6114.2.dta
Data file K:\NewmanPaper\Piliang\3SubProteomes\Piliang3SP\mgf5ppm\ERLIC_3SubProteomes5ppm.mgf

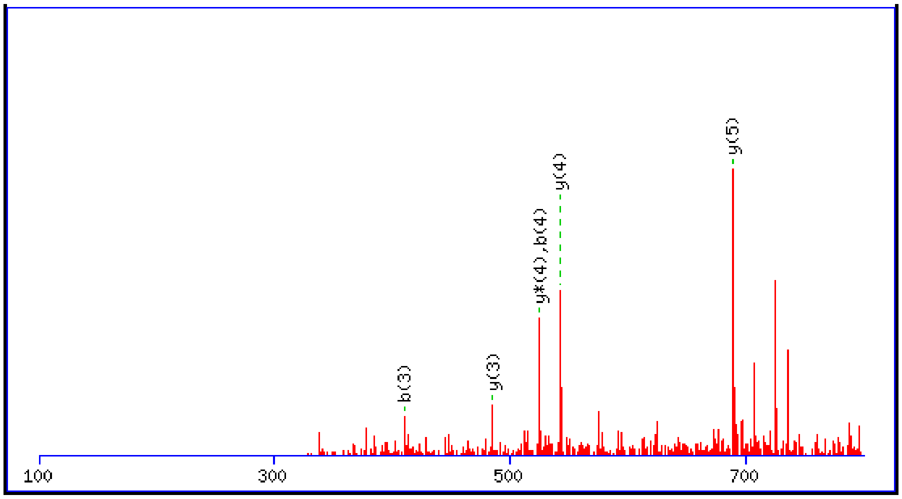


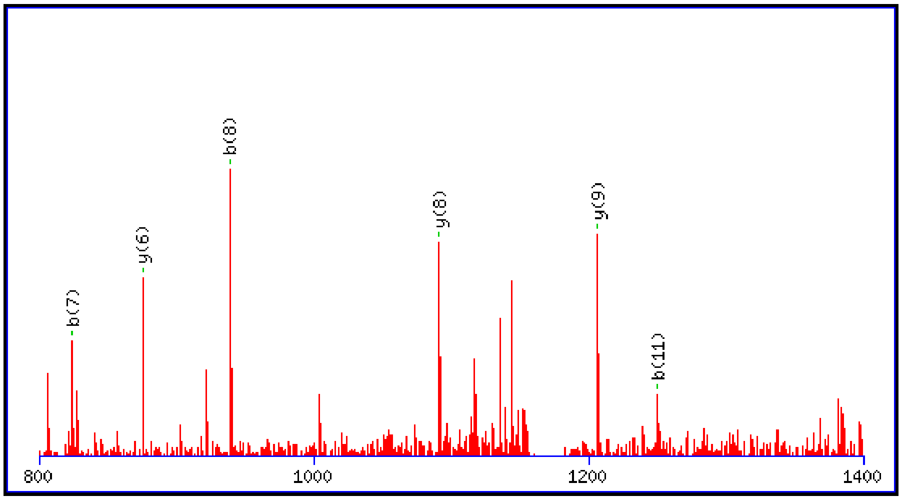


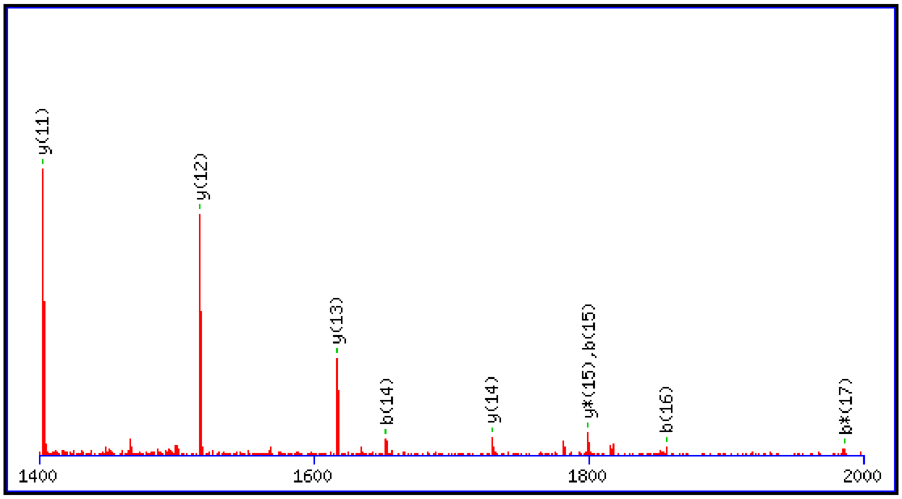


**Monoisotopic mass of neutral peptide Mr(calc):** 2340.0375
**Fixed modifications:** Carbamidomethyl (C)
**Variable modifications:**
**N3** : Deamidated_N (N)
**Ions Score:** 94 **Expect:** 1.3e-007
**Matches (Bold Red):** 21/194 fragment ions using 26 most intense peaks

| # | b | $b^{++}$ | b* | $b^{*++}$ | $b^{0}$ | $b^{0++}$ | Seq. | y | $y^{++}$ | y* | $y^{*++}$ | $y^{0}$ | $y^{0++}$ | # |
|---|---|---|---|---|---|---|---|---|---|---|---|---|---|---|
| 1 | 148.0757 | 74.5415 | | | | | F | | | | | | | 19 |
| 2 | 295.1441 | 148.0757 | | | | | F | 2193.9764 | 1097.4918 | 2176.9498 | 1088.9785 | 2175.9658 | 1088.4865 | 18 |
| 3 | **410.1710** | 205.5892 | 393.1445 | 197.0759 | | | N | 2046.9080 | 1023.9576 | 2029.8814 | 1015.4443 | 2028.8974 | 1014.9523 | 17 |
| 4 | **525.1980** | 263.1026 | 508.1714 | 254.5894 | 507.1874 | 254.0973 | D | 1931.8810 | 966.4441 | 1914.8545 | 957.9309 | 1913.8705 | 957.4389 | 16 |
| 5 | 612.2300 | 306.6186 | 595.2035 | 298.1054 | 594.2194 | 297.6134 | S | 1816.8541 | 908.9307 | **1799.8275** | 900.4174 | 1798.8435 | 899.9254 | 15 |

| | | | | | | | | | | | | | | |
|---|---|---|---|---|---|---|---|---|---|---|---|---|---|---|
| **6** | 725.3141 | 363.1607 | 708.2875 | 354.6474 | 707.3035 | 354.1554 | **I** | **1729.8220** | 865.4147 | 1712.7955 | 856.9014 | 1711.8115 | 856.4094 | **14** |
| **7** | **824.3825** | 412.6949 | 807.3559 | 404.1816 | 806.3719 | 403.6896 | **V** | **1616.7380** | 808.8726 | 1599.7114 | 800.3594 | 1598.7274 | 799.8673 | **13** |
| **8** | **939.4094** | 470.2084 | 922.3829 | 461.6951 | 921.3989 | 461.2031 | **D** | **1517.6696** | 759.3384 | 1500.6430 | 750.8251 | 1499.6590 | 750.3331 | **12** |
| **9** | 1036.4622 | 518.7347 | 1019.4356 | 510.2215 | 1018.4516 | 509.7295 | **P** | **1402.6426** | 701.8250 | 1385.6161 | 693.3117 | 1384.6321 | 692.8197 | **11** |
| **10** | 1135.5306 | 568.2689 | 1118.5041 | 559.7557 | 1117.5200 | 559.2637 | **V** | 1305.5899 | 653.2986 | 1288.5633 | 644.7853 | 1287.5793 | 644.2933 | **10** |
| **11** | **1250.5576** | 625.7824 | 1233.5310 | 617.2691 | 1232.5470 | 616.7771 | **D** | **1206.5215** | 603.7644 | 1189.4949 | 595.2511 | 1188.5109 | 594.7591 | **9** |
| **12** | 1337.5896 | 669.2984 | 1320.5630 | 660.7852 | 1319.5790 | 660.2931 | **S** | **1091.4945** | 546.2509 | 1074.4680 | 537.7376 | 1073.4839 | 537.2456 | **8** |
| **13** | 1466.6322 | 733.8197 | 1449.6056 | 725.3065 | 1448.6216 | 724.8144 | **E** | 1004.4625 | 502.7349 | 987.4359 | 494.2216 | 986.4519 | 493.7296 | **7** |
| **14** | **1652.7115** | 826.8594 | 1635.6849 | 818.3461 | 1634.7009 | 817.8541 | **W** | **875.4199** | 438.2136 | 858.3933 | 429.7003 | | | **6** |
| **15** | **1799.7799** | 900.3936 | 1782.7534 | 891.8803 | 1781.7693 | 891.3883 | **F** | **689.3406** | 345.1739 | 672.3140 | 336.6606 | | | **5** |
| **16** | **1856.8014** | 928.9043 | 1839.7748 | 920.3910 | 1838.7908 | 919.8990 | **G** | **542.2722** | 271.6397 | **525.2456** | 263.1264 | | | **4** |
| **17** | 2003.8698 | 1002.4385 | **1986.8432** | 993.9253 | 1985.8592 | 993.4332 | **F** | **485.2507** | 243.1290 | 468.2241 | 234.6157 | | | **3** |
| **18** | 2166.9331 | 1083.9702 | 2149.9066 | 1075.4569 | 2148.9225 | 1074.9649 | **Y** | 338.1823 | 169.5948 | 321.1557 | 161.0815 | | | **2** |
| **19** | | | | | | | **R** | 175.1190 | 88.0631 | 158.0924 | 79.5498 | | | **1** |

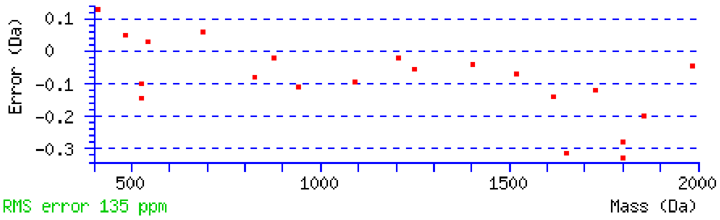


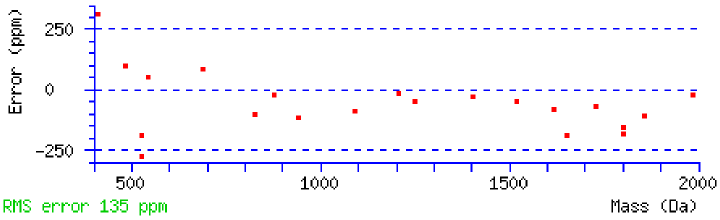


### All matches to this query

| Score | Mr(calc): | Delta | Sequence |
|---|---|---|---|
| 94.0 | 2340.0375 | 0.0078 | FFNDSIVDPVDSEWFGFYR |
| 46.8 | 2339.0535 | 0.9918 | FFNDSIVDPVDSEWFGFYR |
| 11.5 | 2340.0512 | -0.0059 | LLSASSSPTLPPSPGGGGGSKGNR |
| 3.8 | 2340.0341 | 0.0112 | TYWESNGTAGSHFITIRIK |
| 2.9 | 2339.0569 | 0.9884 | RAPQTGIVDECCFRSCDLR |
| 2.1 | 2338.0607 | 1.9846 | APGTLVGQISANDLDSPASPIR |
| 1.9 | 2340.0512 | -0.0060 | NTVNGTGTPVHISTLQVGETR |
| 1.8 | 2340.0512 | -0.0060 | NTVNGTGTPVHISTLQVGETR |
| 1.4 | 2340.0512 | -0.0059 | LLSASSSPTLPPSPGGGGGSKGNR |
| 1.2 | 2340.0552 | -0.0099 | WPEPXALSAAVQAGLQVTGER |

---

Spectrum No: 111; Query: 2233; Rank: 1

---

## Peptide View

MS/MS Fragmentation of **HYSCACPSGWNLSDDSVNCVR**
Found in **IPI00205325**, Tax_Id=10116 Gene_Symbol=Lrp2 Low-density lipoprotein receptor-related protein 2 precursor

Match to Query 2233: 2484.000108 from(1243.007330,2+)
Title: 100101RatKid_NS_deglyco_14.2681.2681.2.dta
Data file K:\NewmanPaper\Piliang\3SubProteomes\Piliang3SP\mgf5ppm\ERLIC_3SubProteomes5ppm.mgf

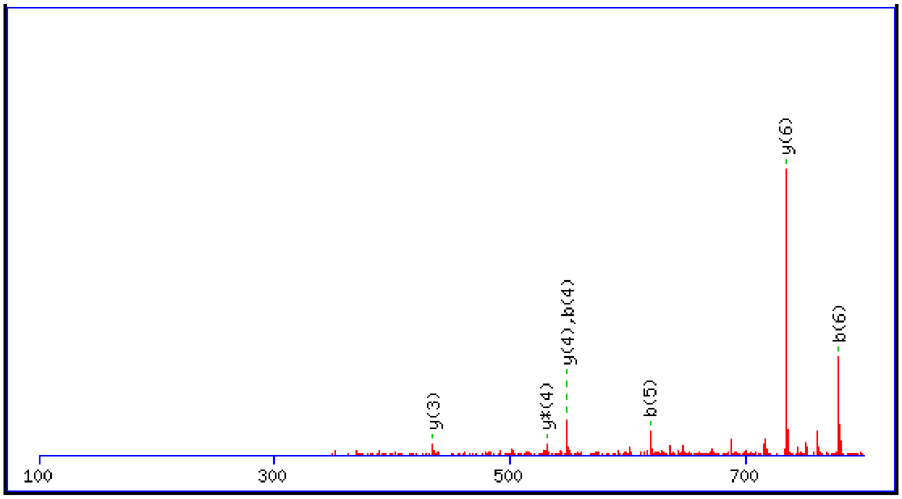


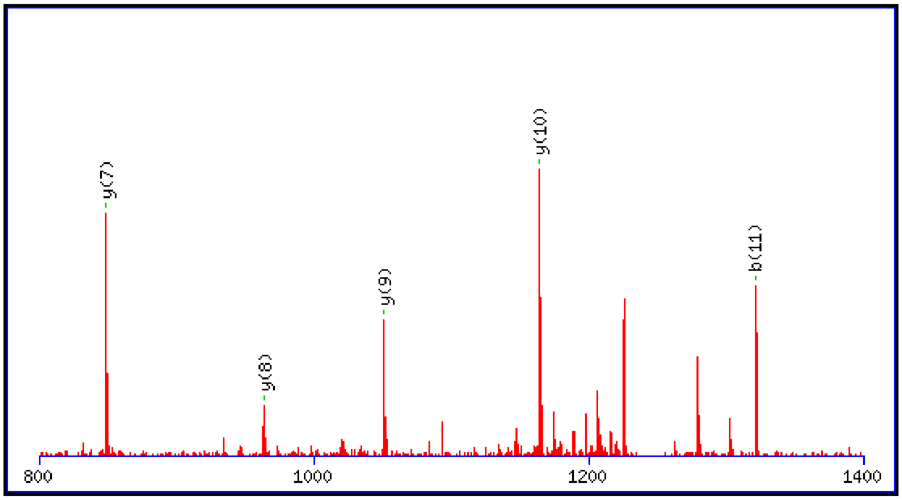


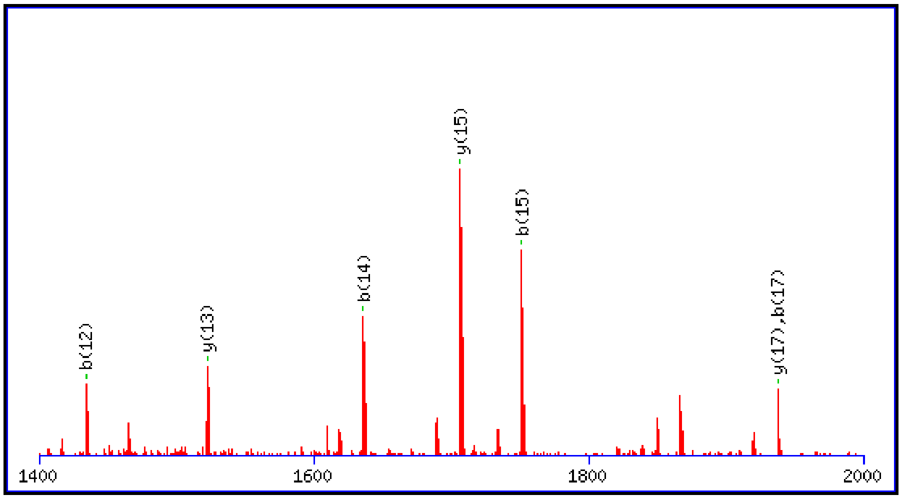


**Monoisotopic mass of neutral peptide Mr(calc):** 2483.9893
**Fixed modifications:** Carbamidomethyl (C)
**Variable modifications:**
**N11** : Deamidated_N (N)
**Ions Score:** 94 **Expect:** 9.8e-008
**Matches (Bold Red):** 19/206 fragment ions using 25 most intense peaks

| # | b | $b^{++}$ | b* | $b^{*++}$ | $b^0$ | $b^{0++}$ | Seq. | y | $y^{++}$ | y* | $y^{*++}$ | $y^0$ | $y^{0++}$ | # |
|---|---|---|---|---|---|---|---|---|---|---|---|---|---|---|
| 1 | 138.0662 | 69.5367 | | | | | H | | | | | | | 21 |
| 2 | 301.1295 | 151.0684 | | | | | Y | 2347.9376 | 1174.4725 | 2330.9111 | 1165.9592 | 2329.9271 | 1165.4672 | 20 |
| 3 | 388.1615 | 194.5844 | | | 370.1510 | 185.5791 | S | 2184.8743 | 1092.9408 | 2167.8478 | 1084.4275 | 2166.8637 | 1083.9355 | 19 |
| 4 | **548.1922** | 274.5997 | | | 530.1816 | 265.5945 | C | 2097.8423 | 1049.4248 | 2080.8157 | 1040.9115 | 2079.8317 | 1040.4195 | 18 |
| 5 | **619.2293** | 310.1183 | | | 601.2187 | 301.1130 | A | **1937.8116** | 969.4095 | 1920.7851 | 960.8962 | 1919.8011 | 960.4042 | 17 |

| | | | | | | | | | | | | | | |
|---|---|---|---|---|---|---|---|---|---|---|---|---|---|---|
| **6** | **779.2600** | 390.1336 | | | 761.2494 | 381.1283 | **C** | 1866.7745 | 933.8909 | 1849.7480 | 925.3776 | 1848.7640 | 924.8856 | **16** |
| **7** | 876.3127 | 438.6600 | | | 858.3022 | 429.6547 | **P** | **1706.7439** | 853.8756 | 1689.7173 | 845.3623 | 1688.7333 | 844.8703 | **15** |
| **8** | 963.3447 | 482.1760 | | | 945.3342 | 473.1707 | **S** | 1609.6911 | 805.3492 | 1592.6646 | 796.8359 | 1591.6805 | 796.3439 | **14** |
| **9** | 1020.3662 | 510.6867 | | | 1002.3556 | 501.6815 | **G** | **1522.6591** | 761.8332 | 1505.6325 | 753.3199 | 1504.6485 | 752.8279 | **13** |
| **10** | 1206.4455 | 603.7264 | | | 1188.4350 | 594.7211 | **W** | 1465.6376 | 733.3224 | 1448.6111 | 724.8092 | 1447.6270 | 724.3172 | **12** |
| **11** | **1321.4725** | 661.2399 | 1304.4459 | 652.7266 | 1303.4619 | 652.2346 | **N** | 1279.5583 | 640.2828 | 1262.5317 | 631.7695 | 1261.5477 | 631.2775 | **11** |
| **12** | **1434.5565** | 717.7819 | 1417.5300 | 709.2686 | 1416.5460 | 708.7766 | **L** | **1164.5314** | 582.7693 | 1147.5048 | 574.2560 | 1146.5208 | 573.7640 | **10** |
| **13** | 1521.5886 | 761.2979 | 1504.5620 | 752.7846 | 1503.5780 | 752.2926 | **S** | **1051.4473** | 526.2273 | 1034.4207 | 517.7140 | 1033.4367 | 517.2220 | **9** |
| **14** | **1636.6155** | 818.8114 | 1619.5889 | 810.2981 | 1618.6049 | 809.8061 | **D** | **964.4153** | 482.7113 | 947.3887 | 474.1980 | 946.4047 | 473.7060 | **8** |
| **15** | **1751.6424** | 876.3249 | 1734.6159 | 867.8116 | 1733.6319 | 867.3196 | **D** | **849.3883** | 425.1978 | 832.3618 | 416.6845 | 831.3778 | 416.1925 | **7** |
| **16** | 1838.6745 | 919.8409 | 1821.6479 | 911.3276 | 1820.6639 | 910.8356 | **S** | **734.3614** | 367.6843 | 717.3348 | 359.1711 | 716.3508 | 358.6790 | **6** |
| **17** | **1937.7429** | 969.3751 | 1920.7163 | 960.8618 | 1919.7323 | 960.3698 | **V** | 647.3294 | 324.1683 | 630.3028 | 315.6550 | | | **5** |
| **18** | 2051.7858 | 1026.3965 | 2034.7593 | 1017.8833 | 2033.7752 | 1017.3913 | **N** | **548.2609** | 274.6341 | **531.2344** | 266.1208 | | | **4** |
| **19** | 2211.8165 | 1106.4119 | 2194.7899 | 1097.8986 | 2193.8059 | 1097.4066 | **C** | **434.2180** | 217.6126 | 417.1915 | 209.0994 | | | **3** |
| **20** | 2310.8849 | 1155.9461 | 2293.8583 | 1147.4328 | 2292.8743 | 1146.9408 | **V** | 274.1874 | 137.5973 | 257.1608 | 129.0840 | | | **2** |
| **21** | | | | | | | **R** | 175.1190 | 88.0631 | 158.0924 | 79.5498 | | | **1** |

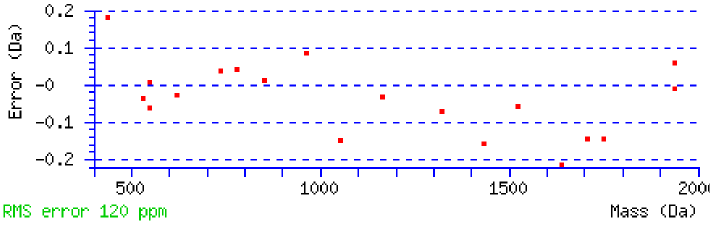


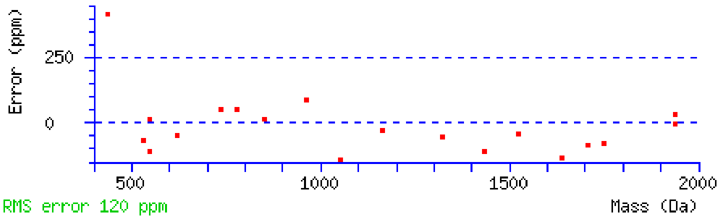


### All matches to this query

| Score | Mr(calc): | Delta | Sequence |
|---|---|---|---|
| 94.0 | 2483.9893 | 0.0108 | HYSCACPSGWNLSDDSVNCVR |
| 59.4 | 2483.9893 | 0.0108 | HYSCACPSGWNLSDDSVNCVR |
| 32.7 | 2483.0053 | 0.9948 | HYSCACPSGWNLSDDSVNCVR |
| 14.4 | 2481.9938 | 2.0063 | TTSSGVGGSTESNTDLFTYRNK |
| 9.6 | 2482.9778 | 1.0223 | TTSSGVGGSTESNTDLFTYRNK |
| 9.1 | 2481.9938 | 2.0063 | TTSSGVGGSTESNTDLFTYRNK |
| 9.1 | 2481.9938 | 2.0063 | TTSSGVGGSTESNTDLFTYRNK |
| 8.5 | 2481.9938 | 2.0063 | TTSSGVGGSTESNTDLFTYRNK |
| 2.5 | 2482.9989 | 1.0012 | TEGSDGAVSLSSADAQDSLEKEK |
| 2.5 | 2481.9938 | 2.0063 | TTSSGVGGSTESNTDLFTYRNK |

---

Spectrum No: 112; Query: 791; Rank: 1

---

## Peptide View

MS/MS Fragmentation of **SEELDFLGNSSLQK**
Found in **IPI00766673**, Tax_Id=10116 Gene_Symbol=Tlr3 similar to toll-like receptor 3

Match to Query 791: 1566.746768 from(784.380660,2+)
Title: 100101RatKid_NS_deglyco_23.3535.3535.2.dta
Data file K:\NewmanPaper\Piliang\3SubProteomes\Piliang3SP\mgf5ppm\ERLIC_3SubProteomes5ppm.mgf

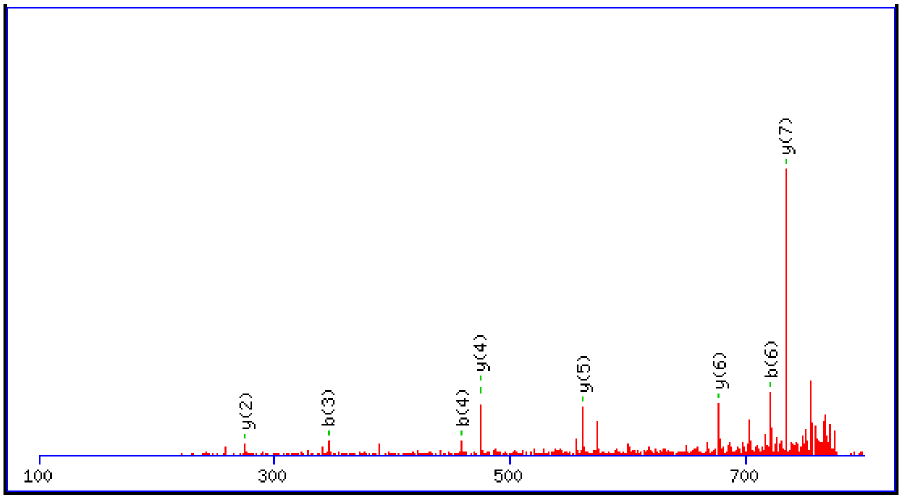


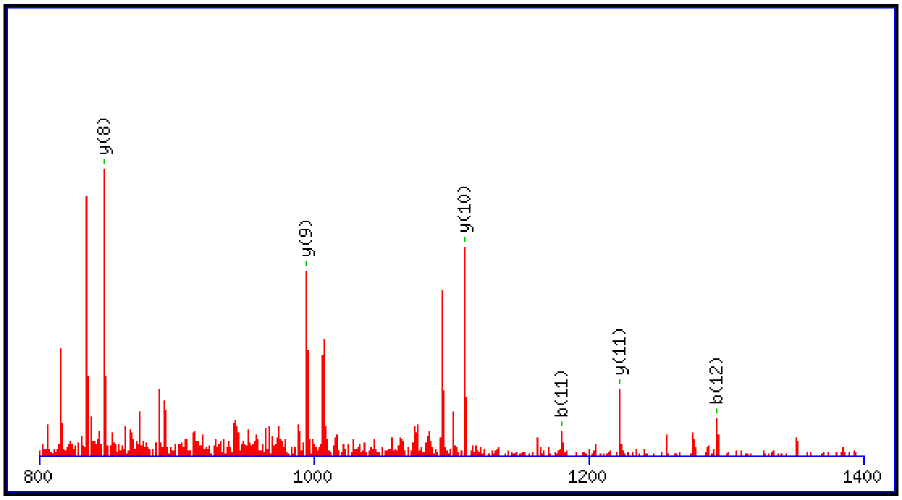


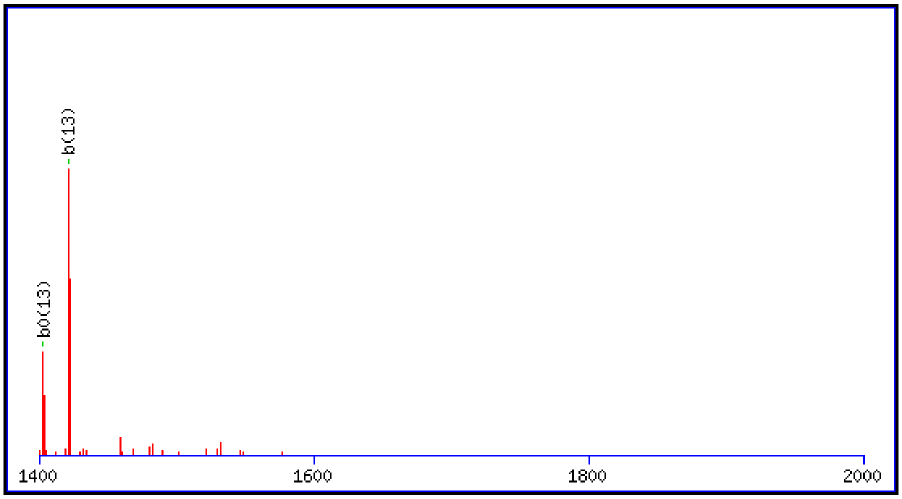


**Monoisotopic mass of neutral peptide Mr(calc):** 1566.7413
**Fixed modifications:** Carbamidomethyl (C)
**Variable modifications:**
**N9** : Deamidated_N (N)
**Ions Score:** 94 **Expect:** 7.3e-008
**Matches (Bold Red):** 16/134 fragment ions using 21 most intense peaks

| # | b | $b^{++}$ | b* | $b^{*++}$ | $b^0$ | $b^{0++}$ | Seq. | y | $y^{++}$ | y* | $y^{*++}$ | $y^0$ | $y^{0++}$ | # |
|---|---|---|---|---|---|---|---|---|---|---|---|---|---|---|
| 1 | 88.0393 | 44.5233 | | | 70.0287 | 35.5180 | S | | | | | | | 14 |
| 2 | 217.0819 | 109.0446 | | | 199.0713 | 100.0393 | E | 1480.7166 | 740.8619 | 1463.6900 | 732.3487 | 1462.7060 | 731.8566 | 13 |
| 3 | **346.1245** | 173.5659 | | | 328.1139 | 164.5606 | E | 1351.6740 | 676.3406 | 1334.6474 | 667.8274 | 1333.6634 | 667.3353 | 12 |
| 4 | **459.2086** | 230.1079 | | | 441.1980 | 221.1026 | L | **1222.6314** | 611.8193 | 1205.6048 | 603.3061 | 1204.6208 | 602.8140 | 11 |
| 5 | 574.2355 | 287.6214 | | | 556.2249 | 278.6161 | D | **1109.5473** | 555.2773 | 1092.5208 | 546.7640 | 1091.5368 | 546.2720 | 10 |

| | | | | | | | | | | | | | | |
|---|---|---|---|---|---|---|---|---|---|---|---|---|---|---|
| **6** | **721.3039** | 361.1556 | | | 703.2933 | 352.1503 | **F** | **994.5204** | 497.7638 | 977.4938 | 489.2506 | 976.5098 | 488.7585 | **9** |
| **7** | 834.3880 | 417.6976 | | | 816.3774 | 408.6923 | **L** | **847.4520** | 424.2296 | 830.4254 | 415.7163 | 829.4414 | 415.2243 | **8** |
| **8** | 891.4094 | 446.2084 | | | 873.3989 | 437.2031 | **G** | **734.3679** | 367.6876 | 717.3414 | 359.1743 | 716.3573 | 358.6823 | **7** |
| **9** | 1006.4364 | 503.7218 | 989.4098 | 495.2086 | 988.4258 | 494.7165 | **N** | **677.3464** | 339.1769 | 660.3199 | 330.6636 | 659.3359 | 330.1716 | **6** |
| **10** | 1093.4684 | 547.2378 | 1076.4419 | 538.7246 | 1075.4578 | 538.2326 | **S** | **562.3195** | 281.6634 | 545.2930 | 273.1501 | 544.3089 | 272.6581 | **5** |
| **11** | **1180.5004** | 590.7539 | 1163.4739 | 582.2406 | 1162.4899 | 581.7486 | **S** | **475.2875** | 238.1474 | 458.2609 | 229.6341 | 457.2769 | 229.1421 | **4** |
| **12** | **1293.5845** | 647.2959 | 1276.5579 | 638.7826 | 1275.5739 | 638.2906 | **L** | 388.2554 | 194.6314 | 371.2289 | 186.1181 | | | **3** |
| **13** | **1421.6431** | 711.3252 | 1404.6165 | 702.8119 | **1403.6325** | 702.3199 | **Q** | **275.1714** | 138.0893 | 258.1448 | 129.5761 | | | **2** |
| **14** | | | | | | | **K** | 147.1128 | 74.0600 | 130.0863 | 65.5468 | | | **1** |

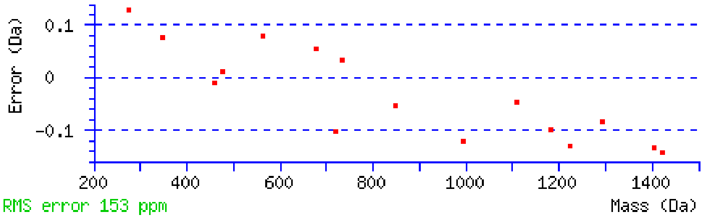


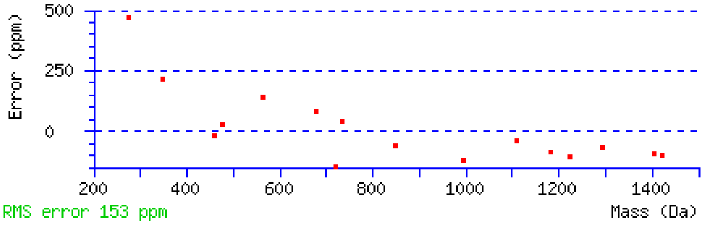


### All matches to this query

| Score | Mr(calc): | Delta | Sequence |
|---|---|---|---|
| 93.9 | 1566.7413 | 0.0055 | SEELDFLGNSSLQK |
| 22.7 | 1565.7573 | 0.9895 | SEELDFLGNSSLQK |
| 10.3 | 1566.7542 | -0.0074 | EESLEVVNSILQK |
| 9.4 | 1566.7460 | 0.0008 | SSVAGCYIRNPTNK |
| 9.3 | 1566.7477 | -0.0009 | SMTVTLGAHNIKAK |
| 9.3 | 1566.7477 | -0.0009 | SMTVTLGAHNIKAK |
| 9.3 | 1566.7477 | -0.0009 | SMTVTLGAHNIKAK |
| 7.7 | 1565.7459 | 1.0009 | ITXLNCIRIHSK |
| 7.1 | 1566.7599 | -0.0132 | YVELTEGVECIQK |
| 6.3 | 1566.7365 | 0.0103 | VIVYSGKATMSTSK |

---

Spectrum No: 113; Query: 2813; Rank: 1

---

## Peptide View

MS/MS Fragmentation of **QAIDDDCNQTGQMTAGLLDWPQGTFASQVTLEGDKVK**
Found in **IPI00364321**, Tax_Id=10116 Gene_Symbol=Etfb Electron transfer flavoprotein subunit beta

Match to Query 2813: 4037.882382 from(1346.968070,3+)
Title: 091008RatKidney_NoSalt_17.5765.5765.3.dta
Data file K:\NewmanPaper\Piliang\3SubProteomes\Piliang3SP\mgf5ppm\ERLIC_3SubProteomes5ppm.mgf

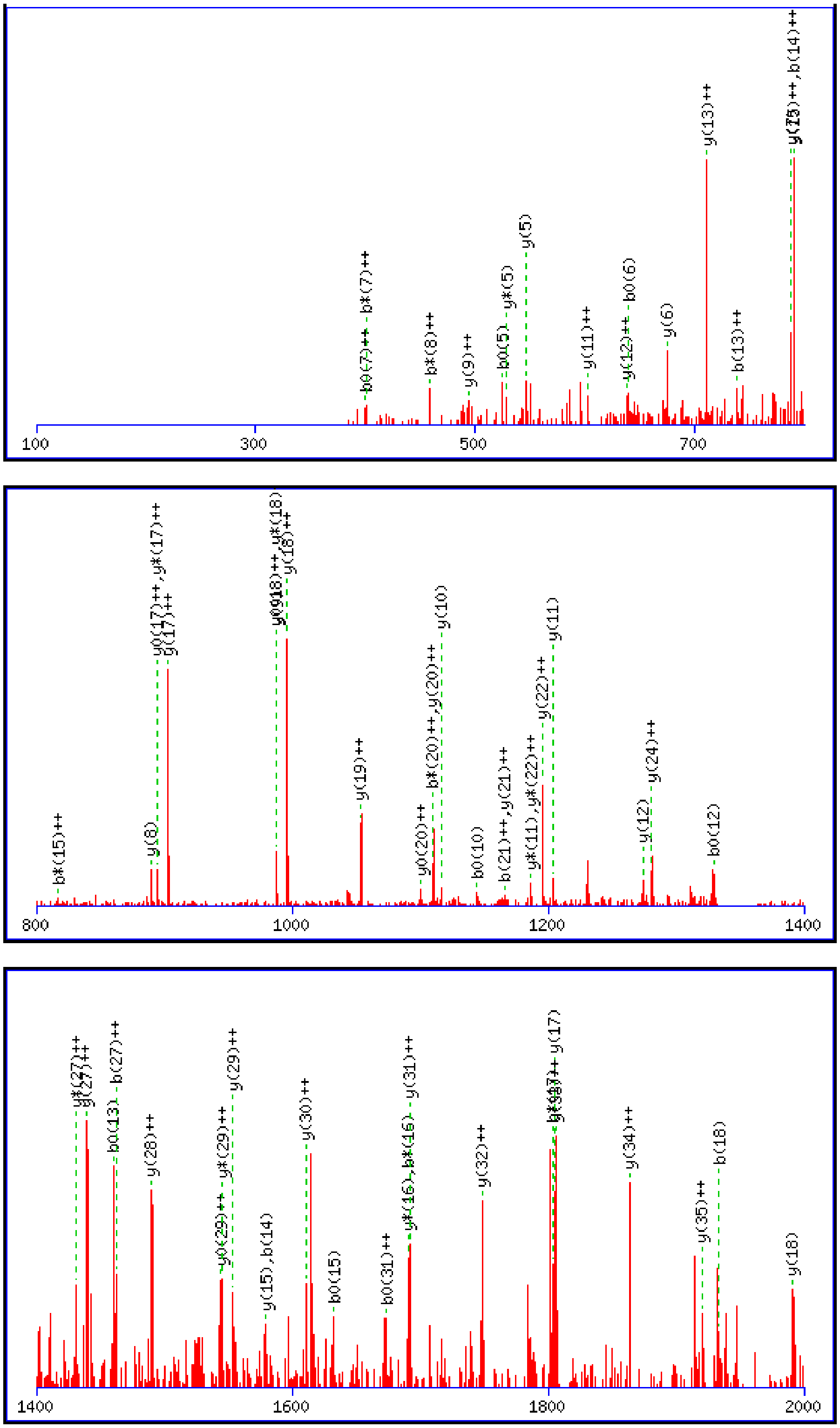


**Monoisotopic mass of neutral peptide Mr(calc):** 4037.8623
**Fixed modifications:** Carbamidomethyl (C)
**Variable modifications:**
**N8** : Deamidated_N (N)
**Ions Score:** 94 **Expect:** 1.8e-007
**Matches (Bold Red):** 64/420 fragment ions using 104 most intense peaks

| # | b | b++ | b* | b*++ | b0 | b0++ | Seq. | y | y++ | y* | y*++ | y0 | y0++ | # |
|---|---|---|---|---|---|---|---|---|---|---|---|---|---|---|
| 1 | 129.0659 | 65.0366 | 112.0393 | 56.5233 | | | Q | | | | | | | 37 |
| 2 | 200.1030 | 100.5551 | 183.0764 | 92.0418 | | | A | 3910.8110 | 1955.9091 | 3893.7844 | 1947.3959 | 3892.8004 | 1946.9038 | 36 |
| 3 | 313.1870 | 157.0972 | 296.1605 | 148.5839 | | | I | 3839.7739 | **1920.3906** | 3822.7473 | 1911.8773 | 3821.7633 | 1911.3853 | 35 |
| 4 | 428.2140 | 214.6106 | 411.1874 | 206.0974 | 410.2034 | 205.6053 | D | 3726.6898 | **1863.8485** | 3709.6632 | 1855.3353 | 3708.6792 | 1854.8433 | 34 |
| 5 | 543.2409 | 272.1241 | 526.2144 | 263.6108 | **525.2304** | 263.1188 | D | 3611.6629 | **1806.3351** | 3594.6363 | 1797.8218 | 3593.6523 | 1797.3298 | 33 |

| | | | | | | | | | | | | | | |
|---|---|---|---|---|---|---|---|---|---|---|---|---|---|---|
| 6 | 658.2679 | 329.6376 | 641.2413 | 321.1243 | 640.2573 | 320.6323 | D | 3496.6359 | 1748.8216 | 3479.6094 | 1740.3083 | 3478.6253 | 1739.8163 | 32 |
| 7 | 818.2985 | 409.6529 | 801.2720 | 401.1396 | 800.2879 | 400.6476 | C | 3381.6090 | 1691.3081 | 3364.5824 | 1682.7948 | 3363.5984 | 1682.3028 | 31 |
| 8 | 933.3254 | 467.1664 | 916.2989 | 458.6531 | 915.3149 | 458.1611 | N | 3221.5783 | 1611.2928 | 3204.5518 | 1602.7795 | 3203.5678 | 1602.2875 | 30 |
| 9 | 1061.3840 | 531.1957 | 1044.3575 | 522.6824 | 1043.3735 | 522.1904 | Q | 3106.5514 | 1553.7793 | 3089.5248 | 1545.2661 | 3088.5408 | 1544.7740 | 29 |
| 10 | 1162.4317 | 581.7195 | 1145.4052 | 573.2062 | 1144.4211 | 572.7142 | T | 2978.4928 | 1489.7500 | 2961.4663 | 1481.2368 | 2960.4822 | 1480.7448 | 28 |
| 11 | 1219.4532 | 610.2302 | 1202.4266 | 601.7169 | 1201.4426 | 601.2249 | G | 2877.4451 | 1439.2262 | 2860.4186 | 1430.7129 | 2859.4346 | 1430.2209 | 27 |
| 12 | 1347.5117 | 674.2595 | 1330.4852 | 665.7462 | 1329.5012 | 665.2542 | Q | 2820.4237 | 1410.7155 | 2803.3971 | 1402.2022 | 2802.4131 | 1401.7102 | 26 |
| 13 | 1478.5522 | 739.7798 | 1461.5257 | 731.2665 | 1460.5417 | 730.7745 | M | 2692.3651 | 1346.6862 | 2675.3385 | 1338.1729 | 2674.3545 | 1337.6809 | 25 |
| 14 | 1579.5999 | 790.3036 | 1562.5734 | 781.7903 | 1561.5893 | 781.2983 | T | 2561.3246 | 1281.1659 | 2544.2980 | 1272.6527 | 2543.3140 | 1272.1607 | 24 |
| 15 | 1650.6370 | 825.8221 | 1633.6105 | 817.3089 | 1632.6265 | 816.8169 | A | 2460.2769 | 1230.6421 | 2443.2504 | 1222.1288 | 2442.2664 | 1221.6368 | 23 |
| 16 | 1707.6585 | 854.3329 | 1690.6319 | 845.8196 | 1689.6479 | 845.3276 | G | 2389.2398 | 1195.1235 | 2372.2133 | 1186.6103 | 2371.2292 | 1186.1183 | 22 |
| 17 | 1820.7426 | 910.8749 | 1803.7160 | 902.3616 | 1802.7320 | 901.8696 | L | 2332.2183 | 1166.6128 | 2315.1918 | 1158.0995 | 2314.2078 | 1157.6075 | 21 |
| 18 | 1933.8266 | 967.4169 | 1916.8001 | 958.9037 | 1915.8160 | 958.4117 | L | 2219.1343 | 1110.0708 | 2202.1077 | 1101.5575 | 2201.1237 | 1101.0655 | 20 |
| 19 | 2048.8536 | 1024.9304 | 2031.8270 | 1016.4171 | 2030.8430 | 1015.9251 | D | 2106.0502 | 1053.5287 | 2089.0237 | 1045.0155 | 2088.0396 | 1044.5235 | 19 |
| 20 | 2234.9329 | 1117.9701 | 2217.9063 | 1109.4568 | 2216.9223 | 1108.9648 | W | 1991.0233 | 996.0153 | 1973.9967 | 987.5020 | 1973.0127 | 987.0100 | 18 |
| 21 | 2331.9856 | 1166.4965 | 2314.9591 | 1157.9832 | 2313.9751 | 1157.4912 | P | 1804.9440 | 902.9756 | 1787.9174 | 894.4623 | 1786.9334 | 893.9703 | 17 |
| 22 | 2460.0442 | 1230.5257 | 2443.0177 | 1222.0125 | 2442.0336 | 1221.5205 | Q | 1707.8912 | 854.4492 | 1690.8646 | 845.9360 | 1689.8806 | 845.4440 | 16 |
| 23 | 2517.0657 | 1259.0365 | 2500.0391 | 1250.5232 | 2499.0551 | 1250.0312 | G | 1579.8326 | 790.4199 | 1562.8061 | 781.9067 | 1561.8220 | 781.4147 | 15 |
| 24 | 2618.1134 | 1309.5603 | 2601.0868 | 1301.0470 | 2600.1028 | 1300.5550 | T | 1522.8112 | 761.9092 | 1505.7846 | 753.3959 | 1504.8006 | 752.9039 | 14 |
| 25 | 2765.1818 | 1383.0945 | 2748.1552 | 1374.5812 | 2747.1712 | 1374.0892 | F | 1421.7635 | 711.3854 | 1404.7369 | 702.8721 | 1403.7529 | 702.3801 | 13 |
| 26 | 2836.2189 | 1418.6131 | 2819.1923 | 1410.0998 | 2818.2083 | 1409.6078 | A | 1274.6951 | 637.8512 | 1257.6685 | 629.3379 | 1256.6845 | 628.8459 | 12 |
| 27 | 2923.2509 | 1462.1291 | 2906.2244 | 1453.6158 | 2905.2403 | 1453.1238 | S | 1203.6579 | 602.3326 | 1186.6314 | 593.8193 | 1185.6474 | 593.3273 | 11 |
| 28 | 3051.3095 | 1526.1584 | 3034.2829 | 1517.6451 | 3033.2989 | 1517.1531 | Q | 1116.6259 | 558.8166 | 1099.5994 | 550.3033 | 1098.6154 | 549.8113 | 10 |
| 29 | 3150.3779 | 1575.6926 | 3133.3514 | 1567.1793 | 3132.3673 | 1566.6873 | V | 988.5673 | 494.7873 | 971.5408 | 486.2740 | 970.5568 | 485.7820 | 9 |
| 30 | 3251.4256 | 1626.2164 | 3234.3990 | 1617.7032 | 3233.4150 | 1617.2111 | T | 889.4989 | 445.2531 | 872.4724 | 436.7398 | 871.4884 | 436.2478 | 8 |
| 31 | 3364.5096 | 1682.7585 | 3347.4831 | 1674.2452 | 3346.4991 | 1673.7532 | L | 788.4512 | 394.7293 | 771.4247 | 386.2160 | 770.4407 | 385.7240 | 7 |
| 32 | 3493.5522 | 1747.2798 | 3476.5257 | 1738.7665 | 3475.5417 | 1738.2745 | E | 675.3672 | 338.1872 | 658.3406 | 329.6740 | 657.3566 | 329.1819 | 6 |
| 33 | 3550.5737 | 1775.7905 | 3533.5472 | 1767.2772 | 3532.5631 | 1766.7852 | G | 546.3246 | 273.6659 | 529.2980 | 265.1527 | 528.3140 | 264.6606 | 5 |
| 34 | 3665.6006 | 1833.3040 | 3648.5741 | 1824.7907 | 3647.5901 | 1824.2987 | D | 489.3031 | 245.1552 | 472.2766 | 236.6419 | 471.2926 | 236.1499 | 4 |
| 35 | 3793.6956 | 1897.3514 | 3776.6691 | 1888.8382 | 3775.6850 | 1888.3462 | K | 374.2762 | 187.6417 | 357.2496 | 179.1285 | | | 3 |
| 36 | 3892.7640 | 1946.8856 | 3875.7375 | 1938.3724 | 3874.7535 | 1937.8804 | V | 246.1812 | 123.5942 | 229.1547 | 115.0810 | | | 2 |
| 37 | | | | | | | K | 147.1128 | 74.0600 | 130.0863 | 65.5468 | | | 1 |

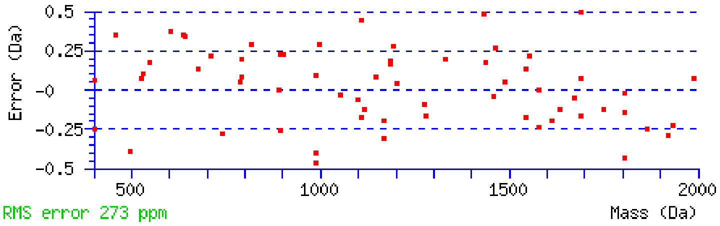


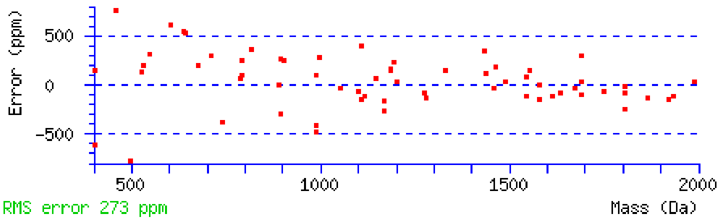


### All matches to this query

| Score | Mr(calc): | Delta | Sequence |
|---|---|---|---|
| 93.8 | 4037.8623 | 0.0201 | QAIDDDCNQTGQMTAGLLDWPQGTFASQVTLEGDKVK |
| 90.4 | 4036.8783 | 1.0041 | QAIDDDCNQTGQMTAGLLDWPQGTFASQVTLEGDKVK |
| 8.1 | 4037.8601 | 0.0222 | ESNTAGNDIFHKFSAFIKNPVPTQDDALYQQLLR |
| 8.1 | 4037.8601 | 0.0222 | ESNTAGNDIFHKFSAFIKNPVPTQDDALYQQLLR |
| 8.1 | 4037.8601 | 0.0222 | ESNTAGNDIFHKFSAFIKNPVPTQDDALYQQLLR |
| 7.8 | 4037.8601 | 0.0222 | ESNTAGNDIFHKFSAFIKNPVPTQDDALYQQLLR |
| 7.5 | 4037.8587 | 0.0236 | AFAEKNNLSFIETSALDSTNVEEAFKNILTEIYR |
| 7.0 | 4036.8389 | 1.0435 | YRVYIVIPLLPGFEGDISTGGGNALQAIMHFNYR |

| | | | |
|---|---|---|---|
| 6.6 | 4037.8587 | 0.0236 | AFAEKNNLSFIETSALDSTNVEEAFKNILTEIYR |
| 6.3 | 4036.9088 | 0.9735 | DNSNIILLGDSQGDLRMADGVANVEHILKIGYLNDR |

---

Spectrum No: 114; Query: 2447; Rank: 1

---

## Peptide View

MS/MS Fragmentation of **HTGPGILSMANAGPNTNGSQFFICTAK**
Found in **IPI00364439**, Tax_Id=10116 Gene_Symbol=RGD1559682_predicted similar to Peptidyl-prolyl cis-trans isomerase A

Match to Query 2447: 2807.290152 from(936.770660,3+)
Title: 091008RatKidney_NH4Format01_10.3633.3633.3.dta
Data file K:\NewmanPaper\Piliang\3SubProteomes\Piliang3SP\mgf5ppm\ERLIC_3SubProteomes5ppm.mgf

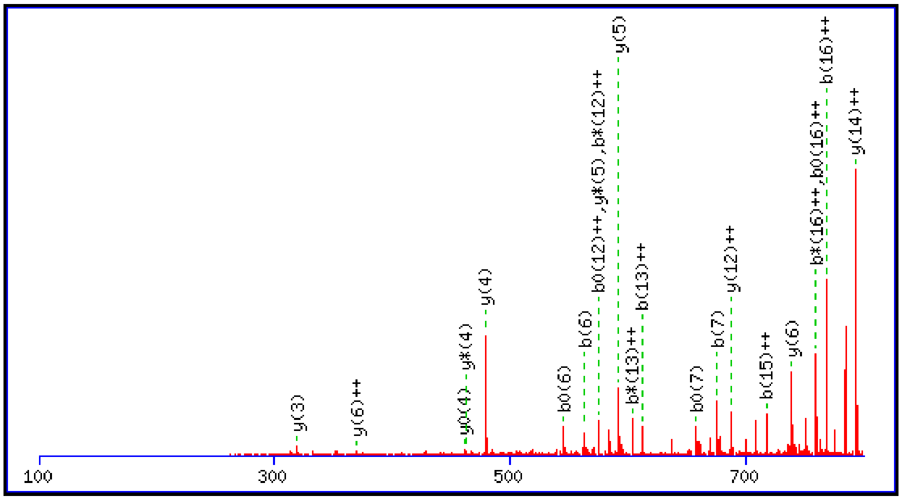


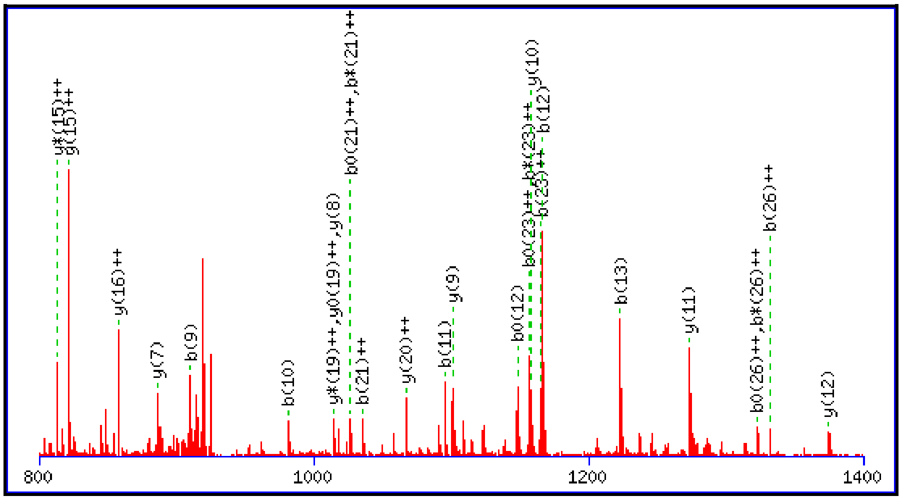

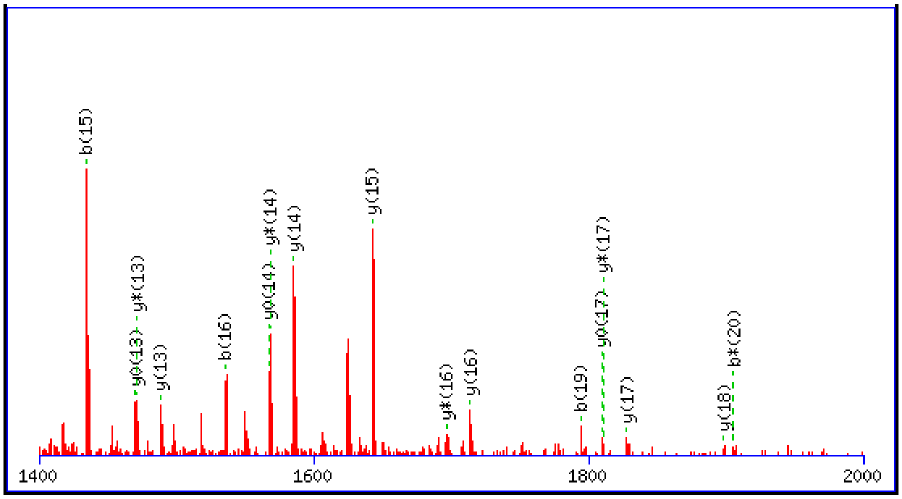


**Monoisotopic mass of neutral peptide Mr(calc):** 2807.3007
**Fixed modifications:** Carbamidomethyl (C)
**Variable modifications:**
**M9** : Oxidation (M)
**N17** : Deamidated_N (N)
**Ions Score:** 94 **Expect:** 1.6e-007
**Matches (Bold Red):** 66/286 fragment ions using 102 most intense peaks

| # | b | $b^{++}$ | b* | $b^{*++}$ | $b^{0}$ | $b^{0++}$ | Seq. | y | $y^{++}$ | y* | $y^{*++}$ | $y^{0}$ | $y^{0++}$ | # |
|---|---|---|---|---|---|---|---|---|---|---|---|---|---|---|
| 1 | 138.0662 | 69.5367 | | | | | H | | | | | | | 27 |
| 2 | 239.1139 | 120.0606 | | | 221.1033 | 111.0553 | T | 2671.2491 | 1336.1282 | 2654.2225 | 1327.6149 | 2653.2385 | 1327.1229 | 26 |
| 3 | 296.1353 | 148.5713 | | | 278.1248 | 139.5660 | G | 2570.2014 | 1285.6043 | 2553.1748 | 1277.0911 | 2552.1908 | 1276.5990 | 25 |
| 4 | 393.1881 | 197.0977 | | | 375.1775 | 188.0924 | P | 2513.1799 | 1257.0936 | 2496.1534 | 1248.5803 | 2495.1694 | 1248.0883 | 24 |
| 5 | 450.2096 | 225.6084 | | | 432.1990 | 216.6031 | G | 2416.1272 | 1208.5672 | 2399.1006 | 1200.0539 | 2398.1166 | 1199.5619 | 23 |
| 6 | **563.2936** | 282.1504 | | | **545.2831** | 273.1452 | I | 2359.1057 | 1180.0565 | 2342.0791 | 1171.5432 | 2341.0951 | 1171.0512 | 22 |
| 7 | **676.3777** | 338.6925 | | | **658.3671** | 329.6872 | L | 2246.0216 | 1123.5145 | 2228.9951 | 1115.0012 | 2228.0111 | 1114.5092 | 21 |
| 8 | 763.4097 | 382.2085 | | | 745.3991 | 373.2032 | S | 2132.9376 | **1066.9724** | 2115.9110 | 1058.4591 | 2114.9270 | 1057.9671 | 20 |
| 9 | **910.4451** | 455.7262 | | | 892.4346 | 446.7209 | M | 2045.9055 | 1023.4564 | 2028.8790 | **1014.9431** | 2027.8950 | **1014.4511** | 19 |
| 10 | **981.4822** | 491.2448 | | | 963.4717 | 482.2395 | A | **1898.8701** | 949.9387 | 1881.8436 | 941.4254 | 1880.8596 | 940.9334 | 18 |
| 11 | **1095.5252** | 548.2662 | 1078.4986 | 539.7529 | 1077.5146 | 539.2609 | N | **1827.8330** | 914.4201 | **1810.8065** | 905.9069 | **1809.8225** | 905.4149 | 17 |
| 12 | **1166.5623** | 583.7848 | 1149.5357 | **575.2715** | **1148.5517** | **574.7795** | A | **1713.7901** | **857.3987** | **1696.7635** | 848.8854 | 1695.7795 | 848.3934 | 16 |
| 13 | **1223.5837** | **612.2955** | 1206.5572 | **603.7822** | 1205.5732 | 603.2902 | G | **1642.7530** | **821.8801** | 1625.7264 | **813.3669** | 1624.7424 | 812.8748 | 15 |
| 14 | 1320.6365 | 660.8219 | 1303.6100 | 652.3086 | 1302.6259 | 651.8166 | P | **1585.7315** | **793.3694** | **1568.7050** | 784.8561 | **1567.7209** | 784.3641 | 14 |
| 15 | **1434.6794** | **717.8434** | 1417.6529 | 709.3301 | 1416.6689 | 708.8381 | N | **1488.6787** | 744.8430 | **1471.6522** | 736.3297 | **1470.6682** | 735.8377 | 13 |
| 16 | **1535.7271** | **768.3672** | 1518.7006 | **759.8539** | 1517.7165 | **759.3619** | T | **1374.6358** | **687.8215** | 1357.6093 | 679.3083 | 1356.6253 | 678.8163 | 12 |
| 17 | 1650.7540 | 825.8807 | 1633.7275 | 817.3674 | 1632.7435 | 816.8754 | N | **1273.5881** | 637.2977 | 1256.5616 | 628.7844 | 1255.5776 | 628.2924 | 11 |
| 18 | 1707.7755 | 854.3914 | 1690.7490 | 845.8781 | 1689.7649 | 845.3861 | G | **1158.5612** | 579.7842 | 1141.5347 | 571.2710 | 1140.5506 | 570.7790 | 10 |
| 19 | **1794.8075** | 897.9074 | 1777.7810 | 889.3941 | 1776.7970 | 888.9021 | S | **1101.5397** | 551.2735 | 1084.5132 | 542.7602 | 1083.5292 | 542.2682 | 9 |
| 20 | 1922.8661 | 961.9367 | **1905.8396** | 953.4234 | 1904.8555 | 952.9314 | Q | **1014.5077** | 507.7575 | 997.4812 | 499.2442 | 996.4972 | 498.7522 | 8 |
| 21 | 2069.9345 | **1035.4709** | 2052.9080 | **1026.9576** | 2051.9240 | **1026.4656** | F | **886.4491** | 443.7282 | 869.4226 | 435.2149 | 868.4386 | 434.7229 | 7 |
| 22 | 2217.0029 | 1109.0051 | 2199.9764 | 1100.4918 | 2198.9924 | 1099.9998 | F | **739.3807** | **370.1940** | 722.3542 | 361.6807 | 721.3702 | 361.1887 | 6 |
| 23 | 2330.0870 | **1165.5471** | 2313.0605 | **1157.0339** | 2312.0764 | **1156.5419** | I | **592.3123** | 296.6598 | **575.2858** | 288.1465 | 574.3017 | 287.6545 | 5 |
| 24 | 2490.1177 | 1245.5625 | 2473.0911 | 1237.0492 | 2472.1071 | 1236.5572 | C | **479.2282** | 240.1178 | **462.2017** | 231.6045 | **461.2177** | 231.1125 | 4 |
| 25 | 2591.1653 | 1296.0863 | 2574.1388 | 1287.5730 | 2573.1548 | 1287.0810 | T | **319.1976** | 160.1024 | 302.1710 | 151.5892 | 301.1870 | 151.0972 | 3 |
| 26 | 2662.2024 | **1331.6049** | 2645.1759 | **1323.0916** | 2644.1919 | **1322.5996** | A | 218.1499 | 109.5786 | 201.1234 | 101.0653 | | | 2 |
| 27 | | | | | | | K | 147.1128 | 74.0600 | 130.0863 | 65.5468 | | | 1 |

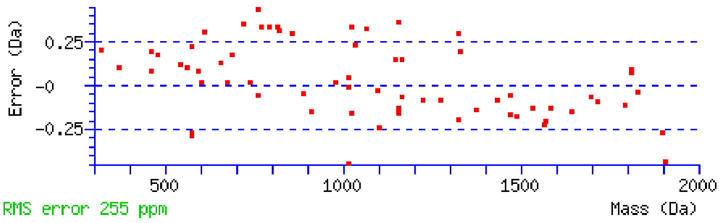


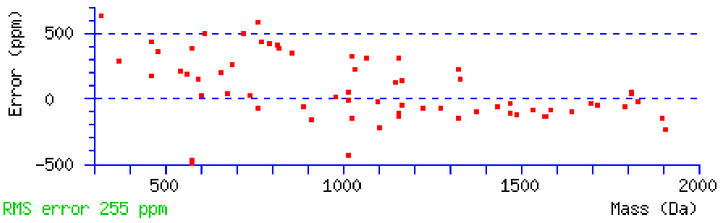


### All matches to this query

| Score | Mr(calc): | Delta | Sequence |
|---|---|---|---|
| 93.7 | 2807.3007 | -0.0106 | HTGPGILSMANAGPNTNGSQFFICTAK |
| 70.9 | 2807.3007 | -0.0106 | HTGPGILSMANAGPNTNGSQFFICTAK |
| 39.0 | 2807.3007 | -0.0106 | HTGPGILSMANAGPNTNGSQFFICTAK |
| 6.1 | 2807.2967 | -0.0066 | DASMLGSLAYNVLLIALCTLYAFK |
| 4.5 | 2805.3060 | 1.9841 | LNSLLKNLKFMNPGGDISLNPSEK |
| 3.8 | 2805.2793 | 2.0109 | QLHLPERSPDDSPPGAPAPGPMSASAK |
| 2.9 | 2807.2919 | -0.0017 | MANYTNLTQGAKEHEEAENITEGKK |
| 1.9 | 2807.2919 | -0.0017 | MANYTNLTQGAKEHEEAENITEGKK |
| 1.4 | 2807.3062 | -0.0160 | QPSASAPAQSSTAPCLSSXPALPRQR |
| 1.4 | 2807.3062 | -0.0160 | QPSASAPAQSSTAPCLSSXPALPRQR |

---

Spectrum No: 115; Query: 1893; Rank: 1

---

## Peptide View

MS/MS Fragmentation of **EGNSALINCSAFGIPDLEYK**
Found in **IPI00188728**, Tax_Id=10116 Gene_Symbol=Mfap3l Microfibrillar-associated protein 3-like precursor

Match to Query 1893: 2199.022968 from(1100.518760,2+)
Title: 100101RatKid_NS_deglyco_27.4337.4337.2.dta
Data file K:\NewmanPaper\Piliang\3SubProteomes\Piliang3SP\mgf5ppm\ERLIC_3SubProteomes5ppm.mgf

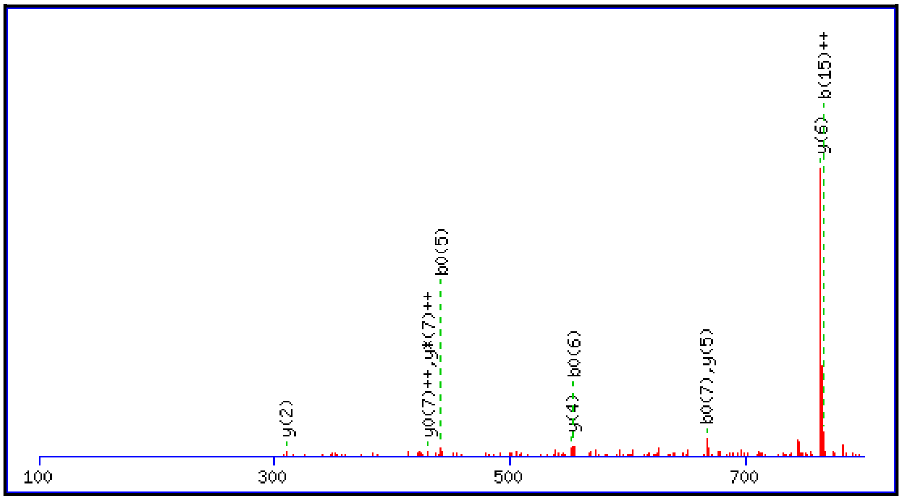

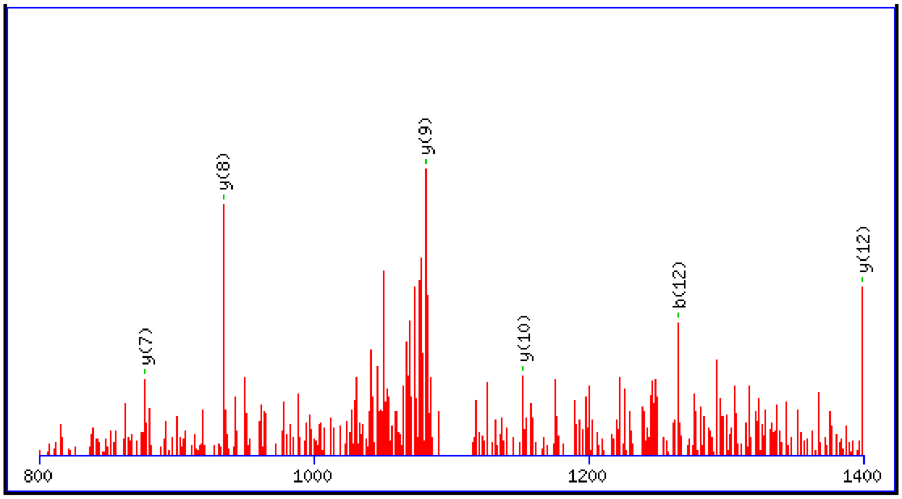


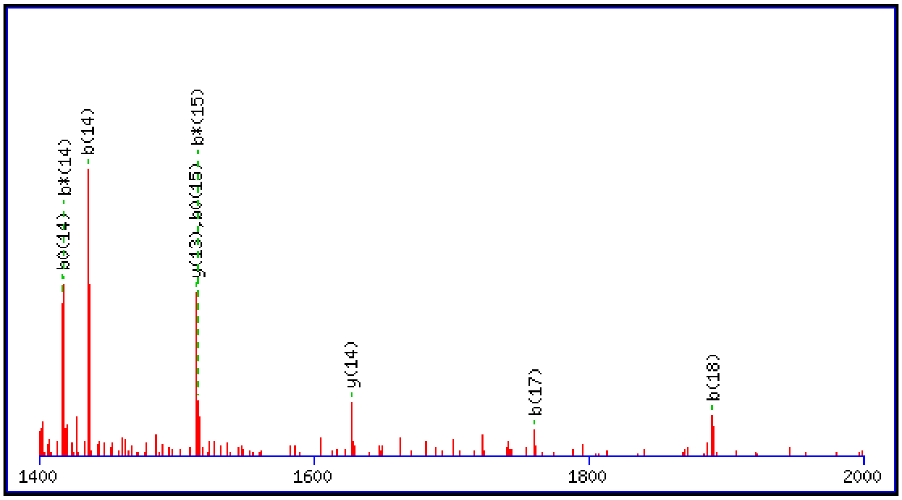


**Monoisotopic mass of neutral peptide Mr(calc):** 2198.0201
**Fixed modifications:** Carbamidomethyl (C)
**Variable modifications:**
**N8** : Deamidated_N (N)
**Ions Score:** 93 **Expect:** 1.3e-007
**Matches (Bold Red):** 25/220 fragment ions using 30 most intense peaks

| # | b | $b^{++}$ | b* | $b^{*++}$ | $b^0$ | $b^{0++}$ | Seq. | y | $y^{++}$ | y* | $y^{*++}$ | $y^0$ | $y^{0++}$ | # |
|---|---|---|---|---|---|---|---|---|---|---|---|---|---|---|
| 1 | 130.0499 | 65.5286 | | | 112.0393 | 56.5233 | E | | | | | | | 20 |
| 2 | 187.0713 | 94.0393 | | | 169.0608 | 85.0340 | G | 2069.9848 | 1035.4961 | 2052.9583 | 1026.9828 | 2051.9743 | 1026.4908 | 19 |
| 3 | 301.1143 | 151.0608 | 284.0877 | 142.5475 | 283.1037 | 142.0555 | N | 2012.9634 | 1006.9853 | 1995.9368 | 998.4720 | 1994.9528 | 997.9800 | 18 |
| 4 | 388.1463 | 194.5768 | 371.1197 | 186.0635 | 370.1357 | 185.5715 | S | 1898.9204 | 949.9639 | 1881.8939 | 941.4506 | 1880.9099 | 940.9586 | 17 |
| 5 | 459.1834 | 230.0953 | 442.1569 | 221.5821 | **441.1728** | 221.0901 | A | 1811.8884 | 906.4478 | 1794.8619 | 897.9346 | 1793.8778 | 897.4426 | 16 |
| 6 | 572.2675 | 286.6374 | 555.2409 | 278.1241 | **554.2569** | 277.6321 | L | 1740.8513 | 870.9293 | 1723.8247 | 862.4160 | 1722.8407 | 861.9240 | 15 |
| 7 | 685.3515 | 343.1794 | 668.3250 | 334.6661 | **667.3410** | 334.1741 | I | **1627.7672** | 814.3873 | 1610.7407 | 805.8740 | 1609.7567 | 805.3820 | 14 |
| 8 | 800.3785 | 400.6929 | 783.3519 | 392.1796 | 782.3679 | 391.6876 | N | **1514.6832** | 757.8452 | 1497.6566 | 749.3319 | 1496.6726 | 748.8399 | 13 |
| 9 | 960.4091 | 480.7082 | 943.3826 | 472.1949 | 942.3986 | 471.7029 | C | **1399.6562** | 700.3318 | 1382.6297 | 691.8185 | 1381.6457 | 691.3265 | 12 |
| 10 | 1047.4411 | 524.2242 | 1030.4146 | 515.7109 | 1029.4306 | 515.2189 | S | 1239.6256 | 620.3164 | 1222.5990 | 611.8032 | 1221.6150 | 611.3111 | 11 |
| 11 | 1118.4783 | 559.7428 | 1101.4517 | 551.2295 | 1100.4677 | 550.7375 | A | **1152.5936** | 576.8004 | 1135.5670 | 568.2871 | 1134.5830 | 567.7951 | 10 |
| 12 | **1265.5467** | 633.2770 | 1248.5201 | 624.7637 | 1247.5361 | 624.2717 | F | **1081.5564** | 541.2819 | 1064.5299 | 532.7686 | 1063.5459 | 532.2766 | 9 |
| 13 | 1322.5681 | 661.7877 | 1305.5416 | 653.2744 | 1304.5576 | 652.7824 | G | **934.4880** | 467.7477 | 917.4615 | 459.2344 | 916.4775 | 458.7424 | 8 |
| 14 | **1435.6522** | 718.3297 | **1418.6257** | 709.8165 | **1417.6416** | 709.3245 | I | **877.4666** | 439.2369 | 860.4400 | **430.7236** | 859.4560 | **430.2316** | 7 |
| 15 | 1532.7050 | **766.8561** | **1515.6784** | 758.3428 | **1514.6944** | 757.8508 | P | **764.3825** | 382.6949 | 747.3559 | 374.1816 | 746.3719 | 373.6896 | 6 |
| 16 | 1647.7319 | 824.3696 | 1630.7054 | 815.8563 | 1629.7213 | 815.3643 | D | **667.3297** | 334.1685 | 650.3032 | 325.6552 | 649.3192 | 325.1632 | 5 |
| 17 | **1760.8160** | 880.9116 | 1743.7894 | 872.3983 | 1742.8054 | 871.9063 | L | **552.3028** | 276.6550 | 535.2762 | 268.1418 | 534.2922 | 267.6498 | 4 |
| 18 | **1889.8586** | 945.4329 | 1872.8320 | 936.9196 | 1871.8480 | 936.4276 | E | 439.2187 | 220.1130 | 422.1922 | 211.5997 | 421.2082 | 211.1077 | 3 |

| | | | | | | | | | | | | | | |
|---|---|---|---|---|---|---|---|---|---|---|---|---|---|---|
| **19** | 2052.9219 | 1026.9646 | 2035.8953 | 1018.4513 | 2034.9113 | 1017.9593 | **Y** | **310.1761** | 155.5917 | 293.1496 | 147.0784 | | | **2** |
| **20** | | | | | | | **K** | 147.1128 | 74.0600 | 130.0863 | 65.5468 | | | **1** |

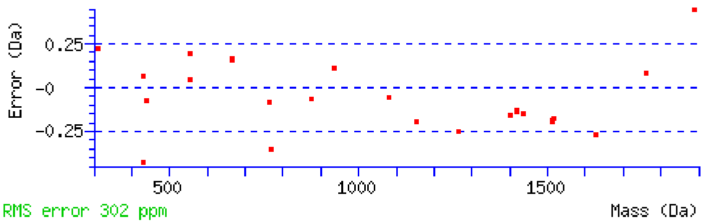


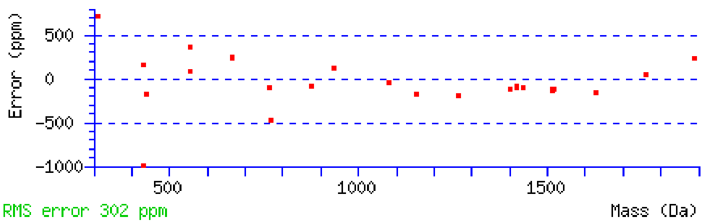


### All matches to this query

| Score | Mr(calc): | Delta | Sequence |
|---|---|---|---|
| 93.1 | 2198.0201 | 1.0029 | EGNSALINCSAFGIPDLEYK |
| 66.8 | 2199.0041 | 0.0188 | EGNSALINCSAFGIPDLEYK |
| 57.1 | 2198.0201 | 1.0029 | EGNSALINCSAFGIPDLEYK |
| 40.3 | 2197.0361 | 1.9869 | EGNSALINCSAFGIPDLEYK |
| 5.4 | 2197.0142 | 2.0087 | LCTVNSVEEKILAAAKYK |
| 4.5 | 2199.0395 | -0.0165 | LSMKPLSDGPPDPKSHELR |
| 3.3 | 2197.0069 | 2.0161 | SRNDLPLLPAQTTATPDVK |
| 3.3 | 2197.0069 | 2.0161 | SRNDLPLLPAQTTATPDVK |
| 3.3 | 2197.0069 | 2.0161 | SRNDLPLLPAQTTATPDVK |
| 0.9 | 2198.0320 | 0.9910 | ATGIRKGISPMNRPPLSDK |

---

Spectrum No: 116; Query: 1378; Rank: 1

---

## Peptide View

MS/MS Fragmentation of **TSLQPMVSALNISLGGTGK**
Found in **IPI00198021**, Tax_Id=10116 Gene_Symbol=Umod Uromodulin precursor

Match to Query 1378: 1873.990808 from(938.002680,2+)
Title: 100101RatKid_NS_deglyco_20.4676.4676.2.dta
Data file K:\NewmanPaper\Piliang\3SubProteomes\Piliang3SP\mgf5ppm\ERLIC_3SubProteomes5ppm.mgf

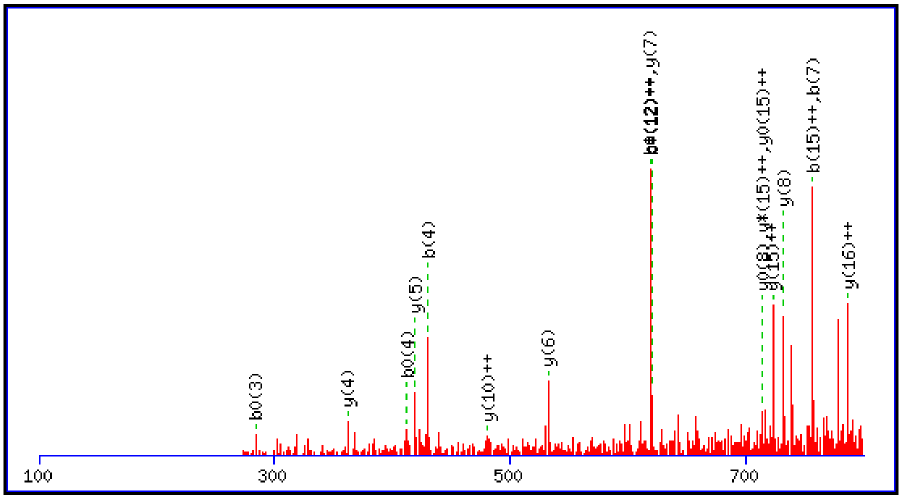

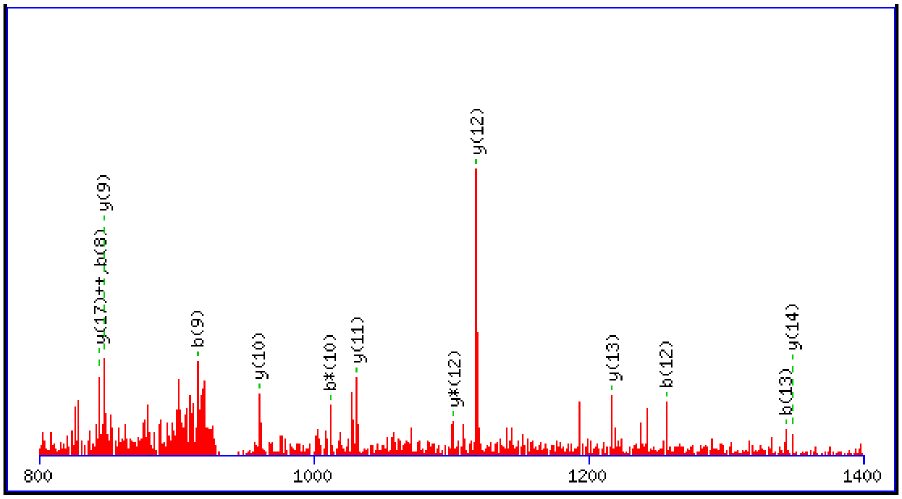


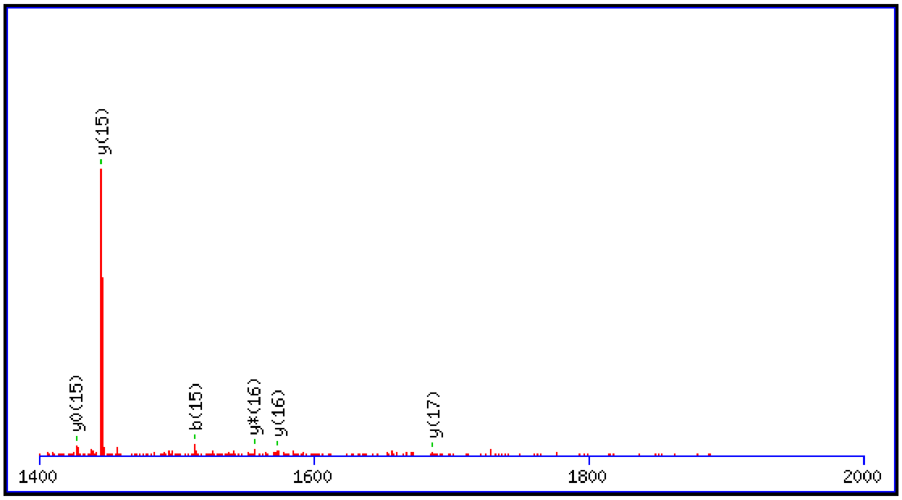


**Monoisotopic mass of neutral peptide Mr(calc):** 1873.9819
**Fixed modifications:** Carbamidomethyl (C)
**Variable modifications:**
**N11** : Deamidated_N (N)
**Ions Score:** 93 **Expect:** 6e-008
**Matches (Bold Red):** 37/206 fragment ions using 54 most intense peaks

| # | b | $b^{++}$ | b* | $b^{*++}$ | $b^{0}$ | $b^{0++}$ | Seq. | y | $y^{++}$ | y* | $y^{*++}$ | $y^{0}$ | $y^{0++}$ | # |
|---|---|---|---|---|---|---|---|---|---|---|---|---|---|---|
| 1 | 102.0550 | 51.5311 | | | 84.0444 | 42.5258 | T | | | | | | | 19 |
| 2 | 189.0870 | 95.0471 | | | 171.0764 | 86.0418 | S | 1773.9415 | 887.4744 | 1756.9150 | 878.9611 | 1755.9309 | 878.4691 | 18 |
| 3 | 302.1710 | 151.5892 | | | **284.1605** | 142.5839 | L | **1686.9095** | **843.9584** | 1669.8829 | 835.4451 | 1668.8989 | 834.9531 | 17 |
| 4 | **430.2296** | 215.6185 | 413.2031 | 207.1052 | **412.2191** | 206.6132 | Q | **1573.8254** | **787.4163** | **1556.7989** | 778.9031 | 1555.8148 | 778.4111 | 16 |
| 5 | 527.2824 | 264.1448 | 510.2558 | 255.6316 | 509.2718 | 255.1396 | P | **1445.7668** | **723.3871** | 1428.7403 | **714.8738** | **1427.7563** | **714.3818** | 15 |
| 6 | 658.3229 | 329.6651 | 641.2963 | 321.1518 | 640.3123 | 320.6598 | M | **1348.7141** | 674.8607 | 1331.6875 | 666.3474 | 1330.7035 | 665.8554 | 14 |
| 7 | **757.3913** | 379.1993 | 740.3647 | 370.6860 | 739.3807 | 370.1940 | V | **1217.6736** | 609.3404 | 1200.6470 | 600.8272 | 1199.6630 | 600.3351 | 13 |
| 8 | **844.4233** | 422.7153 | 827.3968 | 414.2020 | 826.4128 | 413.7100 | S | **1118.6052** | 559.8062 | **1101.5786** | 551.2929 | 1100.5946 | 550.8009 | 12 |
| 9 | **915.4604** | 458.2339 | 898.4339 | 449.7206 | 897.4499 | 449.2286 | A | **1031.5731** | 516.2902 | 1014.5466 | 507.7769 | 1013.5626 | 507.2849 | 11 |
| 10 | 1028.5445 | 514.7759 | **1011.5179** | 506.2626 | 1010.5339 | 505.7706 | L | **960.5360** | **480.7717** | 943.5095 | 472.2584 | 942.5255 | 471.7664 | 10 |
| 11 | 1143.5714 | 572.2894 | 1126.5449 | 563.7761 | 1125.5609 | 563.2841 | N | **847.4520** | 424.2296 | 830.4254 | 415.7163 | 829.4414 | 415.2243 | 9 |
| 12 | **1256.6555** | 628.8314 | 1239.6289 | **620.3181** | 1238.6449 | **619.8261** | I | **732.4250** | 366.7162 | 715.3985 | 358.2029 | **714.4145** | 357.7109 | 8 |
| 13 | **1343.6875** | 672.3474 | 1326.6610 | 663.8341 | 1325.6770 | 663.3421 | S | **619.3410** | 310.1741 | 602.3144 | 301.6608 | 601.3304 | 301.1688 | 7 |
| 14 | 1456.7716 | 728.8894 | 1439.7450 | 720.3762 | 1438.7610 | 719.8841 | L | **532.3089** | 266.6581 | 515.2824 | 258.1448 | 514.2984 | 257.6528 | 6 |
| 15 | **1513.7930** | **757.4002** | 1496.7665 | 748.8869 | 1495.7825 | 748.3949 | G | **419.2249** | 210.1161 | 402.1983 | 201.6028 | 401.2143 | 201.1108 | 5 |
| 16 | 1570.8145 | 785.9109 | 1553.7880 | 777.3976 | 1552.8039 | 776.9056 | G | **362.2034** | 181.6053 | 345.1769 | 173.0921 | 344.1928 | 172.6001 | 4 |
| 17 | 1671.8622 | 836.4347 | 1654.8356 | 827.9215 | 1653.8516 | 827.4295 | T | 305.1819 | 153.0946 | 288.1554 | 144.5813 | 287.1714 | 144.0893 | 3 |
| 18 | 1728.8837 | 864.9455 | 1711.8571 | 856.4322 | 1710.8731 | 855.9402 | G | 204.1343 | 102.5708 | 187.1077 | 94.0575 | | | 2 |

| | | | | | | | | | | | | | | |
|---|---|---|---|---|---|---|---|---|---|---|---|---|---|---|
| 19 | | | | | | | K | 147.1128 | 74.0600 | 130.0863 | 65.5468 | | | 1 |

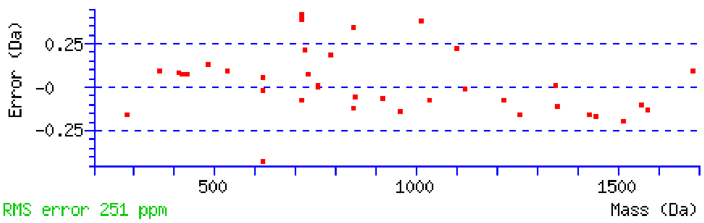


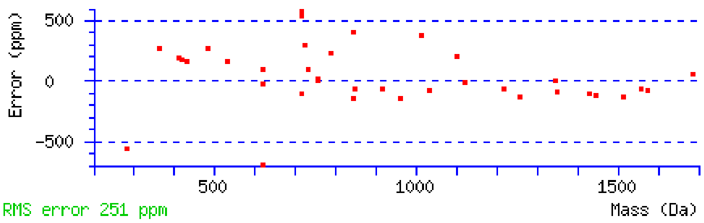


### All matches to this query

| Score | Mr(calc): | Delta | Sequence |
|---|---|---|---|
| 92.8 | 1873.9819 | 0.0089 | TSLQPMVSALNISLGGTGK |
| 21.0 | 1872.9979 | 0.9929 | TSLQPMVSALNISLGGTGK |
| 6.0 | 1872.9913 | 0.9995 | QNGKLCLVIQVANMRK |
| 1.1 | 1871.9788 | 2.0120 | NGKGAGTHCLKLNSFLR |

---

Spectrum No: 117; Query: 763; Rank: 1

---

## Peptide View

MS/MS Fragmentation of **NGSEADIDESLYSR**
Found in **IPI00368347**, Tax_Id=10116 Gene_Symbol=Ube1x Ubiquitin-activating enzyme E1, Chr X

Match to Query 763: 1555.665028 from(778.839790,2+)
Title: 091008RatKidney_NoSalt_23.1775.1775.2.dta
Data file K:\NewmanPaper\Piliang\3SubProteomes\Piliang3SP\mgf5ppm\ERLIC_3SubProteomes5ppm.mgf

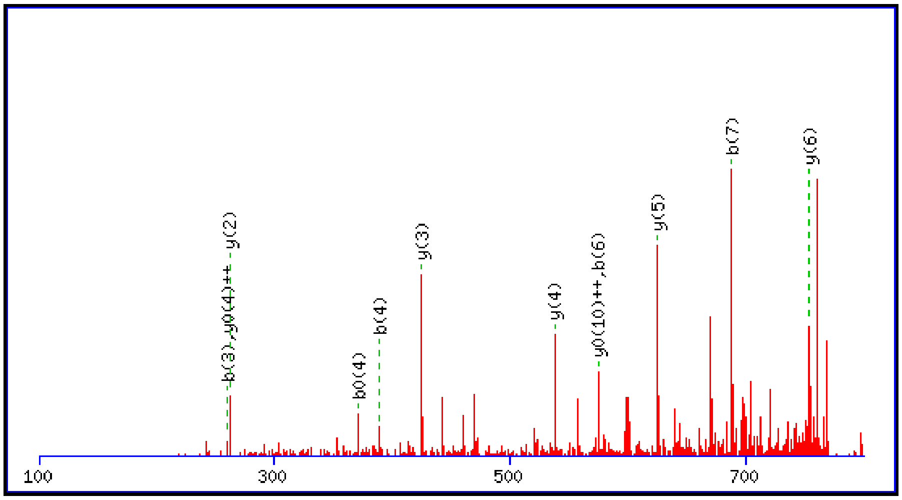

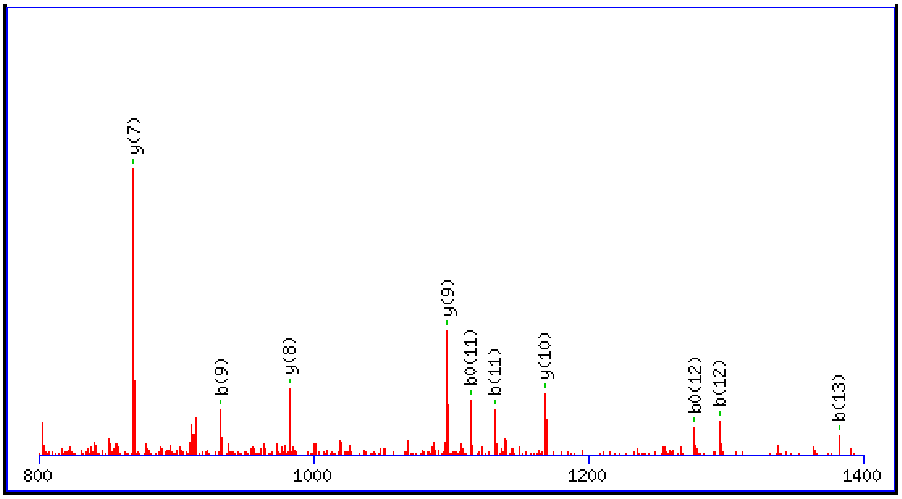


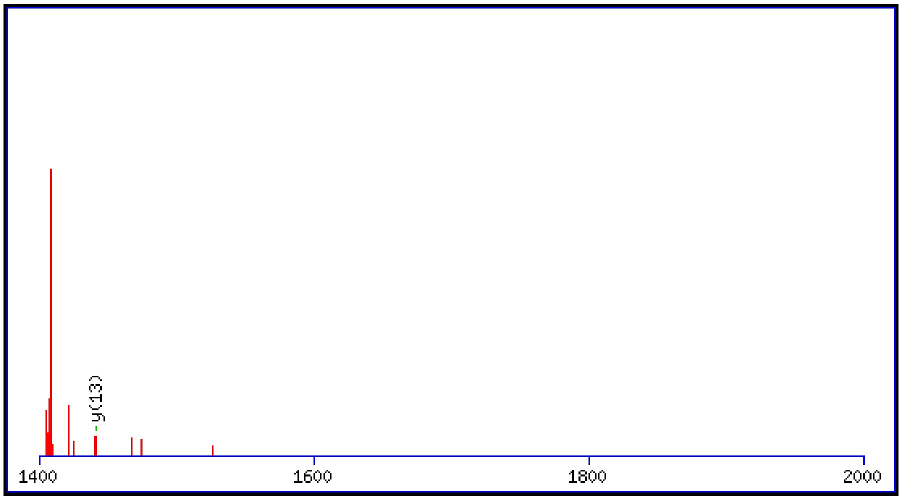


**Monoisotopic mass of neutral peptide Mr(calc):** 1555.6638
**Fixed modifications:** Carbamidomethyl (C)
**Variable modifications:**
**N1** : Deamidated_N (N)
**Ions Score:** 93 **Expect:** 7.6e-008
**Matches (Bold Red):** 23/150 fragment ions using 31 most intense peaks

| # | b | $b^{++}$ | b* | $b^{*++}$ | $b^0$ | $b^{0++}$ | Seq. | y | $y^{++}$ | y* | $y^{*++}$ | $y^0$ | $y^{0++}$ | # |
|---|---|---|---|---|---|---|---|---|---|---|---|---|---|---|
| 1 | 116.0342 | 58.5207 | 99.0077 | 50.0075 | | | N | | | | | | | 14 |
| 2 | 173.0557 | 87.0315 | 156.0291 | 78.5182 | | | G | **1441.6441** | 721.3257 | 1424.6176 | 712.8124 | 1423.6336 | 712.3204 | 13 |
| 3 | **260.0877** | 130.5475 | 243.0612 | 122.0342 | 242.0771 | 121.5422 | S | 1384.6227 | 692.8150 | 1367.5961 | 684.3017 | 1366.6121 | 683.8097 | 12 |
| 4 | **389.1303** | 195.0688 | 372.1037 | 186.5555 | **371.1197** | 186.0635 | E | 1297.5907 | 649.2990 | 1280.5641 | 640.7857 | 1279.5801 | 640.2937 | 11 |
| 5 | 460.1674 | 230.5873 | 443.1409 | 222.0741 | 442.1568 | 221.5821 | A | **1168.5481** | 584.7777 | 1151.5215 | 576.2644 | 1150.5375 | **575.7724** | 10 |
| 6 | **575.1944** | 288.1008 | 558.1678 | 279.5875 | 557.1838 | 279.0955 | D | **1097.5109** | 549.2591 | 1080.4844 | 540.7458 | 1079.5004 | 540.2538 | 9 |
| 7 | **688.2784** | 344.6428 | 671.2519 | 336.1296 | 670.2679 | 335.6376 | I | **982.4840** | 491.7456 | 965.4575 | 483.2324 | 964.4734 | 482.7404 | 8 |
| 8 | 803.3054 | 402.1563 | 786.2788 | 393.6430 | 785.2948 | 393.1510 | D | **869.3999** | 435.2036 | 852.3734 | 426.6903 | 851.3894 | 426.1983 | 7 |
| 9 | **932.3480** | 466.6776 | 915.3214 | 458.1643 | 914.3374 | 457.6723 | E | **754.3730** | 377.6901 | 737.3464 | 369.1769 | 736.3624 | 368.6849 | 6 |
| 10 | 1019.3800 | 510.1936 | 1002.3534 | 501.6804 | 1001.3694 | 501.1883 | S | **625.3304** | 313.1688 | 608.3039 | 304.6556 | 607.3198 | 304.1636 | 5 |
| 11 | **1132.4640** | 566.7357 | 1115.4375 | 558.2224 | **1114.4535** | 557.7304 | L | **538.2984** | 269.6528 | 521.2718 | 261.1396 | 520.2878 | **260.6475** | 4 |
| 12 | **1295.5274** | 648.2673 | 1278.5008 | 639.7541 | **1277.5168** | 639.2620 | Y | **425.2143** | 213.1108 | 408.1878 | 204.5975 | 407.2037 | 204.1055 | 3 |
| 13 | **1382.5594** | 691.7833 | 1365.5329 | 683.2701 | 1364.5488 | 682.7781 | S | **262.1510** | 131.5791 | 245.1244 | 123.0659 | 244.1404 | 122.5738 | 2 |
| 14 | | | | | | | R | 175.1190 | 88.0631 | 158.0924 | 79.5498 | | | 1 |

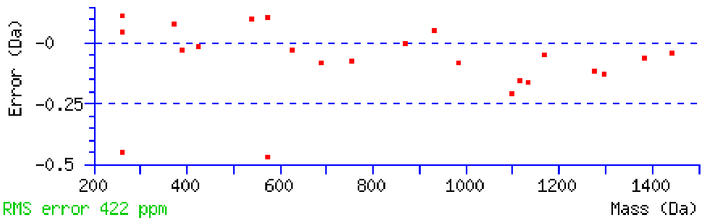


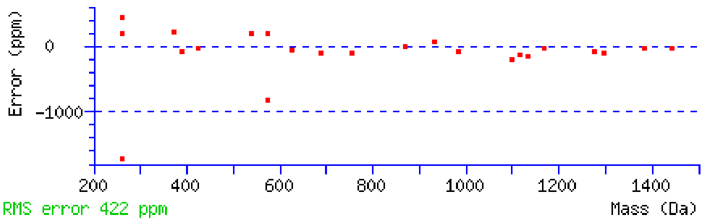


### All matches to this query

| Score | Mr(calc): | Delta | Sequence |
|---|---|---|---|
| 92.8 | 1555.6638 | 0.0013 | NGSEADIDESLYSR |
| 16.4 | 1553.6507 | 2.0144 | MKMFESLDSSATK |
| 16.3 | 1555.6784 | -0.0134 | QLETSETSSMGQSR |
| 14.6 | 1555.6780 | -0.0130 | YQPRDEEVRER |
| 13.6 | 1553.6507 | 2.0144 | MKMFESLDSSATK |
| 13.6 | 1553.6507 | 2.0144 | MKMFESLDSSATK |
| 7.9 | 1555.6799 | -0.0149 | TLNMDMFTWEPR |
| 6.3 | 1555.6603 | 0.0047 | SHRLGGTMPFDSR |
| 4.8 | 1555.6780 | -0.0130 | KESNEGRHYISR |
| 3.9 | 1554.6637 | 1.0013 | TAMGPSSTTNLIEGP |

---

Spectrum No: 118; Query: 2252; Rank: 1

---

## Peptide View

MS/MS Fragmentation of **YGMQNSGNEAAWNYTLEQYQK**
Found in **IPI00327398**, Tax_Id=10116 Gene_Symbol=Enpep Isoform 1 of Glutamyl aminopeptidase

Match to Query 2252: 2511.072288 from(1256.543420,2+)
Title: 100101RatKid_NS_deglyco_21.3338.3338.2.dta
Data file K:\NewmanPaper\Piliang\3SubProteomes\Piliang3SP\mgf5ppm\ERLIC_3SubProteomes5ppm.mgf

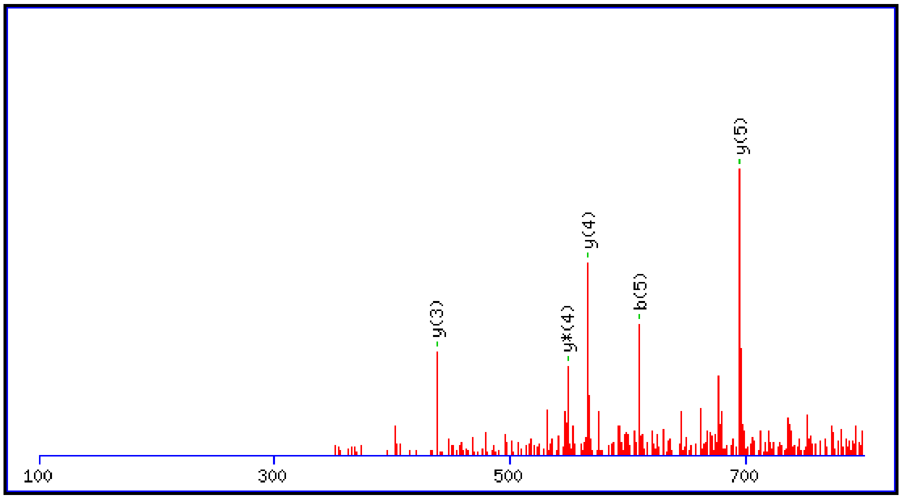

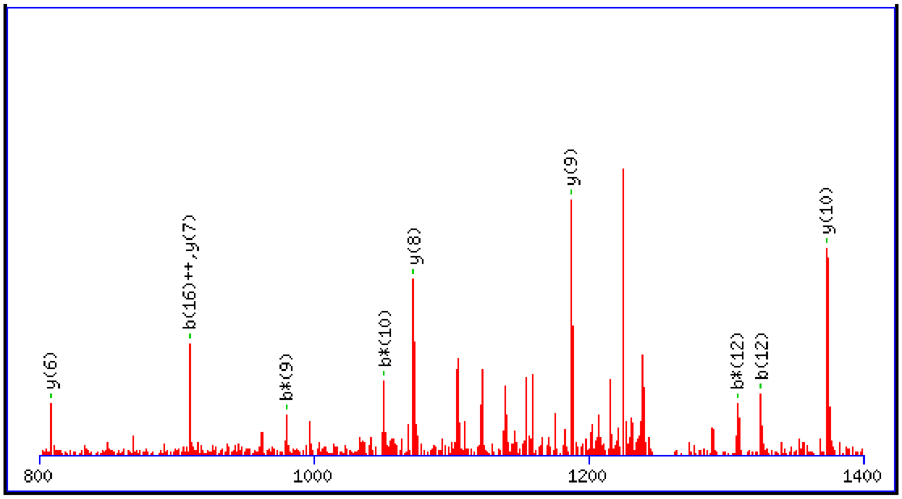


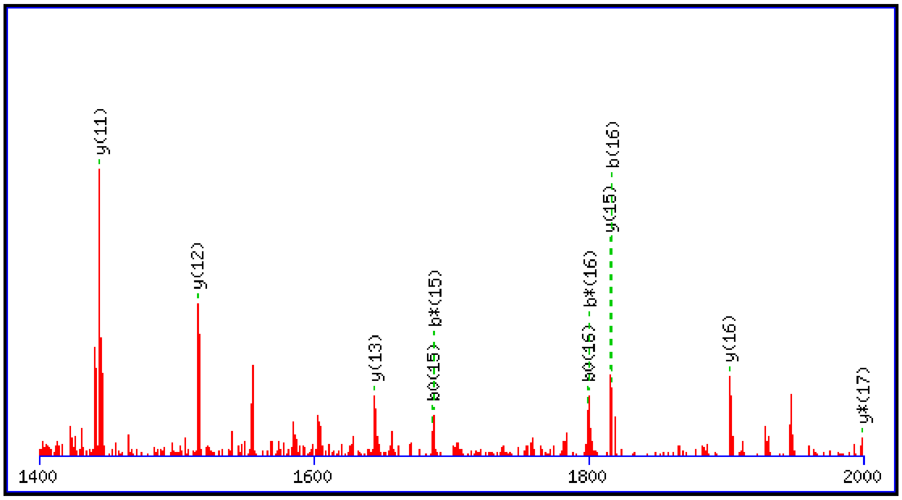


**Monoisotopic mass of neutral peptide Mr(calc):** 2511.0648
**Fixed modifications:** Carbamidomethyl (C)
**Variable modifications:**
**M3** : Oxidation (M)
**N13** : Deamidated_N (N)
**Ions Score:** 93 **Expect:** 1.5e-007
**Matches (Bold Red):** 26/216 fragment ions using 41 most intense peaks

| # | b | $b^{++}$ | b* | $b^{*++}$ | $b^{0}$ | $b^{0++}$ | Seq. | y | $y^{++}$ | y* | $y^{*++}$ | $y^{0}$ | $y^{0++}$ | # |
|---|---|---|---|---|---|---|---|---|---|---|---|---|---|---|
| 1 | 164.0706 | 82.5389 | | | | | Y | | | | | | | 21 |
| 2 | 221.0921 | 111.0497 | | | | | G | 2349.0088 | 1175.0080 | 2331.9823 | 1166.4948 | 2330.9983 | 1166.0028 | 20 |
| 3 | 368.1275 | 184.5674 | | | | | M | 2291.9874 | 1146.4973 | 2274.9608 | 1137.9840 | 2273.9768 | 1137.4920 | 19 |
| 4 | 496.1861 | 248.5967 | 479.1595 | 240.0834 | | | Q | 2144.9519 | 1072.9796 | 2127.9254 | 1064.4663 | 2126.9414 | 1063.9743 | 18 |
| 5 | **610.2290** | 305.6181 | 593.2024 | 297.1049 | | | N | 2016.8934 | 1008.9503 | **1999.8668** | 1000.4370 | 1998.8828 | 999.9450 | 17 |
| 6 | 697.2610 | 349.1341 | 680.2345 | 340.6209 | 679.2504 | 340.1289 | S | **1902.8504** | 951.9289 | 1885.8239 | 943.4156 | 1884.8399 | 942.9236 | 16 |
| 7 | 754.2825 | 377.6449 | 737.2559 | 369.1316 | 736.2719 | 368.6396 | G | **1815.8184** | 908.4128 | 1798.7919 | 899.8996 | 1797.8079 | 899.4076 | 15 |
| 8 | 868.3254 | 434.6663 | 851.2988 | 426.1531 | 850.3148 | 425.6611 | N | 1758.7970 | 879.9021 | 1741.7704 | 871.3888 | 1740.7864 | 870.8968 | 14 |
| 9 | 997.3680 | 499.1876 | **980.3414** | 490.6744 | 979.3574 | 490.1824 | E | **1644.7540** | 822.8807 | 1627.7275 | 814.3674 | 1626.7435 | 813.8754 | 13 |
| 10 | 1068.4051 | 534.7062 | **1051.3786** | 526.1929 | 1050.3945 | 525.7009 | A | **1515.7114** | 758.3594 | 1498.6849 | 749.8461 | 1497.7009 | 749.3541 | 12 |
| 11 | 1139.4422 | 570.2247 | 1122.4157 | 561.7115 | 1121.4317 | 561.2195 | A | **1444.6743** | 722.8408 | 1427.6478 | 714.3275 | 1426.6638 | 713.8355 | 11 |
| 12 | **1325.5215** | 663.2644 | **1308.4950** | 654.7511 | 1307.5110 | 654.2591 | W | **1373.6372** | 687.3222 | 1356.6107 | 678.8090 | 1355.6266 | 678.3170 | 10 |
| 13 | 1440.5485 | 720.7779 | 1423.5219 | 712.2646 | 1422.5379 | 711.7726 | N | **1187.5579** | 594.2826 | 1170.5313 | 585.7693 | 1169.5473 | 585.2773 | 9 |
| 14 | 1603.6118 | 802.3095 | 1586.5852 | 793.7963 | 1585.6012 | 793.3043 | Y | **1072.5310** | 536.7691 | 1055.5044 | 528.2558 | 1054.5204 | 527.7638 | 8 |
| 15 | 1704.6595 | 852.8334 | **1687.6329** | 844.3201 | **1686.6489** | 843.8281 | T | **909.4676** | 455.2375 | 892.4411 | 446.7242 | 891.4571 | 446.2322 | 7 |
| 16 | **1817.7435** | **909.3754** | **1800.7170** | 900.8621 | **1799.7330** | 900.3701 | L | **808.4199** | 404.7136 | 791.3934 | 396.2003 | 790.4094 | 395.7083 | 6 |
| 17 | 1946.7861 | 973.8967 | 1929.7596 | 965.3834 | 1928.7756 | 964.8914 | E | **695.3359** | 348.1716 | 678.3093 | 339.6583 | 677.3253 | 339.1663 | 5 |
| 18 | 2074.8447 | 1037.9260 | 2057.8182 | 1029.4127 | 2056.8341 | 1028.9207 | Q | **566.2933** | 283.6503 | **549.2667** | 275.1370 | | | 4 |

| | | | | | | | | | | | | | | |
|---|---|---|---|---|---|---|---|---|---|---|---|---|---|---|
| 19 | 2237.9080 | 1119.4577 | 2220.8815 | 1110.9444 | 2219.8975 | 1110.4524 | Y | 438.2347 | 219.6210 | 421.2082 | 211.1077 | | | 3 |
| 20 | 2365.9666 | 1183.4869 | 2348.9401 | 1174.9737 | 2347.9561 | 1174.4817 | Q | 275.1714 | 138.0893 | 258.1448 | 129.5761 | | | 2 |
| 21 | | | | | | | K | 147.1128 | 74.0600 | 130.0863 | 65.5468 | | | 1 |

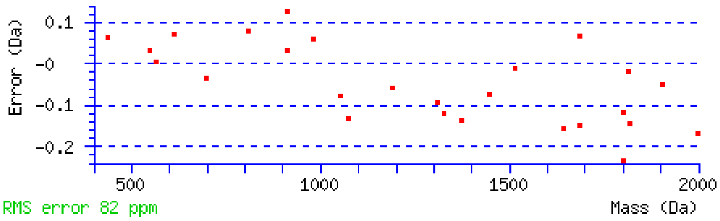


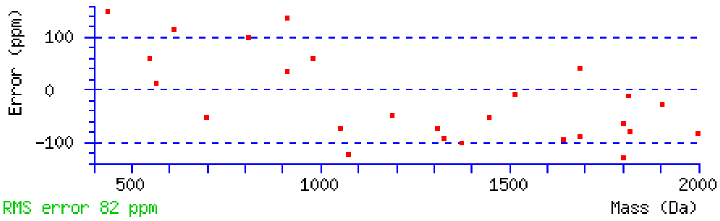


### All matches to this query

| Score | Mr(calc): | Delta | Sequence |
|---|---|---|---|
| 92.8 | 2511.0648 | 0.0075 | YGMQNSGNEAAWNYTLEQYQK |
| 41.6 | 2511.0648 | 0.0075 | YGMQNSGNEAAWNYTLEQYQK |
| 19.8 | 2511.0648 | 0.0075 | YGMQNSGNEAAWNYTLEQYQK |
| 17.9 | 2510.0808 | 0.9915 | YGMQNSGNEAAWNYTLEQYQK |
| 1.9 | 2511.0913 | -0.0190 | KGYFGGGTAAVYLAGEDFYIPR |
| 1.0 | 2511.0809 | -0.0086 | ELQGTGSQLGGTTGEKLPAGRSK |
| 1.0 | 2511.0809 | -0.0086 | ELQGTGSQLGGTTGEKLPAGRSK |
| 0.8 | 2511.0551 | 0.0172 | EGESYNESTVNGTPVNHQALER |

---

Spectrum No: 119; Query: 1997; Rank: 1

---

## Peptide View

MS/MS Fragmentation of **IVNDNPYGNGTAIFTTNGAIAR**
Found in **IPI00205018**, Tax_Id=10116 Gene_Symbol=Aldh6a1 Methylmalonate-semialdehyde dehydrogenase [acylating], mitochondrial precursor

Match to Query 1997: 2279.128108 from(1140.571330,2+)
Title: 100101RatKid_NS_deglyco_09.3724.3724.2.dta
Data file K:\NewmanPaper\Piliang\3SubProteomes\Piliang3SP\mgf5ppm\ERLIC_3SubProteomes5ppm.mgf

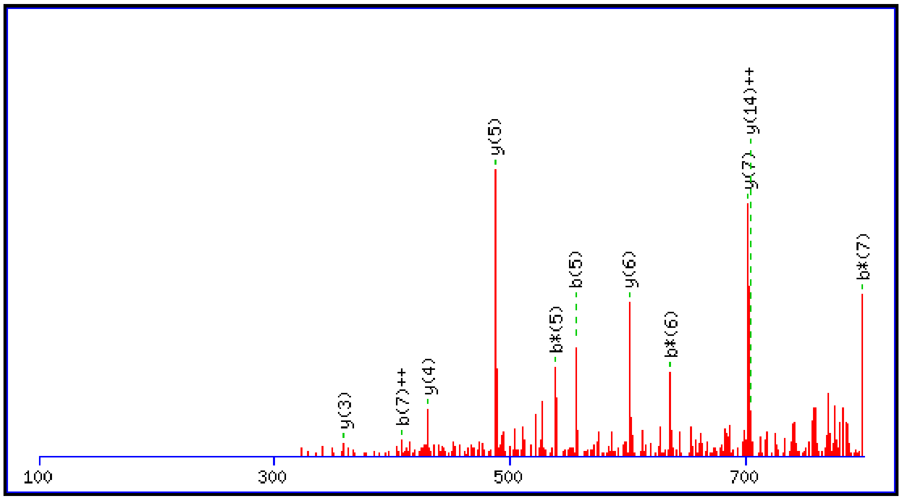

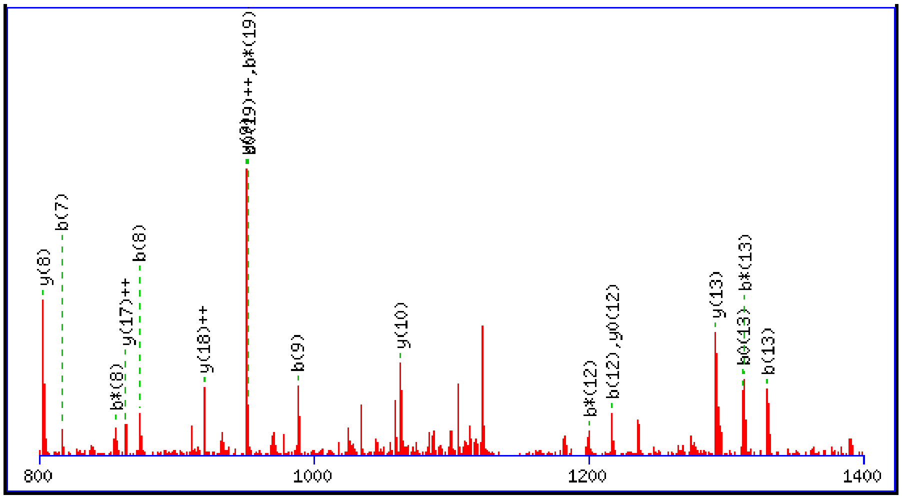


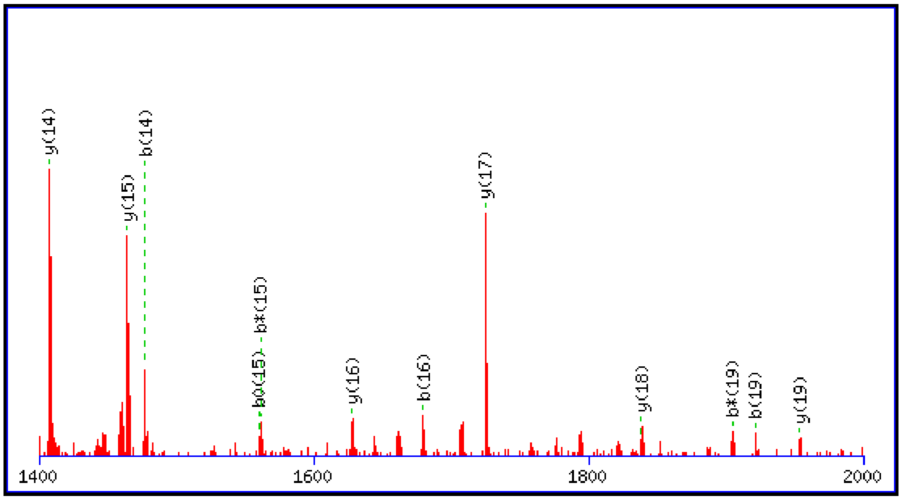


**Monoisotopic mass of neutral peptide Mr(calc):** 2279.1182
**Fixed modifications:** Carbamidomethyl (C)
**Variable modifications:**
**N9** : Deamidated_N (N)
**Ions Score:** 93 **Expect:** 1.2e-007
**Matches (Bold Red):** 41/230 fragment ions using 78 most intense peaks

| # | b | $b^{++}$ | b* | $b^{*++}$ | $b^0$ | $b^{0++}$ | Seq. | y | $y^{++}$ | y* | $y^{*++}$ | $y^0$ | $y^{0++}$ | # |
|---|---|---|---|---|---|---|---|---|---|---|---|---|---|---|
| 1 | 114.0913 | 57.5493 | | | | | I | | | | | | | 22 |
| 2 | 213.1598 | 107.0835 | | | | | V | 2167.0414 | 1084.0244 | 2150.0149 | 1075.5111 | 2149.0309 | 1075.0191 | 21 |
| 3 | 327.2027 | 164.1050 | 310.1761 | 155.5917 | | | N | 2067.9730 | 1034.4901 | 2050.9465 | 1025.9769 | 2049.9625 | 1025.4849 | 20 |
| 4 | 442.2296 | 221.6185 | 425.2031 | 213.1052 | 424.2191 | 212.6132 | D | **1953.9301** | 977.4687 | 1936.9035 | 968.9554 | 1935.9195 | 968.4634 | 19 |
| 5 | **556.2726** | 278.6399 | **539.2460** | 270.1266 | 538.2620 | 269.6346 | N | **1838.9031** | **919.9552** | 1821.8766 | 911.4419 | 1820.8926 | 910.9499 | 18 |
| 6 | 653.3253 | 327.1663 | **636.2988** | 318.6530 | 635.3148 | 318.1610 | P | **1724.8602** | **862.9337** | 1707.8337 | 854.4205 | 1706.8497 | 853.9285 | 17 |
| 7 | **816.3886** | **408.6980** | **799.3621** | 400.1847 | 798.3781 | 399.6927 | Y | **1627.8075** | 814.4074 | 1610.7809 | 805.8941 | 1609.7969 | 805.4021 | 16 |
| 8 | **873.4101** | 437.2087 | **856.3836** | 428.6954 | 855.3995 | 428.2034 | G | **1464.7441** | 732.8757 | 1447.7176 | 724.3624 | 1446.7336 | 723.8704 | 15 |
| 9 | **988.4370** | 494.7222 | 971.4105 | 486.2089 | 970.4265 | 485.7169 | N | **1407.7227** | **704.3650** | 1390.6961 | 695.8517 | 1389.7121 | 695.3597 | 14 |
| 10 | 1045.4585 | 523.2329 | 1028.4320 | 514.7196 | 1027.4479 | 514.2276 | G | **1292.6957** | 646.8515 | 1275.6692 | 638.3382 | 1274.6852 | 637.8462 | 13 |
| 11 | 1146.5062 | 573.7567 | 1129.4796 | 565.2435 | 1128.4956 | 564.7514 | T | 1235.6743 | 618.3408 | 1218.6477 | 609.8275 | **1217.6637** | 609.3355 | 12 |
| 12 | **1217.5433** | 609.2753 | **1200.5168** | 600.7620 | 1199.5327 | 600.2700 | A | 1134.6266 | 567.8169 | 1117.6000 | 559.3037 | 1116.6160 | 558.8116 | 11 |
| 13 | **1330.6274** | 665.8173 | **1313.6008** | 657.3040 | **1312.6168** | 656.8120 | I | **1063.5895** | 532.2984 | 1046.5629 | 523.7851 | 1045.5789 | 523.2931 | 10 |
| 14 | **1477.6958** | 739.3515 | 1460.6692 | 730.8383 | 1459.6852 | 730.3462 | F | **950.5054** | 475.7563 | 933.4789 | 467.2431 | 932.4948 | 466.7511 | 9 |
| 15 | 1578.7435 | 789.8754 | **1561.7169** | 781.3621 | **1560.7329** | 780.8701 | T | **803.4370** | 402.2221 | 786.4104 | 393.7089 | 785.4264 | 393.2169 | 8 |
| 16 | **1679.7911** | 840.3992 | 1662.7646 | 831.8859 | 1661.7806 | 831.3939 | T | **702.3893** | 351.6983 | 685.3628 | 343.1850 | 684.3787 | 342.6930 | 7 |
| 17 | 1793.8341 | 897.4207 | 1776.8075 | 888.9074 | 1775.8235 | 888.4154 | N | **601.3416** | 301.1745 | 584.3151 | 292.6612 | | | 6 |
| 18 | 1850.8555 | 925.9314 | 1833.8290 | 917.4181 | 1832.8450 | 916.9261 | G | **487.2987** | 244.1530 | 470.2722 | 235.6397 | | | 5 |
| | | | | | | | | | | | | | | |

| | | | | | | | | | | | | | | |
|---|---|---|---|---|---|---|---|---|---|---|---|---|---|---|
| **19** | **1921.8926** | 961.4500 | **1904.8661** | **952.9367** | 1903.8821 | **952.4447** | **A** | **430.2772** | 215.6423 | 413.2507 | 207.1290 | | | **4** |
| **20** | 2034.9767 | 1017.9920 | 2017.9502 | 1009.4787 | 2016.9661 | 1008.9867 | **I** | **359.2401** | 180.1237 | 342.2136 | 171.6104 | | | **3** |
| **21** | 2106.0138 | 1053.5105 | 2088.9873 | 1044.9973 | 2088.0033 | 1044.5053 | **A** | 246.1561 | 123.5817 | 229.1295 | 115.0684 | | | **2** |
| **22** | | | | | | | **R** | 175.1190 | 88.0631 | 158.0924 | 79.5498 | | | **1** |

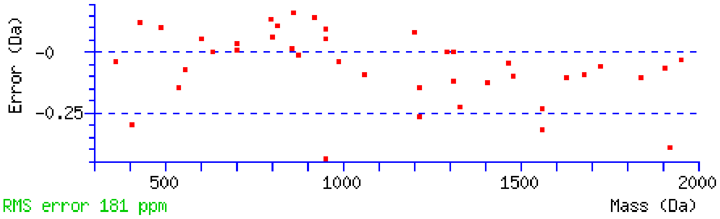


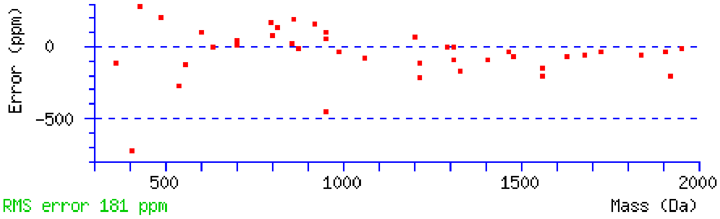


### All matches to this query

| Score | Mr(calc): | Delta | Sequence |
|---|---|---|---|
| 92.7 | 2279.1182 | 0.0099 | IVNDNPYGNGTAIFTTNGAIAR |
| 78.6 | 2279.1182 | 0.0099 | IVNDNPYGNGTAIFTTNGAIAR |
| 42.9 | 2279.1182 | 0.0099 | IVNDNPYGNGTAIFTTNGAIAR |
| 32.0 | 2279.1182 | 0.0099 | IVNDNPYGNGTAIFTTNGAIAR |
| 19.8 | 2278.1342 | 0.9939 | IVNDNPYGNGTAIFTTNGAIAR |
| 2.5 | 2278.1090 | 1.0191 | SGANDQETLADNIRAFSRWK |
| 1.2 | 2278.1407 | 0.9874 | ARSLVMFFLPKLDYMVPR |

---

Spectrum No: 120; Query: 914; Rank: 1

---

## Peptide View

MS/MS Fragmentation of **DYGGNCLIYDADNK**
Found in **IPI00371386**, Tax_Id=10116 Gene_Symbol=Ptdss2_predicted phosphatidylserine synthase 2

Match to Query 914: 1617.665548 from(809.840050,2+)
Title: 100101RatKid_NS_deglyco_21.2307.2307.2.dta
Data file K:\NewmanPaper\Piliang\3SubProteomes\Piliang3SP\mgf5ppm\ERLIC_3SubProteomes5ppm.mgf

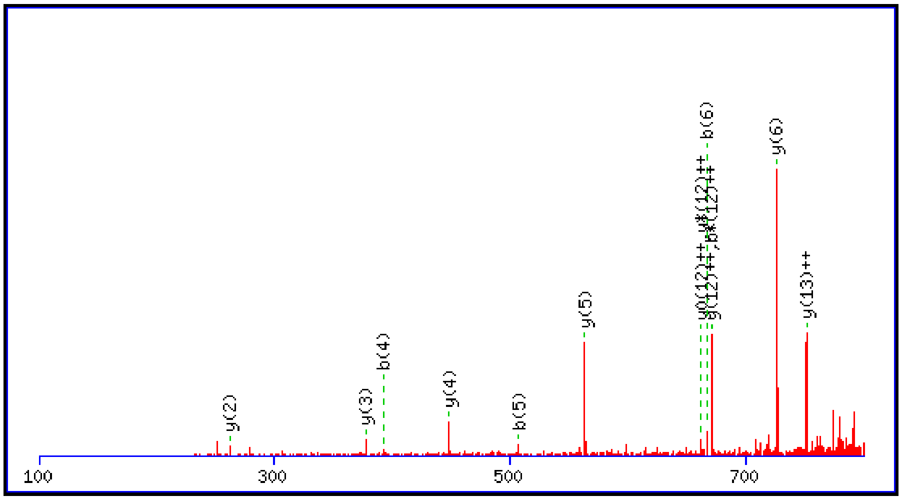

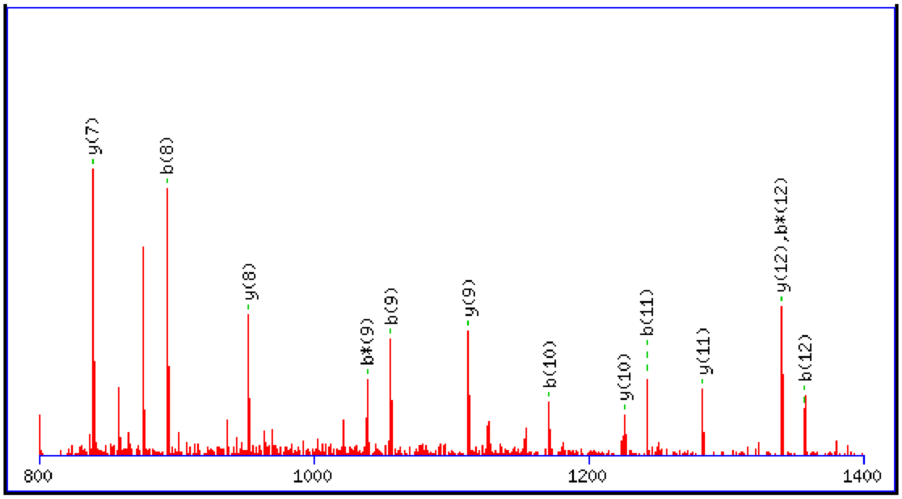


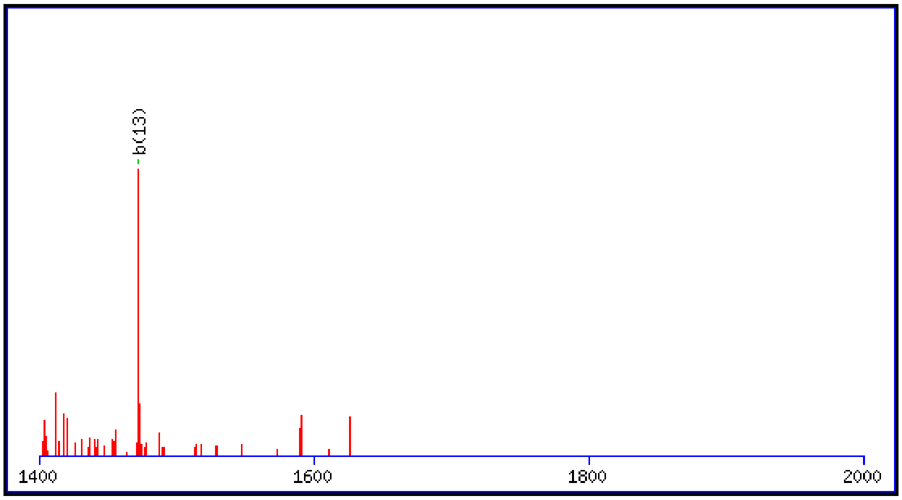


**Monoisotopic mass of neutral peptide Mr(calc):** 1617.6617
**Fixed modifications:** Carbamidomethyl (C)
**Variable modifications:**
**N13** : Deamidated_N (N)
**Ions Score:** 93 **Expect:** 7e-008
**Matches (Bold Red):** 27/144 fragment ions using 34 most intense peaks

| # | b | b$^{++}$ | b* | b*$^{++}$ | b$^{0}$ | b$^{0++}$ | Seq. | y | y$^{++}$ | y* | y*$^{++}$ | y$^{0}$ | y$^{0++}$ | # |
|---|---|---|---|---|---|---|---|---|---|---|---|---|---|---|
| 1 | 116.0342 | 58.5207 | | | 98.0237 | 49.5155 | D | | | | | | | 14 |
| 2 | 279.0975 | 140.0524 | | | 261.0870 | 131.0471 | Y | 1503.6420 | **752.3247** | 1486.6155 | 743.8114 | 1485.6315 | 743.3194 | 13 |
| 3 | 336.1190 | 168.5631 | | | 318.1084 | 159.5579 | G | **1340.5787** | **670.7930** | 1323.5522 | **662.2797** | 1322.5681 | **661.7877** | 12 |
| 4 | **393.1405** | 197.0739 | | | 375.1299 | 188.0686 | G | **1283.5572** | 642.2823 | 1266.5307 | 633.7690 | 1265.5467 | 633.2770 | 11 |
| 5 | **507.1834** | 254.0953 | 490.1569 | 245.5821 | 489.1728 | 245.0901 | N | **1226.5358** | 613.7715 | 1209.5092 | 605.2583 | 1208.5252 | 604.7662 | 10 |
| 6 | **667.2141** | 334.1107 | 650.1875 | 325.5974 | 649.2035 | 325.1054 | C | **1112.4928** | 556.7501 | 1095.4663 | 548.2368 | 1094.4823 | 547.7448 | 9 |
| 7 | 780.2981 | 390.6527 | 763.2716 | 382.1394 | 762.2876 | 381.6474 | L | **952.4622** | 476.7347 | 935.4356 | 468.2215 | 934.4516 | 467.7295 | 8 |
| 8 | **893.3822** | 447.1947 | 876.3556 | 438.6815 | 875.3716 | 438.1894 | I | **839.3781** | 420.1927 | 822.3516 | 411.6794 | 821.3676 | 411.1874 | 7 |
| 9 | **1056.4455** | 528.7264 | **1039.4190** | 520.2131 | 1038.4349 | 519.7211 | Y | **726.2941** | 363.6507 | 709.2675 | 355.1374 | 708.2835 | 354.6454 | 6 |
| 10 | **1171.4725** | 586.2399 | 1154.4459 | 577.7266 | 1153.4619 | 577.2346 | D | **563.2307** | 282.1190 | 546.2042 | 273.6057 | 545.2202 | 273.1137 | 5 |
| 11 | **1242.5096** | 621.7584 | 1225.4830 | 613.2451 | 1224.4990 | 612.7531 | A | **448.2038** | 224.6055 | 431.1772 | 216.0923 | 430.1932 | 215.6003 | 4 |
| 12 | **1357.5365** | 679.2719 | **1340.5100** | **670.7586** | 1339.5259 | 670.2666 | D | **377.1667** | 189.0870 | 360.1401 | 180.5737 | 359.1561 | 180.0817 | 3 |
| 13 | **1472.5634** | 736.7854 | 1455.5369 | 728.2721 | 1454.5529 | 727.7801 | N | **262.1397** | 131.5735 | 245.1132 | 123.0602 | | | 2 |
| 14 | | | | | | | K | 147.1128 | 74.0600 | 130.0863 | 65.5468 | | | 1 |

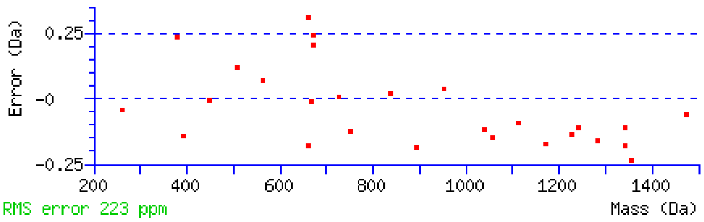


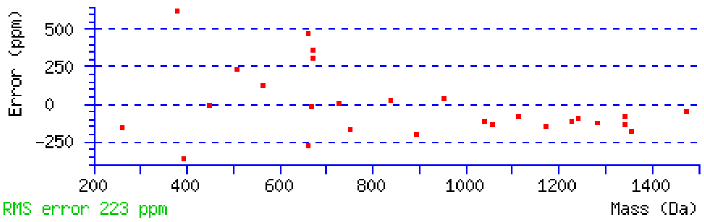


### All matches to this query

| Score | Mr(calc): | Delta | Sequence |
|---|---|---|---|
| 92.7 | 1617.6617 | 0.0039 | DYGGNCLIYDADNK |
| 40.3 | 1616.6777 | 0.9879 | DYGGNCLIYDADNK |
| 26.1 | 1617.6617 | 0.0039 | DYGGNCLIYDADNK |
| 12.8 | 1617.6623 | 0.0032 | ASPNGCLQLNGTVK |
| 9.9 | 1615.6745 | 1.9910 | DCMEEISLFTENK |
| 4.1 | 1617.6682 | -0.0026 | NYENLDQLSYDNK |
| 3.6 | 1617.6519 | 0.0137 | VSNSEEGSRSKNDK |
| 3.6 | 1617.6519 | 0.0137 | VSNSEEGSRSKNDK |
| 2.5 | 1617.6746 | -0.0090 | KLGEMWSSTAADDK |
| 2.5 | 1617.6746 | -0.0090 | KLGEMWSSTAADDK |

---

Spectrum No: 121; Query: 2766; Rank: 1

---

## Peptide View

MS/MS Fragmentation of **CISPLYVCDGDKDCADGSDEAGCVLNCTSAQFK**
Found in **IPI00205325**, Tax_Id=10116 Gene_Symbol=Lrp2 Low-density lipoprotein receptor-related protein 2 precursor

Match to Query 2766: 3712.499112 from(1238.506980,3+)
Title: 091008RatKidney_NoSalt_26.3560.3560.3.dta
Data file K:\NewmanPaper\Piliang\3SubProteomes\Piliang3SP\mgf5ppm\ERLIC_3SubProteomes5ppm.mgf

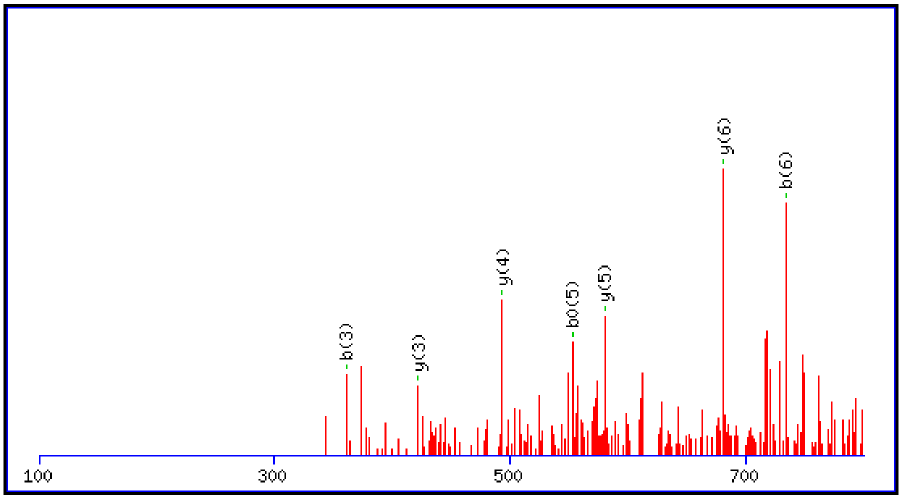

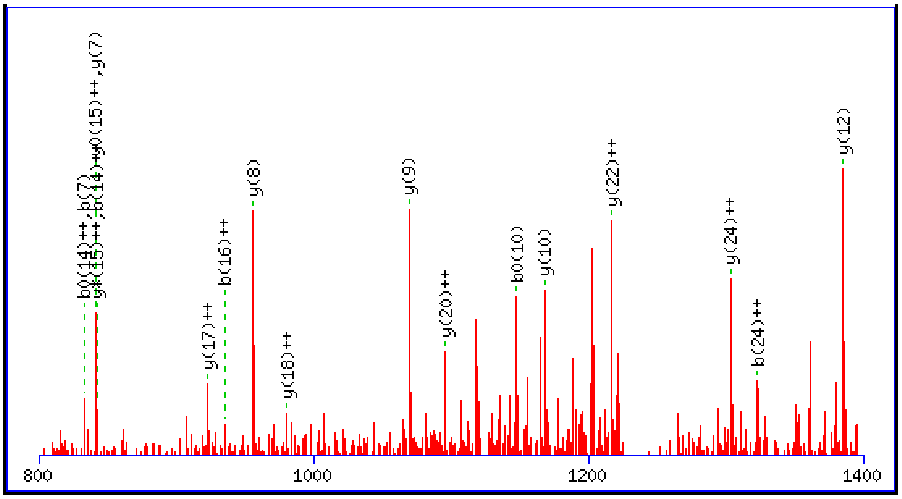


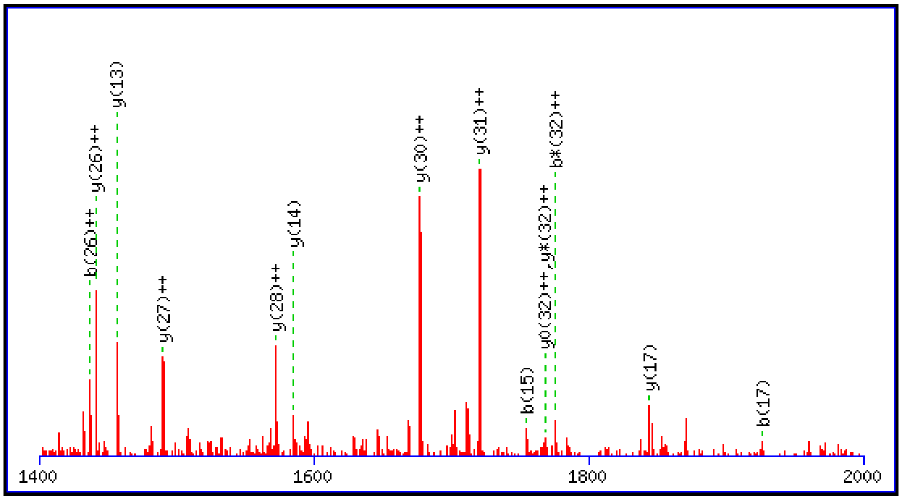


**Monoisotopic mass of neutral peptide Mr(calc):** 3712.5096
**Fixed modifications:** Carbamidomethyl (C)
**Variable modifications:**
**N26** : Deamidated_N (N)
**Ions Score:** 93 **Expect:** 2.2e-007
**Matches (Bold Red):** 39/350 fragment ions using 55 most intense peaks

| # | b | $b^{++}$ | b* | $b^{*++}$ | $b^{0}$ | $b^{0++}$ | Seq. | y | $y^{++}$ | y* | $y^{*++}$ | $y^{0}$ | $y^{0++}$ | # |
|---|---|---|---|---|---|---|---|---|---|---|---|---|---|---|
| 1 | 161.0379 | 81.0226 | | | | | C | | | | | | | 33 |
| 2 | 274.1220 | 137.5646 | | | | | I | 3553.4862 | 1777.2467 | 3536.4597 | **1768.7335** | 3535.4756 | **1768.2415** | 32 |
| 3 | **361.1540** | 181.0806 | | | 343.1435 | 172.0754 | S | 3440.4021 | **1720.7047** | 3423.3756 | 1712.1914 | 3422.3916 | 1711.6994 | 31 |
| 4 | 458.2068 | 229.6070 | | | 440.1962 | 220.6017 | P | 3353.3701 | **1677.1887** | 3336.3436 | 1668.6754 | 3335.3596 | 1668.1834 | 30 |
| 5 | 571.2908 | 286.1491 | | | **553.2803** | 277.1438 | L | 3256.3174 | 1628.6623 | 3239.2908 | 1620.1490 | 3238.3068 | 1619.6570 | 29 |
| 6 | **734.3542** | 367.6807 | | | 716.3436 | 358.6754 | Y | 3143.2333 | **1572.1203** | 3126.2067 | 1563.6070 | 3125.2227 | 1563.1150 | 28 |
| 7 | **833.4226** | 417.2149 | | | 815.4120 | 408.2096 | V | 2980.1700 | **1490.5886** | 2963.1434 | 1482.0753 | 2962.1594 | 1481.5833 | 27 |
| 8 | 993.4532 | 497.2303 | | | 975.4427 | 488.2250 | C | 2881.1015 | **1441.0544** | 2864.0750 | 1432.5411 | 2863.0910 | 1432.0491 | 26 |
| 9 | 1108.4802 | 554.7437 | | | 1090.4696 | 545.7384 | D | 2721.0709 | 1361.0391 | 2704.0443 | 1352.5258 | 2703.0603 | 1352.0338 | 25 |
| 10 | 1165.5016 | 583.2545 | | | **1147.4911** | 574.2492 | G | 2606.0440 | **1303.5256** | 2589.0174 | 1295.0123 | 2588.0334 | 1294.5203 | 24 |
| 11 | 1280.5286 | 640.7679 | | | 1262.5180 | 631.7626 | D | 2549.0225 | 1275.0149 | 2531.9959 | 1266.5016 | 2531.0119 | 1266.0096 | 23 |
| 12 | 1408.6236 | 704.8154 | 1391.5970 | 696.3021 | 1390.6130 | 695.8101 | K | 2433.9955 | **1217.5014** | 2416.9690 | 1208.9881 | 2415.9850 | 1208.4961 | 22 |
| 13 | 1523.6505 | 762.3289 | 1506.6239 | 753.8156 | 1505.6399 | 753.3236 | D | 2305.9006 | 1153.4539 | 2288.8740 | 1144.9407 | 2287.8900 | 1144.4486 | 21 |
| 14 | 1683.6811 | **842.3442** | 1666.6546 | 833.8309 | 1665.6706 | **833.3389** | C | 2190.8736 | **1095.9405** | 2173.8471 | 1087.4272 | 2172.8631 | 1086.9352 | 20 |
| 15 | **1754.7183** | 877.8628 | 1737.6917 | 869.3495 | 1736.7077 | 868.8575 | A | 2030.8430 | 1015.9251 | 2013.8164 | 1007.4119 | 2012.8324 | 1006.9199 | 19 |
| 16 | 1869.7452 | **935.3762** | 1852.7187 | 926.8630 | 1851.7346 | 926.3710 | D | 1959.8059 | **980.4066** | 1942.7793 | 971.8933 | 1941.7953 | 971.4013 | 18 |
| 17 | **1926.7667** | 963.8870 | 1909.7401 | 955.3737 | 1908.7561 | 954.8817 | G | **1844.7789** | **922.8931** | 1827.7524 | 914.3798 | 1826.7684 | 913.8878 | 17 |
| 18 | 2013.7987 | 1007.4030 | 1996.7721 | 998.8897 | 1995.7881 | 998.3977 | S | 1787.7575 | 894.3824 | 1770.7309 | 885.8691 | 1769.7469 | 885.3771 | 16 |
| | | | | | | | | | | | | | | |

| | | | | | | | | | | | | | | |
|---|---|---|---|---|---|---|---|---|---|---|---|---|---|---|
| **19** | 2128.8256 | 1064.9165 | 2111.7991 | 1056.4032 | 2110.8151 | 1055.9112 | **D** | 1700.7254 | 850.8664 | 1683.6989 | **842.3531** | 1682.7149 | **841.8611** | **15** |
| **20** | 2257.8682 | 1129.4378 | 2240.8417 | 1120.9245 | 2239.8577 | 1120.4325 | **E** | **1585.6985** | 793.3529 | 1568.6720 | 784.8396 | 1567.6879 | 784.3476 | **14** |
| **21** | 2328.9053 | 1164.9563 | 2311.8788 | 1156.4430 | 2310.8948 | 1155.9510 | **A** | **1456.6559** | 728.8316 | 1439.6294 | 720.3183 | 1438.6453 | 719.8263 | **13** |
| **22** | 2385.9268 | 1193.4670 | 2368.9003 | 1184.9538 | 2367.9162 | 1184.4618 | **G** | **1385.6188** | 693.3130 | 1368.5922 | 684.7998 | 1367.6082 | 684.3078 | **12** |
| **23** | 2545.9575 | 1273.4824 | 2528.9309 | 1264.9691 | 2527.9469 | 1264.4771 | **C** | 1328.5973 | 664.8023 | 1311.5708 | 656.2890 | 1310.5868 | 655.7970 | **11** |
| **24** | 2645.0259 | **1323.0166** | 2627.9993 | 1314.5033 | 2627.0153 | 1314.0113 | **V** | **1168.5667** | 584.7870 | 1151.5401 | 576.2737 | 1150.5561 | 575.7817 | **10** |
| **25** | 2758.1099 | 1379.5586 | 2741.0834 | 1371.0453 | 2740.0994 | 1370.5533 | **L** | **1069.4983** | 535.2528 | 1052.4717 | 526.7395 | 1051.4877 | 526.2475 | **9** |
| **26** | 2873.1369 | **1437.0721** | 2856.1103 | 1428.5588 | 2855.1263 | 1428.0668 | **N** | **956.4142** | 478.7107 | 939.3877 | 470.1975 | 938.4036 | 469.7055 | **8** |
| **27** | 3033.1675 | 1517.0874 | 3016.1410 | 1508.5741 | 3015.1570 | 1508.0821 | **C** | **841.3873** | 421.1973 | 824.3607 | 412.6840 | 823.3767 | 412.1920 | **7** |
| **28** | 3134.2152 | 1567.6112 | 3117.1886 | 1559.0980 | 3116.2046 | 1558.6060 | **T** | **681.3566** | 341.1819 | 664.3301 | 332.6687 | 663.3461 | 332.1767 | **6** |
| **29** | 3221.2472 | 1611.1272 | 3204.2207 | 1602.6140 | 3203.2367 | 1602.1220 | **S** | **580.3089** | 290.6581 | 563.2824 | 282.1448 | 562.2984 | 281.6528 | **5** |
| **30** | 3292.2843 | 1646.6458 | 3275.2578 | 1638.1325 | 3274.2738 | 1637.6405 | **A** | **493.2769** | 247.1421 | 476.2504 | 238.6288 | | | **4** |
| **31** | 3420.3429 | 1710.6751 | 3403.3164 | 1702.1618 | 3402.3324 | 1701.6698 | **Q** | **422.2398** | 211.6235 | 405.2132 | 203.1103 | | | **3** |
| **32** | 3567.4113 | 1784.2093 | 3550.3848 | **1775.6960** | 3549.4008 | 1775.2040 | **F** | 294.1812 | 147.5942 | 277.1547 | 139.0810 | | | **2** |
| **33** | | | | | | | **K** | 147.1128 | 74.0600 | 130.0863 | 65.5468 | | | **1** |

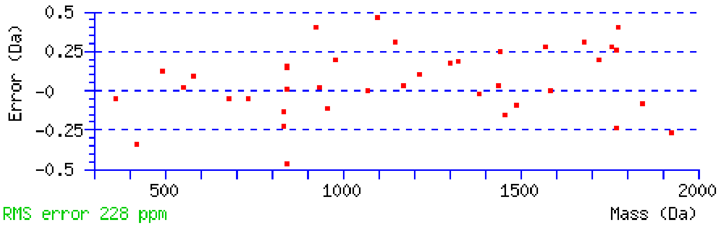


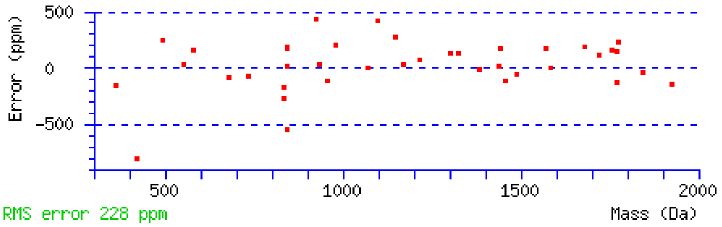


### All matches to this query

| Score | Mr(calc): | Delta | Sequence |
|---|---|---|---|
| 92.6 | 3712.5096 | -0.0105 | CISPLYVCDGDKDCADGSDEAGCVLNCTSAQFK |
| 3.5 | 3710.4940 | 2.0051 | ELPGAKASDDSAVPLTSLAGEAAGASEGTSAGNHPR |
| 2.1 | 3711.4733 | 1.0258 | NAGIYNDGKDGQDWEVNYENCYYKTEESNR |
| 2.1 | 3711.4733 | 1.0258 | NAGIYNDGKDGQDWEVNYENCYYKTEESNR |
| 2.1 | 3711.4733 | 1.0258 | NAGIYNDGKDGQDWEVNYENCYYKTEESNR |
| 2.1 | 3711.4733 | 1.0258 | NAGIYNDGKDGQDWEVNYENCYYKTEESNR |
| 1.9 | 3711.4733 | 1.0258 | NAGIYNDGKDGQDWEVNYENCYYKTEESNR |
| 1.9 | 3711.4733 | 1.0258 | NAGIYNDGKDGQDWEVNYENCYYKTEESNR |
| 1.9 | 3711.4733 | 1.0258 | NAGIYNDGKDGQDWEVNYENCYYKTEESNR |
| 1.9 | 3711.4733 | 1.0258 | NAGIYNDGKDGQDWEVNYENCYYKTEESNR |

---

Spectrum No: 122; Query: 2778; Rank: 1

---

## Peptide View

MS/MS Fragmentation of **MLVSNIDLGPTILDLAGYDLNKTQMDGTSLLPILK**
Found in **IPI00366226**, Tax_Id=10116 Gene_Symbol=Gns Glucosamine (N-acetyl)-6-sulfatase

Match to Query 2778: 3772.991622 from(1258.671150,3+)
Title: 091008RatKidney_NoSalt_15.7354.7354.3.dta
Data file K:\NewmanPaper\Piliang\3SubProteomes\Piliang3SP\mgf5ppm\ERLIC_3SubProteomes5ppm.mgf

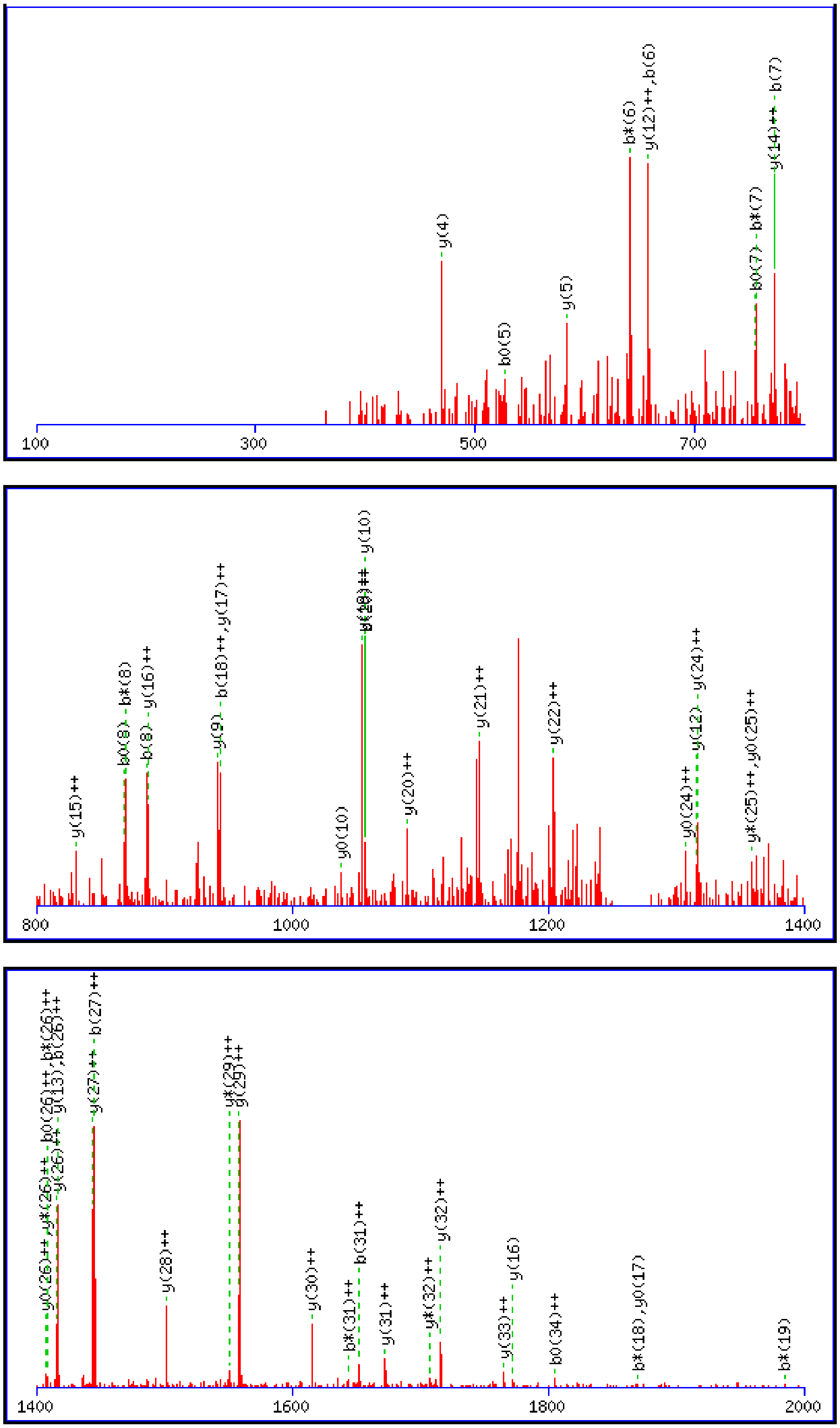


**Monoisotopic mass of neutral peptide Mr(calc):** 3772.9882
**Fixed modifications:** Carbamidomethyl (C)
**Variable modifications:**
**N21** : Deamidated_N (N)
**Ions Score:** 93 **Expect:** 3.9e-008
**Matches (Bold Red):** 54/382 fragment ions using 96 most intense peaks

| # | b | b++ | b* | b*++ | b0 | b0++ | Seq. | y | y++ | y* | y*++ | y0 | y0++ | # |
|---|---|---|---|---|---|---|---|---|---|---|---|---|---|---|
| 1 | 132.0478 | 66.5275 | | | | | M | | | | | | | 35 |
| 2 | 245.1318 | 123.0696 | | | | | L | 3642.9550 | 1821.9812 | 3625.9285 | 1813.4679 | 3624.9445 | 1812.9759 | 34 |
| 3 | 344.2002 | 172.6038 | | | | | V | 3529.8710 | **1765.4391** | 3512.8444 | 1756.9259 | 3511.8604 | 1756.4338 | 33 |
| 4 | 431.2323 | 216.1198 | | | 413.2217 | 207.1145 | S | 3430.8026 | **1715.9049** | 3413.7760 | **1707.3916** | 3412.7920 | 1706.8996 | 32 |
| 5 | 545.2752 | 273.1412 | 528.2486 | 264.6280 | **527.2646** | 264.1360 | N | 3343.7705 | **1672.3889** | 3326.7440 | 1663.8756 | 3325.7600 | 1663.3836 | 31 |

| | | | | | | | | | | | | | | |
|---|---|---|---|---|---|---|---|---|---|---|---|---|---|---|
| 6 | **658.3593** | 329.6833 | **641.3327** | 321.1700 | 640.3487 | 320.6780 | I | 3229.7276 | **1615.3674** | 3212.7011 | 1606.8542 | 3211.7170 | 1606.3622 | 30 |
| 7 | **773.3862** | 387.1967 | **756.3597** | 378.6835 | **755.3756** | 378.1915 | D | 3116.6435 | **1558.8254** | 3099.6170 | **1550.3121** | 3098.6330 | 1549.8201 | 29 |
| 8 | **886.4703** | 443.7388 | **869.4437** | 435.2255 | **868.4597** | 434.7335 | L | 3001.6166 | **1501.3119** | 2984.5900 | 1492.7987 | 2983.6060 | 1492.3067 | 28 |
| 9 | 943.4917 | 472.2495 | 926.4652 | 463.7362 | 925.4812 | 463.2442 | G | 2888.5325 | **1444.7699** | 2871.5060 | 1436.2566 | 2870.5220 | 1435.7646 | 27 |
| 10 | 1040.5445 | 520.7759 | 1023.5179 | 512.2626 | 1022.5339 | 511.7706 | P | 2831.5111 | **1416.2592** | 2814.4845 | **1407.7459** | 2813.5005 | **1407.2539** | 26 |
| 11 | 1141.5922 | 571.2997 | 1124.5656 | 562.7864 | 1123.5816 | 562.2944 | T | 2734.4583 | 1367.7328 | 2717.4318 | **1359.2195** | 2716.4477 | **1358.7275** | 25 |
| 12 | 1254.6762 | 627.8418 | 1237.6497 | 619.3285 | 1236.6657 | 618.8365 | I | 2633.4106 | **1317.2090** | 2616.3841 | 1308.6957 | 2615.4001 | **1308.2037** | 24 |
| 13 | 1367.7603 | 684.3838 | 1350.7338 | 675.8705 | 1349.7497 | 675.3785 | L | 2520.3266 | 1260.6669 | 2503.3000 | 1252.1536 | 2502.3160 | 1251.6616 | 23 |
| 14 | 1482.7872 | 741.8973 | 1465.7607 | 733.3840 | 1464.7767 | 732.8920 | D | 2407.2425 | **1204.1249** | 2390.2160 | 1195.6116 | 2389.2319 | 1195.1196 | 22 |
| 15 | 1595.8713 | 798.4393 | 1578.8448 | 789.9260 | 1577.8607 | 789.4340 | L | 2292.2156 | **1146.6114** | 2275.1890 | 1138.0981 | 2274.2050 | 1137.6061 | 21 |
| 16 | 1666.9084 | 833.9578 | 1649.8819 | 825.4446 | 1648.8979 | 824.9526 | A | 2179.1315 | **1090.0694** | 2162.1049 | 1081.5561 | 2161.1209 | 1081.0641 | 20 |
| 17 | 1723.9299 | 862.4686 | 1706.9033 | 853.9553 | 1705.9193 | 853.4633 | G | 2108.0944 | **1054.5508** | 2091.0678 | 1046.0376 | 2090.0838 | 1045.5455 | 19 |
| 18 | 1886.9932 | **944.0002** | **1869.9667** | 935.4870 | 1868.9826 | 934.9950 | Y | 2051.0729 | 1026.0401 | 2034.0464 | 1017.5268 | 2033.0623 | 1017.0348 | 18 |
| 19 | 2002.0202 | 1001.5137 | **1984.9936** | 993.0004 | 1984.0096 | 992.5084 | D | 1888.0096 | **944.5084** | 1870.9830 | 935.9952 | **1869.9990** | 935.5031 | 17 |
| 20 | 2115.1042 | **1058.0557** | 2098.0777 | 1049.5425 | 2097.0937 | 1049.0505 | L | **1772.9826** | **886.9950** | 1755.9561 | 878.4817 | 1754.9721 | 877.9897 | 16 |
| 21 | 2230.1312 | 1115.5692 | 2213.1046 | 1107.0559 | 2212.1206 | 1106.5639 | N | 1659.8986 | **830.4529** | 1642.8720 | 821.9397 | 1641.8880 | 821.4476 | 15 |
| 22 | 2358.2261 | 1179.6167 | 2341.1996 | 1171.1034 | 2340.2156 | 1170.6114 | K | 1544.8716 | **772.9395** | 1527.8451 | 764.4262 | 1526.8611 | 763.9342 | 14 |
| 23 | 2459.2738 | 1230.1405 | 2442.2472 | 1221.6273 | 2441.2632 | 1221.1353 | T | **1416.7767** | 708.8920 | 1399.7501 | 700.3787 | 1398.7661 | 699.8867 | 13 |
| 24 | 2587.3324 | 1294.1698 | 2570.3058 | 1285.6566 | 2569.3218 | 1285.1645 | Q | **1315.7290** | **658.3681** | 1298.7025 | 649.8549 | 1297.7184 | 649.3629 | 12 |
| 25 | 2718.3729 | 1359.6901 | 2701.3463 | 1351.1768 | 2700.3623 | 1350.6848 | M | 1187.6704 | 594.3388 | 1170.6439 | 585.8256 | 1169.6599 | 585.3336 | 11 |
| 26 | 2833.3998 | **1417.2035** | 2816.3733 | **1408.6903** | 2815.3892 | **1408.1983** | D | **1056.6299** | 528.8186 | 1039.6034 | 520.3053 | **1038.6194** | 519.8133 | 10 |
| 27 | 2890.4213 | **1445.7143** | 2873.3947 | 1437.2010 | 2872.4107 | 1436.7090 | G | **941.6030** | 471.3051 | 924.5764 | 462.7919 | 923.5924 | 462.2999 | 9 |
| 28 | 2991.4689 | 1496.2381 | 2974.4424 | 1487.7248 | 2973.4584 | 1487.2328 | T | 884.5815 | 442.7944 | 867.5550 | 434.2811 | 866.5710 | 433.7891 | 8 |
| 29 | 3078.5010 | 1539.7541 | 3061.4744 | 1531.2409 | 3060.4904 | 1530.7488 | S | 783.5339 | 392.2706 | 766.5073 | 383.7573 | 765.5233 | 383.2653 | 7 |
| 30 | 3191.5850 | 1596.2962 | 3174.5585 | 1587.7829 | 3173.5745 | 1587.2909 | L | 696.5018 | 348.7546 | 679.4753 | 340.2413 | | | 6 |
| 31 | 3304.6691 | **1652.8382** | 3287.6426 | **1644.3249** | 3286.6585 | 1643.8329 | L | **583.4178** | 292.2125 | 566.3912 | 283.6992 | | | 5 |
| 32 | 3401.7219 | 1701.3646 | 3384.6953 | 1692.8513 | 3383.7113 | 1692.3593 | P | **470.3337** | 235.6705 | 453.3071 | 227.1572 | | | 4 |
| 33 | 3514.8059 | 1757.9066 | 3497.7794 | 1749.3933 | 3496.7954 | 1748.9013 | I | 373.2809 | 187.1441 | 356.2544 | 178.6308 | | | 3 |
| 34 | 3627.8900 | 1814.4486 | 3610.8634 | 1805.9354 | 3609.8794 | **1805.4434** | L | 260.1969 | 130.6021 | 243.1703 | 122.0888 | | | 2 |
| 35 | | | | | | | K | 147.1128 | 74.0600 | 130.0863 | 65.5468 | | | 1 |

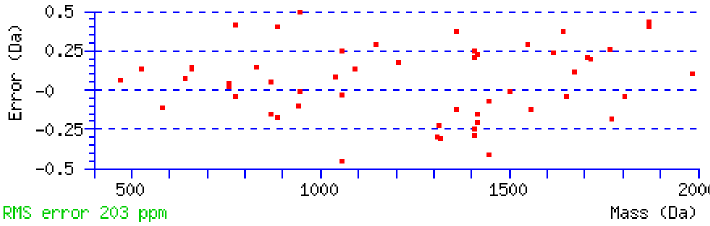


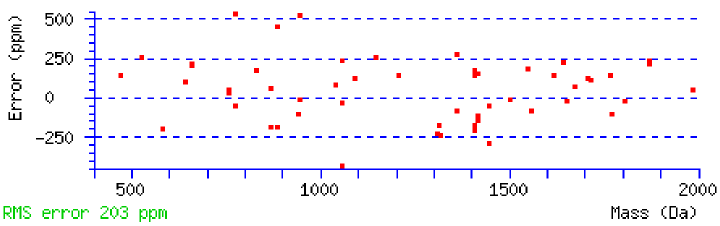


### All matches to this query

| Score | Mr(calc): | Delta | Sequence |
|---|---|---|---|
| 92.6 | 3772.9882 | 0.0034 | MLVSNIDLGPTILDLAGYDLNKTQMDGTSLLPILK |
| 39.0 | 3772.9882 | 0.0034 | MLVSNIDLGPTILDLAGYDLNKTQMDGTSLLPILK |
| 18.5 | 3772.0042 | 0.9874 | MLVSNIDLGPTILDLAGYDLNKTQMDGTSLLPILK |
| 6.2 | 3772.9783 | 0.0133 | EKKDLGTLGYVLGVTMTVIIIAIGVGIVLGYTYK |
| 6.2 | 3772.9783 | 0.0133 | EKKDLGTLGYVLGVTMTVIIIAIGVGIVLGYTYK |
| 2.5 | 3770.9762 | 2.0154 | ITDVQLAIFANMLGVSLFLLVVLYHYVAVNNPK |
| 1.8 | 3772.9783 | 0.0133 | EKKDLGTLGYVLGVTMTVIIIAIGVGIVLGYTYK |
| 1.8 | 3772.9783 | 0.0133 | EKKDLGTLGYVLGVTMTVIIIAIGVGIVLGYTYK |
| 1.8 | 3772.9783 | 0.0133 | EKKDLGTLGYVLGVTMTVIIIAIGVGIVLGYTYK |
| 1.8 | 3772.9783 | 0.0133 | EKKDLGTLGYVLGVTMTVIIIAIGVGIVLGYTYK |

Spectrum No: 123; Query: 491; Rank: 1

## Peptide View

MS/MS Fragmentation of **ALGYENATQALSR**
Found in **IPI00199448**, Tax_Id=10116 Gene_Symbol=Lgals3bp Mama

Match to Query 491: 1393.685488 from(697.850020,2+)
Title: 100101RatKid_NS_deglyco_22.2533.2533.2.dta
Data file K:\NewmanPaper\Piliang\3SubProteomes\Piliang3SP\mgf5ppm\ERLIC_3SubProteomes5ppm.mgf

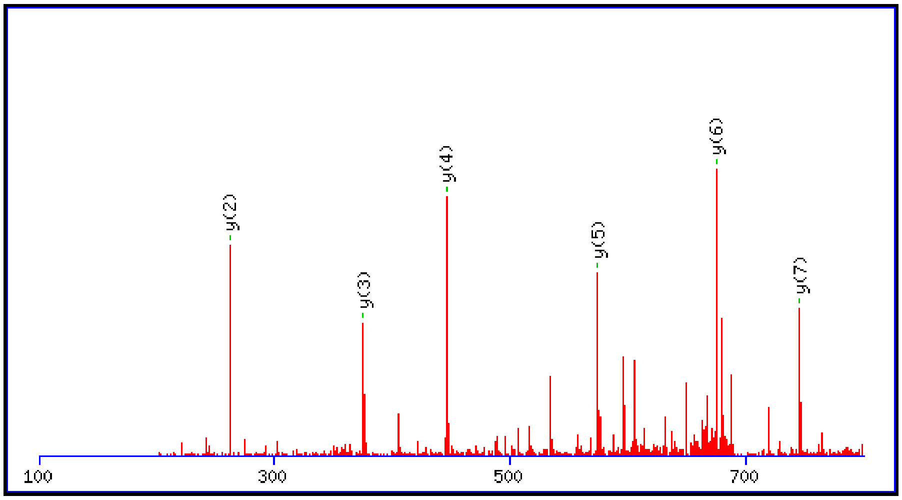


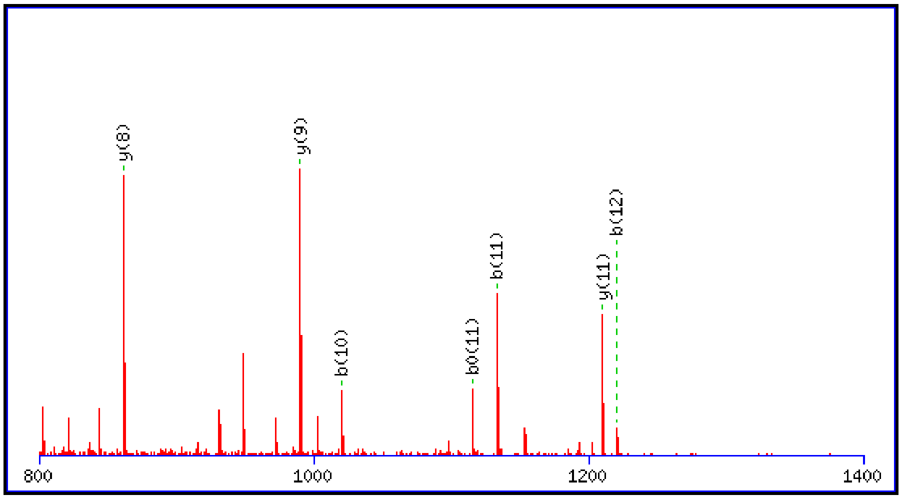

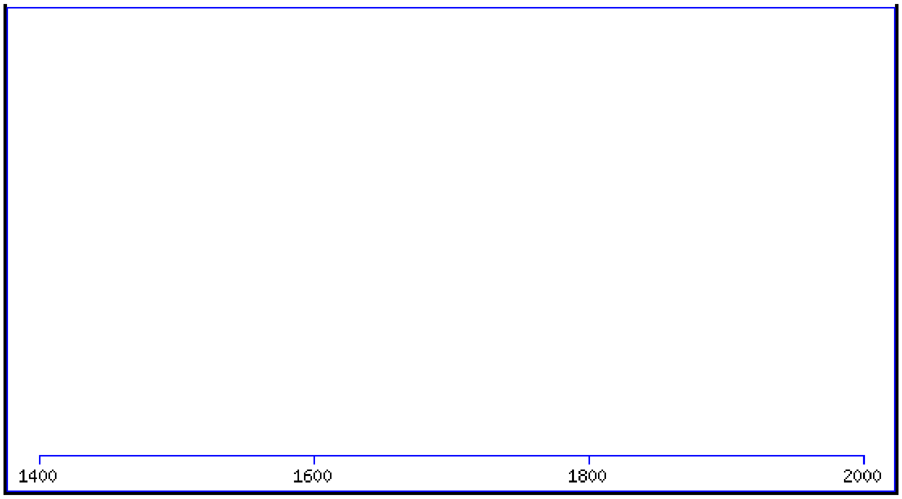


**Monoisotopic mass of neutral peptide Mr(calc):** 1393.6837
**Fixed modifications:** Carbamidomethyl (C)
**Variable modifications:**
**N6** : Deamidated_N (N)
**Ions Score:** 93 **Expect:** 7.8e-008
**Matches (Bold Red):** 13/124 fragment ions using 17 most intense peaks

| # | b | $b^{++}$ | b* | $b^{*++}$ | $b^{0}$ | $b^{0++}$ | Seq. | y | $y^{++}$ | y* | $y^{*++}$ | $y^{0}$ | $y^{0++}$ | # |
|---|---|---|---|---|---|---|---|---|---|---|---|---|---|---|
| 1 | 72.0444 | 36.5258 | | | | | A | | | | | | | 13 |
| 2 | 185.1285 | 93.0679 | | | | | L | 1323.6539 | 662.3306 | 1306.6274 | 653.8173 | 1305.6434 | 653.3253 | 12 |
| 3 | 242.1499 | 121.5786 | | | | | G | **1210.5699** | 605.7886 | 1193.5433 | 597.2753 | 1192.5593 | 596.7833 | 11 |
| 4 | 405.2132 | 203.1103 | | | | | Y | 1153.5484 | 577.2778 | 1136.5218 | 568.7646 | 1135.5378 | 568.2725 | 10 |
| 5 | 534.2558 | 267.6316 | | | 516.2453 | 258.6263 | E | **990.4851** | 495.7462 | 973.4585 | 487.2329 | 972.4745 | 486.7409 | 9 |
| 6 | 649.2828 | 325.1450 | 632.2562 | 316.6318 | 631.2722 | 316.1397 | N | **861.4425** | 431.2249 | 844.4159 | 422.7116 | 843.4319 | 422.2196 | 8 |
| 7 | 720.3199 | 360.6636 | 703.2933 | 352.1503 | 702.3093 | 351.6583 | A | **746.4155** | 373.7114 | 729.3890 | 365.1981 | 728.4050 | 364.7061 | 7 |
| 8 | 821.3676 | 411.1874 | 804.3410 | 402.6741 | 803.3570 | 402.1821 | T | **675.3784** | 338.1928 | 658.3519 | 329.6796 | 657.3679 | 329.1876 | 6 |
| 9 | 949.4261 | 475.2167 | 932.3996 | 466.7034 | 931.4156 | 466.2114 | Q | **574.3307** | 287.6690 | 557.3042 | 279.1557 | 556.3202 | 278.6637 | 5 |
| 10 | **1020.4633** | 510.7353 | 1003.4367 | 502.2220 | 1002.4527 | 501.7300 | A | **446.2722** | 223.6397 | 429.2456 | 215.1264 | 428.2616 | 214.6344 | 4 |
| 11 | **1133.5473** | 567.2773 | 1116.5208 | 558.7640 | **1115.5368** | 558.2720 | L | **375.2350** | 188.1212 | 358.2085 | 179.6079 | 357.2245 | 179.1159 | 3 |
| 12 | **1220.5794** | 610.7933 | 1203.5528 | 602.2800 | 1202.5688 | 601.7880 | S | **262.1510** | 131.5791 | 245.1244 | 123.0659 | 244.1404 | 122.5738 | 2 |
| 13 | | | | | | | R | 175.1190 | 88.0631 | 158.0924 | 79.5498 | | | 1 |

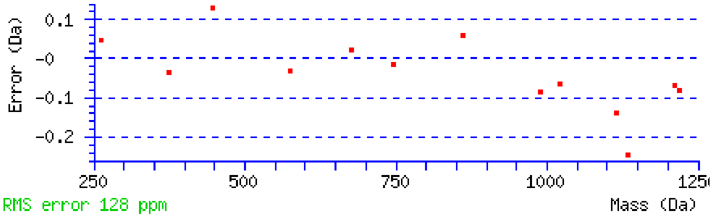


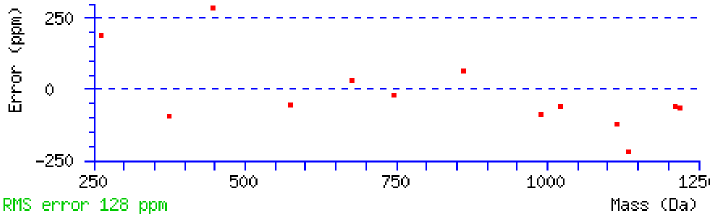


### All matches to this query

| Score | Mr(calc): | Delta | Sequence |
|---|---|---|---|
| 92.5 | 1393.6837 | 0.0018 | ALGYENATQALSR |
| 8.1 | 1393.6949 | -0.0095 | IQENYNTRLSR |
| 7.9 | 1393.6949 | -0.0095 | IQENYNTRLSR |
| 6.1 | 1393.6827 | 0.0028 | NSQQRRVALDK |
| 5.5 | 1393.6741 | 0.0114 | LTPEKEEILNK |
| 5.5 | 1393.6827 | 0.0028 | ASPSNARKQISR |
| 5.5 | 1393.6827 | 0.0028 | ASPSNARKQISR |
| 4.0 | 1393.6936 | -0.0081 | SDATASISLSSNLK |
| | | | |

| | | | |
|---|---|---|---|
| 3.6 | 1393.6759 | 0.0096 | AGTSTLTECVINK |
| 2.9 | 1393.6837 | 0.0018 | YQQLEEASASLR |

---

Spectrum No: 124; Query: 1184; Rank: 1

---

## Peptide View

MS/MS Fragmentation of **DAGYTNATTTDQHGLAK**
Found in **IPI00201262**, Tax_Id=10116 Gene_Symbol=LOC297568;Mug2;Mug1 Alpha-1-inhibitor 3 precursor

Match to Query 1184: 1763.798508 from(882.906530,2+)
Title: 091008RatKidney_NH4Format01_26.609.609.2.dta
Data file K:\NewmanPaper\Piliang\3SubProteomes\Piliang3SP\mgf5ppm\ERLIC_3SubProteomes5ppm.mgf

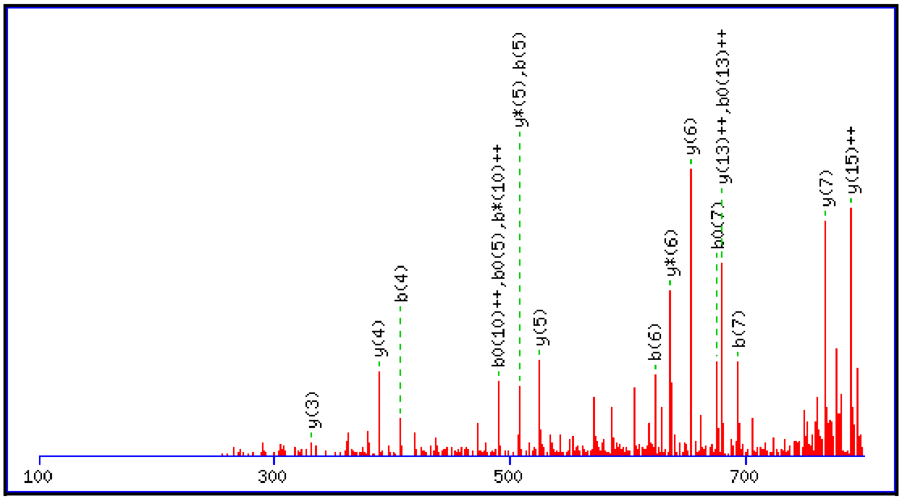


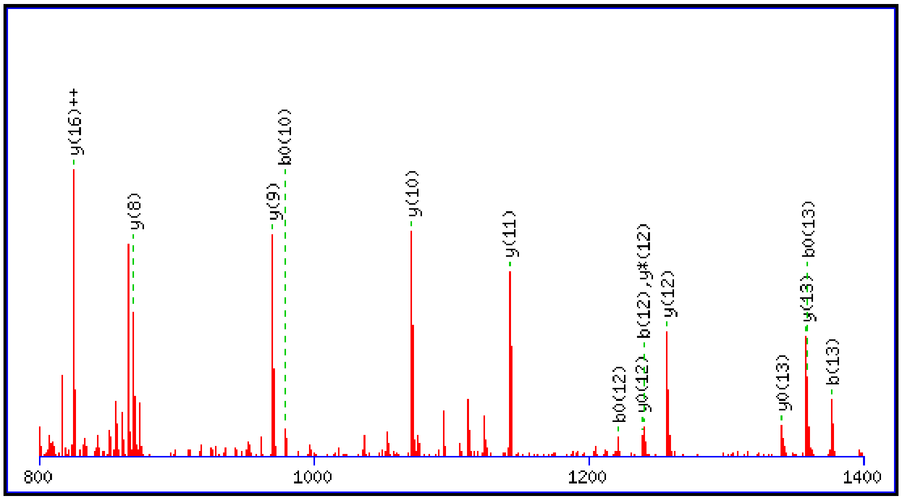

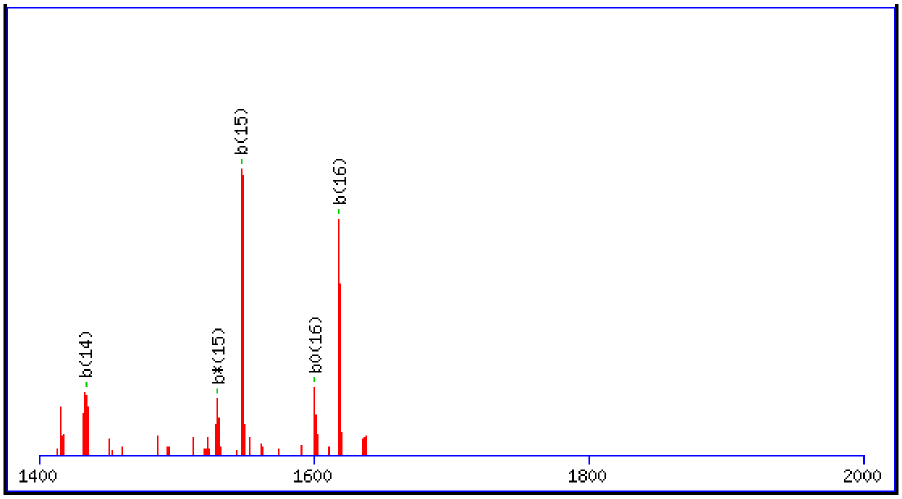


**Monoisotopic mass of neutral peptide Mr(calc):** 1763.7962
**Fixed modifications:** Carbamidomethyl (C)
**Variable modifications:**
**N6** : Deamidated_N (N)
**Ions Score:** 92 **Expect:** 1.3e-007
**Matches (Bold Red):** 38/170 fragment ions using 50 most intense peaks

| # | b | b$^{++}$ | b* | b*$^{++}$ | b$^{0}$ | b$^{0++}$ | Seq. | y | y$^{++}$ | y* | y*$^{++}$ | y$^{0}$ | y$^{0++}$ | # |
|---|---|---|---|---|---|---|---|---|---|---|---|---|---|---|
| 1 | 116.0342 | 58.5207 | | | 98.0237 | 49.5155 | D | | | | | | | 17 |
| 2 | 187.0713 | 94.0393 | | | 169.0608 | 85.0340 | A | 1649.7766 | **825.3919** | 1632.7500 | 816.8786 | 1631.7660 | 816.3866 | 16 |
| 3 | 244.0928 | 122.5500 | | | 226.0822 | 113.5448 | G | 1578.7394 | **789.8734** | 1561.7129 | 781.3601 | 1560.7289 | 780.8681 | 15 |
| 4 | **407.1561** | 204.0817 | | | 389.1456 | 195.0764 | Y | 1521.7180 | 761.3626 | 1504.6914 | 752.8494 | 1503.7074 | 752.3573 | 14 |
| 5 | **508.2038** | 254.6055 | | | **490.1932** | 245.6003 | T | **1358.6546** | **679.8310** | 1341.6281 | 671.3177 | **1340.6441** | 670.8257 | 13 |
| 6 | **623.2307** | 312.1190 | 606.2042 | 303.6057 | 605.2202 | 303.1137 | N | **1257.6070** | 629.3071 | **1240.5804** | 620.7938 | **1239.5964** | 620.3018 | 12 |
| 7 | **694.2679** | 347.6376 | 677.2413 | 339.1243 | **676.2573** | 338.6323 | A | **1142.5800** | 571.7937 | 1125.5535 | 563.2804 | 1124.5695 | 562.7884 | 11 |
| 8 | 795.3155 | 398.1614 | 778.2890 | 389.6481 | 777.3050 | 389.1561 | T | **1071.5429** | 536.2751 | 1054.5164 | 527.7618 | 1053.5324 | 527.2698 | 10 |
| 9 | 896.3632 | 448.6852 | 879.3367 | 440.1720 | 878.3526 | 439.6800 | T | **970.4952** | 485.7513 | 953.4687 | 477.2380 | 952.4847 | 476.7460 | 9 |
| 10 | 997.4109 | 499.2091 | 980.3843 | **490.6958** | **979.4003** | **490.2038** | T | **869.4476** | 435.2274 | 852.4210 | 426.7141 | 851.4370 | 426.2221 | 8 |
| 11 | 1112.4378 | 556.7226 | 1095.4113 | 548.2093 | 1094.4273 | 547.7173 | D | **768.3999** | 384.7036 | 751.3733 | 376.1903 | 750.3893 | 375.6983 | 7 |
| 12 | **1240.4964** | 620.7518 | 1223.4699 | 612.2386 | **1222.4858** | 611.7466 | Q | **653.3729** | 327.1901 | **636.3464** | 318.6768 | | | 6 |
| 13 | **1377.5553** | 689.2813 | 1360.5288 | 680.7680 | **1359.5448** | **680.2760** | H | **525.3144** | 263.1608 | **508.2878** | 254.6475 | | | 5 |
| 14 | **1434.5768** | 717.7920 | 1417.5502 | 709.2788 | 1416.5662 | 708.7867 | G | **388.2554** | 194.6314 | 371.2289 | 186.1181 | | | 4 |
| 15 | **1547.6609** | 774.3341 | **1530.6343** | 765.8208 | 1529.6503 | 765.3288 | L | **331.2340** | 166.1206 | 314.2074 | 157.6074 | | | 3 |
| 16 | **1618.6980** | 809.8526 | 1601.6714 | 801.3393 | **1600.6874** | 800.8473 | A | 218.1499 | 109.5786 | 201.1234 | 101.0653 | | | 2 |
| 17 | | | | | | | K | 147.1128 | 74.0600 | 130.0863 | 65.5468 | | | 1 |

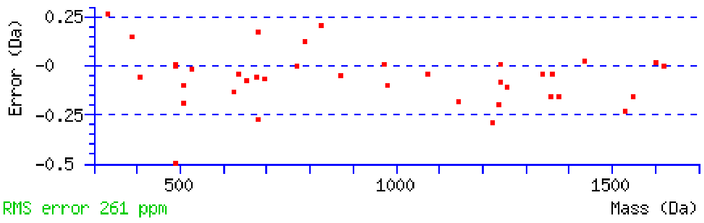


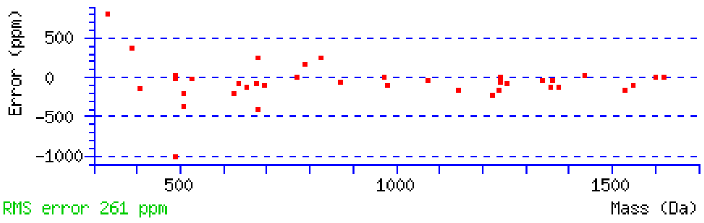


## All matches to this query

| Score | Mr(calc): | Delta | Sequence |
|---|---|---|---|
| 92.0 | 1763.7962 | 0.0023 | DAGYTNATTTDQHGLAK |
| 48.1 | 1762.8122 | 0.9863 | DAGYTNATTTDQHGLAK |
| 7.0 | 1762.7791 | 1.0194 | KSTKQTELTPEDVK |
| 6.1 | 1763.8008 | -0.0023 | EGPDRSVSRLTFLK |
| | | | |

| | | | |
|---|---|---|---|
| 5.0 | 1763.8043 | -0.0058 | MNETMATDSPRRPSR |
| 4.7 | 1763.8043 | -0.0058 | MNETMATDSPRRPSR |
| 3.2 | 1762.7888 | 1.0097 | IPFSMENLKVNFNK |
| 2.5 | 1763.7987 | -0.0002 | GATSKEVMCFNAKLK |
| 2.4 | 1762.7862 | 1.0123 | SDSSLFMFRSHNKK |
| 2.0 | 1762.7815 | 1.0170 | IISVEYDTSLSDWR |

---

Spectrum No: 125; Query: 837; Rank: 1

---

## Peptide View

MS/MS Fragmentation of **TEPPLNASAGDQEEK**
Found in **IPI00210975**, Tax_Id=10116 Gene_Symbol=Hyou1 Hypoxia up-regulated protein 1 precursor

Match to Query 837: 1585.705668 from(793.860110,2+)
Title: 091008RatKidney_NH4Format01_25.789.789.2.dta
Data file K:\NewmanPaper\Piliang\3SubProteomes\Piliang3SP\mgf5ppm\ERLIC_3SubProteomes5ppm.mgf

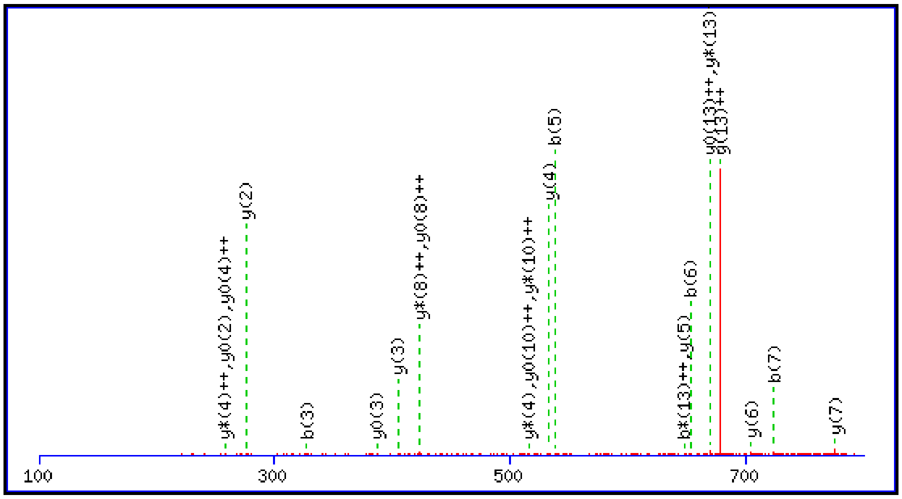


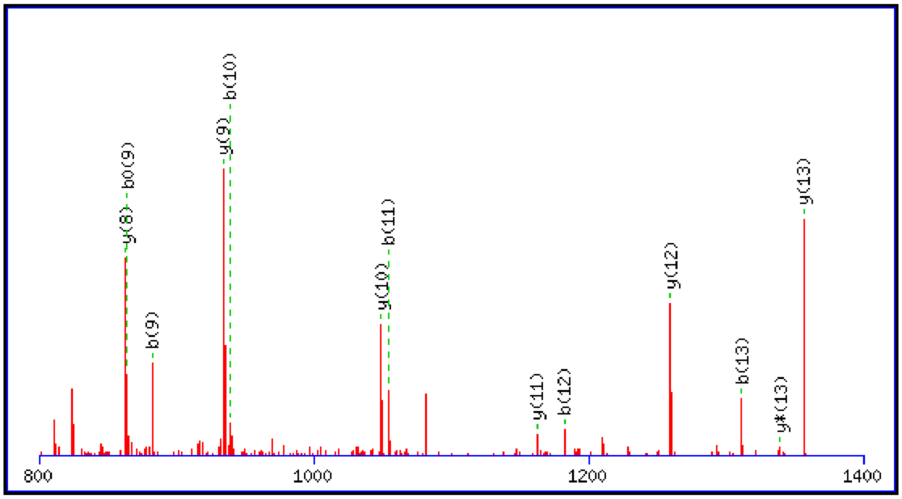

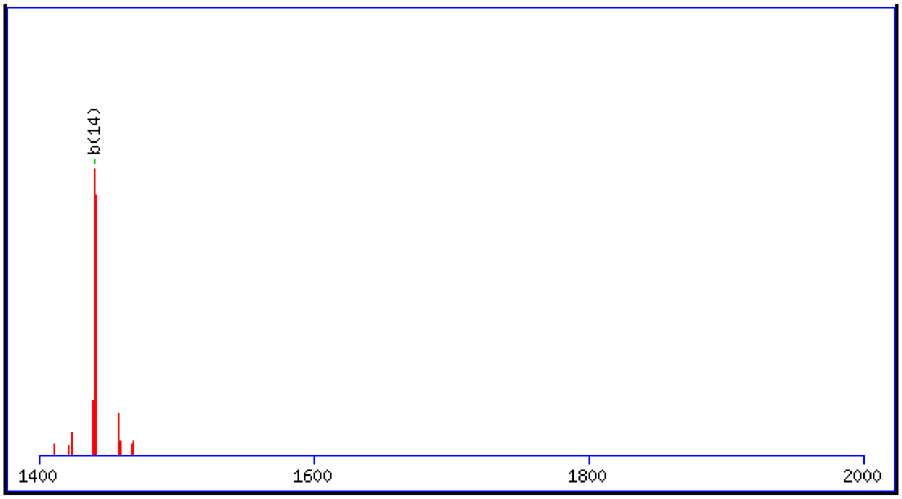


**Monoisotopic mass of neutral peptide Mr(calc):** 1585.7107
**Fixed modifications:** Carbamidomethyl (C)
**Variable modifications:**
**N6** : Deamidated_N (N)
**Ions Score:** 92 **Expect:** 1.1e-007
**Matches (Bold Red):** 37/156 fragment ions using 42 most intense peaks

| # | b | $b^{++}$ | b* | $b^{*++}$ | $b^0$ | $b^{0++}$ | Seq. | y | $y^{++}$ | y* | $y^{*++}$ | $y^0$ | $y^{0++}$ | # |
|---|---|---|---|---|---|---|---|---|---|---|---|---|---|---|
| 1 | 102.0550 | 51.5311 | | | 84.0444 | 42.5258 | T | | | | | | | 15 |
| 2 | 231.0975 | 116.0524 | | | 213.0870 | 107.0471 | E | 1485.6704 | 743.3388 | 1468.6438 | 734.8255 | 1467.6598 | 734.3335 | 14 |
| 3 | **328.1503** | 164.5788 | | | 310.1397 | 155.5735 | P | **1356.6278** | **678.8175** | **1339.6012** | **670.3042** | 1338.6172 | **669.8122** | 13 |
| 4 | 425.2031 | 213.1052 | | | 407.1925 | 204.0999 | P | **1259.5750** | 630.2911 | 1242.5484 | 621.7779 | 1241.5644 | 621.2859 | 12 |
| 5 | **538.2871** | 269.6472 | | | 520.2766 | 260.6419 | L | **1162.5222** | 581.7648 | 1145.4957 | 573.2515 | 1144.5117 | 572.7595 | 11 |
| 6 | **653.3141** | 327.1607 | 636.2875 | 318.6474 | 635.3035 | 318.1554 | N | **1049.4382** | 525.2227 | 1032.4116 | **516.7094** | 1031.4276 | **516.2174** | 10 |
| 7 | **724.3512** | 362.6792 | 707.3246 | 354.1660 | 706.3406 | 353.6740 | A | **934.4112** | 467.7093 | 917.3847 | 459.1960 | 916.4007 | 458.7040 | 9 |
| 8 | 811.3832 | 406.1952 | 794.3567 | 397.6820 | 793.3727 | 397.1900 | S | **863.3741** | 432.1907 | 846.3476 | **423.6774** | 845.3636 | **423.1854** | 8 |
| 9 | **882.4203** | 441.7138 | 865.3938 | 433.2005 | **864.4098** | 432.7085 | A | **776.3421** | 388.6747 | 759.3155 | 380.1614 | 758.3315 | 379.6694 | 7 |
| 10 | **939.4418** | 470.2245 | 922.4152 | 461.7113 | 921.4312 | 461.2193 | G | **705.3050** | 353.1561 | 688.2784 | 344.6429 | 687.2944 | 344.1508 | 6 |
| 11 | **1054.4687** | 527.7380 | 1037.4422 | 519.2247 | 1036.4582 | 518.7327 | D | **648.2835** | 324.6454 | 631.2570 | 316.1321 | 630.2729 | 315.6401 | 5 |
| 12 | **1182.5273** | 591.7673 | 1165.5008 | 583.2540 | 1164.5168 | 582.7620 | Q | **533.2566** | 267.1319 | **516.2300** | **258.6186** | 515.2460 | **258.1266** | 4 |
| 13 | **1311.5699** | 656.2886 | 1294.5434 | **647.7753** | 1293.5593 | 647.2833 | E | **405.1980** | 203.1026 | 388.1714 | 194.5894 | **387.1874** | 194.0974 | 3 |
| 14 | **1440.6125** | 720.8099 | 1423.5860 | 712.2966 | 1422.6019 | 711.8046 | E | **276.1554** | 138.5813 | 259.1288 | 130.0681 | **258.1448** | 129.5761 | 2 |
| 15 | | | | | | | K | 147.1128 | 74.0600 | 130.0863 | 65.5468 | | | 1 |

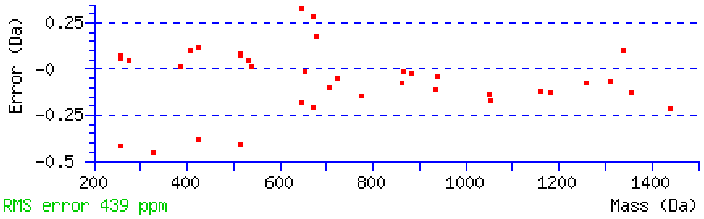


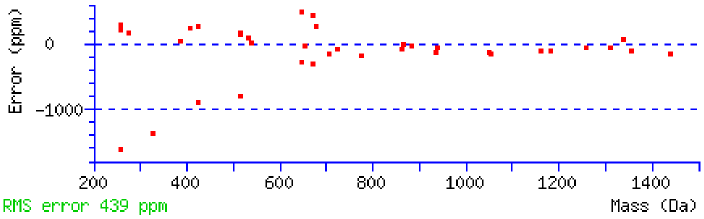


## All matches to this query

| Score | Mr(calc): | Delta | Sequence |
|---|---|---|---|
| 91.7 | 1585.7107 | -0.0051 | TEPPLNASAGDQEEK |
| 15.1 | 1584.7007 | 1.0050 | MSSKNYASHPAAIK |
| 11.8 | 1585.7059 | -0.0002 | VLDCDTISQAKEK |
| 11.4 | 1585.6943 | 0.0114 | ETRYTVVNLFGK |
| 10.8 | 1585.7059 | -0.0002 | VLDCDTISQAKEK |
| 10.2 | 1585.7004 | 0.0053 | VLCEVFSNVNMEK |
| | | | |

| | | | |
|---|---|---|---|
| 9.7 | 1585.7137 | -0.0080 | QTEFAPESGKREK |
| 9.4 | 1583.6942 | 2.0114 | ECPEATFAIPIEK |
| 9.2 | 1584.6903 | 1.0153 | NPPDLEEEETQER |
| 9.0 | 1584.7146 | 0.9910 | MPEFLEDPSVLTK |

---

Spectrum No: 126; Query: 2730; Rank: 1

---

## Peptide View

MS/MS Fragmentation of **ETIGPQQGCGGYLTEDNQSFVSPDSDSNGRYDK**
Found in **IPI00196620**, Tax_Id=10116 Gene_Symbol=Cubn Cubilin precursor

Match to Query 2730: 3621.559902 from(1208.193910,3+)
Title: 091008RatKidney_NoSalt_20.2568.2568.3.dta
Data file K:\NewmanPaper\Piliang\3SubProteomes\Piliang3SP\mgf5ppm\ERLIC_3SubProteomes5ppm.mgf

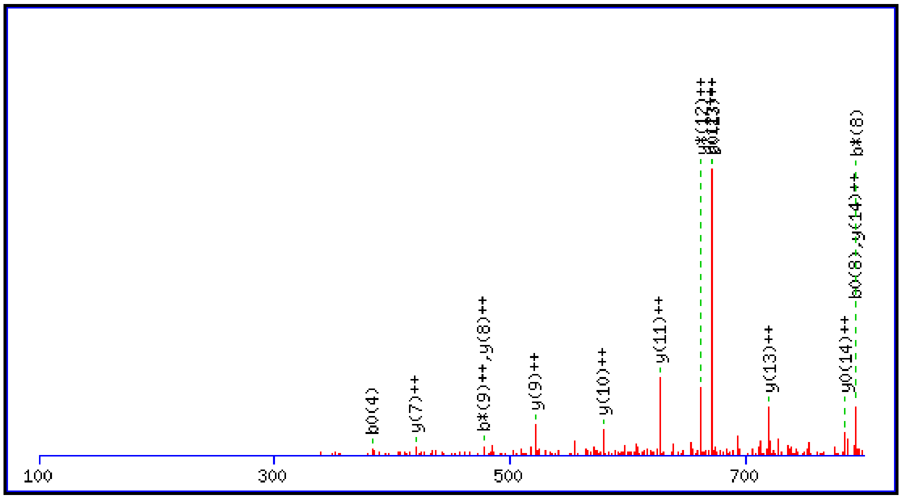


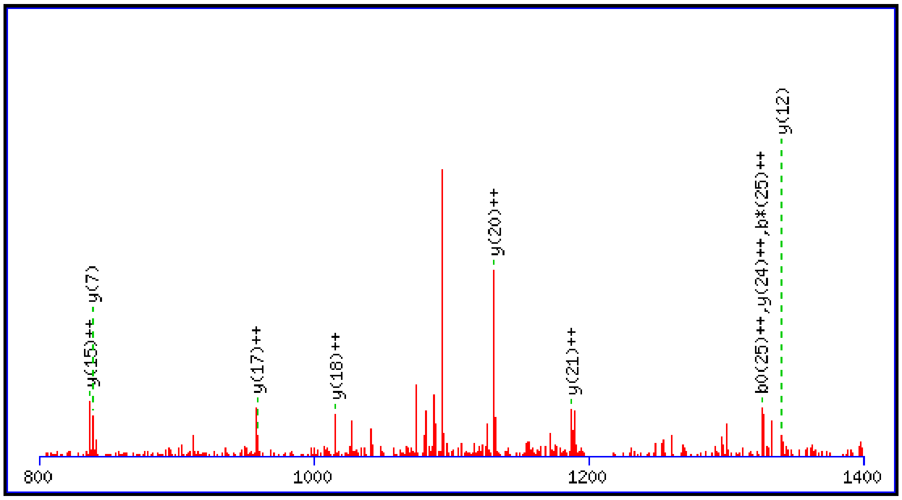

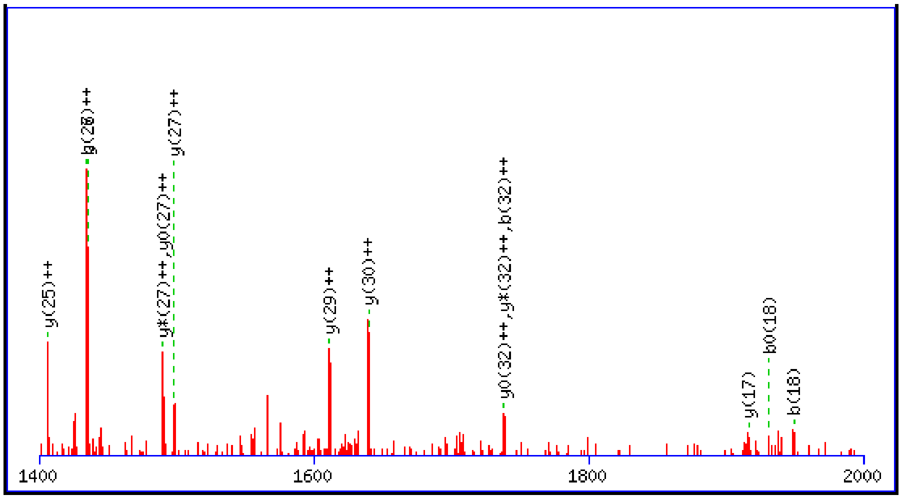


**Monoisotopic mass of neutral peptide Mr(calc):** 3621.5438
**Fixed modifications:** Carbamidomethyl (C)
**Variable modifications:**
**N17** : Deamidated_N (N)
**Ions Score:** 92 **Expect:** 3.3e-007
**Matches (Bold Red):** 39/372 fragment ions using 69 most intense peaks

| # | b | $b^{++}$ | b* | $b^{*++}$ | $b^0$ | $b^{0++}$ | Seq. | y | $y^{++}$ | y* | $y^{*++}$ | $y^0$ | $y^{0++}$ | # |
|---|---|---|---|---|---|---|---|---|---|---|---|---|---|---|
| 1 | 130.0499 | 65.5286 | | | 112.0393 | 56.5233 | E | | | | | | | 33 |
| 2 | 231.0975 | 116.0524 | | | 213.0870 | 107.0471 | T | 3493.5085 | 1747.2579 | 3476.4819 | **1738.7446** | 3475.4979 | **1738.2526** | 32 |
| 3 | 344.1816 | 172.5944 | | | 326.1710 | 163.5892 | I | 3392.4608 | 1696.7340 | 3375.4342 | 1688.2208 | 3374.4502 | 1687.7287 | 31 |
| 4 | 401.2031 | 201.1052 | | | **383.1925** | 192.0999 | G | 3279.3767 | **1640.1920** | 3262.3502 | 1631.6787 | 3261.3661 | 1631.1867 | 30 |
| 5 | 498.2558 | 249.6316 | | | 480.2453 | 240.6263 | P | 3222.3553 | **1611.6813** | 3205.3287 | 1603.1680 | 3204.3447 | 1602.6760 | 29 |
| 6 | 626.3144 | 313.6608 | 609.2879 | 305.1476 | 608.3039 | 304.6556 | Q | 3125.3025 | 1563.1549 | 3108.2759 | 1554.6416 | 3107.2919 | 1554.1496 | 28 |
| 7 | 754.3730 | 377.6901 | 737.3464 | 369.1769 | 736.3624 | 368.6849 | Q | 2997.2439 | **1499.1256** | 2980.2174 | **1490.6123** | 2979.2333 | **1490.1203** | 27 |
| 8 | 811.3945 | 406.2009 | **794.3679** | 397.6876 | **793.3839** | 397.1956 | G | 2869.1853 | **1435.0963** | 2852.1588 | 1426.5830 | 2851.1748 | 1426.0910 | 26 |
| 9 | 971.4251 | 486.2162 | 954.3986 | **477.7029** | 953.4145 | 477.2109 | C | 2812.1639 | **1406.5856** | 2795.1373 | 1398.0723 | 2794.1533 | 1397.5803 | 25 |
| 10 | 1028.4466 | 514.7269 | 1011.4200 | 506.2137 | 1010.4360 | 505.7216 | G | 2652.1332 | **1326.5702** | 2635.1067 | 1318.0570 | 2634.1227 | 1317.5650 | 24 |
| 11 | 1085.4680 | 543.2377 | 1068.4415 | 534.7244 | 1067.4575 | 534.2324 | G | 2595.1118 | 1298.0595 | 2578.0852 | 1289.5462 | 2577.1012 | 1289.0542 | 23 |
| 12 | 1248.5314 | 624.7693 | 1231.5048 | 616.2560 | 1230.5208 | 615.7640 | Y | 2538.0903 | 1269.5488 | 2521.0637 | 1261.0355 | 2520.0797 | 1260.5435 | 22 |
| 13 | 1361.6154 | 681.3114 | 1344.5889 | 672.7981 | 1343.6049 | **672.3061** | L | 2375.0270 | **1188.0171** | 2358.0004 | 1179.5038 | 2357.0164 | 1179.0118 | 21 |
| 14 | 1462.6631 | 731.8352 | 1445.6366 | 723.3219 | 1444.6525 | 722.8299 | T | 2261.9429 | **1131.4751** | 2244.9163 | 1122.9618 | 2243.9323 | 1122.4698 | 20 |
| 15 | 1591.7057 | 796.3565 | 1574.6792 | 787.8432 | 1573.6951 | 787.3512 | E | 2160.8952 | 1080.9512 | 2143.8687 | 1072.4380 | 2142.8847 | 1071.9460 | 19 |
| 16 | 1706.7326 | 853.8700 | 1689.7061 | 845.3567 | 1688.7221 | 844.8647 | D | 2031.8526 | **1016.4300** | 2014.8261 | 1007.9167 | 2013.8421 | 1007.4247 | 18 |
| 17 | 1821.7596 | 911.3834 | 1804.7330 | 902.8702 | 1803.7490 | 902.3781 | N | **1916.8257** | **958.9165** | 1899.7991 | 950.4032 | 1898.8151 | 949.9112 | 17 |
| 18 | **1949.8182** | 975.4127 | 1932.7916 | 966.8994 | **1931.8076** | 966.4074 | Q | 1801.7987 | 901.4030 | 1784.7722 | 892.8897 | 1783.7882 | 892.3977 | 16 |
| 19 | 2036.8502 | 1018.9287 | 2019.8236 | 1010.4155 | 2018.8396 | 1009.9234 | S | 1673.7402 | **837.3737** | 1656.7136 | 828.8604 | 1655.7296 | 828.3684 | 15 |
| 20 | 2183.9186 | 1092.4629 | 2166.8921 | 1083.9497 | 2165.9080 | 1083.4577 | F | 1586.7081 | **793.8577** | 1569.6816 | 785.3444 | 1568.6976 | **784.8524** | 14 |
| 21 | 2282.9870 | 1141.9971 | 2265.9605 | 1133.4839 | 2264.9764 | 1132.9919 | V | 1439.6397 | **720.3235** | 1422.6132 | 711.8102 | 1421.6292 | 711.3182 | 13 |
| 22 | 2370.0190 | 1185.5132 | 2352.9925 | 1176.9999 | 2352.0085 | 1176.5079 | S | **1340.5713** | **670.7893** | 1323.5448 | **662.2760** | 1322.5607 | 661.7840 | 12 |
| 23 | 2467.0718 | 1234.0395 | 2450.0453 | 1225.5263 | 2449.0612 | 1225.0343 | P | 1253.5393 | **627.2733** | 1236.5127 | 618.7600 | 1235.5287 | 618.2680 | 11 |
| 24 | 2582.0987 | 1291.5530 | 2565.0722 | 1283.0397 | 2564.0882 | 1282.5477 | D | 1156.4865 | **578.7469** | 1139.4600 | 570.2336 | 1138.4760 | 569.7416 | 10 |
| 25 | 2669.1308 | 1335.0690 | 2652.1042 | **1326.5558** | 2651.1202 | **1326.0637** | S | 1041.4596 | **521.2334** | 1024.4330 | 512.7202 | 1023.4490 | 512.2281 | 9 |
| 26 | 2784.1577 | 1392.5825 | 2767.1312 | 1384.0692 | 2766.1472 | 1383.5772 | D | 954.4275 | **477.7174** | 937.4010 | 469.2041 | 936.4170 | 468.7121 | 8 |
| 27 | 2871.1897 | **1436.0985** | 2854.1632 | 1427.5852 | 2853.1792 | 1427.0932 | S | **839.4006** | **420.2039** | 822.3741 | 411.6907 | 821.3900 | 411.1987 | 7 |
| 28 | 2985.2327 | 1493.1200 | 2968.2061 | 1484.6067 | 2967.2221 | 1484.1147 | N | 752.3686 | 376.6879 | 735.3420 | 368.1747 | 734.3580 | 367.6826 | 6 |
| 29 | 3042.2541 | 1521.6307 | 3025.2276 | 1513.1174 | 3024.2436 | 1512.6254 | G | 638.3257 | 319.6665 | 621.2991 | 311.1532 | 620.3151 | 310.6612 | 5 |
| 30 | 3198.3553 | 1599.6813 | 3181.3287 | 1591.1680 | 3180.3447 | 1590.6760 | R | 581.3042 | 291.1557 | 564.2776 | 282.6425 | 563.2936 | 282.1504 | 4 |
| 31 | 3361.4186 | 1681.2129 | 3344.3920 | 1672.6997 | 3343.4080 | 1672.2076 | Y | 425.2031 | 213.1052 | 408.1765 | 204.5919 | 407.1925 | 204.0999 | 3 |
| 32 | 3476.4455 | **1738.7264** | 3459.4190 | 1730.2131 | 3458.4350 | 1729.7211 | D | 262.1397 | 131.5735 | 245.1132 | 123.0602 | 244.1292 | 122.5682 | 2 |

| | | | | | | | | | | | | | |
|---|---|---|---|---|---|---|---|---|---|---|---|---|---|
| 33 | | | | | | | K | 147.1128 | 74.0600 | 130.0863 | 65.5468 | | 1 |

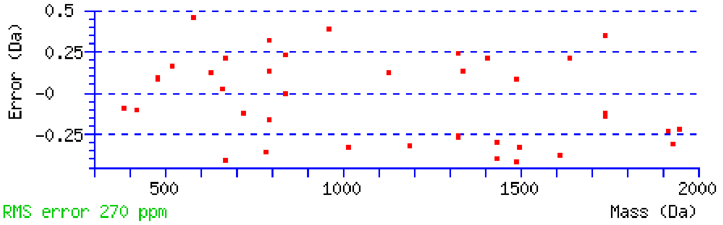


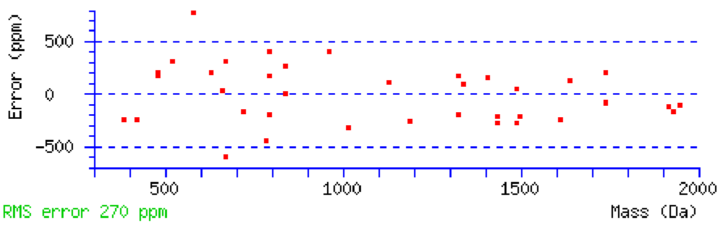


**All matches to this query**

| Score | Mr(calc): | Delta | Sequence |
|---|---|---|---|
| 91.6 | 3621.5438 | 0.0161 | ETIGPQQGCGGYLTEDNQSFVSPDSDSNGRYDK |
| 88.5 | 3621.5438 | 0.0161 | ETIGPQQGCGGYLTEDNQSFVSPDSDSNGRYDK |
| 85.7 | 3620.5598 | 1.0001 | ETIGPQQGCGGYLTEDNQSFVSPDSDSNGRYDK |
| 9.1 | 3621.5791 | -0.0192 | DLTRLQPQNLVGTSTVNSDSPASDPTVFHKR |
| 6.8 | 3621.5791 | -0.0192 | DLTRLQPQNLVGTSTVNSDSPASDPTVFHKR |
| 6.1 | 3620.5823 | 0.9776 | DWYEQASPAPLLVNPEALEPSLSVNGSDGMFK |
| 4.0 | 3619.5388 | 2.0211 | LYSAVKEDELMEFSDLESDSEEKPCIKPR |
| 3.8 | 3619.5651 | 1.9948 | QNPAEGLQTLGAQMQGGFGGGNSQLPKTDGGSETK |
| 3.1 | 3620.5674 | 0.9925 | WTGTGERGYMISAGARATGTAQTFIHIPSTAR |
| 3.1 | 3620.5674 | 0.9925 | WTGTGERGYMISAGARATGTAQTFIHIPSTAR |

---

Spectrum No: 127; Query: 1780; Rank: 1

---

## Peptide View

MS/MS Fragmentation of **NGSLILVQDSDSDDNSLLSK**
Found in **IPI00363032**, Tax_Id=10116 Gene_Symbol=Usp40 similar to Ubiquitin carboxyl-terminal hydrolase 40

Match to Query 1780: 2120.016628 from(1061.015590,2+)
Title: 100101RatKid_NS_deglyco_20.3794.3794.2.dta
Data file K:\NewmanPaper\Piliang\3SubProteomes\Piliang3SP\mgf5ppm\ERLIC_3SubProteomes5ppm.mgf

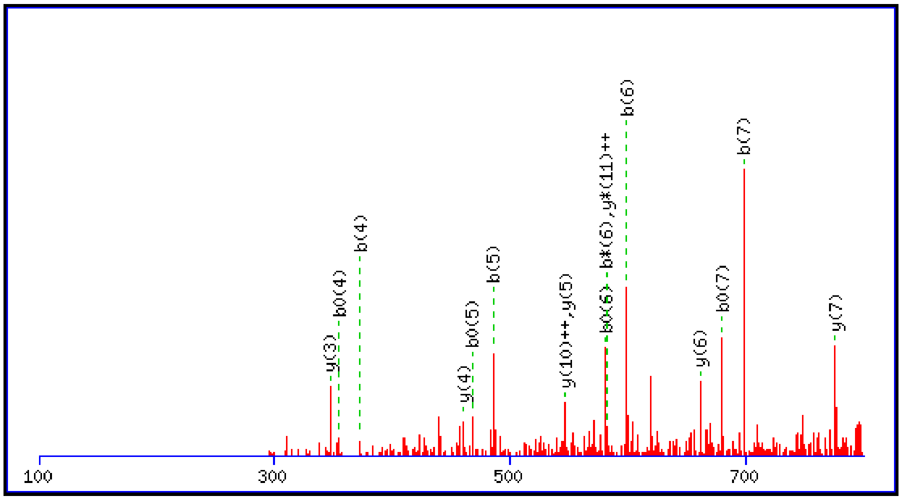

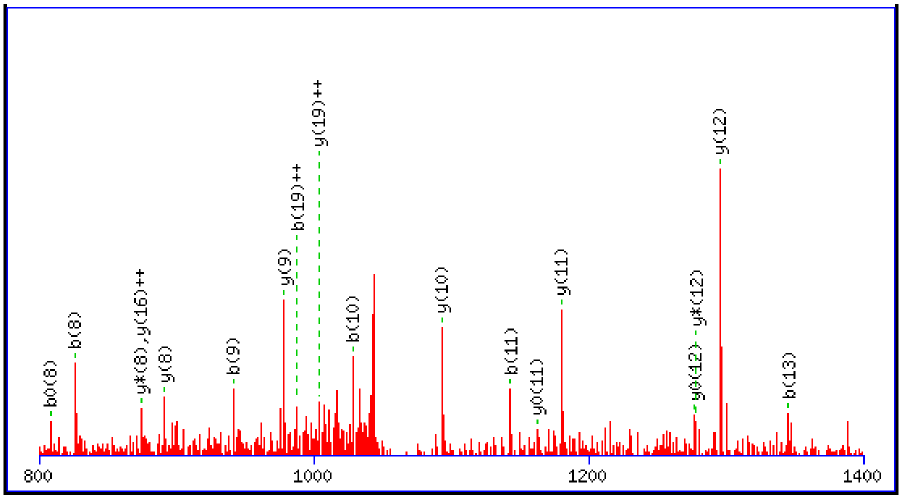


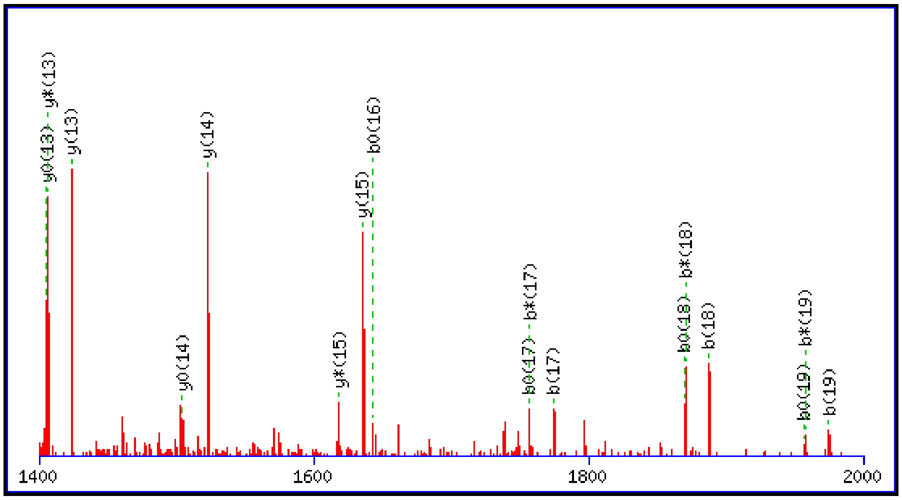


**Monoisotopic mass of neutral peptide Mr(calc):** 2120.0121
**Fixed modifications:** Carbamidomethyl (C)
**Variable modifications:**
**N1** : Deamidated_N (N)
**Ions Score:** 92 **Expect:** 1.7e-007
**Matches (Bold Red):** 51/222 fragment ions using 78 most intense peaks

| # | b | b++ | b* | b*++ | b0 | b0++ | Seq. | y | y++ | y* | y*++ | y0 | y0++ | # |
|---|---|---|---|---|---|---|---|---|---|---|---|---|---|---|
| 1 | 116.0342 | 58.5207 | 99.0077 | 50.0075 | | | N | | | | | | | 20 |
| 2 | 173.0557 | 87.0315 | 156.0291 | 78.5182 | | | G | 2005.9924 | **1003.4998** | 1988.9659 | 994.9866 | 1987.9819 | 994.4946 | 19 |
| 3 | 260.0877 | 130.5475 | 243.0612 | 122.0342 | 242.0771 | 121.5422 | S | 1948.9710 | 974.9891 | 1931.9444 | 966.4758 | 1930.9604 | 965.9838 | 18 |
| 4 | **373.1718** | 187.0895 | 356.1452 | 178.5762 | **355.1612** | 178.0842 | L | 1861.9389 | 931.4731 | 1844.9124 | 922.9598 | 1843.9284 | 922.4678 | 17 |
| 5 | **486.2558** | 243.6316 | 469.2293 | 235.1183 | **468.2453** | 234.6263 | I | 1748.8549 | **874.9311** | 1731.8283 | 866.4178 | 1730.8443 | 865.9258 | 16 |
| 6 | **599.3399** | 300.1736 | **582.3133** | 291.6603 | **581.3293** | 291.1683 | L | **1635.7708** | 818.3890 | **1618.7443** | 809.8758 | 1617.7602 | 809.3838 | 15 |
| 7 | **698.4083** | 349.7078 | 681.3818 | 341.1945 | **680.3977** | 340.7025 | V | **1522.6867** | 761.8470 | 1505.6602 | 753.3337 | **1504.6762** | 752.8417 | 14 |
| 8 | **826.4669** | 413.7371 | 809.4403 | 405.2238 | **808.4563** | 404.7318 | Q | **1423.6183** | 712.3128 | **1406.5918** | 703.7995 | **1405.6078** | 703.3075 | 13 |
| 9 | **941.4938** | 471.2506 | 924.4673 | 462.7373 | 923.4833 | 462.2453 | D | **1295.5597** | 648.2835 | **1278.5332** | 639.7702 | **1277.5492** | 639.2782 | 12 |
| 10 | **1028.5259** | 514.7666 | 1011.4993 | 506.2533 | 1010.5153 | 505.7613 | S | **1180.5328** | 590.7700 | 1163.5063 | **582.2568** | **1162.5222** | 581.7648 | 11 |
| 11 | **1143.5528** | 572.2800 | 1126.5263 | 563.7668 | 1125.5422 | 563.2748 | D | **1093.5008** | **547.2540** | 1076.4742 | 538.7407 | 1075.4902 | 538.2487 | 10 |
| 12 | 1230.5848 | 615.7961 | 1213.5583 | 607.2828 | 1212.5743 | 606.7908 | S | **978.4738** | 489.7406 | 961.4473 | 481.2273 | 960.4633 | 480.7353 | 9 |
| 13 | **1345.6118** | 673.3095 | 1328.5852 | 664.7963 | 1327.6012 | 664.3042 | D | **891.4418** | 446.2245 | **874.4153** | 437.7113 | 873.4312 | 437.2193 | 8 |
| 14 | 1460.6387 | 730.8230 | 1443.6122 | 722.3097 | 1442.6282 | 721.8177 | D | **776.4149** | 388.7111 | 759.3883 | 380.1978 | 758.4043 | 379.7058 | 7 |
| 15 | 1574.6816 | 787.8445 | 1557.6551 | 779.3312 | 1556.6711 | 778.8392 | N | **661.3879** | 331.1976 | 644.3614 | 322.6843 | 643.3774 | 322.1923 | 6 |
| 16 | 1661.7137 | 831.3605 | 1644.6871 | 822.8472 | **1643.7031** | 822.3552 | S | **547.3450** | 274.1761 | 530.3184 | 265.6629 | 529.3344 | 265.1708 | 5 |
| 17 | **1774.7977** | 887.9025 | **1757.7712** | 879.3892 | **1756.7872** | 878.8972 | L | **460.3130** | 230.6601 | 443.2864 | 222.1468 | 442.3024 | 221.6548 | 4 |
| 18 | **1887.8818** | 944.4445 | **1870.8553** | 935.9313 | **1869.8712** | 935.4393 | L | **347.2289** | 174.1181 | 330.2023 | 165.6048 | 329.2183 | 165.1128 | 3 |

| | | | | | | | | | | | | | | |
|---|---|---|---|---|---|---|---|---|---|---|---|---|---|---|
| 19 | 1974.9138 | 987.9606 | 1957.8873 | 979.4473 | 1956.9033 | 978.9553 | S | 234.1448 | 117.5761 | 217.1183 | 109.0628 | 216.1343 | 108.5708 | 2 |
| 20 | | | | | | | K | 147.1128 | 74.0600 | 130.0863 | 65.5468 | | | 1 |

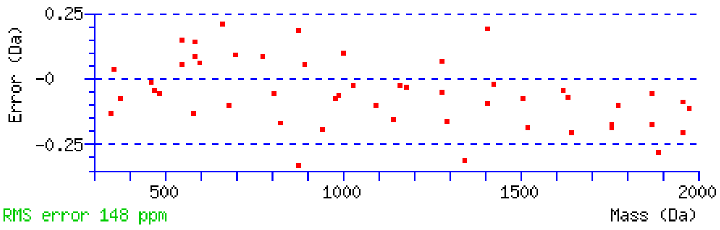


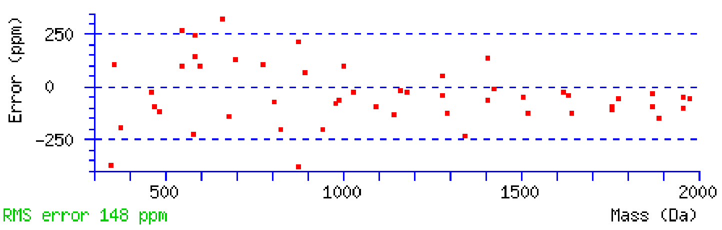


### All matches to this query

| Score | Mr(calc): | Delta | Sequence |
|---|---|---|---|
| 91.5 | 2120.0121 | 0.0046 | NGSLILVQDSDSDDNSLLSK |
| 52.0 | 2119.0281 | 0.9886 | NGSLILVQDSDSDDNSLLSK |
| 25.3 | 2120.0121 | 0.0046 | NGSLILVQDSDSDDNSLLSK |
| 8.8 | 2119.9989 | 0.0177 | INRIEEEKISMASLLSK |
| 6.7 | 2118.0051 | 2.0116 | ALADIVIPQEYGITKAEK |
| 5.1 | 2120.0207 | -0.0041 | FKASLSENVLGSPKELLK |
| 3.8 | 2120.0191 | -0.0025 | ERPDTGVYVKDLSSFVTK |
| 3.8 | 2120.0191 | -0.0025 | ERPDTGVYVKDLSSFVTK |
| 2.8 | 2120.0282 | -0.0116 | MKLAQQYVMTSLQQEYK |
| 1.4 | 2120.0198 | -0.0031 | RLLTDDEVLAVTRHCSR |

---

Spectrum No: 128; Query: 2400; Rank: 1

---

## Peptide View

MS/MS Fragmentation of **NGTNLDWATAETLALGSLLAQGFNVR**
Found in **IPI00364925**, Tax_Id=10116 Gene_Symbol=Dhtkd1 Probable 2-oxoglutarate dehydrogenase E1 component DHKTD1, mitochondrial precursor

Match to Query 2400: 2732.376192 from(911.799340,3+)
Title: 091008RatKidney_NH4Format02_14.5093.5093.3.dta
Data file K:\NewmanPaper\Piliang\3SubProteomes\Piliang3SP\mgf5ppm\ERLIC_3SubProteomes5ppm.mgf

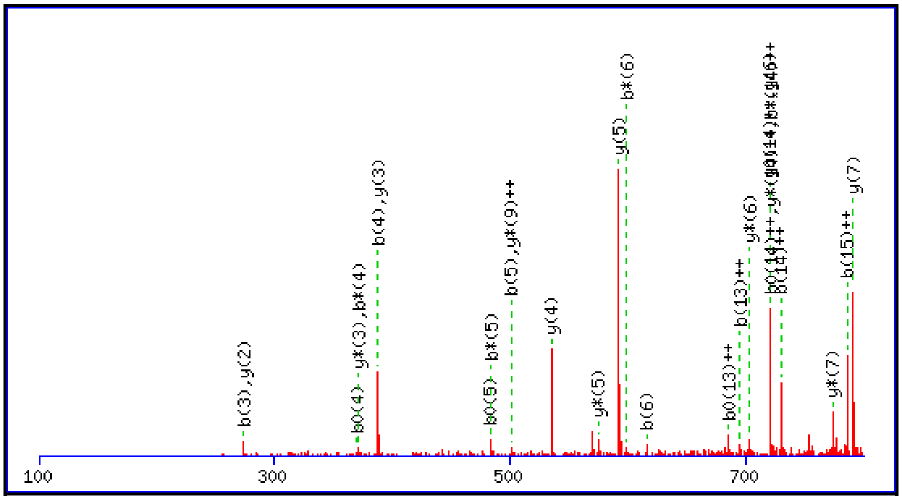

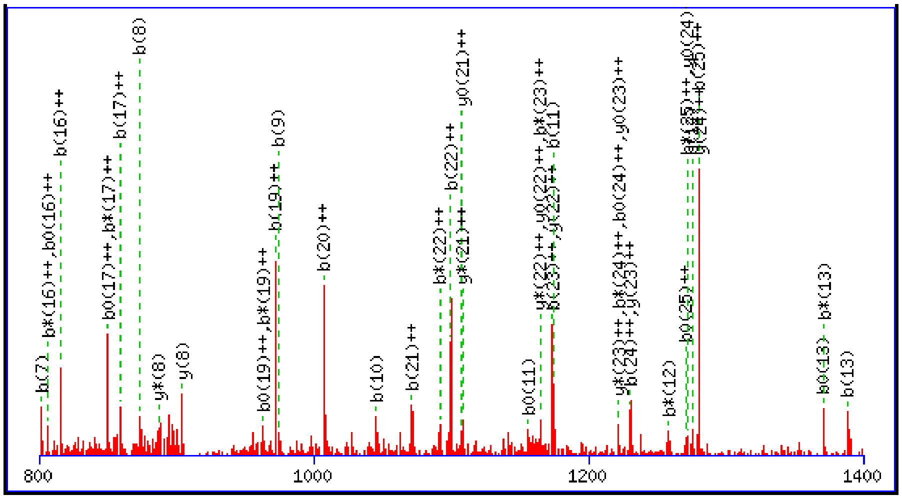


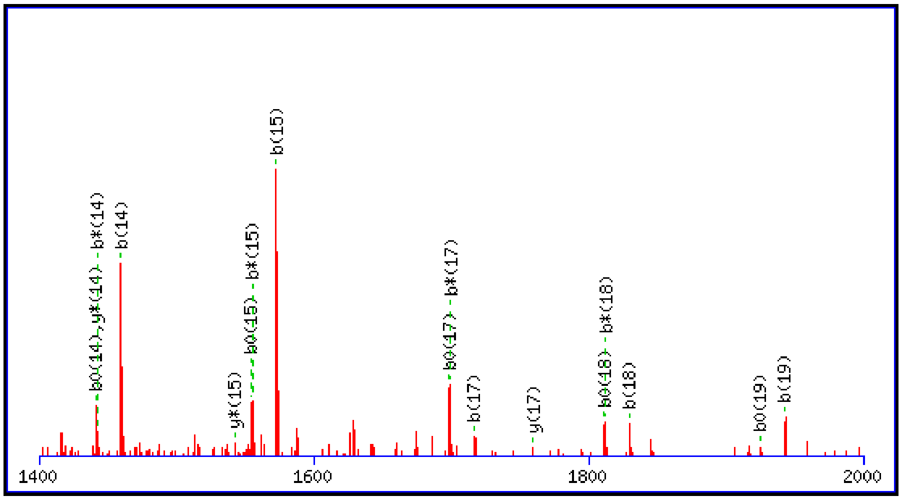


**Monoisotopic mass of neutral peptide Mr(calc):** 2732.3769
**Fixed modifications:** Carbamidomethyl (C)
**Variable modifications:**
**N1** : Deamidated_N (N)
**Ions Score:** 91 **Expect:** 1.3e-007
**Matches (Bold Red):** 89/278 fragment ions using 144 most intense peaks

| # | b | $b^{++}$ | b* | $b^{*++}$ | $b^0$ | $b^{0++}$ | Seq. | y | $y^{++}$ | y* | $y^{*++}$ | $y^0$ | $y^{0++}$ | # |
|---|---|---|---|---|---|---|---|---|---|---|---|---|---|---|
| 1 | 116.0342 | 58.5207 | 99.0077 | 50.0075 | | | N | | | | | | | 26 |
| 2 | 173.0557 | 87.0315 | 156.0291 | 78.5182 | | | G | 2618.3573 | 1309.6823 | 2601.3307 | 1301.1690 | 2600.3467 | 1300.6770 | 25 |
| 3 | **274.1034** | 137.5553 | 257.0768 | 129.0420 | 256.0928 | 128.5500 | T | 2561.3358 | **1281.1716** | 2544.3093 | 1272.6583 | 2543.3253 | **1272.1663** | 24 |
| 4 | **388.1463** | 194.5768 | **371.1197** | 186.0635 | **370.1357** | 185.5715 | N | 2460.2882 | **1230.6477** | 2443.2616 | **1222.1344** | 2442.2776 | **1221.6424** | 23 |
| 5 | **501.2303** | 251.1188 | **484.2038** | 242.6055 | **483.2198** | 242.1135 | L | 2346.2452 | **1173.6262** | 2329.2187 | **1165.1130** | 2328.2347 | **1164.6210** | 22 |
| 6 | **616.2573** | 308.6323 | **599.2307** | 300.1190 | 598.2467 | 299.6270 | D | 2233.1612 | 1117.0842 | 2216.1346 | **1108.5709** | 2215.1506 | **1108.0789** | 21 |
| 7 | **802.3366** | 401.6719 | 785.3101 | 393.1587 | 784.3260 | 392.6667 | W | 2118.1342 | 1059.5707 | 2101.1077 | 1051.0575 | 2100.1237 | 1050.5655 | 20 |
| 8 | **873.3737** | 437.1905 | 856.3472 | 428.6772 | 855.3632 | 428.1852 | A | 1932.0549 | 966.5311 | 1915.0284 | 958.0178 | 1914.0443 | 957.5258 | 19 |
| 9 | **974.4214** | 487.7143 | 957.3948 | 479.2011 | 956.4108 | 478.7091 | T | 1861.0178 | 931.0125 | 1843.9912 | 922.4993 | 1843.0072 | 922.0073 | 18 |
| 10 | **1045.4585** | 523.2329 | 1028.4320 | 514.7196 | 1027.4479 | 514.2276 | A | **1759.9701** | 880.4887 | 1742.9436 | 871.9754 | 1741.9595 | 871.4834 | 17 |
| 11 | **1174.5011** | 587.7542 | 1157.4746 | 579.2409 | **1156.4905** | 578.7489 | E | 1688.9330 | 844.9701 | 1671.9064 | 836.4569 | 1670.9224 | 835.9649 | 16 |
| 12 | **1275.5488** | 638.2780 | **1258.5222** | 629.7648 | 1257.5382 | 629.2727 | T | 1559.8904 | 780.4488 | **1542.8639** | 771.9356 | 1541.8798 | 771.4436 | 15 |
| 13 | **1388.6328** | **694.8201** | **1371.6063** | 686.3068 | **1370.6223** | **685.8148** | L | 1458.8427 | 729.9250 | **1441.8162** | **721.4117** | 1440.8322 | **720.9197** | 14 |
| 14 | **1459.6700** | **730.3386** | **1442.6434** | **721.8253** | **1441.6594** | **721.3333** | A | 1345.7587 | 673.3830 | 1328.7321 | 664.8697 | 1327.7481 | 664.3777 | 13 |
| 15 | **1572.7540** | **786.8806** | **1555.7275** | 778.3674 | **1554.7435** | 777.8754 | L | 1274.7215 | 637.8644 | 1257.6950 | 629.3511 | 1256.7110 | 628.8591 | 12 |
| 16 | 1629.7755 | **815.3914** | 1612.7489 | **806.8781** | 1611.7649 | **806.3861** | G | 1161.6375 | 581.3224 | 1144.6109 | 572.8091 | 1143.6269 | 572.3171 | 11 |
| 17 | **1716.8075** | **858.9074** | **1699.7810** | **850.3941** | **1698.7969** | **849.9021** | S | 1104.6160 | 552.8116 | 1087.5895 | 544.2984 | 1086.6055 | 543.8064 | 10 |
| 18 | **1829.8916** | 915.4494 | **1812.8650** | 906.9362 | **1811.8810** | 906.4441 | L | 1017.5840 | 509.2956 | 1000.5574 | **500.7824** | | | 9 |
| | | | | | | | | | | | | | | |

| | | | | | | | | | | | | | | |
|---|---|---|---|---|---|---|---|---|---|---|---|---|---|---|
| **19** | **1942.9756** | **971.9915** | 1925.9491 | **963.4782** | **1924.9651** | **962.9862** | **L** | **904.4999** | 452.7536 | **887.4734** | 444.2403 | | | **8** |
| **20** | 2014.0128 | **1007.5100** | 1996.9862 | 998.9967 | 1996.0022 | 998.5047 | **A** | **791.4159** | 396.2116 | **774.3893** | 387.6983 | | | **7** |
| **21** | 2142.0713 | **1071.5393** | 2125.0448 | 1063.0260 | 2124.0608 | 1062.5340 | **Q** | **720.3787** | 360.6930 | **703.3522** | 352.1797 | | | **6** |
| **22** | 2199.0928 | **1100.0500** | 2182.0662 | **1091.5368** | 2181.0822 | 1091.0448 | **G** | **592.3202** | 296.6637 | **575.2936** | 288.1504 | | | **5** |
| **23** | 2346.1612 | **1173.5842** | 2329.1347 | **1165.0710** | 2328.1506 | 1164.5790 | **F** | **535.2987** | 268.1530 | 518.2722 | 259.6397 | | | **4** |
| **24** | 2460.2041 | **1230.6057** | 2443.1776 | **1222.0924** | 2442.1936 | **1221.6004** | **N** | **388.2303** | 194.6188 | **371.2037** | 186.1055 | | | **3** |
| **25** | 2559.2726 | **1280.1399** | 2542.2460 | **1271.6266** | 2541.2620 | **1271.1346** | **V** | **274.1874** | 137.5973 | 257.1608 | 129.0840 | | | **2** |
| **26** | | | | | | | **R** | 175.1190 | 88.0631 | 158.0924 | 79.5498 | | | **1** |

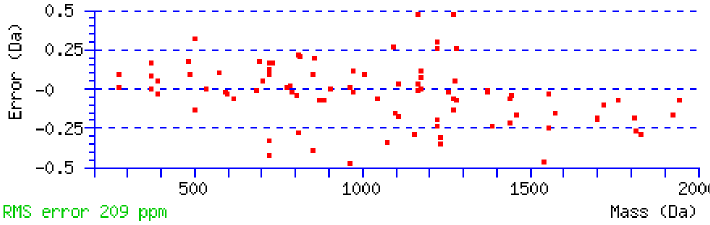


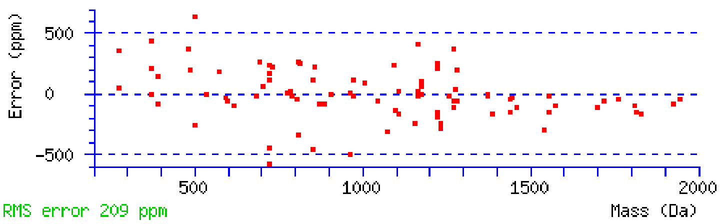


### All matches to this query

| Score | Mr(calc): | Delta | Sequence |
|---|---|---|---|
| 91.5 | 2732.3769 | -0.0007 | NGTNLDWATAETLALGSLLAQGFNVR |
| 83.9 | 2732.3769 | -0.0007 | NGTNLDWATAETLALGSLLAQGFNVR |
| 24.9 | 2731.3929 | 0.9833 | NGTNLDWATAETLALGSLLAQGFNVR |
| 9.7 | 2732.3769 | -0.0007 | NGTNLDWATAETLALGSLLAQGFNVR |
| 5.5 | 2732.3981 | -0.0219 | SRSTNVKQFIAPEAGEGVSLQLGNTK |
| 3.9 | 2732.3816 | -0.0054 | GESLWLNLRNRISQQMDGLAPYR |
| 2.9 | 2730.3653 | 2.0109 | YSHFPDFSYSASSKIIISDVPSRK |
| 2.2 | 2732.3938 | -0.0176 | LWASAFGGEIKSIAAKYSGSQLLQK |
| 2.2 | 2732.3938 | -0.0176 | LWASAFGGEIKSIAAKYSGSQLLQK |
| 2.1 | 2732.3608 | 0.0154 | ASLSALTMNTIVFNKLGGAVIFDDR |

---

Spectrum No: 129; Query: 1279; Rank: 1

---

## Peptide View

MS/MS Fragmentation of **NETLGGTCLNVGCIPSK**
Found in **IPI00365545**, Tax_Id=10116 Gene_Symbol=Dld Dihydrolipoyl dehydrogenase, mitochondrial precursor

Match to Query 1279: 1819.853708 from(910.934130,2+)
Title: 100101RatKid_NS_deglyco_12.3268.3268.2.dta
Data file K:\NewmanPaper\Piliang\3SubProteomes\Piliang3SP\mgf5ppm\ERLIC_3SubProteomes5ppm.mgf

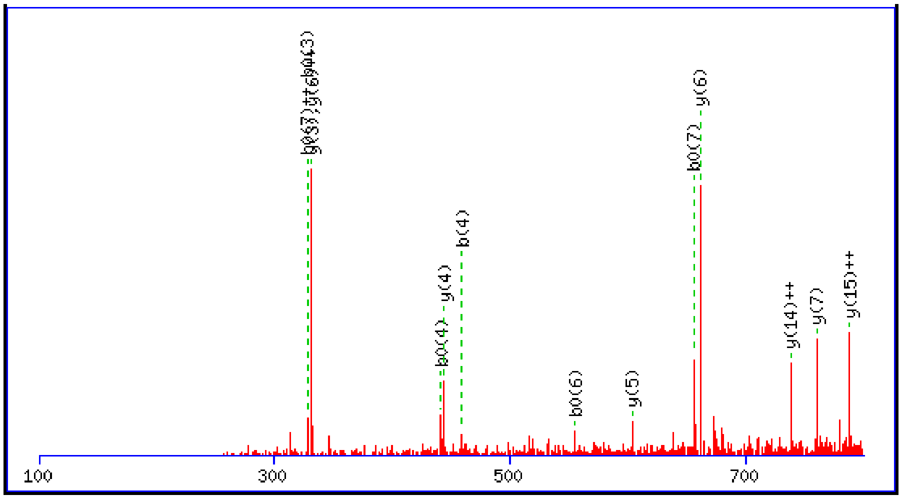


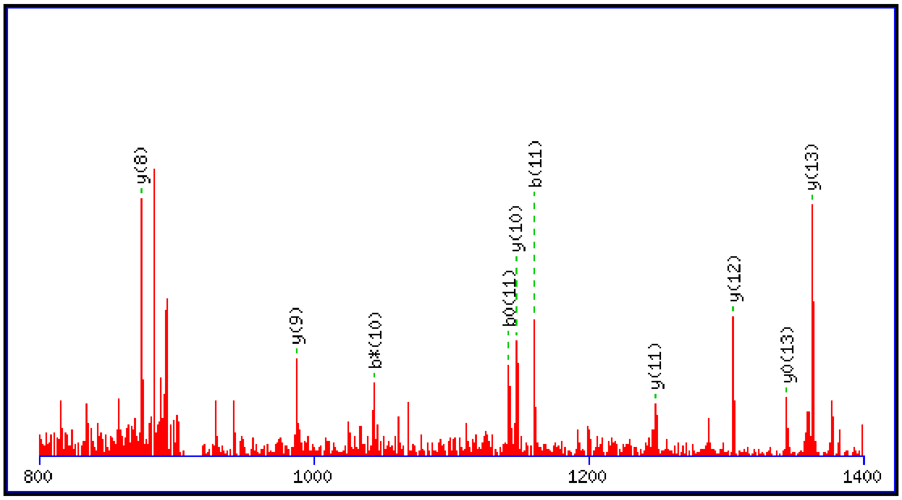


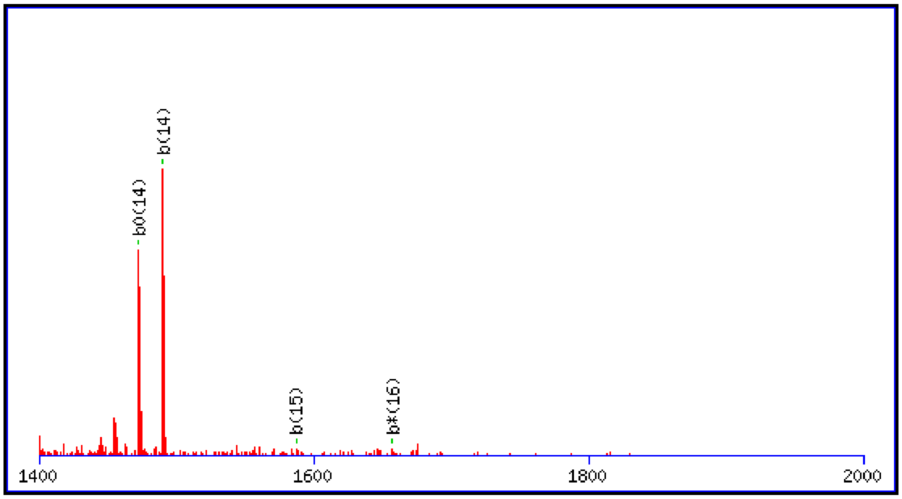


**Monoisotopic mass of neutral peptide Mr(calc):** 1819.8444
**Fixed modifications:** Carbamidomethyl (C)
**Variable modifications:**
**N1** : Deamidated_N (N)
**Ions Score:** 91 **Expect:** 1.6e-007
**Matches (Bold Red):** 28/188 fragment ions using 43 most intense peaks

| # | b | $b^{++}$ | b* | $b^{*++}$ | $b^{0}$ | $b^{0++}$ | Seq. | y | $y^{++}$ | y* | $y^{*++}$ | $y^{0}$ | $y^{0++}$ | # |
|---|---|---|---|---|---|---|---|---|---|---|---|---|---|---|
| 1 | 116.0342 | 58.5207 | 99.0077 | 50.0075 | | | N | | | | | | | 17 |
| 2 | 245.0768 | 123.0420 | 228.0503 | 114.5288 | 227.0662 | 114.0368 | E | 1705.8248 | 853.4160 | 1688.7982 | 844.9027 | 1687.8142 | 844.4107 | 16 |
| 3 | 346.1245 | 173.5659 | 329.0979 | 165.0526 | **328.1139** | 164.5606 | T | 1576.7822 | **788.8947** | 1559.7556 | 780.3815 | 1558.7716 | 779.8894 | 15 |
| 4 | **459.2085** | 230.1079 | 442.1820 | 221.5946 | **441.1980** | 221.1026 | L | 1475.7345 | **738.3709** | 1458.7079 | 729.8576 | 1457.7239 | 729.3656 | 14 |
| 5 | 516.2300 | 258.6186 | 499.2035 | 250.1054 | 498.2194 | 249.6134 | G | **1362.6504** | 681.8289 | 1345.6239 | 673.3156 | **1344.6399** | 672.8236 | 13 |

| | | | | | | | | | | | | | | |
|---|---|---|---|---|---|---|---|---|---|---|---|---|---|---|
| **6** | 573.2515 | 287.1294 | 556.2249 | 278.6161 | **555.2409** | 278.1241 | **G** | **1305.6290** | 653.3181 | 1288.6024 | 644.8048 | 1287.6184 | 644.3128 | **12** |
| **7** | 674.2992 | 337.6532 | 657.2726 | 329.1399 | **656.2886** | **328.6479** | **T** | **1248.6075** | 624.8074 | 1231.5810 | 616.2941 | 1230.5969 | 615.8021 | **11** |
| **8** | 834.3298 | 417.6685 | 817.3033 | 409.1553 | 816.3192 | 408.6633 | **C** | **1147.5598** | 574.2836 | 1130.5333 | 565.7703 | 1129.5493 | 565.2783 | **10** |
| **9** | 947.4139 | 474.2106 | 930.3873 | 465.6973 | 929.4033 | 465.2053 | **L** | **987.5292** | 494.2682 | 970.5026 | 485.7550 | 969.5186 | 485.2629 | **9** |
| **10** | 1061.4568 | 531.2320 | **1044.4302** | 522.7188 | 1043.4462 | 522.2268 | **N** | **874.4451** | 437.7262 | 857.4186 | 429.2129 | 856.4345 | 428.7209 | **8** |
| **11** | **1160.5252** | 580.7662 | 1143.4987 | 572.2530 | **1142.5146** | 571.7610 | **V** | **760.4022** | 380.7047 | 743.3756 | 372.1915 | 742.3916 | 371.6994 | **7** |
| **12** | 1217.5467 | 609.2770 | 1200.5201 | 600.7637 | 1199.5361 | 600.2717 | **G** | **661.3338** | **331.1705** | 644.3072 | 322.6573 | 643.3232 | 322.1652 | **6** |
| **13** | 1377.5773 | 689.2923 | 1360.5508 | 680.7790 | 1359.5668 | 680.2870 | **C** | **604.3123** | 302.6598 | 587.2858 | 294.1465 | 586.3017 | 293.6545 | **5** |
| **14** | **1490.6614** | 745.8343 | 1473.6348 | 737.3211 | **1472.6508** | 736.8290 | **I** | **444.2817** | 222.6445 | 427.2551 | 214.1312 | 426.2711 | 213.6392 | **4** |
| **15** | **1587.7142** | 794.3607 | 1570.6876 | 785.8474 | 1569.7036 | 785.3554 | **P** | **331.1976** | 166.1024 | 314.1710 | 157.5892 | 313.1870 | 157.0972 | **3** |
| **16** | 1674.7462 | 837.8767 | **1657.7196** | 829.3635 | 1656.7356 | 828.8714 | **S** | 234.1448 | 117.5761 | 217.1183 | 109.0628 | 216.1343 | 108.5708 | **2** |
| **17** | | | | | | | **K** | 147.1128 | 74.0600 | 130.0863 | 65.5468 | | | **1** |

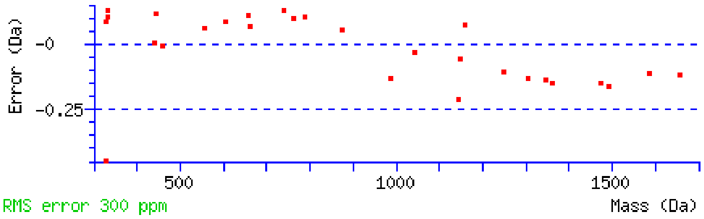


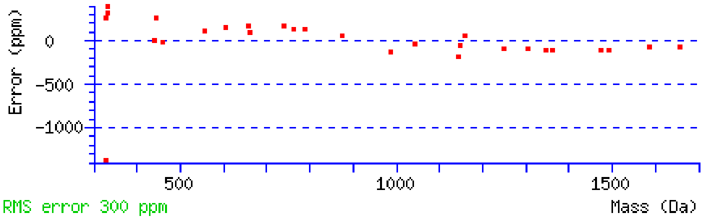


### All matches to this query

| Score | Mr(calc): | Delta | Sequence |
|---|---|---|---|
| 91.3 | 1819.8444 | 0.0093 | NETLGGTCLNVGCIPSK |
| 62.4 | 1818.8604 | 0.9933 | NETLGGTCLNVGCIPSK |
| 47.3 | 1819.8444 | 0.0093 | NETLGGTCLNVGCIPSK |
| 8.5 | 1819.8594 | -0.0057 | ATSSATTLASTPKLRR |
| 8.1 | 1819.8651 | -0.0114 | SKMYLDLLSQSQRR |
| 8.1 | 1819.8651 | -0.0114 | SKMYLDLLSQSQRR |
| 5.8 | 1819.8687 | -0.0150 | NEITTLNEEDSISNLK |
| 5.1 | 1819.8594 | -0.0057 | ATSSATTLASTPKLRR |
| 5.0 | 1819.8594 | -0.0057 | ATSSATTLASTPKLRR |
| 4.7 | 1817.8325 | 2.0212 | NLTLESRVQELQTK |

---

Spectrum No: 130; Query: 1490; Rank: 1

---

## Peptide View

MS/MS Fragmentation of **LQVELDSVTGLLNQSDSK**
Found in **IPI00209113**, Tax_Id=10116 Gene_Symbol=Myh9 Myosin-9

Match to Query 1490: 1945.981668 from(973.998110,2+)
Title: 100101RatKid_NS_deglyco_14.4576.4576.2.dta
Data file K:\NewmanPaper\Piliang\3SubProteomes\Piliang3SP\mgf5ppm\ERLIC_3SubProteomes5ppm.mgf

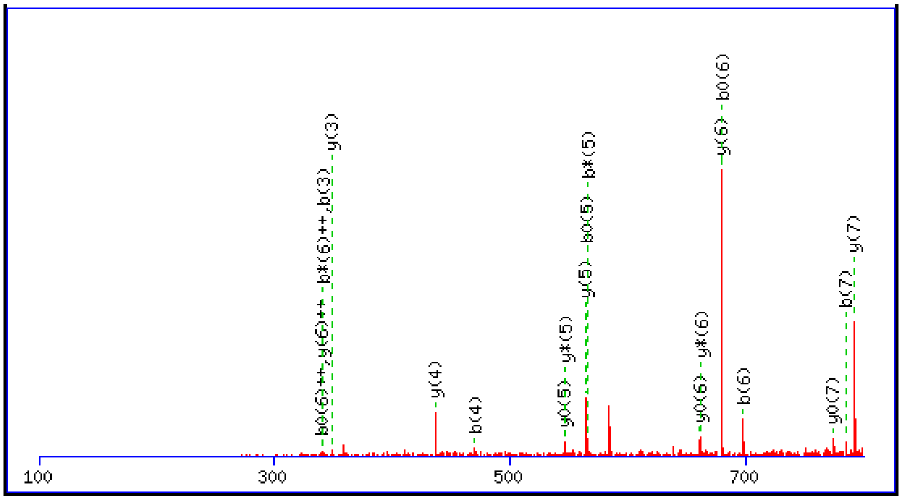


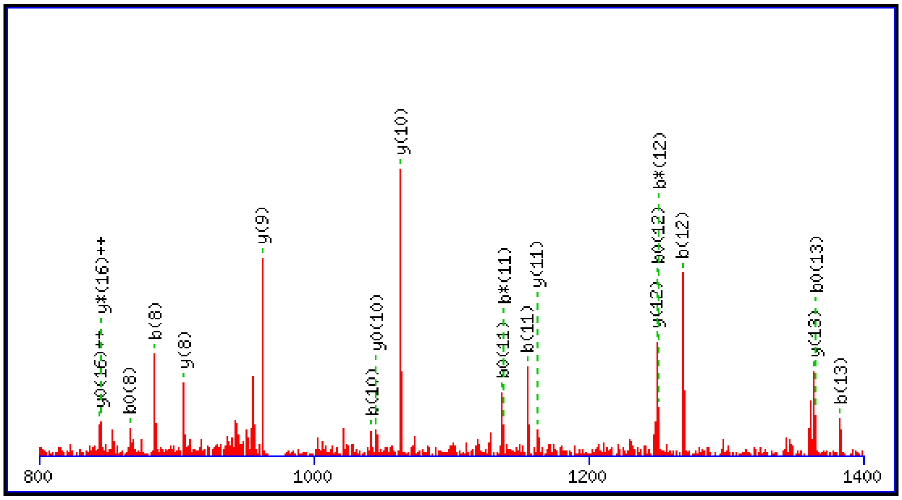


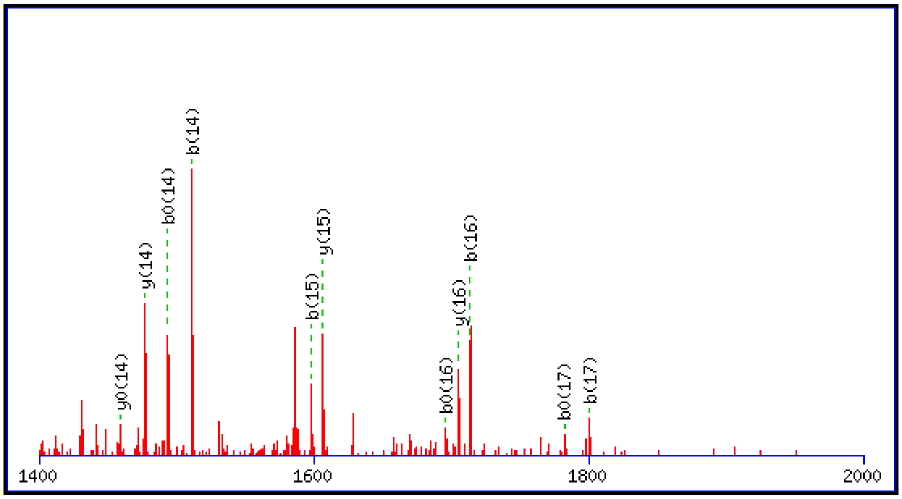


**Monoisotopic mass of neutral peptide Mr(calc):** 1945.9844
**Fixed modifications:** Carbamidomethyl (C)
**Variable modifications:**
**N13** : Deamidated_N (N)
**Ions Score:** 91 **Expect:** 1.5e-007
**Matches (Bold Red):** 51/194 fragment ions using 87 most intense peaks

| # | b | $b^{++}$ | b* | $b^{*++}$ | $b^0$ | $b^{0++}$ | Seq. | y | $y^{++}$ | y* | $y^{*++}$ | $y^0$ | $y^{0++}$ | # |
|---|---|---|---|---|---|---|---|---|---|---|---|---|---|---|
| 1 | 114.0913 | 57.5493 | | | | | L | | | | | | | 18 |
| 2 | 242.1499 | 121.5786 | 225.1234 | 113.0653 | | | Q | 1833.9076 | 917.4574 | 1816.8811 | 908.9442 | 1815.8971 | 908.4522 | 17 |
| 3 | **341.2183** | 171.1128 | 324.1918 | 162.5995 | | | V | **1705.8490** | 853.4282 | 1688.8225 | **844.9149** | 1687.8385 | **844.4229** | 16 |
| 4 | **470.2609** | 235.6341 | 453.2344 | 227.1208 | 452.2504 | 226.6288 | E | **1606.7806** | 803.8940 | 1589.7541 | 795.3807 | 1588.7701 | 794.8887 | 15 |
| 5 | 583.3450 | 292.1761 | **566.3184** | 283.6629 | **565.3344** | 283.1709 | L | **1477.7380** | 739.3727 | 1460.7115 | 730.8594 | **1459.7275** | 730.3674 | 14 |

| | | | | | | | | | | | | | |
|---|---|---|---|---|---|---|---|---|---|---|---|---|---|
| **6** | **698.3719** | 349.6896 | 681.3454 | **341.1763** | **680.3614** | **340.6843** | **D** | **1364.6540** | 682.8306 | 1347.6274 | 674.3173 | 1346.6434 | 673.8253 | **13** |
| **7** | **785.4040** | 393.2056 | 768.3774 | 384.6923 | 767.3934 | 384.2003 | **S** | **1249.6270** | 625.3172 | 1232.6005 | 616.8039 | 1231.6165 | 616.3119 | **12** |
| **8** | **884.4724** | 442.7398 | 867.4458 | 434.2266 | **866.4618** | 433.7345 | **V** | **1162.5950** | 581.8011 | 1145.5685 | 573.2879 | 1144.5844 | 572.7959 | **11** |
| **9** | 985.5201 | 493.2637 | 968.4935 | 484.7504 | 967.5095 | 484.2584 | **T** | **1063.5266** | 532.2669 | 1046.5000 | 523.7537 | **1045.5160** | 523.2616 | **10** |
| **10** | **1042.5415** | 521.7744 | 1025.5150 | 513.2611 | 1024.5310 | 512.7691 | **G** | **962.4789** | 481.7431 | 945.4524 | 473.2298 | 944.4683 | 472.7378 | **9** |
| **11** | **1155.6256** | 578.3164 | **1138.5990** | 569.8032 | **1137.6150** | 569.3111 | **L** | **905.4574** | 453.2324 | 888.4309 | 444.7191 | 887.4469 | 444.2271 | **8** |
| **12** | **1268.7096** | 634.8585 | **1251.6831** | 626.3452 | **1250.6991** | 625.8532 | **L** | **792.3734** | 396.6903 | 775.3468 | 388.1771 | **774.3628** | 387.6850 | **7** |
| **13** | **1383.7366** | 692.3719 | 1366.7100 | 683.8587 | **1365.7260** | 683.3666 | **N** | **679.2893** | **340.1483** | **662.2628** | 331.6350 | **661.2788** | 331.1430 | **6** |
| **14** | **1511.7952** | 756.4012 | 1494.7686 | 747.8879 | **1493.7846** | 747.3959 | **Q** | **564.2624** | 282.6348 | **547.2358** | 274.1216 | **546.2518** | 273.6295 | **5** |
| **15** | **1598.8272** | 799.9172 | 1581.8006 | 791.4040 | 1580.8166 | 790.9119 | **S** | **436.2038** | 218.6055 | 419.1773 | 210.0923 | 418.1932 | 209.6003 | **4** |
| **16** | **1713.8541** | 857.4307 | 1696.8276 | 848.9174 | **1695.8436** | 848.4254 | **D** | **349.1718** | 175.0895 | 332.1452 | 166.5763 | 331.1612 | 166.0842 | **3** |
| **17** | **1800.8862** | 900.9467 | 1783.8596 | 892.4334 | **1782.8756** | 891.9414 | **S** | 234.1448 | 117.5761 | 217.1183 | 109.0628 | 216.1343 | 108.5708 | **2** |
| **18** | | | | | | | **K** | 147.1128 | 74.0600 | 130.0863 | 65.5468 | | | **1** |

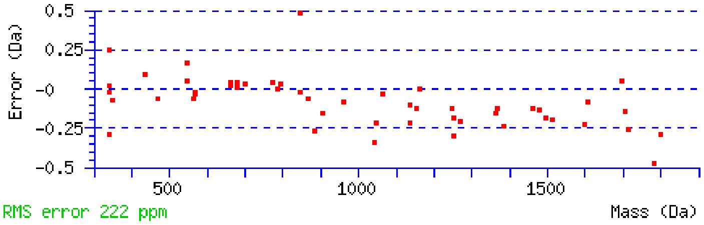


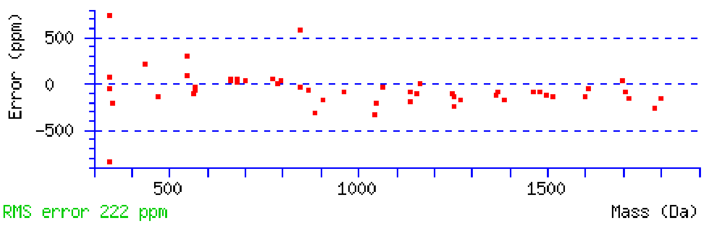


### All matches to this query

| Score | Mr(calc): | Delta | Sequence |
|---|---|---|---|
| 90.7 | 1945.9844 | -0.0027 | LQVELDSVTGLLNQSDSK |
| 0.6 | 1945.9666 | 0.0151 | ELQVLDGANIQSIMASEK |

Spectrum No: 131; Query: 1112; Rank: 1

## Peptide View

MS/MS Fragmentation of **NYTDDAIETDDLTIK**
Found in **IPI00215243**, Tax_Id=10116 Gene_Symbol=Psma7 Isoform RC6-IL of Proteasome subunit alpha type-7

Match to Query 1112: 1726.785648 from(864.400100,2+)
Title: 100101RatKid_NS_deglyco_20.3282.3282.2.dta
Data file K:\NewmanPaper\Piliang\3SubProteomes\Piliang3SP\mgf5ppm\ERLIC_3SubProteomes5ppm.mgf

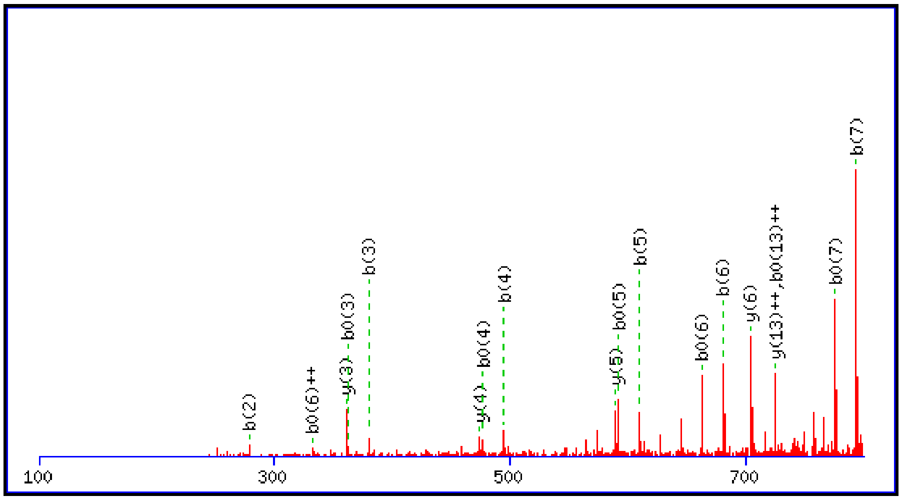

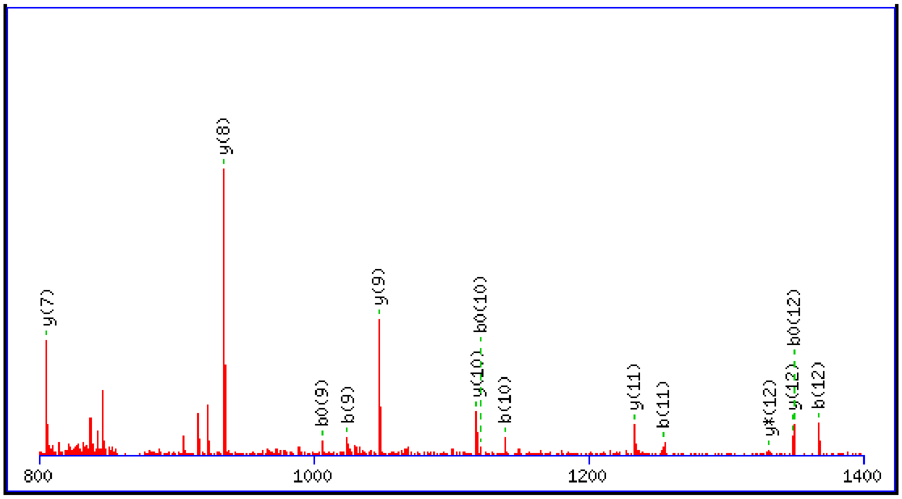


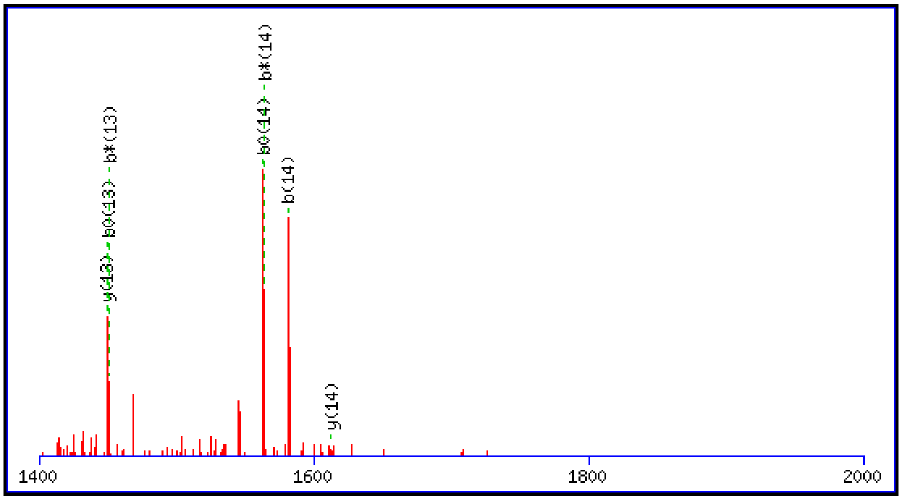


**Monoisotopic mass of neutral peptide Mr(calc):** 1726.7785
**Fixed modifications:** Carbamidomethyl (C)
**Variable modifications:**
**N1** : Deamidated_N (N)
**Ions Score:** 91 **Expect:** 1.8e-007
**Matches (Bold Red):** 39/160 fragment ions using 59 most intense peaks

| # | b | $b^{++}$ | b* | $b^{*++}$ | $b^0$ | $b^{0++}$ | Seq. | y | $y^{++}$ | y* | $y^{*++}$ | $y^0$ | $y^{0++}$ | # |
|---|---|---|---|---|---|---|---|---|---|---|---|---|---|---|
| 1 | 116.0342 | 58.5207 | 99.0077 | 50.0075 | | | N | | | | | | | 15 |
| 2 | **279.0975** | 140.0524 | 262.0710 | 131.5391 | | | Y | **1612.7588** | 806.8831 | 1595.7323 | 798.3698 | 1594.7483 | 797.8778 | 14 |
| 3 | **380.1452** | 190.5762 | 363.1187 | 182.0630 | **362.1347** | 181.5710 | T | **1449.6955** | **725.3514** | 1432.6690 | 716.8381 | 1431.6849 | 716.3461 | 13 |
| 4 | **495.1722** | 248.0897 | 478.1456 | 239.5764 | **477.1616** | 239.0844 | D | **1348.6478** | 674.8276 | **1331.6213** | 666.3143 | 1330.6373 | 665.8223 | 12 |
| 5 | **610.1991** | 305.6032 | 593.1726 | 297.0899 | **592.1885** | 296.5979 | D | **1233.6209** | 617.3141 | 1216.5943 | 608.8008 | 1215.6103 | 608.3088 | 11 |
| 6 | **681.2362** | 341.1217 | 664.2097 | 332.6085 | **663.2257** | **332.1165** | A | **1118.5939** | 559.8006 | 1101.5674 | 551.2873 | 1100.5834 | 550.7953 | 10 |
| 7 | **794.3203** | 397.6638 | 777.2937 | 389.1505 | **776.3097** | 388.6585 | I | **1047.5568** | 524.2821 | 1030.5303 | 515.7688 | 1029.5463 | 515.2768 | 9 |
| 8 | 923.3629 | 462.1851 | 906.3363 | 453.6718 | 905.3523 | 453.1798 | E | **934.4728** | 467.7400 | 917.4462 | 459.2267 | 916.4622 | 458.7347 | 8 |
| 9 | **1024.4106** | 512.7089 | 1007.3840 | 504.1956 | **1006.4000** | 503.7036 | T | **805.4302** | 403.2187 | 788.4036 | 394.7055 | 787.4196 | 394.2134 | 7 |
| 10 | **1139.4375** | 570.2224 | 1122.4110 | 561.7091 | **1121.4269** | 561.2171 | D | **704.3825** | 352.6949 | 687.3559 | 344.1816 | 686.3719 | 343.6896 | 6 |
| 11 | **1254.4644** | 627.7359 | 1237.4379 | 619.2226 | 1236.4539 | 618.7306 | D | **589.3556** | 295.1814 | 572.3290 | 286.6681 | 571.3450 | 286.1761 | 5 |
| 12 | **1367.5485** | 684.2779 | 1350.5220 | 675.7646 | **1349.5379** | 675.2726 | L | **474.3286** | 237.6679 | 457.3021 | 229.1547 | 456.3180 | 228.6627 | 4 |
| 13 | 1468.5962 | 734.8017 | **1451.5696** | 726.2885 | **1450.5856** | **725.7964** | T | **361.2445** | 181.1259 | 344.2180 | 172.6126 | 343.2340 | 172.1206 | 3 |
| 14 | **1581.6802** | 791.3438 | **1564.6537** | 782.8305 | **1563.6697** | 782.3385 | I | 260.1969 | 130.6021 | 243.1703 | 122.0888 | | | 2 |
| 15 | | | | | | | K | 147.1128 | 74.0600 | 130.0863 | 65.5468 | | | 1 |

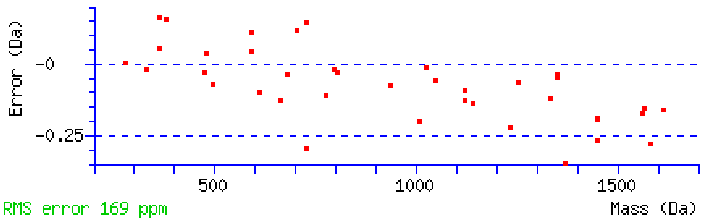


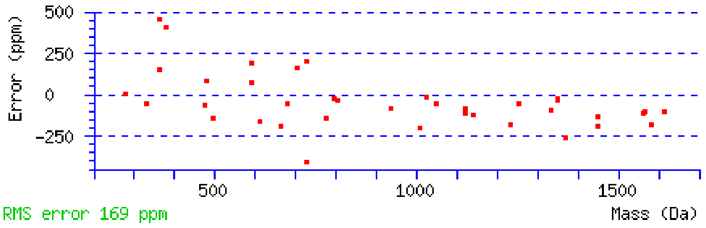


### All matches to this query

| Score | Mr(calc): | Delta | Sequence |
|---|---|---|---|
| 90.6 | 1726.7785 | 0.0072 | NYTDDAIETDDLTIK |
| 48.8 | 1725.7945 | 0.9912 | NYTDDAIETDDLTIK |
| 4.6 | 1726.7990 | -0.0134 | LPRRTHSNMLTIK |
| 4.4 | 1726.7709 | 0.0147 | RMGPGATAGGAEKSNVK |
| 4.3 | 1724.7706 | 2.0151 | QHQTPVYLGATAGMR |
| 3.6 | 1725.7723 | 1.0133 | AASTNHLDTFDLSVR |
| 1.2 | 1726.7749 | 0.0108 | APTYVKETLLKPK |
| 1.0 | 1725.7767 | 1.0090 | MEKAEESEANIFAEK |
| 0.6 | 1725.7949 | 0.9907 | NYXSKLSWIHPLK |
| 0.6 | 1725.7949 | 0.9907 | NYXSKLSWIHPLK |

---

Spectrum No: 132; Query: 1220; Rank: 1

---

## Peptide View

MS/MS Fragmentation of **EETLLYDNATSSVADR**
Found in **IPI00206434**, Tax_Id=10116 Gene_Symbol=Lnpep Leucyl-cystinyl aminopeptidase

Match to Query 1220: 1783.813088 from(892.913820,2+)
Title: 100101RatKid_NS_deglyco_26.2553.2553.2.dta
Data file K:\NewmanPaper\Piliang\3SubProteomes\Piliang3SP\mgf5ppm\ERLIC_3SubProteomes5ppm.mgf

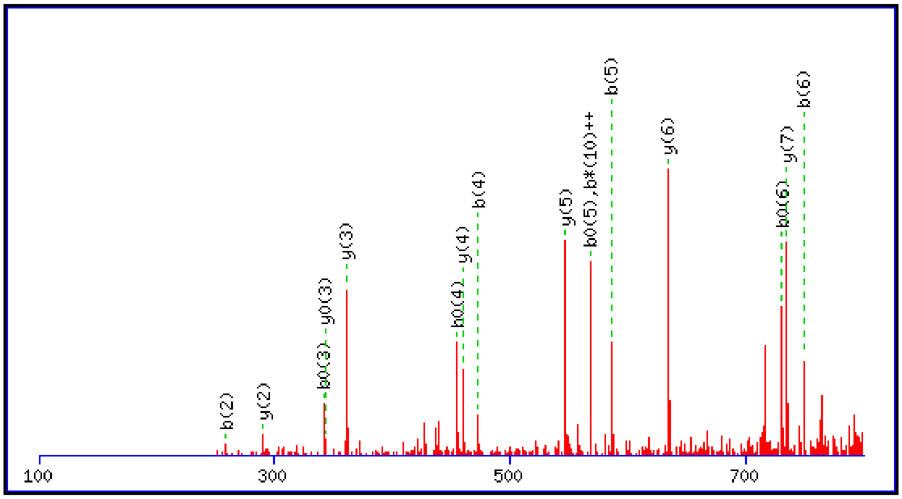

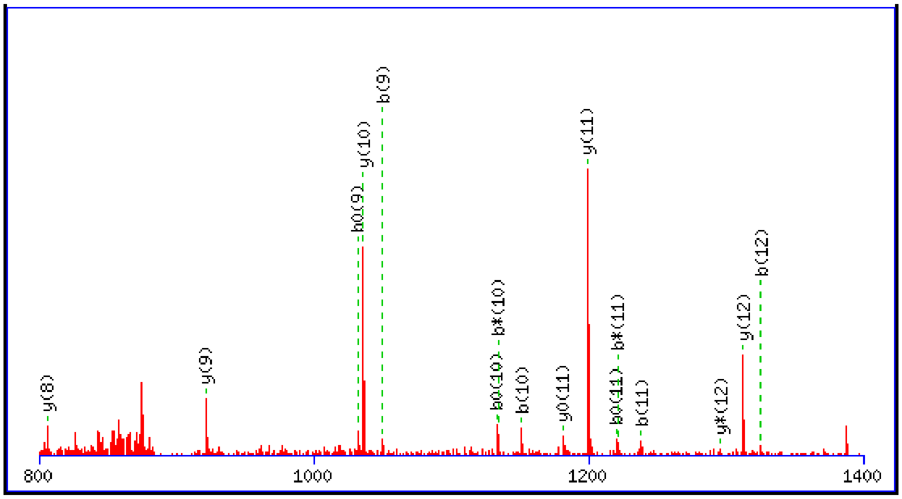


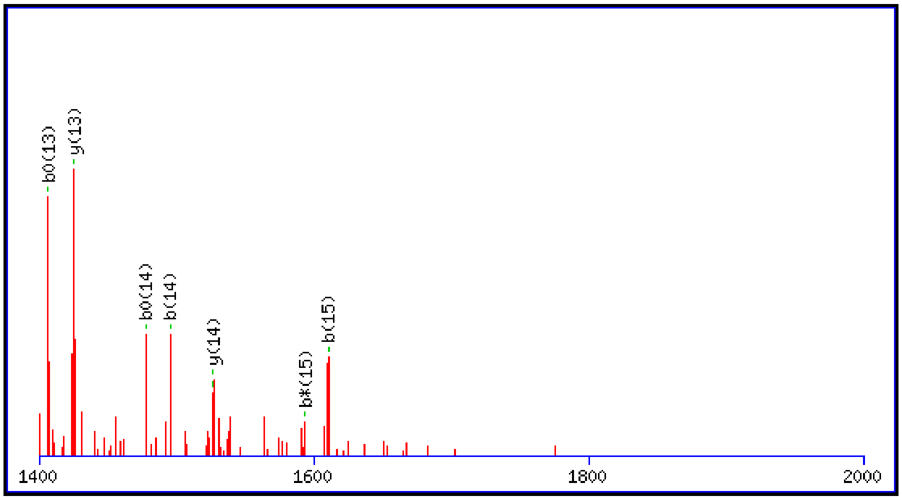


**Monoisotopic mass of neutral peptide Mr(calc):** 1783.8112
**Fixed modifications:** Carbamidomethyl (C)
**Variable modifications:**
**N8** : Deamidated_N (N)
**Ions Score:** 91 **Expect:** 2e-007
**Matches (Bold Red):** 39/164 fragment ions using 72 most intense peaks

| # | b | $b^{++}$ | b* | $b^{*++}$ | $b^{0}$ | $b^{0++}$ | Seq. | y | $y^{++}$ | y* | $y^{*++}$ | $y^{0}$ | $y^{0++}$ | # |
|---|---|---|---|---|---|---|---|---|---|---|---|---|---|---|
| 1 | 130.0499 | 65.5286 | | | 112.0393 | 56.5233 | E | | | | | | | 16 |
| 2 | **259.0925** | 130.0499 | | | 241.0819 | 121.0446 | E | 1655.7759 | 828.3916 | 1638.7493 | 819.8783 | 1637.7653 | 819.3863 | 15 |
| 3 | 360.1401 | 180.5737 | | | **342.1296** | 171.5684 | T | **1526.7333** | 763.8703 | 1509.7067 | 755.3570 | 1508.7227 | 754.8650 | 14 |
| 4 | **473.2242** | 237.1157 | | | **455.2136** | 228.1105 | L | **1425.6856** | 713.3464 | 1408.6591 | 704.8332 | 1407.6750 | 704.3412 | 13 |
| 5 | **586.3083** | 293.6578 | | | **568.2977** | 284.6525 | L | **1312.6015** | 656.8044 | **1295.5750** | 648.2911 | 1294.5910 | 647.7991 | 12 |
| 6 | **749.3716** | 375.1894 | | | **731.3610** | 366.1842 | Y | **1199.5175** | 600.2624 | 1182.4909 | 591.7491 | **1181.5069** | 591.2571 | 11 |
| 7 | 864.3985 | 432.7029 | | | 846.3880 | 423.6976 | D | **1036.4542** | 518.7307 | 1019.4276 | 510.2174 | 1018.4436 | 509.7254 | 10 |
| 8 | 979.4255 | 490.2164 | 962.3989 | 481.7031 | 961.4149 | 481.2111 | N | **921.4272** | 461.2172 | 904.4007 | 452.7040 | 903.4166 | 452.2120 | 9 |
| 9 | **1050.4626** | 525.7349 | 1033.4360 | 517.2217 | **1032.4520** | 516.7297 | A | **806.4003** | 403.7038 | 789.3737 | 395.1905 | 788.3897 | 394.6985 | 8 |
| 10 | **1151.5103** | 576.2588 | **1134.4837** | **567.7455** | **1133.4997** | 567.2535 | T | **735.3632** | 368.1852 | 718.3366 | 359.6719 | 717.3526 | 359.1799 | 7 |
| 11 | **1238.5423** | 619.7748 | **1221.5157** | 611.2615 | **1220.5317** | 610.7695 | S | **634.3155** | 317.6614 | 617.2889 | 309.1481 | 616.3049 | 308.6561 | 6 |
| 12 | **1325.5743** | 663.2908 | 1308.5478 | 654.7775 | 1307.5638 | 654.2855 | S | **547.2835** | 274.1454 | 530.2569 | 265.6321 | 529.2729 | 265.1401 | 5 |
| 13 | 1424.6427 | 712.8250 | 1407.6162 | 704.3117 | **1406.6322** | 703.8197 | V | **460.2514** | 230.6293 | 443.2249 | 222.1161 | 442.2409 | 221.6241 | 4 |
| 14 | **1495.6799** | 748.3436 | 1478.6533 | 739.8303 | **1477.6693** | 739.3383 | A | **361.1830** | 181.0951 | 344.1565 | 172.5819 | **343.1724** | 172.0899 | 3 |
| 15 | **1610.7068** | 805.8570 | **1593.6802** | 797.3438 | 1592.6962 | 796.8518 | D | **290.1459** | 145.5766 | 273.1193 | 137.0633 | 272.1353 | 136.5713 | 2 |
| 16 | | | | | | | R | 175.1190 | 88.0631 | 158.0924 | 79.5498 | | | 1 |

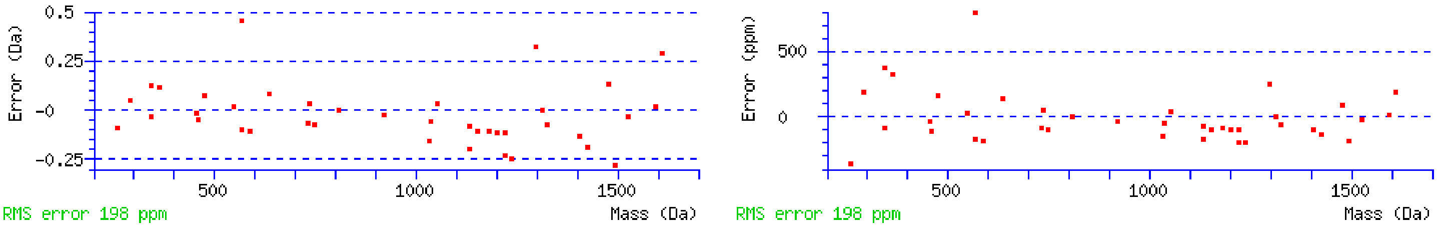


### All matches to this query

| Score | Mr(calc): | Delta | Sequence |
|---|---|---|---|
| 90.6 | 1783.8112 | 0.0019 | EETLLYDNATSSVADR |
| 29.7 | 1782.8272 | 0.9859 | EETLLYDNATSSVADR |
| 5.4 | 1781.8057 | 2.0074 | DLESRQRSLEQANR |
| 3.7 | 1783.8271 | -0.0140 | YCIKSNSXGEGARSER |
| 3.5 | 1783.8270 | -0.0140 | SAPVINNGSPTILGKR |
| 2.5 | 1783.8270 | -0.0140 | SAPVINNGSPTILGKR |
| 2.5 | 1783.8270 | -0.0140 | SAPVINNGSPTILGKR |
| 2.5 | 1783.8270 | -0.0140 | SAPVINNGSPTILGKR |
| 2.1 | 1783.8047 | 0.0084 | YDSVNEKLSDSPSMGR |
| 1.4 | 1783.8029 | 0.0102 | EIKYTSSVKYDSER |

---

Spectrum No: 133; Query: 1716; Rank: 1

---

## Peptide View

MS/MS Fragmentation of **ESTGAQVQVAGDMLPNSTER**
Found in **IPI00464574**, Tax_Id=10116 Gene_Symbol=Pcbp3 Poly(RC) binding protein 3

Match to Query 1716: 2089.968028 from(1045.991290,2+)
Title: 100101RatKid_NS_deglyco_15.2810.2810.2.dta
Data file K:\NewmanPaper\Piliang\3SubProteomes\Piliang3SP\mgf5ppm\ERLIC_3SubProteomes5ppm.mgf

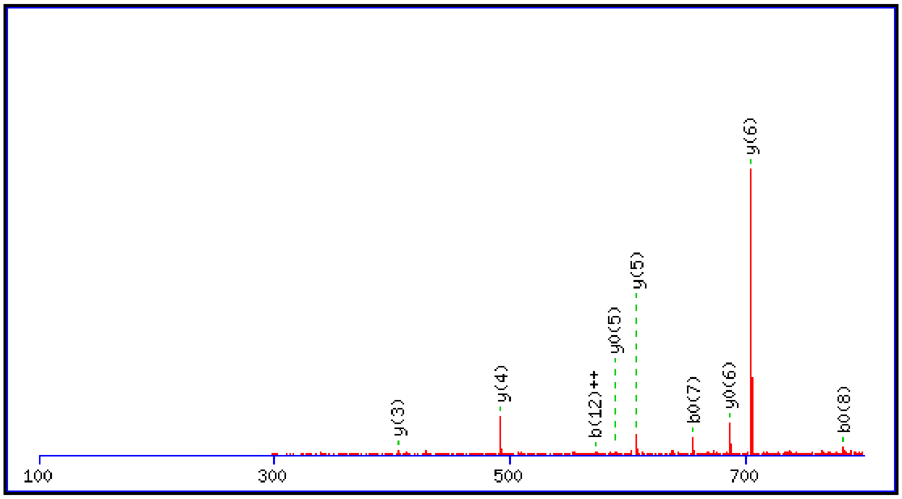

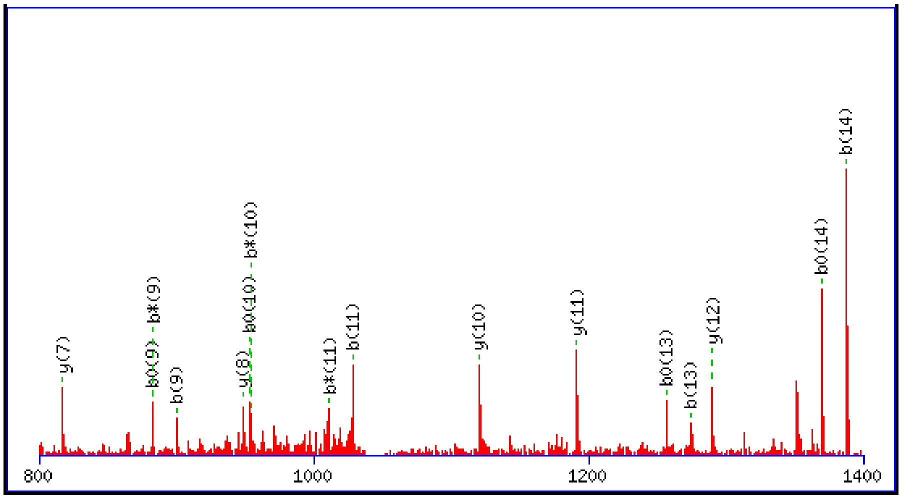


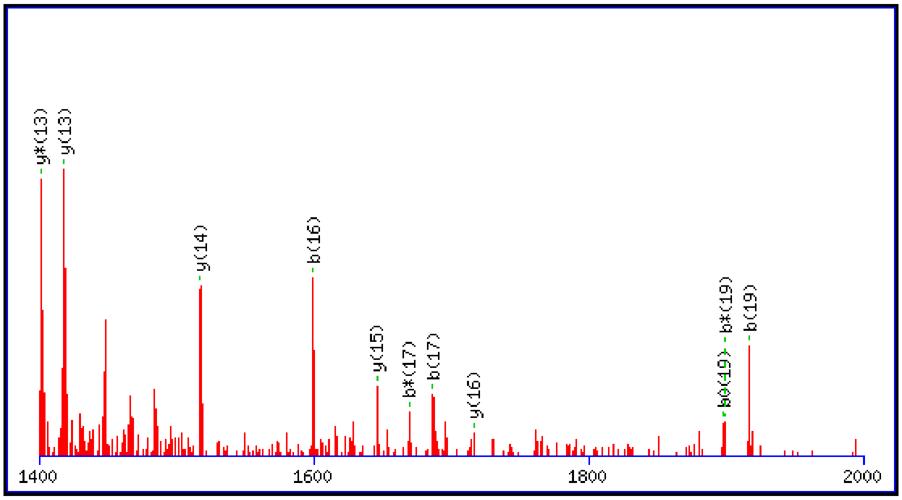


**Monoisotopic mass of neutral peptide Mr(calc):** 2089.9586
**Fixed modifications:** Carbamidomethyl (C)
**Variable modifications:**
**N16** : Deamidated_N (N)
**Ions Score:** 91 **Expect:** 2.1e-007
**Matches (Bold Red):** 36/216 fragment ions using 61 most intense peaks

| # | b | $b^{++}$ | $b^{*}$ | $b^{*++}$ | $b^{0}$ | $b^{0++}$ | Seq. | y | $y^{++}$ | $y^{*}$ | $y^{*++}$ | $y^{0}$ | $y^{0++}$ | # |
|---|---|---|---|---|---|---|---|---|---|---|---|---|---|---|
| 1 | 130.0499 | 65.5286 | | | 112.0393 | 56.5233 | E | | | | | | | 20 |
| 2 | 217.0819 | 109.0446 | | | 199.0713 | 100.0393 | S | 1961.9233 | 981.4653 | 1944.8967 | 972.9520 | 1943.9127 | 972.4600 | 19 |
| 3 | 318.1296 | 159.5684 | | | 300.1190 | 150.5631 | T | 1874.8913 | 937.9493 | 1857.8647 | 929.4360 | 1856.8807 | 928.9440 | 18 |
| 4 | 375.1510 | 188.0792 | | | 357.1405 | 179.0739 | G | 1773.8436 | 887.4254 | 1756.8170 | 878.9122 | 1755.8330 | 878.4201 | 17 |
| 5 | 446.1882 | 223.5977 | | | 428.1776 | 214.5924 | A | **1716.8221** | 858.9147 | 1699.7956 | 850.4014 | 1698.8116 | 849.9094 | 16 |
| 6 | 574.2467 | 287.6270 | 557.2202 | 279.1137 | 556.2362 | 278.6217 | Q | **1645.7850** | 823.3961 | 1628.7585 | 814.8829 | 1627.7744 | 814.3909 | 15 |
| 7 | 673.3151 | 337.1612 | 656.2886 | 328.6479 | **655.3046** | 328.1559 | V | **1517.7264** | 759.3669 | 1500.6999 | 750.8536 | 1499.7159 | 750.3616 | 14 |
| 8 | 801.3737 | 401.1905 | 784.3472 | 392.6772 | **783.3632** | 392.1852 | Q | **1418.6580** | 709.8326 | **1401.6315** | 701.3194 | 1400.6474 | 700.8274 | 13 |
| 9 | **900.4421** | 450.7247 | **883.4156** | 442.2114 | **882.4316** | 441.7194 | V | **1290.5994** | 645.8034 | 1273.5729 | 637.2901 | 1272.5889 | 636.7981 | 12 |
| 10 | 971.4793 | 486.2433 | **954.4527** | 477.7300 | **953.4687** | 477.2380 | A | **1191.5310** | 596.2691 | 1174.5045 | 587.7559 | 1173.5205 | 587.2639 | 11 |
| 11 | **1028.5007** | 514.7540 | **1011.4742** | 506.2407 | 1010.4902 | 505.7487 | G | **1120.4939** | 560.7506 | 1103.4674 | 552.2373 | 1102.4833 | 551.7453 | 10 |
| 12 | 1143.5277 | **572.2675** | 1126.5011 | 563.7542 | 1125.5171 | 563.2622 | D | 1063.4724 | 532.2399 | 1046.4459 | 523.7266 | 1045.4619 | 523.2346 | 9 |
| 13 | **1274.5681** | 637.7877 | 1257.5416 | 629.2744 | **1256.5576** | 628.7824 | M | **948.4455** | 474.7264 | 931.4190 | 466.2131 | 930.4349 | 465.7211 | 8 |
| 14 | **1387.6522** | 694.3297 | 1370.6257 | 685.8165 | **1369.6416** | 685.3245 | L | **817.4050** | 409.2061 | 800.3785 | 400.6929 | 799.3945 | 400.2009 | 7 |
| 15 | 1484.7050 | 742.8561 | 1467.6784 | 734.3428 | 1466.6944 | 733.8508 | P | **704.3210** | 352.6641 | 687.2944 | 344.1508 | **686.3104** | 343.6588 | 6 |
| 16 | **1599.7319** | 800.3696 | 1582.7054 | 791.8563 | 1581.7213 | 791.3643 | N | **607.2682** | 304.1377 | 590.2416 | 295.6245 | **589.2576** | 295.1324 | 5 |
| 17 | **1686.7639** | 843.8856 | **1669.7374** | 835.3723 | 1668.7534 | 834.8803 | S | **492.2413** | 246.6243 | 475.2147 | 238.1110 | 474.2307 | 237.6190 | 4 |
| 18 | 1787.8116 | 894.4094 | 1770.7851 | 885.8962 | 1769.8011 | 885.4042 | T | **405.2092** | 203.1082 | 388.1827 | 194.5950 | 387.1987 | 194.1030 | 3 |

| | | | | | | | | | | | | | |
|---|---|---|---|---|---|---|---|---|---|---|---|---|---|
| 19 | 1916.8542 | 958.9307 | 1899.8277 | 950.4175 | 1898.8436 | 949.9255 | E | 304.1615 | 152.5844 | 287.1350 | 144.0711 | 286.1510 | 143.5791 | 2 |
| 20 | | | | | | | R | 175.1190 | 88.0631 | 158.0924 | 79.5498 | | | 1 |

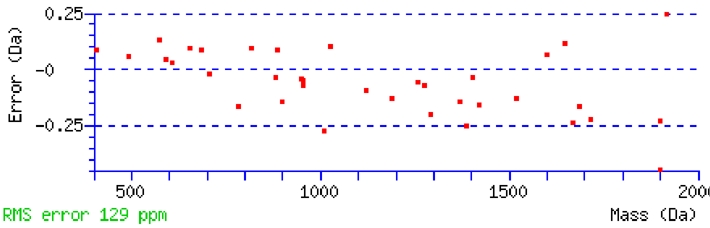


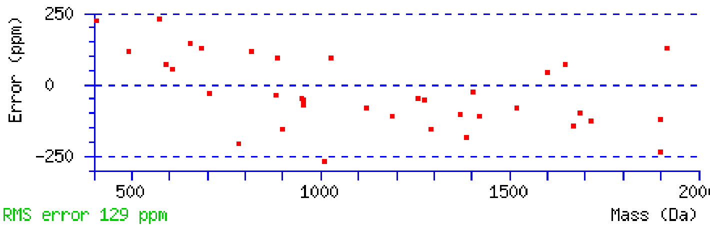


### All matches to this query

| Score | Mr(calc): | Delta | Sequence |
|---|---|---|---|
| 90.6 | 2089.9586 | 0.0094 | ESTGAQVQVAGDMLPNSTER |
| 25.0 | 2088.9746 | 0.9934 | ESTGAQVQVAGDMLPNSTER |
| 15.3 | 2089.9593 | 0.0088 | NIYTPPDSSTSFIQDYSR |
| 10.2 | 2089.9601 | 0.0080 | SVGLMFKNSVYVTFLPK |
| 4.0 | 2087.9458 | 2.0222 | KPSVPSTAPPGVKNLLSR |
| 3.3 | 2089.9476 | 0.0204 | GRSHNAPCINTAAVAASRR |
| 3.0 | 2089.9577 | 0.0103 | MSEMELKAFGSGVDIKPR |
| 3.0 | 2089.9577 | 0.0103 | MSEMELKAFGSGVDIKPR |
| 2.9 | 2089.9786 | -0.0105 | DAGFTVEDICMLRHRDR |
| 2.6 | 2089.9745 | -0.0065 | ATGTAHFFNFLLNTTDYR |

---

Spectrum No: 134; Query: 1565; Rank: 1

---

## Peptide View

MS/MS Fragmentation of **VNASTTDPNSTVEQSALTR**
Found in **IPI00365542**, Tax_Id=10116 Gene_Symbol=Lamb1_predicted laminin, beta 1

Match to Query 1565: 1991.937868 from(996.976210,2+)
Title: 100101RatKid_NS_deglyco_21.1843.1843.2.dta
Data file K:\NewmanPaper\Piliang\3SubProteomes\Piliang3SP\mgf5ppm\ERLIC_3SubProteomes5ppm.mgf

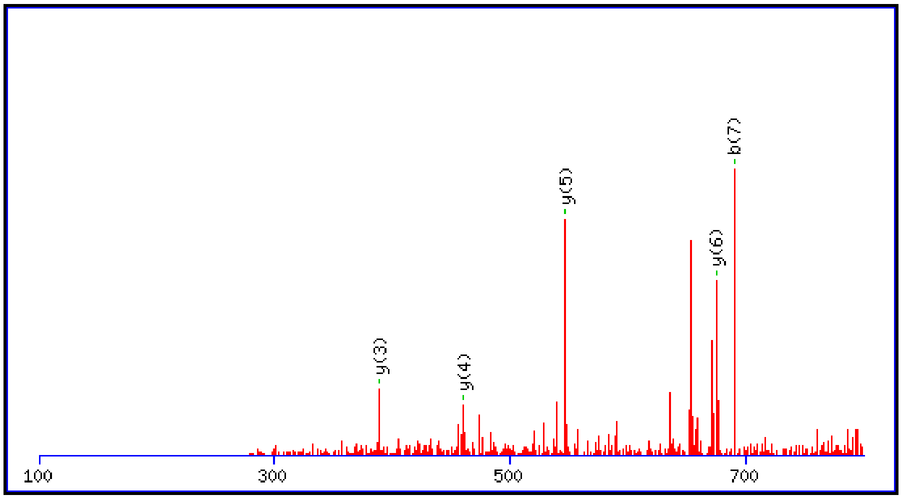

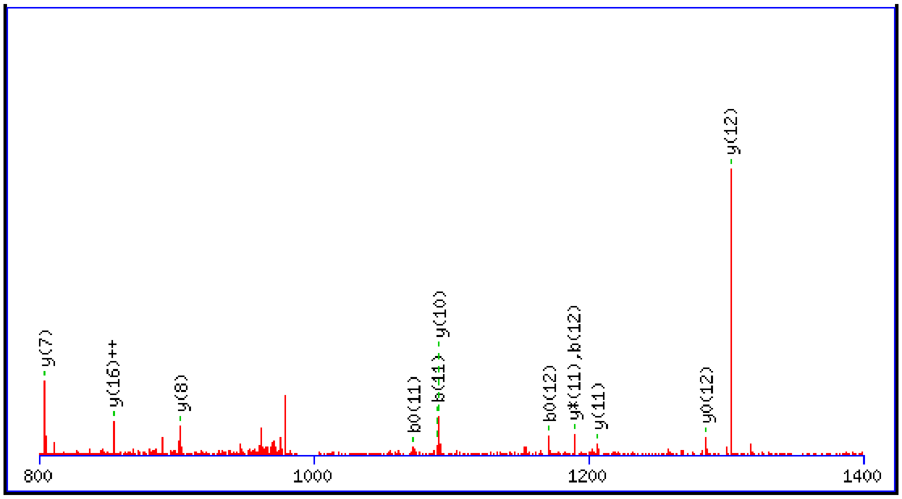


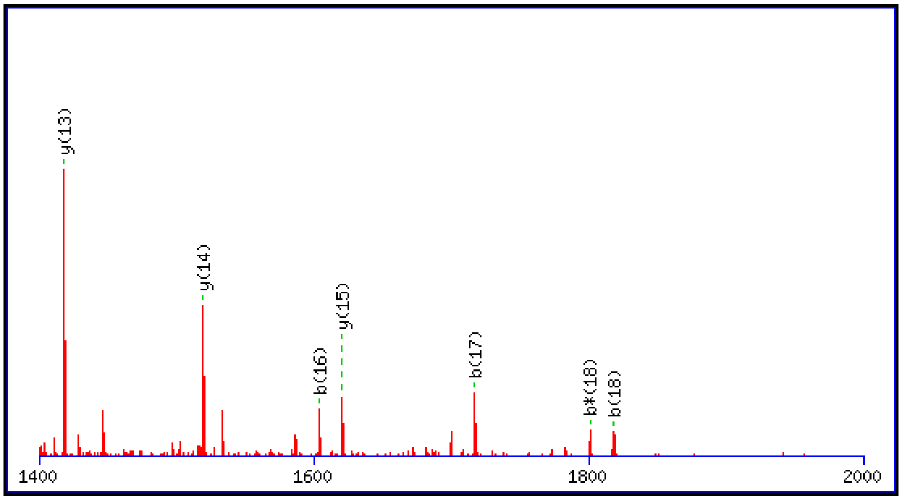


**Monoisotopic mass of neutral peptide Mr(calc):** 1991.9283
**Fixed modifications:** Carbamidomethyl (C)
**Variable modifications:**
**N2** : Deamidated_N (N)
**N9** : Deamidated_N (N)
**Ions Score:** 90 **Expect:** 2.2e-007
**Matches (Bold Red):** 24/206 fragment ions using 37 most intense peaks

| # | b | $b^{++}$ | b* | $b^{*++}$ | $b^{0}$ | $b^{0++}$ | Seq. | y | $y^{++}$ | y* | $y^{*++}$ | $y^{0}$ | $y^{0++}$ | # |
|---|---|---|---|---|---|---|---|---|---|---|---|---|---|---|
| 1 | 100.0757 | 50.5415 | | | | | V | | | | | | | 19 |
| 2 | 215.1026 | 108.0550 | 198.0761 | 99.5417 | | | N | 1893.8672 | 947.4372 | 1876.8407 | 938.9240 | 1875.8566 | 938.4320 | 18 |
| 3 | 286.1397 | 143.5735 | 269.1132 | 135.0602 | | | A | 1778.8403 | 889.9238 | 1761.8137 | 881.4105 | 1760.8297 | 880.9185 | 17 |
| 4 | 373.1718 | 187.0895 | 356.1452 | 178.5762 | 355.1612 | 178.0842 | S | 1707.8032 | **854.4052** | 1690.7766 | 845.8919 | 1689.7926 | 845.3999 | 16 |
| 5 | 474.2194 | 237.6134 | 457.1929 | 229.1001 | 456.2089 | 228.6081 | T | **1620.7711** | 810.8892 | 1603.7446 | 802.3759 | 1602.7606 | 801.8839 | 15 |
| 6 | 575.2671 | 288.1372 | 558.2406 | 279.6239 | 557.2566 | 279.1319 | T | **1519.7235** | 760.3654 | 1502.6969 | 751.8521 | 1501.7129 | 751.3601 | 14 |
| 7 | **690.2941** | 345.6507 | 673.2675 | 337.1374 | 672.2835 | 336.6454 | D | **1418.6758** | 709.8415 | 1401.6492 | 701.3282 | 1400.6652 | 700.8362 | 13 |
| 8 | 787.3468 | 394.1771 | 770.3203 | 385.6638 | 769.3363 | 385.1718 | P | **1303.6488** | 652.3281 | 1286.6223 | 643.8148 | **1285.6383** | 643.3228 | 12 |
| 9 | 902.3738 | 451.6905 | 885.3472 | 443.1772 | 884.3632 | 442.6852 | N | **1206.5961** | 603.8017 | **1189.5695** | 595.2884 | 1188.5855 | 594.7964 | 11 |
| 10 | 989.4058 | 495.2065 | 972.3792 | 486.6933 | 971.3952 | 486.2013 | S | **1091.5691** | 546.2882 | 1074.5426 | 537.7749 | 1073.5586 | 537.2829 | 10 |
| 11 | **1090.4535** | 545.7304 | 1073.4269 | 537.2171 | **1072.4429** | 536.7251 | T | 1004.5371 | 502.7722 | 987.5106 | 494.2589 | 986.5265 | 493.7669 | 9 |
| 12 | **1189.5219** | 595.2646 | 1172.4953 | 586.7513 | **1171.5113** | 586.2593 | V | **903.4894** | 452.2483 | 886.4629 | 443.7351 | 885.4789 | 443.2431 | 8 |
| 13 | 1318.5645 | 659.7859 | 1301.5379 | 651.2726 | 1300.5539 | 650.7806 | E | **804.4210** | 402.7141 | 787.3945 | 394.2009 | 786.4104 | 393.7089 | 7 |
| 14 | 1446.6231 | 723.8152 | 1429.5965 | 715.3019 | 1428.6125 | 714.8099 | Q | **675.3784** | 338.1928 | 658.3519 | 329.6796 | 657.3679 | 329.1876 | 6 |
| 15 | 1533.6551 | 767.3312 | 1516.6285 | 758.8179 | 1515.6445 | 758.3259 | S | **547.3198** | 274.1636 | 530.2933 | 265.6503 | 529.3093 | 265.1583 | 5 |
| 16 | **1604.6922** | 802.8497 | 1587.6657 | 794.3365 | 1586.6816 | 793.8445 | A | **460.2878** | 230.6475 | 443.2613 | 222.1343 | 442.2772 | 221.6423 | 4 |
| 17 | **1717.7763** | 859.3918 | 1700.7497 | 850.8785 | 1699.7657 | 850.3865 | L | **389.2507** | 195.1290 | 372.2241 | 186.6157 | 371.2401 | 186.1237 | 3 |
| 18 | **1818.8239** | 909.9156 | **1801.7974** | 901.4023 | 1800.8134 | 900.9103 | T | 276.1666 | 138.5870 | 259.1401 | 130.0737 | 258.1561 | 129.5817 | 2 |

| 19 | | | | | | | R | 175.1190 | 88.0631 | 158.0924 | 79.5498 | | | 1 |
|---|---|---|---|---|---|---|---|---|---|---|---|---|---|---|

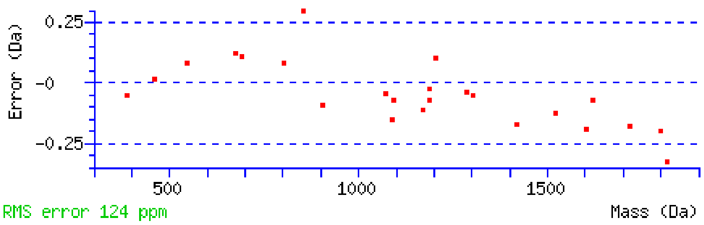


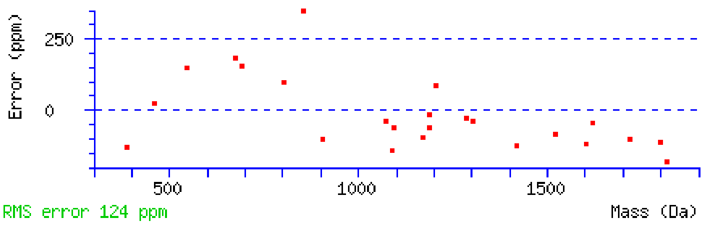


**All matches to this query**

| Score | Mr(calc): | Delta | Sequence |
|---|---|---|---|
| 90.5 | 1991.9283 | 0.0095 | VNASTTDPNSTVEQSALTR |
| 64.4 | 1990.9443 | 0.9935 | VNASTTDPNSTVEQSALTR |
| 16.0 | 1989.9296 | 2.0083 | SPSTYNALESGLTSLTIR |
| 13.6 | 1989.9296 | 2.0083 | SPSTYNALESGLTSLTIR |
| 13.2 | 1990.9443 | 0.9935 | VNASTTDPNSTVEQSALTR |
| 11.4 | 1991.9201 | 0.0178 | QNSESAVSSTVNPITIHK |
| 11.4 | 1991.9201 | 0.0178 | QNSESAVSSTVNPITIHK |
| 10.4 | 1989.9296 | 2.0083 | SPSTYNALESGLTSLTIR |
| 9.7 | 1989.9296 | 2.0083 | SPSTYNALESGLTSLTIR |
| 8.2 | 1989.9296 | 2.0083 | SPSTYNALESGLTSLTIR |

---

Spectrum No: 135; Query: 1932; Rank: 1

---

## Peptide View

MS/MS Fragmentation of **AGFAQCICPTLTCPEANSTK**
Found in **IPI00188885**, Tax_Id=10116 Gene_Symbol=Agrn Isoform 1 of Agrin precursor

Match to Query 1932: 2225.983168 from(1113.998860,2+)
Title: 100101RatKid_NS_deglyco_21.2898.2898.2.dta
Data file K:\NewmanPaper\Piliang\3SubProteomes\Piliang3SP\mgf5ppm\ERLIC_3SubProteomes5ppm.mgf

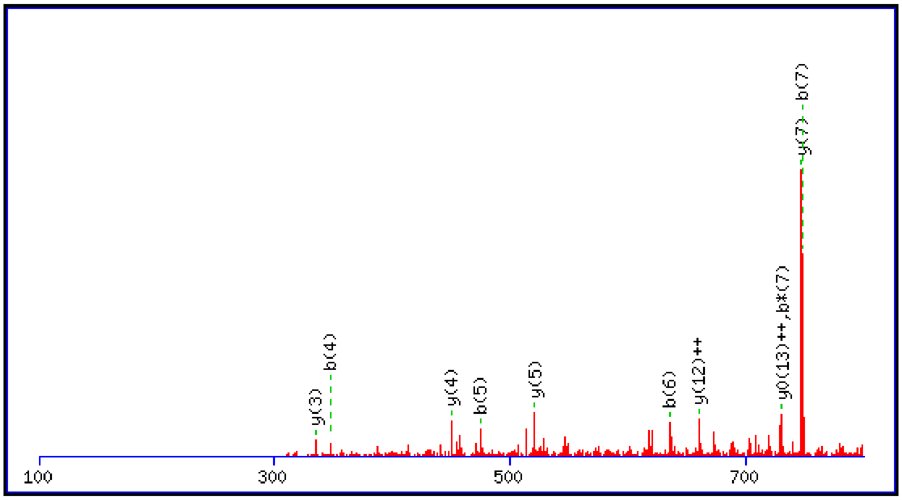

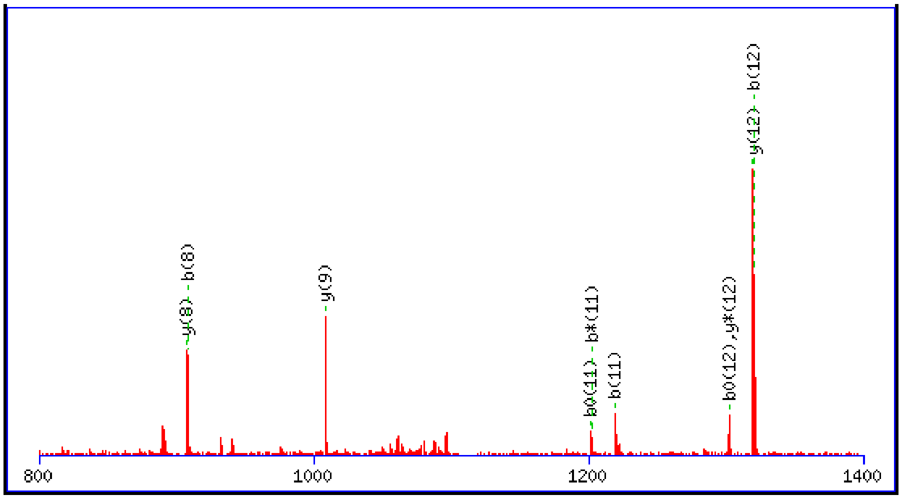


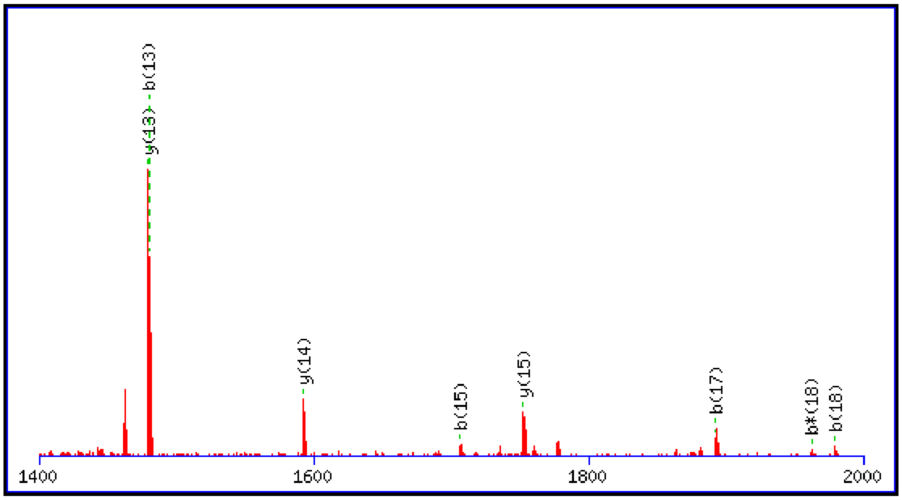


**Monoisotopic mass of neutral peptide Mr(calc):** 2225.9755
**Fixed modifications:** Carbamidomethyl (C)
**Variable modifications:**
**N17** : Deamidated_N (N)
**Ions Score:** 90 **Expect:** 2.6e-007
**Matches (Bold Red):** 29/200 fragment ions using 39 most intense peaks

| # | b | $b^{++}$ | b* | $b^{*++}$ | $b^0$ | $b^{0++}$ | Seq. | y | $y^{++}$ | y* | $y^{*++}$ | $y^0$ | $y^{0++}$ | # |
|---|---|---|---|---|---|---|---|---|---|---|---|---|---|---|
| 1 | 72.0444 | 36.5258 | | | | | A | | | | | | | 20 |
| 2 | 129.0659 | 65.0366 | | | | | G | 2155.9457 | 1078.4765 | 2138.9191 | 1069.9632 | 2137.9351 | 1069.4712 | 19 |
| 3 | 276.1343 | 138.5708 | | | | | F | 2098.9242 | 1049.9657 | 2081.8977 | 1041.4525 | 2080.9137 | 1040.9605 | 18 |
| 4 | **347.1714** | 174.0893 | | | | | A | 1951.8558 | 976.4315 | 1934.8293 | 967.9183 | 1933.8452 | 967.4263 | 17 |
| 5 | **475.2300** | 238.1186 | 458.2034 | 229.6053 | | | Q | 1880.8187 | 940.9130 | 1863.7921 | 932.3997 | 1862.8081 | 931.9077 | 16 |
| 6 | **635.2606** | 318.1339 | 618.2341 | 309.6207 | | | C | **1752.7601** | 876.8837 | 1735.7336 | 868.3704 | 1734.7496 | 867.8784 | 15 |
| 7 | **748.3447** | 374.6760 | **731.3181** | 366.1627 | | | I | **1592.7295** | 796.8684 | 1575.7029 | 788.3551 | 1574.7189 | 787.8631 | 14 |
| 8 | **908.3753** | 454.6913 | 891.3488 | 446.1780 | | | C | **1479.6454** | 740.3263 | 1462.6189 | 731.8131 | 1461.6348 | **731.3211** | 13 |
| 9 | 1005.4281 | 503.2177 | 988.4015 | 494.7044 | | | P | **1319.6148** | **660.3110** | **1302.5882** | 651.7977 | 1301.6042 | 651.3057 | 12 |
| 10 | 1106.4758 | 553.7415 | 1089.4492 | 545.2282 | 1088.4652 | 544.7362 | T | 1222.5620 | 611.7846 | 1205.5354 | 603.2714 | 1204.5514 | 602.7794 | 11 |
| 11 | **1219.5598** | 610.2836 | **1202.5333** | 601.7703 | **1201.5493** | 601.2783 | L | 1121.5143 | 561.2608 | 1104.4878 | 552.7475 | 1103.5037 | 552.2555 | 10 |
| 12 | **1320.6075** | 660.8074 | 1303.5810 | 652.2941 | **1302.5969** | 651.8021 | T | **1008.4302** | 504.7188 | 991.4037 | 496.2055 | 990.4197 | 495.7135 | 9 |
| 13 | **1480.6382** | 740.8227 | 1463.6116 | 732.3094 | 1462.6276 | 731.8174 | C | **907.3826** | 454.1949 | 890.3560 | 445.6816 | 889.3720 | 445.1896 | 8 |
| 14 | 1577.6909 | 789.3491 | 1560.6644 | 780.8358 | 1559.6804 | 780.3438 | P | **747.3519** | 374.1796 | 730.3254 | 365.6663 | 729.3414 | 365.1743 | 7 |
| 15 | **1706.7335** | 853.8704 | 1689.7070 | 845.3571 | 1688.7229 | 844.8651 | E | 650.2992 | 325.6532 | 633.2726 | 317.1399 | 632.2886 | 316.6479 | 6 |
| 16 | 1777.7706 | 889.3890 | 1760.7441 | 880.8757 | 1759.7601 | 880.3837 | A | **521.2566** | 261.1319 | 504.2300 | 252.6186 | 503.2460 | 252.1266 | 5 |
| 17 | **1892.7976** | 946.9024 | 1875.7710 | 938.3891 | 1874.7870 | 937.8971 | N | **450.2194** | 225.6134 | 433.1929 | 217.1001 | 432.2089 | 216.6081 | 4 |
| 18 | **1979.8296** | 990.4184 | **1962.8030** | 981.9052 | 1961.8190 | 981.4132 | S | **335.1925** | 168.0999 | 318.1660 | 159.5866 | 317.1819 | 159.0946 | 3 |

| | | | | | | | | | | | | | | |
|---|---|---|---|---|---|---|---|---|---|---|---|---|---|---|
| **19** | 2080.8773 | 1040.9423 | 2063.8507 | 1032.4290 | 2062.8667 | 1031.9370 | **T** | 248.1605 | 124.5839 | 231.1339 | 116.0706 | 230.1499 | 115.5786 | **2** |
| **20** | | | | | | | **K** | 147.1128 | 74.0600 | 130.0863 | 65.5468 | | | **1** |

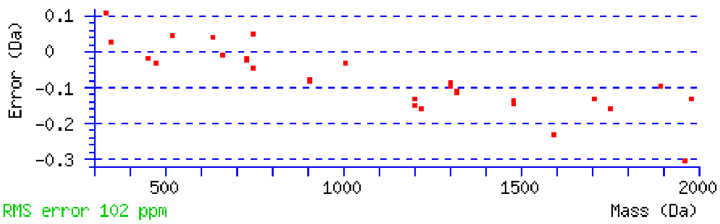


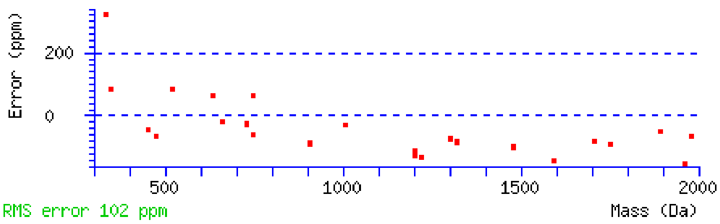


### All matches to this query

| Score | Mr(calc): | Delta | Sequence |
|---|---|---|---|
| 90.3 | 2225.9755 | 0.0076 | AGFAQCICPTLTCPEANSTK |
| 34.3 | 2224.9915 | 0.9917 | AGFAQCICPTLTCPEANSTK |
| 9.1 | 2225.9720 | 0.0111 | NAWEESQITLFLDILMK |
| 5.9 | 2225.9892 | -0.0061 | LMENRSCDQELSEKGTEGK |
| 4.0 | 2223.9660 | 2.0172 | FSNWTNGAFLAQGSLLNMR |
| 1.9 | 2224.9581 | 1.0250 | TNPGCPAEVRLGMGHCFVK |
| 0.5 | 2223.9829 | 2.0003 | EYLKNFPLLTKYCGYR |
| 0.3 | 2224.9956 | 0.9875 | SLAEMLMSKNTRTSDTLSK |
| 0.2 | 2224.9612 | 1.0220 | THYGQKAILFLPLPVSSD |
| 0.2 | 2224.9612 | 1.0220 | THYGQKAILFLPLPVSSD |

---

Spectrum No: 136; Query: 1438; Rank: 1

---

## Peptide View

MS/MS Fragmentation of **NPCTSEQNCTSPFSYK**
Found in **IPI00191681**, Tax_Id=10116 Gene_Symbol=Itgb1 Integrin beta-1 precursor

Match to Query 1438: 1919.774568 from(960.894560,2+)
Title: 100101RatKid_NS_deglyco_23.1805.1805.2.dta
Data file K:\NewmanPaper\Piliang\3SubProteomes\Piliang3SP\mgf5ppm\ERLIC_3SubProteomes5ppm.mgf

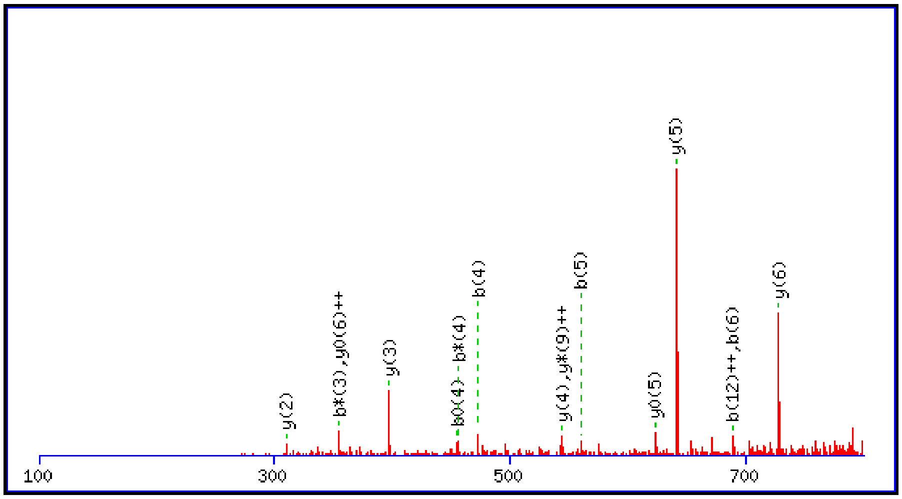

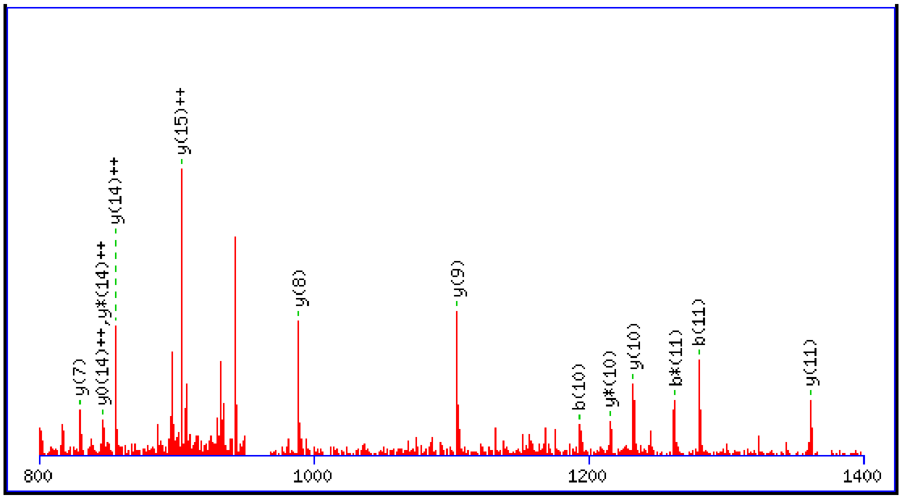


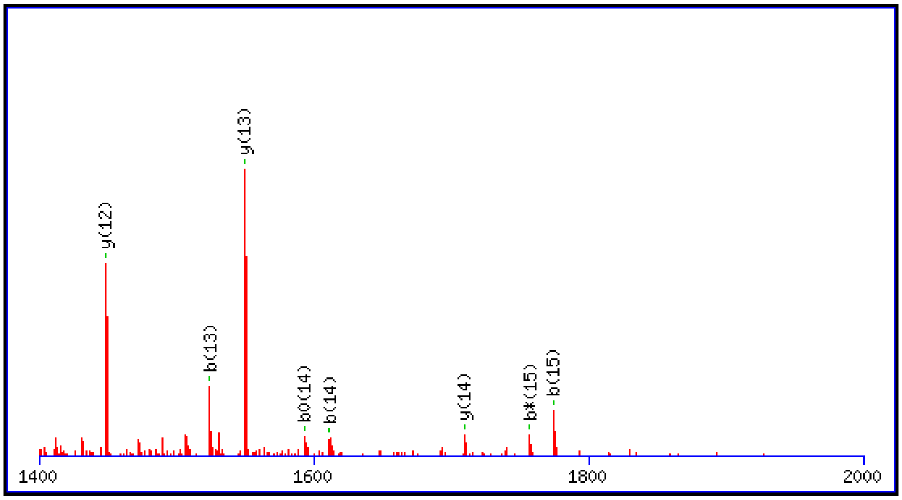


**Monoisotopic mass of neutral peptide Mr(calc):** 1919.7666
**Fixed modifications:** Carbamidomethyl (C)
**Variable modifications:**
**N8** : Deamidated_N (N)
**Ions Score:** 90 **Expect:** 1.9e-007
**Matches (Bold Red):** 36/170 fragment ions using 59 most intense peaks

| # | b | $b^{++}$ | b* | $b^{*++}$ | $b^{0}$ | $b^{0++}$ | Seq. | y | $y^{++}$ | y* | $y^{*++}$ | $y^{0}$ | $y^{0++}$ | # |
|---|---|---|---|---|---|---|---|---|---|---|---|---|---|---|
| 1 | 115.0502 | 58.0287 | 98.0237 | 49.5155 | | | N | | | | | | | 16 |
| 2 | 212.1030 | 106.5551 | 195.0764 | 98.0418 | | | P | 1806.7309 | **903.8691** | 1789.7044 | 895.3558 | 1788.7204 | 894.8638 | 15 |
| 3 | 372.1336 | 186.5704 | **355.1071** | 178.0572 | | | C | **1709.6782** | **855.3427** | 1692.6516 | **846.8294** | 1691.6676 | **846.3374** | 14 |
| 4 | **473.1813** | 237.0943 | **456.1547** | 228.5810 | **455.1707** | 228.0890 | T | **1549.6475** | 775.3274 | 1532.6210 | 766.8141 | 1531.6369 | 766.3221 | 13 |
| 5 | **560.2133** | 280.6103 | 543.1868 | 272.0970 | 542.2028 | 271.6050 | S | **1448.5998** | 724.8036 | 1431.5733 | 716.2903 | 1430.5893 | 715.7983 | 12 |
| 6 | **689.2559** | 345.1316 | 672.2294 | 336.6183 | 671.2454 | 336.1263 | E | **1361.5678** | 681.2875 | 1344.5413 | 672.7743 | 1343.5572 | 672.2823 | 11 |
| 7 | 817.3145 | 409.1609 | 800.2879 | 400.6476 | 799.3039 | 400.1556 | Q | **1232.5252** | 616.7662 | **1215.4987** | 608.2530 | 1214.5146 | 607.7610 | 10 |
| 8 | 932.3414 | 466.6744 | 915.3149 | 458.1611 | 914.3309 | 457.6691 | N | **1104.4666** | 552.7370 | 1087.4401 | **544.2237** | 1086.4561 | 543.7317 | 9 |
| 9 | 1092.3721 | 546.6897 | 1075.3455 | 538.1764 | 1074.3615 | 537.6844 | C | **989.4397** | 495.2235 | 972.4131 | 486.7102 | 971.4291 | 486.2182 | 8 |
| 10 | **1193.4198** | 597.2135 | 1176.3932 | 588.7002 | 1175.4092 | 588.2082 | T | **829.4090** | 415.2082 | 812.3825 | 406.6949 | 811.3985 | 406.2029 | 7 |
| 11 | **1280.4518** | 640.7295 | **1263.4252** | 632.2163 | 1262.4412 | 631.7242 | S | **728.3614** | 364.6843 | 711.3348 | 356.1710 | 710.3508 | **355.6790** | 6 |
| 12 | 1377.5046 | **689.2559** | 1360.4780 | 680.7426 | 1359.4940 | 680.2506 | P | **641.3293** | 321.1683 | 624.3028 | 312.6550 | **623.3188** | 312.1630 | 5 |
| 13 | **1524.5730** | 762.7901 | 1507.5464 | 754.2768 | 1506.5624 | 753.7848 | F | **544.2766** | 272.6419 | 527.2500 | 264.1287 | 526.2660 | 263.6366 | 4 |
| 14 | **1611.6050** | 806.3061 | 1594.5784 | 797.7929 | **1593.5944** | 797.3009 | S | **397.2082** | 199.1077 | 380.1816 | 190.5944 | 379.1976 | 190.1024 | 3 |
| 15 | **1774.6683** | 887.8378 | **1757.6418** | 879.3245 | 1756.6578 | 878.8325 | Y | **310.1761** | 155.5917 | 293.1496 | 147.0784 | | | 2 |
| 16 | | | | | | | K | 147.1128 | 74.0600 | 130.0863 | 65.5468 | | | 1 |

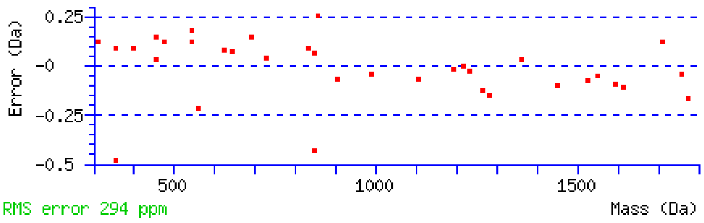


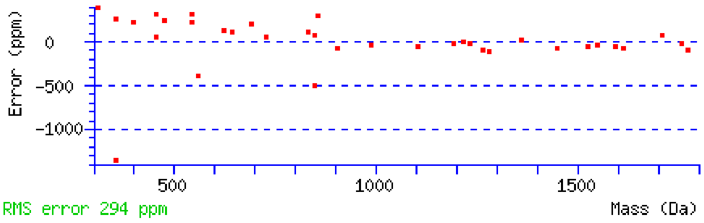


### All matches to this query

| Score | Mr(calc): | Delta | Sequence |
|---|---|---|---|
| 89.8 | 1919.7666 | 0.0080 | NPCTSEQNCTSPFSYK |
| 22.8 | 1918.7826 | 0.9920 | NPCTSEQNCTSPFSYK |
| 20.1 | 1919.7666 | 0.0080 | NPCTSEQNCTSPFSYK |
| 6.9 | 1919.7614 | 0.0131 | KTSNVQSTAGMSVRAK |
| 6.9 | 1919.7614 | 0.0131 | KTSNVQSTAGMSVRAK |
| 4.8 | 1919.7614 | 0.0131 | KTSNVQSTAGMSVRAK |
| 4.2 | 1919.7802 | -0.0056 | ATELSEEAQLEAVGDAK |
| 1.3 | 1919.7720 | 0.0026 | ASGPGTPPAAEPSVLTTK |
| 0.7 | 1919.7614 | 0.0131 | KTSNVQSTAGMSVRAK |
| 0.7 | 1919.7614 | 0.0131 | KTSNVQSTAGMSVRAK |

---

Spectrum No: 137; Query: 2806; Rank: 1

---

## Peptide View

MS/MS Fragmentation of **NGTPGGSFVSPSPLSTSSPILSADSTSVGSFPSAGSSDQGPR**
Found in **IPI00362755**, Tax_Id=10116 Gene_Symbol=Tns similar to tensin

Match to Query 2806: 3980.847432 from(1327.956420,3+)
Title: 100101RatKid_NS_deglyco_08.4830.4830.3.dta
Data file K:\NewmanPaper\Piliang\3SubProteomes\Piliang3SP\mgf5ppm\ERLIC_3SubProteomes5ppm.mgf

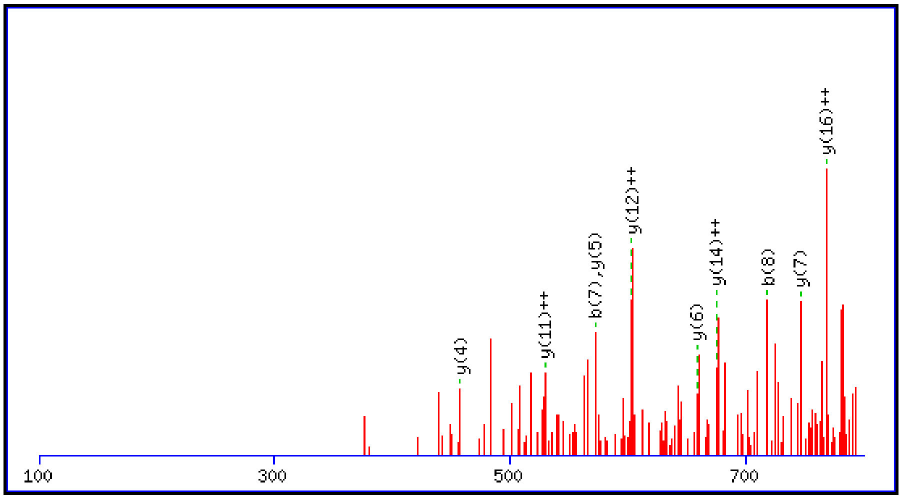

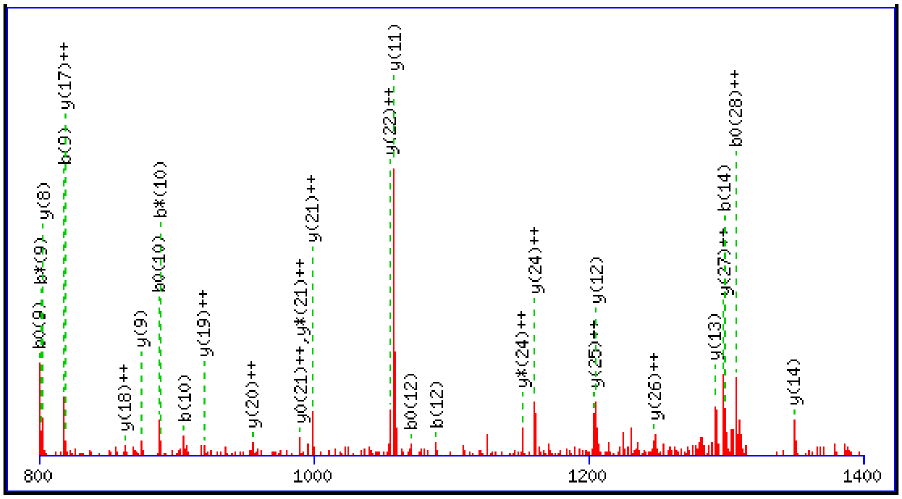


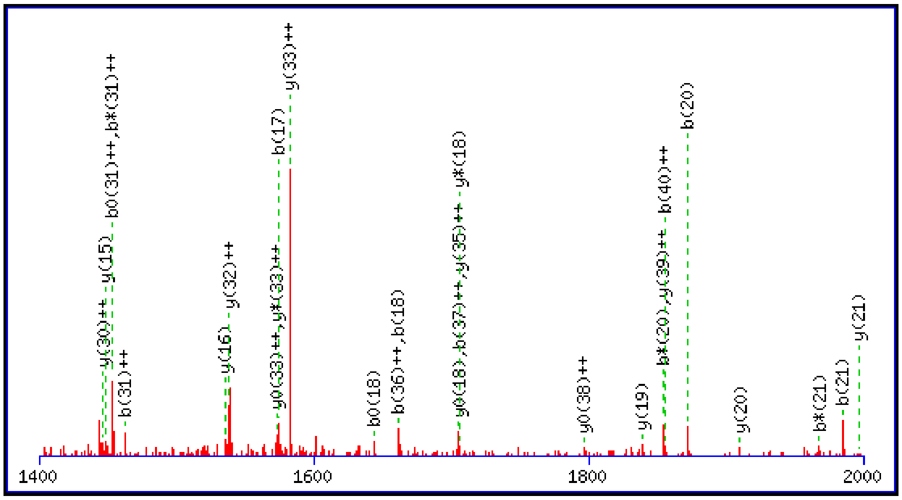


**Monoisotopic mass of neutral peptide Mr(calc):** 3979.8560
**Fixed modifications:** Carbamidomethyl (C)
**Variable modifications:**
**N1** : Deamidated_N (N)
**Ions Score:** 90 **Expect:** 4.5e-007
**Matches (Bold Red):** 67/480 fragment ions using 122 most intense peaks

| # | b | b++ | b* | b*++ | b0 | b0++ | Seq. | y | y++ | y* | y*++ | y0 | y0++ | # |
|---|---|---|---|---|---|---|---|---|---|---|---|---|---|---|
| 1 | 116.0342 | 58.5207 | 99.0077 | 50.0075 | | | N | | | | | | | 42 |
| 2 | 173.0557 | 87.0315 | 156.0291 | 78.5182 | | | G | 3865.8363 | 1933.4218 | 3848.8097 | 1924.9085 | 3847.8257 | 1924.4165 | 41 |
| 3 | 274.1034 | 137.5553 | 257.0768 | 129.0420 | 256.0928 | 128.5500 | T | 3808.8148 | 1904.9110 | 3791.7882 | 1896.3978 | 3790.8042 | 1895.9058 | 40 |
| 4 | 371.1561 | 186.0817 | 354.1296 | 177.5684 | 353.1456 | 177.0764 | P | 3707.7671 | **1854.3872** | 3690.7406 | 1845.8739 | 3689.7566 | 1845.3819 | 39 |
| 5 | 428.1776 | 214.5924 | 411.1510 | 206.0792 | 410.1670 | 205.5871 | G | 3610.7144 | 1805.8608 | 3593.6878 | 1797.3475 | 3592.7038 | **1796.8555** | 38 |
| 6 | 485.1990 | 243.1032 | 468.1725 | 234.5899 | 467.1885 | 234.0979 | G | 3553.6929 | 1777.3501 | 3536.6663 | 1768.8368 | 3535.6823 | 1768.3448 | 37 |
| 7 | **572.2311** | 286.6192 | 555.2045 | 278.1059 | 554.2205 | 277.6139 | S | 3496.6714 | 1748.8394 | 3479.6449 | 1740.3261 | 3478.6609 | 1739.8341 | 36 |
| 8 | **719.2995** | 360.1534 | 702.2729 | 351.6401 | 701.2889 | 351.1481 | F | 3409.6394 | **1705.3233** | 3392.6128 | 1696.8101 | 3391.6288 | 1696.3181 | 35 |
| 9 | **818.3679** | 409.6876 | **801.3414** | 401.1743 | **800.3573** | 400.6823 | V | 3262.5710 | 1631.7891 | 3245.5444 | 1623.2759 | 3244.5604 | 1622.7838 | 34 |
| 10 | **905.3999** | 453.2036 | **888.3734** | 444.6903 | **887.3894** | 444.1983 | S | 3163.5026 | **1582.2549** | 3146.4760 | **1573.7416** | 3145.4920 | **1573.2496** | 33 |
| 11 | 1002.4527 | 501.7300 | 985.4261 | 493.2167 | 984.4421 | 492.7247 | P | 3076.4705 | **1538.7389** | 3059.4440 | 1530.2256 | 3058.4600 | 1529.7336 | 32 |
| 12 | **1089.4847** | 545.2460 | 1072.4582 | 536.7327 | **1071.4742** | 536.2407 | S | 2979.4178 | 1490.2125 | 2962.3912 | 1481.6993 | 2961.4072 | 1481.2072 | 31 |
| 13 | 1186.5375 | 593.7724 | 1169.5109 | 585.2591 | 1168.5269 | 584.7671 | P | 2892.3857 | **1446.6965** | 2875.3592 | 1438.1832 | 2874.3752 | 1437.6912 | 30 |
| 14 | **1299.6216** | 650.3144 | 1282.5950 | 641.8011 | 1281.6110 | 641.3091 | L | 2795.3330 | 1398.1701 | 2778.3064 | 1389.6569 | 2777.3224 | 1389.1648 | 29 |
| 15 | 1386.6536 | 693.8304 | 1369.6270 | 685.3172 | 1368.6430 | 684.8251 | S | 2682.2489 | 1341.6281 | 2665.2224 | 1333.1148 | 2664.2384 | 1332.6228 | 28 |
| 16 | 1487.7013 | 744.3543 | 1470.6747 | 735.8410 | 1469.6907 | 735.3490 | T | 2595.2169 | **1298.1121** | 2578.1903 | 1289.5988 | 2577.2063 | 1289.1068 | 27 |
| 17 | **1574.7333** | 787.8703 | 1557.7067 | 779.3570 | 1556.7227 | 778.8650 | S | 2494.1692 | **1247.5882** | 2477.1427 | 1239.0750 | 2476.1586 | 1238.5830 | 26 |
| 18 | **1661.7653** | 831.3863 | 1644.7388 | 822.8730 | **1643.7547** | 822.3810 | S | 2407.1372 | **1204.0722** | 2390.1106 | 1195.5590 | 2389.1266 | 1195.0669 | 25 |
| | | | | | | | | | | | | | | |

| | | | | | | | | | | | | | | |
|---|---|---|---|---|---|---|---|---|---|---|---|---|---|---|
| 19 | 1758.8181 | 879.9127 | 1741.7915 | 871.3994 | 1740.8075 | 870.9074 | P | 2320.1052 | 1160.5562 | 2303.0786 | 1152.0429 | 2302.0946 | 1151.5509 | 24 |
| 20 | 1871.9021 | 936.4547 | 1854.8756 | 927.9414 | 1853.8916 | 927.4494 | I | 2223.0524 | 1112.0298 | 2206.0258 | 1103.5166 | 2205.0418 | 1103.0246 | 23 |
| 21 | 1984.9862 | 992.9967 | 1967.9597 | 984.4835 | 1966.9756 | 983.9915 | L | 2109.9683 | 1055.4878 | 2092.9418 | 1046.9745 | 2091.9578 | 1046.4825 | 22 |
| 22 | 2072.0182 | 1036.5128 | 2054.9917 | 1027.9995 | 2054.0077 | 1027.5075 | S | 1996.8843 | 998.9458 | 1979.8577 | 990.4325 | 1978.8737 | 989.9405 | 21 |
| 23 | 2143.0553 | 1072.0313 | 2126.0288 | 1063.5180 | 2125.0448 | 1063.0260 | A | 1909.8522 | 955.4298 | 1892.8257 | 946.9165 | 1891.8417 | 946.4245 | 20 |
| 24 | 2258.0823 | 1129.5448 | 2241.0557 | 1121.0315 | 2240.0717 | 1120.5395 | D | 1838.8151 | 919.9112 | 1821.7886 | 911.3979 | 1820.8046 | 910.9059 | 19 |
| 25 | 2345.1143 | 1173.0608 | 2328.0878 | 1164.5475 | 2327.1038 | 1164.0555 | S | 1723.7882 | 862.3977 | 1706.7616 | 853.8845 | 1705.7776 | 853.3924 | 18 |
| 26 | 2446.1620 | 1223.5846 | 2429.1354 | 1215.0714 | 2428.1514 | 1214.5794 | T | 1636.7562 | 818.8817 | 1619.7296 | 810.3684 | 1618.7456 | 809.8764 | 17 |
| 27 | 2533.1940 | 1267.1007 | 2516.1675 | 1258.5874 | 2515.1835 | 1258.0954 | S | 1535.7085 | 768.3579 | 1518.6819 | 759.8446 | 1517.6979 | 759.3526 | 16 |
| 28 | 2632.2624 | 1316.6349 | 2615.2359 | 1308.1216 | 2614.2519 | 1307.6296 | V | 1448.6764 | 724.8419 | 1431.6499 | 716.3286 | 1430.6659 | 715.8366 | 15 |
| 29 | 2689.2839 | 1345.1456 | 2672.2574 | 1336.6323 | 2671.2733 | 1336.1403 | G | 1349.6080 | 675.3077 | 1332.5815 | 666.7944 | 1331.5975 | 666.3024 | 14 |
| 30 | 2776.3159 | 1388.6616 | 2759.2894 | 1380.1483 | 2758.3054 | 1379.6563 | S | 1292.5866 | 646.7969 | 1275.5600 | 638.2836 | 1274.5760 | 637.7916 | 13 |
| 31 | 2923.3843 | 1462.1958 | 2906.3578 | 1453.6825 | 2905.3738 | 1453.1905 | F | 1205.5545 | 603.2809 | 1188.5280 | 594.7676 | 1187.5440 | 594.2756 | 12 |
| 32 | 3020.4371 | 1510.7222 | 3003.4106 | 1502.2089 | 3002.4265 | 1501.7169 | P | 1058.4861 | 529.7467 | 1041.4596 | 521.2334 | 1040.4756 | 520.7414 | 11 |
| 33 | 3107.4691 | 1554.2382 | 3090.4426 | 1545.7249 | 3089.4586 | 1545.2329 | S | 961.4334 | 481.2203 | 944.4068 | 472.7070 | 943.4228 | 472.2150 | 10 |
| 34 | 3178.5063 | 1589.7568 | 3161.4797 | 1581.2435 | 3160.4957 | 1580.7515 | A | 874.4013 | 437.7043 | 857.3748 | 429.1910 | 856.3908 | 428.6990 | 9 |
| 35 | 3235.5277 | 1618.2675 | 3218.5012 | 1609.7542 | 3217.5172 | 1609.2622 | G | 803.3642 | 402.1857 | 786.3377 | 393.6725 | 785.3537 | 393.1805 | 8 |
| 36 | 3322.5597 | 1661.7835 | 3305.5332 | 1653.2702 | 3304.5492 | 1652.7782 | S | 746.3428 | 373.6750 | 729.3162 | 365.1617 | 728.3322 | 364.6697 | 7 |
| 37 | 3409.5918 | 1705.2995 | 3392.5652 | 1696.7862 | 3391.5812 | 1696.2942 | S | 659.3107 | 330.1590 | 642.2842 | 321.6457 | 641.3002 | 321.1537 | 6 |
| 38 | 3524.6187 | 1762.8130 | 3507.5922 | 1754.2997 | 3506.6081 | 1753.8077 | D | 572.2787 | 286.6430 | 555.2522 | 278.1297 | 554.2681 | 277.6377 | 5 |
| 39 | 3652.6773 | 1826.8423 | 3635.6507 | 1818.3290 | 3634.6667 | 1817.8370 | Q | 457.2518 | 229.1295 | 440.2252 | 220.6162 | | | 4 |
| 40 | 3709.6988 | 1855.3530 | 3692.6722 | 1846.8397 | 3691.6882 | 1846.3477 | G | 329.1932 | 165.1002 | 312.1666 | 156.5870 | | | 3 |
| 41 | 3806.7515 | 1903.8794 | 3789.7250 | 1895.3661 | 3788.7410 | 1894.8741 | P | 272.1717 | 136.5895 | 255.1452 | 128.0762 | | | 2 |
| 42 | | | | | | | R | 175.1190 | 88.0631 | 158.0924 | 79.5498 | | | 1 |

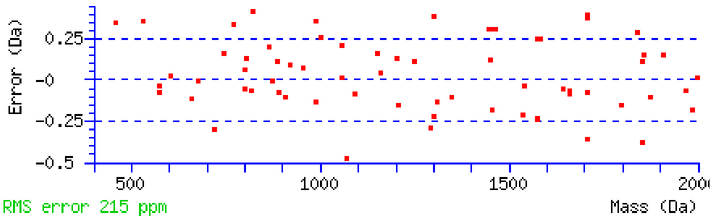


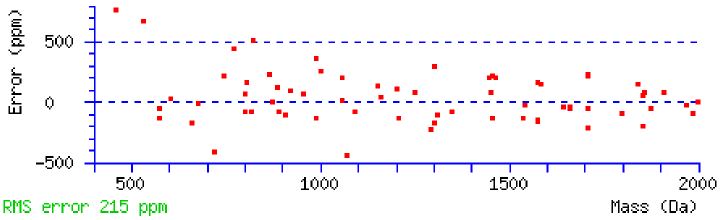


### All matches to this query

| Score | Mr(calc): | Delta | Sequence |
|---|---|---|---|
| 89.7 | 3979.8560 | 0.9915 | NGTPGGSFVSPSPLSTSSPILSADSTSVGSFPSAGSSDQGPR |
| 71.4 | 3978.8720 | 1.9755 | NGTPGGSFVSPSPLSTSSPILSADSTSVGSFPSAGSSDQGPR |
| 8.4 | 3978.8178 | 2.0297 | HYLTAFYVVECIAMSNSMINTLCFVTVRNNTSK |
| 7.3 | 3978.8489 | 1.9986 | MVIIDHGLQTLTHEVIVPHSGWEREPNEDSKPR |
| 7.3 | 3978.8489 | 1.9986 | MVIIDHGLQTLTHEVIVPHSGWEREPNEDSKPR |
| 6.5 | 3979.8774 | 0.9700 | YSNDVTSLPFLLEILTVLPEEVHSRSLRIGANR |
| 6.5 | 3979.8774 | 0.9700 | YSNDVTSLPFLLEILTVLPEEVHSRSLRIGANR |
| 6.0 | 3979.8815 | 0.9660 | LAAVSDENLGNITSVVEAIPEFHKKPSLLSFWTK |
| 5.6 | 3978.8605 | 1.9870 | SSYFVNSTFPVTLASGQESFLGQLPLYSSGYADPLR |
| 4.3 | 3980.8503 | -0.0029 | FSSGSNIPKKSVVMQSPGTSYSSWEPVISAQEVDTR |

---

Spectrum No: 138; Query: 649; Rank: 1

---

## Peptide View

MS/MS Fragmentation of **LINDYVSNQTQGK**
Found in **IPI00200591**, Tax_Id=10116 Gene_Symbol=LOC299282 Serine protease inhibitor A3L precursor

Match to Query 649: 1479.728028 from(740.871290,2+)
Title: 091008RatKidney_NoSalt_28.1315.1315.2.dta
Data file K:\NewmanPaper\Piliang\3SubProteomes\Piliang3SP\mgf5ppm\ERLIC_3SubProteomes5ppm.mgf

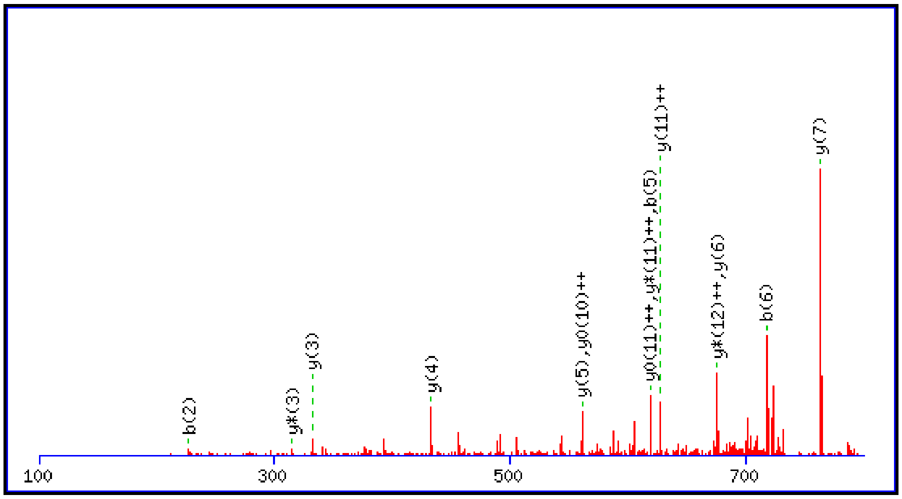


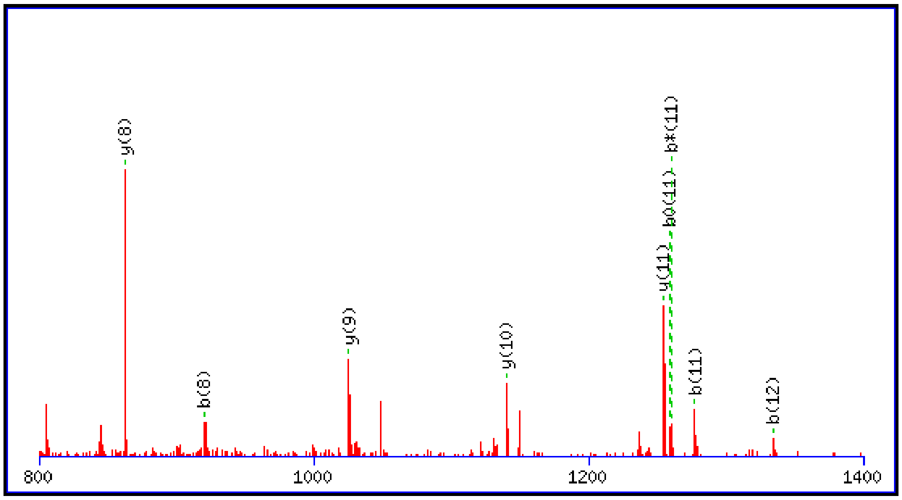


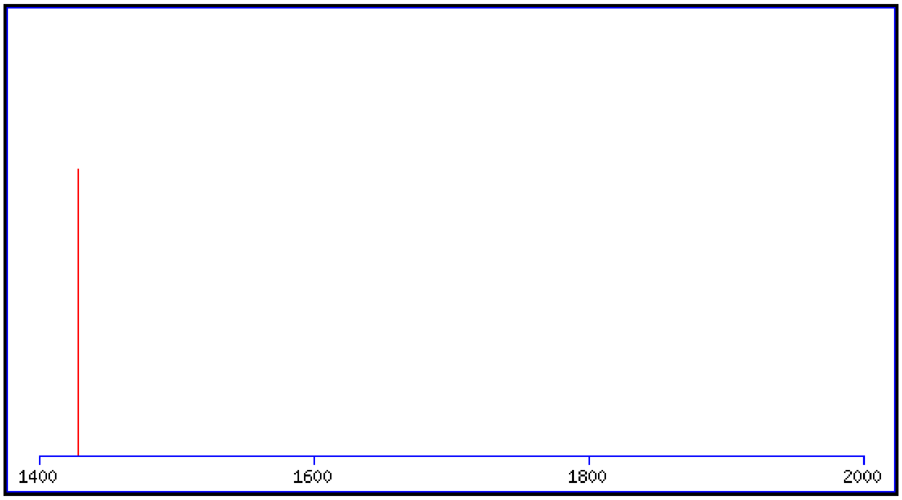


**Monoisotopic mass of neutral peptide Mr(calc):** 1479.7205
**Fixed modifications:** Carbamidomethyl (C)
**Variable modifications:**
**N8** : Deamidated_N (N)
**Ions Score:** 90 **Expect:** 1.6e-007
**Matches (Bold Red):** 23/128 fragment ions using 25 most intense peaks

| # | b | $b^{++}$ | b* | $b^{*++}$ | $b^0$ | $b^{0++}$ | Seq. | y | $y^{++}$ | y* | $y^{*++}$ | $y^0$ | $y^{0++}$ | # |
|---|---|---|---|---|---|---|---|---|---|---|---|---|---|---|

| | | | | | | | | | | | | | | |
|---|---|---|---|---|---|---|---|---|---|---|---|---|---|---|
| 1 | 114.0913 | 57.5493 | | | | | L | | | | | | | 13 |
| 2 | 227.1754 | 114.0913 | | | | | I | 1367.6437 | 684.3255 | 1350.6172 | 675.8122 | 1349.6332 | 675.3202 | 12 |
| 3 | 341.2183 | 171.1128 | 324.1918 | 162.5995 | | | N | 1254.5597 | 627.7835 | 1237.5331 | 619.2702 | 1236.5491 | 618.7782 | 11 |
| 4 | 456.2453 | 228.6263 | 439.2187 | 220.1130 | 438.2347 | 219.6210 | D | 1140.5168 | 570.7620 | 1123.4902 | 562.2487 | 1122.5062 | 561.7567 | 10 |
| 5 | 619.3086 | 310.1579 | 602.2821 | 301.6447 | 601.2980 | 301.1527 | Y | 1025.4898 | 513.2485 | 1008.4633 | 504.7353 | 1007.4792 | 504.2433 | 9 |
| 6 | 718.3770 | 359.6921 | 701.3505 | 351.1789 | 700.3665 | 350.6869 | V | 862.4265 | 431.7169 | 845.3999 | 423.2036 | 844.4159 | 422.7116 | 8 |
| 7 | 805.4090 | 403.2082 | 788.3825 | 394.6949 | 787.3985 | 394.2029 | S | 763.3581 | 382.1827 | 746.3315 | 373.6694 | 745.3475 | 373.1774 | 7 |
| 8 | 920.4360 | 460.7216 | 903.4094 | 452.2084 | 902.4254 | 451.7163 | N | 676.3260 | 338.6667 | 659.2995 | 330.1534 | 658.3155 | 329.6614 | 6 |
| 9 | 1048.4946 | 524.7509 | 1031.4680 | 516.2376 | 1030.4840 | 515.7456 | Q | 561.2991 | 281.1532 | 544.2726 | 272.6399 | 543.2885 | 272.1479 | 5 |
| 10 | 1149.5422 | 575.2748 | 1132.5157 | 566.7615 | 1131.5317 | 566.2695 | T | 433.2405 | 217.1239 | 416.2140 | 208.6106 | 415.2300 | 208.1186 | 4 |
| 11 | 1277.6008 | 639.3040 | 1260.5743 | 630.7908 | 1259.5903 | 630.2988 | Q | 332.1928 | 166.6001 | 315.1663 | 158.0868 | | | 3 |
| 12 | 1334.6223 | 667.8148 | 1317.5957 | 659.3015 | 1316.6117 | 658.8095 | G | 204.1343 | 102.5708 | 187.1077 | 94.0575 | | | 2 |
| 13 | | | | | | | K | 147.1128 | 74.0600 | 130.0863 | 65.5468 | | | 1 |

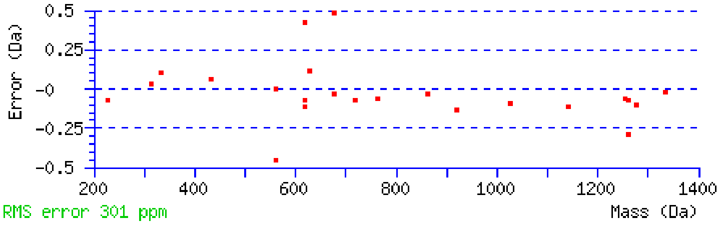


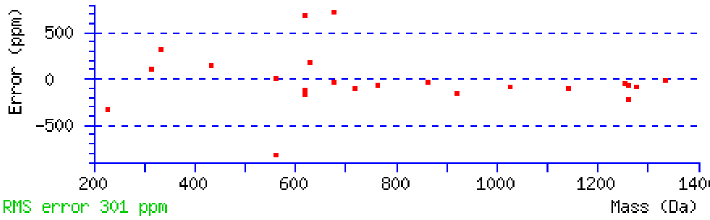


### All matches to this query

| Score | Mr(calc): | Delta | Sequence |
|---|---|---|---|
| 89.5 | 1479.7205 | 0.0075 | LINDYVSNQTQGK |
| 36.6 | 1479.7205 | 0.0075 | LINDYVSNQTQGK |
| 8.2 | 1478.7365 | 0.9915 | LINDYVSNQTQGK |
| 7.5 | 1479.7334 | -0.0053 | KNPDSLELIRSK |
| 6.0 | 1479.7425 | -0.0145 | QMTLLDMAKGTQK |
| 4.4 | 1479.7309 | -0.0029 | LLSQGLAWPKCK |
| 4.0 | 1479.7334 | -0.0054 | QGGGGVGTILSNVLK |
| 3.1 | 1479.7334 | -0.0053 | KNPDSLELIRSK |
| 1.8 | 1479.7425 | -0.0145 | QMTLLDMAKGTQK |
| 1.0 | 1479.7205 | 0.0075 | FEETLKGDEREK |

Spectrum No: 139; Query: 1998; Rank: 1

## Peptide View

MS/MS Fragmentation of **KYPVPFQNENVTAAFVELGR**
Found in **IPI00213569**, Tax_Id=10116 Gene_Symbol=Ugt1a1 UDP-glucuronosyltransferase 1-1 precursor

Match to Query 1998: 2279.158328 from(1140.586440,2+)
Title: 091008RatKidney_NH4Format01_23.3987.3987.2.dta
Data file K:\NewmanPaper\Piliang\3SubProteomes\Piliang3SP\mgf5ppm\ERLIC_3SubProteomes5ppm.mgf

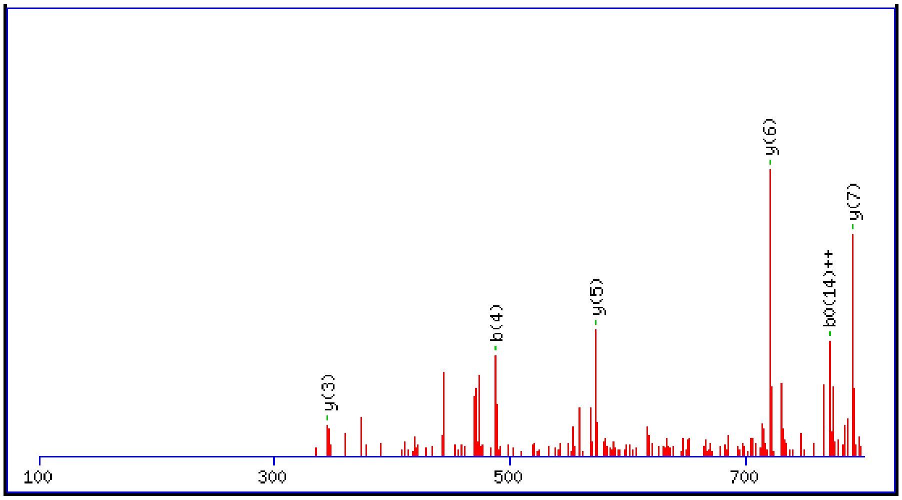


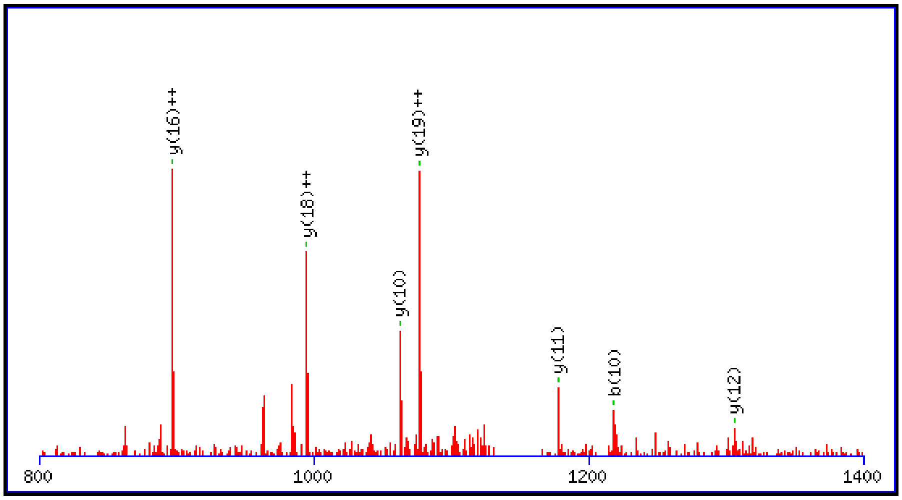


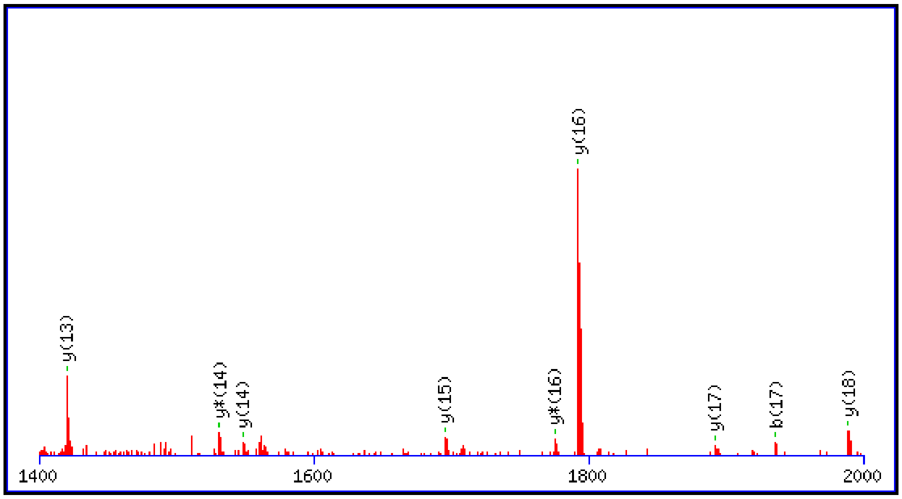


**Monoisotopic mass of neutral peptide Mr(calc):** 2279.1586
**Fixed modifications:** Carbamidomethyl (C)
**Variable modifications:**
**N10** : Deamidated_N (N)
**Ions Score:** 90 **Expect:** 1.8e-007
**Matches (Bold Red):** 22/206 fragment ions using 45 most intense peaks

| # | b | b++ | b* | b*++ | b0 | b0++ | Seq. | y | y++ | y* | y*++ | y0 | y0++ | # |
|---|---|---|---|---|---|---|---|---|---|---|---|---|---|---|
| 1 | 129.1022 | 65.0548 | 112.0757 | 56.5415 | | | K | | | | | | | 20 |
| 2 | 292.1656 | 146.5864 | 275.1390 | 138.0731 | | | Y | 2152.0709 | **1076.5391** | 2135.0444 | 1068.0258 | 2134.0604 | 1067.5338 | 19 |
| 3 | 389.2183 | 195.1128 | 372.1918 | 186.5995 | | | P | **1989.0076** | **995.0074** | 1971.9811 | 986.4942 | 1970.9970 | 986.0022 | 18 |
| 4 | **488.2867** | 244.6470 | 471.2602 | 236.1337 | | | V | **1891.9548** | 946.4811 | 1874.9283 | 937.9678 | 1873.9443 | 937.4758 | 17 |
| 5 | 585.3395 | 293.1734 | 568.3130 | 284.6601 | | | P | **1792.8864** | **896.9469** | **1775.8599** | 888.4336 | 1774.8759 | 887.9416 | 16 |

| | | | | | | | | | | | | | | |
|---|---|---|---|---|---|---|---|---|---|---|---|---|---|---|
| 6 | 732.4079 | 366.7076 | 715.3814 | 358.1943 | | | F | **1695.8337** | 848.4205 | 1678.8071 | 839.9072 | 1677.8231 | 839.4152 | 15 |
| 7 | 860.4665 | 430.7369 | 843.4400 | 422.2236 | | | Q | **1548.7653** | 774.8863 | **1531.7387** | 766.3730 | 1530.7547 | 765.8810 | 14 |
| 8 | 974.5094 | 487.7584 | 957.4829 | 479.2451 | | | N | **1420.7067** | 710.8570 | 1403.6801 | 702.3437 | 1402.6961 | 701.8517 | 13 |
| 9 | 1103.5520 | 552.2796 | 1086.5255 | 543.7664 | 1085.5415 | 543.2744 | E | **1306.6638** | 653.8355 | 1289.6372 | 645.3222 | 1288.6532 | 644.8302 | 12 |
| 10 | **1218.5790** | 609.7931 | 1201.5524 | 601.2798 | 1200.5684 | 600.7878 | N | **1177.6212** | 589.3142 | 1160.5946 | 580.8009 | 1159.6106 | 580.3089 | 11 |
| 11 | 1317.6474 | 659.3273 | 1300.6208 | 650.8140 | 1299.6368 | 650.3220 | V | **1062.5942** | 531.8007 | 1045.5677 | 523.2875 | 1044.5837 | 522.7955 | 10 |
| 12 | 1418.6951 | 709.8512 | 1401.6685 | 701.3379 | 1400.6845 | 700.8459 | T | 963.5258 | 482.2665 | 946.4993 | 473.7533 | 945.5152 | 473.2613 | 9 |
| 13 | 1489.7322 | 745.3697 | 1472.7056 | 736.8564 | 1471.7216 | 736.3644 | A | 862.4781 | 431.7427 | 845.4516 | 423.2294 | 844.4676 | 422.7374 | 8 |
| 14 | 1560.7693 | 780.8883 | 1543.7427 | 772.3750 | 1542.7587 | **771.8830** | A | **791.4410** | 396.2241 | 774.4145 | 387.7109 | 773.4305 | 387.2189 | 7 |
| 15 | 1707.8377 | 854.4225 | 1690.8111 | 845.9092 | 1689.8271 | 845.4172 | F | **720.4039** | 360.7056 | 703.3774 | 352.1923 | 702.3933 | 351.7003 | 6 |
| 16 | 1806.9061 | 903.9567 | 1789.8796 | 895.4434 | 1788.8955 | 894.9514 | V | **573.3355** | 287.1714 | 556.3089 | 278.6581 | 555.3249 | 278.1661 | 5 |
| 17 | **1935.9487** | 968.4780 | 1918.9222 | 959.9647 | 1917.9381 | 959.4727 | E | 474.2671 | 237.6372 | 457.2405 | 229.1239 | 456.2565 | 228.6319 | 4 |
| 18 | 2049.0328 | 1025.0200 | 2032.0062 | 1016.5067 | 2031.0222 | 1016.0147 | L | **345.2245** | 173.1159 | 328.1979 | 164.6026 | | | 3 |
| 19 | 2106.0542 | 1053.5308 | 2089.0277 | 1045.0175 | 2088.0437 | 1044.5255 | G | 232.1404 | 116.5738 | 215.1139 | 108.0606 | | | 2 |
| 20 | | | | | | | R | 175.1190 | 88.0631 | 158.0924 | 79.5498 | | | 1 |

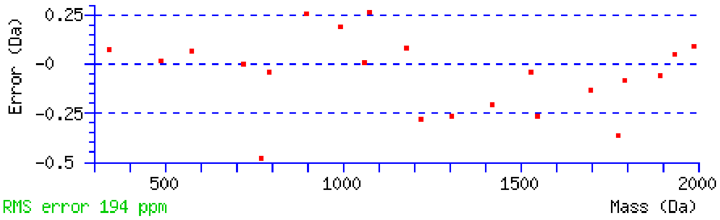


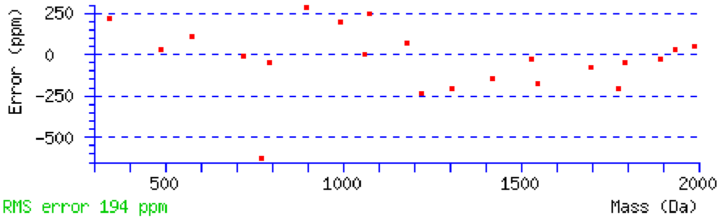


### All matches to this query

| Score | Mr(calc): | Delta | Sequence |
|---|---|---|---|
| 89.6 | 2279.1586 | -0.0003 | KYPVPFQNENVTAAFVELGR |
| 63.8 | 2279.1586 | -0.0003 | KYPVPFQNENVTAAFVELGR |
| 4.9 | 2277.1366 | 2.0218 | ATATPVKASAPSANTKASATQPK |
| 2.0 | 2277.1366 | 2.0218 | ATATPVKASAPSANTKASATQPK |
| 1.6 | 2279.1433 | 0.0150 | DAGENNFINVDLKLARDVFK |
| 1.3 | 2277.1518 | 2.0065 | TIINQVLSQTQIEFASHLR |
| 0.5 | 2279.1490 | 0.0093 | KVFLNKAADFIEELSSLFK |
| 0.4 | 2279.1490 | 0.0093 | KVFLNKAADFIEELSSLFK |
| 0.3 | 2279.1433 | 0.0150 | DAGENNFINVDLKLARDVFK |

---

Spectrum No: 140; Query: 1103; Rank: 1

---

## Peptide View

MS/MS Fragmentation of **VLPFNVTDYCQLVR**
Found in **IPI00204006**, Tax_Id=10116 Gene_Symbol=Fbn1 Fibrillin-1

Match to Query 1103: 1723.856768 from(862.935660,2+)
Title: 091008RatKidney_NH4Format01_23.3770.3770.2.dta
Data file K:\NewmanPaper\Piliang\3SubProteomes\Piliang3SP\mgf5ppm\ERLIC_3SubProteomes5ppm.mgf

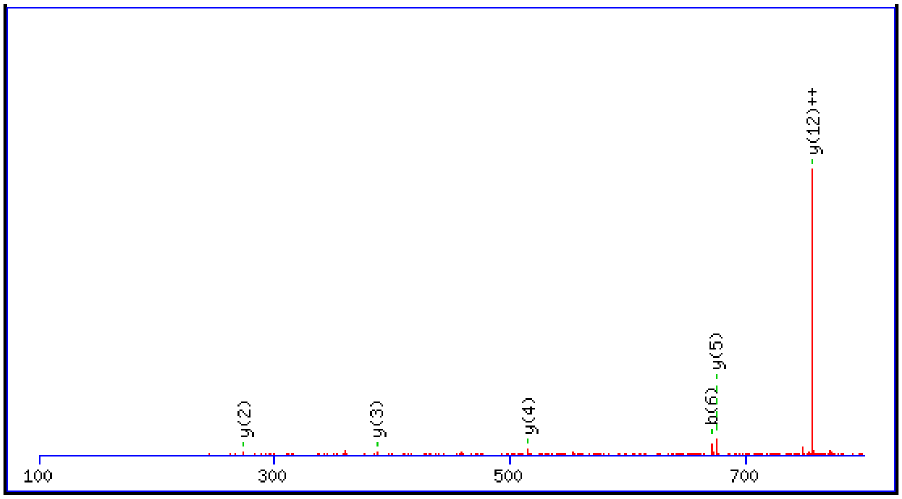


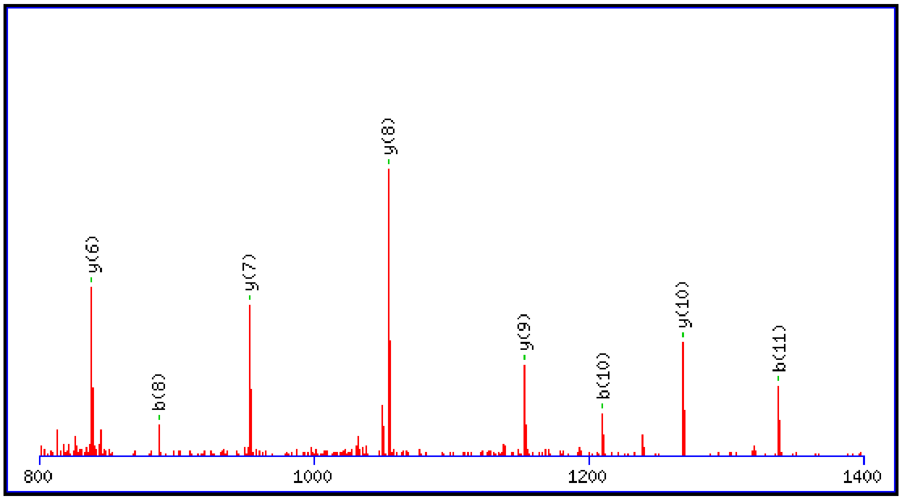


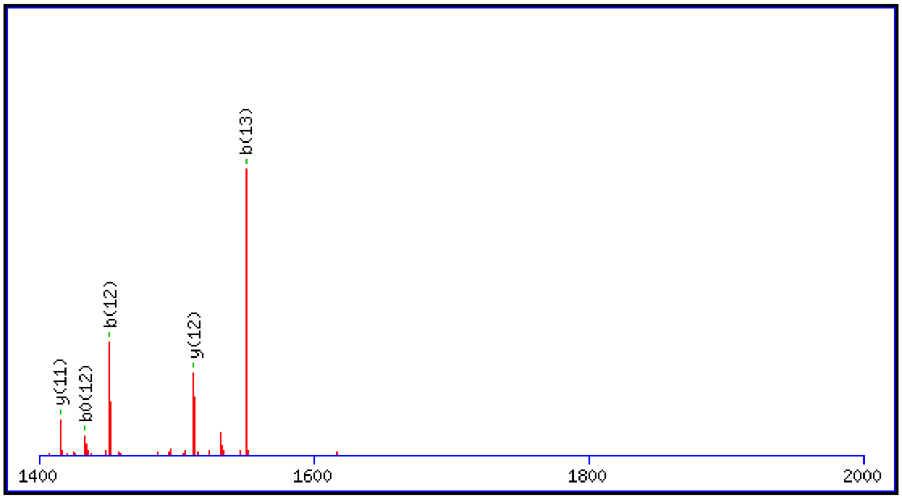


**Monoisotopic mass of neutral peptide Mr(calc):** 1723.8604
**Fixed modifications:** Carbamidomethyl (C)
**Variable modifications:**
**N5** : Deamidated_N (N)
**Ions Score:** 89 **Expect:** 2.1e-007
**Matches (Bold Red):** 19/124 fragment ions using 30 most intense peaks

| # | b | b$^{++}$ | b* | b*$^{++}$ | b$^{0}$ | b$^{0++}$ | Seq. | y | y$^{++}$ | y* | y*$^{++}$ | y$^{0}$ | y$^{0++}$ | # |
|---|---|---|---|---|---|---|---|---|---|---|---|---|---|---|
| 1 | 100.0757 | 50.5415 | | | | | V | | | | | | | 14 |
| 2 | 213.1598 | 107.0835 | | | | | L | 1625.7992 | 813.4032 | 1608.7727 | 804.8900 | 1607.7886 | 804.3980 | 13 |
| 3 | 310.2125 | 155.6099 | | | | | P | **1512.7151** | **756.8612** | 1495.6886 | 748.3479 | 1494.7046 | 747.8559 | 12 |
| 4 | 457.2809 | 229.1441 | | | | | F | **1415.6624** | 708.3348 | 1398.6358 | 699.8215 | 1397.6518 | 699.3295 | 11 |
| 5 | 572.3079 | 286.6576 | 555.2813 | 278.1443 | | | N | **1268.5940** | 634.8006 | 1251.5674 | 626.2873 | 1250.5834 | 625.7953 | 10 |

| | | | | | | | | | | | | | | |
|---|---|---|---|---|---|---|---|---|---|---|---|---|---|---|
| **6** | **671.3763** | 336.1918 | 654.3497 | 327.6785 | | | **V** | **1153.5670** | 577.2871 | 1136.5405 | 568.7739 | 1135.5565 | 568.2819 | **9** |
| **7** | 772.4240 | 386.7156 | 755.3974 | 378.2023 | 754.4134 | 377.7103 | **T** | **1054.4986** | 527.7529 | 1037.4721 | 519.2397 | 1036.4880 | 518.7477 | **8** |
| **8** | **887.4509** | 444.2291 | 870.4244 | 435.7158 | 869.4403 | 435.2238 | **D** | **953.4509** | 477.2291 | 936.4244 | 468.7158 | 935.4404 | 468.2238 | **7** |
| **9** | 1050.5142 | 525.7608 | 1033.4877 | 517.2475 | 1032.5037 | 516.7555 | **Y** | **838.4240** | 419.7156 | 821.3974 | 411.2024 | | | **6** |
| **10** | **1210.5449** | 605.7761 | 1193.5183 | 597.2628 | 1192.5343 | 596.7708 | **C** | **675.3607** | 338.1840 | 658.3341 | 329.6707 | | | **5** |
| **11** | **1338.6035** | 669.8054 | 1321.5769 | 661.2921 | 1320.5929 | 660.8001 | **Q** | **515.3300** | 258.1686 | 498.3035 | 249.6554 | | | **4** |
| **12** | **1451.6875** | 726.3474 | 1434.6610 | 717.8341 | **1433.6770** | 717.3421 | **L** | **387.2714** | 194.1394 | 370.2449 | 185.6261 | | | **3** |
| **13** | **1550.7559** | 775.8816 | 1533.7294 | 767.3683 | 1532.7454 | 766.8763 | **V** | **274.1874** | 137.5973 | 257.1608 | 129.0840 | | | **2** |
| **14** | | | | | | | **R** | 175.1190 | 88.0631 | 158.0924 | 79.5498 | | | **1** |

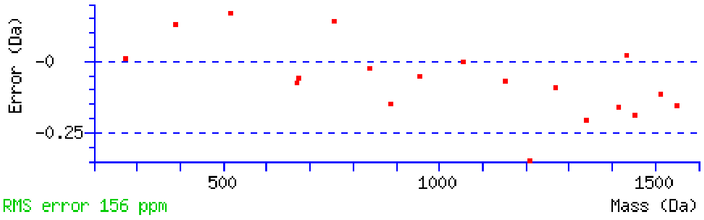


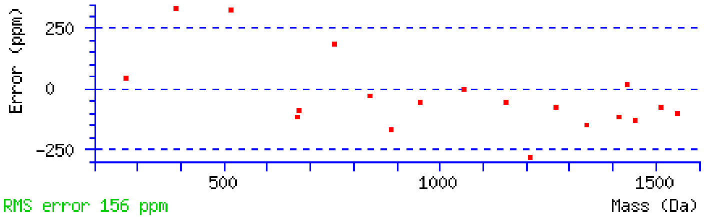


### All matches to this query

| Score | Mr(calc): | Delta | Sequence |
|---|---|---|---|
| 89.3 | 1723.8604 | -0.0036 | VLPFNVTDYCQLVR |
| 13.3 | 1723.8645 | -0.0077 | VISQTNLITTVTPEK |
| 8.5 | 1723.8463 | 0.0105 | IPKASAKFYATIVR |
| 8.4 | 1723.8514 | 0.0053 | LCLSPLTNLCGKLR |
| 8.2 | 1723.8514 | 0.0053 | LCLSPLTNLCGKLR |
| 5.7 | 1723.8645 | -0.0077 | VISQTNLITTVTPEK |
| 5.0 | 1722.8665 | 0.9902 | LVAGELRLSKEGTDR |
| 3.6 | 1722.8665 | 0.9902 | GAGTLDGTLIRNIVSR |
| 3.5 | 1723.8553 | 0.0015 | MRKPDGSTVTQRIR |
| 3.5 | 1723.8553 | 0.0015 | MRKPDGSTVTQRIR |

---

Spectrum No: 141; Query: 469; Rank: 1

---

## Peptide View

MS/MS Fragmentation of **SAVFTVENGSTIR**
Found in **IPI00214373**, Tax_Id=10116 Gene_Symbol=Aadat Kynurenine/alpha-aminoadipate aminotransferase mitochondrial precursor

Match to Query 469: 1380.694828 from(691.354690,2+)
Title: 091008RatKidney_NoSalt_08.1562.1562.2.dta
Data file K:\NewmanPaper\Piliang\3SubProteomes\Piliang3SP\mgf5ppm\ERLIC_3SubProteomes5ppm.mgf

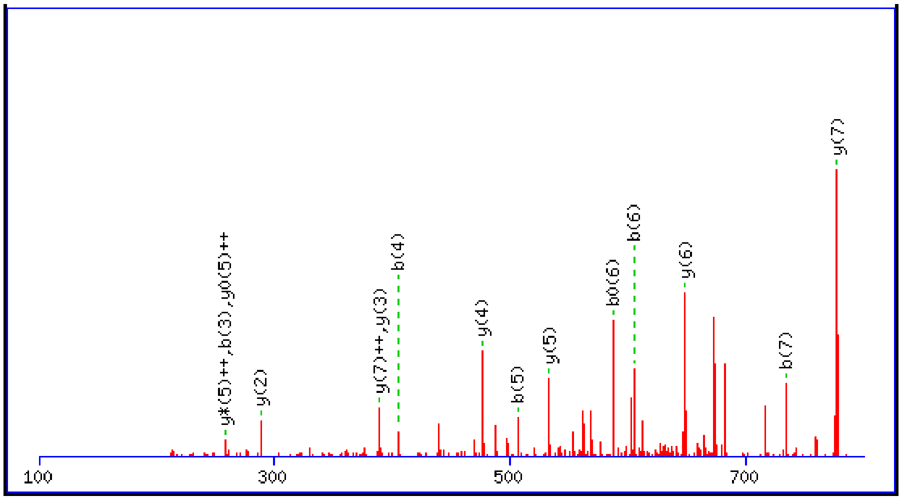


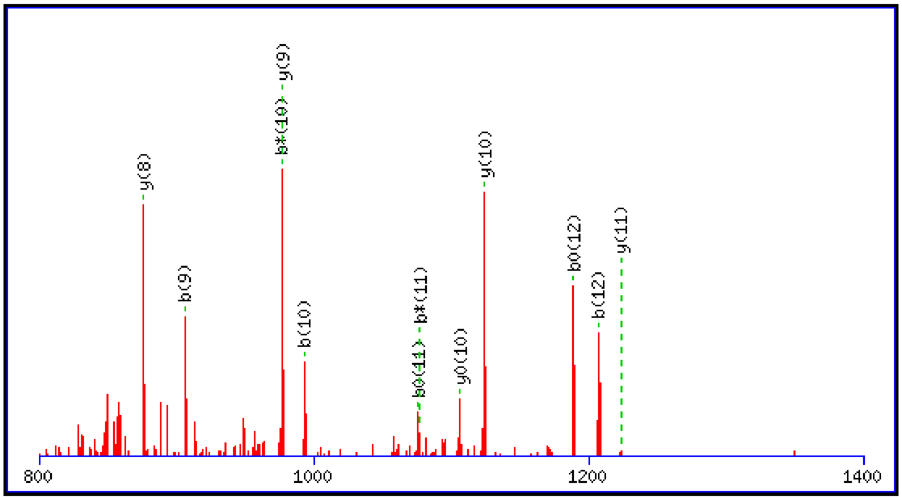


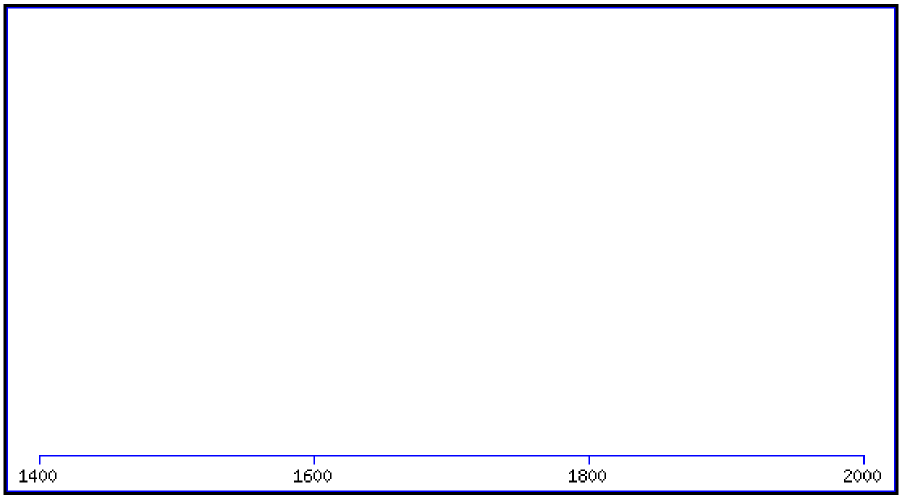


**Monoisotopic mass of neutral peptide Mr(calc):** 1380.6885
**Fixed modifications:** Carbamidomethyl (C)
**Variable modifications:**
**N8** : Deamidated_N (N)
**Ions Score:** 89 **Expect:** 1.7e-007
**Matches (Bold Red):** 27/126 fragment ions using 35 most intense peaks

| # | b | $b^{++}$ | b* | $b^{*++}$ | $b^{0}$ | $b^{0++}$ | Seq. | y | $y^{++}$ | y* | $y^{*++}$ | $y^{0}$ | $y^{0++}$ | # |
|---|---|---|---|---|---|---|---|---|---|---|---|---|---|---|
| 1 | 88.0393 | 44.5233 | | | 70.0287 | 35.5180 | S | | | | | | | 13 |
| 2 | 159.0764 | 80.0418 | | | 141.0659 | 71.0366 | A | 1294.6638 | 647.8355 | 1277.6372 | 639.3222 | 1276.6532 | 638.8302 | 12 |
| 3 | **258.1448** | 129.5761 | | | 240.1343 | 120.5708 | V | **1223.6266** | 612.3170 | 1206.6001 | 603.8037 | 1205.6161 | 603.3117 | 11 |
| 4 | **405.2132** | 203.1103 | | | 387.2027 | 194.1050 | F | **1124.5582** | 562.7827 | 1107.5317 | 554.2695 | **1106.5477** | 553.7775 | 10 |
| 5 | **506.2609** | 253.6341 | | | 488.2504 | 244.6288 | T | **977.4898** | 489.2485 | 960.4633 | 480.7353 | 959.4792 | 480.2433 | 9 |

| | | | | | | | | | | | | | | |
|---|---|---|---|---|---|---|---|---|---|---|---|---|---|---|
| **6** | **605.3293** | 303.1683 | | | **587.3188** | 294.1630 | **V** | **876.4421** | 438.7247 | 859.4156 | 430.2114 | 858.4316 | 429.7194 | **8** |
| **7** | **734.3719** | 367.6896 | | | 716.3614 | 358.6843 | **E** | **777.3737** | **389.1905** | 760.3472 | 380.6772 | 759.3632 | 380.1852 | **7** |
| **8** | 849.3989 | 425.2031 | 832.3723 | 416.6898 | 831.3883 | 416.1978 | **N** | **648.3311** | 324.6692 | 631.3046 | 316.1559 | 630.3206 | 315.6639 | **6** |
| **9** | **906.4203** | 453.7138 | 889.3938 | 445.2005 | 888.4098 | 444.7085 | **G** | **533.3042** | 267.1557 | 516.2776 | **258.6425** | 515.2936 | **258.1504** | **5** |
| **10** | **993.4524** | 497.2298 | **976.4258** | 488.7165 | 975.4418 | 488.2245 | **S** | **476.2827** | 238.6450 | 459.2562 | 230.1317 | 458.2722 | 229.6397 | **4** |
| **11** | 1094.5000 | 547.7537 | **1077.4735** | 539.2404 | **1076.4895** | 538.7484 | **T** | **389.2507** | 195.1290 | 372.2241 | 186.6157 | 371.2401 | 186.1237 | **3** |
| **12** | **1207.5841** | 604.2957 | 1190.5576 | 595.7824 | **1189.5735** | 595.2904 | **I** | **288.2030** | 144.6051 | 271.1765 | 136.0919 | | | **2** |
| **13** | | | | | | | **R** | 175.1190 | 88.0631 | 158.0924 | 79.5498 | | | **1** |

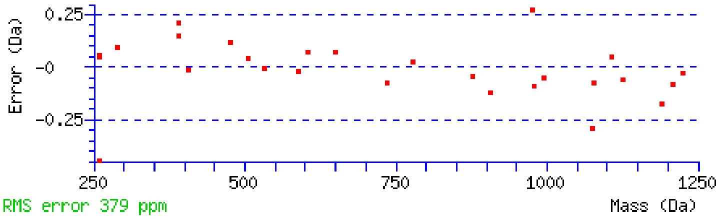


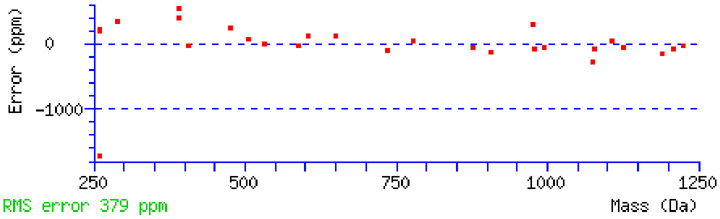


### All matches to this query

| Score | Mr(calc): | Delta | Sequence |
|---|---|---|---|
| 89.3 | 1380.6885 | 0.0063 | SAVFTVENGSTIR |
| 39.7 | 1379.7045 | 0.9903 | SAVFTVENGSTIR |
| 12.2 | 1380.6820 | 0.0129 | DNSFRICVLTR |
| 10.8 | 1380.6997 | -0.0049 | EKFQSRTSEAAK |
| 10.2 | 1380.7071 | -0.0123 | MESKEEFVKVR |
| 9.0 | 1380.6867 | 0.0082 | QERHMIMHKR |
| 8.7 | 1380.6820 | 0.0129 | RTFKEGMDELR |
| 8.5 | 1380.6915 | 0.0034 | TGRLPEAAFLAR |
| 8.1 | 1380.6845 | 0.0103 | SSVDSKRSSVDSK |
| 7.4 | 1380.6932 | 0.0016 | MLNTGLHNPNVR |

---

Spectrum No: 142; Query: 675; Rank: 1

---

## Peptide View

MS/MS Fragmentation of **TVANLSGCEQVDSK**
Found in **IPI00557299**, Tax_Id=10116 Gene_Symbol=LOC498940 Similar to Carboxylesterase 2

Match to Query 675: 1507.684948 from(754.849750,2+)
Title: 100101RatKid_NS_deglyco_24.1123.1123.2.dta
Data file K:\NewmanPaper\Piliang\3SubProteomes\Piliang3SP\mgf5ppm\ERLIC_3SubProteomes5ppm.mgf

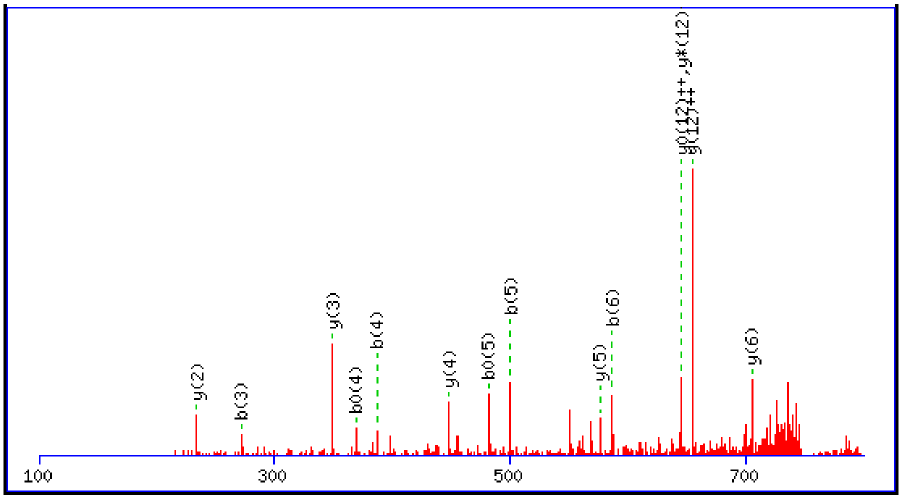


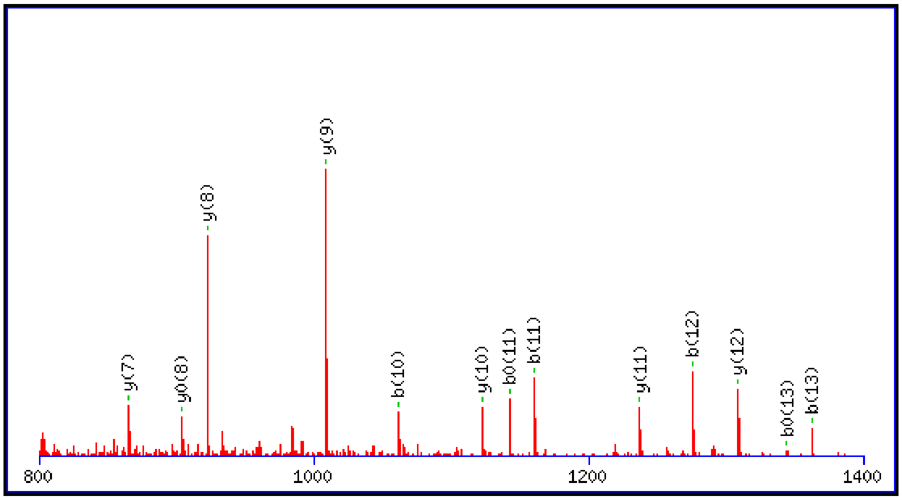


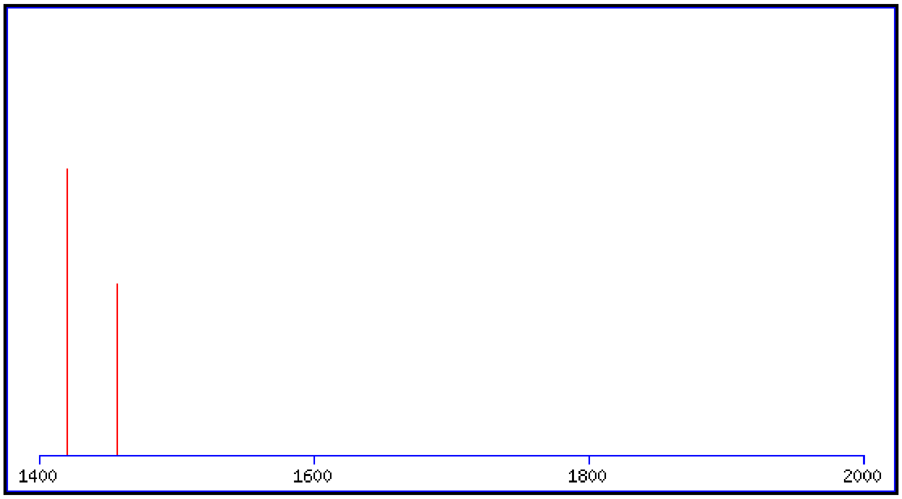


**Monoisotopic mass of neutral peptide Mr(calc):** 1507.6824
**Fixed modifications:** Carbamidomethyl (C)
**Variable modifications:**
**N4** : Deamidated_N (N)
**Ions Score:** 89 **Expect:** 2e-007
**Matches (Bold Red):** 27/148 fragment ions using 41 most intense peaks

| # | b | $b^{++}$ | b* | $b^{*++}$ | $b^0$ | $b^{0++}$ | Seq. | y | $y^{++}$ | y* | $y^{*++}$ | $y^0$ | $y^{0++}$ | # |
|---|---|---|---|---|---|---|---|---|---|---|---|---|---|---|
| 1 | 102.0550 | 51.5311 | | | 84.0444 | 42.5258 | T | | | | | | | 14 |
| 2 | 201.1234 | 101.0653 | | | 183.1128 | 92.0600 | V | 1407.6420 | 704.3247 | 1390.6155 | 695.8114 | 1389.6315 | 695.3194 | 13 |
| 3 | **272.1605** | 136.5839 | | | 254.1499 | 127.5786 | A | **1308.5736** | **654.7904** | 1291.5471 | **646.2772** | 1290.5631 | **645.7852** | 12 |
| 4 | **387.1874** | 194.0973 | 370.1609 | 185.5841 | **369.1769** | 185.0921 | N | **1237.5365** | 619.2719 | 1220.5100 | 610.7586 | 1219.5259 | 610.2666 | 11 |
| 5 | **500.2715** | 250.6394 | 483.2449 | 242.1261 | **482.2609** | 241.6341 | L | **1122.5096** | 561.7584 | 1105.4830 | 553.2451 | 1104.4990 | 552.7531 | 10 |

| | | | | | | | | | | | | | | |
|---|---|---|---|---|---|---|---|---|---|---|---|---|---|---|
| **6** | **587.3035** | 294.1554 | 570.2770 | 285.6421 | 569.2929 | 285.1501 | **S** | **1009.4255** | 505.2164 | 992.3990 | 496.7031 | 991.4149 | 496.2111 | **9** |
| **7** | 644.3250 | 322.6661 | 627.2984 | 314.1529 | 626.3144 | 313.6608 | **G** | **922.3935** | 461.7004 | 905.3669 | 453.1871 | **904.3829** | 452.6951 | **8** |
| **8** | 804.3556 | 402.6815 | 787.3291 | 394.1682 | 786.3451 | 393.6762 | **C** | **865.3720** | 433.1896 | 848.3455 | 424.6764 | 847.3614 | 424.1844 | **7** |
| **9** | 933.3982 | 467.2027 | 916.3717 | 458.6895 | 915.3877 | 458.1975 | **E** | **705.3414** | 353.1743 | 688.3148 | 344.6610 | 687.3308 | 344.1690 | **6** |
| **10** | **1061.4568** | 531.2320 | 1044.4302 | 522.7188 | 1043.4462 | 522.2268 | **Q** | **576.2988** | 288.6530 | 559.2722 | 280.1397 | 558.2882 | 279.6477 | **5** |
| **11** | **1160.5252** | 580.7662 | 1143.4987 | 572.2530 | **1142.5146** | 571.7610 | **V** | **448.2402** | 224.6237 | 431.2136 | 216.1105 | 430.2296 | 215.6185 | **4** |
| **12** | **1275.5522** | 638.2797 | 1258.5256 | 629.7664 | 1257.5416 | 629.2744 | **D** | **349.1718** | 175.0895 | 332.1452 | 166.5763 | 331.1612 | 166.0842 | **3** |
| **13** | **1362.5842** | 681.7957 | 1345.5576 | 673.2825 | **1344.5736** | 672.7904 | **S** | **234.1448** | 117.5761 | 217.1183 | 109.0628 | 216.1343 | 108.5708 | **2** |
| **14** | | | | | | | **K** | 147.1128 | 74.0600 | 130.0863 | 65.5468 | | | **1** |

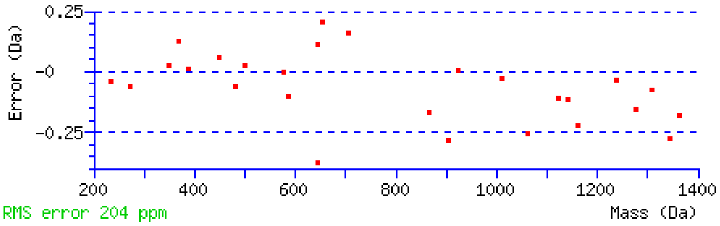


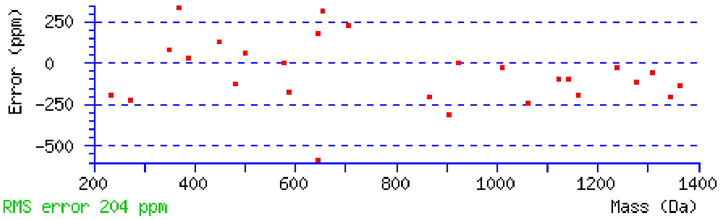


### All matches to this query

| Score | Mr(calc): | Delta | Sequence |
|---|---|---|---|
| 89.2 | 1507.6824 | 0.0025 | TVANLSGCEQVDSK |
| 6.5 | 1507.6782 | 0.0067 | CVLPYTQFSVSK |
| 6.0 | 1507.6960 | -0.0110 | GGSFPLSIVSSYSK |
| 6.0 | 1507.6960 | -0.0110 | GGSFPLSIVSSYSK |
| 6.0 | 1507.6960 | -0.0110 | GGSFPLSIVSSYSK |
| 5.1 | 1507.6960 | -0.0110 | GGSFPLSIVSSYSK |
| 4.6 | 1507.6937 | -0.0087 | VCASDTRAGIEDSK |
| 2.3 | 1506.6666 | 1.0183 | GAAVSLNIRTMSK |
| 2.0 | 1507.6919 | -0.0070 | VNTASILGHSSTQL |
| 0.4 | 1507.6807 | 0.0043 | AEESLPLPAAESSK |

---

Spectrum No: 143; Query: 2794; Rank: 1

---

## Peptide View

MS/MS Fragmentation of **VSGILFNDEMDDFSSPNFTNQFGVAPSPANFIKPGK**
Found in **IPI00206254**, Tax_Id=10116 Gene_Symbol=Ggt1 Gamma-glutamyltranspeptidase 1 precursor

Match to Query 2794: 3903.826362 from(1302.282730,3+)
Title: 100101RatKid_NS_deglyco_19.5285.5285.3.dta
Data file K:\NewmanPaper\Piliang\3SubProteomes\Piliang3SP\mgf5ppm\ERLIC_3SubProteomes5ppm.mgf

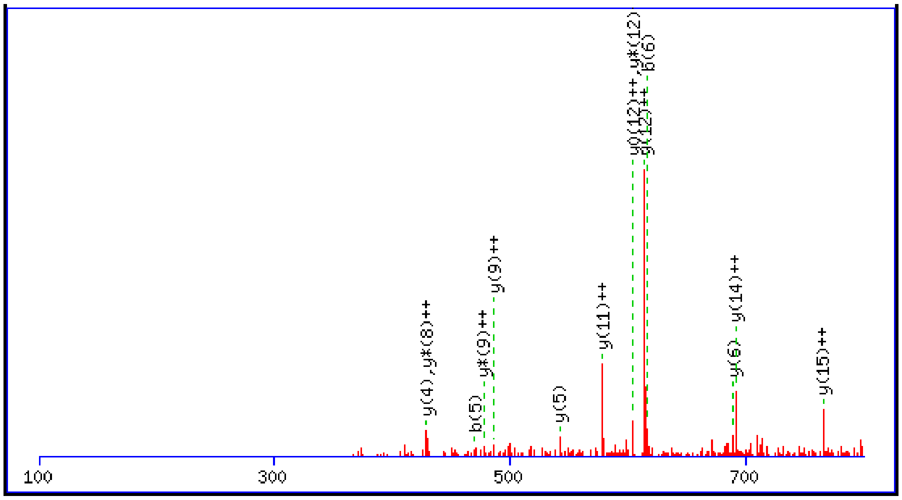


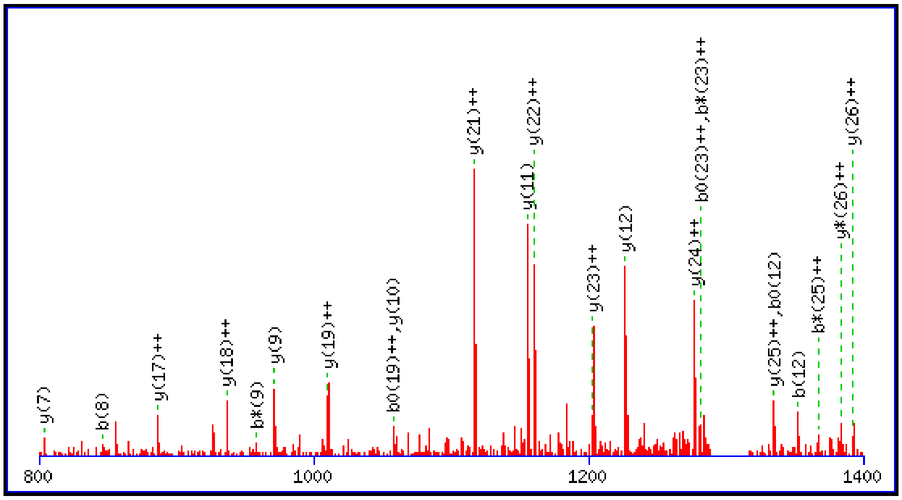


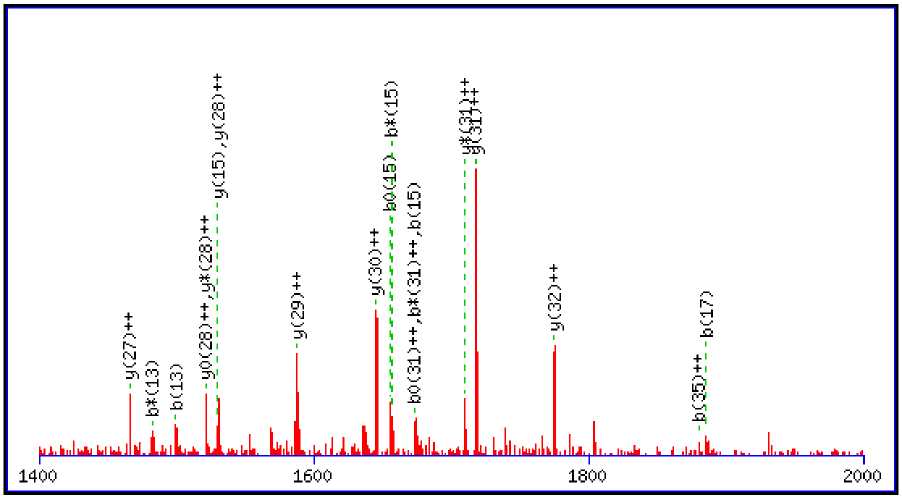


**Monoisotopic mass of neutral peptide Mr(calc):** 3903.8302
**Fixed modifications:** Carbamidomethyl (C)
**Variable modifications:**
**M10** : Oxidation (M)
**N17** : Deamidated_N (N)
**Ions Score:** 89 **Expect:** 5.5e-007
**Matches (Bold Red):** 56/388 fragment ions using 110 most intense peaks

| # | b | b++ | b* | b*++ | b0 | b0++ | Seq. | y | y++ | y* | y*++ | y0 | y0++ | # |
|---|---|---|---|---|---|---|---|---|---|---|---|---|---|---|
| 1 | 100.0757 | 50.5415 | | | | | V | | | | | | | 36 |
| 2 | 187.1077 | 94.0575 | | | 169.0972 | 85.0522 | S | 3805.7690 | 1903.3882 | 3788.7425 | 1894.8749 | 3787.7585 | 1894.3829 | 35 |
| 3 | 244.1292 | 122.5682 | | | 226.1186 | 113.5629 | G | 3718.7370 | 1859.8721 | 3701.7105 | 1851.3589 | 3700.7264 | 1850.8669 | 34 |
| 4 | 357.2132 | 179.1103 | | | 339.2027 | 170.1050 | I | 3661.7155 | 1831.3614 | 3644.6890 | 1822.8481 | 3643.7050 | 1822.3561 | 33 |

| | | | | | | | | | | | | | | |
|---|---|---|---|---|---|---|---|---|---|---|---|---|---|---|
| 5 | **470.2973** | 235.6523 | | | 452.2867 | 226.6470 | L | 3548.6315 | **1774.8194** | 3531.6049 | 1766.3061 | 3530.6209 | 1765.8141 | 32 |
| 6 | **617.3657** | 309.1865 | | | 599.3552 | 300.1812 | F | 3435.5474 | **1718.2773** | 3418.5209 | **1709.7641** | 3417.5368 | 1709.2721 | 31 |
| 7 | 731.4087 | 366.2080 | 714.3821 | 357.6947 | 713.3981 | 357.2027 | N | 3288.4790 | **1644.7431** | 3271.4524 | 1636.2299 | 3270.4684 | 1635.7379 | 30 |
| 8 | **846.4356** | 423.7214 | 829.4090 | 415.2082 | 828.4250 | 414.7162 | D | 3174.4361 | **1587.7217** | 3157.4095 | 1579.2084 | 3156.4255 | 1578.7164 | 29 |
| 9 | 975.4782 | 488.2427 | **958.4516** | 479.7295 | 957.4676 | 479.2374 | E | 3059.4091 | **1530.2082** | 3042.3826 | **1521.6949** | 3041.3986 | **1521.2029** | 28 |
| 10 | 1122.5136 | 561.7604 | 1105.4870 | 553.2472 | 1104.5030 | 552.7552 | M | 2930.3665 | **1465.6869** | 2913.3400 | 1457.1736 | 2912.3560 | 1456.6816 | 27 |
| 11 | 1237.5405 | 619.2739 | 1220.5140 | 610.7606 | 1219.5300 | 610.2686 | D | 2783.3311 | **1392.1692** | 2766.3046 | **1383.6559** | 2765.3206 | 1383.1639 | 26 |
| 12 | **1352.5675** | 676.7874 | 1335.5409 | 668.2741 | **1334.5569** | 667.7821 | D | 2668.3042 | **1334.6557** | 2651.2776 | 1326.1425 | 2650.2936 | 1325.6504 | 25 |
| 13 | **1499.6359** | 750.3216 | **1482.6093** | 741.8083 | 1481.6253 | 741.3163 | F | 2553.2772 | **1277.1423** | 2536.2507 | 1268.6290 | 2535.2667 | 1268.1370 | 24 |
| 14 | 1586.6679 | 793.8376 | 1569.6414 | 785.3243 | 1568.6574 | 784.8323 | S | 2406.2088 | **1203.6081** | 2389.1823 | 1195.0948 | 2388.1983 | 1194.6028 | 23 |
| 15 | **1673.6999** | 837.3536 | **1656.6734** | 828.8403 | **1655.6894** | 828.3483 | S | 2319.1768 | **1160.0920** | 2302.1503 | 1151.5788 | 2301.1662 | 1151.0868 | 22 |
| 16 | 1770.7527 | 885.8800 | 1753.7262 | 877.3667 | 1752.7421 | 876.8747 | P | 2232.1448 | **1116.5760** | 2215.1182 | 1108.0628 | 2214.1342 | 1107.5707 | 21 |
| 17 | **1885.7796** | 943.3935 | 1868.7531 | 934.8802 | 1867.7691 | 934.3882 | N | 2135.0920 | 1068.0496 | 2118.0655 | 1059.5364 | 2117.0814 | 1059.0444 | 20 |
| 18 | 2032.8481 | 1016.9277 | 2015.8215 | 1008.4144 | 2014.8375 | 1007.9224 | F | 2020.0651 | **1010.5362** | 2003.0385 | 1002.0229 | 2002.0545 | 1001.5309 | 19 |
| 19 | 2133.8957 | 1067.4515 | 2116.8692 | 1058.9382 | 2115.8852 | **1058.4462** | T | 1872.9967 | **937.0020** | 1855.9701 | 928.4887 | 1854.9861 | 927.9967 | 18 |
| 20 | 2247.9387 | 1124.4730 | 2230.9121 | 1115.9597 | 2229.9281 | 1115.4677 | N | 1771.9490 | **886.4781** | 1754.9224 | 877.9649 | 1753.9384 | 877.4728 | 17 |
| 21 | 2375.9972 | 1188.5023 | 2358.9707 | 1179.9890 | 2357.9867 | 1179.4970 | Q | 1657.9061 | 829.4567 | 1640.8795 | 820.9434 | 1639.8955 | 820.4514 | 16 |
| 22 | 2523.0657 | 1262.0365 | 2506.0391 | 1253.5232 | 2505.0551 | 1253.0312 | F | **1529.8475** | **765.4274** | 1512.8209 | 756.9141 | 1511.8369 | 756.4221 | 15 |
| 23 | 2580.0871 | 1290.5472 | 2563.0606 | **1282.0339** | 2562.0766 | **1281.5419** | G | 1382.7791 | **691.8932** | 1365.7525 | 683.3799 | 1364.7685 | 682.8879 | 14 |
| 24 | 2679.1555 | 1340.0814 | 2662.1290 | 1331.5681 | 2661.1450 | 1331.0761 | V | 1325.7576 | 663.3824 | 1308.7310 | 654.8692 | 1307.7470 | 654.3772 | 13 |
| 25 | 2750.1927 | 1375.6000 | 2733.1661 | **1367.0867** | 2732.1821 | 1366.5947 | A | **1226.6892** | **613.8482** | 1209.6626 | **605.3350** | 1208.6786 | **604.8429** | 12 |
| 26 | 2847.2454 | 1424.1263 | 2830.2189 | 1415.6131 | 2829.2348 | 1415.1211 | P | **1155.6521** | **578.3297** | 1138.6255 | 569.8164 | 1137.6415 | 569.3244 | 11 |
| 27 | 2934.2774 | 1467.6424 | 2917.2509 | 1459.1291 | 2916.2669 | 1458.6371 | S | **1058.5993** | 529.8033 | 1041.5728 | 521.2900 | 1040.5887 | 520.7980 | 10 |
| 28 | 3031.3302 | 1516.1687 | 3014.3037 | 1507.6555 | 3013.3196 | 1507.1635 | P | **971.5673** | **486.2873** | 954.5407 | **477.7740** | | | 9 |
| 29 | 3102.3673 | 1551.6873 | 3085.3408 | 1543.1740 | 3084.3568 | 1542.6820 | A | 874.5145 | 437.7609 | 857.4880 | **429.2476** | | | 8 |
| 30 | 3216.4102 | 1608.7088 | 3199.3837 | 1600.1955 | 3198.3997 | 1599.7035 | N | **803.4774** | 402.2423 | 786.4509 | 393.7291 | | | 7 |
| 31 | 3363.4787 | 1682.2430 | 3346.4521 | **1673.7297** | 3345.4681 | **1673.2377** | F | **689.4345** | 345.2209 | 672.4079 | 336.7076 | | | 6 |
| 32 | 3476.5627 | 1738.7850 | 3459.5362 | 1730.2717 | 3458.5522 | 1729.7797 | I | **542.3661** | 271.6867 | 525.3395 | 263.1734 | | | 5 |
| 33 | 3604.6577 | 1802.8325 | 3587.6311 | 1794.3192 | 3586.6471 | 1793.8272 | K | **429.2820** | 215.1446 | 412.2554 | 206.6314 | | | 4 |
| 34 | 3701.7105 | 1851.3589 | 3684.6839 | 1842.8456 | 3683.6999 | 1842.3536 | P | 301.1870 | 151.0972 | 284.1605 | 142.5839 | | | 3 |
| 35 | 3758.7319 | **1879.8696** | 3741.7054 | 1871.3563 | 3740.7214 | 1870.8643 | G | 204.1343 | 102.5708 | 187.1077 | 94.0575 | | | 2 |
| 36 | | | | | | | K | 147.1128 | 74.0600 | 130.0863 | 65.5468 | | | 1 |

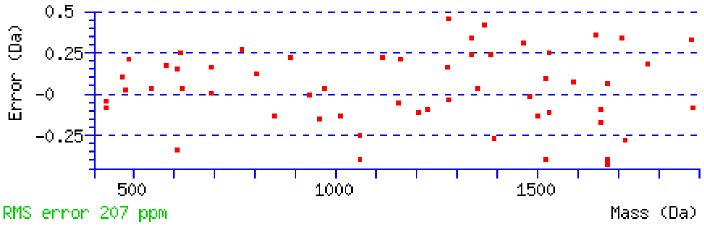


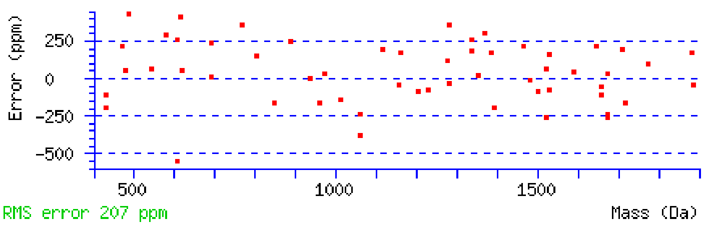


### All matches to this query

| Score | Mr(calc): | Delta | Sequence |
|---|---|---|---|
| 89.1 | 3903.8302 | -0.0038 | VSGILFNDEMDDFSSPNFTNQFGVAPSPANFIKPGK |
| 88.9 | 3903.8302 | -0.0038 | VSGILFNDEMDDFSSPNFTNQFGVAPSPANFIKPGK |
| 87.7 | 3903.8302 | -0.0038 | VSGILFNDEMDDFSSPNFTNQFGVAPSPANFIKPGK |
| 39.0 | 3903.8302 | -0.0038 | VSGILFNDEMDDFSSPNFTNQFGVAPSPANFIKPGK |
| 30.4 | 3902.8462 | 0.9802 | VSGILFNDEMDDFSSPNFTNQFGVAPSPANFIKPGK |
| 7.3 | 3901.7841 | 2.0422 | ELQNEVEKQMNMSPPPGNAGPVIMSLEEKMEADAR |
| 6.5 | 3903.8148 | 0.0116 | IALYIGNLTWWTTDEDLTEAVHSLGVNDILEIK |
| 5.5 | 3903.8424 | -0.0160 | QLLDMGGIYDTERNVWKSIQDLSLIAACTPSAGGK |

| | | | |
|---|---|---|---|
| 5.5 | 3903.8424 | -0.0160 | QLLDMGGIYDTERNVWKSIQDLSLIAACTPSAGGK |
| 4.8 | 3901.7849 | 2.0415 | MDPRTPYRPPAAGVGAVPAAAAGSVGAAASLGNATRGTR |

---

Spectrum No: 144; Query: 1392; Rank: 1

---

## Peptide View

MS/MS Fragmentation of **KQEEFDIANNGSSQANK**
Found in **IPI00213579**, Tax_Id=10116 Gene_Symbol=Tpp2 Tripeptidyl-peptidase 2

Match to Query 1392: 1879.856128 from(940.935340,2+)
Title: 091008RatKidney_NH4Format01_19.893.893.2.dta
Data file K:\NewmanPaper\Piliang\3SubProteomes\Piliang3SP\mgf5ppm\ERLIC_3SubProteomes5ppm.mgf

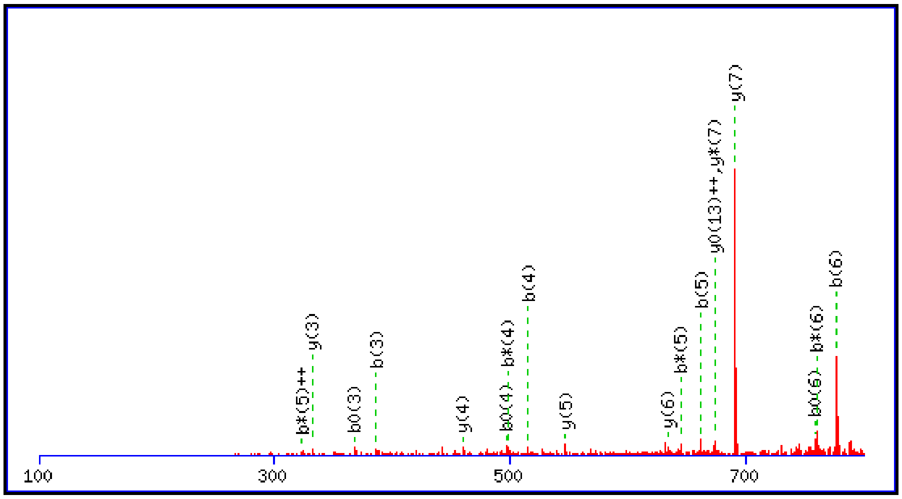


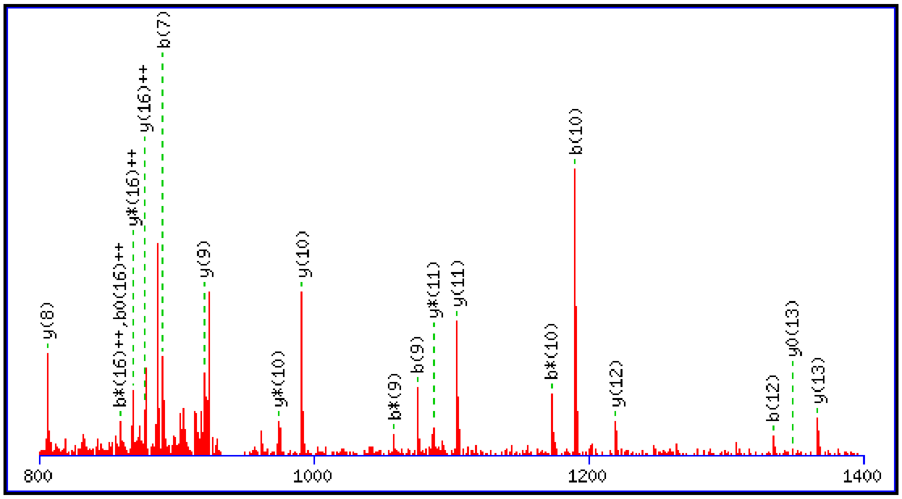

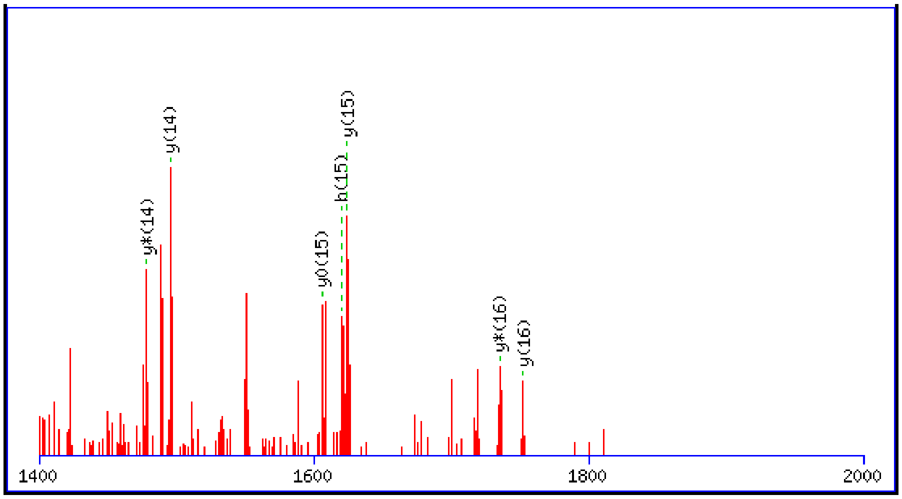


**Monoisotopic mass of neutral peptide Mr(calc):** 1879.8548
**Fixed modifications:** Carbamidomethyl (C)
**Variable modifications:**
**N10** : Deamidated_N (N)
**Ions Score:** 89 **Expect:** 2.8e-007
**Matches (Bold Red):** 44/180 fragment ions using 84 most intense peaks

| # | b | $b^{++}$ | b* | $b^{*++}$ | $b^{0}$ | $b^{0++}$ | Seq. | y | $y^{++}$ | y* | $y^{*++}$ | $y^{0}$ | $y^{0++}$ | # |
|---|---|---|---|---|---|---|---|---|---|---|---|---|---|---|
| 1 | 129.1022 | 65.0548 | 112.0757 | 56.5415 | | | K | | | | | | | 17 |
| 2 | 257.1608 | 129.0840 | 240.1343 | 120.5708 | | | Q | **1752.7671** | **876.8872** | **1735.7406** | **868.3739** | 1734.7565 | 867.8819 | 16 |
| 3 | **386.2034** | 193.6053 | 369.1769 | 185.0921 | **368.1928** | 184.6001 | E | **1624.7085** | 812.8579 | 1607.6820 | 804.3446 | **1606.6980** | 803.8526 | 15 |
| 4 | **515.2460** | 258.1266 | **498.2195** | 249.6134 | **497.2354** | 249.1214 | E | **1495.6659** | 748.3366 | **1478.6394** | 739.8233 | 1477.6554 | 739.3313 | 14 |
| 5 | **662.3144** | 331.6608 | **645.2879** | **323.1476** | 644.3039 | 322.6556 | F | **1366.6233** | 683.8153 | 1349.5968 | 675.3020 | **1348.6128** | **674.8100** | 13 |
| 6 | **777.3414** | 389.1743 | **760.3148** | 380.6610 | **759.3308** | 380.1690 | D | **1219.5549** | 610.2811 | 1202.5284 | 601.7678 | 1201.5444 | 601.2758 | 12 |
| 7 | **890.4254** | 445.7164 | 873.3989 | 437.2031 | 872.4149 | 436.7111 | I | **1104.5280** | 552.7676 | **1087.5014** | 544.2544 | 1086.5174 | 543.7623 | 11 |
| 8 | 961.4625 | 481.2349 | 944.4360 | 472.7216 | 943.4520 | 472.2296 | A | **991.4439** | 496.2256 | **974.4174** | 487.7123 | 973.4334 | 487.2203 | 10 |
| 9 | **1075.5055** | 538.2564 | **1058.4789** | 529.7431 | 1057.4949 | 529.2511 | N | **920.4068** | 460.7070 | 903.3803 | 452.1938 | 902.3962 | 451.7018 | 9 |
| 10 | **1190.5324** | 595.7698 | **1173.5059** | 587.2566 | 1172.5218 | 586.7646 | N | **806.3639** | 403.6856 | 789.3373 | 395.1723 | 788.3533 | 394.6803 | 8 |
| 11 | 1247.5539 | 624.2806 | 1230.5273 | 615.7673 | 1229.5433 | 615.2753 | G | **691.3369** | 346.1721 | **674.3104** | 337.6588 | 673.3264 | 337.1668 | 7 |
| 12 | **1334.5859** | 667.7966 | 1317.5593 | 659.2833 | 1316.5753 | 658.7913 | S | **634.3155** | 317.6614 | 617.2889 | 309.1481 | 616.3049 | 308.6561 | 6 |
| 13 | 1421.6179 | 711.3126 | 1404.5914 | 702.7993 | 1403.6074 | 702.3073 | S | **547.2835** | 274.1454 | 530.2569 | 265.6321 | 529.2729 | 265.1401 | 5 |
| 14 | 1549.6765 | 775.3419 | 1532.6500 | 766.8286 | 1531.6659 | 766.3366 | Q | **460.2514** | 230.6293 | 443.2249 | 222.1161 | | | 4 |
| 15 | **1620.7136** | 810.8604 | 1603.6871 | 802.3472 | 1602.7030 | 801.8552 | A | **332.1928** | 166.6001 | 315.1663 | 158.0868 | | | 3 |
| 16 | 1734.7565 | 867.8819 | 1717.7300 | **859.3686** | 1716.7460 | **858.8766** | N | 261.1557 | 131.0815 | 244.1292 | 122.5682 | | | 2 |
| 17 | | | | | | | K | 147.1128 | 74.0600 | 130.0863 | 65.5468 | | | 1 |

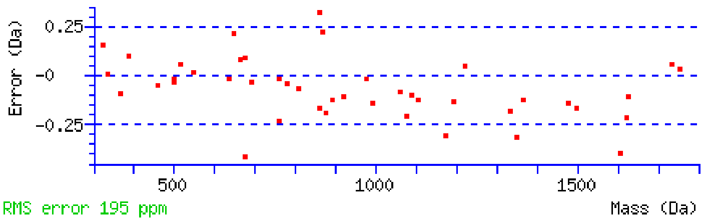


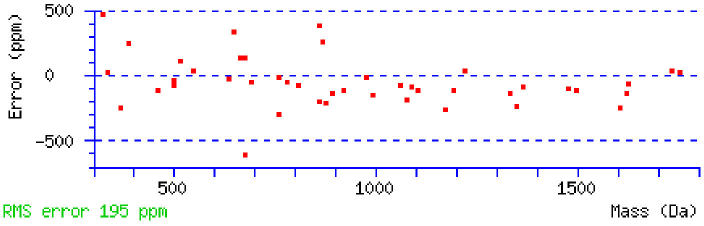


## All matches to this query

| Score | Mr(calc): | Delta | Sequence |
|---|---|---|---|
| 89.0 | 1879.8548 | 0.0014 | KQEEFDIANNGSSQANK |
| 76.2 | 1879.8548 | 0.0014 | KQEEFDIANNGSSQANK |
| 39.5 | 1879.8548 | 0.0014 | KQEEFDIANNGSSQANK |
| 16.7 | 1878.8707 | 0.9854 | KQEEFDIANNGSSQANK |
| | | | |

| | | | |
|---|---|---|---|
| 10.4 | 1879.8427 | 0.0134 | TGFEFNIMVVGQSGLGK |
| 4.0 | 1879.8638 | -0.0077 | ATVPLQSNVIEASPDMK |
| 2.8 | 1879.8717 | -0.0155 | VTQYKNGKDSLYAQGK |
| 2.5 | 1877.8594 | 1.9967 | RMNKVGSESLNFTVAK |
| 1.4 | 1879.8638 | -0.0077 | ATVPLQSNVIEASPDMK |
| 0.0 | 1878.8360 | 1.0201 | DHTVSRLEEANNALTK |

---

Spectrum No: 145; Query: 625; Rank: 1

---

## Peptide View

MS/MS Fragmentation of **IDVNSWIENFTK**
Found in **IPI00391769**, Tax_Id=10116 Gene_Symbol=Cacna2d1 Voltage-gated calcium channel alpha2/delta-1 subunit

Match to Query 625: 1465.711508 from(733.863030,2+)
Title: 091008RatKidney_NoSalt_20.5733.5733.2.dta
Data file K:\NewmanPaper\Piliang\3SubProteomes\Piliang3SP\mgf5ppm\ERLIC_3SubProteomes5ppm.mgf

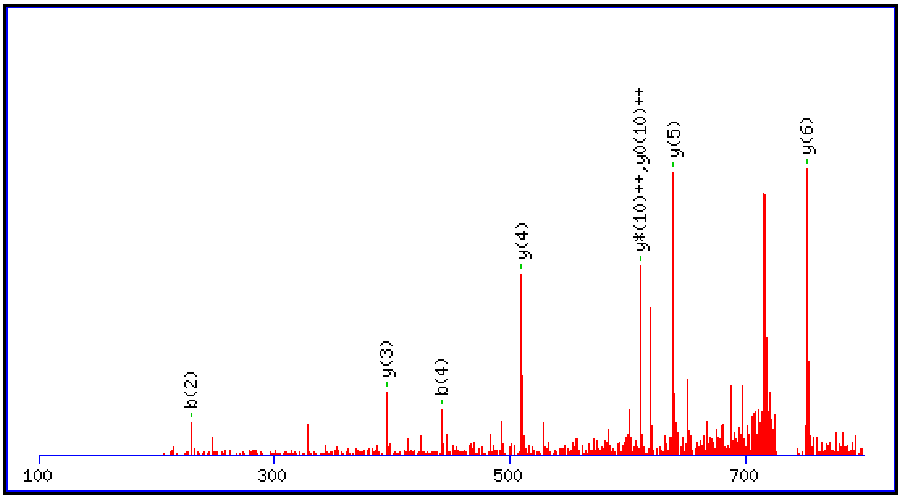


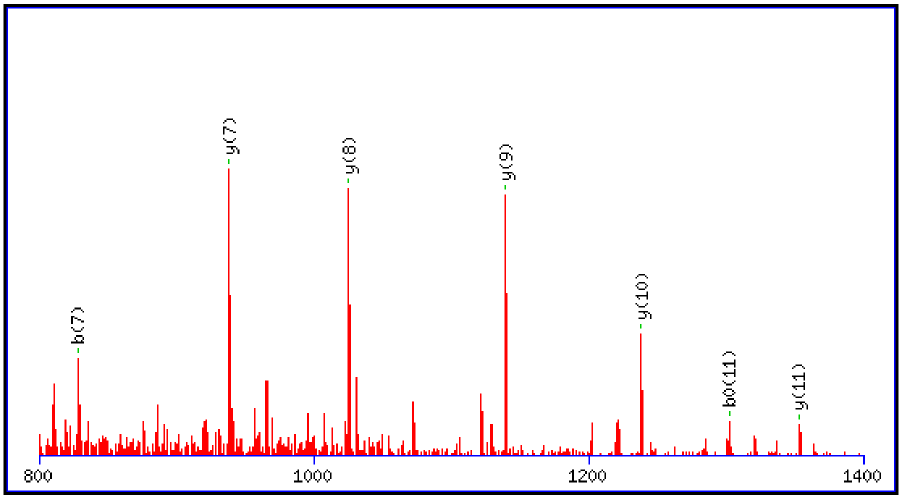

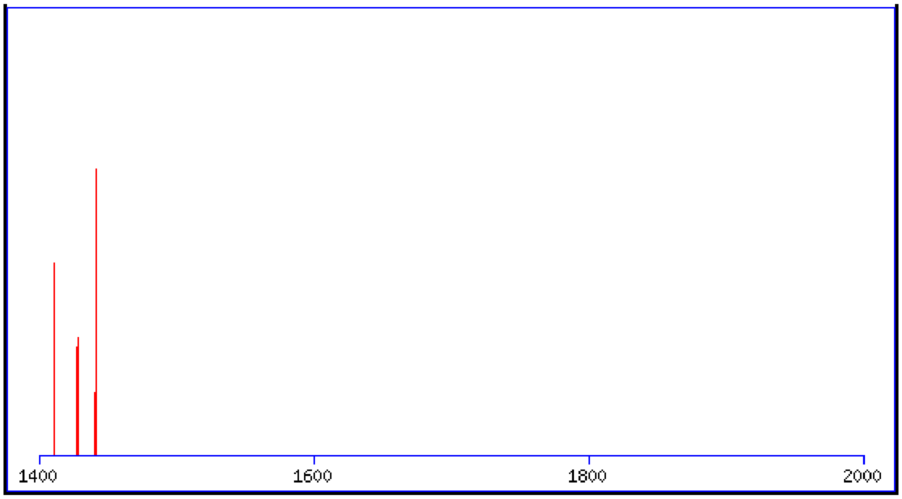


**Monoisotopic mass of neutral peptide Mr(calc):** 1465.7089
**Fixed modifications:** Carbamidomethyl (C)
**Variable modifications:**
**N9** : Deamidated_N (N)
**Ions Score:** 89 **Expect:** 2.2e-007
**Matches (Bold Red):** 15/122 fragment ions using 21 most intense peaks

| # | b | b++ | b* | b*++ | b0 | b0++ | Seq. | y | y++ | y* | y*++ | y0 | y0++ | # |
|---|---|---|---|---|---|---|---|---|---|---|---|---|---|---|
| 1 | 114.0913 | 57.5493 | | | | | I | | | | | | | 12 |
| 2 | **229.1183** | 115.0628 | | | 211.1077 | 106.0575 | D | **1353.6321** | 677.3197 | 1336.6056 | 668.8064 | 1335.6216 | 668.3144 | 11 |
| 3 | 328.1867 | 164.5970 | | | 310.1761 | 155.5917 | V | **1238.6052** | 619.8062 | 1221.5786 | **611.2929** | 1220.5946 | **610.8009** | 10 |
| 4 | **442.2296** | 221.6185 | 425.2031 | 213.1052 | 424.2191 | 212.6132 | N | **1139.5368** | 570.2720 | 1122.5102 | 561.7587 | 1121.5262 | 561.2667 | 9 |
| 5 | 529.2617 | 265.1345 | 512.2351 | 256.6212 | 511.2511 | 256.1292 | S | **1025.4938** | 513.2506 | 1008.4673 | 504.7373 | 1007.4833 | 504.2453 | 8 |
| 6 | 715.3410 | 358.1741 | 698.3144 | 349.6608 | 697.3304 | 349.1688 | W | **938.4618** | 469.7345 | 921.4353 | 461.2213 | 920.4512 | 460.7293 | 7 |
| 7 | **828.4250** | 414.7162 | 811.3985 | 406.2029 | 810.4145 | 405.7109 | I | **752.3825** | 376.6949 | 735.3559 | 368.1816 | 734.3719 | 367.6896 | 6 |
| 8 | 957.4676 | 479.2374 | 940.4411 | 470.7242 | 939.4571 | 470.2322 | E | **639.2984** | 320.1529 | 622.2719 | 311.6396 | 621.2879 | 311.1476 | 5 |
| 9 | 1072.4946 | 536.7509 | 1055.4680 | 528.2376 | 1054.4840 | 527.7456 | N | **510.2558** | 255.6316 | 493.2293 | 247.1183 | 492.2453 | 246.6263 | 4 |
| 10 | 1219.5630 | 610.2851 | 1202.5364 | 601.7718 | 1201.5524 | 601.2798 | F | **395.2289** | 198.1181 | 378.2023 | 189.6048 | 377.2183 | 189.1128 | 3 |
| 11 | 1320.6107 | 660.8090 | 1303.5841 | 652.2957 | **1302.6001** | 651.8037 | T | 248.1605 | 124.5839 | 231.1339 | 116.0706 | 230.1499 | 115.5786 | 2 |
| 12 | | | | | | | K | 147.1128 | 74.0600 | 130.0863 | 65.5468 | | | 1 |

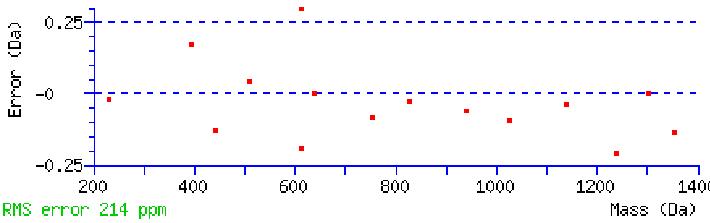


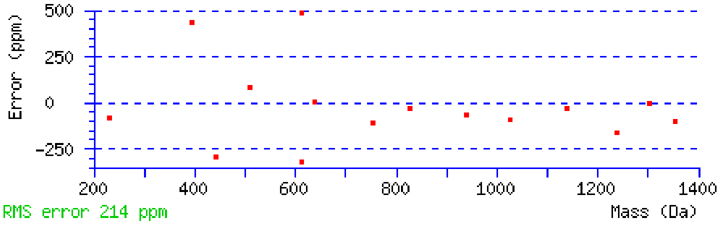


## All matches to this query

| Score | Mr(calc): | Delta | Sequence |
|---|---|---|---|
| 88.9 | 1465.7089 | 0.0026 | IDVNSWIENFTK |
| 19.6 | 1465.7089 | 0.0026 | IDVNSWIENFTK |
| 11.7 | 1465.6983 | 0.0132 | YVSELTLVRVK |
| 7.3 | 1465.7191 | -0.0076 | LNVKWGRSQAAR |
| 5.2 | 1465.7177 | -0.0062 | TKPSNAVTPEKSK |
| 4.1 | 1465.7161 | -0.0046 | LDNGQPVSAPSEPR |
| 2.8 | 1465.7000 | 0.0115 | KVCNVAPIAGETK |
| 1.3 | 1464.7086 | 1.0030 | LNNSLLPRSQSR |

Spectrum No: 146; Query: 2505; Rank: 1

# Peptide View

MS/MS Fragmentation of **KGAGLENPVAVTIFFGANDSTLKDENPK**
Found in **IPI00421610**, Tax_Id=10116 Gene_Symbol=Harpb64 Isoamyl acetate-hydrolyzing esterase 1 homolog

Match to Query 2505: 2932.485042 from(978.502290,3+)
Title: 091008RatKidney_NH4Format01_09.4337.4337.3.dta
Data file K:\NewmanPaper\Piliang\3SubProteomes\Piliang3SP\mgf5ppm\ERLIC_3SubProteomes5ppm.mgf

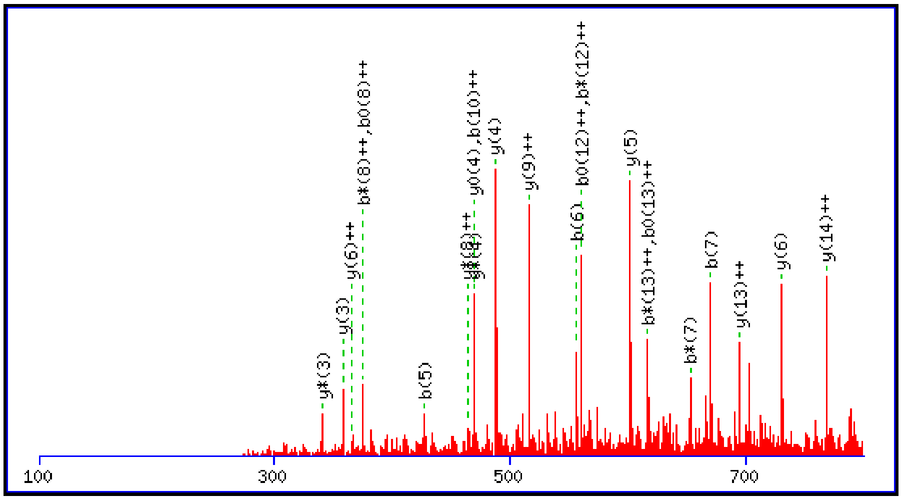


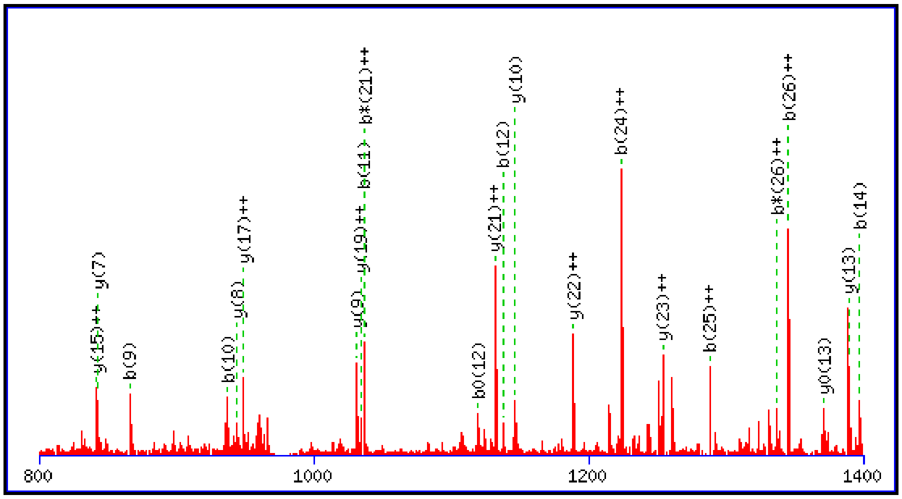


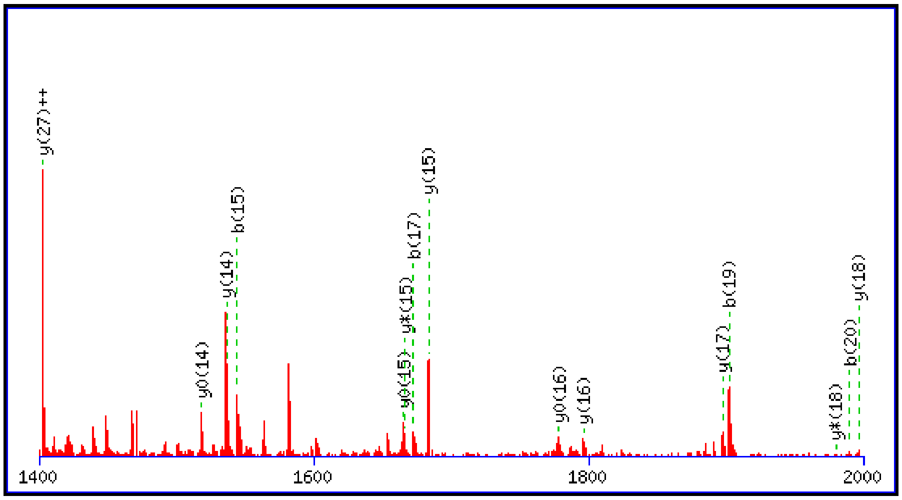


**Monoisotopic mass of neutral peptide Mr(calc):** 2932.4818

**Fixed modifications:** Carbamidomethyl (C)
**Variable modifications:**
**N18** : Deamidated_N (N)
**Ions Score:** 89 **Expect:** 2.3e-007
**Matches (Bold Red):** 61/308 fragment ions using 104 most intense peaks

| # | b | $b^{++}$ | b* | $b^{*++}$ | $b^0$ | $b^{0++}$ | Seq. | y | $y^{++}$ | y* | $y^{*++}$ | $y^0$ | $y^{0++}$ | # |
|---|---|---|---|---|---|---|---|---|---|---|---|---|---|---|
| 1 | 129.1022 | 65.0548 | 112.0757 | 56.5415 | | | K | | | | | | | 28 |
| 2 | 186.1237 | 93.5655 | 169.0972 | 85.0522 | | | G | 2805.3941 | **1403.2007** | 2788.3676 | 1394.6874 | 2787.3836 | 1394.1954 | 27 |
| 3 | 257.1608 | 129.0840 | 240.1343 | 120.5708 | | | A | 2748.3727 | 1374.6900 | 2731.3461 | 1366.1767 | 2730.3621 | 1365.6847 | 26 |
| 4 | 314.1823 | 157.5948 | 297.1557 | 149.0815 | | | G | 2677.3355 | 1339.1714 | 2660.3090 | 1330.6581 | 2659.3250 | 1330.1661 | 25 |
| 5 | **427.2663** | 214.1368 | 410.2398 | 205.6235 | | | L | 2620.3141 | 1310.6607 | 2603.2875 | 1302.1474 | 2602.3035 | 1301.6554 | 24 |
| 6 | **556.3089** | 278.6581 | 539.2824 | 270.1448 | 538.2984 | 269.6528 | E | 2507.2300 | **1254.1186** | 2490.2035 | 1245.6054 | 2489.2195 | 1245.1134 | 23 |
| 7 | **670.3519** | 335.6796 | **653.3253** | 327.1663 | 652.3413 | 326.6743 | N | 2378.1874 | **1189.5974** | 2361.1609 | 1181.0841 | 2360.1769 | 1180.5921 | 22 |
| 8 | 767.4046 | 384.2060 | 750.3781 | **375.6927** | 749.3941 | **375.2007** | P | 2264.1445 | **1132.5759** | 2247.1179 | 1124.0626 | 2246.1339 | 1123.5706 | 21 |
| 9 | **866.4730** | 433.7402 | 849.4465 | 425.2269 | 848.4625 | 424.7349 | V | 2167.0917 | 1084.0495 | 2150.0652 | 1075.5362 | 2149.0812 | 1075.0442 | 20 |
| 10 | **937.5102** | **469.2587** | 920.4836 | 460.7454 | 919.4996 | 460.2534 | A | 2068.0233 | **1034.5153** | 2050.9968 | 1026.0020 | 2050.0128 | 1025.5100 | 19 |
| 11 | **1036.5786** | 518.7929 | 1019.5520 | 510.2796 | 1018.5680 | 509.7876 | V | **1996.9862** | 998.9967 | **1979.9597** | 990.4835 | 1978.9756 | 989.9915 | 18 |
| 12 | **1137.6263** | 569.3168 | 1120.5997 | **560.8035** | **1119.6157** | **560.3115** | T | **1897.9178** | **949.4625** | 1880.8912 | 940.9493 | 1879.9072 | 940.4573 | 17 |
| 13 | 1250.7103 | 625.8588 | 1233.6838 | **617.3455** | 1232.6997 | **616.8535** | I | **1796.8701** | 898.9387 | 1779.8436 | 890.4254 | **1778.8595** | 889.9334 | 16 |
| 14 | **1397.7787** | 699.3930 | 1380.7522 | 690.8797 | 1379.7682 | 690.3877 | F | **1683.7861** | **842.3967** | **1666.7595** | 833.8834 | **1665.7755** | 833.3914 | 15 |
| 15 | **1544.8471** | 772.9272 | 1527.8206 | 764.4139 | 1526.8366 | 763.9219 | F | **1536.7176** | **768.8625** | 1519.6911 | 760.3492 | **1518.7071** | 759.8572 | 14 |
| 16 | 1601.8686 | 801.4379 | 1584.8421 | 792.9247 | 1583.8580 | 792.4327 | G | **1389.6492** | **695.3282** | 1372.6227 | 686.8150 | **1371.6387** | 686.3230 | 13 |
| 17 | **1672.9057** | 836.9565 | 1655.8792 | 828.4432 | 1654.8952 | 827.9512 | A | 1332.6278 | 666.8175 | 1315.6012 | 658.3042 | 1314.6172 | 657.8122 | 12 |
| 18 | 1787.9327 | 894.4700 | 1770.9061 | 885.9567 | 1769.9221 | 885.4647 | N | 1261.5906 | 631.2990 | 1244.5641 | 622.7857 | 1243.5801 | 622.2937 | 11 |
| 19 | **1902.9596** | 951.9834 | 1885.9331 | 943.4702 | 1884.9490 | 942.9782 | D | **1146.5637** | 573.7855 | 1129.5372 | 565.2722 | 1128.5531 | 564.7802 | 10 |
| 20 | **1989.9916** | 995.4995 | 1972.9651 | 986.9862 | 1971.9811 | 986.4942 | S | **1031.5368** | **516.2720** | 1014.5102 | 507.7587 | 1013.5262 | 507.2667 | 9 |
| 21 | 2091.0393 | 1046.0233 | 2074.0128 | **1037.5100** | 2073.0287 | 1037.0180 | T | **944.5047** | 472.7560 | 927.4782 | **464.2427** | 926.4942 | 463.7507 | 8 |
| 22 | 2204.1234 | 1102.5653 | 2187.0968 | 1094.0520 | 2186.1128 | 1093.5600 | L | **843.4571** | 422.2322 | 826.4305 | 413.7189 | 825.4465 | 413.2269 | 7 |
| 23 | 2332.2183 | 1166.6128 | 2315.1918 | 1158.0995 | 2314.2078 | 1157.6075 | K | **730.3730** | **365.6901** | 713.3464 | 357.1769 | 712.3624 | 356.6849 | 6 |
| 24 | 2447.2453 | **1224.1263** | 2430.2187 | 1215.6130 | 2429.2347 | 1215.1210 | D | **602.2780** | 301.6427 | 585.2515 | 293.1294 | 584.2675 | 292.6374 | 5 |
| 25 | 2576.2879 | **1288.6476** | 2559.2613 | 1280.1343 | 2558.2773 | 1279.6423 | E | **487.2511** | 244.1292 | **470.2245** | 235.6159 | **469.2405** | 235.1239 | 4 |
| 26 | 2690.3308 | **1345.6690** | 2673.3042 | **1337.1558** | 2672.3202 | 1336.6638 | N | **358.2085** | 179.6079 | **341.1819** | 171.0946 | | | 3 |
| 27 | 2787.3836 | 1394.1954 | 2770.3570 | 1385.6821 | 2769.3730 | 1385.1901 | P | 244.1656 | 122.5864 | 227.1390 | 114.0731 | | | 2 |
| 28 | | | | | | | K | 147.1128 | 74.0600 | 130.0863 | 65.5468 | | | 1 |

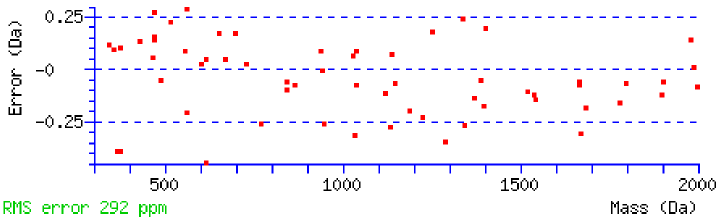


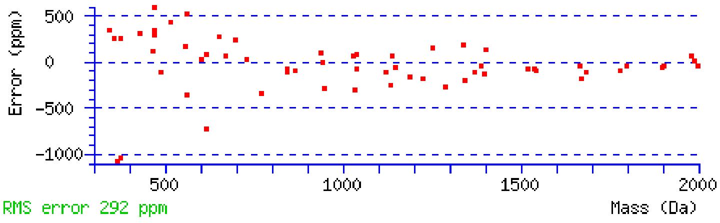


**All matches to this query**

| Score | Mr(calc): | Delta | Sequence |
|---|---|---|---|
| 88.8 | 2932.4818 | 0.0032 | KGAGLENPVAVTIFFGANDSTLKDENPK |
| 84.7 | 2932.4818 | 0.0032 | KGAGLENPVAVTIFFGANDSTLKDENPK |
| 84.0 | 2931.4978 | 0.9873 | KGAGLENPVAVTIFFGANDSTLKDENPK |
| 71.1 | 2932.4818 | 0.0032 | KGAGLENPVAVTIFFGANDSTLKDENPK |

Spectrum No: 147; Query: 2012; Rank: 1

# Peptide View

MS/MS Fragmentation of **VGPVDPSLNTTYVFFDTFFK**
Found in **IPI00464518**, Tax_Id=10116 Gene_Symbol=Hexb Beta-hexosaminidase beta chain precursor

Match to Query 2012: 2294.119908 from(1148.067230,2+)
Title: 091008RatKidney_NoSalt_14.7271.7271.2.dta
Data file K:\NewmanPaper\Piliang\3SubProteomes\Piliang3SP\mgf5ppm\ERLIC_3SubProteomes5ppm.mgf

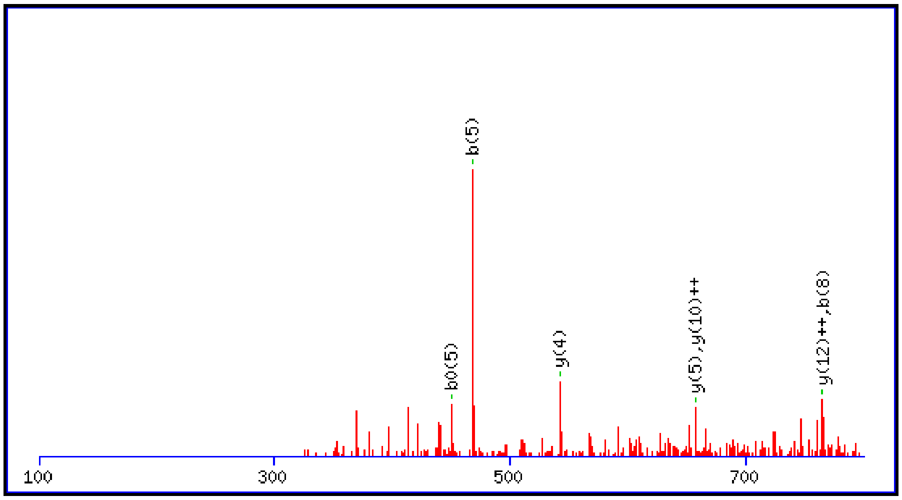


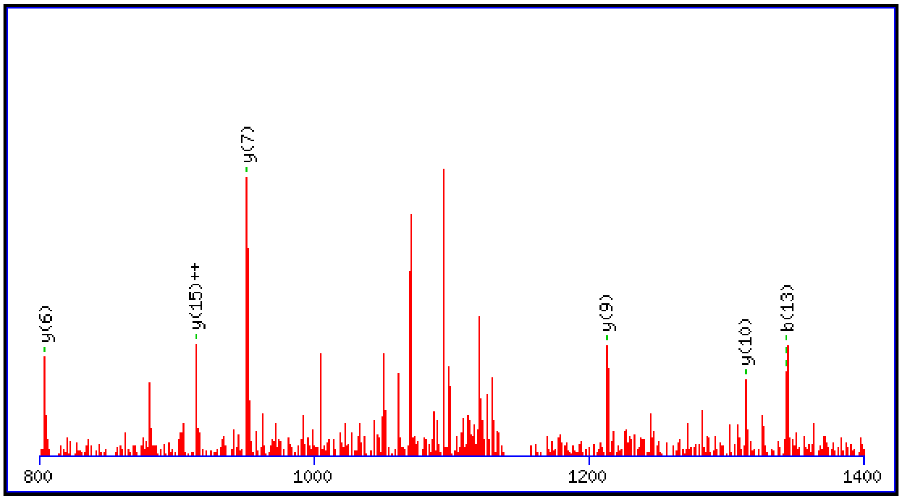


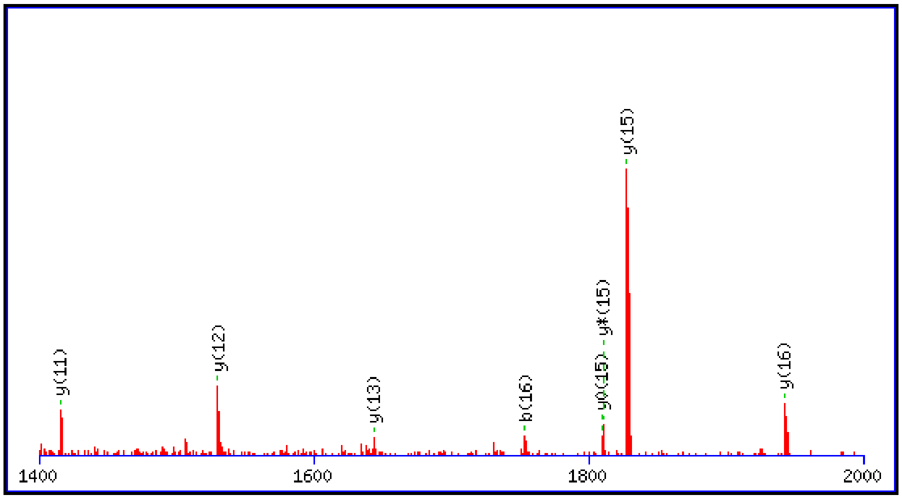


**Monoisotopic mass of neutral peptide Mr(calc):** 2294.1147
**Fixed modifications:** Carbamidomethyl (C)
**Variable modifications:**
**N9** : Deamidated_N (N)

**Ions Score:** 89 **Expect:** 3.2e-007
**Matches (Bold Red):** 21/198 fragment ions using 30 most intense peaks

| # | b | b++ | b* | b*++ | b0 | b0++ | Seq. | y | y++ | y* | y*++ | y0 | y0++ | # |
|---|---|---|---|---|---|---|---|---|---|---|---|---|---|---|
| 1 | 100.0757 | 50.5415 | | | | | V | | | | | | | 20 |
| 2 | 157.0972 | 79.0522 | | | | | G | 2196.0536 | 1098.5304 | 2179.0270 | 1090.0171 | 2178.0430 | 1089.5251 | 19 |
| 3 | 254.1499 | 127.5786 | | | | | P | 2139.0321 | 1070.0197 | 2122.0055 | 1061.5064 | 2121.0215 | 1061.0144 | 18 |
| 4 | 353.2183 | 177.1128 | | | | | V | 2041.9793 | 1021.4933 | 2024.9528 | 1012.9800 | 2023.9688 | 1012.4880 | 17 |
| 5 | **468.2453** | 234.6263 | | | **450.2347** | 225.6210 | D | **1942.9109** | 971.9591 | 1925.8844 | 963.4458 | 1924.9004 | 962.9538 | 16 |
| 6 | 565.2980 | 283.1527 | | | 547.2875 | 274.1474 | P | **1827.8840** | **914.4456** | **1810.8574** | 905.9324 | **1809.8734** | 905.4403 | 15 |
| 7 | 652.3301 | 326.6687 | | | 634.3195 | 317.6634 | S | 1730.8312 | 865.9192 | 1713.8047 | 857.4060 | 1712.8206 | 856.9140 | 14 |
| 8 | **765.4141** | 383.2107 | | | 747.4036 | 374.2054 | L | **1643.7992** | 822.4032 | 1626.7726 | 813.8900 | 1625.7886 | 813.3979 | 13 |
| 9 | 880.4411 | 440.7242 | 863.4145 | 432.2109 | 862.4305 | 431.7189 | N | **1530.7151** | **765.8612** | 1513.6886 | 757.3479 | 1512.7046 | 756.8559 | 12 |
| 10 | 981.4887 | 491.2480 | 964.4622 | 482.7347 | 963.4782 | 482.2427 | T | **1415.6882** | 708.3477 | 1398.6616 | 699.8345 | 1397.6776 | 699.3424 | 11 |
| 11 | 1082.5364 | 541.7719 | 1065.5099 | 533.2586 | 1064.5259 | 532.7666 | T | **1314.6405** | **657.8239** | 1297.6140 | 649.3106 | 1296.6299 | 648.8186 | 10 |
| 12 | 1245.5998 | 623.3035 | 1228.5732 | 614.7902 | 1227.5892 | 614.2982 | Y | **1213.5928** | 607.3001 | 1196.5663 | 598.7868 | 1195.5823 | 598.2948 | 9 |
| 13 | **1344.6682** | 672.8377 | 1327.6416 | 664.3244 | 1326.6576 | 663.8324 | V | 1050.5295 | 525.7684 | 1033.5029 | 517.2551 | 1032.5189 | 516.7631 | 8 |
| 14 | 1491.7366 | 746.3719 | 1474.7100 | 737.8587 | 1473.7260 | 737.3666 | F | **951.4611** | 476.2342 | 934.4345 | 467.7209 | 933.4505 | 467.2289 | 7 |
| 15 | 1638.8050 | 819.9061 | 1621.7784 | 811.3929 | 1620.7944 | 810.9009 | F | **804.3927** | 402.7000 | 787.3661 | 394.1867 | 786.3821 | 393.6947 | 6 |
| 16 | **1753.8319** | 877.4196 | 1736.8054 | 868.9063 | 1735.8214 | 868.4143 | D | **657.3243** | 329.1658 | 640.2977 | 320.6525 | 639.3137 | 320.1605 | 5 |
| 17 | 1854.8796 | 927.9434 | 1837.8531 | 919.4302 | 1836.8691 | 918.9382 | T | **542.2973** | 271.6523 | 525.2708 | 263.1390 | 524.2867 | 262.6470 | 4 |
| 18 | 2001.9480 | 1001.4777 | 1984.9215 | 992.9644 | 1983.9375 | 992.4724 | F | 441.2496 | 221.1285 | 424.2231 | 212.6152 | | | 3 |
| 19 | 2149.0164 | 1075.0119 | 2131.9899 | 1066.4986 | 2131.0059 | 1066.0066 | F | 294.1812 | 147.5942 | 277.1547 | 139.0810 | | | 2 |
| 20 | | | | | | | K | 147.1128 | 74.0600 | 130.0863 | 65.5468 | | | 1 |

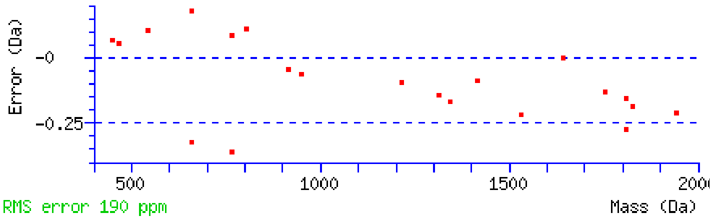


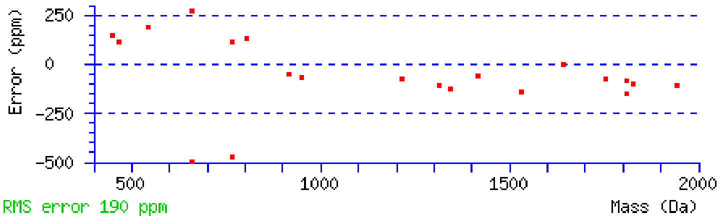


### All matches to this query

| Score | Mr(calc): | Delta | Sequence |
|---|---|---|---|
| 88.7 | 2294.1147 | 0.0052 | VGPVDPSLNTTYVFFDTFFK |
| 28.6 | 2293.1307 | 0.9892 | VGPVDPSLNTTYVFFDTFFK |

---

Spectrum No: 148; Query: 1406; Rank: 1

---

## Peptide View

MS/MS Fragmentation of **GTAGNALMEGASQLVGENR**
Found in **IPI00205389**, Tax_Id=10116 Gene_Symbol=Fgb Isoform 1 of Fibrinogen beta chain precursor

Match to Query 1406: 1890.872308 from(946.443430,2+)
Title: 091008RatKidney_NH4Format01_27.2142.2142.2.dta
Data file K:\NewmanPaper\Piliang\3SubProteomes\Piliang3SP\mgf5ppm\ERLIC_3SubProteomes5ppm.mgf

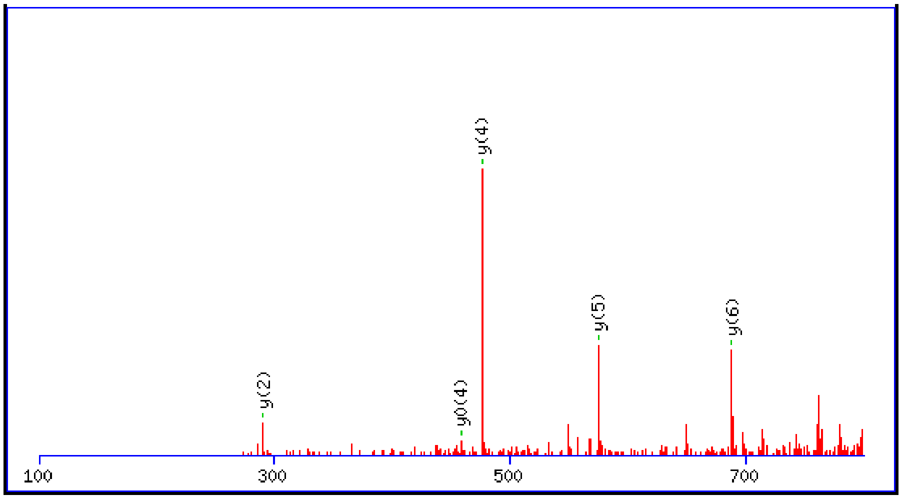


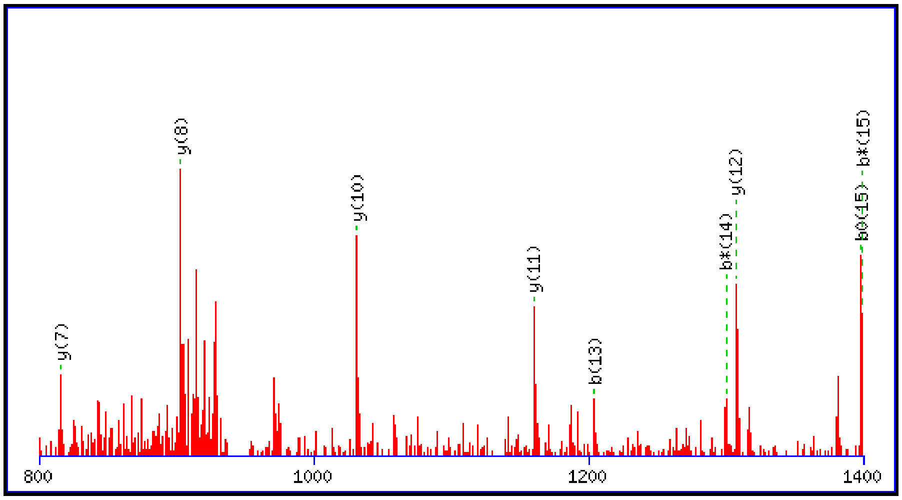


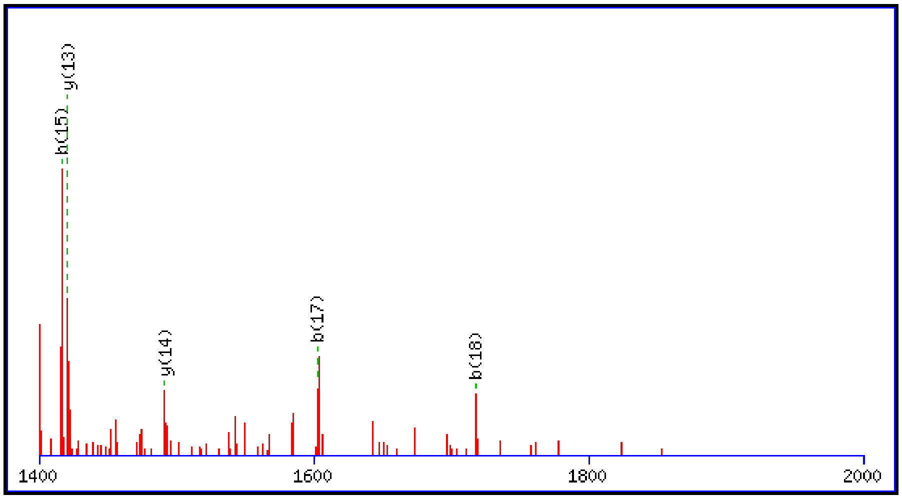


**Monoisotopic mass of neutral peptide Mr(calc):** 1890.8741
**Fixed modifications:** Carbamidomethyl (C)
**Variable modifications:**
**M8** : Oxidation (M)
**N18** : Deamidated_N (N)
**Ions Score:** 89 **Expect:** 3e-007
**Matches (Bold Red):** 19/202 fragment ions using 30 most intense peaks

| # | b | $b^{++}$ | b* | $b^{*++}$ | $b^0$ | $b^{0++}$ | Seq. | y | $y^{++}$ | y* | $y^{*++}$ | $y^0$ | $y^{0++}$ | # |
|---|---|---|---|---|---|---|---|---|---|---|---|---|---|---|
| 1 | 58.0287 | 29.5180 | | | | | G | | | | | | | 19 |
| 2 | 159.0764 | 80.0418 | | | 141.0659 | 71.0366 | T | 1834.8600 | 917.9336 | 1817.8334 | 909.4203 | 1816.8494 | 908.9283 | 18 |
| 3 | 230.1135 | 115.5604 | | | 212.1030 | 106.5551 | A | 1733.8123 | 867.4098 | 1716.7857 | 858.8965 | 1715.8017 | 858.4045 | 17 |
| 4 | 287.1350 | 144.0711 | | | 269.1244 | 135.0659 | G | 1662.7752 | 831.8912 | 1645.7486 | 823.3780 | 1644.7646 | 822.8859 | 16 |
| | | | | | | | | | | | | | | |

| | | | | | | | | | | | | | | |
|---|---|---|---|---|---|---|---|---|---|---|---|---|---|---|
| **5** | 401.1779 | 201.0926 | 384.1514 | 192.5793 | 383.1674 | 192.0873 | **N** | 1605.7537 | 803.3805 | 1588.7272 | 794.8672 | 1587.7431 | 794.3752 | **15** |
| **6** | 472.2150 | 236.6112 | 455.1885 | 228.0979 | 454.2045 | 227.6059 | **A** | **1491.7108** | 746.3590 | 1474.6842 | 737.8458 | 1473.7002 | 737.3537 | **14** |
| **7** | 585.2991 | 293.1532 | 568.2726 | 284.6399 | 567.2885 | 284.1479 | **L** | **1420.6737** | 710.8405 | 1403.6471 | 702.3272 | 1402.6631 | 701.8352 | **13** |
| **8** | 732.3345 | 366.6709 | 715.3080 | 358.1576 | 714.3239 | 357.6656 | **M** | **1307.5896** | 654.2984 | 1290.5631 | 645.7852 | 1289.5790 | 645.2932 | **12** |
| **9** | 861.3771 | 431.1922 | 844.3506 | 422.6789 | 843.3665 | 422.1869 | **E** | **1160.5542** | 580.7807 | 1143.5277 | 572.2675 | 1142.5436 | 571.7755 | **11** |
| **10** | 918.3986 | 459.7029 | 901.3720 | 451.1896 | 900.3880 | 450.6976 | **G** | **1031.5116** | 516.2594 | 1014.4851 | 507.7462 | 1013.5010 | 507.2542 | **10** |
| **11** | 989.4357 | 495.2215 | 972.4091 | 486.7082 | 971.4251 | 486.2162 | **A** | 974.4901 | 487.7487 | 957.4636 | 479.2354 | 956.4796 | 478.7434 | **9** |
| **12** | 1076.4677 | 538.7375 | 1059.4412 | 530.2242 | 1058.4571 | 529.7322 | **S** | **903.4530** | 452.2302 | 886.4265 | 443.7169 | 885.4425 | 443.2249 | **8** |
| **13** | **1204.5263** | 602.7668 | 1187.4997 | 594.2535 | 1186.5157 | 593.7615 | **Q** | **816.4210** | 408.7141 | 799.3945 | 400.2009 | 798.4104 | 399.7089 | **7** |
| **14** | 1317.6103 | 659.3088 | **1300.5838** | 650.7955 | 1299.5998 | 650.3035 | **L** | **688.3624** | 344.6848 | 671.3359 | 336.1716 | 670.3519 | 335.6796 | **6** |
| **15** | **1416.6788** | 708.8430 | **1399.6522** | 700.3297 | **1398.6682** | 699.8377 | **V** | **575.2784** | 288.1428 | 558.2518 | 279.6295 | 557.2678 | 279.1375 | **5** |
| **16** | 1473.7002 | 737.3538 | 1456.6737 | 728.8405 | 1455.6897 | 728.3485 | **G** | **476.2099** | 238.6086 | 459.1834 | 230.0953 | **458.1994** | 229.6033 | **4** |
| **17** | **1602.7428** | 801.8750 | 1585.7163 | 793.3618 | 1584.7323 | 792.8698 | **E** | 419.1885 | 210.0979 | 402.1619 | 201.5846 | 401.1779 | 201.0926 | **3** |
| **18** | **1717.7698** | 859.3885 | 1700.7432 | 850.8752 | 1699.7592 | 850.3832 | **N** | **290.1459** | 145.5766 | 273.1193 | 137.0633 | | | **2** |
| **19** | | | | | | | **R** | 175.1190 | 88.0631 | 158.0924 | 79.5498 | | | **1** |

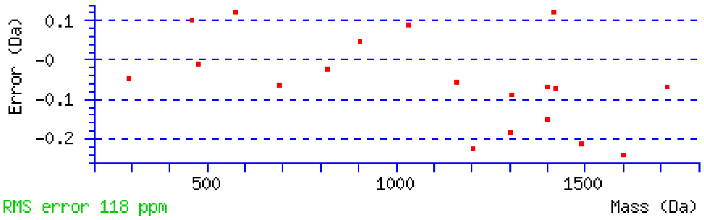


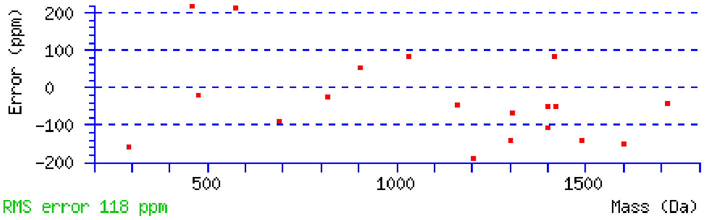


### All matches to this query

| Score | Mr(calc): | Delta | Sequence |
|---|---|---|---|
| 88.7 | 1890.8741 | -0.0018 | GTAGNALMEGASQLVGENR |
| 10.7 | 1889.8594 | 1.0129 | VRTMFSEQQLGELEK |
| 3.0 | 1890.8625 | 0.0098 | AFRSSTIQDQQLFDR |
| 3.0 | 1890.8625 | 0.0098 | AFRSSTIQDQQLFDR |
| 2.4 | 1890.8625 | 0.0098 | AFRSSTIQDQQLFDR |
| 1.0 | 1889.8611 | 1.0112 | EHMDEVCSSQLLTSVR |

---

Spectrum No: 149; Query: 2629; Rank: 1

---

## Peptide View

MS/MS Fragmentation of **TSLDWWTTDTCNMINGTDGDSFHPLISK**
Found in **IPI00231478**, Tax_Id=10116 Gene_Symbol=Scarb2 Lysosome membrane protein 2

Match to Query 2629: 3212.404452 from(1071.808760,3+)
Title: 091008RatKidney_NoSalt_20.5734.5734.3.dta
Data file K:\NewmanPaper\Piliang\3SubProteomes\Piliang3SP\mgf5ppm\ERLIC_3SubProteomes5ppm.mgf

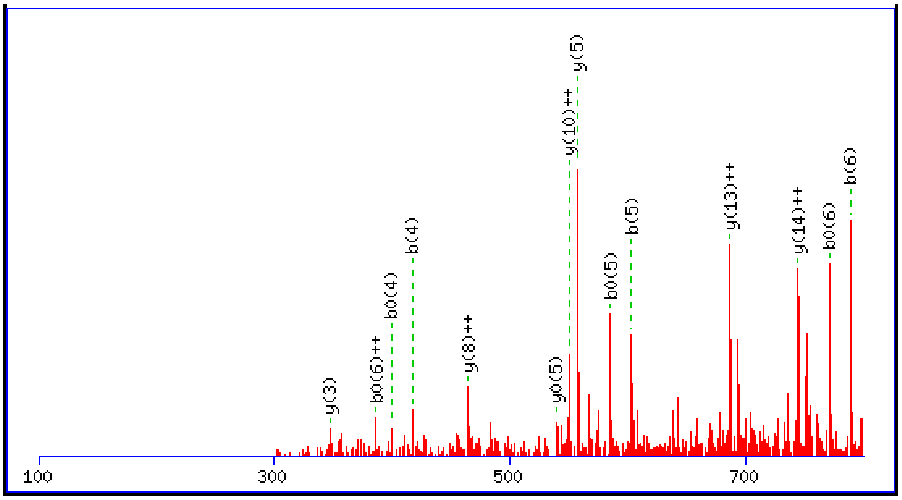


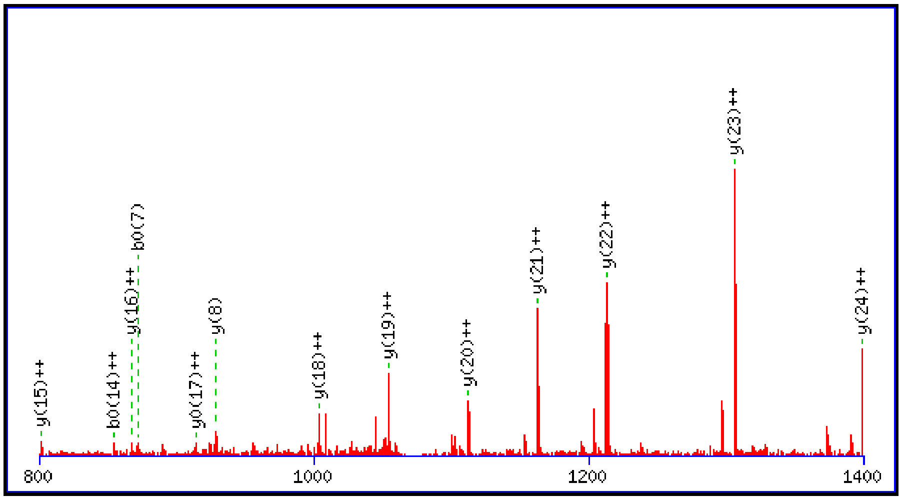


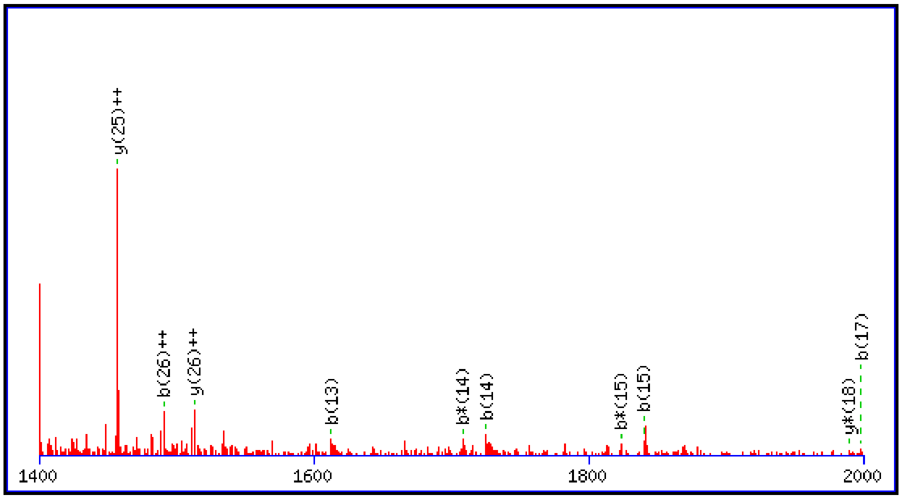


**Monoisotopic mass of neutral peptide Mr(calc):** 3212.4067
**Fixed modifications:** Carbamidomethyl (C)
**Variable modifications:**
**N15** : Deamidated_N (N)
**Ions Score:** 88 **Expect:** 6.2e-007
**Matches (Bold Red):** 37/300 fragment ions using 65 most intense peaks

| # | b | $b^{++}$ | b* | $b^{*++}$ | $b^0$ | $b^{0++}$ | Seq. | y | $y^{++}$ | y* | $y^{*++}$ | $y^0$ | $y^{0++}$ | # |
|---|---|---|---|---|---|---|---|---|---|---|---|---|---|---|
| 1 | 102.0550 | 51.5311 | | | 84.0444 | 42.5258 | T | | | | | | | 28 |
| 2 | 189.0870 | 95.0471 | | | 171.0764 | 86.0418 | S | 3112.3663 | 1556.6868 | 3095.3397 | 1548.1735 | 3094.3557 | 1547.6815 | 27 |
| 3 | 302.1710 | 151.5892 | | | 284.1605 | 142.5839 | L | 3025.3342 | **1513.1708** | 3008.3077 | 1504.6575 | 3007.3237 | 1504.1655 | 26 |
| 4 | **417.1980** | 209.1026 | | | **399.1874** | 200.0974 | D | 2912.2502 | **1456.6287** | 2895.2236 | 1448.1155 | 2894.2396 | 1447.6234 | 25 |
| 5 | **603.2773** | 302.1423 | | | **585.2667** | 293.1370 | W | 2797.2232 | **1399.1153** | 2780.1967 | 1390.6020 | 2779.2127 | 1390.1100 | 24 |

| | | | | | | | | | | | | | | |
|---|---|---|---|---|---|---|---|---|---|---|---|---|---|---|
| 6 | 789.3566 | 395.1819 | | | 771.3461 | 386.1767 | W | 2611.1439 | 1306.0756 | 2594.1174 | 1297.5623 | 2593.1334 | 1297.0703 | 23 |
| 7 | 890.4043 | 445.7058 | | | 872.3937 | 436.7005 | T | 2425.0646 | 1213.0359 | 2408.0381 | 1204.5227 | 2407.0540 | 1204.0307 | 22 |
| 8 | 991.4520 | 496.2296 | | | 973.4414 | 487.2243 | T | 2324.0169 | 1162.5121 | 2306.9904 | 1153.9988 | 2306.0064 | 1153.5068 | 21 |
| 9 | 1106.4789 | 553.7431 | | | 1088.4684 | 544.7378 | D | 2222.9693 | 1111.9883 | 2205.9427 | 1103.4750 | 2204.9587 | 1102.9830 | 20 |
| 10 | 1207.5266 | 604.2669 | | | 1189.5160 | 595.2617 | T | 2107.9423 | 1054.4748 | 2090.9158 | 1045.9615 | 2089.9317 | 1045.4695 | 19 |
| 11 | 1367.5572 | 684.2823 | | | 1349.5467 | 675.2770 | C | 2006.8946 | 1003.9510 | 1989.8681 | 995.4377 | 1988.8841 | 994.9457 | 18 |
| 12 | 1481.6002 | 741.3037 | 1464.5736 | 732.7904 | 1463.5896 | 732.2984 | N | 1846.8640 | 923.9356 | 1829.8374 | 915.4224 | 1828.8534 | 914.9303 | 17 |
| 13 | 1612.6407 | 806.8240 | 1595.6141 | 798.3107 | 1594.6301 | 797.8187 | M | 1732.8211 | 866.9142 | 1715.7945 | 858.4009 | 1714.8105 | 857.9089 | 16 |
| 14 | 1725.7247 | 863.3660 | 1708.6982 | 854.8527 | 1707.7142 | 854.3607 | I | 1601.7806 | 801.3939 | 1584.7540 | 792.8806 | 1583.7700 | 792.3886 | 15 |
| 15 | 1840.7517 | 920.8795 | 1823.7251 | 912.3662 | 1822.7411 | 911.8742 | N | 1488.6965 | 744.8519 | 1471.6700 | 736.3386 | 1470.6859 | 735.8466 | 14 |
| 16 | 1897.7731 | 949.3902 | 1880.7466 | 940.8769 | 1879.7626 | 940.3849 | G | 1373.6696 | 687.3384 | 1356.6430 | 678.8251 | 1355.6590 | 678.3331 | 13 |
| 17 | 1998.8208 | 999.9140 | 1981.7943 | 991.4008 | 1980.8102 | 990.9088 | T | 1316.6481 | 658.8277 | 1299.6216 | 650.3144 | 1298.6375 | 649.8224 | 12 |
| 18 | 2113.8477 | 1057.4275 | 2096.8212 | 1048.9142 | 2095.8372 | 1048.4222 | D | 1215.6004 | 608.3039 | 1198.5739 | 599.7906 | 1197.5899 | 599.2986 | 11 |
| 19 | 2170.8692 | 1085.9382 | 2153.8427 | 1077.4250 | 2152.8586 | 1076.9330 | G | 1100.5735 | 550.7904 | 1083.5469 | 542.2771 | 1082.5629 | 541.7851 | 10 |
| 20 | 2285.8961 | 1143.4517 | 2268.8696 | 1134.9384 | 2267.8856 | 1134.4464 | D | 1043.5520 | 522.2796 | 1026.5255 | 513.7664 | 1025.5415 | 513.2744 | 9 |
| 21 | 2372.9282 | 1186.9677 | 2355.9016 | 1178.4545 | 2354.9176 | 1177.9624 | S | 928.5251 | 464.7662 | 911.4985 | 456.2529 | 910.5145 | 455.7609 | 8 |
| 22 | 2519.9966 | 1260.5019 | 2502.9700 | 1251.9887 | 2501.9860 | 1251.4967 | F | 841.4931 | 421.2502 | 824.4665 | 412.7369 | 823.4825 | 412.2449 | 7 |
| 23 | 2657.0555 | 1329.0314 | 2640.0290 | 1320.5181 | 2639.0449 | 1320.0261 | H | 694.4246 | 347.7160 | 677.3981 | 339.2027 | 676.4141 | 338.7107 | 6 |
| 24 | 2754.1083 | 1377.5578 | 2737.0817 | 1369.0445 | 2736.0977 | 1368.5525 | P | 557.3657 | 279.1865 | 540.3392 | 270.6732 | 539.3552 | 270.1812 | 5 |
| 25 | 2867.1923 | 1434.0998 | 2850.1658 | 1425.5865 | 2849.1818 | 1425.0945 | L | 460.3130 | 230.6601 | 443.2864 | 222.1468 | 442.3024 | 221.6548 | 4 |
| 26 | 2980.2764 | 1490.6418 | 2963.2498 | 1482.1286 | 2962.2658 | 1481.6366 | I | 347.2289 | 174.1181 | 330.2023 | 165.6048 | 329.2183 | 165.1128 | 3 |
| 27 | 3067.3084 | 1534.1578 | 3050.2819 | 1525.6446 | 3049.2979 | 1525.1526 | S | 234.1448 | 117.5761 | 217.1183 | 109.0628 | 216.1343 | 108.5708 | 2 |
| 28 | | | | | | | K | 147.1128 | 74.0600 | 130.0863 | 65.5468 | | | 1 |

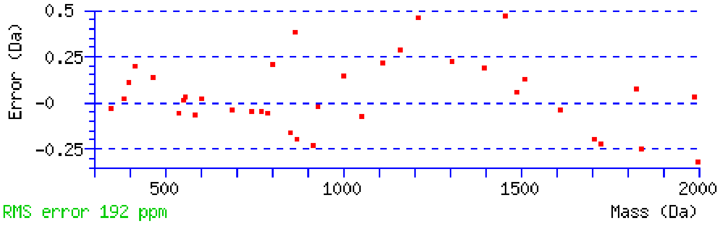


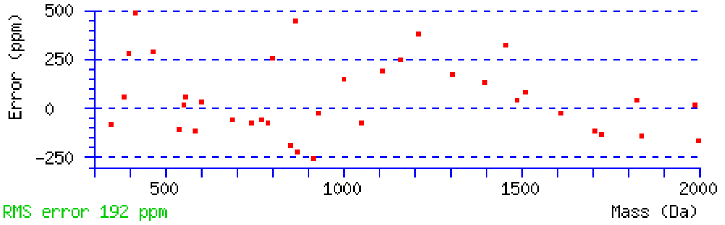


**All matches to this query**

| Score | Mr(calc): | Delta | Sequence |
|---|---|---|---|
| 88.4 | 3212.4067 | -0.0023 | TSLDWWTTDTCNMINGTDGDSFHPLISK |
| 73.0 | 3212.4067 | -0.0023 | TSLDWWTTDTCNMINGTDGDSFHPLISK |
| 13.8 | 3211.4227 | 0.9818 | TSLDWWTTDTCNMINGTDGDSFHPLISK |
| 3.5 | 3211.4002 | 1.0042 | GSFSGRLSPAYSLGSLTGASPRQSPHAQR |
| 3.4 | 3212.4080 | -0.0035 | NAININELFIEISRRLPSTDASPASGGK |
| 1.8 | 3212.4308 | -0.0263 | GLEWMGVMWSGGNTDYNSALKSRLSISR |
| 1.2 | 3212.4113 | -0.0068 | MIYLVTEYASGGEIFDHLVAHGRMAEK |
| 0.8 | 3212.4308 | -0.0263 | GLEWMGVMWSGGNTDYNSALKSRLSISR |
| 0.6 | 3212.4308 | -0.0263 | GLEWMGVMWSGGNTDYNSALKSRLSISR |
| 0.4 | 3210.3698 | 2.0346 | IGSENNMTSCHRPVCRKEGVLLDYSK |

---

Spectrum No: 150; Query: 1128; Rank: 1

---

## Peptide View

MS/MS Fragmentation of **VISNNCTSYGVLDIGK**

Found in **IPI00231197**, Tax_Id=10116 Gene_Symbol=Cd36 Platelet glycoprotein 4

Match to Query 1128: 1739.842028 from(870.928290,2+)
Title: 100101RatKid_NS_deglyco_25.2853.2853.2.dta
Data file K:\NewmanPaper\Piliang\3SubProteomes\Piliang3SP\mgf5ppm\ERLIC_3SubProteomes5ppm.mgf

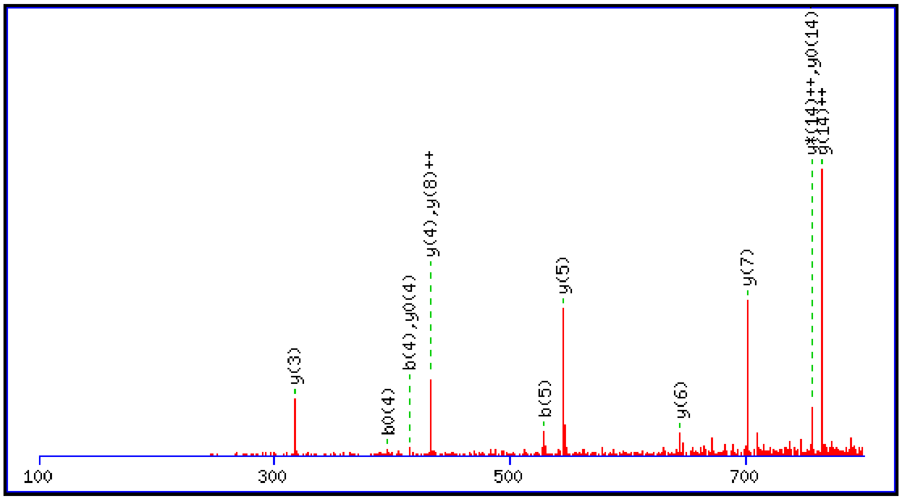


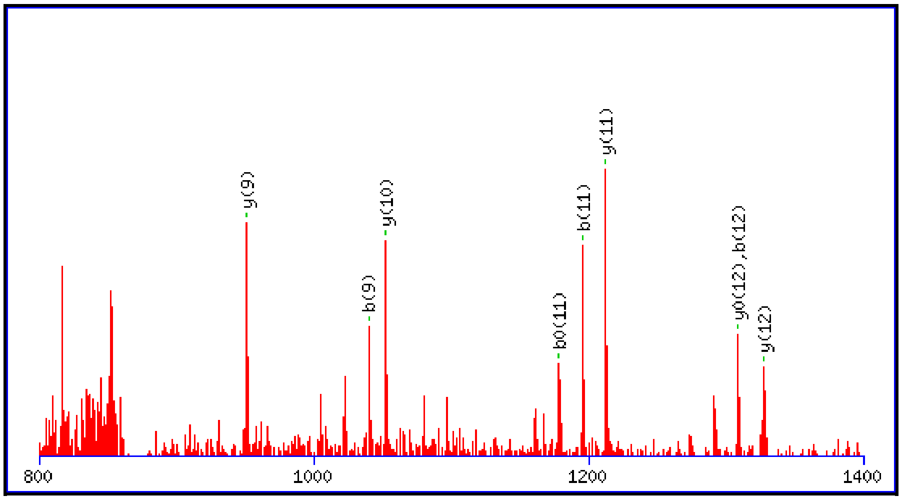


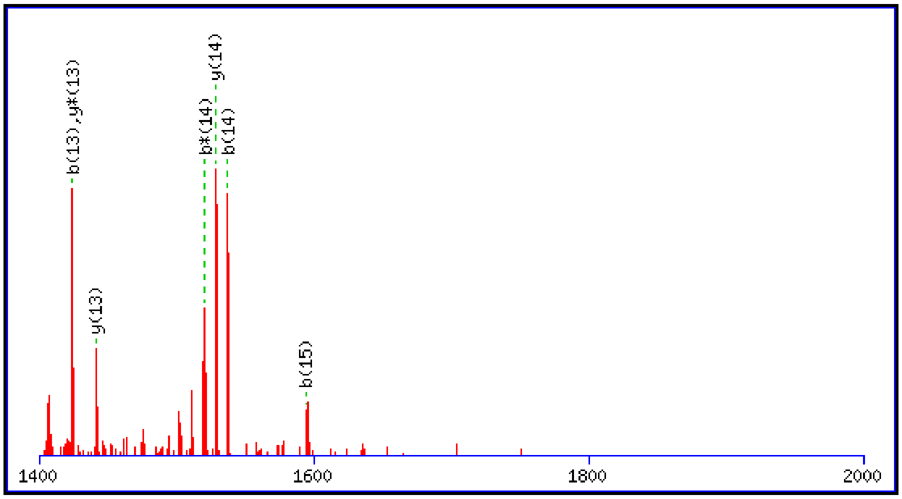


**Monoisotopic mass of neutral peptide Mr(calc):** 1739.8400
**Fixed modifications:** Carbamidomethyl (C)
**Variable modifications:**
**N5** : Deamidated_N (N)
**Ions Score:** 88 **Expect:** 2.8e-007
**Matches (Bold Red):** 29/164 fragment ions using 39 most intense peaks

| # | b | b$^{++}$ | b* | b*$^{++}$ | b$^{0}$ | b$^{0++}$ | Seq. | y | y$^{++}$ | y* | y*$^{++}$ | y$^{0}$ | y$^{0++}$ | # |
|---|---|---|---|---|---|---|---|---|---|---|---|---|---|---|
| **1** | 100.0757 | 50.5415 | | | | | **V** | | | | | | | **16** |

| | | | | | | | | | | | | | | |
|---|---|---|---|---|---|---|---|---|---|---|---|---|---|---|
| 2 | 213.1598 | 107.0835 | | | | | I | 1641.7789 | 821.3931 | 1624.7523 | 812.8798 | 1623.7683 | 812.3878 | 15 |
| 3 | 300.1918 | 150.5995 | | | 282.1812 | 141.5942 | S | 1528.6948 | 764.8510 | 1511.6682 | 756.3378 | 1510.6842 | 755.8458 | 14 |
| 4 | 414.2347 | 207.6210 | 397.2082 | 199.1077 | 396.2241 | 198.6157 | N | 1441.6628 | 721.3350 | 1424.6362 | 712.8217 | 1423.6522 | 712.3297 | 13 |
| 5 | 529.2616 | 265.1345 | 512.2351 | 256.6212 | 511.2511 | 256.1292 | N | 1327.6198 | 664.3136 | 1310.5933 | 655.8003 | 1309.6093 | 655.3083 | 12 |
| 6 | 689.2923 | 345.1498 | 672.2657 | 336.6365 | 671.2817 | 336.1445 | C | 1212.5929 | 606.8001 | 1195.5664 | 598.2868 | 1194.5823 | 597.7948 | 11 |
| 7 | 790.3400 | 395.6736 | 773.3134 | 387.1604 | 772.3294 | 386.6683 | T | 1052.5623 | 526.7848 | 1035.5357 | 518.2715 | 1034.5517 | 517.7795 | 10 |
| 8 | 877.3720 | 439.1896 | 860.3455 | 430.6764 | 859.3614 | 430.1844 | S | 951.5146 | 476.2609 | 934.4880 | 467.7477 | 933.5040 | 467.2556 | 9 |
| 9 | 1040.4353 | 520.7213 | 1023.4088 | 512.2080 | 1022.4248 | 511.7160 | Y | 864.4825 | 432.7449 | 847.4560 | 424.2316 | 846.4720 | 423.7396 | 8 |
| 10 | 1097.4568 | 549.2320 | 1080.4302 | 540.7188 | 1079.4462 | 540.2268 | G | 701.4192 | 351.2132 | 684.3927 | 342.7000 | 683.4087 | 342.2080 | 7 |
| 11 | 1196.5252 | 598.7662 | 1179.4987 | 590.2530 | 1178.5146 | 589.7610 | V | 644.3978 | 322.7025 | 627.3712 | 314.1892 | 626.3872 | 313.6972 | 6 |
| 12 | 1309.6093 | 655.3083 | 1292.5827 | 646.7950 | 1291.5987 | 646.3030 | L | 545.3293 | 273.1683 | 528.3028 | 264.6550 | 527.3188 | 264.1630 | 5 |
| 13 | 1424.6362 | 712.8217 | 1407.6097 | 704.3085 | 1406.6257 | 703.8165 | D | 432.2453 | 216.6263 | 415.2187 | 208.1130 | 414.2347 | 207.6210 | 4 |
| 14 | 1537.7203 | 769.3638 | 1520.6937 | 760.8505 | 1519.7097 | 760.3585 | I | 317.2183 | 159.1128 | 300.1918 | 150.5995 | | | 3 |
| 15 | 1594.7417 | 797.8745 | 1577.7152 | 789.3612 | 1576.7312 | 788.8692 | G | 204.1343 | 102.5708 | 187.1077 | 94.0575 | | | 2 |
| 16 | | | | | | | K | 147.1128 | 74.0600 | 130.0863 | 65.5468 | | | 1 |

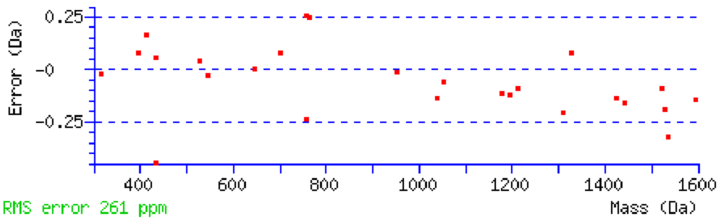


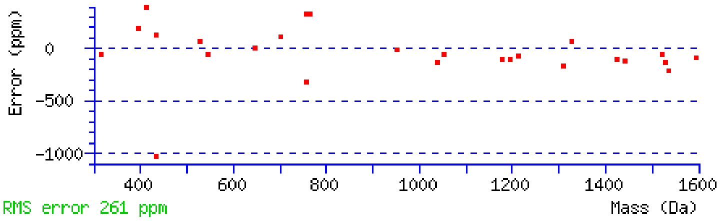


### All matches to this query

| Score | Mr(calc): | Delta | Sequence |
|---|---|---|---|
| 88.2 | 1739.8400 | 0.0020 | VISNNCTSYGVLDIGK |
| 74.1 | 1739.8400 | 0.0020 | VISNNCTSYGVLDIGK |
| 39.8 | 1738.8560 | 0.9860 | VISNNCTSYGVLDIGK |
| 6.6 | 1739.8502 | -0.0082 | MRKPDGSTVTQRIR |
| 5.6 | 1739.8502 | -0.0082 | MRKPDGSTVTQRIR |
| 5.6 | 1739.8502 | -0.0082 | MRKPDGSTVTQRIR |
| 1.9 | 1738.8291 | 1.0129 | SSPPPPLPAEERPGTK |
| 0.3 | 1738.8443 | 0.9977 | LHLWTNGSVAYSIAK |
| 0.1 | 1739.8593 | -0.0173 | IKSEIEEELKSLDK |

---

Spectrum No: 151; Query: 2462; Rank: 1

---

## Peptide View

MS/MS Fragmentation of **TVVTEAGNLLKDNATEEEILHYLEK**
Found in **IPI00195160**, Tax_Id=10116 Gene_Symbol=Psap Sulfated glycoprotein 1 precursor

Match to Query 2462: 2829.433782 from(944.151870,3+)
Title: 091008RatKidney_NH4Format01_18.5332.5332.3.dta
Data file K:\NewmanPaper\Piliang\3SubProteomes\Piliang3SP\mgf5ppm\ERLIC_3SubProteomes5ppm.mgf

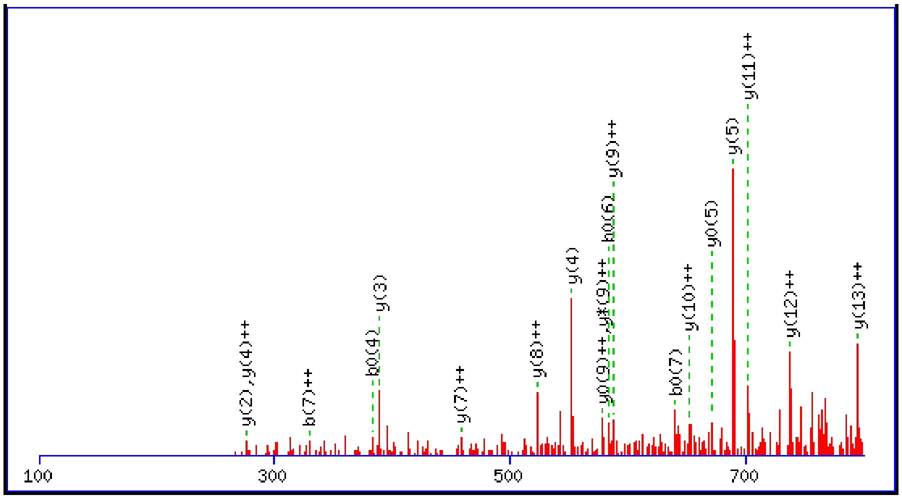


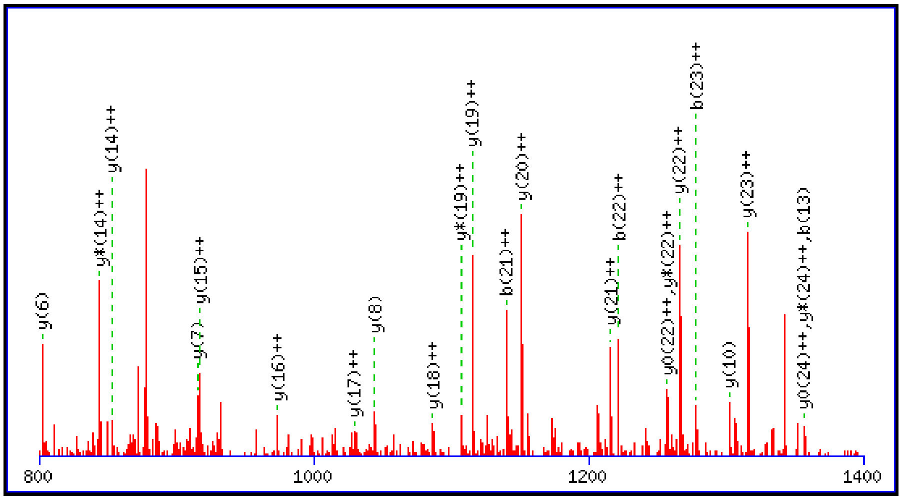


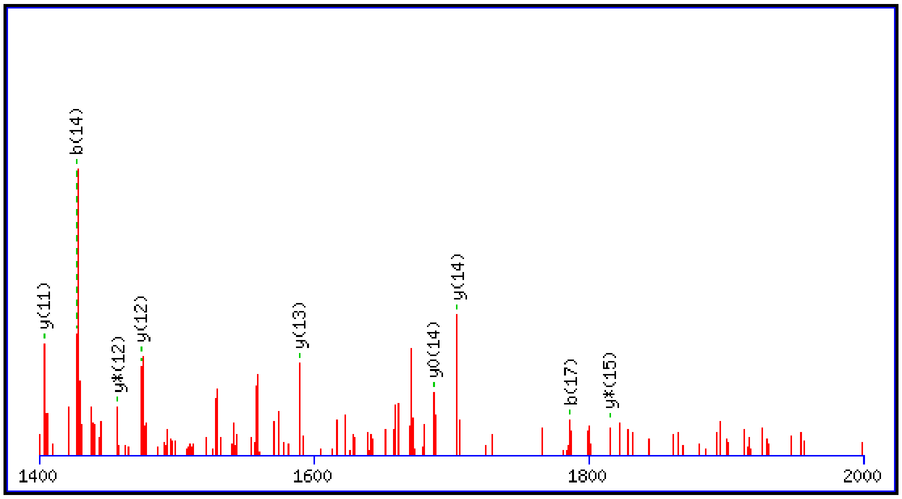


**Monoisotopic mass of neutral peptide Mr(calc):** 2829.4283
**Fixed modifications:** Carbamidomethyl (C)
**Variable modifications:**
**N13** : Deamidated_N (N)
**Ions Score:** 88 **Expect:** 2.7e-007
**Matches (Bold Red):** 52/272 fragment ions using 96 most intense peaks

| # | b | b++ | b* | b*++ | b0 | b0++ | Seq. | y | y++ | y* | y*++ | y0 | y0++ | # |
|---|---|---|---|---|---|---|---|---|---|---|---|---|---|---|
| 1 | 102.0550 | 51.5311 | | | 84.0444 | 42.5258 | T | | | | | | | 25 |
| 2 | 201.1234 | 101.0653 | | | 183.1128 | 92.0600 | V | 2729.3880 | 1365.1976 | 2712.3614 | **1356.6844** | 2711.3774 | **1356.1923** | 24 |
| 3 | 300.1918 | 150.5995 | | | 282.1812 | 141.5942 | V | 2630.3196 | **1315.6634** | 2613.2930 | 1307.1501 | 2612.3090 | 1306.6581 | 23 |
| 4 | 401.2395 | 201.1234 | | | **383.2289** | 192.1181 | T | 2531.2511 | **1266.1292** | 2514.2246 | **1257.6159** | 2513.2406 | **1257.1239** | 22 |
| 5 | 530.2821 | 265.6447 | | | 512.2715 | 256.6394 | E | 2430.2035 | **1215.6054** | 2413.1769 | 1207.0921 | 2412.1929 | 1206.6001 | 21 |

| | | | | | | | | | | | | | | |
|---|---|---|---|---|---|---|---|---|---|---|---|---|---|---|
| 6 | 601.3192 | 301.1632 | | | **583.3086** | 292.1579 | A | 2301.1609 | **1151.0841** | 2284.1343 | 1142.5708 | 2283.1503 | 1142.0788 | 20 |
| 7 | 658.3406 | **329.6740** | | | **640.3301** | 320.6687 | G | 2230.1238 | **1115.5655** | 2213.0972 | **1107.0522** | 2212.1132 | 1106.5602 | 19 |
| 8 | 772.3836 | 386.6954 | 755.3570 | 378.1821 | 754.3730 | 377.6901 | N | 2173.1023 | **1087.0548** | 2156.0758 | 1078.5415 | 2155.0917 | 1078.0495 | 18 |
| 9 | 885.4676 | 443.2375 | 868.4411 | 434.7242 | 867.4571 | 434.2322 | L | 2059.0594 | **1030.0333** | 2042.0328 | 1021.5200 | 2041.0488 | 1021.0280 | 17 |
| 10 | 998.5517 | 499.7795 | 981.5251 | 491.2662 | 980.5411 | 490.7742 | L | 1945.9753 | **973.4913** | 1928.9488 | 964.9780 | 1927.9647 | 964.4860 | 16 |
| 11 | 1126.6467 | 563.8270 | 1109.6201 | 555.3137 | 1108.6361 | 554.8217 | K | 1832.8912 | **916.9493** | **1815.8647** | 908.4360 | 1814.8807 | 907.9440 | 15 |
| 12 | 1241.6736 | 621.3404 | 1224.6470 | 612.8272 | 1223.6630 | 612.3352 | D | **1704.7963** | **852.9018** | 1687.7697 | **844.3885** | **1686.7857** | 843.8965 | 14 |
| 13 | **1356.7005** | 678.8539 | 1339.6740 | 670.3406 | 1338.6900 | 669.8486 | N | **1589.7693** | **795.3883** | 1572.7428 | 786.8750 | 1571.7588 | 786.3830 | 13 |
| 14 | **1427.7376** | 714.3725 | 1410.7111 | 705.8592 | 1409.7271 | 705.3672 | A | **1474.7424** | **737.8748** | **1457.7159** | 729.3616 | 1456.7318 | 728.8696 | 12 |
| 15 | 1528.7853 | 764.8963 | 1511.7588 | 756.3830 | 1510.7748 | 755.8910 | T | **1403.7053** | **702.3563** | 1386.6787 | 693.8430 | 1385.6947 | 693.3510 | 11 |
| 16 | 1657.8279 | 829.4176 | 1640.8014 | 820.9043 | 1639.8174 | 820.4123 | E | **1302.6576** | **651.8324** | 1285.6311 | 643.3192 | 1284.6470 | 642.8272 | 10 |
| 17 | **1786.8705** | 893.9389 | 1769.8440 | 885.4256 | 1768.8599 | 884.9336 | E | 1173.6150 | **587.3111** | 1156.5885 | **578.7979** | 1155.6045 | **578.3059** | 9 |
| 18 | 1915.9131 | 958.4602 | 1898.8866 | 949.9469 | 1897.9025 | 949.4549 | E | **1044.5724** | **522.7898** | 1027.5459 | 514.2766 | 1026.5619 | 513.7846 | 8 |
| 19 | 2028.9972 | 1015.0022 | 2011.9706 | 1006.4889 | 2010.9866 | 1005.9969 | I | **915.5298** | **458.2686** | 898.5033 | 449.7553 | 897.5193 | 449.2633 | 7 |
| 20 | 2142.0812 | 1071.5443 | 2125.0547 | 1063.0310 | 2124.0707 | 1062.5390 | L | **802.4458** | 401.7265 | 785.4192 | 393.2132 | 784.4352 | 392.7212 | 6 |
| 21 | 2279.1401 | **1140.0737** | 2262.1136 | 1131.5604 | 2261.1296 | 1131.0684 | H | **689.3617** | 345.1845 | 672.3352 | 336.6712 | **671.3511** | 336.1792 | 5 |
| 22 | 2442.2035 | **1221.6054** | 2425.1769 | 1213.0921 | 2424.1929 | 1212.6001 | Y | **552.3028** | **276.6550** | 535.2762 | 268.1418 | 534.2922 | 267.6498 | 4 |
| 23 | 2555.2875 | **1278.1474** | 2538.2610 | 1269.6341 | 2537.2770 | 1269.1421 | L | **389.2395** | 195.1234 | 372.2129 | 186.6101 | 371.2289 | 186.1181 | 3 |
| 24 | 2684.3301 | 1342.6687 | 2667.3036 | 1334.1554 | 2666.3196 | 1333.6634 | E | **276.1554** | 138.5813 | 259.1288 | 130.0681 | 258.1448 | 129.5761 | 2 |
| 25 | | | | | | | K | 147.1128 | 74.0600 | 130.0863 | 65.5468 | | | 1 |

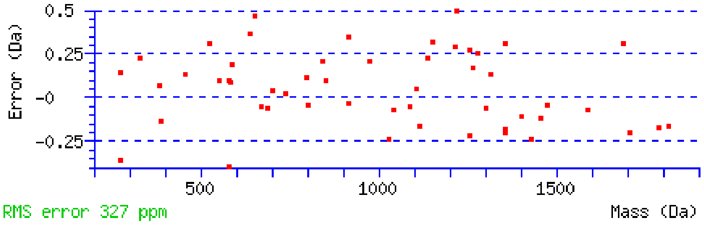


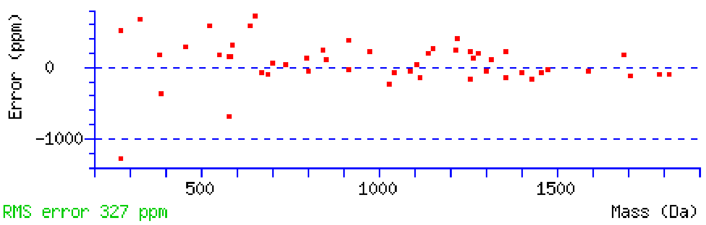


### All matches to this query

| Score | Mr(calc): | Delta | Sequence |
|---|---|---|---|
| 88.0 | 2829.4283 | 0.0055 | TVVTEAGNLLKDNATEEEILHYLEK |
| 48.1 | 2829.4283 | 0.0055 | TVVTEAGNLLKDNATEEEILHYLEK |
| 18.8 | 2828.4443 | 0.9895 | TVVTEAGNLLKDNATEEEILHYLEK |

---

Spectrum No: 152; Query: 1811; Rank: 1

---

## Peptide View

MS/MS Fragmentation of **GQIIGISCQSVNGTAPITYR**
Found in **IPI00206467**, Tax_Id=10116 Gene_Symbol=Pecam Platelet endothelial cell adhesion molecule precursor

Match to Query 1811: 2135.075068 from(1068.544810,2+)
Title: 100101RatKid_NS_deglyco_21.3138.3138.2.dta
Data file K:\NewmanPaper\Piliang\3SubProteomes\Piliang3SP\mgf5ppm\ERLIC_3SubProteomes5ppm.mgf

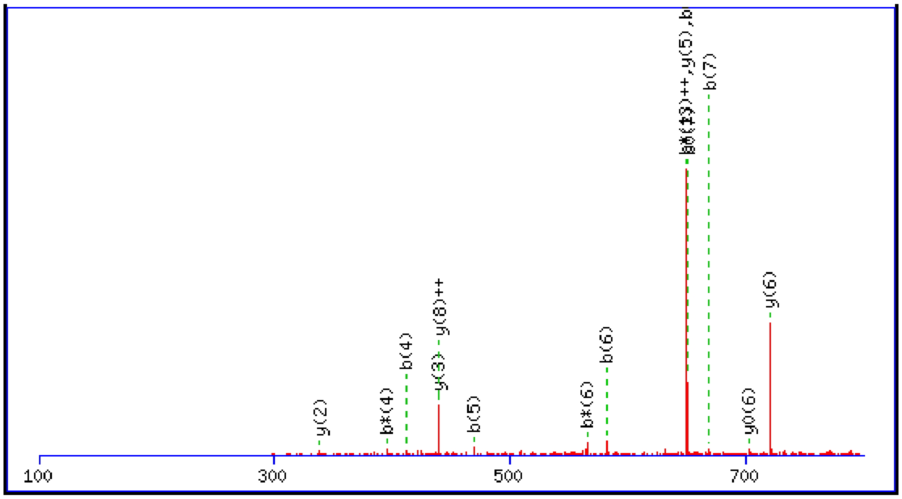


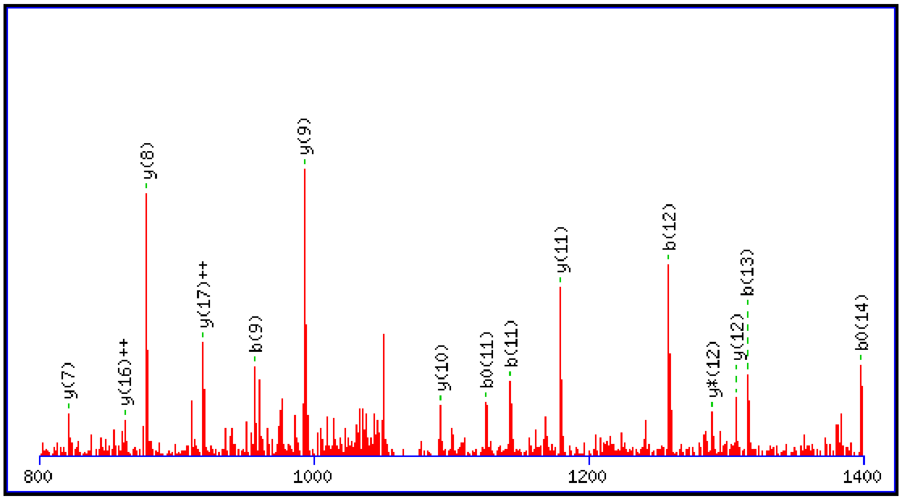


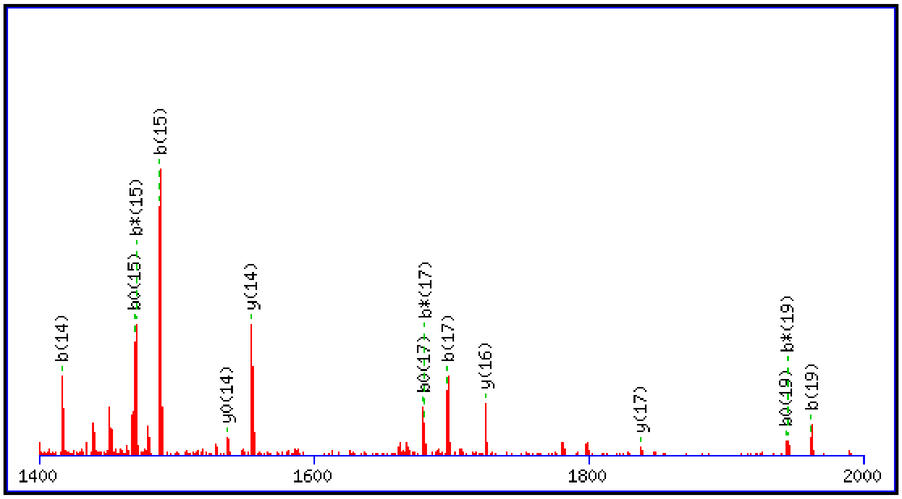


**Monoisotopic mass of neutral peptide Mr(calc):** 2135.0681
**Fixed modifications:** Carbamidomethyl (C)
**Variable modifications:**
**N12** : Deamidated_N (N)
**Ions Score:** 88 **Expect:** 3.2e-007
**Matches (Bold Red):** 44/210 fragment ions using 73 most intense peaks

| # | b | $b^{++}$ | b* | $b^{*++}$ | $b^0$ | $b^{0++}$ | Seq. | y | $y^{++}$ | y* | $y^{*++}$ | $y^0$ | $y^{0++}$ | # |
|---|---|---|---|---|---|---|---|---|---|---|---|---|---|---|
| 1 | 58.0287 | 29.5180 | | | | | G | | | | | | | 20 |
| 2 | 186.0873 | 93.5473 | 169.0608 | 85.0340 | | | Q | 2079.0539 | 1040.0306 | 2062.0274 | 1031.5173 | 2061.0433 | 1031.0253 | 19 |
| 3 | 299.1714 | 150.0893 | 282.1448 | 141.5761 | | | I | 1950.9953 | 976.0013 | 1933.9688 | 967.4880 | 1932.9848 | 966.9960 | 18 |
| 4 | **412.2554** | 206.6314 | **395.2289** | 198.1181 | | | I | **1837.9113** | **919.4593** | 1820.8847 | 910.9460 | 1819.9007 | 910.4540 | 17 |
| 5 | **469.2769** | 235.1421 | 452.2504 | 226.6288 | | | G | **1724.8272** | **862.9172** | 1707.8007 | 854.4040 | 1706.8166 | 853.9120 | 16 |

| | | | | | | | | | | | | | |
|---|---|---|---|---|---|---|---|---|---|---|---|---|---|
| **6** | **582.3610** | 291.6841 | **565.3344** | 283.1709 | | | **I** | 1667.8057 | 834.4065 | 1650.7792 | 825.8932 | 1649.7952 | 825.4012 | **15** |
| **7** | **669.3930** | 335.2001 | 652.3665 | 326.6869 | **651.3824** | 326.1949 | **S** | **1554.7217** | 777.8645 | 1537.6951 | 769.3512 | **1536.7111** | 768.8592 | **14** |
| **8** | 829.4237 | 415.2155 | 812.3971 | 406.7022 | 811.4131 | 406.2102 | **C** | 1467.6897 | 734.3485 | 1450.6631 | 725.8352 | 1449.6791 | 725.3432 | **13** |
| **9** | **957.4822** | 479.2448 | 940.4557 | 470.7315 | 939.4717 | 470.2395 | **Q** | **1307.6590** | 654.3331 | **1290.6325** | 645.8199 | 1289.6484 | 645.3279 | **12** |
| **10** | 1044.5143 | 522.7608 | 1027.4877 | 514.2475 | 1026.5037 | 513.7555 | **S** | **1179.6004** | 590.3038 | 1162.5739 | 581.7906 | 1161.5899 | 581.2986 | **11** |
| **11** | **1143.5827** | 572.2950 | 1126.5561 | 563.7817 | **1125.5721** | 563.2897 | **V** | **1092.5684** | 546.7878 | 1075.5418 | 538.2746 | 1074.5578 | 537.7826 | **10** |
| **12** | **1258.6096** | 629.8084 | 1241.5831 | 621.2952 | 1240.5990 | 620.8032 | **N** | **993.5000** | 497.2536 | 976.4734 | 488.7404 | 975.4894 | 488.2483 | **9** |
| **13** | **1315.6311** | 658.3192 | 1298.6045 | **649.8059** | 1297.6205 | **649.3139** | **G** | **878.4730** | **439.7402** | 861.4465 | 431.2269 | 860.4625 | 430.7349 | **8** |
| **14** | **1416.6788** | 708.8430 | 1399.6522 | 700.3297 | **1398.6682** | 699.8377 | **T** | **821.4516** | 411.2294 | 804.4250 | 402.7162 | 803.4410 | 402.2241 | **7** |
| **15** | **1487.7159** | 744.3616 | **1470.6893** | 735.8483 | **1469.7053** | 735.3563 | **A** | **720.4039** | 360.7056 | 703.3774 | 352.1923 | **702.3933** | 351.7003 | **6** |
| **16** | 1584.7686 | 792.8880 | 1567.7421 | 784.3747 | 1566.7581 | 783.8827 | **P** | **649.3668** | 325.1870 | 632.3402 | 316.6738 | 631.3562 | 316.1817 | **5** |
| **17** | **1697.8527** | 849.4300 | **1680.8261** | 840.9167 | **1679.8421** | 840.4247 | **I** | 552.3140 | 276.6607 | 535.2875 | 268.1474 | 534.3035 | 267.6554 | **4** |
| **18** | 1798.9004 | 899.9538 | 1781.8738 | 891.4405 | 1780.8898 | 890.9485 | **T** | **439.2300** | 220.1186 | 422.2034 | 211.6053 | 421.2194 | 211.1133 | **3** |
| **19** | **1961.9637** | 981.4855 | **1944.9372** | 972.9722 | **1943.9531** | 972.4802 | **Y** | **338.1823** | 169.5948 | 321.1557 | 161.0815 | | | **2** |
| **20** | | | | | | | **R** | 175.1190 | 88.0631 | 158.0924 | 79.5498 | | | **1** |

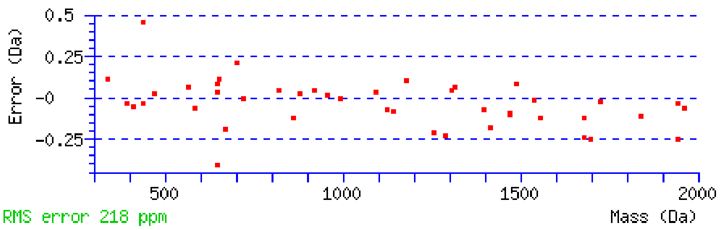


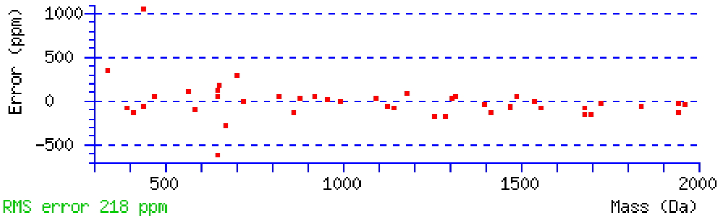


**All matches to this query**

| Score | Mr(calc): | Delta | Sequence |
|---|---|---|---|
| 88.0 | 2135.0681 | 0.0070 | GQIIGISCQSVNGTAPITYR |
| 22.7 | 2134.0841 | 0.9910 | GQIIGISCQSVNGTAPITYR |
| 0.2 | 2135.0697 | 0.0054 | LEPVLPSVAALSSAGTMERK |
| 0.2 | 2135.0697 | 0.0054 | LEPVLPSVAALSSAGTMERK |

---

Spectrum No: 153; Query: 937; Rank: 1

---

## Peptide View

MS/MS Fragmentation of **VDLEDFENNTAYAK**
Found in **IPI00369539**, Tax_Id=10116 Gene_Symbol=Mfap4 Microfibrillar-associated protein 4

Match to Query 937: 1628.722468 from(815.368510,2+)
Title: 100101RatKid_NS_deglyco_26.2984.2984.2.dta
Data file K:\NewmanPaper\Piliang\3SubProteomes\Piliang3SP\mgf5ppm\ERLIC_3SubProteomes5ppm.mgf

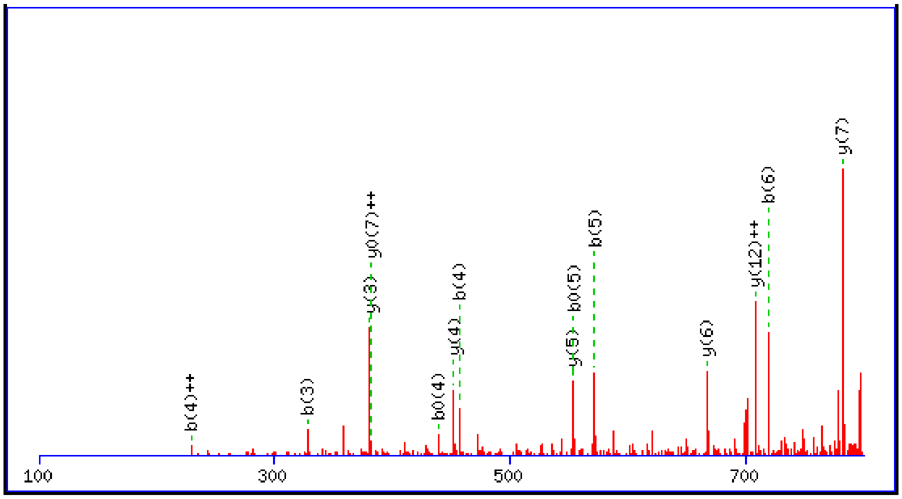


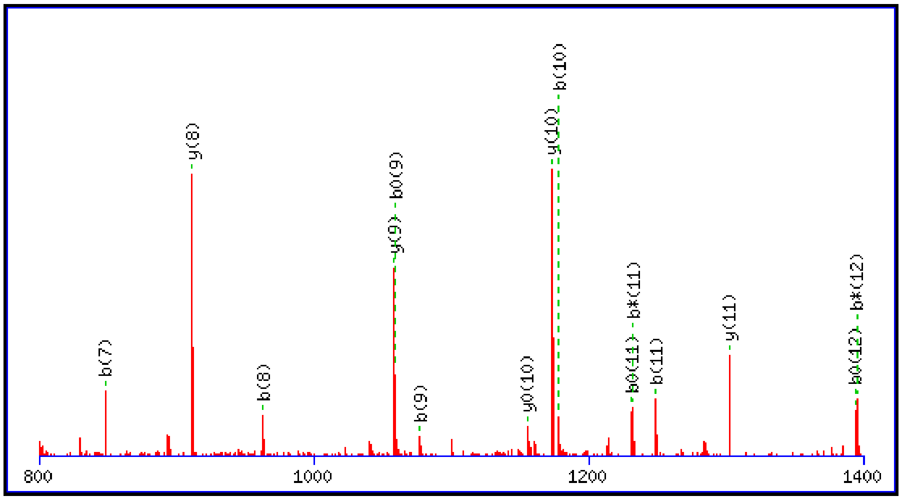


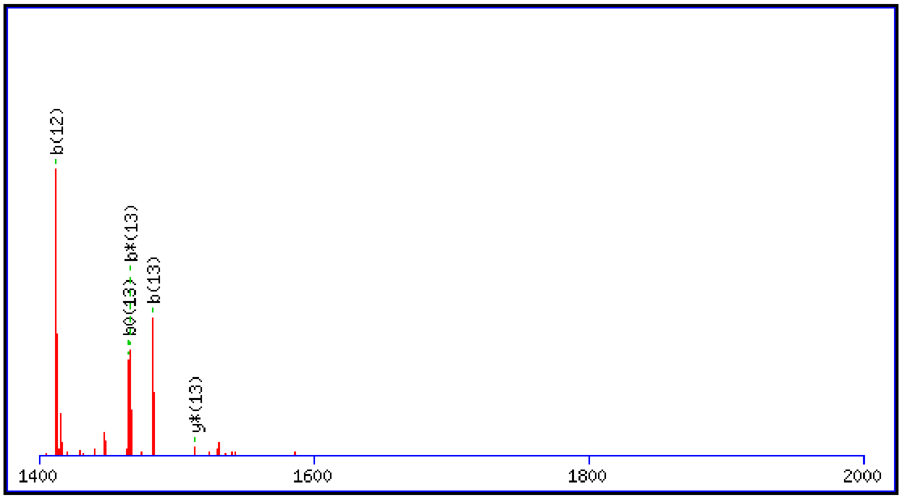


**Monoisotopic mass of neutral peptide Mr(calc):** 1628.7206
**Fixed modifications:** Carbamidomethyl (C)
**Variable modifications:**
**N8** : Deamidated_N (N)
**Ions Score:** 88 **Expect:** 3.4e-007
**Matches (Bold Red):** 34/132 fragment ions using 48 most intense peaks

| # | b | $b^{++}$ | b* | $b^{*++}$ | $b^0$ | $b^{0++}$ | Seq. | y | $y^{++}$ | y* | $y^{*++}$ | $y^0$ | $y^{0++}$ | # |
|---|---|---|---|---|---|---|---|---|---|---|---|---|---|---|
| 1 | 100.0757 | 50.5415 | | | | | V | | | | | | | 14 |
| 2 | 215.1026 | 108.0550 | | | 197.0921 | 99.0497 | D | 1530.6595 | 765.8334 | **1513.6329** | 757.3201 | 1512.6489 | 756.8281 | 13 |
| 3 | **328.1867** | 164.5970 | | | 310.1761 | 155.5917 | L | 1415.6325 | **708.3199** | 1398.6060 | 699.8066 | 1397.6219 | 699.3146 | 12 |
| 4 | **457.2293** | **229.1183** | | | **439.2187** | 220.1130 | E | **1302.5484** | 651.7779 | 1285.5219 | 643.2646 | 1284.5379 | 642.7726 | 11 |
| 5 | **572.2562** | 286.6318 | | | **554.2457** | 277.6265 | D | **1173.5059** | 587.2566 | 1156.4793 | 578.7433 | **1155.4953** | 578.2513 | 10 |

| | | | | | | | | | | | | | | |
|---|---|---|---|---|---|---|---|---|---|---|---|---|---|---|
| **6** | **719.3246** | 360.1660 | | | 701.3141 | 351.1607 | **F** | **1058.4789** | 529.7431 | 1041.4524 | 521.2298 | 1040.4683 | 520.7378 | **9** |
| **7** | **848.3672** | 424.6873 | | | 830.3567 | 415.6820 | **E** | **911.4105** | 456.2089 | 894.3839 | 447.6956 | 893.3999 | 447.2036 | **8** |
| **8** | **963.3942** | 482.2007 | 946.3676 | 473.6875 | 945.3836 | 473.1954 | **N** | **782.3679** | 391.6876 | 765.3414 | 383.1743 | 764.3573 | **382.6823** | **7** |
| **9** | **1077.4371** | 539.2222 | 1060.4106 | 530.7089 | **1059.4265** | 530.2169 | **N** | **667.3410** | 334.1741 | 650.3144 | 325.6608 | 649.3304 | 325.1688 | **6** |
| **10** | **1178.4848** | 589.7460 | 1161.4582 | 581.2328 | 1160.4742 | 580.7407 | **T** | **553.2980** | 277.1527 | 536.2715 | 268.6394 | 535.2875 | 268.1474 | **5** |
| **11** | **1249.5219** | 625.2646 | **1232.4953** | 616.7513 | **1231.5113** | 616.2593 | **A** | **452.2504** | 226.6288 | 435.2238 | 218.1155 | | | **4** |
| **12** | **1412.5852** | 706.7963 | **1395.5587** | 698.2830 | **1394.5747** | 697.7910 | **Y** | **381.2132** | 191.1103 | 364.1867 | 182.5970 | | | **3** |
| **13** | **1483.6223** | 742.3148 | **1466.5958** | 733.8015 | **1465.6118** | 733.3095 | **A** | 218.1499 | 109.5786 | 201.1234 | 101.0653 | | | **2** |
| **14** | | | | | | | **K** | 147.1128 | 74.0600 | 130.0863 | 65.5468 | | | **1** |

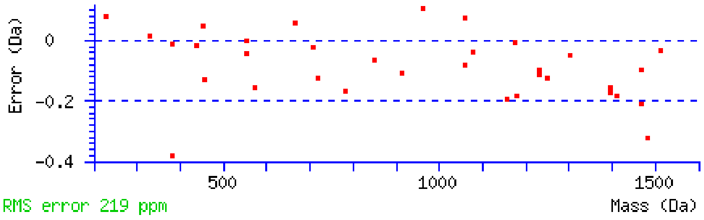


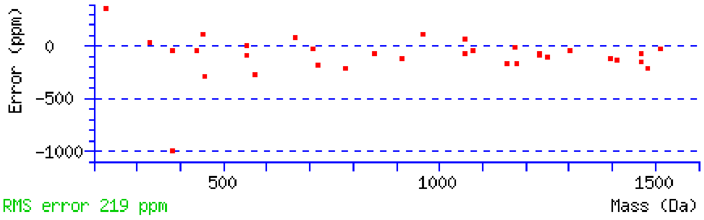


### All matches to this query

| Score | Mr(calc): | Delta | Sequence |
|---|---|---|---|
| 87.8 | 1628.7206 | 0.0019 | VDLEDFENNTAYAK |
| 85.7 | 1628.7206 | 0.0019 | VDLEDFENNTAYAK |
| 9.4 | 1628.7083 | 0.0141 | SQPDFGSEIVTGNAK |
| 7.2 | 1627.7131 | 1.0094 | KVVEDEVYSESHK |
| 7.1 | 1628.7294 | -0.0070 | ADVNGDLNTVSTKSK |
| 5.7 | 1628.7382 | -0.0157 | NNVVNTMRLWSAK |
| 5.6 | 1628.7157 | 0.0068 | THNTLYTPEMLTK |
| 2.4 | 1627.7131 | 1.0094 | KVVEDEVYSESHK |
| 2.4 | 1627.7131 | 1.0094 | KVVEDEVYSESHK |
| 0.6 | 1627.7065 | 1.0159 | MSPTERLEHAFSK |

---

Spectrum No: 154; Query: 1765; Rank: 1

---

## Peptide View

MS/MS Fragmentation of **QQSQEDGDYSPNGSAQPGDK**
Found in **IPI00364925**, Tax_Id=10116 Gene_Symbol=Dhtkd1 Probable 2-oxoglutarate dehydrogenase E1 component DHKTD1, mitochondrial precursor

Match to Query 1765: 2107.862948 from(1054.938750,2+)
Title: 100101RatKid_NS_deglyco_21.670.670.2.dta
Data file K:\NewmanPaper\Piliang\3SubProteomes\Piliang3SP\mgf5ppm\ERLIC_3SubProteomes5ppm.mgf

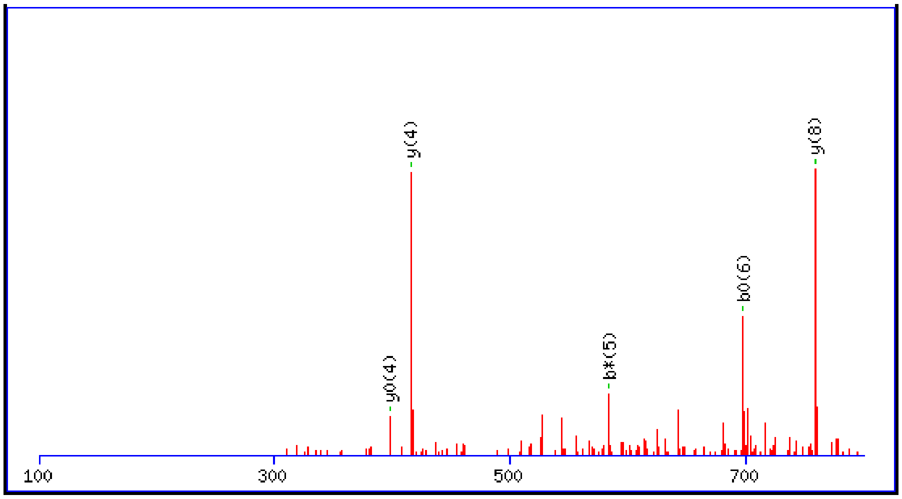


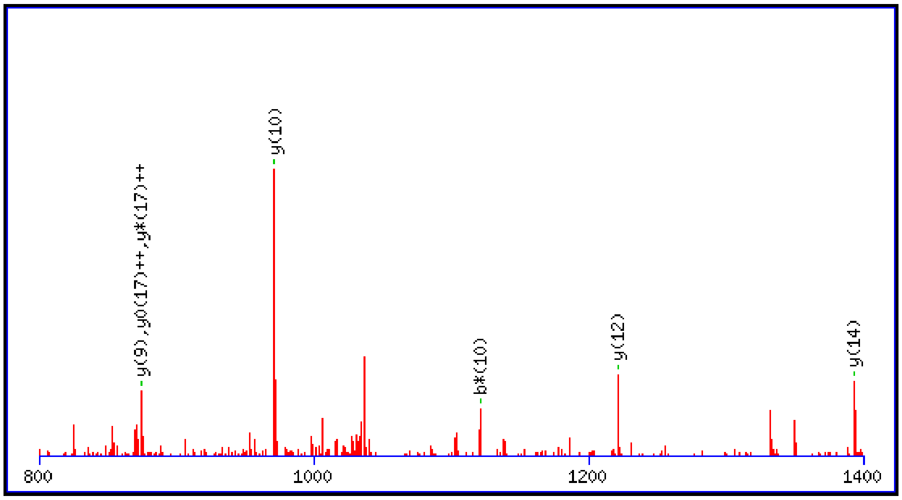


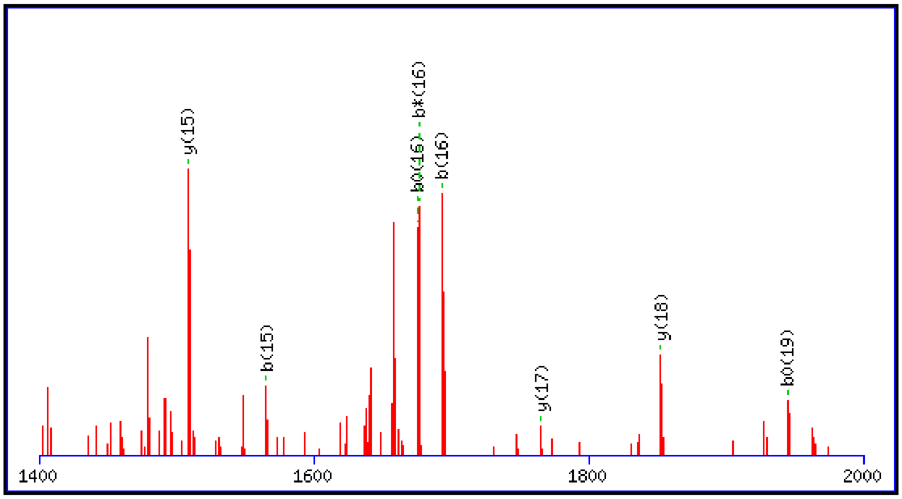


**Monoisotopic mass of neutral peptide Mr(calc):** 2107.8566
**Fixed modifications:** Carbamidomethyl (C)
**Variable modifications:**
**N12** : Deamidated_N (N)
**Ions Score:** 88 **Expect:** 3.8e-007
**Matches (Bold Red):** 20/222 fragment ions using 28 most intense peaks

| # | b | $b^{++}$ | b* | $b^{*++}$ | $b^0$ | $b^{0++}$ | Seq. | y | $y^{++}$ | y* | $y^{*++}$ | $y^0$ | $y^{0++}$ | # |
|---|---|---|---|---|---|---|---|---|---|---|---|---|---|---|
| 1 | 129.0659 | 65.0366 | 112.0393 | 56.5233 | | | Q | | | | | | | 20 |
| 2 | 257.1244 | 129.0659 | 240.0979 | 120.5526 | | | Q | 1980.8053 | 990.9063 | 1963.7788 | 982.3930 | 1962.7948 | 981.9010 | 19 |
| 3 | 344.1565 | 172.5819 | 327.1299 | 164.0686 | 326.1459 | 163.5766 | S | **1852.7468** | 926.8770 | 1835.7202 | 918.3637 | 1834.7362 | 917.8717 | 18 |
| 4 | 472.2150 | 236.6112 | 455.1885 | 228.0979 | 454.2045 | 227.6059 | Q | **1765.7147** | 883.3610 | 1748.6882 | **874.8477** | 1747.7042 | **874.3557** | 17 |
| 5 | 601.2576 | 301.1325 | **584.2311** | 292.6192 | 583.2471 | 292.1272 | E | 1637.6562 | 819.3317 | 1620.6296 | 810.8184 | 1619.6456 | 810.3264 | 16 |

| | | | | | | | | | | | | | | |
|---|---|---|---|---|---|---|---|---|---|---|---|---|---|---|
| **6** | 716.2846 | 358.6459 | 699.2580 | 350.1327 | **698.2740** | 349.6406 | **D** | **1508.6136** | 754.8104 | 1491.5870 | 746.2971 | 1490.6030 | 745.8051 | **15** |
| **7** | 773.3060 | 387.1567 | 756.2795 | 378.6434 | 755.2955 | 378.1514 | **G** | **1393.5866** | 697.2969 | 1376.5601 | 688.7837 | 1375.5761 | 688.2917 | **14** |
| **8** | 888.3330 | 444.6701 | 871.3064 | 436.1569 | 870.3224 | 435.6648 | **D** | 1336.5652 | 668.7862 | 1319.5386 | 660.2729 | 1318.5546 | 659.7809 | **13** |
| **9** | 1051.3963 | 526.2018 | 1034.3698 | 517.6885 | 1033.3857 | 517.1965 | **Y** | **1221.5382** | 611.2727 | 1204.5117 | 602.7595 | 1203.5277 | 602.2675 | **12** |
| **10** | 1138.4283 | 569.7178 | **1121.4018** | 561.2045 | 1120.4178 | 560.7125 | **S** | 1058.4749 | 529.7411 | 1041.4483 | 521.2278 | 1040.4643 | 520.7358 | **11** |
| **11** | 1235.4811 | 618.2442 | 1218.4546 | 609.7309 | 1217.4705 | 609.2389 | **P** | **971.4429** | 486.2251 | 954.4163 | 477.7118 | 953.4323 | 477.2198 | **10** |
| **12** | 1350.5080 | 675.7577 | 1333.4815 | 667.2444 | 1332.4975 | 666.7524 | **N** | **874.3901** | 437.6987 | 857.3635 | 429.1854 | 856.3795 | 428.6934 | **9** |
| **13** | 1407.5295 | 704.2684 | 1390.5030 | 695.7551 | 1389.5189 | 695.2631 | **G** | **759.3632** | 380.1852 | 742.3366 | 371.6719 | 741.3526 | 371.1799 | **8** |
| **14** | 1494.5615 | 747.7844 | 1477.5350 | 739.2711 | 1476.5510 | 738.7791 | **S** | 702.3417 | 351.6745 | 685.3151 | 343.1612 | 684.3311 | 342.6692 | **7** |
| **15** | **1565.5986** | 783.3030 | 1548.5721 | 774.7897 | 1547.5881 | 774.2977 | **A** | 615.3097 | 308.1585 | 598.2831 | 299.6452 | 597.2991 | 299.1532 | **6** |
| **16** | **1693.6572** | 847.3322 | **1676.6307** | 838.8190 | **1675.6467** | 838.3270 | **Q** | 544.2726 | 272.6399 | 527.2460 | 264.1266 | 526.2620 | 263.6346 | **5** |
| **17** | 1790.7100 | 895.8586 | 1773.6834 | 887.3454 | 1772.6994 | 886.8533 | **P** | **416.2140** | 208.6106 | 399.1874 | 200.0974 | **398.2034** | 199.6053 | **4** |
| **18** | 1847.7315 | 924.3694 | 1830.7049 | 915.8561 | 1829.7209 | 915.3641 | **G** | 319.1612 | 160.0842 | 302.1347 | 151.5710 | 301.1506 | 151.0790 | **3** |
| **19** | 1962.7584 | 981.8828 | 1945.7318 | 973.3696 | **1944.7478** | 972.8776 | **D** | 262.1397 | 131.5735 | 245.1132 | 123.0602 | 244.1292 | 122.5682 | **2** |
| **20** | | | | | | | **K** | 147.1128 | 74.0600 | 130.0863 | 65.5468 | | | **1** |

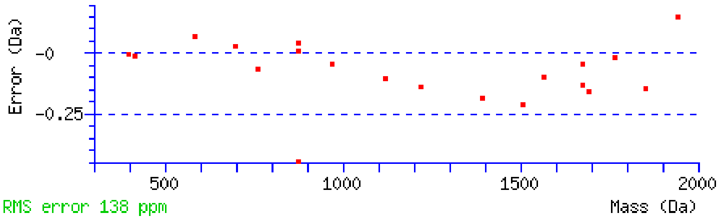


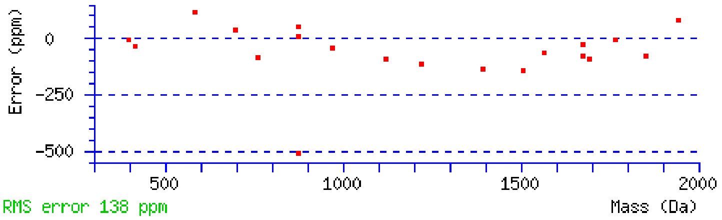


**All matches to this query**

| Score | Mr(calc): | Delta | Sequence |
|---|---|---|---|
| 87.6 | 2107.8566 | 0.0063 | QQSQEDGDYSPNGSAQPGDK |
| 2.7 | 2107.8815 | -0.0186 | TGDMVLKSVNGSYTIRK |
| 2.7 | 2107.8815 | -0.0186 | TGDMVLKSVNGSYTIRK |
| 2.4 | 2107.8815 | -0.0186 | TGDMVLKSVNGSYTIRK |
| 2.4 | 2107.8815 | -0.0186 | TGDMVLKSVNGSYTIRK |
| 0.4 | 2106.8726 | 0.9903 | QQSQEDGDYSPNGSAQPGDK |
| 0.2 | 2105.8643 | 1.9987 | STTNPGSFVLTGMHNGQAK |
| 0.2 | 2105.8643 | 1.9987 | STTNPGSFVLTGMHNGQAK |
| 0.2 | 2105.8643 | 1.9987 | STTNPGSFVLTGMHNGQAK |
| 0.2 | 2105.8643 | 1.9987 | STTNPGSFVLTGMHNGQAK |

---

Spectrum No: 155; Query: 874; Rank: 1

---

## Peptide View

MS/MS Fragmentation of **CSGDGLDSDGNQTLR**
Found in **IPI00364764**, Tax_Id=10116 Gene_Symbol=RGD1559717_predicted similar to R17.3

Match to Query 874: 1594.655628 from(798.335090,2+)
Title: 100101RatKid_NS_deglyco_24.1065.1065.2.dta
Data file K:\NewmanPaper\Piliang\3SubProteomes\Piliang3SP\mgf5ppm\ERLIC_3SubProteomes5ppm.mgf

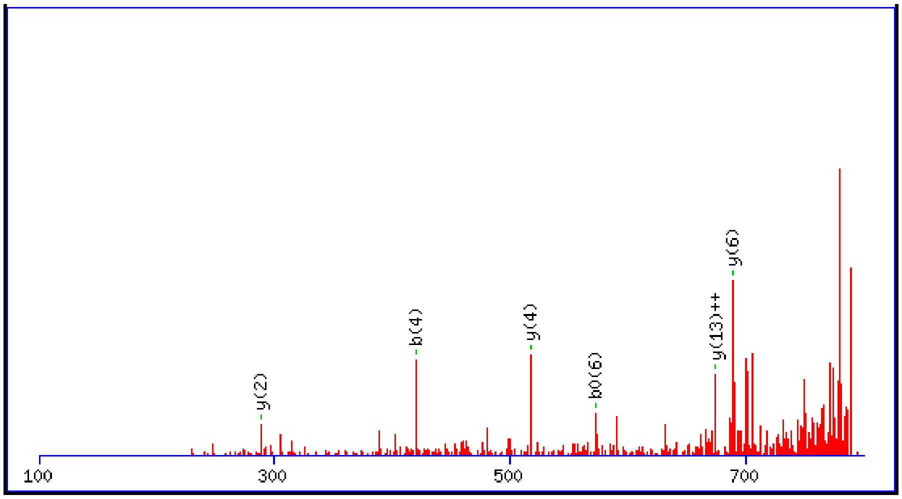


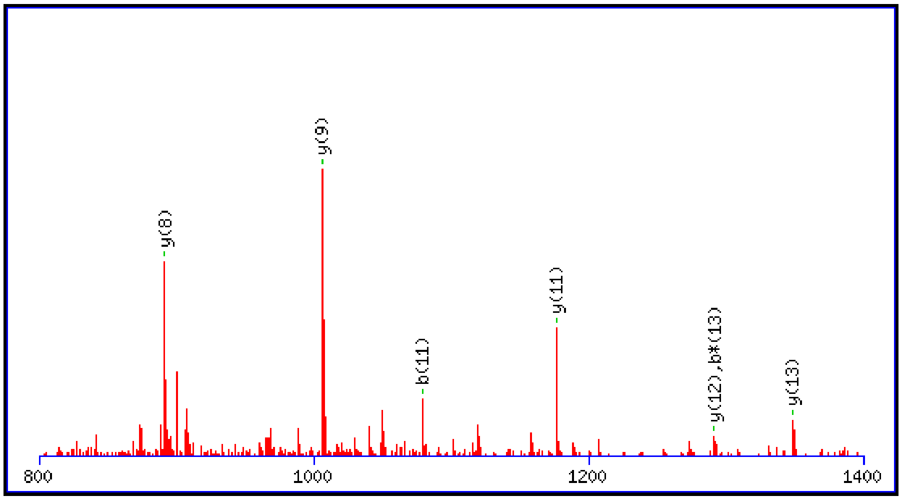


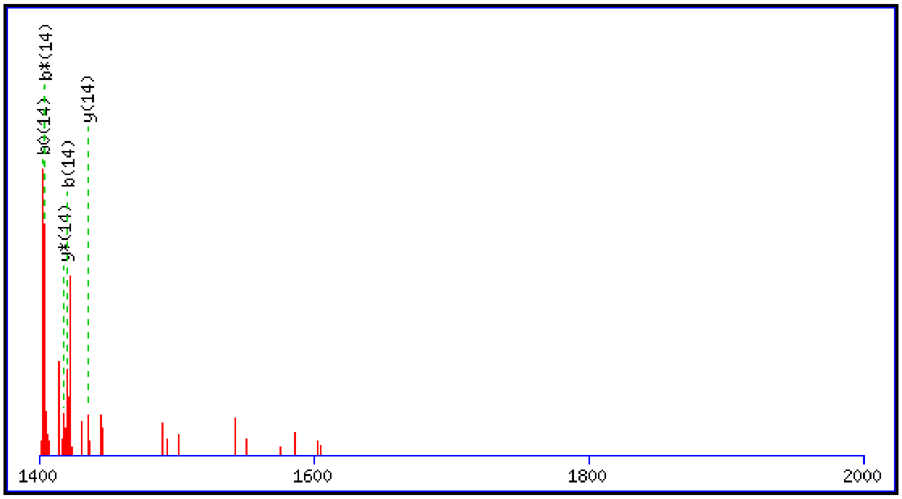


**Monoisotopic mass of neutral peptide Mr(calc):** 1594.6529
**Fixed modifications:** Carbamidomethyl (C)
**Variable modifications:**
**N11** : Deamidated_N (N)
**Ions Score:** 88 **Expect:** 2.3e-007
**Matches (Bold Red):** 18/142 fragment ions using 34 most intense peaks

| # | b | $b^{++}$ | b* | $b^{*++}$ | $b^{0}$ | $b^{0++}$ | Seq. | y | $y^{++}$ | y* | $y^{*++}$ | $y^{0}$ | $y^{0++}$ | # |
|---|---|---|---|---|---|---|---|---|---|---|---|---|---|---|
| 1 | 161.0379 | 81.0226 | | | | | C | | | | | | | 15 |
| 2 | 248.0700 | 124.5386 | | | 230.0594 | 115.5333 | S | **1435.6295** | 718.3184 | **1418.6030** | 709.8051 | 1417.6190 | 709.3131 | 14 |
| 3 | 305.0914 | 153.0493 | | | 287.0809 | 144.0441 | G | **1348.5975** | **674.8024** | 1331.5710 | 666.2891 | 1330.5870 | 665.7971 | 13 |
| 4 | **420.1184** | 210.5628 | | | 402.1078 | 201.5575 | D | **1291.5761** | 646.2917 | 1274.5495 | 637.7784 | 1273.5655 | 637.2864 | 12 |
| 5 | 477.1398 | 239.0735 | | | 459.1293 | 230.0683 | G | **1176.5491** | 588.7782 | 1159.5226 | 580.2649 | 1158.5385 | 579.7729 | 11 |

| | | | | | | | | | | | | | | |
|---|---|---|---|---|---|---|---|---|---|---|---|---|---|---|
| **6** | 590.2239 | 295.6156 | | | **572.2133** | 286.6103 | **L** | 1119.5277 | 560.2675 | 1102.5011 | 551.7542 | 1101.5171 | 551.2622 | **10** |
| **7** | 705.2508 | 353.1291 | | | 687.2403 | 344.1238 | **D** | **1006.4436** | 503.7254 | 989.4170 | 495.2122 | 988.4330 | 494.7201 | **9** |
| **8** | 792.2829 | 396.6451 | | | 774.2723 | 387.6398 | **S** | **891.4166** | 446.2120 | 874.3901 | 437.6987 | 873.4061 | 437.2067 | **8** |
| **9** | 907.3098 | 454.1585 | | | 889.2992 | 445.1533 | **D** | 804.3846 | 402.6959 | 787.3581 | 394.1827 | 786.3741 | 393.6907 | **7** |
| **10** | 964.3313 | 482.6693 | | | 946.3207 | 473.6640 | **G** | **689.3577** | 345.1825 | 672.3311 | 336.6692 | 671.3471 | 336.1772 | **6** |
| **11** | **1079.3582** | 540.1827 | 1062.3317 | 531.6695 | 1061.3476 | 531.1775 | **N** | 632.3362 | 316.6717 | 615.3097 | 308.1585 | 614.3256 | 307.6665 | **5** |
| **12** | 1207.4168 | 604.2120 | 1190.3902 | 595.6988 | 1189.4062 | 595.2067 | **Q** | **517.3093** | 259.1583 | 500.2827 | 250.6450 | 499.2987 | 250.1530 | **4** |
| **13** | 1308.4645 | 654.7359 | **1291.4379** | 646.2226 | 1290.4539 | 645.7306 | **T** | 389.2507 | 195.1290 | 372.2241 | 186.6157 | 371.2401 | 186.1237 | **3** |
| **14** | **1421.5485** | 711.2779 | **1404.5220** | 702.7646 | **1403.5380** | 702.2726 | **L** | **288.2030** | 144.6051 | 271.1765 | 136.0919 | | | **2** |
| **15** | | | | | | | **R** | 175.1190 | 88.0631 | 158.0924 | 79.5498 | | | **1** |

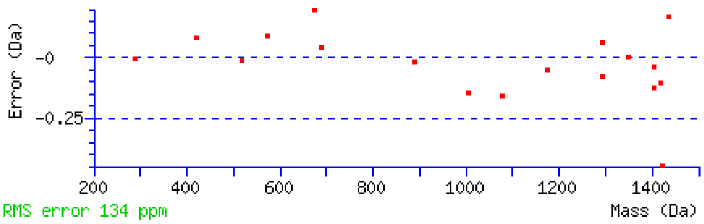


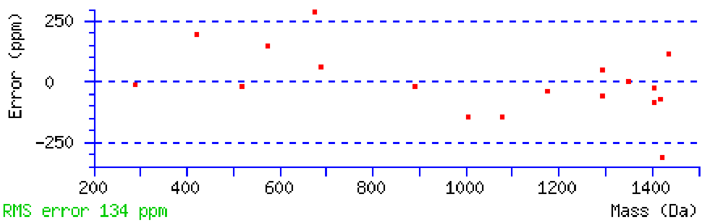


### All matches to this query

| Score | Mr(calc): | Delta | Sequence |
|---|---|---|---|
| 87.6 | 1594.6529 | 0.0027 | CSGDGLDSDGNQTLR |

---

Spectrum No: 156; Query: 1367; Rank: 1

---

## Peptide View

MS/MS Fragmentation of **VGLIGSCTNSSYEDMGR**
Found in **IPI00421539**, Tax_Id=10116 Gene_Symbol=Aco2 Aconitate hydratase, mitochondrial precursor

Match to Query 1367: 1861.776928 from(931.895740,2+)
Title: 091008RatKidney_NH4Format01_14.1882.1882.2.dta
Data file K:\NewmanPaper\Piliang\3SubProteomes\Piliang3SP\mgf5ppm\ERLIC_3SubProteomes5ppm.mgf

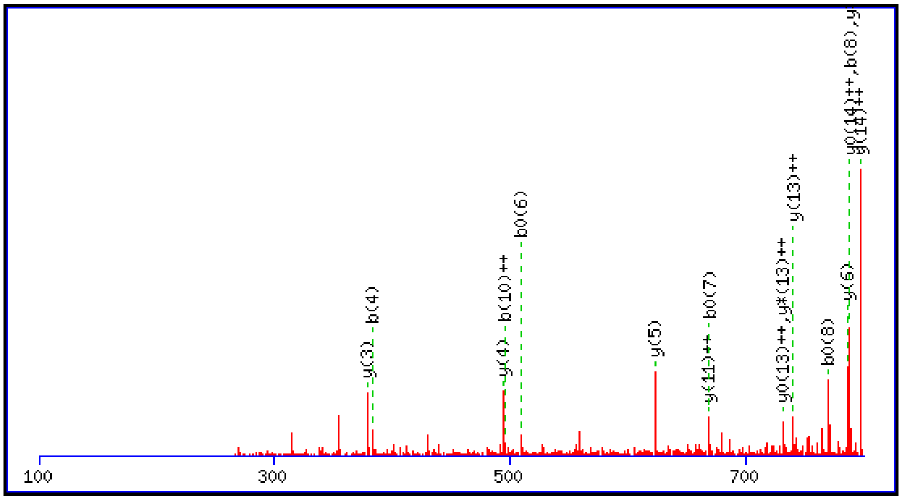

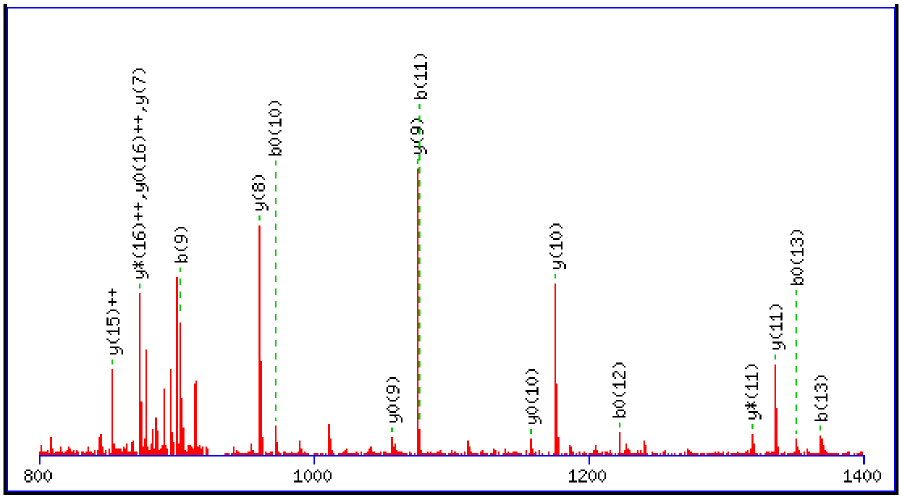


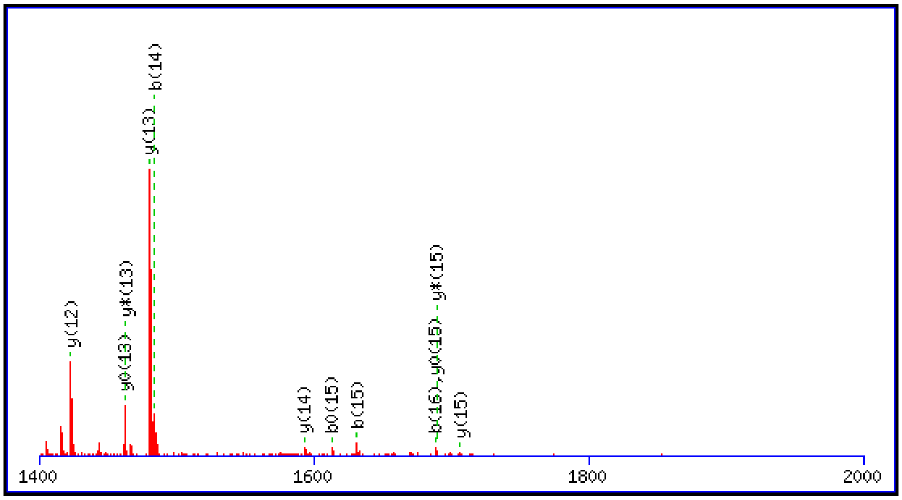


**Monoisotopic mass of neutral peptide Mr(calc):** 1861.7822
**Fixed modifications:** Carbamidomethyl (C)
**Variable modifications:**
**N9** : Deamidated_N (N)
**M15** : Oxidation (M)
**Ions Score:** 87 **Expect:** 3.3e-007
**Matches (Bold Red):** 46/160 fragment ions using 77 most intense peaks

| # | b | $b^{++}$ | b* | $b^{*++}$ | $b^{0}$ | $b^{0++}$ | Seq. | y | $y^{++}$ | y* | $y^{*++}$ | $y^{0}$ | $y^{0++}$ | # |
|---|---|---|---|---|---|---|---|---|---|---|---|---|---|---|
| 1 | 100.0757 | 50.5415 | | | | | V | | | | | | | 17 |
| 2 | 157.0972 | 79.0522 | | | | | G | 1763.7211 | 882.3642 | 1746.6945 | **873.8509** | 1745.7105 | **873.3589** | 16 |
| 3 | 270.1812 | 135.5942 | | | | | L | **1706.6996** | **853.8535** | **1689.6731** | 845.3402 | **1688.6891** | 844.8482 | 15 |
| 4 | **383.2653** | 192.1363 | | | | | I | **1593.6156** | **797.3114** | 1576.5890 | **788.7981** | 1575.6050 | **788.3061** | 14 |
| 5 | 440.2867 | 220.6470 | | | | | G | **1480.5315** | **740.7694** | **1463.5049** | **732.2561** | **1462.5209** | **731.7641** | 13 |
| 6 | 527.3188 | 264.1630 | | | **509.3082** | 255.1577 | S | **1423.5100** | 712.2587 | 1406.4835 | 703.7454 | 1405.4995 | 703.2534 | 12 |
| 7 | 687.3494 | 344.1783 | | | **669.3389** | 335.1731 | C | **1336.4780** | **668.7426** | **1319.4515** | 660.2294 | 1318.4674 | 659.7374 | 11 |
| 8 | **788.3971** | 394.7022 | | | **770.3865** | 385.6969 | T | **1176.4474** | 588.7273 | 1159.4208 | 580.2140 | **1158.4368** | 579.7220 | 10 |
| 9 | **903.4240** | 452.2157 | 886.3975 | 443.7024 | 885.4135 | 443.2104 | N | **1075.3997** | 538.2035 | 1058.3731 | 529.6902 | **1057.3891** | 529.1982 | 9 |
| 10 | 990.4561 | **495.7317** | 973.4295 | 487.2184 | **972.4455** | 486.7264 | S | **960.3727** | 480.6900 | 943.3462 | 472.1767 | 942.3622 | 471.6847 | 8 |
| 11 | **1077.4881** | 539.2477 | 1060.4615 | 530.7344 | 1059.4775 | 530.2424 | S | **873.3407** | 437.1740 | 856.3142 | 428.6607 | 855.3301 | 428.1687 | 7 |
| 12 | 1240.5514 | 620.7793 | 1223.5249 | 612.2661 | **1222.5409** | 611.7741 | Y | **786.3087** | 393.6580 | 769.2821 | 385.1447 | 768.2981 | 384.6527 | 6 |
| 13 | **1369.5940** | 685.3006 | 1352.5675 | 676.7874 | **1351.5835** | 676.2954 | E | **623.2454** | 312.1263 | 606.2188 | 303.6130 | 605.2348 | 303.1210 | 5 |
| 14 | **1484.6210** | 742.8141 | 1467.5944 | 734.3008 | 1466.6104 | 733.8088 | D | **494.2028** | 247.6050 | 477.1762 | 239.0917 | 476.1922 | 238.5997 | 4 |
| 15 | **1631.6564** | 816.3318 | 1614.6298 | 807.8185 | **1613.6458** | 807.3265 | M | **379.1758** | 190.0915 | 362.1493 | 181.5783 | | | 3 |
| 16 | **1688.6778** | 844.8426 | 1671.6513 | 836.3293 | 1670.6673 | 835.8373 | G | 232.1404 | 116.5738 | 215.1139 | 108.0606 | | | 2 |
| 17 | | | | | | | R | 175.1190 | 88.0631 | 158.0924 | 79.5498 | | | 1 |

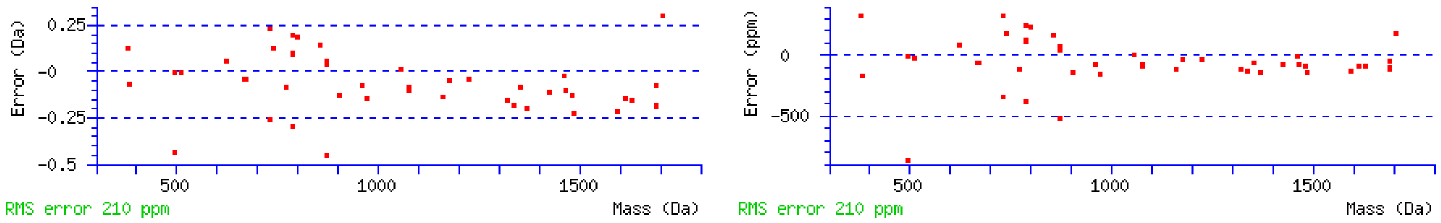


### All matches to this query

| Score | Mr(calc): | Delta | Sequence |
|---|---|---|---|
| 87.5 | 1861.7822 | -0.0053 | VGLIGSCTNSSYEDMGR |

---

Spectrum No: 157; Query: 2292; Rank: 1

---

## Peptide View

MS/MS Fragmentation of **DLNISLDTDAPLSVGKDYGVSAYR**
Found in **IPI00326990**, Tax_Id=10116 Gene_Symbol=Slc15a2 Oligopeptide transporter, kidney isoform

Match to Query 2292: 2569.263972 from(857.428600,3+)
Title: 100101RatKid_NS_deglyco_24.4219.4219.3.dta
Data file K:\NewmanPaper\Piliang\3SubProteomes\Piliang3SP\mgf5ppm\ERLIC_3SubProteomes5ppm.mgf

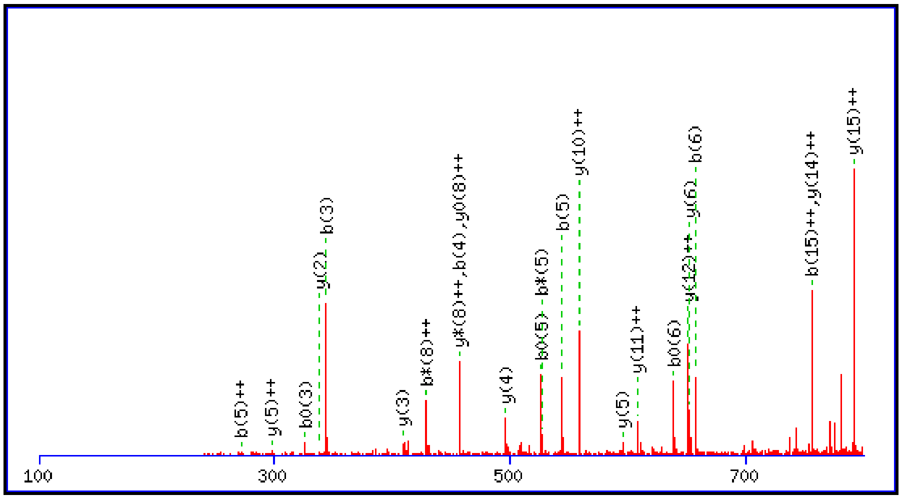


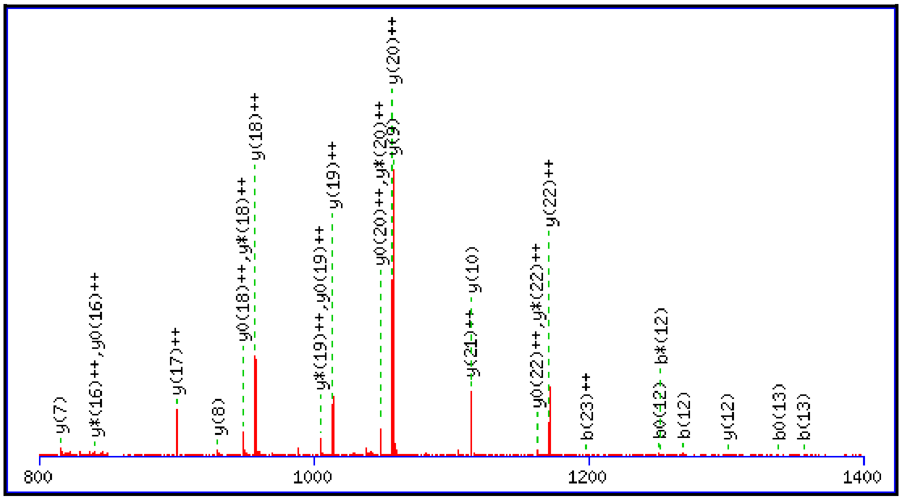

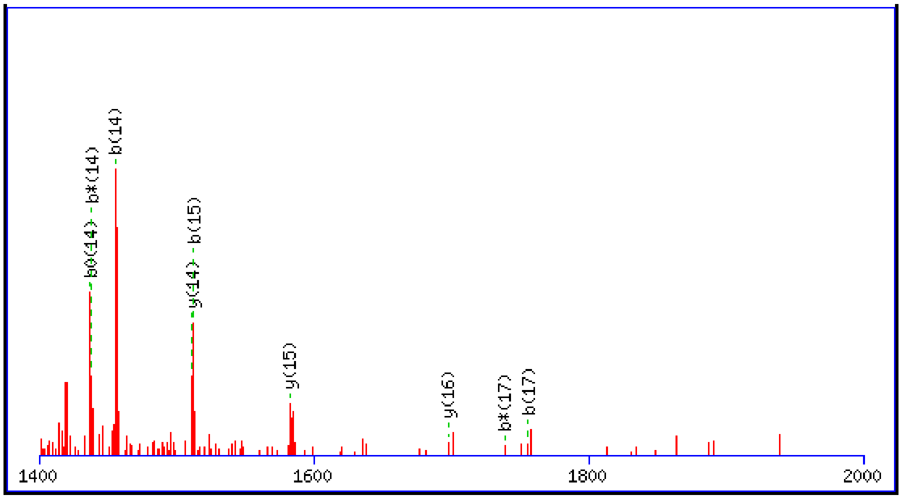


**Monoisotopic mass of neutral peptide Mr(calc):** 2569.2548
**Fixed modifications:** Carbamidomethyl (C)
**Variable modifications:**
**N3** : Deamidated_N (N)
**Ions Score:** 87 **Expect:** 4.6e-007
**Matches (Bold Red):** 60/266 fragment ions using 103 most intense peaks

| # | b | $b^{++}$ | b* | $b^{*++}$ | $b^0$ | $b^{0++}$ | Seq. | y | $y^{++}$ | y* | $y^{*++}$ | $y^0$ | $y^{0++}$ | # |
|---|---|---|---|---|---|---|---|---|---|---|---|---|---|---|
| 1 | 116.0342 | 58.5207 | | | 98.0237 | 49.5155 | D | | | | | | | 24 |
| 2 | 229.1183 | 115.0628 | | | 211.1077 | 106.0575 | L | 2455.2351 | 1228.1212 | 2438.2086 | 1219.6079 | 2437.2245 | 1219.1159 | 23 |
| 3 | **344.1452** | 172.5762 | 327.1187 | 164.0630 | **326.1347** | 163.5710 | N | 2342.1510 | **1171.5792** | 2325.1245 | **1163.0659** | 2324.1405 | **1162.5739** | 22 |
| 4 | **457.2293** | 229.1183 | 440.2027 | 220.6050 | 439.2187 | 220.1130 | I | 2227.1241 | **1114.0657** | 2210.0976 | 1105.5524 | 2209.1135 | 1105.0604 | 21 |
| 5 | **544.2613** | **272.6343** | **527.2348** | 264.1210 | **526.2507** | 263.6290 | S | 2114.0400 | **1057.5237** | 2097.0135 | **1049.0104** | 2096.0295 | **1048.5184** | 20 |
| 6 | **657.3454** | 329.1763 | 640.3188 | 320.6631 | **639.3348** | 320.1710 | L | 2027.0080 | **1014.0076** | 2009.9815 | **1005.4944** | 2008.9974 | **1005.0024** | 19 |
| 7 | 772.3723 | 386.6898 | 755.3458 | 378.1765 | 754.3618 | 377.6845 | D | 1913.9239 | **957.4656** | 1896.8974 | **948.9523** | 1895.9134 | **948.4603** | 18 |
| 8 | 873.4200 | 437.2136 | 856.3934 | **428.7004** | 855.4094 | 428.2084 | T | 1798.8970 | **899.9521** | 1781.8705 | 891.4389 | 1780.8864 | 890.9469 | 17 |
| 9 | 988.4469 | 494.7271 | 971.4204 | 486.2138 | 970.4364 | 485.7218 | D | **1697.8493** | 849.4283 | 1680.8228 | **840.9150** | 1679.8388 | **840.4230** | 16 |
| 10 | 1059.4841 | 530.2457 | 1042.4575 | 521.7324 | 1041.4735 | 521.2404 | A | **1582.8224** | **791.9148** | 1565.7958 | 783.4016 | 1564.8118 | 782.9095 | 15 |
| 11 | 1156.5368 | 578.7720 | 1139.5103 | 570.2588 | 1138.5263 | 569.7668 | P | **1511.7853** | **756.3963** | 1494.7587 | 747.8830 | 1493.7747 | 747.3910 | 14 |
| 12 | **1269.6209** | 635.3141 | **1252.5943** | 626.8008 | **1251.6103** | 626.3088 | L | 1414.7325 | 707.8699 | 1397.7060 | 699.3566 | 1396.7219 | 698.8646 | 13 |
| 13 | **1356.6529** | 678.8301 | 1339.6264 | 670.3168 | **1338.6423** | 669.8248 | S | **1301.6484** | **651.3279** | 1284.6219 | 642.8146 | 1283.6379 | 642.3226 | 12 |
| 14 | **1455.7213** | 728.3643 | **1438.6948** | 719.8510 | **1437.7108** | 719.3590 | V | 1214.6164 | **607.8118** | 1197.5899 | 599.2986 | 1196.6058 | 598.8066 | 11 |
| 15 | **1512.7428** | **756.8750** | 1495.7162 | 748.3618 | 1494.7322 | 747.8697 | G | **1115.5480** | **558.2776** | 1098.5215 | 549.7644 | 1097.5374 | 549.2724 | 10 |
| 16 | 1640.8378 | 820.9225 | 1623.8112 | 812.4092 | 1622.8272 | 811.9172 | K | **1058.5265** | 529.7669 | 1041.5000 | 521.2536 | 1040.5160 | 520.7616 | 9 |
| 17 | **1755.8647** | 878.4360 | **1738.8381** | 869.9227 | 1737.8541 | 869.4307 | D | **930.4316** | 465.7194 | 913.4050 | **457.2062** | 912.4210 | **456.7141** | 8 |
| 18 | 1918.9280 | 959.9676 | 1901.9015 | 951.4544 | 1900.9175 | 950.9624 | Y | **815.4046** | 408.2060 | 798.3781 | 399.6927 | 797.3941 | 399.2007 | 7 |
| 19 | 1975.9495 | 988.4784 | 1958.9229 | 979.9651 | 1957.9389 | 979.4731 | G | **652.3413** | 326.6743 | 635.3148 | 318.1610 | 634.3307 | 317.6690 | 6 |
| 20 | 2075.0179 | 1038.0126 | 2057.9914 | 1029.4993 | 2057.0073 | 1029.0073 | V | **595.3198** | **298.1636** | 578.2933 | 289.6503 | 577.3093 | 289.1583 | 5 |
| 21 | 2162.0499 | 1081.5286 | 2145.0234 | 1073.0153 | 2144.0394 | 1072.5233 | S | **496.2514** | 248.6293 | 479.2249 | 240.1161 | 478.2409 | 239.6241 | 4 |
| 22 | 2233.0870 | 1117.0472 | 2216.0605 | 1108.5339 | 2215.0765 | 1108.0419 | A | **409.2194** | 205.1133 | 392.1928 | 196.6001 | | | 3 |
| 23 | 2396.1504 | **1198.5788** | 2379.1238 | 1190.0655 | 2378.1398 | 1189.5735 | Y | **338.1823** | 169.5948 | 321.1557 | 161.0815 | | | 2 |
| 24 | | | | | | | R | 175.1190 | 88.0631 | 158.0924 | 79.5498 | | | 1 |

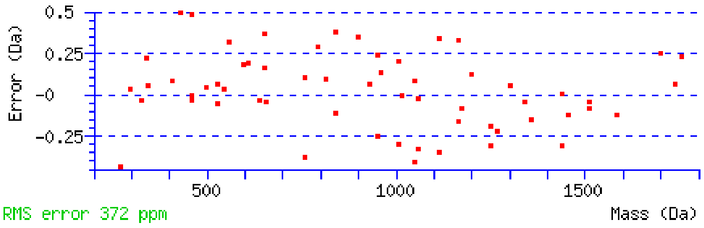


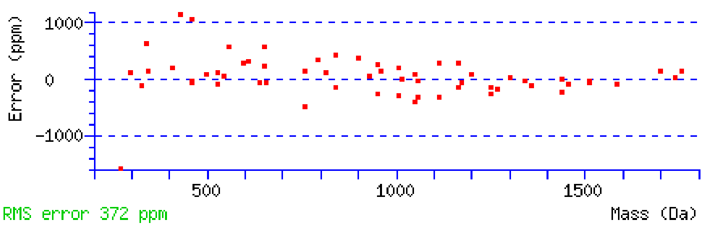

### All matches to this query

| Score | Mr(calc): | Delta | Sequence |
|---|---|---|---|
| 87.2 | 2569.2548 | 0.0092 | DLNISLDTDAPLSVGKDYGVSAYR |
| 54.2 | 2568.2708 | 0.9932 | DLNISLDTDAPLSVGKDYGVSAYR |
| 6.1 | 2568.2585 | 1.0055 | NIDRTDLQTITNDPRLVFTVR |
| 4.7 | 2567.2649 | 1.9991 | DKPTETLLNTVKDNLPIQIQK |
| 3.5 | 2568.2479 | 1.0160 | TQSLVCPETVSRVSSVLNRNSR |
| 2.5 | 2568.2585 | 1.0055 | NIDRTDLQTITNDPRLVFTVR |
| 2.5 | 2568.2672 | 0.9968 | IIITNTNCWSPRKAQSRPAFK |
| 2.5 | 2569.2512 | 0.0128 | IIITNTNCWSPRKAQSRPAFK |
| 2.3 | 2569.2693 | -0.0054 | VTINDLNENSVTLIQKNCHLNK |
| 2.1 | 2567.2745 | 1.9895 | NIDRTDLQTITNDPRLVFTVR |

Spectrum No: 158; Query: 1009; Rank: 1

## Peptide View

MS/MS Fragmentation of **FSLVNDSEGWLCIK**
Found in **IPI00471800**, Tax_Id=10116 Gene_Symbol=Cdh16 Cadherin 16

Match to Query 1009: 1667.791228 from(834.902890,2+)
Title: 100101RatKid_NS_deglyco_25.5196.5196.2.dta
Data file K:\NewmanPaper\Piliang\3SubProteomes\Piliang3SP\mgf5ppm\ERLIC_3SubProteomes5ppm.mgf

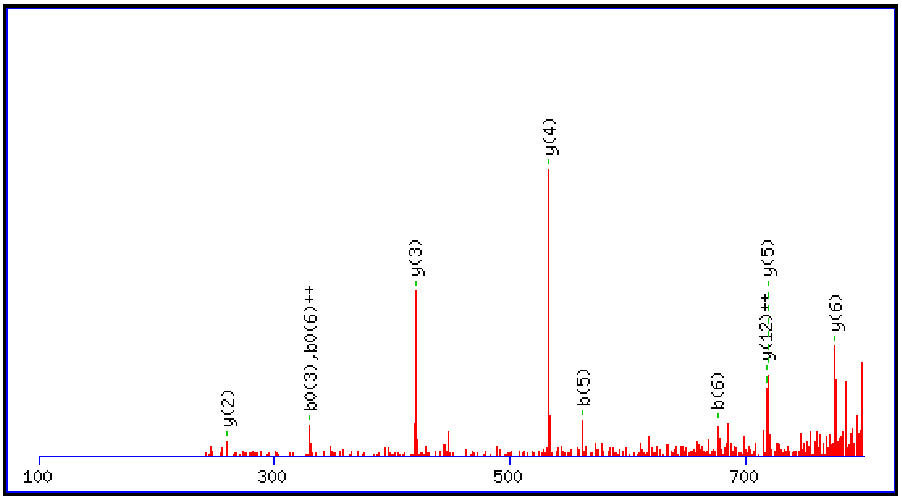


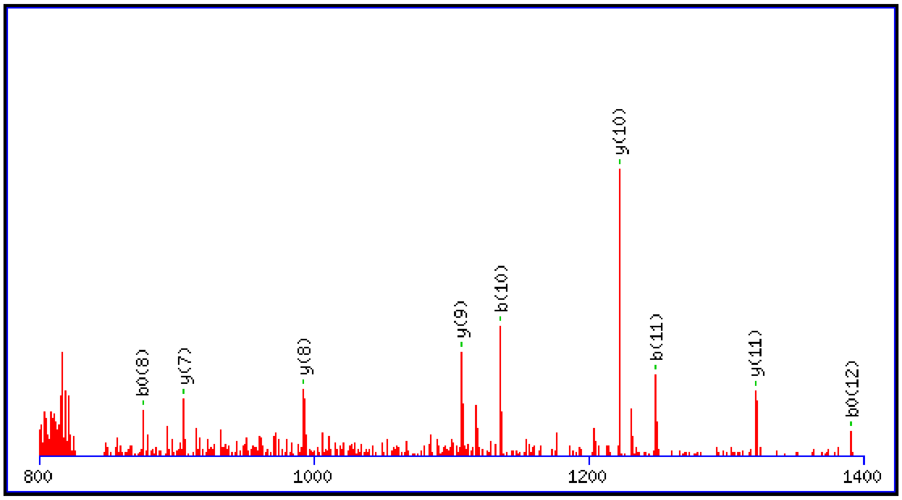

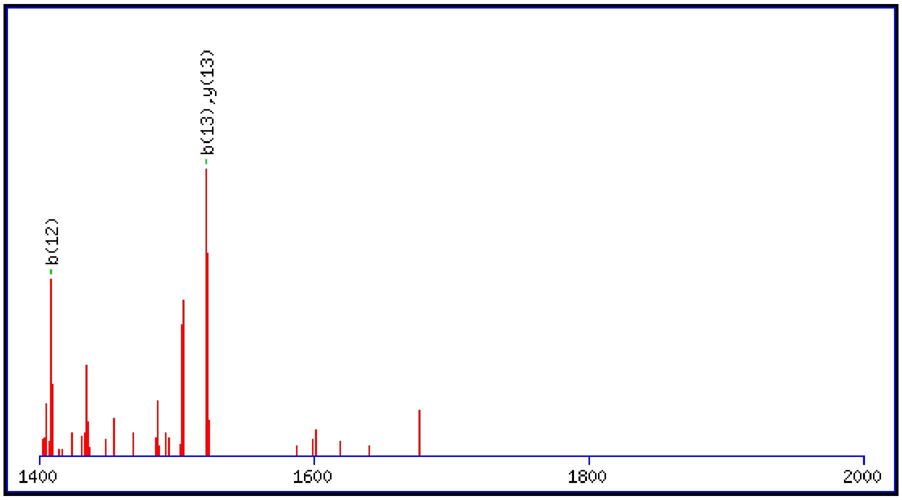


**Monoisotopic mass of neutral peptide Mr(calc):** 1667.7865
**Fixed modifications:** Carbamidomethyl (C)
**Variable modifications:**
**N5** : Deamidated_N (N)
**Ions Score:** 87 **Expect:** 3.7e-007
**Matches (Bold Red):** 22/134 fragment ions using 37 most intense peaks

| # | b | $b^{++}$ | b* | $b^{*++}$ | $b^{0}$ | $b^{0++}$ | Seq. | y | $y^{++}$ | y* | $y^{*++}$ | $y^{0}$ | $y^{0++}$ | # |
|---|---|---|---|---|---|---|---|---|---|---|---|---|---|---|
| 1 | 148.0757 | 74.5415 | | | | | F | | | | | | | 14 |
| 2 | 235.1077 | 118.0575 | | | 217.0972 | 109.0522 | S | **1521.7254** | 761.3663 | 1504.6988 | 752.8530 | 1503.7148 | 752.3610 | 13 |
| 3 | 348.1918 | 174.5995 | | | **330.1812** | 165.5942 | L | 1434.6933 | **717.8503** | 1417.6668 | 709.3370 | 1416.6828 | 708.8450 | 12 |
| 4 | 447.2602 | 224.1337 | | | 429.2496 | 215.1285 | V | **1321.6093** | 661.3083 | 1304.5827 | 652.7950 | 1303.5987 | 652.3030 | 11 |
| 5 | **562.2871** | 281.6472 | 545.2606 | 273.1339 | 544.2766 | 272.6419 | N | **1222.5409** | 611.7741 | 1205.5143 | 603.2608 | 1204.5303 | 602.7688 | 10 |
| 6 | **677.3141** | 339.1607 | 660.2875 | 330.6474 | 659.3035 | **330.1554** | D | **1107.5139** | 554.2606 | 1090.4874 | 545.7473 | 1089.5034 | 545.2553 | 9 |
| 7 | 764.3461 | 382.6767 | 747.3196 | 374.1634 | 746.3355 | 373.6714 | S | **992.4870** | 496.7471 | 975.4604 | 488.2339 | 974.4764 | 487.7418 | 8 |
| 8 | 893.3887 | 447.1980 | 876.3621 | 438.6847 | **875.3781** | 438.1927 | E | **905.4550** | 453.2311 | 888.4284 | 444.7178 | 887.4444 | 444.2258 | 7 |
| 9 | 950.4102 | 475.7087 | 933.3836 | 467.1954 | 932.3996 | 466.7034 | G | **776.4124** | 388.7098 | 759.3858 | 380.1965 | | | 6 |
| 10 | **1136.4895** | 568.7484 | 1119.4629 | 560.2351 | 1118.4789 | 559.7431 | W | **719.3909** | 360.1991 | 702.3643 | 351.6858 | | | 5 |
| 11 | **1249.5735** | 625.2904 | 1232.5470 | 616.7771 | 1231.5630 | 616.2851 | L | **533.3116** | 267.1594 | 516.2850 | 258.6462 | | | 4 |
| 12 | **1409.6042** | 705.3057 | 1392.5776 | 696.7925 | **1391.5936** | 696.3004 | C | **420.2275** | 210.6174 | 403.2010 | 202.1041 | | | 3 |
| 13 | **1522.6882** | 761.8478 | 1505.6617 | 753.3345 | 1504.6777 | 752.8425 | I | **260.1969** | 130.6021 | 243.1703 | 122.0888 | | | 2 |
| 14 | | | | | | | K | 147.1128 | 74.0600 | 130.0863 | 65.5468 | | | 1 |

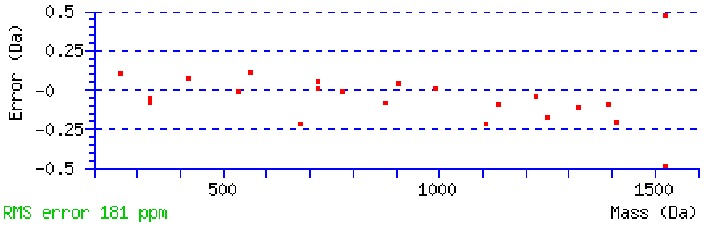


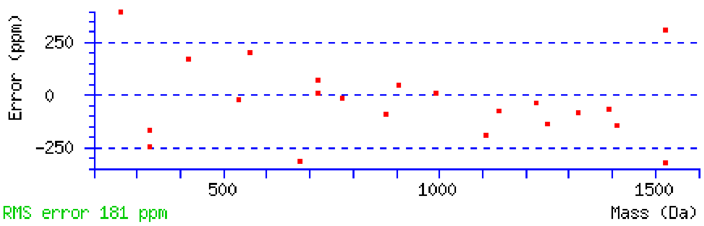


### All matches to this query

| Score | Mr(calc): | Delta | Sequence |
|---|---|---|---|
| 87.1 | 1667.7865 | 0.0047 | FSLVNDSEGWLCIK |
| 28.7 | 1666.8025 | 0.9887 | FSLVNDSEGWLCIK |
| 10.8 | 1666.7832 | 1.0080 | SLAMLGSSEDNTALSR |
| 8.8 | 1666.7953 | 0.9959 | EMDCSVLKRLMNR |
| 7.7 | 1667.7841 | 0.0071 | GILCDVTLIAEEQK |
| 7.1 | 1666.7872 | 1.0040 | EEADSFNQKMVQLK |
| 7.0 | 1667.7920 | -0.0008 | TKPSLSFHTGIGSEK |
| | | | |

| | | | |
|---|---|---|---|
| 7.0 | 1667.7920 | -0.0008 | TKPSLSFHTGIGSEK |
| 5.8 | 1667.7920 | -0.0008 | TKPSLSFHTGIGSEK |
| 5.8 | 1667.7920 | -0.0008 | TKPSLSFHTGIGSEK |

---

Spectrum No: 159; Query: 2479; Rank: 1

---

## Peptide View

MS/MS Fragmentation of **GNSMEEILEGLKFNLTETPETEIHR**
Found in **IPI00211075**, Tax_Id=10116 Gene_Symbol=Serpina3n Serine protease inhibitor A3N precursor

Match to Query 2479: 2887.396092 from(963.472640,3+)
Title: 100101RatKid_NS_deglyco_23.5737.5737.3.dta
Data file K:\NewmanPaper\Piliang\3SubProteomes\Piliang3SP\mgf5ppm\ERLIC_3SubProteomes5ppm.mgf

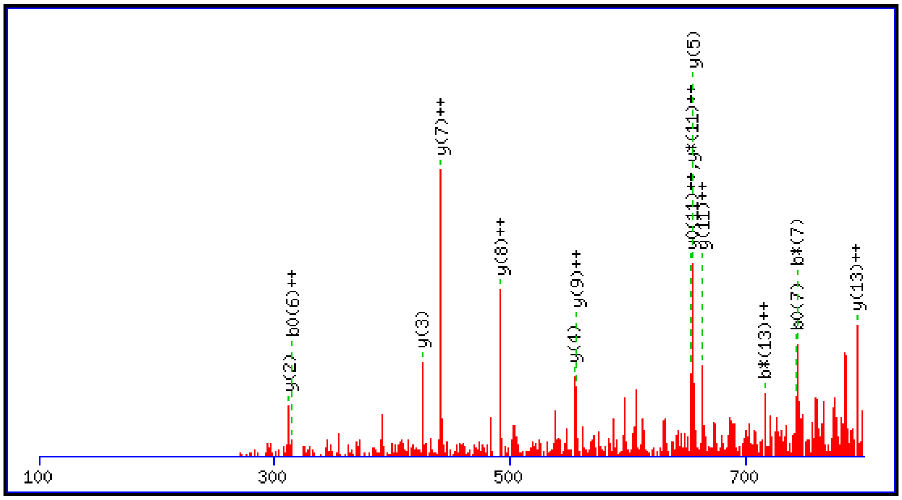


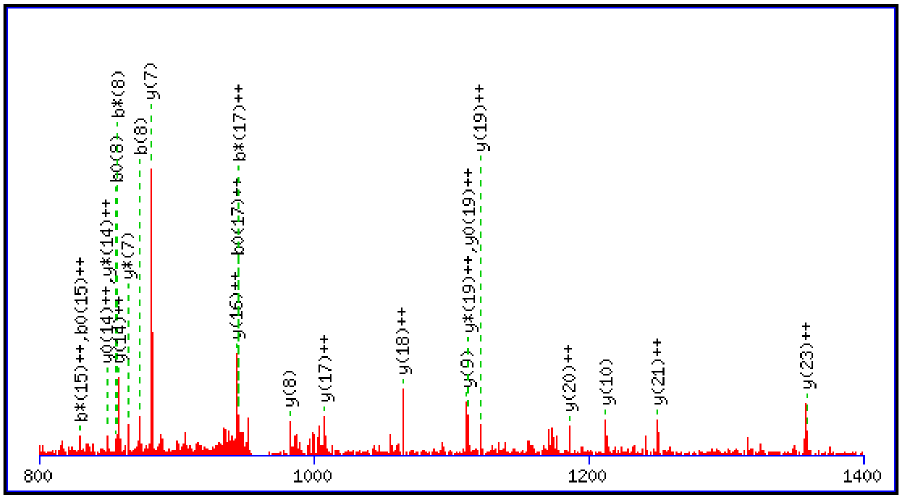

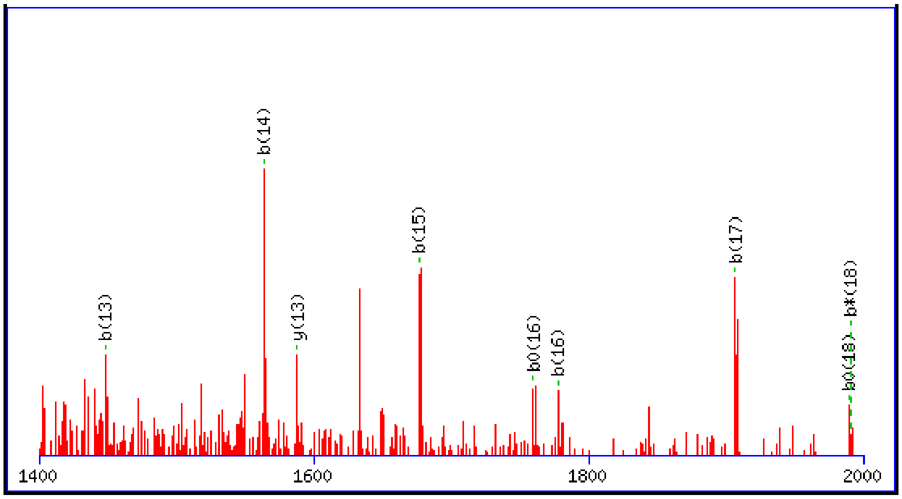


**Monoisotopic mass of neutral peptide Mr(calc):** 2887.3909
**Fixed modifications:** Carbamidomethyl (C)
**Variable modifications:**
**N14** : Deamidated_N (N)
**Ions Score:** 87 **Expect:** 5.9e-007
**Matches (Bold Red):** 48/276 fragment ions using 65 most intense peaks

| # | b | $b^{++}$ | b* | $b^{*++}$ | $b^0$ | $b^{0++}$ | Seq. | y | $y^{++}$ | y* | $y^{*++}$ | $y^0$ | $y^{0++}$ | # |
|---|---|---|---|---|---|---|---|---|---|---|---|---|---|---|
| 1 | 58.0287 | 29.5180 | | | | | G | | | | | | | 25 |
| 2 | 172.0717 | 86.5395 | 155.0451 | 78.0262 | | | N | 2831.3768 | 1416.1920 | 2814.3502 | 1407.6787 | 2813.3662 | 1407.1867 | 24 |
| 3 | 259.1037 | 130.0555 | 242.0771 | 121.5422 | 241.0931 | 121.0502 | S | 2717.3338 | **1359.1706** | 2700.3073 | 1350.6573 | 2699.3233 | 1350.1653 | 23 |
| 4 | 390.1442 | 195.5757 | 373.1176 | 187.0625 | 372.1336 | 186.5704 | M | 2630.3018 | 1315.6545 | 2613.2753 | 1307.1413 | 2612.2912 | 1306.6493 | 22 |
| 5 | 519.1868 | 260.0970 | 502.1602 | 251.5838 | 501.1762 | 251.0917 | E | 2499.2613 | **1250.1343** | 2482.2348 | 1241.6210 | 2481.2508 | 1241.1290 | 21 |
| 6 | 648.2294 | 324.6183 | 631.2028 | 316.1050 | 630.2188 | **315.6130** | E | 2370.2187 | **1185.6130** | 2353.1922 | 1177.0997 | 2352.2082 | 1176.6077 | 20 |
| 7 | 761.3134 | 381.1604 | **744.2869** | 372.6471 | **743.3029** | 372.1551 | I | 2241.1761 | **1121.0917** | 2224.1496 | **1112.5784** | 2223.1656 | **1112.0864** | 19 |
| 8 | **874.3975** | 437.7024 | **857.3709** | 429.1891 | **856.3869** | 428.6971 | L | 2128.0921 | **1064.5497** | 2111.0655 | 1056.0364 | 2110.0815 | 1055.5444 | 18 |
| 9 | 1003.4401 | 502.2237 | 986.4135 | 493.7104 | 985.4295 | 493.2184 | E | 2015.0080 | **1008.0076** | 1997.9815 | 999.4944 | 1996.9974 | 999.0024 | 17 |
| 10 | 1060.4616 | 530.7344 | 1043.4350 | 522.2211 | 1042.4510 | 521.7291 | G | 1885.9654 | **943.4863** | 1868.9389 | 934.9731 | 1867.9548 | 934.4811 | 16 |
| 11 | 1173.5456 | 587.2764 | 1156.5191 | 578.7632 | 1155.5351 | 578.2712 | L | 1828.9439 | 914.9756 | 1811.9174 | 906.4623 | 1810.9334 | 905.9703 | 15 |
| 12 | 1301.6406 | 651.3239 | 1284.6140 | 642.8107 | 1283.6300 | 642.3186 | K | 1715.8599 | **858.4336** | 1698.8333 | **849.9203** | 1697.8493 | **849.4283** | 14 |
| 13 | **1448.7090** | 724.8581 | 1431.6824 | **716.3449** | 1430.6984 | 715.8529 | F | **1587.7649** | **794.3861** | 1570.7384 | 785.8728 | 1569.7544 | 785.3808 | 13 |
| 14 | **1563.7359** | 782.3716 | 1546.7094 | 773.8583 | 1545.7254 | 773.3663 | N | 1440.6965 | 720.8519 | 1423.6700 | 712.3386 | 1422.6859 | 711.8466 | 12 |
| 15 | **1676.8200** | 838.9136 | 1659.7934 | **830.4004** | 1658.8094 | **829.9084** | L | 1325.6696 | **663.3384** | 1308.6430 | **654.8251** | 1307.6590 | **654.3331** | 11 |
| 16 | **1777.8677** | 889.4375 | 1760.8411 | 880.9242 | **1759.8571** | 880.4322 | T | **1212.5855** | 606.7964 | 1195.5590 | 598.2831 | 1194.5749 | 597.7911 | 10 |
| 17 | **1906.9103** | 953.9588 | 1889.8837 | **945.4455** | 1888.8997 | **944.9535** | E | **1111.5378** | **556.2726** | 1094.5113 | 547.7593 | 1093.5273 | 547.2673 | 9 |
| 18 | 2007.9579 | 1004.4826 | **1990.9314** | 995.9693 | **1989.9474** | 995.4773 | T | **982.4952** | **491.7513** | 965.4687 | 483.2380 | 964.4847 | 482.7460 | 8 |
| 19 | 2105.0107 | 1053.0090 | 2087.9842 | 1044.4957 | 2087.0001 | 1044.0037 | P | **881.4476** | **441.2274** | **864.4210** | 432.7141 | 863.4370 | 432.2221 | 7 |
| 20 | 2234.0533 | 1117.5303 | 2217.0268 | 1109.0170 | 2216.0427 | 1108.5250 | E | 784.3948 | 392.7010 | 767.3682 | 384.1878 | 766.3842 | 383.6958 | 6 |
| 21 | 2335.1010 | 1168.0541 | 2318.0744 | 1159.5409 | 2317.0904 | 1159.0488 | T | **655.3522** | 328.1797 | 638.3257 | 319.6665 | 637.3416 | 319.1745 | 5 |
| 22 | 2464.1436 | 1232.5754 | 2447.1170 | 1224.0621 | 2446.1330 | 1223.5701 | E | **554.3045** | 277.6559 | 537.2780 | 269.1426 | 536.2940 | 268.6506 | 4 |
| 23 | 2577.2276 | 1289.1175 | 2560.2011 | 1280.6042 | 2559.2171 | 1280.1122 | I | **425.2619** | 213.1346 | 408.2354 | 204.6213 | | | 3 |
| 24 | 2714.2865 | 1357.6469 | 2697.2600 | 1349.1336 | 2696.2760 | 1348.6416 | H | **312.1779** | 156.5926 | 295.1513 | 148.0793 | | | 2 |
| 25 | | | | | | | R | 175.1190 | 88.0631 | 158.0924 | 79.5498 | | | 1 |

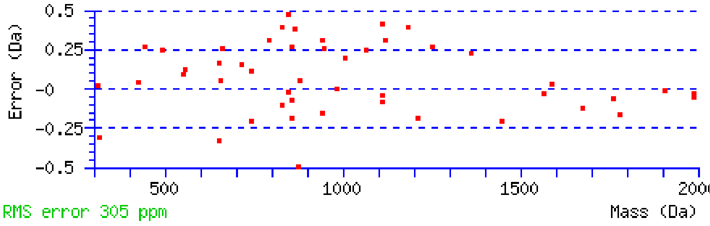


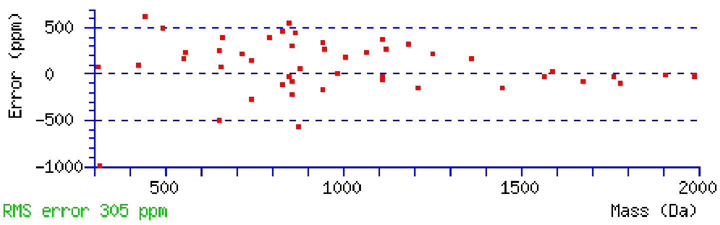

### All matches to this query

| Score | Mr(calc): | Delta | Sequence |
|---|---|---|---|
| 87.1 | 2887.3909 | 0.0052 | GNSMEEILEGLKFNLTETPETEIHR |
| 29.7 | 2887.3909 | 0.0052 | GNSMEEILEGLKFNLTETPETEIHR |
| 19.0 | 2886.4069 | 0.9892 | GNSMEEILEGLKFNLTETPETEIHR |
| 3.8 | 2887.3688 | 0.0273 | HFSAASSLTVHHMLLRIXNGEEISK |
| 3.8 | 2887.3687 | 0.0273 | HFSAASSLTVHHMLLRIXNGEEISK |
| 2.9 | 2885.3711 | 2.0250 | MDSKNVHSPVFLVFPPEIITSEYR |
| 2.2 | 2885.3711 | 2.0250 | MDSKNVHSPVFLVFPPEIITSEYR |

---

Spectrum No: 160; Query: 1590; Rank: 1

---

## Peptide View

MS/MS Fragmentation of **MGAGEALGNASQLFEAWLK**
Found in **IPI00327398**, Tax_Id=10116 Gene_Symbol=Enpep Isoform 1 of Glutamyl aminopeptidase

Match to Query 1590: 2008.964268 from(1005.489410,2+)
Title: 091008RatKidney_NH4Format01_23.4545.4545.2.dta
Data file K:\NewmanPaper\Piliang\3SubProteomes\Piliang3SP\mgf5ppm\ERLIC_3SubProteomes5ppm.mgf

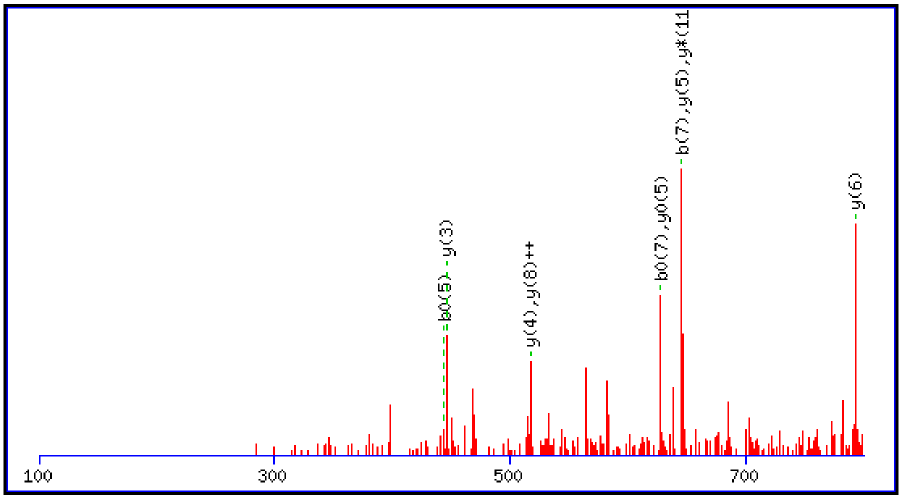


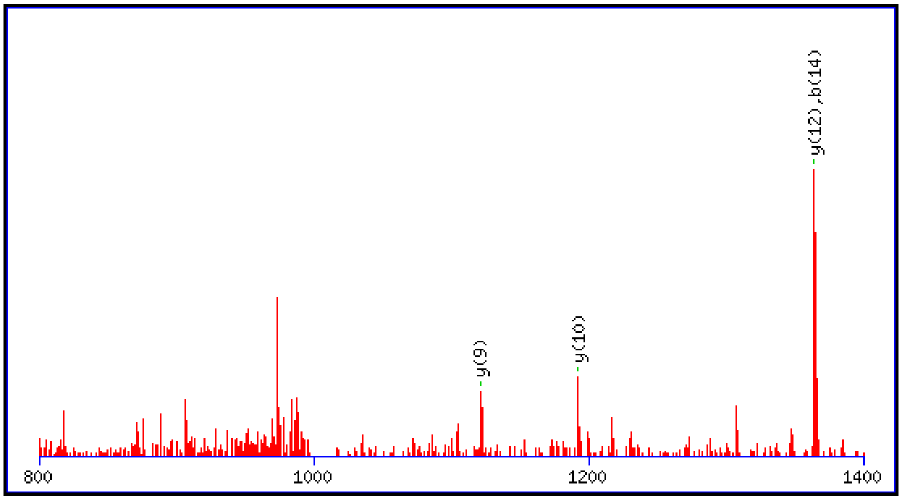

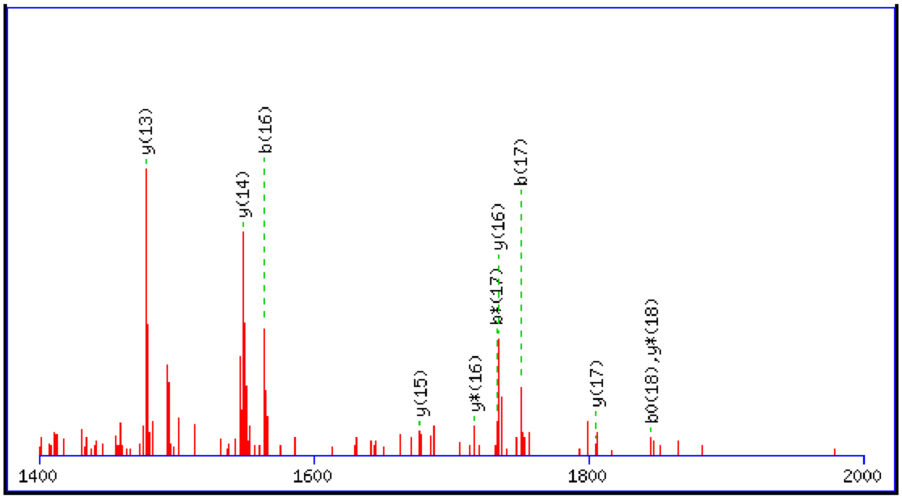


**Monoisotopic mass of neutral peptide Mr(calc):** 2008.9564
**Fixed modifications:** Carbamidomethyl (C)
**Variable modifications:**
**M1** : Oxidation (M)
**N9** : Deamidated_N (N)
**Ions Score:** 87 **Expect:** 4.3e-007
**Matches (Bold Red):** 25/184 fragment ions using 41 most intense peaks

| # | b | $b^{++}$ | b* | $b^{*++}$ | $b^0$ | $b^{0++}$ | Seq. | y | $y^{++}$ | y* | $y^{*++}$ | $y^0$ | $y^{0++}$ | # |
|---|---|---|---|---|---|---|---|---|---|---|---|---|---|---|
| 1 | 148.0427 | 74.5250 | | | | | M | | | | | | | 19 |
| 2 | 205.0641 | 103.0357 | | | | | G | 1862.9283 | 931.9678 | **1845.9018** | 923.4545 | 1844.9177 | 922.9625 | 18 |
| 3 | 276.1013 | 138.5543 | | | | | A | **1805.9068** | 903.4571 | 1788.8803 | 894.9438 | 1787.8963 | 894.4518 | 17 |
| 4 | 333.1227 | 167.0650 | | | | | G | **1734.8697** | 867.9385 | **1717.8432** | 859.4252 | 1716.8592 | 858.9332 | 16 |
| 5 | 462.1653 | 231.5863 | | | **444.1547** | 222.5810 | E | **1677.8483** | 839.4278 | 1660.8217 | 830.9145 | 1659.8377 | 830.4225 | 15 |
| 6 | 533.2024 | 267.1049 | | | 515.1919 | 258.0996 | A | **1548.8057** | 774.9065 | 1531.7791 | 766.3932 | 1530.7951 | 765.9012 | 14 |
| 7 | **646.2865** | 323.6469 | | | **628.2759** | 314.6416 | L | **1477.7686** | 739.3879 | 1460.7420 | 730.8746 | 1459.7580 | 730.3826 | 13 |
| 8 | 703.3080 | 352.1576 | | | 685.2974 | 343.1523 | G | **1364.6845** | 682.8459 | 1347.6579 | 674.3326 | 1346.6739 | 673.8406 | 12 |
| 9 | 818.3349 | 409.6711 | 801.3083 | 401.1578 | 800.3243 | 400.6658 | N | 1307.6630 | 654.3351 | 1290.6365 | **645.8219** | 1289.6525 | 645.3299 | 11 |
| 10 | 889.3720 | 445.1896 | 872.3455 | 436.6764 | 871.3614 | 436.1844 | A | **1192.6361** | 596.8217 | 1175.6095 | 588.3084 | 1174.6255 | 587.8164 | 10 |
| 11 | 976.4040 | 488.7057 | 959.3775 | 480.1924 | 958.3935 | 479.7004 | S | **1121.5990** | 561.3031 | 1104.5724 | 552.7898 | 1103.5884 | 552.2978 | 9 |
| 12 | 1104.4626 | 552.7349 | 1087.4361 | 544.2217 | 1086.4520 | 543.7297 | Q | 1034.5669 | **517.7871** | 1017.5404 | 509.2738 | 1016.5564 | 508.7818 | 8 |
| 13 | 1217.5467 | 609.2770 | 1200.5201 | 600.7637 | 1199.5361 | 600.2717 | L | 906.5084 | 453.7578 | 889.4818 | 445.2445 | 888.4978 | 444.7525 | 7 |
| 14 | **1364.6151** | 682.8112 | 1347.5885 | 674.2979 | 1346.6045 | 673.8059 | F | **793.4243** | 397.2158 | 776.3978 | 388.7025 | 775.4137 | 388.2105 | 6 |
| 15 | 1493.6577 | 747.3325 | 1476.6311 | 738.8192 | 1475.6471 | 738.3272 | E | **646.3559** | 323.6816 | 629.3293 | 315.1683 | **628.3453** | 314.6763 | 5 |
| 16 | **1564.6948** | 782.8510 | 1547.6682 | 774.3378 | 1546.6842 | 773.8458 | A | **517.3133** | 259.1603 | 500.2867 | 250.6470 | | | 4 |
| 17 | **1750.7741** | 875.8907 | **1733.7476** | 867.3774 | 1732.7635 | 866.8854 | W | **446.2762** | 223.6417 | 429.2496 | 215.1285 | | | 3 |
| 18 | 1863.8582 | 932.4327 | 1846.8316 | 923.9195 | **1845.8476** | 923.4274 | L | 260.1969 | 130.6021 | 243.1703 | 122.0888 | | | 2 |
| 19 | | | | | | | K | 147.1128 | 74.0600 | 130.0863 | 65.5468 | | | 1 |

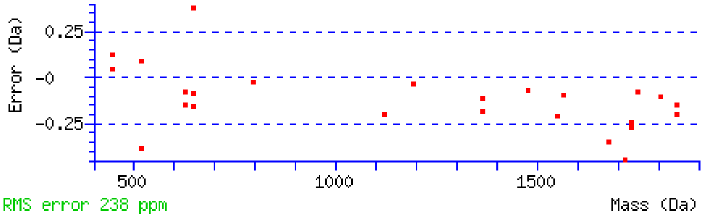


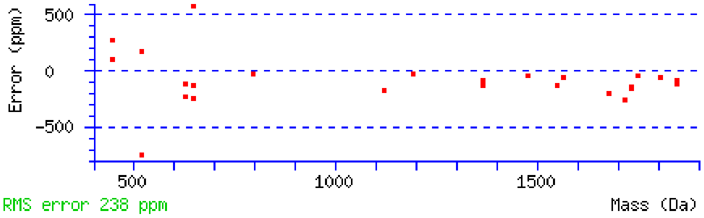


### All matches to this query

| Score | Mr(calc): | Delta | Sequence |
|---|---|---|---|
| 87.1 | 2008.9564 | 0.0079 | MGAGEALGNASQLFEAWLK |
| 17.9 | 2007.9724 | 0.9919 | MGAGEALGNASQLFEAWLK |

| | | | |
|---|---|---|---|
| 5.3 | 2008.9458 | 0.0185 | CVSFILRATVGSLLGEK |
| 2.6 | 2008.9619 | 0.0024 | RQSSQWDPVSLISNALK |
| 1.6 | 2007.9554 | 1.0089 | GNAYGFKISSLNKIADTK |
| 1.6 | 2007.9554 | 1.0089 | GNAYGFKISSLNKIADTK |
| 1.4 | 2006.9400 | 2.0242 | TLKTNTVGTLNMLELAK |
| 0.4 | 2007.9796 | 0.9846 | TCGDVLGNRKGDGYQVLR |

---

Spectrum No: 161; Query: 362; Rank: 1

---

## Peptide View

MS/MS Fragmentation of **ILNETLYENAK**
Found in **IPI00195931**, Tax_Id=10116 Gene_Symbol=Tspan8 D6.1A protein

Match to Query 362: 1307.663228 from(654.838890,2+)
Title: 091008RatKidney_NoSalt_37.1416.1416.2.dta
Data file K:\NewmanPaper\Piliang\3SubProteomes\Piliang3SP\mgf5ppm\ERLIC_3SubProteomes5ppm.mgf

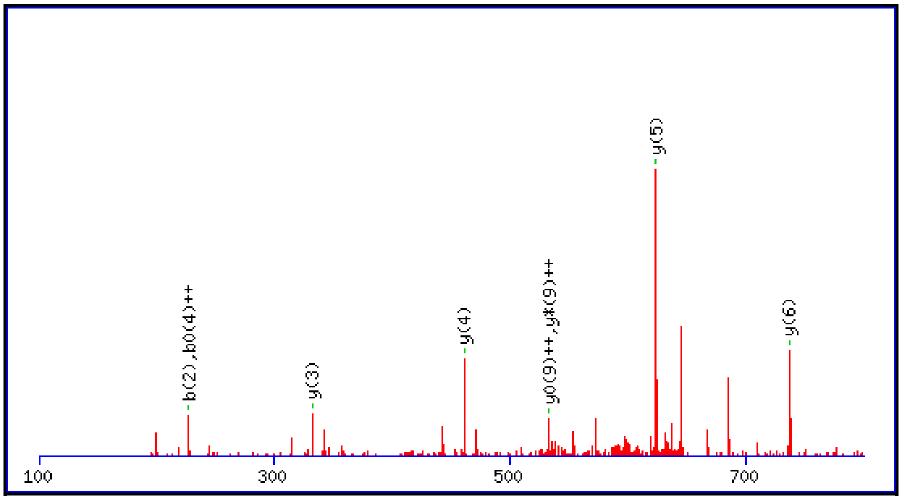


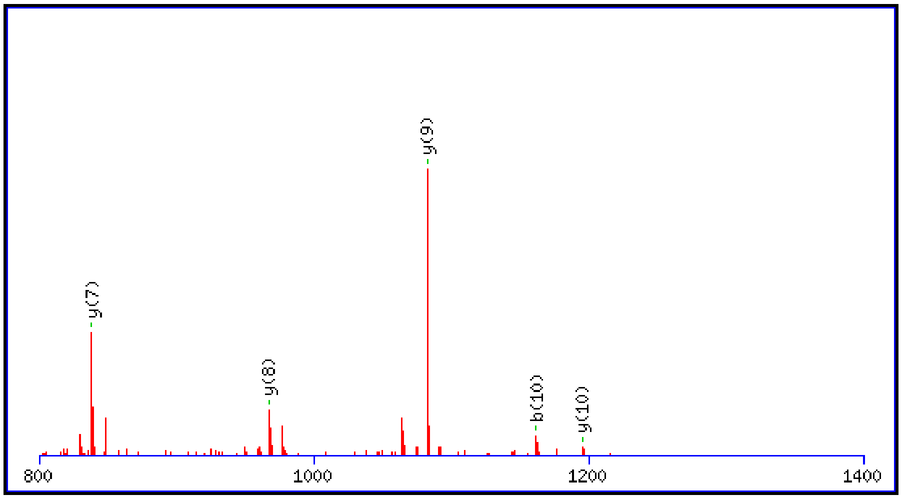

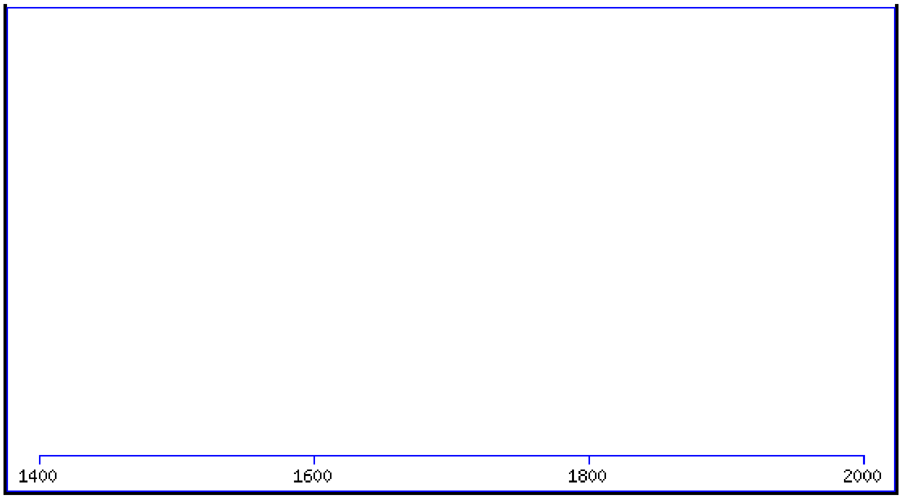


**Monoisotopic mass of neutral peptide Mr(calc):** 1307.6608
**Fixed modifications:** Carbamidomethyl (C)
**Variable modifications:**
**N3** : Deamidated_N (N)
**Ions Score:** 87 **Expect:** 2.4e-007
**Matches (Bold Red):** 13/104 fragment ions using 16 most intense peaks

| # | b | $b^{++}$ | b* | $b^{*++}$ | $b^{0}$ | $b^{0++}$ | Seq. | y | $y^{++}$ | y* | $y^{*++}$ | $y^{0}$ | $y^{0++}$ | # |
|---|---|---|---|---|---|---|---|---|---|---|---|---|---|---|
| 1 | 114.0913 | 57.5493 | | | | | I | | | | | | | 11 |
| 2 | **227.1754** | 114.0913 | | | | | L | **1195.5841** | 598.2957 | 1178.5576 | 589.7824 | 1177.5735 | 589.2904 | 10 |
| 3 | 342.2023 | 171.6048 | 325.1758 | 163.0915 | | | N | **1082.5000** | 541.7537 | 1065.4735 | **533.2404** | 1064.4895 | **532.7484** | 9 |
| 4 | 471.2449 | 236.1261 | 454.2184 | 227.6128 | 453.2344 | **227.1208** | E | **967.4731** | 484.2402 | 950.4466 | 475.7269 | 949.4625 | 475.2349 | 8 |
| 5 | 572.2926 | 286.6499 | 555.2661 | 278.1367 | 554.2820 | 277.6447 | T | **838.4305** | 419.7189 | 821.4040 | 411.2056 | 820.4199 | 410.7136 | 7 |
| 6 | 685.3767 | 343.1920 | 668.3501 | 334.6787 | 667.3661 | 334.1867 | L | **737.3828** | 369.1951 | 720.3563 | 360.6818 | 719.3723 | 360.1898 | 6 |
| 7 | 848.4400 | 424.7236 | 831.4135 | 416.2104 | 830.4294 | 415.7184 | Y | **624.2988** | 312.6530 | 607.2722 | 304.1397 | 606.2882 | 303.6477 | 5 |
| 8 | 977.4826 | 489.2449 | 960.4560 | 480.7317 | 959.4720 | 480.2397 | E | **461.2354** | 231.1214 | 444.2089 | 222.6081 | 443.2249 | 222.1161 | 4 |
| 9 | 1091.5255 | 546.2664 | 1074.4990 | 537.7531 | 1073.5150 | 537.2611 | N | **332.1928** | 166.6001 | 315.1663 | 158.0868 | | | 3 |
| 10 | **1162.5626** | 581.7850 | 1145.5361 | 573.2717 | 1144.5521 | 572.7797 | A | 218.1499 | 109.5786 | 201.1234 | 101.0653 | | | 2 |
| 11 | | | | | | | K | 147.1128 | 74.0600 | 130.0863 | 65.5468 | | | 1 |

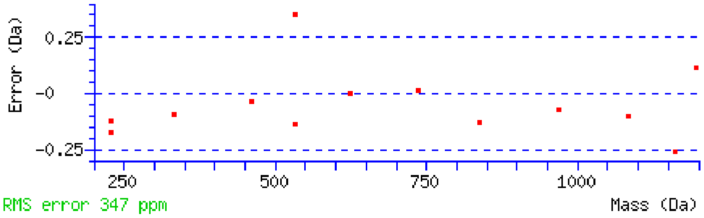


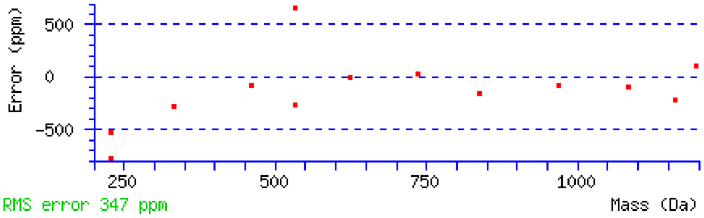


### All matches to this query

| Score | Mr(calc): | Delta | Sequence |
|---|---|---|---|
| 86.9 | 1307.6608 | 0.0024 | ILNETLYENAK |
| 28.8 | 1307.6608 | 0.0024 | ILNETLYENAK |
| 11.1 | 1307.6609 | 0.0024 | ILEDEGLYTQK |
| 10.4 | 1307.6526 | 0.0106 | IINVIHYINK |
| 7.4 | 1307.6755 | -0.0122 | LLSREISSMEK |
| 6.6 | 1307.6533 | 0.0099 | SRTXLRPVPR |
| 4.2 | 1307.6526 | 0.0106 | IIYIVHDEVK |
| 3.9 | 1307.6721 | -0.0089 | ITKQGNIYNEK |
| 3.7 | 1307.6639 | -0.0006 | IISGQNFPKPK |
| 3.7 | 1307.6761 | -0.0129 | ILYXEEKEAK |

Spectrum No: 162; Query: 1584; Rank: 1

# Peptide View

MS/MS Fragmentation of **ATDDIIVNWVNGTLSEAGK**
Found in **IPI00210234**, Tax_Id=10116 Gene_Symbol=Pls3 Plastin-3

Match to Query 1584: 2002.992748 from(1002.503650,2+)
Title: 100101RatKid_NS_deglyco_13.4731.4731.2.dta
Data file K:\NewmanPaper\Piliang\3SubProteomes\Piliang3SP\mgf5ppm\ERLIC_3SubProteomes5ppm.mgf

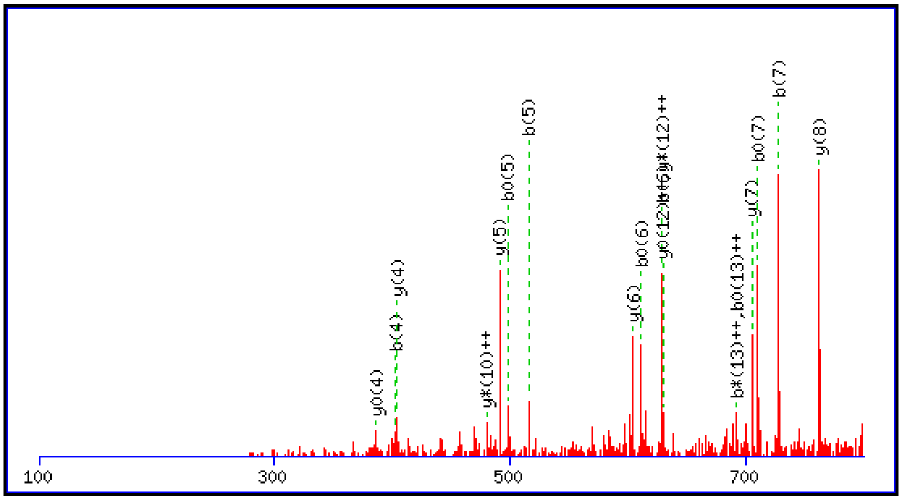


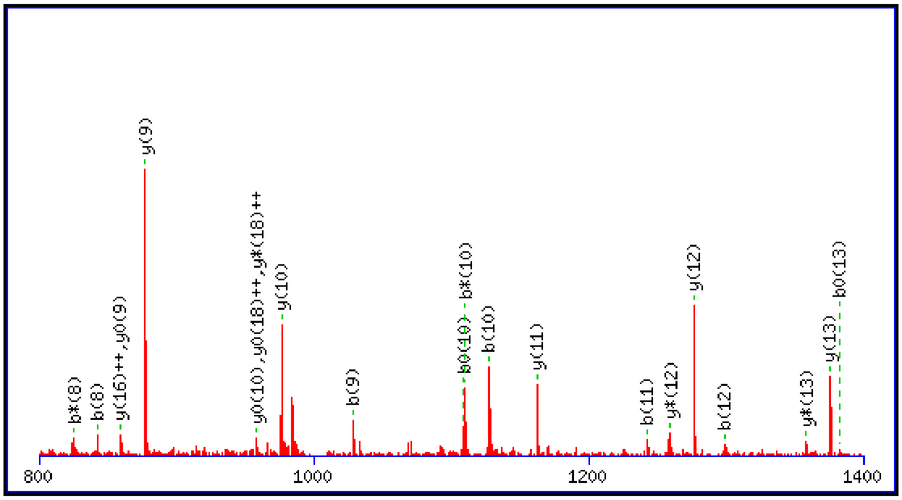


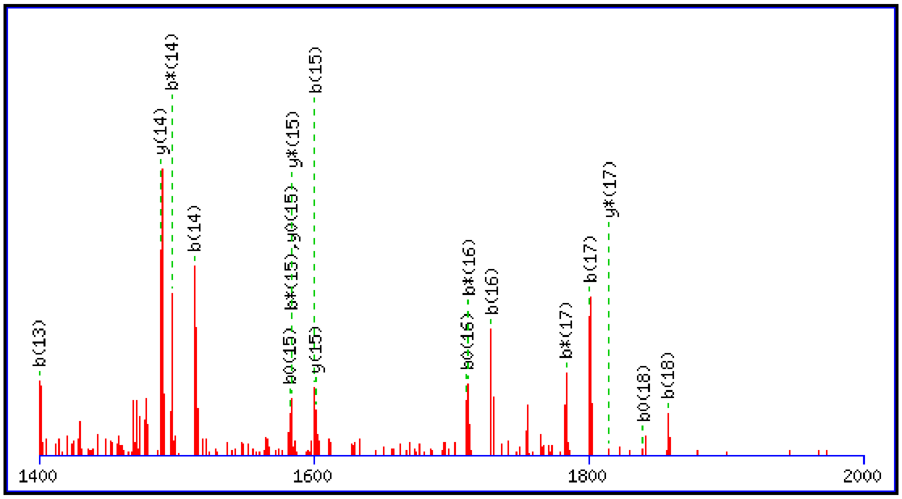

**Monoisotopic mass of neutral peptide Mr(calc):** 2002.9847
**Fixed modifications:** Carbamidomethyl (C)
**Variable modifications:**
**N11** : Deamidated_N (N)
**Ions Score:** 87 **Expect:** 4.3e-007
**Matches (Bold Red):** 57/194 fragment ions using 99 most intense peaks

| # | b | $b^{++}$ | b* | $b^{*++}$ | $b^0$ | $b^{0++}$ | Seq. | y | $y^{++}$ | y* | $y^{*++}$ | $y^0$ | $y^{0++}$ | # |
|---|---|---|---|---|---|---|---|---|---|---|---|---|---|---|
| 1 | 72.0444 | 36.5258 | | | | | A | | | | | | | 19 |
| 2 | 173.0921 | 87.0497 | | | 155.0815 | 78.0444 | T | 1932.9549 | 966.9811 | 1915.9284 | **958.4678** | 1914.9443 | **957.9758** | 18 |
| 3 | 288.1190 | 144.5631 | | | 270.1084 | 135.5579 | D | 1831.9072 | 916.4573 | **1814.8807** | 907.9440 | 1813.8967 | 907.4520 | 17 |
| 4 | **403.1460** | 202.0766 | | | 385.1354 | 193.0713 | D | 1716.8803 | **858.9438** | 1699.8537 | 850.4305 | 1698.8697 | 849.9385 | 16 |
| 5 | **516.2300** | 258.6186 | | | **498.2195** | 249.6134 | I | **1601.8533** | 801.4303 | **1584.8268** | 792.9170 | **1583.8428** | 792.4250 | 15 |
| 6 | **629.3141** | 315.1607 | | | **611.3035** | 306.1554 | I | **1488.7693** | 744.8883 | 1471.7427 | 736.3750 | 1470.7587 | 735.8830 | 14 |
| 7 | **728.3825** | 364.6949 | | | **710.3719** | 355.6896 | V | **1375.6852** | 688.3462 | **1358.6587** | 679.8330 | 1357.6746 | 679.3410 | 13 |
| 8 | **842.4254** | 421.7164 | **825.3989** | 413.2031 | 824.4149 | 412.7111 | N | **1276.6168** | 638.8120 | **1259.5903** | **630.2988** | 1258.6062 | **629.8068** | 12 |
| 9 | **1028.5047** | 514.7560 | 1011.4782 | 506.2427 | 1010.4942 | 505.7507 | W | **1162.5739** | 581.7906 | 1145.5473 | 573.2773 | 1144.5633 | 572.7853 | 11 |
| 10 | **1127.5732** | 564.2902 | **1110.5466** | 555.7769 | **1109.5626** | 555.2849 | V | **976.4946** | 488.7509 | 959.4680 | **480.2376** | **958.4840** | 479.7456 | 10 |
| 11 | **1242.6001** | 621.8037 | 1225.5735 | 613.2904 | 1224.5895 | 612.7984 | N | **877.4261** | 439.2167 | 860.3996 | 430.7034 | **859.4156** | 430.2114 | 9 |
| 12 | **1299.6216** | 650.3144 | 1282.5950 | 641.8011 | 1281.6110 | 641.3091 | G | **762.3992** | 381.7032 | 745.3727 | 373.1900 | 744.3886 | 372.6980 | 8 |
| 13 | **1400.6692** | 700.8383 | 1383.6427 | **692.3250** | **1382.6587** | **691.8330** | T | **705.3777** | 353.1925 | 688.3512 | 344.6792 | 687.3672 | 344.1872 | 7 |
| 14 | **1513.7533** | 757.3803 | **1496.7267** | 748.8670 | 1495.7427 | 748.3750 | L | **604.3301** | 302.6687 | 587.3035 | 294.1554 | 586.3195 | 293.6634 | 6 |
| 15 | **1600.7853** | 800.8963 | **1583.7588** | 792.3830 | **1582.7748** | 791.8910 | S | **491.2460** | 246.1266 | 474.2195 | 237.6134 | 473.2354 | 237.1214 | 5 |
| 16 | **1729.8279** | 865.4176 | **1712.8014** | 856.9043 | **1711.8174** | 856.4123 | E | **404.2140** | 202.6106 | 387.1874 | 194.0974 | **386.2034** | 193.6053 | 4 |
| 17 | **1800.8650** | 900.9362 | **1783.8385** | 892.4229 | 1782.8545 | 891.9309 | A | 275.1714 | 138.0893 | 258.1448 | 129.5761 | | | 3 |
| 18 | **1857.8865** | 929.4469 | 1840.8599 | 920.9336 | **1839.8759** | 920.4416 | G | 204.1343 | 102.5708 | 187.1077 | 94.0575 | | | 2 |
| 19 | | | | | | | K | 147.1128 | 74.0600 | 130.0863 | 65.5468 | | | 1 |

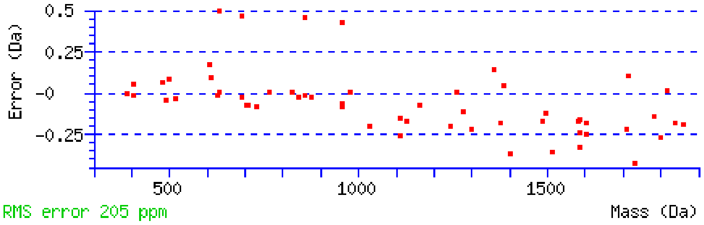


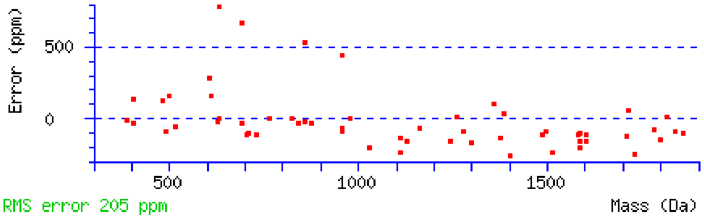


### All matches to this query

| Score | Mr(calc): | Delta | Sequence |
|---|---|---|---|
| 86.9 | 2002.9847 | 0.0080 | ATDDIIVNWVNGTLSEAGK |
| 71.3 | 2002.9847 | 0.0080 | ATDDIIVNWVNGTLSEAGK |
| 29.2 | 2002.0007 | 0.9920 | ATDDIIVNWVNGTLSEAGK |
| 6.4 | 2002.9773 | 0.0154 | WGIILMMRYPEVQKK |
| 5.3 | 2003.0000 | -0.0073 | FTFFADVLSVDNREKSK |

---

Spectrum No: 163; Query: 347; Rank: 1

---

## Peptide View

MS/MS Fragmentation of **LDNYSTQELGR**
Found in **IPI00204348**, Tax_Id=10116 Gene_Symbol=Nt5e 5'-nucleotidase precursor

Match to Query 347: 1295.601928 from(648.808240,2+)
Title: 100101RatKid_NS_deglyco_26.1488.1488.2.dta
Data file K:\NewmanPaper\Piliang\3SubProteomes\Piliang3SP\mgf5ppm\ERLIC_3SubProteomes5ppm.mgf

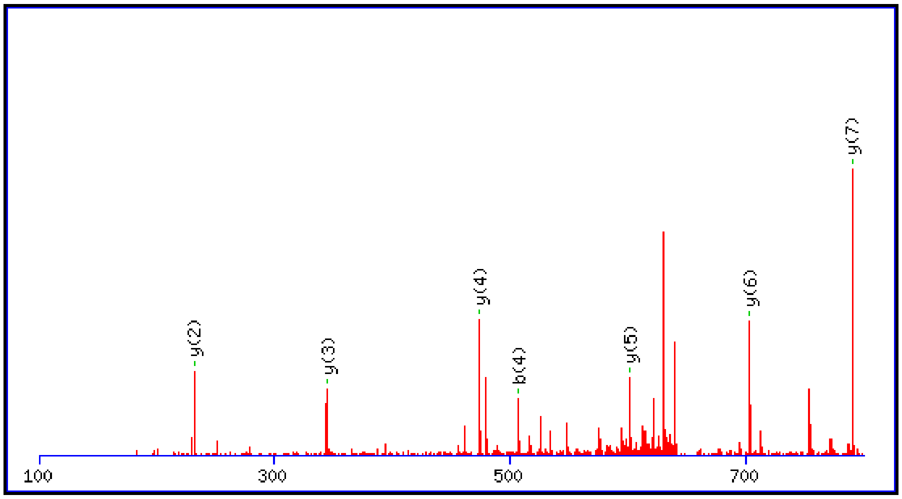


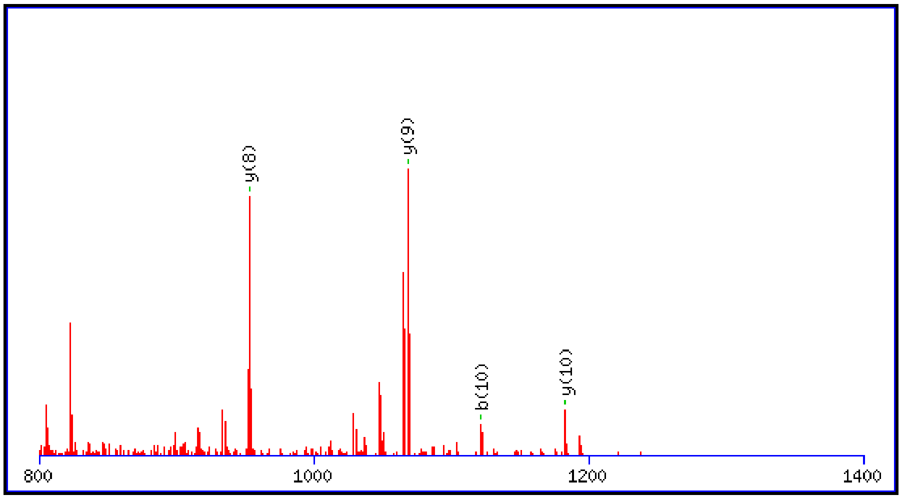


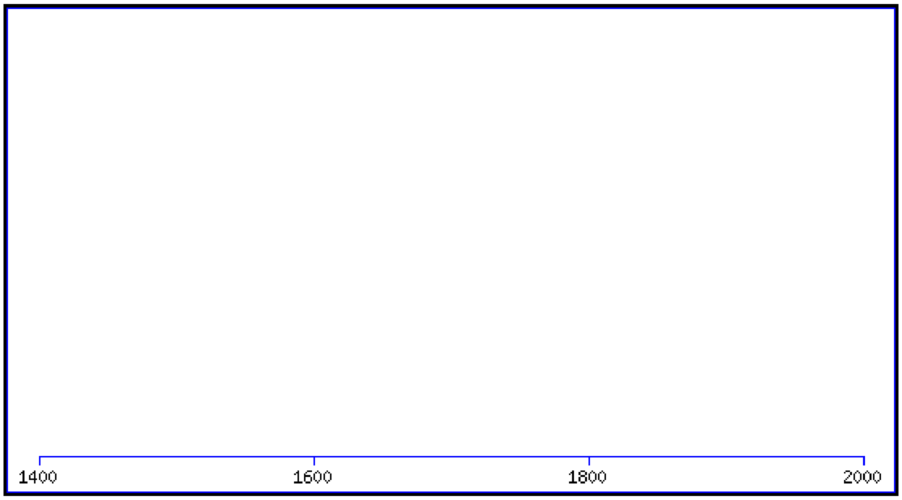


**Monoisotopic mass of neutral peptide Mr(calc):** 1295.5993
**Fixed modifications:** Carbamidomethyl (C)
**Variable modifications:**
**N3** : Deamidated_N (N)
**Ions Score:** 87 **Expect:** 2.9e-007
**Matches (Bold Red):** 11/108 fragment ions using 15 most intense peaks

| # | b | $b^{++}$ | b* | $b^{*++}$ | $b^0$ | $b^{0++}$ | Seq. | y | $y^{++}$ | y* | $y^{*++}$ | $y^0$ | $y^{0++}$ | # |
|---|---|---|---|---|---|---|---|---|---|---|---|---|---|---|
| 1 | 114.0913 | 57.5493 | | | | | L | | | | | | | 11 |
| 2 | 229.1183 | 115.0628 | | | 211.1077 | 106.0575 | D | **1183.5226** | 592.2649 | 1166.4960 | 583.7516 | 1165.5120 | 583.2596 | 10 |
| 3 | 344.1452 | 172.5762 | 327.1187 | 164.0630 | 326.1347 | 163.5710 | N | **1068.4956** | 534.7514 | 1051.4691 | 526.2382 | 1050.4851 | 525.7462 | 9 |
| 4 | **507.2085** | 254.1079 | 490.1820 | 245.5946 | 489.1980 | 245.1026 | Y | **953.4687** | 477.2380 | 936.4421 | 468.7247 | 935.4581 | 468.2327 | 8 |
| 5 | 594.2406 | 297.6239 | 577.2140 | 289.1107 | 576.2300 | 288.6186 | S | **790.4054** | 395.7063 | 773.3788 | 387.1930 | 772.3948 | 386.7010 | 7 |

| | | | | | | | | | | | | | | |
|---|---|---|---|---|---|---|---|---|---|---|---|---|---|---|
| **6** | 695.2883 | 348.1478 | 678.2617 | 339.6345 | 677.2777 | 339.1425 | **T** | **703.3733** | 352.1903 | 686.3468 | 343.6770 | 685.3628 | 343.1850 | **6** |
| **7** | 823.3468 | 412.1771 | 806.3203 | 403.6638 | 805.3363 | 403.1718 | **Q** | **602.3257** | 301.6665 | 585.2991 | 293.1532 | 584.3151 | 292.6612 | **5** |
| **8** | 952.3894 | 476.6984 | 935.3629 | 468.1851 | 934.3789 | 467.6931 | **E** | **474.2671** | 237.6372 | 457.2405 | 229.1239 | 456.2565 | 228.6319 | **4** |
| **9** | 1065.4735 | 533.2404 | 1048.4469 | 524.7271 | 1047.4629 | 524.2351 | **L** | **345.2245** | 173.1159 | 328.1979 | 164.6026 | | | **3** |
| **10** | **1122.4950** | 561.7511 | 1105.4684 | 553.2378 | 1104.4844 | 552.7458 | **G** | **232.1404** | 116.5738 | 215.1139 | 108.0606 | | | **2** |
| **11** | | | | | | | **R** | 175.1190 | 88.0631 | 158.0924 | 79.5498 | | | **1** |

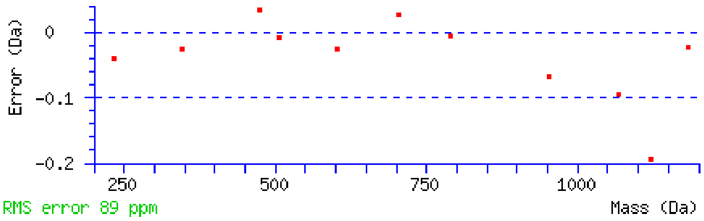


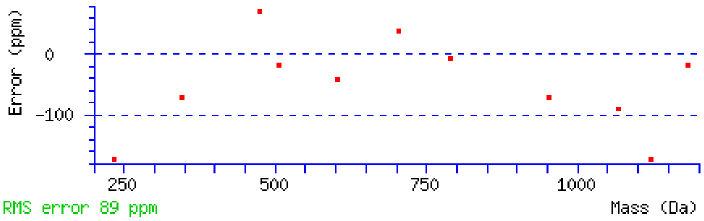


### All matches to this query

| Score | Mr(calc): | Delta | Sequence |
|---|---|---|---|
| 86.6 | 1295.5993 | 0.0026 | LDNYSTQELGR |
| 13.8 | 1295.6064 | -0.0044 | LSGWLXEIGR |
| 8.1 | 1295.5983 | 0.0036 | GEKGQSRELGR |
| 6.7 | 1295.5983 | 0.0036 | LETRAAAQGSGR |
| 6.0 | 1295.5993 | 0.0026 | SYSLENEEAVR |
| 5.7 | 1295.5983 | 0.0036 | DDLTREVRGR |
| 4.3 | 1295.6115 | -0.0095 | MIVHMPPDQGR |
| 4.3 | 1295.6002 | 0.0017 | VEFEICVMGGR |
| 3.3 | 1295.6057 | -0.0038 | MQQSNVALLGR |
| 3.1 | 1295.5993 | 0.0026 | LHDNPLTDENK |

---

Spectrum No: 164; Query: 1354; Rank: 1

---

## Peptide View

MS/MS Fragmentation of **EGTEISHVLLCNVTQR**
Found in **IPI00191929**, Tax_Id=10116 Gene_Symbol=Tmem27 Collectrin precursor

Match to Query 1354: 1855.917148 from(928.965850,2+)
Title: 100101RatKid_NS_deglyco_19.3401.3401.2.dta
Data file K:\NewmanPaper\Piliang\3SubProteomes\Piliang3SP\mgf5ppm\ERLIC_3SubProteomes5ppm.mgf

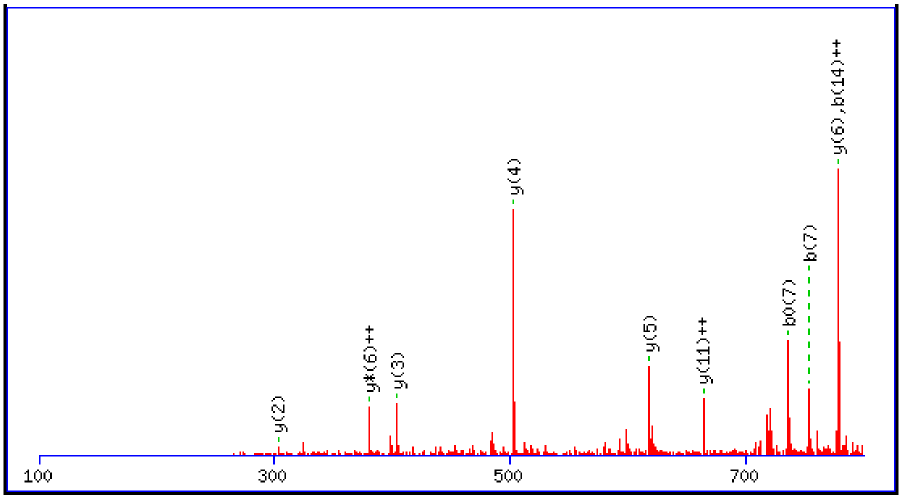


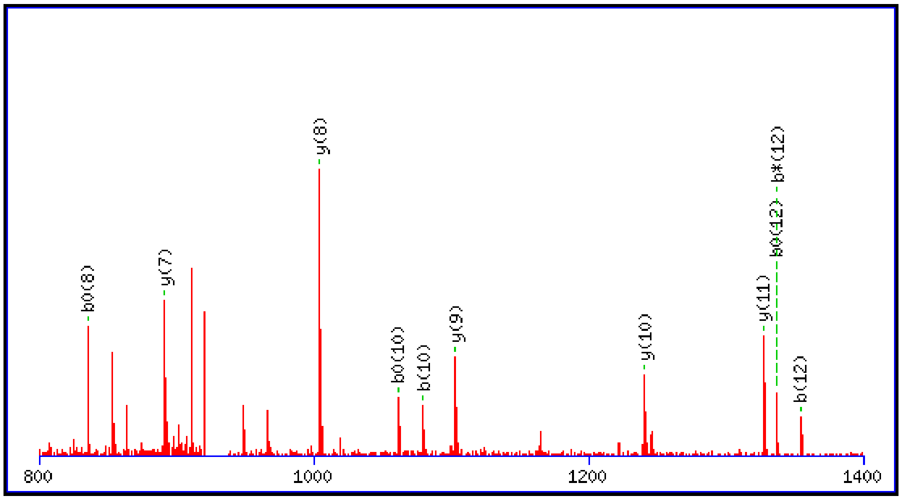


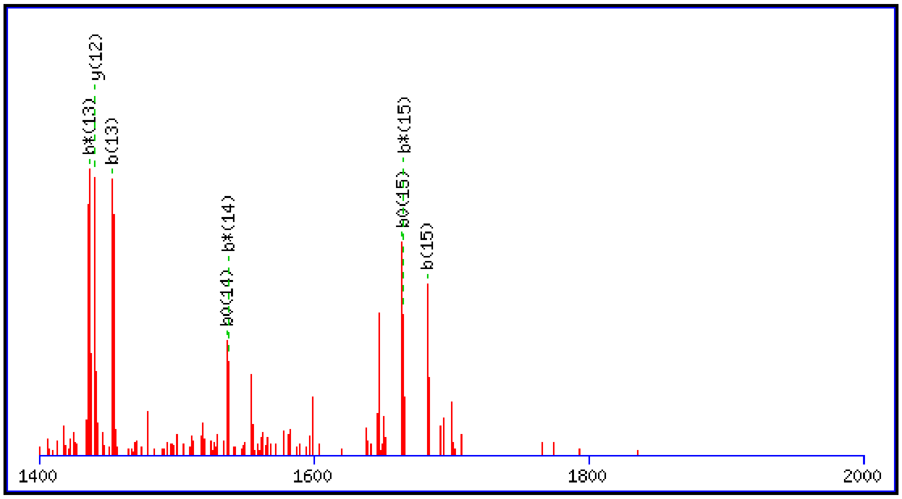


**Monoisotopic mass of neutral peptide Mr(calc):** 1855.9098
**Fixed modifications:** Carbamidomethyl (C)
**Variable modifications:**
**N12** : Deamidated_N (N)
**Ions Score:** 86 **Expect:** 4.1e-007
**Matches (Bold Red):** 29/154 fragment ions using 45 most intense peaks

| # | b | $b^{++}$ | b* | $b^{*++}$ | $b^{0}$ | $b^{0++}$ | Seq. | y | $y^{++}$ | y* | $y^{*++}$ | $y^{0}$ | $y^{0++}$ | # |
|---|---|---|---|---|---|---|---|---|---|---|---|---|---|---|
| 1 | 130.0499 | 65.5286 | | | 112.0393 | 56.5233 | E | | | | | | | 16 |
| 2 | 187.0713 | 94.0393 | | | 169.0608 | 85.0340 | G | 1727.8745 | 864.4409 | 1710.8479 | 855.9276 | 1709.8639 | 855.4356 | 15 |
| 3 | 288.1190 | 144.5631 | | | 270.1084 | 135.5579 | T | 1670.8530 | 835.9302 | 1653.8265 | 827.4169 | 1652.8425 | 826.9249 | 14 |
| 4 | 417.1616 | 209.0844 | | | 399.1510 | 200.0792 | E | 1569.8053 | 785.4063 | 1552.7788 | 776.8930 | 1551.7948 | 776.4010 | 13 |
| 5 | 530.2457 | 265.6265 | | | 512.2351 | 256.6212 | I | **1440.7628** | 720.8850 | 1423.7362 | 712.3717 | 1422.7522 | 711.8797 | 12 |

| | | | | | | | | | | | | | | |
|---|---|---|---|---|---|---|---|---|---|---|---|---|---|---|
| 6 | 617.2777 | 309.1425 | | | 599.2671 | 300.1372 | S | **1327.6787** | **664.3430** | 1310.6521 | 655.8297 | 1309.6681 | 655.3377 | 11 |
| 7 | **754.3366** | 377.6719 | | | **736.3260** | 368.6667 | H | **1240.6467** | 620.8270 | 1223.6201 | 612.3137 | 1222.6361 | 611.8217 | 10 |
| 8 | 853.4050 | 427.2061 | | | **835.3945** | 418.2009 | V | **1103.5878** | 552.2975 | 1086.5612 | 543.7842 | 1085.5772 | 543.2922 | 9 |
| 9 | 966.4891 | 483.7482 | | | 948.4785 | 474.7429 | L | **1004.5193** | 502.7633 | 987.4928 | 494.2500 | 986.5088 | 493.7580 | 8 |
| 10 | **1079.5732** | 540.2902 | | | **1061.5626** | 531.2849 | L | **891.4353** | 446.2213 | 874.4087 | 437.7080 | 873.4247 | 437.2160 | 7 |
| 11 | 1239.6038 | 620.3055 | | | 1221.5932 | 611.3003 | C | **778.3512** | 389.6792 | 761.3247 | **381.1660** | 760.3406 | 380.6740 | 6 |
| 12 | **1354.6307** | 677.8190 | **1337.6042** | 669.3057 | **1336.6202** | 668.8137 | N | **618.3206** | 309.6639 | 601.2940 | 301.1506 | 600.3100 | 300.6586 | 5 |
| 13 | **1453.6991** | 727.3532 | **1436.6726** | 718.8399 | 1435.6886 | 718.3479 | V | **503.2936** | 252.1504 | 486.2671 | 243.6372 | 485.2831 | 243.1452 | 4 |
| 14 | 1554.7468 | **777.8771** | **1537.7203** | 769.3638 | **1536.7363** | 768.8718 | T | **404.2252** | 202.6162 | 387.1987 | 194.1030 | 386.2146 | 193.6110 | 3 |
| 15 | **1682.8054** | 841.9063 | **1665.7789** | 833.3931 | **1664.7948** | 832.9011 | Q | **303.1775** | 152.0924 | 286.1510 | 143.5791 | | | 2 |
| 16 | | | | | | | R | 175.1190 | 88.0631 | 158.0924 | 79.5498 | | | 1 |

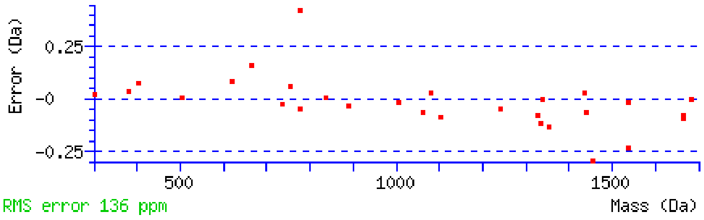


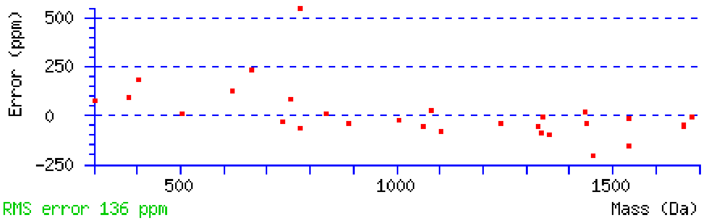


**All matches to this query**

| Score | Mr(calc): | Delta | Sequence |
|---|---|---|---|
| 86.2 | 1855.9098 | 0.0073 | EGTEISHVLLCNVTQR |
| 7.0 | 1854.9258 | 0.9914 | EGTEISHVLLCNVTQR |
| 4.9 | 1855.9114 | 0.0057 | RTVETEAVQMLKDIK |
| 4.2 | 1855.9114 | 0.0057 | RTVETEAVQMLKDIK |
| 2.5 | 1855.9305 | -0.0134 | LADFGVSAKNTRTIQR |
| 0.3 | 1855.9073 | 0.0098 | TKAMVACYPGNGLGYVR |

---

Spectrum No: 165; Query: 2721; Rank: 1

---

## Peptide View

MS/MS Fragmentation of **QADEDIQGTQNLLTSIESETAASEETLTNASQR**
Found in **IPI00365542**, Tax_Id=10116 Gene_Symbol=Lamb1_predicted laminin, beta 1

Match to Query 2721: 3550.647942 from(1184.556590,3+)
Title: 100101RatKid_NS_deglyco_25.5516.5516.3.dta
Data file K:\NewmanPaper\Piliang\3SubProteomes\Piliang3SP\mgf5ppm\ERLIC_3SubProteomes5ppm.mgf

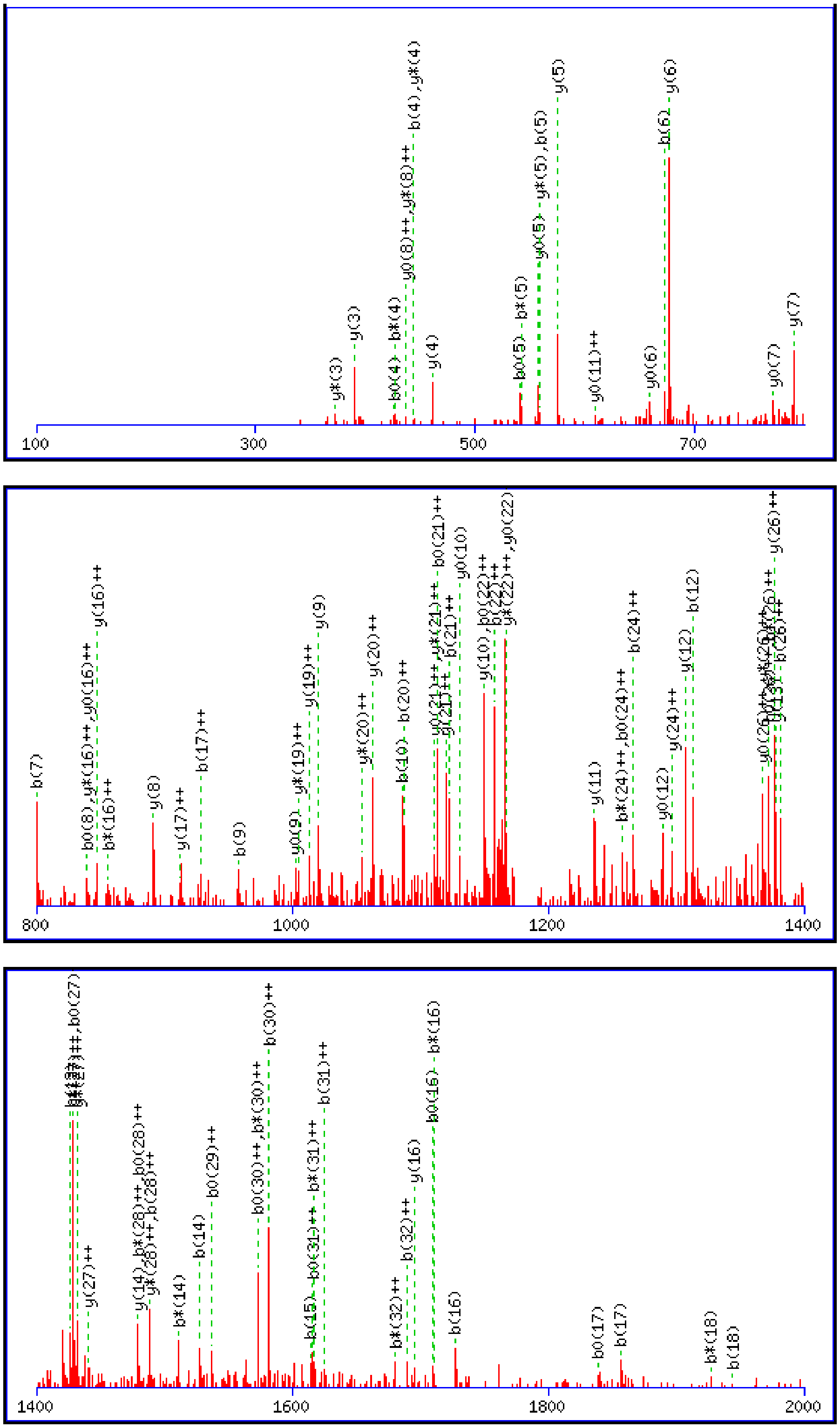


**Monoisotopic mass of neutral peptide Mr(calc):** 3550.6394
**Fixed modifications:** Carbamidomethyl (C)
**Variable modifications:**
**N29** : Deamidated_N (N)
**Ions Score:** 86 **Expect:** 9.8e-007
**Matches (Bold Red):** 95/376 fragment ions using 163 most intense peaks

| # | b | $b^{++}$ | b* | $b^{*++}$ | $b^0$ | $b^{0++}$ | Seq. | y | $y^{++}$ | y* | $y^{*++}$ | $y^0$ | $y^{0++}$ | # |
|---|---|---|---|---|---|---|---|---|---|---|---|---|---|---|
| 1 | 129.0659 | 65.0366 | 112.0393 | 56.5233 | | | Q | | | | | | | 33 |
| 2 | 200.1030 | 100.5551 | 183.0764 | 92.0418 | | | A | 3423.5881 | 1712.2977 | 3406.5616 | 1703.7844 | 3405.5776 | 1703.2924 | 32 |
| 3 | 315.1299 | 158.0686 | 298.1034 | 149.5553 | 297.1193 | 149.0633 | D | 3352.5510 | 1676.7792 | 3335.5245 | 1668.2659 | 3334.5405 | 1667.7739 | 31 |
| 4 | **444.1725** | 222.5899 | **427.1460** | 214.0766 | **426.1619** | 213.5846 | E | 3237.5241 | 1619.2657 | 3220.4975 | 1610.7524 | 3219.5135 | 1610.2604 | 30 |
| 5 | **559.1994** | 280.1034 | **542.1729** | 271.5901 | **541.1889** | 271.0981 | D | 3108.4815 | 1554.7444 | 3091.4549 | 1546.2311 | 3090.4709 | 1545.7391 | 29 |

| | | | | | | | | | | | | | | |
|---|---|---|---|---|---|---|---|---|---|---|---|---|---|---|
| **6** | **672.2835** | 336.6454 | 655.2570 | 328.1321 | 654.2729 | 327.6401 | **I** | 2993.4546 | 1497.2309 | 2976.4280 | **1488.7176** | 2975.4440 | 1488.2256 | **28** |
| **7** | **800.3421** | 400.6747 | 783.3155 | 392.1614 | 782.3315 | 391.6694 | **Q** | 2880.3705 | **1440.6889** | 2863.3439 | **1432.1756** | 2862.3599 | 1431.6836 | **27** |
| **8** | 857.3636 | 429.1854 | 840.3370 | 420.6721 | **839.3530** | 420.1801 | **G** | 2752.3119 | **1376.6596** | 2735.2854 | **1368.1463** | 2734.3013 | **1367.6543** | **26** |
| **9** | **958.4112** | 479.7093 | 941.3847 | 471.1960 | 940.4007 | 470.7040 | **T** | 2695.2904 | 1348.1489 | 2678.2639 | 1339.6356 | 2677.2799 | 1339.1436 | **25** |
| **10** | **1086.4698** | 543.7385 | 1069.4433 | 535.2253 | 1068.4592 | 534.7333 | **Q** | 2594.2428 | **1297.6250** | 2577.2162 | 1289.1117 | 2576.2322 | 1288.6197 | **24** |
| **11** | 1200.5127 | 600.7600 | 1183.4862 | 592.2467 | 1182.5022 | 591.7547 | **N** | 2466.1842 | 1233.5957 | 2449.1576 | 1225.0825 | 2448.1736 | 1224.5905 | **23** |
| **12** | **1313.5968** | 657.3020 | 1296.5703 | 648.7888 | 1295.5862 | 648.2968 | **L** | 2352.1413 | 1176.5743 | 2335.1147 | **1168.0610** | 2334.1307 | **1167.5690** | **22** |
| **13** | **1426.6809** | 713.8441 | 1409.6543 | 705.3308 | 1408.6703 | 704.8388 | **L** | 2239.0572 | **1120.0322** | 2222.0307 | **1111.5190** | 2221.0466 | **1111.0270** | **21** |
| **14** | **1527.7285** | 764.3679 | **1510.7020** | 755.8546 | 1509.7180 | 755.3626 | **T** | 2125.9731 | **1063.4902** | 2108.9466 | **1054.9769** | 2107.9626 | 1054.4849 | **20** |
| **15** | **1614.7606** | 807.8839 | 1597.7340 | 799.3706 | 1596.7500 | 798.8786 | **S** | 2024.9255 | **1012.9664** | 2007.8989 | **1004.4531** | 2006.9149 | 1003.9611 | **19** |
| **16** | **1727.8446** | 864.4260 | **1710.8181** | **855.9127** | **1709.8341** | 855.4207 | **I** | 1937.8934 | 969.4504 | 1920.8669 | 960.9371 | 1919.8829 | 960.4451 | **18** |
| **17** | **1856.8872** | **928.9473** | 1839.8607 | 920.4340 | **1838.8767** | 919.9420 | **E** | 1824.8094 | **912.9083** | 1807.7828 | 904.3950 | 1806.7988 | 903.9030 | **17** |
| **18** | **1943.9193** | 972.4633 | **1926.8927** | 963.9500 | 1925.9087 | 963.4580 | **S** | **1695.7668** | **848.3870** | 1678.7402 | **839.8737** | 1677.7562 | **839.3817** | **16** |
| **19** | 2072.9619 | 1036.9846 | 2055.9353 | 1028.4713 | 2054.9513 | 1027.9793 | **E** | 1608.7347 | 804.8710 | 1591.7082 | 796.3577 | 1590.7242 | 795.8657 | **15** |
| **20** | 2174.0095 | **1087.5084** | 2156.9830 | 1078.9951 | 2155.9990 | 1078.5031 | **T** | **1479.6922** | 740.3497 | 1462.6656 | 731.8364 | 1461.6816 | 731.3444 | **14** |
| **21** | 2245.0466 | **1123.0270** | 2228.0201 | 1114.5137 | 2227.0361 | **1114.0217** | **A** | **1378.6445** | 689.8259 | 1361.6179 | 681.3126 | 1360.6339 | 680.8206 | **13** |
| **22** | 2316.0838 | **1158.5455** | 2299.0572 | 1150.0322 | 2298.0732 | **1149.5402** | **A** | **1307.6074** | 654.3073 | 1290.5808 | 645.7940 | **1289.5968** | 645.3020 | **12** |
| **23** | 2403.1158 | 1202.0615 | 2386.0892 | 1193.5483 | 2385.1052 | 1193.0562 | **S** | **1236.5702** | 618.7888 | 1219.5437 | 610.2755 | 1218.5597 | **609.7835** | **11** |
| **24** | 2532.1584 | **1266.5828** | 2515.1318 | **1258.0696** | 2514.1478 | **1257.5775** | **E** | **1149.5382** | 575.2727 | 1132.5117 | 566.7595 | **1131.5277** | 566.2675 | **10** |
| **25** | 2661.2010 | 1331.1041 | 2644.1744 | 1322.5908 | 2643.1904 | 1322.0988 | **E** | **1020.4956** | 510.7514 | 1003.4691 | 502.2382 | **1002.4851** | 501.7462 | **9** |
| **26** | 2762.2487 | **1381.6280** | 2745.2221 | **1373.1147** | 2744.2381 | **1372.6227** | **T** | **891.4530** | 446.2302 | 874.4265 | **437.7169** | 873.4425 | **437.2249** | **8** |
| **27** | 2875.3327 | 1438.1700 | 2858.3062 | **1429.6567** | 2857.3221 | **1429.1647** | **L** | **790.4054** | 395.7063 | 773.3788 | 387.1930 | **772.3948** | 386.7010 | **7** |
| **28** | 2976.3804 | **1488.6938** | 2959.3538 | **1480.1806** | 2958.3698 | **1479.6886** | **T** | **677.3213** | 339.1643 | 660.2947 | 330.6510 | **659.3107** | 330.1590 | **6** |
| **29** | 3091.4073 | 1546.2073 | 3074.3808 | 1537.6940 | 3073.3968 | **1537.2020** | **N** | **576.2736** | 288.6404 | **559.2471** | 280.1272 | **558.2630** | 279.6352 | **5** |
| **30** | 3162.4444 | **1581.7259** | 3145.4179 | **1573.2126** | 3144.4339 | **1572.7206** | **A** | **461.2467** | 231.1270 | **444.2201** | 222.6137 | 443.2361 | 222.1217 | **4** |
| **31** | 3249.4765 | **1625.2419** | 3232.4499 | **1616.7286** | 3231.4659 | **1616.2366** | **S** | **390.2096** | 195.6084 | **373.1830** | 187.0951 | 372.1990 | 186.6031 | **3** |
| **32** | 3377.5350 | **1689.2712** | 3360.5085 | **1680.7579** | 3359.5245 | 1680.2659 | **Q** | 303.1775 | 152.0924 | 286.1510 | 143.5791 | | | **2** |
| **33** | | | | | | | **R** | 175.1190 | 88.0631 | 158.0924 | 79.5498 | | | **1** |

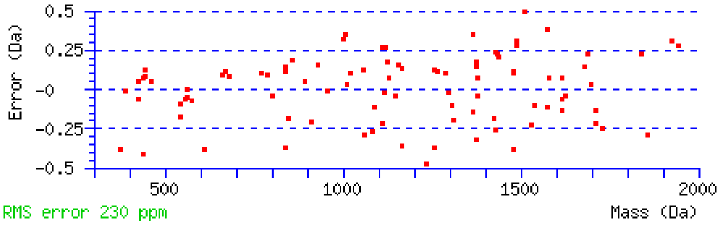


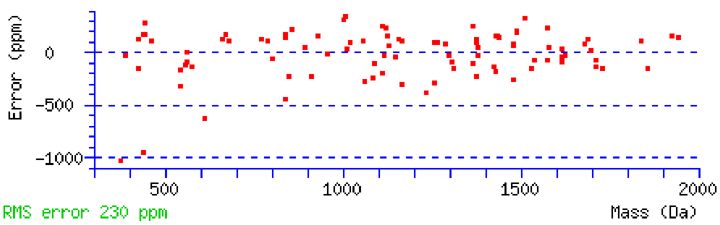


### All matches to this query

| Score | Mr(calc): | Delta | Sequence |
|---|---|---|---|
| 86.1 | 3550.6394 | 0.0085 | QADEDIQGTQNLLTSIESETAASEETLTNASQR |
| 47.2 | 3549.6554 | 0.9925 | QADEDIQGTQNLLTSIESETAASEETLTNASQR |
| 37.5 | 3550.6394 | 0.0085 | QADEDIQGTQNLLTSIESETAASEETLTNASQR |
| 4.0 | 3549.6316 | 1.0164 | VVSAGNLLSHVGHTILGMNTVQLYMKVPGSR |
| 4.0 | 3550.6278 | 0.0201 | MNELALVSVSDRYGSGSIYTLLPPMSFGSKR |
| 4.0 | 3550.6278 | 0.0201 | MNELALVSVSDRYGSGSIYTLLPPMSFGSKR |
| 3.3 | 3550.6278 | 0.0201 | MNELALVSVSDRYGSGSIYTLLPPMSFGSKR |
| 2.8 | 3550.6278 | 0.0201 | MNELALVSVSDRYGSGSIYTLLPPMSFGSKR |
| 2.8 | 3550.6278 | 0.0201 | MNELALVSVSDRYGSGSIYTLLPPMSFGSKR |
| 2.0 | 3550.6528 | -0.0049 | SAIIETYVMDSRIDVSVAAGHTDRSGLGSVLR |

Spectrum No: 166; Query: 900; Rank: 1

## Peptide View

MS/MS Fragmentation of **KLINDYVSNQTQGK**
Found in **IPI00200591**, Tax_Id=10116 Gene_Symbol=LOC299282 Serine protease inhibitor A3L precursor

Match to Query 900: 1607.816888 from(804.915720,2+)
Title: 091008RatKidney_NoSalt_32.1068.1068.2.dta
Data file K:\NewmanPaper\Piliang\3SubProteomes\Piliang3SP\mgf5ppm\ERLIC_3SubProteomes5ppm.mgf

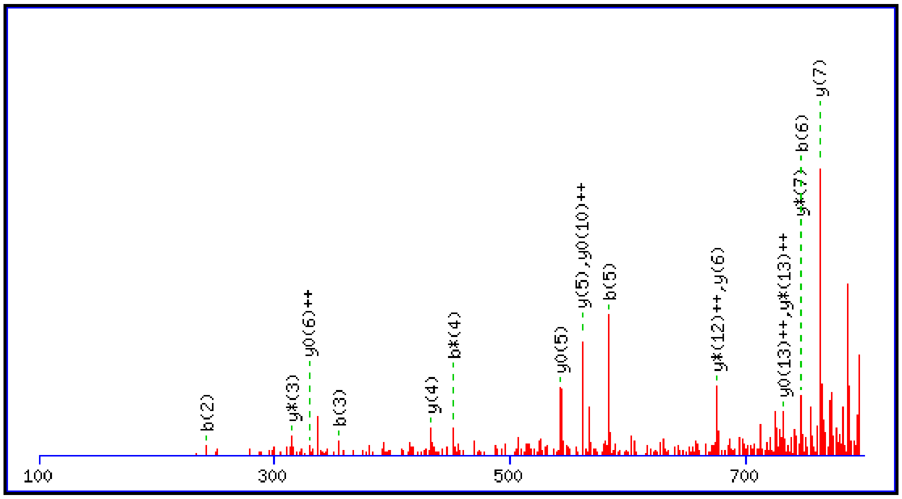


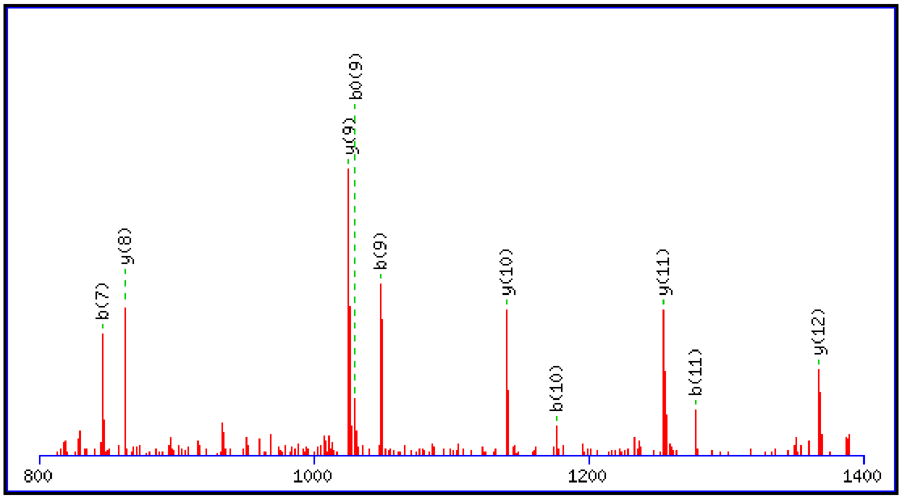


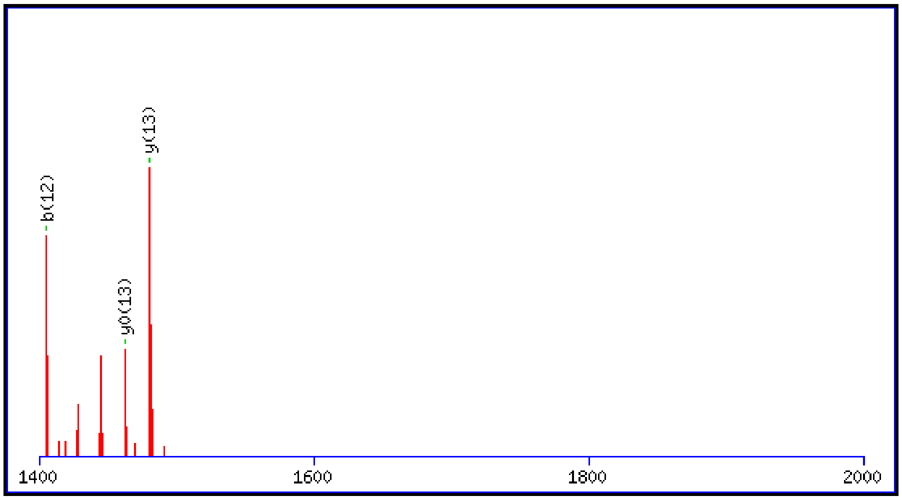


**Monoisotopic mass of neutral peptide Mr(calc):** 1607.8155
**Fixed modifications:** Carbamidomethyl (C)

**Variable modifications:**
**N9** : Deamidated_N (N)
**Ions Score:** 86 **Expect:** 3.6e-007
**Matches (Bold Red):** 32/142 fragment ions using 42 most intense peaks

| # | b | $b^{++}$ | $b^{*}$ | $b^{*++}$ | $b^{0}$ | $b^{0++}$ | Seq. | y | $y^{++}$ | $y^{*}$ | $y^{*++}$ | $y^{0}$ | $y^{0++}$ | # |
|---|---|---|---|---|---|---|---|---|---|---|---|---|---|---|
| 1 | 129.1022 | 65.0548 | 112.0757 | 56.5415 | | | K | | | | | | | 14 |
| 2 | **242.1863** | 121.5968 | 225.1598 | 113.0835 | | | L | **1480.7278** | 740.8675 | 1463.7013 | **732.3543** | **1462.7172** | **731.8623** | 13 |
| 3 | **355.2704** | 178.1388 | 338.2438 | 169.6255 | | | I | **1367.6437** | 684.3255 | 1350.6172 | **675.8122** | 1349.6332 | 675.3202 | 12 |
| 4 | 469.3133 | 235.1603 | **452.2867** | 226.6470 | | | N | **1254.5597** | 627.7835 | 1237.5331 | 619.2702 | 1236.5491 | 618.7782 | 11 |
| 5 | **584.3402** | 292.6738 | 567.3137 | 284.1605 | 566.3297 | 283.6685 | D | **1140.5168** | 570.7620 | 1123.4902 | 562.2487 | 1122.5062 | **561.7567** | 10 |
| 6 | **747.4036** | 374.2054 | 730.3770 | 365.6921 | 729.3930 | 365.2001 | Y | **1025.4898** | 513.2485 | 1008.4633 | 504.7353 | 1007.4792 | 504.2433 | 9 |
| 7 | **846.4720** | 423.7396 | 829.4454 | 415.2264 | 828.4614 | 414.7343 | V | **862.4265** | 431.7169 | 845.3999 | 423.2036 | 844.4159 | 422.7116 | 8 |
| 8 | 933.5040 | 467.2556 | 916.4775 | 458.7424 | 915.4934 | 458.2504 | S | **763.3581** | 382.1827 | **746.3315** | 373.6694 | 745.3475 | 373.1774 | 7 |
| 9 | **1048.5309** | 524.7691 | 1031.5044 | 516.2558 | **1030.5204** | 515.7638 | N | **676.3260** | 338.6667 | 659.2995 | 330.1534 | 658.3155 | **329.6614** | 6 |
| 10 | **1176.5895** | 588.7984 | 1159.5630 | 580.2851 | 1158.5790 | 579.7931 | Q | **561.2991** | 281.1532 | 544.2726 | 272.6399 | **543.2885** | 272.1479 | 5 |
| 11 | **1277.6372** | 639.3222 | 1260.6107 | 630.8090 | 1259.6266 | 630.3170 | T | **433.2405** | 217.1239 | 416.2140 | 208.6106 | 415.2300 | 208.1186 | 4 |
| 12 | **1405.6958** | 703.3515 | 1388.6692 | 694.8383 | 1387.6852 | 694.3462 | Q | 332.1928 | 166.6001 | **315.1663** | 158.0868 | | | 3 |
| 13 | **1462.7172** | **731.8623** | 1445.6907 | 723.3490 | 1444.7067 | 722.8570 | G | 204.1343 | 102.5708 | 187.1077 | 94.0575 | | | 2 |
| 14 | | | | | | | K | 147.1128 | 74.0600 | 130.0863 | 65.5468 | | | 1 |

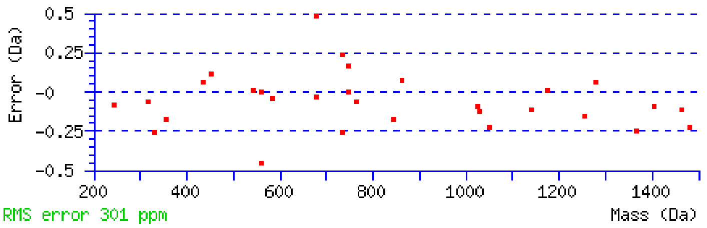


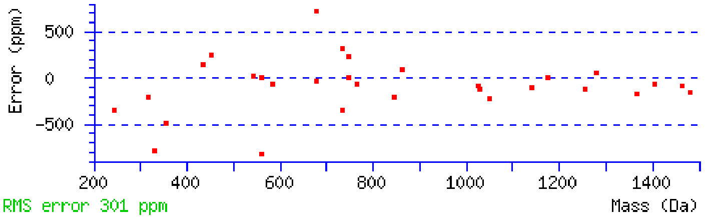


### All matches to this query

| Score | Mr(calc): | Delta | Sequence |
|---|---|---|---|
| 85.8 | 1607.8155 | 0.0014 | KLINDYVSNQTQGK |
| 35.3 | 1607.8155 | 0.0014 | KLINDYVSNQTQGK |
| 7.8 | 1607.8073 | 0.0096 | TQLIFGSVTNIHAK |
| 7.2 | 1607.8158 | 0.0011 | QLIRSSPAAHHRR |
| 7.2 | 1607.8158 | 0.0011 | QLIRSSPAAHHRR |
| 7.1 | 1607.8072 | 0.0097 | QLLAPGNSAGAFLIR |
| 5.8 | 1607.8276 | -0.0107 | KLVENGGMFVCKAR |
| 3.9 | 1607.8130 | 0.0039 | LGALNCTAFSIQWK |
| 3.1 | 1607.8283 | -0.0114 | EKAILQATLREEK |
| 1.8 | 1607.8283 | -0.0115 | KLQTLDLRDNALK |

---

Spectrum No: 167; Query: 934; Rank: 1

---

## Peptide View

MS/MS Fragmentation of **NPDINMTFEQVYR**
Found in **IPI00325312**, Tax_Id=10116 Gene_Symbol=Prom1 prominin 1 isoform 1

Match to Query 934: 1627.733728 from(814.874140,2+)
Title: 091008RatKidney_NoSalt_26.3064.3064.2.dta
Data file K:\NewmanPaper\Piliang\3SubProteomes\Piliang3SP\mgf5ppm\ERLIC_3SubProteomes5ppm.mgf

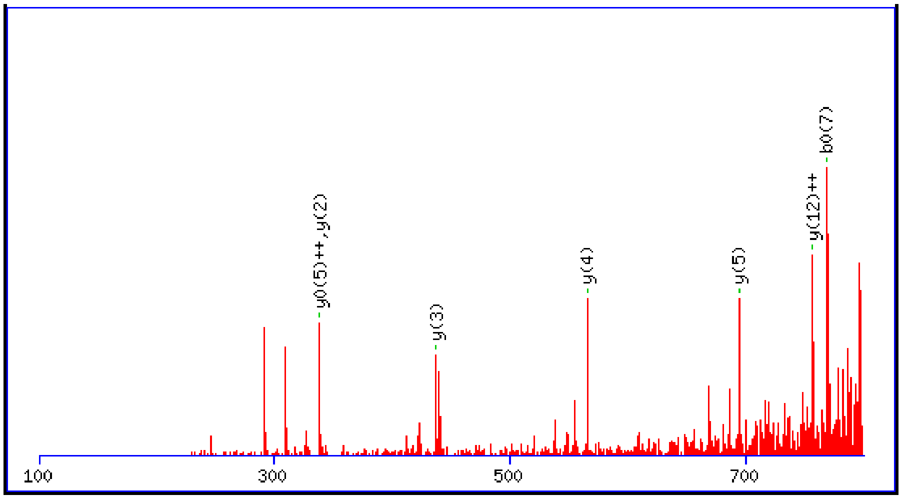


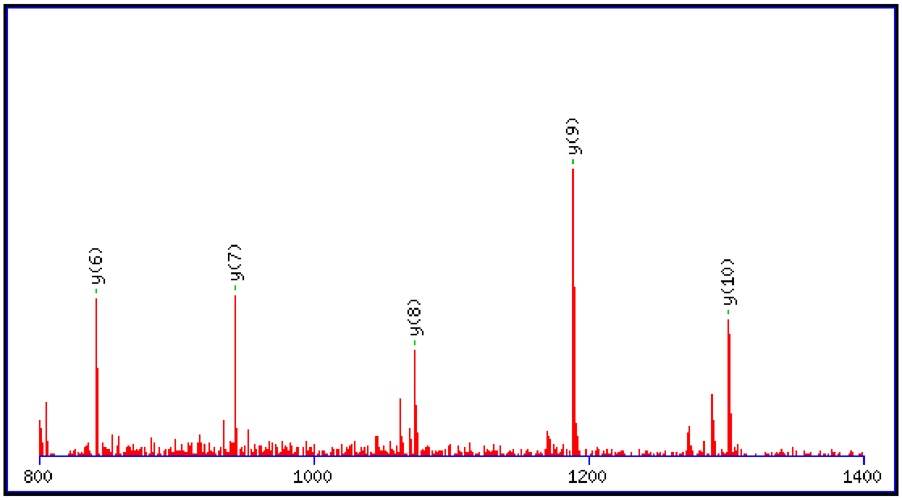


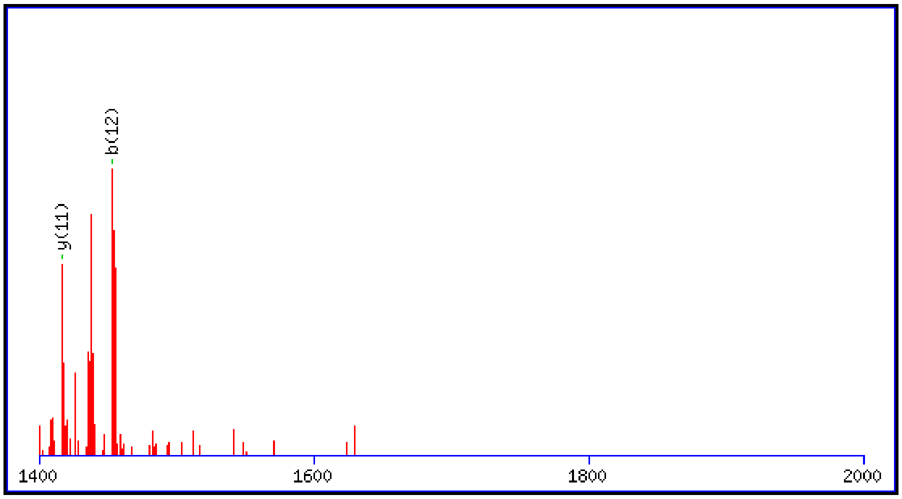


**Monoisotopic mass of neutral peptide Mr(calc):** 1626.7348
**Fixed modifications:** Carbamidomethyl (C)
**Variable modifications:**
**N5** : Deamidated_N (N)
**Ions Score:** 86 **Expect:** 4.7e-007
**Matches (Bold Red):** 14/132 fragment ions using 22 most intense peaks

| # | b | $b^{++}$ | b* | $b^{*++}$ | $b^0$ | $b^{0++}$ | Seq. | y | $y^{++}$ | y* | $y^{*++}$ | $y^0$ | $y^{0++}$ | # |
|---|---|---|---|---|---|---|---|---|---|---|---|---|---|---|
| 1 | 115.0502 | 58.0287 | 98.0237 | 49.5155 | | | N | | | | | | | 13 |
| 2 | 212.1030 | 106.5551 | 195.0764 | 98.0418 | | | P | 1513.6992 | **757.3532** | 1496.6726 | 748.8399 | 1495.6886 | 748.3479 | 12 |
| 3 | 327.1299 | 164.0686 | 310.1034 | 155.5553 | 309.1193 | 155.0633 | D | **1416.6464** | 708.8268 | 1399.6198 | 700.3136 | 1398.6358 | 699.8215 | 11 |
| 4 | 440.2140 | 220.6106 | 423.1874 | 212.0974 | 422.2034 | 211.6053 | I | **1301.6194** | 651.3134 | 1284.5929 | 642.8001 | 1283.6089 | 642.3081 | 10 |
| 5 | 555.2409 | 278.1241 | 538.2144 | 269.6108 | 537.2303 | 269.1188 | N | **1188.5354** | 594.7713 | 1171.5088 | 586.2581 | 1170.5248 | 585.7660 | 9 |

| | | | | | | | | | | | | | | |
|---|---|---|---|---|---|---|---|---|---|---|---|---|---|---|
| **6** | 686.2814 | 343.6443 | 669.2548 | 335.1311 | 668.2708 | 334.6391 | **M** | **1073.5084** | 537.2579 | 1056.4819 | 528.7446 | 1055.4979 | 528.2526 | **8** |
| **7** | 787.3291 | 394.1682 | 770.3025 | 385.6549 | **769.3185** | 385.1629 | **T** | **942.4680** | 471.7376 | 925.4414 | 463.2243 | 924.4574 | 462.7323 | **7** |
| **8** | 934.3975 | 467.7024 | 917.3709 | 459.1891 | 916.3869 | 458.6971 | **F** | **841.4203** | 421.2138 | 824.3937 | 412.7005 | 823.4097 | 412.2085 | **6** |
| **9** | 1063.4401 | 532.2237 | 1046.4135 | 523.7104 | 1045.4295 | 523.2184 | **E** | **694.3519** | 347.6796 | 677.3253 | 339.1663 | 676.3413 | **338.6743** | **5** |
| **10** | 1191.4987 | 596.2530 | 1174.4721 | 587.7397 | 1173.4881 | 587.2477 | **Q** | **565.3093** | 283.1583 | 548.2827 | 274.6450 | | | **4** |
| **11** | 1290.5671 | 645.7872 | 1273.5405 | 637.2739 | 1272.5565 | 636.7819 | **V** | **437.2507** | 219.1290 | 420.2241 | 210.6157 | | | **3** |
| **12** | **1453.6304** | 727.3188 | 1436.6039 | 718.8056 | 1435.6198 | 718.3136 | **Y** | **338.1823** | 169.5948 | 321.1557 | 161.0815 | | | **2** |
| **13** | | | | | | | **R** | 175.1190 | 88.0631 | 158.0924 | 79.5498 | | | **1** |

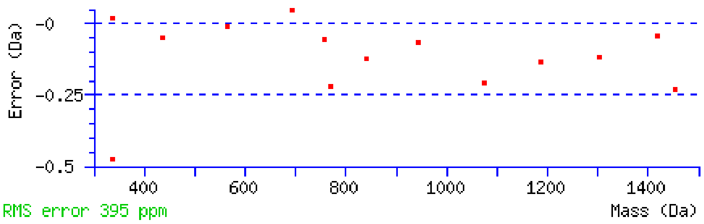


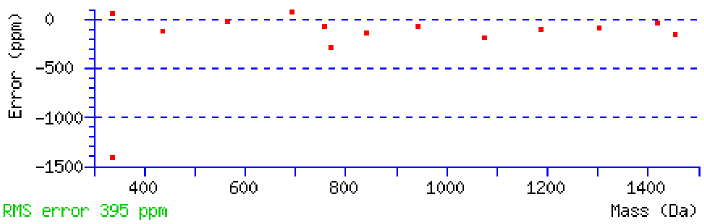


### All matches to this query

| Score | Mr(calc): | Delta | Sequence |
|---|---|---|---|
| 85.8 | 1626.7348 | 0.9989 | NPDINMTFEQVYR |
| 85.1 | 1627.7188 | 0.0149 | NPDINMTFEQVYR |
| 39.2 | 1626.7348 | 0.9989 | NPDINMTFEQVYR |
| 5.8 | 1627.7290 | 0.0047 | NRSACTYLASHLR |
| 5.0 | 1627.7243 | 0.0094 | VLDINDNAPTFSSR |
| 5.0 | 1627.7243 | 0.0094 | VLDINDNAPTFSSR |
| 4.5 | 1627.7243 | 0.0094 | VLDINDNAPTFSSR |
| 1.2 | 1627.7274 | 0.0063 | GSFGTAVRHCDEHR |
| 0.5 | 1627.7178 | 0.0160 | GFSQLSCLKSHER |

---

Spectrum No: 168; Query: 1642; Rank: 1

---

## Peptide View

MS/MS Fragmentation of **SGPSDEGEEEMEDGTVTNGS**
Found in **IPI00192279**, Tax_Id=10116 Gene_Symbol=- 28 kDa protein

Match to Query 1642: 2042.731788 from(1022.373170,2+)
Title: 091008RatKidney_NoSalt_31.613.613.2.dta
Data file K:\NewmanPaper\Piliang\3SubProteomes\Piliang3SP\mgf5ppm\ERLIC_3SubProteomes5ppm.mgf

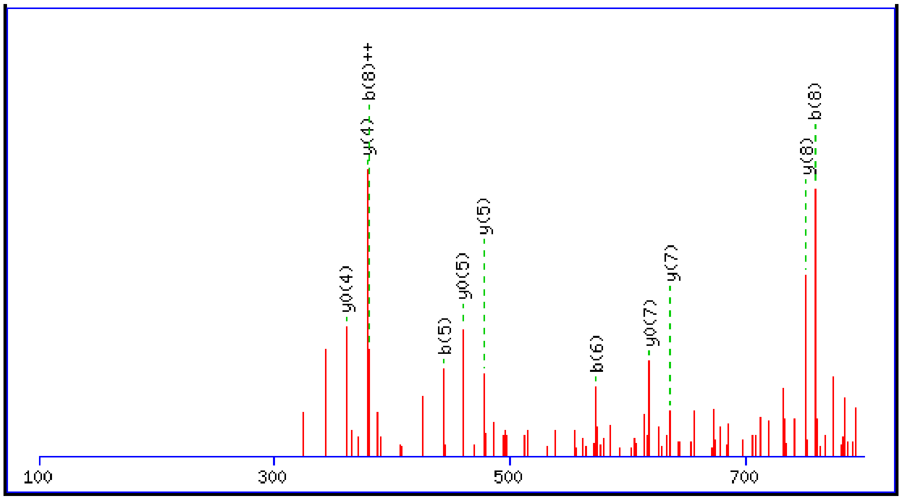


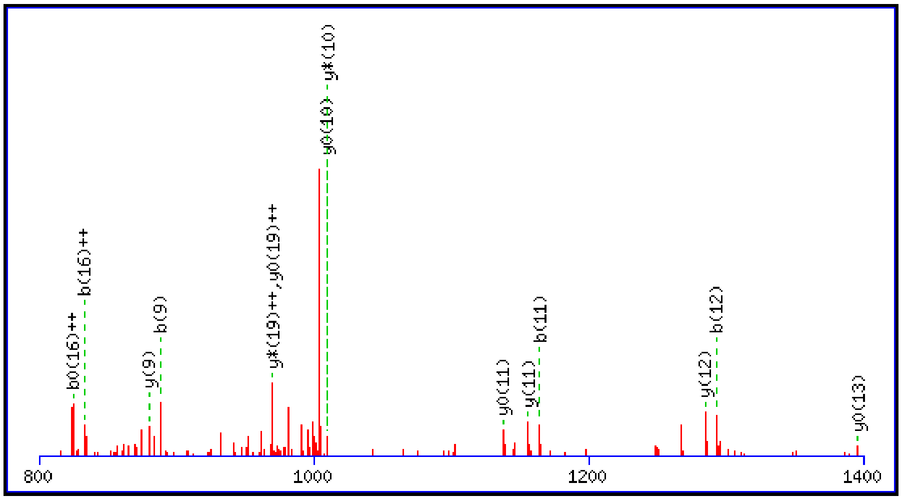


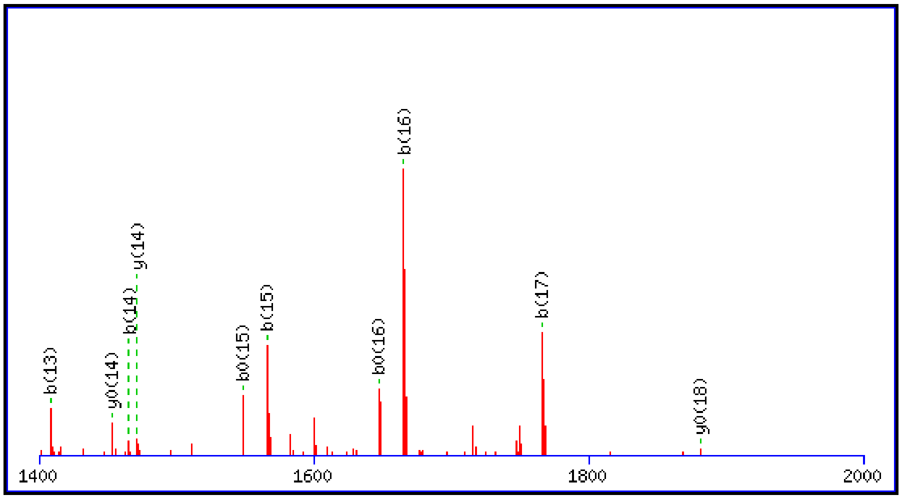


**Monoisotopic mass of neutral peptide Mr(calc):** 2042.7382
**Fixed modifications:** Carbamidomethyl (C)
**Variable modifications:**
**M11** : Oxidation (M)
**N18** : Deamidated_N (N)
**Ions Score:** 86 **Expect:** 2.7e-007
**Matches (Bold Red):** 37/190 fragment ions using 56 most intense peaks

| # | b | $b^{++}$ | b* | $b^{*++}$ | $b^{0}$ | $b^{0++}$ | Seq. | y | $y^{++}$ | y* | $y^{*++}$ | $y^{0}$ | $y^{0++}$ | # |
|---|---|---|---|---|---|---|---|---|---|---|---|---|---|---|
| 1 | 88.0393 | 44.5233 | | | 70.0287 | 35.5180 | S | | | | | | | 20 |
| 2 | 145.0608 | 73.0340 | | | 127.0502 | 64.0287 | G | 1956.7135 | 978.8604 | 1939.6869 | **970.3471** | 1938.7029 | **969.8551** | 19 |
| 3 | 242.1135 | 121.5604 | | | 224.1030 | 112.5551 | P | 1899.6920 | 950.3496 | 1882.6655 | 941.8364 | **1881.6815** | 941.3444 | 18 |
| 4 | 329.1456 | 165.0764 | | | 311.1350 | 156.0711 | S | 1802.6393 | 901.8233 | 1785.6127 | 893.3100 | 1784.6287 | 892.8180 | 17 |
| | | | | | | | | | | | | | | |

| | | | | | | | | | | | | | | |
|---|---|---|---|---|---|---|---|---|---|---|---|---|---|---|
| **5** | **444.1725** | 222.5899 | | | 426.1619 | 213.5846 | **D** | 1715.6072 | 858.3073 | 1698.5807 | 849.7940 | 1697.5967 | 849.3020 | **16** |
| **6** | **573.2151** | 287.1112 | | | 555.2045 | 278.1059 | **E** | 1600.5803 | 800.7938 | 1583.5537 | 792.2805 | 1582.5697 | 791.7885 | **15** |
| **7** | 630.2366 | 315.6219 | | | 612.2260 | 306.6166 | **G** | **1471.5377** | 736.2725 | 1454.5111 | 727.7592 | **1453.5271** | 727.2672 | **14** |
| **8** | **759.2792** | **380.1432** | | | 741.2686 | 371.1379 | **E** | 1414.5162 | 707.7618 | 1397.4897 | 699.2485 | **1396.5057** | 698.7565 | **13** |
| **9** | **888.3217** | 444.6645 | | | 870.3112 | 435.6592 | **E** | **1285.4736** | 643.2405 | 1268.4471 | 634.7272 | 1267.4631 | 634.2352 | **12** |
| **10** | 1017.3643 | 509.1858 | | | 999.3538 | 500.1805 | **E** | **1156.4310** | 578.7192 | 1139.4045 | 570.2059 | **1138.4205** | 569.7139 | **11** |
| **11** | **1164.3997** | 582.7035 | | | 1146.3892 | 573.6982 | **M** | 1027.3884 | 514.1979 | **1010.3619** | 505.6846 | **1009.3779** | 505.1926 | **10** |
| **12** | **1293.4423** | 647.2248 | | | 1275.4318 | 638.2195 | **E** | **880.3530** | 440.6802 | 863.3265 | 432.1669 | 862.3425 | 431.6749 | **9** |
| **13** | **1408.4693** | 704.7383 | | | 1390.4587 | 695.7330 | **D** | **751.3104** | 376.1589 | 734.2839 | 367.6456 | 733.2999 | 367.1536 | **8** |
| **14** | **1465.4907** | 733.2490 | | | 1447.4802 | 724.2437 | **G** | **636.2835** | 318.6454 | 619.2570 | 310.1321 | **618.2729** | 309.6401 | **7** |
| **15** | **1566.5384** | 783.7728 | | | **1548.5279** | 774.7676 | **T** | 579.2620 | 290.1347 | 562.2355 | 281.6214 | 561.2515 | 281.1294 | **6** |
| **16** | **1665.6068** | **833.3071** | | | **1647.5963** | **824.3018** | **V** | **478.2144** | 239.6108 | 461.1878 | 231.0975 | **460.2038** | 230.6055 | **5** |
| **17** | **1766.6545** | 883.8309 | | | 1748.6440 | 874.8256 | **T** | **379.1459** | 190.0766 | 362.1194 | 181.5633 | **361.1354** | 181.0713 | **4** |
| **18** | **1881.6815** | 941.3444 | 1864.6549 | 932.8311 | 1863.6709 | 932.3391 | **N** | 278.0983 | 139.5528 | 261.0717 | 131.0395 | 260.0877 | 130.5475 | **3** |
| **19** | 1938.7029 | **969.8551** | 1921.6764 | 961.3418 | 1920.6924 | 960.8498 | **G** | 163.0713 | 82.0393 | | | 145.0608 | 73.0340 | **2** |
| **20** | | | | | | | **S** | 106.0499 | 53.5286 | | | 88.0393 | 44.5233 | **1** |

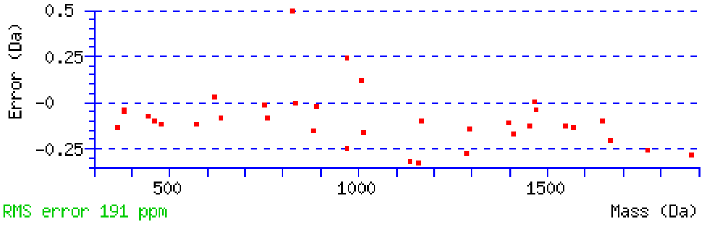


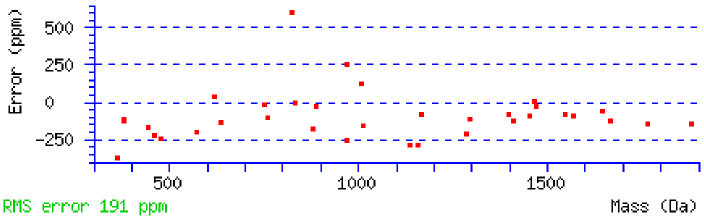


### All matches to this query

| Score | Mr(calc): | Delta | Sequence |
|---|---|---|---|
| 85.7 | 2042.7382 | -0.0064 | SGPSDEGEEEMEDGTVTNGS |

---

Spectrum No: 169; Query: 1975; Rank: 1

---

## Peptide View

MS/MS Fragmentation of **ETQSPAPLQNSSGDPAEEESPV**
Found in **IPI00214787**, Tax_Id=10116 Gene_Symbol=Slc16a1 Monocarboxylate transporter 1

Match to Query 1975: 2268.997148 from(1135.505850,2+)
Title: 100101RatKid_NS_deglyco_22.2416.2416.2.dta
Data file K:\NewmanPaper\Piliang\3SubProteomes\Piliang3SP\mgf5ppm\ERLIC_3SubProteomes5ppm.mgf

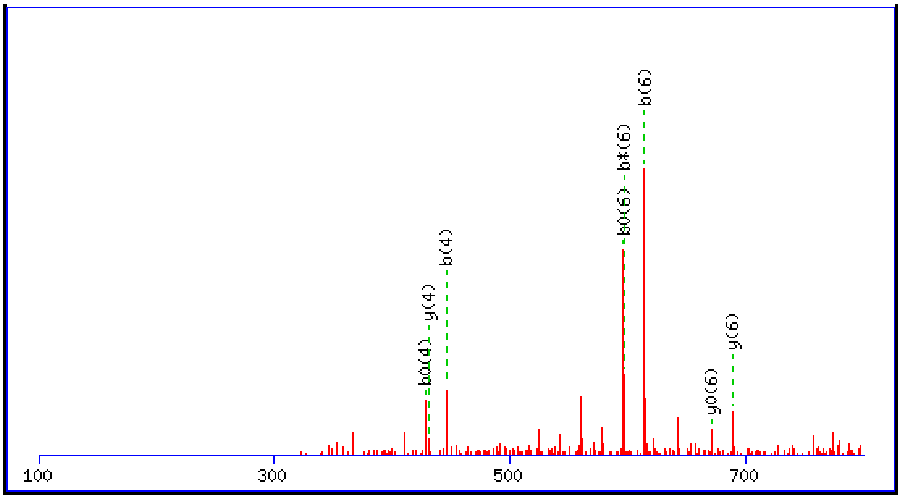


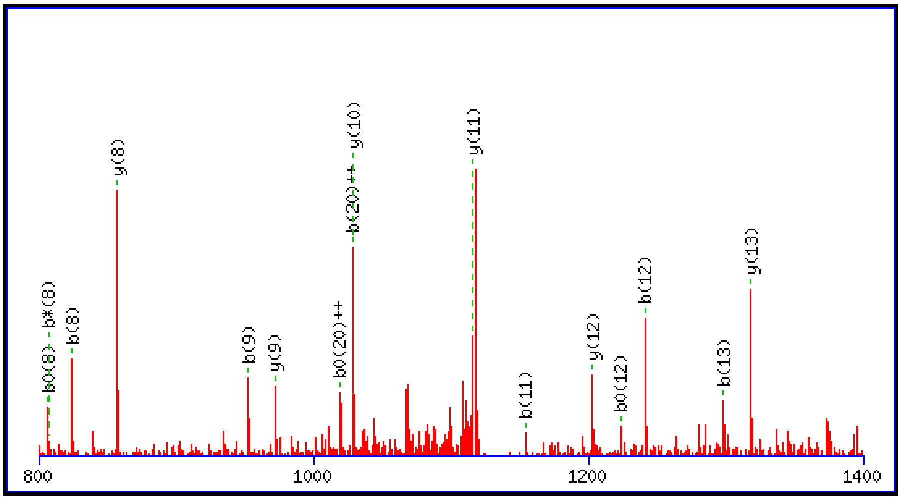


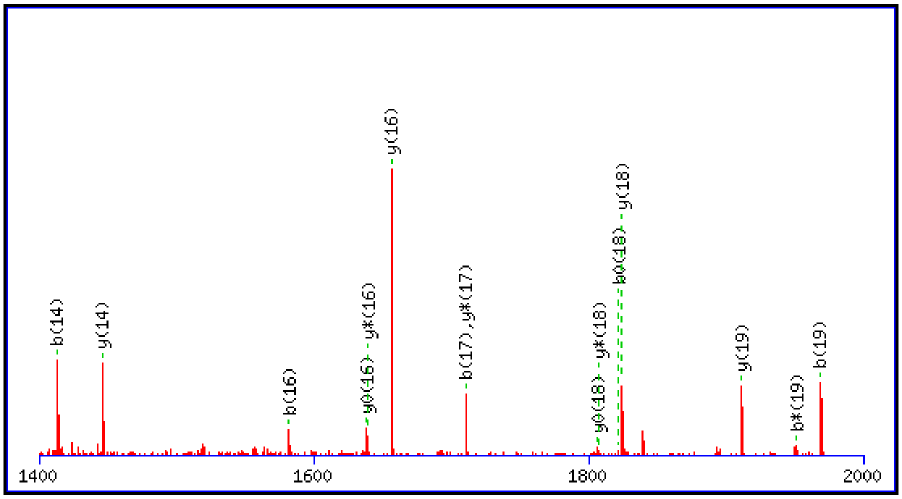


**Monoisotopic mass of neutral peptide Mr(calc):** 2268.9870
**Fixed modifications:** Carbamidomethyl (C)
**Variable modifications:**
**N10** : Deamidated_N (N)
**Ions Score:** 86 **Expect:** 8e-007
**Matches (Bold Red):** 39/220 fragment ions using 61 most intense peaks

| # | b | $b^{++}$ | b* | $b^{*++}$ | $b^0$ | $b^{0++}$ | Seq. | y | $y^{++}$ | y* | $y^{*++}$ | $y^0$ | $y^{0++}$ | # |
|---|---|---|---|---|---|---|---|---|---|---|---|---|---|---|
| 1 | 130.0499 | 65.5286 | | | 112.0393 | 56.5233 | E | | | | | | | 22 |
| 2 | 231.0975 | 116.0524 | | | 213.0870 | 107.0471 | T | 2140.9517 | 1070.9795 | 2123.9251 | 1062.4662 | 2122.9411 | 1061.9742 | 21 |
| 3 | 359.1561 | 180.0817 | 342.1296 | 171.5684 | 341.1456 | 171.0764 | Q | 2039.9040 | 1020.4556 | 2022.8774 | 1011.9424 | 2021.8934 | 1011.4504 | 20 |
| 4 | **446.1882** | 223.5977 | 429.1616 | 215.0844 | **428.1776** | 214.5924 | S | **1911.8454** | 956.4263 | 1894.8189 | 947.9131 | 1893.8349 | 947.4211 | 19 |
| 5 | 543.2409 | 272.1241 | 526.2144 | 263.6108 | 525.2304 | 263.1188 | P | **1824.8134** | 912.9103 | **1807.7868** | 904.3971 | **1806.8028** | 903.9050 | 18 |

| | | | | | | | | | | | | | | |
|---|---|---|---|---|---|---|---|---|---|---|---|---|---|---|
| 6 | 614.2780 | 307.6427 | 597.2515 | 299.1294 | 596.2675 | 298.6374 | A | 1727.7606 | 864.3839 | 1710.7341 | 855.8707 | 1709.7501 | 855.3787 | 17 |
| 7 | 711.3308 | 356.1690 | 694.3042 | 347.6558 | 693.3202 | 347.1638 | P | 1656.7235 | 828.8654 | 1639.6970 | 820.3521 | 1638.7129 | 819.8601 | 16 |
| 8 | 824.4149 | 412.7111 | 807.3883 | 404.1978 | 806.4043 | 403.7058 | L | 1559.6707 | 780.3390 | 1542.6442 | 771.8257 | 1541.6602 | 771.3337 | 15 |
| 9 | 952.4734 | 476.7404 | 935.4469 | 468.2271 | 934.4629 | 467.7351 | Q | 1446.5867 | 723.7970 | 1429.5601 | 715.2837 | 1428.5761 | 714.7917 | 14 |
| 10 | 1067.5004 | 534.2538 | 1050.4738 | 525.7406 | 1049.4898 | 525.2485 | N | 1318.5281 | 659.7677 | 1301.5016 | 651.2544 | 1300.5175 | 650.7624 | 13 |
| 11 | 1154.5324 | 577.7698 | 1137.5059 | 569.2566 | 1136.5218 | 568.7646 | S | 1203.5012 | 602.2542 | | | 1185.4906 | 593.2489 | 12 |
| 12 | 1241.5644 | 621.2859 | 1224.5379 | 612.7726 | 1223.5539 | 612.2806 | S | 1116.4691 | 558.7382 | | | 1098.4586 | 549.7329 | 11 |
| 13 | 1298.5859 | 649.7966 | 1281.5593 | 641.2833 | 1280.5753 | 640.7913 | G | 1029.4371 | 515.2222 | | | 1011.4265 | 506.2169 | 10 |
| 14 | 1413.6128 | 707.3101 | 1396.5863 | 698.7968 | 1395.6023 | 698.3048 | D | 972.4156 | 486.7115 | | | 954.4051 | 477.7062 | 9 |
| 15 | 1510.6656 | 755.8364 | 1493.6391 | 747.3232 | 1492.6550 | 746.8312 | P | 857.3887 | 429.1980 | | | 839.3781 | 420.1927 | 8 |
| 16 | 1581.7027 | 791.3550 | 1564.6762 | 782.8417 | 1563.6922 | 782.3497 | A | 760.3359 | 380.6716 | | | 742.3254 | 371.6663 | 7 |
| 17 | 1710.7453 | 855.8763 | 1693.7188 | 847.3630 | 1692.7347 | 846.8710 | E | 689.2988 | 345.1531 | | | 671.2883 | 336.1478 | 6 |
| 18 | 1839.7879 | 920.3976 | 1822.7614 | 911.8843 | 1821.7773 | 911.3923 | E | 560.2562 | 280.6318 | | | 542.2457 | 271.6265 | 5 |
| 19 | 1968.8305 | 984.9189 | 1951.8039 | 976.4056 | 1950.8199 | 975.9136 | E | 431.2136 | 216.1105 | | | 413.2031 | 207.1052 | 4 |
| 20 | 2055.8625 | 1028.4349 | 2038.8360 | 1019.9216 | 2037.8520 | 1019.4296 | S | 302.1710 | 151.5892 | | | 284.1605 | 142.5839 | 3 |
| 21 | 2152.9153 | 1076.9613 | 2135.8887 | 1068.4480 | 2134.9047 | 1067.9560 | P | 215.1390 | 108.0731 | | | | | 2 |
| 22 | | | | | | | V | 118.0863 | 59.5468 | | | | | 1 |

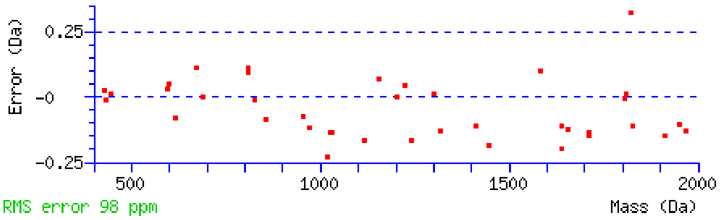


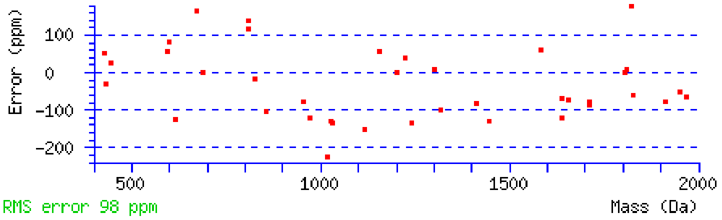


### All matches to this query

| Score | Mr(calc): | Delta | Sequence |
|---|---|---|---|
| 85.7 | 2268.9870 | 0.0102 | ETQSPAPLQNSSGDPAEEESPV |
| 22.9 | 2268.0030 | 0.9942 | ETQSPAPLQNSSGDPAEEESPV |
| 6.8 | 2268.9821 | 0.0151 | NQGQSNLKDTYMLSSTVSSK |
| 4.5 | 2267.9729 | 1.0243 | AKEPVSATILQTDQDLTAK |
| 3.6 | 2267.9981 | 0.9991 | NQGQSNLKDTYMLSSTVSSK |
| 2.5 | 2267.9729 | 1.0243 | AKEPVSATILQTDQDLTAK |
| 2.5 | 2267.9729 | 1.0243 | AKEPVSATILQTDQDLTAK |
| 1.5 | 2269.0089 | -0.0117 | HARRPAAPGCSPSRSLFSR |
| 0.7 | 2269.0089 | -0.0117 | HARRPAAPGCSPSRSLFSR |
| 0.6 | 2269.0089 | -0.0117 | HARRPAAPGCSPSRSLFSR |

---

Spectrum No: 170; Query: 831; Rank: 1

---

## Peptide View

MS/MS Fragmentation of **CISDLTLNVSTTEK**
Found in **IPI00324585**, Tax_Id=10116 Gene_Symbol=Itga1 Integrin alpha-1 precursor

Match to Query 831: 1580.767528 from(791.391040,2+)
Title: 100101RatKid_NS_deglyco_15.3149.3149.2.dta
Data file K:\NewmanPaper\Piliang\3SubProteomes\Piliang3SP\mgf5ppm\ERLIC_3SubProteomes5ppm.mgf

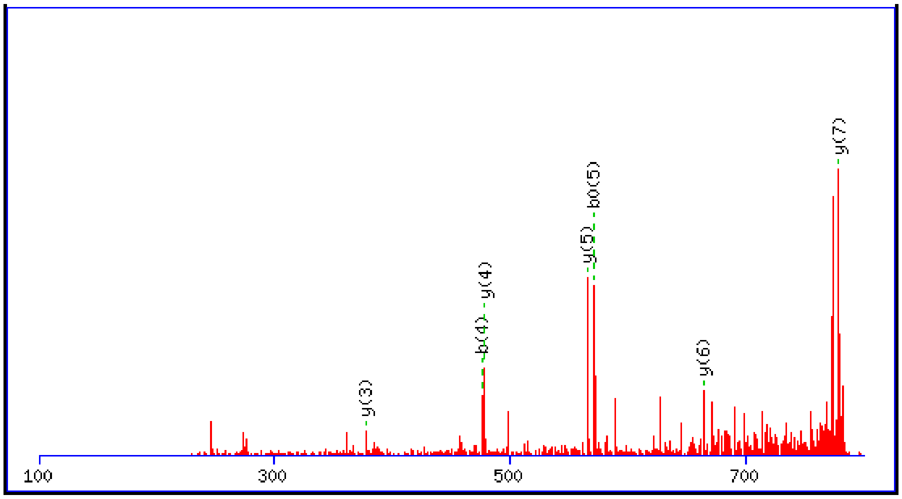


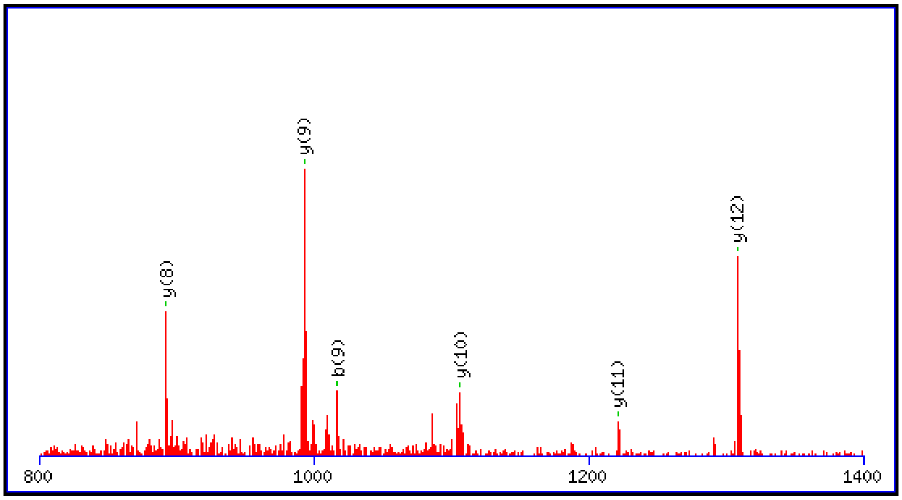


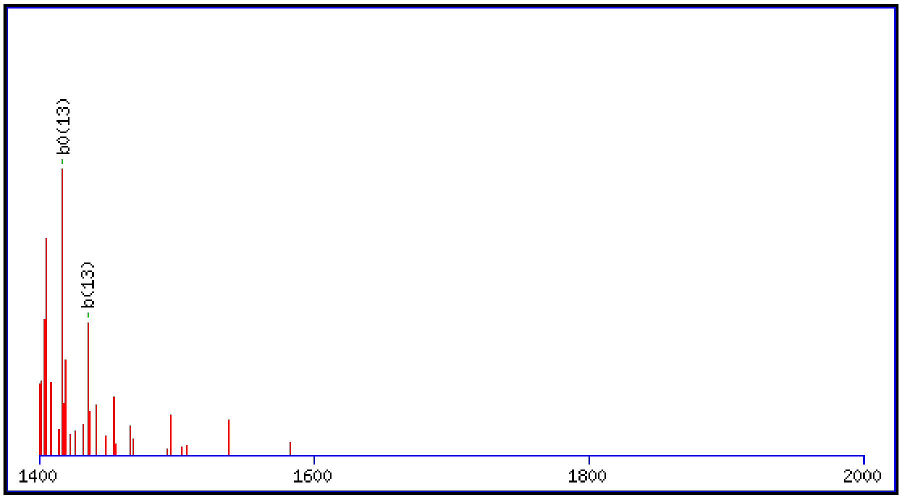


**Monoisotopic mass of neutral peptide Mr(calc):** 1580.7603
**Fixed modifications:** Carbamidomethyl (C)
**Variable modifications:**
**N8** : Deamidated_N (N)
**Ions Score:** 86 **Expect:** 4.2e-007
**Matches (Bold Red):** 15/136 fragment ions using 29 most intense peaks

| # | b | b++ | b* | b*++ | b0 | b0++ | Seq. | y | y++ | y* | y*++ | y0 | y0++ | # |
|---|---|---|---|---|---|---|---|---|---|---|---|---|---|---|
| 1 | 161.0379 | 81.0226 | | | | | C | | | | | | | 14 |
| 2 | 274.1220 | 137.5646 | | | | | I | 1421.7370 | 711.3721 | 1404.7104 | 702.8589 | 1403.7264 | 702.3668 | 13 |
| 3 | 361.1540 | 181.0806 | | | 343.1435 | 172.0754 | S | **1308.6529** | 654.8301 | 1291.6264 | 646.3168 | 1290.6423 | 645.8248 | 12 |
| 4 | **476.1810** | 238.5941 | | | 458.1704 | 229.5888 | D | **1221.6209** | 611.3141 | 1204.5943 | 602.8008 | 1203.6103 | 602.3088 | 11 |
| 5 | 589.2650 | 295.1362 | | | **571.2545** | 286.1309 | L | **1106.5939** | 553.8006 | 1089.5674 | 545.2873 | 1088.5834 | 544.7953 | 10 |

| | | | | | | | | | | | | | | |
|---|---|---|---|---|---|---|---|---|---|---|---|---|---|---|
| 6 | 690.3127 | 345.6600 | | | 672.3021 | 336.6547 | T | 993.5099 | 497.2586 | 976.4833 | 488.7453 | 975.4993 | 488.2533 | 9 |
| 7 | 803.3968 | 402.2020 | | | 785.3862 | 393.1967 | L | 892.4622 | 446.7347 | 875.4356 | 438.2215 | 874.4516 | 437.7295 | 8 |
| 8 | 918.4237 | 459.7155 | 901.3972 | 451.2022 | 900.4131 | 450.7102 | N | 779.3781 | 390.1927 | 762.3516 | 381.6794 | 761.3676 | 381.1874 | 7 |
| 9 | 1017.4921 | 509.2497 | 1000.4656 | 500.7364 | 999.4816 | 500.2444 | V | 664.3512 | 332.6792 | 647.3246 | 324.1660 | 646.3406 | 323.6740 | 6 |
| 10 | 1104.5241 | 552.7657 | 1087.4976 | 544.2524 | 1086.5136 | 543.7604 | S | 565.2828 | 283.1450 | 548.2562 | 274.6318 | 547.2722 | 274.1397 | 5 |
| 11 | 1205.5718 | 603.2896 | 1188.5453 | 594.7763 | 1187.5613 | 594.2843 | T | 478.2508 | 239.6290 | 461.2242 | 231.1157 | 460.2402 | 230.6237 | 4 |
| 12 | 1306.6195 | 653.8134 | 1289.5930 | 645.3001 | 1288.6089 | 644.8081 | T | 377.2031 | 189.1052 | 360.1765 | 180.5919 | 359.1925 | 180.0999 | 3 |
| 13 | 1435.6621 | 718.3347 | 1418.6355 | 709.8214 | 1417.6515 | 709.3294 | E | 276.1554 | 138.5813 | 259.1288 | 130.0681 | 258.1448 | 129.5761 | 2 |
| 14 | | | | | | | K | 147.1128 | 74.0600 | 130.0863 | 65.5468 | | | 1 |

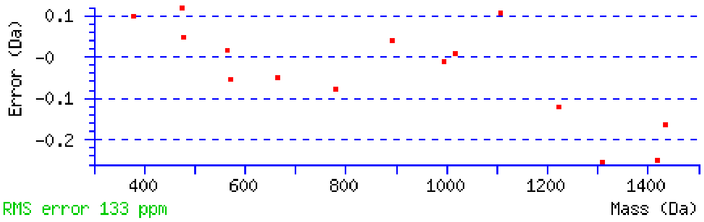


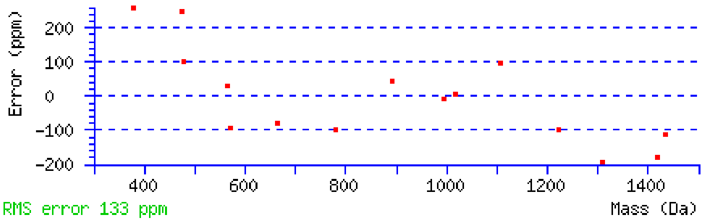


### All matches to this query

| Score | Mr(calc): | Delta | Sequence |
|---|---|---|---|
| 85.6 | 1580.7603 | 0.0072 | CISDLTLNVSTTEK |
| 25.8 | 1579.7763 | 0.9912 | CISDLTLNVSTTEK |
| 9.7 | 1580.7794 | -0.0119 | DSAIGQSPSVHNLEK |
| 9.3 | 1580.7603 | 0.0072 | GLKDMELDTSSIEK |
| 6.9 | 1580.7521 | 0.0155 | MPQLLNSILNVNK |
| 6.8 | 1578.7654 | 2.0021 | LDSPGNLDTLQAKK |
| 3.3 | 1580.7745 | -0.0070 | NLSMKARPEVNVK |
| 2.9 | 1579.7746 | 0.9929 | AEIVPVTTTVLDDK |
| 2.8 | 1579.7746 | 0.9929 | AEIVPVTTTVLDDK |
| 1.6 | 1579.7607 | 1.0069 | GLITIAAQELSDNR |

---

Spectrum No: 171; Query: 703; Rank: 1

---

## Peptide View

MS/MS Fragmentation of **LGALNSSLLLLEDR**
Found in **IPI00199867**, Tax_Id=10116 Gene_Symbol=Emilin1_predicted elastin microfibril interfacer 1

Match to Query 703: 1513.832828 from(757.923690,2+)
Title: 091008RatKidney_NH4Format01_23.3825.3825.2.dta
Data file K:\NewmanPaper\Piliang\3SubProteomes\Piliang3SP\mgf5ppm\ERLIC_3SubProteomes5ppm.mgf

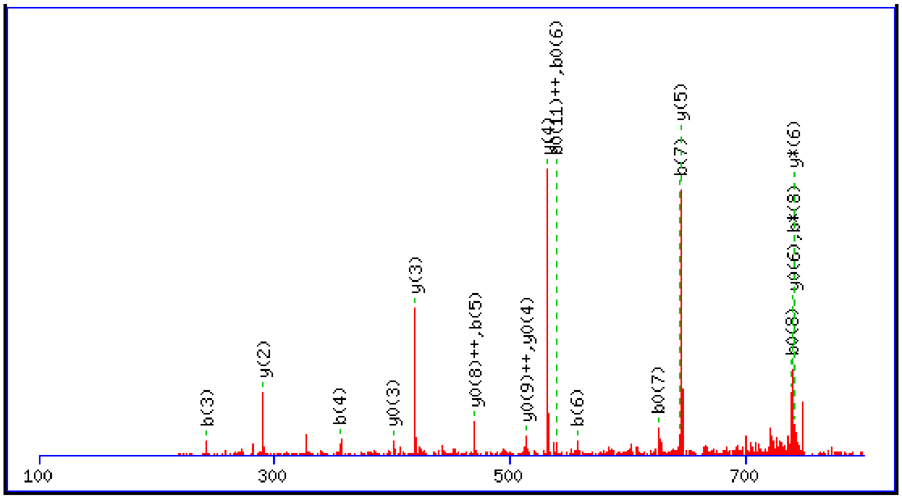


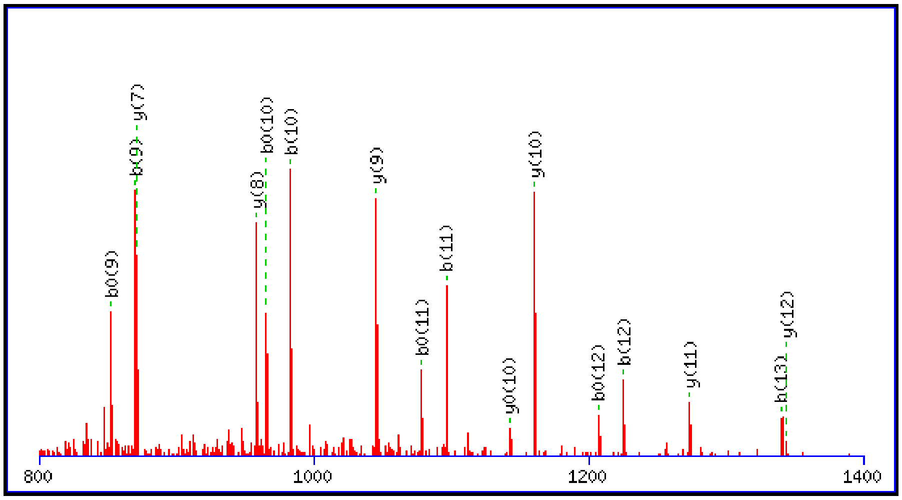


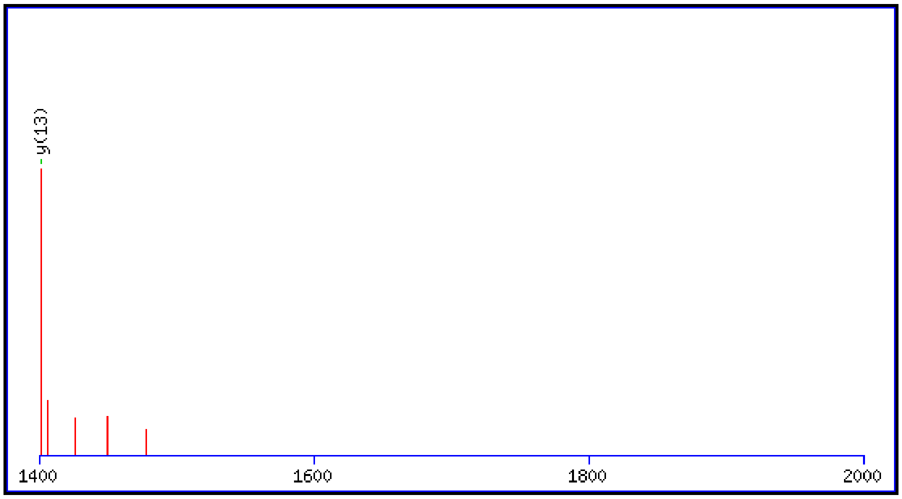


**Monoisotopic mass of neutral peptide Mr(calc):** 1513.8351
**Fixed modifications:** Carbamidomethyl (C)
**Variable modifications:**
**N5** : Deamidated_N (N)
**Ions Score:** 86 **Expect:** 1.9e-007
**Matches (Bold Red):** 37/136 fragment ions using 53 most intense peaks

| # | b | $b^{++}$ | b* | $b^{*++}$ | $b^0$ | $b^{0++}$ | Seq. | y | $y^{++}$ | y* | $y^{*++}$ | $y^0$ | $y^{0++}$ | # |
|---|---|---|---|---|---|---|---|---|---|---|---|---|---|---|
| 1 | 114.0913 | 57.5493 | | | | | L | | | | | | | 14 |
| 2 | 171.1128 | 86.0600 | | | | | G | **1401.7584** | 701.3828 | 1384.7318 | 692.8696 | 1383.7478 | 692.3775 | 13 |
| 3 | **242.1499** | 121.5786 | | | | | A | **1344.7369** | 672.8721 | 1327.7104 | 664.3588 | 1326.7263 | 663.8668 | 12 |
| 4 | **355.2340** | 178.1206 | | | | | L | **1273.6998** | 637.3535 | 1256.6733 | 628.8403 | 1255.6892 | 628.3483 | 11 |
| 5 | **470.2609** | 235.6341 | 453.2344 | 227.1208 | | | N | **1160.6157** | 580.8115 | 1143.5892 | 572.2982 | **1142.6052** | 571.8062 | 10 |

| | | | | | | | | | | | | | | |
|---|---|---|---|---|---|---|---|---|---|---|---|---|---|---|
| **6** | **557.2929** | 279.1501 | 540.2664 | 270.6368 | **539.2824** | 270.1448 | **S** | **1045.5888** | 523.2980 | 1028.5623 | 514.7848 | 1027.5782 | **514.2928** | **9** |
| **7** | **644.3250** | 322.6661 | 627.2984 | 314.1529 | **626.3144** | 313.6608 | **S** | **958.5568** | 479.7820 | 941.5302 | 471.2687 | 940.5462 | **470.7767** | **8** |
| **8** | 757.4090 | 379.2082 | **740.3825** | 370.6949 | **739.3985** | 370.2029 | **L** | **871.5247** | 436.2660 | 854.4982 | 427.7527 | 853.5142 | 427.2607 | **7** |
| **9** | **870.4931** | 435.7502 | 853.4666 | 427.2369 | **852.4825** | 426.7449 | **L** | 758.4407 | 379.7240 | **741.4141** | 371.2107 | **740.4301** | 370.7187 | **6** |
| **10** | **983.5772** | 492.2922 | 966.5506 | 483.7789 | **965.5666** | 483.2869 | **L** | **645.3566** | 323.1819 | 628.3301 | 314.6687 | 627.3461 | 314.1767 | **5** |
| **11** | **1096.6612** | 548.8343 | 1079.6347 | 540.3210 | **1078.6507** | **539.8290** | **L** | **532.2726** | 266.6399 | 515.2460 | 258.1266 | **514.2620** | 257.6346 | **4** |
| **12** | **1225.7038** | 613.3555 | 1208.6773 | 604.8423 | **1207.6933** | 604.3503 | **E** | **419.1885** | 210.0979 | 402.1619 | 201.5846 | **401.1779** | 201.0926 | **3** |
| **13** | **1340.7308** | 670.8690 | 1323.7042 | 662.3557 | 1322.7202 | 661.8637 | **D** | **290.1459** | 145.5766 | 273.1193 | 137.0633 | 272.1353 | 136.5713 | **2** |
| **14** | | | | | | | **R** | 175.1190 | 88.0631 | 158.0924 | 79.5498 | | | **1** |

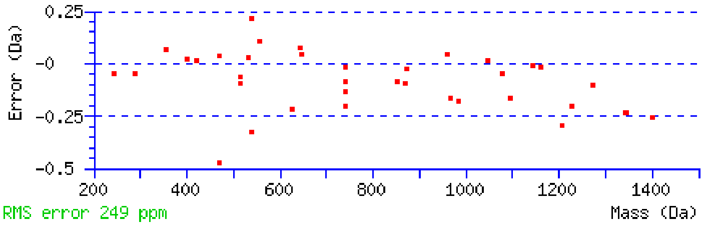


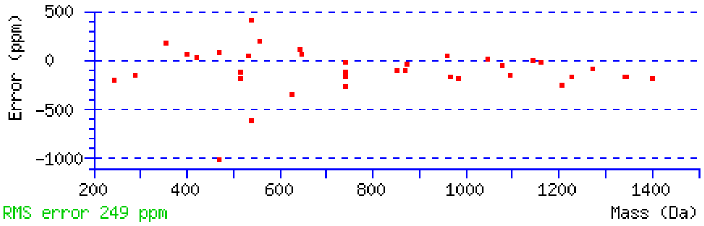


### All matches to this query

| Score | Mr(calc): | Delta | Sequence |
|---|---|---|---|
| 85.5 | 1513.8351 | -0.0023 | LGALNSSLLLLEDR |
| 26.3 | 1513.8212 | 0.0116 | IQDLENRLTKQR |
| 10.9 | 1513.8324 | 0.0004 | KNLISSXLQRGQR |
| 5.7 | 1513.8212 | 0.0116 | LGAVAGANLSQLASSR |
| 5.2 | 1513.8351 | -0.0023 | LQKVSENALPSSLK |
| 2.5 | 1512.8412 | 0.9916 | KLLSDFQHIGSIR |
| 1.3 | 1511.8321 | 2.0008 | GHREYVQINKIR |
| 0.9 | 1512.8218 | 1.0111 | GIALLSPHPFLLR |
| 0.1 | 1513.8212 | 0.0116 | AITSLRANGVEINR |

---

Spectrum No: 172; Query: 291; Rank: 1

---

## Peptide View

MS/MS Fragmentation of **LANTSMFNNSK**
Found in **IPI00206254**, Tax_Id=10116 Gene_Symbol=Ggt1 Gamma-glutamyltranspeptidase 1 precursor

Match to Query 291: 1243.538748 from(622.776650,2+)
Title: 091008RatKidney_NH4Format01_28.597.597.2.dta
Data file K:\NewmanPaper\Piliang\3SubProteomes\Piliang3SP\mgf5ppm\ERLIC_3SubProteomes5ppm.mgf

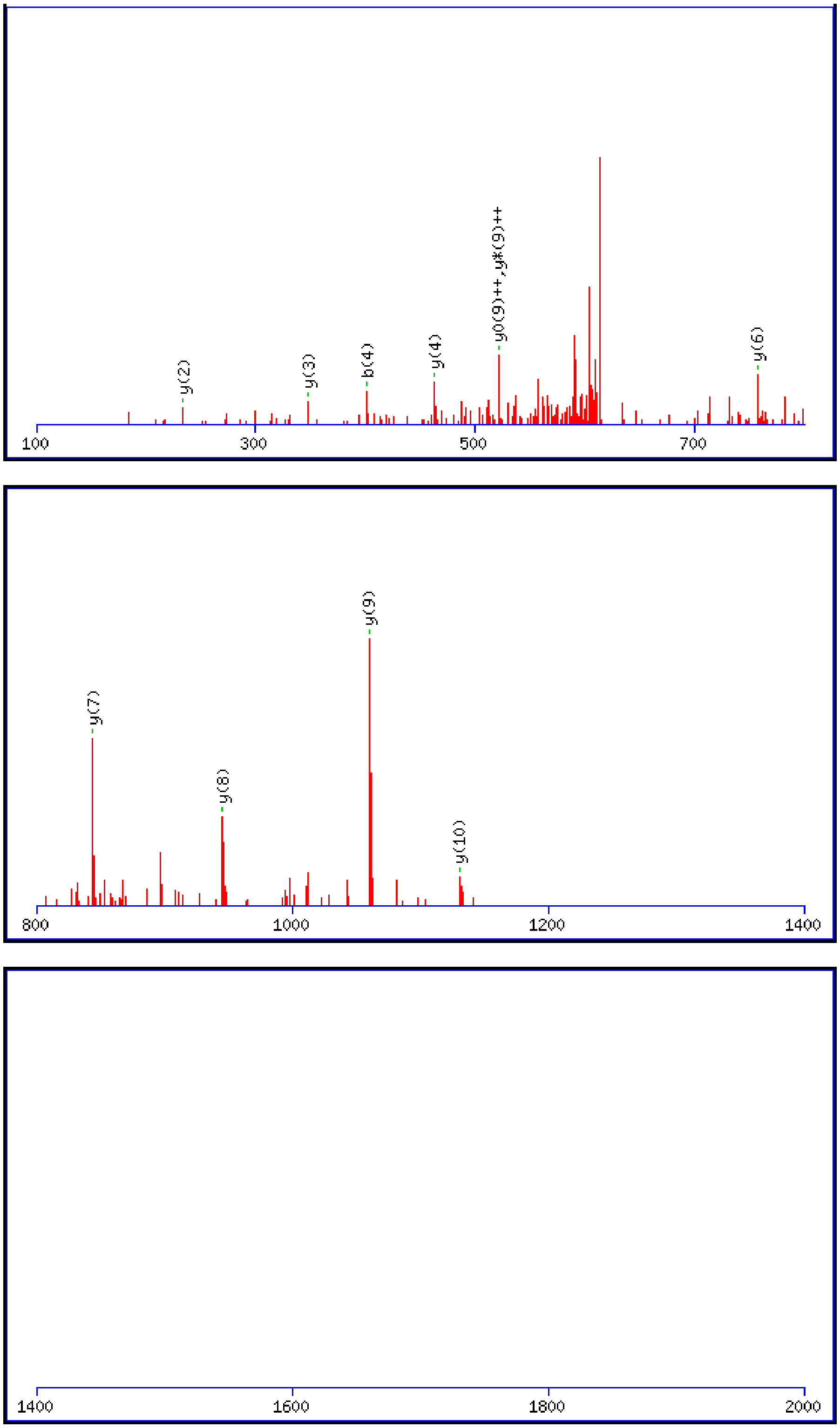


**Monoisotopic mass of neutral peptide Mr(calc):** 1243.5390
**Fixed modifications:** Carbamidomethyl (C)
**Variable modifications:**
**N3** : Deamidated_N (N)
**M6** : Oxidation (M)
**N8** : Deamidated_N (N)
**Ions Score:** 85 **Expect:** 3.3e-007
**Matches (Bold Red):** 11/108 fragment ions using 15 most intense peaks

| # | b | $b^{++}$ | b* | $b^{*++}$ | $b^{0}$ | $b^{0++}$ | Seq. | y | $y^{++}$ | y* | $y^{*++}$ | $y^{0}$ | $y^{0++}$ | # |
|---|---|---|---|---|---|---|---|---|---|---|---|---|---|---|
| 1 | 114.0913 | 57.5493 | | | | | L | | | | | | | 11 |
| 2 | 185.1285 | 93.0679 | | | | | A | **1131.4623** | 566.2348 | 1114.4357 | 557.7215 | 1113.4517 | 557.2295 | 10 |
| 3 | 300.1554 | 150.5813 | 283.1288 | 142.0681 | | | N | **1060.4252** | 530.7162 | 1043.3986 | **522.2029** | 1042.4146 | **521.7109** | 9 |
| 4 | **401.2031** | 201.1052 | 384.1765 | 192.5919 | 383.1925 | 192.0999 | T | **945.3982** | 473.2027 | 928.3717 | 464.6895 | 927.3877 | 464.1975 | 8 |

| | | | | | | | | | | | | | | |
|---|---|---|---|---|---|---|---|---|---|---|---|---|---|---|
| **5** | 488.2351 | 244.6212 | 471.2085 | 236.1079 | 470.2245 | 235.6159 | **S** | **844.3505** | 422.6789 | 827.3240 | 414.1656 | 826.3400 | 413.6736 | **7** |
| **6** | 635.2705 | 318.1389 | 618.2440 | 309.6256 | 617.2599 | 309.1336 | **M** | **757.3185** | 379.1629 | 740.2920 | 370.6496 | 739.3079 | 370.1576 | **6** |
| **7** | 782.3389 | 391.6731 | 765.3124 | 383.1598 | 764.3284 | 382.6678 | **F** | 610.2831 | 305.6452 | 593.2566 | 297.1319 | 592.2725 | 296.6399 | **5** |
| **8** | 897.3659 | 449.1866 | 880.3393 | 440.6733 | 879.3553 | 440.1813 | **N** | **463.2147** | 232.1110 | 446.1881 | 223.5977 | 445.2041 | 223.1057 | **4** |
| **9** | 1011.4088 | 506.2080 | 994.3822 | 497.6948 | 993.3982 | 497.2027 | **N** | **348.1878** | 174.5975 | 331.1612 | 166.0842 | 330.1772 | 165.5922 | **3** |
| **10** | 1098.4408 | 549.7240 | 1081.4143 | 541.2108 | 1080.4302 | 540.7188 | **S** | **234.1448** | 117.5761 | 217.1183 | 109.0628 | 216.1343 | 108.5708 | **2** |
| **11** | | | | | | | **K** | 147.1128 | 74.0600 | 130.0863 | 65.5468 | | | **1** |

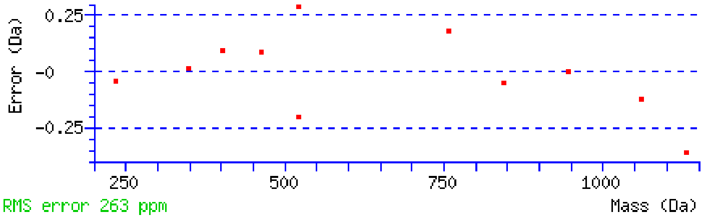


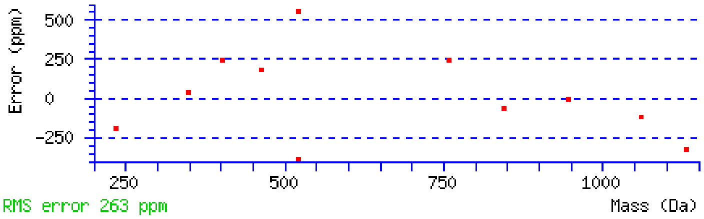


**All matches to this query**

| Score | Mr(calc): | Delta | Sequence |
|---|---|---|---|
| 85.4 | 1243.5390 | -0.0003 | LANTSMFNNSK |
| 65.5 | 1243.5390 | -0.0003 | LANTSMFNNSK |
| 23.7 | 1243.5390 | -0.0003 | LANTSMFNNSK |
| 4.0 | 1243.5485 | -0.0098 | IATEAIENFR |
| 3.5 | 1243.5454 | -0.0066 | ICGEASMKIR |
| 3.2 | 1243.5268 | 0.0120 | LNAMNISETR |
| 0.1 | 1243.5394 | -0.0006 | LAHARGCHSR |

---

Spectrum No: 173; Query: 723; Rank: 1

---

## Peptide View

MS/MS Fragmentation of **EDNEGVFNGSWGGR**
Found in **IPI00208917**, Tax_Id=10116 Gene_Symbol=Aldh7a1 similar to aldehyde dehydrogenase family 7, member A1

Match to Query 723: 1523.624948 from(762.819750,2+)
Title: 091008RatKidney_NoSalt_21.2096.2096.2.dta
Data file K:\NewmanPaper\Piliang\3SubProteomes\Piliang3SP\mgf5ppm\ERLIC_3SubProteomes5ppm.mgf

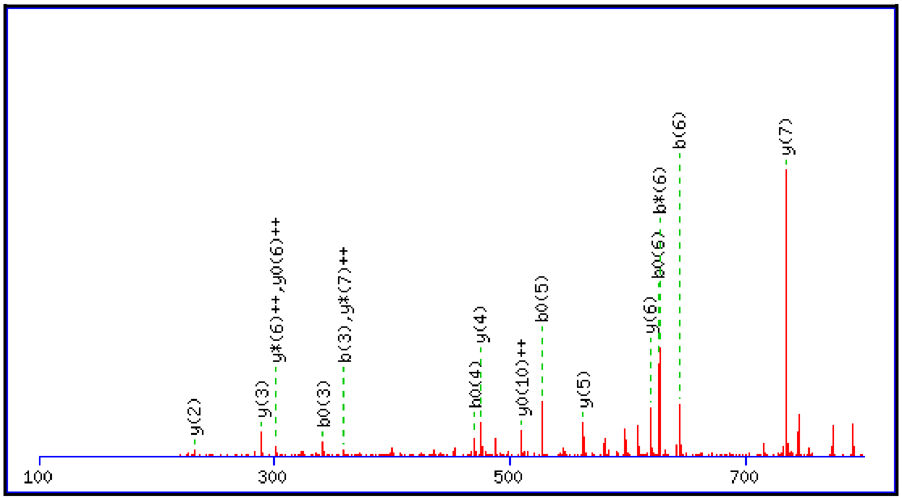

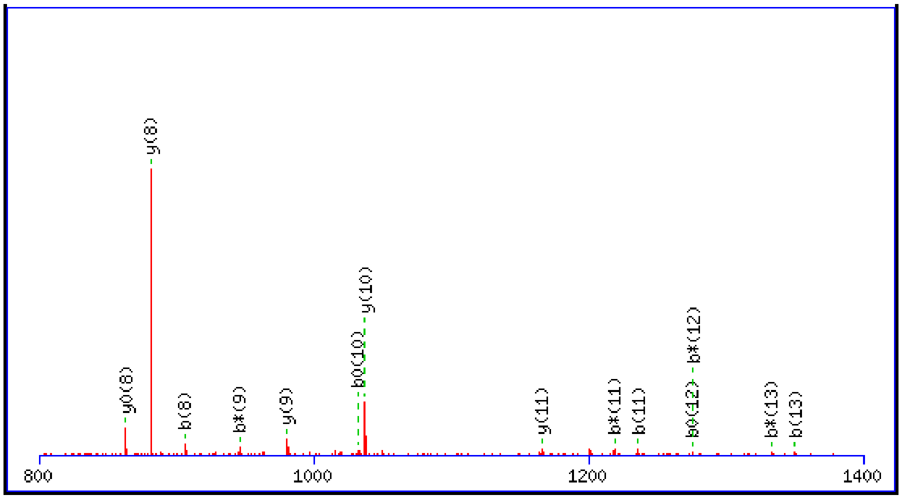


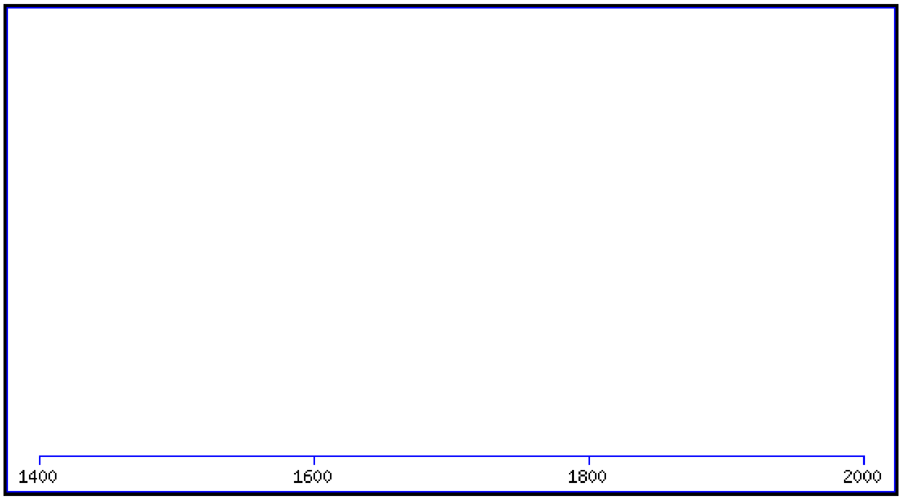


**Monoisotopic mass of neutral peptide Mr(calc):** 1523.6277
**Fixed modifications:** Carbamidomethyl (C)
**Variable modifications:**
**N8** : Deamidated_N (N)
**Ions Score:** 85 **Expect:** 3.9e-007
**Matches (Bold Red):** 31/144 fragment ions using 39 most intense peaks

| # | b | $b^{++}$ | b* | $b^{*++}$ | $b^{0}$ | $b^{0++}$ | Seq. | y | $y^{++}$ | y* | $y^{*++}$ | $y^{0}$ | $y^{0++}$ | # |
|---|---|---|---|---|---|---|---|---|---|---|---|---|---|---|
| 1 | 130.0499 | 65.5286 | | | 112.0393 | 56.5233 | E | | | | | | | 14 |
| 2 | 245.0768 | 123.0420 | | | 227.0662 | 114.0368 | D | 1395.5924 | 698.2998 | 1378.5658 | 689.7866 | 1377.5818 | 689.2945 | 13 |
| 3 | **359.1197** | 180.0635 | 342.0932 | 171.5502 | **341.1092** | 171.0582 | N | 1280.5654 | 640.7864 | 1263.5389 | 632.2731 | 1262.5549 | 631.7811 | 12 |
| 4 | 488.1623 | 244.5848 | 471.1358 | 236.0715 | **470.1518** | 235.5795 | E | **1166.5225** | 583.7649 | 1149.4960 | 575.2516 | 1148.5119 | 574.7596 | 11 |
| 5 | 545.1838 | 273.0955 | 528.1572 | 264.5823 | **527.1732** | 264.0903 | G | **1037.4799** | 519.2436 | 1020.4534 | 510.7303 | 1019.4693 | **510.2383** | 10 |
| 6 | **644.2522** | 322.6297 | **627.2257** | 314.1165 | **626.2416** | 313.6245 | V | **980.4584** | 490.7329 | 963.4319 | 482.2196 | 962.4479 | 481.7276 | 9 |
| 7 | 791.3206 | 396.1640 | 774.2941 | 387.6507 | 773.3101 | 387.1587 | F | **881.3900** | 441.1987 | 864.3635 | 432.6854 | **863.3795** | 432.1934 | 8 |
| 8 | **906.3476** | 453.6774 | 889.3210 | 445.1641 | 888.3370 | 444.6721 | N | **734.3216** | 367.6644 | 717.2951 | **359.1512** | 716.3111 | 358.6592 | 7 |
| 9 | 963.3690 | 482.1882 | **946.3425** | 473.6749 | 945.3585 | 473.1829 | G | **619.2947** | 310.1510 | 602.2681 | **301.6377** | 601.2841 | **301.1457** | 6 |
| 10 | 1050.4011 | 525.7042 | 1033.3745 | 517.1909 | **1032.3905** | 516.6989 | S | **562.2732** | 281.6402 | 545.2467 | 273.1270 | 544.2627 | 272.6350 | 5 |
| 11 | **1236.4804** | 618.7438 | **1219.4538** | 610.2305 | 1218.4698 | 609.7385 | W | **475.2412** | 238.1242 | 458.2146 | 229.6110 | | | 4 |
| 12 | 1293.5018 | 647.2546 | **1276.4753** | 638.7413 | **1275.4913** | 638.2493 | G | **289.1619** | 145.0846 | 272.1353 | 136.5713 | | | 3 |
| 13 | **1350.5233** | 675.7653 | **1333.4967** | 667.2520 | 1332.5127 | 666.7600 | G | **232.1404** | 116.5738 | 215.1139 | 108.0606 | | | 2 |
| 14 | | | | | | | R | 175.1190 | 88.0631 | 158.0924 | 79.5498 | | | 1 |

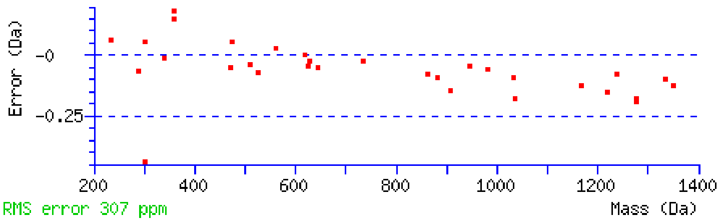


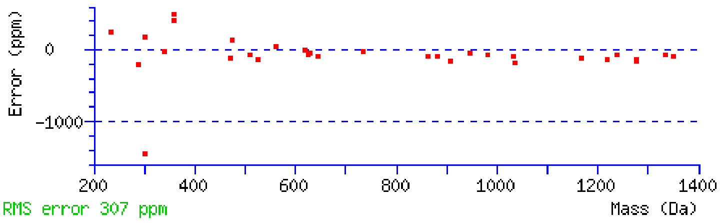


### All matches to this query

| Score | Mr(calc): | Delta | Sequence |
|---|---|---|---|
| 85.2 | 1523.6277 | -0.0028 | EDNEGVFNGSWGGR |
| 15.6 | 1523.6277 | -0.0028 | EDNEGVFNGSWGGR |
| 9.1 | 1523.6365 | -0.0116 | GQSSNAANVQQPSR |
| 8.9 | 1523.6130 | 0.0119 | QRKSGSTSSSPSR |
| 7.5 | 1523.6130 | 0.0119 | QRKSGSTSSSPSR |
| 5.1 | 1523.6365 | -0.0116 | GQSSNAANVQQPSR |
| 3.6 | 1523.6351 | -0.0101 | NGTMTIDWNEWR |
| 3.5 | 1523.6365 | -0.0116 | GQSSNAANVQQPSR |
| 3.3 | 1523.6365 | -0.0116 | GQSSNAANVQQPSR |
| 2.6 | 1521.6137 | 2.0113 | STSYSSPSTXPSR |

---

Spectrum No: 174; Query: 2590; Rank: 1

---

## Peptide View

MS/MS Fragmentation of **RNENFSSGPSFETISASGLNAALAHYSPTK**
Found in **IPI00197684**, Tax_Id=10116 Gene_Symbol=Xpnpep2 Membrane-bound aminopeptidase P

Match to Query 2590: 3153.511662 from(1052.177830,3+)
Title: 100101RatKid_NS_deglyco_13.4729.4729.3.dta
Data file K:\NewmanPaper\Piliang\3SubProteomes\Piliang3SP\mgf5ppm\ERLIC_3SubProteomes5ppm.mgf

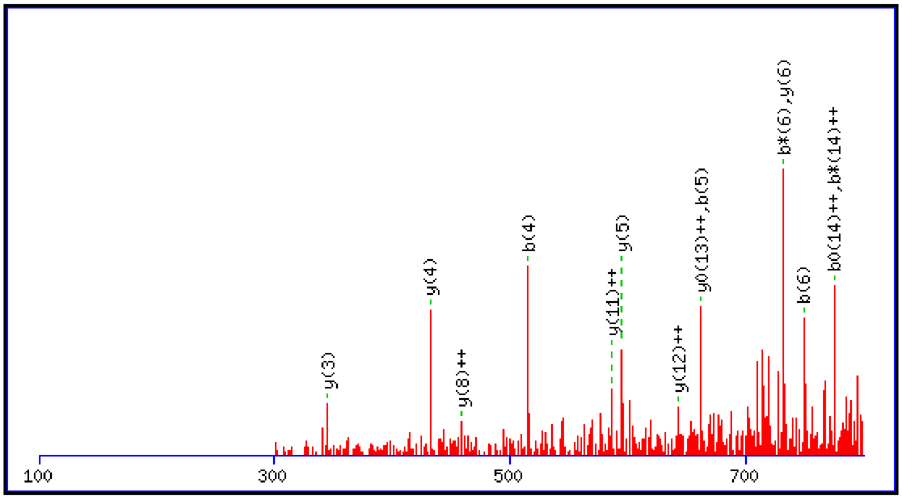

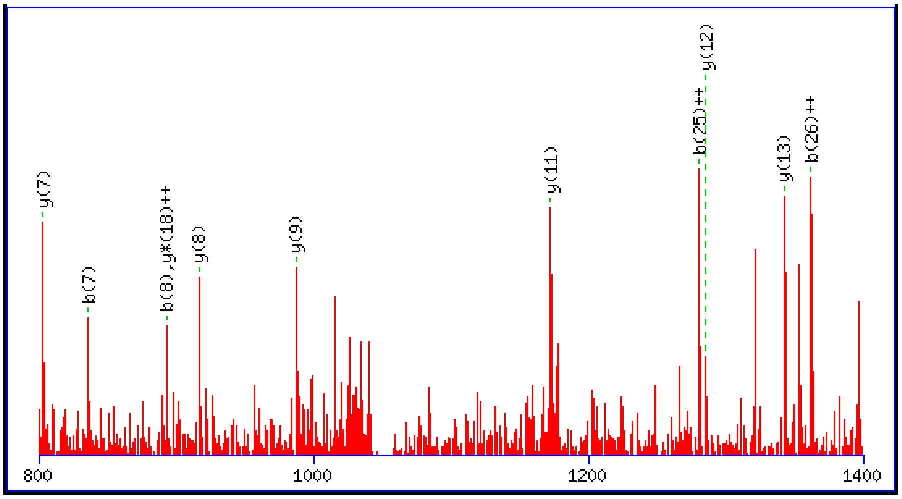


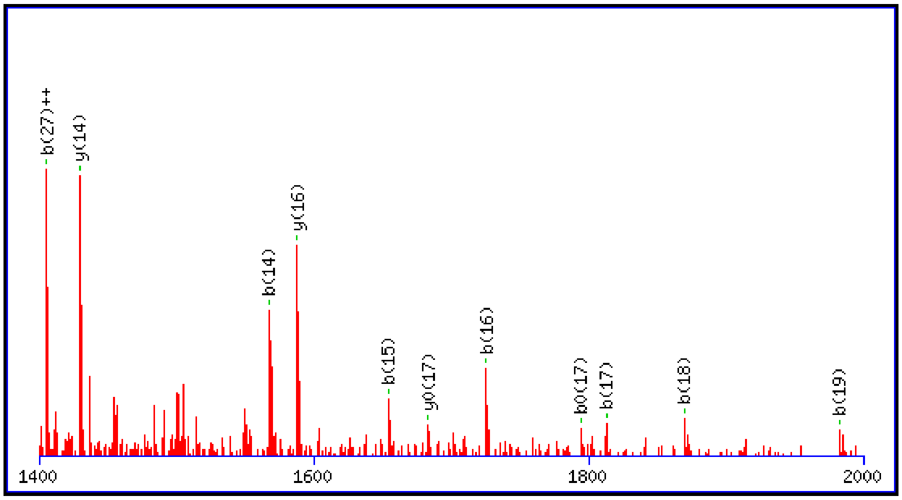


**Monoisotopic mass of neutral peptide Mr(calc):** 3153.5003
**Fixed modifications:** Carbamidomethyl (C)
**Variable modifications:**
**N4** : Deamidated_N (N)
**Ions Score:** 85 **Expect:** 9.9e-007
**Matches (Bold Red):** 36/342 fragment ions using 54 most intense peaks

| # | b | $b^{++}$ | b* | $b^{*++}$ | $b^{0}$ | $b^{0++}$ | Seq. | y | $y^{++}$ | y* | $y^{*++}$ | $y^{0}$ | $y^{0++}$ | # |
|---|---|---|---|---|---|---|---|---|---|---|---|---|---|---|
| 1 | 157.1084 | 79.0578 | 140.0818 | 70.5446 | | | R | | | | | | | 30 |
| 2 | 271.1513 | 136.0793 | 254.1248 | 127.5660 | | | N | 2998.4065 | 1499.7069 | 2981.3799 | 1491.1936 | 2980.3959 | 1490.7016 | 29 |
| 3 | 400.1939 | 200.6006 | 383.1674 | 192.0873 | 382.1833 | 191.5953 | E | 2884.3636 | 1442.6854 | 2867.3370 | 1434.1721 | 2866.3530 | 1433.6801 | 28 |
| 4 | **515.2208** | 258.1141 | 498.1943 | 249.6008 | 497.2103 | 249.1088 | N | 2755.3210 | 1378.1641 | 2738.2944 | 1369.6508 | 2737.3104 | 1369.1588 | 27 |
| 5 | **662.2893** | 331.6483 | 645.2627 | 323.1350 | 644.2787 | 322.6430 | F | 2640.2940 | 1320.6506 | 2623.2675 | 1312.1374 | 2622.2835 | 1311.6454 | 26 |
| 6 | **749.3213** | 375.1643 | **732.2947** | 366.6510 | 731.3107 | 366.1590 | S | 2493.2256 | 1247.1164 | 2476.1991 | 1238.6032 | 2475.2150 | 1238.1112 | 25 |
| 7 | **836.3533** | 418.6803 | 819.3268 | 410.1670 | 818.3427 | 409.6750 | S | 2406.1936 | 1203.6004 | 2389.1670 | 1195.0872 | 2388.1830 | 1194.5951 | 24 |
| 8 | **893.3748** | 447.1910 | 876.3482 | 438.6778 | 875.3642 | 438.1857 | G | 2319.1616 | 1160.0844 | 2302.1350 | 1151.5711 | 2301.1510 | 1151.0791 | 23 |
| 9 | 990.4275 | 495.7174 | 973.4010 | 487.2041 | 972.4170 | 486.7121 | P | 2262.1401 | 1131.5737 | 2245.1135 | 1123.0604 | 2244.1295 | 1122.5684 | 22 |
| 10 | 1077.4596 | 539.2334 | 1060.4330 | 530.7201 | 1059.4490 | 530.2281 | S | 2165.0873 | 1083.0473 | 2148.0608 | 1074.5340 | 2147.0768 | 1074.0420 | 21 |
| 11 | 1224.5280 | 612.7676 | 1207.5014 | 604.2544 | 1206.5174 | 603.7623 | F | 2078.0553 | 1039.5313 | 2061.0287 | 1031.0180 | 2060.0447 | 1030.5260 | 20 |
| 12 | 1353.5706 | 677.2889 | 1336.5440 | 668.7757 | 1335.5600 | 668.2836 | E | 1930.9869 | 965.9971 | 1913.9603 | 957.4838 | 1912.9763 | 956.9918 | 19 |
| 13 | 1454.6183 | 727.8128 | 1437.5917 | 719.2995 | 1436.6077 | 718.8075 | T | 1801.9443 | 901.4758 | 1784.9177 | **892.9625** | 1783.9337 | 892.4705 | 18 |
| 14 | **1567.7023** | 784.3548 | 1550.6758 | **775.8415** | 1549.6918 | **775.3495** | I | 1700.8966 | 850.9519 | 1683.8701 | 842.4387 | **1682.8860** | 841.9467 | 17 |
| 15 | **1654.7343** | 827.8708 | 1637.7078 | 819.3575 | 1636.7238 | 818.8655 | S | **1587.8125** | 794.4099 | 1570.7860 | 785.8966 | 1569.8020 | 785.4046 | 16 |
| 16 | **1725.7715** | 863.3894 | 1708.7449 | 854.8761 | 1707.7609 | 854.3841 | A | 1500.7805 | 750.8939 | 1483.7540 | 742.3806 | 1482.7700 | 741.8886 | 15 |
| 17 | **1812.8035** | 906.9054 | 1795.7769 | 898.3921 | **1794.7929** | 897.9001 | S | **1429.7434** | 715.3753 | 1412.7169 | 706.8621 | 1411.7328 | 706.3701 | 14 |
| 18 | **1869.8250** | 935.4161 | 1852.7984 | 926.9028 | 1851.8144 | 926.4108 | G | **1342.7114** | 671.8593 | 1325.6848 | 663.3461 | 1324.7008 | **662.8540** | 13 |
| | | | | | | | | | | | | | | |

| | | | | | | | | | | | | | | |
|---|---|---|---|---|---|---|---|---|---|---|---|---|---|---|
| **19** | **1982.9090** | 991.9581 | 1965.8825 | 983.4449 | 1964.8985 | 982.9529 | **L** | **1285.6899** | **643.3486** | 1268.6634 | 634.8353 | 1267.6793 | 634.3433 | **12** |
| **20** | 2096.9519 | 1048.9796 | 2079.9254 | 1040.4663 | 2078.9414 | 1039.9743 | **N** | **1172.6058** | **586.8066** | 1155.5793 | 578.2933 | 1154.5953 | 577.8013 | **11** |
| **21** | 2167.9891 | 1084.4982 | 2150.9625 | 1075.9849 | 2149.9785 | 1075.4929 | **A** | 1058.5629 | 529.7851 | 1041.5364 | 521.2718 | 1040.5524 | 520.7798 | **10** |
| **22** | 2239.0262 | 1120.0167 | 2221.9996 | 1111.5034 | 2221.0156 | 1111.0114 | **A** | **987.5258** | 494.2665 | 970.4993 | 485.7533 | 969.5152 | 485.2613 | **9** |
| **23** | 2352.1102 | 1176.5588 | 2335.0837 | 1168.0455 | 2334.0997 | 1167.5535 | **L** | **916.4887** | **458.7480** | 899.4621 | 450.2347 | 898.4781 | 449.7427 | **8** |
| **24** | 2423.1474 | 1212.0773 | 2406.1208 | 1203.5640 | 2405.1368 | 1203.0720 | **A** | **803.4046** | 402.2060 | 786.3781 | 393.6927 | 785.3941 | 393.2007 | **7** |
| **25** | 2560.2063 | **1280.6068** | 2543.1797 | 1272.0935 | 2542.1957 | 1271.6015 | **H** | **732.3675** | 366.6874 | 715.3410 | 358.1741 | 714.3570 | 357.6821 | **6** |
| **26** | 2723.2696 | **1362.1384** | 2706.2430 | 1353.6252 | 2705.2590 | 1353.1332 | **Y** | **595.3086** | 298.1579 | 578.2821 | 289.6447 | 577.2980 | 289.1527 | **5** |
| **27** | 2810.3016 | **1405.6544** | 2793.2751 | 1397.1412 | 2792.2911 | 1396.6492 | **S** | **432.2453** | 216.6263 | 415.2187 | 208.1130 | 414.2347 | 207.6210 | **4** |
| **28** | 2907.3544 | 1454.1808 | 2890.3278 | 1445.6676 | 2889.3438 | 1445.1755 | **P** | **345.2132** | 173.1103 | 328.1867 | 164.5970 | 327.2027 | 164.1050 | **3** |
| **29** | 3008.4021 | 1504.7047 | 2991.3755 | 1496.1914 | 2990.3915 | 1495.6994 | **T** | 248.1605 | 124.5839 | 231.1339 | 116.0706 | 230.1499 | 115.5786 | **2** |
| **30** | | | | | | | **K** | 147.1128 | 74.0600 | 130.0863 | 65.5468 | | | **1** |

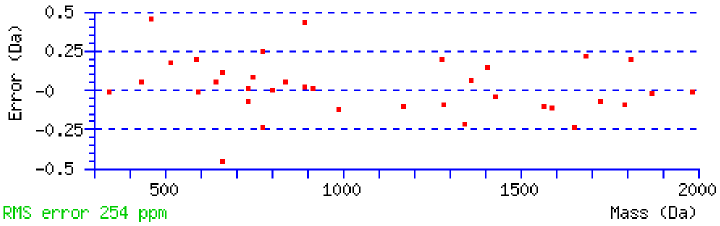


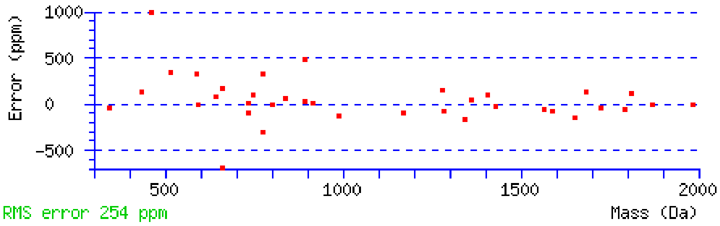


### All matches to this query

| Score | Mr(calc): | Delta | Sequence |
|---|---|---|---|
| 85.2 | 3153.5003 | 0.0114 | RNENFSSGPSFETISASGLNAALAHYSPTK |
| 85.2 | 3153.5003 | 0.0114 | RNENFSSGPSFETISASGLNAALAHYSPTK |
| 39.5 | 3153.5003 | 0.0114 | RNENFSSGPSFETISASGLNAALAHYSPTK |
| 39.3 | 3152.5163 | 0.9954 | RNENFSSGPSFETISASGLNAALAHYSPTK |
| 0.9 | 3152.4930 | 1.0186 | EAASLTAAEPAPTKSIIAADSLTAAEPAPTK |
| 0.4 | 3152.4807 | 1.0309 | TFSWASVTSKNLPPSGAVPVTGTPPHMVK |
| 0.1 | 3151.5278 | 1.9839 | LSLRYTNKXRPLEEQSVSEVGCCSPVK |
| 0.1 | 3151.5278 | 1.9839 | LSLRYTNKXRPLEEQSVSEVGCCSPVK |

---

Spectrum No: 175; Query: 535; Rank: 1

---

## Peptide View

MS/MS Fragmentation of **IIYSIVQTNCSK**
Found in **IPI00327182**, Tax_Id=10116 Gene_Symbol=Kng1 T-kininogen 1 precursor

Match to Query 535: 1425.721968 from(713.868260,2+)
Title: 091008RatKidney_NoSalt_37.1417.1417.2.dta
Data file K:\NewmanPaper\Piliang\3SubProteomes\Piliang3SP\mgf5ppm\ERLIC_3SubProteomes5ppm.mgf

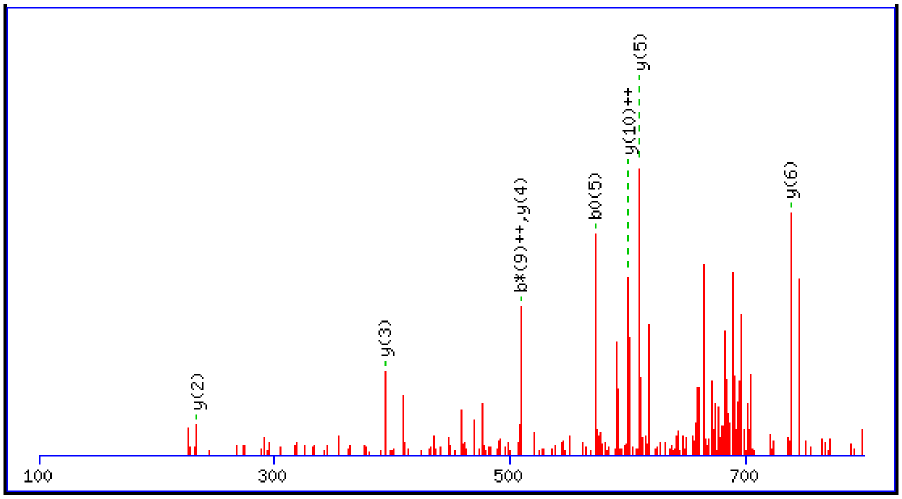


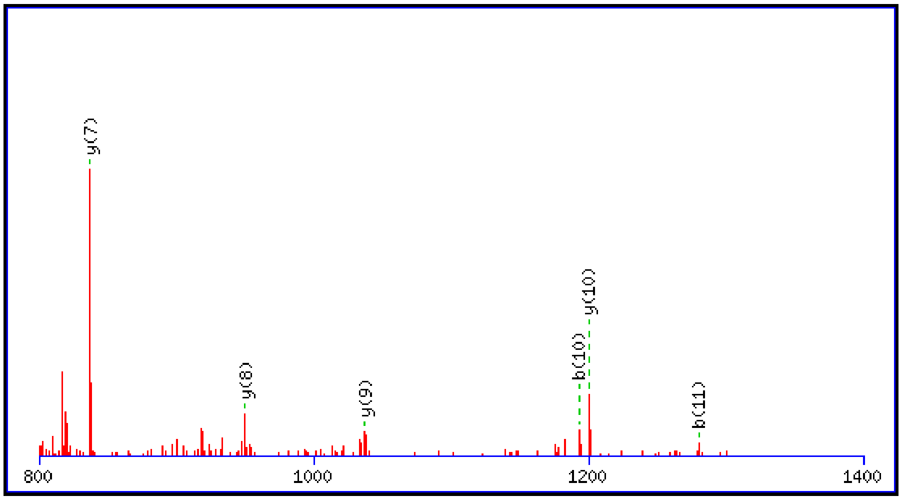


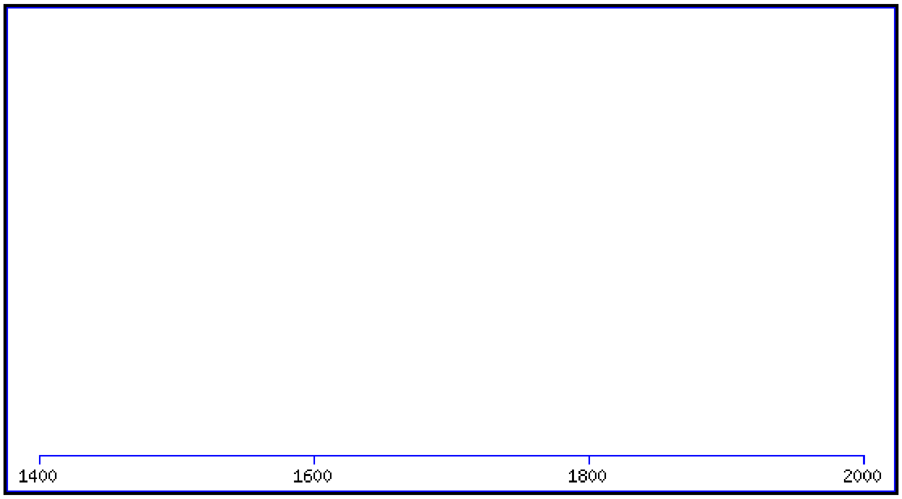


**Monoisotopic mass of neutral peptide Mr(calc):** 1425.7173
**Fixed modifications:** Carbamidomethyl (C)
**Variable modifications:**
**N9** : Deamidated_N (N)
**Ions Score:** 85 **Expect:** 3.9e-007
**Matches (Bold Red):** 14/112 fragment ions using 16 most intense peaks

| # | b | $b^{++}$ | $b^{*}$ | $b^{*++}$ | $b^{0}$ | $b^{0++}$ | Seq. | y | $y^{++}$ | $y^{*}$ | $y^{*++}$ | $y^{0}$ | $y^{0++}$ | # |
|---|---|---|---|---|---|---|---|---|---|---|---|---|---|---|
| 1 | 114.0913 | 57.5493 | | | | | I | | | | | | | 12 |
| 2 | 227.1754 | 114.0913 | | | | | I | 1313.6406 | 657.3239 | 1296.6140 | 648.8107 | 1295.6300 | 648.3186 | 11 |
| 3 | 390.2387 | 195.6230 | | | | | Y | **1200.5565** | **600.7819** | 1183.5300 | 592.2686 | 1182.5459 | 591.7766 | 10 |
| 4 | 477.2708 | 239.1390 | | | 459.2602 | 230.1337 | S | **1037.4932** | 519.2502 | 1020.4666 | 510.7370 | 1019.4826 | 510.2449 | 9 |
| 5 | 590.3548 | 295.6811 | | | **572.3443** | 286.6758 | I | **950.4612** | 475.7342 | 933.4346 | 467.2209 | 932.4506 | 466.7289 | 8 |

| | | | | | | | | | | | | | |
|---|---|---|---|---|---|---|---|---|---|---|---|---|---|
| 6 | 689.4232 | 345.2153 | | | 671.4127 | 336.2100 | V | **837.3771** | 419.1922 | 820.3505 | 410.6789 | 819.3665 | 410.1869 | 7 |
| 7 | 817.4818 | 409.2445 | 800.4553 | 400.7313 | 799.4713 | 400.2393 | Q | **738.3087** | 369.6580 | 721.2821 | 361.1447 | 720.2981 | 360.6527 | 6 |
| 8 | 918.5295 | 459.7684 | 901.5029 | 451.2551 | 900.5189 | 450.7631 | T | **610.2501** | 305.6287 | 593.2235 | 297.1154 | 592.2395 | 296.6234 | 5 |
| 9 | 1033.5564 | 517.2819 | 1016.5299 | **508.7686** | 1015.5459 | 508.2766 | N | **509.2024** | 255.1048 | 492.1759 | 246.5916 | 491.1919 | 246.0996 | 4 |
| 10 | **1193.5871** | 597.2972 | 1176.5605 | 588.7839 | 1175.5765 | 588.2919 | C | **394.1755** | 197.5914 | 377.1489 | 189.0781 | 376.1649 | 188.5861 | 3 |
| 11 | **1280.6191** | 640.8132 | 1263.5926 | 632.2999 | 1262.6085 | 631.8079 | S | **234.1448** | 117.5761 | 217.1183 | 109.0628 | 216.1343 | 108.5708 | 2 |
| 12 | | | | | | | K | 147.1128 | 74.0600 | 130.0863 | 65.5468 | | | 1 |

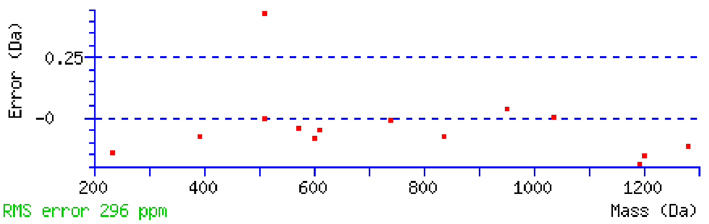


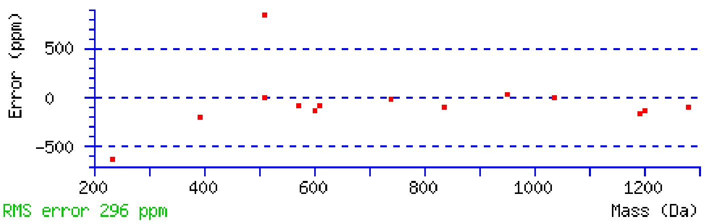


### All matches to this query

| Score | Mr(calc): | Delta | Sequence |
|---|---|---|---|
| 84.8 | 1425.7173 | 0.0046 | IIYSIVQTNCSK |
| 12.1 | 1424.7177 | 1.0043 | RSIWDVISKNK |
| 11.6 | 1423.7194 | 2.0025 | TYEDQLSEAKIK |
| 4.3 | 1425.7228 | -0.0009 | KPSKSEAKGTVSK |
| 1.4 | 1424.7201 | 1.0019 | GFPPNPNILDWR |
| 1.2 | 1423.7130 | 2.0090 | LNSCLVYVDVSR |
| 1.0 | 1425.7228 | -0.0009 | KPSKSEAKGTVSK |
| 0.9 | 1424.7115 | 1.0104 | KMTLSLADRCSK |
| 0.5 | 1425.7228 | -0.0009 | SLLSLLQSRDSK |

---

Spectrum No: 176; Query: 699; Rank: 1

---

## Peptide View

MS/MS Fragmentation of **FINDYVSNQTQGK**
Found in **IPI00200593**, Tax_Id=10116 Gene_Symbol=Serpina3k Serine protease inhibitor A3K precursor

Match to Query 699: 1513.706228 from(757.860390,2+)
Title: 091008RatKidney_NoSalt_34.1214.1214.2.dta
Data file K:\NewmanPaper\Piliang\3SubProteomes\Piliang3SP\mgf5ppm\ERLIC_3SubProteomes5ppm.mgf

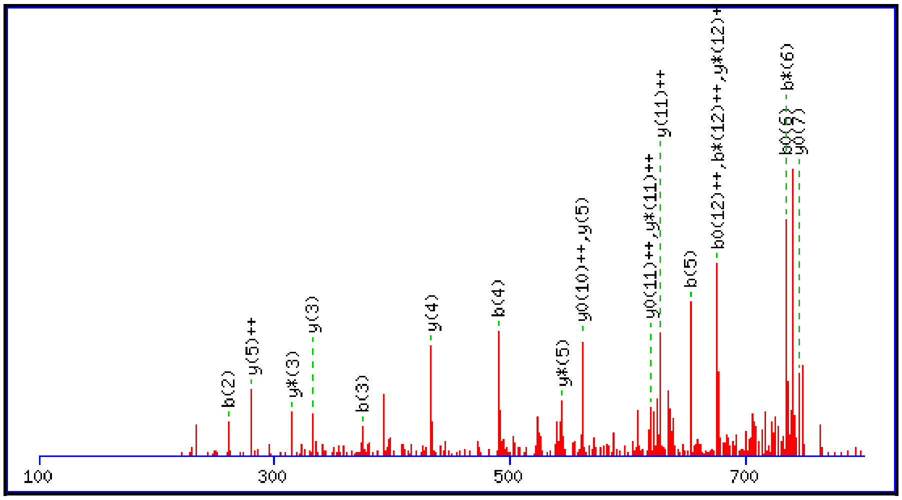

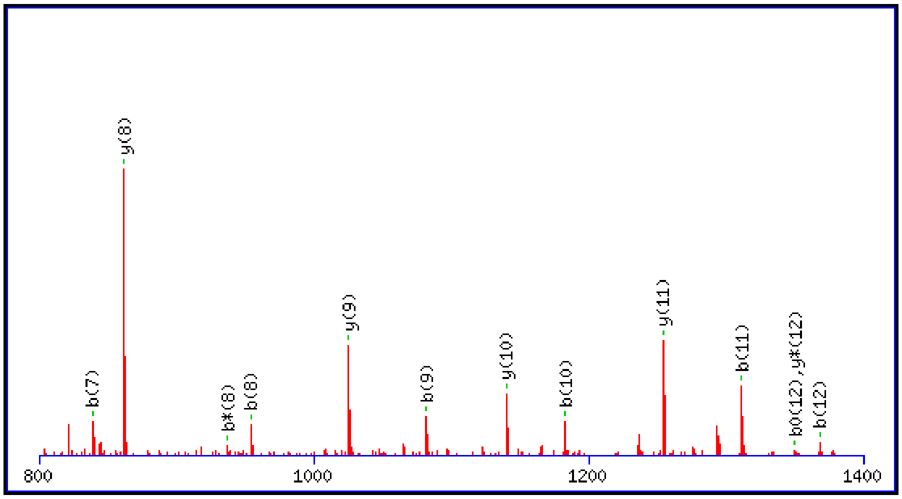


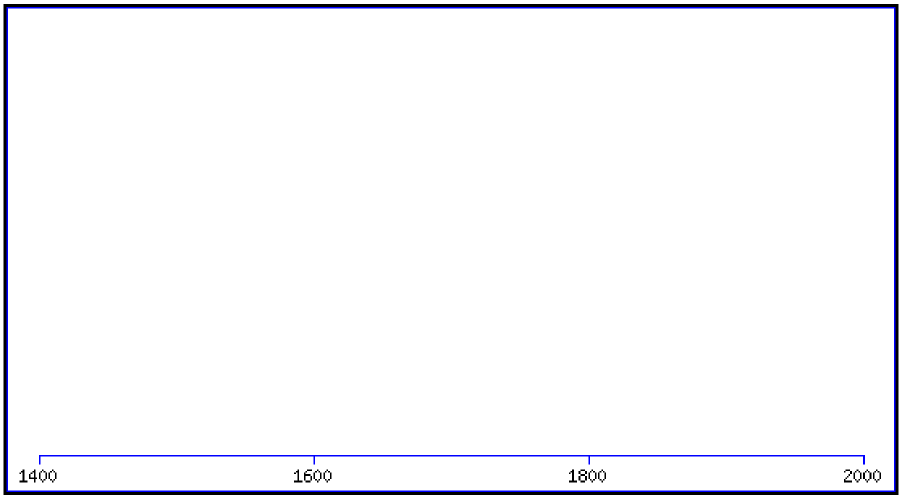


**Monoisotopic mass of neutral peptide Mr(calc):** 1513.7049
**Fixed modifications:** Carbamidomethyl (C)
**Variable modifications:**
**N8** : Deamidated_N (N)
**Ions Score:** 85 **Expect:** 5.4e-007
**Matches (Bold Red):** 34/128 fragment ions using 42 most intense peaks

| # | b | $b^{++}$ | b* | $b^{*++}$ | $b^0$ | $b^{0++}$ | Seq. | y | $y^{++}$ | y* | $y^{*++}$ | $y^0$ | $y^{0++}$ | # |
|---|---|---|---|---|---|---|---|---|---|---|---|---|---|---|
| 1 | 148.0757 | 74.5415 | | | | | F | | | | | | | 13 |
| 2 | **261.1598** | 131.0835 | | | | | I | 1367.6437 | 684.3255 | **1350.6172** | **675.8122** | 1349.6332 | 675.3202 | 12 |
| 3 | **375.2027** | 188.1050 | 358.1761 | 179.5917 | | | N | **1254.5597** | **627.7835** | 1237.5331 | **619.2702** | 1236.5491 | **618.7782** | 11 |
| 4 | **490.2296** | 245.6185 | 473.2031 | 237.1052 | 472.2191 | 236.6132 | D | **1140.5168** | 570.7620 | 1123.4902 | 562.2487 | 1122.5062 | **561.7567** | 10 |
| 5 | **653.2930** | 327.1501 | 636.2664 | 318.6368 | 635.2824 | 318.1448 | Y | **1025.4898** | 513.2485 | 1008.4633 | 504.7353 | 1007.4792 | 504.2433 | 9 |
| 6 | 752.3614 | 376.6843 | **735.3348** | 368.1710 | **734.3508** | 367.6790 | V | **862.4265** | 431.7169 | 845.3999 | 423.2036 | 844.4159 | 422.7116 | 8 |
| 7 | **839.3934** | 420.2003 | 822.3668 | 411.6871 | 821.3828 | 411.1951 | S | 763.3581 | 382.1827 | 746.3315 | 373.6694 | **745.3475** | 373.1774 | 7 |
| 8 | **954.4203** | 477.7138 | **937.3938** | 469.2005 | 936.4098 | 468.7085 | N | **676.3260** | 338.6667 | 659.2995 | 330.1534 | 658.3155 | 329.6614 | 6 |
| 9 | **1082.4789** | 541.7431 | 1065.4524 | 533.2298 | 1064.4683 | 532.7378 | Q | **561.2991** | **281.1532** | **544.2726** | 272.6399 | 543.2885 | 272.1479 | 5 |
| 10 | **1183.5266** | 592.2669 | 1166.5000 | 583.7537 | 1165.5160 | 583.2616 | T | **433.2405** | 217.1239 | 416.2140 | 208.6106 | 415.2300 | 208.1186 | 4 |
| 11 | **1311.5852** | 656.2962 | 1294.5586 | 647.7829 | 1293.5746 | 647.2909 | Q | **332.1928** | 166.6001 | **315.1663** | 158.0868 | | | 3 |
| 12 | **1368.6066** | 684.8070 | 1351.5801 | **676.2937** | **1350.5961** | **675.8017** | G | 204.1343 | 102.5708 | 187.1077 | 94.0575 | | | 2 |
| 13 | | | | | | | K | 147.1128 | 74.0600 | 130.0863 | 65.5468 | | | 1 |

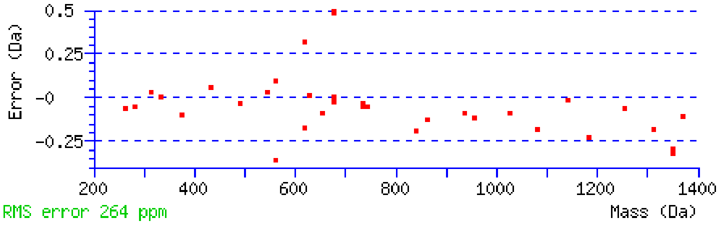


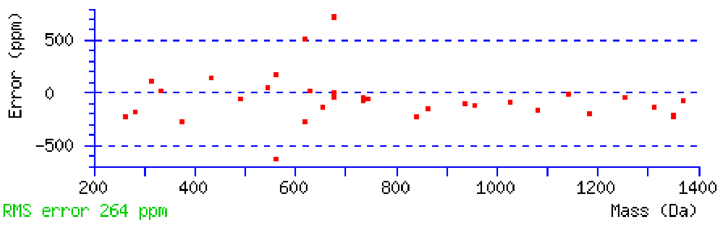


### All matches to this query

| Score | Mr(calc): | Delta | Sequence |
|---|---|---|---|
| 84.7 | 1513.7049 | 0.0013 | FINDYVSNQTQGK |
| 36.8 | 1513.7049 | 0.0013 | FINDYVSNQTQGK |
| 11.9 | 1513.7153 | -0.0090 | FLNWIKTTMHK |
| 11.9 | 1513.7153 | -0.0090 | FLNWIKTTMHK |
| 11.9 | 1513.7057 | 0.0005 | AMRMADFWLTEK |
| 10.9 | 1513.7211 | -0.0149 | KISCTNANLVKGK |
| 9.2 | 1513.7137 | -0.0075 | NVKGTQASKSTQGK |
| 7.2 | 1513.7055 | 0.0007 | DEIRHMRANAER |
| 7.1 | 1513.6983 | 0.0079 | KGNSWMGLSYNNK |
| 6.7 | 1511.7072 | 1.9990 | TETTKDMGRCLGK |

---

Spectrum No: 177; Query: 1351; Rank: 1

---

## Peptide View

MS/MS Fragmentation of **ANVQFGENGTTISAVTNK**
Found in **IPI00231478**, Tax_Id=10116 Gene_Symbol=Scarb2 Lysosome membrane protein 2

Match to Query 1351: 1850.907328 from(926.460940,2+)
Title: 100101RatKid_NS_deglyco_23.2492.2492.2.dta
Data file K:\NewmanPaper\Piliang\3SubProteomes\Piliang3SP\mgf5ppm\ERLIC_3SubProteomes5ppm.mgf

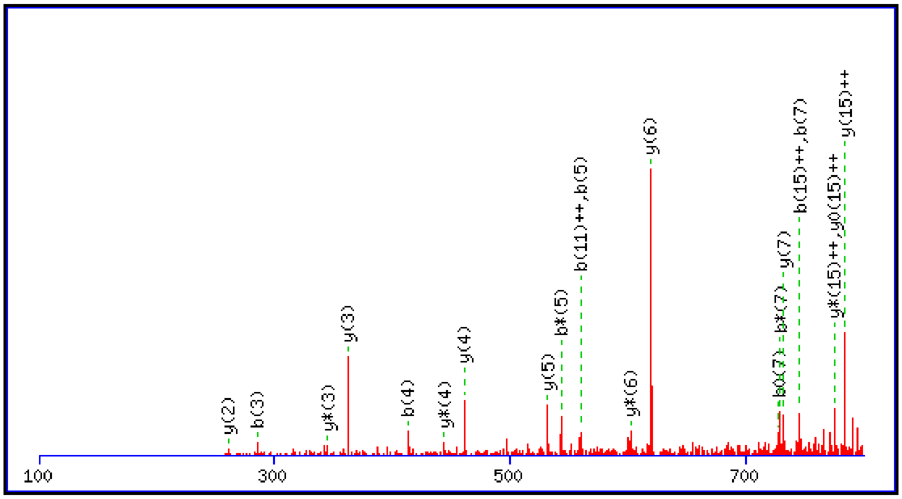

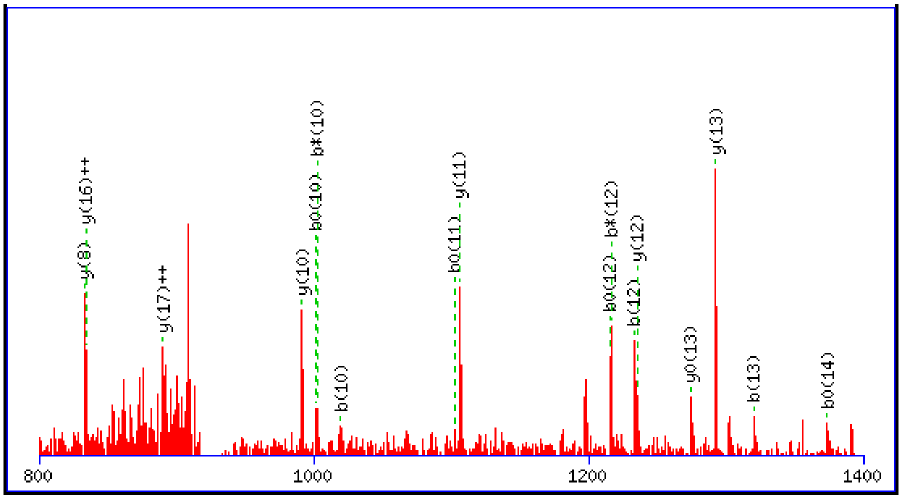


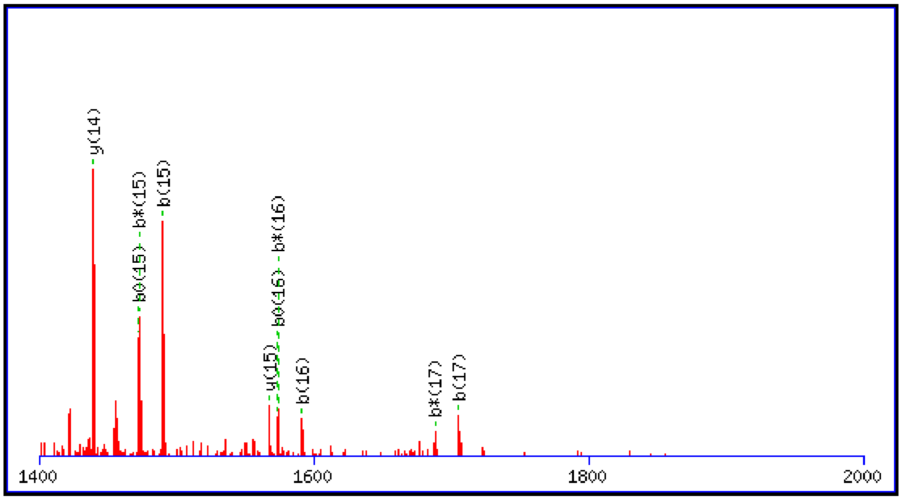


**Monoisotopic mass of neutral peptide Mr(calc):** 1850.9010
**Fixed modifications:** Carbamidomethyl (C)
**Variable modifications:**
**N8** : Deamidated_N (N)
**Ions Score:** 85 **Expect:** 7.5e-007
**Matches (Bold Red):** 48/186 fragment ions using 75 most intense peaks

| # | b | $b^{++}$ | b* | $b^{*++}$ | $b^{0}$ | $b^{0++}$ | Seq. | y | $y^{++}$ | y* | $y^{*++}$ | $y^{0}$ | $y^{0++}$ | # |
|---|---|---|---|---|---|---|---|---|---|---|---|---|---|---|
| 1 | 72.0444 | 36.5258 | | | | | A | | | | | | | 18 |
| 2 | 186.0873 | 93.5473 | 169.0608 | 85.0340 | | | N | 1780.8712 | **890.9392** | 1763.8446 | 882.4260 | 1762.8606 | 881.9339 | 17 |
| 3 | **285.1557** | 143.0815 | 268.1292 | 134.5682 | | | V | 1666.8283 | **833.9178** | 1649.8017 | 825.4045 | 1648.8177 | 824.9125 | 16 |
| 4 | **413.2143** | 207.1108 | 396.1878 | 198.5975 | | | Q | **1567.7598** | **784.3836** | 1550.7333 | **775.8703** | 1549.7493 | **775.3783** | 15 |
| 5 | **560.2827** | 280.6450 | **543.2562** | 272.1317 | | | F | **1439.7013** | 720.3543 | 1422.6747 | 711.8410 | 1421.6907 | 711.3490 | 14 |
| 6 | 617.3042 | 309.1557 | 600.2776 | 300.6425 | | | G | **1292.6328** | 646.8201 | 1275.6063 | 638.3068 | **1274.6223** | 637.8148 | 13 |
| 7 | **746.3468** | 373.6770 | **729.3202** | 365.1638 | **728.3362** | 364.6717 | E | **1235.6114** | 618.3093 | 1218.5848 | 609.7961 | 1217.6008 | 609.3040 | 12 |
| 8 | 861.3737 | 431.1905 | 844.3472 | 422.6772 | 843.3632 | 422.1852 | N | **1106.5688** | 553.7880 | 1089.5422 | 545.2748 | 1088.5582 | 544.7827 | 11 |
| 9 | 918.3952 | 459.7012 | 901.3686 | 451.1880 | 900.3846 | 450.6959 | G | **991.5419** | 496.2746 | 974.5153 | 487.7613 | 973.5313 | 487.2693 | 10 |
| 10 | **1019.4429** | 510.2251 | **1002.4163** | 501.7118 | **1001.4323** | 501.2198 | T | 934.5204 | 467.7638 | 917.4938 | 459.2506 | 916.5098 | 458.7585 | 9 |
| 11 | 1120.4905 | **560.7489** | 1103.4640 | 552.2356 | **1102.4800** | 551.7436 | T | **833.4727** | 417.2400 | 816.4462 | 408.7267 | 815.4621 | 408.2347 | 8 |
| 12 | **1233.5746** | 617.2909 | **1216.5481** | 608.7777 | **1215.5640** | 608.2857 | I | **732.4250** | 366.7162 | 715.3985 | 358.2029 | 714.4145 | 357.7109 | 7 |
| 13 | **1320.6066** | 660.8070 | 1303.5801 | 652.2937 | 1302.5961 | 651.8017 | S | **619.3410** | 310.1741 | **602.3144** | 301.6608 | 601.3304 | 301.1688 | 6 |
| 14 | 1391.6437 | 696.3255 | 1374.6172 | 687.8122 | **1373.6332** | 687.3202 | A | **532.3089** | 266.6581 | 515.2824 | 258.1448 | 514.2984 | 257.6528 | 5 |
| 15 | **1490.7122** | **745.8597** | **1473.6856** | 737.3464 | **1472.7016** | 736.8544 | V | **461.2718** | 231.1396 | **444.2453** | 222.6263 | 443.2613 | 222.1343 | 4 |
| 16 | **1591.7598** | 796.3836 | **1574.7333** | 787.8703 | **1573.7493** | 787.3783 | T | **362.2034** | 181.6053 | **345.1769** | 173.0921 | 344.1928 | 172.6001 | 3 |
| 17 | **1705.8028** | 853.4050 | **1688.7762** | 844.8917 | 1687.7922 | 844.3997 | N | **261.1557** | 131.0815 | 244.1292 | 122.5682 | | | 2 |
| 18 | | | | | | | K | 147.1128 | 74.0600 | 130.0863 | 65.5468 | | | 1 |

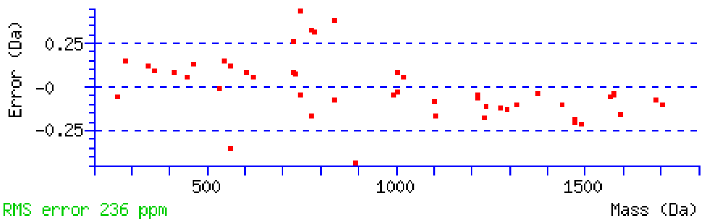


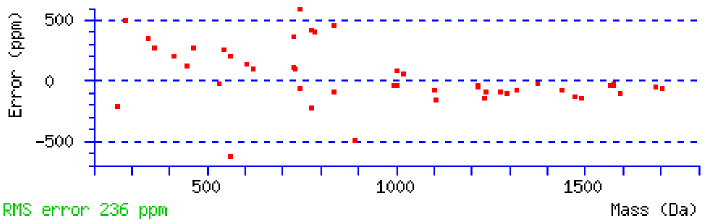


### All matches to this query

| Score | Mr(calc): | Delta | Sequence |
|---|---|---|---|
| 84.5 | 1850.9010 | 0.0063 | ANVQFGENGTTISAVTNK |
| 40.7 | 1850.9010 | 0.0063 | ANVQFGENGTTISAVTNK |
| 36.6 | 1850.9010 | 0.0063 | ANVQFGENGTTISAVTNK |
| 32.1 | 1849.9170 | 0.9903 | ANVQFGENGTTISAVTNK |
| 2.3 | 1849.9138 | 0.9936 | SQSKITRLITAVVMK |
| 0.6 | 1849.8905 | 1.0168 | ADNDDSLLTDISSSKLR |

---

Spectrum No: 178; Query: 1359; Rank: 1

---

## Peptide View

MS/MS Fragmentation of **DGSQNFNQTWENYEK**
Found in **IPI00198903**, Tax_Id=10116 Gene_Symbol=Angptl3 Angiopoietin-like 3

Match to Query 1359: 1859.762548 from(930.888550,2+)
Title: 100101RatKid_NS_deglyco_26.2770.2770.2.dta
Data file K:\NewmanPaper\Piliang\3SubProteomes\Piliang3SP\mgf5ppm\ERLIC_3SubProteomes5ppm.mgf

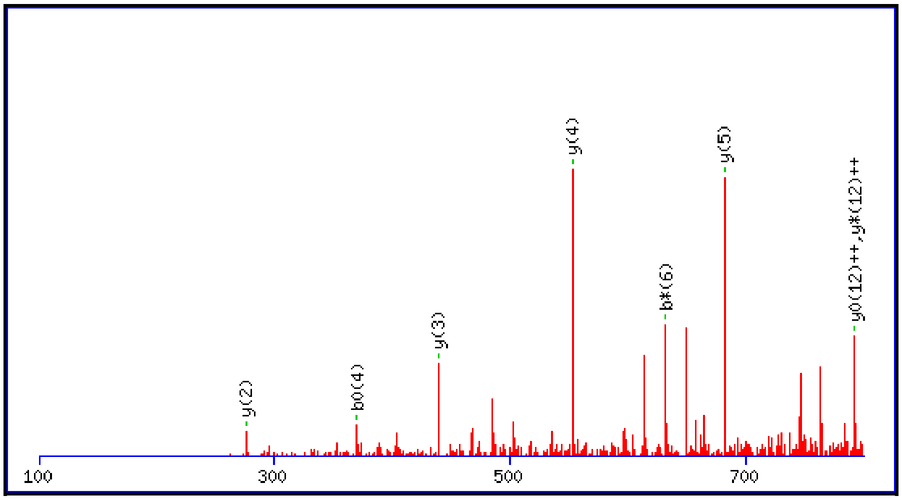

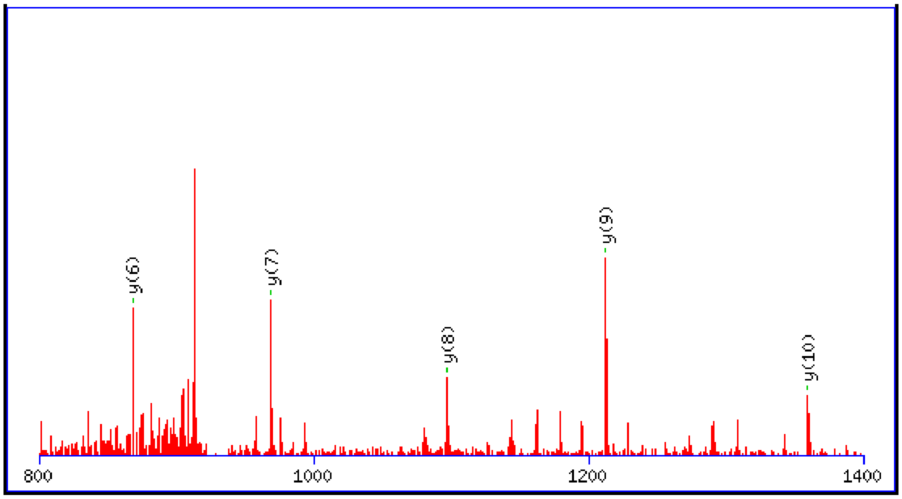


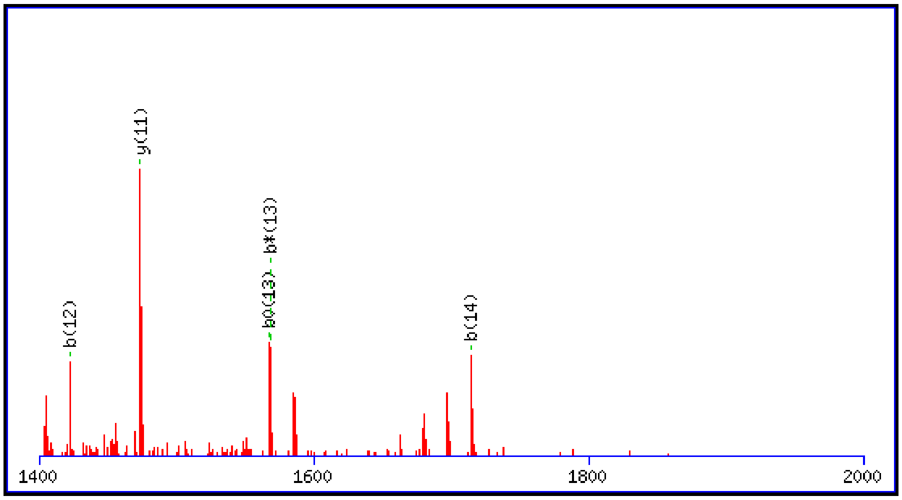


**Monoisotopic mass of neutral peptide Mr(calc):** 1859.7598
**Fixed modifications:** Carbamidomethyl (C)
**Variable modifications:**
**N7** : Deamidated_N (N)
**Ions Score:** 85 **Expect:** 6.3e-007
**Matches (Bold Red):** 18/160 fragment ions using 31 most intense peaks

| # | b | b++ | b* | b*++ | b0 | b0++ | Seq. | y | y++ | y* | y*++ | y0 | y0++ | # |
|---|---|---|---|---|---|---|---|---|---|---|---|---|---|---|
| 1 | 116.0342 | 58.5207 | | | 98.0237 | 49.5155 | D | | | | | | | 15 |
| 2 | 173.0557 | 87.0315 | | | 155.0451 | 78.0262 | G | 1745.7402 | 873.3737 | 1728.7136 | 864.8604 | 1727.7296 | 864.3684 | 14 |
| 3 | 260.0877 | 130.5475 | | | 242.0771 | 121.5422 | S | 1688.7187 | 844.8630 | 1671.6922 | 836.3497 | 1670.7081 | 835.8577 | 13 |
| 4 | 388.1463 | 194.5768 | 371.1197 | 186.0635 | **370.1357** | 185.5715 | Q | 1601.6867 | 801.3470 | 1584.6601 | **792.8337** | 1583.6761 | **792.3417** | 12 |
| 5 | 502.1892 | 251.5982 | 485.1627 | 243.0850 | 484.1787 | 242.5930 | N | **1473.6281** | 737.3177 | 1456.6015 | 728.8044 | 1455.6175 | 728.3124 | 11 |
| 6 | 649.2576 | 325.1325 | **632.2311** | 316.6192 | 631.2471 | 316.1272 | F | **1359.5852** | 680.2962 | 1342.5586 | 671.7829 | 1341.5746 | 671.2909 | 10 |
| 7 | 764.2846 | 382.6459 | 747.2580 | 374.1326 | 746.2740 | 373.6406 | N | **1212.5168** | 606.7620 | 1195.4902 | 598.2487 | 1194.5062 | 597.7567 | 9 |
| 8 | 892.3431 | 446.6752 | 875.3166 | 438.1619 | 874.3326 | 437.6699 | Q | **1097.4898** | 549.2485 | 1080.4633 | 540.7353 | 1079.4793 | 540.2433 | 8 |
| 9 | 993.3908 | 497.1990 | 976.3643 | 488.6858 | 975.3803 | 488.1938 | T | **969.4312** | 485.2193 | 952.4047 | 476.7060 | 951.4207 | 476.2140 | 7 |
| 10 | 1179.4701 | 590.2387 | 1162.4436 | 581.7254 | 1161.4596 | 581.2334 | W | **868.3836** | 434.6954 | 851.3570 | 426.1821 | 850.3730 | 425.6901 | 6 |
| 11 | 1308.5127 | 654.7600 | 1291.4862 | 646.2467 | 1290.5022 | 645.7547 | E | **682.3042** | 341.6558 | 665.2777 | 333.1425 | 664.2937 | 332.6505 | 5 |
| 12 | **1422.5557** | 711.7815 | 1405.5291 | 703.2682 | 1404.5451 | 702.7762 | N | **553.2617** | 277.1345 | 536.2351 | 268.6212 | 535.2511 | 268.1292 | 4 |
| 13 | 1585.6190 | 793.3131 | **1568.5924** | 784.7999 | **1567.6084** | 784.3078 | Y | **439.2187** | 220.1130 | 422.1922 | 211.5997 | 421.2082 | 211.1077 | 3 |
| 14 | **1714.6616** | 857.8344 | 1697.6350 | 849.3212 | 1696.6510 | 848.8291 | E | **276.1554** | 138.5813 | 259.1288 | 130.0681 | 258.1448 | 129.5761 | 2 |
| 15 | | | | | | | K | 147.1128 | 74.0600 | 130.0863 | 65.5468 | | | 1 |

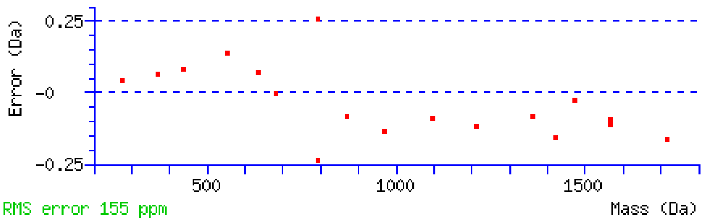


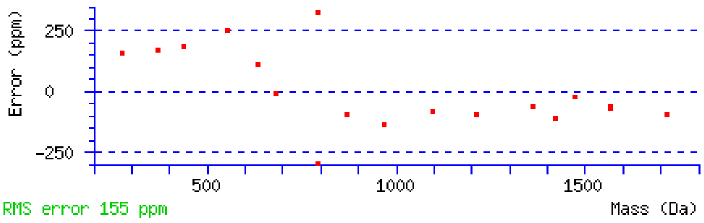


### All matches to this query

| Score | Mr(calc): | Delta | Sequence |
|---|---|---|---|
| 84.5 | 1859.7598 | 0.0027 | DGSQNFNQTWENYEK |
| 51.6 | 1859.7598 | 0.0027 | DGSQNFNQTWENYEK |
| 43.7 | 1859.7598 | 0.0027 | DGSQNFNQTWENYEK |
| 23.3 | 1858.7758 | 0.9867 | DGSQNFNQTWENYEK |
| 8.0 | 1859.7484 | 0.0141 | QACFASCSGYFAXRK |
| 3.7 | 1859.7808 | -0.0182 | MYQGHMQGKGSRAADK |
| 2.8 | 1859.7743 | -0.0118 | GSDIFEYNSKGLLTR |
| 2.3 | 1858.7498 | 1.0127 | AWENGEGGSWIWXTR |
| 1.8 | 1859.7509 | 0.0116 | DSSMNNLKSYPXSHR |
| 1.3 | 1859.7526 | 0.0100 | EDCNVHSAASILASVK |

---

Spectrum No: 179; Query: 824; Rank: 1

---

## Peptide View

MS/MS Fragmentation of **MIENGSLSFLPTLR**
Found in **IPI00191090**, Tax_Id=10116 Gene_Symbol=Bgn Biglycan precursor

Match to Query 824: 1577.818168 from(789.916360,2+)
Title: 100101RatKid_NS_deglyco_19.4623.4623.2.dta
Data file K:\NewmanPaper\Piliang\3SubProteomes\Piliang3SP\mgf5ppm\ERLIC_3SubProteomes5ppm.mgf

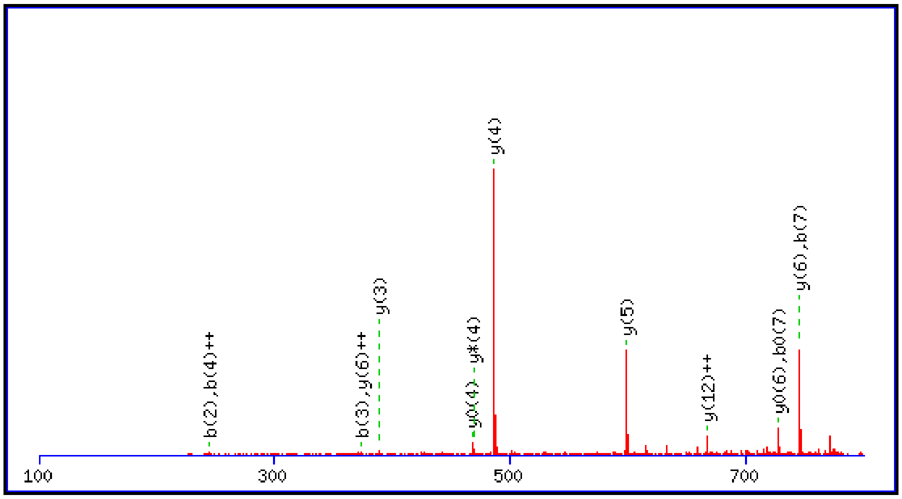

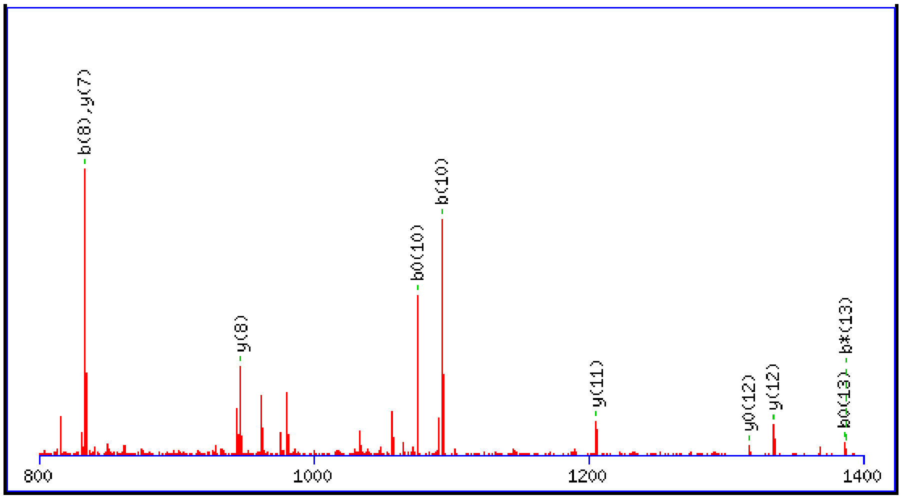


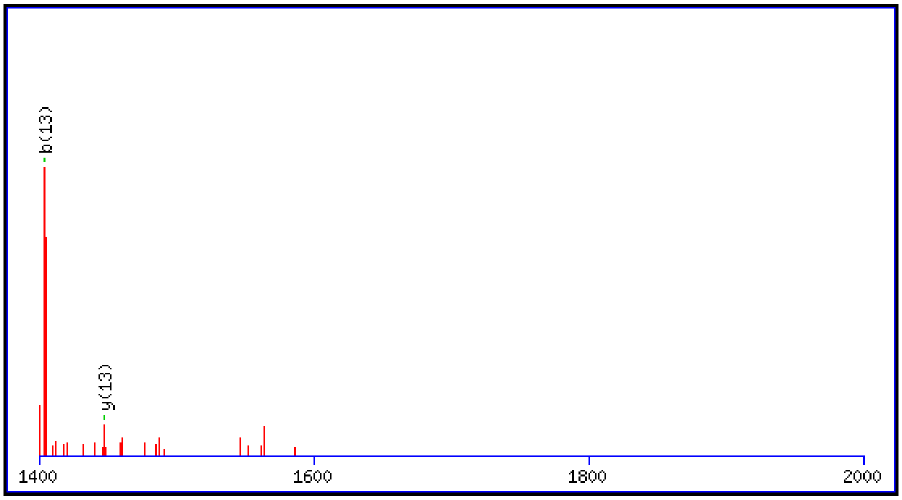


**Monoisotopic mass of neutral peptide Mr(calc):** 1577.8123
**Fixed modifications:** Carbamidomethyl (C)
**Variable modifications:**
**N4** : Deamidated_N (N)
**Ions Score:** 84 **Expect:** 5.1e-007
**Matches (Bold Red):** 26/142 fragment ions using 30 most intense peaks

| # | b | $b^{++}$ | b* | $b^{*++}$ | $b^{0}$ | $b^{0++}$ | Seq. | y | $y^{++}$ | y* | $y^{*++}$ | $y^{0}$ | $y^{0++}$ | # |
|---|---|---|---|---|---|---|---|---|---|---|---|---|---|---|
| 1 | 132.0478 | 66.5275 | | | | | M | | | | | | | 14 |
| 2 | **245.1318** | 123.0696 | | | | | I | **1447.7791** | 724.3932 | 1430.7526 | 715.8799 | 1429.7685 | 715.3879 | 13 |
| 3 | **374.1744** | 187.5908 | | | 356.1639 | 178.5856 | E | **1334.6951** | **667.8512** | 1317.6685 | 659.3379 | **1316.6845** | 658.8459 | 12 |
| 4 | 489.2014 | **245.1043** | 472.1748 | 236.5910 | 471.1908 | 236.0990 | N | **1205.6525** | 603.3299 | 1188.6259 | 594.8166 | 1187.6419 | 594.3246 | 11 |
| 5 | 546.2228 | 273.6150 | 529.1963 | 265.1018 | 528.2123 | 264.6098 | G | 1090.6255 | 545.8164 | 1073.5990 | 537.3031 | 1072.6150 | 536.8111 | 10 |
| 6 | 633.2548 | 317.1311 | 616.2283 | 308.6178 | 615.2443 | 308.1258 | S | 1033.6041 | 517.3057 | 1016.5775 | 508.7924 | 1015.5935 | 508.3004 | 9 |
| 7 | **746.3389** | 373.6731 | 729.3124 | 365.1598 | **728.3283** | 364.6678 | L | **946.5720** | 473.7897 | 929.5455 | 465.2764 | 928.5615 | 464.7844 | 8 |
| 8 | **833.3709** | 417.1891 | 816.3444 | 408.6758 | 815.3604 | 408.1838 | S | **833.4880** | 417.2476 | 816.4614 | 408.7343 | 815.4774 | 408.2423 | 7 |
| 9 | 980.4394 | 490.7233 | 963.4128 | 482.2100 | 962.4288 | 481.7180 | F | **746.4559** | **373.7316** | 729.4294 | 365.2183 | **728.4454** | 364.7263 | 6 |
| 10 | **1093.5234** | 547.2653 | 1076.4969 | 538.7521 | **1075.5129** | 538.2601 | L | **599.3875** | 300.1974 | 582.3610 | 291.6841 | 581.3770 | 291.1921 | 5 |
| 11 | 1190.5762 | 595.7917 | 1173.5496 | 587.2785 | 1172.5656 | 586.7864 | P | **486.3035** | 243.6554 | **469.2769** | 235.1421 | **468.2929** | 234.6501 | 4 |
| 12 | 1291.6239 | 646.3156 | 1274.5973 | 637.8023 | 1273.6133 | 637.3103 | T | **389.2507** | 195.1290 | 372.2241 | 186.6157 | 371.2401 | 186.1237 | 3 |
| 13 | **1404.7079** | 702.8576 | **1387.6814** | 694.3443 | **1386.6974** | 693.8523 | L | 288.2030 | 144.6051 | 271.1765 | 136.0919 | | | 2 |
| 14 | | | | | | | R | 175.1190 | 88.0631 | 158.0924 | 79.5498 | | | 1 |

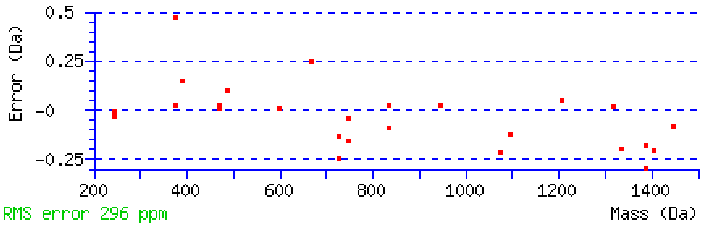


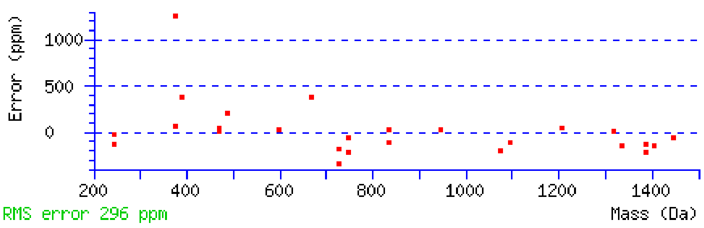


### All matches to this query

| Score | Mr(calc): | Delta | Sequence |
|---|---|---|---|
| 84.3 | 1577.8123 | 0.0059 | MIENGSLSFLPTLR |
| 33.0 | 1576.8283 | 0.9899 | MIENGSLSFLPTLR |
| 8.6 | 1577.8161 | 0.0021 | IEELNRYRDTLR |
| 7.8 | 1577.8309 | -0.0128 | FVPCISKMINLQK |
| 7.3 | 1577.8300 | -0.0118 | KNEYLLSLLAEER |
| 5.1 | 1577.8178 | 0.0004 | VKVDSELQLIQAR |
| 4.5 | 1577.8039 | 0.0143 | ATNSIIKGPAQSRR |
| 4.4 | 1577.8195 | -0.0013 | MLKAESSGERTTLR |
| 3.2 | 1575.8178 | 2.0004 | MLLDNTALLGLGSNK |
| 3.1 | 1577.8202 | -0.0020 | VGNLTPHLHNIFSK |

---

Spectrum No: 180; Query: 2275; Rank: 1

---

## Peptide View

MS/MS Fragmentation of **NGSYCVLQMDQSYKPDENEVR**
Found in **IPI00393333**, Tax_Id=10116 Gene_Symbol=Atic Bifunctional purine biosynthesis protein PURH

Match to Query 2275: 2532.090012 from(845.037280,3+)
Title: 091008RatKidney_NoSalt_18.2538.2538.3.dta
Data file K:\NewmanPaper\Piliang\3SubProteomes\Piliang3SP\mgf5ppm\ERLIC_3SubProteomes5ppm.mgf

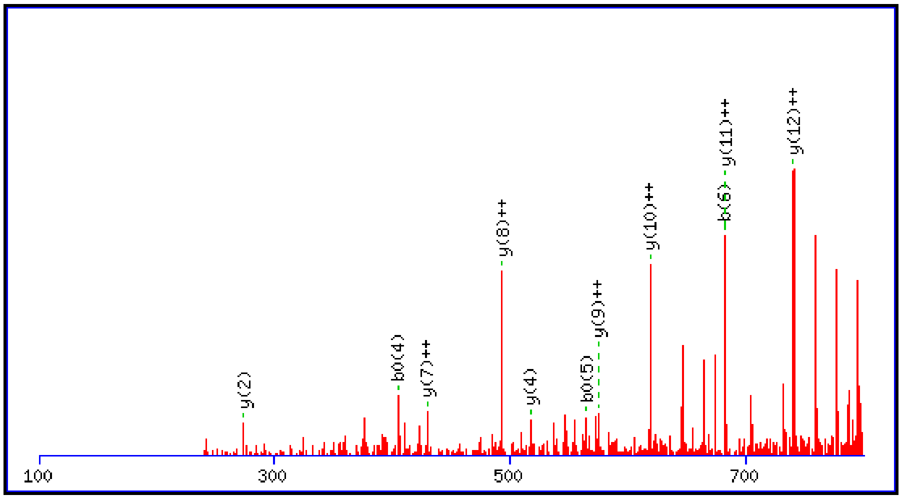

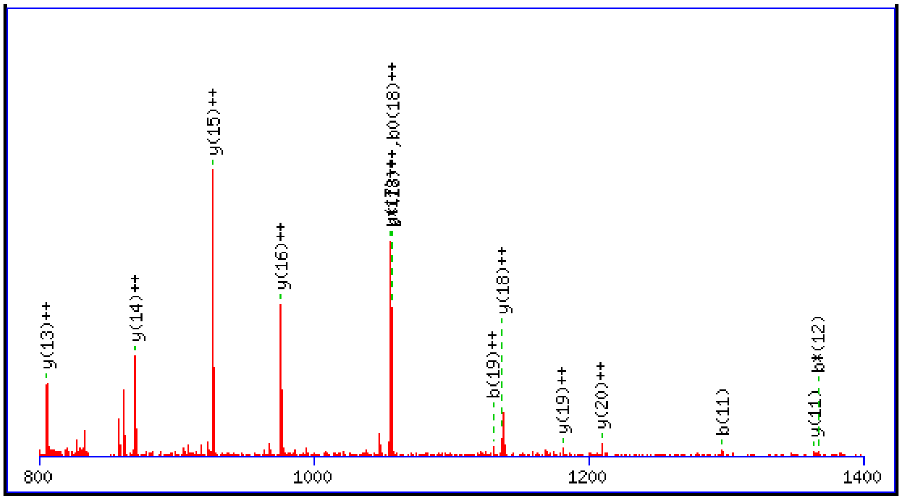


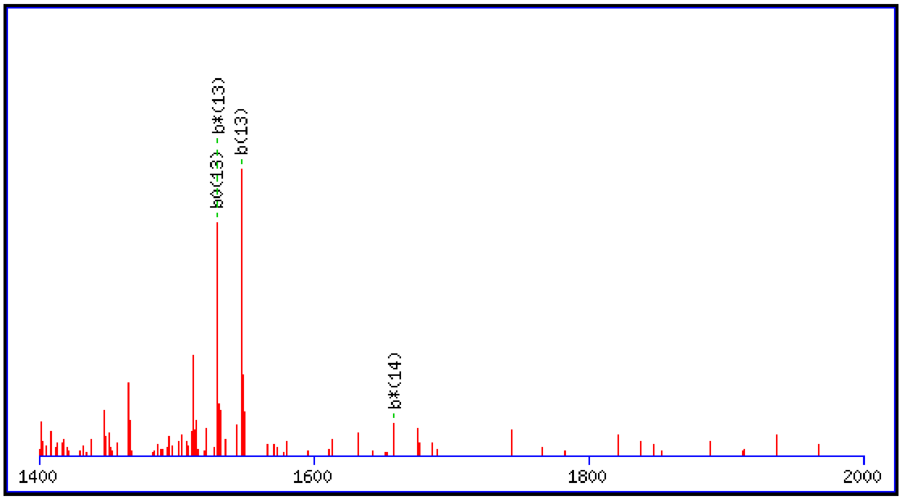


**Monoisotopic mass of neutral peptide Mr(calc):** 2532.0897
**Fixed modifications:** Carbamidomethyl (C)
**Variable modifications:**
**N1** : Deamidated_N (N)
**Ions Score:** 84 **Expect:** 1.3e-006
**Matches (Bold Red):** 29/232 fragment ions using 52 most intense peaks

| # | b | b++ | b* | b*++ | b0 | b0++ | Seq. | y | y++ | y* | y*++ | y0 | y0++ | # |
|---|---|---|---|---|---|---|---|---|---|---|---|---|---|---|
| 1 | 116.0342 | 58.5207 | 99.0077 | 50.0075 | | | N | | | | | | | 21 |
| 2 | 173.0557 | 87.0315 | 156.0291 | 78.5182 | | | G | 2418.0700 | **1209.5387** | 2401.0435 | 1201.0254 | 2400.0595 | 1200.5334 | 20 |
| 3 | 260.0877 | 130.5475 | 243.0612 | 122.0342 | 242.0771 | 121.5422 | S | 2361.0486 | **1181.0279** | 2344.0220 | 1172.5147 | 2343.0380 | 1172.0226 | 19 |
| 4 | 423.1510 | 212.0792 | 406.1245 | 203.5659 | **405.1405** | 203.0739 | Y | 2274.0165 | **1137.5119** | 2256.9900 | 1128.9986 | 2256.0060 | 1128.5066 | 18 |
| 5 | 583.1817 | 292.0945 | 566.1551 | 283.5812 | **565.1711** | 283.0892 | C | 2110.9532 | **1055.9802** | 2093.9267 | 1047.4670 | 2092.9427 | 1046.9750 | 17 |
| 6 | **682.2501** | 341.6287 | 665.2235 | 333.1154 | 664.2395 | 332.6234 | V | 1950.9226 | **975.9649** | 1933.8960 | 967.4516 | 1932.9120 | 966.9596 | 16 |
| 7 | 795.3342 | 398.1707 | 778.3076 | 389.6574 | 777.3236 | 389.1654 | L | 1851.8542 | **926.4307** | 1834.8276 | 917.9174 | 1833.8436 | 917.4254 | 15 |
| 8 | 923.3927 | 462.2000 | 906.3662 | 453.6867 | 905.3822 | 453.1947 | Q | 1738.7701 | **869.8887** | 1721.7435 | 861.3754 | 1720.7595 | 860.8834 | 14 |
| 9 | 1054.4332 | 527.7202 | 1037.4067 | 519.2070 | 1036.4227 | 518.7150 | M | 1610.7115 | **805.8594** | 1593.6850 | 797.3461 | 1592.7009 | 796.8541 | 13 |
| 10 | 1169.4602 | 585.2337 | 1152.4336 | 576.7204 | 1151.4496 | 576.2284 | D | 1479.6710 | **740.3392** | 1462.6445 | 731.8259 | 1461.6605 | 731.3339 | 12 |
| 11 | **1297.5187** | 649.2630 | 1280.4922 | 640.7497 | 1279.5082 | 640.2577 | Q | **1364.6441** | **682.8257** | 1347.6175 | 674.3124 | 1346.6335 | 673.8204 | 11 |
| 12 | 1384.5508 | 692.7790 | **1367.5242** | 684.2657 | 1366.5402 | 683.7737 | S | 1236.5855 | **618.7964** | 1219.5590 | 610.2831 | 1218.5749 | 609.7911 | 10 |
| 13 | **1547.6141** | 774.3107 | **1530.5876** | 765.7974 | **1529.6035** | 765.3054 | Y | 1149.5535 | **575.2804** | 1132.5269 | 566.7671 | 1131.5429 | 566.2751 | 9 |
| 14 | 1675.7091 | 838.3582 | **1658.6825** | 829.8449 | 1657.6985 | 829.3529 | K | 986.4901 | **493.7487** | 969.4636 | 485.2354 | 968.4796 | 484.7434 | 8 |
| 15 | 1772.7618 | 886.8846 | 1755.7353 | 878.3713 | 1754.7513 | 877.8793 | P | 858.3952 | **429.7012** | 841.3686 | 421.1880 | 840.3846 | 420.6959 | 7 |
| 16 | 1887.7888 | 944.3980 | 1870.7622 | 935.8847 | 1869.7782 | 935.3927 | D | 761.3424 | 381.1748 | 744.3159 | 372.6616 | 743.3319 | 372.1696 | 6 |
| 17 | 2016.8314 | 1008.9193 | 1999.8048 | 1000.4060 | 1998.8208 | 999.9140 | E | 646.3155 | 323.6614 | 629.2889 | 315.1481 | 628.3049 | 314.6561 | 5 |
| 18 | 2130.8743 | 1065.9408 | 2113.8477 | **1057.4275** | 2112.8637 | **1056.9355** | N | **517.2729** | 259.1401 | 500.2463 | 250.6268 | 499.2623 | 250.1348 | 4 |

| | | | | | | | | | | | | | | |
|---|---|---|---|---|---|---|---|---|---|---|---|---|---|---|
| **19** | 2259.9169 | **1130.4621** | 2242.8903 | 1121.9488 | 2241.9063 | 1121.4568 | **E** | 403.2300 | 202.1186 | 386.2034 | 193.6053 | 385.2194 | 193.1133 | **3** |
| **20** | 2358.9853 | 1179.9963 | 2341.9587 | 1171.4830 | 2340.9747 | 1170.9910 | **V** | **274.1874** | 137.5973 | 257.1608 | 129.0840 | | | **2** |
| **21** | | | | | | | **R** | 175.1190 | 88.0631 | 158.0924 | 79.5498 | | | **1** |

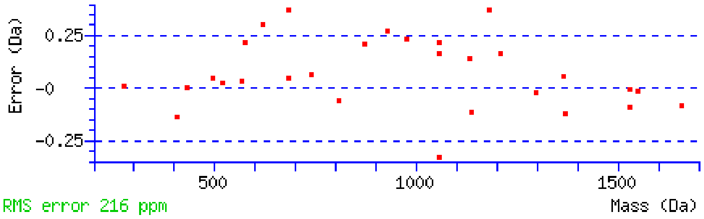


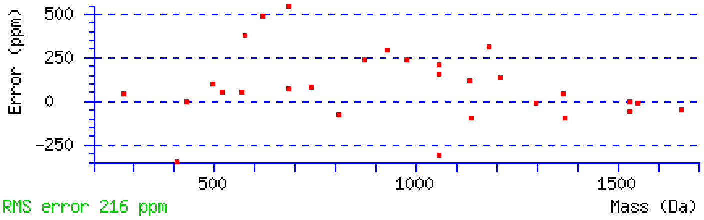


**All matches to this query**

| Score | Mr(calc): | Delta | Sequence |
|---|---|---|---|
| 84.3 | 2532.0897 | 0.0003 | NGSYCVLQMDQSYKPDENEVR |
| 66.0 | 2532.0897 | 0.0003 | NGSYCVLQMDQSYKPDENEVR |
| 62.3 | 2531.1057 | 0.9843 | NGSYCVLQMDQSYKPDENEVR |
| 1.6 | 2531.0934 | 0.9966 | MANRGATRPNGPNTGNKICQFK |
| 1.5 | 2532.0700 | 0.0200 | VSHVCSQTNGSSAQQASAPPAPDR |
| 1.5 | 2532.0700 | 0.0200 | VSHVCSQTNGSSAQQASAPPAPDR |
| 1.1 | 2532.0951 | -0.0051 | LFNSLLLGPTASNSKTEGSSLR |
| 0.9 | 2532.1047 | -0.0146 | LNQAAISQAFSNALHSLDGATSR |
| 0.7 | 2531.0860 | 1.0040 | VSHVCSQTNGSSAQQASAPPAPDR |
| 0.5 | 2531.0860 | 1.0040 | VSHVCSQTNGSSAQQASAPPAPDR |

---

Spectrum No: 181; Query: 1903; Rank: 1

---

## Peptide View

MS/MS Fragmentation of **TNSSFVQGLVDHVKEDCDR**
Found in **IPI00195160**, Tax_Id=10116 Gene_Symbol=Psap Sulfated glycoprotein 1 precursor

Match to Query 1903: 2205.996428 from(1104.005490,2+)
Title: 091008RatKidney_NH4Format02_24.3964.3964.2.dta
Data file K:\NewmanPaper\Piliang\3SubProteomes\Piliang3SP\mgf5ppm\ERLIC_3SubProteomes5ppm.mgf

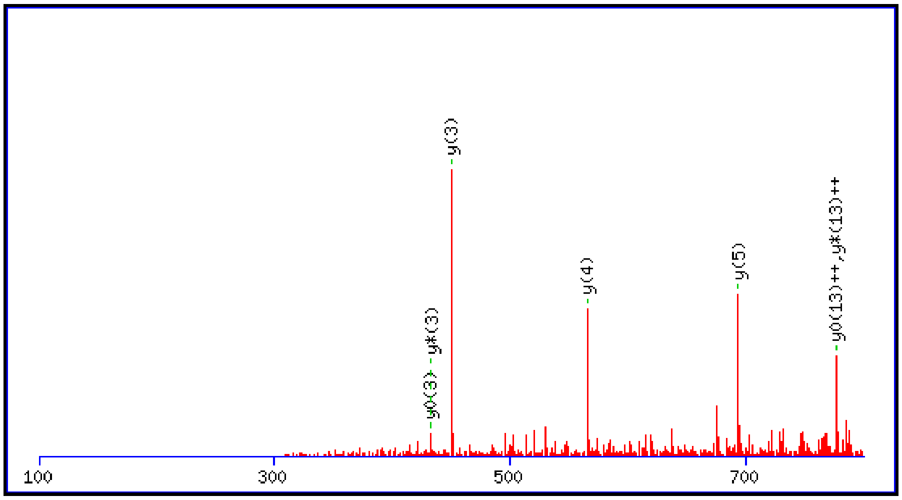

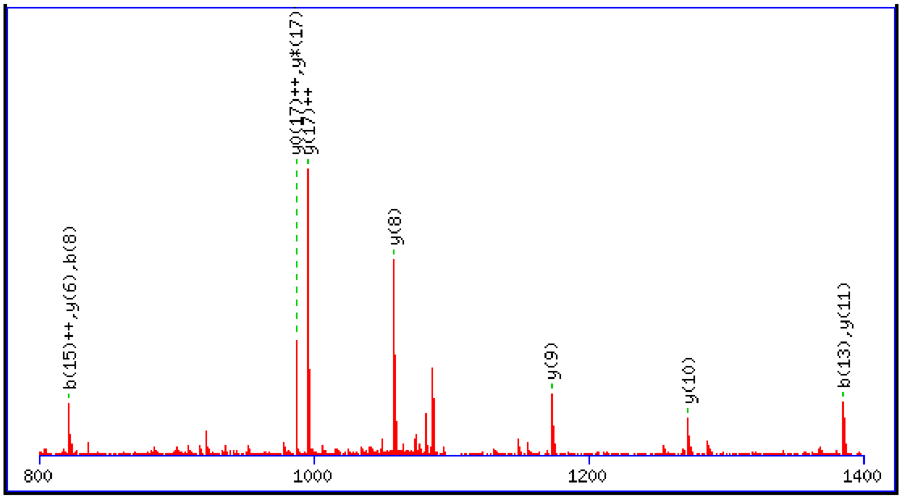


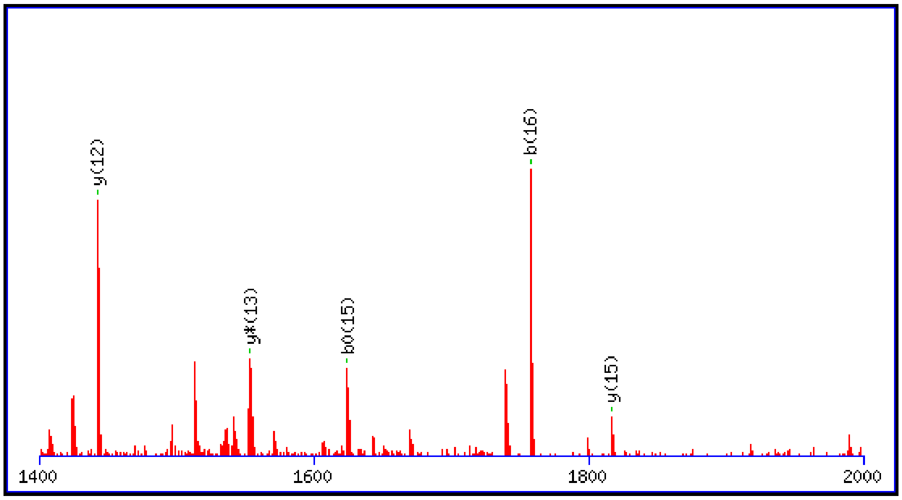


**Monoisotopic mass of neutral peptide Mr(calc):** 2205.9961
**Fixed modifications:** Carbamidomethyl (C)
**Variable modifications:**
**N2** : Deamidated_N (N)
**Ions Score:** 84 **Expect:** 1e-006
**Matches (Bold Red):** 23/212 fragment ions using 30 most intense peaks

| # | b | $b^{++}$ | b* | $b^{*++}$ | $b^{0}$ | $b^{0++}$ | Seq. | y | $y^{++}$ | y* | $y^{*++}$ | $y^{0}$ | $y^{0++}$ | # |
|---|---|---|---|---|---|---|---|---|---|---|---|---|---|---|
| 1 | 102.0550 | 51.5311 | | | 84.0444 | 42.5258 | T | | | | | | | 19 |
| 2 | 217.0819 | 109.0446 | 200.0553 | 100.5313 | 199.0713 | 100.0393 | N | 2105.9557 | 1053.4815 | 2088.9291 | 1044.9682 | 2087.9451 | 1044.4762 | 18 |
| 3 | 304.1139 | 152.5606 | 287.0874 | 144.0473 | 286.1034 | 143.5553 | S | 1990.9287 | **995.9680** | 1973.9022 | **987.4547** | 1972.9182 | **986.9627** | 17 |
| 4 | 391.1459 | 196.0766 | 374.1194 | 187.5633 | 373.1354 | 187.0713 | S | 1903.8967 | 952.4520 | 1886.8701 | 943.9387 | 1885.8861 | 943.4467 | 16 |
| 5 | 538.2144 | 269.6108 | 521.1878 | 261.0975 | 520.2038 | 260.6055 | F | **1816.8647** | 908.9360 | 1799.8381 | 900.4227 | 1798.8541 | 899.9307 | 15 |
| 6 | 637.2828 | 319.1450 | 620.2562 | 310.6318 | 619.2722 | 310.1397 | V | 1669.7962 | 835.4018 | 1652.7697 | 826.8885 | 1651.7857 | 826.3965 | 14 |
| 7 | 765.3414 | 383.1743 | 748.3148 | 374.6610 | 747.3308 | 374.1690 | Q | 1570.7278 | 785.8676 | **1553.7013** | **777.3543** | 1552.7173 | **776.8623** | 13 |
| 8 | **822.3628** | 411.6850 | 805.3363 | 403.1718 | 804.3523 | 402.6798 | G | **1442.6693** | 721.8383 | 1425.6427 | 713.3250 | 1424.6587 | 712.8330 | 12 |
| 9 | 935.4469 | 468.2271 | 918.4203 | 459.7138 | 917.4363 | 459.2218 | L | **1385.6478** | 693.3275 | 1368.6212 | 684.8143 | 1367.6372 | 684.3223 | 11 |
| 10 | 1034.5153 | 517.7613 | 1017.4887 | 509.2480 | 1016.5047 | 508.7560 | V | **1272.5637** | 636.7855 | 1255.5372 | 628.2722 | 1254.5532 | 627.7802 | 10 |
| 11 | 1149.5422 | 575.2748 | 1132.5157 | 566.7615 | 1131.5317 | 566.2695 | D | **1173.4953** | 587.2513 | 1156.4688 | 578.7380 | 1155.4847 | 578.2460 | 9 |
| 12 | 1286.6012 | 643.8042 | 1269.5746 | 635.2909 | 1268.5906 | 634.7989 | H | **1058.4684** | 529.7378 | 1041.4418 | 521.2245 | 1040.4578 | 520.7325 | 8 |
| 13 | **1385.6696** | 693.3384 | 1368.6430 | 684.8251 | 1367.6590 | 684.3331 | V | 921.4095 | 461.2084 | 904.3829 | 452.6951 | 903.3989 | 452.2031 | 7 |
| 14 | 1513.7645 | 757.3859 | 1496.7380 | 748.8726 | 1495.7540 | 748.3806 | K | **822.3410** | 411.6742 | 805.3145 | 403.1609 | 804.3305 | 402.6689 | 6 |
| 15 | 1642.8071 | **821.9072** | 1625.7806 | 813.3939 | **1624.7966** | 812.9019 | E | **694.2461** | 347.6267 | 677.2195 | 339.1134 | 676.2355 | 338.6214 | 5 |
| 16 | **1757.8341** | 879.4207 | 1740.8075 | 870.9074 | 1739.8235 | 870.4154 | D | **565.2035** | 283.1054 | 548.1769 | 274.5921 | 547.1929 | 274.1001 | 4 |
| 17 | 1917.8647 | 959.4360 | 1900.8382 | 950.9227 | 1899.8541 | 950.4307 | C | **450.1765** | 225.5919 | **433.1500** | 217.0786 | **432.1660** | 216.5866 | 3 |
| 18 | 2032.8917 | 1016.9495 | 2015.8651 | 1008.4362 | 2014.8811 | 1007.9442 | D | 290.1459 | 145.5766 | 273.1193 | 137.0633 | 272.1353 | 136.5713 | 2 |

| | | | | | | | | | | | | | | |
|---|---|---|---|---|---|---|---|---|---|---|---|---|---|---|
| 19 | | | | | | | R | 175.1190 | 88.0631 | 158.0924 | 79.5498 | | | 1 |

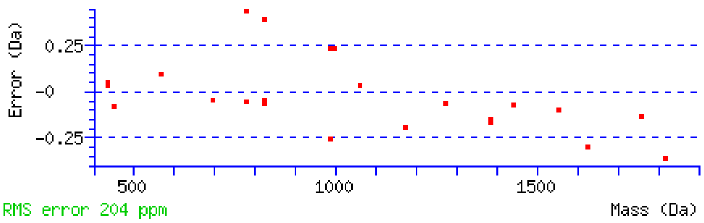


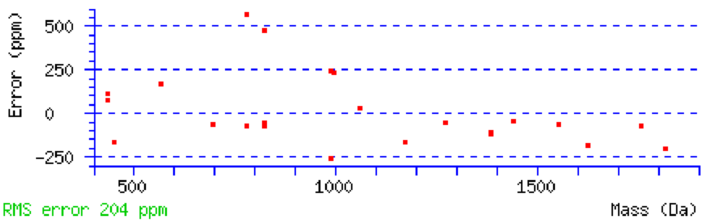


### All matches to this query

| Score | Mr(calc): | Delta | Sequence |
|---|---|---|---|
| 84.2 | 2205.9961 | 0.0004 | TNSSFVQGLVDHVKEDCDR |
| 75.9 | 2205.0121 | 0.9844 | TNSSFVQGLVDHVKEDCDR |
| 4.7 | 2203.9816 | 2.0148 | TFKAWEGLHENSVRLTR |
| 3.8 | 2203.9763 | 2.0201 | LENLTDTTVAQKAGGPSSVR |
| 2.6 | 2204.9966 | 0.9999 | EYPQPMGQGVPSLEFRYK |
| 0.9 | 2204.9724 | 1.0240 | TVAMPVRVVSVEENCTVR |
| 0.3 | 2205.9933 | 0.0031 | IASSVQQRVRVHSSDSYK |
| 0.3 | 2205.9933 | 0.0031 | IASSVQQRVRVHSSDSYK |
| 0.3 | 2203.9884 | 2.0080 | LSAITMPSVARHTMQTIR |
| 0.2 | 2204.9724 | 1.0240 | TVAMPVRVVSVEENCTVR |

---

Spectrum No: 182; Query: 211; Rank: 1

---

## Peptide View

MS/MS Fragmentation of **ISSNETEIMR**
Found in **IPI00370427**, Tax_Id=10116 Gene_Symbol=Tinag Tubulointerstitial nephritis antigen

Match to Query 211: 1195.535988 from(598.775270,2+)
Title: 091008RatKidney_NH4Format01_24.609.609.2.dta
Data file K:\NewmanPaper\Piliang\3SubProteomes\Piliang3SP\mgf5ppm\ERLIC_3SubProteomes5ppm.mgf

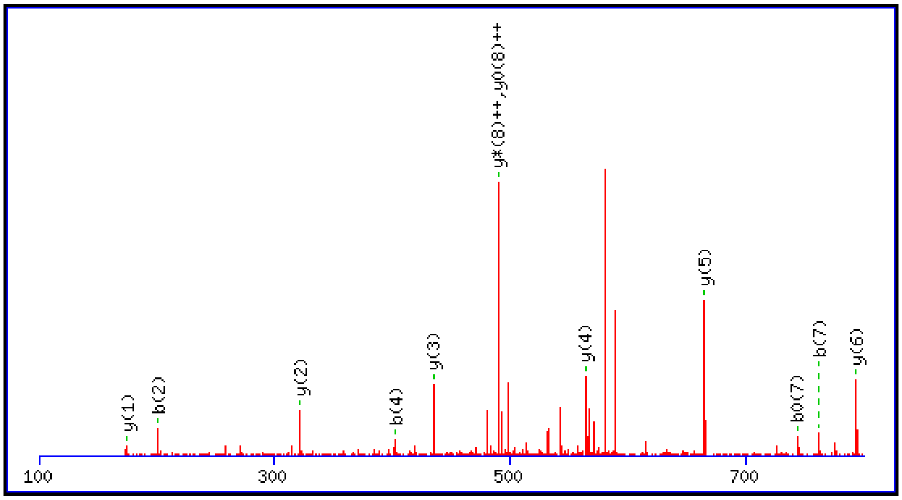

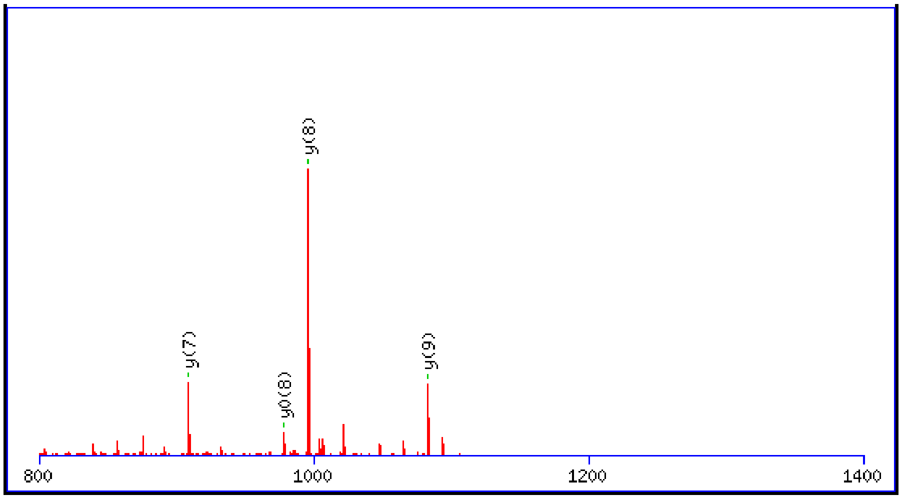


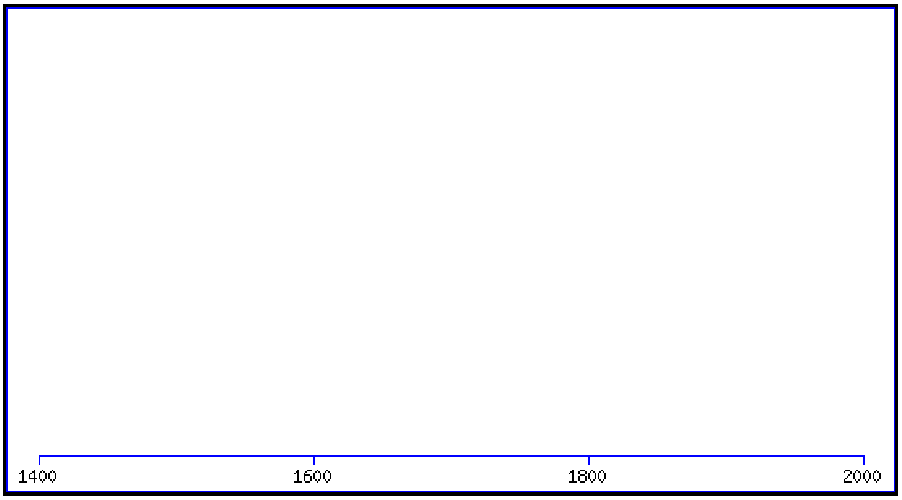


**Monoisotopic mass of neutral peptide Mr(calc):** 1195.5390
**Fixed modifications:** Carbamidomethyl (C)
**Variable modifications:**
**N4** : Deamidated_N (N)
**M9** : Oxidation (M)
**Ions Score:** 84 **Expect:** 4.4e-007
**Matches (Bold Red):** 16/94 fragment ions using 22 most intense peaks

| # | b | $b^{++}$ | b* | $b^{*++}$ | $b^{0}$ | $b^{0++}$ | Seq. | y | $y^{++}$ | y* | $y^{*++}$ | $y^{0}$ | $y^{0++}$ | # |
|---|---|---|---|---|---|---|---|---|---|---|---|---|---|---|
| 1 | 114.0913 | 57.5493 | | | | | I | | | | | | | 10 |
| 2 | **201.1234** | 101.0653 | | | 183.1128 | 92.0600 | S | **1083.4623** | 542.2348 | 1066.4357 | 533.7215 | 1065.4517 | 533.2295 | 9 |
| 3 | 288.1554 | 144.5813 | | | 270.1448 | 135.5761 | S | **996.4302** | 498.7188 | 979.4037 | **490.2055** | **978.4197** | **489.7135** | 8 |
| 4 | **403.1823** | 202.0948 | 386.1558 | 193.5815 | 385.1718 | 193.0895 | N | **909.3982** | 455.2027 | 892.3717 | 446.6895 | 891.3877 | 446.1975 | 7 |
| 5 | 532.2249 | 266.6161 | 515.1984 | 258.1028 | 514.2144 | 257.6108 | E | **794.3713** | 397.6893 | 777.3447 | 389.1760 | 776.3607 | 388.6840 | 6 |
| 6 | 633.2726 | 317.1399 | 616.2461 | 308.6267 | 615.2620 | 308.1347 | T | **665.3287** | 333.1680 | 648.3021 | 324.6547 | 647.3181 | 324.1627 | 5 |
| 7 | **762.3152** | 381.6612 | 745.2886 | 373.1480 | **744.3046** | 372.6560 | E | **564.2810** | 282.6441 | 547.2545 | 274.1309 | 546.2704 | 273.6389 | 4 |
| 8 | 875.3993 | 438.2033 | 858.3727 | 429.6900 | 857.3887 | 429.1980 | I | **435.2384** | 218.1228 | 418.2119 | 209.6096 | | | 3 |
| 9 | 1022.4347 | 511.7210 | 1005.4081 | 503.2077 | 1004.4241 | 502.7157 | M | **322.1544** | 161.5808 | 305.1278 | 153.0675 | | | 2 |
| 10 | | | | | | | R | **175.1190** | 88.0631 | 158.0924 | 79.5498 | | | 1 |

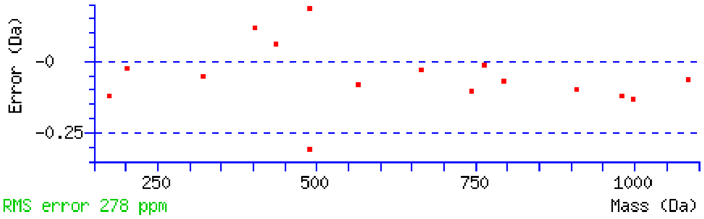


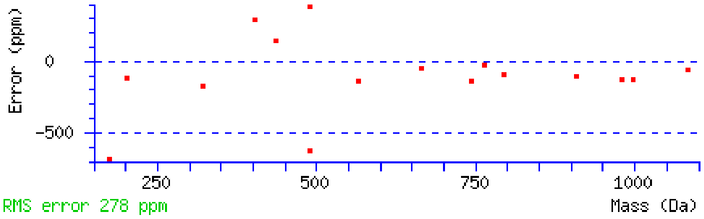

### All matches to this query

| Score | Mr(calc): | Delta | Sequence |
|---|---|---|---|
| 84.1 | 1195.5390 | -0.0030 | ISSNETEIMR |
| 12.0 | 1195.5357 | 0.0003 | INSEFNSDLR |
| 11.3 | 1194.5282 | 1.0078 | QASPTEVVER |
| 10.4 | 1195.5292 | 0.0068 | EGTFPMTQNR |
| 9.7 | 1195.5347 | 0.0013 | DGSAGLGRDLR |
| 9.4 | 1195.5365 | -0.0006 | MGSEMEPLFR |
| 9.2 | 1195.5258 | 0.0102 | ISWYDNENR |
| 8.4 | 1195.5258 | 0.0102 | SSFGLHNDYR |
| 5.8 | 1195.5387 | -0.0027 | TGTVAFHDIR |
| 5.5 | 1195.5478 | -0.0118 | SLWCGMGTIR |

---

Spectrum No: 183; Query: 464; Rank: 1

---

## Peptide View

MS/MS Fragmentation of **CNVSEGVAQCTR**
Found in **IPI00231767**, Tax_Id=10116 Gene_Symbol=Tpi1 Triosephosphate isomerase

Match to Query 464: 1380.580568 from(691.297560,2+)
Title: 100101RatKid_NS_deglyco_12.862.862.2.dta
Data file K:\NewmanPaper\Piliang\3SubProteomes\Piliang3SP\mgf5ppm\ERLIC_3SubProteomes5ppm.mgf

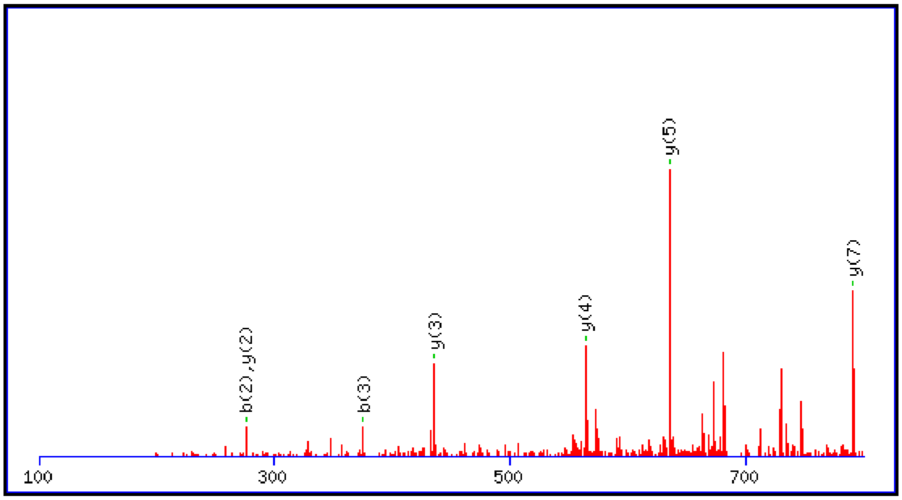


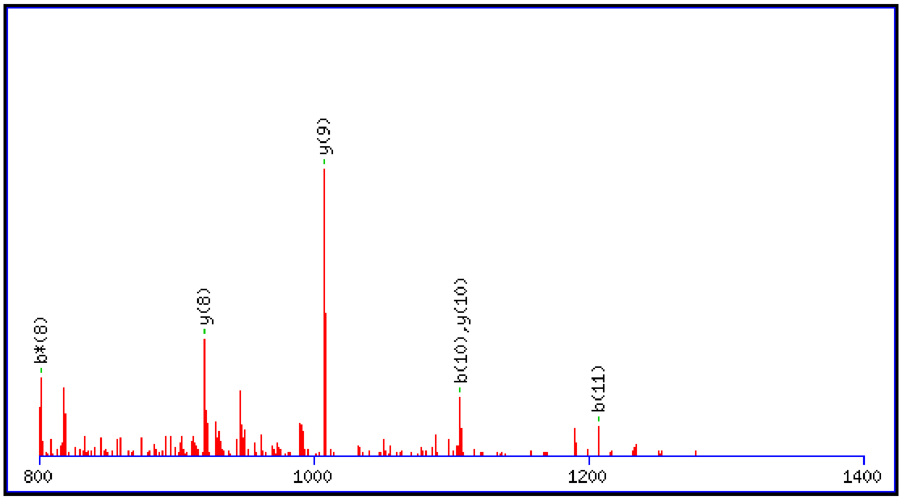

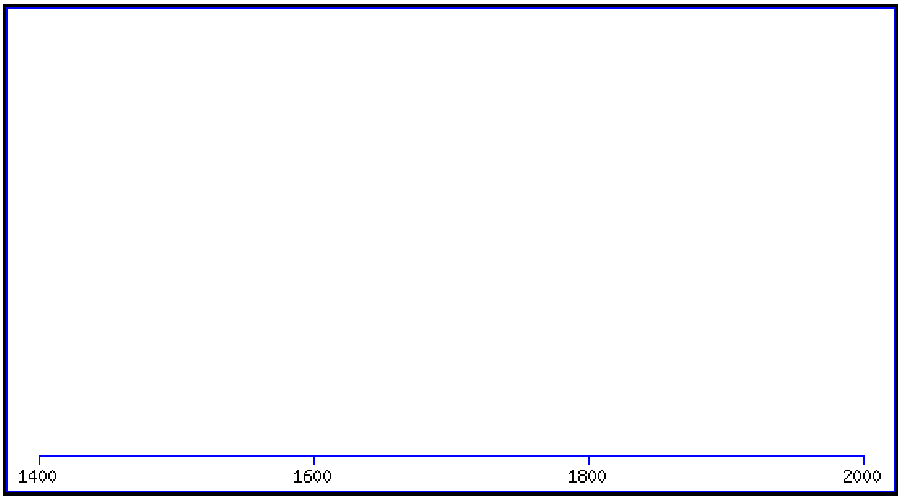


**Monoisotopic mass of neutral peptide Mr(calc):** 1380.5762
**Fixed modifications:** Carbamidomethyl (C)
**Variable modifications:**
**N2** : Deamidated_N (N)
**Ions Score:** 84 **Expect:** 4.4e-007
**Matches (Bold Red):** 13/122 fragment ions using 13 most intense peaks

| # | b | b$^{++}$ | b* | b*$^{++}$ | b$^{0}$ | b$^{0++}$ | Seq. | y | y$^{++}$ | y* | y*$^{++}$ | y$^{0}$ | y$^{0++}$ | # |
|---|---|---|---|---|---|---|---|---|---|---|---|---|---|---|
| 1 | 161.0379 | 81.0226 | | | | | C | | | | | | | 12 |
| 2 | **276.0649** | 138.5361 | 259.0383 | 130.0228 | | | N | 1221.5528 | 611.2800 | 1204.5263 | 602.7668 | 1203.5423 | 602.2748 | 11 |
| 3 | **375.1333** | 188.0703 | 358.1067 | 179.5570 | | | V | **1106.5259** | 553.7666 | 1089.4993 | 545.2533 | 1088.5153 | 544.7613 | 10 |
| 4 | 462.1653 | 231.5863 | 445.1388 | 223.0730 | 444.1547 | 222.5810 | S | **1007.4575** | 504.2324 | 990.4309 | 495.7191 | 989.4469 | 495.2271 | 9 |
| 5 | 591.2079 | 296.1076 | 574.1813 | 287.5943 | 573.1973 | 287.1023 | E | **920.4254** | 460.7164 | 903.3989 | 452.2031 | 902.4149 | 451.7111 | 8 |
| 6 | 648.2294 | 324.6183 | 631.2028 | 316.1050 | 630.2188 | 315.6130 | G | **791.3828** | 396.1951 | 774.3563 | 387.6818 | 773.3723 | 387.1898 | 7 |
| 7 | 747.2978 | 374.1525 | 730.2712 | 365.6393 | 729.2872 | 365.1472 | V | 734.3614 | 367.6843 | 717.3348 | 359.1711 | 716.3508 | 358.6790 | 6 |
| 8 | 818.3349 | 409.6711 | **801.3083** | 401.1578 | 800.3243 | 400.6658 | A | **635.2930** | 318.1501 | 618.2664 | 309.6368 | 617.2824 | 309.1448 | 5 |
| 9 | 946.3935 | 473.7004 | 929.3669 | 465.1871 | 928.3829 | 464.6951 | Q | **564.2559** | 282.6316 | 547.2293 | 274.1183 | 546.2453 | 273.6263 | 4 |
| 10 | **1106.4241** | 553.7157 | 1089.3976 | 545.2024 | 1088.4136 | 544.7104 | C | **436.1973** | 218.6023 | 419.1707 | 210.0890 | 418.1867 | 209.5970 | 3 |
| 11 | **1207.4718** | 604.2395 | 1190.4452 | 595.7263 | 1189.4612 | 595.2343 | T | **276.1666** | 138.5870 | 259.1401 | 130.0737 | 258.1561 | 129.5817 | 2 |
| 12 | | | | | | | R | 175.1190 | 88.0631 | 158.0924 | 79.5498 | | | 1 |

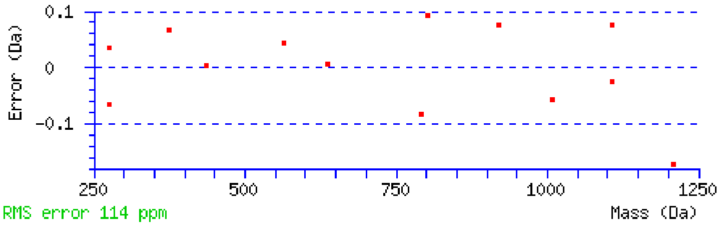


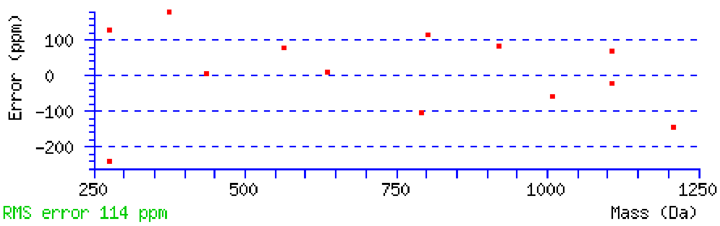


### All matches to this query

| Score | Mr(calc): | Delta | Sequence |
|---|---|---|---|
| 84.1 | 1380.5762 | 0.0044 | CNVSEGVAQCTR |
| 11.5 | 1380.5785 | 0.0021 | MQVLYEFEAR |
| 8.1 | 1380.5836 | -0.0030 | MQNSTPMVCQGK |
| 6.5 | 1380.5671 | 0.0135 | NESHSNGTVTVR |
| 6.0 | 1380.5671 | 0.0135 | NESHSNGTVTVR |
| 4.5 | 1380.5840 | -0.0034 | KFVNQTVTER |
| 3.3 | 1380.5827 | -0.0021 | DSDGGRIEEMEK |
| 3.2 | 1380.5940 | -0.0134 | DGSACVEGSGSSLR |
| 3.1 | 1379.5680 | 1.0126 | TMGYSQEIVEK |
| | | | |

| | | | |
|---|---|---|---|
| 2.7 | 1379.5809 | 0.9997 | MLLQSSVSVEK |

Spectrum No: 184; Query: 511; Rank: 1

## Peptide View

MS/MS Fragmentation of **SFSLISGSINQTR**
Found in **IPI00372786**, Tax_Id=10116 Gene_Symbol=Nid2 similar to nidogen 2 isoform 2

Match to Query 511: 1409.717088 from(705.865820,2+)
Title: 100101RatKid_NS_deglyco_16.3339.3339.2.dta
Data file K:\NewmanPaper\Piliang\3SubProteomes\Piliang3SP\mgf5ppm\ERLIC_3SubProteomes5ppm.mgf

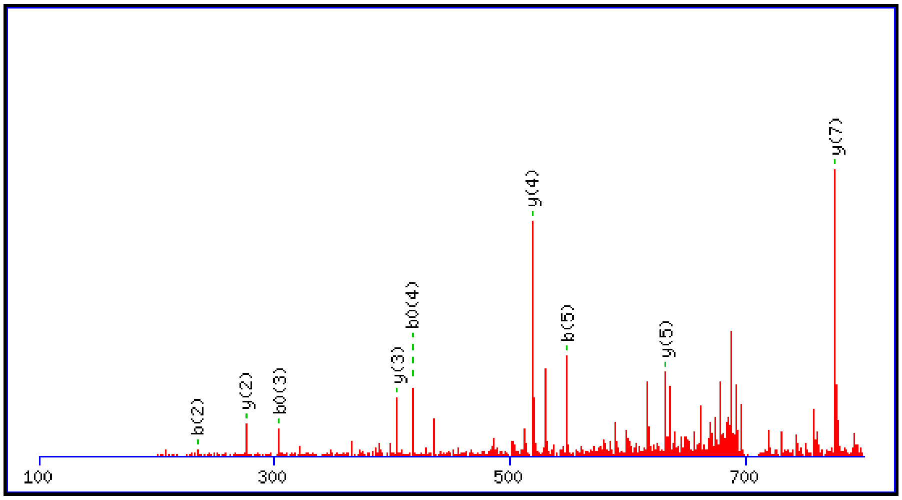


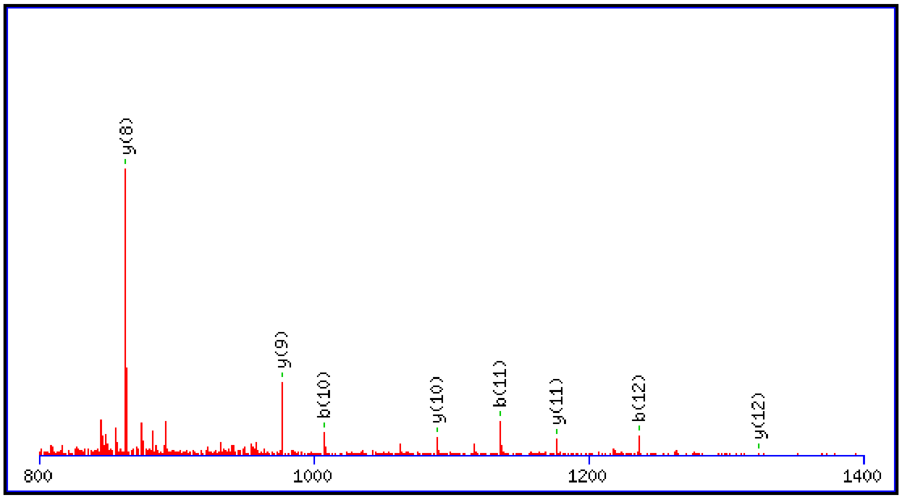

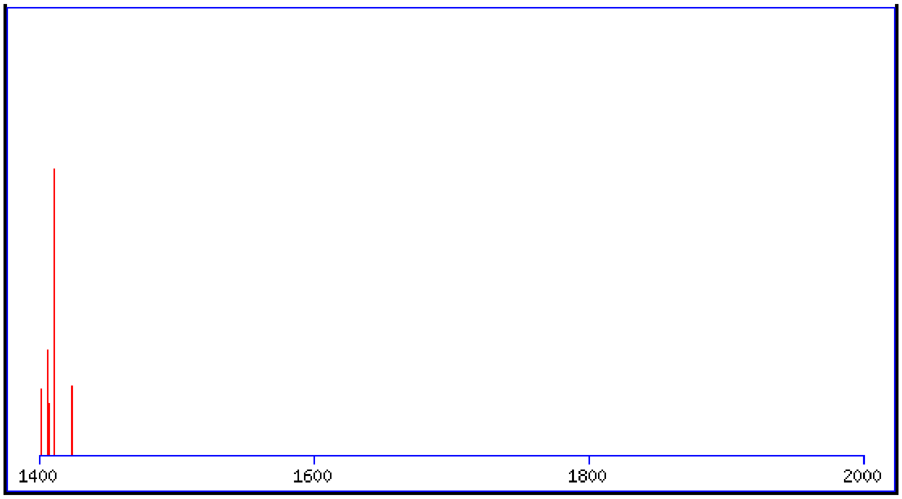


**Monoisotopic mass of neutral peptide Mr(calc):** 1409.7150
**Fixed modifications:** Carbamidomethyl (C)
**Variable modifications:**
**N10** : Deamidated_N (N)
**Ions Score:** 84 **Expect:** 5.1e-007
**Matches (Bold Red):** 17/124 fragment ions using 35 most intense peaks

| # | b | $b^{++}$ | b* | $b^{*++}$ | $b^0$ | $b^{0++}$ | Seq. | y | $y^{++}$ | y* | $y^{*++}$ | $y^0$ | $y^{0++}$ | # |
|---|---|---|---|---|---|---|---|---|---|---|---|---|---|---|
| 1 | 88.0393 | 44.5233 | | | 70.0287 | 35.5180 | S | | | | | | | 13 |
| 2 | **235.1077** | 118.0575 | | | 217.0972 | 109.0522 | F | **1323.6903** | 662.3488 | 1306.6637 | 653.8355 | 1305.6797 | 653.3435 | 12 |
| 3 | 322.1397 | 161.5735 | | | **304.1292** | 152.5682 | S | **1176.6219** | 588.8146 | 1159.5953 | 580.3013 | 1158.6113 | 579.8093 | 11 |
| 4 | 435.2238 | 218.1155 | | | **417.2132** | 209.1103 | L | **1089.5899** | 545.2986 | 1072.5633 | 536.7853 | 1071.5793 | 536.2933 | 10 |
| 5 | **548.3079** | 274.6576 | | | 530.2973 | 265.6523 | I | **976.5058** | 488.7565 | 959.4792 | 480.2433 | 958.4952 | 479.7513 | 9 |
| 6 | 635.3399 | 318.1736 | | | 617.3293 | 309.1683 | S | **863.4217** | 432.2145 | 846.3952 | 423.7012 | 845.4112 | 423.2092 | 8 |
| 7 | 692.3614 | 346.6843 | | | 674.3508 | 337.6790 | G | **776.3897** | 388.6985 | 759.3632 | 380.1852 | 758.3791 | 379.6932 | 7 |
| 8 | 779.3934 | 390.2003 | | | 761.3828 | 381.1951 | S | 719.3682 | 360.1878 | 702.3417 | 351.6745 | 701.3577 | 351.1825 | 6 |
| 9 | 892.4775 | 446.7424 | | | 874.4669 | 437.7371 | I | **632.3362** | 316.6717 | 615.3097 | 308.1585 | 614.3256 | 307.6665 | 5 |
| 10 | **1007.5044** | 504.2558 | 990.4778 | 495.7426 | 989.4938 | 495.2506 | N | **519.2521** | 260.1297 | 502.2256 | 251.6164 | 501.2416 | 251.1244 | 4 |
| 11 | **1135.5630** | 568.2851 | 1118.5364 | 559.7718 | 1117.5524 | 559.2798 | Q | **404.2252** | 202.6162 | 387.1987 | 194.1030 | 386.2146 | 193.6110 | 3 |
| 12 | **1236.6107** | 618.8090 | 1219.5841 | 610.2957 | 1218.6001 | 609.8037 | T | **276.1666** | 138.5870 | 259.1401 | 130.0737 | 258.1561 | 129.5817 | 2 |
| 13 | | | | | | | R | 175.1190 | 88.0631 | 158.0924 | 79.5498 | | | 1 |

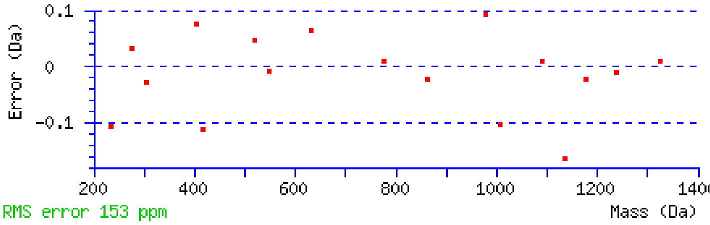


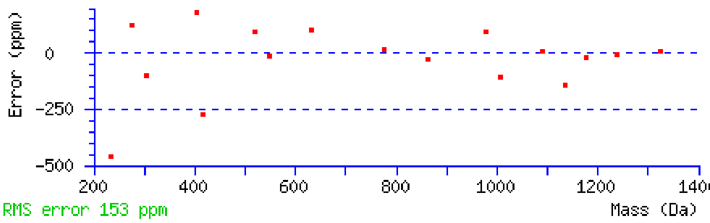


### All matches to this query

| Score | Mr(calc): | Delta | Sequence |
|---|---|---|---|
| 84.0 | 1409.7150 | 0.0020 | SFSLISGSINQTR |
| 5.2 | 1409.7224 | -0.0053 | NEASALLYLLMR |
| 3.6 | 1409.7263 | -0.0092 | SVSQETFRISTR |
| 3.0 | 1409.7303 | -0.0132 | GLSNFYPDLTKR |
| 2.0 | 1409.7038 | 0.0133 | TVFSDSQLSGLEK |
| 1.5 | 1409.7108 | 0.0063 | KDIKFITYFR |
| 1.0 | 1409.7159 | 0.0012 | KFMEKLDACIR |
| 1.0 | 1409.7085 | 0.0086 | NMSIIDAFRSTR |
| | | | |

| | | | |
|---|---|---|---|
| 0.5 | 1409.7085 | 0.0086 | MSSLPKPDGHGIR |

Spectrum No: 185; Query: 621; Rank: 1

## Peptide View

MS/MS Fragmentation of **QSNGSIAIISSMAGK**
Found in **IPI00211096**, Tax_Id=10116 Gene_Symbol=Hsd11b1 Isoform 11-HSD1A of Corticosteroid 11-beta-dehydrogenase isozyme 1

Match to Query 621: 1463.732428 from(732.873490,2+)
Title: 100101RatKid_NS_deglyco_14.3201.3201.2.dta
Data file K:\NewmanPaper\Piliang\3SubProteomes\Piliang3SP\mgf5ppm\ERLIC_3SubProteomes5ppm.mgf

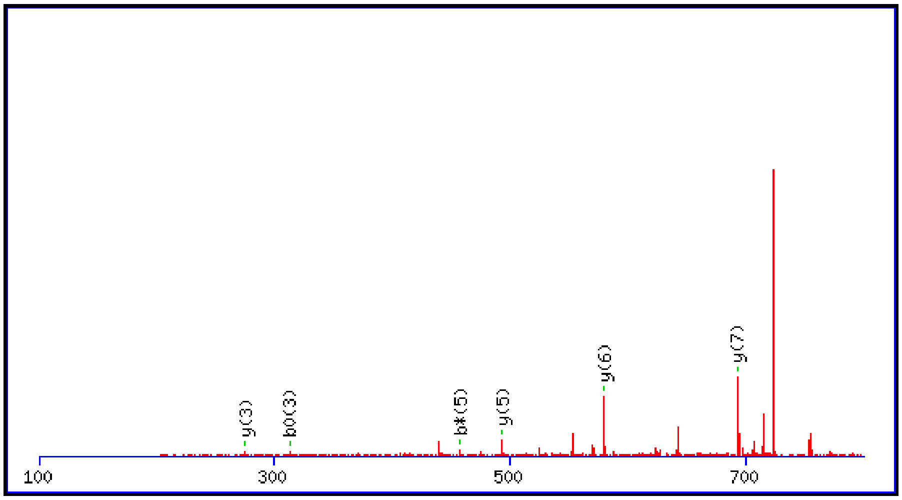


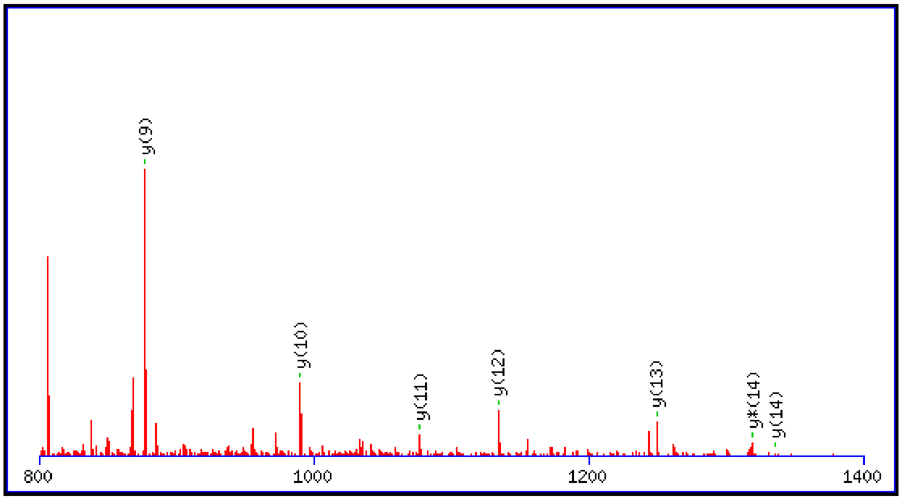

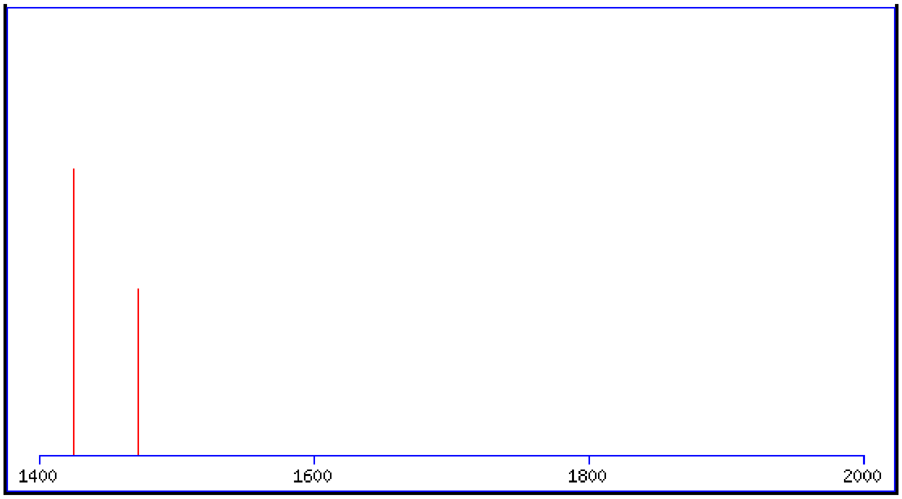


**Monoisotopic mass of neutral peptide Mr(calc):** 1463.7290
**Fixed modifications:** Carbamidomethyl (C)
**Variable modifications:**
**N3** : Deamidated_N (N)
**Ions Score:** 84 **Expect:** 5.6e-007
**Matches (Bold Red):** 13/158 fragment ions using 22 most intense peaks

| # | b | $b^{++}$ | b* | $b^{*++}$ | $b^0$ | $b^{0++}$ | Seq. | y | $y^{++}$ | y* | $y^{*++}$ | $y^0$ | $y^{0++}$ | # |
|---|---|---|---|---|---|---|---|---|---|---|---|---|---|---|
| 1 | 129.0659 | 65.0366 | 112.0393 | 56.5233 | | | Q | | | | | | | 15 |
| 2 | 216.0979 | 108.5526 | 199.0713 | 100.0393 | 198.0873 | 99.5473 | S | **1336.6777** | 668.8425 | **1319.6511** | 660.3292 | 1318.6671 | 659.8372 | 14 |
| 3 | 331.1248 | 166.0660 | 314.0983 | 157.5528 | **313.1143** | 157.0608 | N | **1249.6457** | 625.3265 | 1232.6191 | 616.8132 | 1231.6351 | 616.3212 | 13 |
| 4 | 388.1463 | 194.5768 | 371.1197 | 186.0635 | 370.1357 | 185.5715 | G | **1134.6187** | 567.8130 | 1117.5922 | 559.2997 | 1116.6082 | 558.8077 | 12 |
| 5 | 475.1783 | 238.0928 | **458.1518** | 229.5795 | 457.1677 | 229.0875 | S | **1077.5973** | 539.3023 | 1060.5707 | 530.7890 | 1059.5867 | 530.2970 | 11 |
| 6 | 588.2624 | 294.6348 | 571.2358 | 286.1216 | 570.2518 | 285.6295 | I | **990.5652** | 495.7863 | 973.5387 | 487.2730 | 972.5547 | 486.7810 | 10 |
| 7 | 659.2995 | 330.1534 | 642.2729 | 321.6401 | 641.2889 | 321.1481 | A | **877.4812** | 439.2442 | 860.4546 | 430.7309 | 859.4706 | 430.2389 | 9 |
| 8 | 772.3836 | 386.6954 | 755.3570 | 378.1821 | 754.3730 | 377.6901 | I | 806.4441 | 403.7257 | 789.4175 | 395.2124 | 788.4335 | 394.7204 | 8 |
| 9 | 885.4676 | 443.2374 | 868.4411 | 434.7242 | 867.4571 | 434.2322 | I | **693.3600** | 347.1836 | 676.3334 | 338.6704 | 675.3494 | 338.1783 | 7 |
| 10 | 972.4996 | 486.7535 | 955.4731 | 478.2402 | 954.4891 | 477.7482 | S | **580.2759** | 290.6416 | 563.2494 | 282.1283 | 562.2654 | 281.6363 | 6 |
| 11 | 1059.5317 | 530.2695 | 1042.5051 | 521.7562 | 1041.5211 | 521.2642 | S | **493.2439** | 247.1256 | 476.2173 | 238.6123 | 475.2333 | 238.1203 | 5 |
| 12 | 1190.5722 | 595.7897 | 1173.5456 | 587.2764 | 1172.5616 | 586.7844 | M | 406.2119 | 203.6096 | 389.1853 | 195.0963 | | | 4 |
| 13 | 1261.6093 | 631.3083 | 1244.5827 | 622.7950 | 1243.5987 | 622.3030 | A | **275.1714** | 138.0893 | 258.1448 | 129.5761 | | | 3 |
| 14 | 1318.6307 | 659.8190 | 1301.6042 | 651.3057 | 1300.6202 | 650.8137 | G | 204.1343 | 102.5708 | 187.1077 | 94.0575 | | | 2 |
| 15 | | | | | | | K | 147.1128 | 74.0600 | 130.0863 | 65.5468 | | | 1 |

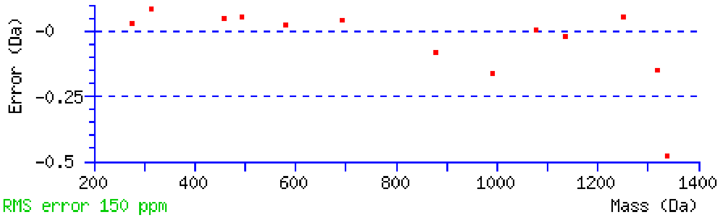


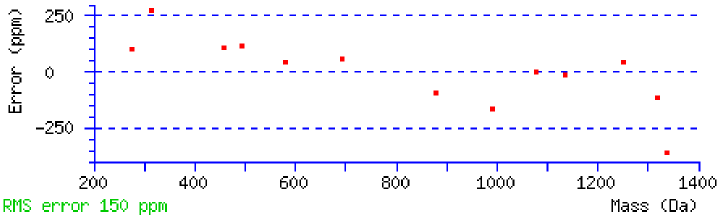


### All matches to this query

| Score | Mr(calc): | Delta | Sequence |
|---|---|---|---|
| 84.0 | 1463.7290 | 0.0035 | QSNGSIAIISSMAGK |
| 14.9 | 1462.7272 | 1.0053 | RMEEKPLSSMQK |
| 8.4 | 1463.7425 | -0.0101 | YEFLTKNTLKK |
| 8.4 | 1463.7425 | -0.0101 | YEFLTKNTLKK |
| 5.7 | 1462.7337 | 0.9987 | KAEEQLALATNMK |
| 1.0 | 1463.7442 | -0.0118 | LKEVGYSTHMVGK |
| | | | |

| | | | |
|---|---|---|---|
| 0.8 | 1463.7319 | 0.0005 | HLLMSIDQRKK |
| 0.8 | 1462.7238 | 1.0086 | KQNAPMTLEEFR |
| 0.6 | 1463.7385 | -0.0061 | ATVPALQQKTEAK |
| 0.6 | 1463.7231 | 0.0093 | WVNQLKYHMTK |

---

Spectrum No: 186; Query: 1102; Rank: 1

---

## Peptide View

MS/MS Fragmentation of **VFDKEGNGTVMGAEIR**
Found in **IPI00365944**, Tax_Id=10116 Gene_Symbol=Myl6_predicted Myosin light polypeptide 6

Match to Query 1102: 1722.830128 from(862.422340,2+)
Title: 100101RatKid_NS_deglyco_09.2141.2141.2.dta
Data file K:\NewmanPaper\Piliang\3SubProteomes\Piliang3SP\mgf5ppm\ERLIC_3SubProteomes5ppm.mgf

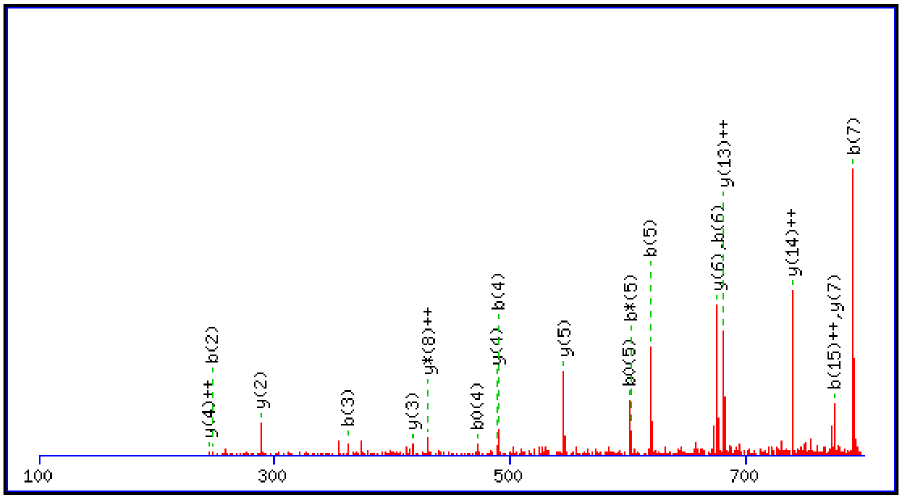


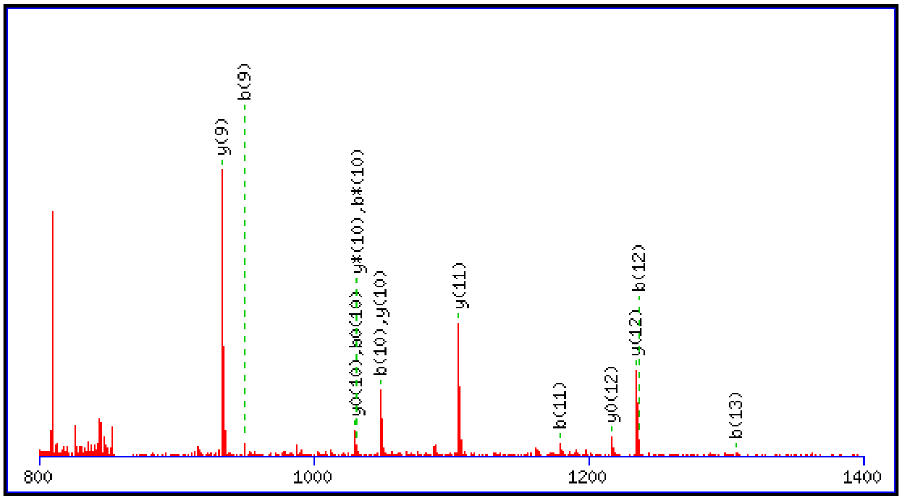

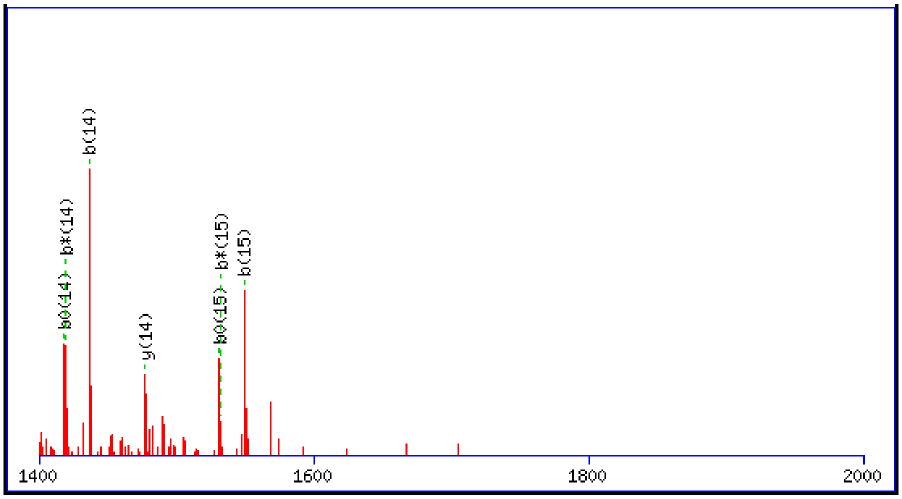


**Monoisotopic mass of neutral peptide Mr(calc):** 1722.8247
**Fixed modifications:** Carbamidomethyl (C)
**Variable modifications:**
**N7** : Deamidated_N (N)
**Ions Score:** 84 **Expect:** 8.8e-007
**Matches (Bold Red):** 41/166 fragment ions using 73 most intense peaks

| # | b | $b^{++}$ | $b^{*}$ | $b^{*++}$ | $b^{0}$ | $b^{0++}$ | Seq. | y | $y^{++}$ | $y^{*}$ | $y^{*++}$ | $y^{0}$ | $y^{0++}$ | # |
|---|---|---|---|---|---|---|---|---|---|---|---|---|---|---|
| 1 | 100.0757 | 50.5415 | | | | | V | | | | | | | 16 |
| 2 | **247.1441** | 124.0757 | | | | | F | 1624.7635 | 812.8854 | 1607.7370 | 804.3721 | 1606.7530 | 803.8801 | 15 |
| 3 | **362.1710** | 181.5892 | | | 344.1605 | 172.5839 | D | **1477.6951** | **739.3512** | 1460.6686 | 730.8379 | 1459.6846 | 730.3459 | 14 |
| 4 | **490.2660** | 245.6366 | 473.2395 | 237.1234 | **472.2554** | 236.6314 | K | 1362.6682 | **681.8377** | 1345.6416 | 673.3245 | 1344.6576 | 672.8324 | 13 |
| 5 | **619.3086** | 310.1579 | **602.2821** | 301.6447 | **601.2980** | 301.1527 | E | **1234.5732** | 617.7902 | 1217.5467 | 609.2770 | **1216.5627** | 608.7850 | 12 |
| 6 | **676.3301** | 338.6687 | 659.3035 | 330.1554 | 658.3195 | 329.6634 | G | **1105.5306** | 553.2690 | 1088.5041 | 544.7557 | 1087.5201 | 544.2637 | 11 |
| 7 | **791.3570** | 396.1821 | 774.3305 | 387.6689 | 773.3464 | 387.1769 | N | **1048.5092** | 524.7582 | **1031.4826** | 516.2449 | **1030.4986** | 515.7529 | 10 |
| 8 | 848.3785 | 424.6929 | 831.3519 | 416.1796 | 830.3679 | 415.6876 | G | **933.4822** | 467.2448 | 916.4557 | 458.7315 | 915.4717 | 458.2395 | 9 |
| 9 | **949.4261** | 475.2167 | 932.3996 | 466.7034 | 931.4156 | 466.2114 | T | 876.4608 | 438.7340 | 859.4342 | **430.2207** | 858.4502 | 429.7287 | 8 |
| 10 | **1048.4946** | 524.7509 | **1031.4680** | 516.2376 | **1030.4840** | 515.7456 | V | **775.4131** | 388.2102 | 758.3865 | 379.6969 | 757.4025 | 379.2049 | 7 |
| 11 | **1179.5350** | 590.2712 | 1162.5085 | 581.7579 | 1161.5245 | 581.2659 | M | **676.3447** | 338.6760 | 659.3181 | 330.1627 | 658.3341 | 329.6707 | 6 |
| 12 | **1236.5565** | 618.7819 | 1219.5300 | 610.2686 | 1218.5459 | 609.7766 | G | **545.3042** | 273.1557 | 528.2776 | 264.6425 | 527.2936 | 264.1504 | 5 |
| 13 | **1307.5936** | 654.3004 | 1290.5671 | 645.7872 | 1289.5831 | 645.2952 | A | **488.2827** | **244.6450** | 471.2562 | 236.1317 | 470.2722 | 235.6397 | 4 |
| 14 | **1436.6362** | 718.8217 | **1419.6097** | 710.3085 | **1418.6257** | 709.8165 | E | **417.2456** | 209.1264 | 400.2191 | 200.6132 | 399.2350 | 200.1212 | 3 |
| 15 | **1549.7203** | **775.3638** | **1532.6937** | 766.8505 | **1531.7097** | 766.3585 | I | **288.2030** | 144.6051 | 271.1765 | 136.0919 | | | 2 |
| 16 | | | | | | | R | 175.1190 | 88.0631 | 158.0924 | 79.5498 | | | 1 |

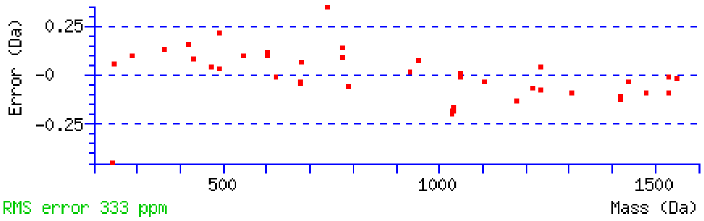


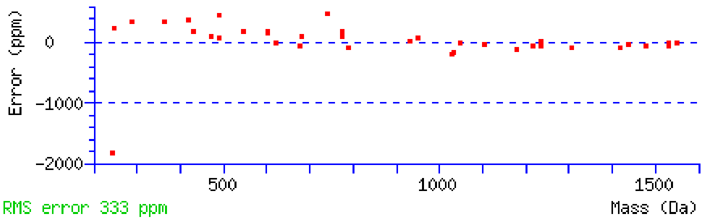


### All matches to this query

| Score | Mr(calc): | Delta | Sequence |
|---|---|---|---|
| 83.9 | 1722.8247 | 0.0055 | VFDKEGNGTVMGAEIR |
| 83.9 | 1722.8247 | 0.0055 | VFDKEGNGTVMGAELR |
| 24.7 | 1721.8407 | 0.9895 | VFDKEGNGTVMGAEIR |
| 24.7 | 1721.8407 | 0.9895 | VFDKEGNGTVMGAELR |
| 12.1 | 1722.8213 | 0.0088 | NEDLFSQPVSFNGIR |
| | | | |

| | | | |
|---|---|---|---|
| 7.3 | 1722.8280 | 0.0021 | DKEVAALTSQMDMLR |
| 7.3 | 1722.8280 | 0.0021 | DKEVAALTSQMDMLR |
| 7.0 | 1722.8414 | -0.0113 | GTAQGTPRNSXGTVLR |
| 6.1 | 1722.8276 | 0.0025 | NKYHPDLRMVTIR |
| 6.0 | 1722.8230 | 0.0072 | WDDDSTIVTPIILR |

---

Spectrum No: 187; Query: 1102; Rank: 1

---

## Peptide View

MS/MS Fragmentation of **VFDKEGNGTVMGAEIR**
Found in **IPI00365944**, Tax_Id=10116 Gene_Symbol=Myl6_predicted Myosin light polypeptide 6

Match to Query 1102: 1722.830128 from(862.422340,2+)
Title: 100101RatKid_NS_deglyco_09.2141.2141.2.dta
Data file K:\NewmanPaper\Piliang\3SubProteomes\Piliang3SP\mgf5ppm\ERLIC_3SubProteomes5ppm.mgf

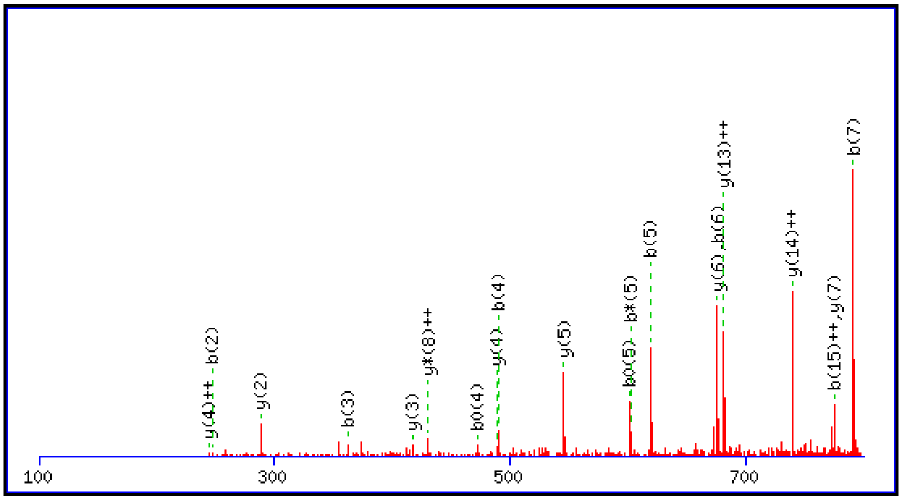


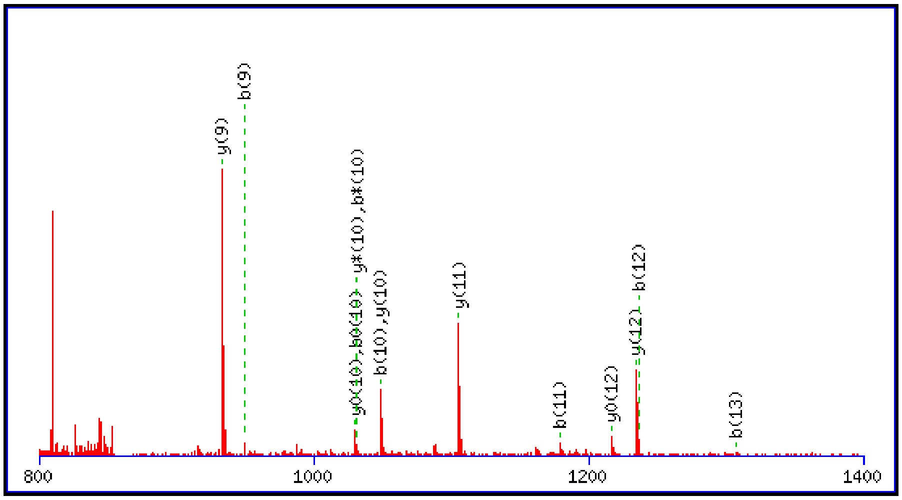

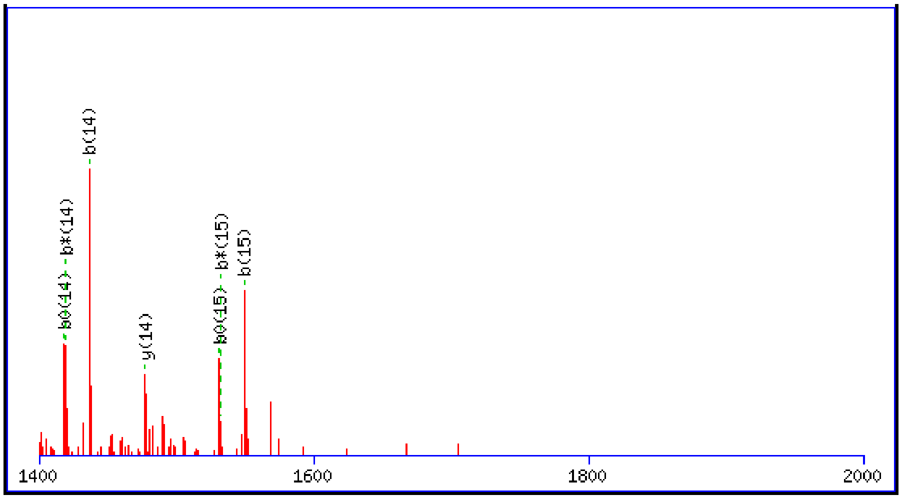


**Monoisotopic mass of neutral peptide Mr(calc):** 1722.8247
**Fixed modifications:** Carbamidomethyl (C)
**Variable modifications:**
**N7** : Deamidated_N (N)
**Ions Score:** 84 **Expect:** 8.8e-007
**Matches (Bold Red):** 41/166 fragment ions using 73 most intense peaks

| # | b | $b^{++}$ | b* | $b^{*++}$ | $b^0$ | $b^{0++}$ | Seq. | y | $y^{++}$ | y* | $y^{*++}$ | $y^0$ | $y^{0++}$ | # |
|---|---|---|---|---|---|---|---|---|---|---|---|---|---|---|
| 1 | 100.0757 | 50.5415 | | | | | V | | | | | | | 16 |
| 2 | **247.1441** | 124.0757 | | | | | F | 1624.7635 | 812.8854 | 1607.7370 | 804.3721 | 1606.7530 | 803.8801 | 15 |
| 3 | **362.1710** | 181.5892 | | | 344.1605 | 172.5839 | D | **1477.6951** | **739.3512** | 1460.6686 | 730.8379 | 1459.6846 | 730.3459 | 14 |
| 4 | **490.2660** | 245.6366 | 473.2395 | 237.1234 | **472.2554** | 236.6314 | K | 1362.6682 | **681.8377** | 1345.6416 | 673.3245 | 1344.6576 | 672.8324 | 13 |
| 5 | **619.3086** | 310.1579 | **602.2821** | 301.6447 | **601.2980** | 301.1527 | E | **1234.5732** | 617.7902 | 1217.5467 | 609.2770 | **1216.5627** | 608.7850 | 12 |
| 6 | **676.3301** | 338.6687 | 659.3035 | 330.1554 | 658.3195 | 329.6634 | G | **1105.5306** | 553.2690 | 1088.5041 | 544.7557 | 1087.5201 | 544.2637 | 11 |
| 7 | **791.3570** | 396.1821 | 774.3305 | 387.6689 | 773.3464 | 387.1769 | N | **1048.5092** | 524.7582 | **1031.4826** | 516.2449 | **1030.4986** | 515.7529 | 10 |
| 8 | 848.3785 | 424.6929 | 831.3519 | 416.1796 | 830.3679 | 415.6876 | G | **933.4822** | 467.2448 | 916.4557 | 458.7315 | 915.4717 | 458.2395 | 9 |
| 9 | **949.4261** | 475.2167 | 932.3996 | 466.7034 | 931.4156 | 466.2114 | T | 876.4608 | 438.7340 | 859.4342 | **430.2207** | 858.4502 | 429.7287 | 8 |
| 10 | **1048.4946** | 524.7509 | **1031.4680** | 516.2376 | **1030.4840** | 515.7456 | V | **775.4131** | 388.2102 | 758.3865 | 379.6969 | 757.4025 | 379.2049 | 7 |
| 11 | **1179.5350** | 590.2712 | 1162.5085 | 581.7579 | 1161.5245 | 581.2659 | M | **676.3447** | 338.6760 | 659.3181 | 330.1627 | 658.3341 | 329.6707 | 6 |
| 12 | **1236.5565** | 618.7819 | 1219.5300 | 610.2686 | 1218.5459 | 609.7766 | G | **545.3042** | 273.1557 | 528.2776 | 264.6425 | 527.2936 | 264.1504 | 5 |
| 13 | **1307.5936** | 654.3004 | 1290.5671 | 645.7872 | 1289.5831 | 645.2952 | A | **488.2827** | **244.6450** | 471.2562 | 236.1317 | 470.2722 | 235.6397 | 4 |
| 14 | **1436.6362** | 718.8217 | **1419.6097** | 710.3085 | **1418.6257** | 709.8165 | E | **417.2456** | 209.1264 | 400.2191 | 200.6132 | 399.2350 | 200.1212 | 3 |
| 15 | **1549.7203** | **775.3638** | **1532.6937** | 766.8505 | **1531.7097** | 766.3585 | I | **288.2030** | 144.6051 | 271.1765 | 136.0919 | | | 2 |
| 16 | | | | | | | R | 175.1190 | 88.0631 | 158.0924 | 79.5498 | | | 1 |

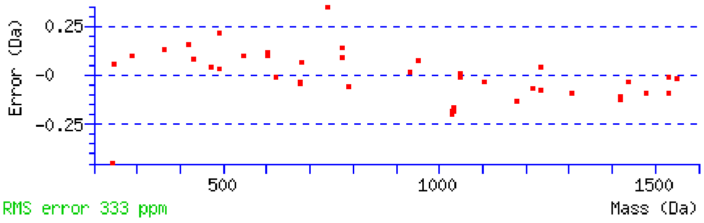


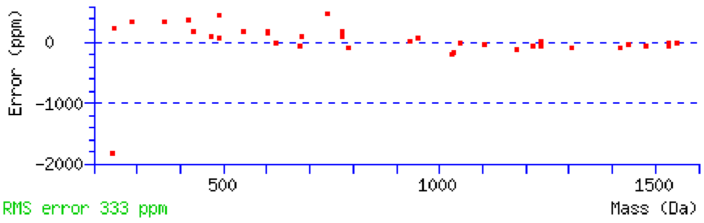


### All matches to this query

| Score | Mr(calc): | Delta | Sequence |
|---|---|---|---|
| 83.9 | 1722.8247 | 0.0055 | VFDKEGNGTVMGAEIR |
| 83.9 | 1722.8247 | 0.0055 | VFDKEGNGTVMGAELR |
| 24.7 | 1721.8407 | 0.9895 | VFDKEGNGTVMGAEIR |
| 24.7 | 1721.8407 | 0.9895 | VFDKEGNGTVMGAELR |
| 12.1 | 1722.8213 | 0.0088 | NEDLFSQPVSFNGIR |
| | | | |

| | | | |
|---|---|---|---|
| 7.3 | 1722.8280 | 0.0021 | DKEVAALTSQMDMLR |
| 7.3 | 1722.8280 | 0.0021 | DKEVAALTSQMDMLR |
| 7.0 | 1722.8414 | -0.0113 | GTAQGTPRNSXGTVLR |
| 6.1 | 1722.8276 | 0.0025 | NKYHPDLRMVTIR |
| 6.0 | 1722.8230 | 0.0072 | WDDDSTIVTPIILR |

---

Spectrum No: 188; Query: 650; Rank: 1

---

## Peptide View

MS/MS Fragmentation of **VGPNGTLFVVDAYK**
Found in **IPI00382223**, Tax_Id=10116 Gene_Symbol=RGD1308874 Ab2-305

Match to Query 650: 1479.756968 from(740.885760,2+)
Title: 091008RatKidney_NH4Format01_23.2960.2960.2.dta
Data file K:\NewmanPaper\Piliang\3SubProteomes\Piliang3SP\mgf5ppm\ERLIC_3SubProteomes5ppm.mgf

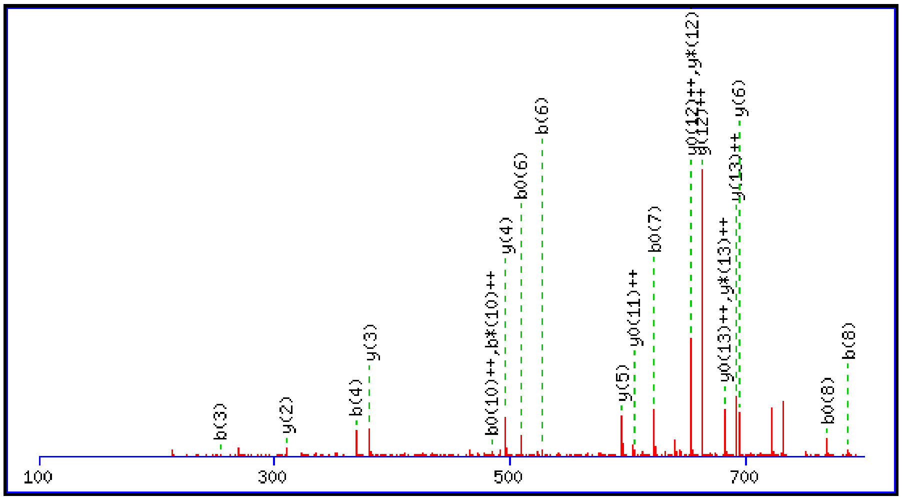


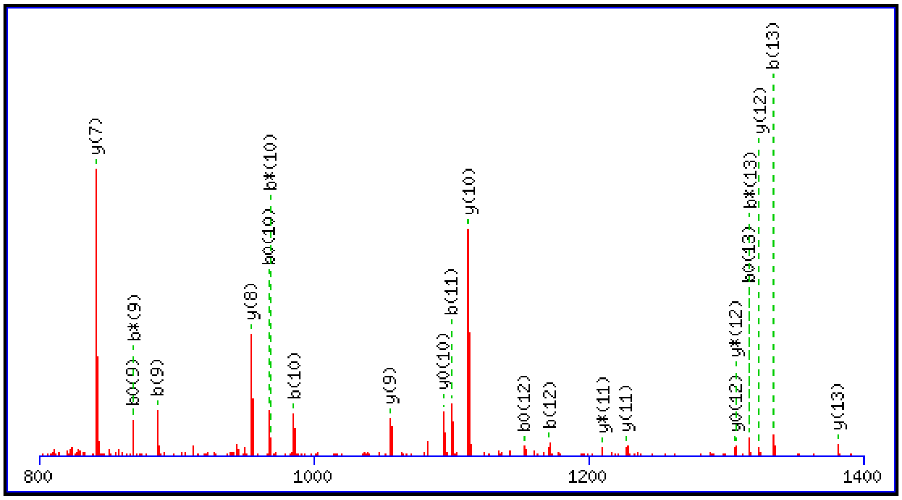

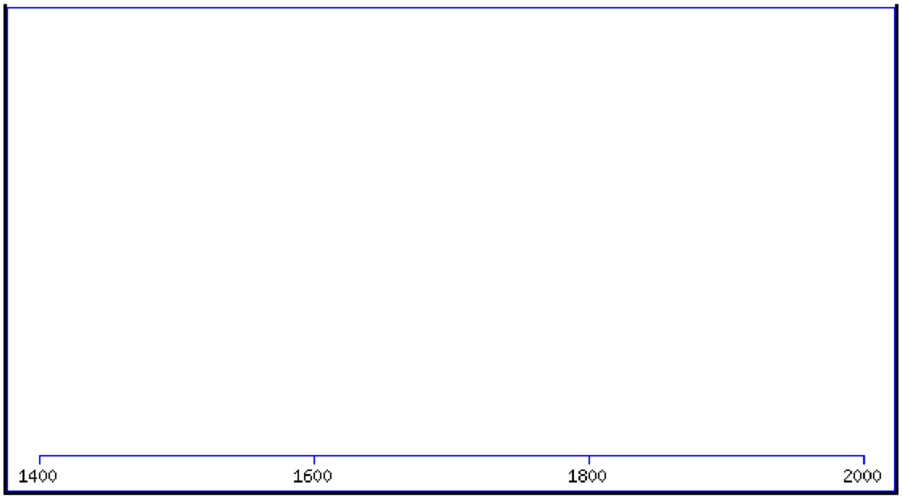


**Monoisotopic mass of neutral peptide Mr(calc):** 1479.7610
**Fixed modifications:** Carbamidomethyl (C)
**Variable modifications:**
**N4** : Deamidated_N (N)
**Ions Score:** 84 **Expect:** 6.5e-007
**Matches (Bold Red):** 44/134 fragment ions using 72 most intense peaks

| # | b | b++ | b* | b*++ | b0 | b0++ | Seq. | y | y++ | y* | y*++ | y0 | y0++ | # |
|---|---|---|---|---|---|---|---|---|---|---|---|---|---|---|
| 1 | 100.0757 | 50.5415 | | | | | V | | | | | | | 14 |
| 2 | 157.0972 | 79.0522 | | | | | G | **1381.6998** | **691.3535** | 1364.6733 | **682.8403** | 1363.6892 | **682.3483** | 13 |
| 3 | **254.1499** | 127.5786 | | | | | P | **1324.6783** | **662.8428** | **1307.6518** | **654.3295** | **1306.6678** | **653.8375** | 12 |
| 4 | **369.1769** | 185.0921 | 352.1503 | 176.5788 | | | N | **1227.6256** | 614.3164 | **1210.5990** | 605.8032 | 1209.6150 | **605.3111** | 11 |
| 5 | 426.1983 | 213.6028 | 409.1718 | 205.0895 | | | G | **1112.5986** | 556.8030 | 1095.5721 | 548.2897 | **1094.5881** | 547.7977 | 10 |
| 6 | **527.2460** | 264.1266 | 510.2194 | 255.6134 | **509.2354** | 255.1214 | T | **1055.5772** | 528.2922 | 1038.5506 | 519.7790 | 1037.5666 | 519.2869 | 9 |
| 7 | 640.3301 | 320.6687 | 623.3035 | 312.1554 | **622.3195** | 311.6634 | L | **954.5295** | 477.7684 | 937.5029 | 469.2551 | 936.5189 | 468.7631 | 8 |
| 8 | **787.3985** | 394.2029 | 770.3719 | 385.6896 | **769.3879** | 385.1976 | F | **841.4454** | 421.2264 | 824.4189 | 412.7131 | 823.4349 | 412.2211 | 7 |
| 9 | **886.4669** | 443.7371 | **869.4403** | 435.2238 | **868.4563** | 434.7318 | V | **694.3770** | 347.6921 | 677.3505 | 339.1789 | 676.3665 | 338.6869 | 6 |
| 10 | **985.5353** | 493.2713 | **968.5088** | **484.7580** | **967.5247** | **484.2660** | V | **595.3086** | 298.1579 | 578.2821 | 289.6447 | 577.2980 | 289.1527 | 5 |
| 11 | **1100.5622** | 550.7848 | 1083.5357 | 542.2715 | 1082.5517 | 541.7795 | D | **496.2402** | 248.6237 | 479.2136 | 240.1105 | 478.2296 | 239.6185 | 4 |
| 12 | **1171.5994** | 586.3033 | 1154.5728 | 577.7900 | **1153.5888** | 577.2980 | A | **381.2132** | 191.1103 | 364.1867 | 182.5970 | | | 3 |
| 13 | **1334.6627** | 667.8350 | **1317.6361** | 659.3217 | **1316.6521** | 658.8297 | Y | **310.1761** | 155.5917 | 293.1496 | 147.0784 | | | 2 |
| 14 | | | | | | | K | 147.1128 | 74.0600 | 130.0863 | 65.5468 | | | 1 |

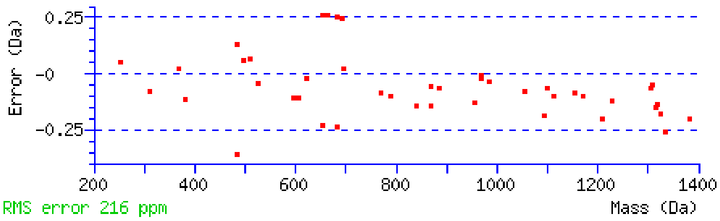


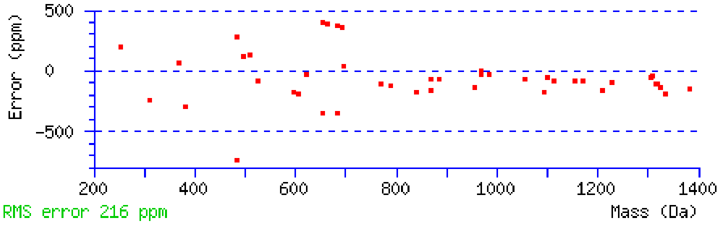


### All matches to this query

| Score | Mr(calc): | Delta | Sequence |
|---|---|---|---|
| 83.9 | 1479.7610 | -0.0040 | VGPNGTLFVVDAYK |
| 9.7 | 1479.7538 | 0.0032 | RLQTICLSGTGMK |
| 6.7 | 1479.7711 | -0.0142 | RLGTLAFTTRHK |
| 6.3 | 1479.7711 | -0.0142 | RLGTLAFTTRHK |
| 5.6 | 1479.7711 | -0.0142 | RLGTLAFTTRHK |
| 2.0 | 1479.7446 | 0.0124 | RLEAELEAVSRK |
| 1.4 | 1477.7541 | 2.0028 | LRPTGIISADVEK |
| | | | |

| | | | |
|---|---|---|---|
| 0.2 | 1478.7412 | 1.0157 | RLPSAAGPGPGCPSR |

---

Spectrum No: 189; Query: 776; Rank: 1

---

## Peptide View

MS/MS Fragmentation of **ITDIENGTFANIPR**
Found in **IPI00365784**, Tax_Id=10116 Gene_Symbol=LOC306805 Similar to asporin

Match to Query 776: 1560.777088 from(781.395820,2+)
Title: 091008RatKidney_NoSalt_26.2794.2794.2.dta
Data file K:\NewmanPaper\Piliang\3SubProteomes\Piliang3SP\mgf5ppm\ERLIC_3SubProteomes5ppm.mgf

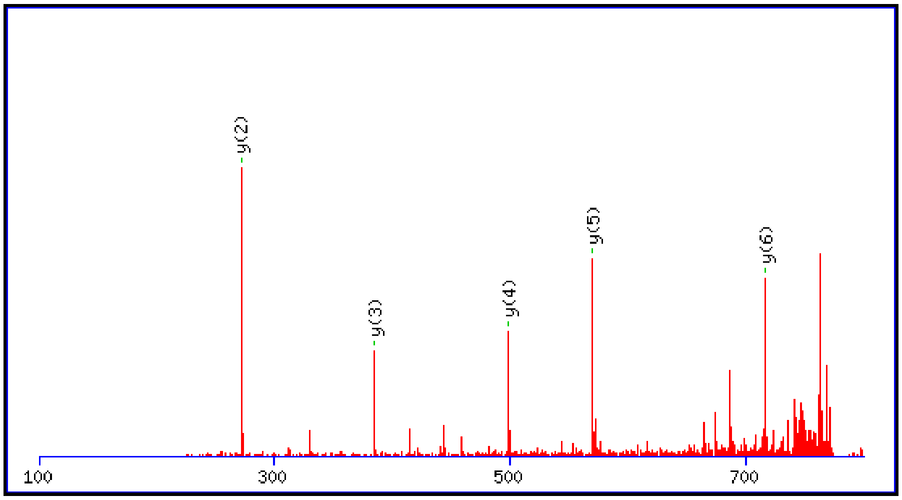


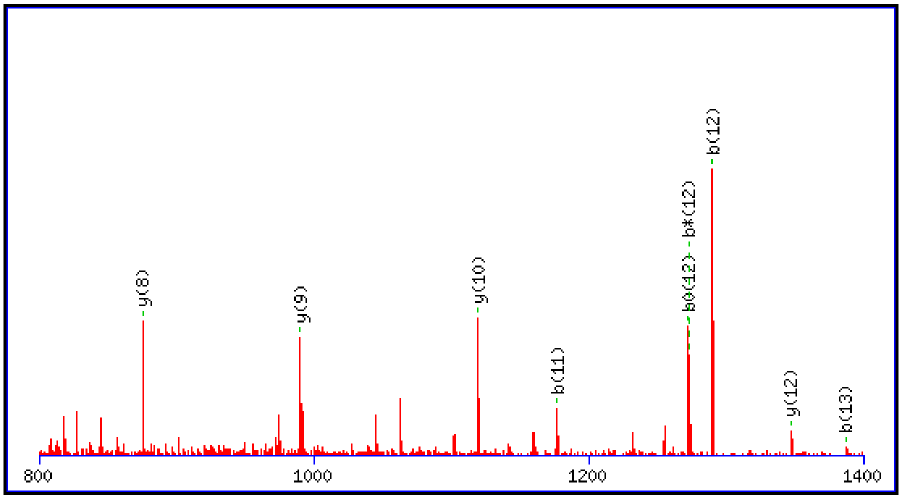

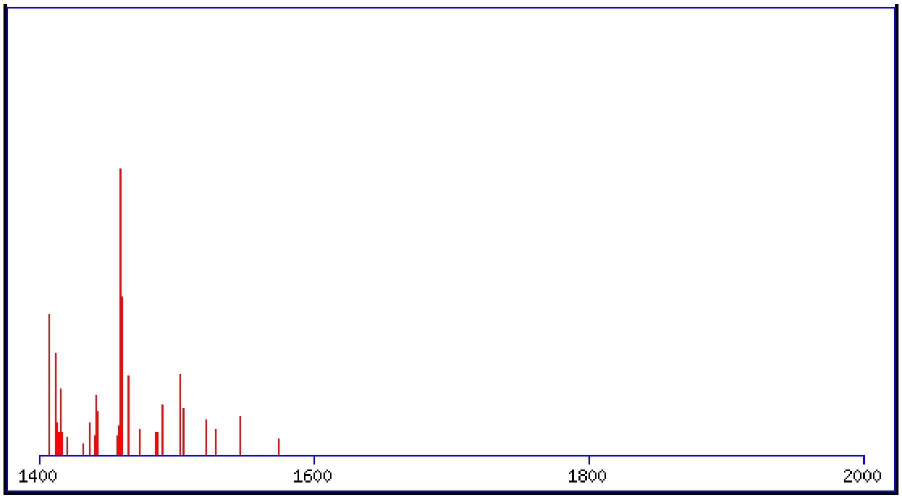


**Monoisotopic mass of neutral peptide Mr(calc):** 1560.7784
**Fixed modifications:** Carbamidomethyl (C)
**Variable modifications:**
**N6** : Deamidated_N (N)
**Ions Score:** 84 **Expect:** 7.1e-007
**Matches (Bold Red):** 14/132 fragment ions using 26 most intense peaks

| # | b | $b^{++}$ | $b^{*}$ | $b^{*++}$ | $b^{0}$ | $b^{0++}$ | Seq. | y | $y^{++}$ | $y^{*}$ | $y^{*++}$ | $y^{0}$ | $y^{0++}$ | # |
|---|---|---|---|---|---|---|---|---|---|---|---|---|---|---|
| 1 | 114.0913 | 57.5493 | | | | | I | | | | | | | 14 |
| 2 | 215.1390 | 108.0731 | | | 197.1285 | 99.0679 | T | 1448.7016 | 724.8544 | 1431.6750 | 716.3412 | 1430.6910 | 715.8492 | 13 |
| 3 | 330.1660 | 165.5866 | | | 312.1554 | 156.5813 | D | **1347.6539** | 674.3306 | 1330.6274 | 665.8173 | 1329.6433 | 665.3253 | 12 |
| 4 | 443.2500 | 222.1287 | | | 425.2395 | 213.1234 | I | 1232.6270 | 616.8171 | 1215.6004 | 608.3038 | 1214.6164 | 607.8118 | 11 |
| 5 | 572.2926 | 286.6499 | | | 554.2821 | 277.6447 | E | **1119.5429** | 560.2751 | 1102.5164 | 551.7618 | 1101.5323 | 551.2698 | 10 |
| 6 | 687.3196 | 344.1634 | 670.2930 | 335.6501 | 669.3090 | 335.1581 | N | **990.5003** | 495.7538 | 973.4738 | 487.2405 | 972.4897 | 486.7485 | 9 |
| 7 | 744.3410 | 372.6741 | 727.3145 | 364.1609 | 726.3305 | 363.6689 | G | **875.4734** | 438.2403 | 858.4468 | 429.7271 | 857.4628 | 429.2350 | 8 |
| 8 | 845.3887 | 423.1980 | 828.3621 | 414.6847 | 827.3781 | 414.1927 | T | 818.4519 | 409.7296 | 801.4254 | 401.2163 | 800.4413 | 400.7243 | 7 |
| 9 | 992.4571 | 496.7322 | 975.4306 | 488.2189 | 974.4465 | 487.7269 | F | **717.4042** | 359.2058 | 700.3777 | 350.6925 | | | 6 |
| 10 | 1063.4942 | 532.2508 | 1046.4677 | 523.7375 | 1045.4837 | 523.2455 | A | **570.3358** | 285.6715 | 553.3093 | 277.1583 | | | 5 |
| 11 | **1177.5372** | 589.2722 | 1160.5106 | 580.7589 | 1159.5266 | 580.2669 | N | **499.2987** | 250.1530 | 482.2722 | 241.6397 | | | 4 |
| 12 | **1290.6212** | 645.8142 | **1273.5947** | 637.3010 | **1272.6107** | 636.8090 | I | **385.2558** | 193.1315 | 368.2292 | 184.6183 | | | 3 |
| 13 | **1387.6740** | 694.3406 | 1370.6474 | 685.8274 | 1369.6634 | 685.3353 | P | **272.1717** | 136.5895 | 255.1452 | 128.0762 | | | 2 |
| 14 | | | | | | | R | 175.1190 | 88.0631 | 158.0924 | 79.5498 | | | 1 |

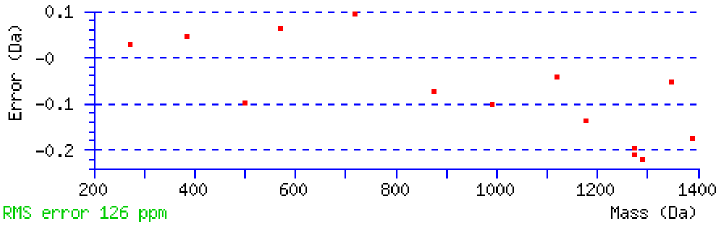


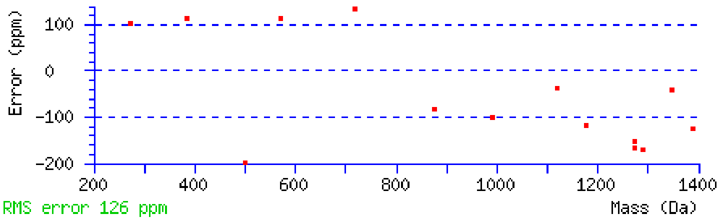


### All matches to this query

| Score | Mr(calc): | Delta | Sequence |
|---|---|---|---|
| 83.5 | 1560.7784 | -0.0013 | ITDIENGTFANIPR |
| 38.6 | 1560.7784 | -0.0013 | ITDIENGTFANIPR |
| 6.3 | 1560.7631 | 0.0140 | NSLKENSTSLNQPK |
| 4.1 | 1558.7579 | 2.0192 | ATGGPLKSMTLFTR |
| 3.0 | 1560.7661 | 0.0110 | TIIEYRSQKTSR |
| 3.0 | 1560.7661 | 0.0110 | TIIEYRSQKTSR |
| 2.5 | 1559.7878 | 0.9893 | RLXHTTMSNPAAAK |
| | | | |

| | | | |
|---|---|---|---|
| 1.9 | 1560.7864 | -0.0093 | DRARTQMMAEKPK |
| 1.0 | 1558.7634 | 2.0137 | RLQGDKNGNMRPR |
| 0.9 | 1560.7644 | 0.0127 | SPSWTKTRAEQNR |

---

Spectrum No: 190; Query: 1421; Rank: 1

---

## Peptide View

MS/MS Fragmentation of **TILVDNNTWNNTHISR**
Found in **IPI00560990**, Tax_Id=10116 Gene_Symbol=Stt3a_predicted similar to Oligosaccharyl transferase STT3 subunit homolog

Match to Query 1421: 1897.934048 from(949.974300,2+)
Title: 100101RatKid_NS_deglyco_12.2555.2555.2.dta
Data file K:\NewmanPaper\Piliang\3SubProteomes\Piliang3SP\mgf5ppm\ERLIC_3SubProteomes5ppm.mgf

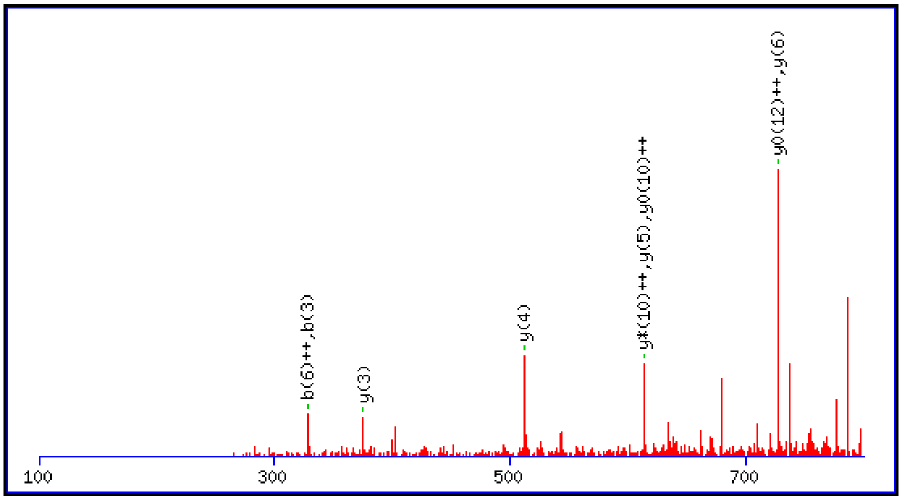


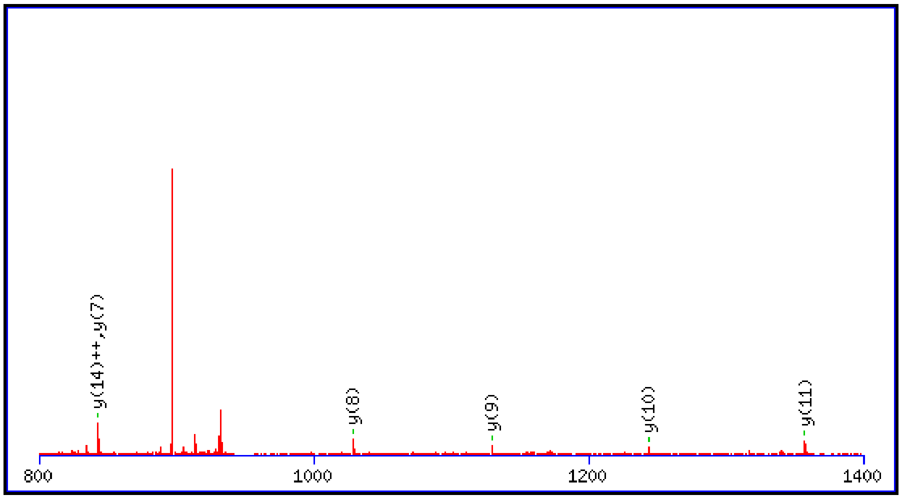

Mascot Search Results: Peptide View

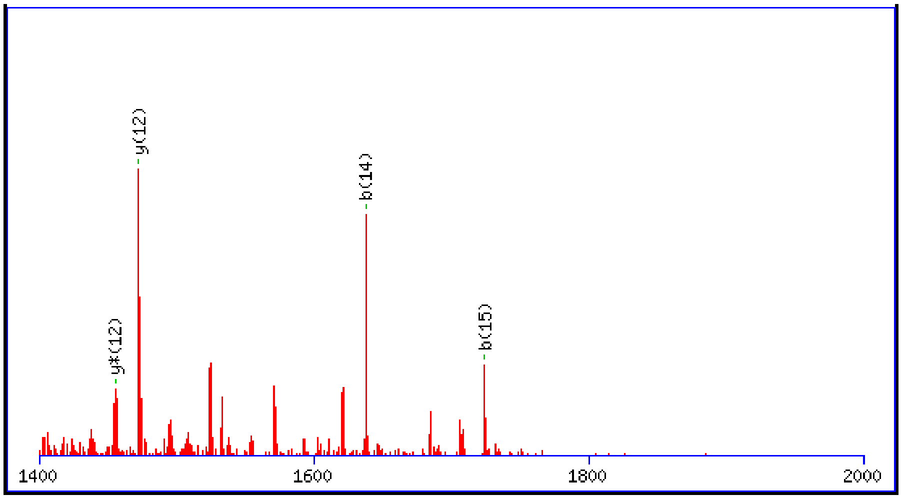


**Monoisotopic mass of neutral peptide Mr(calc):** 1897.9282
**Fixed modifications:** Carbamidomethyl (C)
**Variable modifications:**
**N10** : Deamidated_N (N)
**Ions Score:** 83 **Expect:** 8.3e-007
**Matches (Bold Red):** 19/168 fragment ions using 29 most intense peaks

| # | b | b++ | b* | b*++ | b0 | b0++ | Seq. | y | y++ | y* | y*++ | y0 | y0++ | # |
|---|---|---|---|---|---|---|---|---|---|---|---|---|---|---|
| 1 | 102.0550 | 51.5311 | | | 84.0444 | 42.5258 | T | | | | | | | 16 |
| 2 | 215.1390 | 108.0731 | | | 197.1285 | 99.0679 | I | 1797.8878 | 899.4476 | 1780.8613 | 890.9343 | 1779.8773 | 890.4423 | 15 |
| 3 | **328.2231** | 164.6152 | | | 310.2125 | 155.6099 | L | 1684.8038 | **842.9055** | 1667.7772 | 834.3922 | 1666.7932 | 833.9002 | 14 |
| 4 | 427.2915 | 214.1494 | | | 409.2809 | 205.1441 | V | 1571.7197 | 786.3635 | 1554.6932 | 777.8502 | 1553.7091 | 777.3582 | 13 |
| 5 | 542.3184 | 271.6629 | | | 524.3079 | 262.6576 | D | **1472.6513** | 736.8293 | **1455.6247** | 728.3160 | 1454.6407 | **727.8240** | 12 |
| 6 | 656.3614 | **328.6843** | 639.3348 | 320.1710 | 638.3508 | 319.6790 | N | **1357.6243** | 679.3158 | 1340.5978 | 670.8025 | 1339.6138 | 670.3105 | 11 |
| 7 | 770.4043 | 385.7058 | 753.3777 | 377.1925 | 752.3937 | 376.7005 | N | **1243.5814** | 622.2943 | 1226.5549 | **613.7811** | 1225.5709 | **613.2891** | 10 |
| 8 | 871.4520 | 436.2296 | 854.4254 | 427.7163 | 853.4414 | 427.2243 | T | **1129.5385** | 565.2729 | 1112.5119 | 556.7596 | 1111.5279 | 556.2676 | 9 |
| 9 | 1057.5313 | 529.2693 | 1040.5047 | 520.7560 | 1039.5207 | 520.2640 | W | **1028.4908** | 514.7490 | 1011.4643 | 506.2358 | 1010.4802 | 505.7438 | 8 |
| 10 | 1172.5582 | 586.7827 | 1155.5317 | 578.2695 | 1154.5477 | 577.7775 | N | **842.4115** | 421.7094 | 825.3849 | 413.1961 | 824.4009 | 412.7041 | 7 |
| 11 | 1286.6011 | 643.8042 | 1269.5746 | 635.2909 | 1268.5906 | 634.7989 | N | **727.3846** | 364.1959 | 710.3580 | 355.6826 | 709.3740 | 355.1906 | 6 |
| 12 | 1387.6488 | 694.3281 | 1370.6223 | 685.8148 | 1369.6383 | 685.3228 | T | **613.3416** | 307.1745 | 596.3151 | 298.6612 | 595.3311 | 298.1692 | 5 |
| 13 | 1524.7077 | 762.8575 | 1507.6812 | 754.3442 | 1506.6972 | 753.8522 | H | **512.2940** | 256.6506 | 495.2674 | 248.1373 | 494.2834 | 247.6453 | 4 |
| 14 | **1637.7918** | 819.3995 | 1620.7653 | 810.8863 | 1619.7812 | 810.3943 | I | **375.2350** | 188.1212 | 358.2085 | 179.6079 | 357.2245 | 179.1159 | 3 |
| 15 | **1724.8238** | 862.9156 | 1707.7973 | 854.4023 | 1706.8133 | 853.9103 | S | 262.1510 | 131.5791 | 245.1244 | 123.0659 | 244.1404 | 122.5738 | 2 |
| 16 | | | | | | | R | 175.1190 | 88.0631 | 158.0924 | 79.5498 | | | 1 |

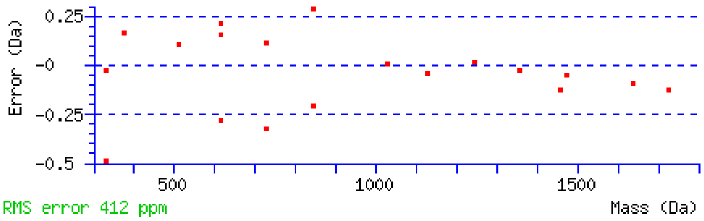


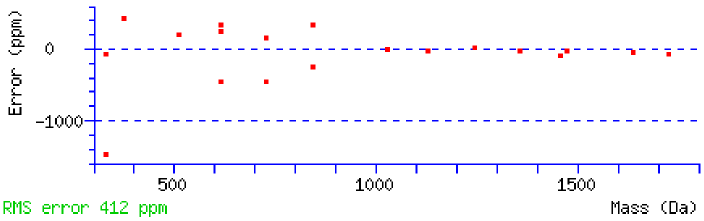


### All matches to this query

| Score | Mr(calc): | Delta | Sequence |
|---|---|---|---|
| 83.5 | 1897.9282 | 0.0058 | TILVDNNTWNNTHISR |
| 71.5 | 1897.9282 | 0.0058 | TILVDNNTWNNTHISR |
| 40.7 | 1897.9282 | 0.0058 | TILVDNNTWNNTHISR |
| 30.9 | 1897.9282 | 0.0058 | TILVDNNTWNNTHISR |
| 7.0 | 1897.9519 | -0.0178 | VDISMGLICVRLLSSR |
| | | | |

| | | | |
|---|---|---|---|
| 3.8 | 1896.9337 | 1.0004 | QRLNGQCXNKSETLHR |
| 3.7 | 1896.9234 | 1.0107 | VLNASAIPEGQFIDSKK |
| 3.1 | 1895.9434 | 1.9906 | GSDSLSILFSYVFILR |
| 2.9 | 1897.9186 | 0.0154 | LLIYNTNNLQTGIPSR |
| 2.9 | 1897.9186 | 0.0154 | LLIYNTNNLQTGIPSR |

Spectrum No: 191; Query: 1111; Rank: 1

## Peptide View

MS/MS Fragmentation of **SCVTITDAFPQNMSR**
Found in **IPI00370450**, Tax_Id=10116 Gene_Symbol=Plxnb2 plexin B2

Match to Query 1111: 1726.768668 from(864.391610,2+)
Title: 100101RatKid_NS_deglyco_25.2968.2968.2.dta
Data file K:\NewmanPaper\Piliang\3SubProteomes\Piliang3SP\mgf5ppm\ERLIC_3SubProteomes5ppm.mgf

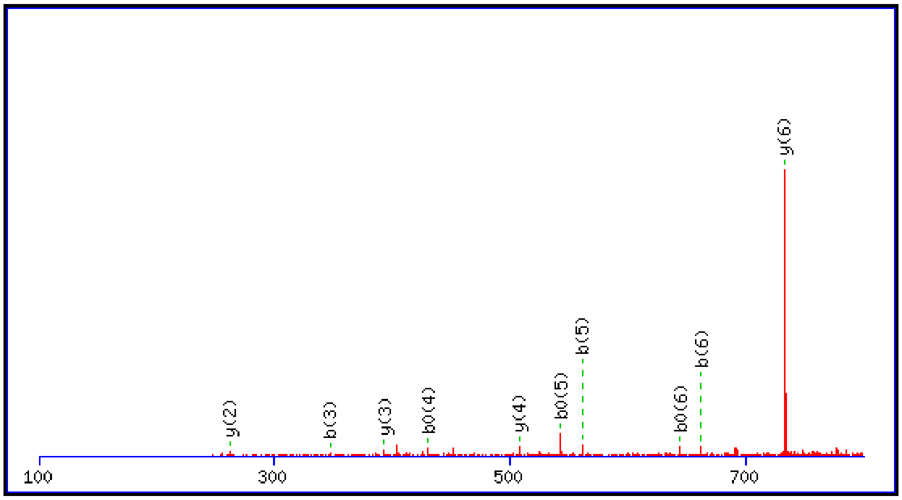


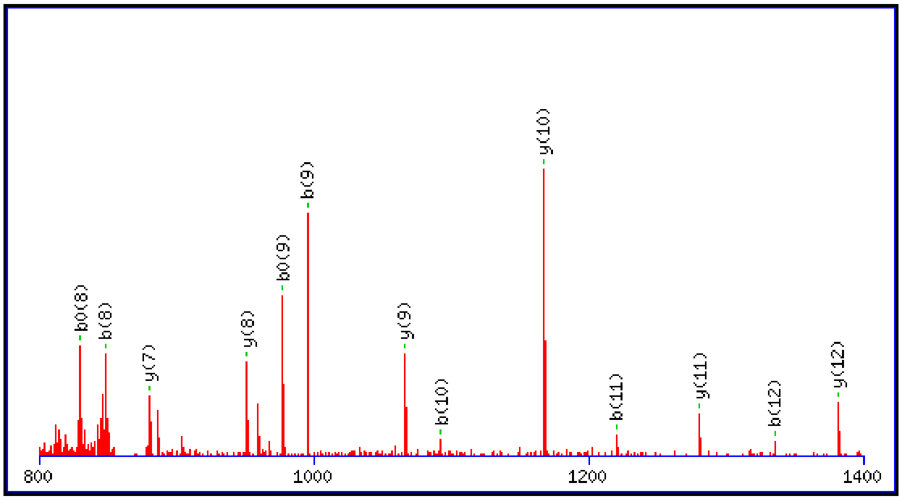

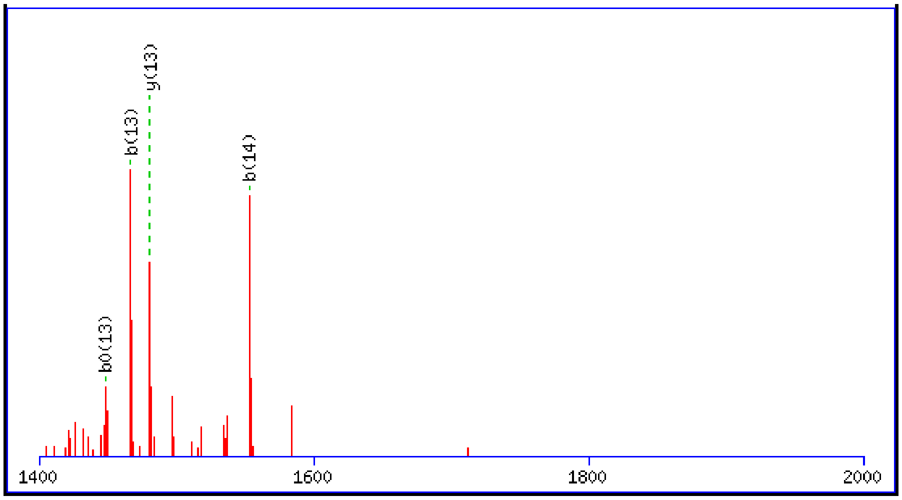


**Monoisotopic mass of neutral peptide Mr(calc):** 1726.7655
**Fixed modifications:** Carbamidomethyl (C)
**Variable modifications:**
**N12** : Deamidated_N (N)
**Ions Score:** 83 **Expect:** 9.6e-007
**Matches (Bold Red):** 27/146 fragment ions using 45 most intense peaks

| # | b | $b^{++}$ | b* | $b^{*++}$ | $b^0$ | $b^{0++}$ | Seq. | y | $y^{++}$ | y* | $y^{*++}$ | $y^0$ | $y^{0++}$ | # |
|---|---|---|---|---|---|---|---|---|---|---|---|---|---|---|
| 1 | 88.0393 | 44.5233 | | | 70.0287 | 35.5180 | S | | | | | | | 15 |
| 2 | 248.0700 | 124.5386 | | | 230.0594 | 115.5333 | C | 1640.7407 | 820.8740 | 1623.7142 | 812.3607 | 1622.7301 | 811.8687 | 14 |
| 3 | **347.1384** | 174.0728 | | | 329.1278 | 165.0675 | V | **1480.7100** | 740.8587 | 1463.6835 | 732.3454 | 1462.6995 | 731.8534 | 13 |
| 4 | 448.1860 | 224.5967 | | | **430.1755** | 215.5914 | T | **1381.6416** | 691.3245 | 1364.6151 | 682.8112 | 1363.6311 | 682.3192 | 12 |
| 5 | **561.2701** | 281.1387 | | | **543.2595** | 272.1334 | I | **1280.5940** | 640.8006 | 1263.5674 | 632.2873 | 1262.5834 | 631.7953 | 11 |
| 6 | **662.3178** | 331.6625 | | | **644.3072** | 322.6573 | T | **1167.5099** | 584.2586 | 1150.4833 | 575.7453 | 1149.4993 | 575.2533 | 10 |
| 7 | 777.3447 | 389.1760 | | | 759.3342 | 380.1707 | D | **1066.4622** | 533.7347 | 1049.4357 | 525.2215 | 1048.4516 | 524.7295 | 9 |
| 8 | **848.3818** | 424.6946 | | | **830.3713** | 415.6893 | A | **951.4353** | 476.2213 | 934.4087 | 467.7080 | 933.4247 | 467.2160 | 8 |
| 9 | **995.4503** | 498.2288 | | | **977.4397** | 489.2235 | F | **880.3982** | 440.7027 | 863.3716 | 432.1894 | 862.3876 | 431.6974 | 7 |
| 10 | **1092.5030** | 546.7551 | | | 1074.4925 | 537.7499 | P | **733.3297** | 367.1685 | 716.3032 | 358.6552 | 715.3192 | 358.1632 | 6 |
| 11 | **1220.5616** | 610.7844 | 1203.5351 | 602.2712 | 1202.5510 | 601.7792 | Q | 636.2770 | 318.6421 | 619.2504 | 310.1289 | 618.2664 | 309.6368 | 5 |
| 12 | **1335.5885** | 668.2979 | 1318.5620 | 659.7846 | 1317.5780 | 659.2926 | N | **508.2184** | 254.6128 | 491.1919 | 246.0996 | 490.2078 | 245.6076 | 4 |
| 13 | **1466.6290** | 733.8181 | 1449.6025 | 725.3049 | **1448.6185** | 724.8129 | M | **393.1915** | 197.0994 | 376.1649 | 188.5861 | 375.1809 | 188.0941 | 3 |
| 14 | **1553.6611** | 777.3342 | 1536.6345 | 768.8209 | 1535.6505 | 768.3289 | S | **262.1510** | 131.5791 | 245.1244 | 123.0659 | 244.1404 | 122.5738 | 2 |
| 15 | | | | | | | R | 175.1190 | 88.0631 | 158.0924 | 79.5498 | | | 1 |

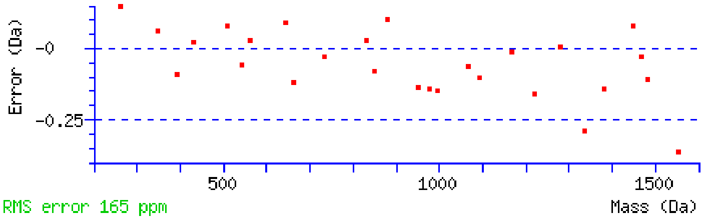


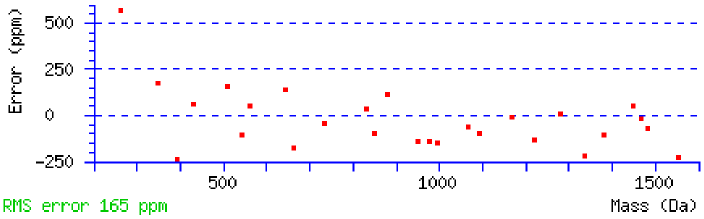


### All matches to this query

| Score | Mr(calc): | Delta | Sequence |
|---|---|---|---|
| 83.2 | 1726.7655 | 0.0032 | SCVTITDAFPQNMSR |
| 20.3 | 1725.7814 | 0.9872 | SCVTITDAFPQNMSR |
| 6.4 | 1726.7758 | -0.0071 | YRSAGSEETSESPTAR |
| 3.2 | 1726.7727 | -0.0040 | ASAKCGTGGKQMEGXSR |
| 3.2 | 1726.7644 | 0.0043 | NTMEKLRCVHTSR |
| 2.6 | 1726.7603 | 0.0083 | FYQDTISALNFTAR |
| | | | |

| | | | |
|---|---|---|---|
| 2.6 | 1726.7603 | 0.0083 | FYQDTISALNFTAR |
| 2.5 | 1724.7535 | 2.0151 | SLKPESGGNHIADLK |
| 2.1 | 1726.7610 | 0.0076 | MPVSRGRDSYGGPPR |
| 1.8 | 1726.7767 | -0.0080 | DGMNIVEAMERFGSR |

---

Spectrum No: 192; Query: 263; Rank: 1

---

## Peptide View

MS/MS Fragmentation of **LDFIVLNETR**
Found in **IPI00208422**, Tax_Id=10116 Gene_Symbol=Dpp4 Dipeptidyl peptidase 4

Match to Query 263: 1219.648868 from(610.831710,2+)
Title: 100101RatKid_NS_deglyco_12.4324.4324.2.dta
Data file K:\NewmanPaper\Piliang\3SubProteomes\Piliang3SP\mgf5ppm\ERLIC_3SubProteomes5ppm.mgf

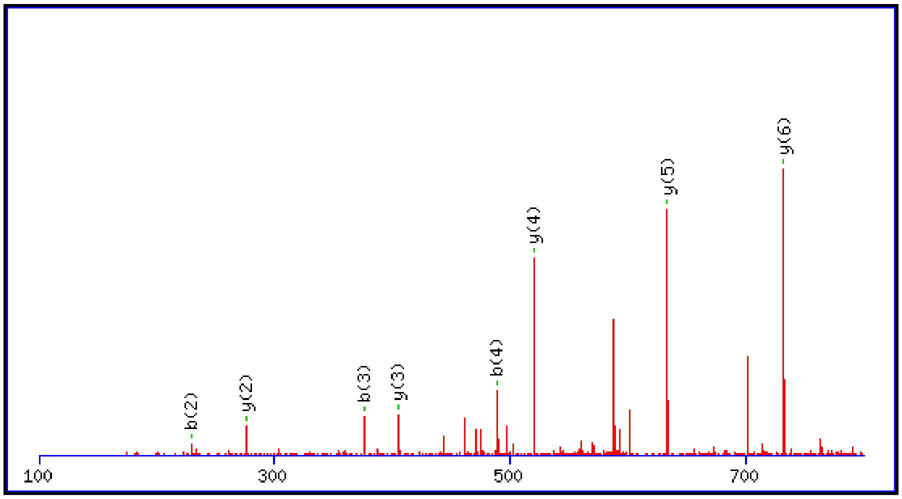


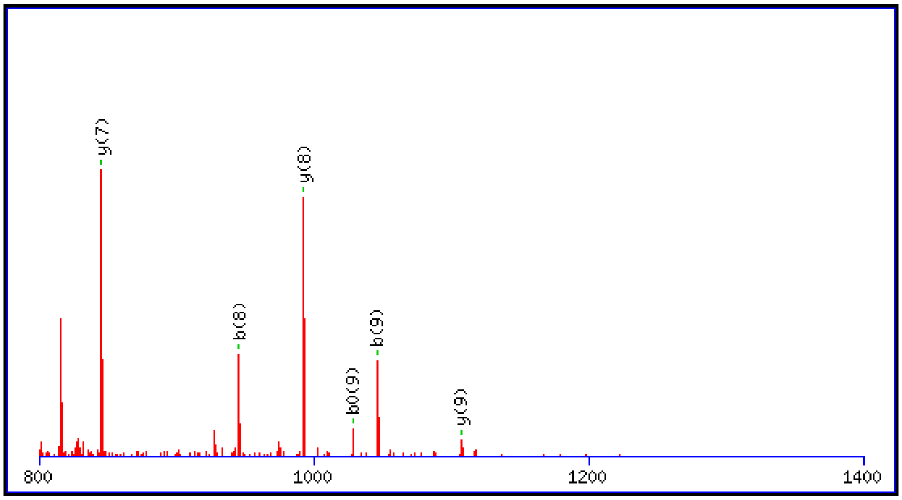

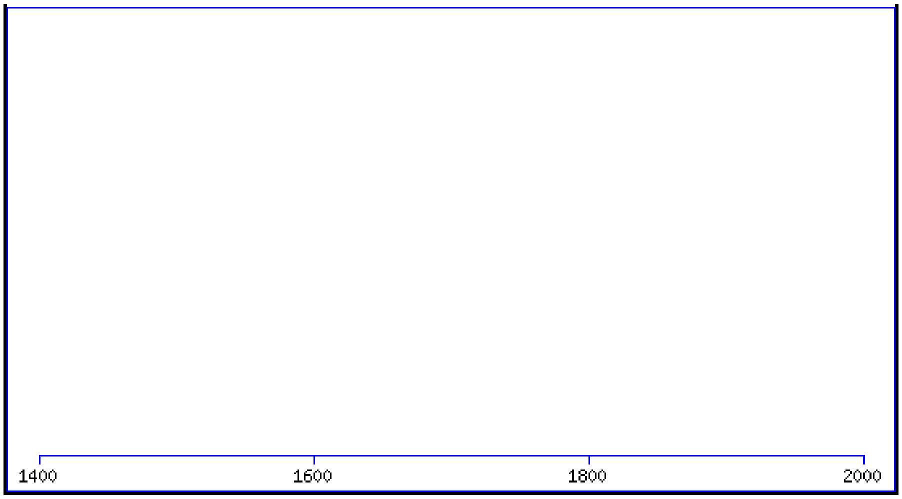


**Monoisotopic mass of neutral peptide Mr(calc):** 1219.6448
**Fixed modifications:** Carbamidomethyl (C)
**Variable modifications:**
**N7** : Deamidated_N (N)
**Ions Score:** 83 **Expect:** 6.1e-007
**Matches (Bold Red):** 14/92 fragment ions using 24 most intense peaks

| # | b | $b^{++}$ | b* | $b^{*++}$ | $b^{0}$ | $b^{0++}$ | Seq. | y | $y^{++}$ | y* | $y^{*++}$ | $y^{0}$ | $y^{0++}$ | # |
|---|---|---|---|---|---|---|---|---|---|---|---|---|---|---|
| 1 | 114.0913 | 57.5493 | | | | | L | | | | | | | 10 |
| 2 | **229.1183** | 115.0628 | | | 211.1077 | 106.0575 | D | **1107.5681** | 554.2877 | 1090.5415 | 545.7744 | 1089.5575 | 545.2824 | 9 |
| 3 | **376.1867** | 188.5970 | | | 358.1761 | 179.5917 | F | **992.5411** | 496.7742 | 975.5146 | 488.2609 | 974.5306 | 487.7689 | 8 |
| 4 | **489.2708** | 245.1390 | | | 471.2602 | 236.1337 | I | **845.4727** | 423.2400 | 828.4462 | 414.7267 | 827.4621 | 414.2347 | 7 |
| 5 | 588.3392 | 294.6732 | | | 570.3286 | 285.6679 | V | **732.3886** | 366.6980 | 715.3621 | 358.1847 | 714.3781 | 357.6927 | 6 |
| 6 | 701.4232 | 351.2153 | | | 683.4127 | 342.2100 | L | **633.3202** | 317.1638 | 616.2937 | 308.6505 | 615.3097 | 308.1585 | 5 |
| 7 | 816.4502 | 408.7287 | 799.4236 | 400.2155 | 798.4396 | 399.7234 | N | **520.2362** | 260.6217 | 503.2096 | 252.1084 | 502.2256 | 251.6164 | 4 |
| 8 | **945.4928** | 473.2500 | 928.4662 | 464.7367 | 927.4822 | 464.2447 | E | **405.2092** | 203.1082 | 388.1827 | 194.5950 | 387.1987 | 194.1030 | 3 |
| 9 | **1046.5404** | 523.7739 | 1029.5139 | 515.2606 | **1028.5299** | 514.7686 | T | **276.1666** | 138.5870 | 259.1401 | 130.0737 | 258.1561 | 129.5817 | 2 |
| 10 | | | | | | | R | 175.1190 | 88.0631 | 158.0924 | 79.5498 | | | 1 |

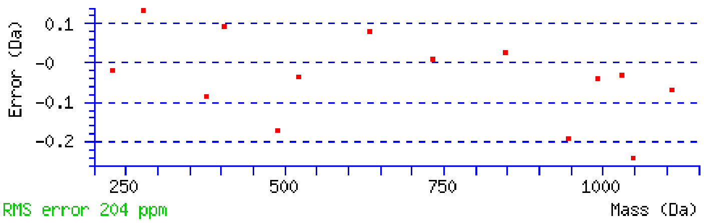


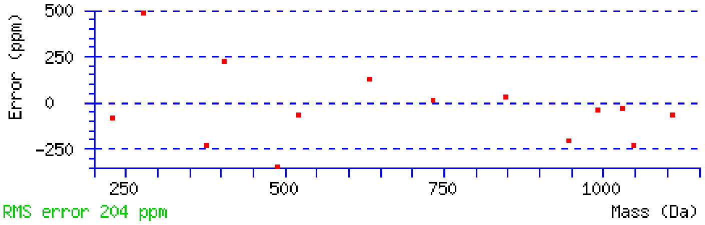


## All matches to this query

| Score | Mr(calc): | Delta | Sequence |
|---|---|---|---|
| 83.1 | 1219.6448 | 0.0040 | LDFIVLNETR |
| 9.9 | 1218.6431 | 1.0058 | YQPVVDLVMR |
| 4.9 | 1218.6456 | 1.0033 | TSSVLEGSALQK |
| 4.0 | 1219.6482 | 0.0007 | NLEIKKSMEK |
| 2.5 | 1218.6469 | 1.0020 | NLQNFITGRR |
| 2.0 | 1218.6397 | 1.0092 | NLFLSNVFHK |
| 1.4 | 1218.6413 | 1.0075 | VKFYIDVKK |
| 0.8 | 1217.6438 | 2.0051 | LMDDLLNKTR |
| 0.8 | 1219.6448 | 0.0040 | KFDQLLAEEK |
| 0.6 | 1219.6449 | 0.0040 | VQSGVVINFEK |

Spectrum No: 193; Query: 784; Rank: 1

## Peptide View

MS/MS Fragmentation of **TCNPETFPLNESR**
Found in **IPI00366496**, Tax_Id=10116 Gene_Symbol=Slc44a2_predicted similar to CTL2 gene

Match to Query 784: 1564.680988 from(783.347770,2+)
Title: 091008RatKidney_NH4Format01_27.1596.1596.2.dta
Data file K:\NewmanPaper\Piliang\3SubProteomes\Piliang3SP\mgf5ppm\ERLIC_3SubProteomes5ppm.mgf

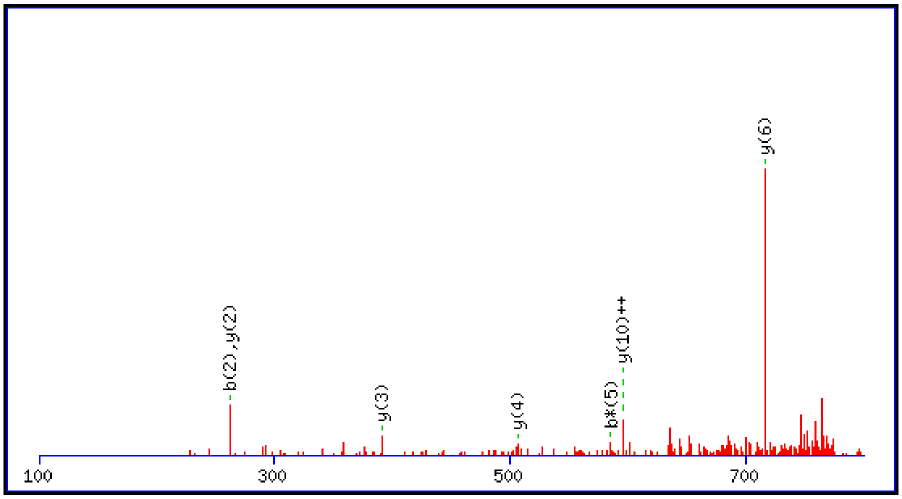


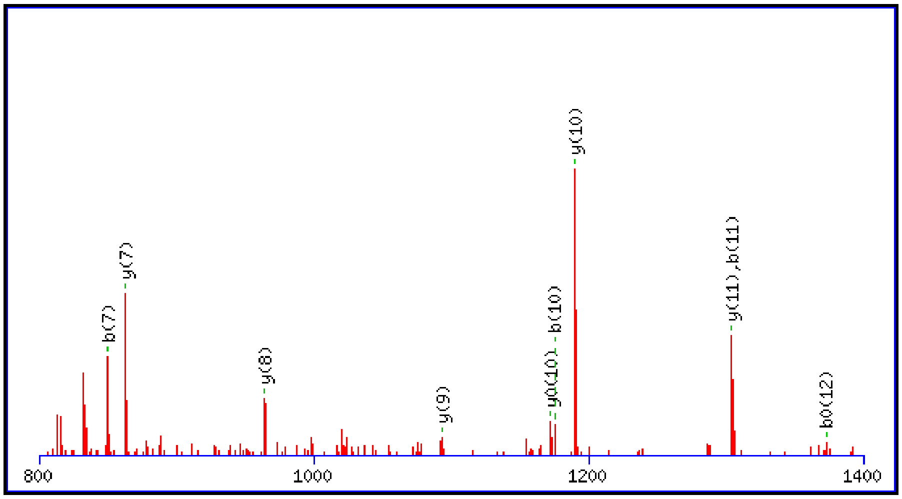


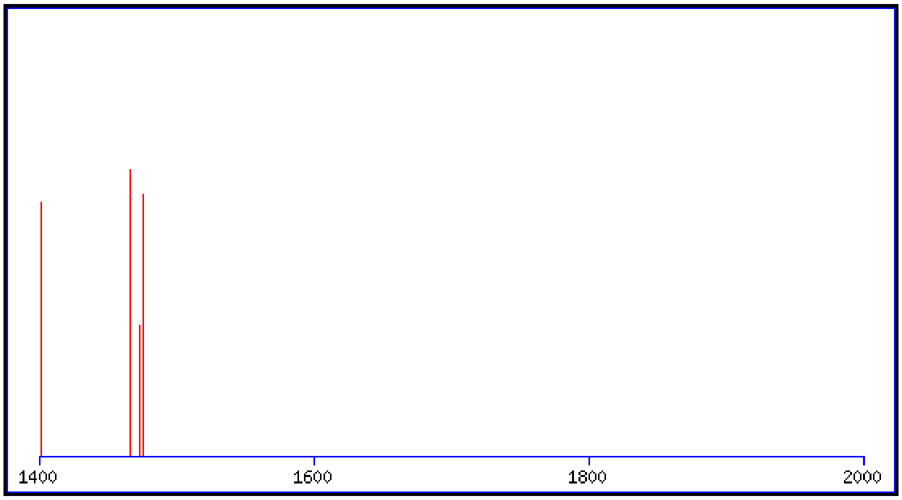


**Monoisotopic mass of neutral peptide Mr(calc):** 1564.6828

**Fixed modifications:** Carbamidomethyl (C)
**Variable modifications:**
**N10** : Deamidated_N (N)
**Ions Score:** 83 **Expect:** 7.9e-007
**Matches (Bold Red):** 17/138 fragment ions using 24 most intense peaks

| # | b | b++ | b* | b*++ | b0 | b0++ | Seq. | y | y++ | y* | y*++ | y0 | y0++ | # |
|---|---|---|---|---|---|---|---|---|---|---|---|---|---|---|
| 1 | 102.0550 | 51.5311 | | | 84.0444 | 42.5258 | T | | | | | | | 13 |
| 2 | **262.0856** | 131.5464 | | | 244.0750 | 122.5412 | C | 1464.6424 | 732.8248 | 1447.6158 | 724.3115 | 1446.6318 | 723.8195 | 12 |
| 3 | 376.1285 | 188.5679 | 359.1020 | 180.0546 | 358.1180 | 179.5626 | N | **1304.6117** | 652.8095 | 1287.5852 | 644.2962 | 1286.6011 | 643.8042 | 11 |
| 4 | 473.1813 | 237.0943 | 456.1547 | 228.5810 | 455.1707 | 228.0890 | P | **1190.5688** | **595.7880** | 1173.5422 | 587.2748 | **1172.5582** | 586.7827 | 10 |
| 5 | 602.2239 | 301.6156 | **585.1973** | 293.1023 | 584.2133 | 292.6103 | E | **1093.5160** | 547.2616 | 1076.4895 | 538.7484 | 1075.5055 | 538.2564 | 9 |
| 6 | 703.2716 | 352.1394 | 686.2450 | 343.6261 | 685.2610 | 343.1341 | T | **964.4734** | 482.7404 | 947.4469 | 474.2271 | 946.4629 | 473.7351 | 8 |
| 7 | **850.3400** | 425.6736 | 833.3134 | 417.1604 | 832.3294 | 416.6683 | F | **863.4258** | 432.2165 | 846.3992 | 423.7032 | 845.4152 | 423.2112 | 7 |
| 8 | 947.3927 | 474.2000 | 930.3662 | 465.6867 | 929.3822 | 465.1947 | P | **716.3573** | 358.6823 | 699.3308 | 350.1690 | 698.3468 | 349.6770 | 6 |
| 9 | 1060.4768 | 530.7420 | 1043.4503 | 522.2288 | 1042.4662 | 521.7368 | L | 619.3046 | 310.1559 | 602.2780 | 301.6426 | 601.2940 | 301.1506 | 5 |
| 10 | **1175.5037** | 588.2555 | 1158.4772 | 579.7422 | 1157.4932 | 579.2502 | N | **506.2205** | 253.6139 | 489.1940 | 245.1006 | 488.2099 | 244.6086 | 4 |
| 11 | **1304.5463** | 652.7768 | 1287.5198 | 644.2635 | 1286.5358 | 643.7715 | E | **391.1936** | 196.1004 | 374.1670 | 187.5871 | 373.1830 | 187.0951 | 3 |
| 12 | 1391.5784 | 696.2928 | 1374.5518 | 687.7795 | **1373.5678** | 687.2875 | S | **262.1510** | 131.5791 | 245.1244 | 123.0659 | 244.1404 | 122.5738 | 2 |
| 13 | | | | | | | R | 175.1190 | 88.0631 | 158.0924 | 79.5498 | | | 1 |

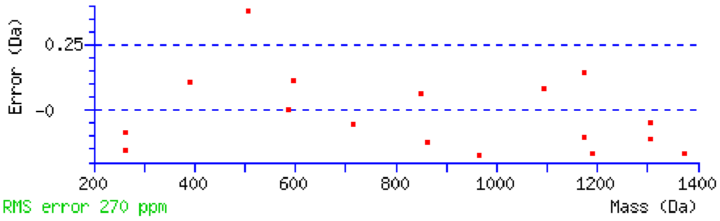


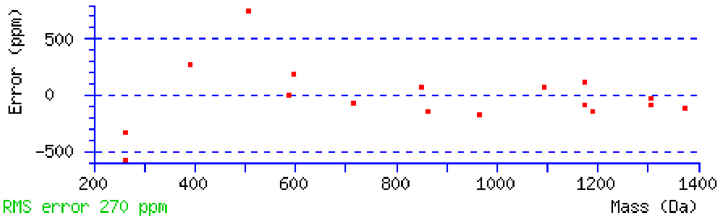


### All matches to this query

| Score | Mr(calc): | Delta | Sequence |
|---|---|---|---|
| 83.0 | 1564.6828 | -0.0018 | TCNPETFPLNESR |
| 10.1 | 1564.6884 | -0.0074 | EYTVDMPFKINK |
| 9.1 | 1564.6810 | -0.0000 | QTYEQANKIFDK |
| 9.1 | 1564.6810 | -0.0000 | QTYEQANKIFDK |
| 7.0 | 1562.6800 | 2.0010 | CYLKLSSNGSPTR |
| 7.0 | 1562.6800 | 2.0010 | CYLKLSSNGSPTR |
| 6.8 | 1562.6654 | 2.0156 | SRYAELDFEPEK |
| 4.1 | 1564.6658 | 0.0152 | LSSAYSLSASEQDK |
| 3.2 | 1564.6828 | -0.0018 | TCNPETFPLNESR |
| 2.9 | 1563.6698 | 1.0112 | DEEVAAFCHMLDK |

---

Spectrum No: 194; Query: 1561; Rank: 1

---

## Peptide View

MS/MS Fragmentation of **VNASTTDPNSTVEQSALTR**
Found in **IPI00365542**, Tax_Id=10116 Gene_Symbol=Lamb1_predicted laminin, beta 1

Match to Query 1561: 1990.942648 from(996.478600,2+)
Title: 091008RatKidney_NoSalt_22.1376.1376.2.dta
Data file K:\NewmanPaper\Piliang\3SubProteomes\Piliang3SP\mgf5ppm\ERLIC_3SubProteomes5ppm.mgf

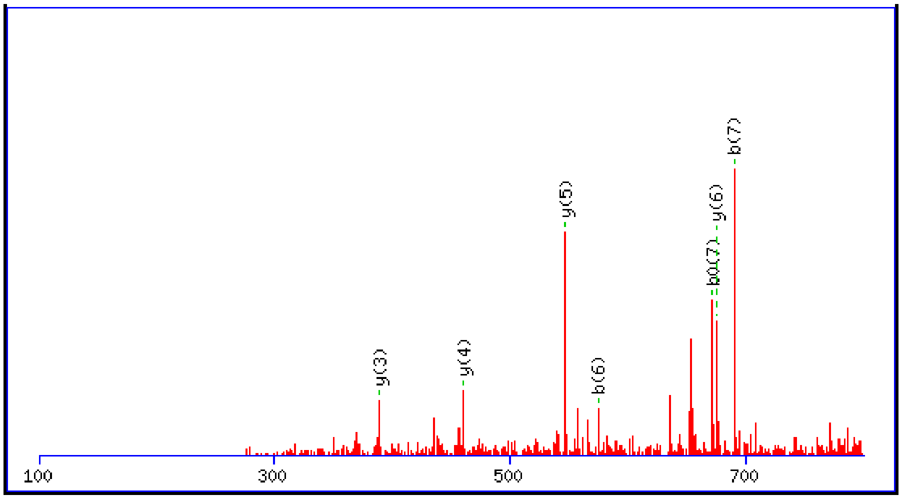


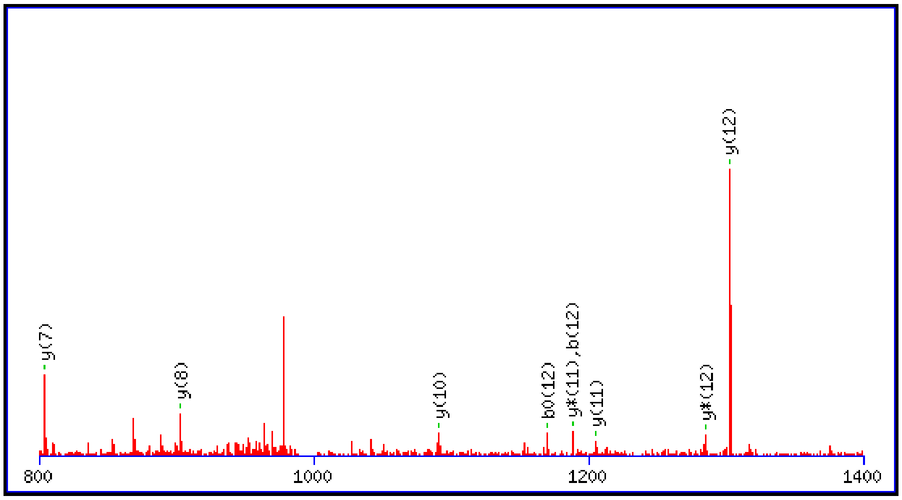


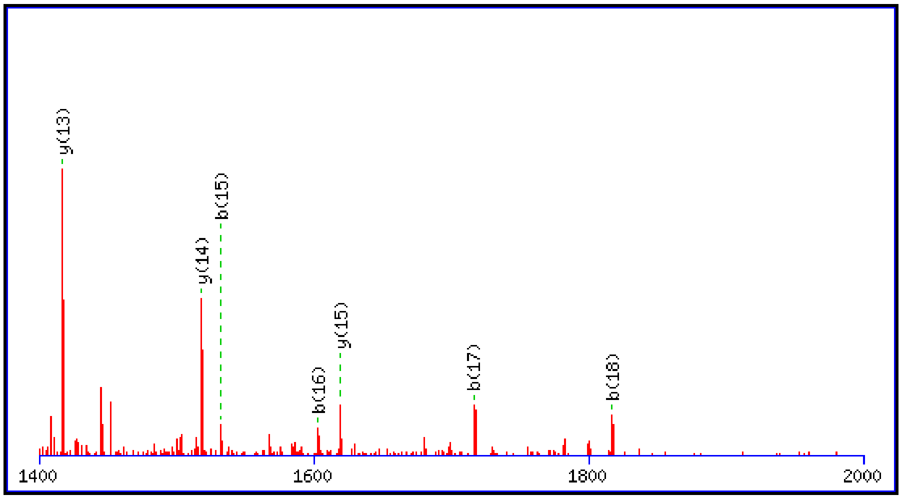


**Monoisotopic mass of neutral peptide Mr(calc):** 1990.9443
**Fixed modifications:** Carbamidomethyl (C)
**Variable modifications:**
**N2** : Deamidated_N (N)
**Ions Score:** 83 **Expect:** 1.2e-006
**Matches (Bold Red):** 23/206 fragment ions using 43 most intense peaks

| # | b | $b^{++}$ | b* | $b^{*++}$ | $b^{0}$ | $b^{0++}$ | Seq. | y | $y^{++}$ | y* | $y^{*++}$ | $y^{0}$ | $y^{0++}$ | # |
|---|---|---|---|---|---|---|---|---|---|---|---|---|---|---|
| 1 | 100.0757 | 50.5415 | | | | | V | | | | | | | 19 |
| 2 | 215.1026 | 108.0550 | 198.0761 | 99.5417 | | | N | 1892.8832 | 946.9452 | 1875.8567 | 938.4320 | 1874.8726 | 937.9400 | 18 |
| 3 | 286.1397 | 143.5735 | 269.1132 | 135.0602 | | | A | 1777.8563 | 889.4318 | 1760.8297 | 880.9185 | 1759.8457 | 880.4265 | 17 |
| 4 | 373.1718 | 187.0895 | 356.1452 | 178.5762 | 355.1612 | 178.0842 | S | 1706.8191 | 853.9132 | 1689.7926 | 845.3999 | 1688.8086 | 844.9079 | 16 |
| 5 | 474.2194 | 237.6134 | 457.1929 | 229.1001 | 456.2089 | 228.6081 | T | **1619.7871** | 810.3972 | 1602.7606 | 801.8839 | 1601.7766 | 801.3919 | 15 |

| | | | | | | | | | | | | | | |
|---|---|---|---|---|---|---|---|---|---|---|---|---|---|---|
| **6** | **575.2671** | 288.1372 | 558.2406 | 279.6239 | 557.2566 | 279.1319 | **T** | **1518.7394** | 759.8734 | 1501.7129 | 751.3601 | 1500.7289 | 750.8681 | **14** |
| **7** | **690.2941** | 345.6507 | 673.2675 | 337.1374 | **672.2835** | 336.6454 | **D** | **1417.6918** | 709.3495 | 1400.6652 | 700.8362 | 1399.6812 | 700.3442 | **13** |
| **8** | 787.3468 | 394.1771 | 770.3203 | 385.6638 | 769.3363 | 385.1718 | **P** | **1302.6648** | 651.8360 | **1285.6383** | 643.3228 | 1284.6543 | 642.8308 | **12** |
| **9** | 901.3898 | 451.1985 | 884.3632 | 442.6852 | 883.3792 | 442.1932 | **N** | **1205.6121** | 603.3097 | **1188.5855** | 594.7964 | 1187.6015 | 594.3044 | **11** |
| **10** | 988.4218 | 494.7145 | 971.3952 | 486.2013 | 970.4112 | 485.7092 | **S** | **1091.5691** | 546.2882 | 1074.5426 | 537.7749 | 1073.5586 | 537.2829 | **10** |
| **11** | 1089.4695 | 545.2384 | 1072.4429 | 536.7251 | 1071.4589 | 536.2331 | **T** | 1004.5371 | 502.7722 | 987.5106 | 494.2589 | 986.5265 | 493.7669 | **9** |
| **12** | **1188.5379** | 594.7726 | 1171.5113 | 586.2593 | **1170.5273** | 585.7673 | **V** | **903.4894** | 452.2483 | 886.4629 | 443.7351 | 885.4789 | 443.2431 | **8** |
| **13** | 1317.5805 | 659.2939 | 1300.5539 | 650.7806 | 1299.5699 | 650.2886 | **E** | **804.4210** | 402.7141 | 787.3945 | 394.2009 | 786.4104 | 393.7089 | **7** |
| **14** | 1445.6391 | 723.3232 | 1428.6125 | 714.8099 | 1427.6285 | 714.3179 | **Q** | **675.3784** | 338.1928 | 658.3519 | 329.6796 | 657.3679 | 329.1876 | **6** |
| **15** | **1532.6711** | 766.8392 | 1515.6445 | 758.3259 | 1514.6605 | 757.8339 | **S** | **547.3198** | 274.1636 | 530.2933 | 265.6503 | 529.3093 | 265.1583 | **5** |
| **16** | **1603.7082** | 802.3577 | 1586.6816 | 793.8445 | 1585.6976 | 793.3525 | **A** | **460.2878** | 230.6475 | 443.2613 | 222.1343 | 442.2772 | 221.6423 | **4** |
| **17** | **1716.7923** | 858.8998 | 1699.7657 | 850.3865 | 1698.7817 | 849.8945 | **L** | **389.2507** | 195.1290 | 372.2241 | 186.6157 | 371.2401 | 186.1237 | **3** |
| **18** | **1817.8399** | 909.4236 | 1800.8134 | 900.9103 | 1799.8294 | 900.4183 | **T** | 276.1666 | 138.5870 | 259.1401 | 130.0737 | 258.1561 | 129.5817 | **2** |
| **19** | | | | | | | **R** | 175.1190 | 88.0631 | 158.0924 | 79.5498 | | | **1** |

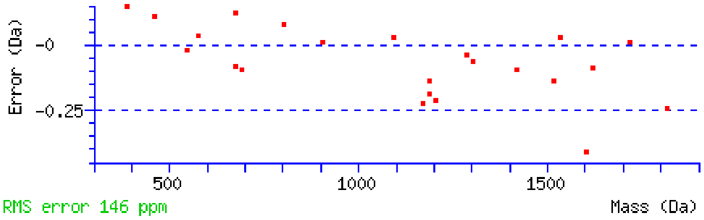


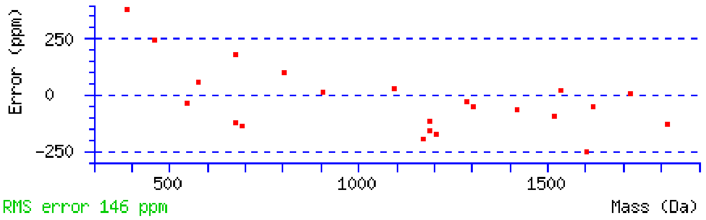


### All matches to this query

| Score | Mr(calc): | Delta | Sequence |
|---|---|---|---|
| 83.0 | 1990.9443 | -0.0017 | VNASTTDPNSTVEQSALTR |
| 56.2 | 1990.9443 | -0.0017 | VNASTTDPNSTVEQSALTR |
| 19.9 | 1990.9249 | 0.0178 | SDTGLYSITAVNNLGTASK |
| 19.9 | 1990.9249 | 0.0178 | SDTGLYSITAVNNLGTASK |
| 13.3 | 1990.9249 | 0.0178 | SDTGLYSITAVNNLGTASK |
| 11.9 | 1990.9249 | 0.0178 | SDTGLYSITAVNNLGTASK |
| 11.9 | 1990.9249 | 0.0178 | SDTGLYSITAVNNLGTASK |
| 3.7 | 1990.9408 | 0.0019 | LLCESSNRALSLQHAGR |
| 1.8 | 1990.9458 | -0.0032 | QMVYFAISDQAFNIATR |
| 1.3 | 1990.9297 | 0.0129 | IDMYAMGLVLWELVSR |

---

Spectrum No: 195; Query: 146; Rank: 1

---

## Peptide View

MS/MS Fragmentation of **ALNVTLSSMGR**
Found in **IPI00213036**, Tax_Id=10116 Gene_Symbol=C4a Complement C4 precursor

Match to Query 146: 1148.585048 from(575.299800,2+)
Title: 091008RatKidney_NoSalt_30.1930.1930.2.dta
Data file K:\NewmanPaper\Piliang\3SubProteomes\Piliang3SP\mgf5ppm\ERLIC_3SubProteomes5ppm.mgf

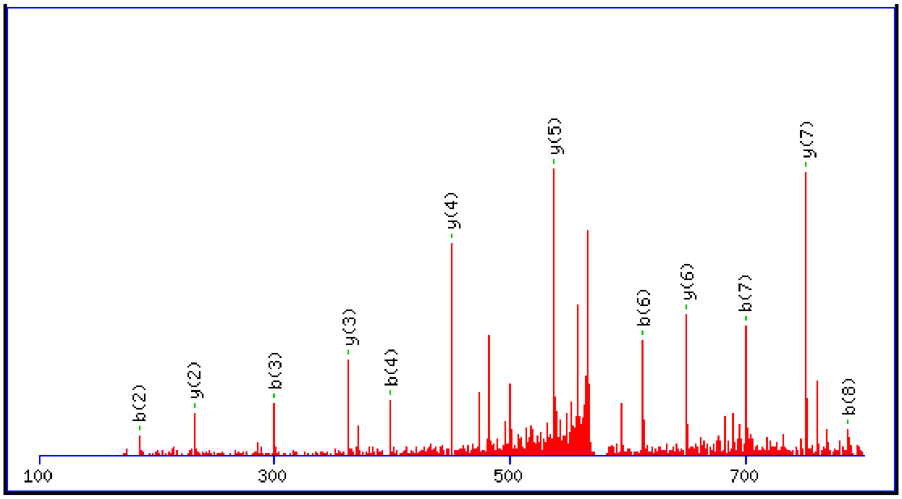


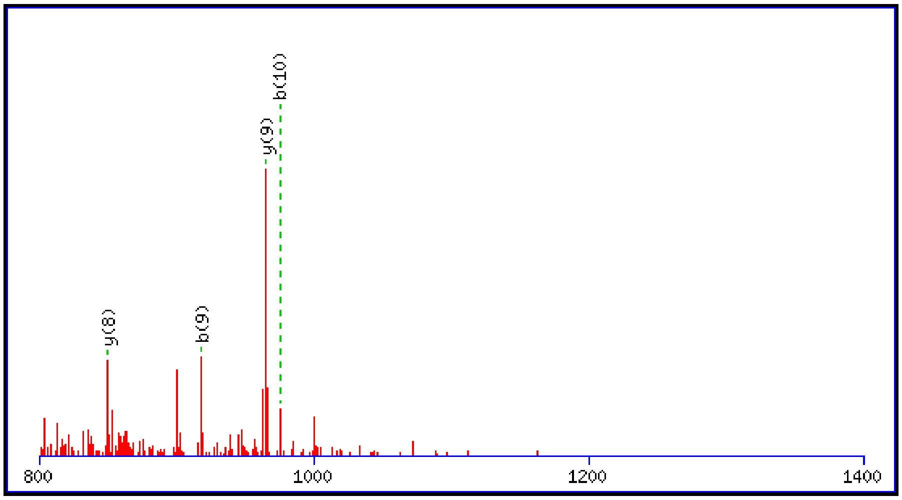


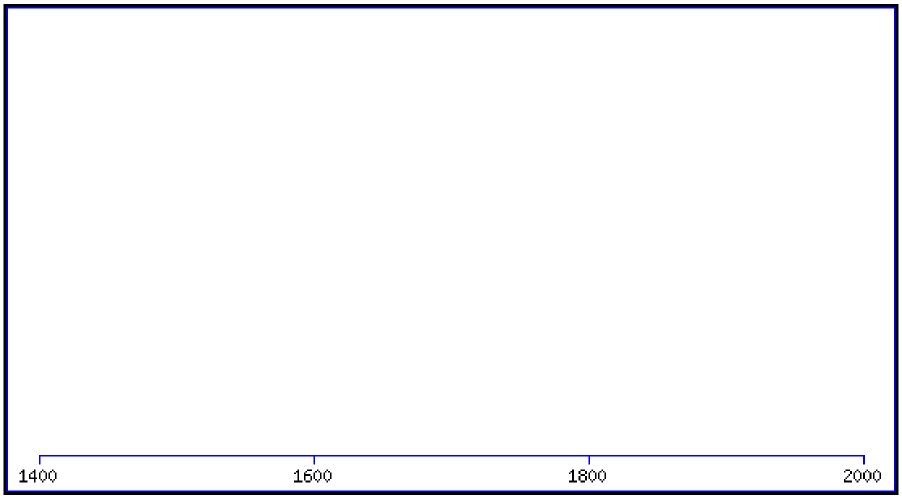


**Monoisotopic mass of neutral peptide Mr(calc):** 1148.5859
**Fixed modifications:** Carbamidomethyl (C)
**Variable modifications:**
**N3** : Deamidated_N (N)
**Ions Score:** 83 **Expect:** 7e-007
**Matches (Bold Red):** 16/102 fragment ions using 23 most intense peaks

| # | b | $b^{++}$ | b* | $b^{*++}$ | $b^0$ | $b^{0++}$ | Seq. | y | $y^{++}$ | y* | $y^{*++}$ | $y^0$ | $y^{0++}$ | # |
|---|---|---|---|---|---|---|---|---|---|---|---|---|---|---|
| 1 | 72.0444 | 36.5258 | | | | | A | | | | | | | 11 |
| 2 | **185.1285** | 93.0679 | | | | | L | 1078.5561 | 539.7817 | 1061.5296 | 531.2684 | 1060.5455 | 530.7764 | 10 |
| 3 | **300.1554** | 150.5813 | 283.1288 | 142.0681 | | | N | **965.4721** | 483.2397 | 948.4455 | 474.7264 | 947.4615 | 474.2344 | 9 |
| 4 | **399.2238** | 200.1155 | 382.1973 | 191.6023 | | | V | **850.4451** | 425.7262 | 833.4186 | 417.2129 | 832.4345 | 416.7209 | 8 |
| 5 | 500.2715 | 250.6394 | 483.2449 | 242.1261 | 482.2609 | 241.6341 | T | **751.3767** | 376.1920 | 734.3502 | 367.6787 | 733.3661 | 367.1867 | 7 |

| | | | | | | | | | | | | | | |
|---|---|---|---|---|---|---|---|---|---|---|---|---|---|---|
| 6 | 613.3555 | 307.1814 | 596.3290 | 298.6681 | 595.3450 | 298.1761 | L | 650.3290 | 325.6681 | 633.3025 | 317.1549 | 632.3185 | 316.6629 | 6 |
| 7 | 700.3876 | 350.6974 | 683.3610 | 342.1842 | 682.3770 | 341.6921 | S | 537.2450 | 269.1261 | 520.2184 | 260.6128 | 519.2344 | 260.1208 | 5 |
| 8 | 787.4196 | 394.2134 | 770.3931 | 385.7002 | 769.4090 | 385.2082 | S | 450.2129 | 225.6101 | 433.1864 | 217.0968 | 432.2024 | 216.6048 | 4 |
| 9 | 918.4601 | 459.7337 | 901.4335 | 451.2204 | 900.4495 | 450.7284 | M | 363.1809 | 182.0941 | 346.1544 | 173.5808 | | | 3 |
| 10 | 975.4816 | 488.2444 | 958.4550 | 479.7311 | 957.4710 | 479.2391 | G | 232.1404 | 116.5738 | 215.1139 | 108.0606 | | | 2 |
| 11 | | | | | | | R | 175.1190 | 88.0631 | 158.0924 | 79.5498 | | | 1 |

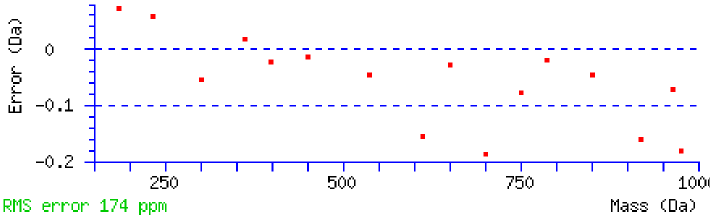


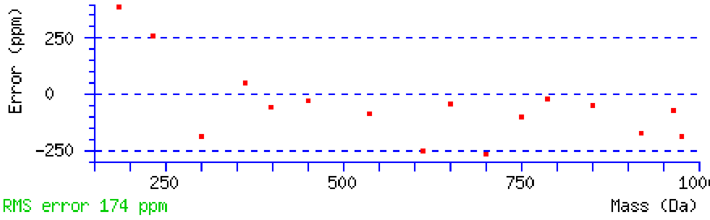


**All matches to this query**

| Score | Mr(calc): | Delta | Sequence |
|---|---|---|---|
| 83.0 | 1148.5859 | -0.0009 | ALNVTLSSMGR |
| 7.5 | 1148.5839 | 0.0011 | ALDRHWHSK |
| 6.7 | 1147.5751 | 1.0100 | ALSGQLPAVGR |
| 6.5 | 1148.5938 | -0.0087 | ALDPAEQHLR |
| 6.0 | 1148.5859 | -0.0009 | VNREIVSGMK |
| 5.8 | 1147.5833 | 1.0018 | VRELNSSNTK |
| 5.6 | 1148.5760 | 0.0090 | MASPLRFDGR |
| 4.5 | 1146.5768 | 2.0082 | SSPDILSDVSK |
| 3.2 | 1146.5854 | 1.9997 | GGGSKRTQGSGR |
| 3.0 | 1148.5794 | 0.0056 | NKDMKVAMGR |

---

Spectrum No: 196; Query: 488; Rank: 1

---

## Peptide View

MS/MS Fragmentation of **AANYTSSLNLPDK**
Found in **IPI00366011**, Tax_Id=10116 Gene_Symbol=Sema4d_predicted similar to semaphorin 4D

Match to Query 488: 1393.677128 from(697.845840,2+)
Title: 100101RatKid_NS_deglyco_25.2166.2166.2.dta
Data file K:\NewmanPaper\Piliang\3SubProteomes\Piliang3SP\mgf5ppm\ERLIC_3SubProteomes5ppm.mgf

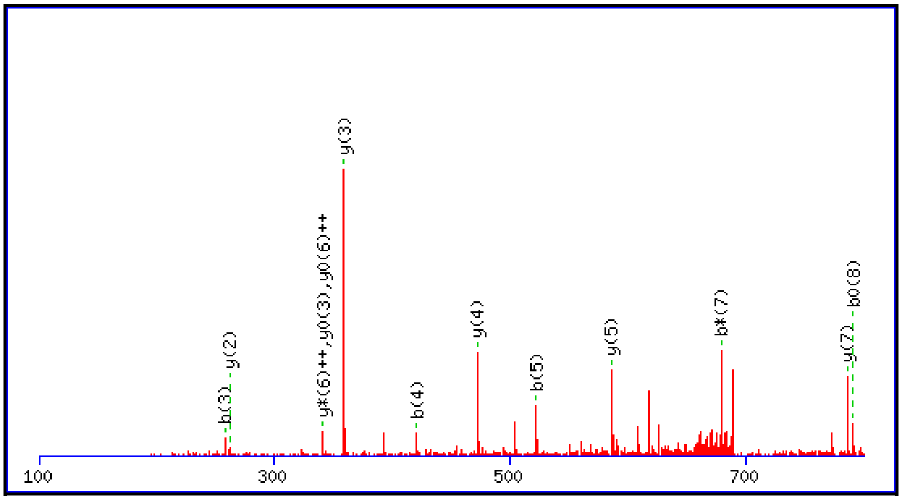

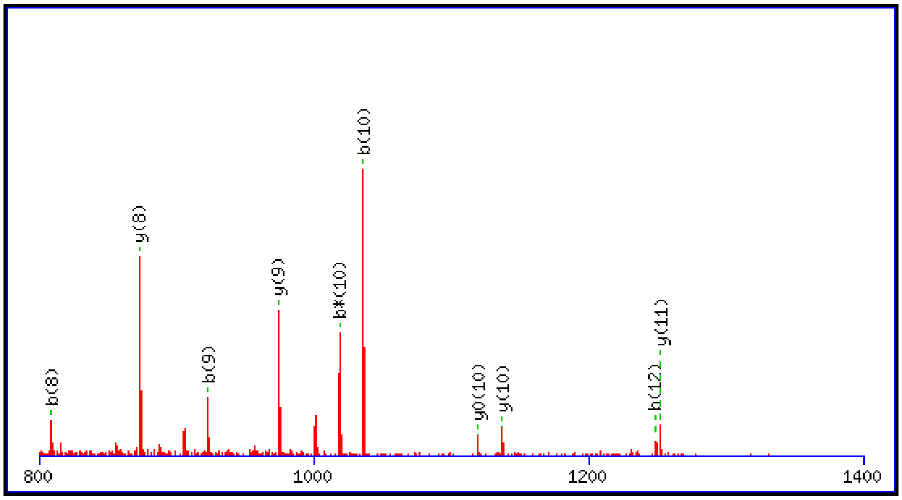


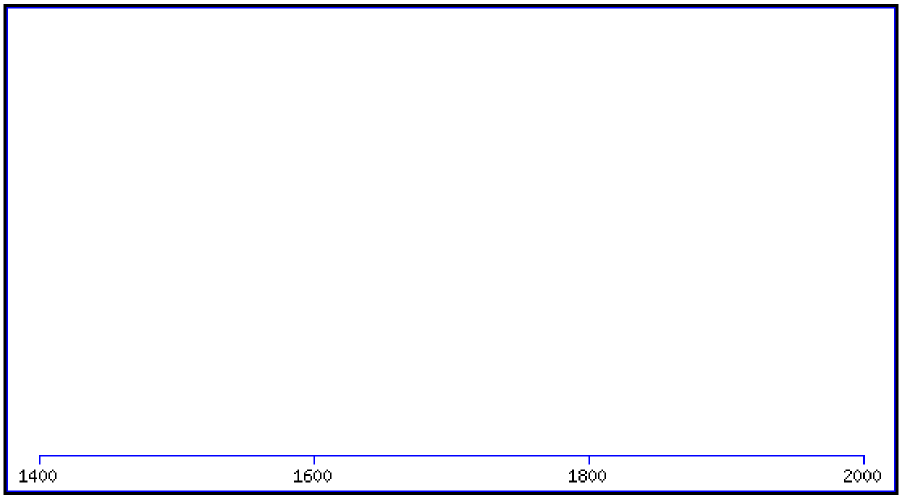


**Monoisotopic mass of neutral peptide Mr(calc):** 1393.6725
**Fixed modifications:** Carbamidomethyl (C)
**Variable modifications:**
**N3** : Deamidated_N (N)
**Ions Score:** 83 **Expect:** 7.5e-007
**Matches (Bold Red):** 23/130 fragment ions using 31 most intense peaks

| # | b | $b^{++}$ | b* | $b^{*++}$ | $b^0$ | $b^{0++}$ | Seq. | y | $y^{++}$ | y* | $y^{*++}$ | $y^0$ | $y^{0++}$ | # |
|---|---|---|---|---|---|---|---|---|---|---|---|---|---|---|
| 1 | 72.0444 | 36.5258 | | | | | A | | | | | | | 13 |
| 2 | 143.0815 | 72.0444 | | | | | A | 1323.6427 | 662.3250 | 1306.6161 | 653.8117 | 1305.6321 | 653.3197 | 12 |
| 3 | **258.1084** | 129.5579 | 241.0819 | 121.0446 | | | N | **1252.6056** | 626.8064 | 1235.5790 | 618.2931 | 1234.5950 | 617.8011 | 11 |
| 4 | **421.1718** | 211.0895 | 404.1452 | 202.5762 | | | Y | **1137.5786** | 569.2930 | 1120.5521 | 560.7797 | **1119.5681** | 560.2877 | 10 |
| 5 | **522.2194** | 261.6134 | 505.1929 | 253.1001 | 504.2089 | 252.6081 | T | **974.5153** | 487.7613 | 957.4888 | 479.2480 | 956.5047 | 478.7560 | 9 |
| 6 | 609.2515 | 305.1294 | 592.2249 | 296.6161 | 591.2409 | 296.1241 | S | **873.4676** | 437.2374 | 856.4411 | 428.7242 | 855.4571 | 428.2322 | 8 |
| 7 | 696.2835 | 348.6454 | **679.2570** | 340.1321 | 678.2729 | 339.6401 | S | **786.4356** | 393.7214 | 769.4090 | 385.2082 | 768.4250 | 384.7162 | 7 |
| 8 | **809.3676** | 405.1874 | 792.3410 | 396.6741 | **791.3570** | 396.1821 | L | 699.4036 | 350.2054 | 682.3770 | **341.6921** | 681.3930 | **341.2001** | 6 |
| 9 | **923.4105** | 462.2089 | 906.3839 | 453.6956 | 905.3999 | 453.2036 | N | **586.3195** | 293.6634 | 569.2930 | 285.1501 | 568.3089 | 284.6581 | 5 |
| 10 | **1036.4946** | 518.7509 | **1019.4680** | 510.2376 | 1018.4840 | 509.7456 | L | **472.2766** | 236.6419 | 455.2500 | 228.1287 | 454.2660 | 227.6366 | 4 |
| 11 | 1133.5473 | 567.2773 | 1116.5208 | 558.7640 | 1115.5368 | 558.2720 | P | **359.1925** | 180.0999 | 342.1660 | 171.5866 | **341.1819** | 171.0946 | 3 |
| 12 | **1248.5743** | 624.7908 | 1231.5477 | 616.2775 | 1230.5637 | 615.7855 | D | **262.1397** | 131.5735 | 245.1132 | 123.0602 | 244.1292 | 122.5682 | 2 |
| 13 | | | | | | | K | 147.1128 | 74.0600 | 130.0863 | 65.5468 | | | 1 |

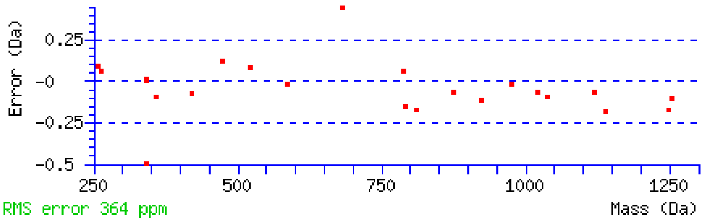


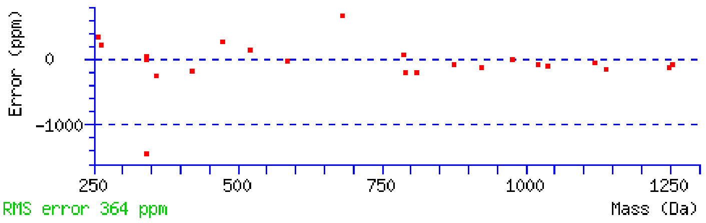


### All matches to this query

| Score | Mr(calc): | Delta | Sequence |
|---|---|---|---|
| 82.9 | 1393.6725 | 0.0046 | AANYTSSLNLPDK |
| 52.1 | 1393.6725 | 0.0046 | AANYTSSLNLPDK |
| 9.6 | 1393.6854 | -0.0083 | EKTLETVPLER |
| 8.9 | 1393.6837 | -0.0066 | GFGNIATNEDTKK |
| 8.4 | 1393.6715 | 0.0057 | VQSKSAQARDPK |
| 7.4 | 1392.6853 | 0.9918 | MLVIEQCKNSR |
| 2.8 | 1393.6734 | 0.0037 | CPVMAGGLFSIDK |
| 2.7 | 1393.6698 | 0.0073 | TRTFGNRAXEGR |
| 2.7 | 1393.6715 | 0.0057 | VQSKSAQARDPK |
| 0.4 | 1391.6681 | 2.0091 | DSGNIPEAIASYR |

---

Spectrum No: 197; Query: 659; Rank: 1

---

## Peptide View

MS/MS Fragmentation of **EVMSLLQPLNITK**
Found in **IPI00388265**, Tax_Id=10116 Gene_Symbol=Pgcp 48 kDa protein

Match to Query 659: 1485.812908 from(743.913730,2+)
Title: 100101RatKid_NS_deglyco_09.5128.5128.2.dta
Data file K:\NewmanPaper\Piliang\3SubProteomes\Piliang3SP\mgf5ppm\ERLIC_3SubProteomes5ppm.mgf

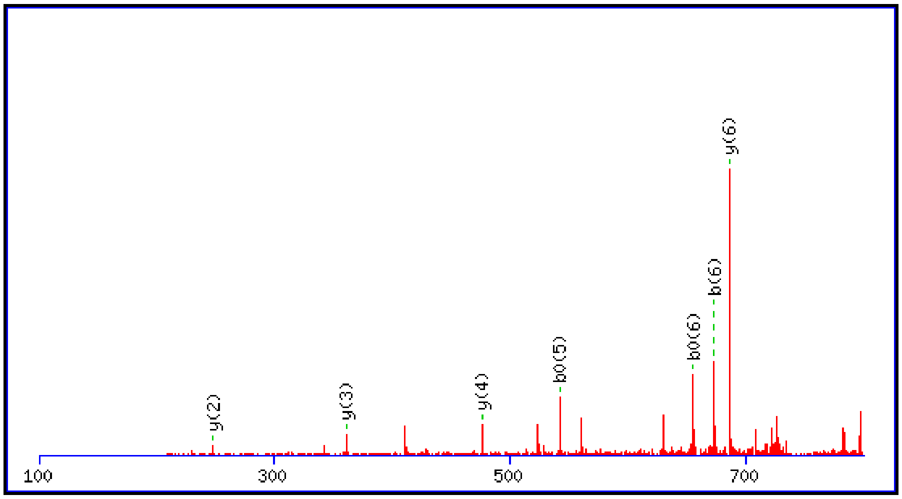

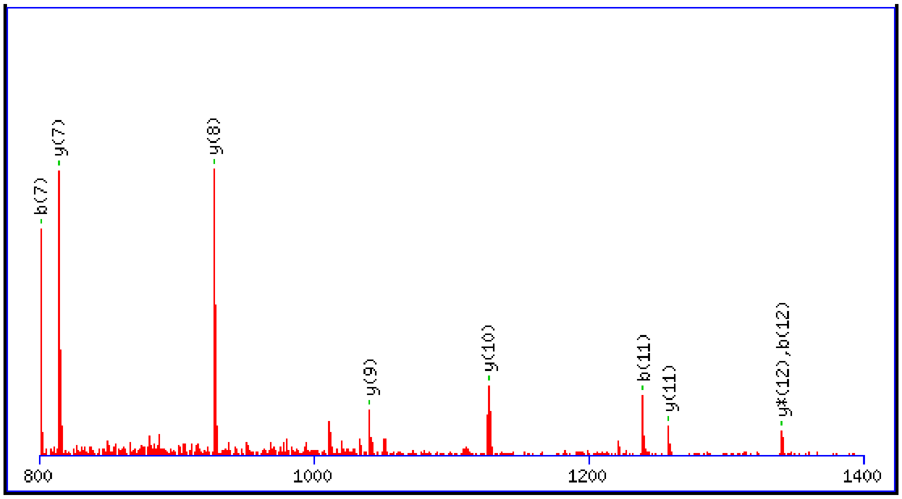


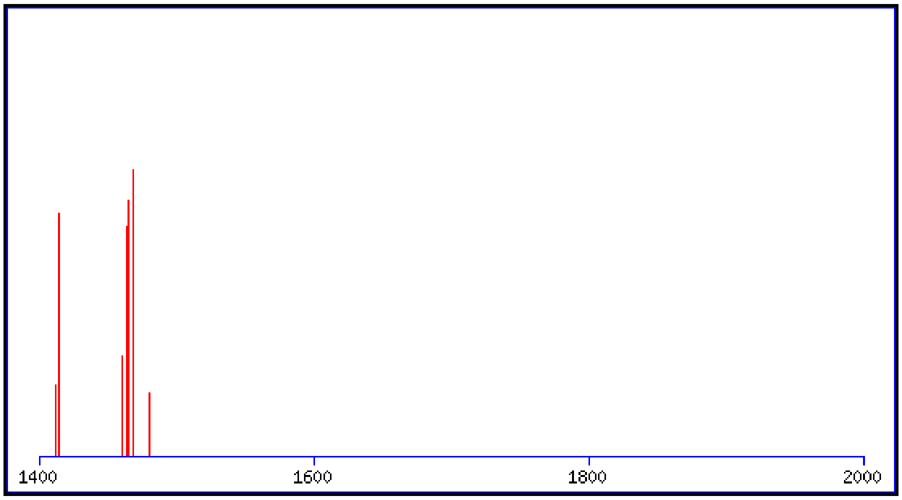


**Monoisotopic mass of neutral peptide Mr(calc):** 1485.8112
**Fixed modifications:** Carbamidomethyl (C)
**Variable modifications:**
**N10** : Deamidated_N (N)
**Ions Score:** 83 **Expect:** 4e-007
**Matches (Bold Red):** 16/130 fragment ions using 20 most intense peaks

| # | b | $b^{++}$ | b* | $b^{*++}$ | $b^0$ | $b^{0++}$ | Seq. | y | $y^{++}$ | y* | $y^{*++}$ | $y^0$ | $y^{0++}$ | # |
|---|---|---|---|---|---|---|---|---|---|---|---|---|---|---|
| 1 | 130.0499 | 65.5286 | | | 112.0393 | 56.5233 | E | | | | | | | 13 |
| 2 | 229.1183 | 115.0628 | | | 211.1077 | 106.0575 | V | 1357.7759 | 679.3916 | **1340.7494** | 670.8783 | 1339.7654 | 670.3863 | 12 |
| 3 | 360.1588 | 180.5830 | | | 342.1482 | 171.5777 | M | **1258.7075** | 629.8574 | 1241.6810 | 621.3441 | 1240.6970 | 620.8521 | 11 |
| 4 | 447.1908 | 224.0990 | | | 429.1802 | 215.0938 | S | **1127.6670** | 564.3372 | 1110.6405 | 555.8239 | 1109.6565 | 555.3319 | 10 |
| 5 | 560.2749 | 280.6411 | | | **542.2643** | 271.6358 | L | **1040.6350** | 520.8211 | 1023.6085 | 512.3079 | 1022.6245 | 511.8159 | 9 |
| 6 | **673.3589** | 337.1831 | | | **655.3484** | 328.1778 | L | **927.5510** | 464.2791 | 910.5244 | 455.7658 | 909.5404 | 455.2738 | 8 |
| 7 | **801.4175** | 401.2124 | 784.3910 | 392.6991 | 783.4069 | 392.2071 | Q | **814.4669** | 407.7371 | 797.4403 | 399.2238 | 796.4563 | 398.7318 | 7 |
| 8 | 898.4703 | 449.7388 | 881.4437 | 441.2255 | 880.4597 | 440.7335 | P | **686.4083** | 343.7078 | 669.3818 | 335.1945 | 668.3977 | 334.7025 | 6 |
| 9 | 1011.5543 | 506.2808 | 994.5278 | 497.7675 | 993.5438 | 497.2755 | L | 589.3555 | 295.1814 | 572.3290 | 286.6681 | 571.3450 | 286.1761 | 5 |
| 10 | 1126.5813 | 563.7943 | 1109.5547 | 555.2810 | 1108.5707 | 554.7890 | N | **476.2715** | 238.6394 | 459.2449 | 230.1261 | 458.2609 | 229.6341 | 4 |
| 11 | **1239.6653** | 620.3363 | 1222.6388 | 611.8230 | 1221.6548 | 611.3310 | I | **361.2445** | 181.1259 | 344.2180 | 172.6126 | 343.2340 | 172.1206 | 3 |
| 12 | **1340.7130** | 670.8601 | 1323.6865 | 662.3469 | 1322.7024 | 661.8549 | T | **248.1605** | 124.5839 | 231.1339 | 116.0706 | 230.1499 | 115.5786 | 2 |
| 13 | | | | | | | K | 147.1128 | 74.0600 | 130.0863 | 65.5468 | | | 1 |

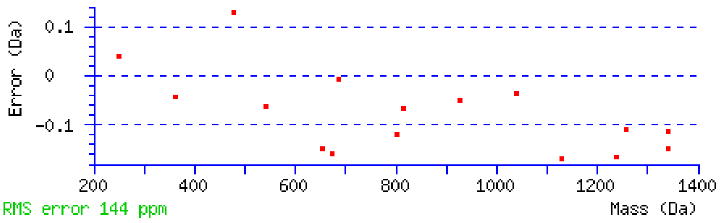


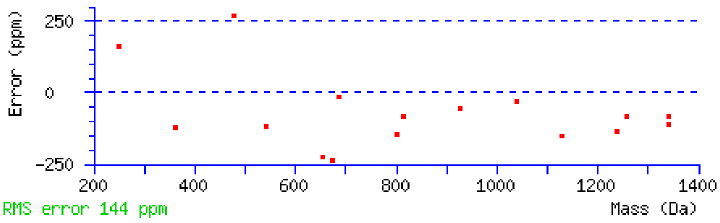


### All matches to this query

| Score | Mr(calc): | Delta | Sequence |
|---|---|---|---|
| 82.9 | 1485.8112 | 0.0017 | EVMSLLQPLNITK |
| 23.1 | 1485.8151 | -0.0022 | DILSRTKEGVGSPK |
| 21.0 | 1485.8191 | -0.0062 | EFNPTKPLLSVNK |
| 16.1 | 1485.8112 | 0.0017 | DLLSKMLVIDPDK |
| 12.8 | 1483.8085 | 2.0045 | MLKSKLNVLTLK |
| 8.1 | 1485.8224 | -0.0095 | VEMALEALRNVIK |
| 6.0 | 1485.8225 | -0.0096 | DLINKMLTINPAK |
| 5.8 | 1485.8151 | -0.0022 | TQKAKQVENVVDK |
| 4.6 | 1484.8198 | 0.9931 | ENETIARLQALVK |
| 4.4 | 1485.8052 | 0.0077 | YPANRAKGKPDAAK |

---

Spectrum No: 198; Query: 2423; Rank: 1

---

## Peptide View

MS/MS Fragmentation of **GRADECALPYLGATCYCDLFCNR**
Found in **IPI00190428**, Tax_Id=10116 Gene_Symbol=Lcn7 Tubulointerstitial nephritis antigen-like precursor

Match to Query 2423: 2782.169292 from(928.397040,3+)
Title: 100101RatKid_NS_deglyco_20.4510.4510.3.dta
Data file K:\NewmanPaper\Piliang\3SubProteomes\Piliang3SP\mgf5ppm\ERLIC_3SubProteomes5ppm.mgf

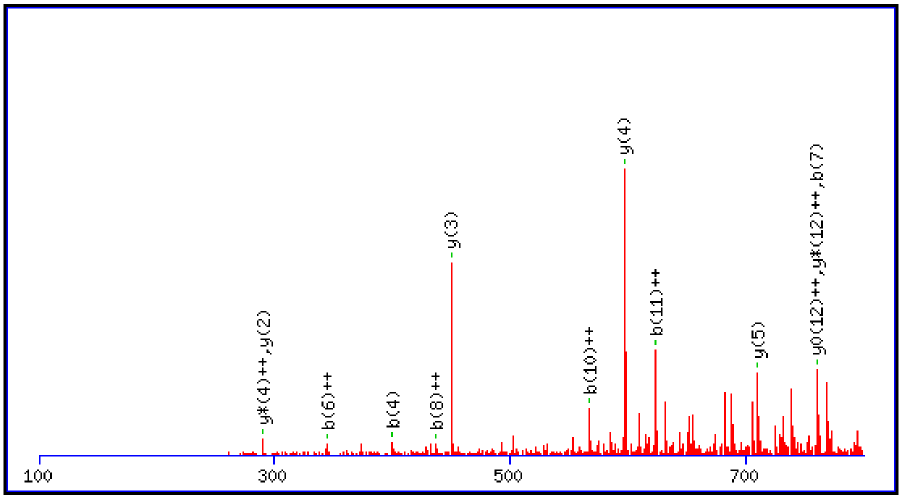

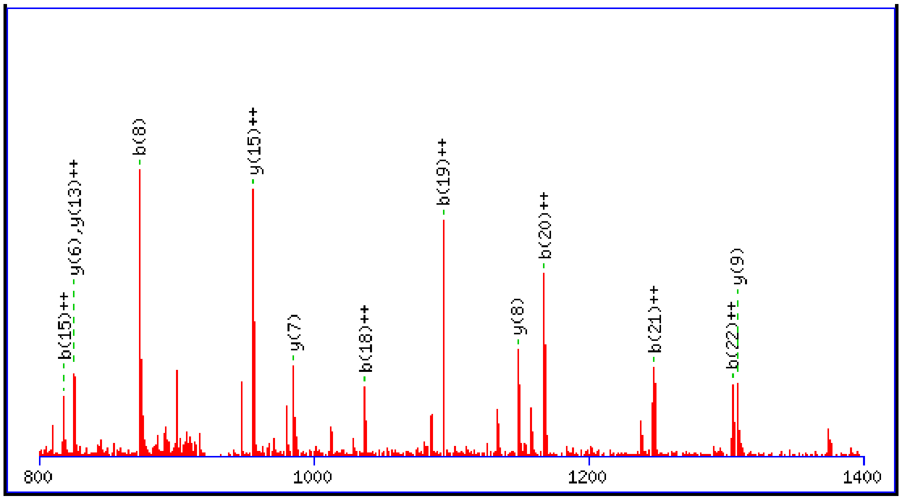


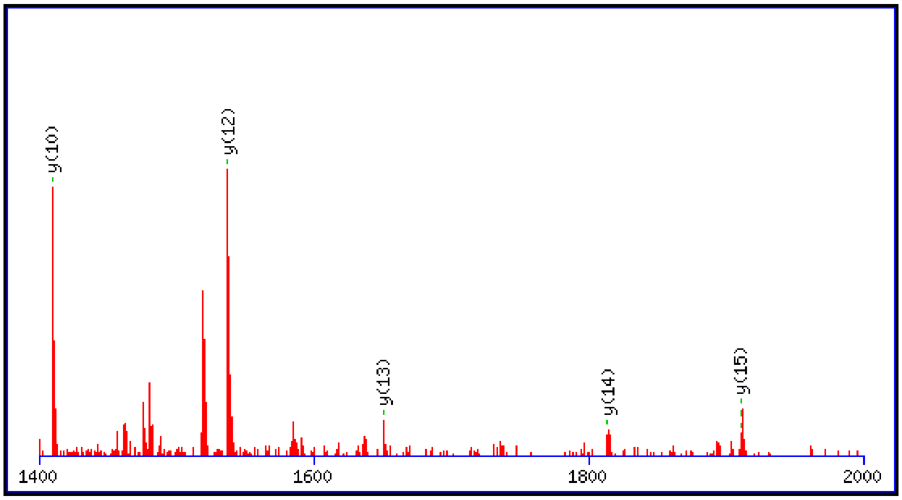


**Monoisotopic mass of neutral peptide Mr(calc):** 2782.1608
**Fixed modifications:** Carbamidomethyl (C)
**Variable modifications:**
**N22** : Deamidated_N (N)
**Ions Score:** 83 **Expect:** 1.8e-006
**Matches (Bold Red):** 31/246 fragment ions using 52 most intense peaks

| # | b | $b^{++}$ | $b^{*}$ | $b^{*++}$ | $b^{0}$ | $b^{0++}$ | Seq. | y | $y^{++}$ | $y^{*}$ | $y^{*++}$ | $y^{0}$ | $y^{0++}$ | # |
|---|---|---|---|---|---|---|---|---|---|---|---|---|---|---|
| 1 | 58.0287 | 29.5180 | | | | | G | | | | | | | 23 |
| 2 | 214.1299 | 107.5686 | 197.1033 | 99.0553 | | | R | 2726.1466 | 1363.5769 | 2709.1200 | 1355.0637 | 2708.1360 | 1354.5716 | 22 |
| 3 | 285.1670 | 143.0871 | 268.1404 | 134.5738 | | | A | 2570.0455 | 1285.5264 | 2553.0189 | 1277.0131 | 2552.0349 | 1276.5211 | 21 |
| 4 | **400.1939** | 200.6006 | 383.1674 | 192.0873 | 382.1833 | 191.5953 | D | 2499.0084 | 1250.0078 | 2481.9818 | 1241.4945 | 2480.9978 | 1241.0025 | 20 |
| 5 | 529.2365 | 265.1219 | 512.2100 | 256.6086 | 511.2259 | 256.1166 | E | 2383.9814 | 1192.4943 | 2366.9549 | 1183.9811 | 2365.9709 | 1183.4891 | 19 |
| 6 | 689.2672 | **345.1372** | 672.2406 | 336.6239 | 671.2566 | 336.1319 | C | 2254.9388 | 1127.9731 | 2237.9123 | 1119.4598 | 2236.9283 | 1118.9678 | 18 |
| 7 | **760.3043** | 380.6558 | 743.2777 | 372.1425 | 742.2937 | 371.6505 | A | 2094.9082 | 1047.9577 | 2077.8816 | 1039.4445 | 2076.8976 | 1038.9524 | 17 |
| 8 | **873.3883** | **437.1978** | 856.3618 | 428.6845 | 855.3778 | 428.1925 | L | 2023.8711 | 1012.4392 | 2006.8445 | 1003.9259 | 2005.8605 | 1003.4339 | 16 |
| 9 | 970.4411 | 485.7242 | 953.4145 | 477.2109 | 952.4305 | 476.7189 | P | **1910.7870** | **955.8971** | 1893.7604 | 947.3839 | 1892.7764 | 946.8919 | 15 |
| 10 | 1133.5044 | **567.2558** | 1116.4779 | 558.7426 | 1115.4939 | 558.2506 | Y | **1813.7342** | 907.3708 | 1796.7077 | 898.8575 | 1795.7237 | 898.3655 | 14 |
| 11 | 1246.5885 | **623.7979** | 1229.5619 | 615.2846 | 1228.5779 | 614.7926 | L | **1650.6709** | **825.8391** | 1633.6444 | 817.3258 | 1632.6603 | 816.8338 | 13 |
| 12 | 1303.6099 | 652.3086 | 1286.5834 | 643.7953 | 1285.5994 | 643.3033 | G | **1537.5868** | 769.2971 | 1520.5603 | **760.7838** | 1519.5763 | **760.2918** | 12 |
| 13 | 1374.6471 | 687.8272 | 1357.6205 | 679.3139 | 1356.6365 | 678.8219 | A | 1480.5654 | 740.7863 | 1463.5388 | 732.2731 | 1462.5548 | 731.7810 | 11 |
| 14 | 1475.6947 | 738.3510 | 1458.6682 | 729.8377 | 1457.6842 | 729.3457 | T | **1409.5283** | 705.2678 | 1392.5017 | 696.7545 | 1391.5177 | 696.2625 | 10 |
| 15 | 1635.7254 | **818.3663** | 1618.6988 | 809.8531 | 1617.7148 | 809.3611 | C | **1308.4806** | 654.7439 | 1291.4540 | 646.2307 | 1290.4700 | 645.7386 | 9 |
| 16 | 1798.7887 | 899.8980 | 1781.7622 | 891.3847 | 1780.7782 | 890.8927 | Y | **1148.4499** | 574.7286 | 1131.4234 | 566.2153 | 1130.4394 | 565.7233 | 8 |
| 17 | 1958.8194 | 979.9133 | 1941.7928 | 971.4000 | 1940.8088 | 970.9080 | C | **985.3866** | 493.1969 | 968.3601 | 484.6837 | 967.3760 | 484.1917 | 7 |
| 18 | 2073.8463 | **1037.4268** | 2056.8198 | 1028.9135 | 2055.8357 | 1028.4215 | D | **825.3560** | 413.1816 | 808.3294 | 404.6683 | 807.3454 | 404.1763 | 6 |

| | | | | | | | | | | | | | | |
|---|---|---|---|---|---|---|---|---|---|---|---|---|---|---|
| **19** | 2186.9304 | **1093.9688** | 2169.9038 | 1085.4556 | 2168.9198 | 1084.9635 | **L** | **710.3290** | 355.6681 | 693.3025 | 347.1549 | | | **5** |
| **20** | 2333.9988 | **1167.5030** | 2316.9722 | 1158.9898 | 2315.9882 | 1158.4978 | **F** | **597.2450** | 299.1261 | 580.2184 | **290.6128** | | | **4** |
| **21** | 2494.0294 | **1247.5184** | 2477.0029 | 1239.0051 | 2476.0189 | 1238.5131 | **C** | **450.1765** | 225.5919 | 433.1500 | 217.0786 | | | **3** |
| **22** | 2609.0564 | **1305.0318** | 2592.0298 | 1296.5186 | 2591.0458 | 1296.0265 | **N** | **290.1459** | 145.5766 | 273.1193 | 137.0633 | | | **2** |
| **23** | | | | | | | **R** | 175.1190 | 88.0631 | 158.0924 | 79.5498 | | | **1** |

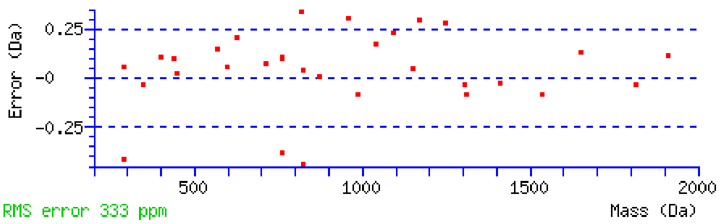


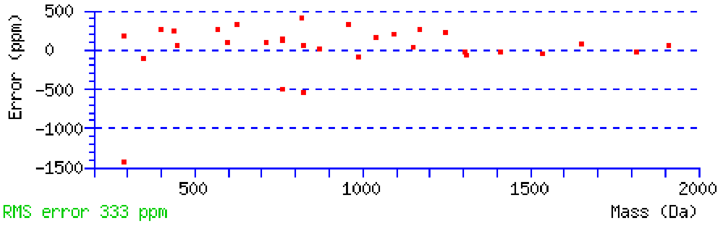


### All matches to this query

| Score | Mr(calc): | Delta | Sequence |
|---|---|---|---|
| 82.8 | 2782.1608 | 0.0085 | GRADECALPYLGATCYCDLFCNR |
| 35.5 | 2781.1768 | 0.9925 | GRADECALPYLGATCYCDLFCNR |
| 6.5 | 2780.1780 | 1.9913 | MVSIFYSIGTPFLNPLIYTLR |
| 4.6 | 2781.1618 | 1.0075 | STRAVFYNSVNGIILVHDLTNK |
| 4.6 | 2781.1618 | 1.0075 | STRAVFYNSVNGIILVHDLTNK |
| 4.0 | 2781.1493 | 1.0200 | DVKEMWNSSTELTEPSPELSLGR |
| 3.3 | 2782.1879 | -0.0187 | SAIFLGPRGSLDLQVMYLDEYR |
| 2.8 | 2780.1780 | 1.9913 | MVSIFYSIGTPFLNPLIYTLR |
| 2.8 | 2780.1780 | 1.9913 | MVSIFYSIGTPFLNPLIYTLR |
| 2.6 | 2782.1879 | -0.0187 | SAIFLGPRGSLDLQVMYLDEYR |

---

Spectrum No: 199; Query: 398; Rank: 1

---

## Peptide View

MS/MS Fragmentation of **LLNLTNDGHSVR**
Found in **IPI00366665**, Tax_Id=10116 Gene_Symbol=Car12 Membrane-bound carbonic anhydrase 12

Match to Query 398: 1338.687488 from(670.351020,2+)
Title: 091008RatKidney_NH4Format01_28.1177.1177.2.dta
Data file K:\NewmanPaper\Piliang\3SubProteomes\Piliang3SP\mgf5ppm\ERLIC_3SubProteomes5ppm.mgf

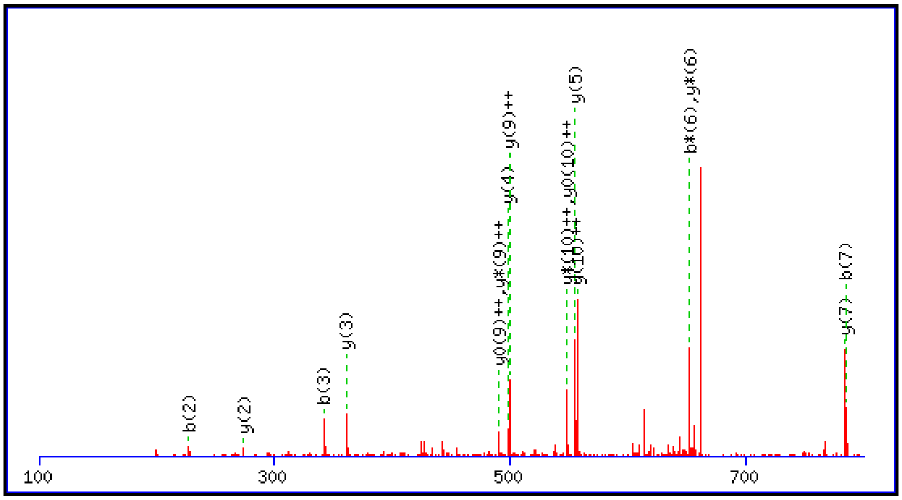

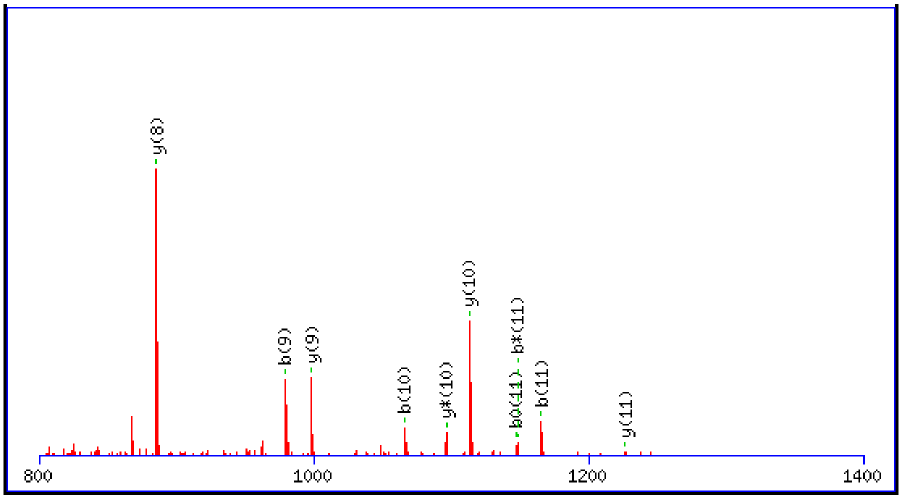


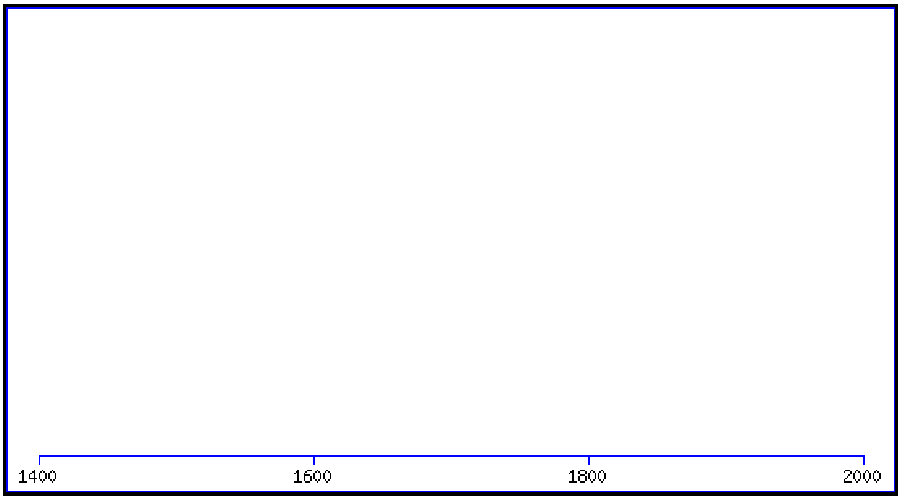


**Monoisotopic mass of neutral peptide Mr(calc):** 1338.6892
**Fixed modifications:** Carbamidomethyl (C)
**Variable modifications:**
**N3** : Deamidated_N (N)
**Ions Score:** 83 **Expect:** 7.4e-007
**Matches (Bold Red):** 26/116 fragment ions using 30 most intense peaks

| # | b | $b^{++}$ | b* | $b^{*++}$ | $b^{0}$ | $b^{0++}$ | Seq. | y | $y^{++}$ | y* | $y^{*++}$ | $y^{0}$ | $y^{0++}$ | # |
|---|---|---|---|---|---|---|---|---|---|---|---|---|---|---|
| 1 | 114.0913 | 57.5493 | | | | | L | | | | | | | 12 |
| 2 | **227.1754** | 114.0913 | | | | | L | **1226.6124** | 613.8098 | 1209.5858 | 605.2966 | 1208.6018 | 604.8045 | 11 |
| 3 | **342.2023** | 171.6048 | 325.1758 | 163.0915 | | | N | **1113.5283** | **557.2678** | **1096.5018** | **548.7545** | 1095.5178 | **548.2625** | 10 |
| 4 | 455.2864 | 228.1468 | 438.2599 | 219.6336 | | | L | **998.5014** | **499.7543** | 981.4748 | **491.2411** | 980.4908 | **490.7490** | 9 |
| 5 | 556.3341 | 278.6707 | 539.3075 | 270.1574 | 538.3235 | 269.6654 | T | **885.4173** | 443.2123 | 868.3908 | 434.6990 | 867.4068 | 434.2070 | 8 |
| 6 | 670.3770 | 335.6921 | **653.3505** | 327.1789 | 652.3664 | 326.6869 | N | **784.3696** | 392.6885 | 767.3431 | 384.1752 | 766.3591 | 383.6832 | 7 |
| 7 | **785.4040** | 393.2056 | 768.3774 | 384.6923 | 767.3934 | 384.2003 | D | 670.3267 | 335.6670 | **653.3002** | 327.1537 | 652.3161 | 326.6617 | 6 |
| 8 | 842.4254 | 421.7163 | 825.3989 | 413.2031 | 824.4149 | 412.7111 | G | **555.2998** | 278.1535 | 538.2732 | 269.6402 | 537.2892 | 269.1482 | 5 |
| 9 | **979.4843** | 490.2458 | 962.4578 | 481.7325 | 961.4738 | 481.2405 | H | **498.2783** | 249.6428 | 481.2518 | 241.1295 | 480.2677 | 240.6375 | 4 |
| 10 | **1066.5164** | 533.7618 | 1049.4898 | 525.2485 | 1048.5058 | 524.7565 | S | **361.2194** | 181.1133 | 344.1928 | 172.6001 | 343.2088 | 172.1081 | 3 |
| 11 | **1165.5848** | 583.2960 | **1148.5582** | 574.7827 | **1147.5742** | 574.2907 | V | **274.1874** | 137.5973 | 257.1608 | 129.0840 | | | 2 |
| 12 | | | | | | | R | 175.1190 | 88.0631 | 158.0924 | 79.5498 | | | 1 |

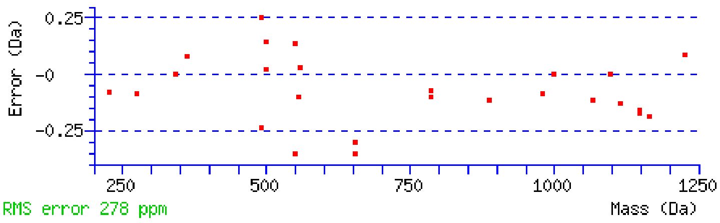


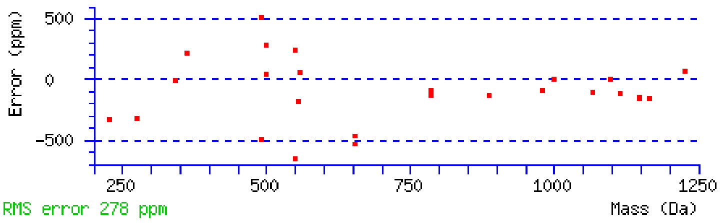


### All matches to this query

| Score | Mr(calc): | Delta | Sequence |
|---|---|---|---|
| 82.7 | 1338.6892 | -0.0017 | LLNLTNDGHSVR |
| 67.4 | 1338.6892 | -0.0017 | LLNLTNDGHSVR |
| 10.9 | 1338.6769 | 0.0106 | LLGNSSRTRVR |
| 9.9 | 1338.6796 | 0.0079 | QDKLISTVEVK |
| 9.2 | 1338.6769 | 0.0106 | IRETARASLSR |
| 8.7 | 1338.6769 | 0.0106 | LLGNSSRTRVR |
| 8.7 | 1338.6769 | 0.0106 | LLGNSSRTRVR |
| 7.7 | 1338.6932 | -0.0057 | LLFSSEKSSWR |
| 7.7 | 1338.6820 | 0.0055 | LLDFVYNVDNK |
| 7.1 | 1338.6853 | 0.0022 | IDILINYAMTR |

---

Spectrum No: 200; Query: 570; Rank: 1

---

## Peptide View

MS/MS Fragmentation of **AFNLTWISTDFK**
Found in **IPI00197592**, Tax_Id=10116 Gene_Symbol=F3 Tissue factor precursor

Match to Query 570: 1442.710708 from(722.362630,2+)
Title: 100101RatKid_NS_deglyco_21.5229.5229.2.dta
Data file K:\NewmanPaper\Piliang\3SubProteomes\Piliang3SP\mgf5ppm\ERLIC_3SubProteomes5ppm.mgf

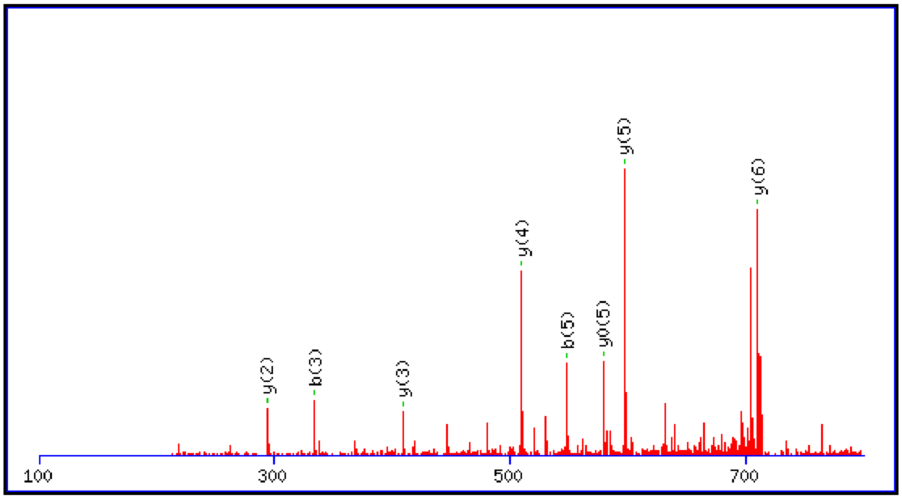

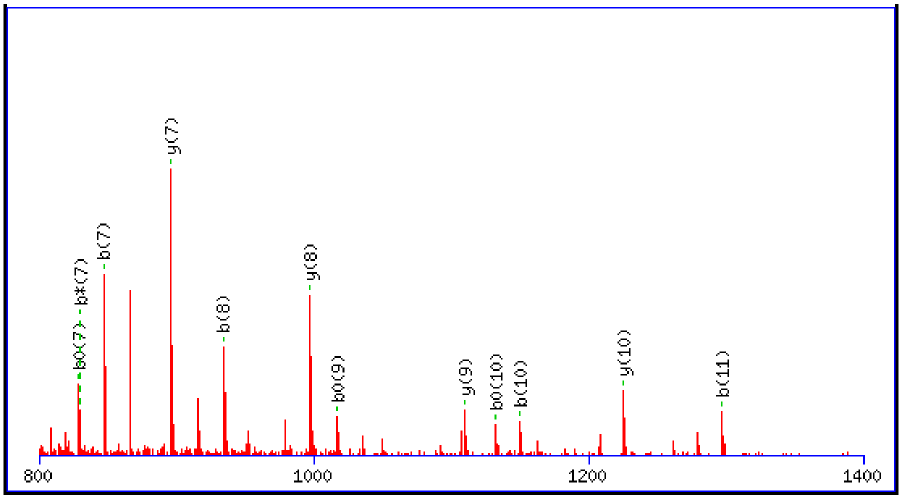


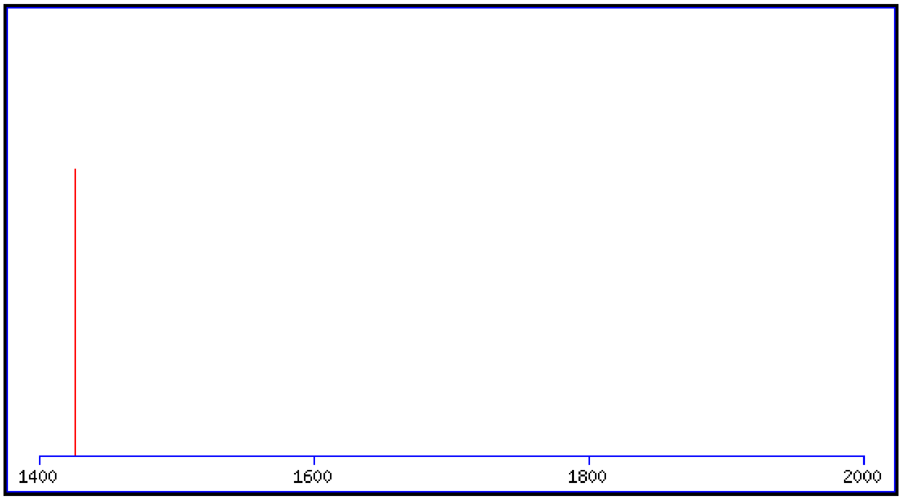


**Monoisotopic mass of neutral peptide Mr(calc):** 1442.7082
**Fixed modifications:** Carbamidomethyl (C)
**Variable modifications:**
**N3** : Deamidated_N (N)
**Ions Score:** 83 **Expect:** 7.9e-007
**Matches (Bold Red):** 20/116 fragment ions using 33 most intense peaks

| # | b | $b^{++}$ | b* | $b^{*++}$ | $b^0$ | $b^{0++}$ | Seq. | y | $y^{++}$ | y* | $y^{*++}$ | $y^0$ | $y^{0++}$ | # |
|---|---|---|---|---|---|---|---|---|---|---|---|---|---|---|
| 1 | 72.0444 | 36.5258 | | | | | A | | | | | | | 12 |
| 2 | 219.1128 | 110.0600 | | | | | F | 1372.6783 | 686.8428 | 1355.6518 | 678.3295 | 1354.6678 | 677.8375 | 11 |
| 3 | **334.1397** | 167.5735 | 317.1132 | 159.0602 | | | N | **1225.6099** | 613.3086 | 1208.5834 | 604.7953 | 1207.5994 | 604.3033 | 10 |
| 4 | 447.2238 | 224.1155 | 430.1973 | 215.6023 | | | L | **1110.5830** | 555.7951 | 1093.5564 | 547.2819 | 1092.5724 | 546.7898 | 9 |
| 5 | **548.2715** | 274.6394 | 531.2449 | 266.1261 | 530.2609 | 265.6341 | T | **997.4989** | 499.2531 | 980.4724 | 490.7398 | 979.4884 | 490.2478 | 8 |
| 6 | 734.3508 | 367.6790 | 717.3242 | 359.1658 | 716.3402 | 358.6738 | W | **896.4512** | 448.7293 | 879.4247 | 440.2160 | 878.4407 | 439.7240 | 7 |
| 7 | **847.4349** | 424.2211 | **830.4083** | 415.7078 | **829.4243** | 415.2158 | I | **710.3719** | 355.6896 | 693.3454 | 347.1763 | 692.3614 | 346.6843 | 6 |
| 8 | **934.4669** | 467.7371 | 917.4403 | 459.2238 | 916.4563 | 458.7318 | S | **597.2879** | 299.1476 | 580.2613 | 290.6343 | **579.2773** | 290.1423 | 5 |
| 9 | 1035.5146 | 518.2609 | 1018.4880 | 509.7476 | **1017.5040** | 509.2556 | T | **510.2558** | 255.6316 | 493.2293 | 247.1183 | 492.2453 | 246.6263 | 4 |
| 10 | **1150.5415** | 575.7744 | 1133.5150 | 567.2611 | **1132.5309** | 566.7691 | D | **409.2082** | 205.1077 | 392.1816 | 196.5944 | 391.1976 | 196.1024 | 3 |
| 11 | **1297.6099** | 649.3086 | 1280.5834 | 640.7953 | 1279.5994 | 640.3033 | F | **294.1812** | 147.5942 | 277.1547 | 139.0810 | | | 2 |
| 12 | | | | | | | K | 147.1128 | 74.0600 | 130.0863 | 65.5468 | | | 1 |

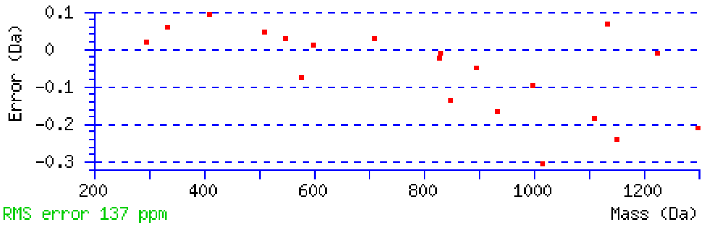


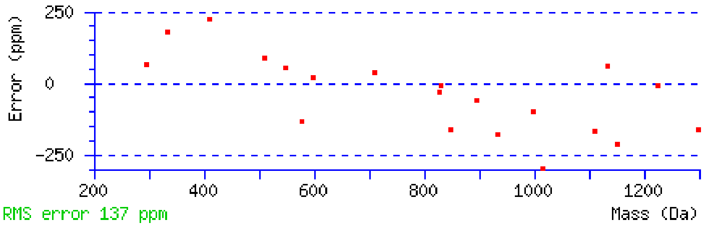


### All matches to this query

| Score | Mr(calc): | Delta | Sequence |
|---|---|---|---|
| 82.5 | 1442.7082 | 0.0025 | AFNLTWISTDFK |
| 7.5 | 1442.7179 | -0.0072 | GSLKMWLKCIK |
| 5.6 | 1442.7065 | 0.0042 | RRLNXSSSIVMK |
| 5.6 | 1442.7065 | 0.0042 | RRLNXSSSIVMK |
| 5.4 | 1442.7228 | -0.0121 | WMLTTVPHESVK |
| 5.3 | 1442.7122 | -0.0015 | VARSPAMNSVHMK |
| 4.8 | 1442.7065 | 0.0042 | RRLNXSSSIVMK |
| 1.5 | 1442.7058 | 0.0049 | LSLLGGDSVDIFK |
| 1.4 | 1442.7228 | -0.0121 | GAFGKVYLAQDMK |

**Mascot:** http://www.matrixscience.com/

# Mascot Search Results

Results Generated by a Script Modified from Mascot Pepitde View
by Newman Sze, School of Biological Sciences, Nanyang Technological University

---

Spectrum No: 201; Query: 957; Rank: 1

---

## Peptide View

MS/MS Fragmentation of **NPDINMTFEQVYR**
Found in **IPI00325312**, Tax_Id=10116 Gene_Symbol=Prom1 prominin 1 isoform 1

Match to Query 957: 1642.735788 from(822.375170,2+)
Title: 100101RatKid_NS_deglyco_21.3048.3048.2.dta
Data file K:\NewmanPaper\Piliang\3SubProteomes\Piliang3SP\mgf5ppm\ERLIC_3SubProteomes5ppm.mgf

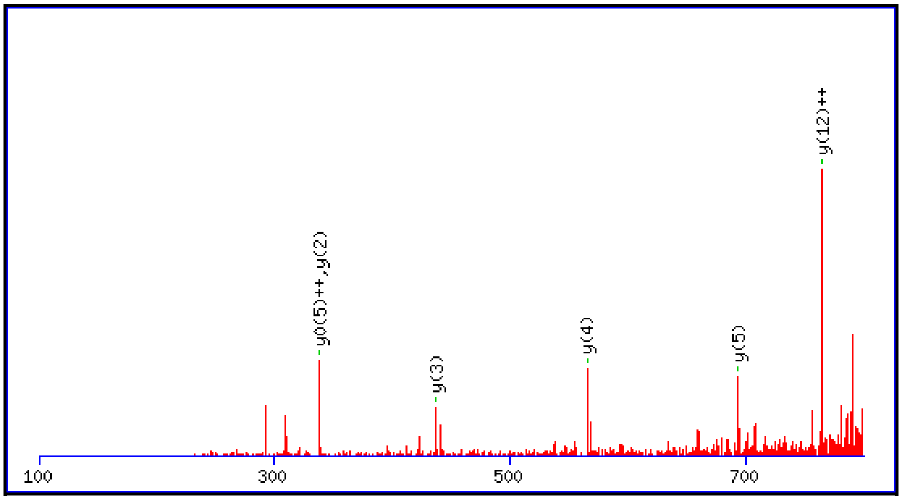


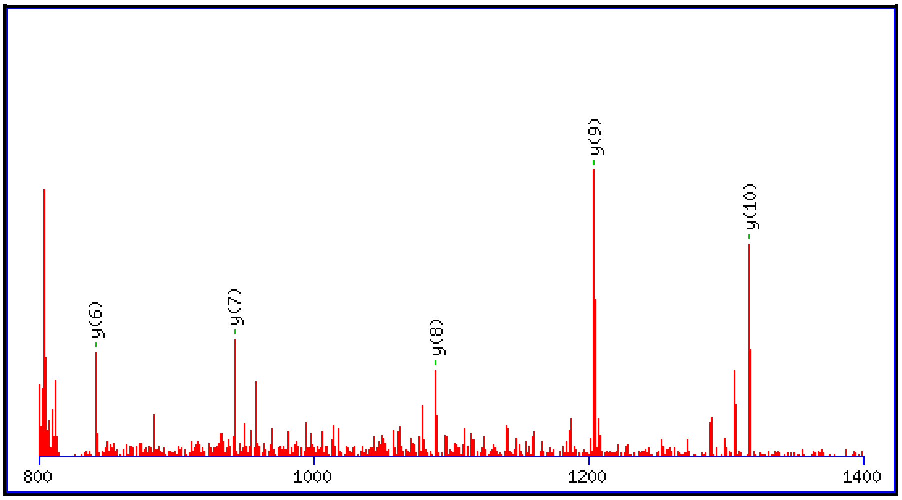

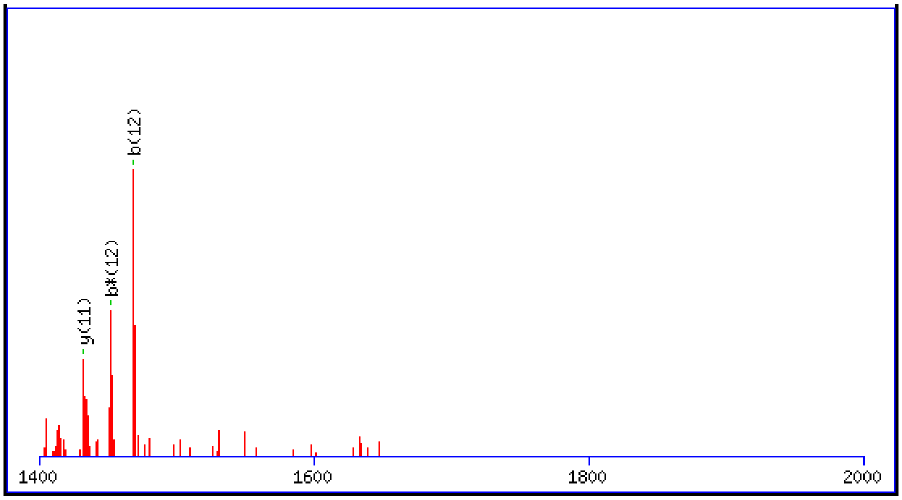


**Monoisotopic mass of neutral peptide Mr(calc):** 1642.7297
**Fixed modifications:** Carbamidomethyl (C)
**Variable modifications:**
**N5** : Deamidated_N (N)
**M6** : Oxidation (M)
**Ions Score:** 82 **Expect:** 1.1e-006
**Matches (Bold Red):** 14/132 fragment ions using 22 most intense peaks

| # | b | b++ | b* | b*++ | b0 | b0++ | Seq. | y | y++ | y* | y*++ | y0 | y0++ | # |
|---|---|---|---|---|---|---|---|---|---|---|---|---|---|---|
| 1 | 115.0502 | 58.0287 | 98.0237 | 49.5155 | | | N | | | | | | | 13 |
| 2 | 212.1030 | 106.5551 | 195.0764 | 98.0418 | | | P | 1529.6941 | **765.3507** | 1512.6675 | 756.8374 | 1511.6835 | 756.3454 | 12 |
| 3 | 327.1299 | 164.0686 | 310.1034 | 155.5553 | 309.1193 | 155.0633 | D | **1432.6413** | 716.8243 | 1415.6148 | 708.3110 | 1414.6307 | 707.8190 | 11 |
| 4 | 440.2140 | 220.6106 | 423.1874 | 212.0974 | 422.2034 | 211.6053 | I | **1317.6144** | 659.3108 | 1300.5878 | 650.7975 | 1299.6038 | 650.3055 | 10 |
| 5 | 555.2409 | 278.1241 | 538.2144 | 269.6108 | 537.2303 | 269.1188 | N | **1204.5303** | 602.7688 | 1187.5038 | 594.2555 | 1186.5197 | 593.7635 | 9 |
| 6 | 702.2763 | 351.6418 | 685.2498 | 343.1285 | 684.2657 | 342.6365 | M | **1089.5034** | 545.2553 | 1072.4768 | 536.7420 | 1071.4928 | 536.2500 | 8 |
| 7 | 803.3240 | 402.1656 | 786.2974 | 393.6524 | 785.3134 | 393.1604 | T | **942.4680** | 471.7376 | 925.4414 | 463.2243 | 924.4574 | 462.7323 | 7 |
| 8 | 950.3924 | 475.6998 | 933.3659 | 467.1866 | 932.3818 | 466.6946 | F | **841.4203** | 421.2138 | 824.3937 | 412.7005 | 823.4097 | 412.2085 | 6 |
| 9 | 1079.4350 | 540.2211 | 1062.4085 | 531.7079 | 1061.4244 | 531.2159 | E | **694.3519** | 347.6796 | 677.3253 | 339.1663 | 676.3413 | **338.6743** | 5 |
| 10 | 1207.4936 | 604.2504 | 1190.4670 | 595.7372 | 1189.4830 | 595.2451 | Q | **565.3093** | 283.1583 | 548.2827 | 274.6450 | | | 4 |
| 11 | 1306.5620 | 653.7846 | 1289.5354 | 645.2714 | 1288.5514 | 644.7794 | V | **437.2507** | 219.1290 | 420.2241 | 210.6157 | | | 3 |
| 12 | **1469.6253** | 735.3163 | **1452.5988** | 726.8030 | 1451.6148 | 726.3110 | Y | **338.1823** | 169.5948 | 321.1557 | 161.0815 | | | 2 |
| 13 | | | | | | | R | 175.1190 | 88.0631 | 158.0924 | 79.5498 | | | 1 |

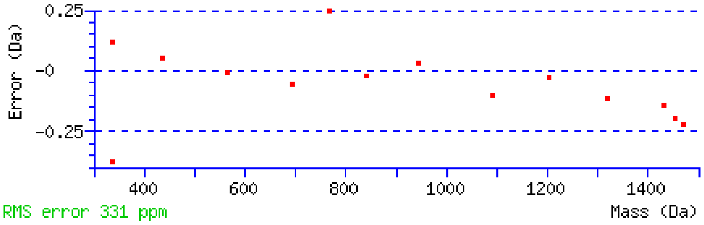


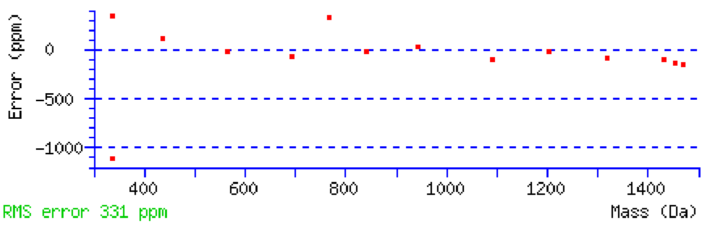


**All matches to this query**

| Score | Mr(calc): | Delta | Sequence |
|---|---|---|---|
| 82.4 | 1642.7297 | 0.0061 | NPDINMTFEQVYR |
| 35.6 | 1642.7297 | 0.0061 | NPDINMTFEQVYR |
| 35.3 | 1641.7457 | 0.9901 | NPDINMTFEQVYR |
| 0.1 | 1641.7334 | 1.0024 | LPNNFDIESAMRR |

Spectrum No: 202; Query: 546; Rank: 1

# Peptide View

MS/MS Fragmentation of **VNDNKTAAEEALR**
Found in **IPI00363849**, Tax_Id=10116 Gene_Symbol=Lamc1 similar to Laminin gamma-1 chain precursor

Match to Query 546: 1430.703908 from(716.359230,2+)
Title: 100101RatKid_NS_deglyco_21.969.969.2.dta
Data file K:\NewmanPaper\Piliang\3SubProteomes\Piliang3SP\mgf5ppm\ERLIC_3SubProteomes5ppm.mgf

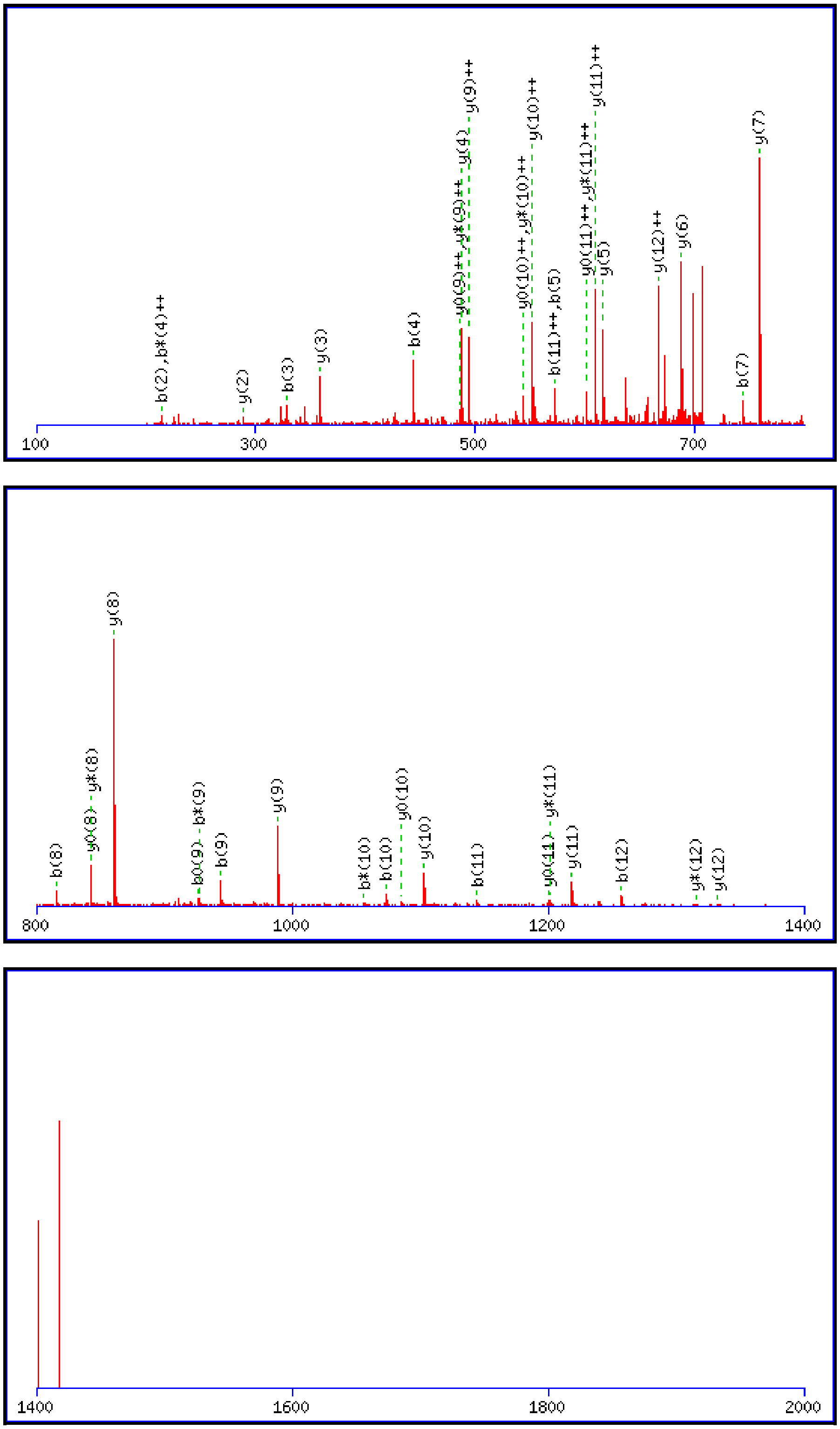


**Monoisotopic mass of neutral peptide Mr(calc):** 1430.7001
**Fixed modifications:** Carbamidomethyl (C)
**Variable modifications:**
**N4** : Deamidated_N (N)
**Ions Score:** 82 **Expect:** 7.6e-007
**Matches (Bold Red):** 42/132 fragment ions using 68 most intense peaks

| # | b | $b^{++}$ | b* | $b^{*++}$ | $b^{0}$ | $b^{0++}$ | Seq. | y | $y^{++}$ | y* | $y^{*++}$ | $y^{0}$ | $y^{0++}$ | # |
|---|---|---|---|---|---|---|---|---|---|---|---|---|---|---|
| 1 | 100.0757 | 50.5415 | | | | | V | | | | | | | 13 |
| 2 | **214.1186** | 107.5629 | 197.0921 | 99.0497 | | | N | **1332.6390** | **666.8231** | **1315.6124** | 658.3099 | 1314.6284 | 657.8179 | 12 |
| 3 | **329.1456** | 165.0764 | 312.1190 | 156.5631 | 311.1350 | 156.0711 | D | **1218.5961** | **609.8017** | **1201.5695** | **601.2884** | **1200.5855** | **600.7964** | 11 |
| 4 | **444.1725** | 222.5899 | 427.1459 | **214.0766** | 426.1619 | 213.5846 | N | **1103.5691** | **552.2882** | 1086.5426 | **543.7749** | **1085.5586** | **543.2829** | 10 |
| 5 | **572.2675** | 286.6374 | 555.2409 | 278.1241 | 554.2569 | 277.6321 | K | **988.5422** | **494.7747** | 971.5156 | **486.2615** | 970.5316 | **485.7694** | 9 |
| 6 | 673.3151 | 337.1612 | 656.2886 | 328.6479 | 655.3046 | 328.1559 | T | **860.4472** | 430.7272 | **843.4207** | 422.2140 | **842.4367** | 421.7220 | 8 |
| 7 | **744.3523** | 372.6798 | 727.3257 | 364.1665 | 726.3417 | 363.6745 | A | **759.3995** | 380.2034 | 742.3730 | 371.6901 | 741.3890 | 371.1981 | 7 |
| 8 | **815.3894** | 408.1983 | 798.3628 | 399.6850 | 797.3788 | 399.1930 | A | **688.3624** | 344.6849 | 671.3359 | 336.1716 | 670.3519 | 335.6796 | 6 |
| 9 | **944.4320** | 472.7196 | **927.4054** | 464.2063 | **926.4214** | 463.7143 | E | **617.3253** | 309.1663 | 600.2988 | 300.6530 | 599.3148 | 300.1610 | 5 |
| 10 | **1073.4746** | 537.2409 | **1056.4480** | 528.7276 | 1055.4640 | 528.2356 | E | **488.2827** | 244.6450 | 471.2562 | 236.1317 | 470.2722 | 235.6397 | 4 |
| 11 | **1144.5117** | **572.7595** | 1127.4851 | 564.2462 | 1126.5011 | 563.7542 | A | **359.2401** | 180.1237 | 342.2136 | 171.6104 | | | 3 |
| 12 | **1257.5957** | 629.3015 | 1240.5692 | 620.7882 | 1239.5852 | 620.2962 | L | **288.2030** | 144.6051 | 271.1765 | 136.0919 | | | 2 |
| 13 | | | | | | | R | 175.1190 | 88.0631 | 158.0924 | 79.5498 | | | 1 |

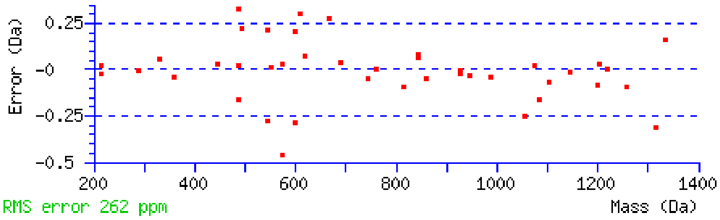


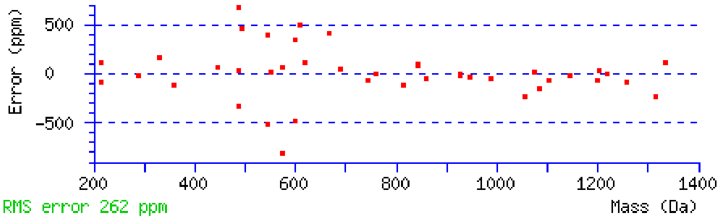


**All matches to this query**

| Score | Mr(calc): | Delta | Sequence |
|---|---|---|---|
| 82.4 | 1430.7001 | 0.0038 | VNDNKTAAEEALR |
| 49.9 | 1430.7001 | 0.0038 | VNDNKTAAEEALR |
| 7.5 | 1430.7048 | -0.0009 | GRNCTEKPTNVR |
| 7.2 | 1428.6861 | 2.0178 | LTVXDKLSETTR |
| 7.2 | 1428.6861 | 2.0178 | LTVXDKLSETTR |
| 5.9 | 1430.7161 | -0.0122 | SRAACVXTRPSGR |
| 5.8 | 1430.6975 | 0.0064 | VLPYILPSTLR |
| 5.6 | 1430.6959 | 0.0080 | VINNFISAGFIR |
| 5.5 | 1430.7089 | -0.0050 | VSRTHFDPMGIR |
| 5.3 | 1430.6975 | 0.0064 | VLPYILPSTLR |

---

Spectrum No: 203; Query: 750; Rank: 1

---

## Peptide View

MS/MS Fragmentation of **DLLTNQSDFSGNTK**
Found in **IPI00210824**, Tax_Id=10116 Gene_Symbol=Serpina6 Corticosteroid-binding globulin precursor

Match to Query 750: 1539.702488 from(770.858520,2+)
Title: 091008RatKidney_NoSalt_41.1427.1427.2.dta
Data file K:\NewmanPaper\Piliang\3SubProteomes\Piliang3SP\mgf5ppm\ERLIC_3SubProteomes5ppm.mgf

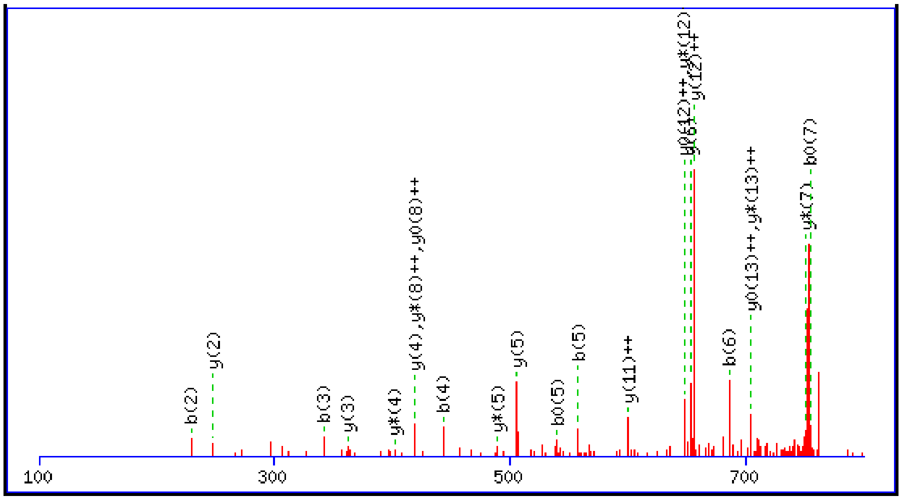


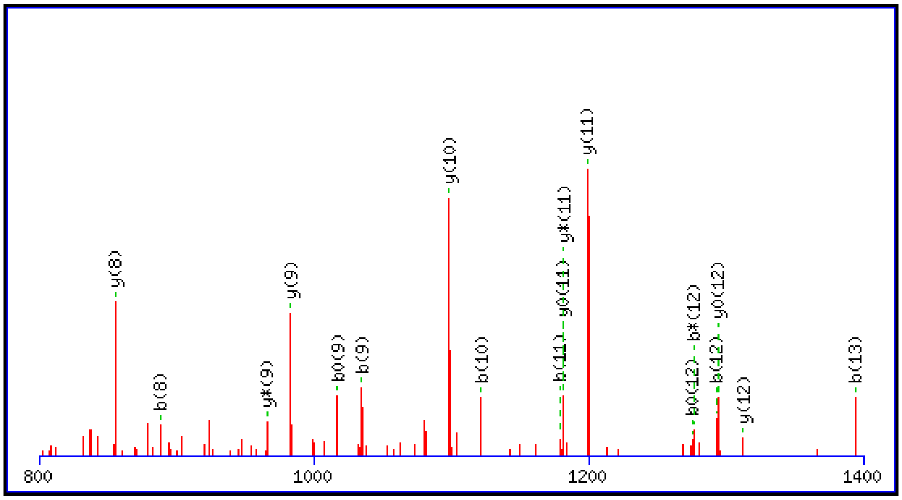


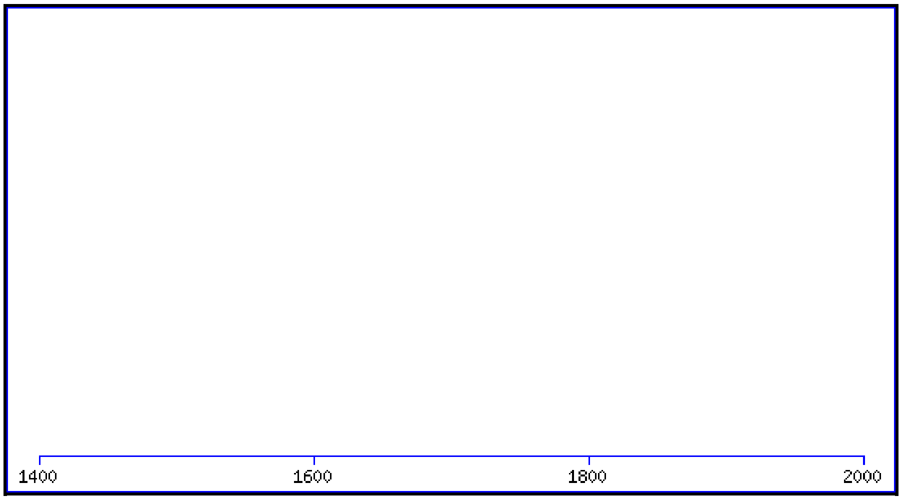


**Monoisotopic mass of neutral peptide Mr(calc):** 1539.7053
**Fixed modifications:** Carbamidomethyl (C)
**Variable modifications:**
**N5** : Deamidated_N (N)
**Ions Score:** 82 **Expect:** 9.8e-007
**Matches (Bold Red):** 41/146 fragment ions using 57 most intense peaks

| # | b | $b^{++}$ | b* | $b^{*++}$ | $b^0$ | $b^{0++}$ | Seq. | y | $y^{++}$ | y* | $y^{*++}$ | $y^0$ | $y^{0++}$ | # |
|---|---|---|---|---|---|---|---|---|---|---|---|---|---|---|
| 1 | 116.0342 | 58.5207 | | | 98.0237 | 49.5155 | D | | | | | | | 14 |
| 2 | **229.1183** | 115.0628 | | | 211.1077 | 106.0575 | L | 1425.6856 | 713.3464 | 1408.6591 | **704.8332** | 1407.6750 | **704.3412** | 13 |
| 3 | **342.2023** | 171.6048 | | | 324.1918 | 162.5995 | L | **1312.6015** | **656.8044** | 1295.5750 | **648.2911** | **1294.5910** | **647.7991** | 12 |
| 4 | **443.2500** | 222.1287 | | | 425.2395 | 213.1234 | T | **1199.5175** | **600.2624** | **1182.4909** | 591.7491 | **1181.5069** | 591.2571 | 11 |
| 5 | **558.2770** | 279.6421 | 541.2504 | 271.1288 | **540.2664** | 270.6368 | N | **1098.4698** | 549.7385 | 1081.4433 | 541.2253 | 1080.4592 | 540.7333 | 10 |

| | | | | | | | | | | | | | | |
|---|---|---|---|---|---|---|---|---|---|---|---|---|---|---|
| **6** | **686.3355** | 343.6714 | 669.3090 | 335.1581 | 668.3250 | 334.6661 | **Q** | **983.4429** | 492.2251 | **966.4163** | 483.7118 | 965.4323 | 483.2198 | **9** |
| **7** | 773.3676 | 387.1874 | 756.3410 | 378.6741 | **755.3570** | 378.1821 | **S** | **855.3843** | 428.1958 | 838.3577 | **419.6825** | 837.3737 | **419.1905** | **8** |
| **8** | **888.3945** | 444.7009 | 871.3680 | 436.1876 | 870.3839 | 435.6956 | **D** | 768.3523 | 384.6798 | **751.3257** | 376.1665 | 750.3417 | 375.6745 | **7** |
| **9** | **1035.4629** | 518.2351 | 1018.4364 | 509.7218 | **1017.4524** | 509.2298 | **F** | **653.3253** | 327.1663 | 636.2988 | 318.6530 | 635.3148 | 318.1610 | **6** |
| **10** | **1122.4950** | 561.7511 | 1105.4684 | 553.2378 | 1104.4844 | 552.7458 | **S** | **506.2569** | 253.6321 | **489.2304** | 245.1188 | 488.2463 | 244.6268 | **5** |
| **11** | **1179.5164** | 590.2618 | 1162.4899 | 581.7486 | 1161.5059 | 581.2566 | **G** | **419.2249** | 210.1161 | **402.1983** | 201.6028 | 401.2143 | 201.1108 | **4** |
| **12** | **1293.5593** | 647.2833 | **1276.5328** | 638.7700 | **1275.5488** | 638.2780 | **N** | **362.2034** | 181.6053 | 345.1769 | 173.0921 | 344.1928 | 172.6001 | **3** |
| **13** | **1394.6070** | 697.8071 | 1377.5805 | 689.2939 | 1376.5965 | 688.8019 | **T** | **248.1605** | 124.5839 | 231.1339 | 116.0706 | 230.1499 | 115.5786 | **2** |
| **14** | | | | | | | **K** | 147.1128 | 74.0600 | 130.0863 | 65.5468 | | | **1** |

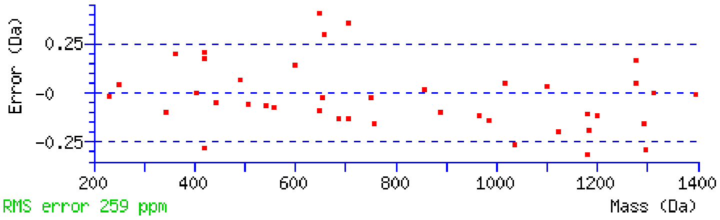


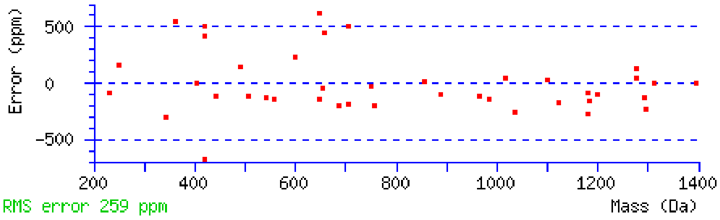


### All matches to this query

| Score | Mr(calc): | Delta | Sequence |
|---|---|---|---|
| 82.3 | 1539.7053 | -0.0028 | DLLTNQSDFSGNTK |
| 34.4 | 1539.7053 | -0.0028 | DLLTNQSDFSGNTK |
| 14.5 | 1539.7043 | -0.0018 | EVLTGSRGDXSLGGR |
| 13.7 | 1539.7083 | -0.0058 | GVGQETLLWNTSR |
| 13.4 | 1539.7043 | -0.0018 | EVLTGSRGDXSLGGR |
| 11.5 | 1538.6959 | 1.0066 | FITDKHFYFNK |
| 8.0 | 1539.7069 | -0.0044 | DIINNTSLAELEK |
| 8.0 | 1539.7069 | -0.0044 | DIINNTSLAELEK |
| 7.5 | 1539.7083 | -0.0058 | GVGQETLLWNTSR |
| 7.4 | 1539.7043 | -0.0018 | EVLTGSRGDXSLGGR |

---

Spectrum No: 204; Query: 1947; Rank: 1

---

## Peptide View

MS/MS Fragmentation of **EDSCQLNYSEGPCLGMQQK**
Found in **IPI00210900**, Tax_Id=10116 Gene_Symbol=Ambp AMBP protein precursor

Match to Query 1947: 2243.923648 from(1122.969100,2+)
Title: 100101RatKid_NS_deglyco_24.2363.2363.2.dta
Data file K:\NewmanPaper\Piliang\3SubProteomes\Piliang3SP\mgf5ppm\ERLIC_3SubProteomes5ppm.mgf

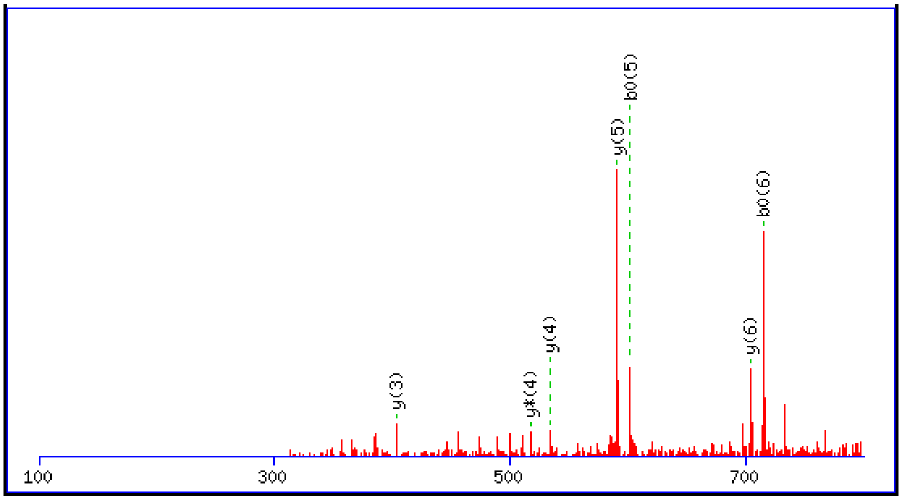


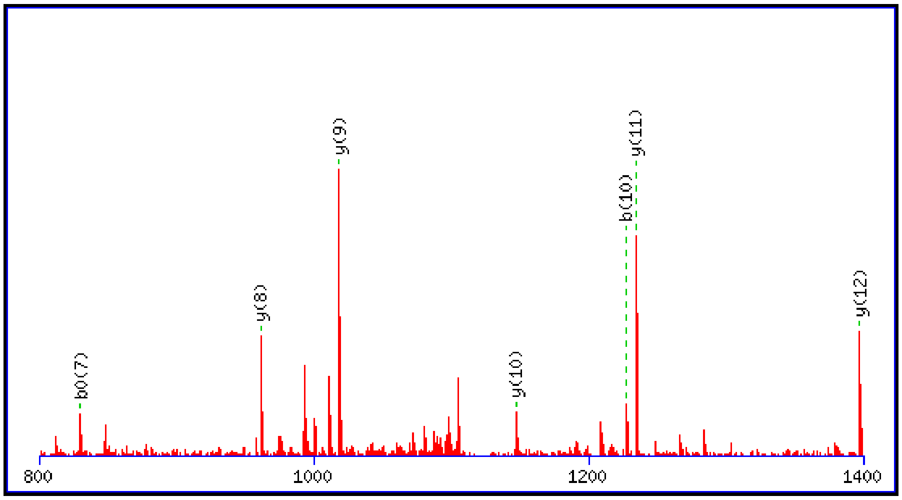


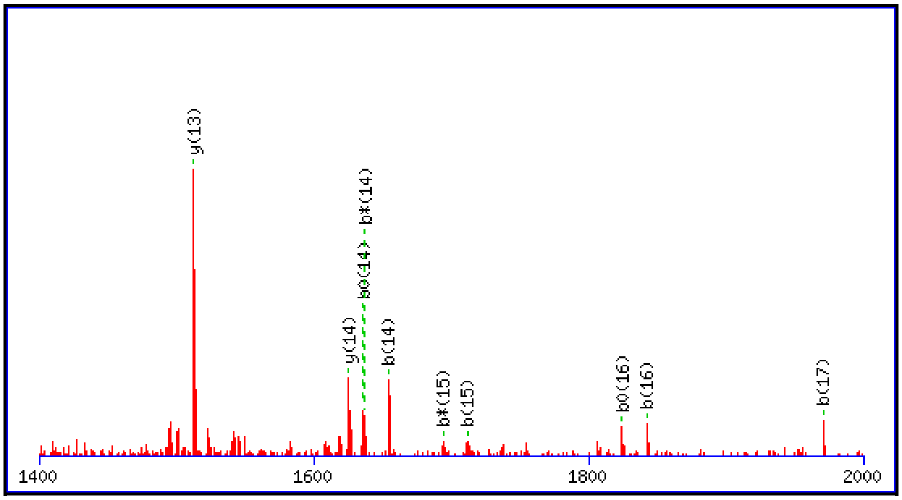


**Monoisotopic mass of neutral peptide Mr(calc):** 2243.9133
**Fixed modifications:** Carbamidomethyl (C)
**Variable modifications:**
**N7** : Deamidated_N (N)
**Ions Score:** 82 **Expect:** 1.3e-006
**Matches (Bold Red):** 24/190 fragment ions using 35 most intense peaks

| # | b | $b^{++}$ | b* | $b^{*++}$ | $b^0$ | $b^{0++}$ | Seq. | y | $y^{++}$ | y* | $y^{*++}$ | $y^0$ | $y^{0++}$ | # |
|---|---|---|---|---|---|---|---|---|---|---|---|---|---|---|
| 1 | 130.0499 | 65.5286 | | | 112.0393 | 56.5233 | E | | | | | | | 19 |
| 2 | 245.0768 | 123.0420 | | | 227.0662 | 114.0368 | D | 2115.8780 | 1058.4426 | 2098.8514 | 1049.9294 | 2097.8674 | 1049.4374 | 18 |
| 3 | 332.1088 | 166.5581 | | | 314.0983 | 157.5528 | S | 2000.8511 | 1000.9292 | 1983.8245 | 992.4159 | 1982.8405 | 991.9239 | 17 |
| 4 | 492.1395 | 246.5734 | | | 474.1289 | 237.5681 | C | 1913.8190 | 957.4132 | 1896.7925 | 948.8999 | 1895.8085 | 948.4079 | 16 |
| 5 | 620.1981 | 310.6027 | 603.1715 | 302.0894 | **602.1875** | 301.5974 | Q | 1753.7884 | 877.3978 | 1736.7618 | 868.8846 | 1735.7778 | 868.3925 | 15 |

| | | | | | | | | | | | | | | |
|---|---|---|---|---|---|---|---|---|---|---|---|---|---|---|
| **6** | 733.2821 | 367.1447 | 716.2556 | 358.6314 | 715.2716 | 358.1394 | **L** | 1625.7298 | 813.3685 | 1608.7033 | 804.8553 | 1607.7192 | 804.3633 | **14** |
| **7** | 848.3091 | 424.6582 | 831.2825 | 416.1449 | 830.2985 | 415.6529 | **N** | 1512.6457 | 756.8265 | 1495.6192 | 748.3132 | 1494.6352 | 747.8212 | **13** |
| **8** | 1011.3724 | 506.1898 | 994.3458 | 497.6766 | 993.3618 | 497.1846 | **Y** | 1397.6188 | 699.3130 | 1380.5923 | 690.7998 | 1379.6082 | 690.3078 | **12** |
| **9** | 1098.4044 | 549.7059 | 1081.3779 | 541.1926 | 1080.3939 | 540.7006 | **S** | 1234.5555 | 617.7814 | 1217.5289 | 609.2681 | 1216.5449 | 608.7761 | **11** |
| **10** | 1227.4470 | 614.2271 | 1210.4205 | 605.7139 | 1209.4365 | 605.2219 | **E** | 1147.5234 | 574.2654 | 1130.4969 | 565.7521 | 1129.5129 | 565.2601 | **10** |
| **11** | 1284.4685 | 642.7379 | 1267.4419 | 634.2246 | 1266.4579 | 633.7326 | **G** | 1018.4809 | 509.7441 | 1001.4543 | 501.2308 | | | **9** |
| **12** | 1381.5212 | 691.2643 | 1364.4947 | 682.7510 | 1363.5107 | 682.2590 | **P** | 961.4594 | 481.2333 | 944.4328 | 472.7201 | | | **8** |
| **13** | 1541.5519 | 771.2796 | 1524.5253 | 762.7663 | 1523.5413 | 762.2743 | **C** | 864.4066 | 432.7069 | 847.3801 | 424.1937 | | | **7** |
| **14** | 1654.6360 | 827.8216 | 1637.6094 | 819.3083 | 1636.6254 | 818.8163 | **L** | 704.3760 | 352.6916 | 687.3494 | 344.1784 | | | **6** |
| **15** | 1711.6574 | 856.3322 | 1694.6309 | 847.8191 | 1693.6469 | 847.3271 | **G** | 591.2919 | 296.1496 | 574.2654 | 287.6363 | | | **5** |
| **16** | 1842.6979 | 921.8526 | 1825.6714 | 913.3393 | 1824.6873 | 912.8473 | **M** | 534.2704 | 267.6389 | 517.2439 | 259.1256 | | | **4** |
| **17** | 1970.7565 | 985.8819 | 1953.7299 | 977.3686 | 1952.7459 | 976.8766 | **Q** | 403.2300 | 202.1186 | 386.2034 | 193.6053 | | | **3** |
| **18** | 2098.8151 | 1049.9112 | 2081.7885 | 1041.3979 | 2080.8045 | 1040.9059 | **Q** | 275.1714 | 138.0893 | 258.1448 | 129.5761 | | | **2** |
| **19** | | | | | | | **K** | 147.1128 | 74.0600 | 130.0863 | 65.5468 | | | **1** |

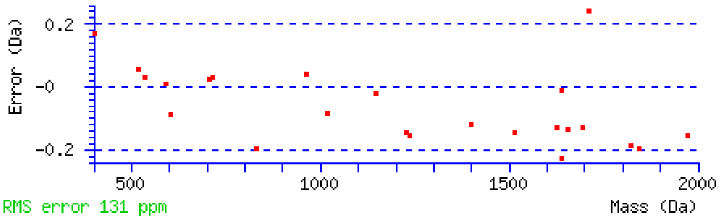


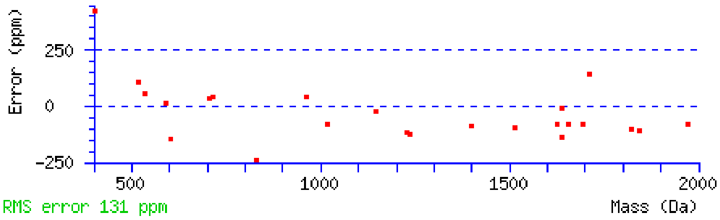


### All matches to this query

| Score | Mr(calc): | Delta | Sequence |
|---|---|---|---|
| 82.3 | 2243.9133 | 0.0103 | EDSCQLNYSEGPCLGMQQK |
| 40.8 | 2242.9293 | 0.9944 | EDSCQLNYSEGPCLGMQQK |
| 9.3 | 2241.9245 | 1.9991 | SPGHXWEIPSLQSPYSQR |
| 4.8 | 2241.9001 | 2.0236 | DDKNTMTDSTILLEDVQK |
| 4.0 | 2241.9245 | 1.9991 | SPGHXWEIPSLQSPYSQR |
| 3.8 | 2241.9175 | 2.0061 | NPQVASTWNEVNTNSNTQR |
| 2.5 | 2241.9175 | 2.0061 | NPQVASTWNEVNTNSNTQR |
| 2.5 | 2241.9175 | 2.0061 | NPQVASTWNEVNTNSNTQR |
| 1.5 | 2241.9295 | 1.9941 | GSPIRITPDFSPETMKAR |
| 0.3 | 2242.9079 | 1.0157 | AHSIPSEKDTGPQTGAGAGMR |

---

Spectrum No: 205; Query: 945; Rank: 1

---

## Peptide View

MS/MS Fragmentation of **DDALLETTDGFNTTK**
Found in **IPI00212546**, Tax_Id=10116 Gene_Symbol=Emb Embigin precursor

Match to Query 945: 1640.743508 from(821.379030,2+)
Title: 100101RatKid_NS_deglyco_28.2626.2626.2.dta
Data file K:\NewmanPaper\Piliang\3SubProteomes\Piliang3SP\mgf5ppm\ERLIC_3SubProteomes5ppm.mgf

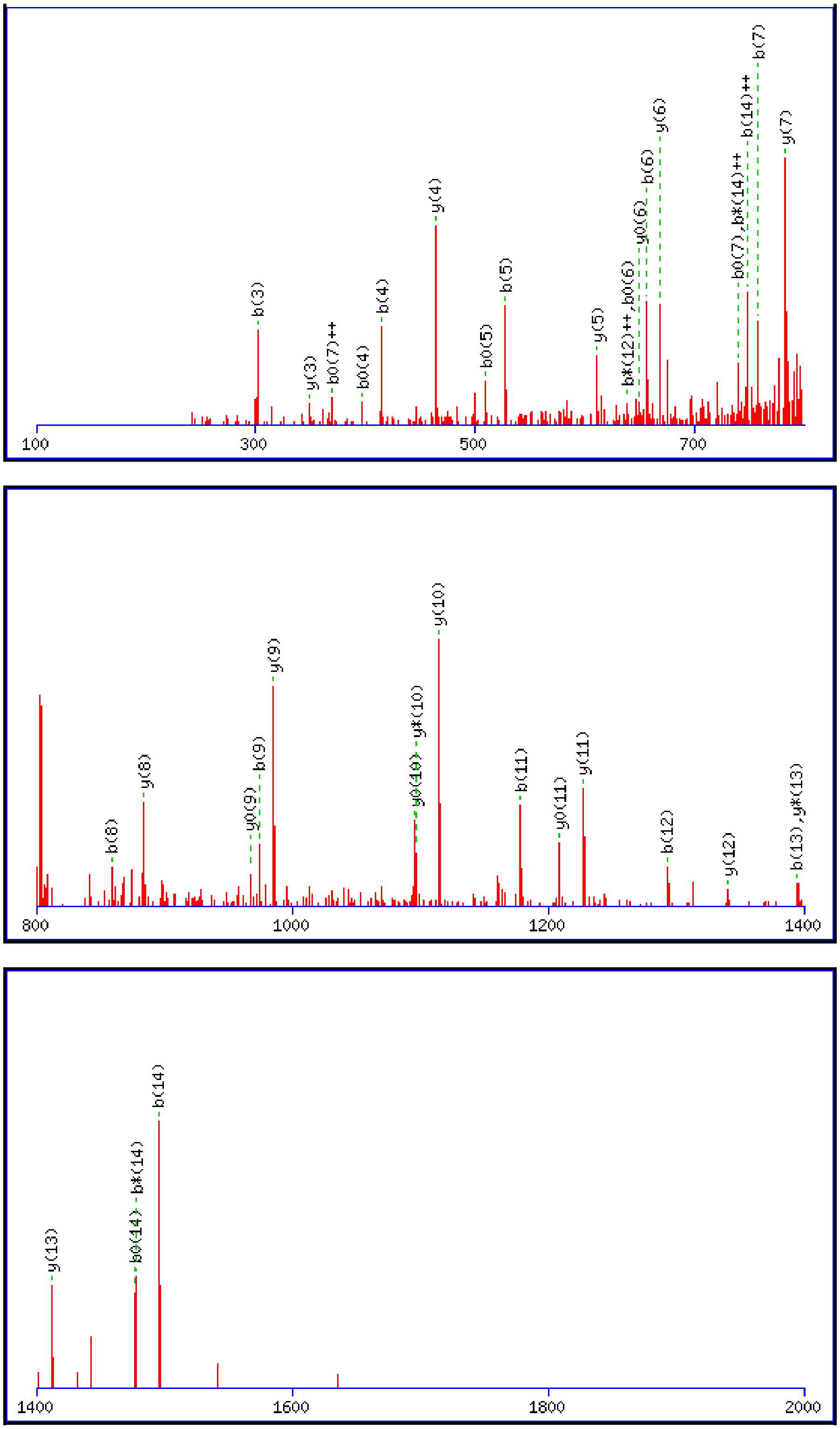


**Monoisotopic mass of neutral peptide Mr(calc):** 1640.7417
**Fixed modifications:** Carbamidomethyl (C)
**Variable modifications:**
**N12** : Deamidated_N (N)
**Ions Score:** 82 **Expect:** 1.1e-006
**Matches (Bold Red):** 38/144 fragment ions using 60 most intense peaks

| # | b | $b^{++}$ | b* | $b^{*++}$ | $b^0$ | $b^{0++}$ | Seq. | y | $y^{++}$ | y* | $y^{*++}$ | $y^0$ | $y^{0++}$ | # |
|---|---|---|---|---|---|---|---|---|---|---|---|---|---|---|
| 1 | 116.0342 | 58.5207 | | | 98.0237 | 49.5155 | D | | | | | | | 15 |
| 2 | 231.0612 | 116.0342 | | | 213.0506 | 107.0289 | D | 1526.7221 | 763.8647 | 1509.6955 | 755.3514 | 1508.7115 | 754.8594 | 14 |
| 3 | **302.0983** | 151.5528 | | | 284.0877 | 142.5475 | A | **1411.6951** | 706.3512 | **1394.6686** | 697.8379 | 1393.6845 | 697.3459 | 13 |
| 4 | **415.1823** | 208.0948 | | | **397.1718** | 199.0895 | L | **1340.6580** | 670.8326 | 1323.6314 | 662.3194 | 1322.6474 | 661.8274 | 12 |
| 5 | **528.2664** | 264.6368 | | | **510.2558** | 255.6316 | L | **1227.5739** | 614.2906 | 1210.5474 | 605.7773 | **1209.5634** | 605.2853 | 11 |

| | | | | | | | | | | | | | | |
|---|---|---|---|---|---|---|---|---|---|---|---|---|---|---|
| 6 | 657.3090 | 329.1581 | | | 639.2984 | 320.1529 | E | 1114.4899 | 557.7486 | 1097.4633 | 549.2353 | 1096.4793 | 548.7433 | 10 |
| 7 | 758.3567 | 379.6820 | | | 740.3461 | 370.6767 | T | 985.4473 | 493.2273 | 968.4207 | 484.7140 | 967.4367 | 484.2220 | 9 |
| 8 | 859.4044 | 430.2058 | | | 841.3938 | 421.2005 | T | 884.3996 | 442.7034 | 867.3730 | 434.1902 | 866.3890 | 433.6982 | 8 |
| 9 | 974.4313 | 487.7193 | | | 956.4207 | 478.7140 | D | 783.3519 | 392.1796 | 766.3254 | 383.6663 | 765.3414 | 383.1743 | 7 |
| 10 | 1031.4528 | 516.2300 | | | 1013.4422 | 507.2247 | G | 668.3250 | 334.6661 | 651.2984 | 326.1529 | 650.3144 | 325.6608 | 6 |
| 11 | 1178.5212 | 589.7642 | | | 1160.5106 | 580.7589 | F | 611.3035 | 306.1554 | 594.2770 | 297.6421 | 593.2929 | 297.1501 | 5 |
| 12 | 1293.5481 | 647.2777 | 1276.5216 | 638.7644 | 1275.5375 | 638.2724 | N | 464.2351 | 232.6212 | 447.2085 | 224.1079 | 446.2245 | 223.6159 | 4 |
| 13 | 1394.5958 | 697.8015 | 1377.5692 | 689.2883 | 1376.5852 | 688.7963 | T | 349.2082 | 175.1077 | 332.1816 | 166.5944 | 331.1976 | 166.1024 | 3 |
| 14 | 1495.6435 | 748.3254 | 1478.6169 | 739.8121 | 1477.6329 | 739.3201 | T | 248.1605 | 124.5839 | 231.1339 | 116.0706 | 230.1499 | 115.5786 | 2 |
| 15 | | | | | | | K | 147.1128 | 74.0600 | 130.0863 | 65.5468 | | | 1 |

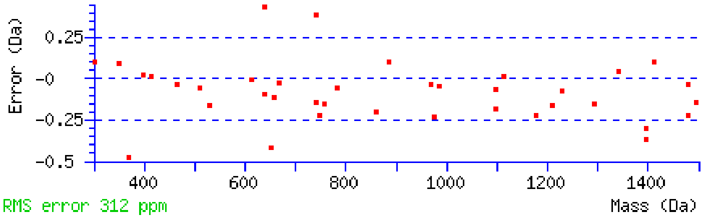


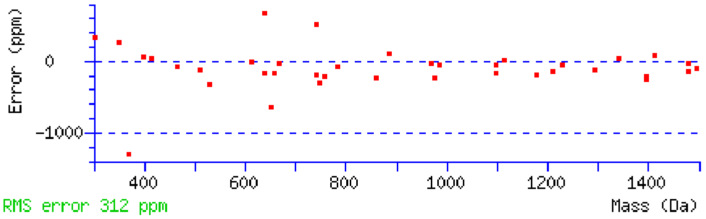


### All matches to this query

| Score | Mr(calc): | Delta | Sequence |
|---|---|---|---|
| 82.2 | 1640.7417 | 0.0018 | DDALLETTDGFNTTK |
| 4.7 | 1639.7542 | 0.9893 | KTNLFSHVASMGPR |
| 3.7 | 1640.7546 | -0.0111 | VLSLDTNITNQVNK |
| 3.5 | 1640.7407 | 0.0028 | RNGLEGKQTTASADL |
| 3.5 | 1640.7407 | 0.0028 | RNGLEGKQTTASADL |
| 3.1 | 1640.7308 | 0.0127 | GYRPENSTAVTGGPR |
| 2.2 | 1640.7308 | 0.0127 | GYRPENSTAVTGGPR |
| 1.1 | 1638.7324 | 2.0111 | KANLHSSLENMGLK |
| 1.1 | 1638.7324 | 2.0111 | KANLHSSLENMGLK |

---

Spectrum No: 206; Query: 2749; Rank: 1

---

## Peptide View

MS/MS Fragmentation of **VFSTDINGLNTQEILNVSVDTPENLAVDWINNK**
Found in **IPI00205325**, Tax_Id=10116 Gene_Symbol=Lrp2 Low-density lipoprotein receptor-related protein 2 precursor

Match to Query 2749: 3672.825342 from(1225.282390,3+)
Title: 100101RatKid_NS_deglyco_16.6878.6878.3.dta
Data file K:\NewmanPaper\Piliang\3SubProteomes\Piliang3SP\mgf5ppm\ERLIC_3SubProteomes5ppm.mgf

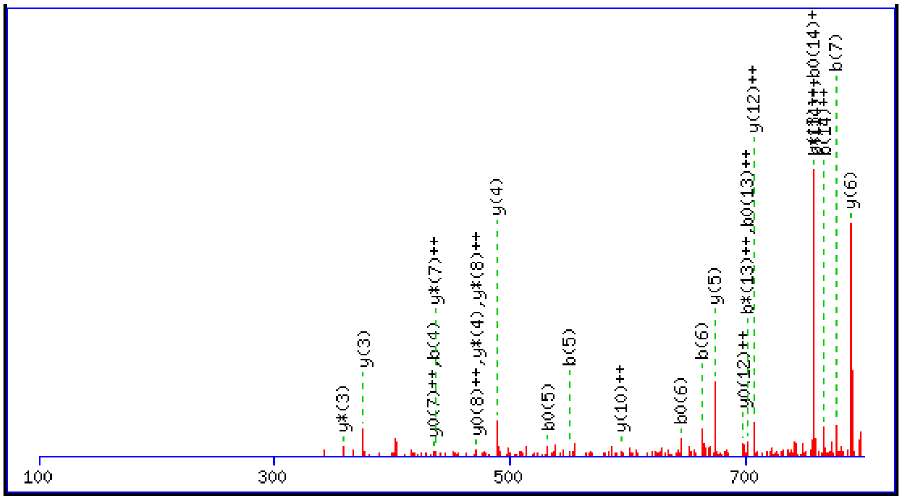


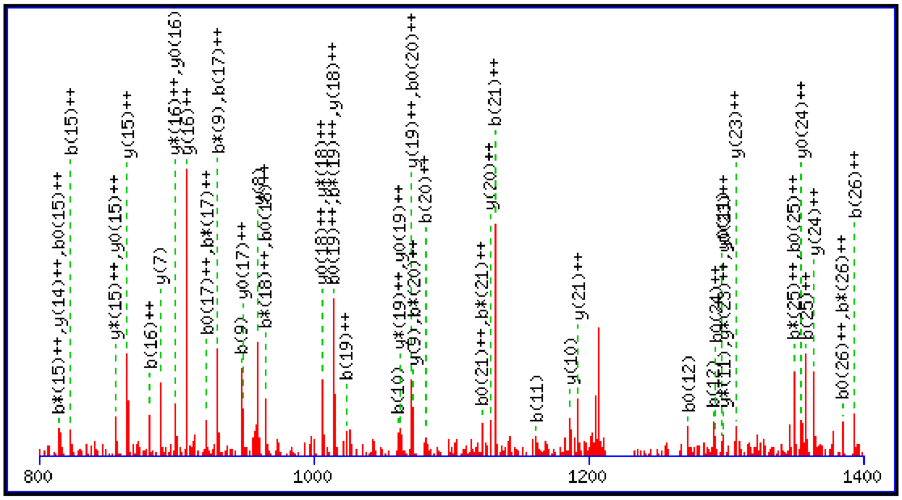


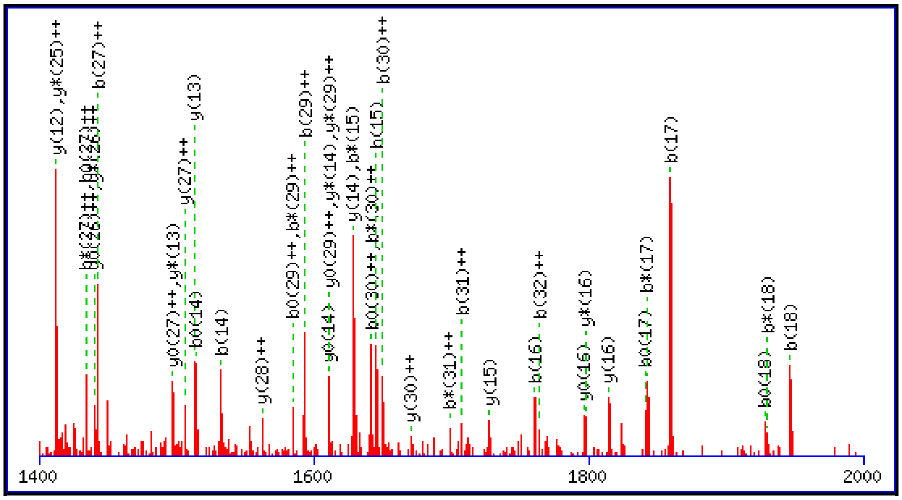


**Monoisotopic mass of neutral peptide Mr(calc):** 3672.8159
**Fixed modifications:** Carbamidomethyl (C)
**Variable modifications:**
**N16** : Deamidated_N (N)
**Ions Score:** 82 **Expect:** 1.4e-006
**Matches (Bold Red):** 125/358 fragment ions using 169 most intense peaks

| # | b | $b^{++}$ | b* | $b^{*++}$ | $b^0$ | $b^{0++}$ | Seq. | y | $y^{++}$ | y* | $y^{*++}$ | $y^0$ | $y^{0++}$ | # |
|---|---|---|---|---|---|---|---|---|---|---|---|---|---|---|
| 1 | 100.0757 | 50.5415 | | | | | V | | | | | | | 33 |
| 2 | 247.1441 | 124.0757 | | | | | F | 3574.7548 | 1787.8810 | 3557.7282 | 1779.3677 | 3556.7442 | 1778.8757 | 32 |
| 3 | 334.1761 | 167.5917 | | | 316.1656 | 158.5864 | S | 3427.6863 | 1714.3468 | 3410.6598 | 1705.8335 | 3409.6758 | 1705.3415 | 31 |
| 4 | **435.2238** | 218.1155 | | | 417.2132 | 209.1103 | T | 3340.6543 | **1670.8308** | 3323.6278 | 1662.3175 | 3322.6437 | 1661.8255 | 30 |
| 5 | **550.2508** | 275.6290 | | | **532.2402** | 266.6237 | D | 3239.6066 | 1620.3070 | 3222.5801 | **1611.7937** | 3221.5961 | **1611.3017** | 29 |

| | | | | | | | | | | | | | | |
|---|---|---|---|---|---|---|---|---|---|---|---|---|---|---|
| 6 | 663.3348 | 332.1710 | | | 645.3243 | 323.1658 | I | 3124.5797 | 1562.7935 | 3107.5531 | 1554.2802 | 3106.5691 | 1553.7882 | 28 |
| 7 | 777.3777 | 389.1925 | 760.3512 | 380.6792 | 759.3672 | 380.1872 | N | 3011.4956 | 1506.2515 | 2994.4691 | 1497.7382 | 2993.4851 | 1497.2462 | 27 |
| 8 | 834.3992 | 417.7032 | 817.3727 | 409.1900 | 816.3886 | 408.6980 | G | 2897.4527 | 1449.2300 | 2880.4262 | 1440.7167 | 2879.4421 | 1440.2247 | 26 |
| 9 | 947.4833 | 474.2453 | 930.4567 | 465.7320 | 929.4727 | 465.2400 | L | 2840.4312 | 1420.7193 | 2823.4047 | 1412.2060 | 2822.4207 | 1411.7140 | 25 |
| 10 | 1061.5262 | 531.2667 | 1044.4997 | 522.7535 | 1043.5156 | 522.2615 | N | 2727.3472 | 1364.1772 | 2710.3206 | 1355.6639 | 2709.3366 | 1355.1719 | 24 |
| 11 | 1162.5739 | 581.7906 | 1145.5473 | 573.2773 | 1144.5633 | 572.7853 | T | 2613.3042 | 1307.1558 | 2596.2777 | 1298.6425 | 2595.2937 | 1298.1505 | 23 |
| 12 | 1290.6325 | 645.8199 | 1273.6059 | 637.3066 | 1272.6219 | 636.8146 | Q | 2512.2566 | 1256.6319 | 2495.2300 | 1248.1186 | 2494.2460 | 1247.6266 | 22 |
| 13 | 1419.6751 | 710.3412 | 1402.6485 | 701.8279 | 1401.6645 | 701.3359 | E | 2384.1980 | 1192.6026 | 2367.1714 | 1184.0894 | 2366.1874 | 1183.5973 | 21 |
| 14 | 1532.7591 | 766.8832 | 1515.7326 | 758.3699 | 1514.7485 | 757.8779 | I | 2255.1554 | 1128.0813 | 2238.1288 | 1119.5681 | 2237.1448 | 1119.0761 | 20 |
| 15 | 1645.8432 | 823.4252 | 1628.8166 | 814.9120 | 1627.8326 | 814.4199 | L | 2142.0713 | 1071.5393 | 2125.0448 | 1063.0260 | 2124.0608 | 1062.5340 | 19 |
| 16 | 1760.8701 | 880.9387 | 1743.8436 | 872.4254 | 1742.8595 | 871.9334 | N | 2028.9873 | 1014.9973 | 2011.9607 | 1006.4840 | 2010.9767 | 1005.9920 | 18 |
| 17 | 1859.9385 | 930.4729 | 1842.9120 | 921.9596 | 1841.9280 | 921.4676 | V | 1913.9603 | 957.4838 | 1896.9338 | 948.9705 | 1895.9498 | 948.4785 | 17 |
| 18 | 1946.9706 | 973.9889 | 1929.9440 | 965.4756 | 1928.9600 | 964.9836 | S | 1814.8919 | 907.9496 | 1797.8654 | 899.4363 | 1796.8814 | 898.9443 | 16 |
| 19 | 2046.0390 | 1023.5231 | 2029.0124 | 1015.0098 | 2028.0284 | 1014.5178 | V | 1727.8599 | 864.4336 | 1710.8333 | 855.9203 | 1709.8493 | 855.4283 | 15 |
| 20 | 2161.0659 | 1081.0366 | 2144.0394 | 1072.5233 | 2143.0553 | 1072.0313 | D | 1628.7915 | 814.8994 | 1611.7649 | 806.3861 | 1610.7809 | 805.8941 | 14 |
| 21 | 2262.1136 | 1131.5604 | 2245.0870 | 1123.0472 | 2244.1030 | 1122.5552 | T | 1513.7645 | 757.3859 | 1496.7380 | 748.8726 | 1495.7540 | 748.3806 | 13 |
| 22 | 2359.1664 | 1180.0868 | 2342.1398 | 1171.5735 | 2341.1558 | 1171.0815 | P | 1412.7169 | 706.8621 | 1395.6903 | 698.3488 | 1394.7063 | 697.8568 | 12 |
| 23 | 2488.2089 | 1244.6081 | 2471.1824 | 1236.0948 | 2470.1984 | 1235.6028 | E | 1315.6641 | 658.3357 | 1298.6375 | 649.8224 | 1297.6535 | 649.3304 | 11 |
| 24 | 2602.2519 | 1301.6296 | 2585.2253 | 1293.1163 | 2584.2413 | 1292.6243 | N | 1186.6215 | 593.8144 | 1169.5949 | 585.3011 | 1168.6109 | 584.8091 | 10 |
| 25 | 2715.3359 | 1358.1716 | 2698.3094 | 1349.6583 | 2697.3254 | 1349.1663 | L | 1072.5786 | 536.7929 | 1055.5520 | 528.2796 | 1054.5680 | 527.7876 | 9 |
| 26 | 2786.3731 | 1393.6902 | 2769.3465 | 1385.1769 | 2768.3625 | 1384.6849 | A | 959.4945 | 480.2509 | 942.4680 | 471.7376 | 941.4839 | 471.2456 | 8 |
| 27 | 2885.4415 | 1443.2244 | 2868.4149 | 1434.7111 | 2867.4309 | 1434.2191 | V | 888.4574 | 444.7323 | 871.4308 | 436.2191 | 870.4468 | 435.7271 | 7 |
| 28 | 3000.4684 | 1500.7378 | 2983.4419 | 1492.2246 | 2982.4578 | 1491.7326 | D | 789.3890 | 395.1981 | 772.3624 | 386.6849 | 771.3784 | 386.1928 | 6 |
| 29 | 3186.5477 | 1593.7775 | 3169.5212 | 1585.2642 | 3168.5372 | 1584.7722 | W | 674.3620 | 337.6847 | 657.3355 | 329.1714 | | | 5 |
| 30 | 3299.6318 | 1650.3195 | 3282.6052 | 1641.8063 | 3281.6212 | 1641.3142 | I | 488.2827 | 244.6450 | 471.2562 | 236.1317 | | | 4 |
| 31 | 3413.6747 | 1707.3410 | 3396.6482 | 1698.8277 | 3395.6642 | 1698.3357 | N | 375.1987 | 188.1030 | 358.1721 | 179.5897 | | | 3 |
| 32 | 3527.7176 | 1764.3625 | 3510.6911 | 1755.8492 | 3509.7071 | 1755.3572 | N | 261.1557 | 131.0815 | 244.1292 | 122.5682 | | | 2 |
| 33 | | | | | | | K | 147.1128 | 74.0600 | 130.0863 | 65.5468 | | | 1 |

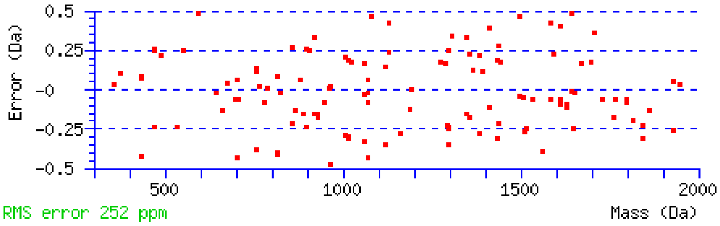


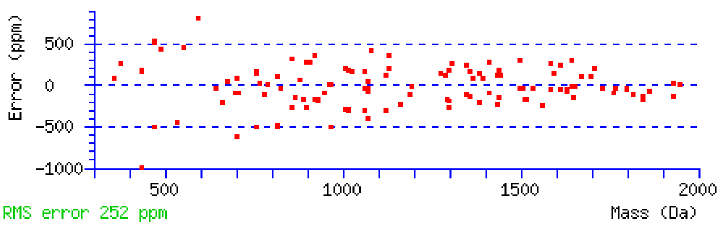


### All matches to this query

| Score | Mr(calc): | Delta | Sequence |
|---|---|---|---|
| 82.1 | 3672.8159 | 0.0095 | VFSTDINGLNTQEILNVSVDTPENLAVDWINNK |
| 70.4 | 3672.8159 | 0.0095 | VFSTDINGLNTQEILNVSVDTPENLAVDWINNK |
| 70.2 | 3672.8159 | 0.0095 | VFSTDINGLNTQEILNVSVDTPENLAVDWINNK |
| 62.6 | 3672.8159 | 0.0095 | VFSTDINGLNTQEILNVSVDTPENLAVDWINNK |
| 59.6 | 3671.8319 | 0.9935 | VFSTDINGLNTQEILNVSVDTPENLAVDWINNK |
| 50.4 | 3672.8159 | 0.0095 | VFSTDINGLNTQEILNVSVDTPENLAVDWINNK |
| 49.1 | 3672.8159 | 0.0095 | VFSTDINGLNTQEILNVSVDTPENLAVDWINNK |
| 8.4 | 3672.8007 | 0.0247 | TILMMGRYVEPIEDVPCGNIVGLVGVDQFLVK |
| 6.3 | 3670.8412 | 1.9842 | LTVQPSAKGFRFTLVSCALSFFLFSFQVHEK |
| 6.3 | 3670.8412 | 1.9842 | LTVQPSAKGFRFTLVSCALSFFLFSFQVHEK |

Spectrum No: 207; Query: 748; Rank: 1

## Peptide View

MS/MS Fragmentation of **IKLDNYSTQELGR**
Found in **IPI00204348**, Tax_Id=10116 Gene_Symbol=Nt5e 5'-nucleotidase precursor

Match to Query 748: 1536.785328 from(769.399940,2+)
Title: 100101RatKid_NS_deglyco_21.2056.2056.2.dta
Data file K:\NewmanPaper\Piliang\3SubProteomes\Piliang3SP\mgf5ppm\ERLIC_3SubProteomes5ppm.mgf

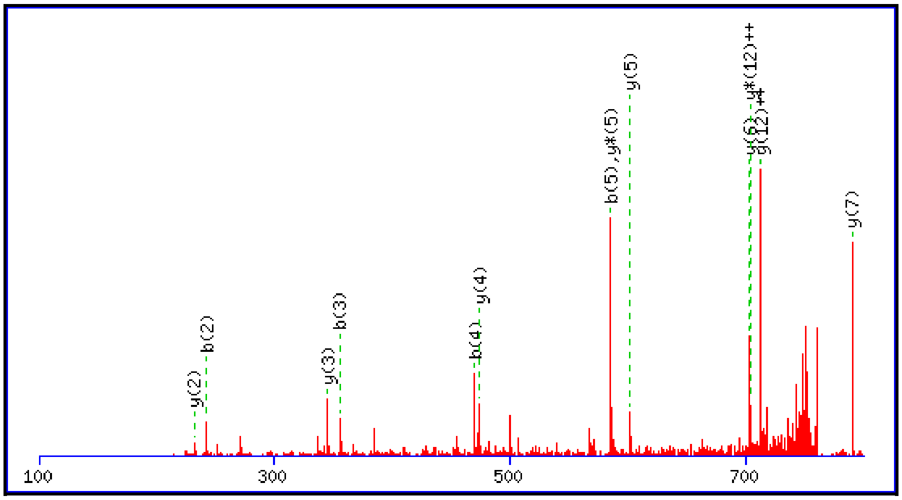


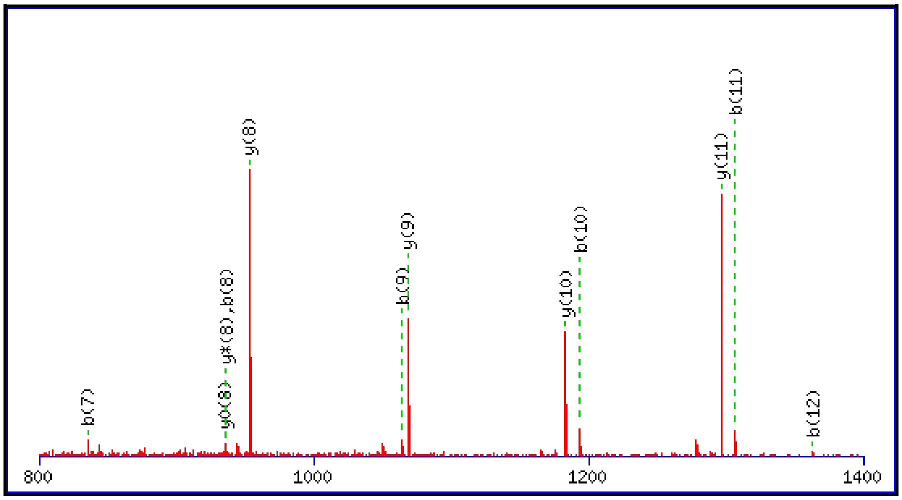


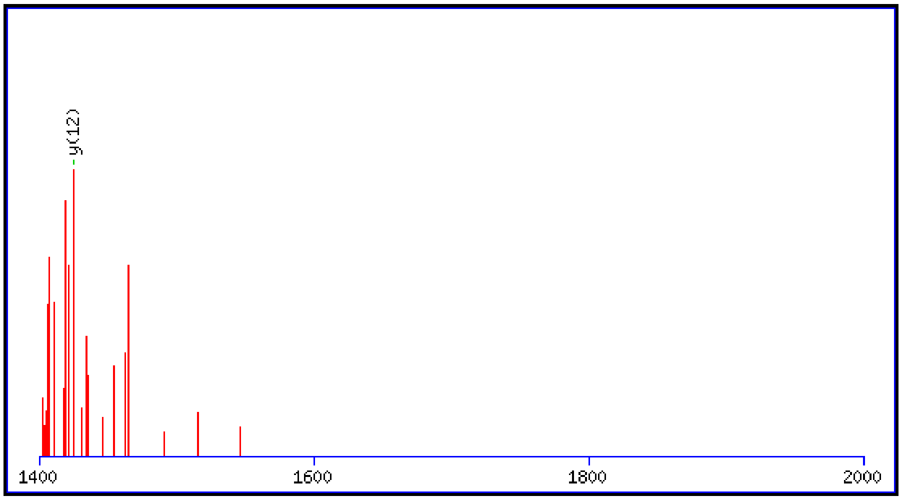


**Monoisotopic mass of neutral peptide Mr(calc):** 1536.7784
**Fixed modifications:** Carbamidomethyl (C)

**Variable modifications:**
**N5** : Deamidated_N (N)
**Ions Score:** 82 **Expect:** 8.6e-007
**Matches (Bold Red):** 26/130 fragment ions using 50 most intense peaks

| # | b | $b^{++}$ | b* | $b^{*++}$ | $b^{0}$ | $b^{0++}$ | Seq. | y | $y^{++}$ | y* | $y^{*++}$ | $y^{0}$ | $y^{0++}$ | # |
|---|---|---|---|---|---|---|---|---|---|---|---|---|---|---|
| 1 | 114.0913 | 57.5493 | | | | | I | | | | | | | 13 |
| 2 | **242.1863** | 121.5968 | 225.1598 | 113.0835 | | | K | **1424.7016** | **712.8544** | 1407.6750 | **704.3412** | 1406.6910 | 703.8492 | 12 |
| 3 | **355.2704** | 178.1388 | 338.2438 | 169.6255 | | | L | **1296.6066** | 648.8070 | 1279.5801 | 640.2937 | 1278.5961 | 639.8017 | 11 |
| 4 | **470.2973** | 235.6523 | 453.2708 | 227.1390 | 452.2867 | 226.6470 | D | **1183.5226** | 592.2649 | 1166.4960 | 583.7516 | 1165.5120 | 583.2596 | 10 |
| 5 | **585.3242** | 293.1658 | 568.2977 | 284.6525 | 567.3137 | 284.1605 | N | **1068.4956** | 534.7514 | 1051.4691 | 526.2382 | 1050.4851 | 525.7462 | 9 |
| 6 | 748.3876 | 374.6974 | 731.3610 | 366.1842 | 730.3770 | 365.6921 | Y | **953.4687** | 477.2380 | **936.4421** | 468.7247 | **935.4581** | 468.2327 | 8 |
| 7 | **835.4196** | 418.2134 | 818.3931 | 409.7002 | 817.4090 | 409.2082 | S | **790.4054** | 395.7063 | 773.3788 | 387.1930 | 772.3948 | 386.7010 | 7 |
| 8 | **936.4673** | 468.7373 | 919.4407 | 460.2240 | 918.4567 | 459.7320 | T | **703.3733** | 352.1903 | 686.3468 | 343.6770 | 685.3628 | 343.1850 | 6 |
| 9 | **1064.5259** | 532.7666 | 1047.4993 | 524.2533 | 1046.5153 | 523.7613 | Q | **602.3257** | 301.6665 | **585.2991** | 293.1532 | 584.3151 | 292.6612 | 5 |
| 10 | **1193.5685** | 597.2879 | 1176.5419 | 588.7746 | 1175.5579 | 588.2826 | E | **474.2671** | 237.6372 | 457.2405 | 229.1239 | 456.2565 | 228.6319 | 4 |
| 11 | **1306.6525** | 653.8299 | 1289.6260 | 645.3166 | 1288.6420 | 644.8246 | L | **345.2245** | 173.1159 | 328.1979 | 164.6026 | | | 3 |
| 12 | **1363.6740** | 682.3406 | 1346.6474 | 673.8274 | 1345.6634 | 673.3353 | G | **232.1404** | 116.5738 | 215.1139 | 108.0606 | | | 2 |
| 13 | | | | | | | R | 175.1190 | 88.0631 | 158.0924 | 79.5498 | | | 1 |

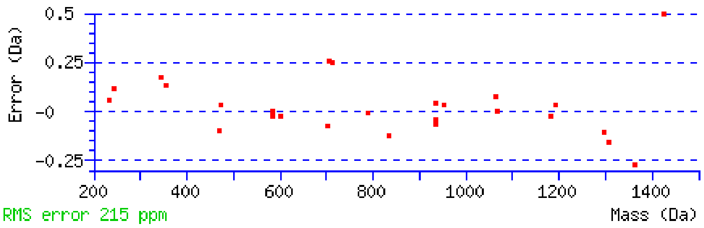


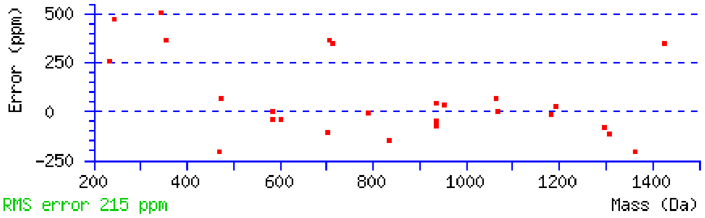


### All matches to this query

| Score | Mr(calc): | Delta | Sequence |
|---|---|---|---|
| 81.9 | 1536.7784 | 0.0070 | IKLDNYSTQELGR |
| 26.9 | 1535.7943 | 0.9910 | IKLDNYSTQELGR |
| 4.6 | 1536.7752 | 0.0102 | IIKNTAMMREAAR |
| 4.6 | 1536.7814 | 0.0040 | KLYVSNLGIGHTR |
| 4.6 | 1536.7814 | 0.0040 | KLYVSNLGIGHTR |
| 2.7 | 1536.7926 | -0.0072 | RPYGRNKPLISR |
| 2.4 | 1536.7913 | -0.0059 | IPLPTTLTSGSKSR |
| 1.1 | 1536.7773 | 0.0080 | KQGRSAINEVVTR |
| 0.9 | 1534.7878 | 1.9975 | KLLIDYDEEELR |
| 0.8 | 1535.7821 | 1.0033 | AALEQRGLAKSGQK |

---

Spectrum No: 208; Query: 183; Rank: 1

---

## Peptide View

MS/MS Fragmentation of **IVDVNLTSEGK**
Found in **IPI00192796**, Tax_Id=10116 Gene_Symbol=RGD1564625_predicted similar to Transmembrane 9 superfamily protein member 3 precursor

Match to Query 183: 1174.604828 from(588.309690,2+)
Title: 091008RatKidney_NoSalt_31.1305.1305.2.dta
Data file K:\NewmanPaper\Piliang\3SubProteomes\Piliang3SP\mgf5ppm\ERLIC_3SubProteomes5ppm.mgf

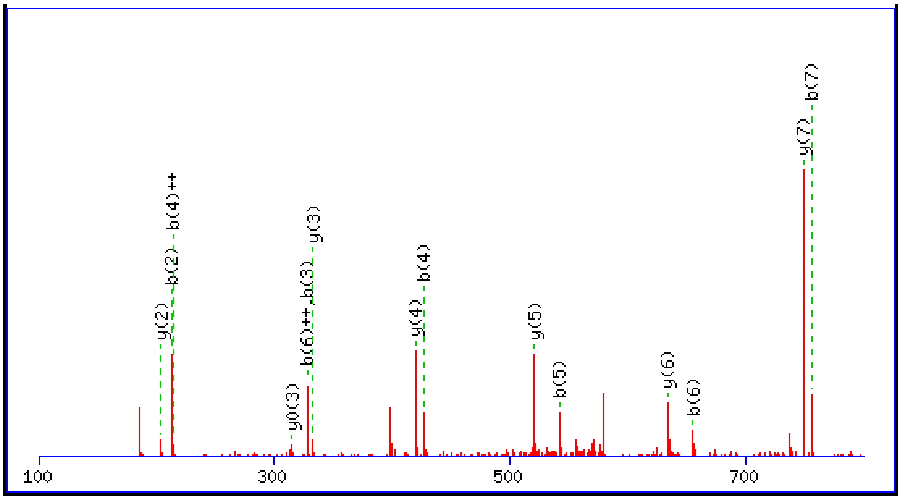


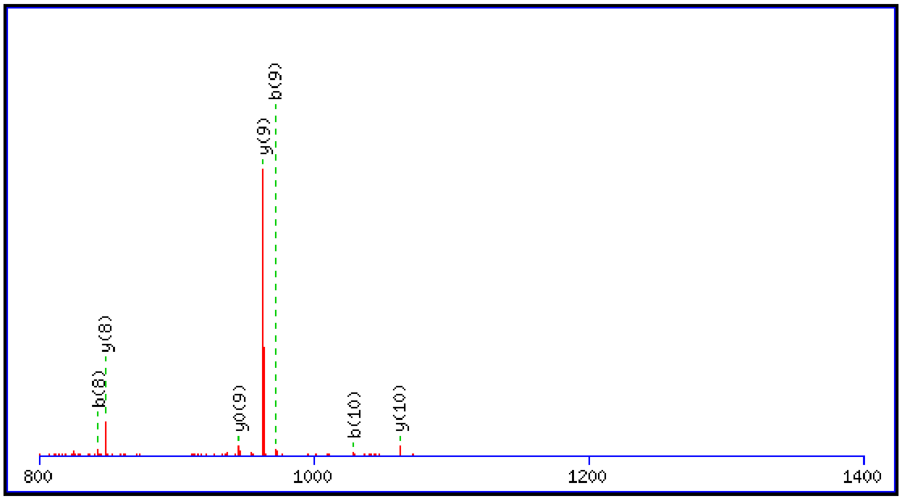


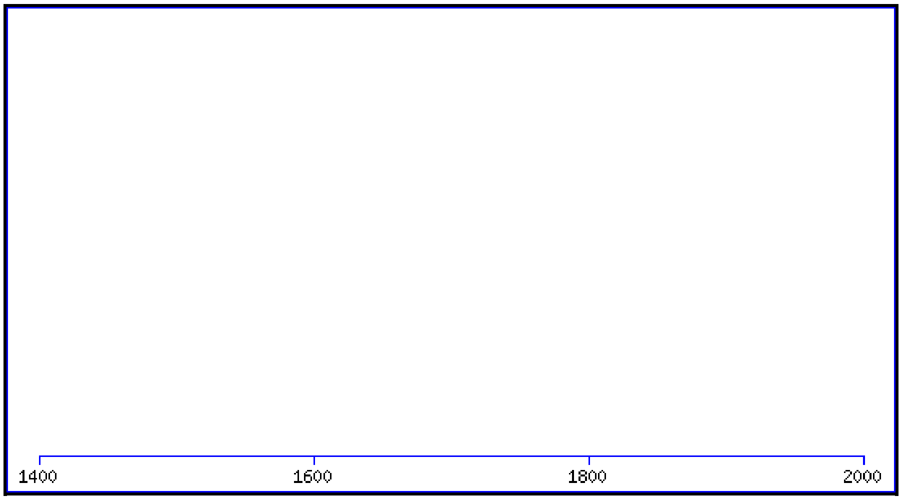


**Monoisotopic mass of neutral peptide Mr(calc):** 1174.6081
**Fixed modifications:** Carbamidomethyl (C)
**Variable modifications:**
**N5** : Deamidated_N (N)
**Ions Score:** 82 **Expect:** 7.1e-007
**Matches (Bold Red):** 22/104 fragment ions using 30 most intense peaks

| # | b | $b^{++}$ | b* | $b^{*++}$ | $b^{0}$ | $b^{0++}$ | Seq. | y | $y^{++}$ | y* | $y^{*++}$ | $y^{0}$ | $y^{0++}$ | # |
|---|---|---|---|---|---|---|---|---|---|---|---|---|---|---|
| 1 | 114.0913 | 57.5493 | | | | | I | | | | | | | 11 |
| 2 | **213.1598** | 107.0835 | | | | | V | **1062.5313** | 531.7693 | 1045.5048 | 523.2560 | 1044.5208 | 522.7640 | 10 |
| 3 | **328.1867** | 164.5970 | | | 310.1761 | 155.5917 | D | **963.4629** | 482.2351 | 946.4364 | 473.7218 | **945.4524** | 473.2298 | 9 |
| 4 | **427.2551** | **214.1312** | | | 409.2445 | 205.1259 | V | **848.4360** | 424.7216 | 831.4094 | 416.2084 | 830.4254 | 415.7163 | 8 |
| 5 | **542.2820** | 271.6447 | 525.2555 | 263.1314 | 524.2715 | 262.6394 | N | **749.3676** | 375.1874 | 732.3410 | 366.6741 | 731.3570 | 366.1821 | 7 |

| | | | | | | | | | | | | | | |
|---|---|---|---|---|---|---|---|---|---|---|---|---|---|---|
| **6** | **655.3661** | **328.1867** | 638.3396 | 319.6734 | 637.3555 | 319.1814 | **L** | **634.3406** | 317.6740 | 617.3141 | 309.1607 | 616.3301 | 308.6687 | **6** |
| **7** | **756.4138** | 378.7105 | 739.3872 | 370.1973 | 738.4032 | 369.7053 | **T** | **521.2566** | 261.1319 | 504.2300 | 252.6186 | 503.2460 | 252.1266 | **5** |
| **8** | **843.4458** | 422.2265 | 826.4193 | 413.7133 | 825.4353 | 413.2213 | **S** | **420.2089** | 210.6081 | 403.1823 | 202.0948 | 402.1983 | 201.6028 | **4** |
| **9** | **972.4884** | 486.7478 | 955.4619 | 478.2346 | 954.4778 | 477.7426 | **E** | **333.1769** | 167.0921 | 316.1503 | 158.5788 | **315.1663** | 158.0868 | **3** |
| **10** | **1029.5099** | 515.2586 | 1012.4833 | 506.7453 | 1011.4993 | 506.2533 | **G** | **204.1343** | 102.5708 | 187.1077 | 94.0575 | | | **2** |
| **11** | | | | | | | **K** | 147.1128 | 74.0600 | 130.0863 | 65.5468 | | | **1** |

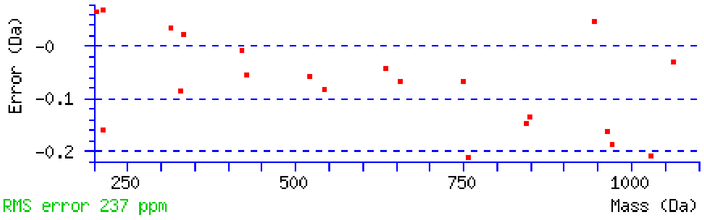


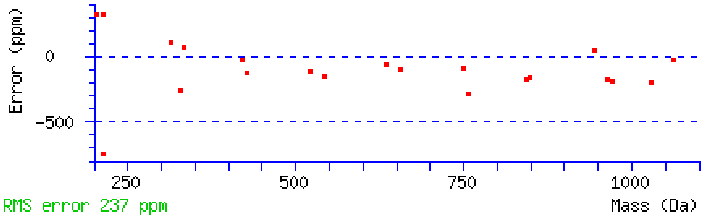


**All matches to this query**

| Score | Mr(calc): | Delta | Sequence |
|---|---|---|---|
| 81.9 | 1174.6081 | -0.0033 | IVDVNLTSEGK |
| 18.2 | 1174.5933 | 0.0115 | LVRGTKYMK |
| 5.4 | 1174.6016 | 0.0032 | LVPTKDTMNR |
| 5.2 | 1174.6127 | -0.0079 | IALKKTITK |
| 5.0 | 1174.6111 | -0.0063 | VLEQHKLTK |
| 5.0 | 1174.6016 | 0.0032 | VLNMAEIQTR |
| 4.0 | 1174.5933 | 0.0115 | LVRGTKYMK |
| 3.6 | 1174.6095 | -0.0046 | LNTGRTEAWK |
| 1.2 | 1174.6016 | 0.0032 | IVQMTEAEVR |
| 0.1 | 1174.6128 | -0.0080 | XQKQLSNCLK |

---

Spectrum No: 209; Query: 448; Rank: 1

---

## Peptide View

MS/MS Fragmentation of **YLNETQQLTEK**
Found in **IPI00201262**, Tax_Id=10116 Gene_Symbol=LOC297568;Mug2;Mug1 Alpha-1-inhibitor 3 precursor

Match to Query 448: 1366.659788 from(684.337170,2+)
Title: 091008RatKidney_NoSalt_26.1200.1200.2.dta
Data file K:\NewmanPaper\Piliang\3SubProteomes\Piliang3SP\mgf5ppm\ERLIC_3SubProteomes5ppm.mgf

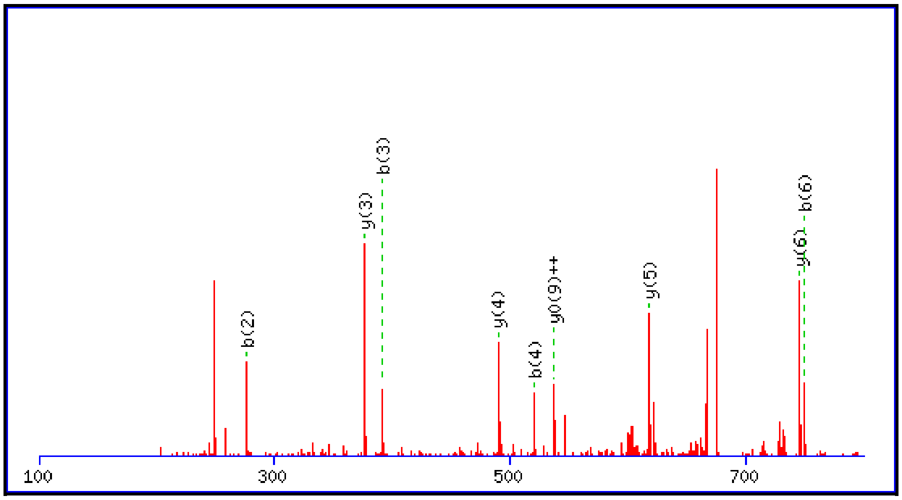

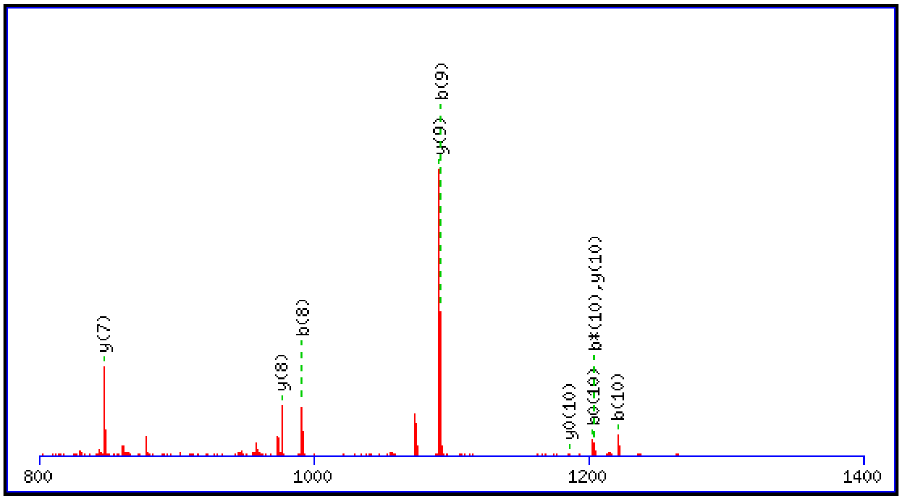


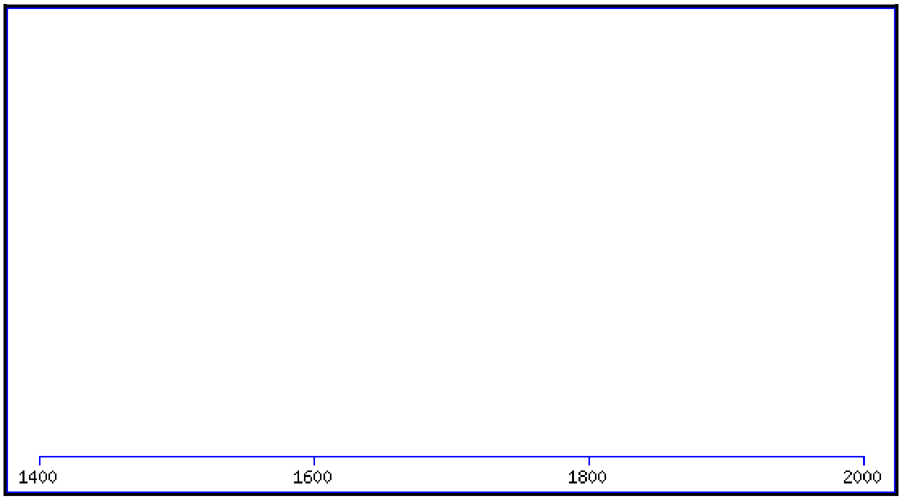


**Monoisotopic mass of neutral peptide Mr(calc):** 1366.6616
**Fixed modifications:** Carbamidomethyl (C)
**Variable modifications:**
**N3** : Deamidated_N (N)
**Ions Score:** 82 **Expect:** 1e-006
**Matches (Bold Red):** 19/108 fragment ions using 23 most intense peaks

| # | b | $b^{++}$ | b* | $b^{*++}$ | $b^0$ | $b^{0++}$ | Seq. | y | $y^{++}$ | y* | $y^{*++}$ | $y^0$ | $y^{0++}$ | # |
|---|---|---|---|---|---|---|---|---|---|---|---|---|---|---|
| 1 | 164.0706 | 82.5389 | | | | | Y | | | | | | | 11 |
| 2 | **277.1547** | 139.0810 | | | | | L | **1204.6056** | 602.8064 | 1187.5790 | 594.2931 | **1186.5950** | 593.8011 | 10 |
| 3 | **392.1816** | 196.5944 | 375.1551 | 188.0812 | | | N | **1091.5215** | 546.2644 | 1074.4950 | 537.7511 | 1073.5109 | **537.2591** | 9 |
| 4 | **521.2242** | 261.1157 | 504.1976 | 252.6025 | 503.2136 | 252.1105 | E | **976.4946** | 488.7509 | 959.4680 | 480.2376 | 958.4840 | 479.7456 | 8 |
| 5 | 622.2719 | 311.6396 | 605.2453 | 303.1263 | 604.2613 | 302.6343 | T | **847.4520** | 424.2296 | 830.4254 | 415.7164 | 829.4414 | 415.2243 | 7 |
| 6 | **750.3305** | 375.6689 | 733.3039 | 367.1556 | 732.3199 | 366.6636 | Q | **746.4043** | 373.7058 | 729.3777 | 365.1925 | 728.3937 | 364.7005 | 6 |
| 7 | 878.3890 | 439.6982 | 861.3625 | 431.1849 | 860.3785 | 430.6929 | Q | **618.3457** | 309.6765 | 601.3192 | 301.1632 | 600.3352 | 300.6712 | 5 |
| 8 | **991.4731** | 496.2402 | 974.4465 | 487.7269 | 973.4625 | 487.2349 | L | **490.2871** | 245.6472 | 473.2606 | 237.1339 | 472.2766 | 236.6419 | 4 |
| 9 | **1092.5208** | 546.7640 | 1075.4942 | 538.2508 | 1074.5102 | 537.7587 | T | **377.2031** | 189.1052 | 360.1765 | 180.5919 | 359.1925 | 180.0999 | 3 |
| 10 | **1221.5634** | 611.2853 | **1204.5368** | 602.7720 | **1203.5528** | 602.2800 | E | 276.1554 | 138.5813 | 259.1288 | 130.0681 | 258.1448 | 129.5761 | 2 |
| 11 | | | | | | | K | 147.1128 | 74.0600 | 130.0863 | 65.5468 | | | 1 |

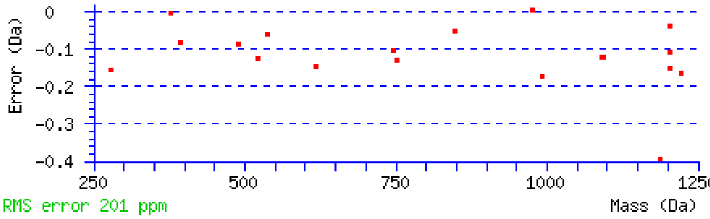


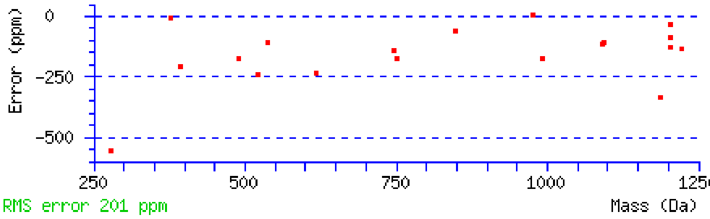

### All matches to this query

| Score | Mr(calc): | Delta | Sequence |
|---|---|---|---|
| 81.8 | 1366.6616 | -0.0018 | YLNETQQLTEK |
| 14.6 | 1366.6728 | -0.0130 | YITENNRSEIK |
| 13.3 | 1366.6680 | -0.0082 | ITDNLLQLMAR |
| 12.2 | 1366.6728 | -0.0130 | YITENNRSEIK |
| 9.7 | 1366.6517 | 0.0081 | ALFTSGWNNTEK |
| 7.9 | 1365.6524 | 1.0074 | YNPNNSSAKITR |
| 7.7 | 1366.6517 | 0.0081 | NNSTWFKLNDK |
| 7.1 | 1365.6524 | 1.0074 | YNPNNSSAKITR |
| 7.1 | 1366.6477 | 0.0121 | STSFNRVANNEK |
| 6.9 | 1365.6524 | 1.0074 | YNPNNSSAKITR |

---

Spectrum No: 210; Query: 668; Rank: 1

---

## Peptide View

MS/MS Fragmentation of **DASTQLSTNLTSVR**
Found in **IPI00325312**, Tax_Id=10116 Gene_Symbol=Prom1 prominin 1 isoform 1

Match to Query 668: 1492.739668 from(747.377110,2+)
Title: 100101RatKid_NS_deglyco_24.2656.2656.2.dta
Data file K:\NewmanPaper\Piliang\3SubProteomes\Piliang3SP\mgf5ppm\ERLIC_3SubProteomes5ppm.mgf

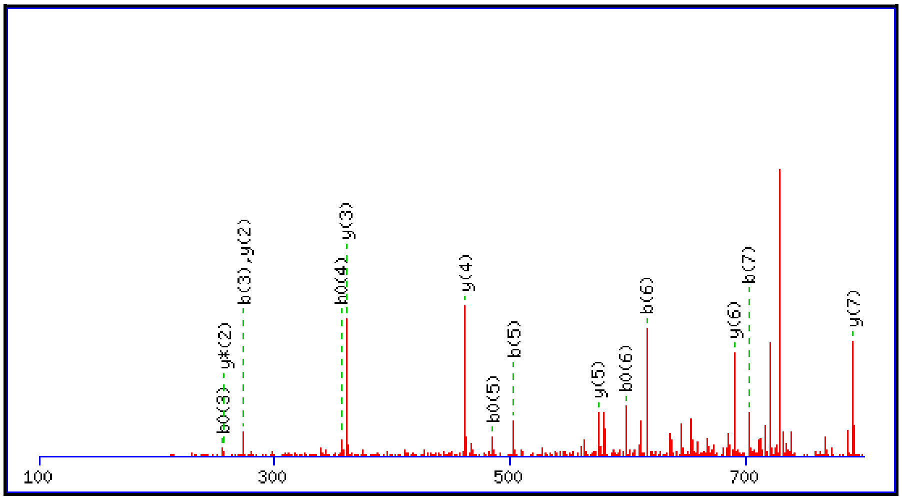

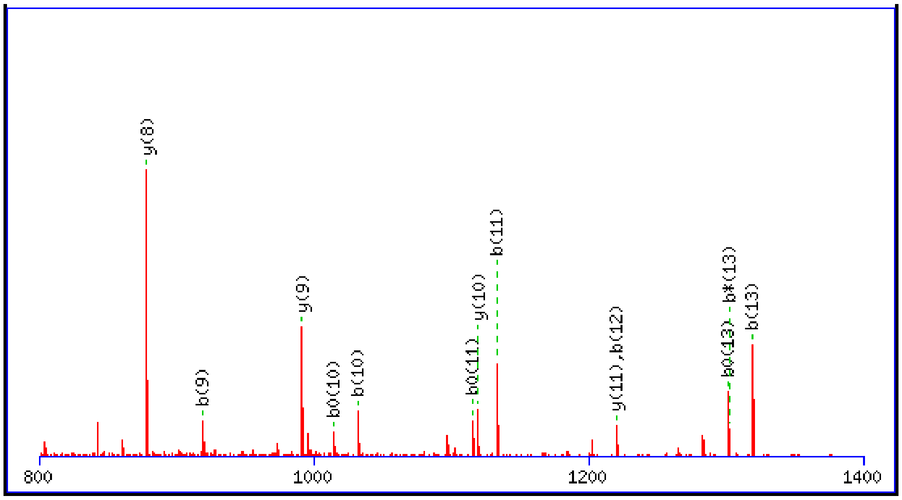


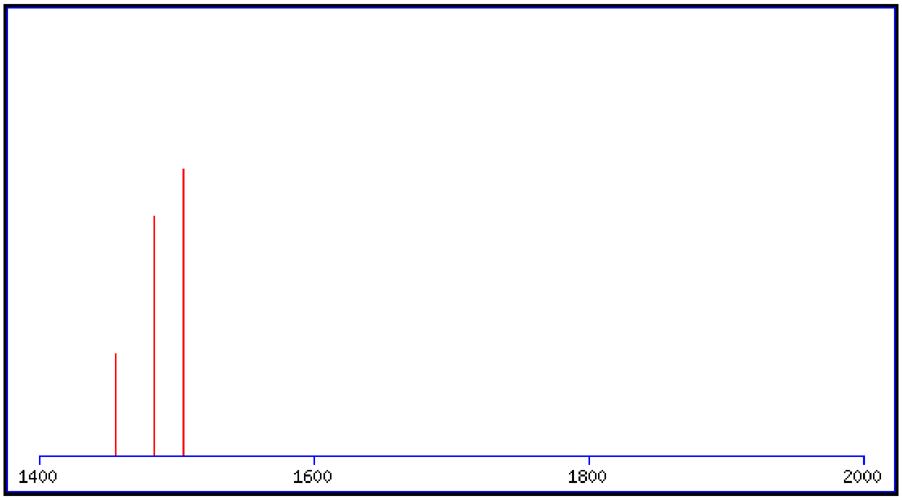


**Monoisotopic mass of neutral peptide Mr(calc):** 1492.7369
**Fixed modifications:** Carbamidomethyl (C)
**Variable modifications:**
**N9** : Deamidated_N (N)
**Ions Score:** 82 **Expect:** 1.3e-006
**Matches (Bold Red):** 28/144 fragment ions using 50 most intense peaks

| # | b | $b^{++}$ | b* | $b^{*++}$ | $b^0$ | $b^{0++}$ | Seq. | y | $y^{++}$ | y* | $y^{*++}$ | $y^0$ | $y^{0++}$ | # |
|---|---|---|---|---|---|---|---|---|---|---|---|---|---|---|
| 1 | 116.0342 | 58.5207 | | | 98.0237 | 49.5155 | D | | | | | | | 14 |
| 2 | 187.0713 | 94.0393 | | | 169.0608 | 85.0340 | A | 1378.7172 | 689.8623 | 1361.6907 | 681.3490 | 1360.7067 | 680.8570 | 13 |
| 3 | **274.1034** | 137.5553 | | | **256.0928** | 128.5500 | S | 1307.6801 | 654.3437 | 1290.6536 | 645.8304 | 1289.6696 | 645.3384 | 12 |
| 4 | 375.1510 | 188.0792 | | | **357.1405** | 179.0739 | T | **1220.6481** | 610.8277 | 1203.6216 | 602.3144 | 1202.6375 | 601.8224 | 11 |
| 5 | **503.2096** | 252.1084 | 486.1831 | 243.5952 | **485.1991** | 243.1032 | Q | **1119.6004** | 560.3038 | 1102.5739 | 551.7906 | 1101.5899 | 551.2986 | 10 |
| 6 | **616.2937** | 308.6505 | 599.2671 | 300.1372 | **598.2831** | 299.6452 | L | **991.5418** | 496.2746 | 974.5153 | 487.7613 | 973.5313 | 487.2693 | 9 |
| 7 | **703.3257** | 352.1665 | 686.2992 | 343.6532 | 685.3151 | 343.1612 | S | **878.4578** | 439.7325 | 861.4312 | 431.2193 | 860.4472 | 430.7272 | 8 |
| 8 | 804.3734 | 402.6903 | 787.3468 | 394.1771 | 786.3628 | 393.6851 | T | **791.4258** | 396.2165 | 774.3992 | 387.7032 | 773.4152 | 387.2112 | 7 |
| 9 | **919.4003** | 460.2038 | 902.3738 | 451.6905 | 901.3898 | 451.1985 | N | **690.3781** | 345.6927 | 673.3515 | 337.1794 | 672.3675 | 336.6874 | 6 |
| 10 | **1032.4844** | 516.7458 | 1015.4578 | 508.2326 | **1014.4738** | 507.7405 | L | **575.3511** | 288.1792 | 558.3246 | 279.6659 | 557.3406 | 279.1739 | 5 |
| 11 | **1133.5321** | 567.2697 | 1116.5055 | 558.7564 | **1115.5215** | 558.2644 | T | **462.2671** | 231.6372 | 445.2405 | 223.1239 | 444.2565 | 222.6319 | 4 |
| 12 | **1220.5641** | 610.7857 | 1203.5375 | 602.2724 | 1202.5535 | 601.7804 | S | **361.2194** | 181.1133 | 344.1928 | 172.6001 | 343.2088 | 172.1081 | 3 |
| 13 | **1319.6325** | 660.3199 | **1302.6060** | 651.8066 | **1301.6219** | 651.3146 | V | **274.1874** | 137.5973 | **257.1608** | 129.0840 | | | 2 |
| 14 | | | | | | | R | 175.1190 | 88.0631 | 158.0924 | 79.5498 | | | 1 |

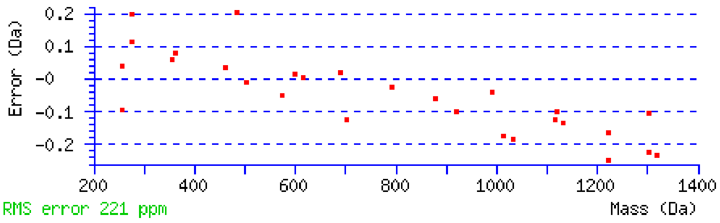


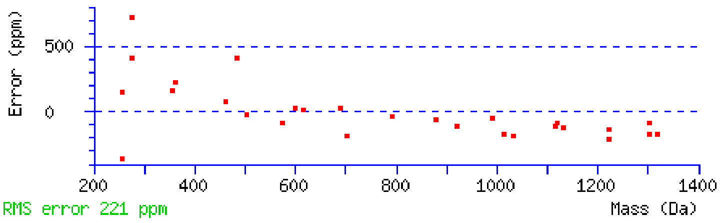


### All matches to this query

| Score | Mr(calc): | Delta | Sequence |
|---|---|---|---|
| 81.8 | 1492.7369 | 0.0028 | DASTQLSTNLTSVR |
| 11.7 | 1492.7286 | 0.0110 | AENGLGSPAIKSIR |
| 7.2 | 1492.7522 | -0.0125 | VSENPYTSGIIASR |
| 6.6 | 1492.7422 | -0.0026 | AAAAWEERFSSLR |
| 6.0 | 1492.7490 | -0.0093 | CLSGSLQVMNKTR |
| 6.0 | 1492.7344 | 0.0053 | NFAKKEMGTPDVR |
| 5.6 | 1492.7286 | 0.0110 | EREVPISLEVSR |
| 5.0 | 1490.7283 | 2.0114 | YTVGLRNXFSVK |
| 5.0 | 1490.7220 | 2.0177 | MALSSLLLLATAK |
| 4.1 | 1491.7219 | 1.0178 | TATSWSSGXRTWR |

---

Spectrum No: 211; Query: 762; Rank: 1

---

## Peptide View

MS/MS Fragmentation of **LEDPTVLQVVNVTK**
Found in **IPI00360957**, Tax_Id=10116 Gene_Symbol=Slc10a5 Sodium/bile acid cotransporter 5 precursor

Match to Query 762: 1554.852028 from(778.433290,2+)
Title: 100101RatKid_NS_deglyco_25.3485.3485.2.dta
Data file K:\NewmanPaper\Piliang\3SubProteomes\Piliang3SP\mgf5ppm\ERLIC_3SubProteomes5ppm.mgf

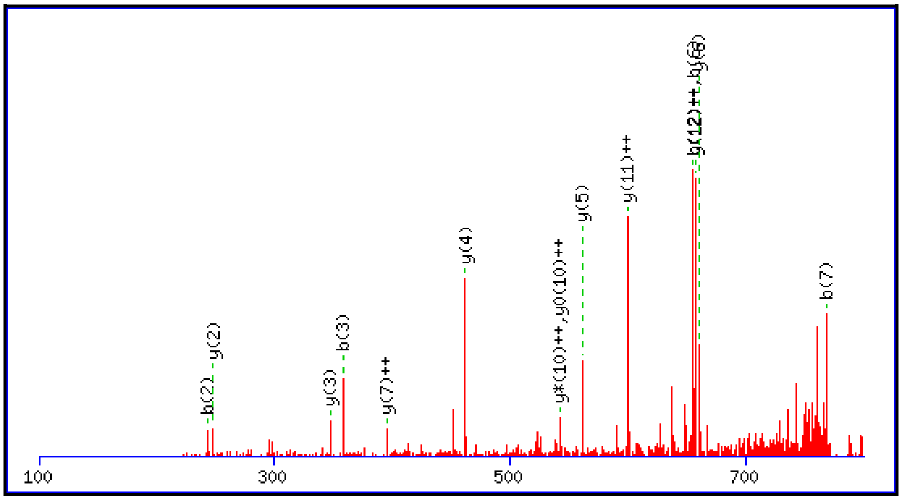

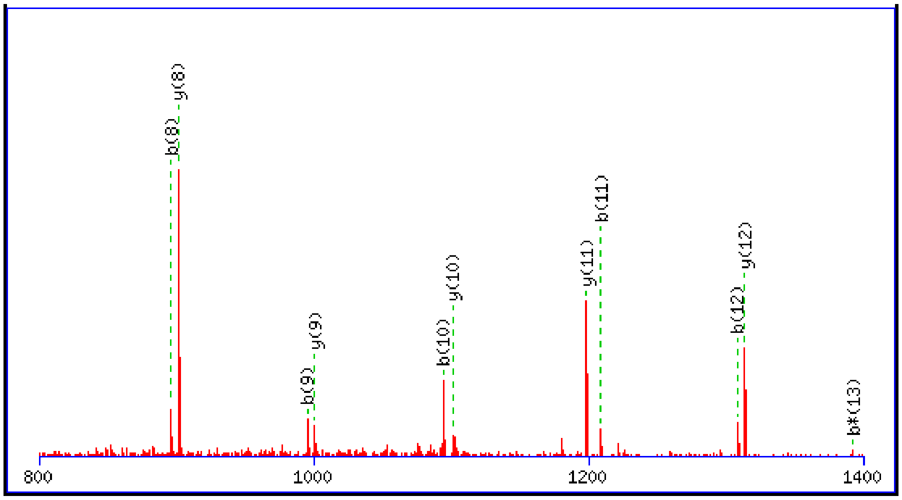


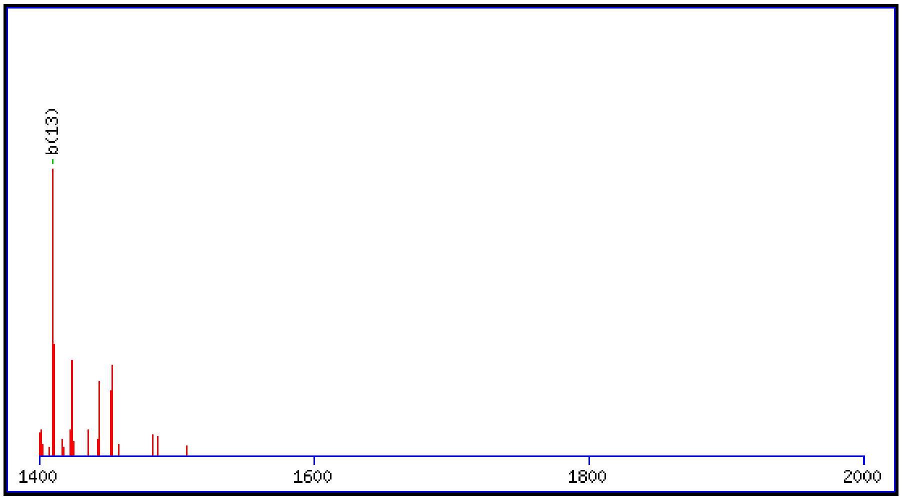


**Monoisotopic mass of neutral peptide Mr(calc):** 1554.8505
**Fixed modifications:** Carbamidomethyl (C)
**Variable modifications:**
**N11** : Deamidated_N (N)
**Ions Score:** 82 **Expect:** 4.7e-007
**Matches (Bold Red):** 27/138 fragment ions using 43 most intense peaks

| # | b | b++ | b* | b*++ | b0 | b0++ | Seq. | y | y++ | y* | y*++ | y0 | y0++ | # |
|---|---|---|---|---|---|---|---|---|---|---|---|---|---|---|
| 1 | 114.0913 | 57.5493 | | | | | L | | | | | | | 14 |
| 2 | **243.1339** | 122.0706 | | | 225.1234 | 113.0653 | E | 1442.7737 | 721.8905 | 1425.7471 | 713.3772 | 1424.7631 | 712.8852 | 13 |
| 3 | **358.1609** | 179.5841 | | | 340.1503 | 170.5788 | D | **1313.7311** | **657.3692** | 1296.7046 | 648.8559 | 1295.7205 | 648.3639 | 12 |
| 4 | 455.2136 | 228.1105 | | | 437.2031 | 219.1052 | P | **1198.7042** | **599.8557** | 1181.6776 | 591.3424 | 1180.6936 | 590.8504 | 11 |
| 5 | 556.2613 | 278.6343 | | | 538.2508 | 269.6290 | T | **1101.6514** | 551.3293 | 1084.6248 | **542.8161** | 1083.6408 | **542.3241** | 10 |
| 6 | **655.3297** | 328.1685 | | | 637.3192 | 319.1632 | V | **1000.6037** | 500.8055 | 983.5772 | 492.2922 | 982.5932 | 491.8002 | 9 |
| 7 | **768.4138** | 384.7105 | | | 750.4032 | 375.7053 | L | **901.5353** | 451.2713 | 884.5088 | 442.7580 | 883.5247 | 442.2660 | 8 |
| 8 | **896.4724** | 448.7398 | 879.4458 | 440.2266 | 878.4618 | 439.7345 | Q | 788.4512 | **394.7293** | 771.4247 | 386.2160 | 770.4407 | 385.7240 | 7 |
| 9 | **995.5408** | 498.2740 | 978.5142 | 489.7608 | 977.5302 | 489.2688 | V | **660.3927** | 330.7000 | 643.3661 | 322.1867 | 642.3821 | 321.6947 | 6 |
| 10 | **1094.6092** | 547.8082 | 1077.5827 | 539.2950 | 1076.5986 | 538.8030 | V | **561.3242** | 281.1658 | 544.2977 | 272.6525 | 543.3137 | 272.1605 | 5 |
| 11 | **1209.6361** | 605.3217 | 1192.6096 | 596.8084 | 1191.6256 | 596.3164 | N | **462.2558** | 231.6316 | 445.2293 | 223.1183 | 444.2453 | 222.6263 | 4 |
| 12 | **1308.7046** | **654.8559** | 1291.6780 | 646.3426 | 1290.6940 | 645.8506 | V | **347.2289** | 174.1181 | 330.2023 | 165.6048 | 329.2183 | 165.1128 | 3 |
| 13 | **1409.7522** | 705.3798 | **1392.7257** | 696.8665 | 1391.7417 | 696.3745 | T | **248.1605** | 124.5839 | 231.1339 | 116.0706 | 230.1499 | 115.5786 | 2 |
| 14 | | | | | | | K | 147.1128 | 74.0600 | 130.0863 | 65.5468 | | | 1 |

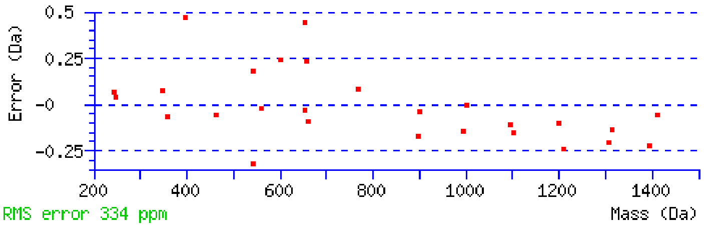


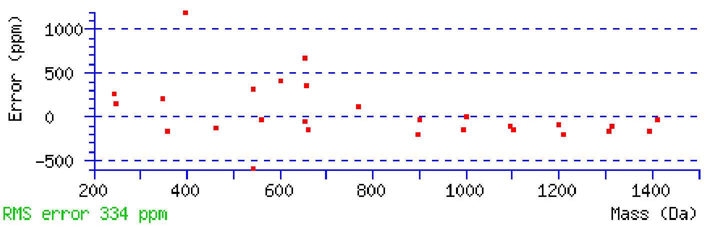


### All matches to this query

| Score | Mr(calc): | Delta | Sequence |
|---|---|---|---|
| 81.8 | 1554.8505 | 0.0015 | LEDPTVLQVVNVTK |
| 3.5 | 1552.8514 | 2.0006 | AFHLPSWKLANLR |
| 1.8 | 1552.8491 | 2.0030 | RTLIALVQVTFGR |
| 1.8 | 1554.8365 | 0.0155 | IVNAPQNNKNLTTK |
| 1.8 | 1554.8365 | 0.0155 | IVNAPQNNKNLTTK |
| 1.8 | 1554.8365 | 0.0155 | IVNAPQNNKNLTTK |
| 1.7 | 1553.8413 | 1.0107 | NKIPTVGEADELLR |
| 0.9 | 1554.8365 | 0.0155 | IVNAPQNNKNLTTK |

---

Spectrum No: 212; Query: 1003; Rank: 1

---

## Peptide View

MS/MS Fragmentation of **MSGDWELEVNGTEAK**
Found in **IPI00361686**, Tax_Id=10116 Gene_Symbol=C1qbp Complement component 1 Q subcomponent-binding protein, mitochondrial precursor

Match to Query 1003: 1665.726188 from(833.870370,2+)
Title: 100101RatKid_NS_deglyco_14.2914.2914.2.dta
Data file K:\NewmanPaper\Piliang\3SubProteomes\Piliang3SP\mgf5ppm\ERLIC_3SubProteomes5ppm.mgf

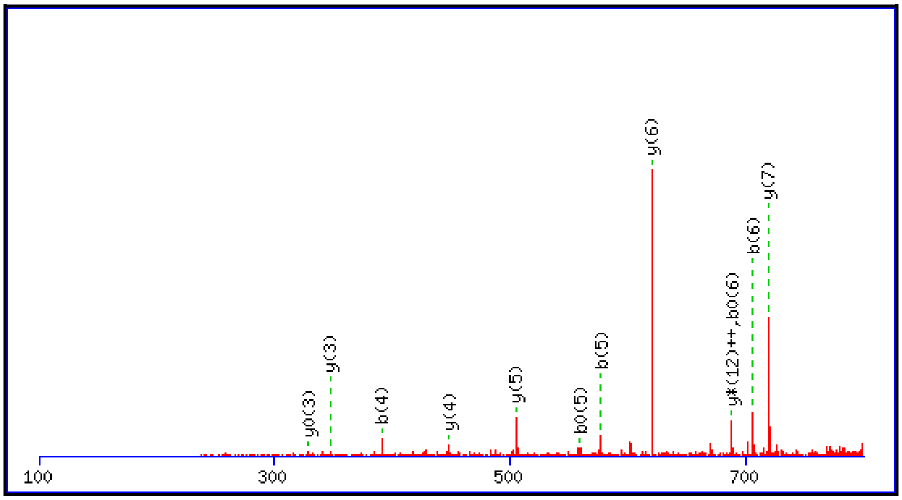

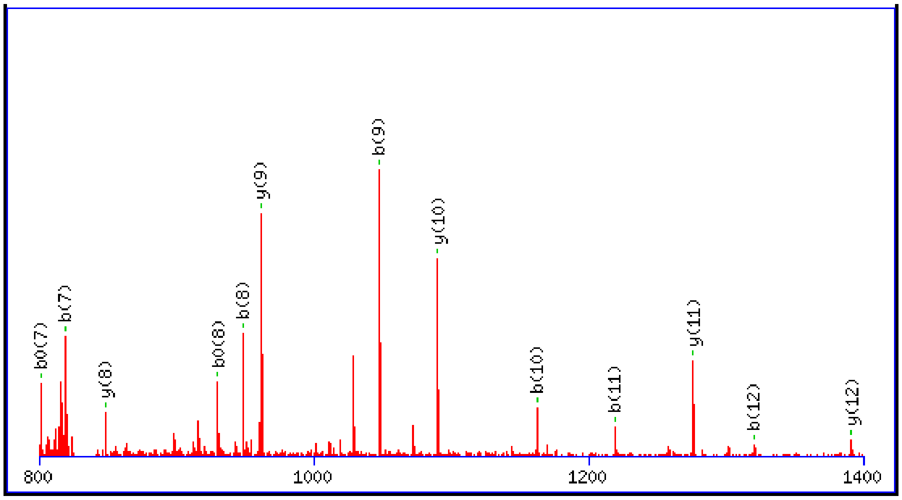


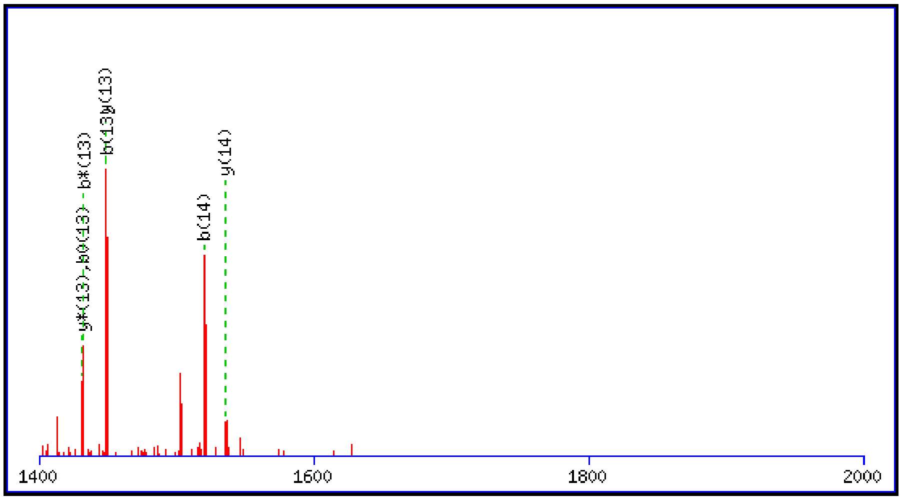


**Monoisotopic mass of neutral peptide Mr(calc):** 1665.7192
**Fixed modifications:** Carbamidomethyl (C)
**Variable modifications:**
**N10** : Deamidated_N (N)
**Ions Score:** 82 **Expect:** 1.1e-006
**Matches (Bold Red):** 32/144 fragment ions using 51 most intense peaks

| # | b | $b^{++}$ | b* | $b^{*++}$ | $b^0$ | $b^{0++}$ | Seq. | y | $y^{++}$ | y* | $y^{*++}$ | $y^0$ | $y^{0++}$ | # |
|---|---|---|---|---|---|---|---|---|---|---|---|---|---|---|
| 1 | 132.0478 | 66.5275 | | | | | M | | | | | | | 15 |
| 2 | 219.0798 | 110.0435 | | | 201.0692 | 101.0382 | S | **1535.6860** | 768.3466 | 1518.6595 | 759.8334 | 1517.6754 | 759.3414 | 14 |
| 3 | 276.1013 | 138.5543 | | | 258.0907 | 129.5490 | G | **1448.6540** | 724.8306 | **1431.6274** | 716.3174 | 1430.6434 | 715.8253 | 13 |
| 4 | **391.1282** | 196.0677 | | | 373.1176 | 187.0625 | D | **1391.6325** | 696.3199 | 1374.6060 | **687.8066** | 1373.6219 | 687.3146 | 12 |
| 5 | **577.2075** | 289.1074 | | | **559.1969** | 280.1021 | W | **1276.6056** | 638.8064 | 1259.5790 | 630.2931 | 1258.5950 | 629.8011 | 11 |
| 6 | **706.2501** | 353.6287 | | | **688.2395** | 344.6234 | E | **1090.5263** | 545.7668 | 1073.4997 | 537.2535 | 1072.5157 | 536.7615 | 10 |
| 7 | **819.3342** | 410.1707 | | | **801.3236** | 401.1654 | L | **961.4837** | 481.2455 | 944.4571 | 472.7322 | 943.4731 | 472.2402 | 9 |
| 8 | **948.3768** | 474.6920 | | | **930.3662** | 465.6867 | E | **848.3996** | 424.7034 | 831.3730 | 416.1902 | 830.3890 | 415.6982 | 8 |
| 9 | **1047.4452** | 524.2262 | | | 1029.4346 | 515.2209 | V | **719.3570** | 360.1821 | 702.3305 | 351.6689 | 701.3464 | 351.1769 | 7 |
| 10 | **1162.4721** | 581.7397 | 1145.4456 | 573.2264 | 1144.4615 | 572.7344 | N | **620.2886** | 310.6479 | 603.2620 | 302.1347 | 602.2780 | 301.6427 | 6 |
| 11 | **1219.4936** | 610.2504 | 1202.4670 | 601.7372 | 1201.4830 | 601.2451 | G | **505.2617** | 253.1345 | 488.2351 | 244.6212 | 487.2511 | 244.1292 | 5 |
| 12 | **1320.5413** | 660.7743 | 1303.5147 | 652.2610 | 1302.5307 | 651.7690 | T | **448.2402** | 224.6237 | 431.2136 | 216.1105 | 430.2296 | 215.6185 | 4 |
| 13 | **1449.5838** | 725.2956 | **1432.5573** | 716.7823 | **1431.5733** | 716.2903 | E | **347.1925** | 174.0999 | 330.1660 | 165.5866 | **329.1819** | 165.0946 | 3 |
| 14 | **1520.6210** | 760.8141 | 1503.5944 | 752.3008 | 1502.6104 | 751.8088 | A | 218.1499 | 109.5786 | 201.1234 | 101.0653 | | | 2 |
| 15 | | | | | | | K | 147.1128 | 74.0600 | 130.0863 | 65.5468 | | | 1 |

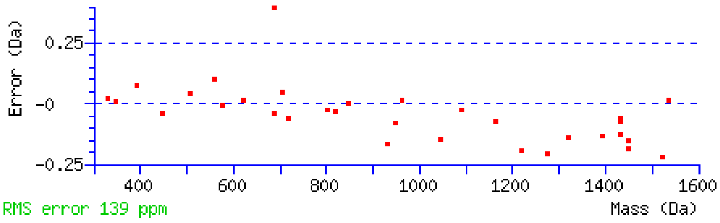


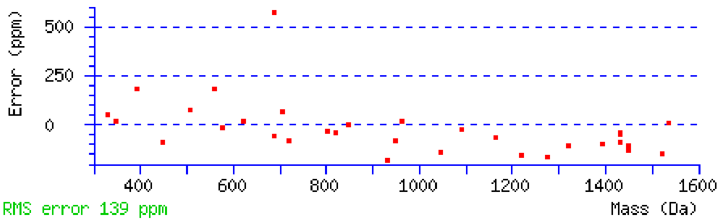


### All matches to this query

| Score | Mr(calc): | Delta | Sequence |
|---|---|---|---|
| 81.7 | 1665.7192 | 0.0070 | MSGDWELEVNGTEAK |
| 28.3 | 1664.7352 | 0.9910 | MSGDWELEVNGTEAK |
| 9.3 | 1665.7151 | 0.0111 | EENCSKAQESLENK |
| 6.7 | 1665.7164 | 0.0098 | KHSAILASPNPDEK |
| 6.4 | 1665.7205 | 0.0057 | FTVHTKSSLPNFK |
| 6.3 | 1665.7143 | 0.0119 | MSEKFEVTNLNMK |
| 5.9 | 1665.7205 | 0.0057 | FTVHTKSSLPNFK |
| 5.8 | 1665.7124 | 0.0138 | TDRSKQQSQTVTK |
| 2.5 | 1664.7311 | 0.9951 | EENCSKAQESLENK |
| 1.5 | 1663.7350 | 1.9912 | MFSENKENVKVMK |

Spectrum No: 213; Query: 2670; Rank: 1

## Peptide View

MS/MS Fragmentation of **RGVDVSQVAWQSQGDTPCSCCIVNNSNGSR**
Found in **IPI00211989**, Tax_Id=10116 Gene_Symbol=Khk Isoform C of Ketohexokinase

Match to Query 2670: 3339.435792 from(1114.152540,3+)
Title: 100101RatKid_NS_deglyco_08.2862.2862.3.dta
Data file K:\NewmanPaper\Piliang\3SubProteomes\Piliang3SP\mgf5ppm\ERLIC_3SubProteomes5ppm.mgf

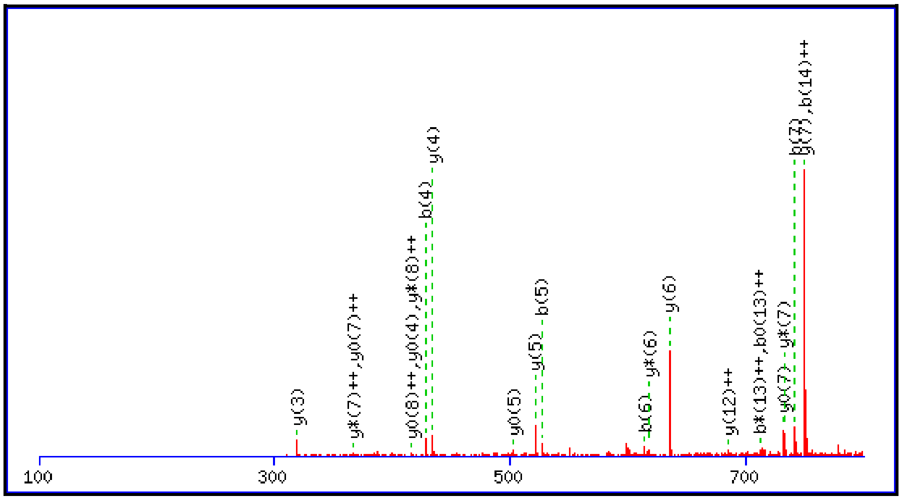

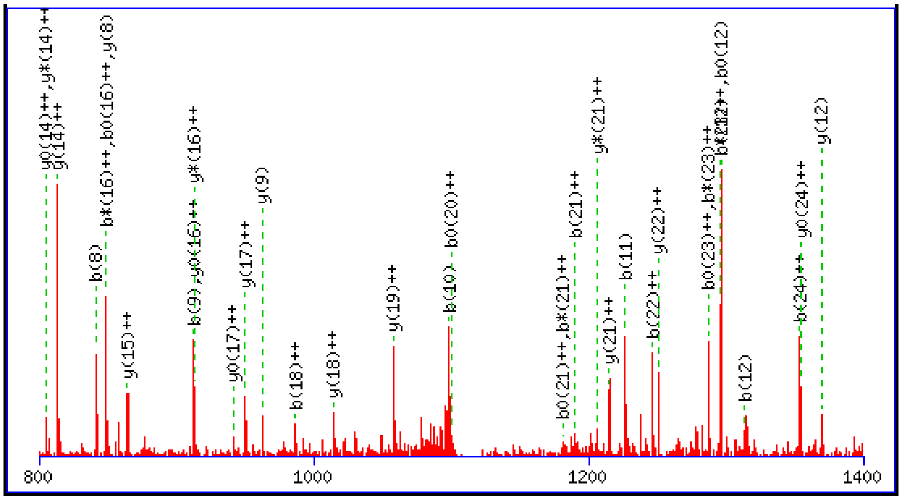


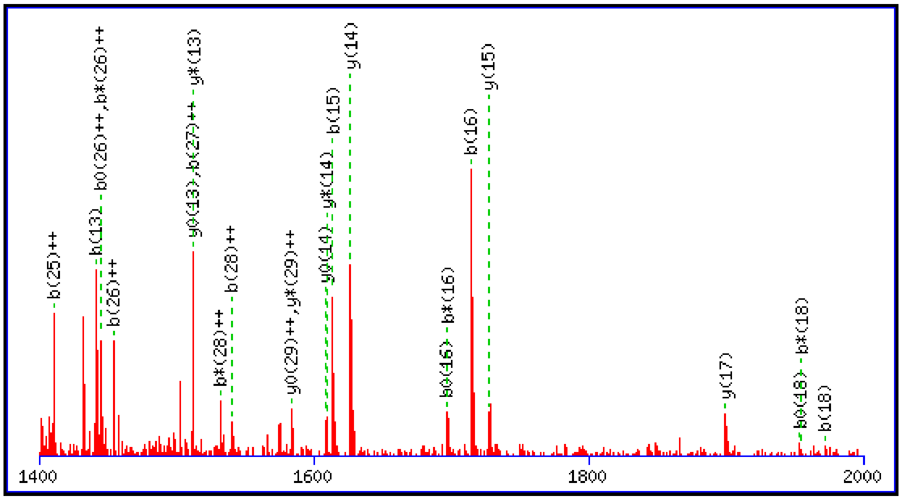


**Monoisotopic mass of neutral peptide Mr(calc):** 3339.4303
**Fixed modifications:** Carbamidomethyl (C)
**Variable modifications:**
**N24** : Deamidated_N (N)
**N27** : Deamidated_N (N)
**Ions Score:** 82 **Expect:** 3.1e-006
**Matches (Bold Red):** 83/340 fragment ions using 128 most intense peaks

| # | b | $b^{++}$ | b* | $b^{*++}$ | $b^0$ | $b^{0++}$ | Seq. | y | $y^{++}$ | y* | $y^{*++}$ | $y^0$ | $y^{0++}$ | # |
|---|---|---|---|---|---|---|---|---|---|---|---|---|---|---|
| 1 | 157.1084 | 79.0578 | 140.0818 | 70.5446 | | | R | | | | | | | 30 |
| 2 | 214.1299 | 107.5686 | 197.1033 | 99.0553 | | | G | 3184.3364 | 1592.6719 | 3167.3099 | **1584.1586** | 3166.3259 | **1583.6666** | 29 |
| 3 | 313.1983 | 157.1028 | 296.1717 | 148.5895 | | | V | 3127.3150 | 1564.1611 | 3110.2884 | 1555.6479 | 3109.3044 | 1555.1558 | 28 |
| 4 | **428.2252** | 214.6162 | 411.1987 | 206.1030 | 410.2146 | 205.6110 | D | 3028.2466 | 1514.6269 | 3011.2200 | 1506.1136 | 3010.2360 | 1505.6216 | 27 |
| 5 | **527.2936** | 264.1504 | 510.2671 | 255.6372 | 509.2831 | 255.1452 | V | 2913.2196 | 1457.1134 | 2896.1931 | 1448.6002 | 2895.2091 | 1448.1082 | 26 |
| 6 | **614.3257** | 307.6665 | 597.2991 | 299.1532 | 596.3151 | 298.6612 | S | 2814.1512 | 1407.5792 | 2797.1247 | 1399.0660 | 2796.1406 | 1398.5740 | 25 |
| 7 | **742.3842** | 371.6958 | 725.3577 | 363.1825 | 724.3737 | 362.6905 | Q | 2727.1192 | 1364.0632 | 2710.0926 | 1355.5500 | 2709.1086 | **1355.0579** | 24 |
| 8 | **841.4526** | 421.2300 | 824.4261 | 412.7167 | 823.4421 | 412.2247 | V | 2599.0606 | 1300.0339 | 2582.0340 | 1291.5207 | 2581.0500 | 1291.0287 | 23 |
| 9 | **912.4898** | 456.7485 | 895.4632 | 448.2352 | 894.4792 | 447.7432 | A | 2499.9922 | **1250.4997** | 2482.9656 | 1241.9865 | 2481.9816 | 1241.4944 | 22 |
| 10 | **1098.5691** | 549.7882 | 1081.5425 | 541.2749 | 1080.5585 | 540.7829 | W | 2428.9551 | **1214.9812** | 2411.9285 | **1206.4679** | 2410.9445 | 1205.9759 | 21 |
| 11 | **1226.6276** | 613.8175 | 1209.6011 | 605.3042 | 1208.6171 | 604.8122 | Q | 2242.8758 | 1121.9415 | 2225.8492 | 1113.4282 | 2224.8652 | 1112.9362 | 20 |
| 12 | **1313.6597** | 657.3335 | **1296.6331** | 648.8202 | **1295.6491** | 648.3282 | S | 2114.8172 | **1057.9122** | 2097.7906 | 1049.3990 | 2096.8066 | 1048.9069 | 19 |
| 13 | **1441.7183** | 721.3628 | 1424.6917 | **712.8495** | 1423.7077 | **712.3575** | Q | 2027.7852 | **1014.3962** | 2010.7586 | 1005.8829 | 2009.7746 | 1005.3909 | 18 |
| 14 | 1498.7397 | **749.8735** | 1481.7132 | 741.3602 | 1480.7292 | 740.8682 | G | **1899.7266** | **950.3669** | 1882.7000 | 941.8537 | 1881.7160 | **941.3616** | 17 |
| 15 | **1613.7667** | 807.3870 | 1596.7401 | 798.8737 | 1595.7561 | 798.3817 | D | 1842.7051 | 921.8562 | 1825.6786 | **913.3429** | 1824.6945 | **912.8509** | 16 |
| 16 | **1714.8143** | 857.9108 | **1697.7878** | **849.3975** | **1696.8038** | **848.9055** | T | **1727.6782** | **864.3427** | 1710.6516 | 855.8294 | 1709.6676 | 855.3374 | 15 |
| 17 | 1811.8671 | 906.4372 | 1794.8406 | 897.9239 | 1793.8565 | 897.4319 | P | **1626.6305** | **813.8189** | **1609.6039** | **805.3056** | **1608.6199** | **804.8136** | 14 |
| 18 | **1971.8978** | **986.4525** | **1954.8712** | 977.9392 | **1953.8872** | 977.4472 | C | 1529.5777 | 765.2925 | **1512.5512** | 756.7792 | **1511.5672** | 756.2872 | 13 |

| | | | | | | | | | | | | | | |
|---|---|---|---|---|---|---|---|---|---|---|---|---|---|---|
| **19** | 2058.9298 | 1029.9685 | 2041.9032 | 1021.4553 | 2040.9192 | 1020.9632 | **S** | **1369.5471** | **685.2772** | 1352.5205 | 676.7639 | 1351.5365 | 676.2719 | **12** |
| **20** | 2218.9604 | 1109.9839 | 2201.9339 | 1101.4706 | 2200.9499 | **1100.9786** | **C** | 1282.5150 | 641.7612 | 1265.4885 | 633.2479 | 1264.5045 | 632.7559 | **11** |
| **21** | 2378.9911 | **1189.9992** | 2361.9645 | **1181.4859** | 2360.9805 | **1180.9939** | **C** | 1122.4844 | 561.7458 | 1105.4578 | 553.2326 | 1104.4738 | 552.7406 | **10** |
| **22** | 2492.0751 | **1246.5412** | 2475.0486 | 1238.0279 | 2474.0646 | 1237.5359 | **I** | **962.4537** | 481.7305 | 945.4272 | 473.2172 | 944.4432 | 472.7252 | **9** |
| **23** | 2591.1436 | **1296.0754** | 2574.1170 | **1287.5621** | 2573.1330 | **1287.0701** | **V** | **849.3697** | 425.1885 | 832.3431 | **416.6752** | 831.3591 | **416.1832** | **8** |
| **24** | 2706.1705 | **1353.5889** | 2689.1439 | 1345.0756 | 2688.1599 | 1344.5836 | **N** | **750.3013** | 375.6543 | **733.2747** | **367.1410** | **732.2907** | **366.6490** | **7** |
| **25** | 2820.2134 | **1410.6103** | 2803.1869 | 1402.0971 | 2802.2029 | 1401.6051 | **N** | **635.2743** | 318.1408 | **618.2478** | 309.6275 | 617.2638 | 309.1355 | **6** |
| **26** | 2907.2454 | **1454.1264** | 2890.2189 | **1445.6131** | 2889.2349 | **1445.1211** | **S** | **521.2314** | 261.1193 | 504.2049 | 252.6061 | **503.2208** | 252.1141 | **5** |
| **27** | 3022.2724 | **1511.6398** | 3005.2458 | 1503.1266 | 3004.2618 | 1502.6345 | **N** | **434.1994** | 217.6033 | 417.1728 | 209.0901 | **416.1888** | 208.5980 | **4** |
| **28** | 3079.2938 | **1540.1506** | 3062.2673 | **1531.6373** | 3061.2833 | 1531.1453 | **G** | **319.1724** | 160.0899 | 302.1459 | 151.5766 | 301.1619 | 151.0846 | **3** |
| **29** | 3166.3259 | **1583.6666** | 3149.2993 | 1575.1533 | 3148.3153 | 1574.6613 | **S** | 262.1510 | 131.5791 | 245.1244 | 123.0659 | 244.1404 | 122.5738 | **2** |
| **30** | | | | | | | **R** | 175.1190 | 88.0631 | 158.0924 | 79.5498 | | | **1** |

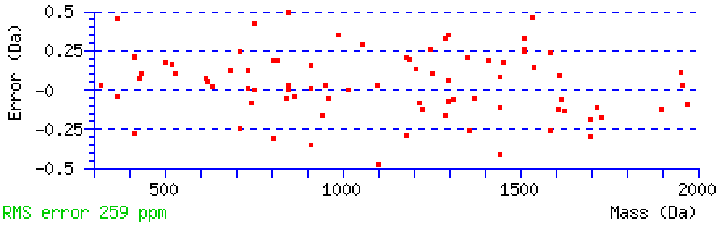


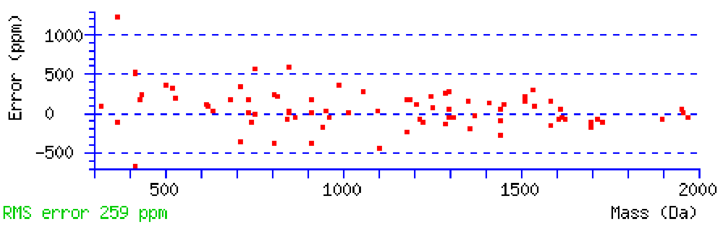


### All matches to this query

| Score | Mr(calc): | Delta | Sequence |
|---|---|---|---|
| 81.6 | 3339.4303 | 0.0055 | RGVDVSQVAWQSQGDTPCSCCIVNNSNGSR |
| 79.7 | 3339.4303 | 0.0055 | RGVDVSQVAWQSQGDTPCSCCIVNNSNGSR |
| 76.3 | 3339.4303 | 0.0055 | RGVDVSQVAWQSQGDTPCSCCIVNNSNGSR |
| 44.2 | 3338.4463 | 0.9895 | RGVDVSQVAWQSQGDTPCSCCIVNNSNGSR |
| 39.6 | 3338.4463 | 0.9895 | RGVDVSQVAWQSQGDTPCSCCIVNNSNGSR |
| 34.8 | 3338.4463 | 0.9895 | RGVDVSQVAWQSQGDTPCSCCIVNNSNGSR |
| 32.1 | 3337.4623 | 1.9735 | RGVDVSQVAWQSQGDTPCSCCIVNNSNGSR |
| 13.9 | 3337.4561 | 1.9797 | EEALQGVNVTIIDQDTTLSSLAITNQCDK |
| 13.9 | 3337.4561 | 1.9797 | EEALQGVNVTIIDQDTTLSSLAITNQCDK |
| 13.9 | 3337.4561 | 1.9797 | EEALQGVNVTIIDQDTTLSSLAITNQCDK |

---

Spectrum No: 214; Query: 908; Rank: 1

---

## Peptide View

MS/MS Fragmentation of **FENQTCFPLPDSR**
Found in **IPI00190776**, Tax_Id=10116 Gene_Symbol=Cspg2 Isoform V0 of Versican core protein precursor (Fragment)

Match to Query 908: 1610.706608 from(806.360580,2+)
Title: 100101RatKid_NS_deglyco_24.2754.2754.2.dta
Data file K:\NewmanPaper\Piliang\3SubProteomes\Piliang3SP\mgf5ppm\ERLIC_3SubProteomes5ppm.mgf

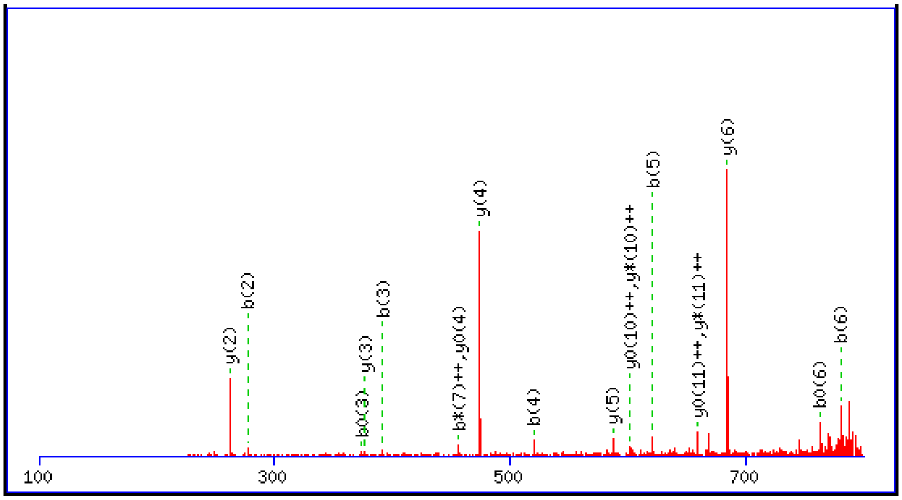


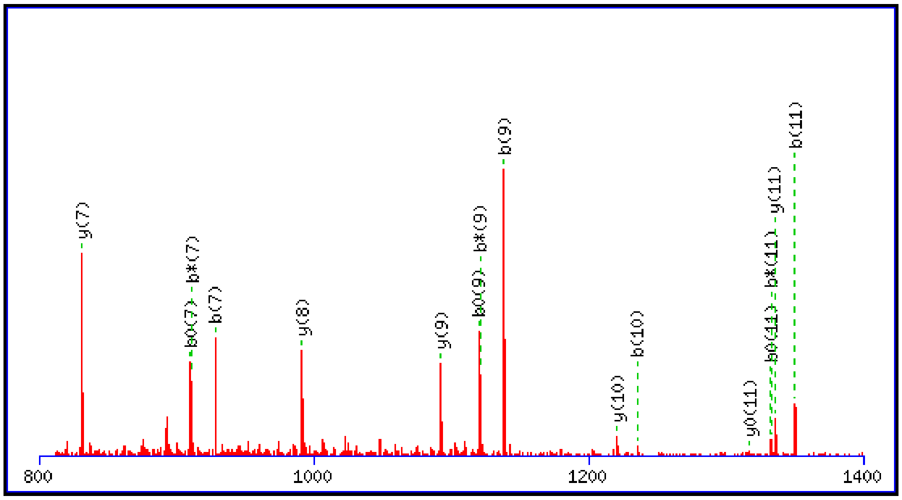


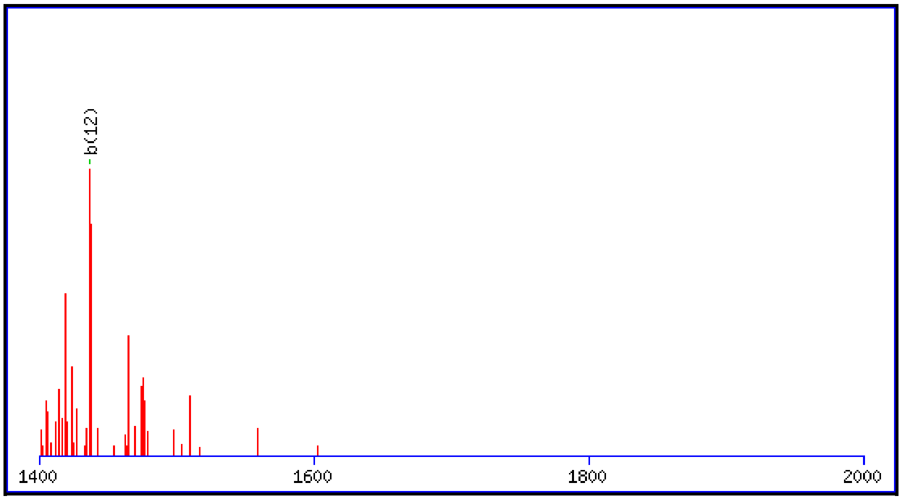


**Monoisotopic mass of neutral peptide Mr(calc):** 1610.7035
**Fixed modifications:** Carbamidomethyl (C)
**Variable modifications:**
**N3** : Deamidated_N (N)
**Ions Score:** 81 **Expect:** 1.3e-006
**Matches (Bold Red):** 35/136 fragment ions using 50 most intense peaks

| # | b | $b^{++}$ | b* | $b^{*++}$ | $b^{0}$ | $b^{0++}$ | Seq. | y | $y^{++}$ | y* | $y^{*++}$ | $y^{0}$ | $y^{0++}$ | # |
|---|---|---|---|---|---|---|---|---|---|---|---|---|---|---|
| 1 | 148.0757 | 74.5415 | | | | | F | | | | | | | 13 |
| 2 | **277.1183** | 139.0628 | | | 259.1077 | 130.0575 | E | 1464.6424 | 732.8248 | 1447.6158 | 724.3115 | 1446.6318 | 723.8195 | 12 |
| 3 | **392.1452** | 196.5762 | 375.1187 | 188.0630 | **374.1347** | 187.5710 | N | **1335.5998** | 668.3035 | 1318.5732 | **659.7902** | **1317.5892** | **659.2982** | 11 |
| 4 | **520.2038** | 260.6055 | 503.1772 | 252.0923 | 502.1932 | 251.6003 | Q | **1220.5728** | 610.7901 | 1203.5463 | **602.2768** | 1202.5623 | **601.7848** | 10 |
| 5 | **621.2515** | 311.1294 | 604.2249 | 302.6161 | 603.2409 | 302.1241 | T | **1092.5143** | 546.7608 | 1075.4877 | 538.2475 | 1074.5037 | 537.7555 | 9 |

| | | | | | | | | | | | | | | |
|---|---|---|---|---|---|---|---|---|---|---|---|---|---|---|
| **6** | **781.2821** | 391.1447 | 764.2556 | 382.6314 | **763.2716** | 382.1394 | **C** | **991.4666** | 496.2369 | 974.4400 | 487.7237 | 973.4560 | 487.2316 | **8** |
| **7** | **928.3505** | 464.6789 | **911.3240** | **456.1656** | **910.3400** | 455.6736 | **F** | **831.4359** | 416.2216 | 814.4094 | 407.7083 | 813.4254 | 407.2163 | **7** |
| **8** | 1025.4033 | 513.2053 | 1008.3768 | 504.6920 | 1007.3927 | 504.2000 | **P** | **684.3675** | 342.6874 | 667.3410 | 334.1741 | 666.3569 | 333.6821 | **6** |
| **9** | **1138.4874** | 569.7473 | **1121.4608** | 561.2340 | **1120.4768** | 560.7420 | **L** | **587.3148** | 294.1610 | 570.2882 | 285.6477 | 569.3042 | 285.1557 | **5** |
| **10** | **1235.5401** | 618.2737 | 1218.5136 | 609.7604 | 1217.5296 | 609.2684 | **P** | **474.2307** | 237.6190 | 457.2041 | 229.1057 | **456.2201** | 228.6137 | **4** |
| **11** | **1350.5671** | 675.7872 | **1333.5405** | 667.2739 | **1332.5565** | 666.7819 | **D** | **377.1779** | 189.0926 | 360.1514 | 180.5793 | 359.1674 | 180.0873 | **3** |
| **12** | **1437.5991** | 719.3032 | 1420.5726 | 710.7899 | 1419.5885 | 710.2979 | **S** | **262.1510** | 131.5791 | 245.1244 | 123.0659 | 244.1404 | 122.5738 | **2** |
| **13** | | | | | | | **R** | 175.1190 | 88.0631 | 158.0924 | 79.5498 | | | **1** |

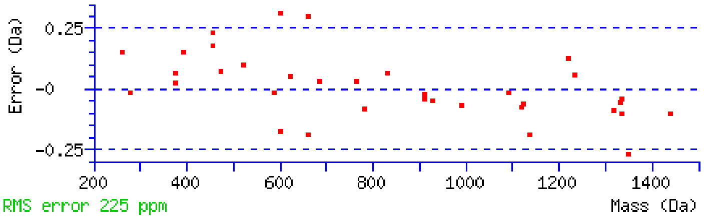


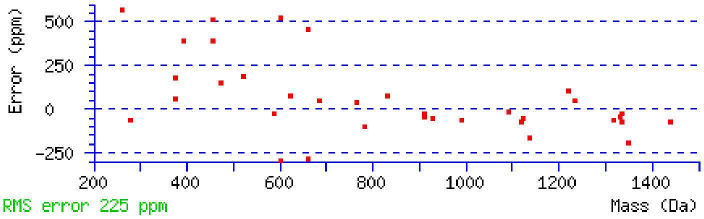


### All matches to this query

| Score | Mr(calc): | Delta | Sequence |
|---|---|---|---|
| 81.5 | 1610.7035 | 0.0031 | FENQTCFPLPDSR |
| 23.7 | 1610.7028 | 0.0038 | EMDQTMAANAQKNK |
| 10.4 | 1610.7076 | -0.0010 | TEKLNDELQSPEK |
| 9.5 | 1610.7141 | -0.0075 | SMPSASSCSTRTPSR |
| 3.7 | 1610.7049 | 0.0017 | RSASPPPAASSSSSSR |
| 2.7 | 1610.7049 | 0.0017 | RSASPPPAASSSSSSR |
| 2.7 | 1610.7049 | 0.0017 | RSASPPPAASSSSSSR |
| 2.0 | 1610.7141 | -0.0075 | MTKSNGEEPRMGSR |
| 1.5 | 1610.7076 | -0.0010 | KEDPEDEKSLVDK |
| 0.5 | 1610.7214 | -0.0148 | LMKTMKPETTKK |

---

Spectrum No: 215; Query: 2522; Rank: 1

---

## Peptide View

MS/MS Fragmentation of **LQAALDNEAGGRPAMEPGNGSLDLGGDAAGR**
Found in **IPI00210090**, Tax_Id=10116 Gene_Symbol=Hnrpu SP120

Match to Query 2522: 2980.403352 from(994.475060,3+)
Title: 100101RatKid_NS_deglyco_12.3060.3060.3.dta
Data file K:\NewmanPaper\Piliang\3SubProteomes\Piliang3SP\mgf5ppm\ERLIC_3SubProteomes5ppm.mgf

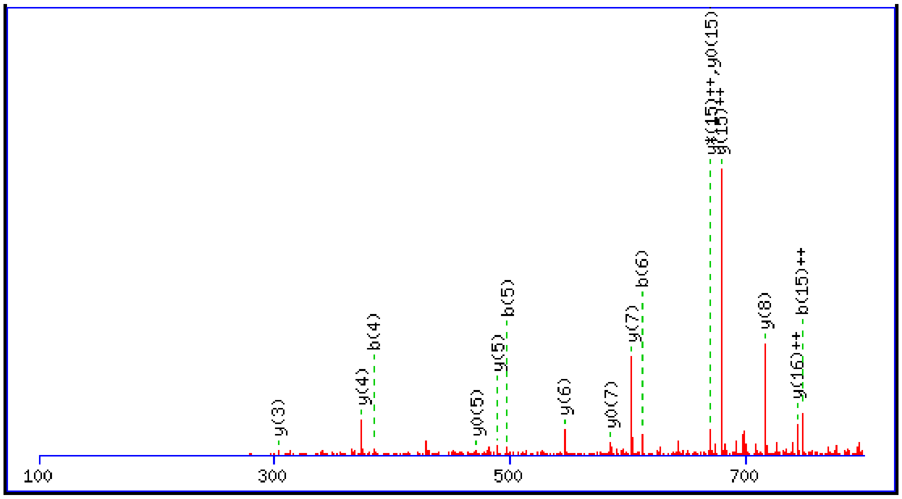


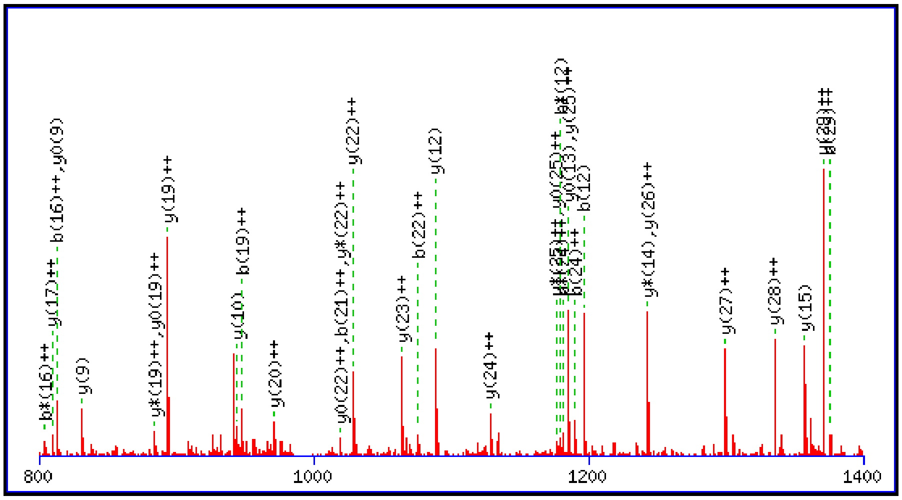


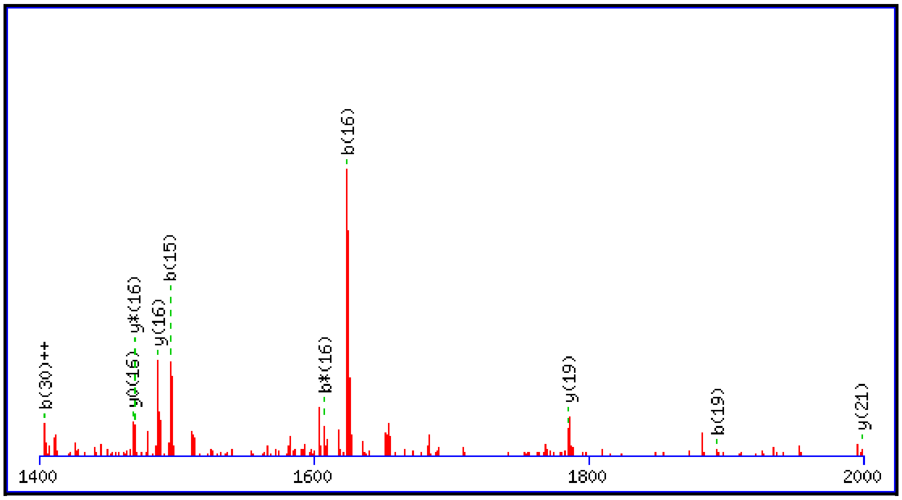


**Monoisotopic mass of neutral peptide Mr(calc):** 2980.3944
**Fixed modifications:** Carbamidomethyl (C)
**Variable modifications:**
**N19** : Deamidated_N (N)
**Ions Score:** 81 **Expect:** 2.6e-006
**Matches (Bold Red):** 60/340 fragment ions using 111 most intense peaks

| # | b | b++ | b* | b*++ | b0 | b0++ | Seq. | y | y++ | y* | y*++ | y0 | y0++ | # |
|---|---|---|---|---|---|---|---|---|---|---|---|---|---|---|
| 1 | 114.0913 | 57.5493 | | | | | L | | | | | | | 31 |
| 2 | 242.1499 | 121.5786 | 225.1234 | 113.0653 | | | Q | 2868.3177 | 1434.6625 | 2851.2911 | 1426.1492 | 2850.3071 | 1425.6572 | 30 |
| 3 | 313.1870 | 157.0972 | 296.1605 | 148.5839 | | | A | 2740.2591 | **1370.6332** | 2723.2326 | 1362.1199 | 2722.2485 | 1361.6279 | 29 |
| 4 | **384.2241** | 192.6157 | 367.1976 | 184.1024 | | | A | 2669.2220 | **1335.1146** | 2652.1954 | 1326.6014 | 2651.2114 | 1326.1094 | 28 |
| 5 | **497.3082** | 249.1577 | 480.2817 | 240.6445 | | | L | 2598.1849 | **1299.5961** | 2581.1583 | 1291.0828 | 2580.1743 | 1290.5908 | 27 |

| | | | | | | | | | | | | | | |
|---|---|---|---|---|---|---|---|---|---|---|---|---|---|---|
| 6 | **612.3352** | 306.6712 | 595.3086 | 298.1579 | 594.3246 | 297.6659 | D | 2485.1008 | **1243.0540** | 2468.0743 | 1234.5408 | 2467.0902 | 1234.0488 | 26 |
| 7 | 726.3781 | 363.6927 | 709.3515 | 355.1794 | 708.3675 | 354.6874 | N | 2370.0739 | **1185.5406** | 2353.0473 | **1177.0273** | 2352.0633 | **1176.5353** | 25 |
| 8 | 855.4207 | 428.2140 | 838.3941 | 419.7007 | 837.4101 | 419.2087 | E | 2256.0309 | **1128.5191** | 2239.0044 | 1120.0058 | 2238.0204 | 1119.5138 | 24 |
| 9 | 926.4578 | 463.7325 | 909.4312 | 455.2193 | 908.4472 | 454.7272 | A | 2126.9884 | **1063.9978** | 2109.9618 | 1055.4845 | 2108.9778 | 1054.9925 | 23 |
| 10 | 983.4793 | 492.2433 | 966.4527 | 483.7300 | 965.4687 | 483.2380 | G | 2055.9512 | **1028.4793** | 2038.9247 | **1019.9660** | 2037.9407 | **1019.4740** | 22 |
| 11 | 1040.5007 | 520.7540 | 1023.4742 | 512.2407 | 1022.4901 | 511.7487 | G | **1998.9298** | 999.9685 | 1981.9032 | 991.4552 | 1980.9192 | 990.9632 | 21 |
| 12 | **1196.6018** | 598.8046 | **1179.5753** | 590.2913 | 1178.5913 | 589.7993 | R | 1941.9083 | **971.4578** | 1924.8818 | 962.9445 | 1923.8977 | 962.4525 | 20 |
| 13 | 1293.6546 | 647.3309 | 1276.6280 | 638.8177 | 1275.6440 | 638.3257 | P | **1785.8072** | **893.4072** | 1768.7806 | **884.8940** | 1767.7966 | **884.4020** | 19 |
| 14 | 1364.6917 | 682.8495 | 1347.6652 | 674.3362 | 1346.6811 | 673.8442 | A | 1688.7544 | 844.8809 | 1671.7279 | 836.3676 | 1670.7439 | 835.8756 | 18 |
| 15 | **1495.7322** | **748.3697** | 1478.7056 | 739.8565 | 1477.7216 | 739.3644 | M | 1617.7173 | **809.3623** | 1600.6908 | 800.8490 | 1599.7068 | 800.3570 | 17 |
| 16 | **1624.7748** | **812.8910** | **1607.7482** | **804.3778** | 1606.7642 | 803.8857 | E | **1486.6768** | **743.8421** | **1469.6503** | 735.3288 | **1468.6663** | 734.8368 | 16 |
| 17 | 1721.8275 | 861.4174 | 1704.8010 | 852.9041 | 1703.8170 | 852.4121 | P | **1357.6342** | **679.3208** | 1340.6077 | **670.8075** | 1339.6237 | **670.3155** | 15 |
| 18 | 1778.8490 | 889.9281 | 1761.8225 | 881.4149 | 1760.8384 | 880.9229 | G | 1260.5815 | 630.7944 | **1243.5549** | 622.2811 | 1242.5709 | 621.7891 | 14 |
| 19 | **1893.8759** | **947.4416** | 1876.8494 | 938.9283 | 1875.8654 | 938.4363 | N | 1203.5600 | 602.2836 | 1186.5335 | 593.7704 | **1185.5494** | 593.2784 | 13 |
| 20 | 1950.8974 | 975.9523 | 1933.8709 | 967.4391 | 1932.8868 | 966.9471 | G | **1088.5331** | 544.7702 | 1071.5065 | 536.2569 | 1070.5225 | 535.7649 | 12 |
| 21 | 2037.9294 | **1019.4684** | 2020.9029 | 1010.9551 | 2019.9189 | 1010.4631 | S | 1031.5116 | 516.2594 | 1014.4851 | 507.7462 | 1013.5010 | 507.2542 | 11 |
| 22 | 2151.0135 | **1076.0104** | 2133.9870 | 1067.4971 | 2133.0029 | 1067.0051 | L | **944.4796** | 472.7434 | 927.4530 | 464.2302 | 926.4690 | 463.7381 | 10 |
| 23 | 2266.0404 | 1133.5239 | 2249.0139 | 1125.0106 | 2248.0299 | 1124.5186 | D | **831.3955** | 416.2014 | 814.3690 | 407.6881 | **813.3850** | 407.1961 | 9 |
| 24 | 2379.1245 | **1190.0659** | 2362.0980 | **1181.5526** | 2361.1139 | 1181.0606 | L | **716.3686** | 358.6879 | 699.3420 | 350.1747 | 698.3580 | 349.6826 | 8 |
| 25 | 2436.1460 | 1218.5766 | 2419.1194 | 1210.0633 | 2418.1354 | 1209.5713 | G | **603.2845** | 302.1459 | 586.2580 | 293.6326 | **585.2740** | 293.1406 | 7 |
| 26 | 2493.1674 | 1247.0874 | 2476.1409 | 1238.5741 | 2475.1569 | 1238.0821 | G | **546.2631** | 273.6352 | 529.2365 | 265.1219 | 528.2525 | 264.6299 | 6 |
| 27 | 2608.1944 | 1304.6008 | 2591.1678 | 1296.0876 | 2590.1838 | 1295.5955 | D | **489.2416** | 245.1244 | 472.2150 | 236.6112 | **471.2310** | 236.1191 | 5 |
| 28 | 2679.2315 | 1340.1194 | 2662.2049 | 1331.6061 | 2661.2209 | 1331.1141 | A | **374.2146** | 187.6110 | 357.1881 | 179.0977 | | | 4 |
| 29 | 2750.2686 | **1375.6379** | 2733.2421 | 1367.1247 | 2732.2580 | 1366.6327 | A | **303.1775** | 152.0924 | 286.1510 | 143.5791 | | | 3 |
| 30 | 2807.2901 | **1404.1487** | 2790.2635 | 1395.6354 | 2789.2795 | 1395.1434 | G | 232.1404 | 116.5738 | 215.1139 | 108.0606 | | | 2 |
| 31 | | | | | | | R | 175.1190 | 88.0631 | 158.0924 | 79.5498 | | | 1 |

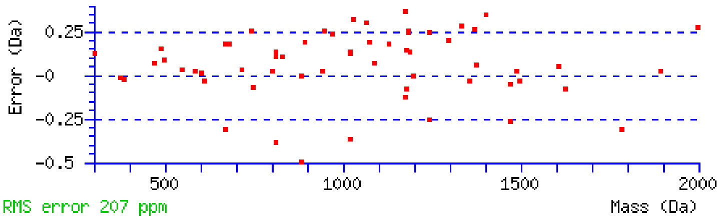


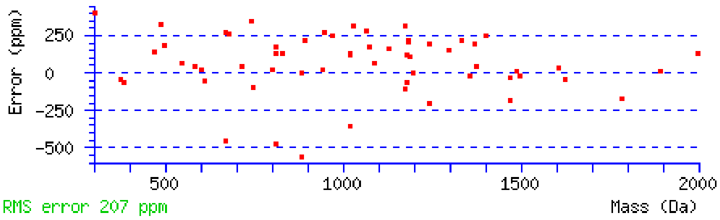


### All matches to this query

| Score | Mr(calc): | Delta | Sequence |
|---|---|---|---|
| 81.3 | 2980.3944 | 0.0089 | LQAALDNEAGGRPAMEPGNGSLDLGGDAAGR |
| 33.5 | 2980.3944 | 0.0089 | LQAALDNEAGGRPAMEPGNGSLDLGGDAAGR |
| 27.0 | 2979.4104 | 0.9929 | LQAALDNEAGGRPAMEPGNGSLDLGGDAAGR |
| 3.9 | 2980.4147 | -0.0114 | VQCAARAVNTNGNEGLELMSPIVTIAR |
| 3.5 | 2980.3808 | 0.0225 | GSASLNLCIKETLRLRPPIMTMMR |
| 3.5 | 2980.3808 | 0.0225 | GSASLNLCIKETLRLRPPIMTMMR |
| 3.5 | 2980.3808 | 0.0225 | GSASLNLCIKETLRLRPPIMTMMR |
| 3.3 | 2980.4147 | -0.0114 | VQCAARAVNTNGNEGLELMSPIVTIAR |
| 3.1 | 2980.4147 | -0.0114 | VQCAARAVNTNGNEGLELMSPIVTIAR |
| 2.9 | 2980.4147 | -0.0114 | VQCAARAVNTNGNEGLELMSPIVTIAR |

---

Spectrum No: 216; Query: 2380; Rank: 1

---

# Peptide View

MS/MS Fragmentation of **FQISPQLQFSPEEVLGMVLNYSR**
Found in **IPI00210975**, Tax_Id=10116 Gene_Symbol=Hyou1 Hypoxia up-regulated protein 1 precursor

Match to Query 2380: 2682.335442 from(895.119090,3+)
Title: 091008RatKidney_NoSalt_18.6599.6599.3.dta
Data file K:\NewmanPaper\Piliang\3SubProteomes\Piliang3SP\mgf5ppm\ERLIC_3SubProteomes5ppm.mgf

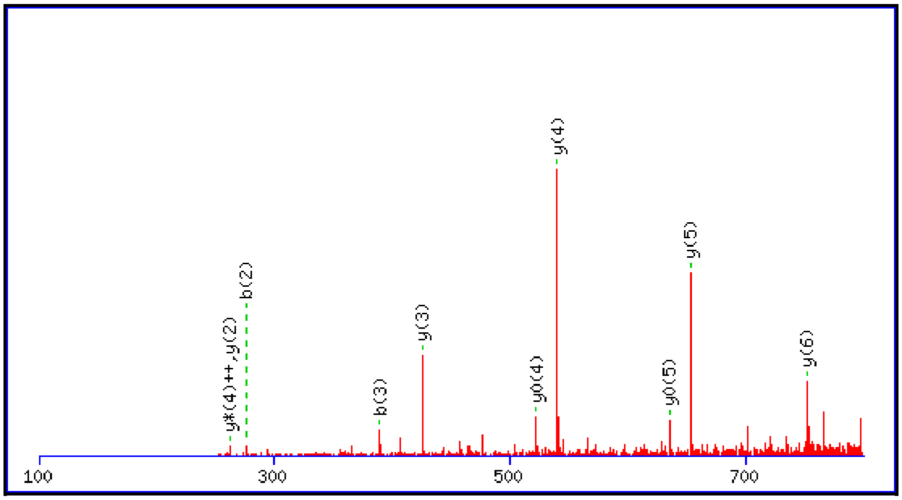


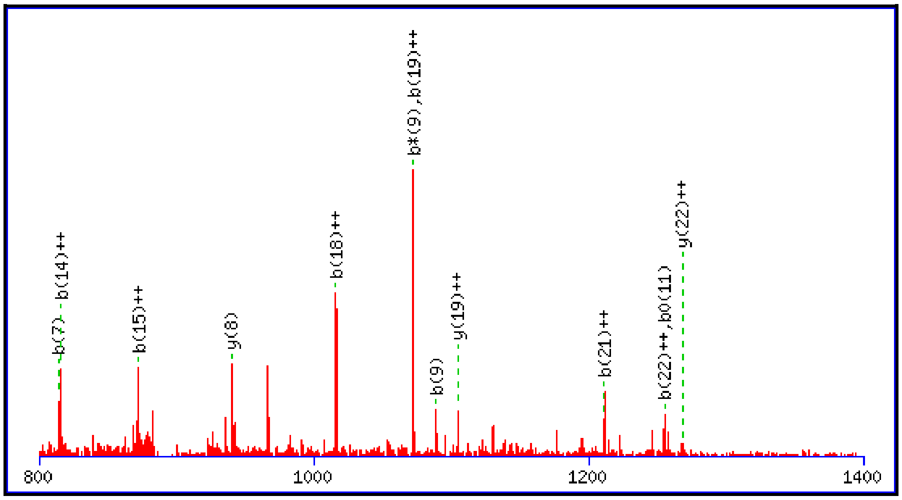


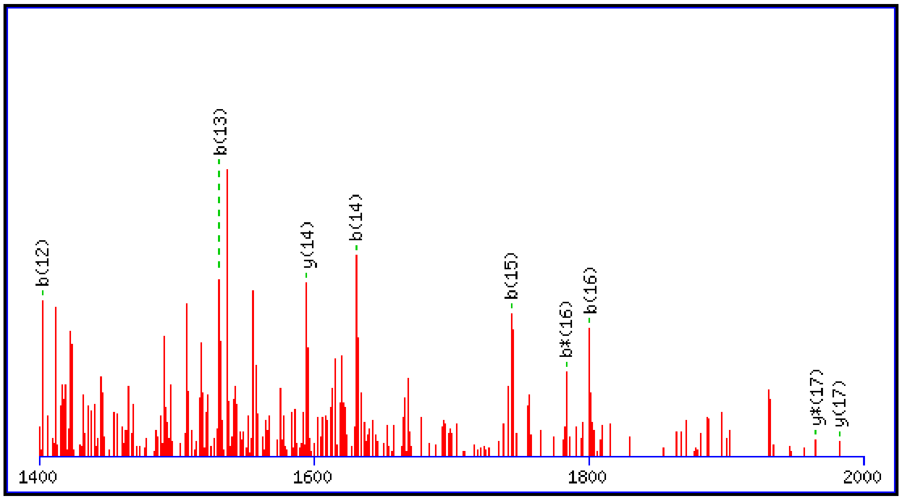


**Monoisotopic mass of neutral peptide Mr(calc):** 2682.3363
**Fixed modifications:** Carbamidomethyl (C)
**Variable modifications:**
**N20** : Deamidated_N (N)
**Ions Score:** 81 **Expect:** 1.6e-006

**Matches (Bold Red): 32/254 fragment ions using 41 most intense peaks**

| # | b | b++ | b* | b*++ | b0 | b0++ | Seq. | y | y++ | y* | y*++ | y0 | y0++ | # |
|---|---|---|---|---|---|---|---|---|---|---|---|---|---|---|
| 1 | 148.0757 | 74.5415 | | | | | F | | | | | | | 23 |
| 2 | **276.1343** | 138.5708 | 259.1077 | 130.0575 | | | Q | 2536.2752 | **1268.6412** | 2519.2486 | 1260.1280 | 2518.2646 | 1259.6360 | 22 |
| 3 | **389.2183** | 195.1128 | 372.1918 | 186.5995 | | | I | 2408.2166 | 1204.6119 | 2391.1901 | 1196.0987 | 2390.2061 | 1195.6067 | 21 |
| 4 | 476.2504 | 238.6288 | 459.2238 | 230.1155 | 458.2398 | 229.6235 | S | 2295.1326 | 1148.0699 | 2278.1060 | 1139.5566 | 2277.1220 | 1139.0646 | 20 |
| 5 | 573.3031 | 287.1552 | 556.2766 | 278.6419 | 555.2926 | 278.1499 | P | 2208.1005 | **1104.5539** | 2191.0740 | 1096.0406 | 2190.0900 | 1095.5486 | 19 |
| 6 | 701.3617 | 351.1845 | 684.3352 | 342.6712 | 683.3511 | 342.1792 | Q | 2111.0478 | 1056.0275 | 2094.0212 | 1047.5142 | 2093.0372 | 1047.0222 | 18 |
| 7 | **814.4458** | 407.7265 | 797.4192 | 399.2132 | 796.4352 | 398.7212 | L | **1982.9892** | 991.9982 | **1965.9626** | 983.4850 | 1964.9786 | 982.9929 | 17 |
| 8 | 942.5043 | 471.7558 | 925.4778 | 463.2425 | 924.4938 | 462.7505 | Q | 1869.9051 | 935.4562 | 1852.8786 | 926.9429 | 1851.8946 | 926.4509 | 16 |
| 9 | **1089.5728** | 545.2900 | **1072.5462** | 536.7767 | 1071.5622 | 536.2847 | F | 1741.8465 | 871.4269 | 1724.8200 | 862.9136 | 1723.8360 | 862.4216 | 15 |
| 10 | 1176.6048 | 588.8060 | 1159.5782 | 580.2928 | 1158.5942 | 579.8007 | S | **1594.7781** | 797.8927 | 1577.7516 | 789.3794 | 1576.7676 | 788.8874 | 14 |
| 11 | 1273.6576 | 637.3324 | 1256.6310 | 628.8191 | **1255.6470** | 628.3271 | P | 1507.7461 | 754.3767 | 1490.7196 | 745.8634 | 1489.7355 | 745.3714 | 13 |
| 12 | **1402.7001** | 701.8537 | 1385.6736 | 693.3404 | 1384.6896 | 692.8484 | E | 1410.6933 | 705.8503 | 1393.6668 | 697.3370 | 1392.6828 | 696.8450 | 12 |
| 13 | **1531.7427** | 766.3750 | 1514.7162 | 757.8617 | 1513.7322 | 757.3697 | E | 1281.6507 | 641.3290 | 1264.6242 | 632.8157 | 1263.6402 | 632.3237 | 11 |
| 14 | **1630.8112** | **815.9092** | 1613.7846 | 807.3959 | 1612.8006 | 806.9039 | V | 1152.6081 | 576.8077 | 1135.5816 | 568.2944 | 1134.5976 | 567.8024 | 10 |
| 15 | **1743.8952** | **872.4512** | 1726.8687 | 863.9380 | 1725.8846 | 863.4460 | L | 1053.5397 | 527.2735 | 1036.5132 | 518.7602 | 1035.5292 | 518.2682 | 9 |
| 16 | **1800.9167** | 900.9620 | **1783.8901** | 892.4487 | 1782.9061 | 891.9567 | G | **940.4557** | 470.7315 | 923.4291 | 462.2182 | 922.4451 | 461.7262 | 8 |
| 17 | 1931.9572 | 966.4822 | 1914.9306 | 957.9689 | 1913.9466 | 957.4769 | M | 883.4342 | 442.2207 | 866.4077 | 433.7075 | 865.4236 | 433.2155 | 7 |
| 18 | 2031.0256 | **1016.0164** | 2013.9990 | 1007.5032 | 2013.0150 | 1007.0111 | V | **752.3937** | 376.7005 | 735.3672 | 368.1872 | 734.3832 | 367.6952 | 6 |
| 19 | 2144.1096 | **1072.5585** | 2127.0831 | 1064.0452 | 2126.0991 | 1063.5532 | L | **653.3253** | 327.1663 | 636.2988 | 318.6530 | **635.3147** | 318.1610 | 5 |
| 20 | 2259.1366 | 1130.0719 | 2242.1100 | 1121.5587 | 2241.1260 | 1121.0666 | N | **540.2412** | 270.6243 | 523.2147 | **262.1110** | **522.2307** | 261.6190 | 4 |
| 21 | 2422.1999 | **1211.6036** | 2405.1734 | 1203.0903 | 2404.1893 | 1202.5983 | Y | **425.2143** | 213.1108 | 408.1878 | 204.5975 | 407.2037 | 204.1055 | 3 |
| 22 | 2509.2319 | **1255.1196** | 2492.2054 | 1246.6063 | 2491.2214 | 1246.1143 | S | **262.1510** | 131.5791 | 245.1244 | 123.0659 | 244.1404 | 122.5738 | 2 |
| 23 | | | | | | | R | 175.1190 | 88.0631 | 158.0924 | 79.5498 | | | 1 |

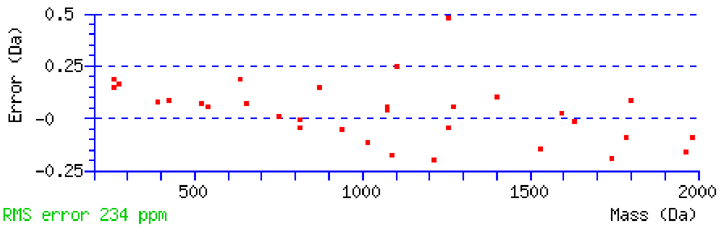


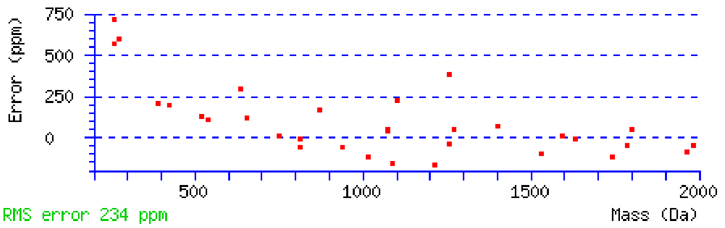


## All matches to this query

| Score | Mr(calc): | Delta | Sequence |
|---|---|---|---|
| 81.3 | 2682.3363 | -0.0009 | FQISPQLQFSPEEVLGMVLNYSR |
| 55.5 | 2681.3523 | 0.9831 | FQISPQLQFSPEEVLGMVLNYSR |
| 5.4 | 2680.3077 | 2.0277 | DTVKVATVTGVGLAALGLIGVMLSR |
| 5.3 | 2680.3077 | 2.0277 | DTVKVATVTGVGLAALGLIGVMLSR |
| 5.3 | 2680.3077 | 2.0277 | DTVKVATVTGVGLAALGLIGVMLSR |
| 2.8 | 2681.3516 | 0.9838 | VKTMNDLSSQLSPLDLHPSPKMSR |
| 2.3 | 2680.3151 | 2.0203 | LKXGLLPTLPNTPMNTTLMDSCR |
| 2.1 | 2681.3060 | 1.0294 | YTFKMNPGDVITFDNWRLLHGR |
| 2.0 | 2682.3295 | 0.0059 | NALEVDLGYLIITAARRLHEVR |
| 1.4 | 2681.3191 | 1.0164 | TISLGAGDRQVIQTPLVDSLPVSR |

Spectrum No: 217; Query: 499; Rank: 1

# Peptide View

MS/MS Fragmentation of **SWPAVGNCTAALR**
Found in **IPI00195516**, Tax_Id=10116 Gene_Symbol=Hpx Hemopexin precursor

Match to Query 499: 1402.662628 from(702.338590,2+)
Title: 091008RatKidney_NoSalt_30.2182.2182.2.dta
Data file K:\NewmanPaper\Piliang\3SubProteomes\Piliang3SP\mgf5ppm\ERLIC_3SubProteomes5ppm.mgf

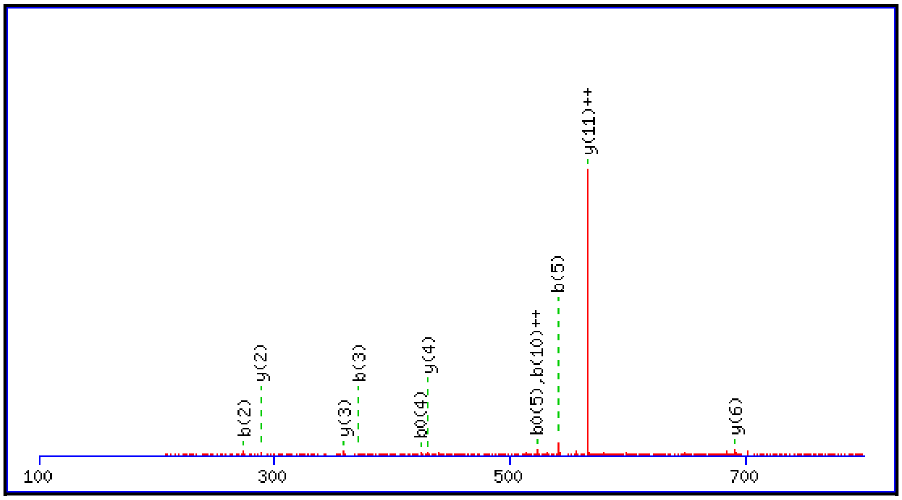


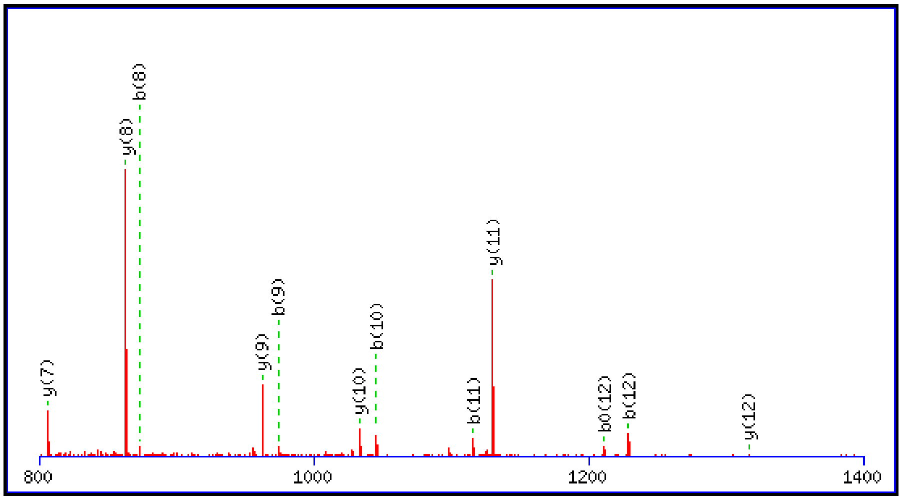


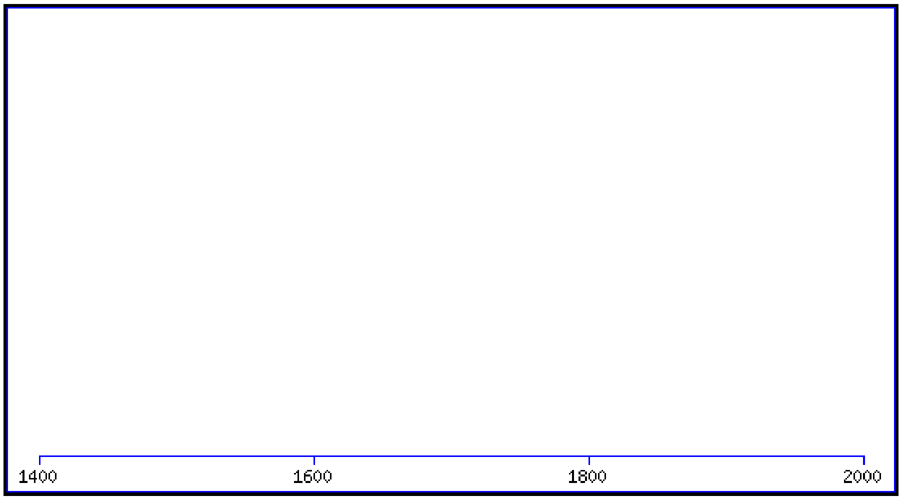


**Monoisotopic mass of neutral peptide Mr(calc):** 1402.6663
**Fixed modifications:** Carbamidomethyl (C)
**Variable modifications:**
**N7** : Deamidated_N (N)
**Ions Score:** 81 **Expect:** 1.1e-006
**Matches (Bold Red):** 23/124 fragment ions using 37 most intense peaks

| # | b | $b^{++}$ | b* | $b^{*++}$ | $b^0$ | $b^{0++}$ | Seq. | y | $y^{++}$ | y* | $y^{*++}$ | $y^0$ | $y^{0++}$ | # |
|---|---|---|---|---|---|---|---|---|---|---|---|---|---|---|
| 1 | 88.0393 | 44.5233 | | | 70.0287 | 35.5180 | S | | | | | | | 13 |
| 2 | **274.1186** | 137.5629 | | | 256.1081 | 128.5577 | W | **1316.6416** | 658.8244 | 1299.6150 | 650.3112 | 1298.6310 | 649.8191 | 12 |
| 3 | **371.1714** | 186.0893 | | | 353.1608 | 177.0840 | P | **1130.5623** | **565.7848** | 1113.5357 | 557.2715 | 1112.5517 | 556.7795 | 11 |
| 4 | 442.2085 | 221.6079 | | | **424.1979** | 212.6026 | A | **1033.5095** | 517.2584 | 1016.4830 | 508.7451 | 1015.4989 | 508.2531 | 10 |
| 5 | **541.2769** | 271.1421 | | | **523.2663** | 262.1368 | V | **962.4724** | 481.7398 | 945.4458 | 473.2266 | 944.4618 | 472.7345 | 9 |
| 6 | 598.2984 | 299.6528 | | | 580.2878 | 290.6475 | G | **863.4040** | 432.2056 | 846.3774 | 423.6923 | 845.3934 | 423.2003 | 8 |
| 7 | 713.3253 | 357.1663 | 696.2988 | 348.6530 | 695.3147 | 348.1610 | N | **806.3825** | 403.6949 | 789.3560 | 395.1816 | 788.3719 | 394.6896 | 7 |
| 8 | **873.3560** | 437.1816 | 856.3294 | 428.6683 | 855.3454 | 428.1763 | C | **691.3556** | 346.1814 | 674.3290 | 337.6681 | 673.3450 | 337.1761 | 6 |
| 9 | **974.4036** | 487.7055 | 957.3771 | 479.1922 | 956.3931 | 478.7002 | T | 531.3249 | 266.1661 | 514.2984 | 257.6528 | 513.3144 | 257.1608 | 5 |
| 10 | **1045.4408** | **523.2240** | 1028.4142 | 514.7107 | 1027.4302 | 514.2187 | A | **430.2772** | 215.6423 | 413.2507 | 207.1290 | | | 4 |
| 11 | **1116.4779** | 558.7426 | 1099.4513 | 550.2293 | 1098.4673 | 549.7373 | A | **359.2401** | 180.1237 | 342.2136 | 171.6104 | | | 3 |
| 12 | **1229.5619** | 615.2846 | 1212.5354 | 606.7713 | **1211.5514** | 606.2793 | L | **288.2030** | 144.6051 | 271.1765 | 136.0919 | | | 2 |
| 13 | | | | | | | R | 175.1190 | 88.0631 | 158.0924 | 79.5498 | | | 1 |

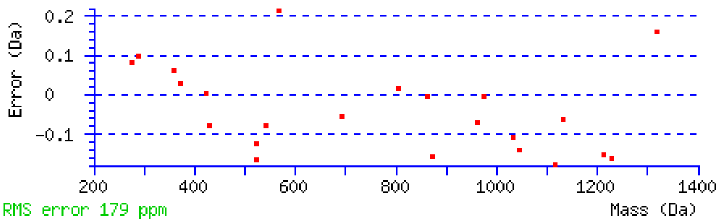


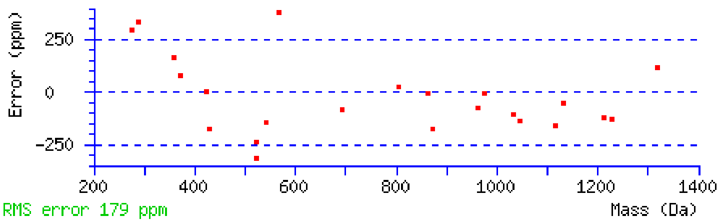


**All matches to this query**

| Score | Mr(calc): | Delta | Sequence |
|---|---|---|---|
| 81.2 | 1402.6663 | -0.0037 | SWPAVGNCTAALR |
| 16.8 | 1402.6688 | -0.0061 | SGENAANIASELAR |
| 12.5 | 1402.6688 | -0.0061 | SGENAANIASELAR |
| 10.7 | 1400.6523 | 2.0103 | XIDSLIGPPPPR |
| 10.4 | 1401.6530 | 1.0096 | LRLGSGSNGLLR |
| 9.0 | 1401.6571 | 1.0055 | YVKTHQVIVR |
| 8.7 | 1402.6646 | -0.0020 | SDAFVPFSIGKR |
| 8.2 | 1401.6653 | 0.9973 | HLSPLTVNSPTR |
| 8.2 | 1401.6653 | 0.9973 | HLSPLTVNSPTR |
| 8.1 | 1401.6653 | 0.9973 | SGKQYSIEAALR |

---

Spectrum No: 218; Query: 165; Rank: 1

---

## Peptide View

MS/MS Fragmentation of **ALNVTLSSMGR**
Found in **IPI00213036**, Tax_Id=10116 Gene_Symbol=C4a Complement C4 precursor

Match to Query 165: 1164.577928 from(583.296240,2+)
Title: 091008RatKidney_NH4Format01_30.1114.1114.2.dta
Data file K:\NewmanPaper\Piliang\3SubProteomes\Piliang3SP\mgf5ppm\ERLIC_3SubProteomes5ppm.mgf

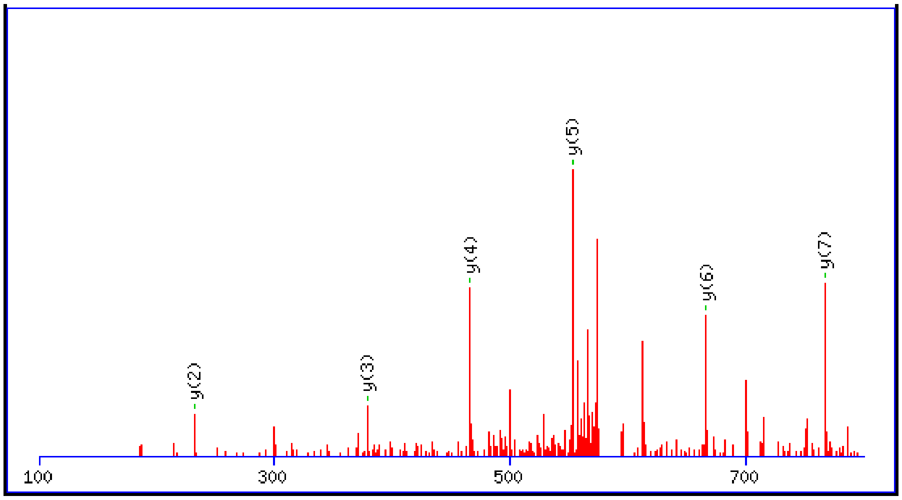


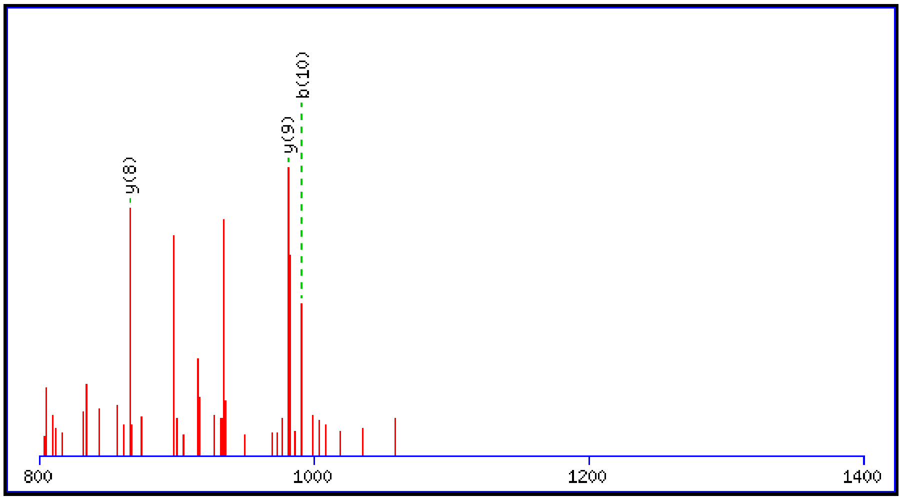


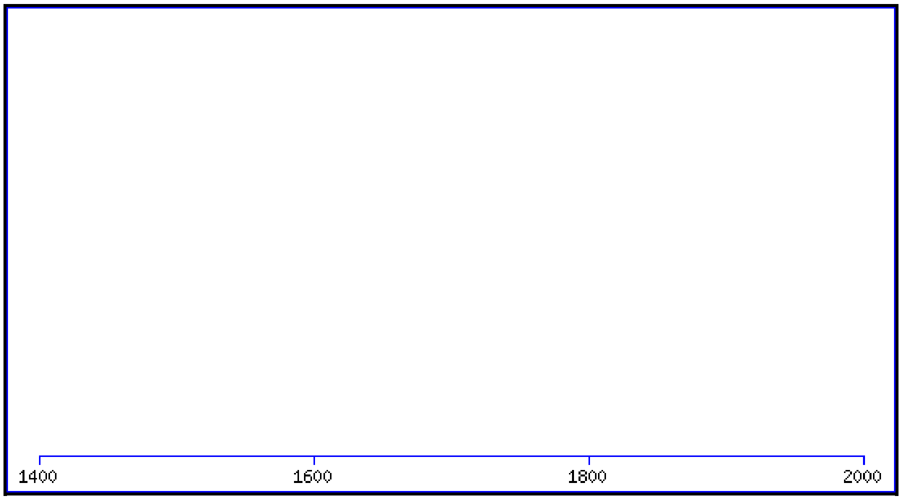


**Monoisotopic mass of neutral peptide Mr(calc):** 1164.5809
**Fixed modifications:** Carbamidomethyl (C)
**Variable modifications:**
**N3** : Deamidated_N (N)
**M9** : Oxidation (M)
**Ions Score:** 81 **Expect:** 8.6e-007
**Matches (Bold Red):** 9/102 fragment ions using 12 most intense peaks

| # | b | b++ | b* | b*++ | b0 | b0++ | Seq. | y | y++ | y* | y*++ | y0 | y0++ | # |
|---|---|---|---|---|---|---|---|---|---|---|---|---|---|---|
| 1 | 72.0444 | 36.5258 | | | | | A | | | | | | | 11 |
| 2 | 185.1285 | 93.0679 | | | | | L | 1094.5510 | 547.7792 | 1077.5245 | 539.2659 | 1076.5405 | 538.7739 | 10 |
| 3 | 300.1554 | 150.5813 | 283.1288 | 142.0681 | | | N | **981.4670** | 491.2371 | 964.4404 | 482.7238 | 963.4564 | 482.2318 | 9 |
| 4 | 399.2238 | 200.1155 | 382.1973 | 191.6023 | | | V | **866.4400** | 433.7237 | 849.4135 | 425.2104 | 848.4295 | 424.7184 | 8 |
| | | | | | | | | | | | | | | |

| | | | | | | | | | | | | | | |
|---|---|---|---|---|---|---|---|---|---|---|---|---|---|---|
| **5** | 500.2715 | 250.6394 | 483.2449 | 242.1261 | 482.2609 | 241.6341 | **T** | **767.3716** | 384.1894 | 750.3451 | 375.6762 | 749.3611 | 375.1842 | **7** |
| **6** | 613.3555 | 307.1814 | 596.3290 | 298.6681 | 595.3450 | 298.1761 | **L** | **666.3239** | 333.6656 | 649.2974 | 325.1523 | 648.3134 | 324.6603 | **6** |
| **7** | 700.3876 | 350.6974 | 683.3610 | 342.1842 | 682.3770 | 341.6921 | **S** | **553.2399** | 277.1236 | 536.2133 | 268.6103 | 535.2293 | 268.1183 | **5** |
| **8** | 787.4196 | 394.2134 | 770.3931 | 385.7002 | 769.4090 | 385.2082 | **S** | **466.2078** | 233.6076 | 449.1813 | 225.0943 | 448.1973 | 224.6023 | **4** |
| **9** | 934.4550 | 467.7311 | 917.4285 | 459.2179 | 916.4444 | 458.7259 | **M** | **379.1758** | 190.0915 | 362.1493 | 181.5783 | | | **3** |
| **10** | **991.4765** | 496.2419 | 974.4499 | 487.7286 | 973.4659 | 487.2366 | **G** | **232.1404** | 116.5738 | 215.1139 | 108.0606 | | | **2** |
| **11** | | | | | | | **R** | 175.1190 | 88.0631 | 158.0924 | 79.5498 | | | **1** |

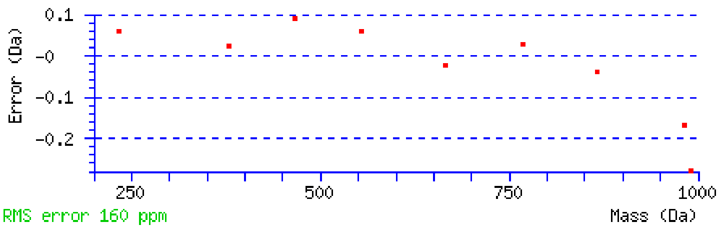


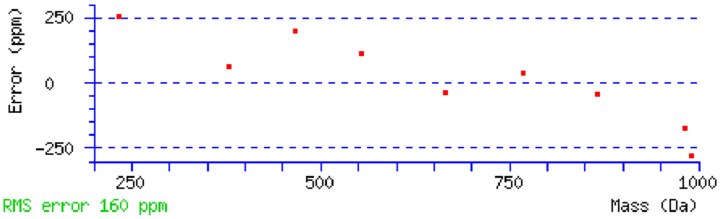


### All matches to this query

| Score | Mr(calc): | Delta | Sequence |
|---|---|---|---|
| 81.2 | 1164.5809 | -0.0029 | ALNVTLSSMGR |
| 5.7 | 1164.5775 | 0.0005 | SPSEYKLEGR |
| 5.3 | 1164.5743 | 0.0036 | NKDMKVAMGR |
| 5.0 | 1164.5693 | 0.0087 | XVPGLWTGSR |
| 5.0 | 1164.5693 | 0.0087 | XVPGLWTGSR |
| 2.2 | 1162.5747 | 2.0032 | KTSGPPKGPSK |
| 1.4 | 1164.5764 | 0.0015 | SRSQPVAKGR |

---

Spectrum No: 219; Query: 1482; Rank: 1

---

## Peptide View

MS/MS Fragmentation of **LNLTAAQLSQLEGLLQAR**
Found in **IPI00199867**, Tax_Id=10116 Gene_Symbol=Emilin1_predicted elastin microfibril interfacer 1

Match to Query 1482: 1939.078812 from(647.366880,3+)
Title: 100101RatKid_NS_deglyco_20.5386.5386.3.dta
Data file K:\NewmanPaper\Piliang\3SubProteomes\Piliang3SP\mgf5ppm\ERLIC_3SubProteomes5ppm.mgf

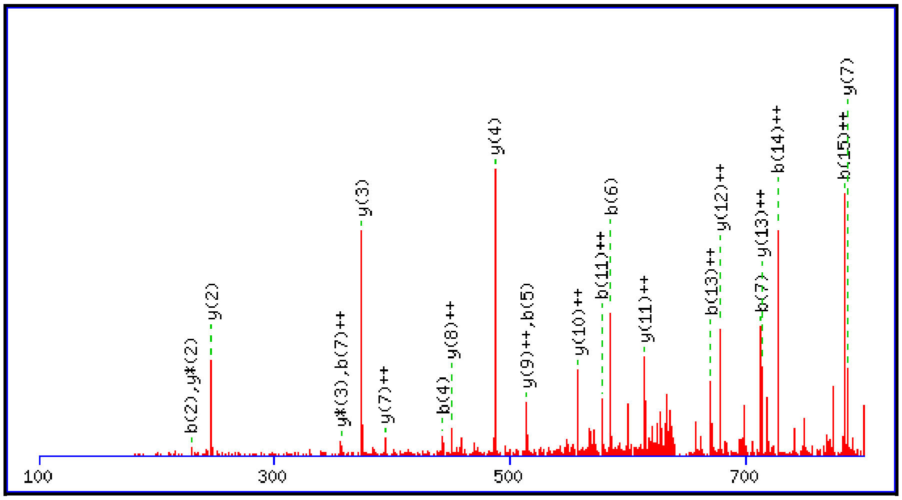

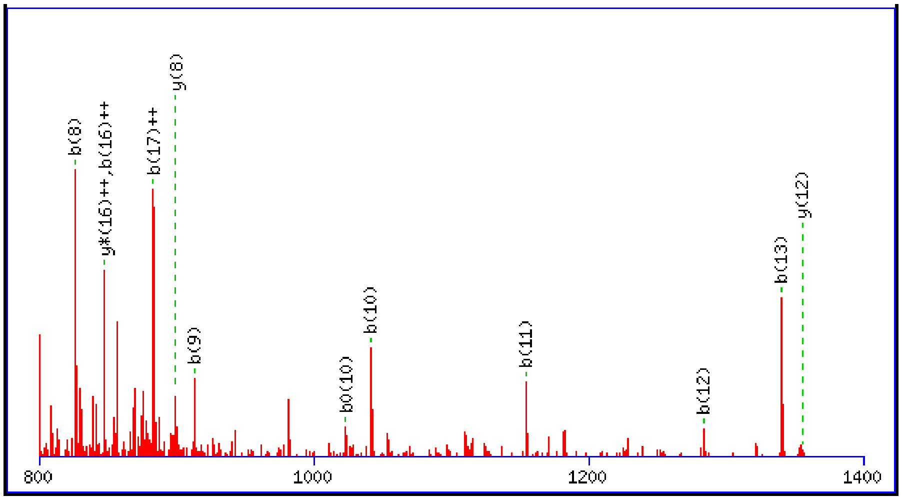


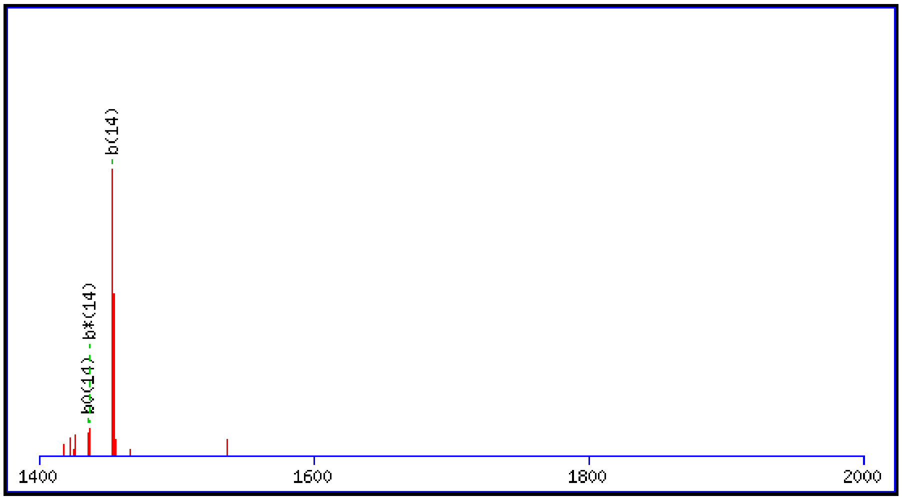


**Monoisotopic mass of neutral peptide Mr(calc):** 1939.0738
**Fixed modifications:** Carbamidomethyl (C)
**Variable modifications:**
**N2** : Deamidated_N (N)
**Ions Score:** 81 **Expect:** 3.1e-007
**Matches (Bold Red):** 38/184 fragment ions using 57 most intense peaks

| # | b | b++ | b* | b*++ | b0 | b0++ | Seq. | y | y++ | y* | y*++ | y0 | y0++ | # |
|---|---|---|---|---|---|---|---|---|---|---|---|---|---|---|
| 1 | 114.0913 | 57.5493 | | | | | L | | | | | | | 18 |
| 2 | **229.1183** | 115.0628 | 212.0917 | 106.5495 | | | N | 1826.9970 | 914.0022 | 1809.9705 | 905.4889 | 1808.9865 | 904.9969 | 17 |
| 3 | 342.2023 | 171.6048 | 325.1758 | 163.0915 | | | L | 1711.9701 | 856.4887 | 1694.9436 | **847.9754** | 1693.9595 | 847.4834 | 16 |
| 4 | **443.2500** | 222.1286 | 426.2235 | 213.6154 | 425.2395 | 213.1234 | T | 1598.8860 | 799.9467 | 1581.8595 | 791.4334 | 1580.8755 | 790.9414 | 15 |
| 5 | **514.2871** | 257.6472 | 497.2606 | 249.1339 | 496.2766 | 248.6419 | A | 1497.8384 | 749.4228 | 1480.8118 | 740.9095 | 1479.8278 | 740.4175 | 14 |
| 6 | **585.3242** | 293.1658 | 568.2977 | 284.6525 | 567.3137 | 284.1605 | A | 1426.8013 | **713.9043** | 1409.7747 | 705.3910 | 1408.7907 | 704.8990 | 13 |
| 7 | **713.3828** | **357.1951** | 696.3563 | 348.6818 | 695.3723 | 348.1898 | Q | **1355.7641** | **678.3857** | 1338.7376 | 669.8724 | 1337.7536 | 669.3804 | 12 |
| 8 | **826.4669** | 413.7371 | 809.4403 | 405.2238 | 808.4563 | 404.7318 | L | 1227.7056 | **614.3564** | 1210.6790 | 605.8431 | 1209.6950 | 605.3511 | 11 |
| 9 | **913.4989** | 457.2531 | 896.4724 | 448.7398 | 895.4884 | 448.2478 | S | 1114.6215 | **557.8144** | 1097.5949 | 549.3011 | 1096.6109 | 548.8091 | 10 |
| 10 | **1041.5575** | 521.2824 | 1024.5309 | 512.7691 | **1023.5469** | 512.2771 | Q | 1027.5895 | **514.2984** | 1010.5629 | 505.7851 | 1009.5789 | 505.2931 | 9 |
| 11 | **1154.6416** | **577.8244** | 1137.6150 | 569.3111 | 1136.6310 | 568.8191 | L | **899.5309** | **450.2691** | 882.5043 | 441.7558 | 881.5203 | 441.2638 | 8 |
| 12 | **1283.6842** | 642.3457 | 1266.6576 | 633.8324 | 1265.6736 | 633.3404 | E | **786.4468** | **393.7271** | 769.4203 | 385.2138 | 768.4363 | 384.7218 | 7 |
| 13 | **1340.7056** | **670.8564** | 1323.6791 | 662.3432 | 1322.6951 | 661.8512 | G | 657.4042 | 329.2058 | 640.3777 | 320.6925 | | | 6 |
| 14 | **1453.7897** | **727.3985** | **1436.7631** | 718.8852 | **1435.7791** | 718.3932 | L | 600.3828 | 300.6950 | 583.3562 | 292.1817 | | | 5 |
| 15 | 1566.8737 | **783.9405** | 1549.8472 | 775.4272 | 1548.8632 | 774.9352 | L | **487.2987** | 244.1530 | 470.2722 | 235.6397 | | | 4 |
| 16 | 1694.9323 | **847.9698** | 1677.9058 | 839.4565 | 1676.9218 | 838.9645 | Q | **374.2146** | 187.6110 | **357.1881** | 179.0977 | | | 3 |
| 17 | 1765.9694 | **883.4884** | 1748.9429 | 874.9751 | 1747.9589 | 874.4831 | A | **246.1561** | 123.5817 | **229.1295** | 115.0684 | | | 2 |
| 18 | | | | | | | R | 175.1190 | 88.0631 | 158.0924 | 79.5498 | | | 1 |

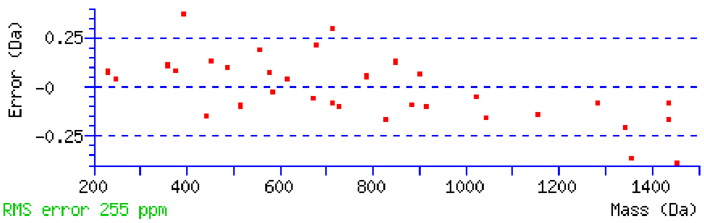


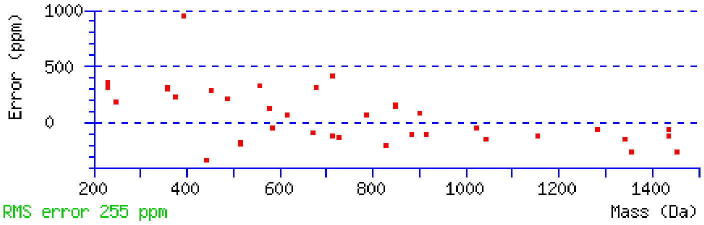


### All matches to this query

| Score | Mr(calc): | Delta | Sequence |
|---|---|---|---|
| 81.1 | 1939.0738 | 0.0050 | LNLTAAQLSQLEGLLQAR |
| 47.4 | 1938.0898 | 0.9890 | LNLTAAQLSQLEGLLQAR |
| 8.4 | 1938.0734 | 1.0054 | WNPSLLGRPLGMLRRR |
| 6.4 | 1939.0924 | -0.0136 | AQSSLKINTPPLLLRXK |
| 6.1 | 1937.0894 | 1.9895 | WNPSLLGRPLGMLRRR |
| 5.9 | 1938.0720 | 1.0068 | MGIAQKENINQKPVVLR |
| 5.9 | 1938.0720 | 1.0068 | MGIAQKENINQKPVVLR |
| 5.3 | 1938.0808 | 0.9980 | ILFIRMLHNKHVMVR |
| 3.6 | 1939.0648 | 0.0140 | ILFIRMLHNKHVMVR |
| 2.4 | 1939.0930 | -0.0142 | YIYPRKANGIYIINLK |

---

Spectrum No: 220; Query: 1983; Rank: 1

---

## Peptide View

MS/MS Fragmentation of **NGSCGVSYIAQEPGNYEVSIK**
Found in **IPI00373752**, Tax_Id=10116 Gene_Symbol=Flnb_predicted filamin, beta

Match to Query 1983: 2272.037728 from(1137.026140,2+)
Title: 091008RatKidney_NH4Format01_18.3482.3482.2.dta
Data file K:\NewmanPaper\Piliang\3SubProteomes\Piliang3SP\mgf5ppm\ERLIC_3SubProteomes5ppm.mgf

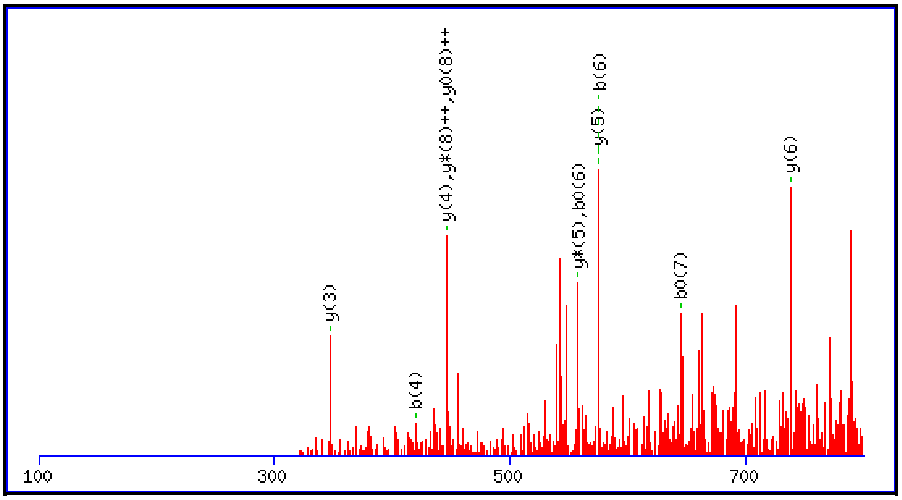

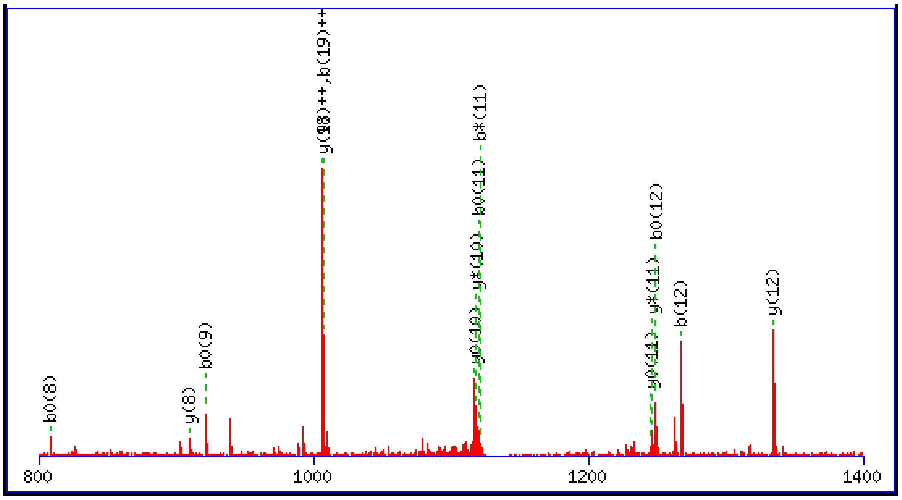


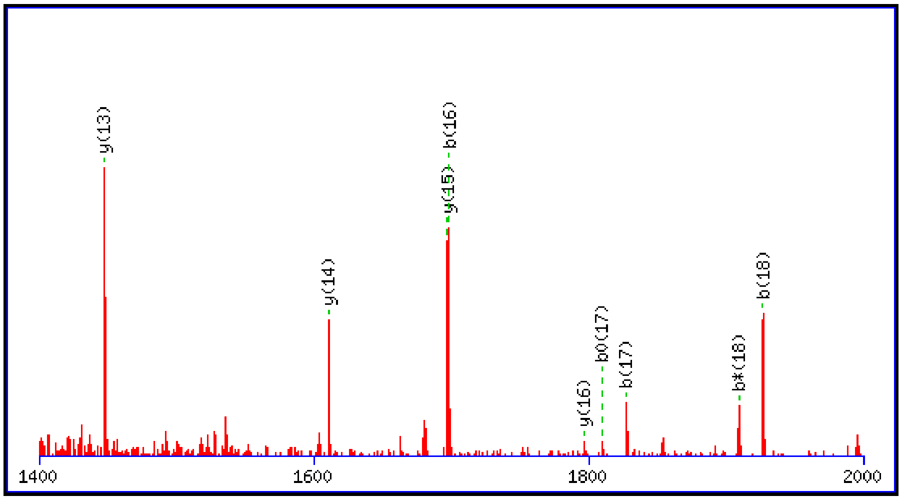


**Monoisotopic mass of neutral peptide Mr(calc):** 2272.0318
**Fixed modifications:** Carbamidomethyl (C)
**Variable modifications:**
**N1** : Deamidated_N (N)
**Ions Score:** 81 **Expect:** 2.2e-006
**Matches (Bold Red):** 35/232 fragment ions using 51 most intense peaks

| # | b | b$^{++}$ | b* | b*$^{++}$ | b$^{0}$ | b$^{0++}$ | Seq. | y | y$^{++}$ | y* | y*$^{++}$ | y$^{0}$ | y$^{0++}$ | # |
|---|---|---|---|---|---|---|---|---|---|---|---|---|---|---|
| 1 | 116.0342 | 58.5207 | 99.0077 | 50.0075 | | | N | | | | | | | 21 |
| 2 | 173.0557 | 87.0315 | 156.0291 | 78.5182 | | | G | 2158.0121 | 1079.5097 | 2140.9856 | 1070.9964 | 2140.0015 | 1070.5044 | 20 |
| 3 | 260.0877 | 130.5475 | 243.0612 | 122.0342 | 242.0771 | 121.5422 | S | 2100.9906 | 1050.9990 | 2083.9641 | 1042.4857 | 2082.9801 | 1041.9937 | 19 |
| 4 | **420.1184** | 210.5628 | 403.0918 | 202.0495 | 402.1078 | 201.5575 | C | 2013.9586 | **1007.4829** | 1996.9321 | 998.9697 | 1995.9481 | 998.4777 | 18 |
| 5 | 477.1398 | 239.0735 | 460.1133 | 230.5603 | 459.1293 | 230.0683 | G | 1853.9280 | 927.4676 | 1836.9014 | 918.9543 | 1835.9174 | 918.4623 | 17 |
| 6 | **576.2082** | 288.6078 | 559.1817 | 280.0945 | **558.1977** | 279.6025 | V | **1796.9065** | 898.9569 | 1779.8800 | 890.4436 | 1778.8959 | 889.9516 | 16 |
| 7 | 663.2403 | 332.1238 | 646.2137 | 323.6105 | **645.2297** | 323.1185 | S | **1697.8381** | 849.4227 | 1680.8115 | 840.9094 | 1679.8275 | 840.4174 | 15 |
| 8 | 826.3036 | 413.6554 | 809.2770 | 405.1422 | **808.2930** | 404.6501 | Y | **1610.8061** | 805.9067 | 1593.7795 | 797.3934 | 1592.7955 | 796.9014 | 14 |
| 9 | 939.3877 | 470.1975 | 922.3611 | 461.6842 | **921.3771** | 461.1922 | I | **1447.7427** | 724.3750 | 1430.7162 | 715.8617 | 1429.7322 | 715.3697 | 13 |
| 10 | 1010.4248 | 505.7160 | 993.3982 | 497.2027 | 992.4142 | 496.7107 | A | **1334.6587** | 667.8330 | 1317.6321 | 659.3197 | 1316.6481 | 658.8277 | 12 |
| 11 | 1138.4833 | 569.7453 | **1121.4568** | 561.2320 | **1120.4728** | 560.7400 | Q | 1263.6216 | 632.3144 | **1246.5950** | 623.8011 | **1245.6110** | 623.3091 | 11 |
| 12 | **1267.5259** | 634.2666 | 1250.4994 | 625.7533 | **1249.5154** | 625.2613 | E | 1135.5630 | 568.2851 | **1118.5364** | 559.7719 | **1117.5524** | 559.2798 | 10 |
| 13 | 1364.5787 | 682.7930 | 1347.5522 | 674.2797 | 1346.5681 | 673.7877 | P | **1006.5204** | 503.7638 | 989.4938 | 495.2506 | 988.5098 | 494.7585 | 9 |
| 14 | 1421.6002 | 711.3037 | 1404.5736 | 702.7904 | 1403.5896 | 702.2984 | G | **909.4676** | 455.2374 | 892.4411 | **446.7242** | 891.4571 | **446.2322** | 8 |
| 15 | 1535.6431 | 768.3252 | 1518.6165 | 759.8119 | 1517.6325 | 759.3199 | N | 852.4462 | 426.7267 | 835.4196 | 418.2134 | 834.4356 | 417.7214 | 7 |
| 16 | **1698.7064** | 849.8568 | 1681.6799 | 841.3436 | 1680.6959 | 840.8516 | Y | **738.4032** | 369.7053 | 721.3767 | 361.1920 | 720.3927 | 360.7000 | 6 |
| 17 | **1827.7490** | 914.3781 | 1810.7225 | 905.8649 | **1809.7384** | 905.3729 | E | **575.3399** | 288.1736 | **558.3134** | 279.6603 | 557.3293 | 279.1683 | 5 |
| 18 | **1926.8174** | 963.9124 | **1909.7909** | 955.3991 | 1908.8069 | 954.9071 | V | **446.2973** | 223.6523 | 429.2708 | 215.1390 | 428.2867 | 214.6470 | 4 |
| | | | | | | | | | | | | | | |

| | | | | | | | | | | | | | | |
|---|---|---|---|---|---|---|---|---|---|---|---|---|---|---|
| **19** | 2013.8495 | **1007.4284** | 1996.8229 | 998.9151 | 1995.8389 | 998.4231 | **S** | **347.2289** | 174.1181 | 330.2023 | 165.6048 | 329.2183 | 165.1128 | **3** |
| **20** | 2126.9335 | 1063.9704 | 2109.9070 | 1055.4571 | 2108.9230 | 1054.9651 | **I** | 260.1969 | 130.6021 | 243.1703 | 122.0888 | | | **2** |
| **21** | | | | | | | **K** | 147.1128 | 74.0600 | 130.0863 | 65.5468 | | | **1** |

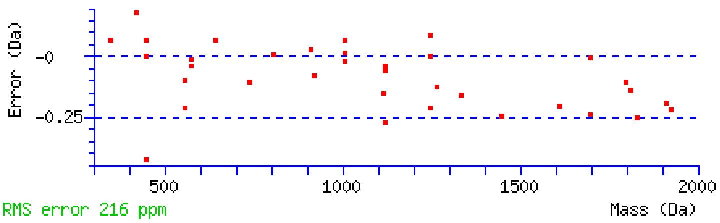


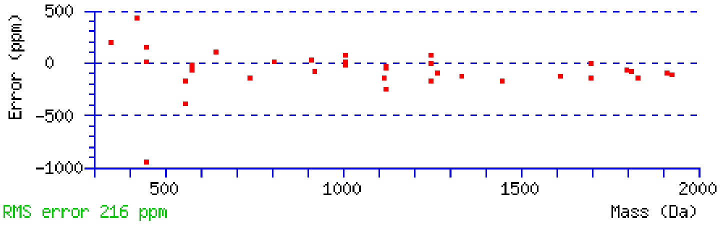


### All matches to this query

| Score | Mr(calc): | Delta | Sequence |
|---|---|---|---|
| 81.0 | 2272.0318 | 0.0060 | NGSCGVSYIAQEPGNYEVSIK |
| 64.3 | 2271.0477 | 0.9900 | NGSCGVSYIAQEPGNYEVSIK |
| 45.2 | 2272.0318 | 0.0060 | NGSCGVSYIAQEPGNYEVSIK |
| 3.5 | 2271.0186 | 1.0191 | LNSFNLIPAVGLNVYSMLK |
| 3.5 | 2272.0582 | -0.0205 | ECSFWKWTTNXRSLNSNK |
| 3.5 | 2272.0582 | -0.0205 | ECSFWKWTTNXRSLNSNK |
| 3.2 | 2272.0419 | -0.0041 | ASXTVSLLSQSPRHPLKPK |
| 1.2 | 2272.0534 | -0.0156 | RIVYFMEGRDSMQLIYR |
| 0.3 | 2272.0208 | 0.0169 | QAFQIGSPWRTMDASERGR |

---

Spectrum No: 221; Query: 1618; Rank: 1

---

## Peptide View

MS/MS Fragmentation of **SGPSDEGEEEMEDGTVTNGS**
Found in **IPI00192279**, Tax_Id=10116 Gene_Symbol=- 28 kDa protein

Match to Query 1618: 2026.740048 from(1014.377300,2+)
Title: 091008RatKidney_NoSalt_29.1279.1279.2.dta
Data file K:\NewmanPaper\Piliang\3SubProteomes\Piliang3SP\mgf5ppm\ERLIC_3SubProteomes5ppm.mgf

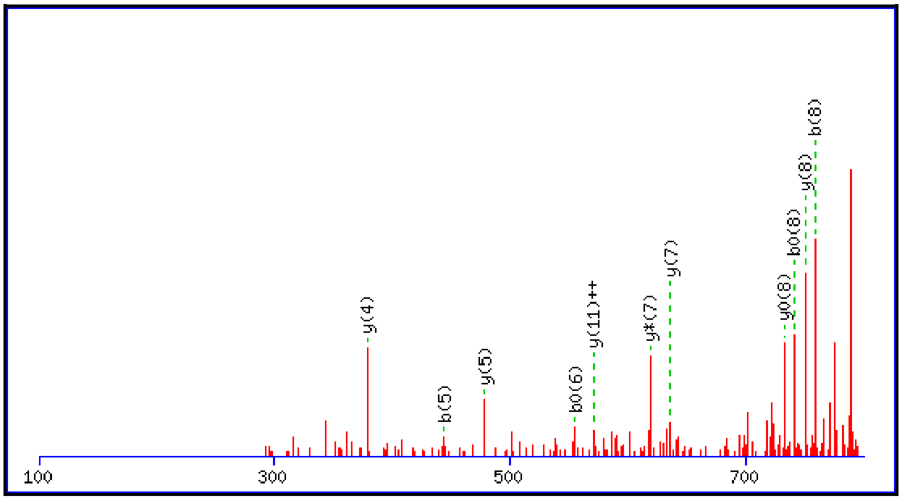

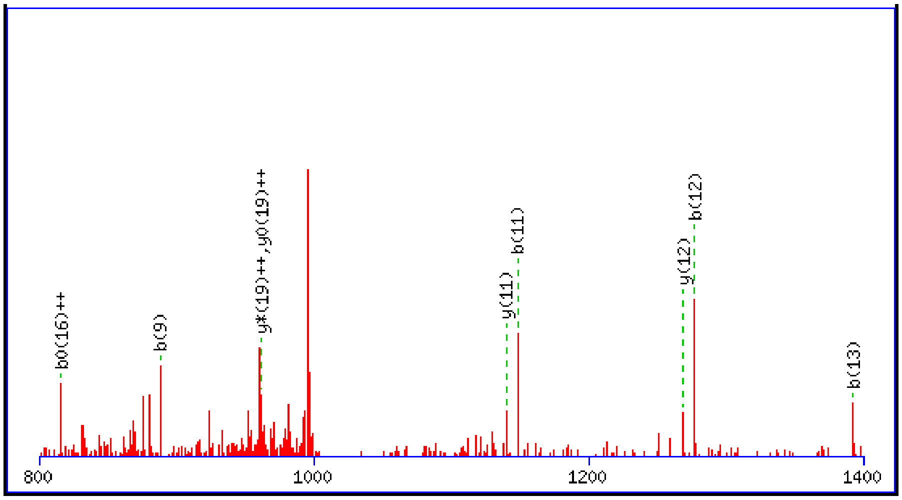


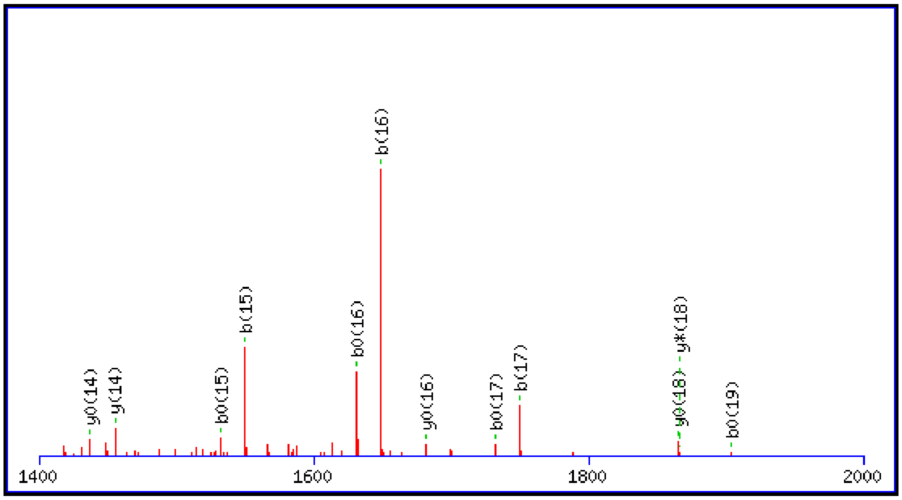


**Monoisotopic mass of neutral peptide Mr(calc):** 2026.7433
**Fixed modifications:** Carbamidomethyl (C)
**Variable modifications:**
**N18** : Deamidated_N (N)
**Ions Score:** 81 **Expect:** 8.7e-007
**Matches (Bold Red):** 34/190 fragment ions using 41 most intense peaks

| # | b | $b^{++}$ | b* | $b^{*++}$ | $b^{0}$ | $b^{0++}$ | Seq. | y | $y^{++}$ | y* | $y^{*++}$ | $y^{0}$ | $y^{0++}$ | # |
|---|---|---|---|---|---|---|---|---|---|---|---|---|---|---|
| 1 | 88.0393 | 44.5233 | | | 70.0287 | 35.5180 | S | | | | | | | 20 |
| 2 | 145.0608 | 73.0340 | | | 127.0502 | 64.0287 | G | 1940.7186 | 970.8629 | 1923.6920 | **962.3496** | 1922.7080 | **961.8576** | 19 |
| 3 | 242.1135 | 121.5604 | | | 224.1030 | 112.5551 | P | 1883.6971 | 942.3522 | **1866.6705** | 933.8389 | **1865.6865** | 933.3469 | 18 |
| 4 | 329.1456 | 165.0764 | | | 311.1350 | 156.0711 | S | 1786.6443 | 893.8258 | 1769.6178 | 885.3125 | 1768.6338 | 884.8205 | 17 |
| 5 | **444.1725** | 222.5899 | | | 426.1619 | 213.5846 | D | 1699.6123 | 850.3098 | 1682.5858 | 841.7965 | **1681.6017** | 841.3045 | 16 |
| 6 | 573.2151 | 287.1112 | | | **555.2045** | 278.1059 | E | 1584.5854 | 792.7963 | 1567.5588 | 784.2830 | 1566.5748 | 783.7910 | 15 |
| 7 | 630.2366 | 315.6219 | | | 612.2260 | 306.6166 | G | **1455.5428** | 728.2750 | 1438.5162 | 719.7617 | **1437.5322** | 719.2697 | 14 |
| 8 | **759.2792** | 380.1432 | | | **741.2686** | 371.1379 | E | 1398.5213 | 699.7643 | 1381.4948 | 691.2510 | 1380.5107 | 690.7590 | 13 |
| 9 | **888.3217** | 444.6645 | | | 870.3112 | 435.6592 | E | **1269.4787** | 635.2430 | 1252.4522 | 626.7297 | 1251.4681 | 626.2377 | 12 |
| 10 | 1017.3643 | 509.1858 | | | 999.3538 | 500.1805 | E | **1140.4361** | **570.7217** | 1123.4096 | 562.2084 | 1122.4256 | 561.7164 | 11 |
| 11 | **1148.4048** | 574.7061 | | | 1130.3943 | 565.7008 | M | 1011.3935 | 506.2004 | 994.3670 | 497.6871 | 993.3830 | 497.1951 | 10 |
| 12 | **1277.4474** | 639.2273 | | | 1259.4369 | 630.2221 | E | 880.3530 | 440.6802 | 863.3265 | 432.1669 | 862.3425 | 431.6749 | 9 |
| 13 | **1392.4744** | 696.7408 | | | 1374.4638 | 687.7355 | D | **751.3104** | 376.1589 | 734.2839 | 367.6456 | **733.2999** | 367.1536 | 8 |
| 14 | 1449.4958 | 725.2516 | | | 1431.4853 | 716.2463 | G | **636.2835** | 318.6454 | **619.2570** | 310.1321 | 618.2729 | 309.6401 | 7 |
| 15 | **1550.5435** | 775.7754 | | | **1532.5329** | 766.7701 | T | 579.2620 | 290.1347 | 562.2355 | 281.6214 | 561.2515 | 281.1294 | 6 |
| 16 | **1649.6119** | 825.3096 | | | **1631.6014** | **816.3043** | V | **478.2144** | 239.6108 | 461.1878 | 231.0975 | 460.2038 | 230.6055 | 5 |
| 17 | **1750.6596** | 875.8334 | | | **1732.6490** | 866.8282 | T | **379.1459** | 190.0766 | 362.1194 | 181.5633 | 361.1354 | 181.0713 | 4 |
| 18 | **1865.6865** | 933.3469 | 1848.6600 | 924.8336 | 1847.6760 | 924.3416 | N | 278.0983 | 139.5528 | 261.0717 | 131.0395 | 260.0877 | 130.5475 | 3 |

| | | | | | | | | | | | | | | |
|---|---|---|---|---|---|---|---|---|---|---|---|---|---|---|
| **19** | 1922.7080 | **961.8576** | 1905.6814 | 953.3444 | **1904.6974** | 952.8524 | **G** | 163.0713 | 82.0393 | | | 145.0608 | 73.0340 | **2** |
| **20** | | | | | | | **S** | 106.0499 | 53.5286 | | | 88.0393 | 44.5233 | **1** |

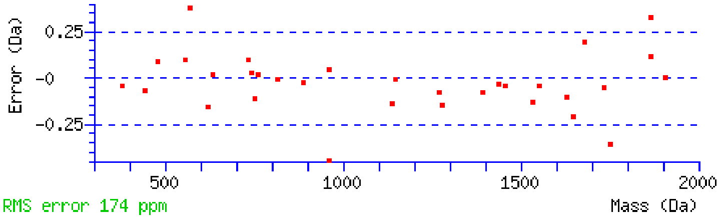


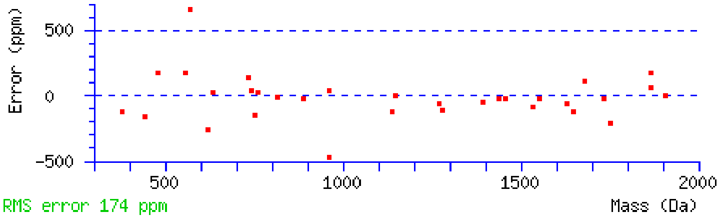


**All matches to this query**

| Score | Mr(calc): | Delta | Sequence |
|---|---|---|---|
| 80.9 | 2026.7433 | -0.0033 | SGPSDEGEEEMEDGTVTNGS |
| 1.7 | 2026.7575 | -0.0174 | DGSGQVPSTAESSPIVEK |

---

Spectrum No: 222; Query: 2058; Rank: 1

---

## Peptide View

MS/MS Fragmentation of **TDTTCLNNTEISENGSDLSQK**
Found in **IPI00372146**, Tax_Id=10116 Gene_Symbol=Rai14 Isoform 2 of Ankycorbin

Match to Query 2058: 2327.017968 from(1164.516260,2+)
Title: 100101RatKid_NS_deglyco_08.2154.2154.2.dta
Data file K:\NewmanPaper\Piliang\3SubProteomes\Piliang3SP\mgf5ppm\ERLIC_3SubProteomes5ppm.mgf

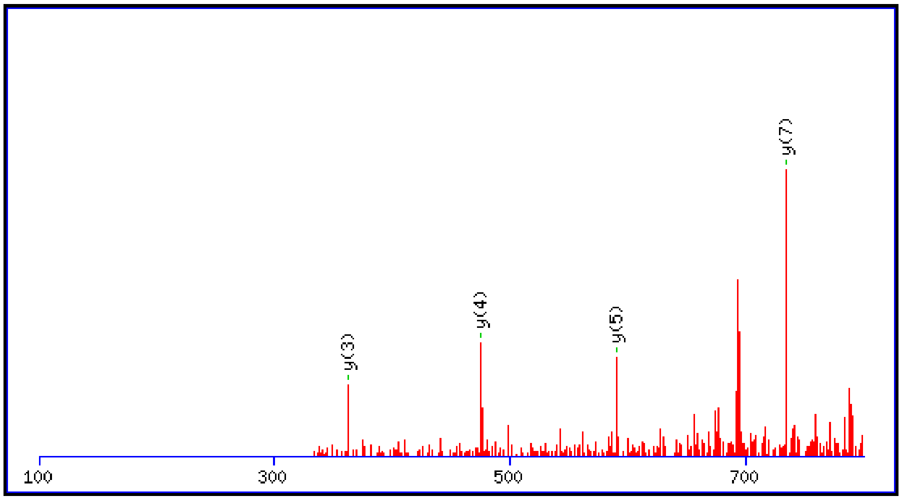

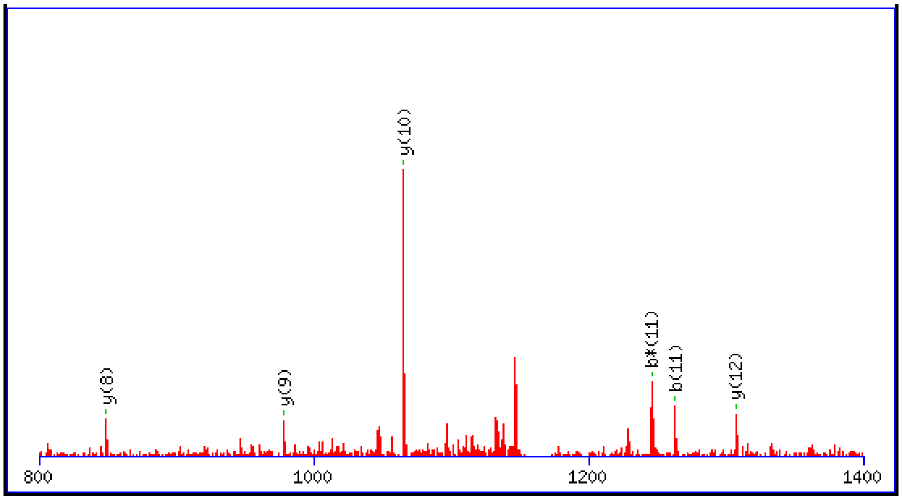


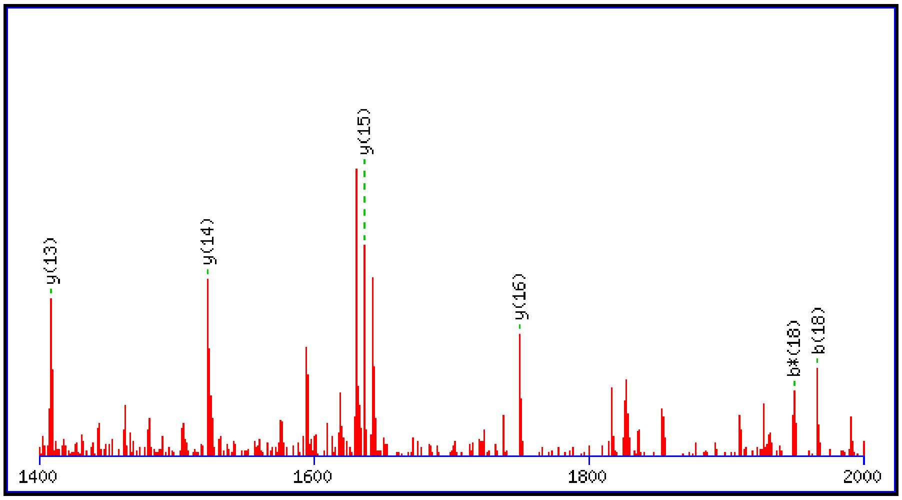


**Monoisotopic mass of neutral peptide Mr(calc):** 2327.0071
**Fixed modifications:** Carbamidomethyl (C)
**Variable modifications:**
**N14** : Deamidated_N (N)
**Ions Score:** 81 **Expect:** 2.4e-006
**Matches (Bold Red):** 16/224 fragment ions using 27 most intense peaks

| # | b | $b^{++}$ | b* | $b^{*++}$ | $b^0$ | $b^{0++}$ | Seq. | y | $y^{++}$ | y* | $y^{*++}$ | $y^0$ | $y^{0++}$ | # |
|---|---|---|---|---|---|---|---|---|---|---|---|---|---|---|
| 1 | 102.0550 | 51.5311 | | | 84.0444 | 42.5258 | T | | | | | | | 21 |
| 2 | 217.0819 | 109.0446 | | | 199.0713 | 100.0393 | D | 2226.9667 | 1113.9870 | 2209.9401 | 1105.4737 | 2208.9561 | 1104.9817 | 20 |
| 3 | 318.1296 | 159.5684 | | | 300.1190 | 150.5631 | T | 2111.9397 | 1056.4735 | 2094.9132 | 1047.9602 | 2093.9292 | 1047.4682 | 19 |
| 4 | 419.1773 | 210.0923 | | | 401.1667 | 201.0870 | T | 2010.8920 | 1005.9497 | 1993.8655 | 997.4364 | 1992.8815 | 996.9444 | 18 |
| 5 | 579.2079 | 290.1076 | | | 561.1973 | 281.1023 | C | 1909.8444 | 955.4258 | 1892.8178 | 946.9125 | 1891.8338 | 946.4205 | 17 |
| 6 | 692.2920 | 346.6496 | | | 674.2814 | 337.6443 | L | **1749.8137** | 875.4105 | 1732.7872 | 866.8972 | 1731.8032 | 866.4052 | 16 |
| 7 | 806.3349 | 403.6711 | 789.3083 | 395.1578 | 788.3243 | 394.6658 | N | **1636.7297** | 818.8685 | 1619.7031 | 810.3552 | 1618.7191 | 809.8632 | 15 |
| 8 | 920.3778 | 460.6925 | 903.3513 | 452.1793 | 902.3673 | 451.6873 | N | **1522.6867** | 761.8470 | 1505.6602 | 753.3337 | 1504.6762 | 752.8417 | 14 |
| 9 | 1021.4255 | 511.2164 | 1004.3990 | 502.7031 | 1003.4149 | 502.2111 | T | **1408.6438** | 704.8255 | 1391.6173 | 696.3123 | 1390.6332 | 695.8203 | 13 |
| 10 | 1150.4681 | 575.7377 | 1133.4415 | 567.2244 | 1132.4575 | 566.7324 | E | **1307.5961** | 654.3017 | 1290.5696 | 645.7884 | 1289.5856 | 645.2964 | 12 |
| 11 | **1263.5522** | 632.2797 | **1246.5256** | 623.7664 | 1245.5416 | 623.2744 | I | 1178.5535 | 589.7804 | 1161.5270 | 581.2671 | 1160.5430 | 580.7751 | 11 |
| 12 | 1350.5842 | 675.7957 | 1333.5576 | 667.2825 | 1332.5736 | 666.7904 | S | **1065.4695** | 533.2384 | 1048.4429 | 524.7251 | 1047.4589 | 524.2331 | 10 |
| 13 | 1479.6268 | 740.3170 | 1462.6002 | 731.8038 | 1461.6162 | 731.3117 | E | **978.4374** | 489.7224 | 961.4109 | 481.2091 | 960.4269 | 480.7171 | 9 |
| 14 | 1594.6537 | 797.8305 | 1577.6272 | 789.3172 | 1576.6432 | 788.8252 | N | **849.3948** | 425.2011 | 832.3683 | 416.6878 | 831.3843 | 416.1958 | 8 |
| 15 | 1651.6752 | 826.3412 | 1634.6486 | 817.8280 | 1633.6646 | 817.3359 | G | **734.3679** | 367.6876 | 717.3414 | 359.1743 | 716.3573 | 358.6823 | 7 |
| 16 | 1738.7072 | 869.8572 | 1721.6807 | 861.3440 | 1720.6966 | 860.8520 | S | 677.3464 | 339.1769 | 660.3199 | 330.6636 | 659.3359 | 330.1716 | 6 |
| 17 | 1853.7342 | 927.3707 | 1836.7076 | 918.8574 | 1835.7236 | 918.3654 | D | **590.3144** | 295.6608 | 573.2879 | 287.1476 | 572.3039 | 286.6556 | 5 |
| 18 | **1966.8182** | 983.9127 | **1949.7917** | 975.3995 | 1948.8076 | 974.9075 | L | **475.2875** | 238.1474 | 458.2609 | 229.6341 | 457.2769 | 229.1421 | 4 |

| | | | | | | | | | | | | | |
|---|---|---|---|---|---|---|---|---|---|---|---|---|---|
| 19 | 2053.8502 | 1027.4288 | 2036.8237 | 1018.9155 | 2035.8397 | 1018.4235 | S | 362.2034 | 181.6053 | 345.1769 | 173.0921 | 344.1928 | 172.6001 | 3 |
| 20 | 2181.9088 | 1091.4580 | 2164.8823 | 1082.9448 | 2163.8983 | 1082.4528 | Q | 275.1714 | 138.0893 | 258.1448 | 129.5761 | | | 2 |
| 21 | | | | | | | K | 147.1128 | 74.0600 | 130.0863 | 65.5468 | | | 1 |

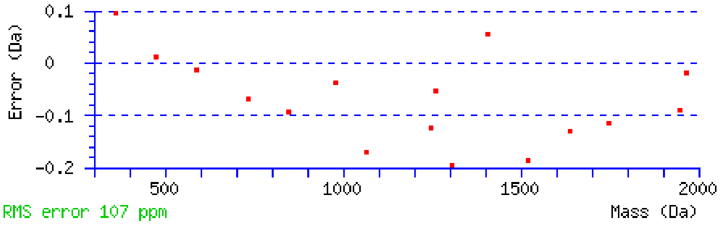


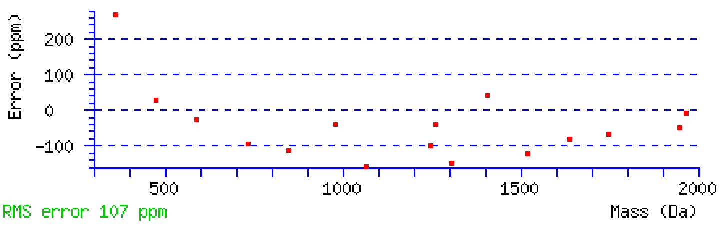


**All matches to this query**

| Score | Mr(calc): | Delta | Sequence |
|---|---|---|---|
| 80.9 | 2327.0071 | 0.0109 | TDTTCLNNTEISENGSDLSQK |
| 30.2 | 2327.0071 | 0.0109 | TDTTCLNNTEISENGSDLSQK |
| 22.6 | 2327.0071 | 0.0109 | TDTTCLNNTEISENGSDLSQK |
| 7.5 | 2326.0231 | 0.9949 | TDTTCLNNTEISENGSDLSQK |
| 2.2 | 2327.0222 | -0.0042 | SYLSLEVLDLSSNNITEIR |
| 2.2 | 2327.0222 | -0.0042 | SYLSLEVLDLSSNNITEIR |
| 2.2 | 2327.0222 | -0.0042 | SYLSLEVLDLSSNNITEIR |
| 0.5 | 2326.0337 | 0.9843 | GWRNHSFVTEFILLGFSR |
| 0.3 | 2327.0340 | -0.0161 | VVYPNNSMCHQDWKKVSR |

---

Spectrum No: 223; Query: 1066; Rank: 1

---

## Peptide View

MS/MS Fragmentation of **DNATEEEILHYLEK**
Found in **IPI00195160**, Tax_Id=10116 Gene_Symbol=Psap Sulfated glycoprotein 1 precursor

Match to Query 1066: 1703.789948 from(852.902250,2+)
Title: 091008RatKidney_NH4Format02_25.4379.4379.2.dta
Data file K:\NewmanPaper\Piliang\3SubProteomes\Piliang3SP\mgf5ppm\ERLIC_3SubProteomes5ppm.mgf

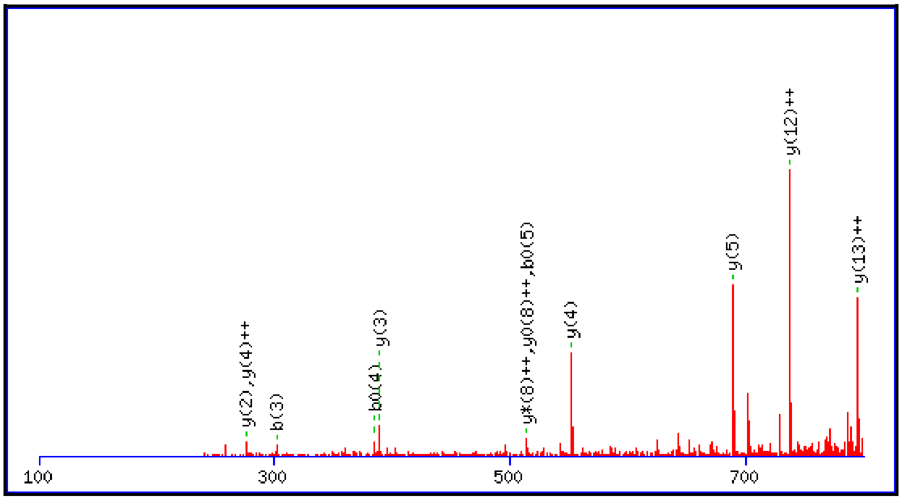

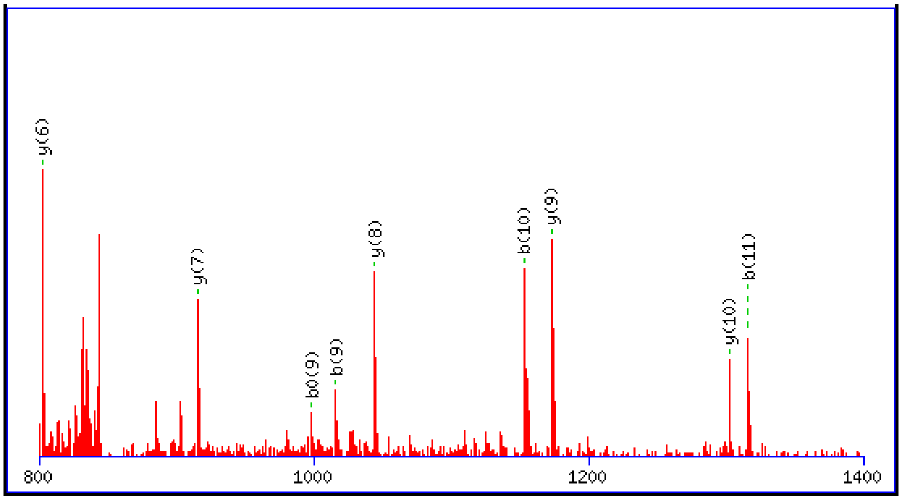


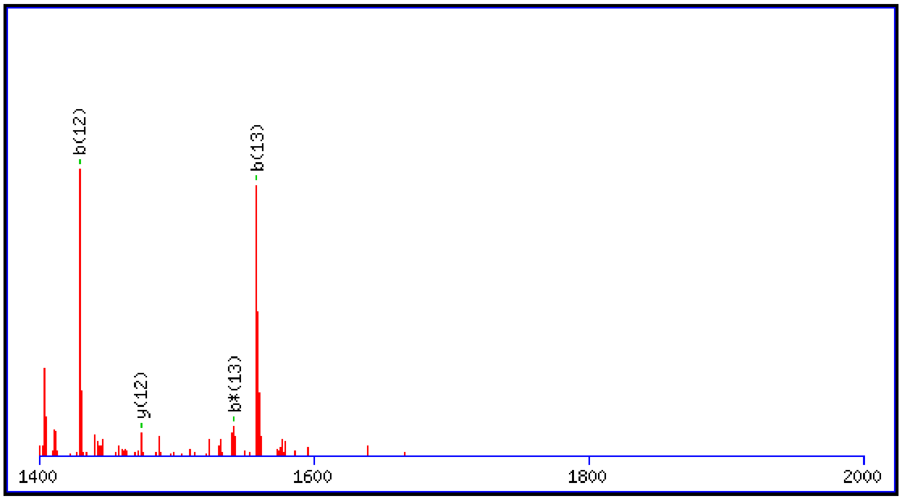


**Monoisotopic mass of neutral peptide Mr(calc):** 1703.7890
**Fixed modifications:** Carbamidomethyl (C)
**Variable modifications:**
**N2** : Deamidated_N (N)
**Ions Score:** 81 **Expect:** 1.6e-006
**Matches (Bold Red):** 25/152 fragment ions using 44 most intense peaks

| # | b | $b^{++}$ | b* | $b^{*++}$ | $b^{0}$ | $b^{0++}$ | Seq. | y | $y^{++}$ | y* | $y^{*++}$ | $y^{0}$ | $y^{0++}$ | # |
|---|---|---|---|---|---|---|---|---|---|---|---|---|---|---|
| 1 | 116.0342 | 58.5207 | | | 98.0237 | 49.5155 | D | | | | | | | 14 |
| 2 | 231.0612 | 116.0342 | 214.0346 | 107.5209 | 213.0506 | 107.0289 | N | 1589.7693 | **795.3883** | 1572.7428 | 786.8750 | 1571.7588 | 786.3830 | 13 |
| 3 | **302.0983** | 151.5528 | 285.0717 | 143.0395 | 284.0877 | 142.5475 | A | **1474.7424** | **737.8748** | 1457.7159 | 729.3616 | 1456.7318 | 728.8696 | 12 |
| 4 | 403.1459 | 202.0766 | 386.1194 | 193.5633 | **385.1354** | 193.0713 | T | 1403.7053 | 702.3563 | 1386.6787 | 693.8430 | 1385.6947 | 693.3510 | 11 |
| 5 | 532.1885 | 266.5979 | 515.1620 | 258.0846 | **514.1780** | 257.5926 | E | **1302.6576** | 651.8324 | 1285.6311 | 643.3192 | 1284.6470 | 642.8272 | 10 |
| 6 | 661.2311 | 331.1192 | 644.2046 | 322.6059 | 643.2206 | 322.1139 | E | **1173.6150** | 587.3111 | 1156.5885 | 578.7979 | 1155.6045 | 578.3059 | 9 |
| 7 | 790.2737 | 395.6405 | 773.2472 | 387.1272 | 772.2632 | 386.6352 | E | **1044.5724** | 522.7898 | 1027.5459 | **514.2766** | 1026.5619 | **513.7846** | 8 |
| 8 | 903.3578 | 452.1825 | 886.3312 | 443.6693 | 885.3472 | 443.1773 | I | **915.5298** | 458.2686 | 898.5033 | 449.7553 | 897.5193 | 449.2633 | 7 |
| 9 | **1016.4419** | 508.7246 | 999.4153 | 500.2113 | **998.4313** | 499.7193 | L | **802.4458** | 401.7265 | 785.4192 | 393.2132 | 784.4352 | 392.7212 | 6 |
| 10 | **1153.5008** | 577.2540 | 1136.4742 | 568.7407 | 1135.4902 | 568.2487 | H | **689.3617** | 345.1845 | 672.3352 | 336.6712 | 671.3511 | 336.1792 | 5 |
| 11 | **1316.5641** | 658.7857 | 1299.5375 | 650.2724 | 1298.5535 | 649.7804 | Y | **552.3028** | **276.6550** | 535.2762 | 268.1418 | 534.2922 | 267.6498 | 4 |
| 12 | **1429.6482** | 715.3277 | 1412.6216 | 706.8144 | 1411.6376 | 706.3224 | L | **389.2395** | 195.1234 | 372.2129 | 186.6101 | 371.2289 | 186.1181 | 3 |
| 13 | **1558.6908** | 779.8490 | **1541.6642** | 771.3357 | 1540.6802 | 770.8437 | E | **276.1554** | 138.5813 | 259.1288 | 130.0681 | 258.1448 | 129.5761 | 2 |
| 14 | | | | | | | K | 147.1128 | 74.0600 | 130.0863 | 65.5468 | | | 1 |

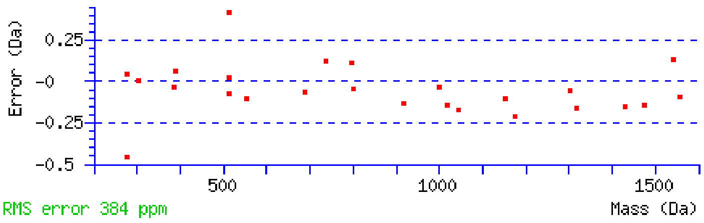


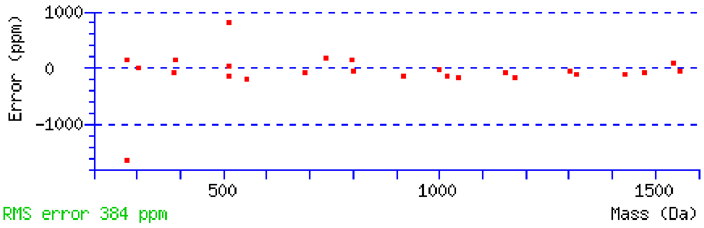


### All matches to this query

| Score | Mr(calc): | Delta | Sequence |
|---|---|---|---|
| 80.9 | 1703.7890 | 0.0010 | DNATEEEILHYLEK |
| 14.1 | 1703.8011 | -0.0111 | YIVRNTMNCYLEK |
| 14.1 | 1703.8011 | -0.0111 | YIVRNTMNCYLEK |
| 10.1 | 1703.7987 | -0.0088 | KTSVADSMLRMLEK |
| 10.1 | 1703.7987 | -0.0088 | KTSVADSMLRMLEK |
| 5.6 | 1703.7987 | -0.0088 | KTSVADSMLRMLEK |
| 5.6 | 1703.7987 | -0.0088 | KTSVADSMLRMLEK |
| 5.0 | 1703.7896 | 0.0003 | SLVKSAEGSTVPLTR |
| 5.0 | 1703.7896 | 0.0003 | SLVKSAEGSTVPLTR |
| 5.0 | 1703.7896 | 0.0003 | SLVKSAEGSTVPLTR |

---

Spectrum No: 224; Query: 2105; Rank: 1

---

## Peptide View

MS/MS Fragmentation of **FVLLMTNCYATPSSNSTDPVK**
Found in **IPI00198021**, Tax_Id=10116 Gene_Symbol=Umod Uromodulin precursor

Match to Query 2105: 2361.094468 from(1181.554510,2+)
Title: 100101RatKid_NS_deglyco_27.2959.2959.2.dta
Data file K:\NewmanPaper\Piliang\3SubProteomes\Piliang3SP\mgf5ppm\ERLIC_3SubProteomes5ppm.mgf

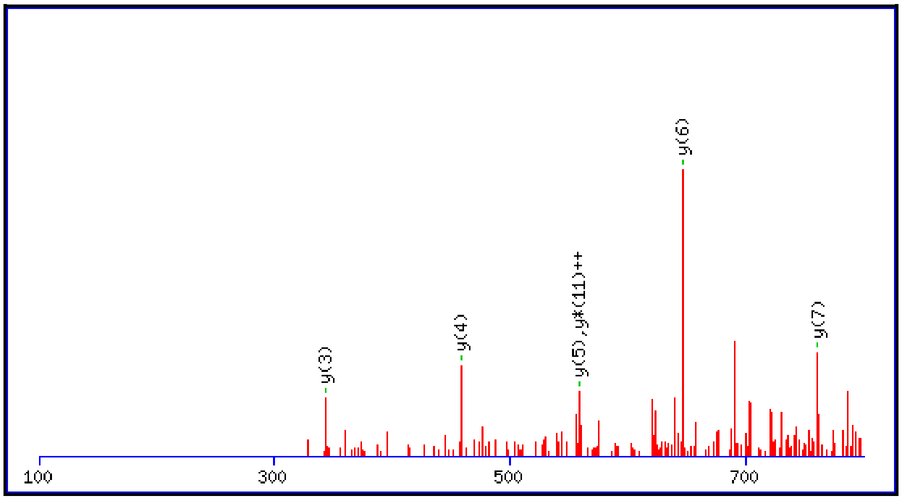

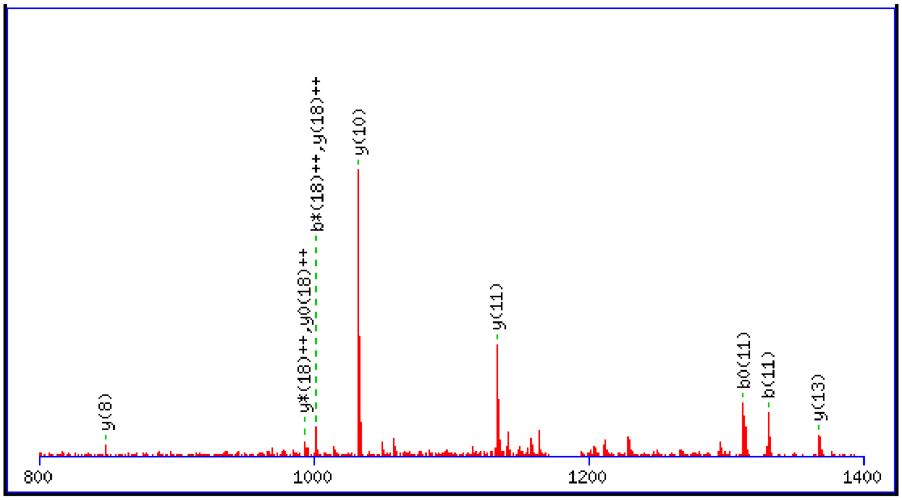


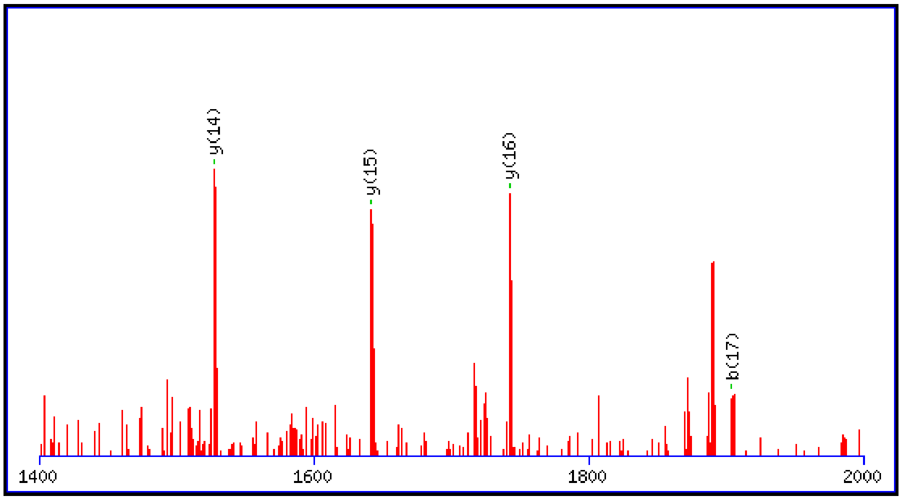


**Monoisotopic mass of neutral peptide Mr(calc):** 2361.0869
**Fixed modifications:** Carbamidomethyl (C)
**Variable modifications:**
**M5** : Oxidation (M)
**N15** : Deamidated_N (N)
**Ions Score:** 81 **Expect:** 2.7e-006
**Matches (Bold Red):** 20/212 fragment ions using 30 most intense peaks

| # | b | $b^{++}$ | b* | $b^{*++}$ | $b^{0}$ | $b^{0++}$ | Seq. | y | $y^{++}$ | y* | $y^{*++}$ | $y^{0}$ | $y^{0++}$ | # |
|---|---|---|---|---|---|---|---|---|---|---|---|---|---|---|
| 1 | 148.0757 | 74.5415 | | | | | F | | | | | | | 21 |
| 2 | 247.1441 | 124.0757 | | | | | V | 2215.0257 | 1108.0165 | 2197.9992 | 1099.5032 | 2197.0151 | 1099.0112 | 20 |
| 3 | 360.2282 | 180.6177 | | | | | L | 2115.9573 | 1058.4823 | 2098.9307 | 1049.9690 | 2097.9467 | 1049.4770 | 19 |
| 4 | 473.3122 | 237.1598 | | | | | L | 2002.8732 | **1001.9403** | 1985.8467 | **993.4270** | 1984.8627 | **992.9350** | 18 |
| 5 | 620.3476 | 310.6775 | | | | | M | 1889.7892 | 945.3982 | 1872.7626 | 936.8849 | 1871.7786 | 936.3929 | 17 |
| 6 | 721.3953 | 361.2013 | | | 703.3847 | 352.1960 | T | **1742.7538** | 871.8805 | 1725.7272 | 863.3672 | 1724.7432 | 862.8752 | 16 |
| 7 | 835.4382 | 418.2228 | 818.4117 | 409.7095 | 817.4277 | 409.2175 | N | **1641.7061** | 821.3567 | 1624.6795 | 812.8434 | 1623.6955 | 812.3514 | 15 |
| 8 | 995.4689 | 498.2381 | 978.4423 | 489.7248 | 977.4583 | 489.2328 | C | **1527.6632** | 764.3352 | 1510.6366 | 755.8219 | 1509.6526 | 755.3299 | 14 |
| 9 | 1158.5322 | 579.7697 | 1141.5057 | 571.2565 | 1140.5217 | 570.7645 | Y | **1367.6325** | 684.3199 | 1350.6060 | 675.8066 | 1349.6219 | 675.3146 | 13 |
| 10 | 1229.5693 | 615.2883 | 1212.5428 | 606.7750 | 1211.5588 | 606.2830 | A | 1204.5692 | 602.7882 | 1187.5426 | 594.2750 | 1186.5586 | 593.7829 | 12 |
| 11 | **1330.6170** | 665.8121 | 1313.5905 | 657.2989 | **1312.6064** | 656.8069 | T | **1133.5321** | 567.2697 | 1116.5055 | **558.7564** | 1115.5215 | 558.2644 | 11 |
| 12 | 1427.6698 | 714.3385 | 1410.6432 | 705.8253 | 1409.6592 | 705.3332 | P | **1032.4844** | 516.7458 | 1015.4578 | 508.2326 | 1014.4738 | 507.7405 | 10 |
| 13 | 1514.7018 | 757.8545 | 1497.6753 | 749.3413 | 1496.6912 | 748.8493 | S | 935.4316 | 468.2195 | 918.4051 | 459.7062 | 917.4211 | 459.2142 | 9 |
| 14 | 1601.7338 | 801.3706 | 1584.7073 | 792.8573 | 1583.7233 | 792.3653 | S | **848.3996** | 424.7034 | 831.3730 | 416.1902 | 830.3890 | 415.6982 | 8 |
| 15 | 1716.7608 | 858.8840 | 1699.7342 | 850.3707 | 1698.7502 | 849.8787 | N | **761.3676** | 381.1874 | 744.3410 | 372.6741 | 743.3570 | 372.1821 | 7 |
| 16 | 1803.7928 | 902.4000 | 1786.7662 | 893.8868 | 1785.7822 | 893.3948 | S | **646.3406** | 323.6740 | 629.3141 | 315.1607 | 628.3301 | 314.6687 | 6 |
| 17 | **1904.8405** | 952.9239 | 1887.8139 | 944.4106 | 1886.8299 | 943.9186 | T | **559.3086** | 280.1579 | 542.2821 | 271.6447 | 541.2980 | 271.1527 | 5 |
| 18 | 2019.8674 | 1010.4373 | 2002.8409 | **1001.9241** | 2001.8569 | 1001.4321 | D | **458.2609** | 229.6341 | 441.2344 | 221.1208 | 440.2504 | 220.6288 | 4 |

| | | | | | | | | | | | | | | |
|---|---|---|---|---|---|---|---|---|---|---|---|---|---|---|
| **19** | 2116.9202 | 1058.9637 | 2099.8936 | 1050.4505 | 2098.9096 | 1049.9584 | **P** | 343.2340 | 172.1206 | 326.2074 | 163.6074 | | | **3** |
| **20** | 2215.9886 | 1108.4979 | 2198.9620 | 1099.9847 | 2197.9780 | 1099.4927 | **V** | 246.1812 | 123.5942 | 229.1547 | 115.0810 | | | **2** |
| **21** | | | | | | | **K** | 147.1128 | 74.0600 | 130.0863 | 65.5468 | | | **1** |

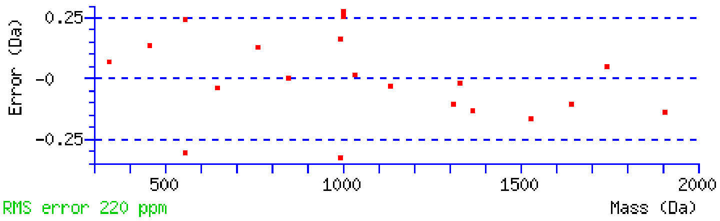


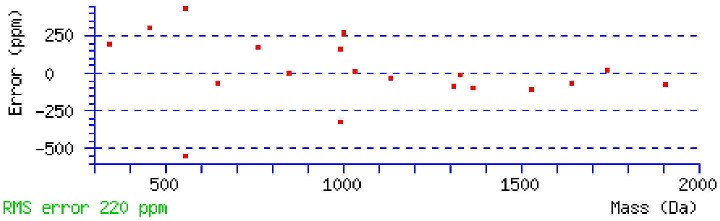


### All matches to this query

| Score | Mr(calc): | Delta | Sequence |
|---|---|---|---|
| 80.7 | 2361.0869 | 0.0076 | FVLLMTNCYATPSSNSTDPVK |
| 23.4 | 2361.0869 | 0.0076 | FVLLMTNCYATPSSNSTDPVK |
| 8.2 | 2361.1109 | -0.0165 | NLMSMELTDTDIRPKFNLK |
| 8.0 | 2361.1109 | -0.0165 | NLMSMELTDTDIRPKFNLK |
| 7.0 | 2360.1028 | 0.9916 | FVLLMTNCYATPSSNSTDPVK |
| 3.8 | 2361.1046 | -0.0101 | SAANWNTTGEVVVSMEPEVPVK |
| 2.2 | 2359.0723 | 2.0222 | KNSSAEKSVVSLAGQAPHFSR |
| 0.6 | 2361.0907 | 0.0038 | MNFDHKNETLSISVQPGEGNK |
| 0.6 | 2360.0728 | 1.0217 | VSTDGADNRDSLDPQALSDEVR |
| 0.1 | 2361.1109 | -0.0165 | NLMSMELTDTDIRPKFNLK |

---

Spectrum No: 225; Query: 913; Rank: 1

---

## Peptide View

MS/MS Fragmentation of **DMSDGFISNLTIQR**
Found in **IPI00209863**, Tax_Id=10116 Gene_Symbol=P4ha1 Prolyl 4-hydroxylase subunit alpha-1 precursor

Match to Query 913: 1612.745328 from(807.379940,2+)
Title: 100101RatKid_NS_deglyco_19.3844.3844.2.dta
Data file K:\NewmanPaper\Piliang\3SubProteomes\Piliang3SP\mgf5ppm\ERLIC_3SubProteomes5ppm.mgf

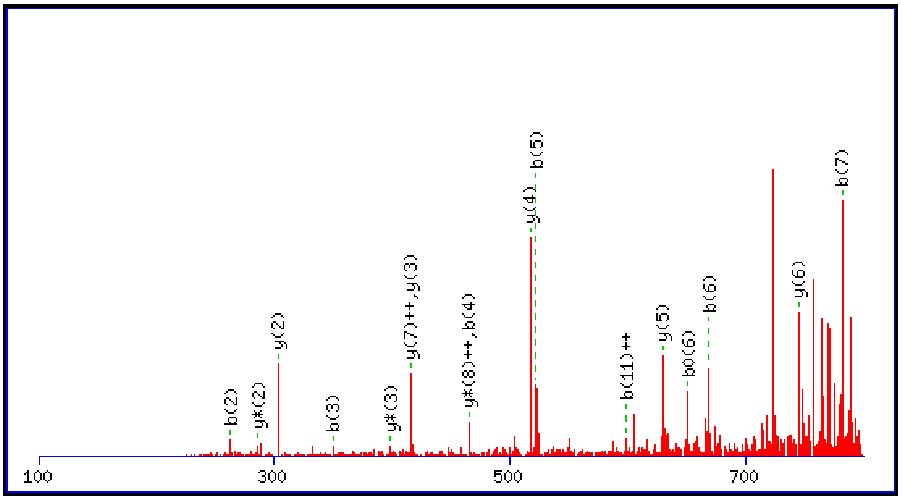

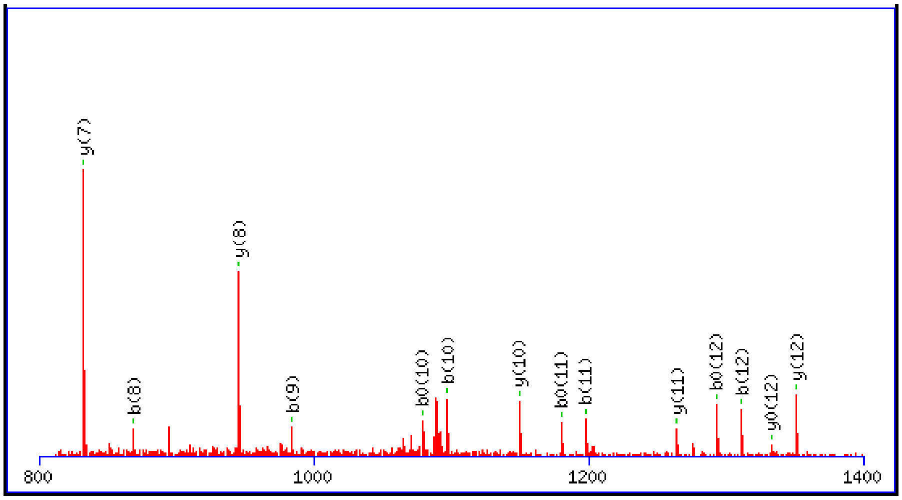


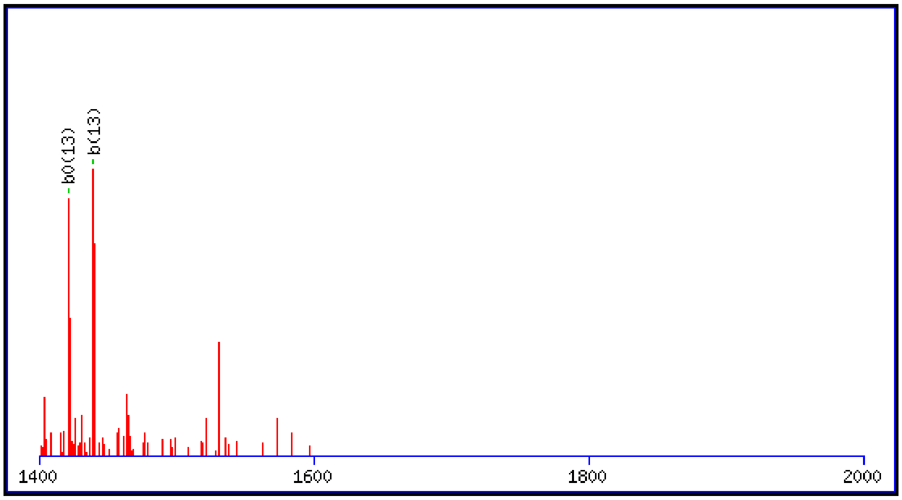


**Monoisotopic mass of neutral peptide Mr(calc):** 1612.7403
**Fixed modifications:** Carbamidomethyl (C)
**Variable modifications:**
**M2** : Oxidation (M)
**N9** : Deamidated_N (N)
**Ions Score:** 81 **Expect:** 1.6e-006
**Matches (Bold Red):** 33/134 fragment ions using 58 most intense peaks

| # | b | $b^{++}$ | b* | $b^{*++}$ | $b^{0}$ | $b^{0++}$ | Seq. | y | $y^{++}$ | y* | $y^{*++}$ | $y^{0}$ | $y^{0++}$ | # |
|---|---|---|---|---|---|---|---|---|---|---|---|---|---|---|
| 1 | 116.0342 | 58.5207 | | | 98.0237 | 49.5155 | D | | | | | | | 14 |
| 2 | **263.0696** | 132.0384 | | | 245.0591 | 123.0332 | M | 1498.7206 | 749.8639 | 1481.6941 | 741.3507 | 1480.7101 | 740.8587 | 13 |
| 3 | **350.1017** | 175.5545 | | | 332.0911 | 166.5492 | S | **1351.6852** | 676.3462 | 1334.6587 | 667.8330 | **1333.6746** | 667.3410 | 12 |
| 4 | **465.1286** | 233.0679 | | | 447.1180 | 224.0627 | D | **1264.6532** | 632.8302 | 1247.6266 | 624.3170 | 1246.6426 | 623.8249 | 11 |
| 5 | **522.1501** | 261.5787 | | | 504.1395 | 252.5734 | G | **1149.6262** | 575.3168 | 1132.5997 | 566.8035 | 1131.6157 | 566.3115 | 10 |
| 6 | **669.2185** | 335.1129 | | | **651.2079** | 326.1076 | F | 1092.6048 | 546.8060 | 1075.5782 | 538.2928 | 1074.5942 | 537.8007 | 9 |
| 7 | **782.3025** | 391.6549 | | | 764.2920 | 382.6496 | I | **945.5364** | 473.2718 | 928.5098 | **464.7585** | 927.5258 | 464.2665 | 8 |
| 8 | **869.3346** | 435.1709 | | | 851.3240 | 426.1656 | S | **832.4523** | **416.7298** | 815.4258 | 408.2165 | 814.4417 | 407.7245 | 7 |
| 9 | **984.3615** | 492.6844 | 967.3350 | 484.1711 | 966.3509 | 483.6791 | N | **745.4203** | 373.2138 | 728.3937 | 364.7005 | 727.4097 | 364.2085 | 6 |
| 10 | **1097.4456** | 549.2264 | 1080.4190 | 540.7131 | **1079.4350** | 540.2211 | L | **630.3933** | 315.7003 | 613.3668 | 307.1870 | 612.3828 | 306.6950 | 5 |
| 11 | **1198.4932** | **599.7503** | 1181.4667 | 591.2370 | **1180.4827** | 590.7450 | T | **517.3093** | 259.1583 | 500.2827 | 250.6450 | 499.2987 | 250.1530 | 4 |
| 12 | **1311.5773** | 656.2923 | 1294.5508 | 647.7790 | **1293.5667** | 647.2870 | I | **416.2616** | 208.6344 | **399.2350** | 200.1212 | | | 3 |
| 13 | **1439.6359** | 720.3216 | 1422.6093 | 711.8083 | **1421.6253** | 711.3163 | Q | **303.1775** | 152.0924 | **286.1510** | 143.5791 | | | 2 |
| 14 | | | | | | | R | 175.1190 | 88.0631 | 158.0924 | 79.5498 | | | 1 |

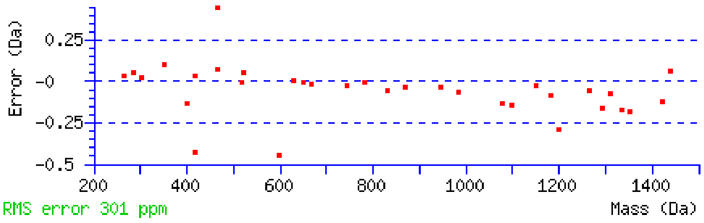


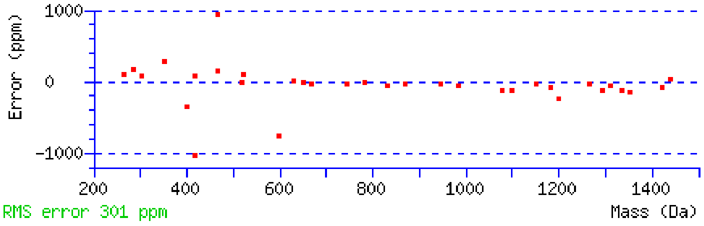


### All matches to this query

| Score | Mr(calc): | Delta | Sequence |
|---|---|---|---|
| 80.7 | 1612.7403 | 0.0050 | DMSDGFISNLTIQR |
| 29.8 | 1611.7563 | 0.9891 | DMSDGFISNLTIQR |
| 10.5 | 1612.7457 | -0.0004 | ADAADGSLASSATIRK |
| 7.8 | 1612.7354 | 0.0099 | MMALQTDIVDLQR |
| 7.3 | 1612.7529 | -0.0075 | SFMNQSHLQTHQR |
| 6.7 | 1612.7419 | 0.0034 | SAEPMGITLKLNDK |
| 3.0 | 1612.7403 | 0.0050 | LLDDSEYTCTIQR |
| 2.1 | 1611.7440 | 1.0014 | QEISEINRMIQR |
| 0.4 | 1611.7450 | 1.0003 | MAEGELNIDSFITR |
| 0.3 | 1612.7596 | -0.0143 | SELKEDSVEELKK |

---

Spectrum No: 226; Query: 2454; Rank: 1

---

## Peptide View

MS/MS Fragmentation of **DLVVGLYSQWMNNSDNNPIHPNLR**
Found in **IPI00230862**, Tax_Id=10116 Gene_Symbol=Anpep Aminopeptidase N

Match to Query 2454: 2812.319232 from(938.447020,3+)
Title: 091008RatKidney_NoSalt_27.4170.4170.3.dta
Data file K:\NewmanPaper\Piliang\3SubProteomes\Piliang3SP\mgf5ppm\ERLIC_3SubProteomes5ppm.mgf

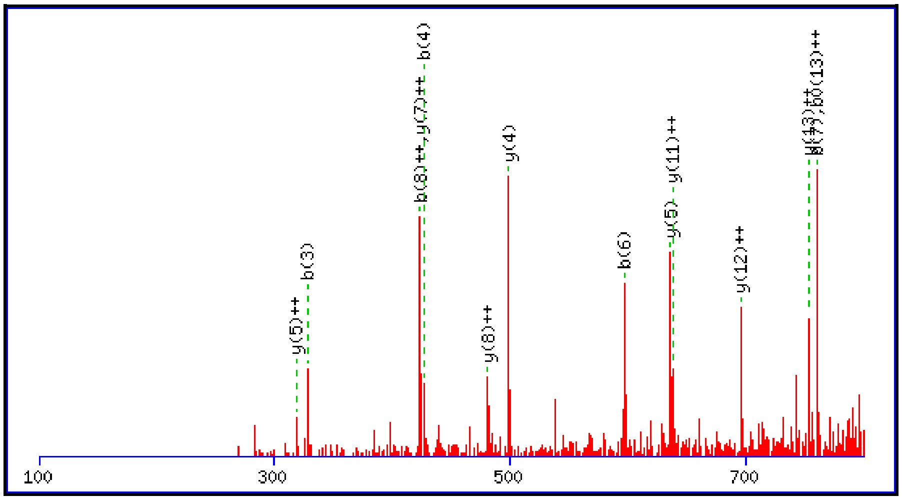

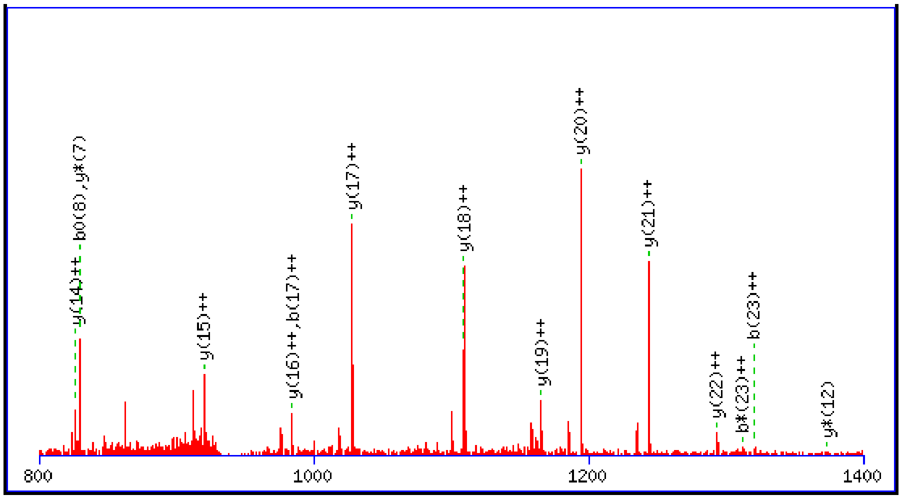


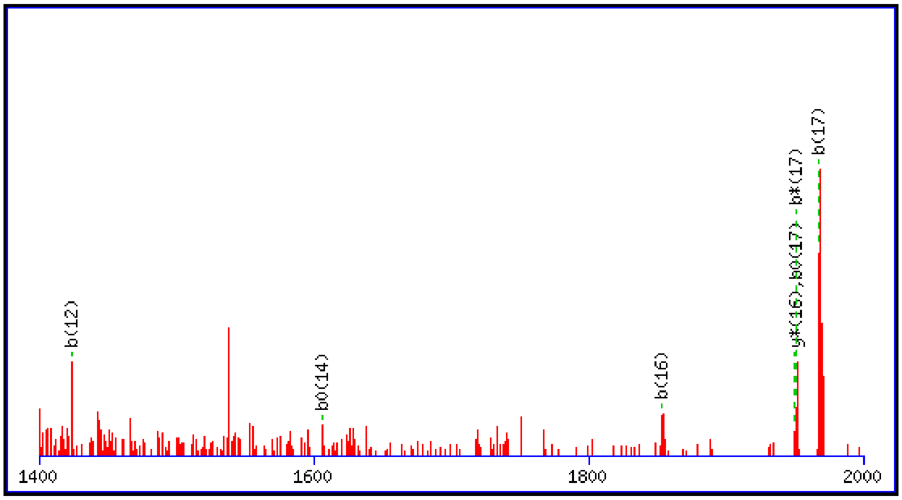


**Monoisotopic mass of neutral peptide Mr(calc):** 2812.3239
**Fixed modifications:** Carbamidomethyl (C)
**Variable modifications:**
**M11** : Oxidation (M)
**N12** : Deamidated_N (N)
**Ions Score:** 80 **Expect:** 3e-006
**Matches (Bold Red):** 36/242 fragment ions using 64 most intense peaks

| # | b | $b^{++}$ | b* | $b^{*++}$ | $b^0$ | $b^{0++}$ | Seq. | y | $y^{++}$ | y* | $y^{*++}$ | $y^0$ | $y^{0++}$ | # |
|---|---|---|---|---|---|---|---|---|---|---|---|---|---|---|
| 1 | 116.0342 | 58.5207 | | | 98.0237 | 49.5155 | D | | | | | | | 24 |
| 2 | 229.1183 | 115.0628 | | | 211.1077 | 106.0575 | L | 2698.3042 | 1349.6557 | 2681.2777 | 1341.1425 | 2680.2936 | 1340.6505 | 23 |
| 3 | **328.1867** | 164.5970 | | | 310.1761 | 155.5917 | V | 2585.2201 | **1293.1137** | 2568.1936 | 1284.6004 | 2567.2096 | 1284.1084 | 22 |
| 4 | **427.2551** | 214.1312 | | | 409.2445 | 205.1259 | V | 2486.1517 | **1243.5795** | 2469.1252 | 1235.0662 | 2468.1412 | 1234.5742 | 21 |
| 5 | 484.2766 | 242.6419 | | | 466.2660 | 233.6366 | G | 2387.0833 | **1194.0453** | 2370.0568 | 1185.5320 | 2369.0727 | 1185.0400 | 20 |
| 6 | **597.3606** | 299.1840 | | | 579.3501 | 290.1787 | L | 2330.0619 | **1165.5346** | 2313.0353 | 1157.0213 | 2312.0513 | 1156.5293 | 19 |
| 7 | **760.4240** | 380.7156 | | | 742.4134 | 371.7103 | Y | 2216.9778 | **1108.9925** | 2199.9512 | 1100.4793 | 2198.9672 | 1099.9872 | 18 |
| 8 | 847.4560 | **424.2316** | | | **829.4454** | 415.2264 | S | 2053.9145 | **1027.4609** | 2036.8879 | 1018.9476 | 2035.9039 | 1018.4556 | 17 |
| 9 | 975.5146 | 488.2609 | 958.4880 | 479.7477 | 957.5040 | 479.2556 | Q | 1966.8824 | **983.9449** | **1949.8559** | 975.4316 | 1948.8719 | 974.9396 | 16 |
| 10 | 1161.5939 | 581.3006 | 1144.5673 | 572.7873 | 1143.5833 | 572.2953 | W | 1838.8239 | **919.9156** | 1821.7973 | 911.4023 | 1820.8133 | 910.9103 | 15 |
| 11 | 1308.6293 | 654.8183 | 1291.6027 | 646.3050 | 1290.6187 | 645.8130 | M | 1652.7445 | **826.8759** | 1635.7180 | 818.3626 | 1634.7340 | 817.8706 | 14 |
| 12 | **1423.6562** | 712.3318 | 1406.6297 | 703.8185 | 1405.6457 | 703.3265 | N | 1505.7091 | **753.3582** | 1488.6826 | 744.8449 | 1487.6986 | 744.3529 | 13 |
| 13 | 1537.6992 | 769.3532 | 1520.6726 | 760.8399 | 1519.6886 | **760.3479** | N | 1390.6822 | **695.8447** | **1373.6557** | 687.3315 | 1372.6716 | 686.8395 | 12 |
| 14 | 1624.7312 | 812.8692 | 1607.7046 | 804.3560 | **1606.7206** | 803.8639 | S | 1276.6393 | **638.8233** | 1259.6127 | 630.3100 | 1258.6287 | 629.8180 | 11 |
| 15 | 1739.7581 | 870.3827 | 1722.7316 | 861.8694 | 1721.7476 | 861.3774 | D | 1189.6072 | 595.3073 | 1172.5807 | 586.7940 | 1171.5967 | 586.3020 | 10 |
| 16 | **1853.8011** | 927.4042 | 1836.7745 | 918.8909 | 1835.7905 | 918.3989 | N | 1074.5803 | 537.7938 | 1057.5538 | 529.2805 | | | 9 |
| 17 | **1967.8440** | **984.4256** | **1950.8174** | 975.9124 | **1949.8334** | 975.4203 | N | 960.5374 | **480.7723** | 943.5108 | 472.2591 | | | 8 |
| 18 | 2064.8967 | 1032.9520 | 2047.8702 | 1024.4387 | 2046.8862 | 1023.9467 | P | 846.4944 | **423.7509** | **829.4679** | 415.2376 | | | 7 |

| | | | | | | | | | | | | | | |
|---|---|---|---|---|---|---|---|---|---|---|---|---|---|---|
| **19** | 2177.9808 | 1089.4940 | 2160.9543 | 1080.9808 | 2159.9702 | 1080.4888 | **I** | 749.4417 | 375.2245 | 732.4151 | 366.7112 | | | **6** |
| **20** | 2315.0397 | 1158.0235 | 2298.0132 | 1149.5102 | 2297.0292 | 1149.0182 | **H** | **636.3576** | **318.6824** | 619.3311 | 310.1692 | | | **5** |
| **21** | 2412.0925 | 1206.5499 | 2395.0659 | 1198.0366 | 2394.0819 | 1197.5446 | **P** | **499.2987** | 250.1530 | 482.2722 | 241.6397 | | | **4** |
| **22** | 2526.1354 | 1263.5713 | 2509.1089 | 1255.0581 | 2508.1248 | 1254.5661 | **N** | 402.2459 | 201.6266 | 385.2194 | 193.1133 | | | **3** |
| **23** | 2639.2195 | **1320.1134** | 2622.1929 | **1311.6001** | 2621.2089 | 1311.1081 | **L** | 288.2030 | 144.6051 | 271.1765 | 136.0919 | | | **2** |
| **24** | | | | | | | **R** | 175.1190 | 88.0631 | 158.0924 | 79.5498 | | | **1** |

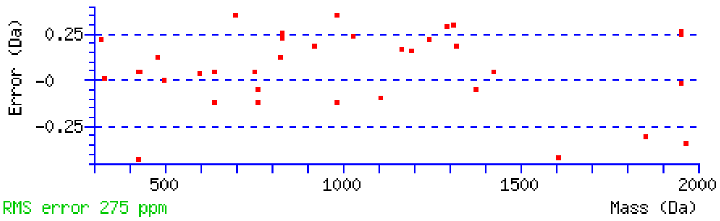


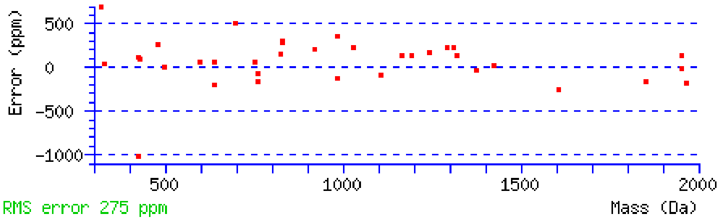


### All matches to this query

| Score | Mr(calc): | Delta | Sequence |
|---|---|---|---|
| 80.4 | 2812.3239 | -0.0046 | DLVVGLYSQWMNNSDNNPIHPNLR |
| 78.4 | 2812.3239 | -0.0046 | DLVVGLYSQWMNNSDNNPIHPNLR |
| 67.5 | 2812.3239 | -0.0046 | DLVVGLYSQWMNNSDNNPIHPNLR |
| 65.6 | 2812.3239 | -0.0046 | DLVVGLYSQWMNNSDNNPIHPNLR |
| 65.4 | 2812.3239 | -0.0046 | DLVVGLYSQWMNNSDNNPIHPNLR |
| 9.2 | 2811.3398 | 0.9794 | DLVVGLYSQWMNNSDNNPIHPNLR |
| 3.4 | 2812.3382 | -0.0190 | VPQRTSSIATALNTSGAGGSRPAQAVR |
| 2.2 | 2811.3051 | 1.0141 | LHNGGDVTFHDVLSMLSYRSVDIR |
| 1.2 | 2811.3133 | 1.0059 | LLVVDFSSQALFQDKNSSQVLGEK |

---

Spectrum No: 227; Query: 359; Rank: 1

---

## Peptide View

MS/MS Fragmentation of **LVNLTFLDLTR**
Found in **IPI00360317**, Tax_Id=10116 Gene_Symbol=Cd180_predicted CD180 antigen

Match to Query 359: 1304.734928 from(653.374740,2+)
Title: 100101RatKid_NS_deglyco_20.4985.4985.2.dta
Data file K:\NewmanPaper\Piliang\3SubProteomes\Piliang3SP\mgf5ppm\ERLIC_3SubProteomes5ppm.mgf

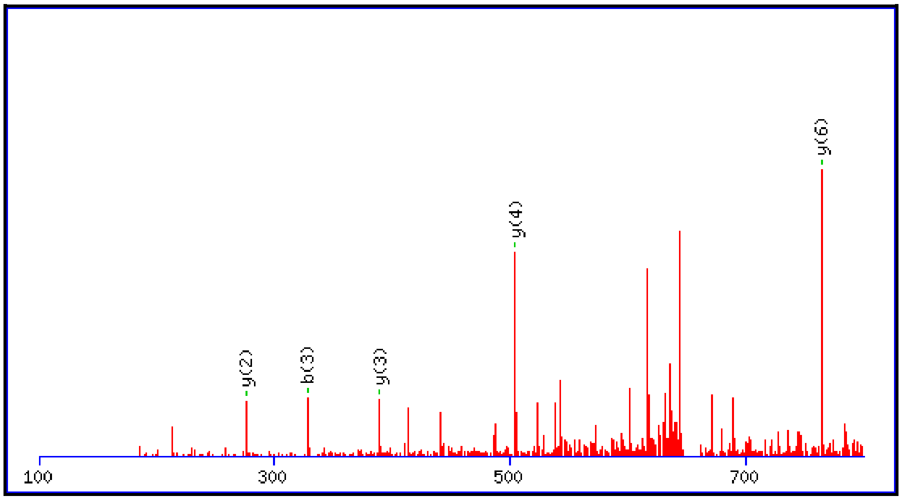

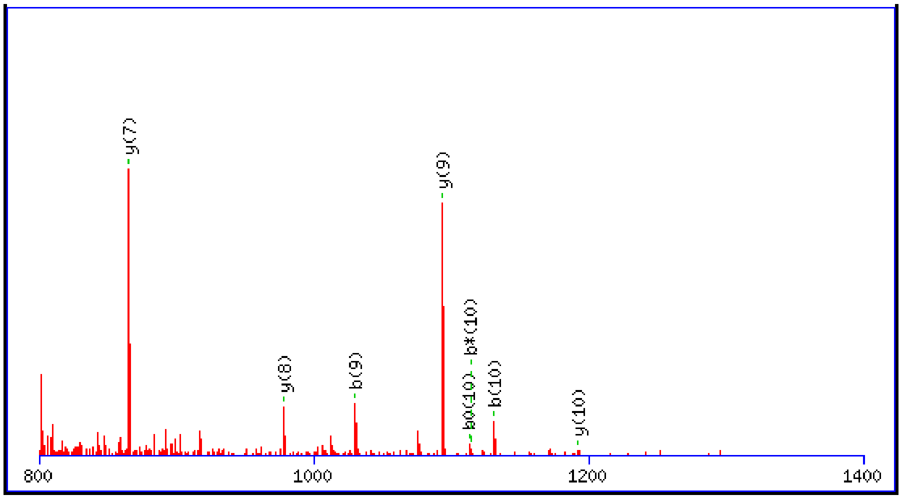


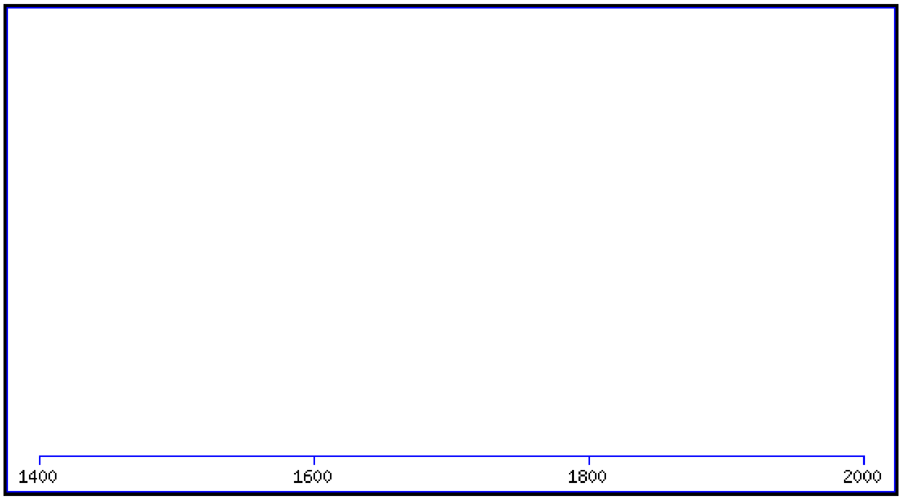


**Monoisotopic mass of neutral peptide Mr(calc):** 1304.7340
**Fixed modifications:** Carbamidomethyl (C)
**Variable modifications:**
**N3** : Deamidated_N (N)
**Ions Score:** 80 **Expect:** 5.2e-007
**Matches (Bold Red):** 13/106 fragment ions using 21 most intense peaks

| # | b | $b^{++}$ | b* | $b^{*++}$ | $b^{0}$ | $b^{0++}$ | Seq. | y | $y^{++}$ | y* | $y^{*++}$ | $y^{0}$ | $y^{0++}$ | # |
|---|---|---|---|---|---|---|---|---|---|---|---|---|---|---|
| 1 | 114.0913 | 57.5493 | | | | | L | | | | | | | 11 |
| 2 | 213.1598 | 107.0835 | | | | | V | **1192.6572** | 596.8322 | 1175.6307 | 588.3190 | 1174.6466 | 587.8270 | 10 |
| 3 | **328.1867** | 164.5970 | 311.1601 | 156.0837 | | | N | **1093.5888** | 547.2980 | 1076.5622 | 538.7848 | 1075.5782 | 538.2928 | 9 |
| 4 | 441.2708 | 221.1390 | 424.2442 | 212.6257 | | | L | **978.5619** | 489.7846 | 961.5353 | 481.2713 | 960.5513 | 480.7793 | 8 |
| 5 | 542.3184 | 271.6629 | 525.2919 | 263.1496 | 524.3079 | 262.6576 | T | **865.4778** | 433.2425 | 848.4512 | 424.7293 | 847.4672 | 424.2373 | 7 |
| 6 | 689.3868 | 345.1971 | 672.3603 | 336.6838 | 671.3763 | 336.1918 | F | **764.4301** | 382.7187 | 747.4036 | 374.2054 | 746.4196 | 373.7134 | 6 |
| 7 | 802.4709 | 401.7391 | 785.4444 | 393.2258 | 784.4603 | 392.7338 | L | 617.3617 | 309.1845 | 600.3352 | 300.6712 | 599.3511 | 300.1792 | 5 |
| 8 | 917.4979 | 459.2526 | 900.4713 | 450.7393 | 899.4873 | 450.2473 | D | **504.2776** | 252.6425 | 487.2511 | 244.1292 | 486.2671 | 243.6372 | 4 |
| 9 | **1030.5819** | 515.7946 | 1013.5554 | 507.2813 | 1012.5714 | 506.7893 | L | **389.2507** | 195.1290 | 372.2241 | 186.6157 | 371.2401 | 186.1237 | 3 |
| 10 | **1131.6296** | 566.3184 | **1114.6030** | 557.8052 | **1113.6190** | 557.3132 | T | **276.1666** | 138.5870 | 259.1401 | 130.0737 | 258.1561 | 129.5817 | 2 |
| 11 | | | | | | | R | 175.1190 | 88.0631 | 158.0924 | 79.5498 | | | 1 |

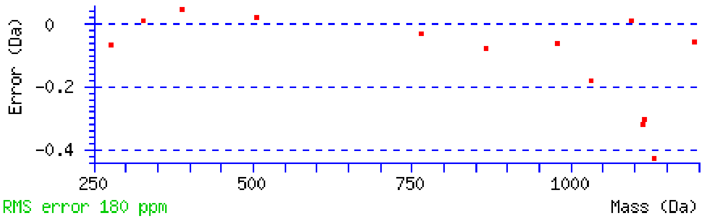


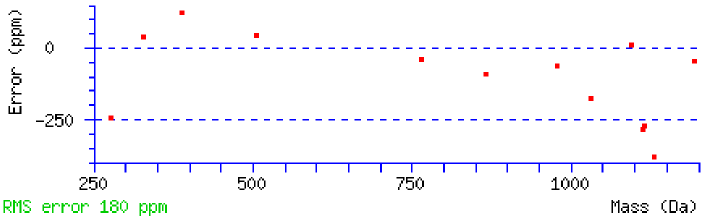

### All matches to this query

| Score | Mr(calc): | Delta | Sequence |
|---|---|---|---|
| 80.2 | 1304.7340 | 0.0009 | LVNLTFLDLTR |
| 6.1 | 1304.7241 | 0.0109 | QKAIDLLYWR |
| 5.0 | 1304.7374 | -0.0024 | LITLMGQKTSLT |
| 4.7 | 1304.7452 | -0.0103 | IVNFVVNSKKR |
| 2.8 | 1304.7452 | -0.0103 | SLGQIYRVLEK |
| 2.0 | 1304.7313 | 0.0037 | ERKPYRSLTR |

---

Spectrum No: 228; Query: 792; Rank: 1

---

## Peptide View

MS/MS Fragmentation of **VVNVSELYGTPCTK**
Found in **IPI00206668**, Tax_Id=10116 Gene_Symbol=Entpd1 Ectonucleoside triphosphate diphosphohydrolase 1

Match to Query 792: 1566.761568 from(784.388060,2+)
Title: 091008RatKidney_NoSalt_30.1984.1984.2.dta
Data file K:\NewmanPaper\Piliang\3SubProteomes\Piliang3SP\mgf5ppm\ERLIC_3SubProteomes5ppm.mgf

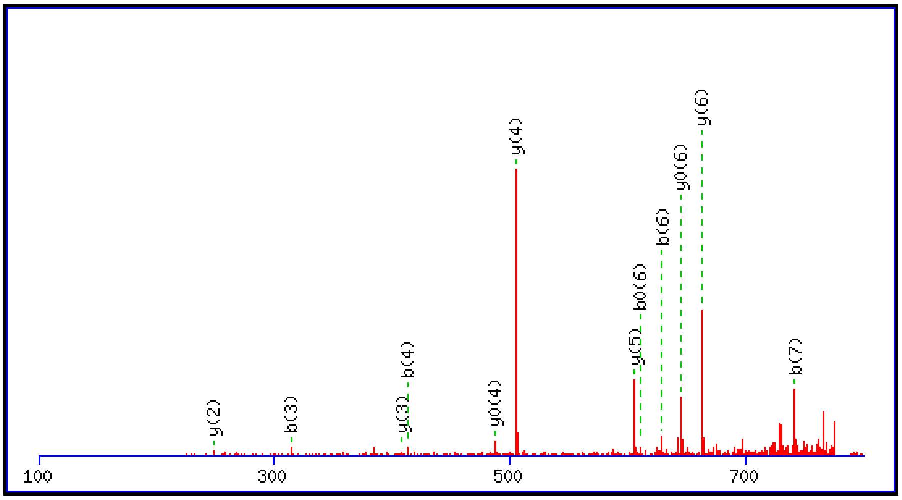


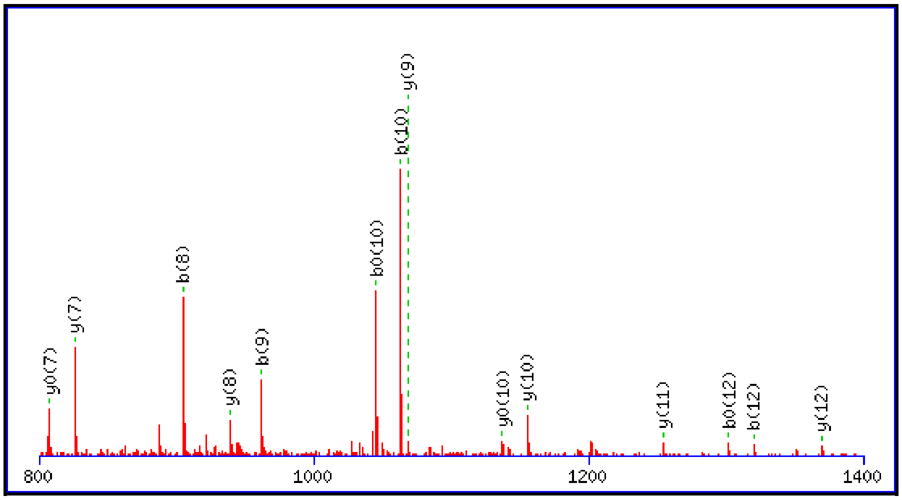

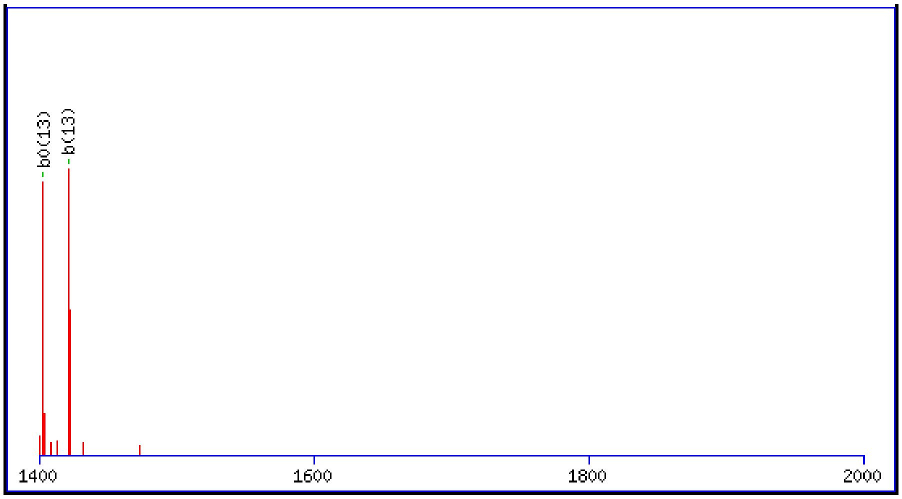


**Monoisotopic mass of neutral peptide Mr(calc):** 1566.7600
**Fixed modifications:** Carbamidomethyl (C)
**Variable modifications:**
**N3** : Deamidated_N (N)
**Ions Score:** 80 **Expect:** 1.7e-006
**Matches (Bold Red):** 28/142 fragment ions using 42 most intense peaks

| # | b | $b^{++}$ | b* | $b^{*++}$ | $b^0$ | $b^{0++}$ | Seq. | y | $y^{++}$ | y* | $y^{*++}$ | $y^0$ | $y^{0++}$ | # |
|---|---|---|---|---|---|---|---|---|---|---|---|---|---|---|
| 1 | 100.0757 | 50.5415 | | | | | V | | | | | | | 14 |
| 2 | 199.1441 | 100.0757 | | | | | V | 1468.6988 | 734.8530 | 1451.6723 | 726.3398 | 1450.6883 | 725.8478 | 13 |
| 3 | **314.1710** | 157.5892 | 297.1445 | 149.0759 | | | N | **1369.6304** | 685.3188 | 1352.6039 | 676.8056 | 1351.6198 | 676.3136 | 12 |
| 4 | **413.2395** | 207.1234 | 396.2129 | 198.6101 | | | V | **1254.6035** | 627.8054 | 1237.5769 | 619.2921 | 1236.5929 | 618.8001 | 11 |
| 5 | 500.2715 | 250.6394 | 483.2449 | 242.1261 | 482.2609 | 241.6341 | S | **1155.5351** | 578.2712 | 1138.5085 | 569.7579 | **1137.5245** | 569.2659 | 10 |
| 6 | **629.3141** | 315.1607 | 612.2875 | 306.6474 | **611.3035** | 306.1554 | E | **1068.5030** | 534.7552 | 1051.4765 | 526.2419 | 1050.4925 | 525.7499 | 9 |
| 7 | **742.3981** | 371.7027 | 725.3716 | 363.1894 | 724.3876 | 362.6974 | L | **939.4604** | 470.2339 | 922.4339 | 461.7206 | 921.4499 | 461.2286 | 8 |
| 8 | **905.4615** | 453.2344 | 888.4349 | 444.7211 | 887.4509 | 444.2291 | Y | **826.3764** | 413.6918 | 809.3498 | 405.1785 | **808.3658** | 404.6865 | 7 |
| 9 | **962.4829** | 481.7451 | 945.4564 | 473.2318 | 944.4724 | 472.7398 | G | **663.3130** | 332.1602 | 646.2865 | 323.6469 | **645.3025** | 323.1549 | 6 |
| 10 | **1063.5306** | 532.2689 | 1046.5041 | 523.7557 | **1045.5200** | 523.2637 | T | **606.2916** | 303.6494 | 589.2650 | 295.1362 | 588.2810 | 294.6441 | 5 |
| 11 | 1160.5834 | 580.7953 | 1143.5568 | 572.2821 | 1142.5728 | 571.7900 | P | **505.2439** | 253.1256 | 488.2173 | 244.6123 | **487.2333** | 244.1203 | 4 |
| 12 | **1320.6140** | 660.8107 | 1303.5875 | 652.2974 | **1302.6035** | 651.8054 | C | **408.1911** | 204.5992 | 391.1646 | 196.0859 | 390.1806 | 195.5939 | 3 |
| 13 | **1421.6617** | 711.3345 | 1404.6352 | 702.8212 | **1403.6511** | 702.3292 | T | **248.1605** | 124.5839 | 231.1339 | 116.0706 | 230.1499 | 115.5786 | 2 |
| 14 | | | | | | | K | 147.1128 | 74.0600 | 130.0863 | 65.5468 | | | 1 |

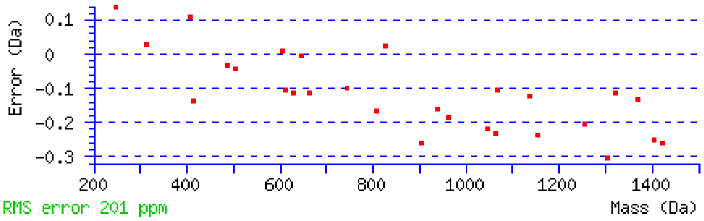


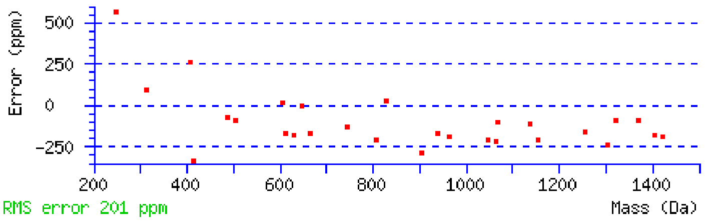


### All matches to this query

| Score | Mr(calc): | Delta | Sequence |
|---|---|---|---|
| 80.2 | 1566.7600 | 0.0016 | VVNVSELYGTPCTK |
| 7.4 | 1566.7599 | 0.0016 | YVELTEGVECIQK |
| 3.0 | 1566.7654 | -0.0039 | KGTEKQITPETQK |
| 2.9 | 1566.7712 | -0.0096 | ILYNTSQECLAVR |
| 1.1 | 1566.7542 | 0.0074 | DDPNSILLLGSKSK |
| 0.6 | 1566.7668 | -0.0052 | RQAFGKPRPTSDK |
| 0.6 | 1566.7668 | -0.0052 | RQAFGKPRPTSDK |

Spectrum No: 229; Query: 969; Rank: 1

# Peptide View

MS/MS Fragmentation of **NLQVYNATSNSLTVK**
Found in **IPI00189714**, Tax_Id=10116 Gene_Symbol=Col12a1 similar to Collagen alpha-1(XII) chain precursor

Match to Query 969: 1651.840148 from(826.927350,2+)
Title: 091008RatKidney_NH4Format01_23.2126.2126.2.dta
Data file K:\NewmanPaper\Piliang\3SubProteomes\Piliang3SP\mgf5ppm\ERLIC_3SubProteomes5ppm.mgf

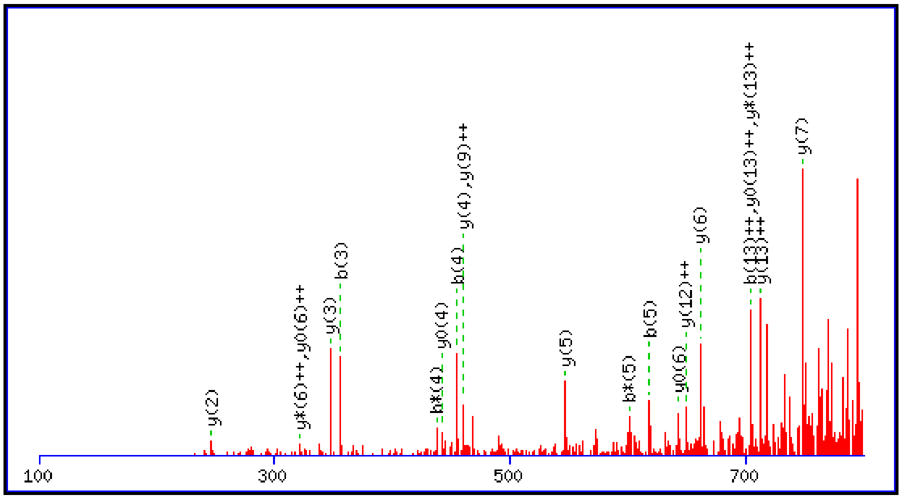


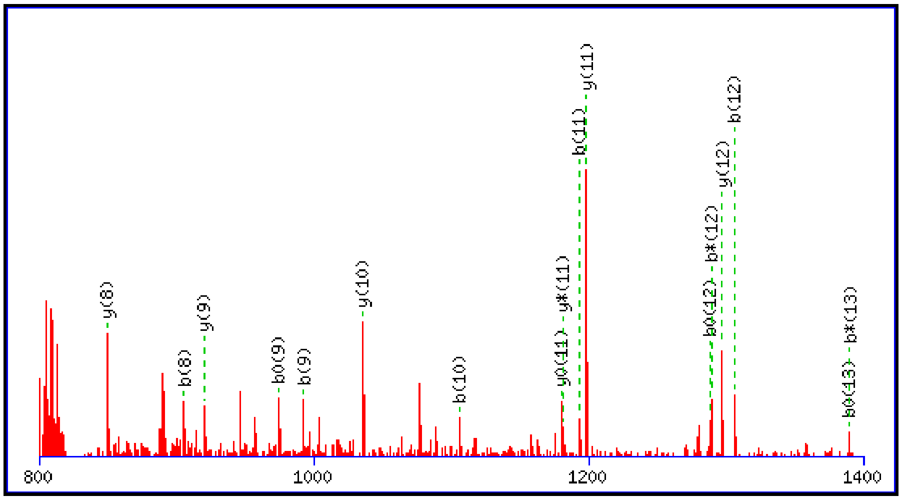


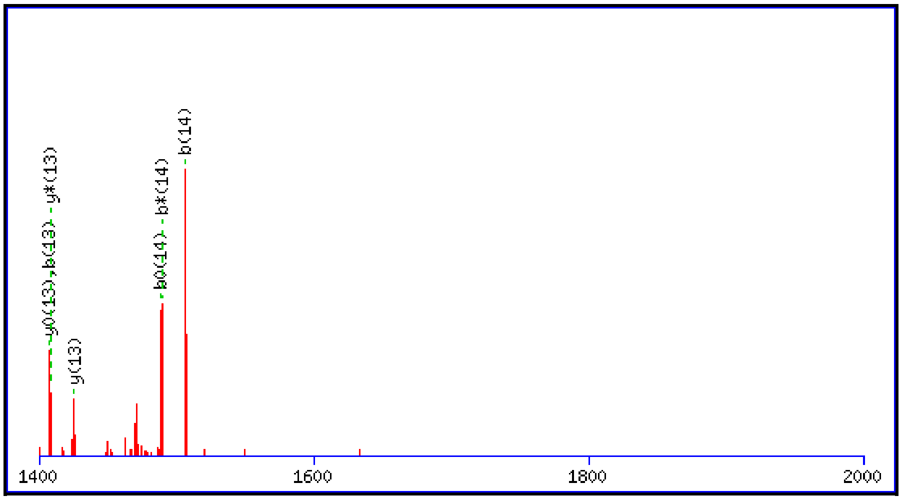

**Monoisotopic mass of neutral peptide Mr(calc):** 1651.8417
**Fixed modifications:** Carbamidomethyl (C)
**Variable modifications:**
**N6** : Deamidated_N (N)
**Ions Score:** 80 **Expect:** 1.6e-006
**Matches (Bold Red):** 45/150 fragment ions using 70 most intense peaks

| # | b | $b^{++}$ | b* | $b^{*++}$ | $b^{0}$ | $b^{0++}$ | Seq. | y | $y^{++}$ | y* | $y^{*++}$ | $y^{0}$ | $y^{0++}$ | # |
|---|---|---|---|---|---|---|---|---|---|---|---|---|---|---|
| 1 | 115.0502 | 58.0287 | 98.0237 | 49.5155 | | | N | | | | | | | 15 |
| 2 | 228.1343 | 114.5708 | 211.1077 | 106.0575 | | | L | 1538.8061 | 769.9067 | 1521.7795 | 761.3934 | 1520.7955 | 760.9014 | 14 |
| 3 | **356.1928** | 178.6001 | 339.1663 | 170.0868 | | | Q | **1425.7220** | **713.3646** | **1408.6954** | **704.8514** | **1407.7114** | **704.3594** | 13 |
| 4 | **455.2613** | 228.1343 | **438.2347** | 219.6210 | | | V | **1297.6634** | **649.3353** | 1280.6369 | 640.8221 | 1279.6529 | 640.3301 | 12 |
| 5 | **618.3246** | 309.6659 | **601.2980** | 301.1527 | | | Y | **1198.5950** | 599.8011 | **1181.5685** | 591.2879 | **1180.5844** | 590.7959 | 11 |
| 6 | 733.3515 | 367.1794 | 716.3250 | 358.6661 | | | N | **1035.5317** | 518.2695 | 1018.5051 | 509.7562 | 1017.5211 | 509.2642 | 10 |
| 7 | 804.3886 | 402.6980 | 787.3621 | 394.1847 | | | A | **920.5047** | **460.7560** | 903.4782 | 452.2427 | 902.4942 | 451.7507 | 9 |
| 8 | **905.4363** | 453.2218 | 888.4098 | 444.7085 | 887.4258 | 444.2165 | T | **849.4676** | 425.2374 | 832.4411 | 416.7242 | 831.4571 | 416.2322 | 8 |
| 9 | **992.4683** | 496.7378 | 975.4418 | 488.2245 | **974.4578** | 487.7325 | S | **748.4199** | 374.7136 | 731.3934 | 366.2003 | 730.4094 | 365.7083 | 7 |
| 10 | **1106.5113** | 553.7593 | 1089.4847 | 545.2460 | 1088.5007 | 544.7540 | N | **661.3879** | 331.1976 | 644.3614 | **322.6843** | **643.3774** | **322.1923** | 6 |
| 11 | **1193.5433** | 597.2753 | 1176.5168 | 588.7620 | 1175.5327 | 588.2700 | S | **547.3450** | 274.1761 | 530.3184 | 265.6629 | 529.3344 | 265.1709 | 5 |
| 12 | **1306.6274** | 653.8173 | **1289.6008** | 645.3040 | **1288.6168** | 644.8120 | L | **460.3130** | 230.6601 | 443.2864 | 222.1468 | **442.3024** | 221.6548 | 4 |
| 13 | **1407.6750** | **704.3412** | **1390.6485** | 695.8279 | **1389.6645** | 695.3359 | T | **347.2289** | 174.1181 | 330.2023 | 165.6048 | 329.2183 | 165.1128 | 3 |
| 14 | **1506.7435** | 753.8754 | **1489.7169** | 745.3621 | **1488.7329** | 744.8701 | V | **246.1812** | 123.5942 | 229.1547 | 115.0810 | | | 2 |
| 15 | | | | | | | K | 147.1128 | 74.0600 | 130.0863 | 65.5468 | | | 1 |

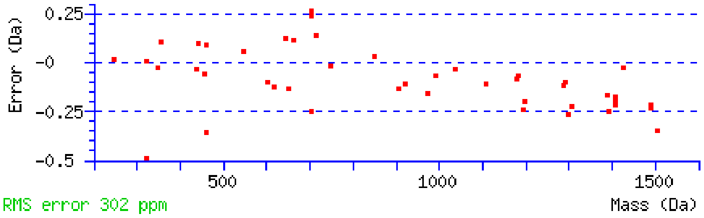


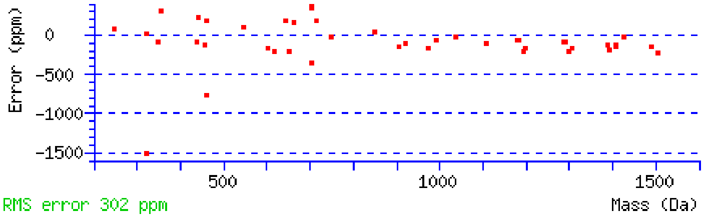


### All matches to this query

| Score | Mr(calc): | Delta | Sequence |
|---|---|---|---|
| 80.0 | 1651.8417 | -0.0015 | NLQVYNATSNSLTVK |
| 42.2 | 1651.8417 | -0.0015 | NLQVYNATSNSLTVK |
| 40.6 | 1651.8417 | -0.0015 | NLQVYNATSNSLTVK |
| 4.7 | 1651.8529 | -0.0127 | EPEKPNIINANREK |
| 3.6 | 1651.8433 | -0.0032 | GITQALNLVDSLSLK |
| 3.2 | 1651.8525 | -0.0123 | LNTLDMDTVKKMVK |
| 0.9 | 1651.8294 | 0.0107 | VKQTKSNVNIGNLR |

---

Spectrum No: 230; Query: 298; Rank: 1

---

## Peptide View

MS/MS Fragmentation of **SCINESAIDSR**
Found in **IPI00231789**, Tax_Id=10116 Gene_Symbol=Mme Neprilysin

Match to Query 298: 1251.539228 from(626.776890,2+)
Title: 091008RatKidney_NH4Format01_31.610.610.2.dta
Data file K:\NewmanPaper\Piliang\3SubProteomes\Piliang3SP\mgf5ppm\ERLIC_3SubProteomes5ppm.mgf

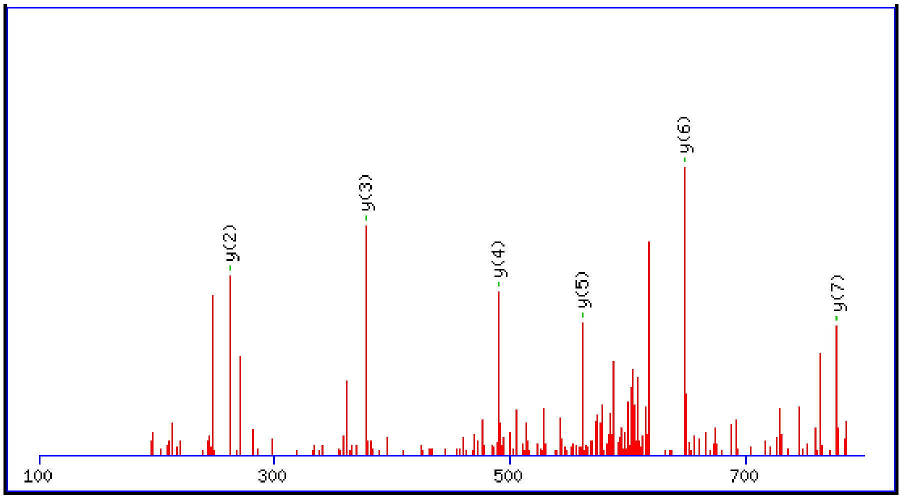


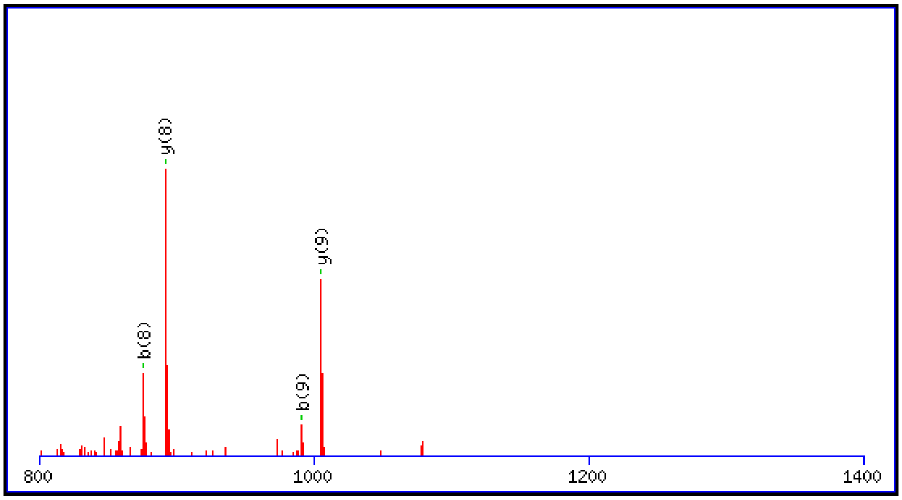


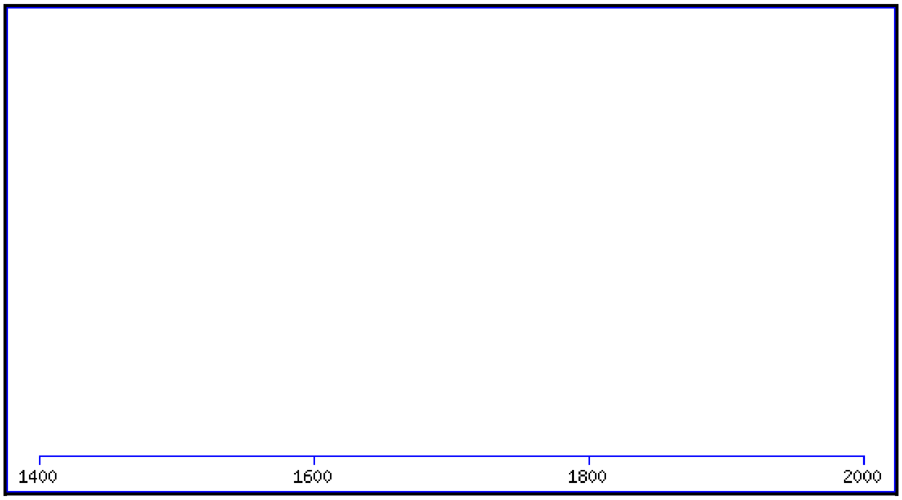


**Monoisotopic mass of neutral peptide Mr(calc):** 1251.5401
**Fixed modifications:** Carbamidomethyl (C)
**Variable modifications:**
**N4** : Deamidated_N (N)
**Ions Score:** 80 **Expect:** 1.1e-006
**Matches (Bold Red):** 10/112 fragment ions using 12 most intense peaks

| # | b | b++ | b* | b*++ | b0 | b0++ | Seq. | y | y++ | y* | y*++ | y0 | y0++ | # |
|---|---|---|---|---|---|---|---|---|---|---|---|---|---|---|
| 1 | 88.0393 | 44.5233 | | | 70.0287 | 35.5180 | S | | | | | | | 11 |
| 2 | 248.0700 | 124.5386 | | | 230.0594 | 115.5333 | C | 1165.5154 | 583.2613 | 1148.4888 | 574.7480 | 1147.5048 | 574.2560 | 10 |
| 3 | 361.1540 | 181.0806 | | | 343.1435 | 172.0754 | I | **1005.4847** | 503.2460 | 988.4582 | 494.7327 | 987.4742 | 494.2407 | 9 |
| 4 | 476.1810 | 238.5941 | 459.1544 | 230.0808 | 458.1704 | 229.5888 | N | **892.4007** | 446.7040 | 875.3741 | 438.1907 | 874.3901 | 437.6987 | 8 |
| 5 | 605.2235 | 303.1154 | 588.1970 | 294.6021 | 587.2130 | 294.1101 | E | **777.3737** | 389.1905 | 760.3472 | 380.6772 | 759.3632 | 380.1852 | 7 |

| | | | | | | | | | | | | | |
|---|---|---|---|---|---|---|---|---|---|---|---|---|---|
| **6** | 692.2556 | 346.6314 | 675.2290 | 338.1182 | 674.2450 | 337.6261 | **S** | **648.3311** | 324.6692 | 631.3046 | 316.1559 | 630.3206 | 315.6639 | **6** |
| **7** | 763.2927 | 382.1500 | 746.2661 | 373.6367 | 745.2821 | 373.1447 | **A** | **561.2991** | 281.1532 | 544.2726 | 272.6399 | 543.2885 | 272.1479 | **5** |
| **8** | **876.3768** | 438.6920 | 859.3502 | 430.1787 | 858.3662 | 429.6867 | **I** | **490.2620** | 245.6346 | 473.2354 | 237.1214 | 472.2514 | 236.6293 | **4** |
| **9** | **991.4037** | 496.2055 | 974.3771 | 487.6922 | 973.3931 | 487.2002 | **D** | **377.1779** | 189.0926 | 360.1514 | 180.5793 | 359.1674 | 180.0873 | **3** |
| **10** | 1078.4357 | 539.7215 | 1061.4092 | 531.2082 | 1060.4252 | 530.7162 | **S** | **262.1510** | 131.5791 | 245.1244 | 123.0659 | 244.1404 | 122.5738 | **2** |
| **11** | | | | | | | **R** | 175.1190 | 88.0631 | 158.0924 | 79.5498 | | | **1** |

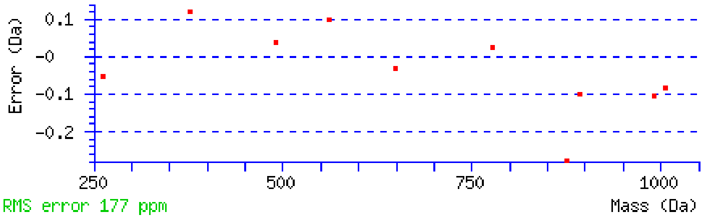


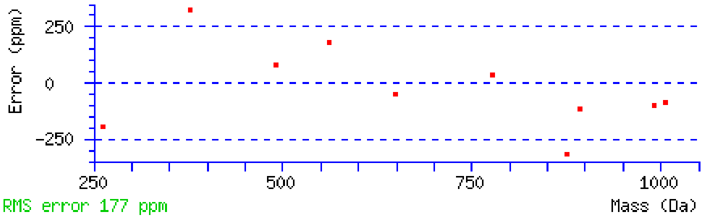


### All matches to this query

| Score | Mr(calc): | Delta | Sequence |
|---|---|---|---|
| 79.8 | 1251.5401 | -0.0009 | SCINESAIDSR |
| 9.9 | 1250.5449 | 0.9944 | LDLMDEGTDAR |
| 8.8 | 1251.5335 | 0.0057 | MVSTILSVSR |
| 8.8 | 1251.5335 | 0.0057 | MVSTILSVSR |
| 8.1 | 1251.5401 | -0.0009 | KMADENSNVSR |
| 8.0 | 1251.5319 | 0.0074 | HIGSCKDLNK |
| 7.9 | 1251.5335 | 0.0057 | MVSTILSVSR |
| 7.9 | 1251.5280 | 0.0112 | EYQMMLLTK |
| 7.0 | 1249.5439 | 1.9953 | SSSSVTSTSSIK |
| 7.0 | 1249.5439 | 1.9953 | SSSSVTSTSSIK |

---

Spectrum No: 231; Query: 135; Rank: 1

---

## Peptide View

MS/MS Fragmentation of **EGNATGHLIGR**
Found in **IPI00196620**, Tax_Id=10116 Gene_Symbol=Cubn Cubilin precursor

Match to Query 135: 1124.558128 from(563.286340,2+)
Title: 100101RatKid_NS_deglyco_20.657.657.2.dta
Data file K:\NewmanPaper\Piliang\3SubProteomes\Piliang3SP\mgf5ppm\ERLIC_3SubProteomes5ppm.mgf

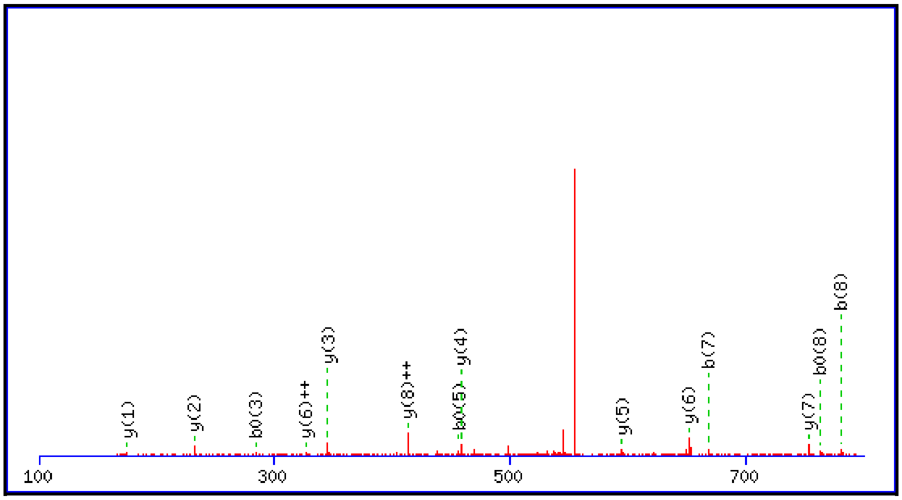

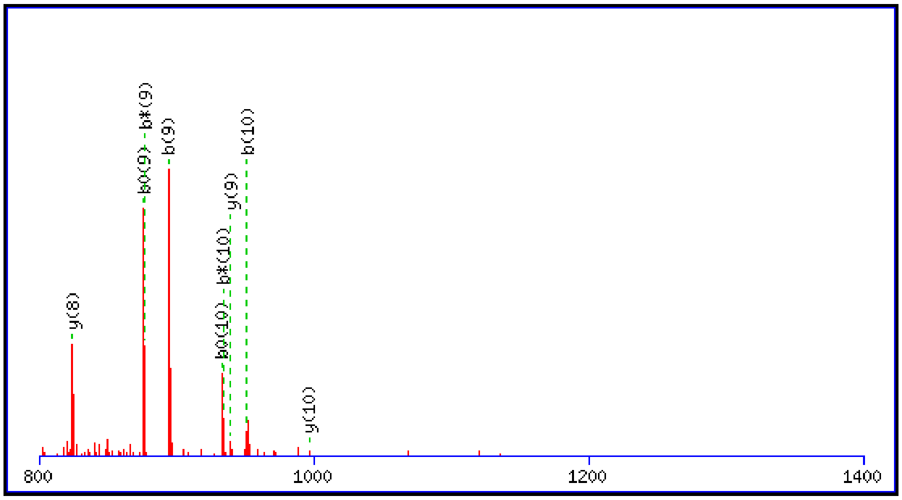


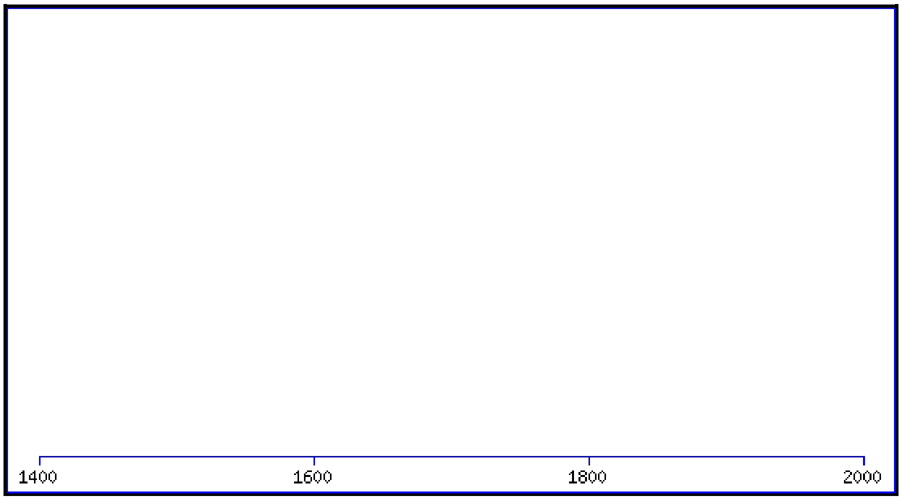


**Monoisotopic mass of neutral peptide Mr(calc):** 1124.5574
**Fixed modifications:** Carbamidomethyl (C)
**Variable modifications:**
**N3** : Deamidated_N (N)
**Ions Score:** 80 **Expect:** 1.2e-006
**Matches (Bold Red):** 23/104 fragment ions using 42 most intense peaks

| # | b | b++ | b* | b*++ | b0 | b0++ | Seq. | y | y++ | y* | y*++ | y0 | y0++ | # |
|---|---|---|---|---|---|---|---|---|---|---|---|---|---|---|
| 1 | 130.0499 | 65.5286 | | | 112.0393 | 56.5233 | E | | | | | | | 11 |
| 2 | 187.0713 | 94.0393 | | | 169.0608 | 85.0340 | G | **996.5221** | 498.7647 | 979.4956 | 490.2514 | 978.5115 | 489.7594 | 10 |
| 3 | 302.0983 | 151.5528 | 285.0717 | 143.0395 | **284.0877** | 142.5475 | N | **939.5006** | 470.2540 | 922.4741 | 461.7407 | 921.4901 | 461.2487 | 9 |
| 4 | 373.1354 | 187.0713 | 356.1088 | 178.5581 | 355.1248 | 178.0660 | A | **824.4737** | **412.7405** | 807.4472 | 404.2272 | 806.4631 | 403.7352 | 8 |
| 5 | 474.1831 | 237.5952 | 457.1565 | 229.0819 | **456.1725** | 228.5899 | T | **753.4366** | 377.2219 | 736.4101 | 368.7087 | 735.4260 | 368.2167 | 7 |
| 6 | 531.2045 | 266.1059 | 514.1780 | 257.5926 | 513.1940 | 257.1006 | G | **652.3889** | **326.6981** | 635.3624 | 318.1848 | | | 6 |
| 7 | **668.2634** | 334.6354 | 651.2369 | 326.1221 | 650.2529 | 325.6301 | H | **595.3675** | 298.1874 | 578.3409 | 289.6741 | | | 5 |
| 8 | **781.3475** | 391.1774 | 764.3210 | 382.6641 | **763.3369** | 382.1721 | L | **458.3085** | 229.6579 | 441.2820 | 221.1446 | | | 4 |
| 9 | **894.4316** | 447.7194 | **877.4050** | 439.2061 | **876.4210** | 438.7141 | I | **345.2245** | 173.1159 | 328.1979 | 164.6026 | | | 3 |
| 10 | **951.4530** | 476.2302 | **934.4265** | 467.7169 | **933.4425** | 467.2249 | G | **232.1404** | 116.5738 | 215.1139 | 108.0606 | | | 2 |
| 11 | | | | | | | R | **175.1190** | 88.0631 | 158.0924 | 79.5498 | | | 1 |

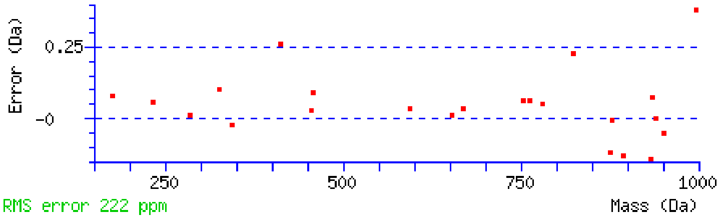


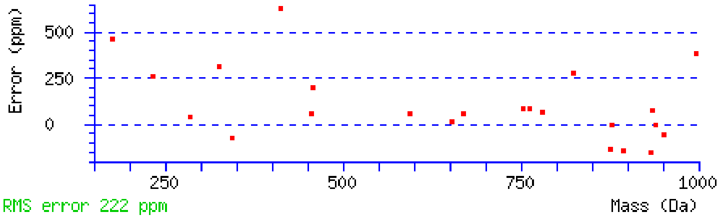

### All matches to this query

| Score | Mr(calc): | Delta | Sequence |
|---|---|---|---|
| 79.7 | 1124.5574 | 0.0007 | EGNATGHLIGR |
| 24.5 | 1124.5526 | 0.0056 | ATCVRLLGR |
| 19.2 | 1124.5526 | 0.0056 | VAMSGRVIGR |
| 17.7 | 1124.5583 | -0.0002 | ACILCVHPR |
| 14.8 | 1123.5611 | 0.9970 | RGSRGSVAVR |
| 13.4 | 1123.5499 | 1.0082 | RGSLGGALTGR |
| 13.3 | 1123.5638 | 0.9943 | ETLGISVGLR |
| 12.9 | 1124.5591 | -0.0009 | NETIKSVVR |
| 12.9 | 1124.5615 | -0.0033 | FTSQPGYIGR |
| 12.6 | 1123.5638 | 0.9943 | QTLDLVLSR |

---

Spectrum No: 232; Query: 206; Rank: 1

---

## Peptide View

MS/MS Fragmentation of **VFGSQNLTTVK**
Found in **IPI00210975**, Tax_Id=10116 Gene_Symbol=Hyou1 Hypoxia up-regulated protein 1 precursor

Match to Query 206: 1193.626748 from(597.820650,2+)
Title: 091008RatKidney_NH4Format01_25.1682.1682.2.dta
Data file K:\NewmanPaper\Piliang\3SubProteomes\Piliang3SP\mgf5ppm\ERLIC_3SubProteomes5ppm.mgf

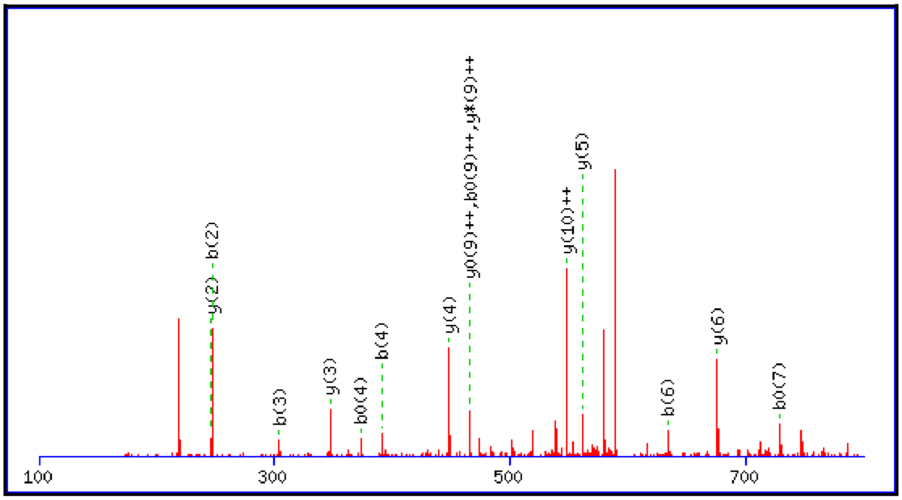

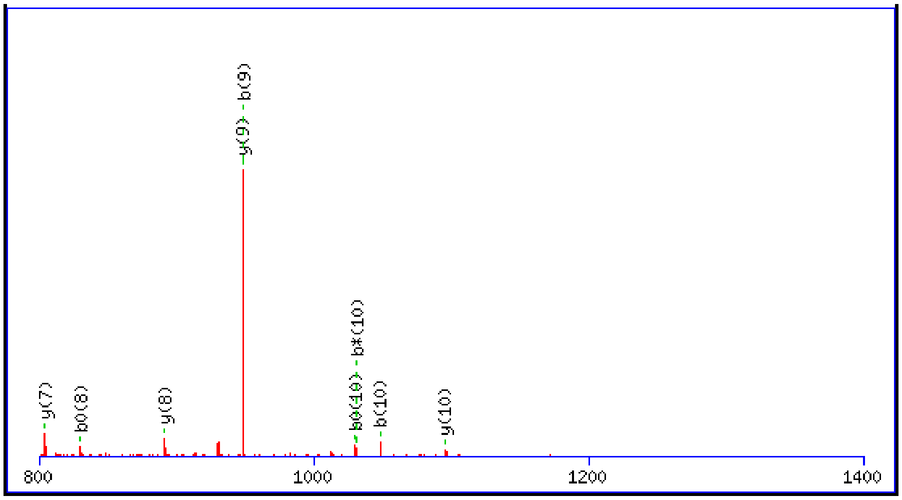


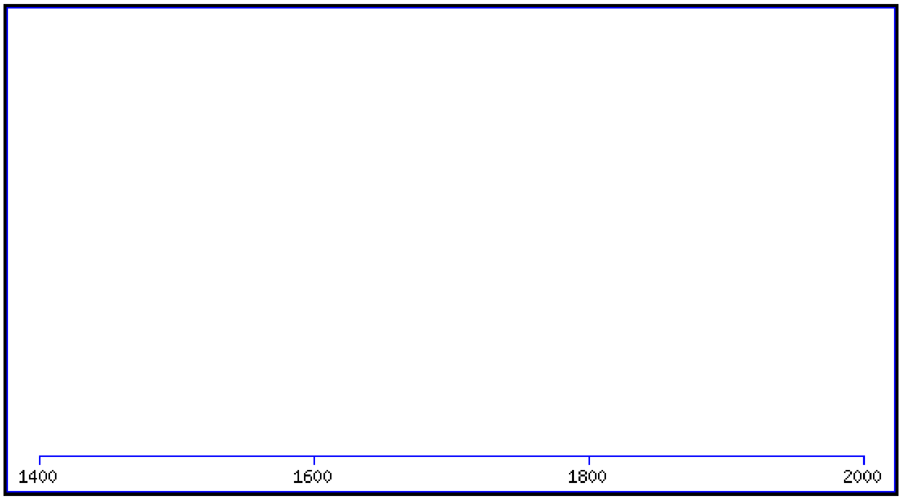


**Monoisotopic mass of neutral peptide Mr(calc):** 1193.6292
**Fixed modifications:** Carbamidomethyl (C)
**Variable modifications:**
**N6** : Deamidated_N (N)
**Ions Score:** 80 **Expect:** 1e-006
**Matches (Bold Red):** 24/102 fragment ions using 32 most intense peaks

| # | b | $b^{++}$ | b* | $b^{*++}$ | $b^0$ | $b^{0++}$ | Seq. | y | $y^{++}$ | y* | $y^{*++}$ | $y^0$ | $y^{0++}$ | # |
|---|---|---|---|---|---|---|---|---|---|---|---|---|---|---|
| 1 | 100.0757 | 50.5415 | | | | | V | | | | | | | 11 |
| 2 | **247.1441** | 124.0757 | | | | | F | **1095.5681** | **548.2877** | 1078.5415 | 539.7744 | 1077.5575 | 539.2824 | 10 |
| 3 | **304.1656** | 152.5864 | | | | | G | **948.4996** | 474.7535 | 931.4731 | **466.2402** | 930.4891 | **465.7482** | 9 |
| 4 | **391.1976** | 196.1024 | | | **373.1870** | 187.0972 | S | **891.4782** | 446.2427 | 874.4516 | 437.7295 | 873.4676 | 437.2374 | 8 |
| 5 | 519.2562 | 260.1317 | 502.2296 | 251.6185 | 501.2456 | 251.1264 | Q | **804.4462** | 402.7267 | 787.4196 | 394.2134 | 786.4356 | 393.7214 | 7 |
| 6 | **634.2831** | 317.6452 | 617.2566 | 309.1319 | 616.2725 | 308.6399 | N | **676.3876** | 338.6974 | 659.3610 | 330.1842 | 658.3770 | 329.6921 | 6 |
| 7 | 747.3672 | 374.1872 | 730.3406 | 365.6740 | **729.3566** | 365.1819 | L | **561.3606** | 281.1840 | 544.3341 | 272.6707 | 543.3501 | 272.1787 | 5 |
| 8 | 848.4149 | 424.7111 | 831.3883 | 416.1978 | **830.4043** | 415.7058 | T | **448.2766** | 224.6419 | 431.2500 | 216.1287 | 430.2660 | 215.6366 | 4 |
| 9 | **949.4625** | 475.2349 | 932.4360 | 466.7216 | 931.4520 | **466.2296** | T | **347.2289** | 174.1181 | 330.2023 | 165.6048 | 329.2183 | 165.1128 | 3 |
| 10 | **1048.5309** | 524.7691 | **1031.5044** | 516.2558 | **1030.5204** | 515.7638 | V | **246.1812** | 123.5942 | 229.1547 | 115.0810 | | | 2 |
| 11 | | | | | | | K | 147.1128 | 74.0600 | 130.0863 | 65.5468 | | | 1 |

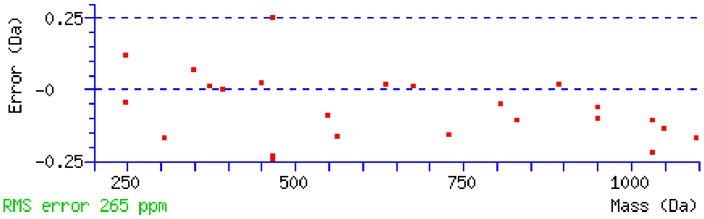


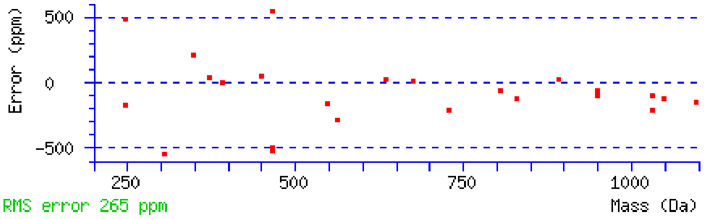

### All matches to this query

| Score | Mr(calc): | Delta | Sequence |
|---|---|---|---|
| 79.7 | 1193.6292 | -0.0025 | VFGSQNLTTVK |
| 10.4 | 1193.6326 | -0.0058 | TVMKTGLDSVK |
| 9.3 | 1191.6169 | 2.0099 | MLTNGSLSEIK |
| 7.5 | 1192.6121 | 1.0146 | MELKQISSNK |
| 7.3 | 1193.6292 | -0.0024 | ANKAVDFTTVK |
| 7.3 | 1192.6162 | 1.0105 | MPTSFVAPTVK |
| 6.5 | 1193.6227 | 0.0041 | VFKKSSPNCK |
| 4.7 | 1193.6326 | -0.0058 | KMASTSVDLVK |
| 4.6 | 1193.6153 | 0.0115 | VQTHLENPTR |
| 4.2 | 1193.6301 | -0.0033 | STKIWMAMVK |

---

Spectrum No: 233; Query: 1677; Rank: 1

---

## Peptide View

MS/MS Fragmentation of **FSPLLYCQSQELPNGSVK**
Found in **IPI00200998**, Tax_Id=10116 Gene_Symbol=Pdzk1 PDZ domain-containing protein 1

Match to Query 1677: 2067.002508 from(1034.508530,2+)
Title: 100101RatKid_NS_deglyco_05.3936.3936.2.dta
Data file K:\NewmanPaper\Piliang\3SubProteomes\Piliang3SP\mgf5ppm\ERLIC_3SubProteomes5ppm.mgf

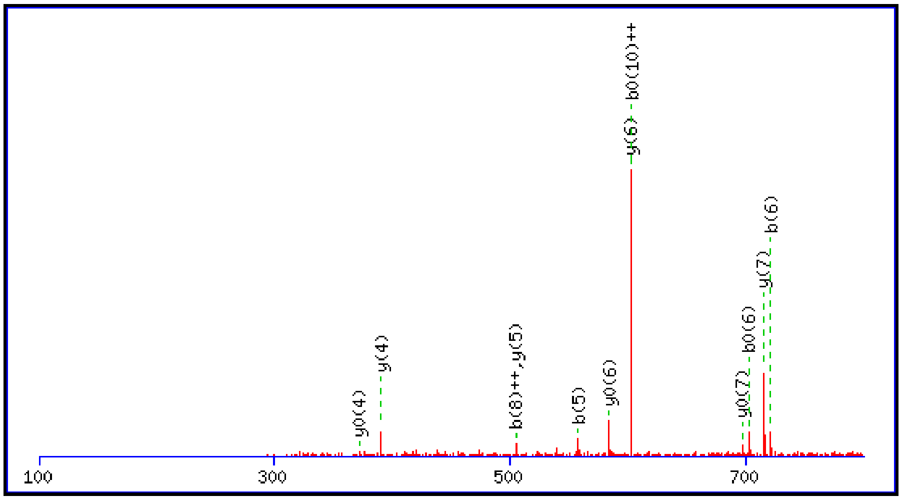


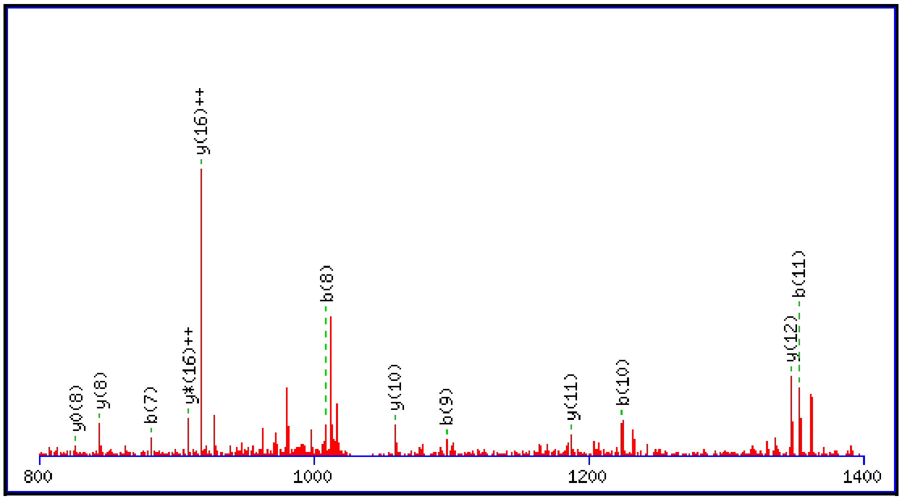

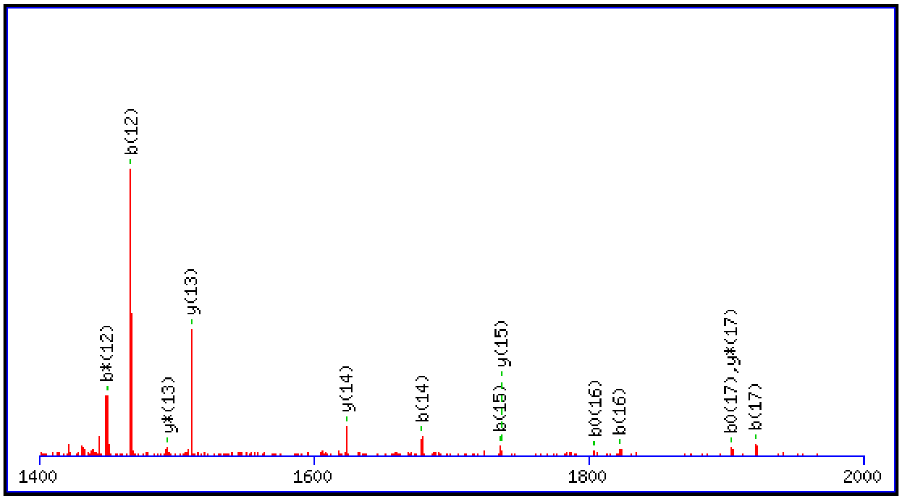


**Monoisotopic mass of neutral peptide Mr(calc):** 2066.9983
**Fixed modifications:** Carbamidomethyl (C)
**Variable modifications:**
**N14** : Deamidated_N (N)
**Ions Score:** 80 **Expect:** 2.8e-006
**Matches (Bold Red):** 37/184 fragment ions using 56 most intense peaks

| # | b | $b^{++}$ | b* | $b^{*++}$ | $b^{0}$ | $b^{0++}$ | Seq. | y | $y^{++}$ | y* | $y^{*++}$ | $y^{0}$ | $y^{0++}$ | # |
|---|---|---|---|---|---|---|---|---|---|---|---|---|---|---|
| 1 | 148.0757 | 74.5415 | | | | | F | | | | | | | 18 |
| 2 | 235.1077 | 118.0575 | | | 217.0972 | 109.0522 | S | 1920.9371 | 960.9722 | **1903.9106** | 952.4589 | 1902.9266 | 951.9669 | 17 |
| 3 | 332.1605 | 166.5839 | | | 314.1499 | 157.5786 | P | 1833.9051 | **917.4562** | 1816.8786 | **908.9429** | 1815.8946 | 908.4509 | 16 |
| 4 | 445.2445 | 223.1259 | | | 427.2340 | 214.1206 | L | **1736.8524** | 868.9298 | 1719.8258 | 860.4165 | 1718.8418 | 859.9245 | 15 |
| 5 | **558.3286** | 279.6679 | | | 540.3180 | 270.6627 | L | **1623.7683** | 812.3878 | 1606.7417 | 803.8745 | 1605.7577 | 803.3825 | 14 |
| 6 | **721.3919** | 361.1996 | | | **703.3814** | 352.1943 | Y | **1510.6842** | 755.8458 | **1493.6577** | 747.3325 | 1492.6737 | 746.8405 | 13 |
| 7 | **881.4226** | 441.2149 | | | 863.4120 | 432.2096 | C | **1347.6209** | 674.3141 | 1330.5944 | 665.8008 | 1329.6103 | 665.3088 | 12 |
| 8 | **1009.4812** | **505.2442** | 992.4546 | 496.7309 | 991.4706 | 496.2389 | Q | **1187.5903** | 594.2988 | 1170.5637 | 585.7855 | 1169.5797 | 585.2935 | 11 |
| 9 | **1096.5132** | 548.7602 | 1079.4866 | 540.2470 | 1078.5026 | 539.7550 | S | **1059.5317** | 530.2695 | 1042.5051 | 521.7562 | 1041.5211 | 521.2642 | 10 |
| 10 | **1224.5718** | 612.7895 | 1207.5452 | 604.2762 | 1206.5612 | **603.7842** | Q | 972.4996 | 486.7535 | 955.4731 | 478.2402 | 954.4891 | 477.7482 | 9 |
| 11 | **1353.6144** | 677.3108 | 1336.5878 | 668.7975 | 1335.6038 | 668.3055 | E | **844.4411** | 422.7242 | 827.4145 | 414.2109 | **826.4305** | 413.7189 | 8 |
| 12 | **1466.6984** | 733.8529 | **1449.6719** | 725.3396 | 1448.6879 | 724.8476 | L | **715.3985** | 358.2029 | 698.3719 | 349.6896 | **697.3879** | 349.1976 | 7 |
| 13 | 1563.7512 | 782.3792 | 1546.7246 | 773.8660 | 1545.7406 | 773.3740 | P | **602.3144** | 301.6608 | 585.2879 | 293.1476 | **584.3038** | 292.6556 | 6 |
| 14 | **1678.7781** | 839.8927 | 1661.7516 | 831.3794 | 1660.7676 | 830.8874 | N | **505.2616** | 253.1345 | 488.2351 | 244.6212 | 487.2511 | 244.1292 | 5 |
| 15 | **1735.7996** | 868.4034 | 1718.7730 | 859.8902 | 1717.7890 | 859.3982 | G | **390.2347** | 195.6210 | 373.2082 | 187.1077 | **372.2241** | 186.6157 | 4 |
| 16 | **1822.8316** | 911.9194 | 1805.8051 | 903.4062 | **1804.8211** | 902.9142 | S | 333.2132 | 167.1103 | 316.1867 | 158.5970 | 315.2027 | 158.1050 | 3 |
| 17 | **1921.9000** | 961.4537 | 1904.8735 | 952.9404 | **1903.8895** | 952.4484 | V | 246.1812 | 123.5942 | 229.1547 | 115.0810 | | | 2 |
| 18 | | | | | | | K | 147.1128 | 74.0600 | 130.0863 | 65.5468 | | | 1 |

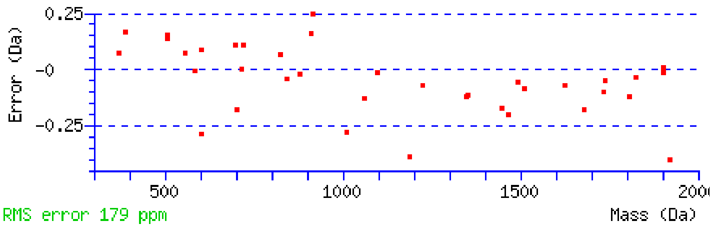


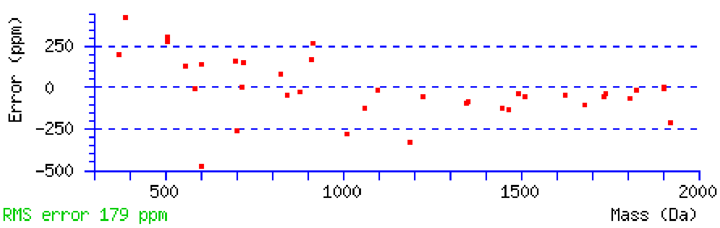


## All matches to this query

| Score | Mr(calc): | Delta | Sequence |
|---|---|---|---|
| 79.6 | 2066.9983 | 0.0042 | FSPLLYCQSQELPNGSVK |
| 18.9 | 2066.0143 | 0.9882 | FSPLLYCQSQELPNGSVK |
| 10.0 | 2067.0169 | -0.0144 | GDWNKLGILDMFLGCIAK |

| | | | |
|---|---|---|---|
| 4.4 | 2067.0016 | 0.0009 | EAMCIGAEVYDNLKNVIK |
| 4.0 | 2066.9925 | 0.0100 | QVLNFTLEDVLLPQSDR |
| 2.5 | 2065.9905 | 1.0120 | ISAAGNSGNESGVRELLRR |
| 1.3 | 2066.9981 | 0.0044 | EASREAESHPVTDGLRDAK |
| 0.9 | 2066.9972 | 0.0053 | RKILQAFPDMHNSSISK |
| 0.9 | 2066.9972 | 0.0053 | RKILQAFPDMHNSSISK |
| 0.9 | 2067.0181 | -0.0156 | VFQECGTPHPVQSRNRR |

Spectrum No: 234; Query: 1540; Rank: 1

## Peptide View

MS/MS Fragmentation of **TVTNAVVTVPAYFNDSQR**
Found in **IPI00208205**, Tax_Id=10116 Gene_Symbol=Hspa8 Heat shock cognate 71 kDa protein

Match to Query 1540: 1981.967128 from(991.990840,2+)
Title: 091008RatKidney_NH4Format02_22.3468.3468.2.dta
Data file K:\NewmanPaper\Piliang\3SubProteomes\Piliang3SP\mgf5ppm\ERLIC_3SubProteomes5ppm.mgf

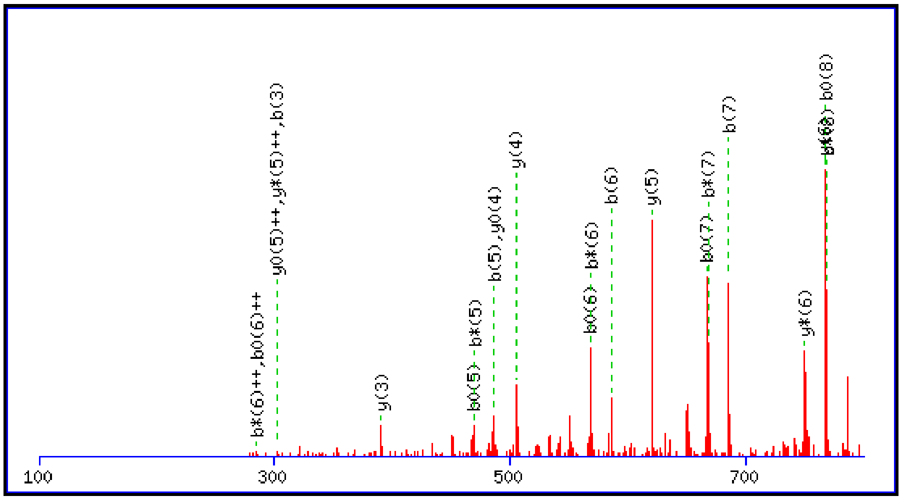


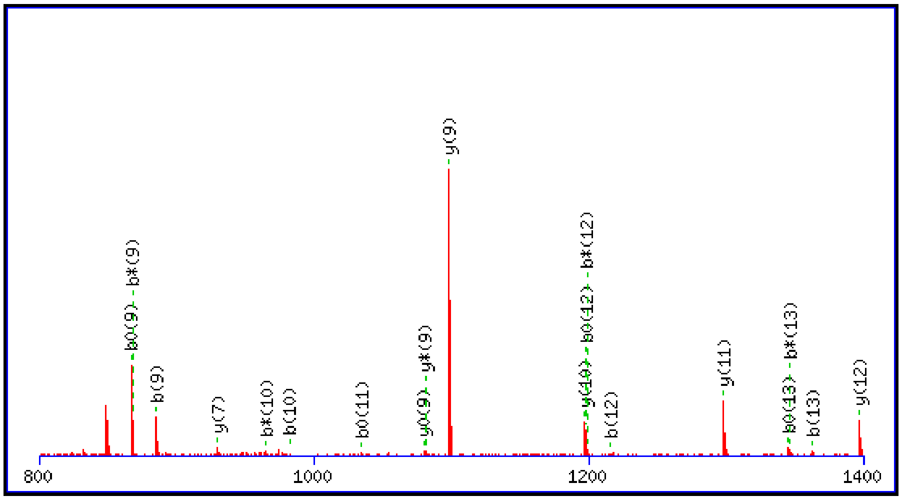

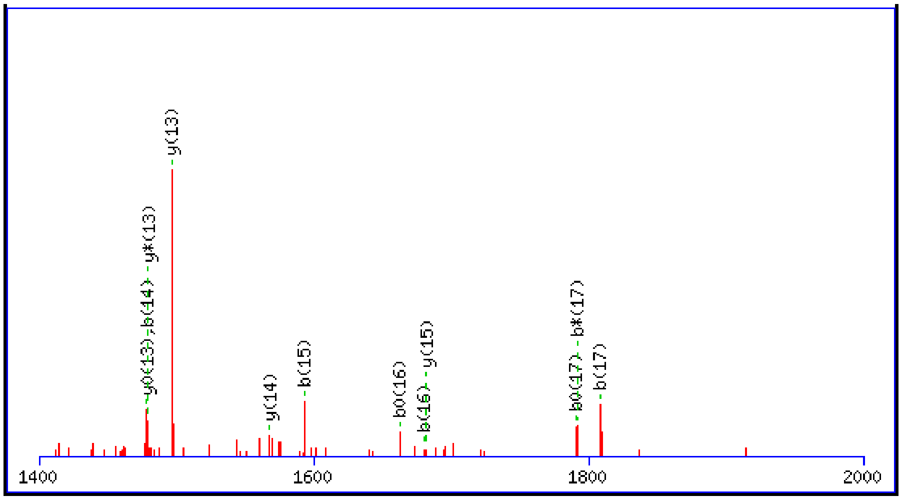


**Monoisotopic mass of neutral peptide Mr(calc):** 1981.9745
**Fixed modifications:** Carbamidomethyl (C)
**Variable modifications:**
**N14** : Deamidated_N (N)
**Ions Score:** 80 **Expect:** 2.3e-006
**Matches (Bold Red):** 53/194 fragment ions using 86 most intense peaks

| # | b | $b^{++}$ | b* | $b^{*++}$ | $b^{0}$ | $b^{0++}$ | Seq. | y | $y^{++}$ | y* | $y^{*++}$ | $y^{0}$ | $y^{0++}$ | # |
|---|---|---|---|---|---|---|---|---|---|---|---|---|---|---|
| 1 | 102.0550 | 51.5311 | | | 84.0444 | 42.5258 | T | | | | | | | 18 |
| 2 | 201.1234 | 101.0653 | | | 183.1128 | 92.0600 | V | 1881.9341 | 941.4707 | 1864.9076 | 932.9574 | 1863.9235 | 932.4654 | 17 |
| 3 | **302.1710** | 151.5892 | | | 284.1605 | 142.5839 | T | 1782.8657 | 891.9365 | 1765.8392 | 883.4232 | 1764.8551 | 882.9312 | 16 |
| 4 | 416.2140 | 208.6106 | 399.1874 | 200.0974 | 398.2034 | 199.6053 | N | **1681.8180** | 841.4126 | 1664.7915 | 832.8994 | 1663.8075 | 832.4074 | 15 |
| 5 | **487.2511** | 244.1292 | **470.2245** | 235.6159 | **469.2405** | 235.1239 | A | **1567.7751** | 784.3912 | 1550.7485 | 775.8779 | 1549.7645 | 775.3859 | 14 |
| 6 | **586.3195** | 293.6634 | **569.2930** | **285.1501** | **568.3089** | **284.6581** | V | **1496.7380** | 748.8726 | **1479.7114** | 740.3594 | **1478.7274** | 739.8673 | 13 |
| 7 | **685.3879** | 343.1976 | **668.3614** | 334.6843 | **667.3774** | 334.1923 | V | **1397.6696** | 699.3384 | 1380.6430 | 690.8251 | 1379.6590 | 690.3331 | 12 |
| 8 | 786.4356 | 393.7214 | **769.4090** | 385.2082 | **768.4250** | 384.7162 | T | **1298.6012** | 649.8042 | 1281.5746 | 641.2909 | 1280.5906 | 640.7989 | 11 |
| 9 | **885.5040** | 443.2556 | **868.4775** | 434.7424 | **867.4934** | 434.2504 | V | **1197.5535** | 599.2804 | 1180.5269 | 590.7671 | 1179.5429 | 590.2751 | 10 |
| 10 | **982.5568** | 491.7820 | **965.5302** | 483.2688 | 964.5462 | 482.7767 | P | **1098.4851** | 549.7462 | **1081.4585** | 541.2329 | **1080.4745** | 540.7409 | 9 |
| 11 | 1053.5939 | 527.3006 | 1036.5673 | 518.7873 | **1035.5833** | 518.2953 | A | 1001.4323 | 501.2198 | 984.4057 | 492.7065 | 983.4217 | 492.2145 | 8 |
| 12 | **1216.6572** | 608.8322 | **1199.6307** | 600.3190 | **1198.6467** | 599.8270 | Y | **930.3952** | 465.7012 | 913.3686 | 457.1880 | 912.3846 | 456.6959 | 7 |
| 13 | **1363.7256** | 682.3665 | **1346.6991** | 673.8532 | **1345.7151** | 673.3612 | F | **767.3319** | 384.1696 | **750.3053** | 375.6563 | 749.3213 | 375.1643 | 6 |
| 14 | **1478.7526** | 739.8799 | 1461.7260 | 731.3666 | 1460.7420 | 730.8746 | N | **620.2634** | 310.6354 | 603.2369 | **302.1221** | 602.2529 | **301.6301** | 5 |
| 15 | **1593.7795** | 797.3934 | 1576.7530 | 788.8801 | 1575.7689 | 788.3881 | D | **505.2365** | 253.1219 | 488.2100 | 244.6086 | **487.2259** | 244.1166 | 4 |
| 16 | **1680.8115** | 840.9094 | 1663.7850 | 832.3961 | **1662.8010** | 831.9041 | S | **390.2096** | 195.6084 | 373.1830 | 187.0951 | 372.1990 | 186.6031 | 3 |
| 17 | **1808.8701** | 904.9387 | **1791.8436** | 896.4254 | **1790.8596** | 895.9334 | Q | 303.1775 | 152.0924 | 286.1510 | 143.5791 | | | 2 |
| 18 | | | | | | | R | 175.1190 | 88.0631 | 158.0924 | 79.5498 | | | 1 |

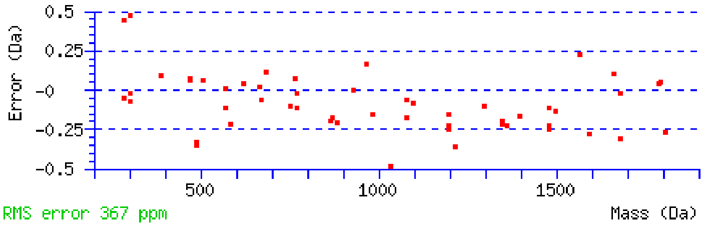


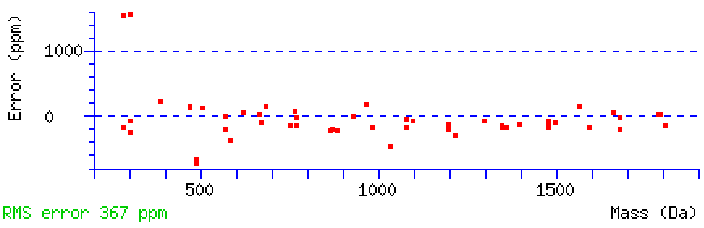


### All matches to this query

| Score | Mr(calc): | Delta | Sequence |
|---|---|---|---|
| 79.5 | 1981.9745 | -0.0074 | TVTNAVVTVPAYFNDSQR |
| 11.2 | 1981.9745 | -0.0074 | TVTNAVVTVPAYFNDSQR |
| 9.2 | 1981.9795 | -0.0124 | CLIVAQKSVTNTTPLEK |
| | | | |

| | | | |
|---|---|---|---|
| 8.0 | 1979.9717 | 1.9954 | EEQVVQVWSKKNIGEK |
| 5.5 | 1981.9795 | -0.0124 | CLIVAQKSVTNTTPLEK |
| 3.7 | 1979.9791 | 1.9880 | ALMQLLVNPFVGPLTNR |
| 3.5 | 1981.9735 | -0.0063 | QNQDRINKTVLYGTPR |
| 3.4 | 1981.9656 | 0.0016 | RKSMEASLTIVPANETR |
| 2.7 | 1981.9735 | -0.0063 | QNQDRINKTVLYGTPR |
| 2.5 | 1981.9730 | -0.0059 | AMDMRSPVLSTLLPSLR |

---

Spectrum No: 235; Query: 1232; Rank: 1

---

## Peptide View

MS/MS Fragmentation of **EEEAIQLDGLNASQIR**
Found in **IPI00365985**, Tax_Id=10116 Gene_Symbol=Tra1_predicted 93 kDa protein

Match to Query 1232: 1785.879948 from(893.947250,2+)
Title: 100101RatKid_NS_deglyco_16.3592.3592.2.dta
Data file K:\NewmanPaper\Piliang\3SubProteomes\Piliang3SP\mgf5ppm\ERLIC_3SubProteomes5ppm.mgf

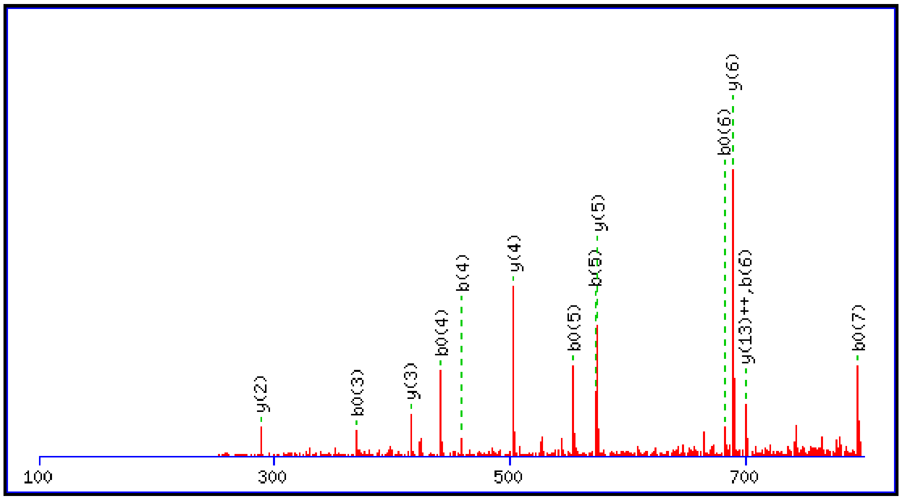


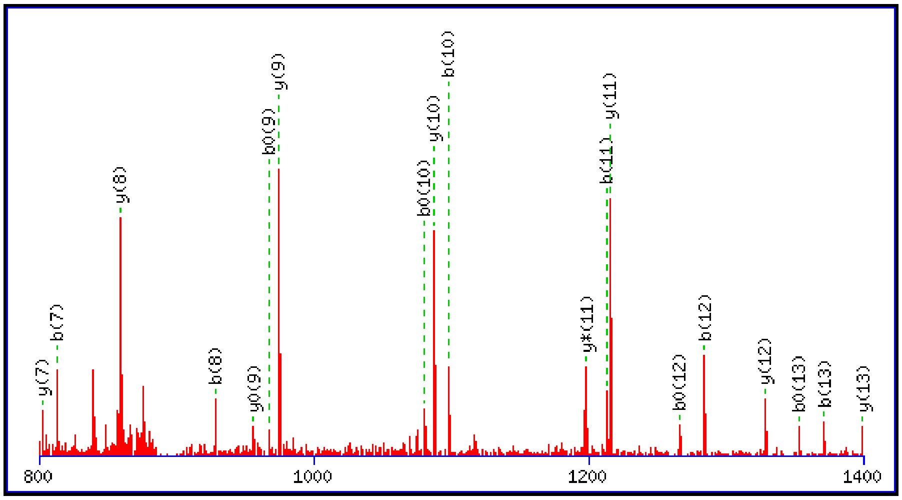

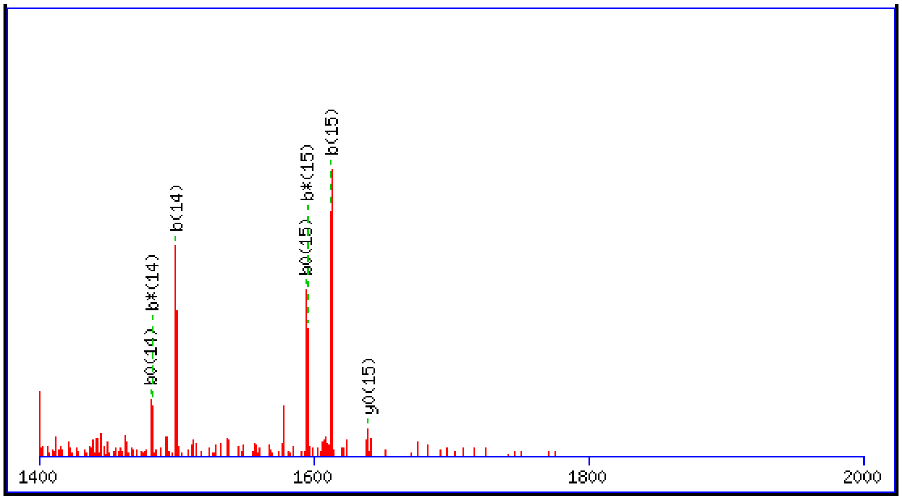


**Monoisotopic mass of neutral peptide Mr(calc):** 1785.8744
**Fixed modifications:** Carbamidomethyl (C)
**Variable modifications:**
**N11** : Deamidated_N (N)
**Ions Score:** 80 **Expect:** 2.1e-006
**Matches (Bold Red):** 40/164 fragment ions using 72 most intense peaks

| # | b | $b^{++}$ | $b^{*}$ | $b^{*++}$ | $b^{0}$ | $b^{0++}$ | Seq. | y | $y^{++}$ | $y^{*}$ | $y^{*++}$ | $y^{0}$ | $y^{0++}$ | # |
|---|---|---|---|---|---|---|---|---|---|---|---|---|---|---|
| 1 | 130.0499 | 65.5286 | | | 112.0393 | 56.5233 | E | | | | | | | 16 |
| 2 | 259.0925 | 130.0499 | | | 241.0819 | 121.0446 | E | 1657.8391 | 829.4232 | 1640.8126 | 820.9099 | **1639.8286** | 820.4179 | 15 |
| 3 | 388.1351 | 194.5712 | | | **370.1245** | 185.5659 | E | 1528.7966 | 764.9019 | 1511.7700 | 756.3886 | 1510.7860 | 755.8966 | 14 |
| 4 | **459.1722** | 230.0897 | | | **441.1616** | 221.0844 | A | **1399.7540** | **700.3806** | 1382.7274 | 691.8673 | 1381.7434 | 691.3753 | 13 |
| 5 | **572.2562** | 286.6318 | | | **554.2457** | 277.6265 | I | **1328.7168** | 664.8621 | 1311.6903 | 656.3488 | 1310.7063 | 655.8568 | 12 |
| 6 | **700.3148** | 350.6610 | 683.2883 | 342.1478 | **682.3042** | 341.6558 | Q | **1215.6328** | 608.3200 | **1198.6062** | 599.8068 | 1197.6222 | 599.3147 | 11 |
| 7 | **813.3989** | 407.2031 | 796.3723 | 398.6898 | **795.3883** | 398.1978 | L | **1087.5742** | 544.2907 | 1070.5477 | 535.7775 | 1069.5636 | 535.2855 | 10 |
| 8 | **928.4258** | 464.7165 | 911.3993 | 456.2033 | 910.4153 | 455.7113 | D | **974.4901** | 487.7487 | 957.4636 | 479.2354 | **956.4796** | 478.7434 | 9 |
| 9 | 985.4473 | 493.2273 | 968.4207 | 484.7140 | **967.4367** | 484.2220 | G | **859.4632** | 430.2352 | 842.4367 | 421.7220 | 841.4526 | 421.2300 | 8 |
| 10 | **1098.5313** | 549.7693 | 1081.5048 | 541.2560 | **1080.5208** | 540.7640 | L | **802.4417** | 401.7245 | 785.4152 | 393.2112 | 784.4312 | 392.7192 | 7 |
| 11 | **1213.5583** | 607.2828 | 1196.5317 | 598.7695 | 1195.5477 | 598.2775 | N | **689.3577** | 345.1825 | 672.3311 | 336.6692 | 671.3471 | 336.1772 | 6 |
| 12 | **1284.5954** | 642.8013 | 1267.5688 | 634.2881 | **1266.5848** | 633.7961 | A | **574.3307** | 287.6690 | 557.3042 | 279.1557 | 556.3202 | 278.6637 | 5 |
| 13 | **1371.6274** | 686.3174 | 1354.6009 | 677.8041 | **1353.6169** | 677.3121 | S | **503.2936** | 252.1504 | 486.2671 | 243.6372 | 485.2831 | 243.1452 | 4 |
| 14 | **1499.6860** | 750.3466 | **1482.6595** | 741.8334 | **1481.6754** | 741.3414 | Q | **416.2616** | 208.6344 | 399.2350 | 200.1212 | | | 3 |
| 15 | **1612.7701** | 806.8887 | **1595.7435** | 798.3754 | **1594.7595** | 797.8834 | I | **288.2030** | 144.6051 | 271.1765 | 136.0919 | | | 2 |
| 16 | | | | | | | R | 175.1190 | 88.0631 | 158.0924 | 79.5498 | | | 1 |

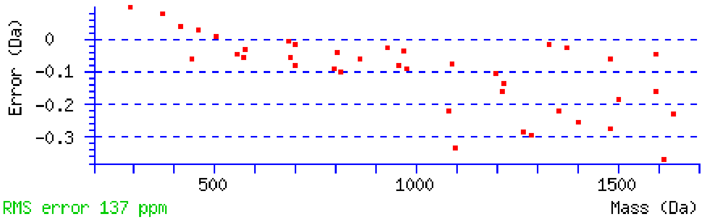


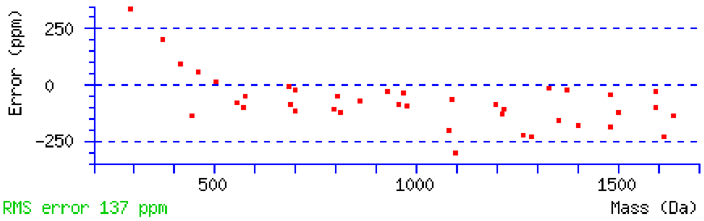


### All matches to this query

| Score | Mr(calc): | Delta | Sequence |
|---|---|---|---|
| 79.5 | 1785.8744 | 0.0055 | EEEAIQLDGLNASQIR |
| 19.1 | 1784.8904 | 0.9895 | EEEAIQLDGLNASQIR |
| 8.3 | 1785.8903 | -0.0104 | ANVTVLDTQIRKLR |
| 0.2 | 1783.8886 | 1.9914 | TQPILDILLKNQTK |
| 0.2 | 1784.8726 | 1.0074 | TQPILDILLKNQTK |

---

Spectrum No: 236; Query: 2668; Rank: 1

---

## Peptide View

MS/MS Fragmentation of **RGVDVSQVAWQSQGDTPCSCCIVNNSNGSR**
Found in **IPI00211989**, Tax_Id=10116 Gene_Symbol=Khk Isoform C of Ketohexokinase

Match to Query 2668: 3338.453682 from(1113.825170,3+)
Title: 100101RatKid_NS_deglyco_14.2737.2737.3.dta
Data file K:\NewmanPaper\Piliang\3SubProteomes\Piliang3SP\mgf5ppm\ERLIC_3SubProteomes5ppm.mgf

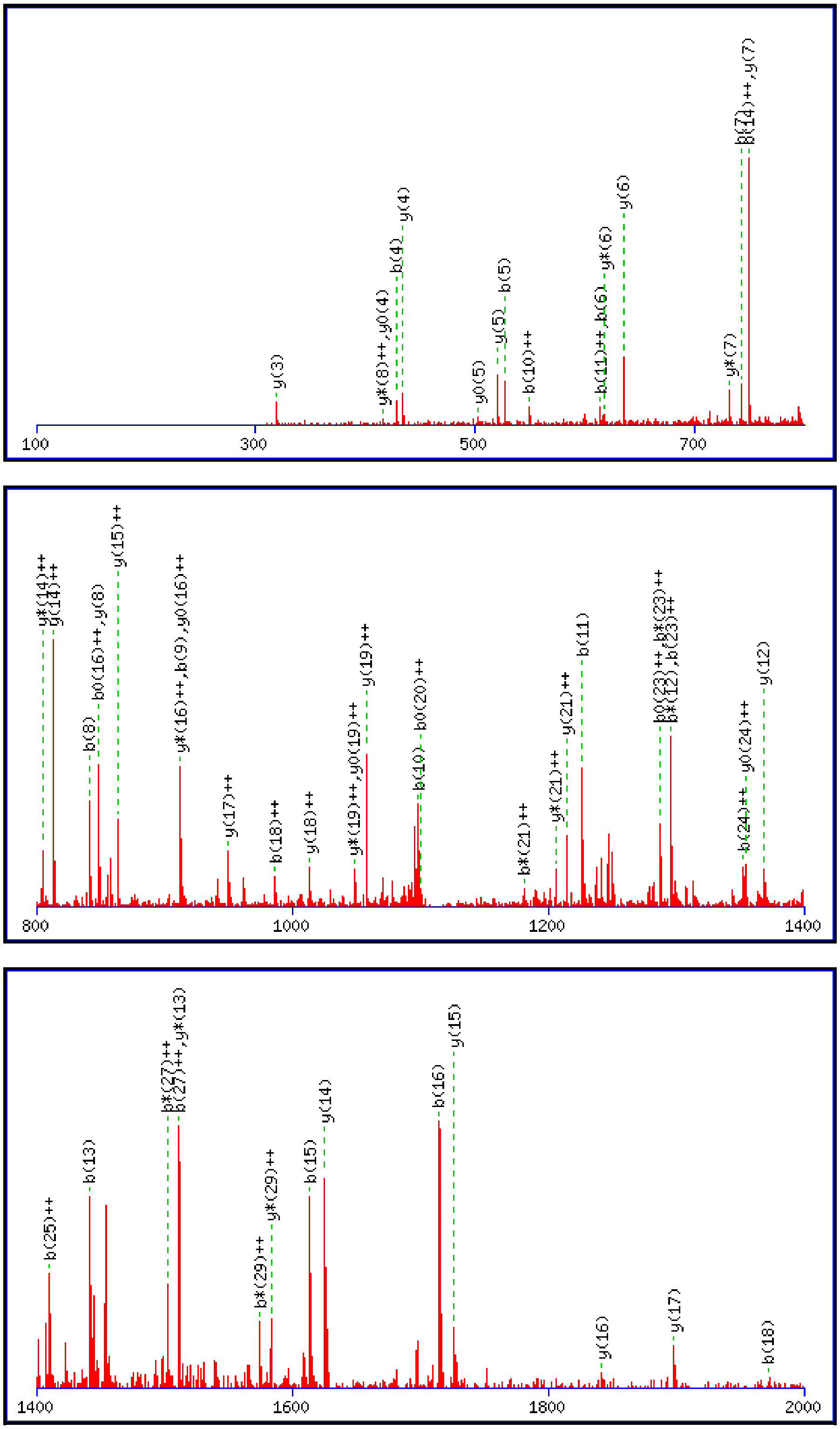

**Monoisotopic mass of neutral peptide Mr(calc):** 3338.4463
**Fixed modifications:** Carbamidomethyl (C)
**Variable modifications:**
**N27** : Deamidated_N (N)
**Ions Score:** 80 **Expect:** 5e-006
**Matches (Bold Red):** 59/340 fragment ions using 81 most intense peaks

| # | b | $b^{++}$ | $b^{*}$ | $b^{*++}$ | $b^{0}$ | $b^{0++}$ | Seq. | y | $y^{++}$ | $y^{*}$ | $y^{*++}$ | $y^{0}$ | $y^{0++}$ | # |
|---|---|---|---|---|---|---|---|---|---|---|---|---|---|---|
| 1 | 157.1084 | 79.0578 | 140.0818 | 70.5446 | | | R | | | | | | | 30 |
| 2 | 214.1299 | 107.5686 | 197.1033 | 99.0553 | | | G | 3183.3524 | 1592.1799 | 3166.3259 | **1583.6666** | 3165.3419 | 1583.1746 | 29 |
| 3 | 313.1983 | 157.1028 | 296.1717 | 148.5895 | | | V | 3126.3310 | 1563.6691 | 3109.3044 | 1555.1558 | 3108.3204 | 1554.6638 | 28 |
| 4 | **428.2252** | 214.6162 | 411.1987 | 206.1030 | 410.2146 | 205.6110 | D | 3027.2626 | 1514.1349 | 3010.2360 | 1505.6216 | 3009.2520 | 1505.1296 | 27 |
| 5 | **527.2936** | 264.1504 | 510.2671 | 255.6372 | 509.2831 | 255.1452 | V | 2912.2356 | 1456.6214 | 2895.2091 | 1448.1082 | 2894.2250 | 1447.6162 | 26 |
| 6 | **614.3257** | 307.6665 | 597.2991 | 299.1532 | 596.3151 | 298.6612 | S | 2813.1672 | 1407.0872 | 2796.1406 | 1398.5740 | 2795.1566 | 1398.0820 | 25 |
| 7 | **742.3842** | 371.6958 | 725.3577 | 363.1825 | 724.3737 | 362.6905 | Q | 2726.1352 | 1363.5712 | 2709.1086 | 1355.0579 | 2708.1246 | **1354.5659** | 24 |
| 8 | **841.4526** | 421.2300 | 824.4261 | 412.7167 | 823.4421 | 412.2247 | V | 2598.0766 | 1299.5419 | 2581.0500 | 1291.0287 | 2580.0660 | 1290.5367 | 23 |
| 9 | **912.4898** | 456.7485 | 895.4632 | 448.2352 | 894.4792 | 447.7432 | A | 2499.0082 | 1250.0077 | 2481.9816 | 1241.4945 | 2480.9976 | 1241.0024 | 22 |
| 10 | **1098.5691** | **549.7882** | 1081.5425 | 541.2749 | 1080.5585 | 540.7829 | W | 2427.9711 | **1214.4892** | 2410.9445 | **1205.9759** | 2409.9605 | 1205.4839 | 21 |
| 11 | **1226.6276** | **613.8175** | 1209.6011 | 605.3042 | 1208.6171 | 604.8122 | Q | 2241.8917 | 1121.4495 | 2224.8652 | 1112.9362 | 2223.8812 | 1112.4442 | 20 |
| 12 | 1313.6597 | 657.3335 | **1296.6331** | 648.8202 | 1295.6491 | 648.3282 | S | 2113.8332 | **1057.4202** | 2096.8066 | **1048.9069** | 2095.8226 | **1048.4149** | 19 |
| 13 | **1441.7183** | 721.3628 | 1424.6917 | 712.8495 | 1423.7077 | 712.3575 | Q | 2026.8011 | **1013.9042** | 2009.7746 | 1005.3909 | 2008.7906 | 1004.8989 | 18 |
| 14 | 1498.7397 | **749.8735** | 1481.7132 | 741.3602 | 1480.7292 | 740.8682 | G | **1898.7426** | **949.8749** | 1881.7160 | 941.3616 | 1880.7320 | 940.8696 | 17 |
| 15 | **1613.7667** | 807.3870 | 1596.7401 | 798.8737 | 1595.7561 | 798.3817 | D | **1841.7211** | 921.3642 | 1824.6946 | **912.8509** | 1823.7105 | **912.3589** | 16 |
| 16 | **1714.8143** | 857.9108 | 1697.7878 | 849.3975 | 1696.8038 | **848.9055** | T | **1726.6942** | **863.8507** | 1709.6676 | 855.3374 | 1708.6836 | 854.8454 | 15 |
| 17 | 1811.8671 | 906.4372 | 1794.8406 | 897.9239 | 1793.8565 | 897.4319 | P | **1625.6465** | **813.3269** | 1608.6199 | **804.8136** | 1607.6359 | 804.3216 | 14 |
| 18 | **1971.8978** | **986.4525** | 1954.8712 | 977.9392 | 1953.8872 | 977.4472 | C | 1528.5937 | 764.8005 | **1511.5672** | 756.2872 | 1510.5831 | 755.7952 | 13 |
| 19 | 2058.9298 | 1029.9685 | 2041.9032 | 1021.4553 | 2040.9192 | 1020.9632 | S | **1368.5631** | 684.7852 | 1351.5365 | 676.2719 | 1350.5525 | 675.7799 | 12 |
| 20 | 2218.9604 | 1109.9839 | 2201.9339 | 1101.4706 | 2200.9499 | **1100.9786** | C | 1281.5310 | 641.2692 | 1264.5045 | 632.7559 | 1263.5205 | 632.2639 | 11 |
| 21 | 2378.9911 | 1189.9992 | 2361.9645 | **1181.4859** | 2360.9805 | 1180.9939 | C | 1121.5004 | 561.2538 | 1104.4738 | 552.7406 | 1103.4898 | 552.2485 | 10 |
| 22 | 2492.0751 | 1246.5412 | 2475.0486 | 1238.0279 | 2474.0646 | 1237.5359 | I | 961.4697 | 481.2385 | 944.4432 | 472.7252 | 943.4592 | 472.2332 | 9 |
| 23 | 2591.1436 | **1296.0754** | 2574.1170 | **1287.5621** | 2573.1330 | **1287.0701** | V | **848.3857** | 424.6965 | 831.3591 | **416.1832** | 830.3751 | 415.6912 | 8 |
| 24 | 2705.1865 | **1353.0969** | 2688.1599 | 1344.5836 | 2687.1759 | 1344.0916 | N | **749.3173** | 375.1623 | **732.2907** | 366.6490 | 731.3067 | 366.1570 | 7 |
| 25 | 2819.2294 | **1410.1183** | 2802.2029 | 1401.6051 | 2801.2188 | 1401.1131 | N | **635.2743** | 318.1408 | **618.2478** | 309.6275 | 617.2638 | 309.1355 | 6 |
| 26 | 2906.2614 | 1453.6344 | 2889.2349 | 1445.1211 | 2888.2509 | 1444.6291 | S | **521.2314** | 261.1193 | 504.2049 | 252.6061 | **503.2208** | 252.1141 | 5 |
| 27 | 3021.2884 | **1511.1478** | 3004.2618 | **1502.6346** | 3003.2778 | 1502.1425 | N | **434.1994** | 217.6033 | 417.1728 | 209.0901 | **416.1888** | 208.5980 | 4 |
| 28 | 3078.3098 | 1539.6586 | 3061.2833 | 1531.1453 | 3060.2993 | 1530.6533 | G | **319.1724** | 160.0899 | 302.1459 | 151.5766 | 301.1619 | 151.0846 | 3 |
| 29 | 3165.3419 | 1583.1746 | 3148.3153 | **1574.6613** | 3147.3313 | 1574.1693 | S | 262.1510 | 131.5791 | 245.1244 | 123.0659 | 244.1404 | 122.5738 | 2 |
| 30 | | | | | | | R | 175.1190 | 88.0631 | 158.0924 | 79.5498 | | | 1 |

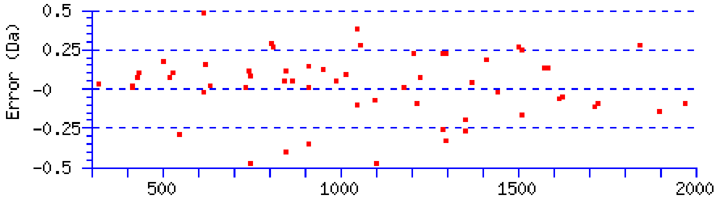


RMS error 232 ppm

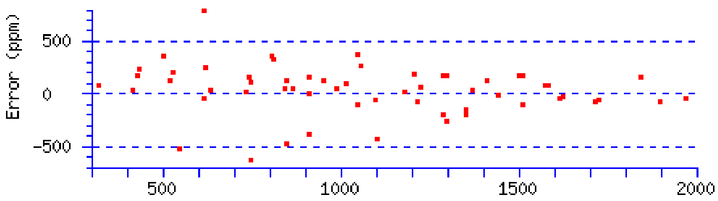


RMS error 232 ppm

## All matches to this query

| Score | Mr(calc): | Delta | Sequence |
|---|---|---|---|
| 79.5 | 3338.4463 | 0.0074 | RGVDVSQVAWQSQGDTPCSCCIVNNSNGSR |
| 71.6 | 3338.4463 | 0.0074 | RGVDVSQVAWQSQGDTPCSCCIVNNSNGSR |
| 68.5 | 3338.4463 | 0.0074 | RGVDVSQVAWQSQGDTPCSCCIVNNSNGSR |
| 39.4 | 3337.4623 | 0.9914 | RGVDVSQVAWQSQGDTPCSCCIVNNSNGSR |
| | | | |

| | | | |
|---|---|---|---|
| 29.2 | 3337.4736 | 0.9801 | VYHCNWLMPSQKASVESCIRVLSDVAK |
| 29.2 | 3337.4736 | 0.9801 | VYHCNWLMPSQKASVESCIRVLSDVAK |
| 15.8 | 3337.4736 | 0.9801 | VYHCNWLMPSQKASVESCIRVLSDVAK |
| 15.8 | 3337.4736 | 0.9801 | VYHCNWLMPSQKASVESCIRVLSDVAK |
| 7.0 | 3337.4736 | 0.9801 | VYHCNWLMPSQKASVESCIRVLSDVAK |
| 6.2 | 3338.4646 | -0.0109 | GEGQNRSPIYSFIGGEHFPRITNVSDATK |

Spectrum No: 237; Query: 152; Rank: 1

## Peptide View

MS/MS Fragmentation of **LNASIADLQSK**
Found in **IPI00190577**, Tax_Id=10116 Gene_Symbol=Lama5 similar to Laminin alpha-5 chain precursor

Match to Query 152: 1159.607608 from(580.811080,2+)
Title: 091008RatKidney_NoSalt_27.1695.1695.2.dta
Data file K:\NewmanPaper\Piliang\3SubProteomes\Piliang3SP\mgf5ppm\ERLIC_3SubProteomes5ppm.mgf

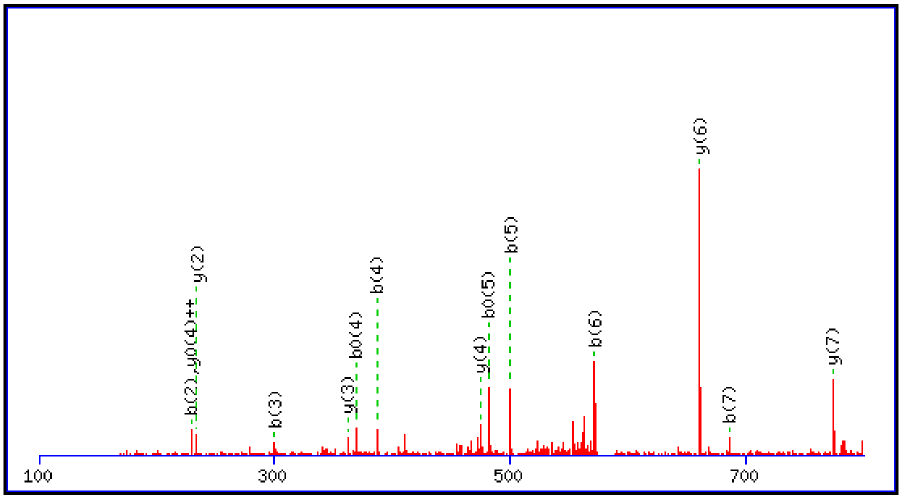


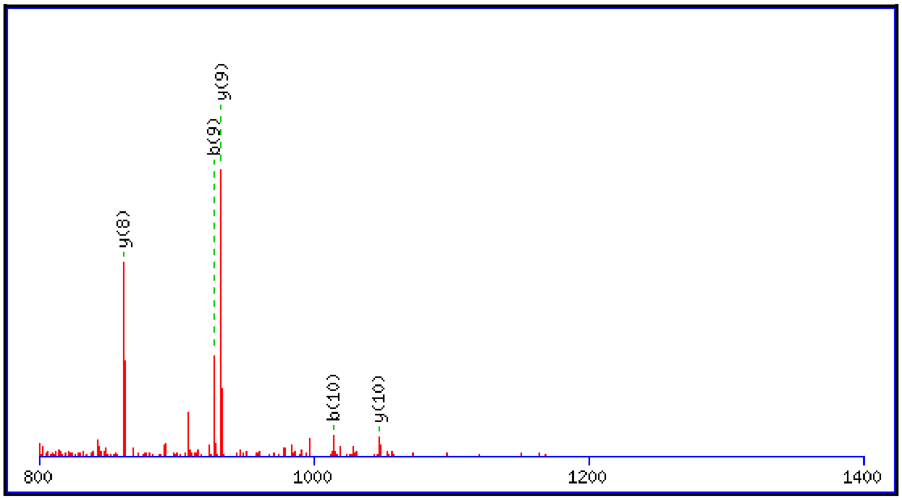

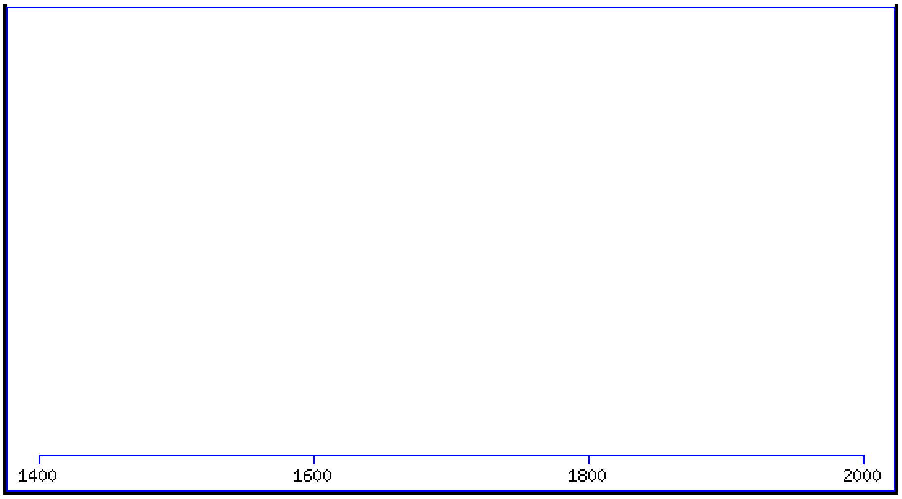


**Monoisotopic mass of neutral peptide Mr(calc):** 1159.6084
**Fixed modifications:** Carbamidomethyl (C)
**Variable modifications:**
**N2** : Deamidated_N (N)
**Ions Score:** 79 **Expect:** 1.3e-006
**Matches (Bold Red):** 19/110 fragment ions using 26 most intense peaks

| # | b | $b^{++}$ | $b^{*}$ | $b^{*++}$ | $b^{0}$ | $b^{0++}$ | Seq. | y | $y^{++}$ | $y^{*}$ | $y^{*++}$ | $y^{0}$ | $y^{0++}$ | # |
|---|---|---|---|---|---|---|---|---|---|---|---|---|---|---|
| 1 | 114.0913 | 57.5493 | | | | | L | | | | | | | 11 |
| 2 | **229.1183** | 115.0628 | 212.0917 | 106.5495 | | | N | **1047.5317** | 524.2695 | 1030.5051 | 515.7562 | 1029.5211 | 515.2642 | 10 |
| 3 | **300.1554** | 150.5813 | 283.1288 | 142.0681 | | | A | **932.5047** | 466.7560 | 915.4782 | 458.2427 | 914.4942 | 457.7507 | 9 |
| 4 | **387.1874** | 194.0973 | 370.1609 | 185.5841 | **369.1769** | 185.0921 | S | **861.4676** | 431.2374 | 844.4411 | 422.7242 | 843.4571 | 422.2322 | 8 |
| 5 | **500.2715** | 250.6394 | 483.2449 | 242.1261 | **482.2609** | 241.6341 | I | **774.4356** | 387.7214 | 757.4090 | 379.2082 | 756.4250 | 378.7162 | 7 |
| 6 | **571.3086** | 286.1579 | 554.2820 | 277.6447 | 553.2980 | 277.1527 | A | **661.3515** | 331.1794 | 644.3250 | 322.6661 | 643.3410 | 322.1741 | 6 |
| 7 | **686.3355** | 343.6714 | 669.3090 | 335.1581 | 668.3250 | 334.6661 | D | 590.3144 | 295.6608 | 573.2879 | 287.1476 | 572.3039 | 286.6556 | 5 |
| 8 | 799.4196 | 400.2134 | 782.3931 | 391.7002 | 781.4090 | 391.2082 | L | **475.2875** | 238.1474 | 458.2609 | 229.6341 | 457.2769 | **229.1421** | 4 |
| 9 | **927.4782** | 464.2427 | 910.4516 | 455.7295 | 909.4676 | 455.2374 | Q | **362.2034** | 181.6053 | 345.1769 | 173.0921 | 344.1928 | 172.6001 | 3 |
| 10 | **1014.5102** | 507.7587 | 997.4837 | 499.2455 | 996.4996 | 498.7535 | S | **234.1448** | 117.5761 | 217.1183 | 109.0628 | 216.1343 | 108.5708 | 2 |
| 11 | | | | | | | K | 147.1128 | 74.0600 | 130.0863 | 65.5468 | | | 1 |

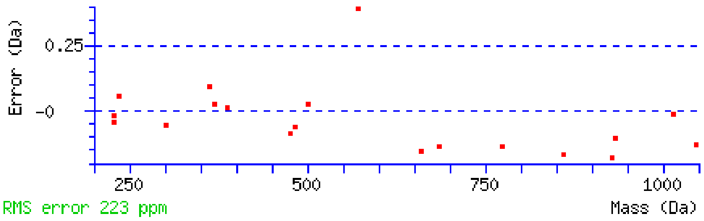


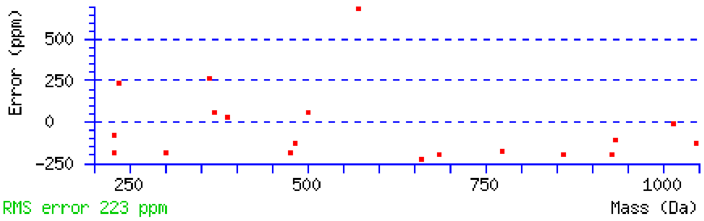


### All matches to this query

| Score | Mr(calc): | Delta | Sequence |
|---|---|---|---|
| 79.3 | 1159.6084 | -0.0008 | LNASIADLQSK |
| 22.4 | 1159.6084 | -0.0008 | LEGSLEQEKK |
| 7.3 | 1159.6019 | 0.0057 | GNLTKHMKSK |
| 6.8 | 1159.6115 | -0.0038 | LHVSSGLLVR |
| 5.8 | 1159.6115 | -0.0038 | LHVSSGLLVR |
| 5.4 | 1159.6019 | 0.0057 | LDSAVQMAGLR |
| 5.1 | 1159.6084 | -0.0008 | GESAILVENTK |
| 4.6 | 1159.6084 | -0.0008 | LEQELKDSAK |
| 3.5 | 1157.6015 | 2.0061 | LVPGVSMWNR |
| 3.3 | 1159.6132 | -0.0056 | LDRPTKQMR |

Spectrum No: 238; Query: 189; Rank: 1

# Peptide View

MS/MS Fragmentation of **ISSNETEIMR**
Found in **IPI00370427**, Tax_Id=10116 Gene_Symbol=Tinag Tubulointerstitial nephritis antigen

Match to Query 189: 1179.545028 from(590.779790,2+)
Title: 100101RatKid_NS_deglyco_23.1241.1241.2.dta
Data file K:\NewmanPaper\Piliang\3SubProteomes\Piliang3SP\mgf5ppm\ERLIC_3SubProteomes5ppm.mgf

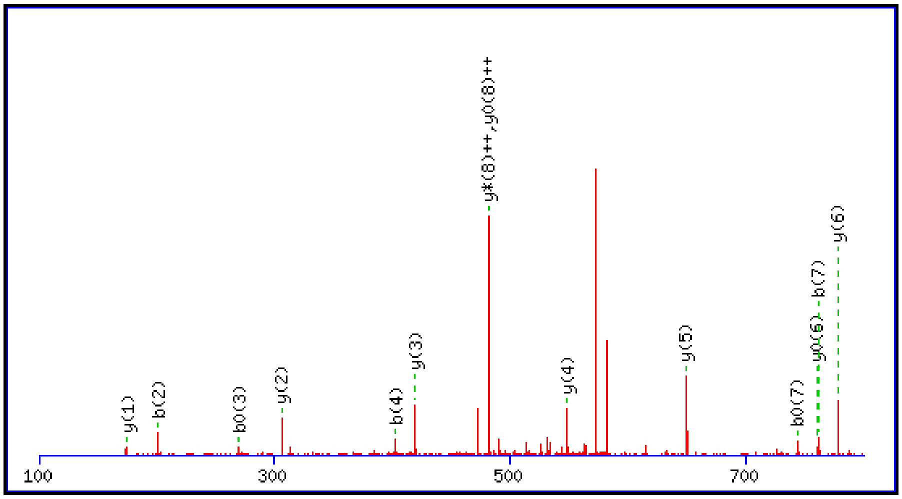


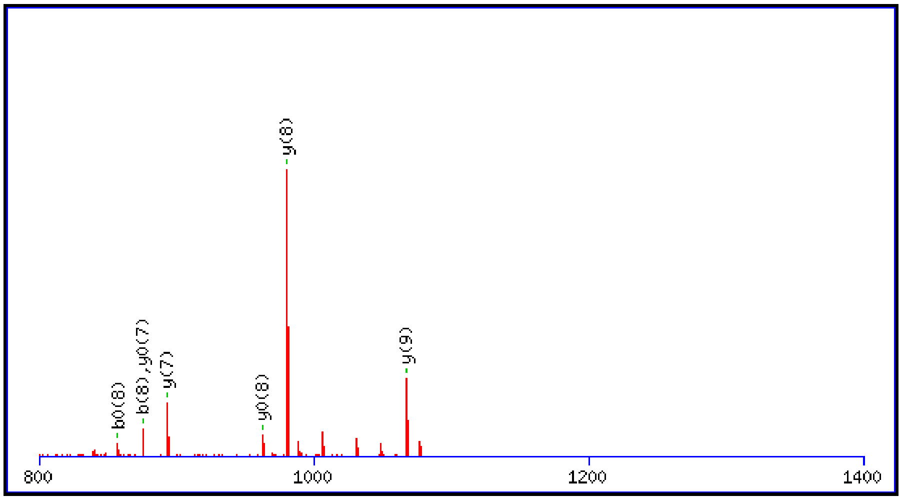


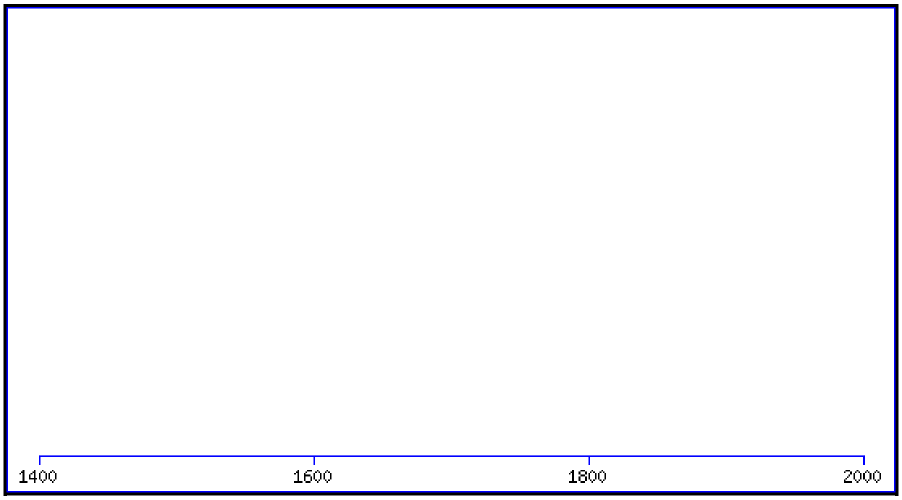

**Monoisotopic mass of neutral peptide Mr(calc):** 1179.5441
**Fixed modifications:** Carbamidomethyl (C)
**Variable modifications:**
**N4** : Deamidated_N (N)
**Ions Score:** 79 **Expect:** 1.3e-006
**Matches (Bold Red):** 21/94 fragment ions using 30 most intense peaks

| # | b | $b^{++}$ | b* | $b^{*++}$ | $b^{0}$ | $b^{0++}$ | Seq. | y | $y^{++}$ | y* | $y^{*++}$ | $y^{0}$ | $y^{0++}$ | # |
|---|---|---|---|---|---|---|---|---|---|---|---|---|---|---|
| 1 | 114.0913 | 57.5493 | | | | | I | | | | | | | 10 |
| 2 | **201.1234** | 101.0653 | | | 183.1128 | 92.0600 | S | **1067.4674** | 534.2373 | 1050.4408 | 525.7240 | 1049.4568 | 525.2320 | 9 |
| 3 | 288.1554 | 144.5813 | | | **270.1448** | 135.5761 | S | **980.4353** | 490.7213 | 963.4088 | **482.2080** | **962.4248** | **481.7160** | 8 |
| 4 | **403.1823** | 202.0948 | 386.1558 | 193.5815 | 385.1718 | 193.0895 | N | **893.4033** | 447.2053 | 876.3768 | 438.6920 | **875.3927** | 438.2000 | 7 |
| 5 | 532.2249 | 266.6161 | 515.1984 | 258.1028 | 514.2144 | 257.6108 | E | **778.3764** | 389.6918 | 761.3498 | 381.1785 | **760.3658** | 380.6865 | 6 |
| 6 | 633.2726 | 317.1399 | 616.2461 | 308.6267 | 615.2620 | 308.1347 | T | **649.3338** | 325.1705 | 632.3072 | 316.6573 | 631.3232 | 316.1652 | 5 |
| 7 | **762.3152** | 381.6612 | 745.2886 | 373.1480 | **744.3046** | 372.6560 | E | **548.2861** | 274.6467 | 531.2595 | 266.1334 | 530.2755 | 265.6414 | 4 |
| 8 | **875.3993** | 438.2033 | 858.3727 | 429.6900 | **857.3887** | 429.1980 | I | **419.2435** | 210.1254 | 402.2170 | 201.6121 | | | 3 |
| 9 | 1006.4397 | 503.7235 | 989.4132 | 495.2102 | 988.4292 | 494.7182 | M | **306.1594** | 153.5834 | 289.1329 | 145.0701 | | | 2 |
| 10 | | | | | | | R | **175.1190** | 88.0631 | 158.0924 | 79.5498 | | | 1 |

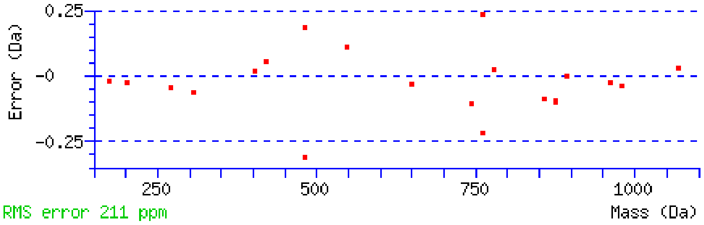


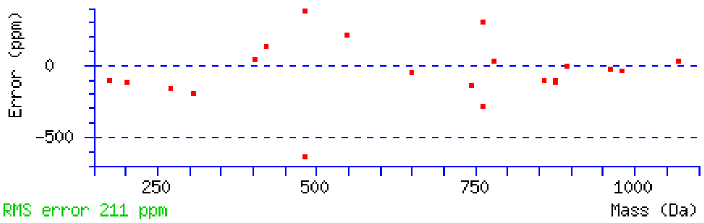


### All matches to this query

| Score | Mr(calc): | Delta | Sequence |
|---|---|---|---|
| 79.3 | 1179.5441 | 0.0009 | ISSNETEIMR |
| 12.9 | 1179.5554 | -0.0103 | ISSGAKDMAER |
| 12.8 | 1179.5520 | -0.0070 | TVPSYNQTNR |
| 12.7 | 1179.5536 | -0.0086 | LSAIPNDTAAK |
| 11.5 | 1179.5520 | -0.0070 | TVPSYNQTNR |
| 11.3 | 1179.5560 | -0.0110 | LWGLYEEDR |
| 9.1 | 1179.5536 | -0.0086 | LASLSDPELR |
| 8.8 | 1179.5397 | 0.0053 | GDDGKPRSLR |
| 8.2 | 1178.5332 | 1.0118 | KASPDPELSR |
| 8.2 | 1179.5488 | -0.0038 | NREMKQAMR |

---

Spectrum No: 239; Query: 943; Rank: 1

---

## Peptide View

MS/MS Fragmentation of **IAVDWESLGYNITR**
Found in **IPI00204137**, Tax_Id=10116 Gene_Symbol=Ptprk Receptor-like protein tyrosine phosphatase kappa extracellular region

Match to Query 943: 1636.811808 from(819.413180,2+)
Title: 100101RatKid_NS_deglyco_25.4471.4471.2.dta
Data file K:\NewmanPaper\Piliang\3SubProteomes\Piliang3SP\mgf5ppm\ERLIC_3SubProteomes5ppm.mgf

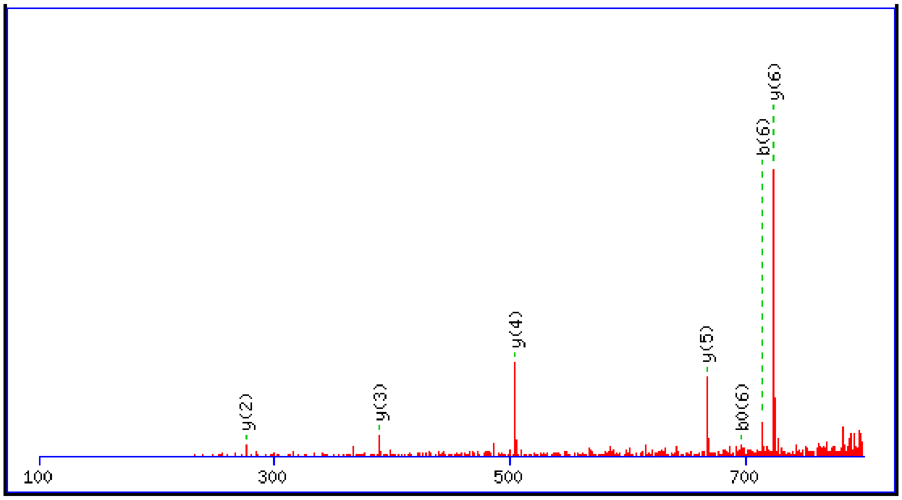


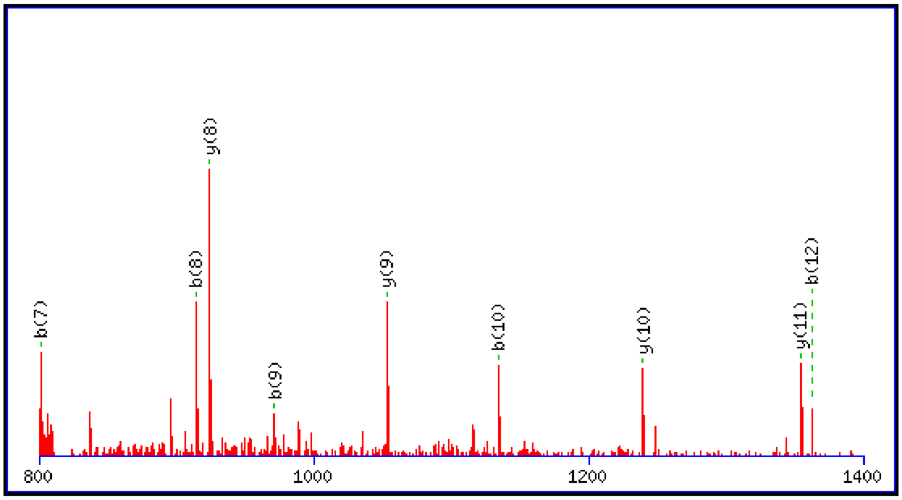


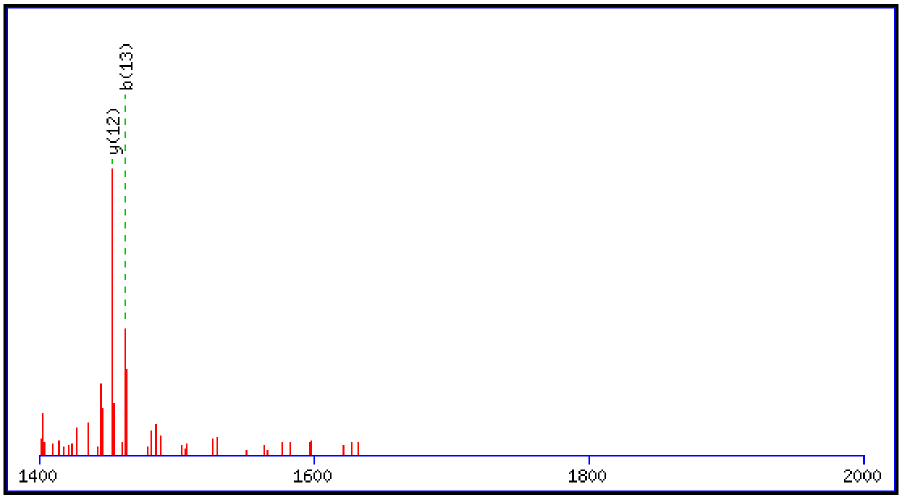


**Monoisotopic mass of neutral peptide Mr(calc):** 1636.8097
**Fixed modifications:** Carbamidomethyl (C)
**Variable modifications:**
**N11** : Deamidated_N (N)
**Ions Score:** 79 **Expect:** 2.2e-006
**Matches (Bold Red):** 18/128 fragment ions using 34 most intense peaks

| # | b | $b^{++}$ | b* | $b^{*++}$ | $b^0$ | $b^{0++}$ | Seq. | y | $y^{++}$ | y* | $y^{*++}$ | $y^0$ | $y^{0++}$ | # |
|---|---|---|---|---|---|---|---|---|---|---|---|---|---|---|
| 1 | 114.0913 | 57.5493 | | | | | I | | | | | | | 14 |
| 2 | 185.1285 | 93.0679 | | | | | A | 1524.7329 | 762.8701 | 1507.7063 | 754.3568 | 1506.7223 | 753.8648 | 13 |
| 3 | 284.1969 | 142.6021 | | | | | V | **1453.6958** | 727.3515 | 1436.6692 | 718.8383 | 1435.6852 | 718.3462 | 12 |
| 4 | 399.2238 | 200.1155 | | | 381.2132 | 191.1103 | D | **1354.6274** | 677.8173 | 1337.6008 | 669.3040 | 1336.6168 | 668.8120 | 11 |
| 5 | 585.3031 | 293.1552 | | | 567.2926 | 284.1499 | W | **1239.6004** | 620.3038 | 1222.5739 | 611.7906 | 1221.5899 | 611.2986 | 10 |

| | | | | | | | | | | | | | | |
|---|---|---|---|---|---|---|---|---|---|---|---|---|---|---|
| **6** | **714.3457** | 357.6765 | | | **696.3352** | 348.6712 | **E** | **1053.5211** | 527.2642 | 1036.4946 | 518.7509 | 1035.5105 | 518.2589 | **9** |
| **7** | **801.3777** | 401.1925 | | | 783.3672 | 392.1872 | **S** | **924.4785** | 462.7429 | 907.4520 | 454.2296 | 906.4680 | 453.7376 | **8** |
| **8** | **914.4618** | 457.7345 | | | 896.4512 | 448.7293 | **L** | 837.4465 | 419.2269 | 820.4199 | 410.7136 | 819.4359 | 410.2216 | **7** |
| **9** | **971.4833** | 486.2453 | | | 953.4727 | 477.2400 | **G** | **724.3624** | 362.6849 | 707.3359 | 354.1716 | 706.3519 | 353.6796 | **6** |
| **10** | **1134.5466** | 567.7769 | | | 1116.5360 | 558.7717 | **Y** | **667.3410** | 334.1741 | 650.3144 | 325.6608 | 649.3304 | 325.1688 | **5** |
| **11** | 1249.5735 | 625.2904 | 1232.5470 | 616.7771 | 1231.5630 | 616.2851 | **N** | **504.2776** | 252.6425 | 487.2511 | 244.1292 | 486.2671 | 243.6372 | **4** |
| **12** | **1362.6576** | 681.8324 | 1345.6311 | 673.3192 | 1344.6470 | 672.8272 | **I** | **389.2507** | 195.1290 | 372.2241 | 186.6157 | 371.2401 | 186.1237 | **3** |
| **13** | **1463.7053** | 732.3563 | 1446.6787 | 723.8430 | 1445.6947 | 723.3510 | **T** | **276.1666** | 138.5870 | 259.1401 | 130.0737 | 258.1561 | 129.5817 | **2** |
| **14** | | | | | | | **R** | 175.1190 | 88.0631 | 158.0924 | 79.5498 | | | **1** |

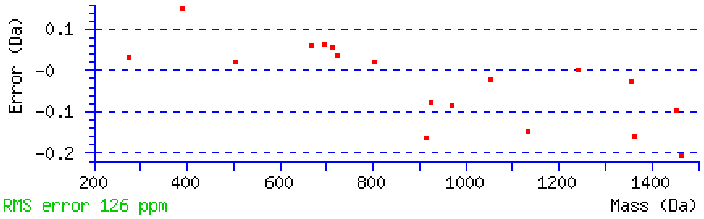


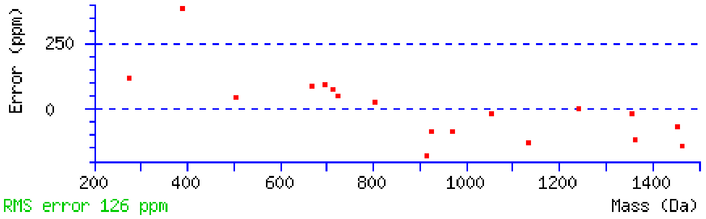


**All matches to this query**

| Score | Mr(calc): | Delta | Sequence |
|---|---|---|---|
| 79.1 | 1636.8097 | 0.0021 | IAVDWESLGYNITR |
| 5.1 | 1636.7977 | 0.0141 | TKLLESEAMNESLR |
| 3.8 | 1636.8130 | -0.0012 | IEVLEEGSFMNLTR |

---

Spectrum No: 240; Query: 1792; Rank: 1

---

## Peptide View

MS/MS Fragmentation of **TLQEDPSWNVTEFHPTPK**
Found in **IPI00230862**, Tax_Id=10116 Gene_Symbol=Anpep Aminopeptidase N

Match to Query 1792: 2125.997248 from(1064.005900,2+)
Title: 091008RatKidney_NoSalt_23.2698.2698.2.dta
Data file K:\NewmanPaper\Piliang\3SubProteomes\Piliang3SP\mgf5ppm\ERLIC_3SubProteomes5ppm.mgf

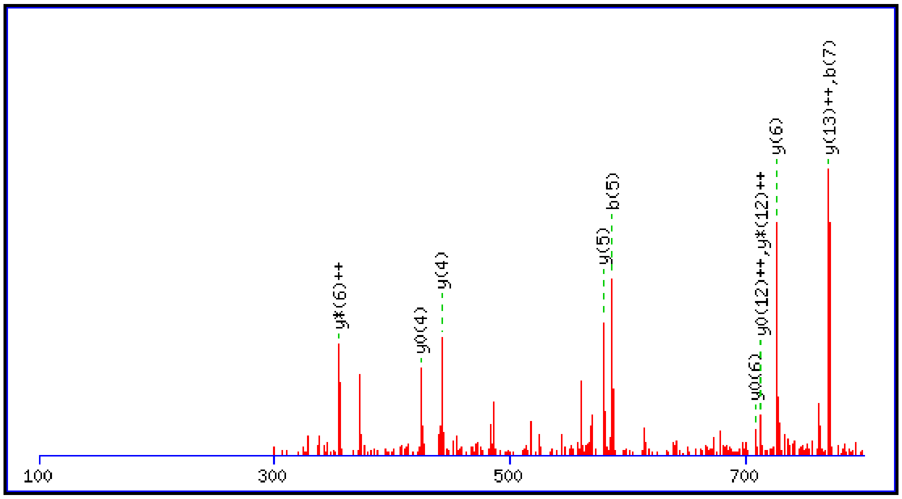

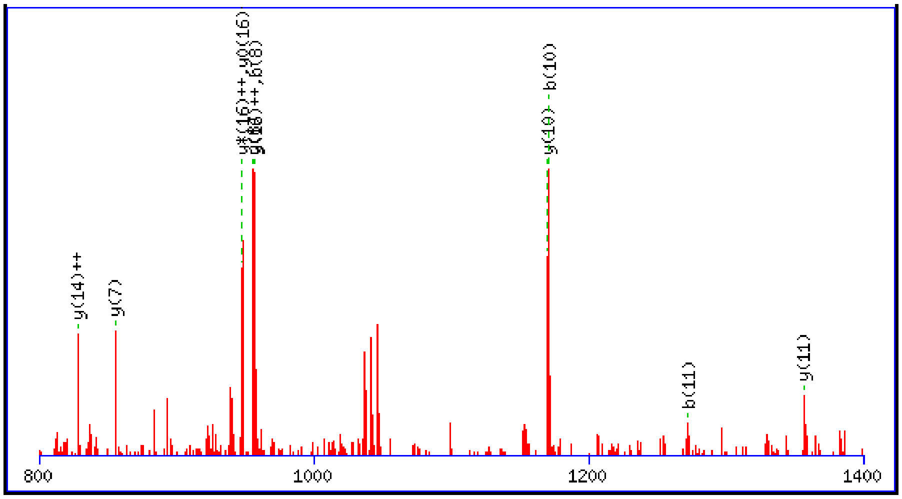


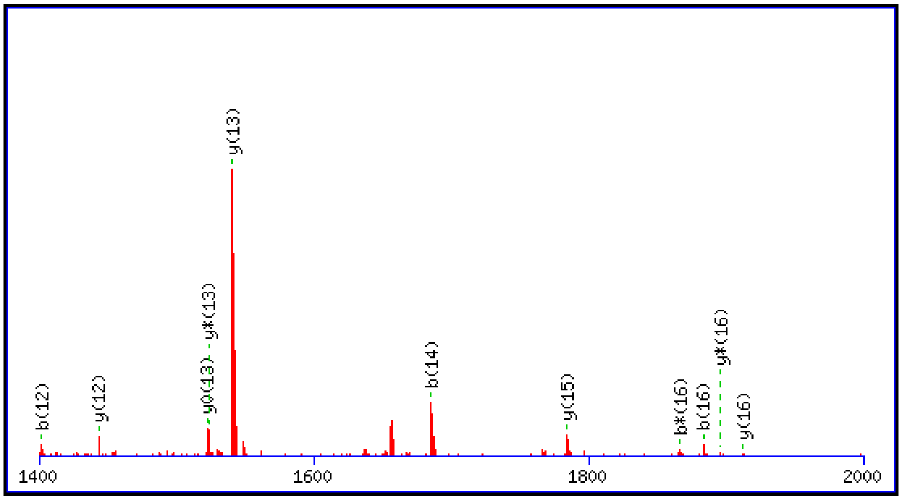


**Monoisotopic mass of neutral peptide Mr(calc):** 2125.9957
**Fixed modifications:** Carbamidomethyl (C)
**Variable modifications:**
**N9** : Deamidated_N (N)
**Ions Score:** 79 **Expect:** 3.3e-006
**Matches (Bold Red):** 33/196 fragment ions using 47 most intense peaks

| # | b | $b^{++}$ | b* | $b^{*++}$ | $b^0$ | $b^{0++}$ | Seq. | y | $y^{++}$ | y* | $y^{*++}$ | $y^0$ | $y^{0++}$ | # |
|---|---|---|---|---|---|---|---|---|---|---|---|---|---|---|
| 1 | 102.0550 | 51.5311 | | | 84.0444 | 42.5258 | T | | | | | | | 18 |
| 2 | 215.1390 | 108.0731 | | | 197.1285 | 99.0679 | L | 2025.9552 | 1013.4813 | 2008.9287 | 1004.9680 | 2007.9447 | 1004.4760 | 17 |
| 3 | 343.1976 | 172.1024 | 326.1710 | 163.5892 | 325.1870 | 163.0972 | Q | **1912.8712** | **956.9392** | **1895.8446** | **948.4260** | 1894.8606 | **947.9339** | 16 |
| 4 | 472.2402 | 236.6237 | 455.2136 | 228.1105 | 454.2296 | 227.6185 | E | **1784.8126** | 892.9099 | 1767.7861 | 884.3967 | 1766.8020 | 883.9047 | 15 |
| 5 | **587.2671** | 294.1372 | 570.2406 | 285.6239 | 569.2566 | 285.1319 | D | 1655.7700 | **828.3886** | 1638.7435 | 819.8754 | 1637.7594 | 819.3834 | 14 |
| 6 | 684.3199 | 342.6636 | 667.2933 | 334.1503 | 666.3093 | 333.6583 | P | **1540.7431** | **770.8752** | **1523.7165** | 762.3619 | **1522.7325** | 761.8699 | 13 |
| 7 | **771.3519** | 386.1796 | 754.3254 | 377.6663 | 753.3414 | 377.1743 | S | **1443.6903** | 722.3488 | 1426.6638 | **713.8355** | 1425.6797 | **713.3435** | 12 |
| 8 | **957.4312** | 479.2193 | 940.4047 | 470.7060 | 939.4207 | 470.2140 | W | **1356.6583** | 678.8328 | 1339.6317 | 670.3195 | 1338.6477 | 669.8275 | 11 |
| 9 | 1072.4582 | 536.7327 | 1055.4316 | 528.2195 | 1054.4476 | 527.7274 | N | **1170.5790** | 585.7931 | 1153.5524 | 577.2798 | 1152.5684 | 576.7878 | 10 |
| 10 | **1171.5266** | 586.2669 | 1154.5000 | 577.7537 | 1153.5160 | 577.2616 | V | 1055.5520 | 528.2796 | 1038.5255 | 519.7664 | 1037.5415 | 519.2744 | 9 |
| 11 | **1272.5743** | 636.7908 | 1255.5477 | 628.2775 | 1254.5637 | 627.7855 | T | **956.4836** | 478.7454 | 939.4571 | 470.2322 | 938.4730 | 469.7402 | 8 |
| 12 | **1401.6169** | 701.3121 | 1384.5903 | 692.7988 | 1383.6063 | 692.3068 | E | **855.4359** | 428.2216 | 838.4094 | 419.7083 | 837.4254 | 419.2163 | 7 |
| 13 | 1548.6853 | 774.8463 | 1531.6587 | 766.3330 | 1530.6747 | 765.8410 | F | **726.3933** | 363.7003 | 709.3668 | **355.1870** | **708.3828** | 354.6950 | 6 |
| 14 | **1685.7442** | 843.3757 | 1668.7176 | 834.8625 | 1667.7336 | 834.3704 | H | **579.3249** | 290.1661 | 562.2984 | 281.6528 | 561.3144 | 281.1608 | 5 |
| 15 | 1782.7970 | 891.9021 | 1765.7704 | 883.3888 | 1764.7864 | 882.8968 | P | **442.2660** | 221.6366 | 425.2395 | 213.1234 | **424.2554** | 212.6314 | 4 |
| 16 | **1883.8446** | 942.4260 | **1866.8181** | 933.9127 | 1865.8341 | 933.4207 | T | 345.2132 | 173.1103 | 328.1867 | 164.5970 | 327.2027 | 164.1050 | 3 |
| 17 | 1980.8974 | 990.9523 | 1963.8708 | 982.4391 | 1962.8868 | 981.9471 | P | 244.1656 | 122.5864 | 227.1390 | 114.0731 | | | 2 |
| 18 | | | | | | | K | 147.1128 | 74.0600 | 130.0863 | 65.5468 | | | 1 |

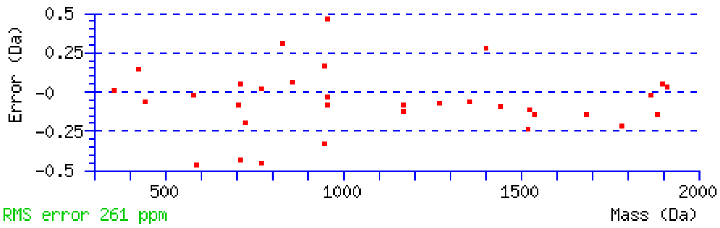


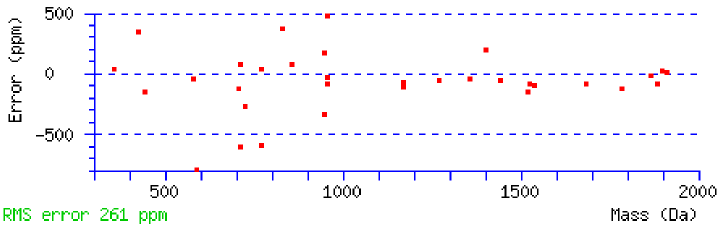


### All matches to this query

| Score | Mr(calc): | Delta | Sequence |
|---|---|---|---|
| 78.9 | 2125.9957 | 0.0016 | TLQEDPSWNVTEFHPTPK |
| 11.9 | 2125.0116 | 0.9856 | TLQEDPSWNVTEFHPTPK |
| 7.6 | 2126.0144 | -0.0171 | KDENLVTGEKQEVSVSVGK |
| 5.2 | 2123.9922 | 2.0050 | IKTMVTEQAAQGSSPPDLR |
| 4.1 | 2123.9922 | 2.0050 | IKTMVTEQAAQGSSPPDLR |
| 3.9 | 2123.9863 | 2.0109 | LQENWMDIGFGILSRHK |
| 3.3 | 2125.9854 | 0.0119 | NQEIEELTKICDELIAK |
| 3.0 | 2125.0013 | 0.9959 | NQEIEELTKICDELIAK |
| 2.2 | 2124.0028 | 1.9945 | LVEDVNPQTFTEPSVQLK |
| 1.9 | 2126.0152 | -0.0180 | LEMLLDIERNSKMAAAPK |

---

Spectrum No: 241; Query: 2656; Rank: 1

---

## Peptide View

MS/MS Fragmentation of **CIPQYWVCDGDADCSDALDELQNCTMR**
Found in **IPI00205325**, Tax_Id=10116 Gene_Symbol=Lrp2 Low-density lipoprotein receptor-related protein 2 precursor

Match to Query 2656: 3292.299762 from(1098.440530,3+)
Title: 100101RatKid_NS_deglyco_24.4619.4619.3.dta
Data file K:\NewmanPaper\Piliang\3SubProteomes\Piliang3SP\mgf5ppm\ERLIC_3SubProteomes5ppm.mgf

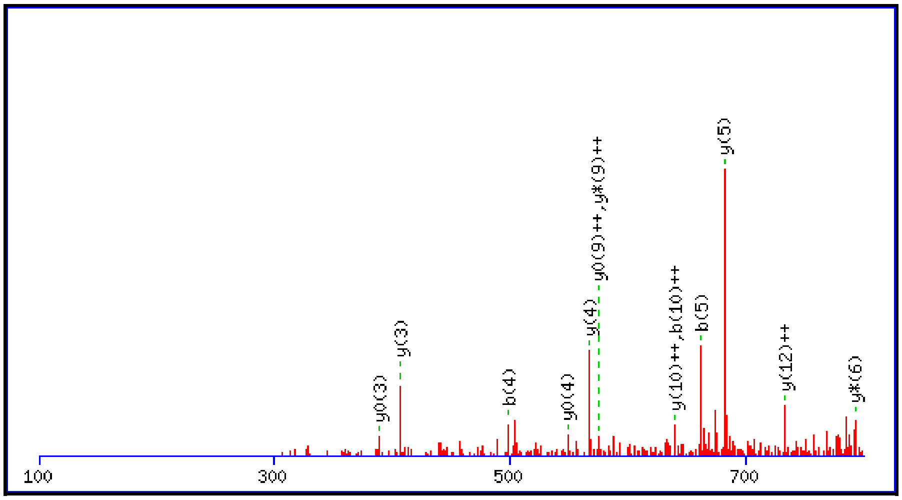

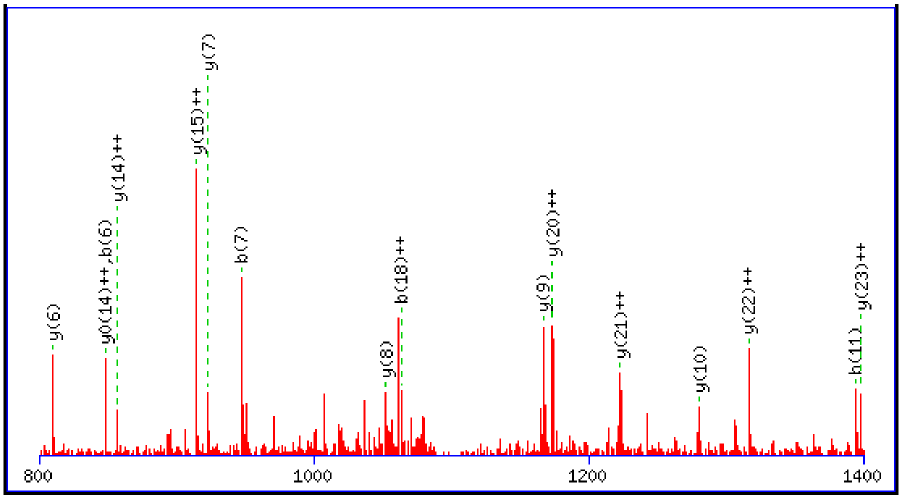


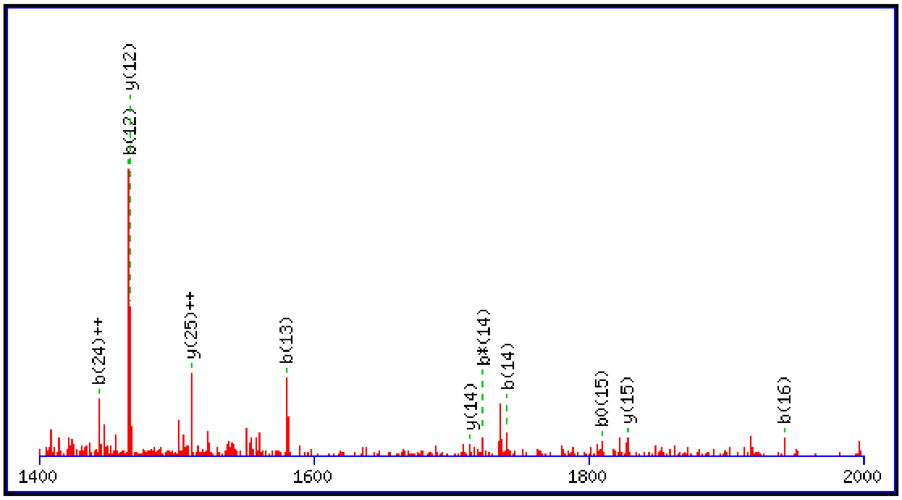


**Monoisotopic mass of neutral peptide Mr(calc):** 3292.2876
**Fixed modifications:** Carbamidomethyl (C)
**Variable modifications:**
**N23** : Deamidated_N (N)
**Ions Score:** 79 **Expect:** 4e-006
**Matches (Bold Red):** 40/286 fragment ions using 55 most intense peaks

| # | b | $b^{++}$ | b* | $b^{*++}$ | $b^{0}$ | $b^{0++}$ | Seq. | y | $y^{++}$ | y* | $y^{*++}$ | $y^{0}$ | $y^{0++}$ | # |
|---|---|---|---|---|---|---|---|---|---|---|---|---|---|---|
| 1 | 161.0379 | 81.0226 | | | | | C | | | | | | | 27 |
| 2 | 274.1220 | 137.5646 | | | | | I | 3133.2642 | 1567.1357 | 3116.2376 | 1558.6225 | 3115.2536 | 1558.1305 | 26 |
| 3 | 371.1748 | 186.0910 | | | | | P | 3020.1801 | **1510.5937** | 3003.1536 | 1502.0804 | 3002.1696 | 1501.5884 | 25 |
| 4 | **499.2333** | 250.1203 | 482.2068 | 241.6070 | | | Q | 2923.1274 | 1462.0673 | 2906.1008 | 1453.5540 | 2905.1168 | 1453.0620 | 24 |
| 5 | **662.2967** | 331.6520 | 645.2701 | 323.1387 | | | Y | 2795.0688 | **1398.0380** | 2778.0422 | 1389.5248 | 2777.0582 | 1389.0327 | 23 |
| 6 | **848.3760** | 424.6916 | 831.3494 | 416.1783 | | | W | 2632.0055 | **1316.5064** | 2614.9789 | 1307.9931 | 2613.9949 | 1307.5011 | 22 |
| 7 | **947.4444** | 474.2258 | 930.4178 | 465.7126 | | | V | 2445.9261 | **1223.4667** | 2428.8996 | 1214.9534 | 2427.9156 | 1214.4614 | 21 |
| 8 | 1107.4750 | 554.2412 | 1090.4485 | 545.7279 | | | C | 2346.8577 | **1173.9325** | 2329.8312 | 1165.4192 | 2328.8472 | 1164.9272 | 20 |
| 9 | 1222.5020 | 611.7546 | 1205.4754 | 603.2414 | 1204.4914 | 602.7493 | D | 2186.8271 | 1093.9172 | 2169.8005 | 1085.4039 | 2168.8165 | 1084.9119 | 19 |
| 10 | 1279.5234 | **640.2654** | 1262.4969 | 631.7521 | 1261.5129 | 631.2601 | G | 2071.8001 | 1036.4037 | 2054.7736 | 1027.8904 | 2053.7896 | 1027.3984 | 18 |
| 11 | **1394.5504** | 697.7788 | 1377.5238 | 689.2656 | 1376.5398 | 688.7735 | D | 2014.7787 | 1007.8930 | 1997.7521 | 999.3797 | 1996.7681 | 998.8877 | 17 |
| 12 | **1465.5875** | 733.2974 | 1448.5610 | 724.7841 | 1447.5769 | 724.2921 | A | 1899.7517 | 950.3795 | 1882.7252 | 941.8662 | 1881.7412 | 941.3742 | 16 |
| 13 | **1580.6144** | 790.8109 | 1563.5879 | 782.2976 | 1562.6039 | 781.8056 | D | **1828.7146** | **914.8609** | 1811.6881 | 906.3477 | 1810.7041 | 905.8557 | 15 |
| 14 | **1740.6451** | 870.8262 | **1723.6185** | 862.3129 | 1722.6345 | 861.8209 | C | **1713.6877** | **857.3475** | 1696.6611 | 848.8342 | 1695.6771 | **848.3422** | 14 |
| 15 | 1827.6771 | 914.3422 | 1810.6506 | 905.8289 | **1809.6666** | 905.3369 | S | 1553.6570 | 777.3322 | 1536.6305 | 768.8189 | 1535.6465 | 768.3269 | 13 |
| 16 | **1942.7041** | 971.8557 | 1925.6775 | 963.3424 | 1924.6935 | 962.8504 | D | **1466.6250** | **733.8161** | 1449.5985 | 725.3029 | 1448.6144 | 724.8109 | 12 |
| 17 | 2013.7412 | 1007.3742 | 1996.7146 | 998.8610 | 1995.7306 | 998.3689 | A | 1351.5981 | 676.3027 | 1334.5715 | 667.7894 | 1333.5875 | 667.2974 | 11 |
| 18 | 2126.8252 | **1063.9163** | 2109.7987 | 1055.4030 | 2108.8147 | 1054.9110 | L | **1280.5609** | **640.7841** | 1263.5344 | 632.2708 | 1262.5504 | 631.7788 | 10 |

| | | | | | | | | | | | | | | |
|---|---|---|---|---|---|---|---|---|---|---|---|---|---|---|
| 19 | 2241.8522 | 1121.4297 | 2224.8256 | 1112.9165 | 2223.8416 | 1112.4244 | D | 1167.4769 | 584.2421 | 1150.4503 | 575.7288 | 1149.4663 | 575.2368 | 9 |
| 20 | 2370.8948 | 1185.9510 | 2353.8682 | 1177.4378 | 2352.8842 | 1176.9457 | E | 1052.4499 | 526.7286 | 1035.4234 | 518.2153 | 1034.4394 | 517.7233 | 8 |
| 21 | 2483.9788 | 1242.4931 | 2466.9523 | 1233.9798 | 2465.9683 | 1233.4878 | L | 923.4073 | 462.2073 | 906.3808 | 453.6940 | 905.3968 | 453.2020 | 7 |
| 22 | 2612.0374 | 1306.5223 | 2595.0109 | 1298.0091 | 2594.0269 | 1297.5171 | Q | 810.3233 | 405.6653 | 793.2967 | 397.1520 | 792.3127 | 396.6600 | 6 |
| 23 | 2727.0644 | 1364.0358 | 2710.0378 | 1355.5225 | 2709.0538 | 1355.0305 | N | 682.2647 | 341.6360 | 665.2382 | 333.1227 | 664.2541 | 332.6307 | 5 |
| 24 | 2887.0950 | 1444.0511 | 2870.0685 | 1435.5379 | 2869.0844 | 1435.0459 | C | 567.2378 | 284.1225 | 550.2112 | 275.6092 | 549.2272 | 275.1172 | 4 |
| 25 | 2988.1427 | 1494.5750 | 2971.1161 | 1486.0617 | 2970.1321 | 1485.5697 | T | 407.2071 | 204.1072 | 390.1806 | 195.5939 | 389.1966 | 195.1019 | 3 |
| 26 | 3119.1832 | 1560.0952 | 3102.1566 | 1551.5819 | 3101.1726 | 1551.0899 | M | 306.1594 | 153.5834 | 289.1329 | 145.0701 | | | 2 |
| 27 | | | | | | | R | 175.1190 | 88.0631 | 158.0924 | 79.5498 | | | 1 |

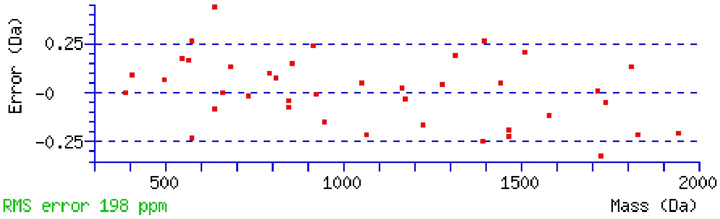


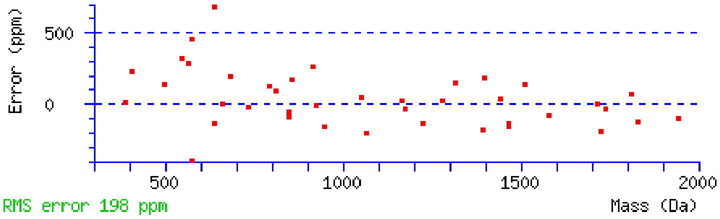


**All matches to this query**

| Score | Mr(calc): | Delta | Sequence |
|---|---|---|---|
| 78.9 | 3292.2876 | 0.0122 | CIPQYWVCDGDADCSDALDELQNCTMR |
| 35.0 | 3291.3036 | 0.9962 | CIPQYWVCDGDADCSDALDELQNCTMR |
| 5.5 | 3290.3167 | 1.9831 | MTLSEIYQWICDNFPYYREAGSGWK |
| 4.8 | 3291.2747 | 1.0251 | LLASFFDRNTLANSCGTGIRSSTNDPR |
| 3.5 | 3292.2722 | 0.0276 | LYNWISETLANARHSDAFLTETVNK |
| 2.1 | 3291.3209 | 0.9789 | SVIGDCTQLLTHYYDDARTMYQVFR |
| 1.0 | 3290.3167 | 1.9831 | MTLSEIYQWICDNFPYYREAGSGWK |
| 0.9 | 3290.3167 | 1.9831 | MTLSEIYQWICDNFPYYREAGSGWK |
| 0.3 | 3291.2958 | 1.0040 | PHQWVEGNIPANSQCTVCHESCGSYHR |
| 0.0 | 3292.2722 | 0.0276 | LYNWISETLANARHSDAFLTETVNK |

---

Spectrum No: 242; Query: 1819; Rank: 1

---

## Peptide View

MS/MS Fragmentation of **LTNQTMGFSFAVEQDIPVK**
Found in **IPI00326140**, Tax_Id=10116 Gene_Symbol=Pzp Alpha-1-macroglobulin precursor

Match to Query 1819: 2141.035388 from(1071.524970,2+)
Title: 100101RatKid_NS_deglyco_27.3729.3729.2.dta
Data file K:\NewmanPaper\Piliang\3SubProteomes\Piliang3SP\mgf5ppm\ERLIC_3SubProteomes5ppm.mgf

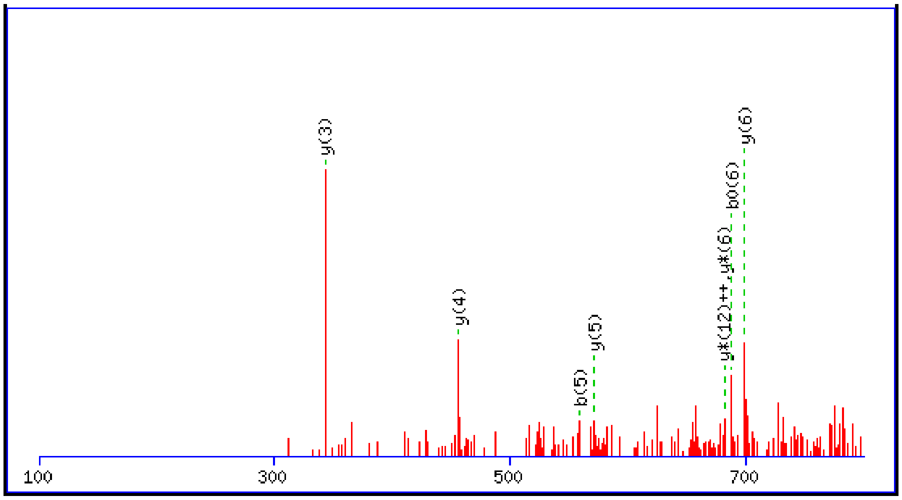


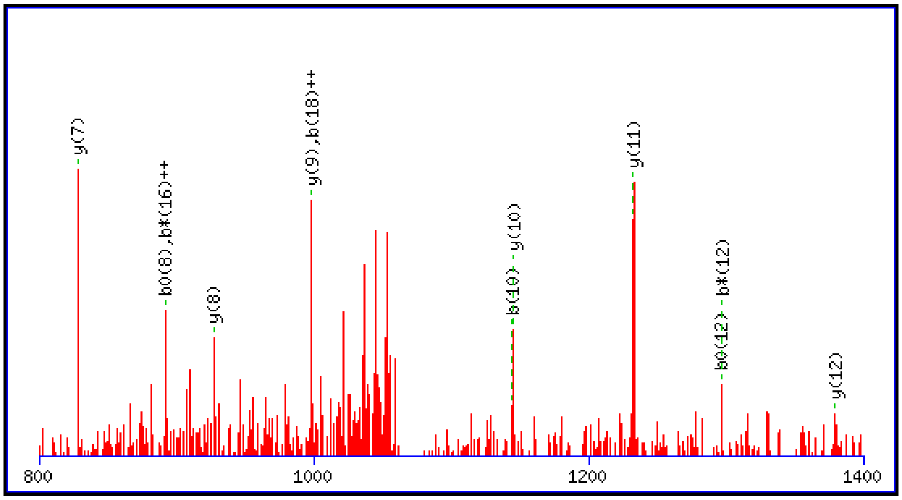


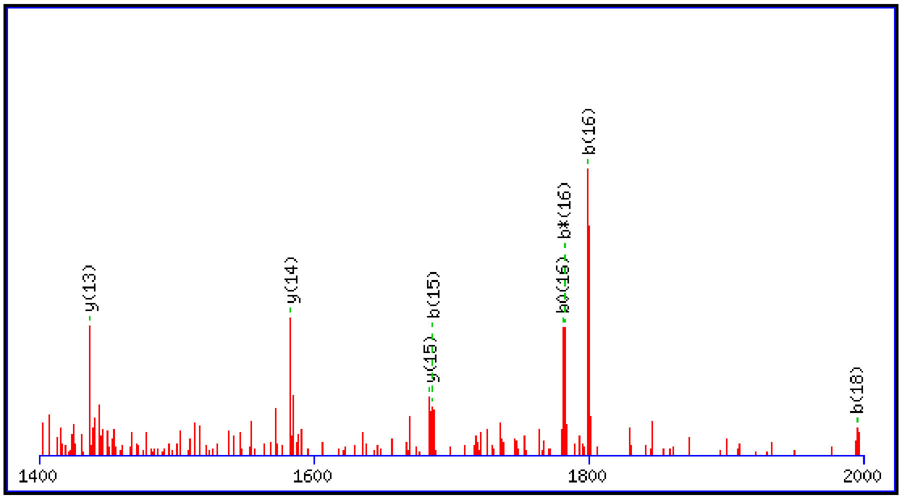


**Monoisotopic mass of neutral peptide Mr(calc):** 2141.0351
**Fixed modifications:** Carbamidomethyl (C)
**Variable modifications:**
**N3** : Deamidated_N (N)
**M6** : Oxidation (M)
**Ions Score:** 79 **Expect:** 3.2e-006
**Matches (Bold Red):** 28/202 fragment ions using 48 most intense peaks

| # | b | $b^{++}$ | b* | $b^{*++}$ | $b^0$ | $b^{0++}$ | Seq. | y | $y^{++}$ | y* | $y^{*++}$ | $y^0$ | $y^{0++}$ | # |
|---|---|---|---|---|---|---|---|---|---|---|---|---|---|---|
| 1 | 114.0913 | 57.5493 | | | | | L | | | | | | | 19 |
| 2 | 215.1390 | 108.0731 | | | 197.1285 | 99.0679 | T | 2028.9583 | 1014.9828 | 2011.9317 | 1006.4695 | 2010.9477 | 1005.9775 | 18 |
| 3 | 330.1660 | 165.5866 | 313.1394 | 157.0733 | 312.1554 | 156.5813 | N | 1927.9106 | 964.4589 | 1910.8841 | 955.9457 | 1909.9000 | 955.4537 | 17 |
| 4 | 458.2245 | 229.6159 | 441.1980 | 221.1026 | 440.2140 | 220.6106 | Q | 1812.8837 | 906.9455 | 1795.8571 | 898.4322 | 1794.8731 | 897.9402 | 16 |

| | | | | | | | | | | | | | | |
|---|---|---|---|---|---|---|---|---|---|---|---|---|---|---|
| 5 | **559.2722** | 280.1397 | 542.2457 | 271.6265 | 541.2616 | 271.1345 | T | **1684.8251** | 842.9162 | 1667.7985 | 834.4029 | 1666.8145 | 833.9109 | 15 |
| 6 | 706.3076 | 353.6574 | 689.2811 | 345.1442 | **688.2971** | 344.6522 | M | **1583.7774** | 792.3923 | 1566.7509 | 783.8791 | 1565.7668 | 783.3871 | 14 |
| 7 | 763.3291 | 382.1682 | 746.3025 | 373.6549 | 745.3185 | 373.1629 | G | **1436.7420** | 718.8746 | 1419.7155 | 710.3614 | 1418.7314 | 709.8694 | 13 |
| 8 | 910.3975 | 455.7024 | 893.3709 | 447.1891 | **892.3869** | 446.6971 | F | **1379.7205** | 690.3639 | 1362.6940 | **681.8506** | 1361.7100 | 681.3586 | 12 |
| 9 | 997.4295 | 499.2184 | 980.4030 | 490.7051 | 979.4190 | 490.2131 | S | **1232.6521** | 616.8297 | 1215.6256 | 608.3164 | 1214.6416 | 607.8244 | 11 |
| 10 | **1144.4979** | 572.7526 | 1127.4714 | 564.2393 | 1126.4874 | 563.7473 | F | **1145.6201** | 573.3137 | 1128.5936 | 564.8004 | 1127.6095 | 564.3084 | 10 |
| 11 | 1215.5351 | 608.2712 | 1198.5085 | 599.7579 | 1197.5245 | 599.2659 | A | **998.5517** | 499.7795 | 981.5251 | 491.2662 | 980.5411 | 490.7742 | 9 |
| 12 | 1314.6035 | 657.8054 | **1297.5769** | 649.2921 | **1296.5929** | 648.8001 | V | **927.5146** | 464.2609 | 910.4880 | 455.7477 | 909.5040 | 455.2556 | 8 |
| 13 | 1443.6461 | 722.3267 | 1426.6195 | 713.8134 | 1425.6355 | 713.3214 | E | **828.4462** | 414.7267 | 811.4196 | 406.2134 | 810.4356 | 405.7214 | 7 |
| 14 | 1571.7046 | 786.3560 | 1554.6781 | 777.8427 | 1553.6941 | 777.3507 | Q | **699.4036** | 350.2054 | **682.3770** | 341.6921 | 681.3930 | 341.2001 | 6 |
| 15 | **1686.7316** | 843.8694 | 1669.7050 | 835.3562 | 1668.7210 | 834.8641 | D | **571.3450** | 286.1761 | 554.3184 | 277.6629 | 553.3344 | 277.1708 | 5 |
| 16 | **1799.8156** | 900.4115 | **1782.7891** | **891.8982** | **1781.8051** | 891.4062 | I | **456.3180** | 228.6627 | 439.2915 | 220.1494 | | | 4 |
| 17 | 1896.8684 | 948.9378 | 1879.8419 | 940.4246 | 1878.8578 | 939.9326 | P | **343.2340** | 172.1206 | 326.2074 | 163.6074 | | | 3 |
| 18 | **1995.9368** | **998.4720** | 1978.9103 | 989.9588 | 1977.9263 | 989.4668 | V | 246.1812 | 123.5942 | 229.1547 | 115.0810 | | | 2 |
| 19 | | | | | | | K | 147.1128 | 74.0600 | 130.0863 | 65.5468 | | | 1 |

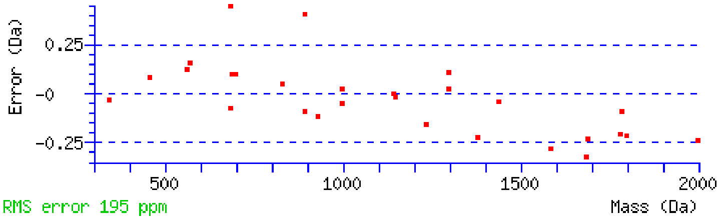


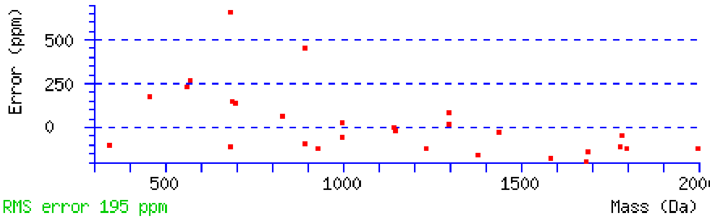


**All matches to this query**

| Score | Mr(calc): | Delta | Sequence |
|---|---|---|---|
| 78.9 | 2141.0351 | 0.0003 | LTNQTMGFSFAVEQDIPVK |
| 57.1 | 2140.0511 | 0.9843 | LTNQTMGFSFAVEQDIPVK |
| 10.7 | 2141.0251 | 0.0103 | KLGANLFTIGFAIYEVPK |
| 3.4 | 2141.0397 | -0.0043 | MNPITLTIIYLTTFKGR |
| 1.1 | 2141.0414 | -0.0060 | GGKPLEMAHKIKTVMFDK |
| 0.3 | 2141.0194 | 0.0160 | VPPPPPTPRDNTEEKYPK |
| 0.0 | 2141.0259 | 0.0095 | TKLPCVSHXCRTGESPAR |
| 0.0 | 2141.0251 | 0.0103 | FXPSLSFKLFNVLKTNK |
| 0.0 | 2141.0251 | 0.0103 | FXPSLSFKLFNVLKTNK |

---

Spectrum No: 243; Query: 1515; Rank: 1

---

## Peptide View

MS/MS Fragmentation of **FSPNLNISESVIPAIIEK**
Found in **IPI00195148**, Tax_Id=10116 Gene_Symbol=Es2 Liver carboxylesterase 1 precursor

Match to Query 1515: 1971.057248 from(986.535900,2+)
Title: 100101RatKid_NS_deglyco_24.4986.4986.2.dta
Data file K:\NewmanPaper\Piliang\3SubProteomes\Piliang3SP\mgf5ppm\ERLIC_3SubProteomes5ppm.mgf

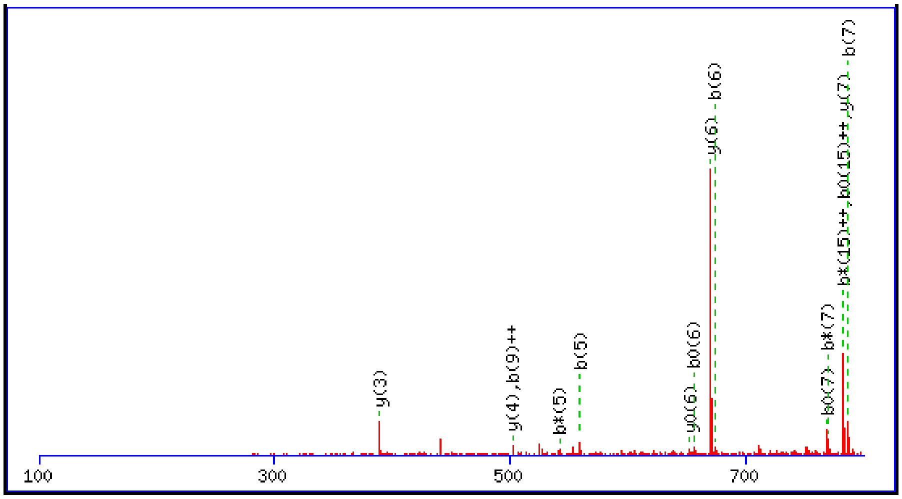


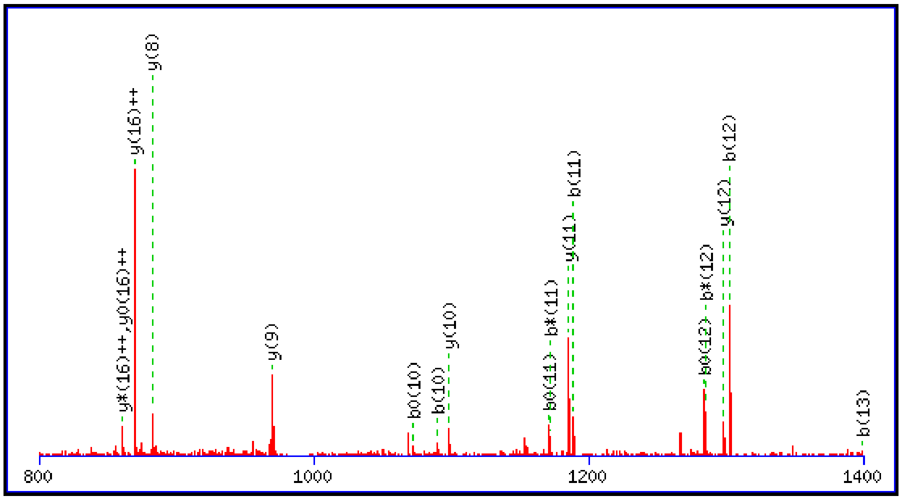


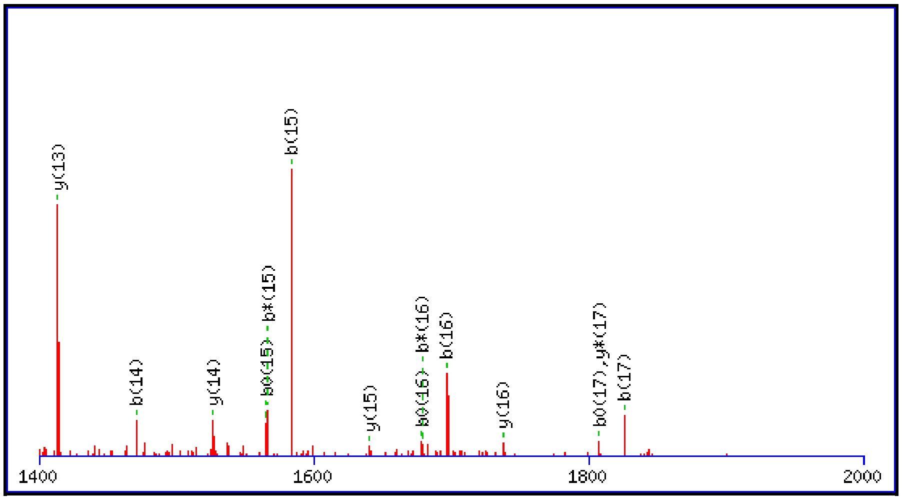


**Monoisotopic mass of neutral peptide Mr(calc):** 1971.0564
**Fixed modifications:** Carbamidomethyl (C)
**Variable modifications:**
**N6** : Deamidated_N (N)
**Ions Score:** 79 **Expect:** 1.6e-006
**Matches (Bold Red):** 46/194 fragment ions using 82 most intense peaks

| # | b | b++ | b* | b*++ | b0 | b0++ | Seq. | y | y++ | y* | y*++ | y0 | y0++ | # |
|---|---|---|---|---|---|---|---|---|---|---|---|---|---|---|
| 1 | 148.0757 | 74.5415 | | | | | F | | | | | | | 18 |
| 2 | 235.1077 | 118.0575 | | | 217.0972 | 109.0522 | S | 1824.9953 | 913.0013 | **1807.9688** | 904.4880 | 1806.9847 | 903.9960 | 17 |
| 3 | 332.1605 | 166.5839 | | | 314.1499 | 157.5786 | P | **1737.9633** | **869.4853** | 1720.9367 | **860.9720** | 1719.9527 | **860.4800** | 16 |
| 4 | 446.2034 | 223.6053 | 429.1769 | 215.0921 | 428.1928 | 214.6001 | N | **1640.9105** | 820.9589 | 1623.8840 | 812.4456 | 1622.9000 | 811.9536 | 15 |
| 5 | **559.2875** | 280.1474 | **542.2609** | 271.6341 | 541.2769 | 271.1421 | L | **1526.8676** | 763.9374 | 1509.8410 | 755.4242 | 1508.8570 | 754.9322 | 14 |

| | | | | | | | | | | | | | | |
|---|---|---|---|---|---|---|---|---|---|---|---|---|---|---|
| 6 | 674.3144 | 337.6608 | 657.2879 | 329.1476 | 656.3038 | 328.6556 | N | 1413.7835 | 707.3954 | 1396.7570 | 698.8821 | 1395.7730 | 698.3901 | 13 |
| 7 | 787.3985 | 394.2029 | 770.3719 | 385.6896 | 769.3879 | 385.1976 | I | 1298.7566 | 649.8819 | 1281.7300 | 641.3687 | 1280.7460 | 640.8767 | 12 |
| 8 | 874.4305 | 437.7189 | 857.4040 | 429.2056 | 856.4199 | 428.7136 | S | 1185.6725 | 593.3399 | 1168.6460 | 584.8266 | 1167.6620 | 584.3346 | 11 |
| 9 | 1003.4731 | 502.2402 | 986.4465 | 493.7269 | 985.4625 | 493.2349 | E | 1098.6405 | 549.8239 | 1081.6140 | 541.3106 | 1080.6299 | 540.8186 | 10 |
| 10 | 1090.5051 | 545.7562 | 1073.4786 | 537.2429 | 1072.4946 | 536.7509 | S | 969.5979 | 485.3026 | 952.5714 | 476.7893 | 951.5873 | 476.2973 | 9 |
| 11 | 1189.5735 | 595.2904 | 1172.5470 | 586.7771 | 1171.5630 | 586.2851 | V | 882.5659 | 441.7866 | 865.5393 | 433.2733 | 864.5553 | 432.7813 | 8 |
| 12 | 1302.6576 | 651.8324 | 1285.6311 | 643.3192 | 1284.6470 | 642.8272 | I | 783.4975 | 392.2524 | 766.4709 | 383.7391 | 765.4869 | 383.2471 | 7 |
| 13 | 1399.7104 | 700.3588 | 1382.6838 | 691.8455 | 1381.6998 | 691.3535 | P | 670.4134 | 335.7103 | 653.3869 | 327.1971 | 652.4028 | 326.7051 | 6 |
| 14 | 1470.7475 | 735.8774 | 1453.7209 | 727.3641 | 1452.7369 | 726.8721 | A | 573.3606 | 287.1840 | 556.3341 | 278.6707 | 555.3501 | 278.1787 | 5 |
| 15 | 1583.8315 | 792.4194 | 1566.8050 | 783.9061 | 1565.8210 | 783.4141 | I | 502.3235 | 251.6654 | 485.2970 | 243.1521 | 484.3130 | 242.6601 | 4 |
| 16 | 1696.9156 | 848.9614 | 1679.8891 | 840.4482 | 1678.9050 | 839.9562 | I | 389.2395 | 195.1234 | 372.2129 | 186.6101 | 371.2289 | 186.1181 | 3 |
| 17 | 1825.9582 | 913.4827 | 1808.9317 | 904.9695 | 1807.9476 | 904.4775 | E | 276.1554 | 138.5813 | 259.1288 | 130.0681 | 258.1448 | 129.5761 | 2 |
| 18 | | | | | | | K | 147.1128 | 74.0600 | 130.0863 | 65.5468 | | | 1 |

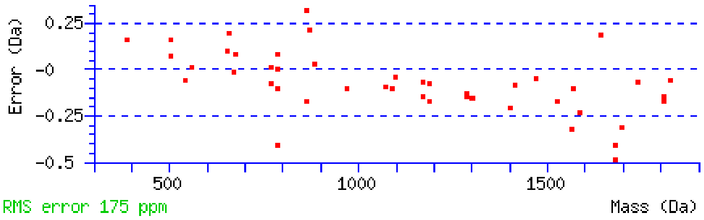


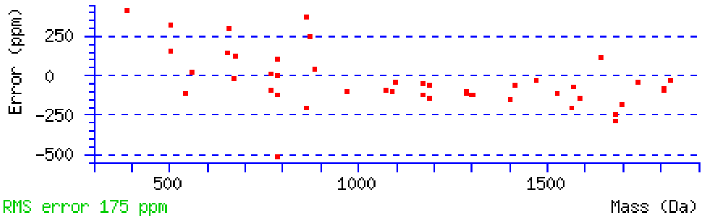


**All matches to this query**

| Score | Mr(calc): | Delta | Sequence |
|---|---|---|---|
| 78.8 | 1971.0564 | 0.0008 | FSPNLNISESVIPAIIEK |
| 58.4 | 1971.0564 | 0.0008 | FSPNLNISESVIPAIIEK |
| 15.2 | 1970.0724 | 0.9848 | FSPNLNISESVIPAIIEK |
| 14.1 | 1971.0636 | -0.0064 | KEQVETSPQASLKTGLQK |
| 8.2 | 1971.0554 | 0.0019 | LHKILGDKVNNTAVIEK |
| 8.2 | 1971.0710 | -0.0138 | SPADIVKNLKESMAVLEK |
| 7.0 | 1971.0398 | 0.0174 | SELSTHQRLHLGKNSHK |
| 6.2 | 1971.0707 | -0.0134 | TLNVKPLVTHRFPLEK |
| 4.8 | 1971.0465 | 0.0107 | FKWPIIAAENEGQGALVK |
| 2.7 | 1971.0524 | 0.0049 | GSLEEAEAERLLLIATEK |

---

Spectrum No: 244; Query: 2769; Rank: 1

---

## Peptide View

MS/MS Fragmentation of **AVATVGPISVAMDASHPSLQFYSSGIYYEPNCSSK**
Found in **IPI00326070**, Tax_Id=10116 Gene_Symbol=Ctsl Cathepsin L1 precursor

Match to Query 2769: 3733.744872 from(1245.588900,3+)
Title: 091008RatKidney_NoSalt_11.5478.5478.3.dta
Data file K:\NewmanPaper\Piliang\3SubProteomes\Piliang3SP\mgf5ppm\ERLIC_3SubProteomes5ppm.mgf

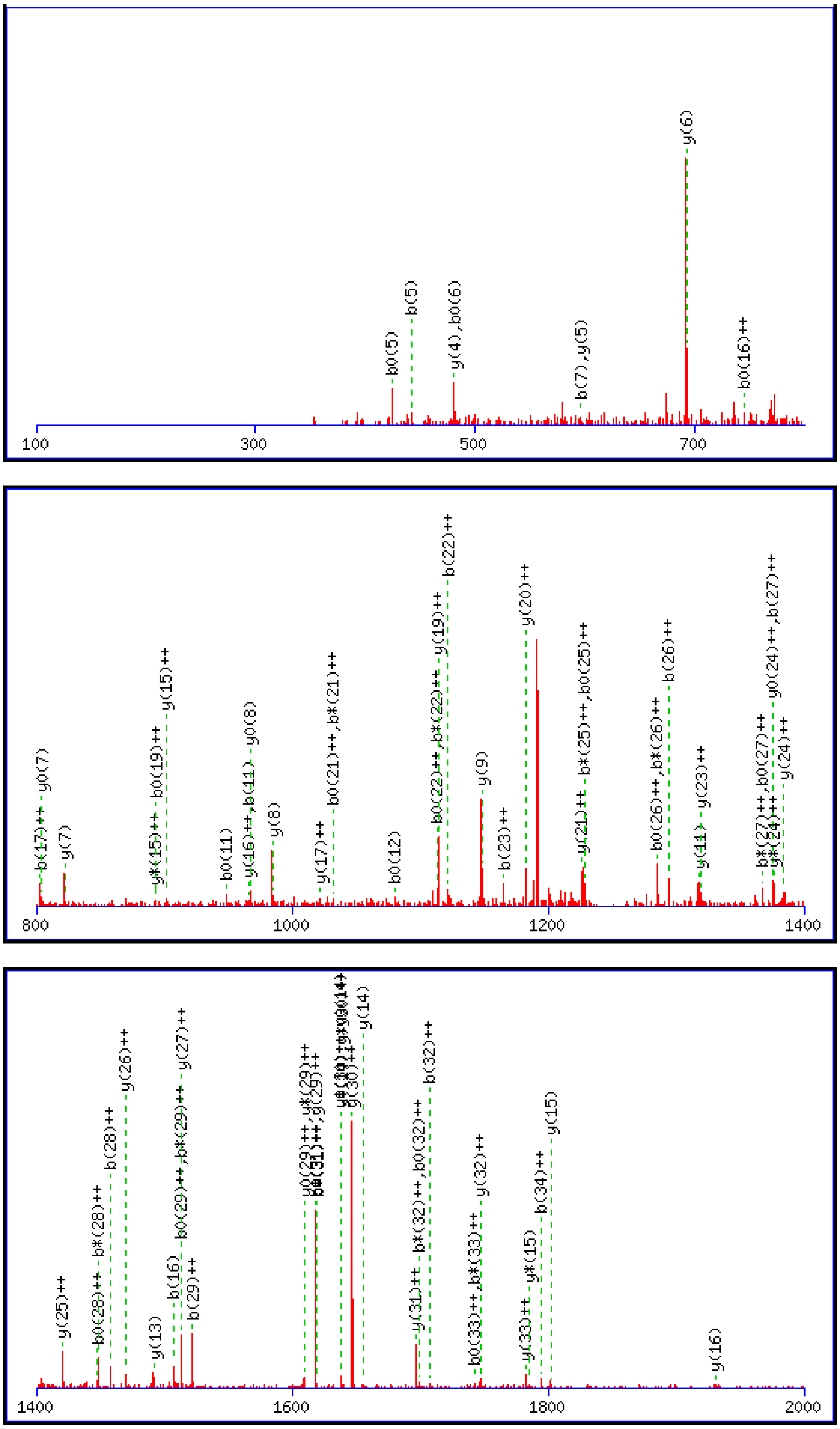


**Monoisotopic mass of neutral peptide Mr(calc):** 3733.7280
**Fixed modifications:** Carbamidomethyl (C)
**Variable modifications:**
**N31** : Deamidated_N (N)
**Ions Score:** 79 **Expect:** 5.6e-006
**Matches (Bold Red):** 78/362 fragment ions using 144 most intense peaks

| # | b | b++ | b* | b*++ | b0 | b0++ | Seq. | y | y++ | y* | y*++ | y0 | y0++ | # |
|---|---|---|---|---|---|---|---|---|---|---|---|---|---|---|
| 1 | 72.0444 | 36.5258 | | | | | A | | | | | | | 35 |
| 2 | 171.1128 | 86.0600 | | | | | V | 3663.6982 | 1832.3527 | 3646.6716 | 1823.8394 | 3645.6876 | 1823.3474 | 34 |
| 3 | 242.1499 | 121.5786 | | | | | A | 3564.6298 | **1782.8185** | 3547.6032 | 1774.3052 | 3546.6192 | 1773.8132 | 33 |
| 4 | 343.1976 | 172.1024 | | | 325.1870 | 163.0972 | T | 3493.5926 | **1747.3000** | 3476.5661 | 1738.7867 | 3475.5821 | 1738.2947 | 32 |
| 5 | **442.2660** | 221.6366 | | | **424.2554** | 212.6314 | V | 3392.5450 | **1696.7761** | 3375.5184 | 1688.2628 | 3374.5344 | 1687.7708 | 31 |

| | | | | | | | | | | | | | | |
|---|---|---|---|---|---|---|---|---|---|---|---|---|---|---|
| 6 | 499.2875 | 250.1474 | | | **481.2769** | 241.1421 | G | 3293.4766 | **1647.2419** | 3276.4500 | **1638.7286** | 3275.4660 | **1638.2366** | 30 |
| 7 | **596.3402** | 298.6738 | | | 578.3297 | 289.6685 | P | 3236.4551 | **1618.7312** | 3219.4285 | **1610.2179** | 3218.4445 | **1609.7259** | 29 |
| 8 | 709.4243 | 355.2158 | | | 691.4137 | 346.2105 | I | 3139.4023 | 1570.2048 | 3122.3758 | 1561.6915 | 3121.3918 | 1561.1995 | 28 |
| 9 | 796.4563 | 398.7318 | | | 778.4458 | 389.7265 | S | 3026.3183 | **1513.6628** | 3009.2917 | 1505.1495 | 3008.3077 | 1504.6575 | 27 |
| 10 | 895.5247 | 448.2660 | | | 877.5142 | 439.2607 | V | 2939.2862 | **1470.1468** | 2922.2597 | 1461.6335 | 2921.2757 | 1461.1415 | 26 |
| 11 | **966.5619** | 483.7846 | | | **948.5513** | 474.7793 | A | 2840.2178 | **1420.6125** | 2823.1913 | 1412.0993 | 2822.2073 | 1411.6073 | 25 |
| 12 | 1097.6023 | 549.3048 | | | **1079.5918** | 540.2995 | M | 2769.1807 | **1385.0940** | 2752.1542 | **1376.5807** | 2751.1701 | **1376.0887** | 24 |
| 13 | 1212.6293 | 606.8183 | | | 1194.6187 | 597.8130 | D | 2638.1402 | **1319.5737** | 2621.1137 | 1311.0605 | 2620.1297 | 1310.5685 | 23 |
| 14 | 1283.6664 | 642.3368 | | | 1265.6558 | 633.3316 | A | 2523.1133 | 1262.0603 | 2506.0867 | 1253.5470 | 2505.1027 | 1253.0550 | 22 |
| 15 | 1370.6984 | 685.8529 | | | 1352.6879 | 676.8476 | S | 2452.0762 | **1226.5417** | 2435.0496 | 1218.0284 | 2434.0656 | 1217.5364 | 21 |
| 16 | **1507.7573** | 754.3823 | | | 1489.7468 | **745.3770** | H | 2365.0441 | **1183.0257** | 2348.0176 | 1174.5124 | 2347.0336 | 1174.0204 | 20 |
| 17 | 1604.8101 | **802.9087** | | | 1586.7995 | 793.9034 | P | 2227.9852 | **1114.4962** | 2210.9587 | 1105.9830 | 2209.9747 | 1105.4910 | 19 |
| 18 | 1691.8421 | 846.4247 | | | 1673.8316 | 837.4194 | S | 2130.9325 | 1065.9699 | 2113.9059 | 1057.4566 | 2112.9219 | 1056.9646 | 18 |
| 19 | 1804.9262 | 902.9667 | | | 1786.9156 | **893.9615** | L | 2043.9004 | **1022.4539** | 2026.8739 | 1013.9406 | 2025.8899 | 1013.4486 | 17 |
| 20 | 1932.9848 | 966.9960 | 1915.9582 | 958.4828 | 1914.9742 | 957.9907 | Q | **1930.8164** | **965.9118** | 1913.7898 | 957.3985 | 1912.8058 | 956.9065 | 16 |
| 21 | 2080.0532 | 1040.5302 | 2063.0266 | **1032.0170** | 2062.0426 | **1031.5249** | F | **1802.7578** | **901.8825** | **1785.7312** | **893.3693** | 1784.7472 | 892.8772 | 15 |
| 22 | 2243.1165 | **1122.0619** | 2226.0900 | **1113.5486** | 2225.1060 | **1113.0566** | Y | **1655.6894** | 828.3483 | **1638.6628** | 819.8351 | **1637.6788** | 819.3430 | 14 |
| 23 | 2330.1485 | **1165.5779** | 2313.1220 | 1157.0646 | 2312.1380 | 1156.5726 | S | **1492.6260** | 746.8167 | 1475.5995 | 738.3034 | 1474.6155 | 737.8114 | 13 |
| 24 | 2417.1806 | 1209.0939 | 2400.1540 | 1200.5807 | 2399.1700 | 1200.0886 | S | 1405.5940 | 703.3006 | 1388.5675 | 694.7874 | 1387.5835 | 694.2954 | 12 |
| 25 | 2474.2020 | 1237.6047 | 2457.1755 | **1229.0914** | 2456.1915 | **1228.5994** | G | **1318.5620** | 659.7846 | 1301.5354 | 651.2714 | 1300.5514 | 650.7793 | 11 |
| 26 | 2587.2861 | **1294.1467** | 2570.2596 | **1285.6334** | 2569.2755 | **1285.1414** | I | 1261.5405 | 631.2739 | 1244.5140 | 622.7606 | 1243.5300 | 622.2686 | 10 |
| 27 | 2750.3494 | **1375.6784** | 2733.3229 | **1367.1651** | 2732.3389 | **1366.6731** | Y | **1148.4565** | 574.7319 | 1131.4299 | 566.2186 | 1130.4459 | 565.7266 | 9 |
| 28 | 2913.4128 | **1457.2100** | 2896.3862 | **1448.6967** | 2895.4022 | **1448.2047** | Y | **985.3931** | 493.2002 | 968.3666 | 484.6869 | **967.3826** | 484.1949 | 8 |
| 29 | 3042.4554 | **1521.7313** | 3025.4288 | **1513.2180** | 3024.4448 | **1512.7260** | E | **822.3298** | 411.6685 | 805.3033 | 403.1553 | **804.3192** | 402.6633 | 7 |
| 30 | 3139.5081 | 1570.2577 | 3122.4816 | 1561.7444 | 3121.4976 | 1561.2524 | P | **693.2872** | 347.1472 | 676.2607 | 338.6340 | 675.2766 | 338.1420 | 6 |
| 31 | 3254.5351 | 1627.7712 | 3237.5085 | **1619.2579** | 3236.5245 | **1618.7659** | N | **596.2344** | 298.6209 | 579.2079 | 290.1076 | 578.2239 | 289.6156 | 5 |
| 32 | 3414.5657 | **1707.7865** | 3397.5392 | **1699.2732** | 3396.5551 | **1698.7812** | C | **481.2075** | 241.1074 | 464.1810 | 232.5941 | 463.1969 | 232.1021 | 4 |
| 33 | 3501.5977 | 1751.3025 | 3484.5712 | **1742.7892** | 3483.5872 | **1742.2972** | S | 321.1769 | 161.0921 | 304.1503 | 152.5788 | 303.1663 | 152.0868 | 3 |
| 34 | 3588.6298 | **1794.8185** | 3571.6032 | 1786.3052 | 3570.6192 | 1785.8132 | S | 234.1448 | 117.5761 | 217.1183 | 109.0628 | 216.1343 | 108.5708 | 2 |
| 35 | | | | | | | K | 147.1128 | 74.0600 | 130.0863 | 65.5468 | | | 1 |

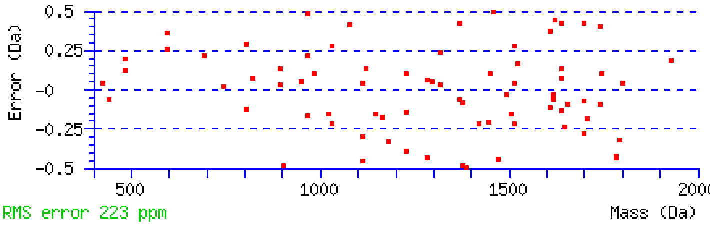


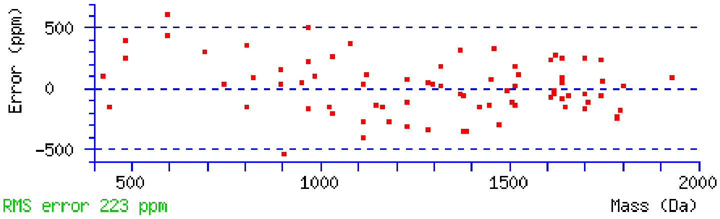


### All matches to this query

| Score | Mr(calc): | Delta | Sequence |
|---|---|---|---|
| 78.8 | 3733.7280 | 0.0169 | AVATVGPISVAMDASHPSLQFYSSGIYYEPNCSSK |
| 68.9 | 3732.7440 | 1.0009 | AVATVGPISVAMDASHPSLQFYSSGIYYEPNCSSK |
| 12.4 | 3733.7706 | -0.0257 | LVGATHIVTGHNADDMAETVLMNFLRGDAGRLAR |
| 8.3 | 3731.7549 | 1.9899 | TLRCYHMWIVMARPACSVPCAVPLLTKXSR |
| 8.3 | 3731.7549 | 1.9899 | TLRCYHMWIVMARPACSVPCAVPLLTKXSR |
| 6.2 | 3731.7380 | 2.0069 | VSSVFYTTIVPMLNPLIYSLRNKDVTLALK |
| 6.0 | 3732.7613 | 0.9835 | ALASGFPHVSAYAHEFNPLSPLYSLDVLADASHR |
| 5.8 | 3731.7582 | 1.9866 | WTAHDVENRVECLYNLLSYVNIDIDPVYLK |
| 5.8 | 3732.7422 | 1.0026 | WTAHDVENRVECLYNLLSYVNIDIDPVYLK |
| 5.8 | 3732.7422 | 1.0026 | WTAHDVENRVECLYNLLSYVNIDIDPVYLK |

Spectrum No: 245; Query: 903; Rank: 1

## Peptide View

MS/MS Fragmentation of **LHNQLLPNTTTVEK**
Found in **IPI00206254**, Tax_Id=10116 Gene_Symbol=Ggt1 Gamma-glutamyltranspeptidase 1 precursor

Match to Query 903: 1607.853148 from(804.933850,2+)
Title: 091008RatKidney_NoSalt_28.1420.1420.2.dta
Data file K:\NewmanPaper\Piliang\3SubProteomes\Piliang3SP\mgf5ppm\ERLIC_3SubProteomes5ppm.mgf

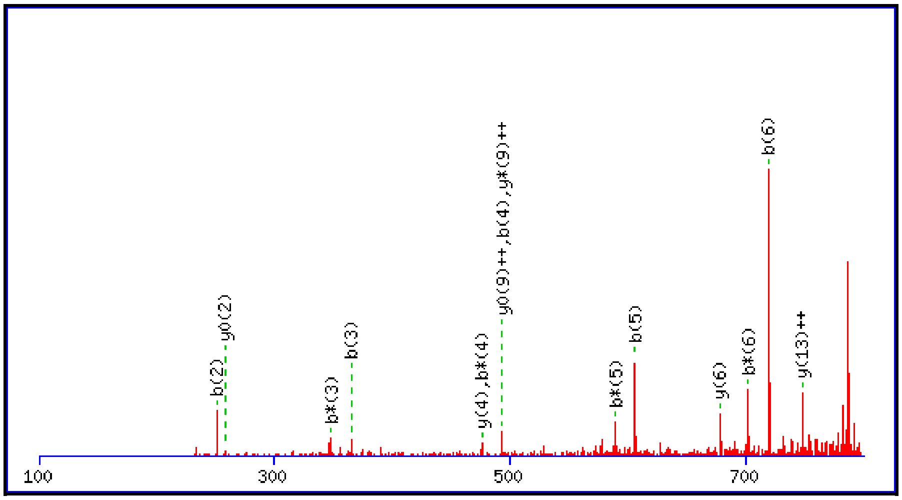


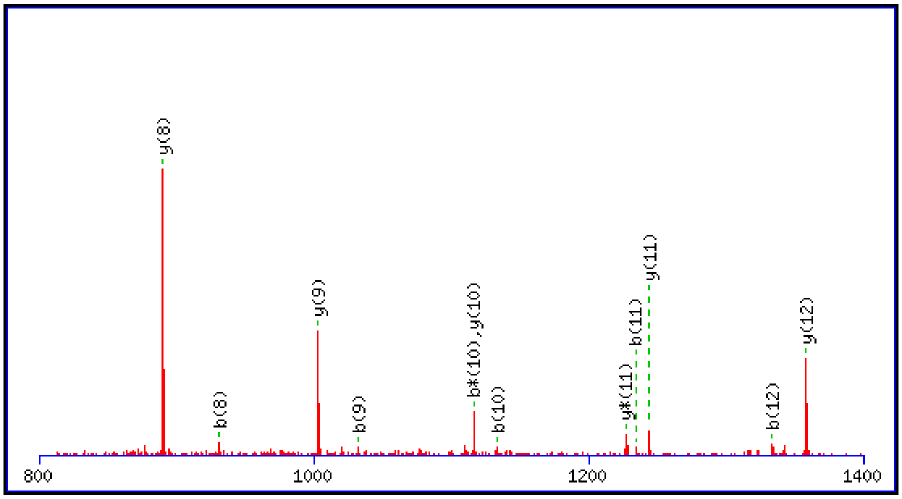

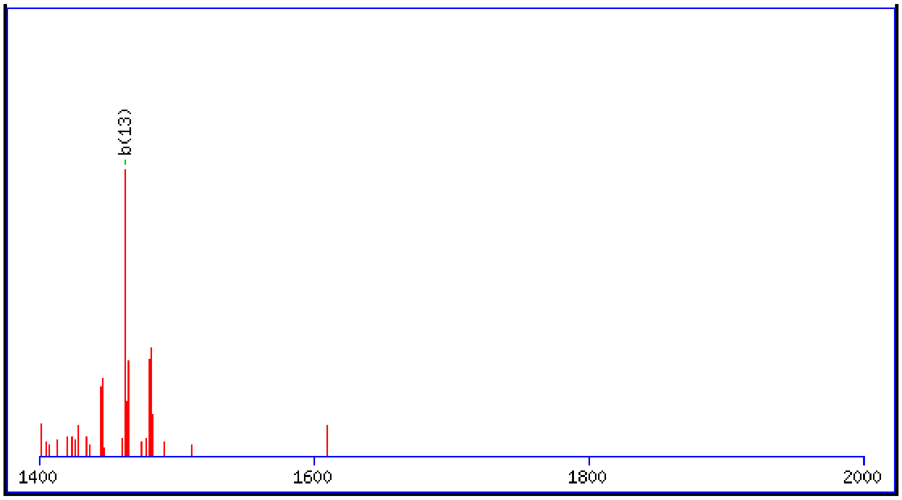


**Monoisotopic mass of neutral peptide Mr(calc):** 1607.8519
**Fixed modifications:** Carbamidomethyl (C)
**Variable modifications:**
**N8** : Deamidated_N (N)
**Ions Score:** 79 **Expect:** 1.4e-006
**Matches (Bold Red):** 28/134 fragment ions using 42 most intense peaks

| # | b | b++ | b* | b*++ | b0 | b0++ | Seq. | y | y++ | y* | y*++ | y0 | y0++ | # |
|---|---|---|---|---|---|---|---|---|---|---|---|---|---|---|
| 1 | 114.0913 | 57.5493 | | | | | L | | | | | | | 14 |
| 2 | **251.1503** | 126.0788 | | | | | H | 1495.7751 | **748.3912** | 1478.7485 | 739.8779 | 1477.7645 | 739.3859 | 13 |
| 3 | **365.1932** | 183.1002 | **348.1666** | 174.5870 | | | N | **1358.7162** | 679.8617 | 1341.6896 | 671.3485 | 1340.7056 | 670.8564 | 12 |
| 4 | **493.2518** | 247.1295 | **476.2252** | 238.6162 | | | Q | **1244.6733** | 622.8403 | **1227.6467** | 614.3270 | 1226.6627 | 613.8350 | 11 |
| 5 | **606.3358** | 303.6715 | **589.3093** | 295.1583 | | | L | **1116.6147** | 558.8110 | 1099.5881 | 550.2977 | 1098.6041 | 549.8057 | 10 |
| 6 | **719.4199** | 360.2136 | **702.3933** | 351.7003 | | | L | **1003.5306** | 502.2689 | 986.5041 | **493.7557** | 985.5200 | **493.2637** | 9 |
| 7 | 816.4726 | 408.7400 | 799.4461 | 400.2267 | | | P | **890.4465** | 445.7269 | 873.4200 | 437.2136 | 872.4360 | 436.7216 | 8 |
| 8 | **931.4996** | 466.2534 | 914.4730 | 457.7402 | | | N | 793.3938 | 397.2005 | 776.3672 | 388.6873 | 775.3832 | 388.1952 | 7 |
| 9 | **1032.5473** | 516.7773 | 1015.5207 | 508.2640 | 1014.5367 | 507.7720 | T | **678.3668** | 339.6871 | 661.3403 | 331.1738 | 660.3563 | 330.6818 | 6 |
| 10 | **1133.5949** | 567.3011 | **1116.5684** | 558.7878 | 1115.5844 | 558.2958 | T | 577.3192 | 289.1632 | 560.2926 | 280.6499 | 559.3086 | 280.1579 | 5 |
| 11 | **1234.6426** | 617.8249 | 1217.6161 | 609.3117 | 1216.6321 | 608.8197 | T | **476.2715** | 238.6394 | 459.2449 | 230.1261 | 458.2609 | 229.6341 | 4 |
| 12 | **1333.7110** | 667.3592 | 1316.6845 | 658.8459 | 1315.7005 | 658.3539 | V | 375.2238 | 188.1155 | 358.1973 | 179.6023 | 357.2132 | 179.1103 | 3 |
| 13 | **1462.7536** | 731.8805 | 1445.7271 | 723.3672 | 1444.7431 | 722.8752 | E | 276.1554 | 138.5813 | 259.1288 | 130.0681 | **258.1448** | 129.5761 | 2 |
| 14 | | | | | | | K | 147.1128 | 74.0600 | 130.0863 | 65.5468 | | | 1 |

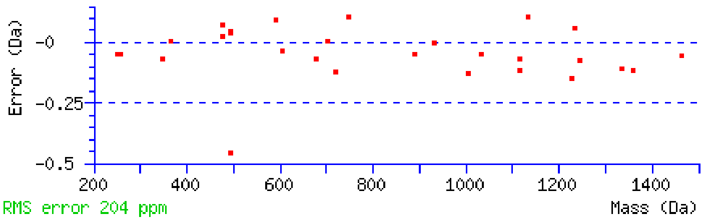


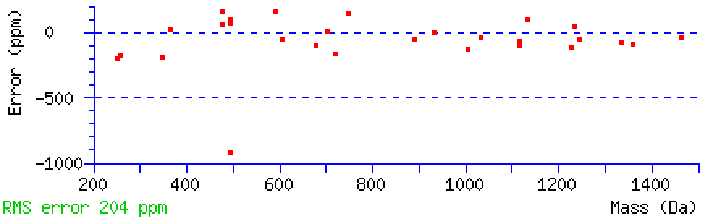


### All matches to this query

| Score | Mr(calc): | Delta | Sequence |
|---|---|---|---|
| 78.7 | 1607.8519 | 0.0013 | LHNQLLPNTTTVEK |
| 39.5 | 1607.8519 | 0.0013 | LHNQLLPNTTTVEK |
| 11.1 | 1607.8592 | -0.0061 | EISLYIPRMLTEK |
| 10.9 | 1607.8420 | 0.0112 | HLHKVFQENNITK |
| 10.9 | 1607.8420 | 0.0112 | HLHKVFQENNITK |
| 5.0 | 1607.8519 | 0.0013 | FSLGSTINVKVGGNSK |
| 3.5 | 1607.8572 | -0.0041 | LHNNLLTWWGNLK |
| | | | |

| | | | |
|---|---|---|---|
| 2.5 | 1607.8645 | -0.0113 | VKHAGGHSPQVNHLK |
| 2.0 | 1607.8413 | 0.0118 | MTQRSSVTIKSGGTR |
| 1.8 | 1607.8453 | 0.0078 | EKAAVFRSMNSALGK |

---

Spectrum No: 246; Query: 2384; Rank: 1

---

## Peptide View

MS/MS Fragmentation of **FQISPQLQFSPEEVLGMVLNYSR**
Found in **IPI00210975**, Tax_Id=10116 Gene_Symbol=Hyou1 Hypoxia up-regulated protein 1 precursor

Match to Query 2384: 2700.336488 from(1351.175520,2+)
Title: 091008RatKidney_NH4Format01_22.4707.4707.2.dta
Data file K:\NewmanPaper\Piliang\3SubProteomes\Piliang3SP\mgf5ppm\ERLIC_3SubProteomes5ppm.mgf

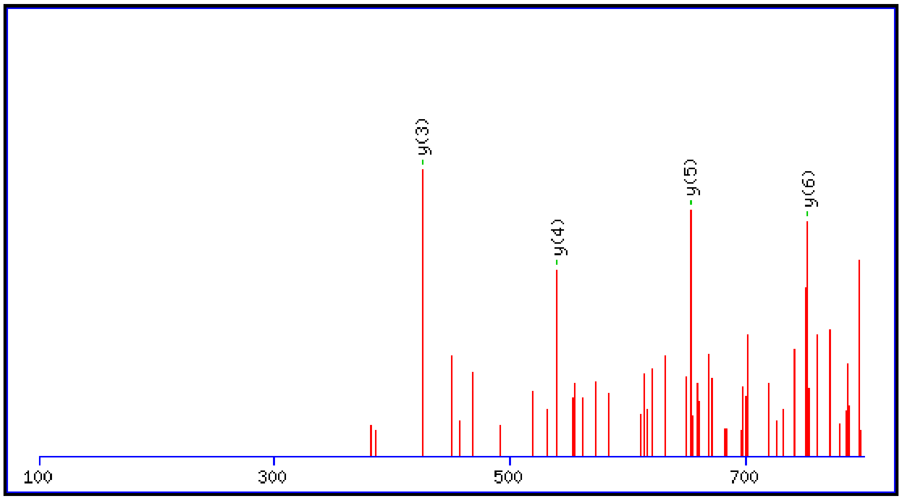


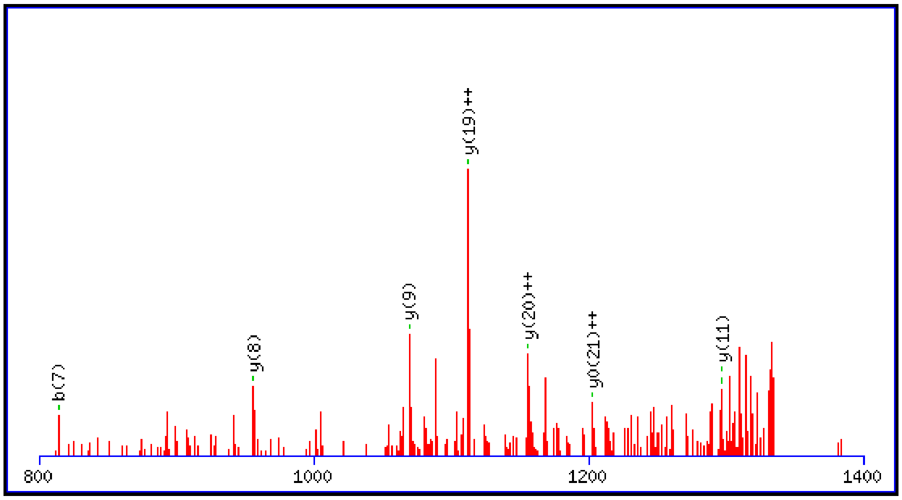

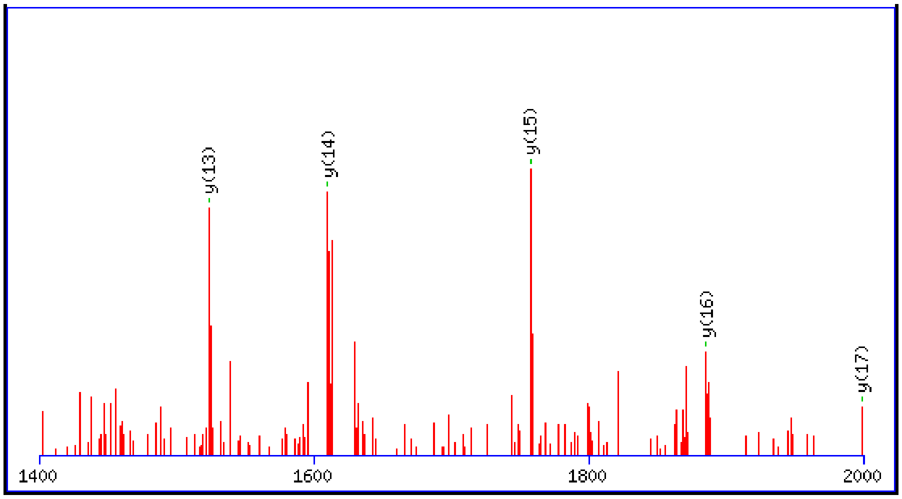


**Monoisotopic mass of neutral peptide Mr(calc):** 2698.3312
**Fixed modifications:** Carbamidomethyl (C)
**Variable modifications:**
**M17** : Oxidation (M)
**N20** : Deamidated_N (N)
**Ions Score:** 79 **Expect:** 3.1e-006
**Matches (Bold Red):** 16/254 fragment ions using 35 most intense peaks

| # | b | $b^{++}$ | b* | $b^{*++}$ | $b^0$ | $b^{0++}$ | Seq. | y | $y^{++}$ | y* | $y^{*++}$ | $y^0$ | $y^{0++}$ | # |
|---|---|---|---|---|---|---|---|---|---|---|---|---|---|---|
| 1 | 148.0757 | 74.5415 | | | | | F | | | | | | | 23 |
| 2 | 276.1343 | 138.5708 | 259.1077 | 130.0575 | | | Q | 2552.2701 | 1276.6387 | 2535.2436 | 1268.1254 | 2534.2595 | 1267.6334 | 22 |
| 3 | 389.2183 | 195.1128 | 372.1918 | 186.5995 | | | I | 2424.2115 | 1212.6094 | 2407.1850 | 1204.0961 | 2406.2010 | **1203.6041** | 21 |
| 4 | 476.2504 | 238.6288 | 459.2238 | 230.1155 | 458.2398 | 229.6235 | S | 2311.1275 | **1156.0674** | 2294.1009 | 1147.5541 | 2293.1169 | 1147.0621 | 20 |
| 5 | 573.3031 | 287.1552 | 556.2766 | 278.6419 | 555.2926 | 278.1499 | P | 2224.0954 | **1112.5514** | 2207.0689 | 1104.0381 | 2206.0849 | 1103.5461 | 19 |
| 6 | 701.3617 | 351.1845 | 684.3352 | 342.6712 | 683.3511 | 342.1792 | Q | 2127.0427 | 1064.0250 | 2110.0161 | 1055.5117 | 2109.0321 | 1055.0197 | 18 |
| 7 | **814.4458** | 407.7265 | 797.4192 | 399.2132 | 796.4352 | 398.7212 | L | **1998.9841** | 999.9957 | 1981.9576 | 991.4824 | 1980.9735 | 990.9904 | 17 |
| 8 | 942.5043 | 471.7558 | 925.4778 | 463.2425 | 924.4938 | 462.7505 | Q | **1885.9000** | 943.4537 | 1868.8735 | 934.9404 | 1867.8895 | 934.4484 | 16 |
| 9 | 1089.5728 | 545.2900 | 1072.5462 | 536.7767 | 1071.5622 | 536.2847 | F | **1757.8415** | 879.4244 | 1740.8149 | 870.9111 | 1739.8309 | 870.4191 | 15 |
| 10 | 1176.6048 | 588.8060 | 1159.5782 | 580.2928 | 1158.5942 | 579.8007 | S | **1610.7730** | 805.8902 | 1593.7465 | 797.3769 | 1592.7625 | 796.8849 | 14 |
| 11 | 1273.6576 | 637.3324 | 1256.6310 | 628.8191 | 1255.6470 | 628.3271 | P | **1523.7410** | 762.3741 | 1506.7145 | 753.8609 | 1505.7305 | 753.3689 | 13 |
| 12 | 1402.7001 | 701.8537 | 1385.6736 | 693.3404 | 1384.6896 | 692.8484 | E | 1426.6883 | 713.8478 | 1409.6617 | 705.3345 | 1408.6777 | 704.8425 | 12 |
| 13 | 1531.7427 | 766.3750 | 1514.7162 | 757.8617 | 1513.7322 | 757.3697 | E | **1297.6457** | 649.3265 | 1280.6191 | 640.8132 | 1279.6351 | 640.3212 | 11 |
| 14 | 1630.8112 | 815.9092 | 1613.7846 | 807.3959 | 1612.8006 | 806.9039 | V | 1168.6031 | 584.8052 | 1151.5765 | 576.2919 | 1150.5925 | 575.7999 | 10 |
| 15 | 1743.8952 | 872.4512 | 1726.8687 | 863.9380 | 1725.8846 | 863.4460 | L | **1069.5347** | 535.2710 | 1052.5081 | 526.7577 | 1051.5241 | 526.2657 | 9 |
| 16 | 1800.9167 | 900.9620 | 1783.8901 | 892.4487 | 1782.9061 | 891.9567 | G | **956.4506** | 478.7289 | 939.4240 | 470.2157 | 938.4400 | 469.7237 | 8 |
| 17 | 1947.9521 | 974.4797 | 1930.9255 | 965.9664 | 1929.9415 | 965.4744 | M | 899.4291 | 450.2182 | 882.4026 | 441.7049 | 881.4186 | 441.2129 | 7 |
| 18 | 2047.0205 | 1024.0139 | 2029.9939 | 1015.5006 | 2029.0099 | 1015.0086 | V | **752.3937** | 376.7005 | 735.3672 | 368.1872 | 734.3832 | 367.6952 | 6 |
| 19 | 2160.1046 | 1080.5559 | 2143.0780 | 1072.0426 | 2142.0940 | 1071.5506 | L | **653.3253** | 327.1663 | 636.2988 | 318.6530 | 635.3147 | 318.1610 | 5 |
| 20 | 2275.1315 | 1138.0694 | 2258.1049 | 1129.5561 | 2257.1209 | 1129.0641 | N | **540.2412** | 270.6243 | 523.2147 | 262.1110 | 522.2307 | 261.6190 | 4 |
| 21 | 2438.1948 | 1219.6011 | 2421.1683 | 1211.0878 | 2420.1843 | 1210.5958 | Y | **425.2143** | 213.1108 | 408.1878 | 204.5975 | 407.2037 | 204.1055 | 3 |
| 22 | 2525.2269 | 1263.1171 | 2508.2003 | 1254.6038 | 2507.2163 | 1254.1118 | S | 262.1510 | 131.5791 | 245.1244 | 123.0659 | 244.1404 | 122.5738 | 2 |
| 23 | | | | | | | R | 175.1190 | 88.0631 | 158.0924 | 79.5498 | | | 1 |

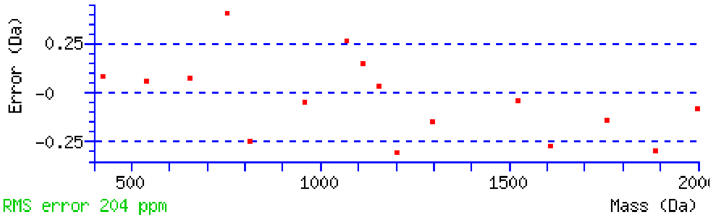


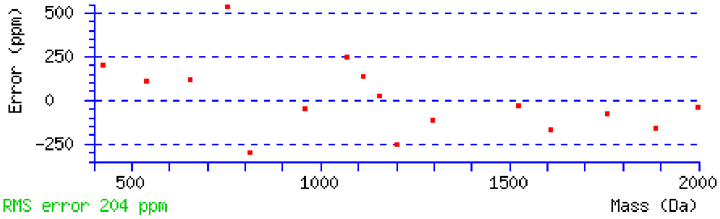

### All matches to this query

| Score | Mr(calc): | Delta | Sequence |
|---|---|---|---|
| 78.7 | 2698.3312 | 2.0053 | FQISPQLQFSPEEVLGMVLNYSR |
| 2.5 | 2699.3241 | 1.0124 | GSITTMLTLAAPQGDGLYKELADVR |
| 2.5 | 2699.3241 | 1.0124 | GSITTMLTLAAPQGDGLYKELADVR |
| 2.5 | 2699.3241 | 1.0124 | GSITTMLTLAAPQGDGLYKELADVR |

---

Spectrum No: 247; Query: 2714; Rank: 1

---

## Peptide View

MS/MS Fragmentation of **SFADENNVFHDPVDGWQNETPSGSLESPAQAR**
Found in **IPI00199445**, Tax_Id=10116 Gene_Symbol=Ank3 ankyrin 3, epithelial isoform 2

Match to Query 2714: 3501.534102 from(1168.185310,3+)
Title: 091008RatKidney_NH4Format02_22.4072.4072.3.dta
Data file K:\NewmanPaper\Piliang\3SubProteomes\Piliang3SP\mgf5ppm\ERLIC_3SubProteomes5ppm.mgf

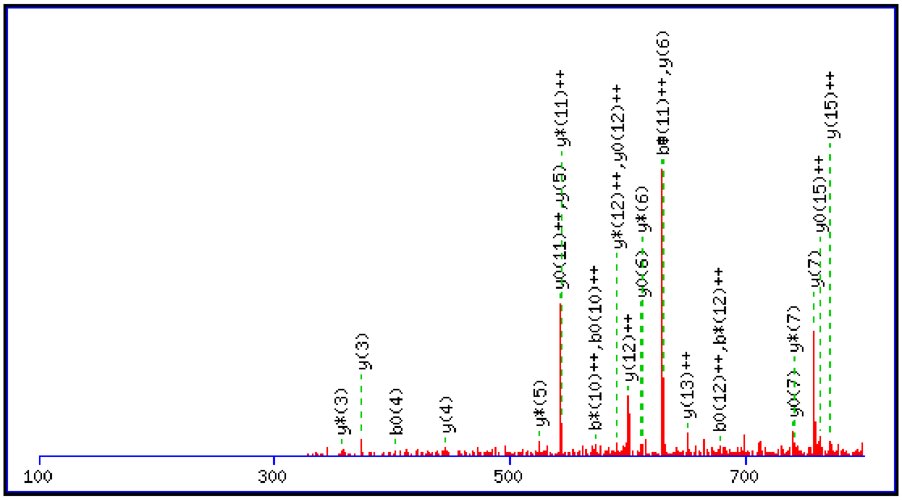


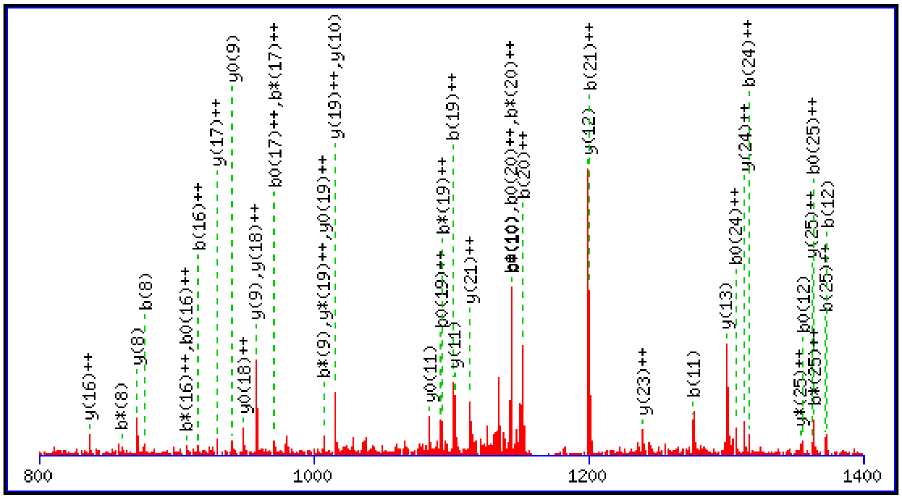

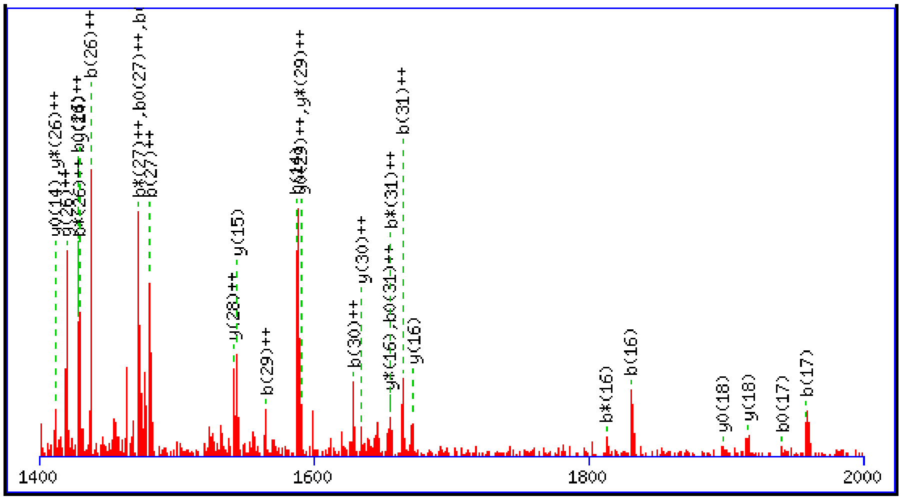


**Monoisotopic mass of neutral peptide Mr(calc):** 3501.5345
**Fixed modifications:** Carbamidomethyl (C)
**Variable modifications:**
**N18** : Deamidated_N (N)
**Ions Score:** 79 **Expect:** 6.5e-006
**Matches (Bold Red):** 101/352 fragment ions using 171 most intense peaks

| # | b | $b^{++}$ | b* | $b^{*++}$ | $b^0$ | $b^{0++}$ | Seq. | y | $y^{++}$ | y* | $y^{*++}$ | $y^0$ | $y^{0++}$ | # |
|---|---|---|---|---|---|---|---|---|---|---|---|---|---|---|
| 1 | 88.0393 | 44.5233 | | | 70.0287 | 35.5180 | S | | | | | | | 32 |
| 2 | 235.1077 | 118.0575 | | | 217.0972 | 109.0522 | F | 3415.5098 | 1708.2585 | 3398.4832 | 1699.7453 | 3397.4992 | 1699.2532 | 31 |
| 3 | 306.1448 | 153.5761 | | | 288.1343 | 144.5708 | A | 3268.4414 | **1634.7243** | 3251.4148 | 1626.2110 | 3250.4308 | 1625.7190 | 30 |
| 4 | 421.1718 | 211.0895 | | | **403.1612** | 202.0842 | D | 3197.4042 | 1599.2058 | 3180.3777 | **1590.6925** | 3179.3937 | **1590.2005** | 29 |
| 5 | 550.2144 | 275.6108 | | | 532.2038 | 266.6055 | E | 3082.3773 | **1541.6923** | 3065.3508 | 1533.1790 | 3064.3667 | 1532.6870 | 28 |
| 6 | 664.2573 | 332.6323 | 647.2307 | 324.1190 | 646.2467 | 323.6270 | N | 2953.3347 | 1477.1710 | 2936.3082 | 1468.6577 | 2935.3241 | 1468.1657 | 27 |
| 7 | 778.3002 | 389.6537 | 761.2737 | 381.1405 | 760.2897 | 380.6485 | N | 2839.2918 | **1420.1495** | 2822.2652 | **1411.6363** | 2821.2812 | 1411.1442 | 26 |
| 8 | **877.3686** | 439.1880 | **860.3421** | 430.6747 | 859.3581 | 430.1827 | V | 2725.2489 | **1363.1281** | 2708.2223 | **1354.6148** | 2707.2383 | 1354.1228 | 25 |
| 9 | 1024.4370 | 512.7222 | **1007.4105** | 504.2089 | 1006.4265 | 503.7169 | F | 2626.1804 | **1313.5939** | 2609.1539 | 1305.0806 | 2608.1699 | 1304.5886 | 24 |
| 10 | 1161.4960 | 581.2516 | **1144.4694** | **572.7383** | **1143.4854** | **572.2463** | H | 2479.1120 | **1240.0597** | 2462.0855 | 1231.5464 | 2461.1015 | 1231.0544 | 23 |
| 11 | **1276.5229** | 638.7651 | 1259.4964 | **630.2518** | 1258.5123 | **629.7598** | D | 2342.0531 | 1171.5302 | 2325.0266 | 1163.0169 | 2324.0426 | 1162.5249 | 22 |
| 12 | **1373.5757** | 687.2915 | 1356.5491 | **678.7782** | **1355.5651** | **678.2862** | P | 2227.0262 | **1114.0167** | 2209.9996 | 1105.5035 | 2209.0156 | 1105.0114 | 21 |
| 13 | **1472.6441** | 736.8257 | 1455.6175 | 728.3124 | 1454.6335 | 727.8204 | V | 2129.9734 | 1065.4903 | 2112.9469 | 1056.9771 | 2111.9628 | 1056.4851 | 20 |
| 14 | **1587.6710** | 794.3392 | 1570.6445 | 785.8259 | 1569.6605 | 785.3339 | D | 2030.9050 | **1015.9561** | 2013.8784 | **1007.4429** | 2012.8944 | **1006.9509** | 19 |
| 15 | 1644.6925 | 822.8499 | 1627.6659 | 814.3366 | 1626.6819 | 813.8446 | G | **1915.8781** | **958.4427** | 1898.8515 | 949.9294 | **1897.8675** | **949.4374** | 18 |
| 16 | **1830.7718** | **915.8895** | **1813.7453** | **907.3763** | 1812.7612 | **906.8843** | W | 1858.8566 | **929.9319** | 1841.8300 | 921.4187 | 1840.8460 | 920.9267 | 17 |
| 17 | **1958.8304** | 979.9188 | 1941.8038 | **971.4056** | **1940.8198** | **970.9135** | Q | **1672.7773** | **836.8923** | **1655.7507** | 828.3790 | 1654.7667 | 827.8870 | 16 |
| 18 | 2073.8573 | 1037.4323 | 2056.8308 | 1028.9190 | 2055.8468 | 1028.4270 | N | **1544.7187** | **772.8630** | 1527.6922 | 764.3497 | 1526.7081 | **763.8577** | 15 |
| 19 | 2202.8999 | **1101.9536** | 2185.8734 | **1093.4403** | 2184.8893 | **1092.9483** | E | **1429.6918** | 715.3495 | 1412.6652 | 706.8362 | **1411.6812** | 706.3442 | 14 |
| 20 | 2303.9476 | **1152.4774** | 2286.9210 | **1143.9642** | 2285.9370 | **1143.4721** | T | **1300.6492** | **650.8282** | 1283.6226 | 642.3149 | 1282.6386 | 641.8229 | 13 |
| 21 | 2401.0004 | **1201.0038** | 2383.9738 | 1192.4905 | 2382.9898 | 1191.9985 | P | **1199.6015** | **600.3044** | 1182.5749 | **591.7911** | 1181.5909 | **591.2991** | 12 |
| 22 | 2488.0324 | 1244.5198 | 2471.0058 | 1236.0066 | 2470.0218 | 1235.5145 | S | **1102.5487** | 551.7780 | 1085.5222 | **543.2647** | **1084.5382** | **542.7727** | 11 |
| 23 | 2545.0538 | 1273.0306 | 2528.0273 | 1264.5173 | 2527.0433 | 1264.0253 | G | **1015.5167** | 508.2620 | 998.4902 | 499.7487 | 997.5061 | 499.2567 | 10 |
| 24 | 2632.0859 | **1316.5466** | 2615.0593 | 1308.0333 | 2614.0753 | **1307.5413** | S | **958.4952** | 479.7513 | 941.4687 | 471.2380 | **940.4847** | 470.7460 | 9 |
| 25 | 2745.1699 | **1373.0886** | 2728.1434 | **1364.5753** | 2727.1594 | **1364.0833** | L | **871.4632** | 436.2352 | 854.4367 | 427.7220 | 853.4526 | 427.2300 | 8 |
| 26 | 2874.2125 | **1437.6099** | 2857.1860 | **1429.0966** | 2856.2020 | **1428.6046** | E | **758.3791** | 379.6932 | **741.3526** | 371.1799 | **740.3686** | 370.6879 | 7 |
| 27 | 2961.2446 | **1481.1259** | 2944.2180 | **1472.6126** | 2943.2340 | **1472.1206** | S | **629.3366** | 315.1719 | **612.3100** | 306.6586 | **611.3260** | 306.1666 | 6 |
| 28 | 3058.2973 | 1529.6523 | 3041.2708 | 1521.1390 | 3040.2868 | 1520.6470 | P | **542.3045** | 271.6559 | **525.2780** | 263.1426 | | | 5 |
| 29 | 3129.3344 | **1565.1709** | 3112.3079 | 1556.6576 | 3111.3239 | 1556.1656 | A | **445.2518** | 223.1295 | 428.2252 | 214.6162 | | | 4 |
| 30 | 3257.3930 | **1629.2001** | 3240.3665 | 1620.6869 | 3239.3824 | 1620.1949 | Q | **374.2146** | 187.6110 | **357.1881** | 179.0977 | | | 3 |
| 31 | 3328.4301 | **1664.7187** | 3311.4036 | **1656.2054** | 3310.4196 | **1655.7134** | A | 246.1561 | 123.5817 | 229.1295 | 115.0684 | | | 2 |
| 32 | | | | | | | R | 175.1190 | 88.0631 | 158.0924 | 79.5498 | | | 1 |

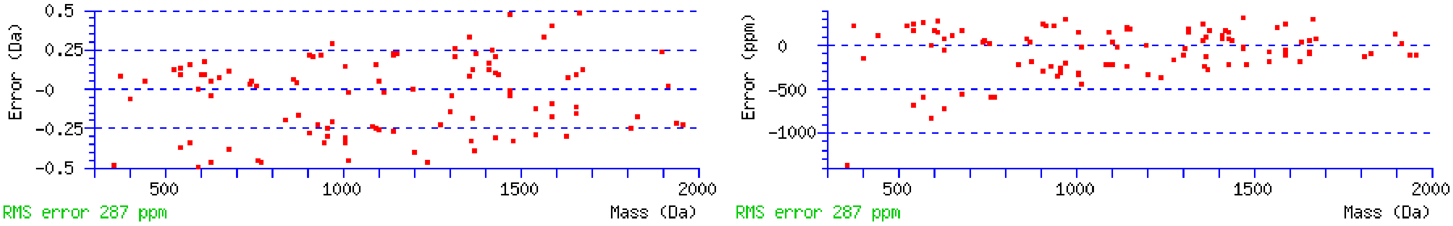


### All matches to this query

| Score | Mr(calc): | Delta | Sequence |
|---|---|---|---|
| 78.5 | 3501.5345 | -0.0004 | SFADENNVFHDPVDGWQNETPSGSLESPAQAR |
| 76.1 | 3501.5345 | -0.0004 | SFADENNVFHDPVDGWQNETPSGSLESPAQAR |
| 75.5 | 3501.5345 | -0.0004 | SFADENNVFHDPVDGWQNETPSGSLESPAQAR |
| 64.9 | 3500.5505 | 0.9836 | SFADENNVFHDPVDGWQNETPSGSLESPAQAR |
| 16.6 | 3501.5454 | -0.0113 | IMPHTEAQIVNMETGELTKLNMPGELCIR |
| 9.6 | 3501.5451 | -0.0110 | CSTEVPFNSWDRLMRHMGLTDNQIQMVR |
| 9.6 | 3499.5222 | 2.0119 | EIQSTWTQQPTANSSYPYVEISLGDVAAQR |
| 9.1 | 3499.5222 | 2.0119 | EIQSTWTQQPTANSSYPYVEISLGDVAAQR |
| 7.7 | 3499.5222 | 2.0119 | EIQSTWTQQPTANSSYPYVEISLGDVAAQR |
| 7.0 | 3501.5201 | 0.0140 | WTPKSPLDPDSGLLSCTLPNGFGGLSGPEGER |

Spectrum No: 248; Query: 1858; Rank: 1

## Peptide View

MS/MS Fragmentation of **SLTQGSMIVGNLAPVNGTSQGK**
Found in **IPI00388323**, Tax_Id=10116 Gene_Symbol=Hspg2 396 kDa protein

Match to Query 1858: 2175.092008 from(1088.553280,2+)
Title: 100101RatKid_NS_deglyco_21.2701.2701.2.dta
Data file K:\NewmanPaper\Piliang\3SubProteomes\Piliang3SP\mgf5ppm\ERLIC_3SubProteomes5ppm.mgf

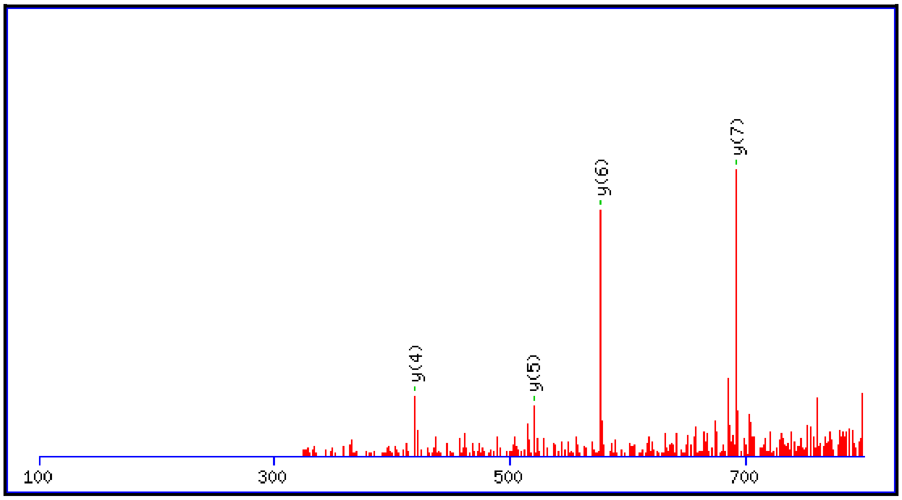

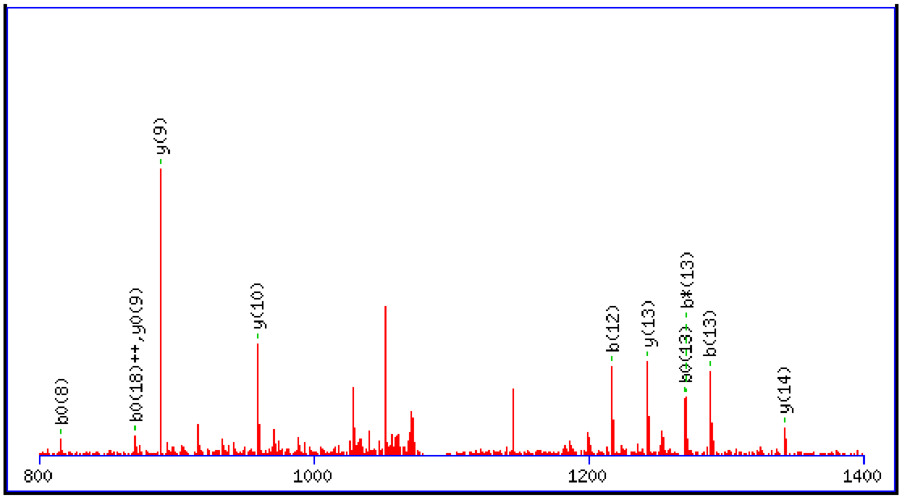


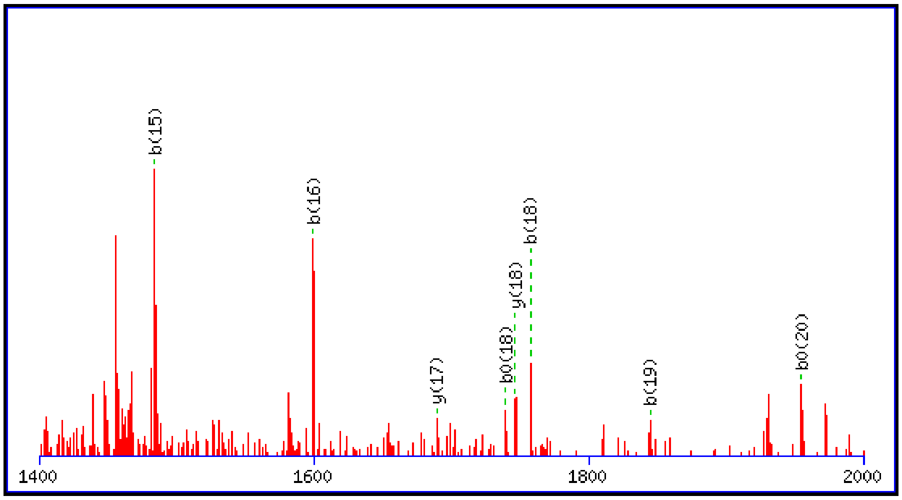


**Monoisotopic mass of neutral peptide Mr(calc):** 2175.0842
**Fixed modifications:** Carbamidomethyl (C)
**Variable modifications:**
**M7** : Oxidation (M)
**N16** : Deamidated_N (N)
**Ions Score:** 78 **Expect:** 2.5e-006
**Matches (Bold Red):** 23/240 fragment ions using 29 most intense peaks

| # | b | $b^{++}$ | b* | $b^{*++}$ | $b^0$ | $b^{0++}$ | Seq. | y | $y^{++}$ | y* | $y^{*++}$ | $y^0$ | $y^{0++}$ | # |
|---|---|---|---|---|---|---|---|---|---|---|---|---|---|---|
| 1 | 88.0393 | 44.5233 | | | 70.0287 | 35.5180 | S | | | | | | | 22 |
| 2 | 201.1234 | 101.0653 | | | 183.1128 | 92.0600 | L | 2089.0594 | 1045.0333 | 2072.0328 | 1036.5201 | 2071.0488 | 1036.0281 | 21 |
| 3 | 302.1710 | 151.5892 | | | 284.1605 | 142.5839 | T | 1975.9753 | 988.4913 | 1958.9488 | 979.9780 | 1957.9648 | 979.4860 | 20 |
| 4 | 430.2296 | 215.6185 | 413.2031 | 207.1052 | 412.2191 | 206.6132 | Q | 1874.9277 | 937.9675 | 1857.9011 | 929.4542 | 1856.9171 | 928.9622 | 19 |
| 5 | 487.2511 | 244.1292 | 470.2245 | 235.6159 | 469.2405 | 235.1239 | G | **1746.8691** | 873.9382 | 1729.8425 | 865.4249 | 1728.8585 | 864.9329 | 18 |
| 6 | 574.2831 | 287.6452 | 557.2566 | 279.1319 | 556.2726 | 278.6399 | S | **1689.8476** | 845.4274 | 1672.8211 | 836.9142 | 1671.8370 | 836.4222 | 17 |
| 7 | 721.3185 | 361.1629 | 704.2920 | 352.6496 | 703.3080 | 352.1576 | M | 1602.8156 | 801.9114 | 1585.7890 | 793.3982 | 1584.8050 | 792.9061 | 16 |
| 8 | 834.4026 | 417.7049 | 817.3760 | 409.1917 | **816.3920** | 408.6996 | I | 1455.7802 | 728.3937 | 1438.7536 | 719.8805 | 1437.7696 | 719.3884 | 15 |
| 9 | 933.4710 | 467.2391 | 916.4444 | 458.7259 | 915.4604 | 458.2339 | V | **1342.6961** | 671.8517 | 1325.6696 | 663.3384 | 1324.6855 | 662.8464 | 14 |
| 10 | 990.4925 | 495.7499 | 973.4659 | 487.2366 | 972.4819 | 486.7446 | G | **1243.6277** | 622.3175 | 1226.6012 | 613.8042 | 1225.6171 | 613.3122 | 13 |
| 11 | 1104.5354 | 552.7713 | 1087.5088 | 544.2581 | 1086.5248 | 543.7661 | N | 1186.6062 | 593.8068 | 1169.5797 | 585.2935 | 1168.5957 | 584.8015 | 12 |
| 12 | **1217.6195** | 609.3134 | 1200.5929 | 600.8001 | 1199.6089 | 600.3081 | L | 1072.5633 | 536.7853 | 1055.5368 | 528.2720 | 1054.5527 | 527.7800 | 11 |
| 13 | **1288.6566** | 644.8319 | **1271.6300** | 636.3186 | **1270.6460** | 635.8266 | A | **959.4792** | 480.2433 | 942.4527 | 471.7300 | 941.4687 | 471.2380 | 10 |
| 14 | 1385.7093 | 693.3583 | 1368.6828 | 684.8450 | 1367.6988 | 684.3530 | P | **888.4421** | 444.7247 | 871.4156 | 436.2114 | **870.4316** | 435.7194 | 9 |
| 15 | **1484.7777** | 742.8925 | 1467.7512 | 734.3792 | 1466.7672 | 733.8872 | V | 791.3894 | 396.1983 | 774.3628 | 387.6850 | 773.3788 | 387.1930 | 8 |
| 16 | **1599.8047** | 800.4060 | 1582.7781 | 791.8927 | 1581.7941 | 791.4007 | N | **692.3210** | 346.6641 | 675.2944 | 338.1508 | 674.3104 | 337.6588 | 7 |
| 17 | 1656.8261 | 828.9167 | 1639.7996 | 820.4034 | 1638.8156 | 819.9114 | G | **577.2940** | 289.1506 | 560.2675 | 280.6374 | 559.2835 | 280.1454 | 6 |
| 18 | **1757.8738** | 879.4406 | 1740.8473 | 870.9273 | **1739.8633** | **870.4353** | T | **520.2726** | 260.6399 | 503.2460 | 252.1266 | 502.2620 | 251.6346 | 5 |

| | | | | | | | | | | | | | | |
|---|---|---|---|---|---|---|---|---|---|---|---|---|---|---|
| **19** | **1844.9059** | 922.9566 | 1827.8793 | 914.4433 | 1826.8953 | 913.9513 | **S** | **419.2249** | 210.1161 | 402.1983 | 201.6028 | 401.2143 | 201.1108 | **4** |
| **20** | 1972.9644 | 986.9859 | 1955.9379 | 978.4726 | **1954.9539** | 977.9806 | **Q** | 332.1928 | 166.6001 | 315.1663 | 158.0868 | | | **3** |
| **21** | 2029.9859 | 1015.4966 | 2012.9593 | 1006.9833 | 2011.9753 | 1006.4913 | **G** | 204.1343 | 102.5708 | 187.1077 | 94.0575 | | | **2** |
| **22** | | | | | | | **K** | 147.1128 | 74.0600 | 130.0863 | 65.5468 | | | **1** |

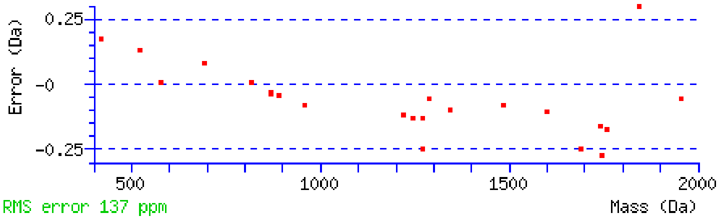


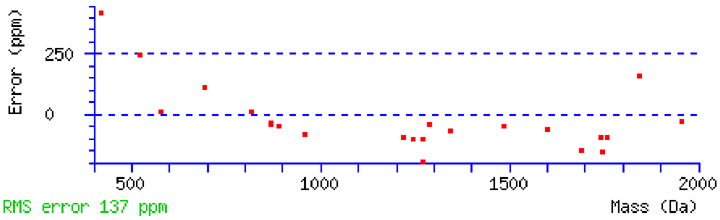


**All matches to this query**

| Score | Mr(calc): | Delta | Sequence |
|---|---|---|---|
| 78.5 | 2175.0842 | 0.0079 | SLTQGSMIVGNLAPVNGTSQGK |
| 31.5 | 2175.0842 | 0.0079 | SLTQGSMIVGNLAPVNGTSQGK |
| 7.9 | 2174.1001 | 0.9919 | SLTQGSMIVGNLAPVNGTSQGK |
| 0.7 | 2175.1072 | -0.0152 | NVAQYGLDPATRYPNLNLR |

---

Spectrum No: 249; Query: 1304; Rank: 1

---

## Peptide View

MS/MS Fragmentation of **LANASLYLAEWQNITK**
Found in **IPI00211616**, Tax_Id=10116 Gene_Symbol=Slc3a2 4F2 cell-surface antigen heavy chain

Match to Query 1304: 1835.931408 from(918.972980,2+)
Title: 100101RatKid_NS_deglyco_24.5037.5037.2.dta
Data file K:\NewmanPaper\Piliang\3SubProteomes\Piliang3SP\mgf5ppm\ERLIC_3SubProteomes5ppm.mgf

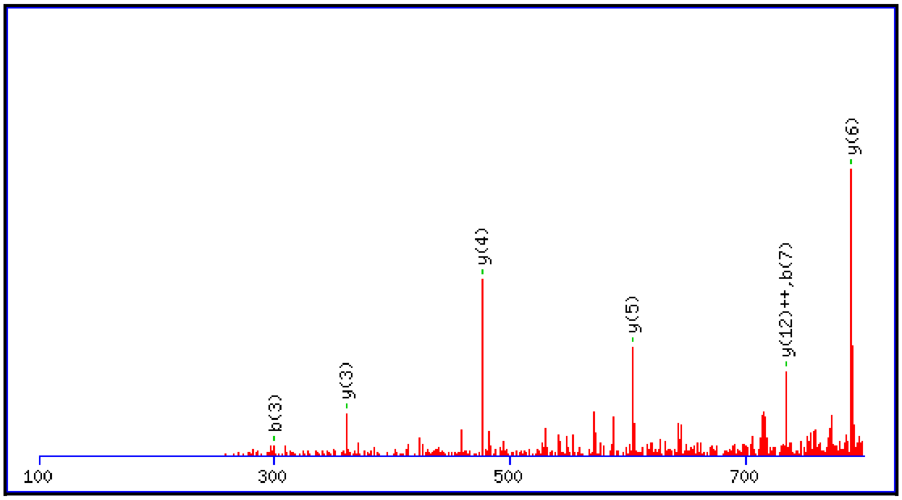

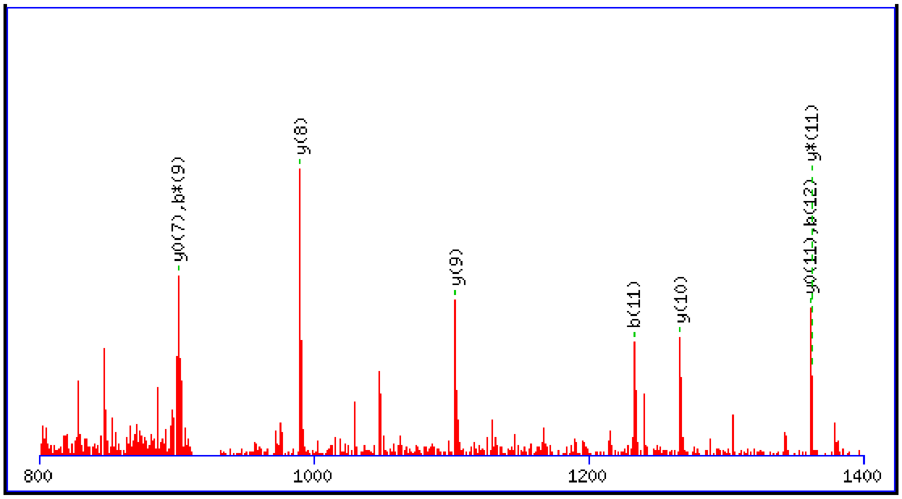


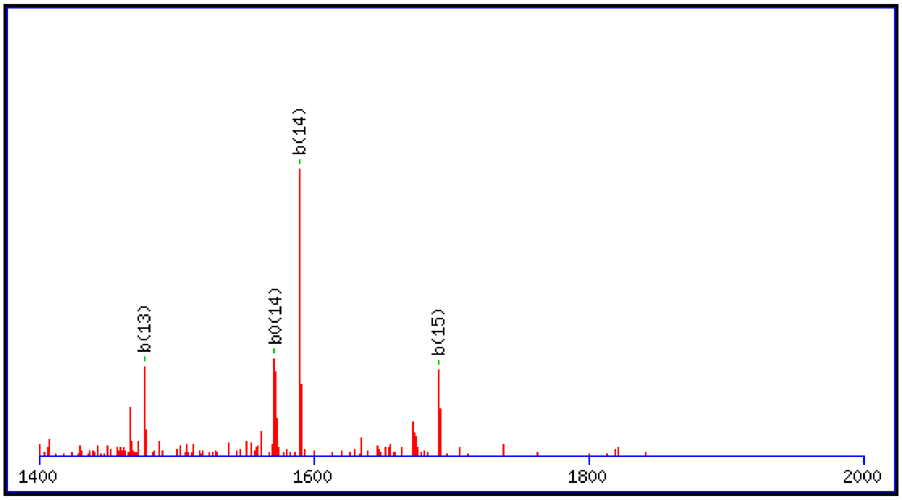


**Monoisotopic mass of neutral peptide Mr(calc):** 1835.9305
**Fixed modifications:** Carbamidomethyl (C)
**Variable modifications:**
**N3** : Deamidated_N (N)
**N13** : Deamidated_N (N)
**Ions Score:** 78 **Expect:** 2.4e-006
**Matches (Bold Red):** 20/166 fragment ions using 22 most intense peaks

| # | b | b$^{++}$ | b* | b*$^{++}$ | b$^{0}$ | b$^{0++}$ | Seq. | y | y$^{++}$ | y* | y*$^{++}$ | y$^{0}$ | y$^{0++}$ | # |
|---|---|---|---|---|---|---|---|---|---|---|---|---|---|---|
| 1 | 114.0913 | 57.5493 | | | | | L | | | | | | | 16 |
| 2 | 185.1285 | 93.0679 | | | | | A | 1723.8537 | 862.4305 | 1706.8272 | 853.9172 | 1705.8432 | 853.4252 | 15 |
| 3 | **300.1554** | 150.5813 | 283.1288 | 142.0681 | | | N | 1652.8166 | 826.9119 | 1635.7901 | 818.3987 | 1634.8061 | 817.9067 | 14 |
| 4 | 371.1925 | 186.0999 | 354.1660 | 177.5866 | | | A | 1537.7897 | 769.3985 | 1520.7631 | 760.8852 | 1519.7791 | 760.3932 | 13 |
| 5 | 458.2245 | 229.6159 | 441.1980 | 221.1026 | 440.2140 | 220.6106 | S | 1466.7526 | **733.8799** | 1449.7260 | 725.3666 | 1448.7420 | 724.8746 | 12 |
| 6 | 571.3086 | 286.1579 | 554.2820 | 277.6447 | 553.2980 | 277.1527 | L | 1379.7205 | 690.3639 | **1362.6940** | 681.8506 | **1361.7100** | 681.3586 | 11 |
| 7 | **734.3719** | 367.6896 | 717.3454 | 359.1763 | 716.3614 | 358.6843 | Y | **1266.6365** | 633.8219 | 1249.6099 | 625.3086 | 1248.6259 | 624.8166 | 10 |
| 8 | 847.4560 | 424.2316 | 830.4294 | 415.7184 | 829.4454 | 415.2264 | L | **1103.5731** | 552.2902 | 1086.5466 | 543.7769 | 1085.5626 | 543.2849 | 9 |
| 9 | 918.4931 | 459.7502 | **901.4666** | 451.2369 | 900.4825 | 450.7449 | A | **990.4891** | 495.7482 | 973.4625 | 487.2349 | 972.4785 | 486.7429 | 8 |
| 10 | 1047.5357 | 524.2715 | 1030.5091 | 515.7582 | 1029.5251 | 515.2662 | E | 919.4520 | 460.2296 | 902.4254 | 451.7163 | **901.4414** | 451.2243 | 7 |
| 11 | **1233.6150** | 617.3111 | 1216.5885 | 608.7979 | 1215.6044 | 608.3059 | W | **790.4094** | 395.7083 | 773.3828 | 387.1951 | 772.3988 | 386.7030 | 6 |
| 12 | **1361.6736** | 681.3404 | 1344.6470 | 672.8272 | 1343.6630 | 672.3351 | Q | **604.3301** | 302.6687 | 587.3035 | 294.1554 | 586.3195 | 293.6634 | 5 |
| 13 | **1476.7005** | 738.8539 | 1459.6740 | 730.3406 | 1458.6900 | 729.8486 | N | **476.2715** | 238.6394 | 459.2449 | 230.1261 | 458.2609 | 229.6341 | 4 |
| 14 | **1589.7846** | 795.3959 | 1572.7580 | 786.8827 | **1571.7740** | 786.3906 | I | **361.2445** | 181.1259 | 344.2180 | 172.6126 | 343.2340 | 172.1206 | 3 |
| 15 | **1690.8323** | 845.9198 | 1673.8057 | 837.4065 | 1672.8217 | 836.9145 | T | 248.1605 | 124.5839 | 231.1339 | 116.0706 | 230.1499 | 115.5786 | 2 |
| 16 | | | | | | | K | 147.1128 | 74.0600 | 130.0863 | 65.5468 | | | 1 |

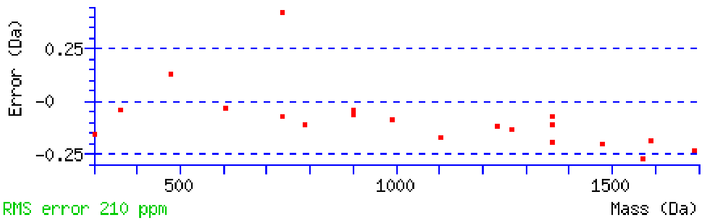


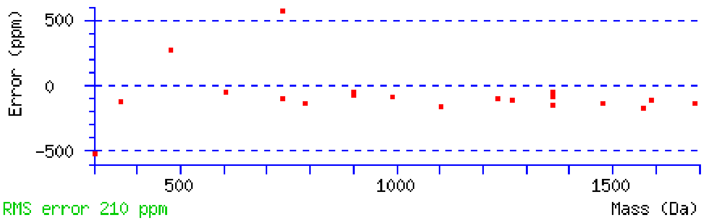


### All matches to this query

| Score | Mr(calc): | Delta | Sequence |
|---|---|---|---|
| 78.3 | 1835.9305 | 0.0009 | LANASLYLAEWQNITK |
| 8.4 | 1835.9394 | -0.0080 | GSTIRQELVLPSXTLK |
| 8.4 | 1835.9394 | -0.0080 | GSTIRQELVLPSXTLK |
| 1.9 | 1835.9281 | 0.0033 | LKEIINNTPLGSSELK |
| 1.9 | 1835.9281 | 0.0033 | LKEIINNTPLGSSELK |
| 0.2 | 1835.9272 | 0.0042 | MVVTRSGSRIQESSQR |
| 0.0 | 1835.9328 | -0.0014 | GMKAGKPVVAASGAGNLTK |

---

Spectrum No: 250; Query: 473; Rank: 1

---

## Peptide View

MS/MS Fragmentation of **SENSSLTLSSSNR**
Found in **IPI00324585**, Tax_Id=10116 Gene_Symbol=Itga1 Integrin alpha-1 precursor

Match to Query 473: 1381.634508 from(691.824530,2+)
Title: 100101RatKid_NS_deglyco_15.1103.1103.2.dta
Data file K:\NewmanPaper\Piliang\3SubProteomes\Piliang3SP\mgf5ppm\ERLIC_3SubProteomes5ppm.mgf

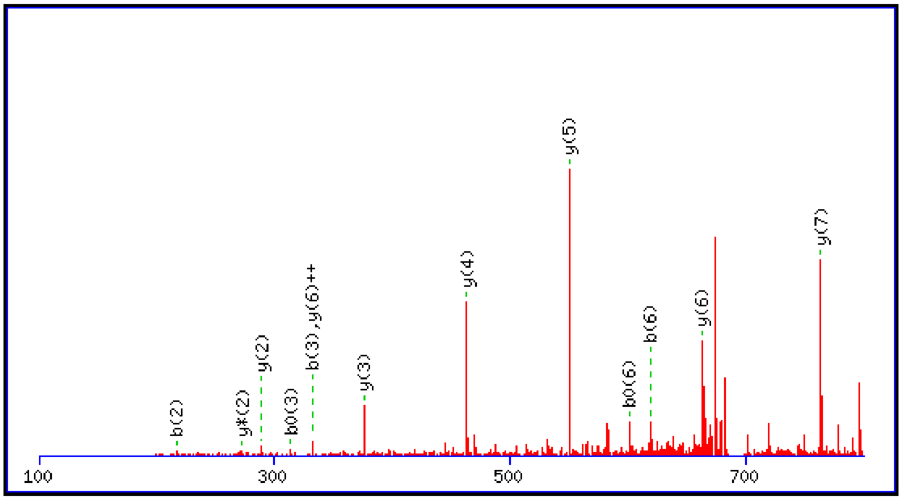

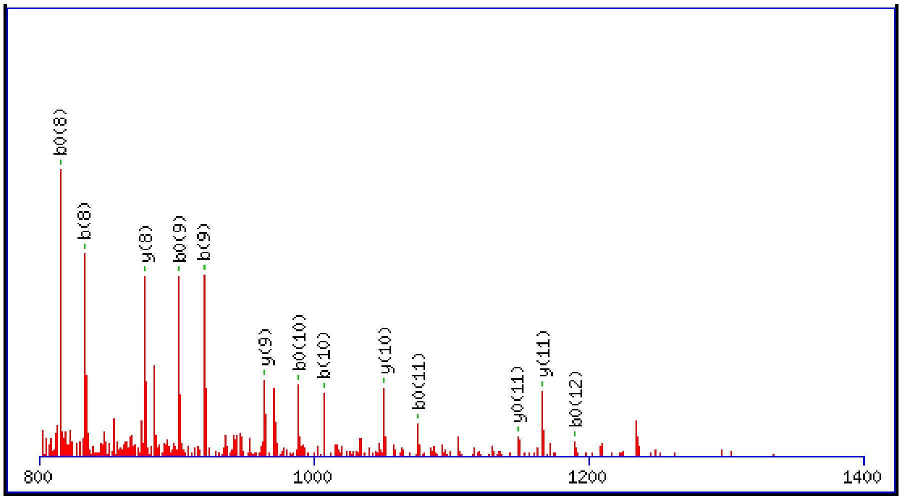


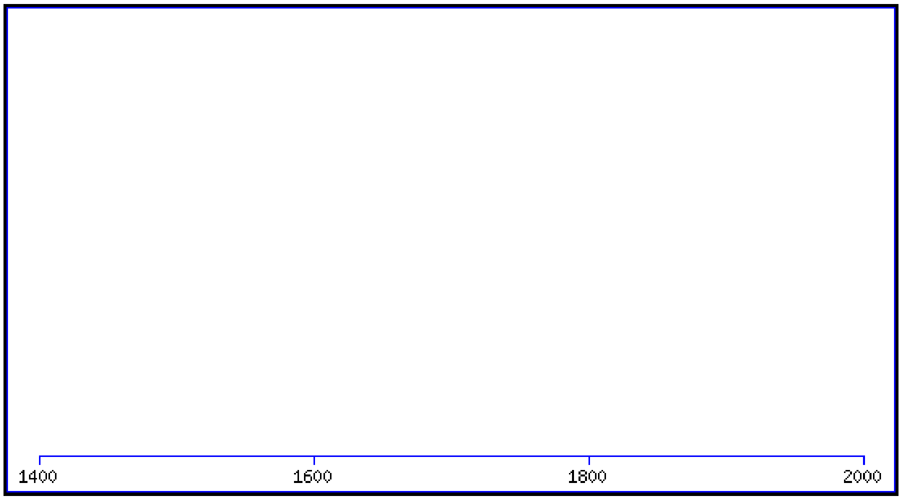


**Monoisotopic mass of neutral peptide Mr(calc):** 1381.6321
**Fixed modifications:** Carbamidomethyl (C)
**Variable modifications:**
**N3** : Deamidated_N (N)
**Ions Score:** 78 **Expect:** 2.1e-006
**Matches (Bold Red):** 26/136 fragment ions using 45 most intense peaks

| # | b | $b^{++}$ | b* | $b^{*++}$ | $b^0$ | $b^{0++}$ | Seq. | y | $y^{++}$ | y* | $y^{*++}$ | $y^0$ | $y^{0++}$ | # |
|---|---|---|---|---|---|---|---|---|---|---|---|---|---|---|
| 1 | 88.0393 | 44.5233 | | | 70.0287 | 35.5180 | S | | | | | | | 13 |
| 2 | **217.0819** | 109.0446 | | | 199.0713 | 100.0393 | E | 1295.6074 | 648.3073 | 1278.5808 | 639.7940 | 1277.5968 | 639.3020 | 12 |
| 3 | **332.1088** | 166.5581 | 315.0823 | 158.0448 | **314.0983** | 157.5528 | N | **1166.5648** | 583.7860 | 1149.5382 | 575.2727 | **1148.5542** | 574.7807 | 11 |
| 4 | 419.1409 | 210.0741 | 402.1143 | 201.5608 | 401.1303 | 201.0688 | S | **1051.5378** | 526.2726 | 1034.5113 | 517.7593 | 1033.5273 | 517.2673 | 10 |
| 5 | 506.1729 | 253.5901 | 489.1463 | 245.0768 | 488.1623 | 244.5848 | S | **964.5058** | 482.7565 | 947.4792 | 474.2433 | 946.4952 | 473.7513 | 9 |
| 6 | **619.2570** | 310.1321 | 602.2304 | 301.6188 | **601.2464** | 301.1268 | L | **877.4738** | 439.2405 | 860.4472 | 430.7272 | 859.4632 | 430.2352 | 8 |
| 7 | 720.3046 | 360.6560 | 703.2781 | 352.1427 | 702.2941 | 351.6507 | T | **764.3897** | 382.6985 | 747.3632 | 374.1852 | 746.3791 | 373.6932 | 7 |
| 8 | **833.3887** | 417.1980 | 816.3621 | 408.6847 | **815.3781** | 408.1927 | L | **663.3420** | **332.1747** | 646.3155 | 323.6614 | 645.3315 | 323.1694 | 6 |
| 9 | **920.4207** | 460.7140 | 903.3942 | 452.2007 | **902.4102** | 451.7087 | S | **550.2580** | 275.6326 | 533.2314 | 267.1193 | 532.2474 | 266.6273 | 5 |
| 10 | **1007.4528** | 504.2300 | 990.4262 | 495.7167 | **989.4422** | 495.2247 | S | **463.2259** | 232.1166 | 446.1994 | 223.6033 | 445.2154 | 223.1113 | 4 |
| 11 | 1094.4848 | 547.7460 | 1077.4582 | 539.2328 | **1076.4742** | 538.7407 | S | **376.1939** | 188.6006 | 359.1674 | 180.0873 | 358.1833 | 179.5953 | 3 |
| 12 | 1208.5277 | 604.7675 | 1191.5012 | 596.2542 | **1190.5171** | 595.7622 | N | **289.1619** | 145.0846 | **272.1353** | 136.5713 | | | 2 |
| 13 | | | | | | | R | 175.1190 | 88.0631 | 158.0924 | 79.5498 | | | 1 |

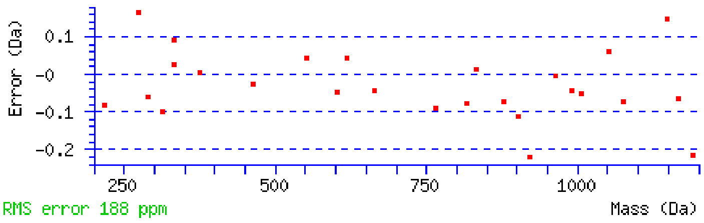


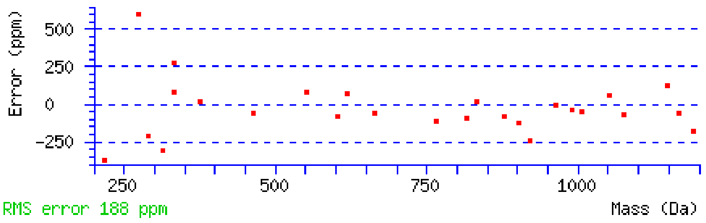


### All matches to this query

| Score | Mr(calc): | Delta | Sequence |
|---|---|---|---|
| 78.3 | 1381.6321 | 0.0024 | SENSSLTLSSSNR |
| 18.3 | 1381.6321 | 0.0024 | SENSSLTLSSSNR |
| 5.9 | 1380.6269 | 1.0076 | SNSFNADRNIDK |
| 5.8 | 1381.6217 | 0.0128 | MSEDSNMEKAIK |
| 5.3 | 1380.6360 | 0.9985 | SPTPVTMEKSPK |
| 5.3 | 1380.6360 | 0.9985 | SPTPVTMEKSPK |
| 3.3 | 1381.6330 | 0.0015 | MDLSGGMLQDKR |

---

Spectrum No: 251; Query: 127; Rank: 1

---

## Peptide View

MS/MS Fragmentation of **LEVLLANVSR**
Found in **IPI00199867**, Tax_Id=10116 Gene_Symbol=Emilin1_predicted elastin microfibril interfacer 1

Match to Query 127: 1113.640448 from(557.827500,2+)
Title: 100101RatKid_NS_deglyco_27.2689.2689.2.dta
Data file K:\NewmanPaper\Piliang\3SubProteomes\Piliang3SP\mgf5ppm\ERLIC_3SubProteomes5ppm.mgf

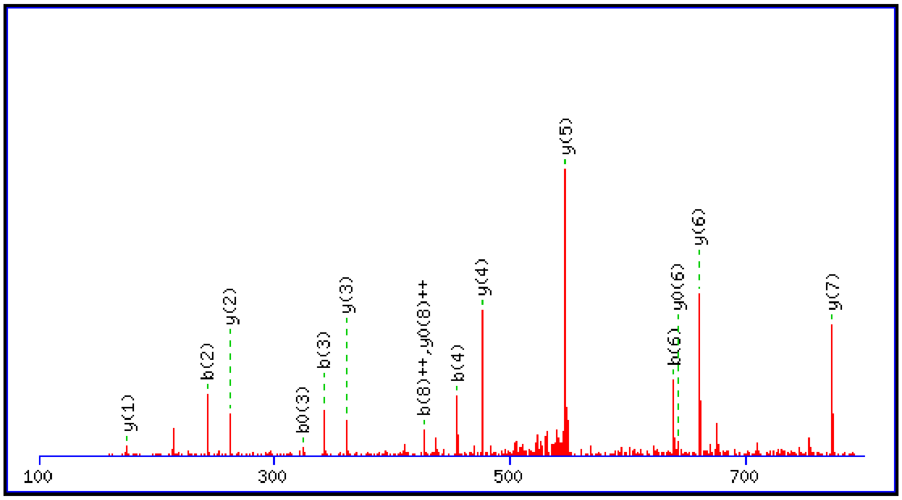

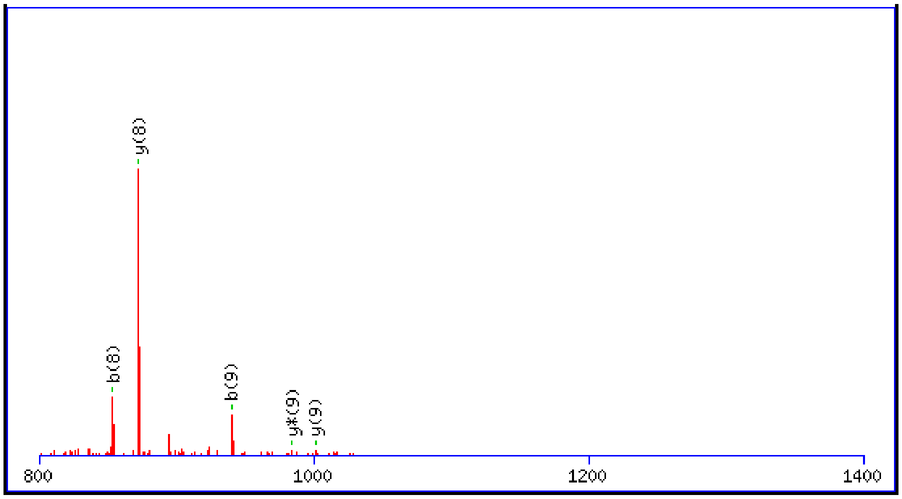


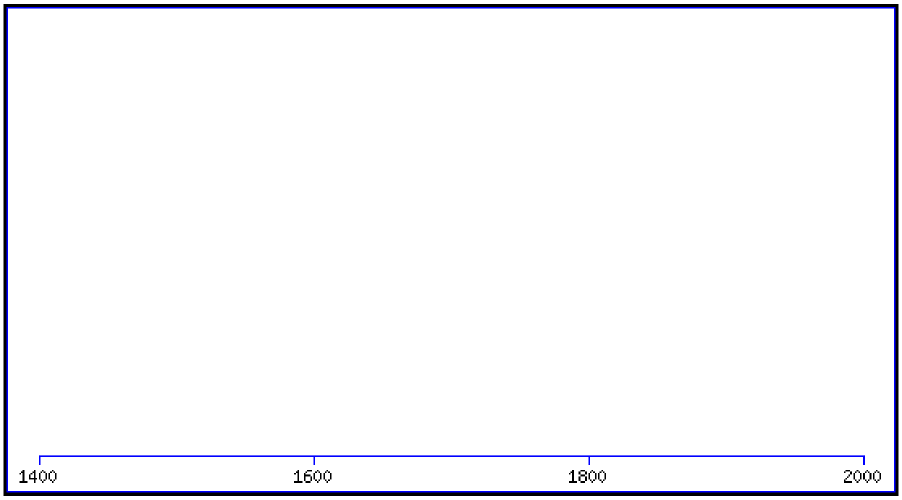


**Monoisotopic mass of neutral peptide Mr(calc):** 1113.6393
**Fixed modifications:** Carbamidomethyl (C)
**Variable modifications:**
**N7** : Deamidated_N (N)
**Ions Score:** 78 **Expect:** 9.3e-007
**Matches (Bold Red):** 20/92 fragment ions using 28 most intense peaks

| # | b | $b^{++}$ | b* | $b^{*++}$ | $b^{0}$ | $b^{0++}$ | Seq. | y | $y^{++}$ | y* | $y^{*++}$ | $y^{0}$ | $y^{0++}$ | # |
|---|---|---|---|---|---|---|---|---|---|---|---|---|---|---|
| 1 | 114.0913 | 57.5493 | | | | | L | | | | | | | 10 |
| 2 | **243.1339** | 122.0706 | | | 225.1234 | 113.0653 | E | **1001.5626** | 501.2849 | **984.5360** | 492.7717 | 983.5520 | 492.2796 | 9 |
| 3 | **342.2023** | 171.6048 | | | **324.1918** | 162.5995 | V | **872.5200** | 436.7636 | 855.4934 | 428.2504 | 854.5094 | **427.7583** | 8 |
| 4 | **455.2864** | 228.1468 | | | 437.2758 | 219.1416 | L | **773.4516** | 387.2294 | 756.4250 | 378.7161 | 755.4410 | 378.2241 | 7 |
| 5 | 568.3705 | 284.6889 | | | 550.3599 | 275.6836 | L | **660.3675** | 330.6874 | 643.3410 | 322.1741 | **642.3569** | 321.6821 | 6 |
| 6 | **639.4076** | 320.2074 | | | 621.3970 | 311.2022 | A | **547.2834** | 274.1454 | 530.2569 | 265.6321 | 529.2729 | 265.1401 | 5 |
| 7 | 754.4345 | 377.7209 | 737.4080 | 369.2076 | 736.4240 | 368.7156 | N | **476.2463** | 238.6268 | 459.2198 | 230.1135 | 458.2358 | 229.6215 | 4 |
| 8 | **853.5029** | **427.2551** | 836.4764 | 418.7418 | 835.4924 | 418.2498 | V | **361.2194** | 181.1133 | 344.1928 | 172.6001 | 343.2088 | 172.1081 | 3 |
| 9 | **940.5350** | 470.7711 | 923.5084 | 462.2578 | 922.5244 | 461.7658 | S | **262.1510** | 131.5791 | 245.1244 | 123.0659 | 244.1404 | 122.5738 | 2 |
| 10 | | | | | | | R | **175.1190** | 88.0631 | 158.0924 | 79.5498 | | | 1 |

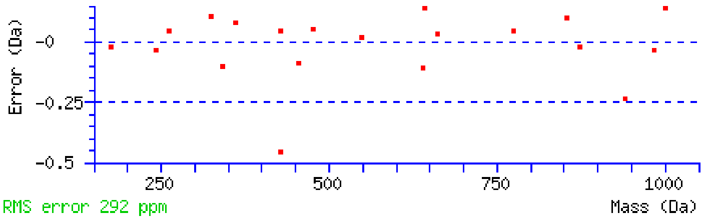


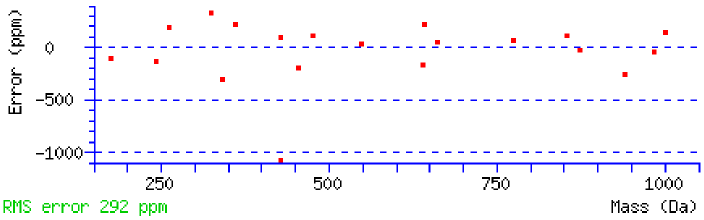


**All matches to this query**

| Score | Mr(calc): | Delta | Sequence |
|---|---|---|---|
| 78.2 | 1113.6393 | 0.0011 | LEVLLANVSR |
| 13.4 | 1113.6393 | 0.0011 | DILLISQNAK |
| 11.1 | 1113.6393 | 0.0011 | LINLVGESLR |
| 11.1 | 1113.6393 | 0.0011 | LLNLNLERK |
| 9.6 | 1113.6506 | -0.0101 | NIVKLAEATR |
| 4.5 | 1113.6394 | 0.0011 | EIVSLLLGDR |
| 4.1 | 1113.6294 | 0.0110 | LQLEKAAWR |
| 4.0 | 1113.6506 | -0.0101 | LQVQERLTK |
| 3.5 | 1112.6414 | 0.9990 | NQVIKAERR |
| 3.4 | 1113.6393 | 0.0011 | VIELENRLK |

---

Spectrum No: 252; Query: 249; Rank: 1

---

## Peptide View

MS/MS Fragmentation of **ELAVAANETAAR**
Found in **IPI00363534**, Tax_Id=10116 Gene_Symbol=Lama1 laminin, alpha 1

Match to Query 249: 1215.606508 from(608.810530,2+)
Title: 091008RatKidney_NH4Format01_25.921.921.2.dta
Data file K:\NewmanPaper\Piliang\3SubProteomes\Piliang3SP\mgf5ppm\ERLIC_3SubProteomes5ppm.mgf

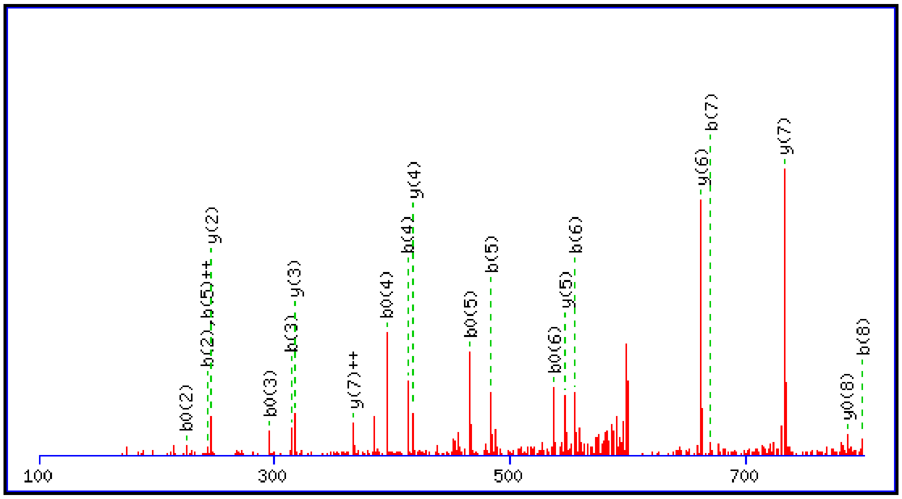


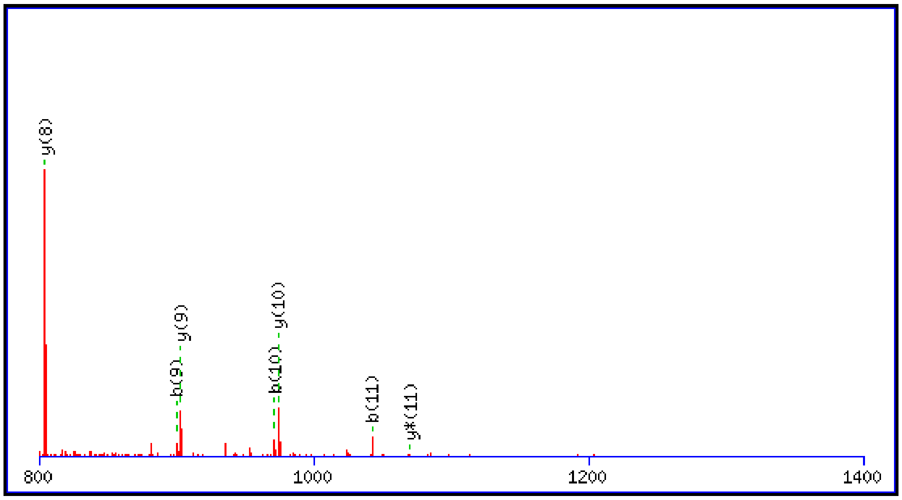

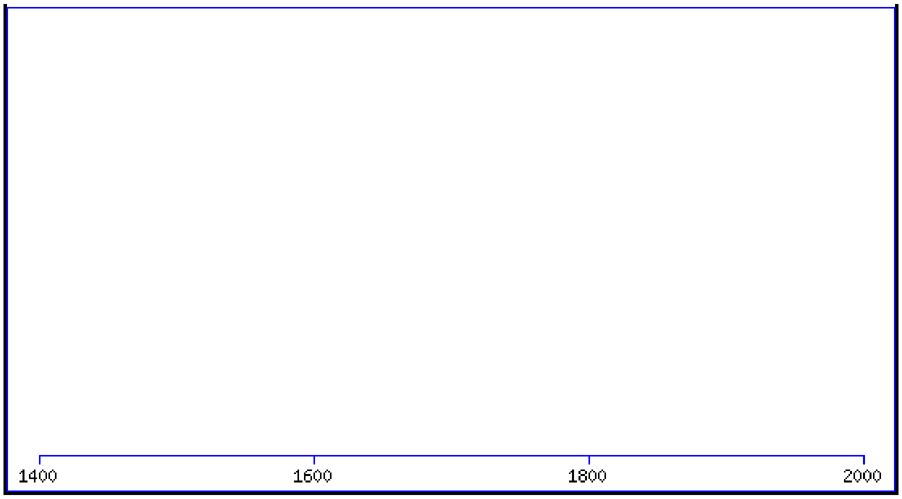


**Monoisotopic mass of neutral peptide Mr(calc):** 1215.6095
**Fixed modifications:** Carbamidomethyl (C)
**Variable modifications:**
**N7** : Deamidated_N (N)
**Ions Score:** 78 **Expect:** 1.8e-006
**Matches (Bold Red):** 28/114 fragment ions using 44 most intense peaks

| # | b | b++ | b* | b*++ | b0 | b0++ | Seq. | y | y++ | y* | y*++ | y0 | y0++ | # |
|---|---|---|---|---|---|---|---|---|---|---|---|---|---|---|
| 1 | 130.0499 | 65.5286 | | | 112.0393 | 56.5233 | E | | | | | | | 12 |
| 2 | **243.1339** | 122.0706 | | | **225.1234** | 113.0653 | L | 1087.5742 | 544.2907 | **1070.5477** | 535.7775 | 1069.5636 | 535.2855 | 11 |
| 3 | **314.1710** | 157.5892 | | | **296.1605** | 148.5839 | A | **974.4901** | 487.7487 | 957.4636 | 479.2354 | 956.4796 | 478.7434 | 10 |
| 4 | **413.2395** | 207.1234 | | | **395.2289** | 198.1181 | V | **903.4530** | 452.2302 | 886.4265 | 443.7169 | 885.4425 | 443.2249 | 9 |
| 5 | **484.2766** | **242.6419** | | | **466.2660** | 233.6366 | A | **804.3846** | 402.6959 | 787.3581 | 394.1827 | **786.3741** | 393.6907 | 8 |
| 6 | **555.3137** | 278.1605 | | | **537.3031** | 269.1552 | A | **733.3475** | **367.1774** | 716.3210 | 358.6641 | 715.3369 | 358.1721 | 7 |
| 7 | **670.3406** | 335.6740 | 653.3141 | 327.1607 | 652.3301 | 326.6687 | N | **662.3104** | 331.6588 | 645.2838 | 323.1456 | 644.2998 | 322.6535 | 6 |
| 8 | **799.3832** | 400.1952 | 782.3567 | 391.6820 | 781.3727 | 391.1900 | E | **547.2835** | 274.1454 | 530.2569 | 265.6321 | 529.2729 | 265.1401 | 5 |
| 9 | **900.4309** | 450.7191 | 883.4043 | 442.2058 | 882.4203 | 441.7138 | T | **418.2409** | 209.6241 | 401.2143 | 201.1108 | 400.2303 | 200.6188 | 4 |
| 10 | **971.4680** | 486.2376 | 954.4415 | 477.7244 | 953.4574 | 477.2324 | A | **317.1932** | 159.1002 | 300.1666 | 150.5870 | | | 3 |
| 11 | **1042.5051** | 521.7562 | 1025.4786 | 513.2429 | 1024.4946 | 512.7509 | A | **246.1561** | 123.5817 | 229.1295 | 115.0684 | | | 2 |
| 12 | | | | | | | R | 175.1190 | 88.0631 | 158.0924 | 79.5498 | | | 1 |

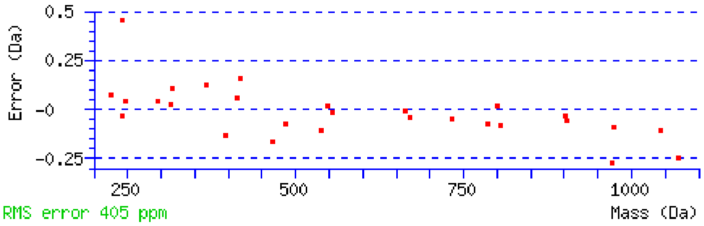


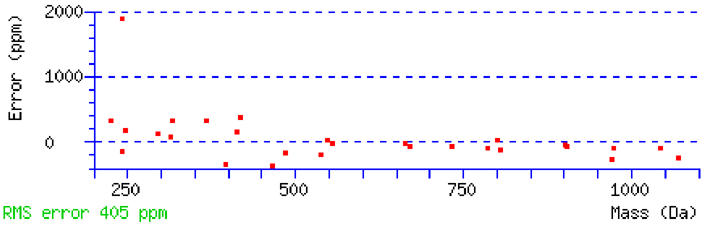


### All matches to this query

| Score | Mr(calc): | Delta | Sequence |
|---|---|---|---|
| 78.2 | 1215.6095 | -0.0030 | ELAVAANETAAR |
| 7.9 | 1215.6013 | 0.0052 | NGITPLHVASK |
| 7.2 | 1215.6095 | -0.0030 | NQLGKNEDGLK |
| 6.1 | 1214.6077 | 0.9988 | TAPAAAVSPMQR |
| 5.1 | 1215.6046 | 0.0019 | RAKLMSTVSK |
| 4.5 | 1213.5979 | 2.0086 | SSPNPFVGSPPK |
| 4.0 | 1214.5982 | 1.0083 | LTSSLLGMVAK |
| 4.0 | 1214.5982 | 1.0083 | LTSSLLGMVAK |
| 3.3 | 1215.6125 | -0.0060 | VKPRHDSGIK |
| | | | |

| | | | |
|---|---|---|---|
| 3.0 | 1215.6141 | -0.0076 | SKPLRKTVK |

---

Spectrum No: 253; Query: 827; Rank: 1

---

## Peptide View

MS/MS Fragmentation of **EAGNITTDGYEILGK**
Found in **IPI00189714**, Tax_Id=10116 Gene_Symbol=Col12a1 similar to Collagen alpha-1(XII) chain precursor

Match to Query 827: 1580.754188 from(791.384370,2+)
Title: 091008RatKidney_NoSalt_27.2733.2733.2.dta
Data file K:\NewmanPaper\Piliang\3SubProteomes\Piliang3SP\mgf5ppm\ERLIC_3SubProteomes5ppm.mgf

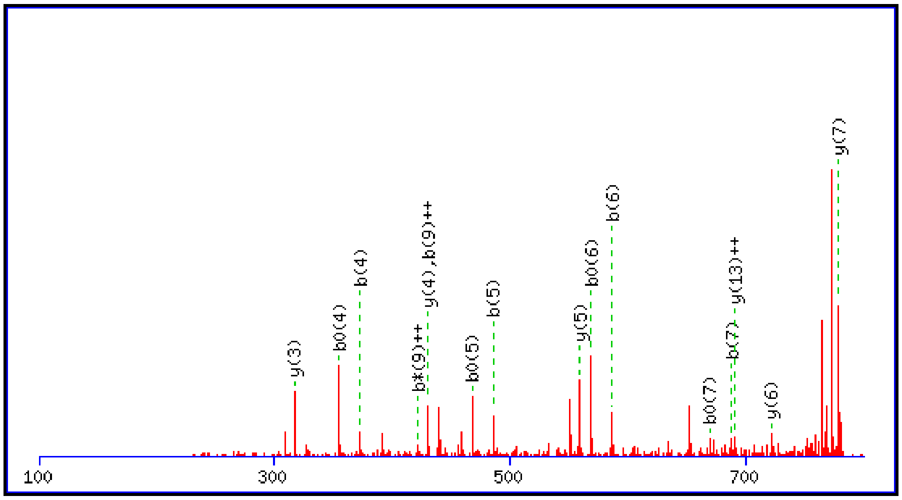


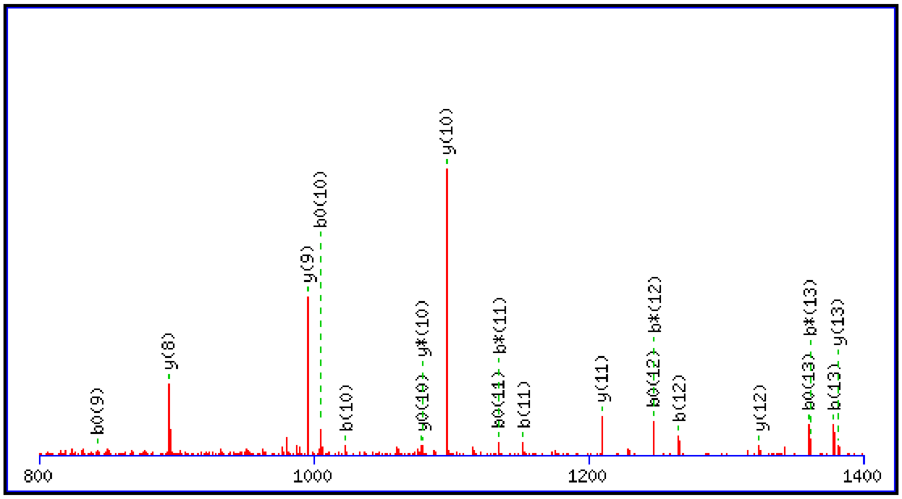

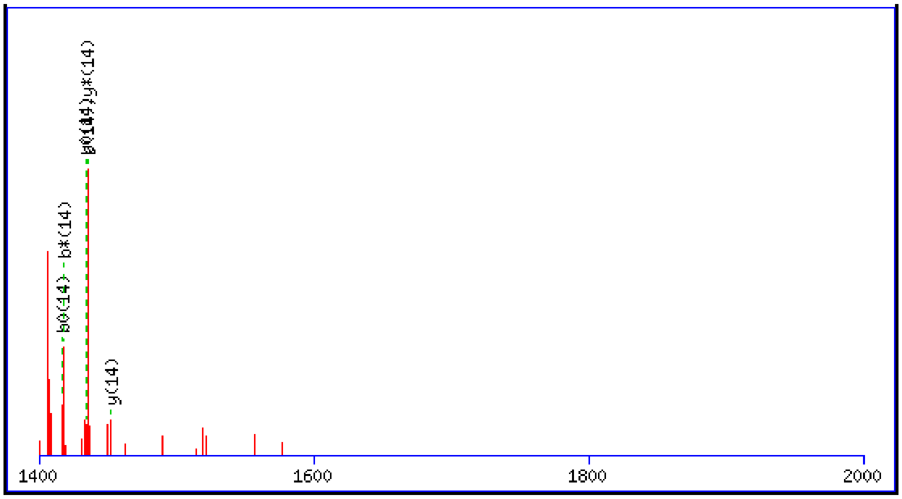


**Monoisotopic mass of neutral peptide Mr(calc):** 1580.7570
**Fixed modifications:** Carbamidomethyl (C)
**Variable modifications:**
**N4** : Deamidated_N (N)
**Ions Score:** 78 **Expect:** 2.7e-006
**Matches (Bold Red):** 42/154 fragment ions using 77 most intense peaks

| # | b | $b^{++}$ | $b^{*}$ | $b^{*++}$ | $b^{0}$ | $b^{0++}$ | Seq. | y | $y^{++}$ | $y^{*}$ | $y^{*++}$ | $y^{0}$ | $y^{0++}$ | # |
|---|---|---|---|---|---|---|---|---|---|---|---|---|---|---|
| 1 | 130.0499 | 65.5286 | | | 112.0393 | 56.5233 | E | | | | | | | 15 |
| 2 | 201.0870 | 101.0471 | | | 183.0764 | 92.0418 | A | **1452.7217** | 726.8645 | **1435.6951** | 718.3512 | **1434.7111** | 717.8592 | 14 |
| 3 | 258.1084 | 129.5579 | | | 240.0979 | 120.5526 | G | **1381.6845** | **691.3459** | 1364.6580 | 682.8326 | 1363.6740 | 682.3406 | 13 |
| 4 | **373.1354** | 187.0713 | 356.1088 | 178.5581 | **355.1248** | 178.0660 | N | **1324.6631** | 662.8352 | 1307.6365 | 654.3219 | 1306.6525 | 653.8299 | 12 |
| 5 | **486.2194** | 243.6134 | 469.1929 | 235.1001 | **468.2089** | 234.6081 | I | **1209.6361** | 605.3217 | 1192.6096 | 596.8084 | 1191.6256 | 596.3164 | 11 |
| 6 | **587.2671** | 294.1372 | 570.2406 | 285.6239 | **569.2566** | 285.1319 | T | **1096.5521** | 548.7797 | **1079.5255** | 540.2664 | **1078.5415** | 539.7744 | 10 |
| 7 | **688.3148** | 344.6610 | 671.2883 | 336.1478 | **670.3042** | 335.6558 | T | **995.5044** | 498.2558 | 978.4779 | 489.7426 | 977.4938 | 489.2506 | 9 |
| 8 | 803.3417 | 402.1745 | 786.3152 | 393.6612 | 785.3312 | 393.1692 | D | **894.4567** | 447.7320 | 877.4302 | 439.2187 | 876.4462 | 438.7267 | 8 |
| 9 | 860.3632 | **430.6852** | 843.3367 | **422.1720** | **842.3526** | 421.6800 | G | **779.4298** | 390.2185 | 762.4032 | 381.7053 | 761.4192 | 381.2132 | 7 |
| 10 | **1023.4265** | 512.2169 | 1006.4000 | 503.7036 | **1005.4160** | 503.2116 | Y | **722.4083** | 361.7078 | 705.3818 | 353.1945 | 704.3978 | 352.7025 | 6 |
| 11 | **1152.4691** | 576.7382 | **1135.4426** | 568.2249 | **1134.4586** | 567.7329 | E | **559.3450** | 280.1761 | 542.3184 | 271.6629 | 541.3344 | 271.1708 | 5 |
| 12 | **1265.5532** | 633.2802 | **1248.5266** | 624.7670 | **1247.5426** | 624.2750 | I | **430.3024** | 215.6548 | 413.2758 | 207.1416 | | | 4 |
| 13 | **1378.6373** | 689.8223 | **1361.6107** | 681.3090 | **1360.6267** | 680.8170 | L | **317.2183** | 159.1128 | 300.1918 | 150.5995 | | | 3 |
| 14 | **1435.6587** | 718.3330 | **1418.6322** | 709.8197 | **1417.6482** | 709.3277 | G | 204.1343 | 102.5708 | 187.1077 | 94.0575 | | | 2 |
| 15 | | | | | | | K | 147.1128 | 74.0600 | 130.0863 | 65.5468 | | | 1 |

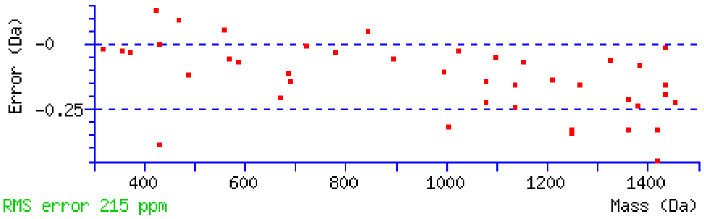


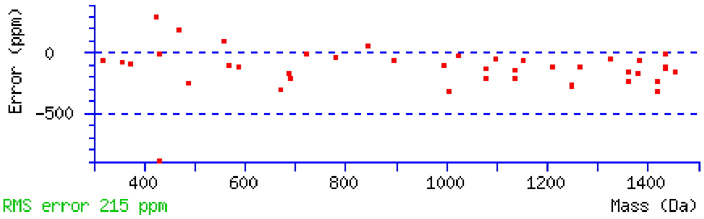


## All matches to this query

| Score | Mr(calc): | Delta | Sequence |
|---|---|---|---|
| 78.1 | 1580.7570 | -0.0028 | EAGNITTDGYEILGK |
| 11.1 | 1580.7575 | -0.0033 | GHFYMHVGSLLLK |
| 5.9 | 1580.7487 | 0.0055 | TPDLDKWEEIKK |
| 5.5 | 1580.7422 | 0.0120 | MTNGVVHANLFGIK |
| 2.7 | 1580.7682 | -0.0140 | ERVPENPSNALPEK |
| 2.5 | 1578.7403 | 2.0139 | AEVAGEEPASKQRK |
| | | | |

| | | | |
|---|---|---|---|
| 2.3 | 1580.7575 | -0.0033 | GHFYMHVGSLLLK |
| 1.8 | 1578.7331 | 2.0211 | FISQETGNLYISK |
| 1.5 | 1580.7521 | 0.0021 | TDLPCDAAIATILK |
| 1.3 | 1578.7473 | 2.0069 | KSTFWRPKTLR |

---

Spectrum No: 254; Query: 487; Rank: 1

---

## Peptide View

MS/MS Fragmentation of **VETGENCTSPAPK**
Found in **IPI00210027**, Tax_Id=10116 Gene_Symbol=Itpr1 Isoform 1 of Inositol 1,4,5-trisphosphate receptor type 1

Match to Query 487: 1389.605288 from(695.809920,2+)
Title: 091008RatKidney_NH4Format01_25.326.326.2.dta
Data file K:\NewmanPaper\Piliang\3SubProteomes\Piliang3SP\mgf5ppm\ERLIC_3SubProteomes5ppm.mgf

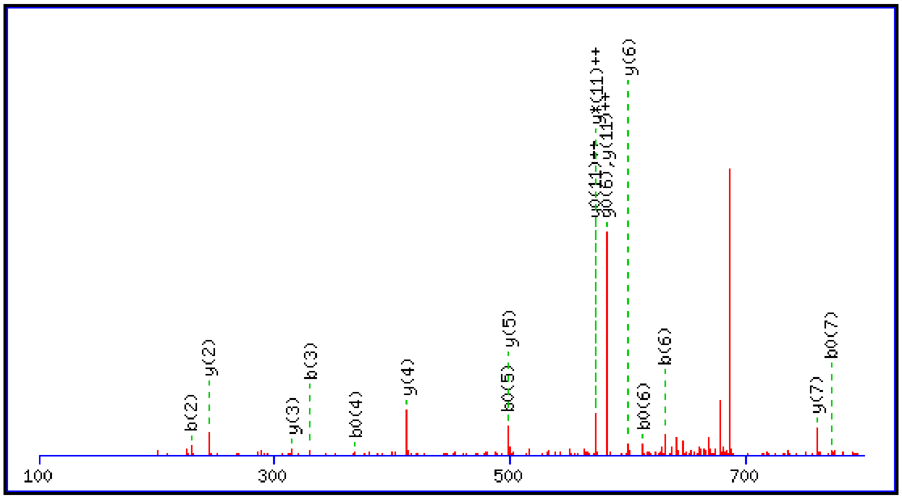


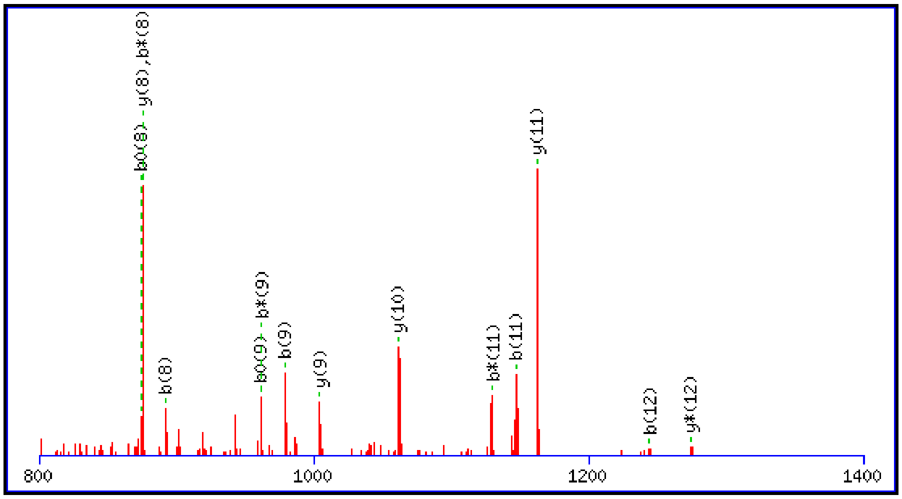

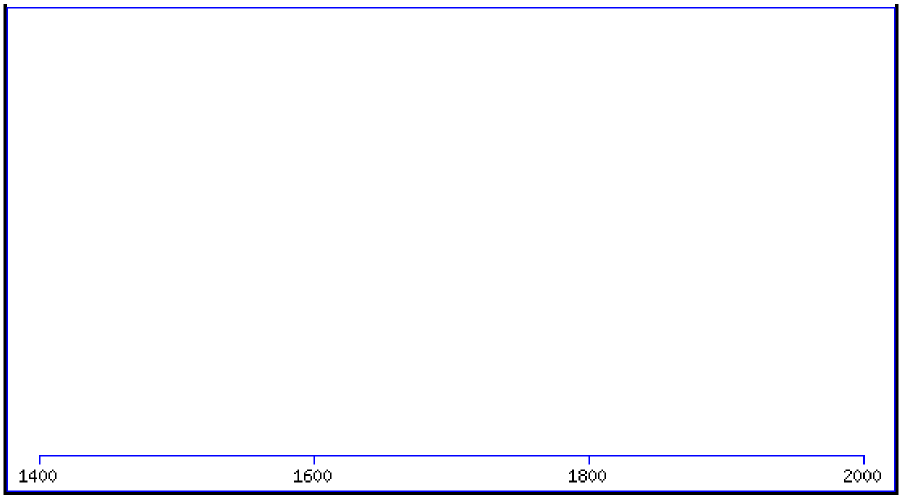


**Monoisotopic mass of neutral peptide Mr(calc):** 1389.6082
**Fixed modifications:** Carbamidomethyl (C)
**Variable modifications:**
**N6** : Deamidated_N (N)
**Ions Score:** 78 **Expect:** 2e-006
**Matches (Bold Red):** 31/124 fragment ions using 46 most intense peaks

| # | b | $b^{++}$ | b* | $b^{*++}$ | $b^0$ | $b^{0++}$ | Seq. | y | $y^{++}$ | y* | $y^{*++}$ | $y^0$ | $y^{0++}$ | # |
|---|---|---|---|---|---|---|---|---|---|---|---|---|---|---|
| 1 | 100.0757 | 50.5415 | | | | | V | | | | | | | 13 |
| 2 | **229.1183** | 115.0628 | | | 211.1077 | 106.0575 | E | 1291.5471 | 646.2772 | **1274.5205** | 637.7639 | 1273.5365 | 637.2719 | 12 |
| 3 | **330.1660** | 165.5866 | | | 312.1554 | 156.5813 | T | **1162.5045** | **581.7559** | 1145.4779 | **573.2426** | 1144.4939 | **572.7506** | 11 |
| 4 | 387.1874 | 194.0974 | | | **369.1769** | 185.0921 | G | **1061.4568** | 531.2320 | 1044.4302 | 522.7188 | 1043.4462 | 522.2268 | 10 |
| 5 | 516.2300 | 258.6186 | | | **498.2195** | 249.6134 | E | **1004.4353** | 502.7213 | 987.4088 | 494.2080 | 986.4248 | 493.7160 | 9 |
| 6 | **631.2570** | 316.1321 | 614.2304 | 307.6188 | **613.2464** | 307.1268 | N | **875.3927** | 438.2000 | 858.3662 | 429.6867 | 857.3822 | 429.1947 | 8 |
| 7 | 791.2876 | 396.1474 | 774.2611 | 387.6342 | **773.2770** | 387.1422 | C | **760.3658** | 380.6865 | 743.3393 | 372.1733 | 742.3552 | 371.6813 | 7 |
| 8 | **892.3353** | 446.6713 | **875.3087** | 438.1580 | **874.3247** | 437.6660 | T | **600.3352** | 300.6712 | 583.3086 | 292.1579 | **582.3246** | 291.6659 | 6 |
| 9 | **979.3673** | 490.1873 | **962.3408** | 481.6740 | **961.3567** | 481.1820 | S | **499.2875** | 250.1474 | 482.2609 | 241.6341 | 481.2769 | 241.1421 | 5 |
| 10 | 1076.4201 | 538.7137 | 1059.3935 | 530.2004 | 1058.4095 | 529.7084 | P | **412.2554** | 206.6314 | 395.2289 | 198.1181 | | | 4 |
| 11 | **1147.4572** | 574.2322 | **1130.4306** | 565.7190 | 1129.4466 | 565.2269 | A | **315.2027** | 158.1050 | 298.1761 | 149.5917 | | | 3 |
| 12 | **1244.5100** | 622.7586 | 1227.4834 | 614.2453 | 1226.4994 | 613.7533 | P | **244.1656** | 122.5864 | 227.1390 | 114.0731 | | | 2 |
| 13 | | | | | | | K | 147.1128 | 74.0600 | 130.0863 | 65.5468 | | | 1 |

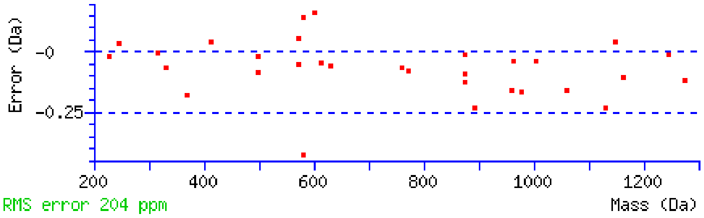


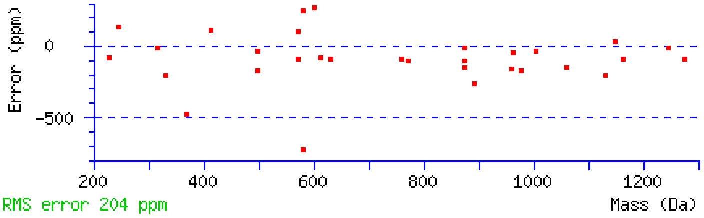


### All matches to this query

| Score | Mr(calc): | Delta | Sequence |
|---|---|---|---|
| 78.1 | 1389.6082 | -0.0029 | VETGENCTSPAPK |
| 14.0 | 1389.5982 | 0.0071 | YAEYSTKKLK |
| 10.9 | 1389.6038 | 0.0015 | RSDLQSTFSGGR |
| 10.9 | 1389.6038 | 0.0015 | RSDLQSTFSGGR |
| 9.8 | 1387.6101 | 1.9952 | KGLKCCASAASR |
| 8.7 | 1388.6081 | 0.9972 | LLASCSADMTIK |
| 7.8 | 1389.5982 | 0.0071 | YAEYSTKKLK |
| 7.8 | 1389.5982 | 0.0071 | YAEYSTKKLK |
| | | | |

| | | | |
|---|---|---|---|
| 7.3 | 1388.6102 | 0.9951 | ASRSPLVTSSPK |
| 6.8 | 1388.6102 | 0.9951 | IISGDIQTVQR |

Spectrum No: 255; Query: 1599; Rank: 1

## Peptide View

MS/MS Fragmentation of **VFADGAPHYVAFYSNVTR**
Found in **IPI00190577**, Tax_Id=10116 Gene_Symbol=Lama5 similar to Laminin alpha-5 chain precursor

Match to Query 1599: 2013.962322 from(672.328050,3+)
Title: 100101RatKid_NS_deglyco_24.3309.3309.3.dta
Data file K:\NewmanPaper\Piliang\3SubProteomes\Piliang3SP\mgf5ppm\ERLIC_3SubProteomes5ppm.mgf

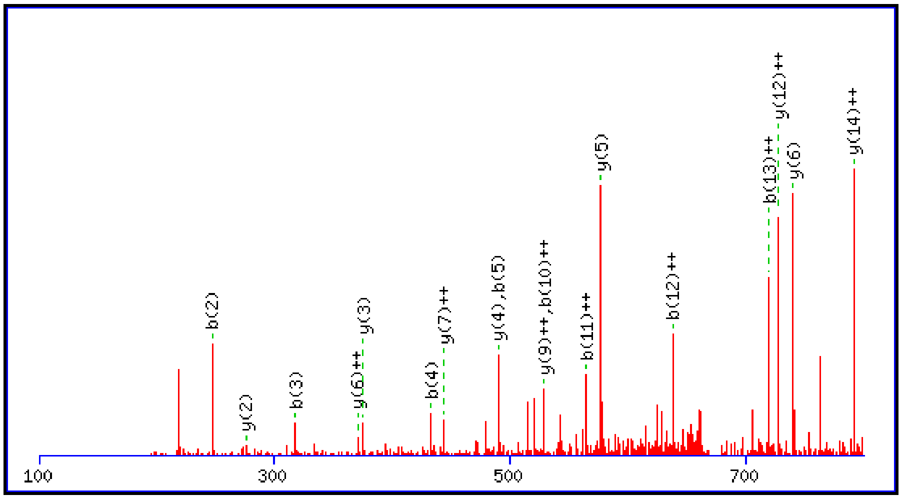


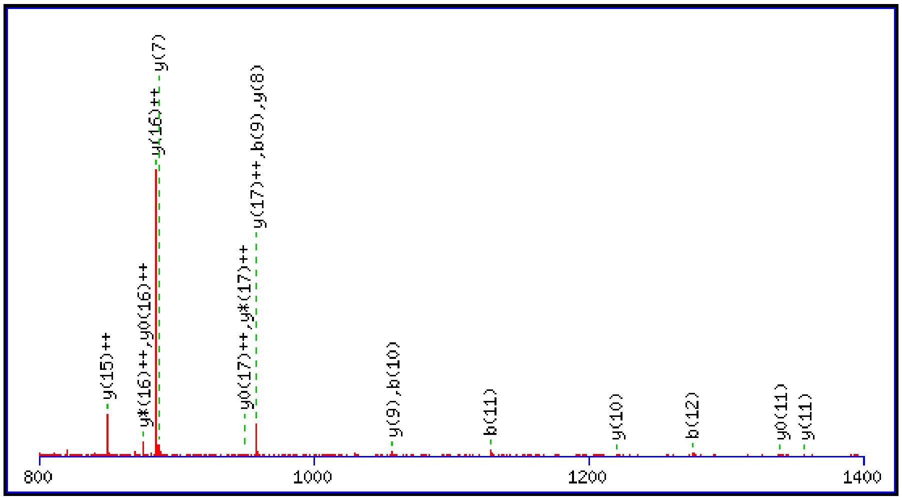

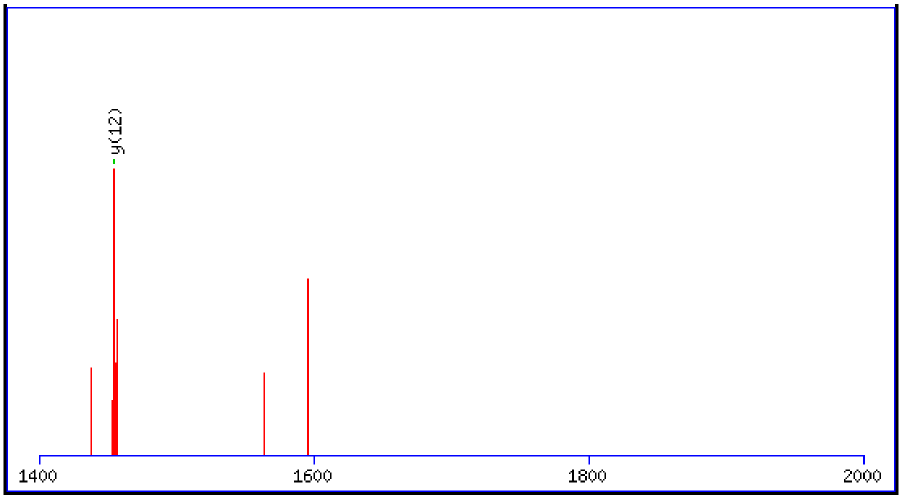


**Monoisotopic mass of neutral peptide Mr(calc):** 2013.9585
**Fixed modifications:** Carbamidomethyl (C)
**Variable modifications:**
**N15** : Deamidated_N (N)
**Ions Score:** 78 **Expect:** 3.6e-006
**Matches (Bold Red):** 36/168 fragment ions using 47 most intense peaks

| # | b | $b^{++}$ | b* | $b^{*++}$ | $b^0$ | $b^{0++}$ | Seq. | y | $y^{++}$ | y* | $y^{*++}$ | $y^0$ | $y^{0++}$ | # |
|---|---|---|---|---|---|---|---|---|---|---|---|---|---|---|
| 1 | 100.0757 | 50.5415 | | | | | V | | | | | | | 18 |
| 2 | **247.1441** | 124.0757 | | | | | F | 1915.8973 | **958.4523** | 1898.8708 | **949.9390** | 1897.8868 | **949.4470** | 17 |
| 3 | **318.1812** | 159.5942 | | | | | A | 1768.8289 | **884.9181** | 1751.8024 | **876.4048** | 1750.8184 | **875.9128** | 16 |
| 4 | **433.2082** | 217.1077 | | | 415.1976 | 208.1024 | D | 1697.7918 | **849.3995** | 1680.7653 | 840.8863 | 1679.7812 | 840.3943 | 15 |
| 5 | **490.2296** | 245.6185 | | | 472.2191 | 236.6132 | G | 1582.7649 | **791.8861** | 1565.7383 | 783.3728 | 1564.7543 | 782.8808 | 14 |
| 6 | 561.2667 | 281.1370 | | | 543.2562 | 272.1317 | A | 1525.7434 | 763.3753 | 1508.7169 | 754.8621 | 1507.7328 | 754.3701 | 13 |
| 7 | 658.3195 | 329.6634 | | | 640.3089 | 320.6581 | P | **1454.7063** | **727.8568** | 1437.6797 | 719.3435 | 1436.6957 | 718.8515 | 12 |
| 8 | 795.3784 | 398.1928 | | | 777.3678 | 389.1876 | H | **1357.6535** | 679.3304 | 1340.6270 | 670.8171 | **1339.6430** | 670.3251 | 11 |
| 9 | **958.4417** | 479.7245 | | | 940.4312 | 470.7192 | Y | **1220.5946** | 610.8009 | 1203.5681 | 602.2877 | 1202.5840 | 601.7957 | 10 |
| 10 | **1057.5102** | **529.2587** | | | 1039.4996 | 520.2534 | V | **1057.5313** | **529.2693** | 1040.5047 | 520.7560 | 1039.5207 | 520.2640 | 9 |
| 11 | **1128.5473** | **564.7773** | | | 1110.5367 | 555.7720 | A | **958.4629** | 479.7351 | 941.4363 | 471.2218 | 940.4523 | 470.7298 | 8 |
| 12 | **1275.6157** | **638.3115** | | | 1257.6051 | 629.3062 | F | **887.4258** | **444.2165** | 870.3992 | 435.7032 | 869.4152 | 435.2112 | 7 |
| 13 | 1438.6790 | **719.8431** | | | 1420.6685 | 710.8379 | Y | **740.3573** | **370.6823** | 723.3308 | 362.1690 | 722.3468 | 361.6770 | 6 |
| 14 | 1525.7110 | 763.3592 | | | 1507.7005 | 754.3539 | S | **577.2940** | 289.1506 | 560.2675 | 280.6374 | 559.2834 | 280.1454 | 5 |
| 15 | 1640.7380 | 820.8726 | 1623.7114 | 812.3594 | 1622.7274 | 811.8673 | N | **490.2620** | 245.6346 | 473.2354 | 237.1214 | 472.2514 | 236.6293 | 4 |
| 16 | 1739.8064 | 870.4068 | 1722.7798 | 861.8936 | 1721.7958 | 861.4016 | V | **375.2350** | 188.1212 | 358.2085 | 179.6079 | 357.2245 | 179.1159 | 3 |
| 17 | 1840.8541 | 920.9307 | 1823.8275 | 912.4174 | 1822.8435 | 911.9254 | T | **276.1666** | 138.5870 | 259.1401 | 130.0737 | 258.1561 | 129.5817 | 2 |
| 18 | | | | | | | R | 175.1190 | 88.0631 | 158.0924 | 79.5498 | | | 1 |

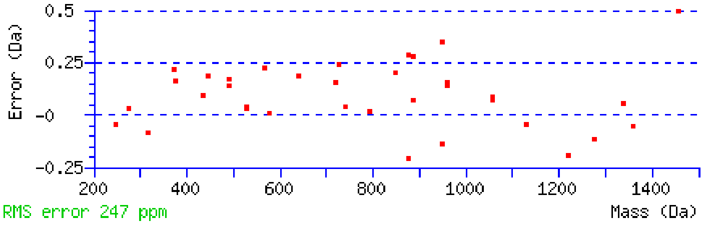


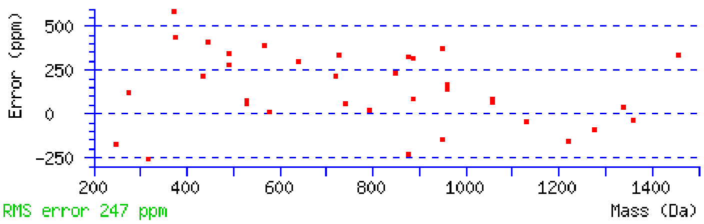


## All matches to this query

| Score | Mr(calc): | Delta | Sequence |
|---|---|---|---|
| 78.1 | 2013.9585 | 0.0038 | VFADGAPHYVAFYSNVTR |
| 30.4 | 2012.9745 | 0.9879 | VFADGAPHYVAFYSNVTR |
| 9.1 | 2011.9656 | 1.9967 | NDVLTLLSSINKDWWK |
| | | | |

| | | | |
|---|---|---|---|
| 6.0 | 2011.9373 | 2.0250 | GQICVVHYTGMLQNGKK |
| 5.7 | 2013.9449 | 0.0175 | FATVGSDKILFHEVNEK |
| 4.6 | 2012.9754 | 0.9869 | MAKRNYVGISEIPPVSR |
| 4.6 | 2012.9754 | 0.9869 | MAKRNYVGISEIPPVSR |
| 4.5 | 2012.9642 | 0.9981 | WMNGSILNTNLLRITGK |
| 4.5 | 2012.9642 | 0.9981 | WMNGSILNTNLLRITGK |
| 4.3 | 2011.9656 | 1.9967 | NDVLTLLSSINKDWWK |

---

Spectrum No: 256; Query: 557; Rank: 1

---

## Peptide View

MS/MS Fragmentation of **YTADPNITSVGPAK**
Found in **IPI00388914**, Tax_Id=10116 Gene_Symbol=Plxnb1_predicted plexin B1

Match to Query 557: 1433.706508 from(717.860530,2+)
Title: 100101RatKid_NS_deglyco_25.1631.1631.2.dta
Data file K:\NewmanPaper\Piliang\3SubProteomes\Piliang3SP\mgf5ppm\ERLIC_3SubProteomes5ppm.mgf

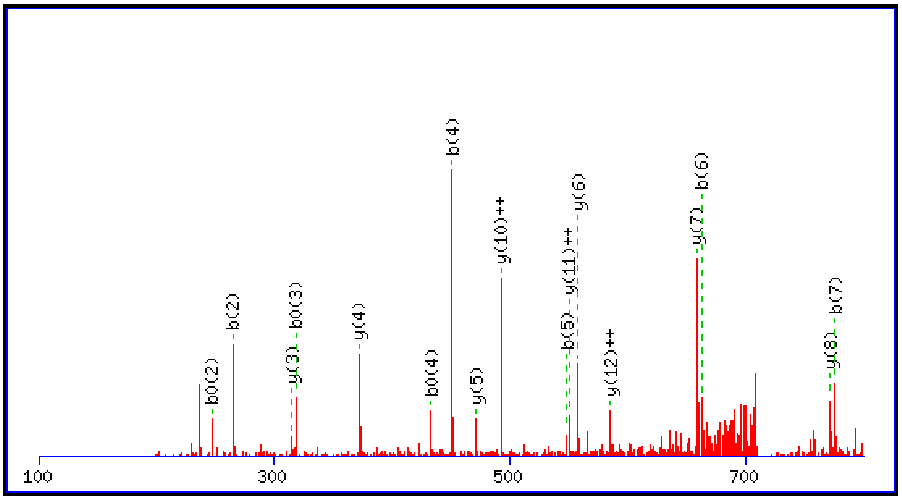


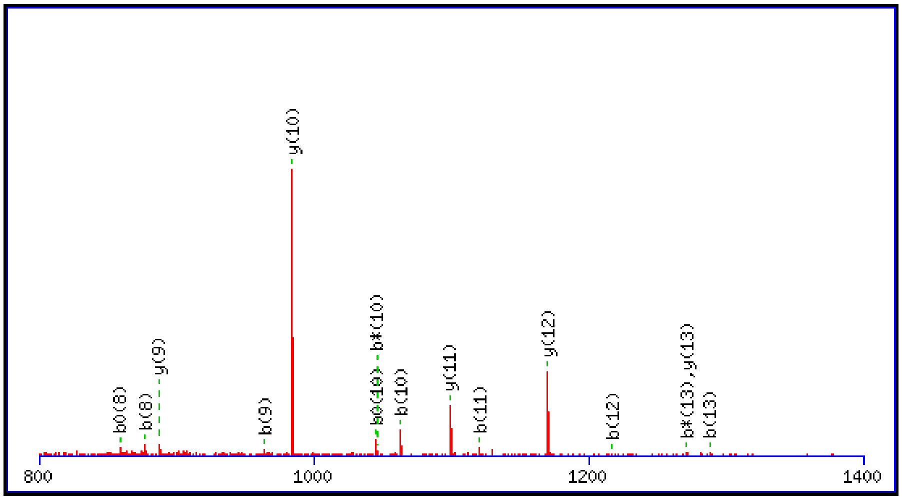

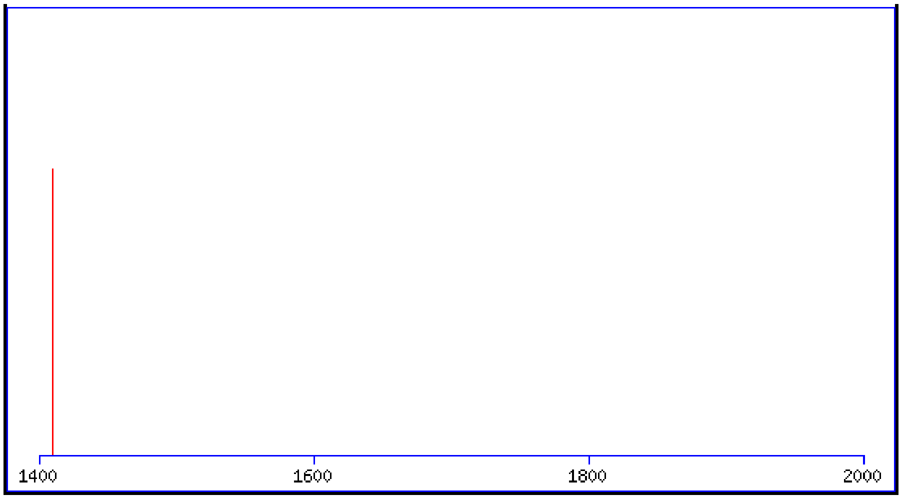


**Monoisotopic mass of neutral peptide Mr(calc):** 1433.7038
**Fixed modifications:** Carbamidomethyl (C)
**Variable modifications:**
**N6** : Deamidated_N (N)
**Ions Score:** 78 **Expect:** 2.5e-006
**Matches (Bold Red):** 32/134 fragment ions using 64 most intense peaks

| # | b | b++ | b* | b*++ | b0 | b0++ | Seq. | y | y++ | y* | y*++ | y0 | y0++ | # |
|---|---|---|---|---|---|---|---|---|---|---|---|---|---|---|
| 1 | 164.0706 | 82.5389 | | | | | Y | | | | | | | 14 |
| 2 | **265.1183** | 133.0628 | | | **247.1077** | 124.0575 | T | **1271.6478** | 636.3275 | 1254.6212 | 627.8142 | 1253.6372 | 627.3222 | 13 |
| 3 | 336.1554 | 168.5813 | | | **318.1448** | 159.5761 | A | **1170.6001** | **585.8037** | 1153.5735 | 577.2904 | 1152.5895 | 576.7984 | 12 |
| 4 | **451.1823** | 226.0948 | | | **433.1718** | 217.0895 | D | **1099.5630** | **550.2851** | 1082.5364 | 541.7719 | 1081.5524 | 541.2798 | 11 |
| 5 | **548.2351** | 274.6212 | | | 530.2245 | 265.6159 | P | **984.5360** | **492.7717** | 967.5095 | 484.2584 | 966.5255 | 483.7664 | 10 |
| 6 | **663.2620** | 332.1347 | 646.2355 | 323.6214 | 645.2515 | 323.1294 | N | **887.4833** | 444.2453 | 870.4567 | 435.7320 | 869.4727 | 435.2400 | 9 |
| 7 | **776.3461** | 388.6767 | 759.3196 | 380.1634 | 758.3355 | 379.6714 | I | **772.4563** | 386.7318 | 755.4298 | 378.2185 | 754.4458 | 377.7265 | 8 |
| 8 | **877.3938** | 439.2005 | 860.3672 | 430.6873 | **859.3832** | 430.1952 | T | **659.3723** | 330.1898 | 642.3457 | 321.6765 | 641.3617 | 321.1845 | 7 |
| 9 | **964.4258** | 482.7165 | 947.3993 | 474.2033 | 946.4152 | 473.7113 | S | **558.3246** | 279.6659 | 541.2980 | 271.1527 | 540.3140 | 270.6606 | 6 |
| 10 | **1063.4942** | 532.2508 | **1046.4677** | 523.7375 | **1045.4837** | 523.2455 | V | **471.2926** | 236.1499 | 454.2660 | 227.6366 | | | 5 |
| 11 | **1120.5157** | 560.7615 | 1103.4891 | 552.2482 | 1102.5051 | 551.7562 | G | **372.2241** | 186.6157 | 355.1976 | 178.1024 | | | 4 |
| 12 | **1217.5685** | 609.2879 | 1200.5419 | 600.7746 | 1199.5579 | 600.2826 | P | **315.2027** | 158.1050 | 298.1761 | 149.5917 | | | 3 |
| 13 | **1288.6056** | 644.8064 | **1271.5790** | 636.2931 | 1270.5950 | 635.8011 | A | 218.1499 | 109.5786 | 201.1234 | 101.0653 | | | 2 |
| 14 | | | | | | | K | 147.1128 | 74.0600 | 130.0863 | 65.5468 | | | 1 |

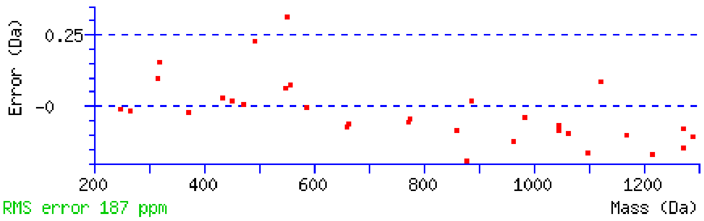


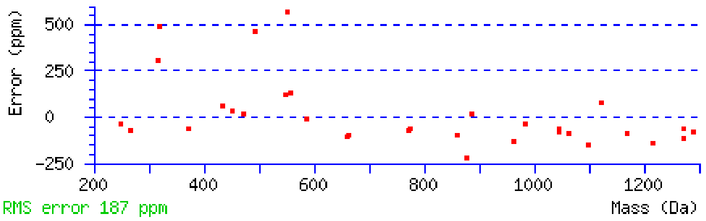


### All matches to this query

| Score | Mr(calc): | Delta | Sequence |
|---|---|---|---|
| 78.0 | 1433.7038 | 0.0027 | YTADPNITSVGPAK |
| 6.1 | 1433.7150 | -0.0085 | VEEPIHATADPQK |
| 3.9 | 1433.7007 | 0.0058 | VQINDMCSVKPK |
| 0.8 | 1433.7068 | -0.0003 | YTVGLRNXFSVK |
| 0.6 | 1433.7072 | -0.0006 | MVNSNLASVEELK |
| 0.3 | 1433.7068 | -0.0003 | SPAPPLPAPPPASR |

Spectrum No: 257; Query: 321; Rank: 1

## Peptide View

MS/MS Fragmentation of **FNDTNVVHTVK**
Found in **IPI00327469**, Tax_Id=10116 Gene_Symbol=Ahsg Alpha-2-HS-glycoprotein precursor

Match to Query 321: 1273.627448 from(637.821000,2+)
Title: 091008RatKidney_NoSalt_29.945.945.2.dta
Data file K:\NewmanPaper\Piliang\3SubProteomes\Piliang3SP\mgf5ppm\ERLIC_3SubProteomes5ppm.mgf

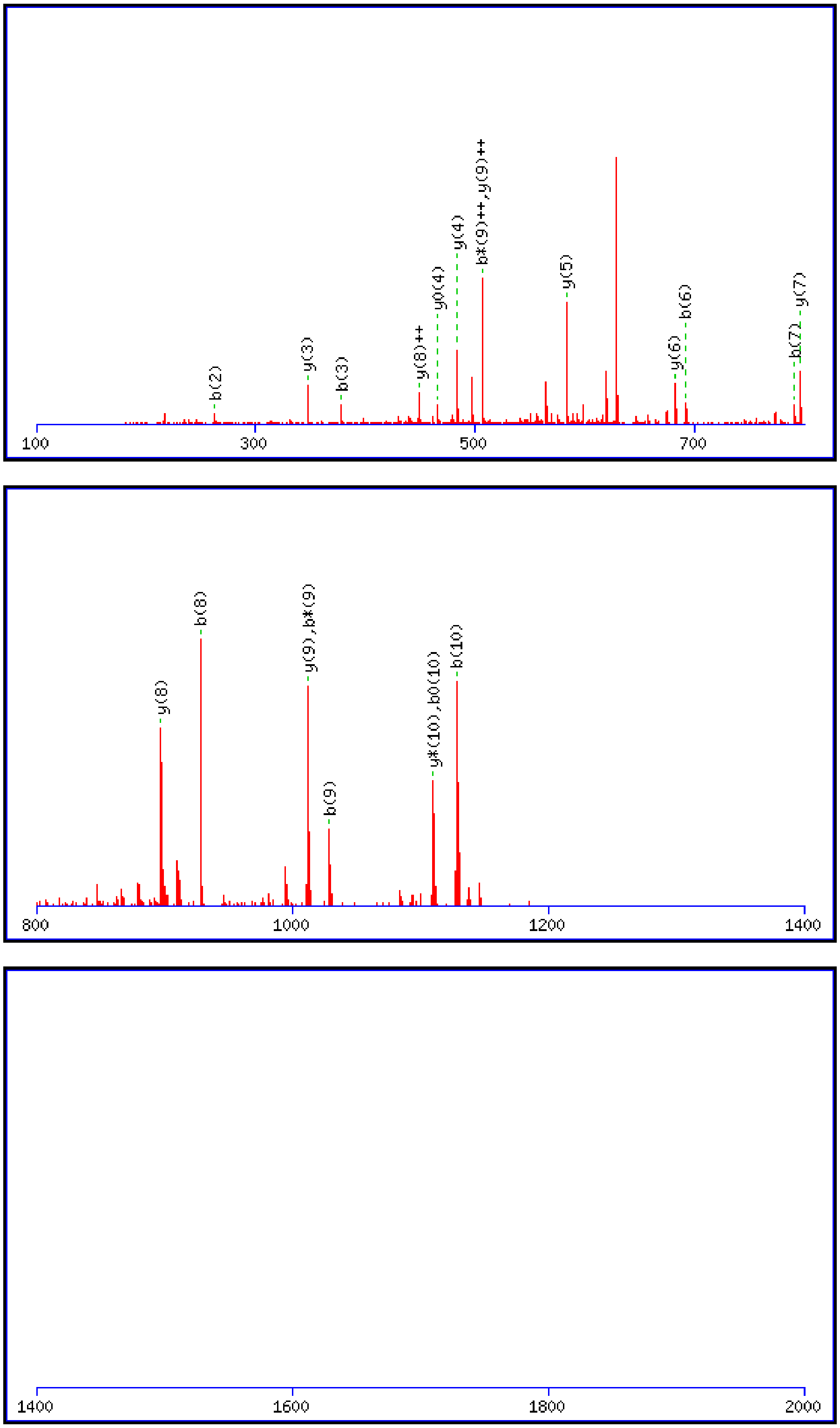


**Monoisotopic mass of neutral peptide Mr(calc):** 1273.6303

**Fixed modifications:** Carbamidomethyl (C)
**Variable modifications:**
**N2** : Deamidated_N (N)
**Ions Score:** 78 **Expect:** 2.1e-006
**Matches (Bold Red):** 21/110 fragment ions using 28 most intense peaks

| # | b | $b^{++}$ | b* | $b^{*++}$ | $b^0$ | $b^{0++}$ | Seq. | y | $y^{++}$ | y* | $y^{*++}$ | $y^0$ | $y^{0++}$ | # |
|---|---|---|---|---|---|---|---|---|---|---|---|---|---|---|
| 1 | 148.0757 | 74.5415 | | | | | F | | | | | | | 11 |
| 2 | **263.1026** | 132.0550 | 246.0761 | 123.5417 | | | N | 1127.5691 | 564.2882 | **1110.5426** | 555.7749 | 1109.5586 | 555.2829 | 10 |
| 3 | **378.1296** | 189.5684 | 361.1030 | 181.0551 | 360.1190 | 180.5631 | D | **1012.5422** | **506.7747** | 995.5156 | 498.2615 | 994.5316 | 497.7694 | 9 |
| 4 | 479.1772 | 240.0923 | 462.1507 | 231.5790 | 461.1667 | 231.0870 | T | **897.5152** | **449.2613** | 880.4887 | 440.7480 | 879.5047 | 440.2560 | 8 |
| 5 | 593.2202 | 297.1137 | 576.1936 | 288.6005 | 575.2096 | 288.1084 | N | **796.4676** | 398.7374 | 779.4410 | 390.2241 | 778.4570 | 389.7321 | 7 |
| 6 | **692.2886** | 346.6479 | 675.2620 | 338.1347 | 674.2780 | 337.6427 | V | **682.4246** | 341.7160 | 665.3981 | 333.2027 | 664.4141 | 332.7107 | 6 |
| 7 | **791.3570** | 396.1821 | 774.3305 | 387.6689 | 773.3464 | 387.1769 | V | **583.3562** | 292.1817 | 566.3297 | 283.6685 | 565.3457 | 283.1765 | 5 |
| 8 | **928.4159** | 464.7116 | 911.3894 | 456.1983 | 910.4053 | 455.7063 | H | **484.2878** | 242.6475 | 467.2613 | 234.1343 | **466.2772** | 233.6423 | 4 |
| 9 | **1029.4636** | 515.2354 | **1012.4370** | **506.7222** | 1011.4530 | 506.2302 | T | **347.2289** | 174.1181 | 330.2023 | 165.6048 | 329.2183 | 165.1128 | 3 |
| 10 | **1128.5320** | 564.7696 | 1111.5055 | 556.2564 | **1110.5214** | 555.7644 | V | 246.1812 | 123.5942 | 229.1547 | 115.0810 | | | 2 |
| 11 | | | | | | | K | 147.1128 | 74.0600 | 130.0863 | 65.5468 | | | 1 |

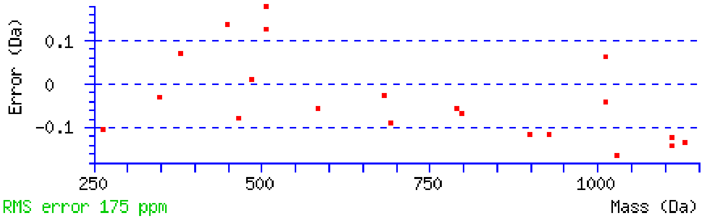


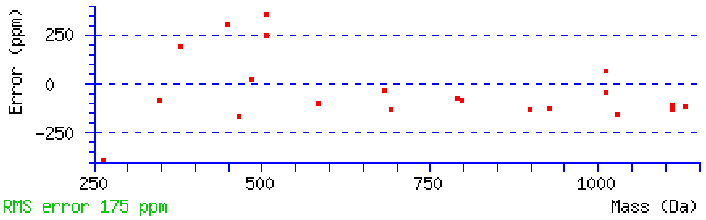


### All matches to this query

| Score | Mr(calc): | Delta | Sequence |
|---|---|---|---|
| 78.0 | 1273.6303 | -0.0028 | FNDTNVVHTVK |
| 59.8 | 1273.6303 | -0.0028 | FNDTNVVHTVK |
| 9.7 | 1273.6150 | 0.0125 | QEAENGALDISK |
| 9.1 | 1273.6263 | 0.0012 | VNLGVDSQGGTAR |
| 7.9 | 1273.6197 | 0.0078 | RNQDRPCLSK |
| 7.2 | 1273.6336 | -0.0062 | TALPNMDREVK |
| 6.2 | 1272.6115 | 1.0159 | ANKAVDFTTVK |
| 6.2 | 1273.6319 | -0.0044 | SLTSLVLYGNK |
| 5.6 | 1273.6401 | -0.0127 | ELISEGADVNVK |
| 5.4 | 1272.6115 | 1.0159 | ANKAVDFTTVK |

---

Spectrum No: 258; Query: 1251; Rank: 1

---

## Peptide View

MS/MS Fragmentation of **ELYESIWQNFTDQK**
Found in **IPI00324300**, Tax_Id=10116 Gene_Symbol=Ace Angiotensin-converting enzyme, somatic isoform precursor

Match to Query 1251: 1800.820408 from(901.417480,2+)
Title: 100101RatKid_NS_deglyco_25.4889.4889.2.dta
Data file K:\NewmanPaper\Piliang\3SubProteomes\Piliang3SP\mgf5ppm\ERLIC_3SubProteomes5ppm.mgf

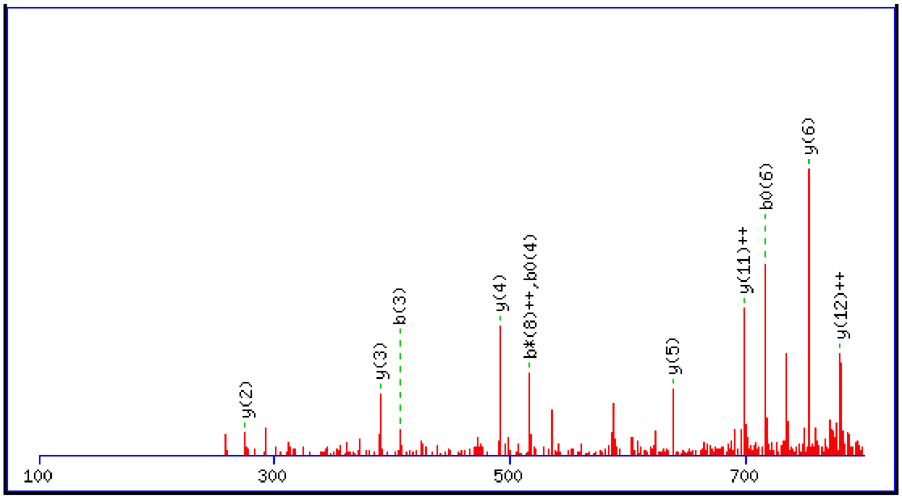


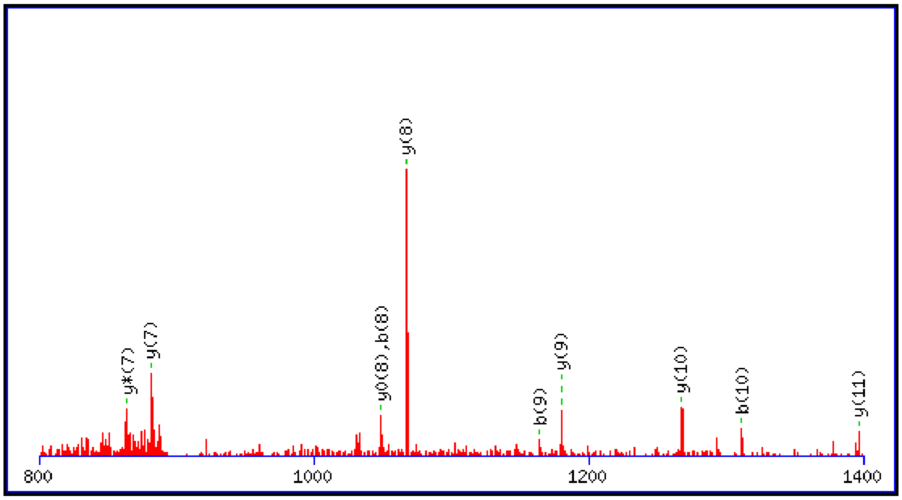


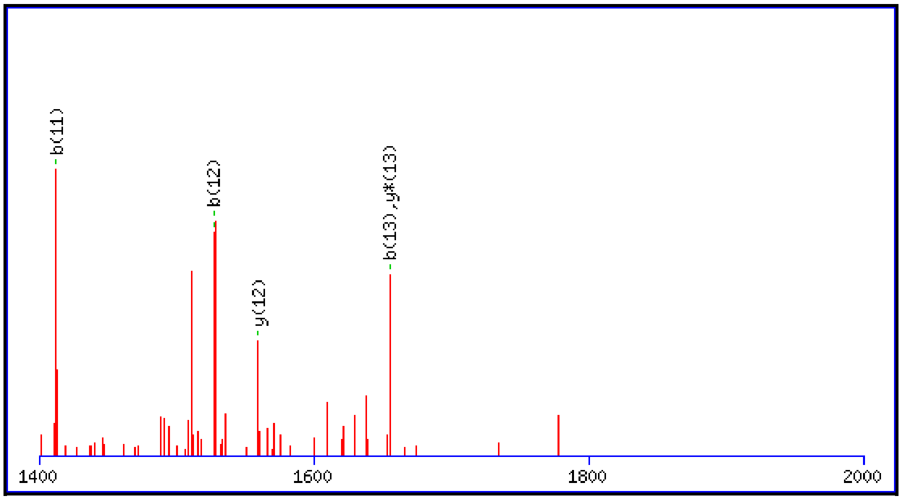


**Monoisotopic mass of neutral peptide Mr(calc):** 1800.8206
**Fixed modifications:** Carbamidomethyl (C)
**Variable modifications:**
**N9** : Deamidated_N (N)
**Ions Score:** 78 **Expect:** 3.9e-006
**Matches (Bold Red):** 26/138 fragment ions using 38 most intense peaks

| # | b | $b^{++}$ | b* | $b^{*++}$ | $b^{0}$ | $b^{0++}$ | Seq. | y | $y^{++}$ | y* | $y^{*++}$ | $y^{0}$ | $y^{0++}$ | # |
|---|---|---|---|---|---|---|---|---|---|---|---|---|---|---|
| 1 | 130.0499 | 65.5286 | | | 112.0393 | 56.5233 | E | | | | | | | 14 |
| 2 | 243.1339 | 122.0706 | | | 225.1234 | 113.0653 | L | 1672.7853 | 836.8963 | **1655.7588** | 828.3830 | 1654.7748 | 827.8910 | 13 |
| 3 | **406.1973** | 203.6023 | | | 388.1867 | 194.5970 | Y | **1559.7013** | **780.3543** | 1542.6747 | 771.8410 | 1541.6907 | 771.3490 | 12 |
| 4 | 535.2399 | 268.1236 | | | **517.2293** | 259.1183 | E | **1396.6379** | **698.8226** | 1379.6114 | 690.3093 | 1378.6274 | 689.8173 | 11 |
| 5 | 622.2719 | 311.6396 | | | 604.2613 | 302.6343 | S | **1267.5953** | 634.3013 | 1250.5688 | 625.7880 | 1249.5848 | 625.2960 | 10 |

| | | | | | | | | | | | | | | |
|---|---|---|---|---|---|---|---|---|---|---|---|---|---|---|
| **6** | 735.3559 | 368.1816 | | | **717.3454** | 359.1763 | **I** | **1180.5633** | 590.7853 | 1163.5368 | 582.2720 | 1162.5527 | 581.7800 | **9** |
| **7** | 921.4353 | 461.2213 | | | 903.4247 | 452.2160 | **W** | **1067.4792** | 534.2433 | 1050.4527 | 525.7300 | **1049.4687** | 525.2380 | **8** |
| **8** | **1049.4938** | 525.2506 | 1032.4673 | **516.7373** | 1031.4833 | 516.2453 | **Q** | **881.3999** | 441.2036 | **864.3734** | 432.6903 | 863.3894 | 432.1983 | **7** |
| **9** | **1164.5208** | 582.7640 | 1147.4942 | 574.2508 | 1146.5102 | 573.7587 | **N** | **753.3414** | 377.1743 | 736.3148 | 368.6610 | 735.3308 | 368.1690 | **6** |
| **10** | **1311.5892** | 656.2982 | 1294.5626 | 647.7850 | 1293.5786 | 647.2929 | **F** | **638.3144** | 319.6608 | 621.2879 | 311.1476 | 620.3039 | 310.6556 | **5** |
| **11** | **1412.6369** | 706.8221 | 1395.6103 | 698.3088 | 1394.6263 | 697.8168 | **T** | **491.2460** | 246.1266 | 474.2195 | 237.6134 | 473.2354 | 237.1214 | **4** |
| **12** | **1527.6638** | 764.3355 | 1510.6373 | 755.8223 | 1509.6532 | 755.3303 | **D** | **390.1983** | 195.6028 | 373.1718 | 187.0895 | 372.1878 | 186.5975 | **3** |
| **13** | **1655.7224** | 828.3648 | 1638.6958 | 819.8516 | 1637.7118 | 819.3595 | **Q** | **275.1714** | 138.0893 | 258.1448 | 129.5761 | | | **2** |
| **14** | | | | | | | **K** | 147.1128 | 74.0600 | 130.0863 | 65.5468 | | | **1** |

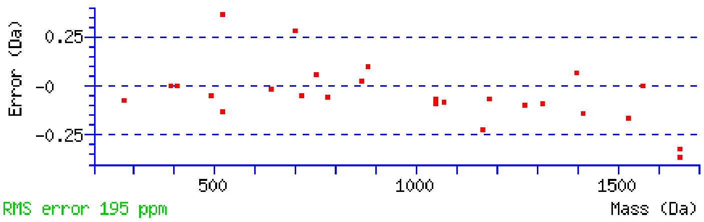


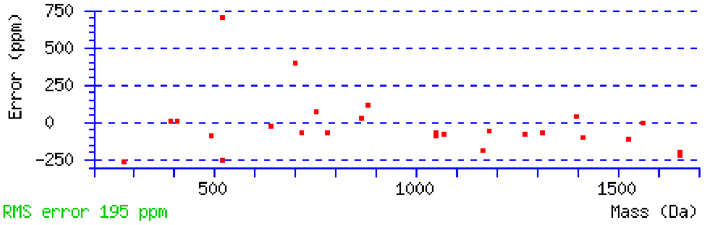


### All matches to this query

| Score | Mr(calc): | Delta | Sequence |
|---|---|---|---|
| 77.9 | 1800.8206 | -0.0002 | ELYESIWQNFTDQK |
| 8.9 | 1798.8284 | 1.9920 | RNAVSELQGELMSIR |
| 8.5 | 1800.8084 | 0.0121 | KHPDASVNFSEFSKK |
| 6.3 | 1800.8035 | 0.0169 | LCNFAFKTKGNLTK |
| 6.2 | 1800.8382 | -0.0178 | PQHAMGDKHFLSLPK |
| 3.0 | 1800.8124 | 0.0080 | VKFSASYFGPEFQSK |
| 1.5 | 1798.8284 | 1.9920 | AARELETLSDMTGGLR |
| 1.3 | 1800.8304 | -0.0100 | CASKSPNPLLTFCVK |
| 0.3 | 1799.8239 | 0.9965 | VVGFMSIPPSENALMK |

---

Spectrum No: 259; Query: 1383; Rank: 1

---

## Peptide View

MS/MS Fragmentation of **GTAGNALMEGASQLVGENR**
Found in **IPI00205389**, Tax_Id=10116 Gene_Symbol=Fgb Isoform 1 of Fibrinogen beta chain precursor

Match to Query 1383: 1874.884648 from(938.449600,2+)
Title: 100101RatKid_NS_deglyco_25.3641.3641.2.dta
Data file K:\NewmanPaper\Piliang\3SubProteomes\Piliang3SP\mgf5ppm\ERLIC_3SubProteomes5ppm.mgf

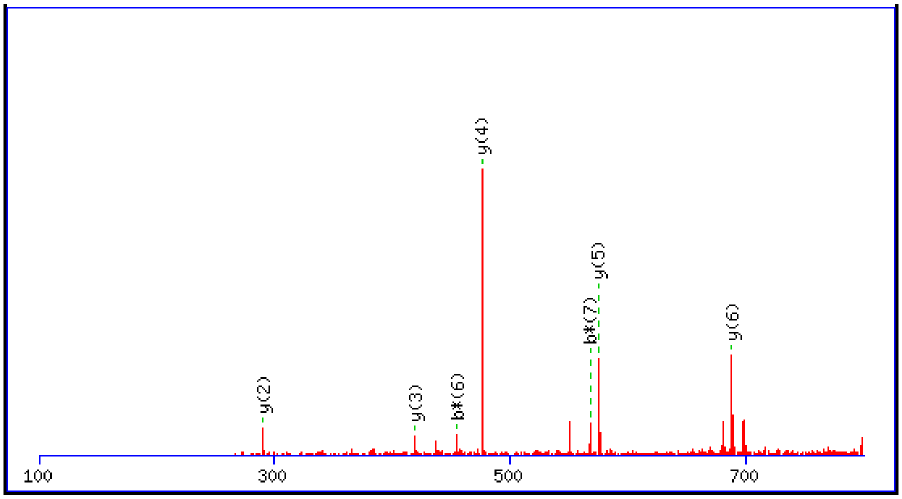


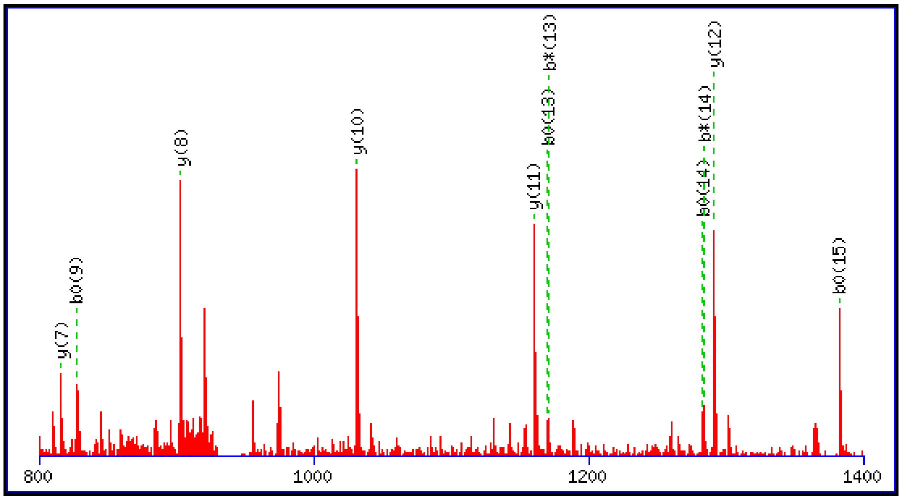


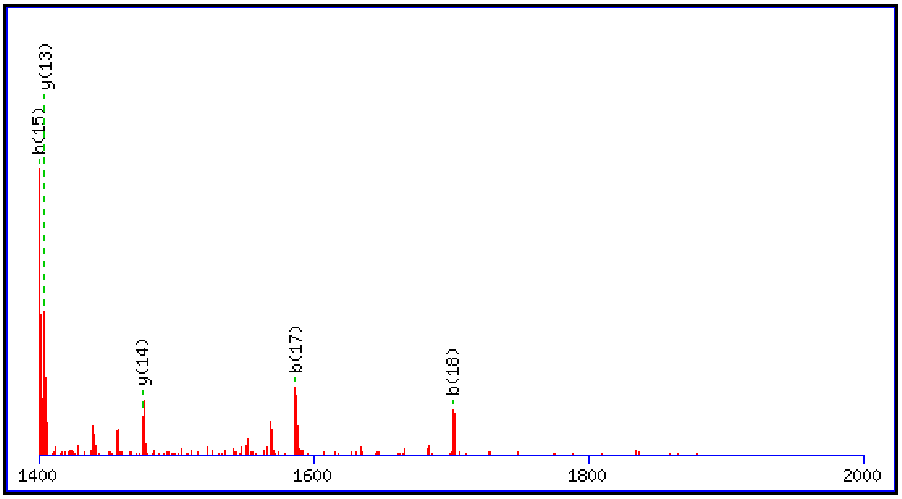


**Monoisotopic mass of neutral peptide Mr(calc):** 1874.8792
**Fixed modifications:** Carbamidomethyl (C)
**Variable modifications:**
**N18** : Deamidated_N (N)
**Ions Score:** 78 **Expect:** 4.1e-006
**Matches (Bold Red):** 23/202 fragment ions using 41 most intense peaks

| # | b | b++ | b* | b*++ | b0 | b0++ | Seq. | y | y++ | y* | y*++ | y0 | y0++ | # |
|---|---|---|---|---|---|---|---|---|---|---|---|---|---|---|
| 1 | 58.0287 | 29.5180 | | | | | G | | | | | | | 19 |
| 2 | 159.0764 | 80.0418 | | | 141.0659 | 71.0366 | T | 1818.8650 | 909.9362 | 1801.8385 | 901.4229 | 1800.8545 | 900.9309 | 18 |
| 3 | 230.1135 | 115.5604 | | | 212.1030 | 106.5551 | A | 1717.8174 | 859.4123 | 1700.7908 | 850.8990 | 1699.8068 | 850.4070 | 17 |
| 4 | 287.1350 | 144.0711 | | | 269.1244 | 135.0659 | G | 1646.7803 | 823.8938 | 1629.7537 | 815.3805 | 1628.7697 | 814.8885 | 16 |
| 5 | 401.1779 | 201.0926 | 384.1514 | 192.5793 | 383.1674 | 192.0873 | N | 1589.7588 | 795.3830 | 1572.7322 | 786.8698 | 1571.7482 | 786.3778 | 15 |

| | | | | | | | | | | | | | | |
|---|---|---|---|---|---|---|---|---|---|---|---|---|---|---|
| 6 | 472.2150 | 236.6112 | **455.1885** | 228.0979 | 454.2045 | 227.6059 | A | **1475.7159** | 738.3616 | 1458.6893 | 729.8483 | 1457.7053 | 729.3563 | 14 |
| 7 | 585.2991 | 293.1532 | **568.2726** | 284.6399 | 567.2885 | 284.1479 | L | **1404.6787** | 702.8430 | 1387.6522 | 694.3297 | 1386.6682 | 693.8377 | 13 |
| 8 | 716.3396 | 358.6734 | 699.3130 | 350.1602 | 698.3290 | 349.6681 | M | **1291.5947** | 646.3010 | 1274.5681 | 637.7877 | 1273.5841 | 637.2957 | 12 |
| 9 | 845.3822 | 423.1947 | 828.3556 | 414.6815 | **827.3716** | 414.1894 | E | **1160.5542** | 580.7807 | 1143.5277 | 572.2675 | 1142.5436 | 571.7755 | 11 |
| 10 | 902.4036 | 451.7055 | 885.3771 | 443.1922 | 884.3931 | 442.7002 | G | **1031.5116** | 516.2594 | 1014.4851 | 507.7462 | 1013.5010 | 507.2542 | 10 |
| 11 | 973.4408 | 487.2240 | 956.4142 | 478.7107 | 955.4302 | 478.2187 | A | 974.4901 | 487.7487 | 957.4636 | 479.2354 | 956.4796 | 478.7434 | 9 |
| 12 | 1060.4728 | 530.7400 | 1043.4462 | 522.2268 | 1042.4622 | 521.7347 | S | **903.4530** | 452.2302 | 886.4265 | 443.7169 | 885.4425 | 443.2249 | 8 |
| 13 | 1188.5314 | 594.7693 | **1171.5048** | 586.2560 | **1170.5208** | 585.7640 | Q | **816.4210** | 408.7141 | 799.3945 | 400.2009 | 798.4104 | 399.7089 | 7 |
| 14 | 1301.6154 | 651.3114 | **1284.5889** | 642.7981 | **1283.6049** | 642.3061 | L | **688.3624** | 344.6848 | 671.3359 | 336.1716 | 670.3519 | 335.6796 | 6 |
| 15 | **1400.6838** | 700.8456 | 1383.6573 | 692.3323 | **1382.6733** | 691.8403 | V | **575.2784** | 288.1428 | 558.2518 | 279.6295 | 557.2678 | 279.1375 | 5 |
| 16 | 1457.7053 | 729.3563 | 1440.6788 | 720.8430 | 1439.6947 | 720.3510 | G | **476.2099** | 238.6086 | 459.1834 | 230.0953 | 458.1994 | 229.6033 | 4 |
| 17 | **1586.7479** | 793.8776 | 1569.7214 | 785.3643 | 1568.7373 | 784.8723 | E | **419.1885** | 210.0979 | 402.1619 | 201.5846 | 401.1779 | 201.0926 | 3 |
| 18 | **1701.7748** | 851.3911 | 1684.7483 | 842.8778 | 1683.7643 | 842.3858 | N | **290.1459** | 145.5766 | 273.1193 | 137.0633 | | | 2 |
| 19 | | | | | | | R | 175.1190 | 88.0631 | 158.0924 | 79.5498 | | | 1 |

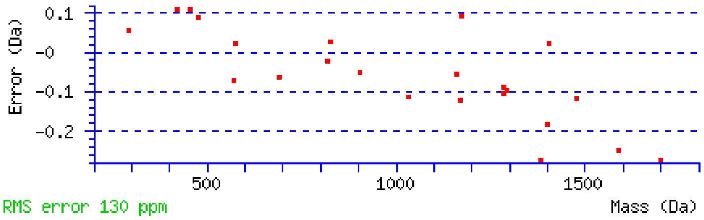


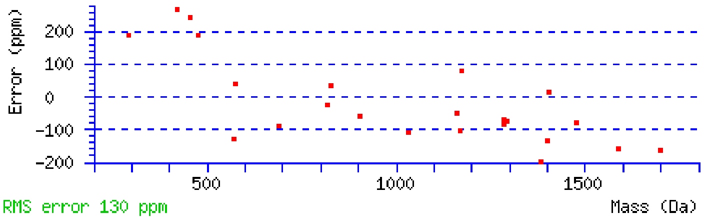


### All matches to this query

| Score | Mr(calc): | Delta | Sequence |
|---|---|---|---|
| 77.7 | 1874.8792 | 0.0054 | GTAGNALMEGASQLVGENR |
| 14.0 | 1873.8952 | 0.9894 | GTAGNALMEGASQLVGENR |
| 3.8 | 1874.8792 | 0.0054 | GTAGNALMEGASQLVGENR |
| 2.1 | 1874.8806 | 0.0041 | MALRPSEGDGSAGRWNR |
| 0.8 | 1873.8645 | 1.0202 | VRTMFSEQQLGELEK |
| 0.6 | 1874.8945 | -0.0098 | VAGAELSVCGNVKDWNR |
| 0.5 | 1874.8999 | -0.0153 | VQAESQKHSLRLEDR |
| 0.0 | 1874.8961 | -0.0115 | ARKVLNDIISTMFNR |
| 0.0 | 1874.8961 | -0.0115 | ARKVLNDIISTMFNR |

---

Spectrum No: 260; Query: 1453; Rank: 1

---

## Peptide View

MS/MS Fragmentation of **KSEANLSALAETHTNIPK**
Found in **IPI00327697**, Tax_Id=10116 Gene_Symbol=Dpep1 Dipeptidase 1 precursor

Match to Query 1453: 1923.995588 from(963.005070,2+)
Title: 091008RatKidney_NH4Format01_23.1733.1733.2.dta
Data file K:\NewmanPaper\Piliang\3SubProteomes\Piliang3SP\mgf5ppm\ERLIC_3SubProteomes5ppm.mgf

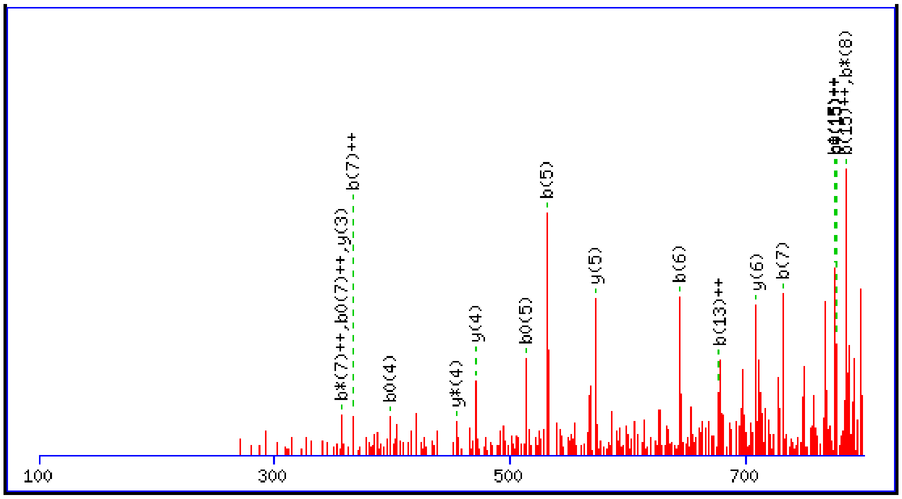


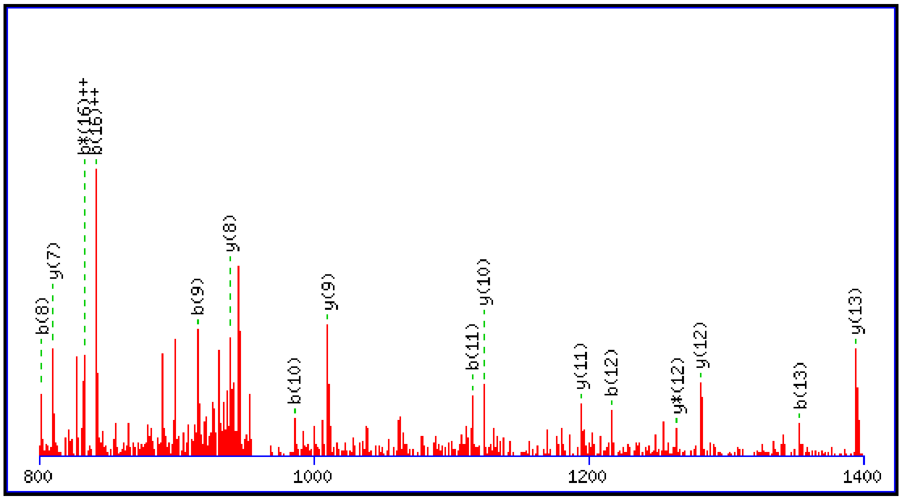


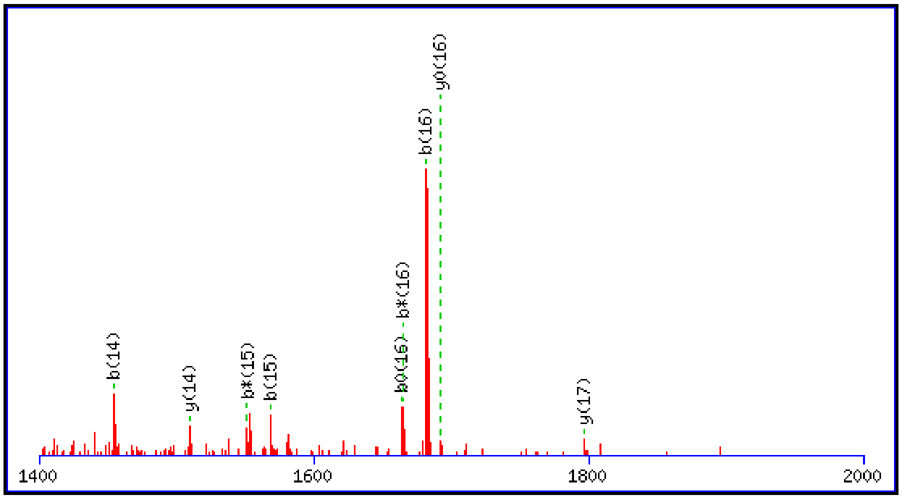


**Monoisotopic mass of neutral peptide Mr(calc):** 1923.9901
**Fixed modifications:** Carbamidomethyl (C)
**Variable modifications:**
**N5** : Deamidated_N (N)
**Ions Score:** 78 **Expect:** 2.4e-006
**Matches (Bold Red):** 43/194 fragment ions using 76 most intense peaks

| # | b | $b^{++}$ | b* | $b^{*++}$ | $b^0$ | $b^{0++}$ | Seq. | y | $y^{++}$ | y* | $y^{*++}$ | $y^0$ | $y^{0++}$ | # |
|---|---|---|---|---|---|---|---|---|---|---|---|---|---|---|
| 1 | 129.1022 | 65.0548 | 112.0757 | 56.5415 | | | K | | | | | | | 18 |
| 2 | 216.1343 | 108.5708 | 199.1077 | 100.0575 | 198.1237 | 99.5655 | S | **1796.9025** | 898.9549 | 1779.8759 | 890.4416 | 1778.8919 | 889.9496 | 17 |
| 3 | 345.1769 | 173.0921 | 328.1503 | 164.5788 | 327.1663 | 164.0868 | E | 1709.8704 | 855.4389 | 1692.8439 | 846.9256 | **1691.8599** | 846.4336 | 16 |
| 4 | 416.2140 | 208.6106 | 399.1874 | 200.0974 | **398.2034** | 199.6053 | A | 1580.8279 | 790.9176 | 1563.8013 | 782.4043 | 1562.8173 | 781.9123 | 15 |
| 5 | **531.2409** | 266.1241 | 514.2144 | 257.6108 | **513.2303** | 257.1188 | N | **1509.7907** | 755.3990 | 1492.7642 | 746.8857 | 1491.7802 | 746.3937 | 14 |

| | | | | | | | | | | | | | | |
|---|---|---|---|---|---|---|---|---|---|---|---|---|---|---|
| **6** | **644.3250** | 322.6661 | 627.2984 | 314.1529 | 626.3144 | 313.6608 | **L** | **1394.7638** | 697.8855 | 1377.7373 | 689.3723 | 1376.7532 | 688.8803 | **13** |
| **7** | **731.3570** | **366.1821** | 714.3305 | **357.6689** | 713.3464 | **357.1769** | **S** | **1281.6797** | 641.3435 | **1264.6532** | 632.8302 | 1263.6692 | 632.3382 | **12** |
| **8** | **802.3941** | 401.7007 | **785.3676** | 393.1874 | 784.3836 | 392.6954 | **A** | **1194.6477** | 597.8275 | 1177.6212 | 589.3142 | 1176.6371 | 588.8222 | **11** |
| **9** | **915.4782** | 458.2427 | 898.4516 | 449.7295 | 897.4676 | 449.2374 | **L** | **1123.6106** | 562.3089 | 1106.5841 | 553.7957 | 1105.6000 | 553.3037 | **10** |
| **10** | **986.5153** | 493.7613 | 969.4887 | 485.2480 | 968.5047 | 484.7560 | **A** | **1010.5265** | 505.7669 | 993.5000 | 497.2536 | 992.5160 | 496.7616 | **9** |
| **11** | **1115.5579** | 558.2826 | 1098.5313 | 549.7693 | 1097.5473 | 549.2773 | **E** | **939.4894** | 470.2483 | 922.4629 | 461.7351 | 921.4789 | 461.2431 | **8** |
| **12** | **1216.6056** | 608.8064 | 1199.5790 | 600.2931 | 1198.5950 | 599.8011 | **T** | **810.4468** | 405.7271 | 793.4203 | 397.2138 | 792.4363 | 396.7218 | **7** |
| **13** | **1353.6645** | **677.3359** | 1336.6379 | 668.8226 | 1335.6539 | 668.3306 | **H** | **709.3991** | 355.2032 | 692.3726 | 346.6899 | 691.3886 | 346.1979 | **6** |
| **14** | **1454.7122** | 727.8597 | 1437.6856 | 719.3464 | 1436.7016 | 718.8544 | **T** | **572.3402** | 286.6738 | 555.3137 | 278.1605 | 554.3297 | 277.6685 | **5** |
| **15** | **1568.7551** | **784.8812** | **1551.7285** | **776.3679** | 1550.7445 | **775.8759** | **N** | **471.2926** | 236.1499 | **454.2660** | 227.6366 | | | **4** |
| **16** | **1681.8391** | **841.4232** | **1664.8126** | **832.9099** | **1663.8286** | 832.4179 | **I** | **357.2496** | 179.1285 | 340.2231 | 170.6152 | | | **3** |
| **17** | 1778.8919 | 889.9496 | 1761.8654 | 881.4363 | 1760.8813 | 880.9443 | **P** | 244.1656 | 122.5864 | 227.1390 | 114.0731 | | | **2** |
| **18** | | | | | | | **K** | 147.1128 | 74.0600 | 130.0863 | 65.5468 | | | **1** |

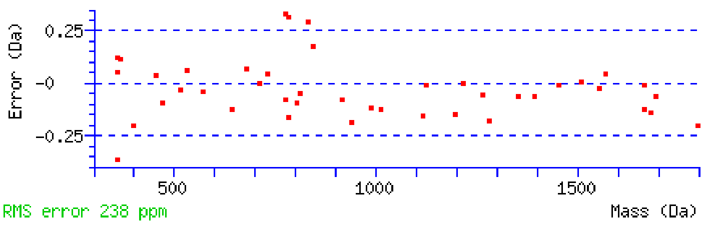


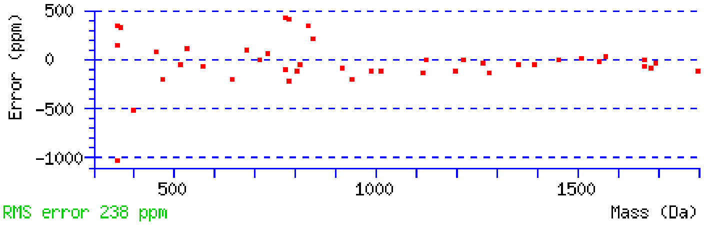


### All matches to this query

| Score | Mr(calc): | Delta | Sequence |
|---|---|---|---|
| 77.6 | 1923.9901 | 0.0055 | KSEANLSALAETHTNIPK |
| 26.4 | 1923.0061 | 0.9895 | KSEANLSALAETHTNIPK |
| 22.6 | 1923.9901 | 0.0055 | KSEANLSALAETHTNIPK |
| 10.2 | 1923.9779 | 0.0177 | TRKLLENSTASIQQQK |
| 10.2 | 1923.9779 | 0.0177 | TRKLLENSTASIQQQK |
| 10.2 | 1923.9779 | 0.0177 | TRKLLENSTASIQQQK |
| 3.3 | 1924.0013 | -0.0058 | LEEDARALINAGSIPAQR |

---

Spectrum No: 261; Query: 1292; Rank: 1

---

## Peptide View

MS/MS Fragmentation of **TSDTGDQTISNGTEANSK**
Found in **IPI00193425**, Tax_Id=10116 Gene_Symbol=Bsg Isoform 2 of Basigin precursor

Match to Query 1292: 1825.789648 from(913.902100,2+)
Title: 100101RatKid_NS_deglyco_23.463.463.2.dta
Data file K:\NewmanPaper\Piliang\3SubProteomes\Piliang3SP\mgf5ppm\ERLIC_3SubProteomes5ppm.mgf

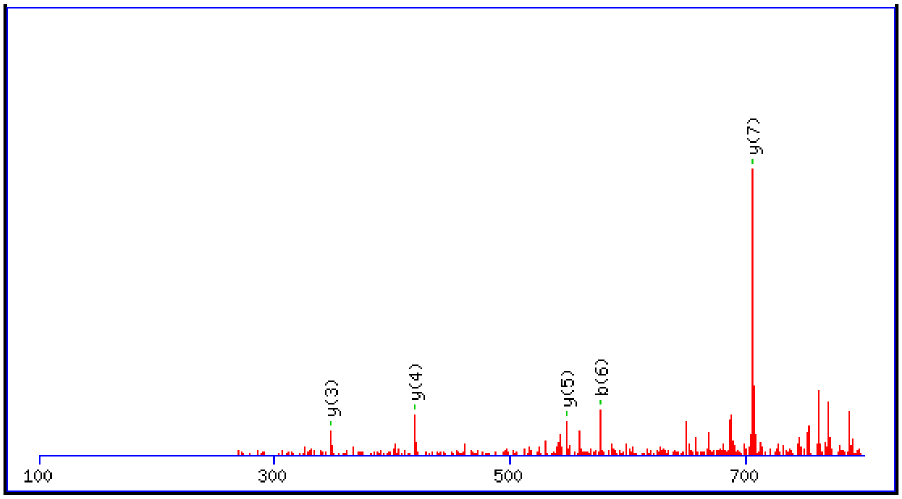


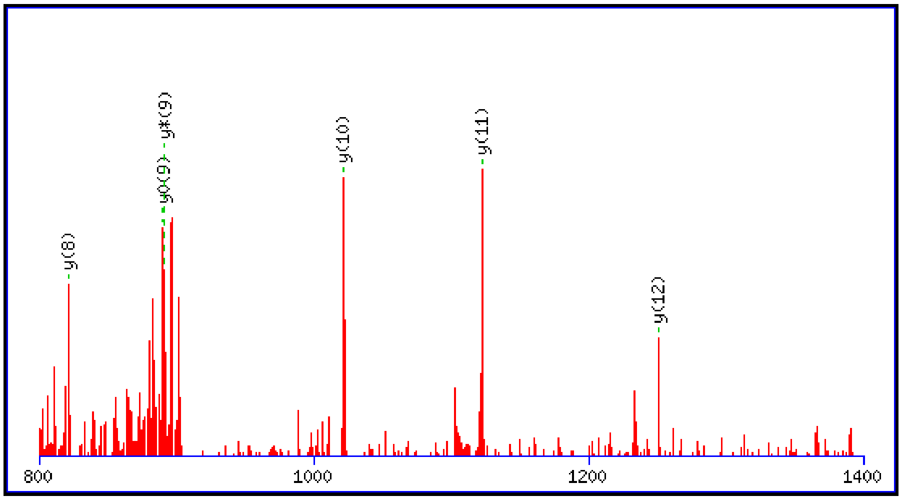


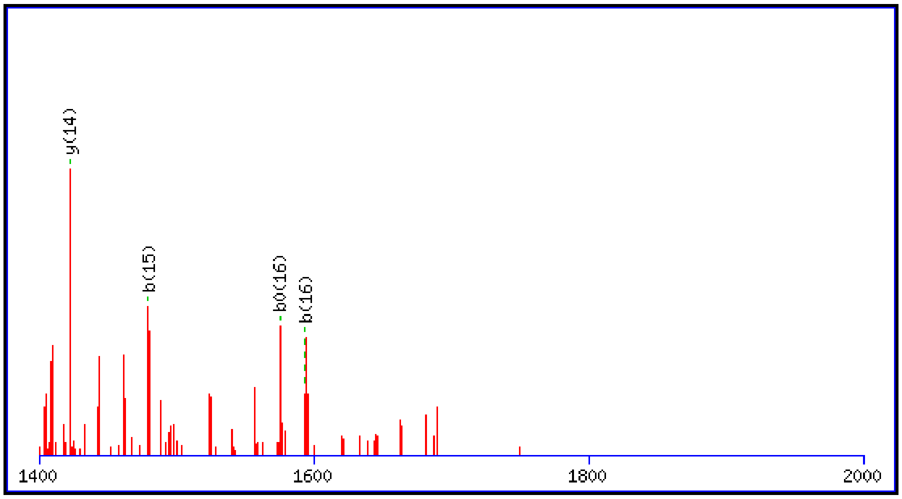


**Monoisotopic mass of neutral peptide Mr(calc):** 1825.7814
**Fixed modifications:** Carbamidomethyl (C)
**Variable modifications:**
**N11** : Deamidated_N (N)
**Ions Score:** 78 **Expect:** 4.3e-006
**Matches (Bold Red):** 15/190 fragment ions using 23 most intense peaks

| # | b | $b^{++}$ | b* | $b^{*++}$ | $b^{0}$ | $b^{0++}$ | Seq. | y | $y^{++}$ | y* | $y^{*++}$ | $y^{0}$ | $y^{0++}$ | # |
|---|---|---|---|---|---|---|---|---|---|---|---|---|---|---|
| 1 | 102.0550 | 51.5311 | | | 84.0444 | 42.5258 | T | | | | | | | 18 |
| 2 | 189.0870 | 95.0471 | | | 171.0764 | 86.0418 | S | 1725.7410 | 863.3741 | 1708.7144 | 854.8608 | 1707.7304 | 854.3688 | 17 |
| 3 | 304.1139 | 152.5606 | | | 286.1034 | 143.5553 | D | 1638.7089 | 819.8581 | 1621.6824 | 811.3448 | 1620.6984 | 810.8528 | 16 |
| 4 | 405.1616 | 203.0844 | | | 387.1510 | 194.0792 | T | 1523.6820 | 762.3446 | 1506.6554 | 753.8314 | 1505.6714 | 753.3393 | 15 |
| 5 | 462.1831 | 231.5952 | | | 444.1725 | 222.5899 | G | **1422.6343** | 711.8208 | 1405.6078 | 703.3075 | 1404.6237 | 702.8155 | 14 |

| | | | | | | | | | | | | | | |
|---|---|---|---|---|---|---|---|---|---|---|---|---|---|---|
| **6** | **577.2100** | 289.1086 | | | 559.1994 | 280.1034 | **D** | 1365.6128 | 683.3101 | 1348.5863 | 674.7968 | 1347.6023 | 674.3048 | **13** |
| **7** | 705.2686 | 353.1379 | 688.2420 | 344.6247 | 687.2580 | 344.1327 | **Q** | **1250.5859** | 625.7966 | 1233.5593 | 617.2833 | 1232.5753 | 616.7913 | **12** |
| **8** | 806.3163 | 403.6618 | 789.2897 | 395.1485 | 788.3057 | 394.6565 | **T** | **1122.5273** | 561.7673 | 1105.5008 | 553.2540 | 1104.5168 | 552.7620 | **11** |
| **9** | 919.4003 | 460.2038 | 902.3738 | 451.6905 | 901.3898 | 451.1985 | **I** | **1021.4796** | 511.2435 | 1004.4531 | 502.7302 | 1003.4691 | 502.2382 | **10** |
| **10** | 1006.4324 | 503.7198 | 989.4058 | 495.2065 | 988.4218 | 494.7145 | **S** | 908.3956 | 454.7014 | **891.3690** | 446.1882 | **890.3850** | 445.6961 | **9** |
| **11** | 1121.4593 | 561.2333 | 1104.4327 | 552.7200 | 1103.4487 | 552.2280 | **N** | **821.3635** | 411.1854 | 804.3370 | 402.6721 | 803.3530 | 402.1801 | **8** |
| **12** | 1178.4808 | 589.7440 | 1161.4542 | 581.2307 | 1160.4702 | 580.7387 | **G** | **706.3366** | 353.6719 | 689.3101 | 345.1587 | 688.3260 | 344.6667 | **7** |
| **13** | 1279.5284 | 640.2679 | 1262.5019 | 631.7546 | 1261.5179 | 631.2626 | **T** | 649.3151 | 325.1612 | 632.2886 | 316.6479 | 631.3046 | 316.1559 | **6** |
| **14** | 1408.5710 | 704.7892 | 1391.5445 | 696.2759 | 1390.5605 | 695.7839 | **E** | **548.2675** | 274.6374 | 531.2409 | 266.1241 | 530.2569 | 265.6321 | **5** |
| **15** | **1479.6081** | 740.3077 | 1462.5816 | 731.7944 | 1461.5976 | 731.3024 | **A** | **419.2249** | 210.1161 | 402.1983 | 201.6028 | 401.2143 | 201.1108 | **4** |
| **16** | **1593.6511** | 797.3292 | 1576.6245 | 788.8159 | **1575.6405** | 788.3239 | **N** | **348.1878** | 174.5975 | 331.1612 | 166.0842 | 330.1772 | 165.5922 | **3** |
| **17** | 1680.6831 | 840.8452 | 1663.6566 | 832.3319 | 1662.6725 | 831.8399 | **S** | 234.1448 | 117.5761 | 217.1183 | 109.0628 | 216.1343 | 108.5708 | **2** |
| **18** | | | | | | | **K** | 147.1128 | 74.0600 | 130.0863 | 65.5468 | | | **1** |

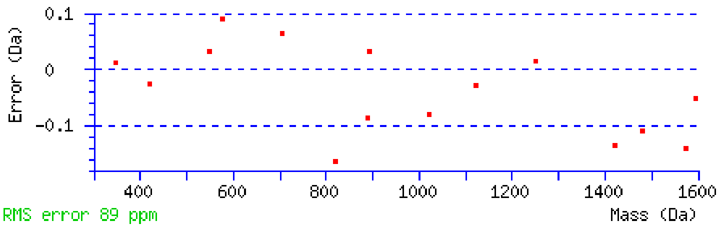


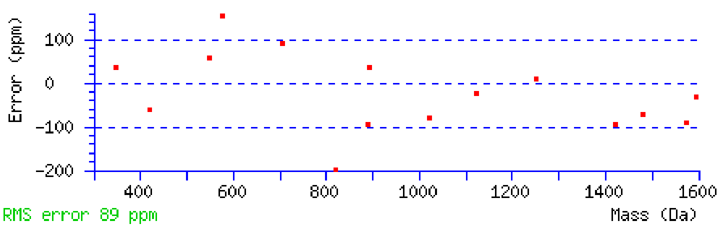


### All matches to this query

| Score | Mr(calc): | Delta | Sequence |
|---|---|---|---|
| 77.6 | 1825.7814 | 0.0083 | TSDTGDQTISNGTEANSK |
| 46.3 | 1825.7814 | 0.0083 | TSDTGDQTISNGTEANSK |
| 23.8 | 1824.7973 | 0.9923 | TSDTGDQTISNGTEANSK |
| 3.5 | 1825.8039 | -0.0143 | MRLEDFNNNINNSLK |

---

Spectrum No: 262; Query: 2148; Rank: 1

---

## Peptide View

MS/MS Fragmentation of **DNLTLWTSDTQGDEAEAGEGGEN**
Found in **IPI00324893**, Tax_Id=10116 Gene_Symbol=Ywhaz 14-3-3 protein zeta/delta

Match to Query 2148: 2408.982568 from(1205.498560,2+)
Title: 091008RatKidney_NoSalt_30.2811.2811.2.dta
Data file K:\NewmanPaper\Piliang\3SubProteomes\Piliang3SP\mgf5ppm\ERLIC_3SubProteomes5ppm.mgf

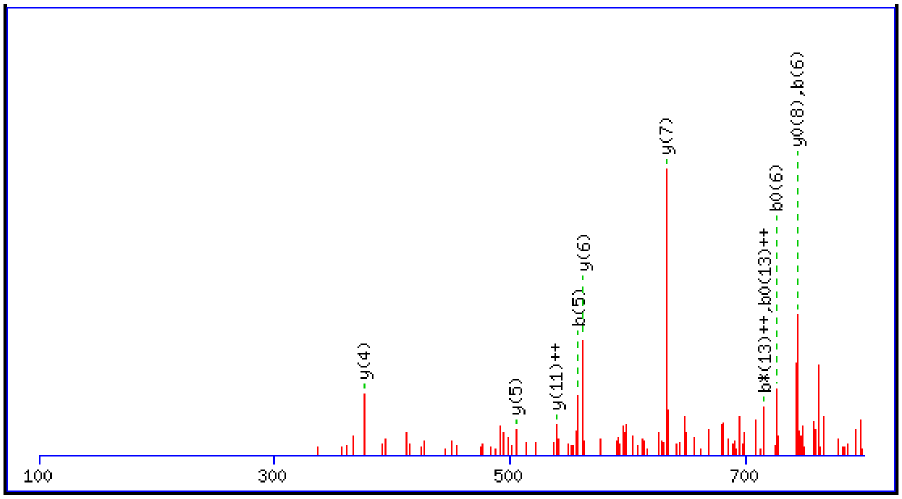


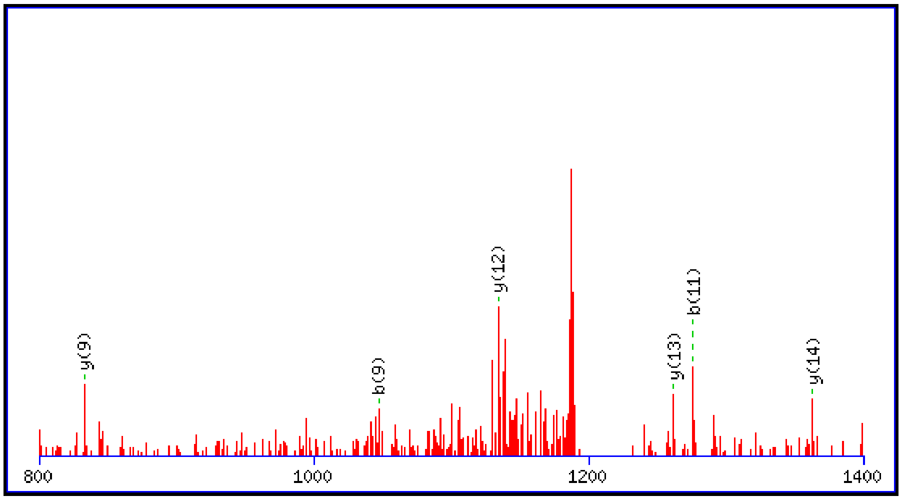


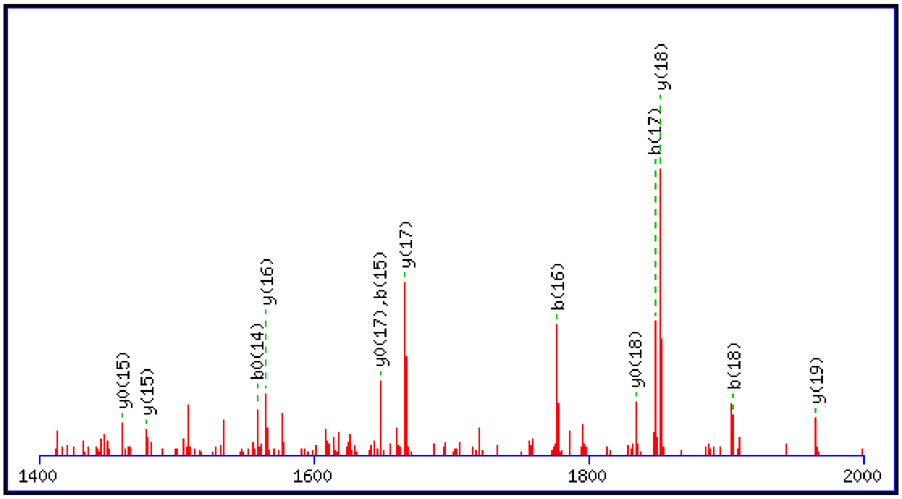


**Monoisotopic mass of neutral peptide Mr(calc):** 2408.9728
**Fixed modifications:** Carbamidomethyl (C)
**Variable modifications:**
**N2** : Deamidated_N (N)
**Ions Score:** 78 **Expect:** 4.6e-006
**Matches (Bold Red):** 30/260 fragment ions using 49 most intense peaks

| # | b | b++ | b* | b*++ | b0 | b0++ | Seq. | y | y++ | y* | y*++ | y0 | y0++ | # |
|---|---|---|---|---|---|---|---|---|---|---|---|---|---|---|
| 1 | 116.0342 | 58.5207 | | | 98.0237 | 49.5155 | D | | | | | | | 23 |
| 2 | 231.0612 | 116.0342 | 214.0346 | 107.5209 | 213.0506 | 107.0289 | N | 2294.9531 | 1147.9802 | 2277.9266 | 1139.4669 | 2276.9426 | 1138.9749 | 22 |
| 3 | 344.1452 | 172.5762 | 327.1187 | 164.0630 | 326.1347 | 163.5710 | L | 2179.9262 | 1090.4667 | 2162.8996 | 1081.9535 | 2161.9156 | 1081.4615 | 21 |
| 4 | 445.1929 | 223.1001 | 428.1663 | 214.5868 | 427.1823 | 214.0948 | T | 2066.8421 | 1033.9247 | 2049.8156 | 1025.4114 | 2048.8316 | 1024.9194 | 20 |
| 5 | **558.2770** | 279.6421 | 541.2504 | 271.1288 | 540.2664 | 270.6368 | L | **1965.7945** | 983.4009 | 1948.7679 | 974.8876 | 1947.7839 | 974.3956 | 19 |

| | | | | | | | | | | | | | | |
|---|---|---|---|---|---|---|---|---|---|---|---|---|---|---|
| 6 | **744.3563** | 372.6818 | 727.3297 | 364.1685 | **726.3457** | 363.6765 | W | **1852.7104** | 926.8588 | 1835.6838 | 918.3456 | **1834.6998** | 917.8535 | 18 |
| 7 | 845.4040 | 423.2056 | 828.3774 | 414.6923 | 827.3934 | 414.2003 | T | **1666.6311** | 833.8192 | 1649.6045 | 825.3059 | **1648.6205** | 824.8139 | 17 |
| 8 | 932.4360 | 466.7216 | 915.4094 | 458.2084 | 914.4254 | 457.7163 | S | **1565.5834** | 783.2953 | 1548.5568 | 774.7821 | 1547.5728 | 774.2901 | 16 |
| 9 | **1047.4629** | 524.2351 | 1030.4364 | 515.7218 | 1029.4524 | 515.2298 | D | **1478.5514** | 739.7793 | 1461.5248 | 731.2660 | **1460.5408** | 730.7740 | 15 |
| 10 | 1148.5106 | 574.7589 | 1131.4841 | 566.2457 | 1130.5000 | 565.7537 | T | **1363.5244** | 682.2658 | 1346.4979 | 673.7526 | 1345.5139 | 673.2606 | 14 |
| 11 | **1276.5692** | 638.7882 | 1259.5426 | 630.2750 | 1258.5586 | 629.7829 | Q | **1262.4767** | 631.7420 | 1245.4502 | 623.2287 | 1244.4662 | 622.7367 | 13 |
| 12 | 1333.5906 | 667.2990 | 1316.5641 | 658.7857 | 1315.5801 | 658.2937 | G | **1134.4182** | 567.7127 | 1117.3916 | 559.1994 | 1116.4076 | 558.7074 | 12 |
| 13 | 1448.6176 | 724.8124 | 1431.5910 | **716.2992** | 1430.6070 | **715.8071** | D | 1077.3967 | **539.2020** | 1060.3702 | 530.6887 | 1059.3861 | 530.1967 | 11 |
| 14 | 1577.6602 | 789.3337 | 1560.6336 | 780.8205 | **1559.6496** | 780.3284 | E | 962.3698 | 481.6885 | 945.3432 | 473.1752 | 944.3592 | 472.6832 | 10 |
| 15 | **1648.6973** | 824.8523 | 1631.6707 | 816.3390 | 1630.6867 | 815.8470 | A | **833.3272** | 417.1672 | 816.3006 | 408.6539 | 815.3166 | 408.1619 | 9 |
| 16 | **1777.7399** | 889.3736 | 1760.7133 | 880.8603 | 1759.7293 | 880.3683 | E | 762.2901 | 381.6487 | 745.2635 | 373.1354 | **744.2795** | 372.6434 | 8 |
| 17 | **1848.7770** | 924.8921 | 1831.7505 | 916.3789 | 1830.7664 | 915.8869 | A | **633.2475** | 317.1274 | 616.2209 | 308.6141 | 615.2369 | 308.1221 | 7 |
| 18 | **1905.7985** | 953.4029 | 1888.7719 | 944.8896 | 1887.7879 | 944.3976 | G | **562.2103** | 281.6088 | 545.1838 | 273.0955 | 544.1998 | 272.6035 | 6 |
| 19 | 2034.8411 | 1017.9242 | 2017.8145 | 1009.4109 | 2016.8305 | 1008.9189 | E | **505.1889** | 253.0981 | 488.1623 | 244.5848 | 487.1783 | 244.0928 | 5 |
| 20 | 2091.8625 | 1046.4349 | 2074.8360 | 1037.9216 | 2073.8520 | 1037.4296 | G | **376.1463** | 188.5768 | 359.1197 | 180.0635 | 358.1357 | 179.5715 | 4 |
| 21 | 2148.8840 | 1074.9456 | 2131.8574 | 1066.4324 | 2130.8734 | 1065.9403 | G | 319.1248 | 160.0661 | 302.0983 | 151.5528 | 301.1143 | 151.0608 | 3 |
| 22 | 2277.9266 | 1139.4669 | 2260.9000 | 1130.9537 | 2259.9160 | 1130.4616 | E | 262.1034 | 131.5553 | 245.0768 | 123.0420 | 244.0928 | 122.5500 | 2 |
| 23 | | | | | | | N | 133.0608 | 67.0340 | 116.0342 | 58.5207 | | | 1 |

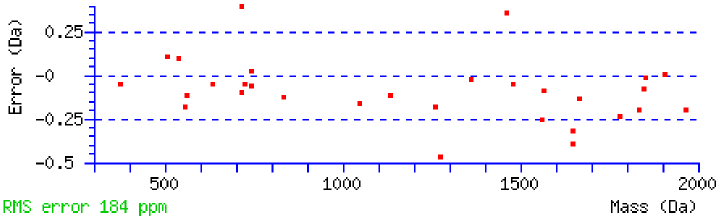


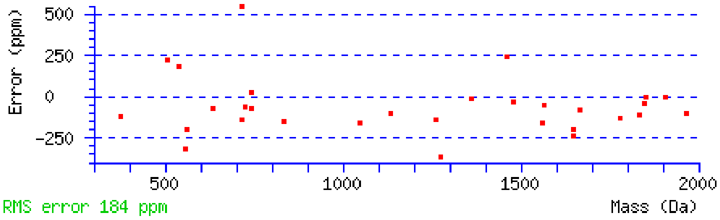


### All matches to this query

| Score | Mr(calc): | Delta | Sequence |
|---|---|---|---|
| 77.5 | 2408.9728 | 0.0098 | DNLTLWTSDTQGDEAEAGEGGEN |
| 68.2 | 2407.9888 | 0.9938 | DNLTLWTSDTQGDEAEAGEGGEN |
| 8.8 | 2408.9728 | 0.0098 | DNLTLWTSDTQGDEAEAGEGGEN |
| 0.6 | 2408.9709 | 0.0117 | VMSSIGNELANSVWEEGSQGR |

---

Spectrum No: 263; Query: 2546; Rank: 1

---

## Peptide View

MS/MS Fragmentation of **CISIDKVCDGVPDCPEGDDENNVTSGR**
Found in **IPI00205325**, Tax_Id=10116 Gene_Symbol=Lrp2 Low-density lipoprotein receptor-related protein 2 precursor

Match to Query 2546: 3008.257362 from(1003.759730,3+)
Title: 100101RatKid_NS_deglyco_23.2435.2435.3.dta
Data file K:\NewmanPaper\Piliang\3SubProteomes\Piliang3SP\mgf5ppm\ERLIC_3SubProteomes5ppm.mgf

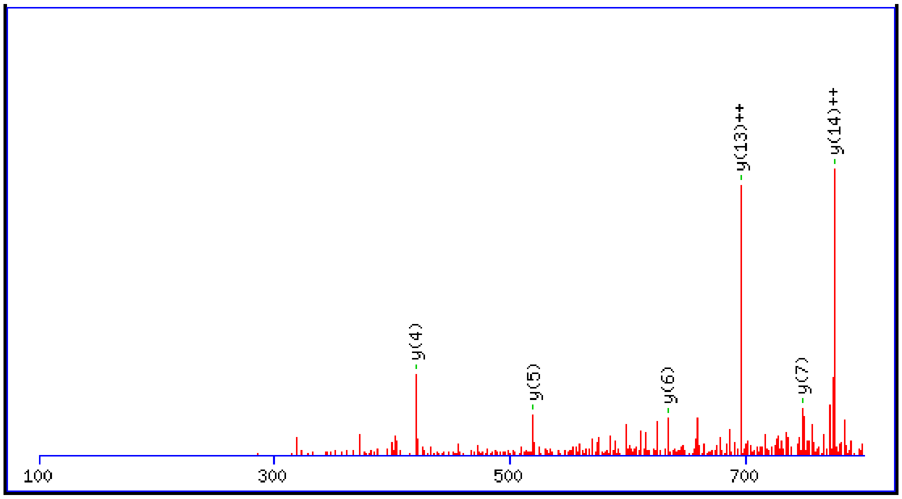


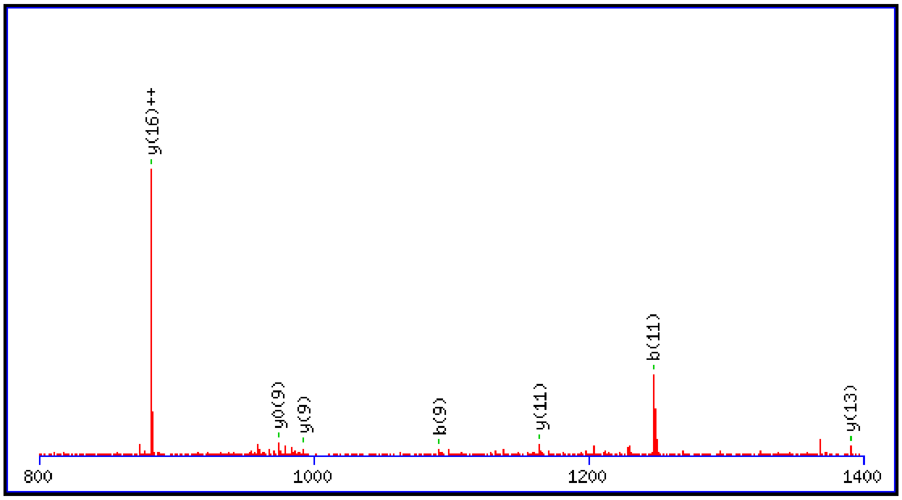


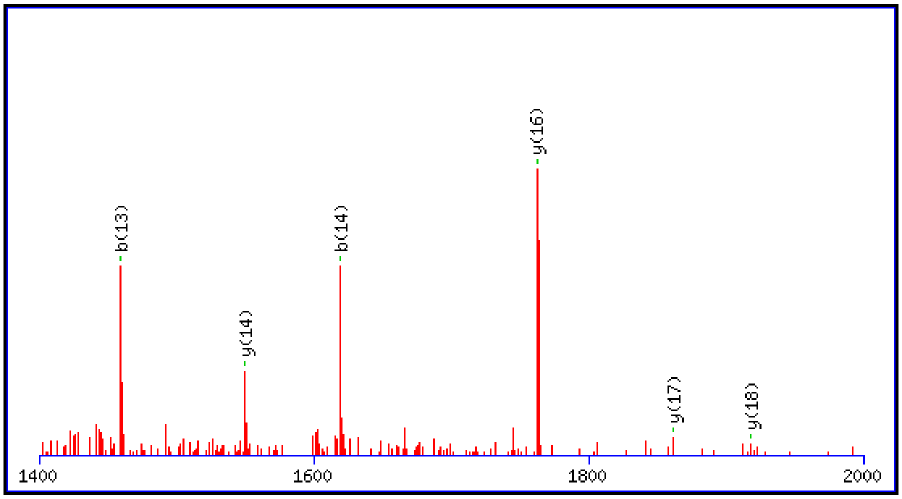


**Monoisotopic mass of neutral peptide Mr(calc):** 3008.2434
**Fixed modifications:** Carbamidomethyl (C)
**Variable modifications:**
**N22** : Deamidated_N (N)
**Ions Score:** 77 **Expect:** 7e-006
**Matches (Bold Red):** 19/294 fragment ions using 34 most intense peaks

| # | b | b++ | b* | b*++ | b0 | b0++ | Seq. | y | y++ | y* | y*++ | y0 | y0++ | # |
|---|---|---|---|---|---|---|---|---|---|---|---|---|---|---|
| 1 | 161.0379 | 81.0226 | | | | | C | | | | | | | 27 |
| 2 | 274.1220 | 137.5646 | | | | | I | 2849.2200 | 1425.1136 | 2832.1935 | 1416.6004 | 2831.2094 | 1416.1084 | 26 |
| 3 | 361.1540 | 181.0806 | | | 343.1435 | 172.0754 | S | 2736.1359 | 1368.5716 | 2719.1094 | 1360.0583 | 2718.1254 | 1359.5663 | 25 |
| 4 | 474.2381 | 237.6227 | | | 456.2275 | 228.6174 | I | 2649.1039 | 1325.0556 | 2632.0774 | 1316.5423 | 2631.0933 | 1316.0503 | 24 |
| 5 | 589.2650 | 295.1362 | | | 571.2545 | 286.1309 | D | 2536.0198 | 1268.5136 | 2518.9933 | 1260.0003 | 2518.0093 | 1259.5083 | 23 |

| | | | | | | | | | | | | | | |
|---|---|---|---|---|---|---|---|---|---|---|---|---|---|---|
| 6 | 717.3600 | 359.1836 | 700.3334 | 350.6704 | 699.3494 | 350.1783 | K | 2420.9929 | 1211.0001 | 2403.9664 | 1202.4868 | 2402.9823 | 1201.9948 | 22 |
| 7 | 816.4284 | 408.7178 | 799.4019 | 400.2046 | 798.4178 | 399.7126 | V | 2292.8979 | 1146.9526 | 2275.8714 | 1138.4393 | 2274.8874 | 1137.9473 | 21 |
| 8 | 976.4591 | 488.7332 | 959.4325 | 480.2199 | 958.4485 | 479.7279 | C | 2193.8295 | 1097.4184 | 2176.8030 | 1088.9051 | 2175.8190 | 1088.4131 | 20 |
| 9 | **1091.4860** | 546.2466 | 1074.4594 | 537.7334 | 1073.4754 | 537.2414 | D | 2033.7989 | 1017.4031 | 2016.7723 | 1008.8898 | 2015.7883 | 1008.3978 | 19 |
| 10 | 1148.5075 | 574.7574 | 1131.4809 | 566.2441 | 1130.4969 | 565.7521 | G | **1918.7719** | 959.8896 | 1901.7454 | 951.3763 | 1900.7614 | 950.8843 | 18 |
| 11 | **1247.5759** | 624.2916 | 1230.5493 | 615.7783 | 1229.5653 | 615.2863 | V | **1861.7505** | 931.3789 | 1844.7239 | 922.8656 | 1843.7399 | 922.3736 | 17 |
| 12 | 1344.6286 | 672.8180 | 1327.6021 | 664.3047 | 1326.6181 | 663.8127 | P | **1762.6821** | **881.8447** | 1745.6555 | 873.3314 | 1744.6715 | 872.8394 | 16 |
| 13 | **1459.6556** | 730.3314 | 1442.6290 | 721.8182 | 1441.6450 | 721.3261 | D | 1665.6293 | 833.3183 | 1648.6027 | 824.8050 | 1647.6187 | 824.3130 | 15 |
| 14 | **1619.6862** | 810.3468 | 1602.6597 | 801.8335 | 1601.6757 | 801.3415 | C | **1550.6023** | **775.8048** | 1533.5758 | 767.2915 | 1532.5918 | 766.7995 | 14 |
| 15 | 1716.7390 | 858.8731 | 1699.7124 | 850.3599 | 1698.7284 | 849.8679 | P | **1390.5717** | **695.7895** | 1373.5452 | 687.2762 | 1372.5611 | 686.7842 | 13 |
| 16 | 1845.7816 | 923.3944 | 1828.7550 | 914.8812 | 1827.7710 | 914.3891 | E | 1293.5189 | 647.2631 | 1276.4924 | 638.7498 | 1275.5084 | 638.2578 | 12 |
| 17 | 1902.8030 | 951.9052 | 1885.7765 | 943.3919 | 1884.7925 | 942.8999 | G | **1164.4763** | 582.7418 | 1147.4498 | 574.2285 | 1146.4658 | 573.7365 | 11 |
| 18 | 2017.8300 | 1009.4186 | 2000.8034 | 1000.9054 | 1999.8194 | 1000.4134 | D | 1107.4549 | 554.2311 | 1090.4283 | 545.7178 | 1089.4443 | 545.2258 | 10 |
| 19 | 2132.8569 | 1066.9321 | 2115.8304 | 1058.4188 | 2114.8464 | 1057.9268 | D | **992.4279** | 496.7176 | 975.4014 | 488.2043 | **974.4174** | 487.7123 | 9 |
| 20 | 2261.8995 | 1131.4534 | 2244.8730 | 1122.9401 | 2243.8890 | 1122.4481 | E | 877.4010 | 439.2041 | 860.3744 | 430.6909 | 859.3904 | 430.1989 | 8 |
| 21 | 2375.9425 | 1188.4749 | 2358.9159 | 1179.9616 | 2357.9319 | 1179.4696 | N | **748.3584** | 374.6828 | 731.3319 | 366.1696 | 730.3478 | 365.6776 | 7 |
| 22 | 2490.9694 | 1245.9883 | 2473.9428 | 1237.4751 | 2472.9588 | 1236.9831 | N | **634.3155** | 317.6614 | 617.2889 | 309.1481 | 616.3049 | 308.6561 | 6 |
| 23 | 2590.0378 | 1295.5225 | 2573.0113 | 1287.0093 | 2572.0272 | 1286.5173 | V | **519.2885** | 260.1479 | 502.2620 | 251.6346 | 501.2780 | 251.1426 | 5 |
| 24 | 2691.0855 | 1346.0464 | 2674.0589 | 1337.5331 | 2673.0749 | 1337.0411 | T | **420.2201** | 210.6137 | 403.1936 | 202.1004 | 402.2096 | 201.6084 | 4 |
| 25 | 2778.1175 | 1389.5624 | 2761.0910 | 1381.0491 | 2760.1069 | 1380.5571 | S | 319.1724 | 160.0899 | 302.1459 | 151.5766 | 301.1619 | 151.0846 | 3 |
| 26 | 2835.1390 | 1418.0731 | 2818.1124 | 1409.5599 | 2817.1284 | 1409.0678 | G | 232.1404 | 116.5738 | 215.1139 | 108.0606 | | | 2 |
| 27 | | | | | | | R | 175.1190 | 88.0631 | 158.0924 | 79.5498 | | | 1 |

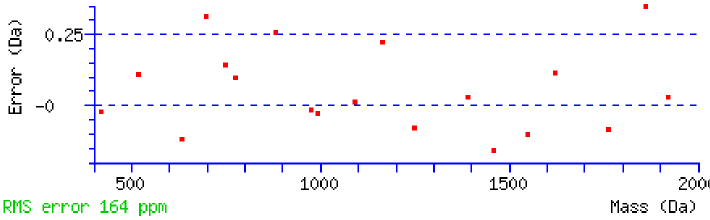


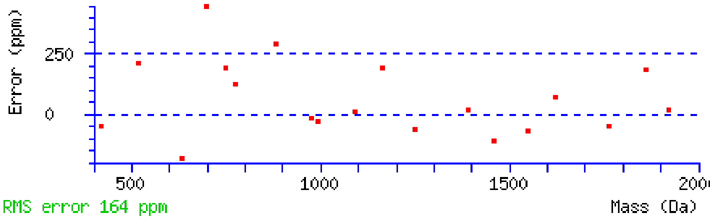


**All matches to this query**

| Score | Mr(calc): | Delta | Sequence |
|---|---|---|---|
| 77.4 | 3008.2434 | 0.0140 | CISIDKVCDGVPDCPEGDDENNVTSGR |
| 63.3 | 3008.2434 | 0.0140 | CISIDKVCDGVPDCPEGDDENNVTSGR |
| 9.8 | 3007.2759 | 0.9815 | VHTGEKPYHCEACGKSFSQSANLIK |
| 9.8 | 3007.2759 | 0.9815 | VHTGEKPYHCEACGKSFSQSANLIK |
| 8.3 | 3007.2759 | 0.9815 | VHTGEKPYHCEACGKSFSQSANLIK |
| 7.5 | 3008.2602 | -0.0029 | EEMELSVLMSQRWDSDTEEQCAKK |
| 5.2 | 3008.2316 | 0.0257 | EQINALTSFLDASLVYSPEPSMANR |
| 5.2 | 3008.2316 | 0.0257 | EQINALTSFLDASLVYSPEPSMANR |
| 5.2 | 3008.2316 | 0.0257 | EQINALTSFLDASLVYSPEPSMANR |
| 5.0 | 3007.2828 | 0.9746 | KSSDTVTGTPAPVQDENNTMAFMQTPK |

---

Spectrum No: 264; Query: 679; Rank: 1

---

## Peptide View

MS/MS Fragmentation of **NRTDVEYELDEK**
Found in **IPI00327143**, Tax_Id=10116 Gene_Symbol=Alpl Alkaline phosphatase, tissue-nonspecific isozyme precursor

Match to Query 679: 1510.680008 from(756.347280,2+)
Title: 091008RatKidney_NH4Format01_27.1105.1105.2.dta
Data file K:\NewmanPaper\Piliang\3SubProteomes\Piliang3SP\mgf5ppm\ERLIC_3SubProteomes5ppm.mgf

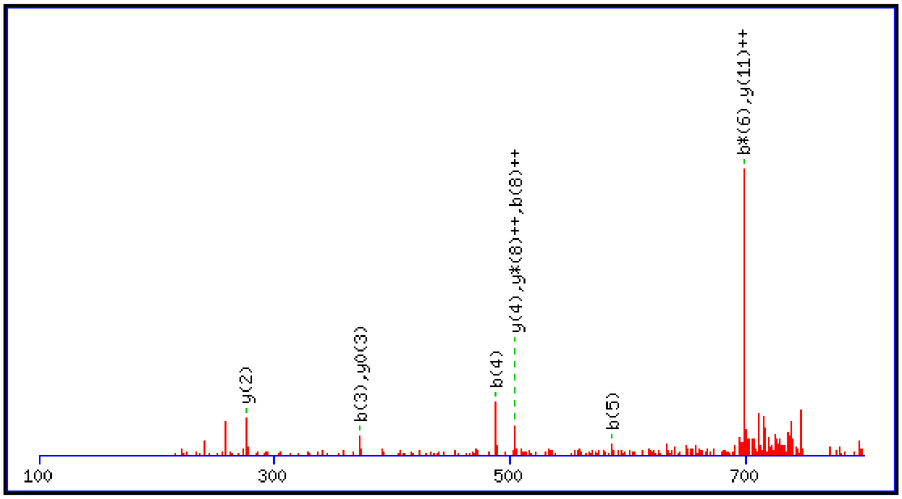


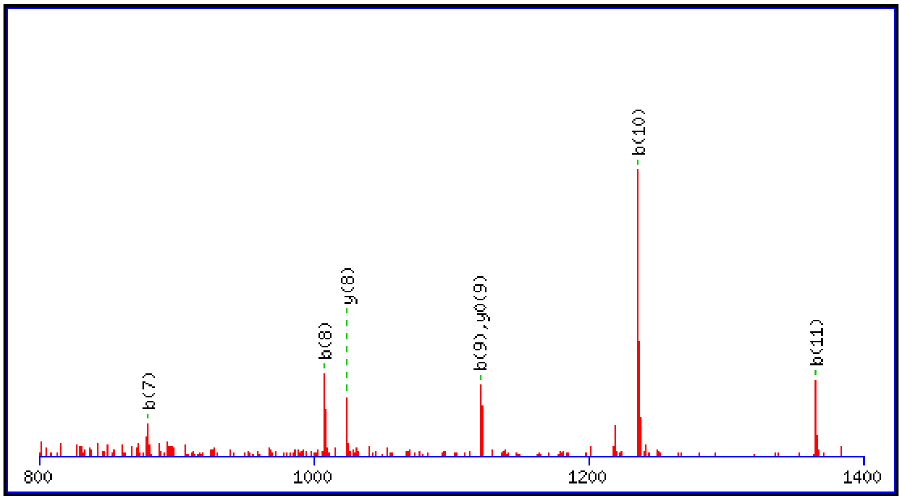


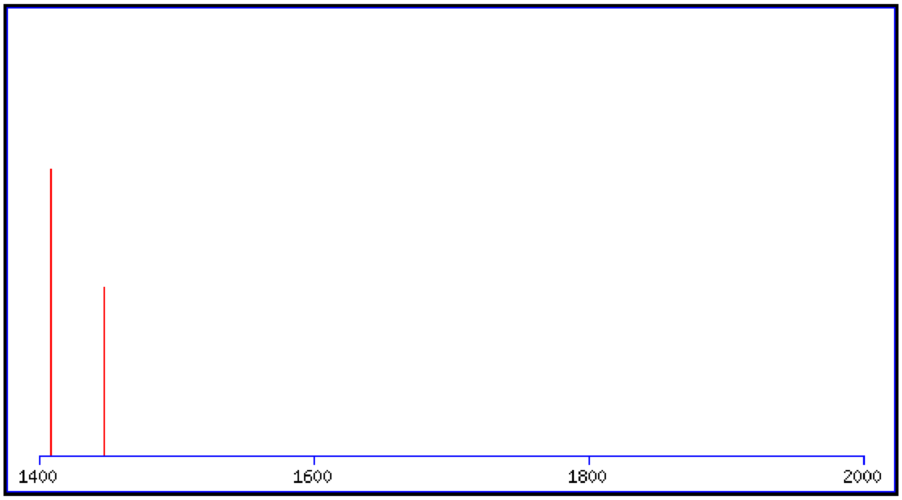


**Monoisotopic mass of neutral peptide Mr(calc):** 1510.6787
**Fixed modifications:** Carbamidomethyl (C)
**Variable modifications:**
**N1** : Deamidated_N (N)
**Ions Score:** 77 **Expect:** 2.7e-006
**Matches (Bold Red):** 17/126 fragment ions using 24 most intense peaks

| # | b | b++ | b* | b*++ | b0 | b0++ | Seq. | y | y++ | y* | y*++ | y0 | y0++ | # |
|---|---|---|---|---|---|---|---|---|---|---|---|---|---|---|
| 1 | 116.0342 | 58.5207 | 99.0077 | 50.0075 | | | N | | | | | | | 12 |
| 2 | 272.1353 | 136.5713 | 255.1088 | 128.0580 | | | R | 1396.6591 | **698.8332** | 1379.6325 | 690.3199 | 1378.6485 | 689.8279 | 11 |

| | | | | | | | | | | | | | | |
|---|---|---|---|---|---|---|---|---|---|---|---|---|---|---|
| **3** | **373.1830** | 187.0951 | 356.1565 | 178.5819 | 355.1724 | 178.0899 | **T** | 1240.5580 | 620.7826 | 1223.5314 | 612.2693 | 1222.5474 | 611.7773 | **10** |
| **4** | **488.2099** | 244.6086 | 471.1834 | 236.0953 | 470.1994 | 235.6033 | **D** | 1139.5103 | 570.2588 | 1122.4837 | 561.7455 | **1121.4997** | 561.2535 | **9** |
| **5** | **587.2784** | 294.1428 | 570.2518 | 285.6295 | 569.2678 | 285.1375 | **V** | **1024.4833** | 512.7453 | 1007.4568 | **504.2320** | 1006.4728 | 503.7400 | **8** |
| **6** | 716.3210 | 358.6641 | **699.2944** | 350.1508 | 698.3104 | 349.6588 | **E** | 925.4149 | 463.2111 | 908.3884 | 454.6978 | 907.4044 | 454.2058 | **7** |
| **7** | **879.3843** | 440.1958 | 862.3577 | 431.6825 | 861.3737 | 431.1905 | **Y** | 796.3723 | 398.6898 | 779.3458 | 390.1765 | 778.3618 | 389.6845 | **6** |
| **8** | **1008.4269** | **504.7171** | 991.4003 | 496.2038 | 990.4163 | 495.7118 | **E** | 633.3090 | 317.1581 | 616.2824 | 308.6449 | 615.2984 | 308.1529 | **5** |
| **9** | **1121.5109** | 561.2591 | 1104.4844 | 552.7458 | 1103.5004 | 552.2538 | **L** | **504.2664** | 252.6368 | 487.2399 | 244.1236 | 486.2558 | 243.6316 | **4** |
| **10** | **1236.5379** | 618.7726 | 1219.5113 | 610.2593 | 1218.5273 | 609.7673 | **D** | 391.1823 | 196.0948 | 374.1558 | 187.5815 | **373.1718** | 187.0895 | **3** |
| **11** | **1365.5805** | 683.2939 | 1348.5539 | 674.7806 | 1347.5699 | 674.2886 | **E** | **276.1554** | 138.5813 | 259.1288 | 130.0681 | 258.1448 | 129.5761 | **2** |
| **12** | | | | | | | **K** | 147.1128 | 74.0600 | 130.0863 | 65.5468 | | | **1** |

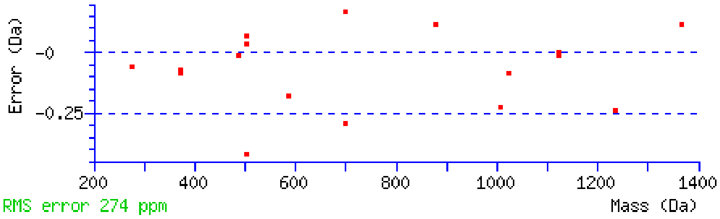


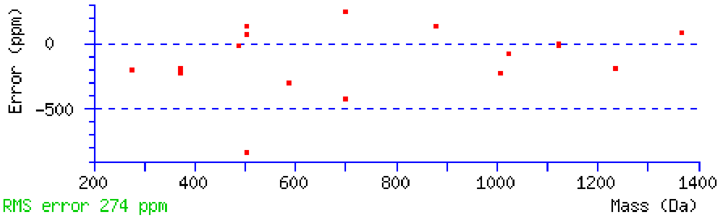


### All matches to this query

| Score | Mr(calc): | Delta | Sequence |
|---|---|---|---|
| 77.4 | 1510.6787 | 0.0013 | NRTDVEYELDEK |
| 17.4 | 1510.6680 | 0.0121 | IYNYNFLMPTR |
| 17.2 | 1510.6778 | 0.0022 | DKMYNLYIVEK |
| 16.8 | 1510.6778 | 0.0022 | DKMYNLYIVEK |
| 16.5 | 1510.6778 | 0.0022 | DYEMKFTKIEK |
| 14.9 | 1510.6777 | 0.0023 | KTGGSGERNATPEK |
| 14.7 | 1509.6657 | 1.0143 | GSEDEDKFDPLMK |
| 12.9 | 1510.6745 | 0.0055 | FFSVFKGNINEK |
| 12.9 | 1510.6916 | -0.0116 | TIETQAVNALDEK |
| 12.9 | 1510.6916 | -0.0116 | TIETQAVNALDEK |

---

Spectrum No: 265; Query: 712; Rank: 1

---

## Peptide View

MS/MS Fragmentation of **CCGASNYTDWER**
Found in **IPI00231686**, Tax_Id=10116 Gene_Symbol=Cd63 CD63 antigen

Match to Query 712: 1518.552548 from(760.283550,2+)
Title: 100101RatKid_NS_deglyco_26.1908.1908.2.dta
Data file K:\NewmanPaper\Piliang\3SubProteomes\Piliang3SP\mgf5ppm\ERLIC_3SubProteomes5ppm.mgf

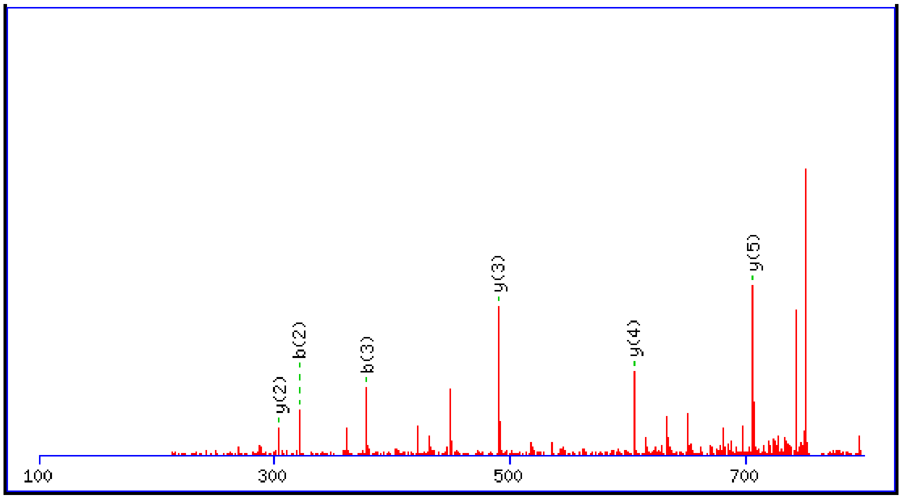


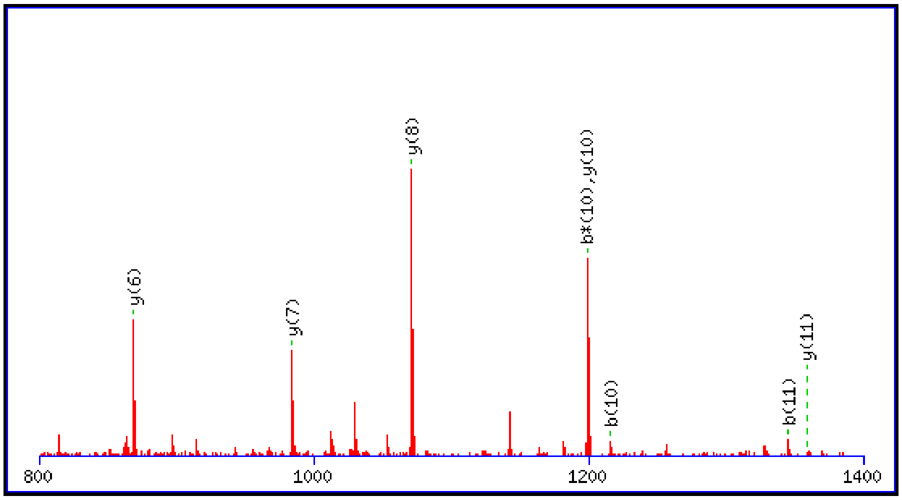


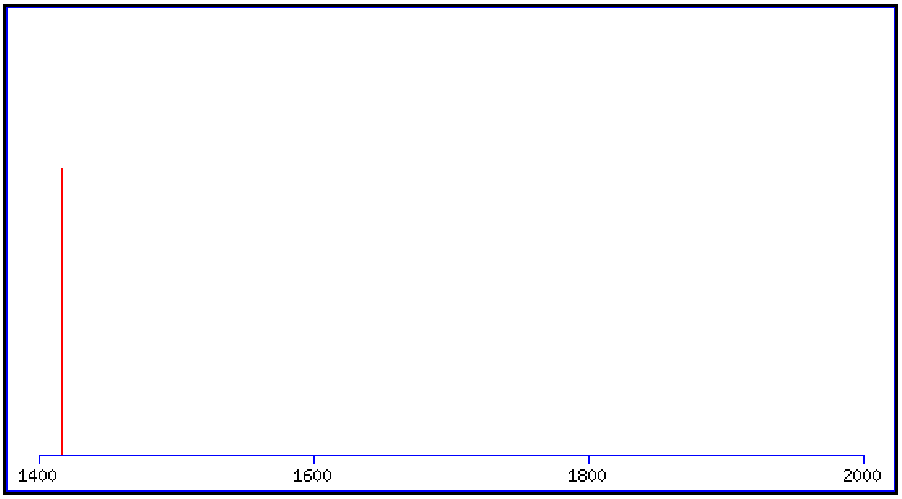


**Monoisotopic mass of neutral peptide Mr(calc):** 1518.5504
**Fixed modifications:** Carbamidomethyl (C)
**Variable modifications:**
**N6** : Deamidated_N (N)
**Ions Score:** 77 **Expect:** 1.3e-006
**Matches (Bold Red):** 14/112 fragment ions using 28 most intense peaks

| # | b | $b^{++}$ | b* | $b^{*++}$ | $b^{0}$ | $b^{0++}$ | Seq. | y | $y^{++}$ | y* | $y^{*++}$ | $y^{0}$ | $y^{0++}$ | # |
|---|---|---|---|---|---|---|---|---|---|---|---|---|---|---|
| 1 | 161.0379 | 81.0226 | | | | | C | | | | | | | 12 |
| 2 | **321.0686** | 161.0379 | | | | | C | **1359.5270** | 680.2671 | 1342.5005 | 671.7539 | 1341.5164 | 671.2619 | 11 |
| 3 | **378.0900** | 189.5487 | | | | | G | **1199.4964** | 600.2518 | 1182.4698 | 591.7385 | 1181.4858 | 591.2465 | 10 |
| 4 | 449.1272 | 225.0672 | | | | | A | 1142.4749 | 571.7411 | 1125.4483 | 563.2278 | 1124.4643 | 562.7358 | 9 |
| 5 | 536.1592 | 268.5832 | | | 518.1486 | 259.5779 | S | **1071.4378** | 536.2225 | 1054.4112 | 527.7093 | 1053.4272 | 527.2172 | 8 |

| | | | | | | | | | | | | | | |
|---|---|---|---|---|---|---|---|---|---|---|---|---|---|---|
| 6 | 651.1861 | 326.0967 | 634.1596 | 317.5834 | 633.1756 | 317.0914 | N | **984.4057** | 492.7065 | 967.3792 | 484.1932 | 966.3952 | 483.7012 | 7 |
| 7 | 814.2494 | 407.6284 | 797.2229 | 399.1151 | 796.2389 | 398.6231 | Y | **869.3788** | 435.1930 | 852.3523 | 426.6798 | 851.3682 | 426.1878 | 6 |
| 8 | 915.2971 | 458.1522 | 898.2706 | 449.6389 | 897.2866 | 449.1469 | T | **706.3155** | 353.6614 | 689.2889 | 345.1481 | 688.3049 | 344.6561 | 5 |
| 9 | 1030.3241 | 515.6657 | 1013.2975 | 507.1524 | 1012.3135 | 506.6604 | D | **605.2678** | 303.1375 | 588.2413 | 294.6243 | 587.2572 | 294.1323 | 4 |
| 10 | **1216.4034** | 608.7053 | **1199.3768** | 600.1921 | 1198.3928 | 599.7000 | W | **490.2409** | 245.6241 | 473.2143 | 237.1108 | 472.2303 | 236.6188 | 3 |
| 11 | **1345.4460** | 673.2266 | 1328.4194 | 664.7134 | 1327.4354 | 664.2213 | E | **304.1615** | 152.5844 | 287.1350 | 144.0711 | 286.1510 | 143.5791 | 2 |
| 12 | | | | | | | R | 175.1190 | 88.0631 | 158.0924 | 79.5498 | | | 1 |

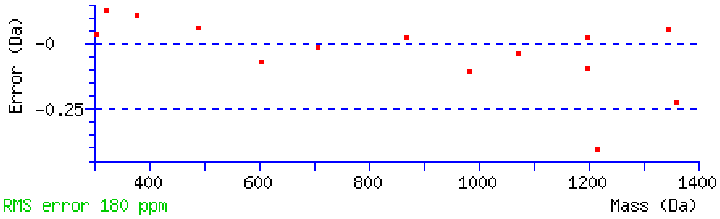


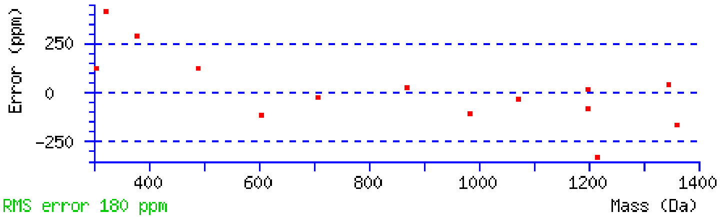


### All matches to this query

| Score | Mr(calc): | Delta | Sequence |
|---|---|---|---|
| 77.3 | 1518.5504 | 0.0022 | CCGASNYTDWER |
| 5.3 | 1518.5541 | -0.0016 | DVPHSNRYPTSS |
| 4.2 | 1518.5479 | 0.0046 | TASSVIELTCAK |
| 1.5 | 1518.5450 | 0.0075 | ESTSDMMDGTEER |
| 1.1 | 1518.5388 | 0.0137 | ETSEQHESKER |
| 1.0 | 1518.5462 | 0.0063 | TKSSVNNMSSFR |

---

Spectrum No: 266; Query: 2289; Rank: 1

---

## Peptide View

MS/MS Fragmentation of **ADECALPYLGATCYCDLFCNR**
Found in **IPI00190428**, Tax_Id=10116 Gene_Symbol=Lcn7 Tubulointerstitial nephritis antigen-like precursor

Match to Query 2289: 2569.049608 from(1285.532080,2+)
Title: 100101RatKid_NS_deglyco_23.5020.5020.2.dta
Data file K:\NewmanPaper\Piliang\3SubProteomes\Piliang3SP\mgf5ppm\ERLIC_3SubProteomes5ppm.mgf

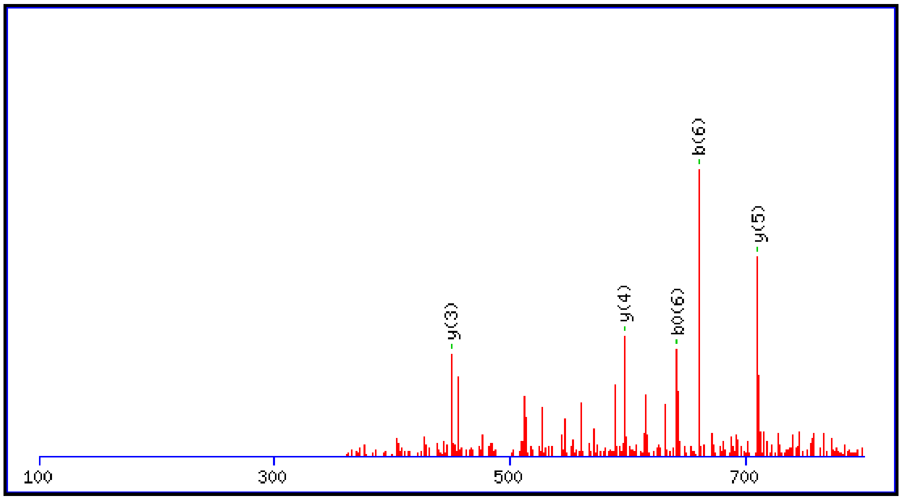

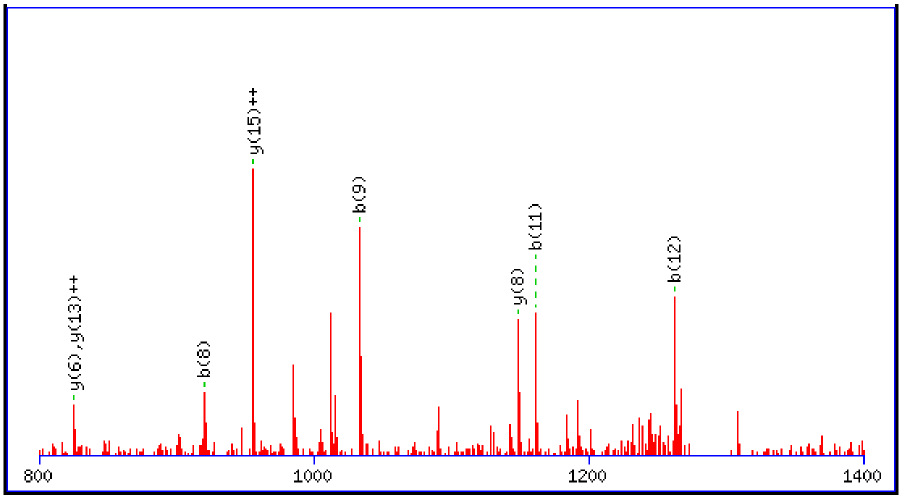


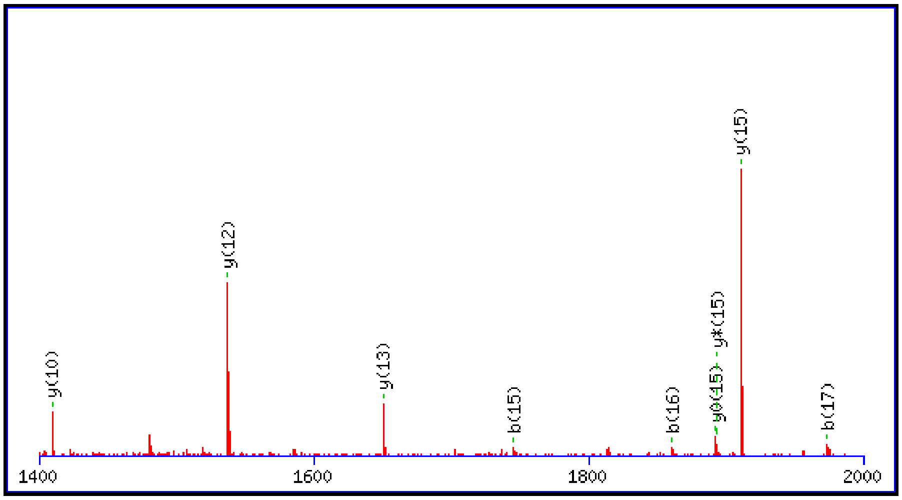


**Monoisotopic mass of neutral peptide Mr(calc):** 2569.0382
**Fixed modifications:** Carbamidomethyl (C)
**Variable modifications:**
**N20** : Deamidated_N (N)
**Ions Score:** 77 **Expect:** 4.9e-006
**Matches (Bold Red):** 22/190 fragment ions using 30 most intense peaks

| # | b | b$^{++}$ | b* | b*$^{++}$ | b$^{0}$ | b$^{0++}$ | Seq. | y | y$^{++}$ | y* | y*$^{++}$ | y$^{0}$ | y$^{0++}$ | # |
|---|---|---|---|---|---|---|---|---|---|---|---|---|---|---|
| 1 | 72.0444 | 36.5258 | | | | | A | | | | | | | 21 |
| 2 | 187.0713 | 94.0393 | | | 169.0608 | 85.0340 | D | 2499.0084 | 1250.0078 | 2481.9818 | 1241.4945 | 2480.9978 | 1241.0025 | 20 |
| 3 | 316.1139 | 158.5606 | | | 298.1034 | 149.5553 | E | 2383.9814 | 1192.4943 | 2366.9549 | 1183.9811 | 2365.9709 | 1183.4891 | 19 |
| 4 | 476.1446 | 238.5759 | | | 458.1340 | 229.5706 | C | 2254.9388 | 1127.9731 | 2237.9123 | 1119.4598 | 2236.9283 | 1118.9678 | 18 |
| 5 | 547.1817 | 274.0945 | | | 529.1711 | 265.0892 | A | 2094.9082 | 1047.9577 | 2077.8816 | 1039.4445 | 2076.8976 | 1038.9524 | 17 |
| 6 | **660.2658** | 330.6365 | | | **642.2552** | 321.6312 | L | 2023.8711 | 1012.4392 | 2006.8445 | 1003.9259 | 2005.8605 | 1003.4339 | 16 |
| 7 | 757.3185 | 379.1629 | | | 739.3080 | 370.1576 | P | **1910.7870** | **955.8971** | **1893.7604** | 947.3839 | **1892.7764** | 946.8919 | 15 |
| 8 | **920.3818** | 460.6946 | | | 902.3713 | 451.6893 | Y | 1813.7342 | 907.3708 | 1796.7077 | 898.8575 | 1795.7237 | 898.3655 | 14 |
| 9 | **1033.4659** | 517.2366 | | | 1015.4553 | 508.2313 | L | **1650.6709** | **825.8391** | 1633.6444 | 817.3258 | 1632.6603 | 816.8338 | 13 |
| 10 | 1090.4874 | 545.7473 | | | 1072.4768 | 536.7420 | G | **1537.5868** | 769.2971 | 1520.5603 | 760.7838 | 1519.5763 | 760.2918 | 12 |
| 11 | **1161.5245** | 581.2659 | | | 1143.5139 | 572.2606 | A | 1480.5654 | 740.7863 | 1463.5388 | 732.2731 | 1462.5548 | 731.7810 | 11 |
| 12 | **1262.5722** | 631.7897 | | | 1244.5616 | 622.7844 | T | **1409.5283** | 705.2678 | 1392.5017 | 696.7545 | 1391.5177 | 696.2625 | 10 |
| 13 | 1422.6028 | 711.8050 | | | 1404.5923 | 702.7998 | C | 1308.4806 | 654.7439 | 1291.4540 | 646.2307 | 1290.4700 | 645.7386 | 9 |
| 14 | 1585.6661 | 793.3367 | | | 1567.6556 | 784.3314 | Y | **1148.4499** | 574.7286 | 1131.4234 | 566.2153 | 1130.4394 | 565.7233 | 8 |
| 15 | **1745.6968** | 873.3520 | | | 1727.6862 | 864.3468 | C | 985.3866 | 493.1969 | 968.3601 | 484.6837 | 967.3760 | 484.1917 | 7 |
| 16 | **1860.7237** | 930.8655 | | | 1842.7132 | 921.8602 | D | **825.3560** | 413.1816 | 808.3294 | 404.6683 | 807.3454 | 404.1763 | 6 |
| 17 | **1973.8078** | 987.4075 | | | 1955.7972 | 978.4023 | L | **710.3290** | 355.6681 | 693.3025 | 347.1549 | | | 5 |
| 18 | 2120.8762 | 1060.9417 | | | 2102.8656 | 1051.9365 | F | **597.2450** | 299.1261 | 580.2184 | 290.6128 | | | 4 |
| | | | | | | | | | | | | | | |

| | | | | | | | | | | | | | | |
|---|---|---|---|---|---|---|---|---|---|---|---|---|---|---|
| **19** | 2280.9069 | 1140.9571 | | | 2262.8963 | 1131.9518 | **C** | **450.1765** | 225.5919 | 433.1500 | 217.0786 | | | **3** |
| **20** | 2395.9338 | 1198.4705 | 2378.9073 | 1189.9573 | 2377.9232 | 1189.4653 | **N** | 290.1459 | 145.5766 | 273.1193 | 137.0633 | | | **2** |
| **21** | | | | | | | **R** | 175.1190 | 88.0631 | 158.0924 | 79.5498 | | | **1** |

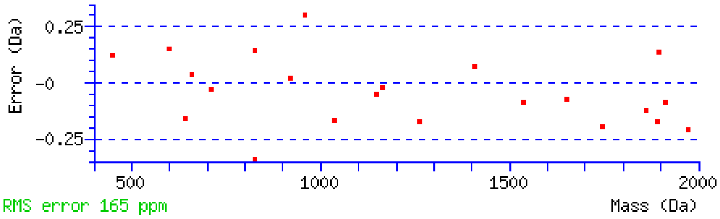


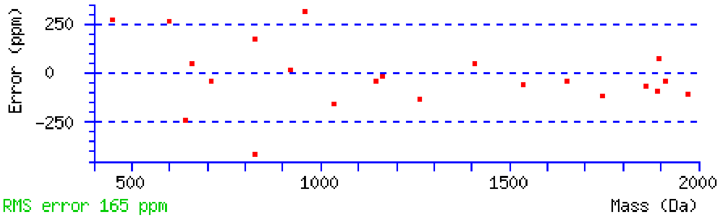


### All matches to this query

| Score | Mr(calc): | Delta | Sequence |
|---|---|---|---|
| 77.3 | 2569.0382 | 0.0114 | ADECALPYLGATCYCDLFCNR |
| 23.1 | 2568.0542 | 0.9954 | ADECALPYLGATCYCDLFCNR |
| 5.0 | 2569.0286 | 0.0210 | VTILIYGLHHNPSYWPNPK |
| 3.5 | 2569.0726 | -0.0230 | ATNDSVVADKSCEKIHFIAPK |
| 3.4 | 2569.0379 | 0.0117 | LEEACGMGEAPTKSSRTWQQK |
| 3.4 | 2569.0379 | 0.0117 | LEEACGMGEAPTKSSRTWQQK |
| 3.4 | 2569.0379 | 0.0117 | LEEACGMGEAPTKSSRTWQQK |
| 2.5 | 2569.0358 | 0.0138 | EDGEDQDDDQMSRGTKSPTEGEK |
| 1.7 | 2569.0493 | 0.0003 | ESLQNGNLNDPSSVESSNGQWPK |
| 1.6 | 2569.0286 | 0.0210 | VTILIYGLHHNPSYWPNPK |

---

Spectrum No: 267; Query: 710; Rank: 1

---

## Peptide View

MS/MS Fragmentation of **LWNSTFLEEYSK**
Found in **IPI00205166**, Tax_Id=10116 Gene_Symbol=Itga6 similar to integrin alpha 6 isoform 2

Match to Query 710: 1516.714388 from(759.364470,2+)
Title: 100101RatKid_NS_deglyco_14.4548.4548.2.dta
Data file K:\NewmanPaper\Piliang\3SubProteomes\Piliang3SP\mgf5ppm\ERLIC_3SubProteomes5ppm.mgf

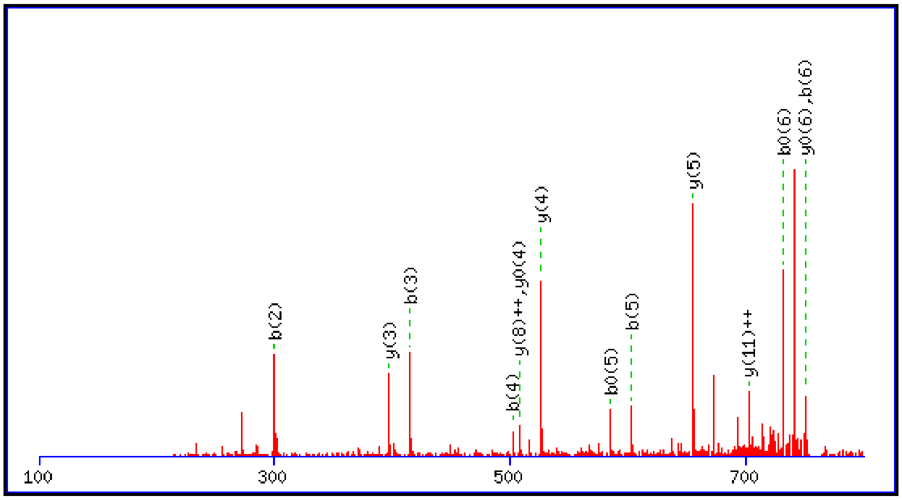

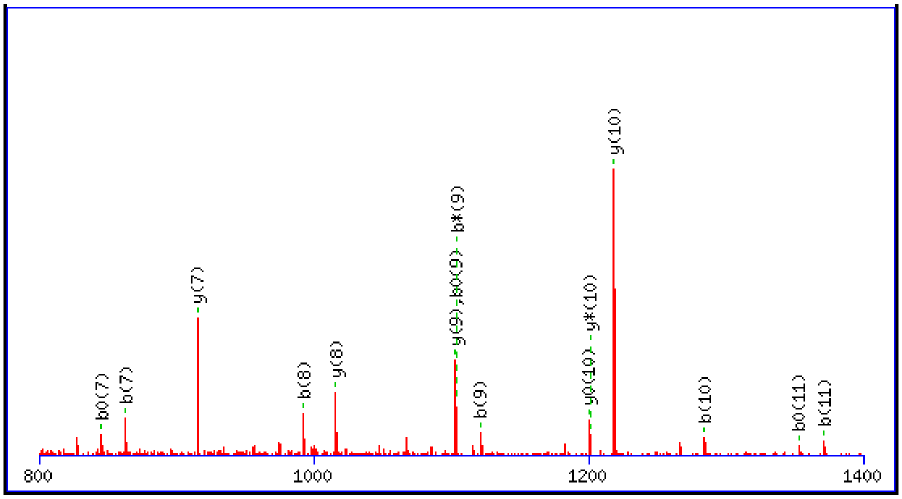


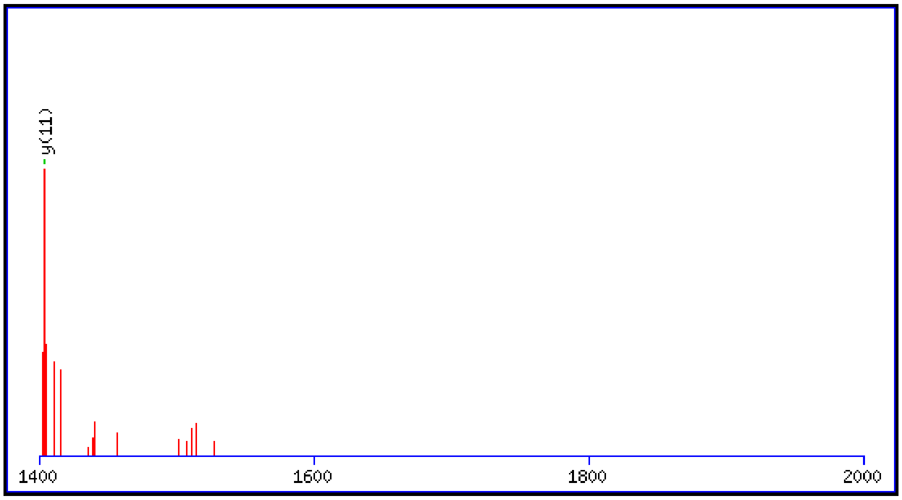


**Monoisotopic mass of neutral peptide Mr(calc):** 1516.7085
**Fixed modifications:** Carbamidomethyl (C)
**Variable modifications:**
**N3** : Deamidated_N (N)
**Ions Score:** 77 **Expect:** 3e-006
**Matches (Bold Red):** 30/120 fragment ions using 44 most intense peaks

| # | b | b++ | b* | b*++ | b0 | b0++ | Seq. | y | y++ | y* | y*++ | y0 | y0++ | # |
|---|---|---|---|---|---|---|---|---|---|---|---|---|---|---|
| 1 | 114.0913 | 57.5493 | | | | | L | | | | | | | 12 |
| 2 | **300.1707** | 150.5890 | | | | | W | **1404.6318** | **702.8195** | 1387.6052 | 694.3063 | 1386.6212 | 693.8142 | 11 |
| 3 | **415.1976** | 208.1024 | 398.1710 | 199.5892 | | | N | **1218.5525** | 609.7799 | **1201.5259** | 601.2666 | **1200.5419** | 600.7746 | 10 |
| 4 | **502.2296** | 251.6184 | 485.2031 | 243.1052 | 484.2191 | 242.6132 | S | **1103.5255** | 552.2664 | 1086.4990 | 543.7531 | 1085.5150 | 543.2611 | 9 |
| 5 | **603.2773** | 302.1423 | 586.2507 | 293.6290 | **585.2667** | 293.1370 | T | **1016.4935** | **508.7504** | 999.4670 | 500.2371 | 998.4829 | 499.7451 | 8 |
| 6 | **750.3457** | 375.6765 | 733.3192 | 367.1632 | **732.3351** | 366.6712 | F | **915.4458** | 458.2266 | 898.4193 | 449.7133 | 897.4353 | 449.2213 | 7 |
| 7 | **863.4298** | 432.2185 | 846.4032 | 423.7053 | **845.4192** | 423.2132 | L | 768.3774 | 384.6923 | 751.3509 | 376.1791 | **750.3668** | 375.6871 | 6 |
| 8 | **992.4724** | 496.7398 | 975.4458 | 488.2265 | 974.4618 | 487.7345 | E | **655.2933** | 328.1503 | 638.2668 | 319.6370 | 637.2828 | 319.1450 | 5 |
| 9 | **1121.5150** | 561.2611 | **1104.4884** | 552.7478 | **1103.5044** | 552.2558 | E | **526.2508** | 263.6290 | 509.2242 | 255.1157 | **508.2402** | 254.6237 | 4 |
| 10 | **1284.5783** | 642.7928 | 1267.5517 | 634.2795 | 1266.5677 | 633.7875 | Y | **397.2082** | 199.1077 | 380.1816 | 190.5944 | 379.1976 | 190.1024 | 3 |
| 11 | **1371.6103** | 686.3088 | 1354.5838 | 677.7955 | **1353.5998** | 677.3035 | S | 234.1448 | 117.5761 | 217.1183 | 109.0628 | 216.1343 | 108.5708 | 2 |
| 12 | | | | | | | K | 147.1128 | 74.0600 | 130.0863 | 65.5468 | | | 1 |

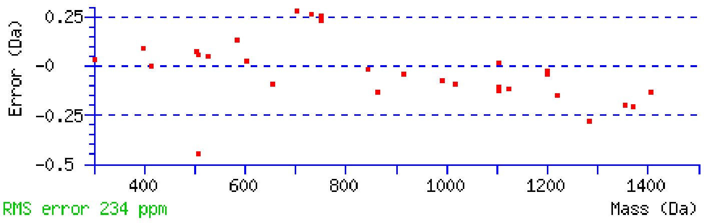


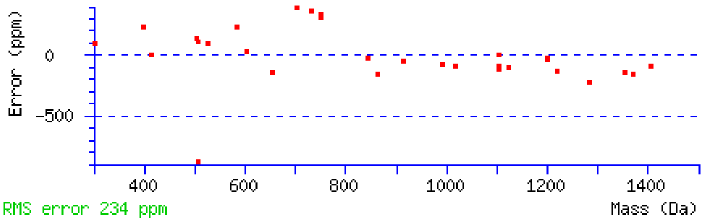


### All matches to this query

| Score | Mr(calc): | Delta | Sequence |
|---|---|---|---|
| 77.3 | 1516.7085 | 0.0058 | LWNSTFLEEYSK |
| 21.9 | 1515.7245 | 0.9899 | LWNSTFLEEYSK |
| 5.1 | 1516.7035 | 0.0109 | IGEERNSALSPHK |
| 3.2 | 1516.7158 | -0.0014 | TANKVDPEHTDYK |
| 3.0 | 1516.7232 | -0.0088 | VAEVIYCSFGGTSK |
| 2.2 | 1514.6977 | 2.0167 | AELSDSETTARKK |
| 1.2 | 1516.7109 | 0.0035 | SLVQEMVGSFGKR |
| 1.0 | 1514.6977 | 2.0167 | AELSDSETTARKK |

---

Spectrum No: 268; Query: 1401; Rank: 1

---

## Peptide View

MS/MS Fragmentation of **NNLTDPSLSEMVEVALR**
Found in **IPI00327143**, Tax_Id=10116 Gene_Symbol=Alpl Alkaline phosphatase, tissue-nonspecific isozyme precursor

Match to Query 1401: 1887.933428 from(944.973990,2+)
Title: 100101RatKid_NS_deglyco_23.5490.5490.2.dta
Data file K:\NewmanPaper\Piliang\3SubProteomes\Piliang3SP\mgf5ppm\ERLIC_3SubProteomes5ppm.mgf

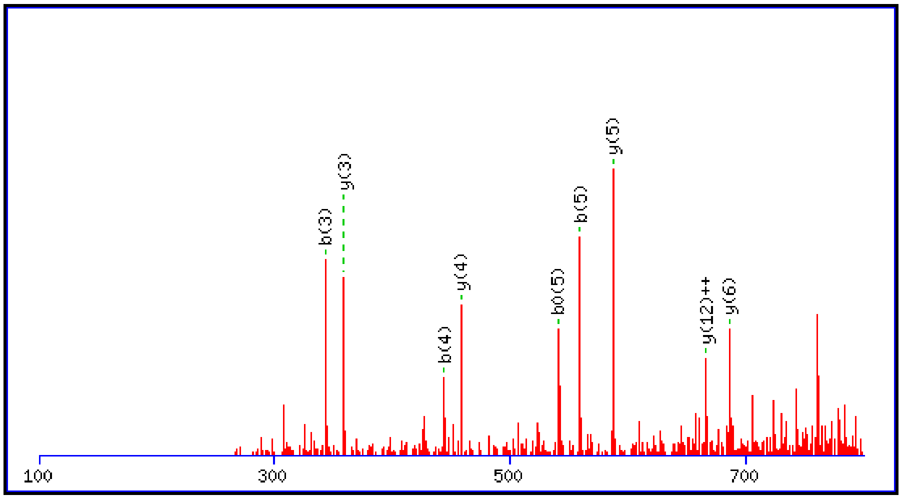

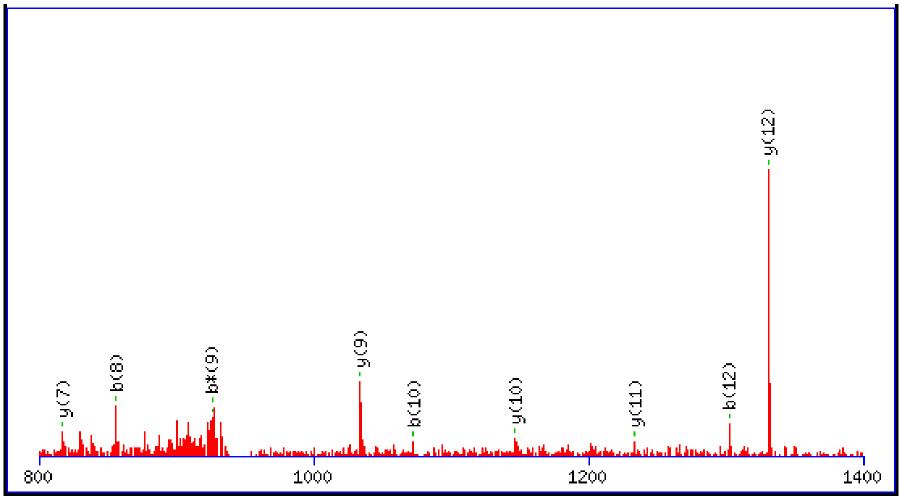


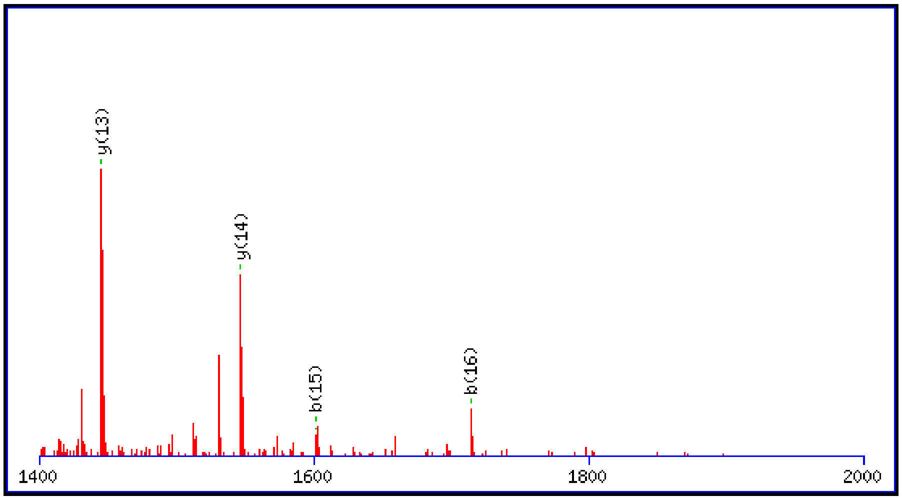


**Monoisotopic mass of neutral peptide Mr(calc):** 1887.9248
**Fixed modifications:** Carbamidomethyl (C)
**Variable modifications:**
**N2** : Deamidated_N (N)
**Ions Score:** 77 **Expect:** 4e-006
**Matches (Bold Red):** 22/178 fragment ions using 40 most intense peaks

| # | b | $b^{++}$ | b* | $b^{*++}$ | $b^{0}$ | $b^{0++}$ | Seq. | y | $y^{++}$ | y* | $y^{*++}$ | $y^{0}$ | $y^{0++}$ | # |
|---|---|---|---|---|---|---|---|---|---|---|---|---|---|---|
| 1 | 115.0502 | 58.0287 | 98.0237 | 49.5155 | | | N | | | | | | | 17 |
| 2 | 230.0771 | 115.5422 | 213.0506 | 107.0289 | | | N | 1774.8891 | 887.9482 | 1757.8626 | 879.4349 | 1756.8786 | 878.9429 | 16 |
| 3 | **343.1612** | 172.0842 | 326.1347 | 163.5710 | | | L | 1659.8622 | 830.4347 | 1642.8357 | 821.9215 | 1641.8516 | 821.4295 | 15 |
| 4 | **444.2089** | 222.6081 | 427.1823 | 214.0948 | 426.1983 | 213.6028 | T | **1546.7781** | 773.8927 | 1529.7516 | 765.3794 | 1528.7676 | 764.8874 | 14 |
| 5 | **559.2358** | 280.1216 | 542.2093 | 271.6083 | **541.2253** | 271.1163 | D | **1445.7305** | 723.3689 | 1428.7039 | 714.8556 | 1427.7199 | 714.3636 | 13 |
| 6 | 656.2886 | 328.6479 | 639.2620 | 320.1347 | 638.2780 | 319.6427 | P | **1330.7035** | **665.8554** | 1313.6770 | 657.3421 | 1312.6929 | 656.8501 | 12 |
| 7 | 743.3206 | 372.1639 | 726.2941 | 363.6507 | 725.3101 | 363.1587 | S | **1233.6507** | 617.3290 | 1216.6242 | 608.8157 | 1215.6402 | 608.3237 | 11 |
| 8 | **856.4047** | 428.7060 | 839.3781 | 420.1927 | 838.3941 | 419.7007 | L | **1146.6187** | 573.8130 | 1129.5922 | 565.2997 | 1128.6082 | 564.8077 | 10 |
| 9 | 943.4367 | 472.2220 | **926.4102** | 463.7087 | 925.4261 | 463.2167 | S | **1033.5347** | 517.2710 | 1016.5081 | 508.7577 | 1015.5241 | 508.2657 | 9 |
| 10 | **1072.4793** | 536.7433 | 1055.4528 | 528.2300 | 1054.4687 | 527.7380 | E | 946.5026 | 473.7550 | 929.4761 | 465.2417 | 928.4921 | 464.7497 | 8 |
| 11 | 1203.5198 | 602.2635 | 1186.4932 | 593.7503 | 1185.5092 | 593.2582 | M | **817.4600** | 409.2337 | 800.4335 | 400.7204 | 799.4495 | 400.2284 | 7 |
| 12 | **1302.5882** | 651.7977 | 1285.5617 | 643.2845 | 1284.5776 | 642.7925 | V | **686.4196** | 343.7134 | 669.3930 | 335.2001 | 668.4090 | 334.7081 | 6 |
| 13 | 1431.6308 | 716.3190 | 1414.6042 | 707.8058 | 1413.6202 | 707.3138 | E | **587.3511** | 294.1792 | 570.3246 | 285.6659 | 569.3406 | 285.1739 | 5 |
| 14 | 1530.6992 | 765.8532 | 1513.6727 | 757.3400 | 1512.6886 | 756.8480 | V | **458.3085** | 229.6579 | 441.2820 | 221.1446 | | | 4 |
| 15 | **1601.7363** | 801.3718 | 1584.7098 | 792.8585 | 1583.7258 | 792.3665 | A | **359.2401** | 180.1237 | 342.2136 | 171.6104 | | | 3 |
| 16 | **1714.8204** | 857.9138 | 1697.7938 | 849.4006 | 1696.8098 | 848.9085 | L | 288.2030 | 144.6051 | 271.1765 | 136.0919 | | | 2 |
| 17 | | | | | | | R | 175.1190 | 88.0631 | 158.0924 | 79.5498 | | | 1 |

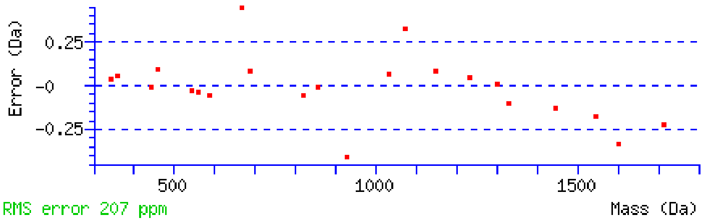


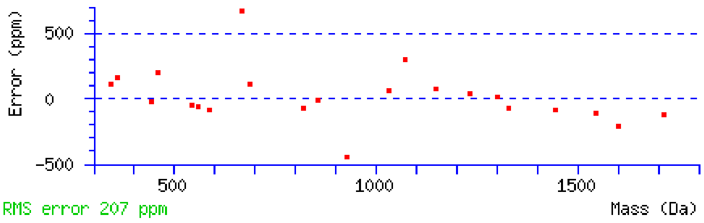


### All matches to this query

| Score | Mr(calc): | Delta | Sequence |
|---|---|---|---|
| 77.2 | 1887.9248 | 0.0087 | NNLTDPSLSEMVEVALR |
| 77.2 | 1887.9248 | 0.0087 | NNLTDPSLSEMVEVALR |
| 52.7 | 1886.9408 | 0.9927 | NNLTDPSLSEMVEVALR |
| 3.4 | 1887.9286 | 0.0049 | LSRSNEKEQLQGLNDR |
| 3.3 | 1887.9149 | 0.0185 | EWDKPSCTVPEVSAKR |
| 1.0 | 1887.9343 | -0.0008 | LTKNIATDSYISVNLR |
| 0.6 | 1887.9455 | -0.0121 | DQTLSPTIISGLHSIAR |
| 0.6 | 1887.9455 | -0.0121 | DQTLSPTIISGLHSIAR |
| 0.3 | 1887.9343 | -0.0008 | SKEVNLHEKANTPVLK |

---

Spectrum No: 269; Query: 1374; Rank: 1

---

## Peptide View

MS/MS Fragmentation of **GQPIPNKNLTVQVNSVR**
Found in **IPI00326140**, Tax_Id=10116 Gene_Symbol=Pzp Alpha-1-macroglobulin precursor

Match to Query 1374: 1864.019788 from(933.017170,2+)
Title: 100101RatKid_NS_deglyco_23.1952.1952.2.dta
Data file K:\NewmanPaper\Piliang\3SubProteomes\Piliang3SP\mgf5ppm\ERLIC_3SubProteomes5ppm.mgf

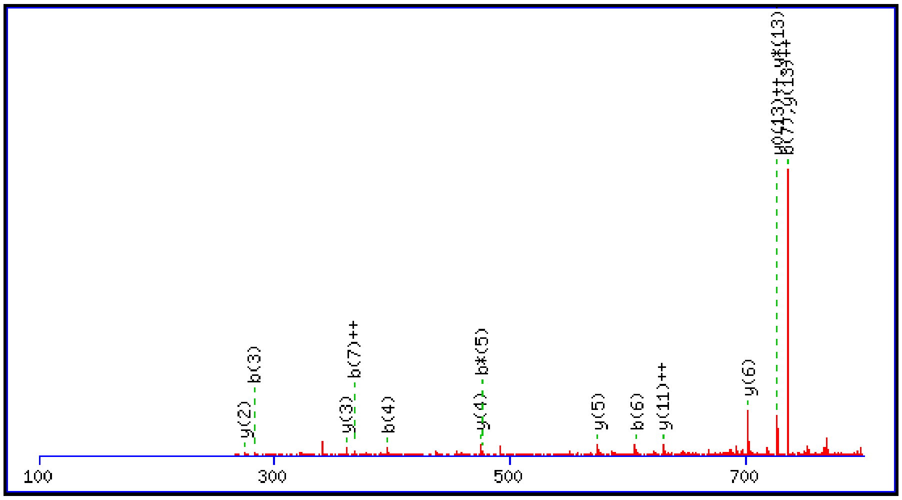

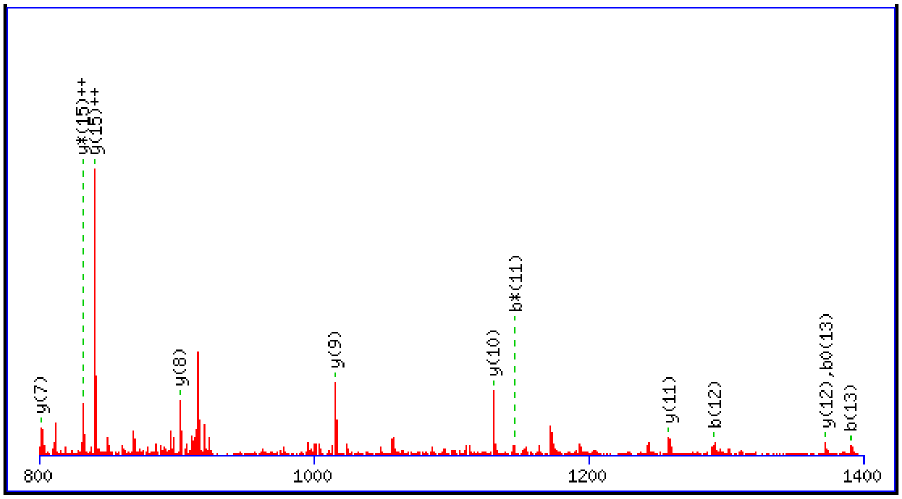


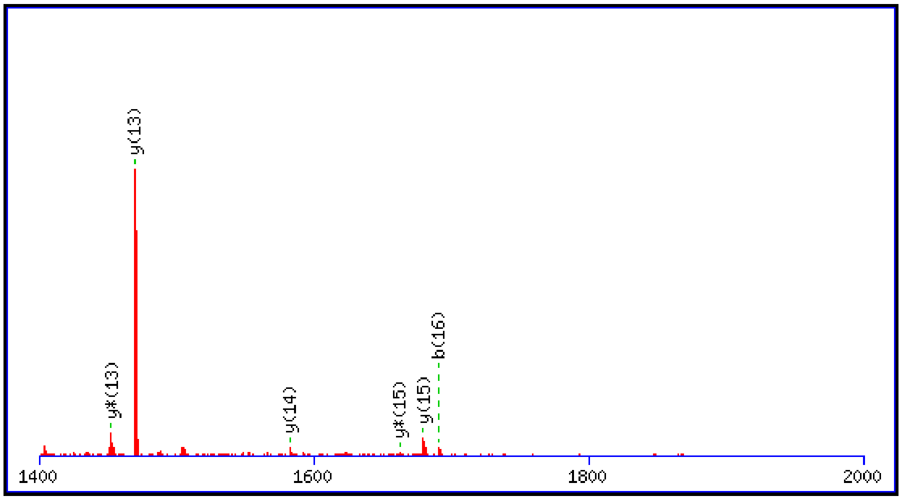


**Monoisotopic mass of neutral peptide Mr(calc):** 1864.0166
**Fixed modifications:** Carbamidomethyl (C)
**Variable modifications:**
**N8** : Deamidated_N (N)
**Ions Score:** 77 **Expect:** 1.2e-006
**Matches (Bold Red):** 33/168 fragment ions using 86 most intense peaks

| # | b | b++ | b* | b*++ | b0 | b0++ | Seq. | y | y++ | y* | y*++ | y0 | y0++ | # |
|---|---|---|---|---|---|---|---|---|---|---|---|---|---|---|
| 1 | 58.0287 | 29.5180 | | | | | G | | | | | | | 17 |
| 2 | 186.0873 | 93.5473 | 169.0608 | 85.0340 | | | Q | 1808.0025 | 904.5049 | 1790.9759 | 895.9916 | 1789.9919 | 895.4996 | 16 |
| 3 | **283.1401** | 142.0737 | 266.1135 | 133.5604 | | | P | **1679.9439** | **840.4756** | **1662.9173** | **831.9623** | 1661.9333 | 831.4703 | 15 |
| 4 | **396.2241** | 198.6157 | 379.1976 | 190.1024 | | | I | **1582.8911** | 791.9492 | 1565.8646 | 783.4359 | 1564.8806 | 782.9439 | 14 |
| 5 | 493.2769 | 247.1421 | **476.2504** | 238.6288 | | | P | **1469.8071** | **735.4072** | **1452.7805** | **726.8939** | 1451.7965 | **726.4019** | 13 |
| 6 | **607.3198** | 304.1636 | 590.2933 | 295.6503 | | | N | **1372.7543** | 686.8808 | 1355.7277 | 678.3675 | 1354.7437 | 677.8755 | 12 |
| 7 | **735.4148** | **368.2110** | 718.3883 | 359.6978 | | | K | **1258.7114** | **629.8593** | 1241.6848 | 621.3460 | 1240.7008 | 620.8540 | 11 |
| 8 | 850.4417 | 425.7245 | 833.4152 | 417.2112 | | | N | **1130.6164** | 565.8118 | 1113.5899 | 557.2986 | 1112.6058 | 556.8066 | 10 |
| 9 | 963.5258 | 482.2665 | 946.4993 | 473.7533 | | | L | **1015.5895** | 508.2984 | 998.5629 | 499.7851 | 997.5789 | 499.2931 | 9 |
| 10 | 1064.5735 | 532.7904 | 1047.5469 | 524.2771 | 1046.5629 | 523.7851 | T | **902.5054** | 451.7563 | 885.4789 | 443.2431 | 884.4948 | 442.7511 | 8 |
| 11 | 1163.6419 | 582.3246 | **1146.6153** | 573.8113 | 1145.6313 | 573.3193 | V | **801.4577** | 401.2325 | 784.4312 | 392.7192 | 783.4472 | 392.2272 | 7 |
| 12 | **1291.7005** | 646.3539 | 1274.6739 | 637.8406 | 1273.6899 | 637.3486 | Q | **702.3893** | 351.6983 | 685.3628 | 343.1850 | 684.3787 | 342.6930 | 6 |
| 13 | **1390.7689** | 695.8881 | 1373.7423 | 687.3748 | **1372.7583** | 686.8828 | V | **574.3307** | 287.6690 | 557.3042 | 279.1557 | 556.3202 | 278.6637 | 5 |
| 14 | 1504.8118 | 752.9095 | 1487.7853 | 744.3963 | 1486.8012 | 743.9043 | N | **475.2623** | 238.1348 | 458.2358 | 229.6215 | 457.2518 | 229.1295 | 4 |
| 15 | 1591.8438 | 796.4256 | 1574.8173 | 787.9123 | 1573.8333 | 787.4203 | S | **361.2194** | 181.1133 | 344.1928 | 172.6001 | 343.2088 | 172.1081 | 3 |
| 16 | **1690.9123** | 845.9598 | 1673.8857 | 837.4465 | 1672.9017 | 836.9545 | V | **274.1874** | 137.5973 | 257.1608 | 129.0840 | | | 2 |
| 17 | | | | | | | R | 175.1190 | 88.0631 | 158.0924 | 79.5498 | | | 1 |

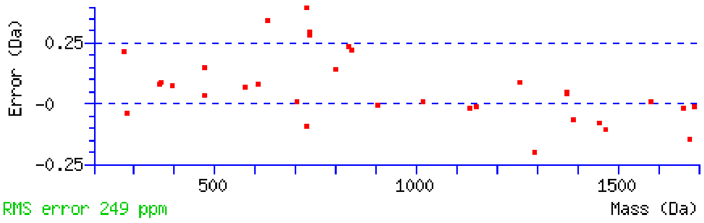


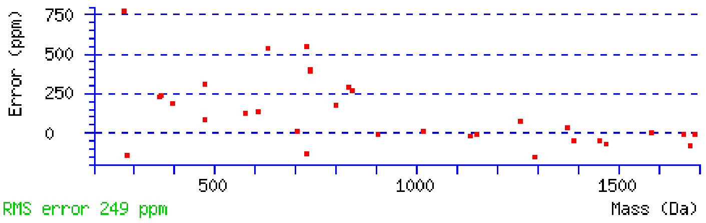


### All matches to this query

| Score | Mr(calc): | Delta | Sequence |
|---|---|---|---|
| 77.0 | 1864.0166 | 0.0031 | GQPIPNKNLTVQVNSVR |
| 61.2 | 1864.0166 | 0.0031 | GQPIPNKNLTVQVNSVR |
| 58.2 | 1864.0166 | 0.0031 | GQPIPNKNLTVQVNSVR |
| 19.9 | 1863.0326 | 0.9871 | GQPIPNKNLTVQVNSVR |
| 2.0 | 1864.0305 | -0.0107 | APLLPSQVNLEDKGALAK |
| 1.5 | 1864.0213 | -0.0015 | AIASHPSAALMALRRGSR |

---

Spectrum No: 270; Query: 2432; Rank: 1

---

## Peptide View

MS/MS Fragmentation of **HTGPGILSMANAGPNTNGSQFFICTAK**
Found in **IPI00364439**, Tax_Id=10116 Gene_Symbol=RGD1559682_predicted similar to Peptidyl-prolyl cis-trans isomerase A

Match to Query 2432: 2791.304982 from(931.442270,3+)
Title: 091008RatKidney_NoSalt_12.4393.4393.3.dta
Data file K:\NewmanPaper\Piliang\3SubProteomes\Piliang3SP\mgf5ppm\ERLIC_3SubProteomes5ppm.mgf

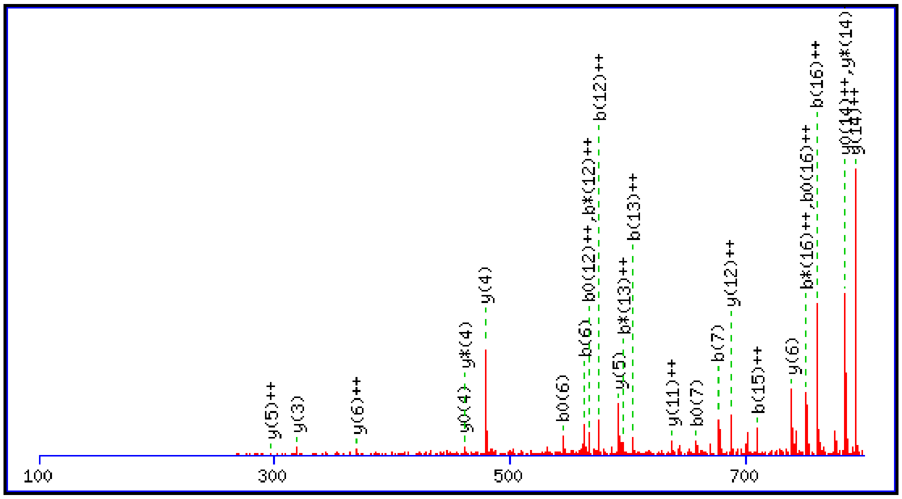

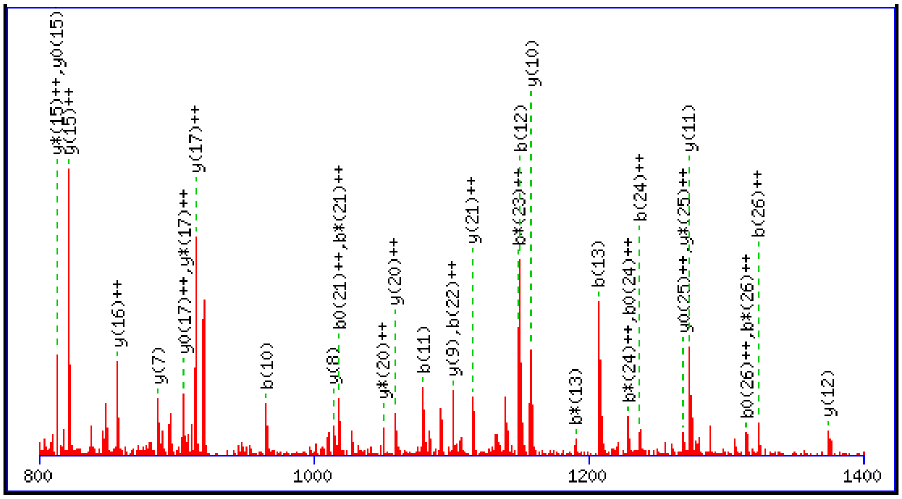


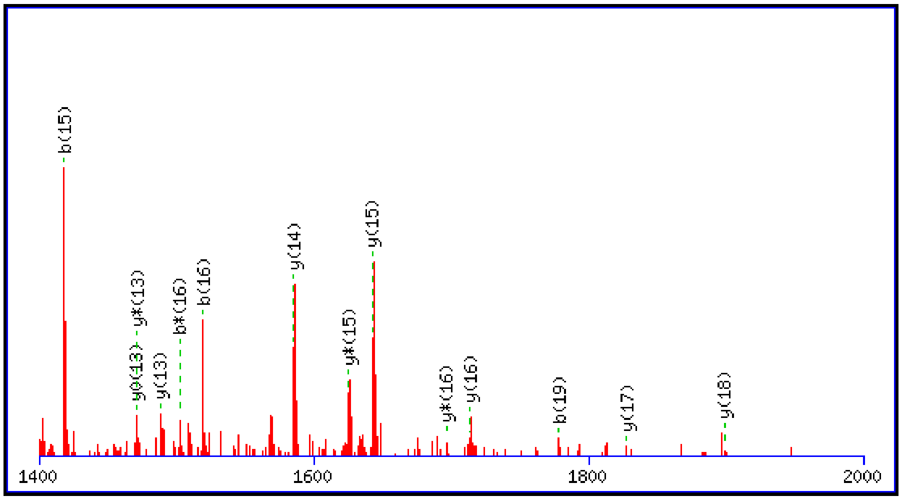


**Monoisotopic mass of neutral peptide Mr(calc):** 2791.3058
**Fixed modifications:** Carbamidomethyl (C)
**Variable modifications:**
**N17** : Deamidated_N (N)
**Ions Score:** 77 **Expect:** 7.1e-006
**Matches (Bold Red):** 73/286 fragment ions using 126 most intense peaks

| # | b | $b^{++}$ | b* | $b^{*++}$ | $b^{0}$ | $b^{0++}$ | Seq. | y | $y^{++}$ | y* | $y^{*++}$ | $y^{0}$ | $y^{0++}$ | # |
|---|---|---|---|---|---|---|---|---|---|---|---|---|---|---|
| 1 | 138.0662 | 69.5367 | | | | | H | | | | | | | 27 |
| 2 | 239.1139 | 120.0606 | | | 221.1033 | 111.0553 | T | 2655.2541 | 1328.1307 | 2638.2276 | 1319.6174 | 2637.2436 | 1319.1254 | 26 |
| 3 | 296.1353 | 148.5713 | | | 278.1248 | 139.5660 | G | 2554.2065 | 1277.6069 | 2537.1799 | **1269.0936** | 2536.1959 | **1268.6016** | 25 |
| 4 | 393.1881 | 197.0977 | | | 375.1775 | 188.0924 | P | 2497.1850 | 1249.0961 | 2480.1585 | 1240.5829 | 2479.1744 | 1240.0909 | 24 |
| 5 | 450.2096 | 225.6084 | | | 432.1990 | 216.6031 | G | 2400.1322 | 1200.5698 | 2383.1057 | 1192.0565 | 2382.1217 | 1191.5645 | 23 |
| 6 | **563.2936** | 282.1504 | | | **545.2831** | 273.1452 | I | 2343.1108 | 1172.0590 | 2326.0842 | 1163.5458 | 2325.1002 | 1163.0537 | 22 |
| 7 | **676.3777** | 338.6925 | | | **658.3671** | 329.6872 | L | 2230.0267 | **1115.5170** | 2213.0002 | 1107.0037 | 2212.0161 | 1106.5117 | 21 |
| 8 | 763.4097 | 382.2085 | | | 745.3991 | 373.2032 | S | 2116.9426 | **1058.9750** | 2099.9161 | **1050.4617** | 2098.9321 | 1049.9697 | 20 |
| 9 | 894.4502 | 447.7287 | | | 876.4396 | 438.7235 | M | 2029.9106 | 1015.4589 | 2012.8841 | 1006.9457 | 2011.9001 | 1006.4537 | 19 |
| 10 | **965.4873** | 483.2473 | | | 947.4767 | 474.2420 | A | **1898.8701** | 949.9387 | 1881.8436 | 941.4254 | 1880.8596 | 940.9334 | 18 |
| 11 | **1079.5302** | 540.2688 | 1062.5037 | 531.7555 | 1061.5197 | 531.2635 | N | **1827.8330** | **914.4201** | 1810.8065 | **905.9069** | 1809.8225 | **905.4149** | 17 |
| 12 | **1150.5674** | **575.7873** | 1133.5408 | **567.2740** | 1132.5568 | **566.7820** | A | **1713.7901** | **857.3987** | **1696.7635** | 848.8854 | 1695.7795 | 848.3934 | 16 |
| 13 | **1207.5888** | **604.2980** | **1190.5623** | **595.7848** | 1189.5783 | 595.2928 | G | **1642.7530** | **821.8801** | **1625.7264** | **813.3669** | 1624.7424 | **812.8748** | 15 |
| 14 | 1304.6416 | 652.8244 | 1287.6150 | 644.3112 | 1286.6310 | 643.8191 | P | **1585.7315** | **793.3694** | 1568.7050 | **784.8561** | 1567.7209 | **784.3641** | 14 |
| 15 | **1418.6845** | **709.8459** | 1401.6580 | 701.3326 | 1400.6739 | 700.8406 | N | **1488.6787** | 744.8430 | **1471.6522** | 736.3297 | **1470.6682** | 735.8377 | 13 |
| 16 | **1519.7322** | **760.3697** | **1502.7056** | **751.8565** | 1501.7216 | **751.3644** | T | **1374.6358** | **687.8215** | 1357.6093 | 679.3083 | 1356.6253 | 678.8163 | 12 |
| 17 | 1634.7591 | 817.8832 | 1617.7326 | 809.3699 | 1616.7486 | 808.8779 | N | **1273.5881** | **637.2977** | 1256.5616 | 628.7844 | 1255.5776 | 628.2924 | 11 |
| 18 | 1691.7806 | 846.3939 | 1674.7540 | 837.8807 | 1673.7700 | 837.3886 | G | **1158.5612** | 579.7842 | 1141.5347 | 571.2710 | 1140.5506 | 570.7790 | 10 |

| | | | | | | | | | | | | | | |
|---|---|---|---|---|---|---|---|---|---|---|---|---|---|---|
| **19** | **1778.8126** | 889.9099 | 1761.7861 | 881.3967 | 1760.8021 | 880.9047 | **S** | **1101.5397** | 551.2735 | 1084.5132 | 542.7602 | 1083.5292 | 542.2682 | **9** |
| **20** | 1906.8712 | 953.9392 | 1889.8446 | 945.4260 | 1888.8606 | 944.9340 | **Q** | **1014.5077** | 507.7575 | 997.4812 | 499.2442 | 996.4972 | 498.7522 | **8** |
| **21** | 2053.9396 | 1027.4734 | 2036.9131 | **1018.9602** | 2035.9290 | **1018.4682** | **F** | **886.4491** | 443.7282 | 869.4226 | 435.2149 | 868.4386 | 434.7229 | **7** |
| **22** | 2201.0080 | **1101.0076** | 2183.9815 | 1092.4944 | 2182.9975 | 1092.0024 | **F** | **739.3807** | **370.1940** | 722.3542 | 361.6807 | 721.3702 | 361.1887 | **6** |
| **23** | 2314.0921 | 1157.5497 | 2297.0655 | **1149.0364** | 2296.0815 | 1148.5444 | **I** | **592.3123** | **296.6598** | 575.2858 | 288.1465 | 574.3017 | 287.6545 | **5** |
| **24** | 2474.1227 | **1237.5650** | 2457.0962 | **1229.0517** | 2456.1122 | **1228.5597** | **C** | **479.2282** | 240.1178 | **462.2017** | 231.6045 | **461.2177** | 231.1125 | **4** |
| **25** | 2575.1704 | 1288.0888 | 2558.1439 | 1279.5756 | 2557.1598 | 1279.0836 | **T** | **319.1976** | 160.1024 | 302.1710 | 151.5892 | 301.1870 | 151.0972 | **3** |
| **26** | 2646.2075 | **1323.6074** | 2629.1810 | **1315.0941** | 2628.1970 | **1314.6021** | **A** | 218.1499 | 109.5786 | 201.1234 | 101.0653 | | | **2** |
| **27** | | | | | | | **K** | 147.1128 | 74.0600 | 130.0863 | 65.5468 | | | **1** |

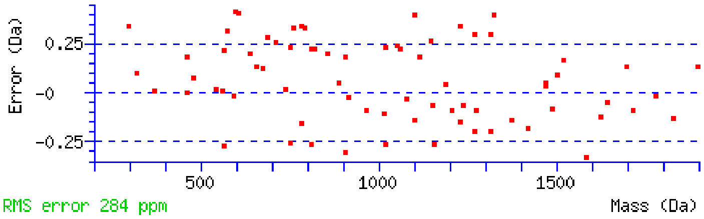


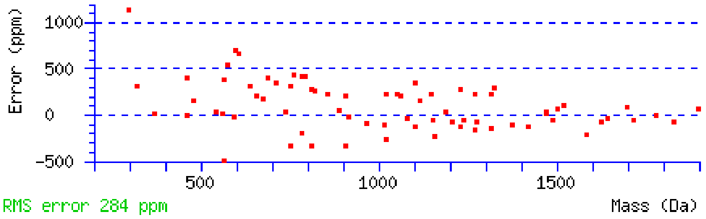


### All matches to this query

| Score | Mr(calc): | Delta | Sequence |
|---|---|---|---|
| 76.9 | 2791.3058 | -0.0008 | HTGPGILSMANAGPNTNGSQFFICTAK |
| 57.4 | 2791.3058 | -0.0008 | HTGPGILSMANAGPNTNGSQFFICTAK |
| 29.3 | 2791.3058 | -0.0008 | HTGPGILSMANAGPNTNGSQFFICTAK |
| 24.7 | 2790.3218 | 0.9832 | HTGPGILSMANAGPNTNGSQFFICTAK |
| 4.2 | 2791.3225 | -0.0175 | QREQQAYMVGLPQDALGQRPSAPR |
| 3.5 | 2790.2894 | 1.0155 | SPAAGGADTAVVEIVSKIKPEVTSPK |
| 3.0 | 2790.3143 | 0.9907 | YLRSLPSPFSPQPNSLSTKALGDR |
| 1.8 | 2789.3017 | 2.0033 | CVSSSAPANTPLAPCPQSEVIIPSVK |
| 1.7 | 2789.3129 | 1.9921 | AGSVELPASSPMPQLPPDTLEMRVR |
| 1.5 | 2789.2721 | 2.0329 | RSSVLVTHAELMPDKTATHEVHR |

---

Spectrum No: 271; Query: 2747; Rank: 1

---

## Peptide View

MS/MS Fragmentation of **LADDVDLEQVANETHGHVGADLAALCSEAALQAIR**
Found in **IPI00212014**, Tax_Id=10116 Gene_Symbol=Vcp Transitional endoplasmic reticulum ATPase

Match to Query 2747: 3672.771372 from(1225.264400,3+)
Title: 091008RatKidney_NoSalt_14.5976.5976.3.dta
Data file K:\NewmanPaper\Piliang\3SubProteomes\Piliang3SP\mgf5ppm\ERLIC_3SubProteomes5ppm.mgf

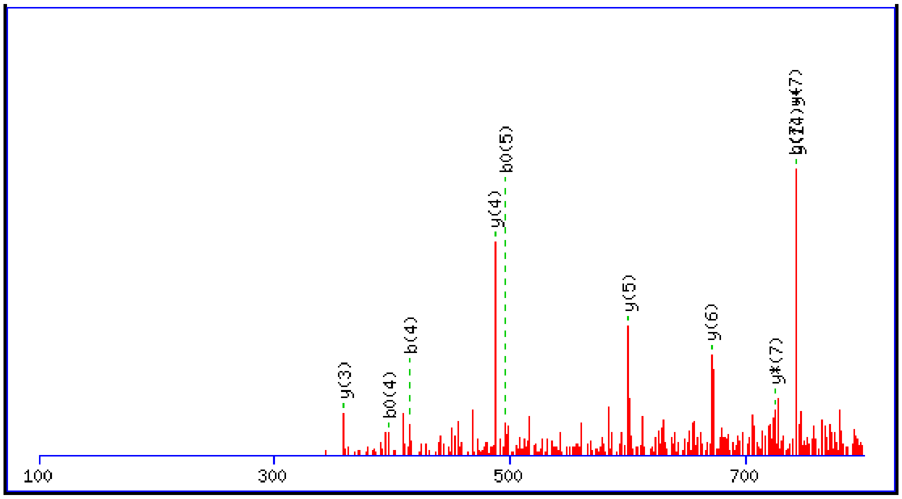


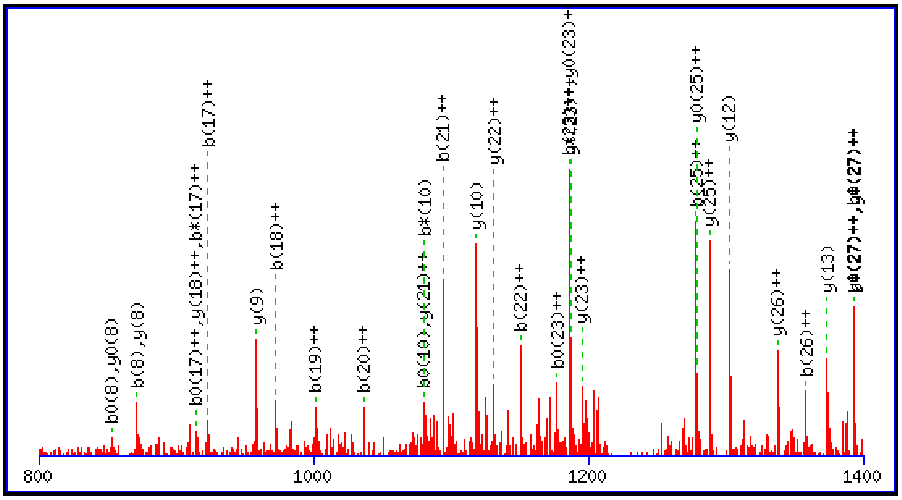


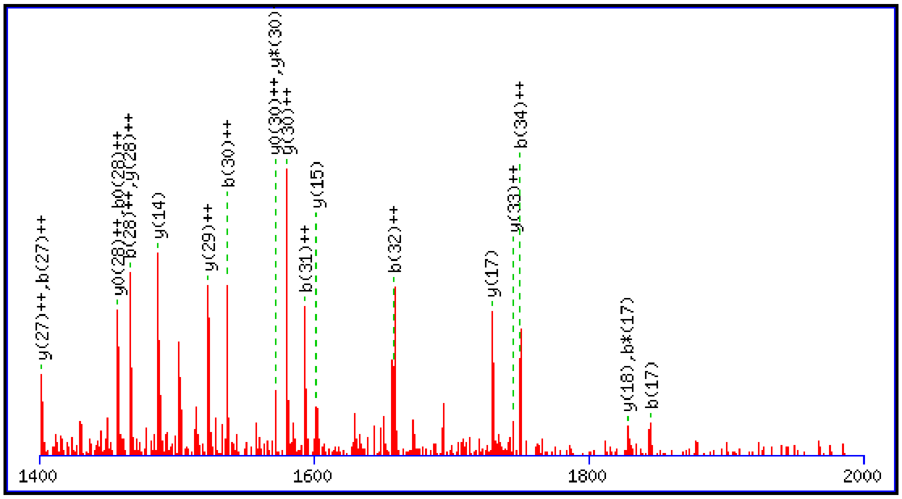


**Monoisotopic mass of neutral peptide Mr(calc):** 3672.7689
**Fixed modifications:** Carbamidomethyl (C)
**Variable modifications:**
**N12** : Deamidated_N (N)
**Ions Score:** 77 **Expect:** 6.5e-006
**Matches (Bold Red):** 67/374 fragment ions using 110 most intense peaks

| # | b | $b^{++}$ | b* | $b^{*++}$ | $b^0$ | $b^{0++}$ | Seq. | y | $y^{++}$ | y* | $y^{*++}$ | $y^0$ | $y^{0++}$ | # |
|---|---|---|---|---|---|---|---|---|---|---|---|---|---|---|
| 1 | 114.0913 | 57.5493 | | | | | L | | | | | | | 35 |
| 2 | 185.1285 | 93.0679 | | | | | A | 3560.6922 | 1780.8497 | 3543.6656 | 1772.3365 | 3542.6816 | 1771.8444 | 34 |
| 3 | 300.1554 | 150.5813 | | | 282.1448 | 141.5761 | D | 3489.6551 | **1745.3312** | 3472.6285 | 1736.8179 | 3471.6445 | 1736.3259 | 33 |
| 4 | **415.1823** | 208.0948 | | | **397.1718** | 199.0895 | D | 3374.6281 | 1687.8177 | 3357.6016 | 1679.3044 | 3356.6176 | 1678.8124 | 32 |
| 5 | 514.2508 | 257.6290 | | | **496.2402** | 248.6237 | V | 3259.6012 | 1630.3042 | 3242.5746 | 1621.7910 | 3241.5906 | 1621.2989 | 31 |

| | | | | | | | | | | | | | | |
|---|---|---|---|---|---|---|---|---|---|---|---|---|---|---|
| 6 | 629.2777 | 315.1425 | | | 611.2671 | 306.1372 | D | 3160.5328 | 1580.7700 | 3143.5062 | 1572.2567 | 3142.5222 | 1571.7647 | 30 |
| 7 | 742.3618 | 371.6845 | | | 724.3512 | 362.6792 | L | 3045.5058 | 1523.2565 | 3028.4793 | 1514.7433 | 3027.4953 | 1514.2513 | 29 |
| 8 | 871.4044 | 436.2058 | | | 853.3938 | 427.2005 | E | 2932.4218 | 1466.7145 | 2915.3952 | 1458.2012 | 2914.4112 | 1457.7092 | 28 |
| 9 | 999.4629 | 500.2351 | 982.4364 | 491.7218 | 981.4524 | 491.2298 | Q | 2803.3792 | 1402.1932 | 2786.3526 | 1393.6799 | 2785.3686 | 1393.1879 | 27 |
| 10 | 1098.5313 | 549.7693 | 1081.5048 | 541.2560 | 1080.5208 | 540.7640 | V | 2675.3206 | 1338.1639 | 2658.2940 | 1329.6507 | 2657.3100 | 1329.1586 | 26 |
| 11 | 1169.5685 | 585.2879 | 1152.5419 | 576.7746 | 1151.5579 | 576.2826 | A | 2576.2522 | 1288.6297 | 2559.2256 | 1280.1164 | 2558.2416 | 1279.6244 | 25 |
| 12 | 1284.5954 | 642.8013 | 1267.5688 | 634.2881 | 1266.5848 | 633.7961 | N | 2505.2151 | 1253.1112 | 2488.1885 | 1244.5979 | 2487.2045 | 1244.1059 | 24 |
| 13 | 1413.6380 | 707.3226 | 1396.6114 | 698.8094 | 1395.6274 | 698.3174 | E | 2390.1881 | 1195.5977 | 2373.1616 | 1187.0844 | 2372.1776 | 1186.5924 | 23 |
| 14 | 1514.6857 | 757.8465 | 1497.6591 | 749.3332 | 1496.6751 | 748.8412 | T | 2261.1455 | 1131.0764 | 2244.1190 | 1122.5631 | 2243.1350 | 1122.0711 | 22 |
| 15 | 1651.7446 | 826.3759 | 1634.7180 | 817.8627 | 1633.7340 | 817.3706 | H | 2160.0978 | 1080.5526 | 2143.0713 | 1072.0393 | 2142.0873 | 1071.5473 | 21 |
| 16 | 1708.7660 | 854.8867 | 1691.7395 | 846.3734 | 1690.7555 | 845.8814 | G | 2023.0389 | 1012.0231 | 2006.0124 | 1003.5098 | 2005.0284 | 1003.0178 | 20 |
| 17 | 1845.8250 | 923.4161 | 1828.7984 | 914.9028 | 1827.8144 | 914.4108 | H | 1966.0175 | 983.5124 | 1948.9909 | 974.9991 | 1948.0069 | 974.5071 | 19 |
| 18 | 1944.8934 | 972.9503 | 1927.8668 | 964.4370 | 1926.8828 | 963.9450 | V | 1828.9586 | 914.9829 | 1811.9320 | 906.4696 | 1810.9480 | 905.9776 | 18 |
| 19 | 2001.9148 | 1001.4611 | 1984.8883 | 992.9478 | 1983.9043 | 992.4558 | G | 1729.8901 | 865.4487 | 1712.8636 | 856.9354 | 1711.8796 | 856.4434 | 17 |
| 20 | 2072.9519 | 1036.9796 | 2055.9254 | 1028.4663 | 2054.9414 | 1027.9743 | A | 1672.8687 | 836.9380 | 1655.8421 | 828.4247 | 1654.8581 | 827.9327 | 16 |
| 21 | 2187.9789 | 1094.4931 | 2170.9523 | 1085.9798 | 2169.9683 | 1085.4878 | D | 1601.8316 | 801.4194 | 1584.8050 | 792.9061 | 1583.8210 | 792.4141 | 15 |
| 22 | 2301.0630 | 1151.0351 | 2284.0364 | 1142.5218 | 2283.0524 | 1142.0298 | L | 1486.8046 | 743.9060 | 1469.7781 | 735.3927 | 1468.7941 | 734.9007 | 14 |
| 23 | 2372.1001 | 1186.5537 | 2355.0735 | 1178.0404 | 2354.0895 | 1177.5484 | A | 1373.7206 | 687.3639 | 1356.6940 | 678.8506 | 1355.7100 | 678.3586 | 13 |
| 24 | 2443.1372 | 1222.0722 | 2426.1106 | 1213.5590 | 2425.1266 | 1213.0669 | A | 1302.6834 | 651.8454 | 1285.6569 | 643.3321 | 1284.6729 | 642.8401 | 12 |
| 25 | 2556.2212 | 1278.6143 | 2539.1947 | 1270.1010 | 2538.2107 | 1269.6090 | L | 1231.6463 | 616.3268 | 1214.6198 | 607.8135 | 1213.6358 | 607.3215 | 11 |
| 26 | 2716.2519 | 1358.6296 | 2699.2253 | 1350.1163 | 2698.2413 | 1349.6243 | C | 1118.5623 | 559.7848 | 1101.5357 | 551.2715 | 1100.5517 | 550.7795 | 10 |
| 27 | 2803.2839 | 1402.1456 | 2786.2574 | 1393.6323 | 2785.2734 | 1393.1403 | S | 958.5316 | 479.7694 | 941.5051 | 471.2562 | 940.5211 | 470.7642 | 9 |
| 28 | 2932.3265 | 1466.6669 | 2915.3000 | 1458.1536 | 2914.3160 | 1457.6616 | E | 871.4996 | 436.2534 | 854.4730 | 427.7402 | 853.4890 | 427.2482 | 8 |
| 29 | 3003.3636 | 1502.1855 | 2986.3371 | 1493.6722 | 2985.3531 | 1493.1802 | A | 742.4570 | 371.7321 | 725.4305 | 363.2189 | | | 7 |
| 30 | 3074.4007 | 1537.7040 | 3057.3742 | 1529.1907 | 3056.3902 | 1528.6987 | A | 671.4199 | 336.2136 | 654.3933 | 327.7003 | | | 6 |
| 31 | 3187.4848 | 1594.2460 | 3170.4583 | 1585.7328 | 3169.4742 | 1585.2408 | L | 600.3828 | 300.6950 | 583.3562 | 292.1817 | | | 5 |
| 32 | 3315.5434 | 1658.2753 | 3298.5168 | 1649.7621 | 3297.5328 | 1649.2700 | Q | 487.2987 | 244.1530 | 470.2722 | 235.6397 | | | 4 |
| 33 | 3386.5805 | 1693.7939 | 3369.5540 | 1685.2806 | 3368.5699 | 1684.7886 | A | 359.2401 | 180.1237 | 342.2136 | 171.6104 | | | 3 |
| 34 | 3499.6646 | 1750.3359 | 3482.6380 | 1741.8226 | 3481.6540 | 1741.3306 | I | 288.2030 | 144.6051 | 271.1765 | 136.0919 | | | 2 |
| 35 | | | | | | | R | 175.1190 | 88.0631 | 158.0924 | 79.5498 | | | 1 |

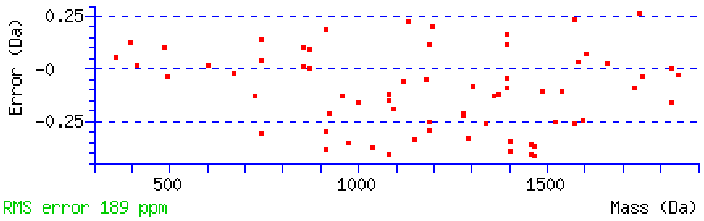


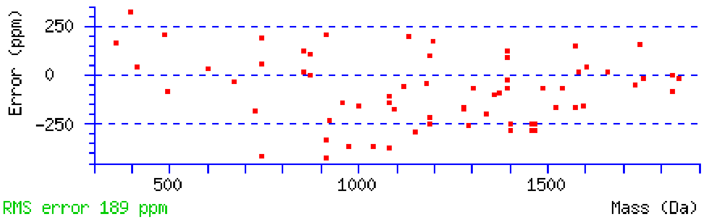


### All matches to this query

| Score | Mr(calc): | Delta | Sequence |
|---|---|---|---|
| 76.9 | 3672.7689 | 0.0024 | LADDVDLEQVANETHGHVGADLAALCSEAALQAIR |
| 67.6 | 3671.7849 | 0.9864 | LADDVDLEQVANETHGHVGADLAALCSEAALQAIR |
| 4.5 | 3671.7758 | 0.9956 | MLLASYSLIVRPATPAPAPQAPATPGAQLAHDMR |
| 4.5 | 3671.7758 | 0.9956 | MLLASYSLIVRPATPAPAPQAPATPGAQLAHDMR |
| 3.8 | 3670.7345 | 2.0369 | AVHTVDFTADKYHVVSGADDYTVKLWDIPNSK |
| 3.6 | 3672.8028 | -0.0314 | TQFVLLNQHNTALLSMLSSAIASEGPRMANFHR |
| 3.6 | 3672.7997 | -0.0284 | TYKLENILPELGIMDVFNTQADLSGIAGAKDVR |
| 2.6 | 3672.7627 | 0.0087 | EEPMTLLGDPSASLTLLSLSSISECNSTRSLLR |
| 2.4 | 3670.7994 | 1.9720 | GTNQLDGMDLLVLLDLIGAANPTFPNFFPKTTR |
| 2.3 | 3670.8005 | 1.9709 | TFPQDHDVYVGKPSLNRPIHASELQSKNRTR |

---

Spectrum No: 272; Query: 2710; Rank: 1

---

## Peptide View

MS/MS Fragmentation of **SLGNVNFSVSVEAQQSPELCGSQVATVPETGRK**
Found in **IPI00212666**, Tax_Id=10116 Gene_Symbol=Mug1 Isoform 1 of Murinoglobulin-1 precursor

Match to Query 2710: 3476.700642 from(1159.907490,3+)
Title: 100101RatKid_NS_deglyco_23.4529.4529.3.dta
Data file K:\NewmanPaper\Piliang\3SubProteomes\Piliang3SP\mgf5ppm\ERLIC_3SubProteomes5ppm.mgf

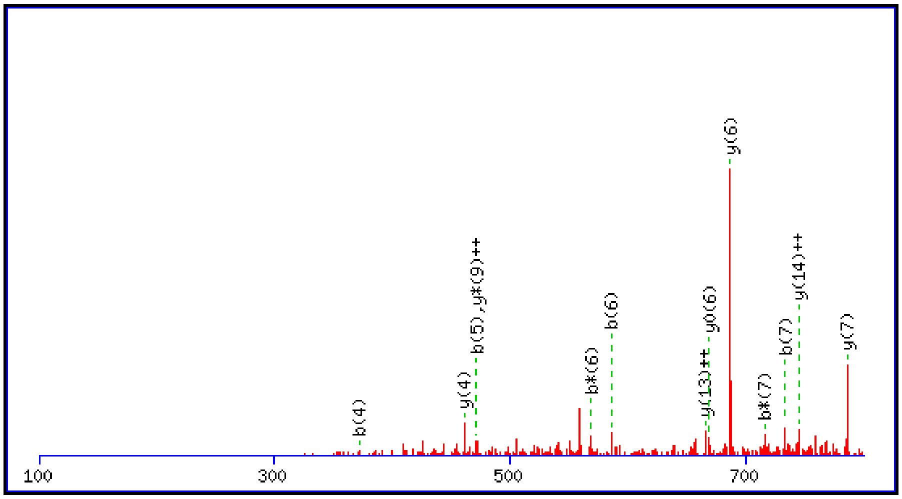


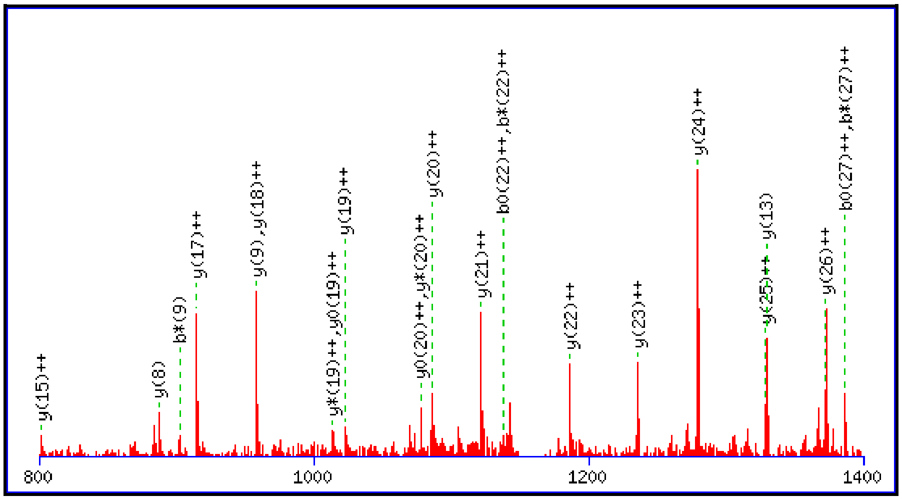

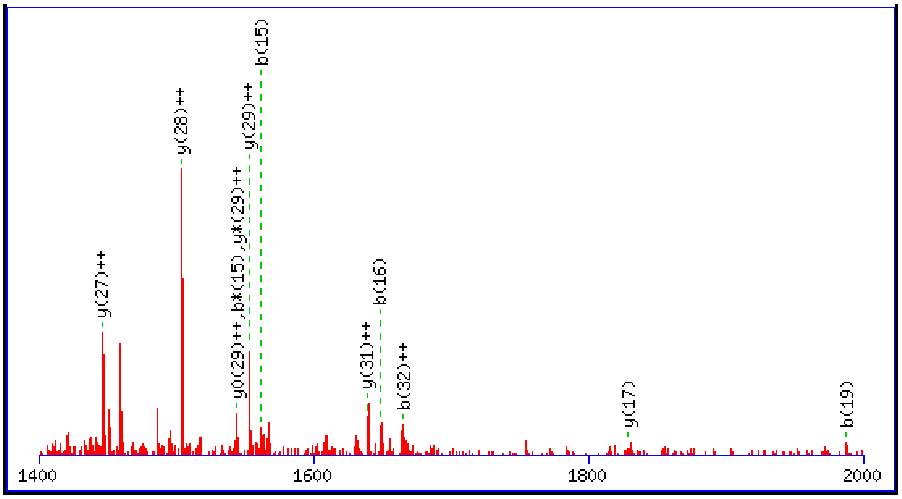


**Monoisotopic mass of neutral peptide Mr(calc):** 3475.6889
**Fixed modifications:** Carbamidomethyl (C)
**Variable modifications:**
**N6** : Deamidated_N (N)
**Ions Score:** 77 **Expect:** 5.9e-006
**Matches (Bold Red):** 48/372 fragment ions using 91 most intense peaks

| # | b | $b^{++}$ | $b^{*}$ | $b^{*++}$ | $b^{0}$ | $b^{0++}$ | Seq. | y | $y^{++}$ | $y^{*}$ | $y^{*++}$ | $y^{0}$ | $y^{0++}$ | # |
|---|---|---|---|---|---|---|---|---|---|---|---|---|---|---|
| 1 | 88.0393 | 44.5233 | | | 70.0287 | 35.5180 | S | | | | | | | 33 |
| 2 | 201.1234 | 101.0653 | | | 183.1128 | 92.0600 | L | 3389.6642 | 1695.3357 | 3372.6376 | 1686.8224 | 3371.6536 | 1686.3304 | 32 |
| 3 | 258.1448 | 129.5761 | | | 240.1343 | 120.5708 | G | 3276.5801 | **1638.7937** | 3259.5536 | 1630.2804 | 3258.5695 | 1629.7884 | 31 |
| 4 | **372.1878** | 186.5975 | 355.1612 | 178.0842 | 354.1772 | 177.5922 | N | 3219.5586 | 1610.2830 | 3202.5321 | 1601.7697 | 3201.5481 | 1601.2777 | 30 |
| 5 | **471.2562** | 236.1317 | 454.2296 | 227.6185 | 453.2456 | 227.1264 | V | 3105.5157 | **1553.2615** | 3088.4892 | **1544.7482** | 3087.5051 | **1544.2562** | 29 |
| 6 | **586.2831** | 293.6452 | **569.2566** | 285.1319 | 568.2725 | 284.6399 | N | 3006.4473 | **1503.7273** | 2989.4208 | 1495.2140 | 2988.4367 | 1494.7220 | 28 |
| 7 | **733.3515** | 367.1794 | **716.3250** | 358.6661 | 715.3410 | 358.1741 | F | 2891.4204 | **1446.2138** | 2874.3938 | 1437.7005 | 2873.4098 | 1437.2085 | 27 |
| 8 | 820.3836 | 410.6954 | 803.3570 | 402.1821 | 802.3730 | 401.6901 | S | 2744.3520 | **1372.6796** | 2727.3254 | 1364.1663 | 2726.3414 | 1363.6743 | 26 |
| 9 | 919.4520 | 460.2296 | **902.4254** | 451.7163 | 901.4414 | 451.2243 | V | 2657.3199 | **1329.1636** | 2640.2934 | 1320.6503 | 2639.3094 | 1320.1583 | 25 |
| 10 | 1006.4840 | 503.7456 | 989.4574 | 495.2324 | 988.4734 | 494.7404 | S | 2558.2515 | **1279.6294** | 2541.2250 | 1271.1161 | 2540.2409 | 1270.6241 | 24 |
| 11 | 1105.5524 | 553.2798 | 1088.5259 | 544.7666 | 1087.5418 | 544.2746 | V | 2471.2195 | **1236.1134** | 2454.1929 | 1227.6001 | 2453.2089 | 1227.1081 | 23 |
| 12 | 1234.5950 | 617.8011 | 1217.5685 | 609.2879 | 1216.5844 | 608.7959 | E | 2372.1511 | **1186.5792** | 2355.1245 | 1178.0659 | 2354.1405 | 1177.5739 | 22 |
| 13 | 1305.6321 | 653.3197 | 1288.6056 | 644.8064 | 1287.6215 | 644.3144 | A | 2243.1085 | **1122.0579** | 2226.0819 | 1113.5446 | 2225.0979 | 1113.0526 | 21 |
| 14 | 1433.6907 | 717.3490 | 1416.6641 | 708.8357 | 1415.6801 | 708.3437 | Q | 2172.0714 | **1086.5393** | 2155.0448 | **1078.0260** | 2154.0608 | **1077.5340** | 20 |
| 15 | **1561.7493** | 781.3783 | **1544.7227** | 772.8650 | 1543.7387 | 772.3730 | Q | 2044.0128 | **1022.5100** | 2026.9862 | **1013.9968** | 2026.0022 | **1013.5047** | 19 |
| 16 | **1648.7813** | 824.8943 | 1631.7547 | 816.3810 | 1630.7707 | 815.8890 | S | 1915.9542 | **958.4807** | 1898.9277 | 949.9675 | 1897.9436 | 949.4755 | 18 |
| 17 | 1745.8341 | 873.4207 | 1728.8075 | 864.9074 | 1727.8235 | 864.4154 | P | **1828.9222** | **914.9647** | 1811.8956 | 906.4515 | 1810.9116 | 905.9594 | 17 |
| 18 | 1874.8767 | 937.9420 | 1857.8501 | 929.4287 | 1856.8661 | 928.9367 | E | 1731.8694 | 866.4383 | 1714.8429 | 857.9251 | 1713.8588 | 857.4331 | 16 |
| 19 | **1987.9607** | 994.4840 | 1970.9342 | 985.9707 | 1969.9502 | 985.4787 | L | 1602.8268 | **801.9170** | 1585.8003 | 793.4038 | 1584.8163 | 792.9118 | 15 |
| 20 | 2147.9914 | 1074.4993 | 2130.9648 | 1065.9860 | 2129.9808 | 1065.4940 | C | 1489.7428 | **745.3750** | 1472.7162 | 736.8617 | 1471.7322 | 736.3697 | 14 |
| 21 | 2205.0128 | 1103.0101 | 2187.9863 | 1094.4968 | 2187.0023 | 1094.0048 | G | **1329.7121** | **665.3597** | 1312.6856 | 656.8464 | 1311.7015 | 656.3544 | 13 |
| 22 | 2292.0449 | 1146.5261 | 2275.0183 | **1138.0128** | 2274.0343 | **1137.5208** | S | 1272.6906 | 636.8490 | 1255.6641 | 628.3357 | 1254.6801 | 627.8437 | 12 |
| 23 | 2420.1034 | 1210.5554 | 2403.0769 | 1202.0421 | 2402.0929 | 1201.5501 | Q | 1185.6586 | 593.3329 | 1168.6321 | 584.8197 | 1167.6480 | 584.3277 | 11 |
| 24 | 2519.1719 | 1260.0896 | 2502.1453 | 1251.5763 | 2501.1613 | 1251.0843 | V | 1057.6000 | 529.3037 | 1040.5735 | 520.7904 | 1039.5895 | 520.2984 | 10 |
| 25 | 2590.2090 | 1295.6081 | 2573.1824 | 1287.0948 | 2572.1984 | 1286.6028 | A | **958.5316** | 479.7694 | 941.5051 | **471.2562** | 940.5211 | 470.7642 | 9 |
| 26 | 2691.2566 | 1346.1320 | 2674.2301 | 1337.6187 | 2673.2461 | 1337.1267 | T | **887.4945** | 444.2509 | 870.4680 | 435.7376 | 869.4839 | 435.2456 | 8 |
| 27 | 2790.3251 | 1395.6662 | 2773.2985 | **1387.1529** | 2772.3145 | **1386.6609** | V | **786.4468** | 393.7271 | 769.4203 | 385.2138 | 768.4363 | 384.7218 | 7 |
| 28 | 2887.3778 | 1444.1925 | 2870.3513 | 1435.6793 | 2869.3673 | 1435.1873 | P | **687.3784** | 344.1928 | 670.3519 | 335.6796 | **669.3678** | 335.1876 | 6 |
| 29 | 3016.4204 | 1508.7138 | 2999.3939 | 1500.2006 | 2998.4099 | 1499.7086 | E | 590.3257 | 295.6665 | 573.2991 | 287.1532 | 572.3151 | 286.6612 | 5 |
| 30 | 3117.4681 | 1559.2377 | 3100.4415 | 1550.7244 | 3099.4575 | 1550.2324 | T | **461.2831** | 231.1452 | 444.2565 | 222.6319 | 443.2725 | 222.1399 | 4 |
| 31 | 3174.4896 | 1587.7484 | 3157.4630 | 1579.2351 | 3156.4790 | 1578.7431 | G | 360.2354 | 180.6213 | 343.2088 | 172.1081 | | | 3 |
| 32 | 3330.5907 | **1665.7990** | 3313.5641 | 1657.2857 | 3312.5801 | 1656.7937 | R | 303.2139 | 152.1106 | 286.1874 | 143.5973 | | | 2 |

| | | | | | | | | | | | | | |
|---|---|---|---|---|---|---|---|---|---|---|---|---|---|
| 33 | | | | | | | K | 147.1128 | 74.0600 | 130.0863 | 65.5468 | | 1 |

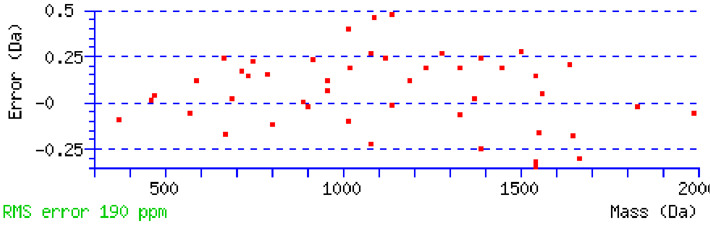


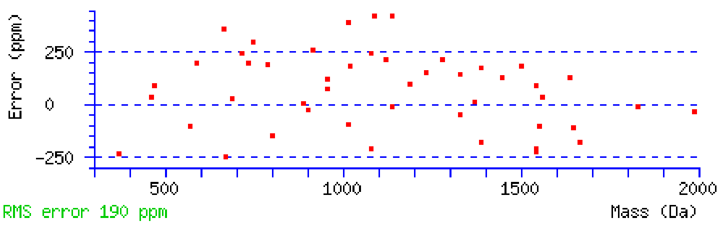


### All matches to this query

| Score | Mr(calc): | Delta | Sequence |
|---|---|---|---|
| 76.9 | 3475.6889 | 1.0117 | SLGNVNFSVSVEAQQSPELCGSQVATVPETGRK |
| 73.9 | 3476.6729 | 0.0277 | SLGNVNFSVSVEAQQSPELCGSQVATVPETGRK |
| 67.0 | 3475.6889 | 1.0117 | SLGNVNFSVSVEAQQSPELCGSQVATVPETGRK |
| 43.9 | 3474.7049 | 1.9957 | SLGNVNFSVSVEAQQSPELCGSQVATVPETGRK |
| 2.9 | 3474.6663 | 2.0344 | ELEVKYASLTDLVNNQSVMITVLEEQCLR |
| 2.9 | 3474.6663 | 2.0344 | ELEVKYASLTDLVNNQSVMITVLEEQCLR |
| 2.8 | 3476.7179 | -0.0172 | RKAFSTCASHFTFVVIGYGTCLFLYVKPK |
| 1.6 | 3474.7105 | 1.9901 | VTAGKDSYLALVDKNIMGYIASLHELATTER |
| 0.8 | 3475.7113 | 0.9894 | QQQASQQASTAADPTLKINPSLTELKALVHK |
| 0.8 | 3475.6711 | 1.0295 | QPCCSNIEKPVVSNIQTRSATVSWALNSAEK |

---

Spectrum No: 273; Query: 1533; Rank: 1

---

## Peptide View

MS/MS Fragmentation of **RQDPVSWNETFEDISR**
Found in **IPI00765413**, Tax_Id=10116 Gene_Symbol=- 58 kDa protein

Match to Query 1533: 1978.906182 from(660.642670,3+)
Title: 100101RatKid_NS_deglyco_21.3546.3546.3.dta
Data file K:\NewmanPaper\Piliang\3SubProteomes\Piliang3SP\mgf5ppm\ERLIC_3SubProteomes5ppm.mgf

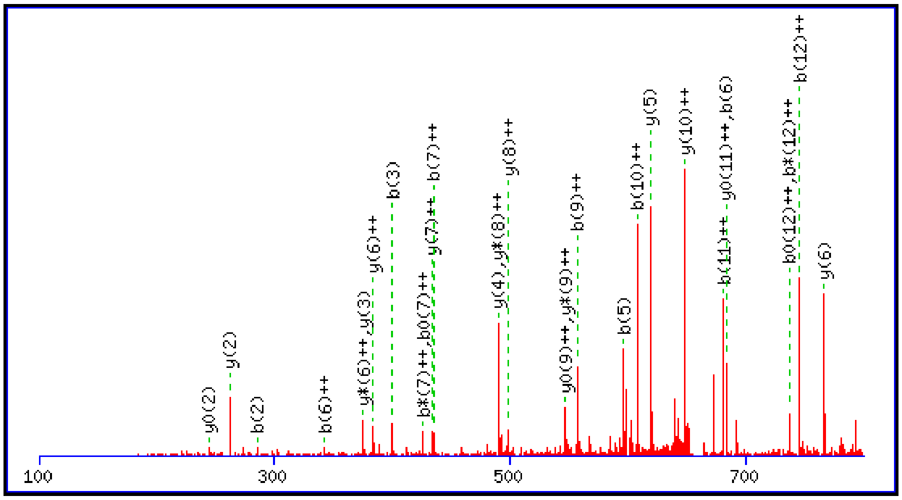

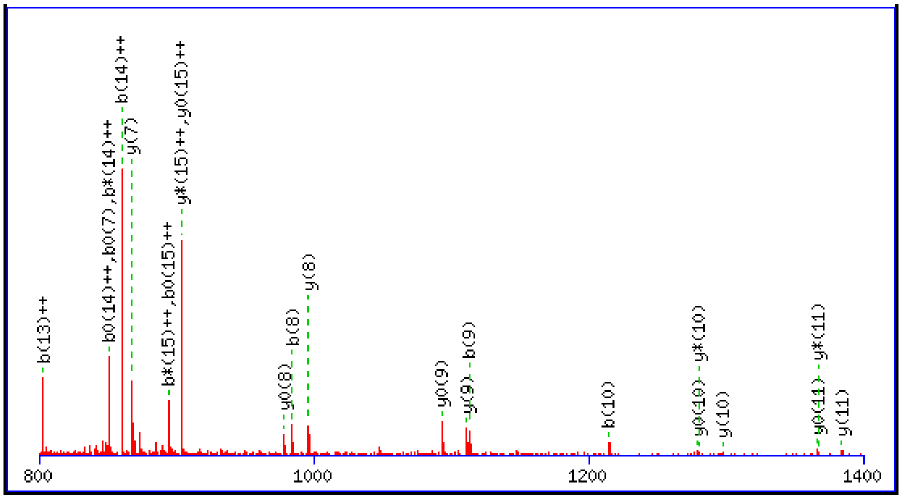


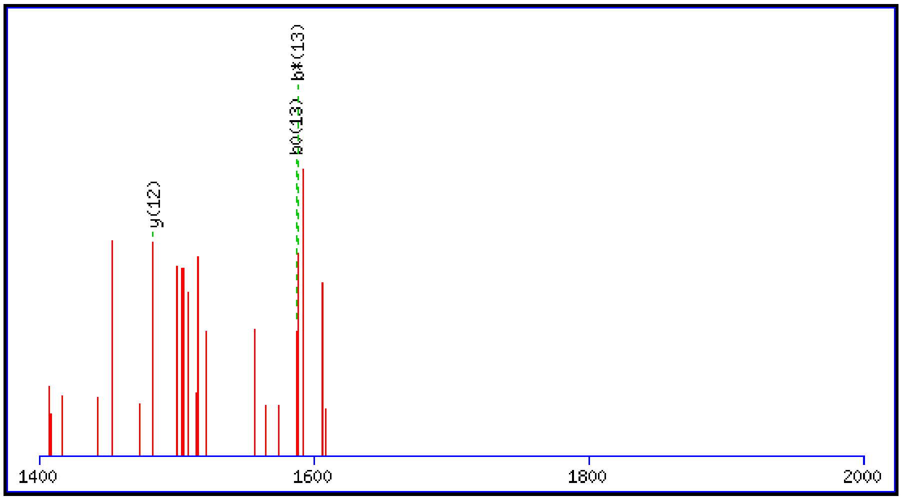


**Monoisotopic mass of neutral peptide Mr(calc):** 1978.9021
**Fixed modifications:** Carbamidomethyl (C)
**Variable modifications:**
**N8** : Deamidated_N (N)
**Ions Score:** 77 **Expect:** 5e-006
**Matches (Bold Red):** 56/174 fragment ions using 64 most intense peaks

| # | b | $b^{++}$ | b* | $b^{*++}$ | $b^0$ | $b^{0++}$ | Seq. | y | $y^{++}$ | y* | $y^{*++}$ | $y^0$ | $y^{0++}$ | # |
|---|---|---|---|---|---|---|---|---|---|---|---|---|---|---|
| 1 | 157.1084 | 79.0578 | 140.0818 | 70.5446 | | | R | | | | | | | 16 |
| 2 | **285.1670** | 143.0871 | 268.1404 | 134.5738 | | | Q | 1823.8082 | 912.4078 | 1806.7817 | **903.8945** | 1805.7977 | **903.4025** | 15 |
| 3 | **400.1939** | 200.6006 | 383.1674 | 192.0873 | 382.1833 | 191.5953 | D | 1695.7497 | 848.3785 | 1678.7231 | 839.8652 | 1677.7391 | 839.3732 | 14 |
| 4 | 497.2467 | 249.1270 | 480.2201 | 240.6137 | 479.2361 | 240.1217 | P | 1580.7227 | 790.8650 | 1563.6962 | 782.3517 | 1562.7122 | 781.8597 | 13 |
| 5 | **596.3151** | 298.6612 | 579.2885 | 290.1479 | 578.3045 | 289.6559 | V | **1483.6700** | 742.3386 | 1466.6434 | 733.8253 | 1465.6594 | 733.3333 | 12 |
| 6 | **683.3471** | **342.1772** | 666.3206 | 333.6639 | 665.3365 | 333.1719 | S | **1384.6015** | 692.8044 | **1367.5750** | 684.2911 | **1366.5910** | **683.7991** | 11 |
| 7 | 869.4264 | **435.2169** | 852.3999 | **426.7036** | **851.4159** | **426.2116** | W | **1297.5695** | **649.2884** | **1280.5430** | 640.7751 | **1279.5590** | 640.2831 | 10 |
| 8 | **984.4534** | 492.7303 | 967.4268 | 484.2170 | 966.4428 | 483.7250 | N | **1111.4902** | 556.2487 | 1094.4637 | **547.7355** | **1093.4796** | **547.2435** | 9 |
| 9 | **1113.4960** | **557.2516** | 1096.4694 | 548.7383 | 1095.4854 | 548.2463 | E | **996.4633** | **498.7353** | 979.4367 | **490.2220** | **978.4527** | 489.7300 | 8 |
| 10 | **1214.5436** | **607.7755** | 1197.5171 | 599.2622 | 1196.5331 | 598.7702 | T | **867.4207** | **434.2140** | 850.3941 | 425.7007 | 849.4101 | 425.2087 | 7 |
| 11 | 1361.6120 | **681.3097** | 1344.5855 | 672.7964 | 1343.6015 | 672.3044 | F | **766.3730** | **383.6901** | 749.3464 | **375.1769** | 748.3624 | 374.6849 | 6 |
| 12 | 1490.6546 | **745.8310** | 1473.6281 | **737.3177** | 1472.6441 | **736.8257** | E | **619.3046** | 310.1559 | 602.2780 | 301.6427 | 601.2940 | 301.1506 | 5 |
| 13 | 1605.6816 | **803.3444** | **1588.6550** | 794.8312 | **1587.6710** | 794.3391 | D | **490.2620** | 245.6346 | 473.2354 | 237.1214 | 472.2514 | 236.6293 | 4 |
| 14 | 1718.7656 | **859.8865** | 1701.7391 | **851.3732** | 1700.7551 | **850.8812** | I | **375.2350** | 188.1212 | 358.2085 | 179.6079 | 357.2245 | 179.1159 | 3 |
| 15 | 1805.7977 | **903.4025** | 1788.7711 | **894.8892** | 1787.7871 | **894.3972** | S | **262.1510** | 131.5791 | 245.1244 | 123.0659 | **244.1404** | 122.5738 | 2 |
| 16 | | | | | | | R | 175.1190 | 88.0631 | 158.0924 | 79.5498 | | | 1 |

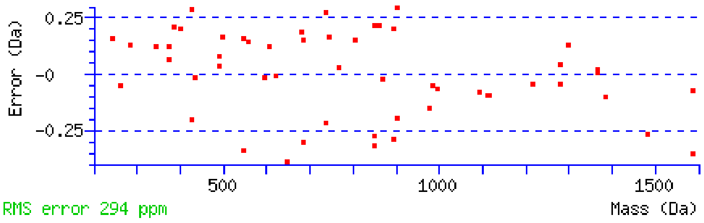


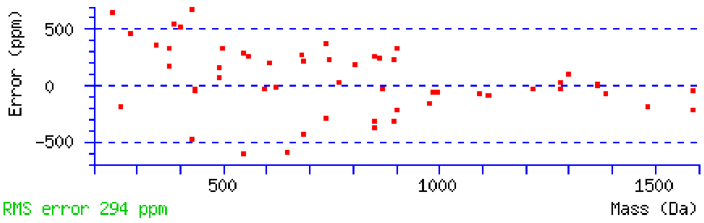


### All matches to this query

| Score | Mr(calc): | Delta | Sequence |
|---|---|---|---|
| 76.8 | 1978.9021 | 0.0041 | RQDPVSWNETFEDISR |
| 29.1 | 1977.9181 | 0.9881 | RQDPVSWNETFEDISR |
| 18.9 | 1978.9046 | 0.0016 | CTLPMFVNLTFREVR |
| 9.4 | 1978.9010 | 0.0052 | THAAAAQDTLTQLEHHR |
| 7.1 | 1978.8938 | 0.0124 | AVKYFWLAASNGDSQSR |
| 4.3 | 1978.9006 | 0.0056 | MPSSIVRMDTSNVPPPR |
| 4.3 | 1978.9006 | 0.0056 | MPSSIVRMDTSNVPPPR |
| 4.3 | 1976.8969 | 2.0093 | SQLDINNKKSVTDSIR |
| 4.0 | 1978.9006 | 0.0056 | MPSSIVRMDTSNVPPPR |
| 4.0 | 1978.9006 | 0.0056 | MPSSIVRMDTSNVPPPR |

---

Spectrum No: 274; Query: 131; Rank: 1

---

## Peptide View

MS/MS Fragmentation of **FNDTEVLQR**
Found in **IPI00358014**, Tax_Id=10116 Gene_Symbol=Btd Biotinidase precursor

Match to Query 131: 1121.534128 from(561.774340,2+)
Title: 091008RatKidney_NH4Format01_24.1462.1462.2.dta
Data file K:\NewmanPaper\Piliang\3SubProteomes\Piliang3SP\mgf5ppm\ERLIC_3SubProteomes5ppm.mgf

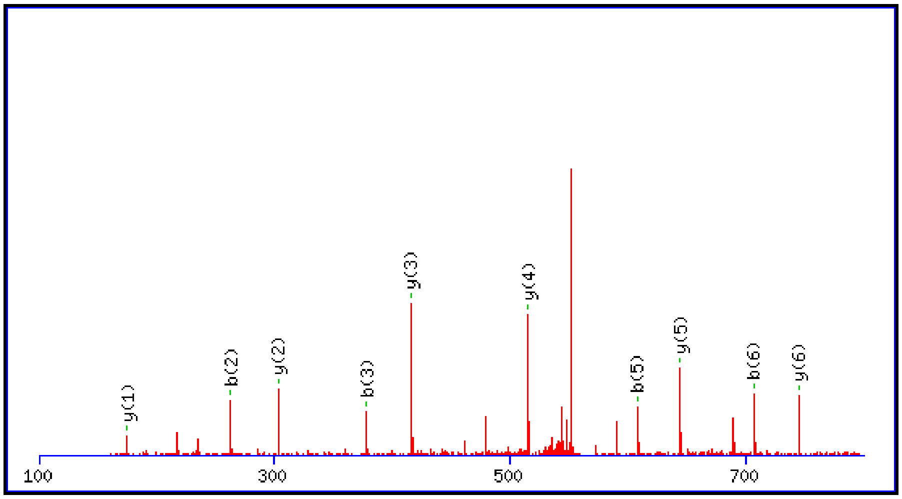

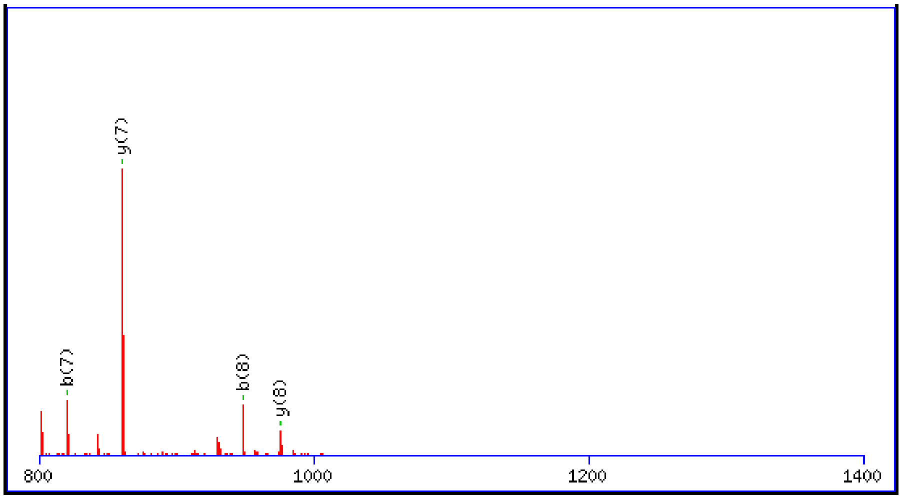


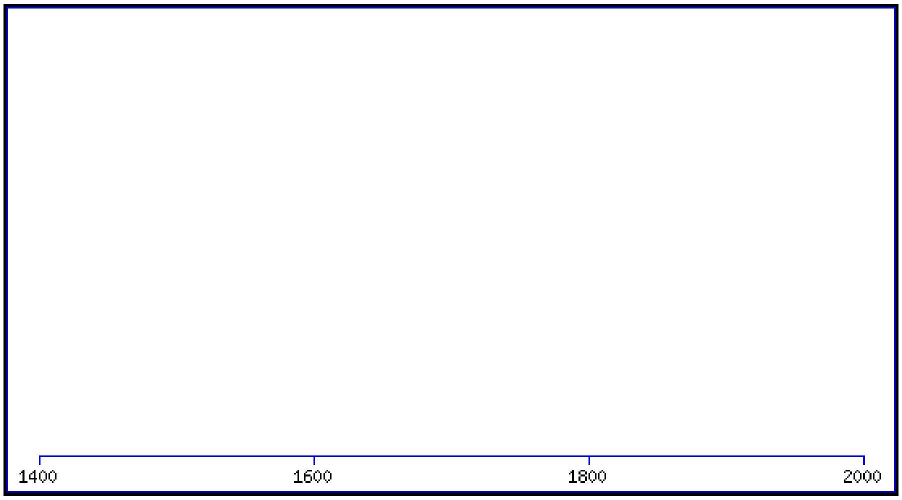


**Monoisotopic mass of neutral peptide Mr(calc):** 1121.5353
**Fixed modifications:** Carbamidomethyl (C)
**Variable modifications:**
**N2** : Deamidated_N (N)
**Ions Score:** 77 **Expect:** 2.3e-006
**Matches (Bold Red):** 14/82 fragment ions using 19 most intense peaks

| # | b | $b^{++}$ | b* | $b^{*++}$ | $b^{0}$ | $b^{0++}$ | Seq. | y | $y^{++}$ | y* | $y^{*++}$ | $y^{0}$ | $y^{0++}$ | # |
|---|---|---|---|---|---|---|---|---|---|---|---|---|---|---|
| 1 | 148.0757 | 74.5415 | | | | | F | | | | | | | 9 |
| 2 | **263.1026** | 132.0550 | 246.0761 | 123.5417 | | | N | **975.4742** | 488.2407 | 958.4476 | 479.7274 | 957.4636 | 479.2354 | 8 |
| 3 | **378.1296** | 189.5684 | 361.1030 | 181.0551 | 360.1190 | 180.5631 | D | **860.4472** | 430.7272 | 843.4207 | 422.2140 | 842.4367 | 421.7220 | 7 |
| 4 | 479.1772 | 240.0923 | 462.1507 | 231.5790 | 461.1667 | 231.0870 | T | **745.4203** | 373.2138 | 728.3937 | 364.7005 | 727.4097 | 364.2085 | 6 |
| 5 | **608.2198** | 304.6136 | 591.1933 | 296.1003 | 590.2093 | 295.6083 | E | **644.3726** | 322.6899 | 627.3461 | 314.1767 | 626.3620 | 313.6847 | 5 |
| 6 | **707.2883** | 354.1478 | 690.2617 | 345.6345 | 689.2777 | 345.1425 | V | **515.3300** | 258.1686 | 498.3035 | 249.6554 | | | 4 |
| 7 | **820.3723** | 410.6898 | 803.3458 | 402.1765 | 802.3618 | 401.6845 | L | **416.2616** | 208.6344 | 399.2350 | 200.1212 | | | 3 |
| 8 | **948.4309** | 474.7191 | 931.4043 | 466.2058 | 930.4203 | 465.7138 | Q | **303.1775** | 152.0924 | 286.1510 | 143.5791 | | | 2 |
| 9 | | | | | | | R | **175.1190** | 88.0631 | 158.0924 | 79.5498 | | | 1 |

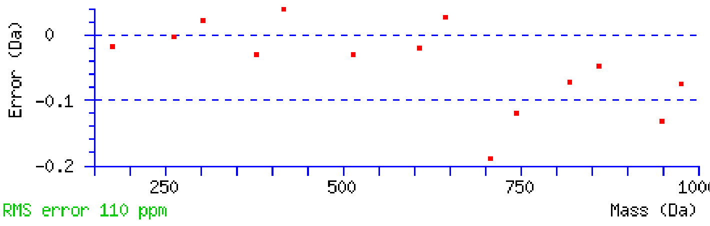


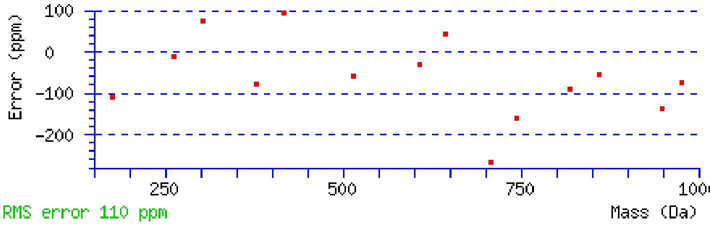


## All matches to this query

| Score | Mr(calc): | Delta | Sequence |
|---|---|---|---|
| 76.8 | 1121.5353 | -0.0012 | FNDTEVLQR |
| 21.4 | 1121.5386 | -0.0045 | EMTEAELKR |
| 20.9 | 1121.5386 | -0.0045 | MNDLLTNRK |
| 13.7 | 1121.5230 | 0.0111 | STTLSHLQR |
| 13.7 | 1121.5230 | 0.0111 | STTLSHLQR |
| 13.7 | 1121.5230 | 0.0111 | STTLSHLQR |
| 13.7 | 1121.5230 | 0.0111 | STTLSHLQR |
| 11.5 | 1121.5353 | -0.0012 | GELDEYIQR |
| 11.1 | 1120.5400 | 0.9941 | LFPGKDNSNK |
| 9.7 | 1121.5326 | 0.0015 | DTHVHRDSR |

---

Spectrum No: 275; Query: 2606; Rank: 1

---

## Peptide View

MS/MS Fragmentation of **GVDVSQVAWQSQGDTPCSCCIVNNSNGSR**
Found in **IPI00211989**, Tax_Id=10116 Gene_Symbol=Khk Isoform C of Ketohexokinase

Match to Query 2606: 3183.333162 from(1062.118330,3+)
Title: 100101RatKid_NS_deglyco_19.3211.3211.3.dta
Data file K:\NewmanPaper\Piliang\3SubProteomes\Piliang3SP\mgf5ppm\ERLIC_3SubProteomes5ppm.mgf

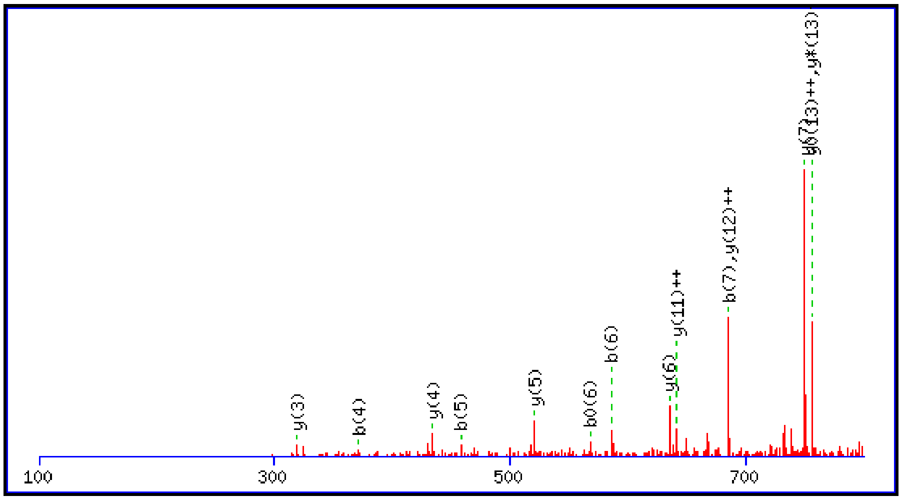


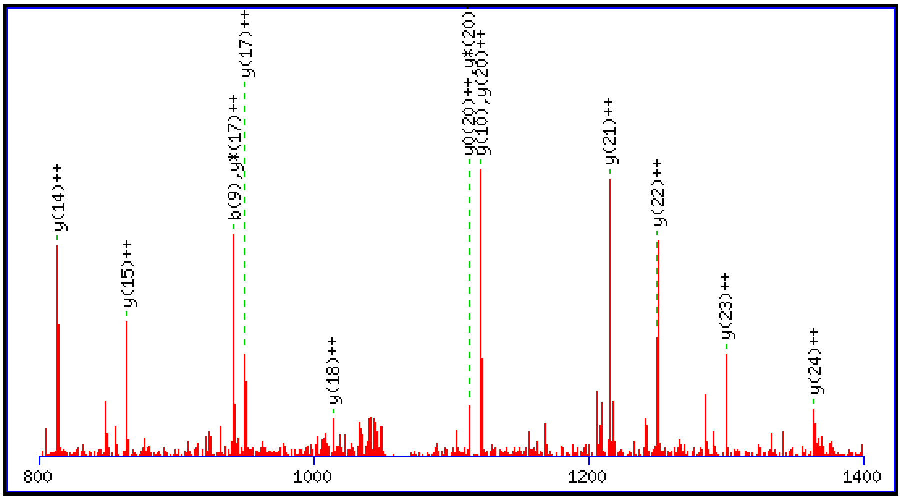

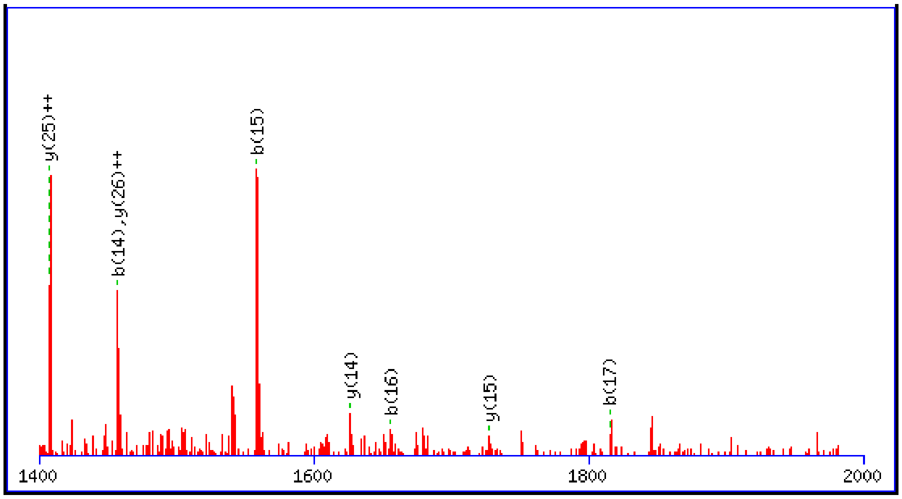


**Monoisotopic mass of neutral peptide Mr(calc):** 3183.3292
**Fixed modifications:** Carbamidomethyl (C)
**Variable modifications:**
**N23** : Deamidated_N (N)
**N26** : Deamidated_N (N)
**Ions Score:** 77 **Expect:** 8.8e-006
**Matches (Bold Red):** 37/320 fragment ions using 54 most intense peaks

| # | b | b$^{++}$ | b* | b*$^{++}$ | b$^{0}$ | b$^{0++}$ | Seq. | y | y$^{++}$ | y* | y*$^{++}$ | y$^{0}$ | y$^{0++}$ | # |
|---|---|---|---|---|---|---|---|---|---|---|---|---|---|---|
| 1 | 58.0287 | 29.5180 | | | | | G | | | | | | | 29 |
| 2 | 157.0972 | 79.0522 | | | | | V | 3127.3150 | 1564.1611 | 3110.2884 | 1555.6479 | 3109.3044 | 1555.1558 | 28 |
| 3 | 272.1241 | 136.5657 | | | 254.1135 | 127.5604 | D | 3028.2466 | 1514.6269 | 3011.2200 | 1506.1136 | 3010.2360 | 1505.6216 | 27 |
| 4 | **371.1925** | 186.0999 | | | 353.1819 | 177.0946 | V | 2913.2196 | **1457.1134** | 2896.1931 | 1448.6002 | 2895.2091 | 1448.1082 | 26 |
| 5 | **458.2245** | 229.6159 | | | 440.2140 | 220.6106 | S | 2814.1512 | **1407.5792** | 2797.1247 | 1399.0660 | 2796.1406 | 1398.5740 | 25 |
| 6 | **586.2831** | 293.6452 | 569.2566 | 285.1319 | **568.2726** | 284.6399 | Q | 2727.1192 | **1364.0632** | 2710.0926 | 1355.5500 | 2709.1086 | 1355.0579 | 24 |
| 7 | **685.3515** | 343.1794 | 668.3250 | 334.6661 | 667.3410 | 334.1741 | V | 2599.0606 | **1300.0339** | 2582.0340 | 1291.5207 | 2581.0500 | 1291.0287 | 23 |
| 8 | **756.3886** | 378.6980 | 739.3621 | 370.1847 | 738.3781 | 369.6927 | A | 2499.9922 | **1250.4997** | 2482.9656 | 1241.9865 | 2481.9816 | 1241.4944 | 22 |
| 9 | **942.4680** | 471.7376 | 925.4414 | 463.2243 | 924.4574 | 462.7323 | W | 2428.9551 | **1214.9812** | 2411.9285 | 1206.4679 | 2410.9445 | 1205.9759 | 21 |
| 10 | 1070.5265 | 535.7669 | 1053.5000 | 527.2536 | 1052.5160 | 526.7616 | Q | 2242.8758 | **1121.9415** | 2225.8492 | **1113.4282** | 2224.8652 | **1112.9362** | 20 |
| 11 | 1157.5586 | 579.2829 | 1140.5320 | 570.7696 | 1139.5480 | 570.2776 | S | 2114.8172 | 1057.9122 | 2097.7906 | 1049.3990 | 2096.8066 | 1048.9069 | 19 |
| 12 | 1285.6171 | 643.3122 | 1268.5906 | 634.7989 | 1267.6066 | 634.3069 | Q | 2027.7852 | **1014.3962** | 2010.7586 | 1005.8829 | 2009.7746 | 1005.3909 | 18 |
| 13 | 1342.6386 | 671.8229 | 1325.6121 | 663.3097 | 1324.6280 | 662.8177 | G | 1899.7266 | **950.3669** | 1882.7000 | **941.8537** | 1881.7160 | 941.3616 | 17 |
| 14 | **1457.6655** | 729.3364 | 1440.6390 | 720.8231 | 1439.6550 | 720.3311 | D | 1842.7051 | 921.8562 | 1825.6786 | 913.3429 | 1824.6945 | 912.8509 | 16 |
| 15 | **1558.7132** | 779.8603 | 1541.6867 | 771.3470 | 1540.7027 | 770.8550 | T | **1727.6782** | **864.3427** | 1710.6516 | 855.8294 | 1709.6676 | 855.3374 | 15 |
| 16 | **1655.7660** | 828.3866 | 1638.7394 | 819.8734 | 1637.7554 | 819.3814 | P | **1626.6305** | **813.8189** | 1609.6039 | 805.3056 | 1608.6199 | 804.8136 | 14 |
| 17 | **1815.7966** | 908.4020 | 1798.7701 | 899.8887 | 1797.7861 | 899.3967 | C | 1529.5777 | 765.2925 | 1512.5512 | **756.7792** | 1511.5672 | **756.2872** | 13 |
| 18 | 1902.8287 | 951.9180 | 1885.8021 | 943.4047 | 1884.8181 | 942.9127 | S | 1369.5471 | **685.2772** | 1352.5205 | 676.7639 | 1351.5365 | 676.2719 | 12 |
| 19 | 2062.8593 | 1031.9333 | 2045.8328 | 1023.4200 | 2044.8488 | 1022.9280 | C | 1282.5150 | **641.7612** | 1265.4885 | 633.2479 | 1264.5045 | 632.7559 | 11 |
| 20 | 2222.8900 | 1111.9486 | 2205.8634 | 1103.4353 | 2204.8794 | 1102.9433 | C | **1122.4844** | 561.7458 | 1105.4578 | 553.2326 | 1104.4738 | 552.7406 | 10 |
| 21 | 2335.9740 | 1168.4907 | 2318.9475 | 1159.9774 | 2317.9635 | 1159.4854 | I | 962.4537 | 481.7305 | 945.4272 | 473.2172 | 944.4432 | 472.7252 | 9 |
| 22 | 2435.0424 | 1218.0249 | 2418.0159 | 1209.5116 | 2417.0319 | 1209.0196 | V | 849.3697 | 425.1885 | 832.3431 | 416.6752 | 831.3591 | 416.1832 | 8 |
| 23 | 2550.0694 | 1275.5383 | 2533.0428 | 1267.0251 | 2532.0588 | 1266.5330 | N | **750.3013** | 375.6543 | 733.2747 | 367.1410 | 732.2907 | 366.6490 | 7 |
| 24 | 2664.1123 | 1332.5598 | 2647.0858 | 1324.0465 | 2646.1017 | 1323.5545 | N | **635.2743** | 318.1408 | 618.2478 | 309.6275 | 617.2638 | 309.1355 | 6 |
| 25 | 2751.1443 | 1376.0758 | 2734.1178 | 1367.5625 | 2733.1338 | 1367.0705 | S | **521.2314** | 261.1193 | 504.2049 | 252.6061 | 503.2208 | 252.1141 | 5 |
| 26 | 2866.1713 | 1433.5893 | 2849.1447 | 1425.0760 | 2848.1607 | 1424.5840 | N | **434.1994** | 217.6033 | 417.1728 | 209.0901 | 416.1888 | 208.5980 | 4 |
| 27 | 2923.1927 | 1462.1000 | 2906.1662 | 1453.5867 | 2905.1822 | 1453.0947 | G | **319.1724** | 160.0899 | 302.1459 | 151.5766 | 301.1619 | 151.0846 | 3 |
| 28 | 3010.2248 | 1505.6160 | 2993.1982 | 1497.1027 | 2992.2142 | 1496.6107 | S | 262.1510 | 131.5791 | 245.1244 | 123.0659 | 244.1404 | 122.5738 | 2 |
| 29 | | | | | | | R | 175.1190 | 88.0631 | 158.0924 | 79.5498 | | | 1 |

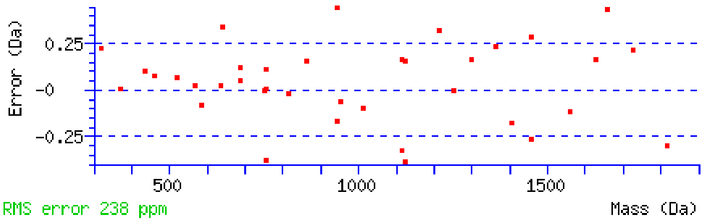


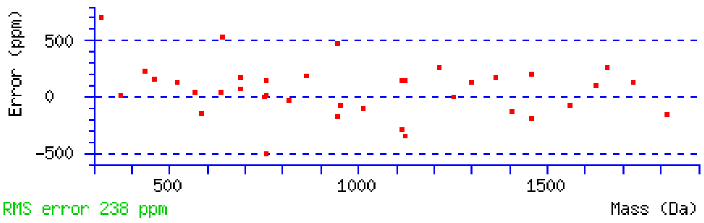


### All matches to this query

| Score | Mr(calc): | Delta | Sequence |
|---|---|---|---|
| 76.7 | 3183.3292 | 0.0040 | GVDVSQVAWQSQGDTPCSCCIVNNSNGSR |
| 68.5 | 3183.3292 | 0.0040 | GVDVSQVAWQSQGDTPCSCCIVNNSNGSR |
| 66.1 | 3183.3292 | 0.0040 | GVDVSQVAWQSQGDTPCSCCIVNNSNGSR |
| 32.4 | 3182.3452 | 0.9880 | GVDVSQVAWQSQGDTPCSCCIVNNSNGSR |
| 21.1 | 3182.3452 | 0.9880 | GVDVSQVAWQSQGDTPCSCCIVNNSNGSR |
| 20.1 | 3182.3452 | 0.9880 | GVDVSQVAWQSQGDTPCSCCIVNNSNGSR |
| 7.5 | 3181.3199 | 2.0133 | ESLSSYEHHVSAEMKSAASEEKSLEEK |
| 7.5 | 3181.3199 | 2.0133 | ESLSSYEHHVSAEMKSAASEEKSLEEK |
| 5.6 | 3183.3015 | 0.0317 | ESNVNYTICQCNHLTHFGVLMDLSR |
| 4.5 | 3183.3600 | -0.0269 | HFKGSLQDEHWTLNDHQIYNKQGAR |

---

Spectrum No: 276; Query: 960; Rank: 1

---

## Peptide View

MS/MS Fragmentation of **DSSGVINVMLNGSEPK**
Found in **IPI00204984**, Tax_Id=10116 Gene_Symbol=Bst1 ADP-ribosyl cyclase 2 precursor

Match to Query 960: 1646.789628 from(824.402090,2+)
Title: 100101RatKid_NS_deglyco_08.3781.3781.2.dta
Data file K:\NewmanPaper\Piliang\3SubProteomes\Piliang3SP\mgf5ppm\ERLIC_3SubProteomes5ppm.mgf

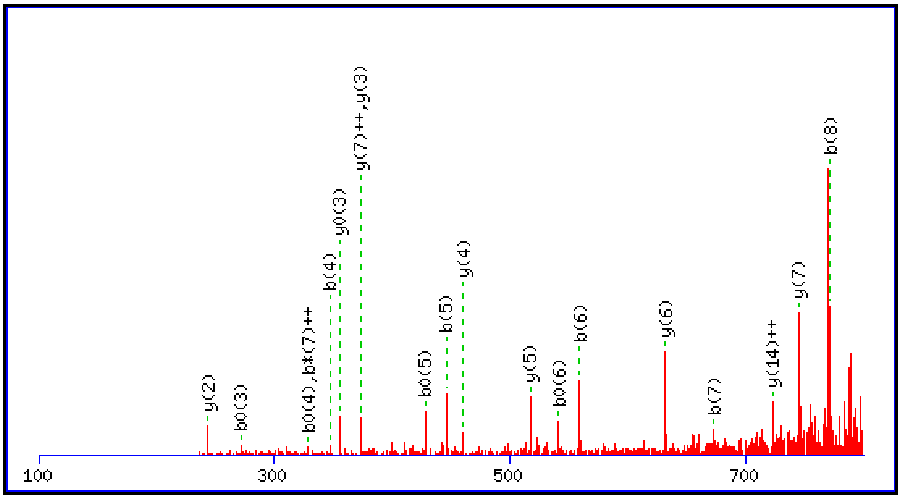

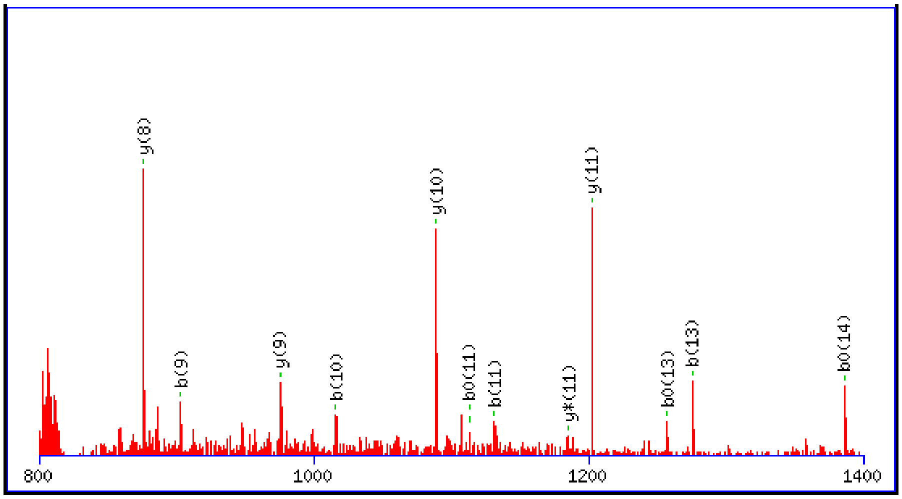


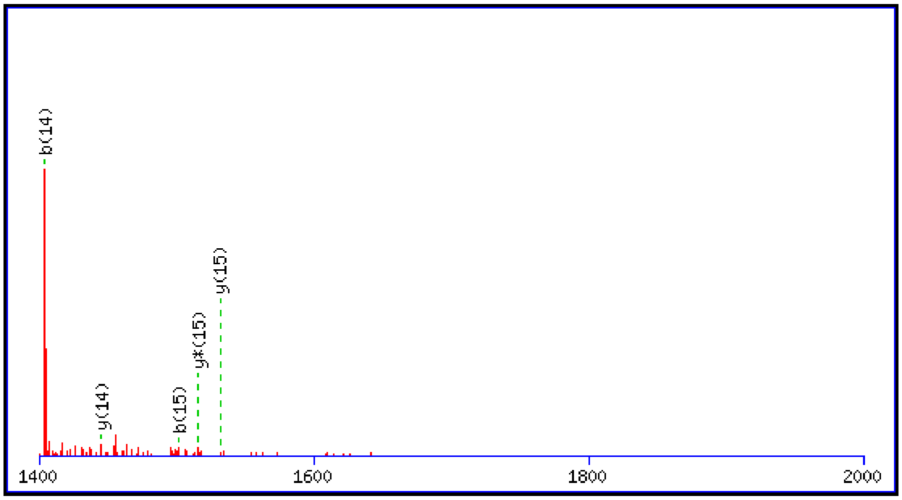


**Monoisotopic mass of neutral peptide Mr(calc):** 1646.7821
**Fixed modifications:** Carbamidomethyl (C)
**Variable modifications:**
**N11** : Deamidated_N (N)
**Ions Score:** 77 **Expect:** 3.9e-006
**Matches (Bold Red):** 36/164 fragment ions using 65 most intense peaks

| # | b | $b^{++}$ | b* | $b^{*++}$ | $b^0$ | $b^{0++}$ | Seq. | y | $y^{++}$ | y* | $y^{*++}$ | $y^0$ | $y^{0++}$ | # |
|---|---|---|---|---|---|---|---|---|---|---|---|---|---|---|
| 1 | 116.0342 | 58.5207 | | | 98.0237 | 49.5155 | D | | | | | | | 16 |
| 2 | 203.0662 | 102.0368 | | | 185.0557 | 93.0315 | S | **1532.7625** | 766.8849 | **1515.7359** | 758.3716 | 1514.7519 | 757.8796 | 15 |
| 3 | 290.0983 | 145.5528 | | | **272.0877** | 136.5475 | S | **1445.7304** | **723.3689** | 1428.7039 | 714.8556 | 1427.7199 | 714.3636 | 14 |
| 4 | **347.1197** | 174.0635 | | | **329.1092** | 165.0582 | G | 1358.6984 | 679.8528 | 1341.6719 | 671.3396 | 1340.6879 | 670.8476 | 13 |
| 5 | **446.1882** | 223.5977 | | | **428.1776** | 214.5924 | V | 1301.6770 | 651.3421 | 1284.6504 | 642.8288 | 1283.6664 | 642.3368 | 12 |
| 6 | **559.2722** | 280.1397 | | | **541.2617** | 271.1345 | I | **1202.6085** | 601.8079 | **1185.5820** | 593.2946 | 1184.5980 | 592.8026 | 11 |
| 7 | **673.3151** | 337.1612 | 656.2886 | **328.6479** | 655.3046 | 328.1559 | N | **1089.5245** | 545.2659 | 1072.4979 | 536.7526 | 1071.5139 | 536.2606 | 10 |
| 8 | **772.3836** | 386.6954 | 755.3570 | 378.1821 | 754.3730 | 377.6901 | V | **975.4816** | 488.2444 | 958.4550 | 479.7311 | 957.4710 | 479.2391 | 9 |
| 9 | **903.4240** | 452.2157 | 886.3975 | 443.7024 | 885.4135 | 443.2104 | M | **876.4131** | 438.7102 | 859.3866 | 430.1969 | 858.4026 | 429.7049 | 8 |
| 10 | **1016.5081** | 508.7577 | 999.4816 | 500.2444 | 998.4975 | 499.7524 | L | **745.3727** | **373.1900** | 728.3461 | 364.6767 | 727.3621 | 364.1847 | 7 |
| 11 | **1131.5350** | 566.2712 | 1114.5085 | 557.7579 | **1113.5245** | 557.2659 | N | **632.2886** | 316.6479 | 615.2620 | 308.1347 | 614.2780 | 307.6427 | 6 |
| 12 | 1188.5565 | 594.7819 | 1171.5300 | 586.2686 | 1170.5459 | 585.7766 | G | **517.2617** | 259.1345 | 500.2351 | 250.6212 | 499.2511 | 250.1292 | 5 |
| 13 | **1275.5885** | 638.2979 | 1258.5620 | 629.7846 | **1257.5780** | 629.2926 | S | **460.2402** | 230.6237 | 443.2136 | 222.1105 | 442.2296 | 221.6185 | 4 |
| 14 | **1404.6311** | 702.8192 | 1387.6046 | 694.3059 | **1386.6206** | 693.8139 | E | **373.2082** | 187.1077 | 356.1816 | 178.5944 | **355.1976** | 178.1024 | 3 |
| 15 | **1501.6839** | 751.3456 | 1484.6573 | 742.8323 | 1483.6733 | 742.3403 | P | **244.1656** | 122.5864 | 227.1390 | 114.0731 | | | 2 |
| 16 | | | | | | | K | 147.1128 | 74.0600 | 130.0863 | 65.5468 | | | 1 |

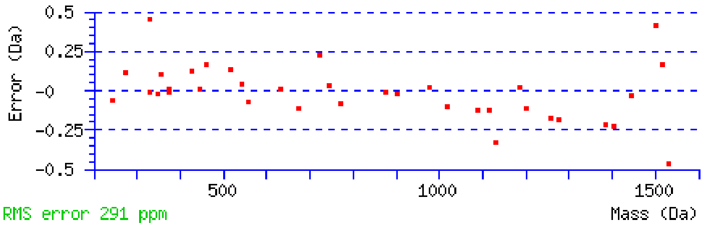


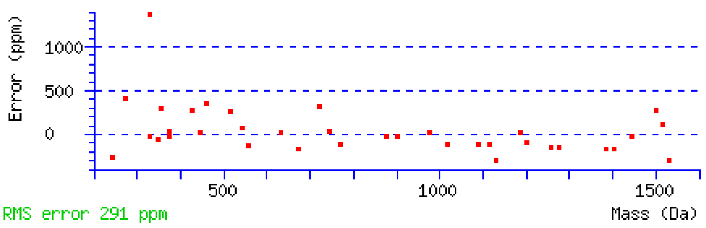


### All matches to this query

| Score | Mr(calc): | Delta | Sequence |
|---|---|---|---|
| 76.7 | 1646.7821 | 0.0075 | DSSGVINVMLNGSEPK |
| 35.2 | 1646.7821 | 0.0075 | DSSGVINVMLNGSEPK |
| 23.4 | 1645.7981 | 0.9915 | DSSGVINVMLNGSEPK |
| 8.5 | 1646.7748 | 0.0148 | STPSSGDVQVTEDAVR |
| 3.9 | 1645.7921 | 0.9975 | RDGVRSGFEEGGAGPR |
| 2.3 | 1646.8004 | -0.0107 | KATGFQRPYKMPK |

---

Spectrum No: 277; Query: 254; Rank: 1

---

## Peptide View

MS/MS Fragmentation of **IEVEANNTVTK**
Found in **IPI00200640**, Tax_Id=10116 Gene_Symbol=Mucdhl Isoform 1 of Mucin and cadherin-like protein precursor

Match to Query 254: 1217.610948 from(609.812750,2+)
Title: 091008RatKidney_NoSalt_31.610.610.2.dta
Data file K:\NewmanPaper\Piliang\3SubProteomes\Piliang3SP\mgf5ppm\ERLIC_3SubProteomes5ppm.mgf

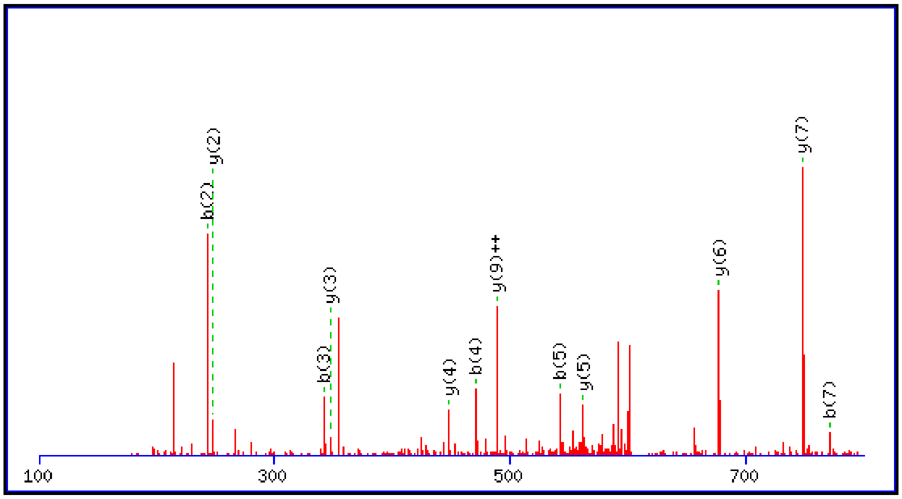

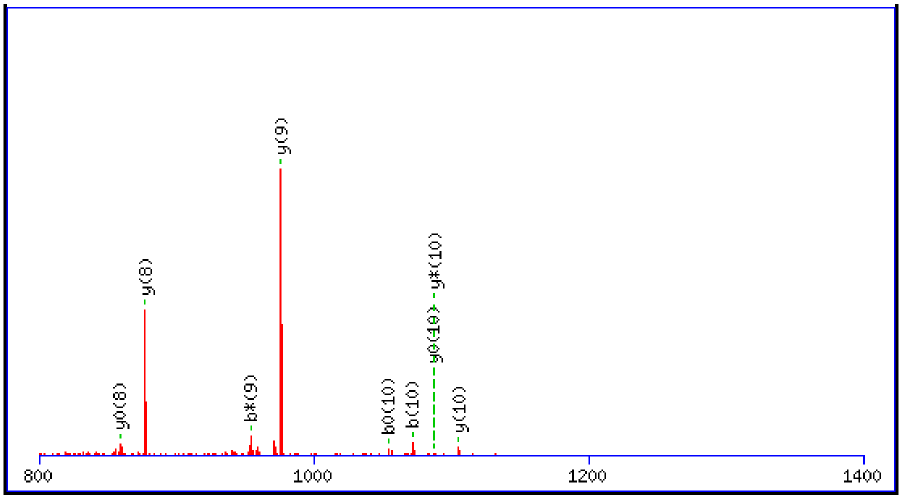


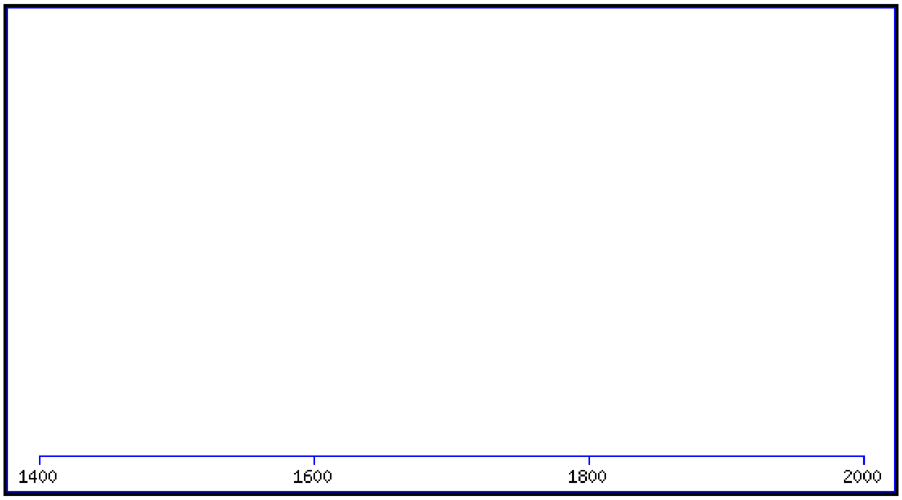


**Monoisotopic mass of neutral peptide Mr(calc):** 1217.6139
**Fixed modifications:** Carbamidomethyl (C)
**Variable modifications:**
**N6** : Deamidated_N (N)
**Ions Score:** 77 **Expect:** 2.9e-006
**Matches (Bold Red):** 21/106 fragment ions using 32 most intense peaks

| # | b | $b^{++}$ | b* | $b^{*++}$ | $b^{0}$ | $b^{0++}$ | Seq. | y | $y^{++}$ | y* | $y^{*++}$ | $y^{0}$ | $y^{0++}$ | # |
|---|---|---|---|---|---|---|---|---|---|---|---|---|---|---|
| 1 | 114.0913 | 57.5493 | | | | | I | | | | | | | 11 |
| 2 | **243.1339** | 122.0706 | | | 225.1234 | 113.0653 | E | **1105.5372** | 553.2722 | **1088.5106** | 544.7589 | **1087.5266** | 544.2669 | 10 |
| 3 | **342.2023** | 171.6048 | | | 324.1918 | 162.5995 | V | **976.4946** | **488.7509** | 959.4680 | 480.2376 | 958.4840 | 479.7456 | 9 |
| 4 | **471.2449** | 236.1261 | | | 453.2344 | 227.1208 | E | **877.4261** | 439.2167 | 860.3996 | 430.7034 | **859.4156** | 430.2114 | 8 |
| 5 | **542.2821** | 271.6447 | | | 524.2715 | 262.6394 | A | **748.3836** | 374.6954 | 731.3570 | 366.1821 | 730.3730 | 365.6901 | 7 |
| 6 | 657.3090 | 329.1581 | 640.2824 | 320.6449 | 639.2984 | 320.1529 | N | **677.3464** | 339.1769 | 660.3199 | 330.6636 | 659.3359 | 330.1716 | 6 |
| 7 | **771.3519** | 386.1796 | 754.3254 | 377.6663 | 753.3414 | 377.1743 | N | **562.3195** | 281.6634 | 545.2930 | 273.1501 | 544.3089 | 272.6581 | 5 |
| 8 | 872.3996 | 436.7034 | 855.3730 | 428.1902 | 854.3890 | 427.6982 | T | **448.2766** | 224.6419 | 431.2500 | 216.1287 | 430.2660 | 215.6366 | 4 |
| 9 | 971.4680 | 486.2376 | **954.4415** | 477.7244 | 953.4574 | 477.2324 | V | **347.2289** | 174.1181 | 330.2023 | 165.6048 | 329.2183 | 165.1128 | 3 |
| 10 | **1072.5157** | 536.7615 | 1055.4891 | 528.2482 | **1054.5051** | 527.7562 | T | **248.1605** | 124.5839 | 231.1339 | 116.0706 | 230.1499 | 115.5786 | 2 |
| 11 | | | | | | | K | 147.1128 | 74.0600 | 130.0863 | 65.5468 | | | 1 |

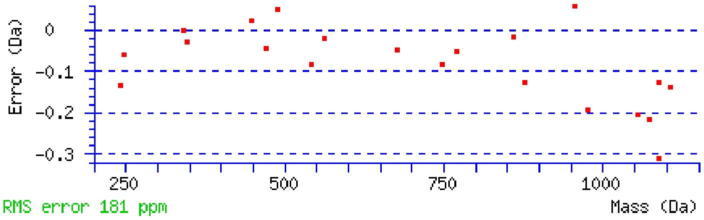


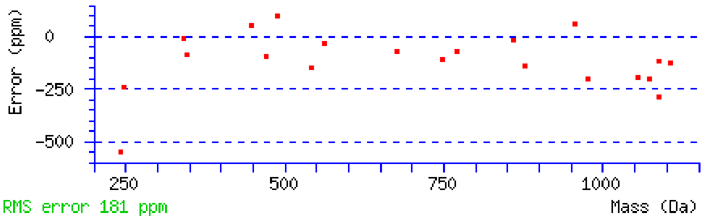

### All matches to this query

| Score | Mr(calc): | Delta | Sequence |
|---|---|---|---|
| 76.6 | 1217.6139 | -0.0030 | IEVEANNTVTK |
| 61.6 | 1217.6139 | -0.0030 | IEVEANNTVTK |
| 18.4 | 1217.6179 | -0.0070 | LEDLNFPEIK |
| 17.4 | 1217.6169 | -0.0060 | NKLDHSAIIK |
| 12.3 | 1217.6193 | -0.0083 | IEDFWGPAKR |
| 9.3 | 1217.6139 | -0.0030 | IDEIASNLQSK |
| 7.8 | 1217.6097 | 0.0012 | DILFYQVIK |
| 7.5 | 1217.6000 | 0.0109 | XNVQTSESGLR |
| 7.1 | 1217.6000 | 0.0109 | LEQTGETERR |
| 5.9 | 1217.6114 | -0.0005 | LEFPSLPQCK |

---

Spectrum No: 278; Query: 927; Rank: 1

---

## Peptide View

MS/MS Fragmentation of **GANLTEDQIFFFPK**
Found in **IPI00373395**, Tax_Id=10116 Gene_Symbol=C8g_predicted 22 kDa protein

Match to Query 927: 1626.793448 from(814.404000,2+)
Title: 091008RatKidney_NoSalt_28.3885.3885.2.dta
Data file K:\NewmanPaper\Piliang\3SubProteomes\Piliang3SP\mgf5ppm\ERLIC_3SubProteomes5ppm.mgf

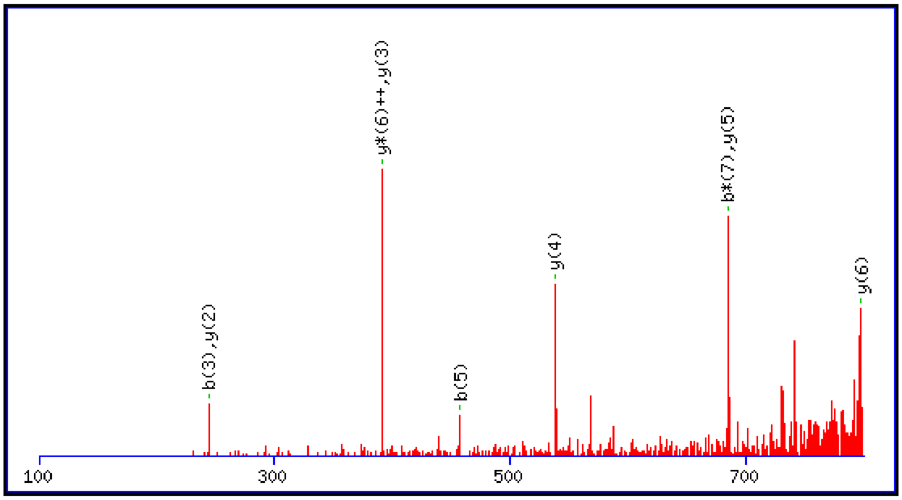


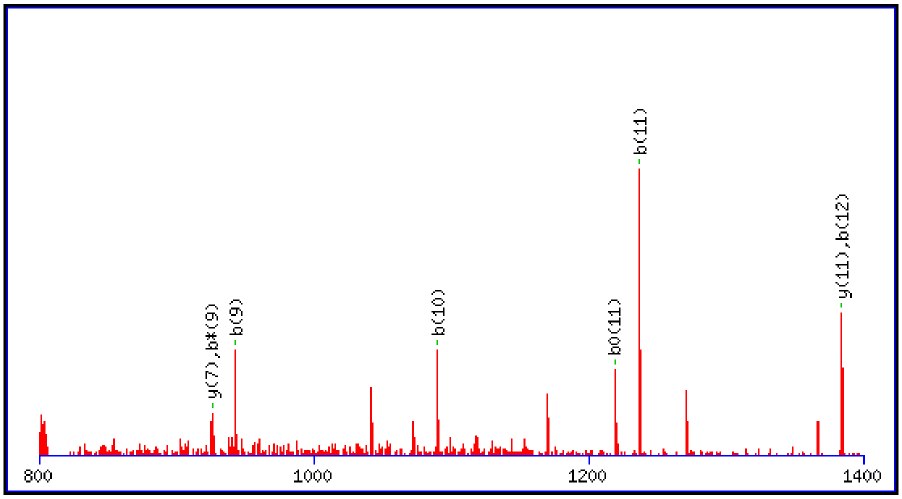

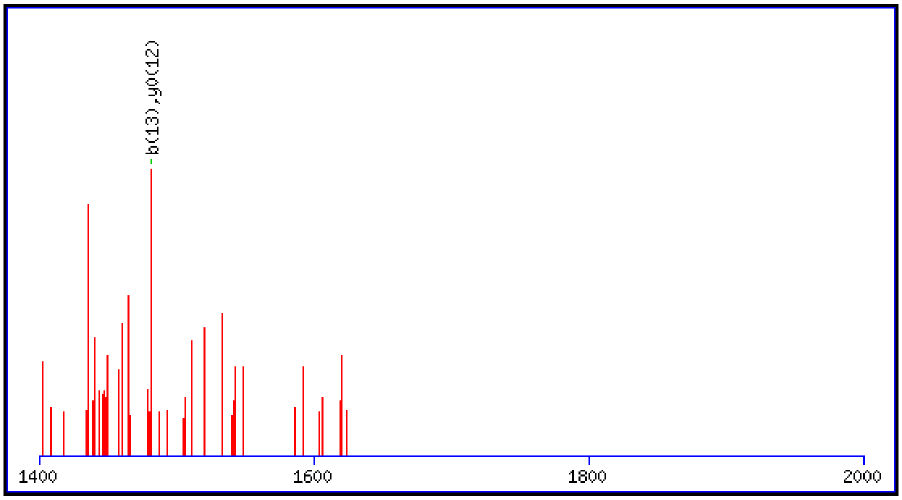


**Monoisotopic mass of neutral peptide Mr(calc):** 1626.7930
**Fixed modifications:** Carbamidomethyl (C)
**Variable modifications:**
**N3** : Deamidated_N (N)
**Ions Score:** 77 **Expect:** 4e-006
**Matches (Bold Red):** 19/130 fragment ions using 15 most intense peaks

| # | b | $b^{++}$ | b* | $b^{*++}$ | $b^{0}$ | $b^{0++}$ | Seq. | y | $y^{++}$ | y* | $y^{*++}$ | $y^{0}$ | $y^{0++}$ | # |
|---|---|---|---|---|---|---|---|---|---|---|---|---|---|---|
| 1 | 58.0287 | 29.5180 | | | | | G | | | | | | | 14 |
| 2 | 129.0659 | 65.0366 | | | | | A | 1570.7788 | 785.8930 | 1553.7522 | 777.3798 | 1552.7682 | 776.8877 | 13 |
| 3 | **244.0928** | 122.5500 | 227.0662 | 114.0368 | | | N | 1499.7417 | 750.3745 | 1482.7151 | 741.8612 | **1481.7311** | 741.3692 | 12 |
| 4 | 357.1769 | 179.0921 | 340.1503 | 170.5788 | | | L | **1384.7147** | 692.8610 | 1367.6882 | 684.3477 | 1366.7042 | 683.8557 | 11 |
| 5 | **458.2245** | 229.6159 | 441.1980 | 221.1026 | 440.2140 | 220.6106 | T | 1271.6307 | 636.3190 | 1254.6041 | 627.8057 | 1253.6201 | 627.3137 | 10 |
| 6 | 587.2671 | 294.1372 | 570.2406 | 285.6239 | 569.2566 | 285.1319 | E | 1170.5830 | 585.7951 | 1153.5564 | 577.2819 | 1152.5724 | 576.7898 | 9 |
| 7 | 702.2941 | 351.6507 | **685.2675** | 343.1374 | 684.2835 | 342.6454 | D | 1041.5404 | 521.2738 | 1024.5138 | 512.7606 | 1023.5298 | 512.2686 | 8 |
| 8 | 830.3526 | 415.6800 | 813.3261 | 407.1667 | 812.3421 | 406.6747 | Q | **926.5135** | 463.7604 | 909.4869 | 455.2471 | | | 7 |
| 9 | **943.4367** | 472.2220 | **926.4102** | 463.7087 | 925.4261 | 463.2167 | I | **798.4549** | 399.7311 | 781.4283 | **391.2178** | | | 6 |
| 10 | **1090.5051** | 545.7562 | 1073.4786 | 537.2429 | 1072.4946 | 536.7509 | F | **685.3708** | 343.1890 | 668.3443 | 334.6758 | | | 5 |
| 11 | **1237.5735** | 619.2904 | 1220.5470 | 610.7771 | **1219.5630** | 610.2851 | F | **538.3024** | 269.6548 | 521.2758 | 261.1416 | | | 4 |
| 12 | **1384.6420** | 692.8246 | 1367.6154 | 684.3113 | 1366.6314 | 683.8193 | F | **391.2340** | 196.1206 | 374.2074 | 187.6074 | | | 3 |
| 13 | **1481.6947** | 741.3510 | 1464.6682 | 732.8377 | 1463.6842 | 732.3457 | P | **244.1656** | 122.5864 | 227.1390 | 114.0731 | | | 2 |
| 14 | | | | | | | K | 147.1128 | 74.0600 | 130.0863 | 65.5468 | | | 1 |

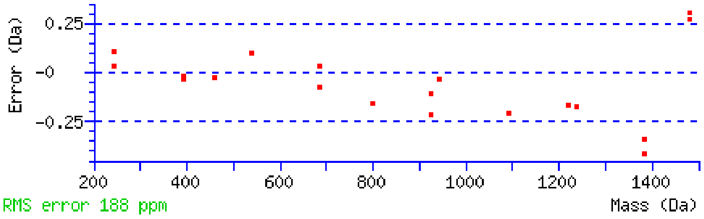


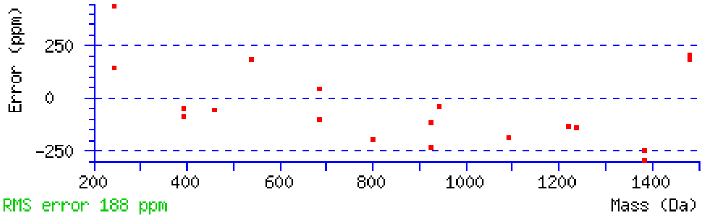


## All matches to this query

| Score | Mr(calc): | Delta | Sequence |
|---|---|---|---|
| 76.5 | 1626.7930 | 0.0005 | GANLTEDQIFFFPK |
| 12.4 | 1626.8018 | -0.0084 | LVVQDKNKYNTPK |
| 12.1 | 1626.8018 | -0.0084 | YLTGLGSSGLGIGPQK |
| 11.5 | 1626.8018 | -0.0084 | LVVQDKNKYNTPK |
| 11.4 | 1626.7930 | 0.0005 | QDPYLVNFFLENK |
| 9.0 | 1626.8018 | -0.0083 | YEILAQKNLQEAK |
| 5.8 | 1626.7807 | 0.0128 | HPAPPPSNYEVLVK |
| | | | |

| | | | |
|---|---|---|---|
| 5.7 | 1625.7814 | 1.0121 | IAGSLIENYPGERK |
| 5.2 | 1626.7841 | 0.0094 | LYTLLNTCIPPSR |
| 4.6 | 1626.7906 | 0.0029 | IGSTVEDNPLYVLK |

---

Spectrum No: 279; Query: 610; Rank: 1

---

## Peptide View

MS/MS Fragmentation of **VWVNSSDGNALIGK**
Found in **IPI00193894**, Tax_Id=10116 Gene_Symbol=Mgam_predicted 198 kDa protein

Match to Query 610: 1459.731028 from(730.872790,2+)
Title: 091008RatKidney_NH4Format01_23.2168.2168.2.dta
Data file K:\NewmanPaper\Piliang\3SubProteomes\Piliang3SP\mgf5ppm\ERLIC_3SubProteomes5ppm.mgf

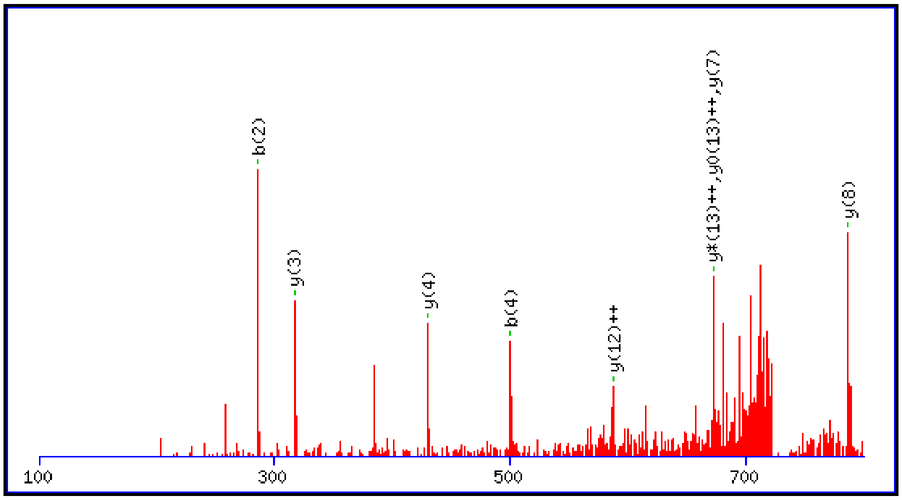


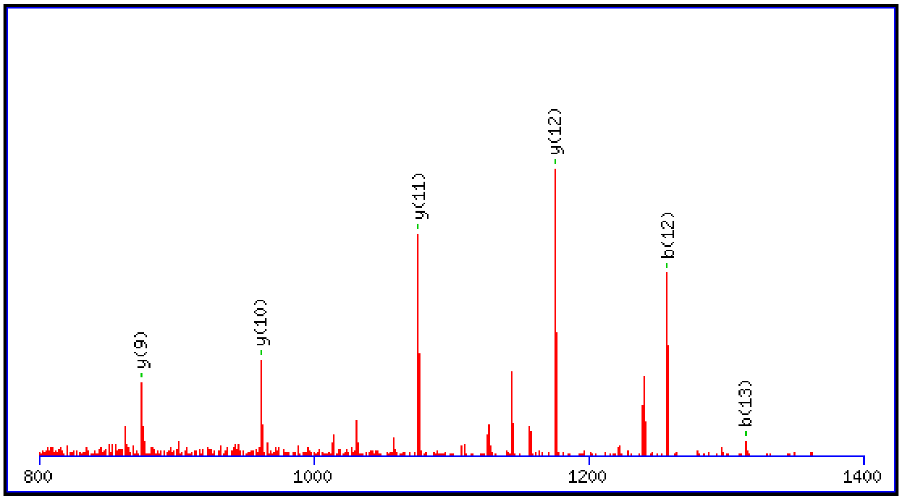

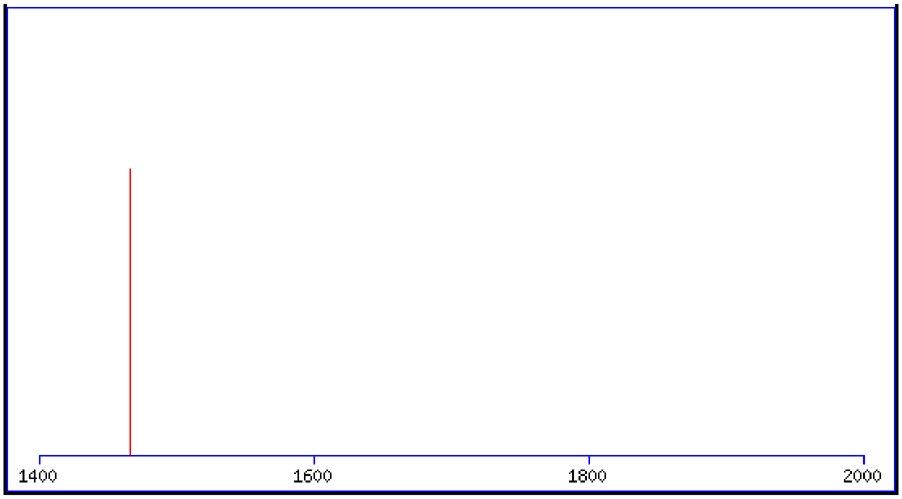


**Monoisotopic mass of neutral peptide Mr(calc):** 1459.7307
**Fixed modifications:** Carbamidomethyl (C)
**Variable modifications:**
**N4** : Deamidated_N (N)
**Ions Score:** 76 **Expect:** 3.3e-006
**Matches (Bold Red):** 15/128 fragment ions using 15 most intense peaks

| # | b | b++ | b* | b*++ | b0 | b0++ | Seq. | y | y++ | y* | y*++ | y0 | y0++ | # |
|---|---|---|---|---|---|---|---|---|---|---|---|---|---|---|
| 1 | 100.0757 | 50.5415 | | | | | V | | | | | | | 14 |
| 2 | **286.1550** | 143.5811 | | | | | W | 1361.6696 | 681.3384 | 1344.6430 | **672.8251** | 1343.6590 | **672.3331** | 13 |
| 3 | 385.2234 | 193.1153 | | | | | V | **1175.5902** | **588.2988** | 1158.5637 | 579.7855 | 1157.5797 | 579.2935 | 12 |
| 4 | **500.2504** | 250.6288 | 483.2238 | 242.1155 | | | N | **1076.5218** | 538.7646 | 1059.4953 | 530.2513 | 1058.5113 | 529.7593 | 11 |
| 5 | 587.2824 | 294.1448 | 570.2558 | 285.6316 | 569.2718 | 285.1395 | S | **961.4949** | 481.2511 | 944.4684 | 472.7378 | 943.4843 | 472.2458 | 10 |
| 6 | 674.3144 | 337.6608 | 657.2879 | 329.1476 | 656.3038 | 328.6556 | S | **874.4629** | 437.7351 | 857.4363 | 429.2218 | 856.4523 | 428.7298 | 9 |
| 7 | 789.3414 | 395.1743 | 772.3148 | 386.6610 | 771.3308 | 386.1690 | D | **787.4308** | 394.2191 | 770.4043 | 385.7058 | 769.4203 | 385.2138 | 8 |
| 8 | 846.3628 | 423.6850 | 829.3363 | 415.1718 | 828.3523 | 414.6798 | G | **672.4039** | 336.7056 | 655.3774 | 328.1923 | | | 7 |
| 9 | 960.4057 | 480.7065 | 943.3792 | 472.1932 | 942.3952 | 471.7012 | N | 615.3824 | 308.1949 | 598.3559 | 299.6816 | | | 6 |
| 10 | 1031.4429 | 516.2251 | 1014.4163 | 507.7118 | 1013.4323 | 507.2198 | A | 501.3395 | 251.1734 | 484.3130 | 242.6601 | | | 5 |
| 11 | 1144.5269 | 572.7671 | 1127.5004 | 564.2538 | 1126.5164 | 563.7618 | L | **430.3024** | 215.6548 | 413.2758 | 207.1416 | | | 4 |
| 12 | **1257.6110** | 629.3091 | 1240.5844 | 620.7959 | 1239.6004 | 620.3038 | I | **317.2183** | 159.1128 | 300.1918 | 150.5995 | | | 3 |
| 13 | **1314.6324** | 657.8199 | 1297.6059 | 649.3066 | 1296.6219 | 648.8146 | G | 204.1343 | 102.5708 | 187.1077 | 94.0575 | | | 2 |
| 14 | | | | | | | K | 147.1128 | 74.0600 | 130.0863 | 65.5468 | | | 1 |

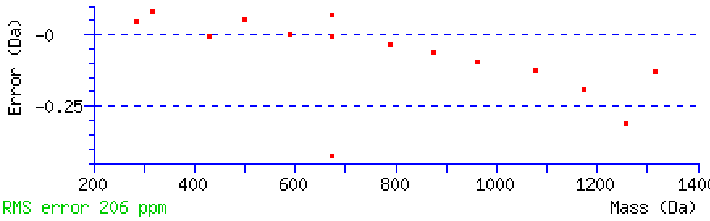


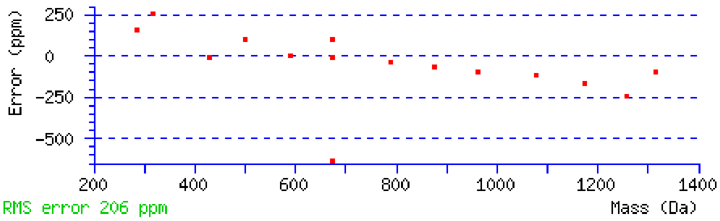


### All matches to this query

| Score | Mr(calc): | Delta | Sequence |
|---|---|---|---|
| 76.4 | 1459.7307 | 0.0003 | VWVNSSDGNALIGK |
| 57.3 | 1459.7307 | 0.0003 | VWVNSSDGNALIGK |
| 11.4 | 1459.7201 | 0.0110 | IASLSRSVVALGK |
| 4.9 | 1459.7225 | 0.0086 | AITAFTAGFVNIR |
| 4.4 | 1459.7200 | 0.0110 | KNLAKSQTLLGK |
| 3.6 | 1457.7110 | 2.0200 | LSTVPSQDAGAQER |
| 3.4 | 1459.7184 | 0.0126 | ANNLSSLSKKYR |
| | | | |

| | | | |
|---|---|---|---|
| 2.1 | 1459.7201 | 0.0110 | IASLSRSVVALGK |
| 1.6 | 1459.7224 | 0.0086 | NGLWGHALLLASK |
| 1.2 | 1459.7225 | 0.0086 | KRDSFLPFSVGK |

Spectrum No: 280; Query: 989; Rank: 1

## Peptide View

MS/MS Fragmentation of **VGVEAFFPNGTQFSR**
Found in **IPI00204808**, Tax_Id=10116 Gene_Symbol=Mep1b Meprin A subunit beta precursor

Match to Query 989: 1655.793008 from(828.903780,2+)
Title: 091008RatKidney_NH4Format01_24.3507.3507.2.dta
Data file K:\NewmanPaper\Piliang\3SubProteomes\Piliang3SP\mgf5ppm\ERLIC_3SubProteomes5ppm.mgf

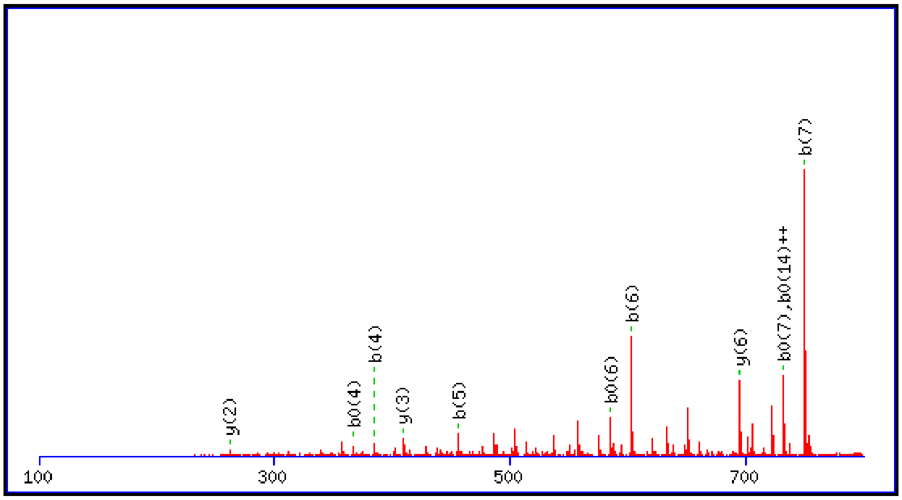


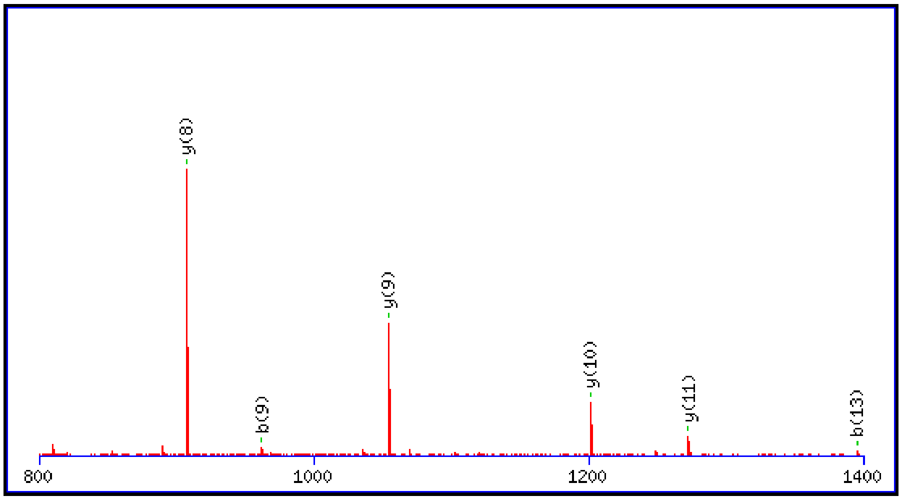

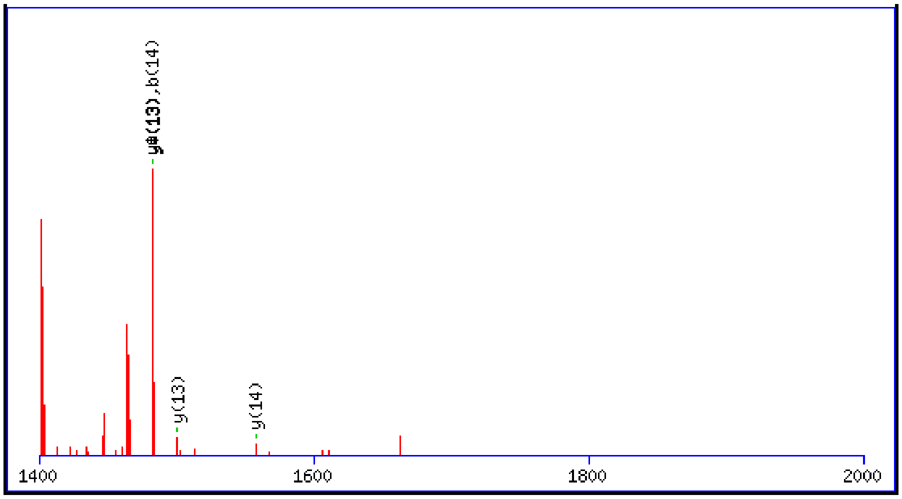


**Monoisotopic mass of neutral peptide Mr(calc):** 1655.7944
**Fixed modifications:** Carbamidomethyl (C)
**Variable modifications:**
**N9** : Deamidated_N (N)
**Ions Score:** 76 **Expect:** 4.2e-006
**Matches (Bold Red):** 22/144 fragment ions using 32 most intense peaks

| # | b | b$^{++}$ | b* | b*$^{++}$ | b$^{0}$ | b$^{0++}$ | Seq. | y | y$^{++}$ | y* | y*$^{++}$ | y$^{0}$ | y$^{0++}$ | # |
|---|---|---|---|---|---|---|---|---|---|---|---|---|---|---|
| 1 | 100.0757 | 50.5415 | | | | | V | | | | | | | 15 |
| 2 | 157.0972 | 79.0522 | | | | | G | **1557.7332** | 779.3703 | 1540.7067 | 770.8570 | 1539.7227 | 770.3650 | 14 |
| 3 | 256.1656 | 128.5864 | | | | | V | **1500.7118** | 750.8595 | **1483.6852** | 742.3462 | **1482.7012** | 741.8542 | 13 |
| 4 | **385.2082** | 193.1077 | | | **367.1976** | 184.1024 | E | 1401.6434 | 701.3253 | 1384.6168 | 692.8120 | 1383.6328 | 692.3200 | 12 |
| 5 | **456.2453** | 228.6263 | | | 438.2347 | 219.6210 | A | **1272.6008** | 636.8040 | 1255.5742 | 628.2907 | 1254.5902 | 627.7987 | 11 |
| 6 | **603.3137** | 302.1605 | | | **585.3031** | 293.1552 | F | **1201.5636** | 601.2855 | 1184.5371 | 592.7722 | 1183.5531 | 592.2802 | 10 |
| 7 | **750.3821** | 375.6947 | | | **732.3715** | 366.6894 | F | **1054.4952** | 527.7513 | 1037.4687 | 519.2380 | 1036.4847 | 518.7460 | 9 |
| 8 | 847.4349 | 424.2211 | | | 829.4243 | 415.2158 | P | **907.4268** | 454.2170 | 890.4003 | 445.7038 | 889.4163 | 445.2118 | 8 |
| 9 | **962.4618** | 481.7345 | 945.4353 | 473.2213 | 944.4512 | 472.7293 | N | 810.3741 | 405.6907 | 793.3475 | 397.1774 | 792.3635 | 396.6854 | 7 |
| 10 | 1019.4833 | 510.2453 | 1002.4567 | 501.7320 | 1001.4727 | 501.2400 | G | **695.3471** | 348.1772 | 678.3206 | 339.6639 | 677.3366 | 339.1719 | 6 |
| 11 | 1120.5309 | 560.7691 | 1103.5044 | 552.2558 | 1102.5204 | 551.7638 | T | 638.3257 | 319.6665 | 621.2991 | 311.1532 | 620.3151 | 310.6612 | 5 |
| 12 | 1248.5895 | 624.7984 | 1231.5630 | 616.2851 | 1230.5790 | 615.7931 | Q | 537.2780 | 269.1426 | 520.2514 | 260.6293 | 519.2674 | 260.1373 | 4 |
| 13 | **1395.6579** | 698.3326 | 1378.6314 | 689.8193 | 1377.6474 | 689.3273 | F | **409.2194** | 205.1133 | 392.1928 | 196.6001 | 391.2088 | 196.1081 | 3 |
| 14 | **1482.6900** | 741.8486 | 1465.6634 | 733.3353 | 1464.6794 | **732.8433** | S | **262.1510** | 131.5791 | 245.1244 | 123.0659 | 244.1404 | 122.5738 | 2 |
| 15 | | | | | | | R | 175.1190 | 88.0631 | 158.0924 | 79.5498 | | | 1 |

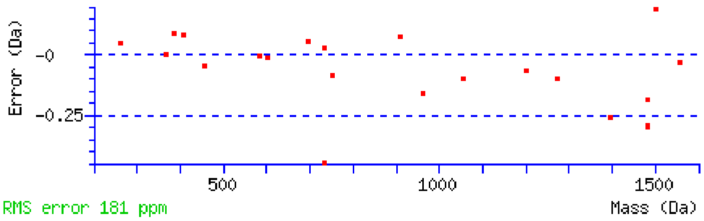


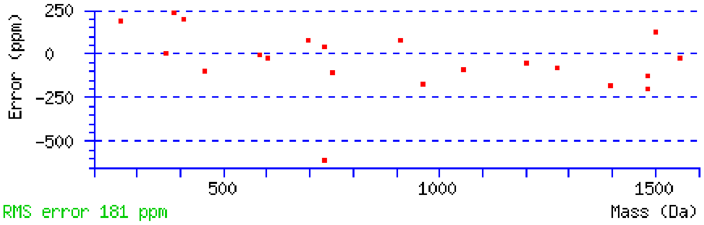


## All matches to this query

| Score | Mr(calc): | Delta | Sequence |
|---|---|---|---|
| 76.4 | 1655.7944 | -0.0014 | VGVEAFFPNGTQFSR |
| 12.3 | 1654.8001 | 0.9929 | RDTMLGVSIVLENK |
| 7.4 | 1654.8001 | 0.9929 | RDTMLGVSIVLENK |
| 6.8 | 1654.7941 | 0.9990 | ATSPFRSPVRGASSR |
| 5.9 | 1654.8039 | 0.9891 | ATLAEVATVSSGSARR |
| 5.5 | 1654.8039 | 0.9891 | ATLAEVATVSSGSARR |
| | | | |

| | | | |
|---|---|---|---|
| 5.5 | 1655.7821 | 0.0109 | SSFASLFSFRRSGK |
| 5.5 | 1655.7821 | 0.0109 | SSFASLFSFRRSGK |
| 4.0 | 1655.7976 | -0.0046 | ATIAAGTSQAGQGSGHSR |
| 2.8 | 1655.8011 | -0.0081 | ANIMRMSDGLFLQK |

---

Spectrum No: 281; Query: 2729; Rank: 1

---

## Peptide View

MS/MS Fragmentation of **GFDTYFGYLLGSEDYYTHEACAPIECLNGTR**
Found in **IPI00198405**, Tax_Id=10116 Gene_Symbol=Arsb Arylsulfatase B

Match to Query 2729: 3620.566542 from(1207.862790,3+)
Title: 100101RatKid_NS_deglyco_09.7047.7047.3.dta
Data file K:\NewmanPaper\Piliang\3SubProteomes\Piliang3SP\mgf5ppm\ERLIC_3SubProteomes5ppm.mgf

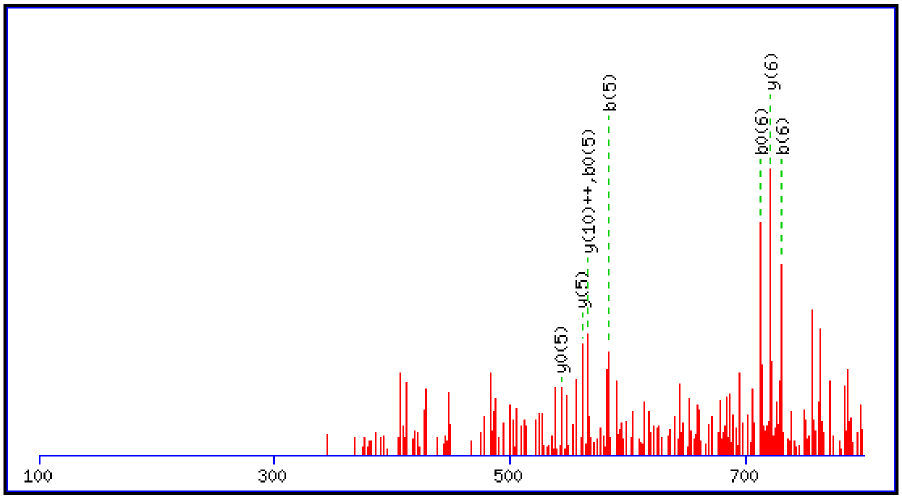


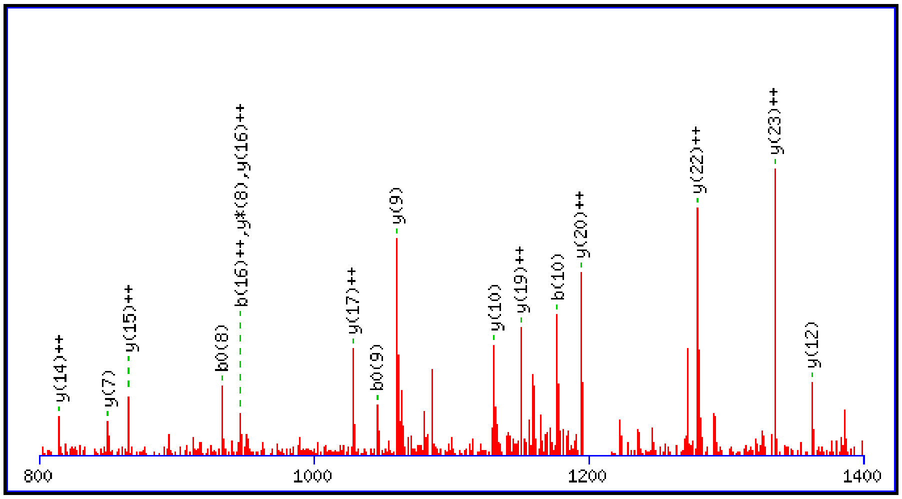

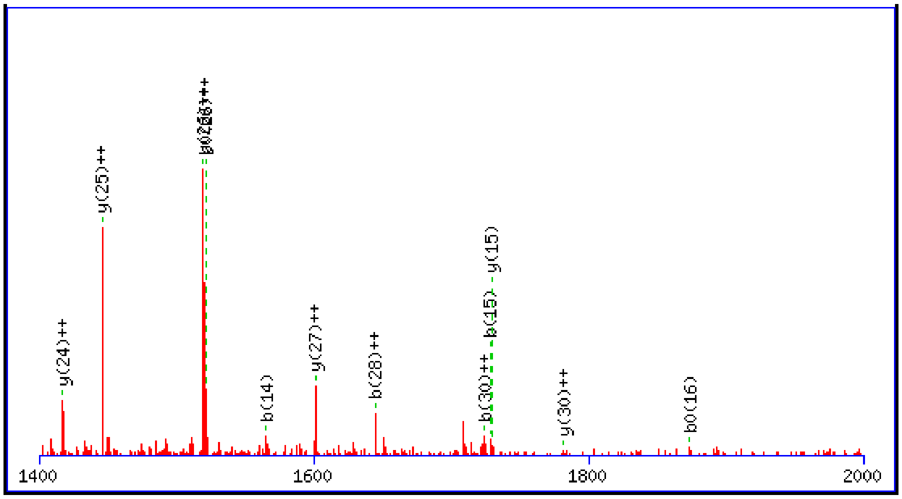


**Monoisotopic mass of neutral peptide Mr(calc):** 3619.5548
**Fixed modifications:** Carbamidomethyl (C)
**Variable modifications:**
**N28** : Deamidated_N (N)
**Ions Score:** 76 **Expect:** 1.1e-005
**Matches (Bold Red):** 37/300 fragment ions using 61 most intense peaks

| # | b | $b^{++}$ | $b^{*}$ | $b^{*++}$ | $b^{0}$ | $b^{0++}$ | Seq. | y | $y^{++}$ | $y^{*}$ | $y^{*++}$ | $y^{0}$ | $y^{0++}$ | # |
|---|---|---|---|---|---|---|---|---|---|---|---|---|---|---|
| 1 | 58.0287 | 29.5180 | | | | | G | | | | | | | 31 |
| 2 | 205.0972 | 103.0522 | | | | | F | 3563.5406 | **1782.2739** | 3546.5141 | 1773.7607 | 3545.5300 | 1773.2687 | 30 |
| 3 | 320.1241 | 160.5657 | | | 302.1135 | 151.5604 | D | 3416.4722 | 1708.7397 | 3399.4456 | 1700.2265 | 3398.4616 | 1699.7345 | 29 |
| 4 | 421.1718 | 211.0895 | | | 403.1612 | 202.0842 | T | 3301.4453 | 1651.2263 | 3284.4187 | 1642.7130 | 3283.4347 | 1642.2210 | 28 |
| 5 | **584.2351** | 292.6212 | | | **566.2245** | 283.6159 | Y | 3200.3976 | **1600.7024** | 3183.3710 | 1592.1892 | 3182.3870 | 1591.6971 | 27 |
| 6 | **731.3035** | 366.1554 | | | **713.2930** | 357.1501 | F | 3037.3342 | **1519.1708** | 3020.3077 | 1510.6575 | 3019.3237 | 1510.1655 | 26 |
| 7 | 788.3250 | 394.6661 | | | 770.3144 | 385.6608 | G | 2890.2658 | **1445.6366** | 2873.2393 | 1437.1233 | 2872.2553 | 1436.6313 | 25 |
| 8 | 951.3883 | 476.1978 | | | **933.3777** | 467.1925 | Y | 2833.2444 | **1417.1258** | 2816.2178 | 1408.6125 | 2815.2338 | 1408.1205 | 24 |
| 9 | 1064.4724 | 532.7398 | | | **1046.4618** | 523.7345 | L | 2670.1810 | **1335.5942** | 2653.1545 | 1327.0809 | 2652.1705 | 1326.5889 | 23 |
| 10 | **1177.5564** | 589.2819 | | | 1159.5459 | 580.2766 | L | 2557.0970 | **1279.0521** | 2540.0704 | 1270.5389 | 2539.0864 | 1270.0468 | 22 |
| 11 | 1234.5779 | 617.7926 | | | 1216.5673 | 608.7873 | G | 2444.0129 | 1222.5101 | 2426.9864 | 1213.9968 | 2426.0023 | 1213.5048 | 21 |
| 12 | 1321.6099 | 661.3086 | | | 1303.5994 | 652.3033 | S | 2386.9914 | **1193.9994** | 2369.9649 | 1185.4861 | 2368.9809 | 1184.9941 | 20 |
| 13 | 1450.6525 | 725.8299 | | | 1432.6420 | 716.8246 | E | 2299.9594 | **1150.4833** | 2282.9329 | 1141.9701 | 2281.9489 | 1141.4781 | 19 |
| 14 | **1565.6795** | 783.3434 | | | 1547.6689 | 774.3381 | D | 2170.9168 | 1085.9621 | 2153.8903 | 1077.4488 | 2152.9063 | 1076.9568 | 18 |
| 15 | **1728.7428** | 864.8750 | | | 1710.7322 | 855.8698 | Y | 2055.8899 | **1028.4486** | 2038.8633 | 1019.9353 | 2037.8793 | 1019.4433 | 17 |
| 16 | 1891.8061 | **946.4067** | | | **1873.7956** | 937.4014 | Y | 1892.8266 | **946.9169** | 1875.8000 | 938.4036 | 1874.8160 | 937.9116 | 16 |
| 17 | 1992.8538 | 996.9305 | | | 1974.8432 | 987.9253 | T | **1729.7632** | **865.3853** | 1712.7367 | 856.8720 | 1711.7527 | 856.3800 | 15 |
| 18 | 2129.9127 | 1065.4600 | | | 2111.9022 | 1056.4547 | H | 1628.7155 | **814.8614** | 1611.6890 | 806.3481 | 1610.7050 | 805.8561 | 14 |
| 19 | 2258.9553 | 1129.9813 | | | 2240.9447 | 1120.9760 | E | 1491.6566 | 746.3320 | 1474.6301 | 737.8187 | 1473.6461 | 737.3267 | 13 |
| 20 | 2329.9924 | 1165.4998 | | | 2311.9819 | 1156.4946 | A | **1362.6140** | 681.8107 | 1345.5875 | 673.2974 | 1344.6035 | 672.8054 | 12 |
| 21 | 2490.0231 | 1245.5152 | | | 2472.0125 | 1236.5099 | C | 1291.5769 | 646.2921 | 1274.5504 | 637.7788 | 1273.5664 | 637.2868 | 11 |
| 22 | 2561.0602 | 1281.0337 | | | 2543.0496 | 1272.0284 | A | **1131.5463** | **566.2768** | 1114.5197 | 557.7635 | 1113.5357 | 557.2715 | 10 |
| 23 | 2658.1130 | 1329.5601 | | | 2640.1024 | 1320.5548 | P | **1060.5092** | 530.7582 | 1043.4826 | 522.2449 | 1042.4986 | 521.7529 | 9 |
| 24 | 2771.1970 | 1386.1021 | | | 2753.1865 | 1377.0969 | I | 963.4564 | 482.2318 | **946.4299** | 473.7186 | 945.4458 | 473.2266 | 8 |
| 25 | 2900.2396 | 1450.6234 | | | 2882.2290 | 1441.6182 | E | **850.3723** | 425.6898 | 833.3458 | 417.1765 | 832.3618 | 416.6845 | 7 |
| 26 | 3060.2703 | 1530.6388 | | | 3042.2597 | **1521.6335** | C | **721.3297** | 361.1685 | 704.3032 | 352.6552 | 703.3192 | 352.1632 | 6 |
| 27 | 3173.3543 | 1587.1808 | | | 3155.3438 | 1578.1755 | L | **561.2991** | 281.1532 | 544.2725 | 272.6399 | **543.2885** | 272.1479 | 5 |
| 28 | 3288.3813 | **1644.6943** | 3271.3547 | 1636.1810 | 3270.3707 | 1635.6890 | N | 448.2150 | 224.6112 | 431.1885 | 216.0979 | 430.2045 | 215.6059 | 4 |
| 29 | 3345.4027 | 1673.2050 | 3328.3762 | 1664.6917 | 3327.3922 | 1664.1997 | G | 333.1881 | 167.0977 | 316.1615 | 158.5844 | 315.1775 | 158.0924 | 3 |
| 30 | 3446.4504 | **1723.7288** | 3429.4239 | 1715.2156 | 3428.4398 | 1714.7236 | T | 276.1666 | 138.5870 | 259.1401 | 130.0737 | 258.1561 | 129.5817 | 2 |
| 31 | | | | | | | R | 175.1190 | 88.0631 | 158.0924 | 79.5498 | | | 1 |

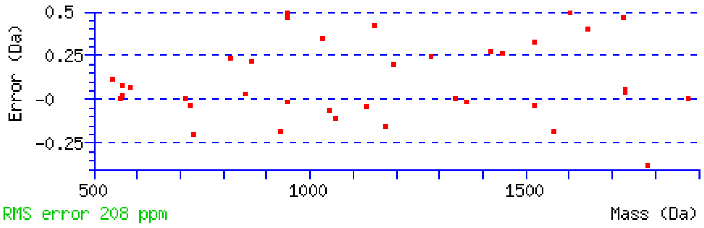


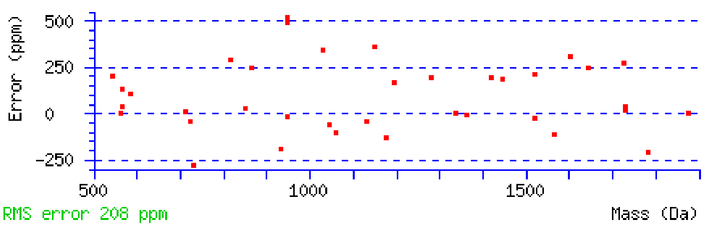


### All matches to this query

| Score | Mr(calc): | Delta | Sequence |
|---|---|---|---|
| 76.4 | 3619.5548 | 1.0117 | GFDTYFGYLLGSEDYYTHEACAPIECLNGTR |
| 6.1 | 3618.5708 | 1.9958 | GFDTYFGYLLGSEDYYTHEACAPIECLNGTR |
| 3.9 | 3620.5604 | 0.0061 | MNNNATDLTTGYLSSAVAILLFGSNFVPLKK |
| 3.9 | 3620.5604 | 0.0061 | MNNNATDLTTGYLSSAVAILLFGSNFVPLKK |
| 2.2 | 3618.5789 | 1.9876 | AEIQCLQERLQAVSEATVQPSLSMDSVVFK |
| 1.3 | 3620.5540 | 0.0125 | TSVVVHCSDGWDRTAQLTSLAMLMLDGYYR |
| 1.3 | 3620.5540 | 0.0125 | TSVVVHCSDGWDRTAQLTSLAMLMLDGYYR |
| 1.3 | 3620.5540 | 0.0125 | TSVVVHCSDGWDRTAQLTSLAMLMLDGYYR |
| 1.3 | 3620.5540 | 0.0125 | TSVVVHCSDGWDRTAQLTSLAMLMLDGYYR |
| 0.6 | 3620.5627 | 0.0039 | LTNAAATSGDGYRGQTSPHTPNEKFYGVTVLK |

---

Spectrum No: 282; Query: 2193; Rank: 1

---

## Peptide View

MS/MS Fragmentation of **KQILEGLEFNLTQIPEADIHK**
Found in **IPI00324019**, Tax_Id=10116 Gene_Symbol=Serpina1 Alpha-1-antiproteinase precursor

Match to Query 2193: 2437.294988 from(1219.654770,2+)
Title: 100101RatKid_NS_deglyco_20.4569.4569.2.dta
Data file K:\NewmanPaper\Piliang\3SubProteomes\Piliang3SP\mgf5ppm\ERLIC_3SubProteomes5ppm.mgf

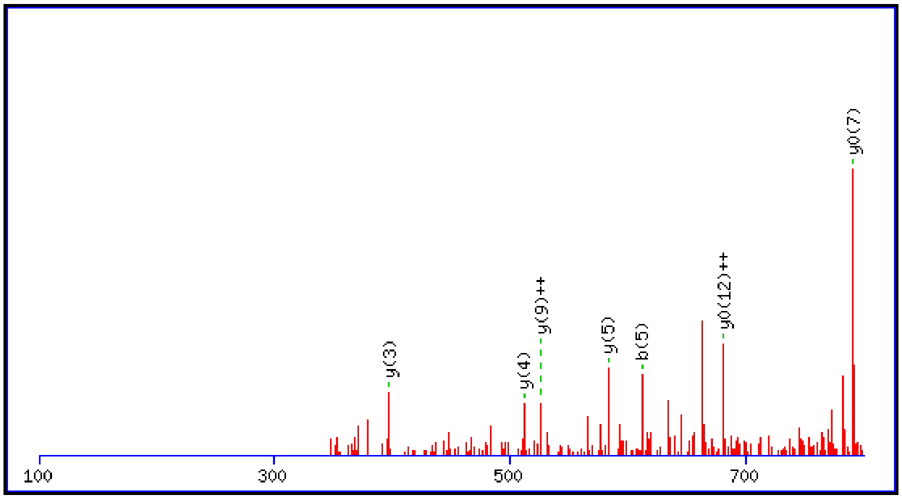

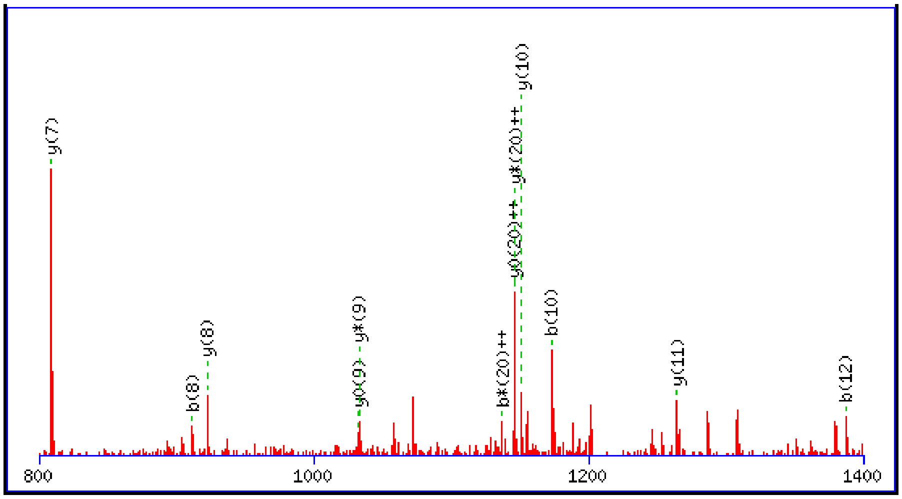


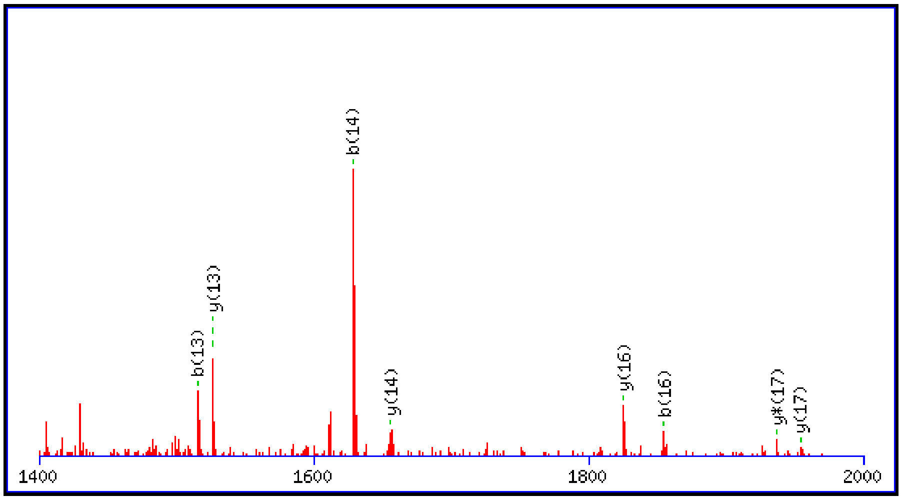


**Monoisotopic mass of neutral peptide Mr(calc):** 2436.2900
**Fixed modifications:** Carbamidomethyl (C)
**Variable modifications:**
**N10** : Deamidated_N (N)
**Ions Score:** 76 **Expect:** 1.9e-006
**Matches (Bold Red):** 28/226 fragment ions using 38 most intense peaks

| # | b | $b^{++}$ | b* | $b^{*++}$ | $b^0$ | $b^{0++}$ | Seq. | y | $y^{++}$ | y* | $y^{*++}$ | $y^0$ | $y^{0++}$ | # |
|---|---|---|---|---|---|---|---|---|---|---|---|---|---|---|
| 1 | 129.1022 | 65.0548 | 112.0757 | 56.5415 | | | K | | | | | | | 21 |
| 2 | 257.1608 | 129.0840 | 240.1343 | 120.5708 | | | Q | 2309.2023 | 1155.1048 | 2292.1758 | **1146.5915** | 2291.1918 | **1146.0995** | 20 |
| 3 | 370.2449 | 185.6261 | 353.2183 | 177.1128 | | | I | 2181.1438 | 1091.0755 | 2164.1172 | 1082.5622 | 2163.1332 | 1082.0702 | 19 |
| 4 | 483.3289 | 242.1681 | 466.3024 | 233.6548 | | | L | 2068.0597 | 1034.5335 | 2051.0332 | 1026.0202 | 2050.0491 | 1025.5282 | 18 |
| 5 | **612.3715** | 306.6894 | 595.3450 | 298.1761 | 594.3610 | 297.6841 | E | **1954.9756** | 977.9915 | **1937.9491** | 969.4782 | 1936.9651 | 968.9862 | 17 |
| 6 | 669.3930 | 335.2001 | 652.3665 | 326.6869 | 651.3824 | 326.1949 | G | **1825.9330** | 913.4702 | 1808.9065 | 904.9569 | 1807.9225 | 904.4649 | 16 |
| 7 | 782.4771 | 391.7422 | 765.4505 | 383.2289 | 764.4665 | 382.7369 | L | 1768.9116 | 884.9594 | 1751.8850 | 876.4462 | 1750.9010 | 875.9541 | 15 |
| 8 | **911.5197** | 456.2635 | 894.4931 | 447.7502 | 893.5091 | 447.2582 | E | **1655.8275** | 828.4174 | 1638.8010 | 819.9041 | 1637.8170 | 819.4121 | 14 |
| 9 | 1058.5881 | 529.7977 | 1041.5615 | 521.2844 | 1040.5775 | 520.7924 | F | **1526.7849** | 763.8961 | 1509.7584 | 755.3828 | 1508.7744 | 754.8908 | 13 |
| 10 | **1173.6150** | 587.3111 | 1156.5885 | 578.7979 | 1155.6044 | 578.3059 | N | 1379.7165 | 690.3619 | 1362.6900 | 681.8486 | 1361.7059 | **681.3566** | 12 |
| 11 | 1286.6991 | 643.8532 | 1269.6725 | 635.3399 | 1268.6885 | 634.8479 | L | **1264.6896** | 632.8484 | 1247.6630 | 624.3352 | 1246.6790 | 623.8431 | 11 |
| 12 | **1387.7468** | 694.3770 | 1370.7202 | 685.8637 | 1369.7362 | 685.3717 | T | **1151.6055** | 576.3064 | 1134.5790 | 567.7931 | 1133.5949 | 567.3011 | 10 |
| 13 | **1515.8053** | 758.4063 | 1498.7788 | 749.8930 | 1497.7948 | 749.4010 | Q | 1050.5578 | **525.7826** | **1033.5313** | 517.2693 | **1032.5473** | 516.7773 | 9 |
| 14 | **1628.8894** | 814.9483 | 1611.8628 | 806.4351 | 1610.8788 | 805.9431 | I | **922.4993** | 461.7533 | 905.4727 | 453.2400 | 904.4887 | 452.7480 | 8 |
| 15 | 1725.9422 | 863.4747 | 1708.9156 | 854.9614 | 1707.9316 | 854.4694 | P | **809.4152** | 405.2112 | 792.3886 | 396.6980 | **791.4046** | 396.2060 | 7 |
| 16 | **1854.9848** | 927.9960 | 1837.9582 | 919.4827 | 1836.9742 | 918.9907 | E | 712.3624 | 356.6849 | 695.3359 | 348.1716 | 694.3519 | 347.6796 | 6 |
| 17 | 1926.0219 | 963.5146 | 1908.9953 | 955.0013 | 1908.0113 | 954.5093 | A | **583.3198** | 292.1636 | 566.2933 | 283.6503 | 565.3093 | 283.1583 | 5 |
| 18 | 2041.0488 | 1021.0280 | 2024.0223 | 1012.5148 | 2023.0382 | 1012.0228 | D | **512.2827** | 256.6450 | 495.2562 | 248.1317 | 494.2722 | 247.6397 | 4 |
| | | | | | | | | | | | | | | |

| | | | | | | | | | | | | | |
|---|---|---|---|---|---|---|---|---|---|---|---|---|---|
| 19 | 2154.1329 | 1077.5701 | 2137.1063 | 1069.0568 | 2136.1223 | 1068.5648 | I | 397.2558 | 199.1315 | 380.2292 | 190.6183 | | | 3 |
| 20 | 2291.1918 | 1146.0995 | 2274.1652 | 1137.5863 | 2273.1812 | 1137.0942 | H | 284.1717 | 142.5895 | 267.1452 | 134.0762 | | | 2 |
| 21 | | | | | | | K | 147.1128 | 74.0600 | 130.0863 | 65.5468 | | | 1 |

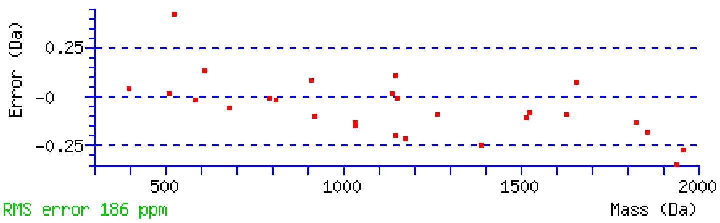


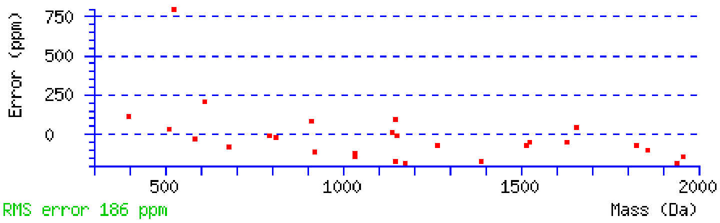


**All matches to this query**

| Score | Mr(calc): | Delta | Sequence |
|---|---|---|---|
| 76.1 | 2436.2900 | 1.0050 | KQILEGLEFNLTQIPEADIHK |
| 25.8 | 2435.3060 | 1.9890 | KQILEGLEFNLTQIPEADIHK |

---

Spectrum No: 283; Query: 1802; Rank: 1

---

## Peptide View

MS/MS Fragmentation of **EGNCSVQSGLTWQDCDFK**
Found in **IPI00327182**, Tax_Id=10116 Gene_Symbol=Kng1 T-kininogen 1 precursor

Match to Query 1802: 2130.869628 from(1066.442090,2+)
Title: 100101RatKid_NS_deglyco_25.2944.2944.2.dta
Data file K:\NewmanPaper\Piliang\3SubProteomes\Piliang3SP\mgf5ppm\ERLIC_3SubProteomes5ppm.mgf

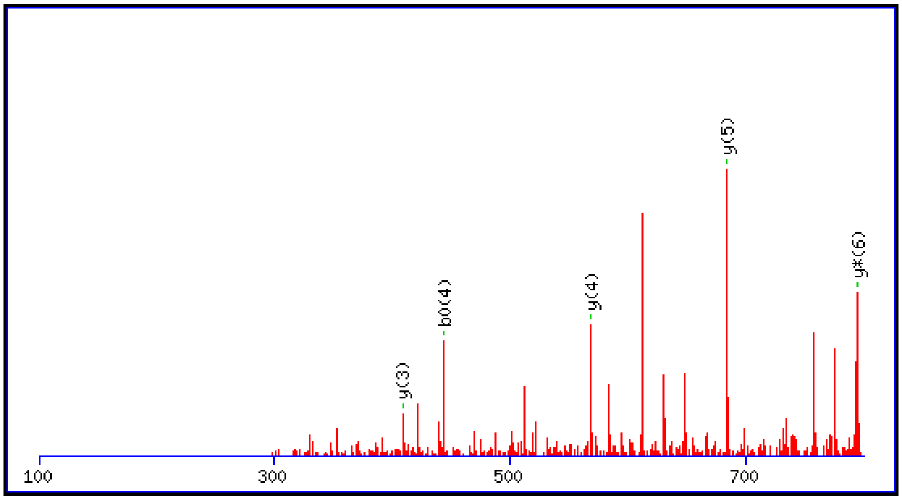

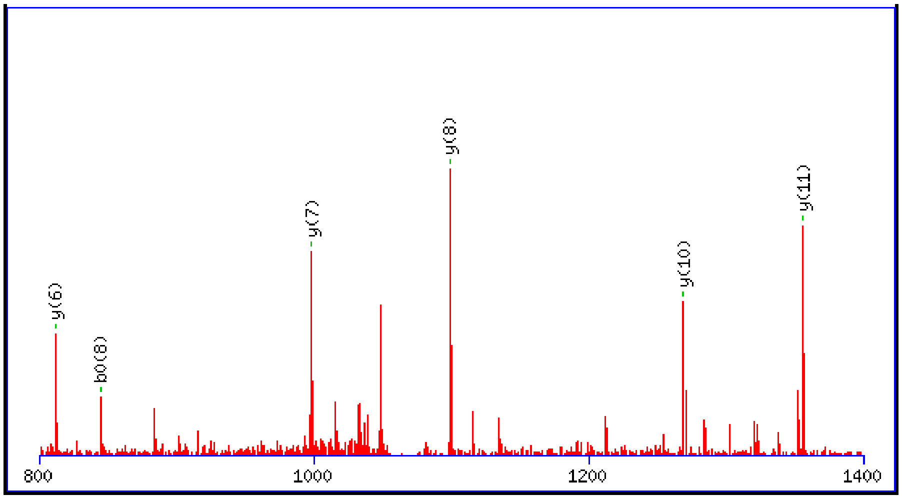


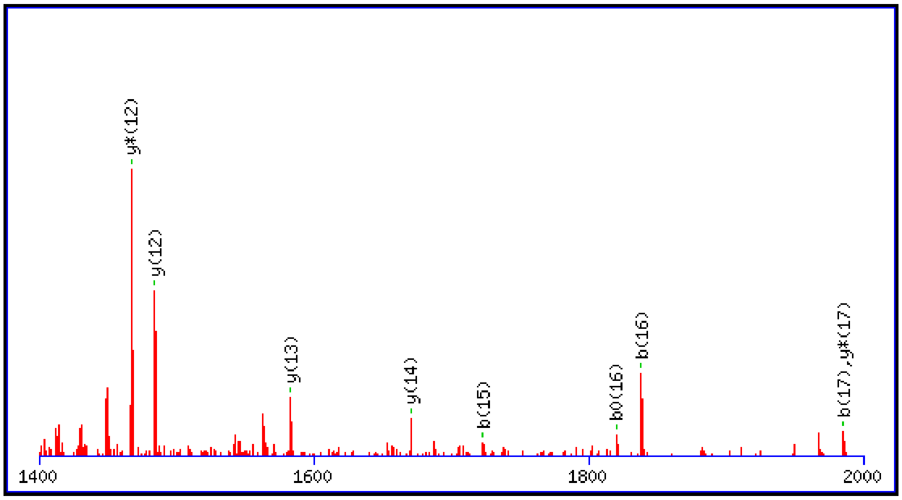


**Monoisotopic mass of neutral peptide Mr(calc):** 2130.8623
**Fixed modifications:** Carbamidomethyl (C)
**Variable modifications:**
**N3** : Deamidated_N (N)
**Ions Score:** 76 **Expect:** 5.1e-006
**Matches (Bold Red):** 20/196 fragment ions using 30 most intense peaks

| # | b | $b^{++}$ | b* | $b^{*++}$ | $b^0$ | $b^{0++}$ | Seq. | y | $y^{++}$ | y* | $y^{*++}$ | $y^0$ | $y^{0++}$ | # |
|---|---|---|---|---|---|---|---|---|---|---|---|---|---|---|
| 1 | 130.0499 | 65.5286 | | | 112.0393 | 56.5233 | E | | | | | | | 18 |
| 2 | 187.0713 | 94.0393 | | | 169.0608 | 85.0340 | G | 2002.8269 | 1001.9171 | **1985.8004** | 993.4038 | 1984.8164 | 992.9118 | 17 |
| 3 | 302.0983 | 151.5528 | 285.0717 | 143.0395 | 284.0877 | 142.5475 | N | 1945.8055 | 973.4064 | 1928.7789 | 964.8931 | 1927.7949 | 964.4011 | 16 |
| 4 | 462.1289 | 231.5681 | 445.1024 | 223.0548 | **444.1184** | 222.5628 | C | 1830.7785 | 915.8929 | 1813.7520 | 907.3796 | 1812.7680 | 906.8876 | 15 |
| 5 | 549.1609 | 275.0841 | 532.1344 | 266.5708 | 531.1504 | 266.0788 | S | **1670.7479** | 835.8776 | 1653.7214 | 827.3643 | 1652.7373 | 826.8723 | 14 |
| 6 | 648.2294 | 324.6183 | 631.2028 | 316.1050 | 630.2188 | 315.6130 | V | **1583.7159** | 792.3616 | 1566.6893 | 783.8483 | 1565.7053 | 783.3563 | 13 |
| 7 | 776.2879 | 388.6476 | 759.2614 | 380.1343 | 758.2774 | 379.6423 | Q | **1484.6475** | 742.8274 | **1467.6209** | 734.3141 | 1466.6369 | 733.8221 | 12 |
| 8 | 863.3200 | 432.1636 | 846.2934 | 423.6503 | **845.3094** | 423.1583 | S | **1356.5889** | 678.7981 | 1339.5623 | 670.2848 | 1338.5783 | 669.7928 | 11 |
| 9 | 920.3414 | 460.6744 | 903.3149 | 452.1611 | 902.3309 | 451.6691 | G | **1269.5569** | 635.2821 | 1252.5303 | 626.7688 | 1251.5463 | 626.2768 | 10 |
| 10 | 1033.4255 | 517.2164 | 1016.3989 | 508.7031 | 1015.4149 | 508.2111 | L | 1212.5354 | 606.7713 | 1195.5088 | 598.2581 | 1194.5248 | 597.7660 | 9 |
| 11 | 1134.4732 | 567.7402 | 1117.4466 | 559.2270 | 1116.4626 | 558.7349 | T | **1099.4513** | 550.2293 | 1082.4248 | 541.7160 | 1081.4408 | 541.2240 | 8 |
| 12 | 1320.5525 | 660.7799 | 1303.5259 | 652.2666 | 1302.5419 | 651.7746 | W | **998.4036** | 499.7055 | 981.3771 | 491.1922 | 980.3931 | 490.7002 | 7 |
| 13 | 1448.6111 | 724.8092 | 1431.5845 | 716.2959 | 1430.6005 | 715.8039 | Q | **812.3243** | 406.6658 | **795.2978** | 398.1525 | 794.3138 | 397.6605 | 6 |
| 14 | 1563.6380 | 782.3226 | 1546.6115 | 773.8094 | 1545.6274 | 773.3174 | D | **684.2658** | 342.6365 | 667.2392 | 334.1232 | 666.2552 | 333.6312 | 5 |
| 15 | **1723.6687** | 862.3380 | 1706.6421 | 853.8247 | 1705.6581 | 853.3327 | C | **569.2388** | 285.1230 | 552.2123 | 276.6098 | 551.2282 | 276.1178 | 4 |
| 16 | **1838.6956** | 919.8514 | 1821.6691 | 911.3382 | **1820.6850** | 910.8462 | D | **409.2082** | 205.1077 | 392.1816 | 196.5944 | 391.1976 | 196.1024 | 3 |
| 17 | **1985.7640** | 993.3856 | 1968.7375 | 984.8724 | 1967.7534 | 984.3804 | F | 294.1812 | 147.5942 | 277.1547 | 139.0810 | | | 2 |
| 18 | | | | | | | K | 147.1128 | 74.0600 | 130.0863 | 65.5468 | | | 1 |

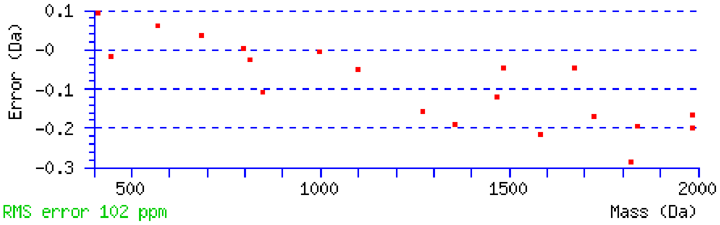


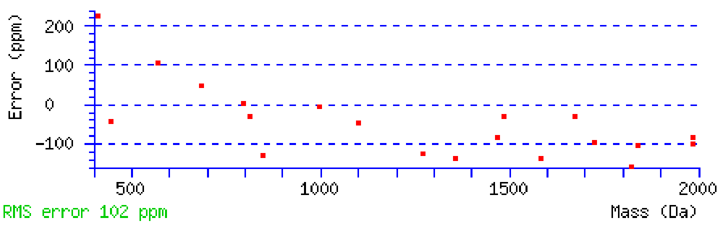


### All matches to this query

| Score | Mr(calc): | Delta | Sequence |
|---|---|---|---|
| 76.1 | 2130.8623 | 0.0073 | EGNCSVQSGLTWQDCDFK |
| 60.9 | 2129.8783 | 0.9913 | EGNCSVQSGLTWQDCDFK |
| 8.2 | 2130.8882 | -0.0185 | SYLIMMLEYFKKDMK |
| 8.2 | 2130.8882 | -0.0185 | SYLIMMLEYFKKDMK |
| 8.2 | 2130.8882 | -0.0185 | SYLIMMLEYFKKDMK |
| 8.2 | 2130.8882 | -0.0185 | SYLIMMLEYFKKDMK |
| 3.6 | 2130.8792 | -0.0096 | YNMVGPCVSTTFQGTNFK |
| 1.3 | 2130.8589 | 0.0107 | KTNNNNNNTTDNLQSINK |
| 0.6 | 2129.8481 | 1.0215 | YSKAYGRMGLALTAMNK |

---

Spectrum No: 284; Query: 1427; Rank: 1

---

## Peptide View

MS/MS Fragmentation of **LHINYNNLTESVGPLPK**
Found in **IPI00206403**, Tax_Id=10116 Gene_Symbol=Lum Lumican precursor

Match to Query 1427: 1908.995448 from(955.505000,2+)
Title: 091008RatKidney_NH4Format01_25.2924.2924.2.dta
Data file K:\NewmanPaper\Piliang\3SubProteomes\Piliang3SP\mgf5ppm\ERLIC_3SubProteomes5ppm.mgf

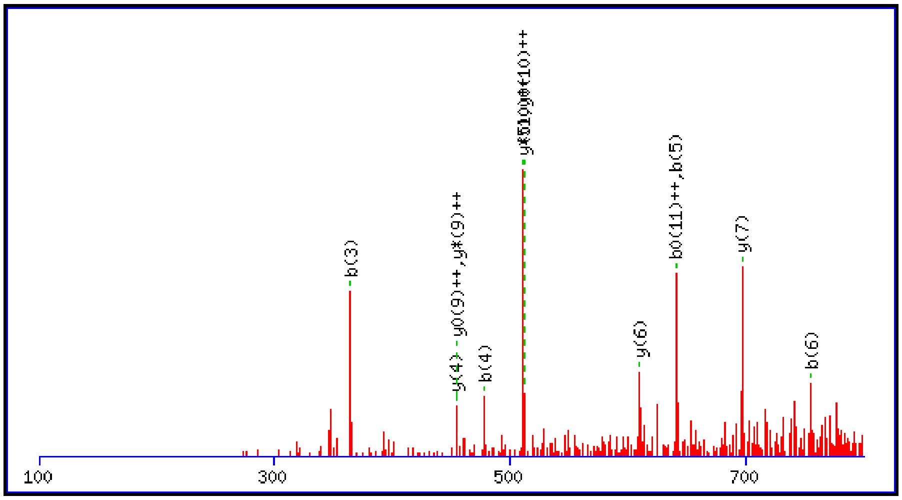

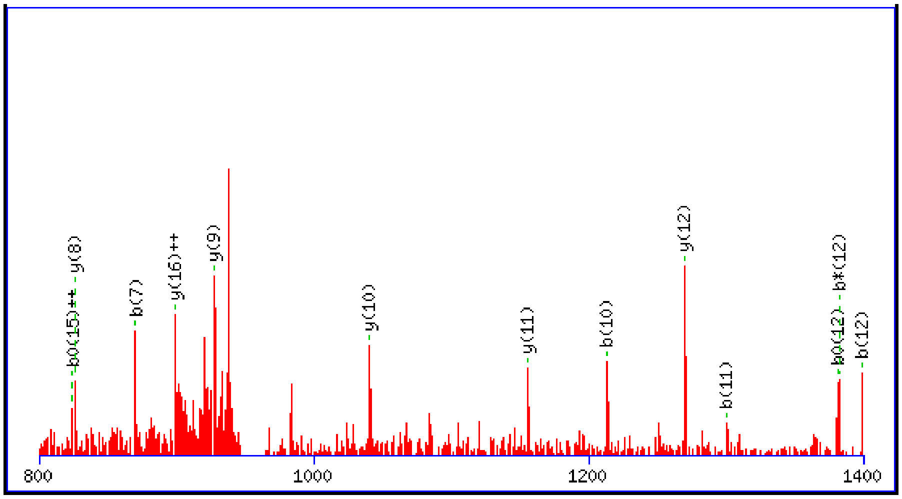


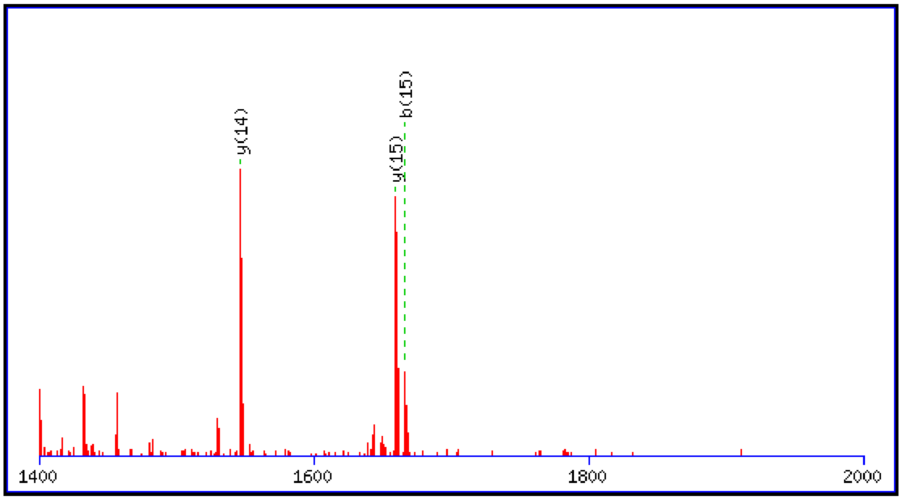


**Monoisotopic mass of neutral peptide Mr(calc):** 1908.9945
**Fixed modifications:** Carbamidomethyl (C)
**Variable modifications:**
**N7** : Deamidated_N (N)
**Ions Score:** 76 **Expect:** 3e-006
**Matches (Bold Red):** 29/158 fragment ions using 46 most intense peaks

| # | b | b$^{++}$ | b* | b*$^{++}$ | b$^{0}$ | b$^{0++}$ | Seq. | y | y$^{++}$ | y* | y*$^{++}$ | y$^{0}$ | y$^{0++}$ | # |
|---|---|---|---|---|---|---|---|---|---|---|---|---|---|---|
| 1 | 114.0913 | 57.5493 | | | | | L | | | | | | | 17 |
| 2 | 251.1503 | 126.0788 | | | | | H | 1796.9177 | **898.9625** | 1779.8912 | 890.4492 | 1778.9072 | 889.9572 | 16 |
| 3 | **364.2343** | 182.6208 | | | | | I | **1659.8588** | 830.4330 | 1642.8323 | 821.9198 | 1641.8483 | 821.4278 | 15 |
| 4 | **478.2772** | 239.6423 | 461.2507 | 231.1290 | | | N | **1546.7748** | 773.8910 | 1529.7482 | 765.3777 | 1528.7642 | 764.8857 | 14 |
| 5 | **641.3406** | 321.1739 | 624.3140 | 312.6606 | | | Y | 1432.7318 | 716.8696 | 1415.7053 | 708.3563 | 1414.7213 | 707.8643 | 13 |
| 6 | **755.3835** | 378.1954 | 738.3569 | 369.6821 | | | N | **1269.6685** | 635.3379 | 1252.6420 | 626.8246 | 1251.6579 | 626.3326 | 12 |
| 7 | **870.4104** | 435.7089 | 853.3839 | 427.1956 | | | N | **1155.6256** | 578.3164 | 1138.5990 | 569.8032 | 1137.6150 | 569.3111 | 11 |
| 8 | 983.4945 | 492.2509 | 966.4679 | 483.7376 | | | L | **1040.5986** | 520.8030 | 1023.5721 | **512.2897** | 1022.5881 | **511.7977** | 10 |
| 9 | 1084.5422 | 542.7747 | 1067.5156 | 534.2615 | 1066.5316 | 533.7694 | T | **927.5146** | 464.2609 | 910.4880 | **455.7477** | 909.5040 | **455.2556** | 9 |
| 10 | **1213.5848** | 607.2960 | 1196.5582 | 598.7827 | 1195.5742 | 598.2907 | E | **826.4669** | 413.7371 | 809.4403 | 405.2238 | 808.4563 | 404.7318 | 8 |
| 11 | **1300.6168** | 650.8120 | 1283.5903 | 642.2988 | 1282.6062 | **641.8068** | S | **697.4243** | 349.2158 | 680.3978 | 340.7025 | 679.4137 | 340.2105 | 7 |
| 12 | **1399.6852** | 700.3462 | **1382.6587** | 691.8330 | **1381.6746** | 691.3410 | V | **610.3923** | 305.6998 | 593.3657 | 297.1865 | | | 6 |
| 13 | 1456.7067 | 728.8570 | 1439.6801 | 720.3437 | 1438.6961 | 719.8517 | G | **511.3239** | 256.1656 | 494.2973 | 247.6523 | | | 5 |
| 14 | 1553.7594 | 777.3834 | 1536.7329 | 768.8701 | 1535.7489 | 768.3781 | P | **454.3024** | 227.6548 | 437.2758 | 219.1416 | | | 4 |
| 15 | **1666.8435** | 833.9254 | 1649.8170 | 825.4121 | 1648.8329 | **824.9201** | L | 357.2496 | 179.1285 | 340.2231 | 170.6152 | | | 3 |
| 16 | 1763.8963 | 882.4518 | 1746.8697 | 873.9385 | 1745.8857 | 873.4465 | P | 244.1656 | 122.5864 | 227.1390 | 114.0731 | | | 2 |
| 17 | | | | | | | K | 147.1128 | 74.0600 | 130.0863 | 65.5468 | | | 1 |

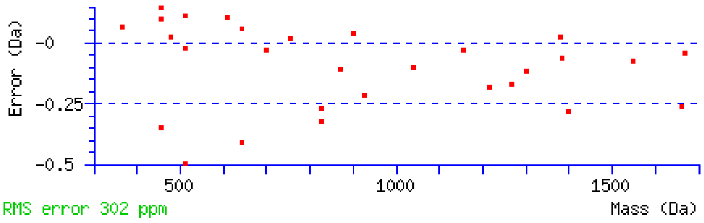


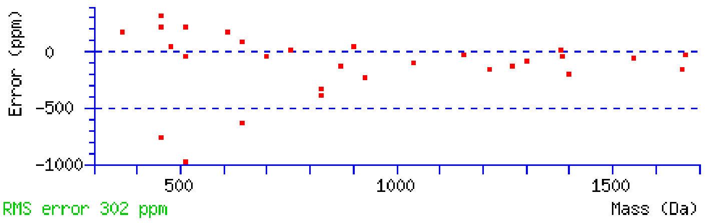


### All matches to this query

| Score | Mr(calc): | Delta | Sequence |
|---|---|---|---|
| 76.1 | 1908.9945 | 0.0010 | LHINYNNLTESVGPLPK |
| 62.9 | 1908.9945 | 0.0010 | LHINYNNLTESVGPLPK |
| 50.6 | 1908.9945 | 0.0010 | LHINYNNLTESVGPLPK |
| 21.2 | 1908.0105 | 0.9850 | LHINYNNLTESVGPLPK |

---

Spectrum No: 285; Query: 1259; Rank: 1

---

## Peptide View

MS/MS Fragmentation of **DLQNFLENVTSSVDVK**
Found in **IPI00565109**, Tax_Id=10116 Gene_Symbol=RGD1307220_predicted similar to alpha 3 type VI collagen isoform 1 precursor

Match to Query 1259: 1807.886168 from(904.950360,2+)
Title: 091008RatKidney_NoSalt_27.5028.5028.2.dta
Data file K:\NewmanPaper\Piliang\3SubProteomes\Piliang3SP\mgf5ppm\ERLIC_3SubProteomes5ppm.mgf

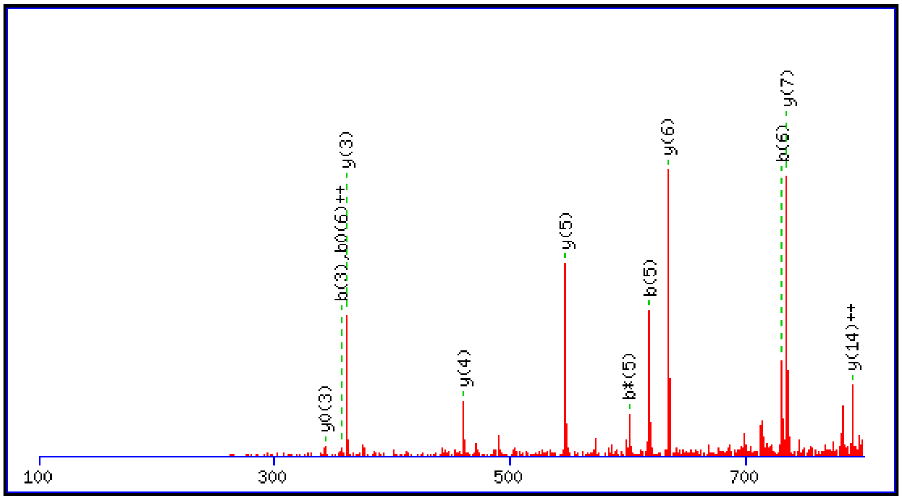

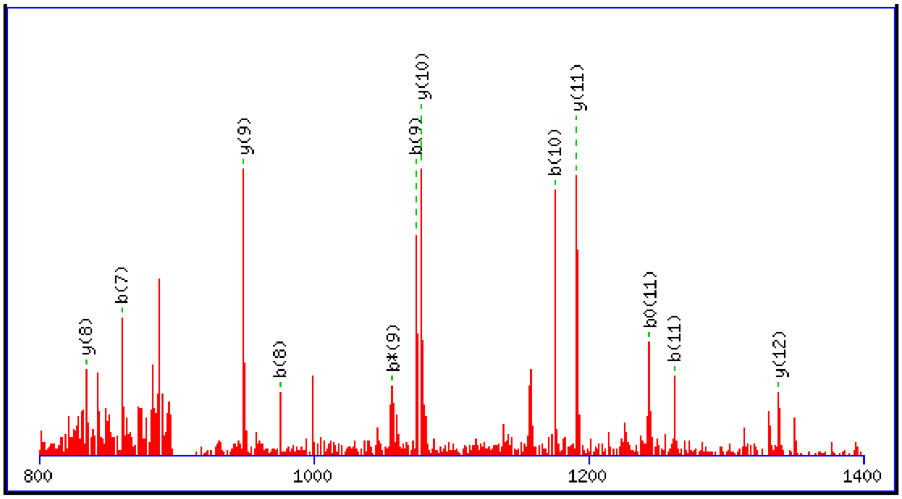


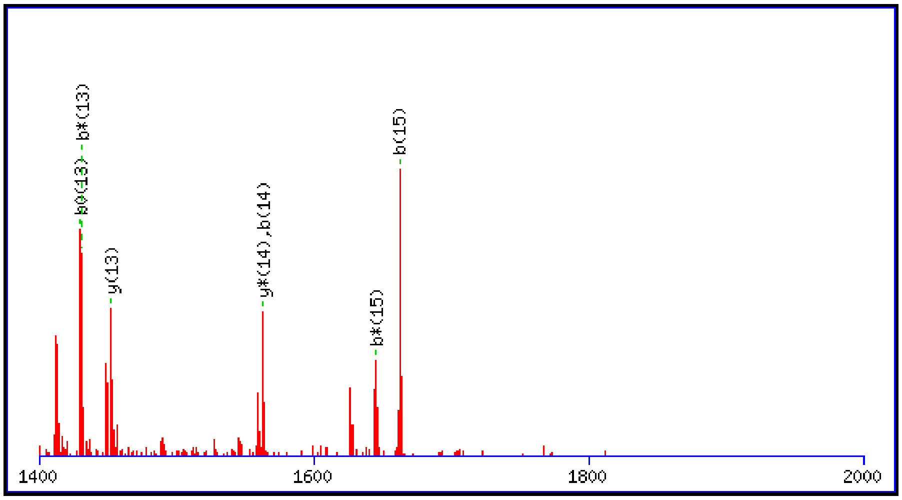


**Monoisotopic mass of neutral peptide Mr(calc):** 1807.8840
**Fixed modifications:** Carbamidomethyl (C)
**Variable modifications:**
**N8** : Deamidated_N (N)
**Ions Score:** 76 **Expect:** 4.6e-006
**Matches (Bold Red):** 31/172 fragment ions using 55 most intense peaks

| # | b | $b^{++}$ | b* | $b^{*++}$ | $b^0$ | $b^{0++}$ | Seq. | y | $y^{++}$ | y* | $y^{*++}$ | $y^0$ | $y^{0++}$ | # |
|---|---|---|---|---|---|---|---|---|---|---|---|---|---|---|
| 1 | 116.0342 | 58.5207 | | | 98.0237 | 49.5155 | D | | | | | | | 16 |
| 2 | 229.1183 | 115.0628 | | | 211.1077 | 106.0575 | L | 1693.8643 | 847.4358 | 1676.8378 | 838.9225 | 1675.8537 | 838.4305 | 15 |
| 3 | **357.1769** | 179.0921 | 340.1503 | 170.5788 | 339.1663 | 170.0868 | Q | 1580.7802 | **790.8938** | **1563.7537** | 782.3805 | 1562.7697 | 781.8885 | 14 |
| 4 | 471.2198 | 236.1135 | 454.1932 | 227.6003 | 453.2092 | 227.1082 | N | **1452.7217** | 726.8645 | 1435.6951 | 718.3512 | 1434.7111 | 717.8592 | 13 |
| 5 | **618.2882** | 309.6477 | **601.2617** | 301.1345 | 600.2776 | 300.6425 | F | **1338.6787** | 669.8430 | 1321.6522 | 661.3297 | 1320.6682 | 660.8377 | 12 |
| 6 | **731.3723** | 366.1898 | 714.3457 | 357.6765 | 713.3617 | **357.1845** | L | **1191.6103** | 596.3088 | 1174.5838 | 587.7955 | 1173.5998 | 587.3035 | 11 |
| 7 | **860.4149** | 430.7111 | 843.3883 | 422.1978 | 842.4043 | 421.7058 | E | **1078.5263** | 539.7668 | 1061.4997 | 531.2535 | 1060.5157 | 530.7615 | 10 |
| 8 | **975.4418** | 488.2245 | 958.4152 | 479.7113 | 957.4312 | 479.2193 | N | **949.4837** | 475.2455 | 932.4571 | 466.7322 | 931.4731 | 466.2402 | 9 |
| 9 | **1074.5102** | 537.7587 | **1057.4837** | 529.2455 | 1056.4996 | 528.7535 | V | **834.4567** | 417.7320 | 817.4302 | 409.2187 | 816.4462 | 408.7267 | 8 |
| 10 | **1175.5579** | 588.2826 | 1158.5313 | 579.7693 | 1157.5473 | 579.2773 | T | **735.3883** | 368.1978 | 718.3618 | 359.6845 | 717.3777 | 359.1925 | 7 |
| 11 | **1262.5899** | 631.7986 | 1245.5634 | 623.2853 | **1244.5794** | 622.7933 | S | **634.3406** | 317.6740 | 617.3141 | 309.1607 | 616.3301 | 308.6687 | 6 |
| 12 | 1349.6219 | 675.3146 | 1332.5954 | 666.8013 | 1331.6114 | 666.3093 | S | **547.3086** | 274.1579 | 530.2821 | 265.6447 | 529.2980 | 265.1527 | 5 |
| 13 | 1448.6904 | 724.8488 | **1431.6638** | 716.3355 | **1430.6798** | 715.8435 | V | **460.2766** | 230.6419 | 443.2500 | 222.1287 | 442.2660 | 221.6366 | 4 |
| 14 | **1563.7173** | 782.3623 | 1546.6908 | 773.8490 | 1545.7067 | 773.3570 | D | **361.2082** | 181.1077 | 344.1816 | 172.5944 | **343.1976** | 172.1024 | 3 |
| 15 | **1662.7857** | 831.8965 | **1645.7592** | 823.3832 | 1644.7752 | 822.8912 | V | 246.1812 | 123.5942 | 229.1547 | 115.0810 | | | 2 |
| 16 | | | | | | | K | 147.1128 | 74.0600 | 130.0863 | 65.5468 | | | 1 |

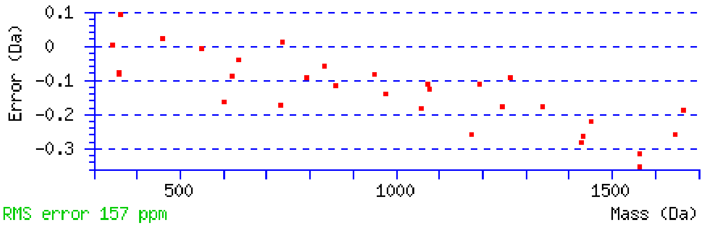


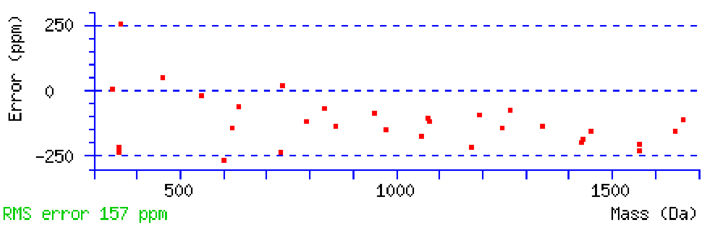


### All matches to this query

| Score | Mr(calc): | Delta | Sequence |
|---|---|---|---|
| 76.1 | 1807.8840 | 0.0022 | DLQNFLENVTSSVDVK |
| 56.5 | 1807.8840 | 0.0022 | DLQNFLENVTSSVDVK |
| 20.2 | 1806.9000 | 0.9862 | DLQNFLENVTSSVDVK |
| 8.5 | 1807.8838 | 0.0024 | LCCLLARASNLPNVK |
| 2.0 | 1807.8869 | -0.0008 | QDQTLELLGPPPPPAR |
| 1.1 | 1806.8933 | 0.9928 | YLIQDSSILQKVLK |

---

Spectrum No: 286; Query: 2009; Rank: 1

---

## Peptide View

MS/MS Fragmentation of **VCDGVPDCPEGDDENNVTSGR**
Found in **IPI00205325**, Tax_Id=10116 Gene_Symbol=Lrp2 Low-density lipoprotein receptor-related protein 2 precursor

Match to Query 2009: 2291.898228 from(1146.956390,2+)
Title: 100101RatKid_NS_deglyco_24.1502.1502.2.dta
Data file K:\NewmanPaper\Piliang\3SubProteomes\Piliang3SP\mgf5ppm\ERLIC_3SubProteomes5ppm.mgf

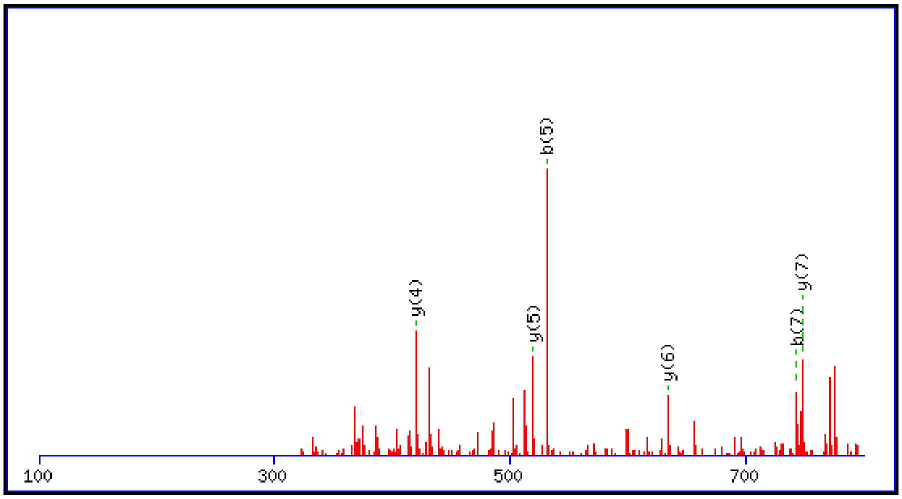

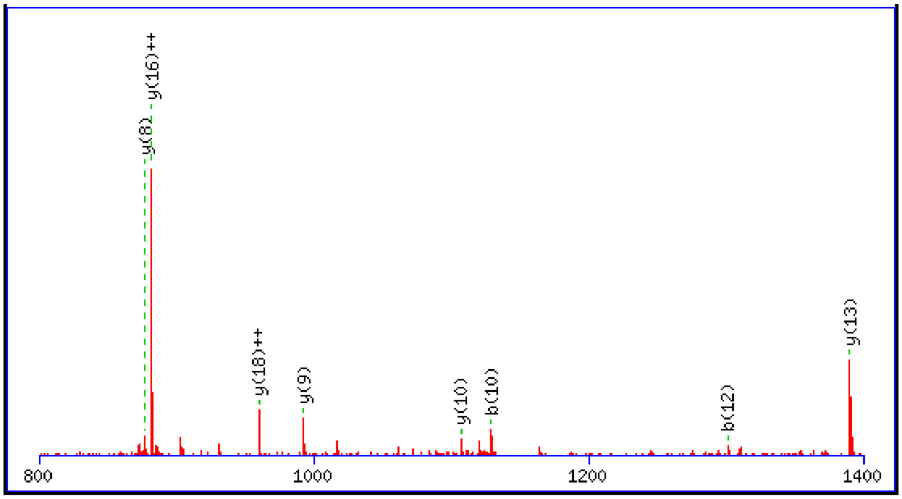


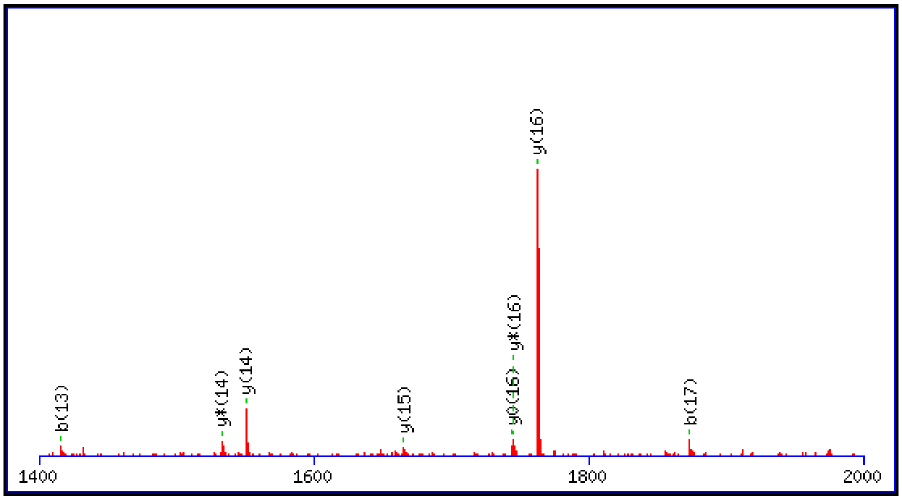


**Monoisotopic mass of neutral peptide Mr(calc):** 2291.8907
**Fixed modifications:** Carbamidomethyl (C)
**Variable modifications:**
**N16** : Deamidated_N (N)
**Ions Score:** 76 **Expect:** 4.6e-006
**Matches (Bold Red):** 22/204 fragment ions using 36 most intense peaks

| # | b | $b^{++}$ | b* | $b^{*++}$ | $b^{0}$ | $b^{0++}$ | Seq. | y | $y^{++}$ | y* | $y^{*++}$ | $y^{0}$ | $y^{0++}$ | # |
|---|---|---|---|---|---|---|---|---|---|---|---|---|---|---|
| 1 | 100.0757 | 50.5415 | | | | | V | | | | | | | 21 |
| 2 | 260.1063 | 130.5568 | | | | | C | 2193.8295 | 1097.4184 | 2176.8030 | 1088.9051 | 2175.8190 | 1088.4131 | 20 |
| 3 | 375.1333 | 188.0703 | | | 357.1227 | 179.0650 | D | 2033.7989 | 1017.4031 | 2016.7723 | 1008.8898 | 2015.7883 | 1008.3978 | 19 |
| 4 | 432.1547 | 216.5810 | | | 414.1442 | 207.5757 | G | 1918.7719 | **959.8896** | 1901.7454 | 951.3763 | 1900.7614 | 950.8843 | 18 |
| 5 | **531.2232** | 266.1152 | | | 513.2126 | 257.1099 | V | 1861.7505 | 931.3789 | 1844.7239 | 922.8656 | 1843.7399 | 922.3736 | 17 |
| 6 | 628.2759 | 314.6416 | | | 610.2654 | 305.6363 | P | **1762.6821** | **881.8447** | **1745.6555** | 873.3314 | **1744.6715** | 872.8394 | 16 |
| 7 | **743.3029** | 372.1551 | | | 725.2923 | 363.1498 | D | **1665.6293** | 833.3183 | 1648.6027 | 824.8050 | 1647.6187 | 824.3130 | 15 |
| 8 | 903.3335 | 452.1704 | | | 885.3230 | 443.1651 | C | **1550.6023** | 775.8048 | **1533.5758** | 767.2915 | 1532.5918 | 766.7995 | 14 |
| 9 | 1000.3863 | 500.6968 | | | 982.3757 | 491.6915 | P | **1390.5717** | 695.7895 | 1373.5452 | 687.2762 | 1372.5611 | 686.7842 | 13 |
| 10 | **1129.4289** | 565.2181 | | | 1111.4183 | 556.2128 | E | 1293.5189 | 647.2631 | 1276.4924 | 638.7498 | 1275.5084 | 638.2578 | 12 |
| 11 | 1186.4503 | 593.7288 | | | 1168.4398 | 584.7235 | G | 1164.4763 | 582.7418 | 1147.4498 | 574.2285 | 1146.4658 | 573.7365 | 11 |
| 12 | **1301.4773** | 651.2423 | | | 1283.4667 | 642.2370 | D | **1107.4549** | 554.2311 | 1090.4283 | 545.7178 | 1089.4443 | 545.2258 | 10 |
| 13 | **1416.5042** | 708.7557 | | | 1398.4937 | 699.7505 | D | **992.4279** | 496.7176 | 975.4014 | 488.2043 | 974.4174 | 487.7123 | 9 |
| 14 | 1545.5468 | 773.2770 | | | 1527.5363 | 764.2718 | E | **877.4010** | 439.2041 | 860.3744 | 430.6909 | 859.3904 | 430.1989 | 8 |
| 15 | 1659.5897 | 830.2985 | 1642.5632 | 821.7852 | 1641.5792 | 821.2932 | N | **748.3584** | 374.6828 | 731.3319 | 366.1696 | 730.3478 | 365.6776 | 7 |
| 16 | 1774.6167 | 887.8120 | 1757.5901 | 879.2987 | 1756.6061 | 878.8067 | N | **634.3155** | 317.6614 | 617.2889 | 309.1481 | 616.3049 | 308.6561 | 6 |
| 17 | **1873.6851** | 937.3462 | 1856.6585 | 928.8329 | 1855.6745 | 928.3409 | V | **519.2885** | 260.1479 | 502.2620 | 251.6346 | 501.2780 | 251.1426 | 5 |
| 18 | 1974.7328 | 987.8700 | 1957.7062 | 979.3567 | 1956.7222 | 978.8647 | T | **420.2201** | 210.6137 | 403.1936 | 202.1004 | 402.2096 | 201.6084 | 4 |

| | | | | | | | | | | | | | | |
|---|---|---|---|---|---|---|---|---|---|---|---|---|---|---|
| **19** | 2061.7648 | 1031.3860 | 2044.7383 | 1022.8728 | 2043.7542 | 1022.3808 | **S** | 319.1724 | 160.0899 | 302.1459 | 151.5766 | 301.1619 | 151.0846 | **3** |
| **20** | 2118.7863 | 1059.8968 | 2101.7597 | 1051.3835 | 2100.7757 | 1050.8915 | **G** | 232.1404 | 116.5738 | 215.1139 | 108.0606 | | | **2** |
| **21** | | | | | | | **R** | 175.1190 | 88.0631 | 158.0924 | 79.5498 | | | **1** |

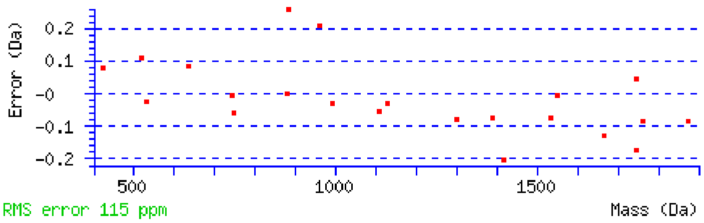


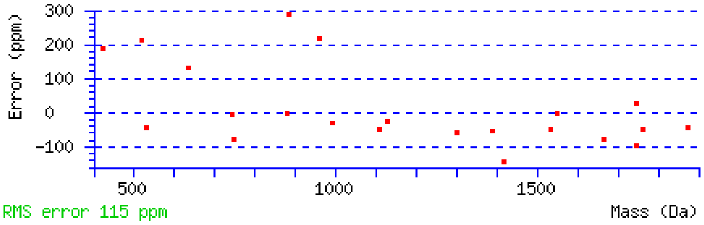


**All matches to this query**

| Score | Mr(calc): | Delta | Sequence |
|---|---|---|---|
| 76.1 | 2291.8907 | 0.0075 | VCDGVPDCPEGDDENNVTSGR |
| 61.0 | 2291.8907 | 0.0075 | VCDGVPDCPEGDDENNVTSGR |
| 6.3 | 2290.9067 | 0.9915 | VCDGVPDCPEGDDENNVTSGR |
| 3.9 | 2290.9082 | 0.9900 | MKPAMETAAEENTEQSQER |
| 2.6 | 2289.9085 | 1.9897 | SPSSDLQSLIQSKAEKPR |
| 2.6 | 2289.9085 | 1.9897 | SPSSDLQSLIQSKAEKPR |
| 2.6 | 2289.9085 | 1.9897 | SPSSDLQSLIQSKAEKPR |
| 2.4 | 2291.8923 | 0.0060 | MKPAMETAAEENTEQSQER |
| 2.1 | 2290.9123 | 0.9859 | WQMMKQXDQELNNPTSDK |
| 2.1 | 2290.9123 | 0.9859 | WQMMKQXDQELNNPTSDK |

---

Spectrum No: 287; Query: 1224; Rank: 1

---

## Peptide View

MS/MS Fragmentation of **LSALDNLLNHSSIFLK**
Found in **IPI00210975**, Tax_Id=10116 Gene_Symbol=Hyou1 Hypoxia up-regulated protein 1 precursor

Match to Query 1224: 1784.962148 from(893.488350,2+)
Title: 091008RatKidney_NH4Format02_24.5410.5410.2.dta
Data file K:\NewmanPaper\Piliang\3SubProteomes\Piliang3SP\mgf5ppm\ERLIC_3SubProteomes5ppm.mgf

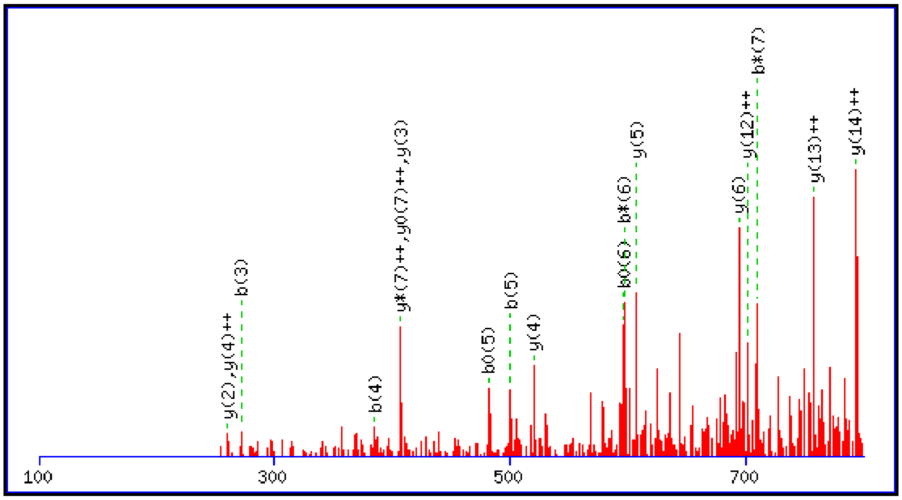

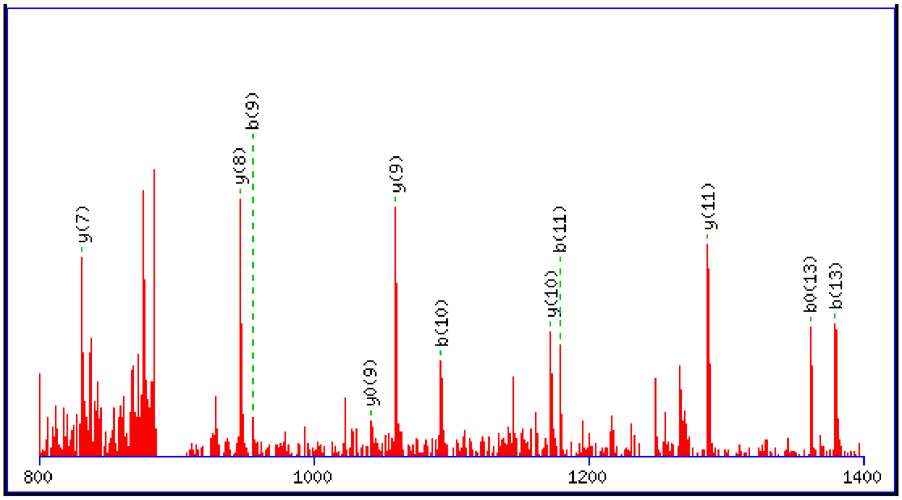


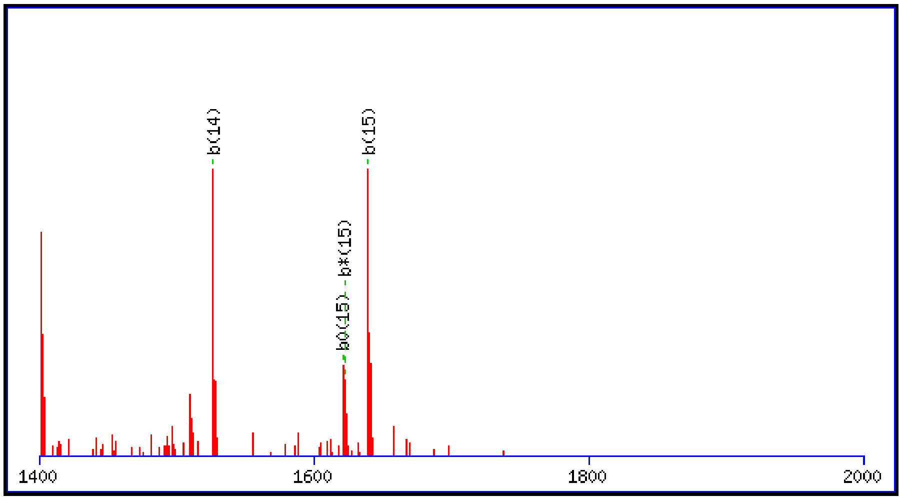


**Monoisotopic mass of neutral peptide Mr(calc):** 1784.9672
**Fixed modifications:** Carbamidomethyl (C)
**Variable modifications:**
**N9** : Deamidated_N (N)
**Ions Score:** 76 **Expect:** 2.3e-006
**Matches (Bold Red):** 33/160 fragment ions using 52 most intense peaks

| # | b | $b^{++}$ | b* | $b^{*++}$ | $b^{0}$ | $b^{0++}$ | Seq. | y | $y^{++}$ | y* | $y^{*++}$ | $y^{0}$ | $y^{0++}$ | # |
|---|---|---|---|---|---|---|---|---|---|---|---|---|---|---|
| 1 | 114.0913 | 57.5493 | | | | | L | | | | | | | 16 |
| 2 | 201.1234 | 101.0653 | | | 183.1128 | 92.0600 | S | 1672.8905 | 836.9489 | 1655.8639 | 828.4356 | 1654.8799 | 827.9436 | 15 |
| 3 | **272.1605** | 136.5839 | | | 254.1499 | 127.5786 | A | 1585.8584 | **793.4329** | 1568.8319 | 784.9196 | 1567.8479 | 784.4276 | 14 |
| 4 | **385.2445** | 193.1259 | | | 367.2340 | 184.1206 | L | 1514.8213 | **757.9143** | 1497.7948 | 749.4010 | 1496.8107 | 748.9090 | 13 |
| 5 | **500.2715** | 250.6394 | | | **482.2609** | 241.6341 | D | 1401.7372 | **701.3723** | 1384.7107 | 692.8590 | 1383.7267 | 692.3670 | 12 |
| 6 | 614.3144 | 307.6608 | **597.2879** | 299.1476 | **596.3039** | 298.6556 | N | **1286.7103** | 643.8588 | 1269.6838 | 635.3455 | 1268.6997 | 634.8535 | 11 |
| 7 | 727.3985 | 364.2029 | **710.3719** | 355.6896 | 709.3879 | 355.1976 | L | **1172.6674** | 586.8373 | 1155.6408 | 578.3241 | 1154.6568 | 577.8320 | 10 |
| 8 | 840.4825 | 420.7449 | 823.4560 | 412.2316 | 822.4720 | 411.7396 | L | **1059.5833** | 530.2953 | 1042.5568 | 521.7820 | **1041.5727** | 521.2900 | 9 |
| 9 | **955.5095** | 478.2584 | 938.4829 | 469.7451 | 937.4989 | 469.2531 | N | **946.4993** | 473.7533 | 929.4727 | 465.2400 | 928.4887 | 464.7480 | 8 |
| 10 | **1092.5684** | 546.7878 | 1075.5418 | 538.2746 | 1074.5578 | 537.7826 | H | **831.4723** | 416.2398 | 814.4458 | **407.7265** | 813.4617 | **407.2345** | 7 |
| 11 | **1179.6004** | 590.3038 | 1162.5739 | 581.7906 | 1161.5899 | 581.2986 | S | **694.4134** | 347.7103 | 677.3869 | 339.1971 | 676.4028 | 338.7051 | 6 |
| 12 | 1266.6324 | 633.8199 | 1249.6059 | 625.3066 | 1248.6219 | 624.8146 | S | **607.3814** | 304.1943 | 590.3548 | 295.6811 | 589.3708 | 295.1890 | 5 |
| 13 | **1379.7165** | 690.3619 | 1362.6900 | 681.8486 | **1361.7059** | 681.3566 | I | **520.3493** | **260.6783** | 503.3228 | 252.1650 | | | 4 |
| 14 | **1526.7849** | 763.8961 | 1509.7584 | 755.3828 | 1508.7744 | 754.8908 | F | **407.2653** | 204.1363 | 390.2387 | 195.6230 | | | 3 |
| 15 | **1639.8690** | 820.4381 | **1622.8424** | 811.9249 | **1621.8584** | 811.4329 | L | **260.1969** | 130.6021 | 243.1703 | 122.0888 | | | 2 |
| 16 | | | | | | | K | 147.1128 | 74.0600 | 130.0863 | 65.5468 | | | 1 |

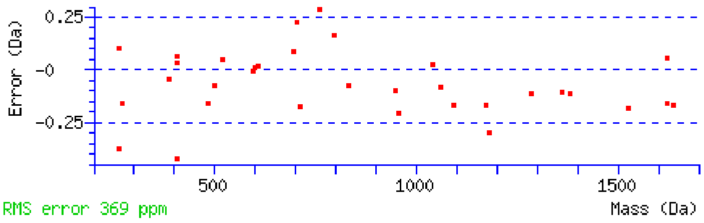


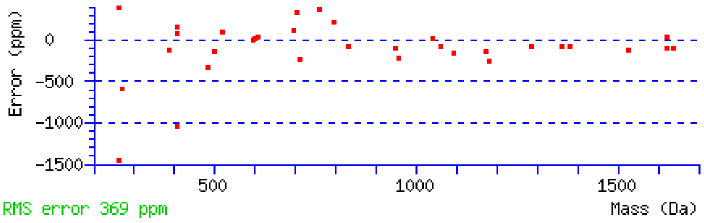


### All matches to this query

| Score | Mr(calc): | Delta | Sequence |
|---|---|---|---|
| 76.0 | 1784.9672 | -0.0051 | LSALDNLLNHSSIFLK |
| 53.9 | 1784.9672 | -0.0051 | LSALDNLLNHSSIFLK |
| 5.6 | 1784.9632 | -0.0010 | EELNKELVQASERLK |
| 4.9 | 1784.9519 | 0.0102 | DINRLLEETQAELLK |
| 4.4 | 1784.9771 | -0.0149 | KVELKENLIAELEEK |
| 3.6 | 1784.9585 | 0.0037 | YPLMVKLNLLLXLK |
| 2.5 | 1784.9454 | 0.0167 | LSSPECLNASLLGGVLR |
| 2.3 | 1784.9785 | -0.0163 | VRELSTSEKHVPYIK |
| 2.3 | 1784.9477 | 0.0144 | VAALLAANSFDIIFLK |
| 1.6 | 1782.9393 | 2.0228 | ASAVSPANLPAVLLQPR |

---

Spectrum No: 288; Query: 920; Rank: 1

---

## Peptide View

MS/MS Fragmentation of **ECLGNCSEPDDPTK**
Found in **IPI00393267**, Tax_Id=10116 Gene_Symbol=Insr Tyrosine-protein kinase receptor

Match to Query 920: 1621.625928 from(811.820240,2+)
Title: 100101RatKid_NS_deglyco_20.879.879.2.dta
Data file K:\NewmanPaper\Piliang\3SubProteomes\Piliang3SP\mgf5ppm\ERLIC_3SubProteomes5ppm.mgf

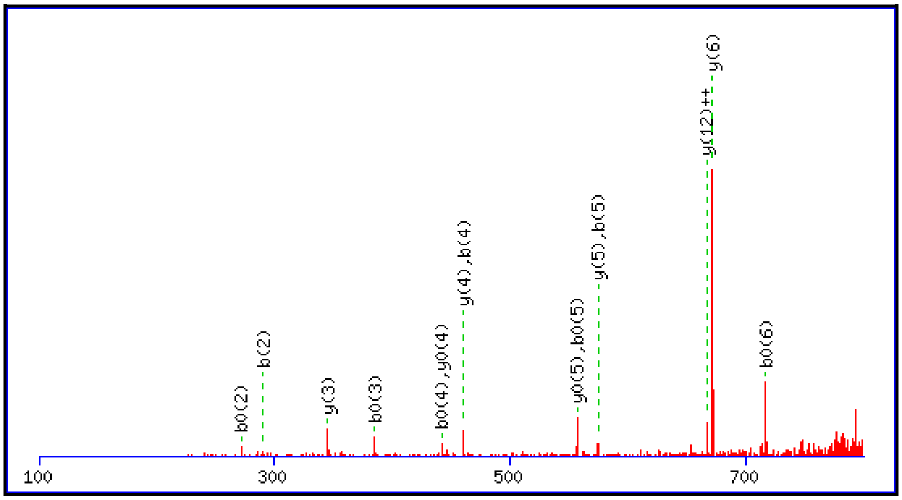

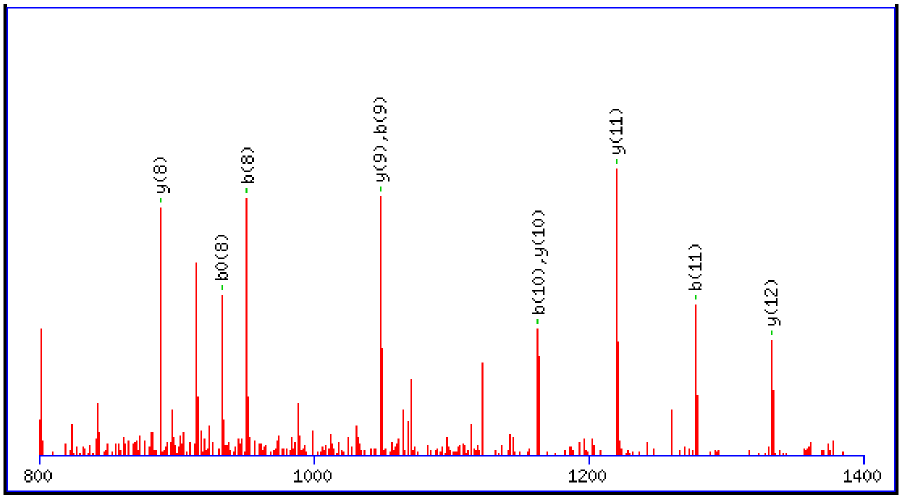


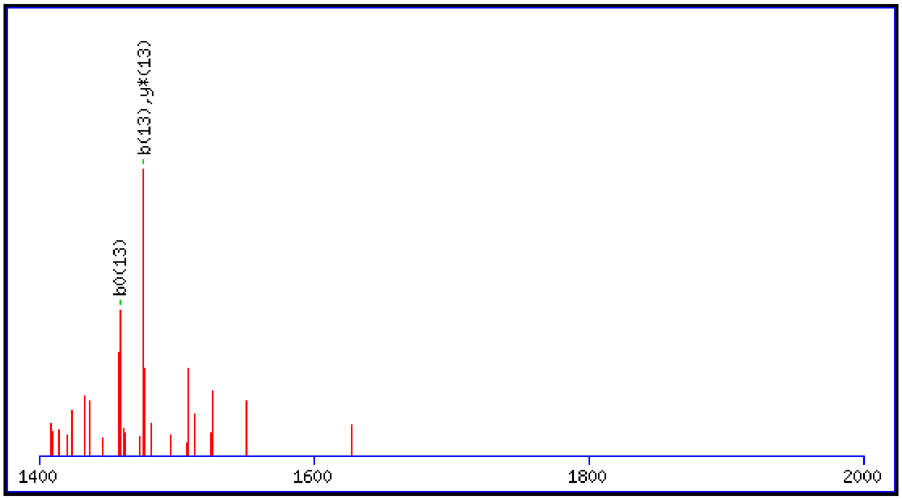


**Monoisotopic mass of neutral peptide Mr(calc):** 1621.6236
**Fixed modifications:** Carbamidomethyl (C)
**Variable modifications:**
**N5** : Deamidated_N (N)
**Ions Score:** 76 **Expect:** 2.7e-006
**Matches (Bold Red):** 28/146 fragment ions using 32 most intense peaks

| # | b | $b^{++}$ | b* | $b^{*++}$ | $b^{0}$ | $b^{0++}$ | Seq. | y | $y^{++}$ | y* | $y^{*++}$ | $y^{0}$ | $y^{0++}$ | # |
|---|---|---|---|---|---|---|---|---|---|---|---|---|---|---|
| 1 | 130.0499 | 65.5286 | | | 112.0393 | 56.5233 | E | | | | | | | 14 |
| 2 | **290.0805** | 145.5439 | | | **272.0700** | 136.5386 | C | 1493.5883 | 747.2978 | **1476.5617** | 738.7845 | 1475.5777 | 738.2925 | 13 |
| 3 | 403.1646 | 202.0859 | | | **385.1540** | 193.0806 | L | **1333.5576** | **667.2825** | 1316.5311 | 658.7692 | 1315.5471 | 658.2772 | 12 |
| 4 | **460.1860** | 230.5967 | | | **442.1755** | 221.5914 | G | **1220.4736** | 610.7404 | 1203.4470 | 602.2271 | 1202.4630 | 601.7351 | 11 |
| 5 | **575.2130** | 288.1101 | 558.1864 | 279.5969 | **557.2024** | 279.1048 | N | **1163.4521** | 582.2297 | 1146.4256 | 573.7164 | 1145.4415 | 573.2244 | 10 |
| 6 | 735.2436 | 368.1255 | 718.2171 | 359.6122 | **717.2331** | 359.1202 | C | **1048.4252** | 524.7162 | 1031.3986 | 516.2029 | 1030.4146 | 515.7109 | 9 |
| 7 | 822.2757 | 411.6415 | 805.2491 | 403.1282 | 804.2651 | 402.6362 | S | **888.3945** | 444.7009 | 871.3680 | 436.1876 | 870.3840 | 435.6956 | 8 |
| 8 | **951.3183** | 476.1628 | 934.2917 | 467.6495 | **933.3077** | 467.1575 | E | 801.3625 | 401.1849 | 784.3359 | 392.6716 | 783.3519 | 392.1796 | 7 |
| 9 | **1048.3710** | 524.6891 | 1031.3445 | 516.1759 | 1030.3605 | 515.6839 | P | **672.3199** | 336.6636 | 655.2933 | 328.1503 | 654.3093 | 327.6583 | 6 |
| 10 | **1163.3980** | 582.2026 | 1146.3714 | 573.6893 | 1145.3874 | 573.1973 | D | **575.2671** | 288.1372 | 558.2406 | 279.6239 | **557.2566** | 279.1319 | 5 |
| 11 | **1278.4249** | 639.7161 | 1261.3984 | 631.2028 | 1260.4143 | 630.7108 | D | **460.2402** | 230.6237 | 443.2136 | 222.1105 | **442.2296** | 221.6185 | 4 |
| 12 | 1375.4777 | 688.2425 | 1358.4511 | 679.7292 | 1357.4671 | 679.2372 | P | **345.2132** | 173.1103 | 328.1867 | 164.5970 | 327.2027 | 164.1050 | 3 |
| 13 | **1476.5253** | 738.7663 | 1459.4988 | 730.2530 | **1458.5148** | 729.7610 | T | 248.1605 | 124.5839 | 231.1339 | 116.0706 | 230.1499 | 115.5786 | 2 |
| 14 | | | | | | | K | 147.1128 | 74.0600 | 130.0863 | 65.5468 | | | 1 |

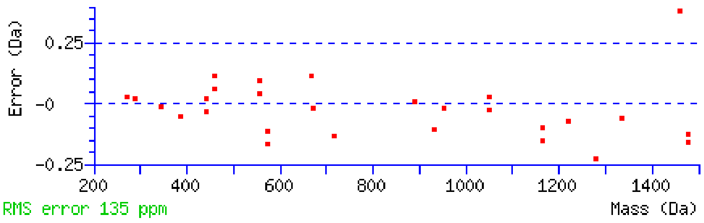


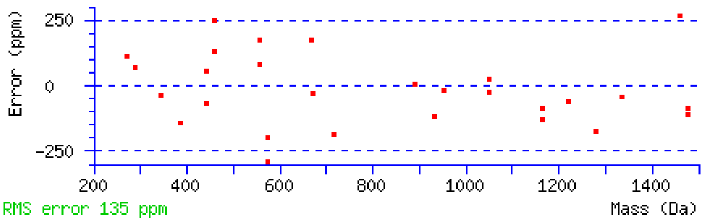


### All matches to this query

| Score | Mr(calc): | Delta | Sequence |
|---|---|---|---|
| 75.9 | 1621.6236 | 0.0023 | ECLGNCSEPDDPTK |
| 8.0 | 1621.6274 | -0.0014 | ASSVGNVADSTEPTK |
| 7.5 | 1621.6274 | -0.0014 | ASSVGNVADSTEPTK |
| 5.7 | 1621.6274 | -0.0014 | ASSVGNVADSTEPTK |
| 5.7 | 1621.6274 | -0.0014 | ASSVGNVADSTEPTK |
| 5.3 | 1621.6274 | -0.0014 | ASSVGNVADSTEPTK |
| 5.3 | 1621.6227 | 0.0032 | DGDEESGNKNENSPK |
| 1.2 | 1621.6274 | -0.0014 | ASSVGNVADSTEPTK |
| 0.7 | 1621.6287 | -0.0027 | EKSHGYQRENSK |
| 0.4 | 1620.6387 | 0.9873 | DGDEESGNKNENSPK |

---

Spectrum No: 289; Query: 1909; Rank: 1

---

## Peptide View

MS/MS Fragmentation of **YHYNGTLLDGTLFDSSYSR**
Found in **IPI00215190**, Tax_Id=10116 Gene_Symbol=Fkbp9 FK506-binding protein 9 precursor

Match to Query 1909: 2209.001208 from(1105.507880,2+)
Title: 100101RatKid_NS_deglyco_13.4624.4624.2.dta
Data file K:\NewmanPaper\Piliang\3SubProteomes\Piliang3SP\mgf5ppm\ERLIC_3SubProteomes5ppm.mgf

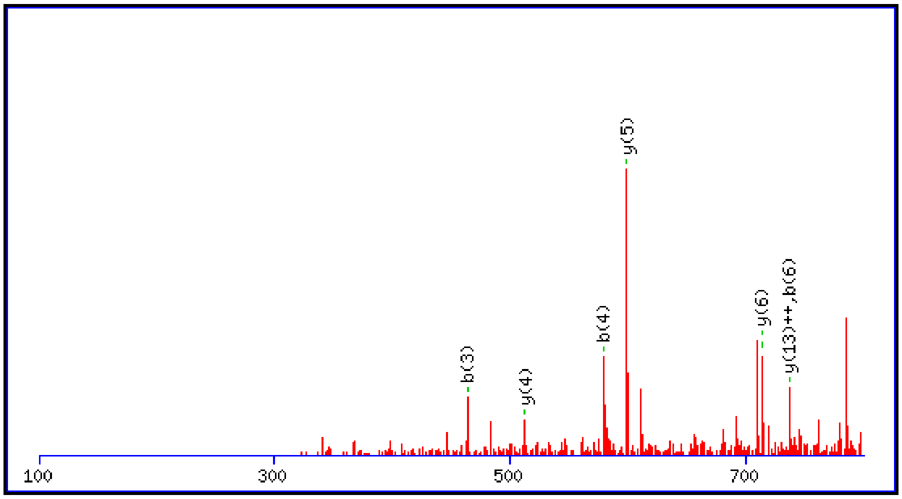

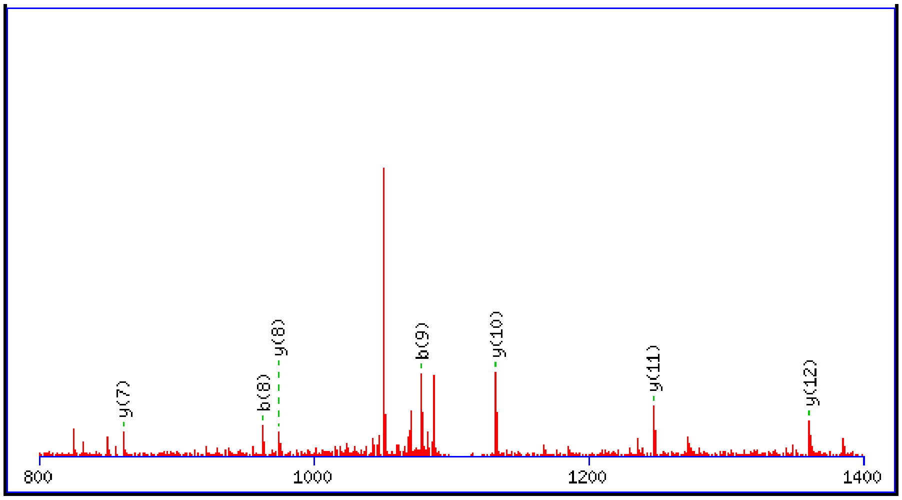


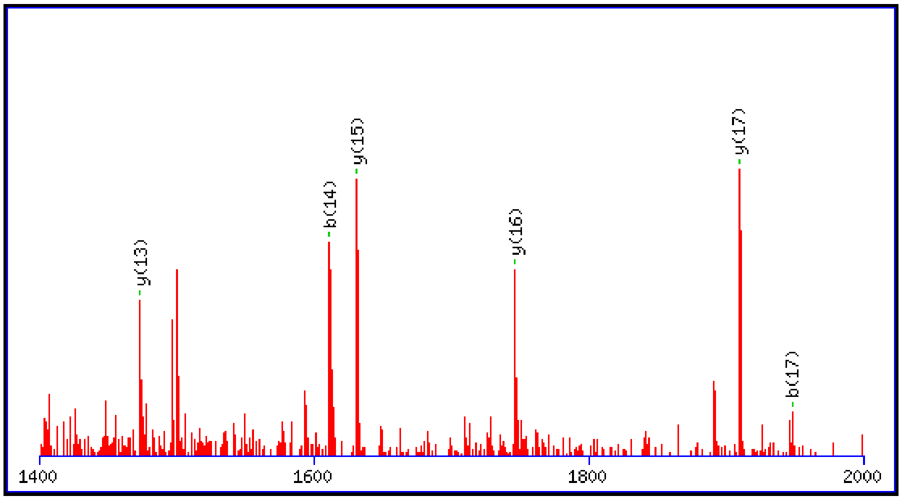


**Monoisotopic mass of neutral peptide Mr(calc):** 2208.9964
**Fixed modifications:** Carbamidomethyl (C)
**Variable modifications:**
**N4** : Deamidated_N (N)
**Ions Score:** 76 **Expect:** 6.9e-006
**Matches (Bold Red):** 20/198 fragment ions using 38 most intense peaks

| # | b | $b^{++}$ | b* | $b^{*++}$ | $b^0$ | $b^{0++}$ | Seq. | y | $y^{++}$ | y* | $y^{*++}$ | $y^0$ | $y^{0++}$ | # |
|---|---|---|---|---|---|---|---|---|---|---|---|---|---|---|
| 1 | 164.0706 | 82.5389 | | | | | Y | | | | | | | 19 |
| 2 | 301.1295 | 151.0684 | | | | | H | 2046.9403 | 1023.9738 | 2029.9138 | 1015.4605 | 2028.9298 | 1014.9685 | 18 |
| 3 | **464.1928** | 232.6001 | | | | | Y | **1909.8814** | 955.4443 | 1892.8549 | 946.9311 | 1891.8708 | 946.4391 | 17 |
| 4 | **579.2198** | 290.1135 | 562.1932 | 281.6003 | | | N | **1746.8181** | 873.9127 | 1729.7915 | 865.3994 | 1728.8075 | 864.9074 | 16 |
| 5 | 636.2412 | 318.6243 | 619.2147 | 310.1110 | | | G | **1631.7911** | 816.3992 | 1614.7646 | 807.8859 | 1613.7806 | 807.3939 | 15 |
| 6 | **737.2889** | 369.1481 | 720.2624 | 360.6348 | 719.2784 | 360.1428 | T | 1574.7697 | 787.8885 | 1557.7431 | 779.3752 | 1556.7591 | 778.8832 | 14 |
| 7 | 850.3730 | 425.6901 | 833.3464 | 417.1769 | 832.3624 | 416.6849 | L | **1473.7220** | **737.3646** | 1456.6955 | 728.8514 | 1455.7114 | 728.3594 | 13 |
| 8 | **963.4571** | 482.2322 | 946.4305 | 473.7189 | 945.4465 | 473.2269 | L | **1360.6379** | 680.8226 | 1343.6114 | 672.3093 | 1342.6274 | 671.8173 | 12 |
| 9 | **1078.4840** | 539.7456 | 1061.4574 | 531.2324 | 1060.4734 | 530.7404 | D | **1247.5539** | 624.2806 | 1230.5273 | 615.7673 | 1229.5433 | 615.2753 | 11 |
| 10 | 1135.5055 | 568.2564 | 1118.4789 | 559.7431 | 1117.4949 | 559.2511 | G | **1132.5269** | 566.7671 | 1115.5004 | 558.2538 | 1114.5164 | 557.7618 | 10 |
| 11 | 1236.5531 | 618.7802 | 1219.5266 | 610.2669 | 1218.5426 | 609.7749 | T | 1075.5055 | 538.2564 | 1058.4789 | 529.7431 | 1057.4949 | 529.2511 | 9 |
| 12 | 1349.6372 | 675.3222 | 1332.6107 | 666.8090 | 1331.6266 | 666.3170 | L | **974.4578** | 487.7325 | 957.4312 | 479.2193 | 956.4472 | 478.7272 | 8 |
| 13 | 1496.7056 | 748.8564 | 1479.6791 | 740.3432 | 1478.6951 | 739.8512 | F | **861.3737** | 431.1905 | 844.3472 | 422.6772 | 843.3632 | 422.1852 | 7 |
| 14 | **1611.7326** | 806.3699 | 1594.7060 | 797.8566 | 1593.7220 | 797.3646 | D | **714.3053** | 357.6563 | 697.2788 | 349.1430 | 696.2947 | 348.6510 | 6 |
| 15 | 1698.7646 | 849.8859 | 1681.7380 | 841.3727 | 1680.7540 | 840.8806 | S | **599.2784** | 300.1428 | 582.2518 | 291.6295 | 581.2678 | 291.1375 | 5 |
| 16 | 1785.7966 | 893.4019 | 1768.7701 | 884.8887 | 1767.7861 | 884.3967 | S | **512.2463** | 256.6268 | 495.2198 | 248.1135 | 494.2358 | 247.6215 | 4 |
| 17 | **1948.8599** | 974.9336 | 1931.8334 | 966.4203 | 1930.8494 | 965.9283 | Y | 425.2143 | 213.1108 | 408.1878 | 204.5975 | 407.2037 | 204.1055 | 3 |
| 18 | 2035.8920 | 1018.4496 | 2018.8654 | 1009.9364 | 2017.8814 | 1009.4443 | S | 262.1510 | 131.5791 | 245.1244 | 123.0659 | 244.1404 | 122.5738 | 2 |

| | | | | | | | | | | | | | | |
|---|---|---|---|---|---|---|---|---|---|---|---|---|---|---|
| 19 | | | | | | | R | 175.1190 | 88.0631 | 158.0924 | 79.5498 | | | 1 |

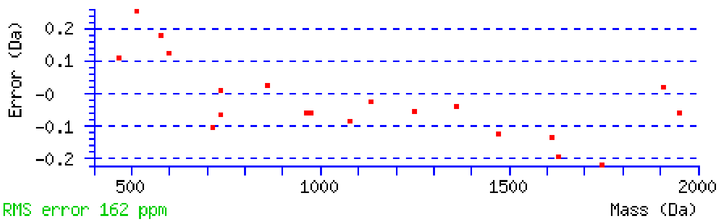


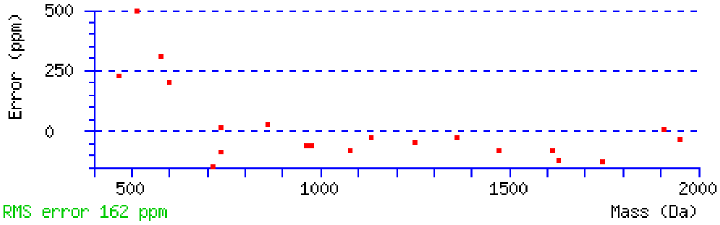


### All matches to this query

| Score | Mr(calc): | Delta | Sequence |
|---|---|---|---|
| 75.9 | 2208.9964 | 0.0048 | YHYNGTLLDGTLFDSSYSR |
| 37.3 | 2208.0124 | 0.9888 | YHYNGTLLDGTLFDSSYSR |
| 2.6 | 2208.9946 | 0.0066 | KRALSISPLSDASLDLQR |
| 1.2 | 2207.0147 | 1.9865 | LSLKGHEDWVTDVAISNDK |
| 0.4 | 2207.9832 | 1.0180 | TIMLLGVSSQKISVRLK |

---

Spectrum No: 290; Query: 231; Rank: 1

---

## Peptide View

MS/MS Fragmentation of **QTAEYAANITK**
Found in **IPI00327398**, Tax_Id=10116 Gene_Symbol=Enpep Isoform 1 of Glutamyl aminopeptidase

Match to Query 231: 1209.588308 from(605.801430,2+)
Title: 091008RatKidney_NoSalt_26.810.810.2.dta
Data file K:\NewmanPaper\Piliang\3SubProteomes\Piliang3SP\mgf5ppm\ERLIC_3SubProteomes5ppm.mgf

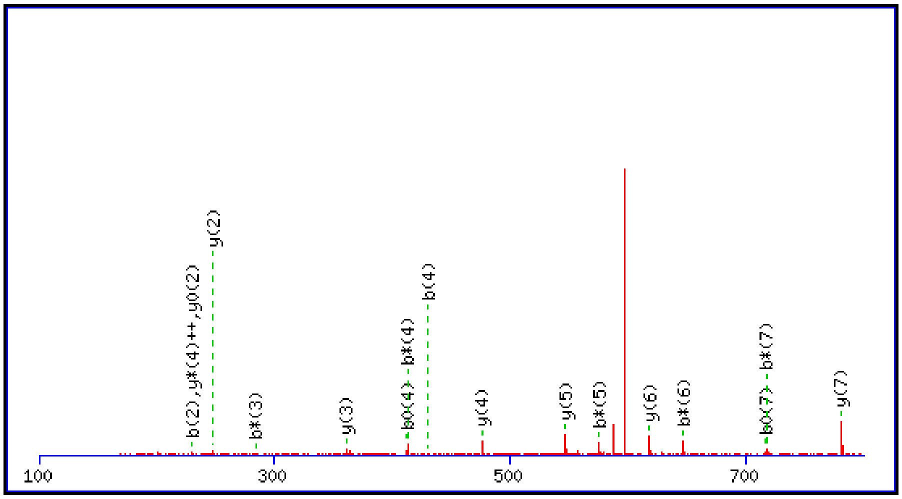

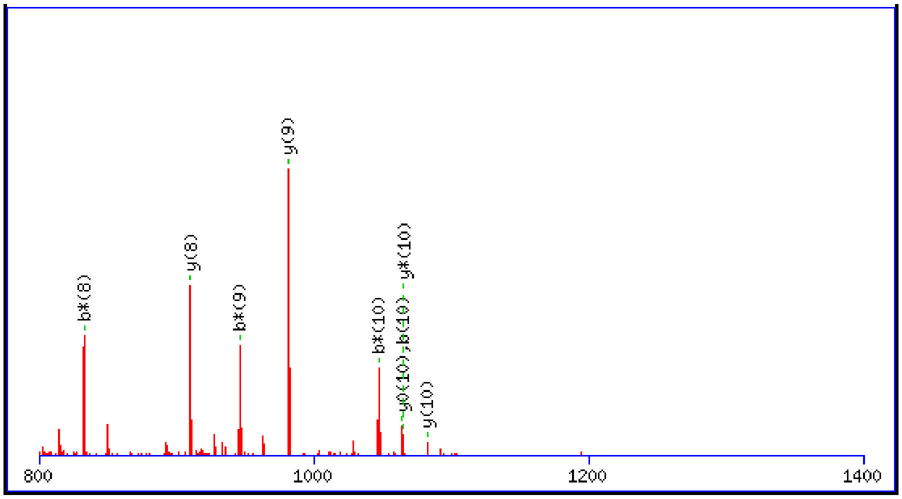


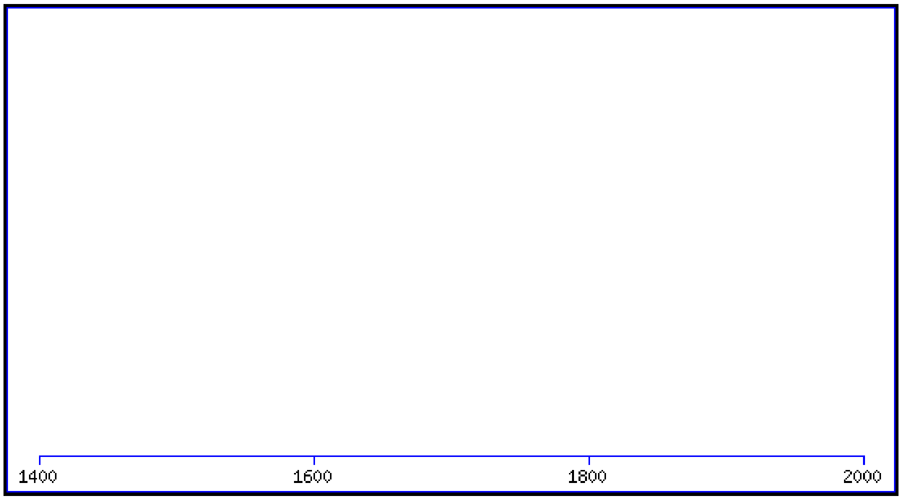


**Monoisotopic mass of neutral peptide Mr(calc):** 1209.5877
**Fixed modifications:** Carbamidomethyl (C)
**Variable modifications:**
**N8** : Deamidated_N (N)
**Ions Score:** 76 **Expect:** 3.7e-006
**Matches (Bold Red):** 26/116 fragment ions using 40 most intense peaks

| # | b | $b^{++}$ | b* | $b^{*++}$ | $b^0$ | $b^{0++}$ | Seq. | y | $y^{++}$ | y* | $y^{*++}$ | $y^0$ | $y^{0++}$ | # |
|---|---|---|---|---|---|---|---|---|---|---|---|---|---|---|
| 1 | 129.0659 | 65.0366 | 112.0393 | 56.5233 | | | Q | | | | | | | 11 |
| 2 | **230.1135** | 115.5604 | 213.0870 | 107.0471 | 212.1030 | 106.5551 | T | **1082.5364** | 541.7719 | **1065.5099** | 533.2586 | **1064.5259** | 532.7666 | 10 |
| 3 | 301.1506 | 151.0790 | **284.1241** | 142.5657 | 283.1401 | 142.0737 | A | **981.4887** | 491.2480 | 964.4622 | 482.7347 | 963.4782 | 482.2427 | 9 |
| 4 | **430.1932** | 215.6003 | **413.1667** | 207.0870 | **412.1827** | 206.5950 | E | **910.4516** | 455.7295 | 893.4251 | 447.2162 | 892.4411 | 446.7242 | 8 |
| 5 | 593.2566 | 297.1319 | **576.2300** | 288.6186 | 575.2460 | 288.1266 | Y | **781.4090** | 391.2082 | 764.3825 | 382.6949 | 763.3985 | 382.2029 | 7 |
| 6 | 664.2937 | 332.6505 | **647.2671** | 324.1372 | 646.2831 | 323.6452 | A | **618.3457** | 309.6765 | 601.3192 | 301.1632 | 600.3351 | 300.6712 | 6 |
| 7 | 735.3308 | 368.1690 | **718.3042** | 359.6558 | **717.3202** | 359.1638 | A | **547.3086** | 274.1579 | 530.2820 | 265.6447 | 529.2980 | 265.1527 | 5 |
| 8 | 850.3577 | 425.6825 | **833.3312** | 417.1692 | 832.3472 | 416.6772 | N | **476.2715** | 238.6394 | 459.2449 | **230.1261** | 458.2609 | 229.6341 | 4 |
| 9 | 963.4418 | 482.2245 | **946.4152** | 473.7113 | 945.4312 | 473.2193 | I | **361.2445** | 181.1259 | 344.2180 | 172.6126 | 343.2340 | 172.1206 | 3 |
| 10 | **1064.4895** | 532.7484 | **1047.4629** | 524.2351 | 1046.4789 | 523.7431 | T | **248.1605** | 124.5839 | 231.1339 | 116.0706 | **230.1499** | 115.5786 | 2 |
| 11 | | | | | | | K | 147.1128 | 74.0600 | 130.0863 | 65.5468 | | | 1 |

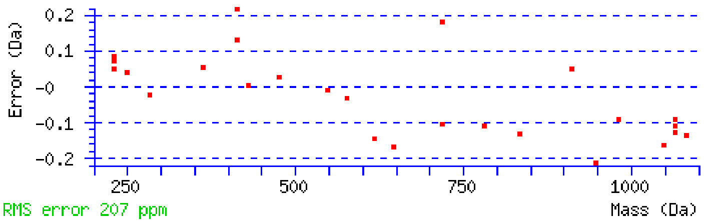


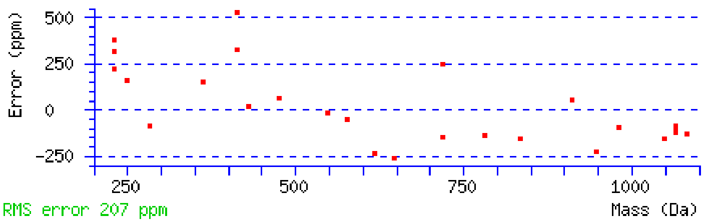

### All matches to this query

| Score | Mr(calc): | Delta | Sequence |
|---|---|---|---|
| 75.9 | 1209.5877 | 0.0006 | QTAEYAANITK |
| 12.0 | 1208.5826 | 1.0057 | DTVLAYWADR |
| 5.9 | 1208.5781 | 1.0102 | MVEVCVESIK |
| 5.0 | 1207.5710 | 2.0173 | EAEATAKRPR |
| 3.5 | 1208.5955 | 0.9928 | DILSVQIXR |
| 3.4 | 1208.5914 | 0.9969 | SETPVTIARR |
| 3.4 | 1208.5914 | 0.9969 | SETPVTIARR |
| 3.4 | 1208.5914 | 0.9969 | SETPVTIARR |
| 3.3 | 1208.5802 | 1.0081 | QVLSGSGGPVTK |
| 3.2 | 1208.5785 | 1.0098 | EVARNQNYSK |

---

Spectrum No: 291; Query: 635; Rank: 1

---

## Peptide View

MS/MS Fragmentation of **VLTNQENPYQNR**
Found in **IPI00215296**, Tax_Id=10116 Gene_Symbol=C1qa Complement C1q subcomponent subunit A precursor

Match to Query 635: 1475.704308 from(738.859430,2+)
Title: 100101RatKid_NS_deglyco_23.986.986.2.dta
Data file K:\NewmanPaper\Piliang\3SubProteomes\Piliang3SP\mgf5ppm\ERLIC_3SubProteomes5ppm.mgf

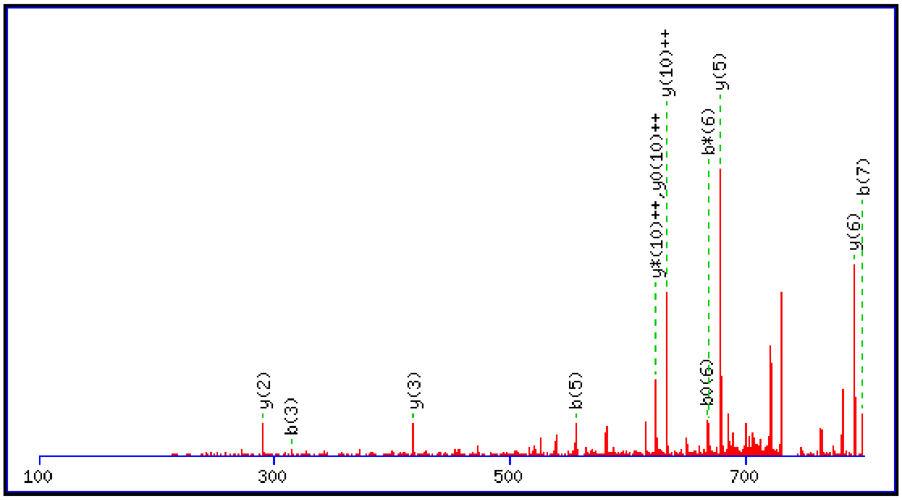


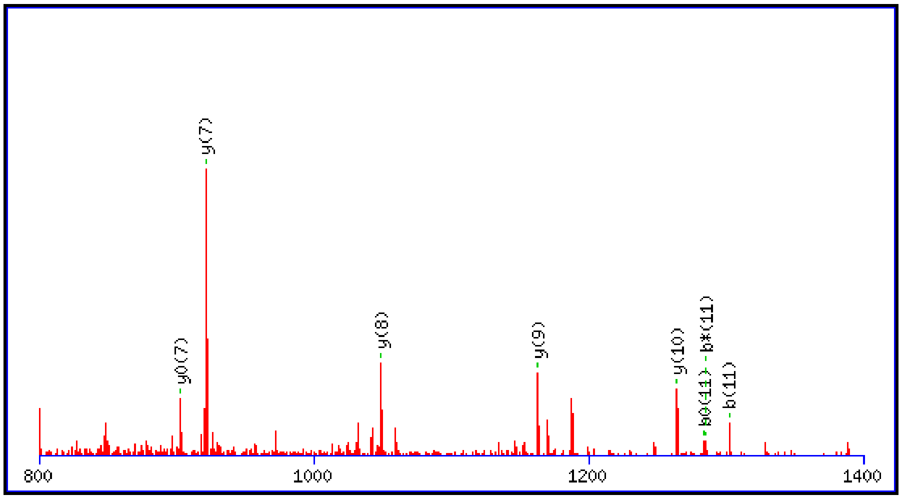

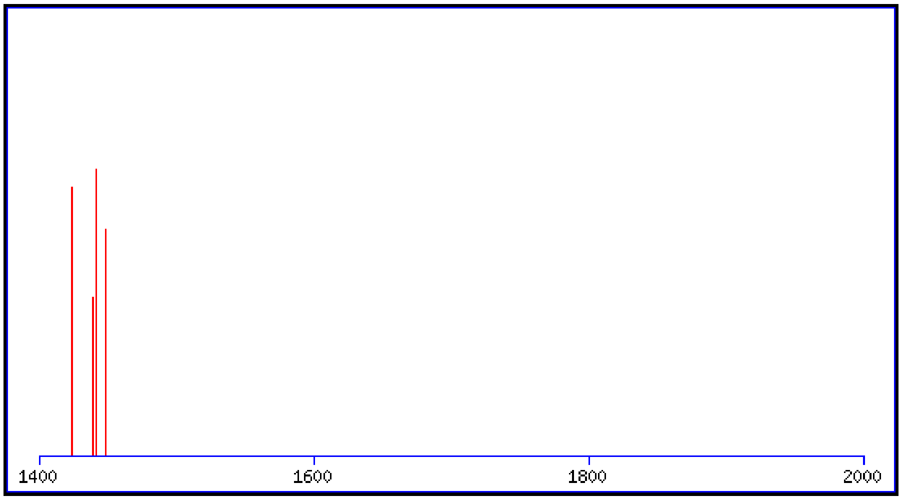


**Monoisotopic mass of neutral peptide Mr(calc):** 1475.7004
**Fixed modifications:** Carbamidomethyl (C)
**Variable modifications:**
**N11** : Deamidated_N (N)
**Ions Score:** 76 **Expect:** 4e-006
**Matches (Bold Red):** 20/110 fragment ions using 33 most intense peaks

| # | b | $b^{++}$ | b* | $b^{*++}$ | $b^0$ | $b^{0++}$ | Seq. | y | $y^{++}$ | y* | $y^{*++}$ | $y^0$ | $y^{0++}$ | # |
|---|---|---|---|---|---|---|---|---|---|---|---|---|---|---|
| 1 | 100.0757 | 50.5415 | | | | | V | | | | | | | 12 |
| 2 | 213.1598 | 107.0835 | | | | | L | 1377.6393 | 689.3233 | 1360.6128 | 680.8100 | 1359.6288 | 680.3180 | 11 |
| 3 | **314.2074** | 157.6074 | | | 296.1969 | 148.6021 | T | **1264.5553** | **632.7813** | 1247.5287 | **624.2680** | 1246.5447 | **623.7760** | 10 |
| 4 | 428.2504 | 214.6288 | 411.2238 | 206.1155 | 410.2398 | 205.6235 | N | **1163.5076** | 582.2574 | 1146.4810 | 573.7442 | 1145.4970 | 573.2521 | 9 |
| 5 | **556.3089** | 278.6581 | 539.2824 | 270.1448 | 538.2984 | 269.6528 | Q | **1049.4647** | 525.2360 | 1032.4381 | 516.7227 | 1031.4541 | 516.2307 | 8 |
| 6 | 685.3515 | 343.1794 | **668.3250** | 334.6661 | **667.3410** | 334.1741 | E | **921.4061** | 461.2067 | 904.3795 | 452.6934 | **903.3955** | 452.2014 | 7 |
| 7 | **799.3945** | 400.2009 | 782.3679 | 391.6876 | 781.3839 | 391.1956 | N | **792.3635** | 396.6854 | 775.3369 | 388.1721 | | | 6 |
| 8 | 896.4472 | 448.7272 | 879.4207 | 440.2140 | 878.4367 | 439.7220 | P | **678.3206** | 339.6639 | 661.2940 | 331.1506 | | | 5 |
| 9 | 1059.5106 | 530.2589 | 1042.4840 | 521.7456 | 1041.5000 | 521.2536 | Y | 581.2678 | 291.1375 | 564.2412 | 282.6243 | | | 4 |
| 10 | 1187.5691 | 594.2882 | 1170.5426 | 585.7749 | 1169.5586 | 585.2829 | Q | **418.2045** | 209.6059 | 401.1779 | 201.0926 | | | 3 |
| 11 | **1302.5961** | 651.8017 | **1285.5695** | 643.2884 | **1284.5855** | 642.7964 | N | **290.1459** | 145.5766 | 273.1193 | 137.0633 | | | 2 |
| 12 | | | | | | | R | 175.1190 | 88.0631 | 158.0924 | 79.5498 | | | 1 |

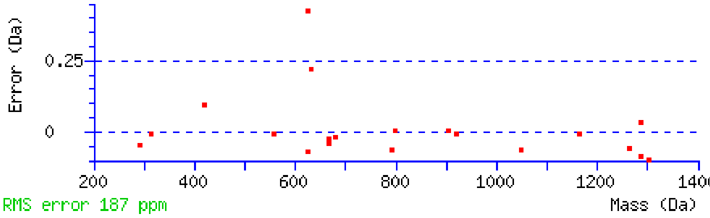


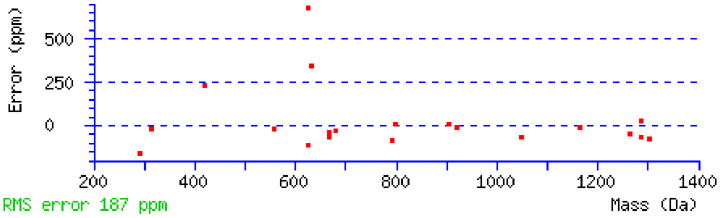


### All matches to this query

| Score | Mr(calc): | Delta | Sequence |
|---|---|---|---|
| 75.8 | 1475.7004 | 0.0039 | VLTNQENPYQNR |
| 28.1 | 1475.7004 | 0.0039 | VLTNQENPYQNR |
| 7.5 | 1475.7133 | -0.0090 | LVSRTASKYTDR |
| 7.4 | 1475.7150 | -0.0107 | VAKVTGGAASKLSK |
| 7.0 | 1475.7133 | -0.0090 | LVSRTASKYTDR |
| 6.5 | 1475.7133 | -0.0090 | LVSRTASKYTDR |
| 5.1 | 1475.7004 | 0.0039 | VLTNQENPYQNR |
| 3.6 | 1475.7150 | -0.0107 | VAKVTGGAASKLSK |
| 1.7 | 1474.6891 | 1.0152 | KMPQYQKELSK |
| | | | |

| | | | |
|---|---|---|---|
| 1.2 | 1473.7106 | 1.9938 | RAPTTALTLRSK |

---

Spectrum No: 292; Query: 863; Rank: 1

---

## Peptide View

MS/MS Fragmentation of **CNTTQGNEVTSILR**
Found in **IPI00327143**, Tax_Id=10116 Gene_Symbol=Alpl Alkaline phosphatase, tissue-nonspecific isozyme precursor

Match to Query 863: 1592.749768 from(797.382160,2+)
Title: 100101RatKid_NS_deglyco_23.2388.2388.2.dta
Data file K:\NewmanPaper\Piliang\3SubProteomes\Piliang3SP\mgf5ppm\ERLIC_3SubProteomes5ppm.mgf

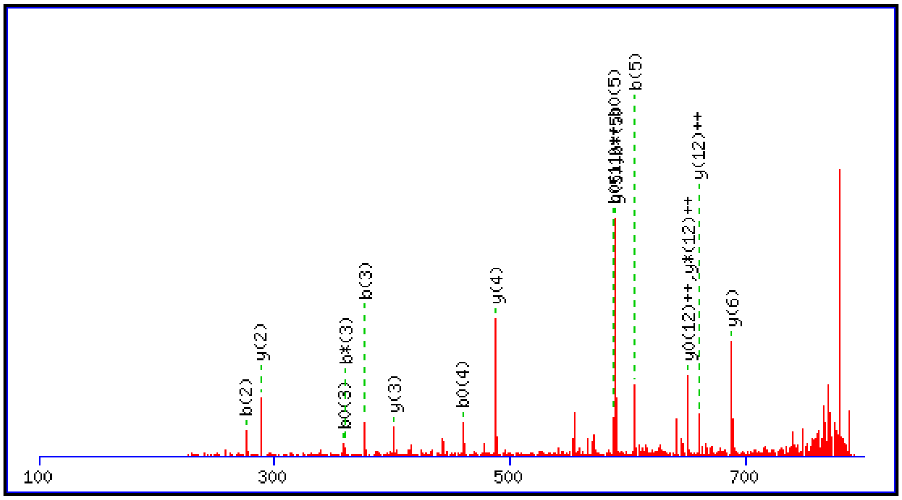


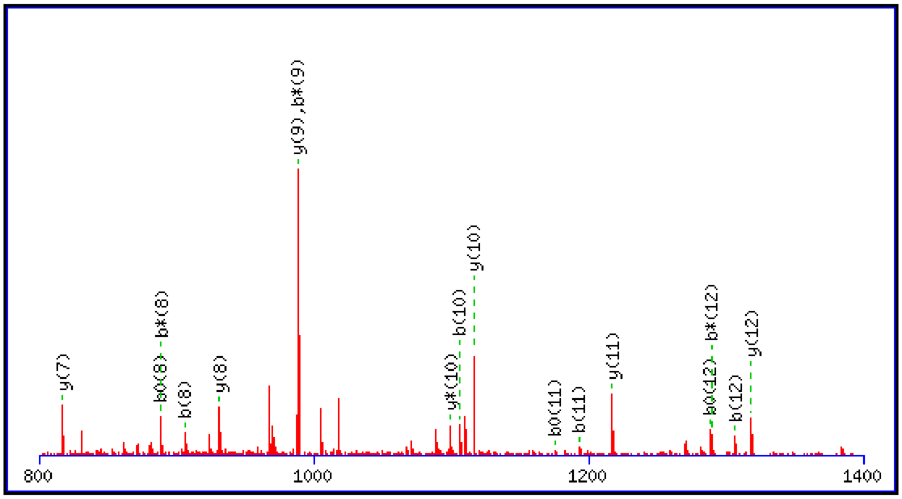

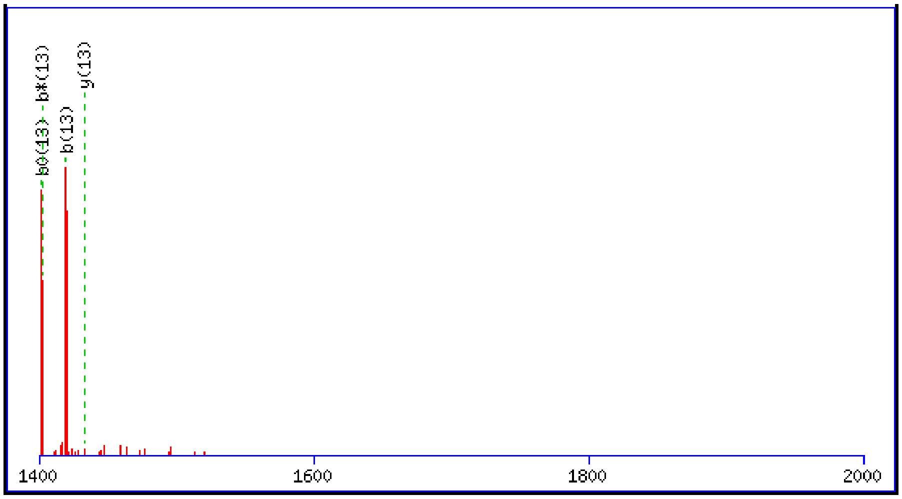


**Monoisotopic mass of neutral peptide Mr(calc):** 1592.7464
**Fixed modifications:** Carbamidomethyl (C)
**Variable modifications:**
**N2** : Deamidated_N (N)
**Ions Score:** 76 **Expect:** 4.9e-006
**Matches (Bold Red):** 38/144 fragment ions using 65 most intense peaks

| # | b | $b^{++}$ | b* | $b^{*++}$ | $b^0$ | $b^{0++}$ | Seq. | y | $y^{++}$ | y* | $y^{*++}$ | $y^0$ | $y^{0++}$ | # |
|---|---|---|---|---|---|---|---|---|---|---|---|---|---|---|
| 1 | 161.0379 | 81.0226 | | | | | C | | | | | | | 14 |
| 2 | **276.0649** | 138.5361 | 259.0383 | 130.0228 | | | N | **1433.7231** | 717.3652 | 1416.6965 | 708.8519 | 1415.7125 | 708.3599 | 13 |
| 3 | **377.1125** | 189.0599 | **360.0860** | 180.5466 | **359.1020** | 180.0546 | T | **1318.6961** | **659.8517** | 1301.6696 | **651.3384** | 1300.6856 | **650.8464** | 12 |
| 4 | 478.1602 | 239.5837 | 461.1337 | 231.0705 | **460.1497** | 230.5785 | T | **1217.6484** | 609.3279 | 1200.6219 | 600.8146 | 1199.6379 | 600.3226 | 11 |
| 5 | **606.2188** | 303.6130 | **589.1922** | 295.0998 | **588.2082** | 294.6078 | Q | **1116.6008** | 558.8040 | **1099.5742** | 550.2907 | 1098.5902 | 549.7987 | 10 |
| 6 | 663.2403 | 332.1238 | 646.2137 | 323.6105 | 645.2297 | 323.1185 | G | **988.5422** | 494.7747 | 971.5156 | 486.2615 | 970.5316 | 485.7694 | 9 |
| 7 | 777.2832 | 389.1452 | 760.2566 | 380.6320 | 759.2726 | 380.1399 | N | **931.5207** | 466.2640 | 914.4942 | 457.7507 | 913.5102 | 457.2587 | 8 |
| 8 | **906.3258** | 453.6665 | **889.2992** | 445.1533 | **888.3152** | 444.6612 | E | **817.4778** | 409.2425 | 800.4512 | 400.7293 | 799.4672 | 400.2373 | 7 |
| 9 | 1005.3942 | 503.2007 | **988.3676** | 494.6875 | 987.3836 | 494.1955 | V | **688.4352** | 344.7212 | 671.4087 | 336.2080 | 670.4246 | 335.7160 | 6 |
| 10 | **1106.4419** | 553.7246 | 1089.4153 | 545.2113 | 1088.4313 | 544.7193 | T | **589.3668** | 295.1870 | 572.3402 | 286.6738 | 571.3562 | 286.1817 | 5 |
| 11 | **1193.4739** | 597.2406 | 1176.4474 | 588.7273 | **1175.4633** | **588.2353** | S | **488.3191** | 244.6632 | 471.2926 | 236.1499 | 470.3085 | 235.6579 | 4 |
| 12 | **1306.5580** | 653.7826 | **1289.5314** | 645.2693 | **1288.5474** | 644.7773 | I | **401.2871** | 201.1472 | 384.2605 | 192.6339 | | | 3 |
| 13 | **1419.6420** | 710.3247 | **1402.6155** | 701.8114 | **1401.6315** | 701.3194 | L | **288.2030** | 144.6051 | 271.1765 | 136.0919 | | | 2 |
| 14 | | | | | | | R | 175.1190 | 88.0631 | 158.0924 | 79.5498 | | | 1 |

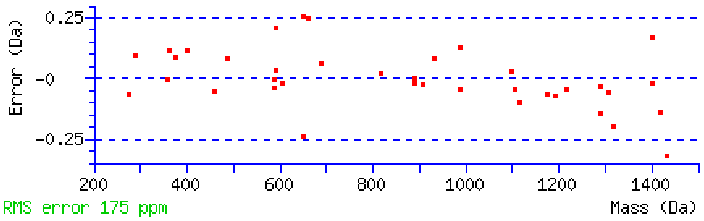


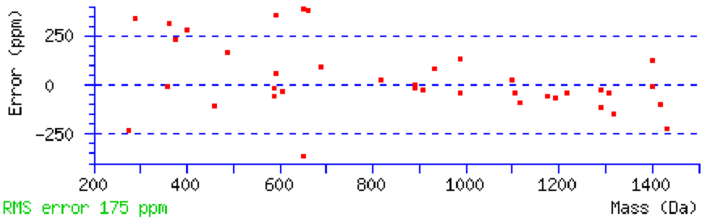


## All matches to this query

| Score | Mr(calc): | Delta | Sequence |
|---|---|---|---|
| 75.7 | 1592.7464 | 0.0033 | CNTTQGNEVTSILR |
| 64.5 | 1592.7464 | 0.0033 | CNTTQGNEVTSILR |
| 12.5 | 1592.7529 | -0.0031 | TATNGISANGVNKSEK |
| 11.8 | 1591.7607 | 0.9891 | ESHVINQVKNSKK |
| 8.7 | 1592.7577 | -0.0079 | TLGESQRTDCSLAR |
| 8.0 | 1592.7633 | -0.0136 | DCTKELLPPTALR |
| 7.7 | 1592.7365 | 0.0132 | NRYGDVPCLDQTR |
| | | | |

| | | | |
|---|---|---|---|
| 7.2 | 1592.7390 | 0.0108 | NRERVESSEETTR |
| 6.7 | 1592.7487 | 0.0011 | IENFTTDYKIIR |
| 6.5 | 1592.7633 | -0.0136 | DCTKELLPPTALR |

Spectrum No: 293; Query: 1140; Rank: 1

## Peptide View

MS/MS Fragmentation of **LTSCATNASMCGDEAR**
Found in **IPI00369995**, Tax_Id=10116 Gene_Symbol=Lrp1 similar to low density lipoprotein receptor-related protein 1

Match to Query 1140: 1743.691808 from(872.853180,2+)
Title: 100101RatKid_NS_deglyco_23.915.915.2.dta
Data file K:\NewmanPaper\Piliang\3SubProteomes\Piliang3SP\mgf5ppm\ERLIC_3SubProteomes5ppm.mgf

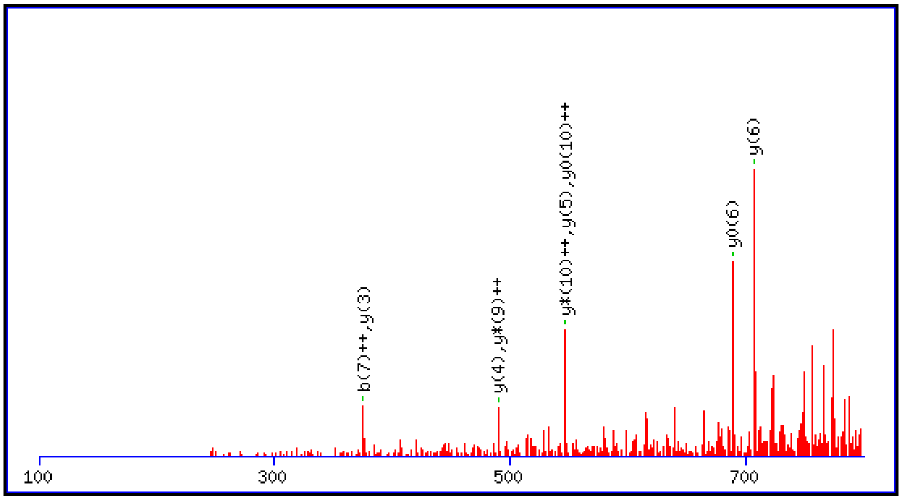


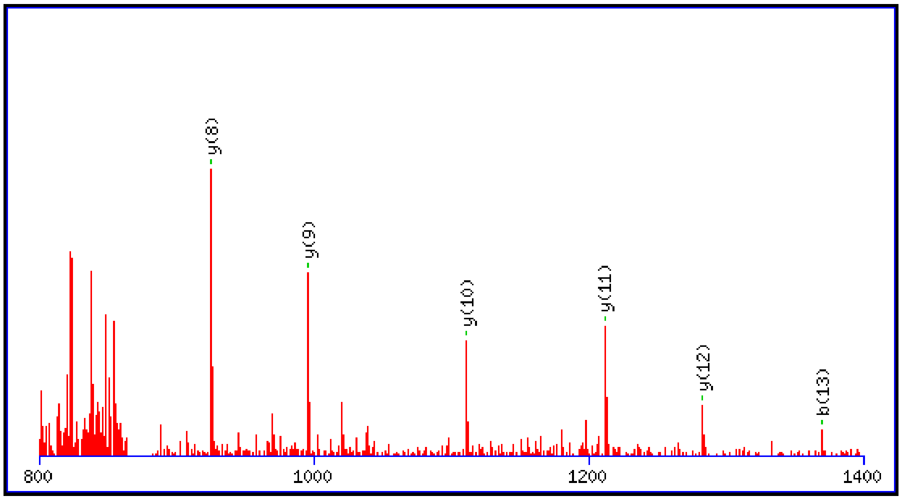

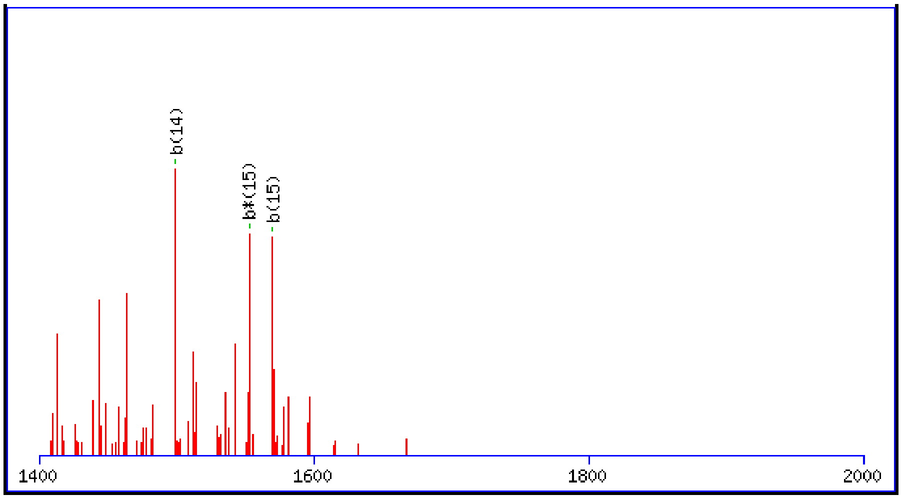


**Monoisotopic mass of neutral peptide Mr(calc):** 1743.6862
**Fixed modifications:** Carbamidomethyl (C)
**Variable modifications:**
**N7** : Deamidated_N (N)
**Ions Score:** 75 **Expect:** 3.7e-006
**Matches (Bold Red):** 18/162 fragment ions using 27 most intense peaks

| # | b | $b^{++}$ | b* | $b^{*++}$ | $b^0$ | $b^{0++}$ | Seq. | y | $y^{++}$ | y* | $y^{*++}$ | $y^0$ | $y^{0++}$ | # |
|---|---|---|---|---|---|---|---|---|---|---|---|---|---|---|
| 1 | 114.0913 | 57.5493 | | | | | L | | | | | | | 16 |
| 2 | 215.1390 | 108.0731 | | | 197.1285 | 99.0679 | T | 1631.6094 | 816.3084 | 1614.5829 | 807.7951 | 1613.5989 | 807.3031 | 15 |
| 3 | 302.1710 | 151.5892 | | | 284.1605 | 142.5839 | S | 1530.5617 | 765.7845 | 1513.5352 | 757.2712 | 1512.5512 | 756.7792 | 14 |
| 4 | 462.2017 | 231.6045 | | | 444.1911 | 222.5992 | C | 1443.5297 | 722.2685 | 1426.5032 | 713.7552 | 1425.5192 | 713.2632 | 13 |
| 5 | 533.2388 | 267.1230 | | | 515.2282 | 258.1178 | A | **1283.4991** | 642.2532 | 1266.4725 | 633.7399 | 1265.4885 | 633.2479 | 12 |
| 6 | 634.2865 | 317.6469 | | | 616.2759 | 308.6416 | T | **1212.4620** | 606.7346 | 1195.4354 | 598.2213 | 1194.4514 | 597.7293 | 11 |
| 7 | 749.3134 | **375.1604** | 732.2869 | 366.6471 | 731.3029 | 366.1551 | N | **1111.4143** | 556.2108 | 1094.3877 | **547.6975** | 1093.4037 | **547.2055** | 10 |
| 8 | 820.3505 | 410.6789 | 803.3240 | 402.1656 | 802.3400 | 401.6736 | A | **996.3873** | 498.6973 | 979.3608 | **490.1840** | 978.3768 | 489.6920 | 9 |
| 9 | 907.3826 | 454.1949 | 890.3560 | 445.6816 | 889.3720 | 445.1896 | S | **925.3502** | 463.1788 | 908.3237 | 454.6655 | 907.3397 | 454.1735 | 8 |
| 10 | 1038.4231 | 519.7152 | 1021.3965 | 511.2019 | 1020.4125 | 510.7099 | M | 838.3182 | 419.6627 | 821.2917 | 411.1495 | 820.3076 | 410.6575 | 7 |
| 11 | 1198.4537 | 599.7305 | 1181.4272 | 591.2172 | 1180.4431 | 590.7252 | C | **707.2777** | 354.1425 | 690.2512 | 345.6292 | **689.2672** | 345.1372 | 6 |
| 12 | 1255.4752 | 628.2412 | 1238.4486 | 619.7279 | 1237.4646 | 619.2359 | G | **547.2471** | 274.1272 | 530.2205 | 265.6139 | 529.2365 | 265.1219 | 5 |
| 13 | **1370.5021** | 685.7547 | 1353.4756 | 677.2414 | 1352.4915 | 676.7494 | D | **490.2256** | 245.6164 | 473.1991 | 237.1032 | 472.2150 | 236.6112 | 4 |
| 14 | **1499.5447** | 750.2760 | 1482.5182 | 741.7627 | 1481.5341 | 741.2707 | E | **375.1987** | 188.1030 | 358.1721 | 179.5897 | 357.1881 | 179.0977 | 3 |
| 15 | **1570.5818** | 785.7945 | **1553.5553** | 777.2813 | 1552.5713 | 776.7893 | A | 246.1561 | 123.5817 | 229.1295 | 115.0684 | | | 2 |
| 16 | | | | | | | R | 175.1190 | 88.0631 | 158.0924 | 79.5498 | | | 1 |

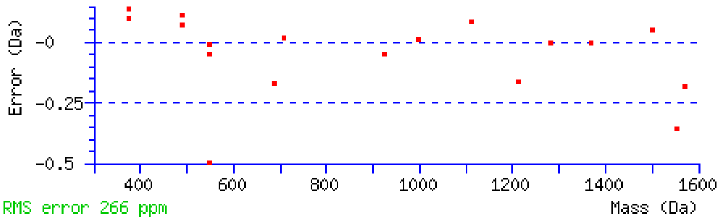


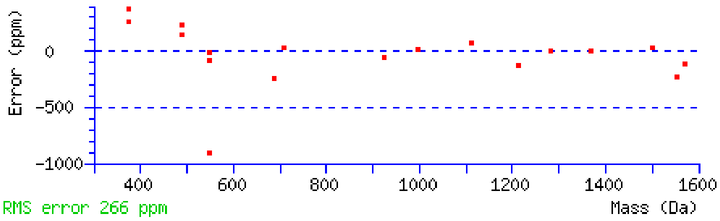


### All matches to this query

| Score | Mr(calc): | Delta | Sequence |
|---|---|---|---|
| 75.5 | 1743.6862 | 0.0056 | LTSCATNASMCGDEAR |
| 24.0 | 1742.7022 | 0.9896 | LTSCATNASMCGDEAR |
| 12.0 | 1743.6828 | 0.0091 | ISTATPYMNGETATK |
| 8.5 | 1742.6801 | 1.0117 | KSAENGIYSVSGDEK |
| 7.5 | 1742.6971 | 0.9947 | HFNSDLMAGDNSGAVK |
| | | | |

| | | | |
|---|---|---|---|
| 0.5 | 1743.7042 | -0.0124 | FFGNSWSETYRNR |
| 0.4 | 1742.6801 | 1.0117 | KSAENGIYSVSGDEK |
| 0.3 | 1741.6783 | 2.0135 | LKSDMXSWTENLR |

Spectrum No: 294; Query: 141; Rank: 1

## Peptide View

MS/MS Fragmentation of **NSSGVILAALGK**
Found in **IPI00363534**, Tax_Id=10116 Gene_Symbol=Lama1 laminin, alpha 1

Match to Query 141: 1129.633408 from(565.823980,2+)
Title: 091008RatKidney_NH4Format01_24.2942.2942.2.dta
Data file K:\NewmanPaper\Piliang\3SubProteomes\Piliang3SP\mgf5ppm\ERLIC_3SubProteomes5ppm.mgf

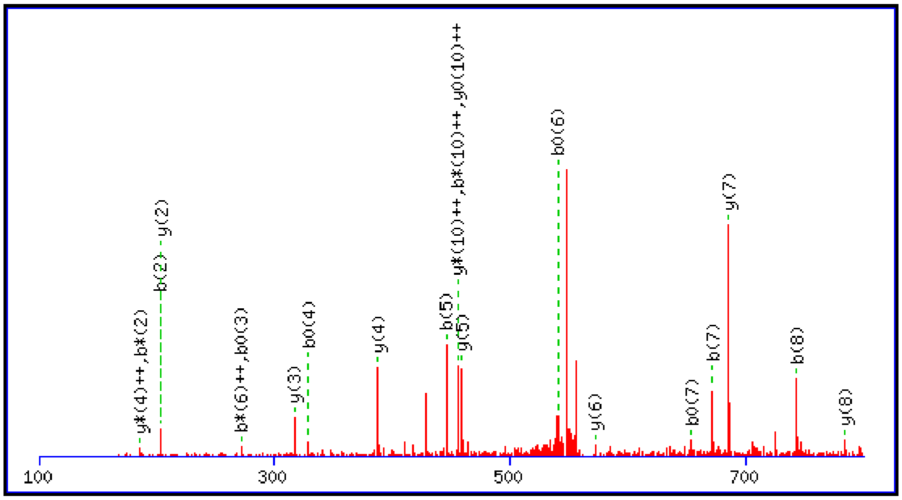


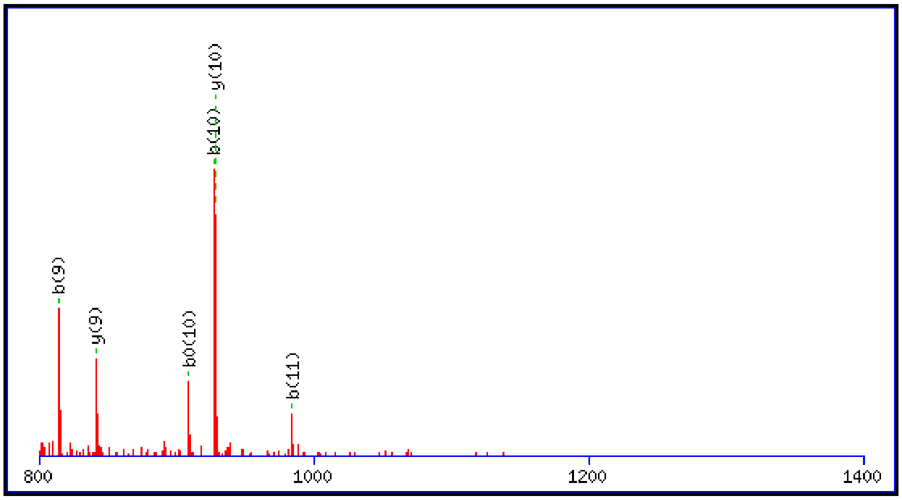

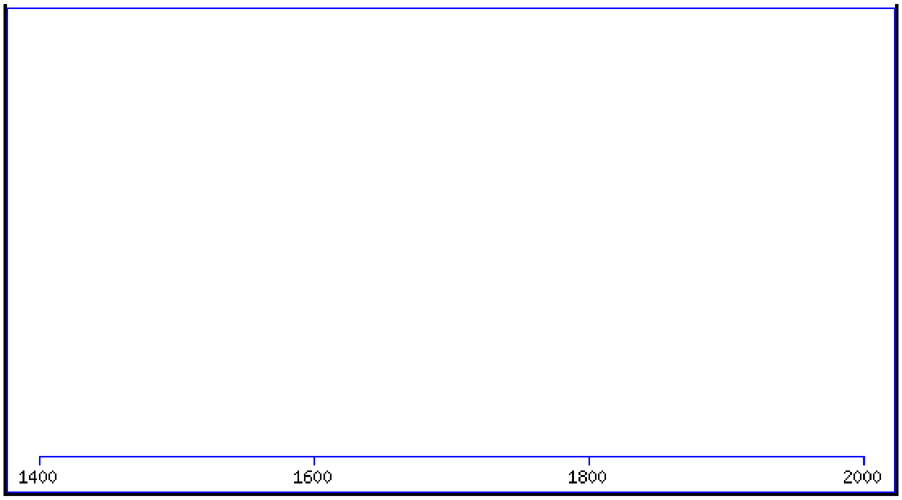


**Monoisotopic mass of neutral peptide Mr(calc):** 1129.6343
**Fixed modifications:** Carbamidomethyl (C)
**Variable modifications:**
**N1** : Deamidated_N (N)
**Ions Score:** 75 **Expect:** 2e-006
**Matches (Bold Red):** 27/112 fragment ions using 34 most intense peaks

| # | b | $b^{++}$ | b* | $b^{*++}$ | $b^0$ | $b^{0++}$ | Seq. | y | $y^{++}$ | y* | $y^{*++}$ | $y^0$ | $y^{0++}$ | # |
|---|---|---|---|---|---|---|---|---|---|---|---|---|---|---|
| 1 | 116.0342 | 58.5207 | 99.0077 | 50.0075 | | | N | | | | | | | 12 |
| 2 | **203.0662** | 102.0368 | **186.0397** | 93.5235 | 185.0557 | 93.0315 | S | 1015.6146 | 508.3109 | 998.5881 | 499.7977 | 997.6041 | 499.3057 | 11 |
| 3 | 290.0983 | 145.5528 | 273.0717 | 137.0395 | **272.0877** | 136.5475 | S | **928.5826** | 464.7949 | 911.5560 | **456.2817** | 910.5720 | **455.7897** | 10 |
| 4 | 347.1197 | 174.0635 | 330.0932 | 165.5502 | **329.1092** | 165.0582 | G | **841.5506** | 421.2789 | 824.5240 | 412.7656 | | | 9 |
| 5 | **446.1881** | 223.5977 | 429.1616 | 215.0844 | 428.1776 | 214.5924 | V | **784.5291** | 392.7682 | 767.5026 | 384.2549 | | | 8 |
| 6 | 559.2722 | 280.1397 | 542.2457 | **271.6265** | **541.2616** | 271.1345 | I | **685.4607** | 343.2340 | 668.4341 | 334.7207 | | | 7 |
| 7 | **672.3563** | 336.6818 | 655.3297 | 328.1685 | **654.3457** | 327.6765 | L | **572.3766** | 286.6920 | 555.3501 | 278.1787 | | | 6 |
| 8 | **743.3934** | 372.2003 | 726.3668 | 363.6871 | 725.3828 | 363.1950 | A | **459.2926** | 230.1499 | 442.2660 | 221.6366 | | | 5 |
| 9 | **814.4305** | 407.7189 | 797.4040 | 399.2056 | 796.4199 | 398.7136 | A | **388.2554** | 194.6314 | 371.2289 | **186.1181** | | | 4 |
| 10 | **927.5146** | 464.2609 | 910.4880 | **455.7476** | **909.5040** | 455.2556 | L | **317.2183** | 159.1128 | 300.1918 | 150.5995 | | | 3 |
| 11 | **984.5360** | 492.7717 | 967.5095 | 484.2584 | 966.5255 | 483.7664 | G | **204.1343** | 102.5708 | 187.1077 | 94.0575 | | | 2 |
| 12 | | | | | | | K | 147.1128 | 74.0600 | 130.0863 | 65.5468 | | | 1 |

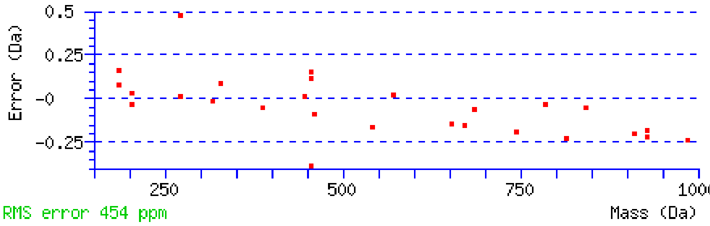


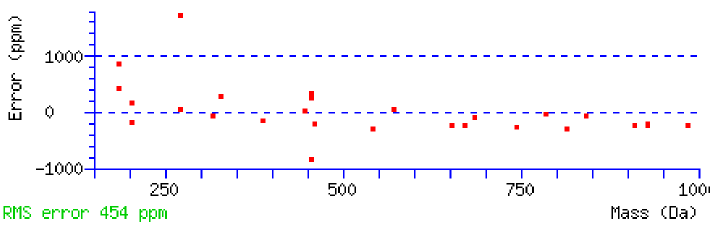


### All matches to this query

| Score | Mr(calc): | Delta | Sequence |
|---|---|---|---|
| 75.5 | 1129.6343 | -0.0009 | NSSGVILAALGK |
| 5.4 | 1129.6356 | -0.0022 | WATVTIQRR |
| 4.9 | 1129.6277 | 0.0057 | MAVANAGQKIK |
| 3.1 | 1128.6291 | 1.0043 | LSGWLXEIGR |
| 2.9 | 1129.6244 | 0.0090 | LKLFGHSGSGK |
| 2.8 | 1128.6390 | 0.9944 | NSGVVILADLK |
| 2.7 | 1128.6390 | 0.9944 | TNELLQLIGK |
| 2.5 | 1128.6251 | 1.0083 | LSQQVTQLGR |
| 2.3 | 1129.6343 | -0.0009 | ENTQVISVLK |
| | | | |

| | | | |
|---|---|---|---|
| 1.9 | 1127.6339 | 1.9995 | AGKWEIPLSK |

---

Spectrum No: 295; Query: 1728; Rank: 1

---

## Peptide View

MS/MS Fragmentation of **IDCYPDEHGVSEANCTAR**
Found in **IPI00193894**, Tax_Id=10116 Gene_Symbol=Mgam_predicted 198 kDa protein

Match to Query 1728: 2093.849508 from(1047.932030,2+)
Title: 100101RatKid_NS_deglyco_21.1319.1319.2.dta
Data file K:\NewmanPaper\Piliang\3SubProteomes\Piliang3SP\mgf5ppm\ERLIC_3SubProteomes5ppm.mgf

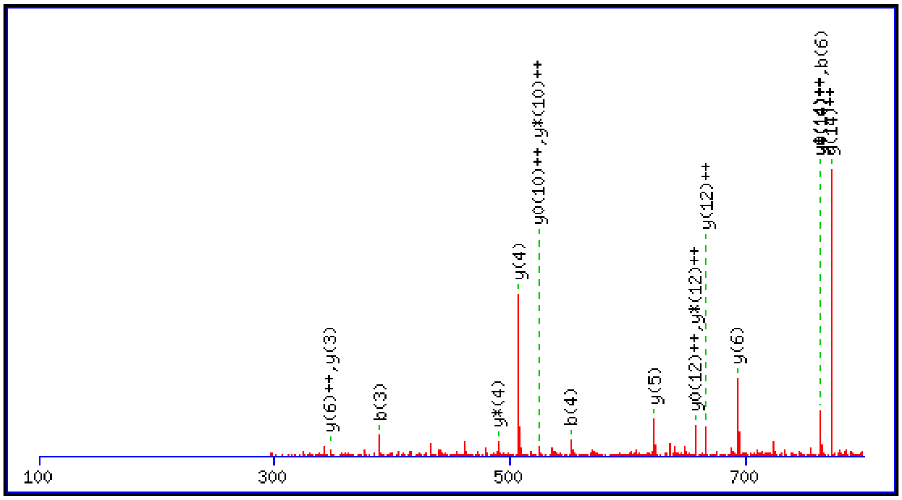


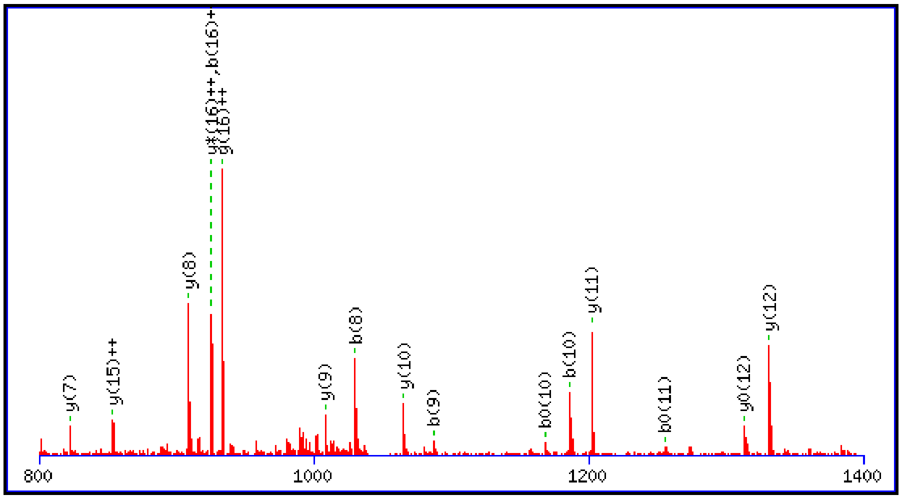

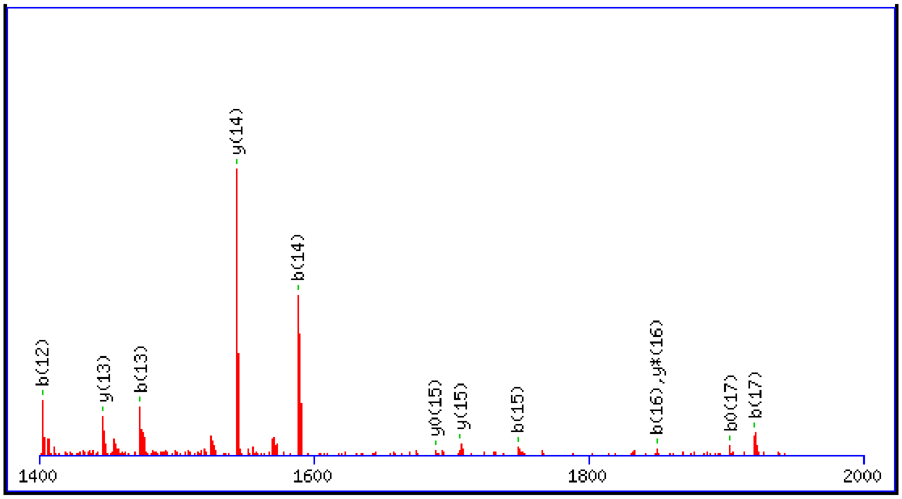


**Monoisotopic mass of neutral peptide Mr(calc):** 2093.8419
**Fixed modifications:** Carbamidomethyl (C)
**Variable modifications:**
**N14** : Deamidated_N (N)
**Ions Score:** 75 **Expect:** 5.7e-006
**Matches (Bold Red):** 46/172 fragment ions using 74 most intense peaks

| # | b | $b^{++}$ | b* | $b^{*++}$ | $b^{0}$ | $b^{0++}$ | Seq. | y | $y^{++}$ | y* | $y^{*++}$ | $y^{0}$ | $y^{0++}$ | # |
|---|---|---|---|---|---|---|---|---|---|---|---|---|---|---|
| 1 | 114.0913 | 57.5493 | | | | | I | | | | | | | 18 |
| 2 | 229.1183 | 115.0628 | | | 211.1077 | 106.0575 | D | 1981.7651 | 991.3862 | 1964.7385 | 982.8729 | 1963.7545 | 982.3809 | 17 |
| 3 | **389.1489** | 195.0781 | | | 371.1384 | 186.0728 | C | 1866.7381 | **933.8727** | **1849.7116** | **925.3594** | 1848.7276 | **924.8674** | 16 |
| 4 | **552.2123** | 276.6098 | | | 534.2017 | 267.6045 | Y | **1706.7075** | **853.8574** | 1689.6809 | 845.3441 | **1688.6969** | 844.8521 | 15 |
| 5 | 649.2650 | 325.1362 | | | 631.2545 | 316.1309 | P | **1543.6442** | **772.3257** | 1526.6176 | **763.8124** | 1525.6336 | **763.3204** | 14 |
| 6 | **764.2920** | 382.6496 | | | 746.2814 | 373.6443 | D | **1446.5914** | 723.7993 | 1429.5648 | 715.2861 | 1428.5808 | 714.7941 | 13 |
| 7 | 893.3346 | 447.1709 | | | 875.3240 | 438.1656 | E | **1331.5644** | **666.2859** | 1314.5379 | **657.7726** | **1313.5539** | **657.2806** | 12 |
| 8 | **1030.3935** | 515.7004 | | | 1012.3829 | 506.6951 | H | **1202.5219** | 601.7646 | 1185.4953 | 593.2513 | 1184.5113 | 592.7593 | 11 |
| 9 | **1087.4149** | 544.2111 | | | 1069.4044 | 535.2058 | G | **1065.4629** | 533.2351 | 1048.4364 | **524.7218** | 1047.4524 | **524.2298** | 10 |
| 10 | **1186.4834** | 593.7453 | | | **1168.4728** | 584.7400 | V | **1008.4415** | 504.7244 | 991.4149 | 496.2111 | 990.4309 | 495.7191 | 9 |
| 11 | 1273.5154 | 637.2613 | | | **1255.5048** | 628.2560 | S | **909.3731** | 455.1902 | 892.3465 | 446.6769 | 891.3625 | 446.1849 | 8 |
| 12 | **1402.5580** | 701.7826 | | | 1384.5474 | 692.7773 | E | **822.3410** | 411.6742 | 805.3145 | 403.1609 | 804.3305 | 402.6689 | 7 |
| 13 | **1473.5951** | 737.3012 | | | 1455.5845 | 728.2959 | A | **693.2984** | **347.1529** | 676.2719 | 338.6396 | 675.2879 | 338.1476 | 6 |
| 14 | **1588.6220** | 794.8146 | 1571.5955 | 786.3014 | 1570.6115 | 785.8094 | N | **622.2613** | 311.6343 | 605.2348 | 303.1210 | 604.2508 | 302.6290 | 5 |
| 15 | **1748.6527** | 874.8300 | 1731.6261 | 866.3167 | 1730.6421 | 865.8247 | C | **507.2344** | 254.1208 | **490.2078** | 245.6076 | 489.2238 | 245.1156 | 4 |
| 16 | **1849.7004** | **925.3538** | 1832.6738 | 916.8405 | 1831.6898 | 916.3485 | T | **347.2037** | 174.1055 | 330.1772 | 165.5922 | 329.1932 | 165.1002 | 3 |
| 17 | **1920.7375** | 960.8724 | 1903.7109 | 952.3591 | **1902.7269** | 951.8671 | A | 246.1561 | 123.5817 | 229.1295 | 115.0684 | | | 2 |
| 18 | | | | | | | R | 175.1190 | 88.0631 | 158.0924 | 79.5498 | | | 1 |

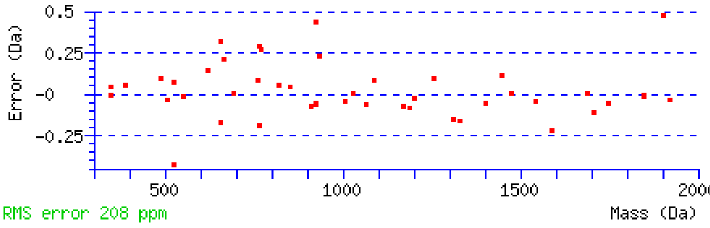


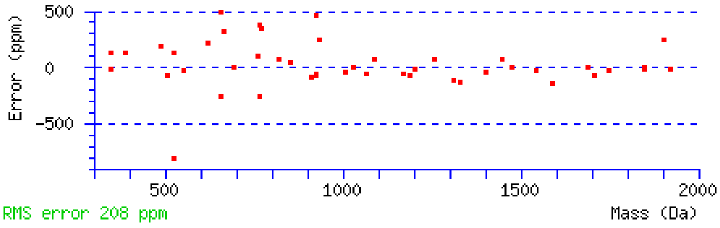


### All matches to this query

| Score | Mr(calc): | Delta | Sequence |
|---|---|---|---|
| 75.4 | 2093.8419 | 0.0076 | IDCYPDEHGVSEANCTAR |
| 7.0 | 2092.8578 | 0.9917 | IDCYPDEHGVSEANCTAR |
| 5.9 | 2092.8373 | 1.0122 | NIRSNYRDMHTHMVK |
| | | | |

| | | | |
|---|---|---|---|
| 5.2 | 2093.8490 | 0.0005 | STSACNRVLNGAVPTDGSK |
| 4.7 | 2093.8548 | -0.0052 | APGAPLDCKETDCGSPSPR |
| 4.1 | 2093.8486 | 0.0009 | TAEPPSPPASRAAYRQR |
| 4.0 | 2091.8407 | 2.0088 | GPAGSQLASXQSNVCLPSK |
| 3.0 | 2093.8490 | 0.0005 | STSACNRVLNGAVPTDGSK |
| 1.8 | 2091.8577 | 1.9918 | KDHSVNKMCASSCDFVK |
| 0.6 | 2092.8669 | 0.9827 | SPDEATAADQESEDDLSASR |

---

Spectrum No: 296; Query: 1864; Rank: 1

---

## Peptide View

MS/MS Fragmentation of **FNEVYDALANAHPNLTVYK**
Found in **IPI00454558**, Tax_Id=10116 Gene_Symbol=Enpp5 Ectonucleotide pyrophosphatase/phosphodiesterase family member 5 precursor

Match to Query 1864: 2180.059448 from(1091.037000,2+)
Title: 091008RatKidney_NH4Format01_18.4100.4100.2.dta
Data file K:\NewmanPaper\Piliang\3SubProteomes\Piliang3SP\mgf5ppm\ERLIC_3SubProteomes5ppm.mgf

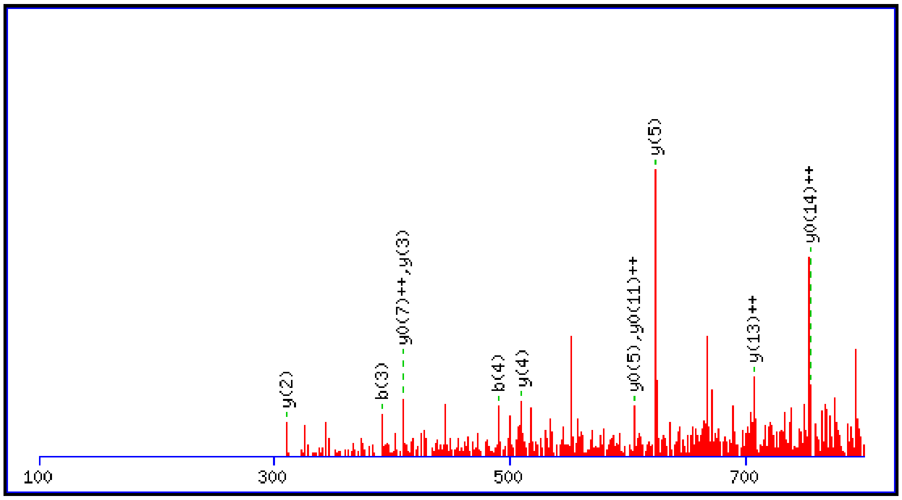


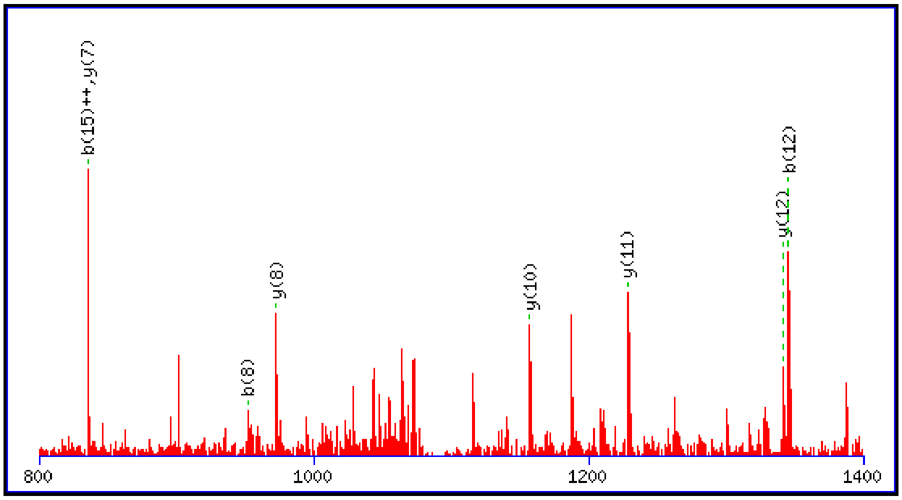

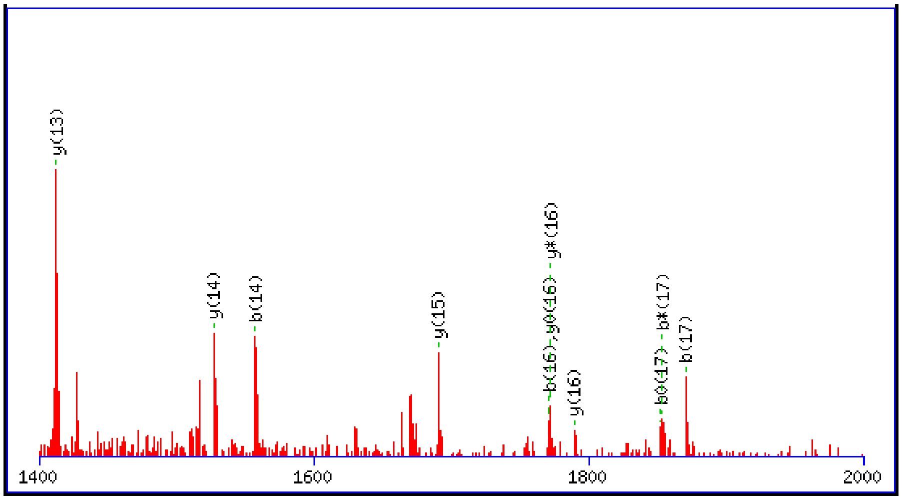


**Monoisotopic mass of neutral peptide Mr(calc):** 2179.0585
**Fixed modifications:** Carbamidomethyl (C)
**Variable modifications:**
**N14** : Deamidated_N (N)
**Ions Score:** 75 **Expect:** 6.7e-006
**Matches (Bold Red):** 30/204 fragment ions using 59 most intense peaks

| # | b | $b^{++}$ | b* | $b^{*++}$ | $b^0$ | $b^{0++}$ | Seq. | y | $y^{++}$ | y* | $y^{*++}$ | $y^0$ | $y^{0++}$ | # |
|---|---|---|---|---|---|---|---|---|---|---|---|---|---|---|
| 1 | 148.0757 | 74.5415 | | | | | F | | | | | | | 19 |
| 2 | 262.1186 | 131.5629 | 245.0921 | 123.0497 | | | N | 2032.9974 | 1017.0024 | 2015.9709 | 1008.4891 | 2014.9869 | 1007.9971 | 18 |
| 3 | **391.1612** | 196.0842 | 374.1347 | 187.5710 | 373.1506 | 187.0790 | E | 1918.9545 | 959.9809 | 1901.9280 | 951.4676 | 1900.9439 | 950.9756 | 17 |
| 4 | **490.2296** | 245.6185 | 473.2031 | 237.1052 | 472.2191 | 236.6132 | V | **1789.9119** | 895.4596 | **1772.8854** | 886.9463 | **1771.9014** | 886.4543 | 16 |
| 5 | 653.2930 | 327.1501 | 636.2664 | 318.6368 | 635.2824 | 318.1448 | Y | **1690.8435** | 845.9254 | 1673.8170 | 837.4121 | 1672.8329 | 836.9201 | 15 |
| 6 | 768.3199 | 384.6636 | 751.2933 | 376.1503 | 750.3093 | 375.6583 | D | **1527.7802** | 764.3937 | 1510.7536 | 755.8805 | 1509.7696 | **755.3884** | 14 |
| 7 | 839.3570 | 420.1821 | 822.3305 | 411.6689 | 821.3464 | 411.1769 | A | **1412.7532** | **706.8803** | 1395.7267 | 698.3670 | 1394.7427 | 697.8750 | 13 |
| 8 | **952.4411** | 476.7242 | 935.4145 | 468.2109 | 934.4305 | 467.7189 | L | **1341.7161** | 671.3617 | 1324.6896 | 662.8484 | 1323.7056 | 662.3564 | 12 |
| 9 | 1023.4782 | 512.2427 | 1006.4516 | 503.7295 | 1005.4676 | 503.2374 | A | **1228.6321** | 614.8197 | 1211.6055 | 606.3064 | 1210.6215 | **605.8144** | 11 |
| 10 | 1137.5211 | 569.2642 | 1120.4946 | 560.7509 | 1119.5106 | 560.2589 | N | **1157.5949** | 579.3011 | 1140.5684 | 570.7878 | 1139.5844 | 570.2958 | 10 |
| 11 | 1208.5582 | 604.7828 | 1191.5317 | 596.2695 | 1190.5477 | 595.7775 | A | 1043.5520 | 522.2796 | 1026.5255 | 513.7664 | 1025.5415 | 513.2744 | 9 |
| 12 | **1345.6171** | 673.3122 | 1328.5906 | 664.7989 | 1327.6066 | 664.3069 | H | **972.5149** | 486.7611 | 955.4884 | 478.2478 | 954.5043 | 477.7558 | 8 |
| 13 | 1442.6699 | 721.8386 | 1425.6434 | 713.3253 | 1424.6593 | 712.8333 | P | **835.4560** | 418.2316 | 818.4294 | 409.7184 | 817.4454 | **409.2264** | 7 |
| 14 | **1557.6968** | 779.3521 | 1540.6703 | 770.8388 | 1539.6863 | 770.3468 | N | 738.4032 | 369.7053 | 721.3767 | 361.1920 | 720.3927 | 360.7000 | 6 |
| 15 | 1670.7809 | **835.8941** | 1653.7544 | 827.3808 | 1652.7703 | 826.8888 | L | **623.3763** | 312.1918 | 606.3497 | 303.6785 | **605.3657** | 303.1865 | 5 |
| 16 | **1771.8286** | 886.4179 | 1754.8020 | 877.9047 | 1753.8180 | 877.4126 | T | **510.2922** | 255.6498 | 493.2657 | 247.1365 | 492.2817 | 246.6445 | 4 |
| 17 | **1870.8970** | 935.9521 | **1853.8704** | 927.4389 | **1852.8864** | 926.9469 | V | **409.2445** | 205.1259 | 392.2180 | 196.6126 | | | 3 |
| 18 | 2033.9603 | 1017.4838 | 2016.9338 | 1008.9705 | 2015.9498 | 1008.4785 | Y | **310.1761** | 155.5917 | 293.1496 | 147.0784 | | | 2 |
| 19 | | | | | | | K | 147.1128 | 74.0600 | 130.0863 | 65.5468 | | | 1 |

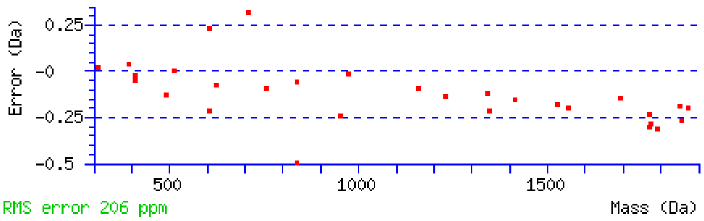


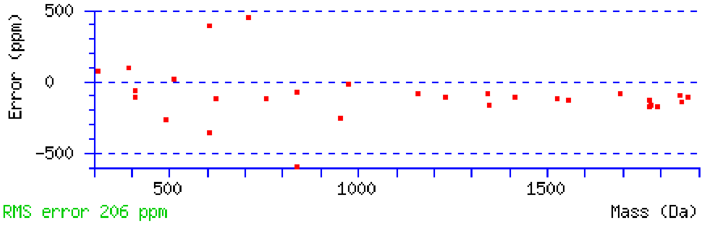


## All matches to this query

| Score | Mr(calc): | Delta | Sequence |
|---|---|---|---|
| 75.4 | 2179.0585 | 1.0009 | FNEVYDALANAHPNLTVYK |
| 71.8 | 2180.0426 | 0.0169 | FNEVYDALANAHPNLTVYK |
| | | | |

| | | | |
|---|---|---|---|
| 61.0 | 2180.0426 | 0.0169 | FNEVYDALANAHPNLTVYK |
| 52.5 | 2179.0585 | 1.0009 | FNEVYDALANAHPNLTVYK |
| 48.4 | 2180.0426 | 0.0169 | FNEVYDALANAHPNLTVYK |
| 13.7 | 2179.0585 | 1.0009 | FNEVYDALANAHPNLTVYK |
| 5.8 | 2178.0745 | 1.9849 | FNEVYDALANAHPNLTVYK |
| 0.9 | 2179.0390 | 1.0204 | VLPIMVRSTAINASRALK |
| 0.4 | 2179.0773 | 0.9822 | SKLEDIANAALAANAVTQVAK |

---

Spectrum No: 297; Query: 630; Rank: 1

---

## Peptide View

MS/MS Fragmentation of **SFSGLLDCGNCSR**
Found in **IPI00391769**, Tax_Id=10116 Gene_Symbol=Cacna2d1 Voltage-gated calcium channel alpha2/delta-1 subunit

Match to Query 630: 1472.606448 from(737.310500,2+)
Title: 100101RatKid_NS_deglyco_23.2554.2554.2.dta
Data file K:\NewmanPaper\Piliang\3SubProteomes\Piliang3SP\mgf5ppm\ERLIC_3SubProteomes5ppm.mgf

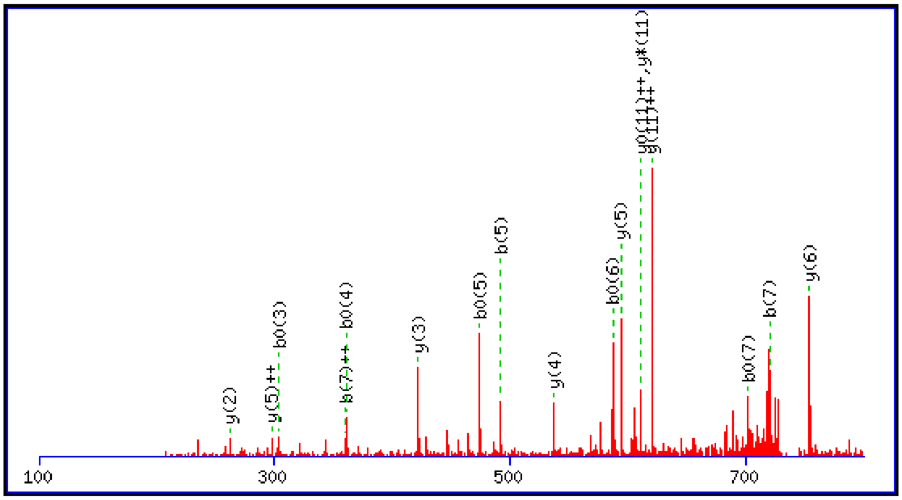


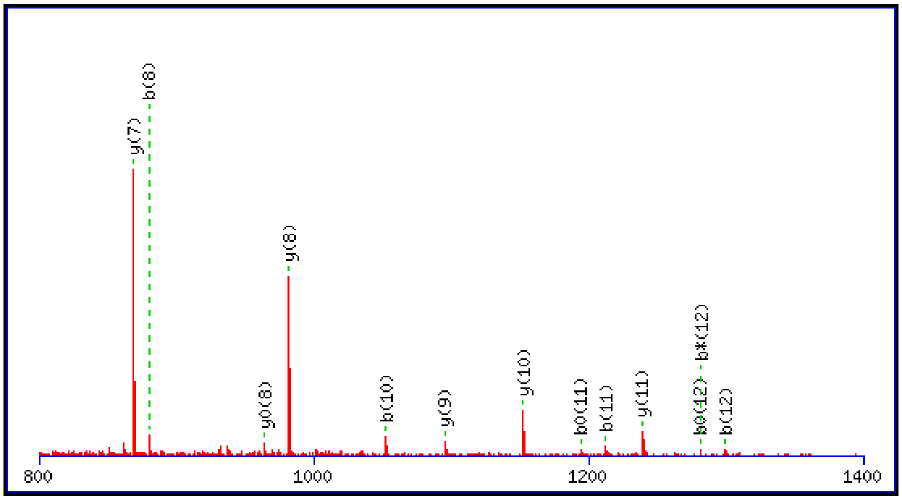

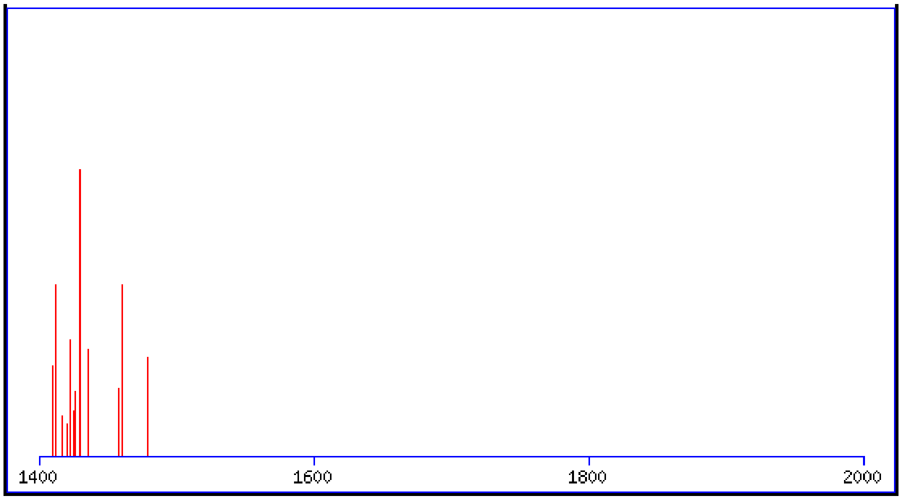


**Monoisotopic mass of neutral peptide Mr(calc):** 1472.6024
**Fixed modifications:** Carbamidomethyl (C)
**Variable modifications:**
**N10** : Deamidated_N (N)
**Ions Score:** 75 **Expect:** 3.8e-006
**Matches (Bold Red):** 30/124 fragment ions using 47 most intense peaks

| # | b | $b^{++}$ | b* | $b^{*++}$ | $b^{0}$ | $b^{0++}$ | Seq. | y | $y^{++}$ | y* | $y^{*++}$ | $y^{0}$ | $y^{0++}$ | # |
|---|---|---|---|---|---|---|---|---|---|---|---|---|---|---|
| 1 | 88.0393 | 44.5233 | | | 70.0287 | 35.5180 | S | | | | | | | 13 |
| 2 | 235.1077 | 118.0575 | | | 217.0972 | 109.0522 | F | 1386.5777 | 693.7925 | 1369.5511 | 685.2792 | 1368.5671 | 684.7872 | 12 |
| 3 | 322.1397 | 161.5735 | | | **304.1292** | 152.5682 | S | **1239.5092** | **620.2583** | 1222.4827 | **611.7450** | 1221.4987 | **611.2530** | 11 |
| 4 | 379.1612 | 190.0842 | | | **361.1506** | 181.0790 | G | **1152.4772** | 576.7422 | 1135.4507 | 568.2290 | 1134.4666 | 567.7370 | 10 |
| 5 | **492.2453** | 246.6263 | | | **474.2347** | 237.6210 | L | **1095.4557** | 548.2315 | 1078.4292 | 539.7182 | 1077.4452 | 539.2262 | 9 |
| 6 | 605.3293 | 303.1683 | | | **587.3188** | 294.1630 | L | **982.3717** | 491.6895 | 965.3451 | 483.1762 | **964.3611** | 482.6842 | 8 |
| 7 | **720.3563** | **360.6818** | | | **702.3457** | 351.6765 | D | **869.2876** | 435.1474 | 852.2611 | 426.6342 | 851.2771 | 426.1422 | 7 |
| 8 | **880.3869** | 440.6971 | | | 862.3764 | 431.6918 | C | **754.2607** | 377.6340 | 737.2341 | 369.1207 | 736.2501 | 368.6287 | 6 |
| 9 | 937.4084 | 469.2078 | | | 919.3978 | 460.2026 | G | **594.2300** | **297.6187** | 577.2035 | 289.1054 | 576.2195 | 288.6134 | 5 |
| 10 | **1052.4353** | 526.7213 | 1035.4088 | 518.2080 | 1034.4248 | 517.7160 | N | **537.2086** | 269.1079 | 520.1820 | 260.5946 | 519.1980 | 260.1026 | 4 |
| 11 | **1212.4660** | 606.7366 | 1195.4394 | 598.2234 | **1194.4554** | 597.7313 | C | **422.1816** | 211.5945 | 405.1551 | 203.0812 | 404.1711 | 202.5892 | 3 |
| 12 | **1299.4980** | 650.2526 | **1282.4715** | 641.7394 | **1281.4874** | 641.2474 | S | **262.1510** | 131.5791 | 245.1244 | 123.0659 | 244.1404 | 122.5738 | 2 |
| 13 | | | | | | | R | 175.1190 | 88.0631 | 158.0924 | 79.5498 | | | 1 |

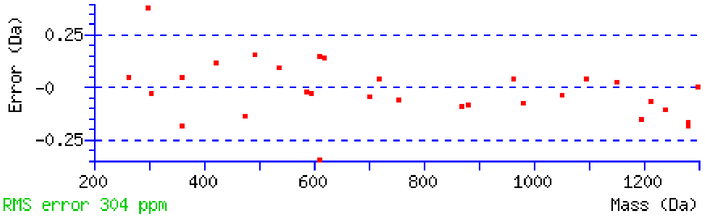


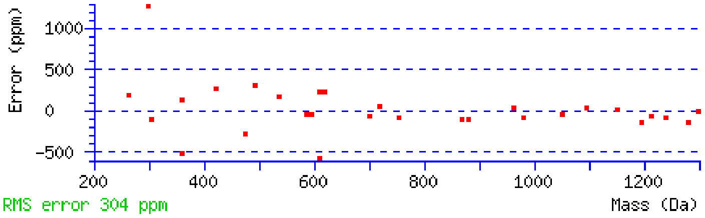


### All matches to this query

| Score | Mr(calc): | Delta | Sequence |
|---|---|---|---|
| 75.3 | 1472.6024 | 0.0040 | SFSGLLDCGNCSR |
| 12.3 | 1472.6119 | -0.0055 | NSPLPNCTYATR |
| 3.2 | 1471.5914 | 1.0150 | ASTAINWLTKK |
| 2.5 | 1471.6093 | 0.9972 | SFSQSSGLNNHSK |
| 2.1 | 1472.6003 | 0.0062 | RSSIWQFFSR |
| 1.5 | 1472.5933 | 0.0132 | SFSQSSGLNNHSK |
| 1.5 | 1472.5933 | 0.0132 | SFSQSSGLNNHSK |
| 1.0 | 1470.5867 | 2.0198 | FQTVESVNLMK |
| | | | |

| | | | |
|---|---|---|---|
| 0.9 | 1470.5859 | 2.0206 | ADEAEANGYSSGSGR |
| 0.6 | 1472.5966 | 0.0098 | ASESPDSAARQMK |

Spectrum No: 298; Query: 1832; Rank: 1

## Peptide View

MS/MS Fragmentation of **SLTFNESYQDVSEIVYGAK**
Found in **IPI00372372**, Tax_Id=10116 Gene_Symbol=Serpinc1 Serine (Or cysteine) peptidase inhibitor, clade C (Antithrombin), member 1

Match to Query 1832: 2150.012528 from(1076.013540,2+)
Title: 100101RatKid_NS_deglyco_24.5051.5051.2.dta
Data file K:\NewmanPaper\Piliang\3SubProteomes\Piliang3SP\mgf5ppm\ERLIC_3SubProteomes5ppm.mgf

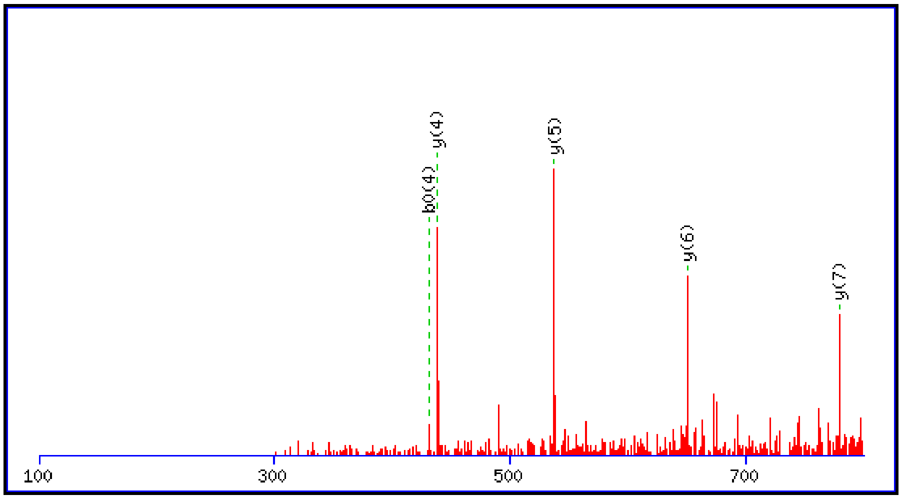


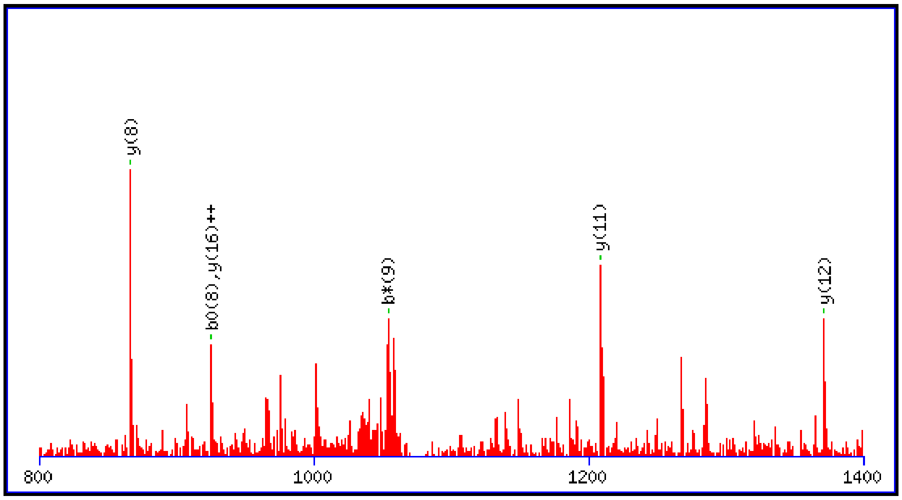

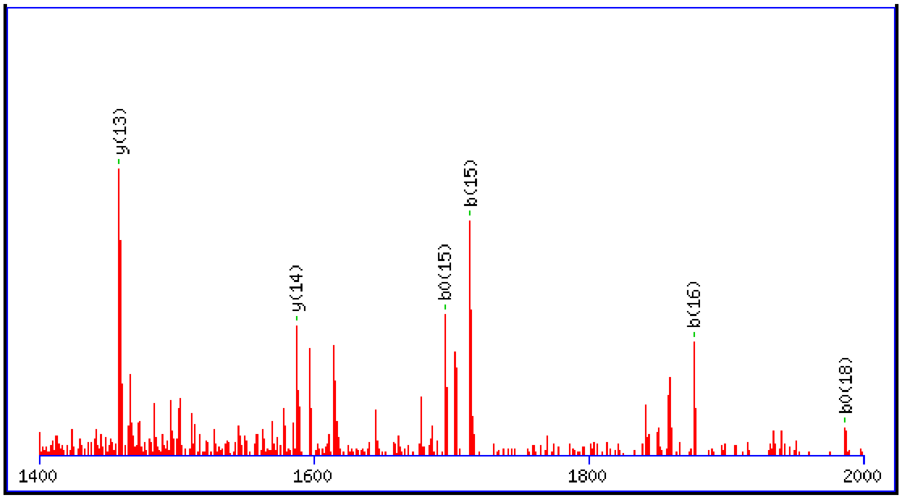


**Monoisotopic mass of neutral peptide Mr(calc):** 2150.0055
**Fixed modifications:** Carbamidomethyl (C)
**Variable modifications:**
**N5** : Deamidated_N (N)
**Ions Score:** 75 **Expect:** 7.8e-006
**Matches (Bold Red):** 17/196 fragment ions using 25 most intense peaks

| # | b | $b^{++}$ | b* | $b^{*++}$ | $b^0$ | $b^{0++}$ | Seq. | y | $y^{++}$ | y* | $y^{*++}$ | $y^0$ | $y^{0++}$ | # |
|---|---|---|---|---|---|---|---|---|---|---|---|---|---|---|
| 1 | 88.0393 | 44.5233 | | | 70.0287 | 35.5180 | S | | | | | | | 19 |
| 2 | 201.1234 | 101.0653 | | | 183.1128 | 92.0600 | L | 2063.9808 | 1032.4940 | 2046.9542 | 1023.9808 | 2045.9702 | 1023.4887 | 18 |
| 3 | 302.1710 | 151.5892 | | | 284.1605 | 142.5839 | T | 1950.8967 | 975.9520 | 1933.8702 | 967.4387 | 1932.8862 | 966.9467 | 17 |
| 4 | 449.2395 | 225.1234 | | | **431.2289** | 216.1181 | F | 1849.8490 | **925.4282** | 1832.8225 | 916.9149 | 1831.8385 | 916.4229 | 16 |
| 5 | 564.2664 | 282.6368 | 547.2398 | 274.1236 | 546.2558 | 273.6316 | N | 1702.7806 | 851.8940 | 1685.7541 | 843.3807 | 1684.7701 | 842.8887 | 15 |
| 6 | 693.3090 | 347.1581 | 676.2824 | 338.6449 | 675.2984 | 338.1529 | E | **1587.7537** | 794.3805 | 1570.7271 | 785.8672 | 1569.7431 | 785.3752 | 14 |
| 7 | 780.3410 | 390.6741 | 763.3145 | 382.1609 | 762.3305 | 381.6689 | S | **1458.7111** | 729.8592 | 1441.6846 | 721.3459 | 1440.7005 | 720.8539 | 13 |
| 8 | 943.4043 | 472.2058 | 926.3778 | 463.6925 | **925.3938** | 463.2005 | Y | **1371.6791** | 686.3432 | 1354.6525 | 677.8299 | 1353.6685 | 677.3379 | 12 |
| 9 | 1071.4629 | 536.2351 | **1054.4364** | 527.7218 | 1053.4524 | 527.2298 | Q | **1208.6157** | 604.8115 | 1191.5892 | 596.2982 | 1190.6052 | 595.8062 | 11 |
| 10 | 1186.4899 | 593.7486 | 1169.4633 | 585.2353 | 1168.4793 | 584.7433 | D | 1080.5572 | 540.7822 | 1063.5306 | 532.2689 | 1062.5466 | 531.7769 | 10 |
| 11 | 1285.5583 | 643.2828 | 1268.5317 | 634.7695 | 1267.5477 | 634.2775 | V | 965.5302 | 483.2688 | 948.5037 | 474.7555 | 947.5197 | 474.2635 | 9 |
| 12 | 1372.5903 | 686.7988 | 1355.5638 | 678.2855 | 1354.5797 | 677.7935 | S | **866.4618** | 433.7345 | 849.4353 | 425.2213 | 848.4512 | 424.7293 | 8 |
| 13 | 1501.6329 | 751.3201 | 1484.6064 | 742.8068 | 1483.6223 | 742.3148 | E | **779.4298** | 390.2185 | 762.4032 | 381.7053 | 761.4192 | 381.2132 | 7 |
| 14 | 1614.7170 | 807.8621 | 1597.6904 | 799.3488 | 1596.7064 | 798.8568 | I | **650.3872** | 325.6972 | 633.3606 | 317.1840 | | | 6 |
| 15 | **1713.7854** | 857.3963 | 1696.7588 | 848.8831 | **1695.7748** | 848.3910 | V | **537.3031** | 269.1552 | 520.2766 | 260.6419 | | | 5 |
| 16 | **1876.8487** | 938.9280 | 1859.8222 | 930.4147 | 1858.8381 | 929.9227 | Y | **438.2347** | 219.6210 | 421.2082 | 211.1077 | | | 4 |
| 17 | 1933.8702 | 967.4387 | 1916.8436 | 958.9255 | 1915.8596 | 958.4334 | G | 275.1714 | 138.0893 | 258.1448 | 129.5761 | | | 3 |
| 18 | 2004.9073 | 1002.9573 | 1987.8807 | 994.4440 | **1986.8967** | 993.9520 | A | 218.1499 | 109.5786 | 201.1234 | 101.0653 | | | 2 |
| 19 | | | | | | | K | 147.1128 | 74.0600 | 130.0863 | 65.5468 | | | 1 |

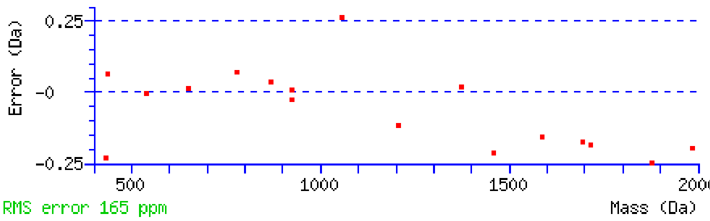


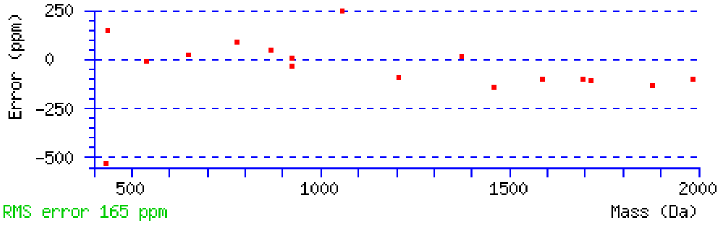


### All matches to this query

| Score | Mr(calc): | Delta | Sequence |
|---|---|---|---|
| 75.2 | 2150.0055 | 0.0070 | SLTFNESYQDVSEIVYGAK |
| 62.9 | 2149.0215 | 0.9910 | SLTFNESYQDVSEIVYGAK |

Spectrum No: 299; Query: 1067; Rank: 1

## Peptide View

MS/MS Fragmentation of **DGWTGEYCNCTTSR**
Found in **IPI00372094**, Tax_Id=10116 Gene_Symbol=Itgb6 Integrin beta-6 precursor

Match to Query 1067: 1706.637188 from(854.325870,2+)
Title: 100101RatKid_NS_deglyco_20.1723.1723.2.dta
Data file K:\NewmanPaper\Piliang\3SubProteomes\Piliang3SP\mgf5ppm\ERLIC_3SubProteomes5ppm.mgf

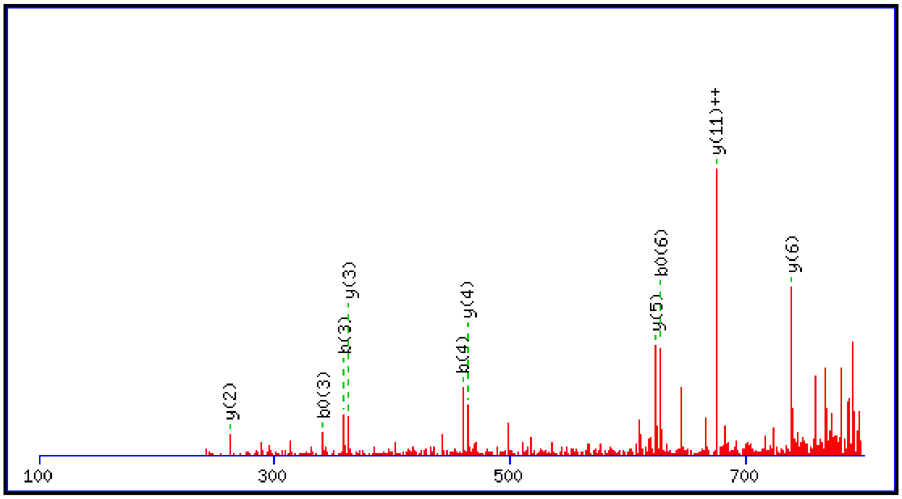


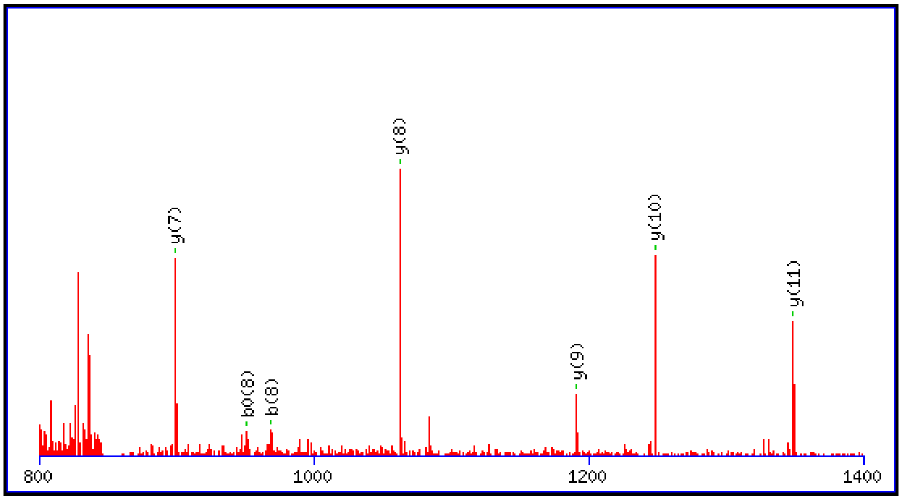


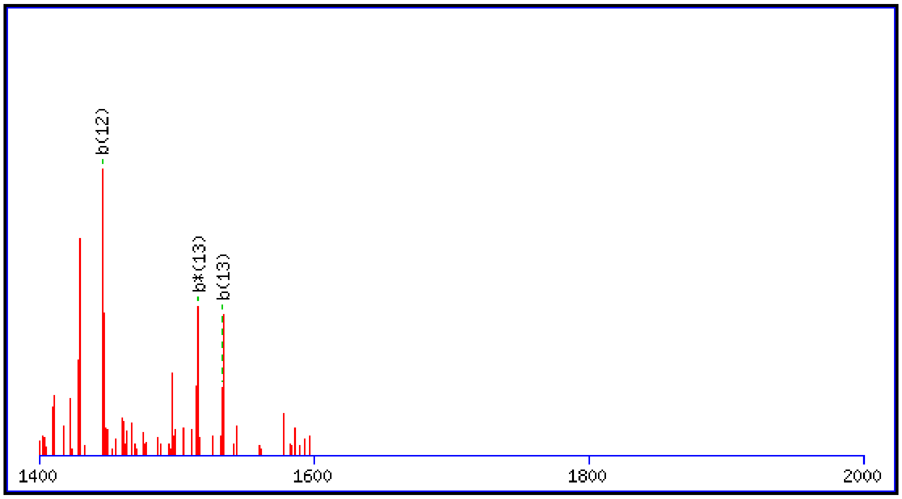

**Monoisotopic mass of neutral peptide Mr(calc):** 1706.6301
**Fixed modifications:** Carbamidomethyl (C)
**Variable modifications:**
**N9** : Deamidated_N (N)
**Ions Score:** 75 **Expect:** 2.9e-006
**Matches (Bold Red):** 20/138 fragment ions using 30 most intense peaks

| # | b | b$^{++}$ | b* | b*$^{++}$ | b$^{0}$ | b$^{0++}$ | Seq. | y | y$^{++}$ | y* | y*$^{++}$ | y$^{0}$ | y$^{0++}$ | # |
|---|---|---|---|---|---|---|---|---|---|---|---|---|---|---|
| 1 | 116.0342 | 58.5207 | | | 98.0237 | 49.5155 | D | | | | | | | 14 |
| 2 | 173.0557 | 87.0315 | | | 155.0451 | 78.0262 | G | 1592.6104 | 796.8088 | 1575.5839 | 788.2956 | 1574.5998 | 787.8036 | 13 |
| 3 | **359.1350** | 180.0711 | | | **341.1244** | 171.0659 | W | 1535.5890 | 768.2981 | 1518.5624 | 759.7848 | 1517.5784 | 759.2928 | 12 |
| 4 | **460.1827** | 230.5950 | | | 442.1721 | 221.5897 | T | **1349.5096** | **675.2585** | 1332.4831 | 666.7452 | 1331.4991 | 666.2532 | 11 |
| 5 | 517.2041 | 259.1057 | | | 499.1936 | 250.1004 | G | **1248.4620** | 624.7346 | 1231.4354 | 616.2213 | 1230.4514 | 615.7293 | 10 |
| 6 | 646.2467 | 323.6270 | | | **628.2362** | 314.6217 | E | **1191.4405** | 596.2239 | 1174.4139 | 587.7106 | 1173.4299 | 587.2186 | 9 |
| 7 | 809.3101 | 405.1587 | | | 791.2995 | 396.1534 | Y | **1062.3979** | 531.7026 | 1045.3714 | 523.1893 | 1044.3873 | 522.6973 | 8 |
| 8 | **969.3407** | 485.1740 | | | **951.3301** | 476.1687 | C | **899.3346** | 450.1709 | 882.3080 | 441.6576 | 881.3240 | 441.1656 | 7 |
| 9 | 1084.3676 | 542.6875 | 1067.3411 | 534.1742 | 1066.3571 | 533.6822 | N | **739.3039** | 370.1556 | 722.2774 | 361.6423 | 721.2934 | 361.1503 | 6 |
| 10 | 1244.3983 | 622.7028 | 1227.3717 | 614.1895 | 1226.3877 | 613.6975 | C | **624.2770** | 312.6421 | 607.2504 | 304.1289 | 606.2664 | 303.6368 | 5 |
| 11 | 1345.4460 | 673.2266 | 1328.4194 | 664.7134 | 1327.4354 | 664.2213 | T | **464.2463** | 232.6268 | 447.2198 | 224.1135 | 446.2358 | 223.6215 | 4 |
| 12 | **1446.4937** | 723.7505 | 1429.4671 | 715.2372 | 1428.4831 | 714.7452 | T | **363.1987** | 182.1030 | 346.1721 | 173.5897 | 345.1881 | 173.0977 | 3 |
| 13 | **1533.5257** | 767.2665 | **1516.4991** | 758.7532 | 1515.5151 | 758.2612 | S | **262.1510** | 131.5791 | 245.1244 | 123.0659 | 244.1404 | 122.5738 | 2 |
| 14 | | | | | | | R | 175.1190 | 88.0631 | 158.0924 | 79.5498 | | | 1 |

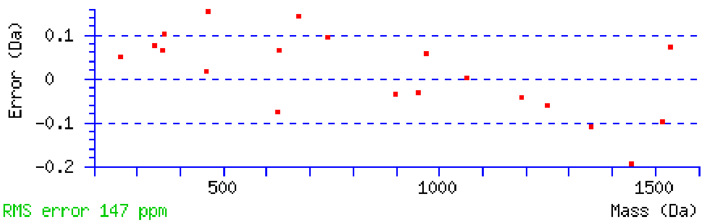


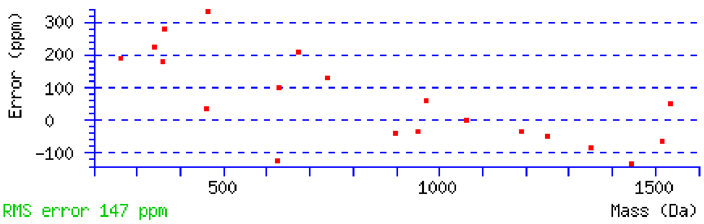


### All matches to this query

| Score | Mr(calc): | Delta | Sequence |
|---|---|---|---|
| 75.2 | 1706.6301 | 0.0071 | DGWTGEYCNCTTSR |
| 21.6 | 1705.6461 | 0.9911 | DGWTGEYCNCTTSR |
| 1.2 | 1706.6355 | 0.0017 | EPNLLSGTAHSVNK |
| 0.2 | 1706.6511 | -0.0139 | NQDTLEPMGPSTIK |

---

Spectrum No: 300; Query: 1203; Rank: 1

---

## Peptide View

MS/MS Fragmentation of **FTCNETTNVIIIHSK**
Found in **IPI00230862**, Tax_Id=10116 Gene_Symbol=Anpep Aminopeptidase N

Match to Query 1203: 1776.874408 from(889.444480,2+)
Title: 100101RatKid_NS_deglyco_23.2169.2169.2.dta
Data file K:\NewmanPaper\Piliang\3SubProteomes\Piliang3SP\mgf5ppm\ERLIC_3SubProteomes5ppm.mgf

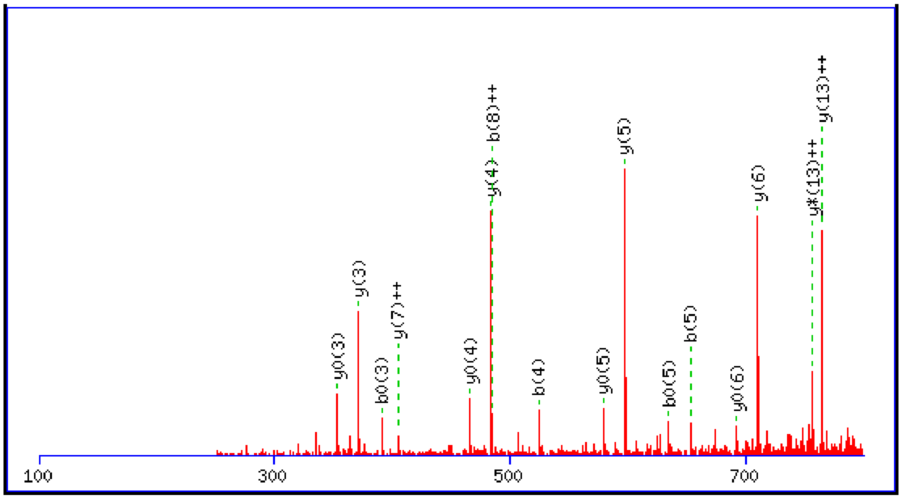


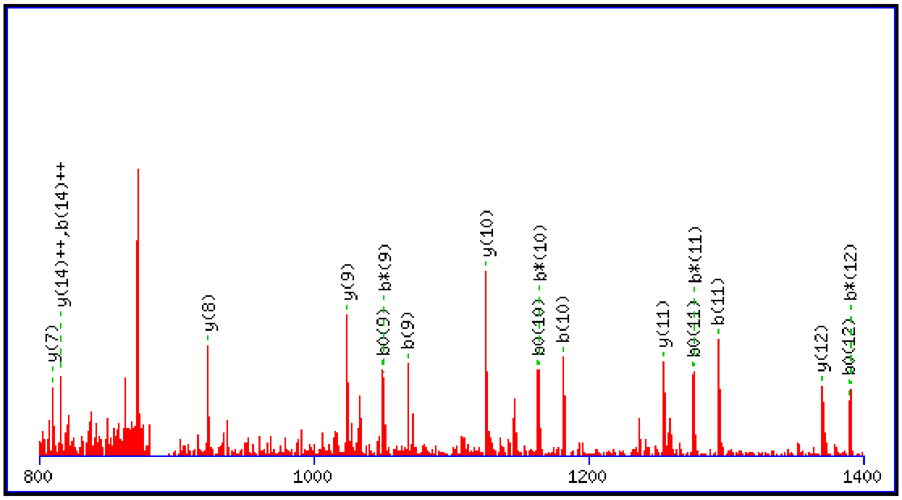


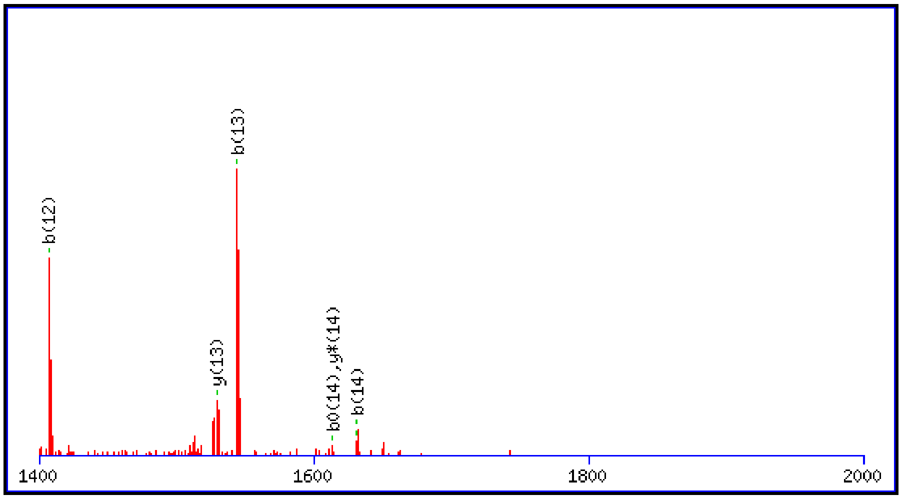


**Monoisotopic mass of neutral peptide Mr(calc):** 1776.8716
**Fixed modifications:** Carbamidomethyl (C)
**Variable modifications:**
**N4** : Deamidated_N (N)
**Ions Score:** 75 **Expect:** 5.4e-006
**Matches (Bold Red):** 41/158 fragment ions using 61 most intense peaks

| # | b | $b^{++}$ | b* | $b^{*++}$ | $b^0$ | $b^{0++}$ | Seq. | y | $y^{++}$ | y* | $y^{*++}$ | $y^0$ | $y^{0++}$ | # |
|---|---|---|---|---|---|---|---|---|---|---|---|---|---|---|
| 1 | 148.0757 | 74.5415 | | | | | F | | | | | | | 15 |
| 2 | 249.1234 | 125.0653 | | | 231.1128 | 116.0600 | T | 1630.8105 | **815.9089** | **1613.7839** | 807.3956 | 1612.7999 | 806.9036 | 14 |
| 3 | 409.1540 | 205.0806 | | | **391.1435** | 196.0754 | C | **1529.7628** | **765.3850** | 1512.7363 | **756.8718** | 1511.7522 | 756.3798 | 13 |
| 4 | **524.1810** | 262.5941 | 507.1544 | 254.0808 | 506.1704 | 253.5888 | N | **1369.7322** | 685.3697 | 1352.7056 | 676.8564 | 1351.7216 | 676.3644 | 12 |
| 5 | **653.2235** | 327.1154 | 636.1970 | 318.6021 | **635.2130** | 318.1101 | E | **1254.7052** | 627.8563 | 1237.6787 | 619.3430 | 1236.6947 | 618.8510 | 11 |

| | | | | | | | | | | | | | | |
|---|---|---|---|---|---|---|---|---|---|---|---|---|---|---|
| **6** | 754.2712 | 377.6393 | 737.2447 | 369.1260 | 736.2607 | 368.6340 | **T** | **1125.6626** | 563.3350 | 1108.6361 | 554.8217 | 1107.6521 | 554.3297 | **10** |
| **7** | 855.3189 | 428.1631 | 838.2924 | 419.6498 | 837.3083 | 419.1578 | **T** | **1024.6150** | 512.8111 | 1007.5884 | 504.2978 | 1006.6044 | 503.8058 | **9** |
| **8** | 969.3618 | **485.1846** | 952.3353 | 476.6713 | 951.3513 | 476.1793 | **N** | **923.5673** | 462.2873 | 906.5407 | 453.7740 | 905.5567 | 453.2820 | **8** |
| **9** | **1068.4302** | 534.7188 | **1051.4037** | 526.2055 | **1050.4197** | 525.7135 | **V** | **809.5243** | **405.2658** | 792.4978 | 396.7525 | 791.5138 | 396.2605 | **7** |
| **10** | **1181.5143** | 591.2608 | **1164.4878** | 582.7475 | **1163.5037** | 582.2555 | **I** | **710.4559** | 355.7316 | 693.4294 | 347.2183 | **692.4454** | 346.7263 | **6** |
| **11** | **1294.5984** | 647.8028 | **1277.5718** | 639.2896 | **1276.5878** | 638.7975 | **I** | **597.3719** | 299.1896 | 580.3453 | 290.6763 | **579.3613** | 290.1843 | **5** |
| **12** | **1407.6824** | 704.3449 | **1390.6559** | 695.8316 | **1389.6719** | 695.3396 | **I** | **484.2878** | 242.6475 | 467.2613 | 234.1343 | **466.2772** | 233.6423 | **4** |
| **13** | **1544.7413** | 772.8743 | 1527.7148 | 764.3610 | 1526.7308 | 763.8690 | **H** | **371.2037** | 186.1055 | 354.1772 | 177.5922 | **353.1932** | 177.1002 | **3** |
| **14** | **1631.7734** | **816.3903** | 1614.7468 | 807.8771 | **1613.7628** | 807.3850 | **S** | 234.1448 | 117.5761 | 217.1183 | 109.0628 | 216.1343 | 108.5708 | **2** |
| **15** | | | | | | | **K** | 147.1128 | 74.0600 | 130.0863 | 65.5468 | | | **1** |

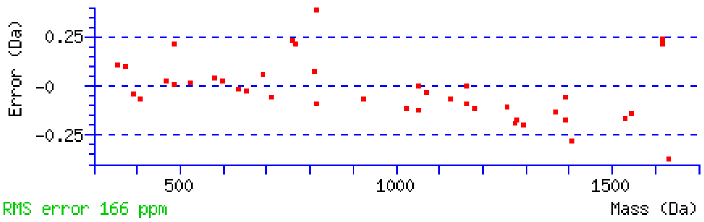


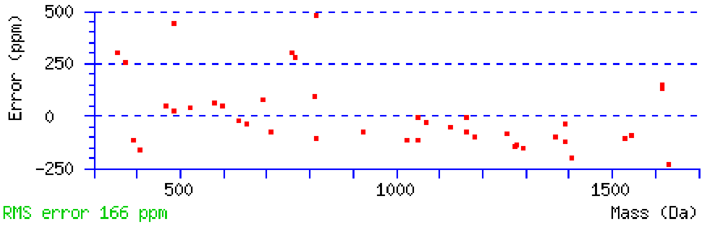


### All matches to this query

| Score | Mr(calc): | Delta | Sequence |
|---|---|---|---|
| 75.1 | 1776.8716 | 0.0028 | FTCNETTNVIIIHSK |
| 55.1 | 1776.8716 | 0.0028 | FTCNETTNVIIIHSK |
| 23.7 | 1775.8876 | 0.9868 | FTCNETTNVIIIHSK |
| 8.8 | 1776.8712 | 0.0032 | FPSGIKHLANYVHSK |
| 8.3 | 1776.8675 | 0.0069 | QIKNLEMERNAESSK |
| 4.7 | 1776.8593 | 0.0151 | NHREDGILTKLICK |
| 4.0 | 1776.8811 | -0.0067 | KIELEDALALGPSWR |
| 4.0 | 1776.8576 | 0.0168 | VGDSNTLIFIRILR |
| 3.5 | 1774.8771 | 1.9973 | LVRDDIPSTQENLMK |
| 3.4 | 1774.8767 | 1.9977 | TKATLIRTTFNWSR |

---

Spectrum No: 301; Query: 1756; Rank: 1

---

## Peptide View

MS/MS Fragmentation of **EGETCGAEDNDTCGVSLYK**
Found in **IPI00365178**, Tax_Id=10116 Gene_Symbol=Fbln2 similar to Fibulin-2 precursor isoform 1

Match to Query 1756: 2104.824708 from(1053.419630,2+)
Title: 100101RatKid_NS_deglyco_26.1813.1813.2.dta
Data file K:\NewmanPaper\Piliang\3SubProteomes\Piliang3SP\mgf5ppm\ERLIC_3SubProteomes5ppm.mgf

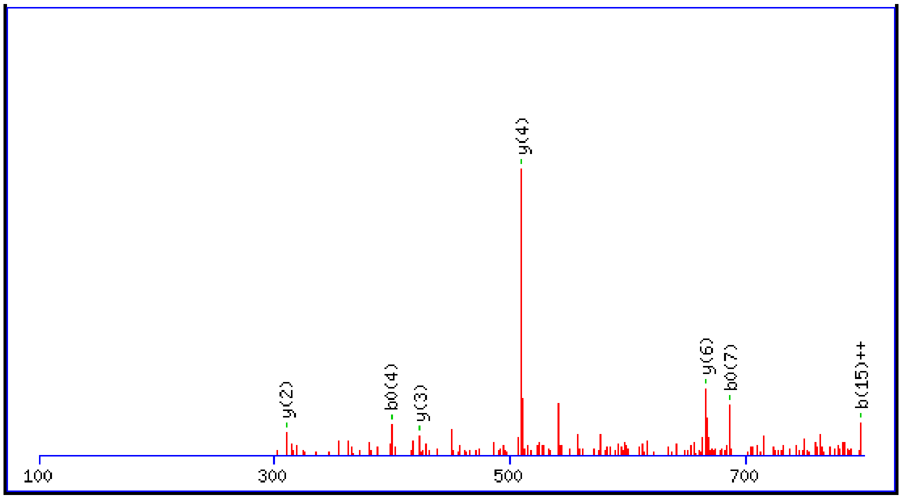


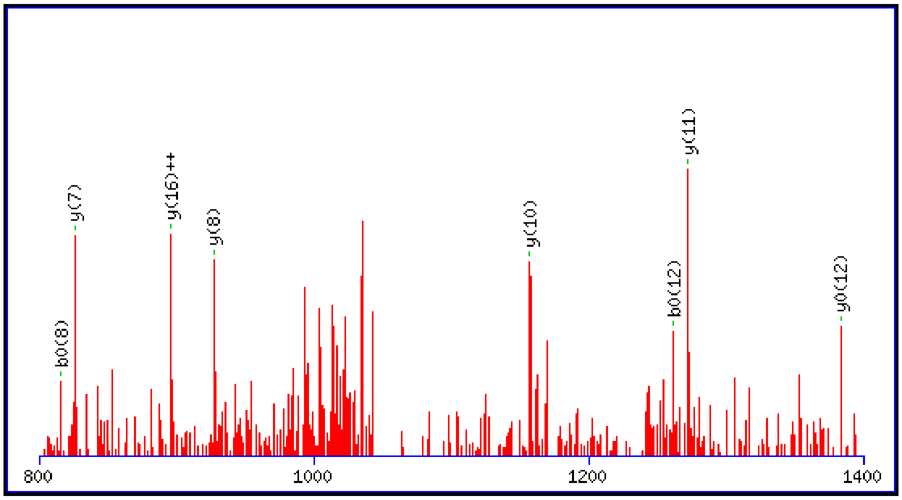


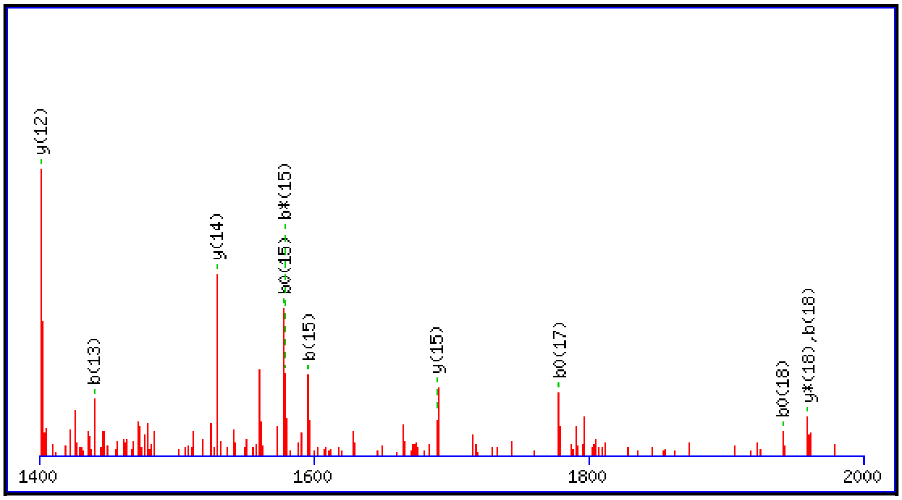


**Monoisotopic mass of neutral peptide Mr(calc):** 2104.8201
**Fixed modifications:** Carbamidomethyl (C)
**Variable modifications:**
**N10** : Deamidated_N (N)
**Ions Score:** 75 **Expect:** 5.3e-006
**Matches (Bold Red):** 26/192 fragment ions using 41 most intense peaks

| # | b | $b^{++}$ | b* | $b^{*++}$ | $b^{0}$ | $b^{0++}$ | Seq. | y | $y^{++}$ | y* | $y^{*++}$ | $y^{0}$ | $y^{0++}$ | # |
|---|---|---|---|---|---|---|---|---|---|---|---|---|---|---|
| **1** | 130.0499 | 65.5286 | | | 112.0393 | 56.5233 | **E** | | | | | | | **19** |
| **2** | 187.0713 | 94.0393 | | | 169.0608 | 85.0340 | **G** | 1976.7848 | 988.8960 | **1959.7583** | 980.3828 | 1958.7742 | 979.8908 | **18** |
| **3** | 316.1139 | 158.5606 | | | 298.1034 | 149.5553 | **E** | 1919.7633 | 960.3853 | 1902.7368 | 951.8720 | 1901.7528 | 951.3800 | **17** |
| **4** | 417.1616 | 209.0844 | | | **399.1510** | 200.0792 | **T** | 1790.7208 | **895.8640** | 1773.6942 | 887.3507 | 1772.7102 | 886.8587 | **16** |
| **5** | 577.1923 | 289.0998 | | | 559.1817 | 280.0945 | **C** | **1689.6731** | 845.3402 | 1672.6465 | 836.8269 | 1671.6625 | 836.3349 | **15** |

| | | | | | | | | | | | | | | |
|---|---|---|---|---|---|---|---|---|---|---|---|---|---|---|
| **6** | 634.2137 | 317.6105 | | | 616.2032 | 308.6052 | **G** | **1529.6424** | 765.3248 | 1512.6159 | 756.8116 | 1511.6319 | 756.3196 | **14** |
| **7** | 705.2508 | 353.1291 | | | **687.2403** | 344.1238 | **A** | 1472.6210 | 736.8141 | 1455.5944 | 728.3008 | 1454.6104 | 727.8088 | **13** |
| **8** | 834.2934 | 417.6504 | | | **816.2829** | 408.6451 | **E** | **1401.5838** | 701.2956 | 1384.5573 | 692.7823 | **1383.5733** | 692.2903 | **12** |
| **9** | 949.3204 | 475.1638 | | | 931.3098 | 466.1585 | **D** | **1272.5413** | 636.7743 | 1255.5147 | 628.2610 | 1254.5307 | 627.7690 | **11** |
| **10** | 1064.3473 | 532.6773 | 1047.3208 | 524.1640 | 1046.3367 | 523.6720 | **N** | **1157.5143** | 579.2608 | 1140.4878 | 570.7475 | 1139.5037 | 570.2555 | **10** |
| **11** | 1179.3742 | 590.1908 | 1162.3477 | 581.6775 | 1161.3637 | 581.1855 | **D** | 1042.4874 | 521.7473 | 1025.4608 | 513.2341 | 1024.4768 | 512.7420 | **9** |
| **12** | 1280.4219 | 640.7146 | 1263.3954 | 632.2013 | **1262.4114** | 631.7093 | **T** | **927.4604** | 464.2339 | 910.4339 | 455.7206 | 909.4499 | 455.2286 | **8** |
| **13** | **1440.4526** | 720.7299 | 1423.4260 | 712.2167 | 1422.4420 | 711.7246 | **C** | **826.4128** | 413.7100 | 809.3862 | 405.1967 | 808.4022 | 404.7047 | **7** |
| **14** | 1497.4740 | 749.2407 | 1480.4475 | 740.7274 | 1479.4635 | 740.2354 | **G** | **666.3821** | 333.6947 | 649.3556 | 325.1814 | 648.3715 | 324.6894 | **6** |
| **15** | **1596.5425** | **798.7749** | **1579.5159** | 790.2616 | **1578.5319** | 789.7696 | **V** | 609.3606 | 305.1840 | 592.3341 | 296.6707 | 591.3501 | 296.1787 | **5** |
| **16** | 1683.5745 | 842.2909 | 1666.5479 | 833.7776 | 1665.5639 | 833.2856 | **S** | **510.2922** | 255.6498 | 493.2657 | 247.1365 | 492.2817 | 246.6445 | **4** |
| **17** | 1796.6585 | 898.8329 | 1779.6320 | 890.3196 | **1778.6480** | 889.8276 | **L** | **423.2602** | 212.1337 | 406.2336 | 203.6205 | | | **3** |
| **18** | **1959.7219** | 980.3646 | 1942.6953 | 971.8513 | **1941.7113** | 971.3593 | **Y** | **310.1761** | 155.5917 | 293.1496 | 147.0784 | | | **2** |
| **19** | | | | | | | **K** | 147.1128 | 74.0600 | 130.0863 | 65.5468 | | | **1** |

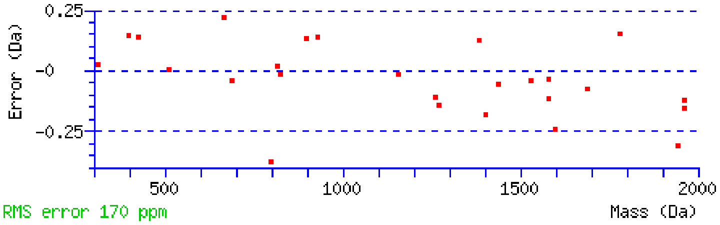


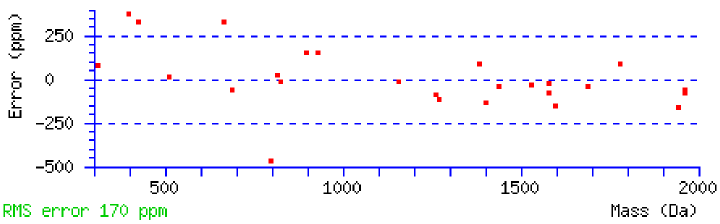


### All matches to this query

| Score | Mr(calc): | Delta | Sequence |
|---|---|---|---|
| 75.1 | 2104.8201 | 0.0046 | EGETCGAEDNDTCGVSLYK |
| 8.7 | 2103.8361 | 0.9886 | EGETCGAEDNDTCGVSLYK |
| 6.2 | 2104.8302 | -0.0055 | KLTSSGCIDDATRGSAVK |
| 6.2 | 2104.8302 | -0.0055 | KLTSSGCIDDATRGSAVK |
| 3.2 | 2104.8073 | 0.0174 | SSSSSQSRSHSGSREHSR |
| 0.3 | 2104.8302 | -0.0055 | KLTSSGCIDDATRGSAVK |

---

Spectrum No: 302; Query: 844; Rank: 1

---

## Peptide View

MS/MS Fragmentation of **FNLTETPETEIHR**
Found in **IPI00211075**, Tax_Id=10116 Gene_Symbol=Serpina3n Serine protease inhibitor A3N precursor

Match to Query 844: 1586.756048 from(794.385300,2+)
Title: 091008RatKidney_NH4Format01_27.1843.1843.2.dta
Data file K:\NewmanPaper\Piliang\3SubProteomes\Piliang3SP\mgf5ppm\ERLIC_3SubProteomes5ppm.mgf

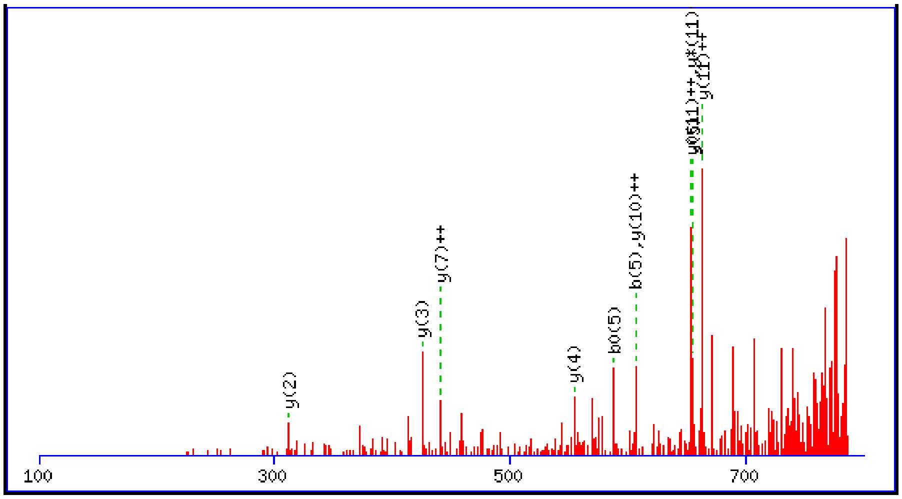


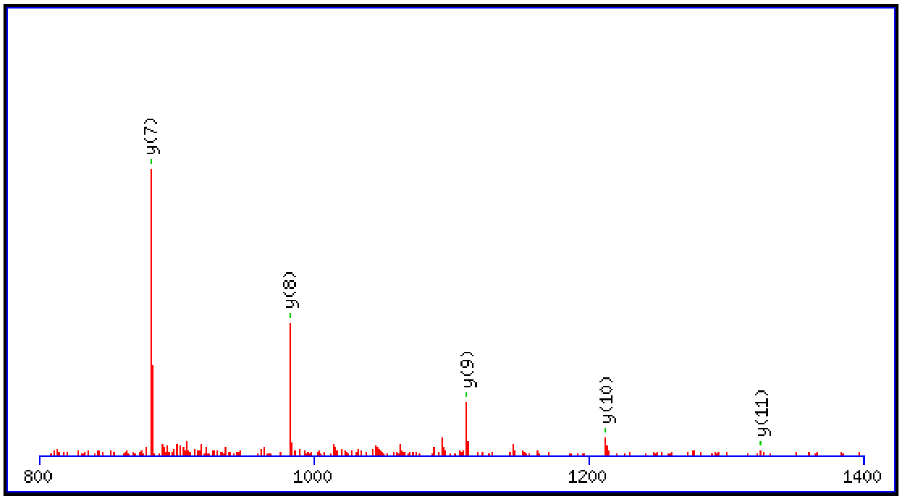


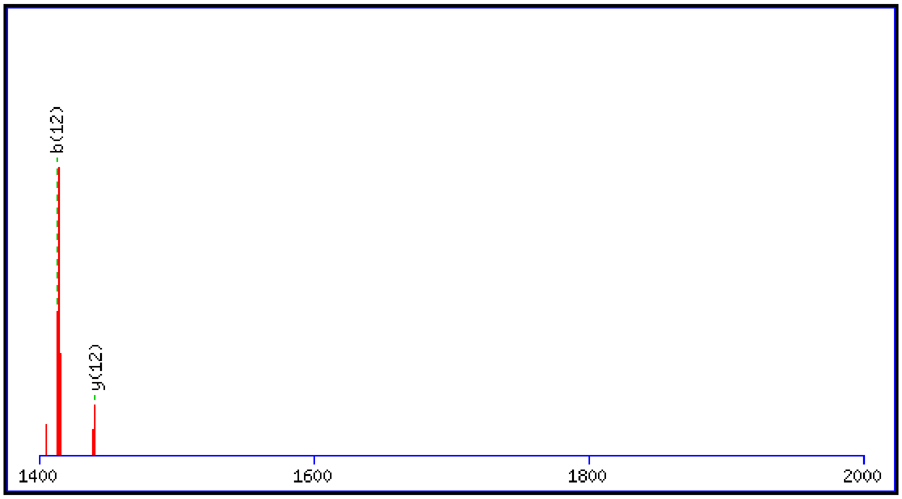


**Monoisotopic mass of neutral peptide Mr(calc):** 1586.7576
**Fixed modifications:** Carbamidomethyl (C)
**Variable modifications:**
**N2** : Deamidated_N (N)
**Ions Score:** 75 **Expect:** 5.2e-006
**Matches (Bold Red):** 18/130 fragment ions using 24 most intense peaks

| # | b | b++ | b* | b*++ | b0 | b0++ | Seq. | y | y++ | y* | y*++ | y0 | y0++ | # |
|---|---|---|---|---|---|---|---|---|---|---|---|---|---|---|
| 1 | 148.0757 | 74.5415 | | | | | F | | | | | | | 13 |
| 2 | 263.1026 | 132.0550 | 246.0761 | 123.5417 | | | N | **1440.6965** | 720.8519 | 1423.6700 | 712.3386 | 1422.6859 | 711.8466 | 12 |
| 3 | 376.1867 | 188.5970 | 359.1601 | 180.0837 | | | L | **1325.6696** | **663.3384** | 1308.6430 | **654.8251** | 1307.6590 | **654.3331** | 11 |
| 4 | 477.2344 | 239.1208 | 460.2078 | 230.6075 | 459.2238 | 230.1155 | T | **1212.5855** | **606.7964** | 1195.5590 | 598.2831 | 1194.5749 | 597.7911 | 10 |
| 5 | **606.2770** | 303.6421 | 589.2504 | 295.1288 | **588.2664** | 294.6368 | E | **1111.5378** | 556.2726 | 1094.5113 | 547.7593 | 1093.5273 | 547.2673 | 9 |

| | | | | | | | | | | | | | | |
|---|---|---|---|---|---|---|---|---|---|---|---|---|---|---|
| **6** | 707.3246 | 354.1660 | 690.2981 | 345.6527 | 689.3141 | 345.1607 | **T** | **982.4952** | 491.7513 | 965.4687 | 483.2380 | 964.4847 | 482.7460 | **8** |
| **7** | 804.3774 | 402.6923 | 787.3509 | 394.1791 | 786.3668 | 393.6871 | **P** | **881.4476** | **441.2274** | 864.4210 | 432.7141 | 863.4370 | 432.2221 | **7** |
| **8** | 933.4200 | 467.2136 | 916.3934 | 458.7004 | 915.4094 | 458.2084 | **E** | 784.3948 | 392.7010 | 767.3682 | 384.1878 | 766.3842 | 383.6958 | **6** |
| **9** | 1034.4677 | 517.7375 | 1017.4411 | 509.2242 | 1016.4571 | 508.7322 | **T** | **655.3522** | 328.1797 | 638.3257 | 319.6665 | 637.3416 | 319.1745 | **5** |
| **10** | 1163.5103 | 582.2588 | 1146.4837 | 573.7455 | 1145.4997 | 573.2535 | **E** | **554.3045** | 277.6559 | 537.2780 | 269.1426 | 536.2940 | 268.6506 | **4** |
| **11** | 1276.5943 | 638.8008 | 1259.5678 | 630.2875 | 1258.5838 | 629.7955 | **I** | **425.2619** | 213.1346 | 408.2354 | 204.6213 | | | **3** |
| **12** | **1413.6532** | 707.3303 | 1396.6267 | 698.8170 | 1395.6427 | 698.3250 | **H** | **312.1779** | 156.5926 | 295.1513 | 148.0793 | | | **2** |
| **13** | | | | | | | **R** | 175.1190 | 88.0631 | 158.0924 | 79.5498 | | | **1** |

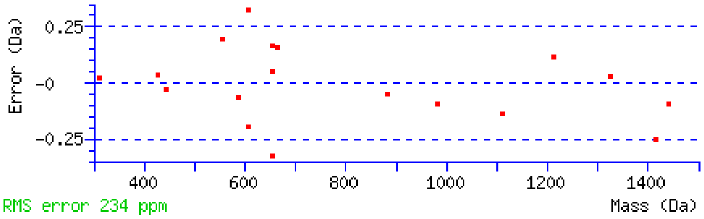


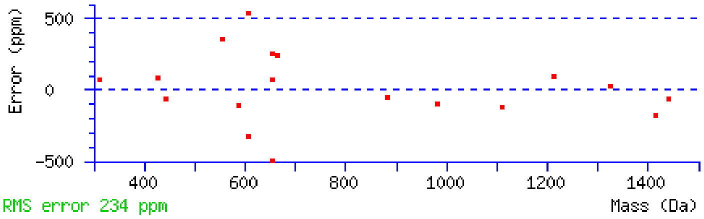


### All matches to this query

| Score | Mr(calc): | Delta | Sequence |
|---|---|---|---|
| 75.0 | 1586.7576 | -0.0016 | FNLTETPETEIHR |
| 17.4 | 1585.7541 | 1.0019 | SVFPLEQAFINNK |
| 17.3 | 1585.7535 | 1.0025 | MVKVSIGLGEGTGSR |
| 14.9 | 1585.7670 | 0.9890 | YLTVKVKASYVR |
| 11.3 | 1586.7471 | 0.0090 | MDPVERRSEAEPR |
| 11.0 | 1584.7548 | 2.0012 | GKSPAQAELSYLNK |
| 11.0 | 1584.7548 | 2.0012 | GKSPAQAELSYLNK |
| 10.9 | 1585.7388 | 1.0172 | GKSPAQAELSYLNK |
| 10.9 | 1585.7388 | 1.0172 | GKSPAQAELSYLNK |
| 9.0 | 1586.7464 | 0.0096 | DINDNPPVFPTTQK |

---

Spectrum No: 303; Query: 728; Rank: 1

---

## Peptide View

MS/MS Fragmentation of **YTGNSSALFILPDK**
Found in **IPI00210091**, Tax_Id=10116 Gene_Symbol=Serpina3m similar to Serine protease inhibitor A3M precursor

Match to Query 728: 1525.768468 from(763.891510,2+)
Title: 091008RatKidney_NoSalt_30.2807.2807.2.dta
Data file K:\NewmanPaper\Piliang\3SubProteomes\Piliang3SP\mgf5ppm\ERLIC_3SubProteomes5ppm.mgf

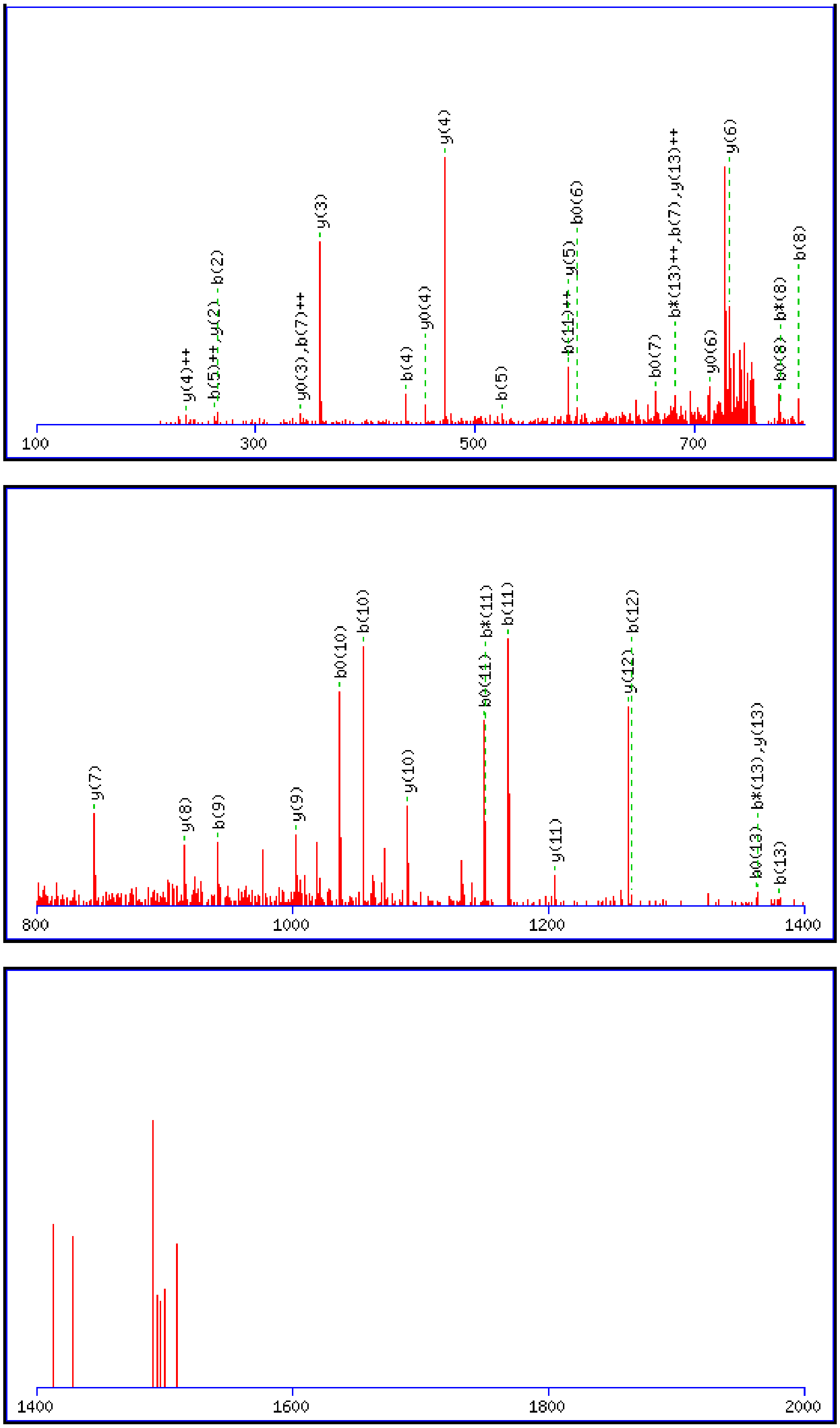


**Monoisotopic mass of neutral peptide Mr(calc):** 1525.7664
**Fixed modifications:** Carbamidomethyl (C)
**Variable modifications:**
**N4** : Deamidated_N (N)
**Ions Score:** 75 **Expect:** 4.6e-006
**Matches (Bold Red):** 40/146 fragment ions using 78 most intense peaks

| # | b | b++ | b* | b*++ | b0 | b0++ | Seq. | y | y++ | y* | y*++ | y0 | y0++ | # |
|---|---|---|---|---|---|---|---|---|---|---|---|---|---|---|
| **1** | 164.0706 | 82.5389 | | | | | **Y** | | | | | | | **14** |
| **2** | **265.1183** | 133.0628 | | | 247.1077 | 124.0575 | **T** | **1363.7104** | **682.3588** | 1346.6838 | 673.8455 | 1345.6998 | 673.3535 | **13** |
| **3** | 322.1397 | 161.5735 | | | 304.1292 | 152.5682 | **G** | **1262.6627** | 631.8350 | 1245.6361 | 623.3217 | 1244.6521 | 622.8297 | **12** |
| **4** | **437.1667** | 219.0870 | 420.1401 | 210.5737 | 419.1561 | 210.0817 | **N** | **1205.6412** | 603.3242 | 1188.6147 | 594.8110 | 1187.6307 | 594.3190 | **11** |
| **5** | **524.1987** | **262.6030** | 507.1722 | 254.0897 | 506.1881 | 253.5977 | **S** | **1090.6143** | 545.8108 | 1073.5877 | 537.2975 | 1072.6037 | 536.8055 | **10** |

| | | | | | | | | | | | | | | |
|---|---|---|---|---|---|---|---|---|---|---|---|---|---|---|
| **6** | 611.2307 | 306.1190 | 594.2042 | 297.6057 | **593.2202** | 297.1137 | **S** | **1003.5823** | 502.2948 | 986.5557 | 493.7815 | 985.5717 | 493.2895 | **9** |
| **7** | **682.2679** | **341.6376** | 665.2413 | 333.1243 | **664.2573** | 332.6323 | **A** | **916.5502** | 458.7788 | 899.5237 | 450.2655 | 898.5397 | 449.7735 | **8** |
| **8** | **795.3519** | 398.1796 | **778.3254** | 389.6663 | **777.3414** | 389.1743 | **L** | **845.5131** | 423.2602 | 828.4866 | 414.7469 | 827.5026 | 414.2549 | **7** |
| **9** | **942.4203** | 471.7138 | 925.3938 | 463.2005 | 924.4098 | 462.7085 | **F** | **732.4291** | 366.7182 | 715.4025 | 358.2049 | **714.4185** | 357.7129 | **6** |
| **10** | **1055.5044** | 528.2558 | 1038.4778 | 519.7426 | **1037.4938** | 519.2506 | **I** | **585.3606** | 293.1840 | 568.3341 | 284.6707 | 567.3501 | 284.1787 | **5** |
| **11** | **1168.5885** | **584.7979** | **1151.5619** | 576.2846 | **1150.5779** | 575.7926 | **L** | **472.2766** | **236.6419** | 455.2500 | 228.1287 | **454.2660** | 227.6366 | **4** |
| **12** | **1265.6412** | 633.3243 | 1248.6147 | 624.8110 | 1247.6307 | 624.3190 | **P** | **359.1925** | 180.0999 | 342.1660 | 171.5866 | **341.1819** | 171.0946 | **3** |
| **13** | **1380.6682** | 690.8377 | **1363.6416** | **682.3244** | **1362.6576** | 681.8324 | **D** | **262.1397** | 131.5735 | 245.1132 | 123.0602 | 244.1292 | 122.5682 | **2** |
| **14** | | | | | | | **K** | 147.1128 | 74.0600 | 130.0863 | 65.5468 | | | **1** |

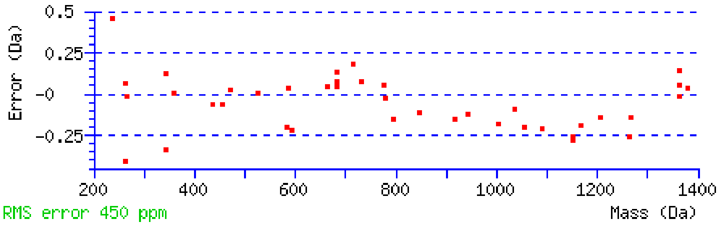


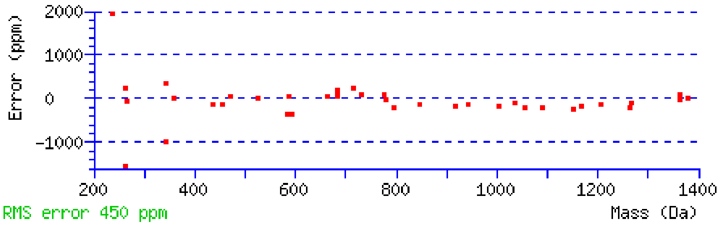


### All matches to this query

| Score | Mr(calc): | Delta | Sequence |
|---|---|---|---|
| 75.0 | 1525.7664 | 0.0021 | YTGNSSALFILPDK |
| 9.7 | 1525.7694 | -0.0009 | KYNWTPALIALR |
| 7.7 | 1525.7694 | -0.0009 | KYNWTPALIALR |
| 7.7 | 1525.7687 | -0.0003 | TKELIGSMKINGR |
| 5.6 | 1523.7596 | 2.0089 | NNELIAIGKGSSLK |
| 5.6 | 1523.7596 | 2.0089 | NNELIAIGKGSSLK |
| 5.6 | 1523.7596 | 2.0089 | NNELIAIGKGSSLK |
| 5.6 | 1523.7596 | 2.0089 | NNELIAIGKGSSLK |
| 3.8 | 1524.7532 | 1.0153 | AATNLENQLHSAQK |
| 3.4 | 1525.7810 | -0.0126 | SSVLVTHAELMPDK |

---

Spectrum No: 304; Query: 2003; Rank: 1

---

## Peptide View

MS/MS Fragmentation of **NVIIWGNHSSTQYPDVNHAK**
Found in **IPI00198717**, Tax_Id=10116 Gene_Symbol=Mdh1 Malate dehydrogenase, cytoplasmic

Match to Query 2003: 2280.099848 from(1141.057200,2+)
Title: 091008RatKidney_NoSalt_06.1125.1125.2.dta
Data file K:\NewmanPaper\Piliang\3SubProteomes\Piliang3SP\mgf5ppm\ERLIC_3SubProteomes5ppm.mgf

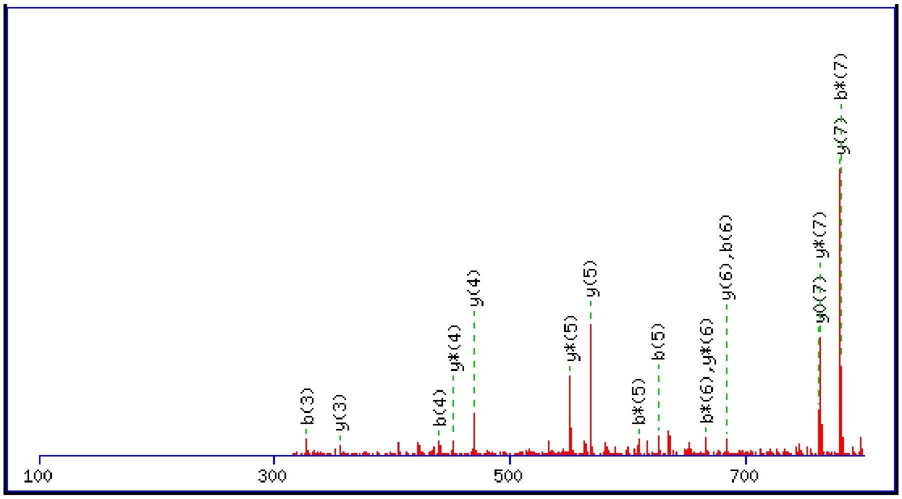


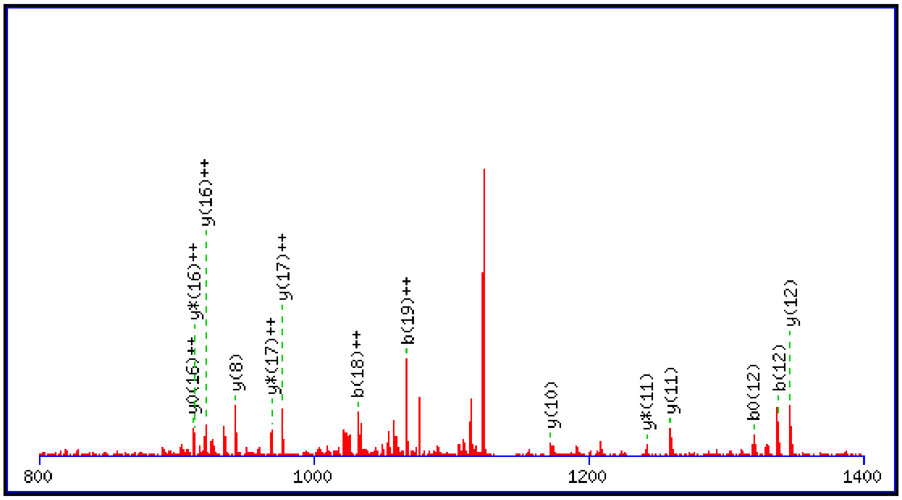


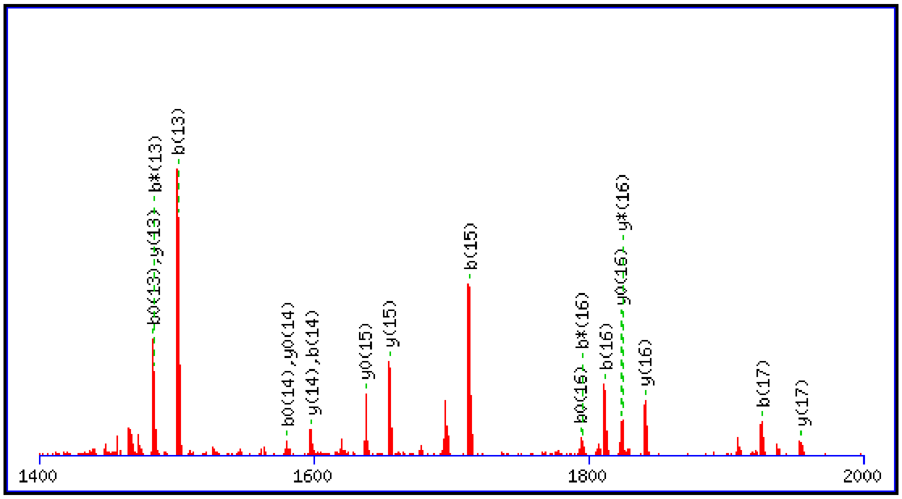


**Monoisotopic mass of neutral peptide Mr(calc):** 2280.0923
**Fixed modifications:** Carbamidomethyl (C)
**Variable modifications:**
**N7** : Deamidated_N (N)
**Ions Score:** 75 **Expect:** 8.8e-006
**Matches (Bold Red):** 50/202 fragment ions using 93 most intense peaks

| # | b | $b^{++}$ | b* | $b^{*++}$ | $b^0$ | $b^{0++}$ | Seq. | y | $y^{++}$ | y* | $y^{*++}$ | $y^0$ | $y^{0++}$ | # |
|---|---|---|---|---|---|---|---|---|---|---|---|---|---|---|
| 1 | 115.0502 | 58.0287 | 98.0237 | 49.5155 | | | N | | | | | | | 20 |
| 2 | 214.1186 | 107.5629 | 197.0921 | 99.0497 | | | V | 2167.0567 | 1084.0320 | 2150.0301 | 1075.5187 | 2149.0461 | 1075.0267 | 19 |
| 3 | **327.2027** | 164.1050 | 310.1761 | 155.5917 | | | I | 2067.9883 | 1034.4978 | 2050.9617 | 1025.9845 | 2049.9777 | 1025.4925 | 18 |
| 4 | **440.2867** | 220.6470 | 423.2602 | 212.1337 | | | I | **1954.9042** | **977.9557** | 1937.8777 | **969.4425** | 1936.8936 | 968.9505 | 17 |
| 5 | **626.3661** | 313.6867 | **609.3395** | 305.1734 | | | W | **1841.8201** | **921.4137** | **1824.7936** | **912.9004** | **1823.8096** | **912.4084** | 16 |

| | | | | | | | | | | | | | | |
|---|---|---|---|---|---|---|---|---|---|---|---|---|---|---|
| **6** | **683.3875** | 342.1974 | **666.3610** | 333.6841 | | | **G** | **1655.7408** | 828.3741 | 1638.7143 | 819.8608 | **1637.7303** | 819.3688 | **15** |
| **7** | 798.4145 | 399.7109 | **781.3879** | 391.1976 | | | **N** | **1598.7194** | 799.8633 | 1581.6928 | 791.3500 | **1580.7088** | 790.8580 | **14** |
| **8** | 935.4734 | 468.2403 | 918.4468 | 459.7270 | | | **H** | **1483.6924** | 742.3499 | 1466.6659 | 733.8366 | 1465.6819 | 733.3446 | **13** |
| **9** | 1022.5054 | 511.7563 | 1005.4788 | 503.2431 | 1004.4948 | 502.7511 | **S** | **1346.6335** | 673.8204 | 1329.6070 | 665.3071 | 1328.6230 | 664.8151 | **12** |
| **10** | 1109.5374 | 555.2724 | 1092.5109 | 546.7591 | 1091.5269 | 546.2671 | **S** | **1259.6015** | 630.3044 | **1242.5749** | 621.7911 | 1241.5909 | 621.2991 | **11** |
| **11** | 1210.5851 | 605.7962 | 1193.5586 | 597.2829 | 1192.5745 | 596.7909 | **T** | **1172.5695** | 586.7884 | 1155.5429 | 578.2751 | 1154.5589 | 577.7831 | **10** |
| **12** | **1338.6437** | 669.8255 | 1321.6171 | 661.3122 | **1320.6331** | 660.8202 | **Q** | 1071.5218 | 536.2645 | 1054.4952 | 527.7513 | 1053.5112 | 527.2592 | **9** |
| **13** | **1501.7070** | 751.3571 | **1484.6805** | 742.8439 | **1483.6964** | 742.3519 | **Y** | **943.4632** | 472.2352 | 926.4367 | 463.7220 | 925.4526 | 463.2300 | **8** |
| **14** | **1598.7598** | 799.8835 | 1581.7332 | 791.3703 | **1580.7492** | 790.8782 | **P** | **780.3999** | 390.7036 | **763.3733** | 382.1903 | **762.3893** | 381.6983 | **7** |
| **15** | **1713.7867** | 857.3970 | 1696.7602 | 848.8837 | 1695.7762 | 848.3917 | **D** | **683.3471** | 342.1772 | **666.3206** | 333.6639 | 665.3365 | 333.1719 | **6** |
| **16** | **1812.8551** | 906.9312 | **1795.8286** | 898.4179 | **1794.8446** | 897.9259 | **V** | **568.3202** | 284.6637 | **551.2936** | 276.1504 | | | **5** |
| **17** | **1926.8981** | 963.9527 | 1909.8715 | 955.4394 | 1908.8875 | 954.9474 | **N** | **469.2518** | 235.1295 | **452.2252** | 226.6162 | | | **4** |
| **18** | 2063.9570 | **1032.4821** | 2046.9304 | 1023.9688 | 2045.9464 | 1023.4768 | **H** | **355.2088** | 178.1081 | 338.1823 | 169.5948 | | | **3** |
| **19** | 2134.9941 | **1068.0007** | 2117.9675 | 1059.4874 | 2116.9835 | 1058.9954 | **A** | 218.1499 | 109.5786 | 201.1234 | 101.0653 | | | **2** |
| **20** | | | | | | | **K** | 147.1128 | 74.0600 | 130.0863 | 65.5468 | | | **1** |

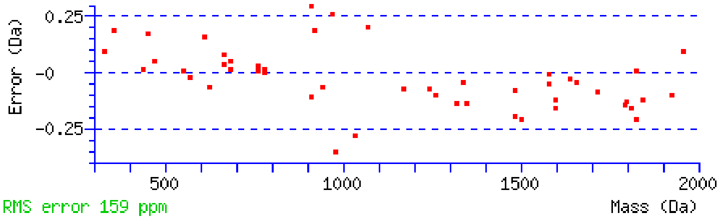


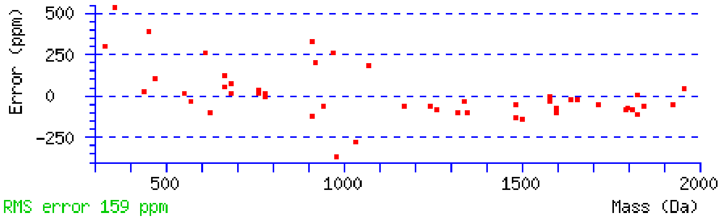


### All matches to this query

| Score | Mr(calc): | Delta | Sequence |
|---|---|---|---|
| 74.9 | 2280.0923 | 0.0075 | NVIIWGNHSSTQYPDVNHAK |
| 68.8 | 2280.0923 | 0.0075 | NVIIWGNHSSTQYPDVNHAK |
| 67.8 | 2279.1083 | 0.9915 | NVIIWGNHSSTQYPDVNHAK |
| 62.2 | 2280.0923 | 0.0075 | NVIIWGNHSSTQYPDVNHAK |
| 4.2 | 2280.0916 | 0.0083 | KLLYERSASLAATEAQLTR |
| 2.6 | 2280.1134 | -0.0136 | NELVESRREAPTQSDVFFR |
| 2.5 | 2280.1102 | -0.0104 | TMANGILKVPAIHVNNSITK |
| 2.1 | 2280.1031 | -0.0032 | GVASGLCVLVSWLTAFVLTK |
| 0.1 | 2280.1030 | -0.0032 | ASKYLHVSLMEPPVVLPLQ |
| 0.1 | 2280.1030 | -0.0032 | ASKYLHVSLMEPPVVLPLQ |

---

Spectrum No: 305; Query: 475; Rank: 1

---

## Peptide View

MS/MS Fragmentation of **NMTLFSDLVAEK**
Found in **IPI00372839**, Tax_Id=10116 Gene_Symbol=Col6a2 similar to procollagen, type VI, alpha 2

Match to Query 475: 1383.657148 from(692.835850,2+)
Title: 091008RatKidney_NH4Format01_26.3262.3262.2.dta
Data file K:\NewmanPaper\Piliang\3SubProteomes\Piliang3SP\mgf5ppm\ERLIC_3SubProteomes5ppm.mgf

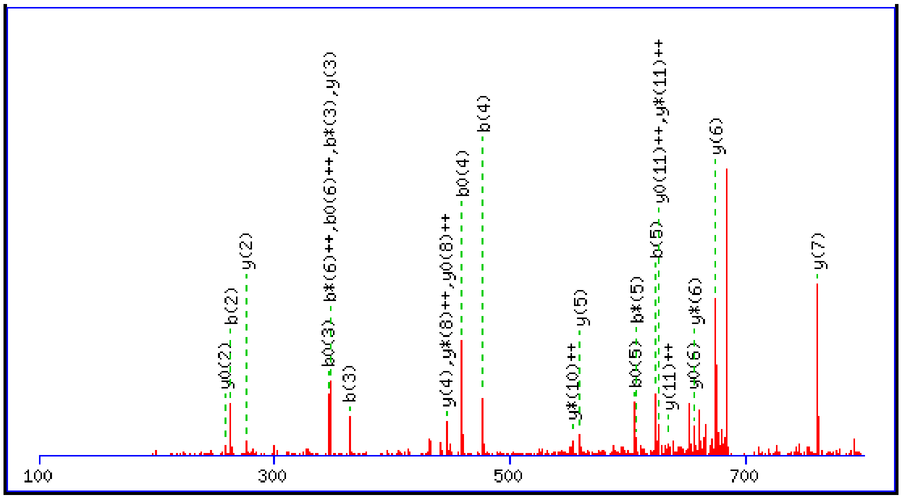


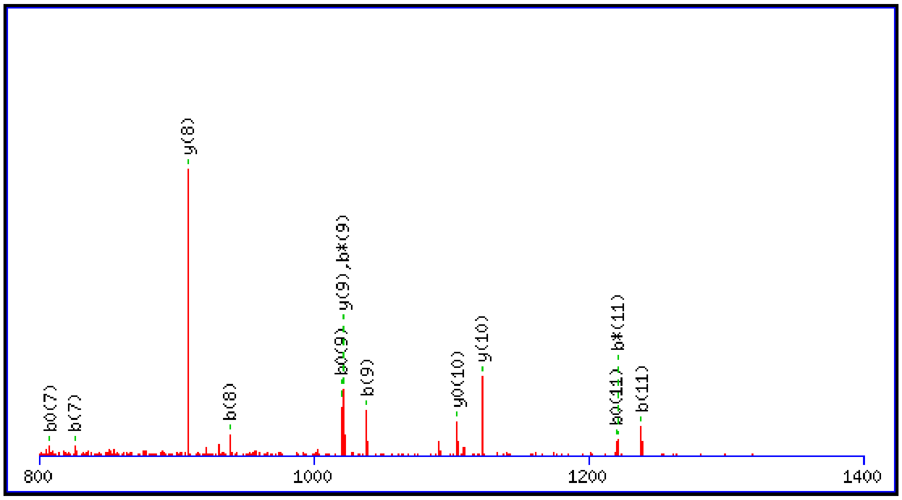


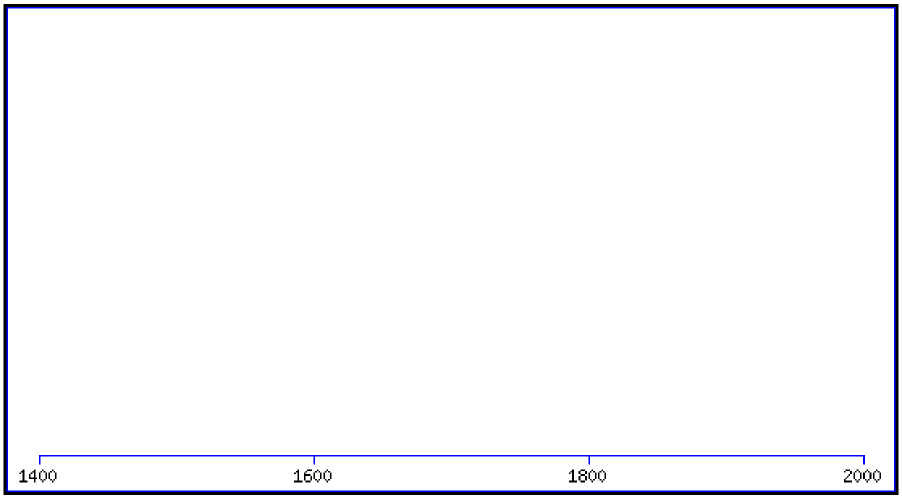


**Monoisotopic mass of neutral peptide Mr(calc):** 1383.6592
**Fixed modifications:** Carbamidomethyl (C)
**Variable modifications:**
**N1** : Deamidated_N (N)
**M2** : Oxidation (M)
**Ions Score:** 75 **Expect:** 5.8e-006
**Matches (Bold Red):** 39/126 fragment ions using 57 most intense peaks

| # | b | b$^{++}$ | b* | b*$^{++}$ | b$^{0}$ | b$^{0++}$ | Seq. | y | y$^{++}$ | y* | y*$^{++}$ | y$^{0}$ | y$^{0++}$ | # |
|---|---|---|---|---|---|---|---|---|---|---|---|---|---|---|
| 1 | 116.0342 | 58.5207 | 99.0077 | 50.0075 | | | N | | | | | | | 12 |
| 2 | **263.0696** | 132.0384 | 246.0431 | 123.5252 | | | M | 1269.6395 | **635.3234** | 1252.6130 | **626.8101** | 1251.6290 | **626.3181** | 11 |
| 3 | **364.1173** | 182.5623 | **347.0907** | 174.0490 | **346.1067** | 173.5570 | T | **1122.6041** | 561.8057 | 1105.5776 | **553.2924** | **1104.5936** | 552.8004 | 10 |
| 4 | **477.2014** | 239.1043 | 460.1748 | 230.5910 | **459.1908** | 230.0990 | L | **1021.5564** | 511.2819 | 1004.5299 | 502.7686 | 1003.5459 | 502.2766 | 9 |

| | | | | | | | | | | | | | | |
|---|---|---|---|---|---|---|---|---|---|---|---|---|---|---|
| **5** | **624.2698** | 312.6385 | **607.2432** | 304.1253 | **606.2592** | 303.6332 | **F** | **908.4724** | 454.7398 | 891.4458 | **446.2266** | 890.4618 | **445.7345** | **8** |
| **6** | 711.3018 | 356.1545 | 694.2753 | **347.6413** | 693.2912 | **347.1493** | **S** | **761.4040** | 381.2056 | 744.3774 | 372.6923 | 743.3934 | 372.2003 | **7** |
| **7** | **826.3287** | 413.6680 | 809.3022 | 405.1547 | **808.3182** | 404.6627 | **D** | **674.3719** | 337.6896 | **657.3454** | 329.1763 | **656.3614** | 328.6843 | **6** |
| **8** | **939.4128** | 470.2100 | 922.3863 | 461.6968 | 921.4022 | 461.2048 | **L** | **559.3450** | 280.1761 | 542.3184 | 271.6629 | 541.3344 | 271.1709 | **5** |
| **9** | **1038.4812** | 519.7442 | **1021.4547** | 511.2310 | **1020.4707** | 510.7390 | **V** | **446.2609** | 223.6341 | 429.2344 | 215.1208 | 428.2504 | 214.6288 | **4** |
| **10** | 1109.5183 | 555.2628 | 1092.4918 | 546.7495 | 1091.5078 | 546.2575 | **A** | **347.1925** | 174.0999 | 330.1660 | 165.5866 | 329.1819 | 165.0946 | **3** |
| **11** | **1238.5609** | 619.7841 | **1221.5344** | 611.2708 | **1220.5504** | 610.7788 | **E** | **276.1554** | 138.5813 | 259.1288 | 130.0681 | **258.1448** | 129.5761 | **2** |
| **12** | | | | | | | **K** | 147.1128 | 74.0600 | 130.0863 | 65.5468 | | | **1** |

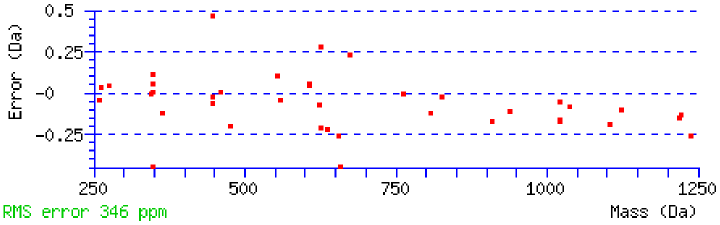


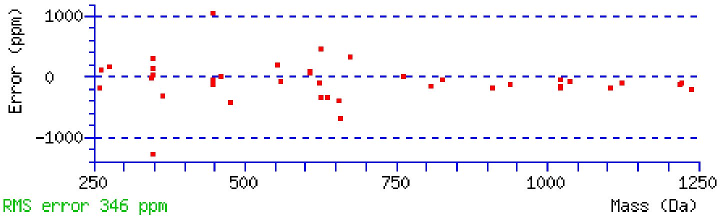


### All matches to this query

| Score | Mr(calc): | Delta | Sequence |
|---|---|---|---|
| 74.9 | 1383.6592 | -0.0020 | NMTLFSDLVAEK |
| 20.7 | 1383.6646 | -0.0075 | SAANKSVSIVAEK |
| 14.8 | 1383.6646 | -0.0075 | SAANKSVSIVAEK |
| 14.8 | 1383.6646 | -0.0075 | SAANKSVSIVAEK |
| 10.7 | 1383.6435 | 0.0136 | SFEGLSKPSPQK |
| 8.1 | 1383.6646 | -0.0075 | KELQSNKESIK |
| 7.9 | 1382.6574 | 0.9997 | IMNTFSVMPSPK |
| 6.4 | 1383.6639 | -0.0067 | VYRGCSCILEK |
| 6.1 | 1382.6534 | 1.0038 | EMAKMVADTISR |
| 5.7 | 1383.6630 | -0.0059 | ANTKGHTDIDSPK |

---

Spectrum No: 306; Query: 559; Rank: 1

---

## Peptide View

MS/MS Fragmentation of **DITNLINNTFIR**
Found in **IPI00373408**, Tax_Id=10116 Gene_Symbol=Sort1 Sortilin precursor

Match to Query 559: 1433.751608 from(717.883080,2+)
Title: 091008RatKidney_NoSalt_27.4439.4439.2.dta
Data file K:\NewmanPaper\Piliang\3SubProteomes\Piliang3SP\mgf5ppm\ERLIC_3SubProteomes5ppm.mgf

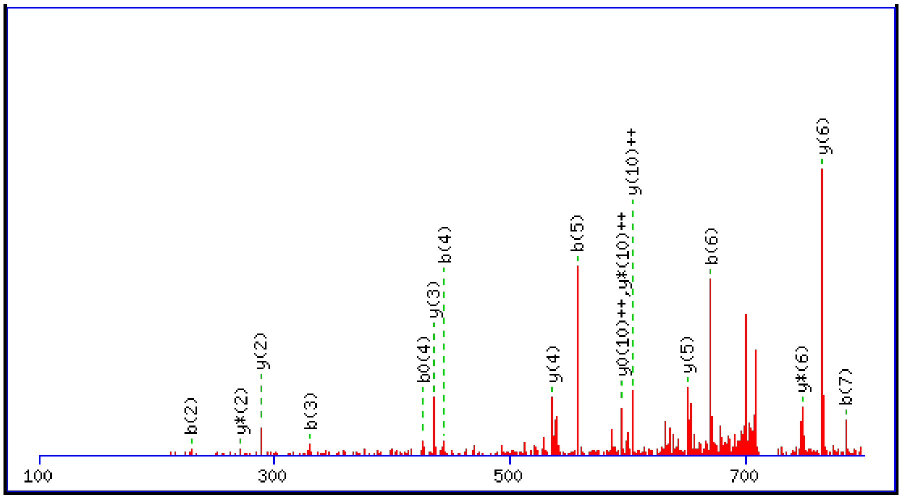


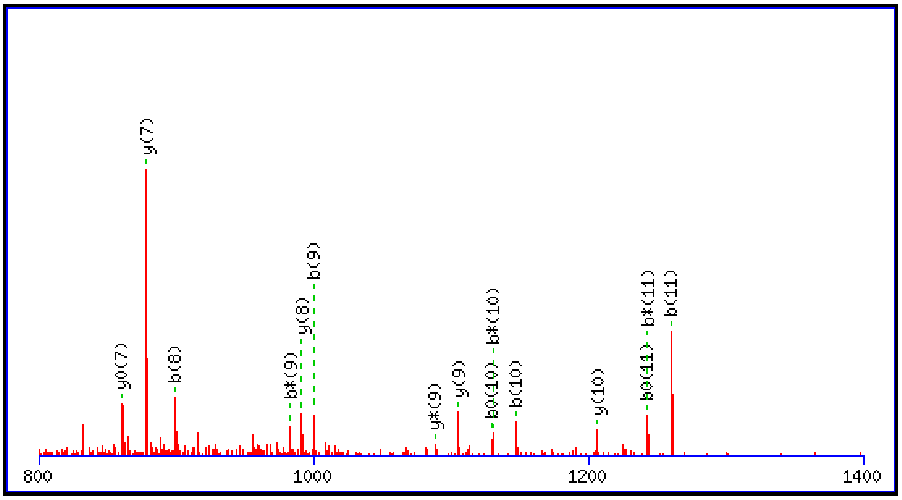


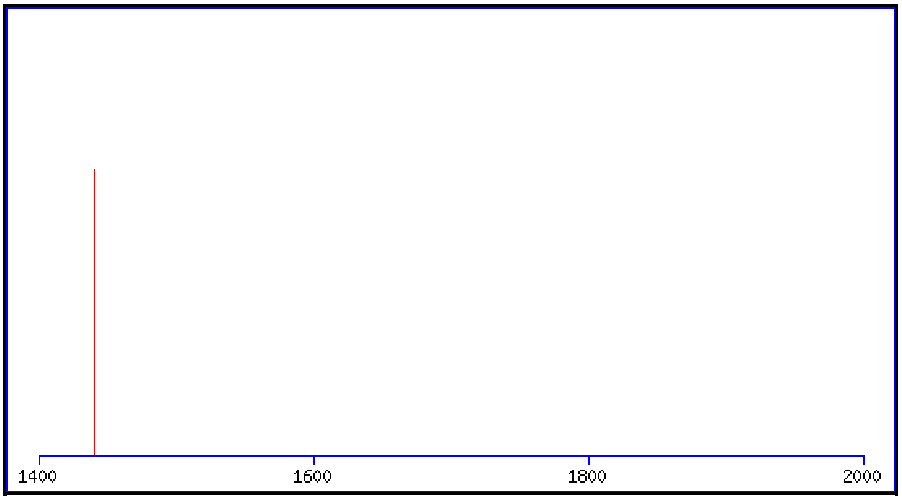


**Monoisotopic mass of neutral peptide Mr(calc):** 1433.7514
**Fixed modifications:** Carbamidomethyl (C)
**Variable modifications:**
**N7** : Deamidated_N (N)
**Ions Score:** 75 **Expect:** 4.1e-006
**Matches (Bold Red):** 32/120 fragment ions using 60 most intense peaks

| # | b | $b^{++}$ | b* | $b^{*++}$ | $b^{0}$ | $b^{0++}$ | Seq. | y | $y^{++}$ | y* | $y^{*++}$ | $y^{0}$ | $y^{0++}$ | # |
|---|---|---|---|---|---|---|---|---|---|---|---|---|---|---|
| 1 | 116.0342 | 58.5207 | | | 98.0237 | 49.5155 | D | | | | | | | 12 |
| 2 | **229.1183** | 115.0628 | | | 211.1077 | 106.0575 | I | 1319.7318 | 660.3695 | 1302.7052 | 651.8562 | 1301.7212 | 651.3642 | 11 |
| 3 | **330.1660** | 165.5866 | | | 312.1554 | 156.5813 | T | **1206.6477** | **603.8275** | 1189.6212 | **595.3142** | 1188.6371 | **594.8222** | 10 |
| 4 | **444.2089** | 222.6081 | 427.1823 | 214.0948 | **426.1983** | 213.6028 | N | **1105.6000** | 553.3037 | **1088.5735** | 544.7904 | 1087.5895 | 544.2984 | 9 |
| 5 | **557.2930** | 279.1501 | 540.2664 | 270.6368 | 539.2824 | 270.1448 | L | **991.5571** | 496.2822 | 974.5306 | 487.7689 | 973.5465 | 487.2769 | 8 |

| | | | | | | | | | | | | | | |
|---|---|---|---|---|---|---|---|---|---|---|---|---|---|---|
| **6** | **670.3770** | 335.6921 | 653.3505 | 327.1789 | 652.3665 | 326.6869 | **I** | **878.4730** | 439.7402 | 861.4465 | 431.2269 | **860.4625** | 430.7349 | **7** |
| **7** | **785.4040** | 393.2056 | 768.3774 | 384.6923 | 767.3934 | 384.2003 | **N** | **765.3890** | 383.1981 | **748.3624** | 374.6848 | 747.3784 | 374.1928 | **6** |
| **8** | **899.4469** | 450.2271 | 882.4203 | 441.7138 | 881.4363 | 441.2218 | **N** | **650.3620** | 325.6847 | 633.3355 | 317.1714 | 632.3515 | 316.6794 | **5** |
| **9** | **1000.4946** | 500.7509 | **983.4680** | 492.2376 | 982.4840 | 491.7456 | **T** | **536.3191** | 268.6632 | 519.2926 | 260.1499 | 518.3085 | 259.6579 | **4** |
| **10** | **1147.5630** | 574.2851 | **1130.5364** | 565.7719 | **1129.5524** | 565.2798 | **F** | **435.2714** | 218.1394 | 418.2449 | 209.6261 | | | **3** |
| **11** | **1260.6470** | 630.8272 | **1243.6205** | 622.3139 | **1242.6365** | 621.8219 | **I** | **288.2030** | 144.6051 | **271.1765** | 136.0919 | | | **2** |
| **12** | | | | | | | **R** | 175.1190 | 88.0631 | 158.0924 | 79.5498 | | | **1** |

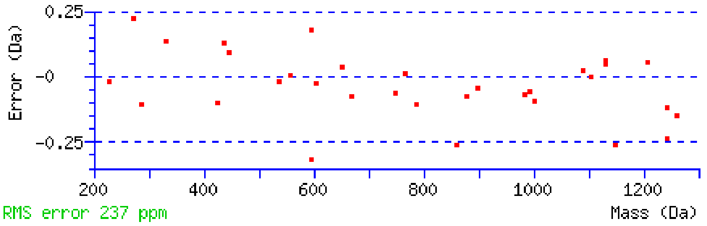


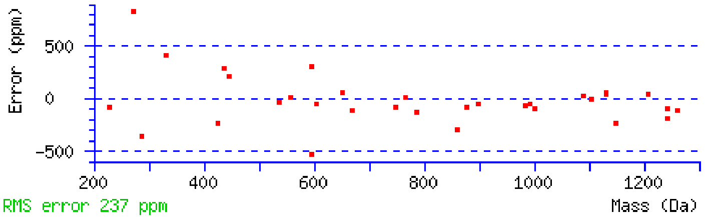


### All matches to this query

| Score | Mr(calc): | Delta | Sequence |
|---|---|---|---|
| 74.7 | 1433.7514 | 0.0002 | DITNLINNTFIR |
| 72.4 | 1433.7514 | 0.0002 | DITNLINNTFIR |
| 33.1 | 1433.7514 | 0.0002 | DITNLINNTFIR |
| 14.2 | 1433.7626 | -0.0110 | GEKELLFNKNSR |
| 12.8 | 1433.7626 | -0.0110 | DLFIDGRSKNIR |
| 7.9 | 1433.7627 | -0.0111 | SISDVASPIRSFR |
| 6.2 | 1433.7402 | 0.0114 | LKDLSLNDPQYK |
| 5.8 | 1433.7392 | 0.0124 | ITIAPDGGVSRLR |
| 4.5 | 1432.7609 | 0.9907 | RGMNIPQPPIGPR |
| 4.0 | 1431.7470 | 2.0046 | GDDGIPGPPGPKGIR |

---

Spectrum No: 307; Query: 780; Rank: 1

---

## Peptide View

MS/MS Fragmentation of **DGNCTSPQALCNAR**
Found in **IPI00205325**, Tax_Id=10116 Gene_Symbol=Lrp2 Low-density lipoprotein receptor-related protein 2 precursor

Match to Query 780: 1563.636468 from(782.825510,2+)
Title: 100101RatKid_NS_deglyco_19.1055.1055.2.dta
Data file K:\NewmanPaper\Piliang\3SubProteomes\Piliang3SP\mgf5ppm\ERLIC_3SubProteomes5ppm.mgf

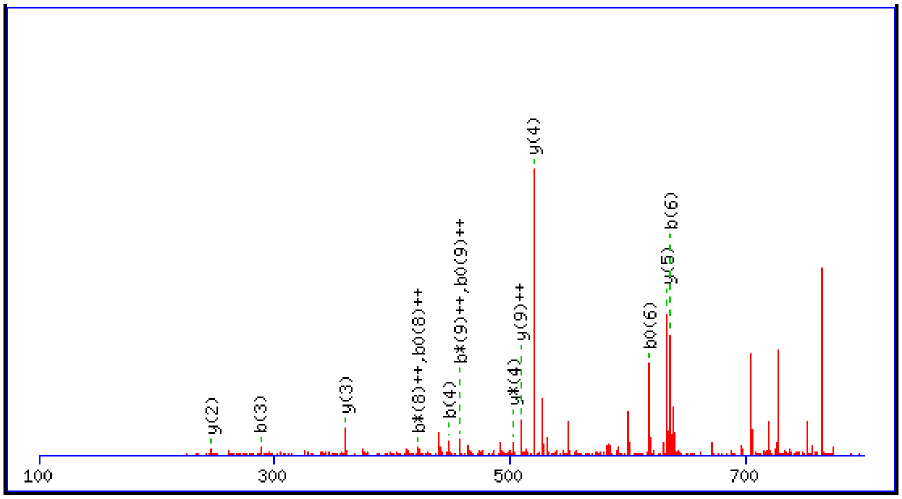


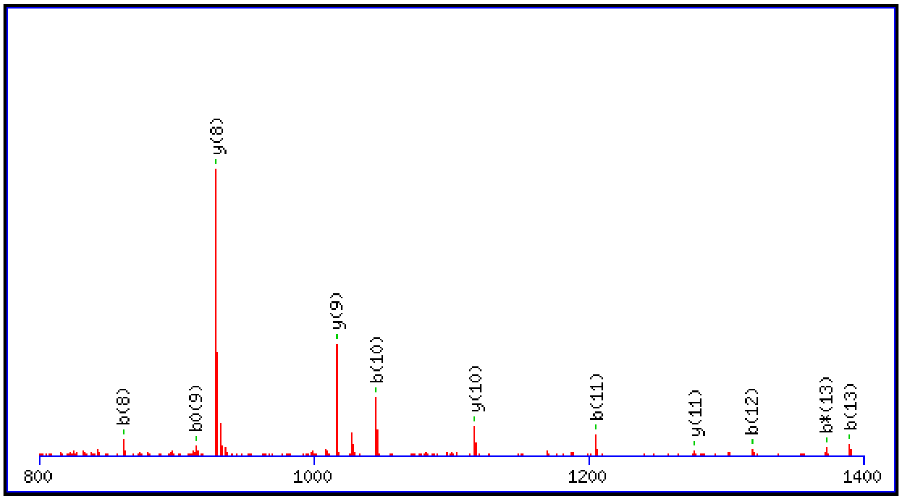


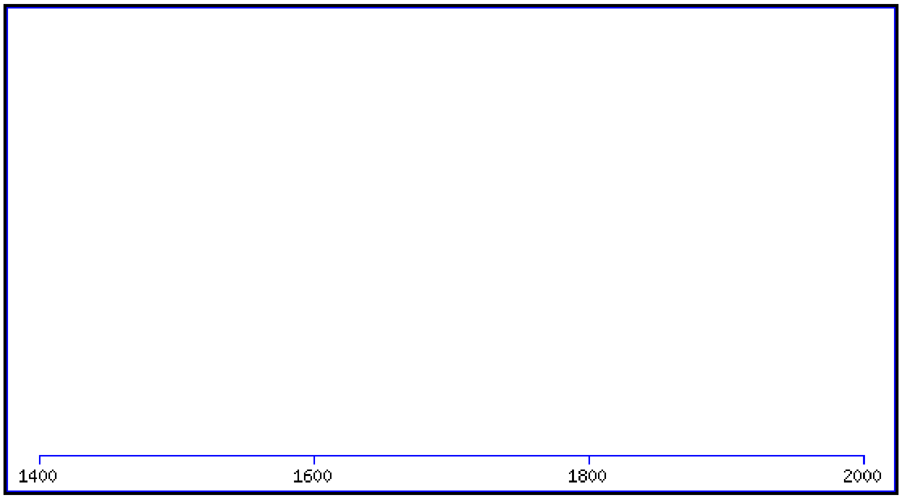


**Monoisotopic mass of neutral peptide Mr(calc):** 1563.6406
**Fixed modifications:** Carbamidomethyl (C)
**Variable modifications:**
**N3** : Deamidated_N (N)
**Ions Score:** 75 **Expect:** 4.2e-006
**Matches (Bold Red):** 25/136 fragment ions using 33 most intense peaks

| # | b | b$^{++}$ | b* | b*$^{++}$ | b$^{0}$ | b$^{0++}$ | Seq. | y | y$^{++}$ | y* | y*$^{++}$ | y$^{0}$ | y$^{0++}$ | # |
|---|---|---|---|---|---|---|---|---|---|---|---|---|---|---|
| 1 | 116.0342 | 58.5207 | | | 98.0237 | 49.5155 | D | | | | | | | 14 |
| 2 | 173.0557 | 87.0315 | | | 155.0451 | 78.0262 | G | 1449.6209 | 725.3141 | 1432.5944 | 716.8008 | 1431.6104 | 716.3088 | 13 |
| 3 | **288.0826** | 144.5449 | 271.0561 | 136.0317 | 270.0721 | 135.5397 | N | 1392.5995 | 696.8034 | 1375.5729 | 688.2901 | 1374.5889 | 687.7981 | 12 |
| 4 | **448.1133** | 224.5603 | 431.0867 | 216.0470 | 430.1027 | 215.5550 | C | **1277.5725** | 639.2899 | 1260.5460 | 630.7766 | 1259.5620 | 630.2846 | 11 |
| 5 | 549.1609 | 275.0841 | 532.1344 | 266.5708 | 531.1504 | 266.0788 | T | **1117.5419** | 559.2746 | 1100.5153 | 550.7613 | 1099.5313 | 550.2693 | 10 |

| | | | | | | | | | | | | | | |
|---|---|---|---|---|---|---|---|---|---|---|---|---|---|---|
| 6 | 636.1930 | 318.6001 | 619.1664 | 310.0869 | 618.1824 | 309.5948 | S | 1016.4942 | 508.7507 | 999.4676 | 500.2375 | 998.4836 | 499.7455 | 9 |
| 7 | 733.2457 | 367.1265 | 716.2192 | 358.6132 | 715.2352 | 358.1212 | P | 929.4622 | 465.2347 | 912.4356 | 456.7214 | | | 8 |
| 8 | 861.3043 | 431.1558 | 844.2778 | 422.6425 | 843.2938 | 422.1505 | Q | 832.4094 | 416.7083 | 815.3828 | 408.1951 | | | 7 |
| 9 | 932.3414 | 466.6744 | 915.3149 | 458.1611 | 914.3309 | 457.6691 | A | 704.3508 | 352.6790 | 687.3243 | 344.1658 | | | 6 |
| 10 | 1045.4255 | 523.2164 | 1028.3989 | 514.7031 | 1027.4149 | 514.2111 | L | 633.3137 | 317.1605 | 616.2872 | 308.6472 | | | 5 |
| 11 | 1205.4561 | 603.2317 | 1188.4296 | 594.7184 | 1187.4456 | 594.2264 | C | 520.2296 | 260.6185 | 503.2031 | 252.1052 | | | 4 |
| 12 | 1319.4991 | 660.2532 | 1302.4725 | 651.7399 | 1301.4885 | 651.2479 | N | 360.1990 | 180.6031 | 343.1724 | 172.0899 | | | 3 |
| 13 | 1390.5362 | 695.7717 | 1373.5096 | 687.2585 | 1372.5256 | 686.7664 | A | 246.1561 | 123.5817 | 229.1295 | 115.0684 | | | 2 |
| 14 | | | | | | | R | 175.1190 | 88.0631 | 158.0924 | 79.5498 | | | 1 |

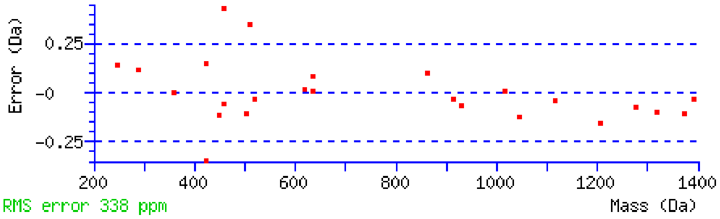


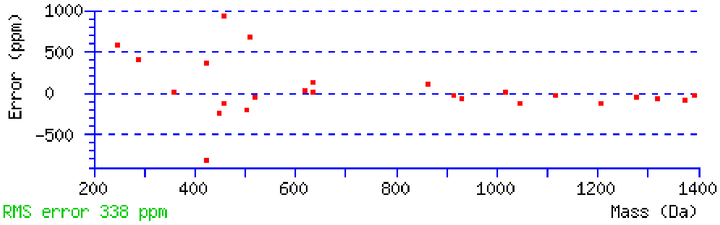


### All matches to this query

| Score | Mr(calc): | Delta | Sequence |
|---|---|---|---|
| 74.7 | 1563.6406 | -0.0041 | DGNCTSPQALCNAR |
| 20.9 | 1563.6283 | 0.0082 | MRSESQRMSNAR |
| 16.5 | 1563.6283 | 0.0082 | MRSESQRMSNAR |
| 13.2 | 1563.6517 | -0.0153 | SPGSESKVLGCKR |
| 11.3 | 1561.6418 | 1.9946 | DKPYACDICGKR |
| 11.2 | 1563.6406 | -0.0041 | DGNCTSPQALCNAR |
| 10.3 | 1563.6355 | 0.0010 | ASSFYGSASSNHLR |
| 4.1 | 1563.6357 | 0.0008 | RDSIQMSMKGCR |
| 3.7 | 1563.6517 | -0.0153 | SPGSESKVLGCKR |
| 3.7 | 1563.6517 | -0.0153 | SPGSESKVLGCKR |

---

Spectrum No: 308; Query: 2316; Rank: 1

---

## Peptide View

MS/MS Fragmentation of **VIDLWDLAQSANFTEKELESFR**
Found in **IPI00364124**, Tax_Id=10116 Gene_Symbol=Lrpap1 Alpha-2-macroglobulin receptor-associated protein precursor

Match to Query 2316: 2611.287612 from(871.436480,3+)
Title: 091008RatKidney_NoSalt_21.6217.6217.3.dta
Data file K:\NewmanPaper\Piliang\3SubProteomes\Piliang3SP\mgf5ppm\ERLIC_3SubProteomes5ppm.mgf

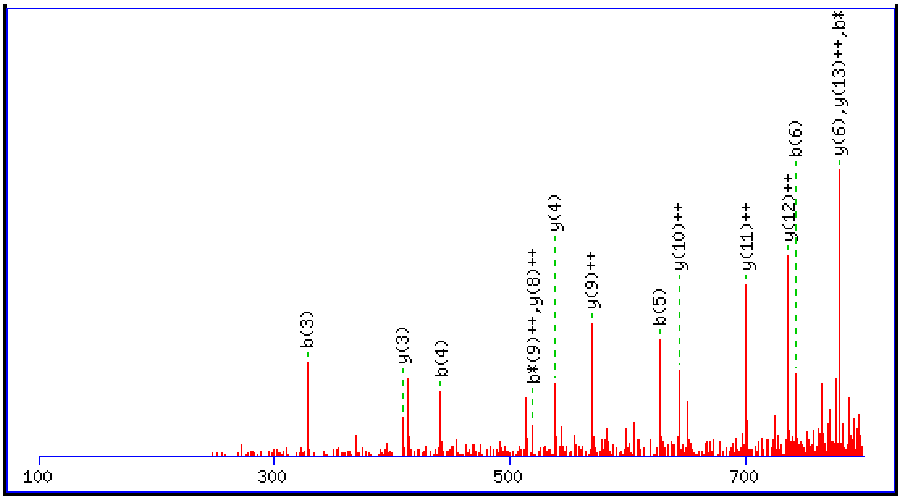


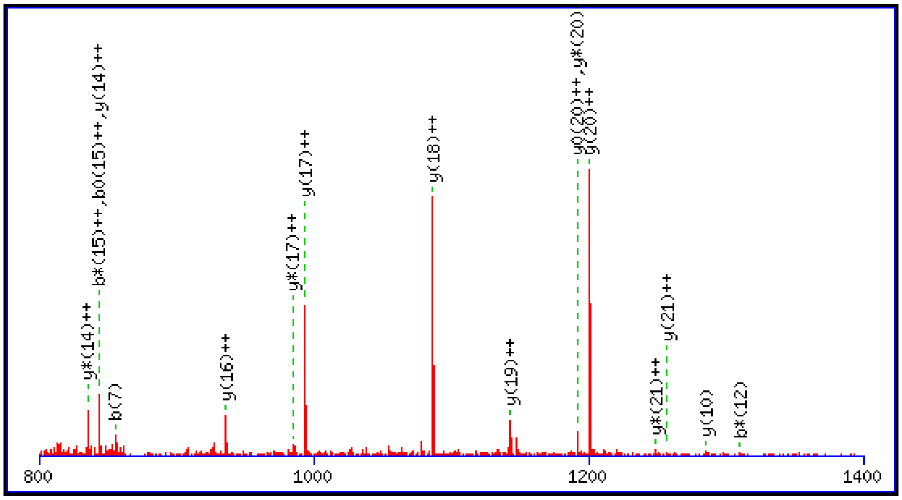


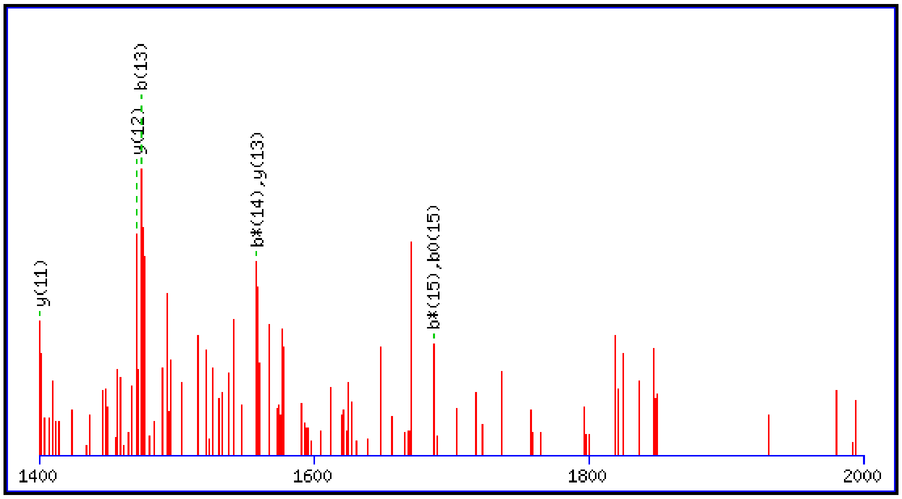


**Monoisotopic mass of neutral peptide Mr(calc):** 2611.2806
**Fixed modifications:** Carbamidomethyl (C)
**Variable modifications:**
**N12** : Deamidated_N (N)
**Ions Score:** 75 **Expect:** 9.3e-006
**Matches (Bold Red):** 39/228 fragment ions using 63 most intense peaks

| # | b | $b^{++}$ | b* | $b^{*++}$ | $b^0$ | $b^{0++}$ | Seq. | y | $y^{++}$ | y* | $y^{*++}$ | $y^0$ | $y^{0++}$ | # |
|---|---|---|---|---|---|---|---|---|---|---|---|---|---|---|
| 1 | 100.0757 | 50.5415 | | | | | V | | | | | | | 22 |
| 2 | 213.1598 | 107.0835 | | | | | I | 2513.2195 | **1257.1134** | 2496.1929 | **1248.6001** | 2495.2089 | 1248.1081 | 21 |
| 3 | **328.1867** | 164.5970 | | | 310.1761 | 155.5917 | D | 2400.1354 | **1200.5713** | 2383.1088 | **1192.0581** | 2382.1248 | **1191.5661** | 20 |
| 4 | **441.2708** | 221.1390 | | | 423.2602 | 212.1337 | L | 2285.1084 | **1143.0579** | 2268.0819 | 1134.5446 | 2267.0979 | 1134.0526 | 19 |
| 5 | **627.3501** | 314.1787 | | | 609.3395 | 305.1734 | W | 2172.0244 | **1086.5158** | 2154.9978 | 1078.0026 | 2154.0138 | 1077.5105 | 18 |

| | | | | | | | | | | | | | | |
|---|---|---|---|---|---|---|---|---|---|---|---|---|---|---|
| **6** | **742.3770** | 371.6921 | | | 724.3665 | 362.6869 | **D** | 1985.9451 | **993.4762** | 1968.9185 | **984.9629** | 1967.9345 | 984.4709 | **17** |
| **7** | **855.4611** | 428.2342 | | | 837.4505 | 419.2289 | **L** | 1870.9181 | **935.9627** | 1853.8916 | 927.4494 | 1852.9076 | 926.9574 | **16** |
| **8** | 926.4982 | 463.7527 | | | 908.4876 | 454.7475 | **A** | 1757.8341 | 879.4207 | 1740.8075 | 870.9074 | 1739.8235 | 870.4154 | **15** |
| **9** | 1054.5568 | 527.7820 | 1037.5302 | **519.2688** | 1036.5462 | 518.7767 | **Q** | 1686.7969 | **843.9021** | 1669.7704 | **835.3888** | 1668.7864 | 834.8968 | **14** |
| **10** | 1141.5888 | 571.2980 | 1124.5623 | 562.7848 | 1123.5782 | 562.2928 | **S** | **1558.7384** | **779.8728** | 1541.7118 | 771.3595 | 1540.7278 | 770.8675 | **13** |
| **11** | 1212.6259 | 606.8166 | 1195.5994 | 598.3033 | 1194.6154 | 597.8113 | **A** | **1471.7063** | **736.3568** | 1454.6798 | 727.8435 | 1453.6958 | 727.3515 | **12** |
| **12** | 1327.6529 | 664.3301 | **1310.6263** | 655.8168 | 1309.6423 | 655.3248 | **N** | **1400.6692** | **700.8383** | 1383.6427 | 692.3250 | 1382.6587 | 691.8330 | **11** |
| **13** | **1474.7213** | 737.8643 | 1457.6947 | 729.3510 | 1456.7107 | 728.8590 | **F** | **1285.6423** | **643.3248** | 1268.6157 | 634.8115 | 1267.6317 | 634.3195 | **10** |
| **14** | 1575.7689 | 788.3881 | **1558.7424** | **779.8748** | 1557.7584 | 779.3828 | **T** | 1138.5739 | **569.7906** | 1121.5473 | 561.2773 | 1120.5633 | 560.7853 | **9** |
| **15** | 1704.8115 | 852.9094 | **1687.7850** | **844.3961** | **1686.8010** | **843.9041** | **E** | 1037.5262 | **519.2667** | 1020.4997 | 510.7535 | 1019.5156 | 510.2615 | **8** |
| **16** | 1832.9065 | 916.9569 | 1815.8800 | 908.4436 | 1814.8959 | 907.9516 | **K** | 908.4836 | 454.7454 | 891.4571 | 446.2322 | 890.4730 | 445.7402 | **7** |
| **17** | 1961.9491 | 981.4782 | 1944.9225 | 972.9649 | 1943.9385 | 972.4729 | **E** | **780.3886** | 390.6980 | 763.3621 | 382.1847 | 762.3781 | 381.6927 | **6** |
| **18** | 2075.0332 | 1038.0202 | 2058.0066 | 1029.5069 | 2057.0226 | 1029.0149 | **L** | 651.3461 | 326.1767 | 634.3195 | 317.6634 | 633.3355 | 317.1714 | **5** |
| **19** | 2204.0758 | 1102.5415 | 2187.0492 | 1094.0282 | 2186.0652 | 1093.5362 | **E** | **538.2620** | 269.6346 | 521.2354 | 261.1214 | 520.2514 | 260.6293 | **4** |
| **20** | 2291.1078 | 1146.0575 | 2274.0812 | 1137.5443 | 2273.0972 | 1137.0522 | **S** | **409.2194** | 205.1133 | 392.1928 | 196.6001 | 391.2088 | 196.1081 | **3** |
| **21** | 2438.1762 | 1219.5917 | 2421.1496 | 1211.0785 | 2420.1656 | 1210.5865 | **F** | 322.1874 | 161.5973 | 305.1608 | 153.0840 | | | **2** |
| **22** | | | | | | | **R** | 175.1190 | 88.0631 | 158.0924 | 79.5498 | | | **1** |

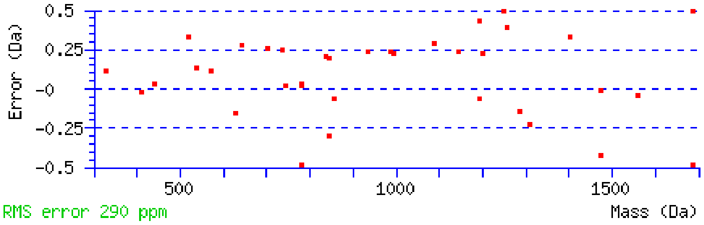


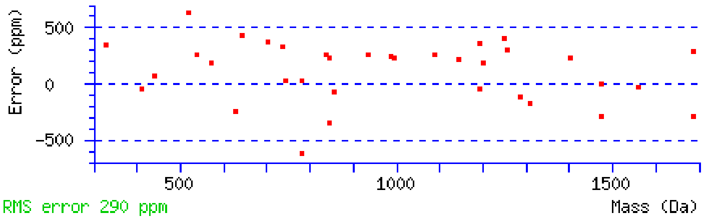


### All matches to this query

| Score | Mr(calc): | Delta | Sequence |
|---|---|---|---|
| 74.6 | 2611.2806 | 0.0070 | VIDLWDLAQSANFTEKELESFR |
| 5.9 | 2610.2966 | 0.9911 | VIDLWDLAQSANFTEKELESFR |
| 5.7 | 2609.2954 | 1.9922 | IPVEANFLYVYSTKPNYYSWR |
| 4.9 | 2611.3002 | -0.0126 | IEGLDSEMIMKPLVTEINAKLR |
| 2.2 | 2611.2975 | -0.0099 | NIDGEALVAIESFYPVFTPPPKK |
| 2.2 | 2611.2975 | -0.0099 | NIDGEALVAIESFYPVFTPPPKK |
| 2.2 | 2610.2794 | 1.0082 | IPVEANFLYVYSTKPNYYSWR |
| 1.7 | 2610.2764 | 1.0112 | VLIEFSLLRLENPDEACAVSQK |
| 1.6 | 2609.2880 | 1.9996 | TLLRAAGPLSPPGAGHTVPGPGVATR |
| 1.5 | 2611.2993 | -0.0117 | KIQELQSEKETLSTQLDLAETK |

---

Spectrum No: 309; Query: 2653; Rank: 1

---

## Peptide View

MS/MS Fragmentation of **KKPTPIQLNPAPDGSAVNGTSSAETNLEALQK**
Found in **IPI00231247**, Tax_Id=10116 Gene_Symbol=Map2k1 Dual specificity mitogen-activated protein kinase kinase 1

Match to Query 2653: 3276.688212 from(1093.236680,3+)
Title: 091008RatKidney_NH4Format01_13.2839.2839.3.dta
Data file K:\NewmanPaper\Piliang\3SubProteomes\Piliang3SP\mgf5ppm\ERLIC_3SubProteomes5ppm.mgf

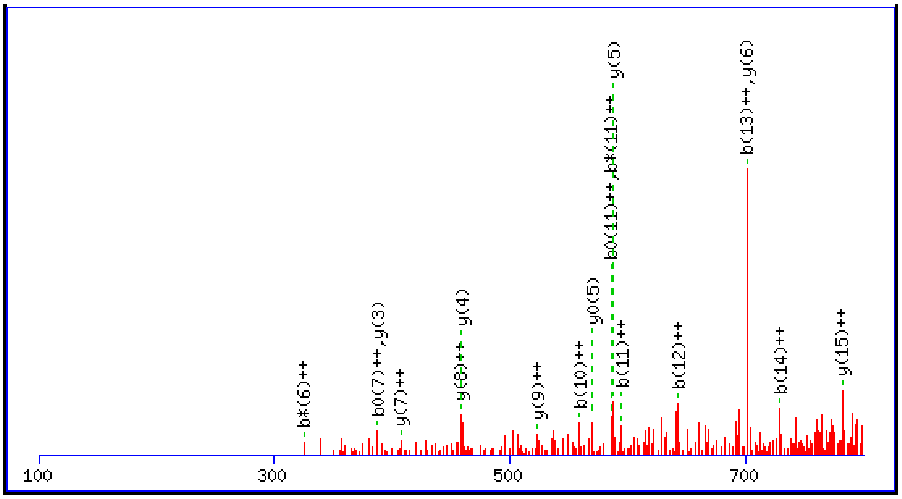


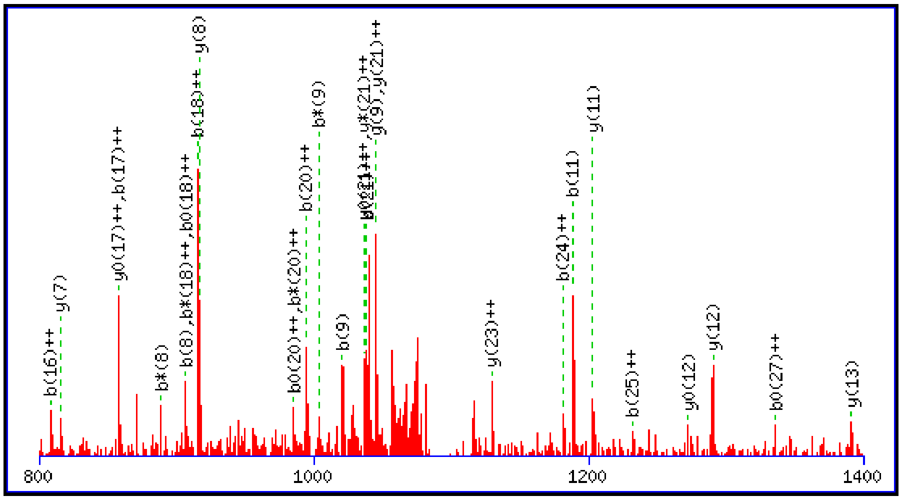


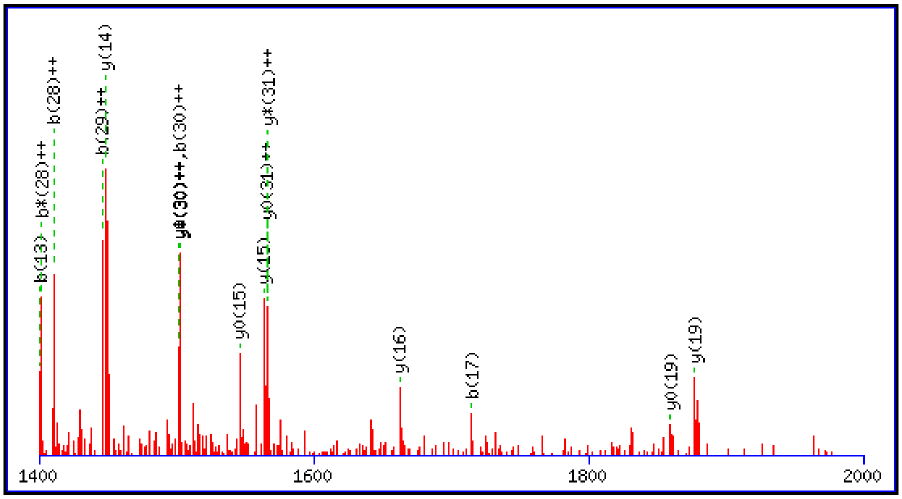


**Monoisotopic mass of neutral peptide Mr(calc):** 3276.6837
**Fixed modifications:** Carbamidomethyl (C)
**Variable modifications:**
**N18** : Deamidated_N (N)
**Ions Score:** 74 **Expect:** 5.2e-006
**Matches (Bold Red):** 64/358 fragment ions using 108 most intense peaks

| # | b | b++ | b* | b*++ | b0 | b0++ | Seq. | y | y++ | y* | y*++ | y0 | y0++ | # |
|---|---|---|---|---|---|---|---|---|---|---|---|---|---|---|
| 1 | 129.1022 | 65.0548 | 112.0757 | 56.5415 | | | K | | | | | | | 32 |
| 2 | 257.1972 | 129.1022 | 240.1707 | 120.5890 | | | K | 3149.5961 | 1575.3017 | 3132.5695 | **1566.7884** | 3131.5855 | **1566.2964** | 31 |
| 3 | 354.2500 | 177.6286 | 337.2234 | 169.1153 | | | P | 3021.5011 | 1511.2542 | 3004.4746 | **1502.7409** | 3003.4905 | **1502.2489** | 30 |
| 4 | 455.2976 | 228.1525 | 438.2711 | 219.6392 | 437.2871 | 219.1472 | T | 2924.4483 | 1462.7278 | 2907.4218 | 1454.2145 | 2906.4378 | 1453.7225 | 29 |
| 5 | 552.3504 | 276.6788 | 535.3239 | 268.1656 | 534.3398 | 267.6736 | P | 2823.4007 | 1412.2040 | 2806.3741 | 1403.6907 | 2805.3901 | 1403.1987 | 28 |

| | | | | | | | | | | | | | | |
|---|---|---|---|---|---|---|---|---|---|---|---|---|---|---|
| 6 | 665.4345 | 333.2209 | 648.4079 | **324.7076** | 647.4239 | 324.2156 | I | 2726.3479 | 1363.6776 | 2709.3214 | 1355.1643 | 2708.3373 | 1354.6723 | 27 |
| 7 | 793.4931 | 397.2502 | 776.4665 | 388.7369 | 775.4825 | **388.2449** | Q | 2613.2638 | 1307.1356 | 2596.2373 | 1298.6223 | 2595.2533 | 1298.1303 | 26 |
| 8 | **906.5771** | 453.7922 | **889.5506** | 445.2789 | 888.5666 | 444.7869 | L | 2485.2053 | 1243.1063 | 2468.1787 | 1234.5930 | 2467.1947 | 1234.1010 | 25 |
| 9 | **1020.6200** | 510.8137 | **1003.5935** | 502.3004 | 1002.6095 | 501.8084 | N | 2372.1212 | 1186.5642 | 2355.0946 | 1178.0510 | 2354.1106 | 1177.5590 | 24 |
| 10 | 1117.6728 | **559.3400** | 1100.6463 | 550.8268 | 1099.6622 | 550.3348 | P | 2258.0783 | **1129.5428** | 2241.0517 | 1121.0295 | 2240.0677 | 1120.5375 | 23 |
| 11 | **1188.7099** | **594.8586** | 1171.6834 | **586.3453** | 1170.6994 | **585.8533** | A | 2161.0255 | 1081.0164 | 2143.9990 | 1072.5031 | 2143.0149 | 1072.0111 | 22 |
| 12 | 1285.7627 | **643.3850** | 1268.7361 | 634.8717 | 1267.7521 | 634.3797 | P | 2089.9884 | **1045.4978** | 2072.9618 | **1036.9846** | 2071.9778 | **1036.4926** | 21 |
| 13 | **1400.7896** | **700.8985** | 1383.7631 | 692.3852 | 1382.7791 | 691.8932 | D | 1992.9356 | 996.9715 | 1975.9091 | 988.4582 | 1974.9251 | 987.9662 | 20 |
| 14 | 1457.8111 | **729.4092** | 1440.7845 | 720.8959 | 1439.8005 | 720.4039 | G | **1877.9087** | 939.4580 | 1860.8821 | 930.9447 | **1859.8981** | 930.4527 | 19 |
| 15 | 1544.8431 | 772.9252 | 1527.8166 | 764.4119 | 1526.8326 | 763.9199 | S | 1820.8872 | 910.9472 | 1803.8607 | 902.4340 | 1802.8767 | 901.9420 | 18 |
| 16 | 1615.8802 | **808.4438** | 1598.8537 | 799.9305 | 1597.8697 | 799.4385 | A | 1733.8552 | 867.4312 | 1716.8286 | 858.9180 | 1715.8446 | **858.4260** | 17 |
| 17 | **1714.9486** | **857.9780** | 1697.9221 | 849.4647 | 1696.9381 | 848.9727 | V | **1662.8181** | 831.9127 | 1645.7915 | 823.3994 | 1644.8075 | 822.9074 | 16 |
| 18 | 1829.9756 | **915.4914** | 1812.9490 | **906.9782** | 1811.9650 | **906.4861** | N | **1563.7497** | **782.3785** | 1546.7231 | 773.8652 | **1545.7391** | 773.3732 | 15 |
| 19 | 1886.9970 | 944.0022 | 1869.9705 | 935.4889 | 1868.9865 | 934.9969 | G | **1448.7227** | 724.8650 | 1431.6962 | 716.3517 | 1430.7122 | 715.8597 | 14 |
| 20 | 1988.0447 | **994.5260** | 1971.0182 | **986.0127** | 1970.0342 | **985.5207** | T | **1391.7013** | 696.3543 | 1374.6747 | 687.8410 | 1373.6907 | 687.3490 | 13 |
| 21 | 2075.0768 | **1038.0420** | 2058.0502 | 1029.5287 | 2057.0662 | 1029.0367 | S | **1290.6536** | 645.8304 | 1273.6270 | 637.3172 | **1272.6430** | 636.8251 | 12 |
| 22 | 2162.1088 | 1081.5580 | 2145.0822 | 1073.0448 | 2144.0982 | 1072.5527 | S | **1203.6216** | 602.3144 | 1186.5950 | 593.8011 | 1185.6110 | 593.3091 | 11 |
| 23 | 2233.1459 | 1117.0766 | 2216.1193 | 1108.5633 | 2215.1353 | 1108.0713 | A | 1116.5895 | 558.7984 | 1099.5630 | 550.2851 | 1098.5790 | 549.7931 | 10 |
| 24 | 2362.1885 | **1181.5979** | 2345.1619 | 1173.0846 | 2344.1779 | 1172.5926 | E | **1045.5524** | **523.2798** | 1028.5259 | 514.7666 | 1027.5419 | 514.2746 | 9 |
| 25 | 2463.2362 | **1232.1217** | 2446.2096 | 1223.6084 | 2445.2256 | 1223.1164 | T | **916.5098** | **458.7585** | 899.4833 | 450.2453 | 898.4993 | 449.7533 | 8 |
| 26 | 2577.2791 | 1289.1432 | 2560.2525 | 1280.6299 | 2559.2685 | 1280.1379 | N | **815.4621** | **408.2347** | 798.4356 | 399.7214 | 797.4516 | 399.2294 | 7 |
| 27 | 2690.3632 | 1345.6852 | 2673.3366 | 1337.1719 | 2672.3526 | **1336.6799** | L | **701.4192** | 351.2132 | 684.3927 | 342.7000 | 683.4087 | 342.2080 | 6 |
| 28 | 2819.4058 | **1410.2065** | 2802.3792 | **1401.6932** | 2801.3952 | 1401.2012 | E | **588.3352** | 294.6712 | 571.3086 | 286.1579 | **570.3246** | 285.6659 | 5 |
| 29 | 2890.4429 | **1445.7251** | 2873.4163 | 1437.2118 | 2872.4323 | 1436.7198 | A | **459.2926** | 230.1499 | 442.2660 | 221.6366 | | | 4 |
| 30 | 3003.5269 | **1502.2671** | 2986.5004 | 1493.7538 | 2985.5164 | 1493.2618 | L | **388.2554** | 194.6314 | 371.2289 | 186.1181 | | | 3 |
| 31 | 3131.5855 | **1566.2964** | 3114.5590 | 1557.7831 | 3113.5749 | 1557.2911 | Q | 275.1714 | 138.0893 | 258.1448 | 129.5761 | | | 2 |
| 32 | | | | | | | K | 147.1128 | 74.0600 | 130.0863 | 65.5468 | | | 1 |

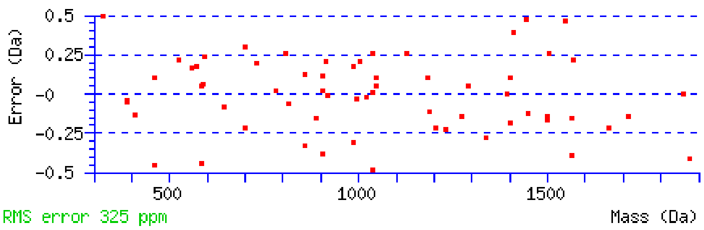


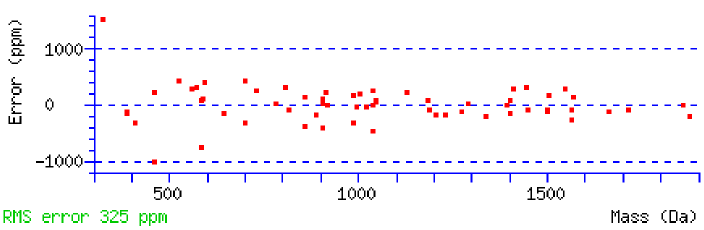


### All matches to this query

| Score | Mr(calc): | Delta | Sequence |
|---|---|---|---|
| 74.4 | 3276.6837 | 0.0045 | KKPTPIQLNPAPDGSAVNGTSSAETNLEALQK |
| 62.9 | 3276.6837 | 0.0045 | KKPTPIQLNPAPDGSAVNGTSSAETNLEALQK |
| 54.6 | 3275.6997 | 0.9885 | KKPTPIQLNPAPDGSAVNGTSSAETNLEALQK |
| 50.4 | 3276.6837 | 0.0045 | KKPTPIQLNPAPDGSAVNGTSSAETNLEALQK |
| 0.9 | 3274.6673 | 2.0210 | MPSLEVPVPKISGPDANVHLKTPNVGISGPK |

---

Spectrum No: 310; Query: 585; Rank: 1

---

## Peptide View

MS/MS Fragmentation of **DEELNCTVVELK**
Found in **IPI00211075**, Tax_Id=10116 Gene_Symbol=Serpina3n Serine protease inhibitor A3N precursor

Match to Query 585: 1448.668568 from(725.341560,2+)
Title: 091008RatKidney_NoSalt_42.1491.1491.2.dta
Data file K:\NewmanPaper\Piliang\3SubProteomes\Piliang3SP\mgf5ppm\ERLIC_3SubProteomes5ppm.mgf

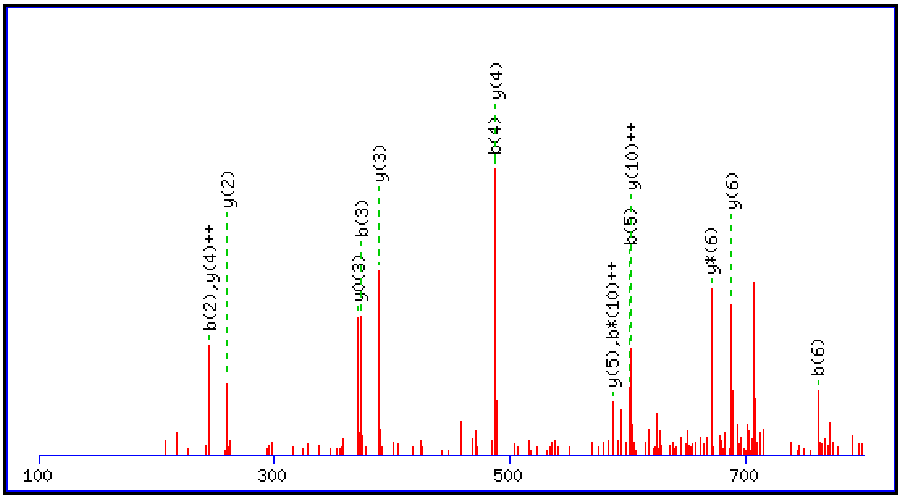


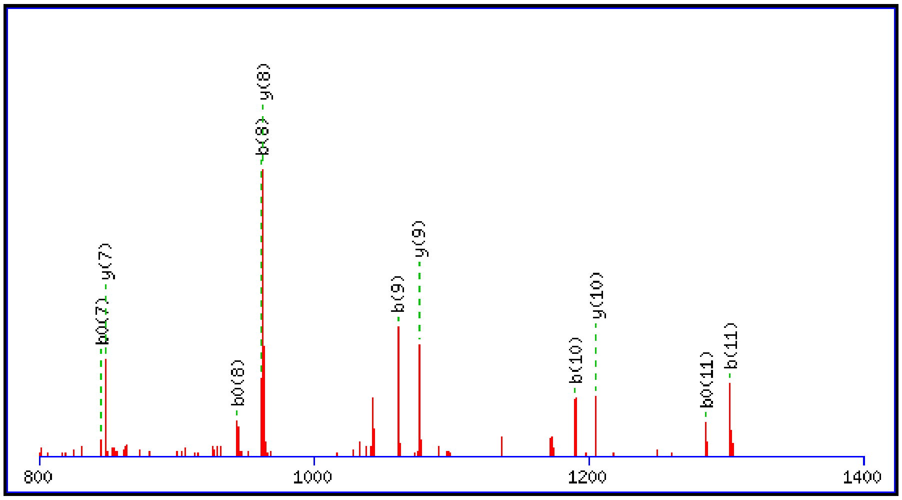


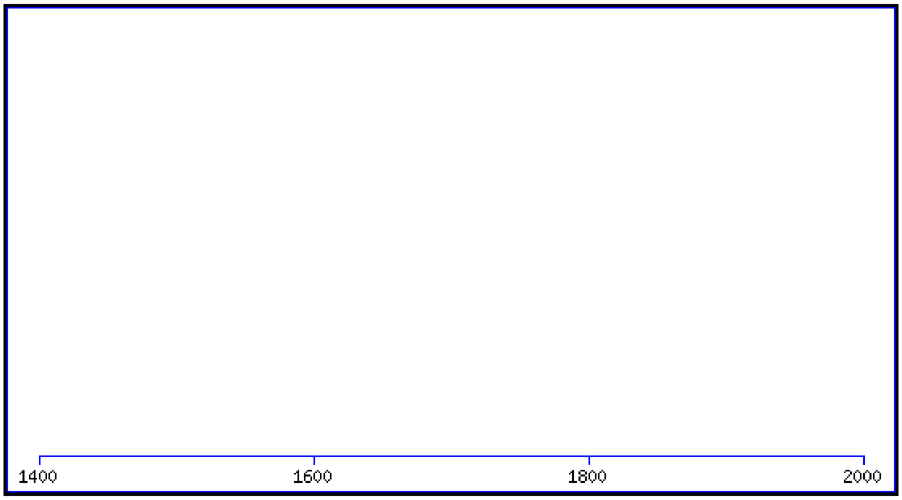


**Monoisotopic mass of neutral peptide Mr(calc):** 1448.6705
**Fixed modifications:** Carbamidomethyl (C)
**Variable modifications:**
**N5** : Deamidated_N (N)
**Ions Score:** 74 **Expect:** 5.5e-006
**Matches (Bold Red):** 26/120 fragment ions using 36 most intense peaks

| # | b | $b^{++}$ | b* | $b^{*++}$ | $b^0$ | $b^{0++}$ | Seq. | y | $y^{++}$ | y* | $y^{*++}$ | $y^0$ | $y^{0++}$ | # |
|---|---|---|---|---|---|---|---|---|---|---|---|---|---|---|
| 1 | 116.0342 | 58.5207 | | | 98.0237 | 49.5155 | D | | | | | | | 12 |
| 2 | **245.0768** | 123.0420 | | | 227.0662 | 114.0368 | E | 1334.6508 | 667.8290 | 1317.6243 | 659.3158 | 1316.6402 | 658.8238 | 11 |

| | | | | | | | | | | | | | | |
|---|---|---|---|---|---|---|---|---|---|---|---|---|---|---|
| **3** | **374.1194** | 187.5633 | | | 356.1088 | 178.5581 | **E** | **1205.6082** | **603.3077** | 1188.5817 | 594.7945 | 1187.5976 | 594.3025 | **10** |
| **4** | **487.2035** | 244.1054 | | | 469.1929 | 235.1001 | **L** | **1076.5656** | 538.7864 | 1059.5391 | 530.2732 | 1058.5551 | 529.7812 | **9** |
| **5** | **602.2304** | 301.6188 | 585.2039 | 293.1056 | 584.2198 | 292.6136 | **N** | **963.4816** | 482.2444 | 946.4550 | 473.7311 | 945.4710 | 473.2391 | **8** |
| **6** | **762.2611** | 381.6342 | 745.2345 | 373.1209 | 744.2505 | 372.6289 | **C** | **848.4546** | 424.7309 | 831.4281 | 416.2177 | 830.4441 | 415.7257 | **7** |
| **7** | 863.3087 | 432.1580 | 846.2822 | 423.6447 | **845.2982** | 423.1527 | **T** | **688.4240** | 344.7156 | **671.3974** | 336.2023 | 670.4134 | 335.7103 | **6** |
| **8** | **962.3771** | 481.6922 | 945.3506 | 473.1789 | **944.3666** | 472.6869 | **V** | **587.3763** | 294.1918 | 570.3497 | 285.6785 | 569.3657 | 285.1865 | **5** |
| **9** | **1061.4456** | 531.2264 | 1044.4190 | 522.7131 | 1043.4350 | 522.2211 | **V** | **488.3079** | **244.6576** | 471.2813 | 236.1443 | 470.2973 | 235.6523 | **4** |
| **10** | **1190.4882** | 595.7477 | 1173.4616 | **587.2344** | 1172.4776 | 586.7424 | **E** | **389.2395** | 195.1234 | 372.2129 | 186.6101 | **371.2289** | 186.1181 | **3** |
| **11** | **1303.5722** | 652.2897 | 1286.5457 | 643.7765 | **1285.5617** | 643.2845 | **L** | **260.1969** | 130.6021 | 243.1703 | 122.0888 | | | **2** |
| **12** | | | | | | | **K** | 147.1128 | 74.0600 | 130.0863 | 65.5468 | | | **1** |

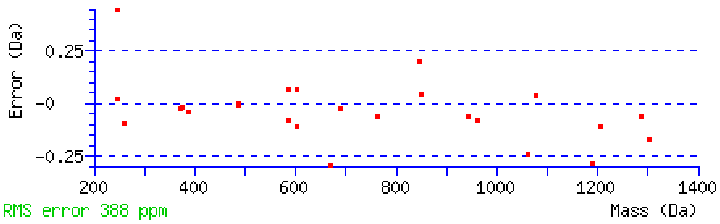


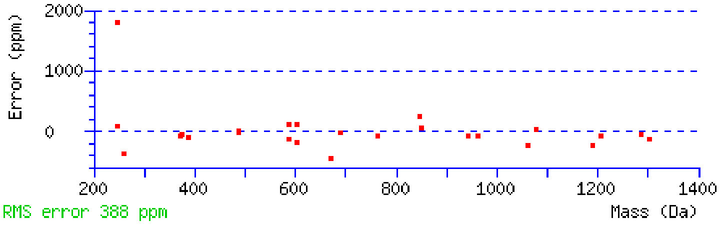


### All matches to this query

| Score | Mr(calc): | Delta | Sequence |
|---|---|---|---|
| 74.4 | 1448.6705 | -0.0019 | DEELNCTVVELK |
| 17.0 | 1448.6783 | -0.0097 | DAGENNFINVDLK |
| 16.8 | 1448.6783 | -0.0097 | DAGENNFINVDLK |
| 15.2 | 1448.6606 | 0.0080 | NEAATAEVWGCLK |
| 15.0 | 1448.6565 | 0.0120 | KMNDSNSSGAGGPVK |
| 12.1 | 1448.6701 | -0.0015 | TYQFLNDIRAK |
| 12.1 | 1448.6701 | -0.0015 | TYQFLNDIRAK |
| 10.8 | 1448.6639 | 0.0046 | QDGAVAMEMQPLK |
| 9.7 | 1448.6588 | 0.0097 | DYFNQTLNILK |
| 8.9 | 1448.6734 | -0.0049 | ILEMEHTQQLK |

---

Spectrum No: 311; Query: 529; Rank: 1

---

## Peptide View

MS/MS Fragmentation of **VIPVCNASLPAQR**
Found in **IPI00192504**, Tax_Id=10116 Gene_Symbol=Mrc2 Macrophage mannose receptor 2 precursor

Match to Query 529: 1424.747568 from(713.381060,2+)
Title: 100101RatKid_NS_deglyco_21.1950.1950.2.dta
Data file K:\NewmanPaper\Piliang\3SubProteomes\Piliang3SP\mgf5ppm\ERLIC_3SubProteomes5ppm.mgf

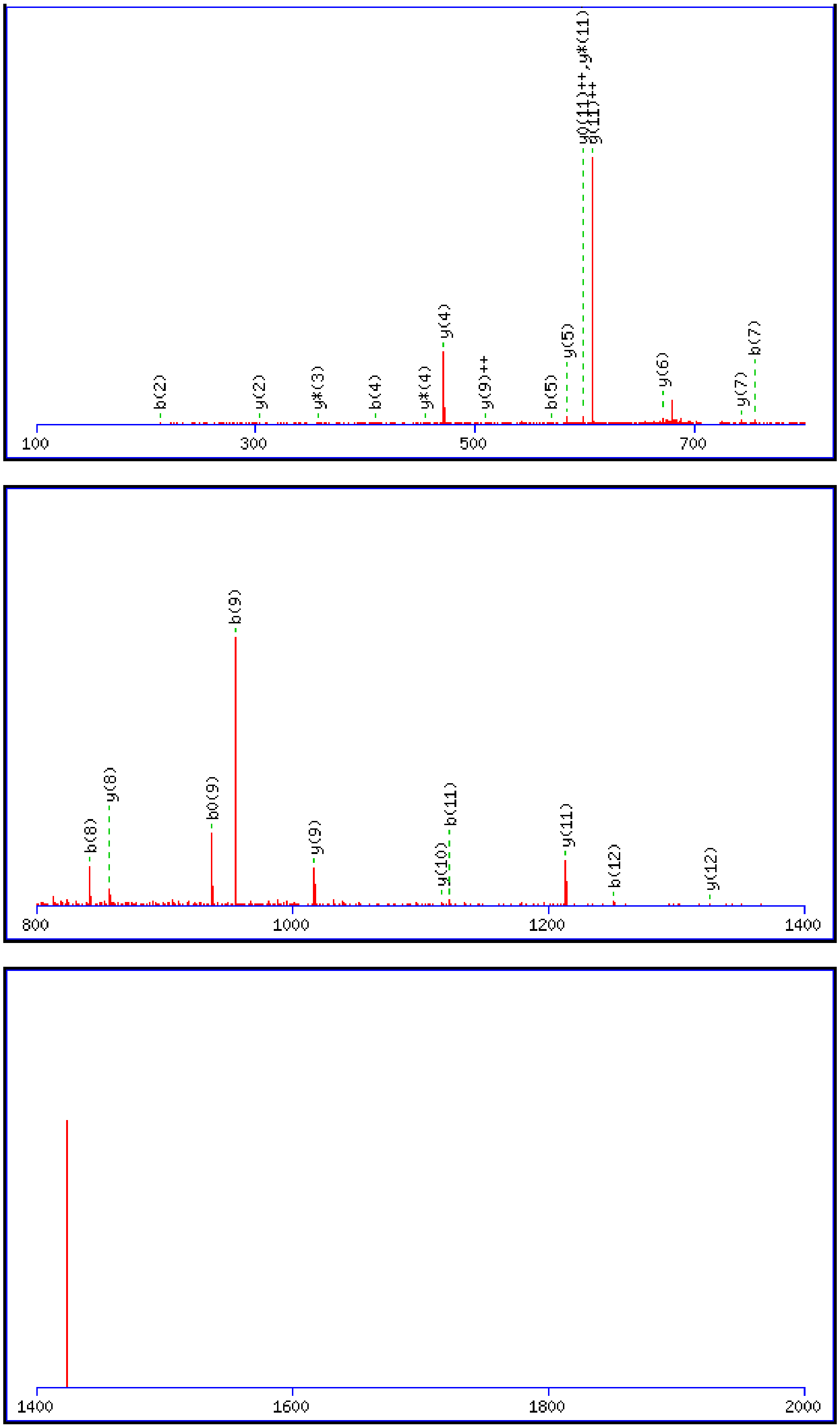


**Monoisotopic mass of neutral peptide Mr(calc):** 1424.7446
**Fixed modifications:** Carbamidomethyl (C)
**Variable modifications:**
**N6** : Deamidated_N (N)
**Ions Score:** 74 **Expect:** 3.5e-006
**Matches (Bold Red):** 25/110 fragment ions using 46 most intense peaks

| # | b | $b^{++}$ | b* | $b^{*++}$ | $b^{0}$ | $b^{0++}$ | Seq. | y | $y^{++}$ | y* | $y^{*++}$ | $y^{0}$ | $y^{0++}$ | # |
|---|---|---|---|---|---|---|---|---|---|---|---|---|---|---|
| 1 | 100.0757 | 50.5415 | | | | | V | | | | | | | 13 |
| 2 | **213.1598** | 107.0835 | | | | | I | **1326.6834** | 663.8454 | 1309.6569 | 655.3321 | 1308.6729 | 654.8401 | 12 |
| 3 | 310.2125 | 155.6099 | | | | | P | **1213.5994** | **607.3033** | 1196.5728 | **598.7901** | 1195.5888 | **598.2980** | 11 |
| 4 | **409.2809** | 205.1441 | | | | | V | **1116.5466** | 558.7769 | 1099.5201 | 550.2637 | 1098.5360 | 549.7717 | 10 |
| 5 | **569.3116** | 285.1594 | | | | | C | **1017.4782** | **509.2427** | 1000.4516 | 500.7295 | 999.4676 | 500.2375 | 9 |

| | | | | | | | | | | | | | | |
|---|---|---|---|---|---|---|---|---|---|---|---|---|---|---|
| **6** | 684.3385 | 342.6729 | 667.3120 | 334.1596 | | | **N** | **857.4475** | 429.2274 | 840.4210 | 420.7141 | 839.4370 | 420.2221 | **8** |
| **7** | **755.3756** | 378.1915 | 738.3491 | 369.6782 | | | **A** | **742.4206** | 371.7139 | 725.3941 | 363.2007 | 724.4100 | 362.7087 | **7** |
| **8** | **842.4077** | 421.7075 | 825.3811 | 413.1942 | 824.3971 | 412.7022 | **S** | **671.3835** | 336.1954 | 654.3570 | 327.6821 | 653.3729 | 327.1901 | **6** |
| **9** | **955.4917** | 478.2495 | 938.4652 | 469.7362 | **937.4812** | 469.2442 | **L** | **584.3515** | 292.6794 | 567.3249 | 284.1661 | | | **5** |
| **10** | 1052.5445 | 526.7759 | 1035.5179 | 518.2626 | 1034.5339 | 517.7706 | **P** | **471.2674** | 236.1373 | **454.2409** | 227.6241 | | | **4** |
| **11** | **1123.5816** | 562.2944 | 1106.5551 | 553.7812 | 1105.5710 | 553.2892 | **A** | 374.2146 | 187.6110 | **357.1881** | 179.0977 | | | **3** |
| **12** | **1251.6402** | 626.3237 | 1234.6136 | 617.8105 | 1233.6296 | 617.3184 | **Q** | **303.1775** | 152.0924 | 286.1510 | 143.5791 | | | **2** |
| **13** | | | | | | | **R** | 175.1190 | 88.0631 | 158.0924 | 79.5498 | | | **1** |

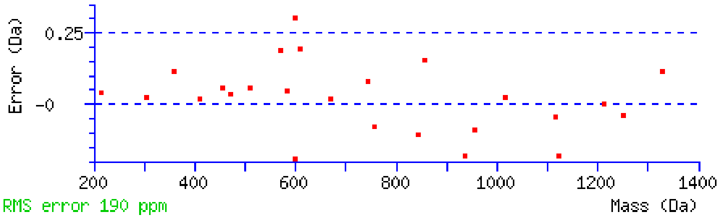


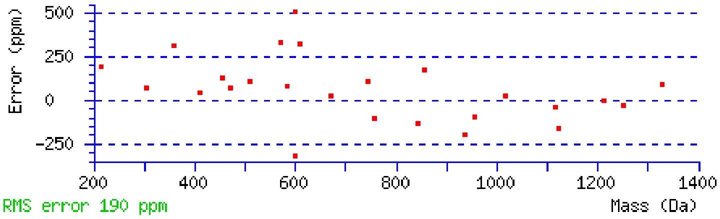


### All matches to this query

| Score | Mr(calc): | Delta | Sequence |
|---|---|---|---|
| 74.4 | 1424.7446 | 0.0030 | VIPVCNASLPAQR |
| 13.3 | 1424.7558 | -0.0082 | NSRLLMSSFSKR |
| 7.9 | 1423.7323 | 1.0152 | LGSKVITDPSLSK |
| 7.9 | 1423.7323 | 1.0152 | LGSKVITDPSLSK |
| 7.9 | 1423.7323 | 1.0152 | LGSKVITDPSLSK |
| 3.6 | 1423.7320 | 1.0156 | NPPVLYHNSRAR |
| 2.8 | 1424.7524 | -0.0049 | IVRDYDVYARR |
| 2.4 | 1423.7436 | 1.0040 | LGLSTAEILSGKR |
| 2.4 | 1423.7337 | 1.0139 | LIASRFKHSGTK |
| 2.4 | 1423.7337 | 1.0139 | LIASRFKHSGTK |

---

Spectrum No: 312; Query: 2024; Rank: 1

---

## Peptide View

MS/MS Fragmentation of **QILEGLEFNLTQIPEADIHK**
Found in **IPI00324019**, Tax_Id=10116 Gene_Symbol=Serpina1 Alpha-1-antiproteinase precursor

Match to Query 2024: 2308.200788 from(1155.107670,2+)
Title: 091008RatKidney_NoSalt_26.4790.4790.2.dta
Data file K:\NewmanPaper\Piliang\3SubProteomes\Piliang3SP\mgf5ppm\ERLIC_3SubProteomes5ppm.mgf

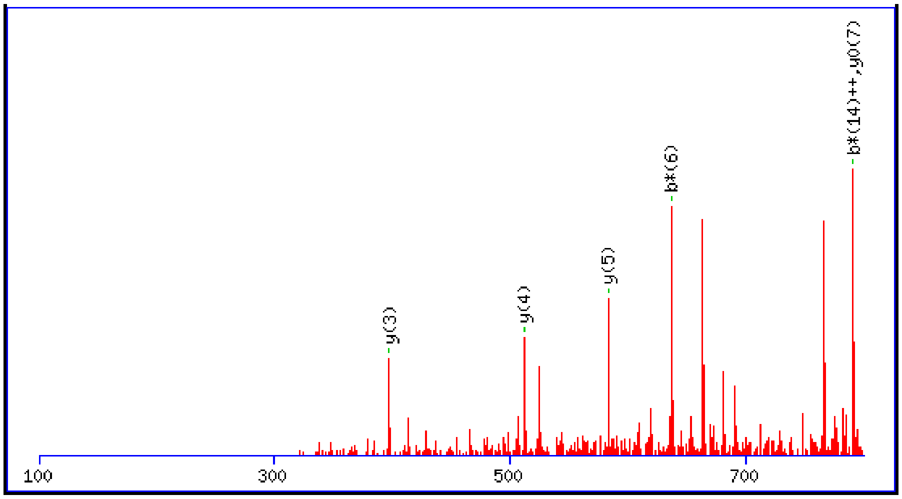


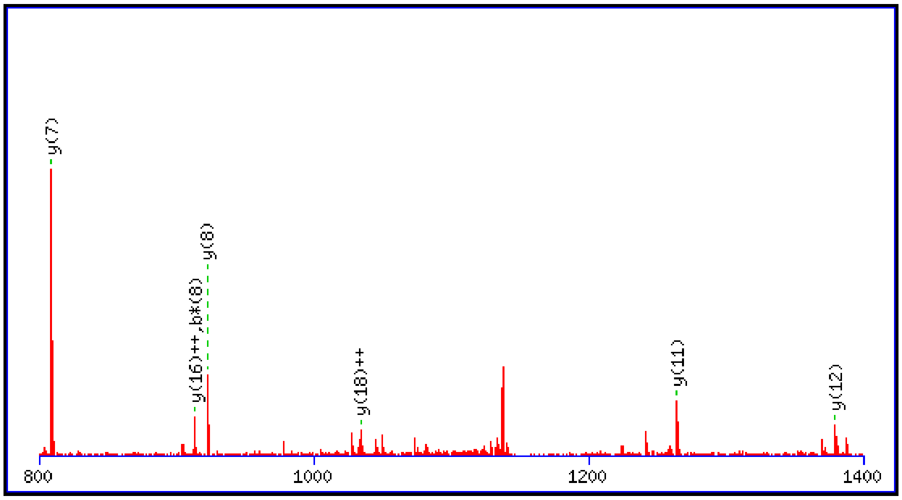


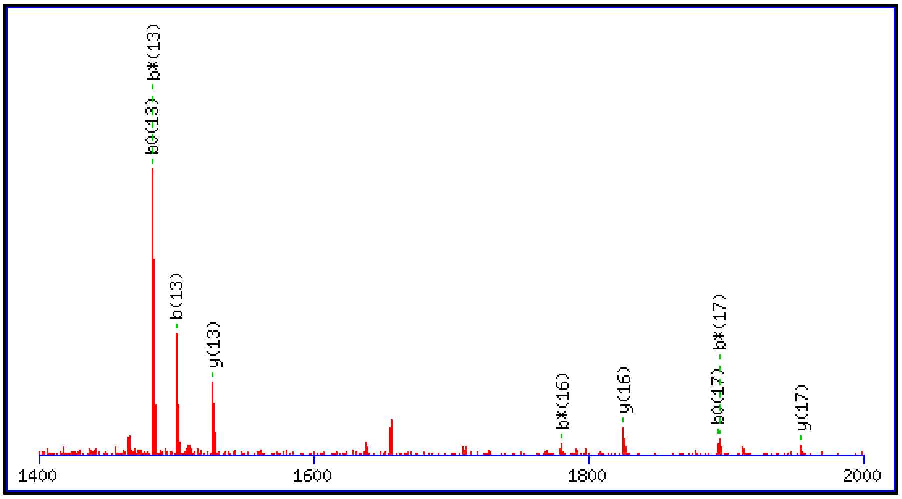


**Monoisotopic mass of neutral peptide Mr(calc):** 2308.1950
**Fixed modifications:** Carbamidomethyl (C)
**Variable modifications:**
**N9** : Deamidated_N (N)
**Ions Score:** 74 **Expect:** 4.4e-006
**Matches (Bold Red):** 22/216 fragment ions using 31 most intense peaks

| # | b | $b^{++}$ | b* | $b^{*++}$ | $b^0$ | $b^{0++}$ | Seq. | y | $y^{++}$ | y* | $y^{*++}$ | $y^0$ | $y^{0++}$ | # |
|---|---|---|---|---|---|---|---|---|---|---|---|---|---|---|
| 1 | 129.0659 | 65.0366 | 112.0393 | 56.5233 | | | Q | | | | | | | 20 |
| 2 | 242.1499 | 121.5786 | 225.1234 | 113.0653 | | | I | 2181.1438 | 1091.0755 | 2164.1172 | 1082.5622 | 2163.1332 | 1082.0702 | 19 |
| 3 | 355.2340 | 178.1206 | 338.2074 | 169.6074 | | | L | 2068.0597 | **1034.5335** | 2051.0332 | 1026.0202 | 2050.0491 | 1025.5282 | 18 |
| 4 | 484.2766 | 242.6419 | 467.2500 | 234.1287 | 466.2660 | 233.6366 | E | **1954.9756** | 977.9915 | 1937.9491 | 969.4782 | 1936.9651 | 968.9862 | 17 |
| 5 | 541.2980 | 271.1527 | 524.2715 | 262.6394 | 523.2875 | 262.1474 | G | **1825.9330** | **913.4702** | 1808.9065 | 904.9569 | 1807.9225 | 904.4649 | 16 |

| | | | | | | | | | | | | | | |
|---|---|---|---|---|---|---|---|---|---|---|---|---|---|---|
| **6** | 654.3821 | 327.6947 | **637.3556** | 319.1814 | 636.3715 | 318.6894 | **L** | 1768.9116 | 884.9594 | 1751.8850 | 876.4462 | 1750.9010 | 875.9541 | **15** |
| **7** | 783.4247 | 392.2160 | 766.3981 | 383.7027 | 765.4141 | 383.2107 | **E** | 1655.8275 | 828.4174 | 1638.8010 | 819.9041 | 1637.8170 | 819.4121 | **14** |
| **8** | 930.4931 | 465.7502 | **913.4666** | 457.2369 | 912.4825 | 456.7449 | **F** | **1526.7849** | 763.8961 | 1509.7584 | 755.3828 | 1508.7744 | 754.8908 | **13** |
| **9** | 1045.5200 | 523.2637 | 1028.4935 | 514.7504 | 1027.5095 | 514.2584 | **N** | **1379.7165** | 690.3619 | 1362.6900 | 681.8486 | 1361.7059 | 681.3566 | **12** |
| **10** | 1158.6041 | 579.8057 | 1141.5776 | 571.2924 | 1140.5935 | 570.8004 | **L** | **1264.6896** | 632.8484 | 1247.6630 | 624.3352 | 1246.6790 | 623.8431 | **11** |
| **11** | 1259.6518 | 630.3295 | 1242.6252 | 621.8163 | 1241.6412 | 621.3243 | **T** | 1151.6055 | 576.3064 | 1134.5790 | 567.7931 | 1133.5949 | 567.3011 | **10** |
| **12** | 1387.7104 | 694.3588 | 1370.6838 | 685.8455 | 1369.6998 | 685.3535 | **Q** | 1050.5578 | 525.7826 | 1033.5313 | 517.2693 | 1032.5473 | 516.7773 | **9** |
| **13** | **1500.7944** | 750.9009 | **1483.7679** | 742.3876 | **1482.7839** | 741.8956 | **I** | **922.4993** | 461.7533 | 905.4727 | 453.2400 | 904.4887 | 452.7480 | **8** |
| **14** | 1597.8472 | 799.4272 | 1580.8206 | **790.9140** | 1579.8366 | 790.4220 | **P** | **809.4152** | 405.2112 | 792.3886 | 396.6980 | **791.4046** | 396.2060 | **7** |
| **15** | 1726.8898 | 863.9485 | 1709.8632 | 855.4353 | 1708.8792 | 854.9432 | **E** | 712.3624 | 356.6849 | 695.3359 | 348.1716 | 694.3519 | 347.6796 | **6** |
| **16** | 1797.9269 | 899.4671 | **1780.9004** | 890.9538 | 1779.9163 | 890.4618 | **A** | **583.3198** | 292.1636 | 566.2933 | 283.6503 | 565.3093 | 283.1583 | **5** |
| **17** | 1912.9538 | 956.9806 | **1895.9273** | 948.4673 | **1894.9433** | 947.9753 | **D** | **512.2827** | 256.6450 | 495.2562 | 248.1317 | 494.2722 | 247.6397 | **4** |
| **18** | 2026.0379 | 1013.5226 | 2009.0114 | 1005.0093 | 2008.0273 | 1004.5173 | **I** | **397.2558** | 199.1315 | 380.2292 | 190.6183 | | | **3** |
| **19** | 2163.0968 | 1082.0520 | 2146.0703 | 1073.5388 | 2145.0863 | 1073.0468 | **H** | 284.1717 | 142.5895 | 267.1452 | 134.0762 | | | **2** |
| **20** | | | | | | | **K** | 147.1128 | 74.0600 | 130.0863 | 65.5468 | | | **1** |

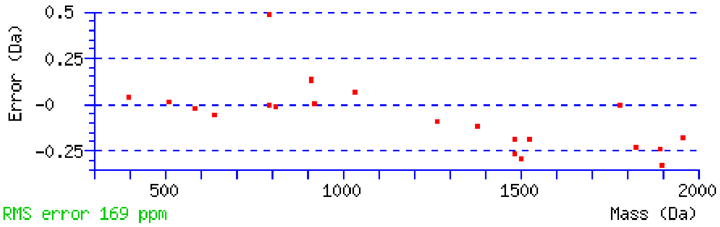


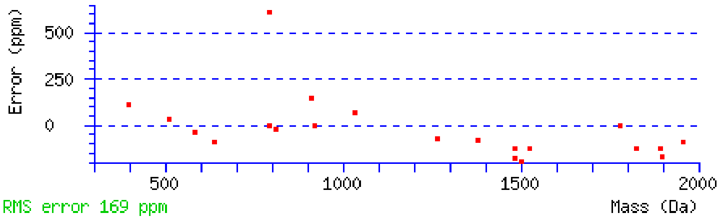


### All matches to this query

| Score | Mr(calc): | Delta | Sequence |
|---|---|---|---|
| 74.3 | 2308.1950 | 0.0057 | QILEGLEFNLTQIPEADIHK |
| 19.4 | 2307.2110 | 0.9898 | QILEGLEFNLTQIPEADIHK |

---

Spectrum No: 313; Query: 293; Rank: 1

---

## Peptide View

MS/MS Fragmentation of **QELSQDNATLK**
Found in **IPI00190577**, Tax_Id=10116 Gene_Symbol=Lama5 similar to Laminin alpha-5 chain precursor

Match to Query 293: 1246.606048 from(624.310300,2+)
Title: 100101RatKid_NS_deglyco_23.839.839.2.dta
Data file K:\NewmanPaper\Piliang\3SubProteomes\Piliang3SP\mgf5ppm\ERLIC_3SubProteomes5ppm.mgf

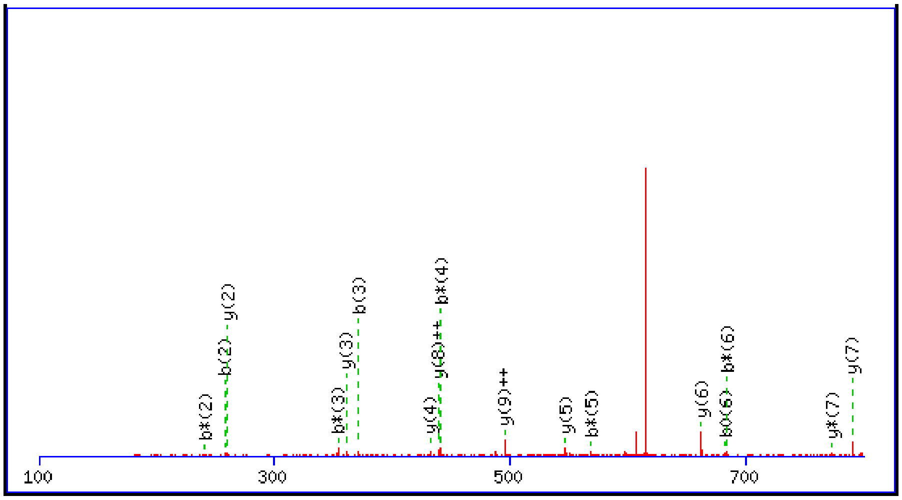


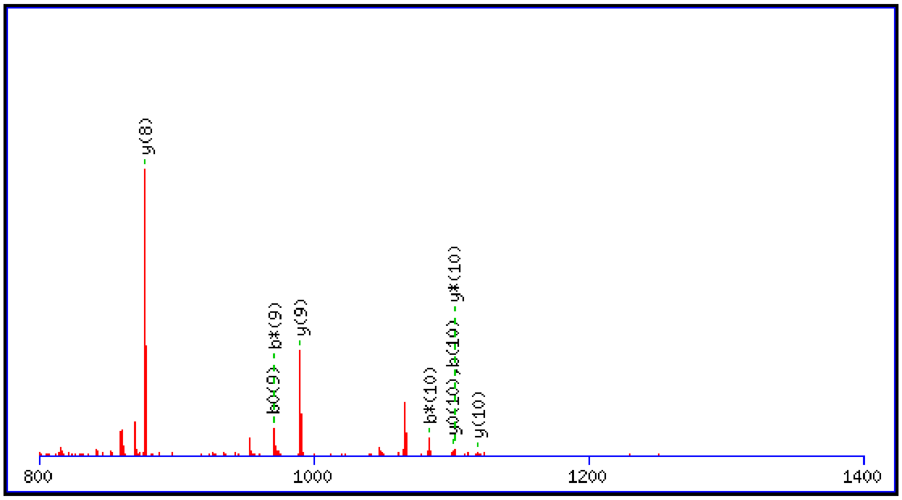


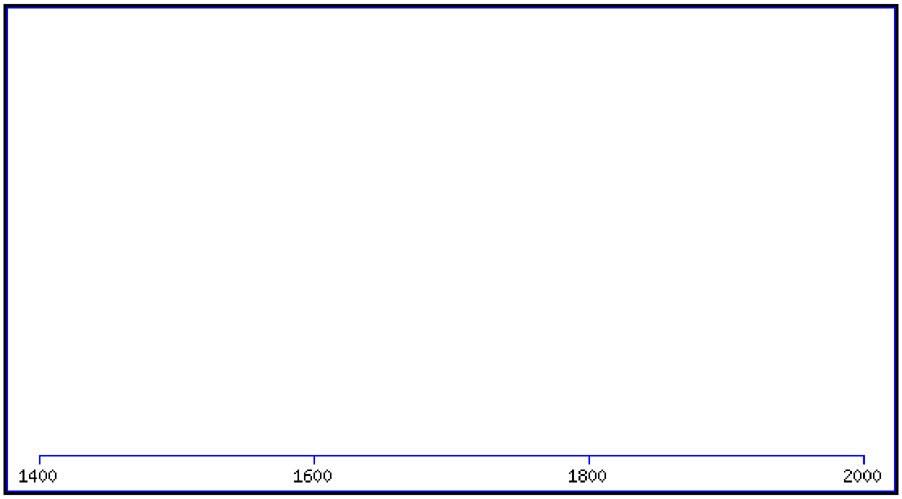


**Monoisotopic mass of neutral peptide Mr(calc):** 1246.6041
**Fixed modifications:** Carbamidomethyl (C)
**Variable modifications:**
**N7** : Deamidated_N (N)
**Ions Score:** 74 **Expect:** 4.9e-006
**Matches (Bold Red):** 26/114 fragment ions using 41 most intense peaks

| # | b | $b^{++}$ | b* | $b^{*++}$ | $b^0$ | $b^{0++}$ | Seq. | y | $y^{++}$ | y* | $y^{*++}$ | $y^0$ | $y^{0++}$ | # |
|---|---|---|---|---|---|---|---|---|---|---|---|---|---|---|
| 1 | 129.0659 | 65.0366 | 112.0393 | 56.5233 | | | Q | | | | | | | 11 |
| 2 | **258.1084** | 129.5579 | **241.0819** | 121.0446 | 240.0979 | 120.5526 | E | **1119.5528** | 560.2800 | **1102.5263** | 551.7668 | **1101.5422** | 551.2748 | 10 |
| 3 | **371.1925** | 186.0999 | **354.1660** | 177.5866 | 353.1819 | 177.0946 | L | **990.5102** | **495.7587** | 973.4837 | 487.2455 | 972.4996 | 486.7535 | 9 |
| 4 | 458.2245 | 229.6159 | **441.1980** | 221.1026 | 440.2140 | 220.6106 | S | **877.4261** | **439.2167** | 860.3996 | 430.7034 | 859.4156 | 430.2114 | 8 |
| 5 | 586.2831 | 293.6452 | **569.2566** | 285.1319 | 568.2726 | 284.6399 | Q | **790.3941** | 395.7007 | **773.3676** | 387.1874 | 772.3836 | 386.6954 | 7 |

| | | | | | | | | | | | | | | |
|---|---|---|---|---|---|---|---|---|---|---|---|---|---|---|
| 6 | 701.3101 | 351.1587 | **684.2835** | 342.6454 | **683.2995** | 342.1534 | D | **662.3355** | 331.6714 | 645.3090 | 323.1581 | 644.3250 | 322.6661 | 6 |
| 7 | 816.3370 | 408.6721 | 799.3104 | 400.1589 | 798.3264 | 399.6669 | N | **547.3086** | 274.1579 | 530.2820 | 265.6447 | 529.2980 | 265.1527 | 5 |
| 8 | 887.3741 | 444.1907 | 870.3476 | 435.6774 | 869.3635 | 435.1854 | A | **432.2817** | 216.6445 | 415.2551 | 208.1312 | 414.2711 | 207.6392 | 4 |
| 9 | 988.4218 | 494.7145 | **971.3952** | 486.2013 | **970.4112** | 485.7093 | T | **361.2445** | 181.1259 | 344.2180 | 172.6126 | 343.2340 | 172.1206 | 3 |
| 10 | **1101.5059** | 551.2566 | **1084.4793** | 542.7433 | 1083.4953 | 542.2513 | L | **260.1969** | 130.6021 | 243.1703 | 122.0888 | | | 2 |
| 11 | | | | | | | K | 147.1128 | 74.0600 | 130.0863 | 65.5468 | | | 1 |

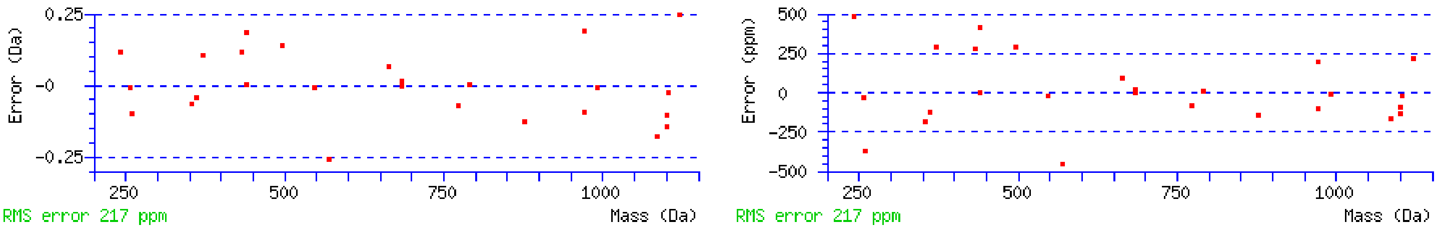


### All matches to this query

| Score | Mr(calc): | Delta | Sequence |
|---|---|---|---|
| 74.2 | 1246.6041 | 0.0020 | QELSQDNATLK |
| 23.3 | 1246.6041 | 0.0020 | AGKLSENPSESK |
| 17.5 | 1246.5958 | 0.0102 | KIESFGSKSGK |
| 12.3 | 1246.6128 | -0.0068 | WALGVMAADASR |
| 12.1 | 1246.6032 | 0.0028 | EKSMATFILK |
| 10.6 | 1246.5958 | 0.0102 | KIESFGSKSGK |
| 7.4 | 1246.6111 | -0.0051 | FKTLFVAGER |
| 6.8 | 1246.6153 | -0.0093 | KQKEQSGGEEK |
| 6.5 | 1246.6111 | -0.0050 | KQNVKYYPK |
| 6.0 | 1246.6153 | -0.0093 | ESSQSSAQPAKK |

---

Spectrum No: 314; Query: 1769; Rank: 1

---

## Peptide View

MS/MS Fragmentation of **AWGTPCELCPPVNTSEYK**
Found in **IPI00204006**, Tax_Id=10116 Gene_Symbol=Fbn1 Fibrillin-1

Match to Query 1769: 2108.927488 from(1055.471020,2+)
Title: 100101RatKid_NS_deglyco_23.3372.3372.2.dta
Data file K:\NewmanPaper\Piliang\3SubProteomes\Piliang3SP\mgf5ppm\ERLIC_3SubProteomes5ppm.mgf

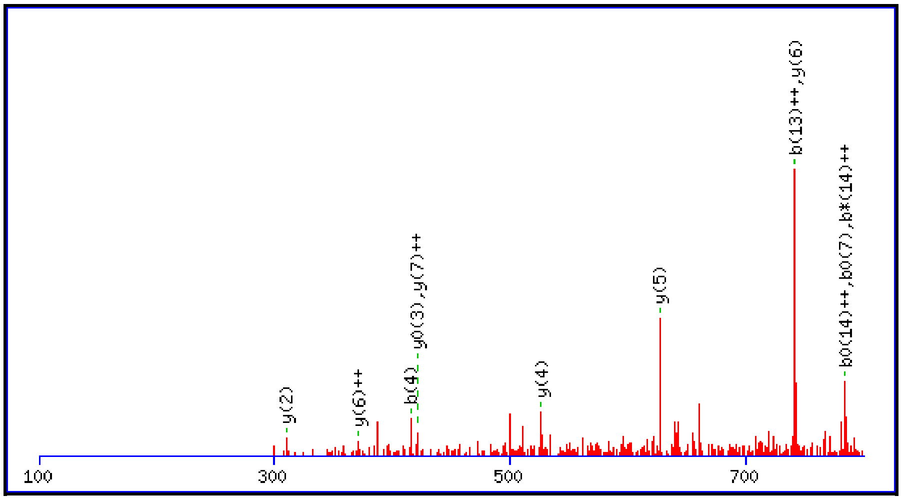

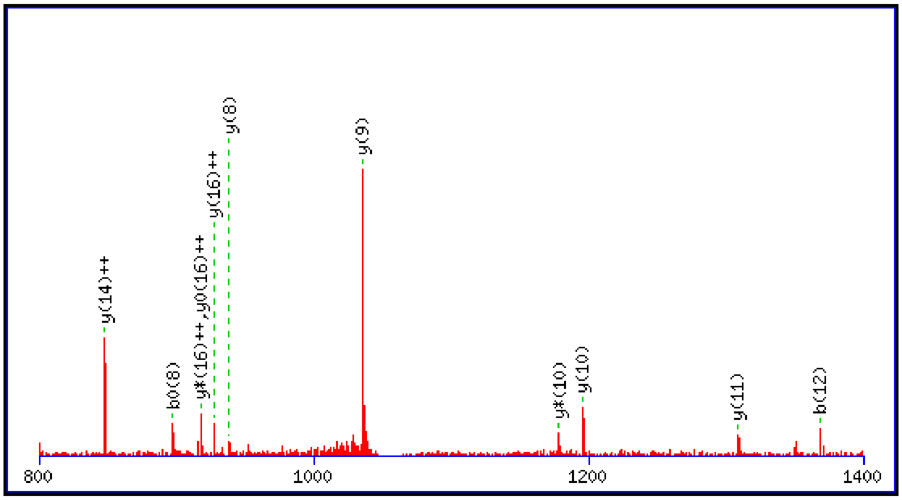


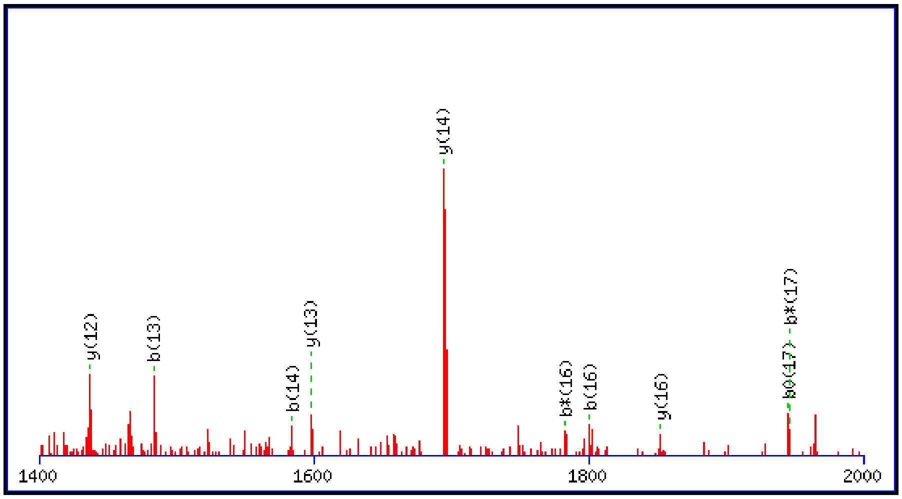


**Monoisotopic mass of neutral peptide Mr(calc):** 2108.9183
**Fixed modifications:** Carbamidomethyl (C)
**Variable modifications:**
**N13** : Deamidated_N (N)
**Ions Score:** 74 **Expect:** 1e-005
**Matches (Bold Red):** 33/170 fragment ions using 57 most intense peaks

| # | b | $b^{++}$ | b* | $b^{*++}$ | $b^0$ | $b^{0++}$ | Seq. | y | $y^{++}$ | y* | $y^{*++}$ | $y^0$ | $y^{0++}$ | # |
|---|---|---|---|---|---|---|---|---|---|---|---|---|---|---|
| 1 | 72.0444 | 36.5258 | | | | | A | | | | | | | 18 |
| 2 | 258.1237 | 129.5655 | | | | | W | 2038.8885 | 1019.9479 | 2021.8619 | 1011.4346 | 2020.8779 | 1010.9426 | 17 |
| 3 | 315.1452 | 158.0762 | | | | | G | **1852.8092** | **926.9082** | 1835.7826 | **918.3950** | 1834.7986 | **917.9029** | 16 |
| 4 | **416.1928** | 208.6001 | | | 398.1823 | 199.5948 | T | 1795.7877 | 898.3975 | 1778.7612 | 889.8842 | 1777.7771 | 889.3922 | 15 |
| 5 | 513.2456 | 257.1264 | | | 495.2350 | 248.1212 | P | **1694.7400** | **847.8737** | 1677.7135 | 839.3604 | 1676.7295 | 838.8684 | 14 |
| 6 | 673.2763 | 337.1418 | | | 655.2657 | 328.1365 | C | **1597.6873** | 799.3473 | 1580.6607 | 790.8340 | 1579.6767 | 790.3420 | 13 |
| 7 | 802.3189 | 401.6631 | | | **784.3083** | 392.6578 | E | **1437.6566** | 719.3319 | 1420.6301 | 710.8187 | 1419.6461 | 710.3267 | 12 |
| 8 | 915.4029 | 458.2051 | | | **897.3924** | 449.1998 | L | **1308.6140** | 654.8107 | 1291.5875 | 646.2974 | 1290.6035 | 645.8054 | 11 |
| 9 | 1075.4336 | 538.2204 | | | 1057.4230 | 529.2151 | C | **1195.5300** | 598.2686 | **1178.5034** | 589.7553 | 1177.5194 | 589.2633 | 10 |
| 10 | 1172.4863 | 586.7468 | | | 1154.4758 | 577.7415 | P | **1035.4993** | 518.2533 | 1018.4728 | 509.7400 | 1017.4887 | 509.2480 | 9 |
| 11 | 1269.5391 | 635.2732 | | | 1251.5285 | 626.2679 | P | **938.4465** | 469.7269 | 921.4200 | 461.2136 | 920.4360 | 460.7216 | 8 |
| 12 | **1368.6075** | 684.8074 | | | 1350.5969 | 675.8021 | V | 841.3938 | **421.2005** | 824.3672 | 412.6873 | 823.3832 | 412.1952 | 7 |
| 13 | **1483.6344** | **742.3209** | 1466.6079 | 733.8076 | 1465.6239 | 733.3156 | N | **742.3254** | **371.6663** | 725.2988 | 363.1530 | 724.3148 | 362.6610 | 6 |
| 14 | **1584.6821** | 792.8447 | 1567.6556 | **784.3314** | 1566.6716 | **783.8394** | T | **627.2984** | 314.1529 | 610.2719 | 305.6396 | 609.2879 | 305.1476 | 5 |
| 15 | 1671.7142 | 836.3607 | 1654.6876 | 827.8474 | 1653.7036 | 827.3554 | S | **526.2508** | 263.6290 | 509.2242 | 255.1157 | 508.2402 | 254.6237 | 4 |
| 16 | **1800.7567** | 900.8820 | **1783.7302** | 892.3687 | 1782.7462 | 891.8767 | E | 439.2187 | 220.1130 | 422.1922 | 211.5997 | **421.2082** | 211.1077 | 3 |
| 17 | 1963.8201 | 982.4137 | **1946.7935** | 973.9004 | **1945.8095** | 973.4084 | Y | **310.1761** | 155.5917 | 293.1496 | 147.0784 | | | 2 |
| 18 | | | | | | | K | 147.1128 | 74.0600 | 130.0863 | 65.5468 | | | 1 |

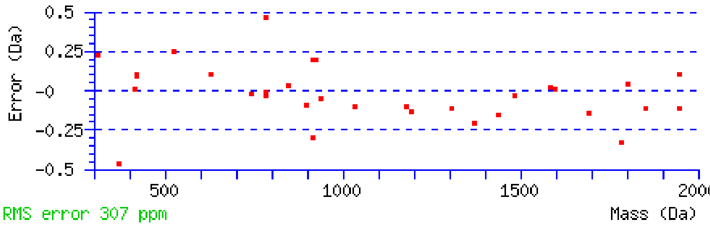


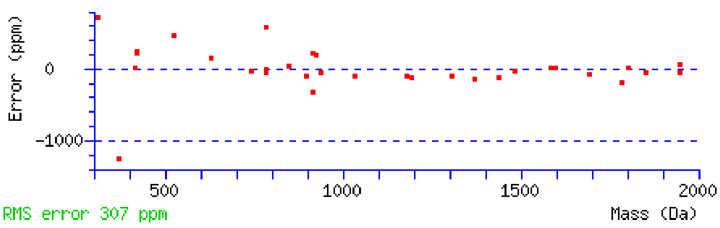


### All matches to this query

| Score | Mr(calc): | Delta | Sequence |
|---|---|---|---|
| 74.1 | 2108.9183 | 0.0092 | AWGTPCELCPPVNTSEYK |
| 3.3 | 2107.9197 | 1.0078 | MSVCTLSVPVSSISPGRR |
| 2.4 | 2107.9197 | 1.0078 | MSVCTLSVPVSSISPGRR |
| 2.4 | 2107.9197 | 1.0078 | MSVCTLSVPVSSISPGRR |
| 2.0 | 2106.9388 | 1.9887 | ERKPNNLSDTVKELFR |
| 1.4 | 2106.9115 | 2.0160 | ALMNTQYGLERIMSDER |
| 1.3 | 2106.9228 | 2.0047 | MSVPCSEYRKTSGLGSPR |
| 0.3 | 2108.9126 | 0.0149 | QFSQLPANVAVCSSETYK |

---

Spectrum No: 315; Query: 1809; Rank: 1

---

## Peptide View

MS/MS Fragmentation of **CDPAIKPHEELLILNLTR**
Found in **IPI00325860**, Tax_Id=10116 Gene_Symbol=Podxl Podocalyxin precursor

Match to Query 1809: 2132.137122 from(711.719650,3+)
Title: 100101RatKid_NS_deglyco_14.4023.4023.3.dta
Data file K:\NewmanPaper\Piliang\3SubProteomes\Piliang3SP\mgf5ppm\ERLIC_3SubProteomes5ppm.mgf

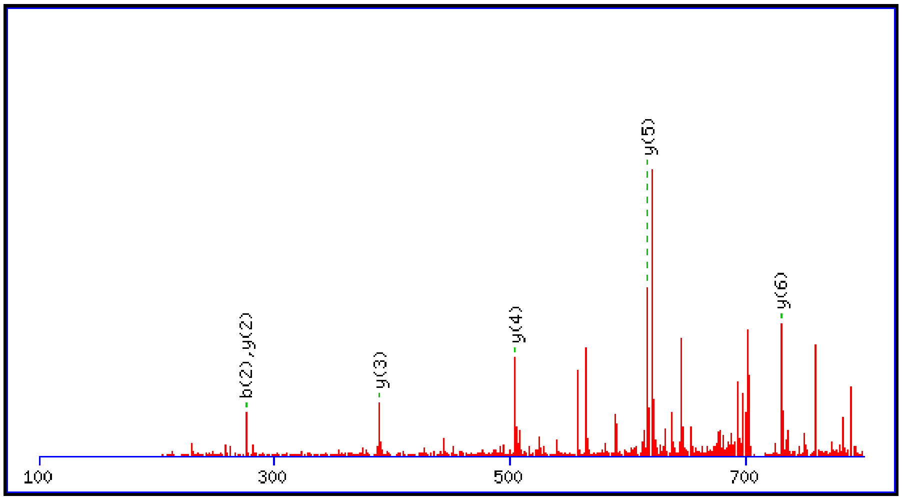

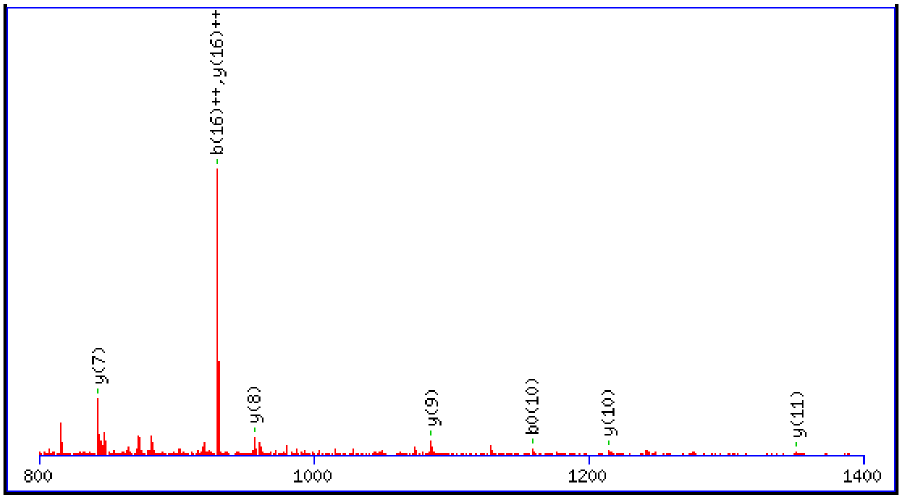


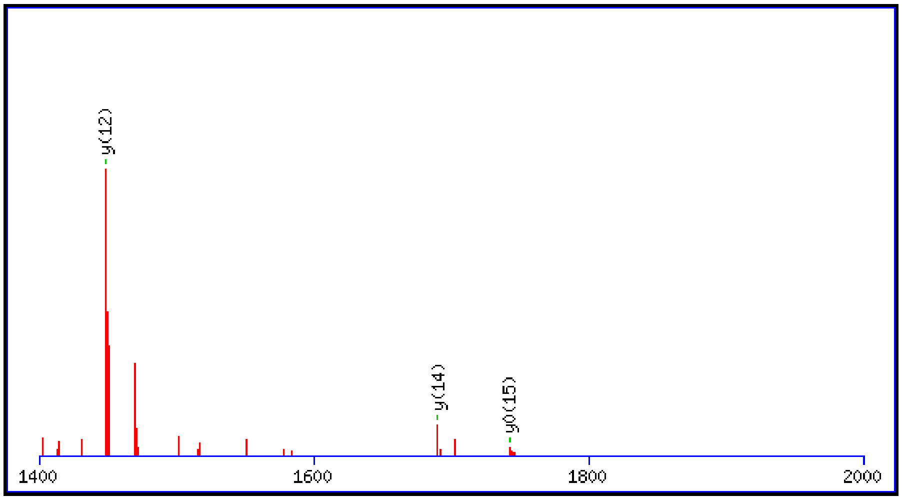


**Monoisotopic mass of neutral peptide Mr(calc):** 2132.1299
**Fixed modifications:** Carbamidomethyl (C)
**Variable modifications:**
**N15** : Deamidated_N (N)
**Ions Score:** 74 **Expect:** 3.9e-006
**Matches (Bold Red):** 17/190 fragment ions using 31 most intense peaks

| # | b | $b^{++}$ | b* | $b^{*++}$ | $b^0$ | $b^{0++}$ | Seq. | y | $y^{++}$ | y* | $y^{*++}$ | $y^0$ | $y^{0++}$ | # |
|---|---|---|---|---|---|---|---|---|---|---|---|---|---|---|
| 1 | 161.0379 | 81.0226 | | | | | C | | | | | | | 18 |
| 2 | **276.0649** | 138.5361 | | | 258.0543 | 129.5308 | D | 1973.1066 | 987.0569 | 1956.0800 | 978.5437 | 1955.0960 | 978.0517 | 17 |
| 3 | 373.1176 | 187.0625 | | | 355.1071 | 178.0572 | P | 1858.0797 | **929.5435** | 1841.0531 | 921.0302 | 1840.0691 | 920.5382 | 16 |
| 4 | 444.1547 | 222.5810 | | | 426.1442 | 213.5757 | A | 1761.0269 | 881.0171 | 1744.0003 | 872.5038 | **1743.0163** | 872.0118 | 15 |
| 5 | 557.2388 | 279.1230 | | | 539.2282 | 270.1178 | I | **1689.9898** | 845.4985 | 1672.9632 | 836.9853 | 1671.9792 | 836.4932 | 14 |
| 6 | 685.3338 | 343.1705 | 668.3072 | 334.6573 | 667.3232 | 334.1652 | K | 1576.9057 | 788.9565 | 1559.8792 | 780.4432 | 1558.8951 | 779.9512 | 13 |
| 7 | 782.3865 | 391.6969 | 765.3600 | 383.1836 | 764.3760 | 382.6916 | P | **1448.8107** | 724.9090 | 1431.7842 | 716.3957 | 1430.8002 | 715.9037 | 12 |
| 8 | 919.4454 | 460.2264 | 902.4189 | 451.7131 | 901.4349 | 451.2211 | H | **1351.7580** | 676.3826 | 1334.7314 | 667.8694 | 1333.7474 | 667.3773 | 11 |
| 9 | 1048.4880 | 524.7477 | 1031.4615 | 516.2344 | 1030.4775 | 515.7424 | E | **1214.6991** | 607.8532 | 1197.6725 | 599.3399 | 1196.6885 | 598.8479 | 10 |
| 10 | 1177.5306 | 589.2690 | 1160.5041 | 580.7557 | **1159.5201** | 580.2637 | E | **1085.6565** | 543.3319 | 1068.6299 | 534.8186 | 1067.6459 | 534.3266 | 9 |
| 11 | 1290.6147 | 645.8110 | 1273.5882 | 637.2977 | 1272.6041 | 636.8057 | L | **956.6139** | 478.8106 | 939.5873 | 470.2973 | 938.6033 | 469.8053 | 8 |
| 12 | 1403.6988 | 702.3530 | 1386.6722 | 693.8397 | 1385.6882 | 693.3477 | L | **843.5298** | 422.2685 | 826.5033 | 413.7553 | 825.5193 | 413.2633 | 7 |
| 13 | 1516.7828 | 758.8951 | 1499.7563 | 750.3818 | 1498.7723 | 749.8898 | I | **730.4458** | 365.7265 | 713.4192 | 357.2132 | 712.4352 | 356.7212 | 6 |
| 14 | 1629.8669 | 815.4371 | 1612.8403 | 806.9238 | 1611.8563 | 806.4318 | L | **617.3617** | 309.1845 | 600.3351 | 300.6712 | 599.3511 | 300.1792 | 5 |
| 15 | 1744.8938 | 872.9506 | 1727.8673 | 864.4373 | 1726.8833 | 863.9453 | N | **504.2776** | 252.6425 | 487.2511 | 244.1292 | 486.2671 | 243.6372 | 4 |
| 16 | 1857.9779 | **929.4926** | 1840.9513 | 920.9793 | 1839.9673 | 920.4873 | L | **389.2507** | 195.1290 | 372.2241 | 186.6157 | 371.2401 | 186.1237 | 3 |
| 17 | 1959.0256 | 980.0164 | 1941.9990 | 971.5031 | 1941.0150 | 971.0111 | T | **276.1666** | 138.5870 | 259.1401 | 130.0737 | 258.1561 | 129.5817 | 2 |
| 18 | | | | | | | R | 175.1190 | 88.0631 | 158.0924 | 79.5498 | | | 1 |

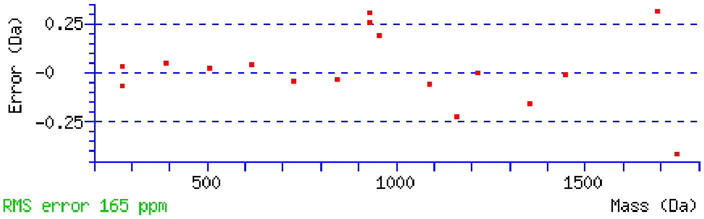


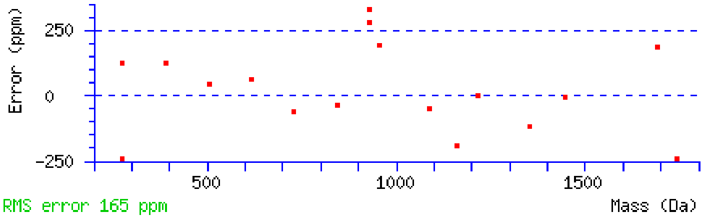


### All matches to this query

| Score | Mr(calc): | Delta | Sequence |
|---|---|---|---|
| 74.1 | 2132.1299 | 0.0072 | CDPAIKPHEELLILNLTR |
| 9.8 | 2131.1459 | 0.9912 | CDPAIKPHEELLILNLTR |
| 5.6 | 2130.1256 | 2.0116 | IPQGTVGQVMLDDRAYLVR |
| 4.5 | 2132.1306 | 0.0065 | VFPVLWTLVSPFINENTR |
| 2.5 | 2132.1378 | -0.0007 | LFNHQEHKVIIVGLDNAGK |
| 1.8 | 2132.1239 | 0.0132 | EFLRARRPPGPDLSPDGPR |
| 0.9 | 2131.1241 | 1.0130 | LKMQIVSLKAGSLVVTLR |
| 0.8 | 2132.1428 | -0.0057 | SVILHLLSQLKLGMDLTR |
| 0.6 | 2132.1306 | 0.0065 | VFPVLWTLVSPFINENTR |
| 0.0 | 2132.1428 | -0.0057 | SVILHLLSQLKLGMDLTR |

---

Spectrum No: 316; Query: 970; Rank: 1

---

## Peptide View

MS/MS Fragmentation of **KIENLEQNISLPVR**
Found in **IPI00400747**, Tax_Id=10116 Gene_Symbol=Man2b1 Mannosidase 2, alpha B1

Match to Query 970: 1652.908908 from(827.461730,2+)
Title: 091008RatKidney_NH4Format01_18.3090.3090.2.dta
Data file K:\NewmanPaper\Piliang\3SubProteomes\Piliang3SP\mgf5ppm\ERLIC_3SubProteomes5ppm.mgf

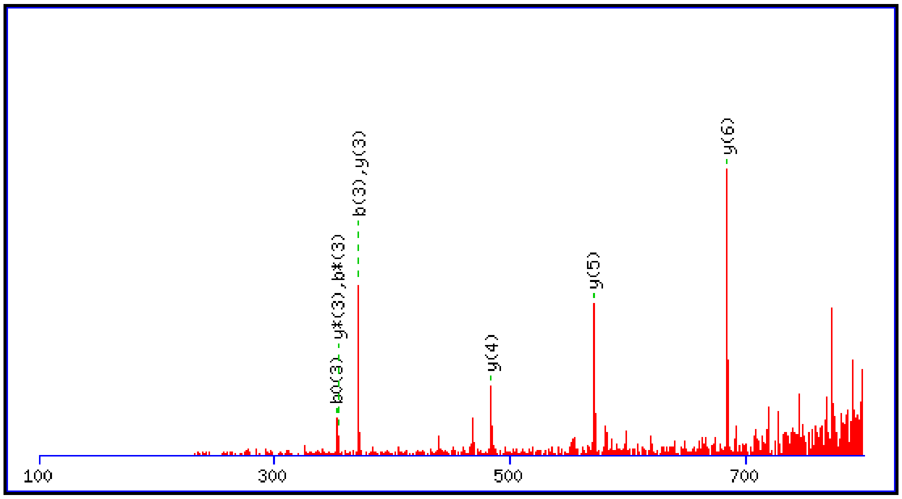

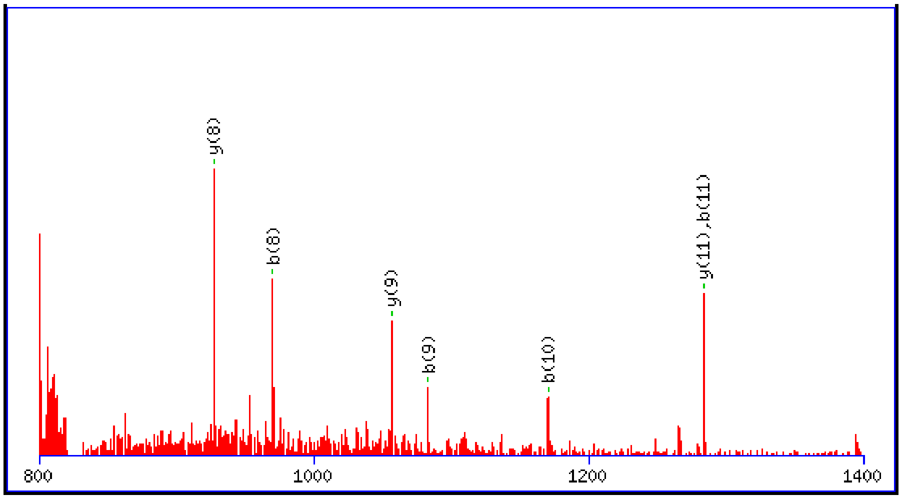


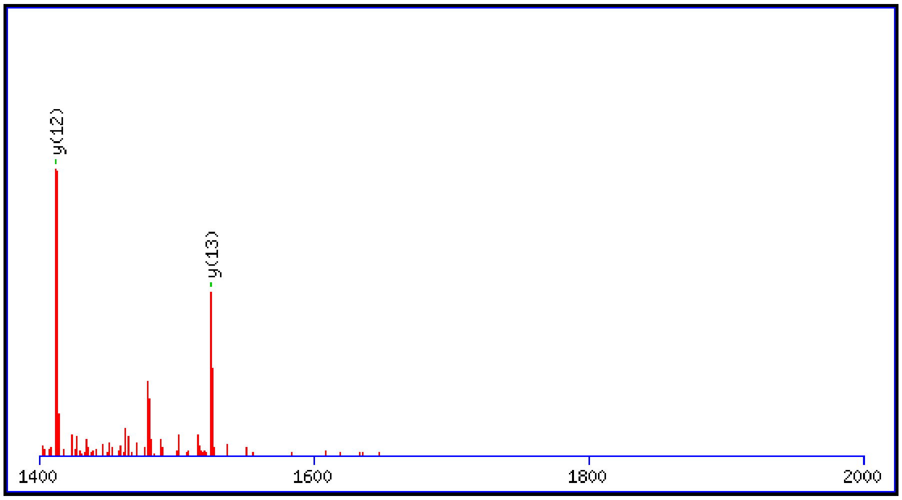


**Monoisotopic mass of neutral peptide Mr(calc):** 1652.9097
**Fixed modifications:** Carbamidomethyl (C)
**Variable modifications:**
**N8** : Deamidated_N (N)
**Ions Score:** 74 **Expect:** 2.5e-006
**Matches (Bold Red):** 17/144 fragment ions using 26 most intense peaks

| # | b | $b^{++}$ | b* | $b^{*++}$ | $b^{0}$ | $b^{0++}$ | Seq. | y | $y^{++}$ | y* | $y^{*++}$ | $y^{0}$ | $y^{0++}$ | # |
|---|---|---|---|---|---|---|---|---|---|---|---|---|---|---|
| 1 | 129.1022 | 65.0548 | 112.0757 | 56.5415 | | | K | | | | | | | 14 |
| 2 | 242.1863 | 121.5968 | 225.1598 | 113.0835 | | | I | **1525.8220** | 763.4147 | 1508.7955 | 754.9014 | 1507.8115 | 754.4094 | 13 |
| 3 | **371.2289** | 186.1181 | **354.2023** | 177.6048 | **353.2183** | 177.1128 | E | **1412.7380** | 706.8726 | 1395.7114 | 698.3594 | 1394.7274 | 697.8673 | 12 |
| 4 | 485.2718 | 243.1395 | 468.2453 | 234.6263 | 467.2613 | 234.1343 | N | **1283.6954** | 642.3513 | 1266.6688 | 633.8381 | 1265.6848 | 633.3460 | 11 |
| 5 | 598.3559 | 299.6816 | 581.3293 | 291.1683 | 580.3453 | 290.6763 | L | 1169.6525 | 585.3299 | 1152.6259 | 576.8166 | 1151.6419 | 576.3246 | 10 |
| 6 | 727.3985 | 364.2029 | 710.3719 | 355.6896 | 709.3879 | 355.1976 | E | **1056.5684** | 528.7878 | 1039.5418 | 520.2746 | 1038.5578 | 519.7826 | 9 |
| 7 | 855.4571 | 428.2322 | 838.4305 | 419.7189 | 837.4465 | 419.2269 | Q | **927.5258** | 464.2665 | 910.4993 | 455.7533 | 909.5152 | 455.2613 | 8 |
| 8 | **970.4840** | 485.7456 | 953.4574 | 477.2324 | 952.4734 | 476.7404 | N | 799.4672 | 400.2372 | 782.4407 | 391.7240 | 781.4567 | 391.2320 | 7 |
| 9 | **1083.5681** | 542.2877 | 1066.5415 | 533.7744 | 1065.5575 | 533.2824 | I | **684.4403** | 342.7238 | 667.4137 | 334.2105 | 666.4297 | 333.7185 | 6 |
| 10 | **1170.6001** | 585.8037 | 1153.5735 | 577.2904 | 1152.5895 | 576.7984 | S | **571.3562** | 286.1817 | 554.3297 | 277.6685 | 553.3457 | 277.1765 | 5 |
| 11 | **1283.6842** | 642.3457 | 1266.6576 | 633.8324 | 1265.6736 | 633.3404 | L | **484.3242** | 242.6657 | 467.2976 | 234.1525 | | | 4 |
| 12 | 1380.7369 | 690.8721 | 1363.7104 | 682.3588 | 1362.7263 | 681.8668 | P | **371.2401** | 186.1237 | **354.2136** | 177.6104 | | | 3 |
| 13 | 1479.8053 | 740.4063 | 1462.7788 | 731.8930 | 1461.7948 | 731.4010 | V | 274.1874 | 137.5973 | 257.1608 | 129.0840 | | | 2 |
| 14 | | | | | | | R | 175.1190 | 88.0631 | 158.0924 | 79.5498 | | | 1 |

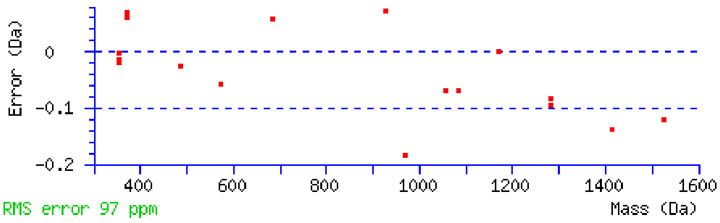


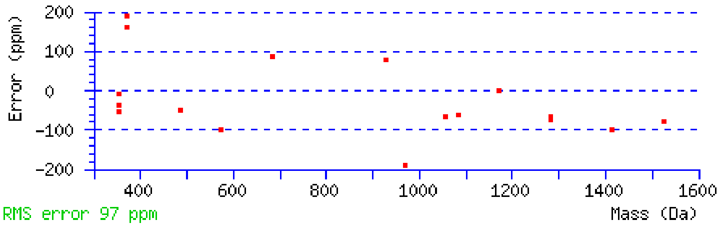


### All matches to this query

| Score | Mr(calc): | Delta | Sequence |
|---|---|---|---|
| 74.1 | 1652.9097 | -0.0008 | KIENLEQNISLPVR |
| 43.4 | 1652.9097 | -0.0008 | KIENLEQNISLPVR |
| 6.1 | 1652.9209 | -0.0120 | KQNLPQNIKIESNK |
| 1.9 | 1652.8958 | 0.0132 | LQLNQSKNNIPRAR |
| 1.8 | 1651.9174 | 0.9915 | LYATVVRKGSLLPR |
| 1.8 | 1652.9209 | -0.0120 | RSAANALEGPAVKIEK |
| 1.8 | 1652.8985 | 0.0104 | KSAPETLTLPDPEKK |
| 1.6 | 1652.8958 | 0.0132 | LQLNQSKNNIPRAR |
| 0.3 | 1652.9097 | -0.0008 | ADLTGISKSPSLHLSK |

---

Spectrum No: 317; Query: 2119; Rank: 1

---

## Peptide View

MS/MS Fragmentation of **VLTLANFTTKDEGDYMCELR**
Found in **IPI00188956**, Tax_Id=10116 Gene_Symbol=Thy1 Thy-1 membrane glycoprotein precursor

Match to Query 2119: 2376.105942 from(793.042590,3+)
Title: 100101RatKid_NS_deglyco_13.4855.4855.3.dta
Data file K:\NewmanPaper\Piliang\3SubProteomes\Piliang3SP\mgf5ppm\ERLIC_3SubProteomes5ppm.mgf

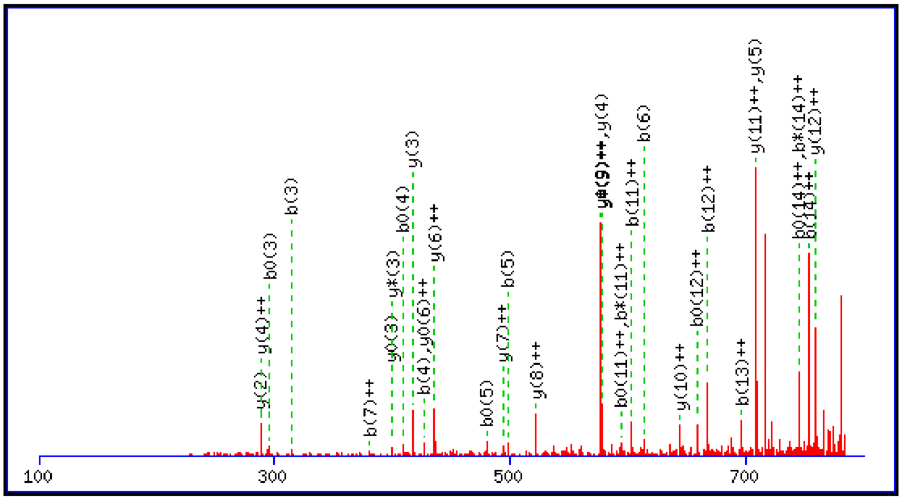

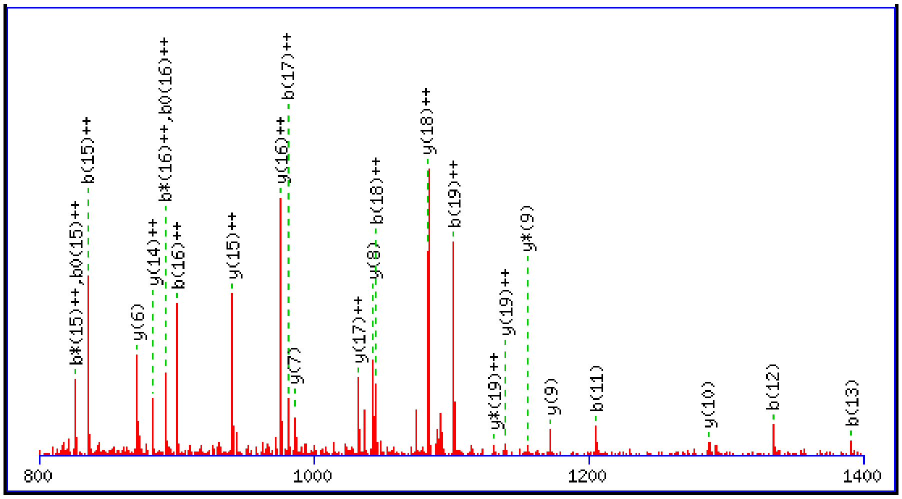


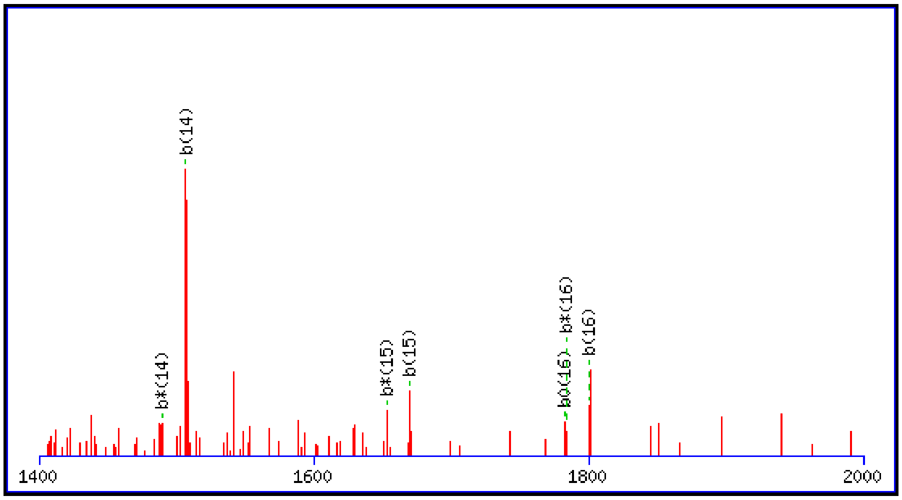


**Monoisotopic mass of neutral peptide Mr(calc):** 2376.0977
**Fixed modifications:** Carbamidomethyl (C)
**Variable modifications:**
**N6** : Deamidated_N (N)
**Ions Score:** 74 **Expect:** 1.1e-005
**Matches (Bold Red):** 65/210 fragment ions using 107 most intense peaks

| # | b | $b^{++}$ | b* | $b^{*++}$ | $b^{0}$ | $b^{0++}$ | Seq. | y | $y^{++}$ | y* | $y^{*++}$ | $y^{0}$ | $y^{0++}$ | # |
|---|---|---|---|---|---|---|---|---|---|---|---|---|---|---|
| 1 | 100.0757 | 50.5415 | | | | | V | | | | | | | 20 |
| 2 | 213.1598 | 107.0835 | | | | | L | 2278.0366 | **1139.5219** | 2261.0101 | **1131.0087** | 2260.0260 | 1130.5167 | 19 |
| 3 | **314.2074** | 157.6074 | | | **296.1969** | 148.6021 | T | 2164.9525 | **1082.9799** | 2147.9260 | 1074.4666 | 2146.9420 | 1073.9746 | 18 |
| 4 | **427.2915** | 214.1494 | | | **409.2809** | 205.1441 | L | 2063.9049 | **1032.4561** | 2046.8783 | 1023.9428 | 2045.8943 | 1023.4508 | 17 |
| 5 | **498.3286** | 249.6679 | | | **480.3180** | 240.6627 | A | 1950.8208 | **975.9140** | 1933.7943 | 967.4008 | 1932.8102 | 966.9088 | 16 |
| 6 | **613.3555** | 307.1814 | 596.3290 | 298.6681 | 595.3450 | 298.1761 | N | 1879.7837 | **940.3955** | 1862.7571 | 931.8822 | 1861.7731 | 931.3902 | 15 |
| 7 | 760.4240 | **380.7156** | 743.3974 | 372.2023 | 742.4134 | 371.7103 | F | 1764.7568 | **882.8820** | 1747.7302 | 874.3687 | 1746.7462 | 873.8767 | 14 |
| 8 | 861.4716 | 431.2395 | 844.4451 | 422.7262 | 843.4611 | 422.2342 | T | 1617.6883 | 809.3478 | 1600.6618 | 800.8345 | 1599.6778 | 800.3425 | 13 |
| 9 | 962.5193 | 481.7633 | 945.4928 | 473.2500 | 944.5088 | 472.7580 | T | 1516.6407 | **758.8240** | 1499.6141 | 750.3107 | 1498.6301 | 749.8187 | 12 |
| 10 | 1090.6143 | 545.8108 | 1073.5877 | 537.2975 | 1072.6037 | 536.8055 | K | 1415.5930 | **708.3001** | 1398.5664 | 699.7869 | 1397.5824 | 699.2948 | 11 |
| 11 | **1205.6412** | **603.3243** | 1188.6147 | **594.8110** | 1187.6307 | **594.3190** | D | **1287.4980** | **644.2526** | 1270.4715 | 635.7394 | 1269.4875 | 635.2474 | 10 |
| 12 | **1334.6838** | **667.8455** | 1317.6573 | 659.3323 | 1316.6733 | **658.8403** | E | **1172.4711** | 586.7392 | **1155.4445** | **578.2259** | 1154.4605 | **577.7339** | 9 |
| 13 | **1391.7053** | **696.3563** | 1374.6787 | 687.8430 | 1373.6947 | 687.3510 | G | **1043.4285** | **522.2179** | 1026.4019 | 513.7046 | 1025.4179 | 513.2126 | 8 |
| 14 | **1506.7322** | **753.8698** | **1489.7057** | **745.3565** | 1488.7217 | **744.8645** | D | **986.4070** | **493.7071** | 969.3805 | 485.1939 | 968.3965 | 484.7019 | 7 |
| 15 | **1669.7956** | **835.4014** | **1652.7690** | **826.8881** | 1651.7850 | **826.3961** | Y | **871.3801** | **436.1937** | 854.3535 | 427.6804 | 853.3695 | **427.1884** | 6 |
| 16 | **1800.8360** | **900.9217** | **1783.8095** | **892.4084** | **1782.8255** | **891.9164** | M | **708.3167** | 354.6620 | 691.2902 | 346.1487 | 690.3062 | 345.6567 | 5 |
| 17 | 1960.8667 | **980.9370** | 1943.8401 | 972.4237 | 1942.8561 | 971.9317 | C | **577.2763** | **289.1418** | 560.2497 | 280.6285 | 559.2657 | 280.1365 | 4 |
| 18 | 2089.9093 | **1045.4583** | 2072.8827 | 1036.9450 | 2071.8987 | 1036.4530 | E | **417.2456** | 209.1264 | **400.2191** | 200.6132 | **399.2350** | 200.1212 | 3 |

| | | | | | | | | | | | | | | |
|---|---|---|---|---|---|---|---|---|---|---|---|---|---|---|
| **19** | 2202.9933 | **1102.0003** | 2185.9668 | 1093.4870 | 2184.9828 | 1092.9950 | **L** | **288.2030** | 144.6051 | 271.1765 | 136.0919 | | | **2** |
| **20** | | | | | | | **R** | 175.1190 | 88.0631 | 158.0924 | 79.5498 | | | **1** |

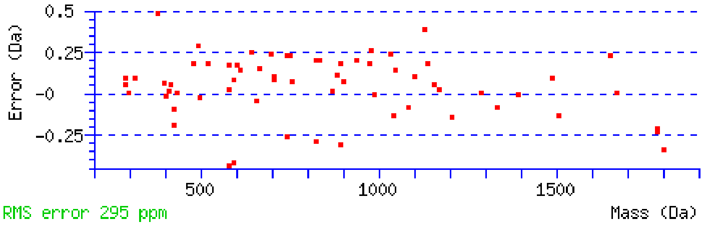


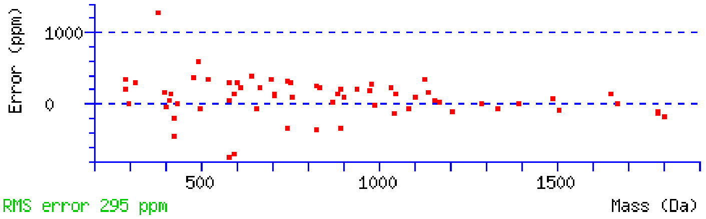


### All matches to this query

| Score | Mr(calc): | Delta | Sequence |
|---|---|---|---|
| 74.0 | 2376.0977 | 0.0082 | VLTLANFTTKDEGDYMCELR |
| 23.8 | 2375.1137 | 0.9922 | VLTLANFTTKDEGDYMCELR |
| 5.9 | 2376.1049 | 0.0010 | XLLDMDTGVTEGGLNVTLTIR |
| 5.1 | 2376.1056 | 0.0003 | GEVYQLDILDTSGNHPFPAMR |
| 4.3 | 2376.0876 | 0.0184 | SSKNHASASSAFNKTALPLTGK |
| 0.1 | 2375.1175 | 0.9885 | SYDYRVSLKTAISTTLELR |
| 0.1 | 2375.1175 | 0.9885 | SYDYRVSLKTAISTTLELR |
| 0.1 | 2375.1175 | 0.9885 | SYDYRVSLKTAISTTLELR |
| 0.1 | 2375.1175 | 0.9885 | SYDYRVSLKTAISTTLELR |
| 0.1 | 2375.1175 | 0.9885 | SYDYRVSLKTAISTTLELR |

---

Spectrum No: 318; Query: 2660; Rank: 1

---

## Peptide View

MS/MS Fragmentation of **CIPQYWVCDGDADCSDALDELQNCTMR**
Found in **IPI00205325**, Tax_Id=10116 Gene_Symbol=Lrp2 Low-density lipoprotein receptor-related protein 2 precursor

Match to Query 2660: 3309.291102 from(1104.104310,3+)
Title: 100101RatKid_NS_deglyco_24.4321.4321.3.dta
Data file K:\NewmanPaper\Piliang\3SubProteomes\Piliang3SP\mgf5ppm\ERLIC_3SubProteomes5ppm.mgf

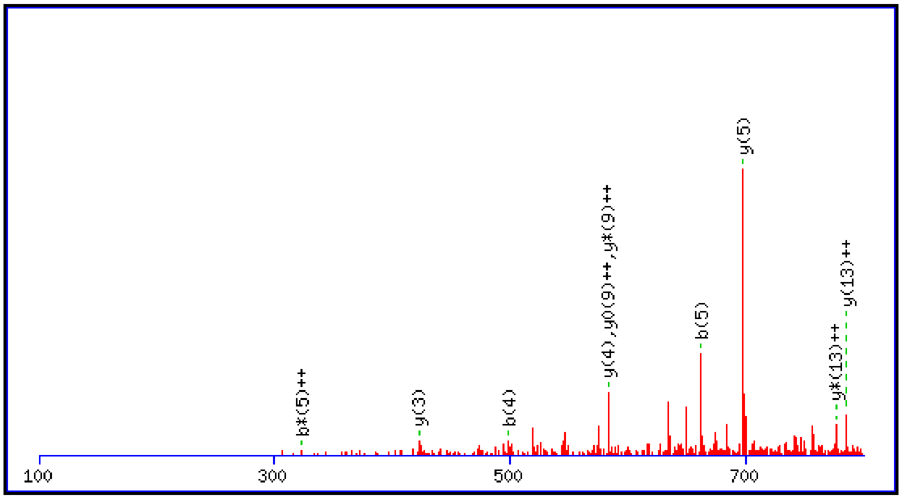

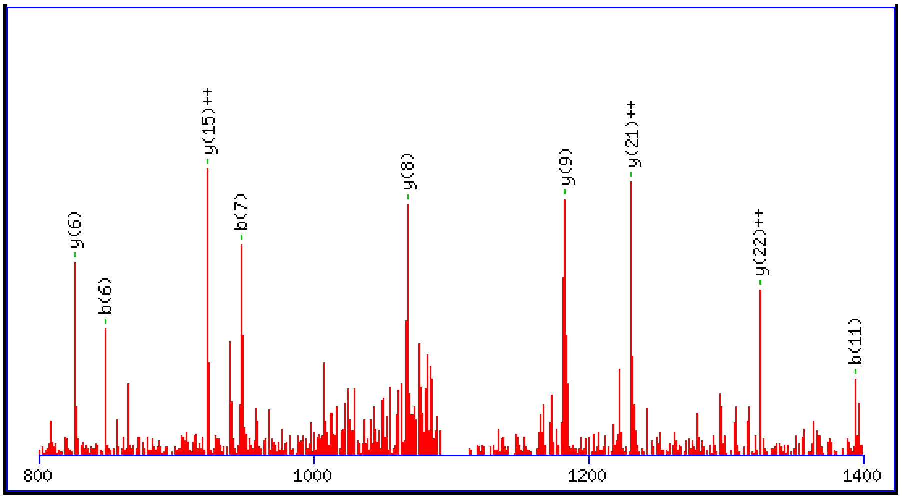


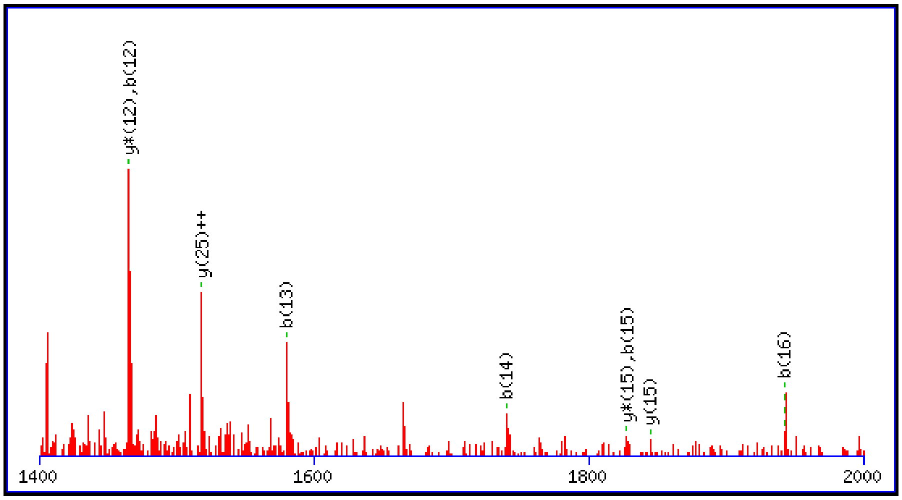


**Monoisotopic mass of neutral peptide Mr(calc):** 3308.2825
**Fixed modifications:** Carbamidomethyl (C)
**Variable modifications:**
**N23** : Deamidated_N (N)
**M26** : Oxidation (M)
**Ions Score:** 74 **Expect:** 1.3e-005
**Matches (Bold Red):** 28/286 fragment ions using 43 most intense peaks

| # | b | $b^{++}$ | b* | $b^{*++}$ | $b^{0}$ | $b^{0++}$ | Seq. | y | $y^{++}$ | y* | $y^{*++}$ | $y^{0}$ | $y^{0++}$ | # |
|---|---|---|---|---|---|---|---|---|---|---|---|---|---|---|
| 1 | 161.0379 | 81.0226 | | | | | C | | | | | | | 27 |
| 2 | 274.1220 | 137.5646 | | | | | I | 3149.2591 | 1575.1332 | 3132.2326 | 1566.6199 | 3131.2485 | 1566.1279 | 26 |
| 3 | 371.1748 | 186.0910 | | | | | P | 3036.1750 | **1518.5912** | 3019.1485 | 1510.0779 | 3018.1645 | 1509.5859 | 25 |
| 4 | **499.2333** | 250.1203 | 482.2068 | 241.6070 | | | Q | 2939.1223 | 1470.0648 | 2922.0957 | 1461.5515 | 2921.1117 | 1461.0595 | 24 |
| 5 | **662.2967** | 331.6520 | 645.2701 | **323.1387** | | | Y | 2811.0637 | 1406.0355 | 2794.0372 | 1397.5222 | 2793.0531 | 1397.0302 | 23 |
| 6 | **848.3760** | 424.6916 | 831.3494 | 416.1783 | | | W | 2648.0004 | **1324.5038** | 2630.9738 | 1315.9906 | 2629.9898 | 1315.4985 | 22 |
| 7 | **947.4444** | 474.2258 | 930.4178 | 465.7126 | | | V | 2461.9211 | **1231.4642** | 2444.8945 | 1222.9509 | 2443.9105 | 1222.4589 | 21 |
| 8 | 1107.4750 | 554.2412 | 1090.4485 | 545.7279 | | | C | 2362.8527 | 1181.9300 | 2345.8261 | 1173.4167 | 2344.8421 | 1172.9247 | 20 |
| 9 | 1222.5020 | 611.7546 | 1205.4754 | 603.2414 | 1204.4914 | 602.7493 | D | 2202.8220 | 1101.9146 | 2185.7955 | 1093.4014 | 2184.8114 | 1092.9094 | 19 |
| 10 | 1279.5234 | 640.2654 | 1262.4969 | 631.7521 | 1261.5129 | 631.2601 | G | 2087.7951 | 1044.4012 | 2070.7685 | 1035.8879 | 2069.7845 | 1035.3959 | 18 |
| 11 | **1394.5504** | 697.7788 | 1377.5238 | 689.2656 | 1376.5398 | 688.7735 | D | 2030.7736 | 1015.8904 | 2013.7470 | 1007.3772 | 2012.7630 | 1006.8852 | 17 |
| 12 | **1465.5875** | 733.2974 | 1448.5610 | 724.7841 | 1447.5769 | 724.2921 | A | 1915.7467 | 958.3770 | 1898.7201 | 949.8637 | 1897.7361 | 949.3717 | 16 |
| 13 | **1580.6144** | 790.8109 | 1563.5879 | 782.2976 | 1562.6039 | 781.8056 | D | **1844.7095** | **922.8584** | **1827.6830** | 914.3451 | 1826.6990 | 913.8531 | 15 |
| 14 | **1740.6451** | 870.8262 | 1723.6185 | 862.3129 | 1722.6345 | 861.8209 | C | 1729.6826 | 865.3449 | 1712.6560 | 856.8317 | 1711.6720 | 856.3397 | 14 |
| 15 | **1827.6771** | 914.3422 | 1810.6506 | 905.8289 | 1809.6666 | 905.3369 | S | 1569.6519 | **785.3296** | 1552.6254 | **776.8163** | 1551.6414 | 776.3243 | 13 |
| 16 | **1942.7041** | 971.8557 | 1925.6775 | 963.3424 | 1924.6935 | 962.8504 | D | 1482.6199 | 741.8136 | **1465.5934** | 733.3003 | 1464.6094 | 732.8083 | 12 |
| 17 | 2013.7412 | 1007.3742 | 1996.7146 | 998.8610 | 1995.7306 | 998.3689 | A | 1367.5930 | 684.3001 | 1350.5664 | 675.7869 | 1349.5824 | 675.2948 | 11 |
| 18 | 2126.8252 | 1063.9163 | 2109.7987 | 1055.4030 | 2108.8147 | 1054.9110 | L | 1296.5559 | 648.7816 | 1279.5293 | 640.2683 | 1278.5453 | 639.7763 | 10 |

| | | | | | | | | | | | | | | |
|---|---|---|---|---|---|---|---|---|---|---|---|---|---|---|
| **19** | 2241.8522 | 1121.4297 | 2224.8256 | 1112.9165 | 2223.8416 | 1112.4244 | **D** | **1183.4718** | 592.2395 | 1166.4452 | **583.7263** | 1165.4612 | **583.2343** | **9** |
| **20** | 2370.8948 | 1185.9510 | 2353.8682 | 1177.4378 | 2352.8842 | 1176.9457 | **E** | **1068.4449** | 534.7261 | 1051.4183 | 526.2128 | 1050.4343 | 525.7208 | **8** |
| **21** | 2483.9788 | 1242.4931 | 2466.9523 | 1233.9798 | 2465.9683 | 1233.4878 | **L** | 939.4023 | 470.2048 | 922.3757 | 461.6915 | 921.3917 | 461.1995 | **7** |
| **22** | 2612.0374 | 1306.5223 | 2595.0109 | 1298.0091 | 2594.0269 | 1297.5171 | **Q** | **826.3182** | 413.6627 | 809.2916 | 405.1495 | 808.3076 | 404.6575 | **6** |
| **23** | 2727.0644 | 1364.0358 | 2710.0378 | 1355.5225 | 2709.0538 | 1355.0305 | **N** | **698.2596** | 349.6334 | 681.2331 | 341.1202 | 680.2491 | 340.6282 | **5** |
| **24** | 2887.0950 | 1444.0511 | 2870.0685 | 1435.5379 | 2869.0844 | 1435.0459 | **C** | **583.2327** | 292.1200 | 566.2061 | 283.6067 | 565.2221 | 283.1147 | **4** |
| **25** | 2988.1427 | 1494.5750 | 2971.1161 | 1486.0617 | 2970.1321 | 1485.5697 | **T** | **423.2020** | 212.1047 | 406.1755 | 203.5914 | 405.1915 | 203.0994 | **3** |
| **26** | 3135.1781 | 1568.0927 | 3118.1515 | 1559.5794 | 3117.1675 | 1559.0874 | **M** | 322.1544 | 161.5808 | 305.1278 | 153.0675 | | | **2** |
| **27** | | | | | | | **R** | 175.1190 | 88.0631 | 158.0924 | 79.5498 | | | **1** |

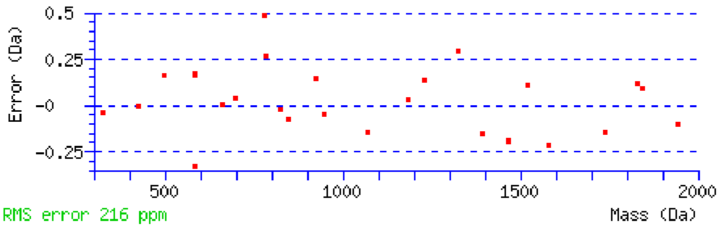


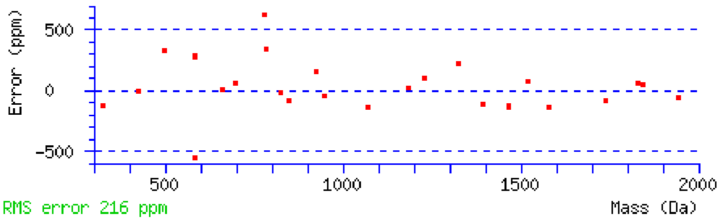


**All matches to this query**

| Score | Mr(calc): | Delta | Sequence |
|---|---|---|---|
| 73.6 | 3308.2825 | 1.0086 | CIPQYWVCDGDADCSDALDELQNCTMR |
| 35.0 | 3307.2985 | 1.9926 | CIPQYWVCDGDADCSDALDELQNCTMR |
| 6.2 | 3307.2760 | 2.0151 | VLFSFPGNQVTSVAGVYFTDVYGGINR |
| 5.4 | 3307.2650 | 2.0261 | NSTTPNAPYQQGMGMPDMLGRMPYAPNK |
| 5.4 | 3307.2650 | 2.0261 | NSTTPNAPYQQGMGMPDMLGRMPYAPNK |
| 2.9 | 3307.2760 | 2.0151 | VLFSFPGNQVTSVAGVYFTDVYGGINR |
| 1.0 | 3308.2600 | 1.0311 | DSYFWIALQDQNNTGEYTWKTMGQR |
| 1.0 | 3307.2650 | 2.0261 | NSTTPNAPYQQGMGMPDMLGRMPYAPNK |
| 1.0 | 3307.2650 | 2.0261 | NSTTPNAPYQQGMGMPDMLGRMPYAPNK |
| 0.9 | 3307.3140 | 1.9771 | ITAHCLAYSNSSVNPIIYAFLSENFR |

---

Spectrum No: 319; Query: 615; Rank: 1

---

## Peptide View

MS/MS Fragmentation of **VSNMTLPASAEVLK**
Found in **IPI00206336**, Tax_Id=10116 Gene_Symbol=Lamp1 Lysosome-associated membrane glycoprotein 1 precursor

Match to Query 615: 1459.760148 from(730.887350,2+)
Title: 100101RatKid_NS_deglyco_25.2839.2839.2.dta
Data file K:\NewmanPaper\Piliang\3SubProteomes\Piliang3SP\mgf5ppm\ERLIC_3SubProteomes5ppm.mgf

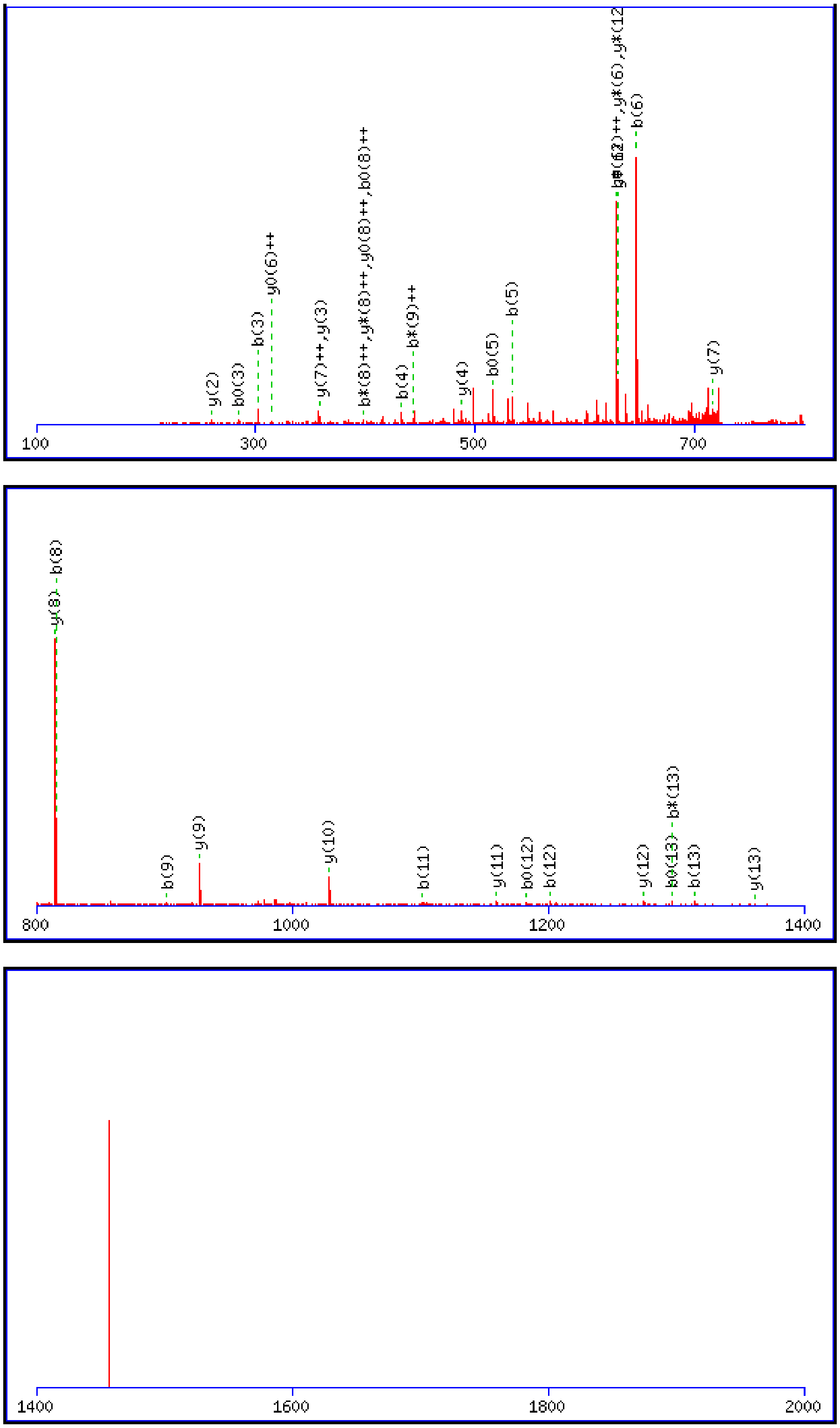


**Monoisotopic mass of neutral peptide Mr(calc):** 1459.7592
**Fixed modifications:** Carbamidomethyl (C)
**Variable modifications:**
**N3** : Deamidated_N (N)
**Ions Score:** 74 **Expect:** 5.3e-006
**Matches (Bold Red):** 36/144 fragment ions using 60 most intense peaks

| # | b | $b^{++}$ | b* | $b^{*++}$ | $b^0$ | $b^{0++}$ | Seq. | y | $y^{++}$ | y* | $y^{*++}$ | $y^0$ | $y^{0++}$ | # |
|---|---|---|---|---|---|---|---|---|---|---|---|---|---|---|
| 1 | 100.0757 | 50.5415 | | | | | V | | | | | | | 14 |
| 2 | 187.1077 | 94.0575 | | | 169.0972 | 85.0522 | S | **1361.6981** | 681.3527 | 1344.6715 | 672.8394 | 1343.6875 | 672.3474 | 13 |
| 3 | **302.1347** | 151.5710 | 285.1081 | 143.0577 | **284.1241** | 142.5657 | N | **1274.6661** | 637.8367 | 1257.6395 | **629.3234** | 1256.6555 | **628.8314** | 12 |
| 4 | **433.1751** | 217.0912 | 416.1486 | 208.5779 | 415.1646 | 208.0859 | M | **1159.6391** | 580.3232 | 1142.6126 | 571.8099 | 1141.6286 | 571.3179 | 11 |
| 5 | **534.2228** | 267.6150 | 517.1963 | 259.1018 | **516.2123** | 258.6098 | T | **1028.5986** | 514.8030 | 1011.5721 | 506.2897 | 1010.5881 | 505.7977 | 10 |

| | | | | | | | | | | | | | | |
|---|---|---|---|---|---|---|---|---|---|---|---|---|---|---|
| 6 | **647.3069** | 324.1571 | **630.2803** | 315.6438 | **629.2963** | 315.1518 | L | **927.5510** | 464.2791 | 910.5244 | 455.7658 | 909.5404 | 455.2738 | 9 |
| 7 | 744.3596 | 372.6835 | 727.3331 | 364.1702 | 726.3491 | 363.6782 | P | **814.4669** | 407.7371 | 797.4403 | **399.2238** | 796.4563 | **398.7318** | 8 |
| 8 | **815.3968** | 408.2020 | 798.3702 | **399.6887** | 797.3862 | **399.1967** | A | **717.4141** | **359.2107** | 700.3876 | 350.6974 | 699.4036 | 350.2054 | 7 |
| 9 | **902.4288** | 451.7180 | 885.4022 | **443.2048** | 884.4182 | 442.7127 | S | 646.3770 | 323.6921 | **629.3505** | 315.1789 | 628.3665 | **314.6869** | 6 |
| 10 | 973.4659 | 487.2366 | 956.4394 | 478.7233 | 955.4553 | 478.2313 | A | 559.3450 | 280.1761 | 542.3184 | 271.6629 | 541.3344 | 271.1709 | 5 |
| 11 | **1102.5085** | 551.7579 | 1085.4819 | 543.2446 | 1084.4979 | 542.7526 | E | **488.3079** | 244.6576 | 471.2813 | 236.1443 | 470.2973 | 235.6523 | 4 |
| 12 | **1201.5769** | 601.2921 | 1184.5504 | 592.7788 | **1183.5663** | 592.2868 | V | **359.2653** | 180.1363 | 342.2387 | 171.6230 | | | 3 |
| 13 | **1314.6610** | 657.8341 | **1297.6344** | 649.3209 | **1296.6504** | 648.8288 | L | **260.1969** | 130.6021 | 243.1703 | 122.0888 | | | 2 |
| 14 | | | | | | | K | 147.1128 | 74.0600 | 130.0863 | 65.5468 | | | 1 |

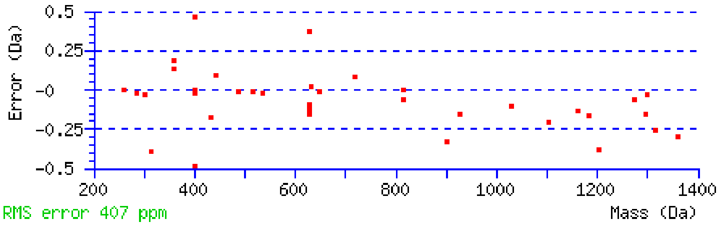


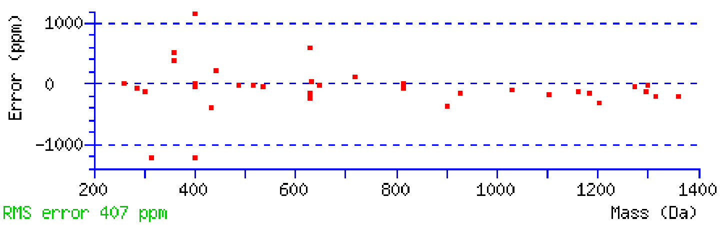


### All matches to this query

| Score | Mr(calc): | Delta | Sequence |
|---|---|---|---|
| 73.6 | 1459.7592 | 0.0009 | VSNMTLPASAEVLK |
| 16.2 | 1459.7671 | -0.0069 | WLGTGNKGSNALLK |
| 14.5 | 1459.7606 | -0.0004 | KSCGXIPGKQAWK |
| 8.9 | 1459.7518 | 0.0084 | KLNESLKQQNEK |
| 8.4 | 1459.7466 | 0.0135 | GFRQASTLCRHK |
| 5.9 | 1458.7467 | 1.0135 | IKDDVGDNWRIK |
| 4.7 | 1459.7671 | -0.0069 | AVLQPHENIVPNK |
| 4.6 | 1459.7558 | 0.0043 | SSYKYDSLLLGSK |
| 4.2 | 1459.7476 | 0.0125 | FTLGKVWITTSK |
| 3.4 | 1459.7476 | 0.0125 | FTLGKVWITTSK |

---

Spectrum No: 320; Query: 352; Rank: 1

---

## Peptide View

MS/MS Fragmentation of **VYNGSVPFEER**
Found in **IPI00339118**, Tax_Id=10116 Gene_Symbol=Enpp1 Isoform 2 of Ectonucleotide pyrophosphatase/phosphodiesterase family member 1

Match to Query 352: 1296.599948 from(649.307250,2+)
Title: 100101RatKid_NS_deglyco_23.2021.2021.2.dta
Data file K:\NewmanPaper\Piliang\3SubProteomes\Piliang3SP\mgf5ppm\ERLIC_3SubProteomes5ppm.mgf

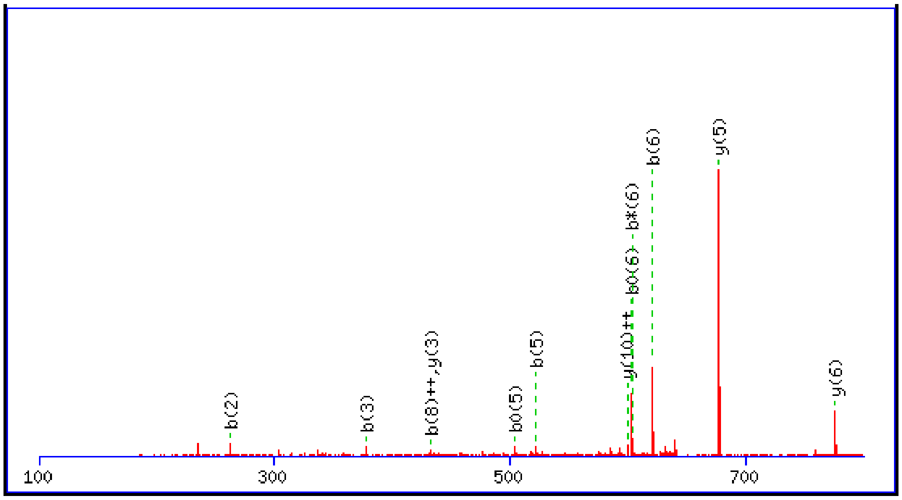


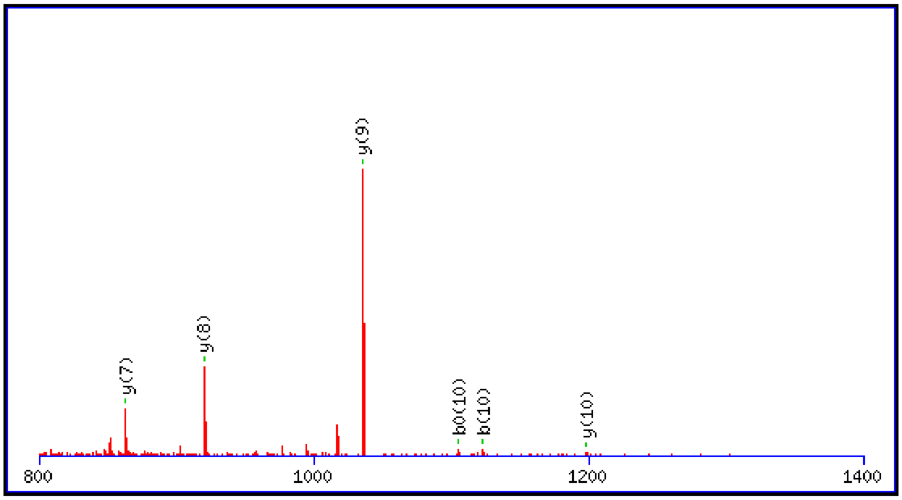


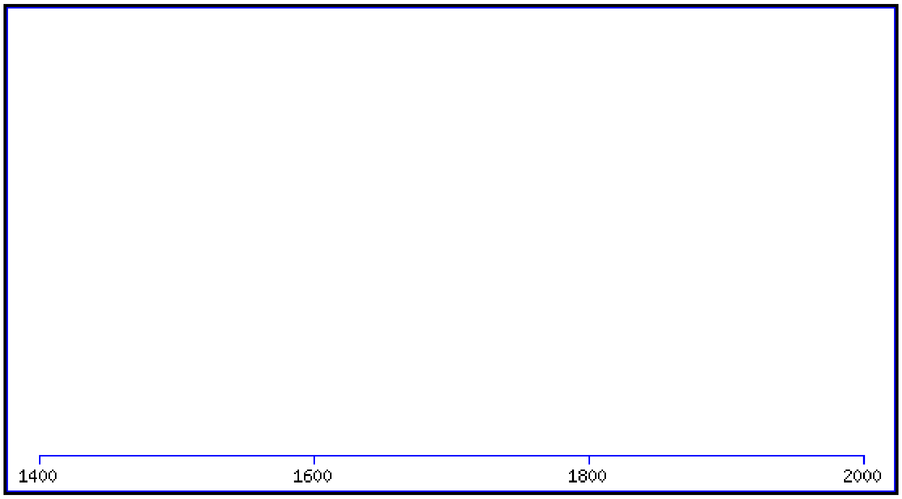


**Monoisotopic mass of neutral peptide Mr(calc):** 1296.5986
**Fixed modifications:** Carbamidomethyl (C)
**Variable modifications:**
**N3** : Deamidated_N (N)
**Ions Score:** 73 **Expect:** 5.9e-006
**Matches (Bold Red):** 18/106 fragment ions using 22 most intense peaks

| # | b | $b^{++}$ | b* | $b^{*++}$ | $b^0$ | $b^{0++}$ | Seq. | y | $y^{++}$ | y* | $y^{*++}$ | $y^0$ | $y^{0++}$ | # |
|---|---|---|---|---|---|---|---|---|---|---|---|---|---|---|
| 1 | 100.0757 | 50.5415 | | | | | V | | | | | | | 11 |
| 2 | **263.1390** | 132.0731 | | | | | Y | **1198.5375** | **599.7724** | 1181.5109 | 591.2591 | 1180.5269 | 590.7671 | 10 |
| 3 | **378.1660** | 189.5866 | 361.1394 | 181.0733 | | | N | **1035.4742** | 518.2407 | 1018.4476 | 509.7274 | 1017.4636 | 509.2354 | 9 |
| 4 | 435.1874 | 218.0973 | 418.1609 | 209.5841 | | | G | **920.4472** | 460.7272 | 903.4207 | 452.2140 | 902.4367 | 451.7220 | 8 |
| 5 | **522.2194** | 261.6134 | 505.1929 | 253.1001 | **504.2089** | 252.6081 | S | **863.4258** | 432.2165 | 846.3992 | 423.7032 | 845.4152 | 423.2112 | 7 |

| | | | | | | | | | | | | | | |
|---|---|---|---|---|---|---|---|---|---|---|---|---|---|---|
| 6 | **621.2879** | 311.1476 | **604.2613** | 302.6343 | **603.2773** | 302.1423 | V | **776.3937** | 388.7005 | 759.3672 | 380.1872 | 758.3832 | 379.6952 | 6 |
| 7 | 718.3406 | 359.6740 | 701.3141 | 351.1607 | 700.3301 | 350.6687 | P | **677.3253** | 339.1663 | 660.2988 | 330.6530 | 659.3148 | 330.1610 | 5 |
| 8 | 865.4090 | **433.2082** | 848.3825 | 424.6949 | 847.3985 | 424.2029 | F | 580.2726 | 290.6399 | 563.2460 | 282.1266 | 562.2620 | 281.6346 | 4 |
| 9 | 994.4516 | 497.7295 | 977.4251 | 489.2162 | 976.4411 | 488.7242 | E | **433.2041** | 217.1057 | 416.1776 | 208.5924 | 415.1936 | 208.1004 | 3 |
| 10 | **1123.4942** | 562.2508 | 1106.4677 | 553.7375 | **1105.4837** | 553.2455 | E | 304.1615 | 152.5844 | 287.1350 | 144.0711 | 286.1510 | 143.5791 | 2 |
| 11 | | | | | | | R | 175.1190 | 88.0631 | 158.0924 | 79.5498 | | | 1 |

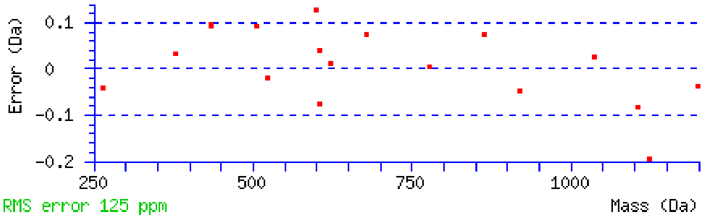


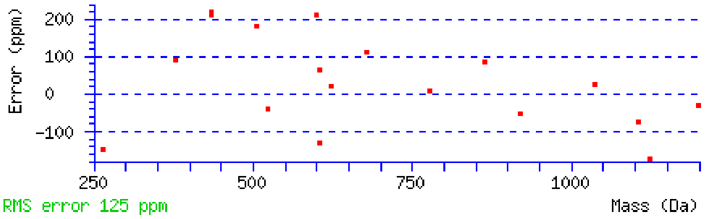


### All matches to this query

| Score | Mr(calc): | Delta | Sequence |
|---|---|---|---|
| 73.5 | 1296.5986 | 0.0013 | VYNGSVPFEER |
| 11.1 | 1296.6020 | -0.0020 | MDVAFLLDNSR |
| 5.0 | 1294.5863 | 2.0136 | WTLSNMLGDNK |
| 4.0 | 1295.5890 | 1.0110 | NMADLMWSTVK |
| 2.7 | 1296.5914 | 0.0085 | SLMGSRAAMDSR |
| 1.9 | 1296.6067 | -0.0067 | WRMMSLSQSR |
| 1.9 | 1296.6067 | -0.0067 | WRMMSLSQSR |
| 1.3 | 1295.5849 | 1.0150 | TMERCNLIDK |
| 1.0 | 1296.5976 | 0.0024 | RGPTANGFLER |
| 0.9 | 1296.5992 | 0.0007 | VLSIQSHVVR |

---

Spectrum No: 321; Query: 1825; Rank: 1

---

## Peptide View

MS/MS Fragmentation of **DCSTNPPRLPVTAVNTTMR**
Found in **IPI00197684**, Tax_Id=10116 Gene_Symbol=Xpnpep2 Membrane-bound aminopeptidase P

Match to Query 1825: 2146.017312 from(716.346380,3+)
Title: 091008RatKidney_NoSalt_25.1872.1872.3.dta
Data file K:\NewmanPaper\Piliang\3SubProteomes\Piliang3SP\mgf5ppm\ERLIC_3SubProteomes5ppm.mgf

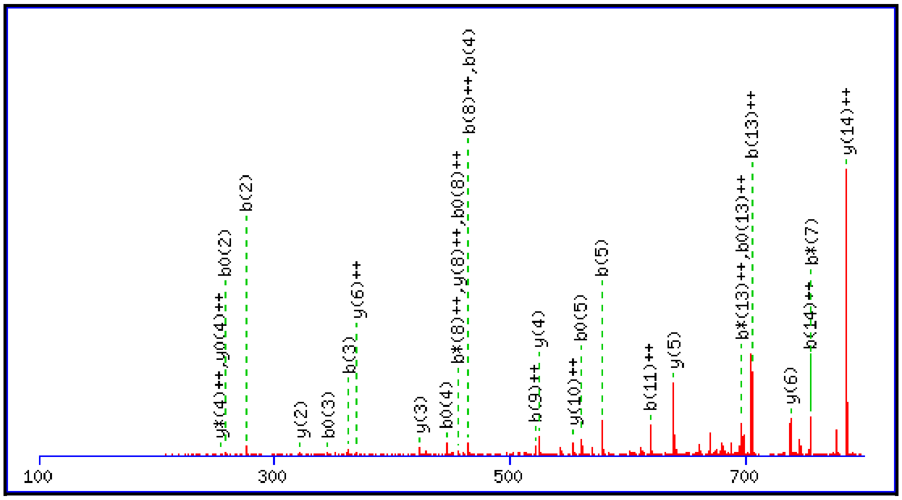

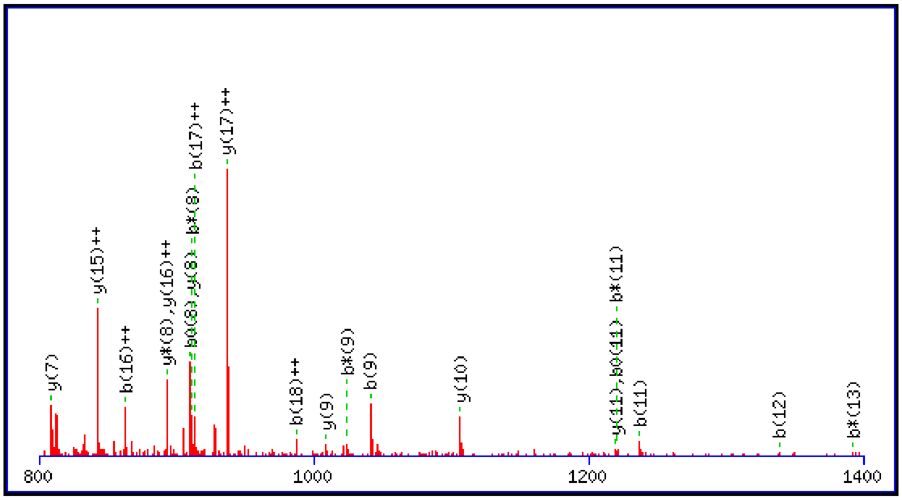


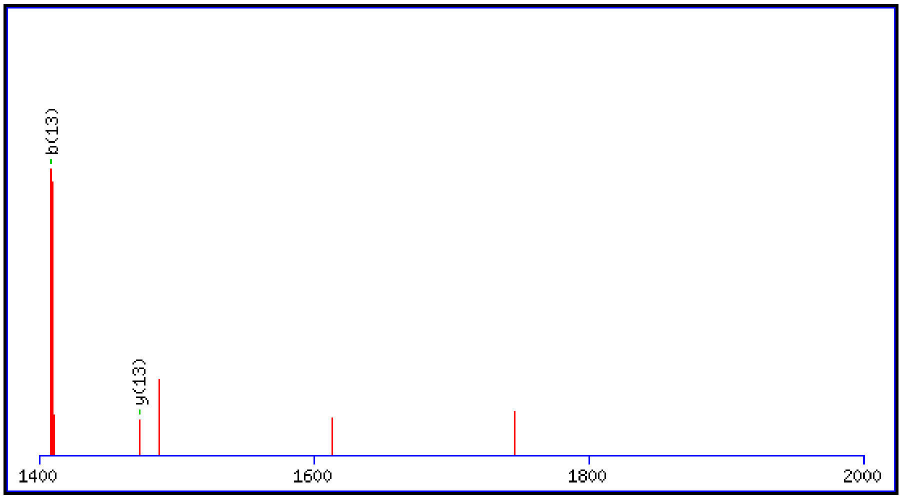


**Monoisotopic mass of neutral peptide Mr(calc):** 2146.0147
**Fixed modifications:** Carbamidomethyl (C)
**Variable modifications:**
**N15** : Deamidated_N (N)
**M18** : Oxidation (M)
**Ions Score:** 73 **Expect:** 1.2e-005
**Matches (Bold Red):** 52/204 fragment ions using 68 most intense peaks

| # | b | $b^{++}$ | b* | $b^{*++}$ | $b^{0}$ | $b^{0++}$ | Seq. | y | $y^{++}$ | y* | $y^{*++}$ | $y^{0}$ | $y^{0++}$ | # |
|---|---|---|---|---|---|---|---|---|---|---|---|---|---|---|
| 1 | 116.0342 | 58.5207 | | | 98.0237 | 49.5155 | D | | | | | | | 19 |
| 2 | **276.0649** | 138.5361 | | | **258.0543** | 129.5308 | C | 2031.9950 | 1016.5011 | 2014.9685 | 1007.9879 | 2013.9845 | 1007.4959 | 18 |
| 3 | **363.0969** | 182.0521 | | | **345.0863** | 173.0468 | S | 1871.9644 | **936.4858** | 1854.9378 | 927.9725 | 1853.9538 | 927.4805 | 17 |
| 4 | **464.1446** | 232.5759 | | | **446.1340** | 223.5706 | T | 1784.9323 | **892.9698** | 1767.9058 | 884.4565 | 1766.9218 | 883.9645 | 16 |
| 5 | **578.1875** | 289.5974 | 561.1610 | 281.0841 | **560.1769** | 280.5921 | N | 1683.8847 | **842.4460** | 1666.8581 | 833.9327 | 1665.8741 | 833.4407 | 15 |
| 6 | 675.2403 | 338.1238 | 658.2137 | 329.6105 | 657.2297 | 329.1185 | P | 1569.8417 | **785.4245** | 1552.8152 | 776.9112 | 1551.8312 | 776.4192 | 14 |
| 7 | 772.2930 | 386.6502 | **755.2665** | 378.1369 | 754.2825 | 377.6449 | P | **1472.7890** | 736.8981 | 1455.7624 | 728.3849 | 1454.7784 | 727.8928 | 13 |
| 8 | 928.3941 | **464.7007** | **911.3676** | **456.1874** | **910.3836** | **455.6954** | R | 1375.7362 | 688.3717 | 1358.7097 | 679.8585 | 1357.7256 | 679.3665 | 12 |
| 9 | **1041.4782** | **521.2427** | **1024.4517** | 512.7295 | 1023.4676 | 512.2375 | L | **1219.6351** | 610.3212 | 1202.6086 | 601.8079 | 1201.6245 | 601.3159 | 11 |
| 10 | 1138.5310 | 569.7691 | 1121.5044 | 561.2558 | 1120.5204 | 560.7638 | P | **1106.5510** | **553.7792** | 1089.5245 | 545.2659 | 1088.5405 | 544.7739 | 10 |
| 11 | **1237.5994** | **619.3033** | **1220.5728** | 610.7901 | **1219.5888** | 610.2980 | V | **1009.4983** | 505.2528 | 992.4717 | 496.7395 | 991.4877 | 496.2475 | 9 |
| 12 | **1338.6471** | 669.8272 | 1321.6205 | 661.3139 | 1320.6365 | 660.8219 | T | **910.4299** | **455.7186** | **893.4033** | 447.2053 | 892.4193 | 446.7133 | 8 |
| 13 | **1409.6842** | **705.3457** | **1392.6576** | **696.8325** | 1391.6736 | **696.3404** | A | **809.3822** | 405.1947 | 792.3556 | 396.6815 | 791.3716 | 396.1894 | 7 |
| 14 | 1508.7526 | **754.8799** | 1491.7260 | 746.3667 | 1490.7420 | 745.8747 | V | **738.3451** | **369.6762** | 721.3185 | 361.1629 | 720.3345 | 360.6709 | 6 |
| 15 | 1623.7795 | 812.3934 | 1606.7530 | 803.8801 | 1605.7690 | 803.3881 | N | **639.2767** | 320.1420 | 622.2501 | 311.6287 | 621.2661 | 311.1367 | 5 |
| 16 | 1724.8272 | **862.9172** | 1707.8007 | 854.4040 | 1706.8166 | 853.9120 | T | **524.2497** | 262.6285 | 507.2232 | **254.1152** | 506.2391 | **253.6232** | 4 |
| 17 | 1825.8749 | **913.4411** | 1808.8483 | 904.9278 | 1807.8643 | 904.4358 | T | **423.2020** | 212.1047 | 406.1755 | 203.5914 | 405.1915 | 203.0994 | 3 |

| | | | | | | | | | | | | | | |
|---|---|---|---|---|---|---|---|---|---|---|---|---|---|---|
| 18 | 1972.9103 | 986.9588 | 1955.8837 | 978.4455 | 1954.8997 | 977.9535 | M | 322.1544 | 161.5808 | 305.1278 | 153.0675 | | | 2 |
| 19 | | | | | | | R | 175.1190 | 88.0631 | 158.0924 | 79.5498 | | | 1 |

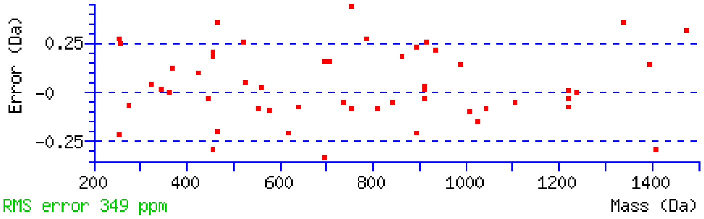


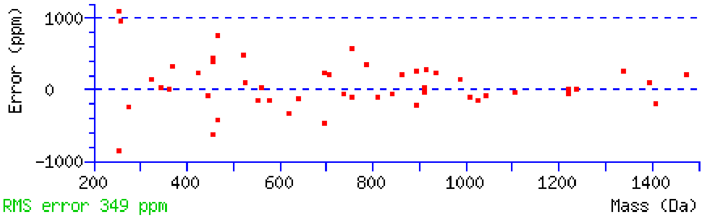


**All matches to this query**

| Score | Mr(calc): | Delta | Sequence |
|---|---|---|---|
| 73.2 | 2146.0147 | 0.0026 | DCSTNPPRLPVTAVNTTMR |
| 41.9 | 2146.0147 | 0.0026 | DCSTNPPRLPVTAVNTTMR |
| 33.1 | 2145.0307 | 0.9866 | DCSTNPPRLPVTAVNTTMR |
| 4.2 | 2145.0078 | 1.0095 | VNCSDKYGTTPLVWAARK |
| 3.9 | 2144.0263 | 1.9910 | DIALEITVTNGPSDPRNPR |
| 3.4 | 2145.0338 | 0.9835 | DAEDGNANFLGKASGVDLVPR |
| 1.8 | 2145.0303 | 0.9870 | HWVQMGGPRVTLGADGRTK |
| 1.7 | 2143.9990 | 2.0183 | LSDSGNYTCMAANIVAKRR |
| 1.6 | 2145.9983 | 0.0190 | SLSLFSNVSQSHGIYIGEK |
| 1.6 | 2144.0276 | 1.9897 | RTRHVHSFSVSLHASSEK |

---

Spectrum No: 322; Query: 1440; Rank: 1

---

## Peptide View

MS/MS Fragmentation of **DAFTNVSSPDAGATLPNDK**
Found in **IPI00369140**, Tax_Id=10116 Gene_Symbol=RGD1309019_predicted 38 kDa protein

Match to Query 1440: 1919.875788 from(960.945170,2+)
Title: 091008RatKidney_NoSalt_32.1881.1881.2.dta
Data file K:\NewmanPaper\Piliang\3SubProteomes\Piliang3SP\mgf5ppm\ERLIC_3SubProteomes5ppm.mgf

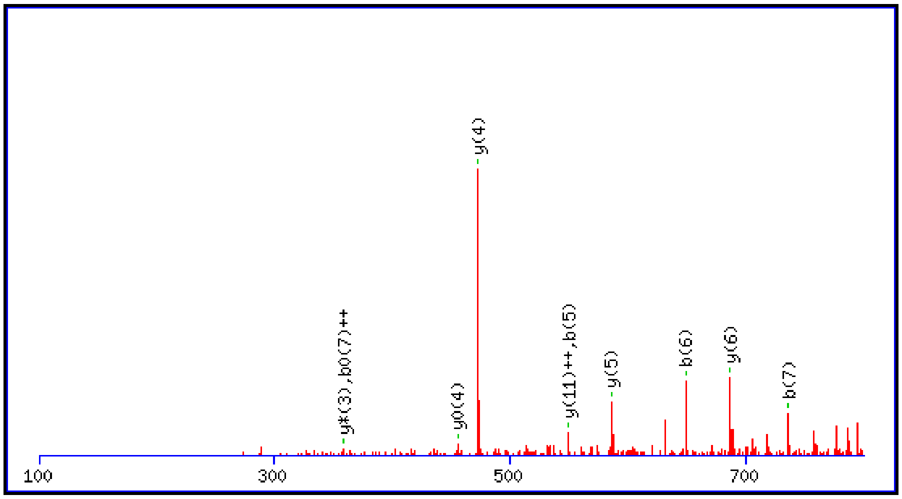

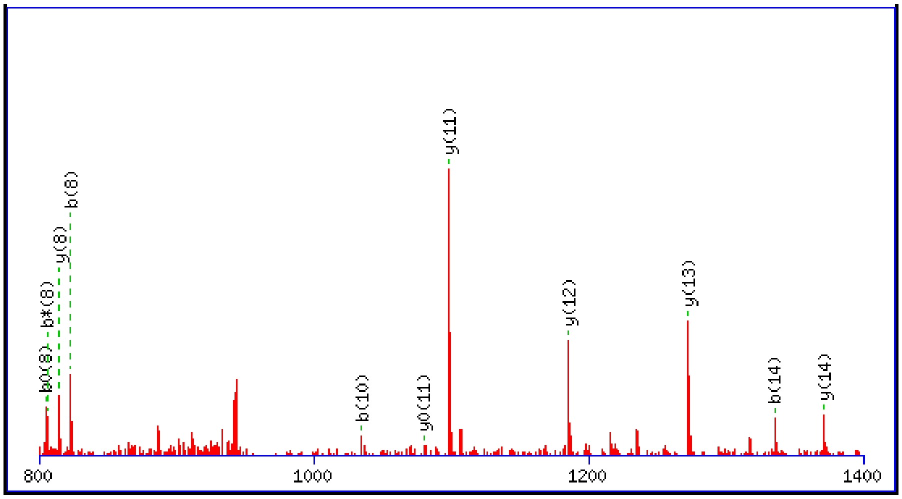


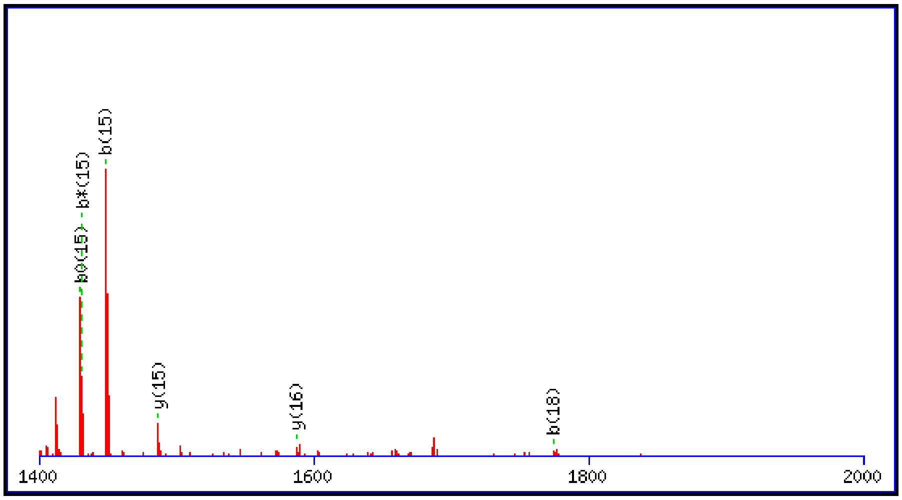


**Monoisotopic mass of neutral peptide Mr(calc):** 1919.8749
**Fixed modifications:** Carbamidomethyl (C)
**Variable modifications:**
**N5** : Deamidated_N (N)
**Ions Score:** 73 **Expect:** 1.1e-005
**Matches (Bold Red):** 27/206 fragment ions using 43 most intense peaks

| # | b | $b^{++}$ | b* | $b^{*++}$ | $b^0$ | $b^{0++}$ | Seq. | y | $y^{++}$ | y* | $y^{*++}$ | $y^0$ | $y^{0++}$ | # |
|---|---|---|---|---|---|---|---|---|---|---|---|---|---|---|
| 1 | 116.0342 | 58.5207 | | | 98.0237 | 49.5155 | D | | | | | | | 19 |
| 2 | 187.0713 | 94.0393 | | | 169.0608 | 85.0340 | A | 1805.8552 | 903.4312 | 1788.8286 | 894.9180 | 1787.8446 | 894.4260 | 18 |
| 3 | 334.1397 | 167.5735 | | | 316.1292 | 158.5682 | F | 1734.8181 | 867.9127 | 1717.7915 | 859.3994 | 1716.8075 | 858.9074 | 17 |
| 4 | 435.1874 | 218.0974 | | | 417.1769 | 209.0921 | T | **1587.7497** | 794.3785 | 1570.7231 | 785.8652 | 1569.7391 | 785.3732 | 16 |
| 5 | **550.2144** | 275.6108 | 533.1878 | 267.0975 | 532.2038 | 266.6055 | N | **1486.7020** | 743.8546 | 1469.6754 | 735.3414 | 1468.6914 | 734.8493 | 15 |
| 6 | **649.2828** | 325.1450 | 632.2562 | 316.6318 | 631.2722 | 316.1397 | V | **1371.6751** | 686.3412 | 1354.6485 | 677.8279 | 1353.6645 | 677.3359 | 14 |
| 7 | **736.3148** | 368.6610 | 719.2883 | 360.1478 | 718.3042 | **359.6558** | S | **1272.6066** | 636.8070 | 1255.5801 | 628.2937 | 1254.5961 | 627.8017 | 13 |
| 8 | **823.3468** | 412.1771 | **806.3203** | 403.6638 | **805.3363** | 403.1718 | S | **1185.5746** | 593.2909 | 1168.5481 | 584.7777 | 1167.5640 | 584.2857 | 12 |
| 9 | 920.3996 | 460.7034 | 903.3730 | 452.1902 | 902.3890 | 451.6982 | P | **1098.5426** | **549.7749** | 1081.5160 | 541.2617 | **1080.5320** | 540.7696 | 11 |
| 10 | **1035.4265** | 518.2169 | 1018.4000 | 509.7036 | 1017.4160 | 509.2116 | D | 1001.4898 | 501.2485 | 984.4633 | 492.7353 | 983.4793 | 492.2433 | 10 |
| 11 | 1106.4637 | 553.7355 | 1089.4371 | 545.2222 | 1088.4531 | 544.7302 | A | 886.4629 | 443.7351 | 869.4363 | 435.2218 | 868.4523 | 434.7298 | 9 |
| 12 | 1163.4851 | 582.2462 | 1146.4586 | 573.7329 | 1145.4746 | 573.2409 | G | **815.4258** | 408.2165 | 798.3992 | 399.7032 | 797.4152 | 399.2112 | 8 |
| 13 | 1234.5222 | 617.7648 | 1217.4957 | 609.2515 | 1216.5117 | 608.7595 | A | 758.4043 | 379.7058 | 741.3777 | 371.1925 | 740.3937 | 370.7005 | 7 |
| 14 | **1335.5699** | 668.2886 | 1318.5434 | 659.7753 | 1317.5593 | 659.2833 | T | **687.3672** | 344.1872 | 670.3406 | 335.6740 | 669.3566 | 335.1819 | 6 |
| 15 | **1448.6540** | 724.8306 | **1431.6274** | 716.3174 | **1430.6434** | 715.8253 | L | **586.3195** | 293.6634 | 569.2930 | 285.1501 | 568.3089 | 284.6581 | 5 |
| 16 | 1545.7067 | 773.3570 | 1528.6802 | 764.8437 | 1527.6962 | 764.3517 | P | **473.2354** | 237.1214 | 456.2089 | 228.6081 | **455.2249** | 228.1161 | 4 |
| 17 | 1659.7497 | 830.3785 | 1642.7231 | 821.8652 | 1641.7391 | 821.3732 | N | 376.1827 | 188.5950 | **359.1561** | 180.0817 | 358.1721 | 179.5897 | 3 |
| 18 | **1774.7766** | 887.8919 | 1757.7501 | 879.3787 | 1756.7660 | 878.8867 | D | 262.1397 | 131.5735 | 245.1132 | 123.0602 | 244.1292 | 122.5682 | 2 |

| | | | | | | | | | | | | | | |
|---|---|---|---|---|---|---|---|---|---|---|---|---|---|---|
| **19** | | | | | | | **K** | 147.1128 | 74.0600 | 130.0863 | 65.5468 | | | **1** |

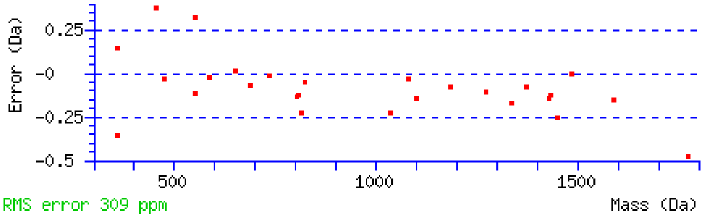


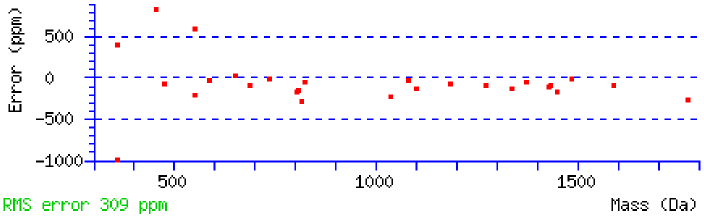


### All matches to this query

| Score | Mr(calc): | Delta | Sequence |
|---|---|---|---|
| 73.0 | 1919.8749 | 0.0009 | DAFTNVSSPDAGATLPNDK |
| 35.7 | 1919.8749 | 0.0009 | DAFTNVSSPDAGATLPNDK |
| 26.3 | 1918.8909 | 0.9849 | DAFTNVSSPDAGATLPNDK |
| 4.9 | 1919.8599 | 0.0159 | RNGSKLSNDHQQNQSK |
| 0.6 | 1919.8740 | 0.0018 | YNNLLLLENQFICGK |
| 0.5 | 1918.8703 | 1.0055 | HETYRSTLKTLPGTR |
| 0.5 | 1918.8703 | 1.0055 | HETYRSTLKTLPGTR |
| 0.2 | 1919.8852 | -0.0094 | MKGGVIYIYPDEAAASR |

---

Spectrum No: 323; Query: 2484; Rank: 1

---

## Peptide View

MS/MS Fragmentation of **GNSMEEILEGLKFNLTETPETEIHR**
Found in **IPI00211075**, Tax_Id=10116 Gene_Symbol=Serpina3n Serine protease inhibitor A3N precursor

Match to Query 2484: 2903.387622 from(968.803150,3+)
Title: 091008RatKidney_NH4Format01_25.3668.3668.3.dta
Data file K:\NewmanPaper\Piliang\3SubProteomes\Piliang3SP\mgf5ppm\ERLIC_3SubProteomes5ppm.mgf

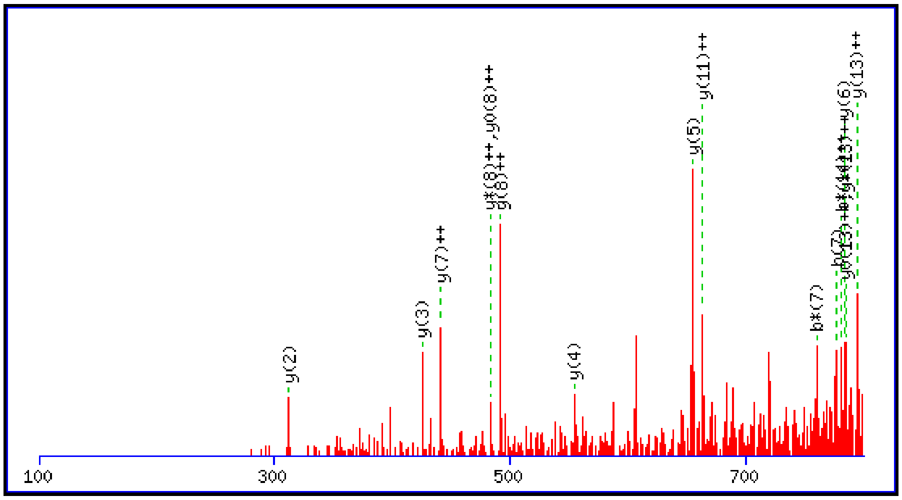

Mascot Search Results: Peptide View

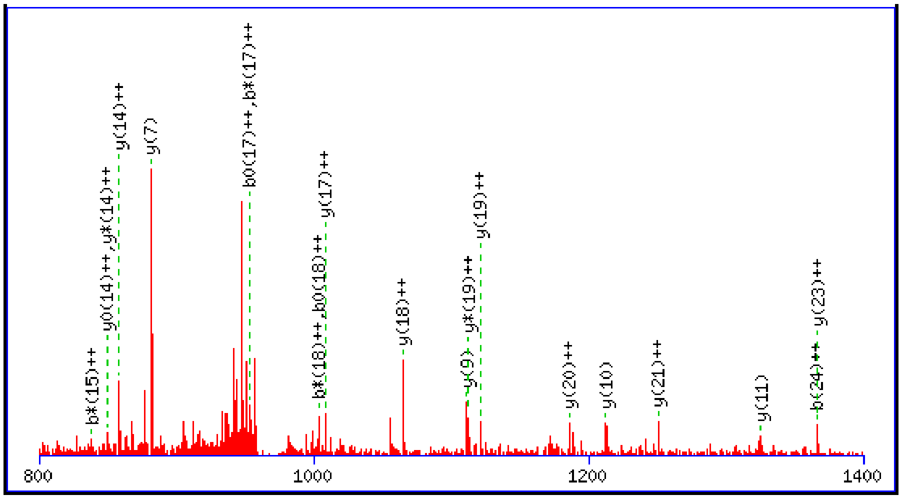


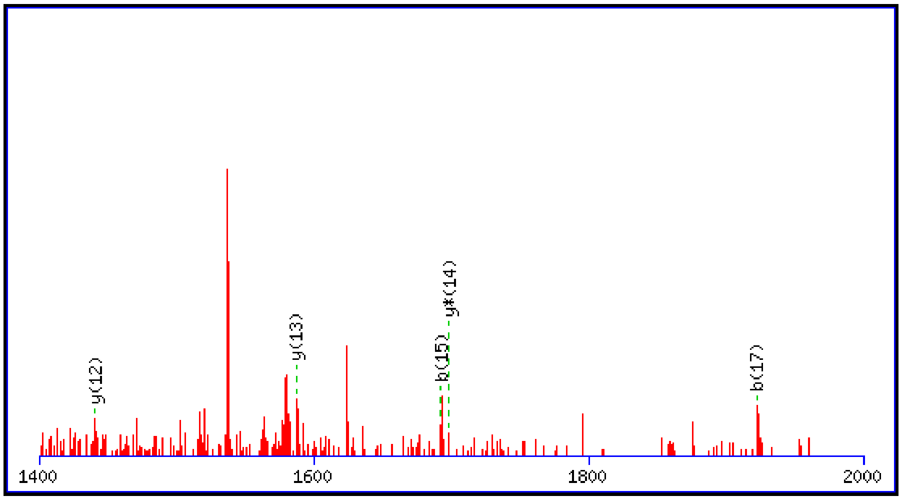


**Monoisotopic mass of neutral peptide Mr(calc):** 2903.3858
**Fixed modifications:** Carbamidomethyl (C)
**Variable modifications:**
**M4** : Oxidation (M)
**N14** : Deamidated_N (N)
**Ions Score:** 73 **Expect:** 1.7e-005
**Matches (Bold Red):** 41/276 fragment ions using 69 most intense peaks

| # | b | $b^{++}$ | b* | $b^{*++}$ | $b^0$ | $b^{0++}$ | Seq. | y | $y^{++}$ | y* | $y^{*++}$ | $y^0$ | $y^{0++}$ | # |
|---|---|---|---|---|---|---|---|---|---|---|---|---|---|---|
| 1 | 58.0287 | 29.5180 | | | | | G | | | | | | | 25 |
| 2 | 172.0717 | 86.5395 | 155.0451 | 78.0262 | | | N | 2847.3717 | 1424.1895 | 2830.3451 | 1415.6762 | 2829.3611 | 1415.1842 | 24 |
| 3 | 259.1037 | 130.0555 | 242.0771 | 121.5422 | 241.0931 | 121.0502 | S | 2733.3288 | **1367.1680** | 2716.3022 | 1358.6547 | 2715.3182 | 1358.1627 | 23 |
| 4 | 406.1391 | 203.5732 | 389.1125 | 195.0599 | 388.1285 | 194.5679 | M | 2646.2967 | 1323.6520 | 2629.2702 | 1315.1387 | 2628.2862 | 1314.6467 | 22 |
| 5 | 535.1817 | 268.0945 | 518.1551 | 259.5812 | 517.1711 | 259.0892 | E | 2499.2613 | **1250.1343** | 2482.2348 | 1241.6210 | 2481.2508 | 1241.1290 | 21 |
| 6 | 664.2243 | 332.6158 | 647.1977 | 324.1025 | 646.2137 | 323.6105 | E | 2370.2187 | **1185.6130** | 2353.1922 | 1177.0997 | 2352.2082 | 1176.6077 | 20 |
| 7 | **777.3083** | 389.1578 | **760.2818** | 380.6445 | 759.2978 | 380.1525 | I | 2241.1761 | **1121.0917** | 2224.1496 | **1112.5784** | 2223.1656 | 1112.0864 | 19 |
| 8 | 890.3924 | 445.6998 | 873.3659 | 437.1866 | 872.3818 | 436.6946 | L | 2128.0921 | **1064.5497** | 2111.0655 | 1056.0364 | 2110.0815 | 1055.5444 | 18 |
| 9 | 1019.4350 | 510.2211 | 1002.4085 | 501.7079 | 1001.4244 | 501.2159 | E | 2015.0080 | **1008.0076** | 1997.9815 | 999.4944 | 1996.9974 | 999.0024 | 17 |
| 10 | 1076.4565 | 538.7319 | 1059.4299 | 530.2186 | 1058.4459 | 529.7266 | G | 1885.9654 | 943.4863 | 1868.9389 | 934.9731 | 1867.9548 | 934.4811 | 16 |
| 11 | 1189.5405 | 595.2739 | 1172.5140 | 586.7606 | 1171.5300 | 586.2686 | L | 1828.9439 | 914.9756 | 1811.9174 | 906.4623 | 1810.9334 | 905.9703 | 15 |
| 12 | 1317.6355 | 659.3214 | 1300.6089 | 650.8081 | 1299.6249 | 650.3161 | K | 1715.8599 | **858.4336** | **1698.8333** | **849.9203** | 1697.8493 | **849.4283** | 14 |
| 13 | 1464.7039 | 732.8556 | 1447.6774 | 724.3423 | 1446.6933 | 723.8503 | F | **1587.7649** | **794.3861** | 1570.7384 | **785.8728** | 1569.7544 | **785.3808** | 13 |
| 14 | 1579.7308 | 790.3691 | 1562.7043 | **781.8558** | 1561.7203 | 781.3638 | N | **1440.6965** | 720.8519 | 1423.6700 | 712.3386 | 1422.6859 | 711.8466 | 12 |
| 15 | **1692.8149** | 846.9111 | 1675.7884 | **838.3978** | 1674.8043 | 837.9058 | L | **1325.6696** | **663.3384** | 1308.6430 | 654.8251 | 1307.6590 | 654.3331 | 11 |
| 16 | 1793.8626 | 897.4349 | 1776.8360 | 888.9217 | 1775.8520 | 888.4297 | T | **1212.5855** | 606.7964 | 1195.5590 | 598.2831 | 1194.5749 | 597.7911 | 10 |
| 17 | **1922.9052** | 961.9562 | 1905.8786 | **953.4430** | 1904.8946 | **952.9509** | E | **1111.5378** | 556.2726 | 1094.5113 | 547.7593 | 1093.5273 | 547.2673 | 9 |
| 18 | 2023.9529 | 1012.4801 | 2006.9263 | **1003.9668** | 2005.9423 | **1003.4748** | T | 982.4952 | **491.7513** | 965.4687 | **483.2380** | 964.4847 | **482.7460** | 8 |

| | | | | | | | | | | | | | | |
|---|---|---|---|---|---|---|---|---|---|---|---|---|---|---|
| 19 | 2121.0056 | 1061.0065 | 2103.9791 | 1052.4932 | 2102.9951 | 1052.0012 | P | 881.4476 | 441.2274 | 864.4210 | 432.7141 | 863.4370 | 432.2221 | 7 |
| 20 | 2250.0482 | 1125.5277 | 2233.0217 | 1117.0145 | 2232.0377 | 1116.5225 | E | 784.3948 | 392.7010 | 767.3682 | 384.1878 | 766.3842 | 383.6958 | 6 |
| 21 | 2351.0959 | 1176.0516 | 2334.0693 | 1167.5383 | 2333.0853 | 1167.0463 | T | 655.3522 | 328.1797 | 638.3257 | 319.6665 | 637.3416 | 319.1745 | 5 |
| 22 | 2480.1385 | 1240.5729 | 2463.1119 | 1232.0596 | 2462.1279 | 1231.5676 | E | 554.3045 | 277.6559 | 537.2780 | 269.1426 | 536.2940 | 268.6506 | 4 |
| 23 | 2593.2226 | 1297.1149 | 2576.1960 | 1288.6016 | 2575.2120 | 1288.1096 | I | 425.2619 | 213.1346 | 408.2354 | 204.6213 | | | 3 |
| 24 | 2730.2815 | 1365.6444 | 2713.2549 | 1357.1311 | 2712.2709 | 1356.6391 | H | 312.1779 | 156.5926 | 295.1513 | 148.0793 | | | 2 |
| 25 | | | | | | | R | 175.1190 | 88.0631 | 158.0924 | 79.5498 | | | 1 |

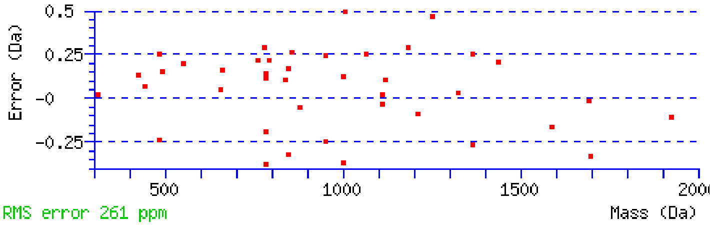


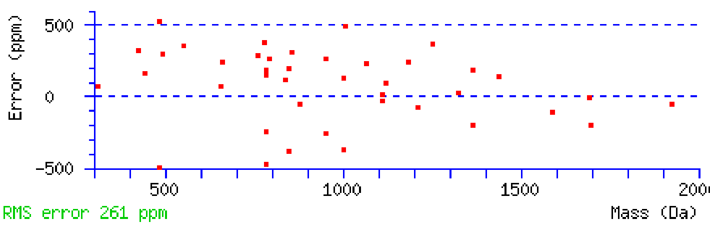


**All matches to this query**

| Score | Mr(calc): | Delta | Sequence |
|---|---|---|---|
| 72.9 | 2903.3858 | 0.0018 | GNSMEEILEGLKFNLTETPETEIHR |
| 14.7 | 2903.3858 | 0.0018 | GNSMEEILEGLKFNLTETPETEIHR |
| 12.5 | 2902.4018 | 0.9858 | GNSMEEILEGLKFNLTETPETEIHR |
| 5.2 | 2901.3787 | 2.0089 | GDTADARPPTPLLPGHPTASSTSIKVR |

---

Spectrum No: 324; Query: 2603; Rank: 1

---

## Peptide View

MS/MS Fragmentation of **GVDVSQVAWQSQGDTPCSCCIVNNSNGSR**
Found in **IPI00211989**, Tax_Id=10116 Gene_Symbol=Khk Isoform C of Ketohexokinase

Match to Query 2603: 3182.360862 from(1061.794230,3+)
Title: 100101RatKid_NS_deglyco_08.3131.3131.3.dta
Data file K:\NewmanPaper\Piliang\3SubProteomes\Piliang3SP\mgf5ppm\ERLIC_3SubProteomes5ppm.mgf

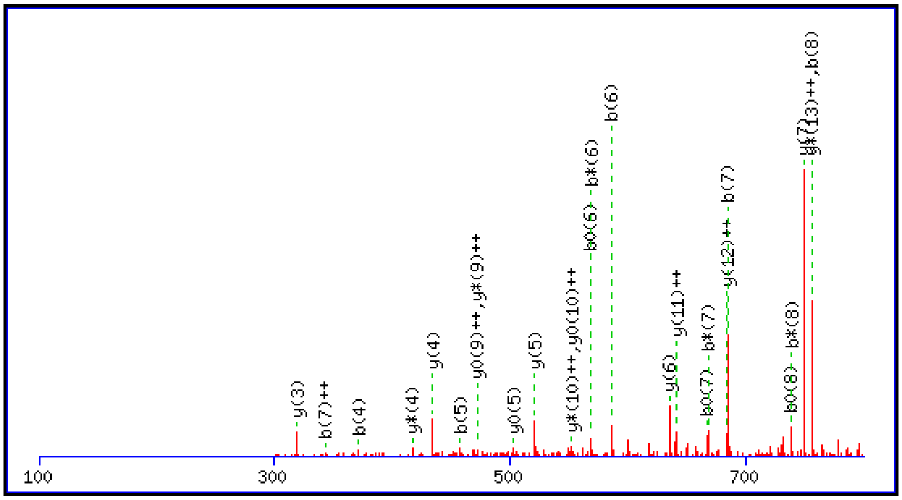

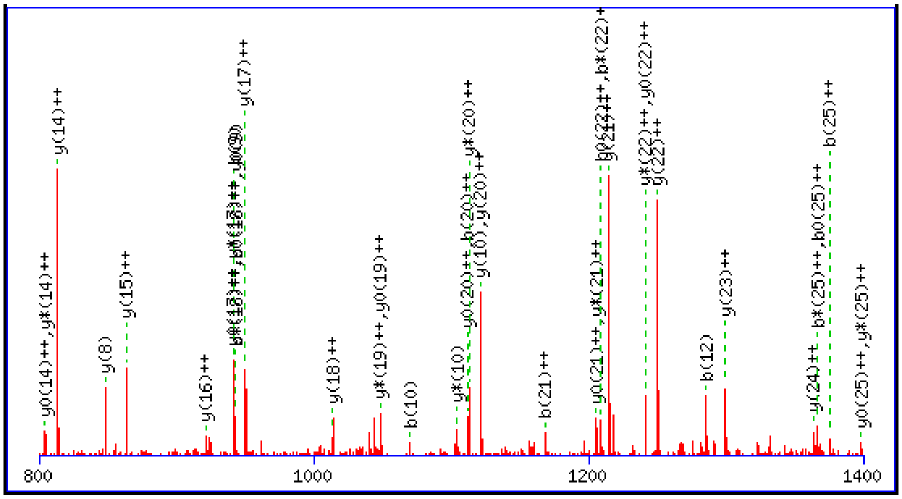


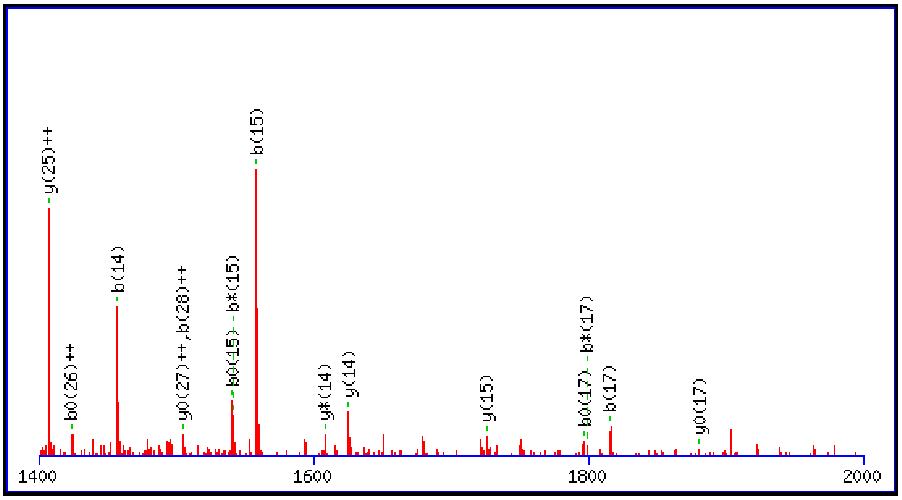


**Monoisotopic mass of neutral peptide Mr(calc):** 3182.3452
**Fixed modifications:** Carbamidomethyl (C)
**Variable modifications:**
**N26** : Deamidated_N (N)
**Ions Score:** 73 **Expect:** 2.3e-005
**Matches (Bold Red):** 81/320 fragment ions using 107 most intense peaks

| # | b | b++ | b* | b*++ | b0 | b0++ | Seq. | y | y++ | y* | y*++ | y0 | y0++ | # |
|---|---|---|---|---|---|---|---|---|---|---|---|---|---|---|
| 1 | 58.0287 | 29.5180 | | | | | G | | | | | | | 29 |
| 2 | 157.0972 | 79.0522 | | | | | V | 3126.3310 | 1563.6691 | 3109.3044 | 1555.1558 | 3108.3204 | 1554.6638 | 28 |
| 3 | 272.1241 | 136.5657 | | | 254.1135 | 127.5604 | D | 3027.2626 | 1514.1349 | 3010.2360 | 1505.6216 | 3009.2520 | **1505.1296** | 27 |
| 4 | **371.1925** | 186.0999 | | | 353.1819 | 177.0946 | V | 2912.2356 | 1456.6214 | 2895.2091 | 1448.1082 | 2894.2250 | 1447.6162 | 26 |
| 5 | **458.2245** | 229.6159 | | | 440.2140 | 220.6106 | S | 2813.1672 | **1407.0872** | 2796.1406 | **1398.5740** | 2795.1566 | **1398.0820** | 25 |
| 6 | **586.2831** | 293.6452 | **569.2566** | 285.1319 | **568.2726** | 284.6399 | Q | 2726.1352 | **1363.5712** | 2709.1086 | 1355.0579 | 2708.1246 | 1354.5659 | 24 |
| 7 | **685.3515** | **343.1794** | **668.3250** | 334.6661 | **667.3410** | 334.1741 | V | 2598.0766 | **1299.5419** | 2581.0500 | 1291.0287 | 2580.0660 | 1290.5367 | 23 |
| 8 | **756.3886** | 378.6980 | **739.3621** | 370.1847 | **738.3781** | 369.6927 | A | 2499.0082 | **1250.0077** | 2481.9816 | **1241.4945** | 2480.9976 | **1241.0024** | 22 |
| 9 | **942.4680** | 471.7376 | 925.4414 | 463.2243 | 924.4574 | 462.7323 | W | 2427.9711 | **1214.4892** | 2410.9445 | **1205.9759** | 2409.9605 | **1205.4839** | 21 |
| 10 | **1070.5265** | 535.7669 | 1053.5000 | 527.2536 | 1052.5160 | 526.7616 | Q | 2241.8917 | **1121.4495** | 2224.8652 | **1112.9362** | 2223.8812 | **1112.4442** | 20 |
| 11 | 1157.5586 | 579.2829 | 1140.5320 | 570.7696 | 1139.5480 | 570.2776 | S | 2113.8332 | 1057.4202 | 2096.8066 | **1048.9069** | 2095.8226 | **1048.4149** | 19 |
| 12 | **1285.6171** | 643.3122 | 1268.5906 | 634.7989 | 1267.6066 | 634.3069 | Q | 2026.8011 | **1013.9042** | 2009.7746 | 1005.3909 | 2008.7906 | 1004.8989 | 18 |
| 13 | 1342.6386 | 671.8229 | 1325.6121 | 663.3097 | 1324.6280 | 662.8177 | G | 1898.7426 | **949.8749** | 1881.7160 | **941.3616** | **1880.7320** | **940.8696** | 17 |
| 14 | **1457.6655** | 729.3364 | 1440.6390 | 720.8231 | 1439.6550 | 720.3311 | D | 1841.7211 | **921.3642** | 1824.6946 | 912.8509 | 1823.7105 | 912.3589 | 16 |
| 15 | **1558.7132** | 779.8603 | **1541.6867** | 771.3470 | **1540.7027** | 770.8550 | T | **1726.6942** | **863.8507** | 1709.6676 | 855.3374 | 1708.6836 | 854.8454 | 15 |
| 16 | 1655.7660 | 828.3866 | 1638.7394 | 819.8734 | 1637.7554 | 819.3814 | P | **1625.6465** | **813.3269** | **1608.6199** | **804.8136** | 1607.6359 | **804.3216** | 14 |
| 17 | **1815.7966** | 908.4020 | **1798.7701** | 899.8887 | **1797.7861** | 899.3967 | C | 1528.5937 | 764.8005 | 1511.5672 | **756.2872** | 1510.5831 | 755.7952 | 13 |
| 18 | 1902.8287 | 951.9180 | 1885.8021 | **943.4047** | 1884.8181 | **942.9127** | S | 1368.5631 | **684.7852** | 1351.5365 | 676.2719 | 1350.5525 | 675.7799 | 12 |

| | | | | | | | | | | | | | | |
|---|---|---|---|---|---|---|---|---|---|---|---|---|---|---|
| **19** | 2062.8593 | 1031.9333 | 2045.8328 | 1023.4200 | 2044.8488 | 1022.9280 | **C** | 1281.5310 | **641.2692** | 1264.5045 | 632.7559 | 1263.5205 | 632.2639 | **11** |
| **20** | 2222.8900 | **1111.9486** | 2205.8634 | 1103.4353 | 2204.8794 | 1102.9433 | **C** | **1121.5004** | 561.2538 | **1104.4738** | **552.7406** | 1103.4898 | **552.2485** | **10** |
| **21** | 2335.9740 | **1168.4907** | 2318.9475 | 1159.9774 | 2317.9635 | 1159.4854 | **I** | 961.4697 | 481.2385 | 944.4432 | **472.7252** | **943.4592** | **472.2332** | **9** |
| **22** | 2435.0424 | 1218.0249 | 2418.0159 | **1209.5116** | 2417.0319 | **1209.0196** | **V** | **848.3857** | 424.6965 | 831.3591 | 416.1832 | 830.3751 | 415.6912 | **8** |
| **23** | 2549.0854 | 1275.0463 | 2532.0588 | 1266.5330 | 2531.0748 | 1266.0410 | **N** | **749.3173** | 375.1623 | 732.2907 | 366.6490 | 731.3067 | 366.1570 | **7** |
| **24** | 2663.1283 | 1332.0678 | 2646.1017 | 1323.5545 | 2645.1177 | 1323.0625 | **N** | **635.2743** | 318.1408 | 618.2478 | 309.6275 | 617.2638 | 309.1355 | **6** |
| **25** | 2750.1603 | **1375.5838** | 2733.1338 | **1367.0705** | 2732.1498 | **1366.5785** | **S** | **521.2314** | 261.1193 | 504.2049 | 252.6061 | **503.2208** | 252.1141 | **5** |
| **26** | 2865.1873 | 1433.0973 | 2848.1607 | 1424.5840 | 2847.1767 | **1424.0920** | **N** | **434.1994** | 217.6033 | **417.1728** | 209.0901 | 416.1888 | 208.5980 | **4** |
| **27** | 2922.2087 | 1461.6080 | 2905.1822 | 1453.0947 | 2904.1982 | 1452.6027 | **G** | **319.1724** | 160.0899 | 302.1459 | 151.5766 | 301.1619 | 151.0846 | **3** |
| **28** | 3009.2408 | **1505.1240** | 2992.2142 | 1496.6107 | 2991.2302 | 1496.1187 | **S** | 262.1510 | 131.5791 | 245.1244 | 123.0659 | 244.1404 | 122.5738 | **2** |
| **29** | | | | | | | **R** | 175.1190 | 88.0631 | 158.0924 | 79.5498 | | | **1** |

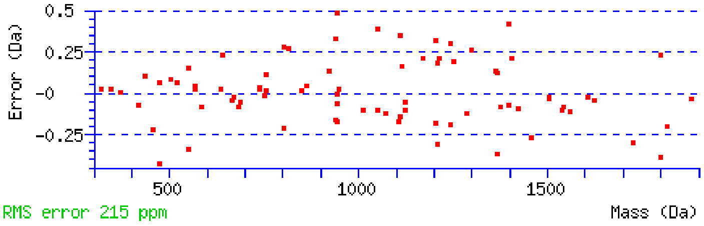


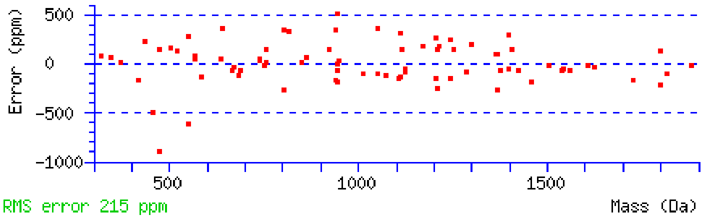


**All matches to this query**

| Score | Mr(calc): | Delta | Sequence |
|---|---|---|---|
| 72.7 | 3182.3452 | 0.0157 | GVDVSQVAWQSQGDTPCSCCIVNNSNGSR |
| 61.7 | 3182.3452 | 0.0157 | GVDVSQVAWQSQGDTPCSCCIVNNSNGSR |
| 60.8 | 3182.3452 | 0.0157 | GVDVSQVAWQSQGDTPCSCCIVNNSNGSR |
| 20.0 | 3181.3612 | 0.9997 | GVDVSQVAWQSQGDTPCSCCIVNNSNGSR |
| 5.2 | 3182.3562 | 0.0047 | SLKEFQENTNRQTVLLQATCLELK |
| 2.3 | 3181.3811 | 0.9798 | QKTNQQIEYLIFVSTGYNQRPLDGK |
| 2.3 | 3181.3811 | 0.9798 | QKTNQQIEYLIFVSTGYNQRPLDGK |
| 2.3 | 3181.3801 | 0.9808 | DEVAHTVTESRVLQNTRHPFLTALK |
| 2.1 | 3182.3742 | -0.0133 | YLVENNANISIANKYDNTCLMIAAYK |
| 2.1 | 3182.3742 | -0.0133 | YLVENNANISIANKYDNTCLMIAAYK |

---

Spectrum No: 325; Query: 2539; Rank: 1

---

## Peptide View

MS/MS Fragmentation of **AFINGTGVETVVSADLPNAHGLAVDWVSR**
Found in **IPI00369995**, Tax_Id=10116 Gene_Symbol=Lrp1 similar to low density lipoprotein receptor-related protein 1

Match to Query 2539: 2996.510232 from(999.844020,3+)
Title: 100101RatKid_NS_deglyco_16.5694.5694.3.dta
Data file K:\NewmanPaper\Piliang\3SubProteomes\Piliang3SP\mgf5ppm\ERLIC_3SubProteomes5ppm.mgf

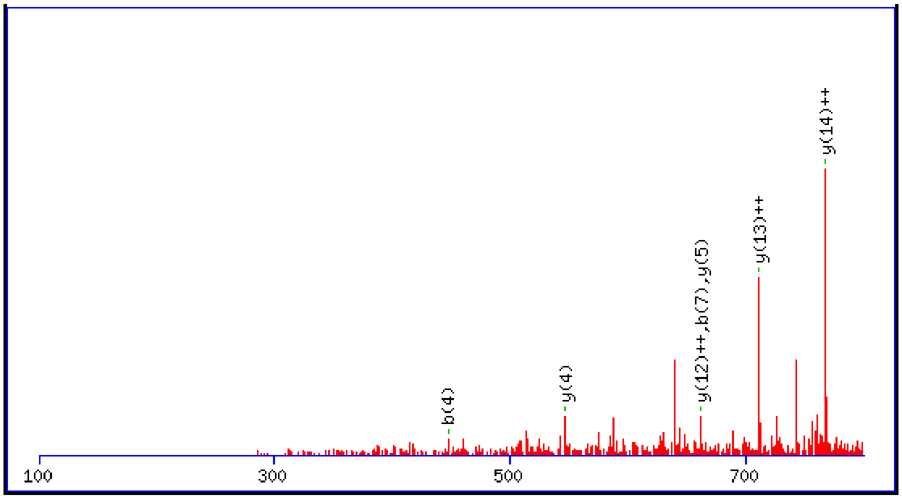


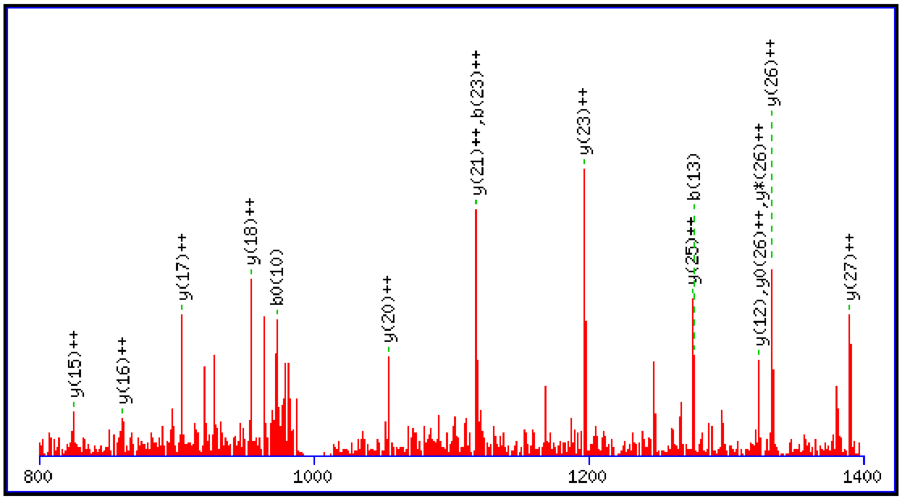


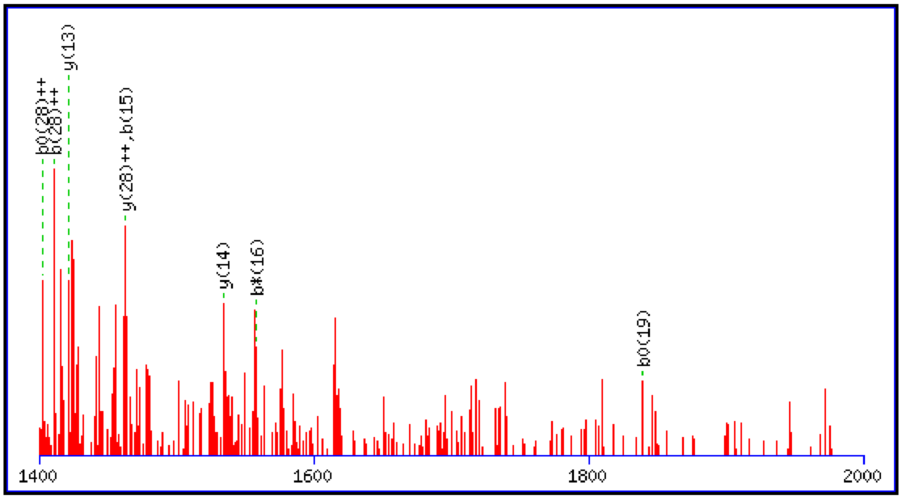


**Monoisotopic mass of neutral peptide Mr(calc):** 2995.5040
**Fixed modifications:** Carbamidomethyl (C)
**Variable modifications:**
**N4** : Deamidated_N (N)
**Ions Score:** 73 **Expect:** 1.1e-005
**Matches (Bold Red):** 31/318 fragment ions using 60 most intense peaks

| # | b | b$^{++}$ | b* | b*$^{++}$ | b$^{0}$ | b$^{0++}$ | Seq. | y | y$^{++}$ | y* | y*$^{++}$ | y$^{0}$ | y$^{0++}$ | # |
|---|---|---|---|---|---|---|---|---|---|---|---|---|---|---|
| 1 | 72.0444 | 36.5258 | | | | | A | | | | | | | 29 |
| 2 | 219.1128 | 110.0600 | | | | | F | 2925.4741 | **1463.2407** | 2908.4476 | 1454.7274 | 2907.4635 | 1454.2354 | 28 |
| 3 | 332.1969 | 166.6021 | | | | | I | 2778.4057 | **1389.7065** | 2761.3791 | 1381.1932 | 2760.3951 | 1380.7012 | 27 |
| 4 | **447.2238** | 224.1155 | 430.1973 | 215.6023 | | | N | 2665.3216 | **1333.1645** | 2648.2951 | **1324.6512** | 2647.3111 | **1324.1592** | 26 |
| 5 | 504.2453 | 252.6263 | 487.2187 | 244.1130 | | | G | 2550.2947 | **1275.6510** | 2533.2681 | 1267.1377 | 2532.2841 | 1266.6457 | 25 |

| | | | | | | | | | | | | | | |
|---|---|---|---|---|---|---|---|---|---|---|---|---|---|---|
| 6 | 605.2929 | 303.1501 | 588.2664 | 294.6368 | 587.2824 | 294.1448 | T | 2493.2732 | 1247.1403 | 2476.2467 | 1238.6270 | 2475.2627 | 1238.1350 | 24 |
| 7 | **662.3144** | 331.6608 | 645.2879 | 323.1476 | 644.3038 | 322.6556 | G | 2392.2255 | **1196.6164** | 2375.1990 | 1188.1031 | 2374.2150 | 1187.6111 | 23 |
| 8 | 761.3828 | 381.1951 | 744.3563 | 372.6818 | 743.3723 | 372.1898 | V | 2335.2041 | 1168.1057 | 2318.1775 | 1159.5924 | 2317.1935 | 1159.1004 | 22 |
| 9 | 890.4254 | 445.7163 | 873.3989 | 437.2031 | 872.4149 | 436.7111 | E | 2236.1357 | **1118.5715** | 2219.1091 | 1110.0582 | 2218.1251 | 1109.5662 | 21 |
| 10 | 991.4731 | 496.2402 | 974.4465 | 487.7269 | **973.4625** | 487.2349 | T | 2107.0931 | **1054.0502** | 2090.0665 | 1045.5369 | 2089.0825 | 1045.0449 | 20 |
| 11 | 1090.5415 | 545.7744 | 1073.5150 | 537.2611 | 1072.5309 | 536.7691 | V | 2006.0454 | 1003.5263 | 1989.0189 | 995.0131 | 1988.0348 | 994.5211 | 19 |
| 12 | 1189.6099 | 595.3086 | 1172.5834 | 586.7953 | 1171.5994 | 586.3033 | V | 1906.9770 | **953.9921** | 1889.9504 | 945.4789 | 1888.9664 | 944.9868 | 18 |
| 13 | **1276.6420** | 638.8246 | 1259.6154 | 630.3113 | 1258.6314 | 629.8193 | S | 1807.9086 | **904.4579** | 1790.8820 | 895.9446 | 1789.8980 | 895.4526 | 17 |
| 14 | 1347.6791 | 674.3432 | 1330.6525 | 665.8299 | 1329.6685 | 665.3379 | A | 1720.8765 | **860.9419** | 1703.8500 | 852.4286 | 1702.8660 | 851.9366 | 16 |
| 15 | **1462.7060** | 731.8566 | 1445.6795 | 723.3434 | 1444.6954 | 722.8514 | D | 1649.8394 | **825.4234** | 1632.8129 | 816.9101 | 1631.8289 | 816.4181 | 15 |
| 16 | 1575.7901 | 788.3987 | **1558.7635** | 779.8854 | 1557.7795 | 779.3934 | L | **1534.8125** | **767.9099** | 1517.7859 | 759.3966 | 1516.8019 | 758.9046 | 14 |
| 17 | 1672.8428 | 836.9251 | 1655.8163 | 828.4118 | 1654.8323 | 827.9198 | P | **1421.7284** | **711.3678** | 1404.7019 | 702.8546 | 1403.7179 | 702.3626 | 13 |
| 18 | 1786.8858 | 893.9465 | 1769.8592 | 885.4332 | 1768.8752 | 884.9412 | N | **1324.6757** | **662.8415** | 1307.6491 | 654.3282 | 1306.6651 | 653.8362 | 12 |
| 19 | 1857.9229 | 929.4651 | 1840.8963 | 920.9518 | **1839.9123** | 920.4598 | A | 1210.6327 | 605.8200 | 1193.6062 | 597.3067 | 1192.6222 | 596.8147 | 11 |
| 20 | 1994.9818 | 997.9945 | 1977.9552 | 989.4813 | 1976.9712 | 988.9893 | H | 1139.5956 | 570.3014 | 1122.5691 | 561.7882 | 1121.5851 | 561.2962 | 10 |
| 21 | 2052.0033 | 1026.5053 | 2034.9767 | 1017.9920 | 2033.9927 | 1017.5000 | G | 1002.5367 | 501.7720 | 985.5102 | 493.2587 | 984.5261 | 492.7667 | 9 |
| 22 | 2165.0873 | 1083.0473 | 2148.0608 | 1074.5340 | 2147.0768 | 1074.0420 | L | 945.5152 | 473.2613 | 928.4887 | 464.7480 | 927.5047 | 464.2560 | 8 |
| 23 | 2236.1244 | **1118.5659** | 2219.0979 | 1110.0526 | 2218.1139 | 1109.5606 | A | 832.4312 | 416.7192 | 815.4046 | 408.2060 | 814.4206 | 407.7139 | 7 |
| 24 | 2335.1928 | 1168.1001 | 2318.1663 | 1159.5868 | 2317.1823 | 1159.0948 | V | 761.3941 | 381.2007 | 744.3675 | 372.6874 | 743.3835 | 372.1954 | 6 |
| 25 | 2450.2198 | 1225.6135 | 2433.1932 | 1217.1003 | 2432.2092 | 1216.6083 | D | **662.3257** | 331.6665 | 645.2991 | 323.1532 | 644.3151 | 322.6612 | 5 |
| 26 | 2636.2991 | 1318.6532 | 2619.2726 | 1310.1399 | 2618.2885 | 1309.6479 | W | **547.2987** | 274.1530 | 530.2722 | 265.6397 | 529.2881 | 265.1477 | 4 |
| 27 | 2735.3675 | 1368.1874 | 2718.3410 | 1359.6741 | 2717.3570 | 1359.1821 | V | 361.2194 | 181.1133 | 344.1928 | 172.6001 | 343.2088 | 172.1081 | 3 |
| 28 | 2822.3995 | **1411.7034** | 2805.3730 | 1403.1901 | 2804.3890 | **1402.6981** | S | 262.1510 | 131.5791 | 245.1244 | 123.0659 | 244.1404 | 122.5738 | 2 |
| 29 | | | | | | | R | 175.1190 | 88.0631 | 158.0924 | 79.5498 | | | 1 |

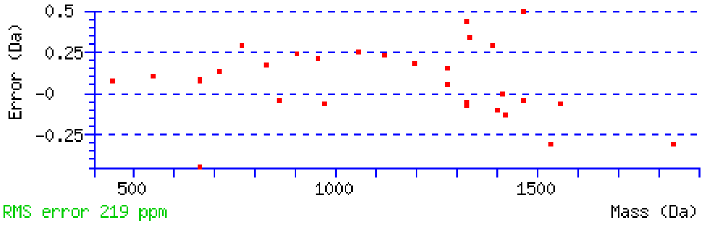


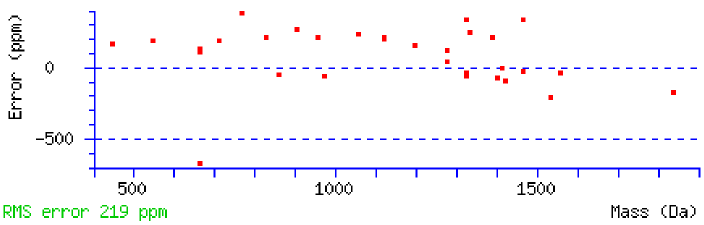


### All matches to this query

| Score | Mr(calc): | Delta | Sequence |
|---|---|---|---|
| 72.7 | 2995.5040 | 1.0063 | AFINGTGVETVVSADLPNAHGLAVDWVSR |
| 49.5 | 2996.4880 | 0.0223 | AFINGTGVETVVSADLPNAHGLAVDWVSR |
| 46.7 | 2995.5040 | 1.0063 | AFINGTGVETVVSADLPNAHGLAVDWVSR |
| 45.8 | 2994.5200 | 1.9903 | AFINGTGVETVVSADLPNAHGLAVDWVSR |
| 6.0 | 2996.5154 | -0.0052 | CIIVGNGGVLANKSLGSRIDDYDIVIR |
| 2.6 | 2994.5144 | 1.9958 | RQVTRATVPTSVQTLGMTPATMTSKPR |
| 1.9 | 2994.5144 | 1.9958 | RQVTRATVPTSVQTLGMTPATMTSKPR |
| 0.1 | 2995.5211 | 0.9891 | AFIVVLGIITAVICPAALCCRDGTLGR |

---

Spectrum No: 326; Query: 1339; Rank: 1

---

## Peptide View

MS/MS Fragmentation of **VGLIGSCTNSSYEDMGR**
Found in **IPI00421539**, Tax_Id=10116 Gene_Symbol=Aco2 Aconitate hydratase, mitochondrial precursor

Match to Query 1339: 1845.790328 from(923.902440,2+)
Title: 091008RatKidney_NoSalt_18.2370.2370.2.dta
Data file K:\NewmanPaper\Piliang\3SubProteomes\Piliang3SP\mgf5ppm\ERLIC_3SubProteomes5ppm.mgf

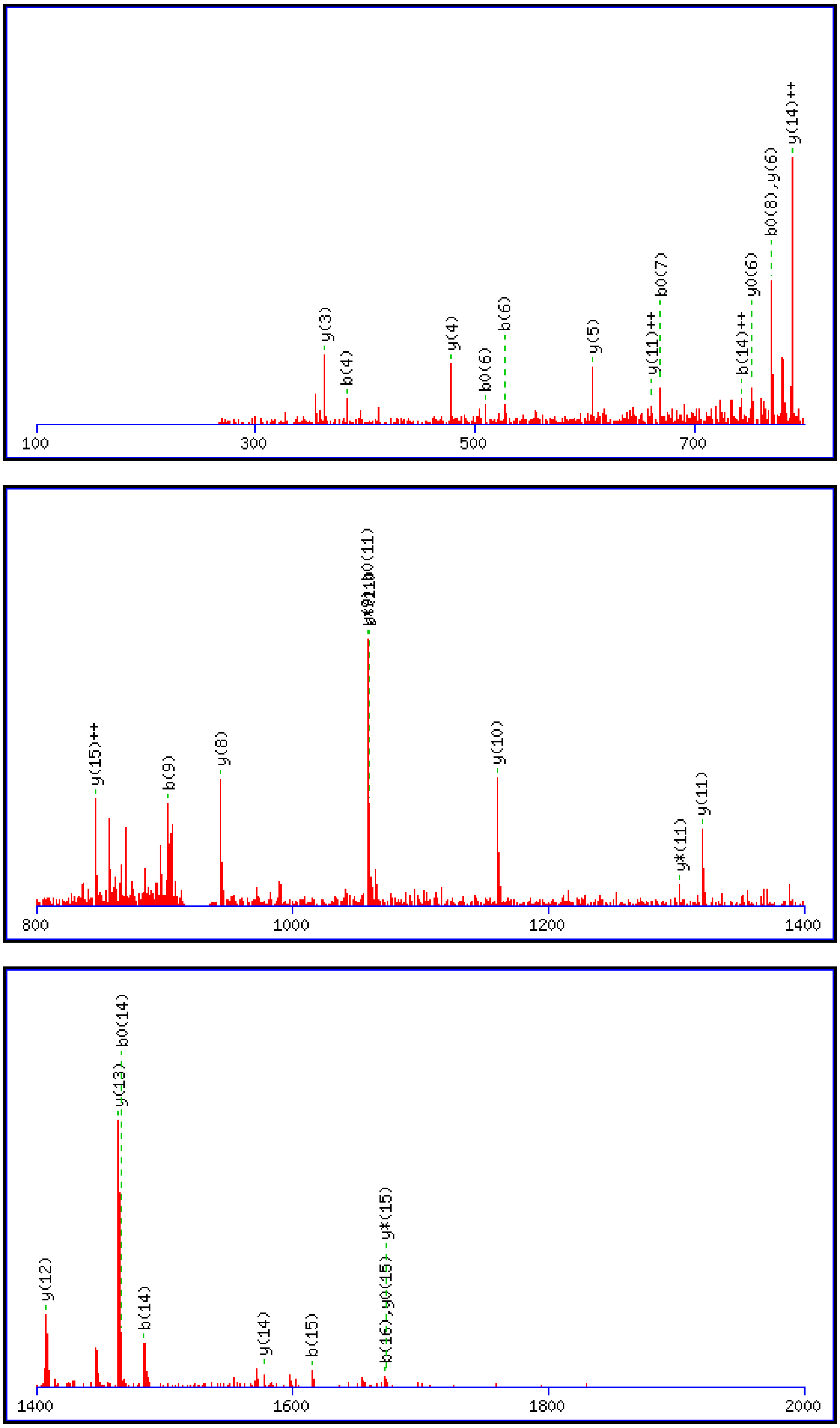


**Monoisotopic mass of neutral peptide Mr(calc):** 1845.7873
**Fixed modifications:** Carbamidomethyl (C)
**Variable modifications:**
**N9** : Deamidated_N (N)
**Ions Score:** 73 **Expect:** 1.1e-005
**Matches (Bold Red):** 31/160 fragment ions using 51 most intense peaks

| # | b | b++ | b* | b*++ | b0 | b0++ | Seq. | y | y++ | y* | y*++ | y0 | y0++ | # |
|---|---|---|---|---|---|---|---|---|---|---|---|---|---|---|
| 1 | 100.0757 | 50.5415 | | | | | V | | | | | | | 17 |
| 2 | 157.0972 | 79.0522 | | | | | G | 1747.7262 | 874.3667 | 1730.6996 | 865.8534 | 1729.7156 | 865.3614 | 16 |

| | | | | | | | | | | | | | | |
|---|---|---|---|---|---|---|---|---|---|---|---|---|---|---|
| 3 | 270.1812 | 135.5942 | | | | | L | 1690.7047 | **845.8560** | **1673.6782** | 837.3427 | **1672.6941** | 836.8507 | 15 |
| 4 | **383.2653** | 192.1363 | | | | | I | **1577.6206** | **789.3140** | 1560.5941 | 780.8007 | 1559.6101 | 780.3087 | 14 |
| 5 | 440.2867 | 220.6470 | | | | | G | **1464.5366** | 732.7719 | 1447.5100 | 724.2587 | 1446.5260 | 723.7666 | 13 |
| 6 | **527.3188** | 264.1630 | | | **509.3082** | 255.1577 | S | **1407.5151** | 704.2612 | 1390.4886 | 695.7479 | 1389.5045 | 695.2559 | 12 |
| 7 | 687.3494 | 344.1783 | | | **669.3389** | 335.1731 | C | **1320.4831** | **660.7452** | **1303.4565** | 652.2319 | 1302.4725 | 651.7399 | 11 |
| 8 | 788.3971 | 394.7022 | | | **770.3865** | 385.6969 | T | **1160.4524** | 580.7299 | 1143.4259 | 572.2166 | 1142.4419 | 571.7246 | 10 |
| 9 | **903.4240** | 452.2157 | 886.3975 | 443.7024 | 885.4135 | 443.2104 | N | **1059.4048** | 530.2060 | 1042.3782 | 521.6927 | 1041.3942 | 521.2007 | 9 |
| 10 | 990.4561 | 495.7317 | 973.4295 | 487.2184 | 972.4455 | 486.7264 | S | **944.3778** | 472.6925 | 927.3513 | 464.1793 | 926.3673 | 463.6873 | 8 |
| 11 | 1077.4881 | 539.2477 | **1060.4615** | 530.7344 | **1059.4775** | 530.2424 | S | 857.3458 | 429.1765 | 840.3192 | 420.6633 | 839.3352 | 420.1713 | 7 |
| 12 | 1240.5514 | 620.7793 | 1223.5249 | 612.2661 | 1222.5409 | 611.7741 | Y | **770.3138** | 385.6605 | 753.2872 | 377.1472 | **752.3032** | 376.6552 | 6 |
| 13 | 1369.5940 | 685.3006 | 1352.5675 | 676.7874 | 1351.5835 | 676.2954 | E | **607.2504** | 304.1289 | 590.2239 | 295.6156 | 589.2399 | 295.1236 | 5 |
| 14 | **1484.6210** | **742.8141** | 1467.5944 | 734.3008 | **1466.6104** | 733.8088 | D | **478.2078** | 239.6076 | 461.1813 | 231.0943 | 460.1973 | 230.6023 | 4 |
| 15 | **1615.6614** | 808.3344 | 1598.6349 | 799.8211 | 1597.6509 | 799.3291 | M | **363.1809** | 182.0941 | 346.1544 | 173.5808 | | | 3 |
| 16 | **1672.6829** | 836.8451 | 1655.6564 | 828.3318 | 1654.6723 | 827.8398 | G | 232.1404 | 116.5738 | 215.1139 | 108.0606 | | | 2 |
| 17 | | | | | | | R | 175.1190 | 88.0631 | 158.0924 | 79.5498 | | | 1 |

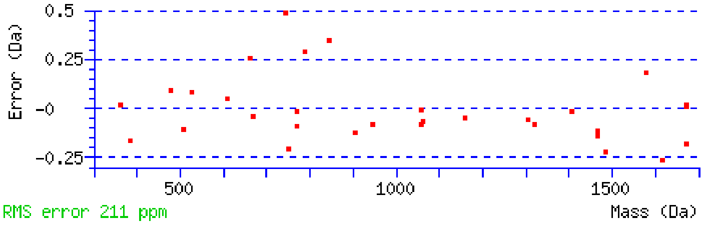


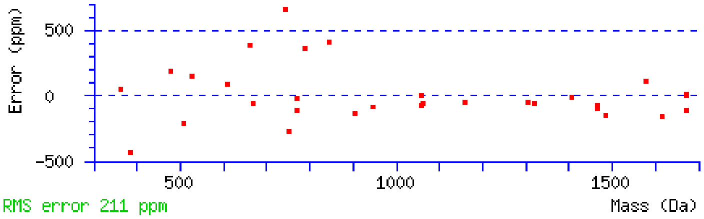


### All matches to this query

| Score | Mr(calc): | Delta | Sequence |
|---|---|---|---|
| 72.7 | 1845.7873 | 0.0030 | VGLIGSCTNSSYEDMGR |
| 7.2 | 1844.8033 | 0.9870 | VGLIGSCTNSSYEDMGR |
| 2.0 | 1844.7859 | 1.0044 | ALLEAGASPNAPNTFGR |
| 1.4 | 1844.7706 | 1.0197 | ANGKPEGKSQEPATSGK |
| 0.2 | 1845.7856 | 0.0048 | QAYTNTPMVDNELLR |

---

Spectrum No: 327; Query: 2652; Rank: 1

---

## Peptide View

MS/MS Fragmentation of **SPLGPHNQTAEQLQTLEQQSISLQQDTER**
Found in **IPI00190577**, Tax_Id=10116 Gene_Symbol=Lama5 similar to Laminin alpha-5 chain precursor

Match to Query 2652: 3276.596262 from(1093.206030,3+)
Title: 100101RatKid_NS_deglyco_19.3885.3885.3.dta
Data file K:\NewmanPaper\Piliang\3SubProteomes\Piliang3SP\mgf5ppm\ERLIC_3SubProteomes5ppm.mgf

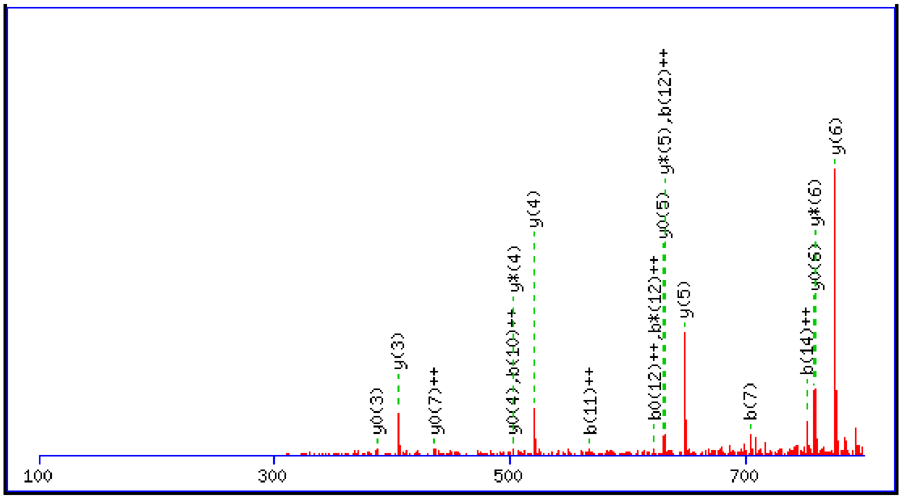


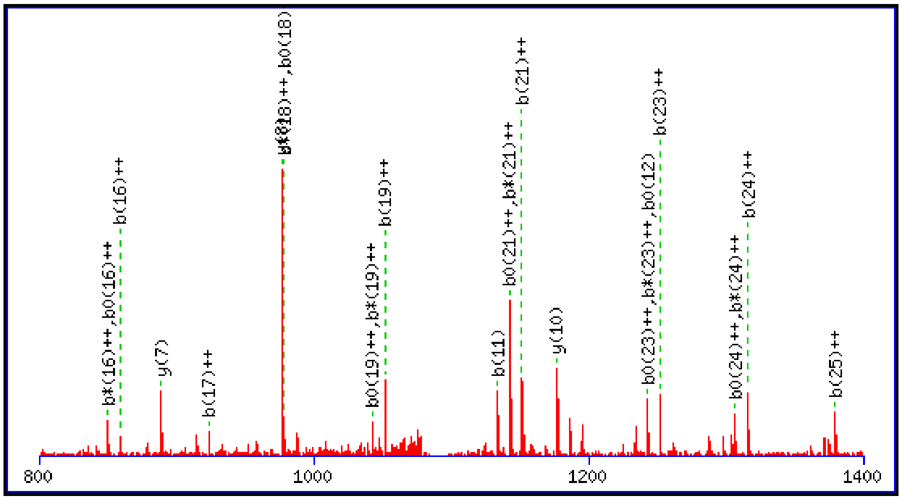


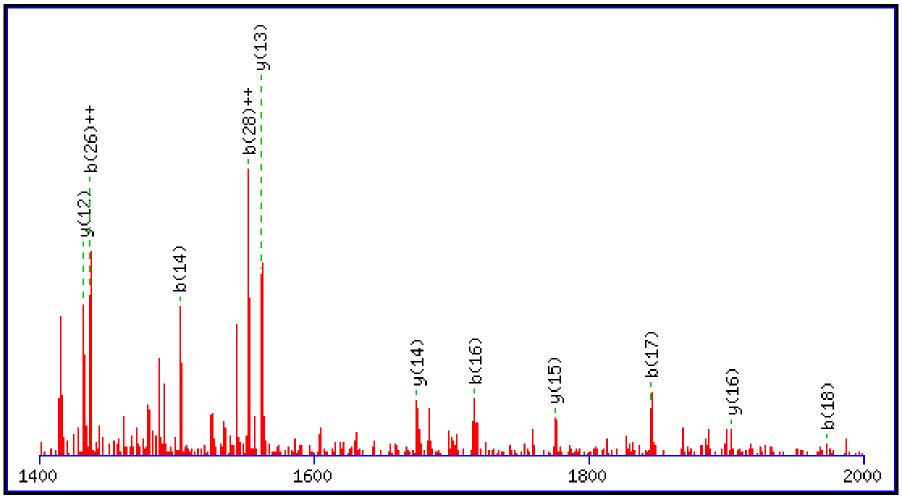


**Monoisotopic mass of neutral peptide Mr(calc):** 3276.5858
**Fixed modifications:** Carbamidomethyl (C)
**Variable modifications:**
**N7** : Deamidated_N (N)
**Ions Score:** 73 **Expect:** 1.7e-005
**Matches (Bold Red):** 54/322 fragment ions using 73 most intense peaks

| # | b | b++ | b* | b*++ | b0 | b0++ | Seq. | y | y++ | y* | y*++ | y0 | y0++ | # |
|---|---|---|---|---|---|---|---|---|---|---|---|---|---|---|
| 1 | 88.0393 | 44.5233 | | | 70.0287 | 35.5180 | S | | | | | | | 29 |
| 2 | 185.0921 | 93.0497 | | | 167.0815 | 84.0444 | P | 3190.5611 | 1595.7842 | 3173.5345 | 1587.2709 | 3172.5505 | 1586.7789 | 28 |
| 3 | 298.1761 | 149.5917 | | | 280.1656 | 140.5864 | L | 3093.5083 | 1547.2578 | 3076.4818 | 1538.7445 | 3075.4978 | 1538.2525 | 27 |
| 4 | 355.1976 | 178.1024 | | | 337.1870 | 169.0972 | G | 2980.4243 | 1490.7158 | 2963.3977 | 1482.2025 | 2962.4137 | 1481.7105 | 26 |
| 5 | 452.2504 | 226.6288 | | | 434.2398 | 217.6235 | P | 2923.4028 | 1462.2050 | 2906.3762 | 1453.6918 | 2905.3922 | 1453.1998 | 25 |

| | | | | | | | | | | | | | | |
|---|---|---|---|---|---|---|---|---|---|---|---|---|---|---|
| 6 | 589.3093 | 295.1583 | | | 571.2987 | 286.1530 | H | 2826.3500 | 1413.6787 | 2809.3235 | 1405.1654 | 2808.3395 | 1404.6734 | 24 |
| 7 | **704.3362** | 352.6717 | 687.3097 | 344.1585 | 686.3256 | 343.6665 | N | 2689.2911 | 1345.1492 | 2672.2646 | 1336.6359 | 2671.2806 | 1336.1439 | 23 |
| 8 | 832.3948 | 416.7010 | 815.3682 | 408.1878 | 814.3842 | 407.6957 | Q | 2574.2642 | 1287.6357 | 2557.2376 | 1279.1225 | 2556.2536 | 1278.6304 | 22 |
| 9 | 933.4425 | 467.2249 | 916.4159 | 458.7116 | 915.4319 | 458.2196 | T | 2446.2056 | 1223.6064 | 2429.1791 | 1215.0932 | 2428.1950 | 1214.6012 | 21 |
| 10 | 1004.4796 | **502.7434** | 987.4530 | 494.2302 | 986.4690 | 493.7381 | A | 2345.1579 | 1173.0826 | 2328.1314 | 1164.5693 | 2327.1474 | 1164.0773 | 20 |
| 11 | **1133.5222** | **567.2647** | 1116.4956 | 558.7514 | 1115.5116 | 558.2594 | E | 2274.1208 | 1137.5640 | 2257.0943 | 1129.0508 | 2256.1102 | 1128.5588 | 19 |
| 12 | 1261.5807 | **631.2940** | 1244.5542 | **622.7807** | **1243.5702** | **622.2887** | Q | 2145.0782 | 1073.0427 | 2128.0517 | 1064.5295 | 2127.0677 | 1064.0375 | 18 |
| 13 | 1374.6648 | 687.8360 | 1357.6383 | 679.3228 | 1356.6542 | 678.8308 | L | 2017.0196 | 1009.0135 | 1999.9931 | 1000.5002 | 1999.0091 | 1000.0082 | 17 |
| 14 | **1502.7234** | **751.8653** | 1485.6968 | 743.3521 | 1484.7128 | 742.8601 | Q | **1903.9356** | 952.4714 | 1886.9090 | 943.9582 | 1885.9250 | 943.4661 | 16 |
| 15 | 1603.7711 | 802.3892 | 1586.7445 | 793.8759 | 1585.7605 | 793.3839 | T | **1775.8770** | 888.4421 | 1758.8505 | 879.9289 | 1757.8664 | 879.4369 | 15 |
| 16 | **1716.8551** | **858.9312** | 1699.8286 | **850.4179** | 1698.8446 | **849.9259** | L | **1674.8293** | 837.9183 | 1657.8028 | 829.4050 | 1656.8188 | 828.9130 | 14 |
| 17 | **1845.8977** | **923.4525** | 1828.8712 | 914.9392 | 1827.8872 | 914.4472 | E | **1561.7453** | 781.3763 | 1544.7187 | 772.8630 | 1543.7347 | 772.3710 | 13 |
| 18 | **1973.9563** | 987.4818 | 1956.9298 | **978.9685** | 1955.9457 | **978.4765** | Q | **1432.7027** | 716.8550 | 1415.6761 | 708.3417 | 1414.6921 | 707.8497 | 12 |
| 19 | 2102.0149 | **1051.5111** | 2084.9883 | **1042.9978** | 2084.0043 | **1042.5058** | Q | 1304.6441 | 652.8257 | 1287.6175 | 644.3124 | 1286.6335 | 643.8204 | 11 |
| 20 | 2189.0469 | 1095.0271 | 2172.0204 | 1086.5138 | 2171.0363 | 1086.0218 | S | **1176.5855** | 588.7964 | 1159.5590 | 580.2831 | 1158.5749 | 579.7911 | 10 |
| 21 | 2302.1310 | **1151.5691** | 2285.1044 | **1143.0559** | 2284.1204 | **1142.5638** | I | 1089.5535 | 545.2804 | 1072.5269 | 536.7671 | 1071.5429 | 536.2751 | 9 |
| 22 | 2389.1630 | 1195.0851 | 2372.1365 | 1186.5719 | 2371.1524 | 1186.0799 | S | **976.4694** | 488.7383 | 959.4429 | 480.2251 | 958.4589 | 479.7331 | 8 |
| 23 | 2502.2471 | **1251.6272** | 2485.2205 | **1243.1139** | 2484.2365 | **1242.6219** | L | **889.4374** | 445.2223 | 872.4108 | 436.7091 | 871.4268 | **436.2170** | 7 |
| 24 | 2630.3056 | **1315.6565** | 2613.2791 | **1307.1432** | 2612.2951 | **1306.6512** | Q | **776.3533** | 388.6803 | **759.3268** | 380.1670 | **758.3428** | 379.6750 | 6 |
| 25 | 2758.3642 | **1379.6858** | 2741.3377 | 1371.1725 | 2740.3537 | 1370.6805 | Q | **648.2947** | 324.6510 | **631.2682** | 316.1377 | **630.2842** | 315.6457 | 5 |
| 26 | 2873.3912 | **1437.1992** | 2856.3646 | 1428.6859 | 2855.3806 | 1428.1939 | D | **520.2362** | 260.6217 | **503.2096** | 252.1084 | **502.2256** | 251.6164 | 4 |
| 27 | 2974.4388 | 1487.7231 | 2957.4123 | 1479.2098 | 2956.4283 | 1478.7178 | T | **405.2092** | 203.1082 | 388.1827 | 194.5950 | **387.1987** | 194.1030 | 3 |
| 28 | 3103.4814 | **1552.2444** | 3086.4549 | 1543.7311 | 3085.4709 | 1543.2391 | E | 304.1615 | 152.5844 | 287.1350 | 144.0711 | 286.1510 | 143.5791 | 2 |
| 29 | | | | | | | R | 175.1190 | 88.0631 | 158.0924 | 79.5498 | | | 1 |

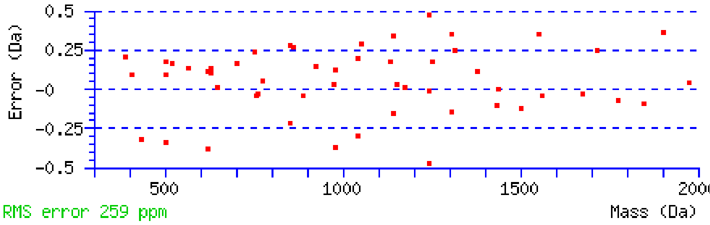


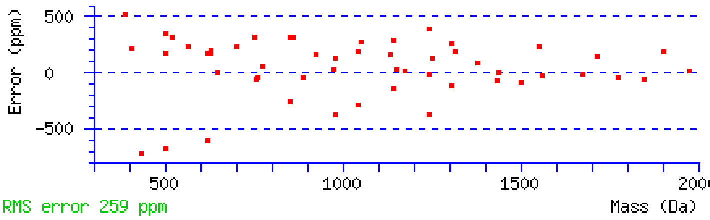


### All matches to this query

| Score | Mr(calc): | Delta | Sequence |
|---|---|---|---|
| 72.6 | 3276.5858 | 0.0104 | SPLGPHNQTAEQLQTLEQQSISLQQDTER |
| 28.8 | 3275.6018 | 0.9945 | SPLGPHNQTAEQLQTLEQQSISLQQDTER |
| 4.0 | 3276.5730 | 0.0233 | MRVCTKWVLLSHWLVLAYMLMVCCK |
| 3.0 | 3276.6059 | -0.0096 | GALGATPQLMAVFGSLSLYALGNSLLLPWR |
| 2.5 | 3276.5944 | 0.0019 | FHIHLYNPDSLIACVLPYHETRVFVR |
| 1.6 | 3274.5883 | 2.0080 | AELQDMCDNPELTALQIALAECQEKLKK |
| 1.4 | 3276.5808 | 0.0155 | YLQKSSISLAAVLPGWLPPPPTPGSPCAGR |
| 1.4 | 3276.5808 | 0.0155 | YLQKSSISLAAVLPGWLPPPPTPGSPCAGR |
| 1.4 | 3276.5808 | 0.0155 | YLQKSSISLAAVLPGWLPPPPTPGSPCAGR |
| 1.0 | 3275.5797 | 1.0166 | GSPTPSGTRPTAGQAAPPSQQPPKPQEQSRR |

---

Spectrum No: 328; Query: 608; Rank: 1

---

## Peptide View

MS/MS Fragmentation of **FVNSTGYLTEAEK**
Found in **IPI00358387**, Tax_Id=10116 Gene_Symbol=Sumf1_predicted sulfatase modifying factor 1

Match to Query 608: 1458.685708 from(730.350130,2+)
Title: 091008RatKidney_NH4Format01_24.2004.2004.2.dta
Data file K:\NewmanPaper\Piliang\3SubProteomes\Piliang3SP\mgf5ppm\ERLIC_3SubProteomes5ppm.mgf

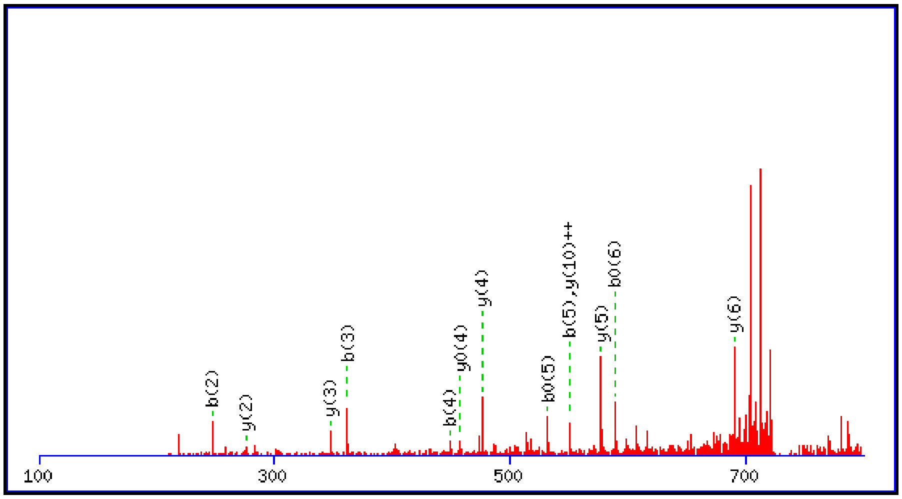


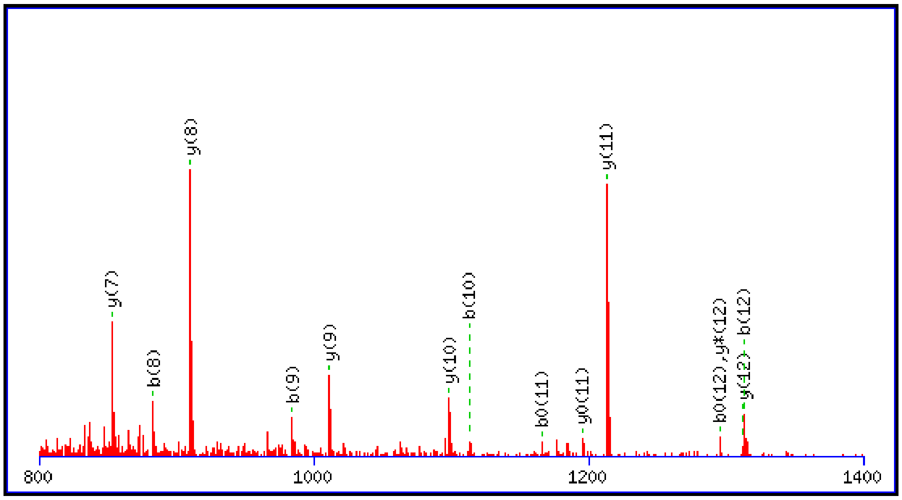


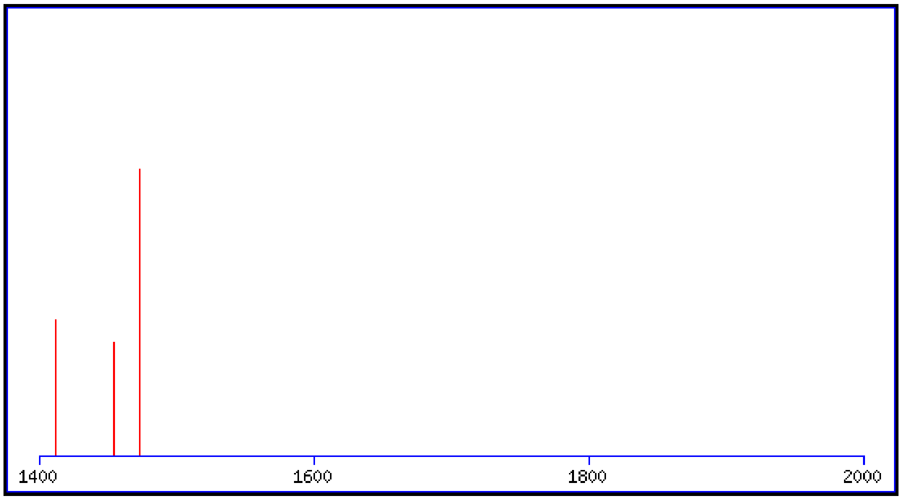


**Monoisotopic mass of neutral peptide Mr(calc):** 1458.6878
**Fixed modifications:** Carbamidomethyl (C)
**Variable modifications:**
**N3** : Deamidated_N (N)
**Ions Score:** 72 **Expect:** 9.3e-006
**Matches (Bold Red):** 27/132 fragment ions using 66 most intense peaks

| # | b | $b^{++}$ | b* | $b^{*++}$ | $b^0$ | $b^{0++}$ | Seq. | y | $y^{++}$ | y* | $y^{*++}$ | $y^0$ | $y^{0++}$ | # |
|---|---|---|---|---|---|---|---|---|---|---|---|---|---|---|

| | | | | | | | | | | | | | | |
|---|---|---|---|---|---|---|---|---|---|---|---|---|---|---|
| 1 | 148.0757 | 74.5415 | | | | | F | | | | | | | 13 |
| 2 | 247.1441 | 124.0757 | | | | | V | 1312.6267 | 656.8170 | 1295.6001 | 648.3037 | 1294.6161 | 647.8117 | 12 |
| 3 | 362.1710 | 181.5892 | 345.1445 | 173.0759 | | | N | 1213.5583 | 607.2828 | 1196.5317 | 598.7695 | 1195.5477 | 598.2775 | 11 |
| 4 | 449.2031 | 225.1052 | 432.1765 | 216.5919 | 431.1925 | 216.0999 | S | 1098.5313 | 549.7693 | 1081.5048 | 541.2560 | 1080.5208 | 540.7640 | 10 |
| 5 | 550.2507 | 275.6290 | 533.2242 | 267.1157 | 532.2402 | 266.6237 | T | 1011.4993 | 506.2533 | 994.4728 | 497.7400 | 993.4888 | 497.2480 | 9 |
| 6 | 607.2722 | 304.1397 | 590.2457 | 295.6265 | 589.2616 | 295.1345 | G | 910.4516 | 455.7295 | 893.4251 | 447.2162 | 892.4411 | 446.7242 | 8 |
| 7 | 770.3355 | 385.6714 | 753.3090 | 377.1581 | 752.3250 | 376.6661 | Y | 853.4302 | 427.2187 | 836.4036 | 418.7055 | 835.4196 | 418.2134 | 7 |
| 8 | 883.4196 | 442.2134 | 866.3931 | 433.7002 | 865.4090 | 433.2082 | L | 690.3668 | 345.6871 | 673.3403 | 337.1738 | 672.3563 | 336.6818 | 6 |
| 9 | 984.4673 | 492.7373 | 967.4407 | 484.2240 | 966.4567 | 483.7320 | T | 577.2828 | 289.1450 | 560.2562 | 280.6318 | 559.2722 | 280.1397 | 5 |
| 10 | 1113.5099 | 557.2586 | 1096.4833 | 548.7453 | 1095.4993 | 548.2533 | E | 476.2351 | 238.6212 | 459.2086 | 230.1079 | 458.2245 | 229.6159 | 4 |
| 11 | 1184.5470 | 592.7771 | 1167.5204 | 584.2639 | 1166.5364 | 583.7719 | A | 347.1925 | 174.0999 | 330.1660 | 165.5866 | 329.1819 | 165.0946 | 3 |
| 12 | 1313.5896 | 657.2984 | 1296.5630 | 648.7852 | 1295.5790 | 648.2931 | E | 276.1554 | 138.5813 | 259.1288 | 130.0681 | 258.1448 | 129.5761 | 2 |
| 13 | | | | | | | K | 147.1128 | 74.0600 | 130.0863 | 65.5468 | | | 1 |

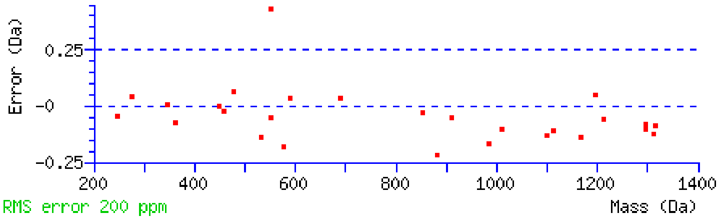


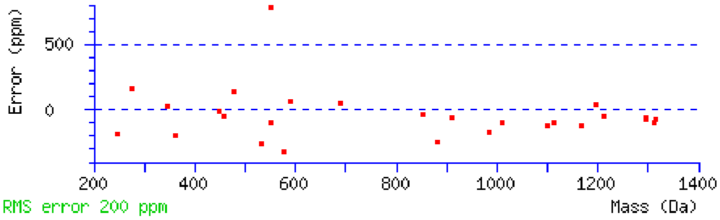


### All matches to this query

| Score | Mr(calc): | Delta | Sequence |
|---|---|---|---|
| 72.5 | 1458.6878 | -0.0021 | FVNSTGYLTEAEK |
| 7.1 | 1458.6990 | -0.0133 | EYRKDYEESIK |
| 6.1 | 1458.6891 | -0.0034 | FNVRNGYGFINR |
| 5.9 | 1456.6671 | 2.0186 | RKQQDGGTTGTTK |
| 5.7 | 1458.6878 | -0.0021 | FEAEYVTEKSEK |
| 3.6 | 1457.6915 | 0.9942 | KYAPSANVTTAQK |
| 3.3 | 1458.6812 | 0.0046 | REGGGGGGGGEGGLQSK |
| 2.7 | 1456.6851 | 2.0006 | FINLQPSTLTDK |
| 0.5 | 1457.6803 | 1.0054 | FDEAALSIQKEK |

---

Spectrum No: 329; Query: 2536; Rank: 1

---

## Peptide View

MS/MS Fragmentation of **LQAALDNEAGGRPAMEPGNGSLDLGGDAAGR**
Found in **IPI00210090**, Tax_Id=10116 Gene_Symbol=Hnrpu SP120

Match to Query 2536: 2996.383962 from(999.801930,3+)
Title: 091008RatKidney_NH4Format02_24.2597.2597.3.dta
Data file K:\NewmanPaper\Piliang\3SubProteomes\Piliang3SP\mgf5ppm\ERLIC_3SubProteomes5ppm.mgf

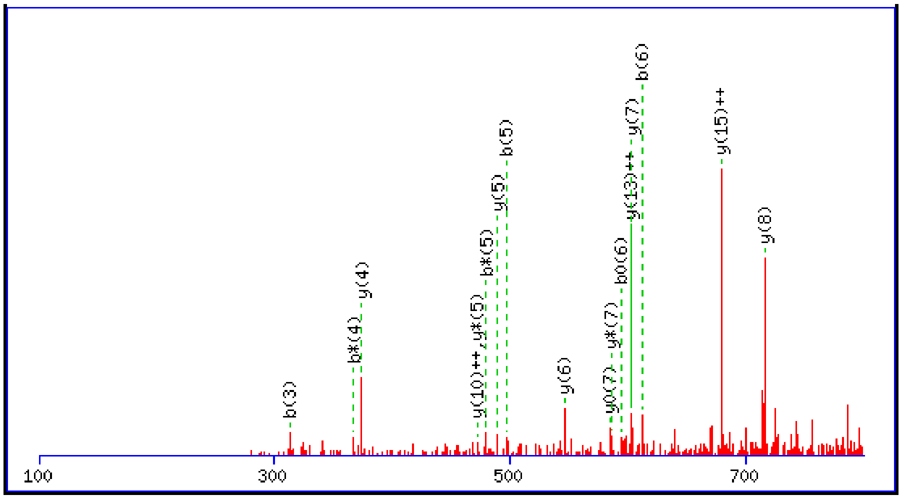


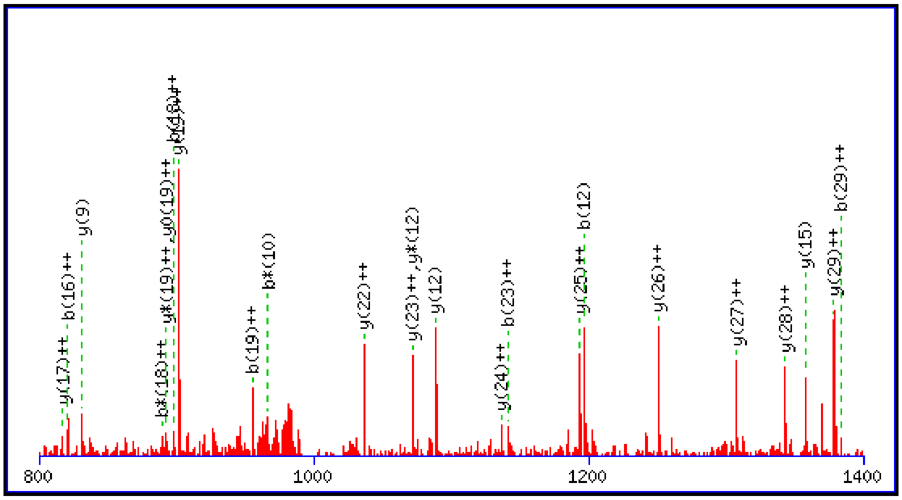


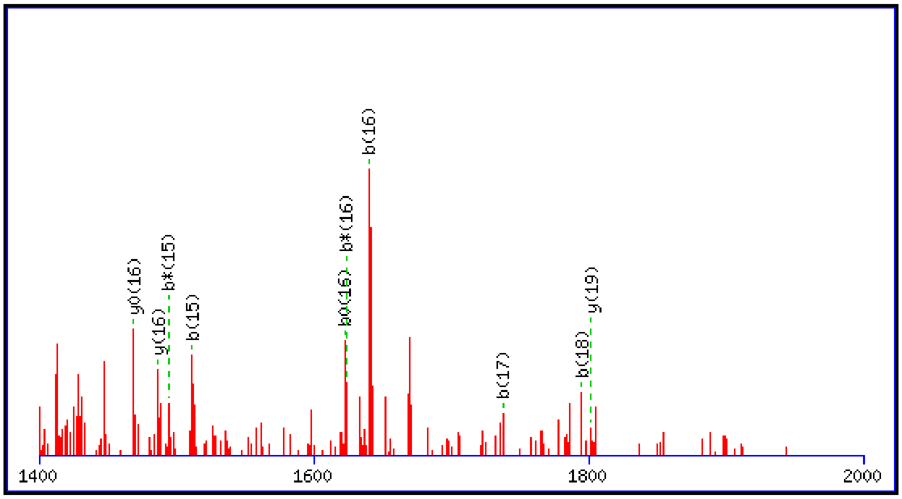


**Monoisotopic mass of neutral peptide Mr(calc):** 2996.3894
**Fixed modifications:** Carbamidomethyl (C)
**Variable modifications:**
**M15** : Oxidation (M)
**N19** : Deamidated_N (N)
**Ions Score:** 72 **Expect:** 2.1e-005
**Matches (Bold Red):** 51/340 fragment ions using 97 most intense peaks

| # | b | $b^{++}$ | b* | $b^{*++}$ | $b^0$ | $b^{0++}$ | Seq. | y | $y^{++}$ | y* | $y^{*++}$ | $y^0$ | $y^{0++}$ | # |
|---|---|---|---|---|---|---|---|---|---|---|---|---|---|---|
| 1 | 114.0913 | 57.5493 | | | | | L | | | | | | | 31 |
| 2 | 242.1499 | 121.5786 | 225.1234 | 113.0653 | | | Q | 2884.3126 | 1442.6599 | 2867.2861 | 1434.1467 | 2866.3020 | 1433.6547 | 30 |
| 3 | **313.1870** | 157.0972 | 296.1605 | 148.5839 | | | A | 2756.2540 | **1378.6307** | 2739.2275 | 1370.1174 | 2738.2435 | 1369.6254 | 29 |
| 4 | 384.2241 | 192.6157 | **367.1976** | 184.1024 | | | A | 2685.2169 | **1343.1121** | 2668.1904 | 1334.5988 | 2667.2063 | 1334.1068 | 28 |

| | | | | | | | | | | | | | | |
|---|---|---|---|---|---|---|---|---|---|---|---|---|---|---|
| 5 | **497.3082** | 249.1577 | **480.2817** | 240.6445 | | | L | 2614.1798 | **1307.5935** | 2597.1532 | 1299.0803 | 2596.1692 | 1298.5883 | 27 |
| 6 | **612.3352** | 306.6712 | 595.3086 | 298.1579 | **594.3246** | 297.6659 | D | 2501.0957 | **1251.0515** | 2484.0692 | 1242.5382 | 2483.0852 | 1242.0462 | 26 |
| 7 | 726.3781 | 363.6927 | 709.3515 | 355.1794 | 708.3675 | 354.6874 | N | 2386.0688 | **1193.5380** | 2369.0422 | 1185.0248 | 2368.0582 | 1184.5328 | 25 |
| 8 | 855.4207 | 428.2140 | 838.3941 | 419.7007 | 837.4101 | 419.2087 | E | 2272.0259 | **1136.5166** | 2254.9993 | 1128.0033 | 2254.0153 | 1127.5113 | 24 |
| 9 | 926.4578 | 463.7325 | 909.4312 | 455.2193 | 908.4472 | 454.7272 | A | 2142.9833 | **1071.9953** | 2125.9567 | 1063.4820 | 2124.9727 | 1062.9900 | 23 |
| 10 | 983.4793 | 492.2433 | **966.4527** | 483.7300 | 965.4687 | 483.2380 | G | 2071.9462 | **1036.4767** | 2054.9196 | 1027.9634 | 2053.9356 | 1027.4714 | 22 |
| 11 | 1040.5007 | 520.7540 | 1023.4742 | 512.2407 | 1022.4901 | 511.7487 | G | 2014.9247 | 1007.9660 | 1997.8981 | 999.4527 | 1996.9141 | 998.9607 | 21 |
| 12 | **1196.6018** | 598.8046 | 1179.5753 | 590.2913 | 1178.5913 | 589.7993 | R | 1957.9032 | 979.4553 | 1940.8767 | 970.9420 | 1939.8927 | 970.4500 | 20 |
| 13 | 1293.6546 | 647.3309 | 1276.6280 | 638.8177 | 1275.6440 | 638.3257 | P | **1801.8021** | **901.4047** | 1784.7756 | **892.8914** | 1783.7916 | **892.3994** | 19 |
| 14 | 1364.6917 | 682.8495 | 1347.6652 | 674.3362 | 1346.6811 | 673.8442 | A | 1704.7494 | 852.8783 | 1687.7228 | 844.3650 | 1686.7388 | 843.8730 | 18 |
| 15 | **1511.7271** | 756.3672 | **1494.7006** | 747.8539 | 1493.7165 | 747.3619 | M | 1633.7122 | **817.3598** | 1616.6857 | 808.8465 | 1615.7017 | 808.3545 | 17 |
| 16 | **1640.7697** | **820.8885** | **1623.7432** | 812.3752 | **1622.7591** | 811.8832 | E | **1486.6768** | 743.8421 | 1469.6503 | 735.3288 | **1468.6663** | 734.8368 | 16 |
| 17 | **1737.8225** | 869.4149 | 1720.7959 | 860.9016 | 1719.8119 | 860.4096 | P | **1357.6342** | **679.3208** | 1340.6077 | 670.8075 | 1339.6237 | 670.3155 | 15 |
| 18 | **1794.8439** | **897.9256** | 1777.8174 | **889.4123** | 1776.8334 | 888.9203 | G | 1260.5815 | 630.7944 | 1243.5549 | 622.2811 | 1242.5709 | 621.7891 | 14 |
| 19 | 1909.8709 | **955.4391** | 1892.8443 | 946.9258 | 1891.8603 | 946.4338 | N | 1203.5600 | **602.2836** | 1186.5335 | 593.7704 | 1185.5494 | 593.2784 | 13 |
| 20 | 1966.8923 | 983.9498 | 1949.8658 | 975.4365 | 1948.8818 | 974.9445 | G | **1088.5331** | 544.7702 | **1071.5065** | 536.2569 | 1070.5225 | 535.7649 | 12 |
| 21 | 2053.9244 | 1027.4658 | 2036.8978 | 1018.9525 | 2035.9138 | 1018.4605 | S | 1031.5116 | 516.2594 | 1014.4851 | 507.7462 | 1013.5010 | 507.2542 | 11 |
| 22 | 2167.0084 | 1084.0078 | 2149.9819 | 1075.4946 | 2148.9979 | 1075.0026 | L | 944.4796 | **472.7434** | 927.4530 | 464.2302 | 926.4690 | 463.7381 | 10 |
| 23 | 2282.0354 | **1141.5213** | 2265.0088 | 1133.0080 | 2264.0248 | 1132.5160 | D | **831.3955** | 416.2014 | 814.3690 | 407.6881 | 813.3850 | 407.1961 | 9 |
| 24 | 2395.1194 | 1198.0634 | 2378.0929 | 1189.5501 | 2377.1089 | 1189.0581 | L | **716.3686** | 358.6879 | 699.3420 | 350.1747 | 698.3580 | 349.6826 | 8 |
| 25 | 2452.1409 | 1226.5741 | 2435.1143 | 1218.0608 | 2434.1303 | 1217.5688 | G | **603.2845** | 302.1459 | **586.2580** | 293.6326 | **585.2740** | 293.1406 | 7 |
| 26 | 2509.1624 | 1255.0848 | 2492.1358 | 1246.5715 | 2491.1518 | 1246.0795 | G | **546.2631** | 273.6352 | 529.2365 | 265.1219 | 528.2525 | 264.6299 | 6 |
| 27 | 2624.1893 | 1312.5983 | 2607.1627 | 1304.0850 | 2606.1787 | 1303.5930 | D | **489.2416** | 245.1244 | **472.2150** | 236.6112 | 471.2310 | 236.1191 | 5 |
| 28 | 2695.2264 | 1348.1168 | 2678.1999 | 1339.6036 | 2677.2158 | 1339.1116 | A | **374.2146** | 187.6110 | 357.1881 | 179.0977 | | | 4 |
| 29 | 2766.2635 | **1383.6354** | 2749.2370 | 1375.1221 | 2748.2530 | 1374.6301 | A | 303.1775 | 152.0924 | 286.1510 | 143.5791 | | | 3 |
| 30 | 2823.2850 | 1412.1461 | 2806.2584 | 1403.6329 | 2805.2744 | 1403.1409 | G | 232.1404 | 116.5738 | 215.1139 | 108.0606 | | | 2 |
| 31 | | | | | | | R | 175.1190 | 88.0631 | 158.0924 | 79.5498 | | | 1 |

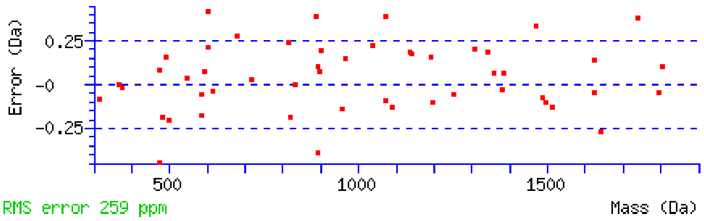


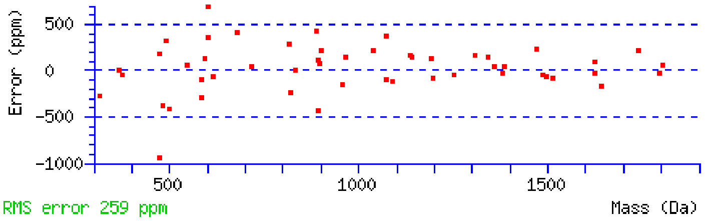


### All matches to this query

| Score | Mr(calc): | Delta | Sequence |
|---|---|---|---|
| 72.4 | 2996.3894 | -0.0054 | LQAALDNEAGGRPAMEPGNGSLDLGGDAAGR |
| 31.1 | 2996.3894 | -0.0054 | LQAALDNEAGGRPAMEPGNGSLDLGGDAAGR |
| 27.9 | 2995.4054 | 0.9786 | LQAALDNEAGGRPAMEPGNGSLDLGGDAAGR |
| 2.4 | 2996.4042 | -0.0203 | SFNQSSHLSHVQAHERIHTGEKPYK |

---

Spectrum No: 330; Query: 115; Rank: 1

---

## Peptide View

MS/MS Fragmentation of **VLTLANFTTK**
Found in **IPI00188956**, Tax_Id=10116 Gene_Symbol=Thy1 Thy-1 membrane glycoprotein precursor

Match to Query 115: 1107.619228 from(554.816890,2+)

Title: 100101RatKid_NS_deglyco_20.3455.3455.2.dta
Data file K:\NewmanPaper\Piliang\3SubProteomes\Piliang3SP\mgf5ppm\ERLIC_3SubProteomes5ppm.mgf

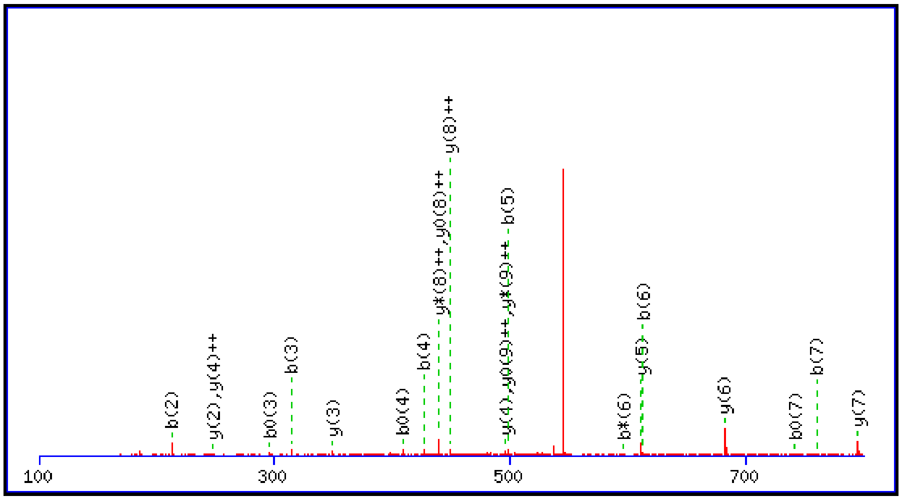


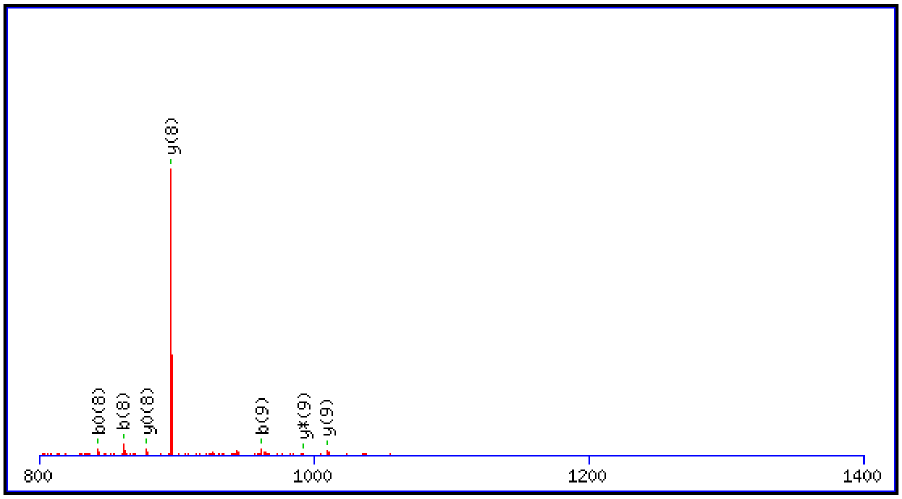


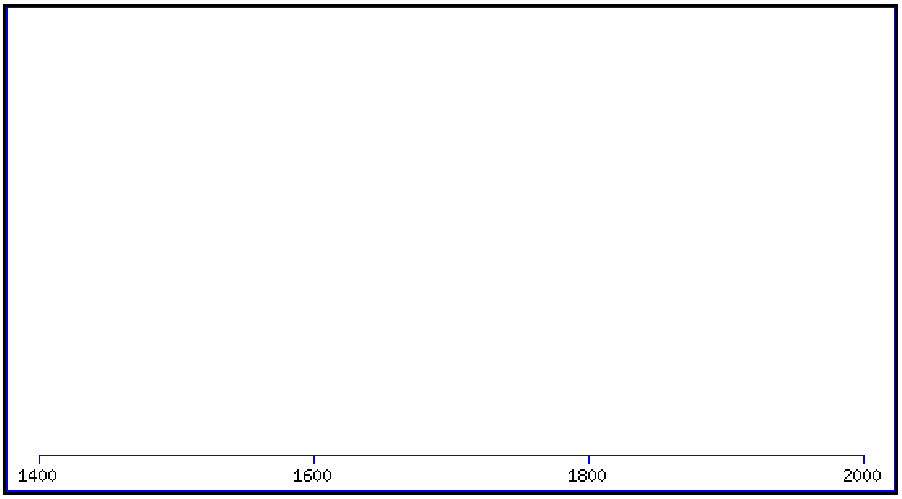


**Monoisotopic mass of neutral peptide Mr(calc):** 1107.6176
**Fixed modifications:** Carbamidomethyl (C)
**Variable modifications:**
**N6** : Deamidated_N (N)
**Ions Score:** 72 **Expect:** 2.9e-006
**Matches (Bold Red):** 29/92 fragment ions using 40 most intense peaks

| # | b | $b^{++}$ | b* | $b^{*++}$ | $b^0$ | $b^{0++}$ | Seq. | y | $y^{++}$ | y* | $y^{*++}$ | $y^0$ | $y^{0++}$ | # |
|---|---|---|---|---|---|---|---|---|---|---|---|---|---|---|
| 1 | 100.0757 | 50.5415 | | | | | V | | | | | | | 10 |
| 2 | **213.1598** | 107.0835 | | | | | L | **1009.5564** | 505.2819 | **992.5299** | **496.7686** | 991.5459 | **496.2766** | 9 |
| 3 | **314.2074** | 157.6074 | | | **296.1969** | 148.6021 | T | **896.4724** | **448.7398** | 879.4458 | **440.2265** | **878.4618** | **439.7345** | 8 |

| | | | | | | | | | | | | | | |
|---|---|---|---|---|---|---|---|---|---|---|---|---|---|---|
| **4** | **427.2915** | 214.1494 | | | **409.2809** | 205.1441 | **L** | **795.4247** | 398.2160 | 778.3981 | 389.7027 | 777.4141 | 389.2107 | **7** |
| **5** | **498.3286** | 249.6679 | | | 480.3180 | 240.6627 | **A** | **682.3406** | 341.6740 | 665.3141 | 333.1607 | 664.3301 | 332.6687 | **6** |
| **6** | **613.3555** | 307.1814 | **596.3290** | 298.6681 | 595.3450 | 298.1761 | **N** | **611.3035** | 306.1554 | 594.2770 | 297.6421 | 593.2929 | 297.1501 | **5** |
| **7** | **760.4240** | 380.7156 | 743.3974 | 372.2023 | **742.4134** | 371.7103 | **F** | **496.2766** | **248.6419** | 479.2500 | 240.1287 | 478.2660 | 239.6366 | **4** |
| **8** | **861.4716** | 431.2395 | 844.4451 | 422.7262 | **843.4611** | 422.2342 | **T** | **349.2082** | 175.1077 | 332.1816 | 166.5944 | 331.1976 | 166.1024 | **3** |
| **9** | **962.5193** | 481.7633 | 945.4928 | 473.2500 | 944.5088 | 472.7580 | **T** | **248.1605** | 124.5839 | 231.1339 | 116.0706 | 230.1499 | 115.5786 | **2** |
| **10** | | | | | | | **K** | 147.1128 | 74.0600 | 130.0863 | 65.5468 | | | **1** |

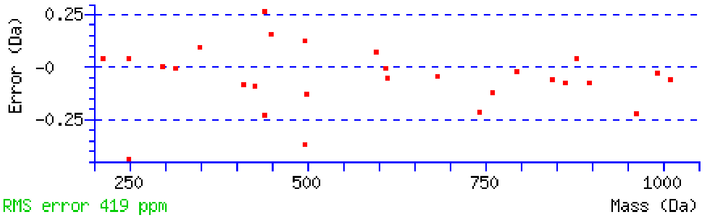


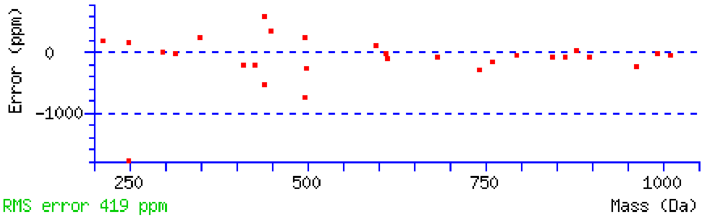


### All matches to this query

| Score | Mr(calc): | Delta | Sequence |
|---|---|---|---|
| 72.3 | 1107.6176 | 0.0017 | VLTLANFTTK |
| 12.3 | 1107.6288 | -0.0096 | LVTFSASQKK |
| 9.3 | 1107.6165 | 0.0027 | NLLGKRISK |
| 8.3 | 1107.6165 | 0.0027 | AKARAILTGK |
| 5.0 | 1107.6189 | 0.0003 | VIHWNSPKK |
| 5.0 | 1107.6111 | 0.0082 | VLIFSQMVR |
| 5.0 | 1107.6111 | 0.0082 | VLIFTQMTR |
| 4.0 | 1106.6158 | 1.0035 | IAQLYMIQK |
| 4.0 | 1106.6084 | 1.0109 | KAILTNQYR |
| 4.0 | 1106.6073 | 1.0119 | VGRLSRLAR |

---

Spectrum No: 331; Query: 2466; Rank: 1

---

## Peptide View

MS/MS Fragmentation of **AFMTVDGQESPSVTVVGNATTLDVERK**
Found in **IPI00363534**, Tax_Id=10116 Gene_Symbol=Lama1 laminin, alpha 1

Match to Query 2466: 2851.398822 from(951.473550,3+)
Title: 100101RatKid_NS_deglyco_19.3739.3739.3.dta
Data file K:\NewmanPaper\Piliang\3SubProteomes\Piliang3SP\mgf5ppm\ERLIC_3SubProteomes5ppm.mgf

Mascot Search Results: Peptide View

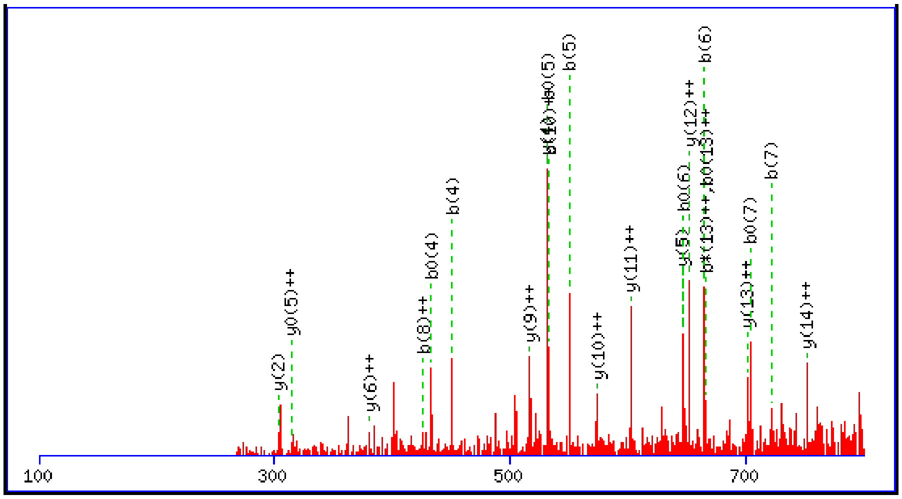


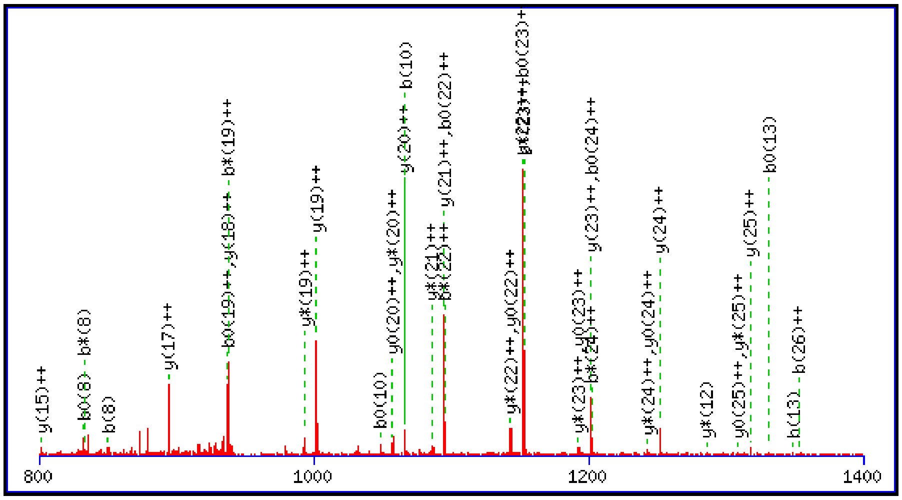


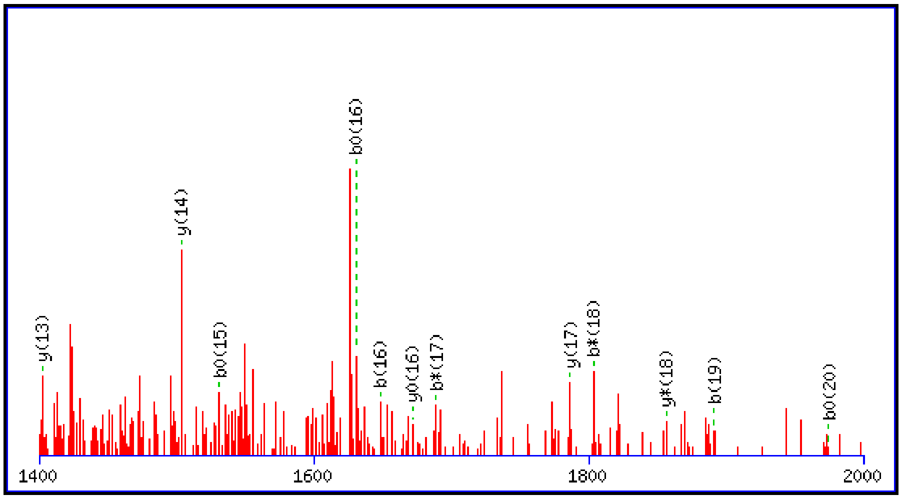


**Monoisotopic mass of neutral peptide Mr(calc):** 2851.3910
**Fixed modifications:** Carbamidomethyl (C)
**Variable modifications:**
**N18** : Deamidated_N (N)
**Ions Score:** 72 **Expect:** 1.5e-005
**Matches (Bold Red):** 74/288 fragment ions using 151 most intense peaks

| # | b | $b^{++}$ | b* | $b^{*++}$ | $b^0$ | $b^{0++}$ | Seq. | y | $y^{++}$ | y* | $y^{*++}$ | $y^0$ | $y^{0++}$ | # |
|---|---|---|---|---|---|---|---|---|---|---|---|---|---|---|
| 1 | 72.0444 | 36.5258 | | | | | A | | | | | | | 27 |
| 2 | 219.1128 | 110.0600 | | | | | F | 2781.3611 | 1391.1842 | 2764.3346 | 1382.6709 | 2763.3505 | 1382.1789 | 26 |
| 3 | 350.1533 | 175.5803 | | | | | M | 2634.2927 | **1317.6500** | 2617.2661 | **1309.1367** | 2616.2821 | **1308.6447** | 25 |
| 4 | **451.2010** | 226.1041 | | | **433.1904** | 217.0988 | T | 2503.2522 | **1252.1297** | 2486.2257 | **1243.6165** | 2485.2416 | **1243.1245** | 24 |
| 5 | **550.2694** | 275.6383 | | | **532.2588** | 266.6330 | V | 2402.2045 | **1201.6059** | 2385.1780 | **1193.0926** | 2384.1940 | **1192.6006** | 23 |

| | | | | | | | | | | | | | | |
|---|---|---|---|---|---|---|---|---|---|---|---|---|---|---|
| 6 | 665.2963 | 333.1518 | | | 647.2858 | 324.1465 | D | 2303.1361 | 1152.0717 | 2286.1096 | 1143.5584 | 2285.1256 | 1143.0664 | 22 |
| 7 | 722.3178 | 361.6625 | | | 704.3072 | 352.6573 | G | 2188.1092 | 1094.5582 | 2171.0826 | 1086.0450 | 2170.0986 | 1085.5529 | 21 |
| 8 | 850.3764 | 425.6918 | 833.3498 | 417.1785 | 832.3658 | 416.6865 | Q | 2131.0877 | 1066.0475 | 2114.0612 | 1057.5342 | 2113.0771 | 1057.0422 | 20 |
| 9 | 979.4190 | 490.2131 | 962.3924 | 481.6998 | 961.4084 | 481.2078 | E | 2003.0291 | 1002.0182 | 1986.0026 | 993.5049 | 1985.0186 | 993.0129 | 19 |
| 10 | 1066.4510 | 533.7291 | 1049.4244 | 525.2159 | 1048.4404 | 524.7238 | S | 1873.9865 | 937.4969 | 1856.9600 | 928.9836 | 1855.9760 | 928.4916 | 18 |
| 11 | 1163.5038 | 582.2555 | 1146.4772 | 573.7422 | 1145.4932 | 573.2502 | P | 1786.9545 | 893.9809 | 1769.9280 | 885.4676 | 1768.9439 | 884.9756 | 17 |
| 12 | 1250.5358 | 625.7715 | 1233.5092 | 617.2583 | 1232.5252 | 616.7662 | S | 1689.9018 | 845.4545 | 1672.8752 | 836.9412 | 1671.8912 | 836.4492 | 16 |
| 13 | 1349.6042 | 675.3057 | 1332.5776 | 666.7925 | 1331.5936 | 666.3005 | V | 1602.8697 | 801.9385 | 1585.8432 | 793.4252 | 1584.8592 | 792.9332 | 15 |
| 14 | 1450.6519 | 725.8296 | 1433.6253 | 717.3163 | 1432.6413 | 716.8243 | T | 1503.8013 | 752.4043 | 1486.7748 | 743.8910 | 1485.7907 | 743.3990 | 14 |
| 15 | 1549.7203 | 775.3638 | 1532.6937 | 766.8505 | 1531.7097 | 766.3585 | V | 1402.7536 | 701.8805 | 1385.7271 | 693.3672 | 1384.7431 | 692.8752 | 13 |
| 16 | 1648.7887 | 824.8980 | 1631.7622 | 816.3847 | 1630.7781 | 815.8927 | V | 1303.6852 | 652.3462 | 1286.6587 | 643.8330 | 1285.6746 | 643.3410 | 12 |
| 17 | 1705.8102 | 853.4087 | 1688.7836 | 844.8954 | 1687.7996 | 844.4034 | G | 1204.6168 | 602.8120 | 1187.5903 | 594.2988 | 1186.6062 | 593.8068 | 11 |
| 18 | 1820.8371 | 910.9222 | 1803.8106 | 902.4089 | 1802.8265 | 901.9169 | N | 1147.5953 | 574.3013 | 1130.5688 | 565.7880 | 1129.5848 | 565.2960 | 10 |
| 19 | 1891.8742 | 946.4407 | 1874.8477 | 937.9275 | 1873.8637 | 937.4355 | A | 1032.5684 | 516.7878 | 1015.5419 | 508.2746 | 1014.5578 | 507.7826 | 9 |
| 20 | 1992.9219 | 996.9646 | 1975.8953 | 988.4513 | 1974.9113 | 987.9593 | T | 961.5313 | 481.2693 | 944.5047 | 472.7560 | 943.5207 | 472.2640 | 8 |
| 21 | 2093.9696 | 1047.4884 | 2076.9430 | 1038.9751 | 2075.9590 | 1038.4831 | T | 860.4836 | 430.7454 | 843.4571 | 422.2322 | 842.4730 | 421.7402 | 7 |
| 22 | 2207.0536 | 1104.0305 | 2190.0271 | 1095.5172 | 2189.0431 | 1095.0252 | L | 759.4359 | 380.2216 | 742.4094 | 371.7083 | 741.4254 | 371.2163 | 6 |
| 23 | 2322.0806 | 1161.5439 | 2305.0540 | 1153.0307 | 2304.0700 | 1152.5386 | D | 646.3519 | 323.6796 | 629.3253 | 315.1663 | 628.3413 | 314.6743 | 5 |
| 24 | 2421.1490 | 1211.0781 | 2404.1224 | 1202.5649 | 2403.1384 | 1202.0729 | V | 531.3249 | 266.1661 | 514.2984 | 257.6528 | 513.3144 | 257.1608 | 4 |
| 25 | 2550.1916 | 1275.5994 | 2533.1650 | 1267.0862 | 2532.1810 | 1266.5941 | E | 432.2565 | 216.6319 | 415.2300 | 208.1186 | 414.2459 | 207.6266 | 3 |
| 26 | 2706.2927 | 1353.6500 | 2689.2661 | 1345.1367 | 2688.2821 | 1344.6447 | R | 303.2139 | 152.1106 | 286.1874 | 143.5973 | | | 2 |
| 27 | | | | | | | K | 147.1128 | 74.0600 | 130.0863 | 65.5468 | | | 1 |

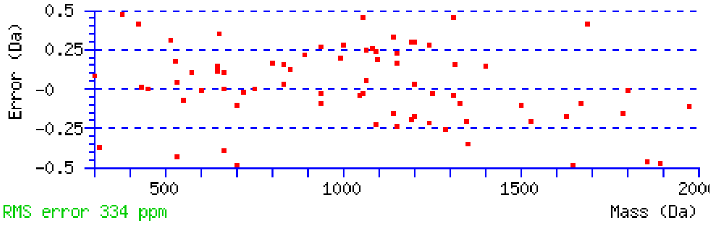


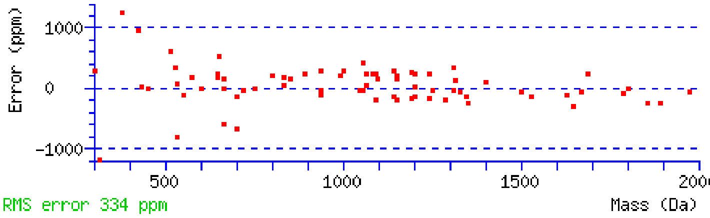


**All matches to this query**

| Score | Mr(calc): | Delta | Sequence |
|---|---|---|---|
| 72.3 | 2851.3910 | 0.0078 | AFMTVDGQESPSVTVVGNATTLDVERK |
| 17.2 | 2850.4070 | 0.9919 | AFMTVDGQESPSVTVVGNATTLDVERK |
| 5.1 | 2851.3844 | 0.0144 | VHTIVISVQHDEEVCLDEMRDALK |
| 1.0 | 2849.3922 | 2.0066 | FVTAKKNGQLAATVETAGDSELFLMK |
| 1.0 | 2849.3922 | 2.0066 | FVTAKKNGQLAATVETAGDSELFLMK |
| 1.0 | 2849.3922 | 2.0066 | FVTAKKNGQLAATVETAGDSELFLMK |
| 0.8 | 2850.3874 | 1.0114 | MITQTSLTPYIASPVSAYQVAKETR |
| 0.4 | 2850.3874 | 1.0114 | MITQTSLTPYIASPVSAYQVAKETR |
| 0.3 | 2851.3762 | 0.0227 | RVMKLSTLSSPSAILWAPDSPPCSR |
| 0.3 | 2851.3762 | 0.0227 | RVMKLSTLSSPSAILWAPDSPPCSR |

---

Spectrum No: 332; Query: 218; Rank: 1

---

## Peptide View

MS/MS Fragmentation of **LPVTAVNTTMR**
Found in **IPI00197684**, Tax_Id=10116 Gene_Symbol=Xpnpep2 Membrane-bound aminopeptidase P

Match to Query 218: 1202.633188 from(602.323870,2+)
Title: 100101RatKid_NS_deglyco_19.1959.1959.2.dta
Data file K:\NewmanPaper\Piliang\3SubProteomes\Piliang3SP\mgf5ppm\ERLIC_3SubProteomes5ppm.mgf

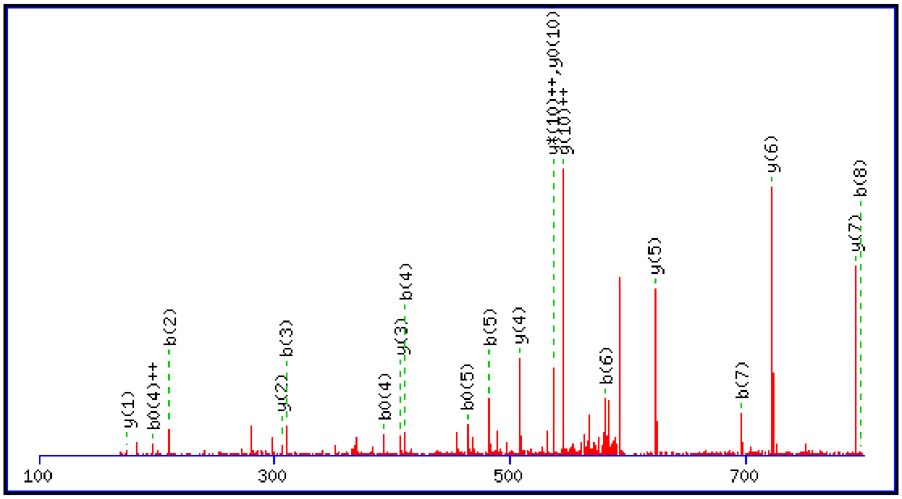


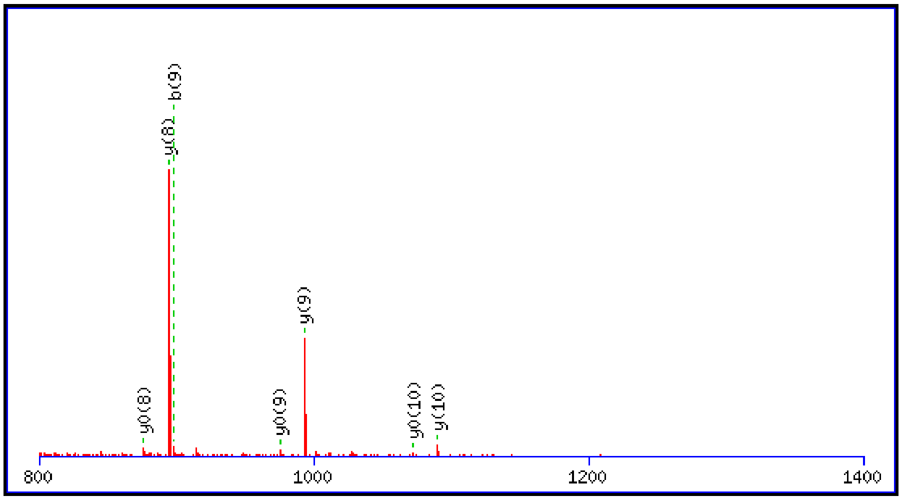


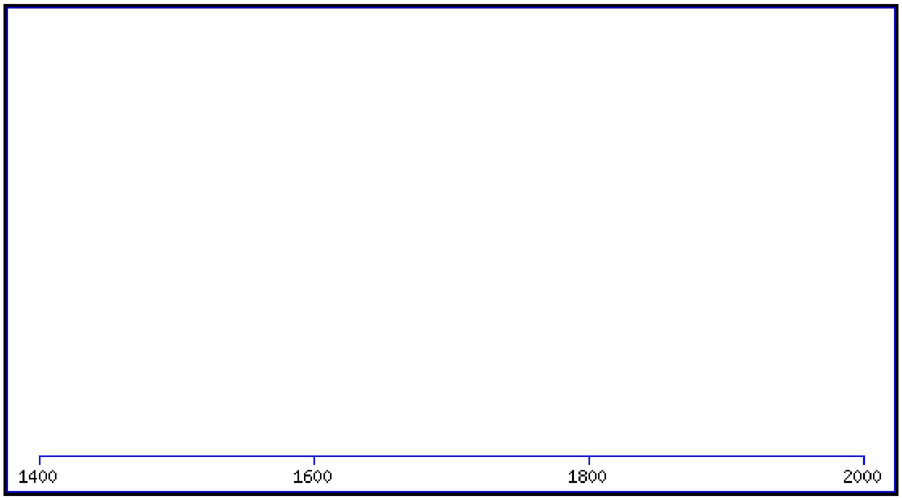


**Monoisotopic mass of neutral peptide Mr(calc):** 1202.6329
**Fixed modifications:** Carbamidomethyl (C)
**Variable modifications:**
**N7** : Deamidated_N (N)
**Ions Score:** 72 **Expect:** 7.2e-006
**Matches (Bold Red):** 27/98 fragment ions using 51 most intense peaks

| # | b | $b^{++}$ | b* | $b^{*++}$ | $b^{0}$ | $b^{0++}$ | Seq. | y | $y^{++}$ | y* | $y^{*++}$ | $y^{0}$ | $y^{0++}$ | # |
|---|---|---|---|---|---|---|---|---|---|---|---|---|---|---|
| 1 | 114.0913 | 57.5493 | | | | | L | | | | | | | 11 |
| 2 | **211.1441** | 106.0757 | | | | | P | **1090.5561** | **545.7817** | 1073.5296 | **537.2684** | **1072.5456** | **536.7764** | 10 |

| | | | | | | | | | | | | | | |
|---|---|---|---|---|---|---|---|---|---|---|---|---|---|---|
| **3** | **310.2125** | 155.6099 | | | | | **V** | **993.5034** | 497.2553 | 976.4768 | 488.7420 | **975.4928** | 488.2500 | **9** |
| **4** | **411.2602** | 206.1337 | | | **393.2496** | **197.1285** | **T** | **894.4349** | 447.7211 | 877.4084 | 439.2078 | **876.4244** | 438.7158 | **8** |
| **5** | **482.2973** | 241.6523 | | | **464.2867** | 232.6470 | **A** | **793.3873** | 397.1973 | 776.3607 | 388.6840 | 775.3767 | 388.1920 | **7** |
| **6** | **581.3657** | 291.1865 | | | 563.3552 | 282.1812 | **V** | **722.3501** | 361.6787 | 705.3236 | 353.1654 | 704.3396 | 352.6734 | **6** |
| **7** | **696.3927** | 348.7000 | 679.3661 | 340.1867 | 678.3821 | 339.6947 | **N** | **623.2817** | 312.1445 | 606.2552 | 303.6312 | 605.2712 | 303.1392 | **5** |
| **8** | **797.4403** | 399.2238 | 780.4138 | 390.7105 | 779.4298 | 390.2185 | **T** | **508.2548** | 254.6310 | 491.2282 | 246.1178 | 490.2442 | 245.6258 | **4** |
| **9** | **898.4880** | 449.7476 | 881.4615 | 441.2344 | 880.4775 | 440.7424 | **T** | **407.2071** | 204.1072 | 390.1806 | 195.5939 | 389.1966 | 195.1019 | **3** |
| **10** | 1029.5285 | 515.2679 | 1012.5020 | 506.7546 | 1011.5179 | 506.2626 | **M** | **306.1594** | 153.5834 | 289.1329 | 145.0701 | | | **2** |
| **11** | | | | | | | **R** | **175.1190** | 88.0631 | 158.0924 | 79.5498 | | | **1** |

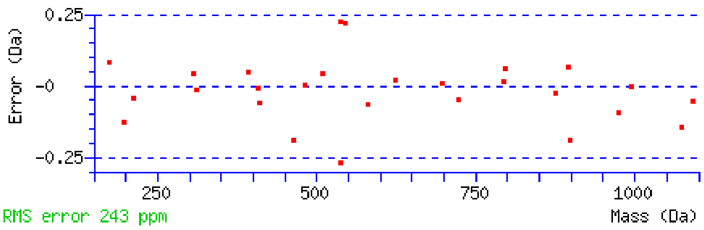


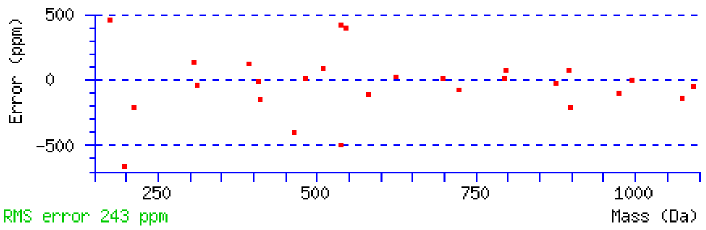


### All matches to this query

| Score | Mr(calc): | Delta | Sequence |
|---|---|---|---|
| 72.2 | 1202.6329 | 0.0003 | LPVTAVNTTMR |
| 4.6 | 1200.6211 | 2.0121 | EAPRKETTGGR |

---

Spectrum No: 333; Query: 923; Rank: 1

---

## Peptide View

MS/MS Fragmentation of **ANTTQPGIIEGGQVLK**
Found in **IPI00764984**, Tax_Id=10116 Gene_Symbol=Itgav_predicted 106 kDa protein

Match to Query 923: 1625.867088 from(813.940820,2+)
Title: 100101RatKid_NS_deglyco_21.2210.2210.2.dta
Data file K:\NewmanPaper\Piliang\3SubProteomes\Piliang3SP\mgf5ppm\ERLIC_3SubProteomes5ppm.mgf

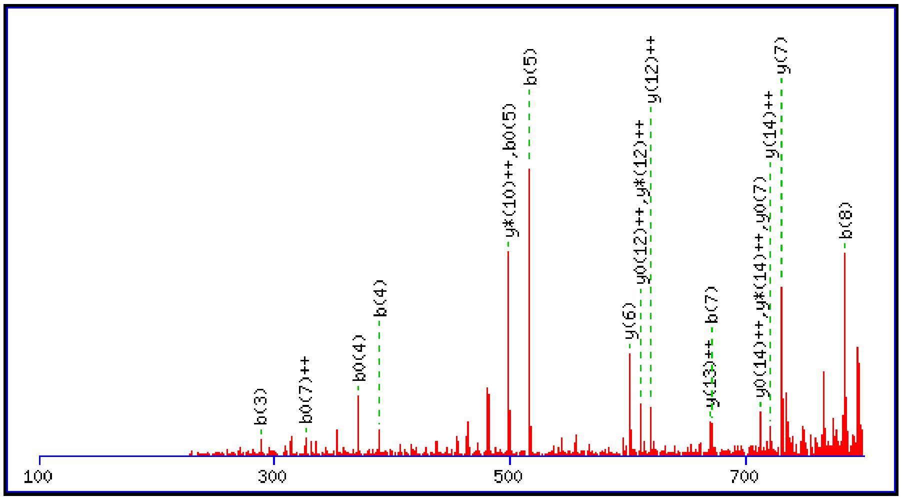

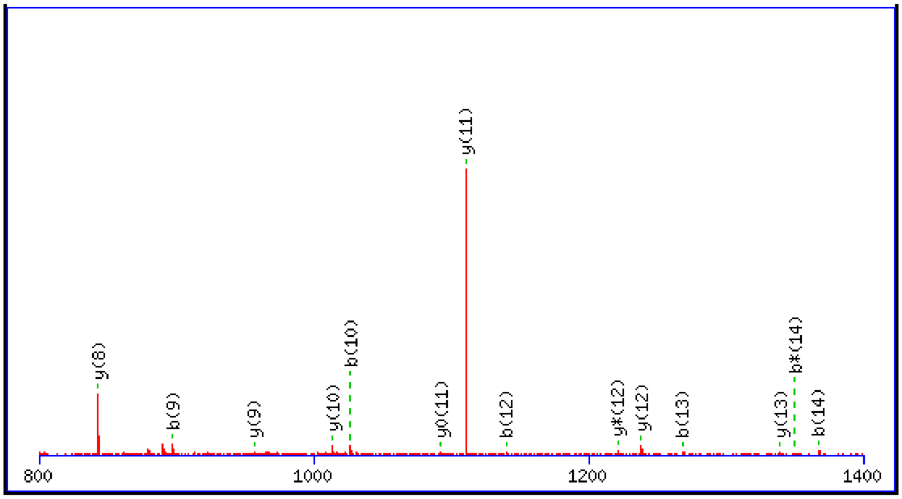


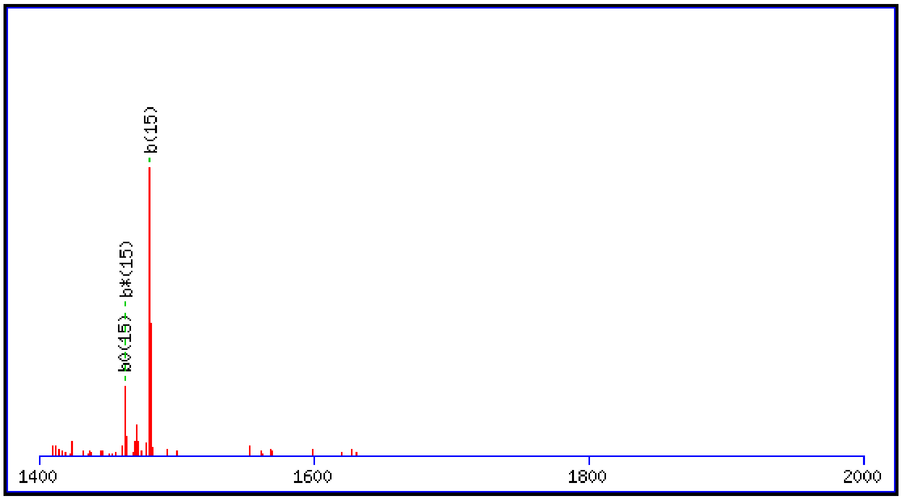


**Monoisotopic mass of neutral peptide Mr(calc):** 1625.8624
**Fixed modifications:** Carbamidomethyl (C)
**Variable modifications:**
**N2** : Deamidated_N (N)
**Ions Score:** 72 **Expect:** 6e-006
**Matches (Bold Red):** 36/162 fragment ions using 58 most intense peaks

| # | b | b$^{++}$ | b* | b*$^{++}$ | b$^{0}$ | b$^{0++}$ | Seq. | y | y$^{++}$ | y* | y*$^{++}$ | y$^{0}$ | y$^{0++}$ | # |
|---|---|---|---|---|---|---|---|---|---|---|---|---|---|---|
| 1 | 72.0444 | 36.5258 | | | | | A | | | | | | | 16 |
| 2 | 187.0713 | 94.0393 | 170.0448 | 85.5260 | | | N | 1555.8326 | 778.4199 | 1538.8061 | 769.9067 | 1537.8220 | 769.4147 | 15 |
| 3 | **288.1190** | 144.5631 | 271.0925 | 136.0499 | 270.1084 | 135.5579 | T | 1440.8057 | **720.9065** | 1423.7791 | **712.3932** | 1422.7951 | **711.9012** | 14 |
| 4 | **389.1667** | 195.0870 | 372.1401 | 186.5737 | **371.1561** | 186.0817 | T | **1339.7580** | **670.3826** | 1322.7314 | 661.8694 | 1321.7474 | 661.3774 | 13 |
| 5 | **517.2253** | 259.1163 | 500.1987 | 250.6030 | **499.2147** | 250.1110 | Q | **1238.7103** | **619.8588** | **1221.6838** | **611.3455** | 1220.6998 | **610.8535** | 12 |
| 6 | 614.2780 | 307.6427 | 597.2515 | 299.1294 | 596.2675 | 298.6374 | P | **1110.6517** | 555.8295 | 1093.6252 | 547.3162 | **1092.6412** | 546.8242 | 11 |
| 7 | **671.2995** | 336.1534 | 654.2729 | 327.6401 | 653.2889 | **327.1481** | G | **1013.5990** | 507.3031 | 996.5724 | **498.7899** | 995.5884 | 498.2978 | 10 |
| 8 | **784.3836** | 392.6954 | 767.3570 | 384.1821 | 766.3730 | 383.6901 | I | **956.5775** | 478.7924 | 939.5510 | 470.2791 | 938.5669 | 469.7871 | 9 |
| 9 | **897.4676** | 449.2374 | 880.4411 | 440.7242 | 879.4571 | 440.2322 | I | **843.4934** | 422.2504 | 826.4669 | 413.7371 | 825.4829 | 413.2451 | 8 |
| 10 | **1026.5102** | 513.7587 | 1009.4837 | 505.2455 | 1008.4996 | 504.7535 | E | **730.4094** | 365.7083 | 713.3828 | 357.1951 | **712.3988** | 356.7030 | 7 |
| 11 | 1083.5317 | 542.2695 | 1066.5051 | 533.7562 | 1065.5211 | 533.2642 | G | **601.3668** | 301.1870 | 584.3402 | 292.6738 | | | 6 |
| 12 | **1140.5531** | 570.7802 | 1123.5266 | 562.2669 | 1122.5426 | 561.7749 | G | 544.3453 | 272.6763 | 527.3188 | 264.1630 | | | 5 |
| 13 | **1268.6117** | 634.8095 | 1251.5852 | 626.2962 | 1250.6012 | 625.8042 | Q | 487.3239 | 244.1656 | 470.2973 | 235.6523 | | | 4 |
| 14 | **1367.6801** | 684.3437 | **1350.6536** | 675.8304 | 1349.6696 | 675.3384 | V | 359.2653 | 180.1363 | 342.2387 | 171.6230 | | | 3 |
| 15 | **1480.7642** | 740.8857 | **1463.7376** | 732.3725 | **1462.7536** | 731.8805 | L | 260.1969 | 130.6021 | 243.1703 | 122.0888 | | | 2 |
| 16 | | | | | | | K | 147.1128 | 74.0600 | 130.0863 | 65.5468 | | | 1 |

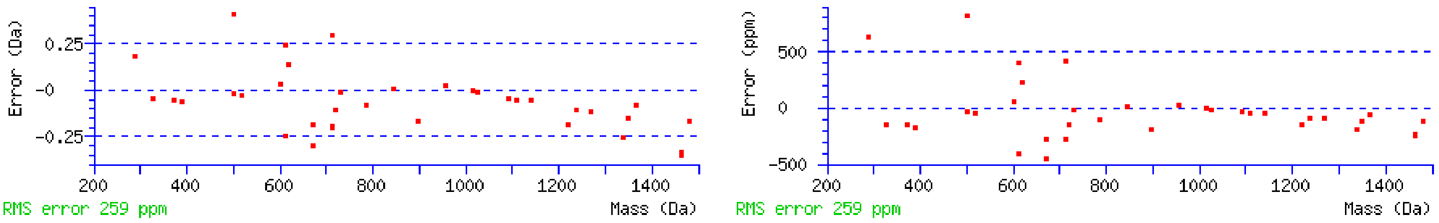


### All matches to this query

| Score | Mr(calc): | Delta | Sequence |
|---|---|---|---|
| 72.2 | 1625.8624 | 0.0046 | ANTTQPGIIEGGQVLK |
| 23.5 | 1624.8784 | 0.9887 | ANTTQPGIIEGGQVLK |
| 13.0 | 1625.8624 | 0.0047 | DINTKLPQAQQELK |
| 12.9 | 1625.8575 | 0.0095 | TRILVTSKMEQLK |
| 12.9 | 1625.8575 | 0.0095 | TRILVTSKMEQLK |
| 5.8 | 1625.8575 | 0.0095 | TRILVTSKMEQLK |
| 5.5 | 1625.8624 | 0.0047 | EGTPSGAAPSSALAAVLK |
| 4.7 | 1625.8624 | 0.0047 | SLDEGAPQDTPKLKK |
| 4.5 | 1624.8798 | 0.9873 | LVHNFTDAVIQGRR |
| 3.6 | 1625.8616 | 0.0055 | IYVVQPKAMKIEK |

---

Spectrum No: 334; Query: 619; Rank: 1

---

## Peptide View

MS/MS Fragmentation of **VEAIGSLANTTMEK**
Found in **IPI00193397**, Tax_Id=10116 Gene_Symbol=Slc34a1 Sodium-dependent phosphate transport protein 2A

Match to Query 619: 1463.719888 from(732.867220,2+)
Title: 100101RatKid_NS_deglyco_27.2072.2072.2.dta
Data file K:\NewmanPaper\Piliang\3SubProteomes\Piliang3SP\mgf5ppm\ERLIC_3SubProteomes5ppm.mgf

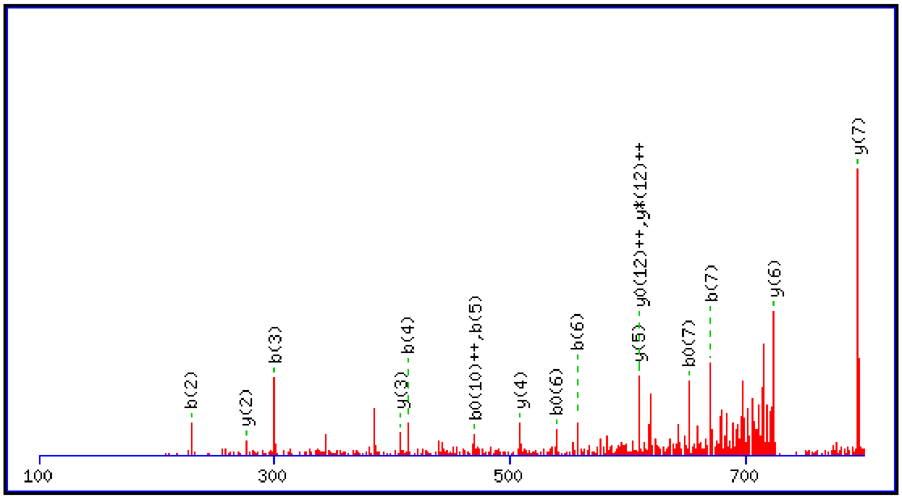

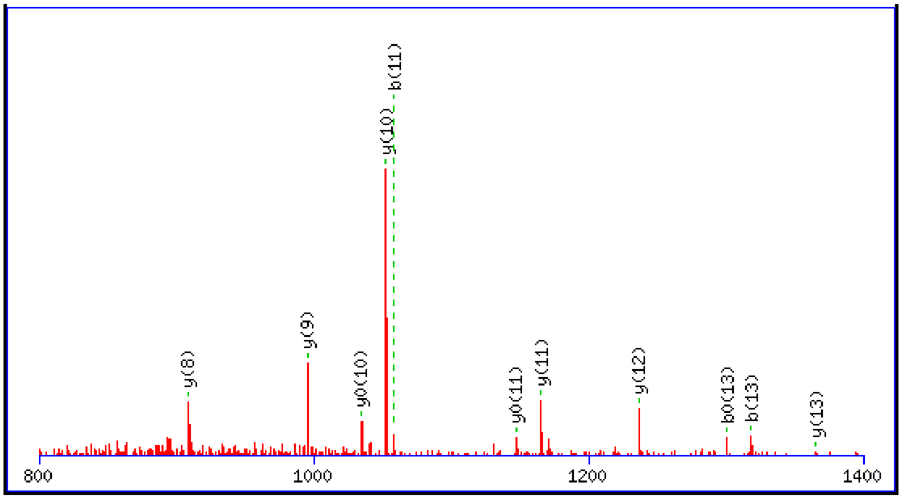


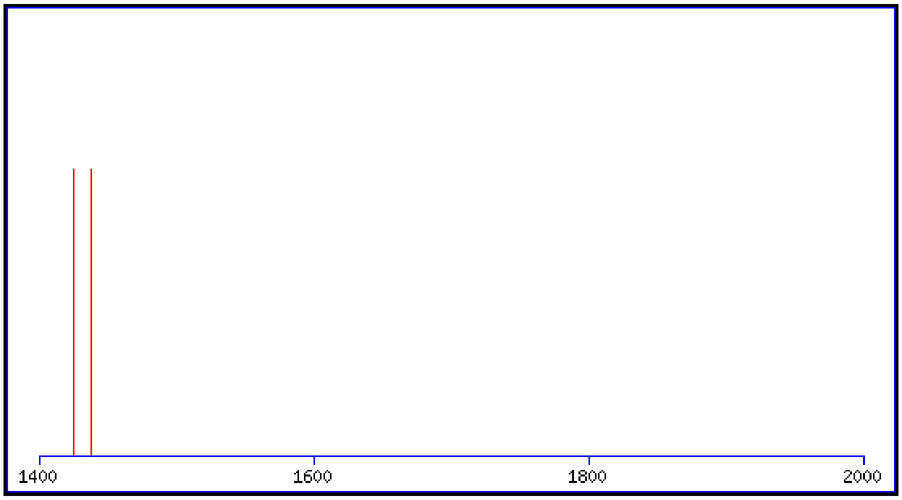


**Monoisotopic mass of neutral peptide Mr(calc):** 1463.7177
**Fixed modifications:** Carbamidomethyl (C)
**Variable modifications:**
**N9** : Deamidated_N (N)
**Ions Score:** 72 **Expect:** 9e-006
**Matches (Bold Red):** 28/136 fragment ions using 47 most intense peaks

| # | b | $b^{++}$ | b* | $b^{*++}$ | $b^0$ | $b^{0++}$ | Seq. | y | $y^{++}$ | y* | $y^{*++}$ | $y^0$ | $y^{0++}$ | # |
|---|---|---|---|---|---|---|---|---|---|---|---|---|---|---|
| 1 | 100.0757 | 50.5415 | | | | | V | | | | | | | 14 |
| 2 | **229.1183** | 115.0628 | | | 211.1077 | 106.0575 | E | **1365.6566** | 683.3319 | 1348.6301 | 674.8187 | 1347.6461 | 674.3267 | 13 |
| 3 | **300.1554** | 150.5813 | | | 282.1448 | 141.5761 | A | **1236.6140** | 618.8107 | 1219.5875 | **610.2974** | 1218.6035 | **609.8054** | 12 |
| 4 | **413.2395** | 207.1234 | | | 395.2289 | 198.1181 | I | **1165.5769** | 583.2921 | 1148.5504 | 574.7788 | **1147.5663** | 574.2868 | 11 |
| 5 | **470.2609** | 235.6341 | | | 452.2504 | 226.6288 | G | **1052.4928** | 526.7501 | 1035.4663 | 518.2368 | **1034.4823** | 517.7448 | 10 |
| 6 | **557.2930** | 279.1501 | | | **539.2824** | 270.1448 | S | **995.4714** | 498.2393 | 978.4448 | 489.7261 | 977.4608 | 489.2340 | 9 |
| 7 | **670.3770** | 335.6921 | | | **652.3665** | 326.6869 | L | **908.4394** | 454.7233 | 891.4128 | 446.2100 | 890.4288 | 445.7180 | 8 |
| 8 | 741.4141 | 371.2107 | | | 723.4036 | 362.2054 | A | **795.3553** | 398.1813 | 778.3287 | 389.6680 | 777.3447 | 389.1760 | 7 |
| 9 | 856.4411 | 428.7242 | 839.4145 | 420.2109 | 838.4305 | 419.7189 | N | **724.3182** | 362.6627 | 707.2916 | 354.1495 | 706.3076 | 353.6574 | 6 |
| 10 | 957.4887 | 479.2480 | 940.4622 | 470.7347 | 939.4782 | **470.2427** | T | **609.2912** | 305.1493 | 592.2647 | 296.6360 | 591.2807 | 296.1440 | 5 |
| 11 | **1058.5364** | 529.7719 | 1041.5099 | 521.2586 | 1040.5259 | 520.7666 | T | **508.2436** | 254.6254 | 491.2170 | 246.1121 | 490.2330 | 245.6201 | 4 |
| 12 | 1189.5769 | 595.2921 | 1172.5504 | 586.7788 | 1171.5663 | 586.2868 | M | **407.1959** | 204.1016 | 390.1693 | 195.5883 | 389.1853 | 195.0963 | 3 |
| 13 | **1318.6195** | 659.8134 | 1301.5930 | 651.3001 | **1300.6089** | 650.8081 | E | **276.1554** | 138.5813 | 259.1288 | 130.0681 | 258.1448 | 129.5761 | 2 |
| 14 | | | | | | | K | 147.1128 | 74.0600 | 130.0863 | 65.5468 | | | 1 |

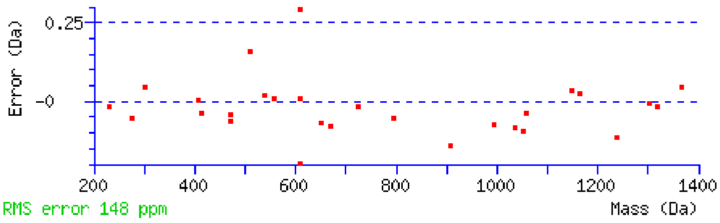


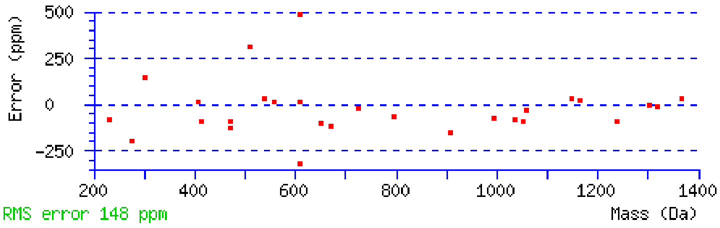


### All matches to this query

| Score | Mr(calc): | Delta | Sequence |
|---|---|---|---|
| 72.2 | 1463.7177 | 0.0022 | VEAIGSLANTTMEK |
| 17.6 | 1463.7133 | 0.0066 | RVSTISALSHEGK |
| 16.2 | 1463.7256 | -0.0057 | NNKLSLQFEEDK |
| 12.7 | 1463.7272 | -0.0074 | IDPVEKLQESVK |
| 11.5 | 1463.7133 | 0.0066 | RVSTISALSHEGK |
| 11.5 | 1463.7133 | 0.0066 | RVSTISALSHEGK |
| 6.3 | 1463.7273 | -0.0074 | EEGPVLVADTVKK |
| 4.7 | 1463.7061 | 0.0138 | DAILKYNVAYSK |
| 4.7 | 1463.7061 | 0.0138 | DAILKYNVAYSK |
| 4.7 | 1463.7061 | 0.0138 | DAILKYNVAYSK |

---

Spectrum No: 335; Query: 2459; Rank: 1

---

## Peptide View

MS/MS Fragmentation of **MCQLYPETFECVTNSSDILQAFR**
Found in **IPI00327697**, Tax_Id=10116 Gene_Symbol=Dpep1 Dipeptidase 1 precursor

Match to Query 2459: 2825.247928 from(1413.631240,2+)
Title: 100101RatKid_NS_deglyco_20.5460.5460.2.dta
Data file K:\NewmanPaper\Piliang\3SubProteomes\Piliang3SP\mgf5ppm\ERLIC_3SubProteomes5ppm.mgf

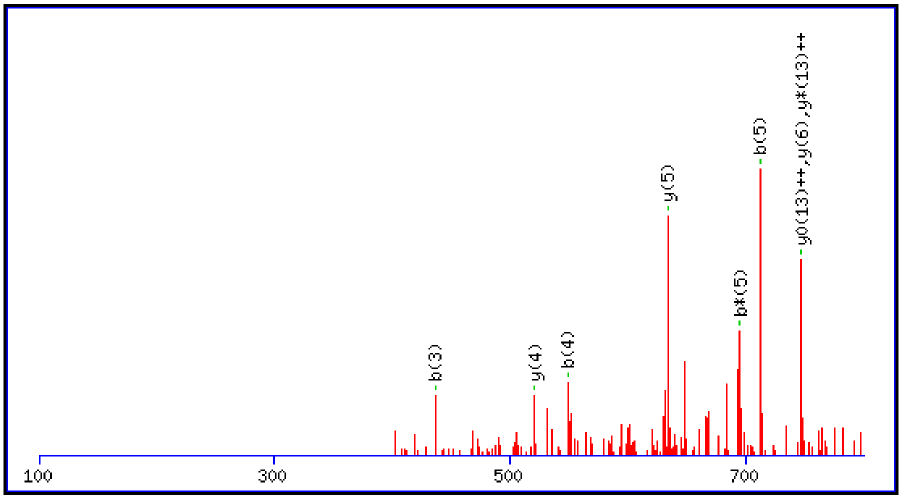

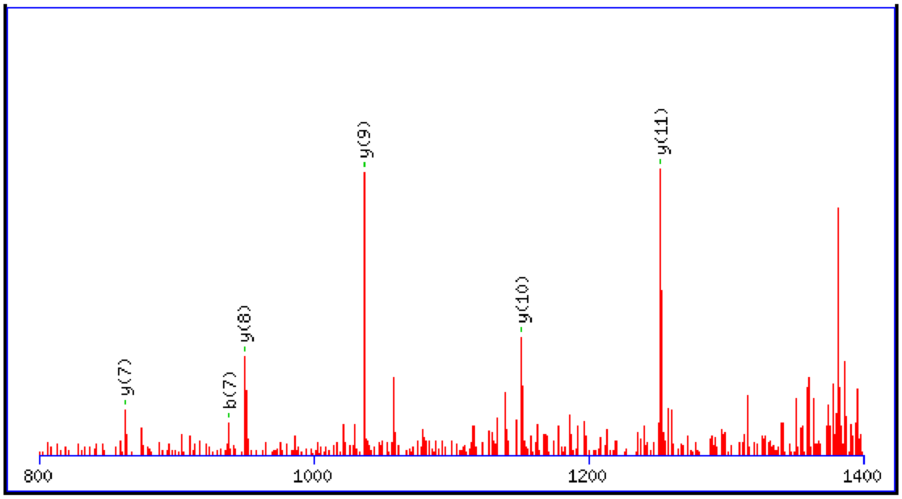


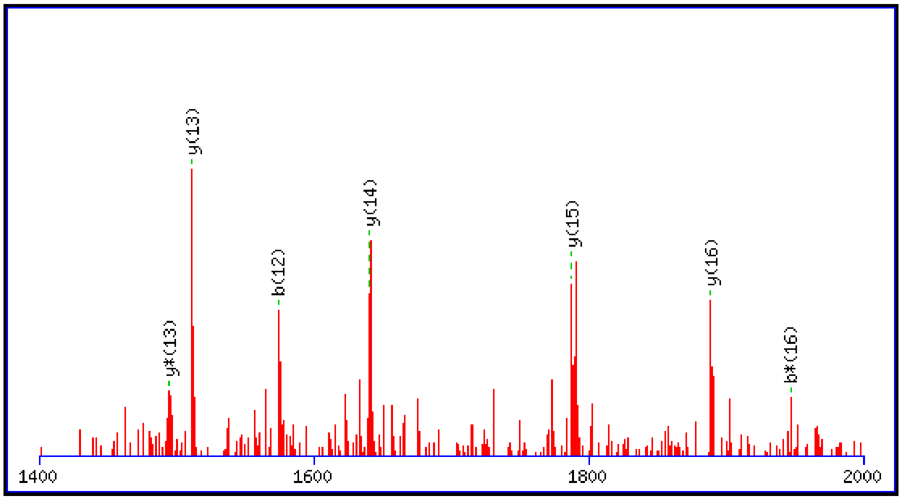


**Monoisotopic mass of neutral peptide Mr(calc):** 2825.2346
**Fixed modifications:** Carbamidomethyl (C)
**Variable modifications:**
**M1** : Oxidation (M)
**N14** : Deamidated_N (N)
**Ions Score:** 72 **Expect:** 2.3e-005
**Matches (Bold Red):** 22/236 fragment ions using 35 most intense peaks

| # | b | $b^{++}$ | b* | $b^{*++}$ | $b^{0}$ | $b^{0++}$ | Seq. | y | $y^{++}$ | y* | $y^{*++}$ | $y^{0}$ | $y^{0++}$ | # |
|---|---|---|---|---|---|---|---|---|---|---|---|---|---|---|
| 1 | 148.0427 | 74.5250 | | | | | M | | | | | | | 23 |
| 2 | 308.0733 | 154.5403 | | | | | C | 2679.2065 | 1340.1069 | 2662.1800 | 1331.5936 | 2661.1960 | 1331.1016 | 22 |
| 3 | **436.1319** | 218.5696 | 419.1054 | 210.0563 | | | Q | 2519.1759 | 1260.0916 | 2502.1493 | 1251.5783 | 2501.1653 | 1251.0863 | 21 |
| 4 | **549.2160** | 275.1116 | 532.1894 | 266.5983 | | | L | 2391.1173 | 1196.0623 | 2374.0907 | 1187.5490 | 2373.1067 | 1187.0570 | 20 |
| 5 | **712.2793** | 356.6433 | **695.2528** | 348.1300 | | | Y | 2278.0332 | 1139.5203 | 2261.0067 | 1131.0070 | 2260.0227 | 1130.5150 | 19 |
| 6 | 809.3321 | 405.1697 | 792.3055 | 396.6564 | | | P | 2114.9699 | 1057.9886 | 2097.9434 | 1049.4753 | 2096.9593 | 1048.9833 | 18 |
| 7 | **938.3747** | 469.6910 | 921.3481 | 461.1777 | 920.3641 | 460.6857 | E | 2017.9171 | 1009.4622 | 2000.8906 | 1000.9489 | 1999.9066 | 1000.4569 | 17 |
| 8 | 1039.4223 | 520.2148 | 1022.3958 | 511.7015 | 1021.4118 | 511.2095 | T | **1888.8745** | 944.9409 | 1871.8480 | 936.4276 | 1870.8640 | 935.9356 | 16 |
| 9 | 1186.4908 | 593.7490 | 1169.4642 | 585.2357 | 1168.4802 | 584.7437 | F | **1787.8269** | 894.4171 | 1770.8003 | 885.9038 | 1769.8163 | 885.4118 | 15 |
| 10 | 1315.5333 | 658.2703 | 1298.5068 | 649.7570 | 1297.5228 | 649.2650 | E | **1640.7585** | 820.8829 | 1623.7319 | 812.3696 | 1622.7479 | 811.8776 | 14 |
| 11 | 1475.5640 | 738.2856 | 1458.5374 | 729.7724 | 1457.5534 | 729.2804 | C | **1511.7159** | 756.3616 | **1494.6893** | **747.8483** | 1493.7053 | **747.3563** | 13 |
| 12 | **1574.6324** | 787.8198 | 1557.6059 | 779.3066 | 1556.6218 | 778.8146 | V | 1351.6852 | 676.3462 | 1334.6587 | 667.8330 | 1333.6746 | 667.3410 | 12 |
| 13 | 1675.6801 | 838.3437 | 1658.6535 | 829.8304 | 1657.6695 | 829.3384 | T | **1252.6168** | 626.8120 | 1235.5903 | 618.2988 | 1234.6062 | 617.8068 | 11 |
| 14 | 1790.7070 | 895.8571 | 1773.6805 | 887.3439 | 1772.6965 | 886.8519 | N | **1151.5691** | 576.2882 | 1134.5426 | 567.7749 | 1133.5586 | 567.2829 | 10 |
| 15 | 1877.7390 | 939.3732 | 1860.7125 | 930.8599 | 1859.7285 | 930.3679 | S | **1036.5422** | 518.7747 | 1019.5156 | 510.2615 | 1018.5316 | 509.7694 | 9 |
| 16 | 1964.7711 | 982.8892 | **1947.7445** | 974.3759 | 1946.7605 | 973.8839 | S | **949.5102** | 475.2587 | 932.4836 | 466.7454 | 931.4996 | 466.2534 | 8 |
| 17 | 2079.7980 | 1040.4026 | 2062.7715 | 1031.8894 | 2061.7875 | 1031.3974 | D | **862.4781** | 431.7427 | 845.4516 | 423.2294 | 844.4676 | 422.7374 | 7 |
| 18 | 2192.8821 | 1096.9447 | 2175.8555 | 1088.4314 | 2174.8715 | 1087.9394 | I | **747.4512** | 374.2292 | 730.4246 | 365.7160 | | | 6 |

| | | | | | | | | | | | | | |
|---|---|---|---|---|---|---|---|---|---|---|---|---|---|
| **19** | 2305.9661 | 1153.4867 | 2288.9396 | 1144.9734 | 2287.9556 | 1144.4814 | **L** | **634.3671** | 317.6872 | 617.3406 | 309.1739 | | | **5** |
| **20** | 2434.0247 | 1217.5160 | 2416.9982 | 1209.0027 | 2416.0142 | 1208.5107 | **Q** | **521.2831** | 261.1452 | 504.2565 | 252.6319 | | | **4** |
| **21** | 2505.0618 | 1253.0346 | 2488.0353 | 1244.5213 | 2487.0513 | 1244.0293 | **A** | 393.2245 | 197.1159 | 376.1979 | 188.6026 | | | **3** |
| **22** | 2652.1303 | 1326.5688 | 2635.1037 | 1318.0555 | 2634.1197 | 1317.5635 | **F** | 322.1874 | 161.5973 | 305.1608 | 153.0840 | | | **2** |
| **23** | | | | | | | **R** | 175.1190 | 88.0631 | 158.0924 | 79.5498 | | | **1** |

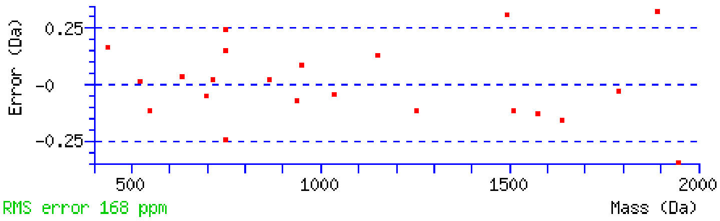


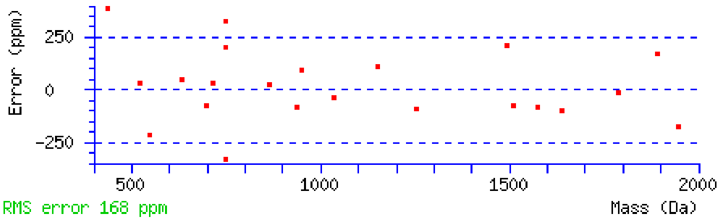


**All matches to this query**

| Score | Mr(calc): | Delta | Sequence |
|---|---|---|---|
| 72.1 | 2825.2346 | 0.0133 | MCQLYPETFECVTNSSDILQAFR |
| 19.9 | 2824.2506 | 0.9973 | MCQLYPETFECVTNSSDILQAFR |
| 2.4 | 2823.2171 | 2.0309 | AQGNNYGQTSNGVAEESPNMLVYRK |
| 2.4 | 2823.2171 | 2.0309 | AQGNNYGQTSNGVAEESPNMLVYRK |
| 1.3 | 2825.2595 | -0.0116 | DSTMNVSQNLYELLPRTSPLKNK |
| 1.3 | 2824.2660 | 0.9819 | QINDIQLSRDMTMFVTASKDNTAK |
| 0.6 | 2825.2595 | -0.0116 | DSTMNVSQNLYELLPRTSPLKNK |

---

Spectrum No: 336; Query: 2167; Rank: 1

---

## Peptide View

MS/MS Fragmentation of **GANSSSQNPLVYLDVGADGQPLGR**
Found in **IPI00201489**, Tax_Id=10116 Gene_Symbol=Ppif Peptidyl-prolyl cis-trans isomerase, mitochondrial precursor

Match to Query 2167: 2415.164248 from(1208.589400,2+)
Title: 091008RatKidney_NH4Format01_12.4414.4414.2.dta
Data file K:\NewmanPaper\Piliang\3SubProteomes\Piliang3SP\mgf5ppm\ERLIC_3SubProteomes5ppm.mgf

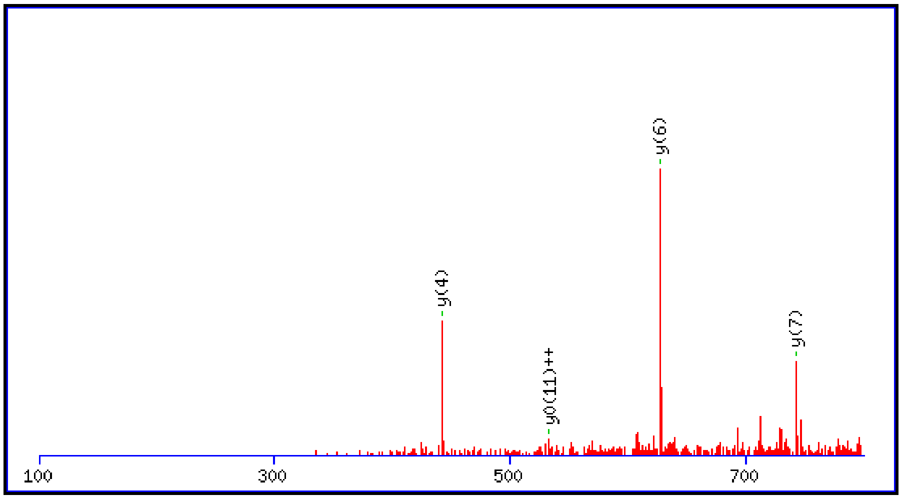

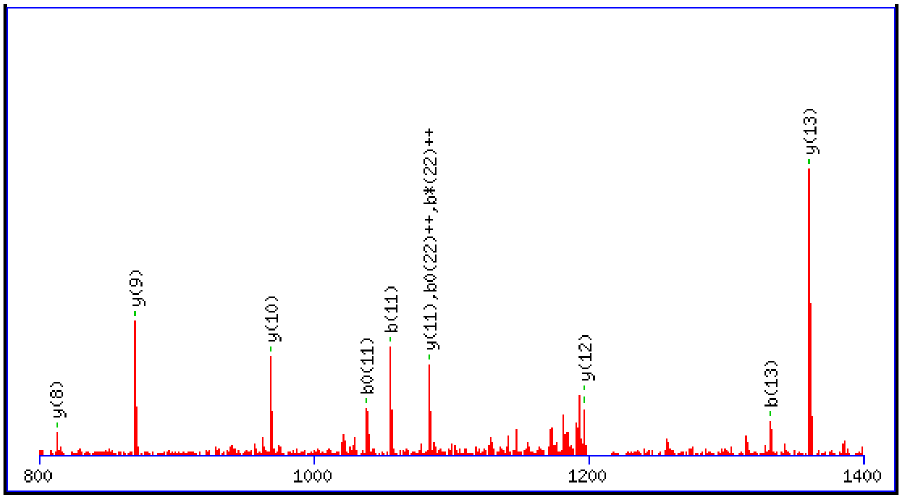


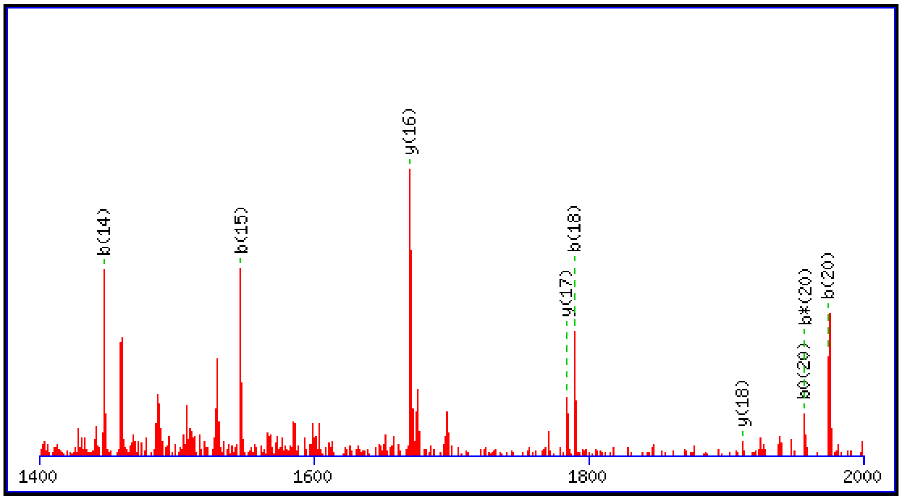


**Monoisotopic mass of neutral peptide Mr(calc):** 2415.1666
**Fixed modifications:** Carbamidomethyl (C)
**Variable modifications:**
**N3** : Deamidated_N (N)
**Ions Score:** 72 **Expect:** 1.7e-005
**Matches (Bold Red):** 24/254 fragment ions using 46 most intense peaks

| # | b | $b^{++}$ | b* | $b^{*++}$ | $b^0$ | $b^{0++}$ | Seq. | y | $y^{++}$ | y* | $y^{*++}$ | $y^0$ | $y^{0++}$ | # |
|---|---|---|---|---|---|---|---|---|---|---|---|---|---|---|
| 1 | 58.0287 | 29.5180 | | | | | G | | | | | | | 24 |
| 2 | 129.0659 | 65.0366 | | | | | A | 2359.1524 | 1180.0799 | 2342.1259 | 1171.5666 | 2341.1419 | 1171.0746 | 23 |
| 3 | 244.0928 | 122.5500 | 227.0662 | 114.0368 | | | N | 2288.1153 | 1144.5613 | 2271.0888 | 1136.0480 | 2270.1048 | 1135.5560 | 22 |
| 4 | 331.1248 | 166.0660 | 314.0983 | 157.5528 | 313.1143 | 157.0608 | S | 2173.0884 | 1087.0478 | 2156.0618 | 1078.5346 | 2155.0778 | 1078.0425 | 21 |
| 5 | 418.1568 | 209.5821 | 401.1303 | 201.0688 | 400.1463 | 200.5768 | S | 2086.0564 | 1043.5318 | 2069.0298 | 1035.0185 | 2068.0458 | 1034.5265 | 20 |
| 6 | 505.1889 | 253.0981 | 488.1623 | 244.5848 | 487.1783 | 244.0928 | S | 1999.0243 | 1000.0158 | 1981.9978 | 991.5025 | 1981.0138 | 991.0105 | 19 |
| 7 | 633.2475 | 317.1274 | 616.2209 | 308.6141 | 615.2369 | 308.1221 | Q | **1911.9923** | 956.4998 | 1894.9658 | 947.9865 | 1893.9817 | 947.4945 | 18 |
| 8 | 747.2904 | 374.1488 | 730.2638 | 365.6356 | 729.2798 | 365.1435 | N | **1783.9337** | 892.4705 | 1766.9072 | 883.9572 | 1765.9232 | 883.4652 | 17 |
| 9 | 844.3431 | 422.6752 | 827.3166 | 414.1619 | 826.3326 | 413.6699 | P | **1669.8908** | 835.4490 | 1652.8643 | 826.9358 | 1651.8802 | 826.4438 | 16 |
| 10 | 957.4272 | 479.2172 | 940.4007 | 470.7040 | 939.4166 | 470.2120 | L | 1572.8380 | 786.9227 | 1555.8115 | 778.4094 | 1554.8275 | 777.9174 | 15 |
| 11 | **1056.4956** | 528.7514 | 1039.4691 | 520.2382 | **1038.4851** | 519.7462 | V | 1459.7540 | 730.3806 | 1442.7274 | 721.8673 | 1441.7434 | 721.3753 | 14 |
| 12 | 1219.5590 | 610.2831 | 1202.5324 | 601.7698 | 1201.5484 | 601.2778 | Y | **1360.6856** | 680.8464 | 1343.6590 | 672.3331 | 1342.6750 | 671.8411 | 13 |
| 13 | **1332.6430** | 666.8251 | 1315.6165 | 658.3119 | 1314.6324 | 657.8199 | L | **1197.6222** | 599.3148 | 1180.5957 | 590.8015 | 1179.6117 | 590.3095 | 12 |
| 14 | **1447.6700** | 724.3386 | 1430.6434 | 715.8253 | 1429.6594 | 715.3333 | D | **1084.5382** | 542.7727 | 1067.5116 | 534.2594 | 1066.5276 | **533.7674** | 11 |
| 15 | **1546.7384** | 773.8728 | 1529.7118 | 765.3595 | 1528.7278 | 764.8675 | V | **969.5112** | 485.2592 | 952.4847 | 476.7460 | 951.5007 | 476.2540 | 10 |
| 16 | 1603.7598 | 802.3836 | 1586.7333 | 793.8703 | 1585.7493 | 793.3783 | G | **870.4428** | 435.7250 | 853.4163 | 427.2118 | 852.4322 | 426.7198 | 9 |
| 17 | 1674.7969 | 837.9021 | 1657.7704 | 829.3888 | 1656.7864 | 828.8968 | A | **813.4213** | 407.2143 | 796.3948 | 398.7010 | 795.4108 | 398.2090 | 8 |
| 18 | **1789.8239** | 895.4156 | 1772.7973 | 886.9023 | 1771.8133 | 886.4103 | D | **742.3842** | 371.6958 | 725.3577 | 363.1825 | 724.3737 | 362.6905 | 7 |

| | | | | | | | | | | | | | | |
|---|---|---|---|---|---|---|---|---|---|---|---|---|---|---|
| 19 | 1846.8454 | 923.9263 | 1829.8188 | 915.4130 | 1828.8348 | 914.9210 | G | **627.3573** | 314.1823 | 610.3307 | 305.6690 | | | 6 |
| 20 | **1974.9039** | 987.9556 | **1957.8774** | 979.4423 | **1956.8934** | 978.9503 | Q | 570.3358 | 285.6715 | 553.3093 | 277.1583 | | | 5 |
| 21 | 2071.9567 | 1036.4820 | 2054.9301 | 1027.9687 | 2053.9461 | 1027.4767 | P | **442.2772** | 221.6423 | 425.2507 | 213.1290 | | | 4 |
| 22 | 2185.0408 | 1093.0240 | 2168.0142 | **1084.5107** | 2167.0302 | **1084.0187** | L | 345.2245 | 173.1159 | 328.1979 | 164.6026 | | | 3 |
| 23 | 2242.0622 | 1121.5348 | 2225.0357 | 1113.0215 | 2224.0517 | 1112.5295 | G | 232.1404 | 116.5738 | 215.1139 | 108.0606 | | | 2 |
| 24 | | | | | | | R | 175.1190 | 88.0631 | 158.0924 | 79.5498 | | | 1 |

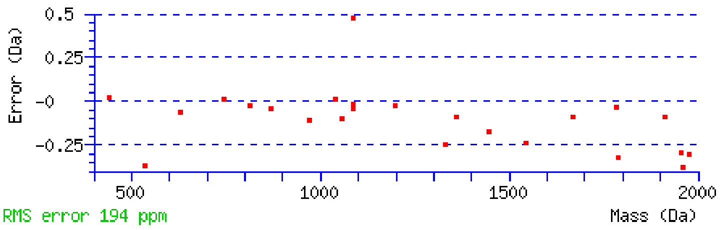


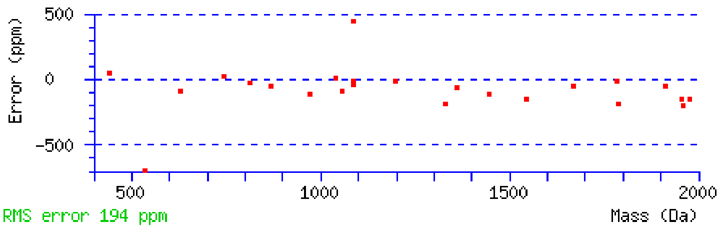


**All matches to this query**

| Score | Mr(calc): | Delta | Sequence |
|---|---|---|---|
| 71.9 | 2415.1666 | -0.0024 | GANSSSQNPLVYLDVGADGQPLGR |
| 57.1 | 2415.1666 | -0.0024 | GANSSSQNPLVYLDVGADGQPLGR |
| 56.1 | 2414.1826 | 0.9816 | GANSSSQNPLVYLDVGADGQPLGR |
| 1.8 | 2413.1752 | 1.9890 | NWWASLEVSSLNSLVQMLKK |
| 1.6 | 2415.1570 | 0.0073 | IAEQVASFQEEKSKLDAEVSK |
| 1.4 | 2414.1592 | 1.0050 | NWWASLEVSSLNSLVQMLKK |
| 0.3 | 2415.1471 | 0.0171 | ETPNPWKETLGPNKIPSGQNK |
| 0.0 | 2415.1835 | -0.0193 | YLTSNVAYGSTGIRDVHLELK |

---

Spectrum No: 337; Query: 236; Rank: 1

---

## Peptide View

MS/MS Fragmentation of **STNSSLTLLFK**
Found in **IPI00196620**, Tax_Id=10116 Gene_Symbol=Cubn Cubilin precursor

Match to Query 236: 1210.645608 from(606.330080,2+)
Title: 091008RatKidney_NoSalt_16.3521.3521.2.dta
Data file K:\NewmanPaper\Piliang\3SubProteomes\Piliang3SP\mgf5ppm\ERLIC_3SubProteomes5ppm.mgf

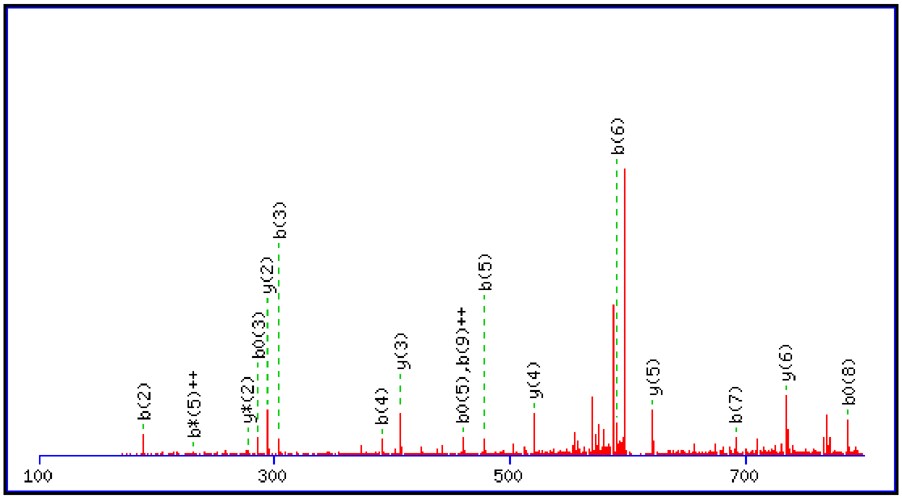

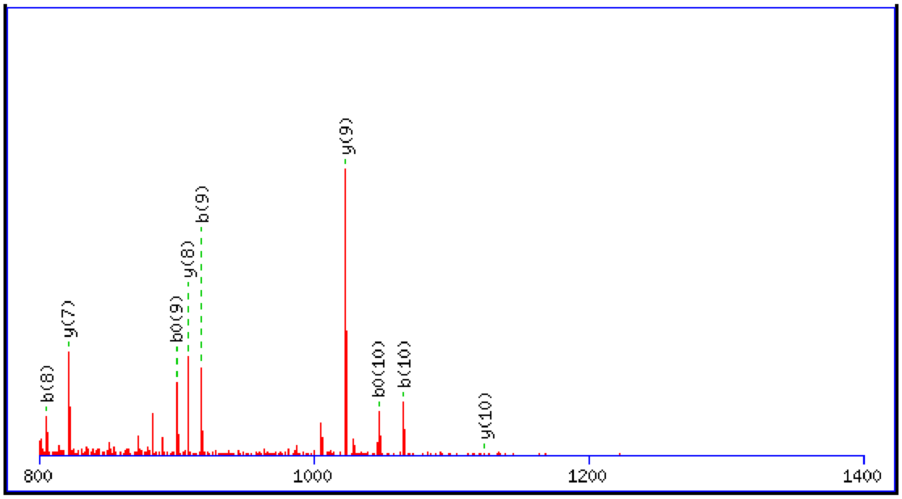


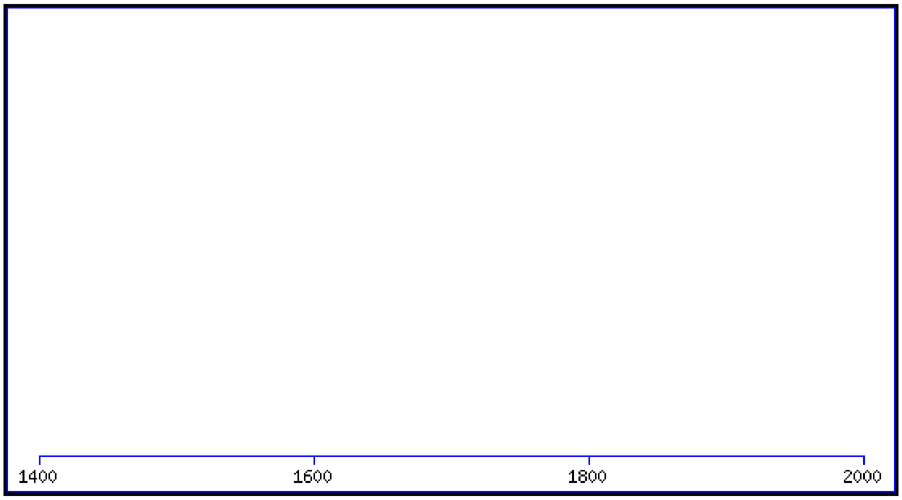


**Monoisotopic mass of neutral peptide Mr(calc):** 1210.6445
**Fixed modifications:** Carbamidomethyl (C)
**Variable modifications:**
**N3** : Deamidated_N (N)
**Ions Score:** 72 **Expect:** 5.5e-006
**Matches (Bold Red):** 26/108 fragment ions using 43 most intense peaks

| # | b | $b^{++}$ | b* | $b^{*++}$ | $b^0$ | $b^{0++}$ | Seq. | y | $y^{++}$ | y* | $y^{*++}$ | $y^0$ | $y^{0++}$ | # |
|---|---|---|---|---|---|---|---|---|---|---|---|---|---|---|
| 1 | 88.0393 | 44.5233 | | | 70.0287 | 35.5180 | S | | | | | | | 11 |
| 2 | **189.0870** | 95.0471 | | | 171.0764 | 86.0418 | T | **1124.6198** | 562.8135 | 1107.5932 | 554.3002 | 1106.6092 | 553.8082 | 10 |
| 3 | **304.1139** | 152.5606 | 287.0874 | 144.0473 | **286.1034** | 143.5553 | N | **1023.5721** | 512.2897 | 1006.5455 | 503.7764 | 1005.5615 | 503.2844 | 9 |
| 4 | **391.1459** | 196.0766 | 374.1194 | 187.5633 | 373.1354 | 187.0713 | S | **908.5451** | 454.7762 | 891.5186 | 446.2629 | 890.5346 | 445.7709 | 8 |
| 5 | **478.1780** | 239.5926 | 461.1514 | **231.0794** | **460.1674** | 230.5873 | S | **821.5131** | 411.2602 | 804.4866 | 402.7469 | 803.5026 | 402.2549 | 7 |
| 6 | **591.2620** | 296.1347 | 574.2355 | 287.6214 | 573.2515 | 287.1294 | L | **734.4811** | 367.7442 | 717.4545 | 359.2309 | 716.4705 | 358.7389 | 6 |
| 7 | **692.3097** | 346.6585 | 675.2832 | 338.1452 | 674.2992 | 337.6532 | T | **621.3970** | 311.2022 | 604.3705 | 302.6889 | 603.3865 | 302.1969 | 5 |
| 8 | **805.3938** | 403.2005 | 788.3672 | 394.6873 | **787.3832** | 394.1952 | L | **520.3493** | 260.6783 | 503.3228 | 252.1650 | | | 4 |
| 9 | **918.4778** | **459.7426** | 901.4513 | 451.2293 | **900.4673** | 450.7373 | L | **407.2653** | 204.1363 | 390.2387 | 195.6230 | | | 3 |
| 10 | **1065.5463** | 533.2768 | 1048.5197 | 524.7635 | **1047.5357** | 524.2715 | F | **294.1812** | 147.5942 | **277.1547** | 139.0810 | | | 2 |
| 11 | | | | | | | K | 147.1128 | 74.0600 | 130.0863 | 65.5468 | | | 1 |

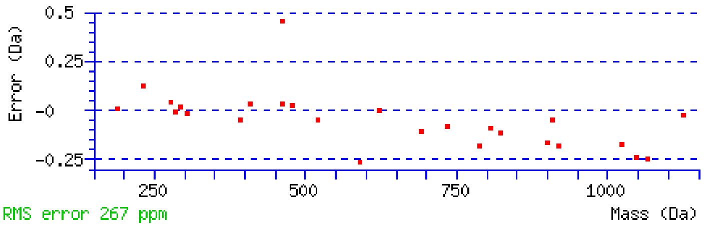


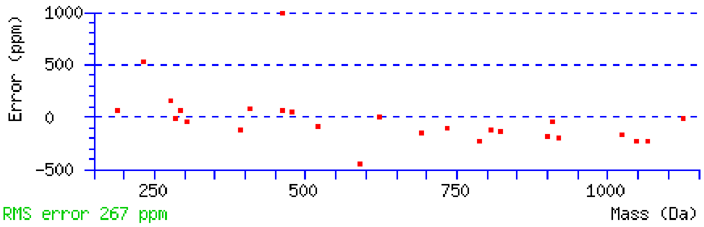

### All matches to this query

| Score | Mr(calc): | Delta | Sequence |
|---|---|---|---|
| 71.8 | 1210.6445 | 0.0011 | STNSSLTLLFK |
| 10.6 | 1210.6492 | -0.0036 | FNSRTKAIMK |
| 8.9 | 1210.6532 | -0.0076 | QCVAFKALFK |
| 7.5 | 1210.6346 | 0.0110 | RFSELGFEVK |
| 7.3 | 1210.6363 | 0.0093 | ITGAPAIIFTK |
| 6.3 | 1209.6370 | 1.0086 | SLLKTKPSEK |
| 5.8 | 1210.6557 | -0.0101 | TSVVLNHLAEK |
| 5.8 | 1209.6394 | 1.0062 | KEPSIFSAGFK |
| 4.3 | 1210.6458 | -0.0002 | EHRVGIPEFK |
| 3.8 | 1210.6454 | 0.0003 | YLVLANMLMK |

---

Spectrum No: 338; Query: 1695; Rank: 1

---

## Peptide View

MS/MS Fragmentation of **EYQLNDSASYYLNDLDR**
Found in **IPI00231726**, Tax_Id=10116 Gene_Symbol=Gnai3 Guanine nucleotide-binding protein G

Match to Query 1695: 2079.903188 from(1040.958870,2+)
Title: 091008RatKidney_NoSalt_21.4116.4116.2.dta
Data file K:\NewmanPaper\Piliang\3SubProteomes\Piliang3SP\mgf5ppm\ERLIC_3SubProteomes5ppm.mgf

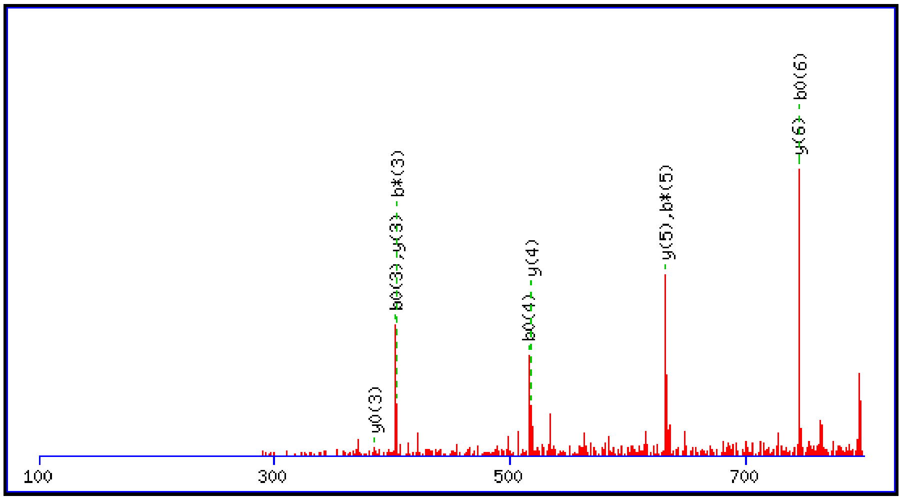


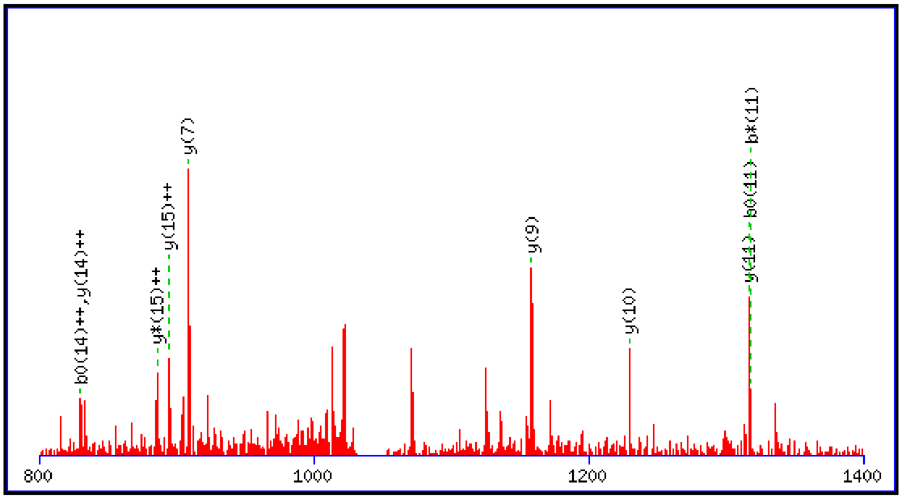

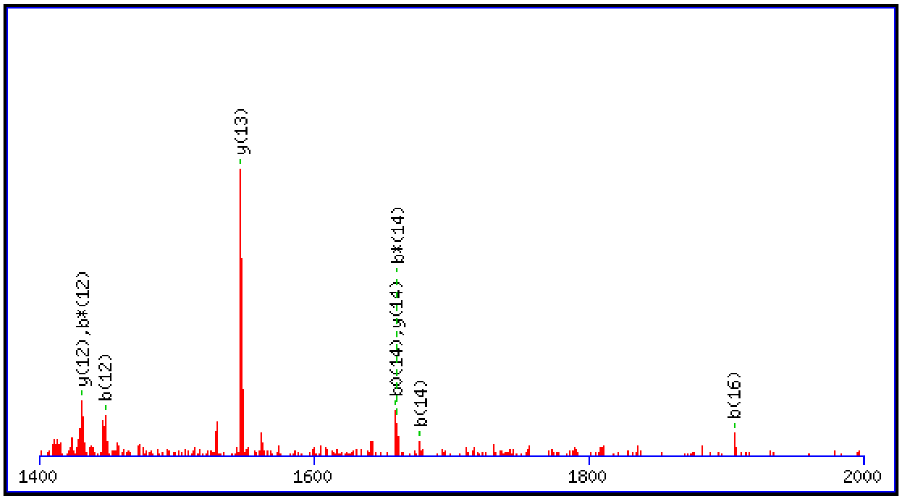


**Monoisotopic mass of neutral peptide Mr(calc):** 2078.9068
**Fixed modifications:** Carbamidomethyl (C)
**Variable modifications:**
**N5** : Deamidated_N (N)
**Ions Score:** 72 **Expect:** 1.6e-005
**Matches (Bold Red):** 29/186 fragment ions using 42 most intense peaks

| # | b | $b^{++}$ | b* | $b^{*++}$ | $b^{0}$ | $b^{0++}$ | Seq. | y | $y^{++}$ | y* | $y^{*++}$ | $y^{0}$ | $y^{0++}$ | # |
|---|---|---|---|---|---|---|---|---|---|---|---|---|---|---|
| 1 | 130.0499 | 65.5286 | | | 112.0393 | 56.5233 | E | | | | | | | 17 |
| 2 | 293.1132 | 147.0602 | | | 275.1026 | 138.0550 | Y | 1950.8716 | 975.9394 | 1933.8450 | 967.4261 | 1932.8610 | 966.9341 | 16 |
| 3 | 421.1718 | 211.0895 | **404.1452** | 202.5763 | **403.1612** | 202.0842 | Q | 1787.8082 | **894.4078** | 1770.7817 | **885.8945** | 1769.7977 | 885.4025 | 15 |
| 4 | 534.2558 | 267.6316 | 517.2293 | 259.1183 | **516.2453** | 258.6263 | L | **1659.7497** | **830.3785** | 1642.7231 | 821.8652 | 1641.7391 | 821.3732 | 14 |
| 5 | 649.2828 | 325.1450 | **632.2562** | 316.6318 | 631.2722 | 316.1397 | N | **1546.6656** | 773.8364 | 1529.6391 | 765.3232 | 1528.6550 | 764.8312 | 13 |
| 6 | 764.3097 | 382.6585 | 747.2832 | 374.1452 | **746.2992** | 373.6532 | D | **1431.6387** | 716.3230 | 1414.6121 | 707.8097 | 1413.6281 | 707.3177 | 12 |
| 7 | 851.3417 | 426.1745 | 834.3152 | 417.6612 | 833.3312 | 417.1692 | S | **1316.6117** | 658.8095 | 1299.5852 | 650.2962 | 1298.6012 | 649.8042 | 11 |
| 8 | 922.3789 | 461.6931 | 905.3523 | 453.1798 | 904.3683 | 452.6878 | A | **1229.5797** | 615.2935 | 1212.5531 | 606.7802 | 1211.5691 | 606.2882 | 10 |
| 9 | 1009.4109 | 505.2091 | 992.3843 | 496.6958 | 991.4003 | 496.2038 | S | **1158.5426** | 579.7749 | 1141.5160 | 571.2617 | 1140.5320 | 570.7696 | 9 |
| 10 | 1172.4742 | 586.7407 | 1155.4477 | 578.2275 | 1154.4637 | 577.7355 | Y | 1071.5106 | 536.2589 | 1054.4840 | 527.7456 | 1053.5000 | 527.2536 | 8 |
| 11 | 1335.5375 | 668.2724 | **1318.5110** | 659.7591 | **1317.5270** | 659.2671 | Y | **908.4472** | 454.7272 | 891.4207 | 446.2140 | 890.4367 | 445.7220 | 7 |
| 12 | **1448.6216** | 724.8144 | **1431.5951** | 716.3012 | 1430.6110 | 715.8092 | L | **745.3839** | 373.1956 | 728.3573 | 364.6823 | 727.3733 | 364.1903 | 6 |
| 13 | 1562.6645 | 781.8359 | 1545.6380 | 773.3226 | 1544.6540 | 772.8306 | N | **632.2998** | 316.6536 | 615.2733 | 308.1403 | 614.2893 | 307.6483 | 5 |
| 14 | **1677.6915** | 839.3494 | **1660.6649** | 830.8361 | **1659.6809** | **830.3441** | D | **518.2569** | 259.6321 | 501.2304 | 251.1188 | 500.2463 | 250.6268 | 4 |
| 15 | 1790.7755 | 895.8914 | 1773.7490 | 887.3781 | 1772.7650 | 886.8861 | L | **403.2300** | 202.1186 | 386.2034 | 193.6053 | **385.2194** | 193.1133 | 3 |
| 16 | **1905.8025** | 953.4049 | 1888.7759 | 944.8916 | 1887.7919 | 944.3996 | D | 290.1459 | 145.5766 | 273.1193 | 137.0633 | 272.1353 | 136.5713 | 2 |
| 17 | | | | | | | R | 175.1190 | 88.0631 | 158.0924 | 79.5498 | | | 1 |

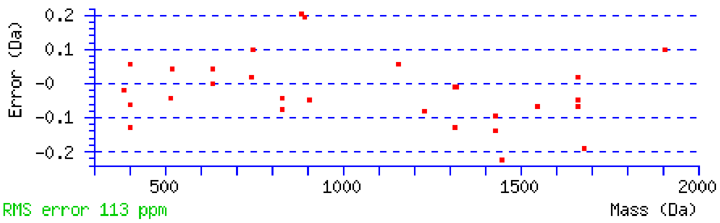


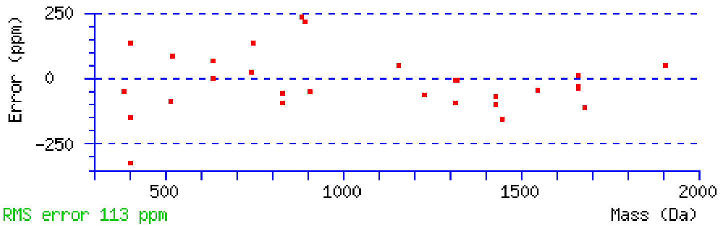


## All matches to this query

| Score | Mr(calc): | Delta | Sequence |
|---|---|---|---|
| 71.7 | 2078.9068 | 0.9963 | EYQLNDSASYYLNDLDR |
| 56.5 | 2079.8909 | 0.0123 | EYQLNDSASYYLNDLDR |
| 50.3 | 2078.9068 | 0.9963 | EYQLNDSASYYLNDLDR |
| 3.6 | 2077.9116 | 1.9916 | EYQLNDSAAYYLNDLER |

| | | | |
|---|---|---|---|
| 3.6 | 2078.9204 | 0.9828 | MEEQNNSAGKELQHKTR |
| 2.6 | 2077.8782 | 2.0250 | KYNISWELGNEPNNYR |
| 1.6 | 2079.8915 | 0.0117 | DSPNQSLLTGYIQTDLR |
| 1.5 | 2079.8915 | 0.0117 | DSPNQSLLTGYIQTDLR |
| 1.5 | 2079.8915 | 0.0117 | DSPNQSLLTGYIQTDLR |

---

Spectrum No: 339; Query: 684; Rank: 1

---

## Peptide View

MS/MS Fragmentation of **TIVYLNGSAQECR**
Found in **IPI00204348**, Tax_Id=10116 Gene_Symbol=Nt5e 5'-nucleotidase precursor

Match to Query 684: 1510.711828 from(756.363190,2+)
Title: 100101RatKid_NS_deglyco_15.2067.2067.2.dta
Data file K:\NewmanPaper\Piliang\3SubProteomes\Piliang3SP\mgf5ppm\ERLIC_3SubProteomes5ppm.mgf

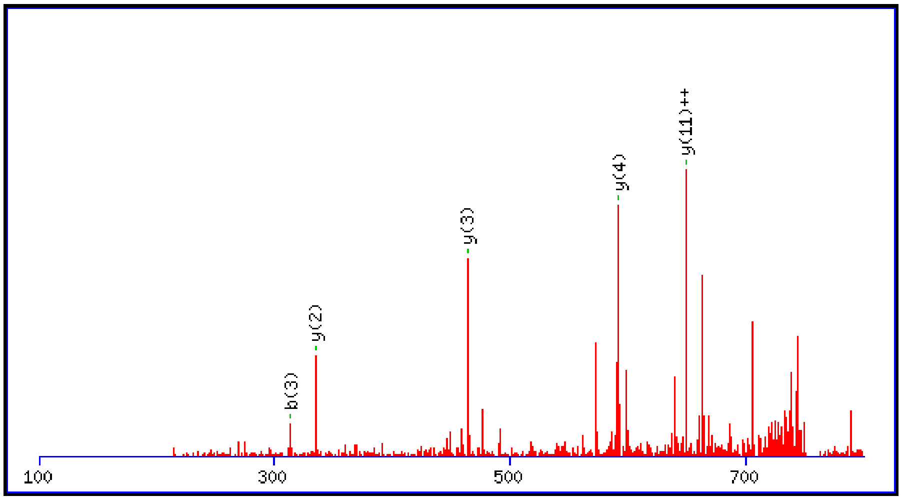


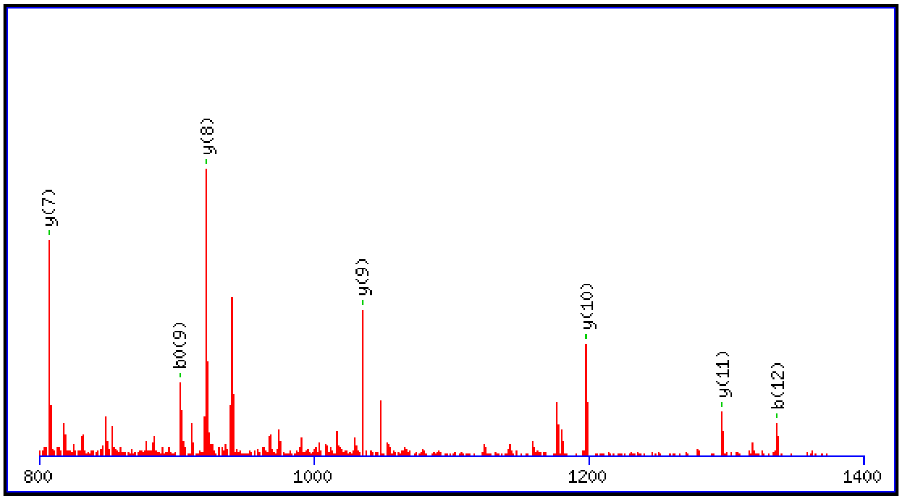

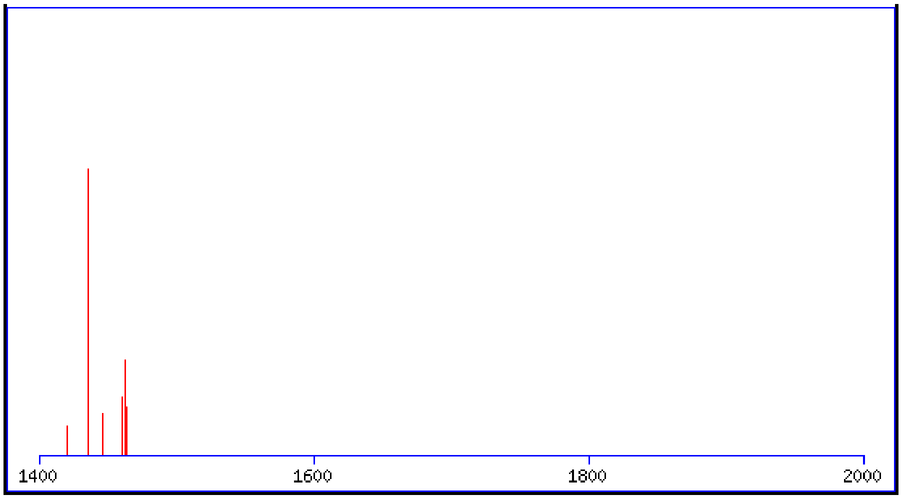


**Monoisotopic mass of neutral peptide Mr(calc):** 1510.7086
**Fixed modifications:** Carbamidomethyl (C)
**Variable modifications:**
**N6** : Deamidated_N (N)
**Ions Score:** 72 **Expect:** 1.2e-005
**Matches (Bold Red):** 12/130 fragment ions using 17 most intense peaks

| # | b | b++ | b* | b*++ | b0 | b0++ | Seq. | y | y++ | y* | y*++ | y0 | y0++ | # |
|---|---|---|---|---|---|---|---|---|---|---|---|---|---|---|
| 1 | 102.0550 | 51.5311 | | | 84.0444 | 42.5258 | T | | | | | | | 13 |
| 2 | 215.1390 | 108.0731 | | | 197.1285 | 99.0679 | I | 1410.6682 | 705.8377 | 1393.6416 | 697.3245 | 1392.6576 | 696.8324 | 12 |
| 3 | **314.2074** | 157.6074 | | | 296.1969 | 148.6021 | V | **1297.5841** | **649.2957** | 1280.5576 | 640.7824 | 1279.5736 | 640.2904 | 11 |
| 4 | 477.2708 | 239.1390 | | | 459.2602 | 230.1337 | Y | **1198.5157** | 599.7615 | 1181.4892 | 591.2482 | 1180.5051 | 590.7562 | 10 |
| 5 | 590.3548 | 295.6811 | | | 572.3443 | 286.6758 | L | **1035.4524** | 518.2298 | 1018.4258 | 509.7166 | 1017.4418 | 509.2245 | 9 |
| 6 | 705.3818 | 353.1945 | 688.3552 | 344.6812 | 687.3712 | 344.1892 | N | **922.3683** | 461.6878 | 905.3418 | 453.1745 | 904.3577 | 452.6825 | 8 |
| 7 | 762.4032 | 381.7053 | 745.3767 | 373.1920 | 744.3927 | 372.7000 | G | **807.3414** | 404.1743 | 790.3148 | 395.6611 | 789.3308 | 395.1690 | 7 |
| 8 | 849.4353 | 425.2213 | 832.4087 | 416.7080 | 831.4247 | 416.2160 | S | 750.3199 | 375.6636 | 733.2934 | 367.1503 | 732.3093 | 366.6583 | 6 |
| 9 | 920.4724 | 460.7398 | 903.4458 | 452.2265 | **902.4618** | 451.7345 | A | 663.2879 | 332.1476 | 646.2613 | 323.6343 | 645.2773 | 323.1423 | 5 |
| 10 | 1048.5309 | 524.7691 | 1031.5044 | 516.2558 | 1030.5204 | 515.7638 | Q | **592.2508** | 296.6290 | 575.2242 | 288.1157 | 574.2402 | 287.6237 | 4 |
| 11 | 1177.5735 | 589.2904 | 1160.5470 | 580.7771 | 1159.5630 | 580.2851 | E | **464.1922** | 232.5997 | 447.1656 | 224.0865 | 446.1816 | 223.5945 | 3 |
| 12 | **1337.6042** | 669.3057 | 1320.5776 | 660.7925 | 1319.5936 | 660.3004 | C | **335.1496** | 168.0784 | 318.1231 | 159.5652 | | | 2 |
| 13 | | | | | | | R | 175.1190 | 88.0631 | 158.0924 | 79.5498 | | | 1 |

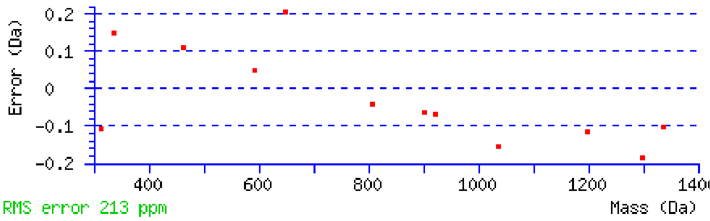


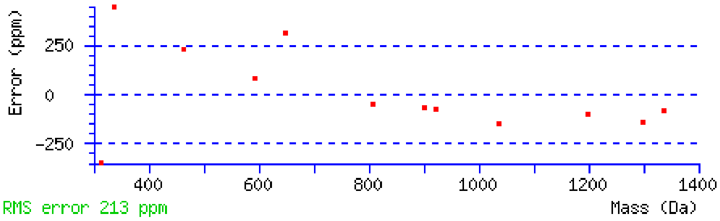


### All matches to this query

| Score | Mr(calc): | Delta | Sequence |
|---|---|---|---|
| 71.6 | 1510.7086 | 0.0033 | TIVYLNGSAQECR |
| 1.6 | 1510.7001 | 0.0117 | ASRLQTDSQGRGR |

---

Spectrum No: 340; Query: 2095; Rank: 1

---

## Peptide View

MS/MS Fragmentation of **EIDRDQLMTVLANVTHLLIR**
Found in **IPI00363534**, Tax_Id=10116 Gene_Symbol=Lama1 laminin, alpha 1

Match to Query 2095: 2350.270992 from(784.430940,3+)
Title: 100101RatKid_NS_deglyco_10.7536.7536.3.dta
Data file K:\NewmanPaper\Piliang\3SubProteomes\Piliang3SP\mgf5ppm\ERLIC_3SubProteomes5ppm.mgf

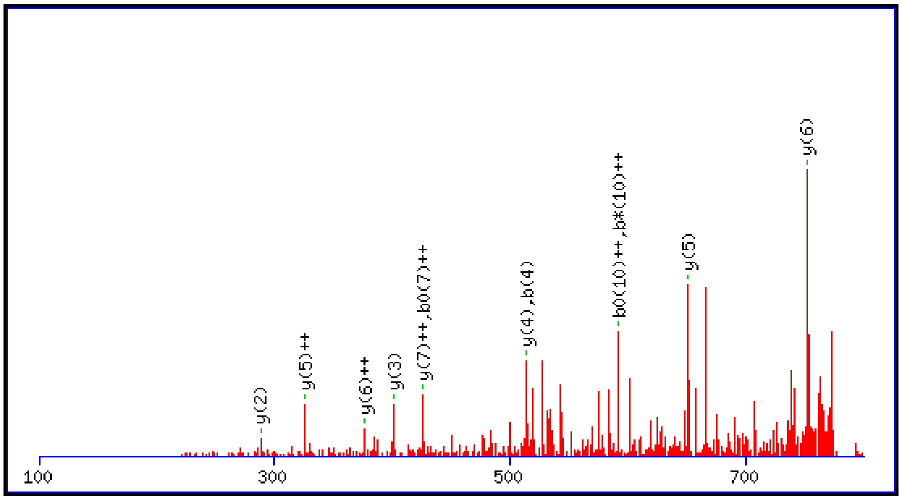


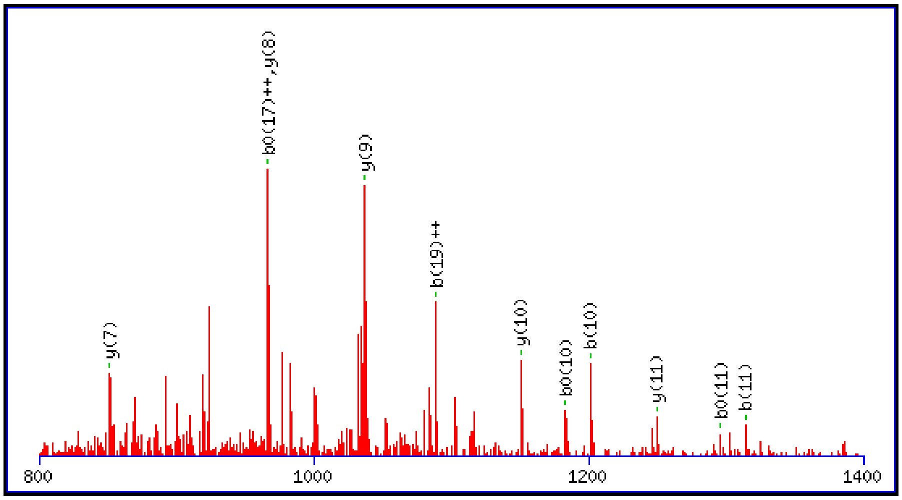


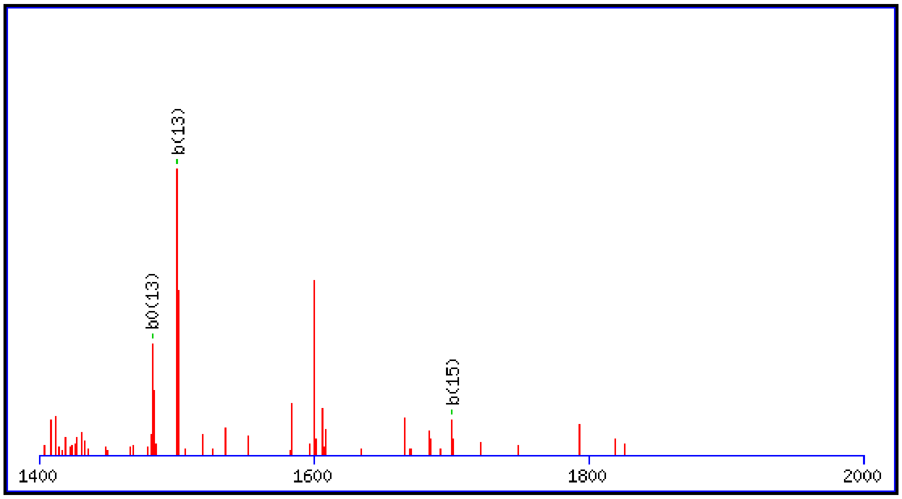


**Monoisotopic mass of neutral peptide Mr(calc):** 2350.2678
**Fixed modifications:** Carbamidomethyl (C)
**Variable modifications:**
**N13** : Deamidated_N (N)
**Ions Score:** 72 **Expect:** 4.2e-006
**Matches (Bold Red):** 26/212 fragment ions using 37 most intense peaks

| # | b | $b^{++}$ | b* | $b^{*++}$ | $b^0$ | $b^{0++}$ | Seq. | y | $y^{++}$ | y* | $y^{*++}$ | $y^0$ | $y^{0++}$ | # |
|---|---|---|---|---|---|---|---|---|---|---|---|---|---|---|

| | | | | | | | | | | | | | | |
|---|---|---|---|---|---|---|---|---|---|---|---|---|---|---|
| 1 | 130.0499 | 65.5286 | | | 112.0393 | 56.5233 | E | | | | | | | 20 |
| 2 | 243.1339 | 122.0706 | | | 225.1234 | 113.0653 | I | 2222.2325 | 1111.6199 | 2205.2060 | 1103.1066 | 2204.2220 | 1102.6146 | 19 |
| 3 | 358.1609 | 179.5841 | | | 340.1503 | 170.5788 | D | 2109.1485 | 1055.0779 | 2092.1219 | 1046.5646 | 2091.1379 | 1046.0726 | 18 |
| 4 | 514.2620 | 257.6346 | 497.2354 | 249.1214 | 496.2514 | 248.6293 | R | 1994.1215 | 997.5644 | 1977.0950 | 989.0511 | 1976.1110 | 988.5591 | 17 |
| 5 | 629.2889 | 315.1481 | 612.2624 | 306.6348 | 611.2784 | 306.1428 | D | 1838.0204 | 919.5139 | 1820.9939 | 911.0006 | 1820.0099 | 910.5086 | 16 |
| 6 | 757.3475 | 379.1774 | 740.3210 | 370.6641 | 739.3369 | 370.1721 | Q | 1722.9935 | 862.0004 | 1705.9669 | 853.4871 | 1704.9829 | 852.9951 | 15 |
| 7 | 870.4316 | 435.7194 | 853.4050 | 427.2061 | 852.4210 | 426.7141 | L | 1594.9349 | 797.9711 | 1577.9084 | 789.4578 | 1576.9243 | 788.9658 | 14 |
| 8 | 1001.4721 | 501.2397 | 984.4455 | 492.7264 | 983.4615 | 492.2344 | M | 1481.8508 | 741.4291 | 1464.8243 | 732.9158 | 1463.8403 | 732.4238 | 13 |
| 9 | 1102.5197 | 551.7635 | 1085.4932 | 543.2502 | 1084.5092 | 542.7582 | T | 1350.8104 | 675.9088 | 1333.7838 | 667.3955 | 1332.7998 | 666.9035 | 12 |
| 10 | 1201.5882 | 601.2977 | 1184.5616 | 592.7844 | 1183.5776 | 592.2924 | V | 1249.7627 | 625.3850 | 1232.7361 | 616.8717 | 1231.7521 | 616.3797 | 11 |
| 11 | 1314.6722 | 657.8397 | 1297.6457 | 649.3265 | 1296.6616 | 648.8345 | L | 1150.6943 | 575.8508 | 1133.6677 | 567.3375 | 1132.6837 | 566.8455 | 10 |
| 12 | 1385.7093 | 693.3583 | 1368.6828 | 684.8450 | 1367.6988 | 684.3530 | A | 1037.6102 | 519.3087 | 1020.5836 | 510.7955 | 1019.5996 | 510.3035 | 9 |
| 13 | 1500.7363 | 750.8718 | 1483.7097 | 742.3585 | 1482.7257 | 741.8665 | N | 966.5731 | 483.7902 | 949.5465 | 475.2769 | 948.5625 | 474.7849 | 8 |
| 14 | 1599.8047 | 800.4060 | 1582.7781 | 791.8927 | 1581.7941 | 791.4007 | V | 851.5461 | 426.2767 | 834.5196 | 417.7634 | 833.5356 | 417.2714 | 7 |
| 15 | 1700.8524 | 850.9298 | 1683.8258 | 842.4165 | 1682.8418 | 841.9245 | T | 752.4777 | 376.7425 | 735.4512 | 368.2292 | 734.4672 | 367.7372 | 6 |
| 16 | 1837.9113 | 919.4593 | 1820.8847 | 910.9460 | 1819.9007 | 910.4540 | H | 651.4301 | 326.2187 | 634.4035 | 317.7054 | | | 5 |
| 17 | 1950.9953 | 976.0013 | 1933.9688 | 967.4880 | 1932.9848 | 966.9960 | L | 514.3711 | 257.6892 | 497.3446 | 249.1759 | | | 4 |
| 18 | 2064.0794 | 1032.5433 | 2047.0528 | 1024.0301 | 2046.0688 | 1023.5381 | L | 401.2871 | 201.1472 | 384.2605 | 192.6339 | | | 3 |
| 19 | 2177.1635 | 1089.0854 | 2160.1369 | 1080.5721 | 2159.1529 | 1080.0801 | I | 288.2030 | 144.6051 | 271.1765 | 136.0919 | | | 2 |
| 20 | | | | | | | R | 175.1190 | 88.0631 | 158.0924 | 79.5498 | | | 1 |

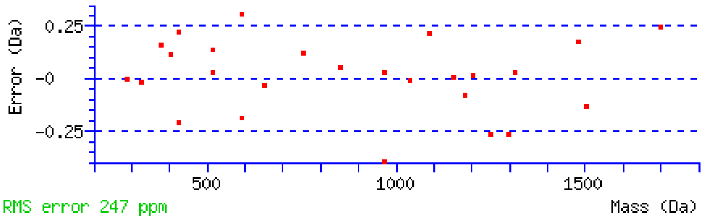


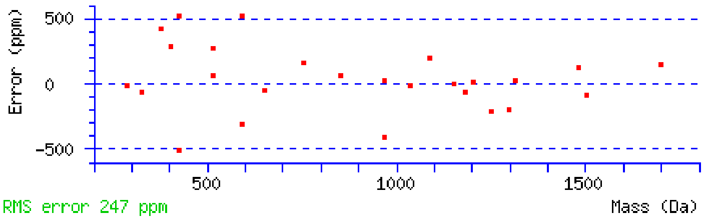


**All matches to this query**

| Score | Mr(calc): | Delta | Sequence |
|---|---|---|---|
| 71.6 | 2350.2678 | 0.0032 | EIDRDQLMTVLANVTHLLIR |
| 19.7 | 2349.2838 | 0.9872 | EIDRDQLMTVLANVTHLLIR |
| 2.8 | 2350.2733 | -0.0023 | STNLLLTRTLSNSLQNVIKR |
| 0.2 | 2350.2930 | -0.0220 | NVVAECLGKLTLIDPETLLPR |

---

Spectrum No: 341; Query: 2832; Rank: 1

---

## Peptide View

MS/MS Fragmentation of **DYGPRPDADSESDNSDNNTIFVQGLGEGVSTDQVGEFFK**
Found in **IPI00364132**, Tax_Id=10116 Gene_Symbol=Taf15_predicted 59 kDa protein

Match to Query 2832: 4206.857172 from(1403.293000,3+)
Title: 091008RatKidney_NoSalt_22.5159.5159.3.dta
Data file K:\NewmanPaper\Piliang\3SubProteomes\Piliang3SP\mgf5ppm\ERLIC_3SubProteomes5ppm.mgf

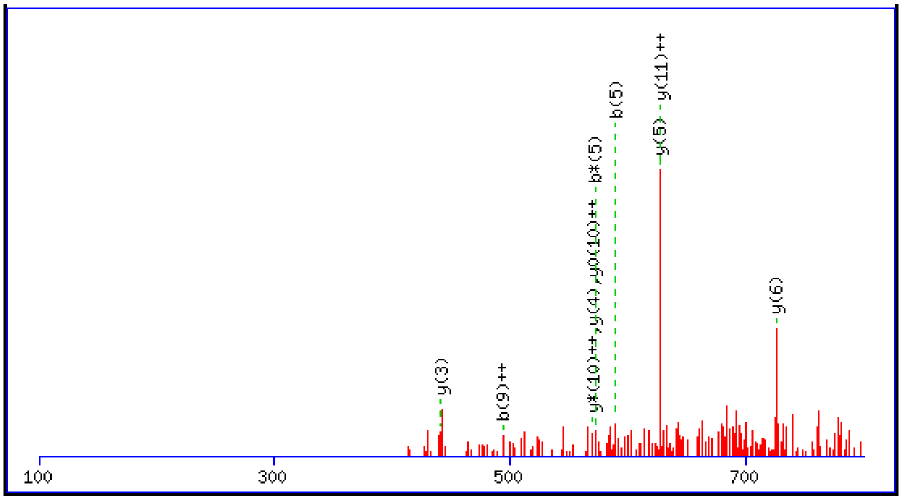


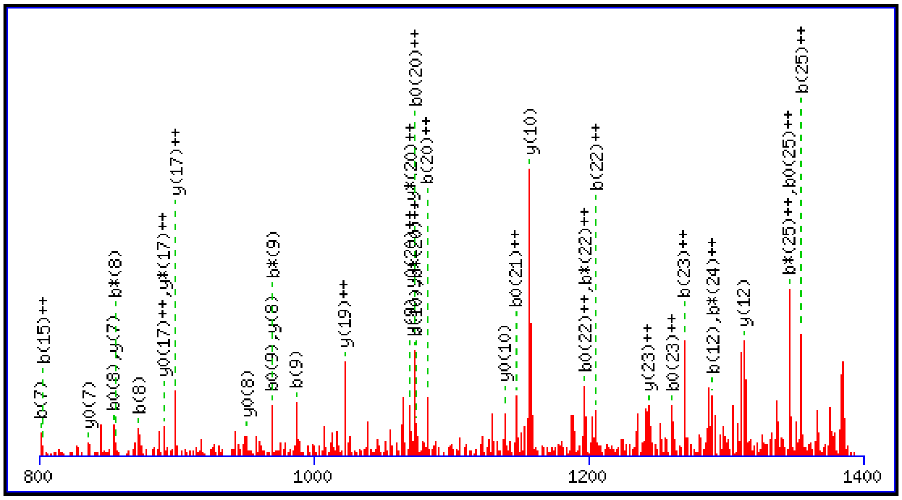


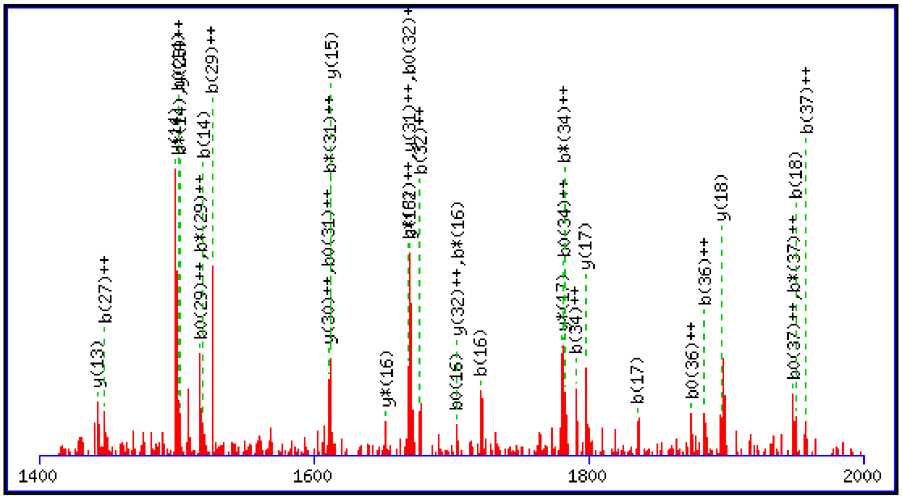


**Monoisotopic mass of neutral peptide Mr(calc):** 4206.8414
**Fixed modifications:** Carbamidomethyl (C)
**Variable modifications:**
**N18** : Deamidated_N (N)
**Ions Score:** 72 **Expect:** 3.6e-005
**Matches (Bold Red):** 85/442 fragment ions using 150 most intense peaks

| # | b | b++ | b* | b*++ | b0 | b0++ | Seq. | y | y++ | y* | y*++ | y0 | y0++ | # |
|---|---|---|---|---|---|---|---|---|---|---|---|---|---|---|
| 1 | 116.0342 | 58.5207 | | | 98.0237 | 49.5155 | D | | | | | | | 39 |
| 2 | 279.0975 | 140.0524 | | | 261.0870 | 131.0471 | Y | 4092.8217 | 2046.9145 | 4075.7952 | 2038.4012 | 4074.8112 | 2037.9092 | 38 |
| 3 | 336.1190 | 168.5631 | | | 318.1084 | 159.5579 | G | 3929.7584 | 1965.3828 | 3912.7318 | 1956.8696 | 3911.7478 | 1956.3776 | 37 |
| 4 | 433.1718 | 217.0895 | | | 415.1612 | 208.0842 | P | 3872.7369 | 1936.8721 | 3855.7104 | 1928.3588 | 3854.7264 | 1927.8668 | 36 |
| 5 | **589.2729** | 295.1401 | **572.2463** | 286.6268 | 571.2623 | 286.1348 | R | 3775.6842 | 1888.3457 | 3758.6576 | 1879.8324 | 3757.6736 | 1879.3404 | 35 |

| | | | | | | | | | | | | | | |
|---|---|---|---|---|---|---|---|---|---|---|---|---|---|---|
| 6 | 686.3257 | 343.6665 | 669.2991 | 335.1532 | 668.3151 | 334.6612 | P | 3619.5831 | 1810.2952 | 3602.5565 | 1801.7819 | 3601.5725 | 1801.2899 | 34 |
| 7 | 801.3526 | 401.1799 | 784.3260 | 392.6667 | 783.3420 | 392.1747 | D | 3522.5303 | 1761.7688 | 3505.5037 | 1753.2555 | 3504.5197 | 1752.7635 | 33 |
| 8 | 872.3897 | 436.6985 | 855.3632 | 428.1852 | 854.3791 | 427.6932 | A | 3407.5034 | 1704.2553 | 3390.4768 | 1695.7420 | 3389.4928 | 1695.2500 | 32 |
| 9 | 987.4167 | 494.2120 | 970.3901 | 485.6987 | 969.4061 | 485.2067 | D | 3336.4662 | 1668.7368 | 3319.4397 | 1660.2235 | 3318.4557 | 1659.7315 | 31 |
| 10 | 1074.4487 | 537.7280 | 1057.4221 | 529.2147 | 1056.4381 | 528.7227 | S | 3221.4393 | 1611.2233 | 3204.4127 | 1602.7100 | 3203.4287 | 1602.2180 | 30 |
| 11 | 1203.4913 | 602.2493 | 1186.4647 | 593.7360 | 1185.4807 | 593.2440 | E | 3134.4073 | 1567.7073 | 3117.3807 | 1559.1940 | 3116.3967 | 1558.7020 | 29 |
| 12 | 1290.5233 | 645.7653 | 1273.4968 | 637.2520 | 1272.5127 | 636.7600 | S | 3005.3647 | 1503.1860 | 2988.3381 | 1494.6727 | 2987.3541 | 1494.1807 | 28 |
| 13 | 1405.5502 | 703.2788 | 1388.5237 | 694.7655 | 1387.5397 | 694.2735 | D | 2918.3326 | 1459.6700 | 2901.3061 | 1451.1567 | 2900.3221 | 1450.6647 | 27 |
| 14 | 1519.5932 | 760.3002 | 1502.5666 | 751.7869 | 1501.5826 | 751.2949 | N | 2803.3057 | 1402.1565 | 2786.2792 | 1393.6432 | 2785.2951 | 1393.1512 | 26 |
| 15 | 1606.6252 | 803.8162 | 1589.5986 | 795.3030 | 1588.6146 | 794.8110 | S | 2689.2628 | 1345.1350 | 2672.2362 | 1336.6218 | 2671.2522 | 1336.1297 | 25 |
| 16 | 1721.6521 | 861.3297 | 1704.6256 | 852.8164 | 1703.6416 | 852.3244 | D | 2602.2307 | 1301.6190 | 2585.2042 | 1293.1057 | 2584.2202 | 1292.6137 | 24 |
| 17 | 1835.6951 | 918.3512 | 1818.6685 | 909.8379 | 1817.6845 | 909.3459 | N | 2487.2038 | 1244.1055 | 2470.1773 | 1235.5923 | 2469.1932 | 1235.1003 | 23 |
| 18 | 1950.7220 | 975.8646 | 1933.6955 | 967.3514 | 1932.7114 | 966.8594 | N | 2373.1609 | 1187.0841 | 2356.1343 | 1178.5708 | 2355.1503 | 1178.0788 | 22 |
| 19 | 2051.7697 | 1026.3885 | 2034.7431 | 1017.8752 | 2033.7591 | 1017.3832 | T | 2258.1339 | 1129.5706 | 2241.1074 | 1121.0573 | 2240.1234 | 1120.5653 | 21 |
| 20 | 2164.8537 | 1082.9305 | 2147.8272 | 1074.4172 | 2146.8432 | 1073.9252 | I | 2157.0863 | 1079.0468 | 2140.0597 | 1070.5335 | 2139.0757 | 1070.0415 | 20 |
| 21 | 2311.9222 | 1156.4647 | 2294.8956 | 1147.9514 | 2293.9116 | 1147.4594 | F | 2044.0022 | 1022.5047 | 2026.9757 | 1013.9915 | 2025.9916 | 1013.4995 | 19 |
| 22 | 2410.9906 | 1205.9989 | 2393.9640 | 1197.4857 | 2392.9800 | 1196.9936 | V | 1896.9338 | 948.9705 | 1879.9072 | 940.4573 | 1878.9232 | 939.9652 | 18 |
| 23 | 2539.0492 | 1270.0282 | 2522.0226 | 1261.5149 | 2521.0386 | 1261.0229 | Q | 1797.8654 | 899.4363 | 1780.8388 | 890.9230 | 1779.8548 | 890.4310 | 17 |
| 24 | 2596.0706 | 1298.5389 | 2579.0441 | 1290.0257 | 2578.0601 | 1289.5337 | G | 1669.8068 | 835.4070 | 1652.7802 | 826.8938 | 1651.7962 | 826.4018 | 16 |
| 25 | 2709.1547 | 1355.0810 | 2692.1281 | 1346.5677 | 2691.1441 | 1346.0757 | L | 1612.7853 | 806.8963 | 1595.7588 | 798.3830 | 1594.7748 | 797.8910 | 15 |
| 26 | 2766.1761 | 1383.5917 | 2749.1496 | 1375.0784 | 2748.1656 | 1374.5864 | G | 1499.7013 | 750.3543 | 1482.6747 | 741.8410 | 1481.6907 | 741.3490 | 14 |
| 27 | 2895.2187 | 1448.1130 | 2878.1922 | 1439.5997 | 2877.2082 | 1439.1077 | E | 1442.6798 | 721.8435 | 1425.6533 | 713.3303 | 1424.6692 | 712.8383 | 13 |
| 28 | 2952.2402 | 1476.6237 | 2935.2137 | 1468.1105 | 2934.2296 | 1467.6185 | G | 1313.6372 | 657.3222 | 1296.6107 | 648.8090 | 1295.6266 | 648.3170 | 12 |
| 29 | 3051.3086 | 1526.1579 | 3034.2821 | 1517.6447 | 3033.2981 | 1517.1527 | V | 1256.6157 | 628.8115 | 1239.5892 | 620.2982 | 1238.6052 | 619.8062 | 11 |
| 30 | 3138.3406 | 1569.6740 | 3121.3141 | 1561.1607 | 3120.3301 | 1560.6687 | S | 1157.5473 | 579.2773 | 1140.5208 | 570.7640 | 1139.5368 | 570.2720 | 10 |
| 31 | 3239.3883 | 1620.1978 | 3222.3618 | 1611.6845 | 3221.3778 | 1611.1925 | T | 1070.5153 | 535.7613 | 1053.4888 | 527.2480 | 1052.5047 | 526.7560 | 9 |
| 32 | 3354.4153 | 1677.7113 | 3337.3887 | 1669.1980 | 3336.4047 | 1668.7060 | D | 969.4676 | 485.2374 | 952.4411 | 476.7242 | 951.4571 | 476.2322 | 8 |
| 33 | 3482.4738 | 1741.7406 | 3465.4473 | 1733.2273 | 3464.4633 | 1732.7353 | Q | 854.4407 | 427.7240 | 837.4141 | 419.2107 | 836.4301 | 418.7187 | 7 |
| 34 | 3581.5423 | 1791.2748 | 3564.5157 | 1782.7615 | 3563.5317 | 1782.2695 | V | 726.3821 | 363.6947 | 709.3556 | 355.1814 | 708.3715 | 354.6894 | 6 |
| 35 | 3638.5637 | 1819.7855 | 3621.5372 | 1811.2722 | 3620.5532 | 1810.7802 | G | 627.3137 | 314.1605 | 610.2871 | 305.6472 | 609.3031 | 305.1552 | 5 |
| 36 | 3767.6063 | 1884.3068 | 3750.5798 | 1875.7935 | 3749.5957 | 1875.3015 | E | 570.2922 | 285.6498 | 553.2657 | 277.1365 | 552.2817 | 276.6445 | 4 |
| 37 | 3914.6747 | 1957.8410 | 3897.6482 | 1949.3277 | 3896.6642 | 1948.8357 | F | 441.2496 | 221.1285 | 424.2231 | 212.6152 | | | 3 |
| 38 | 4061.7431 | 2031.3752 | 4044.7166 | 2022.8619 | 4043.7326 | 2022.3699 | F | 294.1812 | 147.5942 | 277.1547 | 139.0810 | | | 2 |
| 39 | | | | | | | K | 147.1128 | 74.0600 | 130.0863 | 65.5468 | | | 1 |

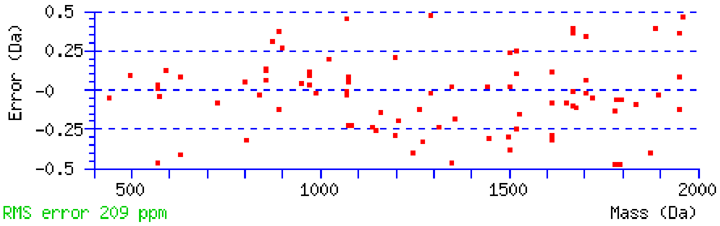


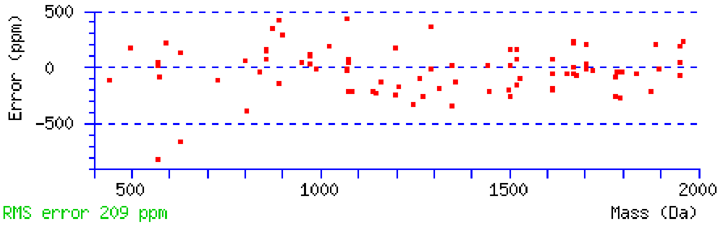


### All matches to this query

| Score | Mr(calc): | Delta | Sequence |
|---|---|---|---|
| 71.6 | 4206.8414 | 0.0157 | DYGPRPDADSESDNSDNNTIFVQGLGEGVSTDQVGEFFK |
| 71.6 | 4206.8414 | 0.0157 | DYGPRPDADSESDNSDNNTIFVQGLGEGVSTDQVGEFFK |
| 66.7 | 4205.8574 | 0.9998 | DYGPRPDADSESDNSDNNTIFVQGLGEGVSTDQVGEFFK |
| 66.2 | 4206.8414 | 0.0157 | DYGPRPDADSESDNSDNNTIFVQGLGEGVSTDQVGEFFK |
| 9.5 | 4205.8209 | 1.0362 | VEIDAINDPFIDLNYMVYMLQYVSTHGKFNGTVK |
| 7.5 | 4206.8718 | -0.0147 | LHTPMYFFLGNFAFLEIWYVSSTAPNMLANILSK |

| | | | |
|---|---|---|---|
| 6.5 | 4205.8209 | 1.0362 | VEIDAINDPFIDLNYMVYMLQYVSTHGKFNGTVK |
| 5.6 | 4206.8718 | -0.0147 | LHTPMYFFLGNFAFLEIWYVSSTAPNMLANILSK |
| 4.9 | 4204.8675 | 1.9897 | EMLPTNPIRLGLALNFSVFHYEIANSPEEAISLAK |
| 4.9 | 4204.8311 | 2.0261 | EMPPTNPIRLGLALNFSVFHYEIANSPEEAISLAK |

---

Spectrum No: 342; Query: 1866; Rank: 1

---

## Peptide View

MS/MS Fragmentation of **RSGPSDEGEEEMEDGTVTNGS**
Found in **IPI00192279**, Tax_Id=10116 Gene_Symbol=- 28 kDa protein

Match to Query 1866: 2182.847308 from(1092.430930,2+)
Title: 100101RatKid_NS_deglyco_24.1138.1138.2.dta
Data file K:\NewmanPaper\Piliang\3SubProteomes\Piliang3SP\mgf5ppm\ERLIC_3SubProteomes5ppm.mgf

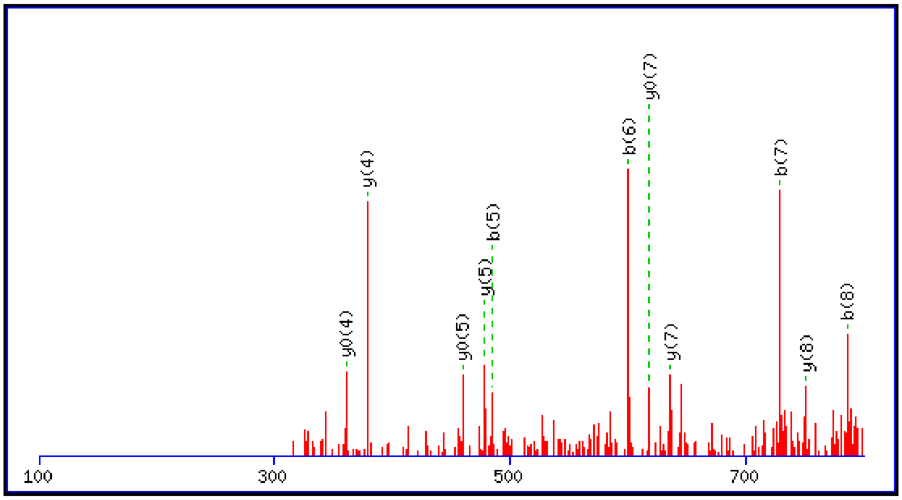


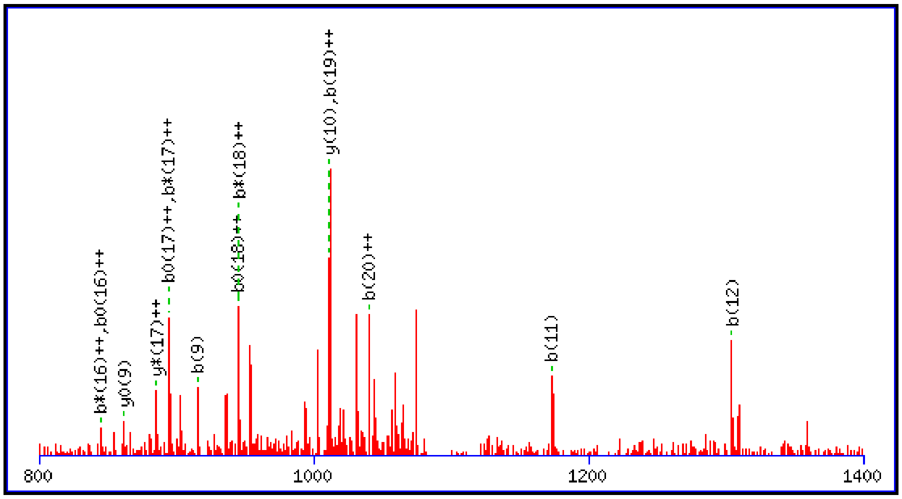

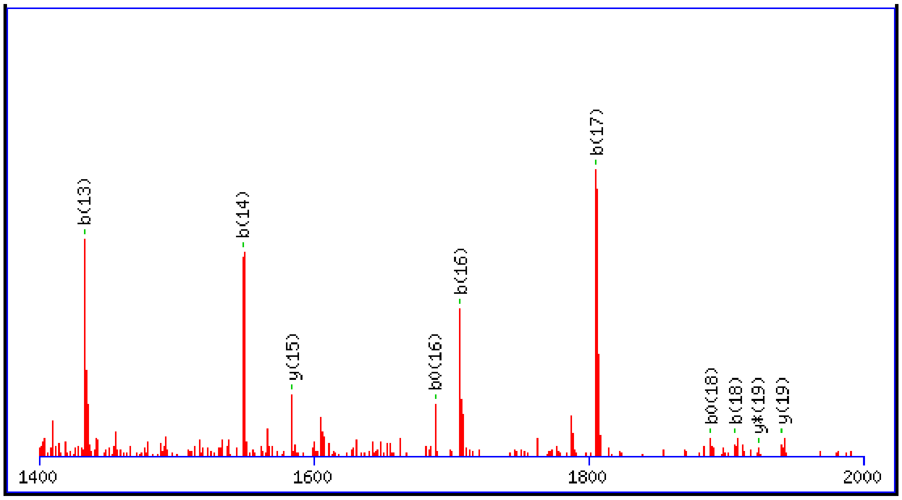


**Monoisotopic mass of neutral peptide Mr(calc):** 2182.8444
**Fixed modifications:** Carbamidomethyl (C)
**Variable modifications:**
**N19** : Deamidated_N (N)
**Ions Score:** 72 **Expect:** 1.3e-005
**Matches (Bold Red):** 35/234 fragment ions using 56 most intense peaks

| # | b | $b^{++}$ | b* | $b^{*++}$ | $b^0$ | $b^{0++}$ | Seq. | y | $y^{++}$ | y* | $y^{*++}$ | $y^0$ | $y^{0++}$ | # |
|---|---|---|---|---|---|---|---|---|---|---|---|---|---|---|
| 1 | 157.1084 | 79.0578 | 140.0818 | 70.5446 | | | R | | | | | | | 21 |
| 2 | 244.1404 | 122.5738 | 227.1139 | 114.0606 | 226.1298 | 113.5686 | S | 2027.7506 | 1014.3789 | 2010.7240 | 1005.8657 | 2009.7400 | 1005.3737 | 20 |
| 3 | 301.1619 | 151.0846 | 284.1353 | 142.5713 | 283.1513 | 142.0793 | G | **1940.7186** | 970.8629 | **1923.6920** | 962.3496 | 1922.7080 | 961.8576 | 19 |
| 4 | 398.2146 | 199.6110 | 381.1881 | 191.0977 | 380.2041 | 190.6057 | P | 1883.6971 | 942.3522 | 1866.6705 | 933.8389 | 1865.6865 | 933.3469 | 18 |
| 5 | **485.2467** | 243.1270 | 468.2201 | 234.6137 | 467.2361 | 234.1217 | S | 1786.6443 | 893.8258 | 1769.6178 | **885.3125** | 1768.6338 | 884.8205 | 17 |
| 6 | **600.2736** | 300.6404 | 583.2471 | 292.1272 | 582.2630 | 291.6352 | D | 1699.6123 | 850.3098 | 1682.5858 | 841.7965 | 1681.6017 | 841.3045 | 16 |
| 7 | **729.3162** | 365.1617 | 712.2897 | 356.6485 | 711.3056 | 356.1565 | E | **1584.5854** | 792.7963 | 1567.5588 | 784.2830 | 1566.5748 | 783.7910 | 15 |
| 8 | **786.3377** | 393.6725 | 769.3111 | 385.1592 | 768.3271 | 384.6672 | G | 1455.5428 | 728.2750 | 1438.5162 | 719.7617 | 1437.5322 | 719.2697 | 14 |
| 9 | **915.3803** | 458.1938 | 898.3537 | 449.6805 | 897.3697 | 449.1885 | E | 1398.5213 | 699.7643 | 1381.4948 | 691.2510 | 1380.5107 | 690.7590 | 13 |
| 10 | 1044.4229 | 522.7151 | 1027.3963 | 514.2018 | 1026.4123 | 513.7098 | E | 1269.4787 | 635.2430 | 1252.4522 | 626.7297 | 1251.4681 | 626.2377 | 12 |
| 11 | **1173.4654** | 587.2364 | 1156.4389 | 578.7231 | 1155.4549 | 578.2311 | E | 1140.4361 | 570.7217 | 1123.4096 | 562.2084 | 1122.4256 | 561.7164 | 11 |
| 12 | **1304.5059** | 652.7566 | 1287.4794 | 644.2433 | 1286.4954 | 643.7513 | M | **1011.3935** | 506.2004 | 994.3670 | 497.6871 | 993.3830 | 497.1951 | 10 |
| 13 | **1433.5485** | 717.2779 | 1416.5220 | 708.7646 | 1415.5380 | 708.2726 | E | 880.3530 | 440.6802 | 863.3265 | 432.1669 | **862.3425** | 431.6749 | 9 |
| 14 | **1548.5755** | 774.7914 | 1531.5489 | 766.2781 | 1530.5649 | 765.7861 | D | **751.3104** | 376.1589 | 734.2839 | 367.6456 | 733.2999 | 367.1536 | 8 |
| 15 | 1605.5969 | 803.3021 | 1588.5704 | 794.7888 | 1587.5864 | 794.2968 | G | **636.2835** | 318.6454 | 619.2570 | 310.1321 | **618.2729** | 309.6401 | 7 |
| 16 | **1706.6446** | 853.8259 | 1689.6181 | **845.3127** | **1688.6340** | **844.8207** | T | 579.2620 | 290.1347 | 562.2355 | 281.6214 | 561.2515 | 281.1294 | 6 |
| 17 | **1805.7130** | 903.3602 | 1788.6865 | **894.8469** | 1787.7025 | **894.3549** | V | **478.2144** | 239.6108 | 461.1878 | 231.0975 | **460.2038** | 230.6055 | 5 |
| 18 | **1906.7607** | 953.8840 | 1889.7342 | **945.3707** | **1888.7501** | **944.8787** | T | **379.1459** | 190.0766 | 362.1194 | 181.5633 | **361.1354** | 181.0713 | 4 |
| 19 | 2021.7876 | **1011.3975** | 2004.7611 | 1002.8842 | 2003.7771 | 1002.3922 | N | 278.0983 | 139.5528 | 261.0717 | 131.0395 | 260.0877 | 130.5475 | 3 |
| 20 | 2078.8091 | **1039.9082** | 2061.7826 | 1031.3949 | 2060.7985 | 1030.9029 | G | 163.0713 | 82.0393 | | | 145.0608 | 73.0340 | 2 |
| 21 | | | | | | | S | 106.0499 | 53.5286 | | | 88.0393 | 44.5233 | 1 |

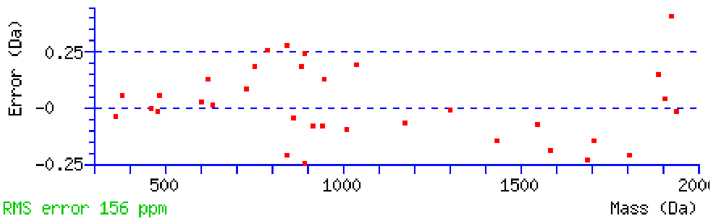


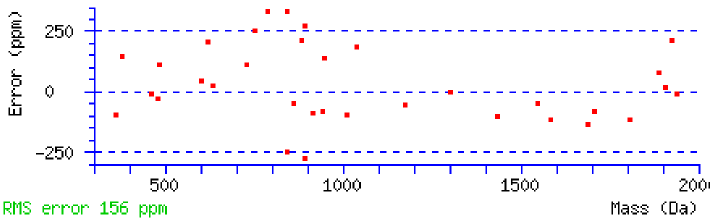


## All matches to this query

| Score | Mr(calc): | Delta | Sequence |
|---|---|---|---|
| | | | |

| | | | |
|---|---|---|---|
| 71.5 | 2182.8444 | 0.0029 | RSGPSDEGEEEMEDGTVTNGS |
| 48.2 | 2181.8604 | 0.9869 | RSGPSDEGEEEMEDGTVTNGS |

---

Spectrum No: 343; Query: 280; Rank: 1

---

## Peptide View

MS/MS Fragmentation of **FVHVNTSAILK**
Found in **IPI00201298**, Tax_Id=10116 Gene_Symbol=Pomt1 Protein O-mannosyl-transferase 1

Match to Query 280: 1228.679892 from(410.567240,3+)
Title: 091008RatKidney_NH4Format01_23.2091.2091.3.dta
Data file K:\NewmanPaper\Piliang\3SubProteomes\Piliang3SP\mgf5ppm\ERLIC_3SubProteomes5ppm.mgf

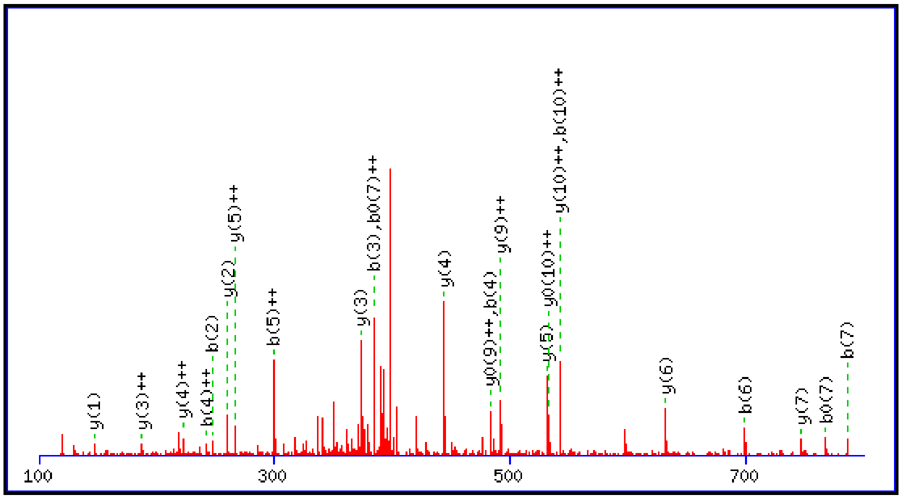


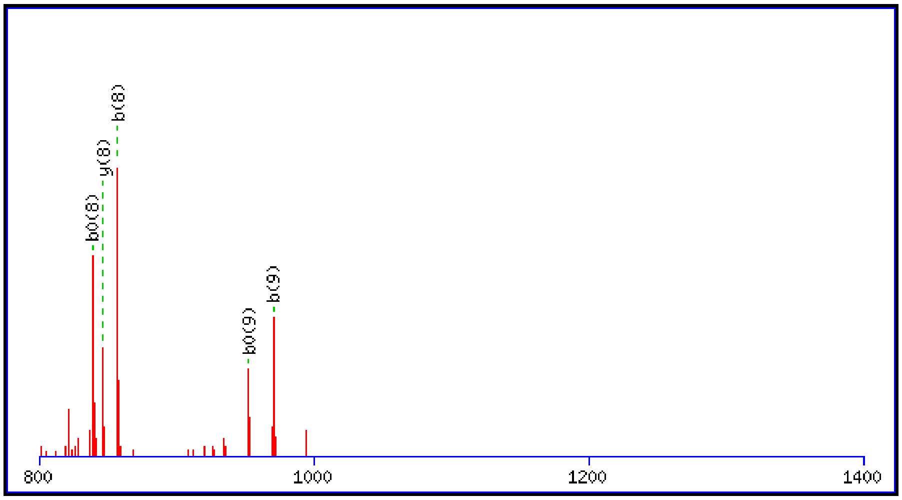

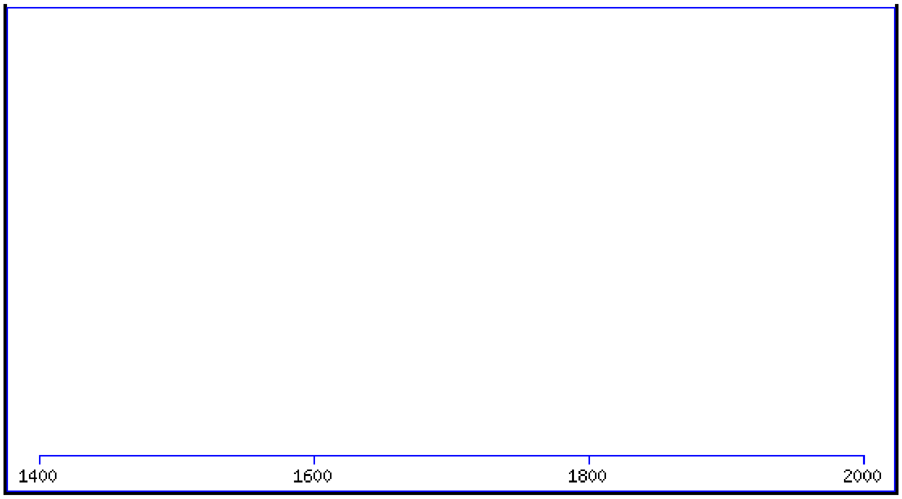


**Monoisotopic mass of neutral peptide Mr(calc):** 1228.6816
**Fixed modifications:** Carbamidomethyl (C)
**Variable modifications:**
**N5** : Deamidated_N (N)
**Ions Score:** 71 **Expect:** 5.3e-006
**Matches (Bold Red):** 29/94 fragment ions using 31 most intense peaks

| # | b | $b^{++}$ | b* | $b^{*++}$ | $b^{0}$ | $b^{0++}$ | Seq. | y | $y^{++}$ | y* | $y^{*++}$ | $y^{0}$ | $y^{0++}$ | # |
|---|---|---|---|---|---|---|---|---|---|---|---|---|---|---|
| 1 | 148.0757 | 74.5415 | | | | | F | | | | | | | 11 |
| 2 | **247.1441** | 124.0757 | | | | | V | 1082.6204 | **541.8139** | 1065.5939 | 533.3006 | 1064.6099 | **532.8086** | 10 |
| 3 | **384.2030** | 192.6051 | | | | | H | 983.5520 | **492.2796** | 966.5255 | 483.7664 | 965.5415 | **483.2744** | 9 |
| 4 | **483.2714** | **242.1394** | | | | | V | **846.4931** | 423.7502 | 829.4666 | 415.2369 | 828.4825 | 414.7449 | 8 |
| 5 | 598.2984 | **299.6528** | 581.2718 | 291.1395 | | | N | **747.4247** | 374.2160 | 730.3981 | 365.7027 | 729.4141 | 365.2107 | 7 |
| 6 | **699.3460** | 350.1767 | 682.3195 | 341.6634 | 681.3355 | 341.1714 | T | **632.3978** | 316.7025 | 615.3712 | 308.1892 | 614.3872 | 307.6972 | 6 |
| 7 | **786.3781** | 393.6927 | 769.3515 | 385.1794 | **768.3675** | **384.6874** | S | **531.3501** | **266.1787** | 514.3235 | 257.6654 | 513.3395 | 257.1734 | 5 |
| 8 | **857.4152** | 429.2112 | 840.3886 | 420.6980 | **839.4046** | 420.2059 | A | **444.3180** | **222.6627** | 427.2915 | 214.1494 | | | 4 |
| 9 | **970.4993** | 485.7533 | 953.4727 | 477.2400 | **952.4887** | 476.7480 | I | **373.2809** | **187.1441** | 356.2544 | 178.6308 | | | 3 |
| 10 | 1083.5833 | **542.2953** | 1066.5568 | 533.7820 | 1065.5728 | 533.2900 | L | **260.1969** | 130.6021 | 243.1703 | 122.0888 | | | 2 |
| 11 | | | | | | | K | **147.1128** | 74.0600 | 130.0863 | 65.5468 | | | 1 |

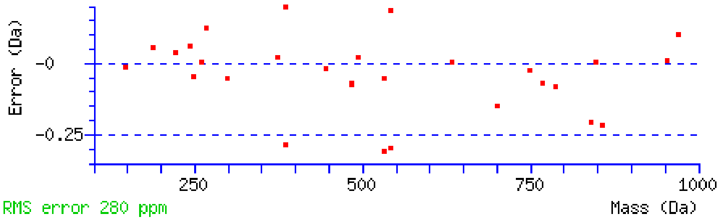


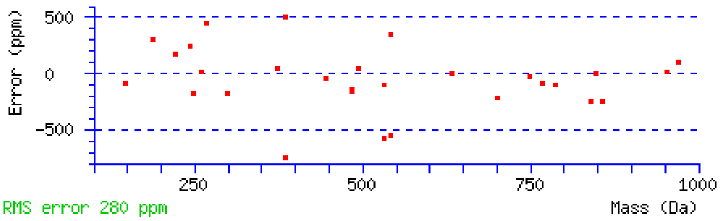


### All matches to this query

| Score | Mr(calc): | Delta | Sequence |
|---|---|---|---|
| 71.5 | 1228.6816 | -0.0017 | FVHVNTSAILK |
| 9.8 | 1227.6823 | 0.9976 | KLGNLAVAADEK |
| 8.3 | 1227.6710 | 1.0089 | EEIKVEKPEK |
| 7.8 | 1227.6757 | 1.0042 | EKSRMSHLIK |
| 7.0 | 1227.6823 | 0.9976 | NATQEAVAALLK |
| 6.8 | 1226.6843 | 1.9956 | QELRQELRR |
| 6.0 | 1228.6887 | -0.0088 | QRSIEAERLK |
| 6.0 | 1228.6888 | -0.0089 | NRLVNQSALSK |
| 4.1 | 1228.6888 | -0.0089 | EVVRQRSDLK |
| 3.3 | 1228.6676 | 0.0123 | NQWLRRNIK |

Spectrum No: 344; Query: 1212; Rank: 1

## Peptide View

MS/MS Fragmentation of **TDYNASVSVPDSSGPER**
Found in **IPI00194974**, Tax_Id=10116 Gene_Symbol=Hnrpk Hnrpk protein

Match to Query 1212: 1780.771268 from(891.392910,2+)
Title: 091008RatKidney_NH4Format01_18.1423.1423.2.dta
Data file K:\NewmanPaper\Piliang\3SubProteomes\Piliang3SP\mgf5ppm\ERLIC_3SubProteomes5ppm.mgf

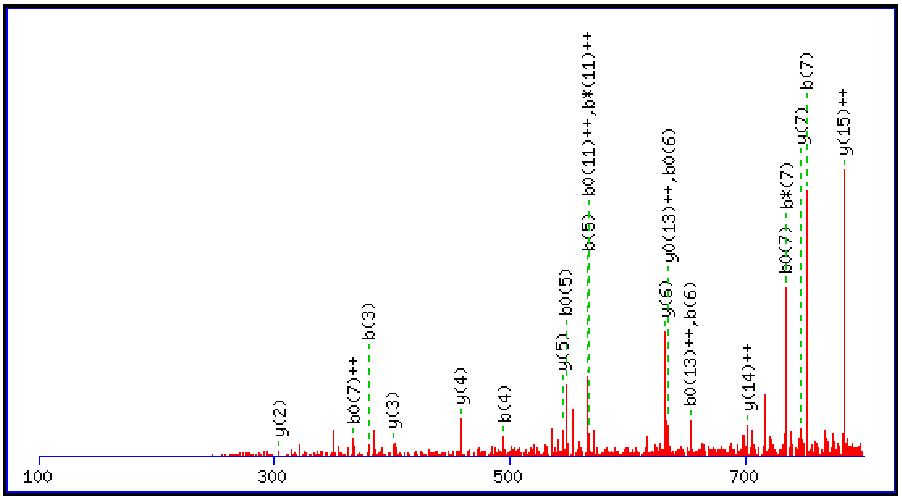


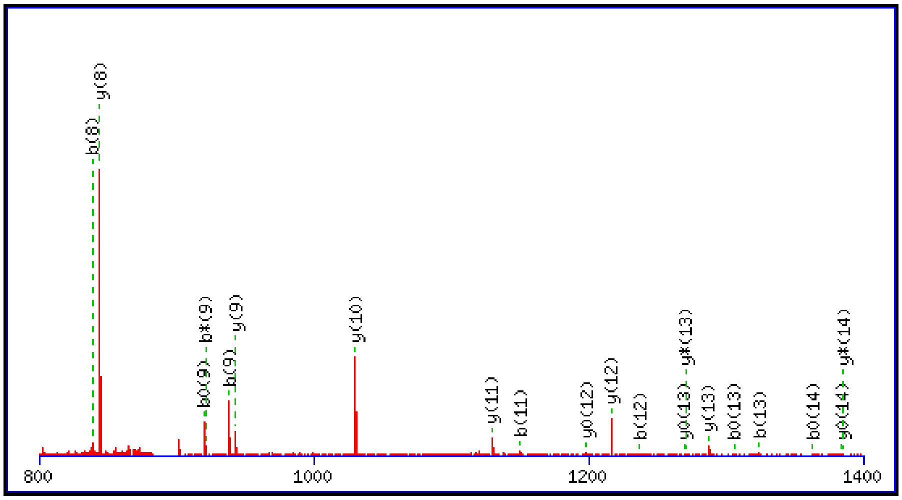


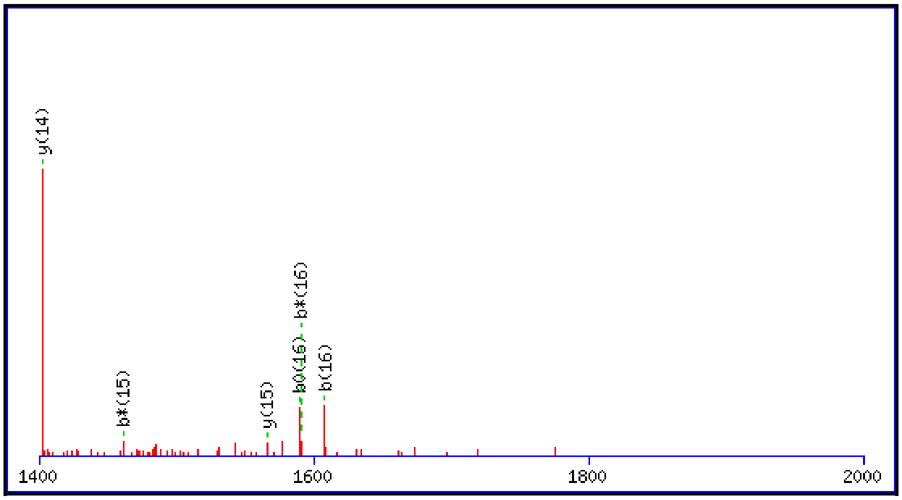

**Monoisotopic mass of neutral peptide Mr(calc):** 1780.7751
**Fixed modifications:** Carbamidomethyl (C)
**Variable modifications:**
**N4** : Deamidated_N (N)
**Ions Score:** 71 **Expect:** 1.5e-005
**Matches (Bold Red):** 48/184 fragment ions using 109 most intense peaks

| # | b | $b^{++}$ | b* | $b^{*++}$ | $b^{0}$ | $b^{0++}$ | Seq. | y | $y^{++}$ | y* | $y^{*++}$ | $y^{0}$ | $y^{0++}$ | # |
|---|---|---|---|---|---|---|---|---|---|---|---|---|---|---|
| 1 | 102.0550 | 51.5311 | | | 84.0444 | 42.5258 | T | | | | | | | 17 |
| 2 | 217.0819 | 109.0446 | | | 199.0713 | 100.0393 | D | 1680.7347 | 840.8710 | 1663.7082 | 832.3577 | 1662.7242 | 831.8657 | 16 |
| 3 | **380.1452** | 190.5763 | | | 362.1347 | 181.5710 | Y | **1565.7078** | **783.3575** | 1548.6813 | 774.8443 | 1547.6972 | 774.3523 | 15 |
| 4 | **495.1722** | 248.0897 | 478.1456 | 239.5764 | 477.1616 | 239.0844 | N | **1402.6445** | **701.8259** | **1385.6179** | 693.3126 | **1384.6339** | 692.8206 | 14 |
| 5 | **566.2093** | 283.6083 | 549.1827 | 275.0950 | **548.1987** | 274.6030 | A | **1287.6175** | 644.3124 | **1270.5910** | 635.7991 | **1269.6070** | **635.3071** | 13 |
| 6 | **653.2413** | 327.1243 | 636.2148 | 318.6110 | **635.2307** | 318.1190 | S | **1216.5804** | 608.7938 | 1199.5539 | 600.2806 | **1198.5699** | 599.7886 | 12 |
| 7 | **752.3097** | 376.6585 | **735.2832** | 368.1452 | **734.2992** | **367.6532** | V | **1129.5484** | 565.2778 | 1112.5218 | 556.7646 | 1111.5378 | 556.2726 | 11 |
| 8 | **839.3417** | 420.1745 | 822.3152 | 411.6612 | 821.3312 | 411.1692 | S | **1030.4800** | 515.7436 | 1013.4534 | 507.2304 | 1012.4694 | 506.7383 | 10 |
| 9 | **938.4102** | 469.7087 | **921.3836** | 461.1954 | **920.3996** | 460.7034 | V | **943.4480** | 472.2276 | 926.4214 | 463.7143 | 925.4374 | 463.2223 | 9 |
| 10 | 1035.4629 | 518.2351 | 1018.4364 | 509.7218 | 1017.4524 | 509.2298 | P | **844.3795** | 422.6934 | 827.3530 | 414.1801 | 826.3690 | 413.6881 | 8 |
| 11 | **1150.4899** | 575.7486 | 1133.4633 | **567.2353** | 1132.4793 | **566.7433** | D | **747.3268** | 374.1670 | 730.3002 | 365.6537 | 729.3162 | 365.1617 | 7 |
| 12 | **1237.5219** | 619.2646 | 1220.4953 | 610.7513 | 1219.5113 | 610.2593 | S | **632.2998** | 316.6536 | 615.2733 | 308.1403 | 614.2893 | 307.6483 | 6 |
| 13 | **1324.5539** | 662.7806 | 1307.5274 | 654.2673 | **1306.5434** | **653.7753** | S | **545.2678** | 273.1375 | 528.2413 | 264.6243 | 527.2572 | 264.1323 | 5 |
| 14 | 1381.5754 | 691.2913 | 1364.5488 | 682.7781 | **1363.5648** | 682.2860 | G | **458.2358** | 229.6215 | 441.2092 | 221.1082 | 440.2252 | 220.6162 | 4 |
| 15 | 1478.6282 | 739.8177 | **1461.6016** | 731.3044 | 1460.6176 | 730.8124 | P | **401.2143** | 201.1108 | 384.1878 | 192.5975 | 383.2037 | 192.1055 | 3 |
| 16 | **1607.6707** | 804.3390 | **1590.6442** | 795.8257 | **1589.6602** | 795.3337 | E | **304.1615** | 152.5844 | 287.1350 | 144.0711 | 286.1510 | 143.5791 | 2 |
| 17 | | | | | | | R | 175.1190 | 88.0631 | 158.0924 | 79.5498 | | | 1 |

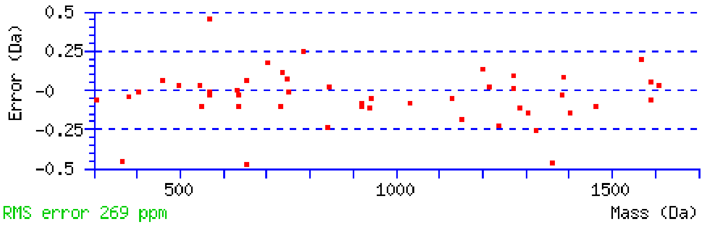


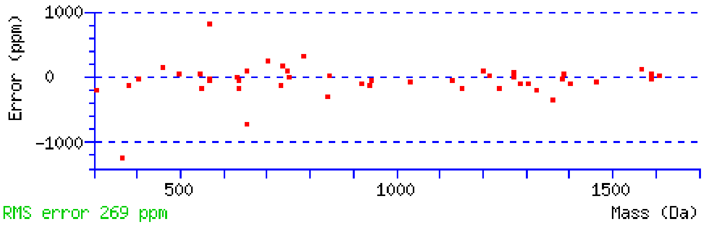


### All matches to this query

| Score | Mr(calc): | Delta | Sequence |
|---|---|---|---|
| 71.5 | 1780.7751 | -0.0039 | TDYNASVSVPDSSGPER |
| 2.7 | 1780.7855 | -0.0142 | IFMQESDASNFLKR |
| 1.6 | 1780.7546 | 0.0167 | RSELYEAEERALR |
| 0.6 | 1779.7553 | 1.0159 | DVTRTNSSNARLTGK |

---

Spectrum No: 345; Query: 655; Rank: 1

---

## Peptide View

MS/MS Fragmentation of **GVVDSDDLPLNVSR**
Found in **IPI00365985**, Tax_Id=10116 Gene_Symbol=Tra1_predicted 93 kDa protein

Match to Query 655: 1485.734668 from(743.874610,2+)
Title: 100101RatKid_NS_deglyco_19.3030.3030.2.dta
Data file K:\NewmanPaper\Piliang\3SubProteomes\Piliang3SP\mgf5ppm\ERLIC_3SubProteomes5ppm.mgf

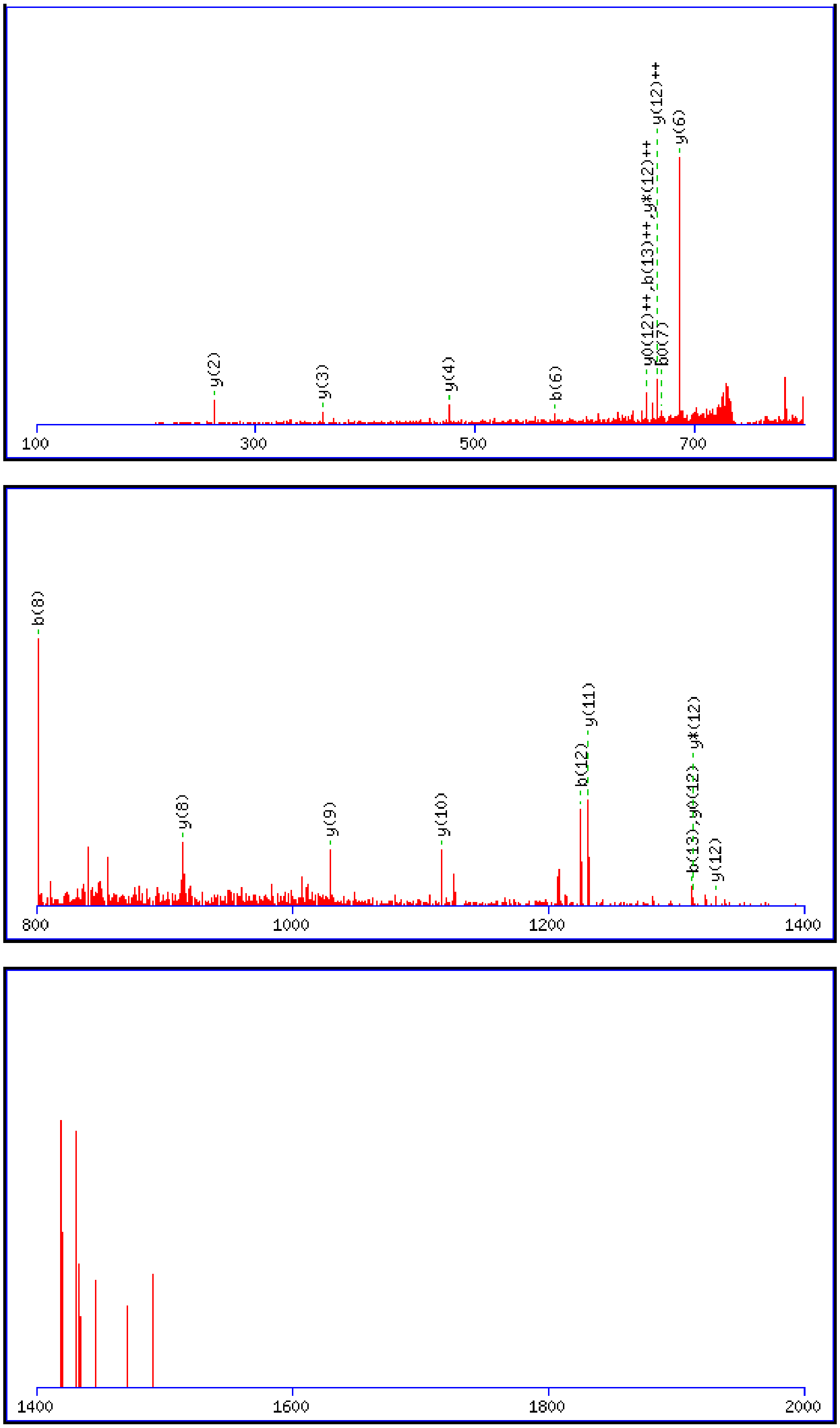


**Monoisotopic mass of neutral peptide Mr(calc):** 1485.7311
**Fixed modifications:** Carbamidomethyl (C)
**Variable modifications:**
**N11** : Deamidated_N (N)
**Ions Score:** 71 **Expect:** 1e-005
**Matches (Bold Red):** 20/128 fragment ions using 33 most intense peaks

| # | b | $b^{++}$ | b* | $b^{*++}$ | $b^0$ | $b^{0++}$ | Seq. | y | $y^{++}$ | y* | $y^{*++}$ | $y^0$ | $y^{0++}$ | # |
|---|---|---|---|---|---|---|---|---|---|---|---|---|---|---|
| 1 | 58.0287 | 29.5180 | | | | | G | | | | | | | 14 |
| 2 | 157.0972 | 79.0522 | | | | | V | 1429.7169 | 715.3621 | 1412.6904 | 706.8488 | 1411.7063 | 706.3568 | 13 |
| 3 | 256.1656 | 128.5864 | | | | | V | **1330.6485** | **665.8279** | **1313.6219** | **657.3146** | **1312.6379** | **656.8226** | 12 |
| 4 | 371.1925 | 186.0999 | | | 353.1819 | 177.0946 | D | **1231.5801** | 616.2937 | 1214.5535 | 607.7804 | 1213.5695 | 607.2884 | 11 |
| 5 | 458.2245 | 229.6159 | | | 440.2140 | 220.6106 | S | **1116.5531** | 558.7802 | 1099.5266 | 550.2669 | 1098.5426 | 549.7749 | 10 |

| | | | | | | | | | | | | | | |
|---|---|---|---|---|---|---|---|---|---|---|---|---|---|---|
| **6** | **573.2515** | 287.1294 | | | 555.2409 | 278.1241 | **D** | **1029.5211** | 515.2642 | 1012.4946 | 506.7509 | 1011.5105 | 506.2589 | **9** |
| **7** | 688.2784 | 344.6429 | | | **670.2679** | 335.6376 | **D** | **914.4942** | 457.7507 | 897.4676 | 449.2374 | 896.4836 | 448.7454 | **8** |
| **8** | **801.3625** | 401.1849 | | | 783.3519 | 392.1796 | **L** | 799.4672 | 400.2372 | 782.4407 | 391.7240 | 781.4567 | 391.2320 | **7** |
| **9** | 898.4153 | 449.7113 | | | 880.4047 | 440.7060 | **P** | **686.3832** | 343.6952 | 669.3566 | 335.1819 | 668.3726 | 334.6899 | **6** |
| **10** | 1011.4993 | 506.2533 | | | 993.4888 | 497.2480 | **L** | 589.3304 | 295.1688 | 572.3038 | 286.6556 | 571.3198 | 286.1636 | **5** |
| **11** | 1126.5263 | 563.7668 | 1109.4997 | 555.2535 | 1108.5157 | 554.7615 | **N** | **476.2463** | 238.6268 | 459.2198 | 230.1135 | 458.2358 | 229.6215 | **4** |
| **12** | **1225.5947** | 613.3010 | 1208.5681 | 604.7877 | 1207.5841 | 604.2957 | **V** | **361.2194** | 181.1133 | 344.1928 | 172.6001 | 343.2088 | 172.1081 | **3** |
| **13** | **1312.6267** | **656.8170** | 1295.6001 | 648.3037 | 1294.6161 | 647.8117 | **S** | **262.1510** | 131.5791 | 245.1244 | 123.0659 | 244.1404 | 122.5738 | **2** |
| **14** | | | | | | | **R** | 175.1190 | 88.0631 | 158.0924 | 79.5498 | | | **1** |

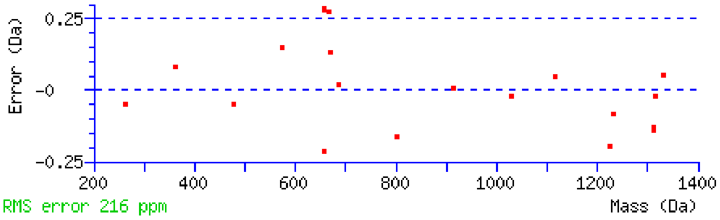


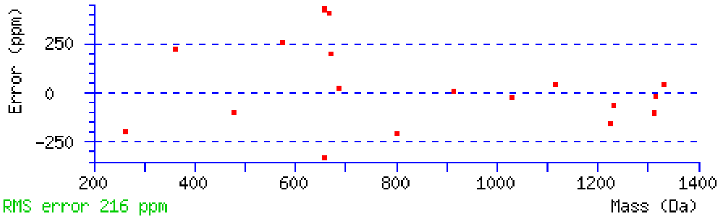


### All matches to this query

| Score | Mr(calc): | Delta | Sequence |
|---|---|---|---|
| 71.4 | 1485.7311 | 0.0036 | GVVDSDDLPLNVSR |
| 7.8 | 1485.7385 | -0.0038 | ILDECGLNLPDVK |
| 7.4 | 1484.7245 | 1.0102 | CTVVCTLVRLGK |
| 6.3 | 1483.7168 | 2.0179 | NGWPQGNXDAVLR |
| 4.2 | 1485.7228 | 0.0118 | NFSLSVNSVAVLR |
| 4.2 | 1485.7228 | 0.0118 | NFSLSVNSVAVLR |
| 4.1 | 1485.7341 | 0.0006 | VKNFAQLTVSGSR |
| 4.1 | 1485.7341 | 0.0006 | VKNFAQLTVSGSR |
| 4.0 | 1484.7300 | 1.0047 | FITEVWHPDVDK |
| 3.3 | 1485.7212 | 0.0135 | EWEERLPADVSR |

---

Spectrum No: 346; Query: 2591; Rank: 1

---

## Peptide View

MS/MS Fragmentation of **DSSDEANCTTLCSQKEFECGSGECILR**
Found in **IPI00205325**, Tax_Id=10116 Gene_Symbol=Lrp2 Low-density lipoprotein receptor-related protein 2 precursor

Match to Query 2591: 3154.275852 from(1052.432560,3+)
Title: 100101RatKid_NS_deglyco_24.2834.2834.3.dta
Data file K:\NewmanPaper\Piliang\3SubProteomes\Piliang3SP\mgf5ppm\ERLIC_3SubProteomes5ppm.mgf

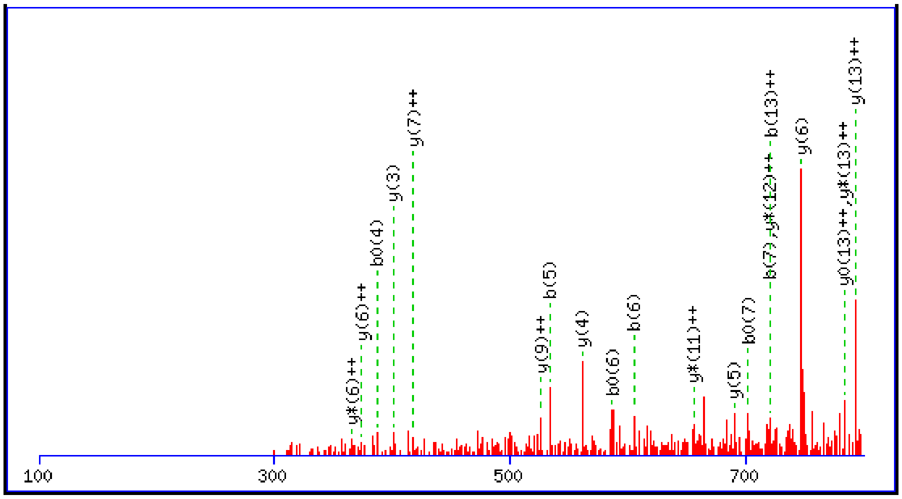


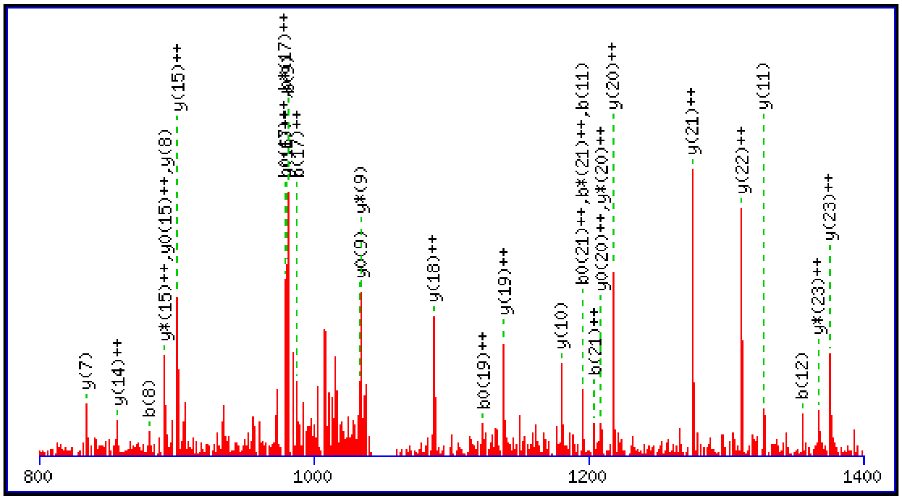


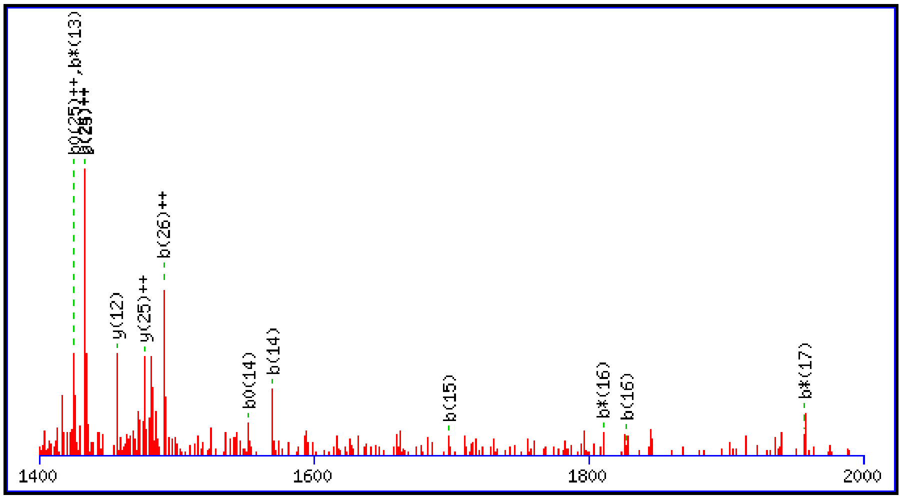


**Monoisotopic mass of neutral peptide Mr(calc):** 3153.2631
**Fixed modifications:** Carbamidomethyl (C)
**Variable modifications:**
**N7** : Deamidated_N (N)
**Ions Score:** 71 **Expect:** 2.6e-005
**Matches (Bold Red):** 65/292 fragment ions using 128 most intense peaks

| # | b | $b^{++}$ | b* | $b^{*++}$ | $b^{0}$ | $b^{0++}$ | Seq. | y | $y^{++}$ | y* | $y^{*++}$ | $y^{0}$ | $y^{0++}$ | # |
|---|---|---|---|---|---|---|---|---|---|---|---|---|---|---|
| 1 | 116.0342 | 58.5207 | | | 98.0237 | 49.5155 | D | | | | | | | 27 |
| 2 | 203.0662 | 102.0368 | | | 185.0557 | 93.0315 | S | 3039.2435 | 1520.1254 | 3022.2169 | 1511.6121 | 3021.2329 | 1511.1201 | 26 |
| 3 | 290.0983 | 145.5528 | | | 272.0877 | 136.5475 | S | 2952.2114 | **1476.6094** | 2935.1849 | 1468.0961 | 2934.2009 | 1467.6041 | 25 |
| 4 | 405.1252 | 203.0662 | | | **387.1147** | 194.0610 | D | 2865.1794 | **1433.0933** | 2848.1529 | 1424.5801 | 2847.1688 | 1424.0881 | 24 |
| 5 | **534.1678** | 267.5875 | | | 516.1572 | 258.5823 | E | 2750.1525 | **1375.5799** | 2733.1259 | **1367.0666** | 2732.1419 | 1366.5746 | 23 |

| | | | | | | | | | | | | | | |
|---|---|---|---|---|---|---|---|---|---|---|---|---|---|---|
| 6 | **605.2049** | 303.1061 | | | **587.1944** | 294.1008 | A | 2621.1099 | **1311.0586** | 2604.0833 | 1302.5453 | 2603.0993 | 1302.0533 | 22 |
| 7 | **720.2319** | 360.6196 | 703.2053 | 352.1063 | **702.2213** | 351.6143 | N | 2550.0728 | **1275.5400** | 2533.0462 | 1267.0267 | 2532.0622 | 1266.5347 | 21 |
| 8 | **880.2625** | 440.6349 | 863.2360 | 432.1216 | 862.2519 | 431.6296 | C | 2435.0458 | **1218.0265** | 2418.0193 | **1209.5133** | 2417.0353 | **1209.0213** | 20 |
| 9 | **981.3102** | 491.1587 | 964.2836 | 482.6455 | 963.2996 | 482.1535 | T | 2275.0152 | **1138.0112** | 2257.9886 | 1129.4979 | 2257.0046 | 1129.0059 | 19 |
| 10 | 1082.3579 | 541.6826 | 1065.3313 | 533.1693 | 1064.3473 | 532.6773 | T | 2173.9675 | **1087.4874** | 2156.9409 | 1078.9741 | 2155.9569 | 1078.4821 | 18 |
| 11 | **1195.4419** | 598.2246 | 1178.4154 | 589.7113 | 1177.4314 | 589.2193 | L | 2072.9198 | 1036.9635 | 2055.8933 | 1028.4503 | 2054.9092 | 1027.9583 | 17 |
| 12 | **1355.4726** | 678.2399 | 1338.4460 | 669.7267 | 1337.4620 | 669.2346 | C | 1959.8357 | **980.4215** | 1942.8092 | 971.9082 | 1941.8252 | 971.4162 | 16 |
| 13 | 1442.5046 | **721.7559** | **1425.4781** | 713.2427 | 1424.4940 | 712.7507 | S | 1799.8051 | **900.4062** | 1782.7785 | **891.8929** | 1781.7945 | **891.4009** | 15 |
| 14 | **1570.5632** | 785.7852 | 1553.5366 | 777.2720 | **1552.5526** | 776.7799 | Q | 1712.7731 | **856.8902** | 1695.7465 | 848.3769 | 1694.7625 | 847.8849 | 14 |
| 15 | **1698.6582** | 849.8327 | 1681.6316 | 841.3194 | 1680.6476 | 840.8274 | K | 1584.7145 | **792.8609** | 1567.6879 | **784.3476** | 1566.7039 | **783.8556** | 13 |
| 16 | **1827.7007** | 914.3540 | **1810.6742** | 905.8407 | 1809.6902 | 905.3487 | E | **1456.6195** | 728.8134 | 1439.5930 | **720.3001** | 1438.6090 | 719.8081 | 12 |
| 17 | 1974.7692 | **987.8882** | **1957.7426** | **979.3749** | 1956.7586 | **978.8829** | F | **1327.5769** | 664.2921 | 1310.5504 | **655.7788** | 1309.5664 | 655.2868 | 11 |
| 18 | 2103.8117 | 1052.4095 | 2086.7852 | 1043.8962 | 2085.8012 | 1043.4042 | E | **1180.5085** | 590.7579 | 1163.4820 | 582.2446 | 1162.4980 | 581.7526 | 10 |
| 19 | 2263.8424 | 1132.4248 | 2246.8158 | 1123.9116 | 2245.8318 | **1123.4196** | C | 1051.4659 | **526.2366** | **1034.4394** | 517.7233 | **1033.4554** | 517.2313 | 9 |
| 20 | 2320.8639 | 1160.9356 | 2303.8373 | 1152.4223 | 2302.8533 | 1151.9303 | G | **891.4353** | 446.2213 | 874.4087 | 437.7080 | 873.4247 | 437.2160 | 8 |
| 21 | 2407.8959 | **1204.4516** | 2390.8693 | **1195.9383** | 2389.8853 | **1195.4463** | S | **834.4138** | **417.7105** | 817.3873 | 409.1973 | 816.4032 | 408.7053 | 7 |
| 22 | 2464.9174 | 1232.9623 | 2447.8908 | 1224.4490 | 2446.9068 | 1223.9570 | G | **747.3818** | **374.1945** | 730.3552 | **365.6813** | 729.3712 | 365.1892 | 6 |
| 23 | 2593.9599 | 1297.4836 | 2576.9334 | 1288.9703 | 2575.9494 | 1288.4783 | E | **690.3603** | 345.6838 | 673.3338 | 337.1705 | 672.3498 | 336.6785 | 5 |
| 24 | 2753.9906 | 1377.4989 | 2736.9640 | 1368.9857 | 2735.9800 | 1368.4937 | C | **561.3177** | 281.1625 | 544.2912 | 272.6492 | | | 4 |
| 25 | 2867.0747 | **1434.0410** | 2850.0481 | **1425.5277** | 2849.0641 | **1425.0357** | I | **401.2871** | 201.1472 | 384.2605 | 192.6339 | | | 3 |
| 26 | 2980.1587 | **1490.5830** | 2963.1322 | 1482.0697 | 2962.1482 | 1481.5777 | L | 288.2030 | 144.6051 | 271.1765 | 136.0919 | | | 2 |
| 27 | | | | | | | R | 175.1190 | 88.0631 | 158.0924 | 79.5498 | | | 1 |

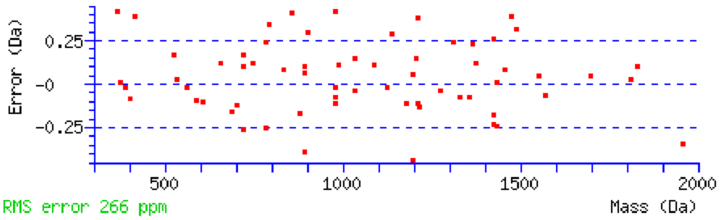


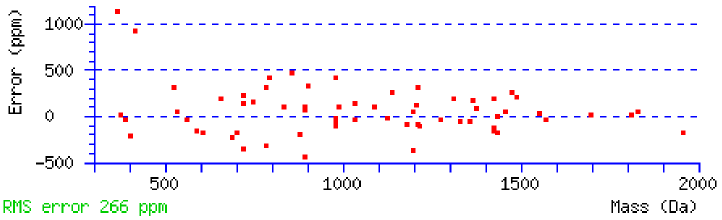


### All matches to this query

| Score | Mr(calc): | Delta | Sequence |
|---|---|---|---|
| 71.4 | 3153.2631 | 1.0127 | DSSDEANCTTLCSQKEFECGSGECILR |
| 46.1 | 3152.2791 | 1.9967 | DSSDEANCTTLCSQKEFECGSGECILR |
| 1.7 | 3153.2986 | 0.9772 | AFSRYSQLISHQSIHIGVKPYDCK |
| 0.8 | 3153.2986 | 0.9772 | AFSRYSQLISHQSIHIGVKPYDCK |
| 0.8 | 3153.2986 | 0.9772 | AFSRYSQLISHQSIHIGVKPYDCK |
| 0.6 | 3152.2543 | 2.0216 | EQKLHQDTATVGNFISTKSIQSDER |
| 0.5 | 3152.2814 | 1.9944 | GFGFVTFENIDDAKDAMMAMNGKSVDGR |

---

Spectrum No: 347; Query: 427; Rank: 1

---

## Peptide View

MS/MS Fragmentation of **LFIVGSNSSSSTR**
Found in **IPI00191738**, Tax_Id=10116 Gene_Symbol=Gclm Glutamate--cysteine ligase regulatory subunit

Match to Query 427: 1354.674808 from(678.344680,2+)
Title: 100101RatKid_NS_deglyco_04.2134.2134.2.dta
Data file K:\NewmanPaper\Piliang\3SubProteomes\Piliang3SP\mgf5ppm\ERLIC_3SubProteomes5ppm.mgf

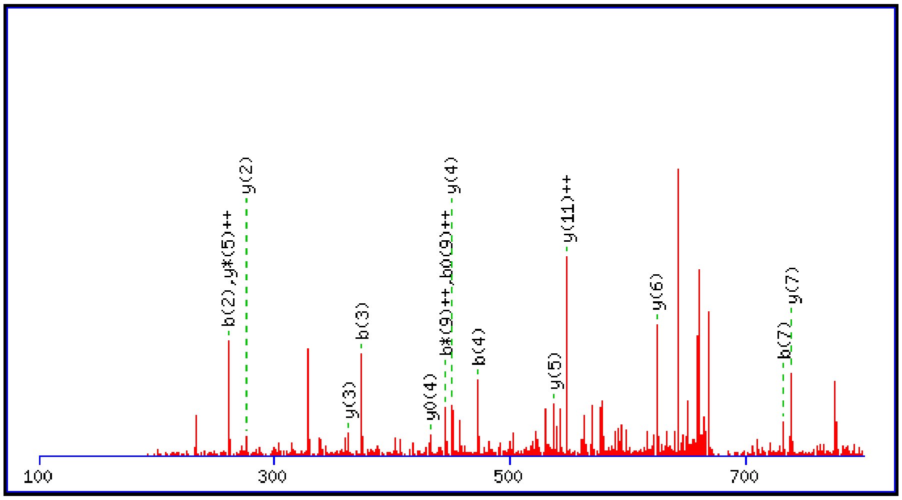


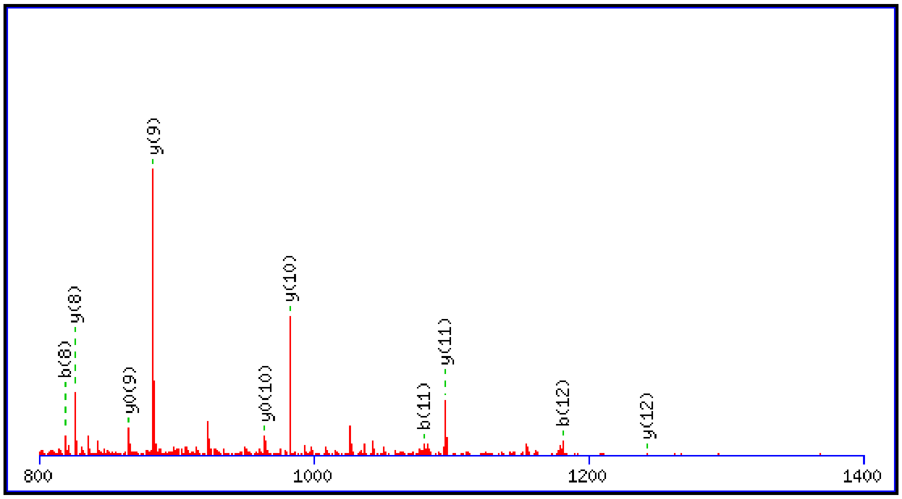


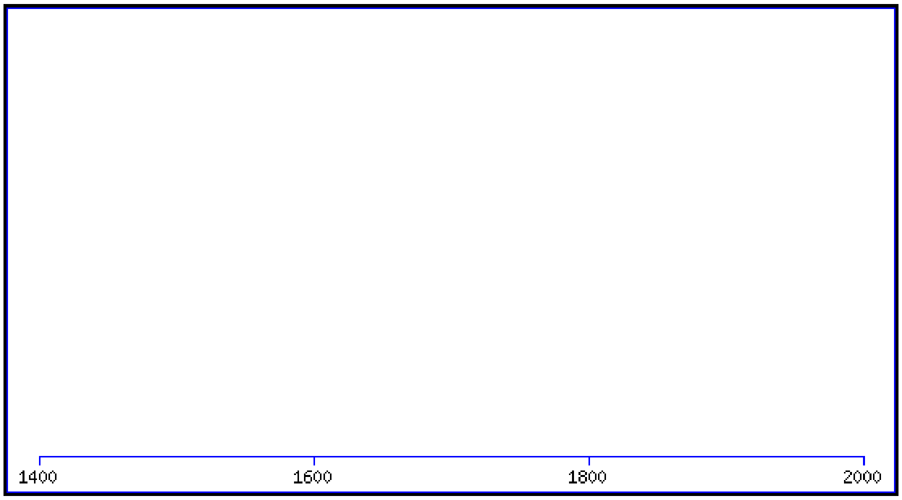


**Monoisotopic mass of neutral peptide Mr(calc):** 1354.6729
**Fixed modifications:** Carbamidomethyl (C)
**Variable modifications:**
**N7** : Deamidated_N (N)
**Ions Score:** 71 **Expect:** 1e-005
**Matches (Bold Red):** 25/120 fragment ions using 51 most intense peaks

| # | b | b++ | b* | b*++ | b0 | b0++ | Seq. | y | y++ | y* | y*++ | y0 | y0++ | # |
|---|---|---|---|---|---|---|---|---|---|---|---|---|---|---|
| 1 | 114.0913 | 57.5493 | | | | | L | | | | | | | 13 |
| 2 | **261.1598** | 131.0835 | | | | | F | **1242.5961** | 621.8017 | 1225.5695 | 613.2884 | 1224.5855 | 612.7964 | 12 |
| 3 | **374.2438** | 187.6255 | | | | | I | **1095.5276** | **548.2675** | 1078.5011 | 539.7542 | 1077.5171 | 539.2622 | 11 |
| 4 | **473.3122** | 237.1598 | | | | | V | **982.4436** | 491.7254 | 965.4170 | 483.2122 | **964.4330** | 482.7201 | 10 |
| 5 | 530.3337 | 265.6705 | | | | | G | **883.3752** | 442.1912 | 866.3486 | 433.6779 | **865.3646** | 433.1859 | 9 |

| | | | | | | | | | | | | | | |
|---|---|---|---|---|---|---|---|---|---|---|---|---|---|---|
| **6** | 617.3657 | 309.1865 | | | 599.3552 | 300.1812 | **S** | **826.3537** | 413.6805 | 809.3272 | 405.1672 | 808.3431 | 404.6752 | **8** |
| **7** | **732.3927** | 366.7000 | 715.3661 | 358.1867 | 714.3821 | 357.6947 | **N** | **739.3217** | 370.1645 | 722.2951 | 361.6512 | 721.3111 | 361.1592 | **7** |
| **8** | **819.4247** | 410.2160 | 802.3981 | 401.7027 | 801.4141 | 401.2107 | **S** | **624.2947** | 312.6510 | 607.2682 | 304.1377 | 606.2842 | 303.6457 | **6** |
| **9** | 906.4567 | 453.7320 | 889.4302 | **445.2187** | 888.4462 | **444.7267** | **S** | **537.2627** | 269.1350 | 520.2362 | **260.6217** | 519.2521 | 260.1297 | **5** |
| **10** | 993.4887 | 497.2480 | 976.4622 | 488.7347 | 975.4782 | 488.2427 | **S** | **450.2307** | 225.6190 | 433.2041 | 217.1057 | **432.2201** | 216.6137 | **4** |
| **11** | **1080.5208** | 540.7640 | 1063.4942 | 532.2507 | 1062.5102 | 531.7587 | **S** | **363.1987** | 182.1030 | 346.1721 | 173.5897 | 345.1881 | 173.0977 | **3** |
| **12** | **1181.5685** | 591.2879 | 1164.5419 | 582.7746 | 1163.5579 | 582.2826 | **T** | **276.1666** | 138.5870 | 259.1401 | 130.0737 | 258.1561 | 129.5817 | **2** |
| **13** | | | | | | | **R** | 175.1190 | 88.0631 | 158.0924 | 79.5498 | | | **1** |

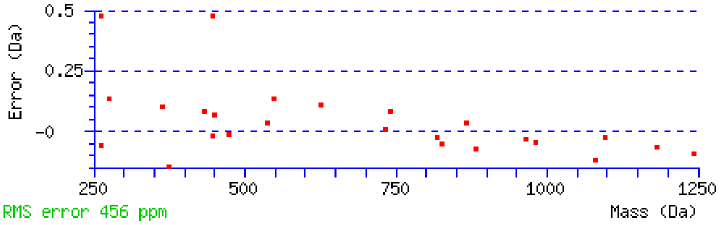


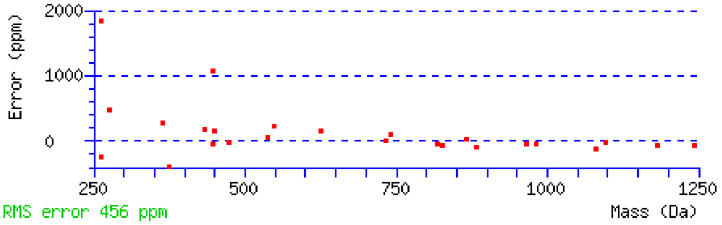


### All matches to this query

| Score | Mr(calc): | Delta | Sequence |
|---|---|---|---|
| 71.4 | 1354.6729 | 0.0020 | LFIVGSNSSSSTR |
| 8.0 | 1354.6663 | 0.0085 | SKFQGATGMLGSR |
| 6.3 | 1354.6679 | 0.0069 | MLIRVANNISK |
| 5.8 | 1353.6653 | 1.0095 | QSQDLSRSLLK |
| 5.0 | 1353.6823 | 0.9925 | MKRNNFSTVNK |
| 4.7 | 1354.6625 | 0.0123 | MILSTISWMGGK |
| 4.6 | 1354.6663 | 0.0085 | MKRNNFSTVNK |
| 4.6 | 1354.6663 | 0.0085 | MKRNNFSTVNK |
| 4.1 | 1354.6728 | 0.0020 | EAKEAPAQPASEK |
| 3.9 | 1353.6806 | 0.9942 | HFISSSRSLLK |

---

Spectrum No: 348; Query: 2212; Rank: 1

---

## Peptide View

MS/MS Fragmentation of **IISANGCKVDNSSLTGESEPQTR**
Found in **IPI00326305**, Tax_Id=10116 Gene_Symbol=Atp1a1 Sodium/potassium-transporting ATPase subunit alpha-1 precursor

Match to Query 2212: 2463.148872 from(822.056900,3+)
Title: 091008RatKidney_NH4Format02_24.1628.1628.3.dta
Data file K:\NewmanPaper\Piliang\3SubProteomes\Piliang3SP\mgf5ppm\ERLIC_3SubProteomes5ppm.mgf

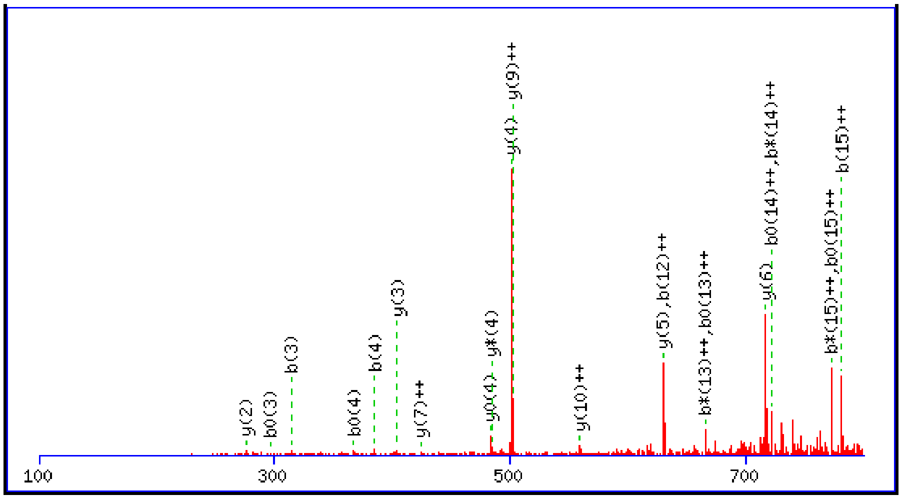


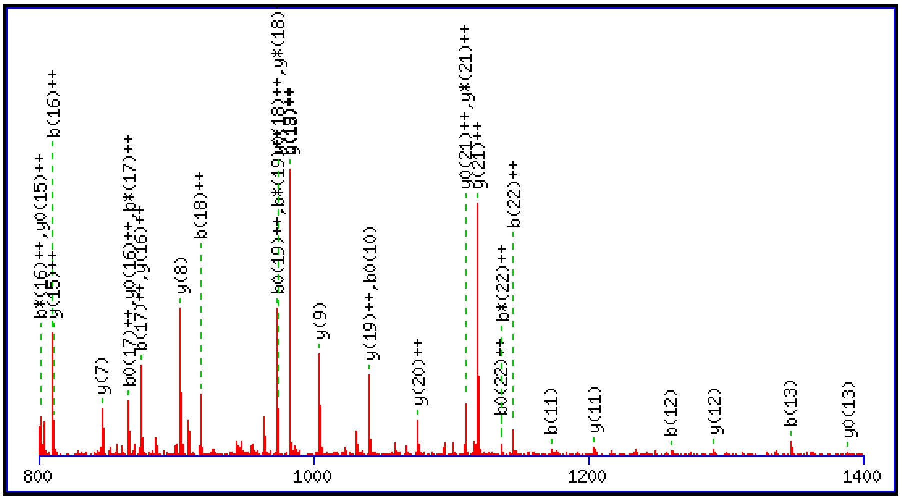


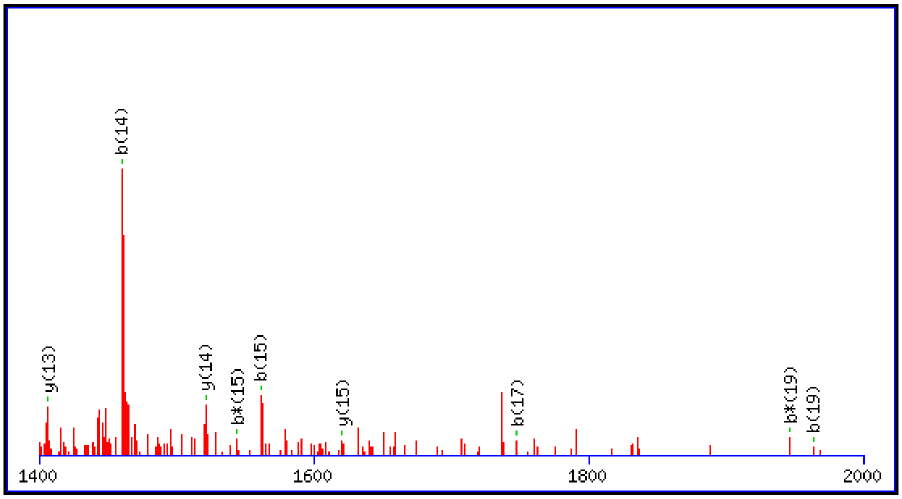


**Monoisotopic mass of neutral peptide Mr(calc):** 2463.1547
**Fixed modifications:** Carbamidomethyl (C)
**Variable modifications:**
**N11** : Deamidated_N (N)
**Ions Score:** 71 **Expect:** 2.3e-005
**Matches (Bold Red):** 65/250 fragment ions using 113 most intense peaks

| # | b | b++ | b* | b*++ | b0 | b0++ | Seq. | y | y++ | y* | y*++ | y0 | y0++ | # |
|---|---|---|---|---|---|---|---|---|---|---|---|---|---|---|
| 1 | 114.0913 | 57.5493 | | | | | I | | | | | | | 23 |
| 2 | 227.1754 | 114.0913 | | | | | I | 2351.0780 | 1176.0426 | 2334.0514 | 1167.5293 | 2333.0674 | 1167.0373 | 22 |
| 3 | **314.2074** | 157.6074 | | | **296.1969** | 148.6021 | S | 2237.9939 | **1119.5006** | 2220.9673 | **1110.9873** | 2219.9833 | **1110.4953** | 21 |
| 4 | **385.2445** | 193.1259 | | | **367.2340** | 184.1206 | A | 2150.9619 | **1075.9846** | 2133.9353 | 1067.4713 | 2132.9513 | 1066.9793 | 20 |
| 5 | 499.2875 | 250.1474 | 482.2609 | 241.6341 | 481.2769 | 241.1421 | N | 2079.9247 | **1040.4660** | 2062.8982 | 1031.9527 | 2061.9142 | 1031.4607 | 19 |

| | | | | | | | | | | | | | | |
|---|---|---|---|---|---|---|---|---|---|---|---|---|---|---|
| 6 | 556.3089 | 278.6581 | 539.2824 | 270.1448 | 538.2984 | 269.6528 | G | 1965.8818 | **983.4445** | 1948.8553 | **974.9313** | 1947.8713 | **974.4393** | 18 |
| 7 | 716.3396 | 358.6734 | 699.3130 | 350.1602 | 698.3290 | 349.6681 | C | 1908.8604 | 954.9338 | 1891.8338 | 946.4205 | 1890.8498 | 945.9285 | 17 |
| 8 | 844.4345 | 422.7209 | 827.4080 | 414.2076 | 826.4240 | 413.7156 | K | 1748.8297 | **874.9185** | 1731.8032 | 866.4052 | 1730.8191 | **865.9132** | 16 |
| 9 | 943.5030 | 472.2551 | 926.4764 | 463.7418 | 925.4924 | 463.2498 | V | **1620.7347** | **810.8710** | 1603.7082 | 802.3577 | 1602.7242 | **801.8657** | 15 |
| 10 | 1058.5299 | 529.7686 | 1041.5034 | 521.2553 | **1040.5193** | 520.7633 | D | **1521.6663** | 761.3368 | 1504.6398 | 752.8235 | 1503.6558 | 752.3315 | 14 |
| 11 | **1173.5568** | 587.2821 | 1156.5303 | 578.7688 | 1155.5463 | 578.2768 | N | **1406.6394** | 703.8233 | 1389.6128 | 695.3101 | **1388.6288** | 694.8180 | 13 |
| 12 | **1260.5889** | **630.7981** | 1243.5623 | 622.2848 | 1242.5783 | 621.7928 | S | **1291.6125** | 646.3099 | 1274.5859 | 637.7966 | 1273.6019 | 637.3046 | 12 |
| 13 | **1347.6209** | 674.3141 | 1330.5943 | **665.8008** | 1329.6103 | **665.3088** | S | **1204.5804** | 602.7938 | 1187.5539 | 594.2806 | 1186.5699 | 593.7886 | 11 |
| 14 | **1460.7050** | 730.8561 | 1443.6784 | **722.3428** | 1442.6944 | **721.8508** | L | 1117.5484 | **559.2778** | 1100.5218 | 550.7646 | 1099.5378 | 550.2726 | 10 |
| 15 | **1561.7526** | **781.3800** | **1544.7261** | **772.8667** | 1543.7421 | **772.3747** | T | **1004.4643** | **502.7358** | 987.4378 | 494.2225 | 986.4538 | 493.7305 | 9 |
| 16 | 1618.7741 | **809.8907** | 1601.7476 | **801.3774** | 1600.7635 | 800.8854 | G | **903.4167** | 452.2120 | 886.3901 | 443.6987 | 885.4061 | 443.2067 | 8 |
| 17 | **1747.8167** | **874.4120** | 1730.7901 | **865.8987** | 1729.8061 | **865.4067** | E | **846.3952** | **423.7012** | 829.3686 | 415.1880 | 828.3846 | 414.6959 | 7 |
| 18 | 1834.8487 | **917.9280** | 1817.8222 | 909.4147 | 1816.8382 | 908.9227 | S | **717.3526** | 359.1799 | 700.3260 | 350.6667 | 699.3420 | 350.1747 | 6 |
| 19 | **1963.8913** | **982.4493** | **1946.8648** | **973.9360** | 1945.8808 | **973.4440** | E | **630.3206** | 315.6639 | 613.2940 | 307.1506 | 612.3100 | 306.6586 | 5 |
| 20 | 2060.9441 | 1030.9757 | 2043.9175 | 1022.4624 | 2042.9335 | 1021.9704 | P | **501.2780** | 251.1426 | **484.2514** | 242.6293 | **483.2674** | 242.1373 | 4 |
| 21 | 2189.0027 | 1095.0050 | 2171.9761 | 1086.4917 | 2170.9921 | 1085.9997 | Q | **404.2252** | 202.6162 | 387.1987 | 194.1030 | 386.2146 | 193.6110 | 3 |
| 22 | 2290.0503 | **1145.5288** | 2273.0238 | **1137.0155** | 2272.0398 | **1136.5235** | T | **276.1666** | 138.5870 | 259.1401 | 130.0737 | 258.1561 | 129.5817 | 2 |
| 23 | | | | | | | R | 175.1190 | 88.0631 | 158.0924 | 79.5498 | | | 1 |

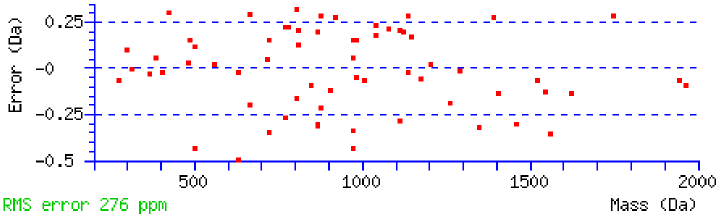


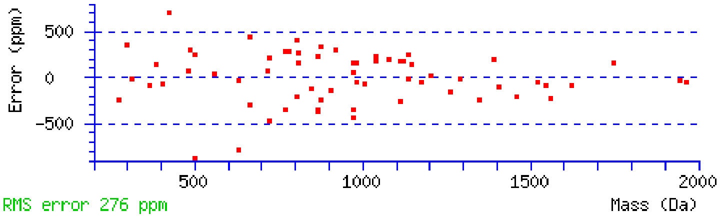


### All matches to this query

| Score | Mr(calc): | Delta | Sequence |
|---|---|---|---|
| 71.2 | 2463.1547 | -0.0059 | IISANGCKVDNSSLTGESEPQTR |
| 54.7 | 2463.1547 | -0.0059 | IISANGCKVDNSSLTGESEPQTR |
| 6.3 | 2461.1325 | 2.0164 | GGAKSIIITTSSVNAPMGVNQEK |
| 1.7 | 2462.1389 | 1.0099 | KNSSRPAVTQGTAPSTAXMRTK |
| 1.7 | 2462.1389 | 1.0099 | KNSSRPAVTQGTAPSTAXMRTK |
| 1.6 | 2461.1186 | 2.0303 | KNSSRPAVTQGTAPSTAXMRTK |
| 1.4 | 2461.1325 | 2.0164 | GGAKSIIITTSSVNAPMGVNQEK |
| 0.8 | 2463.1672 | -0.0183 | LQYLAAQNKGLQTQLSESRR |
| 0.5 | 2461.1501 | 1.9988 | WMSYEERHLLDELYITKR |
| 0.5 | 2461.1501 | 1.9988 | WMSYEERHLLDELYITKR |

---

Spectrum No: 349; Query: 1020; Rank: 1

---

## Peptide View

MS/MS Fragmentation of **TYCSSELVSNCTQK**
Found in **IPI00212725**, Tax_Id=10116 Gene_Symbol=Cd164 Endolyn precursor

Match to Query 1020: 1676.706548 from(839.360550,2+)
Title: 100101RatKid_NS_deglyco_25.1264.1264.2.dta
Data file K:\NewmanPaper\Piliang\3SubProteomes\Piliang3SP\mgf5ppm\ERLIC_3SubProteomes5ppm.mgf

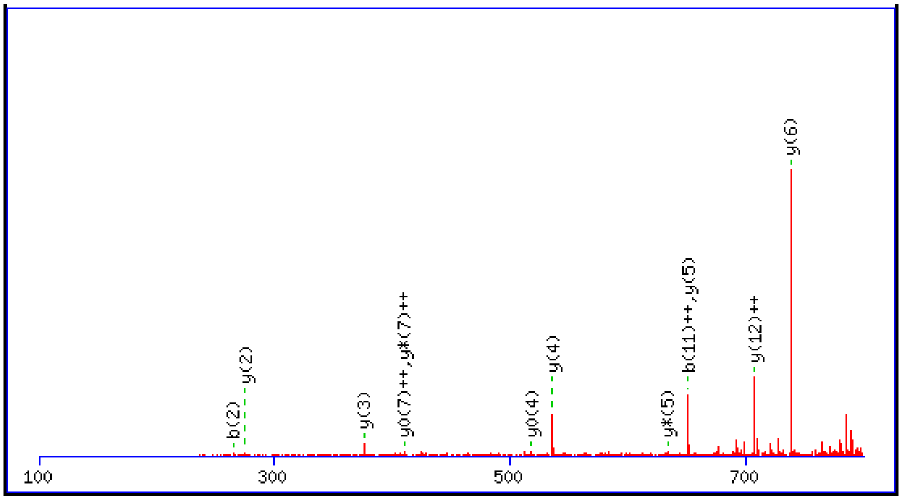


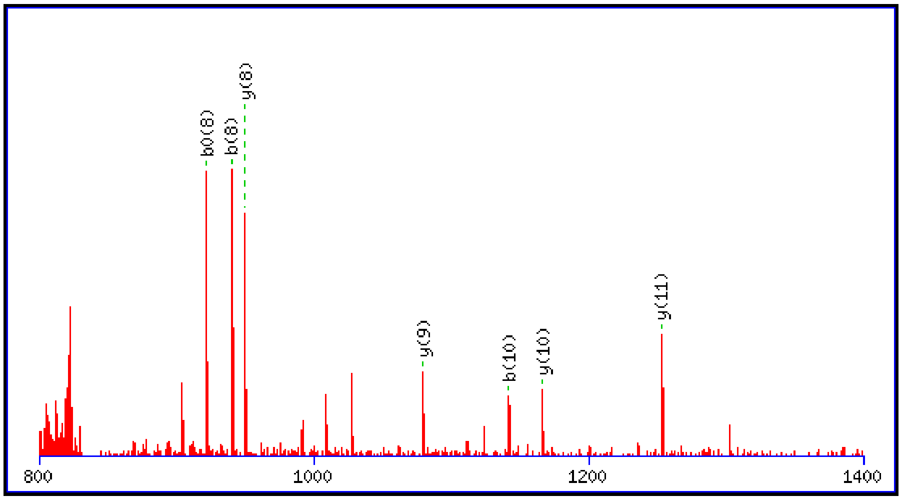


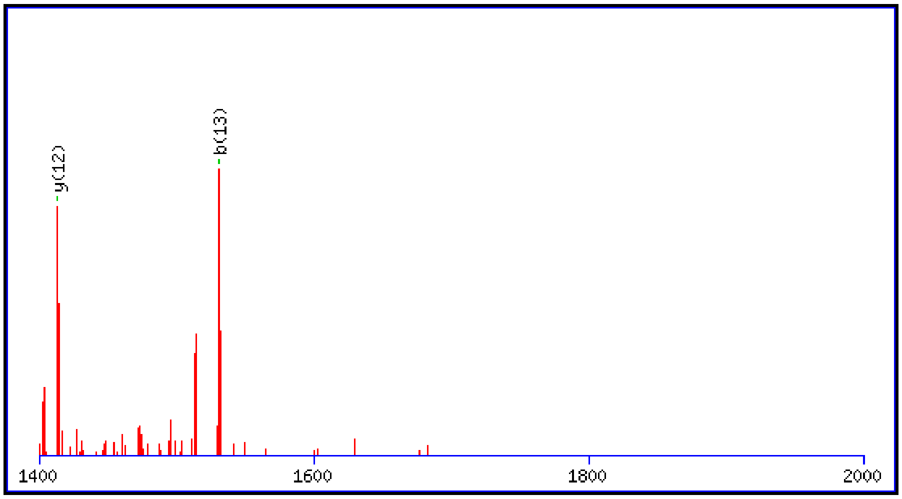


**Monoisotopic mass of neutral peptide Mr(calc):** 1676.7022
**Fixed modifications:** Carbamidomethyl (C)
**Variable modifications:**
**N10** : Deamidated_N (N)
**Ions Score:** 71 **Expect:** 1.4e-005
**Matches (Bold Red):** 21/134 fragment ions using 30 most intense peaks

| # | b | $b^{++}$ | b* | $b^{*++}$ | $b^{0}$ | $b^{0++}$ | Seq. | y | $y^{++}$ | y* | $y^{*++}$ | $y^{0}$ | $y^{0++}$ | # |
|---|---|---|---|---|---|---|---|---|---|---|---|---|---|---|
| 1 | 102.0550 | 51.5311 | | | 84.0444 | 42.5258 | T | | | | | | | 14 |
| 2 | **265.1183** | 133.0628 | | | 247.1077 | 124.0575 | Y | 1576.6618 | 788.8345 | 1559.6352 | 780.3213 | 1558.6512 | 779.8292 | 13 |
| 3 | 425.1489 | 213.0781 | | | 407.1384 | 204.0728 | C | **1413.5985** | **707.3029** | 1396.5719 | 698.7896 | 1395.5879 | 698.2976 | 12 |
| 4 | 512.1810 | 256.5941 | | | 494.1704 | 247.5888 | S | **1253.5678** | 627.2875 | 1236.5413 | 618.7743 | 1235.5572 | 618.2823 | 11 |
| 5 | 599.2130 | 300.1101 | | | 581.2024 | 291.1049 | S | **1166.5358** | 583.7715 | 1149.5092 | 575.2583 | 1148.5252 | 574.7662 | 10 |

| | | | | | | | | | | | | | | |
|---|---|---|---|---|---|---|---|---|---|---|---|---|---|---|
| **6** | 728.2556 | 364.6314 | | | 710.2450 | 355.6261 | **E** | **1079.5037** | 540.2555 | 1062.4772 | 531.7422 | 1061.4932 | 531.2502 | **9** |
| **7** | 841.3396 | 421.1735 | | | 823.3291 | 412.1682 | **L** | **950.4612** | 475.7342 | 933.4346 | 467.2209 | 932.4506 | 466.7289 | **8** |
| **8** | **940.4081** | 470.7077 | | | **922.3975** | 461.7024 | **V** | 837.3771 | 419.1922 | 820.3505 | **410.6789** | 819.3665 | **410.1869** | **7** |
| **9** | 1027.4401 | 514.2237 | | | 1009.4295 | 505.2184 | **S** | **738.3087** | 369.6580 | 721.2821 | 361.1447 | 720.2981 | 360.6527 | **6** |
| **10** | **1142.4670** | 571.7372 | 1125.4405 | 563.2239 | 1124.4565 | 562.7319 | **N** | **651.2766** | 326.1420 | **634.2501** | 317.6287 | 633.2661 | 317.1367 | **5** |
| **11** | 1302.4977 | **651.7525** | 1285.4711 | 643.2392 | 1284.4871 | 642.7472 | **C** | **536.2497** | 268.6285 | 519.2232 | 260.1152 | **518.2391** | 259.6232 | **4** |
| **12** | 1403.5454 | 702.2763 | 1386.5188 | 693.7630 | 1385.5348 | 693.2710 | **T** | **376.2191** | 188.6132 | 359.1925 | 180.0999 | 358.2085 | 179.6079 | **3** |
| **13** | **1531.6039** | 766.3056 | 1514.5774 | 757.7923 | 1513.5934 | 757.3003 | **Q** | **275.1714** | 138.0893 | 258.1448 | 129.5761 | | | **2** |
| **14** | | | | | | | **K** | 147.1128 | 74.0600 | 130.0863 | 65.5468 | | | **1** |

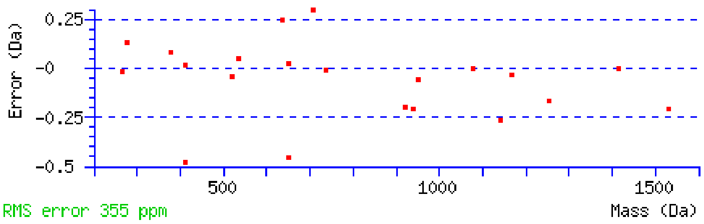


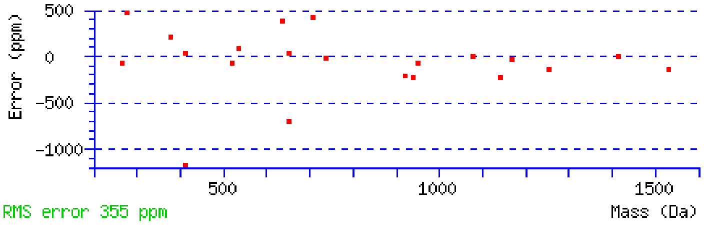


### All matches to this query

| Score | Mr(calc): | Delta | Sequence |
|---|---|---|---|
| 71.2 | 1676.7022 | 0.0044 | TYCSSELVSNCTQK |
| 16.7 | 1675.7182 | 0.9884 | TYCSSELVSNCTQK |
| 2.9 | 1675.6967 | 1.0098 | VKAGNANSKTGEPSR |
| 2.5 | 1676.7117 | -0.0051 | MLNFGASLQQASEGK |
| 1.3 | 1676.6906 | 0.0160 | QMTAQHYVTEFNK |
| 1.0 | 1675.7164 | 0.9901 | SGNSLPLSNSTMFIQ |
| 1.0 | 1675.7164 | 0.9901 | SGNSLPLSNSTMFIQ |
| 1.0 | 1676.7108 | -0.0043 | IMSFLKXVCTQK |
| 0.4 | 1676.7229 | -0.0164 | KIDERMSDAQGGYK |
| 0.1 | 1674.7032 | 2.0033 | STKGPREESTSCTR |

---

Spectrum No: 350; Query: 312; Rank: 1

---

## Peptide View

MS/MS Fragmentation of **MTLSQDNSTLR**
Found in **IPI00199607**, Tax_Id=10116 Gene_Symbol=Ceacam1_v4;Ceacam1_v1;Ceacam1;Ceacam1_v3 carcinoembryonic antigen-related cell adhesion molecule isoform 2

Match to Query 312: 1265.596608 from(633.805580,2+)
Title: 100101RatKid_NS_deglyco_23.1366.1366.2.dta
Data file K:\NewmanPaper\Piliang\3SubProteomes\Piliang3SP\mgf5ppm\ERLIC_3SubProteomes5ppm.mgf

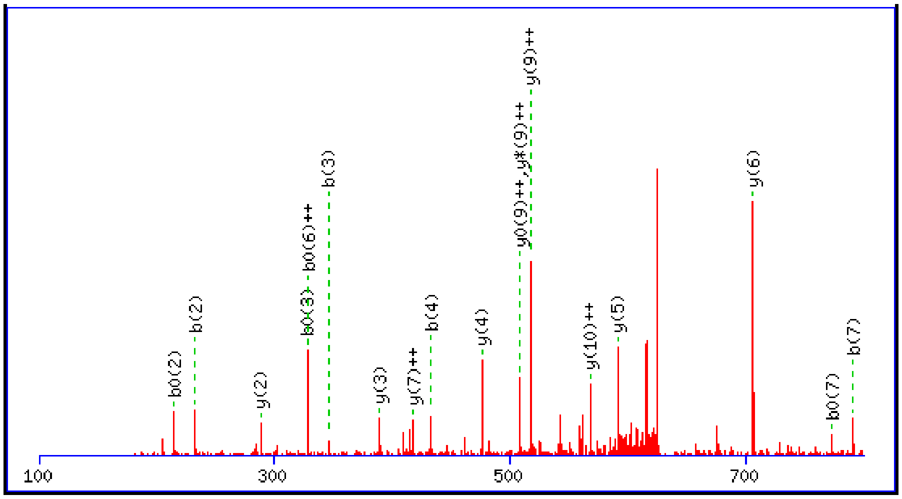


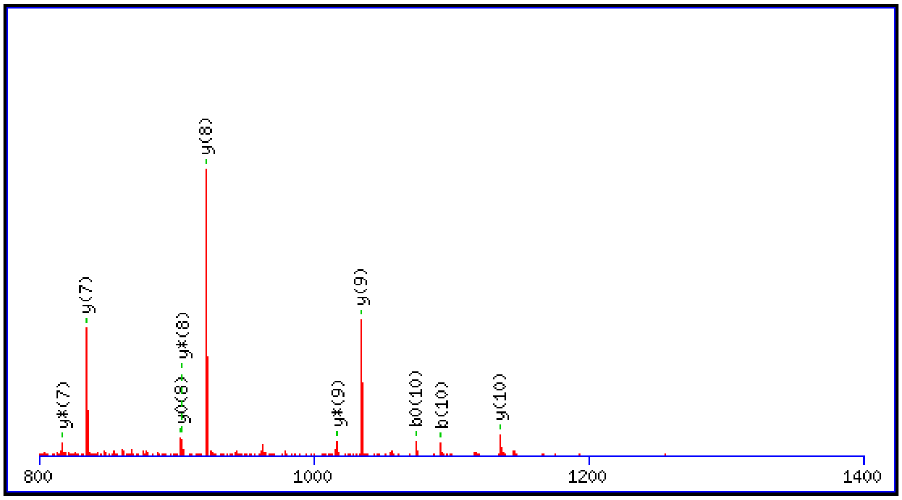


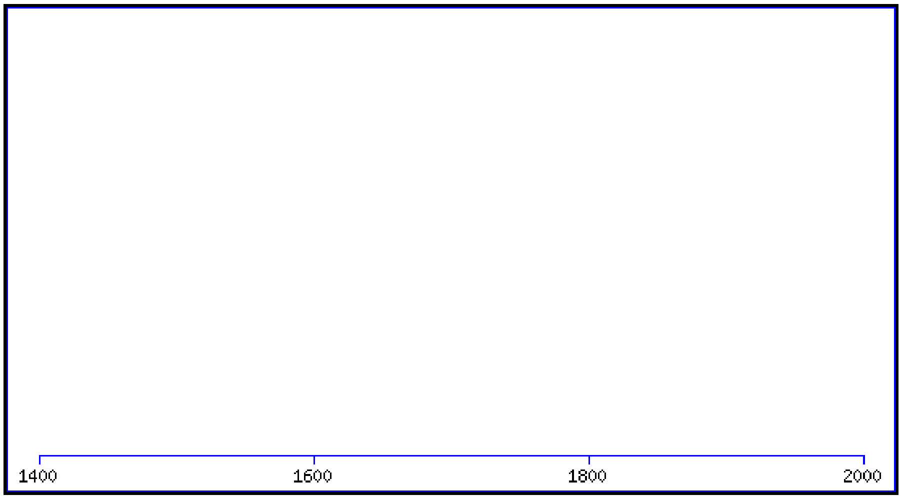


**Monoisotopic mass of neutral peptide Mr(calc):** 1265.5921
**Fixed modifications:** Carbamidomethyl (C)
**Variable modifications:**
**N7** : Deamidated_N (N)
**Ions Score:** 71 **Expect:** 1.1e-005
**Matches (Bold Red):** 28/106 fragment ions using 44 most intense peaks

| # | b | $b^{++}$ | b* | $b^{*++}$ | $b^0$ | $b^{0++}$ | Seq. | y | $y^{++}$ | y* | $y^{*++}$ | $y^0$ | $y^{0++}$ | # |
|---|---|---|---|---|---|---|---|---|---|---|---|---|---|---|
| 1 | 132.0478 | 66.5275 | | | | | M | | | | | | | 11 |
| 2 | **233.0954** | 117.0514 | | | **215.0849** | 108.0461 | T | **1135.5590** | **568.2831** | 1118.5324 | 559.7698 | 1117.5484 | 559.2778 | 10 |
| 3 | **346.1795** | 173.5934 | | | **328.1689** | 164.5881 | L | **1034.5113** | **517.7593** | **1017.4847** | **509.2460** | 1016.5007 | **508.7540** | 9 |
| 4 | **433.2115** | 217.1094 | | | 415.2010 | 208.1041 | S | **921.4272** | 461.2172 | **904.4007** | 452.7040 | **903.4166** | 452.2120 | 8 |
| 5 | 561.2701 | 281.1387 | 544.2436 | 272.6254 | 543.2595 | 272.1334 | Q | **834.3952** | **417.7012** | **817.3686** | 409.1880 | 816.3846 | 408.6959 | 7 |

| | | | | | | | | | | | | | | |
|---|---|---|---|---|---|---|---|---|---|---|---|---|---|---|
| **6** | 676.2971 | 338.6522 | 659.2705 | 330.1389 | 658.2865 | **329.6469** | **D** | **706.3366** | 353.6719 | 689.3101 | 345.1587 | 688.3260 | 344.6667 | **6** |
| **7** | **791.3240** | 396.1656 | 774.2974 | 387.6524 | **773.3134** | 387.1603 | **N** | **591.3097** | 296.1585 | 574.2831 | 287.6452 | 573.2991 | 287.1532 | **5** |
| **8** | 878.3560 | 439.6816 | 861.3295 | 431.1684 | 860.3455 | 430.6764 | **S** | **476.2827** | 238.6450 | 459.2562 | 230.1317 | 458.2722 | 229.6397 | **4** |
| **9** | 979.4037 | 490.2055 | 962.3771 | 481.6922 | 961.3931 | 481.2002 | **T** | **389.2507** | 195.1290 | 372.2241 | 186.6157 | 371.2401 | 186.1237 | **3** |
| **10** | **1092.4878** | 546.7475 | 1075.4612 | 538.2342 | **1074.4772** | 537.7422 | **L** | **288.2030** | 144.6051 | 271.1765 | 136.0919 | | | **2** |
| **11** | | | | | | | **R** | 175.1190 | 88.0631 | 158.0924 | 79.5498 | | | **1** |

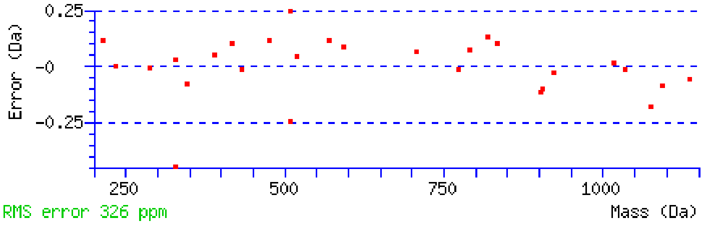


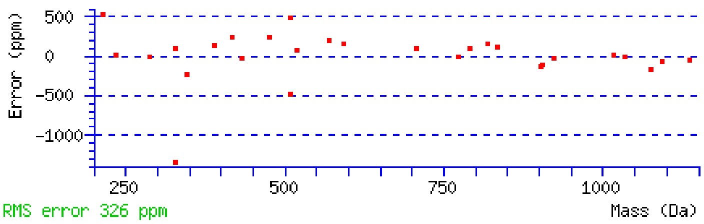


### All matches to this query

| Score | Mr(calc): | Delta | Sequence |
|---|---|---|---|
| 71.1 | 1265.5921 | 0.0045 | MTLSQDNSTLR |
| 13.0 | 1265.5856 | 0.0110 | TGMREEMSLGR |
| 8.4 | 1265.5922 | 0.0044 | VKDDMTEVTGR |
| 5.5 | 1263.5876 | 2.0090 | VNSLSKLKSK |
| 4.0 | 1265.6017 | -0.0050 | QEQLELTLGR |
| 3.5 | 1265.6043 | -0.0077 | MMPGRMVGVTR |
| 3.2 | 1265.5888 | 0.0078 | DGNGYISAAELR |
| 3.0 | 1265.5921 | 0.0045 | TSLMSAESPTSR |
| 2.7 | 1265.5921 | 0.0045 | STLSESMQLDR |
| 2.7 | 1264.5982 | 0.9984 | QAFENGVRMGR |

---

Spectrum No: 351; Query: 1896; Rank: 1

---

## Peptide View

MS/MS Fragmentation of **DLRNEIQVNTSQIPDTVTR**
Found in **IPI00211648**, Tax_Id=10116 Gene_Symbol=Slc3a1 Neutral and basic amino acid transport protein rBAT

Match to Query 1896: 2199.120868 from(1100.567710,2+)
Title: 100101RatKid_NS_deglyco_16.3817.3817.2.dta
Data file K:\NewmanPaper\Piliang\3SubProteomes\Piliang3SP\mgf5ppm\ERLIC_3SubProteomes5ppm.mgf

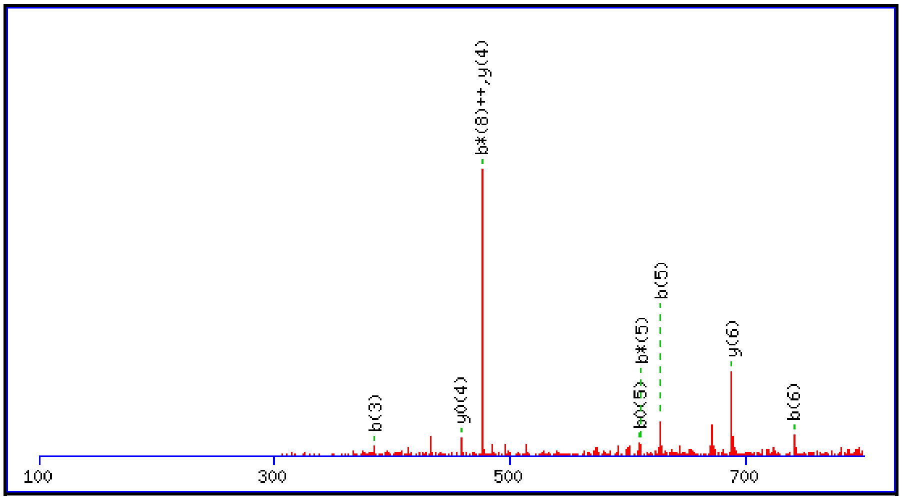

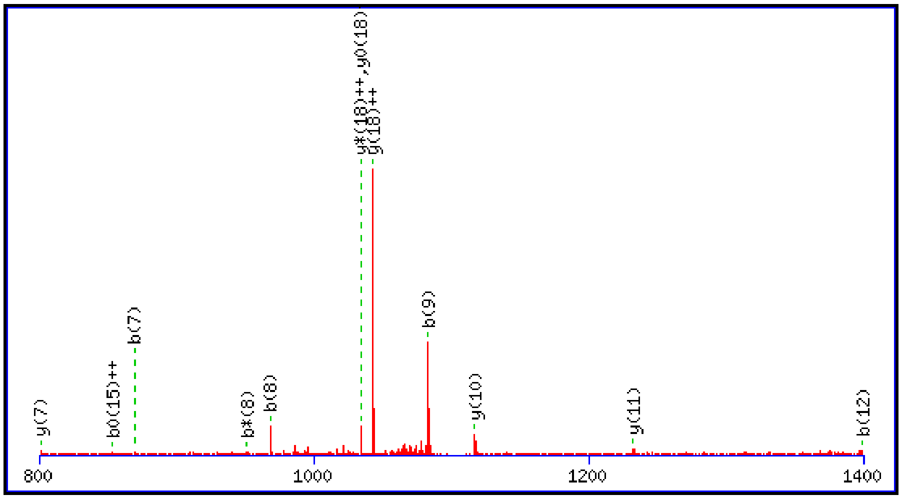


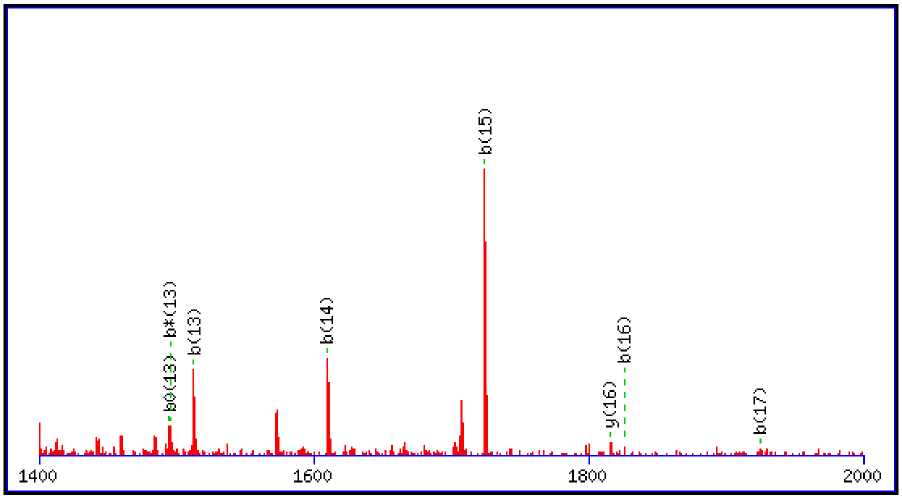


**Monoisotopic mass of neutral peptide Mr(calc):** 2199.1131
**Fixed modifications:** Carbamidomethyl (C)
**Variable modifications:**
**N9** : Deamidated_N (N)
**Ions Score:** 71 **Expect:** 1.2e-005
**Matches (Bold Red):** 29/210 fragment ions using 46 most intense peaks

| # | b | $b^{++}$ | b* | $b^{*++}$ | $b^{0}$ | $b^{0++}$ | Seq. | y | $y^{++}$ | y* | $y^{*++}$ | $y^{0}$ | $y^{0++}$ | # |
|---|---|---|---|---|---|---|---|---|---|---|---|---|---|---|
| 1 | 116.0342 | 58.5207 | | | 98.0237 | 49.5155 | D | | | | | | | 19 |
| 2 | 229.1183 | 115.0628 | | | 211.1077 | 106.0575 | L | 2085.0935 | **1043.0504** | 2068.0669 | **1034.5371** | 2067.0829 | **1034.0451** | 18 |
| 3 | **385.2194** | 193.1133 | 368.1928 | 184.6001 | 367.2088 | 184.1081 | R | 1972.0094 | 986.5083 | 1954.9829 | 977.9951 | 1953.9988 | 977.5031 | 17 |
| 4 | 499.2623 | 250.1348 | 482.2358 | 241.6215 | 481.2518 | 241.1295 | N | **1815.9083** | 908.4578 | 1798.8817 | 899.9445 | 1797.8977 | 899.4525 | 16 |
| 5 | **628.3049** | 314.6561 | **611.2784** | 306.1428 | **610.2943** | 305.6508 | E | 1701.8654 | 851.4363 | 1684.8388 | 842.9230 | 1683.8548 | 842.4310 | 15 |
| 6 | **741.3890** | 371.1981 | 724.3624 | 362.6849 | 723.3784 | 362.1928 | I | 1572.8228 | 786.9150 | 1555.7962 | 778.4017 | 1554.8122 | 777.9097 | 14 |
| 7 | **869.4476** | 435.2274 | 852.4210 | 426.7141 | 851.4370 | 426.2221 | Q | 1459.7387 | 730.3730 | 1442.7122 | 721.8597 | 1441.7281 | 721.3677 | 13 |
| 8 | **968.5160** | 484.7616 | **951.4894** | **476.2483** | 950.5054 | 475.7563 | V | 1331.6801 | 666.3437 | 1314.6536 | 657.8304 | 1313.6696 | 657.3384 | 12 |
| 9 | **1083.5429** | 542.2751 | 1066.5164 | 533.7618 | 1065.5323 | 533.2698 | N | **1232.6117** | 616.8095 | 1215.5852 | 608.2962 | 1214.6012 | 607.8042 | 11 |
| 10 | 1184.5906 | 592.7989 | 1167.5640 | 584.2857 | 1166.5800 | 583.7936 | T | **1117.5848** | 559.2960 | 1100.5582 | 550.7828 | 1099.5742 | 550.2907 | 10 |
| 11 | 1271.6226 | 636.3149 | 1254.5961 | 627.8017 | 1253.6120 | 627.3097 | S | 1016.5371 | 508.7722 | 999.5106 | 500.2589 | 998.5265 | 499.7669 | 9 |
| 12 | **1399.6812** | 700.3442 | 1382.6546 | 691.8310 | 1381.6706 | 691.3390 | Q | 929.5051 | 465.2562 | 912.4785 | 456.7429 | 911.4945 | 456.2509 | 8 |
| 13 | **1512.7653** | 756.8863 | **1495.7387** | 748.3730 | **1494.7547** | 747.8810 | I | **801.4465** | 401.2269 | 784.4199 | 392.7136 | 783.4359 | 392.2216 | 7 |
| 14 | **1609.8180** | 805.4126 | 1592.7915 | 796.8994 | 1591.8075 | 796.4074 | P | **688.3624** | 344.6849 | 671.3359 | 336.1716 | 670.3519 | 335.6796 | 6 |
| 15 | **1724.8450** | 862.9261 | 1707.8184 | 854.4128 | 1706.8344 | **853.9208** | D | 591.3097 | 296.1585 | 574.2831 | 287.6452 | 573.2991 | 287.1532 | 5 |
| 16 | **1825.8926** | 913.4500 | 1808.8661 | 904.9367 | 1807.8821 | 904.4447 | T | **476.2827** | 238.6450 | 459.2562 | 230.1317 | **458.2722** | 229.6397 | 4 |
| 17 | **1924.9611** | 962.9842 | 1907.9345 | 954.4709 | 1906.9505 | 953.9789 | V | 375.2350 | 188.1212 | 358.2085 | 179.6079 | 357.2245 | 179.1159 | 3 |
| 18 | 2026.0087 | 1013.5080 | 2008.9822 | 1004.9947 | 2007.9982 | 1004.5027 | T | 276.1666 | 138.5870 | 259.1401 | 130.0737 | 258.1561 | 129.5817 | 2 |

| 19 | | | | | | | R | 175.1190 | 88.0631 | 158.0924 | 79.5498 | | | 1 |
|---|---|---|---|---|---|---|---|---|---|---|---|---|---|---|

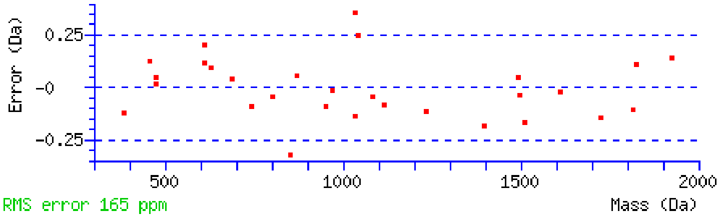


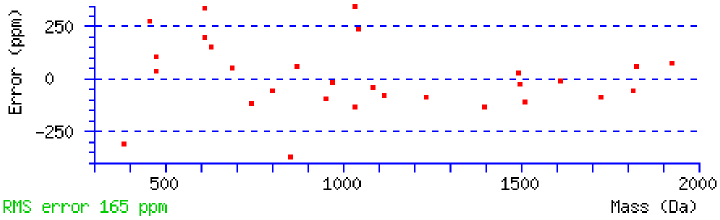


### All matches to this query

| Score | Mr(calc): | Delta | Sequence |
|---|---|---|---|
| 71.0 | 2199.1131 | 0.0077 | DLRNEIQVNTSQIPDTVTR |
| 39.7 | 2199.1131 | 0.0077 | DLRNEIQVNTSQIPDTVTR |
| 11.4 | 2198.1291 | 0.9917 | DLRNEIQVNTSQIPDTVTR |
| 3.9 | 2198.1378 | 0.9831 | LSAKHPHSQTSNSHPLKAMK |
| 2.2 | 2199.1032 | 0.0177 | NDPTYPRPSEPSLERARSK |
| 1.9 | 2199.1405 | -0.0196 | FKTPGQGMVVHLSNPIMRR |
| 1.6 | 2198.1282 | 0.9926 | NLRREEGIYSVLVALMAGK |
| 1.6 | 2198.1282 | 0.9926 | NLRREEGIYSVLVALMAGK |
| 1.4 | 2199.1089 | 0.0120 | NLLPKSLVNGVPWPQEATR |
| 1.3 | 2197.0983 | 2.0226 | LQELLKPSTGIIRTLPQM |

Spectrum No: 352; Query: 1357; Rank: 1

## Peptide View

MS/MS Fragmentation of **MAATFIGNSTAIQELFK**
Found in **IPI00765366**, Tax_Id=10116 Gene_Symbol=Tubb4 tubulin, beta 4

Match to Query 1357: 1858.928848 from(930.471700,2+)
Title: 091008RatKidney_NoSalt_04.4989.4989.2.dta
Data file K:\NewmanPaper\Piliang\3SubProteomes\Piliang3SP\mgf5ppm\ERLIC_3SubProteomes5ppm.mgf

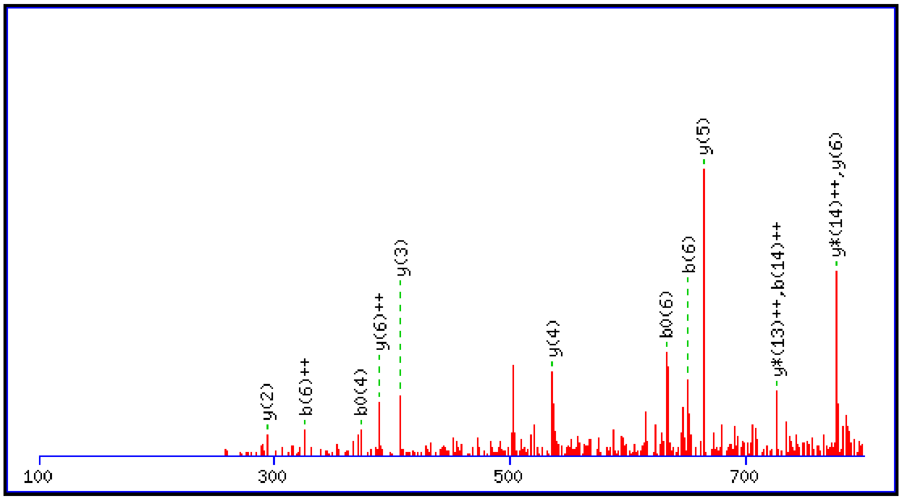

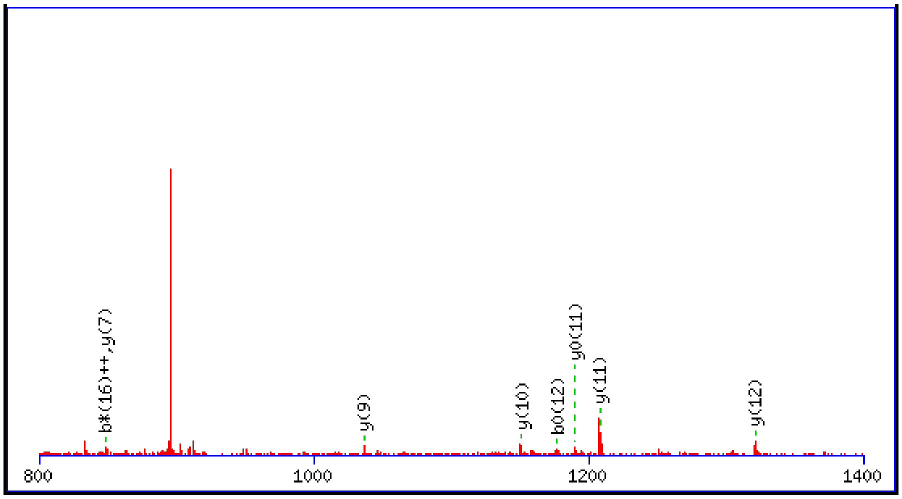


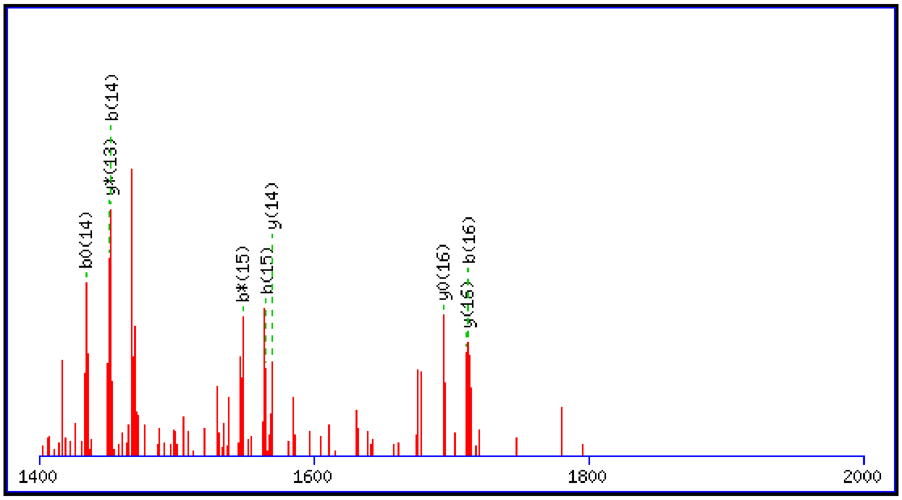


**Monoisotopic mass of neutral peptide Mr(calc):** 1857.9182
**Fixed modifications:** Carbamidomethyl (C)
**Variable modifications:**
**M1** : Oxidation (M)
**N8** : Deamidated_N (N)
**Ions Score:** 71 **Expect:** 1.5e-005
**Matches (Bold Red):** 30/166 fragment ions using 56 most intense peaks

| # | b | $b^{++}$ | b* | $b^{*++}$ | $b^0$ | $b^{0++}$ | Seq. | y | $y^{++}$ | y* | $y^{*++}$ | $y^0$ | $y^{0++}$ | # |
|---|---|---|---|---|---|---|---|---|---|---|---|---|---|---|
| 1 | 148.0427 | 74.5250 | | | | | M | | | | | | | 17 |
| 2 | 219.0798 | 110.0435 | | | | | A | **1711.8901** | 856.4487 | 1694.8636 | 847.9354 | **1693.8796** | 847.4434 | 16 |
| 3 | 290.1169 | 145.5621 | | | | | A | 1640.8530 | 820.9301 | 1623.8265 | 812.4169 | 1622.8424 | 811.9249 | 15 |
| 4 | 391.1646 | 196.0859 | | | **373.1540** | 187.0806 | T | **1569.8159** | 785.4116 | 1552.7893 | **776.8983** | 1551.8053 | 776.4063 | 14 |
| 5 | 538.2330 | 269.6201 | | | 520.2224 | 260.6149 | F | 1468.7682 | 734.8877 | **1451.7417** | **726.3745** | 1450.7577 | 725.8825 | 13 |
| 6 | **651.3171** | **326.1622** | | | **633.3065** | 317.1569 | I | **1321.6998** | 661.3535 | 1304.6733 | 652.8403 | 1303.6892 | 652.3483 | 12 |
| 7 | 708.3385 | 354.6729 | | | 690.3280 | 345.6676 | G | **1208.6157** | 604.8115 | 1191.5892 | 596.2982 | **1190.6052** | 595.8062 | 11 |
| 8 | 823.3655 | 412.1864 | 806.3389 | 403.6731 | 805.3549 | 403.1811 | N | **1151.5943** | 576.3008 | 1134.5677 | 567.7875 | 1133.5837 | 567.2955 | 10 |
| 9 | 910.3975 | 455.7024 | 893.3709 | 447.1891 | 892.3869 | 446.6971 | S | **1036.5673** | 518.7873 | 1019.5408 | 510.2740 | 1018.5568 | 509.7820 | 9 |
| 10 | 1011.4452 | 506.2262 | 994.4186 | 497.7129 | 993.4346 | 497.2209 | T | 949.5353 | 475.2713 | 932.5088 | 466.7580 | 931.5247 | 466.2660 | 8 |
| 11 | 1082.4823 | 541.7448 | 1065.4557 | 533.2315 | 1064.4717 | 532.7395 | A | **848.4876** | 424.7475 | 831.4611 | 416.2342 | 830.4771 | 415.7422 | 7 |
| 12 | 1195.5664 | 598.2868 | 1178.5398 | 589.7735 | **1177.5558** | 589.2815 | I | **777.4505** | **389.2289** | 760.4240 | 380.7156 | 759.4400 | 380.2236 | 6 |
| 13 | 1323.6249 | 662.3161 | 1306.5984 | 653.8028 | 1305.6144 | 653.3108 | Q | **664.3665** | 332.6869 | 647.3399 | 324.1736 | 646.3559 | 323.6816 | 5 |
| 14 | **1452.6675** | **726.8374** | 1435.6410 | 718.3241 | **1434.6570** | 717.8321 | E | **536.3079** | 268.6576 | 519.2813 | 260.1443 | 518.2973 | 259.6523 | 4 |
| 15 | **1565.7516** | 783.3794 | **1548.7250** | 774.8662 | 1547.7410 | 774.3741 | L | **407.2653** | 204.1363 | 390.2387 | 195.6230 | | | 3 |
| 16 | **1712.8200** | 856.9136 | 1695.7935 | **848.4004** | 1694.8094 | 847.9084 | F | **294.1812** | 147.5942 | 277.1547 | 139.0810 | | | 2 |
| 17 | | | | | | | K | 147.1128 | 74.0600 | 130.0863 | 65.5468 | | | 1 |

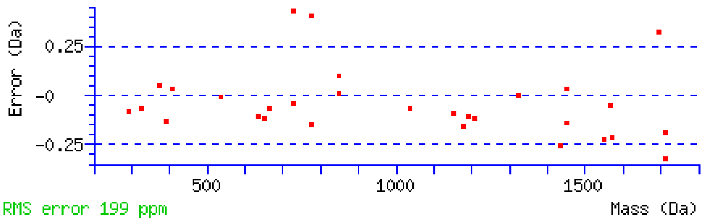


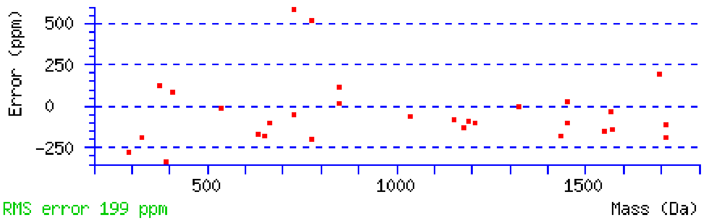


### All matches to this query

| Score | Mr(calc): | Delta | Sequence |
|---|---|---|---|
| 70.8 | 1857.9182 | 1.0106 | MAATFIGNSTAIQELFK |
| 57.1 | 1856.9342 | 1.9946 | MAATFIGNSTAIQELFK |
| 57.1 | 1856.9342 | 1.9946 | MASTFIGNSTAIQELFK |
| 57.1 | 1856.9342 | 1.9946 | MSATFIGNSTAIQELFK |
| 56.9 | 1857.9182 | 1.0106 | MASTFIGNSTAIQELFK |
| 56.9 | 1857.9182 | 1.0106 | MSATFIGNSTAIQELFK |
| 8.8 | 1856.9113 | 2.0175 | FKKNNFIIISNYFK |
| 7.8 | 1856.9205 | 2.0083 | SNKNLLLKSEIEIMK |
| 4.0 | 1856.9258 | 2.0031 | TKNISAISYINQRNR |
| 3.2 | 1856.9206 | 2.0083 | SDEKLIKSIINMEIK |

---

Spectrum No: 353; Query: 264; Rank: 1

---

## Peptide View

MS/MS Fragmentation of **TGNWSALPSCK**
Found in **IPI00195241**, Tax_Id=10116 Gene_Symbol=Apoh Beta-2-glycoprotein 1 precursor

Match to Query 264: 1220.547368 from(611.280960,2+)
Title: 091008RatKidney_NoSalt_28.1494.1494.2.dta
Data file K:\NewmanPaper\Piliang\3SubProteomes\Piliang3SP\mgf5ppm\ERLIC_3SubProteomes5ppm.mgf

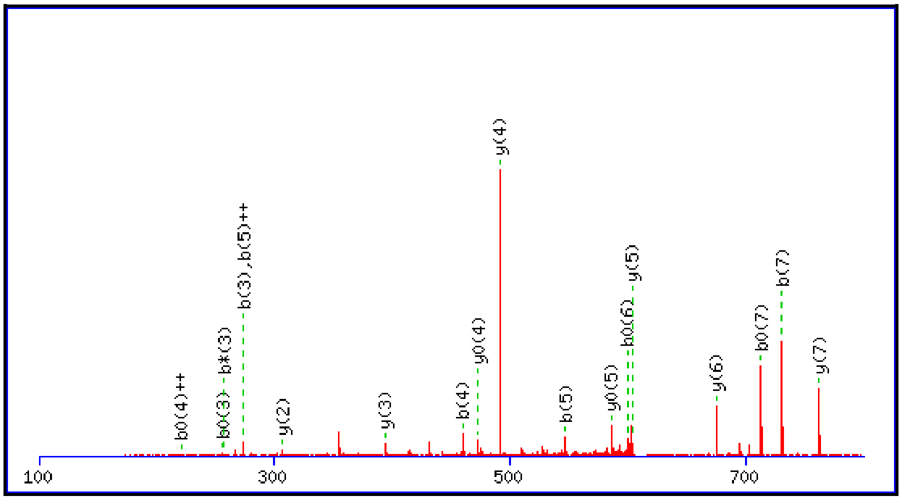

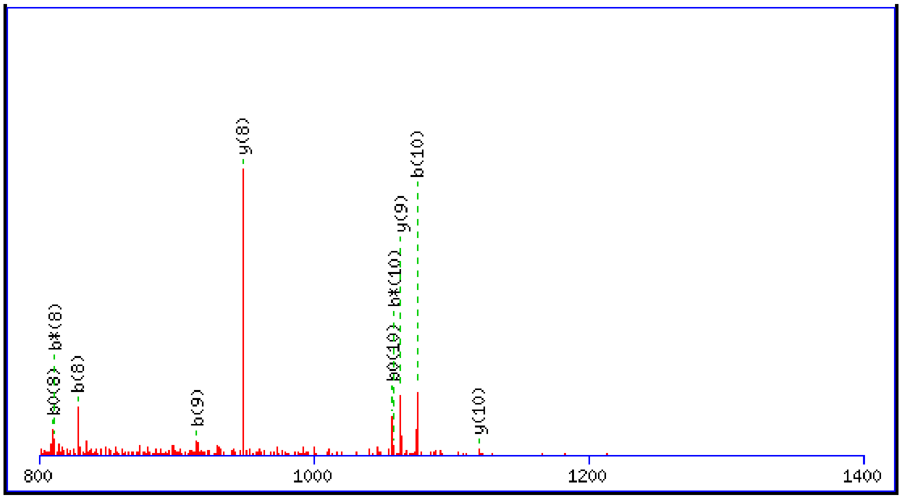


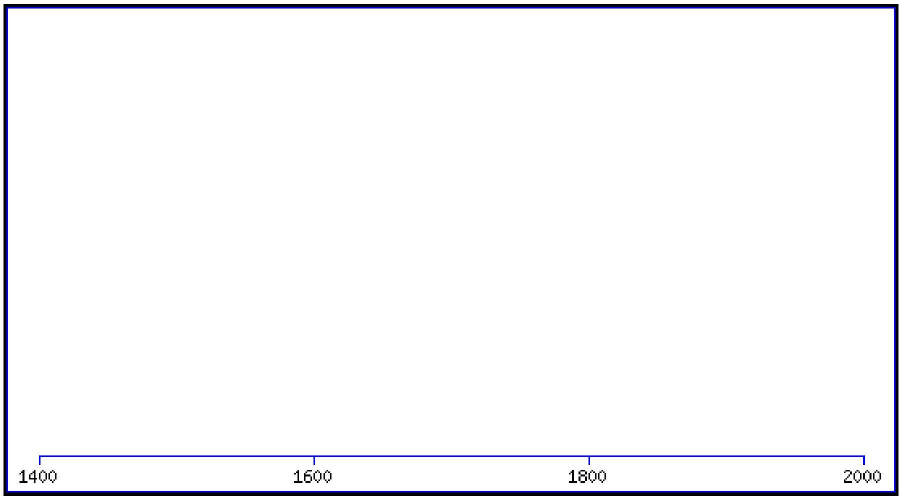


**Monoisotopic mass of neutral peptide Mr(calc):** 1220.5496
**Fixed modifications:** Carbamidomethyl (C)
**Variable modifications:**
**N3** : Deamidated_N (N)
**Ions Score:** 71 **Expect:** 9.4e-006
**Matches (Bold Red):** 28/112 fragment ions using 43 most intense peaks

| # | b | $b^{++}$ | b* | $b^{*++}$ | $b^0$ | $b^{0++}$ | Seq. | y | $y^{++}$ | y* | $y^{*++}$ | $y^0$ | $y^{0++}$ | # |
|---|---|---|---|---|---|---|---|---|---|---|---|---|---|---|
| 1 | 102.0550 | 51.5311 | | | 84.0444 | 42.5258 | T | | | | | | | 11 |
| 2 | 159.0764 | 80.0418 | | | 141.0659 | 71.0366 | G | **1120.5092** | 560.7582 | 1103.4826 | 552.2449 | 1102.4986 | 551.7529 | 10 |
| 3 | **274.1034** | 137.5553 | **257.0768** | 129.0420 | **256.0928** | 128.5500 | N | **1063.4877** | 532.2475 | 1046.4612 | 523.7342 | 1045.4771 | 523.2422 | 9 |
| 4 | **460.1827** | 230.5950 | 443.1561 | 222.0817 | 442.1721 | **221.5897** | W | **948.4608** | 474.7340 | 931.4342 | 466.2207 | 930.4502 | 465.7287 | 8 |
| 5 | **547.2147** | **274.1110** | 530.1881 | 265.5977 | 529.2041 | 265.1057 | S | **762.3815** | 381.6944 | 745.3549 | 373.1811 | 744.3709 | 372.6891 | 7 |
| 6 | 618.2518 | 309.6295 | 601.2253 | 301.1163 | **600.2412** | 300.6243 | A | **675.3494** | 338.1783 | 658.3229 | 329.6651 | 657.3389 | 329.1731 | 6 |
| 7 | **731.3359** | 366.1716 | 714.3093 | 357.6583 | **713.3253** | 357.1663 | L | **604.3123** | 302.6598 | 587.2858 | 294.1465 | **586.3017** | 293.6545 | 5 |
| 8 | **828.3886** | 414.6980 | **811.3621** | 406.1847 | **810.3781** | 405.6927 | P | **491.2282** | 246.1178 | 474.2017 | 237.6045 | **473.2177** | 237.1125 | 4 |
| 9 | **915.4207** | 458.2140 | 898.3941 | 449.7007 | 897.4101 | 449.2087 | S | **394.1755** | 197.5914 | 377.1489 | 189.0781 | 376.1649 | 188.5861 | 3 |
| 10 | **1075.4513** | 538.2293 | **1058.4248** | 529.7160 | **1057.4408** | 529.2240 | C | **307.1435** | 154.0754 | 290.1169 | 145.5621 | | | 2 |
| 11 | | | | | | | K | 147.1128 | 74.0600 | 130.0863 | 65.5468 | | | 1 |

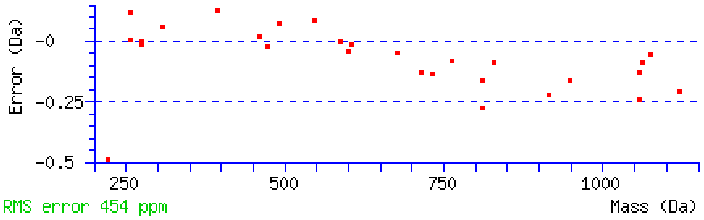


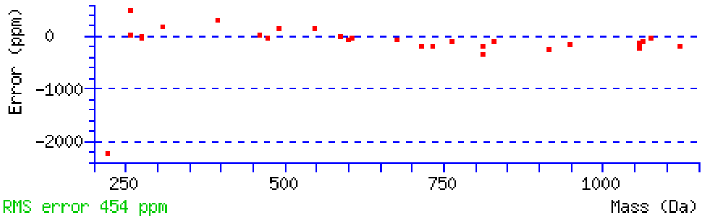

### All matches to this query

| Score | Mr(calc): | Delta | Sequence |
|---|---|---|---|
| 70.6 | 1220.5496 | -0.0022 | TGNWSALPSCK |
| 15.1 | 1220.5594 | -0.0121 | LEELNLNSCK |
| 2.1 | 1220.5455 | 0.0018 | NSADPGTSMGKR |
| 1.8 | 1218.5452 | 2.0022 | DCVNSPSVWR |
| 1.6 | 1220.5455 | 0.0019 | MEGERKNNNK |
| 1.6 | 1220.5455 | 0.0019 | MEGERKNNNK |
| 1.6 | 1220.5455 | 0.0019 | MEGERKNNNK |
| 0.5 | 1220.5520 | -0.0047 | ADLSNDDEKSK |
| 0.5 | 1220.5438 | 0.0036 | KFSSSNKTDK |
| 0.5 | 1220.5438 | 0.0036 | KFSSSNKTDK |

---

Spectrum No: 354; Query: 981; Rank: 1

---

## Peptide View

MS/MS Fragmentation of **SVVTEEFNGSDWER**
Found in **IPI00197723**, Tax_Id=10116 Gene_Symbol=RGD1565681_predicted similar to proline rich 6

Match to Query 981: 1654.719008 from(828.366780,2+)
Title: 100101RatKid_NS_deglyco_15.3315.3315.2.dta
Data file K:\NewmanPaper\Piliang\3SubProteomes\Piliang3SP\mgf5ppm\ERLIC_3SubProteomes5ppm.mgf

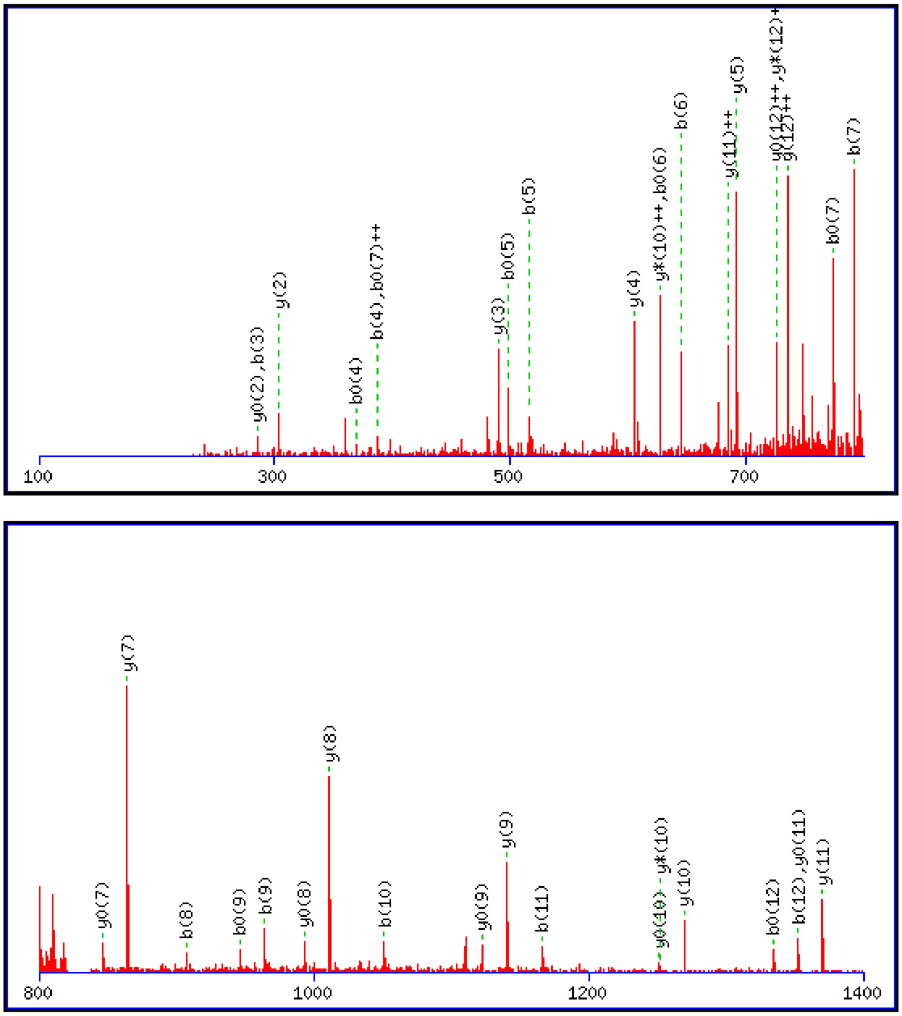

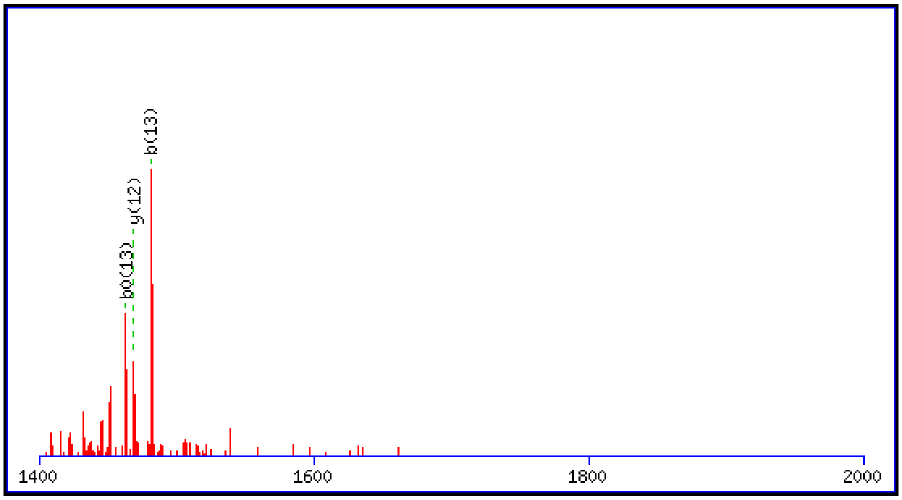


**Monoisotopic mass of neutral peptide Mr(calc):** 1654.7111
**Fixed modifications:** Carbamidomethyl (C)
**Variable modifications:**
**N8** : Deamidated_N (N)
**Ions Score:** 71 **Expect:** 1.5e-005
**Matches (Bold Red):** 41/140 fragment ions using 71 most intense peaks

| # | b | $b^{++}$ | b* | $b^{*++}$ | $b^{0}$ | $b^{0++}$ | Seq. | y | $y^{++}$ | y* | $y^{*++}$ | $y^{0}$ | $y^{0++}$ | # |
|---|---|---|---|---|---|---|---|---|---|---|---|---|---|---|
| 1 | 88.0393 | 44.5233 | | | 70.0287 | 35.5180 | S | | | | | | | 14 |
| 2 | 187.1077 | 94.0575 | | | 169.0972 | 85.0522 | V | 1568.6863 | 784.8468 | 1551.6598 | 776.3335 | 1550.6758 | 775.8415 | 13 |
| 3 | **286.1761** | 143.5917 | | | 268.1656 | 134.5864 | V | **1469.6179** | **735.3126** | 1452.5914 | **726.7993** | 1451.6074 | **726.3073** | 12 |
| 4 | **387.2238** | 194.1155 | | | **369.2132** | 185.1103 | T | **1370.5495** | **685.7784** | 1353.5230 | 677.2651 | **1352.5389** | 676.7731 | 11 |
| 5 | **516.2664** | 258.6368 | | | **498.2558** | 249.6316 | E | **1269.5018** | 635.2546 | **1252.4753** | **626.7413** | **1251.4913** | 626.2493 | 10 |
| 6 | **645.3090** | 323.1581 | | | **627.2984** | 314.1529 | E | **1140.4592** | 570.7333 | 1123.4327 | 562.2200 | **1122.4487** | 561.7280 | 9 |
| 7 | **792.3774** | 396.6923 | | | **774.3668** | **387.6871** | F | **1011.4166** | 506.2120 | 994.3901 | 497.6987 | **993.4061** | 497.2067 | 8 |
| 8 | **907.4043** | 454.2058 | 890.3778 | 445.6925 | 889.3938 | 445.2005 | N | **864.3482** | 432.6778 | 847.3217 | 424.1645 | **846.3377** | 423.6725 | 7 |
| 9 | **964.4258** | 482.7165 | 947.3993 | 474.2033 | **946.4152** | 473.7113 | G | 749.3213 | 375.1643 | 732.2947 | 366.6510 | 731.3107 | 366.1590 | 6 |
| 10 | **1051.4578** | 526.2326 | 1034.4313 | 517.7193 | 1033.4473 | 517.2273 | S | **692.2998** | 346.6536 | 675.2733 | 338.1403 | 674.2893 | 337.6483 | 5 |
| 11 | **1166.4848** | 583.7460 | 1149.4582 | 575.2328 | 1148.4742 | 574.7407 | D | **605.2678** | 303.1375 | 588.2413 | 294.6243 | 587.2572 | 294.1323 | 4 |
| 12 | **1352.5641** | 676.7857 | 1335.5375 | 668.2724 | **1334.5535** | 667.7804 | W | **490.2409** | 245.6241 | 473.2143 | 237.1108 | 472.2303 | 236.6188 | 3 |
| 13 | **1481.6067** | 741.3070 | 1464.5801 | 732.7937 | **1463.5961** | 732.3017 | E | **304.1615** | 152.5844 | 287.1350 | 144.0711 | **286.1510** | 143.5791 | 2 |
| 14 | | | | | | | R | 175.1190 | 88.0631 | 158.0924 | 79.5498 | | | 1 |

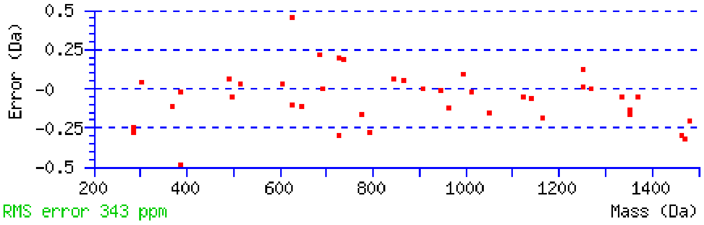


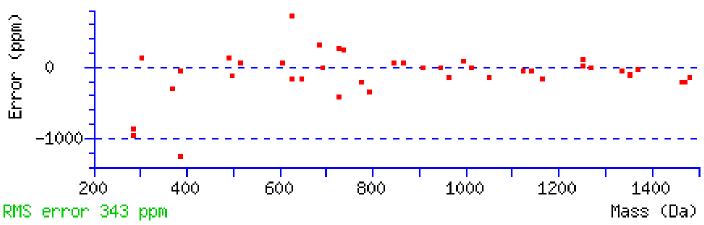


### All matches to this query

| Score | Mr(calc): | Delta | Sequence |
|---|---|---|---|
| 70.6 | 1654.7111 | 0.0079 | SVVTEEFNGSDWER |
| 16.6 | 1653.7271 | 0.9919 | SVVTEEFNGSDWER |
| 6.7 | 1654.7213 | -0.0023 | GRTTHSPYEERSR |
| 6.7 | 1654.7213 | -0.0023 | GRTTHSPYEERSR |
| 6.7 | 1654.7213 | -0.0023 | GRTTHSPYEERSR |
| 3.9 | 1652.7078 | 2.0112 | IATMETMNIFLEK |
| 3.6 | 1652.7253 | 1.9937 | SPISNGTFGFEGHMR |

| | | | |
|---|---|---|---|
| 2.2 | 1652.7078 | 2.0112 | IATMETMNIFLEK |
| 1.6 | 1654.7352 | -0.0162 | SIWEEQINSRDAK |
| 1.4 | 1653.7108 | 1.0083 | SVDSRNRGPDSLNR |

---

Spectrum No: 355; Query: 1907; Rank: 1

---

## Peptide View

MS/MS Fragmentation of **VAANFSTPVISTSGSSDPGQER**
Found in **IPI00391338**, Tax_Id=10116 Gene_Symbol=RGD1562791_predicted similar to ICOS ligand precursor

Match to Query 1907: 2207.040808 from(1104.527680,2+)
Title: 100101RatKid_NS_deglyco_24.2430.2430.2.dta
Data file K:\NewmanPaper\Piliang\3SubProteomes\Piliang3SP\mgf5ppm\ERLIC_3SubProteomes5ppm.mgf

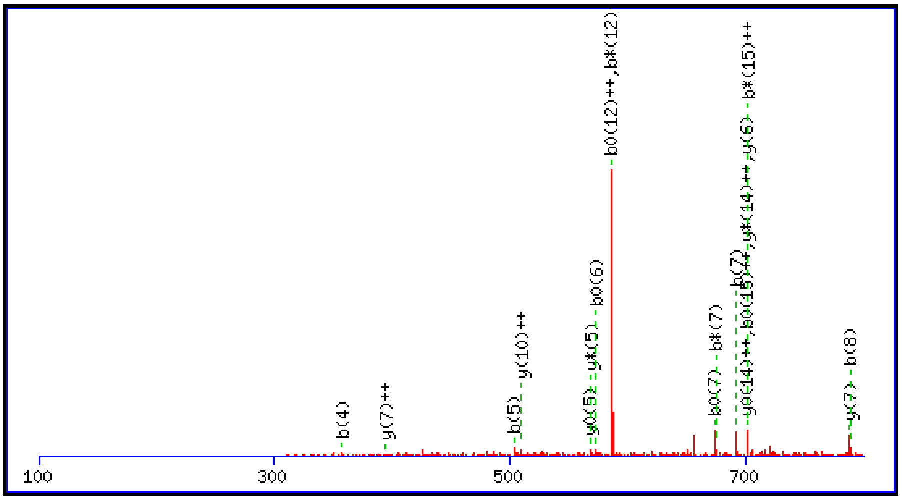


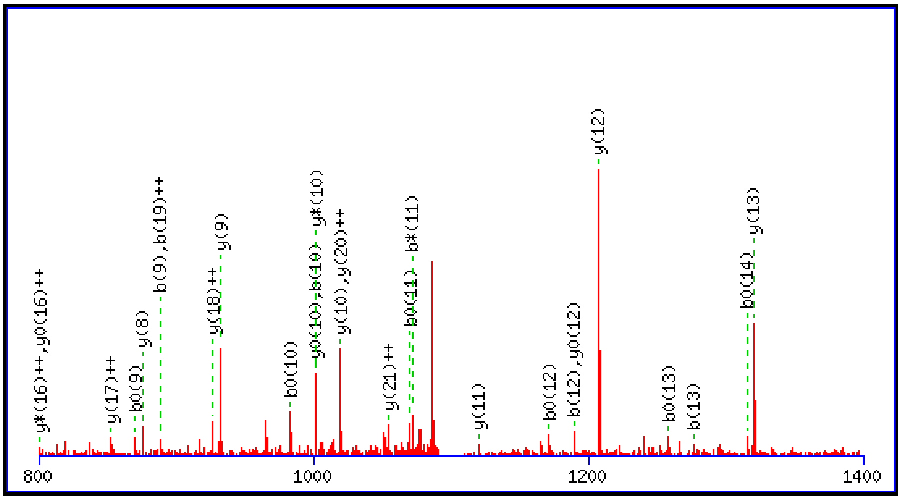

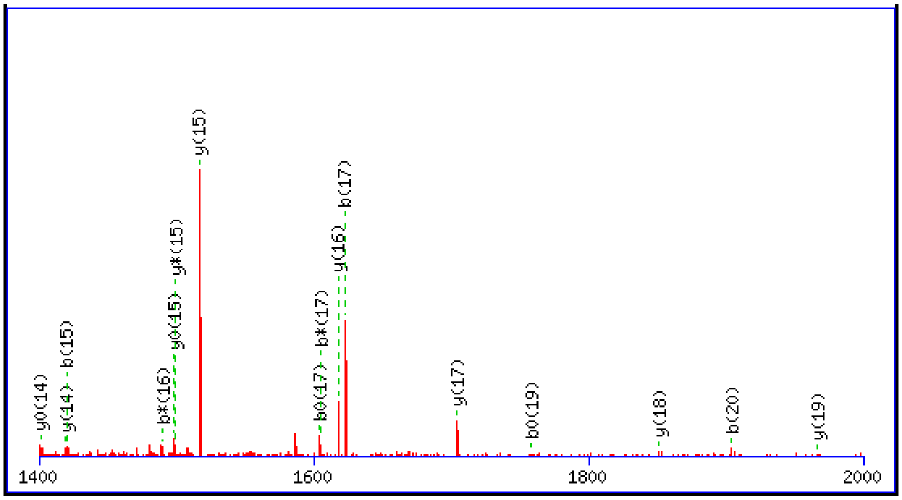


**Monoisotopic mass of neutral peptide Mr(calc):** 2207.0342
**Fixed modifications:** Carbamidomethyl (C)
**Variable modifications:**
**N4** : Deamidated_N (N)
**Ions Score:** 70 **Expect:** 2.5e-005
**Matches (Bold Red):** 63/234 fragment ions using 107 most intense peaks

| # | b | $b^{++}$ | b* | $b^{*++}$ | $b^0$ | $b^{0++}$ | Seq. | y | $y^{++}$ | y* | $y^{*++}$ | $y^0$ | $y^{0++}$ | # |
|---|---|---|---|---|---|---|---|---|---|---|---|---|---|---|
| 1 | 100.0757 | 50.5415 | | | | | V | | | | | | | 22 |
| 2 | 171.1128 | 86.0600 | | | | | A | 2108.9731 | **1054.9902** | 2091.9465 | 1046.4769 | 2090.9625 | 1045.9849 | 21 |
| 3 | 242.1499 | 121.5786 | | | | | A | 2037.9360 | **1019.4716** | 2020.9094 | 1010.9583 | 2019.9254 | 1010.4663 | 20 |
| 4 | **357.1769** | 179.0921 | 340.1503 | 170.5788 | | | N | **1966.8988** | 983.9531 | 1949.8723 | 975.4398 | 1948.8883 | 974.9478 | 19 |
| 5 | **504.2453** | 252.6263 | 487.2187 | 244.1130 | | | F | **1851.8719** | **926.4396** | 1834.8454 | 917.9263 | 1833.8613 | 917.4343 | 18 |
| 6 | 591.2773 | 296.1423 | 574.2507 | 287.6290 | **573.2667** | 287.1370 | S | **1704.8035** | **852.9054** | 1687.7769 | 844.3921 | 1686.7929 | 843.9001 | 17 |
| 7 | **692.3250** | 346.6661 | **675.2984** | 338.1529 | **674.3144** | 337.6608 | T | **1617.7715** | 809.3894 | 1600.7449 | **800.8761** | 1599.7609 | **800.3841** | 16 |
| 8 | **789.3777** | 395.1925 | 772.3512 | 386.6792 | 771.3672 | 386.1872 | P | **1516.7238** | 758.8655 | **1499.6972** | 750.3523 | **1498.7132** | 749.8603 | 15 |
| 9 | **888.4462** | 444.7267 | 871.4196 | 436.2134 | **870.4356** | 435.7214 | V | **1419.6710** | 710.3392 | 1402.6445 | **701.8259** | **1401.6605** | **701.3339** | 14 |
| 10 | **1001.5302** | 501.2687 | 984.5037 | 492.7555 | **983.5197** | 492.2635 | I | **1320.6026** | 660.8049 | 1303.5761 | 652.2917 | 1302.5920 | 651.7997 | 13 |
| 11 | 1088.5622 | 544.7848 | **1071.5357** | 536.2715 | **1070.5517** | 535.7795 | S | **1207.5185** | 604.2629 | 1190.4920 | 595.7496 | **1189.5080** | 595.2576 | 12 |
| 12 | **1189.6099** | 595.3086 | 1172.5834 | **586.7953** | **1171.5994** | **586.3033** | T | **1120.4865** | 560.7469 | 1103.4600 | 552.2336 | 1102.4760 | 551.7416 | 11 |
| 13 | **1276.6420** | 638.8246 | 1259.6154 | 630.3113 | **1258.6314** | 629.8193 | S | **1019.4388** | **510.2231** | **1002.4123** | 501.7098 | **1001.4283** | 501.2178 | 10 |
| 14 | 1333.6634 | 667.3353 | 1316.6369 | 658.8221 | **1315.6529** | 658.3301 | G | **932.4068** | 466.7070 | 915.3803 | 458.1938 | 914.3962 | 457.7018 | 9 |
| 15 | **1420.6954** | 710.8514 | 1403.6689 | **702.3381** | 1402.6849 | **701.8461** | S | **875.3854** | 438.1963 | 858.3588 | 429.6830 | 857.3748 | 429.1910 | 8 |
| 16 | 1507.7275 | 754.3674 | **1490.7009** | 745.8541 | 1489.7169 | 745.3621 | S | **788.3533** | **394.6803** | 771.3268 | 386.1670 | 770.3428 | 385.6750 | 7 |
| 17 | **1622.7544** | 811.8808 | **1605.7279** | 803.3676 | **1604.7439** | 802.8756 | D | **701.3213** | 351.1643 | 684.2947 | 342.6510 | 683.3107 | 342.1590 | 6 |
| 18 | 1719.8072 | 860.4072 | 1702.7806 | 851.8940 | 1701.7966 | 851.4019 | P | **586.2944** | 293.6508 | **569.2678** | 285.1375 | **568.2838** | 284.6455 | 5 |
| 19 | 1776.8286 | **888.9180** | 1759.8021 | 880.4047 | **1758.8181** | 879.9127 | G | 489.2416 | 245.1244 | 472.2150 | 236.6112 | 471.2310 | 236.1191 | 4 |
| 20 | **1904.8872** | 952.9472 | 1887.8607 | 944.4340 | 1886.8767 | 943.9420 | Q | 432.2201 | 216.6137 | 415.1936 | 208.1004 | 414.2096 | 207.6084 | 3 |
| 21 | 2033.9298 | 1017.4685 | 2016.9033 | 1008.9553 | 2015.9192 | 1008.4633 | E | 304.1615 | 152.5844 | 287.1350 | 144.0711 | 286.1510 | 143.5791 | 2 |
| 22 | | | | | | | R | 175.1190 | 88.0631 | 158.0924 | 79.5498 | | | 1 |

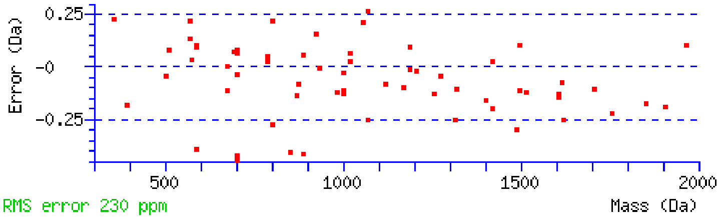


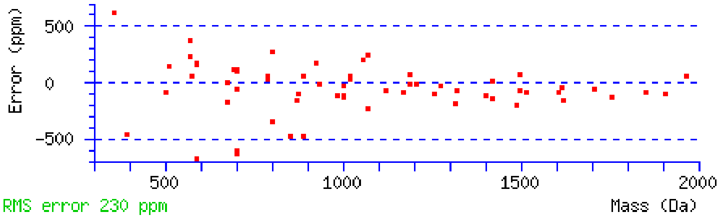


## All matches to this query

| | | | |
|---|---|---|---|

| Score | Mr(calc): | Delta | Sequence |
|---|---|---|---|
| 70.4 | 2207.0342 | 0.0066 | VAANFSTPVISTSGSSDPGQER |
| 43.7 | 2206.0502 | 0.9906 | VAANFSTPVISTSGSSDPGQER |
| 7.1 | 2205.0500 | 1.9908 | MRSTNQSSVSEFLLLGLSR |
| 5.0 | 2206.0469 | 0.9939 | KDIQTISNILISIGRCSK |
| 5.0 | 2206.0469 | 0.9939 | KDIQTISNILISIGRCSK |
| 3.4 | 2205.0483 | 1.9925 | KATSFQLITSLHKESINK |
| 3.4 | 2205.0483 | 1.9925 | KATSFQLITSLHKESINK |
| 3.3 | 2207.0228 | 0.0180 | MARGPGQLSGPRPDTVTMPK |
| 3.3 | 2205.0426 | 1.9982 | QTSSYNIPASASISRTSISR |
| 1.0 | 2205.0219 | 2.0189 | ELDEATESNEAMGREVNALK |

---

Spectrum No: 356; Query: 2597; Rank: 1

---

## Peptide View

MS/MS Fragmentation of **GEDDSWDFGTGAGFFVNATEDPWNTNYR**
Found in **IPI00362395**, Tax_Id=10116 Gene_Symbol=Esd esterase D/formylglutathione hydrolase

Match to Query 2597: 3168.316482 from(1057.112770,3+)
Title: 091008RatKidney_NoSalt_19.6861.6861.3.dta
Data file K:\NewmanPaper\Piliang\3SubProteomes\Piliang3SP\mgf5ppm\ERLIC_3SubProteomes5ppm.mgf

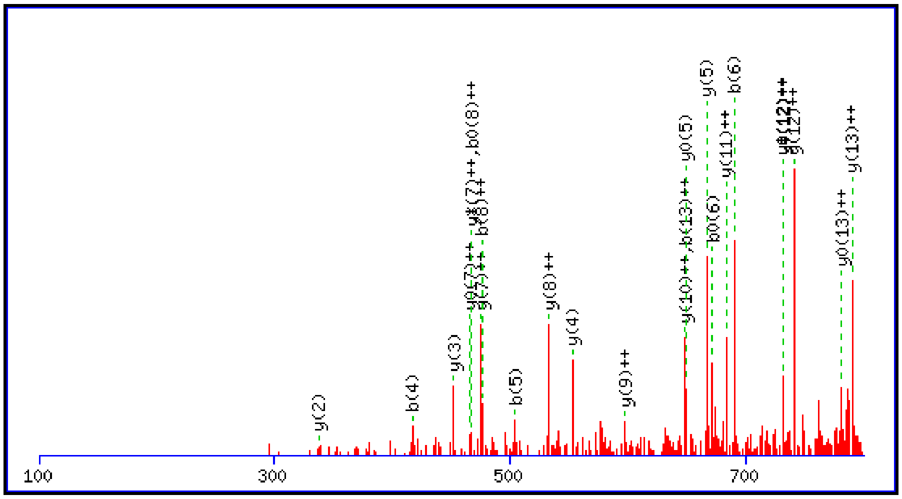


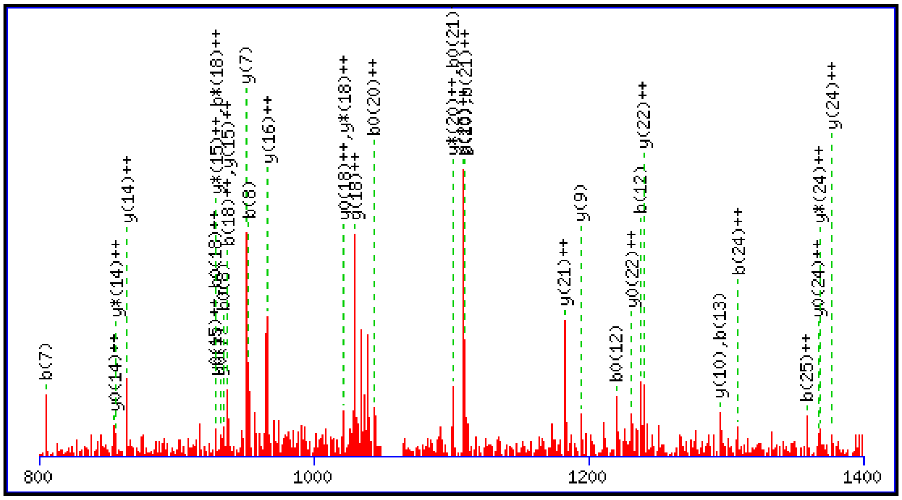

Mascot Search Results: Peptide View

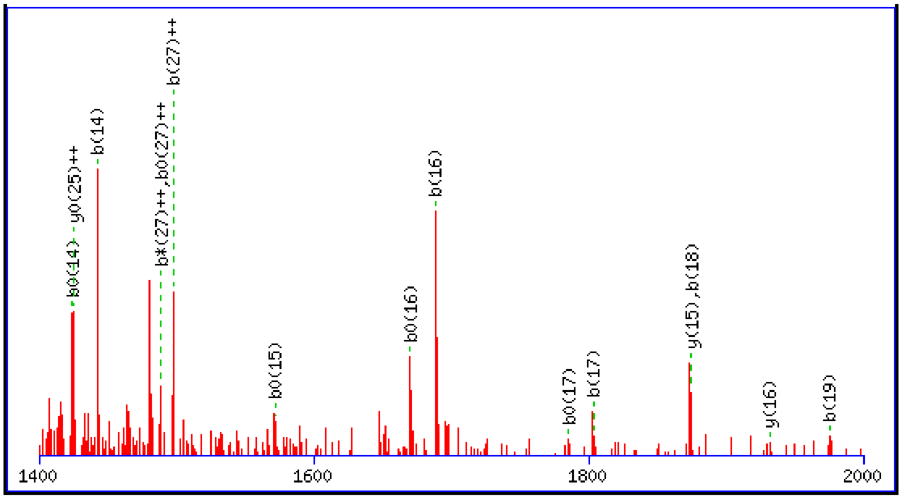


**Monoisotopic mass of neutral peptide Mr(calc):** 3168.3010
**Fixed modifications:** Carbamidomethyl (C)
**Variable modifications:**
**N17** : Deamidated_N (N)
**Ions Score:** 70 **Expect:** 3.5e-005
**Matches (Bold Red):** 76/284 fragment ions using 114 most intense peaks

| # | b | $b^{++}$ | b* | $b^{*++}$ | $b^{0}$ | $b^{0++}$ | Seq. | y | $y^{++}$ | y* | $y^{*++}$ | $y^{0}$ | $y^{0++}$ | # |
|---|---|---|---|---|---|---|---|---|---|---|---|---|---|---|
| 1 | 58.0287 | 29.5180 | | | | | G | | | | | | | 28 |
| 2 | 187.0713 | 94.0393 | | | 169.0608 | 85.0340 | E | 3112.2868 | 1556.6470 | 3095.2602 | 1548.1337 | 3094.2762 | 1547.6417 | 27 |
| 3 | 302.0983 | 151.5528 | | | 284.0877 | 142.5475 | D | 2983.2442 | 1492.1257 | 2966.2176 | 1483.6124 | 2965.2336 | 1483.1204 | 26 |
| 4 | **417.1252** | 209.0662 | | | 399.1147 | 200.0610 | D | 2868.2172 | 1434.6122 | 2851.1907 | 1426.0990 | 2850.2067 | **1425.6070** | 25 |
| 5 | **504.1572** | 252.5823 | | | 486.1467 | 243.5770 | S | 2753.1903 | **1377.0988** | 2736.1637 | **1368.5855** | 2735.1797 | **1368.0935** | 24 |
| 6 | **690.2366** | 345.6219 | | | **672.2260** | 336.6166 | W | 2666.1583 | 1333.5828 | 2649.1317 | 1325.0695 | 2648.1477 | 1324.5775 | 23 |
| 7 | **805.2635** | 403.1354 | | | 787.2529 | 394.1301 | D | 2480.0789 | **1240.5431** | 2463.0524 | 1232.0298 | 2462.0684 | **1231.5378** | 22 |
| 8 | **952.3319** | **476.6696** | | | **934.3214** | **467.6643** | F | 2365.0520 | **1183.0296** | 2348.0254 | 1174.5164 | 2347.0414 | 1174.0244 | 21 |
| 9 | 1009.3534 | 505.1803 | | | 991.3428 | 496.1750 | G | 2217.9836 | **1109.4954** | 2200.9570 | **1100.9822** | 2199.9730 | 1100.4901 | 20 |
| 10 | **1110.4011** | 555.7042 | | | 1092.3905 | 546.6989 | T | 2160.9621 | 1080.9847 | 2143.9356 | 1072.4714 | 2142.9516 | 1071.9794 | 19 |
| 11 | 1167.4225 | 584.2149 | | | 1149.4120 | 575.2096 | G | 2059.9144 | **1030.4609** | 2042.8879 | **1021.9476** | 2041.9039 | **1021.4556** | 18 |
| 12 | **1238.4596** | 619.7335 | | | **1220.4491** | 610.7282 | A | 2002.8930 | 1001.9501 | 1985.8664 | 993.4369 | 1984.8824 | 992.9448 | 17 |
| 13 | **1295.4811** | **648.2442** | | | 1277.4705 | 639.2389 | G | **1931.8559** | **966.4316** | 1914.8293 | 957.9183 | 1913.8453 | 957.4263 | 16 |
| 14 | **1442.5495** | 721.7784 | | | **1424.5390** | 712.7731 | F | **1874.8344** | **937.9208** | 1857.8078 | **929.4076** | 1856.8238 | **928.9156** | 15 |
| 15 | 1589.6179 | 795.3126 | | | **1571.6074** | 786.3073 | F | 1727.7660 | **864.3866** | 1710.7394 | **855.8734** | 1709.7554 | **855.3813** | 14 |
| 16 | **1688.6863** | 844.8468 | | | **1670.6758** | 835.8415 | V | 1580.6976 | **790.8524** | 1563.6710 | 782.3391 | 1562.6870 | **781.8471** | 13 |
| 17 | **1803.7133** | 902.3603 | 1786.6867 | 893.8470 | **1785.7027** | 893.3550 | N | 1481.6292 | **741.3182** | 1464.6026 | **732.8049** | 1463.6186 | **732.3129** | 12 |
| 18 | **1874.7504** | **937.8788** | 1857.7238 | **929.3656** | 1856.7398 | **928.8736** | A | 1366.6022 | **683.8047** | 1349.5757 | 675.2915 | 1348.5917 | 674.7995 | 11 |
| 19 | **1975.7981** | 988.4027 | 1958.7715 | 979.8894 | 1957.7875 | 979.3974 | T | **1295.5651** | **648.2862** | 1278.5386 | 639.7729 | 1277.5545 | 639.2809 | 10 |
| 20 | 2104.8407 | 1052.9240 | 2087.8141 | 1044.4107 | 2086.8301 | **1043.9187** | E | **1194.5174** | **597.7624** | 1177.4909 | 589.2491 | 1176.5069 | 588.7571 | 9 |
| 21 | 2219.8676 | **1110.4374** | 2202.8411 | 1101.9242 | 2201.8570 | **1101.4322** | D | 1065.4748 | **533.2411** | 1048.4483 | 524.7278 | 1047.4643 | 524.2358 | 8 |
| 22 | 2316.9204 | 1158.9638 | 2299.8938 | 1150.4505 | 2298.9098 | 1149.9585 | P | **950.4479** | **475.7276** | 933.4213 | **467.2143** | **932.4373** | **466.7223** | 7 |
| 23 | 2502.9997 | 1252.0035 | 2485.9731 | 1243.4902 | 2484.9891 | 1242.9982 | W | 853.3951 | 427.2012 | 836.3686 | 418.6879 | 835.3846 | 418.1959 | 6 |
| 24 | 2617.0426 | **1309.0249** | 2600.0161 | 1300.5117 | 2599.0320 | 1300.0197 | N | **667.3158** | 334.1615 | 650.2893 | 325.6483 | **649.3052** | 325.1563 | 5 |
| 25 | 2718.0903 | **1359.5488** | 2701.0637 | 1351.0355 | 2700.0797 | 1350.5435 | T | **553.2729** | 277.1401 | 536.2463 | 268.6268 | 535.2623 | 268.1348 | 4 |
| 26 | 2832.1332 | 1416.5702 | 2815.1067 | 1408.0570 | 2814.1227 | 1407.5650 | N | **452.2252** | 226.6162 | 435.1987 | 218.1030 | | | 3 |
| 27 | 2995.1965 | **1498.1019** | 2978.1700 | **1489.5886** | 2977.1860 | **1489.0966** | Y | **338.1823** | 169.5948 | 321.1557 | 161.0815 | | | 2 |
| 28 | | | | | | | R | 175.1190 | 88.0631 | 158.0924 | 79.5498 | | | 1 |

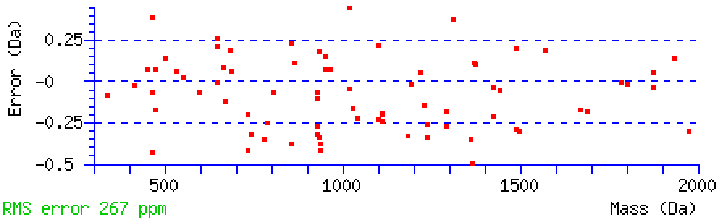


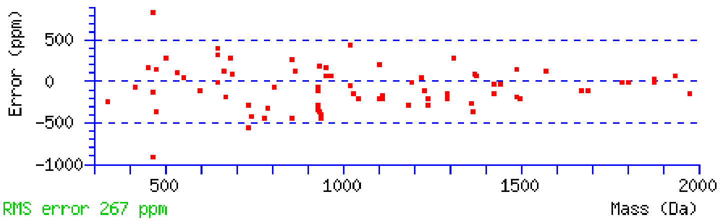


### All matches to this query

| Score | Mr(calc): | Delta | Sequence |
|---|---|---|---|
| 70.3 | 3168.3010 | 0.0155 | GEDDSWDFGTGAGFFVNATEDPWNTNYR |
| 67.8 | 3167.3170 | 0.9995 | GEDDSWDFGTGAGFFVNATEDPWNTNYR |
| 63.5 | 3168.3010 | 0.0155 | GEDDSWDFGTGAGFFVNATEDPWNTNYR |
| 61.2 | 3168.3010 | 0.0155 | GEDDSWDFGTGAGFFVNATEDPWNTNYR |
| 3.6 | 3166.3169 | 1.9996 | SLSSAEDIHTPEQDRPFFSANSVSEQK |
| 2.2 | 3166.3186 | 1.9978 | QNSSSSQGSSSNSGGSSGIPQPPPGMSFYAAK |
| 0.7 | 3167.2931 | 1.0234 | SDNFNTGNMTVLSPYLNTTVLPSSSSSR |
| 0.2 | 3167.2848 | 1.0317 | MAEGERQPPPDSSEETPSATPTFIIPK |

---

Spectrum No: 357; Query: 2731; Rank: 1

---

## Peptide View

MS/MS Fragmentation of **VVNLNGELGDSWIVPLDNLTKEDLDEEEDTHL**
Found in **IPI00325860**, Tax_Id=10116 Gene_Symbol=Podxl Podocalyxin precursor

Match to Query 2731: 3621.724002 from(1208.248610,3+)
Title: 091008RatKidney_NoSalt_19.6408.6408.3.dta
Data file K:\NewmanPaper\Piliang\3SubProteomes\Piliang3SP\mgf5ppm\ERLIC_3SubProteomes5ppm.mgf

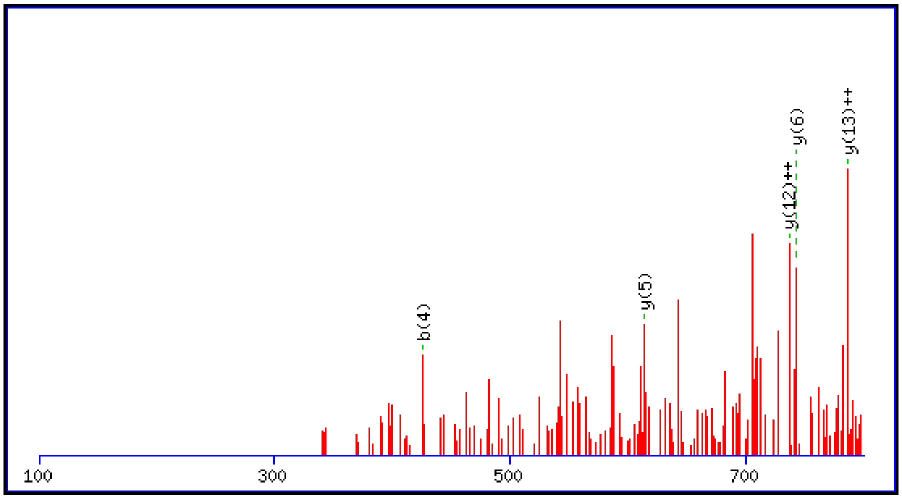

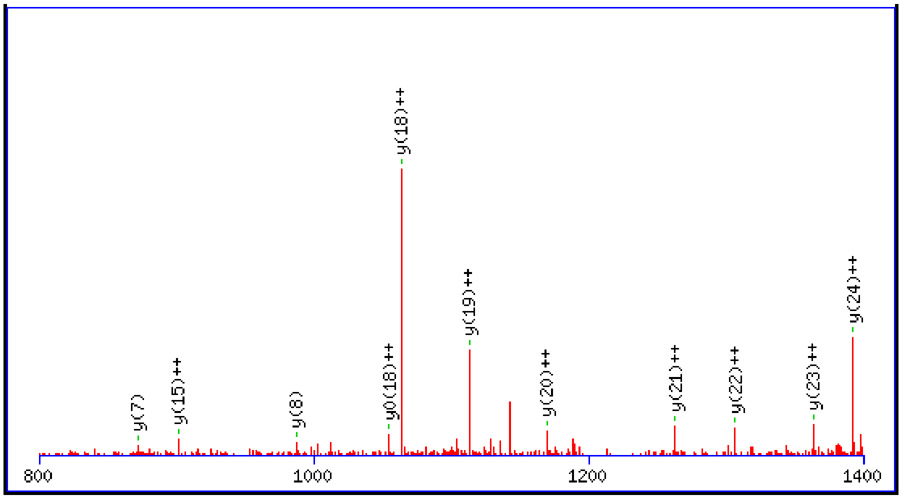


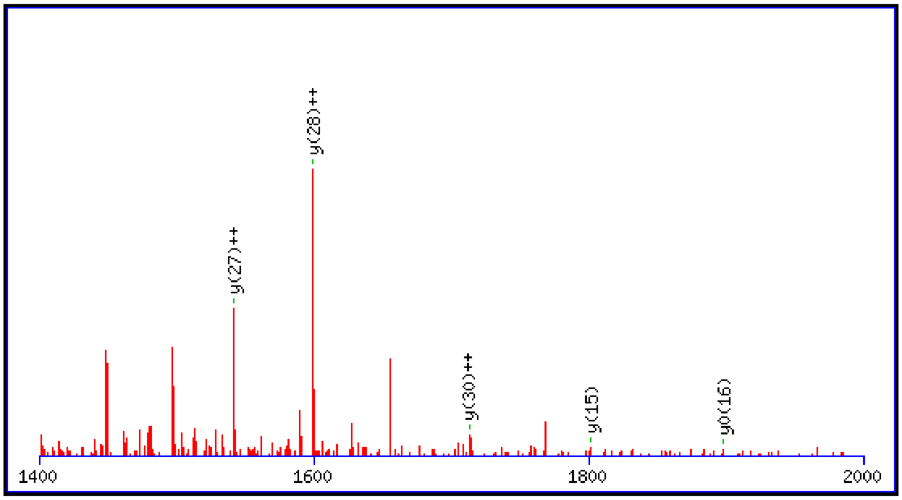


**Monoisotopic mass of neutral peptide Mr(calc):** 3621.7210
**Fixed modifications:** Carbamidomethyl (C)
**Variable modifications:**
**N18** : Deamidated_N (N)
**Ions Score:** 70 **Expect:** 3.6e-005
**Matches (Bold Red):** 21/330 fragment ions using 43 most intense peaks

| # | b | $b^{++}$ | b* | $b^{*++}$ | $b^0$ | $b^{0++}$ | Seq. | y | $y^{++}$ | y* | $y^{*++}$ | $y^0$ | $y^{0++}$ | # |
|---|---|---|---|---|---|---|---|---|---|---|---|---|---|---|
| 1 | 100.0757 | 50.5415 | | | | | V | | | | | | | 32 |
| 2 | 199.1441 | 100.0757 | | | | | V | 3523.6599 | 1762.3336 | 3506.6333 | 1753.8203 | 3505.6493 | 1753.3283 | 31 |
| 3 | 313.1870 | 157.0972 | 296.1605 | 148.5839 | | | N | 3424.5914 | **1712.7994** | 3407.5649 | 1704.2861 | 3406.5809 | 1703.7941 | 30 |
| 4 | **426.2711** | 213.6392 | 409.2445 | 205.1259 | | | L | 3310.5485 | 1655.7779 | 3293.5220 | 1647.2646 | 3292.5379 | 1646.7726 | 29 |
| 5 | 540.3140 | 270.6606 | 523.2875 | 262.1474 | | | N | 3197.4644 | **1599.2359** | 3180.4379 | 1590.7226 | 3179.4539 | 1590.2306 | 28 |
| 6 | 597.3355 | 299.1714 | 580.3089 | 290.6581 | | | G | 3083.4215 | **1542.2144** | 3066.3950 | 1533.7011 | 3065.4110 | 1533.2091 | 27 |
| 7 | 726.3781 | 363.6927 | 709.3515 | 355.1794 | 708.3675 | 354.6874 | E | 3026.4001 | 1513.7037 | 3009.3735 | 1505.1904 | 3008.3895 | 1504.6984 | 26 |
| 8 | 839.4621 | 420.2347 | 822.4356 | 411.7214 | 821.4516 | 411.2294 | L | 2897.3575 | 1449.1824 | 2880.3309 | 1440.6691 | 2879.3469 | 1440.1771 | 25 |
| 9 | 896.4836 | 448.7454 | 879.4571 | 440.2322 | 878.4730 | 439.7402 | G | 2784.2734 | **1392.6403** | 2767.2469 | 1384.1271 | 2766.2628 | 1383.6351 | 24 |
| 10 | 1011.5106 | 506.2589 | 994.4840 | 497.7456 | 993.5000 | 497.2536 | D | 2727.2519 | **1364.1296** | 2710.2254 | 1355.6163 | 2709.2414 | 1355.1243 | 23 |
| 11 | 1098.5426 | 549.7749 | 1081.5160 | 541.2617 | 1080.5320 | 540.7696 | S | 2612.2250 | **1306.6161** | 2595.1984 | 1298.1029 | 2594.2144 | 1297.6109 | 22 |
| 12 | 1284.6219 | 642.8146 | 1267.5953 | 634.3013 | 1266.6113 | 633.8093 | W | 2525.1930 | **1263.1001** | 2508.1664 | 1254.5868 | 2507.1824 | 1254.0948 | 21 |
| 13 | 1397.7060 | 699.3566 | 1380.6794 | 690.8433 | 1379.6954 | 690.3513 | I | 2339.1137 | **1170.0605** | 2322.0871 | 1161.5472 | 2321.1031 | 1161.0552 | 20 |
| 14 | 1496.7744 | 748.8908 | 1479.7478 | 740.3775 | 1478.7638 | 739.8855 | V | 2226.0296 | **1113.5184** | 2209.0030 | 1105.0052 | 2208.0190 | 1104.5131 | 19 |
| 15 | 1593.8271 | 797.4172 | 1576.8006 | 788.9039 | 1575.8166 | 788.4119 | P | 2126.9612 | **1063.9842** | 2109.9346 | 1055.4710 | 2108.9506 | **1054.9789** | 18 |
| 16 | 1706.9112 | 853.9592 | 1689.8846 | 845.4460 | 1688.9006 | 844.9540 | L | 2029.9084 | 1015.4578 | 2012.8819 | 1006.9446 | 2011.8978 | 1006.4526 | 17 |
| 17 | 1821.9381 | 911.4727 | 1804.9116 | 902.9594 | 1803.9276 | 902.4674 | D | 1916.8243 | 958.9158 | 1899.7978 | 950.4025 | **1898.8138** | 949.9105 | 16 |
| 18 | 1936.9651 | 968.9862 | 1919.9385 | 960.4729 | 1918.9545 | 959.9809 | N | **1801.7974** | **901.4023** | 1784.7709 | 892.8891 | 1783.7868 | 892.3971 | 15 |
| | | | | | | | | | | | | | | |

| | | | | | | | | | | | | | | |
|---|---|---|---|---|---|---|---|---|---|---|---|---|---|---|
| **19** | 2050.0491 | 1025.5282 | 2033.0226 | 1017.0149 | 2032.0386 | 1016.5229 | **L** | 1686.7705 | 843.8889 | 1669.7439 | 835.3756 | 1668.7599 | 834.8836 | **14** |
| **20** | 2151.0968 | 1076.0520 | 2134.0703 | 1067.5388 | 2133.0863 | 1067.0468 | **T** | 1573.6864 | **787.3468** | 1556.6599 | 778.8336 | 1555.6758 | 778.3416 | **13** |
| **21** | 2279.1918 | 1140.0995 | 2262.1652 | 1131.5863 | 2261.1812 | 1131.0942 | **K** | 1472.6387 | **736.8230** | 1455.6122 | 728.3097 | 1454.6282 | 727.8177 | **12** |
| **22** | 2408.2344 | 1204.6208 | 2391.2078 | 1196.1076 | 2390.2238 | 1195.6155 | **E** | 1344.5438 | 672.7755 | | | 1326.5332 | 663.7702 | **11** |
| **23** | 2523.2613 | 1262.1343 | 2506.2348 | 1253.6210 | 2505.2508 | 1253.1290 | **D** | 1215.5012 | 608.2542 | | | 1197.4906 | 599.2489 | **10** |
| **24** | 2636.3454 | 1318.6763 | 2619.3188 | 1310.1631 | 2618.3348 | 1309.6710 | **L** | 1100.4742 | 550.7408 | | | 1082.4637 | 541.7355 | **9** |
| **25** | 2751.3723 | 1376.1898 | 2734.3458 | 1367.6765 | 2733.3618 | 1367.1845 | **D** | **987.3902** | 494.1987 | | | 969.3796 | 485.1934 | **8** |
| **26** | 2880.4149 | 1440.7111 | 2863.3884 | 1432.1978 | 2862.4044 | 1431.7058 | **E** | **872.3632** | 436.6852 | | | 854.3527 | 427.6800 | **7** |
| **27** | 3009.4575 | 1505.2324 | 2992.4310 | 1496.7191 | 2991.4469 | 1496.2271 | **E** | **743.3206** | 372.1640 | | | 725.3101 | 363.1587 | **6** |
| **28** | 3138.5001 | 1569.7537 | 3121.4736 | 1561.2404 | 3120.4895 | 1560.7484 | **E** | **614.2780** | 307.6427 | | | 596.2675 | 298.6374 | **5** |
| **29** | 3253.5270 | 1627.2672 | 3236.5005 | 1618.7539 | 3235.5165 | 1618.2619 | **D** | 485.2354 | 243.1214 | | | 467.2249 | 234.1161 | **4** |
| **30** | 3354.5747 | 1677.7910 | 3337.5482 | 1669.2777 | 3336.5642 | 1668.7857 | **T** | 370.2085 | 185.6079 | | | 352.1979 | 176.6026 | **3** |
| **31** | 3491.6336 | 1746.3205 | 3474.6071 | 1737.8072 | 3473.6231 | 1737.3152 | **H** | 269.1608 | 135.0840 | | | | | **2** |
| **32** | | | | | | | **L** | 132.1019 | 66.5546 | | | | | **1** |

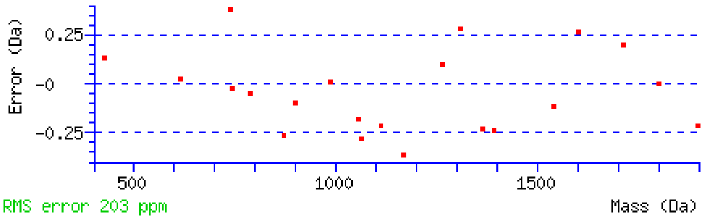


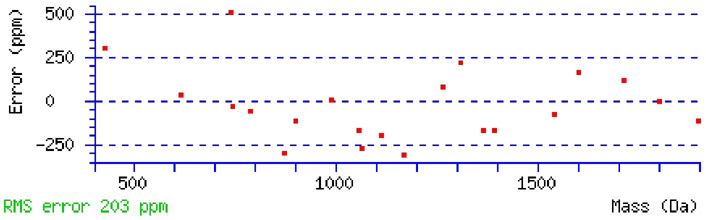


**All matches to this query**

| Score | Mr(calc): | Delta | Sequence |
|---|---|---|---|
| 70.2 | 3621.7210 | 0.0030 | VVNLNGELGDSWIVPLDNLTKEDLDEEEDTHL |
| 66.3 | 3621.7210 | 0.0030 | VVNLNGELGDSWIVPLDNLTKEDLDEEEDTHL |
| 53.0 | 3621.7210 | 0.0030 | VVNLNGELGDSWIVPLDNLTKEDLDEEEDTHL |
| 39.5 | 3620.7370 | 0.9870 | VVNLNGELGDSWIVPLDNLTKEDLDEEEDTHL |
| 6.4 | 3619.7455 | 1.9785 | QDLVDKLISLFYGVLTPMLNPIIYSLRNK |
| 5.3 | 3620.6956 | 1.0284 | DGIKINDSNFLGKILLQVANLNATTLSYTLK |
| 4.6 | 3621.6897 | 0.0343 | SIFLWLGYSNSFFNPLIYTAFNRSYSSAFK |
| 4.2 | 3619.7455 | 1.9785 | QDLVDKLISLFYGVLTPMLNPIIYSLRNK |
| 4.0 | 3620.6956 | 1.0284 | DGIKINDSNFLGKILLQVANLNATTLSYTLK |
| 4.0 | 3620.6956 | 1.0284 | DGIKINDSNFLGKILLQVANLNATTLSYTLK |

---

Spectrum No: 358; Query: 1800; Rank: 1

---

## Peptide View

MS/MS Fragmentation of **DCSTNPPRLPVTAVNTTMR**
Found in **IPI00197684**, Tax_Id=10116 Gene_Symbol=Xpnpep2 Membrane-bound aminopeptidase P

Match to Query 1800: 2130.025962 from(711.015930,3+)
Title: 100101RatKid_NS_deglyco_20.2694.2694.3.dta
Data file K:\NewmanPaper\Piliang\3SubProteomes\Piliang3SP\mgf5ppm\ERLIC_3SubProteomes5ppm.mgf

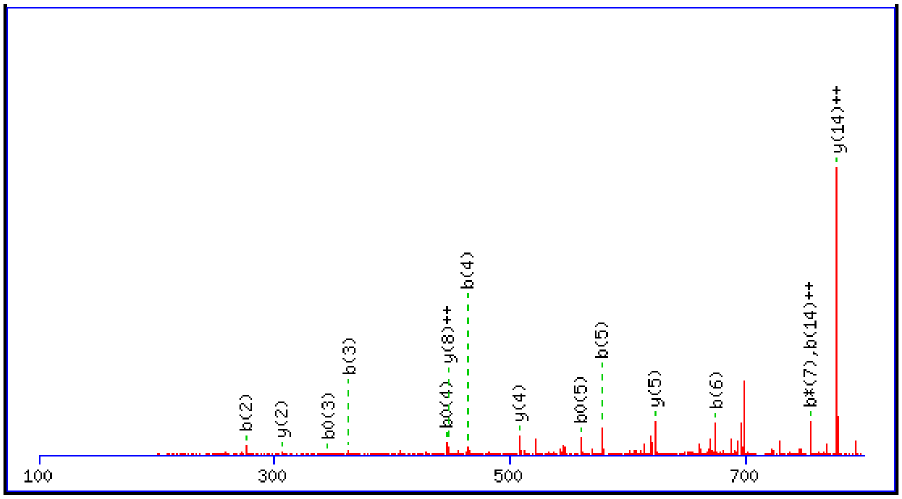


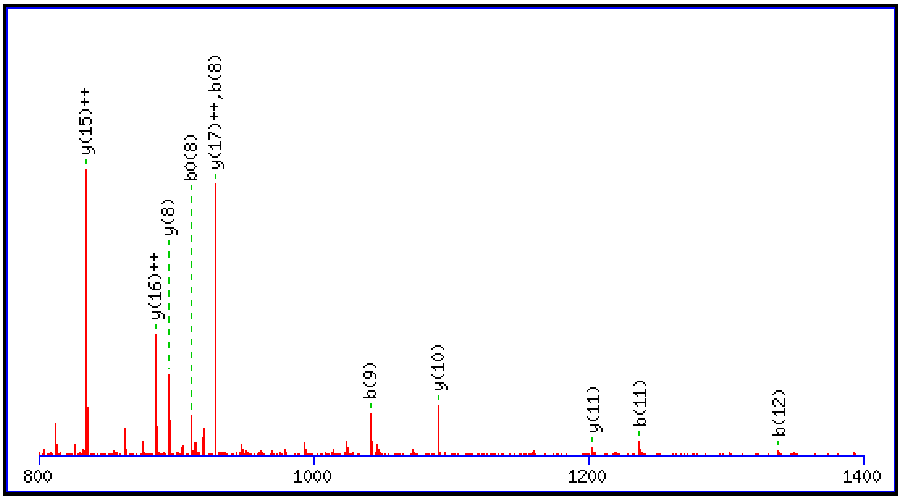


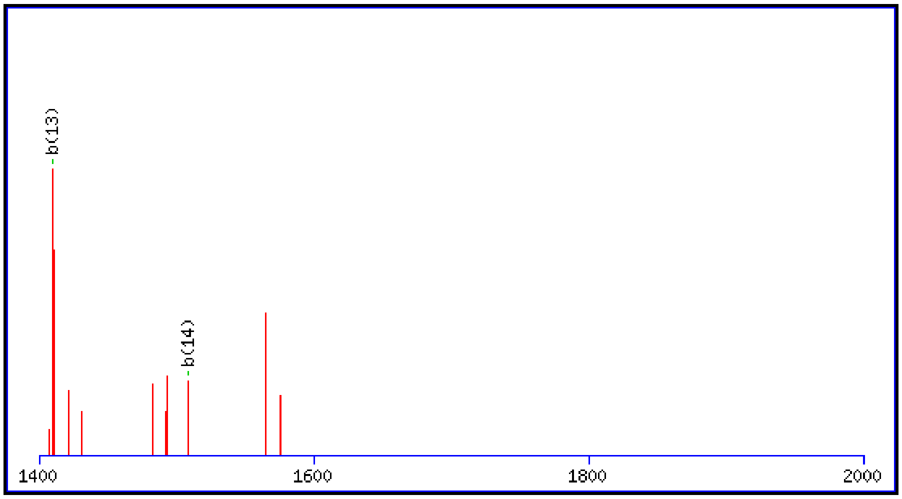


**Monoisotopic mass of neutral peptide Mr(calc):** 2130.0198
**Fixed modifications:** Carbamidomethyl (C)
**Variable modifications:**
**N15** : Deamidated_N (N)
**Ions Score:** 70 **Expect:** 2.3e-005
**Matches (Bold Red):** 28/204 fragment ions using 45 most intense peaks

| # | b | b++ | b* | b*++ | b0 | b0++ | Seq. | y | y++ | y* | y*++ | y0 | y0++ | # |
|---|---|---|---|---|---|---|---|---|---|---|---|---|---|---|
| 1 | 116.0342 | 58.5207 | | | 98.0237 | 49.5155 | D | | | | | | | 19 |
| 2 | **276.0649** | 138.5361 | | | 258.0543 | 129.5308 | C | 2016.0001 | 1008.5037 | 1998.9736 | 999.9904 | 1997.9895 | 999.4984 | 18 |
| 3 | **363.0969** | 182.0521 | | | **345.0863** | 173.0468 | S | 1855.9695 | **928.4884** | 1838.9429 | 919.9751 | 1837.9589 | 919.4831 | 17 |
| 4 | **464.1446** | 232.5759 | | | **446.1340** | 223.5706 | T | 1768.9374 | **884.9724** | 1751.9109 | 876.4591 | 1750.9269 | 875.9671 | 16 |
| 5 | **578.1875** | 289.5974 | 561.1610 | 281.0841 | **560.1769** | 280.5921 | N | 1667.8897 | **834.4485** | 1650.8632 | 825.9352 | 1649.8792 | 825.4432 | 15 |

| | | | | | | | | | | | | | | |
|---|---|---|---|---|---|---|---|---|---|---|---|---|---|---|
| 6 | 675.2403 | 338.1238 | 658.2137 | 329.6105 | 657.2297 | 329.1185 | P | 1553.8468 | 777.4270 | 1536.8203 | 768.9138 | 1535.8363 | 768.4218 | 14 |
| 7 | 772.2930 | 386.6502 | 755.2665 | 378.1369 | 754.2825 | 377.6449 | P | 1456.7941 | 728.9007 | 1439.7675 | 720.3874 | 1438.7835 | 719.8954 | 13 |
| 8 | 928.3941 | 464.7007 | 911.3676 | 456.1874 | 910.3836 | 455.6954 | R | 1359.7413 | 680.3743 | 1342.7147 | 671.8610 | 1341.7307 | 671.3690 | 12 |
| 9 | 1041.4782 | 521.2427 | 1024.4517 | 512.7295 | 1023.4676 | 512.2375 | L | 1203.6402 | 602.3237 | 1186.6136 | 593.8105 | 1185.6296 | 593.3184 | 11 |
| 10 | 1138.5310 | 569.7691 | 1121.5044 | 561.2558 | 1120.5204 | 560.7638 | P | 1090.5561 | 545.7817 | 1073.5296 | 537.2684 | 1072.5456 | 536.7764 | 10 |
| 11 | 1237.5994 | 619.3033 | 1220.5728 | 610.7901 | 1219.5888 | 610.2980 | V | 993.5034 | 497.2553 | 976.4768 | 488.7420 | 975.4928 | 488.2500 | 9 |
| 12 | 1338.6471 | 669.8272 | 1321.6205 | 661.3139 | 1320.6365 | 660.8219 | T | 894.4349 | 447.7211 | 877.4084 | 439.2078 | 876.4244 | 438.7158 | 8 |
| 13 | 1409.6842 | 705.3457 | 1392.6576 | 696.8325 | 1391.6736 | 696.3404 | A | 793.3873 | 397.1973 | 776.3607 | 388.6840 | 775.3767 | 388.1920 | 7 |
| 14 | 1508.7526 | 754.8799 | 1491.7260 | 746.3667 | 1490.7420 | 745.8747 | V | 722.3501 | 361.6787 | 705.3236 | 353.1654 | 704.3396 | 352.6734 | 6 |
| 15 | 1623.7795 | 812.3934 | 1606.7530 | 803.8801 | 1605.7690 | 803.3881 | N | 623.2817 | 312.1445 | 606.2552 | 303.6312 | 605.2712 | 303.1392 | 5 |
| 16 | 1724.8272 | 862.9172 | 1707.8007 | 854.4040 | 1706.8166 | 853.9120 | T | 508.2548 | 254.6310 | 491.2282 | 246.1178 | 490.2442 | 245.6258 | 4 |
| 17 | 1825.8749 | 913.4411 | 1808.8483 | 904.9278 | 1807.8643 | 904.4358 | T | 407.2071 | 204.1072 | 390.1806 | 195.5939 | 389.1966 | 195.1019 | 3 |
| 18 | 1956.9154 | 978.9613 | 1939.8888 | 970.4480 | 1938.9048 | 969.9560 | M | 306.1594 | 153.5834 | 289.1329 | 145.0701 | | | 2 |
| 19 | | | | | | | R | 175.1190 | 88.0631 | 158.0924 | 79.5498 | | | 1 |

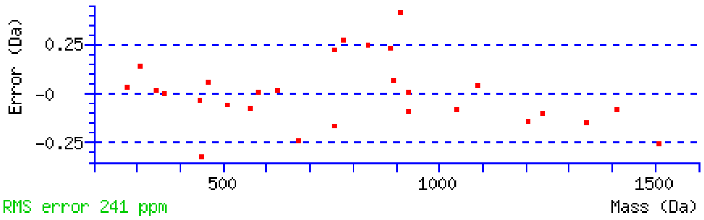


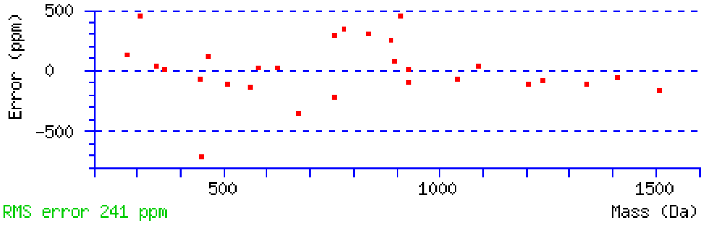


**All matches to this query**

| Score | Mr(calc): | Delta | Sequence |
|---|---|---|---|
| 70.1 | 2130.0198 | 0.0062 | DCSTNPPRLPVTAVNTTMR |
| 52.8 | 2129.0358 | 0.9902 | DCSTNPPRLPVTAVNTTMR |
| 30.8 | 2130.0198 | 0.0062 | DCSTNPPRLPVTAVNTTMR |
| 7.0 | 2130.0064 | 0.0195 | DKVSLAALGGRVFFFSXR |
| 6.8 | 2128.0388 | 1.9872 | HQELLVNLNPMVTPLASR |
| 5.6 | 2129.0201 | 1.0059 | LSYNGRSSVVQCRPLTGR |
| 4.4 | 2128.0388 | 1.9872 | HQELLVNLNPMVTPLASR |
| 3.5 | 2130.0309 | -0.0050 | IRVNSVNPTVVLTDMGKK |
| 3.4 | 2130.0309 | -0.0050 | IRVNSVNPTVVLTDMGKK |
| 3.4 | 2130.0309 | -0.0050 | IRVNSVNPTVVLTDMGKK |

---

Spectrum No: 359; Query: 2621; Rank: 1

---

## Peptide View

MS/MS Fragmentation of **SFSGNASILSPLLQVPDIDGDGAPDLLMLSR**
Found in **IPI00372350**, Tax_Id=10116 Gene_Symbol=Itfg3 Protein ITFG3

Match to Query 2621: 3198.621792 from(1067.214540,3+)
Title: 100101RatKid_NS_deglyco_22.6316.6316.3.dta
Data file K:\NewmanPaper\Piliang\3SubProteomes\Piliang3SP\mgf5ppm\ERLIC_3SubProteomes5ppm.mgf

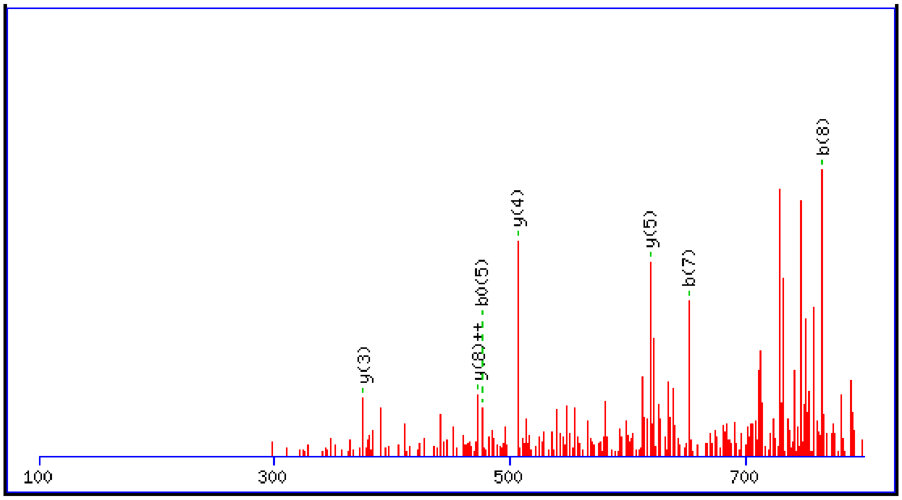


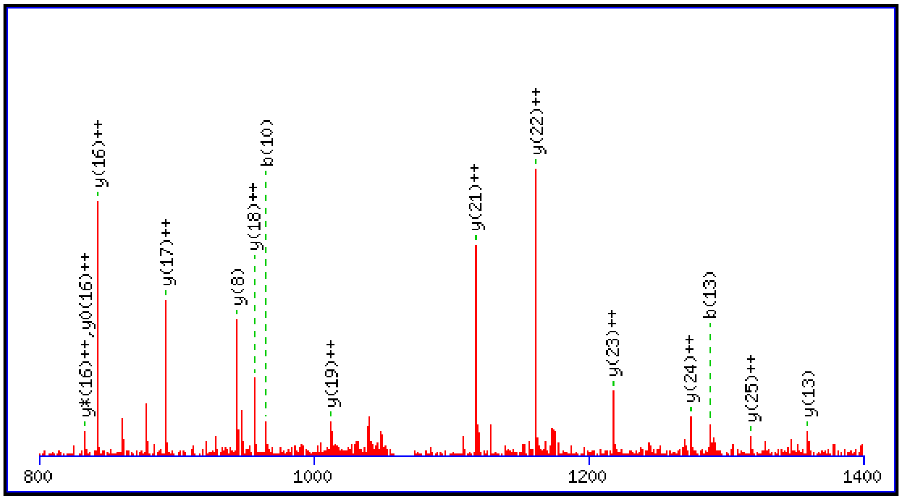


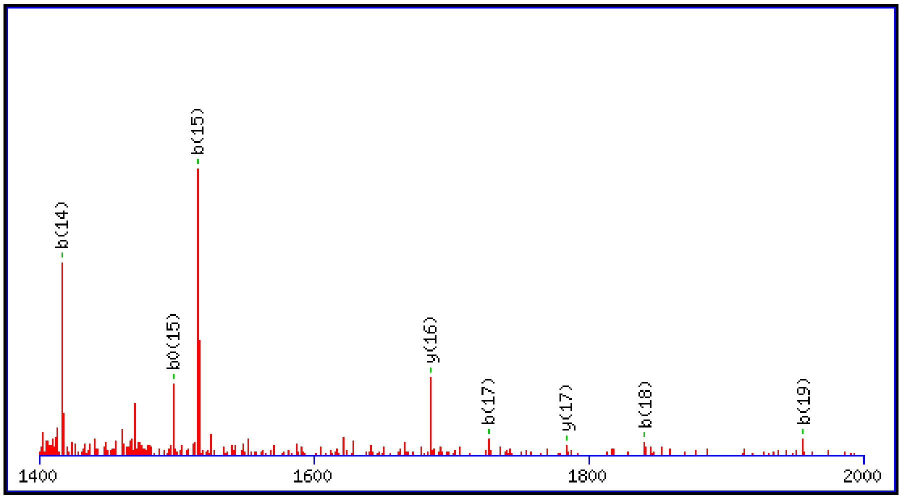


**Monoisotopic mass of neutral peptide Mr(calc):** 3198.6118
**Fixed modifications:** Carbamidomethyl (C)
**Variable modifications:**
**N5** : Deamidated_N (N)
**Ions Score:** 70 **Expect:** 2e-005
**Matches (Bold Red):** 30/350 fragment ions using 42 most intense peaks

| # | b | $b^{++}$ | b* | $b^{*++}$ | $b^0$ | $b^{0++}$ | Seq. | y | $y^{++}$ | y* | $y^{*++}$ | $y^0$ | $y^{0++}$ | # |
|---|---|---|---|---|---|---|---|---|---|---|---|---|---|---|
| 1 | 88.0393 | 44.5233 | | | 70.0287 | 35.5180 | S | | | | | | | 31 |
| 2 | 235.1077 | 118.0575 | | | 217.0972 | 109.0522 | F | 3112.5871 | 1556.7972 | 3095.5605 | 1548.2839 | 3094.5765 | 1547.7919 | 30 |
| 3 | 322.1397 | 161.5735 | | | 304.1292 | 152.5682 | S | 2965.5187 | 1483.2630 | 2948.4921 | 1474.7497 | 2947.5081 | 1474.2577 | 29 |
| 4 | 379.1612 | 190.0842 | | | 361.1506 | 181.0790 | G | 2878.4866 | 1439.7470 | 2861.4601 | 1431.2337 | 2860.4761 | 1430.7417 | 28 |
| 5 | 494.1881 | 247.5977 | 477.1616 | 239.0844 | **476.1776** | 238.5924 | N | 2821.4652 | 1411.2362 | 2804.4386 | 1402.7230 | 2803.4546 | 1402.2309 | 27 |

| | | | | | | | | | | | | | | |
|---|---|---|---|---|---|---|---|---|---|---|---|---|---|---|
| 6 | 565.2253 | 283.1163 | 548.1987 | 274.6030 | 547.2147 | 274.1110 | A | 2706.4382 | 1353.7228 | 2689.4117 | 1345.2095 | 2688.4277 | 1344.7175 | 26 |
| 7 | 652.2573 | 326.6323 | 635.2307 | 318.1190 | 634.2467 | 317.6270 | S | 2635.4011 | 1318.2042 | 2618.3746 | 1309.6909 | 2617.3906 | 1309.1989 | 25 |
| 8 | 765.3414 | 383.1743 | 748.3148 | 374.6610 | 747.3308 | 374.1690 | I | 2548.3691 | 1274.6882 | 2531.3426 | 1266.1749 | 2530.3585 | 1265.6829 | 24 |
| 9 | 878.4254 | 439.7163 | 861.3989 | 431.2031 | 860.4149 | 430.7111 | L | 2435.2850 | 1218.1462 | 2418.2585 | 1209.6329 | 2417.2745 | 1209.1409 | 23 |
| 10 | 965.4574 | 483.2324 | 948.4309 | 474.7191 | 947.4469 | 474.2271 | S | 2322.2010 | 1161.6041 | 2305.1744 | 1153.0909 | 2304.1904 | 1152.5988 | 22 |
| 11 | 1062.5102 | 531.7587 | 1045.4837 | 523.2455 | 1044.4996 | 522.7535 | P | 2235.1689 | 1118.0881 | 2218.1424 | 1109.5748 | 2217.1584 | 1109.0828 | 21 |
| 12 | 1175.5943 | 588.3008 | 1158.5677 | 579.7875 | 1157.5837 | 579.2955 | L | 2138.1162 | 1069.5617 | 2121.0896 | 1061.0485 | 2120.1056 | 1060.5564 | 20 |
| 13 | 1288.6783 | 644.8428 | 1271.6518 | 636.3295 | 1270.6678 | 635.8375 | L | 2025.0321 | 1013.0197 | 2008.0056 | 1004.5064 | 2007.0216 | 1004.0144 | 19 |
| 14 | 1416.7369 | 708.8721 | 1399.7104 | 700.3588 | 1398.7263 | 699.8668 | Q | 1911.9481 | 956.4777 | 1894.9215 | 947.9644 | 1893.9375 | 947.4724 | 18 |
| 15 | 1515.8053 | 758.4063 | 1498.7788 | 749.8930 | 1497.7948 | 749.4010 | V | 1783.8895 | 892.4484 | 1766.8629 | 883.9351 | 1765.8789 | 883.4431 | 17 |
| 16 | 1612.8581 | 806.9327 | 1595.8315 | 798.4194 | 1594.8475 | 797.9274 | P | 1684.8211 | 842.9142 | 1667.7945 | 834.4009 | 1666.8105 | 833.9089 | 16 |
| 17 | 1727.8850 | 864.4462 | 1710.8585 | 855.9329 | 1709.8745 | 855.4409 | D | 1587.7683 | 794.3878 | 1570.7418 | 785.8745 | 1569.7577 | 785.3825 | 15 |
| 18 | 1840.9691 | 920.9882 | 1823.9426 | 912.4749 | 1822.9585 | 911.9829 | I | 1472.7414 | 736.8743 | 1455.7148 | 728.3610 | 1454.7308 | 727.8690 | 14 |
| 19 | 1955.9960 | 978.5017 | 1938.9695 | 969.9884 | 1937.9855 | 969.4964 | D | 1359.6573 | 680.3323 | 1342.6307 | 671.8190 | 1341.6467 | 671.3270 | 13 |
| 20 | 2013.0175 | 1007.0124 | 1995.9910 | 998.4991 | 1995.0069 | 998.0071 | G | 1244.6303 | 622.8188 | 1227.6038 | 614.3055 | 1226.6198 | 613.8135 | 12 |
| 21 | 2128.0444 | 1064.5259 | 2111.0179 | 1056.0126 | 2110.0339 | 1055.5206 | D | 1187.6089 | 594.3081 | 1170.5823 | 585.7948 | 1169.5983 | 585.3028 | 11 |
| 22 | 2185.0659 | 1093.0366 | 2168.0394 | 1084.5233 | 2167.0553 | 1084.0313 | G | 1072.5819 | 536.7946 | 1055.5554 | 528.2813 | 1054.5714 | 527.7893 | 10 |
| 23 | 2256.1030 | 1128.5552 | 2239.0765 | 1120.0419 | 2238.0925 | 1119.5499 | A | 1015.5605 | 508.2839 | 998.5339 | 499.7706 | 997.5499 | 499.2786 | 9 |
| 24 | 2353.1558 | 1177.0815 | 2336.1292 | 1168.5683 | 2335.1452 | 1168.0763 | P | 944.5234 | 472.7653 | 927.4968 | 464.2520 | 926.5128 | 463.7600 | 8 |
| 25 | 2468.1827 | 1234.5950 | 2451.1562 | 1226.0817 | 2450.1722 | 1225.5897 | D | 847.4706 | 424.2389 | 830.4441 | 415.7257 | 829.4600 | 415.2337 | 7 |
| 26 | 2581.2668 | 1291.1370 | 2564.2402 | 1282.6238 | 2563.2562 | 1282.1318 | L | 732.4437 | 366.7255 | 715.4171 | 358.2122 | 714.4331 | 357.7202 | 6 |
| 27 | 2694.3509 | 1347.6791 | 2677.3243 | 1339.1658 | 2676.3403 | 1338.6738 | L | 619.3596 | 310.1834 | 602.3330 | 301.6702 | 601.3490 | 301.1782 | 5 |
| 28 | 2825.3913 | 1413.1993 | 2808.3648 | 1404.6860 | 2807.3808 | 1404.1940 | M | 506.2755 | 253.6414 | 489.2490 | 245.1281 | 488.2650 | 244.6361 | 4 |
| 29 | 2938.4754 | 1469.7413 | 2921.4489 | 1461.2281 | 2920.4648 | 1460.7361 | L | 375.2350 | 188.1212 | 358.2085 | 179.6079 | 357.2245 | 179.1159 | 3 |
| 30 | 3025.5074 | 1513.2574 | 3008.4809 | 1504.7441 | 3007.4969 | 1504.2521 | S | 262.1510 | 131.5791 | 245.1244 | 123.0659 | 244.1404 | 122.5738 | 2 |
| 31 | | | | | | | R | 175.1190 | 88.0631 | 158.0924 | 79.5498 | | | 1 |

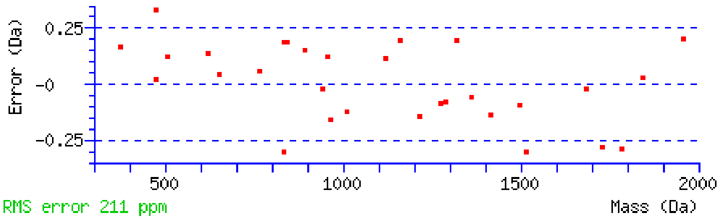


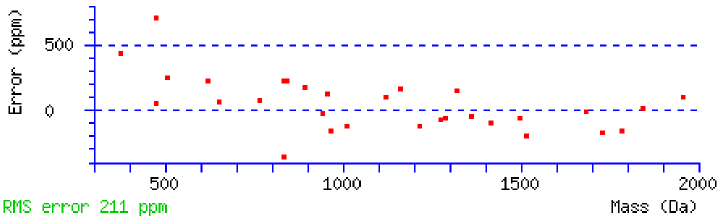


**All matches to this query**

| Score | Mr(calc): | Delta | Sequence |
|---|---|---|---|
| 70.0 | 3198.6118 | 0.0100 | SFSGNASILSPLLQVPDIDGDGAPDLLMLSR |
| 46.1 | 3197.6278 | 0.9940 | SFSGNASILSPLLQVPDIDGDGAPDLLMLSR |
| 12.2 | 3196.6404 | 1.9814 | DRDDVNVEIVFLHNISPNLELEALFKR |
| 7.1 | 3196.6404 | 1.9814 | DRDDVNVEIVFLHNISPNLELEALFKR |
| 6.5 | 3197.6244 | 0.9974 | DRDDVNVEIVFLHNISPNLELEALFKR |
| 4.6 | 3197.6140 | 1.0078 | IVAVFYTLIIPMLNPLIYSLRNTEVK |
| 4.0 | 3198.6400 | -0.0182 | VMKTIPLYNLTGLSVSNGKDQLVVFHTK |
| 3.0 | 3197.6447 | 0.9770 | GVSPEPIHLKVFSPNVVNLTLVDLPGMTK |
| 2.8 | 3196.6049 | 2.0169 | DPLGMGLTIISLGFSALTGLIIGVFVNYR |
| 2.8 | 3196.6049 | 2.0169 | DPLGMGLTIISLGFSALTGLIIGVFVNYR |

Spectrum No: 360; Query: 304; Rank: 1

# Peptide View

MS/MS Fragmentation of **ATYNISLIHPK**
Found in **IPI00327398**, Tax_Id=10116 Gene_Symbol=Enpep Isoform 1 of Glutamyl aminopeptidase

Match to Query 304: 1256.673788 from(629.344170,2+)
Title: 091008RatKidney_NH4Format02_26.2031.2031.2.dta
Data file K:\NewmanPaper\Piliang\3SubProteomes\Piliang3SP\mgf5ppm\ERLIC_3SubProteomes5ppm.mgf

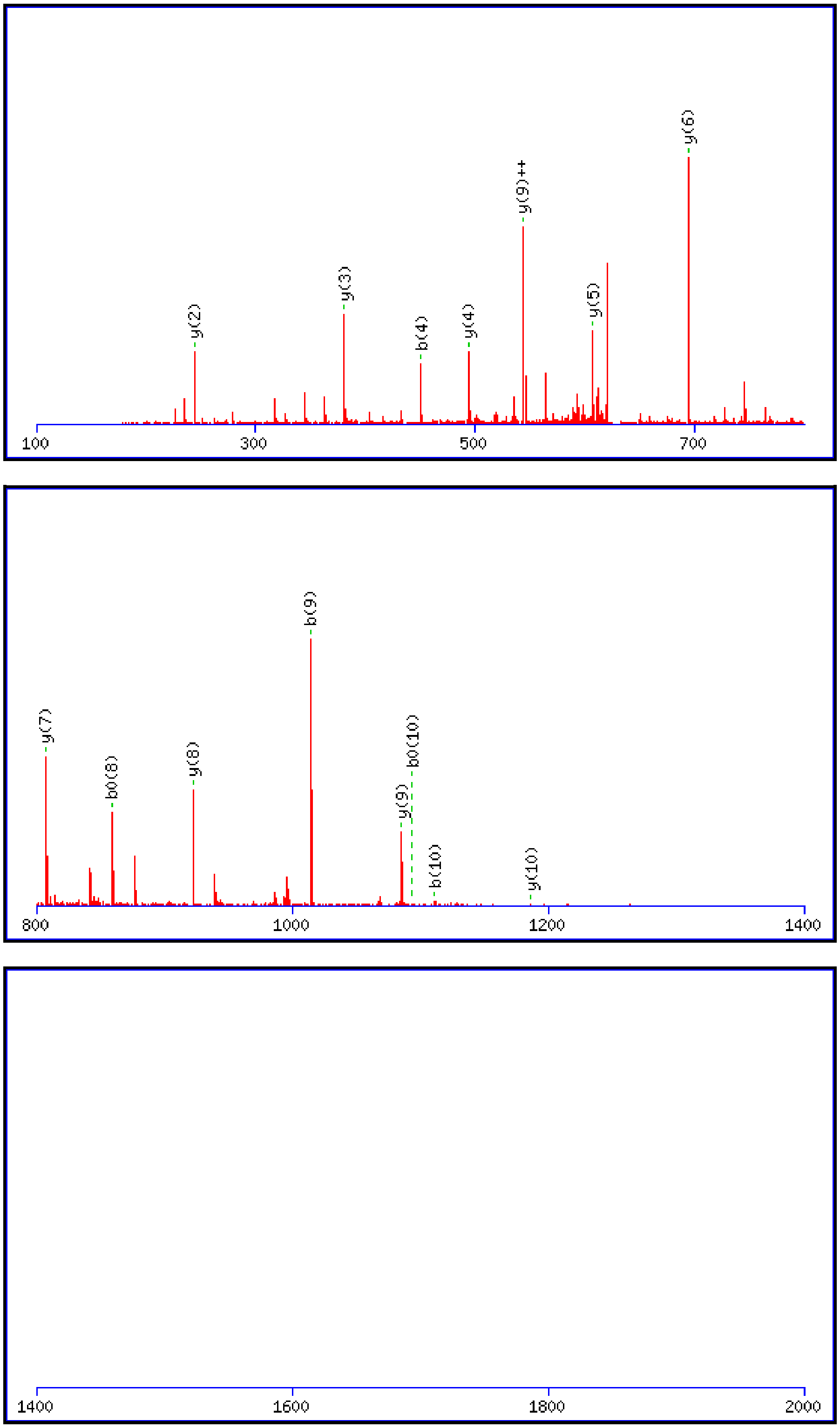


**Monoisotopic mass of neutral peptide Mr(calc):** 1256.6765
**Fixed modifications:** Carbamidomethyl (C)
**Variable modifications:**
**N4** : Deamidated_N (N)
**Ions Score:** 70 **Expect:** 8.5e-006

**Matches (Bold Red): 15/102 fragment ions using 29 most intense peaks**

| # | b | $b^{++}$ | b* | $b^{*++}$ | $b^0$ | $b^{0++}$ | Seq. | y | $y^{++}$ | y* | $y^{*++}$ | $y^0$ | $y^{0++}$ | # |
|---|---|---|---|---|---|---|---|---|---|---|---|---|---|---|
| 1 | 72.0444 | 36.5258 | | | | | A | | | | | | | 11 |
| 2 | 173.0921 | 87.0497 | | | 155.0815 | 78.0444 | T | **1186.6466** | 593.8270 | 1169.6201 | 585.3137 | 1168.6361 | 584.8217 | 10 |
| 3 | 336.1554 | 168.5813 | | | 318.1448 | 159.5761 | Y | **1085.5990** | **543.3031** | 1068.5724 | 534.7898 | 1067.5884 | 534.2978 | 9 |
| 4 | **451.1823** | 226.0948 | 434.1558 | 217.5815 | 433.1718 | 217.0895 | N | **922.5356** | 461.7715 | 905.5091 | 453.2582 | 904.5251 | 452.7662 | 8 |
| 5 | 564.2664 | 282.6368 | 547.2398 | 274.1236 | 546.2558 | 273.6316 | I | **807.5087** | 404.2580 | 790.4822 | 395.7447 | 789.4981 | 395.2527 | 7 |
| 6 | 651.2984 | 326.1529 | 634.2719 | 317.6396 | 633.2879 | 317.1476 | S | **694.4246** | 347.7160 | 677.3981 | 339.2027 | 676.4141 | 338.7107 | 6 |
| 7 | 764.3825 | 382.6949 | 747.3559 | 374.1816 | 746.3719 | 373.6896 | L | **607.3926** | 304.1999 | 590.3661 | 295.6867 | | | 5 |
| 8 | 877.4666 | 439.2369 | 860.4400 | 430.7236 | **859.4560** | 430.2316 | I | **494.3085** | 247.6579 | 477.2820 | 239.1446 | | | 4 |
| 9 | **1014.5255** | 507.7664 | 997.4989 | 499.2531 | 996.5149 | 498.7611 | H | **381.2245** | 191.1159 | 364.1979 | 182.6026 | | | 3 |
| 10 | **1111.5782** | 556.2928 | 1094.5517 | 547.7795 | **1093.5677** | 547.2875 | P | **244.1656** | 122.5864 | 227.1390 | 114.0731 | | | 2 |
| 11 | | | | | | | K | 147.1128 | 74.0600 | 130.0863 | 65.5468 | | | 1 |

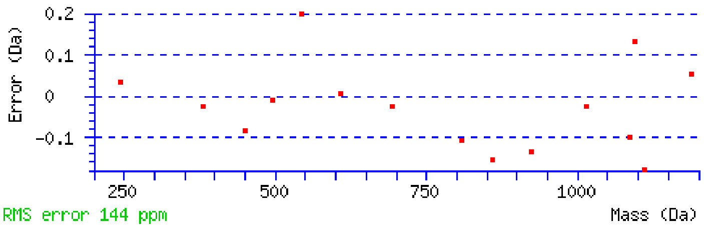


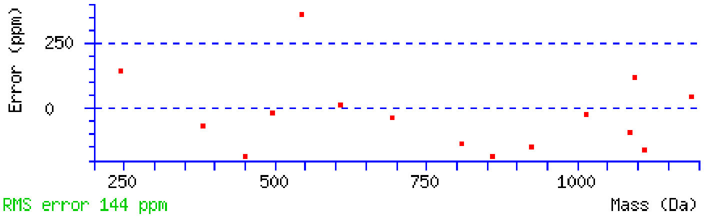


### All matches to this query

| Score | Mr(calc): | Delta | Sequence |
|---|---|---|---|
| 70.0 | 1256.6765 | -0.0027 | ATYNISLIHPK |
| 5.2 | 1256.6765 | -0.0027 | SLIHLDLGFNK |
| 4.7 | 1256.6613 | 0.0125 | TDDTPVVLVGNK |
| 4.4 | 1254.6721 | 2.0017 | YAVDTVLRYR |
| 3.8 | 1256.6837 | -0.0099 | AGDTIRLEAGVR |
| 0.4 | 1256.6837 | -0.0099 | RSSDPALRLDK |

---

Spectrum No: 361; Query: 97; Rank: 1

---

## Peptide View

MS/MS Fragmentation of **IANISMEVGR**
Found in **IPI00363534**, Tax_Id=10116 Gene_Symbol=Lama1 laminin, alpha 1

Match to Query 97: 1089.547568 from(545.781060,2+)
Title: 091008RatKidney_NoSalt_26.1658.1658.2.dta
Data file K:\NewmanPaper\Piliang\3SubProteomes\Piliang3SP\mgf5ppm\ERLIC_3SubProteomes5ppm.mgf

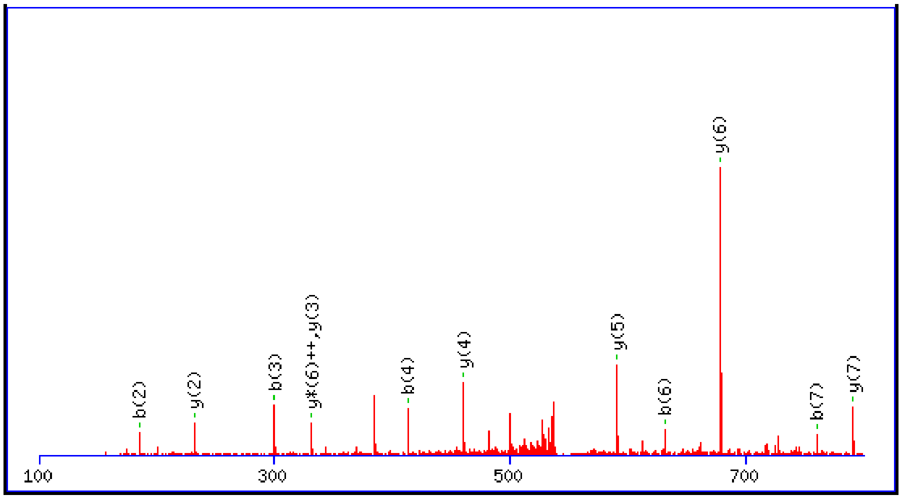


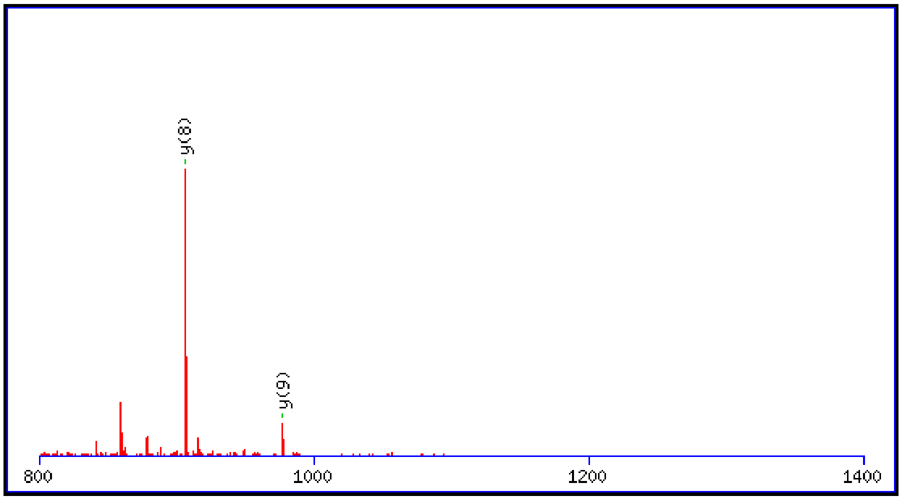


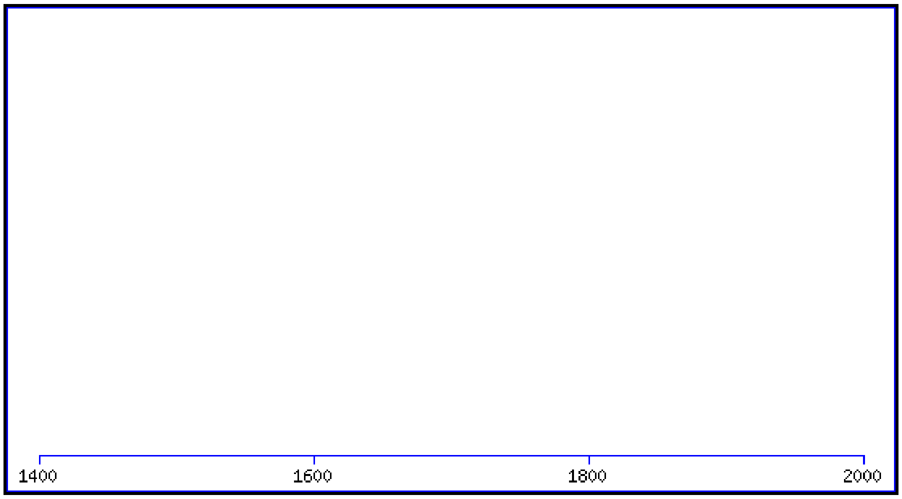


**Monoisotopic mass of neutral peptide Mr(calc):** 1089.5488
**Fixed modifications:** Carbamidomethyl (C)
**Variable modifications:**
**N3** : Deamidated_N (N)
**Ions Score:** 70 **Expect:** 1.3e-005
**Matches (Bold Red):** 14/90 fragment ions using 20 most intense peaks

| # | b | $b^{++}$ | b* | $b^{*++}$ | $b^0$ | $b^{0++}$ | Seq. | y | $y^{++}$ | y* | $y^{*++}$ | $y^0$ | $y^{0++}$ | # |
|---|---|---|---|---|---|---|---|---|---|---|---|---|---|---|
| 1 | 114.0913 | 57.5493 | | | | | I | | | | | | | 10 |
| 2 | **185.1285** | 93.0679 | | | | | A | **977.4721** | 489.2397 | 960.4455 | 480.7264 | 959.4615 | 480.2344 | 9 |
| 3 | **300.1554** | 150.5813 | 283.1288 | 142.0681 | | | N | **906.4349** | 453.7211 | 889.4084 | 445.2078 | 888.4244 | 444.7158 | 8 |
| 4 | **413.2395** | 207.1234 | 396.2129 | 198.6101 | | | I | **791.4080** | 396.2076 | 774.3815 | 387.6944 | 773.3974 | 387.2024 | 7 |
| 5 | 500.2715 | 250.6394 | 483.2449 | 242.1261 | 482.2609 | 241.6341 | S | **678.3239** | 339.6656 | 661.2974 | **331.1523** | 660.3134 | 330.6603 | 6 |

| | | | | | | | | | | | | | | |
|---|---|---|---|---|---|---|---|---|---|---|---|---|---|---|
| **6** | **631.3120** | 316.1596 | 614.2854 | 307.6463 | 613.3014 | 307.1543 | **M** | **591.2919** | 296.1496 | 574.2654 | 287.6363 | 573.2813 | 287.1443 | **5** |
| **7** | **760.3546** | 380.6809 | 743.3280 | 372.1676 | 742.3440 | 371.6756 | **E** | **460.2514** | 230.6293 | 443.2249 | 222.1161 | 442.2409 | 221.6241 | **4** |
| **8** | 859.4230 | 430.2151 | 842.3964 | 421.7019 | 841.4124 | 421.2098 | **V** | **331.2088** | 166.1081 | 314.1823 | 157.5948 | | | **3** |
| **9** | 916.4444 | 458.7259 | 899.4179 | 450.2126 | 898.4339 | 449.7206 | **G** | **232.1404** | 116.5738 | 215.1139 | 108.0606 | | | **2** |
| **10** | | | | | | | **R** | 175.1190 | 88.0631 | 158.0924 | 79.5498 | | | **1** |

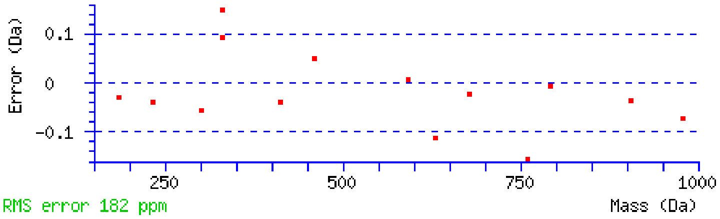


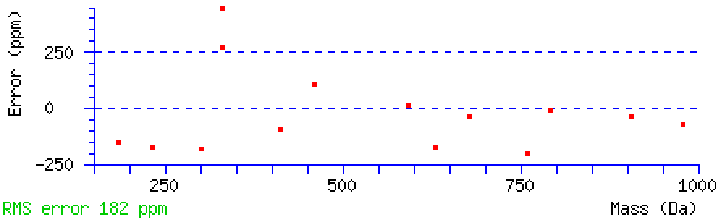


**All matches to this query**

| Score | Mr(calc): | Delta | Sequence |
|---|---|---|---|
| 70.0 | 1089.5488 | -0.0012 | IANISMEVGR |
| 10.8 | 1089.5567 | -0.0091 | IGKYESHTR |
| 9.7 | 1089.5488 | -0.0012 | AIDLENIMR |
| 9.4 | 1089.5389 | 0.0086 | LAQYMAHTR |
| 8.4 | 1089.5488 | -0.0013 | EALVAMGIDR |
| 8.0 | 1089.5528 | -0.0053 | IALNMDWVK |
| 7.1 | 1089.5529 | -0.0053 | ILWAMPSLGS |
| 4.7 | 1089.5567 | -0.0091 | LTSGLHSYGR |
| 4.7 | 1089.5389 | 0.0086 | LAWGMLNGGR |
| 3.0 | 1089.5461 | 0.0014 | RGNTMAERR |

---

Spectrum No: 362; Query: 94; Rank: 1

---

## Peptide View

MS/MS Fragmentation of **DGLALNLTLR**
Found in **IPI00212868**, Tax_Id=10116 Gene_Symbol=Lamb2 Laminin subunit beta-2 precursor

Match to Query 94: 1085.606308 from(543.810430,2+)
Title: 091008RatKidney_NoSalt_18.3269.3269.2.dta
Data file K:\NewmanPaper\Piliang\3SubProteomes\Piliang3SP\mgf5ppm\ERLIC_3SubProteomes5ppm.mgf

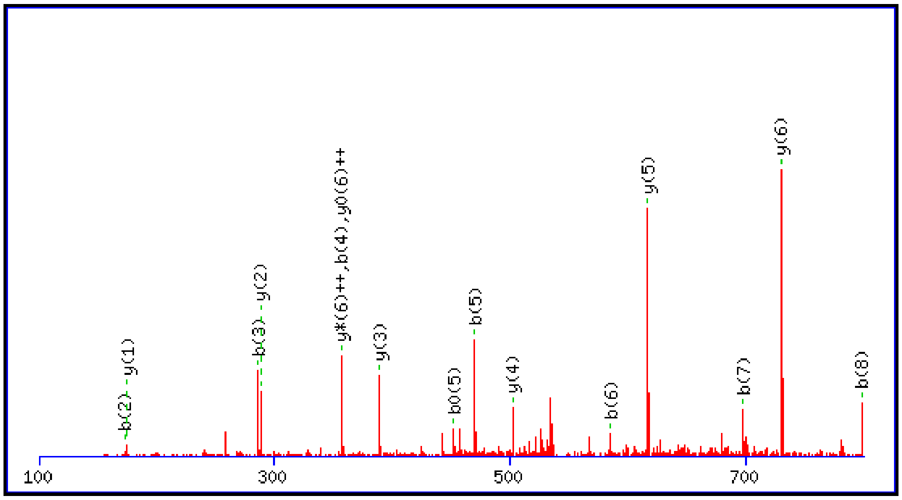

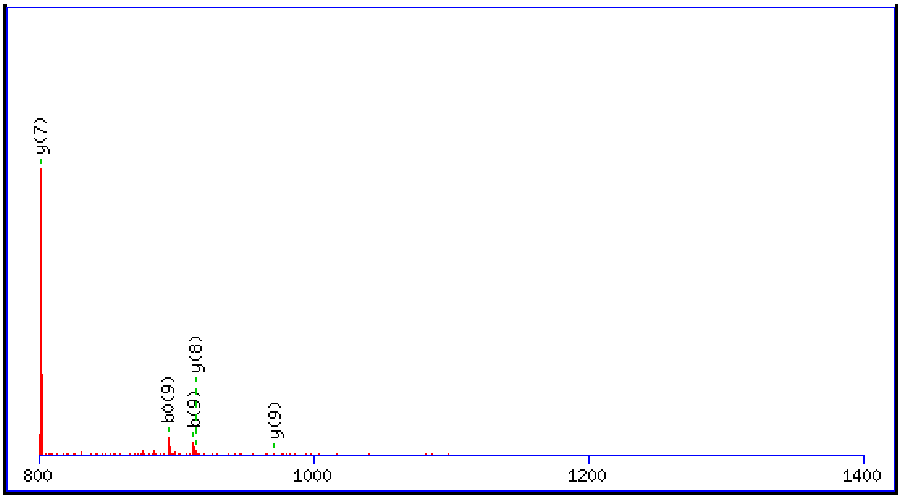


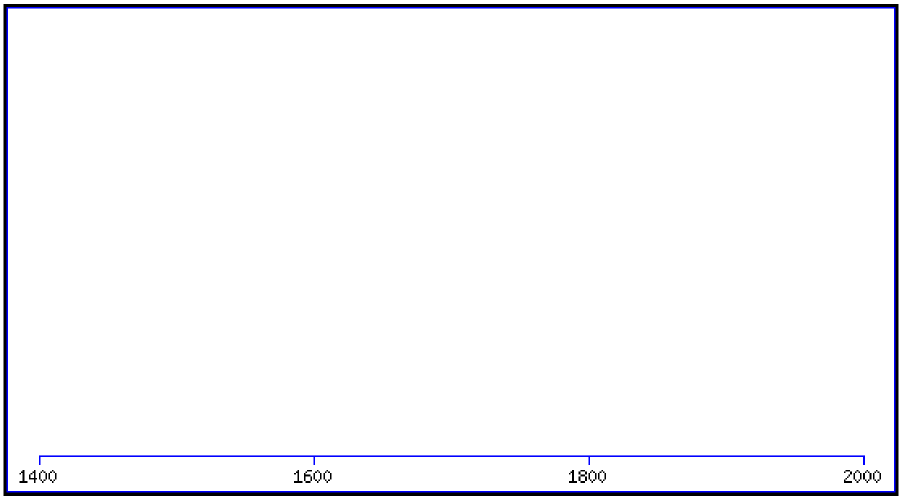


**Monoisotopic mass of neutral peptide Mr(calc):** 1085.6080
**Fixed modifications:** Carbamidomethyl (C)
**Variable modifications:**
**N6** : Deamidated_N (N)
**Ions Score:** 70 **Expect:** 7.5e-006
**Matches (Bold Red):** 21/94 fragment ions using 32 most intense peaks

| # | b | b$^{++}$ | b* | b*$^{++}$ | b$^{0}$ | b$^{0++}$ | Seq. | y | y$^{++}$ | y* | y*$^{++}$ | y$^{0}$ | y$^{0++}$ | # |
|---|---|---|---|---|---|---|---|---|---|---|---|---|---|---|
| 1 | 116.0342 | 58.5207 | | | 98.0237 | 49.5155 | D | | | | | | | 10 |
| 2 | **173.0557** | 87.0315 | | | 155.0451 | 78.0262 | G | **971.5884** | 486.2978 | 954.5619 | 477.7846 | 953.5778 | 477.2926 | 9 |
| 3 | **286.1397** | 143.5735 | | | 268.1292 | 134.5682 | L | **914.5669** | 457.7871 | 897.5404 | 449.2738 | 896.5564 | 448.7818 | 8 |
| 4 | **357.1769** | 179.0921 | | | 339.1663 | 170.0868 | A | **801.4829** | 401.2451 | 784.4563 | 392.7318 | 783.4723 | 392.2398 | 7 |
| 5 | **470.2609** | 235.6341 | | | **452.2504** | 226.6288 | L | **730.4458** | 365.7265 | 713.4192 | **357.2132** | 712.4352 | **356.7212** | 6 |
| 6 | **585.2879** | 293.1476 | 568.2613 | 284.6343 | 567.2773 | 284.1423 | N | **617.3617** | 309.1845 | 600.3351 | 300.6712 | 599.3511 | 300.1792 | 5 |
| 7 | **698.3719** | 349.6896 | 681.3454 | 341.1763 | 680.3614 | 340.6843 | L | **502.3348** | 251.6710 | 485.3082 | 243.1577 | 484.3242 | 242.6657 | 4 |
| 8 | **799.4196** | 400.2134 | 782.3931 | 391.7002 | 781.4090 | 391.2082 | T | **389.2507** | 195.1290 | 372.2241 | 186.6157 | 371.2401 | 186.1237 | 3 |
| 9 | **912.5037** | 456.7555 | 895.4771 | 448.2422 | **894.4931** | 447.7502 | L | **288.2030** | 144.6051 | 271.1765 | 136.0919 | | | 2 |
| 10 | | | | | | | R | **175.1190** | 88.0631 | 158.0924 | 79.5498 | | | 1 |

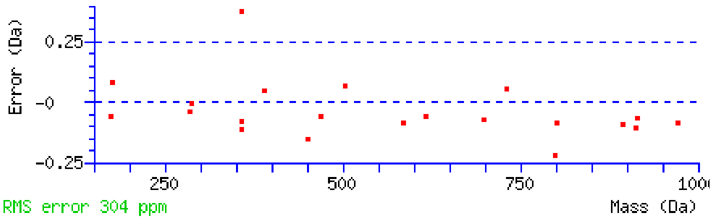


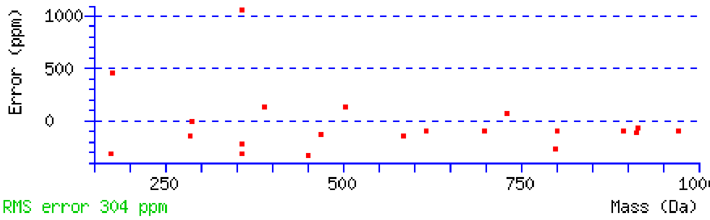


## All matches to this query

| Score | Mr(calc): | Delta | Sequence |
|---|---|---|---|
| 69.9 | 1085.6080 | -0.0017 | DGLALNLTLR |
| 19.0 | 1085.6081 | -0.0018 | LQDELVTLR |
| 16.6 | 1085.6080 | -0.0017 | NLEISILER |
| 14.1 | 1085.5968 | 0.0095 | DQLIEVIEK |
| 14.1 | 1084.5989 | 1.0074 | ARQLEEALR |
| 12.1 | 1084.5989 | 1.0074 | IQNISNLQR |
| 11.0 | 1084.6128 | 0.9935 | ELLITSQPGK |
| 10.7 | 1085.6080 | -0.0017 | ILKNNIDVR |
| 9.3 | 1085.5968 | 0.0095 | QEVDILELK |
| 8.3 | 1083.6036 | 2.0027 | NNASLPLSLR |

---

Spectrum No: 363; Query: 2713; Rank: 1

---

## Peptide View

MS/MS Fragmentation of **SNSTAGELDALQAEAGSLDKTVNELAEQLEFIK**
Found in **IPI00365542**, Tax_Id=10116 Gene_Symbol=Lamb1_predicted laminin, beta 1

Match to Query 2713: 3492.732372 from(1165.251400,3+)
Title: 100101RatKid_NS_deglyco_12.7567.7567.3.dta
Data file K:\NewmanPaper\Piliang\3SubProteomes\Piliang3SP\mgf5ppm\ERLIC_3SubProteomes5ppm.mgf

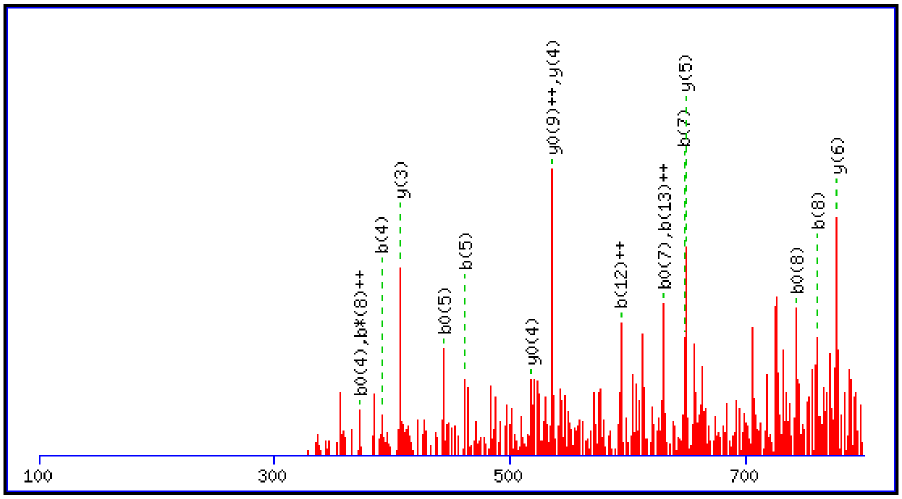


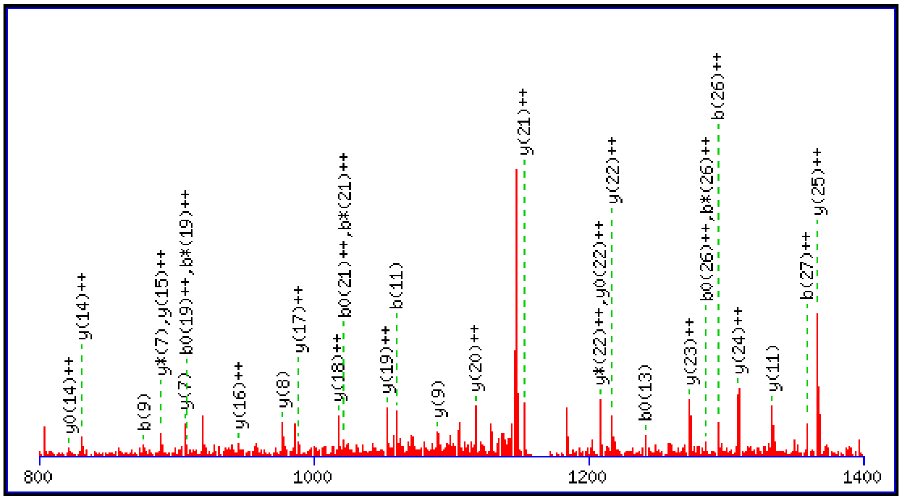

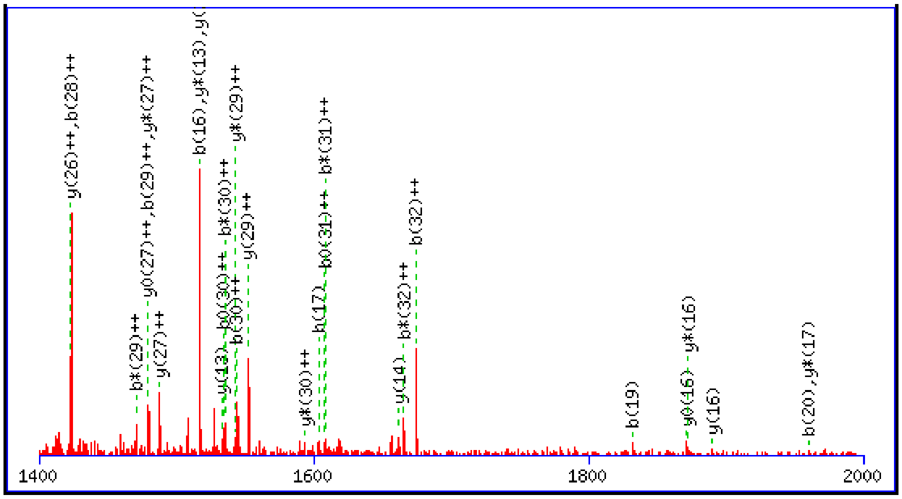


**Monoisotopic mass of neutral peptide Mr(calc):** 3491.7155
**Fixed modifications:** Carbamidomethyl (C)
**Variable modifications:**
**N2** : Deamidated_N (N)
**Ions Score:** 70 **Expect:** 2.4e-005
**Matches (Bold Red):** 77/376 fragment ions using 146 most intense peaks

| # | b | $b^{++}$ | b* | $b^{*++}$ | $b^0$ | $b^{0++}$ | Seq. | y | $y^{++}$ | y* | $y^{*++}$ | $y^0$ | $y^{0++}$ | # |
|---|---|---|---|---|---|---|---|---|---|---|---|---|---|---|
| 1 | 88.0393 | 44.5233 | | | 70.0287 | 35.5180 | S | | | | | | | 33 |
| 2 | 203.0662 | 102.0368 | 186.0397 | 93.5235 | 185.0557 | 93.0315 | N | 3405.6908 | 1703.3490 | 3388.6642 | 1694.8357 | 3387.6802 | 1694.3437 | 32 |
| 3 | 290.0983 | 145.5528 | 273.0717 | 137.0395 | 272.0877 | 136.5475 | S | 3290.6638 | 1645.8356 | 3273.6373 | 1637.3223 | 3272.6533 | 1636.8303 | 31 |
| 4 | **391.1459** | 196.0766 | 374.1194 | 187.5633 | **373.1354** | 187.0713 | T | 3203.6318 | 1602.3195 | 3186.6052 | **1593.8063** | 3185.6212 | 1593.3143 | 30 |
| 5 | **462.1831** | 231.5952 | 445.1565 | 223.0819 | **444.1725** | 222.5899 | A | 3102.5841 | **1551.7957** | 3085.5576 | **1543.2824** | 3084.5736 | 1542.7904 | 29 |
| 6 | 519.2045 | 260.1059 | 502.1780 | 251.5926 | 501.1940 | 251.1006 | G | 3031.5470 | **1516.2771** | 3014.5205 | 1507.7639 | 3013.5364 | 1507.2719 | 28 |
| 7 | **648.2471** | 324.6272 | 631.2206 | 316.1139 | **630.2366** | 315.6219 | E | 2974.5255 | **1487.7664** | 2957.4990 | **1479.2531** | 2956.5150 | **1478.7611** | 27 |
| 8 | **761.3312** | 381.1692 | 744.3046 | **372.6560** | **743.3206** | 372.1639 | L | 2845.4829 | **1423.2451** | 2828.4564 | 1414.7318 | 2827.4724 | 1414.2398 | 26 |
| 9 | **876.3581** | 438.6827 | 859.3316 | 430.1694 | 858.3476 | 429.6774 | D | 2732.3989 | **1366.7031** | 2715.3723 | 1358.1898 | 2714.3883 | 1357.6978 | 25 |
| 10 | 947.3952 | 474.2013 | 930.3687 | 465.6880 | 929.3847 | 465.1960 | A | 2617.3719 | **1309.1896** | 2600.3454 | 1300.6763 | 2599.3614 | 1300.1843 | 24 |
| 11 | **1060.4793** | 530.7433 | 1043.4528 | 522.2300 | 1042.4687 | 521.7380 | L | 2546.3348 | **1273.6711** | 2529.3083 | 1265.1578 | 2528.3243 | 1264.6658 | 23 |
| 12 | 1188.5379 | **594.7726** | 1171.5113 | 586.2593 | 1170.5273 | 585.7673 | Q | 2433.2508 | **1217.1290** | 2416.2242 | **1208.6157** | 2415.2402 | **1208.1237** | 22 |
| 13 | 1259.5750 | **630.2911** | 1242.5484 | 621.7779 | **1241.5644** | 621.2859 | A | 2305.1922 | **1153.0997** | 2288.1656 | 1144.5865 | 2287.1816 | 1144.0944 | 21 |
| 14 | 1388.6176 | 694.8124 | 1371.5910 | 686.2992 | 1370.6070 | 685.8071 | E | 2234.1551 | **1117.5812** | 2217.1285 | 1109.0679 | 2216.1445 | 1108.5759 | 20 |
| 15 | 1459.6547 | 730.3310 | 1442.6282 | 721.8177 | 1441.6441 | 721.3257 | A | 2105.1125 | **1053.0599** | 2088.0859 | 1044.5466 | 2087.1019 | 1044.0546 | 19 |
| 16 | **1516.6762** | 758.8417 | 1499.6496 | 750.3284 | 1498.6656 | 749.8364 | G | 2034.0754 | **1017.5413** | 2017.0488 | 1009.0280 | 2016.0648 | 1008.5360 | 18 |
| 17 | **1603.7082** | 802.3577 | 1586.6816 | 793.8445 | 1585.6976 | 793.3525 | S | 1977.0539 | **989.0306** | **1960.0274** | 980.5173 | 1959.0433 | 980.0253 | 17 |
| 18 | 1716.7923 | 858.8998 | 1699.7657 | 850.3865 | 1698.7817 | 849.8945 | L | **1890.0219** | **945.5146** | **1872.9953** | 937.0013 | **1872.0113** | 936.5093 | 16 |
| 19 | **1831.8192** | 916.4132 | 1814.7927 | **907.9000** | 1813.8086 | **907.4080** | D | 1776.9378 | **888.9725** | 1759.9113 | 880.4593 | 1758.9272 | 879.9673 | 15 |
| 20 | **1959.9142** | 980.4607 | 1942.8876 | 971.9474 | 1941.9036 | 971.4554 | K | **1661.9109** | **831.4591** | 1644.8843 | 822.9458 | 1643.9003 | **822.4538** | 14 |
| 21 | 2060.9618 | 1030.9846 | 2043.9353 | **1022.4713** | 2042.9513 | **1021.9793** | T | **1533.8159** | 767.4116 | **1516.7894** | 758.8983 | 1515.8053 | 758.4063 | 13 |
| 22 | 2160.0303 | 1080.5188 | 2143.0037 | 1072.0055 | 2142.0197 | 1071.5135 | V | 1432.7682 | 716.8877 | 1415.7417 | 708.3745 | 1414.7577 | 707.8825 | 12 |
| 23 | 2274.0732 | 1137.5402 | 2257.0466 | 1129.0270 | 2256.0626 | 1128.5349 | N | **1333.6998** | 667.3535 | 1316.6733 | 658.8403 | 1315.6892 | 658.3483 | 11 |
| 24 | 2403.1158 | 1202.0615 | 2386.0892 | 1193.5483 | 2385.1052 | 1193.0562 | E | 1219.6569 | 610.3321 | 1202.6303 | 601.8188 | 1201.6463 | 601.3268 | 10 |
| 25 | 2516.1998 | 1258.6036 | 2499.1733 | 1250.0903 | 2498.1893 | 1249.5983 | L | **1090.6143** | 545.8108 | 1073.5877 | 537.2975 | 1072.6037 | **536.8055** | 9 |
| 26 | 2587.2370 | **1294.1221** | 2570.2104 | **1285.6088** | 2569.2264 | **1285.1168** | A | **977.5302** | 489.2688 | 960.5037 | 480.7555 | 959.5197 | 480.2635 | 8 |
| 27 | 2716.2795 | **1358.6434** | 2699.2530 | 1350.1301 | 2698.2690 | 1349.6381 | E | **906.4931** | 453.7502 | **889.4666** | 445.2369 | 888.4825 | 444.7449 | 7 |
| 28 | 2844.3381 | **1422.6727** | 2827.3116 | 1414.1594 | 2826.3276 | 1413.6674 | Q | **777.4505** | 389.2289 | 760.4240 | 380.7156 | 759.4400 | 380.2236 | 6 |
| 29 | 2957.4222 | **1479.2147** | 2940.3956 | **1470.7015** | 2939.4116 | 1470.2095 | L | **649.3919** | 325.1996 | 632.3654 | 316.6863 | 631.3814 | 316.1943 | 5 |
| 30 | 3086.4648 | **1543.7360** | 3069.4382 | **1535.2228** | 3068.4542 | **1534.7307** | E | **536.3079** | 268.6576 | 519.2813 | 260.1443 | **518.2973** | 259.6523 | 4 |
| 31 | 3233.5332 | 1617.2702 | 3216.5066 | **1608.7570** | 3215.5226 | **1608.2650** | F | **407.2653** | 204.1363 | 390.2387 | 195.6230 | | | 3 |
| 32 | 3346.6173 | **1673.8123** | 3329.5907 | **1665.2990** | 3328.6067 | 1664.8070 | I | 260.1969 | 130.6021 | 243.1703 | 122.0888 | | | 2 |

| | | | | | | | | | | | | | | |
|---|---|---|---|---|---|---|---|---|---|---|---|---|---|---|
| 33 | | | | | | | K | 147.1128 | 74.0600 | 130.0863 | 65.5468 | | | 1 |

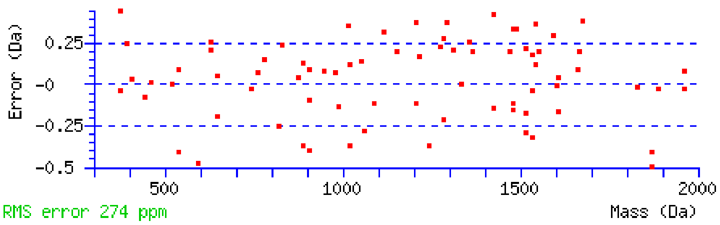


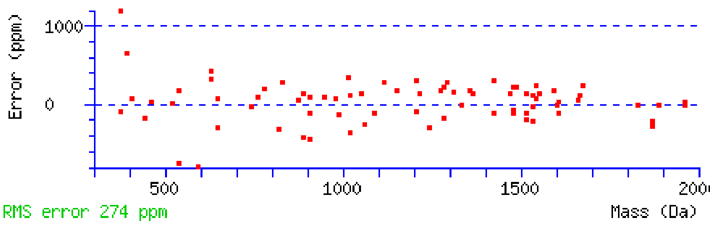


**All matches to this query**

| Score | Mr(calc): | Delta | Sequence |
|---|---|---|---|
| 69.9 | 3491.7155 | 1.0169 | SNSTAGELDALQAEAGSLDKTVNELAEQLEFIK |
| 59.8 | 3492.6995 | 0.0329 | SNSTAGELDALQAEAGSLDKTVNELAEQLEFIK |
| 48.3 | 3490.7314 | 2.0009 | SNSTAGELDALQAEAGSLDKTVNELAEQLEFIK |
| 48.1 | 3491.7155 | 1.0169 | SNSTAGELDALQAEAGSLDKTVNELAEQLEFIK |
| 4.1 | 3490.7311 | 2.0013 | GLNISYLGIQRQVQLLMSFFYEAIRLMK |
| 2.7 | 3491.7631 | 0.9692 | LVAMNMPLNSDGTVMFNATLFALVRTALKIK |
| 2.7 | 3491.7631 | 0.9692 | LVAMNMPLNSDGTVMFNATLFALVRTALKIK |
| 2.4 | 3492.7471 | -0.0148 | LVAMNMPLNSDGTVMFNATLFALVRTALKIK |
| 2.4 | 3492.7471 | -0.0148 | LVAMNMPLNSDGTVMFNATLFALVRTALKIK |
| 2.4 | 3492.7471 | -0.0148 | LVAMNMPLNSDGTVMFNATLFALVRTALKIK |

---

Spectrum No: 364; Query: 1904; Rank: 1

---

## Peptide View

MS/MS Fragmentation of **VNCSTSCAAPEIGGLETPTNK**
Found in **IPI00372952**, Tax_Id=10116 Gene_Symbol=Icam2 Intercellular adhesion molecule 2

Match to Query 1904: 2205.996688 from(1104.005620,2+)
Title: 100101RatKid_NS_deglyco_25.2138.2138.2.dta
Data file K:\NewmanPaper\Piliang\3SubProteomes\Piliang3SP\mgf5ppm\ERLIC_3SubProteomes5ppm.mgf

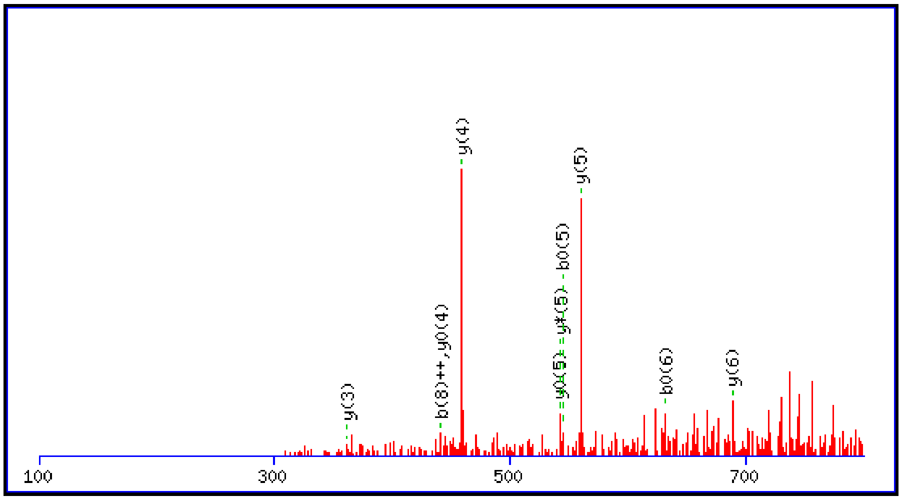

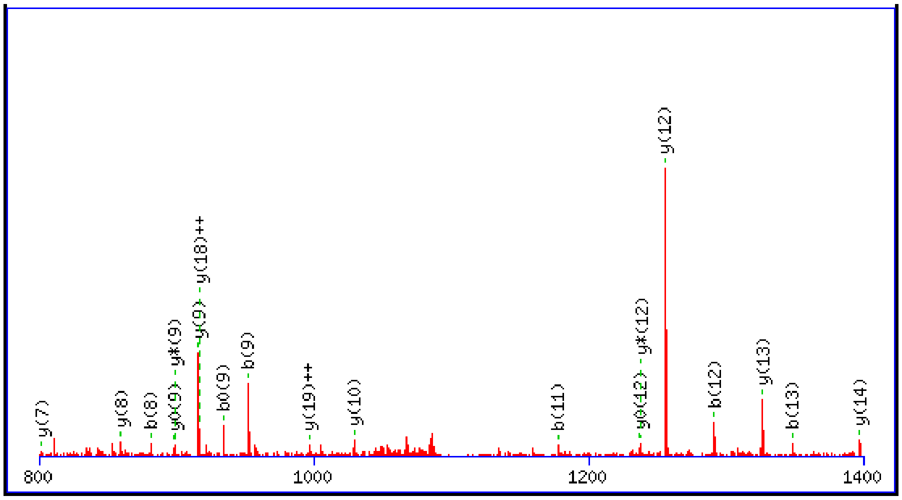


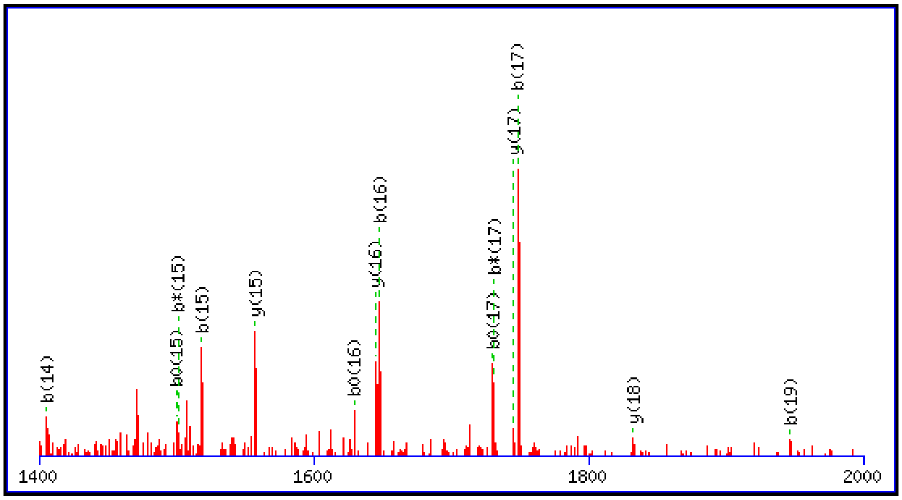


**Monoisotopic mass of neutral peptide Mr(calc):** 2205.9882
**Fixed modifications:** Carbamidomethyl (C)
**Variable modifications:**
**N2** : Deamidated_N (N)
**Ions Score:** 70 **Expect:** 2.9e-005
**Matches (Bold Red):** 43/228 fragment ions using 109 most intense peaks

| # | b | $b^{++}$ | b* | $b^{*++}$ | $b^0$ | $b^{0++}$ | Seq. | y | $y^{++}$ | y* | $y^{*++}$ | $y^0$ | $y^{0++}$ | # |
|---|---|---|---|---|---|---|---|---|---|---|---|---|---|---|
| 1 | 100.0757 | 50.5415 | | | | | V | | | | | | | 21 |
| 2 | 215.1026 | 108.0550 | 198.0761 | 99.5417 | | | N | 2107.9271 | 1054.4672 | 2090.9005 | 1045.9539 | 2089.9165 | 1045.4619 | 20 |
| 3 | 375.1333 | 188.0703 | 358.1067 | 179.5570 | | | C | 1992.9001 | **996.9537** | 1975.8736 | 988.4404 | 1974.8896 | 987.9484 | 19 |
| 4 | 462.1653 | 231.5863 | 445.1388 | 223.0730 | 444.1547 | 222.5810 | S | **1832.8695** | **916.9384** | 1815.8429 | 908.4251 | 1814.8589 | 907.9331 | 18 |
| 5 | 563.2130 | 282.1101 | 546.1864 | 273.5969 | **545.2024** | 273.1048 | T | **1745.8374** | 873.4224 | 1728.8109 | 864.9091 | 1727.8269 | 864.4171 | 17 |
| 6 | 650.2450 | 325.6261 | 633.2185 | 317.1129 | **632.2344** | 316.6209 | S | **1644.7898** | 822.8985 | 1627.7632 | 814.3852 | 1626.7792 | 813.8932 | 16 |
| 7 | 810.2757 | 405.6415 | 793.2491 | 397.1282 | 792.2651 | 396.6362 | C | **1557.7577** | 779.3825 | 1540.7312 | 770.8692 | 1539.7472 | 770.3772 | 15 |
| 8 | **881.3128** | **441.1600** | 864.2862 | 432.6468 | 863.3022 | 432.1547 | A | **1397.7271** | 699.3672 | 1380.7005 | 690.8539 | 1379.7165 | 690.3619 | 14 |
| 9 | **952.3499** | 476.6786 | 935.3233 | 468.1653 | **934.3393** | 467.6733 | A | **1326.6900** | 663.8486 | 1309.6634 | 655.3353 | 1308.6794 | 654.8433 | 13 |
| 10 | 1049.4027 | 525.2050 | 1032.3761 | 516.6917 | 1031.3921 | 516.1997 | P | **1255.6529** | 628.3301 | **1238.6263** | 619.8168 | **1237.6423** | 619.3248 | 12 |
| 11 | **1178.4452** | 589.7263 | 1161.4187 | 581.2130 | 1160.4347 | 580.7210 | E | 1158.6001 | 579.8037 | 1141.5735 | 571.2904 | 1140.5895 | 570.7984 | 11 |
| 12 | **1291.5293** | 646.2683 | 1274.5028 | 637.7550 | 1273.5187 | 637.2630 | I | **1029.5575** | 515.2824 | 1012.5310 | 506.7691 | 1011.5469 | 506.2771 | 10 |
| 13 | **1348.5508** | 674.7790 | 1331.5242 | 666.2657 | 1330.5402 | 665.7737 | G | **916.4734** | 458.7404 | **899.4469** | 450.2271 | **898.4629** | 449.7351 | 9 |
| 14 | **1405.5722** | 703.2898 | 1388.5457 | 694.7765 | 1387.5617 | 694.2845 | G | **859.4520** | 430.2296 | 842.4254 | 421.7164 | 841.4414 | 421.2243 | 8 |
| 15 | **1518.6563** | 759.8318 | **1501.6298** | 751.3185 | **1500.6457** | 750.8265 | L | **802.4305** | 401.7189 | 785.4040 | 393.2056 | 784.4199 | 392.7136 | 7 |
| 16 | **1647.6989** | 824.3531 | 1630.6723 | 815.8398 | **1629.6883** | 815.3478 | E | **689.3464** | 345.1769 | 672.3199 | 336.6636 | 671.3359 | 336.1716 | 6 |
| 17 | **1748.7466** | 874.8769 | **1731.7200** | 866.3636 | **1730.7360** | 865.8716 | T | **560.3039** | 280.6556 | **543.2773** | 272.1423 | **542.2933** | 271.6503 | 5 |
| 18 | 1845.7993 | 923.4033 | 1828.7728 | 914.8900 | 1827.7888 | 914.3980 | P | **459.2562** | 230.1317 | 442.2296 | 221.6185 | **441.2456** | 221.1264 | 4 |

| | | | | | | | | | | | | | | |
|---|---|---|---|---|---|---|---|---|---|---|---|---|---|---|
| 19 | 1946.8470 | 973.9271 | 1929.8205 | 965.4139 | 1928.8364 | 964.9219 | T | 362.2034 | 181.6053 | 345.1769 | 173.0921 | 344.1928 | 172.6001 | 3 |
| 20 | 2060.8899 | 1030.9486 | 2043.8634 | 1022.4353 | 2042.8794 | 1021.9433 | N | 261.1557 | 131.0815 | 244.1292 | 122.5682 | | | 2 |
| 21 | | | | | | | K | 147.1128 | 74.0600 | 130.0863 | 65.5468 | | | 1 |

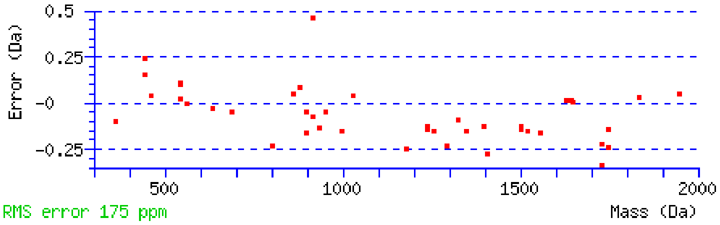


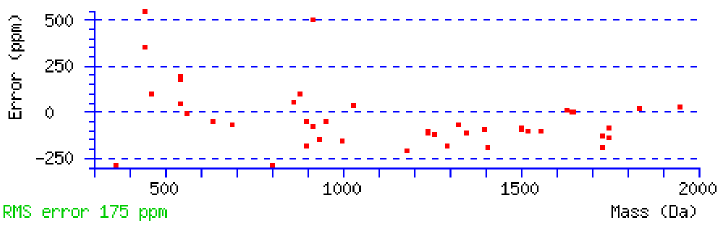


### All matches to this query

| Score | Mr(calc): | Delta | Sequence |
|---|---|---|---|
| 69.8 | 2205.9882 | 0.0085 | VNCSTSCAAPEIGGLETPTNK |
| 44.0 | 2205.0042 | 0.9925 | VNCSTSCAAPEIGGLETPTNK |
| 10.5 | 2205.9882 | 0.0085 | VNCSTSCAAPEIGGLETPTNK |
| 2.0 | 2206.0089 | -0.0122 | MVGANNQKNLTSFKTTAER |
| 1.5 | 2206.0089 | -0.0122 | MVGANNQKNLTSFKTTAER |
| 1.5 | 2206.0089 | -0.0122 | MVGANNQKNLTSFKTTAER |
| 1.3 | 2205.9782 | 0.0185 | VFTTLVLCDKPAVSENPR |
| 1.3 | 2205.9782 | 0.0185 | VFTTLVLCDKPAVSENPR |
| 1.1 | 2206.0042 | -0.0075 | GPSENGELSAPLELGSLVNGVS |
| 0.9 | 2206.0042 | -0.0075 | GPSENGELSAPLELGSLVNGVS |

---

Spectrum No: 365; Query: 2704; Rank: 1

---

## Peptide View

MS/MS Fragmentation of **LLYISPENFQIDNSPNSTAGPSCSLLLEAFR**
Found in **IPI00464518**, Tax_Id=10116 Gene_Symbol=Hexb Beta-hexosaminidase beta chain precursor

Match to Query 2704: 3453.684522 from(1152.235450,3+)
Title: 100101RatKid_NS_deglyco_11.7176.7176.3.dta
Data file K:\NewmanPaper\Piliang\3SubProteomes\Piliang3SP\mgf5ppm\ERLIC_3SubProteomes5ppm.mgf

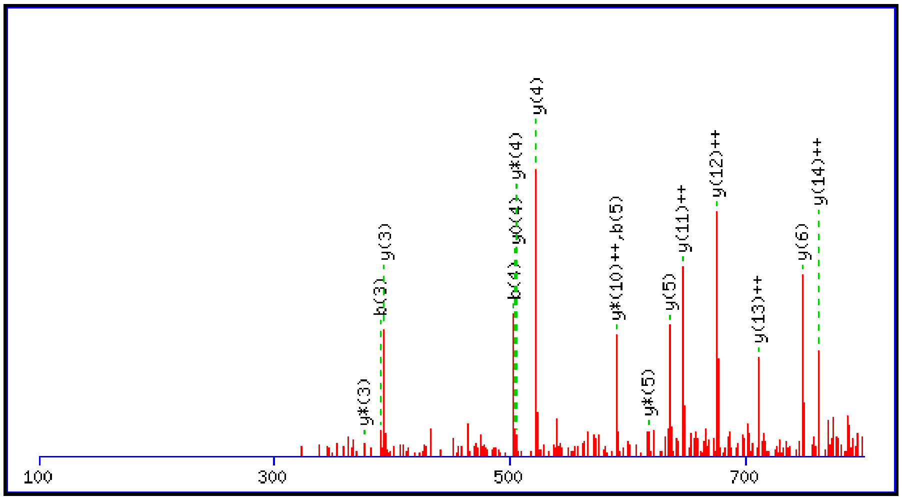

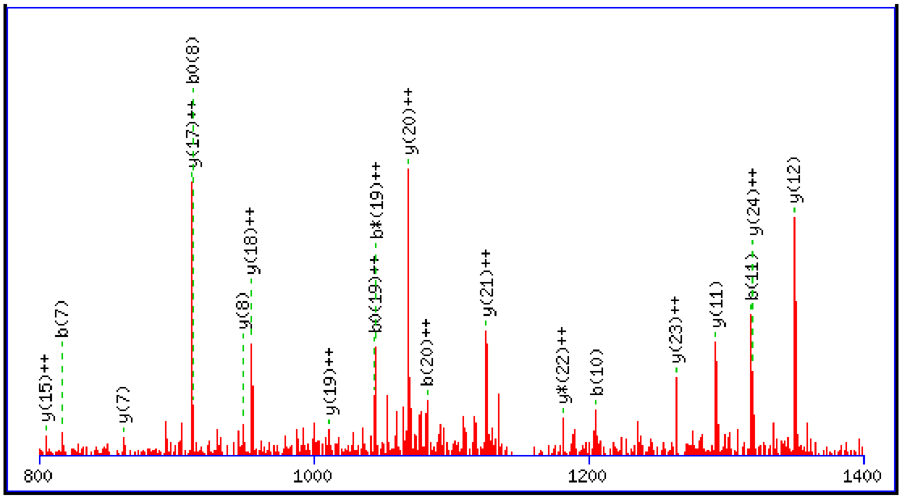


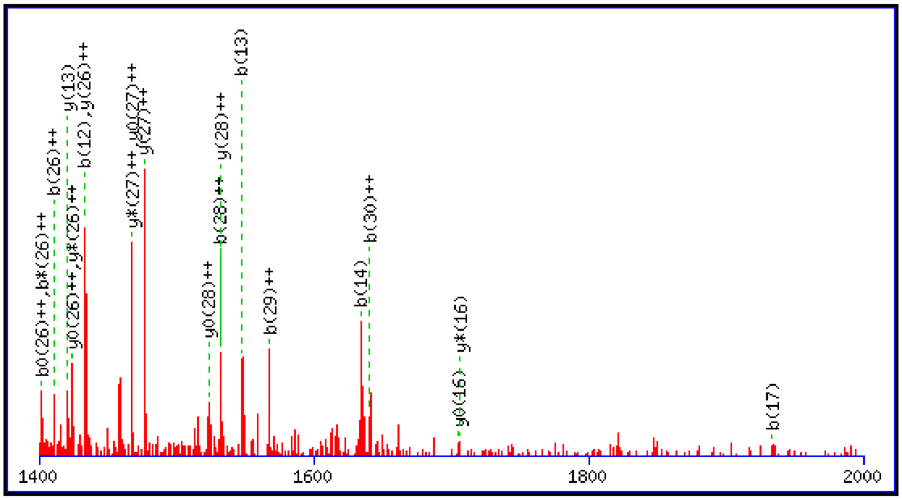


**Monoisotopic mass of neutral peptide Mr(calc):** 3453.6762
**Fixed modifications:** Carbamidomethyl (C)
**Variable modifications:**
**N16** : Deamidated_N (N)
**Ions Score:** 70 **Expect:** 3e-005
**Matches (Bold Red):** 57/332 fragment ions using 113 most intense peaks

| # | b | $b^{++}$ | b* | $b^{*++}$ | $b^0$ | $b^{0++}$ | Seq. | y | $y^{++}$ | y* | $y^{*++}$ | $y^0$ | $y^{0++}$ | # |
|---|---|---|---|---|---|---|---|---|---|---|---|---|---|---|
| 1 | 114.0913 | 57.5493 | | | | | L | | | | | | | 31 |
| 2 | 227.1754 | 114.0913 | | | | | L | 3341.5994 | 1671.3034 | 3324.5729 | 1662.7901 | 3323.5889 | 1662.2981 | 30 |
| 3 | **390.2387** | 195.6230 | | | | | Y | 3228.5154 | 1614.7613 | 3211.4888 | 1606.2481 | 3210.5048 | 1605.7560 | 29 |
| 4 | **503.3228** | 252.1650 | | | | | I | 3065.4520 | **1533.2297** | 3048.4255 | 1524.7164 | 3047.4415 | **1524.2244** | 28 |
| 5 | **590.3548** | 295.6811 | | | 572.3443 | 286.6758 | S | 2952.3680 | **1476.6876** | 2935.3414 | **1468.1744** | 2934.3574 | **1467.6823** | 27 |
| 6 | 687.4076 | 344.2074 | | | 669.3970 | 335.2022 | P | 2865.3360 | **1433.1716** | 2848.3094 | **1424.6583** | 2847.3254 | **1424.1663** | 26 |
| 7 | **816.4502** | 408.7287 | | | 798.4396 | 399.7234 | E | 2768.2832 | 1384.6452 | 2751.2566 | 1376.1320 | 2750.2726 | 1375.6400 | 25 |
| 8 | 930.4931 | 465.7502 | 913.4666 | 457.2369 | **912.4825** | 456.7449 | N | 2639.2406 | **1320.1239** | 2622.2141 | 1311.6107 | 2621.2300 | 1311.1187 | 24 |
| 9 | 1077.5615 | 539.2844 | 1060.5350 | 530.7711 | 1059.5510 | 530.2791 | F | 2525.1977 | **1263.1025** | 2508.1711 | 1254.5892 | 2507.1871 | 1254.0972 | 23 |
| 10 | **1205.6201** | 603.3137 | 1188.5936 | 594.8004 | 1187.6095 | 594.3084 | Q | 2378.1293 | 1189.5683 | 2361.1027 | **1181.0550** | 2360.1187 | 1180.5630 | 22 |
| 11 | **1318.7042** | 659.8557 | 1301.6776 | 651.3424 | 1300.6936 | 650.8504 | I | 2250.0707 | **1125.5390** | 2233.0441 | 1117.0257 | 2232.0601 | 1116.5337 | 21 |
| 12 | **1433.7311** | 717.3692 | 1416.7046 | 708.8559 | 1415.7205 | 708.3639 | D | 2136.9866 | **1068.9969** | 2119.9601 | 1060.4837 | 2118.9761 | 1059.9917 | 20 |
| 13 | **1547.7740** | 774.3907 | 1530.7475 | 765.8774 | 1529.7635 | 765.3854 | N | 2021.9597 | **1011.4835** | 2004.9331 | 1002.9702 | 2003.9491 | 1002.4782 | 19 |
| 14 | **1634.8061** | 817.9067 | 1617.7795 | 809.3934 | 1616.7955 | 808.9014 | S | 1907.9167 | **954.4620** | 1890.8902 | 945.9487 | 1889.9062 | 945.4567 | 18 |
| 15 | 1731.8588 | 866.4331 | 1714.8323 | 857.9198 | 1713.8483 | 857.4278 | P | 1820.8847 | **910.9460** | 1803.8582 | 902.4327 | 1802.8742 | 901.9407 | 17 |
| 16 | 1846.8858 | 923.9465 | 1829.8592 | 915.4332 | 1828.8752 | 914.9412 | N | 1723.8320 | 862.4196 | **1706.8054** | 853.9063 | **1705.8214** | 853.4143 | 16 |
| 17 | **1933.9178** | 967.4625 | 1916.8912 | 958.9493 | 1915.9072 | 958.4573 | S | 1608.8050 | **804.9061** | 1591.7785 | 796.3929 | 1590.7945 | 795.9009 | 15 |
| 18 | 2034.9655 | 1017.9864 | 2017.9389 | 1009.4731 | 2016.9549 | 1008.9811 | T | 1521.7730 | **761.3901** | 1504.7464 | 752.8769 | 1503.7624 | 752.3849 | 14 |
| | | | | | | | | | | | | | | |

| | | | | | | | | | | | | | | |
|---|---|---|---|---|---|---|---|---|---|---|---|---|---|---|
| 19 | 2106.0026 | 1053.5049 | 2088.9760 | **1044.9917** | 2087.9920 | **1044.4996** | A | **1420.7253** | **710.8663** | 1403.6988 | 702.3530 | 1402.7147 | 701.8610 | 13 |
| 20 | 2163.0240 | **1082.0157** | 2145.9975 | 1073.5024 | 2145.0135 | 1073.0104 | G | **1349.6882** | **675.3477** | 1332.6616 | 666.8345 | 1331.6776 | 666.3425 | 12 |
| 21 | 2260.0768 | 1130.5420 | 2243.0503 | 1122.0288 | 2242.0662 | 1121.5368 | P | **1292.6667** | **646.8370** | 1275.6402 | 638.3237 | 1274.6562 | 637.8317 | 11 |
| 22 | 2347.1088 | 1174.0581 | 2330.0823 | 1165.5448 | 2329.0983 | 1165.0528 | S | 1195.6140 | 598.3106 | 1178.5874 | **589.7973** | 1177.6034 | 589.3053 | 10 |
| 23 | 2507.1395 | 1254.0734 | 2490.1129 | 1245.5601 | 2489.1289 | 1245.0681 | C | 1108.5819 | 554.7946 | 1091.5554 | 546.2813 | 1090.5714 | 545.7893 | 9 |
| 24 | 2594.1715 | 1297.5894 | 2577.1450 | 1289.0761 | 2576.1610 | 1288.5841 | S | **948.5513** | 474.7793 | 931.5247 | 466.2660 | 930.5407 | 465.7740 | 8 |
| 25 | 2707.2556 | 1354.1314 | 2690.2290 | 1345.6182 | 2689.2450 | 1345.1261 | L | **861.5193** | 431.2633 | 844.4927 | 422.7500 | 843.5087 | 422.2580 | 7 |
| 26 | 2820.3396 | **1410.6735** | 2803.3131 | **1402.1602** | 2802.3291 | **1401.6682** | L | **748.4352** | 374.7212 | 731.4087 | 366.2080 | 730.4246 | 365.7160 | 6 |
| 27 | 2933.4237 | 1467.2155 | 2916.3972 | 1458.7022 | 2915.4131 | 1458.2102 | L | **635.3511** | 318.1792 | **618.3246** | 309.6659 | 617.3406 | 309.1739 | 5 |
| 28 | 3062.4663 | **1531.7368** | 3045.4398 | 1523.2235 | 3044.4557 | 1522.7315 | E | **522.2671** | 261.6372 | **505.2405** | 253.1239 | **504.2565** | 252.6319 | 4 |
| 29 | 3133.5034 | **1567.2553** | 3116.4769 | 1558.7421 | 3115.4929 | 1558.2501 | A | **393.2245** | 197.1159 | **376.1979** | 188.6026 | | | 3 |
| 30 | 3280.5718 | **1640.7896** | 3263.5453 | 1632.2763 | 3262.5613 | 1631.7843 | F | 322.1874 | 161.5973 | 305.1608 | 153.0840 | | | 2 |
| 31 | | | | | | | R | 175.1190 | 88.0631 | 158.0924 | 79.5498 | | | 1 |

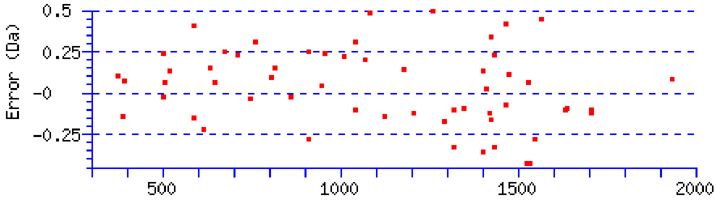


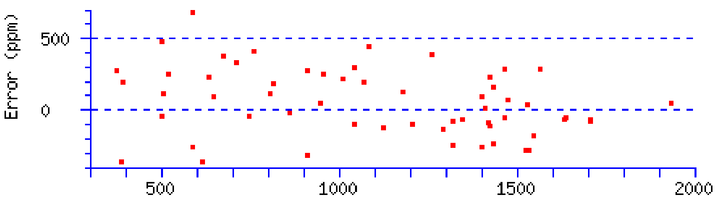


**All matches to this query**

| Score | Mr(calc): | Delta | Sequence |
|---|---|---|---|
| 69.8 | 3453.6762 | 0.0083 | LLYISPENFQIDNSPNSTAGPSCSLLLEAFR |
| 57.6 | 3453.6762 | 0.0083 | LLYISPENFQIDNSPNSTAGPSCSLLLEAFR |
| 44.3 | 3453.6762 | 0.0083 | LLYISPENFQIDNSPNSTAGPSCSLLLEAFR |
| 37.2 | 3452.6922 | 0.9923 | LLYISPENFQIDNSPNSTAGPSCSLLLEAFR |
| 7.2 | 3453.7084 | -0.0239 | LDGHNTFSYISIFTPLWLSLLTLMATTFR |
| 2.8 | 3452.7051 | 0.9794 | DRIIEHLTFANCLSIISNGIPQTLSDLGFK |
| 2.5 | 3453.7133 | -0.0288 | FFFEPVPEFPLNPNFTIVDNKDNTAGIITR |
| 2.5 | 3453.7133 | -0.0288 | FFFEPVPEFPLNPNFTIVDNKDNTAGIITR |
| 2.5 | 3453.7133 | -0.0288 | FFFEPVPEFPLNPNFTIVDNKDNTAGIITR |
| 2.5 | 3453.7133 | -0.0288 | FFFEPVPEFPLNPNFTIVDNKDNTAGIITR |

---

Spectrum No: 366; Query: 1042; Rank: 1

---

## Peptide View

MS/MS Fragmentation of **NNSNDIVNAIMELTM**
Found in **IPI00371266**, Tax_Id=10116 Gene_Symbol=Naca_predicted nascent-polypeptide-associated complex alpha polypeptide

Match to Query 1042: 1694.754408 from(848.384480,2+)
Title: 100101RatKid_NS_deglyco_19.6755.6755.2.dta
Data file K:\NewmanPaper\Piliang\3SubProteomes\Piliang3SP\mgf5ppm\ERLIC_3SubProteomes5ppm.mgf

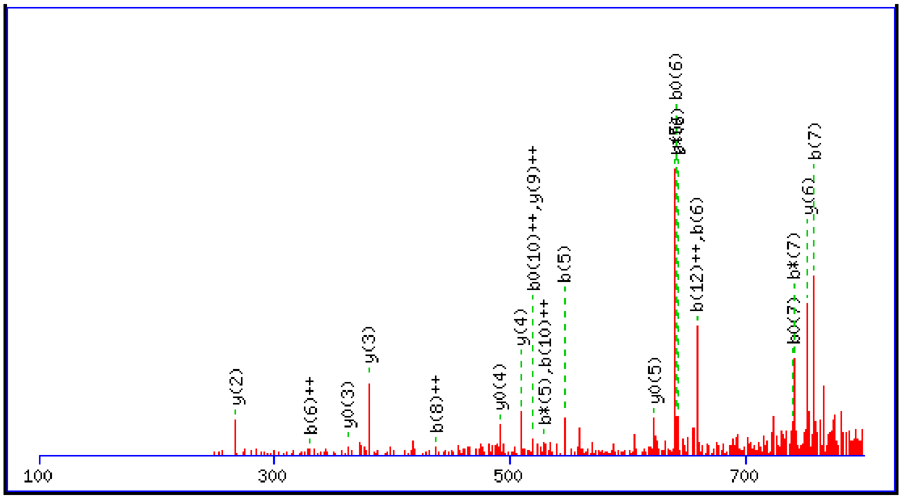


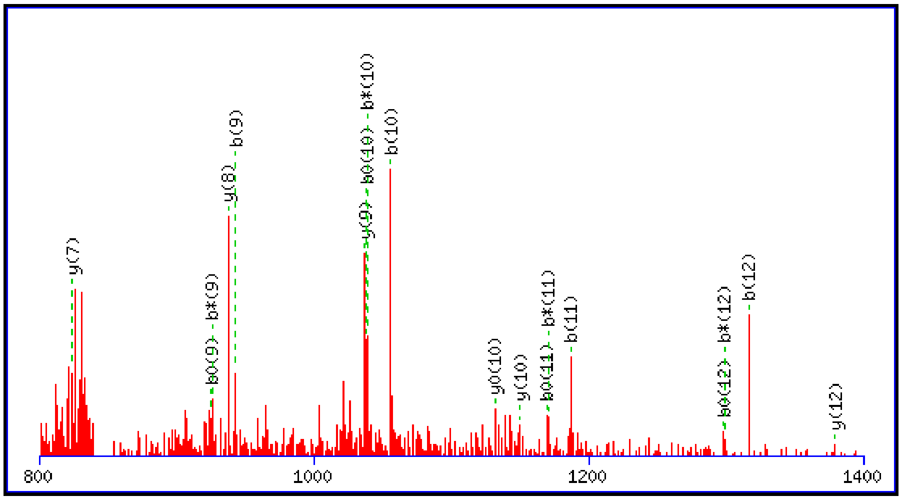


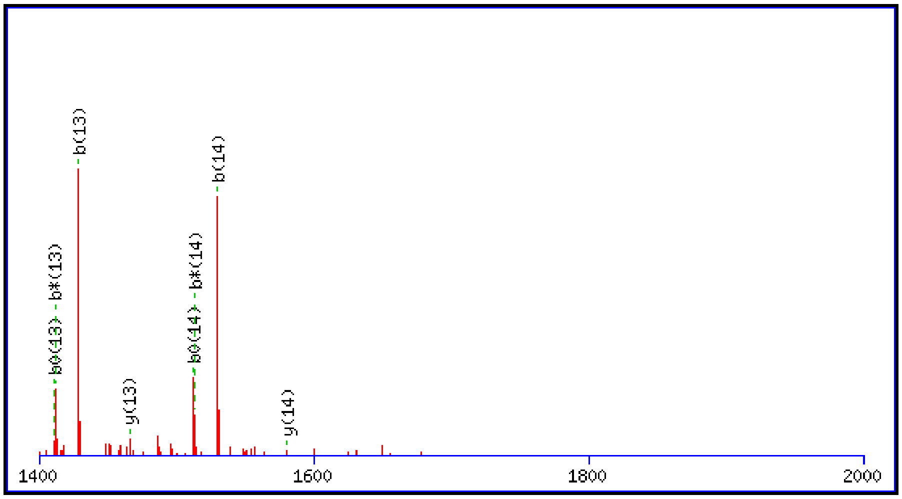


**Monoisotopic mass of neutral peptide Mr(calc):** 1694.7491
**Fixed modifications:** Carbamidomethyl (C)
**Variable modifications:**
**N1** : Deamidated_N (N)
**M15** : Oxidation (M)
**Ions Score:** 70 **Expect:** 2.1e-005
**Matches (Bold Red):** 48/148 fragment ions using 109 most intense peaks

| # | b | $b^{++}$ | b* | $b^{*++}$ | $b^{0}$ | $b^{0++}$ | Seq. | y | $y^{++}$ | y* | $y^{*++}$ | $y^{0}$ | $y^{0++}$ | # |
|---|---|---|---|---|---|---|---|---|---|---|---|---|---|---|
| 1 | 116.0342 | 58.5207 | 99.0077 | 50.0075 | | | N | | | | | | | 15 |
| 2 | 230.0771 | 115.5422 | 213.0506 | 107.0289 | | | N | **1580.7295** | 790.8684 | 1563.7029 | 782.3551 | 1562.7189 | 781.8631 | 14 |
| 3 | 317.1092 | 159.0582 | 300.0826 | 150.5449 | 299.0986 | 150.0529 | S | **1466.6865** | 733.8469 | 1449.6600 | 725.3336 | 1448.6760 | 724.8416 | 13 |
| 4 | 431.1521 | 216.0797 | 414.1255 | 207.5664 | 413.1415 | 207.0744 | N | **1379.6545** | 690.3309 | 1362.6280 | 681.8176 | 1361.6440 | 681.3256 | 12 |
| | | | | | | | | | | | | | | |

| | | | | | | | | | | | | | | |
|---|---|---|---|---|---|---|---|---|---|---|---|---|---|---|
| 5 | **546.1790** | 273.5932 | **529.1525** | 265.0799 | 528.1685 | 264.5879 | D | 1265.6116 | 633.3094 | 1248.5850 | 624.7962 | 1247.6010 | 624.3042 | 11 |
| 6 | **659.2631** | **330.1352** | **642.2366** | 321.6219 | **641.2525** | 321.1299 | I | **1150.5846** | 575.7960 | 1133.5581 | 567.2827 | **1132.5741** | 566.7907 | 10 |
| 7 | **758.3315** | 379.6694 | **741.3050** | 371.1561 | **740.3210** | 370.6641 | V | **1037.5006** | **519.2539** | 1020.4740 | 510.7407 | 1019.4900 | 510.2486 | 9 |
| 8 | 872.3744 | **436.6909** | 855.3479 | 428.1776 | 854.3639 | 427.6856 | N | **938.4322** | 469.7197 | 921.4056 | 461.2064 | 920.4216 | 460.7144 | 8 |
| 9 | **943.4116** | 472.2094 | **926.3850** | 463.6961 | **925.4010** | 463.2041 | A | **824.3892** | 412.6983 | | | 806.3787 | 403.6930 | 7 |
| 10 | **1056.4956** | **528.7514** | **1039.4691** | 520.2382 | **1038.4851** | **519.7462** | I | **753.3521** | 377.1797 | | | 735.3416 | 368.1744 | 6 |
| 11 | **1187.5361** | 594.2717 | **1170.5096** | 585.7584 | **1169.5255** | 585.2664 | M | **640.2681** | 320.6377 | | | **622.2575** | 311.6324 | 5 |
| 12 | **1316.5787** | **658.7930** | **1299.5521** | 650.2797 | **1298.5681** | 649.7877 | E | **509.2276** | 255.1174 | | | **491.2170** | 246.1121 | 4 |
| 13 | **1429.6628** | 715.3350 | **1412.6362** | 706.8217 | **1411.6522** | 706.3297 | L | **380.1850** | 190.5961 | | | **362.1744** | 181.5908 | 3 |
| 14 | **1530.7104** | 765.8589 | **1513.6839** | 757.3456 | **1512.6999** | 756.8536 | T | **267.1009** | 134.0541 | | | 249.0904 | 125.0488 | 2 |
| 15 | | | | | | | M | 166.0532 | 83.5303 | | | | | 1 |

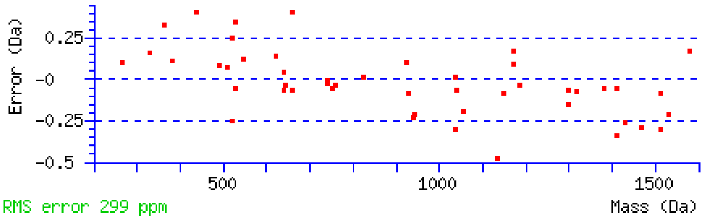


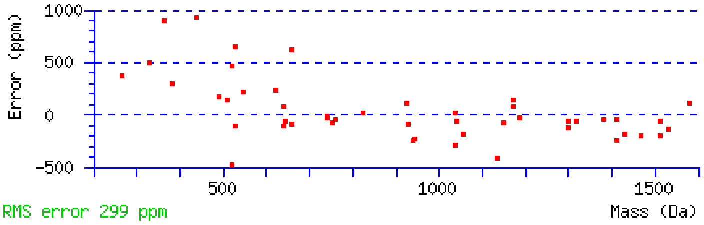


**All matches to this query**

| Score | Mr(calc): | Delta | Sequence |
|---|---|---|---|
| 69.7 | 1694.7491 | 0.0053 | NNSNDIVNAIMELTM |
| 66.7 | 1694.7491 | 0.0053 | NNSNDIVNAIMELTM |
| 66.7 | 1694.7491 | 0.0053 | NNSNDIVNAIMELTM |
| 31.1 | 1693.7651 | 0.9893 | NNSNDIVNAIMELTM |
| 28.6 | 1694.7491 | 0.0053 | NNSNDIVNAIMELTM |
| 24.4 | 1694.7491 | 0.0053 | NNSNDIVNAIMELTM |
| 21.6 | 1694.7491 | 0.0053 | NNSNDIVNAIMELTM |
| 13.8 | 1694.7491 | 0.0053 | NNSNDIVNAIMELTM |
| 9.8 | 1694.7487 | 0.0057 | TCGNLLNPWTPSQK |
| 8.1 | 1693.7651 | 0.9893 | NNSNDIVNAIMELTM |

---

Spectrum No: 367; Query: 1257; Rank: 1

---

## Peptide View

MS/MS Fragmentation of **WNDSPCNQSLPSICK**
Found in **IPI00192504**, Tax_Id=10116 Gene_Symbol=Mrc2 Macrophage mannose receptor 2 precursor

Match to Query 1257: 1805.772588 from(903.893570,2+)
Title: 100101RatKid_NS_deglyco_23.2514.2514.2.dta
Data file K:\NewmanPaper\Piliang\3SubProteomes\Piliang3SP\mgf5ppm\ERLIC_3SubProteomes5ppm.mgf

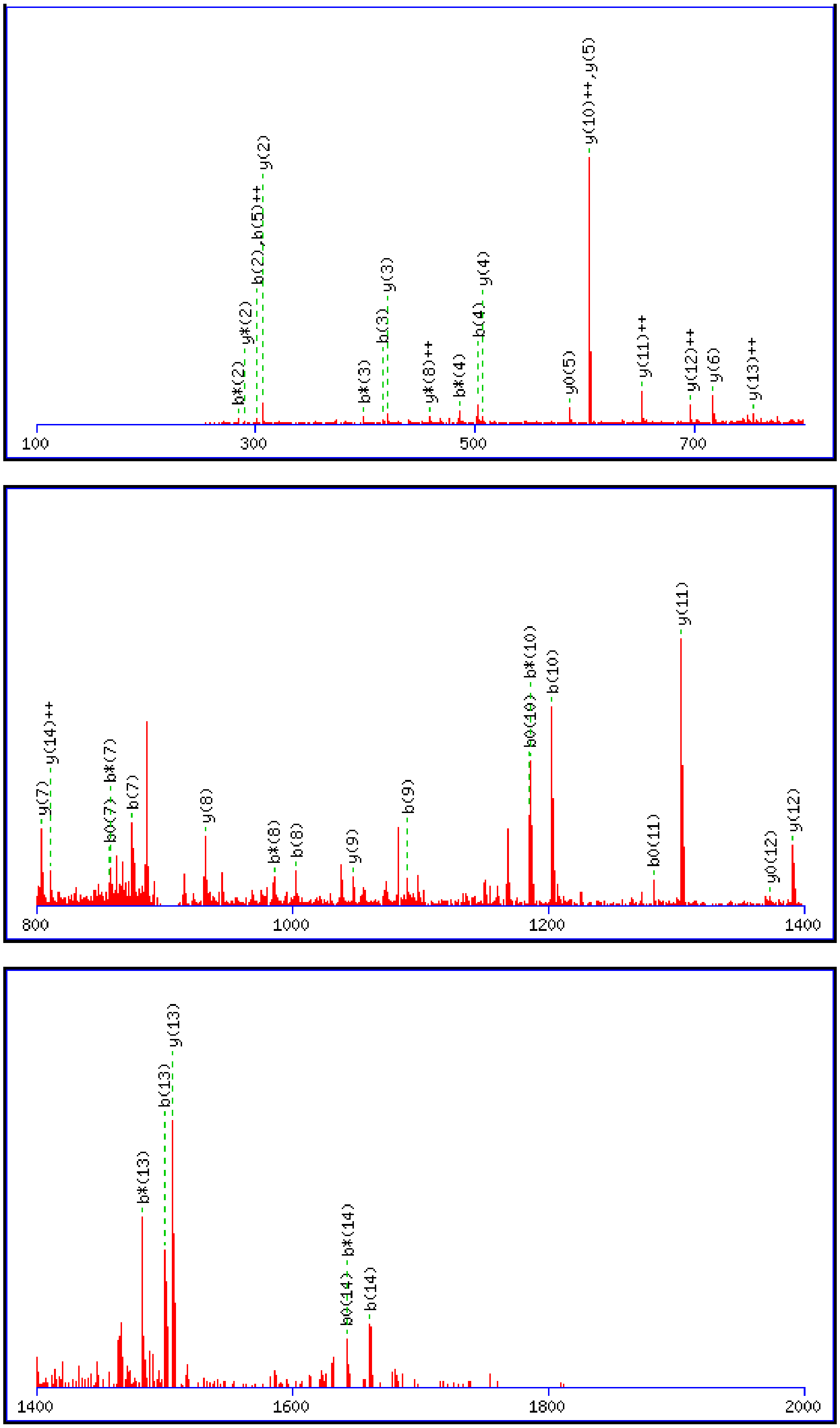


**Monoisotopic mass of neutral peptide Mr(calc):** 1805.7713
**Fixed modifications:** Carbamidomethyl (C)
**Variable modifications:**
**N7** : Deamidated_N (N)
**Ions Score:** 70 **Expect:** 2.2e-005
**Matches (Bold Red):** 42/156 fragment ions using 75 most intense peaks

| # | b | $b^{++}$ | b* | $b^{*++}$ | $b^0$ | $b^{0++}$ | Seq. | y | $y^{++}$ | y* | $y^{*++}$ | $y^0$ | $y^{0++}$ | # |
|---|---|---|---|---|---|---|---|---|---|---|---|---|---|---|
| 1 | 187.0866 | 94.0469 | | | | | W | | | | | | | 15 |
| 2 | **301.1295** | 151.0684 | **284.1030** | 142.5551 | | | N | 1620.6992 | **810.8533** | 1603.6727 | 802.3400 | 1602.6887 | 801.8480 | 14 |
| 3 | **416.1565** | 208.5819 | **399.1299** | 200.0686 | 398.1459 | 199.5766 | D | **1506.6563** | **753.8318** | 1489.6298 | 745.3185 | 1488.6457 | 744.8265 | 13 |
| 4 | **503.1885** | 252.0979 | **486.1619** | 243.5846 | 485.1779 | 243.0926 | S | **1391.6294** | **696.3183** | 1374.6028 | 687.8050 | **1373.6188** | 687.3130 | 12 |
| 5 | 600.2413 | **300.6243** | 583.2147 | 292.1110 | 582.2307 | 291.6190 | P | **1304.5973** | **652.8023** | 1287.5708 | 644.2890 | 1286.5868 | 643.7970 | 11 |

| | | | | | | | | | | | | | | |
|---|---|---|---|---|---|---|---|---|---|---|---|---|---|---|
| **6** | 760.2719 | 380.6396 | 743.2454 | 372.1263 | 742.2613 | 371.6343 | **C** | 1207.5446 | **604.2759** | 1190.5180 | 595.7626 | 1189.5340 | 595.2706 | **10** |
| **7** | **875.2988** | 438.1531 | **858.2723** | 429.6398 | **857.2883** | 429.1478 | **N** | **1047.5139** | 524.2606 | 1030.4874 | 515.7473 | 1029.5034 | 515.2553 | **9** |
| **8** | **1003.3574** | 502.1823 | **986.3309** | 493.6691 | 985.3468 | 493.1771 | **Q** | **932.4870** | 466.7471 | 915.4604 | **458.2339** | 914.4764 | 457.7418 | **8** |
| **9** | **1090.3894** | 545.6984 | 1073.3629 | 537.1851 | 1072.3789 | 536.6931 | **S** | **804.4284** | 402.7178 | 787.4019 | 394.2046 | 786.4178 | 393.7126 | **7** |
| **10** | **1203.4735** | 602.2404 | **1186.4470** | 593.7271 | **1185.4629** | 593.2351 | **L** | **717.3964** | 359.2018 | 700.3698 | 350.6886 | 699.3858 | 350.1965 | **6** |
| **11** | 1300.5263 | 650.7668 | 1283.4997 | 642.2535 | **1282.5157** | 641.7615 | **P** | **604.3123** | 302.6598 | 587.2858 | 294.1465 | **586.3017** | 293.6545 | **5** |
| **12** | 1387.5583 | 694.2828 | 1370.5317 | 685.7695 | 1369.5477 | 685.2775 | **S** | **507.2595** | 254.1334 | 490.2330 | 245.6201 | 489.2490 | 245.1281 | **4** |
| **13** | **1500.6424** | 750.8248 | **1483.6158** | 742.3115 | 1482.6318 | 741.8195 | **I** | **420.2275** | 210.6174 | 403.2010 | 202.1041 | | | **3** |
| **14** | **1660.6730** | 830.8401 | **1643.6465** | 822.3269 | **1642.6624** | 821.8349 | **C** | **307.1435** | 154.0754 | **290.1169** | 145.5621 | | | **2** |
| **15** | | | | | | | **K** | 147.1128 | 74.0600 | 130.0863 | 65.5468 | | | **1** |

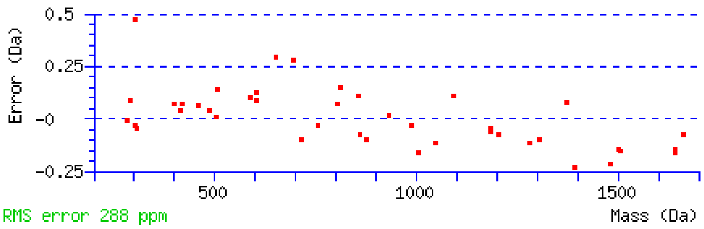


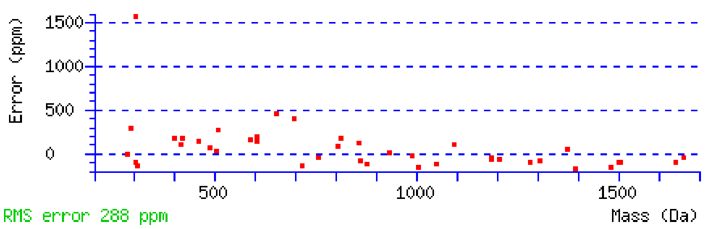


### All matches to this query

| Score | Mr(calc): | Delta | Sequence |
|---|---|---|---|
| 69.6 | 1805.7713 | 0.0013 | WNDSPCNQSLPSICK |
| 23.0 | 1805.7713 | 0.0013 | WNDSPCNQSLPSICK |
| 15.9 | 1804.7872 | 0.9853 | WNDSPCNQSLPSICK |
| 11.0 | 1805.7737 | -0.0011 | LESEGSPETLTNLKK |
| 11.0 | 1805.7737 | -0.0011 | LESEGSPETLTNLKK |
| 11.0 | 1805.7737 | -0.0011 | LESEGSPETLTNLKK |
| 7.9 | 1805.7824 | -0.0098 | SLTSKFMTTFSQLR |
| 6.8 | 1805.7672 | 0.0054 | SMEETRPVPTVKEK |
| 6.8 | 1805.7824 | -0.0098 | SLTSKFMTTFSQLR |
| 6.2 | 1805.7824 | -0.0098 | SLTSKFMTTFSQLR |

---

Spectrum No: 368; Query: 997; Rank: 1

---

## Peptide View

MS/MS Fragmentation of **ANEQVIQNLNQTYK**
Found in **IPI00205333**, Tax_Id=10116 Gene_Symbol=Gpr116 Probable G-protein coupled receptor 116 precursor

Match to Query 997: 1662.824828 from(832.419690,2+)
Title: 100101RatKid_NS_deglyco_25.2260.2260.2.dta
Data file K:\NewmanPaper\Piliang\3SubProteomes\Piliang3SP\mgf5ppm\ERLIC_3SubProteomes5ppm.mgf

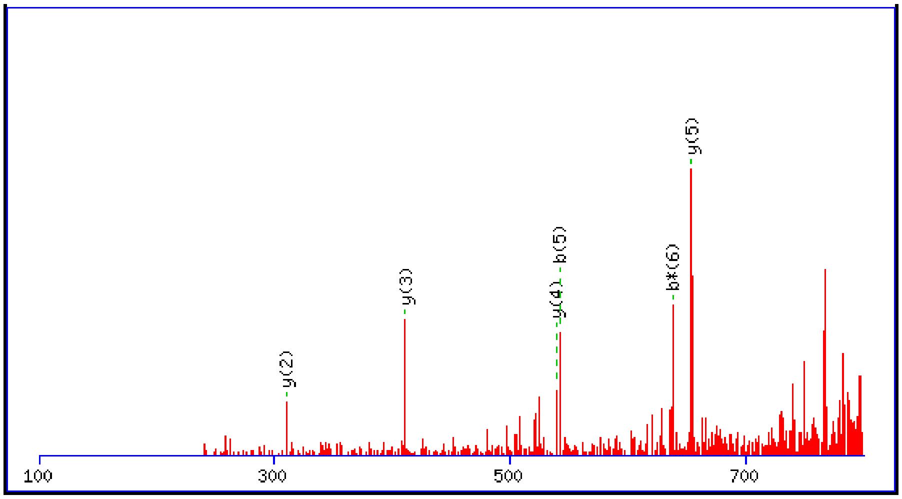


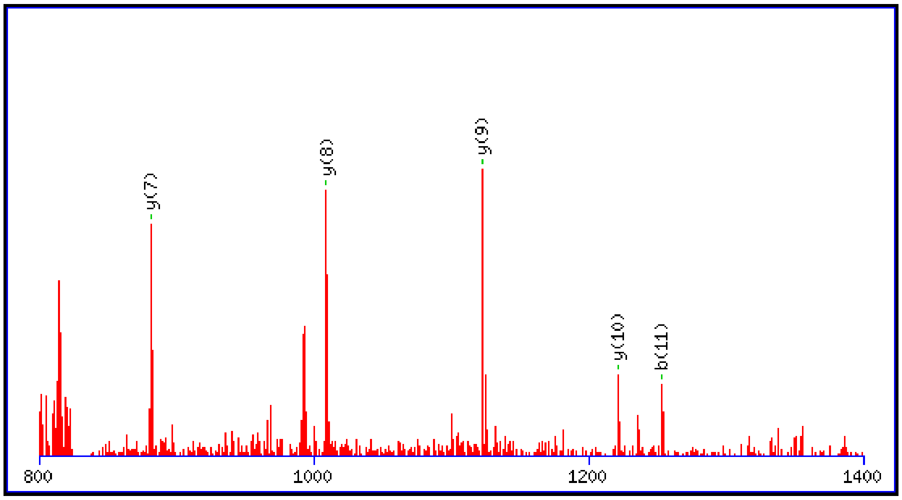


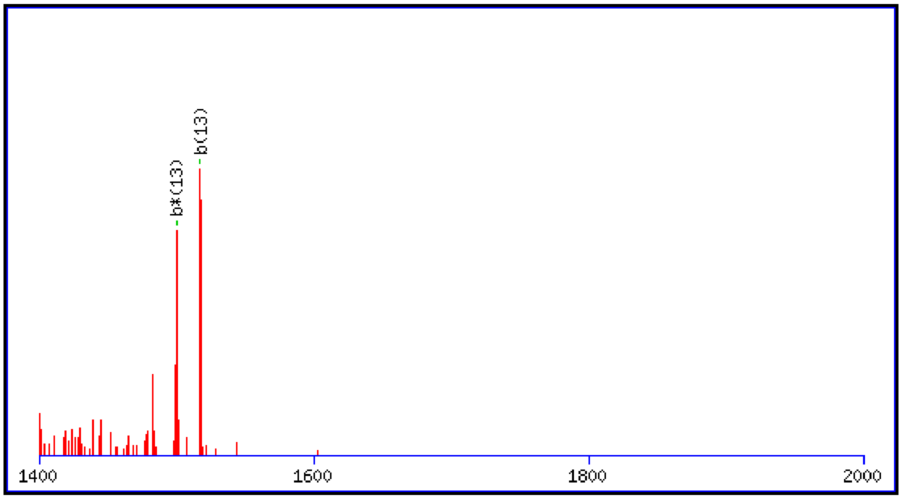


**Monoisotopic mass of neutral peptide Mr(calc):** 1662.8213
**Fixed modifications:** Carbamidomethyl (C)
**Variable modifications:**
**N10** : Deamidated_N (N)
**Ions Score:** 70 **Expect:** 1.9e-005
**Matches (Bold Red):** 13/146 fragment ions using 19 most intense peaks

| # | b | $b^{++}$ | b* | $b^{*++}$ | $b^{0}$ | $b^{0++}$ | Seq. | y | $y^{++}$ | y* | $y^{*++}$ | $y^{0}$ | $y^{0++}$ | # |
|---|---|---|---|---|---|---|---|---|---|---|---|---|---|---|
| 1 | 72.0444 | 36.5258 | | | | | A | | | | | | | 14 |
| 2 | 186.0873 | 93.5473 | 169.0608 | 85.0340 | | | N | 1592.7915 | 796.8994 | 1575.7649 | 788.3861 | 1574.7809 | 787.8941 | 13 |
| 3 | 315.1299 | 158.0686 | 298.1034 | 149.5553 | 297.1193 | 149.0633 | E | 1478.7485 | 739.8779 | 1461.7220 | 731.3646 | 1460.7380 | 730.8726 | 12 |
| 4 | 443.1885 | 222.0979 | 426.1619 | 213.5846 | 425.1779 | 213.0926 | Q | 1349.7060 | 675.3566 | 1332.6794 | 666.8433 | 1331.6954 | 666.3513 | 11 |
| 5 | **542.2569** | 271.6321 | 525.2304 | 263.1188 | 524.2463 | 262.6268 | V | **1221.6474** | 611.3273 | 1204.6208 | 602.8141 | 1203.6368 | 602.3220 | 10 |

| | | | | | | | | | | | | | | |
|---|---|---|---|---|---|---|---|---|---|---|---|---|---|---|
| **6** | 655.3410 | 328.1741 | **638.3144** | 319.6608 | 637.3304 | 319.1688 | **I** | **1122.5790** | 561.7931 | 1105.5524 | 553.2798 | 1104.5684 | 552.7878 | **9** |
| **7** | 783.3995 | 392.2034 | 766.3730 | 383.6901 | 765.3890 | 383.1981 | **Q** | **1009.4949** | 505.2511 | 992.4683 | 496.7378 | 991.4843 | 496.2458 | **8** |
| **8** | 897.4425 | 449.2249 | 880.4159 | 440.7116 | 879.4319 | 440.2196 | **N** | **881.4363** | 441.2218 | 864.4098 | 432.7085 | 863.4258 | 432.2165 | **7** |
| **9** | 1010.5265 | 505.7669 | 993.5000 | 497.2536 | 992.5160 | 496.7616 | **L** | 767.3934 | 384.2003 | 750.3668 | 375.6871 | 749.3828 | 375.1951 | **6** |
| **10** | 1125.5535 | 563.2804 | 1108.5269 | 554.7671 | 1107.5429 | 554.2751 | **N** | **654.3093** | 327.6583 | 637.2828 | 319.1450 | 636.2988 | 318.6530 | **5** |
| **11** | **1253.6120** | 627.3097 | 1236.5855 | 618.7964 | 1235.6015 | 618.3044 | **Q** | **539.2824** | 270.1448 | 522.2558 | 261.6316 | 521.2718 | 261.1396 | **4** |
| **12** | 1354.6597 | 677.8335 | 1337.6332 | 669.3202 | 1336.6492 | 668.8282 | **T** | **411.2238** | 206.1155 | 394.1973 | 197.6023 | 393.2132 | 197.1103 | **3** |
| **13** | **1517.7231** | 759.3652 | **1500.6965** | 750.8519 | 1499.7125 | 750.3599 | **Y** | **310.1761** | 155.5917 | 293.1496 | 147.0784 | | | **2** |
| **14** | | | | | | | **K** | 147.1128 | 74.0600 | 130.0863 | 65.5468 | | | **1** |

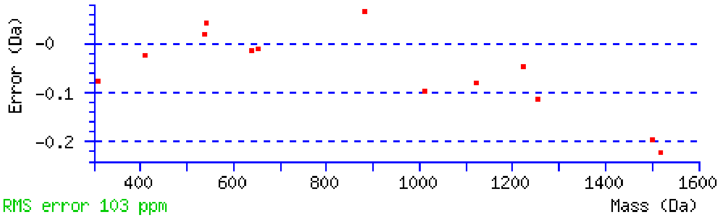


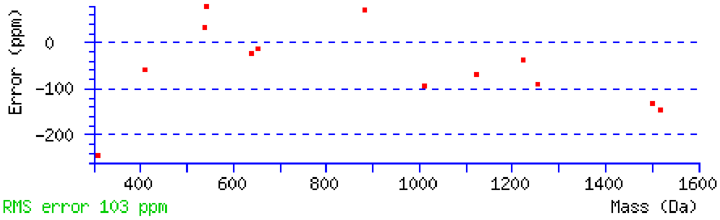


### All matches to this query

| Score | Mr(calc): | Delta | Sequence |
|---|---|---|---|
| 69.6 | 1662.8213 | 0.0036 | ANEQVIQNLNQTYK |
| 53.3 | 1662.8213 | 0.0036 | ANEQVIQNLNQTYK |
| 19.9 | 1661.8373 | 0.9876 | ANEQVIQNLNQTYK |
| 11.6 | 1662.8213 | 0.0036 | ANEQVIQNLNQTYK |
| 8.3 | 1662.8270 | -0.0022 | SPFVVNVAPPLDLSK |
| 6.9 | 1660.8225 | 2.0023 | ALKKVLEDYEAFR |
| 6.2 | 1662.8382 | -0.0134 | HFVTGIIDSLAVSPK |
| 5.5 | 1662.8259 | -0.0011 | VYISELKNIRLR |
| 5.3 | 1662.8101 | 0.0148 | LLVNVDEEDYELGR |
| 3.4 | 1662.8342 | -0.0093 | KTEPAAGKETGAAKPK |

---

Spectrum No: 369; Query: 665; Rank: 1

---

## Peptide View

MS/MS Fragmentation of **ILYGLENATQELK**
Found in **IPI00361301**, Tax_Id=10116 Gene_Symbol=Lama2_predicted similar to Laminin alpha-2 chain precursor

Match to Query 665: 1491.782848 from(746.898700,2+)
Title: 100101RatKid_NS_deglyco_21.3449.3449.2.dta
Data file K:\NewmanPaper\Piliang\3SubProteomes\Piliang3SP\mgf5ppm\ERLIC_3SubProteomes5ppm.mgf

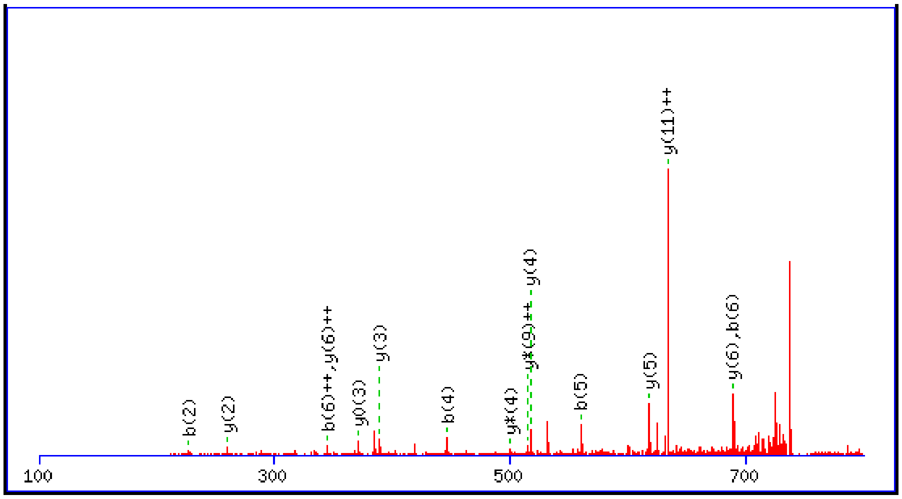


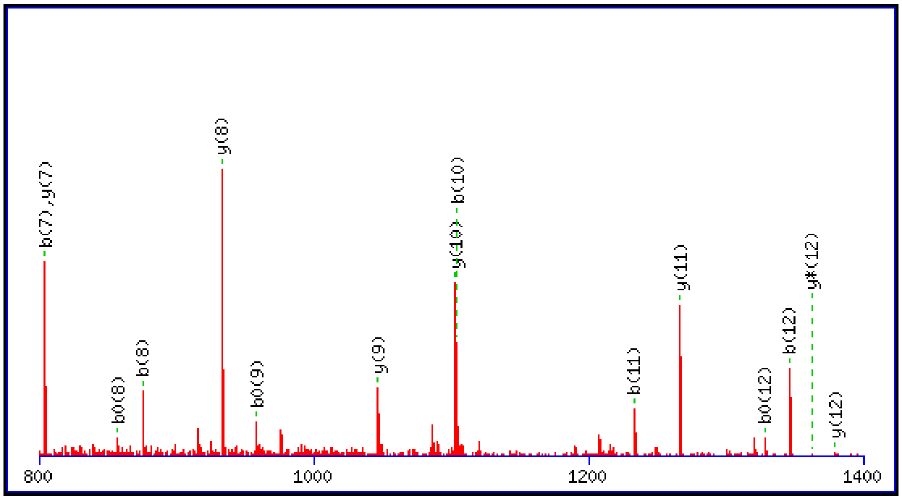


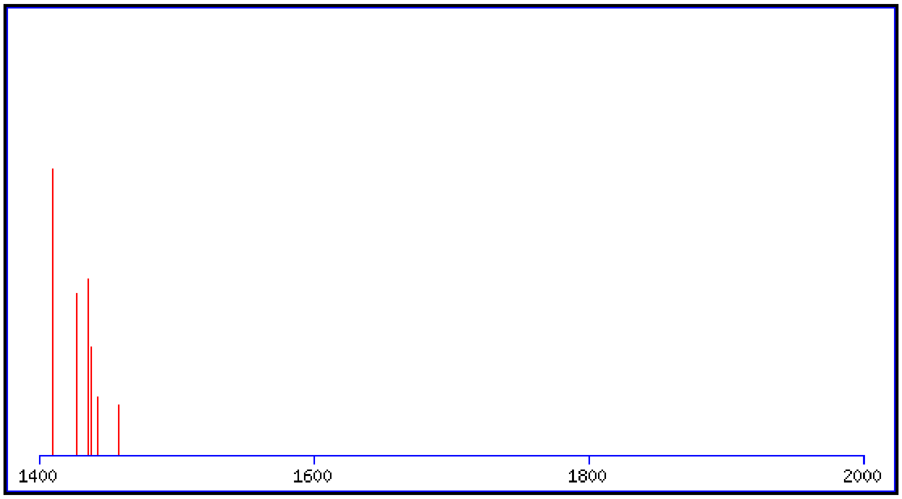


**Monoisotopic mass of neutral peptide Mr(calc):** 1491.7820
**Fixed modifications:** Carbamidomethyl (C)
**Variable modifications:**
**N7** : Deamidated_N (N)
**Ions Score:** 70 **Expect:** 1.3e-005
**Matches (Bold Red):** 30/118 fragment ions using 48 most intense peaks

| # | b | b$^{++}$ | b* | b*$^{++}$ | b$^{0}$ | b$^{0++}$ | Seq. | y | y$^{++}$ | y* | y*$^{++}$ | y$^{0}$ | y$^{0++}$ | # |
|---|---|---|---|---|---|---|---|---|---|---|---|---|---|---|
| 1 | 114.0913 | 57.5493 | | | | | I | | | | | | | 13 |
| 2 | **227.1754** | 114.0913 | | | | | L | **1379.7053** | 690.3563 | **1362.6787** | 681.8430 | 1361.6947 | 681.3510 | 12 |
| 3 | 390.2387 | 195.6230 | | | | | Y | **1266.6212** | **633.8142** | 1249.5947 | 625.3010 | 1248.6107 | 624.8090 | 11 |
| 4 | **447.2602** | 224.1337 | | | | | G | **1103.5579** | 552.2826 | 1086.5313 | 543.7693 | 1085.5473 | 543.2773 | 10 |
| 5 | **560.3443** | 280.6758 | | | | | L | **1046.5364** | 523.7719 | 1029.5099 | **515.2586** | 1028.5259 | 514.7666 | 9 |

| | | | | | | | | | | | | | | |
|---|---|---|---|---|---|---|---|---|---|---|---|---|---|---|
| **6** | **689.3869** | **345.1971** | | | 671.3763 | 336.1918 | **E** | **933.4524** | 467.2298 | 916.4258 | 458.7165 | 915.4418 | 458.2245 | **8** |
| **7** | **804.4138** | 402.7105 | 787.3872 | 394.1973 | 786.4032 | 393.7053 | **N** | **804.4098** | 402.7085 | 787.3832 | 394.1952 | 786.3992 | 393.7032 | **7** |
| **8** | **875.4509** | 438.2291 | 858.4244 | 429.7158 | **857.4403** | 429.2238 | **A** | **689.3828** | **345.1951** | 672.3563 | 336.6818 | 671.3723 | 336.1898 | **6** |
| **9** | 976.4986 | 488.7529 | 959.4720 | 480.2397 | **958.4880** | 479.7476 | **T** | **618.3457** | 309.6765 | 601.3192 | 301.1632 | 600.3352 | 300.6712 | **5** |
| **10** | **1104.5572** | 552.7822 | 1087.5306 | 544.2689 | 1086.5466 | 543.7769 | **Q** | **517.2980** | 259.1527 | **500.2715** | 250.6394 | 499.2875 | 250.1474 | **4** |
| **11** | **1233.5998** | 617.3035 | 1216.5732 | 608.7902 | 1215.5892 | 608.2982 | **E** | **389.2395** | 195.1234 | 372.2129 | 186.6101 | **371.2289** | 186.1181 | **3** |
| **12** | **1346.6838** | 673.8455 | 1329.6573 | 665.3323 | **1328.6733** | 664.8403 | **L** | **260.1969** | 130.6021 | 243.1703 | 122.0888 | | | **2** |
| **13** | | | | | | | **K** | 147.1128 | 74.0600 | 130.0863 | 65.5468 | | | **1** |

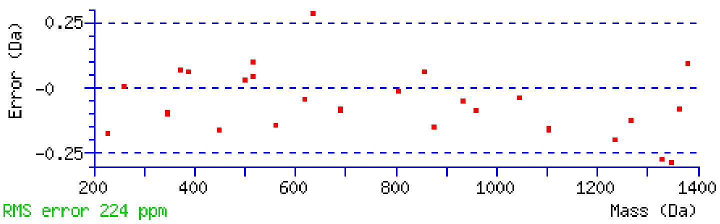


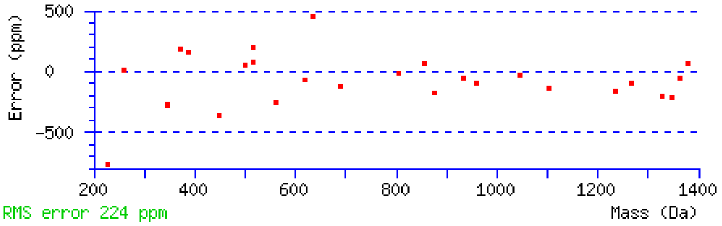


### All matches to this query

| Score | Mr(calc): | Delta | Sequence |
|---|---|---|---|
| 69.5 | 1491.7820 | 0.0008 | ILYGLENATQELK |
| 8.2 | 1491.7722 | 0.0107 | DNSWGKVFGILEK |
| 7.5 | 1491.7821 | 0.0008 | GDNVLINTFSGLLK |
| 5.6 | 1491.7698 | 0.0131 | LLPGLETESKLGR |
| 5.2 | 1491.7868 | -0.0039 | ATSDWKGLRMTVK |
| 4.6 | 1491.7755 | 0.0073 | QCNNVLVYELLK |
| 4.2 | 1491.7933 | -0.0105 | LFKGSQGEDGTLIK |
| 4.1 | 1491.7810 | 0.0018 | LLTRSSTLSGHIK |
| 4.1 | 1491.7810 | 0.0018 | LLTRSSTLSGHIK |
| 4.1 | 1491.7810 | 0.0018 | LLTRSSTLSGHIK |

---

Spectrum No: 370; Query: 2565; Rank: 1

---

## Peptide View

MS/MS Fragmentation of **MGAGEALGNASQLFEAWLKGNESIPVNLR**
Found in **IPI00327398**, Tax_Id=10116 Gene_Symbol=Enpep Isoform 1 of Glutamyl aminopeptidase

Match to Query 2565: 3090.522132 from(1031.181320,3+)
Title: 100101RatKid_NS_deglyco_19.6450.6450.3.dta
Data file K:\NewmanPaper\Piliang\3SubProteomes\Piliang3SP\mgf5ppm\ERLIC_3SubProteomes5ppm.mgf

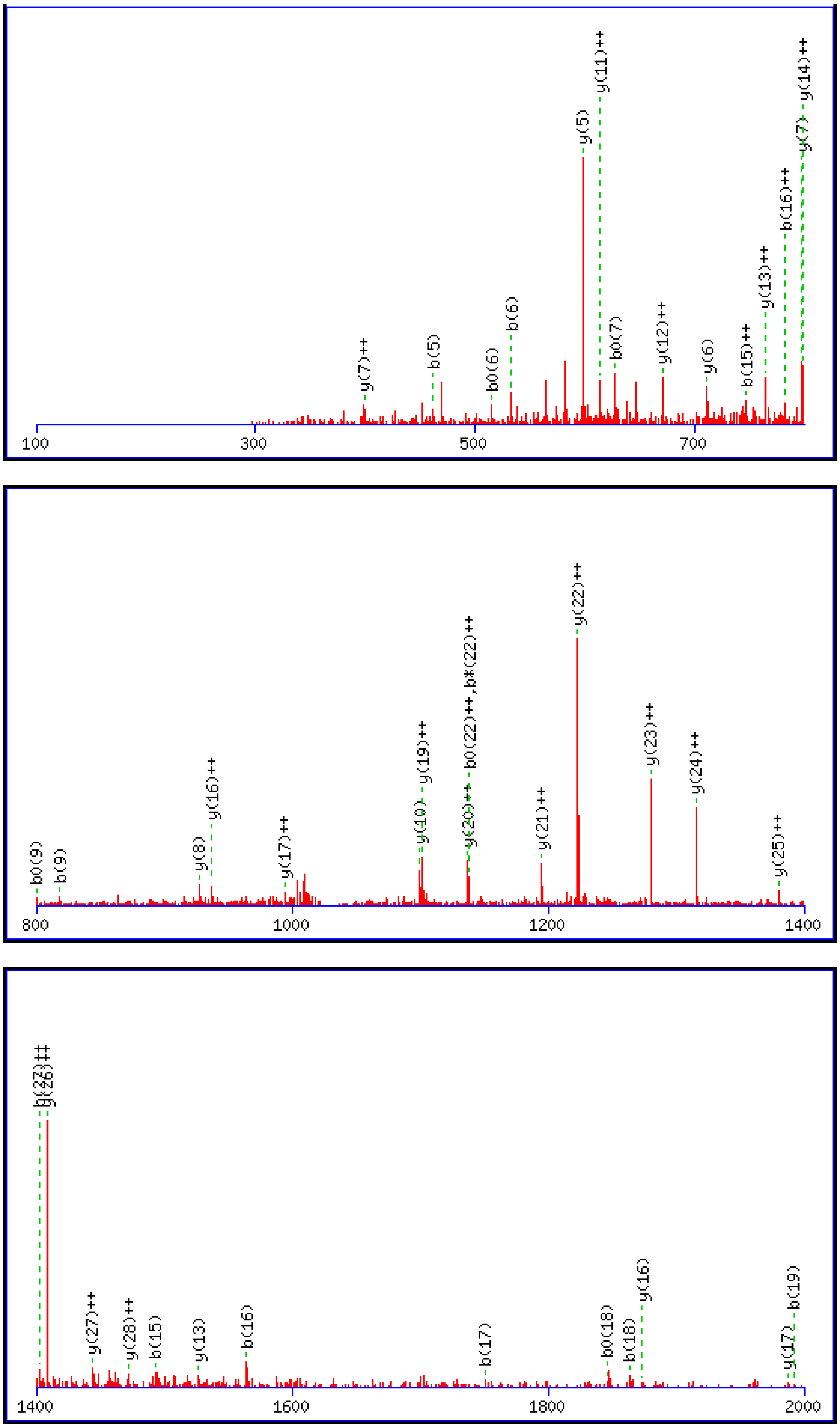

**Monoisotopic mass of neutral peptide Mr(calc):** 3089.5127
**Fixed modifications:** Carbamidomethyl (C)
**Variable modifications:**
**M1** : Oxidation (M)
**N9** : Deamidated_N (N)
**N21** : Deamidated_N (N)
**Ions Score:** 70 **Expect:** 2.9e-005
**Matches (Bold Red):** 42/300 fragment ions using 100 most intense peaks

| # | b | $b^{++}$ | b* | $b^{*++}$ | $b^0$ | $b^{0++}$ | Seq. | y | $y^{++}$ | y* | $y^{*++}$ | $y^0$ | $y^{0++}$ | # |
|---|---|---|---|---|---|---|---|---|---|---|---|---|---|---|
| 1 | 148.0427 | 74.5250 | | | | | M | | | | | | | 29 |
| 2 | 205.0641 | 103.0357 | | | | | G | 2943.4847 | **1472.2460** | 2926.4581 | 1463.7327 | 2925.4741 | 1463.2407 | 28 |
| 3 | 276.1013 | 138.5543 | | | | | A | 2886.4632 | **1443.7352** | 2869.4367 | 1435.2220 | 2868.4526 | 1434.7300 | 27 |
| 4 | 333.1227 | 167.0650 | | | | | G | 2815.4261 | **1408.2167** | 2798.3995 | 1399.7034 | 2797.4155 | 1399.2114 | 26 |

| | | | | | | | | | | | | | | |
|---|---|---|---|---|---|---|---|---|---|---|---|---|---|---|
| 5 | 462.1653 | 231.5863 | | | 444.1547 | 222.5810 | E | 2758.4046 | 1379.7059 | 2741.3781 | 1371.1927 | 2740.3941 | 1370.7007 | 25 |
| 6 | 533.2024 | 267.1049 | | | 515.1919 | 258.0996 | A | 2629.3620 | 1315.1847 | 2612.3355 | 1306.6714 | 2611.3515 | 1306.1794 | 24 |
| 7 | 646.2865 | 323.6469 | | | 628.2759 | 314.6416 | L | 2558.3249 | 1279.6661 | 2541.2984 | 1271.1528 | 2540.3143 | 1270.6608 | 23 |
| 8 | 703.3080 | 352.1576 | | | 685.2974 | 343.1523 | G | 2445.2409 | 1223.1241 | 2428.2143 | 1214.6108 | 2427.2303 | 1214.1188 | 22 |
| 9 | 818.3349 | 409.6711 | 801.3083 | 401.1578 | 800.3243 | 400.6658 | N | 2388.2194 | 1194.6133 | 2371.1928 | 1186.1001 | 2370.2088 | 1185.6080 | 21 |
| 10 | 889.3720 | 445.1896 | 872.3455 | 436.6764 | 871.3614 | 436.1844 | A | 2273.1925 | 1137.0999 | 2256.1659 | 1128.5866 | 2255.1819 | 1128.0946 | 20 |
| 11 | 976.4040 | 488.7057 | 959.3775 | 480.1924 | 958.3935 | 479.7004 | S | 2202.1553 | 1101.5813 | 2185.1288 | 1093.0680 | 2184.1448 | 1092.5760 | 19 |
| 12 | 1104.4626 | 552.7349 | 1087.4361 | 544.2217 | 1086.4520 | 543.7297 | Q | 2115.1233 | 1058.0653 | 2098.0968 | 1049.5520 | 2097.1127 | 1049.0600 | 18 |
| 13 | 1217.5467 | 609.2770 | 1200.5201 | 600.7637 | 1199.5361 | 600.2717 | L | 1987.0647 | 994.0360 | 1970.0382 | 985.5227 | 1969.0542 | 985.0307 | 17 |
| 14 | 1364.6151 | 682.8112 | 1347.5885 | 674.2979 | 1346.6045 | 673.8059 | F | 1873.9807 | 937.4940 | 1856.9541 | 928.9807 | 1855.9701 | 928.4887 | 16 |
| 15 | 1493.6577 | 747.3325 | 1476.6311 | 738.8192 | 1475.6471 | 738.3272 | E | 1726.9123 | 863.9598 | 1709.8857 | 855.4465 | 1708.9017 | 854.9545 | 15 |
| 16 | 1564.6948 | 782.8510 | 1547.6682 | 774.3378 | 1546.6842 | 773.8458 | A | 1597.8697 | 799.4385 | 1580.8431 | 790.9252 | 1579.8591 | 790.4332 | 14 |
| 17 | 1750.7741 | 875.8907 | 1733.7476 | 867.3774 | 1732.7635 | 866.8854 | W | 1526.8325 | 763.9199 | 1509.8060 | 755.4066 | 1508.8220 | 754.9146 | 13 |
| 18 | 1863.8582 | 932.4327 | 1846.8316 | 923.9195 | 1845.8476 | 923.4274 | L | 1340.7532 | 670.8803 | 1323.7267 | 662.3670 | 1322.7427 | 661.8750 | 12 |
| 19 | 1991.9531 | 996.4802 | 1974.9266 | 987.9669 | 1973.9426 | 987.4749 | K | 1227.6692 | 614.3382 | 1210.6426 | 605.8249 | 1209.6586 | 605.3329 | 11 |
| 20 | 2048.9746 | 1024.9909 | 2031.9481 | 1016.4777 | 2030.9640 | 1015.9857 | G | 1099.5742 | 550.2907 | 1082.5477 | 541.7775 | 1081.5636 | 541.2855 | 10 |
| 21 | 2164.0015 | 1082.5044 | 2146.9750 | 1073.9911 | 2145.9910 | 1073.4991 | N | 1042.5527 | 521.7800 | 1025.5262 | 513.2667 | 1024.5422 | 512.7747 | 9 |
| 22 | 2293.0441 | 1147.0257 | 2276.0176 | 1138.5124 | 2275.0336 | 1138.0204 | E | 927.5258 | 464.2665 | 910.4993 | 455.7533 | 909.5152 | 455.2613 | 8 |
| 23 | 2380.0762 | 1190.5417 | 2363.0496 | 1182.0284 | 2362.0656 | 1181.5364 | S | 798.4832 | 399.7452 | 781.4567 | 391.2320 | 780.4726 | 390.7400 | 7 |
| 24 | 2493.1602 | 1247.0837 | 2476.1337 | 1238.5705 | 2475.1497 | 1238.0785 | I | 711.4512 | 356.2292 | 694.4246 | 347.7160 | | | 6 |
| 25 | 2590.2130 | 1295.6101 | 2573.1864 | 1287.0969 | 2572.2024 | 1286.6048 | P | 598.3671 | 299.6872 | 581.3406 | 291.1739 | | | 5 |
| 26 | 2689.2814 | 1345.1443 | 2672.2549 | 1336.6311 | 2671.2708 | 1336.1391 | V | 501.3144 | 251.1608 | 484.2878 | 242.6475 | | | 4 |
| 27 | 2803.3243 | 1402.1658 | 2786.2978 | 1393.6525 | 2785.3138 | 1393.1605 | N | 402.2459 | 201.6266 | 385.2194 | 193.1133 | | | 3 |
| 28 | 2916.4084 | 1458.7078 | 2899.3818 | 1450.1946 | 2898.3978 | 1449.7026 | L | 288.2030 | 144.6051 | 271.1765 | 136.0919 | | | 2 |
| 29 | | | | | | | R | 175.1190 | 88.0631 | 158.0924 | 79.5498 | | | 1 |

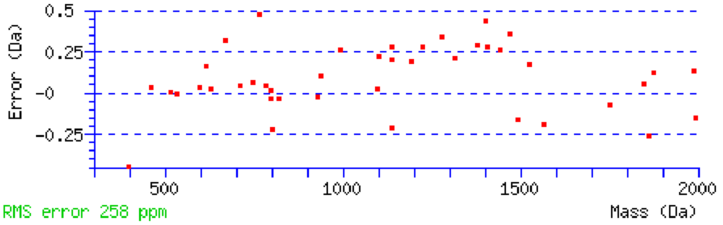


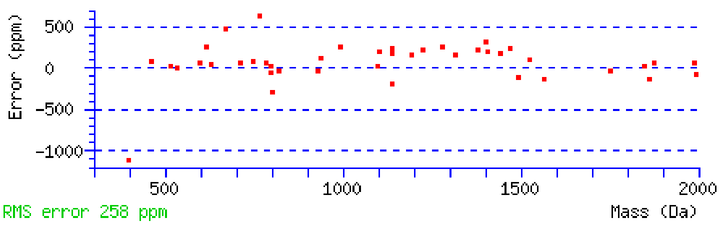


### All matches to this query

| Score | Mr(calc): | Delta | Sequence |
|---|---|---|---|
| 69.5 | 3089.5127 | 1.0094 | MGAGEALGNASQLFEAWLKGNESIPVNLR |
| 67.8 | 3090.4967 | 0.0254 | MGAGEALGNASQLFEAWLKGNESIPVNLR |
| 58.7 | 3089.5127 | 1.0094 | MGAGEALGNASQLFEAWLKGNESIPVNLR |
| 50.0 | 3089.5127 | 1.0094 | MGAGEALGNASQLFEAWLKGNESIPVNLR |
| 9.8 | 3088.5287 | 1.9934 | MGAGEALGNASQLFEAWLKGNESIPVNLR |
| 8.3 | 3088.5036 | 2.0185 | CLSVSPGGQWLASGSDDGTLRLWEVATAR |
| 5.9 | 3088.5287 | 1.9934 | MGAGEALGNASQLFEAWLKGNESIPVNLR |
| 4.5 | 3088.5320 | 1.9901 | ELLILEILQMGLKQISWTEVKNASR |
| 2.5 | 3089.5290 | 0.9931 | IILLSHAKSMSLIEIGNPSQSLENVCR |
| 2.4 | 3089.5290 | 0.9931 | IILLSHAKSMSLIEIGNPSQSLENVCR |

---

Spectrum No: 371; Query: 1988; Rank: 1

---

## Peptide View

MS/MS Fragmentation of **LLNTTDVYLLPSLNPDGFER**
Found in **IPI00213182**, Tax_Id=10116 Gene_Symbol=Cpd Isoform 1 of Carboxypeptidase D precursor

Match to Query 1988: 2277.156328 from(1139.585440,2+)
Title: 091008RatKidney_NoSalt_25.5204.5204.2.dta
Data file K:\NewmanPaper\Piliang\3SubProteomes\Piliang3SP\mgf5ppm\ERLIC_3SubProteomes5ppm.mgf

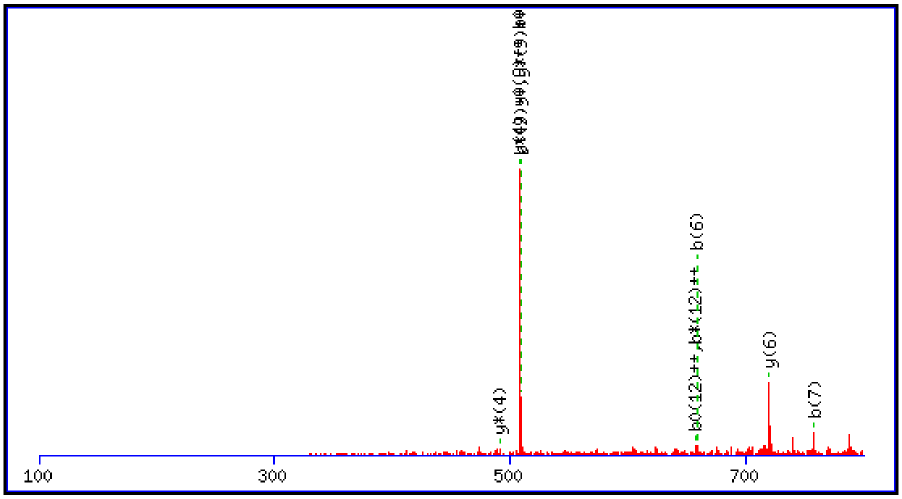


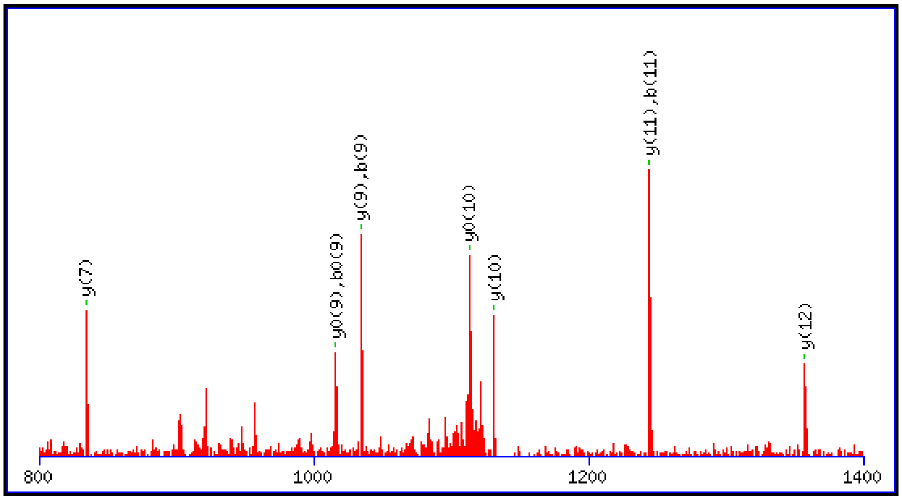


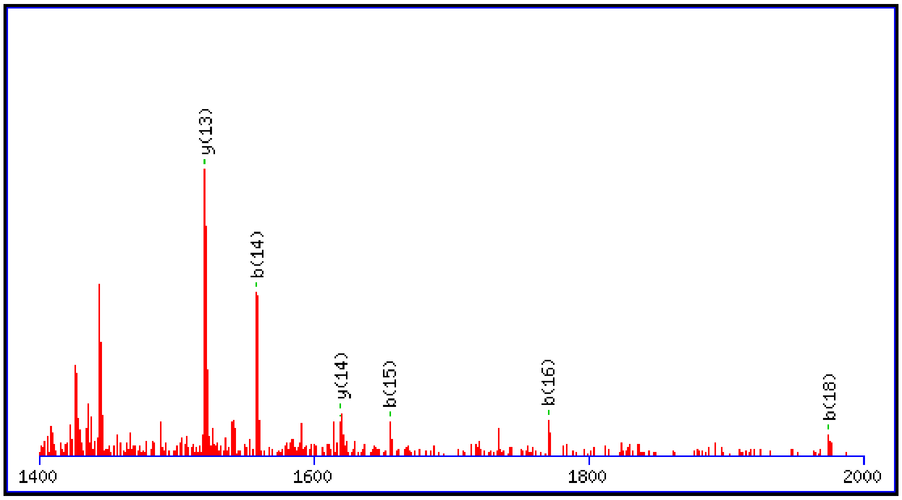


**Monoisotopic mass of neutral peptide Mr(calc):** 2277.1529
**Fixed modifications:** Carbamidomethyl (C)
**Variable modifications:**
**N3** : Deamidated_N (N)
**Ions Score:** 69 **Expect:** 2e-005
**Matches (Bold Red):** 27/216 fragment ions using 40 most intense peaks

| # | b | $b^{++}$ | b* | $b^{*++}$ | $b^{0}$ | $b^{0++}$ | Seq. | y | $y^{++}$ | y* | $y^{*++}$ | $y^{0}$ | $y^{0++}$ | # |
|---|---|---|---|---|---|---|---|---|---|---|---|---|---|---|
| 1 | 114.0913 | 57.5493 | | | | | L | | | | | | | 20 |
| 2 | 227.1754 | 114.0913 | | | | | L | 2165.0761 | 1083.0417 | 2148.0495 | 1074.5284 | 2147.0655 | 1074.0364 | 19 |
| 3 | 342.2023 | 171.6048 | 325.1758 | 163.0915 | | | N | 2051.9920 | 1026.4996 | 2034.9655 | 1017.9864 | 2033.9815 | 1017.4944 | 18 |
| 4 | 443.2500 | 222.1286 | 426.2235 | 213.6154 | 425.2395 | 213.1234 | T | 1936.9651 | 968.9862 | 1919.9385 | 960.4729 | 1918.9545 | 959.9809 | 17 |
| 5 | 544.2977 | 272.6525 | 527.2711 | 264.1392 | 526.2871 | 263.6472 | T | 1835.9174 | 918.4623 | 1818.8909 | 909.9491 | 1817.9068 | 909.4571 | 16 |
| 6 | **659.3246** | 330.1660 | 642.2981 | 321.6527 | 641.3141 | 321.1607 | D | 1734.8697 | 867.9385 | 1717.8432 | 859.4252 | 1716.8592 | 858.9332 | 15 |
| 7 | **758.3931** | 379.7002 | 741.3665 | 371.1869 | 740.3825 | 370.6949 | V | **1619.8428** | 810.4250 | 1602.8162 | 801.9118 | 1601.8322 | 801.4197 | 14 |
| 8 | 921.4564 | 461.2318 | 904.4298 | 452.7186 | 903.4458 | 452.2265 | Y | **1520.7744** | 760.8908 | 1503.7478 | 752.3775 | 1502.7638 | 751.8855 | 13 |
| 9 | **1034.5404** | 517.7739 | 1017.5139 | **509.2606** | **1016.5299** | **508.7686** | L | **1357.7110** | 679.3592 | 1340.6845 | 670.8459 | 1339.7005 | 670.3539 | 12 |
| 10 | 1147.6245 | 574.3159 | 1130.5980 | 565.8026 | 1129.6139 | 565.3106 | L | **1244.6270** | 622.8171 | 1227.6004 | 614.3039 | 1226.6164 | 613.8118 | 11 |
| 11 | **1244.6773** | 622.8423 | 1227.6507 | 614.3290 | 1226.6667 | 613.8370 | P | **1131.5429** | 566.2751 | 1114.5164 | 557.7618 | **1113.5323** | 557.2698 | 10 |
| 12 | 1331.7093 | 666.3583 | 1314.6828 | **657.8450** | 1313.6987 | **657.3530** | S | **1034.4901** | 517.7487 | 1017.4636 | **509.2354** | **1016.4796** | **508.7434** | 9 |
| 13 | 1444.7934 | 722.9003 | 1427.7668 | 714.3870 | 1426.7828 | 713.8950 | L | 947.4581 | 474.2327 | 930.4316 | 465.7194 | 929.4476 | 465.2274 | 8 |
| 14 | **1558.8363** | 779.9218 | 1541.8097 | 771.4085 | 1540.8257 | 770.9165 | N | **834.3741** | 417.6907 | 817.3475 | 409.1774 | 816.3635 | 408.6854 | 7 |
| 15 | **1655.8891** | 828.4482 | 1638.8625 | 819.9349 | 1637.8785 | 819.4429 | P | **720.3311** | 360.6692 | 703.3046 | 352.1559 | 702.3206 | 351.6639 | 6 |
| 16 | **1770.9160** | 885.9616 | 1753.8895 | 877.4484 | 1752.9054 | 876.9564 | D | 623.2784 | 312.1428 | 606.2518 | 303.6295 | 605.2678 | 303.1375 | 5 |
| 17 | 1827.9375 | 914.4724 | 1810.9109 | 905.9591 | 1809.9269 | 905.4671 | G | **508.2514** | 254.6293 | **491.2249** | 246.1161 | 490.2409 | 245.6241 | 4 |
| 18 | **1975.0059** | 988.0066 | 1957.9793 | 979.4933 | 1956.9953 | 979.0013 | F | 451.2300 | 226.1186 | 434.2034 | 217.6053 | 433.2194 | 217.1133 | 3 |
| 19 | 2104.0485 | 1052.5279 | 2087.0219 | 1044.0146 | 2086.0379 | 1043.5226 | E | 304.1615 | 152.5844 | 287.1350 | 144.0711 | 286.1510 | 143.5791 | 2 |
| 20 | | | | | | | R | 175.1190 | 88.0631 | 158.0924 | 79.5498 | | | 1 |

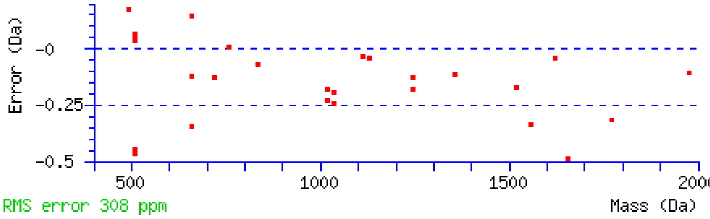


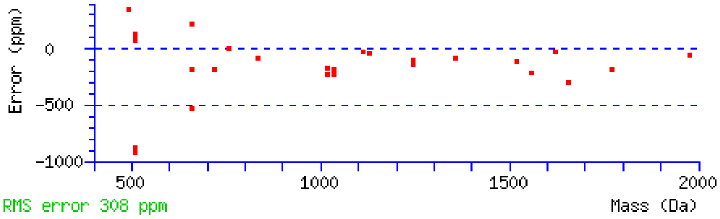


### All matches to this query

| Score | Mr(calc): | Delta | Sequence |
|---|---|---|---|
| 69.4 | 2277.1529 | 0.0035 | LLNTTDVYLLPSLNPDGFER |
| 46.8 | 2276.1689 | 0.9875 | LLNTTDVYLLPSLNPDGFER |
| 40.9 | 2277.1529 | 0.0035 | LLNTTDVYLLPSLNPDGFER |
| 4.8 | 2277.1497 | 0.0066 | MDKSELGCLRAIVLFNPDAK |
| 3.9 | 2275.1660 | 1.9903 | SSIFQRLLHCLLGKAHSTK |
| 3.9 | 2275.1660 | 1.9903 | SSIFQRLLHCLLGKAHSTK |
| 3.8 | 2276.1752 | 0.9811 | VMHRDIKPANVFITATGVVK |
| 1.2 | 2277.1437 | 0.0127 | MVRYPGQSVSTVSPAHYARR |
| 1.0 | 2276.1566 | 0.9998 | LQLGPETLQQDNPKLIYAR |
| 0.9 | 2277.1383 | 0.0181 | LKRASSEDMLNKPGSASSGVAR |

---

Spectrum No: 372; Query: 2104; Rank: 1

---

## Peptide View

MS/MS Fragmentation of **ALNELASQGFPGNATTPSGEQLR**
Found in **IPI00190577**, Tax_Id=10116 Gene_Symbol=Lama5 similar to Laminin alpha-5 chain precursor

Match to Query 2104: 2358.155508 from(1180.085030,2+)
Title: 100101RatKid_NS_deglyco_23.3257.3257.2.dta
Data file K:\NewmanPaper\Piliang\3SubProteomes\Piliang3SP\mgf5ppm\ERLIC_3SubProteomes5ppm.mgf

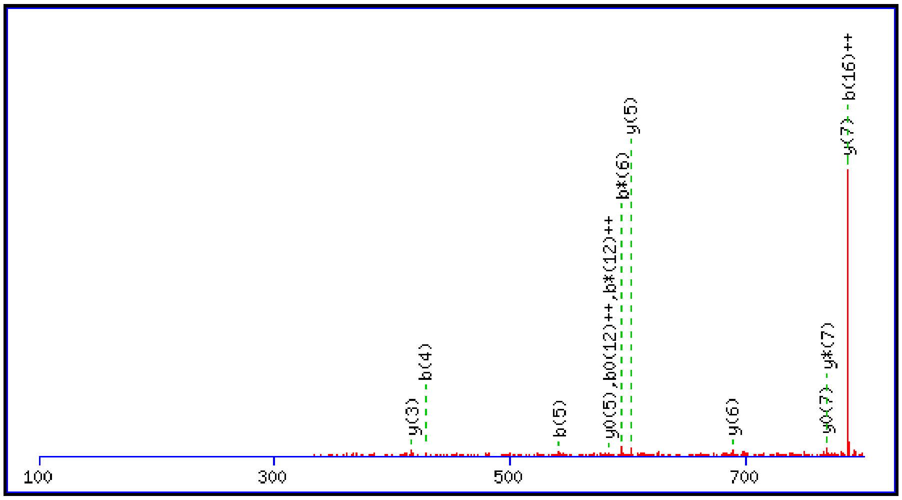


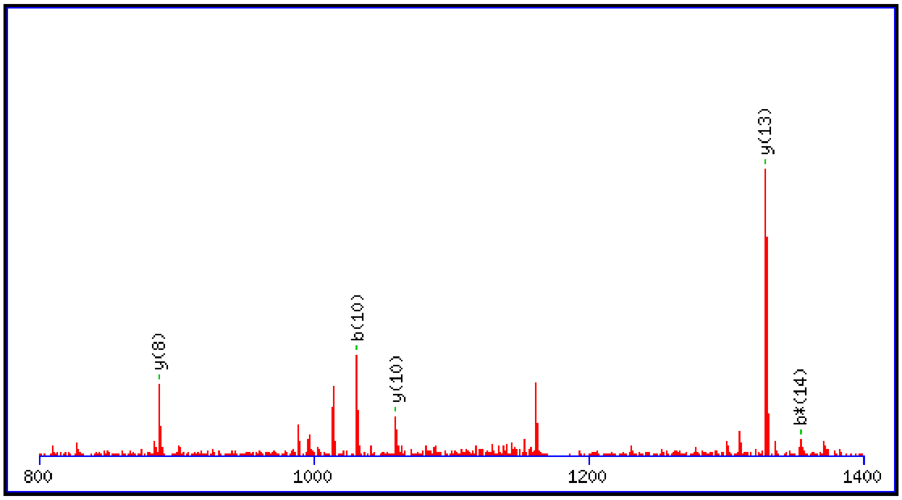


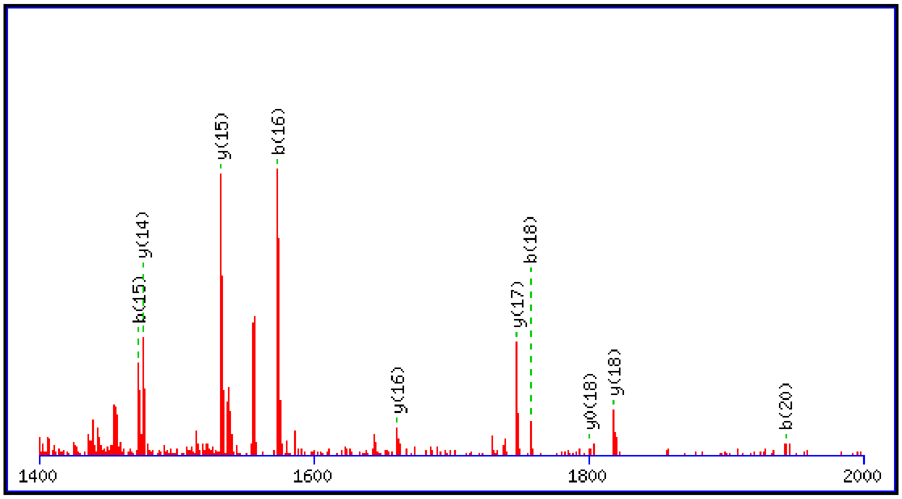


**Monoisotopic mass of neutral peptide Mr(calc):** 2358.1451
**Fixed modifications:** Carbamidomethyl (C)
**Variable modifications:**
**N13** : Deamidated_N (N)
**Ions Score:** 69 **Expect:** 2.6e-005
**Matches (Bold Red):** 28/248 fragment ions using 46 most intense peaks

| # | b | $b^{++}$ | b* | $b^{*++}$ | $b^0$ | $b^{0++}$ | Seq. | y | $y^{++}$ | y* | $y^{*++}$ | $y^0$ | $y^{0++}$ | # |
|---|---|---|---|---|---|---|---|---|---|---|---|---|---|---|
| 1 | 72.0444 | 36.5258 | | | | | A | | | | | | | 23 |
| 2 | 185.1285 | 93.0679 | | | | | L | 2288.1153 | 1144.5613 | 2271.0888 | 1136.0480 | 2270.1048 | 1135.5560 | 22 |

| | | | | | | | | | | | | | | |
|---|---|---|---|---|---|---|---|---|---|---|---|---|---|---|
| 3 | 299.1714 | 150.0893 | 282.1448 | 141.5761 | | | N | 2175.0313 | 1088.0193 | 2158.0047 | 1079.5060 | 2157.0207 | 1079.0140 | 21 |
| 4 | **428.2140** | 214.6106 | 411.1874 | 206.0974 | 410.2034 | 205.6053 | E | 2060.9883 | 1030.9978 | 2043.9618 | 1022.4845 | 2042.9778 | 1021.9925 | 20 |
| 5 | **541.2980** | 271.1527 | 524.2715 | 262.6394 | 523.2875 | 262.1474 | L | 1931.9457 | 966.4765 | 1914.9192 | 957.9632 | 1913.9352 | 957.4712 | 19 |
| 6 | 612.3352 | 306.6712 | **595.3086** | 298.1579 | 594.3246 | 297.6659 | A | **1818.8617** | 909.9345 | 1801.8351 | 901.4212 | **1800.8511** | 900.9292 | 18 |
| 7 | 699.3672 | 350.1872 | 682.3406 | 341.6740 | 681.3566 | 341.1819 | S | **1747.8246** | 874.4159 | 1730.7980 | 865.9026 | 1729.8140 | 865.4106 | 17 |
| 8 | 827.4258 | 414.2165 | 810.3992 | 405.7032 | 809.4152 | 405.2112 | Q | **1660.7925** | 830.8999 | 1643.7660 | 822.3866 | 1642.7820 | 821.8946 | 16 |
| 9 | 884.4472 | 442.7272 | 867.4207 | 434.2140 | 866.4367 | 433.7220 | G | **1532.7340** | 766.8706 | 1515.7074 | 758.3573 | 1514.7234 | 757.8653 | 15 |
| 10 | **1031.5156** | 516.2615 | 1014.4891 | 507.7482 | 1013.5051 | 507.2562 | F | **1475.7125** | 738.3599 | 1458.6859 | 729.8466 | 1457.7019 | 729.3546 | 14 |
| 11 | 1128.5684 | 564.7878 | 1111.5419 | 556.2746 | 1110.5578 | 555.7826 | P | **1328.6441** | 664.8257 | 1311.6175 | 656.3124 | 1310.6335 | 655.8204 | 13 |
| 12 | 1185.5899 | 593.2986 | 1168.5633 | **584.7853** | 1167.5793 | **584.2933** | G | 1231.5913 | 616.2993 | 1214.5648 | 607.7860 | 1213.5808 | 607.2940 | 12 |
| 13 | 1300.6168 | 650.8120 | 1283.5903 | 642.2988 | 1282.6062 | 641.8068 | N | 1174.5699 | 587.7886 | 1157.5433 | 579.2753 | 1156.5593 | 578.7833 | 11 |
| 14 | 1371.6539 | 686.3306 | **1354.6274** | 677.8173 | 1353.6433 | 677.3253 | A | **1059.5429** | 530.2751 | 1042.5164 | 521.7618 | 1041.5324 | 521.2698 | 10 |
| 15 | **1472.7016** | 736.8544 | 1455.6750 | 728.3412 | 1454.6910 | 727.8492 | T | 988.5058 | 494.7565 | 971.4793 | 486.2433 | 970.4952 | 485.7513 | 9 |
| 16 | **1573.7493** | **787.3783** | 1556.7227 | 778.8650 | 1555.7387 | 778.3730 | T | **887.4581** | 444.2327 | 870.4316 | 435.7194 | 869.4476 | 435.2274 | 8 |
| 17 | 1670.8020 | 835.9047 | 1653.7755 | 827.3914 | 1652.7915 | 826.8994 | P | **786.4104** | 393.7089 | **769.3839** | 385.1956 | **768.3999** | 384.7036 | 7 |
| 18 | **1757.8341** | 879.4207 | 1740.8075 | 870.9074 | 1739.8235 | 870.4154 | S | **689.3577** | 345.1825 | 672.3311 | 336.6692 | 671.3471 | 336.1772 | 6 |
| 19 | 1814.8555 | 907.9314 | 1797.8290 | 899.4181 | 1796.8450 | 898.9261 | G | **602.3257** | 301.6665 | 585.2991 | 293.1532 | **584.3151** | 292.6612 | 5 |
| 20 | **1943.8981** | 972.4527 | 1926.8716 | 963.9394 | 1925.8876 | 963.4474 | E | 545.3042 | 273.1557 | 528.2776 | 264.6425 | 527.2936 | 264.1504 | 4 |
| 21 | 2071.9567 | 1036.4820 | 2054.9301 | 1027.9687 | 2053.9461 | 1027.4767 | Q | **416.2616** | 208.6344 | 399.2350 | 200.1212 | | | 3 |
| 22 | 2185.0408 | 1093.0240 | 2168.0142 | 1084.5107 | 2167.0302 | 1084.0187 | L | 288.2030 | 144.6051 | 271.1765 | 136.0919 | | | 2 |
| 23 | | | | | | | R | 175.1190 | 88.0631 | 158.0924 | 79.5498 | | | 1 |

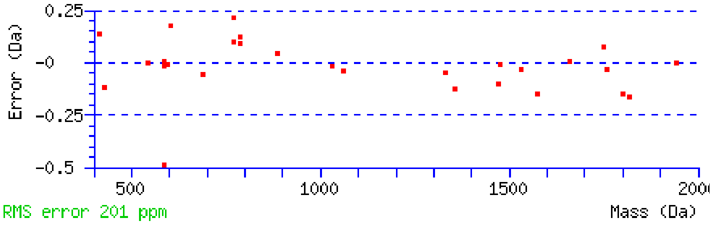


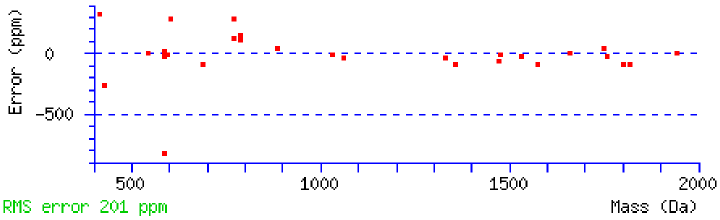


**All matches to this query**

| Score | Mr(calc): | Delta | Sequence |
|---|---|---|---|
| 69.3 | 2358.1451 | 0.0104 | ALNELASQGFPGNATTPSGEQLR |
| 25.9 | 2357.1611 | 0.9944 | ALNELASQGFPGNATTPSGEQLR |
| 23.6 | 2358.1451 | 0.0104 | ALNELASQGFPGNATTPSGEQLR |
| 3.9 | 2358.1402 | 0.0153 | LLGTHPKKEPMEALNTAQGAR |
| 2.0 | 2358.1515 | 0.0040 | LECPIRTYLVTARSAASSGAR |
| 2.0 | 2358.1515 | 0.0040 | LECPIRTYLVTARSAASSGAR |
| 1.4 | 2358.1402 | 0.0153 | SQLSLSMANHDIPPDAVRAKK |
| 0.9 | 2358.1409 | 0.0146 | HEFLIFEDRKFADIGNTVK |
| 0.8 | 2358.1597 | -0.0041 | ELRSTLDSIPSLSLPSSIKR |
| 0.7 | 2358.1354 | 0.0201 | KLVQLLASSPCESVMKRPR |

---

Spectrum No: 373; Query: 169; Rank: 1

---

## Peptide View

MS/MS Fragmentation of **VNETEEGYAR**
Found in **IPI00371511**, Tax_Id=10116 Gene_Symbol=Creg_predicted cellular repressor of E1A-stimulated genes

Match to Query 169: 1167.503068 from(584.758810,2+)

Title: 091008RatKidney_NH4Format01_23.433.433.2.dta
Data file K:\NewmanPaper\Piliang\3SubProteomes\Piliang3SP\mgf5ppm\ERLIC_3SubProteomes5ppm.mgf

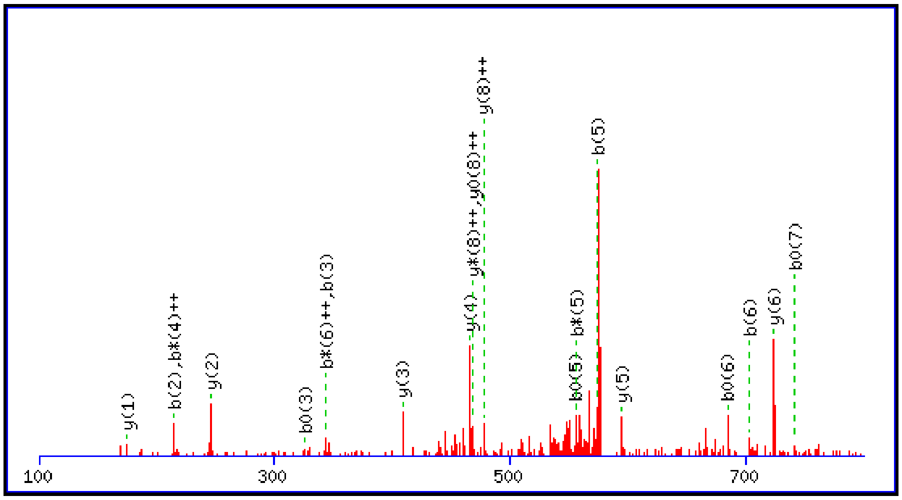


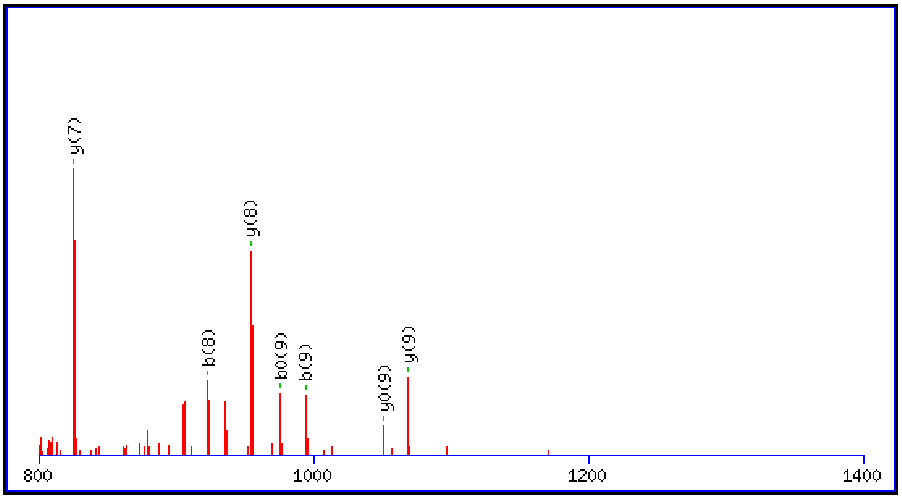


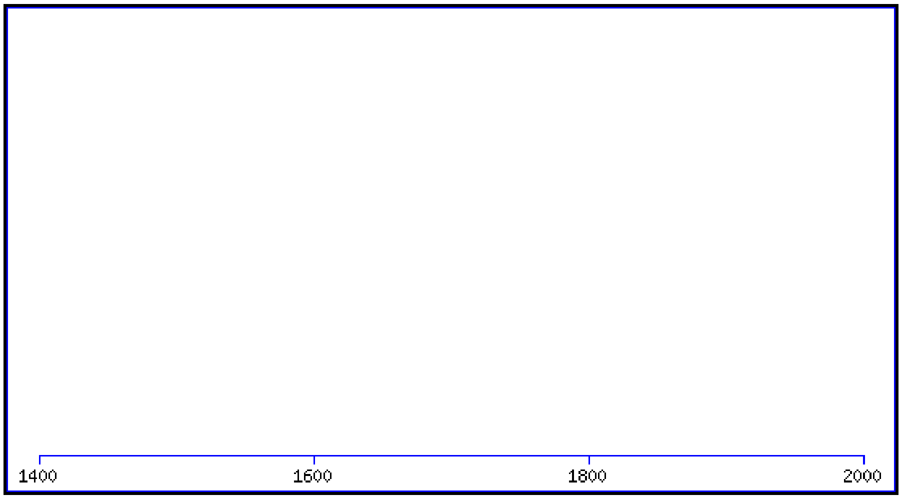


**Monoisotopic mass of neutral peptide Mr(calc):** 1167.5044
**Fixed modifications:** Carbamidomethyl (C)
**Variable modifications:**
**N2** : Deamidated_N (N)
**Ions Score:** 69 **Expect:** 1.1e-005
**Matches (Bold Red):** 27/94 fragment ions using 45 most intense peaks

| # | b | $b^{++}$ | b* | $b^{*++}$ | $b^{0}$ | $b^{0++}$ | Seq. | y | $y^{++}$ | y* | $y^{*++}$ | $y^{0}$ | $y^{0++}$ | # |
|---|---|---|---|---|---|---|---|---|---|---|---|---|---|---|
| 1 | 100.0757 | 50.5415 | | | | | V | | | | | | | 10 |
| 2 | **215.1026** | 108.0550 | 198.0761 | 99.5417 | | | N | **1069.4433** | 535.2253 | 1052.4167 | 526.7120 | **1051.4327** | 526.2200 | 9 |
| 3 | **344.1452** | 172.5762 | 327.1187 | 164.0630 | **326.1347** | 163.5710 | E | **954.4163** | **477.7118** | 937.3898 | **469.1985** | 936.4058 | **468.7065** | 8 |

| | | | | | | | | | | | | | | |
|---|---|---|---|---|---|---|---|---|---|---|---|---|---|---|
| 4 | 445.1929 | 223.1001 | 428.1663 | **214.5868** | 427.1823 | 214.0948 | T | **825.3737** | 413.1905 | 808.3472 | 404.6772 | 807.3632 | 404.1852 | 7 |
| 5 | **574.2355** | 287.6214 | **557.2089** | 279.1081 | **556.2249** | 278.6161 | E | **724.3260** | 362.6667 | 707.2995 | 354.1534 | 706.3155 | 353.6614 | 6 |
| 6 | **703.2781** | 352.1427 | 686.2515 | **343.6294** | **685.2675** | 343.1374 | E | **595.2835** | 298.1454 | 578.2569 | 289.6321 | 577.2729 | 289.1401 | 5 |
| 7 | 760.2995 | 380.6534 | 743.2730 | 372.1401 | **742.2890** | 371.6481 | G | **466.2409** | 233.6241 | 449.2143 | 225.1108 | | | 4 |
| 8 | **923.3629** | 462.1851 | 906.3363 | 453.6718 | 905.3523 | 453.1798 | Y | **409.2194** | 205.1133 | 392.1928 | 196.6001 | | | 3 |
| 9 | **994.4000** | 497.7036 | 977.3734 | 489.1904 | **976.3894** | 488.6984 | A | **246.1561** | 123.5817 | 229.1295 | 115.0684 | | | 2 |
| 10 | | | | | | | R | **175.1190** | 88.0631 | 158.0924 | 79.5498 | | | 1 |

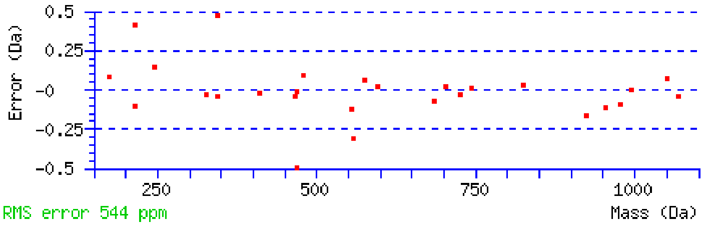


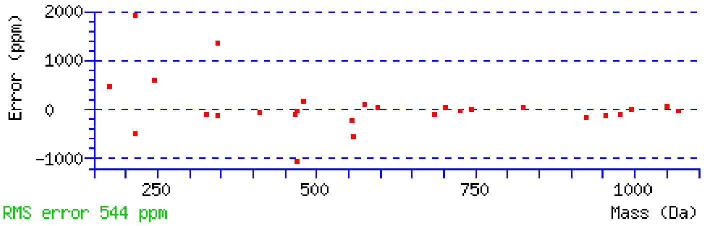


### All matches to this query

| Score | Mr(calc): | Delta | Sequence |
|---|---|---|---|
| 69.2 | 1167.5044 | -0.0013 | VNETEEGYAR |
| 11.1 | 1166.5043 | 0.9988 | DMTEVVHIK |
| 10.1 | 1167.4921 | 0.0109 | AEAGVSEVGGGR |
| 8.0 | 1166.4968 | 1.0062 | NVPDSAKEAR |
| 6.0 | 1167.4921 | 0.0110 | QDVDNASLAR |
| 5.9 | 1167.5033 | -0.0003 | ADGNQLRSAR |
| 5.0 | 1166.4926 | 1.0104 | FLSHKFTK |
| 5.0 | 1167.5107 | -0.0077 | QSIPATMNAR |
| 4.7 | 1167.4951 | 0.0080 | ARTXTRTR |
| 4.6 | 1167.4951 | 0.0080 | LTGRSRYR |

---

Spectrum No: 374; Query: 419; Rank: 1

---

## Peptide View

MS/MS Fragmentation of **DELADEIANSSGK**
Found in **IPI00209113**, Tax_Id=10116 Gene_Symbol=Myh9 Myosin-9

Match to Query 419: 1348.599388 from(675.306970,2+)
Title: 100101RatKid_NS_deglyco_21.1559.1559.2.dta
Data file K:\NewmanPaper\Piliang\3SubProteomes\Piliang3SP\mgf5ppm\ERLIC_3SubProteomes5ppm.mgf

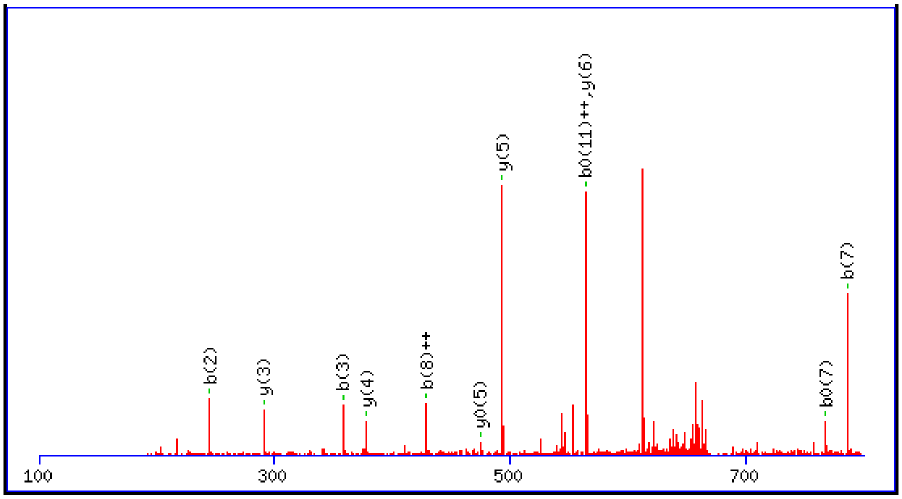


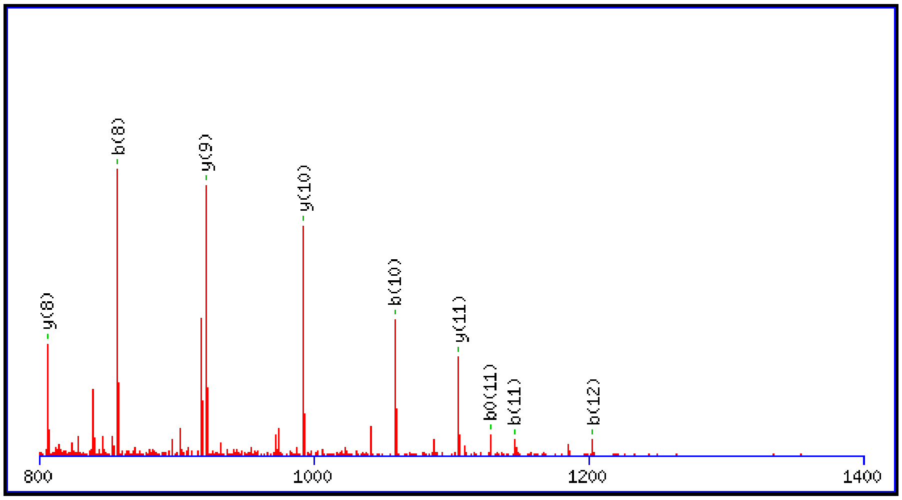


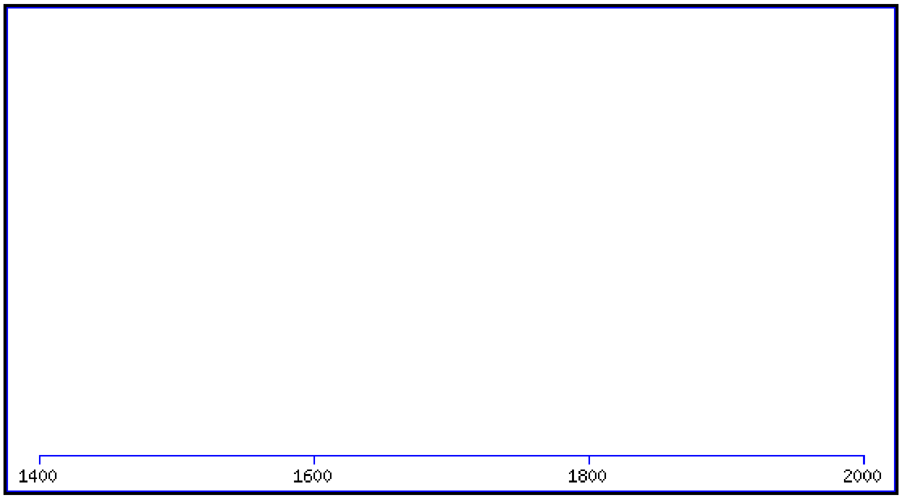


**Monoisotopic mass of neutral peptide Mr(calc):** 1348.5994
**Fixed modifications:** Carbamidomethyl (C)
**Variable modifications:**
**N9** : Deamidated_N (N)
**Ions Score:** 69 **Expect:** 1.6e-005
**Matches (Bold Red):** 21/124 fragment ions using 31 most intense peaks

| # | b | $b^{++}$ | b* | $b^{*++}$ | $b^0$ | $b^{0++}$ | Seq. | y | $y^{++}$ | y* | $y^{*++}$ | $y^0$ | $y^{0++}$ | # |
|---|---|---|---|---|---|---|---|---|---|---|---|---|---|---|
| 1 | 116.0342 | 58.5207 | | | 98.0237 | 49.5155 | D | | | | | | | 13 |
| 2 | **245.0768** | 123.0420 | | | 227.0662 | 114.0368 | E | 1234.5797 | 617.7935 | 1217.5532 | 609.2802 | 1216.5692 | 608.7882 | 12 |
| 3 | **358.1609** | 179.5841 | | | 340.1503 | 170.5788 | L | **1105.5372** | 553.2722 | 1088.5106 | 544.7589 | 1087.5266 | 544.2669 | 11 |
| 4 | **429.1980** | 215.1026 | | | 411.1874 | 206.0974 | A | **992.4531** | 496.7302 | 975.4265 | 488.2169 | 974.4425 | 487.7249 | 10 |
| 5 | 544.2249 | 272.6161 | | | 526.2144 | 263.6108 | D | **921.4160** | 461.2116 | 904.3894 | 452.6984 | 903.4054 | 452.2063 | 9 |

| | | | | | | | | | | | | | | |
|---|---|---|---|---|---|---|---|---|---|---|---|---|---|---|
| **6** | 673.2675 | 337.1374 | | | 655.2570 | 328.1321 | **E** | **806.3890** | 403.6982 | 789.3625 | 395.1849 | 788.3785 | 394.6929 | **8** |
| **7** | **786.3516** | 393.6794 | | | **768.3410** | 384.6742 | **I** | 677.3464 | 339.1769 | 660.3199 | 330.6636 | 659.3359 | 330.1716 | **7** |
| **8** | **857.3887** | **429.1980** | | | 839.3781 | 420.1927 | **A** | **564.2624** | 282.6348 | 547.2358 | 274.1216 | 546.2518 | 273.6295 | **6** |
| **9** | 972.4156 | 486.7115 | 955.3891 | 478.1982 | 954.4051 | 477.7062 | **N** | **493.2253** | 247.1163 | 476.1987 | 238.6030 | **475.2147** | 238.1110 | **5** |
| **10** | **1059.4477** | 530.2275 | 1042.4211 | 521.7142 | 1041.4371 | 521.2222 | **S** | **378.1983** | 189.6028 | 361.1718 | 181.0895 | 360.1878 | 180.5975 | **4** |
| **11** | **1146.4797** | 573.7435 | 1129.4531 | 565.2302 | **1128.4691** | **564.7382** | **S** | **291.1663** | 146.0868 | 274.1397 | 137.5735 | 273.1557 | 137.0815 | **3** |
| **12** | **1203.5012** | 602.2542 | 1186.4746 | 593.7409 | 1185.4906 | 593.2489 | **G** | 204.1343 | 102.5708 | 187.1077 | 94.0575 | | | **2** |
| **13** | | | | | | | **K** | 147.1128 | 74.0600 | 130.0863 | 65.5468 | | | **1** |

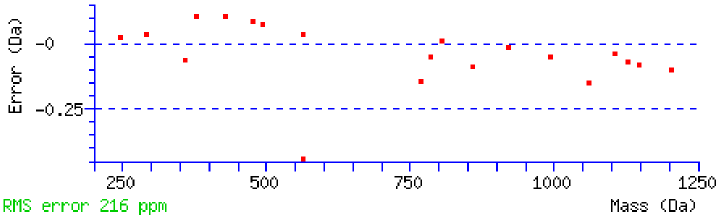


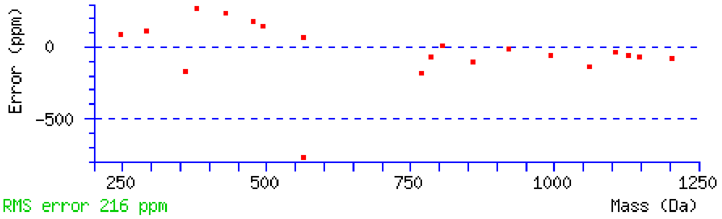


### All matches to this query

| Score | Mr(calc): | Delta | Sequence |
|---|---|---|---|
| 69.2 | 1348.5994 | 0.0000 | DELADEIANSSGK |
| 5.8 | 1347.6071 | 0.9923 | NELTEHAKTPK |
| 5.0 | 1348.6064 | -0.0070 | LEDALYFDRK |
| 4.5 | 1347.5894 | 1.0100 | LSFDKDAMVAR |
| 3.3 | 1348.6097 | -0.0104 | MIYANSKNAIK |
| 3.3 | 1348.6097 | -0.0104 | MIYANSKNAIK |
| 2.4 | 1348.6024 | -0.0030 | ELVSDTNQHVK |
| 2.2 | 1348.5958 | 0.0035 | VSMENRSYRK |
| 1.7 | 1348.5920 | 0.0074 | LYKNCPKAMK |
| 1.7 | 1348.6098 | -0.0104 | TASLFEQKSMK |

---

Spectrum No: 375; Query: 941; Rank: 1

---

## Peptide View

MS/MS Fragmentation of **LATIANFSALGLEPGR**
Found in **IPI00199585**, Tax_Id=10116 Gene_Symbol=Slc22a5 Organic cation/carnitine transporter 2

Match to Query 941: 1629.876588 from(815.945570,2+)
Title: 100101RatKid_NS_deglyco_23.4633.4633.2.dta
Data file K:\NewmanPaper\Piliang\3SubProteomes\Piliang3SP\mgf5ppm\ERLIC_3SubProteomes5ppm.mgf

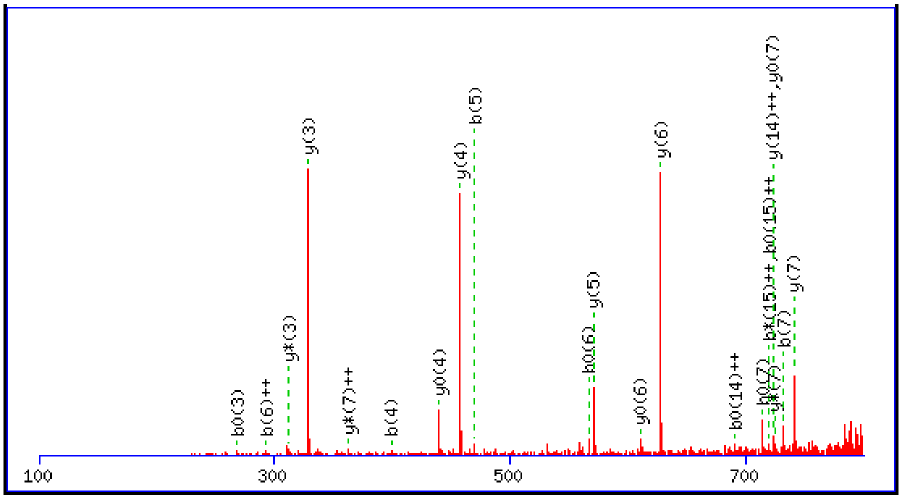


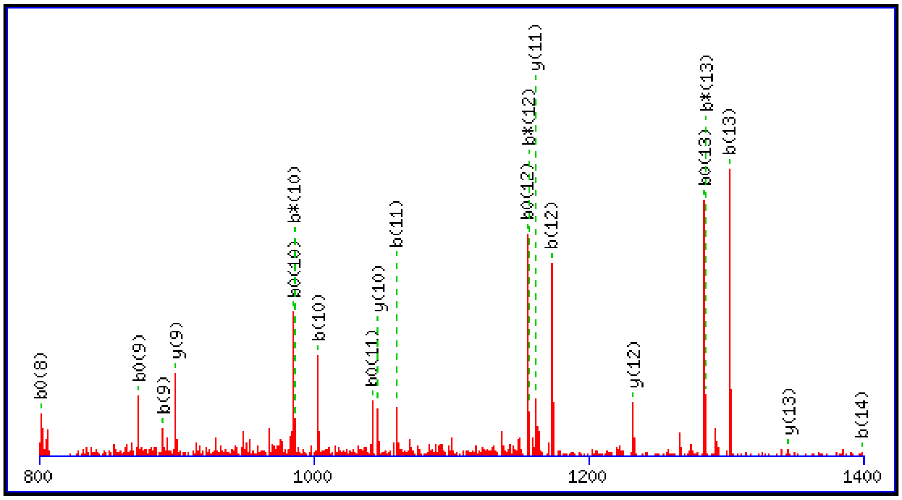


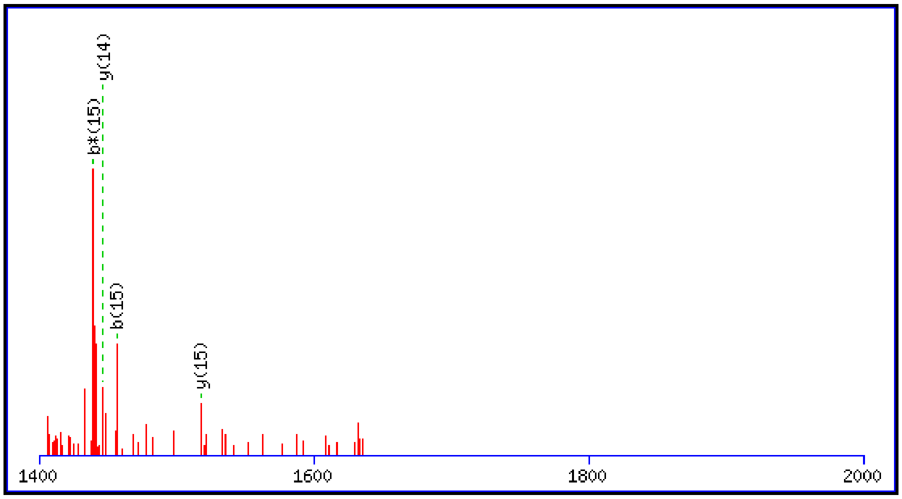


**Monoisotopic mass of neutral peptide Mr(calc):** 1629.8726
**Fixed modifications:** Carbamidomethyl (C)
**Variable modifications:**
**N6** : Deamidated_N (N)
**Ions Score:** 69 **Expect:** 1.2e-005
**Matches (Bold Red):** 46/160 fragment ions using 81 most intense peaks

| # | b | $b^{++}$ | b* | $b^{*++}$ | $b^0$ | $b^{0++}$ | Seq. | y | $y^{++}$ | y* | $y^{*++}$ | $y^0$ | $y^{0++}$ | # |
|---|---|---|---|---|---|---|---|---|---|---|---|---|---|---|
| 1 | 114.0913 | 57.5493 | | | | | L | | | | | | | 16 |
| 2 | 185.1285 | 93.0679 | | | | | A | **1517.7958** | 759.4016 | 1500.7693 | 750.8883 | 1499.7853 | 750.3963 | 15 |
| 3 | 286.1761 | 143.5917 | | | **268.1656** | 134.5864 | T | **1446.7587** | **723.8830** | 1429.7322 | 715.3697 | 1428.7481 | 714.8777 | 14 |
| 4 | **399.2602** | 200.1337 | | | 381.2496 | 191.1285 | I | **1345.7110** | 673.3592 | 1328.6845 | 664.8459 | 1327.7005 | 664.3539 | 13 |
| 5 | **470.2973** | 235.6523 | | | 452.2867 | 226.6470 | A | **1232.6270** | 616.8171 | 1215.6004 | 608.3038 | 1214.6164 | 607.8118 | 12 |

| | | | | | | | | | | | | | | |
|---|---|---|---|---|---|---|---|---|---|---|---|---|---|---|
| 6 | 585.3242 | **293.1658** | 568.2977 | 284.6525 | **567.3137** | 284.1605 | N | **1161.5899** | 581.2986 | 1144.5633 | 572.7853 | 1143.5793 | 572.2933 | 11 |
| 7 | **732.3927** | 366.7000 | 715.3661 | 358.1867 | **714.3821** | 357.6947 | F | **1046.5629** | 523.7851 | 1029.5364 | 515.2718 | 1028.5524 | 514.7798 | 10 |
| 8 | 819.4247 | 410.2160 | 802.3981 | 401.7027 | **801.4141** | 401.2107 | S | **899.4945** | 450.2509 | 882.4680 | 441.7376 | 881.4839 | 441.2456 | 9 |
| 9 | **890.4618** | 445.7345 | 873.4353 | 437.2213 | **872.4512** | 436.7293 | A | 812.4625 | 406.7349 | 795.4359 | 398.2216 | 794.4519 | 397.7296 | 8 |
| 10 | **1003.5459** | 502.2766 | **986.5193** | 493.7633 | **985.5353** | 493.2713 | L | **741.4254** | 371.2163 | **724.3988** | **362.7030** | **723.4148** | 362.2110 | 7 |
| 11 | **1060.5673** | 530.7873 | 1043.5408 | 522.2740 | **1042.5568** | 521.7820 | G | **628.3413** | 314.6743 | 611.3148 | 306.1610 | **610.3307** | 305.6690 | 6 |
| 12 | **1173.6514** | 587.3293 | **1156.6248** | 578.8161 | **1155.6408** | 578.3241 | L | **571.3198** | 286.1636 | 554.2933 | 277.6503 | 553.3093 | 277.1583 | 5 |
| 13 | **1302.6940** | 651.8506 | **1285.6674** | 643.3374 | **1284.6834** | 642.8453 | E | **458.2358** | 229.6215 | 441.2092 | 221.1082 | **440.2252** | 220.6162 | 4 |
| 14 | **1399.7468** | 700.3770 | 1382.7202 | 691.8637 | 1381.7362 | **691.3717** | P | **329.1932** | 165.1002 | **312.1666** | 156.5870 | | | 3 |
| 15 | **1456.7682** | 728.8877 | **1439.7417** | **720.3745** | 1438.7577 | **719.8825** | G | 232.1404 | 116.5738 | 215.1139 | 108.0606 | | | 2 |
| 16 | | | | | | | R | 175.1190 | 88.0631 | 158.0924 | 79.5498 | | | 1 |

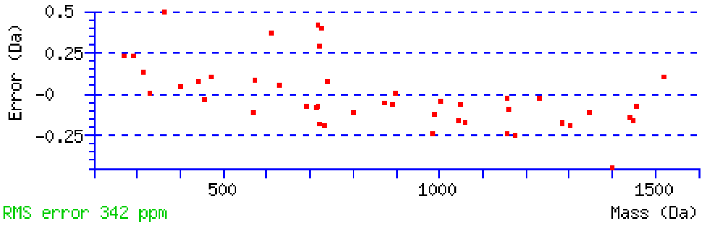


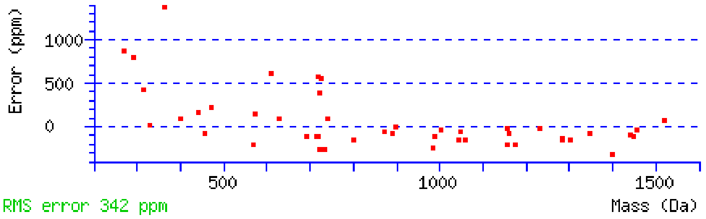


### All matches to this query

| Score | Mr(calc): | Delta | Sequence |
|---|---|---|---|
| 69.1 | 1629.8726 | 0.0040 | LATIANFSALGLEPGR |
| 20.0 | 1628.8886 | 0.9880 | LATIANFSALGLEPGR |
| 7.1 | 1628.8733 | 1.0033 | ETEDLRKVALEAQK |
| 5.5 | 1629.8620 | 0.0146 | EEAGALLSLCKRQR |
| 5.1 | 1629.8798 | -0.0032 | ERNGTLISISQQKR |
| 3.7 | 1629.8686 | 0.0080 | GKANSTGTLVITNPTR |
| 1.7 | 1627.8828 | 1.9938 | TQASKQIMKHLTSR |
| 1.1 | 1628.8725 | 1.0041 | YNVLTLVRMLSLK |
| 0.7 | 1628.8872 | 0.9894 | NLTKDLLNELEVVK |
| 0.7 | 1629.8630 | 0.0136 | YLTLLSDVLVVSTK |

---

Spectrum No: 376; Query: 944; Rank: 1

---

## Peptide View

MS/MS Fragmentation of **CLVPHTVNLSSAWR**
Found in **IPI00199585**, Tax_Id=10116 Gene_Symbol=Slc22a5 Organic cation/carnitine transporter 2

Match to Query 944: 1639.814982 from(547.612270,3+)
Title: 100101RatKid_NS_deglyco_25.3179.3179.3.dta
Data file K:\NewmanPaper\Piliang\3SubProteomes\Piliang3SP\mgf5ppm\ERLIC_3SubProteomes5ppm.mgf

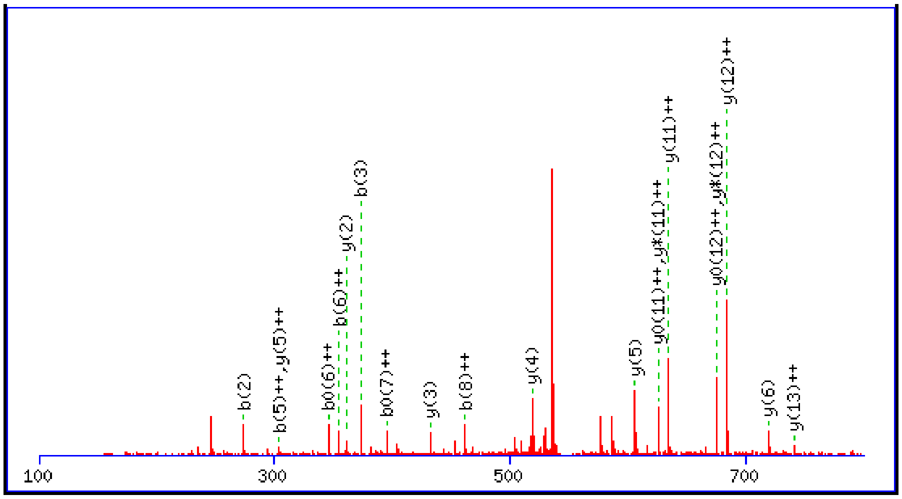


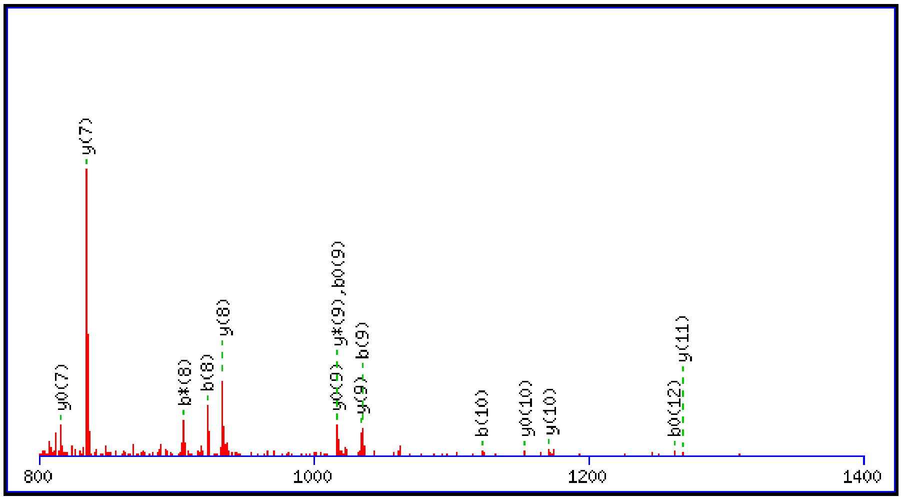


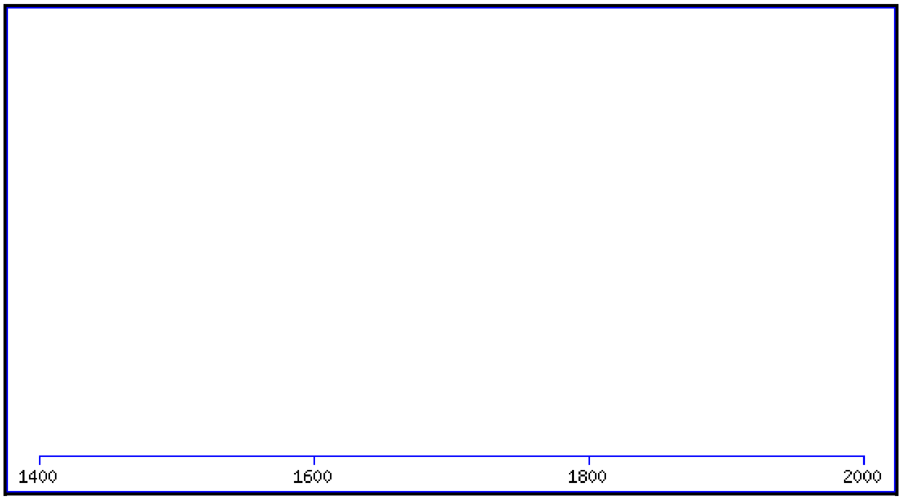


**Monoisotopic mass of neutral peptide Mr(calc):** 1639.8141
**Fixed modifications:** Carbamidomethyl (C)
**Variable modifications:**
**N8** : Deamidated_N (N)
**Ions Score:** 69 **Expect:** 2.1e-005
**Matches (Bold Red):** 35/126 fragment ions using 50 most intense peaks

| # | b | $b^{++}$ | b* | $b^{*++}$ | $b^0$ | $b^{0++}$ | Seq. | y | $y^{++}$ | y* | $y^{*++}$ | $y^0$ | $y^{0++}$ | # |
|---|---|---|---|---|---|---|---|---|---|---|---|---|---|---|
| 1 | 161.0379 | 81.0226 | | | | | C | | | | | | | 14 |
| 2 | **274.1220** | 137.5646 | | | | | L | 1480.7907 | **740.8990** | 1463.7641 | 732.3857 | 1462.7801 | 731.8937 | 13 |
| 3 | **373.1904** | 187.0988 | | | | | V | 1367.7066 | **684.3569** | 1350.6801 | **675.8437** | 1349.6961 | **675.3517** | 12 |
| 4 | 470.2432 | 235.6252 | | | | | P | **1268.6382** | **634.8227** | 1251.6117 | **626.3095** | 1250.6276 | **625.8175** | 11 |
| 5 | 607.3021 | **304.1547** | | | | | H | **1171.5854** | 586.2964 | 1154.5589 | 577.7831 | **1153.5749** | 577.2911 | 10 |

| | | | | | | | | | | | | | | |
|---|---|---|---|---|---|---|---|---|---|---|---|---|---|---|
| **6** | 708.3498 | **354.6785** | | | 690.3392 | **345.6732** | **T** | **1034.5265** | 517.7669 | **1017.5000** | 509.2536 | **1016.5160** | 508.7616 | **9** |
| **7** | 807.4182 | 404.2127 | | | 789.4076 | **395.2074** | **V** | **933.4788** | 467.2431 | 916.4523 | 458.7298 | 915.4683 | 458.2378 | **8** |
| **8** | **922.4451** | **461.7262** | **905.4186** | 453.2129 | 904.4345 | 452.7209 | **N** | **834.4104** | 417.7089 | 817.3839 | 409.1956 | **816.3999** | 408.7036 | **7** |
| **9** | **1035.5292** | 518.2682 | 1018.5026 | 509.7549 | **1017.5186** | 509.2629 | **L** | **719.3835** | 360.1954 | 702.3570 | 351.6821 | 701.3729 | 351.1901 | **6** |
| **10** | **1122.5612** | 561.7842 | 1105.5347 | 553.2710 | 1104.5506 | 552.7790 | **S** | **606.2994** | **303.6534** | 589.2729 | 295.1401 | 588.2889 | 294.6481 | **5** |
| **11** | 1209.5932 | 605.3003 | 1192.5667 | 596.7870 | 1191.5827 | 596.2950 | **S** | **519.2674** | 260.1373 | 502.2409 | 251.6241 | 501.2568 | 251.1321 | **4** |
| **12** | 1280.6303 | 640.8188 | 1263.6038 | 632.3055 | **1262.6198** | 631.8135 | **A** | **432.2354** | 216.6213 | 415.2088 | 208.1081 | | | **3** |
| **13** | 1466.7097 | 733.8585 | 1449.6831 | 725.3452 | 1448.6991 | 724.8532 | **W** | **361.1983** | 181.1028 | 344.1717 | 172.5895 | | | **2** |
| **14** | | | | | | | **R** | 175.1190 | 88.0631 | 158.0924 | 79.5498 | | | **1** |

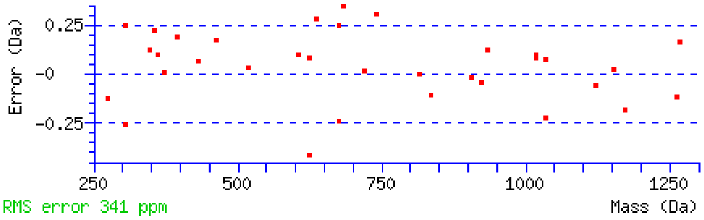


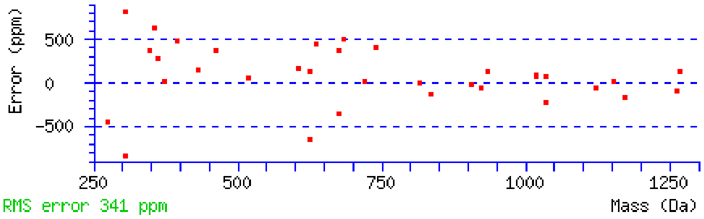


### All matches to this query

| Score | Mr(calc): | Delta | Sequence |
|---|---|---|---|
| 69.0 | 1639.8141 | 0.0009 | CLVPHTVNLSSAWR |
| 5.7 | 1637.8039 | 2.0111 | SFSQSSILIQHRR |
| 5.7 | 1638.7978 | 1.0172 | DQINIETKNKTVR |
| 5.7 | 1639.8294 | -0.0145 | GTDLSLASSVVLRSR |
| 5.7 | 1639.8294 | -0.0145 | GTDLSLASSVVLRSR |
| 4.8 | 1639.8004 | 0.0146 | TCKDGATELLLLAR |
| 3.5 | 1638.8277 | 0.9873 | MTGRGKTKPLNTVR |
| 3.3 | 1639.7988 | 0.0162 | AQDCISQGPLPTPTR |
| 2.5 | 1638.8076 | 1.0074 | CNPYVKTYLLPDR |
| 2.4 | 1639.8239 | -0.0090 | VKGVAYTNTTMLGER |

---

Spectrum No: 377; Query: 2165; Rank: 1

---

## Peptide View

MS/MS Fragmentation of **YRAEYAVANDTGFVDIPQQEK**
Found in **IPI00326070**, Tax_Id=10116 Gene_Symbol=Ctsl Cathepsin L1 precursor

Match to Query 2165: 2414.146062 from(805.722630,3+)
Title: 100101RatKid_NS_deglyco_14.3110.3110.3.dta
Data file K:\NewmanPaper\Piliang\3SubProteomes\Piliang3SP\mgf5ppm\ERLIC_3SubProteomes5ppm.mgf

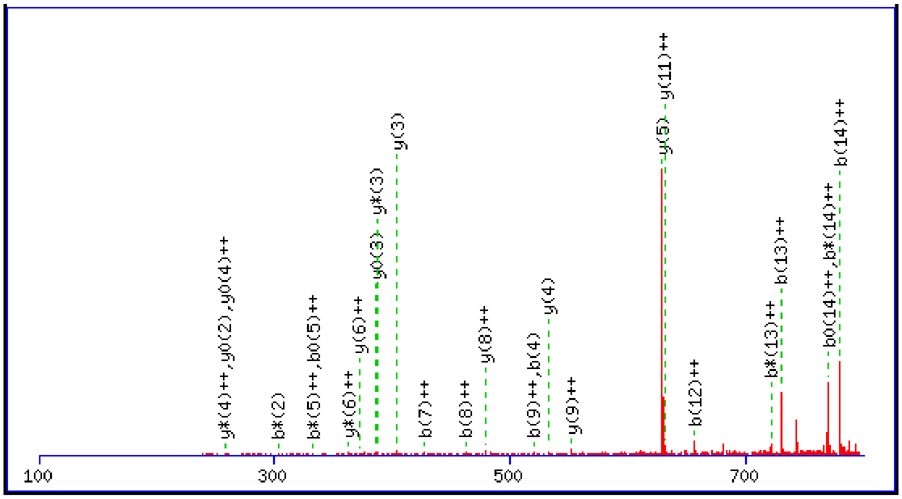


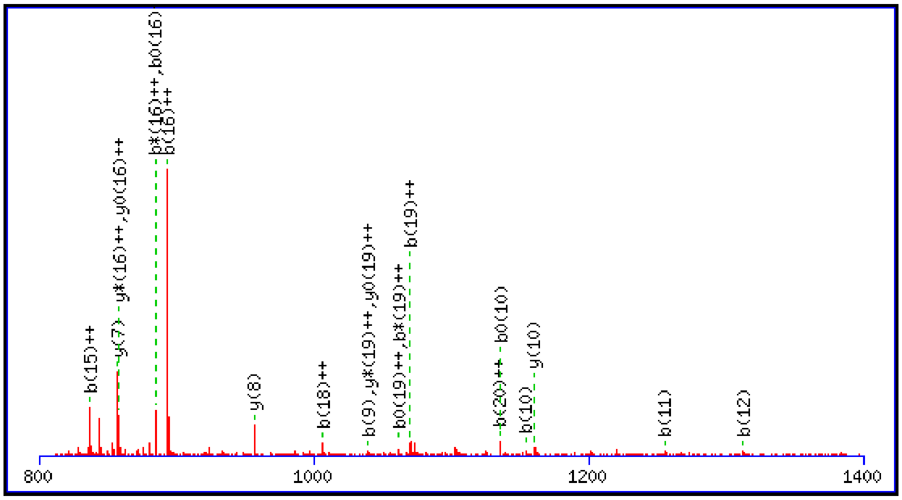


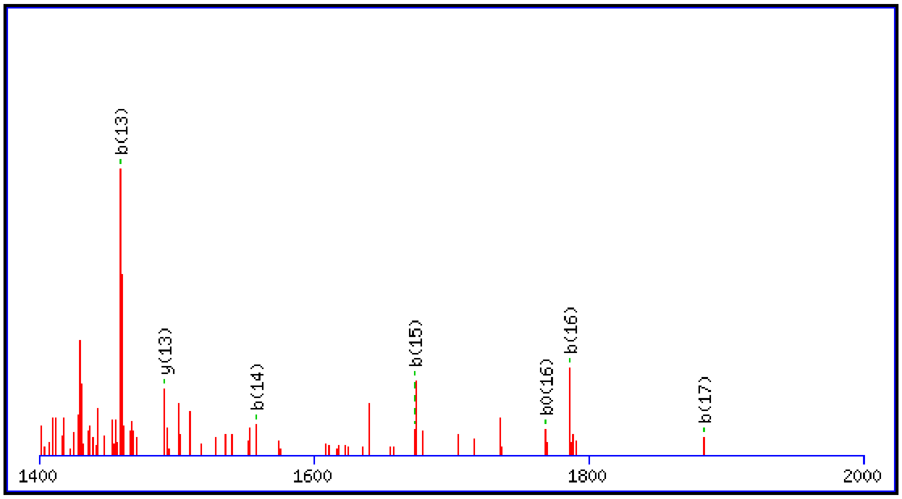


**Monoisotopic mass of neutral peptide Mr(calc):** 2414.1390
**Fixed modifications:** Carbamidomethyl (C)
**Variable modifications:**
**N9** : Deamidated_N (N)
**Ions Score:** 69 **Expect:** 3.7e-005
**Matches (Bold Red):** 54/230 fragment ions using 75 most intense peaks

| # | b | $b^{++}$ | b* | $b^{*++}$ | $b^0$ | $b^{0++}$ | Seq. | y | $y^{++}$ | y* | $y^{*++}$ | $y^0$ | $y^{0++}$ | # |
|---|---|---|---|---|---|---|---|---|---|---|---|---|---|---|
| 1 | 164.0706 | 82.5389 | | | | | Y | | | | | | | 21 |
| 2 | 320.1717 | 160.5895 | **303.1452** | 152.0762 | | | R | 2252.0830 | 1126.5451 | 2235.0564 | 1118.0318 | 2234.0724 | 1117.5398 | 20 |
| 3 | 391.2088 | 196.1081 | 374.1823 | 187.5948 | | | A | 2095.9819 | 1048.4946 | 2078.9553 | **1039.9813** | 2077.9713 | **1039.4893** | 19 |
| 4 | **520.2514** | 260.6293 | 503.2249 | 252.1161 | 502.2409 | 251.6241 | E | 2024.9447 | 1012.9760 | 2007.9182 | 1004.4627 | 2006.9342 | 1003.9707 | 18 |
| 5 | 683.3148 | 342.1610 | 666.2882 | **333.6477** | 665.3042 | **333.1557** | Y | 1895.9021 | 948.4547 | 1878.8756 | 939.9414 | 1877.8916 | 939.4494 | 17 |

| | | | | | | | | | | | | | | |
|---|---|---|---|---|---|---|---|---|---|---|---|---|---|---|
| 6 | 754.3519 | 377.6796 | 737.3253 | 369.1663 | 736.3413 | 368.6743 | A | 1732.8388 | 866.9230 | 1715.8123 | **858.4098** | 1714.8283 | **857.9178** | 16 |
| 7 | 853.4203 | **427.2138** | 836.3937 | 418.7005 | 835.4097 | 418.2085 | V | 1661.8017 | 831.4045 | 1644.7752 | 822.8912 | 1643.7911 | 822.3992 | 15 |
| 8 | 924.4574 | **462.7323** | 907.4308 | 454.2191 | 906.4468 | 453.7271 | A | 1562.7333 | 781.8703 | 1545.7067 | 773.3570 | 1544.7227 | 772.8650 | 14 |
| 9 | **1039.4843** | **520.2458** | 1022.4578 | 511.7325 | 1021.4738 | 511.2405 | N | **1491.6962** | 746.3517 | 1474.6696 | 737.8385 | 1473.6856 | 737.3464 | 13 |
| 10 | **1154.5113** | 577.7593 | 1137.4847 | 569.2460 | **1136.5007** | 568.7540 | D | 1376.6692 | 688.8383 | 1359.6427 | 680.3250 | 1358.6587 | 679.8330 | 12 |
| 11 | **1255.5590** | 628.2831 | 1238.5324 | 619.7698 | 1237.5484 | 619.2778 | T | 1261.6423 | **631.3248** | 1244.6157 | 622.8115 | 1243.6317 | 622.3195 | 11 |
| 12 | **1312.5804** | **656.7938** | 1295.5539 | 648.2806 | 1294.5699 | 647.7886 | G | **1160.5946** | 580.8009 | 1143.5681 | 572.2877 | 1142.5841 | 571.7957 | 10 |
| 13 | **1459.6488** | **730.3281** | 1442.6223 | **721.8148** | 1441.6383 | 721.3228 | F | 1103.5732 | **552.2902** | 1086.5466 | 543.7769 | 1085.5626 | 543.2849 | 9 |
| 14 | **1558.7172** | **779.8623** | 1541.6907 | **771.3490** | 1540.7067 | **770.8570** | V | **956.5047** | **478.7560** | 939.4782 | 470.2427 | 938.4942 | 469.7507 | 8 |
| 15 | **1673.7442** | **837.3757** | 1656.7176 | 828.8625 | 1655.7336 | 828.3704 | D | **857.4363** | 429.2218 | 840.4098 | 420.7085 | 839.4258 | 420.2165 | 7 |
| 16 | **1786.8283** | **893.9178** | 1769.8017 | **885.4045** | **1768.8177** | **884.9125** | I | 742.4094 | **371.7083** | 725.3828 | **363.1951** | 724.3988 | 362.7030 | 6 |
| 17 | **1883.8810** | 942.4441 | 1866.8545 | 933.9309 | 1865.8705 | 933.4389 | P | **629.3253** | 315.1663 | 612.2988 | 306.6530 | 611.3148 | 306.1610 | 5 |
| 18 | 2011.9396 | **1006.4734** | 1994.9130 | 997.9602 | 1993.9290 | 997.4682 | Q | **532.2726** | 266.6399 | 515.2460 | **258.1266** | 514.2620 | **257.6346** | 4 |
| 19 | 2139.9982 | **1070.5027** | 2122.9716 | **1061.9894** | 2121.9876 | **1061.4974** | Q | **404.2140** | 202.6106 | **387.1874** | 194.0974 | **386.2034** | 193.6053 | 3 |
| 20 | 2269.0408 | **1135.0240** | 2252.0142 | 1126.5107 | 2251.0302 | 1126.0187 | E | 276.1554 | 138.5813 | 259.1288 | 130.0681 | **258.1448** | 129.5761 | 2 |
| 21 | | | | | | | K | 147.1128 | 74.0600 | 130.0863 | 65.5468 | | | 1 |

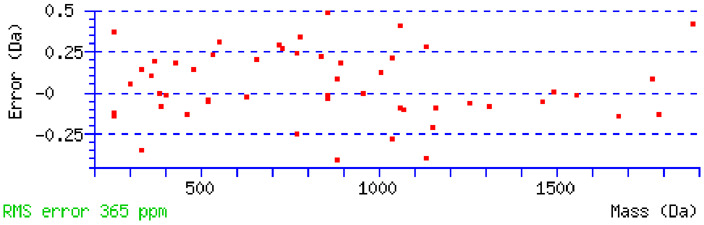


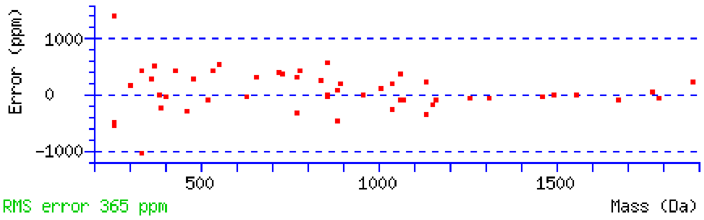


### All matches to this query

| Score | Mr(calc): | Delta | Sequence |
|---|---|---|---|
| 68.9 | 2414.1390 | 0.0071 | YRAEYAVANDTGFVDIPQQEK |
| 12.3 | 2413.1550 | 0.9911 | YRAEYAVANDTGFVDIPQQEK |
| 5.8 | 2414.1631 | -0.0170 | HPGSNSLSGPVKALIAQEWSEK |
| 5.1 | 2414.1284 | 0.0177 | YQANLEITGPKVASPGPQEKK |
| 5.1 | 2414.1284 | 0.0177 | YQANLEITGPKVASPGPQEKK |
| 4.1 | 2412.1488 | 1.9973 | TAQPRHHQDPYKAGLSHYVK |
| 3.9 | 2414.1454 | 0.0007 | GVTILSGQPACGLSLQPEGHWK |
| 3.4 | 2414.1375 | 0.0086 | CQPRLMYPAKLSINIDGETK |
| 3.2 | 2414.1495 | -0.0034 | LNSPTDSTPALLSSAVTPRKAK |
| 3.2 | 2414.1375 | 0.0086 | CQPRLLYPAKLSMNIDGETK |

---

Spectrum No: 378; Query: 767; Rank: 1

---

## Peptide View

MS/MS Fragmentation of **QIDYILGQYNTTK**
Found in **IPI00325312**, Tax_Id=10116 Gene_Symbol=Prom1 prominin 1 isoform 1

Match to Query 767: 1556.775188 from(779.394870,2+)
Title: 100101RatKid_NS_deglyco_25.2958.2958.2.dta
Data file K:\NewmanPaper\Piliang\3SubProteomes\Piliang3SP\mgf5ppm\ERLIC_3SubProteomes5ppm.mgf

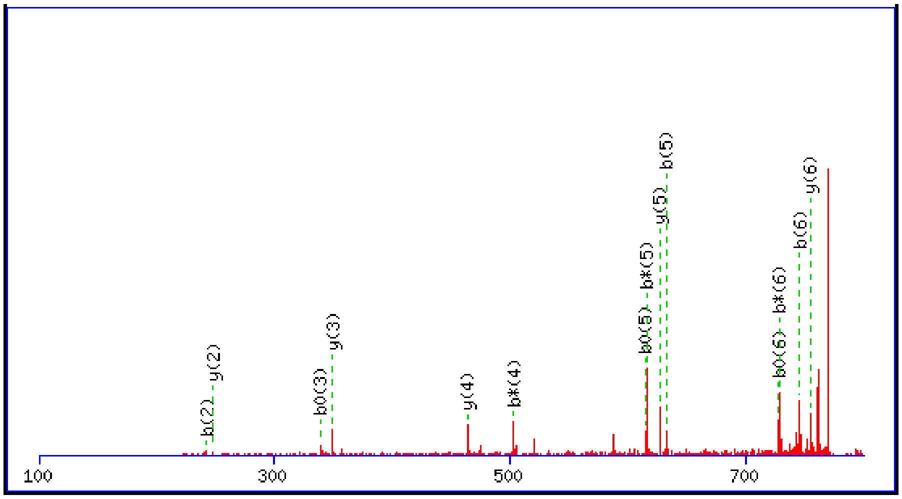


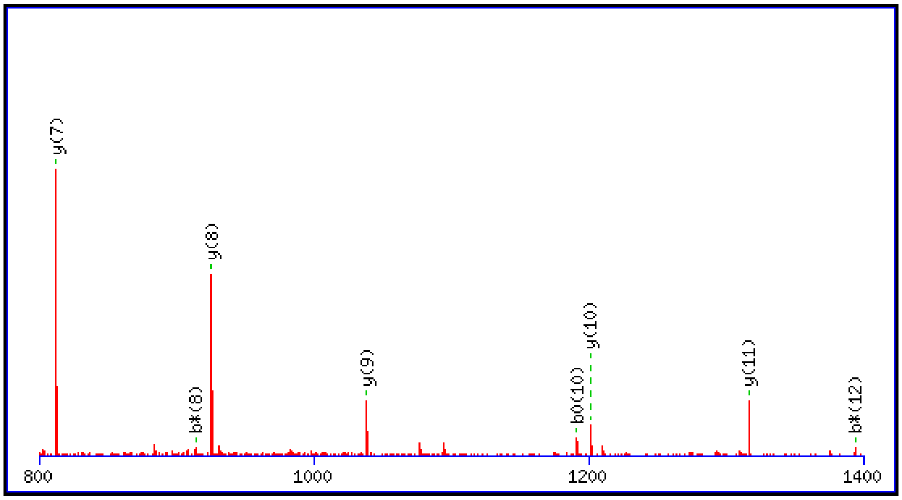


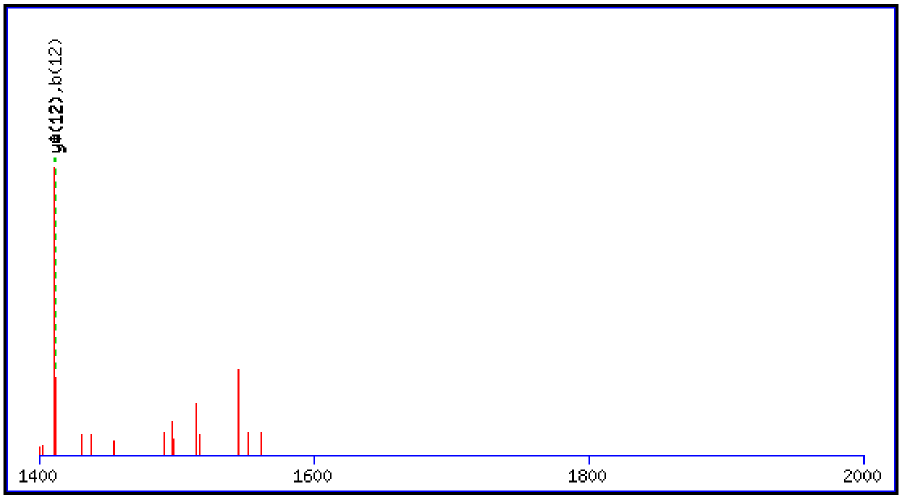


**Monoisotopic mass of neutral peptide Mr(calc):** 1556.7722
**Fixed modifications:** Carbamidomethyl (C)
**Variable modifications:**
**N10** : Deamidated_N (N)
**Ions Score:** 69 **Expect:** 2.2e-005
**Matches (Bold Red):** 25/138 fragment ions using 37 most intense peaks

| # | b | $b^{++}$ | b* | $b^{*++}$ | $b^0$ | $b^{0++}$ | Seq. | y | $y^{++}$ | y* | $y^{*++}$ | $y^0$ | $y^{0++}$ | # |
|---|---|---|---|---|---|---|---|---|---|---|---|---|---|---|
| 1 | 129.0659 | 65.0366 | 112.0393 | 56.5233 | | | Q | | | | | | | 13 |
| 2 | **242.1499** | 121.5786 | 225.1234 | 113.0653 | | | I | 1429.7209 | 715.3641 | **1412.6944** | 706.8508 | **1411.7104** | 706.3588 | 12 |
| 3 | 357.1769 | 179.0921 | 340.1503 | 170.5788 | **339.1663** | 170.0868 | D | **1316.6369** | 658.8221 | 1299.6103 | 650.3088 | 1298.6263 | 649.8168 | 11 |
| 4 | 520.2402 | 260.6237 | **503.2136** | 252.1105 | 502.2296 | 251.6185 | Y | **1201.6099** | 601.3086 | 1184.5834 | 592.7953 | 1183.5994 | 592.3033 | 10 |
| 5 | **633.3243** | 317.1658 | **616.2977** | 308.6525 | **615.3137** | 308.1605 | I | **1038.5466** | 519.7769 | 1021.5200 | 511.2637 | 1020.5360 | 510.7717 | 9 |

| | | | | | | | | | | | | | |
|---|---|---|---|---|---|---|---|---|---|---|---|---|---|
| 6 | 746.4083 | 373.7078 | 729.3818 | 365.1945 | 728.3978 | 364.7025 | L | 925.4625 | 463.2349 | 908.4360 | 454.7216 | 907.4520 | 454.2296 | 8 |
| 7 | 803.4298 | 402.2185 | 786.4032 | 393.7053 | 785.4192 | 393.2132 | G | 812.3785 | 406.6929 | 795.3519 | 398.1796 | 794.3679 | 397.6876 | 7 |
| 8 | 931.4884 | 466.2478 | 914.4618 | 457.7345 | 913.4778 | 457.2425 | Q | 755.3570 | 378.1821 | 738.3305 | 369.6689 | 737.3464 | 369.1769 | 6 |
| 9 | 1094.5517 | 547.7795 | 1077.5251 | 539.2662 | 1076.5411 | 538.7742 | Y | 627.2984 | 314.1529 | 610.2719 | 305.6396 | 609.2879 | 305.1476 | 5 |
| 10 | 1209.5786 | 605.2930 | 1192.5521 | 596.7797 | 1191.5681 | 596.2877 | N | 464.2351 | 232.6212 | 447.2085 | 224.1079 | 446.2245 | 223.6159 | 4 |
| 11 | 1310.6263 | 655.8168 | 1293.5998 | 647.3035 | 1292.6157 | 646.8115 | T | 349.2082 | 175.1077 | 332.1816 | 166.5944 | 331.1976 | 166.1024 | 3 |
| 12 | 1411.6740 | 706.3406 | 1394.6474 | 697.8274 | 1393.6634 | 697.3353 | T | 248.1605 | 124.5839 | 231.1339 | 116.0706 | 230.1499 | 115.5786 | 2 |
| 13 | | | | | | | K | 147.1128 | 74.0600 | 130.0863 | 65.5468 | | | 1 |

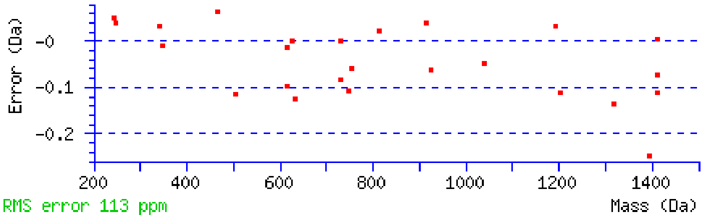


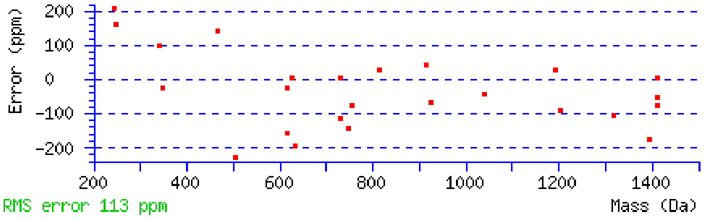


**All matches to this query**

| Score | Mr(calc): | Delta | Sequence |
|---|---|---|---|
| 68.8 | 1556.7722 | 0.0030 | QIDYILGQYNTTK |
| 6.5 | 1556.7712 | 0.0040 | DSRGHLGLVANPLK |
| 4.6 | 1556.7712 | 0.0040 | IKYDAIARQGQSK |
| 3.8 | 1554.7712 | 2.0040 | MAPPSEETPLISQR |
| 2.9 | 1556.7786 | -0.0034 | GPLAVLTCIATHPK |
| 2.8 | 1556.7699 | 0.0053 | TDLTSVLKGNLSTK |
| 2.3 | 1554.7663 | 2.0089 | RMETKPVITCLK |
| 1.8 | 1555.7647 | 1.0105 | ADSFLLERTLPSK |
| 1.8 | 1555.7647 | 1.0105 | ADSFLLERTLPSK |
| 1.0 | 1556.7738 | 0.0014 | ELLNIDLIKDYK |

---

Spectrum No: 379; Query: 422; Rank: 1

---

## Peptide View

MS/MS Fragmentation of **GVAVTNTSQLGFR**
Found in **IPI00192845**, Tax_Id=10116 Gene_Symbol=P2rx4 40 kDa protein

Match to Query 422: 1349.694888 from(675.854720,2+)
Title: 100101RatKid_NS_deglyco_21.2369.2369.2.dta
Data file K:\NewmanPaper\Piliang\3SubProteomes\Piliang3SP\mgf5ppm\ERLIC_3SubProteomes5ppm.mgf

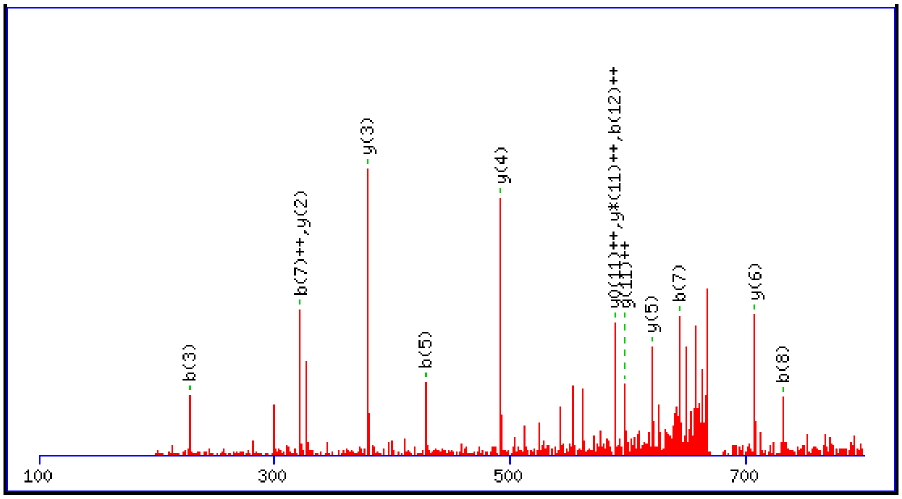


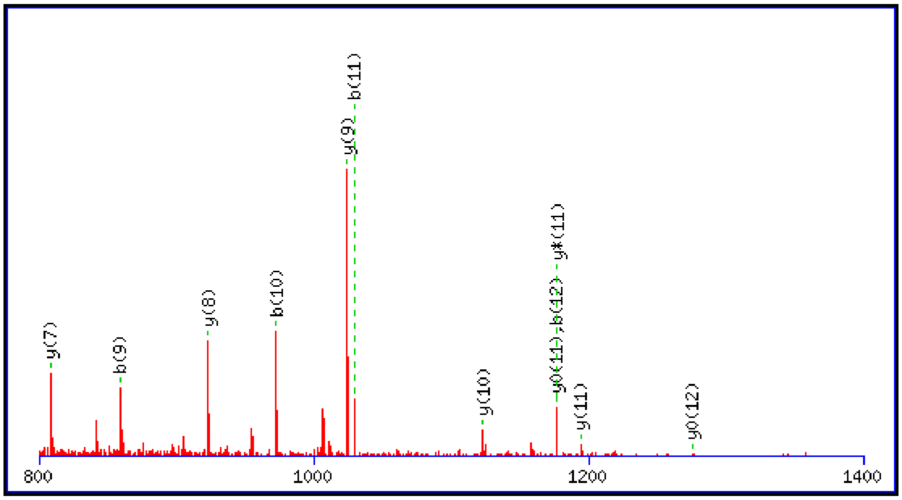


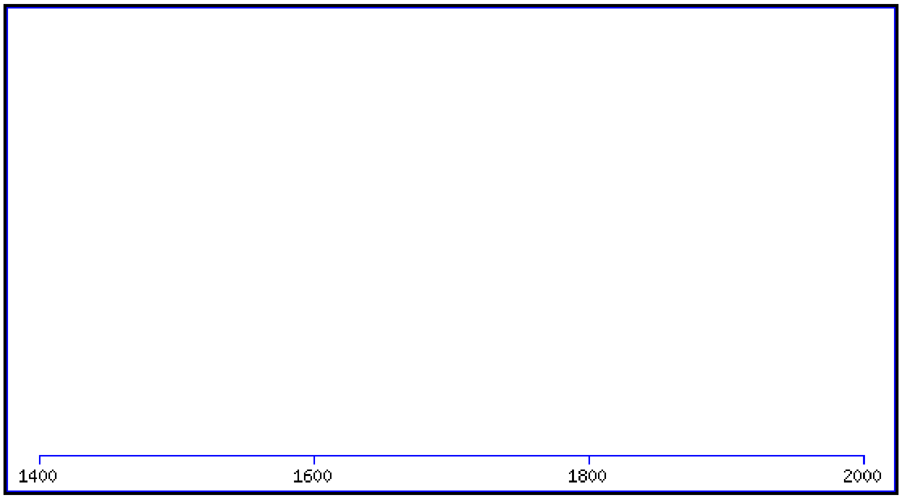


**Monoisotopic mass of neutral peptide Mr(calc):** 1349.6939
**Fixed modifications:** Carbamidomethyl (C)
**Variable modifications:**
**N6** : Deamidated_N (N)
**Ions Score:** 69 **Expect:** 1.7e-005
**Matches (Bold Red):** 26/116 fragment ions using 38 most intense peaks

| # | b | $b^{++}$ | b* | $b^{*++}$ | $b^0$ | $b^{0++}$ | Seq. | y | $y^{++}$ | y* | $y^{*++}$ | $y^0$ | $y^{0++}$ | # |
|---|---|---|---|---|---|---|---|---|---|---|---|---|---|---|
| 1 | 58.0287 | 29.5180 | | | | | G | | | | | | | 13 |
| 2 | 157.0972 | 79.0522 | | | | | V | 1293.6797 | 647.3435 | 1276.6532 | 638.8302 | **1275.6692** | 638.3382 | 12 |
| 3 | **228.1343** | 114.5708 | | | | | A | **1194.6113** | **597.8093** | **1177.5848** | **589.2960** | **1176.6008** | **588.8040** | 11 |
| 4 | 327.2027 | 164.1050 | | | | | V | **1123.5742** | 562.2907 | 1106.5477 | 553.7775 | 1105.5636 | 553.2855 | 10 |
| 5 | **428.2504** | 214.6288 | | | 410.2398 | 205.6235 | T | **1024.5058** | 512.7565 | 1007.4792 | 504.2433 | 1006.4952 | 503.7513 | 9 |

| | | | | | | | | | | | | | | |
|---|---|---|---|---|---|---|---|---|---|---|---|---|---|---|
| **6** | 543.2773 | 272.1423 | 526.2507 | 263.6290 | 525.2667 | 263.1370 | **N** | **923.4581** | 462.2327 | 906.4316 | 453.7194 | 905.4476 | 453.2274 | **8** |
| **7** | **644.3250** | **322.6661** | 627.2984 | 314.1529 | 626.3144 | 313.6608 | **T** | **808.4312** | 404.7192 | 791.4046 | 396.2060 | 790.4206 | 395.7139 | **7** |
| **8** | **731.3570** | 366.1821 | 714.3305 | 357.6689 | 713.3464 | 357.1769 | **S** | **707.3835** | 354.1954 | 690.3570 | 345.6821 | 689.3729 | 345.1901 | **6** |
| **9** | **859.4156** | 430.2114 | 842.3890 | 421.6982 | 841.4050 | 421.2061 | **Q** | **620.3515** | 310.6794 | 603.3249 | 302.1661 | | | **5** |
| **10** | **972.4996** | 486.7535 | 955.4731 | 478.2402 | 954.4891 | 477.7482 | **L** | **492.2929** | 246.6501 | 475.2663 | 238.1368 | | | **4** |
| **11** | **1029.5211** | 515.2642 | 1012.4946 | 506.7509 | 1011.5105 | 506.2589 | **G** | **379.2088** | 190.1081 | 362.1823 | 181.5948 | | | **3** |
| **12** | **1176.5895** | **588.7984** | 1159.5630 | 580.2851 | 1158.5790 | 579.7931 | **F** | **322.1874** | 161.5973 | 305.1608 | 153.0840 | | | **2** |
| **13** | | | | | | | **R** | 175.1190 | 88.0631 | 158.0924 | 79.5498 | | | **1** |

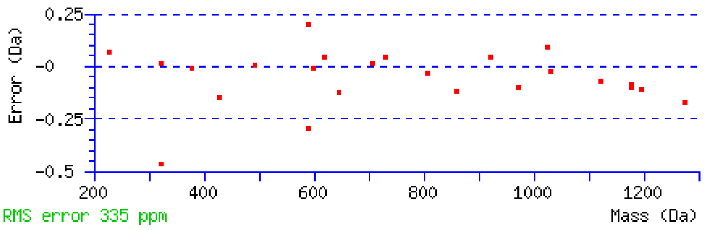


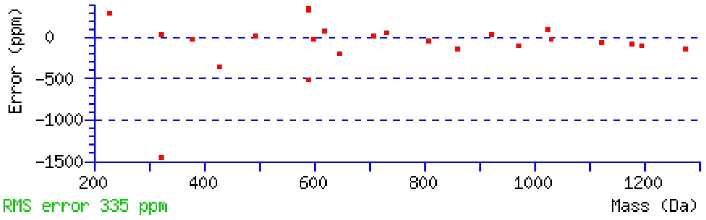


### All matches to this query

| Score | Mr(calc): | Delta | Sequence |
|---|---|---|---|
| 68.6 | 1349.6939 | 0.0009 | GVAVTNTSQLGFR |
| 8.4 | 1349.6874 | 0.0074 | VKVGVNGFGCTGR |
| 6.8 | 1349.6912 | 0.0037 | RAHQLRPNDSR |
| 4.8 | 1349.6907 | 0.0042 | ARALQMEKTMR |
| 1.6 | 1349.6816 | 0.0132 | NLSRVPSQITR |
| 1.6 | 1349.6816 | 0.0132 | NLSRVPSQITR |
| 1.5 | 1349.6939 | 0.0010 | ENVYLTAERVR |
| 1.3 | 1349.7068 | -0.0119 | NLSISVSGLRPK |

---

Spectrum No: 380; Query: 2701; Rank: 1

---

## Peptide View

MS/MS Fragmentation of **ADCLDASDESACPTRFPNGTYCPAAMFECK**
Found in **IPI00205325**, Tax_Id=10116 Gene_Symbol=Lrp2 Low-density lipoprotein receptor-related protein 2 precursor

Match to Query 2701: 3441.379662 from(1148.133830,3+)
Title: 100101RatKid_NS_deglyco_16.4202.4202.3.dta
Data file K:\NewmanPaper\Piliang\3SubProteomes\Piliang3SP\mgf5ppm\ERLIC_3SubProteomes5ppm.mgf

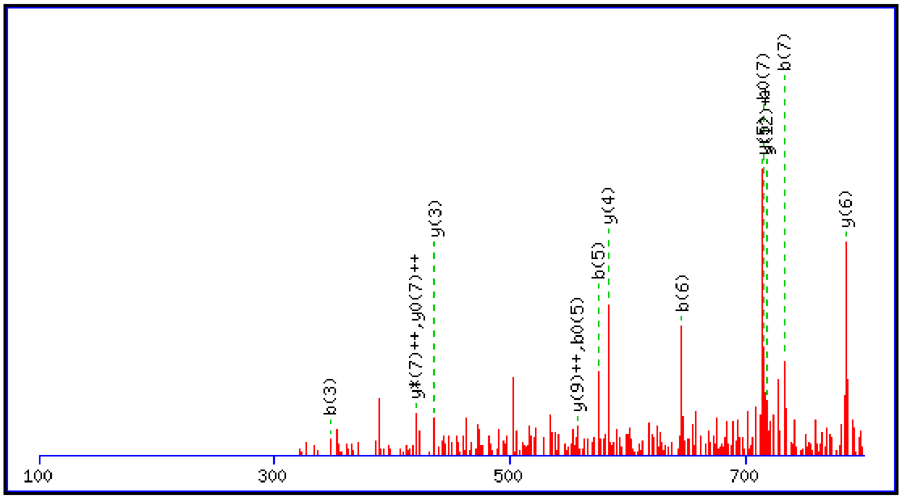

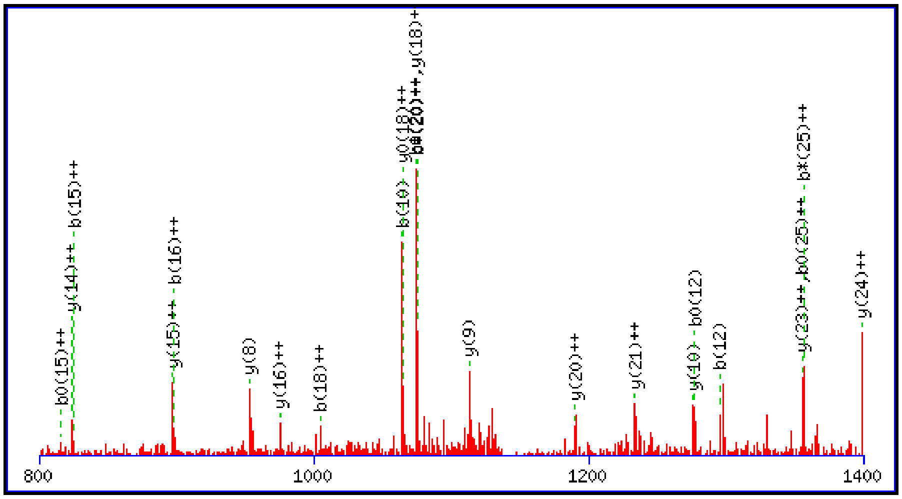


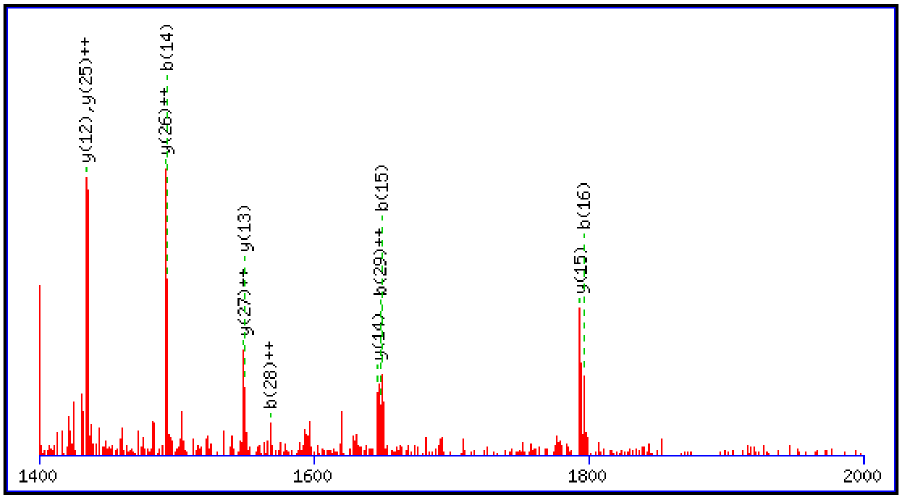


**Monoisotopic mass of neutral peptide Mr(calc):** 3441.3716
**Fixed modifications:** Carbamidomethyl (C)
**Variable modifications:**
**N18** : Deamidated_N (N)
**Ions Score:** 68 **Expect:** 5e-005
**Matches (Bold Red):** 49/314 fragment ions using 89 most intense peaks

| # | b | $b^{++}$ | b* | $b^{*++}$ | $b^{0}$ | $b^{0++}$ | Seq. | y | $y^{++}$ | y* | $y^{*++}$ | $y^{0}$ | $y^{0++}$ | # |
|---|---|---|---|---|---|---|---|---|---|---|---|---|---|---|
| 1 | 72.0444 | 36.5258 | | | | | A | | | | | | | 30 |
| 2 | 187.0713 | 94.0393 | | | 169.0608 | 85.0340 | D | 3371.3418 | 1686.1745 | 3354.3152 | 1677.6613 | 3353.3312 | 1677.1693 | 29 |
| 3 | **347.1020** | 174.0546 | | | 329.0914 | 165.0493 | C | 3256.3149 | 1628.6611 | 3239.2883 | 1620.1478 | 3238.3043 | 1619.6558 | 28 |
| 4 | 460.1860 | 230.5967 | | | 442.1755 | 221.5914 | L | 3096.2842 | **1548.6457** | 3079.2577 | 1540.1325 | 3078.2736 | 1539.6405 | 27 |
| 5 | **575.2130** | 288.1101 | | | **557.2024** | 279.1049 | D | 2983.2001 | **1492.1037** | 2966.1736 | 1483.5904 | 2965.1896 | 1483.0984 | 26 |
| 6 | **646.2501** | 323.6287 | | | 628.2395 | 314.6234 | A | 2868.1732 | **1434.5902** | 2851.1466 | 1426.0770 | 2850.1626 | 1425.5850 | 25 |
| 7 | **733.2821** | 367.1447 | | | **715.2716** | 358.1394 | S | 2797.1361 | **1399.0717** | 2780.1095 | 1390.5584 | 2779.1255 | 1390.0664 | 24 |
| 8 | 848.3091 | 424.6582 | | | 830.2985 | 415.6529 | D | 2710.1041 | **1355.5557** | 2693.0775 | 1347.0424 | 2692.0935 | 1346.5504 | 23 |
| 9 | 977.3517 | 489.1795 | | | 959.3411 | 480.1742 | E | 2595.0771 | 1298.0422 | 2578.0506 | 1289.5289 | 2577.0665 | 1289.0369 | 22 |
| 10 | **1064.3837** | 532.6955 | | | 1046.3731 | 523.6902 | S | 2466.0345 | **1233.5209** | 2449.0080 | 1225.0076 | 2448.0240 | 1224.5156 | 21 |
| 11 | 1135.4208 | 568.2140 | | | 1117.4102 | 559.2088 | A | 2379.0025 | **1190.0049** | 2361.9759 | 1181.4916 | 2360.9919 | 1180.9996 | 20 |
| 12 | **1295.4515** | 648.2294 | | | **1277.4409** | 639.2241 | C | 2307.9654 | 1154.4863 | 2290.9388 | 1145.9731 | 2289.9548 | 1145.4810 | 19 |
| 13 | 1392.5042 | 696.7557 | | | 1374.4937 | 687.7505 | P | 2147.9347 | **1074.4710** | 2130.9082 | 1065.9577 | 2129.9242 | **1065.4657** | 18 |
| 14 | **1493.5519** | 747.2796 | | | 1475.5413 | 738.2743 | T | 2050.8820 | 1025.9446 | 2033.8554 | 1017.4313 | 2032.8714 | 1016.9393 | 17 |
| 15 | **1649.6530** | **825.3301** | 1632.6265 | 816.8169 | 1631.6424 | **816.3249** | R | 1949.8343 | **975.4208** | 1932.8077 | 966.9075 | 1931.8237 | 966.4155 | 16 |
| 16 | **1796.7214** | **898.8644** | 1779.6949 | 890.3511 | 1778.7109 | 889.8591 | F | **1793.7332** | **897.3702** | 1776.7066 | 888.8569 | 1775.7226 | 888.3649 | 15 |
| 17 | 1893.7742 | 947.3907 | 1876.7476 | 938.8775 | 1875.7636 | 938.3855 | P | **1646.6648** | **823.8360** | 1629.6382 | 815.3227 | 1628.6542 | 814.8307 | 14 |
| 18 | 2008.8011 | **1004.9042** | 1991.7746 | 996.3909 | 1990.7906 | 995.8989 | N | **1549.6120** | 775.3096 | 1532.5854 | 766.7964 | 1531.6014 | 766.3044 | 13 |

| | | | | | | | | | | | | | | |
|---|---|---|---|---|---|---|---|---|---|---|---|---|---|---|
| **19** | 2065.8226 | 1033.4149 | 2048.7960 | 1024.9017 | 2047.8120 | 1024.4097 | **G** | **1434.5851** | **717.7962** | 1417.5585 | 709.2829 | 1416.5745 | 708.7909 | **12** |
| **20** | 2166.8703 | 1083.9388 | 2149.8437 | **1075.4255** | 2148.8597 | **1074.9335** | **T** | 1377.5636 | 689.2854 | 1360.5370 | 680.7722 | 1359.5530 | 680.2802 | **11** |
| **21** | 2329.9336 | 1165.4704 | 2312.9070 | 1156.9572 | 2311.9230 | 1156.4652 | **Y** | **1276.5159** | 638.7616 | 1259.4894 | 630.2483 | 1258.5054 | 629.7563 | **10** |
| **22** | 2489.9642 | 1245.4858 | 2472.9377 | 1236.9725 | 2471.9537 | 1236.4805 | **C** | **1113.4526** | **557.2299** | 1096.4260 | 548.7167 | 1095.4420 | 548.2246 | **9** |
| **23** | 2587.0170 | 1294.0121 | 2569.9905 | 1285.4989 | 2569.0064 | 1285.0069 | **P** | **953.4219** | 477.2146 | 936.3954 | 468.7013 | 935.4114 | 468.2093 | **8** |
| **24** | 2658.0541 | 1329.5307 | 2641.0276 | 1321.0174 | 2640.0436 | 1320.5254 | **A** | 856.3692 | 428.6882 | 839.3426 | **420.1750** | 838.3586 | **419.6829** | **7** |
| **25** | 2729.0912 | 1365.0493 | 2712.0647 | **1356.5360** | 2711.0807 | **1356.0440** | **A** | **785.3321** | 393.1697 | 768.3055 | 384.6564 | 767.3215 | 384.1644 | **6** |
| **26** | 2860.1317 | 1430.5695 | 2843.1052 | 1422.0562 | 2842.1212 | 1421.5642 | **M** | **714.2949** | 357.6511 | 697.2684 | 349.1378 | 696.2844 | 348.6458 | **5** |
| **27** | 3007.2001 | 1504.1037 | 2990.1736 | 1495.5904 | 2989.1896 | 1495.0984 | **F** | **583.2545** | 292.1309 | 566.2279 | 283.6176 | 565.2439 | 283.1256 | **4** |
| **28** | 3136.2427 | **1568.6250** | 3119.2162 | 1560.1117 | 3118.2322 | 1559.6197 | **E** | **436.1860** | 218.5967 | 419.1595 | 210.0834 | 418.1755 | 209.5914 | **3** |
| **29** | 3296.2734 | **1648.6403** | 3279.2468 | 1640.1271 | 3278.2628 | 1639.6350 | **C** | 307.1435 | 154.0754 | 290.1169 | 145.5621 | | | **2** |
| **30** | | | | | | | **K** | 147.1128 | 74.0600 | 130.0863 | 65.5468 | | | **1** |

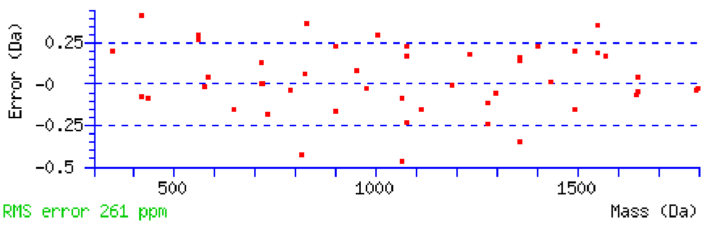


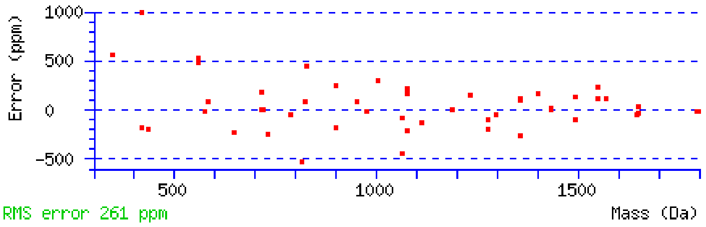


### All matches to this query

| Score | Mr(calc): | Delta | Sequence |
|---|---|---|---|
| 68.4 | 3441.3716 | 0.0080 | ADCLDASDESACPTRFPNGTYCPAAMFECK |
| 39.9 | 3440.3876 | 0.9920 | ADCLDASDESACPTRFPNGTYCPAAMFECK |
| 6.2 | 3439.3487 | 2.0310 | TNISEDREECCTTSKNATQENSGDNIPMR |
| 6.2 | 3439.3487 | 2.0310 | TNISEDREECCTTSKNATQENSGDNIPMR |
| 5.7 | 3439.3487 | 2.0310 | TNISEDREECCTTSKNATQENSGDNIPMR |
| 2.4 | 3440.3443 | 1.0353 | FMKDDISAPPTEGVVYGLYLEGAGWDK |
| 2.1 | 3439.3486 | 2.0310 | GSDELLSGSVLSSPNSNMSGMVVTANGNDSKK |
| 1.9 | 3441.3965 | -0.0168 | TTMNNYLPSNHMTMISQQPNNLGTNSLNK |
| 1.8 | 3441.3965 | -0.0168 | TTMNNYLPSNHMTMISQQPNNLGTNSLNK |
| 1.8 | 3441.4008 | -0.0212 | RWFILTDNCLYYFEFTTVSVWGGGNR |

---

Spectrum No: 381; Query: 898; Rank: 1

---

## Peptide View

MS/MS Fragmentation of **EQLFFNLSDPELR**
Found in **IPI00191766**, Tax_Id=10116 Gene_Symbol=L1cam Isoform 1 of Neural cell adhesion molecule L1 precursor

Match to Query 898: 1607.788048 from(804.901300,2+)
Title: 100101RatKid_NS_deglyco_25.4429.4429.2.dta
Data file K:\NewmanPaper\Piliang\3SubProteomes\Piliang3SP\mgf5ppm\ERLIC_3SubProteomes5ppm.mgf

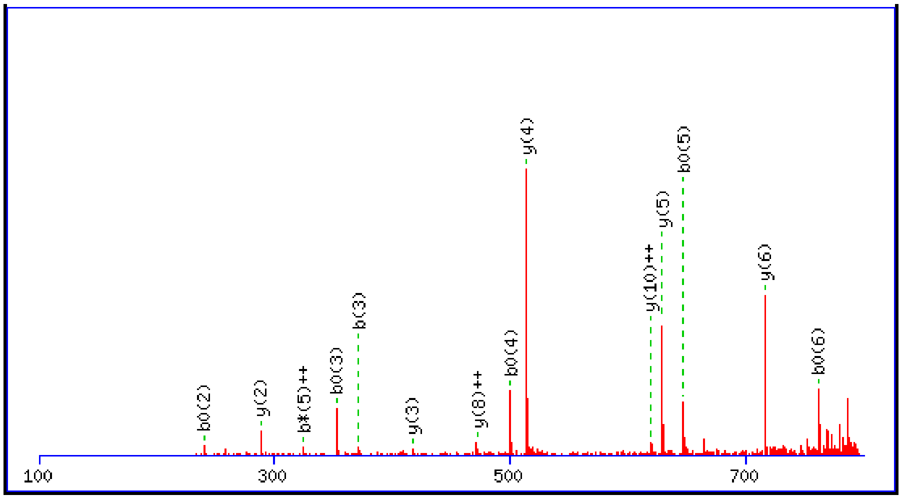


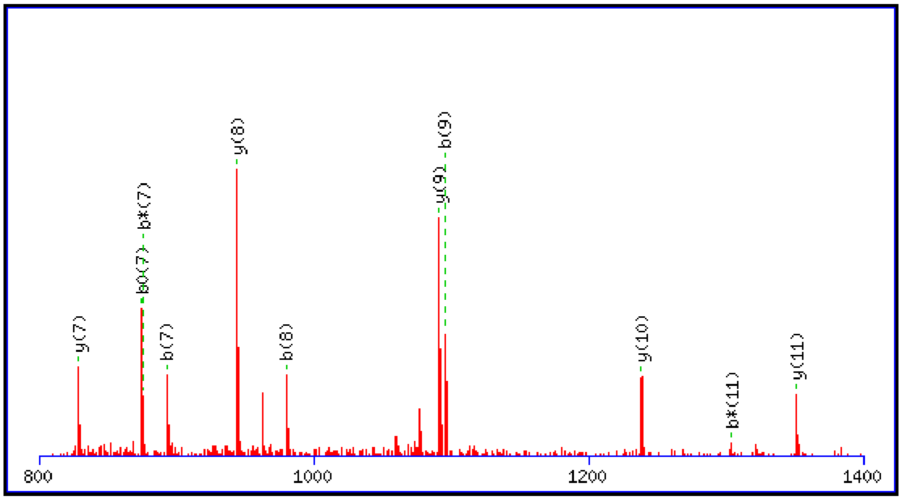


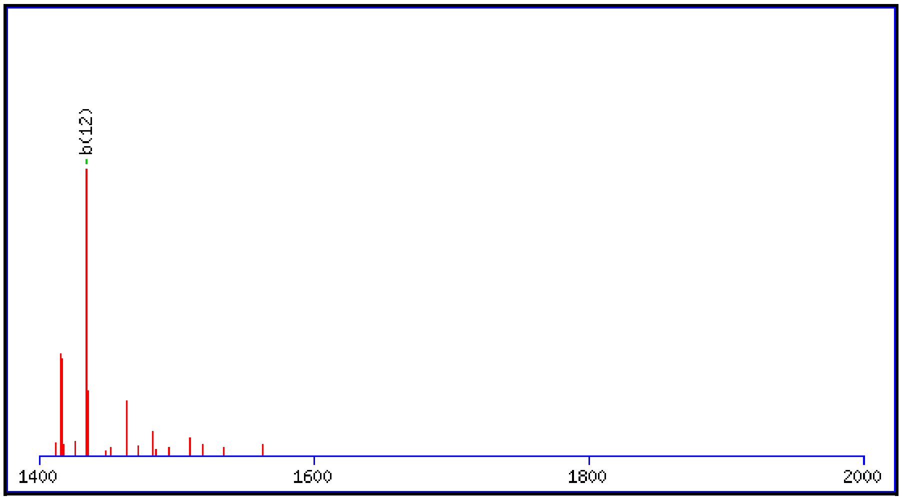


**Monoisotopic mass of neutral peptide Mr(calc):** 1607.7831
**Fixed modifications:** Carbamidomethyl (C)
**Variable modifications:**
**N6** : Deamidated_N (N)
**Ions Score:** 68 **Expect:** 2.5e-005
**Matches (Bold Red):** 26/138 fragment ions using 53 most intense peaks

| # | b | $b^{++}$ | b* | $b^{*++}$ | $b^0$ | $b^{0++}$ | Seq. | y | $y^{++}$ | y* | $y^{*++}$ | $y^0$ | $y^{0++}$ | # |
|---|---|---|---|---|---|---|---|---|---|---|---|---|---|---|
| 1 | 130.0499 | 65.5286 | | | 112.0393 | 56.5233 | E | | | | | | | 13 |
| 2 | 258.1084 | 129.5579 | 241.0819 | 121.0446 | **240.0979** | 120.5526 | Q | 1479.7478 | 740.3775 | 1462.7213 | 731.8643 | 1461.7372 | 731.3723 | 12 |
| 3 | **371.1925** | 186.0999 | 354.1660 | 177.5866 | **353.1819** | 177.0946 | L | **1351.6892** | 676.3483 | 1334.6627 | 667.8350 | 1333.6787 | 667.3430 | 11 |
| 4 | 518.2609 | 259.6341 | 501.2344 | 251.1208 | **500.2504** | 250.6288 | F | **1238.6052** | **619.8062** | 1221.5786 | 611.2929 | 1220.5946 | 610.8009 | 10 |
| 5 | 665.3293 | 333.1683 | 648.3028 | **324.6550** | **647.3188** | 324.1630 | F | **1091.5368** | 546.2720 | 1074.5102 | 537.7587 | 1073.5262 | 537.2667 | 9 |

| | | | | | | | | | | | | | | |
|---|---|---|---|---|---|---|---|---|---|---|---|---|---|---|
| **6** | 780.3563 | 390.6818 | 763.3297 | 382.1685 | **762.3457** | 381.6765 | **N** | **944.4683** | **472.7378** | 927.4418 | 464.2245 | 926.4578 | 463.7325 | **8** |
| **7** | **893.4403** | 447.2238 | **876.4138** | 438.7105 | **875.4298** | 438.2185 | **L** | **829.4414** | 415.2243 | 812.4149 | 406.7111 | 811.4308 | 406.2191 | **7** |
| **8** | **980.4724** | 490.7398 | 963.4458 | 482.2265 | 962.4618 | 481.7345 | **S** | **716.3573** | 358.6823 | 699.3308 | 350.1690 | 698.3468 | 349.6770 | **6** |
| **9** | **1095.4993** | 548.2533 | 1078.4728 | 539.7400 | 1077.4887 | 539.2480 | **D** | **629.3253** | 315.1663 | 612.2988 | 306.6530 | 611.3148 | 306.1610 | **5** |
| **10** | 1192.5521 | 596.7797 | 1175.5255 | 588.2664 | 1174.5415 | 587.7744 | **P** | **514.2984** | 257.6528 | 497.2718 | 249.1396 | 496.2878 | 248.6475 | **4** |
| **11** | 1321.5947 | 661.3010 | **1304.5681** | 652.7877 | 1303.5841 | 652.2957 | **E** | **417.2456** | 209.1264 | 400.2191 | 200.6132 | 399.2350 | 200.1212 | **3** |
| **12** | **1434.6787** | 717.8430 | 1417.6522 | 709.3297 | 1416.6682 | 708.8377 | **L** | **288.2030** | 144.6051 | 271.1765 | 136.0919 | | | **2** |
| **13** | | | | | | | **R** | 175.1190 | 88.0631 | 158.0924 | 79.5498 | | | **1** |

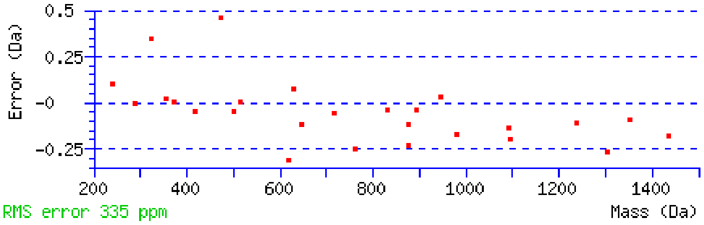


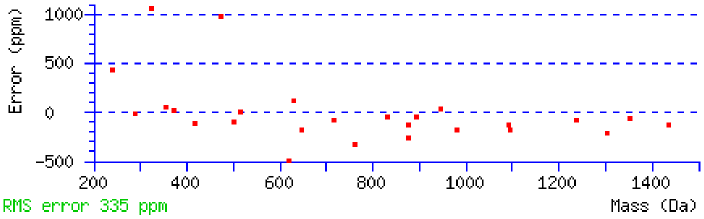


### All matches to this query

| Score | Mr(calc): | Delta | Sequence |
|---|---|---|---|
| 68.3 | 1607.7831 | 0.0049 | EQLFFNLSDPELR |
| 14.6 | 1606.7991 | 0.9889 | EQLFFNLSDPELR |
| 9.7 | 1607.8032 | -0.0151 | EKNRTSSIIPVER |
| 4.6 | 1607.7920 | -0.0039 | GKLAAATSLPEDTVR |
| 4.6 | 1607.7920 | -0.0039 | GKLAAATSLPEDTVR |
| 2.9 | 1606.7773 | 1.0108 | TGVFITLSIVLER |
| 2.0 | 1607.8032 | -0.0151 | EKNRTSSIIPVER |
| 1.9 | 1605.7780 | 2.0100 | QEAVSMAKKNSEQR |
| 0.6 | 1606.7718 | 1.0163 | ISLFADDMIVYLK |
| 0.3 | 1606.7760 | 1.0121 | ETEATNAILMEQIK |

---

Spectrum No: 382; Query: 671; Rank: 1

---

## Peptide View

MS/MS Fragmentation of **EVMSLLQPLNITK**
Found in **IPI00388265**, Tax_Id=10116 Gene_Symbol=Pgcp 48 kDa protein

Match to Query 671: 1501.803448 from(751.909000,2+)
Title: 091008RatKidney_NH4Format01_18.3736.3736.2.dta
Data file K:\NewmanPaper\Piliang\3SubProteomes\Piliang3SP\mgf5ppm\ERLIC_3SubProteomes5ppm.mgf

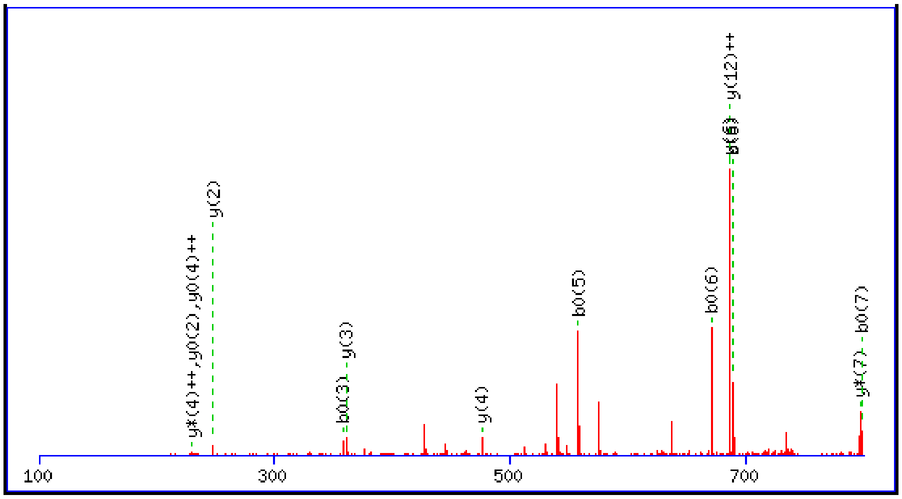


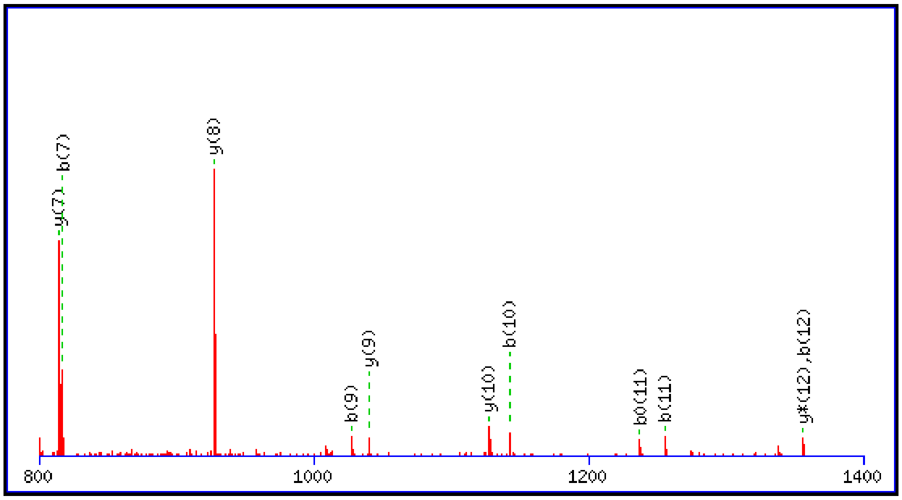


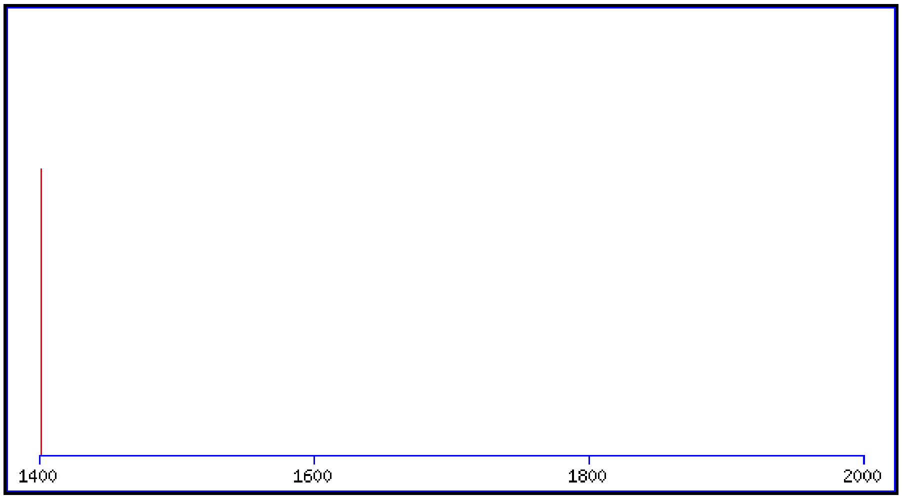


**Monoisotopic mass of neutral peptide Mr(calc):** 1501.8062
**Fixed modifications:** Carbamidomethyl (C)
**Variable modifications:**
**M3** : Oxidation (M)
**N10** : Deamidated_N (N)
**Ions Score:** 68 **Expect:** 1.4e-005
**Matches (Bold Red):** 25/130 fragment ions using 26 most intense peaks

| # | b | $b^{++}$ | b* | $b^{*++}$ | $b^0$ | $b^{0++}$ | Seq. | y | $y^{++}$ | y* | $y^{*++}$ | $y^0$ | $y^{0++}$ | # |
|---|---|---|---|---|---|---|---|---|---|---|---|---|---|---|
| 1 | 130.0499 | 65.5286 | | | 112.0393 | 56.5233 | E | | | | | | | 13 |
| 2 | 229.1183 | 115.0628 | | | 211.1077 | 106.0575 | V | 1373.7709 | **687.3891** | **1356.7443** | 678.8758 | 1355.7603 | 678.3838 | 12 |
| 3 | 376.1537 | 188.5805 | | | **358.1431** | 179.5752 | M | 1274.7024 | 637.8549 | 1257.6759 | 629.3416 | 1256.6919 | 628.8496 | 11 |
| 4 | 463.1857 | 232.0965 | | | 445.1752 | 223.0912 | S | **1127.6670** | 564.3372 | 1110.6405 | 555.8239 | 1109.6565 | 555.3319 | 10 |
| | | | | | | | | | | | | | | |

| | | | | | | | | | | | | | | |
|---|---|---|---|---|---|---|---|---|---|---|---|---|---|---|
| **5** | 576.2698 | 288.6385 | | | **558.2592** | 279.6332 | **L** | **1040.6350** | 520.8211 | 1023.6085 | 512.3079 | 1022.6245 | 511.8159 | **9** |
| **6** | **689.3538** | 345.1806 | | | **671.3433** | 336.1753 | **L** | **927.5510** | 464.2791 | 910.5244 | 455.7658 | 909.5404 | 455.2738 | **8** |
| **7** | **817.4124** | 409.2098 | 800.3859 | 400.6966 | **799.4019** | 400.2046 | **Q** | **814.4669** | 407.7371 | **797.4403** | 399.2238 | 796.4563 | 398.7318 | **7** |
| **8** | 914.4652 | 457.7362 | 897.4386 | 449.2230 | 896.4546 | 448.7309 | **P** | **686.4083** | 343.7078 | 669.3818 | 335.1945 | 668.3977 | 334.7025 | **6** |
| **9** | **1027.5492** | 514.2783 | 1010.5227 | 505.7650 | 1009.5387 | 505.2730 | **L** | 589.3555 | 295.1814 | 572.3290 | 286.6681 | 571.3450 | 286.1761 | **5** |
| **10** | **1142.5762** | 571.7917 | 1125.5496 | 563.2785 | 1124.5656 | 562.7864 | **N** | **476.2715** | 238.6394 | 459.2449 | **230.1261** | 458.2609 | **229.6341** | **4** |
| **11** | **1255.6602** | 628.3338 | 1238.6337 | 619.8205 | **1237.6497** | 619.3285 | **I** | **361.2445** | 181.1259 | 344.2180 | 172.6126 | 343.2340 | 172.1206 | **3** |
| **12** | **1356.7079** | 678.8576 | 1339.6814 | 670.3443 | 1338.6974 | 669.8523 | **T** | **248.1605** | 124.5839 | 231.1339 | 116.0706 | **230.1499** | 115.5786 | **2** |
| **13** | | | | | | | **K** | 147.1128 | 74.0600 | 130.0863 | 65.5468 | | | **1** |

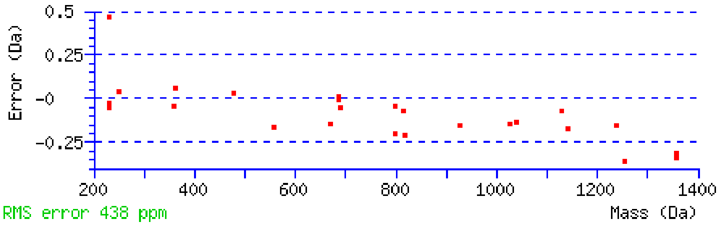


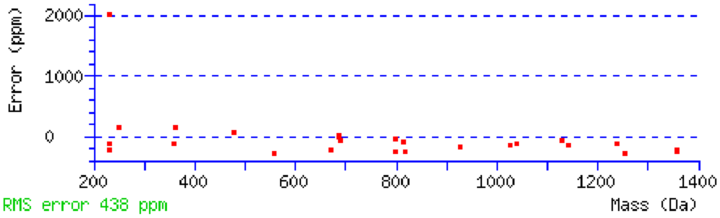


### All matches to this query

| Score | Mr(calc): | Delta | Sequence |
|---|---|---|---|
| 68.3 | 1501.8062 | -0.0027 | EVMSLLQPLNITK |
| 11.6 | 1501.8174 | -0.0139 | MPILSKLSQDINK |
| 7.1 | 1499.8056 | 1.9979 | ELSVEEQIKRNR |
| 6.2 | 1501.7889 | 0.0146 | AFQELLNQVGGLGR |
| 6.1 | 1499.7943 | 2.0091 | QESEPDKLTIGKR |
| 4.8 | 1500.7896 | 1.0138 | GSLLADGRQKDVDK |
| 4.8 | 1501.7988 | 0.0047 | LKIDTDKENAEVK |
| 4.8 | 1501.8062 | -0.0027 | DLLSKMLVIDPDK |
| 4.0 | 1501.8174 | -0.0139 | VEMALEALRNVIK |
| 3.9 | 1501.7889 | 0.0146 | NAGLAFIELVNEGR |

---

Spectrum No: 383; Query: 1697; Rank: 1

---

## Peptide View

MS/MS Fragmentation of **GALLANFLTQGQVCCNGTR**
Found in **IPI00203690**, Tax_Id=10116 Gene_Symbol=Aldh9a1 4-trimethylaminobutyraldehyde dehydrogenase

Match to Query 1697: 2079.986742 from(694.336190,3+)
Title: 091008RatKidney_NH4Format02_21.4235.4235.3.dta
Data file K:\NewmanPaper\Piliang\3SubProteomes\Piliang3SP\mgf5ppm\ERLIC_3SubProteomes5ppm.mgf

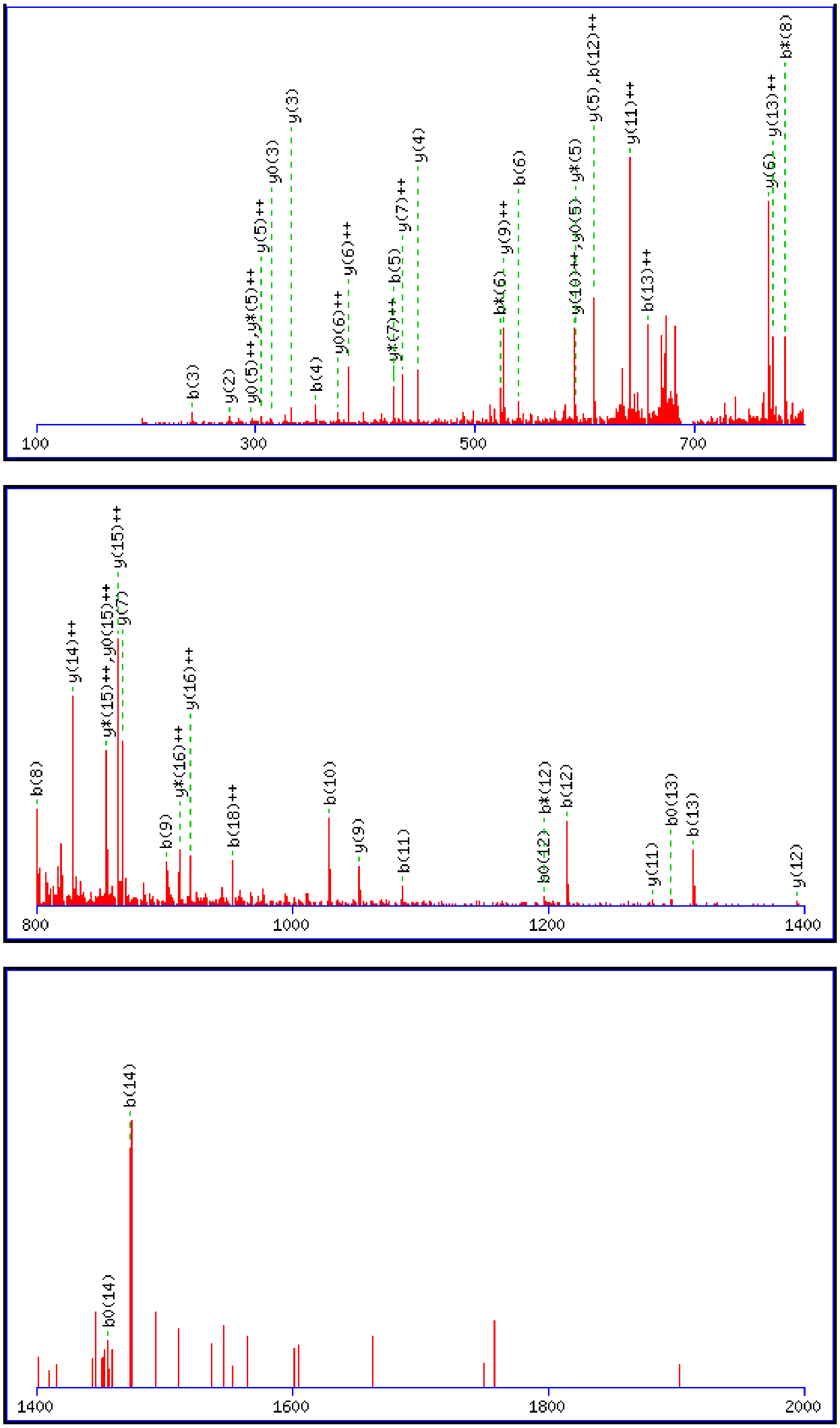


**Monoisotopic mass of neutral peptide Mr(calc):** 2079.9830
**Fixed modifications:** Carbamidomethyl (C)
**Variable modifications:**
**N16** : Deamidated_N (N)
**Ions Score:** 68 **Expect:** 3.8e-005
**Matches (Bold Red):** 49/188 fragment ions using 82 most intense peaks

| # | b | $b^{++}$ | b* | $b^{*++}$ | $b^0$ | $b^{0++}$ | Seq. | y | $y^{++}$ | y* | $y^{*++}$ | $y^0$ | $y^{0++}$ | # |
|---|---|---|---|---|---|---|---|---|---|---|---|---|---|---|
| 1 | 58.0287 | 29.5180 | | | | | G | | | | | | | 19 |
| 2 | 129.0659 | 65.0366 | | | | | A | 2023.9688 | 1012.4880 | 2006.9423 | 1003.9748 | 2005.9582 | 1003.4828 | 18 |
| 3 | **242.1499** | 121.5786 | | | | | L | 1952.9317 | 976.9695 | 1935.9051 | 968.4562 | 1934.9211 | 967.9642 | 17 |
| 4 | **355.2340** | 178.1206 | | | | | L | 1839.8476 | **920.4275** | 1822.8211 | **911.9142** | 1821.8371 | 911.4222 | 16 |
| 5 | **426.2711** | 213.6392 | | | | | A | 1726.7636 | **863.8854** | 1709.7370 | **855.3721** | 1708.7530 | **854.8801** | 15 |

| | | | | | | | | | | | | | | |
|---|---|---|---|---|---|---|---|---|---|---|---|---|---|---|
| **6** | **540.3140** | 270.6606 | **523.2875** | 262.1474 | | | **N** | 1655.7264 | **828.3669** | 1638.6999 | 819.8536 | 1637.7159 | 819.3616 | **14** |
| **7** | 687.3824 | 344.1949 | 670.3559 | 335.6816 | | | **F** | 1541.6835 | **771.3454** | 1524.6570 | 762.8321 | 1523.6730 | 762.3401 | **13** |
| **8** | **800.4665** | 400.7369 | **783.4400** | 392.2236 | | | **L** | **1394.6151** | 697.8112 | 1377.5886 | 689.2979 | 1376.6045 | 688.8059 | **12** |
| **9** | **901.5142** | 451.2607 | 884.4876 | 442.7475 | 883.5036 | 442.2554 | **T** | **1281.5310** | **641.2692** | 1264.5045 | 632.7559 | 1263.5205 | 632.2639 | **11** |
| **10** | **1029.5728** | 515.2900 | 1012.5462 | 506.7767 | 1011.5622 | 506.2847 | **Q** | 1180.4834 | **590.7453** | 1163.4568 | 582.2320 | 1162.4728 | 581.7400 | **10** |
| **11** | **1086.5942** | 543.8007 | 1069.5677 | 535.2875 | 1068.5837 | 534.7955 | **G** | **1052.4248** | **526.7160** | 1035.3982 | 518.2028 | 1034.4142 | 517.7107 | **9** |
| **12** | **1214.6528** | **607.8300** | **1197.6263** | 599.3168 | **1196.6422** | 598.8248 | **Q** | 995.4033 | 498.2053 | 978.3768 | 489.6920 | 977.3928 | 489.2000 | **8** |
| **13** | **1313.7212** | **657.3642** | 1296.6947 | 648.8510 | **1295.7106** | 648.3590 | **V** | **867.3447** | **434.1760** | 850.3182 | **425.6627** | 849.3342 | 425.1707 | **7** |
| **14** | **1473.7519** | 737.3796 | 1456.7253 | 728.8663 | **1455.7413** | 728.3743 | **C** | **768.2763** | **384.6418** | 751.2498 | 376.1285 | 750.2658 | **375.6365** | **6** |
| **15** | 1633.7825 | 817.3949 | 1616.7560 | 808.8816 | 1615.7719 | 808.3896 | **C** | **608.2457** | **304.6265** | **591.2191** | **296.1132** | **590.2351** | **295.6212** | **5** |
| **16** | 1748.8094 | 874.9084 | 1731.7829 | 866.3951 | 1730.7989 | 865.9031 | **N** | **448.2150** | 224.6112 | 431.1885 | 216.0979 | 430.2045 | 215.6059 | **4** |
| **17** | 1805.8309 | 903.4191 | 1788.8044 | 894.9058 | 1787.8203 | 894.4138 | **G** | **333.1881** | 167.0977 | 316.1615 | 158.5844 | **315.1775** | 158.0924 | **3** |
| **18** | 1906.8786 | **953.9429** | 1889.8520 | 945.4297 | 1888.8680 | 944.9377 | **T** | **276.1666** | 138.5870 | 259.1401 | 130.0737 | 258.1561 | 129.5817 | **2** |
| **19** | | | | | | | **R** | 175.1190 | 88.0631 | 158.0924 | 79.5498 | | | **1** |

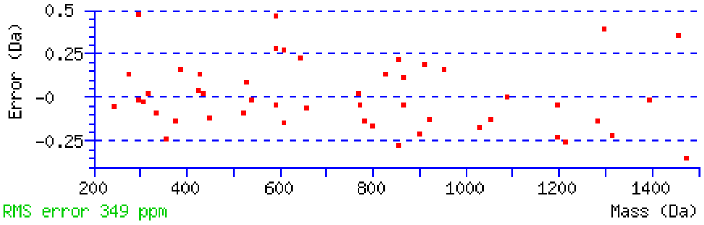


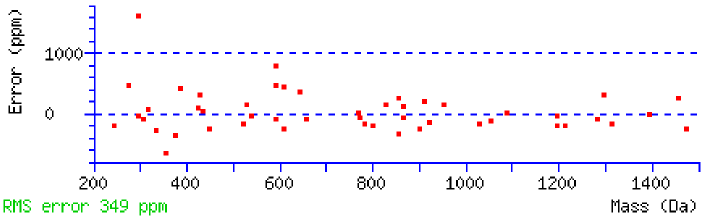


### All matches to this query

| Score | Mr(calc): | Delta | Sequence |
|---|---|---|---|
| 68.3 | 2079.9830 | 0.0037 | GALLANFLTQGQVCCNGTR |
| 30.4 | 2078.9990 | 0.9877 | GALLANFLTQGQVCCNGTR |
| 15.4 | 2079.9830 | 0.0037 | GALLANFLTQGQVCCNGTR |

---

Spectrum No: 384; Query: 1636; Rank: 1

---

## Peptide View

MS/MS Fragmentation of **DEYPSVLMYLNATQPER**
Found in **IPI00207180**, Tax_Id=10116 Gene_Symbol=Slc6a18 Sodium- and chloride-dependent transporter XTRP2

Match to Query 1636: 2041.928328 from(1021.971440,2+)
Title: 091008RatKidney_NoSalt_31.3582.3582.2.dta
Data file K:\NewmanPaper\Piliang\3SubProteomes\Piliang3SP\mgf5ppm\ERLIC_3SubProteomes5ppm.mgf

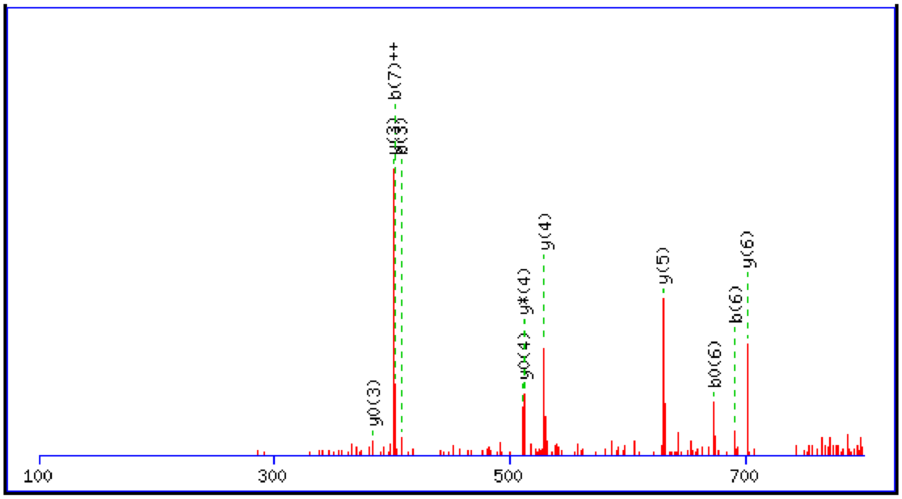


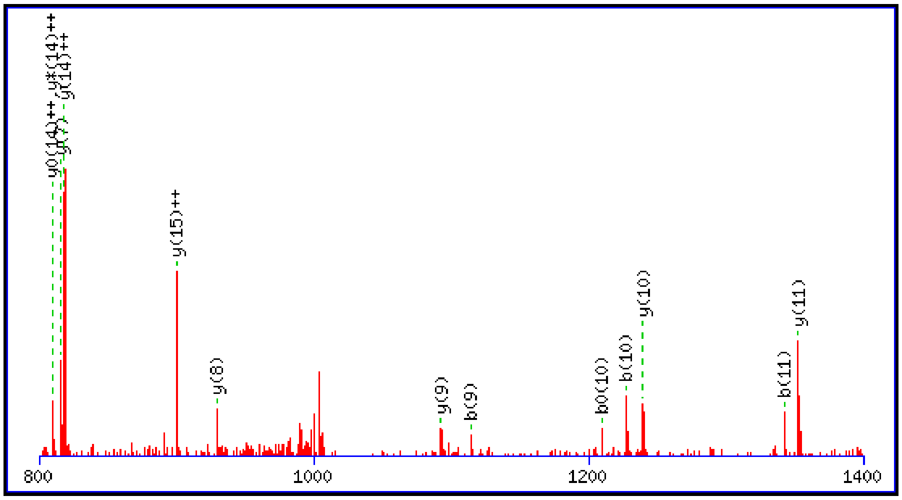


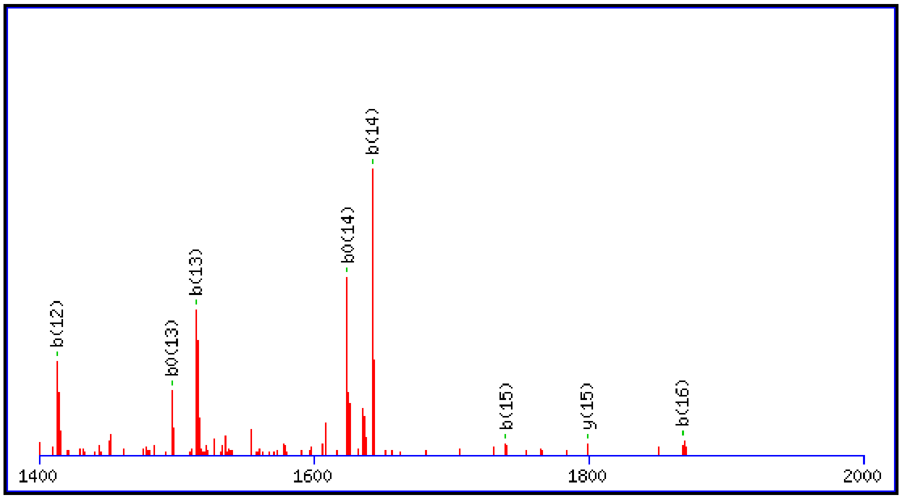


**Monoisotopic mass of neutral peptide Mr(calc):** 2041.9302
**Fixed modifications:** Carbamidomethyl (C)
**Variable modifications:**
**M8** : Oxidation (M)
**N11** : Deamidated_N (N)
**Ions Score:** 68 **Expect:** 3.7e-005
**Matches (Bold Red):** 32/170 fragment ions using 52 most intense peaks

| # | b | $b^{++}$ | b* | $b^{*++}$ | $b^0$ | $b^{0++}$ | Seq. | y | $y^{++}$ | y* | $y^{*++}$ | $y^0$ | $y^{0++}$ | # |
|---|---|---|---|---|---|---|---|---|---|---|---|---|---|---|
| 1 | 116.0342 | 58.5207 | | | 98.0237 | 49.5155 | D | | | | | | | 17 |
| 2 | 245.0768 | 123.0420 | | | 227.0662 | 114.0368 | E | 1927.9106 | 964.4589 | 1910.8841 | 955.9457 | 1909.9000 | 955.4537 | 16 |
| 3 | **408.1401** | 204.5737 | | | 390.1296 | 195.5684 | Y | **1798.8680** | **899.9376** | 1781.8415 | 891.4244 | 1780.8574 | 890.9324 | 15 |
| 4 | 505.1929 | 253.1001 | | | 487.1823 | 244.0948 | P | 1635.8047 | **818.4060** | 1618.7781 | **809.8927** | 1617.7941 | **809.4007** | 14 |
| | | | | | | | | | | | | | | |

| | | | | | | | | | | | | | | |
|---|---|---|---|---|---|---|---|---|---|---|---|---|---|---|
| 5 | 592.2249 | 296.6161 | | | 574.2144 | 287.6108 | S | 1538.7519 | 769.8796 | 1521.7254 | 761.3663 | 1520.7414 | 760.8743 | 13 |
| 6 | **691.2933** | 346.1503 | | | **673.2828** | 337.1450 | V | 1451.7199 | 726.3636 | 1434.6933 | 717.8503 | 1433.7093 | 717.3583 | 12 |
| 7 | 804.3774 | **402.6923** | | | 786.3668 | 393.6871 | L | **1352.6515** | 676.8294 | 1335.6249 | 668.3161 | 1334.6409 | 667.8241 | 11 |
| 8 | 951.4128 | 476.2100 | | | 933.4022 | 467.2048 | M | **1239.5674** | 620.2873 | 1222.5409 | 611.7741 | 1221.5568 | 611.2821 | 10 |
| 9 | **1114.4761** | 557.7417 | | | 1096.4656 | 548.7364 | Y | **1092.5320** | 546.7696 | 1075.5055 | 538.2564 | 1074.5214 | 537.7644 | 9 |
| 10 | **1227.5602** | 614.2837 | | | **1209.5496** | 605.2785 | L | **929.4687** | 465.2380 | 912.4421 | 456.7247 | 911.4581 | 456.2327 | 8 |
| 11 | **1342.5871** | 671.7972 | 1325.5606 | 663.2839 | 1324.5766 | 662.7919 | N | **816.3846** | 408.6959 | 799.3581 | 400.1827 | 798.3741 | 399.6907 | 7 |
| 12 | **1413.6243** | 707.3158 | 1396.5977 | 698.8025 | 1395.6137 | 698.3105 | A | **701.3577** | 351.1825 | 684.3311 | 342.6692 | 683.3471 | 342.1772 | 6 |
| 13 | **1514.6719** | 757.8396 | 1497.6454 | 749.3263 | **1496.6614** | 748.8343 | T | **630.3206** | 315.6639 | 613.2940 | 307.1506 | 612.3100 | 306.6586 | 5 |
| 14 | **1642.7305** | 821.8689 | 1625.7040 | 813.3556 | **1624.7199** | 812.8636 | Q | **529.2729** | 265.1401 | **512.2463** | 256.6268 | **511.2623** | 256.1348 | 4 |
| 15 | **1739.7833** | 870.3953 | 1722.7567 | 861.8820 | 1721.7727 | 861.3900 | P | **401.2143** | 201.1108 | 384.1878 | 192.5975 | **383.2037** | 192.1055 | 3 |
| 16 | **1868.8259** | 934.9166 | 1851.7993 | 926.4033 | 1850.8153 | 925.9113 | E | 304.1615 | 152.5844 | 287.1350 | 144.0711 | 286.1510 | 143.5791 | 2 |
| 17 | | | | | | | R | 175.1190 | 88.0631 | 158.0924 | 79.5498 | | | 1 |

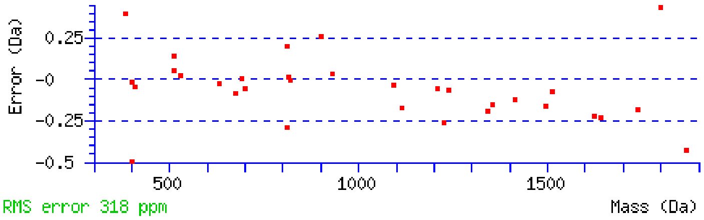


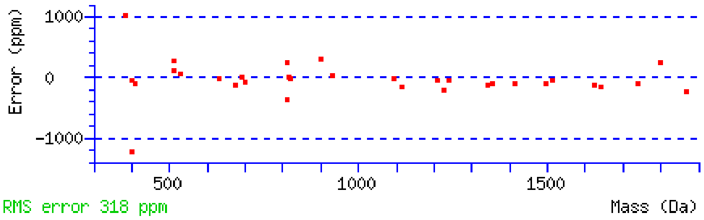


**All matches to this query**

| Score | Mr(calc): | Delta | Sequence |
|---|---|---|---|
| 68.3 | 2041.9302 | -0.0019 | DEYPSVLMYLNATQPER |
| 5.1 | 2041.9357 | -0.0074 | NTPYVNEVSNDEALLKR |
| 2.9 | 2041.9308 | -0.0025 | YMRDTGLTALTNLKLR |
| 2.4 | 2041.9308 | -0.0025 | YMRDTGLTALTNLKLR |
| 2.2 | 2041.9218 | 0.0065 | ATSRLTTDFHNAEKTNR |
| 1.4 | 2041.9317 | -0.0034 | ASLKPSDSPRSVSSENSSK |
| 1.4 | 2041.9317 | -0.0034 | ASLKPSDSPRSVSSENSSK |
| 1.0 | 2039.9109 | 2.0174 | ARPQPPDGGQCRPPGSQR |
| 0.0 | 2041.9375 | -0.0091 | KRENTFPSSSSCSSSPGVK |

---

Spectrum No: 385; Query: 2075; Rank: 1

---

## Peptide View

MS/MS Fragmentation of **VPFNPNDTFESEFYLDEKR**
Found in **IPI00200591**, Tax_Id=10116 Gene_Symbol=LOC299282 Serine protease inhibitor A3L precursor

Match to Query 2075: 2347.067142 from(783.362990,3+)
Title: 091008RatKidney_NoSalt_26.3801.3801.3.dta
Data file K:\NewmanPaper\Piliang\3SubProteomes\Piliang3SP\mgf5ppm\ERLIC_3SubProteomes5ppm.mgf

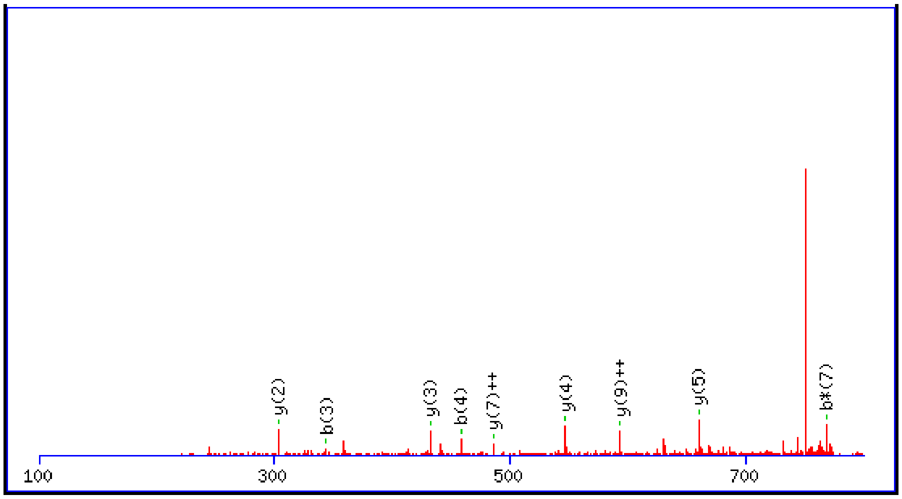


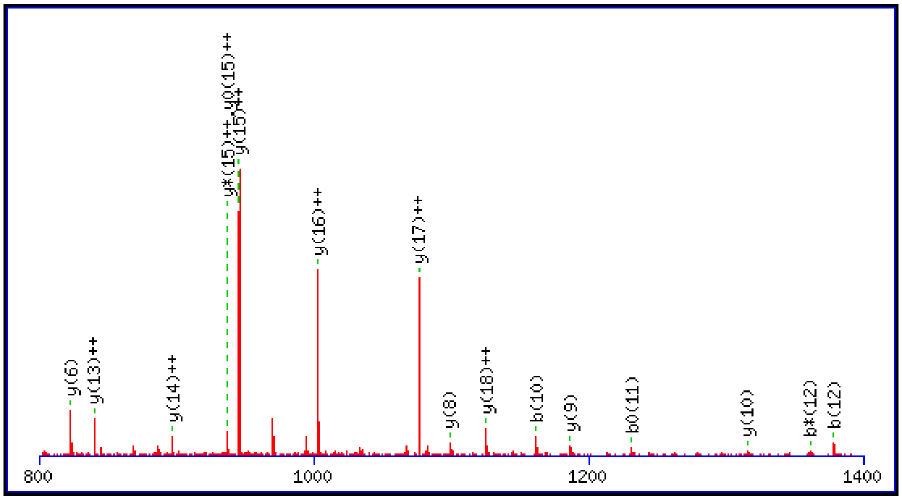


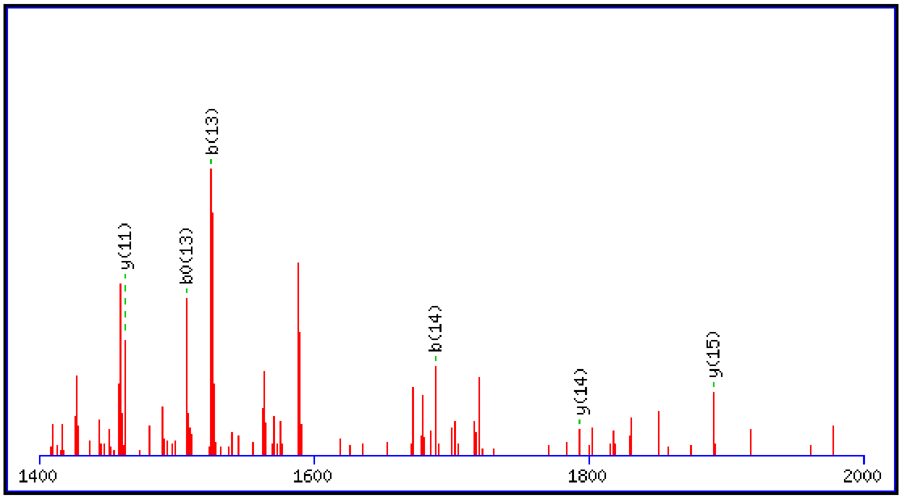


**Monoisotopic mass of neutral peptide Mr(calc):** 2347.0644
**Fixed modifications:** Carbamidomethyl (C)
**Variable modifications:**
**N6** : Deamidated_N (N)
**Ions Score:** 68 **Expect:** 5.4e-005
**Matches (Bold Red):** 31/194 fragment ions using 61 most intense peaks

| # | b | $b^{++}$ | b* | $b^{*++}$ | $b^{0}$ | $b^{0++}$ | Seq. | y | $y^{++}$ | y* | $y^{*++}$ | $y^{0}$ | $y^{0++}$ | # |
|---|---|---|---|---|---|---|---|---|---|---|---|---|---|---|
| 1 | 100.0757 | 50.5415 | | | | | V | | | | | | | 19 |
| 2 | 197.1285 | 99.0679 | | | | | P | 2249.0033 | **1125.0053** | 2231.9768 | 1116.4920 | 2230.9927 | 1116.0000 | 18 |
| 3 | **344.1969** | 172.6021 | | | | | F | 2151.9505 | **1076.4789** | 2134.9240 | 1067.9656 | 2133.9400 | 1067.4736 | 17 |
| 4 | **458.2398** | 229.6235 | 441.2132 | 221.1103 | | | N | 2004.8821 | **1002.9447** | 1987.8556 | 994.4314 | 1986.8716 | 993.9394 | 16 |
| 5 | 555.2926 | 278.1499 | 538.2660 | 269.6366 | | | P | **1890.8392** | **945.9232** | 1873.8127 | **937.4100** | 1872.8286 | **936.9180** | 15 |

| | | | | | | | | | | | | | | |
|---|---|---|---|---|---|---|---|---|---|---|---|---|---|---|
| **6** | 670.3195 | 335.6634 | 653.2929 | 327.1501 | | | **N** | **1793.7864** | **897.3969** | 1776.7599 | 888.8836 | 1775.7759 | 888.3916 | **14** |
| **7** | 785.3464 | 393.1769 | **768.3199** | 384.6636 | 767.3359 | 384.1716 | **D** | 1678.7595 | **839.8834** | 1661.7330 | 831.3701 | 1660.7489 | 830.8781 | **13** |
| **8** | 886.3941 | 443.7007 | 869.3676 | 435.1874 | 868.3836 | 434.6954 | **T** | 1563.7326 | 782.3699 | 1546.7060 | 773.8566 | 1545.7220 | 773.3646 | **12** |
| **9** | 1033.4625 | 517.2349 | 1016.4360 | 508.7216 | 1015.4520 | 508.2296 | **F** | **1462.6849** | 731.8461 | 1445.6583 | 723.3328 | 1444.6743 | 722.8408 | **11** |
| **10** | **1162.5051** | 581.7562 | 1145.4786 | 573.2429 | 1144.4946 | 572.7509 | **E** | **1315.6165** | 658.3119 | 1298.5899 | 649.7986 | 1297.6059 | 649.3066 | **10** |
| **11** | 1249.5372 | 625.2722 | 1232.5106 | 616.7589 | **1231.5266** | 616.2669 | **S** | **1186.5739** | **593.7906** | 1169.5473 | 585.2773 | 1168.5633 | 584.7853 | **9** |
| **12** | **1378.5797** | 689.7935 | **1361.5532** | 681.2802 | 1360.5692 | 680.7882 | **E** | **1099.5419** | 550.2746 | 1082.5153 | 541.7613 | 1081.5313 | 541.2693 | **8** |
| **13** | **1525.6482** | 763.3277 | 1508.6216 | 754.8144 | **1507.6376** | 754.3224 | **F** | 970.4993 | **485.7533** | 953.4727 | 477.2400 | 952.4887 | 476.7480 | **7** |
| **14** | **1688.7115** | 844.8594 | 1671.6849 | 836.3461 | 1670.7009 | 835.8541 | **Y** | **823.4308** | 412.2191 | 806.4043 | 403.7058 | 805.4203 | 403.2138 | **6** |
| **15** | 1801.7956 | 901.4014 | 1784.7690 | 892.8881 | 1783.7850 | 892.3961 | **L** | **660.3675** | 330.6874 | 643.3410 | 322.1741 | 642.3569 | 321.6821 | **5** |
| **16** | 1916.8225 | 958.9149 | 1899.7959 | 950.4016 | 1898.8119 | 949.9096 | **D** | **547.2835** | 274.1454 | 530.2569 | 265.6321 | 529.2729 | 265.1401 | **4** |
| **17** | 2045.8651 | 1023.4362 | 2028.8385 | 1014.9229 | 2027.8545 | 1014.4309 | **E** | **432.2565** | 216.6319 | 415.2300 | 208.1186 | 414.2459 | 207.6266 | **3** |
| **18** | 2173.9601 | 1087.4837 | 2156.9335 | 1078.9704 | 2155.9495 | 1078.4784 | **K** | **303.2139** | 152.1106 | 286.1874 | 143.5973 | | | **2** |
| **19** | | | | | | | **R** | 175.1190 | 88.0631 | 158.0924 | 79.5498 | | | **1** |

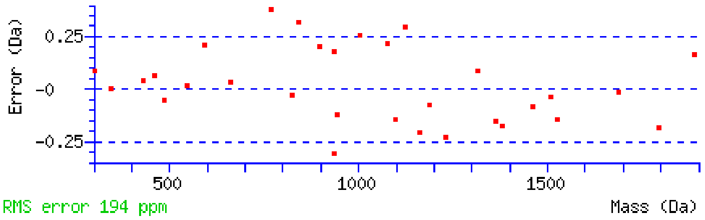


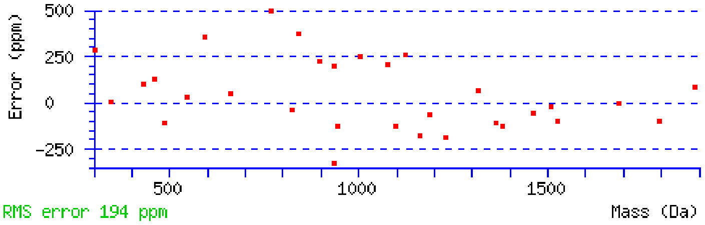


**All matches to this query**

| Score | Mr(calc): | Delta | Sequence |
|---|---|---|---|
| 68.3 | 2347.0644 | 0.0027 | VPFNPNDTFESEFYLDEKR |
| 50.9 | 2347.0644 | 0.0027 | VPFNPNDTFESEFYLDEKR |
| 28.8 | 2346.0804 | 0.9867 | VPFNPNDTFESEFYLDEKR |
| 9.4 | 2346.0748 | 0.9923 | SSPLRASSLTIMATFLGLLI |
| 5.4 | 2347.0862 | -0.0191 | VDENNPEYLRERNMAPDLR |
| 4.3 | 2346.0748 | 0.9923 | SSPLRASSLTIMATFLGLLI |
| 4.3 | 2347.0861 | -0.0190 | VEARTPNPNLLQSEKSLYK |
| 4.3 | 2347.0861 | -0.0190 | VEARTPNPNLLQSEKSLYK |
| 4.3 | 2347.0861 | -0.0190 | VEARTPNPNLLQSEKSLYK |
| 4.3 | 2347.0861 | -0.0190 | VEARTPNPNLLQSEKSLYK |

---

Spectrum No: 386; Query: 636; Rank: 1

---

## Peptide View

MS/MS Fragmentation of **VSNMTLPASAEVLK**
Found in **IPI00206336**, Tax_Id=10116 Gene_Symbol=Lamp1 Lysosome-associated membrane glycoprotein 1 precursor

Match to Query 636: 1475.750968 from(738.882760,2+)
Title: 091008RatKidney_NH4Format01_27.1684.1684.2.dta
Data file K:\NewmanPaper\Piliang\3SubProteomes\Piliang3SP\mgf5ppm\ERLIC_3SubProteomes5ppm.mgf

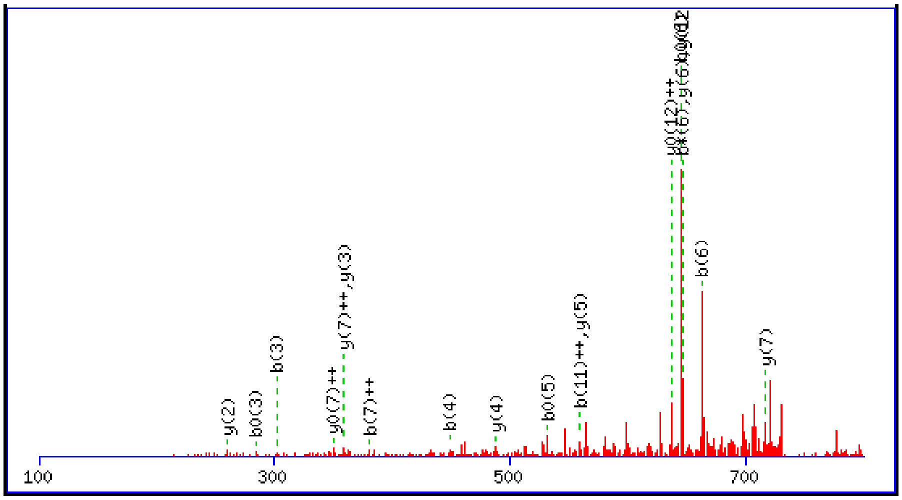


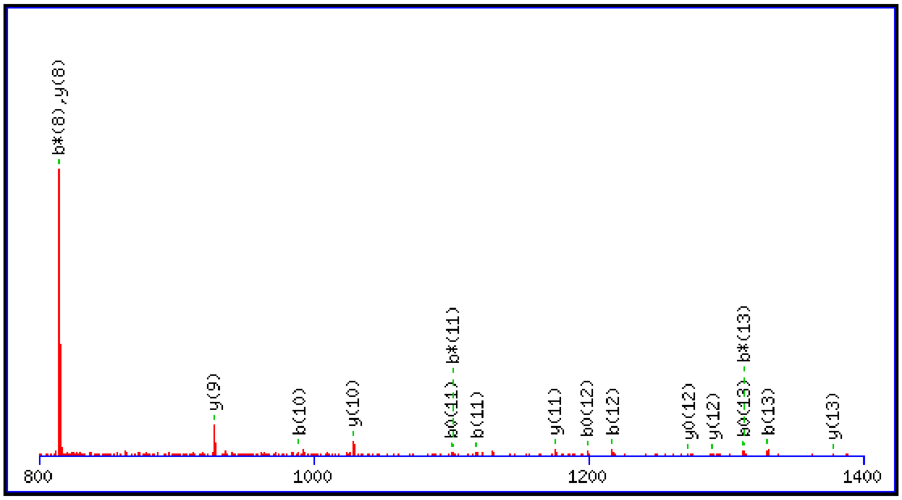


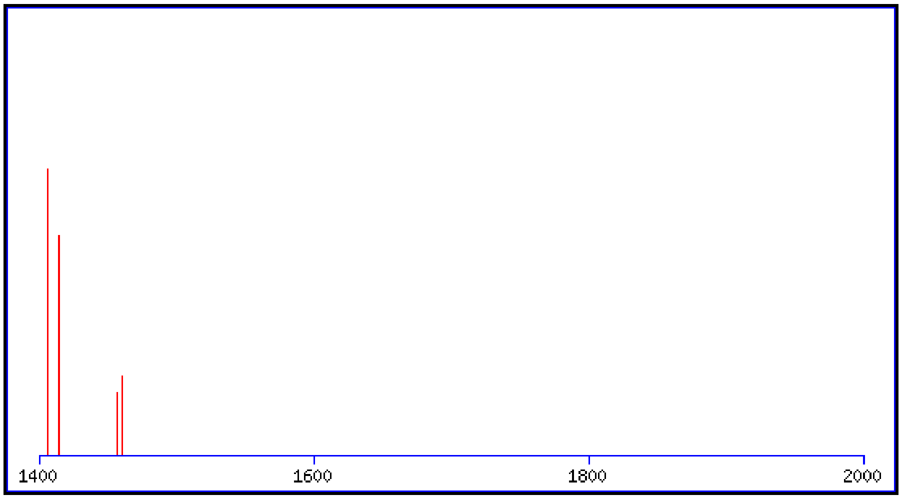


**Monoisotopic mass of neutral peptide Mr(calc):** 1475.7541
**Fixed modifications:** Carbamidomethyl (C)
**Variable modifications:**
**N3** : Deamidated_N (N)
**M4** : Oxidation (M)
**Ions Score:** 68 **Expect:** 2.1e-005
**Matches (Bold Red):** 36/144 fragment ions using 79 most intense peaks

| # | b | b++ | b* | b*++ | $b^0$ | $b^{0++}$ | Seq. | y | y++ | y* | y*++ | $y^0$ | $y^{0++}$ | # |
|---|---|---|---|---|---|---|---|---|---|---|---|---|---|---|
| 1 | 100.0757 | 50.5415 | | | | | V | | | | | | | 14 |
| 2 | 187.1077 | 94.0575 | | | 169.0972 | 85.0522 | S | **1377.6930** | 689.3501 | 1360.6665 | 680.8369 | 1359.6824 | 680.3449 | 13 |
| 3 | **302.1347** | 151.5710 | 285.1081 | 143.0577 | **284.1241** | 142.5657 | N | **1290.6610** | **645.8341** | 1273.6344 | 637.3209 | **1272.6504** | **636.8288** | 12 |
| 4 | **449.1701** | 225.0887 | 432.1435 | 216.5754 | 431.1595 | 216.0834 | M | **1175.6340** | 588.3207 | 1158.6075 | 579.8074 | 1157.6235 | 579.3154 | 11 |

| | | | | | | | | | | | | | | |
|---|---|---|---|---|---|---|---|---|---|---|---|---|---|---|
| **5** | 550.2177 | 275.6125 | 533.1912 | 267.0992 | **532.2072** | 266.6072 | **T** | **1028.5986** | 514.8030 | 1011.5721 | 506.2897 | 1010.5881 | 505.7977 | **10** |
| **6** | **663.3018** | 332.1545 | **646.2753** | 323.6413 | **645.2912** | 323.1493 | **L** | **927.5510** | 464.2791 | 910.5244 | 455.7658 | 909.5404 | 455.2738 | **9** |
| **7** | 760.3546 | **380.6809** | 743.3280 | 372.1676 | 742.3440 | 371.6756 | **P** | **814.4669** | 407.7371 | 797.4403 | 399.2238 | 796.4563 | 398.7318 | **8** |
| **8** | 831.3917 | 416.1995 | **814.3651** | 407.6862 | 813.3811 | 407.1942 | **A** | **717.4141** | **359.2107** | 700.3876 | 350.6974 | 699.4036 | **350.2054** | **7** |
| **9** | 918.4237 | 459.7155 | 901.3972 | 451.2022 | 900.4131 | 450.7102 | **S** | **646.3770** | 323.6921 | 629.3505 | 315.1789 | 628.3665 | 314.6869 | **6** |
| **10** | **989.4608** | 495.2340 | 972.4343 | 486.7208 | 971.4503 | 486.2288 | **A** | **559.3450** | 280.1761 | 542.3184 | 271.6629 | 541.3344 | 271.1709 | **5** |
| **11** | **1118.5034** | **559.7553** | **1101.4769** | 551.2421 | **1100.4928** | 550.7501 | **E** | **488.3079** | 244.6576 | 471.2813 | 236.1443 | 470.2973 | 235.6523 | **4** |
| **12** | **1217.5718** | 609.2896 | 1200.5453 | 600.7763 | **1199.5613** | 600.2843 | **V** | **359.2653** | 180.1363 | 342.2387 | 171.6230 | | | **3** |
| **13** | **1330.6559** | 665.8316 | **1313.6293** | 657.3183 | **1312.6453** | 656.8263 | **L** | **260.1969** | 130.6021 | 243.1703 | 122.0888 | | | **2** |
| **14** | | | | | | | **K** | 147.1128 | 74.0600 | 130.0863 | 65.5468 | | | **1** |

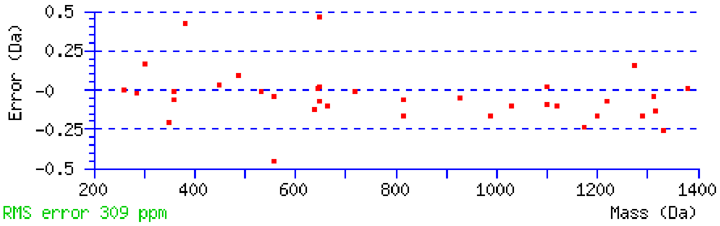


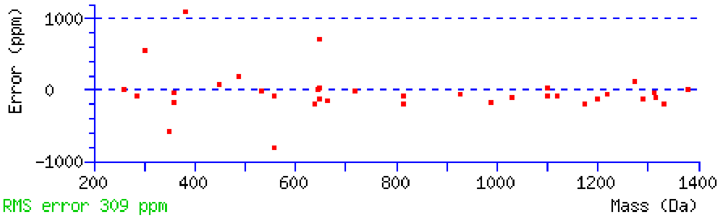


### All matches to this query

| Score | Mr(calc): | Delta | Sequence |
|---|---|---|---|
| 68.3 | 1475.7541 | -0.0032 | VSNMTLPASAEVLK |
| 14.1 | 1475.7468 | 0.0042 | GDSGDTLAATQSLLK |
| 13.7 | 1475.7620 | -0.0110 | GDFDNLSGKGKPLK |
| 4.3 | 1475.7653 | -0.0144 | MAKTQSQPILNTK |
| 3.7 | 1475.7442 | 0.0067 | TSITALAWSPNGMK |
| 2.2 | 1475.7409 | 0.0101 | SVYWENTLHSLK |
| 2.1 | 1475.7620 | -0.0110 | LLSSYVSVHAESGK |
| 2.0 | 1475.7467 | 0.0042 | SVDSRNTLNDLLK |
| 1.5 | 1475.7497 | 0.0012 | GTTILTNLTGLHR |
| 0.8 | 1475.7541 | -0.0031 | MDLVLELKNNASK |

---

Spectrum No: 387; Query: 1630; Rank: 1

---

## Peptide View

MS/MS Fragmentation of **KLHINYNNLTESVGPLPK**
Found in **IPI00206403**, Tax_Id=10116 Gene_Symbol=Lum Lumican precursor

Match to Query 1630: 2037.090908 from(1019.552730,2+)
Title: 091008RatKidney_NH4Format01_25.2595.2595.2.dta
Data file K:\NewmanPaper\Piliang\3SubProteomes\Piliang3SP\mgf5ppm\ERLIC_3SubProteomes5ppm.mgf

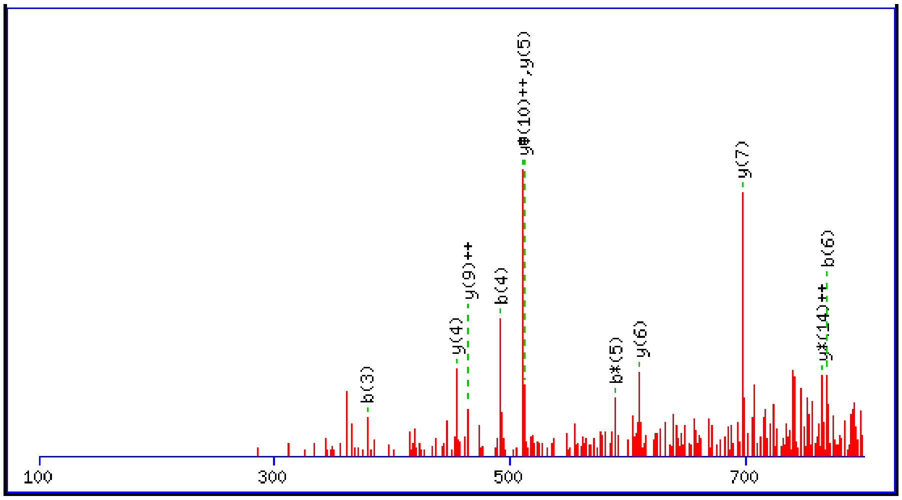


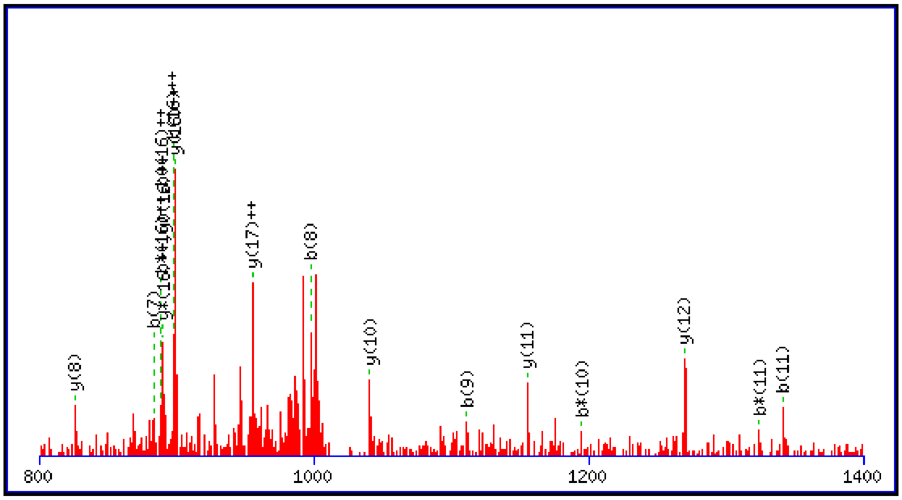


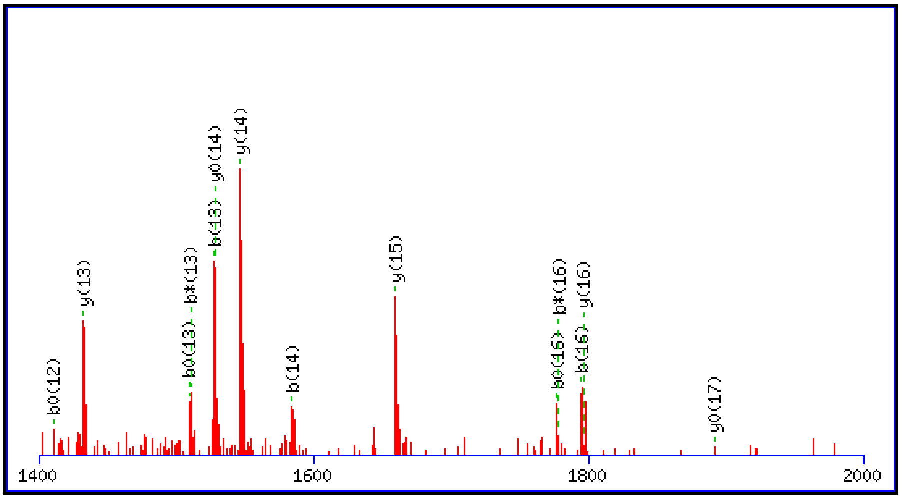


**Monoisotopic mass of neutral peptide Mr(calc):** 2037.0894
**Fixed modifications:** Carbamidomethyl (C)
**Variable modifications:**
**N8** : Deamidated_N (N)
**Ions Score:** 68 **Expect:** 1.2e-005
**Matches (Bold Red):** 44/174 fragment ions using 84 most intense peaks

| # | b | $b^{++}$ | b* | $b^{*++}$ | $b^0$ | $b^{0++}$ | Seq. | y | $y^{++}$ | y* | $y^{*++}$ | $y^0$ | $y^{0++}$ | # |
|---|---|---|---|---|---|---|---|---|---|---|---|---|---|---|
| 1 | 129.1022 | 65.0548 | 112.0757 | 56.5415 | | | K | | | | | | | 18 |
| 2 | 242.1863 | 121.5968 | 225.1598 | 113.0835 | | | L | 1910.0018 | **955.5045** | 1892.9752 | 946.9913 | **1891.9912** | 946.4993 | 17 |
| 3 | **379.2452** | 190.1262 | 362.2187 | 181.6130 | | | H | **1796.9177** | **898.9625** | 1779.8912 | **890.4492** | 1778.9072 | **889.9572** | 16 |
| 4 | **492.3293** | 246.6683 | 475.3027 | 238.1550 | | | I | **1659.8588** | 830.4330 | 1642.8323 | 821.9198 | 1641.8483 | 821.4278 | 15 |
| 5 | 606.3722 | 303.6897 | **589.3457** | 295.1765 | | | N | **1546.7748** | 773.8910 | 1529.7482 | **765.3777** | **1528.7642** | 764.8857 | 14 |

| | | | | | | | | | | | | | | |
|---|---|---|---|---|---|---|---|---|---|---|---|---|---|---|
| **6** | **769.4355** | 385.2214 | 752.4090 | 376.7081 | | | **Y** | **1432.7318** | 716.8696 | 1415.7053 | 708.3563 | 1414.7213 | 707.8643 | **13** |
| **7** | **883.4785** | 442.2429 | 866.4519 | 433.7296 | | | **N** | **1269.6685** | 635.3379 | 1252.6420 | 626.8246 | 1251.6579 | 626.3326 | **12** |
| **8** | **998.5054** | 499.7563 | 981.4788 | 491.2431 | | | **N** | **1155.6256** | 578.3164 | 1138.5990 | 569.8032 | 1137.6150 | 569.3111 | **11** |
| **9** | **1111.5895** | 556.2984 | 1094.5629 | 547.7851 | | | **L** | **1040.5986** | 520.8030 | 1023.5721 | **512.2897** | 1022.5881 | **511.7977** | **10** |
| **10** | 1212.6371 | 606.8222 | **1195.6106** | 598.3089 | 1194.6266 | 597.8169 | **T** | 927.5146 | **464.2609** | 910.4880 | 455.7477 | 909.5040 | 455.2556 | **9** |
| **11** | **1341.6797** | 671.3435 | **1324.6532** | 662.8302 | 1323.6692 | 662.3382 | **E** | **826.4669** | 413.7371 | 809.4403 | 405.2238 | 808.4563 | 404.7318 | **8** |
| **12** | 1428.7118 | 714.8595 | 1411.6852 | 706.3462 | **1410.7012** | 705.8542 | **S** | **697.4243** | 349.2158 | 680.3978 | 340.7025 | 679.4137 | 340.2105 | **7** |
| **13** | **1527.7802** | 764.3937 | **1510.7536** | 755.8805 | **1509.7696** | 755.3884 | **V** | **610.3923** | 305.6998 | 593.3657 | 297.1865 | | | **6** |
| **14** | **1584.8016** | 792.9045 | 1567.7751 | 784.3912 | 1566.7911 | 783.8992 | **G** | **511.3239** | 256.1656 | 494.2973 | 247.6523 | | | **5** |
| **15** | 1681.8544 | 841.4308 | 1664.8279 | 832.9176 | 1663.8438 | 832.4256 | **P** | **454.3024** | 227.6548 | 437.2758 | 219.1416 | | | **4** |
| **16** | **1794.9385** | **897.9729** | **1777.9119** | **889.4596** | **1776.9279** | **888.9676** | **L** | 357.2496 | 179.1285 | 340.2231 | 170.6152 | | | **3** |
| **17** | **1891.9912** | 946.4993 | 1874.9647 | 937.9860 | 1873.9807 | 937.4940 | **P** | 244.1656 | 122.5864 | 227.1390 | 114.0731 | | | **2** |
| **18** | | | | | | | **K** | 147.1128 | 74.0600 | 130.0863 | 65.5468 | | | **1** |

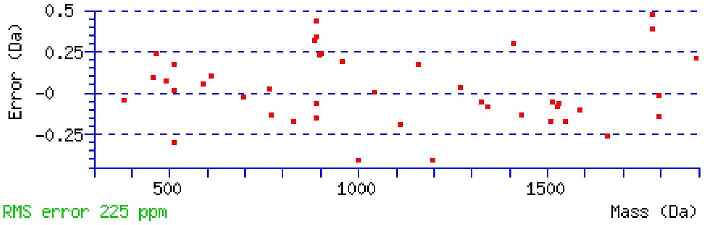


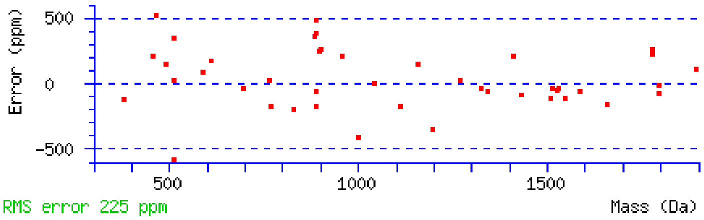


### All matches to this query

| Score | Mr(calc): | Delta | Sequence |
|---|---|---|---|
| 68.3 | 2037.0894 | 0.0015 | KLHINYNNLTESVGPLPK |
| 55.2 | 2037.0894 | 0.0015 | KLHINYNNLTESVGPLPK |
| 43.3 | 2037.0894 | 0.0015 | KLHINYNNLTESVGPLPK |
| 14.9 | 2036.1054 | 0.9855 | KLHINYNNLTESVGPLPK |

---

Spectrum No: 388; Query: 654; Rank: 1

---

## Peptide View

MS/MS Fragmentation of **KYQTLNCSVNVR**
Found in **IPI00205166**, Tax_Id=10116 Gene_Symbol=Itga6 similar to integrin alpha 6 isoform 2

Match to Query 654: 1481.727668 from(741.871110,2+)
Title: 091008RatKidney_NH4Format01_23.978.978.2.dta
Data file K:\NewmanPaper\Piliang\3SubProteomes\Piliang3SP\mgf5ppm\ERLIC_3SubProteomes5ppm.mgf

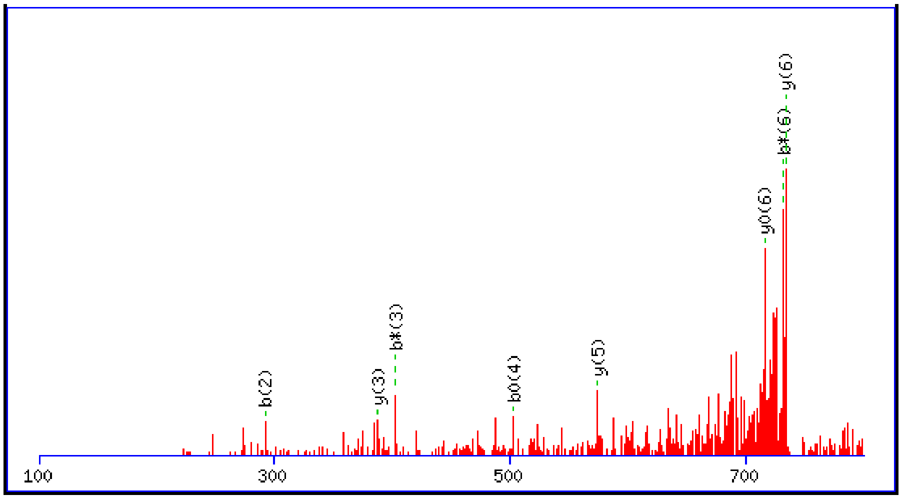


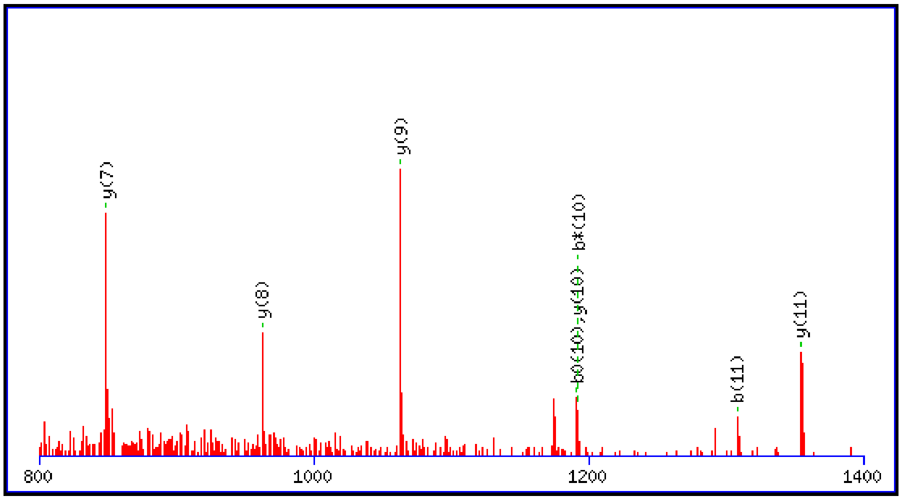


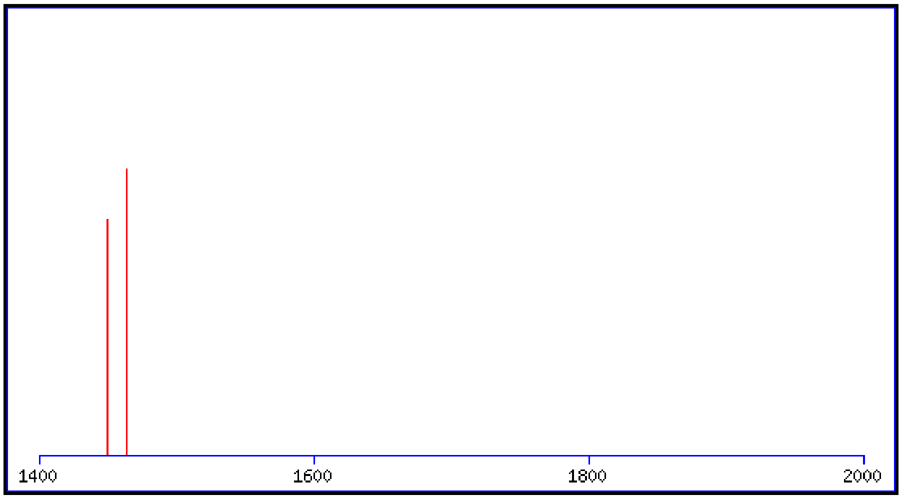


**Monoisotopic mass of neutral peptide Mr(calc):** 1481.7296
**Fixed modifications:** Carbamidomethyl (C)
**Variable modifications:**
**N6** : Deamidated_N (N)
**Ions Score:** 68 **Expect:** 2.3e-005
**Matches (Bold Red):** 16/118 fragment ions using 26 most intense peaks

| # | b | b++ | b* | b*++ | b0 | b0++ | Seq. | y | y++ | y* | y*++ | y0 | y0++ | # |
|---|---|---|---|---|---|---|---|---|---|---|---|---|---|---|
| 1 | 129.1022 | 65.0548 | 112.0757 | 56.5415 | | | K | | | | | | | 12 |
| 2 | **292.1656** | 146.5864 | 275.1390 | 138.0731 | | | Y | **1354.6420** | 677.8246 | 1337.6154 | 669.3113 | 1336.6314 | 668.8193 | 11 |
| 3 | 420.2241 | 210.6157 | **403.1976** | 202.1024 | | | Q | **1191.5786** | 596.2930 | 1174.5521 | 587.7797 | 1173.5681 | 587.2877 | 10 |
| 4 | 521.2718 | 261.1396 | 504.2453 | 252.6263 | **503.2613** | 252.1343 | T | **1063.5201** | 532.2637 | 1046.4935 | 523.7504 | 1045.5095 | 523.2584 | 9 |
| 5 | 634.3559 | 317.6816 | 617.3293 | 309.1683 | 616.3453 | 308.6763 | L | **962.4724** | 481.7398 | 945.4458 | 473.2266 | 944.4618 | 472.7345 | 8 |

| | | | | | | | | | | | | | | |
|---|---|---|---|---|---|---|---|---|---|---|---|---|---|---|
| **6** | 749.3828 | 375.1951 | **732.3563** | 366.6818 | 731.3723 | 366.1898 | **N** | **849.3883** | 425.1978 | 832.3618 | 416.6845 | 831.3778 | 416.1925 | **7** |
| **7** | 909.4135 | 455.2104 | 892.3869 | 446.6971 | 891.4029 | 446.2051 | **C** | **734.3614** | 367.6843 | 717.3348 | 359.1711 | **716.3508** | 358.6790 | **6** |
| **8** | 996.4455 | 498.7264 | 979.4190 | 490.2131 | 978.4349 | 489.7211 | **S** | **574.3307** | 287.6690 | 557.3042 | 279.1557 | 556.3202 | 278.6637 | **5** |
| **9** | 1095.5139 | 548.2606 | 1078.4874 | 539.7473 | 1077.5034 | 539.2553 | **V** | 487.2987 | 244.1530 | 470.2722 | 235.6397 | | | **4** |
| **10** | 1209.5568 | 605.2821 | **1192.5303** | 596.7688 | **1191.5463** | 596.2768 | **N** | **388.2303** | 194.6188 | 371.2037 | 186.1055 | | | **3** |
| **11** | **1308.6253** | 654.8163 | 1291.5987 | 646.3030 | 1290.6147 | 645.8110 | **V** | 274.1874 | 137.5973 | 257.1608 | 129.0840 | | | **2** |
| **12** | | | | | | | **R** | 175.1190 | 88.0631 | 158.0924 | 79.5498 | | | **1** |

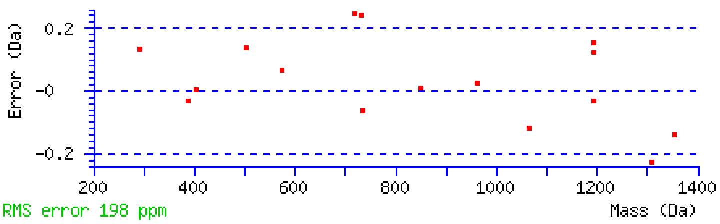


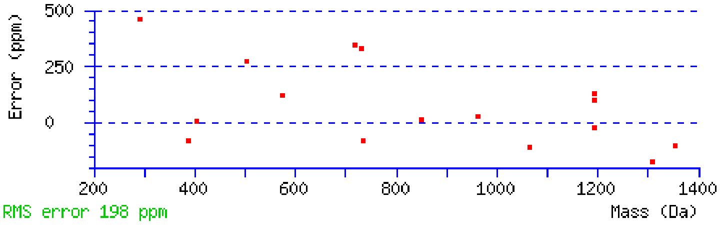


### All matches to this query

| Score | Mr(calc): | Delta | Sequence |
|---|---|---|---|
| 68.3 | 1481.7296 | -0.0020 | KYQTLNCSVNVR |
| 43.2 | 1481.7296 | -0.0020 | KYQTLNCSVNVR |
| 9.0 | 1481.7140 | 0.0137 | GFSAGSARLPGLNR |
| 3.8 | 1480.7305 | 0.9971 | CFVLMDNILNLK |

---

Spectrum No: 389; Query: 603; Rank: 1

---

## Peptide View

MS/MS Fragmentation of **DTNGSQFFITTVK**
Found in **IPI00213644**, Tax_Id=10116 Gene_Symbol=Ppib Peptidyl-prolyl cis-trans isomerase B precursor

Match to Query 603: 1457.696728 from(729.855640,2+)
Title: 091008RatKidney_NH4Format02_21.3308.3308.2.dta
Data file K:\NewmanPaper\Piliang\3SubProteomes\Piliang3SP\mgf5ppm\ERLIC_3SubProteomes5ppm.mgf

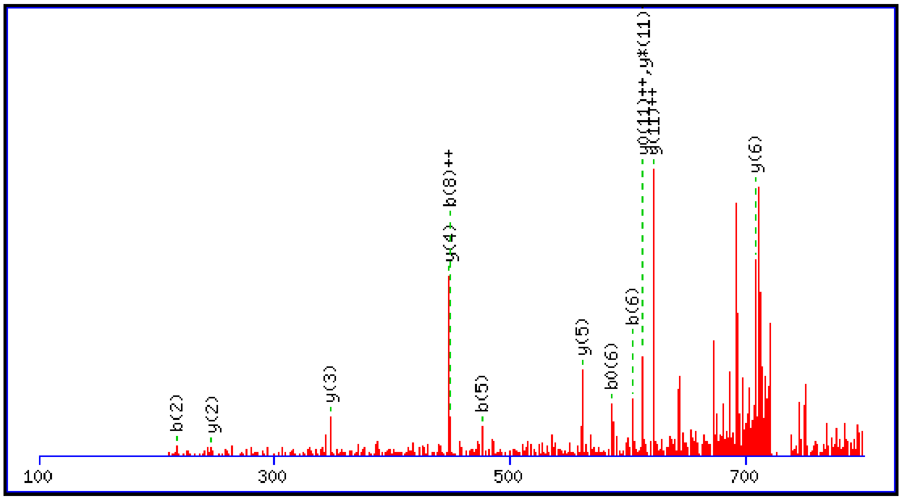

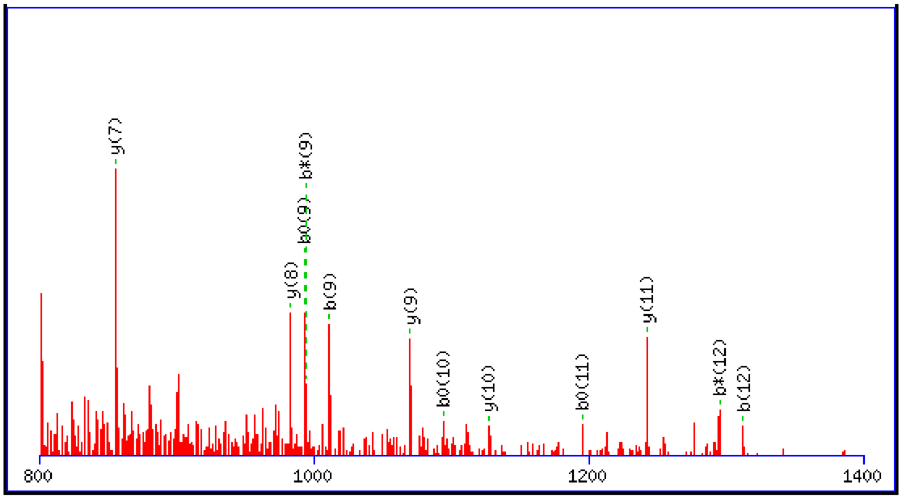


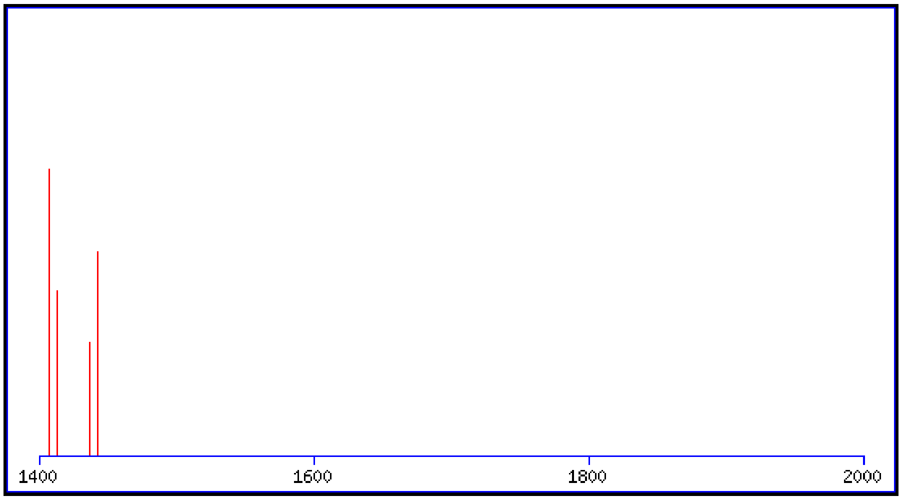


**Monoisotopic mass of neutral peptide Mr(calc):** 1457.7038
**Fixed modifications:** Carbamidomethyl (C)
**Variable modifications:**
**N3** : Deamidated_N (N)
**Ions Score:** 68 **Expect:** 2.5e-005
**Matches (Bold Red):** 25/136 fragment ions using 41 most intense peaks

| # | b | $b^{++}$ | $b^{*}$ | $b^{*++}$ | $b^{0}$ | $b^{0++}$ | Seq. | y | $y^{++}$ | $y^{*}$ | $y^{*++}$ | $y^{0}$ | $y^{0++}$ | # |
|---|---|---|---|---|---|---|---|---|---|---|---|---|---|---|
| 1 | 116.0342 | 58.5207 | | | 98.0237 | 49.5155 | D | | | | | | | 13 |
| 2 | **217.0819** | 109.0446 | | | 199.0713 | 100.0393 | T | 1343.6842 | 672.3457 | 1326.6576 | 663.8324 | 1325.6736 | 663.3404 | 12 |
| 3 | 332.1088 | 166.5581 | 315.0823 | 158.0448 | 314.0983 | 157.5528 | N | **1242.6365** | **621.8219** | 1225.6099 | **613.3086** | 1224.6259 | **612.8166** | 11 |
| 4 | 389.1303 | 195.0688 | 372.1037 | 186.5555 | 371.1197 | 186.0635 | G | **1127.6095** | 564.3084 | 1110.5830 | 555.7951 | 1109.5990 | 555.3031 | 10 |
| 5 | **476.1623** | 238.5848 | 459.1358 | 230.0715 | 458.1518 | 229.5795 | S | **1070.5881** | 535.7977 | 1053.5615 | 527.2844 | 1052.5775 | 526.7924 | 9 |
| 6 | **604.2209** | 302.6141 | 587.1944 | 294.1008 | **586.2103** | 293.6088 | Q | **983.5560** | 492.2817 | 966.5295 | 483.7684 | 965.5455 | 483.2764 | 8 |
| 7 | 751.2893 | 376.1483 | 734.2628 | 367.6350 | 733.2788 | 367.1430 | F | **855.4975** | 428.2524 | 838.4709 | 419.7391 | 837.4869 | 419.2471 | 7 |
| 8 | 898.3577 | **449.6825** | 881.3312 | 441.1692 | 880.3472 | 440.6772 | F | **708.4291** | 354.7182 | 691.4025 | 346.2049 | 690.4185 | 345.7129 | 6 |
| 9 | **1011.4418** | 506.2245 | **994.4152** | 497.7113 | **993.4312** | 497.2193 | I | **561.3606** | 281.1840 | 544.3341 | 272.6707 | 543.3501 | 272.1787 | 5 |
| 10 | 1112.4895 | 556.7484 | 1095.4629 | 548.2351 | **1094.4789** | 547.7431 | T | **448.2766** | 224.6419 | 431.2500 | 216.1287 | 430.2660 | 215.6366 | 4 |
| 11 | 1213.5372 | 607.2722 | 1196.5106 | 598.7589 | **1195.5266** | 598.2669 | T | **347.2289** | 174.1181 | 330.2023 | 165.6048 | 329.2183 | 165.1128 | 3 |
| 12 | **1312.6056** | 656.8064 | **1295.5790** | 648.2931 | 1294.5950 | 647.8011 | V | **246.1812** | 123.5942 | 229.1547 | 115.0810 | | | 2 |
| 13 | | | | | | | K | 147.1128 | 74.0600 | 130.0863 | 65.5468 | | | 1 |

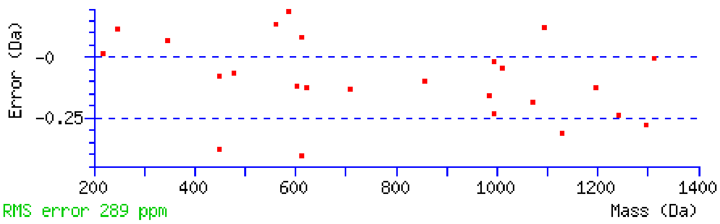


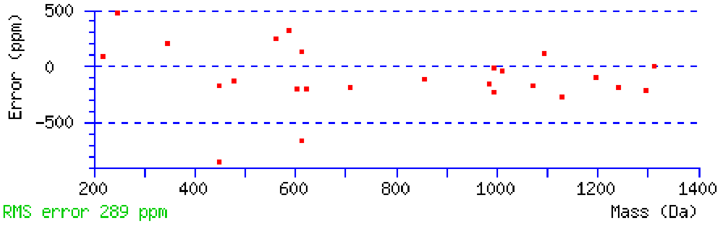


### All matches to this query

| Score | Mr(calc): | Delta | Sequence |
|---|---|---|---|
| 68.3 | 1457.7038 | -0.0071 | DTNGSQFFITTVK |
| 8.7 | 1457.6837 | 0.0131 | KMSEQINTSLVK |
| 8.7 | 1457.6837 | 0.0131 | KMSEQINTSLVK |
| 7.6 | 1457.7038 | -0.0071 | FVNSTGYLTEAEK |
| 7.2 | 1457.7071 | -0.0104 | SEYKKMESEISK |
| 7.0 | 1456.6963 | 1.0004 | GNFLLXLTLTDGR |
| 4.0 | 1457.6972 | -0.0005 | SSILPFTPPIVK |
| 3.0 | 1456.6997 | 0.9971 | ETVQKSVCVLSK |
| 3.0 | 1456.6996 | 0.9971 | KMSEQINTSLVK |
| 0.8 | 1457.7085 | -0.0118 | HLKNSMNGLDWK |

---

Spectrum No: 390; Query: 518; Rank: 1

---

## Peptide View

MS/MS Fragmentation of **FQLLNFSSSELK**
Found in **IPI00464765**, Tax_Id=10116 Gene_Symbol=Igsf4a Immunoglobulin superfamily, member 4A

Match to Query 518: 1412.720528 from(707.367540,2+)
Title: 100101RatKid_NS_deglyco_25.4270.4270.2.dta
Data file K:\NewmanPaper\Piliang\3SubProteomes\Piliang3SP\mgf5ppm\ERLIC_3SubProteomes5ppm.mgf

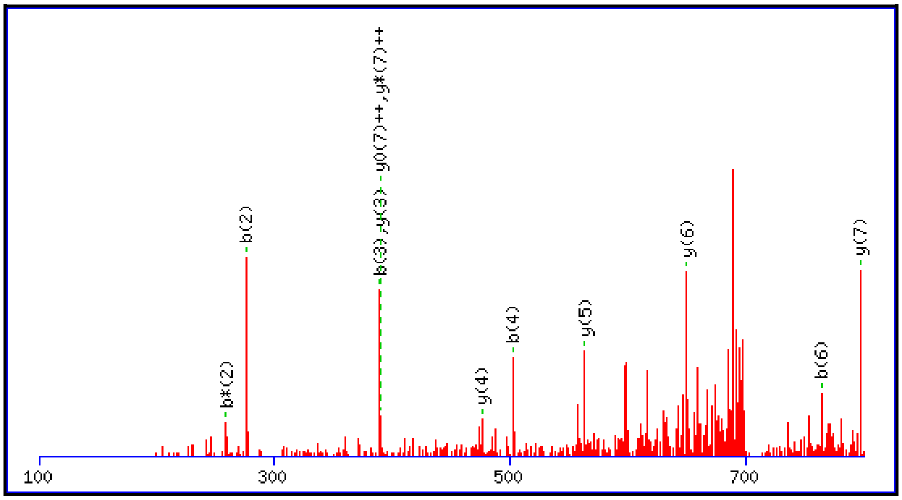

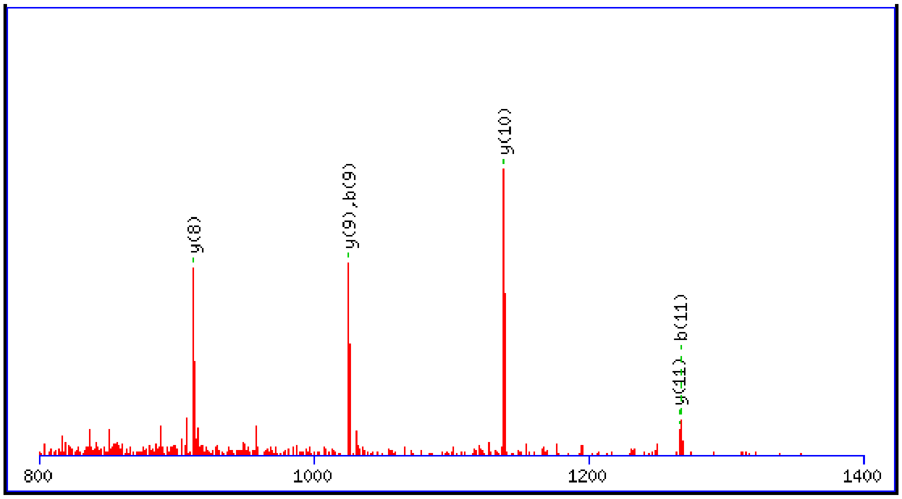


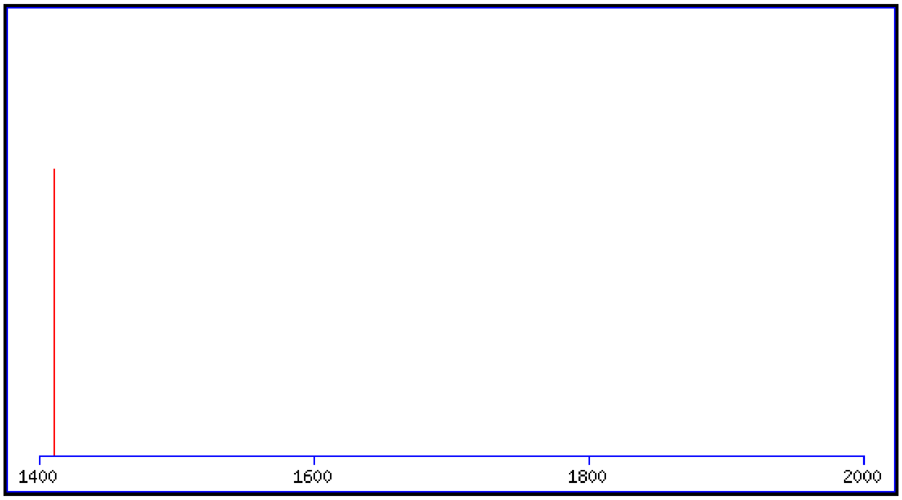


**Monoisotopic mass of neutral peptide Mr(calc):** 1412.7187
**Fixed modifications:** Carbamidomethyl (C)
**Variable modifications:**
**N5** : Deamidated_N (N)
**Ions Score:** 68 **Expect:** 1.8e-005
**Matches (Bold Red):** 18/114 fragment ions using 31 most intense peaks

| # | b | $b^{++}$ | b* | $b^{*++}$ | $b^{0}$ | $b^{0++}$ | Seq. | y | $y^{++}$ | y* | $y^{*++}$ | $y^{0}$ | $y^{0++}$ | # |
|---|---|---|---|---|---|---|---|---|---|---|---|---|---|---|
| 1 | 148.0757 | 74.5415 | | | | | F | | | | | | | 12 |
| 2 | **276.1343** | 138.5708 | **259.1077** | 130.0575 | | | Q | **1266.6576** | 633.8324 | 1249.6311 | 625.3192 | 1248.6470 | 624.8272 | 11 |
| 3 | **389.2183** | 195.1128 | 372.1918 | 186.5995 | | | L | **1138.5990** | 569.8031 | 1121.5725 | 561.2899 | 1120.5885 | 560.7979 | 10 |
| 4 | **502.3024** | 251.6548 | 485.2758 | 243.1416 | | | L | **1025.5150** | 513.2611 | 1008.4884 | 504.7478 | 1007.5044 | 504.2558 | 9 |
| 5 | 617.3293 | 309.1683 | 600.3028 | 300.6550 | | | N | **912.4309** | 456.7191 | 895.4043 | 448.2058 | 894.4203 | 447.7138 | 8 |
| 6 | **764.3977** | 382.7025 | 747.3712 | 374.1892 | | | F | **797.4040** | 399.2056 | 780.3774 | **390.6923** | 779.3934 | **390.2003** | 7 |
| 7 | 851.4298 | 426.2185 | 834.4032 | 417.7053 | 833.4192 | 417.2132 | S | **650.3355** | 325.6714 | 633.3090 | 317.1581 | 632.3250 | 316.6661 | 6 |
| 8 | 938.4618 | 469.7345 | 921.4353 | 461.2213 | 920.4512 | 460.7293 | S | **563.3035** | 282.1554 | 546.2770 | 273.6421 | 545.2930 | 273.1501 | 5 |
| 9 | **1025.4938** | 513.2506 | 1008.4673 | 504.7373 | 1007.4833 | 504.2453 | S | **476.2715** | 238.6394 | 459.2449 | 230.1261 | 458.2609 | 229.6341 | 4 |
| 10 | 1154.5364 | 577.7718 | 1137.5099 | 569.2586 | 1136.5259 | 568.7666 | E | **389.2395** | 195.1234 | 372.2129 | 186.6101 | 371.2289 | 186.1181 | 3 |
| 11 | **1267.6205** | 634.3139 | 1250.5939 | 625.8006 | 1249.6099 | 625.3086 | L | 260.1969 | 130.6021 | 243.1703 | 122.0888 | | | 2 |
| 12 | | | | | | | K | 147.1128 | 74.0600 | 130.0863 | 65.5468 | | | 1 |

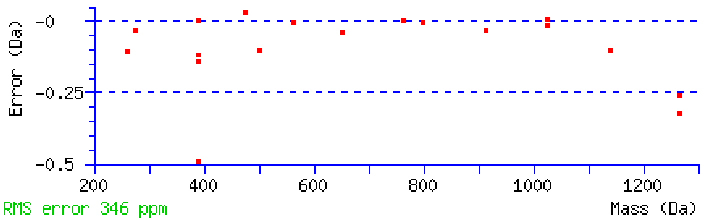


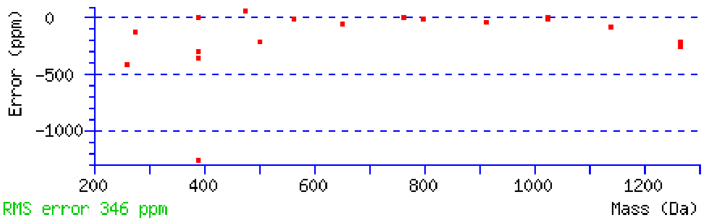


### All matches to this query

| Score | Mr(calc): | Delta | Sequence |
|---|---|---|---|
| 68.2 | 1412.7187 | 0.0018 | FQLLNFSSSELK |
| 29.4 | 1412.7156 | 0.0050 | CMLLAHADLIEK |
| 17.4 | 1412.7334 | -0.0128 | VSIVDLTCHLEK |
| 16.2 | 1412.7300 | -0.0094 | VRVESGYFSLEK |
| 13.7 | 1411.7307 | 0.9899 | NDPNQLNNLIKK |
| 12.6 | 1412.7177 | 0.0028 | KFNATERVLQK |
| 12.1 | 1411.7146 | 1.0060 | KMKLVESQLEK |
| 11.4 | 1411.7307 | 0.9899 | NDPNQLNNLIKK |
| 11.4 | 1411.7307 | 0.9899 | NDPNQLNNLIKK |
| 11.4 | 1411.7152 | 1.0053 | YYYVKNIIKK |

---

Spectrum No: 391; Query: 887; Rank: 1

---

## Peptide View

MS/MS Fragmentation of **DMSDGFISNLTIQR**
Found in **IPI00209863**, Tax_Id=10116 Gene_Symbol=P4ha1 Prolyl 4-hydroxylase subunit alpha-1 precursor

Match to Query 887: 1596.750428 from(799.382490,2+)
Title: 100101RatKid_NS_deglyco_19.4358.4358.2.dta
Data file K:\NewmanPaper\Piliang\3SubProteomes\Piliang3SP\mgf5ppm\ERLIC_3SubProteomes5ppm.mgf

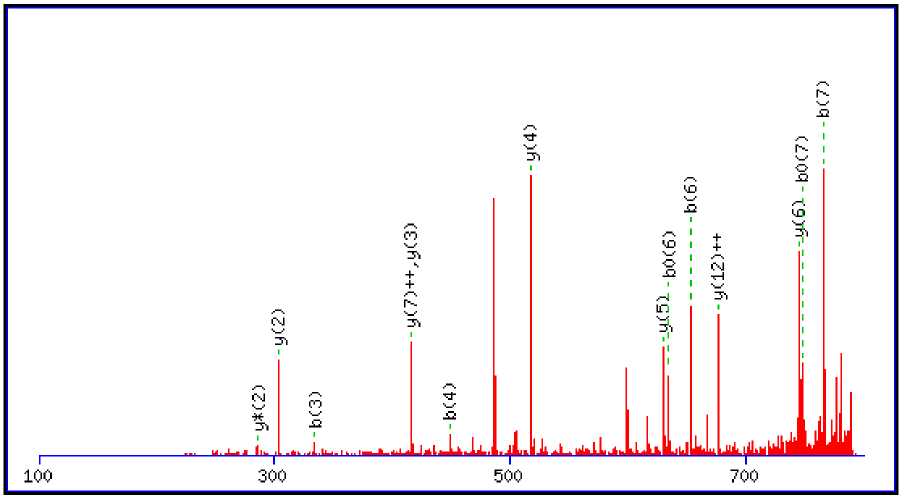

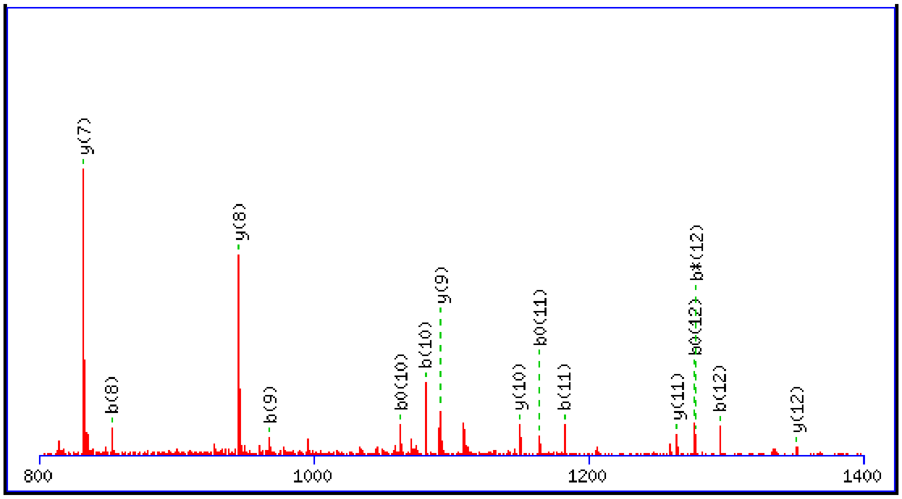


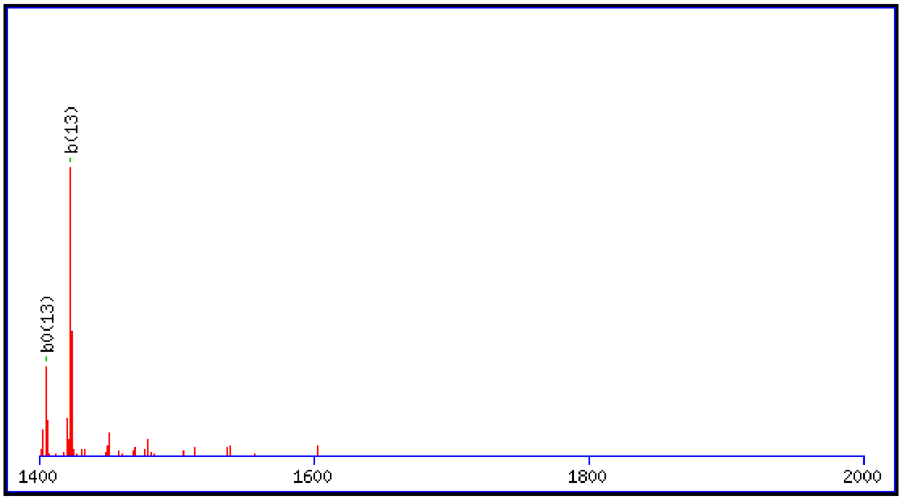


**Monoisotopic mass of neutral peptide Mr(calc):** 1596.7454
**Fixed modifications:** Carbamidomethyl (C)
**Variable modifications:**
**N9** : Deamidated_N (N)
**Ions Score:** 68 **Expect:** 2.7e-005
**Matches (Bold Red):** 31/134 fragment ions using 60 most intense peaks

| # | b | b++ | b* | b*++ | b0 | b0++ | Seq. | y | y++ | y* | y*++ | y0 | y0++ | # |
|---|---|---|---|---|---|---|---|---|---|---|---|---|---|---|
| 1 | 116.0342 | 58.5207 | | | 98.0237 | 49.5155 | D | | | | | | | 14 |
| 2 | 247.0747 | 124.0410 | | | 229.0641 | 115.0357 | M | 1482.7257 | 741.8665 | 1465.6991 | 733.3532 | 1464.7151 | 732.8612 | 13 |
| 3 | **334.1067** | 167.5570 | | | 316.0962 | 158.5517 | S | **1351.6852** | **676.3462** | 1334.6587 | 667.8330 | 1333.6746 | 667.3410 | 12 |
| 4 | **449.1337** | 225.0705 | | | 431.1231 | 216.0652 | D | **1264.6532** | 632.8302 | 1247.6266 | 624.3170 | 1246.6426 | 623.8249 | 11 |
| 5 | 506.1551 | 253.5812 | | | 488.1446 | 244.5759 | G | **1149.6262** | 575.3168 | 1132.5997 | 566.8035 | 1131.6157 | 566.3115 | 10 |
| 6 | **653.2236** | 327.1154 | | | **635.2130** | 318.1101 | F | **1092.6048** | 546.8060 | 1075.5782 | 538.2928 | 1074.5942 | 537.8007 | 9 |
| 7 | **766.3076** | 383.6574 | | | **748.2971** | 374.6522 | I | **945.5364** | 473.2718 | 928.5098 | 464.7585 | 927.5258 | 464.2665 | 8 |
| 8 | **853.3396** | 427.1735 | | | 835.3291 | 418.1682 | S | **832.4523** | **416.7298** | 815.4258 | 408.2165 | 814.4417 | 407.7245 | 7 |
| 9 | **968.3666** | 484.6869 | 951.3400 | 476.1737 | 950.3560 | 475.6816 | N | **745.4203** | 373.2138 | 728.3937 | 364.7005 | 727.4097 | 364.2085 | 6 |
| 10 | **1081.4506** | 541.2290 | 1064.4241 | 532.7157 | **1063.4401** | 532.2237 | L | **630.3933** | 315.7003 | 613.3668 | 307.1870 | 612.3828 | 306.6950 | 5 |
| 11 | **1182.4983** | 591.7528 | 1165.4718 | 583.2395 | **1164.4878** | 582.7475 | T | **517.3093** | 259.1583 | 500.2827 | 250.6450 | 499.2987 | 250.1530 | 4 |
| 12 | **1295.5824** | 648.2948 | **1278.5558** | 639.7816 | **1277.5718** | 639.2895 | I | **416.2616** | 208.6344 | 399.2350 | 200.1212 | | | 3 |
| 13 | **1423.6410** | 712.3241 | 1406.6144 | 703.8108 | **1405.6304** | 703.3188 | Q | **303.1775** | 152.0924 | **286.1510** | 143.5791 | | | 2 |
| 14 | | | | | | | R | 175.1190 | 88.0631 | 158.0924 | 79.5498 | | | 1 |

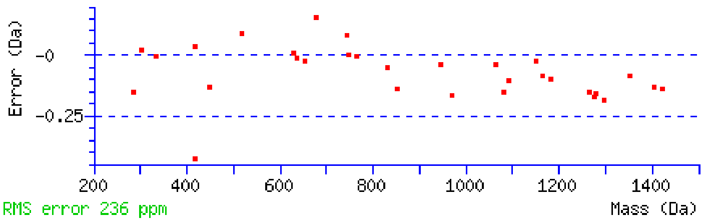


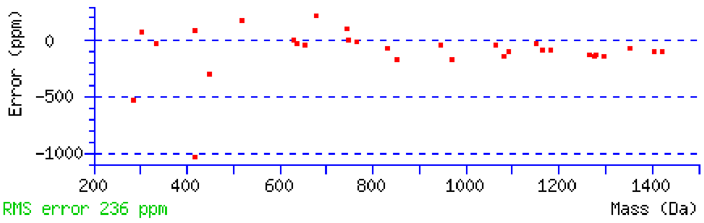


### All matches to this query

| Score | Mr(calc): | Delta | Sequence |
|---|---|---|---|
| 68.2 | 1596.7454 | 0.0051 | DMSDGFISNLTIQR |
| 16.1 | 1595.7614 | 0.9891 | DMSDGFISNLTIQR |
| 8.5 | 1594.7409 | 2.0096 | ITYEPITTTLKR |
| 8.2 | 1596.7443 | 0.0062 | MNLQAQSKAQNKR |
| 7.4 | 1596.7466 | 0.0038 | HGLPFVTLTLPSR |
| 7.4 | 1596.7466 | 0.0038 | HGLPFVTLTLPSR |
| 7.0 | 1594.7521 | 1.9983 | VFVATSSGSIAIRK |
| 6.3 | 1595.7629 | 0.9876 | IQMPLSFMWNXK |
| 6.3 | 1595.7461 | 1.0044 | TDSISIQSMIESNR |
| 6.3 | 1594.7331 | 2.0174 | RSECVSEMLDLEK |

---

Spectrum No: 392; Query: 1294; Rank: 1

---

## Peptide View

MS/MS Fragmentation of **GTFTDCALANMTQQIR**
Found in **IPI00372839**, Tax_Id=10116 Gene_Symbol=Col6a2 similar to procollagen, type VI, alpha 2

Match to Query 1294: 1826.837468 from(914.426010,2+)
Title: 100101RatKid_NS_deglyco_12.4502.4502.2.dta
Data file K:\NewmanPaper\Piliang\3SubProteomes\Piliang3SP\mgf5ppm\ERLIC_3SubProteomes5ppm.mgf

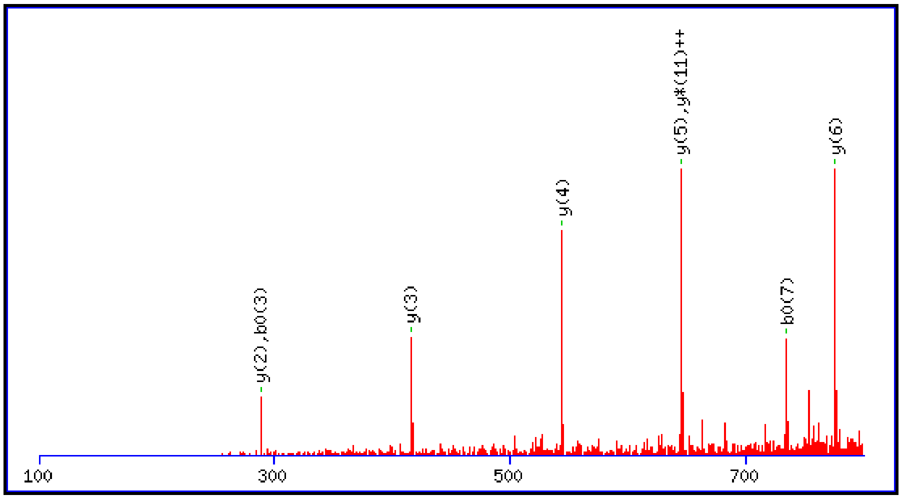

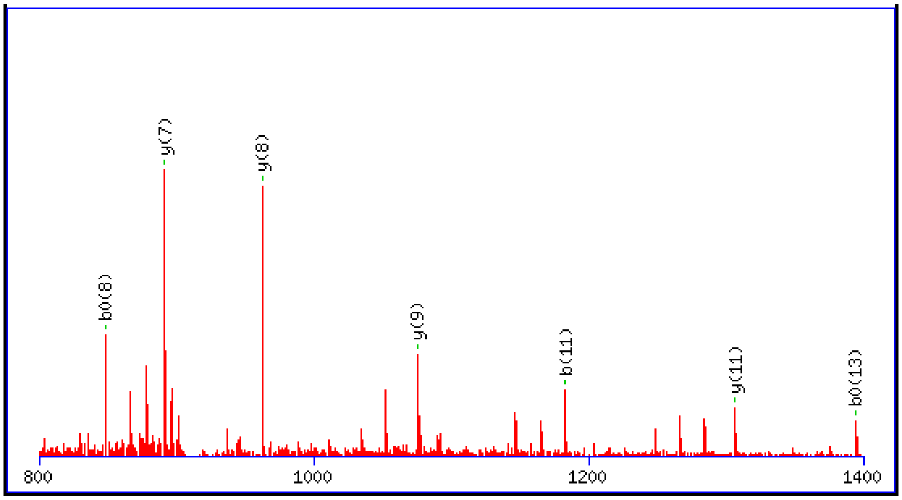


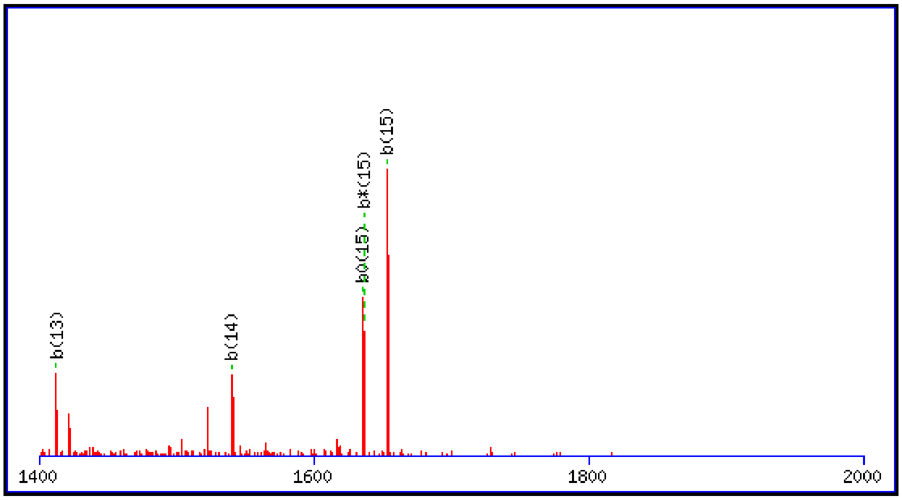


**Monoisotopic mass of neutral peptide Mr(calc):** 1826.8291
**Fixed modifications:** Carbamidomethyl (C)
**Variable modifications:**
**N10** : Deamidated_N (N)
**Ions Score:** 68 **Expect:** 3.4e-005
**Matches (Bold Red):** 20/152 fragment ions using 21 most intense peaks

| # | b | $b^{++}$ | b* | $b^{*++}$ | $b^0$ | $b^{0++}$ | Seq. | y | $y^{++}$ | y* | $y^{*++}$ | $y^0$ | $y^{0++}$ | # |
|---|---|---|---|---|---|---|---|---|---|---|---|---|---|---|
| 1 | 58.0287 | 29.5180 | | | | | G | | | | | | | 16 |
| 2 | 159.0764 | 80.0418 | | | 141.0659 | 71.0366 | T | 1770.8149 | 885.9111 | 1753.7884 | 877.3978 | 1752.8044 | 876.9058 | 15 |
| 3 | 306.1448 | 153.5761 | | | **288.1343** | 144.5708 | F | 1669.7672 | 835.3873 | 1652.7407 | 826.8740 | 1651.7567 | 826.3820 | 14 |
| 4 | 407.1925 | 204.0999 | | | 389.1819 | 195.0946 | T | 1522.6988 | 761.8531 | 1505.6723 | 753.3398 | 1504.6883 | 752.8478 | 13 |
| 5 | 522.2195 | 261.6134 | | | 504.2089 | 252.6081 | D | 1421.6512 | 711.3292 | 1404.6246 | 702.8159 | 1403.6406 | 702.3239 | 12 |
| 6 | 682.2501 | 341.6287 | | | 664.2395 | 332.6234 | C | **1306.6242** | 653.8157 | 1289.5977 | **645.3025** | 1288.6136 | 644.8105 | 11 |
| 7 | 753.2872 | 377.1472 | | | **735.2767** | 368.1420 | A | 1146.5936 | 573.8004 | 1129.5670 | 565.2871 | 1128.5830 | 564.7951 | 10 |
| 8 | 866.3713 | 433.6893 | | | **848.3607** | 424.6840 | L | **1075.5565** | 538.2819 | 1058.5299 | 529.7686 | 1057.5459 | 529.2766 | 9 |
| 9 | 937.4084 | 469.2078 | | | 919.3978 | 460.2026 | A | **962.4724** | 481.7398 | 945.4458 | 473.2266 | 944.4618 | 472.7345 | 8 |
| 10 | 1052.4353 | 526.7213 | 1035.4088 | 518.2080 | 1034.4248 | 517.7160 | N | **891.4353** | 446.2213 | 874.4087 | 437.7080 | 873.4247 | 437.2160 | 7 |
| 11 | **1183.4758** | 592.2415 | 1166.4493 | 583.7283 | 1165.4653 | 583.2363 | M | **776.4083** | 388.7078 | 759.3818 | 380.1945 | 758.3978 | 379.7025 | 6 |
| 12 | 1284.5235 | 642.7654 | 1267.4969 | 634.2521 | 1266.5129 | 633.7601 | T | **645.3679** | 323.1876 | 628.3413 | 314.6743 | 627.3573 | 314.1823 | 5 |
| 13 | **1412.5821** | 706.7947 | 1395.5555 | 698.2814 | **1394.5715** | 697.7894 | Q | **544.3202** | 272.6637 | 527.2936 | 264.1504 | | | 4 |
| 14 | **1540.6407** | 770.8240 | 1523.6141 | 762.3107 | 1522.6301 | 761.8187 | Q | **416.2616** | 208.6344 | 399.2350 | 200.1212 | | | 3 |
| 15 | **1653.7247** | 827.3660 | **1636.6982** | 818.8527 | **1635.7142** | 818.3607 | I | **288.2030** | 144.6051 | 271.1765 | 136.0919 | | | 2 |
| 16 | | | | | | | R | 175.1190 | 88.0631 | 158.0924 | 79.5498 | | | 1 |

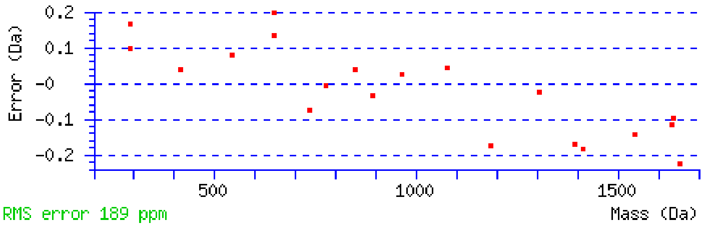


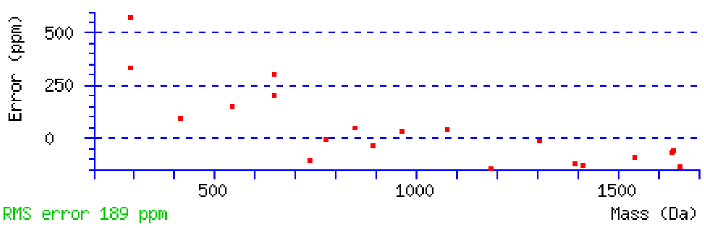


### All matches to this query

| Score | Mr(calc): | Delta | Sequence |
|---|---|---|---|
| 68.1 | 1826.8291 | 0.0084 | GTFTDCALANMTQQIR |
| 14.0 | 1825.8451 | 0.9924 | GTFTDCALANMTQQIR |
| 3.7 | 1826.8556 | -0.0181 | TIFNWTCSCNGAKLR |
| 3.0 | 1826.8291 | 0.0084 | MNERMNTTEIFDGIR |
| 2.5 | 1826.8291 | 0.0084 | MNERMNTTEIFDGIR |
| 1.1 | 1826.8331 | 0.0043 | TLWMPSSTSXWSMLR |
| 0.9 | 1824.8126 | 2.0249 | EGGNFLDTTDTGNSVAAR |
| 0.6 | 1826.8460 | -0.0085 | HYLLVGAGAIGCEMLK |
| 0.3 | 1826.8240 | 0.0135 | NYGKFLEEYTSQLR |
| 0.3 | 1826.8240 | 0.0135 | NYGKFLEEYTSQLR |

---

Spectrum No: 393; Query: 1926; Rank: 1

---

## Peptide View

MS/MS Fragmentation of **KNTTGLPQNILSQVHQLNSK**
Found in **IPI00196620**, Tax_Id=10116 Gene_Symbol=Cubn Cubilin precursor

Match to Query 1926: 2220.184242 from(741.068690,3+)
Title: 091008RatKidney_NH4Format01_23.2589.2589.3.dta
Data file K:\NewmanPaper\Piliang\3SubProteomes\Piliang3SP\mgf5ppm\ERLIC_3SubProteomes5ppm.mgf

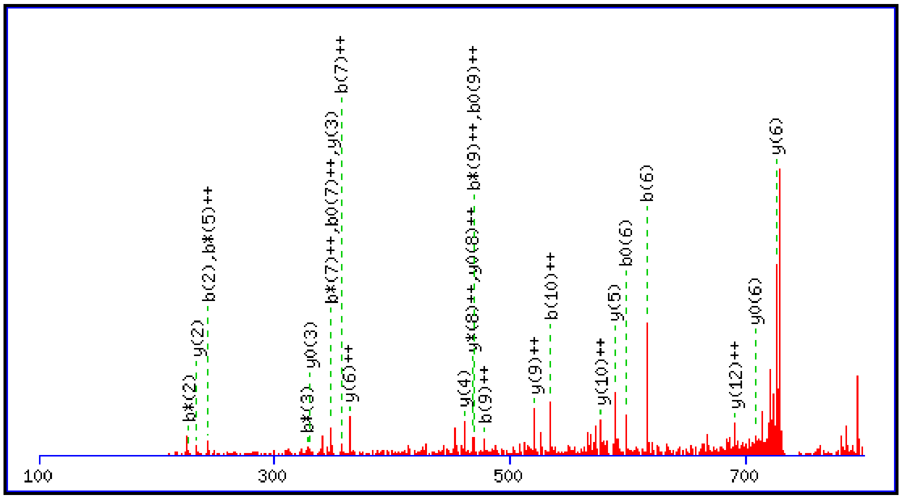

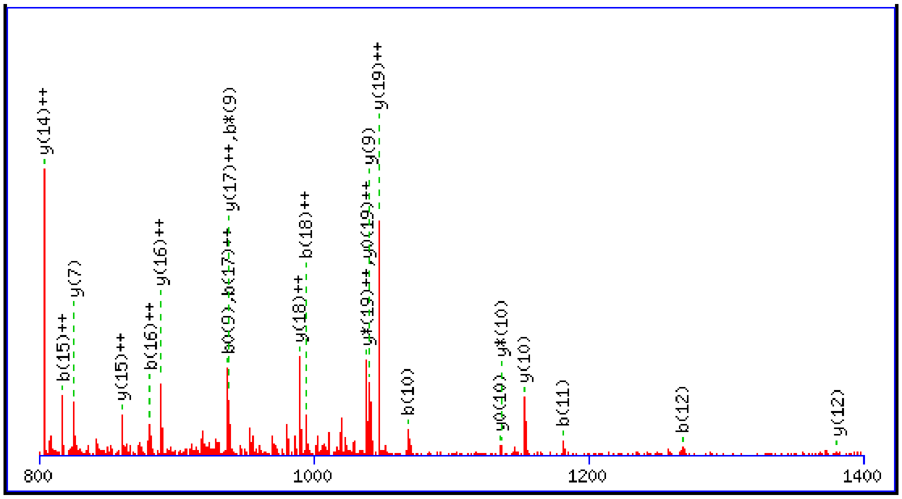


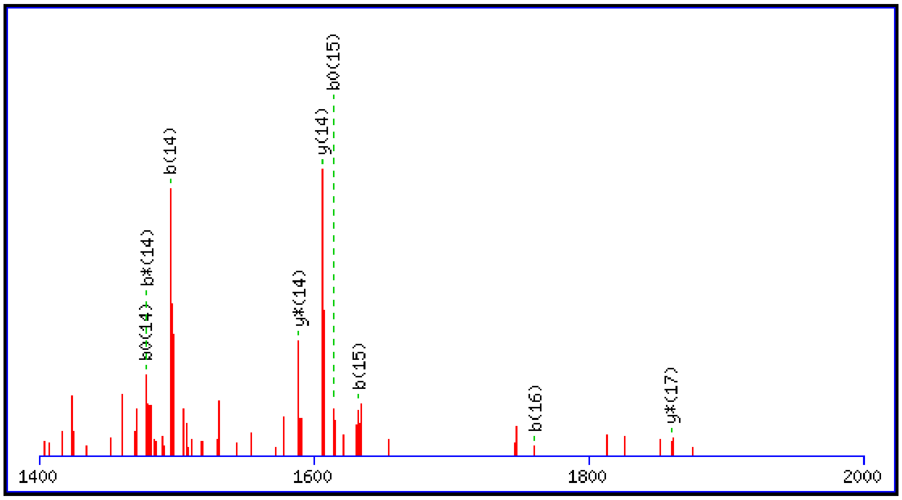


**Monoisotopic mass of neutral peptide Mr(calc):** 2220.1862
**Fixed modifications:** Carbamidomethyl (C)
**Variable modifications:**
**N2** : Deamidated_N (N)
**Ions Score:** 68 **Expect:** 1.4e-005
**Matches (Bold Red):** 59/222 fragment ions using 100 most intense peaks

| # | b | $b^{++}$ | b* | $b^{*++}$ | $b^0$ | $b^{0++}$ | Seq. | y | $y^{++}$ | y* | $y^{*++}$ | $y^0$ | $y^{0++}$ | # |
|---|---|---|---|---|---|---|---|---|---|---|---|---|---|---|
| 1 | 129.1022 | 65.0548 | 112.0757 | 56.5415 | | | K | | | | | | | 20 |
| 2 | **244.1292** | 122.5682 | **227.1026** | 114.0550 | | | N | 2093.0986 | **1047.0529** | 2076.0720 | **1038.5396** | 2075.0880 | **1038.0476** | 19 |
| 3 | 345.1769 | 173.0921 | **328.1503** | 164.5788 | 327.1663 | 164.0868 | T | 1978.0716 | **989.5394** | 1961.0451 | 981.0262 | 1960.0611 | 980.5342 | 18 |
| 4 | 446.2245 | 223.6159 | 429.1980 | 215.1026 | 428.2140 | 214.6106 | T | 1877.0239 | **939.0156** | **1859.9974** | 930.5023 | 1859.0134 | 930.0103 | 17 |
| 5 | 503.2460 | 252.1266 | 486.2194 | **243.6134** | 485.2354 | 243.1214 | G | 1775.9763 | **888.4918** | 1758.9497 | 879.9785 | 1757.9657 | 879.4865 | 16 |
| 6 | **616.3301** | 308.6687 | 599.3035 | 300.1554 | **598.3195** | 299.6634 | L | 1718.9548 | **859.9810** | 1701.9282 | 851.4678 | 1700.9442 | 850.9758 | 15 |
| 7 | 713.3828 | **357.1951** | 696.3563 | **348.6818** | 695.3723 | **348.1898** | P | **1605.8707** | **803.4390** | **1588.8442** | 794.9257 | 1587.8602 | 794.4337 | 14 |
| 8 | 841.4414 | 421.2243 | 824.4149 | 412.7111 | 823.4308 | 412.2191 | Q | 1508.8180 | 754.9126 | 1491.7914 | 746.3993 | 1490.8074 | 745.9073 | 13 |
| 9 | 955.4843 | **478.2458** | **938.4578** | **469.7325** | **937.4738** | **469.2405** | N | **1380.7594** | **690.8833** | 1363.7328 | 682.3701 | 1362.7488 | 681.8780 | 12 |
| 10 | **1068.5684** | **534.7878** | 1051.5418 | 526.2746 | 1050.5578 | 525.7826 | I | 1266.7165 | 633.8619 | 1249.6899 | 625.3486 | 1248.7059 | 624.8566 | 11 |
| 11 | **1181.6525** | 591.3299 | 1164.6259 | 582.8166 | 1163.6419 | 582.3246 | L | **1153.6324** | **577.3198** | **1136.6058** | 568.8066 | **1135.6218** | 568.3146 | 10 |
| 12 | **1268.6845** | 634.8459 | 1251.6579 | 626.3326 | 1250.6739 | 625.8406 | S | **1040.5483** | **520.7778** | 1023.5218 | 512.2645 | 1022.5378 | 511.7725 | 9 |
| 13 | 1396.7431 | 698.8752 | 1379.7165 | 690.3619 | 1378.7325 | 689.8699 | Q | 953.5163 | 477.2618 | 936.4898 | **468.7485** | 935.5057 | **468.2565** | 8 |
| 14 | **1495.8115** | 748.4094 | **1478.7849** | 739.8961 | **1477.8009** | 739.4041 | V | **825.4577** | 413.2325 | 808.4312 | 404.7192 | 807.4472 | 404.2272 | 7 |
| 15 | **1632.8704** | **816.9388** | 1615.8438 | 808.4256 | **1614.8598** | 807.9336 | H | **726.3893** | **363.6983** | 709.3628 | 355.1850 | **708.3787** | 354.6930 | 6 |
| 16 | **1760.9290** | **880.9681** | 1743.9024 | 872.4548 | 1742.9184 | 871.9628 | Q | **589.3304** | 295.1688 | 572.3039 | 286.6556 | 571.3198 | 286.1636 | 5 |
| 17 | 1874.0130 | **937.5102** | 1856.9865 | 928.9969 | 1856.0025 | 928.5049 | L | **461.2718** | 231.1395 | 444.2453 | 222.6263 | 443.2613 | 222.1343 | 4 |
| 18 | 1988.0560 | **994.5316** | 1971.0294 | 986.0183 | 1970.0454 | 985.5263 | N | **348.1878** | 174.5975 | 331.1612 | 166.0842 | **330.1772** | 165.5922 | 3 |
| | | | | | | | | | | | | | | |

| | | | | | | | | | | | | | | |
|---|---|---|---|---|---|---|---|---|---|---|---|---|---|---|
| **19** | 2075.0880 | **1038.0476** | 2058.0614 | 1029.5344 | 2057.0774 | 1029.0423 | **S** | **234.1448** | 117.5761 | 217.1183 | 109.0628 | 216.1343 | 108.5708 | **2** |
| **20** | | | | | | | **K** | 147.1128 | 74.0600 | 130.0863 | 65.5468 | | | **1** |

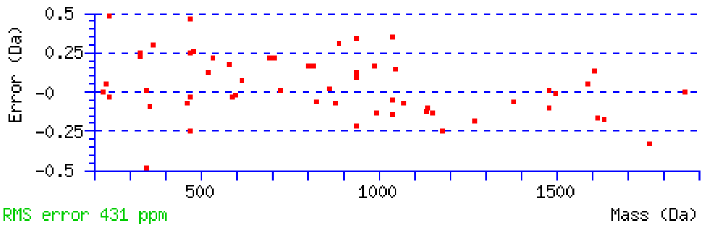


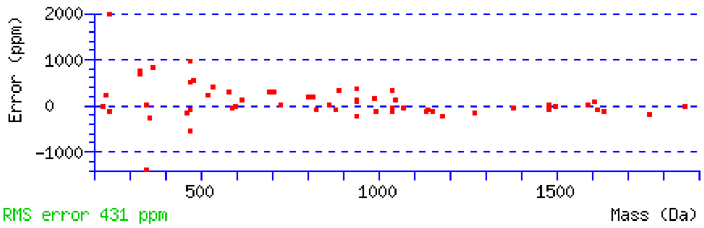


### All matches to this query

| Score | Mr(calc): | Delta | Sequence |
|---|---|---|---|
| 68.1 | 2220.1862 | -0.0020 | KNTTGLPQNILSQVHQLNSK |
| 57.9 | 2220.1862 | -0.0020 | KNTTGLPQNILSQVHQLNSK |
| 37.6 | 2219.2022 | 0.9820 | KNTTGLPQNILSQVHQLNSK |
| 22.7 | 2220.1862 | -0.0020 | KNTTGLPQNILSQVHQLNSK |
| 1.8 | 2218.1786 | 2.0056 | FTVPIPALNNSPQLKEFFR |

---

Spectrum No: 394; Query: 2491; Rank: 1

---

## Peptide View

MS/MS Fragmentation of **ACIENGTSCIDICGEPQFLELMHVK**
Found in **IPI00372804**, Tax_Id=10116 Gene_Symbol=Sccpdh Probable saccharopine dehydrogenase

Match to Query 2491: 2921.307222 from(974.776350,3+)
Title: 091008RatKidney_NH4Format01_09.5044.5044.3.dta
Data file K:\NewmanPaper\Piliang\3SubProteomes\Piliang3SP\mgf5ppm\ERLIC_3SubProteomes5ppm.mgf

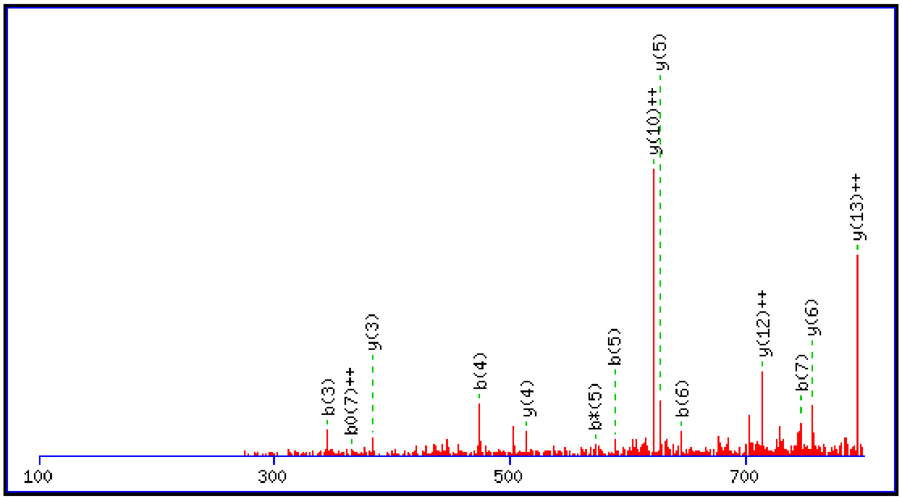

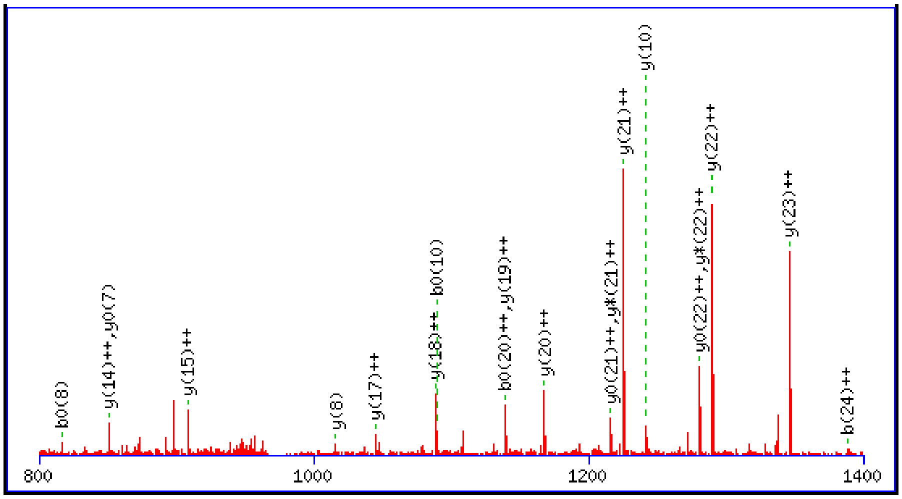


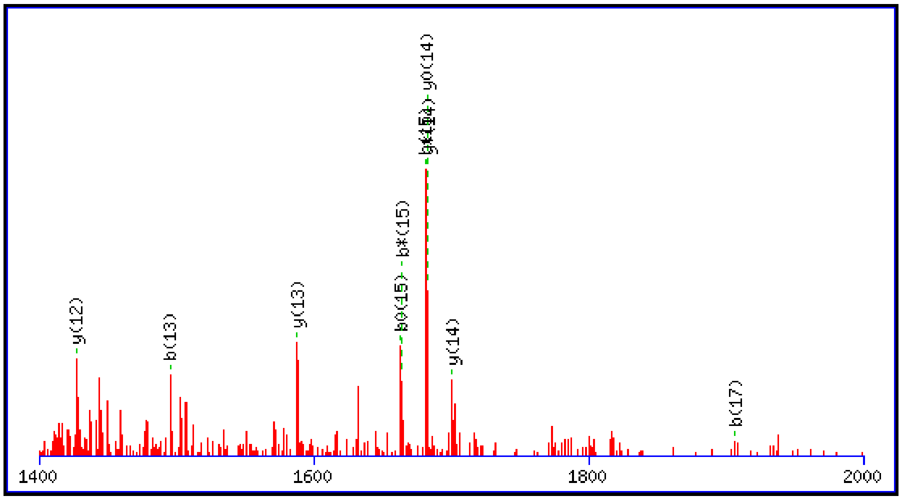


**Monoisotopic mass of neutral peptide Mr(calc):** 2921.3068
**Fixed modifications:** Carbamidomethyl (C)
**Variable modifications:**
**N5** : Deamidated_N (N)
**Ions Score:** 68 **Expect:** 6.1e-005
**Matches (Bold Red):** 44/264 fragment ions using 87 most intense peaks

| # | b | $b^{++}$ | b* | $b^{*++}$ | $b^{0}$ | $b^{0++}$ | Seq. | y | $y^{++}$ | y* | $y^{*++}$ | $y^{0}$ | $y^{0++}$ | # |
|---|---|---|---|---|---|---|---|---|---|---|---|---|---|---|
| 1 | 72.0444 | 36.5258 | | | | | A | | | | | | | 25 |
| 2 | 232.0750 | 116.5412 | | | | | C | 2851.2769 | 1426.1421 | 2834.2504 | 1417.6288 | 2833.2664 | 1417.1368 | 24 |
| 3 | **345.1591** | 173.0832 | | | | | I | 2691.2463 | **1346.1268** | 2674.2197 | 1337.6135 | 2673.2357 | 1337.1215 | 23 |
| 4 | **474.2017** | 237.6045 | | | 456.1911 | 228.5992 | E | 2578.1622 | **1289.5847** | 2561.1357 | **1281.0715** | 2560.1517 | **1280.5795** | 22 |
| 5 | **589.2286** | 295.1180 | **572.2021** | 286.6047 | 571.2181 | 286.1127 | N | 2449.1196 | **1225.0635** | 2432.0931 | **1216.5502** | 2431.1091 | **1216.0582** | 21 |
| 6 | **646.2501** | 323.6287 | 629.2235 | 315.1154 | 628.2395 | 314.6234 | G | 2334.0927 | **1167.5500** | 2317.0661 | 1159.0367 | 2316.0821 | 1158.5447 | 20 |
| 7 | **747.2978** | 374.1525 | 730.2712 | 365.6393 | 729.2872 | **365.1472** | T | 2277.0712 | **1139.0393** | 2260.0447 | 1130.5260 | 2259.0607 | 1130.0340 | 19 |
| 8 | 834.3298 | 417.6685 | 817.3033 | 409.1553 | **816.3192** | 408.6633 | S | 2176.0235 | **1088.5154** | 2158.9970 | 1080.0021 | 2158.0130 | 1079.5101 | 18 |
| 9 | 994.3605 | 497.6839 | 977.3339 | 489.1706 | 976.3499 | 488.6786 | C | 2088.9915 | **1044.9994** | 2071.9650 | 1036.4861 | 2070.9810 | 1035.9941 | 17 |
| 10 | 1107.4445 | 554.2259 | 1090.4180 | 545.7126 | **1089.4340** | 545.2206 | I | 1928.9609 | 964.9841 | 1911.9343 | 956.4708 | 1910.9503 | 955.9788 | 16 |
| 11 | 1222.4715 | 611.7394 | 1205.4449 | 603.2261 | 1204.4609 | 602.7341 | D | 1815.8768 | **908.4420** | 1798.8503 | 899.9288 | 1797.8662 | 899.4368 | 15 |
| 12 | 1335.5555 | 668.2814 | 1318.5290 | 659.7681 | 1317.5450 | 659.2761 | I | **1700.8499** | **850.9286** | **1683.8233** | 842.4153 | **1682.8393** | 841.9233 | 14 |
| 13 | **1495.5862** | 748.2967 | 1478.5596 | 739.7834 | 1477.5756 | 739.2914 | C | **1587.7658** | **794.3865** | 1570.7392 | 785.8733 | 1569.7552 | 785.3813 | 13 |
| 14 | 1552.6076 | 776.8075 | 1535.5811 | 768.2942 | 1534.5971 | 767.8022 | G | **1427.7351** | **714.3712** | 1410.7086 | 705.8579 | 1409.7246 | 705.3659 | 12 |
| 15 | **1681.6502** | 841.3288 | **1664.6237** | 832.8155 | **1663.6397** | 832.3235 | E | 1370.7137 | 685.8605 | 1353.6871 | 677.3472 | 1352.7031 | 676.8552 | 11 |
| 16 | 1778.7030 | 889.8551 | 1761.6764 | 881.3419 | 1760.6924 | 880.8499 | P | **1241.6711** | **621.3392** | 1224.6445 | 612.8259 | 1223.6605 | 612.3339 | 10 |
| 17 | **1906.7616** | 953.8844 | 1889.7350 | 945.3711 | 1888.7510 | 944.8791 | Q | 1144.6183 | 572.8128 | 1127.5918 | 564.2995 | 1126.6078 | 563.8075 | 9 |
| 18 | 2053.8300 | 1027.4186 | 2036.8034 | 1018.9054 | 2035.8194 | 1018.4133 | F | **1016.5598** | 508.7835 | 999.5332 | 500.2702 | 998.5492 | 499.7782 | 8 |

| | | | | | | | | | | | | | |
|---|---|---|---|---|---|---|---|---|---|---|---|---|---|
| 19 | 2166.9140 | 1083.9607 | 2149.8875 | 1075.4474 | 2148.9035 | 1074.9554 | L | 869.4913 | 435.2493 | 852.4648 | 426.7360 | 851.4808 | 426.2440 | 7 |
| 20 | 2295.9566 | 1148.4820 | 2278.9301 | 1139.9687 | 2277.9461 | 1139.4767 | E | 756.4073 | 378.7073 | 739.3807 | 370.1940 | 738.3967 | 369.7020 | 6 |
| 21 | 2409.0407 | 1205.0240 | 2392.0142 | 1196.5107 | 2391.0301 | 1196.0187 | L | 627.3647 | 314.1860 | 610.3381 | 305.6727 | | | 5 |
| 22 | 2540.0812 | 1270.5442 | 2523.0546 | 1262.0310 | 2522.0706 | 1261.5390 | M | 514.2806 | 257.6439 | 497.2541 | 249.1307 | | | 4 |
| 23 | 2677.1401 | 1339.0737 | 2660.1136 | 1330.5604 | 2659.1295 | 1330.0684 | H | 383.2401 | 192.1237 | 366.2136 | 183.6104 | | | 3 |
| 24 | 2776.2085 | 1388.6079 | 2759.1820 | 1380.0946 | 2758.1980 | 1379.6026 | V | 246.1812 | 123.5942 | 229.1547 | 115.0810 | | | 2 |
| 25 | | | | | | | K | 147.1128 | 74.0600 | 130.0863 | 65.5468 | | | 1 |

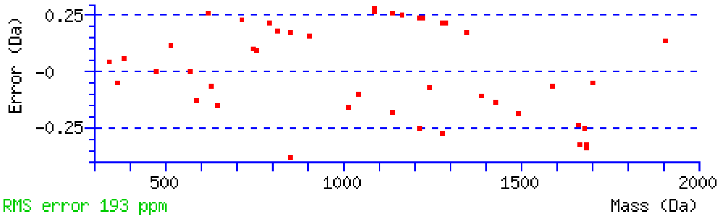


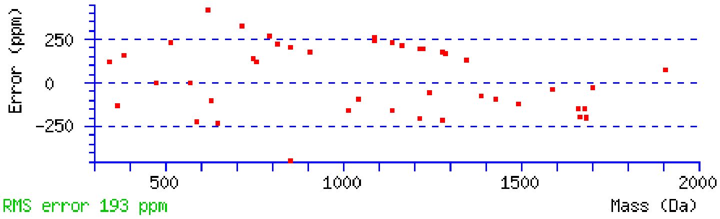


### All matches to this query

| Score | Mr(calc): | Delta | Sequence |
|---|---|---|---|
| 68.1 | 2921.3068 | 0.0005 | ACIENGTSCIDICGEPQFLELMHVK |
| 31.6 | 2920.3228 | 0.9845 | ACIENGTSCIDICGEPQFLELMHVK |
| 12.2 | 2919.3265 | 1.9807 | MYTDKLETVPPLNELTEIPGEDKK |
| 12.1 | 2919.3232 | 1.9840 | DYDVSFGDGVLTIKLGGDLGTYVINK |
| 5.8 | 2920.3330 | 0.9742 | LMHAGVTDSGETVNIGDVSYKLKTPK |
| 5.8 | 2921.3122 | -0.0050 | CPSDPEMLKSSVSSPLHPTMRINLN |
| 5.7 | 2919.3265 | 1.9807 | MYTDKLETVPPLNELTEIPGEDKK |
| 4.4 | 2920.3330 | 0.9742 | LMHAGVTDSGETVNIGDVSYKLKTPK |
| 4.4 | 2919.2949 | 2.0124 | SHWSMDLLQSGPLVKDMNTKSLEK |
| 4.3 | 2919.3160 | 1.9912 | MPNILTSNLKIINEDTNCISPTQK |

Spectrum No: 395; Query: 230; Rank: 1

## Peptide View

MS/MS Fragmentation of **DALQNMSSSLK**
Found in **IPI00325312**, Tax_Id=10116 Gene_Symbol=Prom1 prominin 1 isoform 1

Match to Query 230: 1209.553488 from(605.784020,2+)
Title: 091008RatKidney_NoSalt_32.646.646.2.dta
Data file K:\NewmanPaper\Piliang\3SubProteomes\Piliang3SP\mgf5ppm\ERLIC_3SubProteomes5ppm.mgf

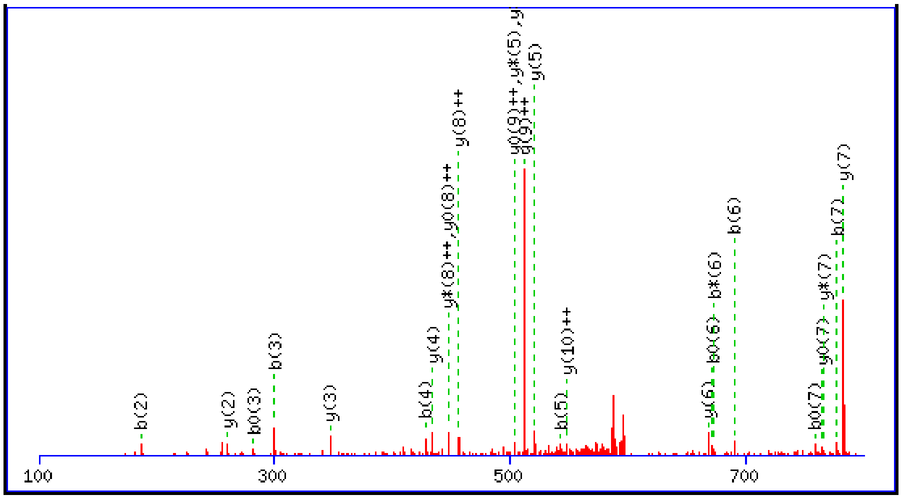


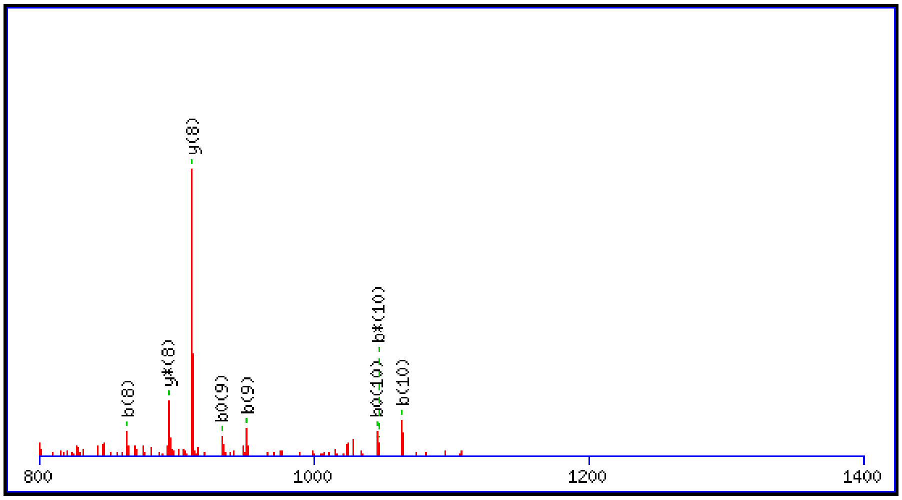


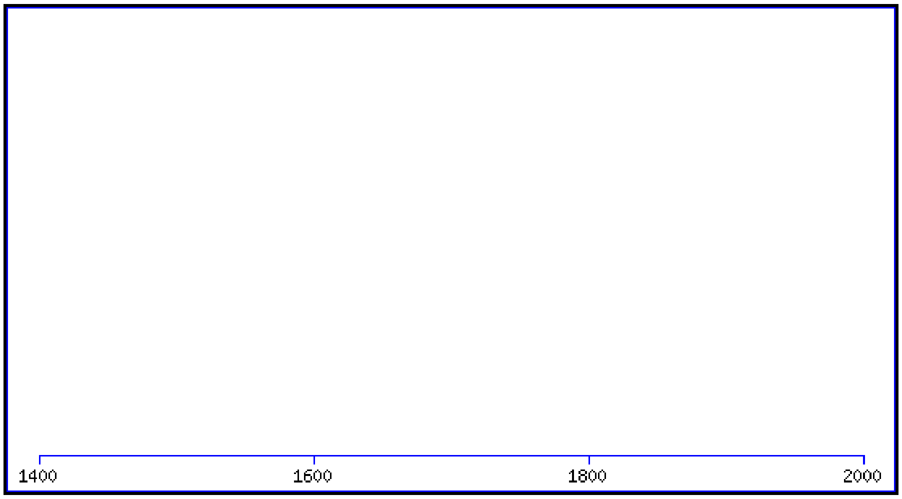


**Monoisotopic mass of neutral peptide Mr(calc):** 1209.5547
**Fixed modifications:** Carbamidomethyl (C)
**Variable modifications:**
**N5** : Deamidated_N (N)
**M6** : Oxidation (M)
**Ions Score:** 68 **Expect:** 2.1e-005
**Matches (Bold Red):** 34/110 fragment ions using 55 most intense peaks

| # | b | $b^{++}$ | b* | $b^{*++}$ | $b^0$ | $b^{0++}$ | Seq. | y | $y^{++}$ | y* | $y^{*++}$ | $y^0$ | $y^{0++}$ | # |
|---|---|---|---|---|---|---|---|---|---|---|---|---|---|---|
| 1 | 116.0342 | 58.5207 | | | 98.0237 | 49.5155 | D | | | | | | | 11 |
| 2 | **187.0713** | 94.0393 | | | 169.0608 | 85.0340 | A | 1095.5350 | **548.2712** | 1078.5085 | 539.7579 | 1077.5245 | 539.2659 | 10 |
| 3 | **300.1554** | 150.5813 | | | **282.1448** | 141.5761 | L | 1024.4979 | **512.7526** | 1007.4714 | **504.2393** | 1006.4874 | **503.7473** | 9 |
| 4 | **428.2140** | 214.6106 | 411.1874 | 206.0974 | 410.2034 | 205.6053 | Q | **911.4139** | **456.2106** | **894.3873** | **447.6973** | 893.4033 | **447.2053** | 8 |
| | | | | | | | | | | | | | | |

| | | | | | | | | | | | | | | |
|---|---|---|---|---|---|---|---|---|---|---|---|---|---|---|
| **5** | **543.2409** | 272.1241 | 526.2144 | 263.6108 | 525.2303 | 263.1188 | **N** | **783.3553** | 392.1813 | **766.3287** | 383.6680 | **765.3447** | 383.1760 | **7** |
| **6** | **690.2763** | 345.6418 | **673.2498** | 337.1285 | **672.2658** | 336.6365 | **M** | **668.3284** | 334.6678 | 651.3018 | 326.1545 | 650.3178 | 325.6625 | **6** |
| **7** | **777.3083** | 389.1578 | 760.2818 | 380.6445 | **759.2978** | 380.1525 | **S** | **521.2930** | 261.1501 | **504.2664** | 252.6368 | 503.2824 | 252.1448 | **5** |
| **8** | **864.3404** | 432.6738 | 847.3138 | 424.1605 | 846.3298 | 423.6685 | **S** | **434.2609** | 217.6341 | 417.2344 | 209.1208 | 416.2504 | 208.6288 | **4** |
| **9** | **951.3724** | 476.1898 | 934.3458 | 467.6766 | **933.3618** | 467.1846 | **S** | **347.2289** | 174.1181 | 330.2023 | 165.6048 | 329.2183 | 165.1128 | **3** |
| **10** | **1064.4565** | 532.7319 | **1047.4299** | 524.2186 | **1046.4459** | 523.7266 | **L** | **260.1969** | 130.6021 | 243.1703 | 122.0888 | | | **2** |
| **11** | | | | | | | **K** | 147.1128 | 74.0600 | 130.0863 | 65.5468 | | | **1** |

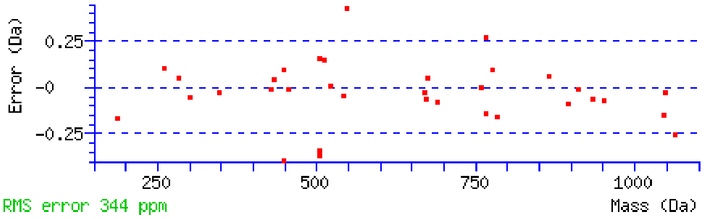


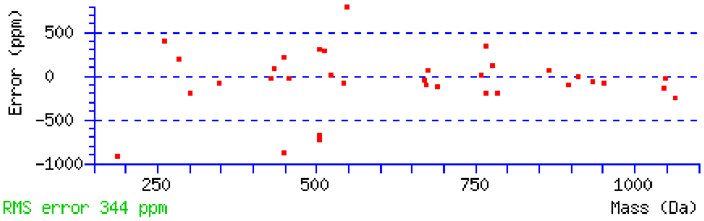


### All matches to this query

| Score | Mr(calc): | Delta | Sequence |
|---|---|---|---|
| 68.0 | 1209.5547 | -0.0012 | DALQNMSSSLK |
| 14.2 | 1209.5522 | 0.0013 | EGIQTCMFPK |
| 7.9 | 1208.5607 | 0.9928 | QLLKYGKIS |
| 6.8 | 1209.5448 | 0.0087 | SVITSIFGVK |
| 6.0 | 1209.5481 | 0.0054 | NAEMKNAMRK |
| 5.9 | 1209.5431 | 0.0104 | SYDYRVSLK |
| 5.2 | 1209.5577 | -0.0042 | QPSKMPNGKK |
| 5.0 | 1209.5431 | 0.0104 | ANLWPAQTTK |
| 5.0 | 1209.5431 | 0.0104 | ANLWPAQTTK |
| 4.5 | 1209.5547 | -0.0012 | GDSSQLMLDTK |

---

Spectrum No: 396; Query: 2028; Rank: 1

---

## Peptide View

MS/MS Fragmentation of **NVTYGTYLDDPDPDDGFNYK**
Found in **IPI00554194**, Tax_Id=10116 Gene_Symbol=Calu calumenin isoform b

Match to Query 2028: 2308.970948 from(1155.492750,2+)
Title: 100101RatKid_NS_deglyco_20.3666.3666.2.dta
Data file K:\NewmanPaper\Piliang\3SubProteomes\Piliang3SP\mgf5ppm\ERLIC_3SubProteomes5ppm.mgf

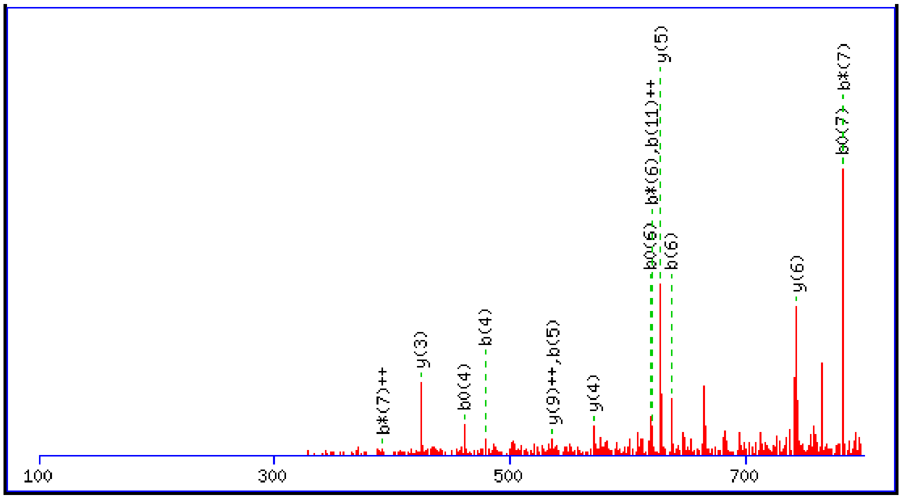


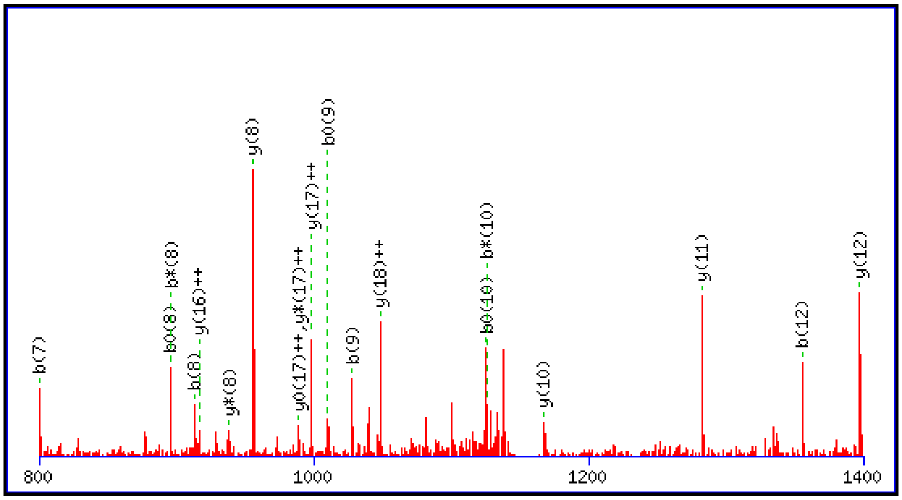


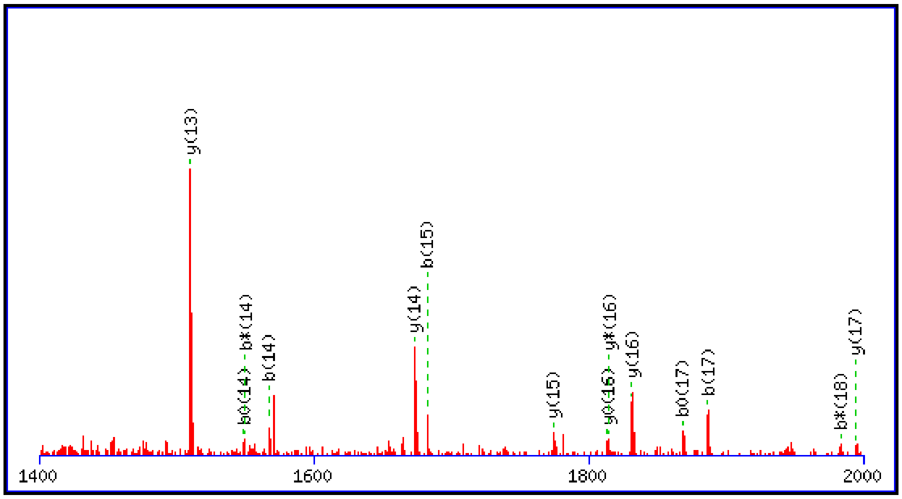


**Monoisotopic mass of neutral peptide Mr(calc):** 2308.9648
**Fixed modifications:** Carbamidomethyl (C)
**Variable modifications:**
**N1** : Deamidated_N (N)
**Ions Score:** 68 **Expect:** 4.5e-005
**Matches (Bold Red):** 48/214 fragment ions using 91 most intense peaks

| # | b | $b^{++}$ | b* | $b^{*++}$ | $b^{0}$ | $b^{0++}$ | Seq. | y | $y^{++}$ | y* | $y^{*++}$ | $y^{0}$ | $y^{0++}$ | # |
|---|---|---|---|---|---|---|---|---|---|---|---|---|---|---|
| 1 | 116.0342 | 58.5207 | 99.0077 | 50.0075 | | | N | | | | | | | 20 |
| 2 | 215.1026 | 108.0550 | 198.0761 | 99.5417 | | | V | 2194.9451 | 1097.9762 | 2177.9186 | 1089.4629 | 2176.9346 | 1088.9709 | 19 |
| 3 | 316.1503 | 158.5788 | 299.1238 | 150.0655 | 298.1397 | 149.5735 | T | 2095.8767 | **1048.4420** | 2078.8502 | 1039.9287 | 2077.8662 | 1039.4367 | 18 |
| 4 | **479.2136** | 240.1105 | 462.1871 | 231.5972 | **461.2031** | 231.1052 | Y | **1994.8290** | **997.9182** | 1977.8025 | **989.4049** | 1976.8185 | **988.9129** | 17 |
| 5 | **536.2351** | 268.6212 | 519.2085 | 260.1079 | 518.2245 | 259.6159 | G | **1831.7657** | **916.3865** | **1814.7392** | 907.8732 | **1813.7552** | 907.3812 | 16 |

| | | | | | | | | | | | | | | |
|---|---|---|---|---|---|---|---|---|---|---|---|---|---|---|
| 6 | 637.2828 | 319.1450 | 620.2562 | 310.6318 | 619.2722 | 310.1397 | T | 1774.7443 | 887.8758 | 1757.7177 | 879.3625 | 1756.7337 | 878.8705 | 15 |
| 7 | 800.3461 | 400.6767 | 783.3196 | 392.1634 | 782.3355 | 391.6714 | Y | 1673.6966 | 837.3519 | 1656.6700 | 828.8387 | 1655.6860 | 828.3466 | 14 |
| 8 | 913.4302 | 457.2187 | 896.4036 | 448.7054 | 895.4196 | 448.2134 | L | 1510.6332 | 755.8203 | 1493.6067 | 747.3070 | 1492.6227 | 746.8150 | 13 |
| 9 | 1028.4571 | 514.7322 | 1011.4306 | 506.2189 | 1010.4465 | 505.7269 | D | 1397.5492 | 699.2782 | 1380.5226 | 690.7650 | 1379.5386 | 690.2729 | 12 |
| 10 | 1143.4841 | 572.2457 | 1126.4575 | 563.7324 | 1125.4735 | 563.2404 | D | 1282.5222 | 641.7648 | 1265.4957 | 633.2515 | 1264.5117 | 632.7595 | 11 |
| 11 | 1240.5368 | 620.7720 | 1223.5103 | 612.2588 | 1222.5263 | 611.7668 | P | 1167.4953 | 584.2513 | 1150.4687 | 575.7380 | 1149.4847 | 575.2460 | 10 |
| 12 | 1355.5638 | 678.2855 | 1338.5372 | 669.7722 | 1337.5532 | 669.2802 | D | 1070.4425 | 535.7249 | 1053.4160 | 527.2116 | 1052.4320 | 526.7196 | 9 |
| 13 | 1452.6165 | 726.8119 | 1435.5900 | 718.2986 | 1434.6060 | 717.8066 | P | 955.4156 | 478.2114 | 938.3890 | 469.6982 | 937.4050 | 469.2061 | 8 |
| 14 | 1567.6435 | 784.3254 | 1550.6169 | 775.8121 | 1549.6329 | 775.3201 | D | 858.3628 | 429.6851 | 841.3363 | 421.1718 | 840.3523 | 420.6798 | 7 |
| 15 | 1682.6704 | 841.8388 | 1665.6439 | 833.3256 | 1664.6598 | 832.8336 | D | 743.3359 | 372.1716 | 726.3093 | 363.6583 | 725.3253 | 363.1663 | 6 |
| 16 | 1739.6919 | 870.3496 | 1722.6653 | 861.8363 | 1721.6813 | 861.3443 | G | 628.3089 | 314.6581 | 611.2824 | 306.1448 | | | 5 |
| 17 | 1886.7603 | 943.8838 | 1869.7337 | 935.3705 | 1868.7497 | 934.8785 | F | 571.2875 | 286.1474 | 554.2609 | 277.6341 | | | 4 |
| 18 | 2000.8032 | 1000.9052 | 1983.7767 | 992.3920 | 1982.7927 | 991.9000 | N | 424.2191 | 212.6132 | 407.1925 | 204.0999 | | | 3 |
| 19 | 2163.8665 | 1082.4369 | 2146.8400 | 1073.9236 | 2145.8560 | 1073.4316 | Y | 310.1761 | 155.5917 | 293.1496 | 147.0784 | | | 2 |
| 20 | | | | | | | K | 147.1128 | 74.0600 | 130.0863 | 65.5468 | | | 1 |

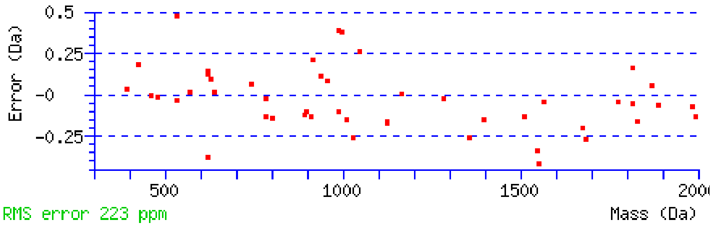


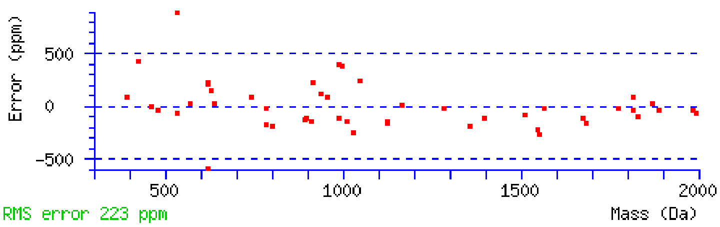


**All matches to this query**

| Score | Mr(calc): | Delta | Sequence |
|---|---|---|---|
| 67.8 | 2308.9648 | 0.0061 | NVTYGTYLDDPDPDDGFNYK |
| 40.6 | 2307.9808 | 0.9901 | NVTYGTYLDDPDPDDGFNYK |
| 26.2 | 2308.9648 | 0.0062 | NATYSYVLDDPDPDDGFNYK |
| 19.2 | 2308.9648 | 0.0061 | NVTYGTYLDDPDPDDGFNYK |
| 19.1 | 2307.9808 | 0.9902 | NATYSYVLDDPDPDDGFNYK |
| 5.5 | 2308.9783 | -0.0073 | SSGVKTLFNPAPAIADLDPR |
| 2.2 | 2308.9646 | 0.0063 | QYWCTFKDTSISCYKSR |
| 2.2 | 2308.9646 | 0.0063 | QYWCTFKDTSISCYKSR |
| 1.1 | 2308.9825 | -0.0115 | SGELRKEINSNTEQLQSSK |
| 1.1 | 2308.9825 | -0.0115 | SGELRKEINSNTEQLQSSK |

---

Spectrum No: 397; Query: 1343; Rank: 1

---

## Peptide View

MS/MS Fragmentation of **TRCNTTQGNEVTSILR**
Found in **IPI00327143**, Tax_Id=10116 Gene_Symbol=Alpl Alkaline phosphatase, tissue-nonspecific isozyme precursor

Match to Query 1343: 1849.899972 from(617.640600,3+)
Title: 100101RatKid_NS_deglyco_19.1947.1947.3.dta
Data file K:\NewmanPaper\Piliang\3SubProteomes\Piliang3SP\mgf5ppm\ERLIC_3SubProteomes5ppm.mgf

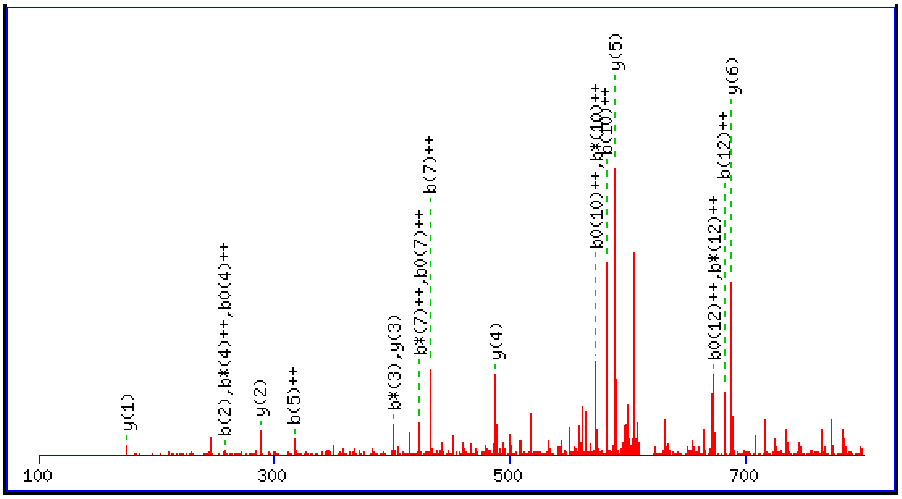


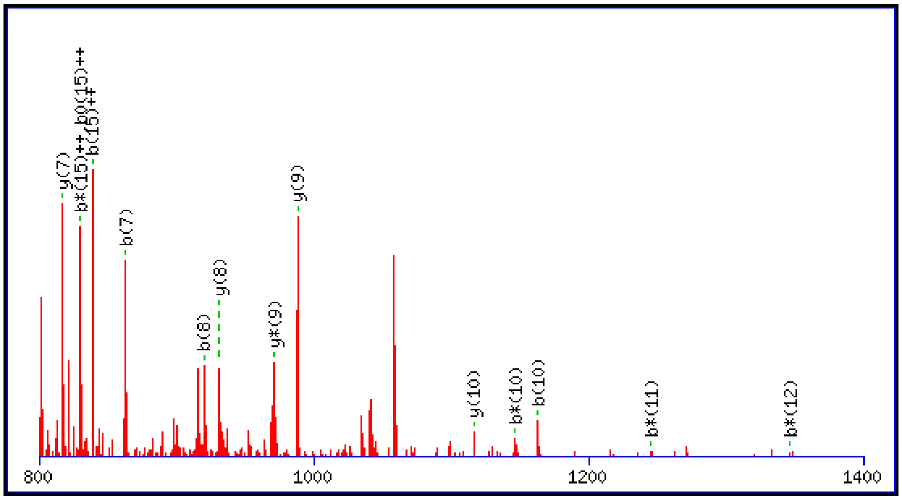


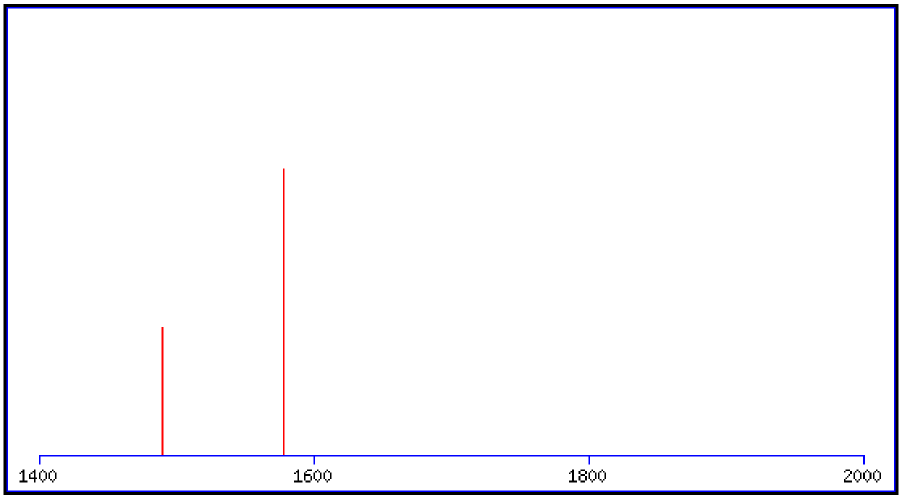


**Monoisotopic mass of neutral peptide Mr(calc):** 1849.8952
**Fixed modifications:** Carbamidomethyl (C)
**Variable modifications:**
**N4** : Deamidated_N (N)
**Ions Score:** 68 **Expect:** 4.2e-005
**Matches (Bold Red):** 34/172 fragment ions using 43 most intense peaks

| # | b | $b^{++}$ | b* | $b^{*++}$ | $b^0$ | $b^{0++}$ | Seq. | y | $y^{++}$ | y* | $y^{*++}$ | $y^0$ | $y^{0++}$ | # |
|---|---|---|---|---|---|---|---|---|---|---|---|---|---|---|
| 1 | 102.0550 | 51.5311 | | | 84.0444 | 42.5258 | T | | | | | | | 16 |
| 2 | **258.1561** | 129.5817 | 241.1295 | 121.0684 | 240.1455 | 120.5764 | R | 1749.8548 | 875.4310 | 1732.8283 | 866.9178 | 1731.8443 | 866.4258 | 15 |
| 3 | 418.1867 | 209.5970 | **401.1602** | 201.0837 | 400.1762 | 200.5917 | C | 1593.7537 | 797.3805 | 1576.7272 | 788.8672 | 1575.7431 | 788.3752 | 14 |
| 4 | 533.2137 | 267.1105 | 516.1871 | **258.5972** | 515.2031 | **258.1052** | N | 1433.7231 | 717.3652 | 1416.6965 | 708.8519 | 1415.7125 | 708.3599 | 13 |
| 5 | 634.2613 | **317.6343** | 617.2348 | 309.1210 | 616.2508 | 308.6290 | T | 1318.6961 | 659.8517 | 1301.6696 | 651.3384 | 1300.6856 | 650.8464 | 12 |

| | | | | | | | | | | | | | |
|---|---|---|---|---|---|---|---|---|---|---|---|---|---|
| 6 | 735.3090 | 368.1581 | 718.2825 | 359.6449 | 717.2984 | 359.1529 | T | 1217.6484 | 609.3279 | 1200.6219 | 600.8146 | 1199.6379 | 600.3226 | 11 |
| 7 | **863.3676** | **432.1874** | 846.3410 | **423.6742** | 845.3570 | **423.1821** | Q | **1116.6008** | 558.8040 | 1099.5742 | 550.2907 | 1098.5902 | 549.7987 | 10 |
| 8 | **920.3891** | 460.6982 | 903.3625 | 452.1849 | 902.3785 | 451.6929 | G | **988.5422** | 494.7747 | **971.5156** | 486.2615 | 970.5316 | 485.7694 | 9 |
| 9 | 1034.4320 | 517.7196 | 1017.4054 | 509.2064 | 1016.4214 | 508.7143 | N | **931.5207** | 466.2640 | 914.4942 | 457.7507 | 913.5102 | 457.2587 | 8 |
| 10 | **1163.4746** | **582.2409** | **1146.4480** | **573.7276** | 1145.4640 | **573.2356** | E | **817.4778** | 409.2425 | 800.4512 | 400.7293 | 799.4672 | 400.2373 | 7 |
| 11 | 1262.5430 | 631.7751 | **1245.5164** | 623.2619 | 1244.5324 | 622.7698 | V | **688.4352** | 344.7212 | 671.4087 | 336.2080 | 670.4246 | 335.7160 | 6 |
| 12 | 1363.5907 | **682.2990** | **1346.5641** | **673.7857** | 1345.5801 | **673.2937** | T | **589.3668** | 295.1870 | 572.3402 | 286.6738 | 571.3562 | 286.1817 | 5 |
| 13 | 1450.6227 | 725.8150 | 1433.5961 | 717.3017 | 1432.6121 | 716.8097 | S | **488.3191** | 244.6632 | 471.2926 | 236.1499 | 470.3085 | 235.6579 | 4 |
| 14 | 1563.7068 | 782.3570 | 1546.6802 | 773.8437 | 1545.6962 | 773.3517 | I | **401.2871** | 201.1472 | 384.2605 | 192.6339 | | | 3 |
| 15 | 1676.7908 | **838.8990** | 1659.7643 | **830.3858** | 1658.7803 | **829.8938** | L | **288.2030** | 144.6051 | 271.1765 | 136.0919 | | | 2 |
| 16 | | | | | | | R | **175.1190** | 88.0631 | 158.0924 | 79.5498 | | | 1 |

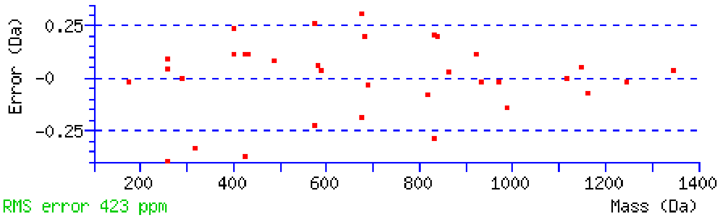


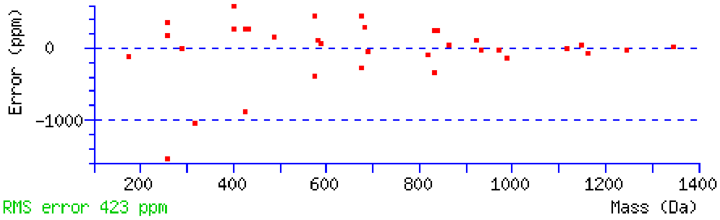


### All matches to this query

| Score | Mr(calc): | Delta | Sequence |
|---|---|---|---|
| 67.8 | 1849.8952 | 0.0048 | TRCNTTQGNEVTSILR |
| 38.4 | 1848.9112 | 0.9888 | TRCNTTQGNEVTSILR |
| 32.4 | 1849.8952 | 0.0048 | TRCNTTQGNEVTSILR |
| 8.2 | 1849.8840 | 0.0160 | DQQNMANGSTVVVTLTR |
| 6.5 | 1848.8931 | 1.0069 | HGRSSVCRFGPLQIR |
| 6.5 | 1848.8931 | 1.0069 | HGRSSVCRFGPLQIR |
| 6.4 | 1849.9118 | -0.0119 | TRIFGCQNPFRQGGGR |
| 4.8 | 1848.8813 | 1.0187 | TGNNSTKAETRKPDNSK |
| 4.6 | 1848.8827 | 1.0173 | DRSTIPGQHGDREEPR |
| 4.6 | 1847.8931 | 2.0069 | IYSNAGEKSFDRLLR |

---

Spectrum No: 398; Query: 725; Rank: 1

---

## Peptide View

MS/MS Fragmentation of **ENGVFEEISNSSGR**
Found in **IPI00326260**, Tax_Id=10116 Gene_Symbol=Sdfr1 Isoform 2 of Neuroplastin precursor

Match to Query 725: 1524.672228 from(763.343390,2+)
Title: 100101RatKid_NS_deglyco_26.2476.2476.2.dta
Data file K:\NewmanPaper\Piliang\3SubProteomes\Piliang3SP\mgf5ppm\ERLIC_3SubProteomes5ppm.mgf

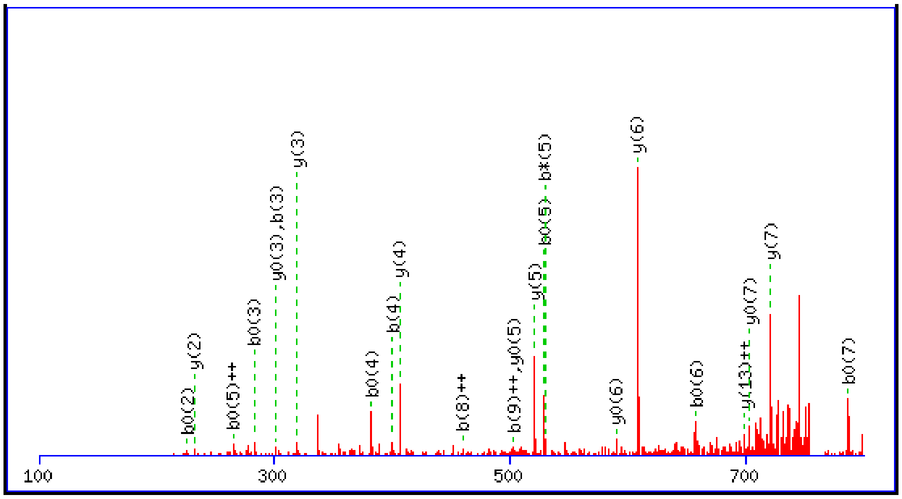


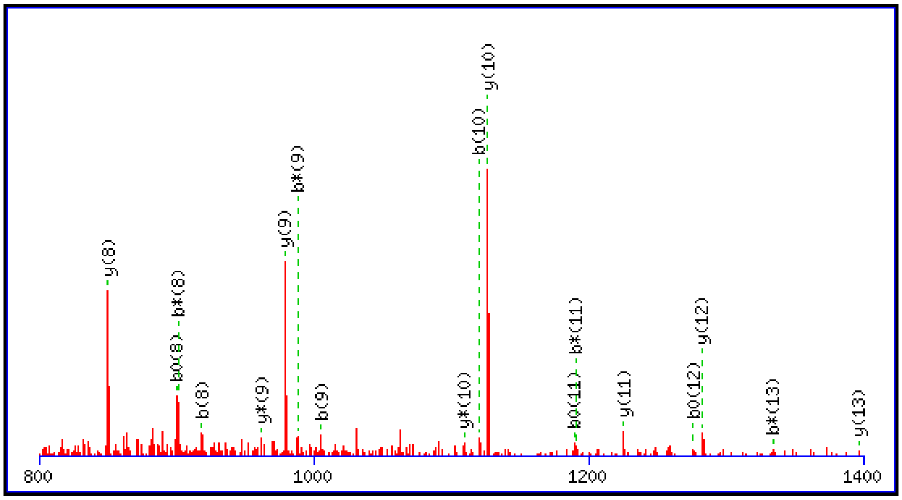


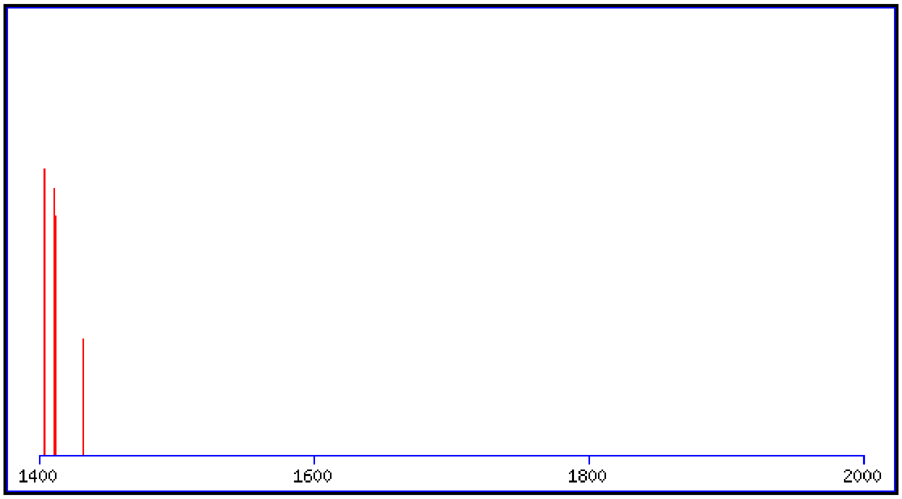


**Monoisotopic mass of neutral peptide Mr(calc):** 1524.6692
**Fixed modifications:** Carbamidomethyl (C)
**Variable modifications:**
**N10** : Deamidated_N (N)
**Ions Score:** 68 **Expect:** 2.5e-005
**Matches (Bold Red):** 41/150 fragment ions using 89 most intense peaks

| # | b | $b^{++}$ | b* | $b^{*++}$ | $b^0$ | $b^{0++}$ | Seq. | y | $y^{++}$ | y* | $y^{*++}$ | $y^0$ | $y^{0++}$ | # |
|---|---|---|---|---|---|---|---|---|---|---|---|---|---|---|
| 1 | 130.0499 | 65.5286 | | | 112.0393 | 56.5233 | E | | | | | | | 14 |
| 2 | 244.0928 | 122.5500 | 227.0662 | 114.0368 | **226.0822** | 113.5448 | N | **1396.6339** | **698.8206** | 1379.6074 | 690.3073 | 1378.6233 | 689.8153 | 13 |
| 3 | **301.1143** | 151.0608 | 284.0877 | 142.5475 | **283.1037** | 142.0555 | G | **1282.5910** | 641.7991 | 1265.5644 | 633.2859 | 1264.5804 | 632.7938 | 12 |
| 4 | **400.1827** | 200.5950 | 383.1561 | 192.0817 | **382.1721** | 191.5897 | V | **1225.5695** | 613.2884 | 1208.5430 | 604.7751 | 1207.5589 | 604.2831 | 11 |
| 5 | 547.2511 | 274.1292 | **530.2245** | 265.6159 | **529.2405** | **265.1239** | F | **1126.5011** | 563.7542 | **1109.4746** | 555.2409 | 1108.4905 | 554.7489 | 10 |

| | | | | | | | | | | | | | | |
|---|---|---|---|---|---|---|---|---|---|---|---|---|---|---|
| **6** | 676.2937 | 338.6505 | 659.2671 | 330.1372 | **658.2831** | 329.6452 | **E** | **979.4327** | 490.2200 | **962.4061** | 481.7067 | 961.4221 | 481.2147 | **9** |
| **7** | 805.3363 | 403.1718 | 788.3097 | 394.6585 | **787.3257** | 394.1665 | **E** | **850.3901** | 425.6987 | 833.3635 | 417.1854 | 832.3795 | 416.6934 | **8** |
| **8** | **918.4203** | **459.7138** | **901.3938** | 451.2005 | **900.4098** | 450.7085 | **I** | **721.3475** | 361.1774 | 704.3210 | 352.6641 | **703.3369** | 352.1721 | **7** |
| **9** | **1005.4524** | **503.2298** | **988.4258** | 494.7165 | 987.4418 | 494.2245 | **S** | **608.2634** | 304.6354 | 591.2369 | 296.1221 | **590.2529** | 295.6301 | **6** |
| **10** | **1120.4793** | 560.7433 | 1103.4528 | 552.2300 | 1102.4687 | 551.7380 | **N** | **521.2314** | 261.1193 | 504.2049 | 252.6061 | **503.2208** | 252.1141 | **5** |
| **11** | 1207.5113 | 604.2593 | **1190.4848** | 595.7460 | **1189.5008** | 595.2540 | **S** | **406.2045** | 203.6059 | 389.1779 | 195.0926 | 388.1939 | 194.6006 | **4** |
| **12** | 1294.5434 | 647.7753 | 1277.5168 | 639.2620 | **1276.5328** | 638.7700 | **S** | **319.1724** | 160.0899 | 302.1459 | 151.5766 | **301.1619** | 151.0846 | **3** |
| **13** | 1351.5648 | 676.2860 | **1334.5383** | 667.7728 | 1333.5543 | 667.2808 | **G** | **232.1404** | 116.5738 | 215.1139 | 108.0606 | | | **2** |
| **14** | | | | | | | **R** | 175.1190 | 88.0631 | 158.0924 | 79.5498 | | | **1** |

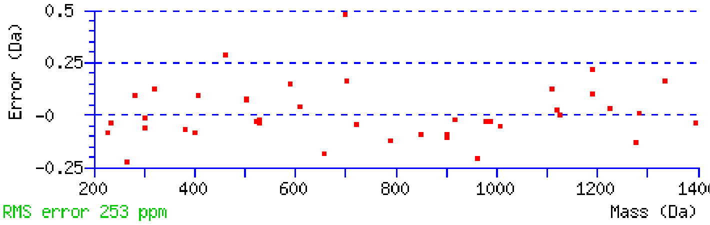


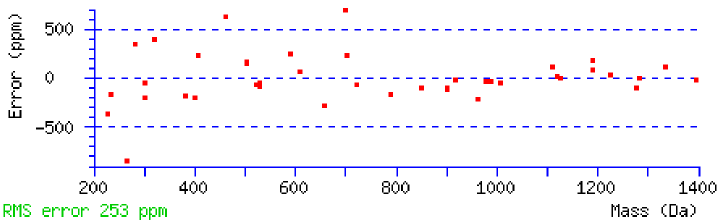


### All matches to this query

| Score | Mr(calc): | Delta | Sequence |
|---|---|---|---|
| 67.8 | 1524.6692 | 0.0030 | ENGVFEEISNSSGR |
| 3.8 | 1523.6664 | 1.0058 | DPSPQTRRTCAR |
| 3.8 | 1523.6664 | 1.0058 | DPSPQTRRTCAR |

---

Spectrum No: 399; Query: 2493; Rank: 1

---

## Peptide View

MS/MS Fragmentation of **VWPDYPNITVDPSLDWDSQVQQYR**
Found in **IPI00191437**, Tax_Id=10116 Gene_Symbol=- 64 kDa protein

Match to Query 2493: 2921.365708 from(1461.690130,2+)
Title: 100101RatKid_NS_deglyco_19.5292.5292.2.dta
Data file K:\NewmanPaper\Piliang\3SubProteomes\Piliang3SP\mgf5ppm\ERLIC_3SubProteomes5ppm.mgf

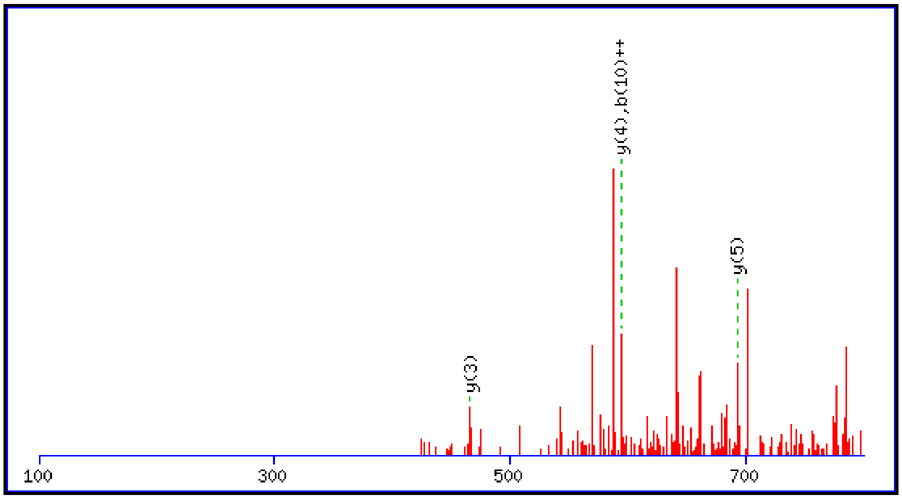

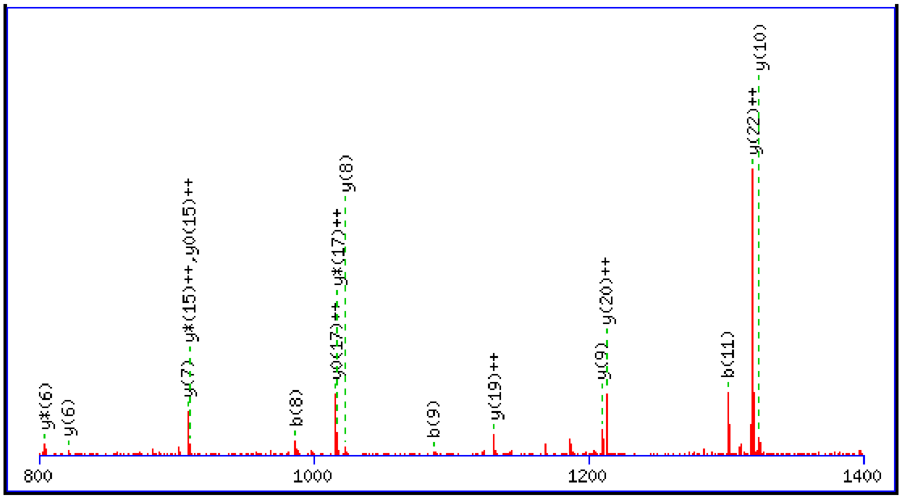


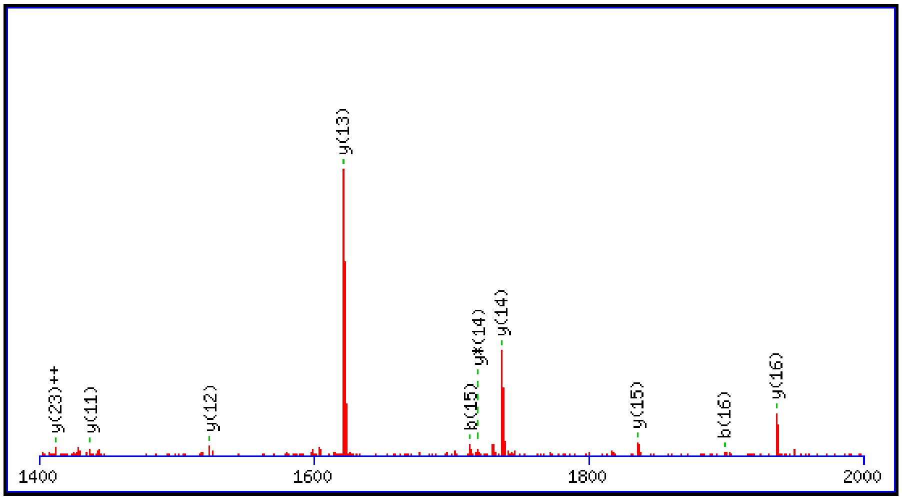


**Monoisotopic mass of neutral peptide Mr(calc):** 2921.3508
**Fixed modifications:** Carbamidomethyl (C)
**Variable modifications:**
**N7** : Deamidated_N (N)
**Ions Score:** 68 **Expect:** 6.2e-005
**Matches (Bold Red):** 30/246 fragment ions using 65 most intense peaks

| # | b | $b^{++}$ | b* | $b^{*++}$ | $b^{0}$ | $b^{0++}$ | Seq. | y | $y^{++}$ | y* | $y^{*++}$ | $y^{0}$ | $y^{0++}$ | # |
|---|---|---|---|---|---|---|---|---|---|---|---|---|---|---|
| 1 | 100.0757 | 50.5415 | | | | | V | | | | | | | 24 |
| 2 | 286.1550 | 143.5811 | | | | | W | 2823.2897 | **1412.1485** | 2806.2631 | 1403.6352 | 2805.2791 | 1403.1432 | 23 |
| 3 | 383.2078 | 192.1075 | | | | | P | 2637.2103 | **1319.1088** | 2620.1838 | 1310.5955 | 2619.1998 | 1310.1035 | 22 |
| 4 | 498.2347 | 249.6210 | | | 480.2241 | 240.6157 | D | 2540.1576 | 1270.5824 | 2523.1310 | 1262.0692 | 2522.1470 | 1261.5771 | 21 |
| 5 | 661.2980 | 331.1527 | | | 643.2875 | 322.1474 | Y | 2425.1306 | **1213.0690** | 2408.1041 | 1204.5557 | 2407.1201 | 1204.0637 | 20 |
| 6 | 758.3508 | 379.6790 | | | 740.3402 | 370.6738 | P | 2262.0673 | **1131.5373** | 2245.0408 | 1123.0240 | 2244.0567 | 1122.5320 | 19 |
| 7 | 873.3777 | 437.1925 | 856.3512 | 428.6792 | 855.3672 | 428.1872 | N | 2165.0145 | 1083.0109 | 2147.9880 | 1074.4976 | 2147.0040 | 1074.0056 | 18 |
| 8 | **986.4618** | 493.7345 | 969.4353 | 485.2213 | 968.4512 | 484.7293 | I | 2049.9876 | 1025.4974 | 2032.9611 | **1016.9842** | 2031.9770 | **1016.4922** | 17 |
| 9 | **1087.5095** | 544.2584 | 1070.4829 | 535.7451 | 1069.4989 | 535.2531 | T | **1936.9035** | 968.9554 | 1919.8770 | 960.4421 | 1918.8930 | 959.9501 | 16 |
| 10 | 1186.5779 | **593.7926** | 1169.5513 | 585.2793 | 1168.5673 | 584.7873 | V | **1835.8559** | 918.4316 | 1818.8293 | **909.9183** | 1817.8453 | **909.4263** | 15 |
| 11 | **1301.6048** | 651.3061 | 1284.5783 | 642.7928 | 1283.5943 | 642.3008 | D | **1736.7875** | 868.8974 | **1719.7609** | 860.3841 | 1718.7769 | 859.8921 | 14 |
| 12 | 1398.6576 | 699.8324 | 1381.6311 | 691.3192 | 1380.6470 | 690.8272 | P | **1621.7605** | 811.3839 | 1604.7340 | 802.8706 | 1603.7499 | 802.3786 | 13 |
| 13 | 1485.6896 | 743.3485 | 1468.6631 | 734.8352 | 1467.6791 | 734.3432 | S | **1524.7077** | 762.8575 | 1507.6812 | 754.3442 | 1506.6972 | 753.8522 | 12 |
| 14 | 1598.7737 | 799.8905 | 1581.7471 | 791.3772 | 1580.7631 | 790.8852 | L | **1437.6757** | 719.3415 | 1420.6492 | 710.8282 | 1419.6652 | 710.3362 | 11 |
| 15 | **1713.8006** | 857.4040 | 1696.7741 | 848.8907 | 1695.7901 | 848.3987 | D | **1324.5917** | 662.7995 | 1307.5651 | 654.2862 | 1306.5811 | 653.7942 | 10 |
| 16 | **1899.8800** | 950.4436 | 1882.8534 | 941.9303 | 1881.8694 | 941.4383 | W | **1209.5647** | 605.2860 | 1192.5382 | 596.7727 | 1191.5541 | 596.2807 | 9 |
| 17 | 2014.9069 | 1007.9571 | 1997.8803 | 999.4438 | 1996.8963 | 998.9518 | D | **1023.4854** | 512.2463 | 1006.4589 | 503.7331 | 1005.4748 | 503.2411 | 8 |
| 18 | 2101.9389 | 1051.4731 | 2084.9124 | 1042.9598 | 2083.9284 | 1042.4678 | S | **908.4585** | 454.7329 | 891.4319 | 446.2196 | 890.4479 | 445.7276 | 7 |
| | | | | | | | | | | | | | | |

| | | | | | | | | | | | | | | |
|---|---|---|---|---|---|---|---|---|---|---|---|---|---|---|
| **19** | 2229.9975 | 1115.5024 | 2212.9710 | 1106.9891 | 2211.9869 | 1106.4971 | **Q** | **821.4264** | 411.2169 | **804.3999** | 402.7036 | | | **6** |
| **20** | 2329.0659 | 1165.0366 | 2312.0394 | 1156.5233 | 2311.0553 | 1156.0313 | **V** | **693.3679** | 347.1876 | 676.3413 | 338.6743 | | | **5** |
| **21** | 2457.1245 | 1229.0659 | 2440.0979 | 1220.5526 | 2439.1139 | 1220.0606 | **Q** | **594.2994** | 297.6534 | 577.2729 | 289.1401 | | | **4** |
| **22** | 2585.1831 | 1293.0952 | 2568.1565 | 1284.5819 | 2567.1725 | 1284.0899 | **Q** | **466.2409** | 233.6241 | 449.2143 | 225.1108 | | | **3** |
| **23** | 2748.2464 | 1374.6268 | 2731.2199 | 1366.1136 | 2730.2358 | 1365.6216 | **Y** | 338.1823 | 169.5948 | 321.1557 | 161.0815 | | | **2** |
| **24** | | | | | | | **R** | 175.1190 | 88.0631 | 158.0924 | 79.5498 | | | **1** |

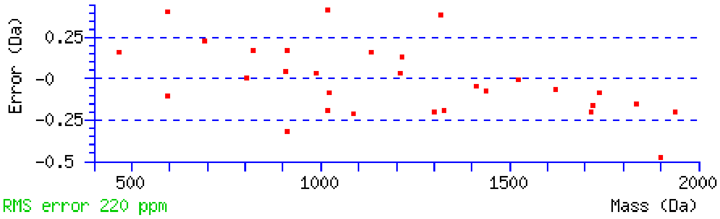


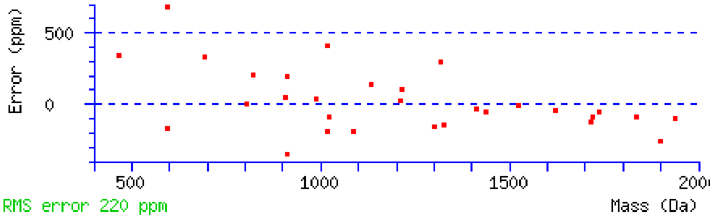


**All matches to this query**

| Score | Mr(calc): | Delta | Sequence |
|---|---|---|---|
| 67.8 | 2921.3508 | 0.0149 | VWPDYPNITVDPSLDWDSQVQQYR |
| 47.4 | 2920.3668 | 0.9989 | VWPDYPNITVDPSLDWDSQVQQYR |
| 10.6 | 2921.3766 | -0.0108 | EFPFPTTITAVRTNVANLSDAALWK |
| 4.0 | 2921.3766 | -0.0108 | EFPFPTTITAVRTNVANLSDAALWK |
| 4.0 | 2921.3766 | -0.0108 | EFPFPTTITAVRTNVANLSDAALWK |
| 3.8 | 2921.3766 | -0.0108 | EFPFPTTITAVRTNVANLSDAALWK |
| 3.8 | 2921.3766 | -0.0108 | EFPFPTTITAVRTNVANLSDAALWK |
| 3.7 | 2920.3449 | 1.0208 | LAYLLQQTDEYVANLTNLVWEHK |
| 3.6 | 2921.3525 | 0.0132 | GTAEGAVDAMLVHGQDAITVSNGGRIMR |
| 2.9 | 2921.3907 | -0.0250 | SLGTTTATISFPHMAMPMLLVLIIR |

---

Spectrum No: 400; Query: 2244; Rank: 1

---

## Peptide View

MS/MS Fragmentation of **WKVPFNPNDTFESEFYLDEK**
Found in **IPI00200591**, Tax_Id=10116 Gene_Symbol=LOC299282 Serine protease inhibitor A3L precursor

Match to Query 2244: 2505.143682 from(836.055170,3+)
Title: 100101RatKid_NS_deglyco_18.5085.5085.3.dta
Data file K:\NewmanPaper\Piliang\3SubProteomes\Piliang3SP\mgf5ppm\ERLIC_3SubProteomes5ppm.mgf

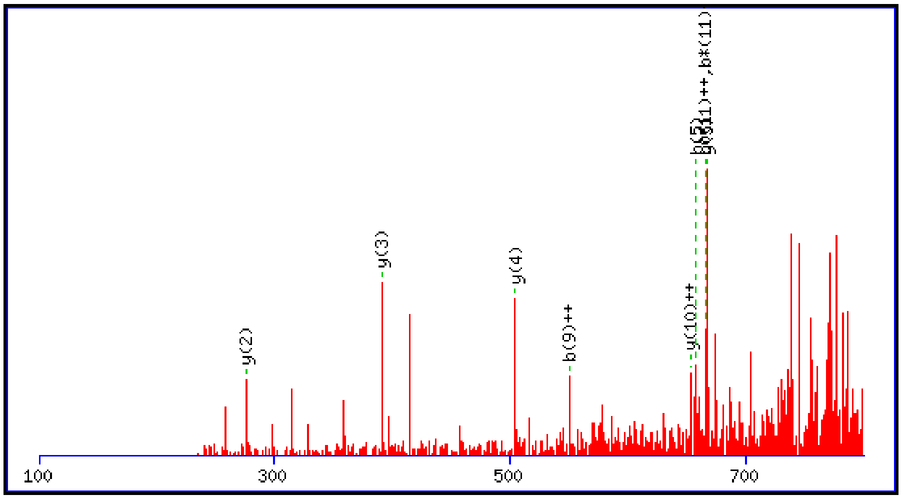

Mascot Search Results: Peptide View

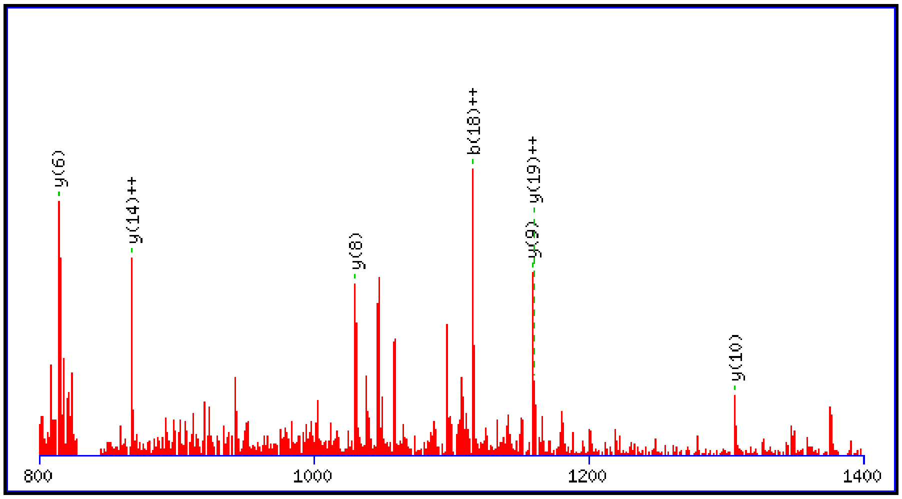


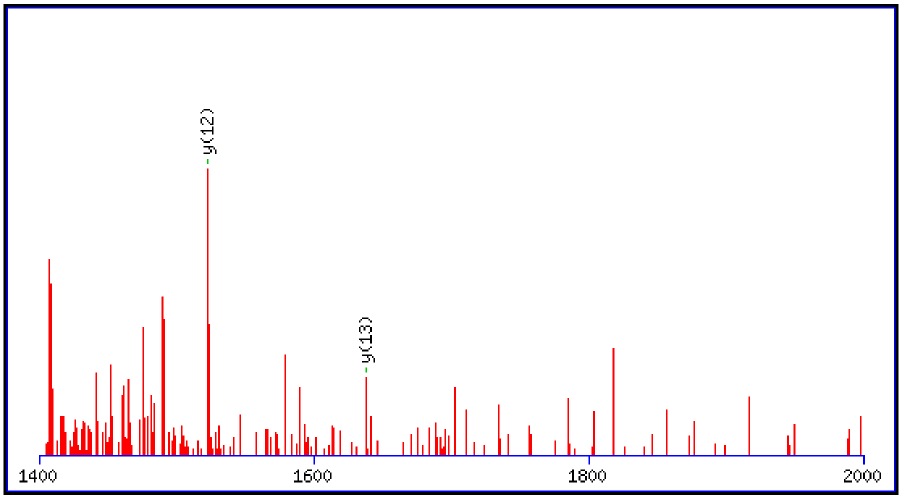


**Monoisotopic mass of neutral peptide Mr(calc):** 2505.1376
**Fixed modifications:** Carbamidomethyl (C)
**Variable modifications:**
**N8** : Deamidated_N (N)
**Ions Score:** 68 **Expect:** 5.6e-005
**Matches (Bold Red):** 18/208 fragment ions using 26 most intense peaks

| # | b | $b^{++}$ | b* | $b^{*++}$ | $b^{0}$ | $b^{0++}$ | Seq. | y | $y^{++}$ | y* | $y^{*++}$ | $y^{0}$ | $y^{0++}$ | # |
|---|---|---|---|---|---|---|---|---|---|---|---|---|---|---|
| 1 | 187.0866 | 94.0469 | | | | | W | | | | | | | 20 |
| 2 | 315.1816 | 158.0944 | 298.1550 | 149.5811 | | | K | 2320.0656 | **1160.5364** | 2303.0390 | 1152.0232 | 2302.0550 | 1151.5311 | 19 |
| 3 | 414.2500 | 207.6286 | 397.2234 | 199.1153 | | | V | 2191.9706 | 1096.4889 | 2174.9441 | 1087.9757 | 2173.9601 | 1087.4837 | 18 |
| 4 | 511.3027 | 256.1550 | 494.2762 | 247.6417 | | | P | 2092.9022 | 1046.9547 | 2075.8757 | 1038.4415 | 2074.8916 | 1037.9495 | 17 |
| 5 | **658.3711** | 329.6892 | 641.3446 | 321.1759 | | | F | 1995.8494 | 998.4284 | 1978.8229 | 989.9151 | 1977.8389 | 989.4231 | 16 |
| 6 | 772.4141 | 386.7107 | 755.3875 | 378.1974 | | | N | 1848.7810 | 924.8942 | 1831.7545 | 916.3809 | 1830.7705 | 915.8889 | 15 |
| 7 | 869.4668 | 435.2371 | 852.4403 | 426.7238 | | | P | 1734.7381 | **867.8727** | 1717.7115 | 859.3594 | 1716.7275 | 858.8674 | 14 |
| 8 | 984.4938 | 492.7505 | 967.4672 | 484.2372 | | | N | **1637.6853** | 819.3463 | 1620.6588 | 810.8330 | 1619.6748 | 810.3410 | 13 |
| 9 | 1099.5207 | **550.2640** | 1082.4942 | 541.7507 | 1081.5101 | 541.2587 | D | **1522.6584** | 761.8328 | 1505.6318 | 753.3196 | 1504.6478 | 752.8276 | 12 |
| 10 | 1200.5684 | 600.7878 | 1183.5418 | 592.2746 | 1182.5578 | 591.7826 | T | 1407.6315 | 704.3194 | 1390.6049 | 695.8061 | 1389.6209 | 695.3141 | 11 |
| 11 | 1347.6368 | 674.3220 | 1330.6103 | **665.8088** | 1329.6262 | **665.3168** | F | **1306.5838** | **653.7955** | 1289.5572 | 645.2823 | 1288.5732 | 644.7902 | 10 |
| 12 | 1476.6794 | 738.8433 | 1459.6529 | 730.3301 | 1458.6688 | 729.8381 | E | **1159.5154** | 580.2613 | 1142.4888 | 571.7480 | 1141.5048 | 571.2560 | 9 |
| 13 | 1563.7114 | 782.3594 | 1546.6849 | 773.8461 | 1545.7009 | 773.3541 | S | **1030.4728** | 515.7400 | 1013.4462 | 507.2267 | 1012.4622 | 506.7347 | 8 |
| 14 | 1692.7540 | 846.8806 | 1675.7275 | 838.3674 | 1674.7435 | 837.8754 | E | 943.4407 | 472.2240 | 926.4142 | 463.7107 | 925.4302 | 463.2187 | 7 |
| 15 | 1839.8224 | 920.4149 | 1822.7959 | 911.9016 | 1821.8119 | 911.4096 | F | **814.3981** | 407.7027 | 797.3716 | 399.1894 | 796.3876 | 398.6974 | 6 |
| 16 | 2002.8858 | 1001.9465 | 1985.8592 | 993.4332 | 1984.8752 | 992.9412 | Y | **667.3297** | 334.1685 | 650.3032 | 325.6552 | 649.3192 | 325.1632 | 5 |
| 17 | 2115.9698 | 1058.4886 | 2098.9433 | 1049.9753 | 2097.9593 | 1049.4833 | L | **504.2664** | 252.6368 | 487.2399 | 244.1236 | 486.2558 | 243.6316 | 4 |
| 18 | 2230.9968 | **1116.0020** | 2213.9702 | 1107.4887 | 2212.9862 | 1106.9967 | D | **391.1823** | 196.0948 | 374.1558 | 187.5815 | 373.1718 | 187.0895 | 3 |

| | | | | | | | | | | | | | | |
|---|---|---|---|---|---|---|---|---|---|---|---|---|---|---|
| 19 | 2360.0394 | 1180.5233 | 2343.0128 | 1172.0100 | 2342.0288 | 1171.5180 | E | 276.1554 | 138.5813 | 259.1288 | 130.0681 | 258.1448 | 129.5761 | 2 |
| 20 | | | | | | | K | 147.1128 | 74.0600 | 130.0863 | 65.5468 | | | 1 |

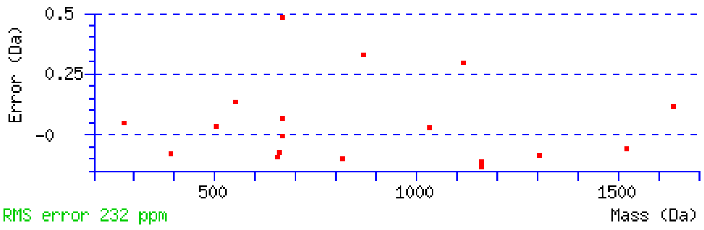


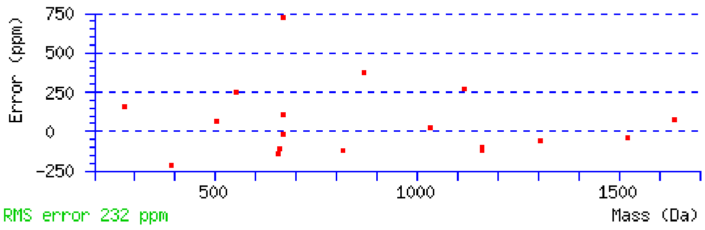


## All matches to this query

| Score | Mr(calc): | Delta | Sequence |
|---|---|---|---|
| 67.7 | 2505.1376 | 0.0061 | WKVPFNPNDTFESEFYLDEK |
| 53.8 | 2505.1376 | 0.0061 | WKVPFNPNDTFESEFYLDEK |
| 46.3 | 2504.1536 | 0.9901 | WKVPFNPNDTFESEFYLDEK |
| 14.8 | 2503.1127 | 2.0309 | TLARKNITMEMESNDTIENMK |
| 1.6 | 2505.1285 | 0.0152 | SSSSGSDDYAYTQALLLHQRAR |
| 0.1 | 2503.1454 | 1.9983 | MAHITINQYLQQVYEAIDTR |

| |
|---|
| **Mascot:** http://www.matrixscience.com/ |

# Mascot Search Results

Results Generated by a Script Modified from Mascot Pepitde View
by Newman Sze, School of Biological Sciences, Nanyang Technological University

---

Spectrum No: 401; Query: 2613; Rank: 1

---

## Peptide View

MS/MS Fragmentation of **VNYEAETWDWIAETLSSNHMNFSSADR**
Found in **IPI00327398**, Tax_Id=10116 Gene_Symbol=Enpep Isoform 1 of Glutamyl aminopeptidase

Match to Query 2613: 3189.371262 from(1064.131030,3+)
Title: 100101RatKid_NS_deglyco_18.6398.6398.3.dta
Data file K:\NewmanPaper\Piliang\3SubProteomes\Piliang3SP\mgf5ppm\ERLIC_3SubProteomes5ppm.mgf

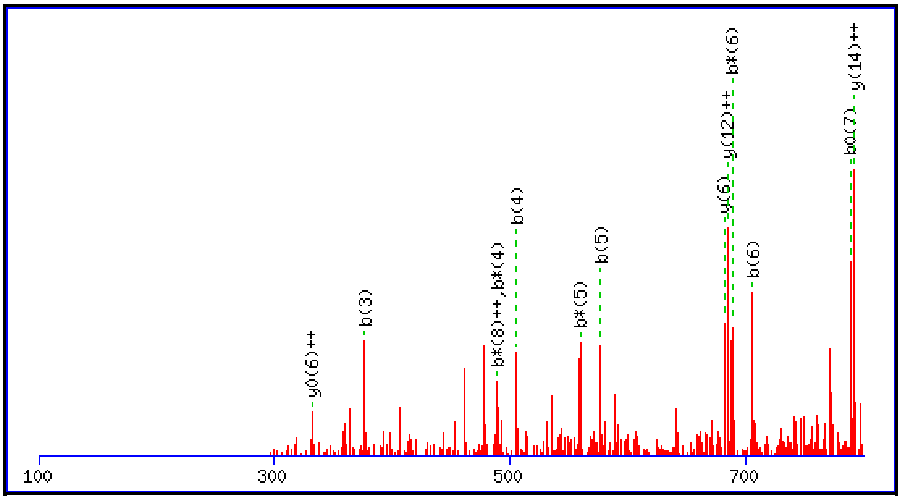


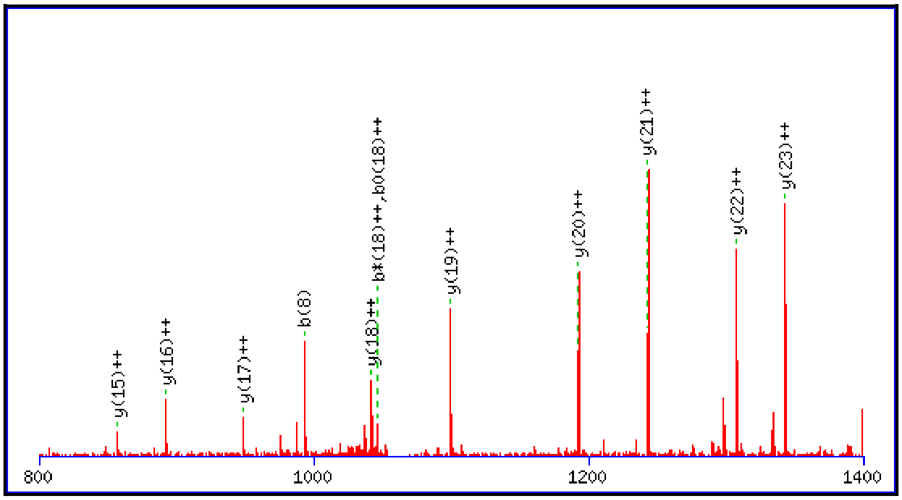

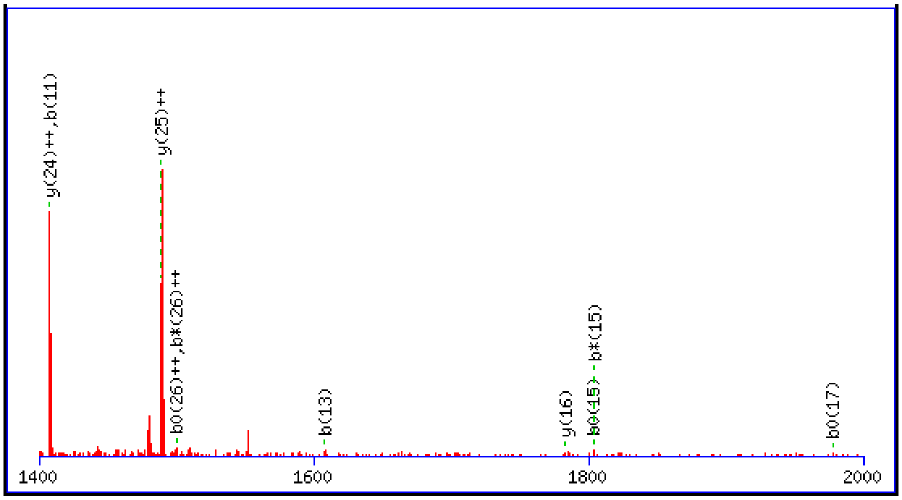


**Monoisotopic mass of neutral peptide Mr(calc):** 3189.3621
**Fixed modifications:** Carbamidomethyl (C)
**Variable modifications:**
**M20** : Oxidation (M)
**N21** : Deamidated_N (N)
**Ions Score:** 68 **Expect:** 7.5e-005
**Matches (Bold Red):** 35/302 fragment ions using 56 most intense peaks

| # | b | $b^{++}$ | b* | $b^{*++}$ | $b^0$ | $b^{0++}$ | Seq. | y | $y^{++}$ | y* | $y^{*++}$ | $y^0$ | $y^{0++}$ | # |
|---|---|---|---|---|---|---|---|---|---|---|---|---|---|---|
| 1 | 100.0757 | 50.5415 | | | | | V | | | | | | | 27 |
| 2 | 214.1186 | 107.5629 | 197.0921 | 99.0497 | | | N | 3091.3010 | 1546.1542 | 3074.2745 | 1537.6409 | 3073.2905 | 1537.1489 | 26 |
| 3 | **377.1819** | 189.0946 | 360.1554 | 180.5813 | | | Y | 2977.2581 | **1489.1327** | 2960.2316 | 1480.6194 | 2959.2475 | 1480.1274 | 25 |
| 4 | **506.2245** | 253.6159 | **489.1980** | 245.1026 | 488.2140 | 244.6106 | E | 2814.1948 | **1407.6010** | 2797.1682 | 1399.0878 | 2796.1842 | 1398.5957 | 24 |
| 5 | **577.2617** | 289.1345 | **560.2351** | 280.6212 | 559.2511 | 280.1292 | A | 2685.1522 | **1343.0797** | 2668.1256 | 1334.5665 | 2667.1416 | 1334.0744 | 23 |
| 6 | **706.3042** | 353.6558 | **689.2777** | 345.1425 | 688.2937 | 344.6505 | E | 2614.1151 | **1307.5612** | 2597.0885 | 1299.0479 | 2596.1045 | 1298.5559 | 22 |
| 7 | 807.3519 | 404.1796 | 790.3254 | 395.6663 | **789.3414** | 395.1743 | T | 2485.0725 | **1243.0399** | 2468.0459 | 1234.5266 | 2467.0619 | 1234.0346 | 21 |
| 8 | **993.4312** | 497.2193 | 976.4047 | **488.7060** | 975.4207 | 488.2140 | W | 2384.0248 | **1192.5160** | 2366.9982 | 1184.0028 | 2366.0142 | 1183.5108 | 20 |
| 9 | 1108.4582 | 554.7327 | 1091.4316 | 546.2195 | 1090.4476 | 545.7274 | D | 2197.9455 | **1099.4764** | 2180.9189 | 1090.9631 | 2179.9349 | 1090.4711 | 19 |
| 10 | 1294.5375 | 647.7724 | 1277.5109 | 639.2591 | 1276.5269 | 638.7671 | W | 2082.9185 | **1041.9629** | 2065.8920 | 1033.4496 | 2064.9080 | 1032.9576 | 18 |
| 11 | **1407.6216** | 704.3144 | 1390.5950 | 695.8011 | 1389.6110 | 695.3091 | I | 1896.8392 | **948.9233** | 1879.8127 | 940.4100 | 1878.8287 | 939.9180 | 17 |
| 12 | 1478.6587 | 739.8330 | 1461.6321 | 731.3197 | 1460.6481 | 730.8277 | A | **1783.7552** | **892.3812** | 1766.7286 | 883.8679 | 1765.7446 | 883.3759 | 16 |
| 13 | **1607.7013** | 804.3543 | 1590.6747 | 795.8410 | 1589.6907 | 795.3490 | E | 1712.7180 | **856.8627** | 1695.6915 | 848.3494 | 1694.7075 | 847.8574 | 15 |
| 14 | 1708.7489 | 854.8781 | 1691.7224 | 846.3648 | 1690.7384 | 845.8728 | T | 1583.6755 | **792.3414** | 1566.6489 | 783.8281 | 1565.6649 | 783.3361 | 14 |
| 15 | 1821.8330 | 911.4201 | **1804.8065** | 902.9069 | **1803.8224** | 902.4149 | L | 1482.6278 | 741.8175 | 1465.6012 | 733.3043 | 1464.6172 | 732.8122 | 13 |
| 16 | 1908.8650 | 954.9362 | 1891.8385 | 946.4229 | 1890.8545 | 945.9309 | S | 1369.5437 | **685.2755** | 1352.5172 | 676.7622 | 1351.5331 | 676.2702 | 12 |
| 17 | 1995.8971 | 998.4522 | 1978.8705 | 989.9389 | **1977.8865** | 989.4469 | S | 1282.5117 | 641.7595 | 1265.4851 | 633.2462 | 1264.5011 | 632.7542 | 11 |
| 18 | 2109.9400 | 1055.4736 | 2092.9134 | **1046.9604** | 2091.9294 | **1046.4684** | N | 1195.4797 | 598.2435 | 1178.4531 | 589.7302 | 1177.4691 | 589.2382 | 10 |
| 19 | 2246.9989 | 1124.0031 | 2229.9724 | 1115.4898 | 2228.9883 | 1114.9978 | H | 1081.4367 | 541.2220 | 1064.4102 | 532.7087 | 1063.4262 | 532.2167 | 9 |
| 20 | 2394.0343 | 1197.5208 | 2377.0078 | 1189.0075 | 2376.0237 | 1188.5155 | M | 944.3778 | 472.6925 | 927.3513 | 464.1793 | 926.3673 | 463.6873 | 8 |
| 21 | 2509.0612 | 1255.0343 | 2492.0347 | 1246.5210 | 2491.0507 | 1246.0290 | N | 797.3424 | 399.1748 | 780.3159 | 390.6616 | 779.3318 | 390.1696 | 7 |
| 22 | 2656.1297 | 1328.5685 | 2639.1031 | 1320.0552 | 2638.1191 | 1319.5632 | F | **682.3155** | 341.6614 | 665.2889 | 333.1481 | 664.3049 | **332.6561** | 6 |
| 23 | 2743.1617 | 1372.0845 | 2726.1351 | 1363.5712 | 2725.1511 | 1363.0792 | S | 535.2471 | 268.1272 | 518.2205 | 259.6139 | 517.2365 | 259.1219 | 5 |
| 24 | 2830.1937 | 1415.6005 | 2813.1672 | 1407.0872 | 2812.1831 | 1406.5952 | S | 448.2150 | 224.6112 | 431.1885 | 216.0979 | 430.2045 | 215.6059 | 4 |
| 25 | 2901.2308 | 1451.1191 | 2884.2043 | 1442.6058 | 2883.2203 | 1442.1138 | A | 361.1830 | 181.0951 | 344.1565 | 172.5819 | 343.1724 | 172.0899 | 3 |
| 26 | 3016.2578 | 1508.6325 | 2999.2312 | **1500.1192** | 2998.2472 | **1499.6272** | D | 290.1459 | 145.5766 | 273.1193 | 137.0633 | 272.1353 | 136.5713 | 2 |
| 27 | | | | | | | R | 175.1190 | 88.0631 | 158.0924 | 79.5498 | | | 1 |

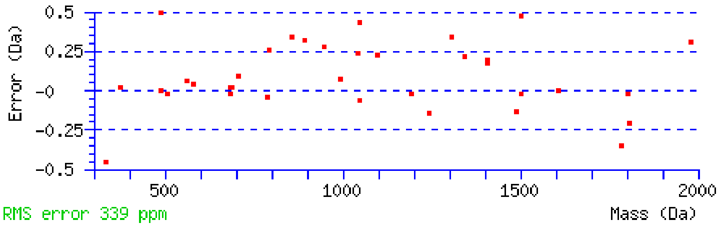


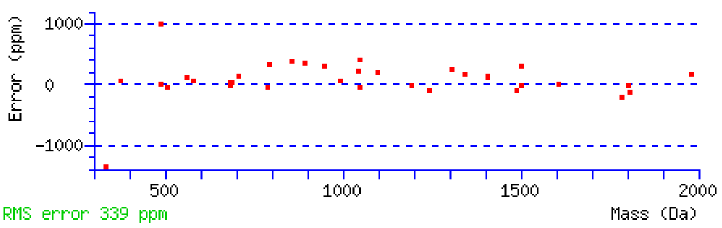


### All matches to this query

| Score | Mr(calc): | Delta | Sequence |
|---|---|---|---|
| 67.7 | 3189.3621 | 0.0091 | VNYEAETWDWIAETLSSNHMNFSSADR |
| 67.7 | 3189.3621 | 0.0091 | VNYEAETWDWIAETLSSNHMNFSSADR |
| 13.5 | 3188.3781 | 0.9931 | VNYEAETWDWIAETLSSNHMNFSSADR |
| 4.8 | 3188.3613 | 1.0100 | MEEAVMSILHNLEMENAEIHENSHKR |
| 4.0 | 3188.3876 | 0.9836 | GPGKPSVASTSGPQSTRTPSHQELKMQR |
| 2.6 | 3187.3758 | 1.9955 | AALIPAGLSDGQFYSPPESEAGSEEEAEEK |
| 2.6 | 3187.3758 | 1.9955 | AALIPAGLSDGQFYSPPESEAGSEEEAEEK |
| 2.6 | 3187.3758 | 1.9955 | AALIPAGLSDGQFYSPPESEAGSEEEAEEK |
| 2.6 | 3187.3475 | 2.0238 | SALLYGTLSSEAPQDGESNQSGYCAMISR |
| 2.6 | 3187.3475 | 2.0238 | SALLYGTLSSEAPQDGESNQSGYCAMISR |

---

Spectrum No: 402; Query: 2382; Rank: 1

---

## Peptide View

MS/MS Fragmentation of **IHVSDQELQSANASVDDSRLEELK**
Found in **IPI00368347**, Tax_Id=10116 Gene_Symbol=Ube1x Ubiquitin-activating enzyme E1, Chr X

Match to Query 2382: 2685.305382 from(896.109070,3+)
Title: 091008RatKidney_NH4Format02_21.2731.2731.3.dta
Data file K:\NewmanPaper\Piliang\3SubProteomes\Piliang3SP\mgf5ppm\ERLIC_3SubProteomes5ppm.mgf

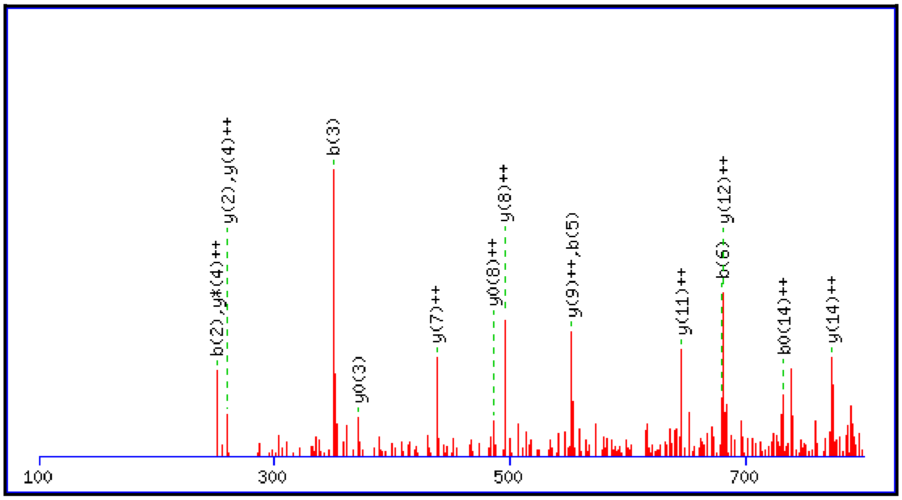

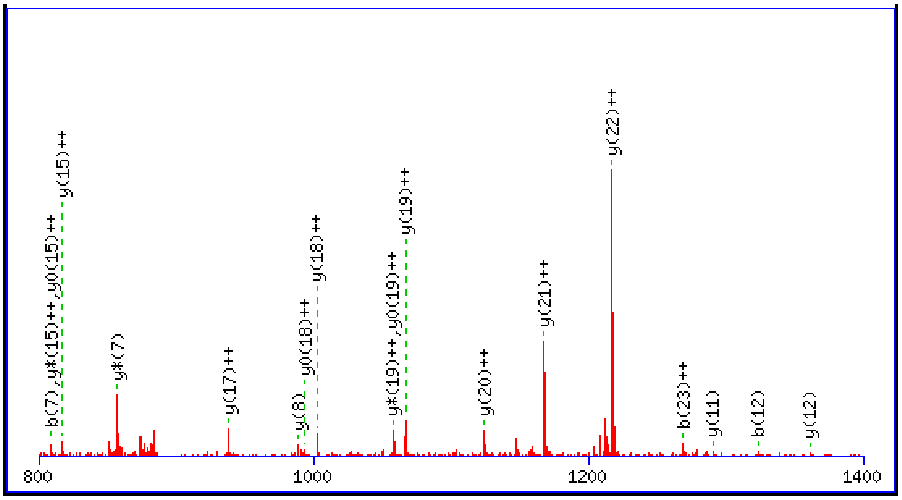


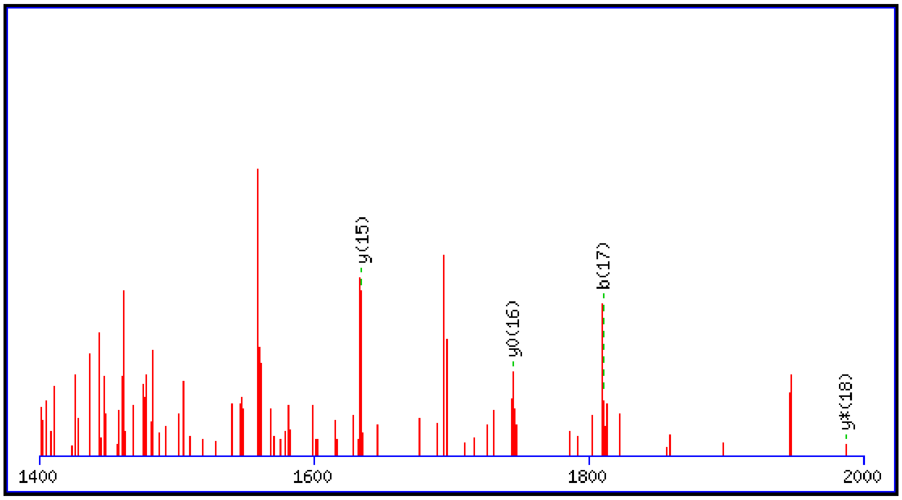


**Monoisotopic mass of neutral peptide Mr(calc):** 2683.2936
**Fixed modifications:** Carbamidomethyl (C)
**Variable modifications:**
**N12** : Deamidated_N (N)
**Ions Score:** 68 **Expect:** 4.7e-005
**Matches (Bold Red):** 39/256 fragment ions using 75 most intense peaks

| # | b | $b^{++}$ | b* | $b^{*++}$ | $b^{0}$ | $b^{0++}$ | Seq. | y | $y^{++}$ | y* | $y^{*++}$ | $y^{0}$ | $y^{0++}$ | # |
|---|---|---|---|---|---|---|---|---|---|---|---|---|---|---|
| 1 | 114.0913 | 57.5493 | | | | | I | | | | | | | 24 |
| 2 | **251.1503** | 126.0788 | | | | | H | 2571.2169 | 1286.1121 | 2554.1903 | 1277.5988 | 2553.2063 | 1277.1068 | 23 |
| 3 | **350.2187** | 175.6130 | | | | | V | 2434.1580 | **1217.5826** | 2417.1314 | 1209.0694 | 2416.1474 | 1208.5773 | 22 |
| 4 | 437.2507 | 219.1290 | | | 419.2401 | 210.1237 | S | 2335.0896 | **1168.0484** | 2318.0630 | 1159.5351 | 2317.0790 | 1159.0431 | 21 |
| 5 | **552.2776** | 276.6425 | | | 534.2671 | 267.6372 | D | 2248.0575 | **1124.5324** | 2231.0310 | 1116.0191 | 2230.0470 | 1115.5271 | 20 |
| 6 | **680.3362** | 340.6717 | 663.3097 | 332.1585 | 662.3257 | 331.6665 | Q | 2133.0306 | **1067.0189** | 2116.0040 | **1058.5057** | 2115.0200 | **1058.0137** | 19 |
| 7 | **809.3788** | 405.1930 | 792.3523 | 396.6798 | 791.3682 | 396.1878 | E | 2004.9720 | **1002.9896** | **1987.9455** | 994.4764 | 1986.9614 | **993.9844** | 18 |
| 8 | 922.4629 | 461.7351 | 905.4363 | 453.2218 | 904.4523 | 452.7298 | L | 1875.9294 | **938.4683** | 1858.9029 | 929.9551 | 1857.9189 | 929.4631 | 17 |
| 9 | 1050.5215 | 525.7644 | 1033.4949 | 517.2511 | 1032.5109 | 516.7591 | Q | 1762.8454 | 881.9263 | 1745.8188 | 873.4130 | **1744.8348** | 872.9210 | 16 |
| 10 | 1137.5535 | 569.2804 | 1120.5269 | 560.7671 | 1119.5429 | 560.2751 | S | **1634.7868** | **817.8970** | 1617.7602 | **809.3838** | 1616.7762 | **808.8917** | 15 |
| 11 | 1208.5906 | 604.7989 | 1191.5640 | 596.2857 | 1190.5800 | 595.7937 | A | 1547.7547 | **774.3810** | 1530.7282 | 765.8677 | 1529.7442 | 765.3757 | 14 |
| 12 | **1323.6175** | 662.3124 | 1306.5910 | 653.7991 | 1305.6070 | 653.3071 | N | 1476.7176 | 738.8625 | 1459.6911 | 730.3492 | 1458.7071 | 729.8572 | 13 |
| 13 | 1394.6546 | 697.8310 | 1377.6281 | 689.3177 | 1376.6441 | 688.8257 | A | **1361.6907** | **681.3490** | 1344.6642 | 672.8357 | 1343.6801 | 672.3437 | 12 |
| 14 | 1481.6867 | 741.3470 | 1464.6601 | 732.8337 | 1463.6761 | **732.3417** | S | **1290.6536** | **645.8304** | 1273.6270 | 637.3172 | 1272.6430 | 636.8251 | 11 |
| 15 | 1580.7551 | 790.8812 | 1563.7285 | 782.3679 | 1562.7445 | 781.8759 | V | 1203.6216 | 602.3144 | 1186.5950 | 593.8011 | 1185.6110 | 593.3091 | 10 |
| 16 | 1695.7820 | 848.3947 | 1678.7555 | 839.8814 | 1677.7715 | 839.3894 | D | 1104.5531 | **552.7802** | 1087.5266 | 544.2669 | 1086.5426 | 543.7749 | 9 |
| 17 | **1810.8090** | 905.9081 | 1793.7824 | 897.3948 | 1792.7984 | 896.9028 | D | **989.5262** | **495.2667** | 972.4997 | 486.7535 | 971.5156 | **486.2615** | 8 |
| 18 | 1897.8410 | 949.4241 | 1880.8144 | 940.9109 | 1879.8304 | 940.4189 | S | 874.4993 | **437.7533** | **857.4727** | 429.2400 | 856.4887 | 428.7480 | 7 |

| | | | | | | | | | | | | | | |
|---|---|---|---|---|---|---|---|---|---|---|---|---|---|---|
| 19 | 2053.9421 | 1027.4747 | 2036.9156 | 1018.9614 | 2035.9315 | 1018.4694 | R | 787.4672 | 394.2373 | 770.4407 | 385.7240 | 769.4567 | 385.2320 | 6 |
| 20 | 2167.0262 | 1084.0167 | 2149.9996 | 1075.5034 | 2149.0156 | 1075.0114 | L | 631.3661 | 316.1867 | 614.3396 | 307.6734 | 613.3556 | 307.1814 | 5 |
| 21 | 2296.0688 | 1148.5380 | 2279.0422 | 1140.0247 | 2278.0582 | 1139.5327 | E | 518.2821 | **259.6447** | 501.2555 | **251.1314** | 500.2715 | 250.6394 | 4 |
| 22 | 2425.1114 | 1213.0593 | 2408.0848 | 1204.5460 | 2407.1008 | 1204.0540 | E | 389.2395 | 195.1234 | 372.2129 | 186.6101 | **371.2289** | 186.1181 | 3 |
| 23 | 2538.1954 | **1269.6013** | 2521.1689 | 1261.0881 | 2520.1849 | 1260.5961 | L | **260.1969** | 130.6021 | 243.1703 | 122.0888 | | | 2 |
| 24 | | | | | | | K | 147.1128 | 74.0600 | 130.0863 | 65.5468 | | | 1 |

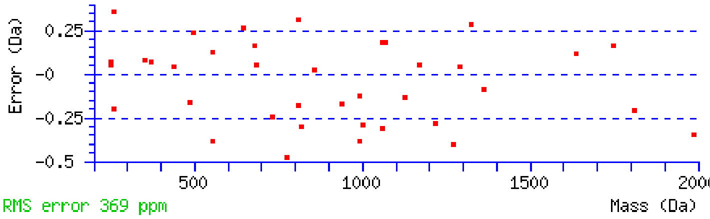


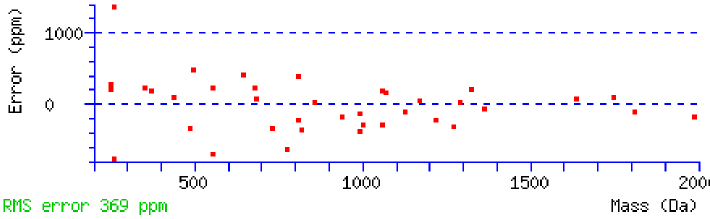


### All matches to this query

| Score | Mr(calc): | Delta | Sequence |
|---|---|---|---|
| 67.6 | 2683.2936 | 2.0117 | IHVSDQELQSANASVDDSRLEELK |
| 8.1 | 2685.2890 | 0.0164 | GANIFLTSSGLIKLGDFGCSVKLK |
| 6.7 | 2685.2890 | 0.0164 | GANIFLTSSGLIKLGDFGCSVKLK |
| 6.7 | 2685.2890 | 0.0164 | GANIFLTSSGLIKLGDFGCSVKLK |
| 5.8 | 2685.3027 | 0.0027 | ITLSQVGDVLRALGTNPTNAEVKK |
| 5.8 | 2685.3027 | 0.0027 | ITLSQVGDVLRALGTNPTNAEVKK |
| 4.4 | 2685.2890 | 0.0164 | GANIFLTSSGLIKLGDFGCSVKLK |
| 4.4 | 2685.2890 | 0.0164 | GANIFLTSSGLIKLGDFGCSVKLK |
| 4.2 | 2685.2890 | 0.0164 | GANIFLTSSGLIKLGDFGCSVKLK |
| 1.2 | 2683.3136 | 1.9918 | TKIDLQHPEGAVEPKSIVGHLQK |

---

Spectrum No: 403; Query: 2358; Rank: 1

---

## Peptide View

MS/MS Fragmentation of **EVLNYTTVPVYLPEITIGAHQGSR**
Found in **IPI00189759**, Tax_Id=10116 Gene_Symbol=LOC316632 NADH dehydrogenase 1 alpha subcomplex 10-like protein

Match to Query 2358: 2657.383008 from(1329.698780,2+)
Title: 091008RatKidney_NoSalt_09.4827.4827.2.dta
Data file K:\NewmanPaper\Piliang\3SubProteomes\Piliang3SP\mgf5ppm\ERLIC_3SubProteomes5ppm.mgf

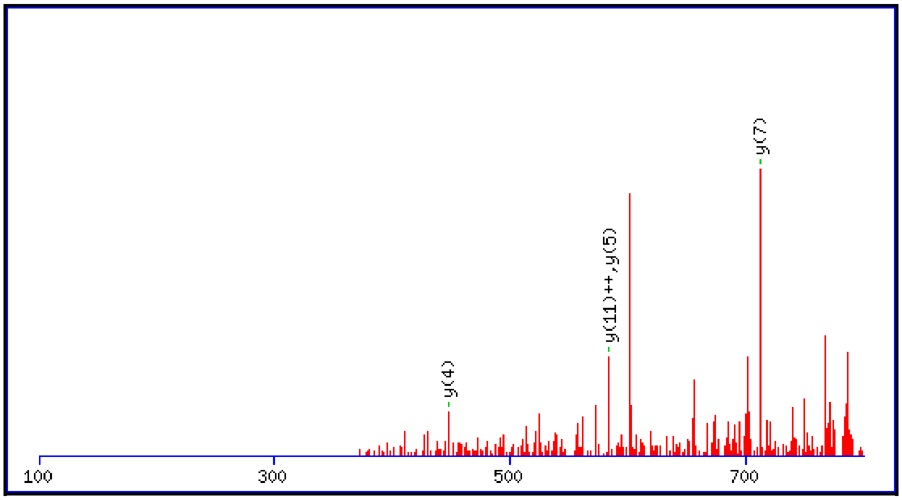

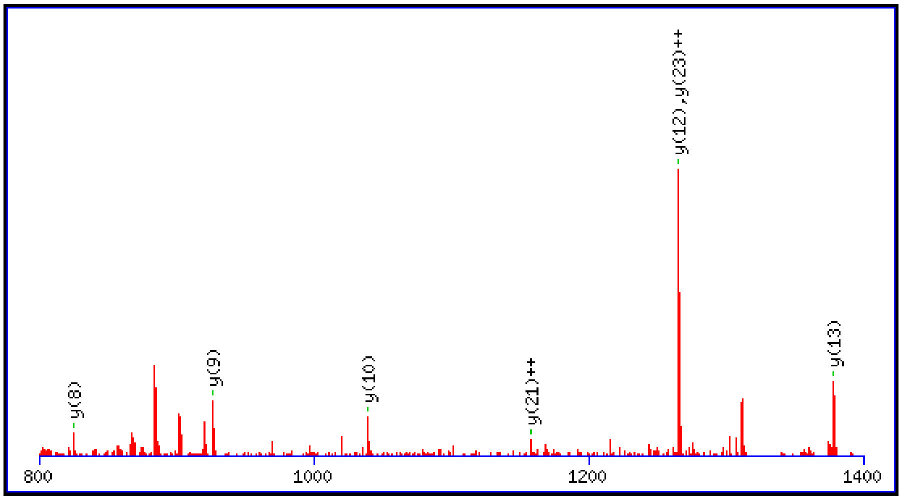


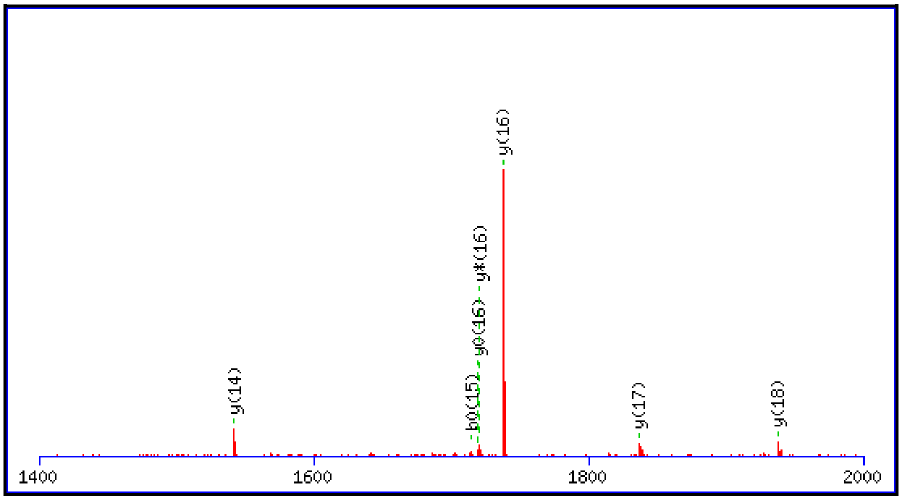


**Monoisotopic mass of neutral peptide Mr(calc):** 2657.3701
**Fixed modifications:** Carbamidomethyl (C)
**Variable modifications:**
**N4** : Deamidated_N (N)
**Ions Score:** 68 **Expect:** 2.1e-005
**Matches (Bold Red):** 18/268 fragment ions using 37 most intense peaks

| # | b | $b^{++}$ | b* | $b^{*++}$ | $b^{0}$ | $b^{0++}$ | Seq. | y | $y^{++}$ | y* | $y^{*++}$ | $y^{0}$ | $y^{0++}$ | # |
|---|---|---|---|---|---|---|---|---|---|---|---|---|---|---|
| 1 | 130.0499 | 65.5286 | | | 112.0393 | 56.5233 | E | | | | | | | 24 |
| 2 | 229.1183 | 115.0628 | | | 211.1077 | 106.0575 | V | 2529.3348 | **1265.1710** | 2512.3082 | 1256.6577 | 2511.3242 | 1256.1657 | 23 |
| 3 | 342.2023 | 171.6048 | | | 324.1918 | 162.5995 | L | 2430.2663 | 1215.6368 | 2413.2398 | 1207.1235 | 2412.2558 | 1206.6315 | 22 |
| 4 | 457.2293 | 229.1183 | 440.2027 | 220.6050 | 439.2187 | 220.1130 | N | 2317.1823 | **1159.0948** | 2300.1557 | 1150.5815 | 2299.1717 | 1150.0895 | 21 |
| 5 | 620.2926 | 310.6499 | 603.2661 | 302.1367 | 602.2820 | 301.6447 | Y | 2202.1553 | 1101.5813 | 2185.1288 | 1093.0680 | 2184.1448 | 1092.5760 | 20 |
| 6 | 721.3403 | 361.1738 | 704.3137 | 352.6605 | 703.3297 | 352.1685 | T | 2039.0920 | 1020.0496 | 2022.0655 | 1011.5364 | 2021.0815 | 1011.0444 | 19 |
| 7 | 822.3880 | 411.6976 | 805.3614 | 403.1843 | 804.3774 | 402.6923 | T | **1938.0443** | 969.5258 | 1921.0178 | 961.0125 | 1920.0338 | 960.5205 | 18 |
| 8 | 921.4564 | 461.2318 | 904.4298 | 452.7186 | 903.4458 | 452.2265 | V | **1836.9967** | 919.0020 | 1819.9701 | 910.4887 | 1818.9861 | 909.9967 | 17 |
| 9 | 1018.5091 | 509.7582 | 1001.4826 | 501.2449 | 1000.4986 | 500.7529 | P | **1737.9282** | 869.4678 | **1720.9017** | 860.9545 | **1719.9177** | 860.4625 | 16 |
| 10 | 1117.5776 | 559.2924 | 1100.5510 | 550.7791 | 1099.5670 | 550.2871 | V | 1640.8755 | 820.9414 | 1623.8489 | 812.4281 | 1622.8649 | 811.9361 | 15 |
| 11 | 1280.6409 | 640.8241 | 1263.6143 | 632.3108 | 1262.6303 | 631.8188 | Y | **1541.8071** | 771.4072 | 1524.7805 | 762.8939 | 1523.7965 | 762.4019 | 14 |
| 12 | 1393.7250 | 697.3661 | 1376.6984 | 688.8528 | 1375.7144 | 688.3608 | L | **1378.7437** | 689.8755 | 1361.7172 | 681.3622 | 1360.7332 | 680.8702 | 13 |
| 13 | 1490.7777 | 745.8925 | 1473.7512 | 737.3792 | 1472.7672 | 736.8872 | P | **1265.6597** | 633.3335 | 1248.6331 | 624.8202 | 1247.6491 | 624.3282 | 12 |
| 14 | 1619.8203 | 810.4138 | 1602.7938 | 801.9005 | 1601.8097 | 801.4085 | E | 1168.6069 | **584.8071** | 1151.5804 | 576.2938 | 1150.5963 | 575.8018 | 11 |
| 15 | 1732.9044 | 866.9558 | 1715.8778 | 858.4426 | **1714.8938** | 857.9505 | I | **1039.5643** | 520.2858 | 1022.5378 | 511.7725 | 1021.5538 | 511.2805 | 10 |
| 16 | 1833.9521 | 917.4797 | 1816.9255 | 908.9664 | 1815.9415 | 908.4744 | T | **926.4803** | 463.7438 | 909.4537 | 455.2305 | 908.4697 | 454.7385 | 9 |
| 17 | 1947.0361 | 974.0217 | 1930.0096 | 965.5084 | 1929.0256 | 965.0164 | I | **825.4326** | 413.2199 | 808.4060 | 404.7067 | 807.4220 | 404.2146 | 8 |
| 18 | 2004.0576 | 1002.5324 | 1987.0310 | 994.0192 | 1986.0470 | 993.5271 | G | **712.3485** | 356.6779 | 695.3220 | 348.1646 | 694.3379 | 347.6726 | 7 |

| | | | | | | | | | | | | | |
|---|---|---|---|---|---|---|---|---|---|---|---|---|---|
| **19** | 2075.0947 | 1038.0510 | 2058.0681 | 1029.5377 | 2057.0841 | 1029.0457 | **A** | 655.3270 | 328.1672 | 638.3005 | 319.6539 | 637.3165 | 319.1619 | **6** |
| **20** | 2212.1536 | 1106.5804 | 2195.1271 | 1098.0672 | 2194.1430 | 1097.5752 | **H** | **584.2899** | 292.6486 | 567.2634 | 284.1353 | 566.2794 | 283.6433 | **5** |
| **21** | 2340.2122 | 1170.6097 | 2323.1856 | 1162.0965 | 2322.2016 | 1161.6044 | **Q** | **447.2310** | 224.1191 | 430.2045 | 215.6059 | 429.2205 | 215.1139 | **4** |
| **22** | 2397.2337 | 1199.1205 | 2380.2071 | 1190.6072 | 2379.2231 | 1190.1152 | **G** | 319.1724 | 160.0899 | 302.1459 | 151.5766 | 301.1619 | 151.0846 | **3** |
| **23** | 2484.2657 | 1242.6365 | 2467.2391 | 1234.1232 | 2466.2551 | 1233.6312 | **S** | 262.1510 | 131.5791 | 245.1244 | 123.0659 | 244.1404 | 122.5738 | **2** |
| **24** | | | | | | | **R** | 175.1190 | 88.0631 | 158.0924 | 79.5498 | | | **1** |

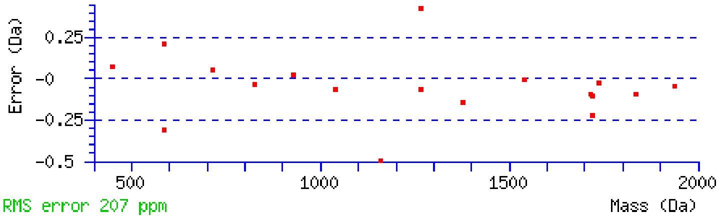


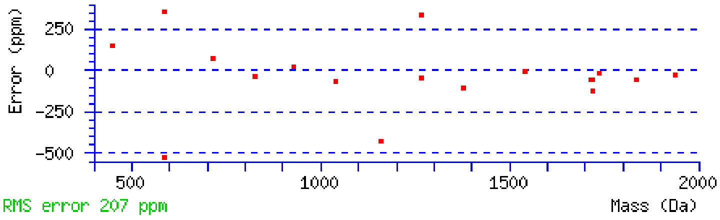


**All matches to this query**

| Score | Mr(calc): | Delta | Sequence |
|---|---|---|---|
| 67.5 | 2657.3701 | 0.0129 | EVLNYTTVPVYLPEITIGAHQGSR |
| 64.1 | 2656.3861 | 0.9969 | EVLNYTTVPVYLPEITIGAHQGSR |
| 0.4 | 2657.3986 | -0.0156 | NYLDLTVTYTTLMILLSRIEER |
| 0.2 | 2655.3779 | 2.0051 | SLGKAVATVSLSVALASVTVRSSGCR |
| 0.2 | 2655.3779 | 2.0051 | SLGKAVATVSLSVALASVTVRSSGCR |
| 0.2 | 2655.3779 | 2.0051 | SLGKAVATVSLSVALASVTVRSSGCR |
| 0.2 | 2655.3779 | 2.0051 | SLGKAVATVSLSVALASVTVRSSGCR |

---

Spectrum No: 404; Query: 2596; Rank: 1

---

## Peptide View

MS/MS Fragmentation of **GYYNQSEGGSHTIQEMFGCDVGSDGSLLR**
Found in **IPI00855224**, Tax_Id=10116 Gene_Symbol=RT1-Aw2 MHC class I RT1.Ab heavy chain precursor

Match to Query 2596: 3164.353422 from(1055.791750,3+)
Title: 100101RatKid_NS_deglyco_20.4320.4320.3.dta
Data file K:\NewmanPaper\Piliang\3SubProteomes\Piliang3SP\mgf5ppm\ERLIC_3SubProteomes5ppm.mgf

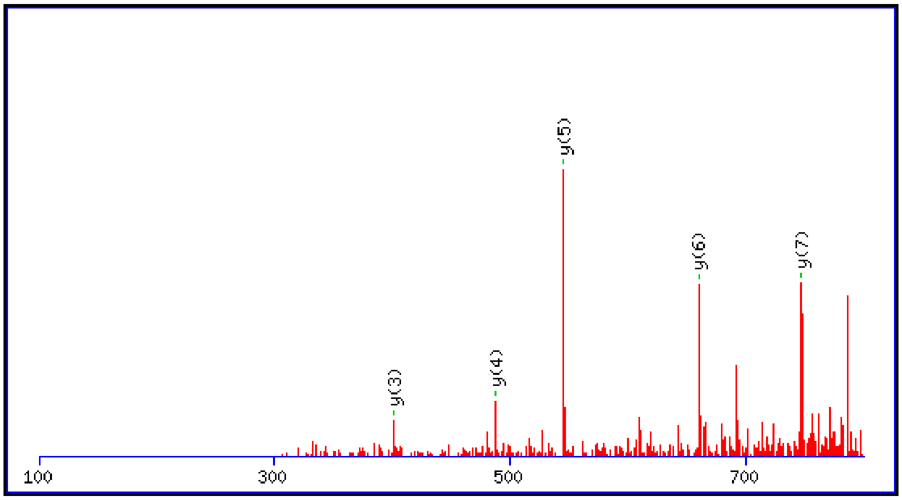

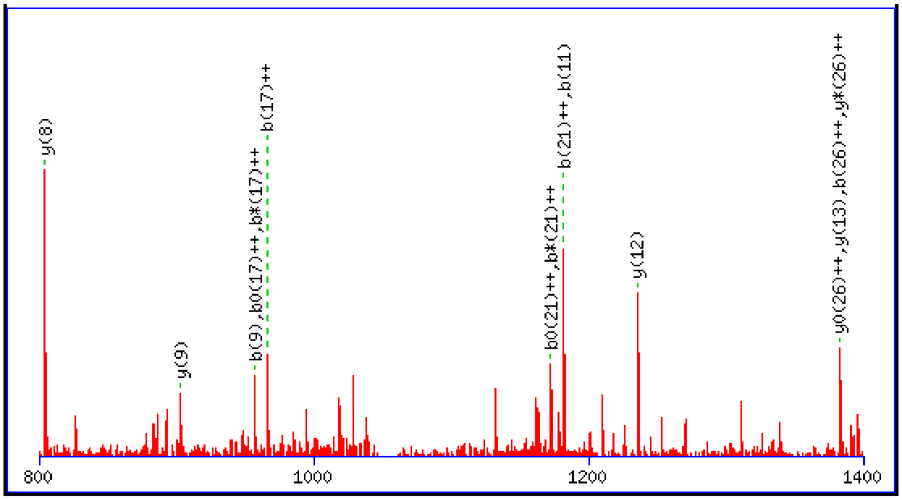


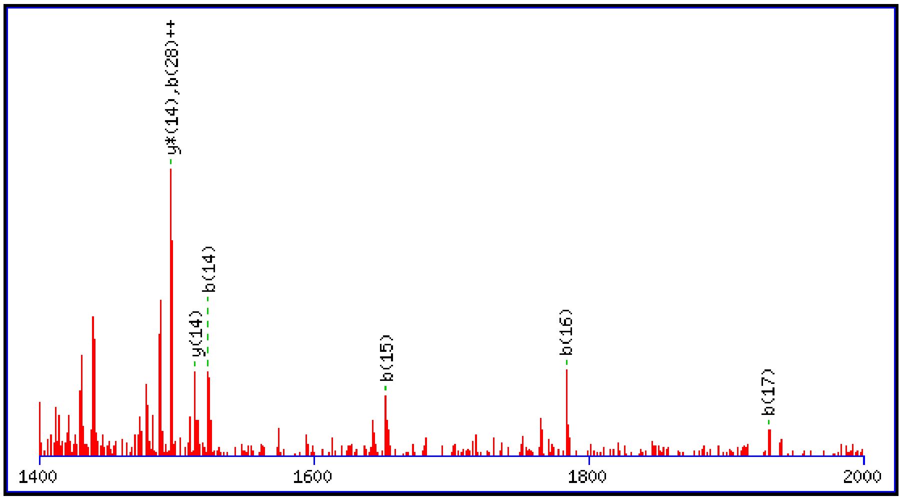


**Monoisotopic mass of neutral peptide Mr(calc):** 3164.3452
**Fixed modifications:** Carbamidomethyl (C)
**Variable modifications:**
**N4** : Deamidated_N (N)
**Ions Score:** 68 **Expect:** 7.2e-005
**Matches (Bold Red):** 27/314 fragment ions using 28 most intense peaks

| # | b | $b^{++}$ | b* | $b^{*++}$ | $b^{0}$ | $b^{0++}$ | Seq. | y | $y^{++}$ | y* | $y^{*++}$ | $y^{0}$ | $y^{0++}$ | # |
|---|---|---|---|---|---|---|---|---|---|---|---|---|---|---|
| 1 | 58.0287 | 29.5180 | | | | | G | | | | | | | 29 |
| 2 | 221.0921 | 111.0497 | | | | | Y | 3108.3310 | 1554.6691 | 3091.3044 | 1546.1558 | 3090.3204 | 1545.6638 | 28 |
| 3 | 384.1554 | 192.5813 | | | | | Y | 2945.2676 | 1473.1374 | 2928.2411 | 1464.6242 | 2927.2571 | 1464.1322 | 27 |
| 4 | 499.1823 | 250.0948 | 482.1558 | 241.5815 | | | N | 2782.2043 | 1391.6058 | 2765.1777 | **1383.0925** | 2764.1937 | **1382.6005** | 26 |
| 5 | 627.2409 | 314.1241 | 610.2144 | 305.6108 | | | Q | 2667.1774 | 1334.0923 | 2650.1508 | 1325.5790 | 2649.1668 | 1325.0870 | 25 |
| 6 | 714.2729 | 357.6401 | 697.2464 | 349.1268 | 696.2624 | 348.6348 | S | 2539.1188 | 1270.0630 | 2522.0922 | 1261.5498 | 2521.1082 | 1261.0577 | 24 |
| 7 | 843.3155 | 422.1614 | 826.2890 | 413.6481 | 825.3050 | 413.1561 | E | 2452.0868 | 1226.5470 | 2435.0602 | 1218.0337 | 2434.0762 | 1217.5417 | 23 |
| 8 | 900.3370 | 450.6721 | 883.3104 | 442.1589 | 882.3264 | 441.6669 | G | 2323.0442 | 1162.0257 | 2306.0176 | 1153.5124 | 2305.0336 | 1153.0204 | 22 |
| 9 | **957.3585** | 479.1829 | 940.3319 | 470.6696 | 939.3479 | 470.1776 | G | 2266.0227 | 1133.5150 | 2248.9961 | 1125.0017 | 2248.0121 | 1124.5097 | 21 |
| 10 | 1044.3905 | 522.6989 | 1027.3639 | 514.1856 | 1026.3799 | 513.6936 | S | 2209.0012 | 1105.0043 | 2191.9747 | 1096.4910 | 2190.9907 | 1095.9990 | 20 |
| 11 | **1181.4494** | 591.2283 | 1164.4229 | 582.7151 | 1163.4388 | 582.2231 | H | 2121.9692 | 1061.4882 | 2104.9427 | 1052.9750 | 2103.9586 | 1052.4830 | 19 |
| 12 | 1282.4971 | 641.7522 | 1265.4705 | 633.2389 | 1264.4865 | 632.7469 | T | 1984.9103 | 992.9588 | 1967.8837 | 984.4455 | 1966.8997 | 983.9535 | 18 |
| 13 | 1395.5811 | 698.2942 | 1378.5546 | 689.7809 | 1377.5706 | 689.2889 | I | 1883.8626 | 942.4349 | 1866.8361 | 933.9217 | 1865.8520 | 933.4297 | 17 |
| 14 | **1523.6397** | 762.3235 | 1506.6132 | 753.8102 | 1505.6292 | 753.3182 | Q | 1770.7785 | 885.8929 | 1753.7520 | 877.3796 | 1752.7680 | 876.8876 | 16 |
| 15 | **1652.6823** | 826.8448 | 1635.6558 | 818.3315 | 1634.6718 | 817.8395 | E | 1642.7200 | 821.8636 | 1625.6934 | 813.3503 | 1624.7094 | 812.8583 | 15 |
| 16 | **1783.7228** | 892.3650 | 1766.6963 | 883.8518 | 1765.7122 | 883.3598 | M | **1513.6774** | 757.3423 | **1496.6508** | 748.8291 | 1495.6668 | 748.3370 | 14 |
| 17 | **1930.7912** | **965.8992** | 1913.7647 | **957.3860** | 1912.7806 | **956.8940** | F | **1382.6369** | 691.8221 | 1365.6103 | 683.3088 | 1364.6263 | 682.8168 | 13 |
| 18 | 1987.8127 | 994.4100 | 1970.7861 | 985.8967 | 1969.8021 | 985.4047 | G | **1235.5685** | 618.2879 | 1218.5419 | 609.7746 | 1217.5579 | 609.2826 | 12 |
| | | | | | | | | | | | | | | |

| | | | | | | | | | | | | | | |
|---|---|---|---|---|---|---|---|---|---|---|---|---|---|---|
| 19 | 2147.8433 | 1074.4253 | 2130.8168 | 1065.9120 | 2129.8328 | 1065.4200 | C | 1178.5470 | 589.7771 | 1161.5205 | 581.2639 | 1160.5364 | 580.7719 | 11 |
| 20 | 2262.8703 | 1131.9388 | 2245.8437 | 1123.4255 | 2244.8597 | 1122.9335 | D | 1018.5164 | 509.7618 | 1001.4898 | 501.2485 | 1000.5058 | 500.7565 | 10 |
| 21 | 2361.9387 | **1181.4730** | 2344.9121 | **1172.9597** | 2343.9281 | **1172.4677** | V | **903.4894** | 452.2483 | 886.4629 | 443.7351 | 885.4789 | 443.2431 | 9 |
| 22 | 2418.9601 | 1209.9837 | 2401.9336 | 1201.4704 | 2400.9496 | 1200.9784 | G | **804.4210** | 402.7141 | 787.3945 | 394.2009 | 786.4104 | 393.7089 | 8 |
| 23 | 2505.9922 | 1253.4997 | 2488.9656 | 1244.9865 | 2487.9816 | 1244.4944 | S | **747.3995** | 374.2034 | 730.3730 | 365.6901 | 729.3890 | 365.1981 | 7 |
| 24 | 2621.0191 | 1311.0132 | 2603.9926 | 1302.4999 | 2603.0086 | 1302.0079 | D | **660.3675** | 330.6874 | 643.3410 | 322.1741 | 642.3570 | 321.6821 | 6 |
| 25 | 2678.0406 | 1339.5239 | 2661.0140 | 1331.0107 | 2660.0300 | 1330.5186 | G | **545.3406** | 273.1739 | 528.3140 | 264.6606 | 527.3300 | 264.1686 | 5 |
| 26 | 2765.0726 | **1383.0399** | 2748.0461 | 1374.5267 | 2747.0620 | 1374.0347 | S | **488.3191** | 244.6632 | 471.2926 | 236.1499 | 470.3085 | 235.6579 | 4 |
| 27 | 2878.1567 | 1439.5820 | 2861.1301 | 1431.0687 | 2860.1461 | 1430.5767 | L | **401.2871** | 201.1472 | 384.2605 | 192.6339 | | | 3 |
| 28 | 2991.2407 | **1496.1240** | 2974.2142 | 1487.6107 | 2973.2302 | 1487.1187 | L | 288.2030 | 144.6051 | 271.1765 | 136.0919 | | | 2 |
| 29 | | | | | | | R | 175.1190 | 88.0631 | 158.0924 | 79.5498 | | | 1 |

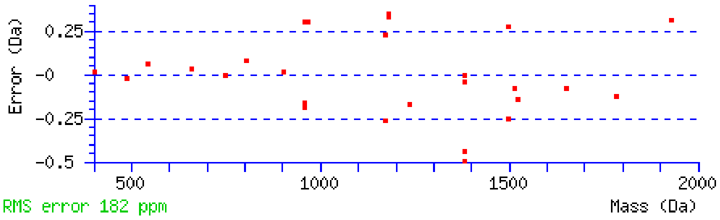


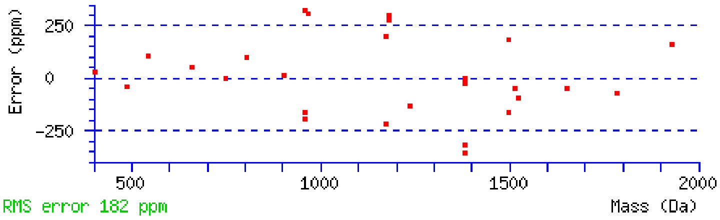


**All matches to this query**

| Score | Mr(calc): | Delta | Sequence |
|---|---|---|---|
| 67.5 | 3164.3452 | 0.0083 | GYYNQSEGGSHTIQEMFGCDVGSDGSLLR |
| 48.4 | 3163.3611 | 0.9923 | GYYNQSEGGSHTIQEMFGCDVGSDGSLLR |
| 43.0 | 3164.3815 | -0.0281 | GYYNQSEGGSHTLQLMYGCDVGSDGSLLR |
| 4.5 | 3164.3518 | 0.0017 | DGSTALMVALDAGQSEIASMLYSRMNIK |
| 4.2 | 3164.3518 | 0.0017 | DGSTALMVALDAGQSEIASMLYSRMNIK |
| 3.9 | 3164.3518 | 0.0017 | DGSTALMVALDAGQSEIASMLYSRMNIK |
| 3.5 | 3164.3518 | 0.0017 | DGSTALMVALDAGQSEIASMLYSRMNIK |
| 2.3 | 3164.3536 | -0.0002 | LMRYHSVSTETPHERNVDHLPGGESR |
| 1.9 | 3162.3163 | 2.0372 | DTAAAVMYTVVTPMLNPFIYSLRNR |
| 1.7 | 3162.3163 | 2.0372 | DTAAAVMYTVVTPMLNPFIYSLRNR |

---

Spectrum No: 405; Query: 355; Rank: 1

---

## Peptide View

MS/MS Fragmentation of **LNAINITSALDR**
Found in **IPI00454453**, Tax_Id=10116 Gene_Symbol=Sgce Epsilon-sarcoglycan

Match to Query 355: 1300.698748 from(651.356650,2+)
Title: 091008RatKidney_NH4Format01_23.2946.2946.2.dta
Data file K:\NewmanPaper\Piliang\3SubProteomes\Piliang3SP\mgf5ppm\ERLIC_3SubProteomes5ppm.mgf

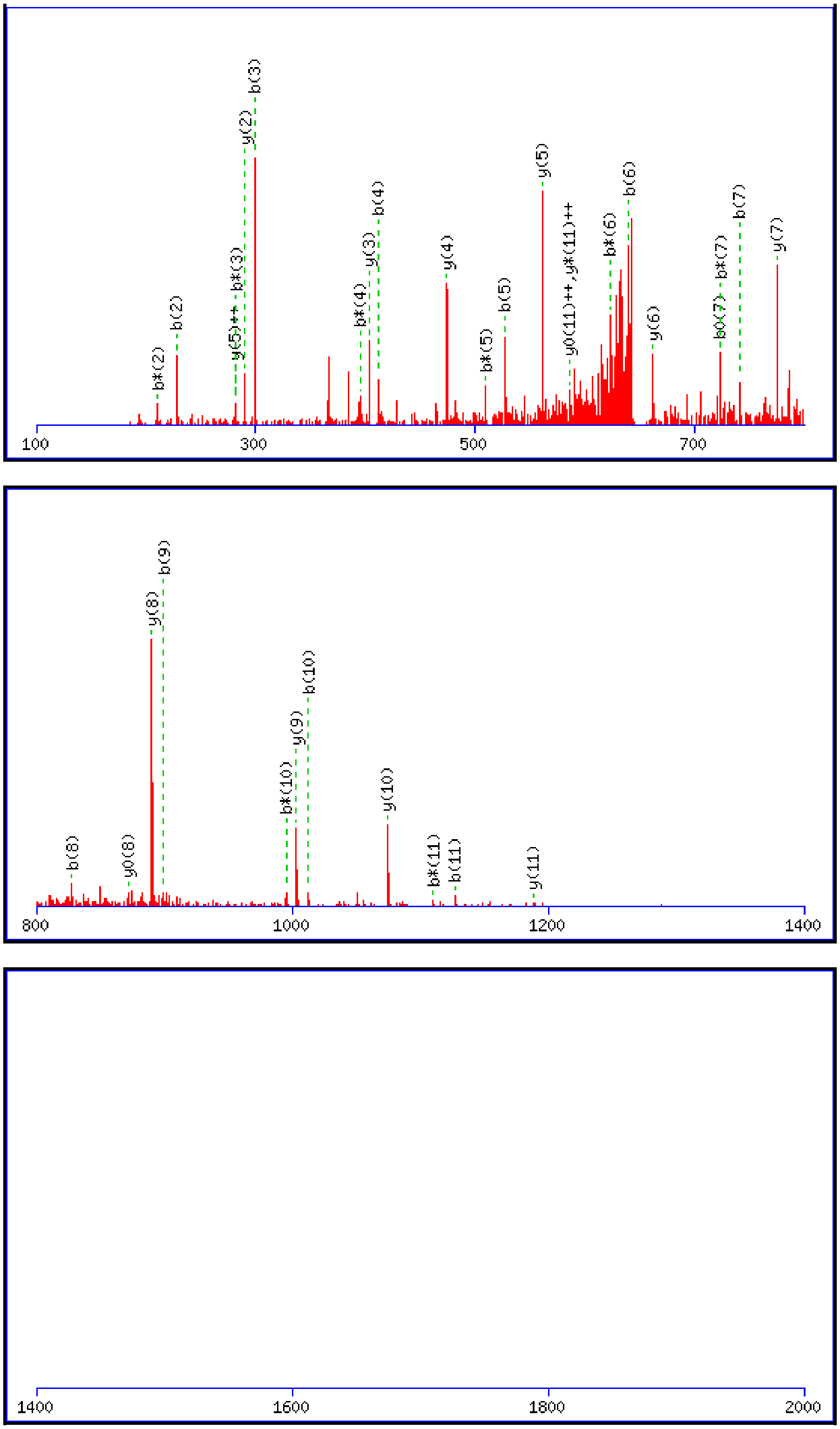


**Monoisotopic mass of neutral peptide Mr(calc):** 1300.6986
**Fixed modifications:** Carbamidomethyl (C)
**Variable modifications:**
**N5** : Deamidated_N (N)
**Ions Score:** 67 **Expect:** 1.8e-005
**Matches (Bold Red):** 33/116 fragment ions using 76 most intense peaks

| # | b | $b^{++}$ | b* | $b^{*++}$ | $b^0$ | $b^{0++}$ | Seq. | y | $y^{++}$ | y* | $y^{*++}$ | $y^0$ | $y^{0++}$ | # |
|---|---|---|---|---|---|---|---|---|---|---|---|---|---|---|
| 1 | 114.0913 | 57.5493 | | | | | L | | | | | | | 12 |
| 2 | **228.1343** | 114.5708 | **211.1077** | 106.0575 | | | N | **1188.6219** | 594.8146 | 1171.5953 | **586.3013** | 1170.6113 | **585.8093** | 11 |
| 3 | **299.1714** | 150.0893 | **282.1448** | 141.5761 | | | A | **1074.5790** | 537.7931 | 1057.5524 | 529.2798 | 1056.5684 | 528.7878 | 10 |
| 4 | **412.2554** | 206.6314 | **395.2289** | 198.1181 | | | I | **1003.5418** | 502.2746 | 986.5153 | 493.7613 | 985.5313 | 493.2693 | 9 |
| 5 | **527.2824** | 264.1448 | **510.2558** | 255.6316 | | | N | **890.4578** | 445.7325 | 873.4312 | 437.2193 | **872.4472** | 436.7272 | 8 |

| | | | | | | | | | | | | | | |
|---|---|---|---|---|---|---|---|---|---|---|---|---|---|---|
| **6** | **640.3664** | 320.6869 | **623.3399** | 312.1736 | | | **I** | **775.4308** | 388.2191 | 758.4043 | 379.7058 | 757.4203 | 379.2138 | **7** |
| **7** | **741.4141** | 371.2107 | **724.3876** | 362.6974 | **723.4036** | 362.2054 | **T** | **662.3468** | 331.6770 | 645.3202 | 323.1638 | 644.3362 | 322.6717 | **6** |
| **8** | **828.4462** | 414.7267 | 811.4196 | 406.2134 | 810.4356 | 405.7214 | **S** | **561.2991** | **281.1532** | 544.2726 | 272.6399 | 543.2885 | 272.1479 | **5** |
| **9** | **899.4833** | 450.2453 | 882.4567 | 441.7320 | 881.4727 | 441.2400 | **A** | **474.2671** | 237.6372 | 457.2405 | 229.1239 | 456.2565 | 228.6319 | **4** |
| **10** | **1012.5673** | 506.7873 | **995.5408** | 498.2740 | 994.5568 | 497.7820 | **L** | **403.2300** | 202.1186 | 386.2034 | 193.6053 | 385.2194 | 193.1133 | **3** |
| **11** | **1127.5943** | 564.3008 | **1110.5677** | 555.7875 | 1109.5837 | 555.2955 | **D** | **290.1459** | 145.5766 | 273.1193 | 137.0633 | 272.1353 | 136.5713 | **2** |
| **12** | | | | | | | **R** | 175.1190 | 88.0631 | 158.0924 | 79.5498 | | | **1** |

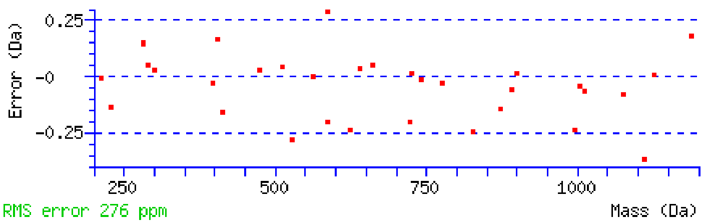


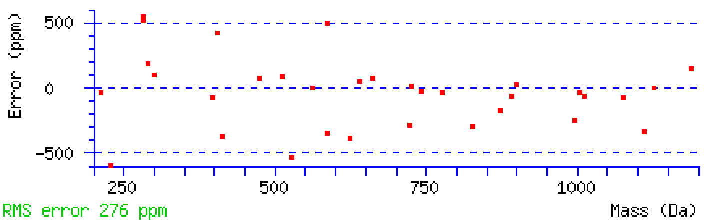


### All matches to this query

| Score | Mr(calc): | Delta | Sequence |
|---|---|---|---|
| 67.4 | 1300.6986 | 0.0001 | LNAINITSALDR |
| 34.6 | 1300.6986 | 0.0001 | LNAINITSALDR |
| 3.8 | 1300.7074 | -0.0086 | WLCARVLLXR |
| 3.8 | 1300.7074 | -0.0086 | WLCARVLLXR |
| 2.8 | 1300.7034 | -0.0046 | AARLQISVCQR |
| 1.5 | 1300.6987 | 0.0001 | VQDDVTKLDLR |
| 1.3 | 1300.6921 | 0.0066 | RLEAGAMVLADR |
| 0.2 | 1300.6987 | 0.0001 | LLLSGTADGADLR |

---

Spectrum No: 406; Query: 242; Rank: 1

---

## Peptide View

MS/MS Fragmentation of **VYVYAVNQTR**
Found in **IPI00324585**, Tax_Id=10116 Gene_Symbol=Itga1 Integrin alpha-1 precursor

Match to Query 242: 1212.615588 from(607.315070,2+)
Title: 100101RatKid_NS_deglyco_20.1583.1583.2.dta
Data file K:\NewmanPaper\Piliang\3SubProteomes\Piliang3SP\mgf5ppm\ERLIC_3SubProteomes5ppm.mgf

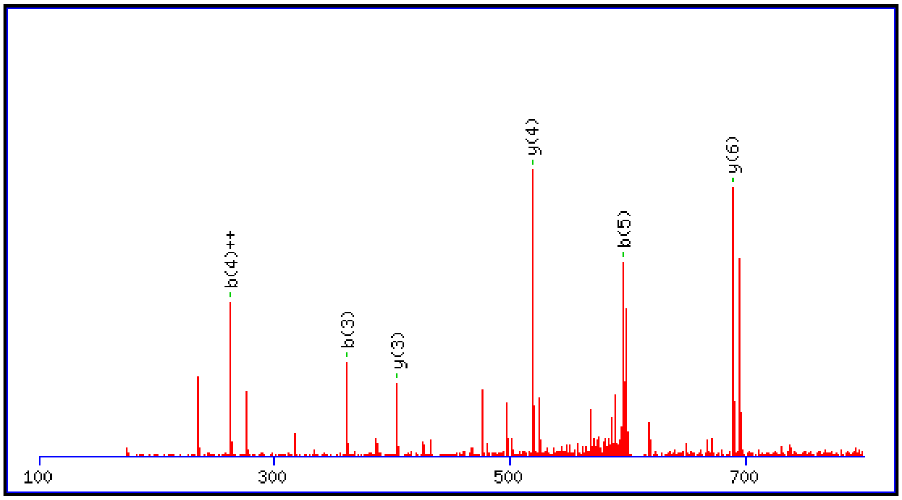

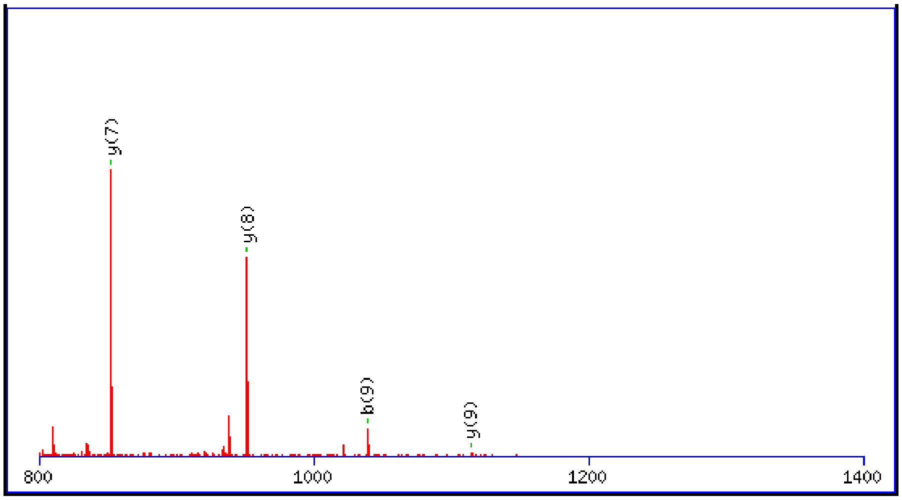


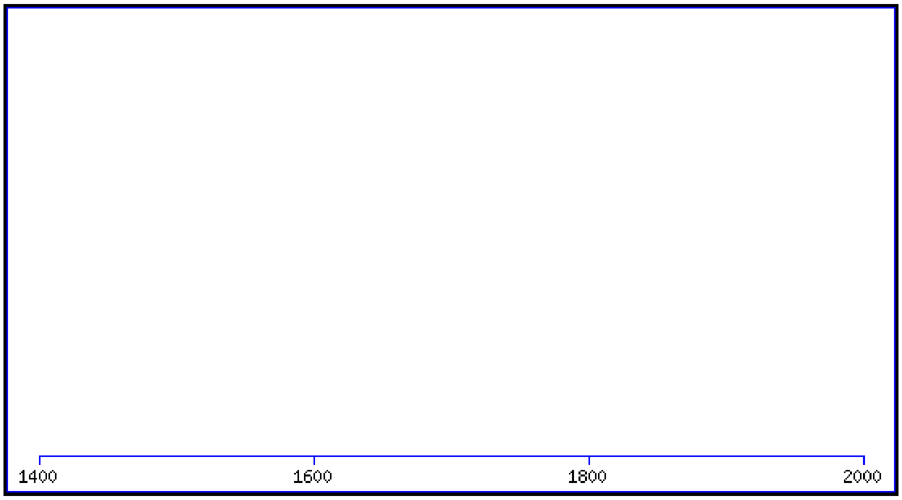


**Monoisotopic mass of neutral peptide Mr(calc):** 1212.6139
**Fixed modifications:** Carbamidomethyl (C)
**Variable modifications:**
**N7** : Deamidated_N (N)
**Ions Score:** 67 **Expect:** 1.8e-005
**Matches (Bold Red):** 11/78 fragment ions using 12 most intense peaks

| # | b | $b^{++}$ | b* | $b^{*++}$ | $b^0$ | $b^{0++}$ | Seq. | y | $y^{++}$ | y* | $y^{*++}$ | $y^0$ | $y^{0++}$ | # |
|---|---|---|---|---|---|---|---|---|---|---|---|---|---|---|
| 1 | 100.0757 | 50.5415 | | | | | V | | | | | | | 10 |
| 2 | **263.1390** | 132.0731 | | | | | Y | **1114.5527** | 557.7800 | 1097.5262 | 549.2667 | 1096.5422 | 548.7747 | 9 |
| 3 | **362.2074** | 181.6074 | | | | | V | **951.4894** | 476.2483 | 934.4629 | 467.7351 | 933.4789 | 467.2431 | 8 |
| 4 | 525.2708 | **263.1390** | | | | | Y | **852.4210** | 426.7141 | 835.3945 | 418.2009 | 834.4104 | 417.7089 | 7 |
| 5 | **596.3079** | 298.6576 | | | | | A | **689.3577** | 345.1825 | 672.3311 | 336.6692 | 671.3471 | 336.1772 | 6 |
| 6 | 695.3763 | 348.1918 | | | | | V | 618.3206 | 309.6639 | 601.2940 | 301.1506 | 600.3100 | 300.6586 | 5 |
| 7 | 810.4032 | 405.7053 | 793.3767 | 397.1920 | | | N | **519.2521** | 260.1297 | 502.2256 | 251.6164 | 501.2416 | 251.1244 | 4 |
| 8 | 938.4618 | 469.7345 | 921.4353 | 461.2213 | | | Q | **404.2252** | 202.6162 | 387.1987 | 194.1030 | 386.2146 | 193.6110 | 3 |
| 9 | **1039.5095** | 520.2584 | 1022.4829 | 511.7451 | 1021.4989 | 511.2531 | T | 276.1666 | 138.5870 | 259.1401 | 130.0737 | 258.1561 | 129.5817 | 2 |
| 10 | | | | | | | R | 175.1190 | 88.0631 | 158.0924 | 79.5498 | | | 1 |

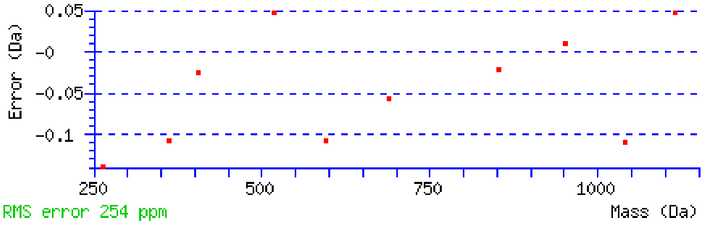


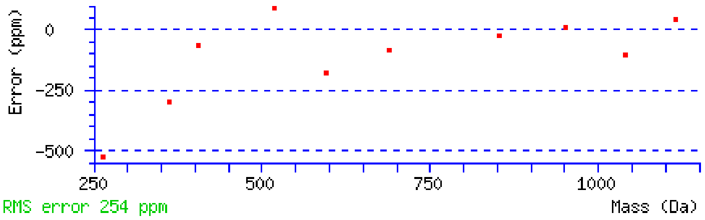


**All matches to this query**

| Score | Mr(calc): | Delta | Sequence |
|---|---|---|---|
| 67.4 | 1212.6139 | 0.0017 | VYVYAVNQTR |
| 17.4 | 1212.6268 | -0.0112 | VYVNGLISLR |
| 7.5 | 1212.6145 | 0.0010 | RCAHXEIKSR |
| 6.7 | 1212.6251 | -0.0095 | VYSGRTFVER |
| 6.2 | 1212.6073 | 0.0082 | KFQYQLACR |
| 5.7 | 1212.6251 | -0.0095 | KFSVEVHDPR |
| 5.3 | 1210.6032 | 2.0124 | MASIVPLKEK |
| 3.6 | 1212.6251 | -0.0095 | VVYSDLHPQR |
| 2.6 | 1210.6054 | 2.0102 | GNSELLRHER |
| 2.2 | 1212.6139 | 0.0017 | FNPVTGEVPPR |

---

Spectrum No: 407; Query: 1039; Rank: 1

---

## Peptide View

MS/MS Fragmentation of **SSSHLPPSSYFNASGR**
Found in **IPI00190499**, Tax_Id=10116 Gene_Symbol=Tpp1 Tripeptidyl-peptidase 1 precursor

Match to Query 1039: 1693.764222 from(565.595350,3+)
Title: 091008RatKidney_NH4Format02_24.1718.1718.3.dta
Data file K:\NewmanPaper\Piliang\3SubProteomes\Piliang3SP\mgf5ppm\ERLIC_3SubProteomes5ppm.mgf

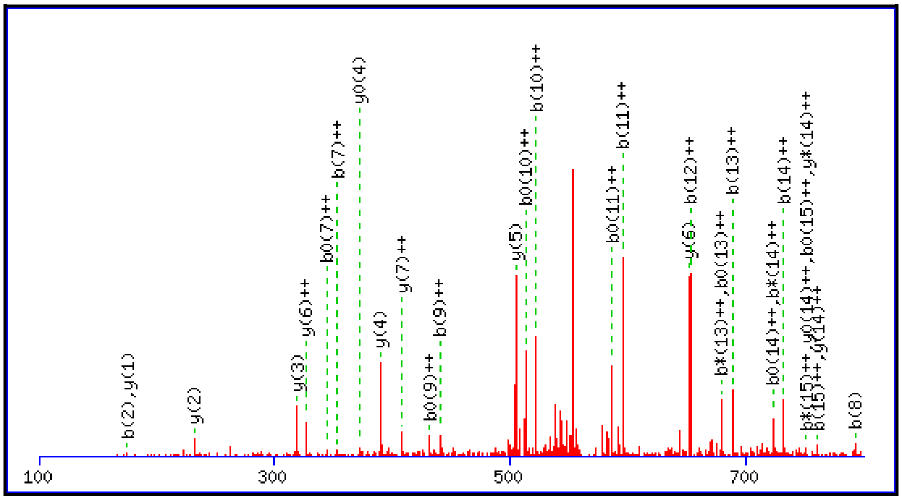


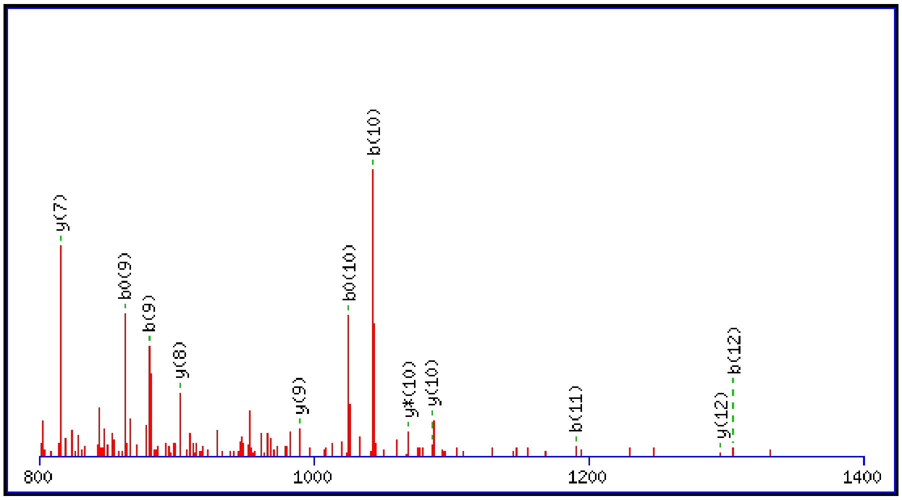

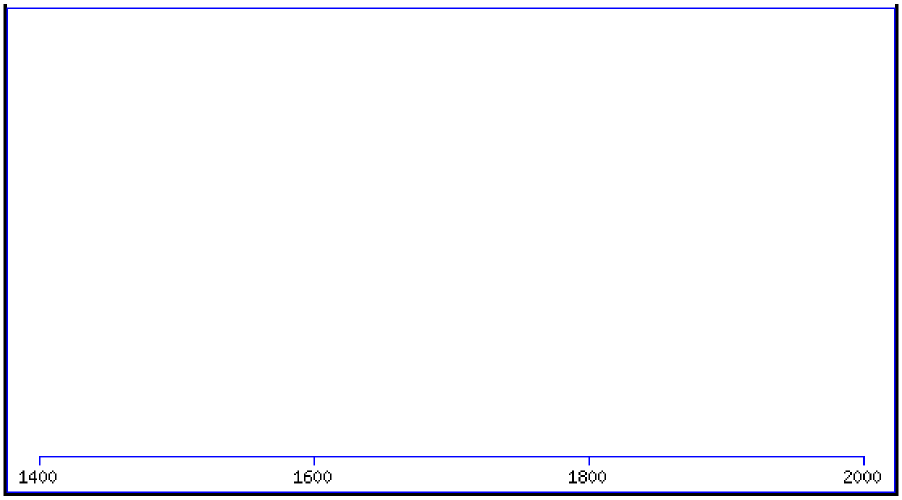


**Monoisotopic mass of neutral peptide Mr(calc):** 1693.7696
**Fixed modifications:** Carbamidomethyl (C)
**Variable modifications:**
**N12** : Deamidated_N (N)
**Ions Score:** 67 **Expect:** 3.8e-005
**Matches (Bold Red):** 44/154 fragment ions using 66 most intense peaks

| # | b | $b^{++}$ | b* | $b^{*++}$ | $b^{0}$ | $b^{0++}$ | Seq. | y | $y^{++}$ | y* | $y^{*++}$ | $y^{0}$ | $y^{0++}$ | # |
|---|---|---|---|---|---|---|---|---|---|---|---|---|---|---|
| 1 | 88.0393 | 44.5233 | | | 70.0287 | 35.5180 | S | | | | | | | 16 |
| 2 | **175.0713** | 88.0393 | | | 157.0608 | 79.0340 | S | 1607.7449 | 804.3761 | 1590.7183 | 795.8628 | 1589.7343 | 795.3708 | 15 |
| 3 | 262.1034 | 131.5553 | | | 244.0928 | 122.5500 | S | 1520.7128 | **760.8601** | 1503.6863 | **752.3468** | 1502.7023 | **751.8548** | 14 |
| 4 | 399.1623 | 200.0848 | | | 381.1517 | 191.0795 | H | 1433.6808 | 717.3440 | 1416.6542 | 708.8308 | 1415.6702 | 708.3388 | 13 |
| 5 | 512.2463 | 256.6268 | | | 494.2358 | 247.6215 | L | **1296.6219** | 648.8146 | 1279.5953 | 640.3013 | 1278.6113 | 639.8093 | 12 |
| 6 | 609.2991 | 305.1532 | | | 591.2885 | 296.1479 | P | 1183.5378 | 592.2725 | 1166.5113 | 583.7593 | 1165.5273 | 583.2673 | 11 |
| 7 | 706.3519 | **353.6796** | | | 688.3413 | **344.6743** | P | **1086.4851** | 543.7462 | **1069.4585** | 535.2329 | 1068.4745 | 534.7409 | 10 |
| 8 | **793.3839** | 397.1956 | | | 775.3733 | 388.1903 | S | **989.4323** | 495.2198 | 972.4057 | 486.7065 | 971.4217 | 486.2145 | 9 |
| 9 | **880.4159** | **440.7116** | | | **862.4054** | **431.7063** | S | **902.4003** | 451.7038 | 885.3737 | 443.1905 | 884.3897 | 442.6985 | 8 |
| 10 | **1043.4792** | **522.2433** | | | **1025.4687** | **513.2380** | Y | **815.3682** | **408.1878** | 798.3417 | 399.6745 | 797.3577 | 399.1825 | 7 |
| 11 | **1190.5477** | **595.7775** | | | 1172.5371 | **586.7722** | F | **652.3049** | **326.6561** | 635.2784 | 318.1428 | 634.2943 | 317.6508 | 6 |
| 12 | **1305.5746** | **653.2909** | 1288.5481 | 644.7777 | 1287.5640 | 644.2857 | N | **505.2365** | 253.1219 | 488.2099 | 244.6086 | 487.2259 | 244.1166 | 5 |
| 13 | 1376.6117 | **688.8095** | 1359.5852 | **680.2962** | 1358.6011 | **679.8042** | A | **390.2096** | 195.6084 | 373.1830 | 187.0951 | **372.1990** | 186.6031 | 4 |
| 14 | 1463.6437 | **732.3255** | 1446.6172 | **723.8122** | 1445.6332 | **723.3202** | S | **319.1724** | 160.0899 | 302.1459 | 151.5766 | 301.1619 | 151.0846 | 3 |
| 15 | 1520.6652 | **760.8362** | 1503.6387 | **752.3230** | 1502.6546 | **751.8310** | G | **232.1404** | 116.5738 | 215.1139 | 108.0606 | | | 2 |
| 16 | | | | | | | R | **175.1190** | 88.0631 | 158.0924 | 79.5498 | | | 1 |

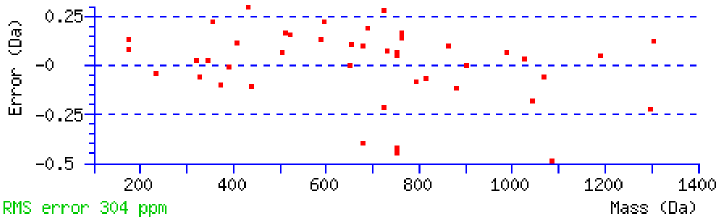


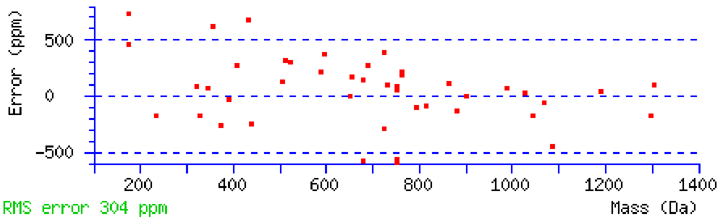


### All matches to this query

| Score | Mr(calc): | Delta | Sequence |
|---|---|---|---|
| 67.3 | 1693.7696 | -0.0054 | SSSHLPPSSYFNASGR |
| 9.3 | 1693.7560 | 0.0082 | NDSVIVADQTPTPTR |
| 7.6 | 1693.7645 | -0.0003 | TGEHNSSGSRTLRGR |
| 6.6 | 1691.7606 | 2.0036 | ILIANTGMDTDKIK |
| 6.5 | 1693.7560 | 0.0082 | NDSVIVADQTPTPTR |
| | | | |

| | | | |
|---|---|---|---|
| 6.1 | 1692.7581 | 1.0062 | QQGQEPSQLNKGSGR |
| 5.1 | 1691.7718 | 1.9924 | LSGMDLINLSKVSR |
| 4.4 | 1692.7581 | 1.0062 | ENGPRAPASGGSLSGTR |
| 4.4 | 1692.7581 | 1.0062 | ENGPRAPASGGSLSGTR |
| 4.2 | 1692.7720 | 0.9922 | NDSVIVADQTPTPTR |

---

Spectrum No: 408; Query: 875; Rank: 1

---

## Peptide View

MS/MS Fragmentation of **DSSKSSNVSLPDCKA**
Found in **IPI00764367**, Tax_Id=10116 Gene_Symbol=LOC686172 similar to RT1 class I, CE1

Match to Query 875: 1594.718548 from(798.366550,2+)
Title: 091008RatKidney_NoSalt_22.1010.1010.2.dta
Data file K:\NewmanPaper\Piliang\3SubProteomes\Piliang3SP\mgf5ppm\ERLIC_3SubProteomes5ppm.mgf

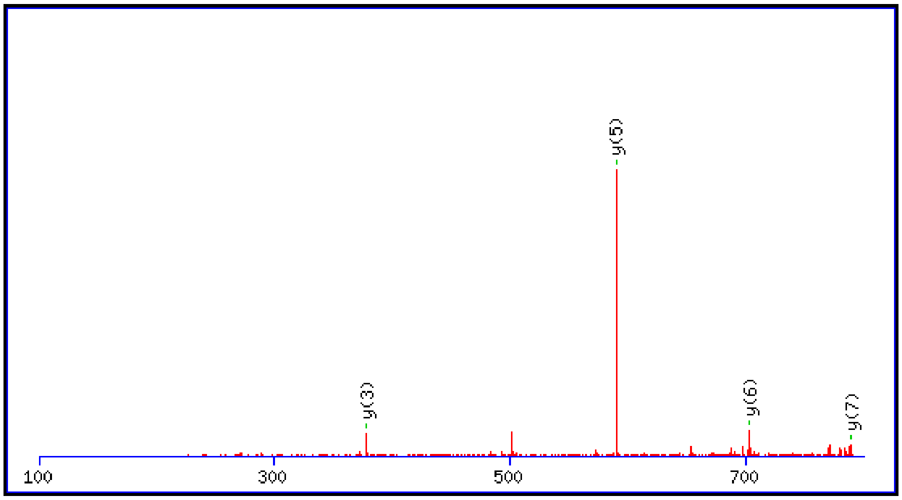


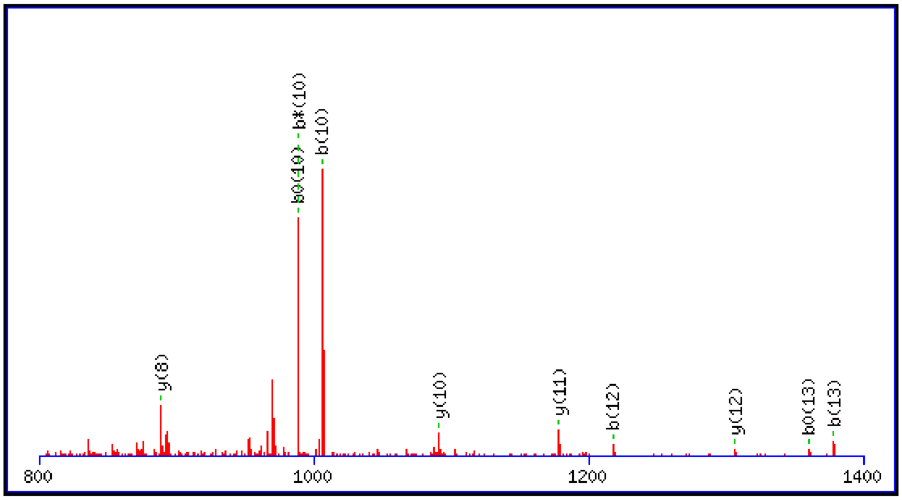

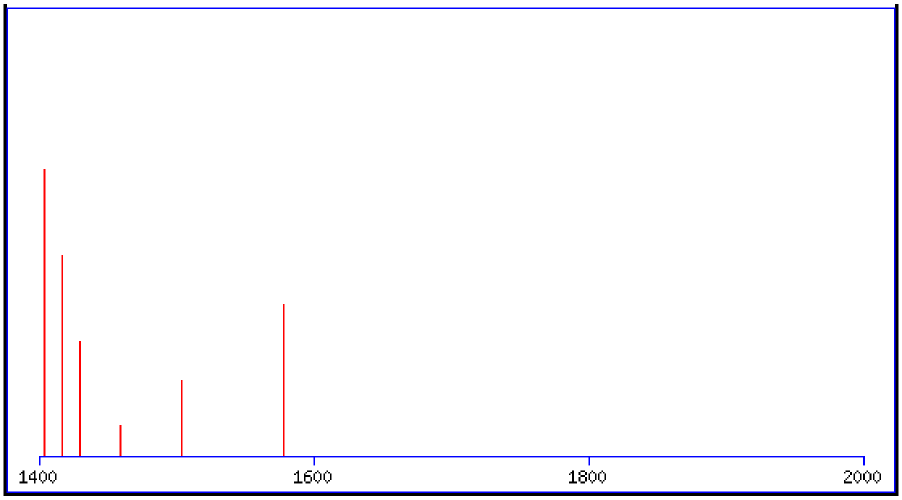


**Monoisotopic mass of neutral peptide Mr(calc):** 1594.7144
**Fixed modifications:** Carbamidomethyl (C)
**Variable modifications:**
**N7** : Deamidated_N (N)
**Ions Score:** 67 **Expect:** 3.4e-005
**Matches (Bold Red):** 14/154 fragment ions using 26 most intense peaks

| # | b | b++ | b* | b*++ | b0 | b0++ | Seq. | y | y++ | y* | y*++ | y0 | y0++ | # |
|---|---|---|---|---|---|---|---|---|---|---|---|---|---|---|
| 1 | 116.0342 | 58.5207 | | | 98.0237 | 49.5155 | D | | | | | | | 15 |
| 2 | 203.0662 | 102.0368 | | | 185.0557 | 93.0315 | S | 1480.6948 | 740.8510 | 1463.6682 | 732.3378 | 1462.6842 | 731.8458 | 14 |
| 3 | 290.0983 | 145.5528 | | | 272.0877 | 136.5475 | S | 1393.6628 | 697.3350 | 1376.6362 | 688.8217 | 1375.6522 | 688.3297 | 13 |
| 4 | 418.1932 | 209.6003 | 401.1667 | 201.0870 | 400.1827 | 200.5950 | K | **1306.6307** | 653.8190 | 1289.6042 | 645.3057 | 1288.6202 | 644.8137 | 12 |
| 5 | 505.2253 | 253.1163 | 488.1987 | 244.6030 | 487.2147 | 244.1110 | S | **1178.5358** | 589.7715 | 1161.5092 | 581.2582 | 1160.5252 | 580.7662 | 11 |
| 6 | 592.2573 | 296.6323 | 575.2307 | 288.1190 | 574.2467 | 287.6270 | S | **1091.5037** | 546.2555 | 1074.4772 | 537.7422 | 1073.4932 | 537.2502 | 10 |
| 7 | 707.2842 | 354.1458 | 690.2577 | 345.6325 | 689.2737 | 345.1405 | N | 1004.4717 | 502.7395 | 987.4452 | 494.2262 | 986.4612 | 493.7342 | 9 |
| 8 | 806.3526 | 403.6800 | 789.3261 | 395.1667 | 788.3421 | 394.6747 | V | **889.4448** | 445.2260 | 872.4182 | 436.7128 | 871.4342 | 436.2207 | 8 |
| 9 | 893.3847 | 447.1960 | 876.3581 | 438.6827 | 875.3741 | 438.1907 | S | **790.3764** | 395.6918 | 773.3498 | 387.1785 | 772.3658 | 386.6865 | 7 |
| 10 | **1006.4687** | 503.7380 | **989.4422** | 495.2247 | **988.4582** | 494.7327 | L | **703.3443** | 352.1758 | 686.3178 | 343.6625 | 685.3338 | 343.1705 | 6 |
| 11 | 1103.5215 | 552.2644 | 1086.4950 | 543.7511 | 1085.5109 | 543.2591 | P | **590.2603** | 295.6338 | 573.2337 | 287.1205 | 572.2497 | 286.6285 | 5 |
| 12 | **1218.5484** | 609.7779 | 1201.5219 | 601.2646 | 1200.5379 | 600.7726 | D | 493.2075 | 247.1074 | 476.1810 | 238.5941 | 475.1969 | 238.1021 | 4 |
| 13 | **1378.5791** | 689.7932 | 1361.5525 | 681.2799 | **1360.5685** | 680.7879 | C | **378.1806** | 189.5939 | 361.1540 | 181.0806 | | | 3 |
| 14 | 1506.6741 | 753.8407 | 1489.6475 | 745.3274 | 1488.6635 | 744.8354 | K | 218.1499 | 109.5786 | 201.1234 | 101.0653 | | | 2 |
| 15 | | | | | | | A | 90.0550 | 45.5311 | | | | | 1 |

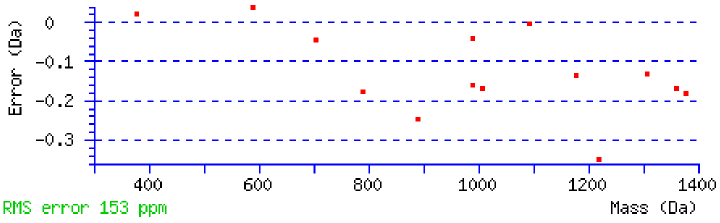


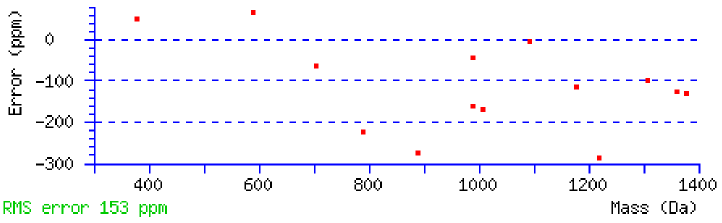


### All matches to this query

| Score | Mr(calc): | Delta | Sequence |
|---|---|---|---|
| 67.2 | 1594.7144 | 0.0041 | DSSKSSNVSLPDCKA |
| 19.2 | 1593.7304 | 0.9881 | DSSKSSNVSLPDCKA |
| 9.1 | 1594.7062 | 0.0124 | FKASRDSTLSEMK |
| 5.4 | 1592.7092 | 2.0094 | NTFREASMSVIMK |
| 4.9 | 1594.7157 | 0.0029 | QLRTKGELSFEK |
| 3.6 | 1594.7270 | -0.0084 | HTLTGHSGKVLSAK |
| | | | |

| | | | |
|---|---|---|---|
| 3.6 | 1594.7270 | -0.0084 | HTLTGHSGKVLSAK |
| 2.2 | 1593.7222 | 0.9964 | IHCSVKAENSVAAK |
| 1.3 | 1592.7130 | 2.0055 | NHRTSQSPXIMLR |
| 1.0 | 1594.7303 | -0.0118 | RAAMSVTLTSVKR |

---

Spectrum No: 409; Query: 2694; Rank: 1

---

## Peptide View

MS/MS Fragmentation of **LTFFNSTLNTSGLSTQGEALPIPGAHRPGVVTK**
Found in **IPI00196725**, Tax_Id=10116 Gene_Symbol=Fthfd 10-formyltetrahydrofolate dehydrogenase

Match to Query 2694: 3410.788932 from(1137.936920,3+)
Title: 091008RatKidney_NoSalt_03.3669.3669.3.dta
Data file K:\NewmanPaper\Piliang\3SubProteomes\Piliang3SP\mgf5ppm\ERLIC_3SubProteomes5ppm.mgf

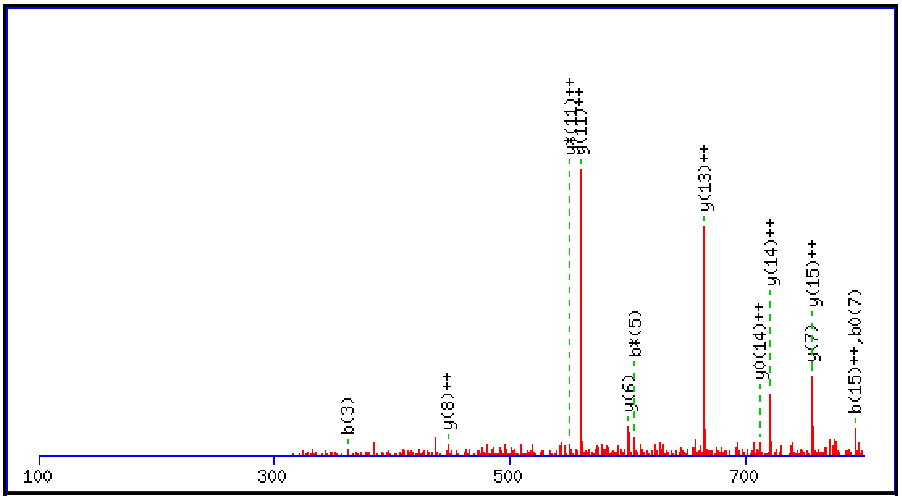


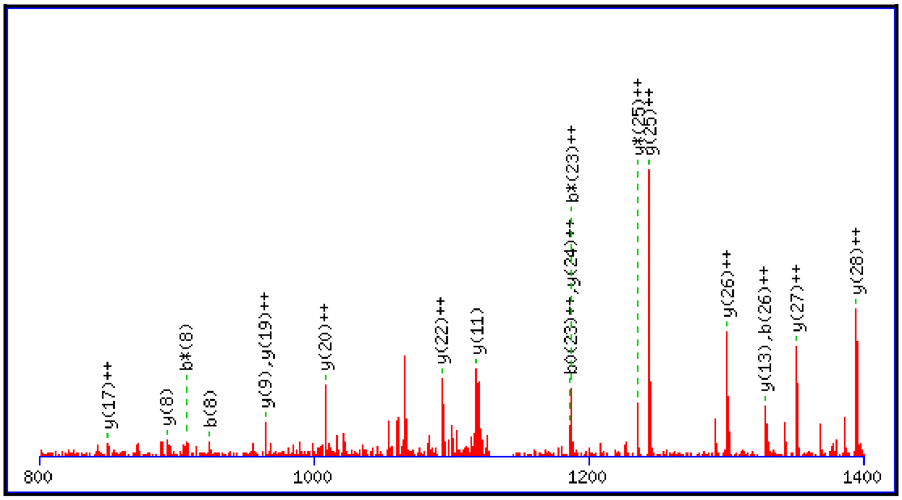

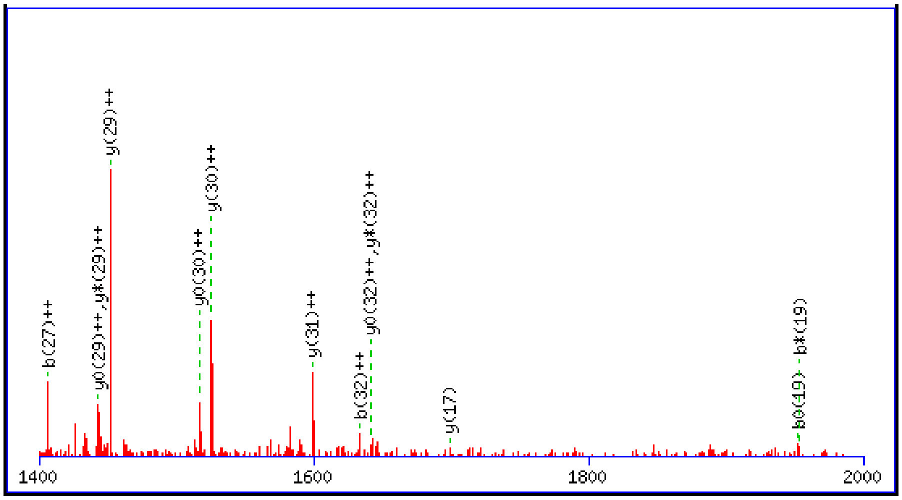


**Monoisotopic mass of neutral peptide Mr(calc):** 3410.7834
**Fixed modifications:** Carbamidomethyl (C)
**Variable modifications:**
**N9** : Deamidated_N (N)
**Ions Score:** 67 **Expect:** 1.6e-005
**Matches (Bold Red):** 45/372 fragment ions using 92 most intense peaks

| # | b | $b^{++}$ | b* | $b^{*++}$ | $b^0$ | $b^{0++}$ | Seq. | y | $y^{++}$ | y* | $y^{*++}$ | $y^0$ | $y^{0++}$ | # |
|---|---|---|---|---|---|---|---|---|---|---|---|---|---|---|
| 1 | 114.0913 | 57.5493 | | | | | L | | | | | | | 33 |
| 2 | 215.1390 | 108.0731 | | | 197.1285 | 99.0679 | T | 3298.7066 | 1649.8570 | 3281.6801 | **1641.3437** | 3280.6961 | **1640.8517** | 32 |
| 3 | **362.2074** | 181.6074 | | | 344.1969 | 172.6021 | F | 3197.6589 | **1599.3331** | 3180.6324 | 1590.8198 | 3179.6484 | 1590.3278 | 31 |
| 4 | 509.2758 | 255.1416 | | | 491.2653 | 246.1363 | F | 3050.5905 | **1525.7989** | 3033.5640 | 1517.2856 | 3032.5800 | **1516.7936** | 30 |
| 5 | 623.3188 | 312.1630 | **606.2922** | 303.6498 | 605.3082 | 303.1577 | N | 2903.5221 | **1452.2647** | 2886.4956 | **1443.7514** | 2885.5116 | **1443.2594** | 29 |
| 6 | 710.3508 | 355.6790 | 693.3243 | 347.1658 | 692.3402 | 346.6738 | S | 2789.4792 | **1395.2432** | 2772.4526 | 1386.7300 | 2771.4686 | 1386.2380 | 28 |
| 7 | 811.3985 | 406.2029 | 794.3719 | 397.6896 | **793.3879** | 397.1976 | T | 2702.4472 | **1351.7272** | 2685.4206 | 1343.2139 | 2684.4366 | 1342.7219 | 27 |
| 8 | **924.4825** | 462.7449 | **907.4560** | 454.2316 | 906.4720 | 453.7396 | L | 2601.3995 | **1301.2034** | 2584.3729 | 1292.6901 | 2583.3889 | 1292.1981 | 26 |
| 9 | 1039.5095 | 520.2584 | 1022.4829 | 511.7451 | 1021.4989 | 511.2531 | N | 2488.3154 | **1244.6613** | 2471.2889 | **1236.1481** | 2470.3049 | 1235.6561 | 25 |
| 10 | 1140.5572 | 570.7822 | 1123.5306 | 562.2689 | 1122.5466 | 561.7769 | T | 2373.2885 | **1187.1479** | 2356.2619 | 1178.6346 | 2355.2779 | 1178.1426 | 24 |
| 11 | 1227.5892 | 614.2982 | 1210.5626 | 605.7850 | 1209.5786 | 605.2929 | S | 2272.2408 | 1136.6240 | 2255.2143 | 1128.1108 | 2254.2302 | 1127.6188 | 23 |
| 12 | 1284.6107 | 642.8090 | 1267.5841 | 634.2957 | 1266.6001 | 633.8037 | G | 2185.2088 | **1093.1080** | 2168.1822 | 1084.5948 | 2167.1982 | 1084.1027 | 22 |
| 13 | 1397.6947 | 699.3510 | 1380.6682 | 690.8377 | 1379.6842 | 690.3457 | L | 2128.1873 | 1064.5973 | 2111.1608 | 1056.0840 | 2110.1768 | 1055.5920 | 21 |
| 14 | 1484.7267 | 742.8670 | 1467.7002 | 734.3537 | 1466.7162 | 733.8617 | S | 2015.1033 | **1008.0553** | 1998.0767 | 999.5420 | 1997.0927 | 999.0500 | 20 |
| 15 | 1585.7744 | **793.3908** | 1568.7479 | 784.8776 | 1567.7639 | 784.3856 | T | 1928.0712 | **964.5392** | 1911.0447 | 956.0260 | 1910.0607 | 955.5340 | 19 |
| 16 | 1713.8330 | 857.4201 | 1696.8065 | 848.9069 | 1695.8224 | 848.4149 | Q | 1827.0235 | 914.0154 | 1809.9970 | 905.5021 | 1809.0130 | 905.0101 | 18 |
| 17 | 1770.8545 | 885.9309 | 1753.8279 | 877.4176 | 1752.8439 | 876.9256 | G | **1698.9650** | **849.9861** | 1681.9384 | 841.4728 | 1680.9544 | 840.9808 | 17 |
| 18 | 1899.8971 | 950.4522 | 1882.8705 | 941.9389 | 1881.8865 | 941.4469 | E | 1641.9435 | 821.4754 | 1624.9170 | 812.9621 | 1623.9329 | 812.4701 | 16 |
| 19 | 1970.9342 | 985.9707 | **1953.9076** | 977.4574 | **1952.9236** | 976.9654 | A | 1512.9009 | **756.9541** | 1495.8744 | 748.4408 | 1494.8903 | 747.9488 | 15 |
| 20 | 2084.0182 | 1042.5128 | 2066.9917 | 1033.9995 | 2066.0077 | 1033.5075 | L | 1441.8638 | **721.4355** | 1424.8372 | 712.9223 | 1423.8532 | **712.4303** | 14 |
| 21 | 2181.0710 | 1091.0391 | 2164.0445 | 1082.5259 | 2163.0604 | 1082.0339 | P | **1328.7797** | **664.8935** | 1311.7532 | 656.3802 | 1310.7692 | 655.8882 | 13 |
| 22 | 2294.1551 | 1147.5812 | 2277.1285 | 1139.0679 | 2276.1445 | 1138.5759 | I | 1231.7270 | 616.3671 | 1214.7004 | 607.8538 | 1213.7164 | 607.3618 | 12 |
| 23 | 2391.2078 | 1196.1076 | 2374.1813 | **1187.5943** | 2373.1973 | **1187.1023** | P | **1118.6429** | **559.8251** | 1101.6164 | **551.3118** | 1100.6323 | 550.8198 | 11 |
| 24 | 2448.2293 | 1224.6183 | 2431.2027 | 1216.1050 | 2430.2187 | 1215.6130 | G | 1021.5901 | 511.2987 | 1004.5636 | 502.7854 | 1003.5796 | 502.2934 | 10 |
| 25 | 2519.2664 | 1260.1368 | 2502.2399 | 1251.6236 | 2501.2558 | 1251.1316 | A | **964.5687** | 482.7880 | 947.5421 | 474.2747 | 946.5581 | 473.7827 | 9 |
| 26 | 2656.3253 | **1328.6663** | 2639.2988 | 1320.1530 | 2638.3148 | 1319.6610 | H | **893.5316** | **447.2694** | 876.5050 | 438.7561 | 875.5210 | 438.2641 | 8 |
| 27 | 2812.4264 | **1406.7169** | 2795.3999 | 1398.2036 | 2794.4159 | 1397.7116 | R | **756.4726** | 378.7400 | 739.4461 | 370.2267 | 738.4621 | 369.7347 | 7 |
| 28 | 2909.4792 | 1455.2432 | 2892.4526 | 1446.7300 | 2891.4686 | 1446.2380 | P | **600.3715** | 300.6894 | 583.3450 | 292.1761 | 582.3610 | 291.6841 | 6 |
| 29 | 2966.5007 | 1483.7540 | 2949.4741 | 1475.2407 | 2948.4901 | 1474.7487 | G | 503.3188 | 252.1630 | 486.2922 | 243.6498 | 485.3082 | 243.1577 | 5 |
| 30 | 3065.5691 | 1533.2882 | 3048.5425 | 1524.7749 | 3047.5585 | 1524.2829 | V | 446.2973 | 223.6523 | 429.2708 | 215.1390 | 428.2867 | 214.6470 | 4 |
| 31 | 3164.6375 | 1582.8224 | 3147.6109 | 1574.3091 | 3146.6269 | 1573.8171 | V | 347.2289 | 174.1181 | 330.2023 | 165.6048 | 329.2183 | 165.1128 | 3 |
| 32 | 3265.6852 | **1633.3462** | 3248.6586 | 1624.8329 | 3247.6746 | 1624.3409 | T | 248.1605 | 124.5839 | 231.1339 | 116.0706 | 230.1499 | 115.5786 | 2 |

| | | | | | | | | | | | | | | |
|---|---|---|---|---|---|---|---|---|---|---|---|---|---|---|
| 33 | | | | | | | K | 147.1128 | 74.0600 | 130.0863 | 65.5468 | | | 1 |

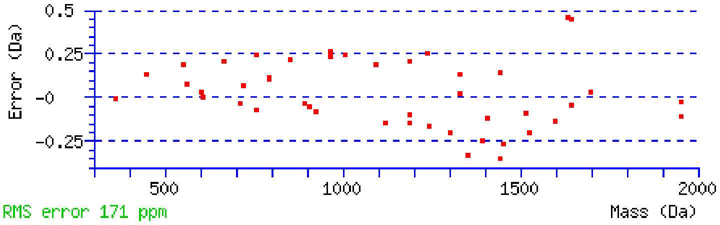


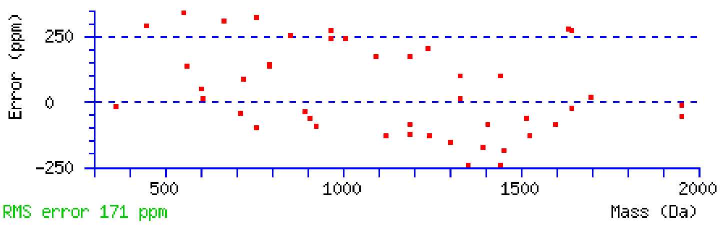


**All matches to this query**

| Score | Mr(calc): | Delta | Sequence |
|---|---|---|---|
| 67.2 | 3410.7834 | 0.0055 | LTFFNSTLNTSGLSTQGEALPIPGAHRPGVVTK |
| 64.8 | 3410.7834 | 0.0055 | LTFFNSTLNTSGLSTQGEALPIPGAHRPGVVTK |
| 64.8 | 3409.7994 | 0.9895 | LTFFNSTLNTSGLSTQGEALPIPGAHRPGVVTK |
| 4.9 | 3408.7500 | 2.0390 | HGLSLLWIWMAELGDGRESNQKLQEEIIK |

---

Spectrum No: 410; Query: 2700; Rank: 1

---

## Peptide View

MS/MS Fragmentation of **EHNFNIYNDSVSDSLNVPYDYTSVMHYSK**
Found in **IPI00204808**, Tax_Id=10116 Gene_Symbol=Mep1b Meprin A subunit beta precursor

Match to Query 2700: 3438.512502 from(1147.178110,3+)
Title: 100101RatKid_NS_deglyco_13.4762.4762.3.dta
Data file K:\NewmanPaper\Piliang\3SubProteomes\Piliang3SP\mgf5ppm\ERLIC_3SubProteomes5ppm.mgf

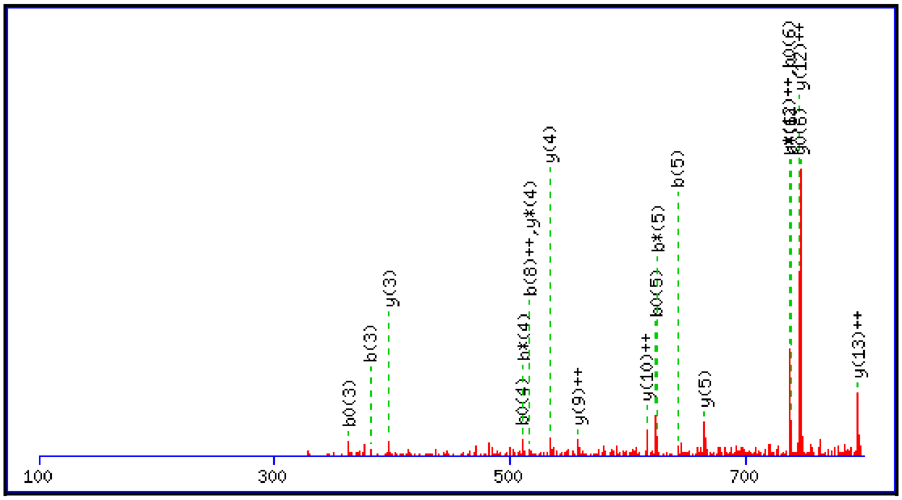

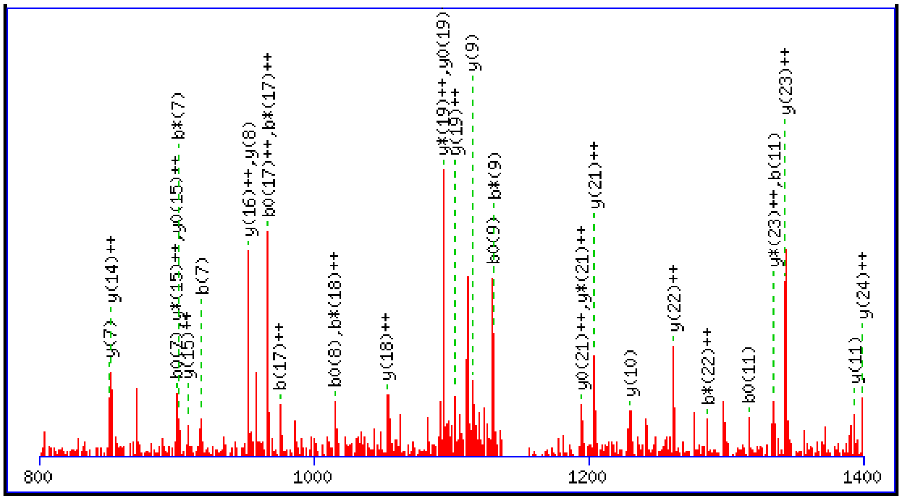


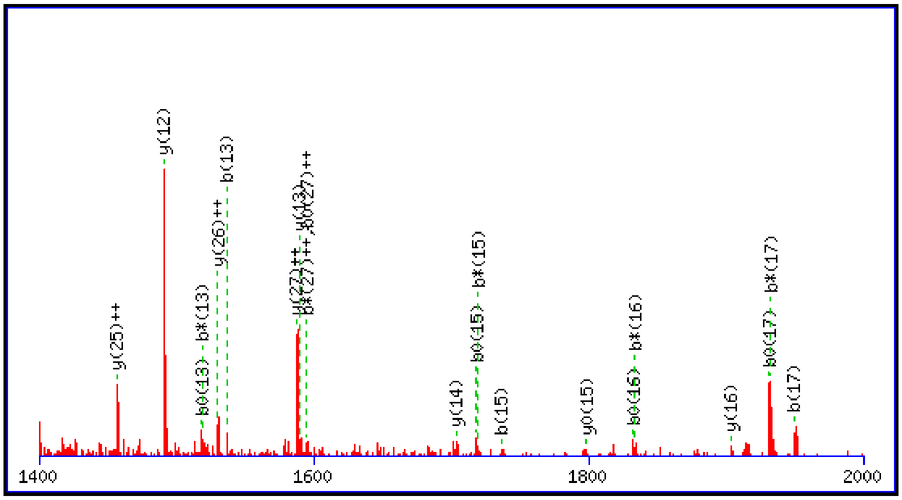


**Monoisotopic mass of neutral peptide Mr(calc):** 3438.4986
**Fixed modifications:** Carbamidomethyl (C)
**Variable modifications:**
**N8** : Deamidated_N (N)
**Ions Score:** 67 **Expect:** 9.1e-005
**Matches (Bold Red):** 75/330 fragment ions using 139 most intense peaks

| # | b | $b^{++}$ | b* | $b^{*++}$ | $b^0$ | $b^{0++}$ | Seq. | y | $y^{++}$ | y* | $y^{*++}$ | $y^0$ | $y^{0++}$ | # |
|---|---|---|---|---|---|---|---|---|---|---|---|---|---|---|
| 1 | 130.0499 | 65.5286 | | | 112.0393 | 56.5233 | E | | | | | | | 29 |
| 2 | 267.1088 | 134.0580 | | | 249.0982 | 125.0527 | H | 3310.4633 | 1655.7353 | 3293.4368 | 1647.2220 | 3292.4528 | 1646.7300 | 28 |
| 3 | **381.1517** | 191.0795 | 364.1252 | 182.5662 | **363.1411** | 182.0742 | N | 3173.4044 | **1587.2059** | 3156.3779 | 1578.6926 | 3155.3939 | 1578.2006 | 27 |
| 4 | 528.2201 | 264.6137 | **511.1936** | 256.1004 | **510.2096** | 255.6084 | F | 3059.3615 | **1530.1844** | 3042.3350 | 1521.6711 | 3041.3509 | 1521.1791 | 26 |
| 5 | **642.2630** | 321.6352 | **625.2365** | 313.1219 | **624.2525** | 312.6299 | N | 2912.2931 | **1456.6502** | 2895.2665 | 1448.1369 | 2894.2825 | 1447.6449 | 25 |
| 6 | 755.3471 | 378.1772 | **738.3206** | 369.6639 | **737.3365** | 369.1719 | I | 2798.2502 | **1399.6287** | 2781.2236 | 1391.1154 | 2780.2396 | 1390.6234 | 24 |
| 7 | **918.4104** | 459.7089 | **901.3839** | 451.1956 | **900.3999** | 450.7036 | Y | 2685.1661 | **1343.0867** | 2668.1395 | **1334.5734** | 2667.1555 | 1334.0814 | 23 |
| 8 | 1033.4374 | **517.2223** | 1016.4108 | 508.7091 | **1015.4268** | 508.2170 | N | 2522.1028 | **1261.5550** | 2505.0762 | 1253.0417 | 2504.0922 | 1252.5497 | 22 |
| 9 | 1148.4643 | 574.7358 | **1131.4378** | 566.2225 | **1130.4538** | 565.7305 | D | 2407.0758 | **1204.0416** | 2390.0493 | **1195.5283** | 2389.0653 | **1195.0363** | 21 |
| 10 | 1235.4963 | 618.2518 | 1218.4698 | 609.7385 | 1217.4858 | 609.2465 | S | 2292.0489 | 1146.5281 | 2275.0223 | 1138.0148 | 2274.0383 | 1137.5228 | 20 |
| 11 | **1334.5648** | 667.7860 | 1317.5382 | 659.2727 | **1316.5542** | 658.7807 | V | 2205.0169 | **1103.0121** | 2187.9903 | **1094.4988** | 2187.0063 | **1094.0068** | 19 |
| 12 | 1421.5968 | 711.3020 | 1404.5702 | 702.7888 | 1403.5862 | 702.2968 | S | 2105.9484 | **1053.4779** | 2088.9219 | 1044.9646 | 2087.9379 | 1044.4726 | 18 |
| 13 | **1536.6237** | 768.8155 | **1519.5972** | 760.3022 | **1518.6132** | 759.8102 | D | 2018.9164 | 1009.9618 | 2001.8899 | 1001.4486 | 2000.9059 | 1000.9566 | 17 |
| 14 | 1623.6558 | 812.3315 | 1606.6292 | 803.8182 | 1605.6452 | 803.3262 | S | **1903.8895** | **952.4484** | 1886.8629 | 943.9351 | 1885.8789 | 943.4431 | 16 |
| 15 | **1736.7398** | 868.8736 | **1719.7133** | 860.3603 | **1718.7293** | 859.8683 | L | 1816.8574 | **908.9324** | 1799.8309 | **900.4191** | **1798.8469** | **899.9271** | 15 |
| 16 | 1850.7828 | 925.8950 | **1833.7562** | 917.3817 | **1832.7722** | 916.8897 | N | **1703.7734** | **852.3903** | 1686.7468 | 843.8771 | 1685.7628 | 843.3850 | 14 |
| 17 | **1949.8512** | **975.4292** | **1932.8246** | **966.9159** | **1931.8406** | **966.4239** | V | **1589.7305** | **795.3689** | 1572.7039 | 786.8556 | 1571.7199 | 786.3636 | 13 |
| 18 | 2046.9039 | 1023.9556 | 2029.8774 | **1015.4423** | 2028.8934 | 1014.9503 | P | **1490.6620** | **745.8347** | 1473.6355 | **737.3214** | 1472.6515 | 736.8294 | 12 |

| | | | | | | | | | | | | |
|---|---|---|---|---|---|---|---|---|---|---|---|---|
| **19** | 2209.9673 | 1105.4873 | 2192.9407 | 1096.9740 | 2191.9567 | 1096.4820 | **Y** | **1393.6093** | 697.3083 | 1376.5827 | 688.7950 | 1375.5987 | 688.3030 | **11** |
| **20** | 2324.9942 | 1163.0007 | 2307.9677 | 1154.4875 | 2306.9836 | 1153.9955 | **D** | **1230.5460** | **615.7766** | 1213.5194 | 607.2633 | 1212.5354 | 606.7713 | **10** |
| **21** | 2488.0575 | 1244.5324 | 2471.0310 | 1236.0191 | 2470.0470 | 1235.5271 | **Y** | **1115.5190** | **558.2631** | 1098.4925 | 549.7499 | 1097.5084 | 549.2579 | **9** |
| **22** | 2589.1052 | 1295.0562 | 2572.0787 | **1286.5430** | 2571.0946 | 1286.0510 | **T** | **952.4557** | 476.7315 | 935.4291 | 468.2182 | 934.4451 | 467.7262 | **8** |
| **23** | 2676.1372 | 1338.5723 | 2659.1107 | 1330.0590 | 2658.1267 | 1329.5670 | **S** | **851.4080** | 426.2076 | 834.3815 | 417.6944 | 833.3974 | 417.2024 | **7** |
| **24** | 2775.2057 | 1388.1065 | 2758.1791 | 1379.5932 | 2757.1951 | 1379.1012 | **V** | 764.3760 | 382.6916 | 747.3494 | 374.1783 | **746.3654** | 373.6863 | **6** |
| **25** | 2906.2461 | 1453.6267 | 2889.2196 | 1445.1134 | 2888.2356 | 1444.6214 | **M** | **665.3076** | 333.1574 | 648.2810 | 324.6441 | 647.2970 | 324.1521 | **5** |
| **26** | 3043.3050 | 1522.1562 | 3026.2785 | 1513.6429 | 3025.2945 | 1513.1509 | **H** | **534.2671** | 267.6372 | **517.2405** | 259.1239 | 516.2565 | 258.6319 | **4** |
| **27** | 3206.3684 | 1603.6878 | 3189.3418 | **1595.1746** | 3188.3578 | **1594.6825** | **Y** | **397.2082** | 199.1077 | 380.1816 | 190.5944 | 379.1976 | 190.1024 | **3** |
| **28** | 3293.4004 | 1647.2038 | 3276.3739 | 1638.6906 | 3275.3898 | 1638.1986 | **S** | 234.1448 | 117.5761 | 217.1183 | 109.0628 | 216.1343 | 108.5708 | **2** |
| **29** | | | | | | | **K** | 147.1128 | 74.0600 | 130.0863 | 65.5468 | | | **1** |

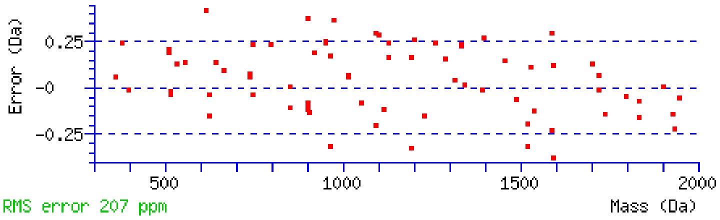


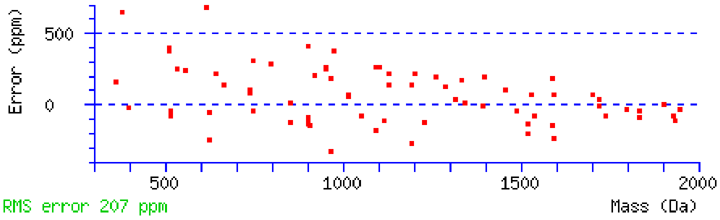


**All matches to this query**

| Score | Mr(calc): | Delta | Sequence |
|---|---|---|---|
| 67.2 | 3438.4986 | 0.0139 | EHNFNIYNDSVSDSLNVPYDYTSVMHYSK |
| 62.6 | 3438.4986 | 0.0139 | EHNFNIYNDSVSDSLNVPYDYTSVMHYSK |
| 47.4 | 3438.4986 | 0.0139 | EHNFNIYNDSVSDSLNVPYDYTSVMHYSK |
| 35.4 | 3438.4986 | 0.0139 | EHNFNIYNDSVSDSLNVPYDYTSVMHYSK |
| 16.3 | 3437.5146 | 0.9979 | EHNFNIYNDSVSDSLNVPYDYTSVMHYSK |
| 12.6 | 3438.5029 | 0.0096 | NPSVSLDLLNLYMVNQISSKKEISETMK |
| 12.6 | 3438.5029 | 0.0096 | NPSVSLDLLNLYMVNQISSKKEISETMK |
| 11.8 | 3437.5189 | 0.9936 | NPSVSLDLLNLYMVNQISSKKEISETMK |
| 7.2 | 3436.5120 | 2.0005 | YLTVAYACVPKNILTAVDPAVANLNPSLK |
| 5.5 | 3437.5189 | 0.9936 | NPSVSLDLLNLYMVNQISSKKEISETMK |

---

Spectrum No: 411; Query: 1839; Rank: 1

---

## Peptide View

MS/MS Fragmentation of **YSNESQDLSVNGYNCTMR**
Found in **IPI00191789**, Tax_Id=10116 Gene_Symbol=Hrg Histidine-rich glycoprotein

Match to Query 1839: 2154.855368 from(1078.434960,2+)
Title: 100101RatKid_NS_deglyco_27.1669.1669.2.dta
Data file K:\NewmanPaper\Piliang\3SubProteomes\Piliang3SP\mgf5ppm\ERLIC_3SubProteomes5ppm.mgf

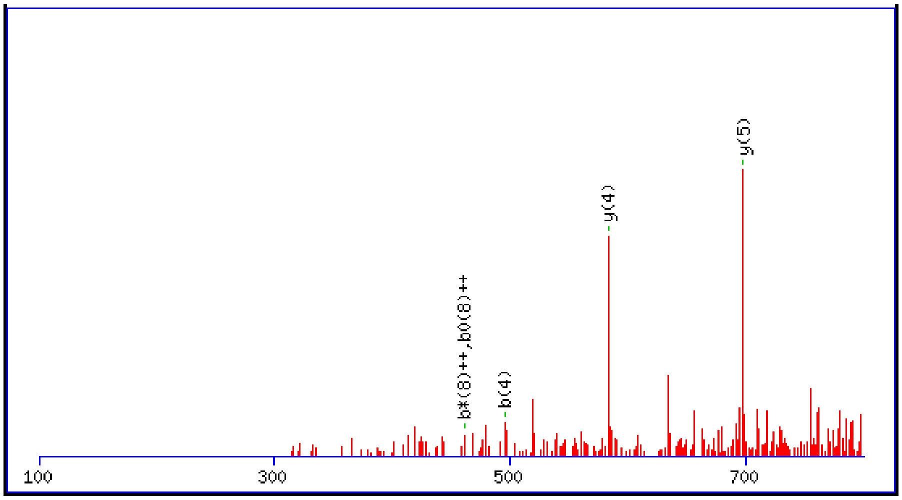


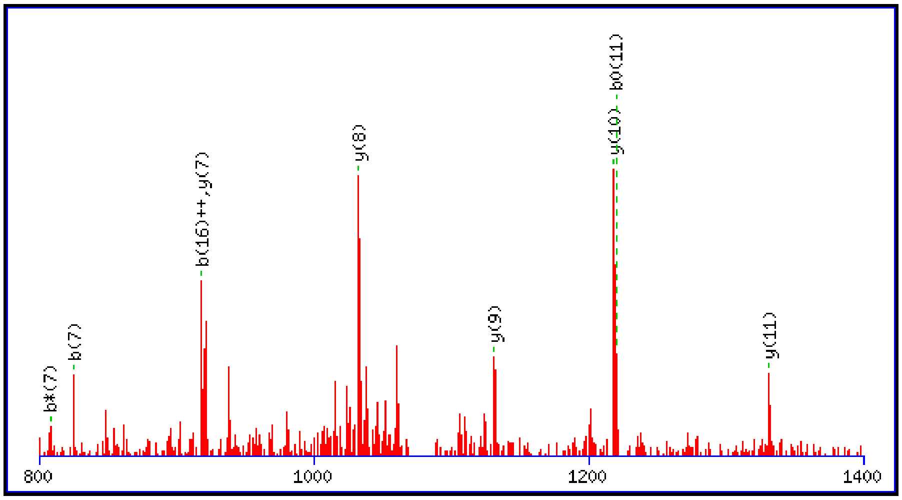


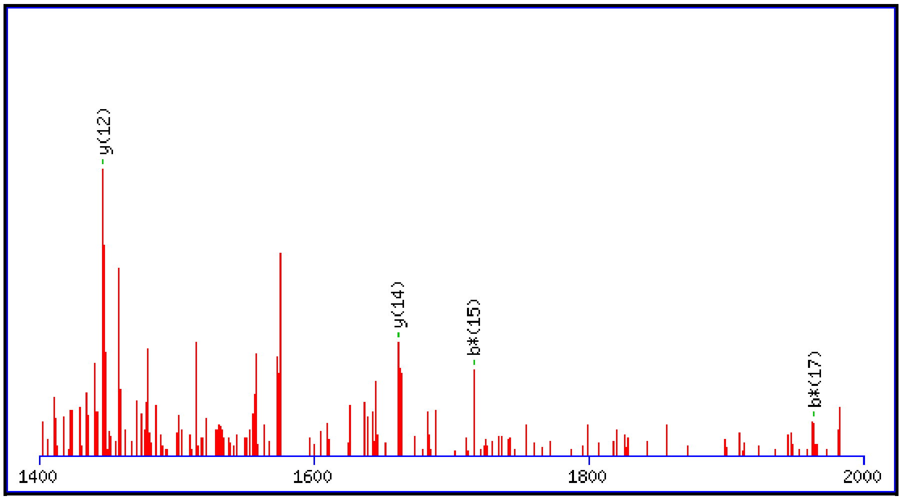


**Monoisotopic mass of neutral peptide Mr(calc):** 2154.8470
**Fixed modifications:** Carbamidomethyl (C)
**Variable modifications:**
**N3** : Deamidated_N (N)
**N14** : Deamidated_N (N)
**M17** : Oxidation (M)
**Ions Score:** 67 **Expect:** 4.4e-005
**Matches (Bold Red):** 18/194 fragment ions using 35 most intense peaks

| # | b | $b^{++}$ | b* | $b^{*++}$ | $b^0$ | $b^{0++}$ | Seq. | y | $y^{++}$ | y* | $y^{*++}$ | $y^0$ | $y^{0++}$ | # |
|---|---|---|---|---|---|---|---|---|---|---|---|---|---|---|
| 1 | 164.0706 | 82.5389 | | | | | Y | | | | | | | 18 |
| 2 | 251.1026 | 126.0550 | | | 233.0921 | 117.0497 | S | 1992.7910 | 996.8991 | 1975.7644 | 988.3858 | 1974.7804 | 987.8938 | 17 |
| 3 | 366.1296 | 183.5684 | 349.1030 | 175.0551 | 348.1190 | 174.5631 | N | 1905.7589 | 953.3831 | 1888.7324 | 944.8698 | 1887.7484 | 944.3778 | 16 |
| 4 | **495.1722** | 248.0897 | 478.1456 | 239.5764 | 477.1616 | 239.0844 | E | 1790.7320 | 895.8696 | 1773.7054 | 887.3564 | 1772.7214 | 886.8643 | 15 |

| | | | | | | | | | | | | | | |
|---|---|---|---|---|---|---|---|---|---|---|---|---|---|---|
| 5 | 582.2042 | 291.6057 | 565.1776 | 283.0925 | 564.1936 | 282.6005 | S | 1661.6894 | 831.3483 | 1644.6628 | 822.8351 | 1643.6788 | 822.3431 | 14 |
| 6 | 710.2628 | 355.6350 | 693.2362 | 347.1217 | 692.2522 | 346.6297 | Q | 1574.6574 | 787.8323 | 1557.6308 | 779.3190 | 1556.6468 | 778.8270 | 13 |
| 7 | 825.2897 | 413.1485 | 808.2632 | 404.6352 | 807.2791 | 404.1432 | D | 1446.5988 | 723.8030 | 1429.5722 | 715.2898 | 1428.5882 | 714.7978 | 12 |
| 8 | 938.3738 | 469.6905 | 921.3472 | 461.1773 | 920.3632 | 460.6852 | L | 1331.5718 | 666.2896 | 1314.5453 | 657.7763 | 1313.5613 | 657.2843 | 11 |
| 9 | 1025.4058 | 513.2065 | 1008.3793 | 504.6933 | 1007.3952 | 504.2013 | S | 1218.4878 | 609.7475 | 1201.4612 | 601.2343 | 1200.4772 | 600.7422 | 10 |
| 10 | 1124.4742 | 562.7407 | 1107.4477 | 554.2275 | 1106.4637 | 553.7355 | V | 1131.4558 | 566.2315 | 1114.4292 | 557.7182 | 1113.4452 | 557.2262 | 9 |
| 11 | 1238.5171 | 619.7622 | 1221.4906 | 611.2489 | 1220.5066 | 610.7569 | N | 1032.3873 | 516.6973 | 1015.3608 | 508.1840 | 1014.3768 | 507.6920 | 8 |
| 12 | 1295.5386 | 648.2729 | 1278.5121 | 639.7597 | 1277.5280 | 639.2677 | G | 918.3444 | 459.6758 | 901.3179 | 451.1626 | 900.3338 | 450.6706 | 7 |
| 13 | 1458.6019 | 729.8046 | 1441.5754 | 721.2913 | 1440.5914 | 720.7993 | Y | 861.3229 | 431.1651 | 844.2964 | 422.6518 | 843.3124 | 422.1598 | 6 |
| 14 | 1573.6289 | 787.3181 | 1556.6023 | 778.8048 | 1555.6183 | 778.3128 | N | 698.2596 | 349.6334 | 681.2331 | 341.1202 | 680.2491 | 340.6282 | 5 |
| 15 | 1733.6595 | 867.3334 | 1716.6330 | 858.8201 | 1715.6490 | 858.3281 | C | 583.2327 | 292.1200 | 566.2061 | 283.6067 | 565.2221 | 283.1147 | 4 |
| 16 | 1834.7072 | 917.8572 | 1817.6807 | 909.3440 | 1816.6966 | 908.8520 | T | 423.2020 | 212.1047 | 406.1755 | 203.5914 | 405.1915 | 203.0994 | 3 |
| 17 | 1981.7426 | 991.3749 | 1964.7161 | 982.8617 | 1963.7320 | 982.3697 | M | 322.1544 | 161.5808 | 305.1278 | 153.0675 | | | 2 |
| 18 | | | | | | | R | 175.1190 | 88.0631 | 158.0924 | 79.5498 | | | 1 |

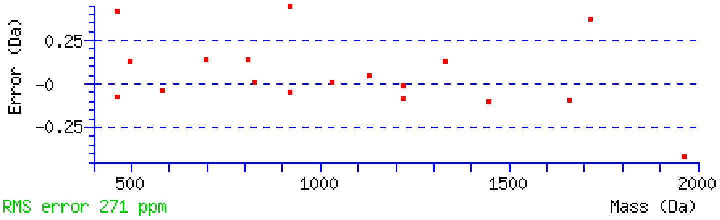


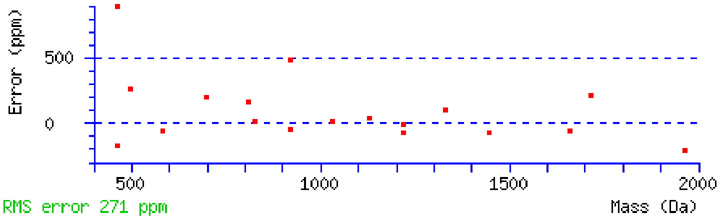


**All matches to this query**

| Score | Mr(calc): | Delta | Sequence |
|---|---|---|---|
| 67.2 | 2154.8470 | 0.0084 | YSNESQDLSVNGYNCTMR |
| 53.1 | 2153.8630 | 0.9924 | YSNESQDLSVNGYNCTMR |
| 40.3 | 2154.8470 | 0.0084 | YSNESQDLSVNGYNCTMR |
| 39.0 | 2154.8470 | 0.0084 | YSNESQDLSVNGYNCTMR |
| 28.7 | 2153.8630 | 0.9924 | YSNESQDLSVNGYNCTMR |
| 3.9 | 2153.8630 | 0.9924 | YSNESQDLSVNGYNCTMR |
| 0.9 | 2153.8493 | 1.0061 | SADEFNCLSCPGSMWSYK |

---

Spectrum No: 412; Query: 1818; Rank: 1

---

## Peptide View

MS/MS Fragmentation of **IVDNGYGYASNGSVYFDTAK**
Found in **IPI00371408**, Tax_Id=10116 Gene_Symbol=Cars_predicted 95 kDa protein

Match to Query 1818: 2140.969808 from(1071.492180,2+)
Title: 100101RatKid_NS_deglyco_13.3388.3388.2.dta
Data file K:\NewmanPaper\Piliang\3SubProteomes\Piliang3SP\mgf5ppm\ERLIC_3SubProteomes5ppm.mgf

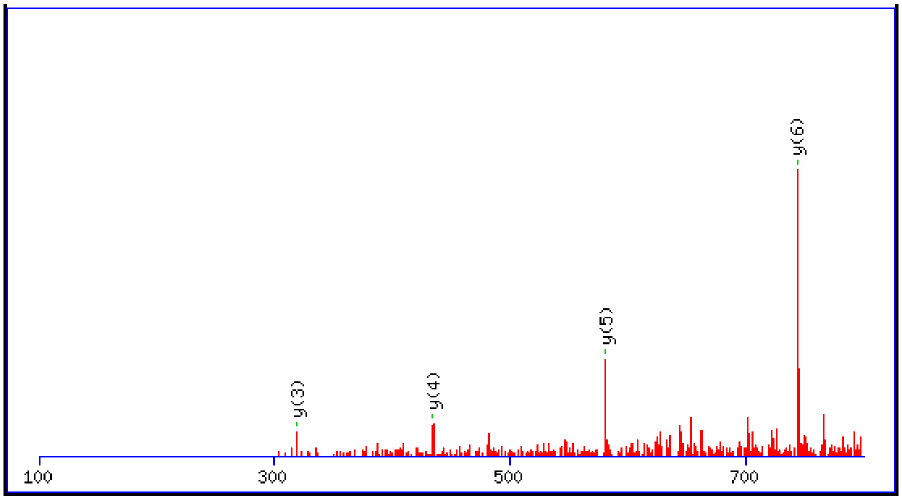


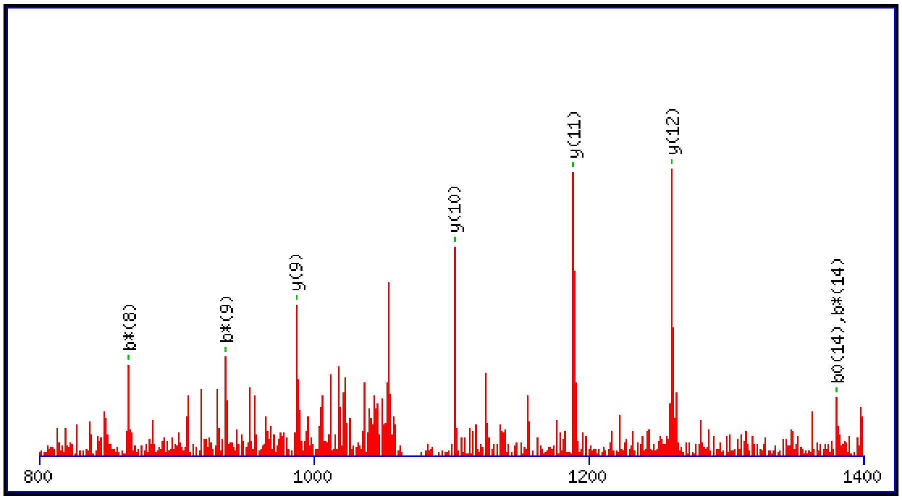


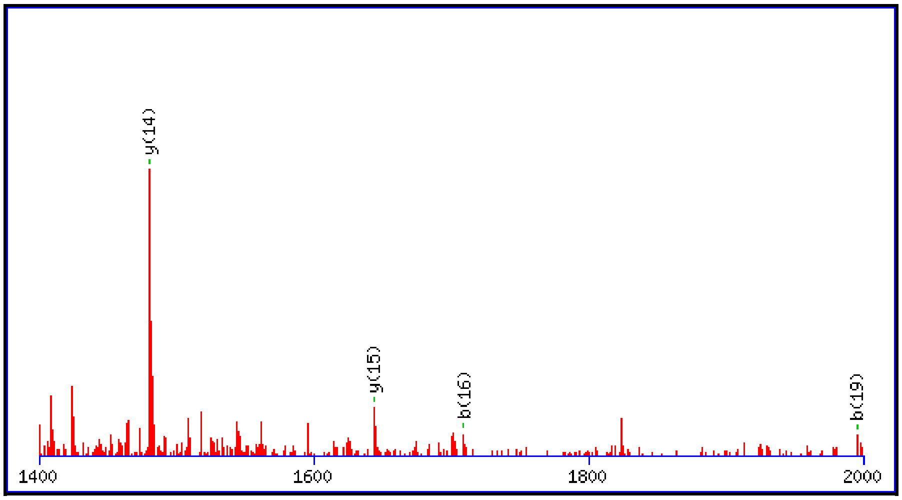


**Monoisotopic mass of neutral peptide Mr(calc):** 2140.9589
**Fixed modifications:** Carbamidomethyl (C)
**Variable modifications:**
**N11** : Deamidated_N (N)
**Ions Score:** 67 **Expect:** 5.1e-005
**Matches (Bold Red):** 16/214 fragment ions using 27 most intense peaks

| # | b | $b^{++}$ | b* | $b^{*++}$ | $b^0$ | $b^{0++}$ | Seq. | y | $y^{++}$ | y* | $y^{*++}$ | $y^0$ | $y^{0++}$ | # |
|---|---|---|---|---|---|---|---|---|---|---|---|---|---|---|
| 1 | 114.0913 | 57.5493 | | | | | I | | | | | | | 20 |
| 2 | 213.1598 | 107.0835 | | | | | V | 2028.8821 | 1014.9447 | 2011.8556 | 1006.4314 | 2010.8716 | 1005.9394 | 19 |
| 3 | 328.1867 | 164.5970 | | | 310.1761 | 155.5917 | D | 1929.8137 | 965.4105 | 1912.7872 | 956.8972 | 1911.8032 | 956.4052 | 18 |
| 4 | 442.2296 | 221.6185 | 425.2031 | 213.1052 | 424.2191 | 212.6132 | N | 1814.7868 | 907.8970 | 1797.7602 | 899.3838 | 1796.7762 | 898.8917 | 17 |
| 5 | 499.2511 | 250.1292 | 482.2245 | 241.6159 | 481.2405 | 241.1239 | G | 1700.7439 | 850.8756 | 1683.7173 | 842.3623 | 1682.7333 | 841.8703 | 16 |

| | | | | | | | | | | | | | | |
|---|---|---|---|---|---|---|---|---|---|---|---|---|---|---|
| 6 | 662.3144 | 331.6608 | 645.2879 | 323.1476 | 644.3039 | 322.6556 | Y | **1643.7224** | 822.3648 | 1626.6958 | 813.8516 | 1625.7118 | 813.3596 | 15 |
| 7 | 719.3359 | 360.1716 | 702.3093 | 351.6583 | 701.3253 | 351.1663 | G | **1480.6591** | 740.8332 | 1463.6325 | 732.3199 | 1462.6485 | 731.8279 | 14 |
| 8 | 882.3992 | 441.7032 | **865.3727** | 433.1900 | 864.3886 | 432.6980 | Y | 1423.6376 | 712.3224 | 1406.6110 | 703.8092 | 1405.6270 | 703.3172 | 13 |
| 9 | 953.4363 | 477.2218 | **936.4098** | 468.7085 | 935.4258 | 468.2165 | A | **1260.5743** | 630.7908 | 1243.5477 | 622.2775 | 1242.5637 | 621.7855 | 12 |
| 10 | 1040.4684 | 520.7378 | 1023.4418 | 512.2245 | 1022.4578 | 511.7325 | S | **1189.5372** | 595.2722 | 1172.5106 | 586.7589 | 1171.5266 | 586.2669 | 11 |
| 11 | 1155.4953 | 578.2513 | 1138.4687 | 569.7380 | 1137.4847 | 569.2460 | N | **1102.5051** | 551.7562 | 1085.4786 | 543.2429 | 1084.4946 | 542.7509 | 10 |
| 12 | 1212.5168 | 606.7620 | 1195.4902 | 598.2487 | 1194.5062 | 597.7567 | G | **987.4782** | 494.2427 | 970.4516 | 485.7295 | 969.4676 | 485.2375 | 9 |
| 13 | 1299.5488 | 650.2780 | 1282.5222 | 641.7648 | 1281.5382 | 641.2727 | S | 930.4567 | 465.7320 | 913.4302 | 457.2187 | 912.4462 | 456.7267 | 8 |
| 14 | 1398.6172 | 699.8122 | **1381.5906** | 691.2990 | **1380.6066** | 690.8070 | V | 843.4247 | 422.2160 | 826.3981 | 413.7027 | 825.4141 | 413.2107 | 7 |
| 15 | 1561.6805 | 781.3439 | 1544.6540 | 772.8306 | 1543.6700 | 772.3386 | Y | **744.3563** | 372.6818 | 727.3297 | 364.1685 | 726.3457 | 363.6765 | 6 |
| 16 | **1708.7489** | 854.8781 | 1691.7224 | 846.3648 | 1690.7384 | 845.8728 | F | **581.2930** | 291.1501 | 564.2664 | 282.6368 | 563.2824 | 282.1448 | 5 |
| 17 | 1823.7759 | 912.3916 | 1806.7493 | 903.8783 | 1805.7653 | 903.3863 | D | **434.2245** | 217.6159 | 417.1980 | 209.1026 | 416.2140 | 208.6106 | 4 |
| 18 | 1924.8236 | 962.9154 | 1907.7970 | 954.4021 | 1906.8130 | 953.9101 | T | **319.1976** | 160.1024 | 302.1710 | 151.5892 | 301.1870 | 151.0972 | 3 |
| 19 | **1995.8607** | 998.4340 | 1978.8341 | 989.9207 | 1977.8501 | 989.4287 | A | 218.1499 | 109.5786 | 201.1234 | 101.0653 | | | 2 |
| 20 | | | | | | | K | 147.1128 | 74.0600 | 130.0863 | 65.5468 | | | 1 |

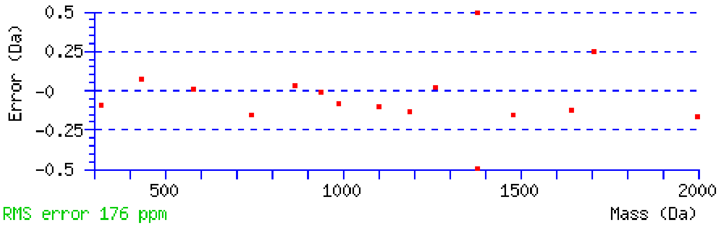


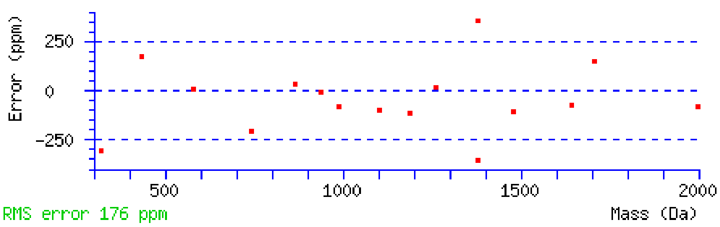


### All matches to this query

| Score | Mr(calc): | Delta | Sequence |
|---|---|---|---|
| 67.2 | 2140.9589 | 0.0109 | IVDNGYGYASNGSVYFDTAK |
| 10.5 | 2140.9589 | 0.0109 | IVDNGYGYASNGSVYFDTAK |
| 2.9 | 2139.9749 | 0.9949 | IVDNGYGYASNGSVYFDTAK |
| 0.6 | 2140.9868 | -0.0170 | SEMKMEIDDLASNVETVSK |
| 0.6 | 2140.9868 | -0.0170 | SEMKMEIDDLASNVETVSK |

---

Spectrum No: 413; Query: 2200; Rank: 1

---

## Peptide View

MS/MS Fragmentation of **EAGSLEPVTVMGTEYVFYNDTK**
Found in **IPI00370450**, Tax_Id=10116 Gene_Symbol=Plxnb2 plexin B2

Match to Query 2200: 2450.124568 from(1226.069560,2+)
Title: 100101RatKid_NS_deglyco_23.4748.4748.2.dta
Data file K:\NewmanPaper\Piliang\3SubProteomes\Piliang3SP\mgf5ppm\ERLIC_3SubProteomes5ppm.mgf

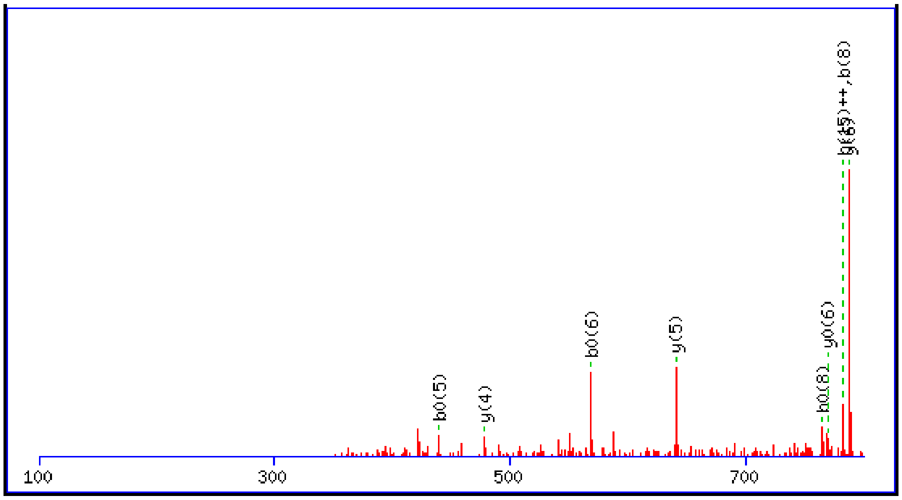


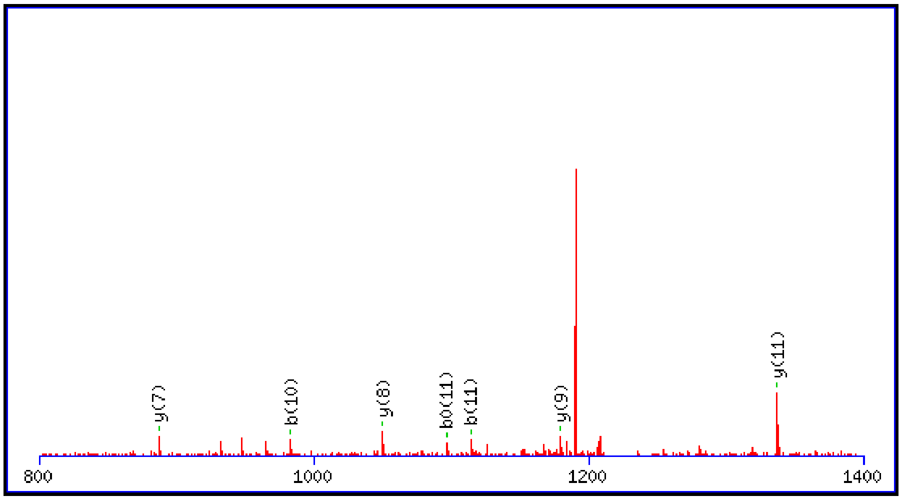


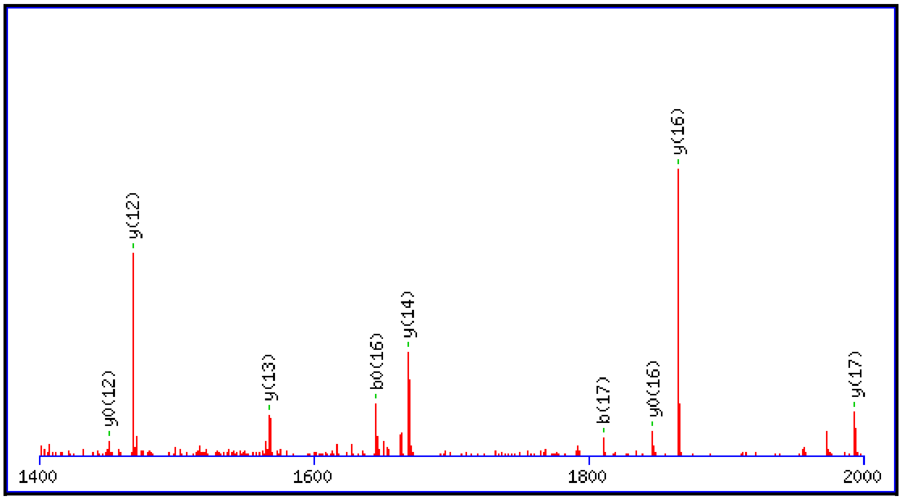


**Monoisotopic mass of neutral peptide Mr(calc):** 2450.1199
**Fixed modifications:** Carbamidomethyl (C)
**Variable modifications:**
**N19** : Deamidated_N (N)
**Ions Score:** 67 **Expect:** 6.6e-005
**Matches (Bold Red):** 25/214 fragment ions using 42 most intense peaks

| # | b | b++ | b* | b*++ | b0 | b0++ | Seq. | y | y++ | y* | y*++ | y0 | y0++ | # |
|---|---|---|---|---|---|---|---|---|---|---|---|---|---|---|
| 1 | 130.0499 | 65.5286 | | | 112.0393 | 56.5233 | E | | | | | | | 22 |
| 2 | 201.0870 | 101.0471 | | | 183.0764 | 92.0418 | A | 2322.0846 | 1161.5459 | 2305.0581 | 1153.0327 | 2304.0740 | 1152.5407 | 21 |
| 3 | 258.1084 | 129.5579 | | | 240.0979 | 120.5526 | G | 2251.0475 | 1126.0274 | 2234.0209 | 1117.5141 | 2233.0369 | 1117.0221 | 20 |
| 4 | 345.1405 | 173.0739 | | | 327.1299 | 164.0686 | S | 2194.0260 | 1097.5167 | 2176.9995 | 1089.0034 | 2176.0155 | 1088.5114 | 19 |
| 5 | 458.2245 | 229.6159 | | | **440.2140** | 220.6106 | L | 2106.9940 | 1054.0006 | 2089.9674 | 1045.4874 | 2088.9834 | 1044.9954 | 18 |

| | | | | | | | | | | | | | | |
|---|---|---|---|---|---|---|---|---|---|---|---|---|---|---|
| **6** | 587.2671 | 294.1372 | | | **569.2566** | 285.1319 | **E** | **1993.9099** | 997.4586 | 1976.8834 | 988.9453 | 1975.8994 | 988.4533 | **17** |
| **7** | 684.3199 | 342.6636 | | | 666.3093 | 333.6583 | **P** | **1864.8673** | 932.9373 | 1847.8408 | 924.4240 | **1846.8568** | 923.9320 | **16** |
| **8** | **783.3883** | 392.1978 | | | **765.3777** | 383.1925 | **V** | 1767.8146 | 884.4109 | 1750.7880 | 875.8977 | 1749.8040 | 875.4056 | **15** |
| **9** | 884.4360 | 442.7216 | | | 866.4254 | 433.7164 | **T** | **1668.7462** | 834.8767 | 1651.7196 | 826.3634 | 1650.7356 | 825.8714 | **14** |
| **10** | **983.5044** | 492.2558 | | | 965.4938 | 483.2506 | **V** | **1567.6985** | 784.3529 | 1550.6719 | 775.8396 | 1549.6879 | 775.3476 | **13** |
| **11** | **1114.5449** | 557.7761 | | | **1096.5343** | 548.7708 | **M** | **1468.6301** | 734.8187 | 1451.6035 | 726.3054 | **1450.6195** | 725.8134 | **12** |
| **12** | 1171.5664 | 586.2868 | | | 1153.5558 | 577.2815 | **G** | **1337.5896** | 669.2984 | 1320.5630 | 660.7852 | 1319.5790 | 660.2931 | **11** |
| **13** | 1272.6140 | 636.8107 | | | 1254.6035 | 627.8054 | **T** | 1280.5681 | 640.7877 | 1263.5416 | 632.2744 | 1262.5576 | 631.7824 | **10** |
| **14** | 1401.6566 | 701.3320 | | | 1383.6461 | 692.3267 | **E** | **1179.5204** | 590.2639 | 1162.4939 | 581.7506 | 1161.5099 | 581.2586 | **9** |
| **15** | 1564.7200 | **782.8636** | | | 1546.7094 | 773.8583 | **Y** | **1050.4778** | 525.7426 | 1033.4513 | 517.2293 | 1032.4673 | 516.7373 | **8** |
| **16** | 1663.7884 | 832.3978 | | | **1645.7778** | 823.3925 | **V** | **887.4145** | 444.2109 | 870.3880 | 435.6976 | 869.4040 | 435.2056 | **7** |
| **17** | **1810.8568** | 905.9320 | | | 1792.8462 | 896.9267 | **F** | **788.3461** | 394.6767 | 771.3196 | 386.1634 | **770.3355** | 385.6714 | **6** |
| **18** | 1973.9201 | 987.4637 | | | 1955.9095 | 978.4584 | **Y** | **641.2777** | 321.1425 | 624.2511 | 312.6292 | 623.2671 | 312.1372 | **5** |
| **19** | 2088.9470 | 1044.9772 | 2071.9205 | 1036.4639 | 2070.9365 | 1035.9719 | **N** | **478.2144** | 239.6108 | 461.1878 | 231.0975 | 460.2038 | 230.6055 | **4** |
| **20** | 2203.9740 | 1102.4906 | 2186.9474 | 1093.9774 | 2185.9634 | 1093.4853 | **D** | 363.1874 | 182.0974 | 346.1609 | 173.5841 | 345.1769 | 173.0921 | **3** |
| **21** | 2305.0217 | 1153.0145 | 2287.9951 | 1144.5012 | 2287.0111 | 1144.0092 | **T** | 248.1605 | 124.5839 | 231.1339 | 116.0706 | 230.1499 | 115.5786 | **2** |
| **22** | | | | | | | **K** | 147.1128 | 74.0600 | 130.0863 | 65.5468 | | | **1** |

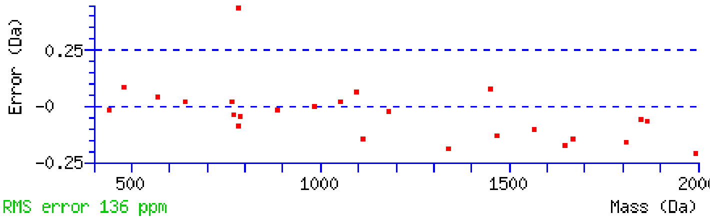


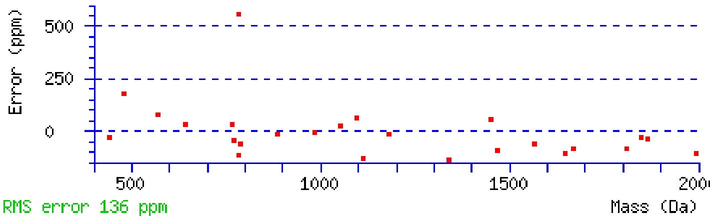


### All matches to this query

| Score | Mr(calc): | Delta | Sequence |
|---|---|---|---|
| 67.1 | 2450.1199 | 0.0046 | EAGSLEPVTVMGTEYVFYNDTK |
| 14.9 | 2449.1359 | 0.9887 | EAGSLEPVTVMGTEYVFYNDTK |
| 7.5 | 2449.1058 | 1.0187 | VWLLEMNSNPALHTNCEVLK |
| 1.6 | 2449.1058 | 1.0187 | VWLLEMNSNPALHTNCEVLK |
| 1.2 | 2449.1058 | 1.0187 | VWLLEMNSNPALHTNCEVLK |
| 1.2 | 2449.1058 | 1.0187 | VWLLEMNSNPALHTNCEVLK |
| 0.8 | 2448.0969 | 2.0277 | QTMFELSKMFLLCLNYWK |
| 0.7 | 2449.1348 | 0.9897 | INNLFGNTRNLLTTDFVQMR |
| 0.7 | 2449.1348 | 0.9897 | INNLFGNTRNLLTTDFVQMR |
| 0.7 | 2450.1049 | 0.0196 | VWIMQNERGISHDDIAQQSK |

---

Spectrum No: 414; Query: 284; Rank: 1

---

## Peptide View

MS/MS Fragmentation of **TDDIINSSGYR**
Found in **IPI00198641**, Tax_Id=10116 Gene_Symbol=LOC246263 Acyl-coenzyme A synthetase ACSM2, mitochondrial precursor

Match to Query 284: 1240.553208 from(621.283880,2+)
Title: 091008RatKidney_NH4Format01_13.1514.1514.2.dta
Data file K:\NewmanPaper\Piliang\3SubProteomes\Piliang3SP\mgf5ppm\ERLIC_3SubProteomes5ppm.mgf

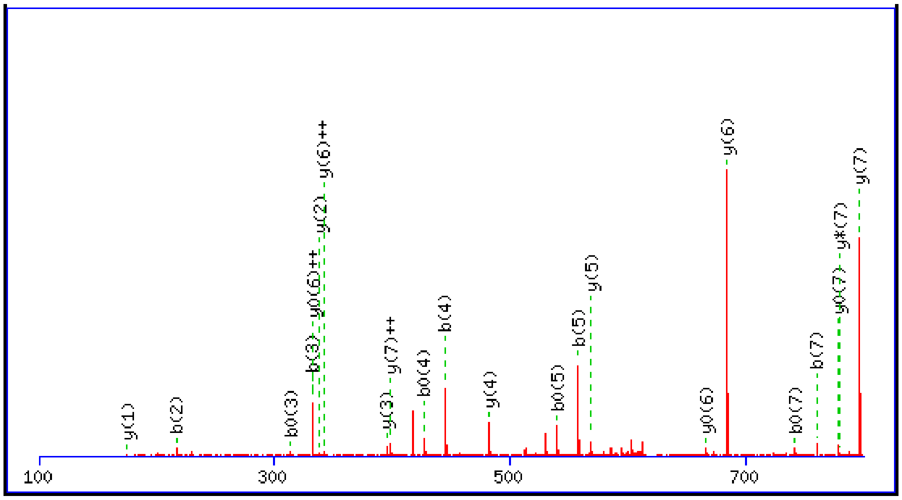


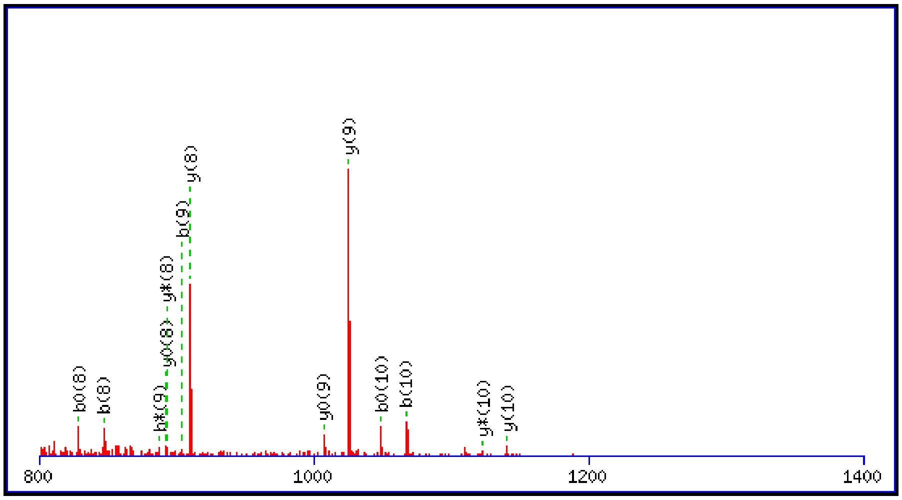


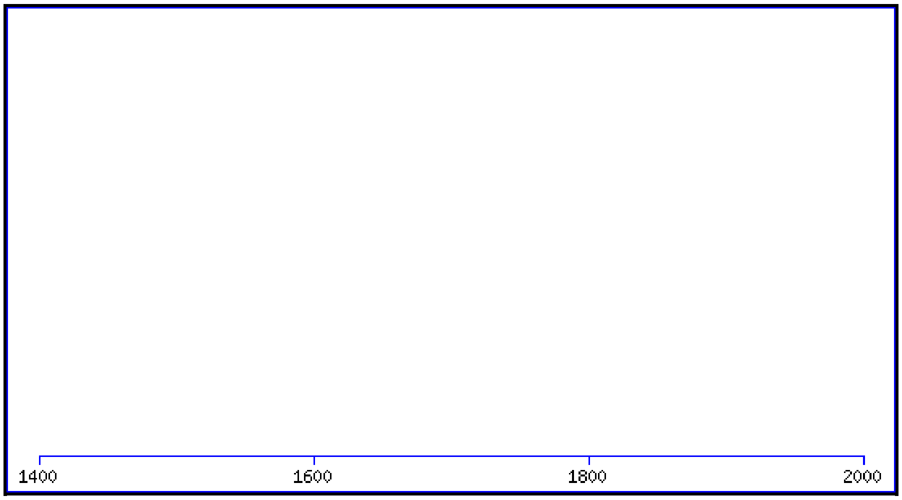


**Monoisotopic mass of neutral peptide Mr(calc):** 1240.5571
**Fixed modifications:** Carbamidomethyl (C)
**Variable modifications:**
**N6** : Deamidated_N (N)
**Ions Score:** 67 **Expect:** 2.6e-005
**Matches (Bold Red):** 35/104 fragment ions using 71 most intense peaks

| # | b | $b^{++}$ | b* | $b^{*++}$ | $b^{0}$ | $b^{0++}$ | Seq. | y | $y^{++}$ | y* | $y^{*++}$ | $y^{0}$ | $y^{0++}$ | # |
|---|---|---|---|---|---|---|---|---|---|---|---|---|---|---|
| **1** | 102.0550 | 51.5311 | | | 84.0444 | 42.5258 | **T** | | | | | | | **11** |
| **2** | **217.0819** | 109.0446 | | | 199.0713 | 100.0393 | **D** | **1140.5168** | 570.7620 | **1123.4902** | 562.2487 | 1122.5062 | 561.7567 | **10** |
| **3** | **332.1088** | 166.5581 | | | **314.0983** | 157.5528 | **D** | **1025.4898** | 513.2485 | 1008.4633 | 504.7353 | **1007.4792** | 504.2433 | **9** |
| **4** | **445.1929** | 223.1001 | | | **427.1823** | 214.0948 | **I** | **910.4629** | 455.7351 | **893.4363** | 447.2218 | **892.4523** | 446.7298 | **8** |
| **5** | **558.2770** | 279.6421 | | | **540.2664** | 270.6368 | **I** | **797.3788** | **399.1930** | **780.3523** | 390.6798 | **779.3682** | 390.1878 | **7** |

| | | | | | | | | | | | | | | |
|---|---|---|---|---|---|---|---|---|---|---|---|---|---|---|
| **6** | 673.3039 | 337.1556 | 656.2774 | 328.6423 | 655.2933 | 328.1503 | **N** | **684.2947** | **342.6510** | 667.2682 | 334.1377 | **666.2842** | **333.6457** | **6** |
| **7** | **760.3359** | 380.6716 | 743.3094 | 372.1583 | **742.3254** | 371.6663 | **S** | **569.2678** | 285.1375 | 552.2413 | 276.6243 | 551.2572 | 276.1323 | **5** |
| **8** | **847.3680** | 424.1876 | 830.3414 | 415.6743 | **829.3574** | 415.1823 | **S** | **482.2358** | 241.6215 | 465.2092 | 233.1082 | 464.2252 | 232.6162 | **4** |
| **9** | **904.3894** | 452.6984 | **887.3629** | 444.1851 | 886.3789 | 443.6931 | **G** | **395.2037** | 198.1055 | 378.1772 | 189.5922 | | | **3** |
| **10** | **1067.4528** | 534.2300 | 1050.4262 | 525.7167 | **1049.4422** | 525.2247 | **Y** | **338.1823** | 169.5948 | 321.1557 | 161.0815 | | | **2** |
| **11** | | | | | | | **R** | **175.1190** | 88.0631 | 158.0924 | 79.5498 | | | **1** |

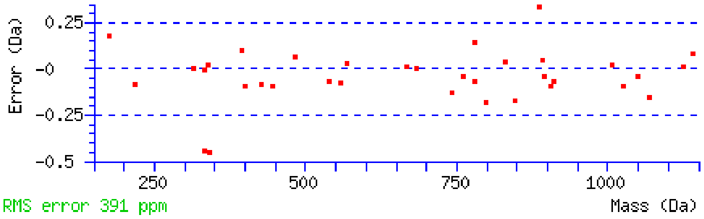


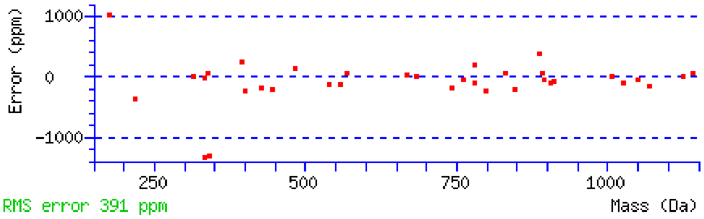


**All matches to this query**

| Score | Mr(calc): | Delta | Sequence |
|---|---|---|---|
| 67.0 | 1240.5571 | -0.0039 | TDDIINSSGYR |
| 14.7 | 1240.5506 | 0.0026 | TDNGGLGYRCK |
| 13.3 | 1240.5449 | 0.0083 | KGESSDLAAQR |
| 8.7 | 1240.5561 | -0.0029 | NKNSLGRSER |
| 8.4 | 1240.5449 | 0.0083 | DSKLNNTNVR |
| 8.4 | 1240.5449 | 0.0083 | DSKLNNTNVR |
| 8.3 | 1240.5561 | -0.0029 | NKNSLGRSER |
| 7.7 | 1240.5449 | 0.0083 | KGESSDLAAQR |
| 7.7 | 1238.5544 | 1.9988 | VDNVNSIIER |
| 6.0 | 1238.5544 | 1.9988 | VDNVNSIIER |

---

Spectrum No: 415; Query: 171; Rank: 1

---

## Peptide View

MS/MS Fragmentation of **VFNTSEDLSR**
Found in **IPI00363534**, Tax_Id=10116 Gene_Symbol=Lama1 laminin, alpha 1

Match to Query 171: 1167.541388 from(584.777970,2+)
Title: 100101RatKid_NS_deglyco_26.1465.1465.2.dta
Data file K:\NewmanPaper\Piliang\3SubProteomes\Piliang3SP\mgf5ppm\ERLIC_3SubProteomes5ppm.mgf

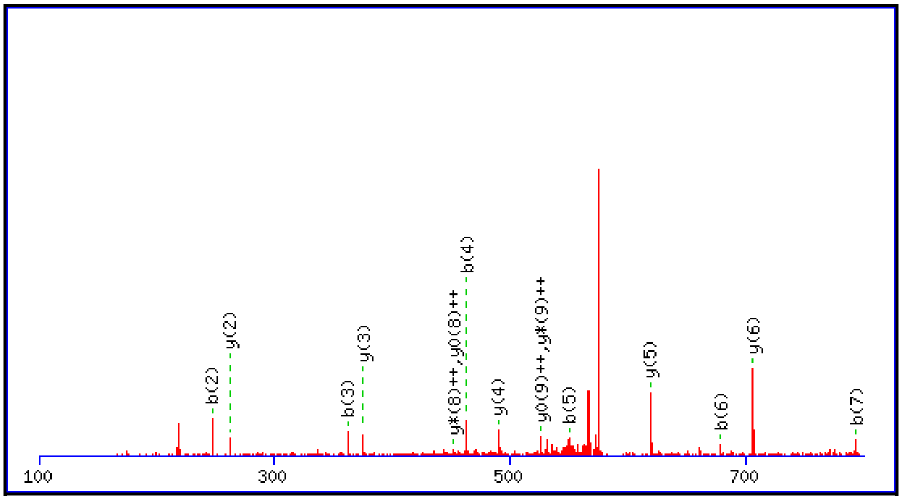

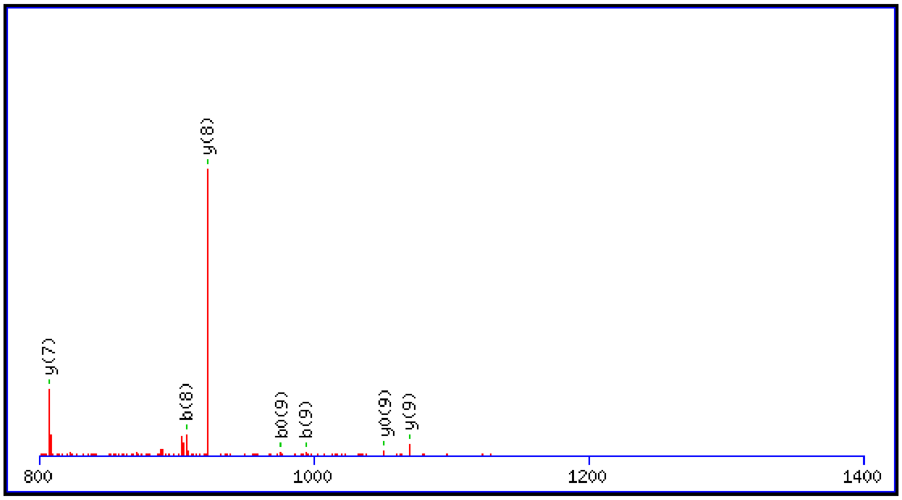


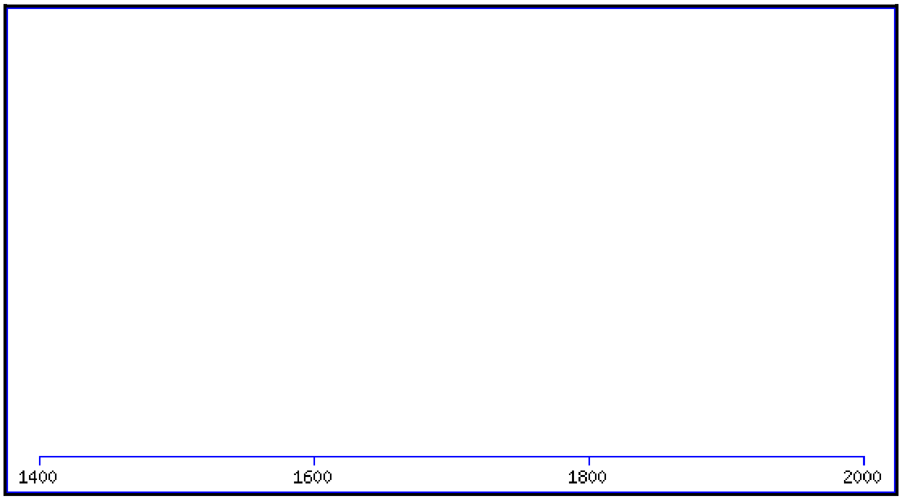


**Monoisotopic mass of neutral peptide Mr(calc):** 1167.5408
**Fixed modifications:** Carbamidomethyl (C)
**Variable modifications:**
**N3** : Deamidated_N (N)
**Ions Score:** 67 **Expect:** 2.5e-005
**Matches (Bold Red):** 22/96 fragment ions using 32 most intense peaks

| # | b | $b^{++}$ | b* | $b^{*++}$ | $b^0$ | $b^{0++}$ | Seq. | y | $y^{++}$ | y* | $y^{*++}$ | $y^0$ | $y^{0++}$ | # |
|---|---|---|---|---|---|---|---|---|---|---|---|---|---|---|
| 1 | 100.0757 | 50.5415 | | | | | V | | | | | | | 10 |
| 2 | **247.1441** | 124.0757 | | | | | F | **1069.4796** | 535.2435 | 1052.4531 | **526.7302** | **1051.4691** | **526.2382** | 9 |
| 3 | **362.1710** | 181.5892 | 345.1445 | 173.0759 | | | N | **922.4112** | 461.7092 | 905.3847 | **453.1960** | 904.4007 | **452.7040** | 8 |
| 4 | **463.2187** | 232.1130 | 446.1922 | 223.5997 | 445.2082 | 223.1077 | T | **807.3843** | 404.1958 | 790.3577 | 395.6825 | 789.3737 | 395.1905 | 7 |
| 5 | **550.2507** | 275.6290 | 533.2242 | 267.1157 | 532.2402 | 266.6237 | S | **706.3366** | 353.6719 | 689.3101 | 345.1587 | 688.3260 | 344.6667 | 6 |
| 6 | **679.2933** | 340.1503 | 662.2668 | 331.6370 | 661.2828 | 331.1450 | E | **619.3046** | 310.1559 | 602.2780 | 301.6427 | 601.2940 | 301.1506 | 5 |
| 7 | **794.3203** | 397.6638 | 777.2937 | 389.1505 | 776.3097 | 388.6585 | D | **490.2620** | 245.6346 | 473.2354 | 237.1214 | 472.2514 | 236.6293 | 4 |
| 8 | **907.4043** | 454.2058 | 890.3778 | 445.6925 | 889.3938 | 445.2005 | L | **375.2350** | 188.1212 | 358.2085 | 179.6079 | 357.2245 | 179.1159 | 3 |
| 9 | **994.4364** | 497.7218 | 977.4098 | 489.2086 | **976.4258** | 488.7165 | S | **262.1510** | 131.5791 | 245.1244 | 123.0659 | 244.1404 | 122.5738 | 2 |
| 10 | | | | | | | R | 175.1190 | 88.0631 | 158.0924 | 79.5498 | | | 1 |

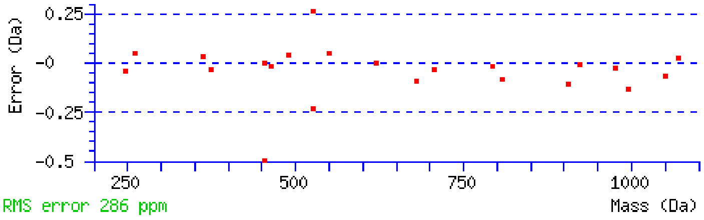


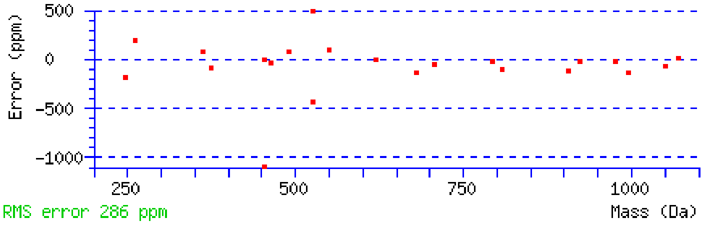

### All matches to this query

| Score | Mr(calc): | Delta | Sequence |
|---|---|---|---|
| 66.9 | 1167.5408 | 0.0006 | VFNTSEDLSR |
| 12.1 | 1167.5383 | 0.0031 | MVQFHFTNK |
| 7.8 | 1167.5450 | -0.0036 | MALLMAEMSR |
| 7.2 | 1165.5285 | 2.0129 | NEDMKSALNK |
| 6.3 | 1165.5285 | 2.0129 | NKMESDGEKK |
| 6.1 | 1165.5285 | 2.0129 | NSDLCIASGTK |
| 5.5 | 1166.5373 | 1.0041 | FELLPDPTR |
| 5.5 | 1167.5397 | 0.0017 | NLSRKDAER |
| 4.9 | 1167.5488 | -0.0075 | MAMSLQGSRR |
| 4.6 | 1167.5424 | -0.0010 | LSVLADEVDK |

---

Spectrum No: 416; Query: 2761; Rank: 1

---

## Peptide View

MS/MS Fragmentation of **RPFGEVYELEIDTLETTCHALDPTPLANCSVR**
Found in **IPI00327469**, Tax_Id=10116 Gene_Symbol=Ahsg Alpha-2-HS-glycoprotein precursor

Match to Query 2761: 3703.759376 from(926.947120,4+)
Title: 100101RatKid_NS_deglyco_20.5565.5565.4.dta
Data file K:\NewmanPaper\Piliang\3SubProteomes\Piliang3SP\mgf5ppm\ERLIC_3SubProteomes5ppm.mgf

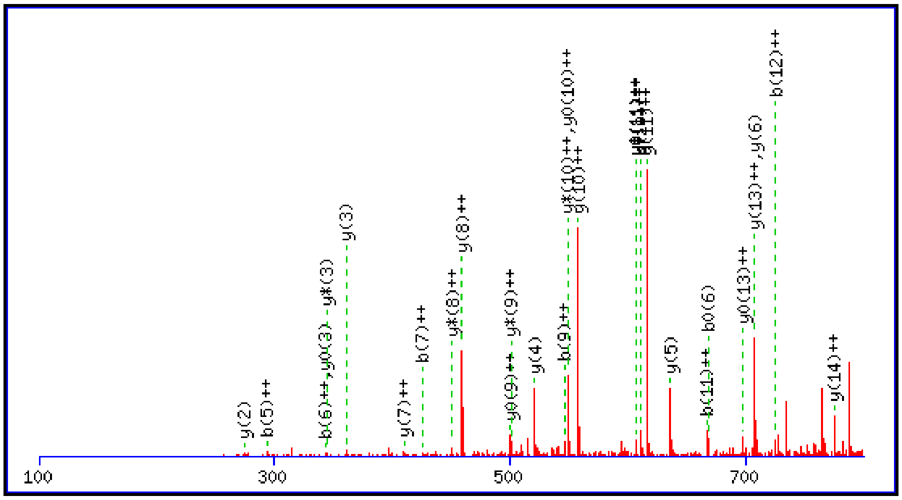


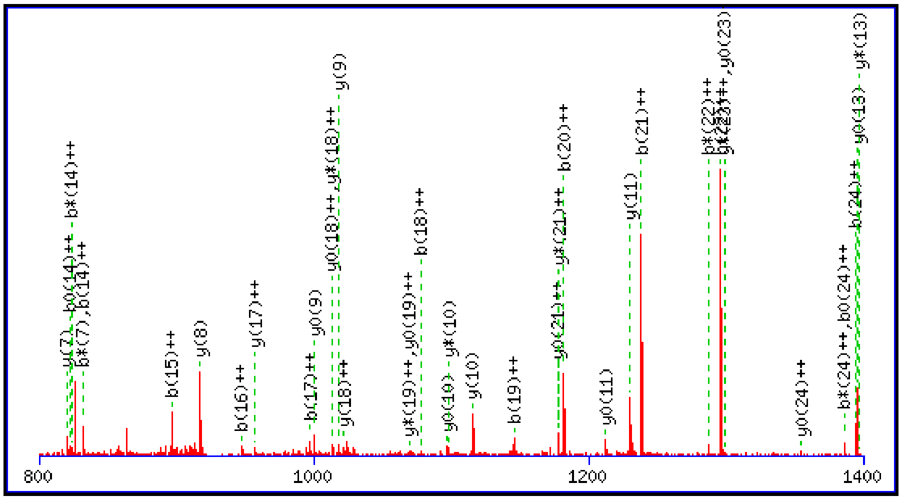

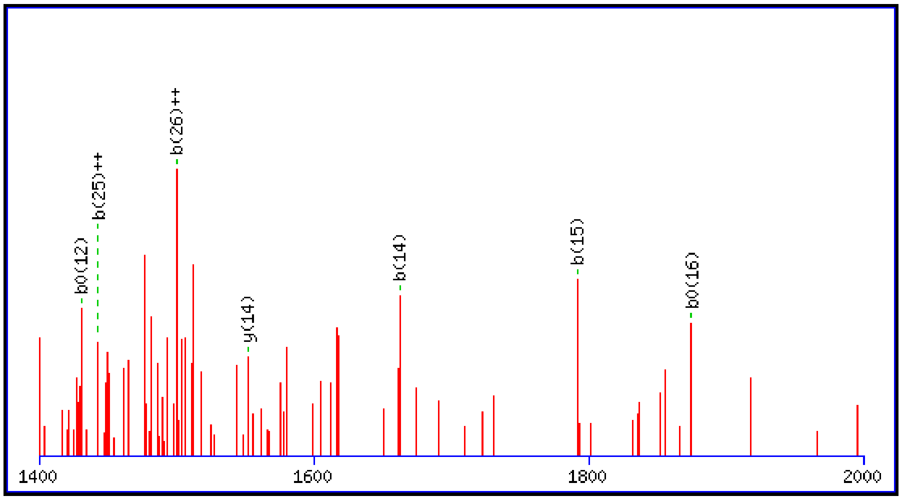


**Monoisotopic mass of neutral peptide Mr(calc):** 3703.7498
**Fixed modifications:** Carbamidomethyl (C)
**Variable modifications:**
**N28** : Deamidated_N (N)
**Ions Score:** 67 **Expect:** 8.1e-005
**Matches (Bold Red):** 74/360 fragment ions using 151 most intense peaks

| # | b | $b^{++}$ | b* | $b^{*++}$ | $b^0$ | $b^{0++}$ | Seq. | y | $y^{++}$ | y* | $y^{*++}$ | $y^0$ | $y^{0++}$ | # |
|---|---|---|---|---|---|---|---|---|---|---|---|---|---|---|
| 1 | 157.1084 | 79.0578 | 140.0818 | 70.5446 | | | R | | | | | | | 32 |
| 2 | 254.1612 | 127.5842 | 237.1346 | 119.0709 | | | P | 3548.6560 | 1774.8316 | 3531.6294 | 1766.3184 | 3530.6454 | 1765.8263 | 31 |
| 3 | 401.2296 | 201.1184 | 384.2030 | 192.6051 | | | F | 3451.6032 | 1726.3052 | 3434.5767 | 1717.7920 | 3433.5926 | 1717.3000 | 30 |
| 4 | 458.2510 | 229.6292 | 441.2245 | 221.1159 | | | G | 3304.5348 | 1652.7710 | 3287.5082 | 1644.2578 | 3286.5242 | 1643.7658 | 29 |
| 5 | 587.2936 | **294.1504** | 570.2671 | 285.6372 | 569.2831 | 285.1452 | E | 3247.5133 | 1624.2603 | 3230.4868 | 1615.7470 | 3229.5028 | 1615.2550 | 28 |
| 6 | 686.3620 | **343.6847** | 669.3355 | 335.1714 | **668.3515** | 334.6794 | V | 3118.4707 | 1559.7390 | 3101.4442 | 1551.2257 | 3100.4602 | 1550.7337 | 27 |
| 7 | 849.4254 | **425.2163** | **832.3988** | 416.7030 | 831.4148 | 416.2110 | Y | 3019.4023 | 1510.2048 | 3002.3758 | 1501.6915 | 3001.3918 | 1501.1995 | 26 |
| 8 | 978.4680 | 489.7376 | 961.4414 | 481.2243 | 960.4574 | 480.7323 | E | 2856.3390 | 1428.6731 | 2839.3124 | 1420.1599 | 2838.3284 | 1419.6679 | 25 |
| 9 | 1091.5520 | **546.2796** | 1074.5255 | 537.7664 | 1073.5415 | 537.2744 | L | 2727.2964 | 1364.1518 | 2710.2699 | 1355.6386 | 2709.2858 | **1355.1466** | 24 |
| 10 | 1220.5946 | **610.8009** | 1203.5681 | 602.2877 | 1202.5841 | 601.7957 | E | 2614.2123 | 1307.6098 | 2597.1858 | **1299.0965** | 2596.2018 | **1298.6045** | 23 |
| 11 | 1333.6787 | **667.3430** | 1316.6521 | 658.8297 | 1315.6681 | 658.3377 | I | 2485.1697 | 1243.0885 | 2468.1432 | 1234.5752 | 2467.1592 | 1234.0832 | 22 |
| 12 | 1448.7056 | **724.8564** | 1431.6791 | 716.3432 | **1430.6951** | 715.8512 | D | 2372.0857 | 1186.5465 | 2355.0591 | **1178.0332** | 2354.0751 | **1177.5412** | 21 |
| 13 | 1549.7533 | 775.3803 | 1532.7268 | 766.8670 | 1531.7427 | 766.3750 | T | 2257.0587 | 1129.0330 | 2240.0322 | 1120.5197 | 2239.0482 | 1120.0277 | 20 |
| 14 | **1662.8374** | **831.9223** | 1645.8108 | **823.4090** | 1644.8268 | **822.9170** | L | 2156.0111 | 1078.5092 | 2138.9845 | **1069.9959** | 2138.0005 | **1069.5039** | 19 |
| 15 | **1791.8800** | **896.4436** | 1774.8534 | 887.9303 | 1773.8694 | 887.4383 | E | 2042.9270 | **1021.9671** | 2025.9004 | **1013.4539** | 2024.9164 | **1012.9619** | 18 |
| 16 | 1892.9276 | **946.9675** | 1875.9011 | 938.4542 | **1874.9171** | 937.9622 | T | 1913.8844 | **957.4458** | 1896.8579 | 948.9326 | 1895.8738 | 948.4406 | 17 |
| 17 | 1993.9753 | **997.4913** | 1976.9488 | 988.9780 | 1975.9648 | 988.4860 | T | 1812.8367 | 906.9220 | 1795.8102 | 898.4087 | 1794.8262 | 897.9167 | 16 |
| 18 | 2154.0060 | **1077.5066** | 2136.9794 | 1068.9933 | 2135.9954 | 1068.5013 | C | 1711.7890 | 856.3982 | 1694.7625 | 847.8849 | 1693.7785 | 847.3929 | 15 |
| 19 | 2291.0649 | **1146.0361** | 2274.0383 | 1137.5228 | 2273.0543 | 1137.0308 | H | **1551.7584** | **776.3828** | 1534.7318 | 767.8696 | 1533.7478 | 767.3776 | 14 |
| 20 | 2362.1020 | **1181.5546** | 2345.0754 | 1173.0414 | 2344.0914 | 1172.5494 | A | 1414.6995 | **707.8534** | **1397.6729** | 699.3401 | **1396.6889** | **698.8481** | 13 |
| 21 | 2475.1861 | **1238.0967** | 2458.1595 | 1229.5834 | 2457.1755 | 1229.0914 | L | 1343.6624 | 672.3348 | 1326.6358 | 663.8215 | 1325.6518 | 663.3295 | 12 |
| 22 | 2590.2130 | **1295.6101** | 2573.1864 | **1287.0969** | 2572.2024 | 1286.6049 | D | **1230.5783** | **615.7928** | 1213.5518 | **607.2795** | **1212.5677** | **606.7875** | 11 |
| 23 | 2687.2658 | 1344.1365 | 2670.2392 | 1335.6232 | 2669.2552 | 1335.1312 | P | **1115.5514** | **558.2793** | **1098.5248** | **549.7660** | **1097.5408** | **549.2740** | 10 |
| 24 | 2788.3134 | **1394.6604** | 2771.2869 | **1386.1471** | 2770.3029 | **1385.6551** | T | **1018.4986** | 509.7529 | 1001.4721 | **501.2397** | **1000.4880** | **500.7477** | 9 |
| 25 | 2885.3662 | **1443.1867** | 2868.3397 | 1434.6735 | 2867.3556 | 1434.1815 | P | **917.4509** | **459.2291** | 900.4244 | **450.7158** | 899.4404 | 450.2238 | 8 |
| 26 | 2998.4503 | **1499.7288** | 2981.4237 | 1491.2155 | 2980.4397 | 1490.7235 | L | **820.3982** | **410.7027** | 803.3716 | 402.1894 | 802.3876 | 401.6974 | 7 |
| 27 | 3069.4874 | 1535.2473 | 3052.4608 | 1526.7341 | 3051.4768 | 1526.2420 | A | **707.3141** | 354.1607 | 690.2875 | 345.6474 | 689.3035 | 345.1554 | 6 |
| 28 | 3184.5143 | 1592.7608 | 3167.4878 | 1584.2475 | 3166.5038 | 1583.7555 | N | **636.2770** | 318.6421 | 619.2504 | 310.1289 | 618.2664 | 309.6368 | 5 |
| 29 | 3344.5450 | 1672.7761 | 3327.5184 | 1664.2628 | 3326.5344 | 1663.7708 | C | **521.2500** | 261.1287 | 504.2235 | 252.6154 | 503.2395 | 252.1234 | 4 |
| 30 | 3431.5770 | 1716.2921 | 3414.5504 | 1707.7789 | 3413.5664 | 1707.2869 | S | **361.2194** | 181.1133 | **344.1928** | 172.6001 | **343.2088** | 172.1081 | 3 |
| 31 | 3530.6454 | 1765.8263 | 3513.6189 | 1757.3131 | 3512.6348 | 1756.8211 | V | **274.1874** | 137.5973 | 257.1608 | 129.0840 | | | 2 |
| 32 | | | | | | | R | 175.1190 | 88.0631 | 158.0924 | 79.5498 | | | 1 |

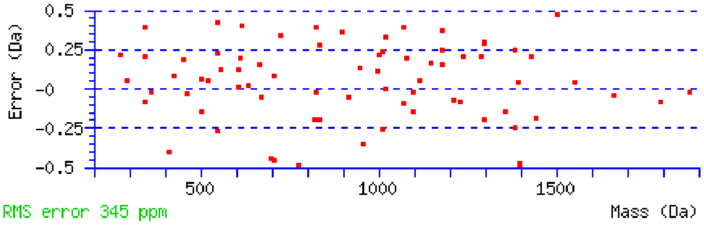


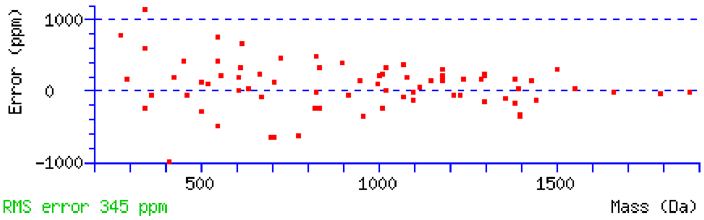


### All matches to this query

| Score | Mr(calc): | Delta | Sequence |
|---|---|---|---|
| 66.9 | 3703.7498 | 0.0096 | RPFGEVYELEIDTLETTCHALDPTPLANCSVR |
| 25.9 | 3702.7658 | 0.9936 | RPFGEVYELEIDTLETTCHALDPTPLANCSVR |
| 6.2 | 3703.7324 | 0.0269 | EVPPSEPVAEPFPSGDIAPPGLISGGKYSQTHRK |
| 6.2 | 3703.7324 | 0.0269 | EVPPSEPVAEPFPSGDIAPPGLISGGKYSQTHRK |
| 5.8 | 3702.7843 | 0.9751 | SCLIAEMIALGLQSYSLVDNLGFNRLLEYLK |
| 5.4 | 3701.7196 | 2.0398 | AADSDLQITNAQTKEEYTDDNALIPKNSSVIVR |
| 4.8 | 3701.7806 | 1.9787 | VARGVSLSTTSLLSGFQAIKLRPNMSGWLSLR |
| 4.7 | 3701.7806 | 1.9787 | VARGVSLSTTSLLSGFQAIKLRPNMSGWLSLR |
| 4.6 | 3703.7745 | -0.0152 | QVYDIAAKFGGLAAGEDNGQRGYLLTYVIAYMR |
| 3.7 | 3702.7412 | 1.0181 | SDGLIGLYQGFGVSVQGIIVYRASYFGAYDTVK |

Spectrum No: 417; Query: 2110; Rank: 1

## Peptide View

MS/MS Fragmentation of **VYINYYDMNAANVGWNGSTFA**
Found in **IPI00230907**, Tax_Id=10116 Gene_Symbol=Mif Macrophage migration inhibitory factor

Match to Query 2110: 2370.021448 from(1186.018000,2+)
Title: 091008RatKidney_NoSalt_18.6295.6295.2.dta
Data file K:\NewmanPaper\Piliang\3SubProteomes\Piliang3SP\mgf5ppm\ERLIC_3SubProteomes5ppm.mgf

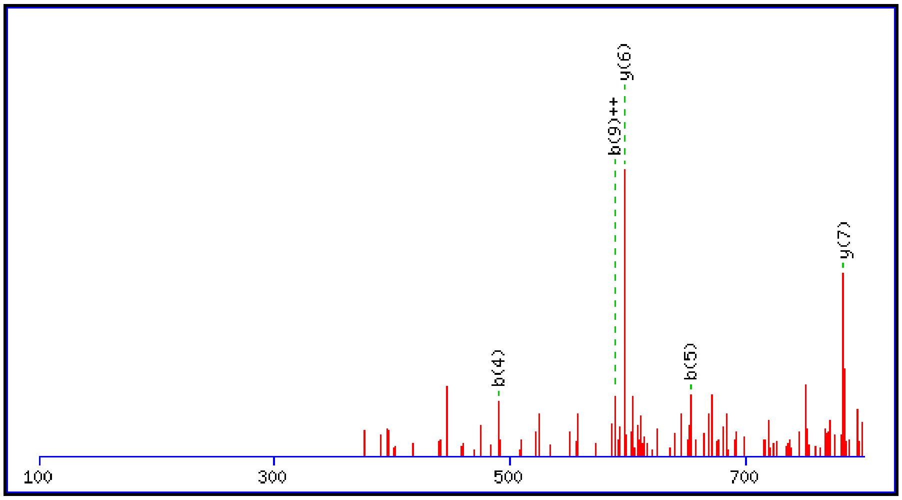

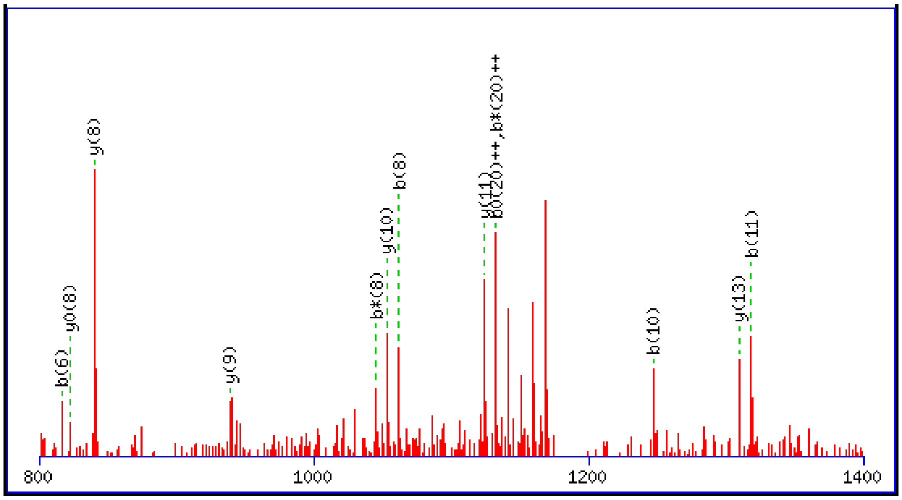


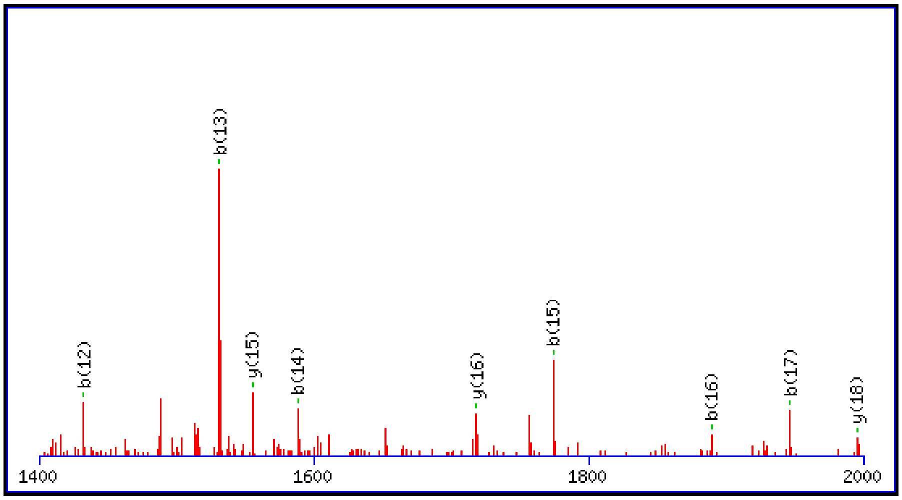


**Monoisotopic mass of neutral peptide Mr(calc):** 2370.0263
**Fixed modifications:** Carbamidomethyl (C)
**Variable modifications:**
**N16** : Deamidated_N (N)
**Ions Score:** 67 **Expect:** 6.3e-005
**Matches (Bold Red):** 27/208 fragment ions using 48 most intense peaks

| # | b | $b^{++}$ | b* | $b^{*++}$ | $b^0$ | $b^{0++}$ | Seq. | y | $y^{++}$ | y* | $y^{*++}$ | $y^0$ | $y^{0++}$ | # |
|---|---|---|---|---|---|---|---|---|---|---|---|---|---|---|
| 1 | 100.0757 | 50.5415 | | | | | V | | | | | | | 21 |
| 2 | 263.1390 | 132.0731 | | | | | Y | 2271.9652 | 1136.4862 | 2254.9386 | 1127.9729 | 2253.9546 | 1127.4809 | 20 |
| 3 | 376.2231 | 188.6152 | | | | | I | 2108.9018 | 1054.9546 | 2091.8753 | 1046.4413 | 2090.8913 | 1045.9493 | 19 |
| 4 | **490.2660** | 245.6366 | 473.2395 | 237.1234 | | | N | **1995.8178** | 998.4125 | 1978.7912 | 989.8992 | 1977.8072 | 989.4072 | 18 |
| 5 | **653.3293** | 327.1683 | 636.3028 | 318.6550 | | | Y | 1881.7748 | 941.3911 | 1864.7483 | 932.8778 | 1863.7643 | 932.3858 | 17 |
| 6 | **816.3927** | 408.7000 | 799.3661 | 400.1867 | | | Y | **1718.7115** | 859.8594 | 1701.6850 | 851.3461 | 1700.7009 | 850.8541 | 16 |
| 7 | 931.4196 | 466.2134 | 914.3931 | 457.7002 | 913.4090 | 457.2082 | D | **1555.6482** | 778.3277 | 1538.6216 | 769.8145 | 1537.6376 | 769.3224 | 15 |
| 8 | **1062.4601** | 531.7337 | **1045.4335** | 523.2204 | 1044.4495 | 522.7284 | M | 1440.6212 | 720.8143 | 1423.5947 | 712.3010 | 1422.6107 | 711.8090 | 14 |
| 9 | 1176.5030 | **588.7551** | 1159.4765 | 580.2419 | 1158.4925 | 579.7499 | N | **1309.5807** | 655.2940 | 1292.5542 | 646.7807 | 1291.5702 | 646.2887 | 13 |
| 10 | **1247.5401** | 624.2737 | 1230.5136 | 615.7604 | 1229.5296 | 615.2684 | A | 1195.5378 | 598.2725 | 1178.5113 | 589.7593 | 1177.5273 | 589.2673 | 12 |
| 11 | **1318.5773** | 659.7923 | 1301.5507 | 651.2790 | 1300.5667 | 650.7870 | A | **1124.5007** | 562.7540 | 1107.4742 | 554.2407 | 1106.4901 | 553.7487 | 11 |
| 12 | **1432.6202** | 716.8137 | 1415.5936 | 708.3005 | 1414.6096 | 707.8084 | N | **1053.4636** | 527.2354 | 1036.4370 | 518.7222 | 1035.4530 | 518.2302 | 10 |
| 13 | **1531.6886** | 766.3479 | 1514.6620 | 757.8347 | 1513.6780 | 757.3427 | V | **939.4207** | 470.2140 | 922.3941 | 461.7007 | 921.4101 | 461.2087 | 9 |
| 14 | **1588.7101** | 794.8587 | 1571.6835 | 786.3454 | 1570.6995 | 785.8534 | G | **840.3523** | 420.6798 | 823.3257 | 412.1665 | **822.3417** | 411.6745 | 8 |
| 15 | **1774.7894** | 887.8983 | 1757.7628 | 879.3850 | 1756.7788 | 878.8930 | W | **783.3308** | 392.1690 | 766.3042 | 383.6558 | 765.3202 | 383.1638 | 7 |
| 16 | **1889.8163** | 945.4118 | 1872.7898 | 936.8985 | 1871.8057 | 936.4065 | N | **597.2515** | 299.1294 | 580.2249 | 290.6161 | 579.2409 | 290.1241 | 6 |
| 17 | **1946.8378** | 973.9225 | 1929.8112 | 965.4092 | 1928.8272 | 964.9172 | G | 482.2245 | 241.6159 | | | 464.2140 | 232.6106 | 5 |
| 18 | 2033.8698 | 1017.4385 | 2016.8432 | 1008.9253 | 2015.8592 | 1008.4333 | S | 425.2031 | 213.1052 | | | 407.1925 | 204.0999 | 4 |

| | | | | | | | | | | | | | | |
|---|---|---|---|---|---|---|---|---|---|---|---|---|---|---|
| 19 | 2134.9175 | 1067.9624 | 2117.8909 | 1059.4491 | 2116.9069 | 1058.9571 | T | 338.1710 | 169.5892 | | | 320.1605 | 160.5839 | 3 |
| 20 | 2281.9859 | 1141.4966 | 2264.9593 | 1132.9833 | 2263.9753 | 1132.4913 | F | 237.1234 | 119.0653 | | | | | 2 |
| 21 | | | | | | | A | 90.0550 | 45.5311 | | | | | 1 |

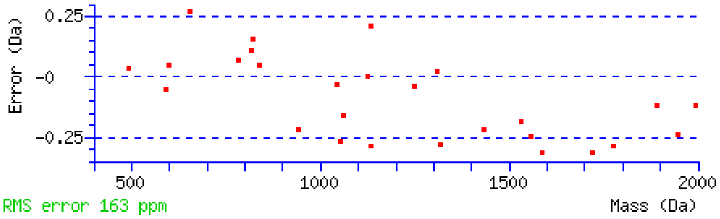


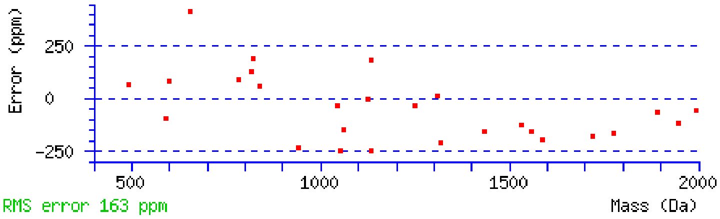


### All matches to this query

| Score | Mr(calc): | Delta | Sequence |
|---|---|---|---|
| 66.8 | 2370.0263 | -0.0048 | VYINYYDMNAANVGWNGSTFA |
| 35.9 | 2370.0263 | -0.0048 | VYINYYDMNAANVGWNGSTFA |
| 34.4 | 2370.0263 | -0.0048 | VYINYYDMNAANVGWNGSTFA |
| 8.6 | 2370.0263 | -0.0048 | VYINYYDMNAANVGWNGSTFA |
| 2.1 | 2369.0337 | 0.9878 | DMITGITLPINNTMKSLWK |
| 2.1 | 2369.0337 | 0.9878 | DMITGITLPINNTMKSLWK |

---

Spectrum No: 418; Query: 1608; Rank: 1

---

## Peptide View

MS/MS Fragmentation of **YQTSSAINGSVIWDRPTK**
Found in **IPI00210872**, Tax_Id=10116 Gene_Symbol=Mep1a Meprin A subunit alpha precursor

Match to Query 1608: 2023.010768 from(1012.512660,2+)
Title: 100101RatKid_NS_deglyco_19.2812.2812.2.dta
Data file K:\NewmanPaper\Piliang\3SubProteomes\Piliang3SP\mgf5ppm\ERLIC_3SubProteomes5ppm.mgf

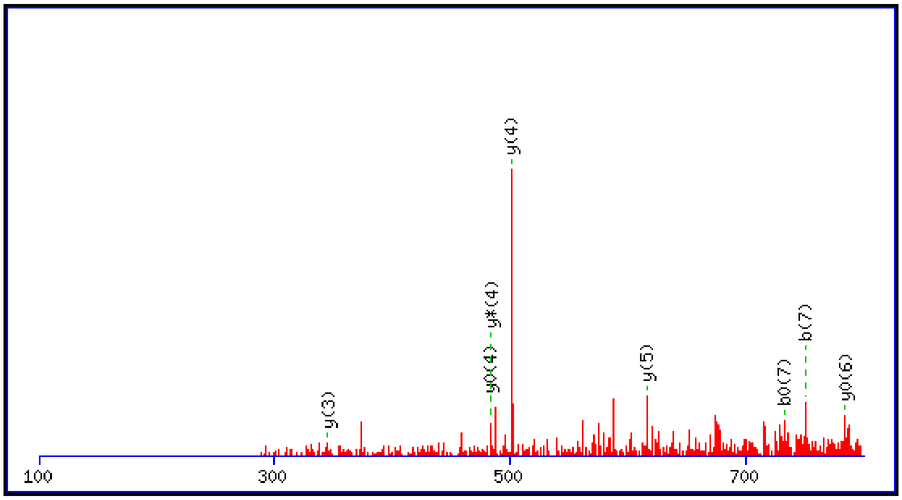

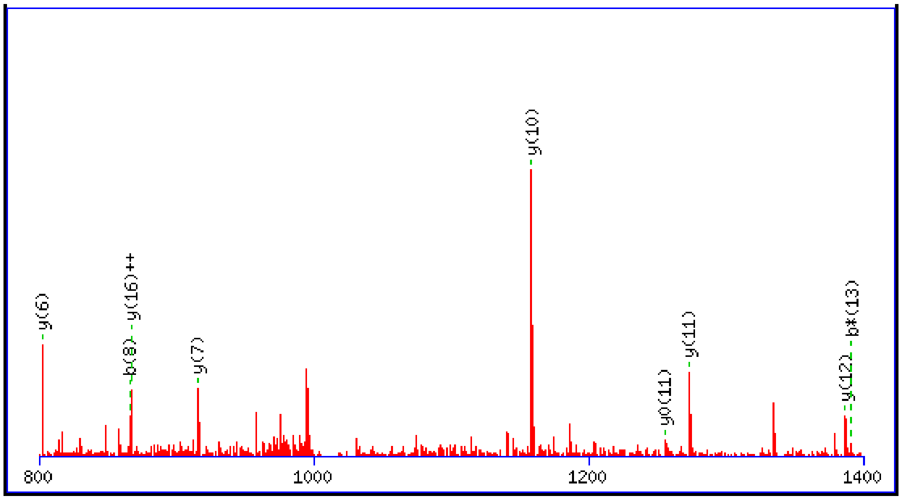


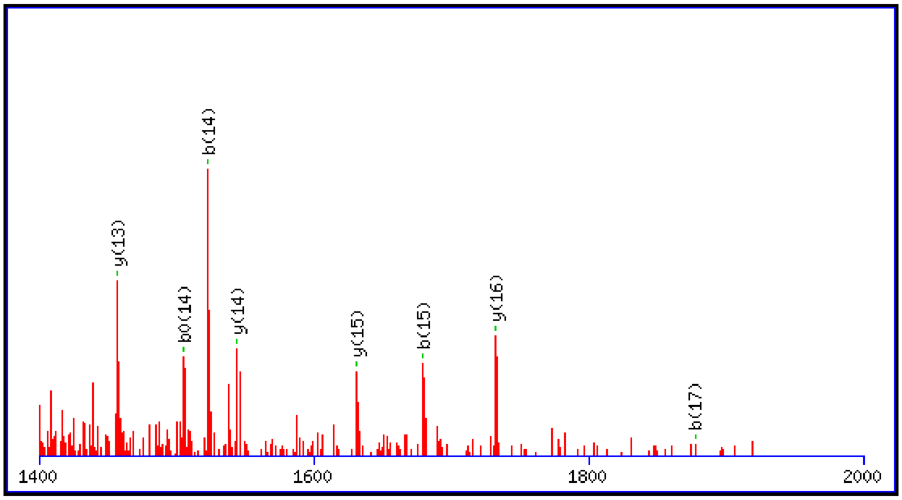


**Monoisotopic mass of neutral peptide Mr(calc):** 2023.0011
**Fixed modifications:** Carbamidomethyl (C)
**Variable modifications:**
**N8** : Deamidated_N (N)
**Ions Score:** 67 **Expect:** 4.4e-005
**Matches (Bold Red):** 25/196 fragment ions using 58 most intense peaks

| # | b | b$^{++}$ | b* | b*$^{++}$ | b$^{0}$ | b$^{0++}$ | Seq. | y | y$^{++}$ | y* | y*$^{++}$ | y$^{0}$ | y$^{0++}$ | # |
|---|---|---|---|---|---|---|---|---|---|---|---|---|---|---|
| 1 | 164.0706 | 82.5389 | | | | | Y | | | | | | | 18 |
| 2 | 292.1292 | 146.5682 | 275.1026 | 138.0550 | | | Q | 1860.9450 | 930.9761 | 1843.9185 | 922.4629 | 1842.9344 | 921.9709 | 17 |
| 3 | 393.1769 | 197.0921 | 376.1503 | 188.5788 | 375.1663 | 188.0868 | T | **1732.8864** | **866.9469** | 1715.8599 | 858.4336 | 1714.8759 | 857.9416 | 16 |
| 4 | 480.2089 | 240.6081 | 463.1823 | 232.0948 | 462.1983 | 231.6028 | S | **1631.8388** | 816.4230 | 1614.8122 | 807.9097 | 1613.8282 | 807.4177 | 15 |
| 5 | 567.2409 | 284.1241 | 550.2144 | 275.6108 | 549.2304 | 275.1188 | S | **1544.8067** | 772.9070 | 1527.7802 | 764.3937 | 1526.7962 | 763.9017 | 14 |
| 6 | 638.2780 | 319.6427 | 621.2515 | 311.1294 | 620.2675 | 310.6374 | A | **1457.7747** | 729.3910 | 1440.7481 | 720.8777 | 1439.7641 | 720.3857 | 13 |
| 7 | **751.3621** | 376.1847 | 734.3355 | 367.6714 | **733.3515** | 367.1794 | I | **1386.7376** | 693.8724 | 1369.7110 | 685.3592 | 1368.7270 | 684.8671 | 12 |
| 8 | **866.3890** | 433.6982 | 849.3625 | 425.1849 | 848.3785 | 424.6929 | N | **1273.6535** | 637.3304 | 1256.6270 | 628.8171 | **1255.6430** | 628.3251 | 11 |
| 9 | 923.4105 | 462.2089 | 906.3839 | 453.6956 | 905.3999 | 453.2036 | G | **1158.6266** | 579.8169 | 1141.6000 | 571.3037 | 1140.6160 | 570.8116 | 10 |
| 10 | 1010.4425 | 505.7249 | 993.4160 | 497.2116 | 992.4320 | 496.7196 | S | 1101.6051 | 551.3062 | 1084.5786 | 542.7929 | 1083.5946 | 542.3009 | 9 |
| 11 | 1109.5109 | 555.2591 | 1092.4844 | 546.7458 | 1091.5004 | 546.2538 | V | 1014.5731 | 507.7902 | 997.5465 | 499.2769 | 996.5625 | 498.7849 | 8 |
| 12 | 1222.5950 | 611.8011 | 1205.5685 | 603.2879 | 1204.5844 | 602.7959 | I | **915.5047** | 458.2560 | 898.4781 | 449.7427 | 897.4941 | 449.2507 | 7 |
| 13 | 1408.6743 | 704.8408 | **1391.6478** | 696.3275 | 1390.6637 | 695.8355 | W | **802.4206** | 401.7139 | 785.3941 | 393.2007 | **784.4100** | 392.7087 | 6 |
| 14 | **1523.7013** | 762.3543 | 1506.6747 | 753.8410 | **1505.6907** | 753.3490 | D | **616.3413** | 308.6743 | 599.3148 | 300.1610 | 598.3307 | 299.6690 | 5 |
| 15 | **1679.8024** | 840.4048 | 1662.7758 | 831.8915 | 1661.7918 | 831.3995 | R | **501.3144** | 251.1608 | **484.2878** | 242.6475 | **483.3038** | 242.1555 | 4 |
| 16 | 1776.8551 | 888.9312 | 1759.8286 | 880.4179 | 1758.8446 | 879.9259 | P | **345.2132** | 173.1103 | 328.1867 | 164.5970 | 327.2027 | 164.1050 | 3 |
| 17 | **1877.9028** | 939.4550 | 1860.8763 | 930.9418 | 1859.8922 | 930.4498 | T | 248.1605 | 124.5839 | 231.1339 | 116.0706 | 230.1499 | 115.5786 | 2 |
| 18 | | | | | | | K | 147.1128 | 74.0600 | 130.0863 | 65.5468 | | | 1 |

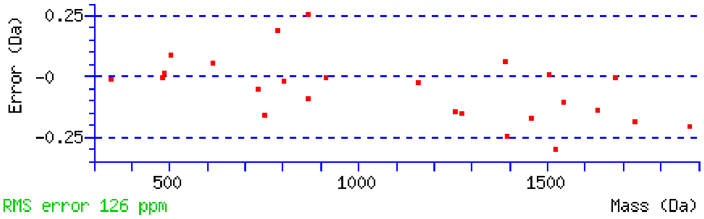


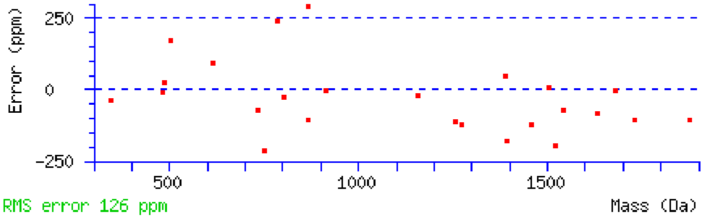


### All matches to this query

| Score | Mr(calc): | Delta | Sequence |
|---|---|---|---|
| 66.7 | 2023.0011 | 0.0097 | YQTSSAINGSVIWDRPTK |
| 12.4 | 2022.0170 | 0.9937 | YQTSSAINGSVIWDRPTK |
| 3.3 | 2022.0048 | 1.0060 | VLKSHGQDYLVGNRLSR |

---

Spectrum No: 419; Query: 1693; Rank: 1

---

## Peptide View

MS/MS Fragmentation of **QLLANSSALVETILGHQER**
Found in **IPI00190577**, Tax_Id=10116 Gene_Symbol=Lama5 similar to Laminin alpha-5 chain precursor

Match to Query 1693: 2079.096762 from(694.039530,3+)
Title: 091008RatKidney_NoSalt_22.5095.5095.3.dta
Data file K:\NewmanPaper\Piliang\3SubProteomes\Piliang3SP\mgf5ppm\ERLIC_3SubProteomes5ppm.mgf

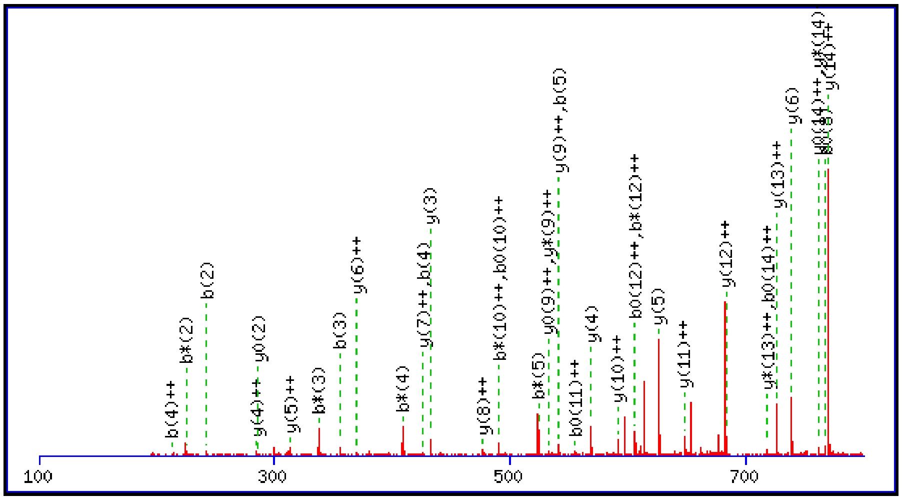


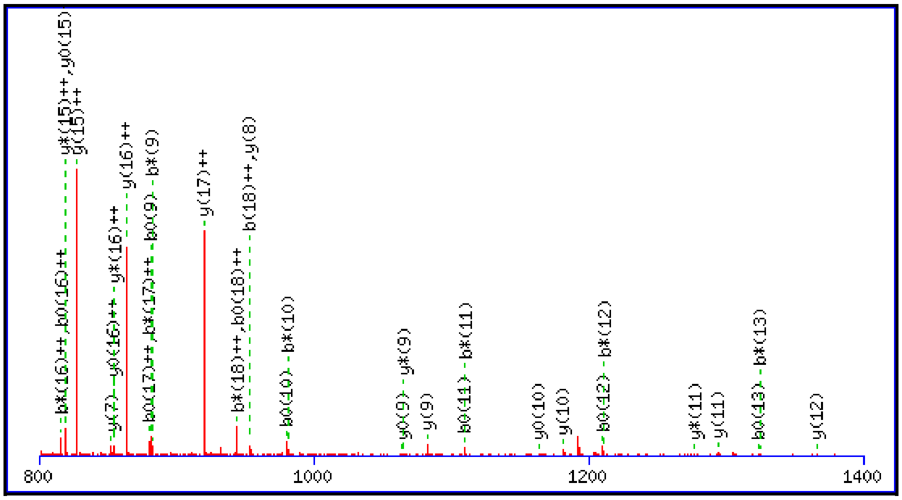

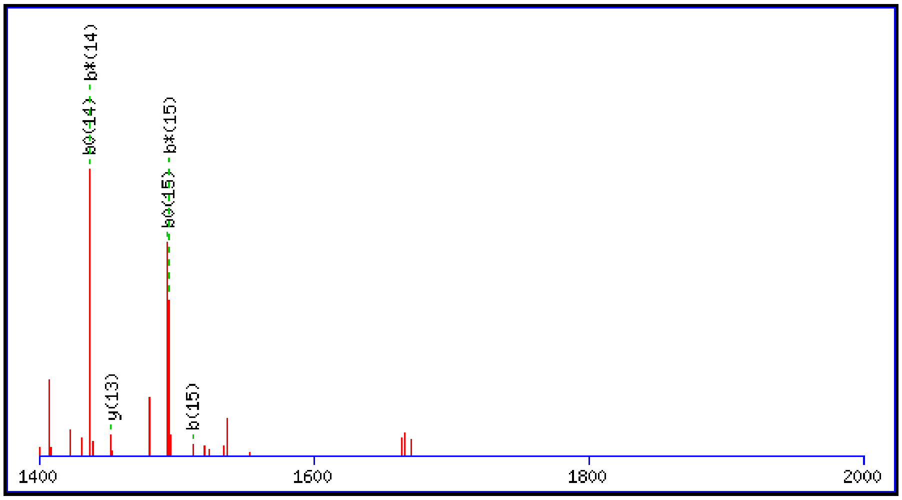


**Monoisotopic mass of neutral peptide Mr(calc):** 2079.0960
**Fixed modifications:** Carbamidomethyl (C)
**Variable modifications:**
**N5** : Deamidated_N (N)
**Ions Score:** 67 **Expect:** 2.2e-005
**Matches (Bold Red):** 77/204 fragment ions using 143 most intense peaks

| # | b | b++ | b* | b*++ | b0 | b0++ | Seq. | y | y++ | y* | y*++ | y0 | y0++ | # |
|---|---|---|---|---|---|---|---|---|---|---|---|---|---|---|
| 1 | 129.0659 | 65.0366 | 112.0393 | 56.5233 | | | Q | | | | | | | 19 |
| 2 | **242.1499** | 121.5786 | **225.1234** | 113.0653 | | | L | 1952.0447 | 976.5260 | 1935.0182 | 968.0127 | 1934.0342 | 967.5207 | 18 |
| 3 | **355.2340** | 178.1206 | **338.2074** | 169.6074 | | | L | 1838.9607 | **919.9840** | 1821.9341 | 911.4707 | 1820.9501 | 910.9787 | 17 |
| 4 | **426.2711** | **213.6392** | **409.2445** | 205.1259 | | | A | 1725.8766 | **863.4419** | 1708.8500 | **854.9287** | 1707.8660 | **854.4367** | 16 |
| 5 | **541.2980** | 271.1527 | **524.2715** | 262.6394 | | | N | 1654.8395 | **827.9234** | 1637.8129 | **819.4101** | 1636.8289 | **818.9181** | 15 |
| 6 | 628.3301 | 314.6687 | 611.3035 | 306.1554 | 610.3195 | 305.6634 | S | 1539.8125 | **770.4099** | 1522.7860 | **761.8966** | 1521.8020 | **761.4046** | 14 |
| 7 | 715.3621 | 358.1847 | 698.3355 | 349.6714 | 697.3515 | 349.1794 | S | **1452.7805** | **726.8939** | 1435.7540 | **718.3806** | 1434.7700 | 717.8886 | 13 |
| 8 | 786.3992 | 393.7032 | 769.3727 | 385.1900 | **768.3886** | 384.6980 | A | **1365.7485** | **683.3779** | 1348.7219 | 674.8646 | 1347.7379 | 674.3726 | 12 |
| 9 | 899.4833 | 450.2453 | **882.4567** | 441.7320 | **881.4727** | 441.2400 | L | **1294.7114** | **647.8593** | **1277.6848** | 639.3461 | 1276.7008 | 638.8540 | 11 |
| 10 | 998.5517 | 499.7795 | **981.5251** | **491.2662** | **980.5411** | **490.7742** | V | **1181.6273** | **591.3173** | 1164.6008 | 582.8040 | **1163.6167** | 582.3120 | 10 |
| 11 | 1127.5943 | 564.3008 | **1110.5677** | 555.7875 | **1109.5837** | **555.2955** | E | **1082.5589** | **541.7831** | **1065.5324** | **533.2698** | **1064.5483** | **532.7778** | 9 |
| 12 | 1228.6420 | 614.8246 | **1211.6154** | **606.3113** | **1210.6314** | **605.8193** | T | **953.5163** | **477.2618** | 936.4898 | 468.7485 | 935.5057 | 468.2565 | 8 |
| 13 | 1341.7260 | 671.3666 | **1324.6995** | 662.8534 | **1323.7155** | 662.3614 | I | **852.4686** | **426.7380** | 835.4421 | 418.2247 | 834.4581 | 417.7327 | 7 |
| 14 | 1454.8101 | 727.9087 | **1437.7835** | 719.3954 | **1436.7995** | **718.9034** | L | **739.3846** | **370.1959** | 722.3580 | 361.6826 | 721.3740 | 361.1906 | 6 |
| 15 | **1511.8315** | 756.4194 | **1494.8050** | 747.9061 | **1493.8210** | 747.4141 | G | **626.3005** | **313.6539** | 609.2739 | 305.1406 | 608.2899 | 304.6486 | 5 |
| 16 | 1648.8905 | 824.9489 | 1631.8639 | **816.4356** | 1630.8799 | **815.9436** | H | **569.2790** | **285.1432** | 552.2525 | 276.6299 | 551.2685 | 276.1379 | 4 |
| 17 | 1776.9490 | 888.9782 | 1759.9225 | **880.4649** | 1758.9385 | **879.9729** | Q | **432.2201** | 216.6137 | 415.1936 | 208.1004 | 414.2096 | 207.6084 | 3 |
| 18 | 1905.9916 | **953.4995** | 1888.9651 | **944.9862** | 1887.9811 | **944.4942** | E | 304.1615 | 152.5844 | 287.1350 | 144.0711 | **286.1510** | 143.5791 | 2 |
| 19 | | | | | | | R | 175.1190 | 88.0631 | 158.0924 | 79.5498 | | | 1 |

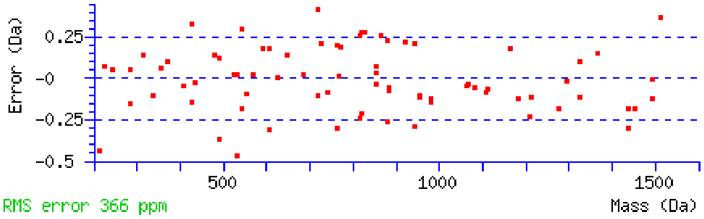


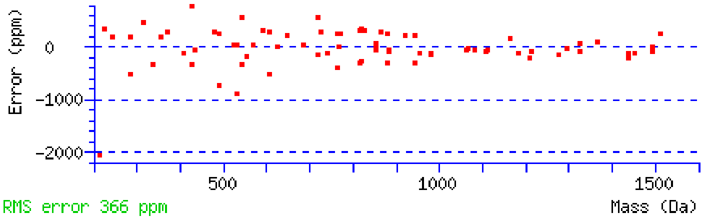


### All matches to this query

| Score | Mr(calc): | Delta | Sequence |
|---|---|---|---|
| 66.7 | 2079.0960 | 0.0008 | QLLANSSALVETILGHQER |
| 41.7 | 2078.1120 | 0.9848 | QLLANSSALVETILGHQER |
| | | | |

| | | | |
|---|---|---|---|
| 3.0 | 2078.0989 | 0.9979 | RIHIIXVTKPLLTARK |
| 0.3 | 2077.0738 | 2.0229 | GASPNVSNVKVETPLHMAAR |

---

Spectrum No: 420; Query: 1263; Rank: 1

---

## Peptide View

MS/MS Fragmentation of **ANEGAIYPDNTTDFQR**
Found in **IPI00325847**, Tax_Id=10116 Gene_Symbol=Cp GPI-anchored ceruloplasmin

Match to Query 1263: 1811.803148 from(906.908850,2+)
Title: 100101RatKid_NS_deglyco_19.2207.2207.2.dta
Data file K:\NewmanPaper\Piliang\3SubProteomes\Piliang3SP\mgf5ppm\ERLIC_3SubProteomes5ppm.mgf

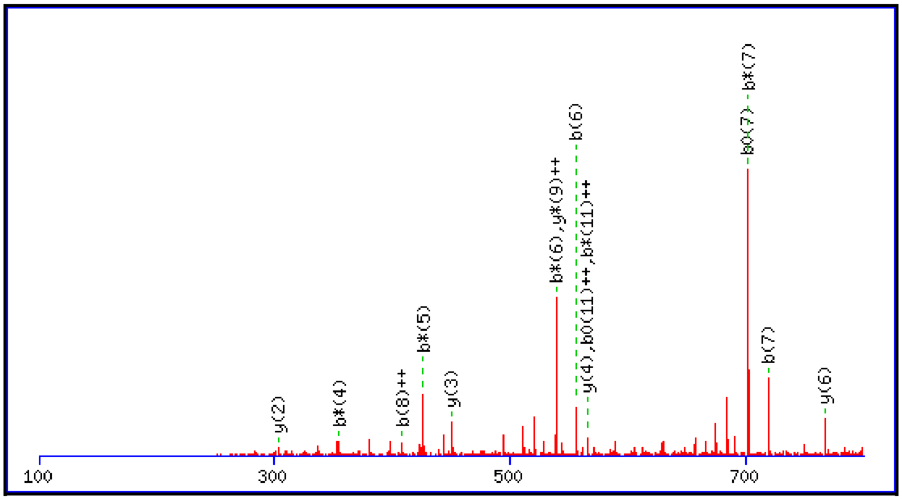


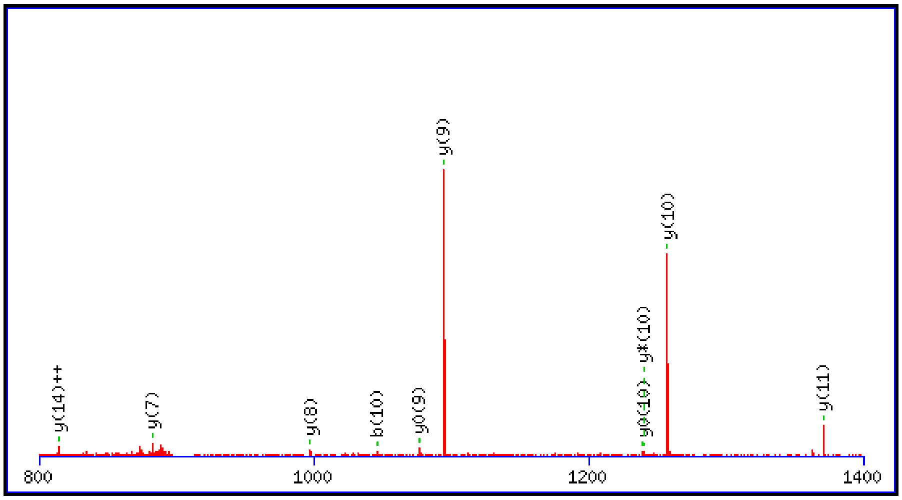

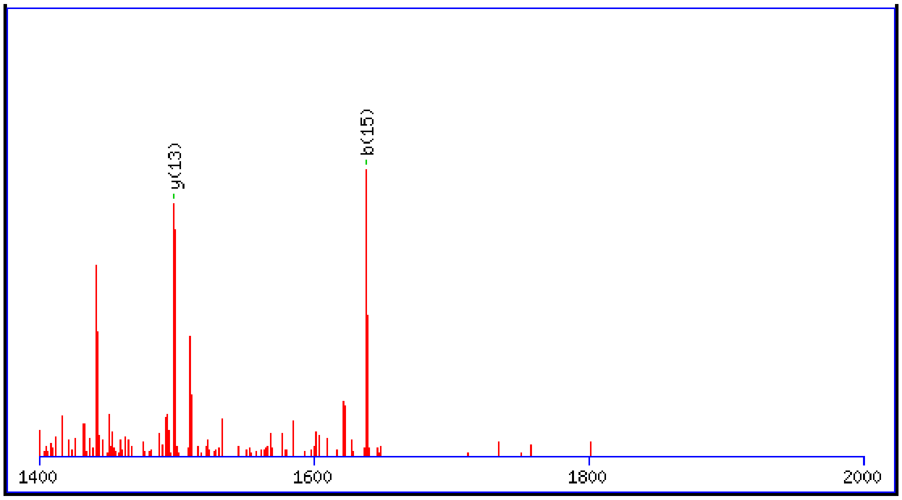


**Monoisotopic mass of neutral peptide Mr(calc):** 1811.7962
**Fixed modifications:** Carbamidomethyl (C)
**Variable modifications:**
**N10** : Deamidated_N (N)
**Ions Score:** 67 **Expect:** 4.5e-005
**Matches (Bold Red):** 27/168 fragment ions using 50 most intense peaks

| # | b | $b^{++}$ | b* | $b^{*++}$ | $b^0$ | $b^{0++}$ | Seq. | y | $y^{++}$ | y* | $y^{*++}$ | $y^0$ | $y^{0++}$ | # |
|---|---|---|---|---|---|---|---|---|---|---|---|---|---|---|
| 1 | 72.0444 | 36.5258 | | | | | A | | | | | | | 16 |
| 2 | 186.0873 | 93.5473 | 169.0608 | 85.0340 | | | N | 1741.7664 | 871.3868 | 1724.7398 | 862.8736 | 1723.7558 | 862.3815 | 15 |
| 3 | 315.1299 | 158.0686 | 298.1034 | 149.5553 | 297.1193 | 149.0633 | E | 1627.7235 | **814.3654** | 1610.6969 | 805.8521 | 1609.7129 | 805.3601 | 14 |
| 4 | 372.1514 | 186.5793 | **355.1248** | 178.0661 | 354.1408 | 177.5740 | G | **1498.6809** | 749.8441 | 1481.6543 | 741.3308 | 1480.6703 | 740.8388 | 13 |
| 5 | 443.1885 | 222.0979 | **426.1619** | 213.5846 | 425.1779 | 213.0926 | A | 1441.6594 | 721.3333 | 1424.6328 | 712.8201 | 1423.6488 | 712.3281 | 12 |
| 6 | **556.2726** | 278.6399 | **539.2460** | 270.1266 | 538.2620 | 269.6346 | I | **1370.6223** | 685.8148 | 1353.5957 | 677.3015 | 1352.6117 | 676.8095 | 11 |
| 7 | **719.3359** | 360.1716 | **702.3093** | 351.6583 | **701.3253** | 351.1663 | Y | **1257.5382** | 629.2727 | **1240.5117** | 620.7595 | **1239.5277** | 620.2675 | 10 |
| 8 | 816.3886 | **408.6980** | 799.3621 | 400.1847 | 798.3781 | 399.6927 | P | **1094.4749** | 547.7411 | 1077.4483 | **539.2278** | **1076.4643** | 538.7358 | 9 |
| 9 | 931.4156 | 466.2114 | 914.3890 | 457.6982 | 913.4050 | 457.2061 | D | **997.4221** | 499.2147 | 980.3956 | 490.7014 | 979.4116 | 490.2094 | 8 |
| 10 | **1046.4425** | 523.7249 | 1029.4160 | 515.2116 | 1028.4320 | 514.7196 | N | **882.3952** | 441.7012 | 865.3686 | 433.1880 | 864.3846 | 432.6959 | 7 |
| 11 | 1147.4902 | 574.2487 | 1130.4637 | **565.7355** | 1129.4796 | **565.2435** | T | **767.3682** | 384.1878 | 750.3417 | 375.6745 | 749.3577 | 375.1825 | 6 |
| 12 | 1248.5379 | 624.7726 | 1231.5113 | 616.2593 | 1230.5273 | 615.7673 | T | 666.3206 | 333.6639 | 649.2940 | 325.1506 | 648.3100 | 324.6586 | 5 |
| 13 | 1363.5648 | 682.2861 | 1346.5383 | 673.7728 | 1345.5543 | 673.2808 | D | **565.2729** | 283.1401 | 548.2463 | 274.6268 | 547.2623 | 274.1348 | 4 |
| 14 | 1510.6332 | 755.8203 | 1493.6067 | 747.3070 | 1492.6227 | 746.8150 | F | **450.2459** | 225.6266 | 433.2194 | 217.1133 | | | 3 |
| 15 | **1638.6918** | 819.8495 | 1621.6653 | 811.3363 | 1620.6813 | 810.8443 | Q | **303.1775** | 152.0924 | 286.1510 | 143.5791 | | | 2 |
| 16 | | | | | | | R | 175.1190 | 88.0631 | 158.0924 | 79.5498 | | | 1 |

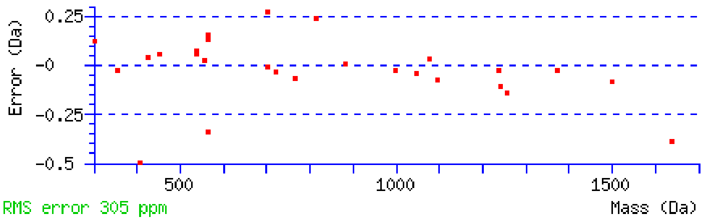


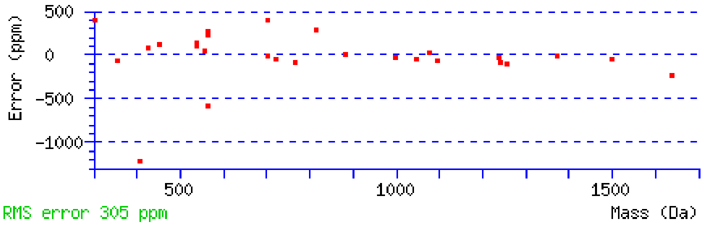


### All matches to this query

| Score | Mr(calc): | Delta | Sequence |
|---|---|---|---|
| 66.6 | 1811.7962 | 0.0069 | ANEGAIYPDNTTDFQR |
| 10.6 | 1811.8050 | -0.0019 | NLSKITNNNLNDSQR |
| 10.6 | 1811.8050 | -0.0019 | NLSKITNNNLNDSQR |
| 9.9 | 1811.8050 | -0.0019 | NLSKITNNNLNDSQR |
| 8.5 | 1811.8050 | -0.0019 | NLSKITNNNLNDSQR |
| | | | |

| | | | |
|---|---|---|---|
| 7.6 | 1811.8050 | -0.0019 | NLSKITNNNLNDSQR |
| 7.6 | 1811.8050 | -0.0019 | NLSKITNNNLNDSQR |
| 7.5 | 1809.7791 | 2.0241 | CQSYQQSTKQLTMK |
| 4.8 | 1811.7962 | 0.0069 | ANEGAIYPDNTTDFQR |
| 4.4 | 1810.8122 | 0.9910 | ANEGAIYPDNTTDFQR |

---

Spectrum No: 421; Query: 995; Rank: 1

---

## Peptide View

MS/MS Fragmentation of **GYLASFEMFNSTFK**
Found in **IPI00400739**, Tax_Id=10116 Gene_Symbol=Entpd5 Ectonucleoside triphosphate diphosphohydrolase 5

Match to Query 995: 1658.732588 from(830.373570,2+)
Title: 091008RatKidney_NH4Format01_24.3877.3877.2.dta
Data file K:\NewmanPaper\Piliang\3SubProteomes\Piliang3SP\mgf5ppm\ERLIC_3SubProteomes5ppm.mgf

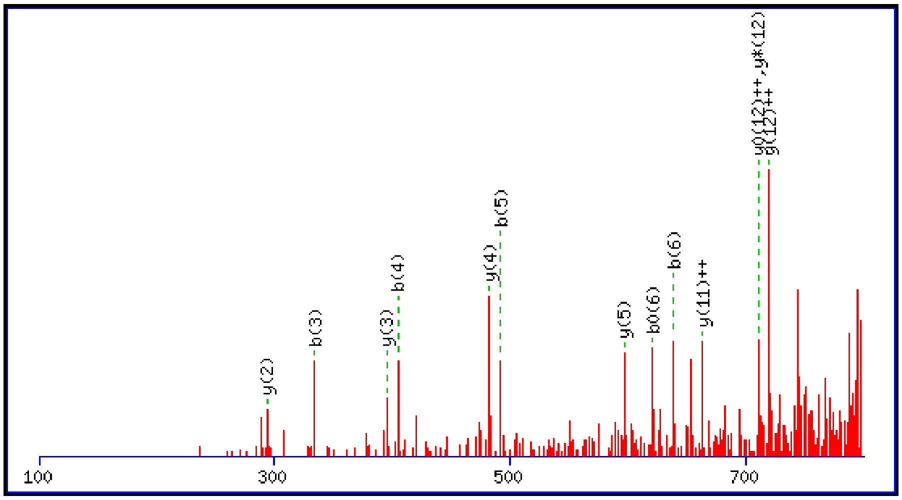


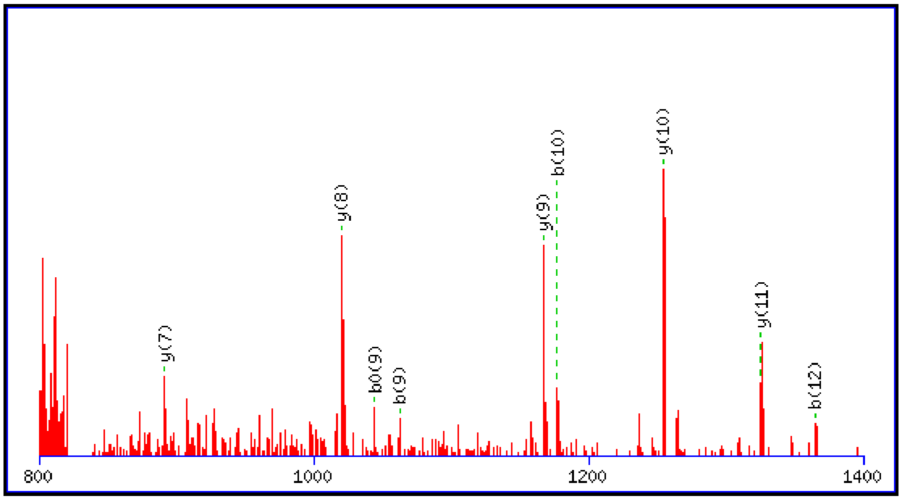

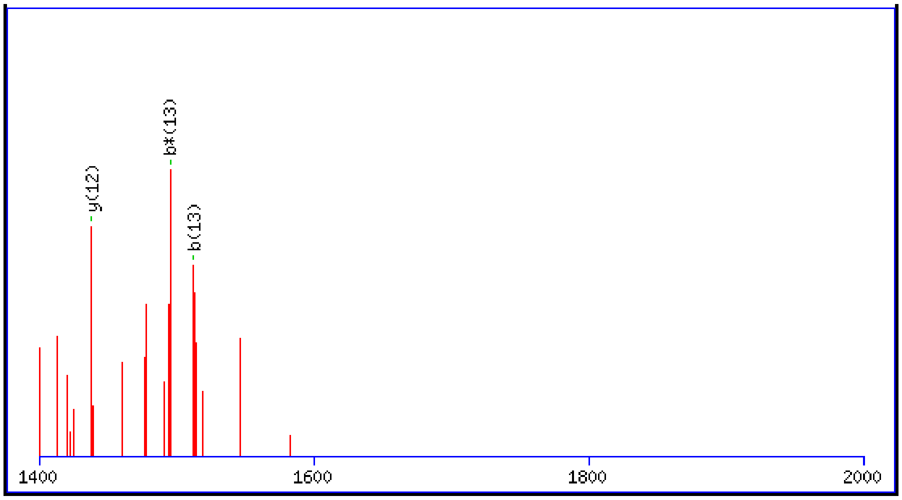


**Monoisotopic mass of neutral peptide Mr(calc):** 1657.7334
**Fixed modifications:** Carbamidomethyl (C)
**Variable modifications:**
**M8** : Oxidation (M)
**N10** : Deamidated_N (N)
**Ions Score:** 66 **Expect:** 4.2e-005
**Matches (Bold Red):** 25/126 fragment ions using 40 most intense peaks

| # | b | $b^{++}$ | b* | $b^{*++}$ | $b^{0}$ | $b^{0++}$ | Seq. | y | $y^{++}$ | y* | $y^{*++}$ | $y^{0}$ | $y^{0++}$ | # |
|---|---|---|---|---|---|---|---|---|---|---|---|---|---|---|
| 1 | 58.0287 | 29.5180 | | | | | G | | | | | | | 14 |
| 2 | 221.0921 | 111.0497 | | | | | Y | 1601.7192 | 801.3632 | 1584.6927 | 792.8500 | 1583.7087 | 792.3580 | 13 |
| 3 | **334.1761** | 167.5917 | | | | | L | **1438.6559** | **719.8316** | 1421.6293 | **711.3183** | 1420.6453 | **710.8263** | 12 |
| 4 | **405.2132** | 203.1103 | | | | | A | **1325.5718** | **663.2896** | 1308.5453 | 654.7763 | 1307.5613 | 654.2843 | 11 |
| 5 | **492.2453** | 246.6263 | | | 474.2347 | 237.6210 | S | **1254.5347** | 627.7710 | 1237.5082 | 619.2577 | 1236.5241 | 618.7657 | 10 |
| 6 | **639.3137** | 320.1605 | | | **621.3031** | 311.1552 | F | **1167.5027** | 584.2550 | 1150.4761 | 575.7417 | 1149.4921 | 575.2497 | 9 |
| 7 | 768.3563 | 384.6818 | | | 750.3457 | 375.6765 | E | **1020.4343** | 510.7208 | 1003.4077 | 502.2075 | 1002.4237 | 501.7155 | 8 |
| 8 | 915.3917 | 458.1995 | | | 897.3811 | 449.1942 | M | **891.3917** | 446.1995 | 874.3651 | 437.6862 | 873.3811 | 437.1942 | 7 |
| 9 | **1062.4601** | 531.7337 | | | **1044.4495** | 522.7284 | F | 744.3563 | 372.6818 | 727.3297 | 364.1685 | 726.3457 | 363.6765 | 6 |
| 10 | **1177.4870** | 589.2472 | 1160.4605 | 580.7339 | 1159.4765 | 580.2419 | N | **597.2879** | 299.1476 | 580.2613 | 290.6343 | 579.2773 | 290.1423 | 5 |
| 11 | 1264.5191 | 632.7632 | 1247.4925 | 624.2499 | 1246.5085 | 623.7579 | S | **482.2609** | 241.6341 | 465.2344 | 233.1208 | 464.2504 | 232.6288 | 4 |
| 12 | **1365.5667** | 683.2870 | 1348.5402 | 674.7737 | 1347.5562 | 674.2817 | T | **395.2289** | 198.1181 | 378.2023 | 189.6048 | 377.2183 | 189.1128 | 3 |
| 13 | **1512.6352** | 756.8212 | **1495.6086** | 748.3079 | 1494.6246 | 747.8159 | F | **294.1812** | 147.5942 | 277.1547 | 139.0810 | | | 2 |
| 14 | | | | | | | K | 147.1128 | 74.0600 | 130.0863 | 65.5468 | | | 1 |

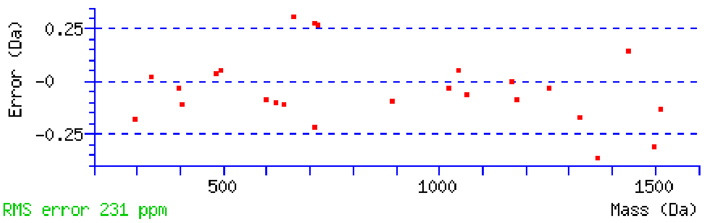


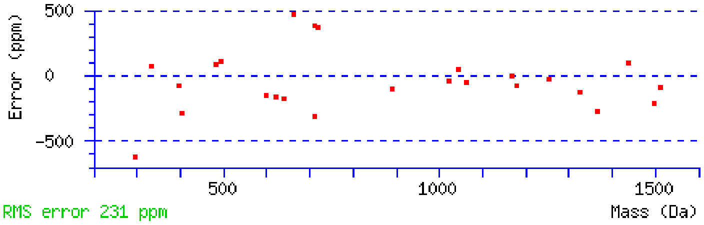


### All matches to this query

| Score | Mr(calc): | Delta | Sequence |
|---|---|---|---|
| 66.4 | 1657.7334 | 0.9992 | GYLASFEMFNSTFK |
| 6.4 | 1657.7211 | 1.0115 | NFAAESFTFLMRK |
| 3.3 | 1657.7382 | 0.9944 | KSTEMRKPESEEK |
| 3.3 | 1657.7382 | 0.9944 | KSTEMRKPESEEK |

Spectrum No: 422; Query: 1139; Rank: 1

## Peptide View

MS/MS Fragmentation of **SCVTITDAFPQNMSR**
Found in **IPI00370450**, Tax_Id=10116 Gene_Symbol=Plxnb2 plexin B2

Match to Query 1139: 1742.763268 from(872.388910,2+)
Title: 100101RatKid_NS_deglyco_25.2413.2413.2.dta
Data file K:\NewmanPaper\Piliang\3SubProteomes\Piliang3SP\mgf5ppm\ERLIC_3SubProteomes5ppm.mgf

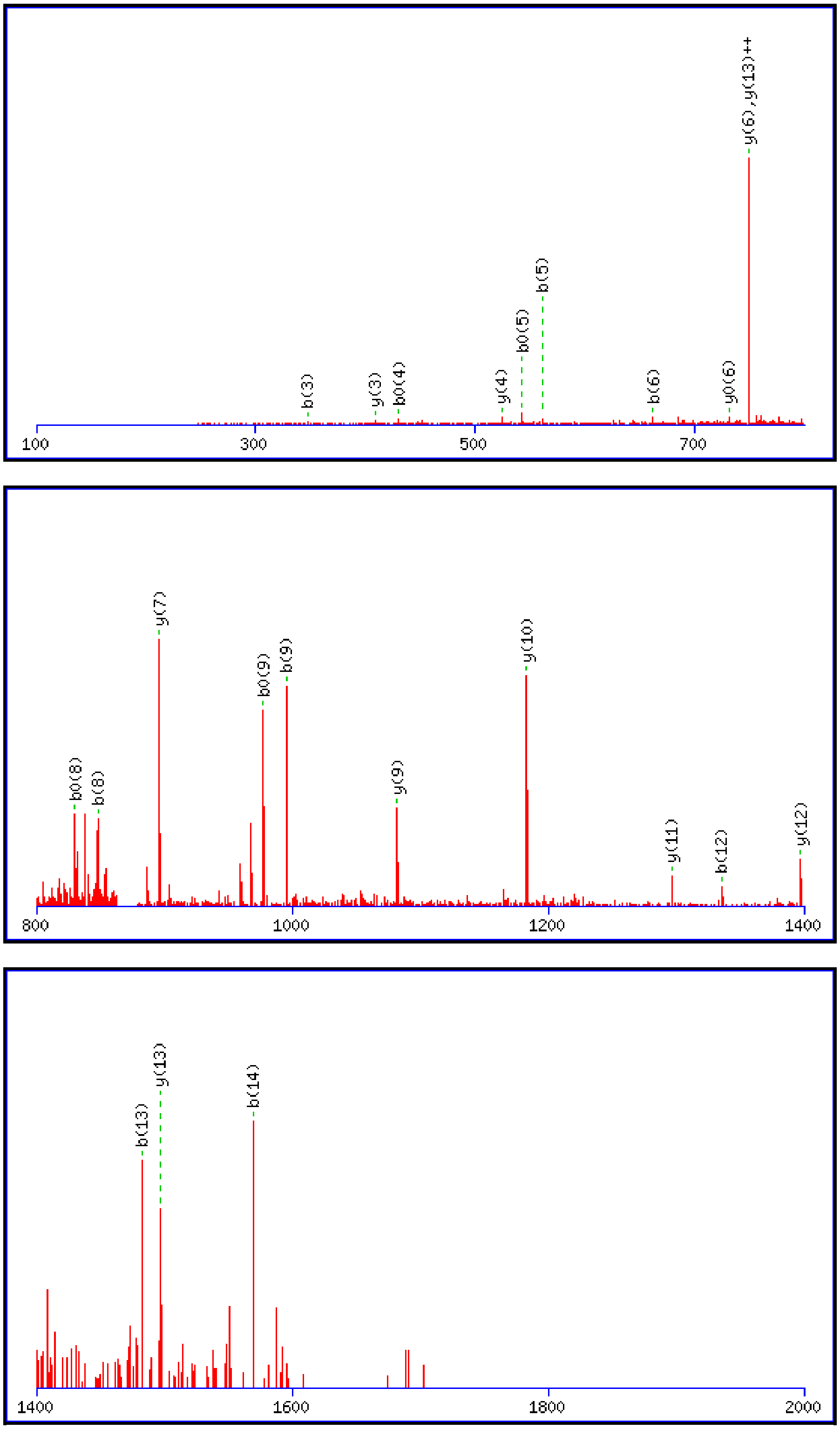


**Monoisotopic mass of neutral peptide Mr(calc):** 1742.7604
**Fixed modifications:** Carbamidomethyl (C)
**Variable modifications:**

```
N12    : Deamidated_N (N)
M13    : Oxidation (M)
Ions Score: 66  Expect: 4.7e-005
Matches (Bold Red): 23/146 fragment ions using 35 most intense peaks
```

| # | b | b++ | b* | b*++ | b0 | b0++ | Seq. | y | y++ | y* | y*++ | y0 | y0++ | # |
|---|---|---|---|---|---|---|---|---|---|---|---|---|---|---|
| 1 | 88.0393 | 44.5233 | | | 70.0287 | 35.5180 | S | | | | | | | 15 |
| 2 | 248.0700 | 124.5386 | | | 230.0594 | 115.5333 | C | 1656.7356 | 828.8714 | 1639.7091 | 820.3582 | 1638.7251 | 819.8662 | 14 |
| 3 | **347.1384** | 174.0728 | | | 329.1278 | 165.0675 | V | **1496.7050** | **748.8561** | 1479.6784 | 740.3428 | 1478.6944 | 739.8508 | 13 |
| 4 | 448.1860 | 224.5967 | | | **430.1755** | 215.5914 | T | **1397.6366** | 699.3219 | 1380.6100 | 690.8086 | 1379.6260 | 690.3166 | 12 |
| 5 | **561.2701** | 281.1387 | | | **543.2595** | 272.1334 | I | **1296.5889** | 648.7981 | 1279.5623 | 640.2848 | 1278.5783 | 639.7928 | 11 |
| 6 | **662.3178** | 331.6625 | | | 644.3072 | 322.6573 | T | **1183.5048** | 592.2560 | 1166.4783 | 583.7428 | 1165.4942 | 583.2508 | 10 |
| 7 | 777.3447 | 389.1760 | | | 759.3342 | 380.1707 | D | **1082.4571** | 541.7322 | 1065.4306 | 533.2189 | 1064.4466 | 532.7269 | 9 |
| 8 | **848.3818** | 424.6946 | | | **830.3713** | 415.6893 | A | 967.4302 | 484.2187 | 950.4036 | 475.7055 | 949.4196 | 475.2135 | 8 |
| 9 | **995.4503** | 498.2288 | | | **977.4397** | 489.2235 | F | **896.3931** | 448.7002 | 879.3665 | 440.1869 | 878.3825 | 439.6949 | 7 |
| 10 | 1092.5030 | 546.7551 | | | 1074.4925 | 537.7499 | P | **749.3247** | 375.1660 | 732.2981 | 366.6527 | **731.3141** | 366.1607 | 6 |
| 11 | 1220.5616 | 610.7844 | 1203.5351 | 602.2712 | 1202.5510 | 601.7792 | Q | 652.2719 | 326.6396 | 635.2453 | 318.1263 | 634.2613 | 317.6343 | 5 |
| 12 | **1335.5885** | 668.2979 | 1318.5620 | 659.7846 | 1317.5780 | 659.2926 | N | **524.2133** | 262.6103 | 507.1868 | 254.0970 | 506.2028 | 253.6050 | 4 |
| 13 | **1482.6239** | 741.8156 | 1465.5974 | 733.3023 | 1464.6134 | 732.8103 | M | **409.1864** | 205.0968 | 392.1598 | 196.5836 | 391.1758 | 196.0915 | 3 |
| 14 | **1569.6560** | 785.3316 | 1552.6294 | 776.8183 | 1551.6454 | 776.3263 | S | 262.1510 | 131.5791 | 245.1244 | 123.0659 | 244.1404 | 122.5738 | 2 |
| 15 | | | | | | | R | 175.1190 | 88.0631 | 158.0924 | 79.5498 | | | 1 |

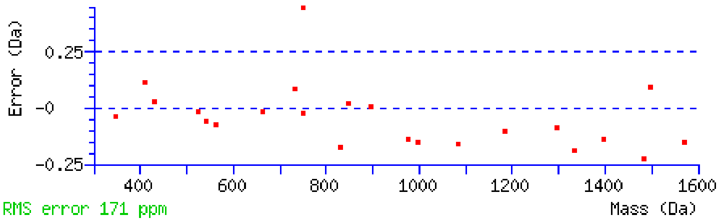


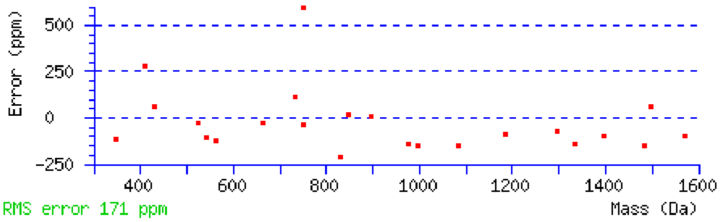


### All matches to this query

| Score | Mr(calc): | Delta | Sequence |
|---|---|---|---|
| 66.3 | 1742.7604 | 0.0029 | SCVTITDAFPQNMSR |
| 8.0 | 1740.7463 | 2.0169 | MKATEEMSDHLPIK |
| 6.5 | 1740.7623 | 2.0010 | MSPARMSPARMSPAR |
| 5.7 | 1740.7623 | 2.0010 | MSPARMSPARMSPAR |
| 4.2 | 1741.7764 | 0.9869 | SCVTITDAFPQNMSR |
| 4.0 | 1740.7623 | 2.0010 | MSPARMSPARMSPAR |
| 3.1 | 1742.7737 | -0.0104 | NSIHFSNSKNSVSSR |
| 2.6 | 1742.7635 | -0.0002 | DVNDWIGPPNNNGVTK |
| 2.4 | 1740.7736 | 1.9896 | DNSLSVSIPQPSPLK |
| 2.4 | 1740.7736 | 1.9896 | DNSLSVSIPQPSPLK |

---

Spectrum No: 423; Query: 2151; Rank: 1

---

## Peptide View

MS/MS Fragmentation of **ASFNITLIHPNNLTALSNMLPK**
Found in **IPI00230862**, Tax_Id=10116 Gene_Symbol=Anpep Aminopeptidase N

Match to Query 2151: 2410.261722 from(804.427850,3+)
Title: 100101RatKid_NS_deglyco_13.6464.6464.3.dta
Data file K:\NewmanPaper\Piliang\3SubProteomes\Piliang3SP\mgf5ppm\ERLIC_3SubProteomes5ppm.mgf

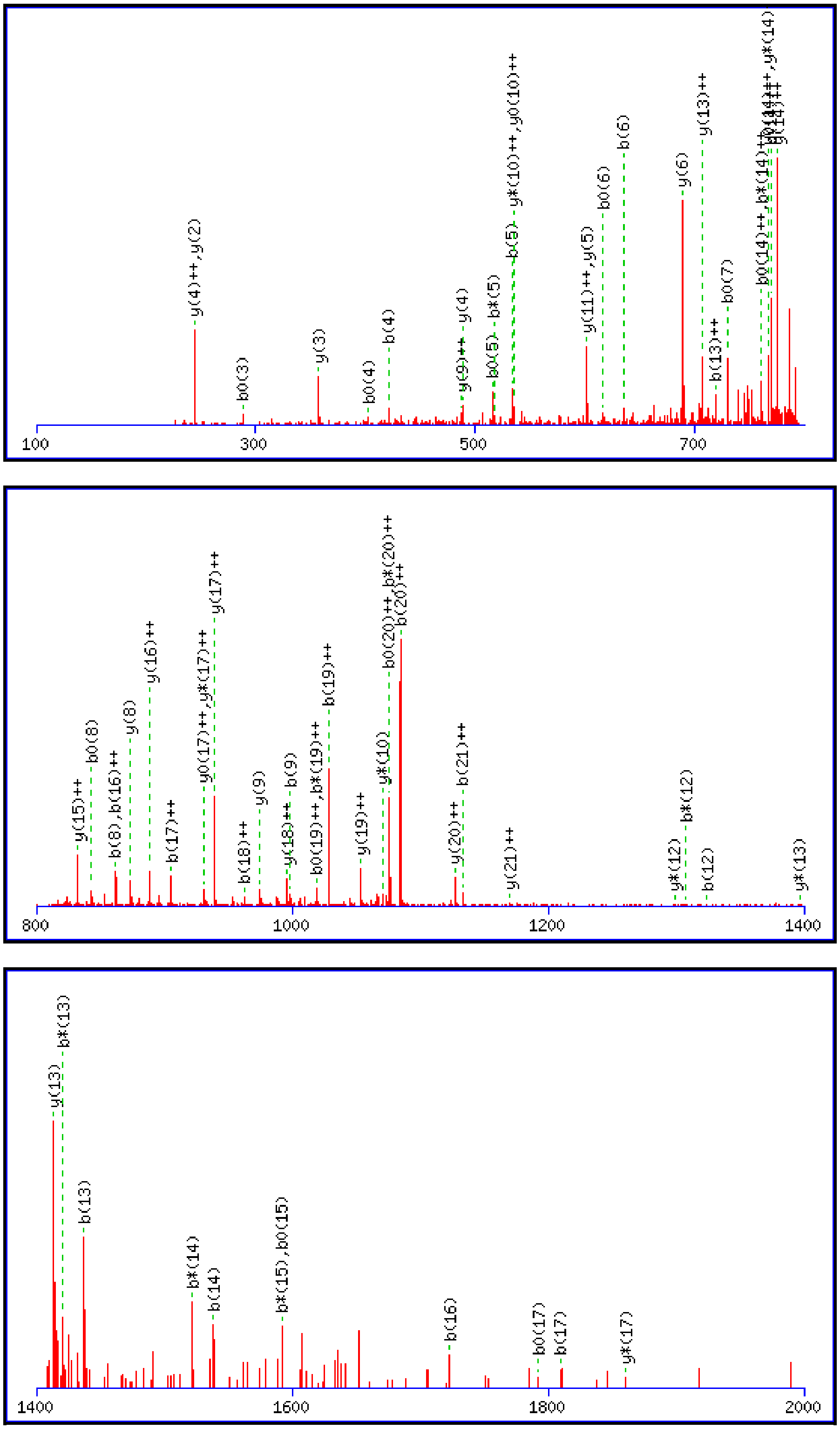


**Monoisotopic mass of neutral peptide Mr(calc):** 2410.2566
**Fixed modifications:** Carbamidomethyl (C)
**Variable modifications:**
**N4** : Deamidated_N (N)
**N12** : Deamidated_N (N)
**Ions Score:** 66 **Expect:** 3e-005
**Matches (Bold Red):** 67/234 fragment ions using 120 most intense peaks

| # | b | $b^{++}$ | b* | $b^{*++}$ | $b^0$ | $b^{0++}$ | Seq. | y | $y^{++}$ | y* | $y^{*++}$ | $y^0$ | $y^{0++}$ | # |
|---|---|---|---|---|---|---|---|---|---|---|---|---|---|---|
| 1 | 72.0444 | 36.5258 | | | | | A | | | | | | | 22 |
| 2 | 159.0764 | 80.0418 | | | 141.0659 | 71.0366 | S | 2340.2268 | **1170.6170** | 2323.2002 | 1162.1038 | 2322.2162 | 1161.6117 | 21 |
| 3 | 306.1448 | 153.5761 | | | **288.1343** | 144.5708 | F | 2253.1948 | **1127.1010** | 2236.1682 | 1118.5877 | 2235.1842 | 1118.0957 | 20 |
| 4 | **421.1718** | 211.0895 | 404.1452 | 202.5762 | **403.1612** | 202.0842 | N | 2106.1263 | **1053.5668** | 2089.0998 | 1045.0535 | 2088.1158 | 1044.5615 | 19 |

| | | | | | | | | | | | | | | |
|---|---|---|---|---|---|---|---|---|---|---|---|---|---|---|
| 5 | **534.2558** | 267.6316 | **517.2293** | 259.1183 | **516.2453** | 258.6263 | I | 1991.0994 | **996.0533** | 1974.0729 | 987.5401 | 1973.0888 | 987.0481 | 18 |
| 6 | **635.3035** | 318.1554 | 618.2770 | 309.6421 | **617.2929** | 309.1501 | T | 1878.0153 | **939.5113** | **1860.9888** | **930.9980** | 1860.0048 | **930.5060** | 17 |
| 7 | 748.3876 | 374.6974 | 731.3610 | 366.1842 | **730.3770** | 365.6921 | L | 1776.9677 | **888.9875** | 1759.9411 | 880.4742 | 1758.9571 | 879.9822 | 16 |
| 8 | **861.4716** | 431.2395 | 844.4451 | 422.7262 | **843.4611** | 422.2342 | I | 1663.8836 | **832.4454** | 1646.8570 | 823.9322 | 1645.8730 | 823.4402 | 15 |
| 9 | **998.5306** | 499.7689 | 981.5040 | 491.2556 | 980.5200 | 490.7636 | H | 1550.7995 | **775.9034** | 1533.7730 | **767.3901** | 1532.7890 | **766.8981** | 14 |
| 10 | 1095.5833 | 548.2953 | 1078.5568 | 539.7820 | 1077.5728 | 539.2900 | P | **1413.7406** | **707.3739** | **1396.7141** | 698.8607 | 1395.7301 | 698.3687 | 13 |
| 11 | 1209.6262 | 605.3168 | 1192.5997 | 596.8035 | 1191.6157 | 596.3115 | N | 1316.6879 | 658.8476 | **1299.6613** | 650.3343 | 1298.6773 | 649.8423 | 12 |
| 12 | **1324.6532** | 662.8302 | **1307.6266** | 654.3170 | 1306.6426 | 653.8249 | N | 1202.6449 | **601.8261** | 1185.6184 | 593.3128 | 1184.6344 | 592.8208 | 11 |
| 13 | **1437.7372** | **719.3723** | **1420.7107** | 710.8590 | 1419.7267 | 710.3670 | L | 1087.6180 | 544.3126 | **1070.5914** | **535.7994** | 1069.6074 | **535.3074** | 10 |
| 14 | **1538.7849** | **769.8961** | **1521.7584** | **761.3828** | 1520.7744 | **760.8908** | T | **974.5339** | **487.7706** | 957.5074 | 479.2573 | 956.5234 | 478.7653 | 9 |
| 15 | 1609.8220 | 805.4147 | **1592.7955** | 796.9014 | **1591.8115** | 796.4094 | A | **873.4863** | 437.2468 | 856.4597 | 428.7335 | 855.4757 | 428.2415 | 8 |
| 16 | **1722.9061** | **861.9567** | 1705.8795 | 853.4434 | 1704.8955 | 852.9514 | L | 802.4491 | 401.7282 | 785.4226 | 393.2149 | 784.4386 | 392.7229 | 7 |
| 17 | **1809.9381** | **905.4727** | 1792.9116 | 896.9594 | **1791.9276** | 896.4674 | S | **689.3651** | 345.1862 | 672.3385 | 336.6729 | 671.3545 | 336.1809 | 6 |
| 18 | 1923.9811 | **962.4942** | 1906.9545 | 953.9809 | 1905.9705 | 953.4889 | N | **602.3330** | 301.6702 | 585.3065 | 293.1569 | | | 5 |
| 19 | 2055.0215 | **1028.0144** | 2037.9950 | **1019.5011** | 2037.0110 | **1019.0091** | M | **488.2901** | **244.6487** | 471.2636 | 236.1354 | | | 4 |
| 20 | 2168.1056 | **1084.5564** | 2151.0791 | **1076.0432** | 2150.0950 | **1075.5512** | L | **357.2496** | 179.1285 | 340.2231 | 170.6152 | | | 3 |
| 21 | 2265.1584 | **1133.0828** | 2248.1318 | 1124.5695 | 2247.1478 | 1124.0775 | P | **244.1656** | 122.5864 | 227.1390 | 114.0731 | | | 2 |
| 22 | | | | | | | K | 147.1128 | 74.0600 | 130.0863 | 65.5468 | | | 1 |

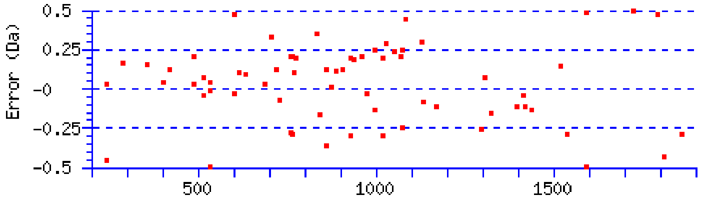


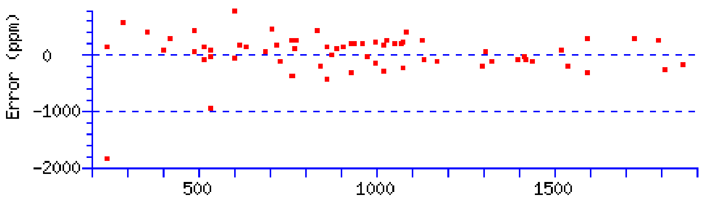


**All matches to this query**

| Score | Mr(calc): | Delta | Sequence |
|---|---|---|---|
| 66.3 | 2410.2566 | 0.0051 | ASFNITLIHPNNLTALSNMLPK |
| 64.8 | 2410.2566 | 0.0051 | ASFNITLIHPNNLTALSNMLPK |
| 59.5 | 2410.2566 | 0.0051 | ASFNITLIHPNNLTALSNMLPK |
| 49.5 | 2410.2566 | 0.0051 | ASFNITLIHPNNLTALSNMLPK |
| 40.3 | 2410.2566 | 0.0051 | ASFNITLIHPNNLTALSNMLPK |
| 40.3 | 2410.2566 | 0.0051 | ASFNITLIHPNNLTALSNMLPK |
| 27.5 | 2409.2726 | 0.9892 | ASFNITLIHPNNLTALSNMLPK |
| 25.5 | 2409.2726 | 0.9892 | ASFNITLIHPNNLTALSNMLPK |
| 25.3 | 2409.2726 | 0.9892 | ASFNITLIHPNNLTALSNMLPK |
| 7.8 | 2409.2726 | 0.9892 | ASFNITLIHPNNLTALSNMLPK |

---

Spectrum No: 424; Query: 805; Rank: 1

---

## Peptide View

MS/MS Fragmentation of **GVFITNETGQPLIGK**
Found in **IPI00400579**, Tax_Id=10116 Gene_Symbol=Gaa Lysosomal alpha-glucosidase precursor

Match to Query 805: 1573.833548 from(787.924050,2+)
Title: 091008RatKidney_NH4Format01_23.2641.2641.2.dta
Data file K:\NewmanPaper\Piliang\3SubProteomes\Piliang3SP\mgf5ppm\ERLIC_3SubProteomes5ppm.mgf

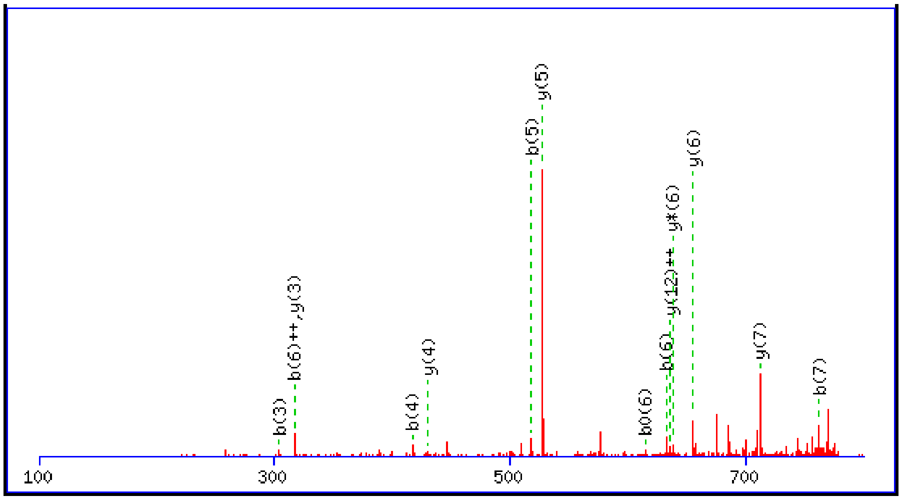


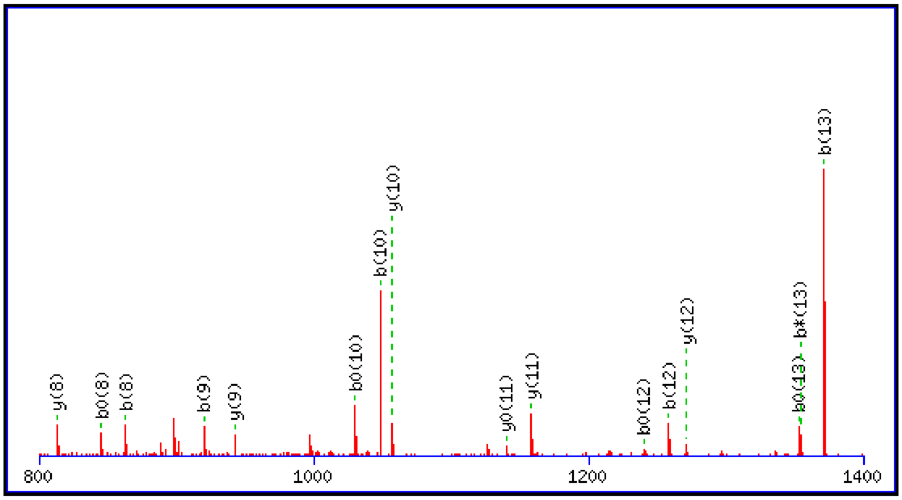


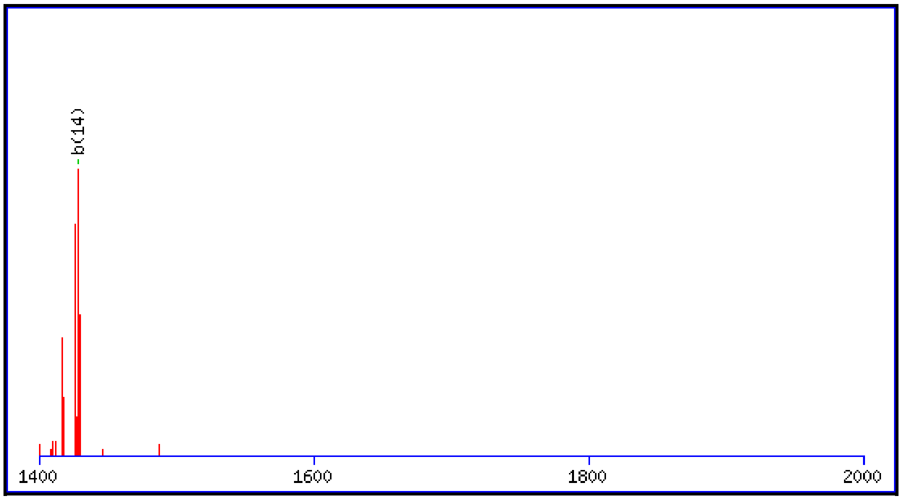


**Monoisotopic mass of neutral peptide Mr(calc):** 1573.8352
**Fixed modifications:** Carbamidomethyl (C)
**Variable modifications:**
**N6** : Deamidated_N (N)
**Ions Score:** 66 **Expect:** 2.7e-005
**Matches (Bold Red):** 31/136 fragment ions using 57 most intense peaks

| # | b | $b^{++}$ | b* | $b^{*++}$ | $b^0$ | $b^{0++}$ | Seq. | y | $y^{++}$ | y* | $y^{*++}$ | $y^0$ | $y^{0++}$ | # |
|---|---|---|---|---|---|---|---|---|---|---|---|---|---|---|
| 1 | 58.0287 | 29.5180 | | | | | G | | | | | | | 15 |
| 2 | 157.0972 | 79.0522 | | | | | V | 1517.8210 | 759.4141 | 1500.7944 | 750.9009 | 1499.8104 | 750.4088 | 14 |
| 3 | **304.1656** | 152.5864 | | | | | F | 1418.7526 | 709.8799 | 1401.7260 | 701.3666 | 1400.7420 | 700.8746 | 13 |
| 4 | **417.2496** | 209.1285 | | | | | I | **1271.6842** | **636.3457** | 1254.6576 | 627.8324 | 1253.6736 | 627.3404 | 12 |
| 5 | **518.2973** | 259.6523 | | | 500.2867 | 250.6470 | T | **1158.6001** | 579.8037 | 1141.5735 | 571.2904 | **1140.5895** | 570.7984 | 11 |

| | | | | | | | | | | | | | |
|---|---|---|---|---|---|---|---|---|---|---|---|---|---|
| **6** | **633.3242** | **317.1658** | 616.2977 | 308.6525 | **615.3137** | 308.1605 | **N** | **1057.5524** | 529.2798 | 1040.5259 | 520.7666 | 1039.5418 | 520.2746 | **10** |
| **7** | **762.3668** | 381.6871 | 745.3403 | 373.1738 | 744.3563 | 372.6818 | **E** | **942.5255** | 471.7664 | 925.4989 | 463.2531 | 924.5149 | 462.7611 | **9** |
| **8** | **863.4145** | 432.2109 | 846.3880 | 423.6976 | **845.4040** | 423.2056 | **T** | **813.4829** | 407.2451 | 796.4563 | 398.7318 | 795.4723 | 398.2398 | **8** |
| **9** | **920.4360** | 460.7216 | 903.4094 | 452.2084 | 902.4254 | 451.7163 | **G** | **712.4352** | 356.7212 | 695.4087 | 348.2080 | | | **7** |
| **10** | **1048.4946** | 524.7509 | 1031.4680 | 516.2376 | **1030.4840** | 515.7456 | **Q** | **655.4137** | 328.2105 | **638.3872** | 319.6972 | | | **6** |
| **11** | 1145.5473 | 573.2773 | 1128.5208 | 564.7640 | 1127.5368 | 564.2720 | **P** | **527.3552** | 264.1812 | 510.3286 | 255.6679 | | | **5** |
| **12** | **1258.6314** | 629.8193 | 1241.6048 | 621.3061 | **1240.6208** | 620.8141 | **L** | **430.3024** | 215.6548 | 413.2758 | 207.1416 | | | **4** |
| **13** | **1371.7155** | 686.3614 | **1354.6889** | 677.8481 | **1353.7049** | 677.3561 | **I** | **317.2183** | 159.1128 | 300.1918 | 150.5995 | | | **3** |
| **14** | **1428.7369** | 714.8721 | 1411.7104 | 706.3588 | 1410.7264 | 705.8668 | **G** | 204.1343 | 102.5708 | 187.1077 | 94.0575 | | | **2** |
| **15** | | | | | | | **K** | 147.1128 | 74.0600 | 130.0863 | 65.5468 | | | **1** |

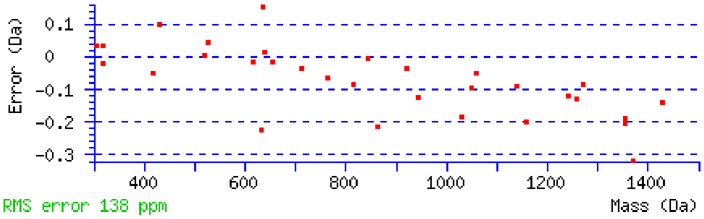


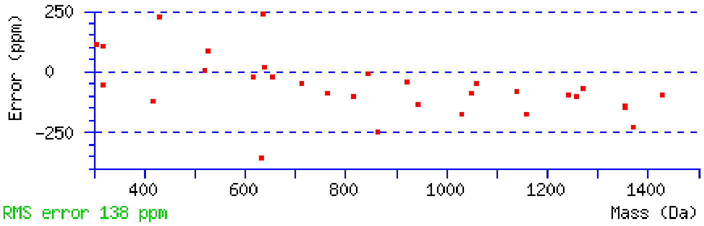


### All matches to this query

| Score | Mr(calc): | Delta | Sequence |
|---|---|---|---|
| 66.1 | 1573.8352 | -0.0016 | GVFITNETGQPLIGK |
| 7.7 | 1573.8327 | 0.0009 | NMKLQPVPWFIGK |
| 5.1 | 1573.8423 | -0.0088 | SLDESTLRKQIER |
| 4.5 | 1573.8199 | 0.0136 | SDLTDKAEVLDQLK |
| 2.5 | 1571.8229 | 2.0107 | EALMPTLGEIKDQK |
| 2.1 | 1571.8229 | 2.0107 | EGKLKPLPGETMEK |
| 1.4 | 1573.8181 | 0.0155 | VRGGRCAILNCLGK |

---

Spectrum No: 425; Query: 1831; Rank: 1

---

## Peptide View

MS/MS Fragmentation of **LWVPNTSFDTASNWNQNR**
Found in **IPI00870509**, Tax_Id=10116 Gene_Symbol=Amn_predicted amnionless

Match to Query 1831: 2149.987028 from(1076.000790,2+)
Title: 100101RatKid_NS_deglyco_14.4243.4243.2.dta
Data file K:\NewmanPaper\Piliang\3SubProteomes\Piliang3SP\mgf5ppm\ERLIC_3SubProteomes5ppm.mgf

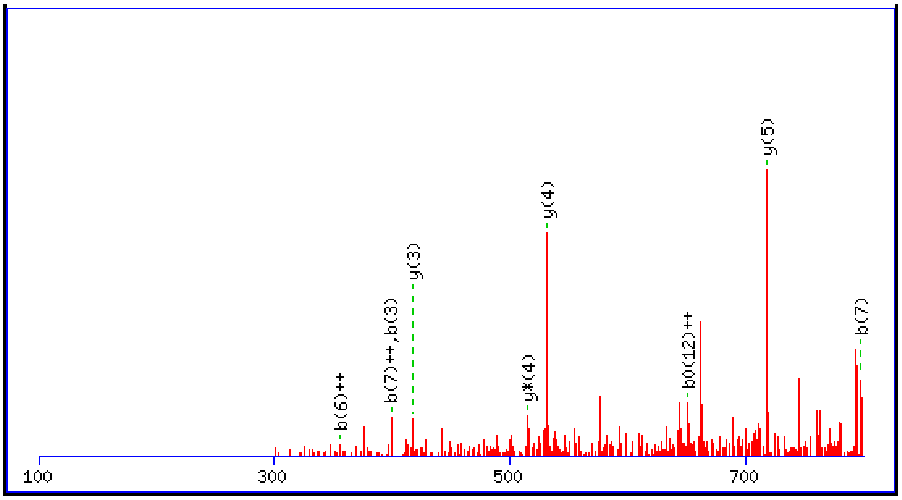


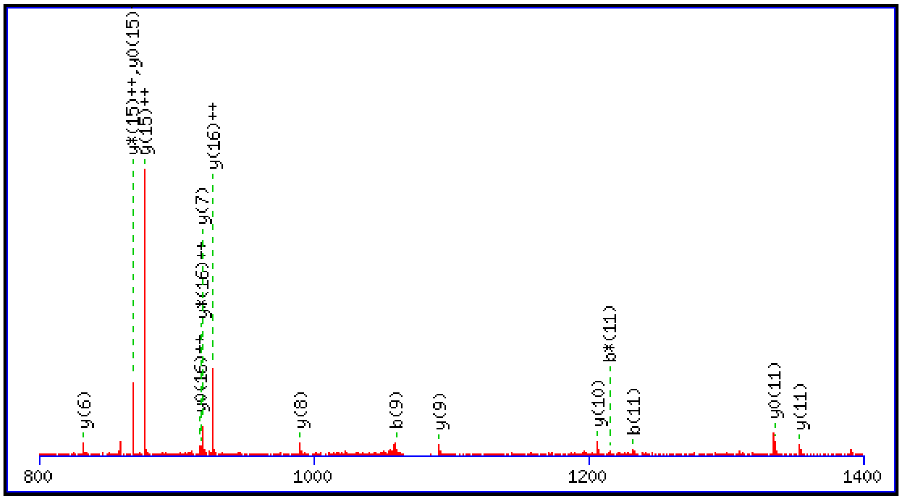


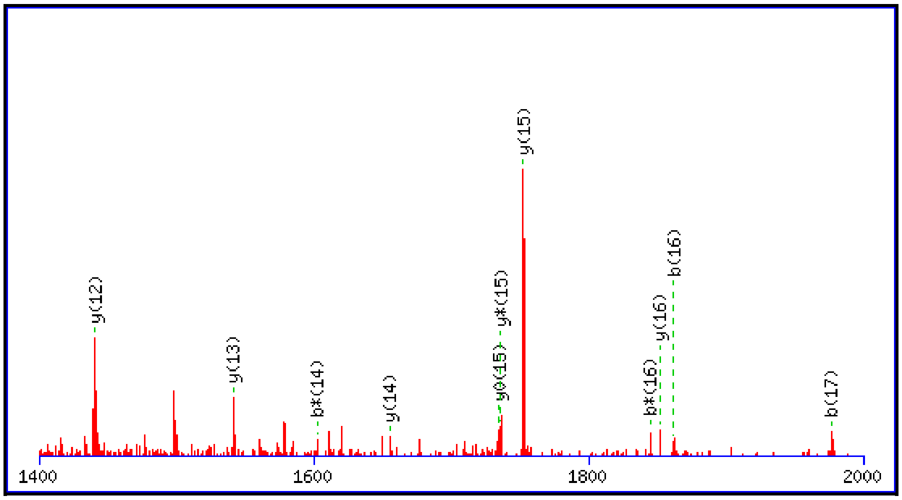


**Monoisotopic mass of neutral peptide Mr(calc):** 2149.9817
**Fixed modifications:** Carbamidomethyl (C)
**Variable modifications:**
**N17** : Deamidated_N (N)
**Ions Score:** 66 **Expect:** 6.6e-005
**Matches (Bold Red):** 36/174 fragment ions using 88 most intense peaks

| # | b | $b^{++}$ | b* | $b^{*++}$ | $b^{0}$ | $b^{0++}$ | Seq. | y | $y^{++}$ | y* | $y^{*++}$ | $y^{0}$ | $y^{0++}$ | # |
|---|---|---|---|---|---|---|---|---|---|---|---|---|---|---|
| 1 | 114.0913 | 57.5493 | | | | | L | | | | | | | 18 |
| 2 | 300.1707 | 150.5890 | | | | | W | 2037.9049 | 1019.4561 | 2020.8784 | 1010.9428 | 2019.8944 | 1010.4508 | 17 |
| 3 | **399.2391** | 200.1232 | | | | | V | **1851.8256** | **926.4164** | 1834.7991 | **917.9032** | 1833.8151 | **917.4112** | 16 |
| 4 | 496.2918 | 248.6496 | | | | | P | **1752.7572** | **876.8822** | **1735.7307** | **868.3690** | **1734.7466** | **867.8770** | 15 |
| 5 | 610.3348 | 305.6710 | 593.3082 | 297.1577 | | | N | **1655.7044** | 828.3559 | 1638.6779 | 819.8426 | 1637.6939 | 819.3506 | 14 |

| | | | | | | | | | | | | | |
|---|---|---|---|---|---|---|---|---|---|---|---|---|---|
| 6 | 711.3824 | 356.1949 | 694.3559 | 347.6816 | 693.3719 | 347.1896 | T | 1541.6615 | 771.3344 | 1524.6350 | 762.8211 | 1523.6510 | 762.3291 | 13 |
| 7 | 798.4145 | 399.7109 | 781.3879 | 391.1976 | 780.4039 | 390.7056 | S | 1440.6138 | 720.8106 | 1423.5873 | 712.2973 | 1422.6033 | 711.8053 | 12 |
| 8 | 945.4829 | 473.2451 | 928.4563 | 464.7318 | 927.4723 | 464.2398 | F | 1353.5818 | 677.2945 | 1336.5553 | 668.7813 | 1335.5712 | 668.2893 | 11 |
| 9 | 1060.5098 | 530.7585 | 1043.4833 | 522.2453 | 1042.4993 | 521.7533 | D | 1206.5134 | 603.7603 | 1189.4868 | 595.2471 | 1188.5028 | 594.7551 | 10 |
| 10 | 1161.5575 | 581.2824 | 1144.5310 | 572.7691 | 1143.5469 | 572.2771 | T | 1091.4865 | 546.2469 | 1074.4599 | 537.7336 | 1073.4759 | 537.2416 | 9 |
| 11 | 1232.5946 | 616.8009 | 1215.5681 | 608.2877 | 1214.5840 | 607.7957 | A | 990.4388 | 495.7230 | 973.4122 | 487.2098 | 972.4282 | 486.7177 | 8 |
| 12 | 1319.6266 | 660.3170 | 1302.6001 | 651.8037 | 1301.6161 | 651.3117 | S | 919.4017 | 460.2045 | 902.3751 | 451.6912 | 901.3911 | 451.1992 | 7 |
| 13 | 1433.6696 | 717.3384 | 1416.6430 | 708.8251 | 1415.6590 | 708.3331 | N | 832.3696 | 416.6885 | 815.3431 | 408.1752 | | | 6 |
| 14 | 1619.7489 | 810.3781 | 1602.7223 | 801.8648 | 1601.7383 | 801.3728 | W | 718.3267 | 359.6670 | 701.3002 | 351.1537 | | | 5 |
| 15 | 1733.7918 | 867.3995 | 1716.7653 | 858.8863 | 1715.7812 | 858.3943 | N | 532.2474 | 266.6273 | 515.2208 | 258.1141 | | | 4 |
| 16 | 1861.8504 | 931.4288 | 1844.8238 | 922.9156 | 1843.8398 | 922.4235 | Q | 418.2045 | 209.6059 | 401.1779 | 201.0926 | | | 3 |
| 17 | 1976.8773 | 988.9423 | 1959.8508 | 980.4290 | 1958.8668 | 979.9370 | N | 290.1459 | 145.5766 | 273.1193 | 137.0633 | | | 2 |
| 18 | | | | | | | R | 175.1190 | 88.0631 | 158.0924 | 79.5498 | | | 1 |

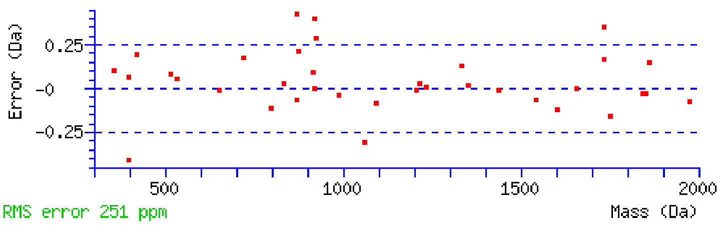


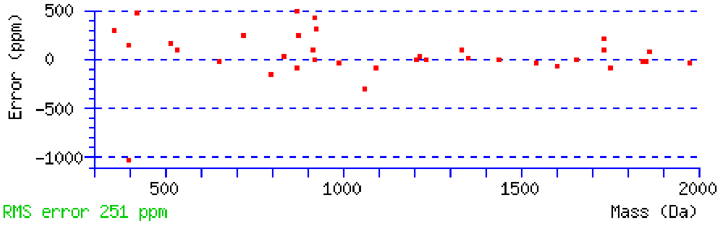


**All matches to this query**

| Score | Mr(calc): | Delta | Sequence |
|---|---|---|---|
| 66.0 | 2149.9817 | 0.0053 | LWVPNTSFDTASNWNQNR |
| 59.6 | 2149.9817 | 0.0053 | LWVPNTSFDTASNWNQNR |
| 48.5 | 2149.9817 | 0.0053 | LWVPNTSFDTASNWNQNR |
| 14.5 | 2149.9817 | 0.0053 | LWVPNTSFDTASNWNQNR |
| 7.1 | 2150.0004 | -0.0134 | RIEELEGQLDQLTQENR |
| 5.9 | 2149.9753 | 0.0117 | RNQKLSSVEEDPGVNQDR |
| 5.9 | 2149.9753 | 0.0117 | RNQKLSSVEEDPGVNQDR |
| 3.8 | 2150.0062 | -0.0191 | KHDEHNEKELDLMINQR |
| 3.5 | 2149.9785 | 0.0085 | WNFLLTAVSKAMTLSHR |
| 3.0 | 2149.9785 | 0.0085 | WNFLLTAVSKAMTLSHR |

---

Spectrum No: 426; Query: 118; Rank: 1

---

## Peptide View

MS/MS Fragmentation of **EYNVSEDTR**
Found in **IPI00200640**, Tax_Id=10116 Gene_Symbol=Mucdhl Isoform 1 of Mucin and cadherin-like protein precursor

Match to Query 118: 1112.462008 from(557.238280,2+)
Title: 091008RatKidney_NoSalt_28.569.569.2.dta
Data file K:\NewmanPaper\Piliang\3SubProteomes\Piliang3SP\mgf5ppm\ERLIC_3SubProteomes5ppm.mgf

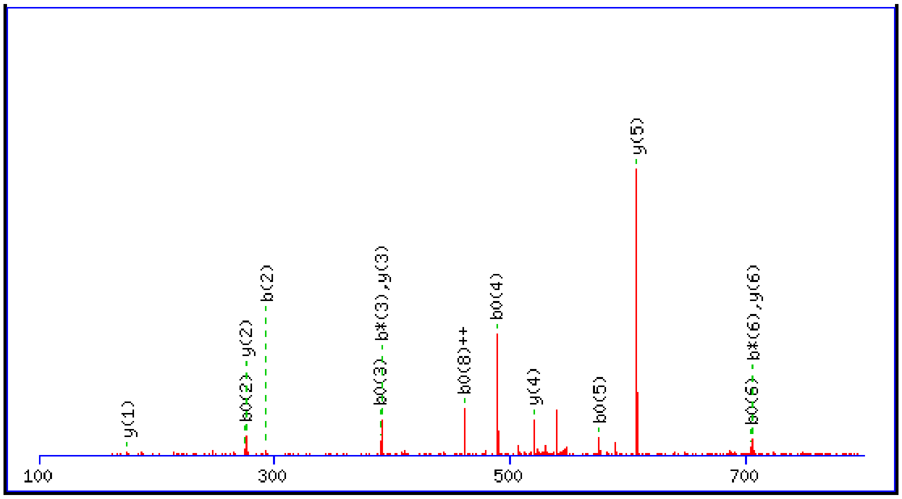


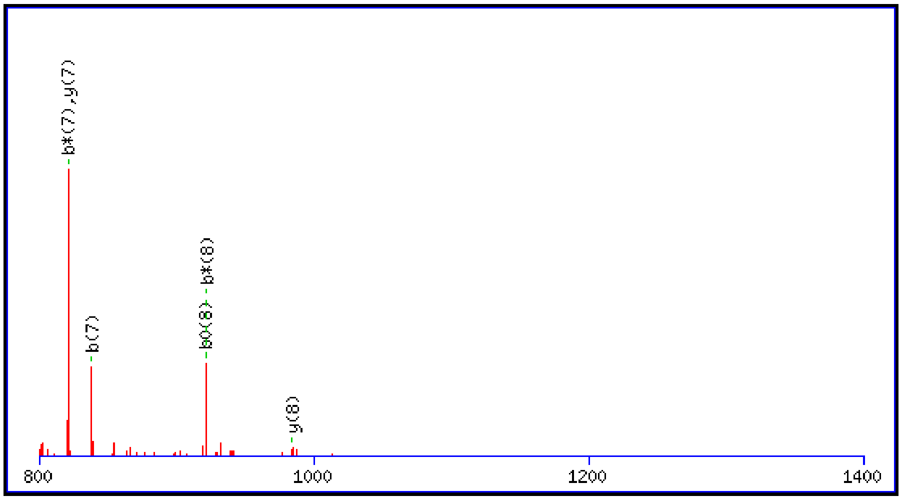


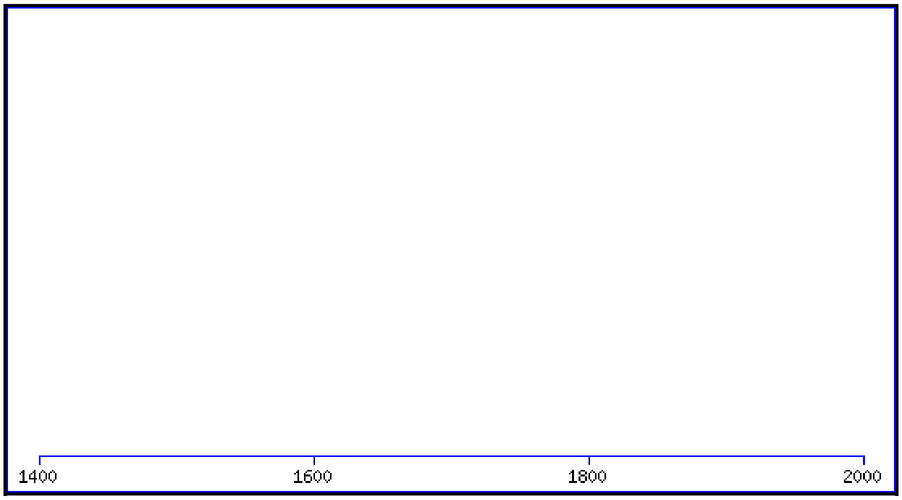


**Monoisotopic mass of neutral peptide Mr(calc):** 1112.4622
**Fixed modifications:** Carbamidomethyl (C)
**Variable modifications:**
**N3** : Deamidated_N (N)
**Ions Score:** 66 **Expect:** 2e-005
**Matches (Bold Red):** 21/90 fragment ions using 27 most intense peaks

| # | b | $b^{++}$ | b* | $b^{*++}$ | $b^0$ | $b^{0++}$ | Seq. | y | $y^{++}$ | y* | $y^{*++}$ | $y^0$ | $y^{0++}$ | # |
|---|---|---|---|---|---|---|---|---|---|---|---|---|---|---|
| 1 | 130.0499 | 65.5286 | | | 112.0393 | 56.5233 | E | | | | | | | 9 |
| 2 | **293.1132** | 147.0602 | | | **275.1026** | 138.0550 | Y | **984.4269** | 492.7171 | 967.4003 | 484.2038 | 966.4163 | 483.7118 | 8 |
| 3 | 408.1401 | 204.5737 | **391.1136** | 196.0604 | **390.1296** | 195.5684 | N | **821.3635** | 411.1854 | 804.3370 | 402.6721 | 803.3530 | 402.1801 | 7 |
| 4 | 507.2085 | 254.1079 | 490.1820 | 245.5946 | **489.1980** | 245.1026 | V | **706.3366** | 353.6719 | 689.3101 | 345.1587 | 688.3260 | 344.6667 | 6 |
| 5 | 594.2406 | 297.6239 | 577.2140 | 289.1107 | **576.2300** | 288.6186 | S | **607.2682** | 304.1377 | 590.2416 | 295.6245 | 589.2576 | 295.1325 | 5 |

| | | | | | | | | | | | | | | |
|---|---|---|---|---|---|---|---|---|---|---|---|---|---|---|
| 6 | 723.2832 | 362.1452 | 706.2566 | 353.6319 | 705.2726 | 353.1399 | E | 520.2362 | 260.6217 | 503.2096 | 252.1084 | 502.2256 | 251.6164 | 4 |
| 7 | 838.3101 | 419.6587 | 821.2836 | 411.1454 | 820.2995 | 410.6534 | D | 391.1936 | 196.1004 | 374.1670 | 187.5872 | 373.1830 | 187.0951 | 3 |
| 8 | 939.3578 | 470.1825 | 922.3312 | 461.6693 | 921.3472 | 461.1773 | T | 276.1666 | 138.5870 | 259.1401 | 130.0737 | 258.1561 | 129.5817 | 2 |
| 9 | | | | | | | R | 175.1190 | 88.0631 | 158.0924 | 79.5498 | | | 1 |

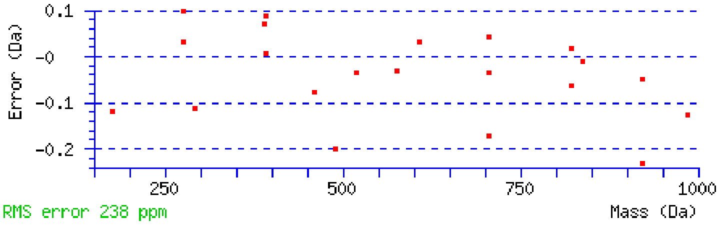


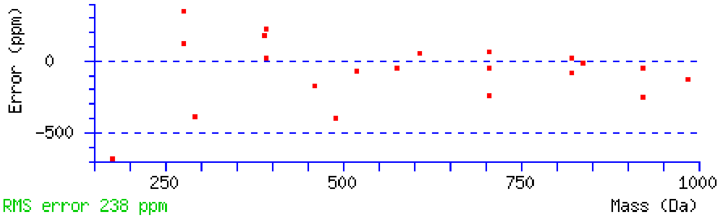


### All matches to this query

| Score | Mr(calc): | Delta | Sequence |
|---|---|---|---|
| 65.9 | 1112.4622 | -0.0002 | EYNVSEDTR |
| 7.8 | 1112.4638 | -0.0018 | EPTTVETEK |
| 6.0 | 1111.4669 | 0.9951 | VEDYTNNQK |
| 5.1 | 1110.4495 | 2.0125 | DQFESHLR |
| 3.1 | 1112.4638 | -0.0018 | EPTTVETEK |
| 2.4 | 1110.4495 | 2.0125 | RYSFSESR |
| 1.8 | 1112.4726 | -0.0106 | EVMFPPGTR |
| 1.7 | 1111.4521 | 1.0099 | YYVREMR |
| 1.7 | 1111.4521 | 1.0099 | YYVREMR |
| 1.4 | 1112.4652 | -0.0032 | GESVFHTTR |

---

Spectrum No: 427; Query: 2760; Rank: 1

---

## Peptide View

MS/MS Fragmentation of **SYLDDGPLQHYIAVSSPTNTTYVVQYALANLTGK**
Found in **IPI00192495**, Tax_Id=10116 Gene_Symbol=Ncstn Isoform 1 of Nicastrin precursor

Match to Query 2760: 3700.833522 from(1234.618450,3+)
Title: 100101RatKid_NS_deglyco_16.6848.6848.3.dta
Data file K:\NewmanPaper\Piliang\3SubProteomes\Piliang3SP\mgf5ppm\ERLIC_3SubProteomes5ppm.mgf

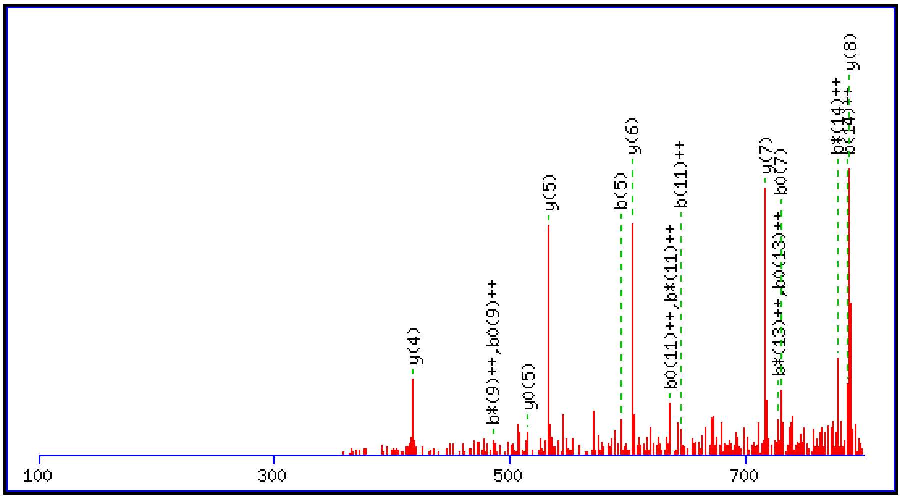

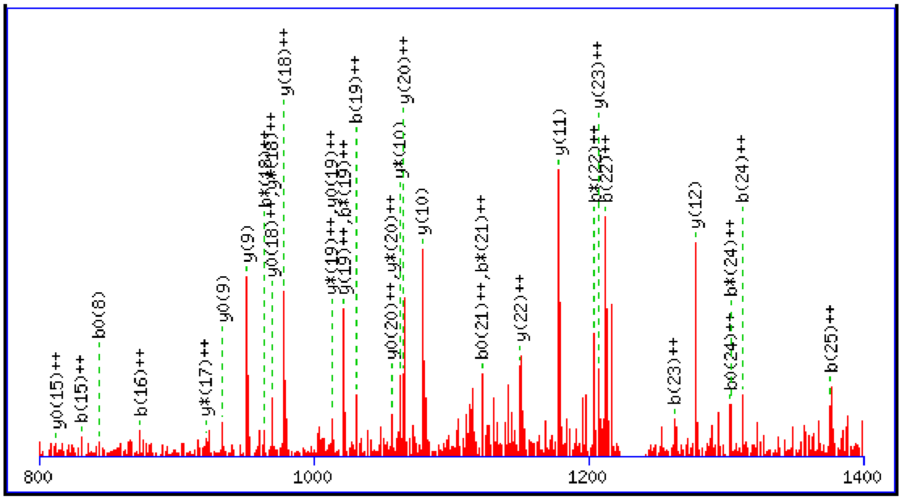


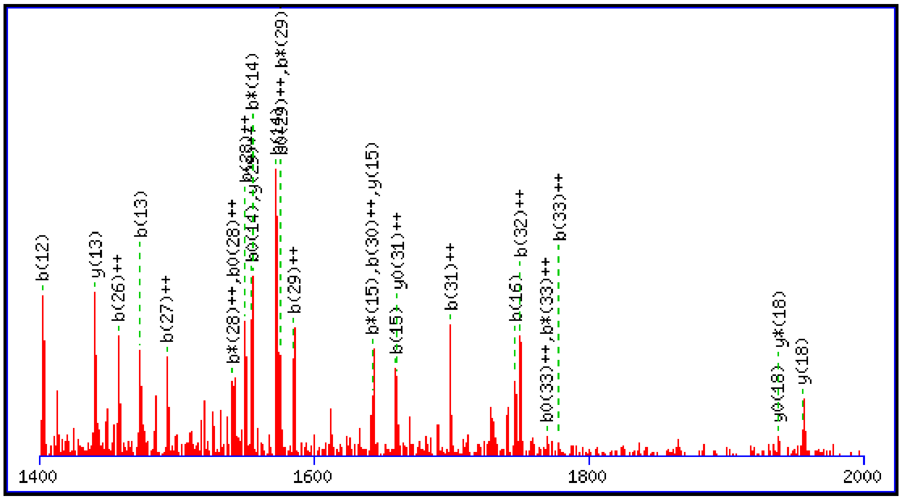


**Monoisotopic mass of neutral peptide Mr(calc):** 3700.8148
**Fixed modifications:** Carbamidomethyl (C)
**Variable modifications:**
**N19** : Deamidated_N (N)
**N30** : Deamidated_N (N)
**Ions Score:** 66 **Expect:** 6.1e-005
**Matches (Bold Red):** 80/376 fragment ions using 145 most intense peaks

| # | b | $b^{++}$ | b* | $b^{*++}$ | $b^{0}$ | $b^{0++}$ | Seq. | y | $y^{++}$ | y* | $y^{*++}$ | $y^{0}$ | $y^{0++}$ | # |
|---|---|---|---|---|---|---|---|---|---|---|---|---|---|---|
| 1 | 88.0393 | 44.5233 | | | 70.0287 | 35.5180 | S | | | | | | | 34 |
| 2 | 251.1026 | 126.0550 | | | 233.0921 | 117.0497 | Y | 3614.7901 | 1807.8987 | 3597.7635 | 1799.3854 | 3596.7795 | 1798.8934 | 33 |
| 3 | 364.1867 | 182.5970 | | | 346.1761 | 173.5917 | L | 3451.7267 | 1726.3670 | 3434.7002 | 1717.8537 | 3433.7162 | 1717.3617 | 32 |
| 4 | 479.2136 | 240.1105 | | | 461.2031 | 231.1052 | D | 3338.6427 | 1669.8250 | 3321.6161 | 1661.3117 | 3320.6321 | **1660.8197** | 31 |
| 5 | **594.2406** | 297.6239 | | | 576.2300 | 288.6186 | D | 3223.6157 | 1612.3115 | 3206.5892 | 1603.7982 | 3205.6052 | 1603.3062 | 30 |
| 6 | 651.2620 | 326.1347 | | | 633.2515 | 317.1294 | G | 3108.5888 | **1554.7980** | 3091.5622 | 1546.2848 | 3090.5782 | 1545.7928 | 29 |
| 7 | 748.3148 | 374.6610 | | | **730.3042** | 365.6558 | P | 3051.5673 | 1526.2873 | 3034.5408 | 1517.7740 | 3033.5568 | 1517.2820 | 28 |
| 8 | 861.3989 | 431.2031 | | | **843.3883** | 422.1978 | L | 2954.5146 | 1477.7609 | 2937.4880 | 1469.2476 | 2936.5040 | 1468.7556 | 27 |
| 9 | 989.4575 | 495.2324 | 972.4309 | **486.7191** | 971.4469 | **486.2271** | Q | 2841.4305 | 1421.2189 | 2824.4040 | 1412.7056 | 2823.4199 | 1412.2136 | 26 |
| 10 | 1126.5164 | 563.7618 | 1109.4898 | 555.2485 | 1108.5058 | 554.7565 | H | 2713.3719 | 1357.1896 | 2696.3454 | 1348.6763 | 2695.3614 | 1348.1843 | 25 |
| 11 | 1289.5797 | **645.2935** | 1272.5531 | **636.7802** | 1271.5691 | **636.2882** | Y | 2576.3130 | 1288.6601 | 2559.2865 | 1280.1469 | 2558.3025 | 1279.6549 | 24 |
| 12 | **1402.6638** | 701.8355 | 1385.6372 | 693.3222 | 1384.6532 | 692.8302 | I | 2413.2497 | **1207.1285** | 2396.2231 | 1198.6152 | 2395.2391 | 1198.1232 | 23 |
| 13 | **1473.7009** | 737.3541 | 1456.6743 | **728.8408** | 1455.6903 | **728.3488** | A | 2300.1656 | **1150.5864** | 2283.1391 | 1142.0732 | 2282.1551 | 1141.5812 | 22 |
| 14 | **1572.7693** | **786.8883** | **1555.7427** | **778.3750** | **1554.7587** | 777.8830 | V | 2229.1285 | 1115.0679 | 2212.1020 | 1106.5546 | 2211.1179 | 1106.0626 | 21 |
| 15 | **1659.8013** | **830.4043** | **1642.7748** | 821.8910 | 1641.7907 | 821.3990 | S | 2130.0601 | **1065.5337** | 2113.0335 | **1057.0204** | 2112.0495 | **1056.5284** | 20 |
| 16 | **1746.8333** | **873.9203** | 1729.8068 | 865.4070 | 1728.8228 | 864.9150 | S | 2043.0281 | **1022.0177** | 2026.0015 | **1013.5044** | 2025.0175 | **1013.0124** | 19 |
| 17 | 1843.8861 | 922.4467 | 1826.8596 | 913.9334 | 1825.8755 | 913.4414 | P | **1955.9960** | **978.5017** | **1938.9695** | **969.9884** | **1937.9855** | **969.4964** | 18 |
| 18 | 1944.9338 | 972.9705 | 1927.9072 | **964.4573** | 1926.9232 | 963.9652 | T | 1858.9433 | 929.9753 | 1841.9167 | **921.4620** | 1840.9327 | 920.9700 | 17 |

| | | | | | | | | | | | | | | |
|---|---|---|---|---|---|---|---|---|---|---|---|---|---|---|
| 19 | 2059.9607 | **1030.4840** | 2042.9342 | **1021.9707** | 2041.9502 | 1021.4787 | N | 1757.8956 | 879.4514 | 1740.8690 | 870.9382 | 1739.8850 | 870.4462 | 16 |
| 20 | 2161.0084 | 1081.0078 | 2143.9819 | 1072.4946 | 2142.9978 | 1072.0026 | T | **1642.8687** | 821.9380 | 1625.8421 | 813.4247 | 1624.8581 | **812.9327** | 15 |
| 21 | 2262.0561 | 1131.5317 | 2245.0295 | **1123.0184** | 2244.0455 | **1122.5264** | T | 1541.8210 | 771.4141 | 1524.7944 | 762.9009 | 1523.8104 | 762.4088 | 14 |
| 22 | 2425.1194 | **1213.0633** | 2408.0929 | **1204.5501** | 2407.1088 | 1204.0581 | Y | **1440.7733** | 720.8903 | 1423.7468 | 712.3770 | 1422.7627 | 711.8850 | 13 |
| 23 | 2524.1878 | **1262.5975** | 2507.1613 | 1254.0843 | 2506.1773 | 1253.5923 | V | **1277.7100** | 639.3586 | 1260.6834 | 630.8454 | 1259.6994 | 630.3533 | 12 |
| 24 | 2623.2562 | **1312.1318** | 2606.2297 | **1303.6185** | 2605.2457 | **1303.1265** | V | **1178.6416** | 589.8244 | 1161.6150 | 581.3111 | 1160.6310 | 580.8191 | 11 |
| 25 | 2751.3148 | **1376.1610** | 2734.2883 | 1367.6478 | 2733.3042 | 1367.1558 | Q | **1079.5731** | 540.2902 | **1062.5466** | 531.7769 | 1061.5626 | 531.2849 | 10 |
| 26 | 2914.3781 | **1457.6927** | 2897.3516 | 1449.1794 | 2896.3676 | 1448.6874 | Y | **951.5146** | 476.2609 | 934.4880 | 467.7476 | **933.5040** | 467.2556 | 9 |
| 27 | 2985.4153 | **1493.2113** | 2968.3887 | 1484.6980 | 2967.4047 | 1484.2060 | A | **788.4512** | 394.7293 | 771.4247 | 386.2160 | 770.4407 | 385.7240 | 8 |
| 28 | 3098.4993 | **1549.7533** | 3081.4728 | **1541.2400** | 3080.4888 | **1540.7480** | L | **717.4141** | 359.2107 | 700.3876 | 350.6974 | 699.4036 | 350.2054 | 7 |
| 29 | 3169.5364 | **1585.2719** | 3152.5099 | **1576.7586** | 3151.5259 | **1576.2666** | A | **604.3301** | 302.6687 | 587.3035 | 294.1554 | 586.3195 | 293.6634 | 6 |
| 30 | 3284.5634 | **1642.7853** | 3267.5368 | 1634.2720 | 3266.5528 | 1633.7800 | N | **533.2929** | 267.1501 | 516.2664 | 258.6368 | **515.2824** | 258.1448 | 5 |
| 31 | 3397.6474 | **1699.3274** | 3380.6209 | 1690.8141 | 3379.6369 | 1690.3221 | L | **418.2660** | 209.6366 | 401.2395 | 201.1234 | 400.2554 | 200.6314 | 4 |
| 32 | 3498.6951 | **1749.8512** | 3481.6686 | 1741.3379 | 3480.6845 | 1740.8459 | T | 305.1819 | 153.0946 | 288.1554 | 144.5813 | 287.1714 | 144.0893 | 3 |
| 33 | 3555.7166 | **1778.3619** | 3538.6900 | **1769.8487** | 3537.7060 | **1769.3566** | G | 204.1343 | 102.5708 | 187.1077 | 94.0575 | | | 2 |
| 34 | | | | | | | K | 147.1128 | 74.0600 | 130.0863 | 65.5468 | | | 1 |

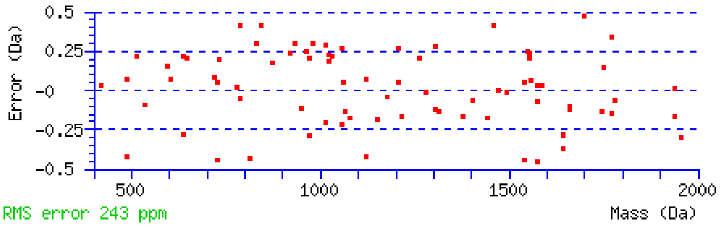


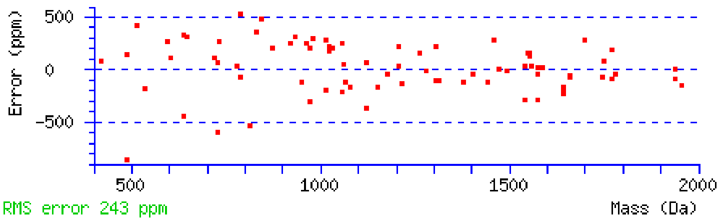


**All matches to this query**

| Score | Mr(calc): | Delta | Sequence |
|---|---|---|---|
| 65.8 | 3700.8148 | 0.0187 | SYLDDGPLQHYIAVSSPTNTTYVVQYALANLTGK |
| 54.4 | 3699.8308 | 1.0027 | SYLDDGPLQHYIAVSSPTNTTYVVQYALANLTGK |
| 27.7 | 3699.8308 | 1.0027 | SYLDDGPLQHYIAVSSPTNTTYVVQYALANLTGK |
| 10.0 | 3699.8493 | 0.9842 | SWCTLGFVRSIALTPQACGALSSPRWITLLMK |
| 3.4 | 3698.8266 | 2.0069 | IKTNVINPAYAAELGQIENSLHMGYSALEIKSK |
| 3.4 | 3698.8266 | 2.0069 | IKTNVINPAYAAELGQIENSLHMGYSALEIKSK |
| 3.0 | 3698.8468 | 1.9867 | SYLDDGPLQHYIAVSSPTNTTYVVQYALANLTGK |
| 2.3 | 3699.8106 | 1.0229 | IKTNVINPAYAAELGQIENSLHMGYSALEIKSK |
| 2.3 | 3699.8106 | 1.0229 | IKTNVINPAYAAELGQIENSLHMGYSALEIKSK |
| 2.0 | 3698.7950 | 2.0385 | ISLPLYSILSALTILGMIMASAFLFFNIKNR |

---

Spectrum No: 428; Query: 1844; Rank: 1

---

## Peptide View

MS/MS Fragmentation of **SLTQGSMIVGNLAPVNGTSQGK**
Found in **IPI00388323**, Tax_Id=10116 Gene_Symbol=Hspg2 396 kDa protein

Match to Query 1844: 2159.095128 from(1080.554840,2+)
Title: 100101RatKid_NS_deglyco_14.3410.3410.2.dta
Data file K:\NewmanPaper\Piliang\3SubProteomes\Piliang3SP\mgf5ppm\ERLIC_3SubProteomes5ppm.mgf

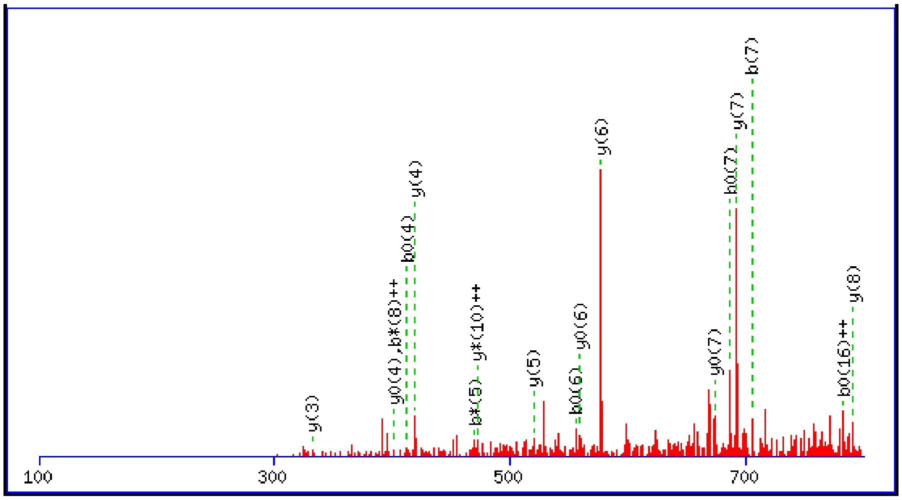


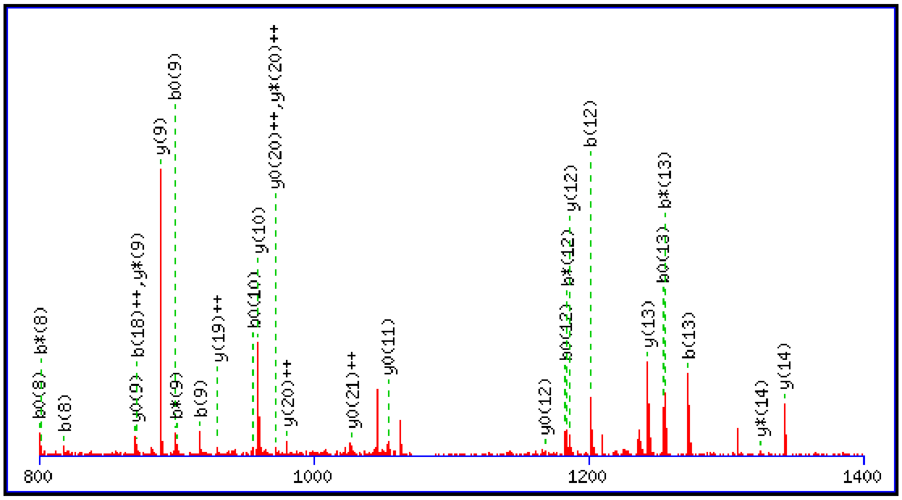


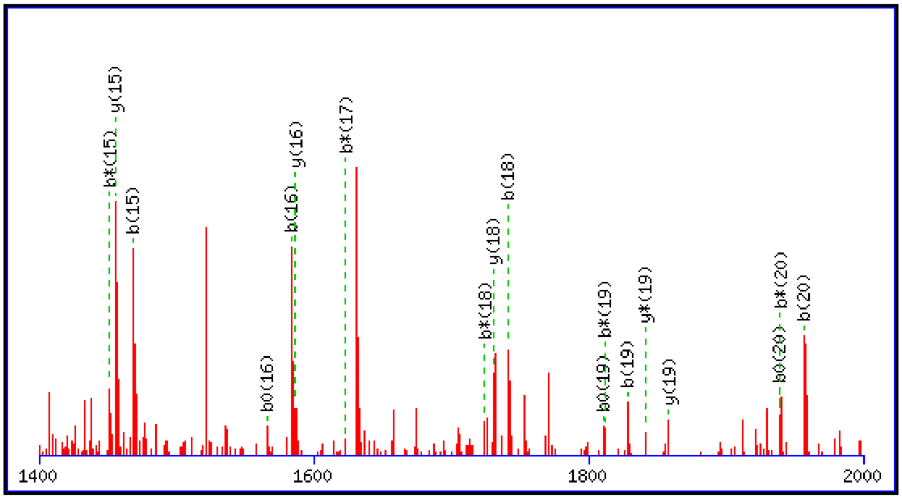


**Monoisotopic mass of neutral peptide Mr(calc):** 2159.0892
**Fixed modifications:** Carbamidomethyl (C)
**Variable modifications:**
**N16** : Deamidated_N (N)
**Ions Score:** 66 **Expect:** 4.4e-005
**Matches (Bold Red):** 64/240 fragment ions using 143 most intense peaks

| # | b | b++ | b* | b*++ | b0 | b0++ | Seq. | y | y++ | y* | y*++ | y0 | y0++ | # |
|---|---|---|---|---|---|---|---|---|---|---|---|---|---|---|
| 1 | 88.0393 | 44.5233 | | | 70.0287 | 35.5180 | S | | | | | | | 22 |
| 2 | 201.1234 | 101.0653 | | | 183.1128 | 92.0600 | L | 2073.0645 | 1037.0359 | 2056.0379 | 1028.5226 | 2055.0539 | **1028.0306** | 21 |
| 3 | 302.1710 | 151.5892 | | | 284.1605 | 142.5839 | T | 1959.9804 | **980.4938** | 1942.9539 | **971.9806** | 1941.9698 | **971.4886** | 20 |
| 4 | 430.2296 | 215.6185 | 413.2031 | 207.1052 | **412.2191** | 206.6132 | Q | **1858.9327** | **929.9700** | **1841.9062** | 921.4567 | 1840.9222 | 920.9647 | 19 |
| 5 | 487.2511 | 244.1292 | **470.2245** | 235.6159 | 469.2405 | 235.1239 | G | **1730.8742** | 865.9407 | 1713.8476 | 857.4274 | 1712.8636 | 856.9354 | 18 |

| | | | | | | | | | | | | | | |
|---|---|---|---|---|---|---|---|---|---|---|---|---|---|---|
| 6 | 574.2831 | 287.6452 | 557.2566 | 279.1319 | **556.2726** | 278.6399 | S | 1673.8527 | 837.4300 | 1656.8261 | 828.9167 | 1655.8421 | 828.4247 | 17 |
| 7 | **705.3236** | 353.1654 | 688.2971 | 344.6522 | **687.3130** | 344.1602 | M | **1586.8207** | 793.9140 | 1569.7941 | 785.4007 | 1568.8101 | 784.9087 | 16 |
| 8 | **818.4077** | 409.7075 | **801.3811** | **401.1942** | **800.3971** | 400.7022 | I | **1455.7802** | 728.3937 | 1438.7536 | 719.8805 | 1437.7696 | 719.3884 | 15 |
| 9 | **917.4761** | 459.2417 | **900.4495** | 450.7284 | **899.4655** | 450.2364 | V | **1342.6961** | 671.8517 | **1325.6696** | 663.3384 | 1324.6855 | 662.8464 | 14 |
| 10 | 974.4975 | 487.7524 | 957.4710 | 479.2391 | **956.4870** | 478.7471 | G | **1243.6277** | 622.3175 | 1226.6012 | 613.8042 | 1225.6171 | 613.3122 | 13 |
| 11 | 1088.5405 | 544.7739 | 1071.5139 | 536.2606 | 1070.5299 | 535.7686 | N | **1186.6062** | 593.8068 | 1169.5797 | 585.2935 | **1168.5957** | 584.8015 | 12 |
| 12 | **1201.6245** | 601.3159 | **1184.5980** | 592.8026 | **1183.6140** | 592.3106 | L | 1072.5633 | 536.7853 | 1055.5368 | 528.2720 | **1054.5527** | 527.7800 | 11 |
| 13 | **1272.6616** | 636.8345 | **1255.6351** | 628.3212 | **1254.6511** | 627.8292 | A | **959.4792** | 480.2433 | 942.4527 | **471.7300** | 941.4687 | 471.2380 | 10 |
| 14 | 1369.7144 | 685.3608 | 1352.6879 | 676.8476 | 1351.7038 | 676.3556 | P | **888.4421** | 444.7247 | **871.4156** | 436.2114 | **870.4316** | 435.7194 | 9 |
| 15 | **1468.7828** | 734.8951 | **1451.7563** | 726.3818 | 1450.7723 | 725.8898 | V | **791.3894** | 396.1983 | 774.3628 | 387.6850 | 773.3788 | 387.1930 | 8 |
| 16 | **1583.8098** | 792.4085 | 1566.7832 | 783.8952 | **1565.7992** | **783.4032** | N | **692.3210** | 346.6641 | 675.2944 | 338.1508 | **674.3104** | 337.6588 | 7 |
| 17 | 1640.8312 | 820.9193 | **1623.8047** | 812.4060 | 1622.8207 | 811.9140 | G | **577.2940** | 289.1506 | 560.2675 | 280.6374 | **559.2835** | 280.1454 | 6 |
| 18 | **1741.8789** | **871.4431** | **1724.8524** | 862.9298 | 1723.8683 | 862.4378 | T | **520.2726** | 260.6399 | 503.2460 | 252.1266 | 502.2620 | 251.6346 | 5 |
| 19 | **1828.9109** | 914.9591 | **1811.8844** | 906.4458 | **1810.9004** | 905.9538 | S | **419.2249** | 210.1161 | 402.1983 | 201.6028 | **401.2143** | 201.1108 | 4 |
| 20 | **1956.9695** | 978.9884 | **1939.9430** | 970.4751 | **1938.9589** | 969.9831 | Q | **332.1928** | 166.6001 | 315.1663 | 158.0868 | | | 3 |
| 21 | 2013.9910 | 1007.4991 | 1996.9644 | 998.9859 | 1995.9804 | 998.4938 | G | 204.1343 | 102.5708 | 187.1077 | 94.0575 | | | 2 |
| 22 | | | | | | | K | 147.1128 | 74.0600 | 130.0863 | 65.5468 | | | 1 |

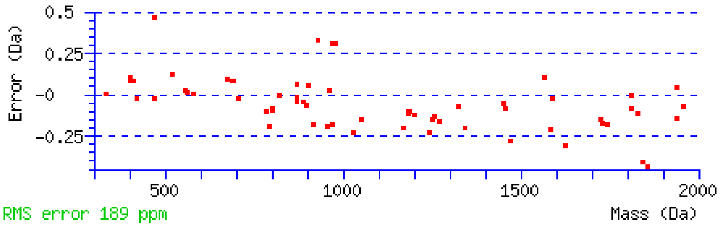


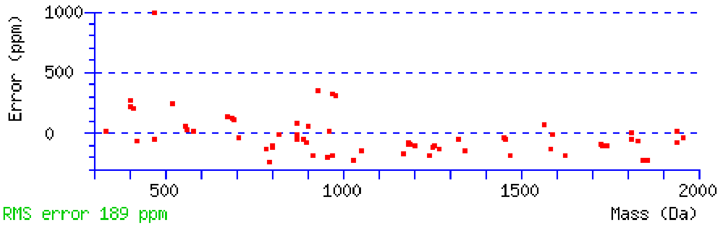


### All matches to this query

| Score | Mr(calc): | Delta | Sequence |
|---|---|---|---|
| 65.8 | 2159.0892 | 0.0059 | SLTQGSMIVGNLAPVNGTSQGK |
| 38.9 | 2159.0892 | 0.0059 | SLTQGSMIVGNLAPVNGTSQGK |
| 6.2 | 2158.1052 | 0.9899 | SLTQGSMIVGNLAPVNGTSQGK |
| 1.1 | 2159.0736 | 0.0216 | SFISVAGTTGISAKSNVARGR |
| 1.1 | 2159.0736 | 0.0216 | SFISVAGTTGISAKSNVARGR |
| 1.1 | 2159.0736 | 0.0216 | SFISVAGTTGISAKSNVARGR |
| 0.8 | 2159.0736 | 0.0216 | SFISVAGTTGISAKSNVARGR |

---

Spectrum No: 429; Query: 2716; Rank: 1

---

## Peptide View

MS/MS Fragmentation of **TVFQKENGTVTAANASTLNDGAAAVVLMTAEAAQR**
Found in **IPI00324302**, Tax_Id=10116 Gene_Symbol=Acat1 Acetyl-CoA acetyltransferase, mitochondrial precursor

Match to Query 2716: 3520.759692 from(1174.593840,3+)
Title: 100101RatKid_NS_deglyco_07.6033.6033.3.dta
Data file K:\NewmanPaper\Piliang\3SubProteomes\Piliang3SP\mgf5ppm\ERLIC_3SubProteomes5ppm.mgf

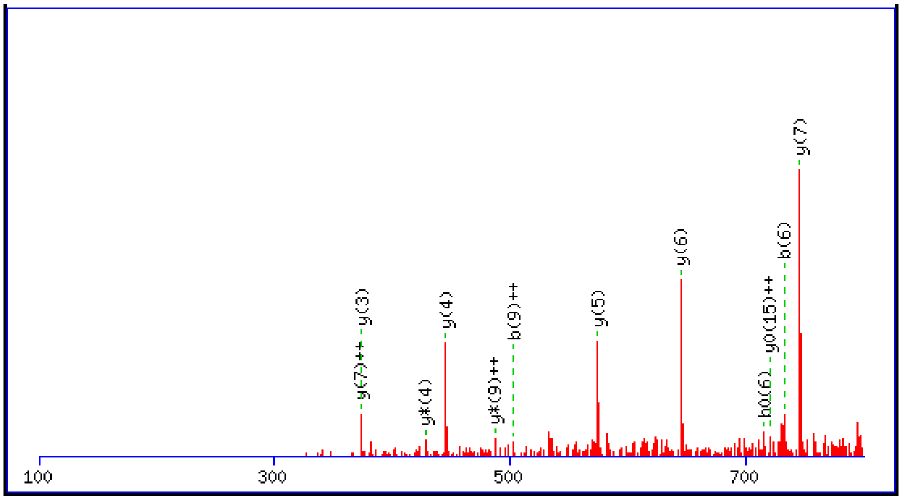


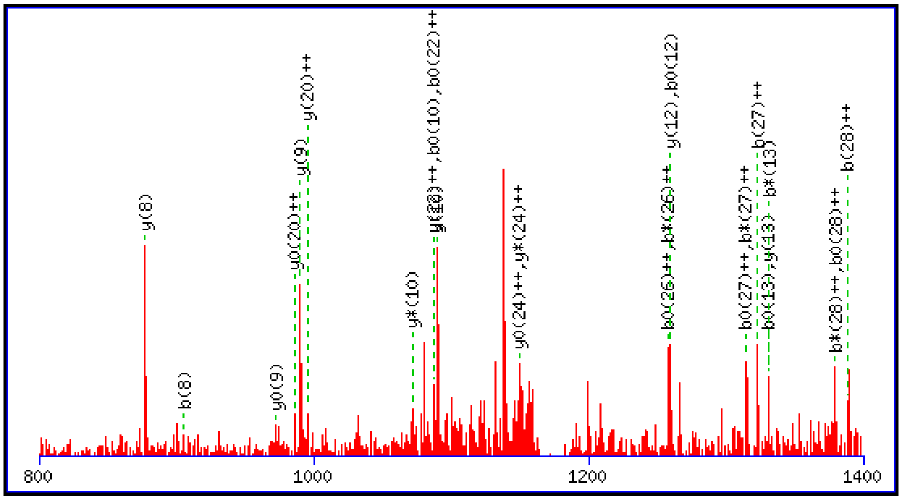


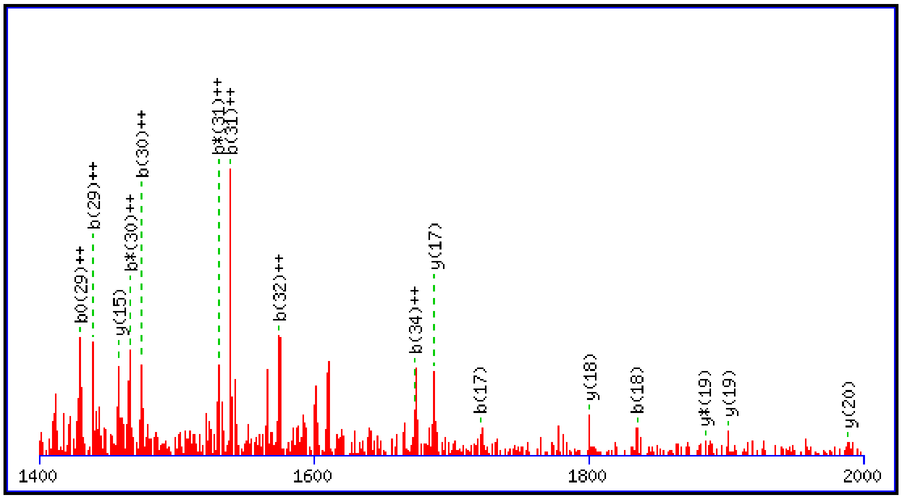


**Monoisotopic mass of neutral peptide Mr(calc):** 3520.7467
**Fixed modifications:** Carbamidomethyl (C)
**Variable modifications:**
**N7** : Deamidated_N (N)
**Ions Score:** 66 **Expect:** 5.7e-005
**Matches (Bold Red):** 54/394 fragment ions using 92 most intense peaks

| # | b | b++ | b* | b*++ | b0 | b0++ | Seq. | y | y++ | y* | y*++ | y0 | y0++ | # |
|---|---|---|---|---|---|---|---|---|---|---|---|---|---|---|
| 1 | 102.0550 | 51.5311 | | | 84.0444 | 42.5258 | T | | | | | | | 35 |
| 2 | 201.1234 | 101.0653 | | | 183.1128 | 92.0600 | V | 3420.7064 | 1710.8568 | 3403.6798 | 1702.3435 | 3402.6958 | 1701.8515 | 34 |
| 3 | 348.1918 | 174.5995 | | | 330.1812 | 165.5942 | F | 3321.6380 | 1661.3226 | 3304.6114 | 1652.8093 | 3303.6274 | 1652.3173 | 33 |
| 4 | 476.2504 | 238.6288 | 459.2238 | 230.1155 | 458.2398 | 229.6235 | Q | 3174.5695 | 1587.7884 | 3157.5430 | 1579.2751 | 3156.5590 | 1578.7831 | 32 |
| 5 | 604.3453 | 302.6763 | 587.3188 | 294.1630 | 586.3348 | 293.6710 | K | 3046.5110 | 1523.7591 | 3029.4844 | 1515.2458 | 3028.5004 | 1514.7538 | 31 |

| | | | | | | | | | | | | | | |
|---|---|---|---|---|---|---|---|---|---|---|---|---|---|---|
| 6 | 733.3879 | 367.1976 | 716.3614 | 358.6843 | 715.3774 | 358.1923 | E | 2918.4160 | 1459.7116 | 2901.3895 | 1451.1984 | 2900.4054 | 1450.7064 | 30 |
| 7 | 848.4149 | 424.7111 | 831.3883 | 416.1978 | 830.4043 | 415.7058 | N | 2789.3734 | 1395.1903 | 2772.3469 | 1386.6771 | 2771.3628 | 1386.1851 | 29 |
| 8 | 905.4363 | 453.2218 | 888.4098 | 444.7085 | 887.4258 | 444.2165 | G | 2674.3465 | 1337.6769 | 2657.3199 | 1329.1636 | 2656.3359 | 1328.6716 | 28 |
| 9 | 1006.4840 | 503.7456 | 989.4574 | 495.2324 | 988.4734 | 494.7404 | T | 2617.3250 | 1309.1661 | 2600.2985 | 1300.6529 | 2599.3144 | 1300.1609 | 27 |
| 10 | 1105.5524 | 553.2798 | 1088.5259 | 544.7666 | 1087.5418 | 544.2746 | V | 2516.2773 | 1258.6423 | 2499.2508 | 1250.1290 | 2498.2668 | 1249.6370 | 26 |
| 11 | 1206.6001 | 603.8037 | 1189.5735 | 595.2904 | 1188.5895 | 594.7984 | T | 2417.2089 | 1209.1081 | 2400.1824 | 1200.5948 | 2399.1984 | 1200.1028 | 25 |
| 12 | 1277.6372 | 639.3222 | 1260.6107 | 630.8090 | 1259.6266 | 630.3170 | A | 2316.1612 | 1158.5843 | 2299.1347 | 1150.0710 | 2298.1507 | 1149.5790 | 24 |
| 13 | 1348.6743 | 674.8408 | 1331.6478 | 666.3275 | 1330.6638 | 665.8355 | A | 2245.1241 | 1123.0657 | 2228.0976 | 1114.5524 | 2227.1136 | 1114.0604 | 23 |
| 14 | 1462.7172 | 731.8623 | 1445.6907 | 723.3490 | 1444.7067 | 722.8570 | N | 2174.0870 | 1087.5471 | 2157.0605 | 1079.0339 | 2156.0764 | 1078.5419 | 22 |
| 15 | 1533.7544 | 767.3808 | 1516.7278 | 758.8675 | 1515.7438 | 758.3755 | A | 2060.0441 | 1030.5257 | 2043.0175 | 1022.0124 | 2042.0335 | 1021.5204 | 21 |
| 16 | 1620.7864 | 810.8968 | 1603.7598 | 802.3836 | 1602.7758 | 801.8915 | S | 1989.0070 | 995.0071 | 1971.9804 | 986.4938 | 1970.9964 | 986.0018 | 20 |
| 17 | 1721.8341 | 861.4207 | 1704.8075 | 852.9074 | 1703.8235 | 852.4154 | T | 1901.9749 | 951.4911 | 1884.9484 | 942.9778 | 1883.9644 | 942.4858 | 19 |
| 18 | 1834.9181 | 917.9627 | 1817.8916 | 909.4494 | 1816.9076 | 908.9574 | L | 1800.9273 | 900.9673 | 1783.9007 | 892.4540 | 1782.9167 | 891.9620 | 18 |
| 19 | 1948.9611 | 974.9842 | 1931.9345 | 966.4709 | 1930.9505 | 965.9789 | N | 1687.8432 | 844.4252 | 1670.8166 | 835.9120 | 1669.8326 | 835.4200 | 17 |
| 20 | 2063.9880 | 1032.4976 | 2046.9615 | 1023.9844 | 2045.9774 | 1023.4924 | D | 1573.8003 | 787.4038 | 1556.7737 | 778.8905 | 1555.7897 | 778.3985 | 16 |
| 21 | 2121.0095 | 1061.0084 | 2103.9829 | 1052.4951 | 2102.9989 | 1052.0031 | G | 1458.7733 | 729.8903 | 1441.7468 | 721.3770 | 1440.7628 | 720.8850 | 15 |
| 22 | 2192.0466 | 1096.5269 | 2175.0200 | 1088.0137 | 2174.0360 | 1087.5216 | A | 1401.7519 | 701.3796 | 1384.7253 | 692.8663 | 1383.7413 | 692.3743 | 14 |
| 23 | 2263.0837 | 1132.0455 | 2246.0571 | 1123.5322 | 2245.0731 | 1123.0402 | A | 1330.7147 | 665.8610 | 1313.6882 | 657.3477 | 1312.7042 | 656.8557 | 13 |
| 24 | 2334.1208 | 1167.5640 | 2317.0943 | 1159.0508 | 2316.1102 | 1158.5588 | A | 1259.6776 | 630.3425 | 1242.6511 | 621.8292 | 1241.6671 | 621.3372 | 12 |
| 25 | 2433.1892 | 1217.0982 | 2416.1627 | 1208.5850 | 2415.1787 | 1208.0930 | V | 1188.6405 | 594.8239 | 1171.6140 | 586.3106 | 1170.6300 | 585.8186 | 11 |
| 26 | 2532.2576 | 1266.6325 | 2515.2311 | 1258.1192 | 2514.2471 | 1257.6272 | V | 1089.5721 | 545.2897 | 1072.5456 | 536.7764 | 1071.5615 | 536.2844 | 10 |
| 27 | 2645.3417 | 1323.1745 | 2628.3151 | 1314.6612 | 2627.3311 | 1314.1692 | L | 990.5037 | 495.7555 | 973.4771 | 487.2422 | 972.4931 | 486.7502 | 9 |
| 28 | 2776.3822 | 1388.6947 | 2759.3556 | 1380.1815 | 2758.3716 | 1379.6894 | M | 877.4196 | 439.2135 | 860.3931 | 430.7002 | 859.4091 | 430.2082 | 8 |
| 29 | 2877.4299 | 1439.2186 | 2860.4033 | 1430.7053 | 2859.4193 | 1430.2133 | T | 746.3791 | 373.6932 | 729.3526 | 365.1799 | 728.3686 | 364.6879 | 7 |
| 30 | 2948.4670 | 1474.7371 | 2931.4404 | 1466.2239 | 2930.4564 | 1465.7318 | A | 645.3315 | 323.1694 | 628.3049 | 314.6561 | 627.3209 | 314.1641 | 6 |
| 31 | 3077.5096 | 1539.2584 | 3060.4830 | 1530.7451 | 3059.4990 | 1530.2531 | E | 574.2944 | 287.6508 | 557.2678 | 279.1375 | 556.2838 | 278.6455 | 5 |
| 32 | 3148.5467 | 1574.7770 | 3131.5201 | 1566.2637 | 3130.5361 | 1565.7717 | A | 445.2518 | 223.1295 | 428.2252 | 214.6162 | | | 4 |
| 33 | 3219.5838 | 1610.2955 | 3202.5572 | 1601.7823 | 3201.5732 | 1601.2903 | A | 374.2146 | 187.6110 | 357.1881 | 179.0977 | | | 3 |
| 34 | 3347.6424 | 1674.3248 | 3330.6158 | 1665.8116 | 3329.6318 | 1665.3195 | Q | 303.1775 | 152.0924 | 286.1510 | 143.5791 | | | 2 |
| 35 | | | | | | | R | 175.1190 | 88.0631 | 158.0924 | 79.5498 | | | 1 |

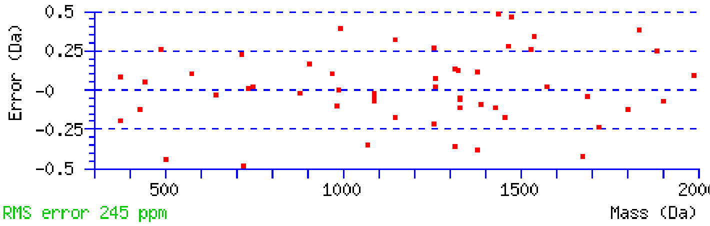


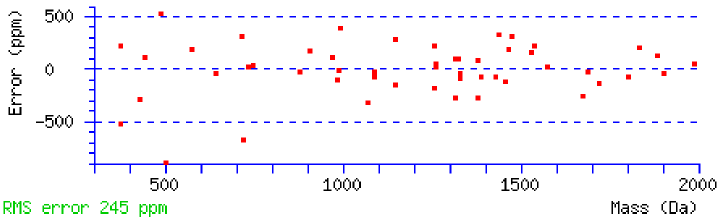


## All matches to this query

| Score | Mr(calc): | Delta | Sequence |
|---|---|---|---|
| 65.7 | 3520.7467 | 0.0129 | TVFQKENGTVTAANASTLNDGAAAVVLMTAEAAQR |
| 53.8 | 3520.7467 | 0.0129 | TVFQKENGTVTAANASTLNDGAAAVVLMTAEAAQR |
| 53.1 | 3519.7627 | 0.9970 | TVFQKENGTVTAANASTLNDGAAAVVLMTAEAAQR |
| 42.3 | 3520.7467 | 0.0129 | TVFQKENGTVTAANASTLNDGAAAVVLMTAEAAQR |
| 3.0 | 3518.7448 | 2.0149 | DIVHLNSDIVTAPAFALLLEFMYEGKLQFK |
| 1.9 | 3520.7870 | -0.0274 | SIPDEGTLAGKINAIQSLLQQIPEPNFILLK |
| 1.8 | 3520.7729 | -0.0132 | SRQSQPVNVGLVDWISLAYQHYAIAVRNTR |
| 1.8 | 3520.7729 | -0.0132 | SRQSQPVNVGLVDWISLAYQHYAIAVRNTR |
| 0.5 | 3519.7462 | 1.0135 | DSPKHPQLVKNSLCAAPRPTALGLLPNSGPPK |

Spectrum No: 430; Query: 1026; Rank: 1

# Peptide View

MS/MS Fragmentation of **MSGDWELEVNGTEAK**
Found in **IPI00361686**, Tax_Id=10116 Gene_Symbol=C1qbp Complement component 1 Q subcomponent-binding protein, mitochondrial precursor

Match to Query 1026: 1681.717848 from(841.866200,2+)
Title: 091008RatKidney_NH4Format01_13.2482.2482.2.dta
Data file K:\NewmanPaper\Piliang\3SubProteomes\Piliang3SP\mgf5ppm\ERLIC_3SubProteomes5ppm.mgf

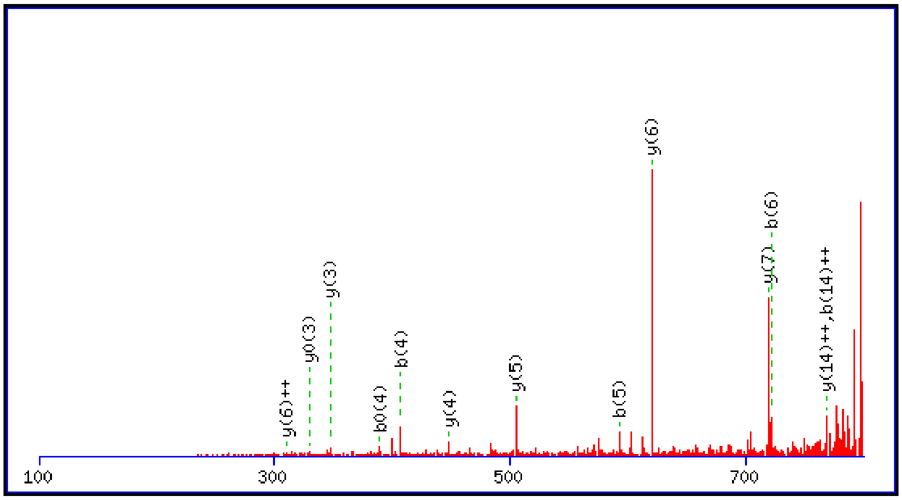


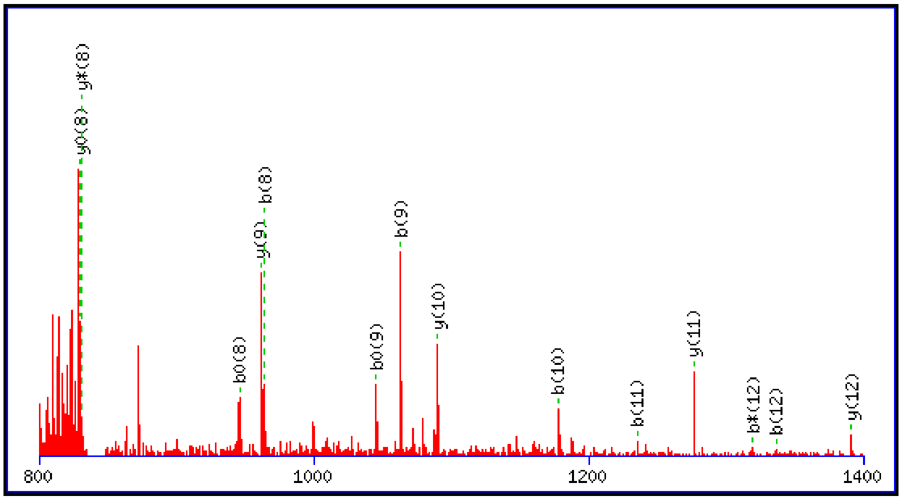


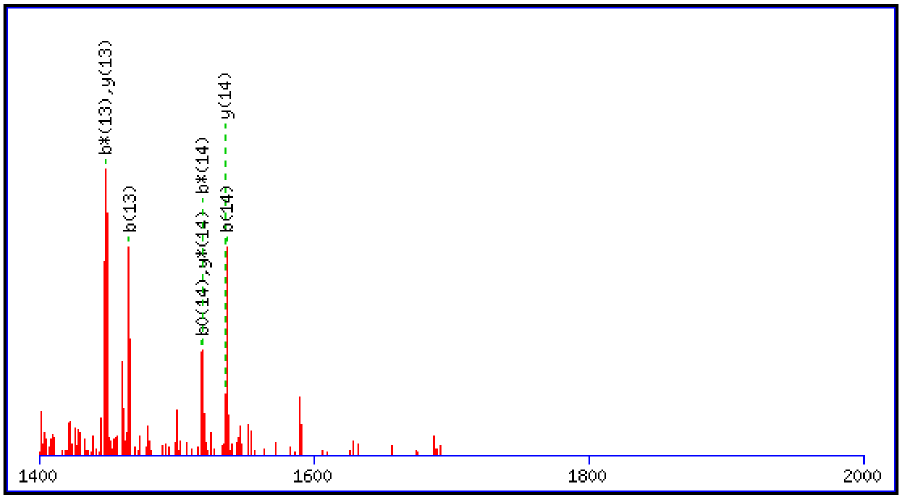

**Monoisotopic mass of neutral peptide Mr(calc):** 1681.7141
**Fixed modifications:** Carbamidomethyl (C)
**Variable modifications:**
**M1** : Oxidation (M)
**N10** : Deamidated_N (N)
**Ions Score:** 66 **Expect:** 4.7e-005
**Matches (Bold Red):** 35/144 fragment ions using 84 most intense peaks

| # | b | $b^{++}$ | b* | $b^{*++}$ | $b^0$ | $b^{0++}$ | Seq. | y | $y^{++}$ | y* | $y^{*++}$ | $y^0$ | $y^{0++}$ | # |
|---|---|---|---|---|---|---|---|---|---|---|---|---|---|---|
| 1 | 148.0427 | 74.5250 | | | | | M | | | | | | | 15 |
| 2 | 235.0747 | 118.0410 | | | 217.0641 | 109.0357 | S | **1535.6860** | **768.3466** | **1518.6595** | 759.8334 | 1517.6754 | 759.3414 | 14 |
| 3 | 292.0962 | 146.5517 | | | 274.0856 | 137.5464 | G | **1448.6540** | 724.8306 | 1431.6274 | 716.3174 | 1430.6434 | 715.8253 | 13 |
| 4 | **407.1231** | 204.0652 | | | **389.1126** | 195.0599 | D | **1391.6325** | 696.3199 | 1374.6060 | 687.8066 | 1373.6219 | 687.3146 | 12 |
| 5 | **593.2024** | 297.1049 | | | 575.1919 | 288.0996 | W | **1276.6056** | 638.8064 | 1259.5790 | 630.2931 | 1258.5950 | 629.8011 | 11 |
| 6 | **722.2450** | 361.6261 | | | 704.2345 | 352.6209 | E | **1090.5263** | 545.7668 | 1073.4997 | 537.2535 | 1072.5157 | 536.7615 | 10 |
| 7 | 835.3291 | 418.1682 | | | 817.3185 | 409.1629 | L | **961.4837** | 481.2455 | 944.4571 | 472.7322 | 943.4731 | 472.2402 | 9 |
| 8 | **964.3717** | 482.6895 | | | **946.3611** | 473.6842 | E | 848.3996 | 424.7034 | **831.3730** | 416.1902 | **830.3890** | 415.6982 | 8 |
| 9 | **1063.4401** | 532.2237 | | | **1045.4295** | 523.2184 | V | **719.3570** | 360.1821 | 702.3305 | 351.6689 | 701.3464 | 351.1769 | 7 |
| 10 | **1178.4670** | 589.7372 | 1161.4405 | 581.2239 | 1160.4565 | 580.7319 | N | **620.2886** | **310.6479** | 603.2620 | 302.1347 | 602.2780 | 301.6427 | 6 |
| 11 | **1235.4885** | 618.2479 | 1218.4619 | 609.7346 | 1217.4779 | 609.2426 | G | **505.2617** | 253.1345 | 488.2351 | 244.6212 | 487.2511 | 244.1292 | 5 |
| 12 | **1336.5362** | 668.7717 | **1319.5096** | 660.2584 | 1318.5256 | 659.7664 | T | **448.2402** | 224.6237 | 431.2136 | 216.1105 | 430.2296 | 215.6185 | 4 |
| 13 | **1465.5788** | 733.2930 | **1448.5522** | 724.7797 | 1447.5682 | 724.2877 | E | **347.1925** | 174.0999 | 330.1660 | 165.5866 | **329.1819** | 165.0946 | 3 |
| 14 | **1536.6159** | **768.8116** | **1519.5893** | 760.2983 | **1518.6053** | 759.8063 | A | 218.1499 | 109.5786 | 201.1234 | 101.0653 | | | 2 |
| 15 | | | | | | | K | 147.1128 | 74.0600 | 130.0863 | 65.5468 | | | 1 |

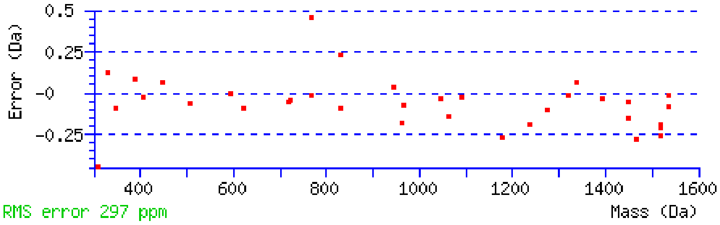


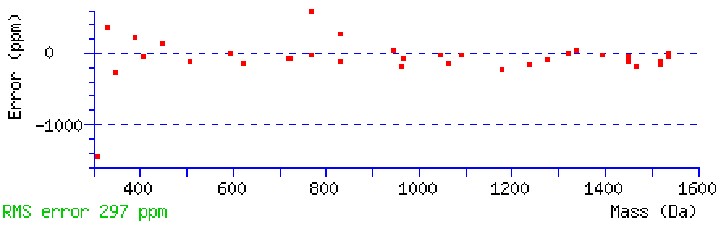


### All matches to this query

| Score | Mr(calc): | Delta | Sequence |
|---|---|---|---|
| 65.6 | 1681.7141 | 0.0037 | MSGDWELEVNGTEAK |
| 14.5 | 1680.7301 | 0.9877 | MSGDWELEVNGTEAK |
| 2.2 | 1681.7059 | 0.0120 | MSVNYAAGLSPYADK |
| 0.3 | 1681.7076 | 0.0103 | VFEECCXTSKEEK |

---

Spectrum No: 431; Query: 271; Rank: 1

---

## Peptide View

MS/MS Fragmentation of **LANTSMFNNSK**
Found in **IPI00206254**, Tax_Id=10116 Gene_Symbol=Ggt1 Gamma-glutamyltranspeptidase 1 precursor

Match to Query 271: 1227.547848 from(614.781200,2+)
Title: 100101RatKid_NS_deglyco_19.1701.1701.2.dta
Data file K:\NewmanPaper\Piliang\3SubProteomes\Piliang3SP\mgf5ppm\ERLIC_3SubProteomes5ppm.mgf

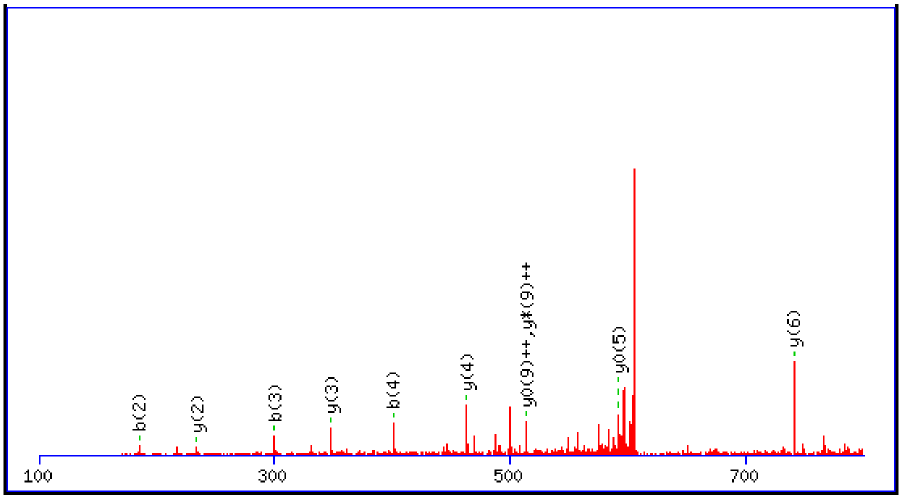


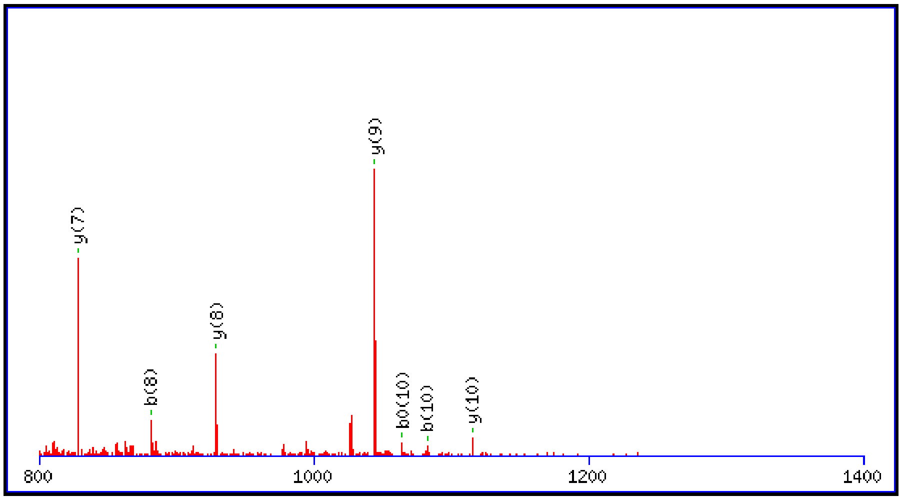


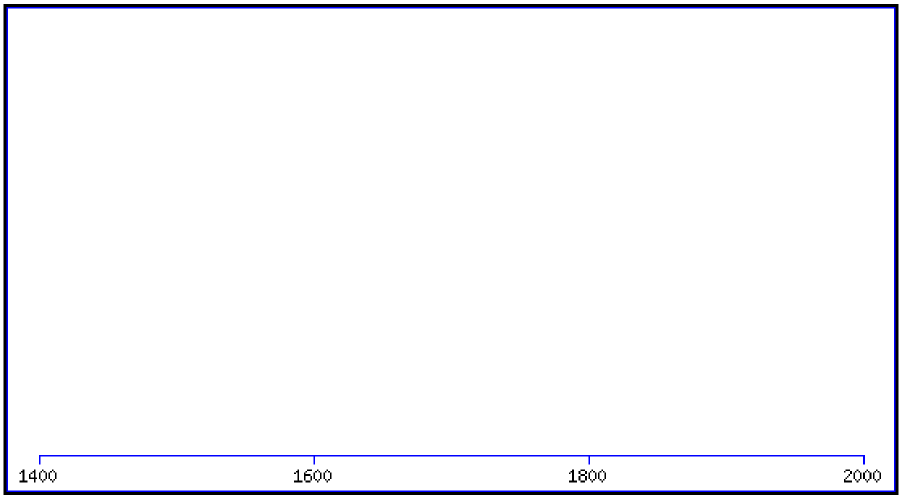


**Monoisotopic mass of neutral peptide Mr(calc):** 1227.5441
**Fixed modifications:** Carbamidomethyl (C)
**Variable modifications:**
**N3** : Deamidated_N (N)
**N8** : Deamidated_N (N)
**Ions Score:** 65 **Expect:** 3.9e-005
**Matches (Bold Red):** 17/108 fragment ions using 29 most intense peaks

| # | b | $b^{++}$ | b* | $b^{*++}$ | $b^0$ | $b^{0++}$ | Seq. | y | $y^{++}$ | y* | $y^{*++}$ | $y^0$ | $y^{0++}$ | # |
|---|---|---|---|---|---|---|---|---|---|---|---|---|---|---|
| 1 | 114.0913 | 57.5493 | | | | | L | | | | | | | 11 |
| 2 | **185.1285** | 93.0679 | | | | | A | **1115.4674** | 558.2373 | 1098.4408 | 549.7240 | 1097.4568 | 549.2320 | 10 |
| 3 | **300.1554** | 150.5813 | 283.1288 | 142.0681 | | | N | **1044.4302** | 522.7188 | 1027.4037 | **514.2055** | 1026.4197 | **513.7135** | 9 |
| 4 | **401.2031** | 201.1052 | 384.1765 | 192.5919 | 383.1925 | 192.0999 | T | **929.4033** | 465.2053 | 912.3768 | 456.6920 | 911.3927 | 456.2000 | 8 |

| | | | | | | | | | | | | | | |
|---|---|---|---|---|---|---|---|---|---|---|---|---|---|---|
| **5** | 488.2351 | 244.6212 | 471.2085 | 236.1079 | 470.2245 | 235.6159 | **S** | **828.3556** | 414.6814 | 811.3291 | 406.1682 | 810.3451 | 405.6762 | **7** |
| **6** | 619.2756 | 310.1414 | 602.2490 | 301.6282 | 601.2650 | 301.1361 | **M** | **741.3236** | 371.1654 | 724.2970 | 362.6522 | 723.3130 | 362.1602 | **6** |
| **7** | 766.3440 | 383.6756 | 749.3174 | 375.1624 | 748.3334 | 374.6704 | **F** | 610.2831 | 305.6452 | 593.2566 | 297.1319 | **592.2725** | 296.6399 | **5** |
| **8** | **881.3709** | 441.1891 | 864.3444 | 432.6758 | 863.3604 | 432.1838 | **N** | **463.2147** | 232.1110 | 446.1881 | 223.5977 | 445.2041 | 223.1057 | **4** |
| **9** | 995.4139 | 498.2106 | 978.3873 | 489.6973 | 977.4033 | 489.2053 | **N** | **348.1878** | 174.5975 | 331.1612 | 166.0842 | 330.1772 | 165.5922 | **3** |
| **10** | **1082.4459** | 541.7266 | 1065.4193 | 533.2133 | **1064.4353** | 532.7213 | **S** | **234.1448** | 117.5761 | 217.1183 | 109.0628 | 216.1343 | 108.5708 | **2** |
| **11** | | | | | | | **K** | 147.1128 | 74.0600 | 130.0863 | 65.5468 | | | **1** |

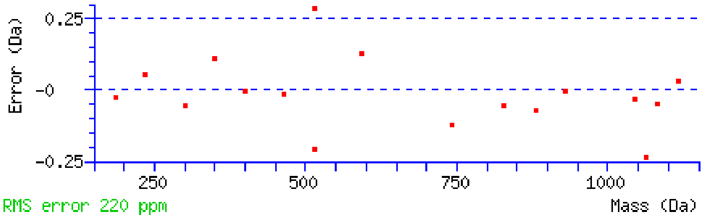


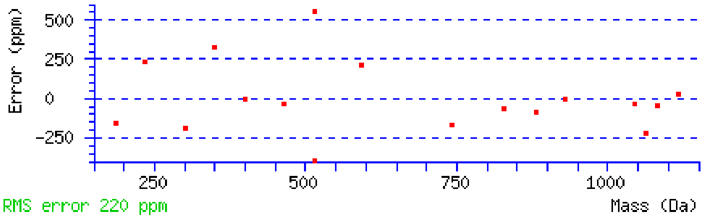


### All matches to this query

| Score | Mr(calc): | Delta | Sequence |
|---|---|---|---|
| 65.5 | 1227.5441 | 0.0037 | LANTSMFNNSK |
| 48.8 | 1227.5441 | 0.0037 | LANTSMFNNSK |
| 39.9 | 1227.5441 | 0.0037 | LANTSMFNNSK |
| 17.4 | 1226.5366 | 1.0112 | MSTSIQPLNR |
| 9.8 | 1227.5511 | -0.0033 | LAFLMHSWK |
| 8.3 | 1226.5366 | 1.0112 | MSTSIQPLNR |
| 7.6 | 1227.5495 | -0.0016 | ALYPXVQSXTK |
| 5.8 | 1227.5537 | -0.0058 | ANLSLGTTGWK |
| 5.8 | 1227.5537 | -0.0058 | ANLSLGTTGWK |
| 5.2 | 1227.5384 | 0.0095 | DEKPLTSGSSK |

---

Spectrum No: 432; Query: 1560; Rank: 1

---

## Peptide View

MS/MS Fragmentation of **ARDAGYTNATTTDQHGLAK**
Found in **IPI00201262**, Tax_Id=10116 Gene_Symbol=LOC297568;Mug2;Mug1 Alpha-1-inhibitor 3 precursor

Match to Query 1560: 1990.938312 from(664.653380,3+)
Title: 100101RatKid_NS_deglyco_23.582.582.3.dta
Data file K:\NewmanPaper\Piliang\3SubProteomes\Piliang3SP\mgf5ppm\ERLIC_3SubProteomes5ppm.mgf

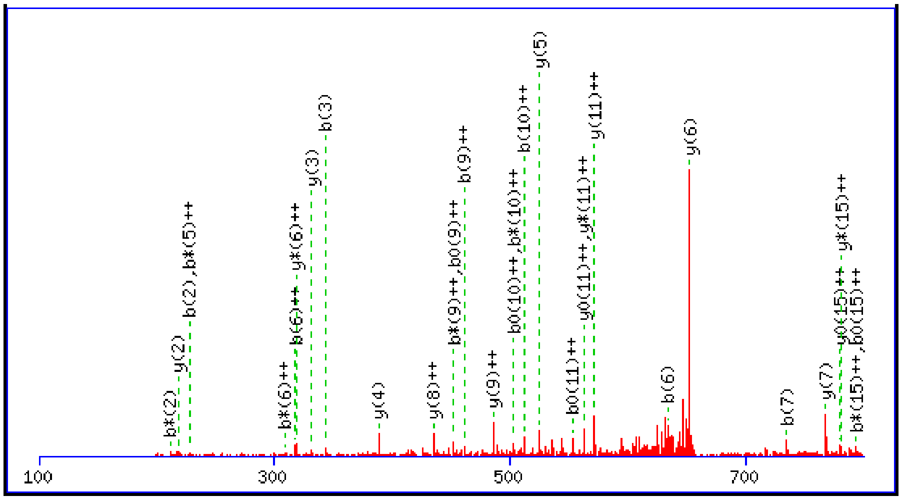


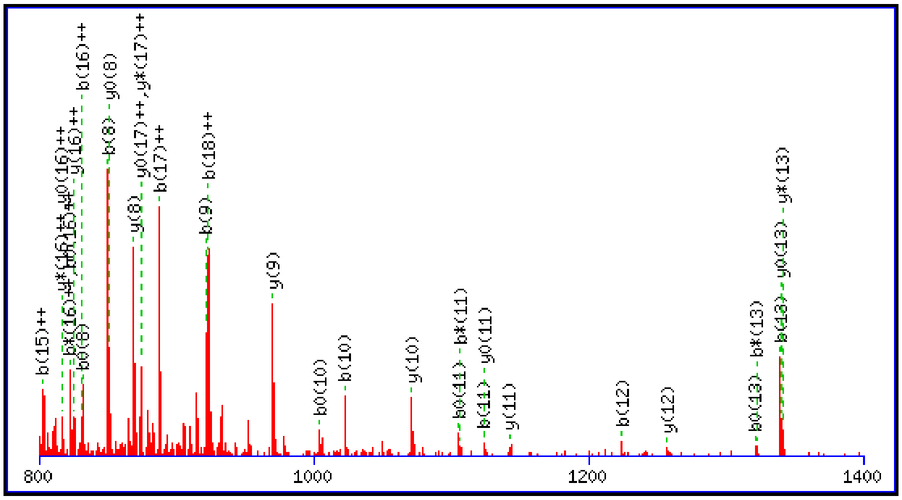


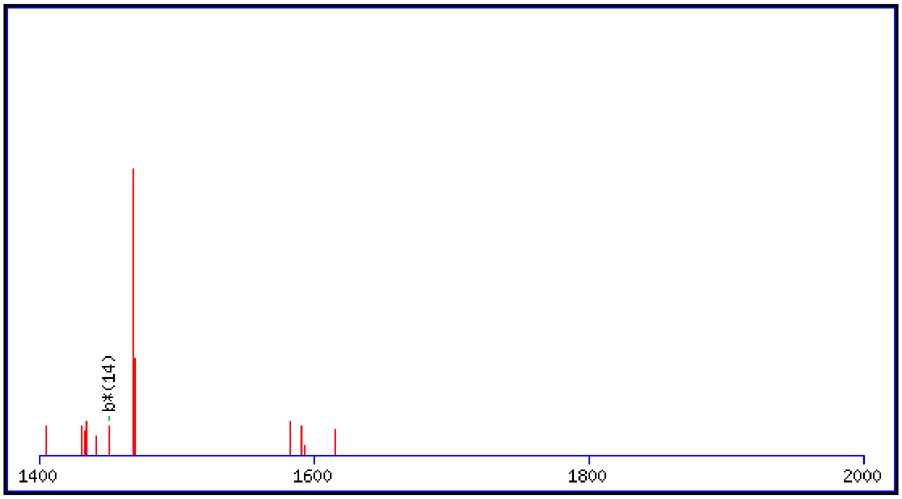


**Monoisotopic mass of neutral peptide Mr(calc):** 1990.9344
**Fixed modifications:** Carbamidomethyl (C)
**Variable modifications:**
**N8** : Deamidated_N (N)
**Ions Score:** 65 **Expect:** 6.9e-005
**Matches (Bold Red):** 64/198 fragment ions using 99 most intense peaks

| # | b | $b^{++}$ | b* | $b^{*++}$ | $b^0$ | $b^{0++}$ | Seq. | y | $y^{++}$ | y* | $y^{*++}$ | $y^0$ | $y^{0++}$ | # |
|---|---|---|---|---|---|---|---|---|---|---|---|---|---|---|
| 1 | 72.0444 | 36.5258 | | | | | A | | | | | | | 19 |
| 2 | **228.1455** | 114.5764 | **211.1190** | 106.0631 | | | R | 1920.9046 | 960.9559 | 1903.8781 | 952.4427 | 1902.8940 | 951.9507 | 18 |
| 3 | **343.1724** | 172.0899 | 326.1459 | 163.5766 | 325.1619 | 163.0846 | D | 1764.8035 | 882.9054 | 1747.7769 | **874.3921** | 1746.7929 | **873.9001** | 17 |
| 4 | 414.2096 | 207.6084 | 397.1830 | 199.0951 | 396.1990 | 198.6031 | A | 1649.7766 | **825.3919** | 1632.7500 | **816.8786** | 1631.7660 | **816.3866** | 16 |
| 5 | 471.2310 | 236.1191 | 454.2045 | **227.6059** | 453.2205 | 227.1139 | G | 1578.7394 | 789.8734 | 1561.7129 | **781.3601** | 1560.7289 | **780.8681** | 15 |

| | | | | | | | | | | | | | | |
|---|---|---|---|---|---|---|---|---|---|---|---|---|---|---|
| 6 | **634.2944** | **317.6508** | 617.2678 | **309.1375** | 616.2838 | 308.6455 | Y | 1521.7180 | 761.3626 | 1504.6914 | 752.8494 | 1503.7074 | 752.3573 | 14 |
| 7 | **735.3420** | 368.1747 | 718.3155 | 359.6614 | 717.3315 | 359.1694 | T | 1358.6546 | 679.8310 | **1341.6281** | 671.3177 | **1340.6441** | 670.8257 | 13 |
| 8 | **850.3690** | 425.6881 | 833.3424 | 417.1748 | **832.3584** | 416.6828 | N | **1257.6070** | 629.3071 | 1240.5804 | 620.7938 | 1239.5964 | 620.3018 | 12 |
| 9 | **921.4061** | **461.2067** | 904.3795 | **452.6934** | 903.3955 | **452.2014** | A | **1142.5800** | **571.7937** | 1125.5535 | **563.2804** | **1124.5695** | **562.7884** | 11 |
| 10 | **1022.4538** | **511.7305** | 1005.4272 | **503.2172** | **1004.4432** | **502.7252** | T | **1071.5429** | 536.2751 | 1054.5164 | 527.7618 | 1053.5324 | 527.2698 | 10 |
| 11 | **1123.5014** | 562.2544 | **1106.4749** | 553.7411 | **1105.4909** | **553.2491** | T | **970.4952** | **485.7513** | 953.4687 | 477.2380 | 952.4847 | 476.7460 | 9 |
| 12 | **1224.5491** | 612.7782 | 1207.5226 | 604.2649 | 1206.5386 | 603.7729 | T | **869.4476** | **435.2274** | 852.4210 | 426.7141 | **851.4370** | 426.2221 | 8 |
| 13 | **1339.5761** | 670.2917 | **1322.5495** | 661.7784 | **1321.5655** | 661.2864 | D | **768.3999** | 384.7036 | 751.3733 | 376.1903 | 750.3893 | 375.6983 | 7 |
| 14 | 1467.6346 | 734.3210 | **1450.6081** | 725.8077 | 1449.6241 | 725.3157 | Q | **653.3729** | 327.1901 | 636.3464 | **318.6768** | | | 6 |
| 15 | 1604.6936 | **802.8504** | 1587.6670 | **794.3371** | 1586.6830 | **793.8451** | H | **525.3144** | 263.1608 | 508.2878 | 254.6475 | | | 5 |
| 16 | 1661.7150 | **831.3611** | 1644.6885 | **822.8479** | 1643.7044 | **822.3559** | G | **388.2554** | 194.6314 | 371.2289 | 186.1181 | | | 4 |
| 17 | 1774.7991 | **887.9032** | 1757.7725 | 879.3899 | 1756.7885 | 878.8979 | L | **331.2340** | 166.1206 | 314.2074 | 157.6074 | | | 3 |
| 18 | 1845.8362 | **923.4217** | 1828.8096 | 914.9085 | 1827.8256 | 914.4165 | A | **218.1499** | 109.5786 | 201.1234 | 101.0653 | | | 2 |
| 19 | | | | | | | K | 147.1128 | 74.0600 | 130.0863 | 65.5468 | | | 1 |

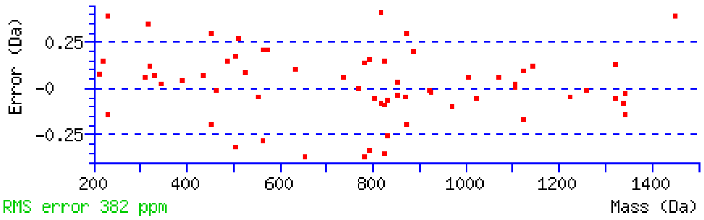


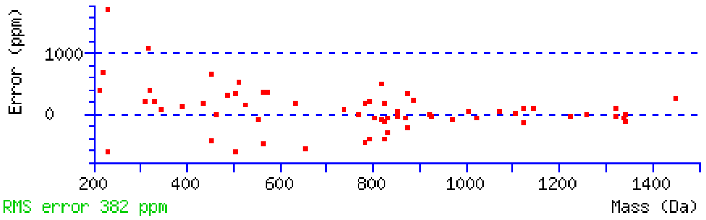


### All matches to this query

| Score | Mr(calc): | Delta | Sequence |
|---|---|---|---|
| 65.5 | 1990.9344 | 0.0039 | ARDAGYTNATTTDQHGLAK |
| 41.4 | 1989.9504 | 0.9879 | ARDAGYTNATTTDQHGLAK |
| 6.2 | 1990.9257 | 0.0126 | EKNLFQKSNIGVEMMK |
| 3.2 | 1990.9401 | -0.0018 | AAKEANIHQFIDSLPEK |
| 2.7 | 1990.9554 | -0.0171 | FPQLSLVPFDHILNDR |
| 2.7 | 1990.9531 | -0.0148 | GAVTGHEECVDALLQHGAK |
| 2.0 | 1990.9257 | 0.0126 | EKNLFQKSNIGVEMMK |
| 1.4 | 1990.9297 | 0.0086 | NIKKSAGAPPSMVGMLFY |
| 1.2 | 1990.9248 | 0.0135 | SLSLKLQDSAVYTERDS |
| 1.1 | 1990.9230 | 0.0153 | HVRNRPXASPTMTMGTAK |

---

Spectrum No: 433; Query: 2452; Rank: 1

---

## Peptide View

MS/MS Fragmentation of **IYTYADTPNDFQLSNFSLPEEDTK**
Found in **IPI00764168**, Tax_Id=10116 Gene_Symbol=LOC684536 similar to Glucosylceramidase precursor (Beta-glucocerebrosidase) (Acid beta-glucosidase) (D-glucosyl-N-acylsphingosine glucohydrolase) isoform 1

Match to Query 2452: 2810.284108 from(1406.149330,2+)
Title: 100101RatKid_NS_deglyco_25.4564.4564.2.dta
Data file K:\NewmanPaper\Piliang\3SubProteomes\Piliang3SP\mgf5ppm\ERLIC_3SubProteomes5ppm.mgf

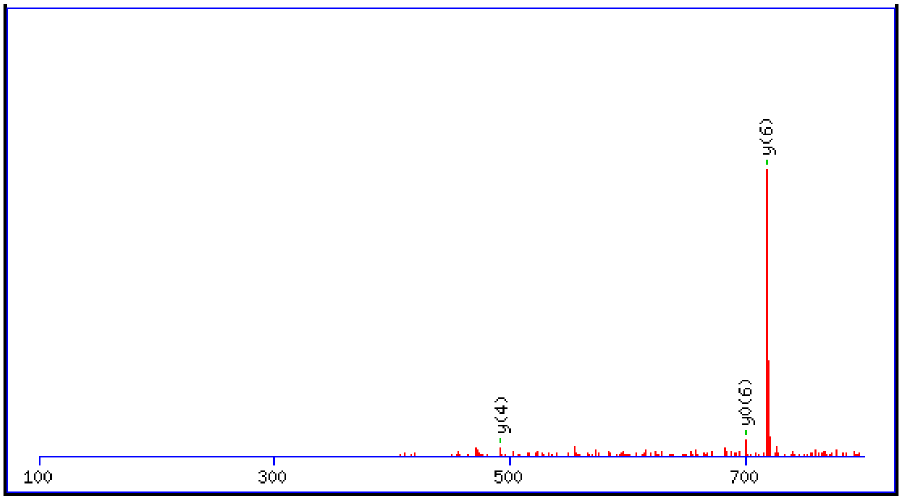


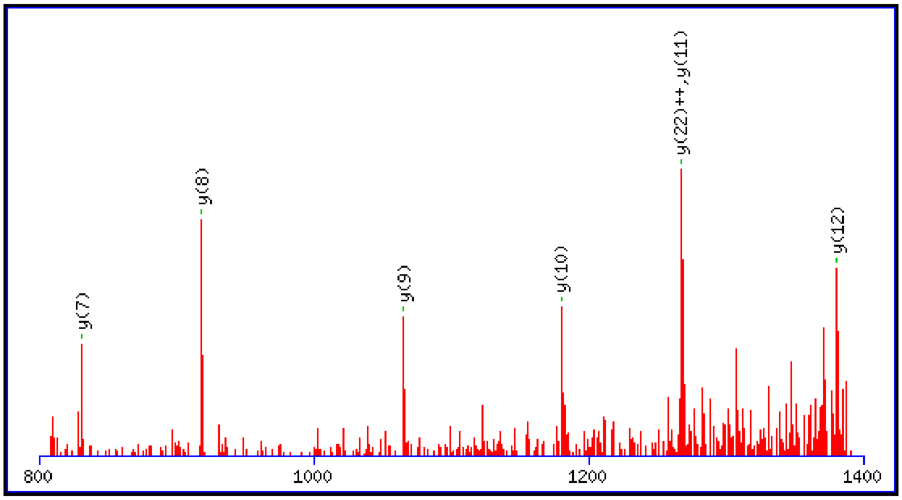


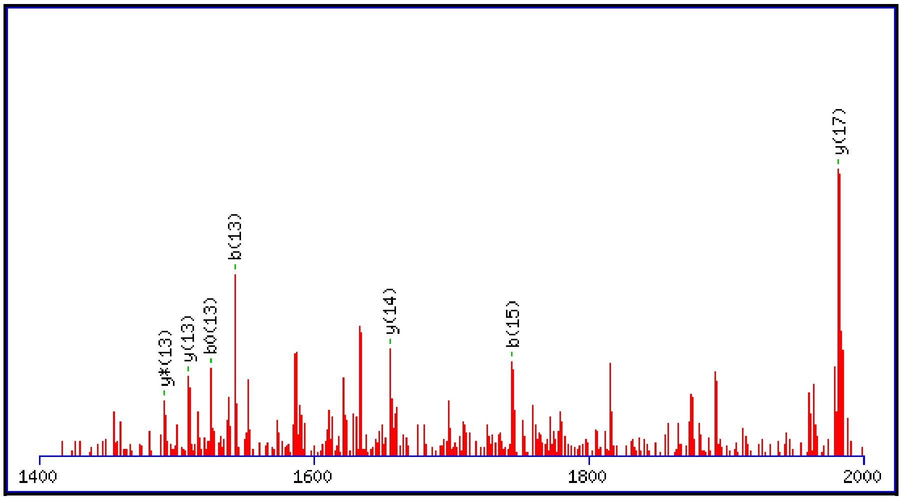


**Monoisotopic mass of neutral peptide Mr(calc):** 2808.2654
**Fixed modifications:** Carbamidomethyl (C)
**Variable modifications:**
**N15** : Deamidated_N (N)
**Ions Score:** 66 **Expect:** 9.9e-005
**Matches (Bold Red):** 17/254 fragment ions using 27 most intense peaks

| # | b | b++ | b* | b*++ | b0 | b0++ | Seq. | y | y++ | y* | y*++ | y0 | y0++ | # |
|---|---|---|---|---|---|---|---|---|---|---|---|---|---|---|
| 1 | 114.0913 | 57.5493 | | | | | I | | | | | | | 24 |
| 2 | 277.1547 | 139.0810 | | | | | Y | 2696.1886 | 1348.5979 | 2679.1621 | 1340.0847 | 2678.1780 | 1339.5927 | 23 |
| 3 | 378.2023 | 189.6048 | | | 360.1918 | 180.5995 | T | 2533.1253 | **1267.0663** | 2516.0987 | 1258.5530 | 2515.1147 | 1258.0610 | 22 |
| 4 | 541.2657 | 271.1365 | | | 523.2551 | 262.1312 | Y | 2432.0776 | 1216.5424 | 2415.0511 | 1208.0292 | 2414.0670 | 1207.5372 | 21 |
| 5 | 612.3028 | 306.6550 | | | 594.2922 | 297.6498 | A | 2269.0143 | 1135.0108 | 2251.9877 | 1126.4975 | 2251.0037 | 1126.0055 | 20 |

| | | | | | | | | | | | | | | |
|---|---|---|---|---|---|---|---|---|---|---|---|---|---|---|
| **6** | 727.3297 | 364.1685 | | | 709.3192 | 355.1632 | **D** | 2197.9772 | 1099.4922 | 2180.9506 | 1090.9789 | 2179.9666 | 1090.4869 | **19** |
| **7** | 828.3774 | 414.6923 | | | 810.3668 | 405.6871 | **T** | 2082.9502 | 1041.9787 | 2065.9237 | 1033.4655 | 2064.9396 | 1032.9735 | **18** |
| **8** | 925.4302 | 463.2187 | | | 907.4196 | 454.2134 | **P** | **1981.9025** | 991.4549 | 1964.8760 | 982.9416 | 1963.8920 | 982.4496 | **17** |
| **9** | 1039.4731 | 520.2402 | 1022.4466 | 511.7269 | 1021.4625 | 511.2349 | **N** | 1884.8498 | 942.9285 | 1867.8232 | 934.4152 | 1866.8392 | 933.9232 | **16** |
| **10** | 1154.5000 | 577.7537 | 1137.4735 | 569.2404 | 1136.4895 | 568.7484 | **D** | 1770.8068 | 885.9071 | 1753.7803 | 877.3938 | 1752.7963 | 876.9018 | **15** |
| **11** | 1301.5685 | 651.2879 | 1284.5419 | 642.7746 | 1283.5579 | 642.2826 | **F** | **1655.7799** | 828.3936 | 1638.7534 | 819.8803 | 1637.7693 | 819.3883 | **14** |
| **12** | 1429.6270 | 715.3172 | 1412.6005 | 706.8039 | 1411.6165 | 706.3119 | **Q** | **1508.7115** | 754.8594 | **1491.6849** | 746.3461 | 1490.7009 | 745.8541 | **13** |
| **13** | **1542.7111** | 771.8592 | 1525.6846 | 763.3459 | **1524.7005** | 762.8539 | **L** | **1380.6529** | 690.8301 | 1363.6264 | 682.3168 | 1362.6423 | 681.8248 | **12** |
| **14** | 1629.7431 | 815.3752 | 1612.7166 | 806.8619 | 1611.7326 | 806.3699 | **S** | **1267.5688** | 634.2881 | 1250.5423 | 625.7748 | 1249.5583 | 625.2828 | **11** |
| **15** | **1744.7701** | 872.8887 | 1727.7435 | 864.3754 | 1726.7595 | 863.8834 | **N** | **1180.5368** | 590.7720 | 1163.5103 | 582.2588 | 1162.5263 | 581.7668 | **10** |
| **16** | 1891.8385 | 946.4229 | 1874.8119 | 937.9096 | 1873.8279 | 937.4176 | **F** | **1065.5099** | 533.2586 | 1048.4833 | 524.7453 | 1047.4993 | 524.2533 | **9** |
| **17** | 1978.8705 | 989.9389 | 1961.8440 | 981.4256 | 1960.8599 | 980.9336 | **S** | **918.4415** | 459.7244 | 901.4149 | 451.2111 | 900.4309 | 450.7191 | **8** |
| **18** | 2091.9546 | 1046.4809 | 2074.9280 | 1037.9676 | 2073.9440 | 1037.4756 | **L** | **831.4094** | 416.2084 | 814.3829 | 407.6951 | 813.3989 | 407.2031 | **7** |
| **19** | 2189.0073 | 1095.0073 | 2171.9808 | 1086.4940 | 2170.9968 | 1086.0020 | **P** | **718.3254** | 359.6663 | 701.2988 | 351.1531 | **700.3148** | 350.6610 | **6** |
| **20** | 2318.0499 | 1159.5286 | 2301.0234 | 1151.0153 | 2300.0394 | 1150.5233 | **E** | 621.2726 | 311.1399 | 604.2461 | 302.6267 | 603.2620 | 302.1347 | **5** |
| **21** | 2447.0925 | 1224.0499 | 2430.0660 | 1215.5366 | 2429.0820 | 1215.0446 | **E** | **492.2300** | 246.6186 | 475.2035 | 238.1054 | 474.2195 | 237.6134 | **4** |
| **22** | 2562.1195 | 1281.5634 | 2545.0929 | 1273.0501 | 2544.1089 | 1272.5581 | **D** | 363.1874 | 182.0974 | 346.1609 | 173.5841 | 345.1769 | 173.0921 | **3** |
| **23** | 2663.1671 | 1332.0872 | 2646.1406 | 1323.5739 | 2645.1566 | 1323.0819 | **T** | 248.1605 | 124.5839 | 231.1339 | 116.0706 | 230.1499 | 115.5786 | **2** |
| **24** | | | | | | | **K** | 147.1128 | 74.0600 | 130.0863 | 65.5468 | | | **1** |

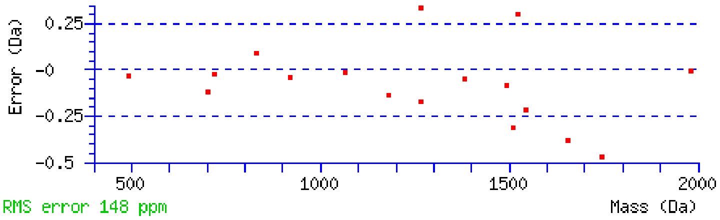


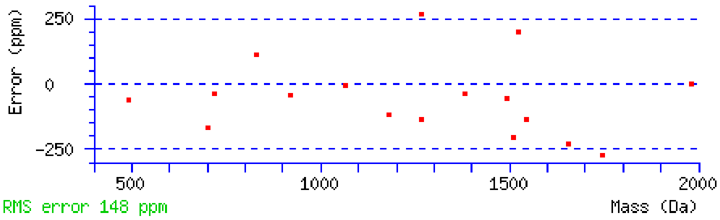


**All matches to this query**

| Score | Mr(calc): | Delta | Sequence |
|---|---|---|---|
| 65.5 | 2808.2654 | 2.0187 | IYTYADTPNDFQLSNFSLPEEDTK |
| 21.5 | 2808.2654 | 2.0187 | IYTYADTPNDFQLSNFSLPEEDTK |
| 5.6 | 2810.2656 | 0.0185 | GLHACNTYMDSLDIVTSKVSSLFR |
| 5.6 | 2810.2656 | 0.0185 | GLHACNTYMDSLDIVTSKVSSLFR |
| 5.6 | 2810.2656 | 0.0185 | GLHACNTYMDSLDIVTSKVSSLFR |
| 5.6 | 2810.2656 | 0.0185 | GLHACNTYMDSLDIVTSKVSSLFR |
| 5.2 | 2809.2583 | 1.0258 | VASMSLMYTVVTPMLNPFIYSLR |
| 5.2 | 2809.2583 | 1.0258 | VASMSLMYTVVTPMLNPFIYSLR |
| 5.2 | 2809.2583 | 1.0258 | VASMSLMYTVVTPMLNPFIYSLR |
| 5.2 | 2809.2583 | 1.0258 | VASMSLMYTVVTPMLNPFIYSLR |

---

Spectrum No: 434; Query: 455; Rank: 1

---

## Peptide View

MS/MS Fragmentation of **NMTLFSDLVAEK**
Found in **IPI00372839**, Tax_Id=10116 Gene_Symbol=Col6a2 similar to procollagen, type VI, alpha 2

Match to Query 455: 1367.668128 from(684.841340,2+)
Title: 100101RatKid_NS_deglyco_21.4679.4679.2.dta
Data file K:\NewmanPaper\Piliang\3SubProteomes\Piliang3SP\mgf5ppm\ERLIC_3SubProteomes5ppm.mgf

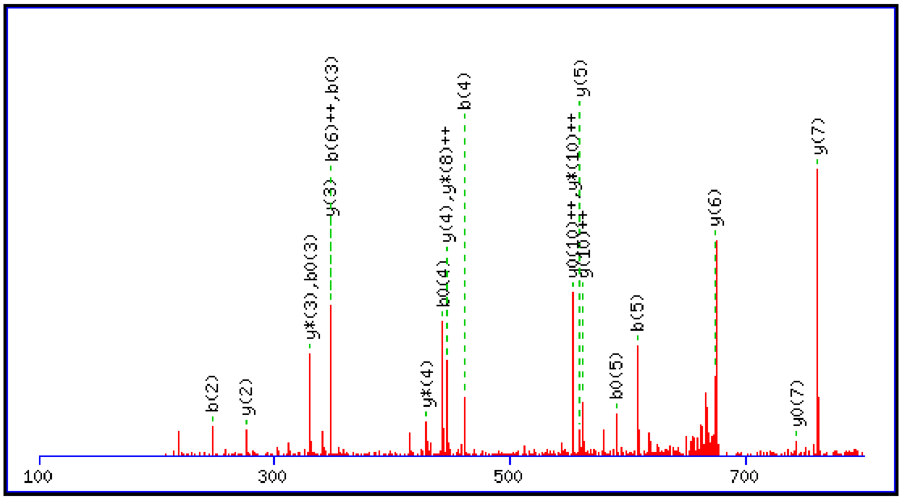


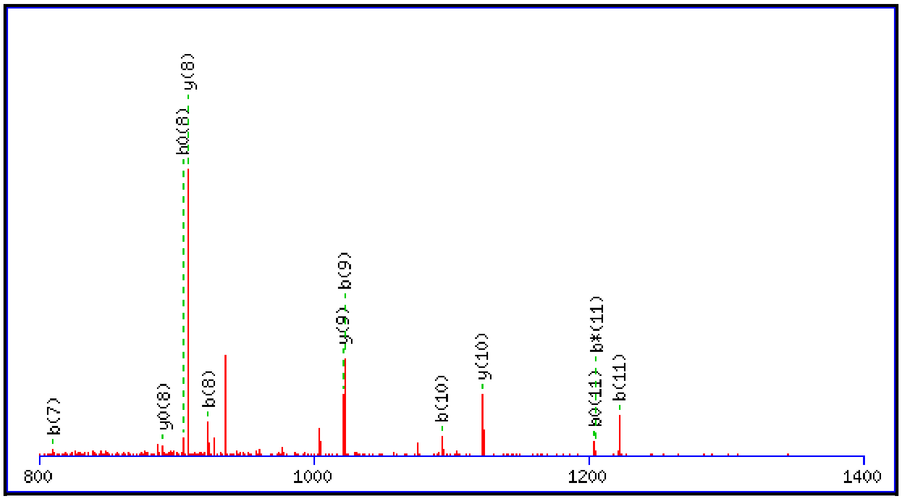


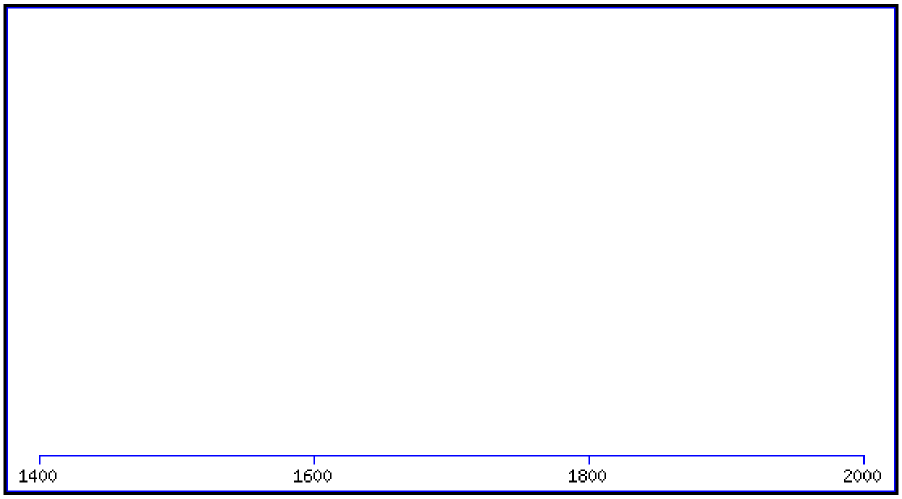


**Monoisotopic mass of neutral peptide Mr(calc):** 1367.6642
**Fixed modifications:** Carbamidomethyl (C)
**Variable modifications:**
**N1** : Deamidated_N (N)
**Ions Score:** 65 **Expect:** 4.1e-005
**Matches (Bold Red):** 33/126 fragment ions using 48 most intense peaks

| # | b | b++ | b* | b*++ | b0 | b0++ | Seq. | y | y++ | y* | y*++ | y0 | y0++ | # |
|---|---|---|---|---|---|---|---|---|---|---|---|---|---|---|
| 1 | 116.0342 | 58.5207 | 99.0077 | 50.0075 | | | N | | | | | | | 12 |
| 2 | **247.0747** | 124.0410 | 230.0481 | 115.5277 | | | M | 1253.6446 | 627.3259 | 1236.6181 | 618.8127 | 1235.6340 | 618.3207 | 11 |
| 3 | **348.1224** | 174.5648 | 331.0958 | 166.0516 | **330.1118** | 165.5595 | T | **1122.6041** | **561.8057** | 1105.5776 | **553.2924** | 1104.5936 | **552.8004** | 10 |
| 4 | **461.2064** | 231.1069 | 444.1799 | 222.5936 | **443.1959** | 222.1016 | L | **1021.5564** | 511.2819 | 1004.5299 | 502.7686 | 1003.5459 | 502.2766 | 9 |
| 5 | **608.2749** | 304.6411 | 591.2483 | 296.1278 | **590.2643** | 295.6358 | F | **908.4724** | 454.7398 | 891.4458 | **446.2266** | **890.4618** | 445.7345 | 8 |

| | | | | | | | | | | | | | | |
|---|---|---|---|---|---|---|---|---|---|---|---|---|---|---|
| **6** | 695.3069 | **348.1571** | 678.2803 | 339.6438 | 677.2963 | 339.1518 | **S** | **761.4040** | 381.2056 | 744.3774 | 372.6923 | **743.3934** | 372.2003 | **7** |
| **7** | **810.3338** | 405.6706 | 793.3073 | 397.1573 | 792.3233 | 396.6653 | **D** | **674.3719** | 337.6896 | 657.3454 | 329.1763 | 656.3614 | 328.6843 | **6** |
| **8** | **923.4179** | 462.2126 | 906.3913 | 453.6993 | **905.4073** | 453.2073 | **L** | **559.3450** | 280.1761 | 542.3184 | 271.6629 | 541.3344 | 271.1708 | **5** |
| **9** | **1022.4863** | 511.7468 | 1005.4598 | 503.2335 | 1004.4757 | 502.7415 | **V** | **446.2609** | 223.6341 | **429.2344** | 215.1208 | 428.2504 | 214.6288 | **4** |
| **10** | **1093.5234** | 547.2653 | 1076.4969 | 538.7521 | 1075.5129 | 538.2601 | **A** | **347.1925** | 174.0999 | **330.1660** | 165.5866 | 329.1819 | 165.0946 | **3** |
| **11** | **1222.5660** | 611.7866 | **1205.5395** | 603.2734 | **1204.5554** | 602.7814 | **E** | **276.1554** | 138.5813 | 259.1288 | 130.0681 | 258.1448 | 129.5761 | **2** |
| **12** | | | | | | | **K** | 147.1128 | 74.0600 | 130.0863 | 65.5468 | | | **1** |

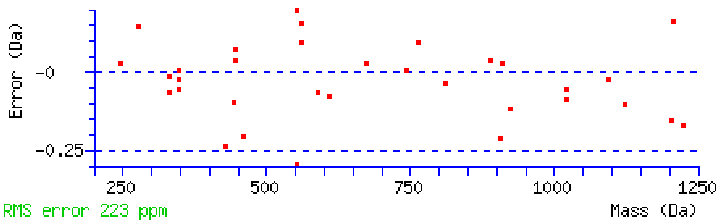


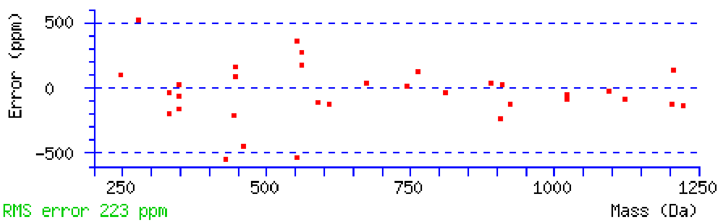


### All matches to this query

| Score | Mr(calc): | Delta | Sequence |
|---|---|---|---|
| 65.4 | 1367.6642 | 0.0039 | NMTLFSDLVAEK |
| 9.9 | 1366.6729 | 0.9953 | HINPSQDDLTVK |
| 8.9 | 1367.6681 | 0.0001 | YGTVRNSAATAEK |
| 8.4 | 1367.6639 | 0.0042 | FTVFFRKSEK |
| 7.8 | 1367.6681 | 0.0000 | EDSQSPGAHLTVK |
| 7.3 | 1366.6625 | 1.0056 | IMNTFSVMPSPK |
| 6.9 | 1367.6795 | -0.0114 | FAEMYQPLLEK |
| 6.9 | 1365.6516 | 2.0165 | AHLTGWLMTLK |
| 5.4 | 1367.6639 | 0.0042 | FTVFFRKSEK |
| 5.0 | 1367.6789 | -0.0107 | QMVTGLMTKAEK |

---

Spectrum No: 435; Query: 73; Rank: 1

---

## Peptide View

MS/MS Fragmentation of **LLNNLTSIK**
Found in **IPI00363849**, Tax_Id=10116 Gene_Symbol=Lamc1 similar to Laminin gamma-1 chain precursor

Match to Query 73: 1015.588508 from(508.801530,2+)
Title: 091008RatKidney_NoSalt_25.2108.2108.2.dta
Data file K:\NewmanPaper\Piliang\3SubProteomes\Piliang3SP\mgf5ppm\ERLIC_3SubProteomes5ppm.mgf

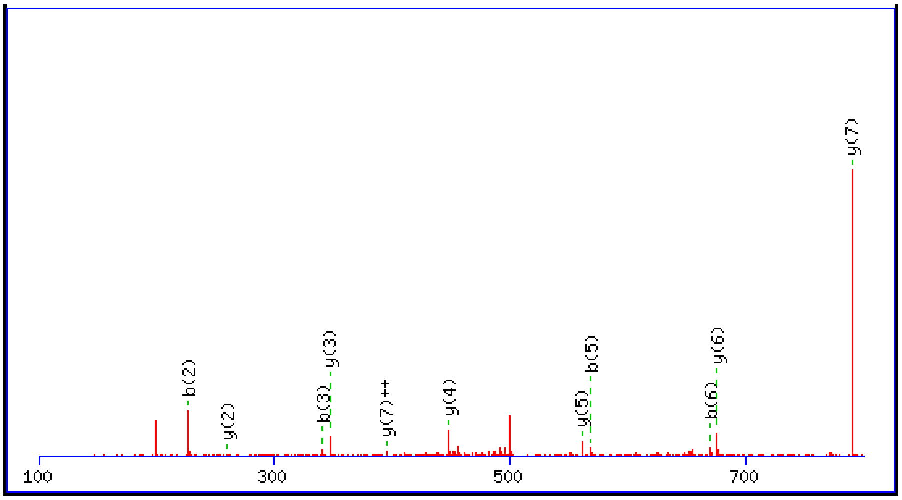


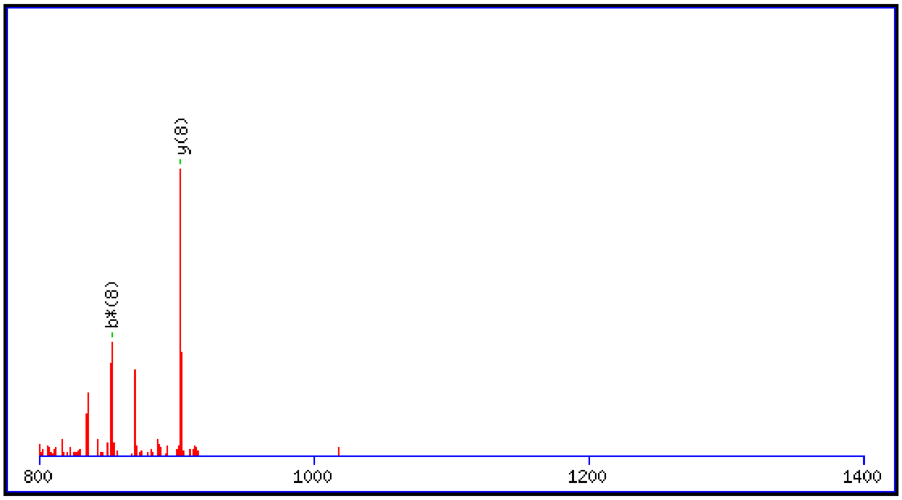


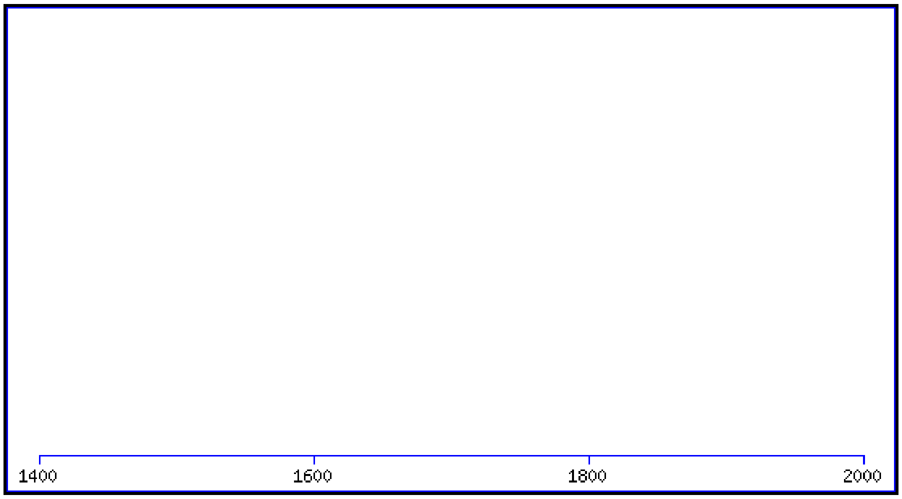


**Monoisotopic mass of neutral peptide Mr(calc):** 1015.5913
**Fixed modifications:** Carbamidomethyl (C)
**Variable modifications:**
**N4** : Deamidated_N (N)
**Ions Score:** 65 **Expect:** 2e-005
**Matches (Bold Red):** 13/78 fragment ions using 17 most intense peaks

| # | b | b++ | b* | b*++ | b0 | b0++ | Seq. | y | y++ | y* | y*++ | y0 | y0++ | # |
|---|---|---|---|---|---|---|---|---|---|---|---|---|---|---|
| 1 | 114.0913 | 57.5493 | | | | | L | | | | | | | 9 |
| 2 | **227.1754** | 114.0913 | | | | | L | **903.5146** | 452.2609 | 886.4880 | 443.7476 | 885.5040 | 443.2556 | 8 |
| 3 | **341.2183** | 171.1128 | 324.1918 | 162.5995 | | | N | **790.4305** | **395.7189** | 773.4040 | 387.2056 | 772.4199 | 386.7136 | 7 |
| 4 | 456.2453 | 228.6263 | 439.2187 | 220.1130 | | | N | **676.3876** | 338.6974 | 659.3610 | 330.1842 | 658.3770 | 329.6921 | 6 |
| 5 | **569.3293** | 285.1683 | 552.3028 | 276.6550 | | | L | **561.3606** | 281.1840 | 544.3341 | 272.6707 | 543.3501 | 272.1787 | 5 |

| | | | | | | | | | | | | | | |
|---|---|---|---|---|---|---|---|---|---|---|---|---|---|---|
| **6** | **670.3770** | 335.6921 | 653.3505 | 327.1789 | 652.3664 | 326.6869 | **T** | **448.2766** | 224.6419 | 431.2500 | 216.1287 | 430.2660 | 215.6366 | **4** |
| **7** | 757.4090 | 379.2082 | 740.3825 | 370.6949 | 739.3985 | 370.2029 | **S** | **347.2289** | 174.1181 | 330.2023 | 165.6048 | 329.2183 | 165.1128 | **3** |
| **8** | 870.4931 | 435.7502 | **853.4666** | 427.2369 | 852.4825 | 426.7449 | **I** | **260.1969** | 130.6021 | 243.1703 | 122.0888 | | | **2** |
| **9** | | | | | | | **K** | 147.1128 | 74.0600 | 130.0863 | 65.5468 | | | **1** |

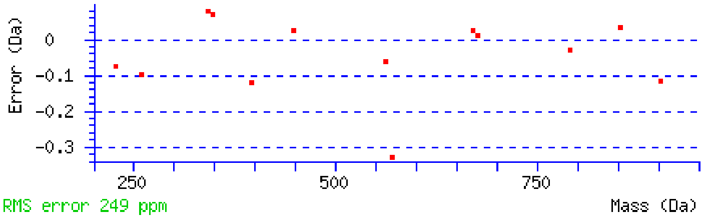


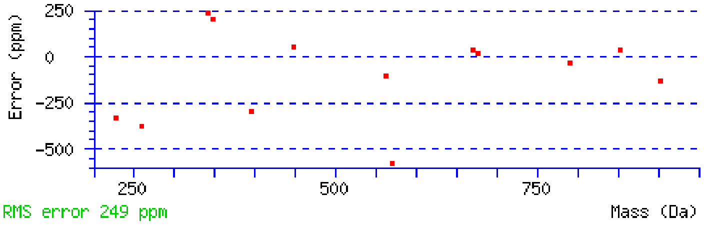


**All matches to this query**

| Score | Mr(calc): | Delta | Sequence |
|---|---|---|---|
| 65.3 | 1015.5913 | -0.0028 | LLNNLTSIK |
| 46.7 | 1015.5913 | -0.0028 | LLNNLTSIK |
| 17.3 | 1015.5913 | -0.0028 | LNILDTLSK |
| 17.1 | 1015.5913 | -0.0028 | LILDSLSQK |
| 9.7 | 1015.5831 | 0.0054 | LIIHLISK |
| 7.9 | 1015.5927 | -0.0042 | TKVHLPGHK |
| 7.8 | 1015.5913 | -0.0028 | DKLVLATEK |
| 7.2 | 1015.5848 | 0.0037 | ILNMIRQK |
| 5.1 | 1015.5913 | -0.0028 | EVSLLQSLK |
| 5.1 | 1015.5814 | 0.0071 | LIAWERTK |

---

Spectrum No: 436; Query: 1081; Rank: 1

---

## Peptide View

MS/MS Fragmentation of **IKTPEKNDTAAEQGGR**
Found in **IPI00778946**, Tax_Id=10116 Gene_Symbol=Itgav_predicted Protein

Match to Query 1081: 1714.848882 from(572.623570,3+)
Title: 100101RatKid_NS_deglyco_23.263.263.3.dta
Data file K:\NewmanPaper\Piliang\3SubProteomes\Piliang3SP\mgf5ppm\ERLIC_3SubProteomes5ppm.mgf

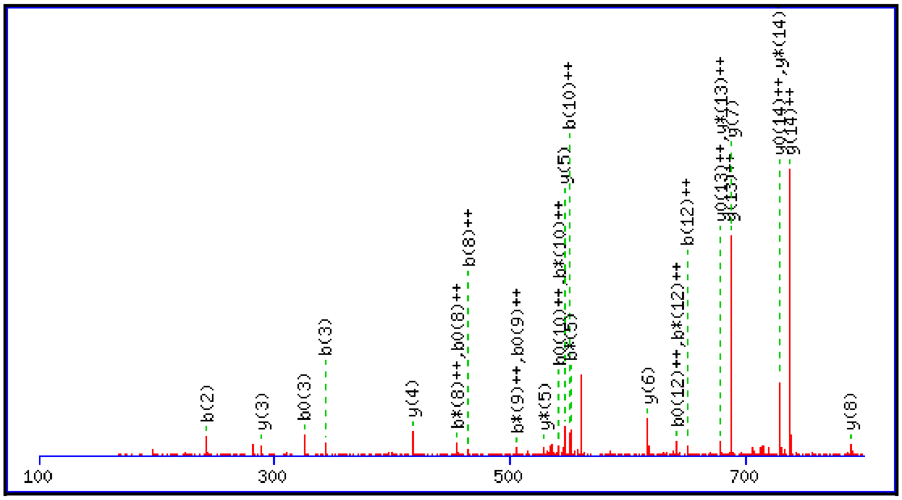

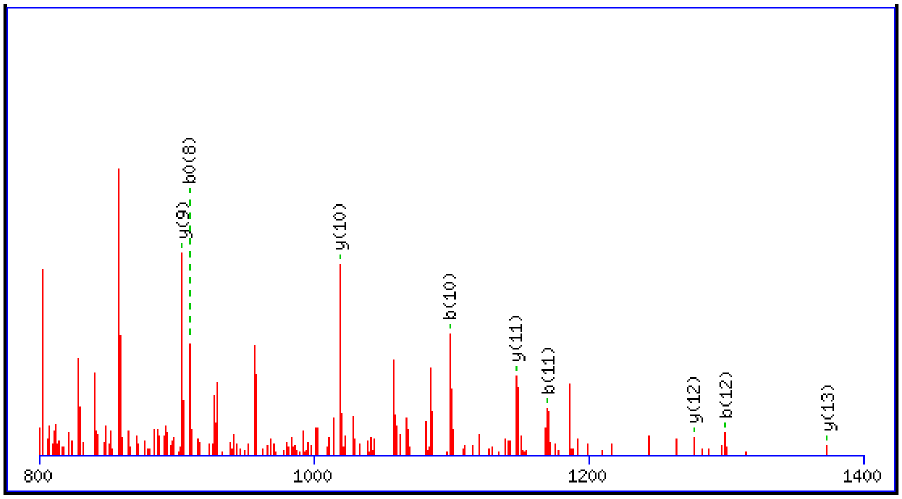


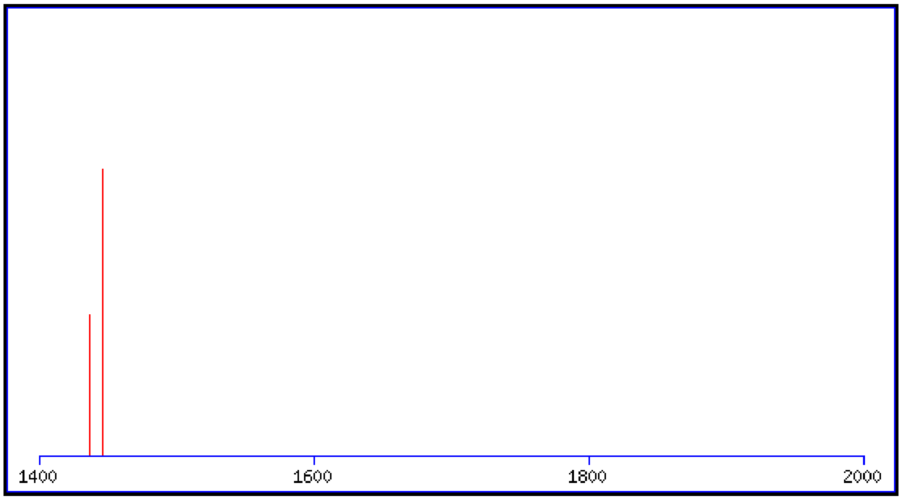


**Monoisotopic mass of neutral peptide Mr(calc):** 1714.8486
**Fixed modifications:** Carbamidomethyl (C)
**Variable modifications:**
**N7** : Deamidated_N (N)
**Ions Score:** 65 **Expect:** 5.2e-005
**Matches (Bold Red):** 37/166 fragment ions using 61 most intense peaks

| # | b | $b^{++}$ | b* | $b^{*++}$ | $b^{0}$ | $b^{0++}$ | Seq. | y | $y^{++}$ | y* | $y^{*++}$ | $y^{0}$ | $y^{0++}$ | # |
|---|---|---|---|---|---|---|---|---|---|---|---|---|---|---|
| 1 | 114.0913 | 57.5493 | | | | | I | | | | | | | 16 |
| 2 | **242.1863** | 121.5968 | 225.1598 | 113.0835 | | | K | 1602.7718 | 801.8895 | 1585.7453 | 793.3763 | 1584.7612 | 792.8843 | 15 |
| 3 | **343.2340** | 172.1206 | 326.2074 | 163.6074 | **325.2234** | 163.1153 | T | 1474.6768 | **737.8421** | 1457.6503 | **729.3288** | 1456.6663 | **728.8368** | 14 |
| 4 | 440.2867 | 220.6470 | 423.2602 | 212.1337 | 422.2762 | 211.6417 | P | **1373.6292** | **687.3182** | 1356.6026 | **678.8049** | 1355.6186 | **678.3129** | 13 |
| 5 | 569.3293 | 285.1683 | **552.3028** | 276.6550 | 551.3188 | 276.1630 | E | **1276.5764** | 638.7918 | 1259.5498 | 630.2786 | 1258.5658 | 629.7866 | 12 |
| 6 | 697.4243 | 349.2158 | 680.3978 | 340.7025 | 679.4137 | 340.2105 | K | **1147.5338** | 574.2705 | 1130.5073 | 565.7573 | 1129.5232 | 565.2653 | 11 |
| 7 | 812.4512 | 406.7293 | 795.4247 | 398.2160 | 794.4407 | 397.7240 | N | **1019.4388** | 510.2231 | 1002.4123 | 501.7098 | 1001.4283 | 501.2178 | 10 |
| 8 | 927.4782 | **464.2427** | 910.4516 | **455.7295** | **909.4676** | **455.2374** | D | **904.4119** | 452.7096 | 887.3854 | 444.1963 | 886.4013 | 443.7043 | 9 |
| 9 | 1028.5259 | 514.7666 | 1011.4993 | **506.2533** | 1010.5153 | **505.7613** | T | **789.3850** | 395.1961 | 772.3584 | 386.6828 | 771.3744 | 386.1908 | 8 |
| 10 | **1099.5630** | **550.2851** | 1082.5364 | **541.7719** | 1081.5524 | **541.2798** | A | **688.3373** | 344.6723 | 671.3107 | 336.1590 | 670.3267 | 335.6670 | 7 |
| 11 | **1170.6001** | 585.8037 | 1153.5735 | 577.2904 | 1152.5895 | 576.7984 | A | **617.3002** | 309.1537 | 600.2736 | 300.6404 | 599.2896 | 300.1484 | 6 |
| 12 | **1299.6427** | **650.3250** | 1282.6161 | **641.8117** | 1281.6321 | **641.3197** | E | **546.2631** | 273.6352 | **529.2365** | 265.1219 | 528.2525 | 264.6299 | 5 |
| 13 | 1427.7013 | 714.3543 | 1410.6747 | 705.8410 | 1409.6907 | 705.3490 | Q | **417.2205** | 209.1139 | 400.1939 | 200.6006 | | | 4 |
| 14 | 1484.7227 | 742.8650 | 1467.6962 | 734.3517 | 1466.7122 | 733.8597 | G | **289.1619** | 145.0846 | 272.1353 | 136.5713 | | | 3 |
| 15 | 1541.7442 | 771.3757 | 1524.7176 | 762.8625 | 1523.7336 | 762.3704 | G | 232.1404 | 116.5738 | 215.1139 | 108.0606 | | | 2 |
| 16 | | | | | | | R | 175.1190 | 88.0631 | 158.0924 | 79.5498 | | | 1 |

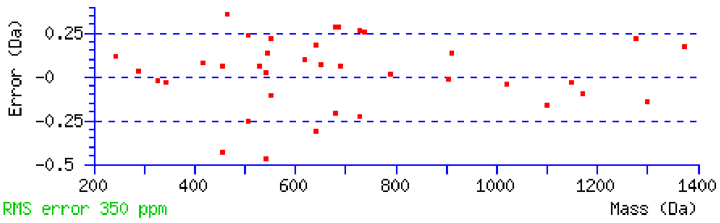


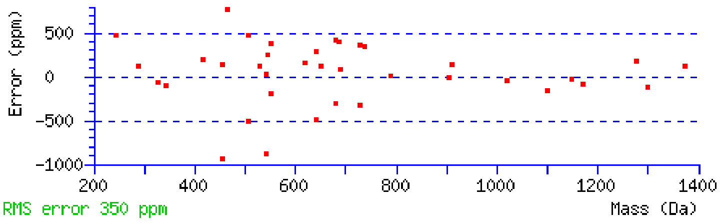


### All matches to this query

| Score | Mr(calc): | Delta | Sequence |
|---|---|---|---|
| 65.2 | 1714.8486 | 0.0003 | IKTPEKNDTAAEQGGR |
| 3.8 | 1712.8400 | 2.0089 | THLQLGSVLYHHTK |
| 3.8 | 1712.8400 | 2.0089 | THLQLGSVLYHHTK |
| 3.8 | 1712.8400 | 2.0089 | THLQLGSVLYHHTK |
| 2.5 | 1713.8451 | 1.0038 | YGTGAVSIQAGSALLAR |
| 2.3 | 1714.8348 | 0.0141 | KITCKNGEWEEPPK |
| 0.9 | 1712.8498 | 1.9991 | EIQPKGRSYELVSK |
| 0.8 | 1713.8450 | 1.0038 | NQLSSYPSAALSKLR |
| 0.2 | 1712.8420 | 2.0069 | QTIIGQPMSVTITTK |
| 0.2 | 1712.8420 | 2.0069 | QTIIGQPMSVTITTK |

---

Spectrum No: 437; Query: 329; Rank: 1

---

## Peptide View

MS/MS Fragmentation of **ELISNASDALDK**
Found in **IPI00365985**, Tax_Id=10116 Gene_Symbol=Tra1_predicted 93 kDa protein

Match to Query 329: 1275.621628 from(638.818090,2+)
Title: 100101RatKid_NS_deglyco_15.2399.2399.2.dta
Data file K:\NewmanPaper\Piliang\3SubProteomes\Piliang3SP\mgf5ppm\ERLIC_3SubProteomes5ppm.mgf

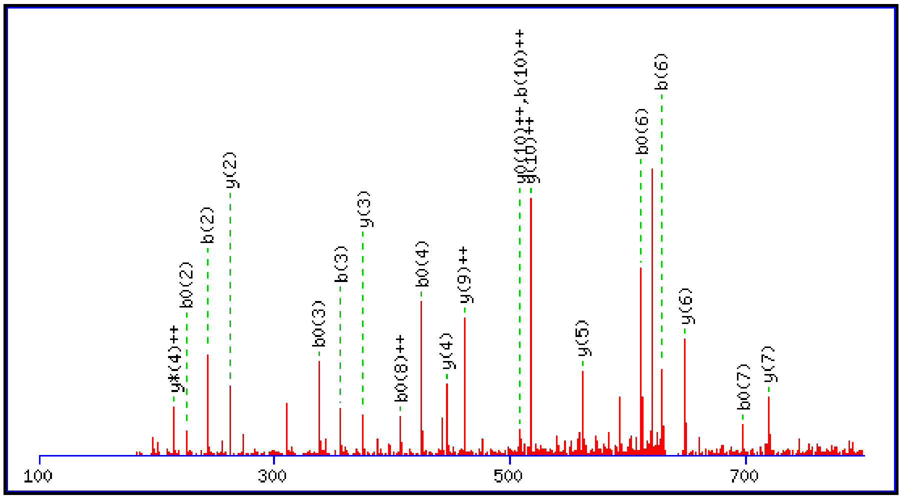

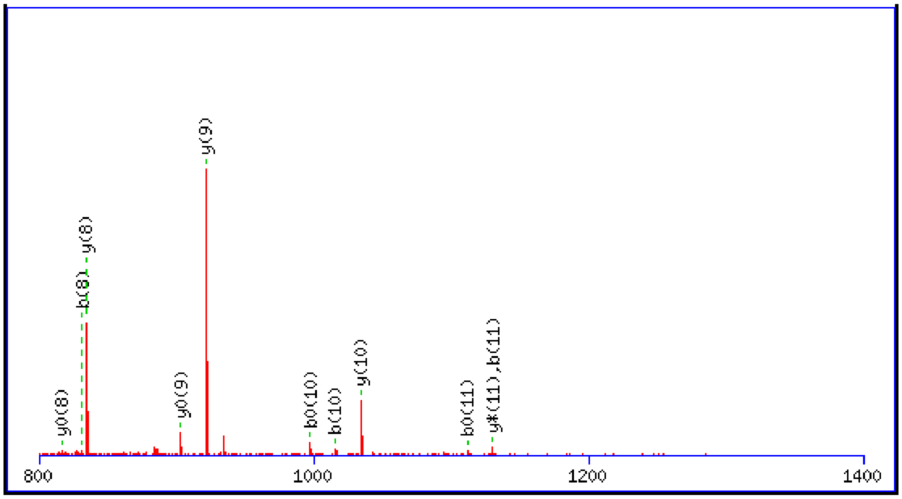


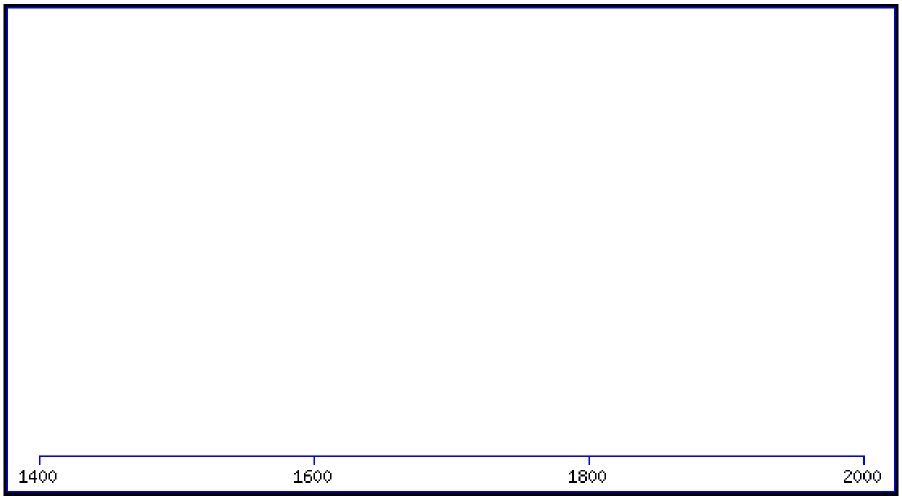


**Monoisotopic mass of neutral peptide Mr(calc):** 1275.6194
**Fixed modifications:** Carbamidomethyl (C)
**Variable modifications:**
**N5** : Deamidated_N (N)
**Ions Score:** 65 **Expect:** 4.2e-005
**Matches (Bold Red):** 31/122 fragment ions using 46 most intense peaks

| # | b | $b^{++}$ | b* | $b^{*++}$ | $b^{0}$ | $b^{0++}$ | Seq. | y | $y^{++}$ | y* | $y^{*++}$ | $y^{0}$ | $y^{0++}$ | # |
|---|---|---|---|---|---|---|---|---|---|---|---|---|---|---|
| 1 | 130.0499 | 65.5286 | | | 112.0393 | 56.5233 | E | | | | | | | 12 |
| 2 | **243.1339** | 122.0706 | | | **225.1234** | 113.0653 | L | 1147.5841 | 574.2957 | **1130.5576** | 565.7824 | 1129.5735 | 565.2904 | 11 |
| 3 | **356.2180** | 178.6126 | | | **338.2074** | 169.6074 | I | **1034.5000** | **517.7537** | 1017.4735 | 509.2404 | 1016.4895 | **508.7484** | 10 |
| 4 | 443.2500 | 222.1287 | | | **425.2395** | 213.1234 | S | **921.4160** | **461.2116** | 904.3894 | 452.6984 | **903.4054** | 452.2063 | 9 |
| 5 | 558.2770 | 279.6421 | 541.2504 | 271.1288 | 540.2664 | 270.6368 | N | **834.3839** | 417.6956 | 817.3574 | 409.1823 | **816.3734** | 408.6903 | 8 |
| 6 | **629.3141** | 315.1607 | 612.2875 | 306.6474 | **611.3035** | 306.1554 | A | **719.3570** | 360.1821 | 702.3305 | 351.6689 | 701.3464 | 351.1769 | 7 |
| 7 | 716.3461 | 358.6767 | 699.3196 | 350.1634 | **698.3355** | 349.6714 | S | **648.3199** | 324.6636 | 631.2933 | 316.1503 | 630.3093 | 315.6583 | 6 |
| 8 | **831.3730** | 416.1902 | 814.3465 | 407.6769 | 813.3625 | **407.1849** | D | **561.2879** | 281.1476 | 544.2613 | 272.6343 | 543.2773 | 272.1423 | 5 |
| 9 | 902.4102 | 451.7087 | 885.3836 | 443.1954 | 884.3996 | 442.7034 | A | **446.2609** | 223.6341 | 429.2344 | **215.1208** | 428.2504 | 214.6288 | 4 |
| 10 | **1015.4942** | **508.2507** | 998.4677 | 499.7375 | **997.4837** | 499.2455 | L | **375.2238** | 188.1155 | 358.1973 | 179.6023 | 357.2132 | 179.1103 | 3 |
| 11 | **1130.5212** | 565.7642 | 1113.4946 | 557.2509 | **1112.5106** | 556.7589 | D | **262.1397** | 131.5735 | 245.1132 | 123.0602 | 244.1292 | 122.5682 | 2 |
| 12 | | | | | | | K | 147.1128 | 74.0600 | 130.0863 | 65.5468 | | | 1 |

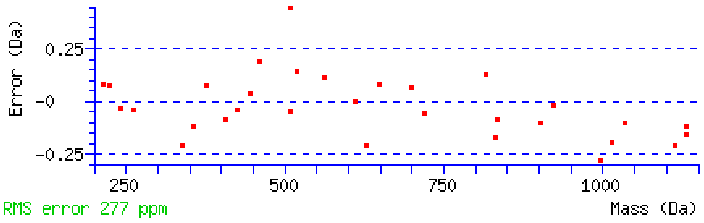


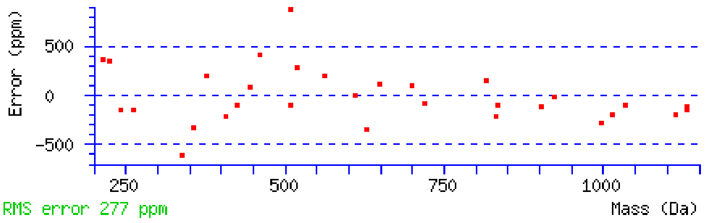


**All matches to this query**

| Score | Mr(calc): | Delta | Sequence |
|---|---|---|---|
| 65.2 | 1275.6194 | 0.0022 | ELISNASDALDK |
| 9.0 | 1275.6306 | -0.0090 | IEISQSDSLER |
| 8.3 | 1275.6264 | -0.0048 | KGGIFSAEFLK |
| 5.9 | 1275.6112 | 0.0105 | FVTLGSETSKK |
| 5.2 | 1275.6320 | -0.0104 | GAVQSEWRTSR |
| 3.8 | 1275.6159 | 0.0058 | HVTSTHMKKK |
| 3.2 | 1275.6129 | 0.0088 | ELALMGETQER |
| 2.1 | 1274.6271 | 0.9945 | LKNISSKYDK |
| 1.3 | 1275.6111 | 0.0105 | LKNISSKYDK |
| 0.8 | 1275.6125 | 0.0091 | KKVHFHGESK |

---

Spectrum No: 438; Query: 507; Rank: 1

---

## Peptide View

MS/MS Fragmentation of **WGEVDLIGNCSR**
Found in **IPI00204451**, Tax_Id=10116 Gene_Symbol=Cfi Complement factor I precursor

Match to Query 507: 1405.632888 from(703.823720,2+)
Title: 100101RatKid_NS_deglyco_28.2758.2758.2.dta
Data file K:\NewmanPaper\Piliang\3SubProteomes\Piliang3SP\mgf5ppm\ERLIC_3SubProteomes5ppm.mgf

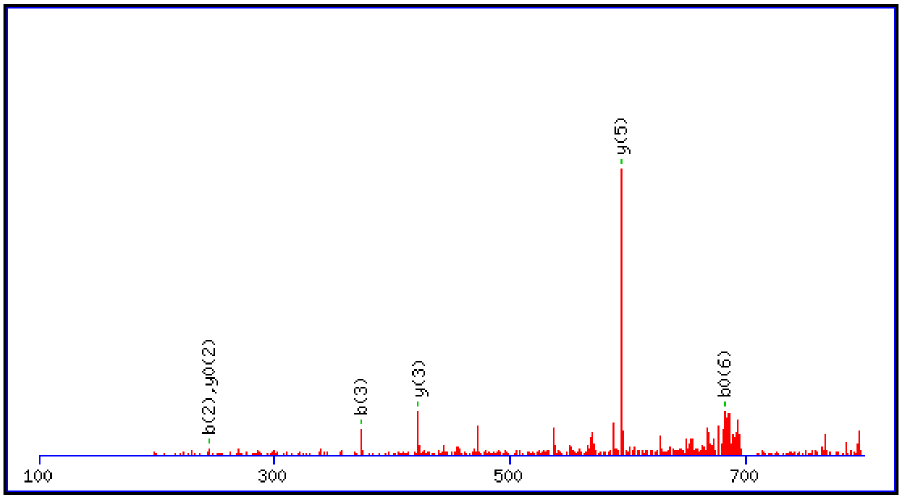

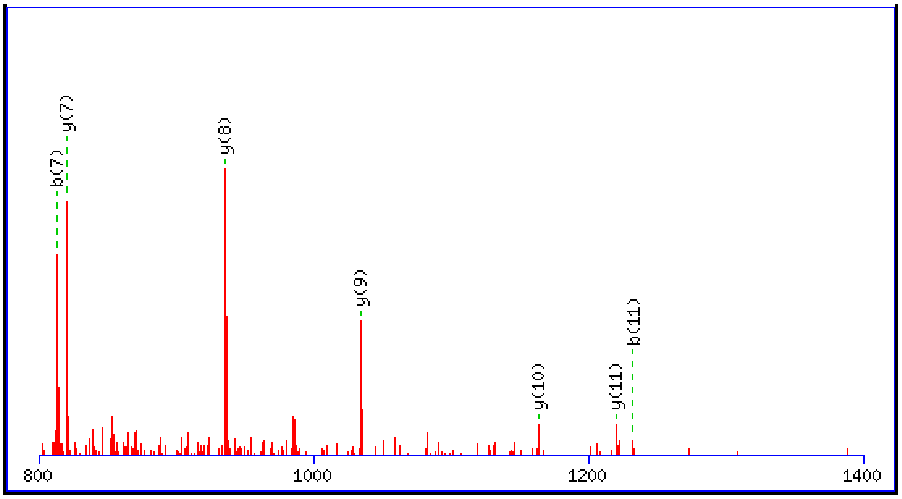


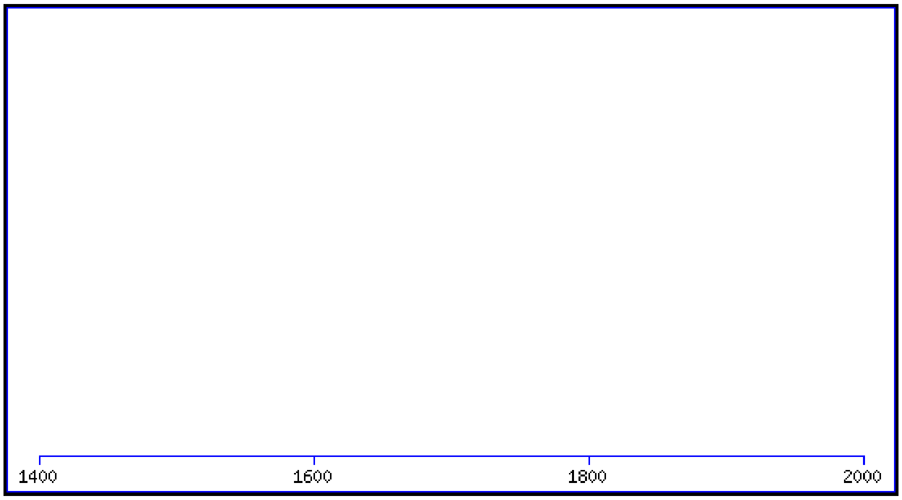


**Monoisotopic mass of neutral peptide Mr(calc):** 1405.6296
**Fixed modifications:** Carbamidomethyl (C)
**Variable modifications:**
**N9** : Deamidated_N (N)
**Ions Score:** 65 **Expect:** 4.8e-005
**Matches (Bold Red):** 13/110 fragment ions using 25 most intense peaks

| # | b | $b^{++}$ | $b^{*}$ | $b^{*++}$ | $b^{0}$ | $b^{0++}$ | Seq. | y | $y^{++}$ | $y^{*}$ | $y^{*++}$ | $y^{0}$ | $y^{0++}$ | # |
|---|---|---|---|---|---|---|---|---|---|---|---|---|---|---|
| 1 | 187.0866 | 94.0469 | | | | | W | | | | | | | 12 |
| 2 | **244.1081** | 122.5577 | | | | | G | **1220.5576** | 610.7824 | 1203.5310 | 602.2691 | 1202.5470 | 601.7771 | 11 |
| 3 | **373.1506** | 187.0790 | | | 355.1401 | 178.0737 | E | **1163.5361** | 582.2717 | 1146.5096 | 573.7584 | 1145.5255 | 573.2664 | 10 |
| 4 | 472.2191 | 236.6132 | | | 454.2085 | 227.6079 | V | **1034.4935** | 517.7504 | 1017.4670 | 509.2371 | 1016.4829 | 508.7451 | 9 |
| 5 | 587.2460 | 294.1266 | | | 569.2354 | 285.1214 | D | **935.4251** | 468.2162 | 918.3986 | 459.7029 | 917.4145 | 459.2109 | 8 |
| 6 | 700.3301 | 350.6687 | | | **682.3195** | 341.6634 | L | **820.3982** | 410.7027 | 803.3716 | 402.1894 | 802.3876 | 401.6974 | 7 |
| 7 | **813.4141** | 407.2107 | | | 795.4036 | 398.2054 | I | 707.3141 | 354.1607 | 690.2875 | 345.6474 | 689.3035 | 345.1554 | 6 |
| 8 | 870.4356 | 435.7214 | | | 852.4250 | 426.7162 | G | **594.2300** | 297.6187 | 577.2035 | 289.1054 | 576.2195 | 288.6134 | 5 |
| 9 | 985.4625 | 493.2349 | 968.4360 | 484.7216 | 967.4520 | 484.2296 | N | 537.2086 | 269.1079 | 520.1820 | 260.5946 | 519.1980 | 260.1026 | 4 |
| 10 | 1145.4932 | 573.2502 | 1128.4666 | 564.7370 | 1127.4826 | 564.2449 | C | **422.1816** | 211.5945 | 405.1551 | 203.0812 | 404.1711 | 202.5892 | 3 |
| 11 | **1232.5252** | 616.7662 | 1215.4987 | 608.2530 | 1214.5146 | 607.7610 | S | 262.1510 | 131.5791 | 245.1244 | 123.0659 | **244.1404** | 122.5738 | 2 |
| 12 | | | | | | | R | 175.1190 | 88.0631 | 158.0924 | 79.5498 | | | 1 |

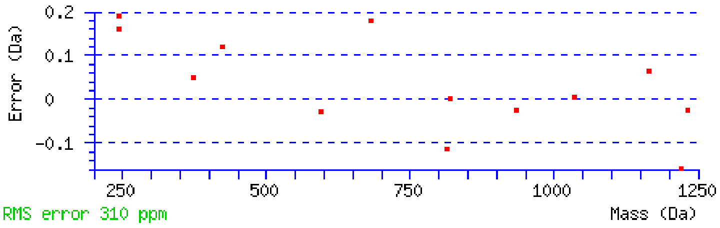


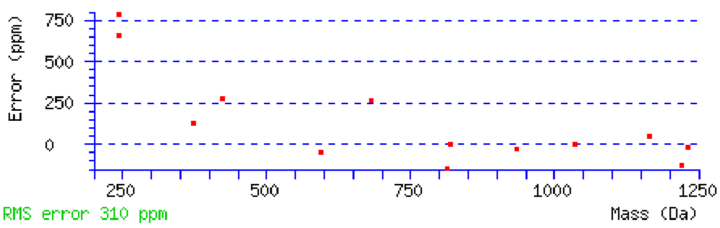


### All matches to this query

| Score | Mr(calc): | Delta | Sequence |
|---|---|---|---|
| 65.0 | 1405.6296 | 0.0033 | WGEVDLIGNCSR |
| 4.1 | 1403.6323 | 2.0006 | RRLVSELESR |
| 1.6 | 1403.6122 | 2.0207 | YEPNKQFLER |

Spectrum No: 439; Query: 1090; Rank: 1

## Peptide View

MS/MS Fragmentation of **IQNYSVMAICDYDK**
Found in **IPI00208422**, Tax_Id=10116 Gene_Symbol=Dpp4 Dipeptidyl peptidase 4

Match to Query 1090: 1719.752648 from(860.883600,2+)
Title: 100101RatKid_NS_deglyco_23.3364.3364.2.dta
Data file K:\NewmanPaper\Piliang\3SubProteomes\Piliang3SP\mgf5ppm\ERLIC_3SubProteomes5ppm.mgf

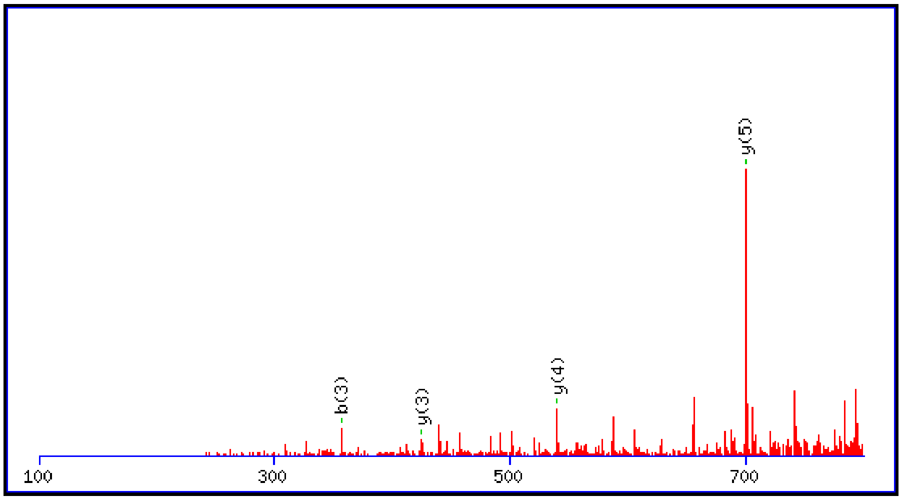


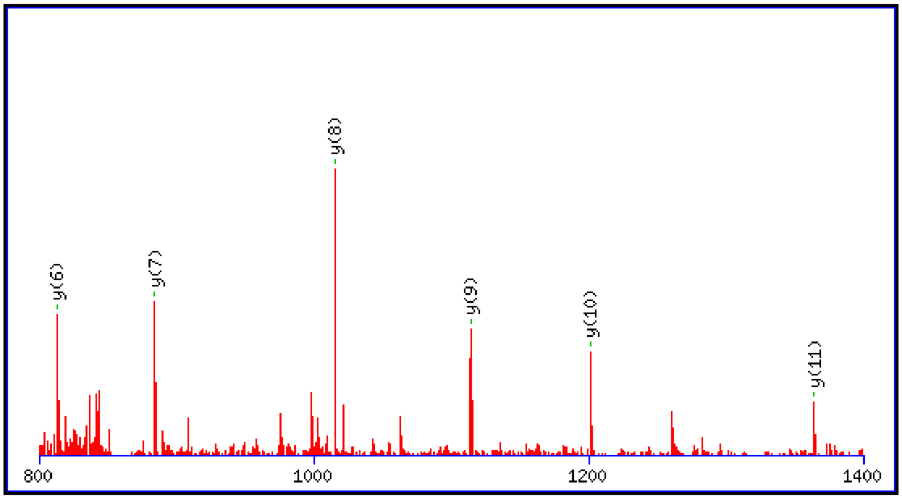

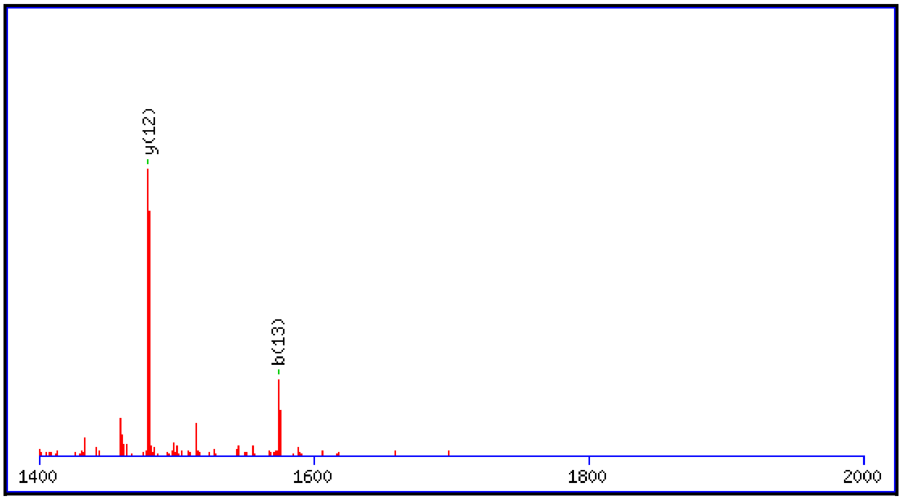


**Monoisotopic mass of neutral peptide Mr(calc):** 1719.7484
**Fixed modifications:** Carbamidomethyl (C)
**Variable modifications:**
**N3** : Deamidated_N (N)
**Ions Score:** 65 **Expect:** 6e-005
**Matches (Bold Red):** 12/144 fragment ions using 21 most intense peaks

| # | b | b++ | b* | b*++ | b0 | b0++ | Seq. | y | y++ | y* | y*++ | y0 | y0++ | # |
|---|---|---|---|---|---|---|---|---|---|---|---|---|---|---|
| 1 | 114.0913 | 57.5493 | | | | | I | | | | | | | 14 |
| 2 | 242.1499 | 121.5786 | 225.1234 | 113.0653 | | | Q | 1607.6716 | 804.3394 | 1590.6451 | 795.8262 | 1589.6611 | 795.3342 | 13 |
| 3 | **357.1769** | 179.0921 | 340.1503 | 170.5788 | | | N | **1479.6130** | 740.3102 | 1462.5865 | 731.7969 | 1461.6025 | 731.3049 | 12 |
| 4 | 520.2402 | 260.6237 | 503.2136 | 252.1105 | | | Y | **1364.5861** | 682.7967 | 1347.5596 | 674.2834 | 1346.5755 | 673.7914 | 11 |
| 5 | 607.2722 | 304.1397 | 590.2457 | 295.6265 | 589.2616 | 295.1345 | S | **1201.5228** | 601.2650 | 1184.4962 | 592.7518 | 1183.5122 | 592.2597 | 10 |
| 6 | 706.3406 | 353.6740 | 689.3141 | 345.1607 | 688.3301 | 344.6687 | V | **1114.4907** | 557.7490 | 1097.4642 | 549.2357 | 1096.4802 | 548.7437 | 9 |
| 7 | 837.3811 | 419.1942 | 820.3546 | 410.6809 | 819.3705 | 410.1889 | M | **1015.4223** | 508.2148 | 998.3958 | 499.7015 | 997.4118 | 499.2095 | 8 |
| 8 | 908.4182 | 454.7128 | 891.3917 | 446.1995 | 890.4077 | 445.7075 | A | **884.3818** | 442.6946 | 867.3553 | 434.1813 | 866.3713 | 433.6893 | 7 |
| 9 | 1021.5023 | 511.2548 | 1004.4757 | 502.7415 | 1003.4917 | 502.2495 | I | **813.3447** | 407.1760 | 796.3182 | 398.6627 | 795.3342 | 398.1707 | 6 |
| 10 | 1181.5329 | 591.2701 | 1164.5064 | 582.7568 | 1163.5224 | 582.2648 | C | **700.2607** | 350.6340 | 683.2341 | 342.1207 | 682.2501 | 341.6287 | 5 |
| 11 | 1296.5599 | 648.7836 | 1279.5333 | 640.2703 | 1278.5493 | 639.7783 | D | **540.2300** | 270.6186 | 523.2035 | 262.1054 | 522.2195 | 261.6134 | 4 |
| 12 | 1459.6232 | 730.3152 | 1442.5967 | 721.8020 | 1441.6126 | 721.3100 | Y | **425.2031** | 213.1052 | 408.1765 | 204.5919 | 407.1925 | 204.0999 | 3 |
| 13 | **1574.6502** | 787.8287 | 1557.6236 | 779.3154 | 1556.6396 | 778.8234 | D | 262.1397 | 131.5735 | 245.1132 | 123.0602 | 244.1292 | 122.5682 | 2 |
| 14 | | | | | | | K | 147.1128 | 74.0600 | 130.0863 | 65.5468 | | | 1 |

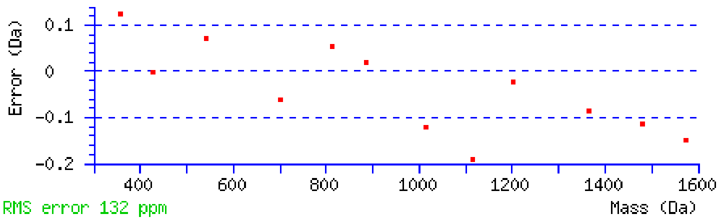


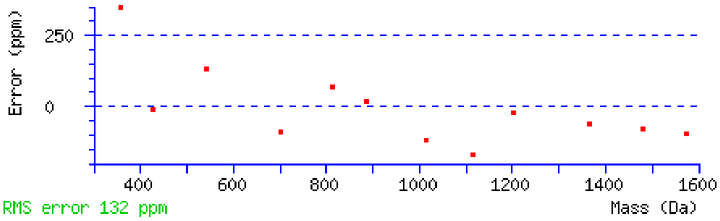


## All matches to this query

| Score | Mr(calc): | Delta | Sequence |
|---|---|---|---|
| 65.0 | 1719.7484 | 0.0043 | IQNYSVMAICDYDK |
| 50.3 | 1718.7644 | 0.9883 | IQNYSVMAICDYDK |
| 5.1 | 1719.7621 | -0.0095 | DPSSQVGACIVNTENK |
| 4.0 | 1719.7634 | -0.0107 | SKPVEWTLTRTDK |
| 3.1 | 1718.7365 | 1.0162 | YPMGRVHVGLSSTR |
| 2.7 | 1719.7634 | -0.0107 | SKPVEWTLTRTDK |
| 2.7 | 1719.7634 | -0.0107 | SKPVEWTLTRTDK |
| | | | |

| | | | |
|---|---|---|---|
| 2.1 | 1718.7365 | 1.0162 | YPMGRVHVGLSSTR |
| 1.7 | 1719.7634 | -0.0107 | SKPVEWTLTRTDK |
| 0.1 | 1719.7522 | 0.0005 | LYTLVLTDPDAPSR |

---

Spectrum No: 440; Query: 598; Rank: 1

---

## Peptide View

MS/MS Fragmentation of **SVLENSTSYEEAK**
Found in **IPI00421601**, Tax_Id=10116 Gene_Symbol=Asah1 N-acylsphingosine amidohydrolase 1

Match to Query 598: 1456.656948 from(729.335750,2+)
Title: 091008RatKidney_NoSalt_21.1079.1079.2.dta
Data file K:\NewmanPaper\Piliang\3SubProteomes\Piliang3SP\mgf5ppm\ERLIC_3SubProteomes5ppm.mgf

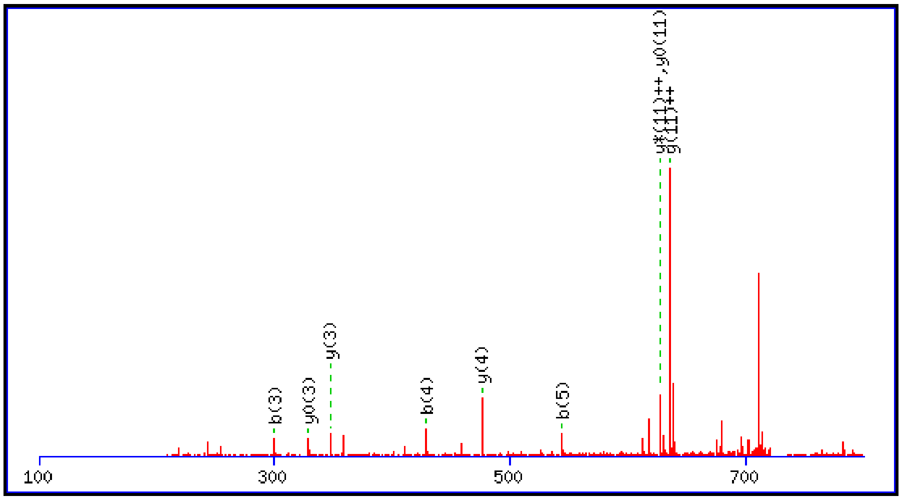


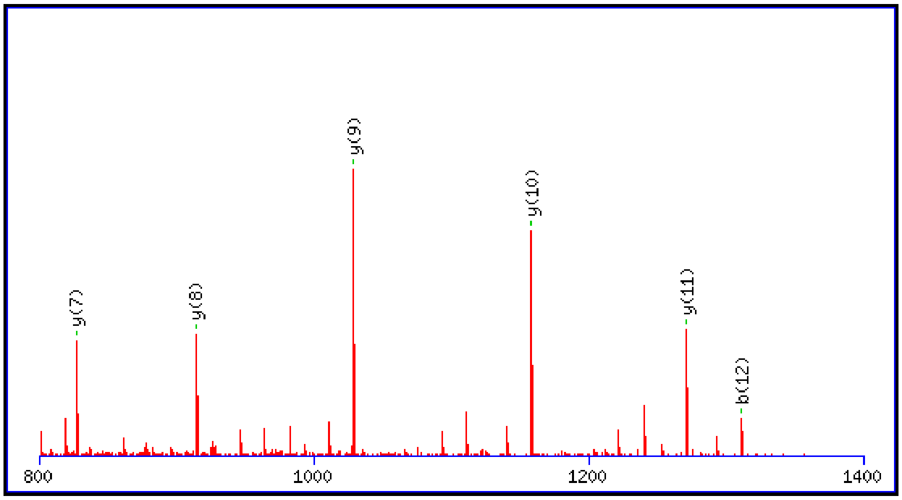

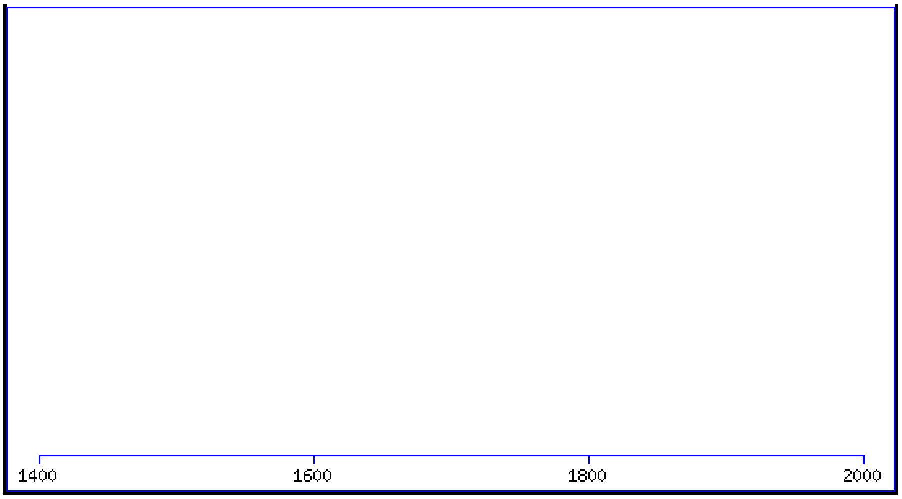


**Monoisotopic mass of neutral peptide Mr(calc):** 1456.6569
**Fixed modifications:** Carbamidomethyl (C)
**Variable modifications:**
**N5** : Deamidated_N (N)
**Ions Score:** 65 **Expect:** 5.2e-005
**Matches (Bold Red):** 15/132 fragment ions using 17 most intense peaks

| # | b | $b^{++}$ | b* | $b^{*++}$ | $b^0$ | $b^{0++}$ | Seq. | y | $y^{++}$ | y* | $y^{*++}$ | $y^0$ | $y^{0++}$ | # |
|---|---|---|---|---|---|---|---|---|---|---|---|---|---|---|
| 1 | 88.0393 | 44.5233 | | | 70.0287 | 35.5180 | S | | | | | | | 13 |
| 2 | 187.1077 | 94.0575 | | | 169.0972 | 85.0522 | V | 1370.6322 | 685.8197 | 1353.6056 | 677.3065 | 1352.6216 | 676.8144 | 12 |
| 3 | **300.1918** | 150.5995 | | | 282.1812 | 141.5942 | L | **1271.5638** | **636.2855** | 1254.5372 | **627.7722** | 1253.5532 | **627.2802** | 11 |
| 4 | **429.2344** | 215.1208 | | | 411.2238 | 206.1155 | E | **1158.4797** | 579.7435 | 1141.4531 | 571.2302 | 1140.4691 | 570.7382 | 10 |
| 5 | **544.2613** | 272.6343 | 527.2348 | 264.1210 | 526.2507 | 263.6290 | N | **1029.4371** | 515.2222 | 1012.4106 | 506.7089 | 1011.4265 | 506.2169 | 9 |
| 6 | 631.2933 | 316.1503 | 614.2668 | 307.6370 | 613.2828 | 307.1450 | S | **914.4102** | 457.7087 | 897.3836 | 449.1954 | 896.3996 | 448.7034 | 8 |
| 7 | 732.3410 | 366.6741 | 715.3145 | 358.1609 | 714.3305 | 357.6689 | T | **827.3781** | 414.1927 | 810.3516 | 405.6794 | 809.3676 | 405.1874 | 7 |
| 8 | 819.3730 | 410.1902 | 802.3465 | 401.6769 | 801.3625 | 401.1849 | S | 726.3305 | 363.6689 | 709.3039 | 355.1556 | 708.3199 | 354.6636 | 6 |
| 9 | 982.4364 | 491.7218 | 965.4098 | 483.2086 | 964.4258 | 482.7165 | Y | 639.2984 | 320.1529 | 622.2719 | 311.6396 | 621.2879 | 311.1476 | 5 |
| 10 | 1111.4790 | 556.2431 | 1094.4524 | 547.7298 | 1093.4684 | 547.2378 | E | **476.2351** | 238.6212 | 459.2086 | 230.1079 | 458.2245 | 229.6159 | 4 |
| 11 | 1240.5216 | 620.7644 | 1223.4950 | 612.2511 | 1222.5110 | 611.7591 | E | **347.1925** | 174.0999 | 330.1660 | 165.5866 | **329.1819** | 165.0946 | 3 |
| 12 | **1311.5587** | 656.2830 | 1294.5321 | 647.7697 | 1293.5481 | 647.2777 | A | 218.1499 | 109.5786 | 201.1234 | 101.0653 | | | 2 |
| 13 | | | | | | | K | 147.1128 | 74.0600 | 130.0863 | 65.5468 | | | 1 |

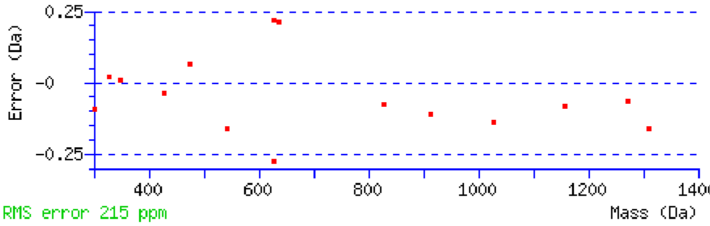


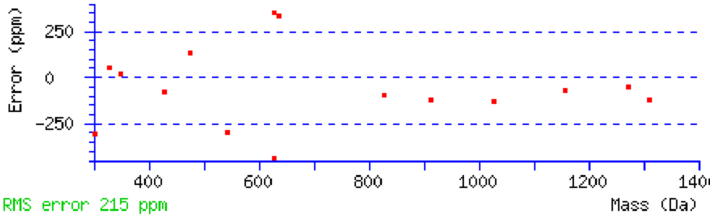


### All matches to this query

| Score | Mr(calc): | Delta | Sequence |
|---|---|---|---|
| 64.9 | 1456.6569 | 0.0001 | SVLENSTSYEEAK |
| 13.7 | 1455.6408 | 1.0161 | RYSGPWXDPRR |
| 13.7 | 1455.6408 | 1.0161 | RYSGPWXDPRR |
| 11.0 | 1456.6438 | 0.0132 | SLLMVTSGYKAK |
| 10.1 | 1456.6520 | 0.0049 | ADLLDTINTCIK |
| 10.0 | 1456.6599 | -0.0030 | SVFPLSVSDSPSR |
| 9.2 | 1456.6438 | 0.0132 | SLLMVTSGYKAK |
| 8.7 | 1456.6599 | -0.0030 | SVFPLSVSDSPSR |
| | | | |

| | | | |
|---|---|---|---|
| 8.7 | 1456.6599 | -0.0030 | SVFPLSVSDSPSR |
| 6.5 | 1456.6520 | 0.0049 | ADLLDTINTCIK |

---

Spectrum No: 441; Query: 1473; Rank: 1

---

## Peptide View

MS/MS Fragmentation of **GHPVDDPTGAFANGSLTFK**
Found in **IPI00196226**, Tax_Id=10116 Gene_Symbol=RGD1303130 Uncharacterized protein C1orf85 homolog precursor

Match to Query 1473: 1930.913982 from(644.645270,3+)
Title: 100101RatKid_NS_deglyco_20.3065.3065.3.dta
Data file K:\NewmanPaper\Piliang\3SubProteomes\Piliang3SP\mgf5ppm\ERLIC_3SubProteomes5ppm.mgf

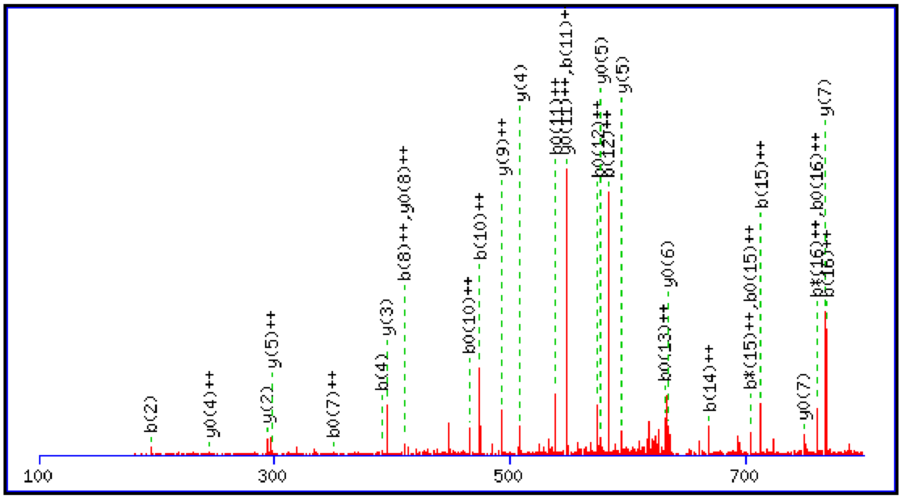


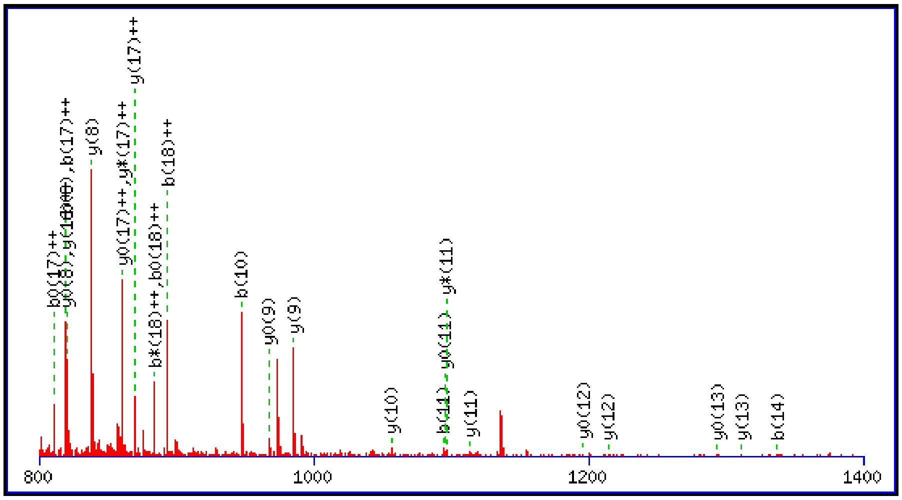

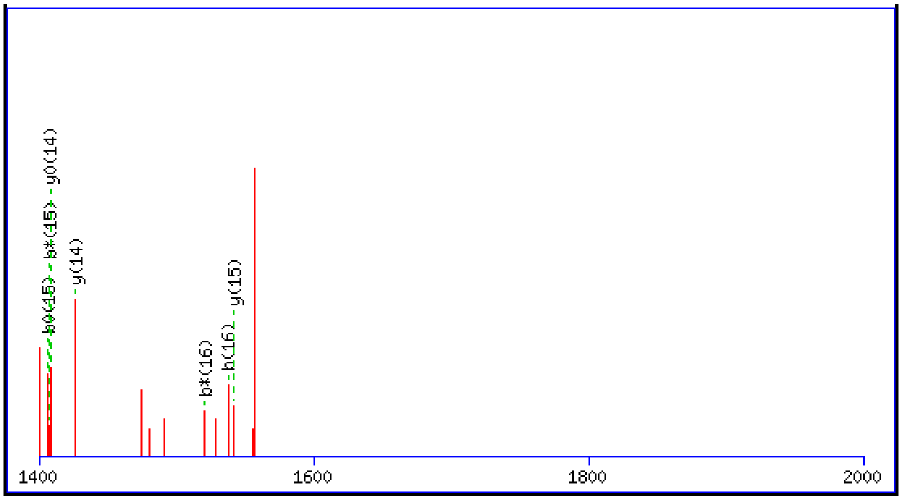


**Monoisotopic mass of neutral peptide Mr(calc):** 1930.9061
**Fixed modifications:** Carbamidomethyl (C)
**Variable modifications:**
**N13** : Deamidated_N (N)
**Ions Score:** 65 **Expect:** 8e-005
**Matches (Bold Red):** 63/180 fragment ions using 116 most intense peaks

| # | b | $b^{++}$ | b* | $b^{*++}$ | $b^0$ | $b^{0++}$ | Seq. | y | $y^{++}$ | y* | $y^{*++}$ | $y^0$ | $y^{0++}$ | # |
|---|---|---|---|---|---|---|---|---|---|---|---|---|---|---|
| 1 | 58.0287 | 29.5180 | | | | | G | | | | | | | 19 |
| 2 | **195.0877** | 98.0475 | | | | | H | 1874.8919 | 937.9496 | 1857.8654 | 929.4363 | 1856.8813 | 928.9443 | 18 |
| 3 | 292.1404 | 146.5738 | | | | | P | 1737.8330 | **869.4201** | 1720.8065 | **860.9069** | 1719.8224 | **860.4149** | 17 |
| 4 | **391.2088** | 196.1081 | | | | | V | 1640.7802 | **820.8938** | 1623.7537 | 812.3805 | 1622.7697 | 811.8885 | 16 |
| 5 | 506.2358 | 253.6215 | | | 488.2252 | 244.6162 | D | **1541.7118** | 771.3596 | 1524.6853 | 762.8463 | 1523.7013 | 762.3543 | 15 |
| 6 | 621.2627 | 311.1350 | | | 603.2522 | 302.1297 | D | **1426.6849** | 713.8461 | 1409.6583 | 705.3328 | **1408.6743** | 704.8408 | 14 |
| 7 | 718.3155 | 359.6614 | | | 700.3049 | **350.6561** | P | **1311.6579** | 656.3326 | 1294.6314 | 647.8193 | **1293.6474** | 647.3273 | 13 |
| 8 | **819.3632** | **410.1852** | | | 801.3526 | 401.1799 | T | **1214.6052** | 607.8062 | 1197.5786 | 599.2930 | **1196.5946** | 598.8009 | 12 |
| 9 | 876.3846 | 438.6959 | | | 858.3741 | 429.6907 | G | **1113.5575** | 557.2824 | **1096.5309** | 548.7691 | **1095.5469** | **548.2771** | 11 |
| 10 | **947.4217** | **474.2145** | | | 929.4112 | **465.2092** | A | **1056.5360** | 528.7717 | 1039.5095 | 520.2584 | 1038.5255 | 519.7664 | 10 |
| 11 | **1094.4902** | **547.7487** | | | 1076.4796 | **538.7434** | F | **985.4989** | **493.2531** | 968.4724 | 484.7398 | **967.4884** | 484.2478 | 9 |
| 12 | 1165.5273 | **583.2673** | | | 1147.5167 | **574.2620** | A | **838.4305** | 419.7189 | 821.4040 | 411.2056 | **820.4199** | **410.7136** | 8 |
| 13 | 1280.5542 | 640.7807 | 1263.5277 | 632.2675 | 1262.5436 | **631.7755** | N | **767.3934** | 384.2003 | 750.3668 | 375.6871 | **749.3828** | 375.1951 | 7 |
| 14 | **1337.5757** | **669.2915** | 1320.5491 | 660.7782 | 1319.5651 | 660.2862 | G | 652.3665 | 326.6869 | 635.3399 | 318.1736 | **634.3559** | 317.6816 | 6 |
| 15 | 1424.6077 | **712.8075** | **1407.5811** | **704.2942** | **1406.5971** | **703.8022** | S | **595.3450** | **298.1761** | 578.3184 | 289.6629 | **577.3344** | 289.1709 | 5 |
| 16 | **1537.6918** | **769.3495** | **1520.6652** | **760.8362** | 1519.6812 | **760.3442** | L | **508.3130** | 254.6601 | 491.2864 | 246.1468 | 490.3024 | **245.6548** | 4 |
| 17 | 1638.7394 | **819.8734** | 1621.7129 | 811.3601 | 1620.7289 | **810.8681** | T | **395.2289** | 198.1181 | 378.2023 | 189.6048 | 377.2183 | 189.1128 | 3 |
| 18 | 1785.8079 | **893.4076** | 1768.7813 | **884.8943** | 1767.7973 | **884.4023** | F | **294.1812** | 147.5942 | 277.1547 | 139.0810 | | | 2 |
| 19 | | | | | | | K | 147.1128 | 74.0600 | 130.0863 | 65.5468 | | | 1 |

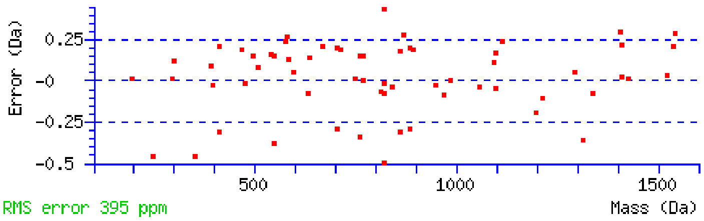


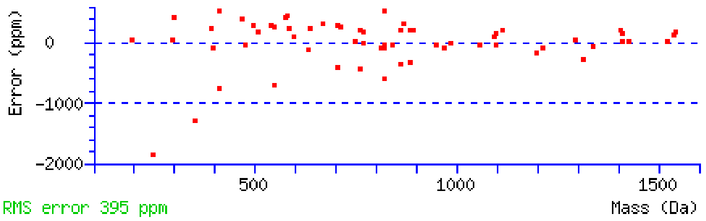


## All matches to this query

| Score | Mr(calc): | Delta | Sequence |
|---|---|---|---|
| 64.8 | 1930.9061 | 0.0079 | GHPVDDPTGAFANGSLTFK |
| 11.5 | 1929.9221 | 0.9919 | GHPVDDPTGAFANGSLTFK |
| | | | |

| | | | |
|---|---|---|---|
| 3.2 | 1928.8993 | 2.0147 | LGQNGTAITFINNNSKR |
| 2.6 | 1930.8996 | 0.0144 | YWCERALDAFSEKTR |
| 1.9 | 1930.9173 | -0.0034 | GHQPEGELVFRGDFNKT |
| 1.8 | 1930.9166 | -0.0026 | GTGALLSSAKGVGAPLSSAK |
| 1.8 | 1930.9166 | -0.0026 | GTGALLSSAKGVGAPLSSAK |
| 1.7 | 1930.9196 | -0.0056 | RELSLSARCHQLEPR |
| 1.7 | 1930.9196 | -0.0056 | RELSLSARCHQLEPR |
| 1.5 | 1928.9146 | 1.9994 | RHDFSLFKLSDSEIR |

---

Spectrum No: 442; Query: 1828; Rank: 1

---

## Peptide View

MS/MS Fragmentation of **AVGKDNFTLIPEGTNGTEER**
Found in **IPI00870112**, Tax_Id=10116 Gene_Symbol=Dpysl2 Dihydropyrimidinase-related protein 2

Match to Query 1828: 2148.032688 from(1075.023620,2+)
Title: 091008RatKidney_NH4Format02_23.2500.2500.2.dta
Data file K:\NewmanPaper\Piliang\3SubProteomes\Piliang3SP\mgf5ppm\ERLIC_3SubProteomes5ppm.mgf

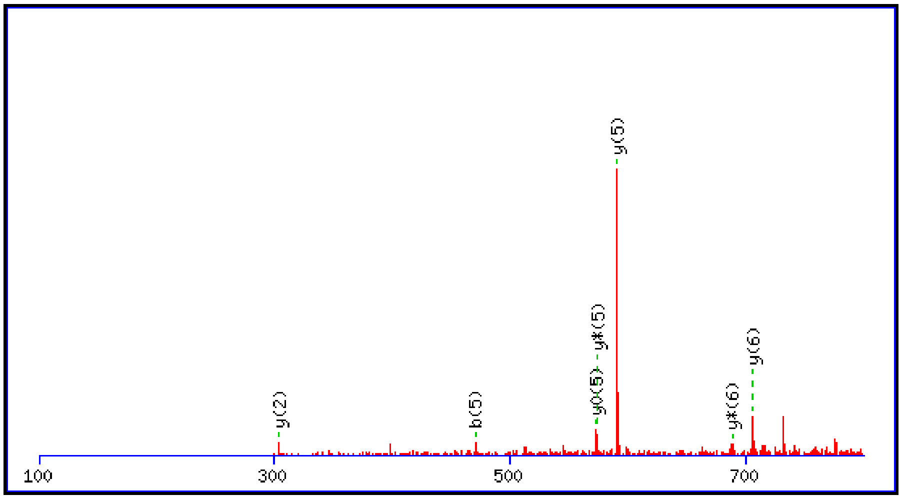


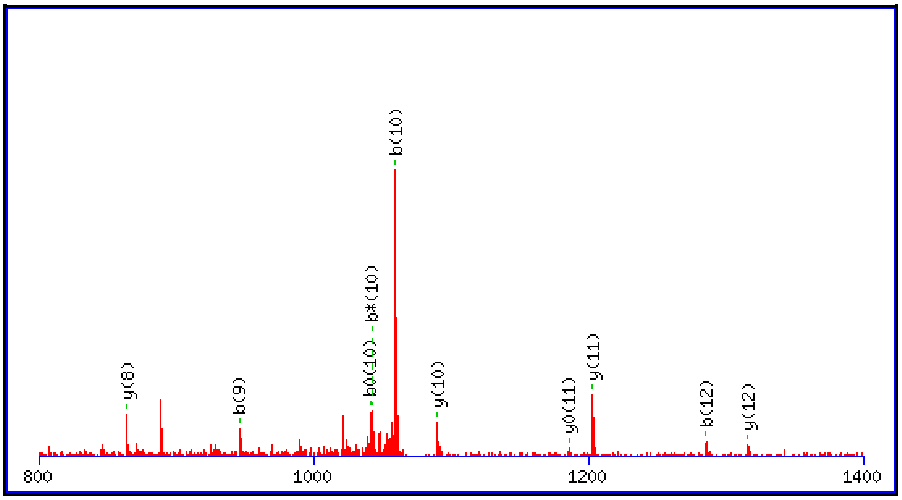

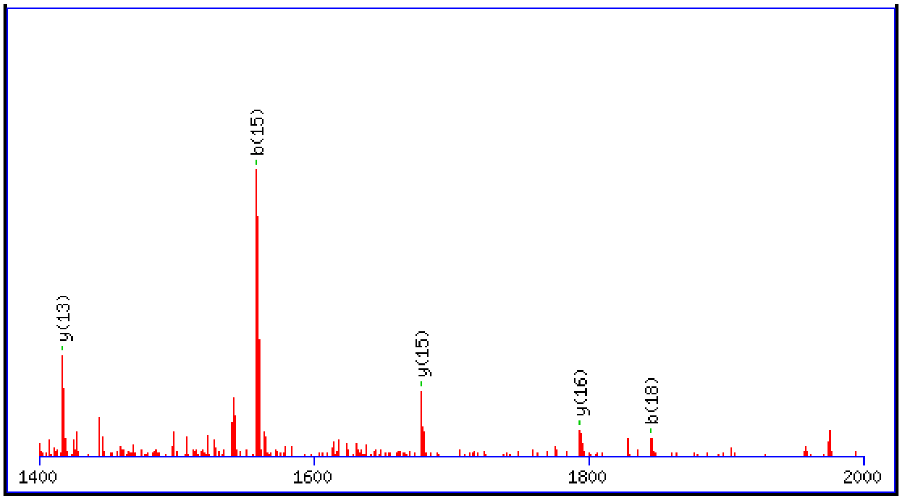


**Monoisotopic mass of neutral peptide Mr(calc):** 2148.0335
**Fixed modifications:** Carbamidomethyl (C)
**Variable modifications:**
**N15** : Deamidated_N (N)
**Ions Score:** 65 **Expect:** 7.9e-005
**Matches (Bold Red):** 22/212 fragment ions using 38 most intense peaks

| # | b | $b^{++}$ | b* | $b^{*++}$ | $b^{0}$ | $b^{0++}$ | Seq. | y | $y^{++}$ | y* | $y^{*++}$ | $y^{0}$ | $y^{0++}$ | # |
|---|---|---|---|---|---|---|---|---|---|---|---|---|---|---|
| 1 | 72.0444 | 36.5258 | | | | | A | | | | | | | 20 |
| 2 | 171.1128 | 86.0600 | | | | | V | 2078.0036 | 1039.5055 | 2060.9771 | 1030.9922 | 2059.9931 | 1030.5002 | 19 |
| 3 | 228.1343 | 114.5708 | | | | | G | 1978.9352 | 989.9713 | 1961.9087 | 981.4580 | 1960.9247 | 980.9660 | 18 |
| 4 | 356.2292 | 178.6183 | 339.2027 | 170.1050 | | | K | 1921.9138 | 961.4605 | 1904.8872 | 952.9472 | 1903.9032 | 952.4552 | 17 |
| 5 | **471.2562** | 236.1317 | 454.2296 | 227.6185 | 453.2456 | 227.1264 | D | **1793.8188** | 897.4130 | 1776.7923 | 888.8998 | 1775.8082 | 888.4078 | 16 |
| 6 | 585.2991 | 293.1532 | 568.2726 | 284.6399 | 567.2885 | 284.1479 | N | **1678.7919** | 839.8996 | 1661.7653 | 831.3863 | 1660.7813 | 830.8943 | 15 |
| 7 | 732.3675 | 366.6874 | 715.3410 | 358.1741 | 714.3569 | 357.6821 | F | 1564.7489 | 782.8781 | 1547.7224 | 774.3648 | 1546.7384 | 773.8728 | 14 |
| 8 | 833.4152 | 417.2112 | 816.3886 | 408.6980 | 815.4046 | 408.2060 | T | **1417.6805** | 709.3439 | 1400.6540 | 700.8306 | 1399.6700 | 700.3386 | 13 |
| 9 | **946.4993** | 473.7533 | 929.4727 | 465.2400 | 928.4887 | 464.7480 | L | **1316.6328** | 658.8201 | 1299.6063 | 650.3068 | 1298.6223 | 649.8148 | 12 |
| 10 | **1059.5833** | 530.2953 | **1042.5568** | 521.7820 | **1041.5728** | 521.2900 | I | **1203.5488** | 602.2780 | 1186.5222 | 593.7648 | **1185.5382** | 593.2727 | 11 |
| 11 | 1156.6361 | 578.8217 | 1139.6095 | 570.3084 | 1138.6255 | 569.8164 | P | **1090.4647** | 545.7360 | 1073.4382 | 537.2227 | 1072.4542 | 536.7307 | 10 |
| 12 | **1285.6787** | 643.3430 | 1268.6521 | 634.8297 | 1267.6681 | 634.3377 | E | 993.4120 | 497.2096 | 976.3854 | 488.6963 | 975.4014 | 488.2043 | 9 |
| 13 | 1342.7001 | 671.8537 | 1325.6736 | 663.3404 | 1324.6896 | 662.8484 | G | **864.3694** | 432.6883 | 847.3428 | 424.1750 | 846.3588 | 423.6830 | 8 |
| 14 | 1443.7478 | 722.3775 | 1426.7213 | 713.8643 | 1425.7373 | 713.3723 | T | 807.3479 | 404.1776 | 790.3213 | 395.6643 | 789.3373 | 395.1723 | 7 |
| 15 | **1558.7748** | 779.8910 | 1541.7482 | 771.3777 | 1540.7642 | 770.8857 | N | **706.3002** | 353.6537 | **689.2737** | 345.1405 | 688.2897 | 344.6485 | 6 |
| 16 | 1615.7962 | 808.4017 | 1598.7697 | 799.8885 | 1597.7857 | 799.3965 | G | **591.2733** | 296.1403 | **574.2467** | 287.6270 | **573.2627** | 287.1350 | 5 |
| 17 | 1716.8439 | 858.9256 | 1699.8174 | 850.4123 | 1698.8333 | 849.9203 | T | 534.2518 | 267.6295 | 517.2253 | 259.1163 | 516.2413 | 258.6243 | 4 |
| 18 | **1845.8865** | 923.4469 | 1828.8599 | 914.9336 | 1827.8759 | 914.4416 | E | 433.2041 | 217.1057 | 416.1776 | 208.5924 | 415.1936 | 208.1004 | 3 |
| 19 | 1974.9291 | 987.9682 | 1957.9025 | 979.4549 | 1956.9185 | 978.9629 | E | **304.1615** | 152.5844 | 287.1350 | 144.0711 | 286.1510 | 143.5791 | 2 |
| 20 | | | | | | | R | 175.1190 | 88.0631 | 158.0924 | 79.5498 | | | 1 |

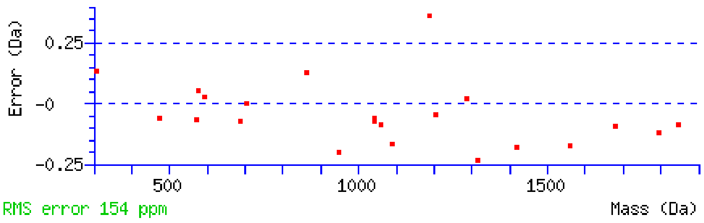


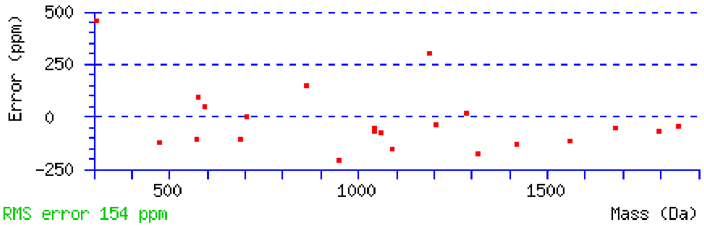


### All matches to this query

| Score | Mr(calc): | Delta | Sequence |
|---|---|---|---|
| 64.7 | 2148.0335 | -0.0008 | AVGKDNFTLIPEGTNGTEER |
| | | | |

| | | | |
|---|---|---|---|
| 20.0 | 2148.0335 | -0.0008 | AVGKDNFTLIPEGTNGTEER |
| 12.6 | 2146.0170 | 2.0157 | FPGMSVLLFLQESLGQER |
| 8.7 | 2147.0495 | 0.9832 | AVGKDNFTLIPEGTNGTEER |
| 3.3 | 2146.0170 | 2.0157 | VRYATWSIIMDSVVPSDK |
| 1.1 | 2148.0229 | 0.0098 | KENVAERALAAXSCTEAESR |
| 0.6 | 2148.0197 | 0.0130 | EGKYIIELAHMIKDNGWD |
| 0.5 | 2148.0171 | 0.0156 | SAACLPSDISWPGTHXKER |
| 0.5 | 2148.0366 | -0.0039 | KFISDIPYLKELFNNMP |
| 0.1 | 2147.0289 | 1.0038 | APQSIELPAVAGHTLTARR |

---

Spectrum No: 443; Query: 1222; Rank: 1

---

## Peptide View

MS/MS Fragmentation of **NGGLGHMNITLLSDLTK**
Found in **IPI00208215**, Tax_Id=10116 Gene_Symbol=Prdx3 Thioredoxin-dependent peroxide reductase, mitochondrial precursor

Match to Query 1222: 1783.921228 from(892.967890,2+)
Title: 091008RatKidney_NH4Format02_17.4082.4082.2.dta
Data file K:\NewmanPaper\Piliang\3SubProteomes\Piliang3SP\mgf5ppm\ERLIC_3SubProteomes5ppm.mgf

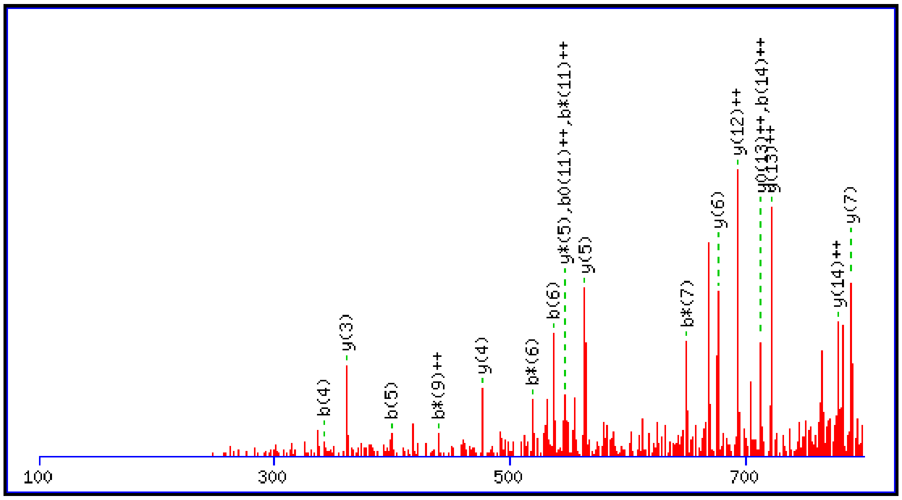


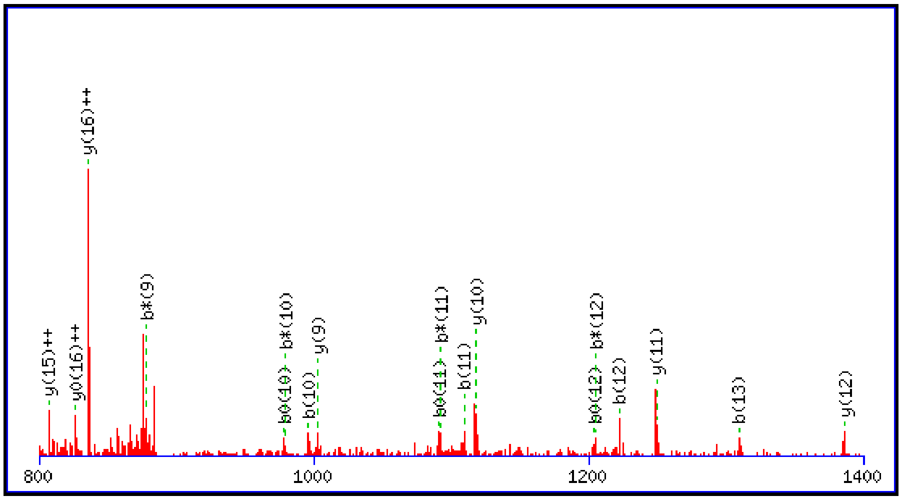

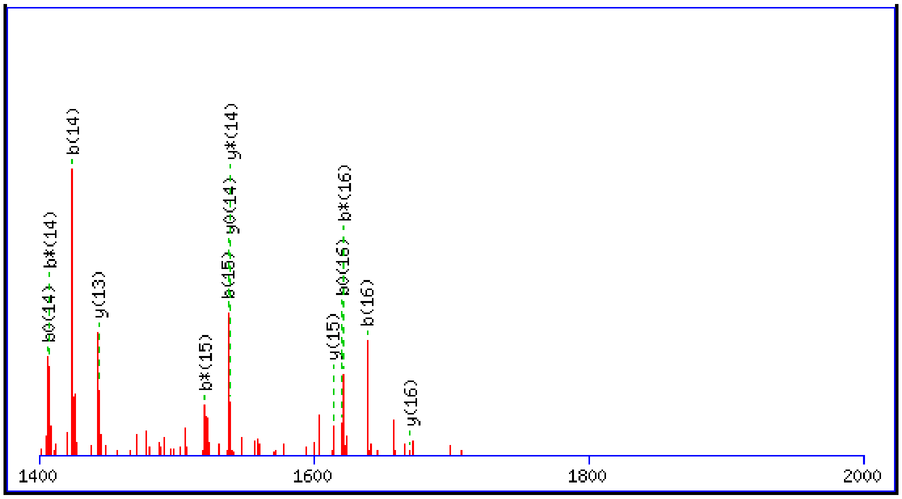


**Monoisotopic mass of neutral peptide Mr(calc):** 1783.9138
**Fixed modifications:** Carbamidomethyl (C)
**Variable modifications:**
**N8** : Deamidated_N (N)
**Ions Score:** 65 **Expect:** 4.5e-005
**Matches (Bold Red):** 50/172 fragment ions using 94 most intense peaks

| # | b | $b^{++}$ | b* | $b^{*++}$ | $b^0$ | $b^{0++}$ | Seq. | y | $y^{++}$ | y* | $y^{*++}$ | $y^0$ | $y^{0++}$ | # |
|---|---|---|---|---|---|---|---|---|---|---|---|---|---|---|
| 1 | 115.0502 | 58.0287 | 98.0237 | 49.5155 | | | N | | | | | | | 17 |
| 2 | 172.0717 | 86.5395 | 155.0451 | 78.0262 | | | G | **1670.8782** | **835.9427** | 1653.8516 | 827.4295 | 1652.8676 | **826.9374** | 16 |
| 3 | 229.0931 | 115.0502 | 212.0666 | 106.5369 | | | G | **1613.8567** | **807.4320** | 1596.8302 | 798.9187 | 1595.8461 | 798.4267 | 15 |
| 4 | **342.1772** | 171.5922 | 325.1506 | 163.0790 | | | L | 1556.8352 | **778.9213** | **1539.8087** | 770.4080 | **1538.8247** | 769.9160 | 14 |
| 5 | **399.1987** | 200.1030 | 382.1721 | 191.5897 | | | G | **1443.7512** | **722.3792** | 1426.7246 | 713.8660 | 1425.7406 | **713.3739** | 13 |
| 6 | **536.2576** | 268.6324 | **519.2310** | 260.1191 | | | H | **1386.7297** | **693.8685** | 1369.7032 | 685.3552 | 1368.7192 | 684.8632 | 12 |
| 7 | 667.2981 | 334.1527 | **650.2715** | 325.6394 | | | M | **1249.6708** | 625.3390 | 1232.6443 | 616.8258 | 1231.6602 | 616.3338 | 11 |
| 8 | 782.3250 | 391.6661 | 765.2984 | 383.1529 | | | N | **1118.6303** | 559.8188 | 1101.6038 | 551.3055 | 1100.6198 | 550.8135 | 10 |
| 9 | 895.4091 | 448.2082 | **878.3825** | **439.6949** | | | I | **1003.6034** | 502.3053 | 986.5768 | 493.7921 | 985.5928 | 493.3001 | 9 |
| 10 | **996.4567** | 498.7320 | **979.4302** | 490.2187 | **978.4462** | 489.7267 | T | 890.5193 | 445.7633 | 873.4928 | 437.2500 | 872.5088 | 436.7580 | 8 |
| 11 | **1109.5408** | 555.2740 | **1092.5142** | **546.7608** | **1091.5302** | **546.2688** | L | **789.4716** | 395.2395 | 772.4451 | 386.7262 | 771.4611 | 386.2342 | 7 |
| 12 | **1222.6249** | 611.8161 | **1205.5983** | 603.3028 | **1204.6143** | 602.8108 | L | **676.3876** | 338.6974 | 659.3610 | 330.1842 | 658.3770 | 329.6921 | 6 |
| 13 | **1309.6569** | 655.3321 | 1292.6303 | 646.8188 | 1291.6463 | 646.3268 | S | **563.3035** | 282.1554 | **546.2770** | 273.6421 | 545.2930 | 273.1501 | 5 |
| 14 | **1424.6838** | **712.8456** | **1407.6573** | 704.3323 | **1406.6733** | 703.8403 | D | **476.2715** | 238.6394 | 459.2449 | 230.1261 | 458.2609 | 229.6341 | 4 |
| 15 | **1537.7679** | 769.3876 | **1520.7413** | 760.8743 | 1519.7573 | 760.3823 | L | **361.2445** | 181.1259 | 344.2180 | 172.6126 | 343.2340 | 172.1206 | 3 |
| 16 | **1638.8156** | 819.9114 | **1621.7890** | 811.3982 | **1620.8050** | 810.9061 | T | 248.1605 | 124.5839 | 231.1339 | 116.0706 | 230.1499 | 115.5786 | 2 |
| 17 | | | | | | | K | 147.1128 | 74.0600 | 130.0863 | 65.5468 | | | 1 |

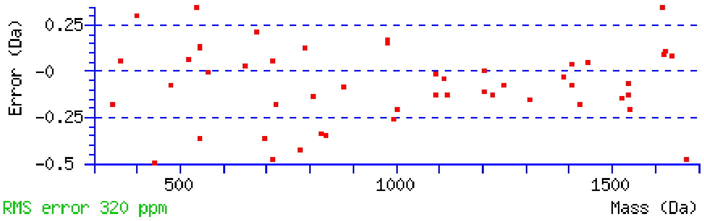


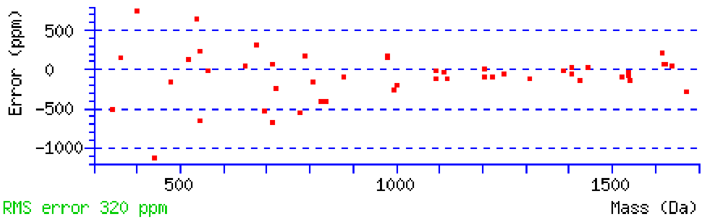


**All matches to this query**

| Score | Mr(calc): | Delta | Sequence |
|---|---|---|---|
| 64.7 | 1783.9138 | 0.0074 | NGGLGHMNITLLSDLTK |
| 58.8 | 1783.9138 | 0.0074 | NGGLGHMNITLLSDLTK |
| 37.4 | 1782.9298 | 0.9914 | NGGLGHMNITLLSDLTK |
| 6.7 | 1783.9039 | 0.0173 | FRGPPGPTPAMGTNASVK |
| | | | |

| | | | |
|---|---|---|---|
| 2.1 | 1783.9046 | 0.0166 | NSYFFRVLFQPHTK |
| 1.0 | 1783.9039 | 0.0173 | GFXLNSCLVYVDVSR |

---

Spectrum No: 444; Query: 367; Rank: 1

---

## Peptide View

MS/MS Fragmentation of **ICGNETLFPIR**
Found in **IPI00196620**, Tax_Id=10116 Gene_Symbol=Cubn Cubilin precursor

Match to Query 367: 1319.651168 from(660.832860,2+)
Title: 091008RatKidney_NH4Format01_25.2714.2714.2.dta
Data file K:\NewmanPaper\Piliang\3SubProteomes\Piliang3SP\mgf5ppm\ERLIC_3SubProteomes5ppm.mgf

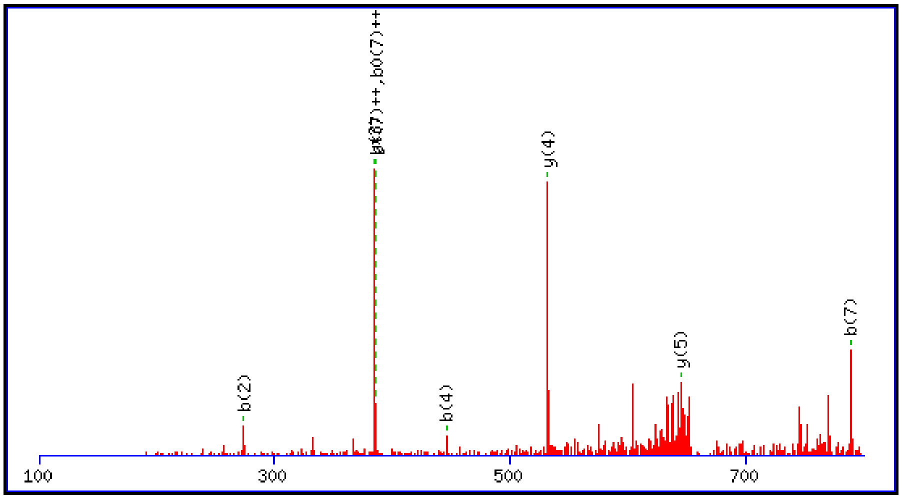


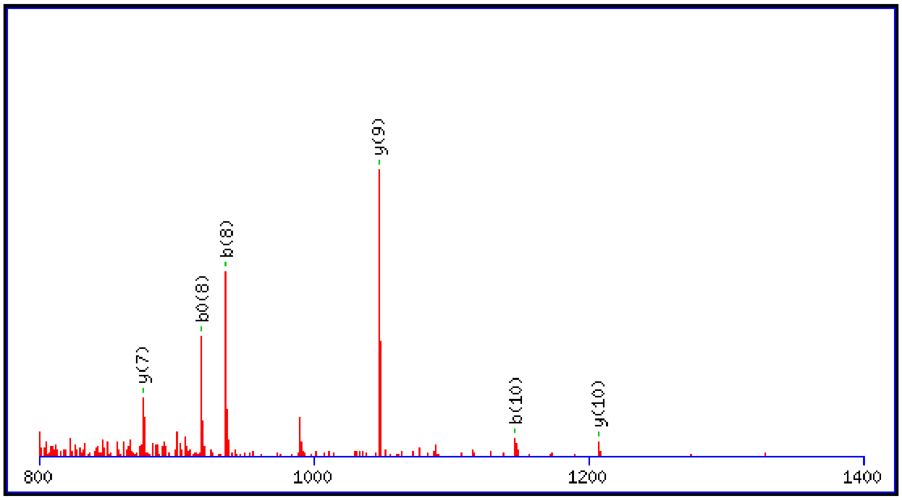

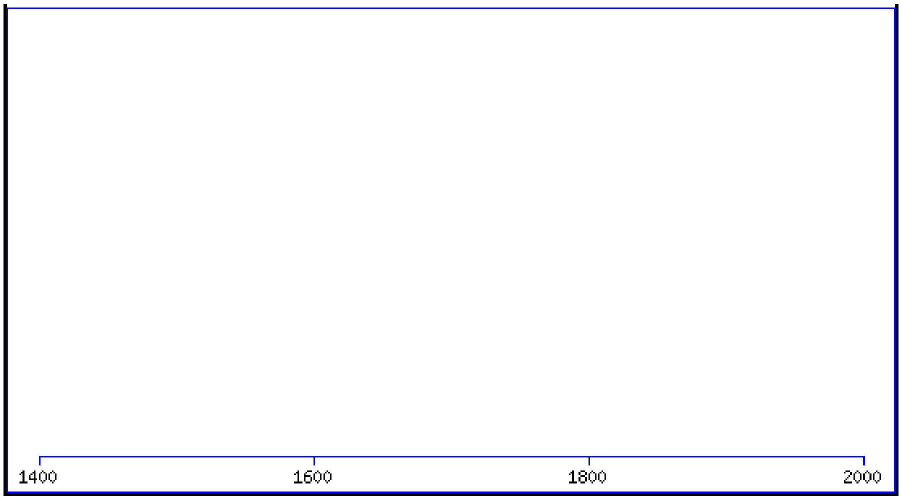


**Monoisotopic mass of neutral peptide Mr(calc):** 1319.6544
**Fixed modifications:** Carbamidomethyl (C)
**Variable modifications:**
**N4** : Deamidated_N (N)
**Ions Score:** 65 **Expect:** 4.5e-005
**Matches (Bold Red):** 14/96 fragment ions using 15 most intense peaks

| # | b | b++ | b* | b*++ | b0 | b0++ | Seq. | y | y++ | y* | y*++ | y0 | y0++ | # |
|---|---|---|---|---|---|---|---|---|---|---|---|---|---|---|
| 1 | 114.0913 | 57.5493 | | | | | I | | | | | | | 11 |
| 2 | **274.1220** | 137.5646 | | | | | C | **1207.5776** | 604.2924 | 1190.5510 | 595.7792 | 1189.5670 | 595.2871 | 10 |
| 3 | 331.1435 | 166.0754 | | | | | G | **1047.5469** | 524.2771 | 1030.5204 | 515.7638 | 1029.5364 | 515.2718 | 9 |
| 4 | **446.1704** | 223.5888 | 429.1438 | 215.0756 | | | N | 990.5255 | 495.7664 | 973.4989 | 487.2531 | 972.5149 | 486.7611 | 8 |
| 5 | 575.2130 | 288.1101 | 558.1864 | 279.5969 | 557.2024 | 279.1048 | E | **875.4985** | 438.2529 | 858.4720 | 429.7396 | 857.4880 | 429.2476 | 7 |
| 6 | 676.2607 | 338.6340 | 659.2341 | 330.1207 | 658.2501 | 329.6287 | T | 746.4559 | 373.7316 | 729.4294 | 365.2183 | 728.4454 | 364.7263 | 6 |
| 7 | **789.3447** | 395.1760 | 772.3182 | **386.6627** | 771.3342 | **386.1707** | L | **645.4083** | 323.2078 | 628.3817 | 314.6945 | | | 5 |
| 8 | **936.4131** | 468.7102 | 919.3866 | 460.1969 | **918.4026** | 459.7049 | F | **532.3242** | 266.6657 | 515.2976 | 258.1525 | | | 4 |
| 9 | 1033.4659 | 517.2366 | 1016.4394 | 508.7233 | 1015.4553 | 508.2313 | P | **385.2558** | 193.1315 | 368.2292 | 184.6183 | | | 3 |
| 10 | **1146.5500** | 573.7786 | 1129.5234 | 565.2653 | 1128.5394 | 564.7733 | I | 288.2030 | 144.6051 | 271.1765 | 136.0919 | | | 2 |
| 11 | | | | | | | R | 175.1190 | 88.0631 | 158.0924 | 79.5498 | | | 1 |

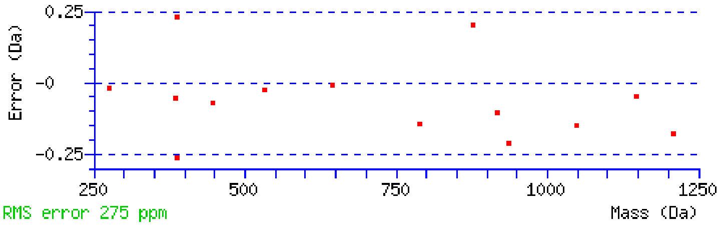


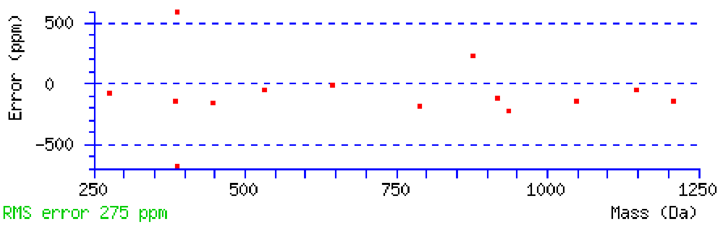


### All matches to this query

| Score | Mr(calc): | Delta | Sequence |
|---|---|---|---|
| 64.5 | 1319.6544 | -0.0032 | ICGNETLFPIR |
| 17.0 | 1319.6622 | -0.0110 | GKQAFEGAPYPR |
| 9.8 | 1319.6391 | 0.0121 | GVIDMGNSLIER |
| 8.4 | 1319.6470 | 0.0042 | NSSAGNFIAPSVR |
| 8.4 | 1319.6470 | 0.0042 | NSSAGNFIAPSVR |
| 5.0 | 1317.6329 | 2.0182 | KQKLQYSTDK |
| 4.3 | 1319.6391 | 0.0121 | LMAENSTTALPR |
| 3.1 | 1319.6469 | 0.0042 | RNFSNSIDLPR |
| 2.5 | 1319.6427 | 0.0084 | IYFRFELPR |
| 2.5 | 1319.6542 | -0.0030 | LRDSENTRSSR |

Spectrum No: 445; Query: 1874; Rank: 1

## Peptide View

MS/MS Fragmentation of **LRNPCTSEQNCTSPFSYK**
Found in **IPI00191681**, Tax_Id=10116 Gene_Symbol=Itgb1 Integrin beta-1 precursor

Match to Query 1874: 2188.960752 from(730.660860,3+)
Title: 100101RatKid_NS_deglyco_20.1833.1833.3.dta
Data file K:\NewmanPaper\Piliang\3SubProteomes\Piliang3SP\mgf5ppm\ERLIC_3SubProteomes5ppm.mgf

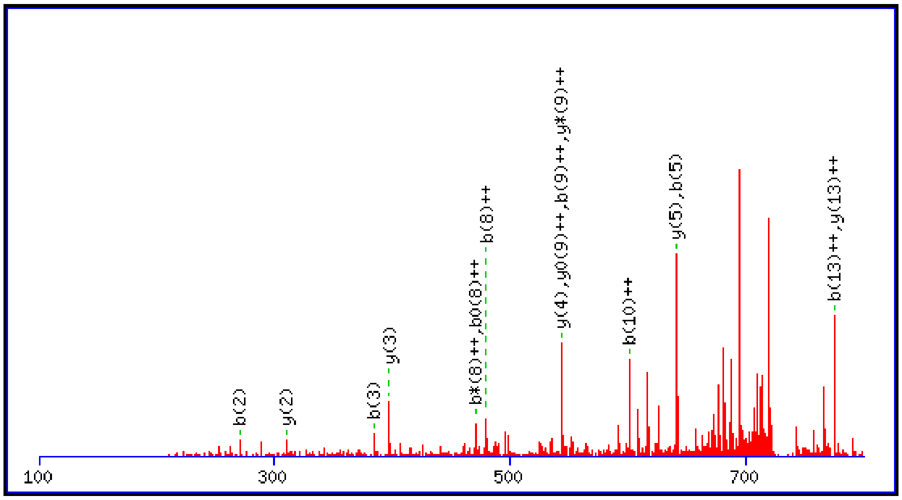


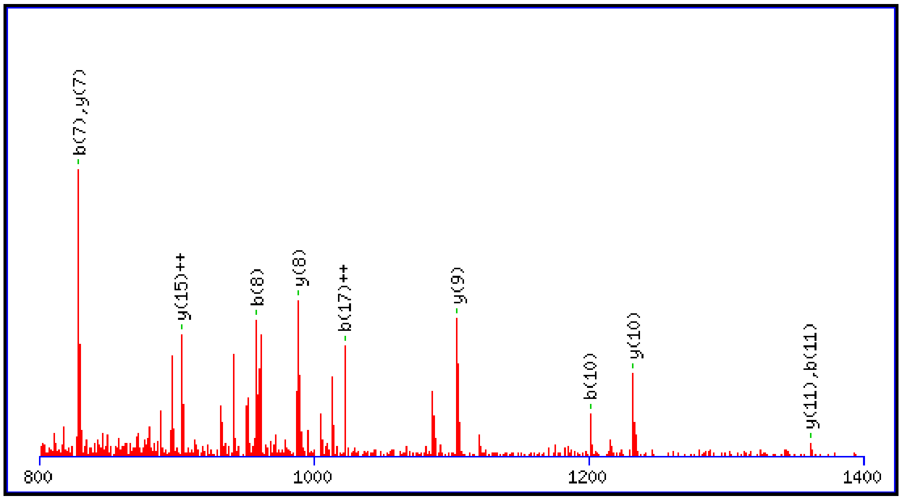


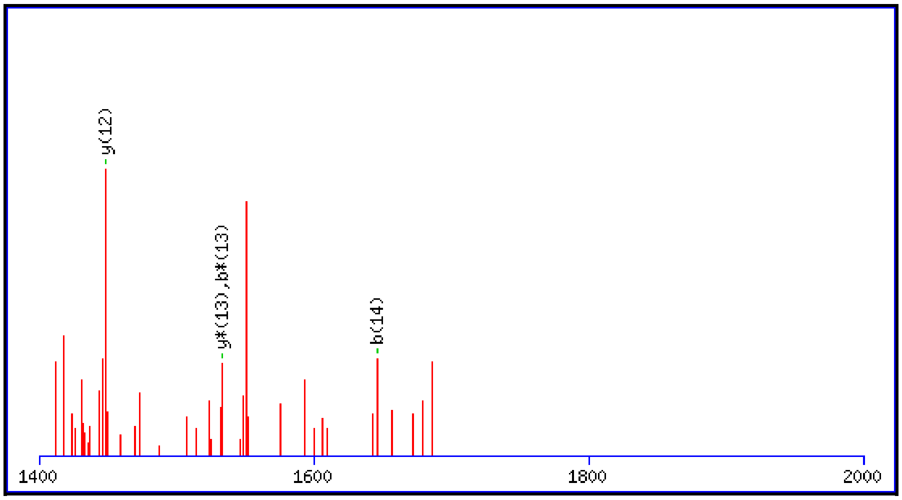

**Monoisotopic mass of neutral peptide Mr(calc):** 2188.9517
**Fixed modifications:** Carbamidomethyl (C)
**Variable modifications:**
**N10** : Deamidated_N (N)
**Ions Score:** 65 **Expect:** 9.2e-005
**Matches (Bold Red):** 31/188 fragment ions using 35 most intense peaks

| # | b | $b^{++}$ | b* | $b^{*++}$ | $b^0$ | $b^{0++}$ | Seq. | y | $y^{++}$ | y* | $y^{*++}$ | $y^0$ | $y^{0++}$ | # |
|---|---|---|---|---|---|---|---|---|---|---|---|---|---|---|
| 1 | 114.0913 | 57.5493 | | | | | L | | | | | | | 18 |
| 2 | **270.1925** | 135.5999 | 253.1659 | 127.0866 | | | R | 2076.8750 | 1038.9411 | 2059.8484 | 1030.4278 | 2058.8644 | 1029.9358 | 17 |
| 3 | **384.2354** | 192.6213 | 367.2088 | 184.1081 | | | N | 1920.7738 | 960.8906 | 1903.7473 | 952.3773 | 1902.7633 | 951.8853 | 16 |
| 4 | 481.2881 | 241.1477 | 464.2616 | 232.6344 | | | P | 1806.7309 | **903.8691** | 1789.7044 | 895.3558 | 1788.7204 | 894.8638 | 15 |
| 5 | **641.3188** | 321.1630 | 624.2922 | 312.6498 | | | C | 1709.6782 | 855.3427 | 1692.6516 | 846.8294 | 1691.6676 | 846.3374 | 14 |
| 6 | 742.3665 | 371.6869 | 725.3399 | 363.1736 | 724.3559 | 362.6816 | T | 1549.6475 | **775.3274** | **1532.6210** | 766.8141 | 1531.6369 | 766.3221 | 13 |
| 7 | **829.3985** | 415.2029 | 812.3719 | 406.6896 | 811.3879 | 406.1976 | S | **1448.5998** | 724.8036 | 1431.5733 | 716.2903 | 1430.5893 | 715.7983 | 12 |
| 8 | **958.4411** | **479.7242** | 941.4145 | **471.2109** | 940.4305 | **470.7189** | E | **1361.5678** | 681.2875 | 1344.5413 | 672.7743 | 1343.5572 | 672.2823 | 11 |
| 9 | 1086.4997 | **543.7535** | 1069.4731 | 535.2402 | 1068.4891 | 534.7482 | Q | **1232.5252** | 616.7662 | 1215.4987 | 608.2530 | 1214.5146 | 607.7610 | 10 |
| 10 | **1201.5266** | **601.2669** | 1184.5001 | 592.7537 | 1183.5160 | 592.2617 | N | **1104.4666** | 552.7370 | 1087.4401 | **544.2237** | 1086.4561 | **543.7317** | 9 |
| 11 | **1361.5573** | 681.2823 | 1344.5307 | 672.7690 | 1343.5467 | 672.2770 | C | **989.4397** | 495.2235 | 972.4131 | 486.7102 | 971.4291 | 486.2182 | 8 |
| 12 | 1462.6049 | 731.8061 | 1445.5784 | 723.2928 | 1444.5944 | 722.8008 | T | **829.4090** | 415.2082 | 812.3825 | 406.6949 | 811.3985 | 406.2029 | 7 |
| 13 | 1549.6370 | **775.3221** | **1532.6104** | 766.8088 | 1531.6264 | 766.3168 | S | 728.3614 | 364.6843 | 711.3348 | 356.1710 | 710.3508 | 355.6790 | 6 |
| 14 | **1646.6897** | 823.8485 | 1629.6632 | 815.3352 | 1628.6792 | 814.8432 | P | **641.3293** | 321.1683 | 624.3028 | 312.6550 | 623.3188 | 312.1630 | 5 |
| 15 | 1793.7581 | 897.3827 | 1776.7316 | 888.8694 | 1775.7476 | 888.3774 | F | **544.2766** | 272.6419 | 527.2500 | 264.1287 | 526.2660 | 263.6366 | 4 |
| 16 | 1880.7902 | 940.8987 | 1863.7636 | 932.3854 | 1862.7796 | 931.8934 | S | **397.2082** | 199.1077 | 380.1816 | 190.5944 | 379.1976 | 190.1024 | 3 |
| 17 | 2043.8535 | **1022.4304** | 2026.8269 | 1013.9171 | 2025.8429 | 1013.4251 | Y | **310.1761** | 155.5917 | 293.1496 | 147.0784 | | | 2 |
| 18 | | | | | | | K | 147.1128 | 74.0600 | 130.0863 | 65.5468 | | | 1 |

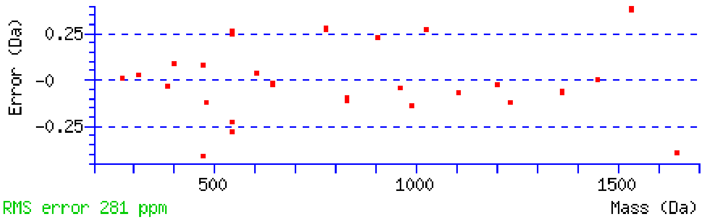


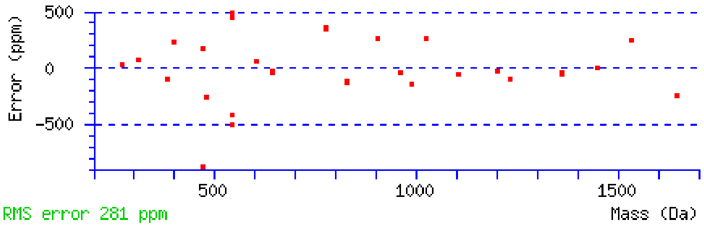


### All matches to this query

| Score | Mr(calc): | Delta | Sequence |
|---|---|---|---|
| 64.5 | 2188.9517 | 0.0090 | LRNPCTSEQNCTSPFSYK |
| 28.5 | 2188.9517 | 0.0090 | LRNPCTSEQNCTSPFSYK |
| 13.1 | 2187.9677 | 0.9930 | LRNPCTSEQNCTSPFSYK |
| 7.0 | 2188.9629 | -0.0022 | TGEMSGPVFTDSGIHIILR |
| 7.0 | 2188.9629 | -0.0022 | TGEMSGPVFTDSGIHIILR |
| 3.5 | 2188.9493 | 0.0115 | SMPVLGSVSSVTKSALNKK |
| 2.8 | 2187.9685 | 0.9923 | VELGQNGEEKSPSSTSHPPK |
| 2.8 | 2187.9685 | 0.9923 | VELGQNGEEKSPSSTSHPPK |
| 1.8 | 2188.9629 | -0.0022 | TGEMSGPVFTDSGIHIILR |
| 1.8 | 2188.9629 | -0.0022 | TGEMSGPVFTDSGIHIILR |

---

Spectrum No: 446; Query: 643; Rank: 1

---

## Peptide View

MS/MS Fragmentation of **EATIVGENETYPR**
Found in **IPI00205606**, Tax_Id=10116 Gene_Symbol=Sel1h Protein sel-1 homolog 1 precursor

Match to Query 643: 1478.694308 from(740.354430,2+)
Title: 100101RatKid_NS_deglyco_08.1864.1864.2.dta
Data file K:\NewmanPaper\Piliang\3SubProteomes\Piliang3SP\mgf5ppm\ERLIC_3SubProteomes5ppm.mgf

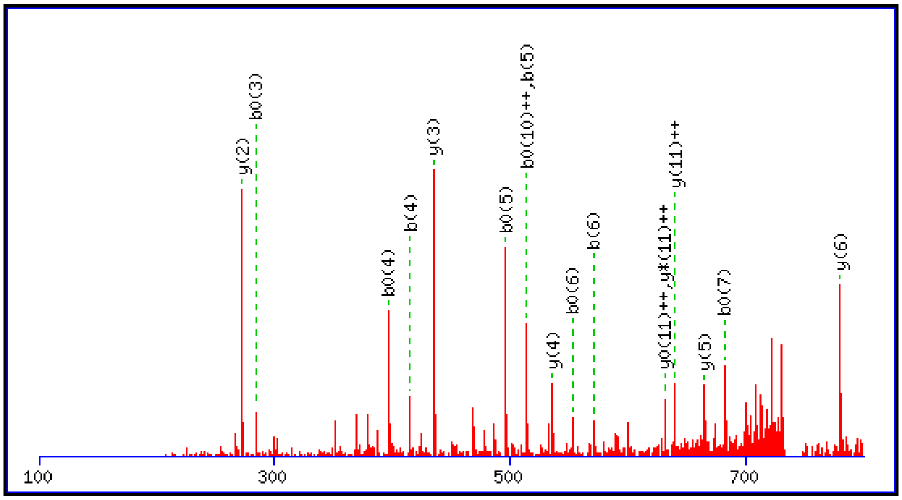


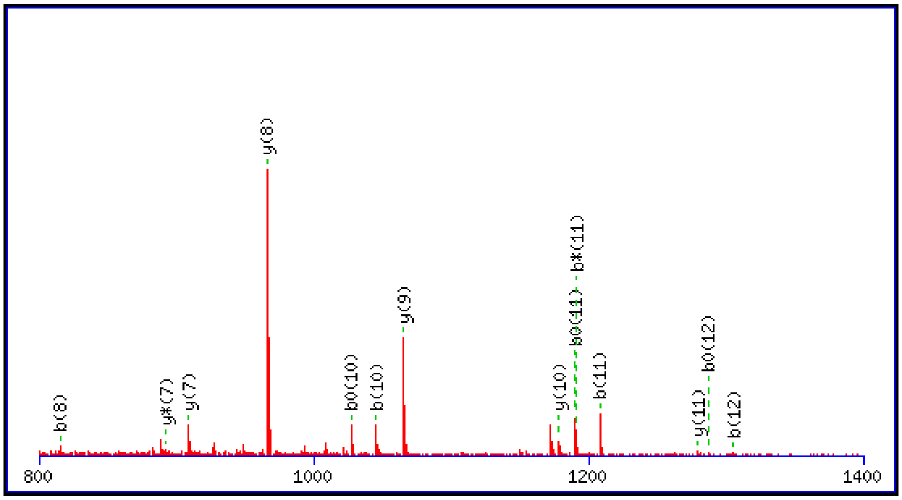


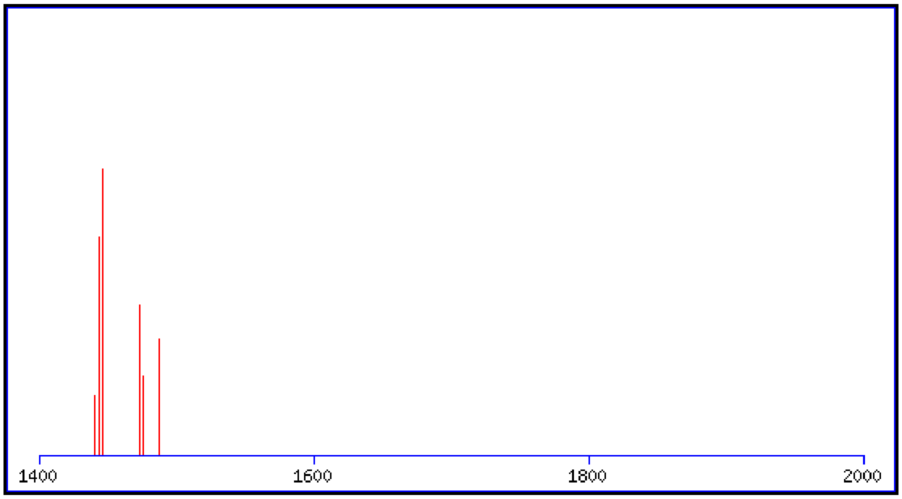


**Monoisotopic mass of neutral peptide Mr(calc):** 1478.6889
**Fixed modifications:** Carbamidomethyl (C)
**Variable modifications:**
**N8** : Deamidated_N (N)
**Ions Score:** 64 **Expect:** 5.9e-005
**Matches (Bold Red):** 31/124 fragment ions using 59 most intense peaks

| # | b | $b^{++}$ | b* | $b^{*++}$ | $b^0$ | $b^{0++}$ | Seq. | y | $y^{++}$ | y* | $y^{*++}$ | $y^0$ | $y^{0++}$ | # |
|---|---|---|---|---|---|---|---|---|---|---|---|---|---|---|
| | | | | | | | | | | | | | | |

| | | | | | | | | | | | | | | |
|---|---|---|---|---|---|---|---|---|---|---|---|---|---|---|
| 1 | 130.0499 | 65.5286 | | | 112.0393 | 56.5233 | E | | | | | | | 13 |
| 2 | 201.0870 | 101.0471 | | | 183.0764 | 92.0418 | A | 1350.6536 | 675.8304 | 1333.6270 | 667.3172 | 1332.6430 | 666.8251 | 12 |
| 3 | 302.1347 | 151.5710 | | | **284.1241** | 142.5657 | T | **1279.6165** | **640.3119** | 1262.5899 | **631.7986** | 1261.6059 | **631.3066** | 11 |
| 4 | **415.2187** | 208.1130 | | | **397.2082** | 199.1077 | I | **1178.5688** | 589.7880 | 1161.5422 | 581.2748 | 1160.5582 | 580.7827 | 10 |
| 5 | **514.2871** | 257.6472 | | | **496.2766** | 248.6419 | V | **1065.4847** | 533.2460 | 1048.4582 | 524.7327 | 1047.4742 | 524.2407 | 9 |
| 6 | **571.3086** | 286.1579 | | | **553.2980** | 277.1527 | G | **966.4163** | 483.7118 | 949.3898 | 475.1985 | 948.4057 | 474.7065 | 8 |
| 7 | 700.3512 | 350.6792 | | | **682.3406** | 341.6740 | E | **909.3948** | 455.2011 | **892.3683** | 446.6878 | 891.3843 | 446.1958 | 7 |
| 8 | **815.3781** | 408.1927 | 798.3516 | 399.6794 | 797.3676 | 399.1874 | N | **780.3523** | 390.6798 | 763.3257 | 382.1665 | 762.3417 | 381.6745 | 6 |
| 9 | 944.4207 | 472.7140 | 927.3942 | 464.2007 | 926.4102 | 463.7087 | E | **665.3253** | 333.1663 | 648.2988 | 324.6530 | 647.3148 | 324.1610 | 5 |
| 10 | **1045.4684** | 523.2378 | 1028.4419 | 514.7246 | **1027.4578** | **514.2326** | T | **536.2827** | 268.6450 | 519.2562 | 260.1317 | 518.2722 | 259.6397 | 4 |
| 11 | **1208.5317** | 604.7695 | **1191.5052** | 596.2562 | **1190.5212** | 595.7642 | Y | **435.2350** | 218.1212 | 418.2085 | 209.6079 | | | 3 |
| 12 | **1305.5845** | 653.2959 | 1288.5579 | 644.7826 | **1287.5739** | 644.2906 | P | **272.1717** | 136.5895 | 255.1452 | 128.0762 | | | 2 |
| 13 | | | | | | | R | 175.1190 | 88.0631 | 158.0924 | 79.5498 | | | 1 |

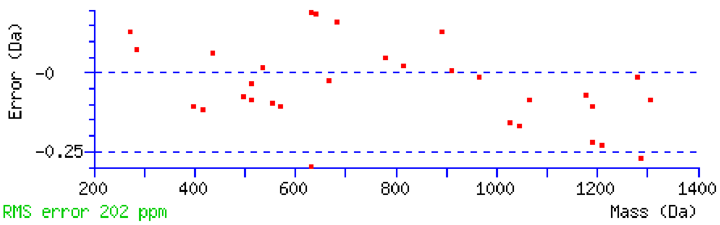


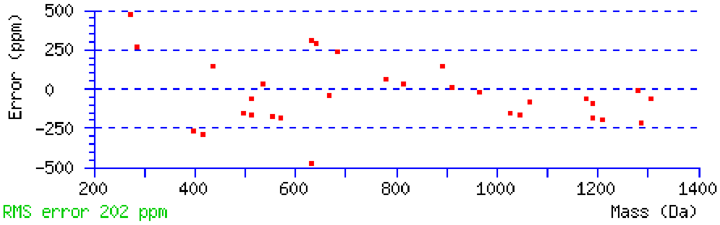


### All matches to this query

| Score | Mr(calc): | Delta | Sequence |
|---|---|---|---|
| 64.4 | 1478.6889 | 0.0054 | EATIVGENETYPR |
| 10.1 | 1477.7049 | 0.9894 | EATIVGENETYPR |
| 8.9 | 1478.7035 | -0.0092 | SLALESSVSSCSPR |
| 6.2 | 1478.6806 | 0.0137 | STLPNPFHLGTSK |
| 6.2 | 1478.6806 | 0.0137 | STLPNPFHLGTSK |
| 4.1 | 1477.6926 | 1.0017 | TKSDAPGQTQLPR |
| 3.0 | 1476.6779 | 2.0165 | LLASGYPLASGIR |
| 3.0 | 1476.6779 | 2.0165 | LLASGYPLASGIR |

---

Spectrum No: 447; Query: 2686; Rank: 1

---

## Peptide View

MS/MS Fragmentation of **SCGECIQAGPNCGWCTNTTFLQEGMPTSAR**
Found in **IPI00191681**, Tax_Id=10116 Gene_Symbol=Itgb1 Integrin beta-1 precursor

Match to Query 2686: 3391.394442 from(1131.472090,3+)
Title: 100101RatKid_NS_deglyco_23.4160.4160.3.dta
Data file K:\NewmanPaper\Piliang\3SubProteomes\Piliang3SP\mgf5ppm\ERLIC_3SubProteomes5ppm.mgf

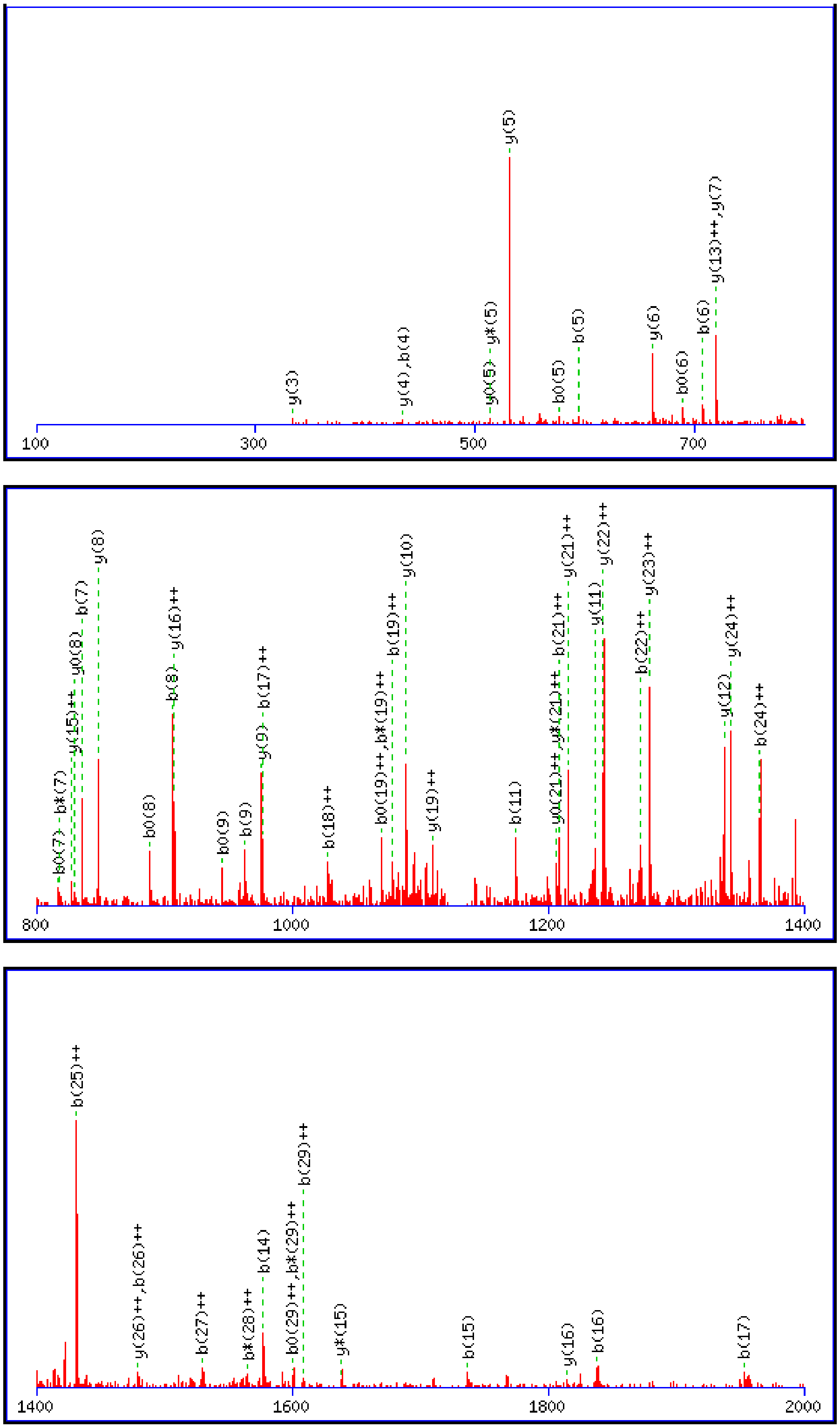


**Monoisotopic mass of neutral peptide Mr(calc):** 3390.3832
**Fixed modifications:** Carbamidomethyl (C)
**Variable modifications:**
**N17** : Deamidated_N (N)
**Ions Score:** 64 **Expect:** 0.00015
**Matches (Bold Red):** 58/332 fragment ions using 104 most intense peaks

| # | b | $b^{++}$ | b* | $b^{*++}$ | $b^{0}$ | $b^{0++}$ | Seq. | y | $y^{++}$ | y* | $y^{*++}$ | $y^{0}$ | $y^{0++}$ | # |
|---|---|---|---|---|---|---|---|---|---|---|---|---|---|---|
| 1 | 88.0393 | 44.5233 | | | 70.0287 | 35.5180 | S | | | | | | | 30 |
| 2 | 248.0700 | 124.5386 | | | 230.0594 | 115.5333 | C | 3304.3584 | 1652.6829 | 3287.3319 | 1644.1696 | 3286.3479 | 1643.6776 | 29 |
| 3 | 305.0914 | 153.0493 | | | 287.0809 | 144.0441 | G | 3144.3278 | 1572.6675 | 3127.3013 | 1564.1543 | 3126.3172 | 1563.6623 | 28 |
| 4 | **434.1340** | 217.5706 | | | 416.1234 | 208.5654 | E | 3087.3063 | 1544.1568 | 3070.2798 | 1535.6435 | 3069.2958 | 1535.1515 | 27 |
| 5 | **594.1647** | 297.5860 | | | **576.1541** | 288.5807 | C | 2958.2637 | **1479.6355** | 2941.2372 | 1471.1222 | 2940.2532 | 1470.6302 | 26 |

| | | | | | | | | | | | | | | |
|---|---|---|---|---|---|---|---|---|---|---|---|---|---|---|
| **6** | **707.2487** | 354.1280 | | | **689.2382** | 345.1227 | **I** | 2798.2331 | 1399.6202 | 2781.2065 | 1391.1069 | 2780.2225 | 1390.6149 | **25** |
| **7** | **835.3073** | 418.1573 | **818.2808** | 409.6440 | **817.2967** | 409.1520 | **Q** | 2685.1490 | **1343.0782** | 2668.1225 | 1334.5649 | 2667.1385 | 1334.0729 | **24** |
| **8** | **906.3444** | 453.6758 | 889.3179 | 445.1626 | **888.3338** | 444.6706 | **A** | 2557.0905 | **1279.0489** | 2540.0639 | 1270.5356 | 2539.0799 | 1270.0436 | **23** |
| **9** | **963.3659** | 482.1866 | 946.3393 | 473.6733 | **945.3553** | 473.1813 | **G** | 2486.0533 | **1243.5303** | 2469.0268 | 1235.0170 | 2468.0428 | 1234.5250 | **22** |
| **10** | 1060.4186 | 530.7130 | 1043.3921 | 522.1997 | 1042.4081 | 521.7077 | **P** | 2429.0319 | **1215.0196** | 2412.0053 | **1206.5063** | 2411.0213 | **1206.0143** | **21** |
| **11** | **1174.4616** | 587.7344 | 1157.4350 | 579.2211 | 1156.4510 | 578.7291 | **N** | 2331.9791 | 1166.4932 | 2314.9526 | 1157.9799 | 2313.9685 | 1157.4879 | **20** |
| **12** | 1334.4922 | 667.7497 | 1317.4657 | 659.2365 | 1316.4817 | 658.7445 | **C** | 2217.9362 | **1109.4717** | 2200.9096 | 1100.9585 | 2199.9256 | 1100.4664 | **19** |
| **13** | 1391.5137 | 696.2605 | 1374.4871 | 687.7472 | 1373.5031 | 687.2552 | **G** | 2057.9055 | 1029.4564 | 2040.8790 | 1020.9431 | 2039.8950 | 1020.4511 | **18** |
| **14** | **1577.5930** | 789.3001 | 1560.5664 | 780.7869 | 1559.5824 | 780.2949 | **W** | 2000.8841 | 1000.9457 | 1983.8575 | 992.4324 | 1982.8735 | 991.9404 | **17** |
| **15** | **1737.6236** | 869.3155 | 1720.5971 | 860.8022 | 1719.6131 | 860.3102 | **C** | **1814.8048** | **907.9060** | 1797.7782 | 899.3927 | 1796.7942 | 898.9007 | **16** |
| **16** | **1838.6713** | 919.8393 | 1821.6448 | 911.3260 | 1820.6608 | 910.8340 | **T** | 1654.7741 | **827.8907** | **1637.7476** | 819.3774 | 1636.7635 | 818.8854 | **15** |
| **17** | **1953.6983** | **977.3528** | 1936.6717 | 968.8395 | 1935.6877 | 968.3475 | **N** | 1553.7264 | 777.3669 | 1536.6999 | 768.8536 | 1535.7159 | 768.3616 | **14** |
| **18** | 2054.7459 | **1027.8766** | 2037.7194 | 1019.3633 | 2036.7354 | 1018.8713 | **T** | 1438.6995 | **719.8534** | 1421.6729 | 711.3401 | 1420.6889 | 710.8481 | **13** |
| **19** | 2155.7936 | **1078.4004** | 2138.7671 | **1069.8872** | 2137.7831 | **1069.3952** | **T** | **1337.6518** | 669.3295 | 1320.6253 | 660.8163 | 1319.6412 | 660.3243 | **12** |
| **20** | 2302.8620 | 1151.9347 | 2285.8355 | 1143.4214 | 2284.8515 | 1142.9294 | **F** | **1236.6041** | 618.8057 | 1219.5776 | 610.2924 | 1218.5936 | 609.8004 | **11** |
| **21** | 2415.9461 | **1208.4767** | 2398.9195 | 1199.9634 | 2397.9355 | 1199.4714 | **L** | **1089.5357** | 545.2715 | 1072.5092 | 536.7582 | 1071.5252 | 536.2662 | **10** |
| **22** | 2544.0047 | **1272.5060** | 2526.9781 | 1263.9927 | 2525.9941 | 1263.5007 | **Q** | **976.4517** | 488.7295 | 959.4251 | 480.2162 | 958.4411 | 479.7242 | **9** |
| **23** | 2673.0473 | 1337.0273 | 2656.0207 | 1328.5140 | 2655.0367 | 1328.0220 | **E** | **848.3931** | 424.7002 | 831.3665 | 416.1869 | **830.3825** | 415.6949 | **8** |
| **24** | 2730.0687 | **1365.5380** | 2713.0422 | 1357.0247 | 2712.0582 | 1356.5327 | **G** | **719.3505** | 360.1789 | 702.3239 | 351.6656 | 701.3399 | 351.1736 | **7** |
| **25** | 2861.1092 | **1431.0582** | 2844.0827 | 1422.5450 | 2843.0987 | 1422.0530 | **M** | **662.3290** | 331.6681 | 645.3025 | 323.1549 | 644.3185 | 322.6629 | **6** |
| **26** | 2958.1620 | **1479.5846** | 2941.1354 | 1471.0714 | 2940.1514 | 1470.5793 | **P** | **531.2885** | 266.1479 | **514.2620** | 257.6346 | **513.2780** | 257.1426 | **5** |
| **27** | 3059.2097 | **1530.1085** | 3042.1831 | 1521.5952 | 3041.1991 | 1521.1032 | **T** | **434.2358** | 217.6215 | 417.2092 | 209.1083 | 416.2252 | 208.6162 | **4** |
| **28** | 3146.2417 | 1573.6245 | 3129.2151 | **1565.1112** | 3128.2311 | 1564.6192 | **S** | **333.1881** | 167.0977 | 316.1615 | 158.5844 | 315.1775 | 158.0924 | **3** |
| **29** | 3217.2788 | **1609.1430** | 3200.2523 | **1600.6298** | 3199.2682 | **1600.1378** | **A** | 246.1561 | 123.5817 | 229.1295 | 115.0684 | | | **2** |
| **30** | | | | | | | **R** | 175.1190 | 88.0631 | 158.0924 | 79.5498 | | | **1** |

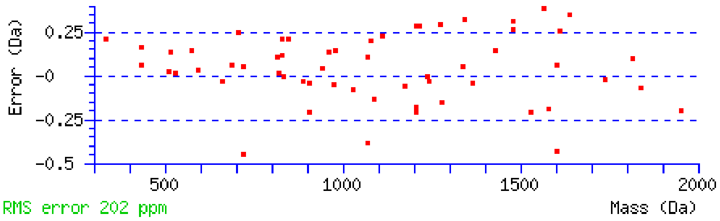


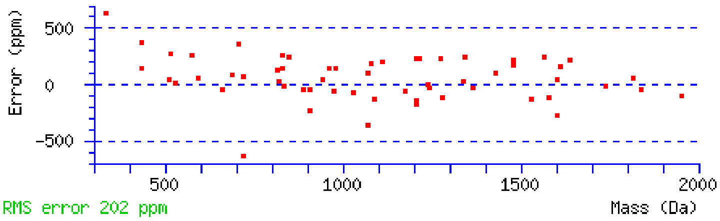


## All matches to this query

| Score | Mr(calc): | Delta | Sequence |
|---|---|---|---|
| 64.4 | 3390.3832 | 1.0112 | SCGECIQAGPNCGWCTNTTFLQEGMPTSAR |
| 50.8 | 3391.3672 | 0.0272 | SCGECIQAGPNCGWCTNTTFLQEGMPTSAR |
| 47.9 | 3390.3832 | 1.0112 | SCGECIQAGPNCGWCTNTTFLQEGMPTSAR |
| 45.9 | 3389.3992 | 1.9952 | SCGECIQAGPNCGWCTNTTFLQEGMPTSAR |
| 4.8 | 3390.3586 | 1.0359 | DGDSTVTGARVSNDCSAGTAGVPALSSSEARR |
| 4.4 | 3390.3804 | 1.0141 | QKPSLGTMPVQVTPPRGTFSPNMGMQPR |
| 4.4 | 3391.3644 | 0.0300 | QKPSLGTMPVQVTPPRGTFSPNMGMQPR |
| 2.8 | 3389.3984 | 1.9960 | IDVASIDMVSEVNMDYTLTMYFQQSWK |
| 2.8 | 3389.3608 | 2.0337 | LYVVGGYDGHTYLNTVESYDAQKDEWK |
| 2.0 | 3390.3868 | 1.0076 | TASWLAAELSKEGHQVALLSGEMMVEQR |

---

Spectrum No: 448; Query: 110; Rank: 1

---

# Peptide View

MS/MS Fragmentation of **AAENFTLLVK**
Found in **IPI00324987**, Tax_Id=10116 Gene_Symbol=P2rx4 P2X purinoceptor 4

Match to Query 110: 1105.602708 from(553.808630,2+)
Title: 091008RatKidney_NH4Format01_24.2623.2623.2.dta
Data file K:\NewmanPaper\Piliang\3SubProteomes\Piliang3SP\mgf5ppm\ERLIC_3SubProteomes5ppm.mgf

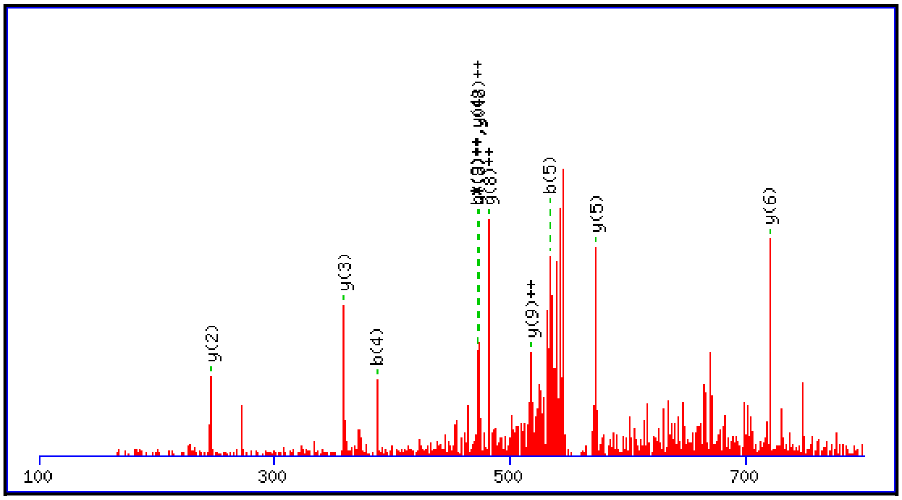


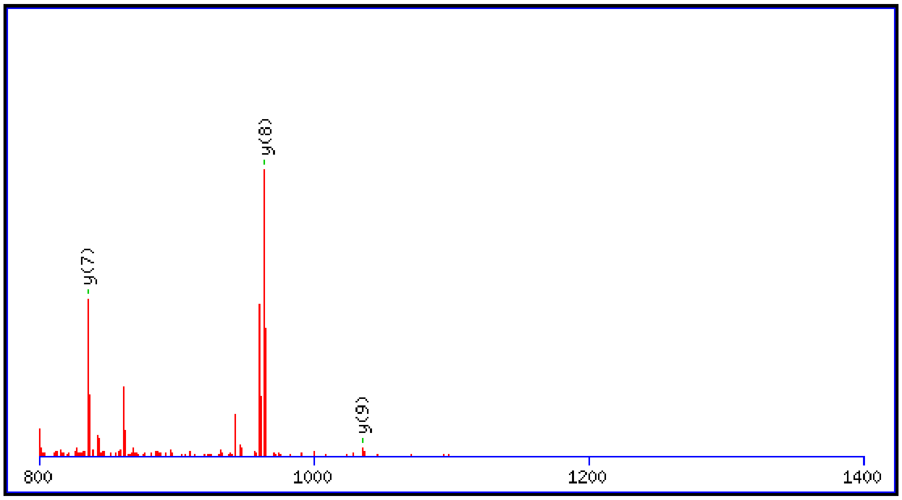


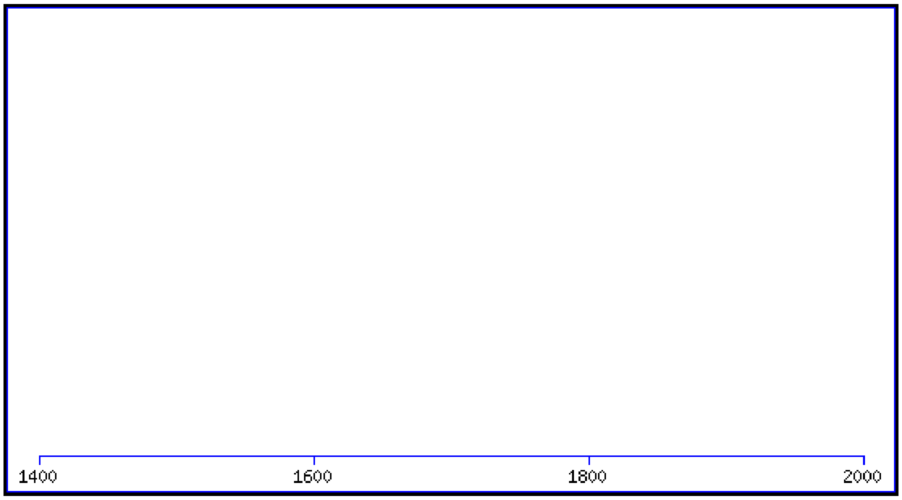


**Monoisotopic mass of neutral peptide Mr(calc):** 1105.6019
**Fixed modifications:** Carbamidomethyl (C)
**Variable modifications:**
**N4** : Deamidated_N (N)
**Ions Score:** 64 **Expect:** 2.8e-005
**Matches (Bold Red):** 15/90 fragment ions using 28 most intense peaks

| # | b | b++ | b* | b*++ | b0 | b0++ | Seq. | y | y++ | y* | y*++ | y0 | y0++ | # |
|---|---|---|---|---|---|---|---|---|---|---|---|---|---|---|
| 1 | 72.0444 | 36.5258 | | | | | A | | | | | | | 10 |
| 2 | 143.0815 | 72.0444 | | | | | A | **1035.5721** | **518.2897** | 1018.5455 | 509.7764 | 1017.5615 | 509.2844 | 9 |
| 3 | 272.1241 | 136.5657 | | | 254.1135 | 127.5604 | E | **964.5350** | **482.7711** | 947.5084 | **474.2578** | 946.5244 | **473.7658** | 8 |
| 4 | **387.1510** | 194.0792 | 370.1245 | 185.5659 | 369.1405 | 185.0739 | N | **835.4924** | 418.2498 | 818.4658 | 409.7366 | 817.4818 | 409.2445 | 7 |
| 5 | **534.2194** | 267.6134 | 517.1929 | 259.1001 | 516.2089 | 258.6081 | F | **720.4654** | 360.7364 | 703.4389 | 352.2231 | 702.4549 | 351.7311 | 6 |
| 6 | 635.2671 | 318.1372 | 618.2406 | 309.6239 | 617.2566 | 309.1319 | T | **573.3970** | 287.2022 | 556.3705 | 278.6889 | 555.3865 | 278.1969 | 5 |
| 7 | 748.3512 | 374.6792 | 731.3246 | 366.1660 | 730.3406 | 365.6740 | L | **472.3493** | 236.6783 | 455.3228 | 228.1650 | | | 4 |
| 8 | 861.4353 | 431.2213 | 844.4087 | 422.7080 | 843.4247 | 422.2160 | L | **359.2653** | 180.1363 | 342.2387 | 171.6230 | | | 3 |
| 9 | 960.5037 | 480.7555 | 943.4771 | **472.2422** | 942.4931 | 471.7502 | V | **246.1812** | 123.5942 | 229.1547 | 115.0810 | | | 2 |
| 10 | | | | | | | K | 147.1128 | 74.0600 | 130.0863 | 65.5468 | | | 1 |

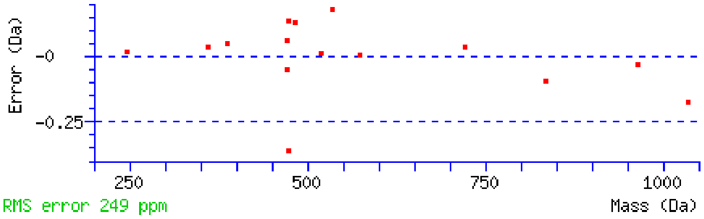


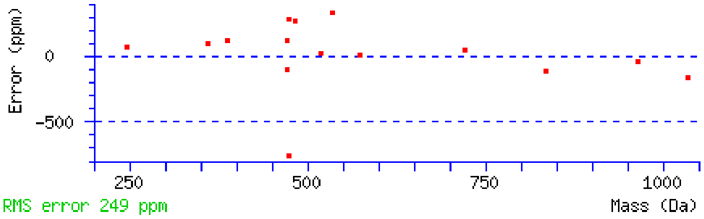


### All matches to this query

| Score | Mr(calc): | Delta | Sequence |
|---|---|---|---|
| 64.4 | 1105.6019 | 0.0008 | AAENFTLLVK |
| 10.9 | 1105.6131 | -0.0104 | TGISKPYNVK |
| 10.4 | 1105.5992 | 0.0035 | APQPGIRNPR |
| 4.0 | 1103.5988 | 2.0039 | NRNFWVLR |
| 3.5 | 1103.5910 | 2.0117 | ARMDVFLPR |
| 3.0 | 1105.5979 | 0.0048 | KETNSKATVK |
| 3.0 | 1103.5863 | 2.0164 | DPGTFDLLVK |
| 2.3 | 1105.6019 | 0.0008 | XSTLPLANVK |
| 0.9 | 1105.5954 | 0.0073 | MFLNLLSPR |
| 0.1 | 1105.6009 | 0.0018 | ARLKTNPVK |

---

Spectrum No: 449; Query: 163; Rank: 1

---

## Peptide View

MS/MS Fragmentation of **VINDTWTWK**
Found in **IPI00210975**, Tax_Id=10116 Gene_Symbol=Hyou1 Hypoxia up-regulated protein 1 precursor

Match to Query 163: 1162.565648 from(582.290100,2+)
Title: 091008RatKidney_NH4Format01_24.2574.2574.2.dta
Data file K:\NewmanPaper\Piliang\3SubProteomes\Piliang3SP\mgf5ppm\ERLIC_3SubProteomes5ppm.mgf

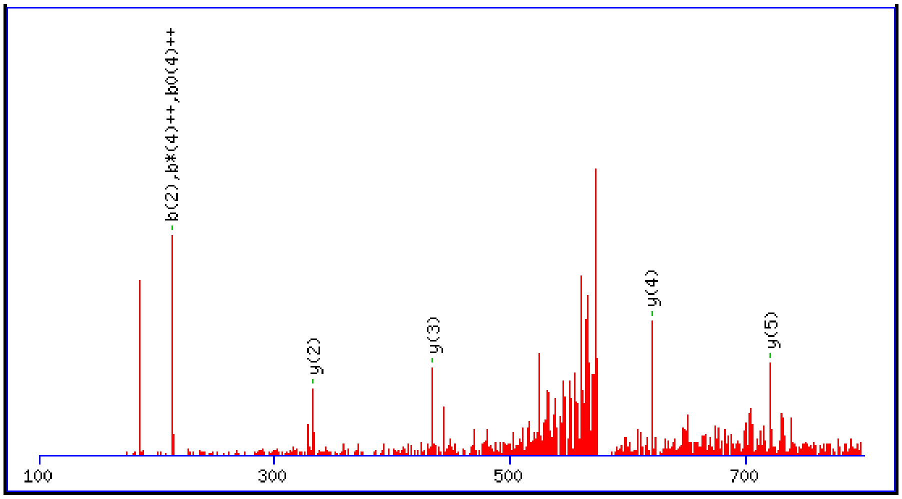


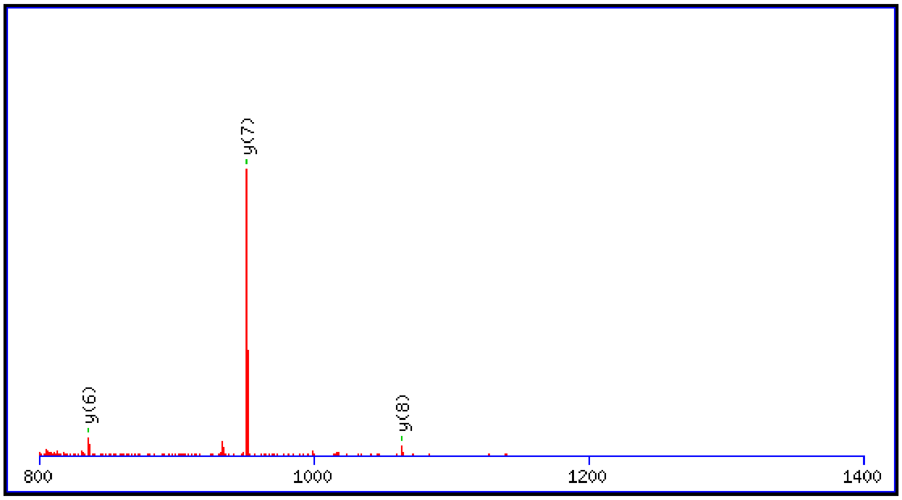


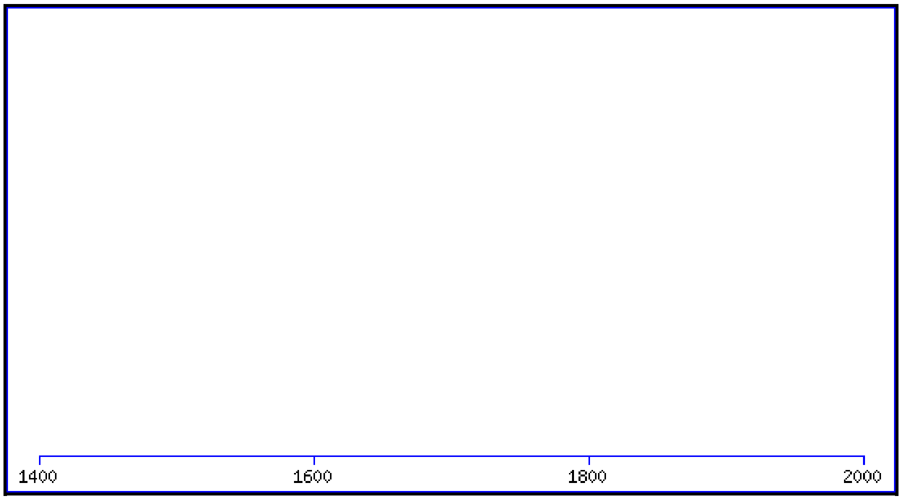


**Monoisotopic mass of neutral peptide Mr(calc):** 1162.5659
**Fixed modifications:** Carbamidomethyl (C)
**Variable modifications:**
**N3** : Deamidated_N (N)
**Ions Score:** 64 **Expect:** 4.6e-005
**Matches (Bold Red):** 10/82 fragment ions using 18 most intense peaks

| # | b | b++ | b* | b*++ | b0 | b0++ | Seq. | y | y++ | y* | y*++ | y0 | y0++ | # |
|---|---|---|---|---|---|---|---|---|---|---|---|---|---|---|
| 1 | 100.0757 | 50.5415 | | | | | V | | | | | | | 9 |
| 2 | **213.1598** | 107.0835 | | | | | I | **1064.5047** | 532.7560 | 1047.4782 | 524.2427 | 1046.4942 | 523.7507 | 8 |
| 3 | 328.1867 | 164.5970 | 311.1601 | 156.0837 | | | N | **951.4207** | 476.2140 | 934.3941 | 467.7007 | 933.4101 | 467.2087 | 7 |
| 4 | 443.2136 | 222.1105 | 426.1871 | **213.5972** | 425.2031 | **213.1052** | D | **836.3937** | 418.7005 | 819.3672 | 410.1872 | 818.3832 | 409.6952 | 6 |
| 5 | 544.2613 | 272.6343 | 527.2348 | 264.1210 | 526.2507 | 263.6290 | T | **721.3668** | 361.1870 | 704.3402 | 352.6738 | 703.3562 | 352.1817 | 5 |

| | | | | | | | | | | | | | | |
|---|---|---|---|---|---|---|---|---|---|---|---|---|---|---|
| **6** | 730.3406 | 365.6740 | 713.3141 | 357.1607 | 712.3301 | 356.6687 | **W** | 620.3191 | 310.6632 | 603.2926 | 302.1499 | 602.3085 | 301.6579 | **4** |
| **7** | 831.3883 | 416.1978 | 814.3618 | 407.6845 | 813.3777 | 407.1925 | **T** | 434.2398 | 217.6235 | 417.2132 | 209.1103 | 416.2292 | 208.6183 | **3** |
| **8** | 1017.4676 | 509.2374 | 1000.4411 | 500.7242 | 999.4571 | 500.2322 | **W** | 333.1921 | 167.0997 | 316.1656 | 158.5864 | | | **2** |
| **9** | | | | | | | **K** | 147.1128 | 74.0600 | 130.0863 | 65.5468 | | | **1** |

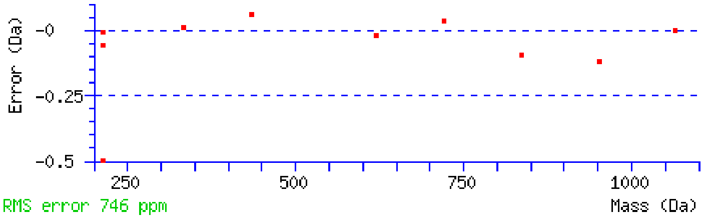


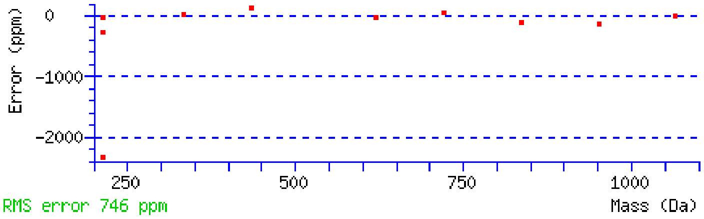


### All matches to this query

| Score | Mr(calc): | Delta | Sequence |
|---|---|---|---|
| 64.4 | 1162.5659 | -0.0002 | VINDTWTWK |
| 9.2 | 1162.5675 | -0.0019 | VISELFFTK |
| 3.1 | 1162.5747 | -0.0091 | VLTLPRSNPS |
| 2.6 | 1162.5635 | 0.0021 | VLPGVDALSNV |
| 2.0 | 1162.5731 | -0.0074 | DPDSFRASIR |
| 2.0 | 1162.5747 | -0.0091 | VIKNTDKHK |
| 2.0 | 1162.5675 | -0.0019 | VISELFFTK |
| 2.0 | 1162.5652 | 0.0004 | VISSQECNVK |
| 2.0 | 1162.5747 | -0.0091 | VLTLPRSNPS |
| 2.0 | 1162.5747 | -0.0091 | VLTLPRSNPS |

---

Spectrum No: 450; Query: 1047; Rank: 1

---

## Peptide View

MS/MS Fragmentation of **KVVNVSELYGTPCTK**
Found in **IPI00206668**, Tax_Id=10116 Gene_Symbol=Entpd1 Ectonucleoside triphosphate diphosphohydrolase 1

Match to Query 1047: 1694.861588 from(848.438070,2+)
Title: 100101RatKid_NS_deglyco_18.2423.2423.2.dta
Data file K:\NewmanPaper\Piliang\3SubProteomes\Piliang3SP\mgf5ppm\ERLIC_3SubProteomes5ppm.mgf

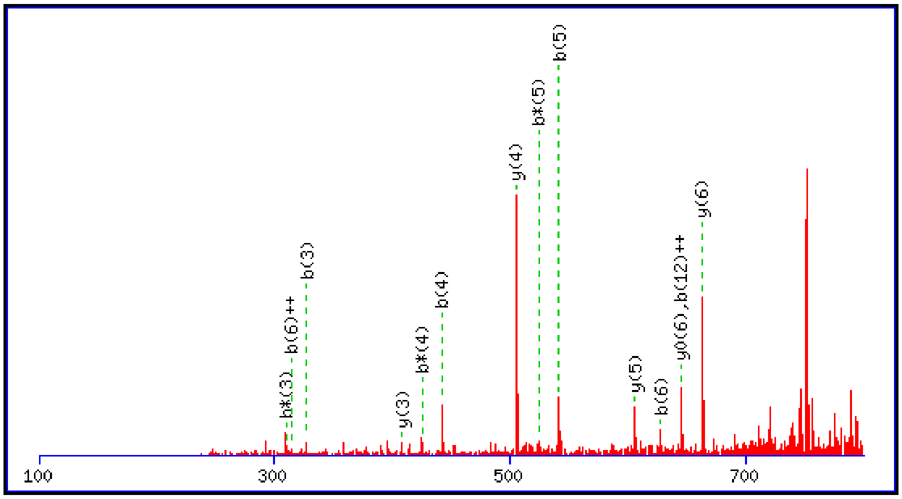

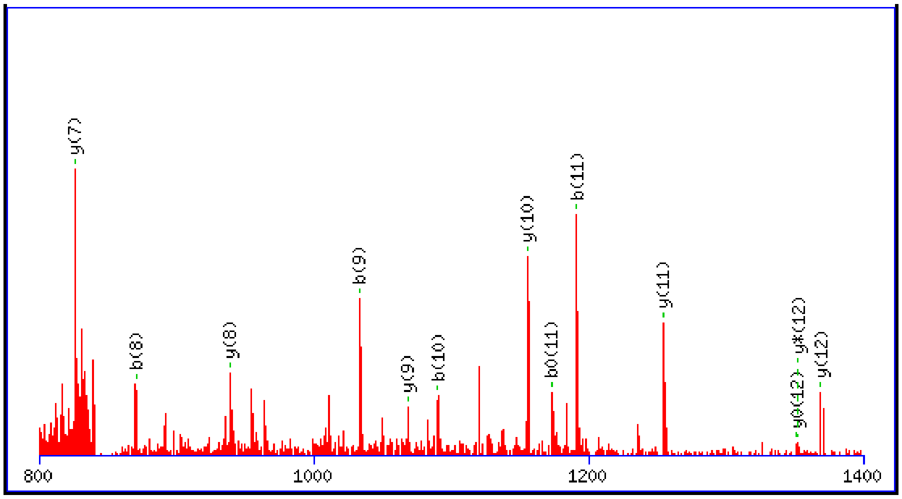


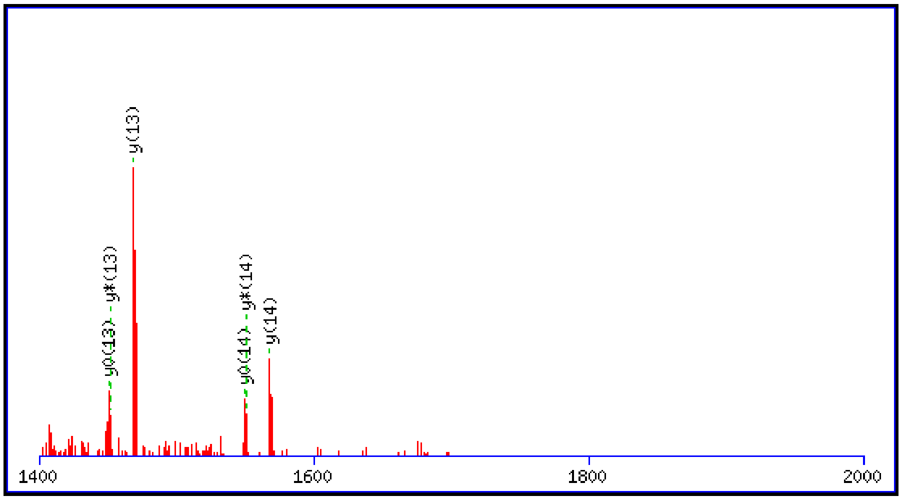


**Monoisotopic mass of neutral peptide Mr(calc):** 1694.8549
**Fixed modifications:** Carbamidomethyl (C)
**Variable modifications:**
**N4** : Deamidated_N (N)
**Ions Score:** 64 **Expect:** 5.7e-005
**Matches (Bold Red):** 34/156 fragment ions using 87 most intense peaks

| # | b | $b^{++}$ | b* | $b^{*++}$ | $b^0$ | $b^{0++}$ | Seq. | y | $y^{++}$ | y* | $y^{*++}$ | $y^0$ | $y^{0++}$ | # |
|---|---|---|---|---|---|---|---|---|---|---|---|---|---|---|
| 1 | 129.1022 | 65.0548 | 112.0757 | 56.5415 | | | K | | | | | | | 15 |
| 2 | 228.1707 | 114.5890 | 211.1441 | 106.0757 | | | V | **1567.7672** | 784.3873 | **1550.7407** | 775.8740 | **1549.7567** | 775.3820 | 14 |
| 3 | **327.2391** | 164.1232 | **310.2125** | 155.6099 | | | V | **1468.6988** | 734.8530 | **1451.6723** | 726.3398 | **1450.6883** | 725.8478 | 13 |
| 4 | **442.2660** | 221.6366 | **425.2395** | 213.1234 | | | N | **1369.6304** | 685.3188 | **1352.6039** | 676.8056 | **1351.6198** | 676.3136 | 12 |
| 5 | **541.3344** | 271.1708 | **524.3079** | 262.6576 | | | V | **1254.6035** | 627.8054 | 1237.5769 | 619.2921 | 1236.5929 | 618.8001 | 11 |
| 6 | **628.3664** | **314.6869** | 611.3399 | 306.1736 | 610.3559 | 305.6816 | S | **1155.5351** | 578.2712 | 1138.5085 | 569.7579 | 1137.5245 | 569.2659 | 10 |
| 7 | 757.4090 | 379.2082 | 740.3825 | 370.6949 | 739.3985 | 370.2029 | E | **1068.5030** | 534.7552 | 1051.4765 | 526.2419 | 1050.4925 | 525.7499 | 9 |
| 8 | **870.4931** | 435.7502 | 853.4666 | 427.2369 | 852.4825 | 426.7449 | L | **939.4604** | 470.2339 | 922.4339 | 461.7206 | 921.4499 | 461.2286 | 8 |
| 9 | **1033.5564** | 517.2819 | 1016.5299 | 508.7686 | 1015.5459 | 508.2766 | Y | **826.3764** | 413.6918 | 809.3498 | 405.1785 | 808.3658 | 404.6865 | 7 |
| 10 | **1090.5779** | 545.7926 | 1073.5513 | 537.2793 | 1072.5673 | 536.7873 | G | **663.3130** | 332.1602 | 646.2865 | 323.6469 | **645.3025** | 323.1549 | 6 |
| 11 | **1191.6256** | 596.3164 | 1174.5990 | 587.8032 | **1173.6150** | 587.3111 | T | **606.2916** | 303.6494 | 589.2650 | 295.1362 | 588.2810 | 294.6441 | 5 |
| 12 | 1288.6783 | **644.8428** | 1271.6518 | 636.3295 | 1270.6678 | 635.8375 | P | **505.2439** | 253.1256 | 488.2173 | 244.6123 | 487.2333 | 244.1203 | 4 |
| 13 | 1448.7090 | 724.8581 | 1431.6824 | 716.3449 | 1430.6984 | 715.8528 | C | **408.1911** | 204.5992 | 391.1646 | 196.0859 | 390.1806 | 195.5939 | 3 |
| 14 | **1549.7567** | 775.3820 | 1532.7301 | 766.8687 | 1531.7461 | 766.3767 | T | 248.1605 | 124.5839 | 231.1339 | 116.0706 | 230.1499 | 115.5786 | 2 |
| 15 | | | | | | | K | 147.1128 | 74.0600 | 130.0863 | 65.5468 | | | 1 |

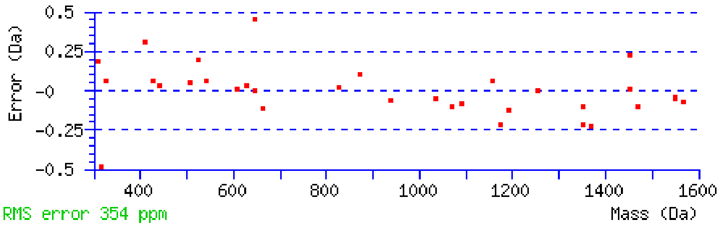


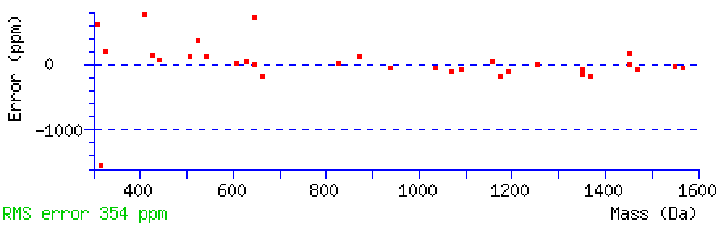


### All matches to this query

| Score | Mr(calc): | Delta | Sequence |
|---|---|---|---|
| 64.3 | 1694.8549 | 0.0067 | KVVNVSELYGTPCTK |
| 16.7 | 1693.8709 | 0.9907 | KVVNVSELYGTPCTK |
| 9.9 | 1694.8505 | 0.0111 | QVVQSSLLGRWGTGK |

---

Spectrum No: 451; Query: 2807; Rank: 1

---

## Peptide View

MS/MS Fragmentation of **ILTNNSQTPILSPQEVVSCSPYAQGCDGGFPYLIAGK**
Found in **IPI00193765**, Tax_Id=10116 Gene_Symbol=Ctsc Dipeptidyl-peptidase 1 precursor

Match to Query 2807: 3981.920616 from(996.487430,4+)
Title: 100101RatKid_NS_deglyco_21.5341.5341.4.dta
Data file K:\NewmanPaper\Piliang\3SubProteomes\Piliang3SP\mgf5ppm\ERLIC_3SubProteomes5ppm.mgf

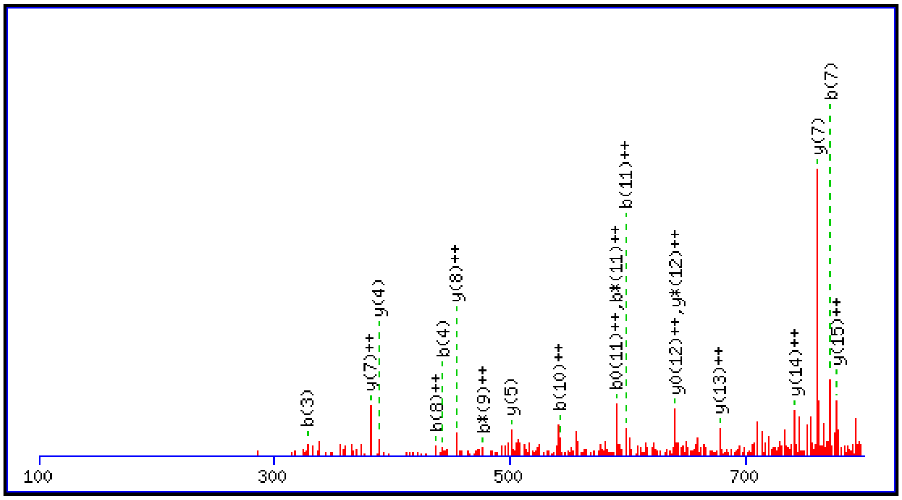


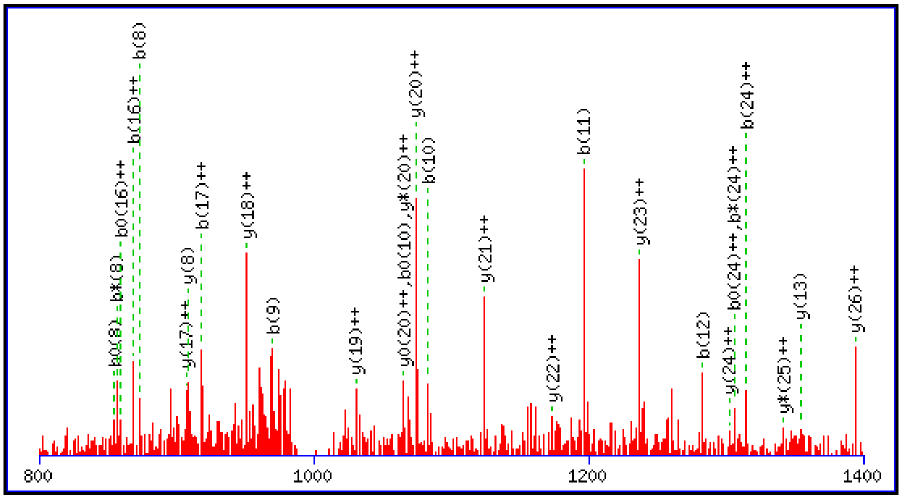

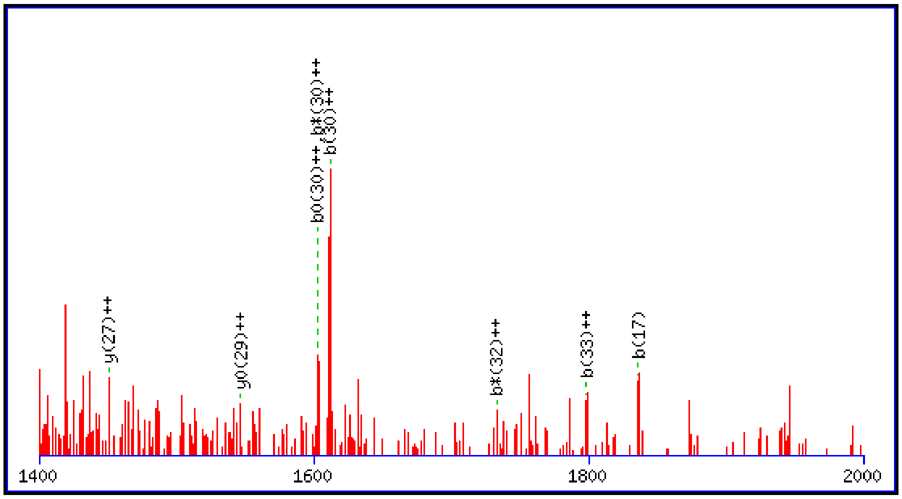


**Monoisotopic mass of neutral peptide Mr(calc):** 3981.9129
**Fixed modifications:** Carbamidomethyl (C)
**Variable modifications:**
**N4** : Deamidated_N (N)
**Ions Score:** 64 **Expect:** 0.00012
**Matches (Bold Red):** 55/402 fragment ions using 111 most intense peaks

| # | b | b$^{++}$ | b* | b*$^{++}$ | b$^{0}$ | b$^{0++}$ | Seq. | y | y$^{++}$ | y* | y*$^{++}$ | y$^{0}$ | y$^{0++}$ | # |
|---|---|---|---|---|---|---|---|---|---|---|---|---|---|---|
| 1 | 114.0913 | 57.5493 | | | | | I | | | | | | | 37 |
| 2 | 227.1754 | 114.0913 | | | | | L | 3869.8361 | 1935.4217 | 3852.8095 | 1926.9084 | 3851.8255 | 1926.4164 | 36 |
| 3 | **328.2231** | 164.6152 | | | 310.2125 | 155.6099 | T | 3756.7520 | 1878.8796 | 3739.7255 | 1870.3664 | 3738.7414 | 1869.8744 | 35 |
| 4 | **443.2500** | 222.1286 | 426.2235 | 213.6154 | 425.2395 | 213.1234 | N | 3655.7043 | 1828.3558 | 3638.6778 | 1819.8425 | 3637.6938 | 1819.3505 | 34 |
| 5 | 557.2929 | 279.1501 | 540.2664 | 270.6368 | 539.2824 | 270.1448 | N | 3540.6774 | 1770.8423 | 3523.6508 | 1762.3291 | 3522.6668 | 1761.8370 | 33 |
| 6 | 644.3250 | 322.6661 | 627.2984 | 314.1529 | 626.3144 | 313.6608 | S | 3426.6345 | 1713.8209 | 3409.6079 | 1705.3076 | 3408.6239 | 1704.8156 | 32 |
| 7 | **772.3836** | 386.6954 | 755.3570 | 378.1821 | 754.3730 | 377.6901 | Q | 3339.6024 | 1670.3049 | 3322.5759 | 1661.7916 | 3321.5919 | 1661.2996 | 31 |
| 8 | **873.4312** | **437.2193** | **856.4047** | 428.7060 | **855.4207** | 428.2140 | T | 3211.5439 | 1606.2756 | 3194.5173 | 1597.7623 | 3193.5333 | 1597.2703 | 30 |
| 9 | **970.4840** | 485.7456 | 953.4574 | **477.2324** | 952.4734 | 476.7404 | P | 3110.4962 | 1555.7517 | 3093.4696 | 1547.2385 | 3092.4856 | **1546.7464** | 29 |
| 10 | **1083.5681** | **542.2877** | 1066.5415 | 533.7744 | **1065.5575** | 533.2824 | I | 3013.4434 | 1507.2253 | 2996.4169 | 1498.7121 | 2995.4328 | 1498.2201 | 28 |
| 11 | **1196.6521** | **598.8297** | 1179.6256 | **590.3164** | 1178.6416 | **589.8244** | L | 2900.3593 | **1450.6833** | 2883.3328 | 1442.1700 | 2882.3488 | 1441.6780 | 27 |
| 12 | **1283.6842** | 642.3457 | 1266.6576 | 633.8324 | 1265.6736 | 633.3404 | S | 2787.2753 | **1394.1413** | 2770.2487 | 1385.6280 | 2769.2647 | 1385.1360 | 26 |
| 13 | 1380.7369 | 690.8721 | 1363.7104 | 682.3588 | 1362.7263 | 681.8668 | P | 2700.2433 | 1350.6253 | 2683.2167 | **1342.1120** | 2682.2327 | 1341.6200 | 25 |
| 14 | 1508.7955 | 754.9014 | 1491.7689 | 746.3881 | 1490.7849 | 745.8961 | Q | 2603.1905 | **1302.0989** | 2586.1639 | 1293.5856 | 2585.1799 | 1293.0936 | 24 |
| 15 | 1637.8381 | 819.4227 | 1620.8115 | 810.9094 | 1619.8275 | 810.4174 | E | 2475.1319 | **1238.0696** | 2458.1054 | 1229.5563 | 2457.1213 | 1229.0643 | 23 |
| 16 | 1736.9065 | **868.9569** | 1719.8800 | 860.4436 | 1718.8959 | **859.9516** | V | 2346.0893 | **1173.5483** | 2329.0628 | 1165.0350 | 2328.0788 | 1164.5430 | 22 |
| 17 | **1835.9749** | **918.4911** | 1818.9484 | 909.9778 | 1817.9643 | 909.4858 | V | 2247.0209 | **1124.0141** | 2229.9944 | 1115.5008 | 2229.0103 | 1115.0088 | 21 |
| 18 | 1923.0069 | 962.0071 | 1905.9804 | 953.4938 | 1904.9964 | 953.0018 | S | 2147.9525 | **1074.4799** | 2130.9259 | **1065.9666** | 2129.9419 | **1065.4746** | 20 |
| 19 | 2083.0376 | 1042.0224 | 2066.0110 | 1033.5092 | 2065.0270 | 1033.0172 | C | 2060.9205 | **1030.9639** | 2043.8939 | 1022.4506 | 2042.9099 | 1021.9586 | 19 |
| 20 | 2170.0696 | 1085.5384 | 2153.0431 | 1077.0252 | 2152.0591 | 1076.5332 | S | 1900.8898 | **950.9485** | 1883.8633 | 942.4353 | 1882.8792 | 941.9433 | 18 |
| 21 | 2267.1224 | 1134.0648 | 2250.0958 | 1125.5516 | 2249.1118 | 1125.0595 | P | 1813.8578 | **907.4325** | 1796.8312 | 898.9193 | 1795.8472 | 898.4272 | 17 |
| 22 | 2430.1857 | 1215.5965 | 2413.1592 | 1207.0832 | 2412.1751 | 1206.5912 | Y | 1716.8050 | 858.9061 | 1699.7785 | 850.3929 | 1698.7945 | 849.9009 | 16 |
| 23 | 2501.2228 | 1251.1151 | 2484.1963 | 1242.6018 | 2483.2123 | 1242.1098 | A | 1553.7417 | **777.3745** | 1536.7151 | 768.8612 | 1535.7311 | 768.3692 | 15 |
| 24 | 2629.2814 | **1315.1443** | 2612.2549 | **1306.6311** | 2611.2708 | **1306.1391** | Q | 1482.7046 | **741.8559** | 1465.6780 | 733.3427 | 1464.6940 | 732.8506 | 14 |
| 25 | 2686.3029 | 1343.6551 | 2669.2763 | 1335.1418 | 2668.2923 | 1334.6498 | G | **1354.6460** | **677.8266** | 1337.6195 | 669.3134 | 1336.6354 | 668.8214 | 13 |
| 26 | 2846.3335 | 1423.6704 | 2829.3070 | 1415.1571 | 2828.3230 | 1414.6651 | C | 1297.6245 | 649.3159 | 1280.5980 | **640.8026** | 1279.6140 | **640.3106** | 12 |
| 27 | 2961.3605 | 1481.1839 | 2944.3339 | 1472.6706 | 2943.3499 | 1472.1786 | D | 1137.5939 | 569.3006 | 1120.5673 | 560.7873 | 1119.5833 | 560.2953 | 11 |
| 28 | 3018.3819 | 1509.6946 | 3001.3554 | 1501.1813 | 3000.3714 | 1500.6893 | G | 1022.5669 | 511.7871 | 1005.5404 | 503.2738 | | | 10 |
| 29 | 3075.4034 | 1538.2053 | 3058.3768 | 1529.6921 | 3057.3928 | 1529.2000 | G | 965.5455 | 483.2764 | 948.5189 | 474.7631 | | | 9 |
| 30 | 3222.4718 | **1611.7395** | 3205.4453 | **1603.2263** | 3204.4612 | **1602.7343** | F | **908.5240** | **454.7656** | 891.4975 | 446.2524 | | | 8 |
| 31 | 3319.5246 | 1660.2659 | 3302.4980 | 1651.7526 | 3301.5140 | 1651.2606 | P | **761.4556** | **381.2314** | 744.4291 | 372.7182 | | | 7 |

| | | | | | | | | | | | | | | |
|---|---|---|---|---|---|---|---|---|---|---|---|---|---|---|
| 32 | 3482.5879 | 1741.7976 | 3465.5613 | 1733.2843 | 3464.5773 | 1732.7923 | Y | 664.4028 | 332.7051 | 647.3763 | 324.1918 | | | 6 |
| 33 | 3595.6720 | 1798.3396 | 3578.6454 | 1789.8263 | 3577.6614 | 1789.3343 | L | 501.3395 | 251.1734 | 484.3130 | 242.6601 | | | 5 |
| 34 | 3708.7560 | 1854.8816 | 3691.7295 | 1846.3684 | 3690.7455 | 1845.8764 | I | 388.2554 | 194.6314 | 371.2289 | 186.1181 | | | 4 |
| 35 | 3779.7931 | 1890.4002 | 3762.7666 | 1881.8869 | 3761.7826 | 1881.3949 | A | 275.1714 | 138.0893 | 258.1448 | 129.5761 | | | 3 |
| 36 | 3836.8146 | 1918.9109 | 3819.7881 | 1910.3977 | 3818.8040 | 1909.9057 | G | 204.1343 | 102.5708 | 187.1077 | 94.0575 | | | 2 |
| 37 | | | | | | | K | 147.1128 | 74.0600 | 130.0863 | 65.5468 | | | 1 |

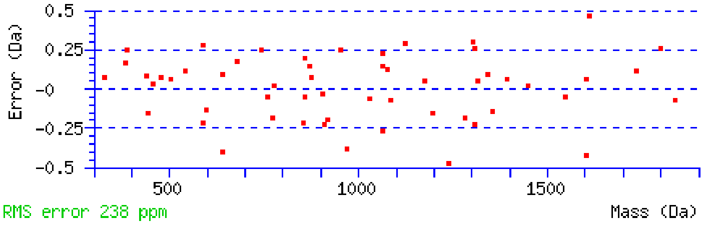


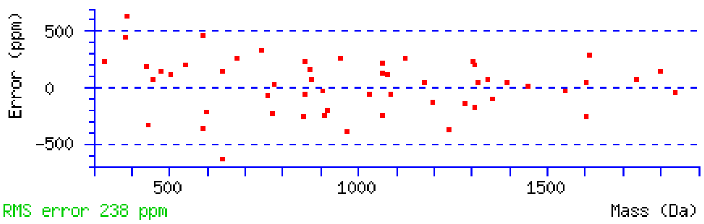


### All matches to this query

| Score | Mr(calc): | Delta | Sequence |
|---|---|---|---|
| 64.3 | 3981.9129 | 0.0078 | ILTNNSQTPILSPQEVVSCSPYAQGCDGGFPYLIAGK |
| 58.0 | 3981.9129 | 0.0078 | ILTNNSQTPILSPQEVVSCSPYAQGCDGGFPYLIAGK |
| 35.7 | 3980.9288 | 0.9918 | ILTNNSQTPILSPQEVVSCSPYAQGCDGGFPYLIAGK |
| 4.7 | 3979.9095 | 2.0111 | SVILDDDVLLILTLDEELSAPLTPSNGLGQEDLPSK |
| 3.6 | 3979.9293 | 1.9914 | NAALSTQLREAATQAQQTLGSTIDKATGTLLYGLASR |
| 1.3 | 3979.8758 | 2.0448 | SQIALKLGVTADDVKNVIIWGNHSSTQYPDVNHAK |
| 1.3 | 3979.8758 | 2.0448 | SQIALKLGVTADDVKNVIIWGNHSSTQYPDVNHAK |
| 1.2 | 3980.8935 | 1.0271 | LFSSLGELSTISAQRSPGGPGGGASYSVRPSGRYPVAR |
| 1.1 | 3980.9227 | 0.9979 | LTFNLTSGNHLGHFIVHPFTGLLTTAKQLDRENK |
| 1.1 | 3980.9227 | 0.9979 | LTFNLTSGNHLGHFIVHPFTGLLTTAKQLDRENK |

---

Spectrum No: 452; Query: 2541; Rank: 1

---

## Peptide View

MS/MS Fragmentation of **NENFSSGPSFETISASGLNAALAHYSPTK**
Found in **IPI00197684**, Tax_Id=10116 Gene_Symbol=Xpnpep2 Membrane-bound aminopeptidase P

Match to Query 2541: 2997.409332 from(1000.143720,3+)
Title: 100101RatKid_NS_deglyco_23.4786.4786.3.dta
Data file K:\NewmanPaper\Piliang\3SubProteomes\Piliang3SP\mgf5ppm\ERLIC_3SubProteomes5ppm.mgf

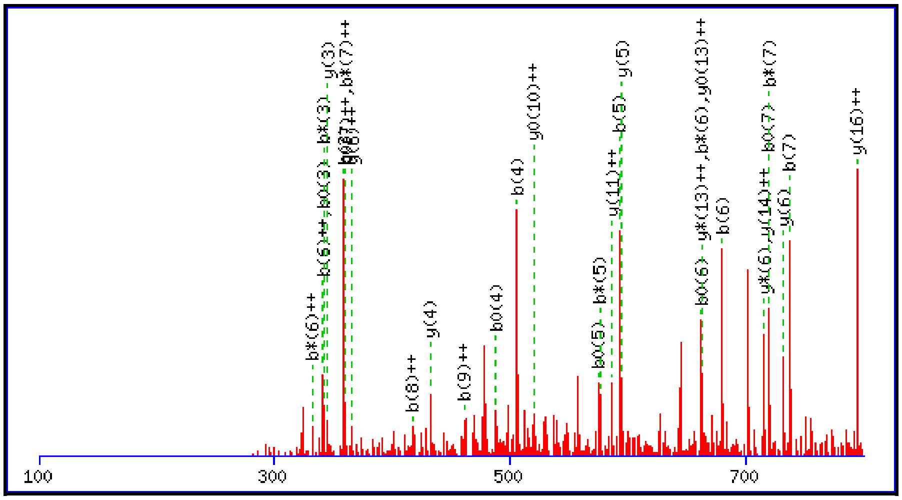

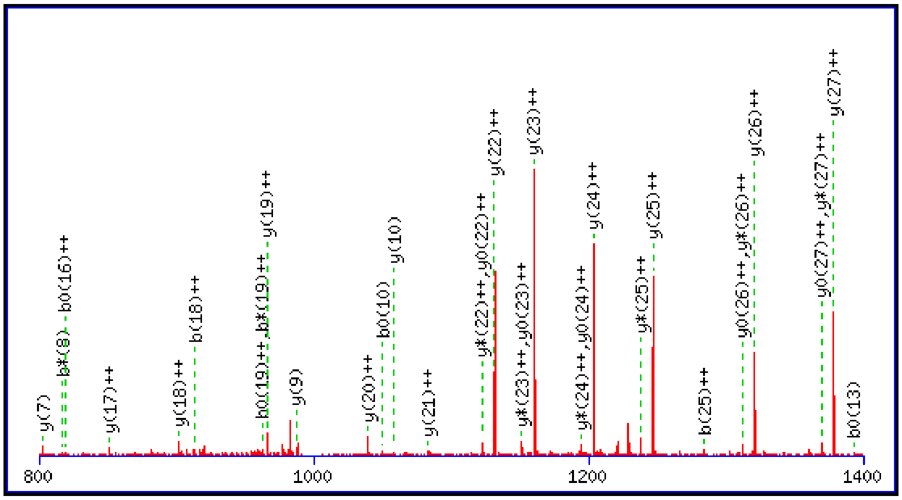


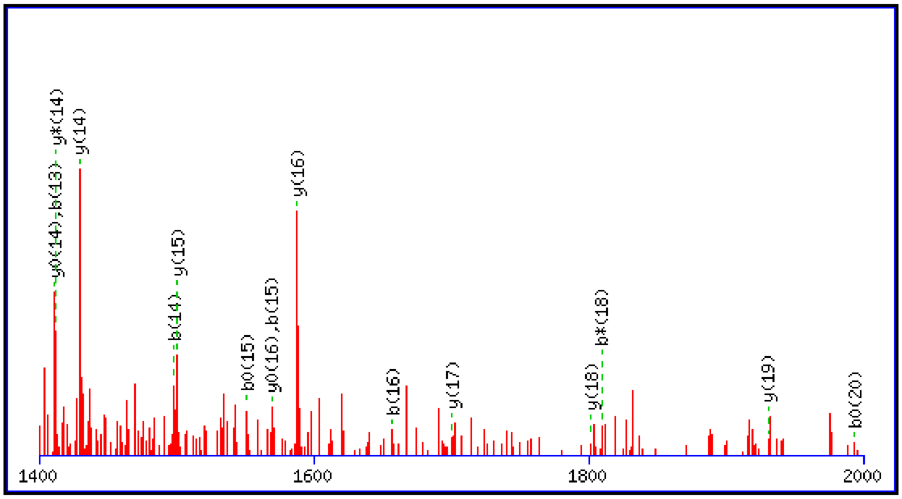


**Monoisotopic mass of neutral peptide Mr(calc):** 2997.3992
**Fixed modifications:** Carbamidomethyl (C)
**Variable modifications:**
**N3** : Deamidated_N (N)
**Ions Score:** 64 **Expect:** 0.00014
**Matches (Bold Red):** 81/332 fragment ions using 173 most intense peaks

| # | b | $b^{++}$ | b* | $b^{*++}$ | $b^0$ | $b^{0++}$ | Seq. | y | $y^{++}$ | y* | $y^{*++}$ | $y^0$ | $y^{0++}$ | # |
|---|---|---|---|---|---|---|---|---|---|---|---|---|---|---|
| 1 | 115.0502 | 58.0287 | 98.0237 | 49.5155 | | | N | | | | | | | 29 |
| 2 | 244.0928 | 122.5500 | 227.0662 | 114.0368 | 226.0822 | 113.5448 | E | 2884.3636 | 1442.6854 | 2867.3370 | 1434.1721 | 2866.3530 | 1433.6801 | 28 |
| 3 | **359.1197** | 180.0635 | **342.0932** | 171.5502 | **341.1092** | 171.0582 | N | 2755.3210 | **1378.1641** | 2738.2944 | **1369.6508** | 2737.3104 | **1369.1588** | 27 |
| 4 | **506.1881** | 253.5977 | 489.1616 | 245.0844 | **488.1776** | 244.5924 | F | 2640.2940 | **1320.6506** | 2623.2675 | **1312.1374** | 2622.2835 | **1311.6454** | 26 |
| 5 | **593.2202** | 297.1137 | **576.1936** | 288.6005 | **575.2096** | 288.1084 | S | 2493.2256 | **1247.1164** | 2476.1991 | **1238.6032** | 2475.2150 | 1238.1112 | 25 |
| 6 | **680.2522** | **340.6297** | **663.2257** | **332.1165** | **662.2416** | 331.6245 | S | 2406.1936 | **1203.6004** | 2389.1670 | **1195.0872** | 2388.1830 | **1194.5951** | 24 |
| 7 | **737.2737** | 369.1405 | **720.2471** | **360.6272** | **719.2631** | **360.1352** | G | 2319.1616 | **1160.0844** | 2302.1350 | **1151.5711** | 2301.1510 | **1151.0791** | 23 |
| 8 | 834.3264 | **417.6669** | **817.2999** | 409.1536 | 816.3159 | 408.6616 | P | 2262.1401 | **1131.5737** | 2245.1135 | **1123.0604** | 2244.1295 | **1122.5684** | 22 |
| 9 | 921.3585 | **461.1829** | 904.3319 | 452.6696 | 903.3479 | 452.1776 | S | 2165.0873 | **1083.0473** | 2148.0608 | 1074.5340 | 2147.0768 | 1074.0420 | 21 |
| 10 | 1068.4269 | 534.7171 | 1051.4003 | 526.2038 | **1050.4163** | 525.7118 | F | 2078.0553 | **1039.5313** | 2061.0287 | 1031.0180 | 2060.0447 | 1030.5260 | 20 |
| 11 | 1197.4695 | 599.2384 | 1180.4429 | 590.7251 | 1179.4589 | 590.2331 | E | **1930.9869** | **965.9971** | 1913.9603 | 957.4838 | 1912.9763 | 956.9918 | 19 |
| 12 | 1298.5171 | 649.7622 | 1281.4906 | 641.2489 | 1280.5066 | 640.7569 | T | **1801.9443** | **901.4758** | 1784.9177 | 892.9625 | 1783.9337 | 892.4705 | 18 |
| 13 | **1411.6012** | 706.3042 | 1394.5747 | 697.7910 | **1393.5906** | 697.2990 | I | **1700.8966** | **850.9519** | 1683.8701 | 842.4387 | 1682.8860 | 841.9467 | 17 |
| 14 | **1498.6332** | 749.8203 | 1481.6067 | 741.3070 | 1480.6227 | 740.8150 | S | **1587.8125** | **794.4099** | 1570.7860 | 785.8966 | **1569.8020** | 785.4046 | 16 |
| 15 | **1569.6703** | 785.3388 | 1552.6438 | 776.8255 | **1551.6598** | 776.3335 | A | **1500.7805** | 750.8939 | 1483.7540 | 742.3806 | 1482.7700 | 741.8886 | 15 |
| 16 | **1656.7024** | 828.8548 | 1639.6758 | 820.3416 | 1638.6918 | **819.8495** | S | **1429.7434** | **715.3753** | **1412.7169** | 706.8621 | **1411.7328** | 706.3701 | 14 |
| 17 | 1713.7238 | 857.3656 | 1696.6973 | 848.8523 | 1695.7133 | 848.3603 | G | 1342.7114 | 671.8593 | 1325.6848 | **663.3461** | 1324.7008 | **662.8540** | 13 |
| 18 | 1826.8079 | **913.9076** | **1809.7814** | 905.3943 | 1808.7973 | 904.9023 | L | 1285.6899 | 643.3486 | 1268.6634 | 634.8353 | 1267.6793 | 634.3433 | 12 |

| | | | | | | | | | | | | | | |
|---|---|---|---|---|---|---|---|---|---|---|---|---|---|---|
| 19 | 1940.8508 | 970.9291 | 1923.8243 | **962.4158** | 1922.8403 | **961.9238** | N | 1172.6058 | **586.8066** | 1155.5793 | 578.2933 | 1154.5953 | 577.8013 | 11 |
| 20 | 2011.8879 | 1006.4476 | 1994.8614 | 997.9343 | **1993.8774** | 997.4423 | A | **1058.5629** | 529.7851 | 1041.5364 | 521.2718 | 1040.5524 | **520.7798** | 10 |
| 21 | 2082.9251 | 1041.9662 | 2065.8985 | 1033.4529 | 2064.9145 | 1032.9609 | A | **987.5258** | 494.2665 | 970.4993 | 485.7533 | 969.5152 | 485.2613 | 9 |
| 22 | 2196.0091 | 1098.5082 | 2178.9826 | 1089.9949 | 2177.9986 | 1089.5029 | L | 916.4887 | 458.7480 | 899.4621 | 450.2347 | 898.4781 | 449.7427 | 8 |
| 23 | 2267.0462 | 1134.0268 | 2250.0197 | 1125.5135 | 2249.0357 | 1125.0215 | A | **803.4046** | 402.2060 | 786.3781 | 393.6927 | 785.3941 | 393.2007 | 7 |
| 24 | 2404.1052 | 1202.5562 | 2387.0786 | 1194.0429 | 2386.0946 | 1193.5509 | H | **732.3675** | **366.6874** | **715.3410** | 358.1741 | 714.3570 | 357.6821 | 6 |
| 25 | 2567.1685 | **1284.0879** | 2550.1419 | 1275.5746 | 2549.1579 | 1275.0826 | Y | **595.3086** | 298.1579 | 578.2821 | 289.6447 | 577.2980 | 289.1527 | 5 |
| 26 | 2654.2005 | 1327.6039 | 2637.1740 | 1319.0906 | 2636.1899 | 1318.5986 | S | **432.2453** | 216.6263 | 415.2187 | 208.1130 | 414.2347 | 207.6210 | 4 |
| 27 | 2751.2533 | 1376.1303 | 2734.2267 | 1367.6170 | 2733.2427 | 1367.1250 | P | **345.2132** | 173.1103 | 328.1867 | 164.5970 | 327.2027 | 164.1050 | 3 |
| 28 | 2852.3010 | 1426.6541 | 2835.2744 | 1418.1408 | 2834.2904 | 1417.6488 | T | 248.1605 | 124.5839 | 231.1339 | 116.0706 | 230.1499 | 115.5786 | 2 |
| 29 | | | | | | | K | 147.1128 | 74.0600 | 130.0863 | 65.5468 | | | 1 |

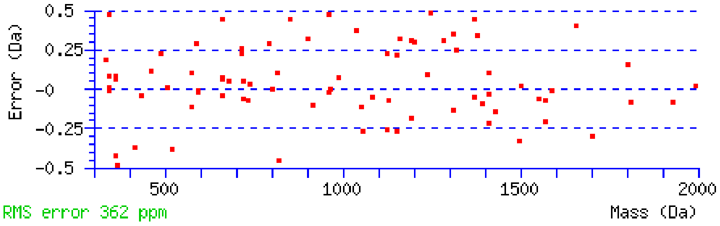


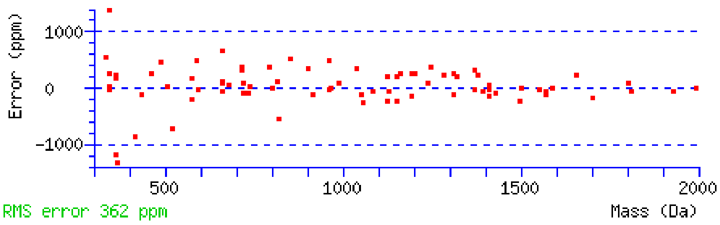


**All matches to this query**

| Score | Mr(calc): | Delta | Sequence |
|---|---|---|---|
| 64.2 | 2997.3992 | 0.0102 | NENFSSGPSFETISASGLNAALAHYSPTK |
| 57.5 | 2997.3992 | 0.0102 | NENFSSGPSFETISASGLNAALAHYSPTK |
| 41.7 | 2997.3992 | 0.0102 | NENFSSGPSFETISASGLNAALAHYSPTK |
| 37.1 | 2996.4152 | 0.9942 | NENFSSGPSFETISASGLNAALAHYSPTK |
| 7.4 | 2995.3798 | 2.0296 | GGVMGAPSTRAEECTACKTLDLGTGEAR |
| 4.6 | 2997.3868 | 0.0225 | EFVAQPNCQQLLASRWYDEFPGWR |
| 3.5 | 2995.3782 | 2.0311 | STECLSCLILSPNGSPKPVMASYLIY |
| 3.5 | 2995.3782 | 2.0311 | STECLSCLILSPNGSPKPVMASYLIY |
| 3.5 | 2995.3782 | 2.0311 | STECLSCLILSPNGSPKPVMASYLIY |
| 2.6 | 2997.3919 | 0.0174 | VVATNPIGTGDPSIPSRMIRTNEAVPK |

---

Spectrum No: 453; Query: 2548; Rank: 1

---

## Peptide View

MS/MS Fragmentation of **AGVLADDHLIEVNGENVENASHEEVVEK**
Found in **IPI00200998**, Tax_Id=10116 Gene_Symbol=Pdzk1 PDZ domain-containing protein 1

Match to Query 2548: 3016.438332 from(1006.486720,3+)
Title: 100101RatKid_NS_deglyco_12.3427.3427.3.dta
Data file K:\NewmanPaper\Piliang\3SubProteomes\Piliang3SP\mgf5ppm\ERLIC_3SubProteomes5ppm.mgf

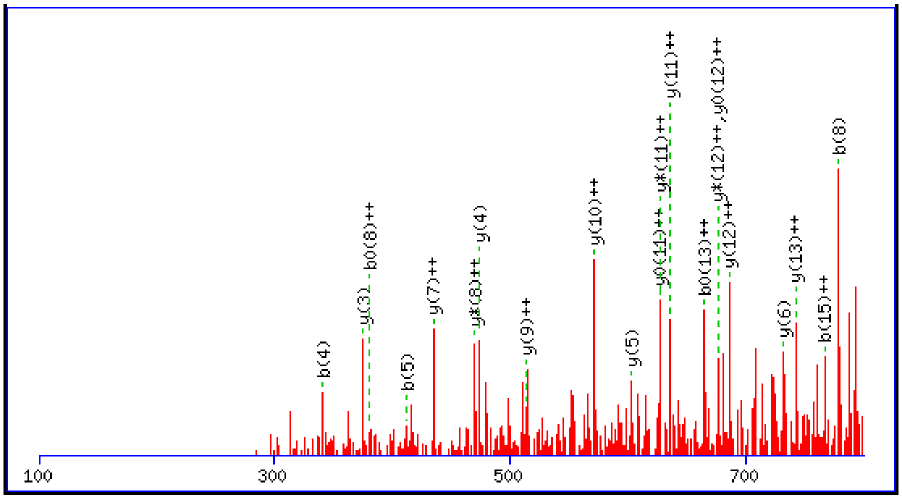


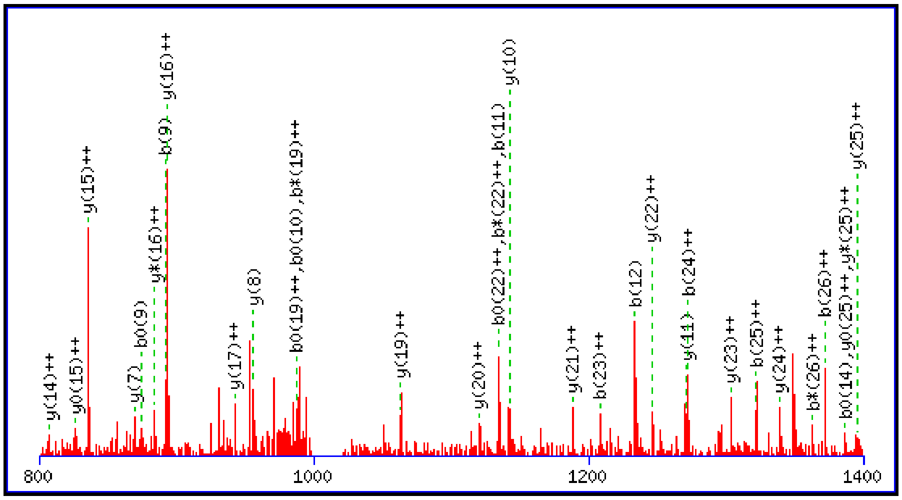


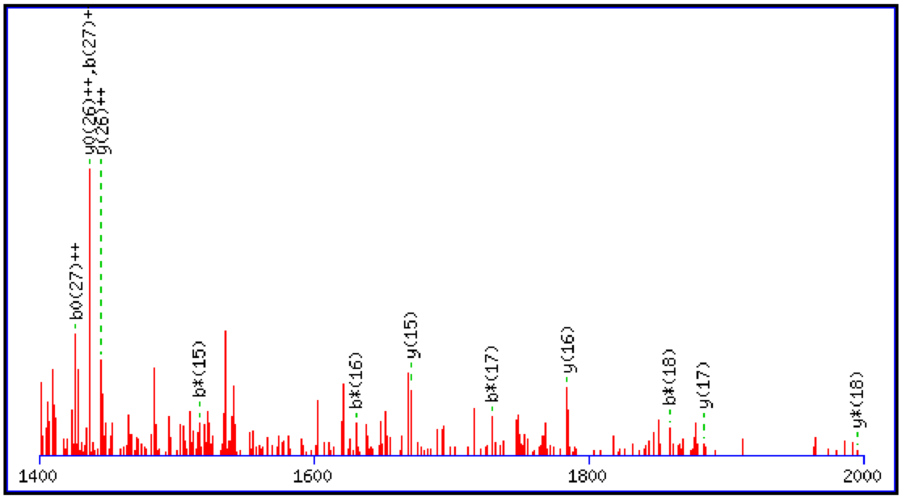


**Monoisotopic mass of neutral peptide Mr(calc):** 3016.4261
**Fixed modifications:** Carbamidomethyl (C)
**Variable modifications:**
**N19** : Deamidated_N (N)
**Ions Score:** 64 **Expect:** 0.00012
**Matches (Bold Red):** 68/288 fragment ions using 165 most intense peaks

| # | b | $b^{++}$ | b* | $b^{*++}$ | $b^0$ | $b^{0++}$ | Seq. | y | $y^{++}$ | y* | $y^{*++}$ | $y^0$ | $y^{0++}$ | # |
|---|---|---|---|---|---|---|---|---|---|---|---|---|---|---|
| 1 | 72.0444 | 36.5258 | | | | | A | | | | | | | 28 |
| 2 | 129.0659 | 65.0366 | | | | | G | 2946.3963 | 1473.7018 | 2929.3698 | 1465.1885 | 2928.3857 | 1464.6965 | 27 |
| 3 | 228.1343 | 114.5708 | | | | | V | 2889.3748 | **1445.1911** | 2872.3483 | **1436.6778** | 2871.3643 | **1436.1858** | 26 |
| 4 | **341.2183** | 171.1128 | | | | | L | 2790.3064 | **1395.6569** | 2773.2799 | **1387.1436** | 2772.2959 | **1386.6516** | 25 |
| 5 | **412.2554** | 206.6314 | | | | | A | 2677.2224 | **1339.1148** | 2660.1958 | 1330.6015 | 2659.2118 | 1330.1095 | 24 |

| | | | | | | | | | | | | | | |
|---|---|---|---|---|---|---|---|---|---|---|---|---|---|---|
| 6 | 527.2824 | 264.1448 | | | 509.2718 | 255.1396 | D | 2606.1853 | 1303.5963 | 2589.1587 | 1295.0830 | 2588.1747 | 1294.5910 | 23 |
| 7 | 642.3093 | 321.6583 | | | 624.2988 | 312.6530 | D | 2491.1583 | 1246.0828 | 2474.1318 | 1237.5695 | 2473.1477 | 1237.0775 | 22 |
| 8 | 779.3682 | 390.1878 | | | 761.3577 | 381.1825 | H | 2376.1314 | 1188.5693 | 2359.1048 | 1180.0560 | 2358.1208 | 1179.5640 | 21 |
| 9 | 892.4523 | 446.7298 | | | 874.4417 | 437.7245 | L | 2239.0725 | 1120.0399 | 2222.0459 | 1111.5266 | 2221.0619 | 1111.0346 | 20 |
| 10 | 1005.5364 | 503.2718 | | | 987.5258 | 494.2665 | I | 2125.9884 | 1063.4978 | 2108.9618 | 1054.9846 | 2107.9778 | 1054.4926 | 19 |
| 11 | 1134.5790 | 567.7931 | | | 1116.5684 | 558.7878 | E | 2012.9043 | 1006.9558 | 1995.8778 | 998.4425 | 1994.8938 | 997.9505 | 18 |
| 12 | 1233.6474 | 617.3273 | | | 1215.6368 | 608.3220 | V | 1883.8617 | 942.4345 | 1866.8352 | 933.9212 | 1865.8512 | 933.4292 | 17 |
| 13 | 1347.6903 | 674.3488 | 1330.6638 | 665.8355 | 1329.6797 | 665.3435 | N | 1784.7933 | 892.9003 | 1767.7668 | 884.3870 | 1766.7828 | 883.8950 | 16 |
| 14 | 1404.7118 | 702.8595 | 1387.6852 | 694.3462 | 1386.7012 | 693.8542 | G | 1670.7504 | 835.8788 | 1653.7238 | 827.3656 | 1652.7398 | 826.8736 | 15 |
| 15 | 1533.7544 | 767.3808 | 1516.7278 | 758.8675 | 1515.7438 | 758.3755 | E | 1613.7289 | 807.3681 | 1596.7024 | 798.8548 | 1595.7184 | 798.3628 | 14 |
| 16 | 1647.7973 | 824.4023 | 1630.7707 | 815.8890 | 1629.7867 | 815.3970 | N | 1484.6863 | 742.8468 | 1467.6598 | 734.3335 | 1466.6758 | 733.8415 | 13 |
| 17 | 1746.8657 | 873.9365 | 1729.8392 | 865.4232 | 1728.8551 | 864.9312 | V | 1370.6434 | 685.8253 | 1353.6169 | 677.3121 | 1352.6328 | 676.8201 | 12 |
| 18 | 1875.9083 | 938.4578 | 1858.8817 | 929.9445 | 1857.8977 | 929.4525 | E | 1271.5750 | 636.2911 | 1254.5484 | 627.7779 | 1253.5644 | 627.2859 | 11 |
| 19 | 1990.9352 | 995.9713 | 1973.9087 | 987.4580 | 1972.9247 | 986.9660 | N | 1142.5324 | 571.7698 | 1125.5059 | 563.2566 | 1124.5218 | 562.7646 | 10 |
| 20 | 2061.9723 | 1031.4898 | 2044.9458 | 1022.9765 | 2043.9618 | 1022.4845 | A | 1027.5055 | 514.2564 | 1010.4789 | 505.7431 | 1009.4949 | 505.2511 | 9 |
| 21 | 2149.0044 | 1075.0058 | 2131.9778 | 1066.4926 | 2130.9938 | 1066.0005 | S | 956.4684 | 478.7378 | 939.4418 | 470.2245 | 938.4578 | 469.7325 | 8 |
| 22 | 2286.0633 | 1143.5353 | 2269.0367 | 1135.0220 | 2268.0527 | 1134.5300 | H | 869.4363 | 435.2218 | 852.4098 | 426.7085 | 851.4258 | 426.2165 | 7 |
| 23 | 2415.1059 | 1208.0566 | 2398.0793 | 1199.5433 | 2397.0953 | 1199.0513 | E | 732.3774 | 366.6923 | 715.3509 | 358.1791 | 714.3668 | 357.6871 | 6 |
| 24 | 2544.1485 | 1272.5779 | 2527.1219 | 1264.0646 | 2526.1379 | 1263.5726 | E | 603.3348 | 302.1710 | 586.3083 | 293.6578 | 585.3243 | 293.1658 | 5 |
| 25 | 2643.2169 | 1322.1121 | 2626.1903 | 1313.5988 | 2625.2063 | 1313.1068 | V | 474.2922 | 237.6498 | 457.2657 | 229.1365 | 456.2817 | 228.6445 | 4 |
| 26 | 2742.2853 | 1371.6463 | 2725.2588 | 1363.1330 | 2724.2747 | 1362.6410 | V | 375.2238 | 188.1155 | 358.1973 | 179.6023 | 357.2132 | 179.1103 | 3 |
| 27 | 2871.3279 | 1436.1676 | 2854.3013 | 1427.6543 | 2853.3173 | 1427.1623 | E | 276.1554 | 138.5813 | 259.1288 | 130.0681 | 258.1448 | 129.5761 | 2 |
| 28 | | | | | | | K | 147.1128 | 74.0600 | 130.0863 | 65.5468 | | | 1 |

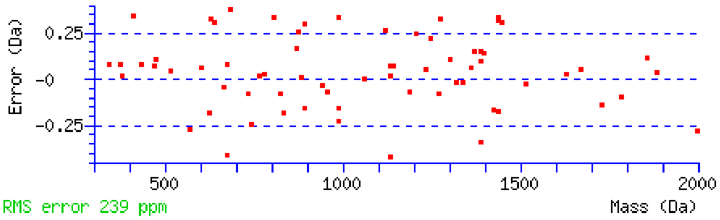


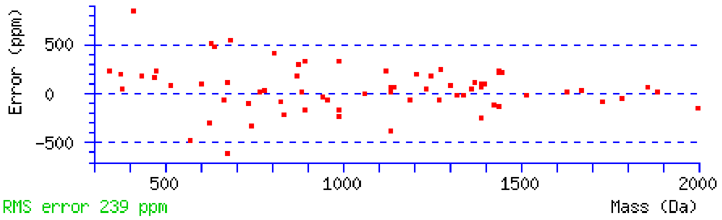


### All matches to this query

| Score | Mr(calc): | Delta | Sequence |
|---|---|---|---|
| 64.1 | 3016.4261 | 0.0122 | AGVLADDHLIEVNGENVENASHEEVVEK |
| 61.3 | 3016.4261 | 0.0122 | AGVLADDHLIEVNGENVENASHEEVVEK |
| 58.1 | 3016.4261 | 0.0122 | AGVLADDHLIEVNGENVENASHEEVVEK |
| 22.6 | 3015.4421 | 0.9962 | AGVLADDHLIEVNGENVENASHEEVVEK |
| 4.2 | 3016.4091 | 0.0292 | ISLFGNDVLRKMSIIQIMIDNYQK |
| 4.0 | 3016.4253 | 0.0131 | MTTDTGTIPFAPNLEDINNILESKFR |
| 4.0 | 3016.4253 | 0.0131 | MTTDTGTIPFAPNLEDINNILESKFR |
| 4.0 | 3016.4253 | 0.0131 | MTTDTGTIPFAPNLEDINNILESKFR |
| 4.0 | 3016.4253 | 0.0131 | MTTDTGTIPFAPNLEDINNILESKFR |
| 3.7 | 3016.4091 | 0.0292 | ISLFGNDVLRKMSIIQIMIDNYQK |

Spectrum No: 454; Query: 319; Rank: 1

## Peptide View

MS/MS Fragmentation of **ALSPNSTISSAPK**

Found in **IPI00208721**, Tax_Id=10116 Gene_Symbol=RGD1563141_predicted 78 kDa protein

Match to Query 319: 1272.652628 from(637.333590,2+)
Title: 091008RatKidney_NH4Format01_24.1085.1085.2.dta
Data file K:\NewmanPaper\Piliang\3SubProteomes\Piliang3SP\mgf5ppm\ERLIC_3SubProteomes5ppm.mgf

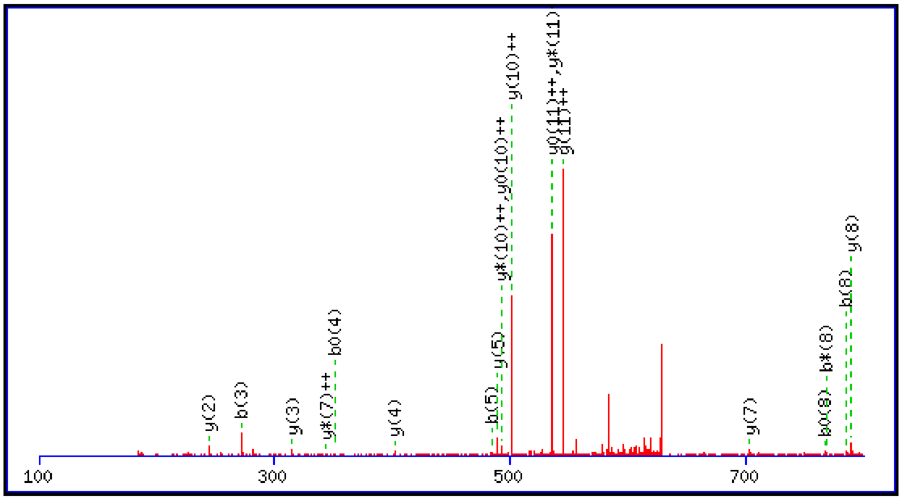


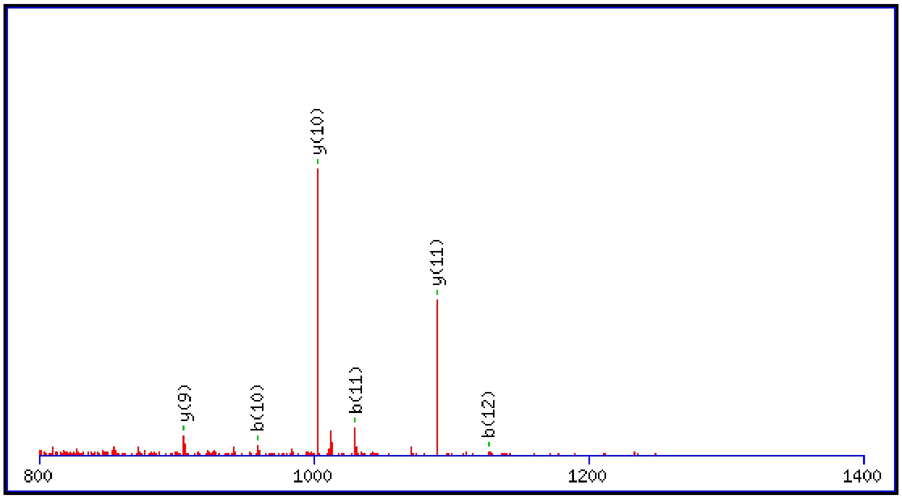


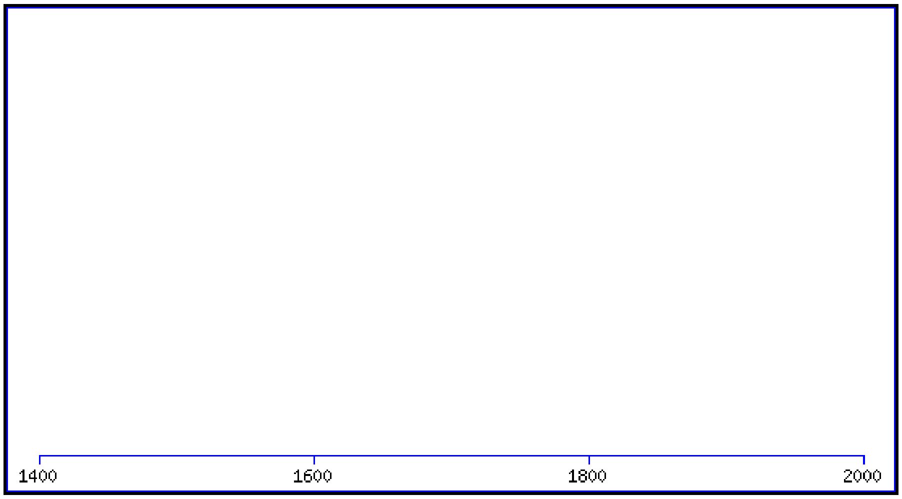


**Monoisotopic mass of neutral peptide Mr(calc):** 1272.6561
**Fixed modifications:** Carbamidomethyl (C)
**Variable modifications:**
**N5** : Deamidated_N (N)
**Ions Score:** 64 **Expect:** 4.4e-005
**Matches (Bold Red):** 25/126 fragment ions using 43 most intense peaks

| # | b | b++ | b* | b*++ | b0 | b0++ | Seq. | y | y++ | y* | y*++ | y0 | y0++ | # |
|---|---|---|---|---|---|---|---|---|---|---|---|---|---|---|
| 1 | 72.0444 | 36.5258 | | | | | A | | | | | | | 13 |

| | | | | | | | | | | | | | | |
|---|---|---|---|---|---|---|---|---|---|---|---|---|---|---|
| **2** | 185.1285 | 93.0679 | | | | | **L** | 1202.6263 | 601.8168 | 1185.5998 | 593.3035 | 1184.6157 | 592.8115 | **12** |
| **3** | **272.1605** | 136.5839 | | | 254.1499 | 127.5786 | **S** | **1089.5422** | **545.2748** | 1072.5157 | **536.7615** | 1071.5317 | **536.2695** | **11** |
| **4** | 369.2132 | 185.1103 | | | **351.2027** | 176.1050 | **P** | **1002.5102** | **501.7587** | 985.4837 | **493.2455** | 984.4996 | **492.7535** | **10** |
| **5** | **484.2402** | 242.6237 | 467.2136 | 234.1105 | 466.2296 | 233.6184 | **N** | **905.4574** | 453.2324 | 888.4309 | 444.7191 | 887.4469 | 444.2271 | **9** |
| **6** | 571.2722 | 286.1397 | 554.2457 | 277.6265 | 553.2616 | 277.1345 | **S** | **790.4305** | 395.7189 | 773.4040 | 387.2056 | 772.4199 | 386.7136 | **8** |
| **7** | 672.3199 | 336.6636 | 655.2933 | 328.1503 | 654.3093 | 327.6583 | **T** | **703.3985** | 352.2029 | 686.3719 | **343.6896** | 685.3879 | 343.1976 | **7** |
| **8** | **785.4040** | 393.2056 | **768.3774** | 384.6923 | **767.3934** | 384.2003 | **I** | 602.3508 | 301.6790 | 585.3243 | 293.1658 | 584.3402 | 292.6738 | **6** |
| **9** | 872.4360 | 436.7216 | 855.4094 | 428.2084 | 854.4254 | 427.7163 | **S** | **489.2667** | 245.1370 | 472.2402 | 236.6237 | 471.2562 | 236.1317 | **5** |
| **10** | **959.4680** | 480.2376 | 942.4415 | 471.7244 | 941.4574 | 471.2324 | **S** | **402.2347** | 201.6210 | 385.2082 | 193.1077 | 384.2241 | 192.6157 | **4** |
| **11** | **1030.5051** | 515.7562 | 1013.4786 | 507.2429 | 1012.4946 | 506.7509 | **A** | **315.2027** | 158.1050 | 298.1761 | 149.5917 | | | **3** |
| **12** | **1127.5579** | 564.2826 | 1110.5313 | 555.7693 | 1109.5473 | 555.2773 | **P** | **244.1656** | 122.5864 | 227.1390 | 114.0731 | | | **2** |
| **13** | | | | | | | **K** | 147.1128 | 74.0600 | 130.0863 | 65.5468 | | | **1** |

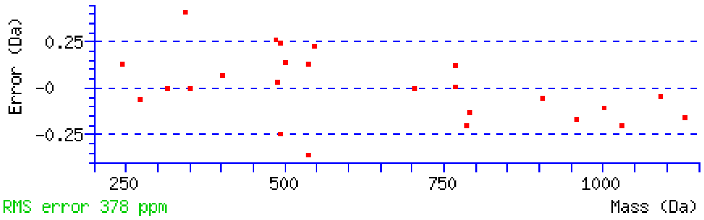


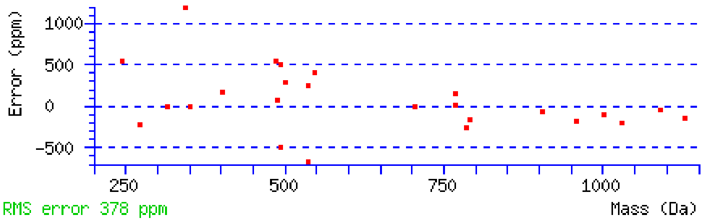


### All matches to this query

| Score | Mr(calc): | Delta | Sequence |
|---|---|---|---|
| 64.1 | 1272.6561 | -0.0035 | ALSPNSTISSAPK |
| 8.9 | 1272.6462 | 0.0064 | LADELSGWVQR |
| 8.8 | 1272.6591 | -0.0065 | GTLKRFSSLGK |
| 6.9 | 1272.6509 | 0.0017 | SPGNKCRLXR |
| 5.9 | 1271.6387 | 1.0139 | NRPNLHILSK |
| 5.5 | 1272.6536 | -0.0010 | LAVTALCWNPK |
| 2.6 | 1272.6575 | -0.0048 | SPKYHSSSKPR |
| 1.0 | 1272.6422 | 0.0104 | LANQKSSNPTGR |
| 0.3 | 1272.6422 | 0.0104 | AIERERDTVQG |

---

Spectrum No: 455; Query: 2302; Rank: 1

---

## Peptide View

MS/MS Fragmentation of **LANASLYLAEWQNITKNFSEDR**
Found in **IPI00211616**, Tax_Id=10116 Gene_Symbol=Slc3a2 4F2 cell-surface antigen heavy chain

Match to Query 2302: 2585.232462 from(862.751430,3+)
Title: 100101RatKid_NS_deglyco_22.5849.5849.3.dta
Data file K:\NewmanPaper\Piliang\3SubProteomes\Piliang3SP\mgf5ppm\ERLIC_3SubProteomes5ppm.mgf

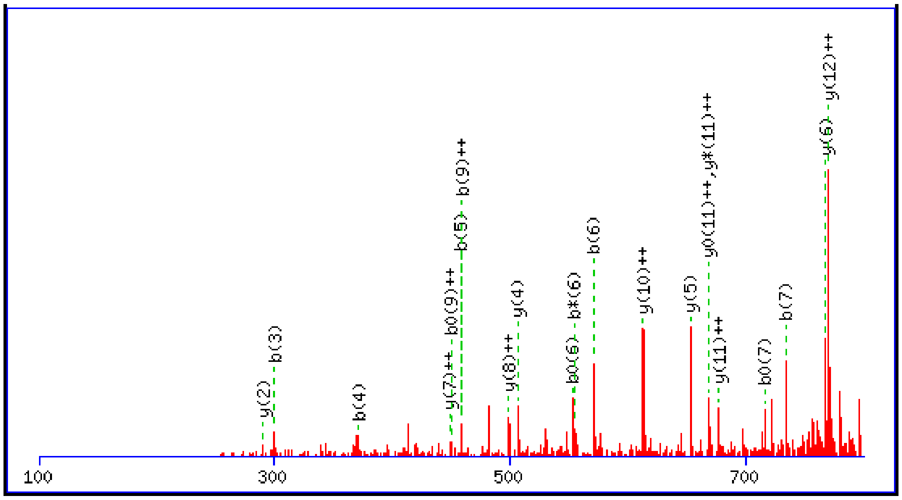


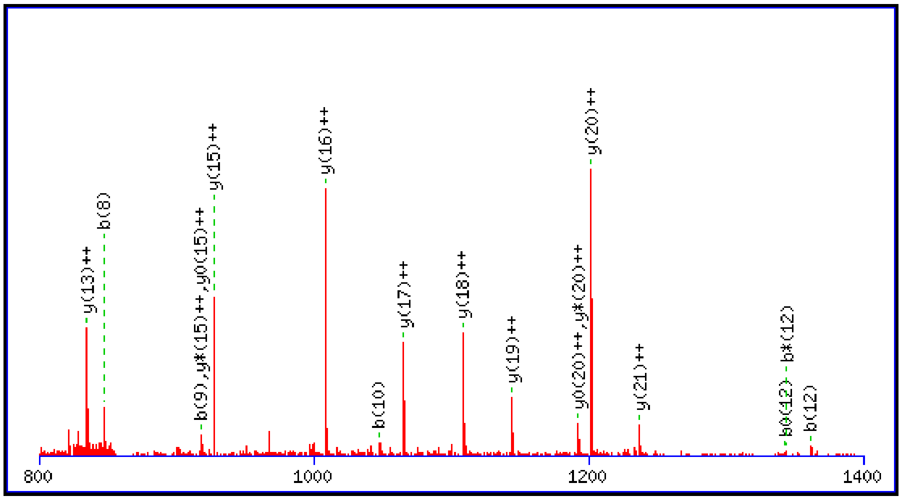


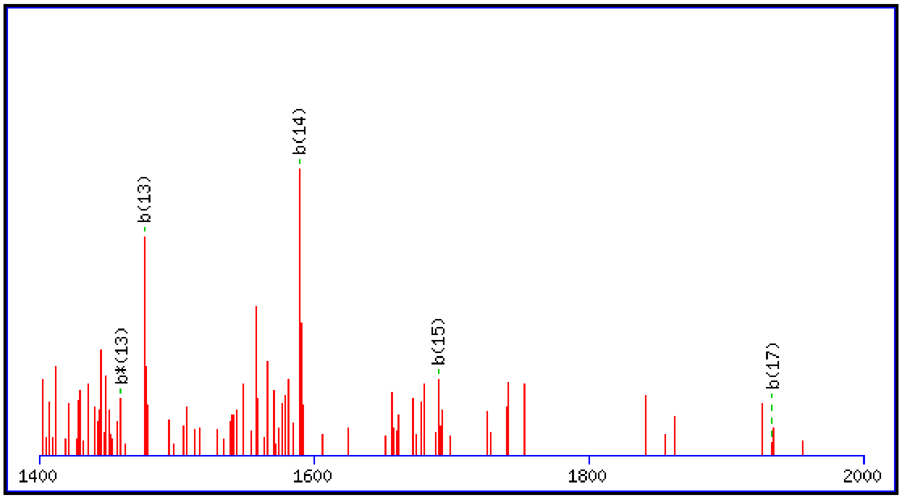


**Monoisotopic mass of neutral peptide Mr(calc):** 2585.2285
**Fixed modifications:** Carbamidomethyl (C)
**Variable modifications:**
**N3** : Deamidated_N (N)
**N13** : Deamidated_N (N)
**N17** : Deamidated_N (N)
**Ions Score:** 64 **Expect:** 0.00011
**Matches (Bold Red):** 44/238 fragment ions using 86 most intense peaks

| # | b | $b^{++}$ | b* | $b^{*++}$ | $b^0$ | $b^{0++}$ | Seq. | y | $y^{++}$ | y* | $y^{*++}$ | $y^0$ | $y^{0++}$ | # |
|---|---|---|---|---|---|---|---|---|---|---|---|---|---|---|
| 1 | 114.0913 | 57.5493 | | | | | L | | | | | | | 22 |
| 2 | 185.1285 | 93.0679 | | | | | A | 2473.1518 | **1237.0795** | 2456.1252 | 1228.5662 | 2455.1412 | 1228.0742 | 21 |
| 3 | **300.1554** | 150.5813 | 283.1288 | 142.0681 | | | N | 2402.1146 | **1201.5610** | 2385.0881 | **1193.0477** | 2384.1041 | **1192.5557** | 20 |
| 4 | **371.1925** | 186.0999 | 354.1660 | 177.5866 | | | A | 2287.0877 | **1144.0475** | 2270.0612 | 1135.5342 | 2269.0771 | 1135.0422 | 19 |

| | | | | | | | | | | | | | | |
|---|---|---|---|---|---|---|---|---|---|---|---|---|---|---|
| 5 | **458.2245** | 229.6159 | 441.1980 | 221.1026 | 440.2140 | 220.6106 | S | 2216.0506 | **1108.5289** | 2199.0240 | 1100.0157 | 2198.0400 | 1099.5237 | 18 |
| 6 | **571.3086** | 286.1579 | **554.2820** | 277.6447 | **553.2980** | 277.1527 | L | 2129.0186 | **1065.0129** | 2111.9920 | 1056.4996 | 2111.0080 | 1056.0076 | 17 |
| 7 | **734.3719** | 367.6896 | 717.3454 | 359.1763 | **716.3614** | 358.6843 | Y | 2015.9345 | **1008.4709** | 1998.9079 | 999.9576 | 1997.9239 | 999.4656 | 16 |
| 8 | **847.4560** | 424.2316 | 830.4294 | 415.7184 | 829.4454 | 415.2264 | L | 1852.8712 | **926.9392** | 1835.8446 | **918.4259** | 1834.8606 | **917.9339** | 15 |
| 9 | **918.4931** | **459.7502** | 901.4666 | 451.2369 | 900.4825 | **450.7449** | A | 1739.7871 | 870.3972 | 1722.7606 | 861.8839 | 1721.7765 | 861.3919 | 14 |
| 10 | **1047.5357** | 524.2715 | 1030.5091 | 515.7582 | 1029.5251 | 515.2662 | E | 1668.7500 | **834.8786** | 1651.7234 | 826.3654 | 1650.7394 | 825.8734 | 13 |
| 11 | 1233.6150 | 617.3111 | 1216.5885 | 608.7979 | 1215.6044 | 608.3059 | W | 1539.7074 | **770.3573** | 1522.6808 | 761.8441 | 1521.6968 | 761.3521 | 12 |
| 12 | **1361.6736** | 681.3404 | **1344.6470** | 672.8272 | **1343.6630** | 672.3351 | Q | 1353.6281 | **677.3177** | 1336.6015 | **668.8044** | 1335.6175 | **668.3124** | 11 |
| 13 | **1476.7005** | 738.8539 | **1459.6740** | 730.3406 | 1458.6900 | 729.8486 | N | 1225.5695 | **613.2884** | 1208.5430 | 604.7751 | 1207.5589 | 604.2831 | 10 |
| 14 | **1589.7846** | 795.3959 | 1572.7580 | 786.8827 | 1571.7740 | 786.3906 | I | 1110.5426 | 555.7749 | 1093.5160 | 547.2616 | 1092.5320 | 546.7696 | 9 |
| 15 | **1690.8323** | 845.9198 | 1673.8057 | 837.4065 | 1672.8217 | 836.9145 | T | 997.4585 | **499.2329** | 980.4320 | 490.7196 | 979.4479 | 490.2276 | 8 |
| 16 | 1818.9272 | 909.9673 | 1801.9007 | 901.4540 | 1800.9167 | 900.9620 | K | 896.4108 | **448.7091** | 879.3843 | 440.1958 | 878.4003 | 439.7038 | 7 |
| 17 | **1933.9542** | 967.4807 | 1916.9276 | 958.9674 | 1915.9436 | 958.4754 | N | **768.3159** | 384.6616 | 751.2893 | 376.1483 | 750.3053 | 375.6563 | 6 |
| 18 | 2081.0226 | 1041.0149 | 2063.9960 | 1032.5017 | 2063.0120 | 1032.0096 | F | **653.2889** | 327.1481 | 636.2624 | 318.6348 | 635.2784 | 318.1428 | 5 |
| 19 | 2168.0546 | 1084.5309 | 2151.0281 | 1076.0177 | 2150.0440 | 1075.5257 | S | **506.2205** | 253.6139 | 489.1940 | 245.1006 | 488.2100 | 244.6086 | 4 |
| 20 | 2297.0972 | 1149.0522 | 2280.0707 | 1140.5390 | 2279.0866 | 1140.0470 | E | 419.1885 | 210.0979 | 402.1619 | 201.5846 | 401.1779 | 201.0926 | 3 |
| 21 | 2412.1241 | 1206.5657 | 2395.0976 | 1198.0524 | 2394.1136 | 1197.5604 | D | **290.1459** | 145.5766 | 273.1193 | 137.0633 | 272.1353 | 136.5713 | 2 |
| 22 | | | | | | | R | 175.1190 | 88.0631 | 158.0924 | 79.5498 | | | 1 |

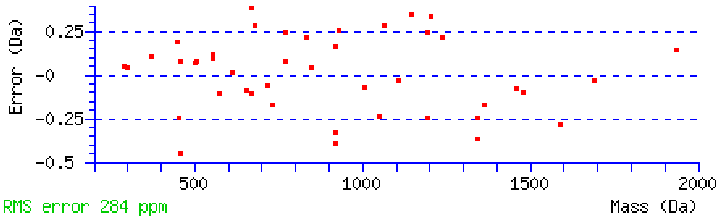


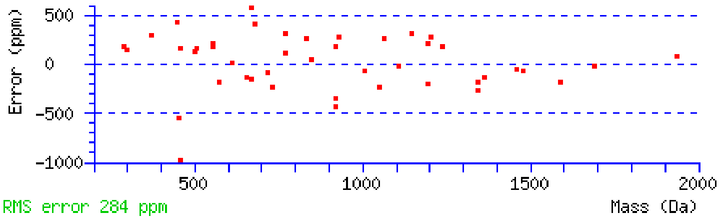


### All matches to this query

| Score | Mr(calc): | Delta | Sequence |
|---|---|---|---|
| 64.1 | 2585.2285 | 0.0040 | LANASLYLAEWQNITKNFSEDR |
| 31.3 | 2584.2445 | 0.9880 | LANASLYLAEWQNITKNFSEDR |
| 27.4 | 2584.2445 | 0.9880 | LANASLYLAEWQNITKNFSEDR |
| 27.1 | 2584.2445 | 0.9880 | LANASLYLAEWQNITKNFSEDR |
| 1.8 | 2583.2023 | 2.0302 | KGTELLLGVDALGLHIYDPENR |
| 0.2 | 2583.2459 | 1.9866 | DIKDTTVGTLSQRITNQVHGLK |
| 0.1 | 2584.2329 | 0.9995 | QALQRHVLSCAARSNLQDSPPR |

---

Spectrum No: 456; Query: 999; Rank: 1

---

## Peptide View

MS/MS Fragmentation of **WSTGLAHNSSAITYR**
Found in **IPI00358382**, Tax_Id=10116 Gene_Symbol=C8a_predicted complement component 8, alpha polypeptide

Match to Query 999: 1663.800728 from(832.907640,2+)
Title: 100101RatKid_NS_deglyco_23.2136.2136.2.dta
Data file K:\NewmanPaper\Piliang\3SubProteomes\Piliang3SP\mgf5ppm\ERLIC_3SubProteomes5ppm.mgf

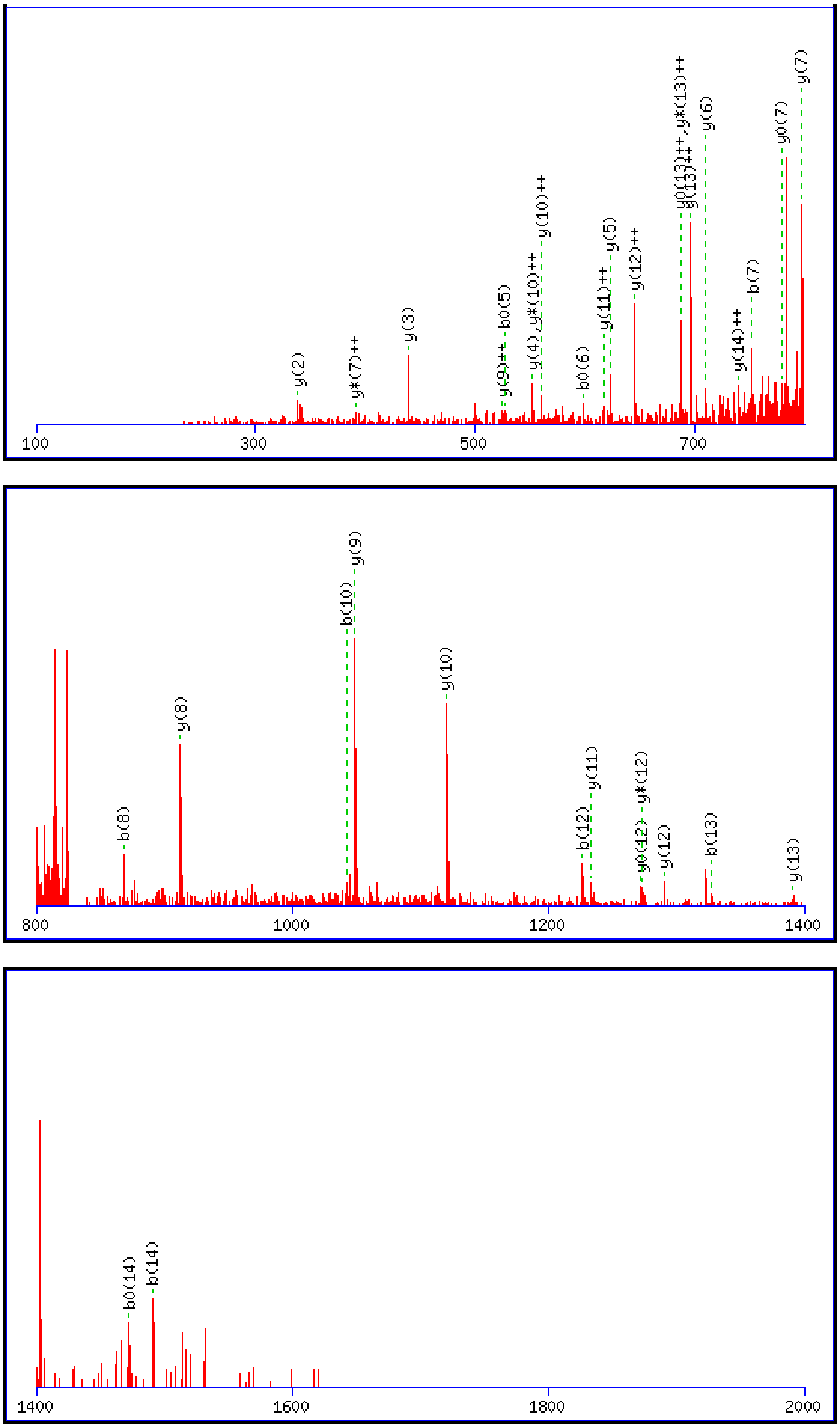


**Monoisotopic mass of neutral peptide Mr(calc):** 1663.7954
**Fixed modifications:** Carbamidomethyl (C)
**Variable modifications:**
**N8** : Deamidated_N (N)
**Ions Score:** 64 **Expect:** 7.4e-005
**Matches (Bold Red):** 34/148 fragment ions using 85 most intense peaks

| # | b | b$^{++}$ | b* | b*$^{++}$ | b$^{0}$ | b$^{0++}$ | Seq. | y | y$^{++}$ | y* | y*$^{++}$ | y$^{0}$ | y$^{0++}$ | # |
|---|---|---|---|---|---|---|---|---|---|---|---|---|---|---|
| 1 | 187.0866 | 94.0469 | | | | | W | | | | | | | 15 |
| 2 | 274.1186 | 137.5629 | | | 256.1081 | 128.5577 | S | 1478.7234 | **739.8653** | 1461.6968 | 731.3521 | 1460.7128 | 730.8601 | 14 |
| 3 | 375.1663 | 188.0868 | | | 357.1557 | 179.0815 | T | **1391.6914** | **696.3493** | 1374.6648 | **687.8360** | 1373.6808 | **687.3440** | 13 |
| 4 | 432.1878 | 216.5975 | | | 414.1772 | 207.5922 | G | **1290.6437** | **645.8255** | **1273.6171** | 637.3122 | **1272.6331** | 636.8202 | 12 |
| 5 | 545.2718 | 273.1396 | | | **527.2613** | 264.1343 | L | **1233.6222** | **617.3147** | 1216.5957 | 608.8015 | 1215.6117 | 608.3095 | 11 |

| | | | | | | | | | | | | | | |
|---|---|---|---|---|---|---|---|---|---|---|---|---|---|---|
| 6 | 616.3089 | 308.6581 | | | 598.2984 | 299.6528 | A | 1120.5382 | 560.7727 | 1103.5116 | 552.2594 | 1102.5276 | 551.7674 | 10 |
| 7 | 753.3678 | 377.1876 | | | 735.3573 | 368.1823 | H | 1049.5010 | 525.2542 | 1032.4745 | 516.7409 | 1031.4905 | 516.2489 | 9 |
| 8 | 868.3948 | 434.7010 | 851.3682 | 426.1878 | 850.3842 | 425.6957 | N | 912.4421 | 456.7247 | 895.4156 | 448.2114 | 894.4316 | 447.7194 | 8 |
| 9 | 955.4268 | 478.2170 | 938.4003 | 469.7038 | 937.4162 | 469.2118 | S | 797.4152 | 399.2112 | 780.3886 | 390.6980 | 779.4046 | 390.2060 | 7 |
| 10 | 1042.4588 | 521.7331 | 1025.4323 | 513.2198 | 1024.4483 | 512.7278 | S | 710.3832 | 355.6952 | 693.3566 | 347.1819 | 692.3726 | 346.6899 | 6 |
| 11 | 1113.4960 | 557.2516 | 1096.4694 | 548.7383 | 1095.4854 | 548.2463 | A | 623.3511 | 312.1792 | 606.3246 | 303.6659 | 605.3406 | 303.1739 | 5 |
| 12 | 1226.5800 | 613.7936 | 1209.5535 | 605.2804 | 1208.5695 | 604.7884 | I | 552.3140 | 276.6607 | 535.2875 | 268.1474 | 534.3035 | 267.6554 | 4 |
| 13 | 1327.6277 | 664.3175 | 1310.6012 | 655.8042 | 1309.6171 | 655.3122 | T | 439.2300 | 220.1186 | 422.2034 | 211.6053 | 421.2194 | 211.1133 | 3 |
| 14 | 1490.6910 | 745.8492 | 1473.6645 | 737.3359 | 1472.6805 | 736.8439 | Y | 338.1823 | 169.5948 | 321.1557 | 161.0815 | | | 2 |
| 15 | | | | | | | R | 175.1190 | 88.0631 | 158.0924 | 79.5498 | | | 1 |

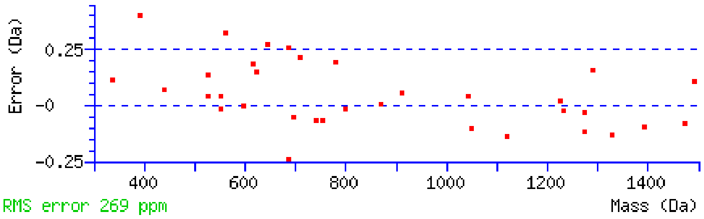


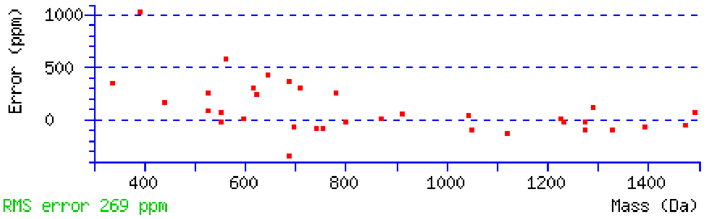


### All matches to this query

| Score | Mr(calc): | Delta | Sequence |
|---|---|---|---|
| 64.1 | 1663.7954 | 0.0053 | WSTGLAHNSSAITYR |
| 5.9 | 1663.7963 | 0.0044 | GGFCNFMHLKPISR |
| 5.8 | 1663.7970 | 0.0037 | QYQNILKSGTLYR |
| 5.0 | 1662.7991 | 1.0016 | SPRSPGKSPPPSHPR |
| 4.7 | 1662.8130 | 0.9877 | QYQNILKSGTLYR |
| 4.0 | 1662.7991 | 1.0016 | SPRSPGKSPPPSHPR |
| 2.8 | 1663.8070 | -0.0062 | GDVDISLPKVEGDLK |
| 2.8 | 1663.8070 | -0.0062 | GDVDLSLPKVEGDLK |
| 0.9 | 1663.7978 | 0.0030 | RMXRDGAHVVISSR |

---

Spectrum No: 457; Query: 238; Rank: 1

---

## Peptide View

MS/MS Fragmentation of **MLEAYNLTEK**
Found in **IPI00189714**, Tax_Id=10116 Gene_Symbol=Col12a1 similar to Collagen alpha-1(XII) chain precursor

Match to Query 238: 1211.574308 from(606.794430,2+)
Title: 091008RatKidney_NoSalt_25.1910.1910.2.dta
Data file K:\NewmanPaper\Piliang\3SubProteomes\Piliang3SP\mgf5ppm\ERLIC_3SubProteomes5ppm.mgf

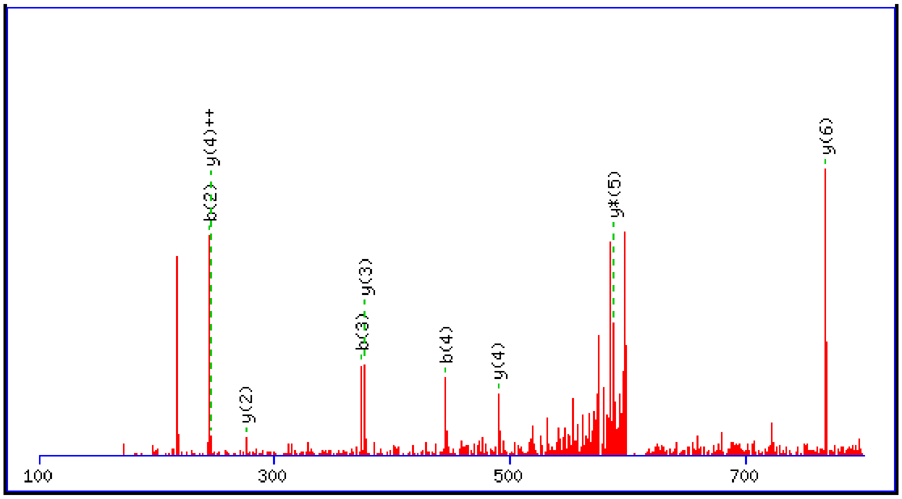


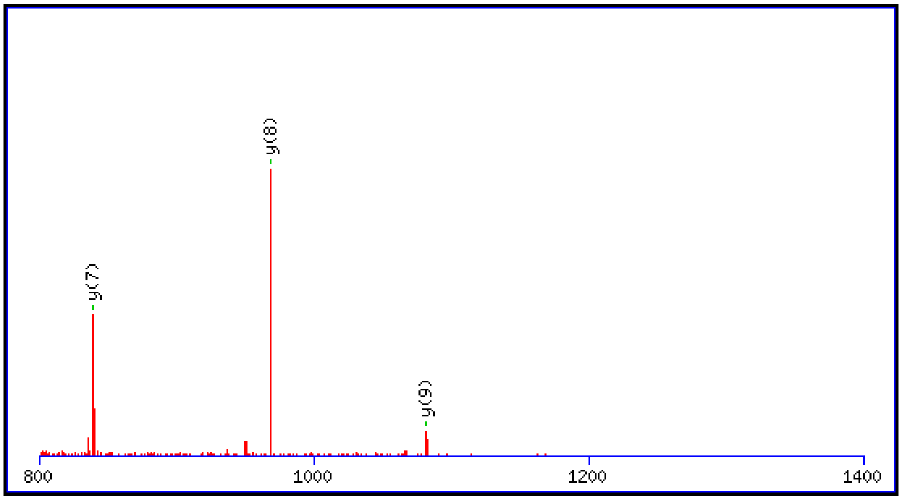


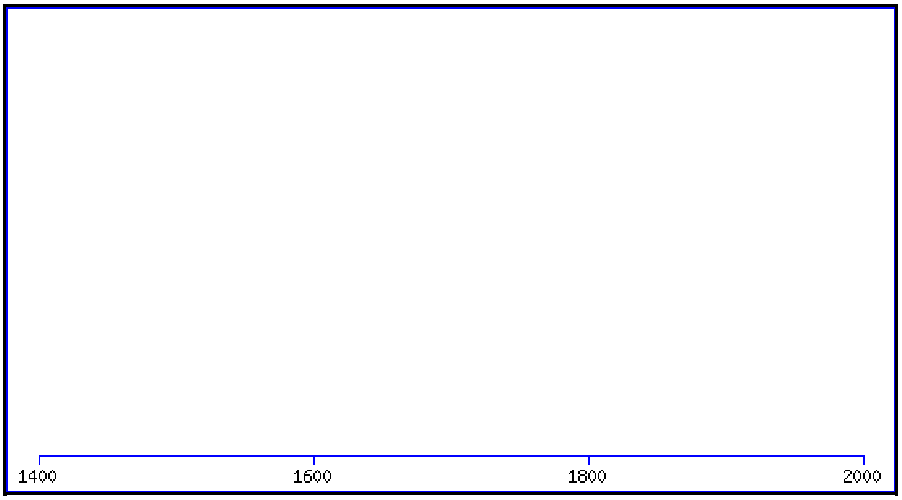


**Monoisotopic mass of neutral peptide Mr(calc):** 1211.5743
**Fixed modifications:** Carbamidomethyl (C)
**Variable modifications:**
**N6** : Deamidated_N (N)
**Ions Score:** 64 **Expect:** 5.6e-005
**Matches (Bold Red):** 12/92 fragment ions using 20 most intense peaks

| # | b | b++ | b* | b*++ | b0 | b0++ | Seq. | y | y++ | y* | y*++ | y0 | y0++ | # |
|---|---|---|---|---|---|---|---|---|---|---|---|---|---|---|
| 1 | 132.0478 | 66.5275 | | | | | M | | | | | | | 10 |
| 2 | **245.1318** | 123.0696 | | | | | L | **1081.5412** | 541.2742 | 1064.5146 | 532.7610 | 1063.5306 | 532.2689 | 9 |
| 3 | **374.1744** | 187.5908 | | | 356.1639 | 178.5856 | E | **968.4571** | 484.7322 | 951.4306 | 476.2189 | 950.4465 | 475.7269 | 8 |
| 4 | **445.2115** | 223.1094 | | | 427.2010 | 214.1041 | A | **839.4145** | 420.2109 | 822.3880 | 411.6976 | 821.4040 | 411.2056 | 7 |
| 5 | 608.2749 | 304.6411 | | | 590.2643 | 295.6358 | Y | **768.3774** | 384.6923 | 751.3509 | 376.1791 | 750.3668 | 375.6871 | 6 |

| | | | | | | | | | | | | | | |
|---|---|---|---|---|---|---|---|---|---|---|---|---|---|---|
| **6** | 723.3018 | 362.1545 | 706.2752 | 353.6413 | 705.2912 | 353.1493 | **N** | 605.3141 | 303.1607 | **588.2875** | 294.6474 | 587.3035 | 294.1554 | **5** |
| **7** | 836.3859 | 418.6966 | 819.3593 | 410.1833 | 818.3753 | 409.6913 | **L** | **490.2871** | **245.6472** | 473.2606 | 237.1339 | 472.2766 | 236.6419 | **4** |
| **8** | 937.4335 | 469.2204 | 920.4070 | 460.7071 | 919.4230 | 460.2151 | **T** | **377.2031** | 189.1052 | 360.1765 | 180.5919 | 359.1925 | 180.0999 | **3** |
| **9** | 1066.4761 | 533.7417 | 1049.4496 | 525.2284 | 1048.4656 | 524.7364 | **E** | **276.1554** | 138.5813 | 259.1288 | 130.0681 | 258.1448 | 129.5761 | **2** |
| **10** | | | | | | | **K** | 147.1128 | 74.0600 | 130.0863 | 65.5468 | | | **1** |

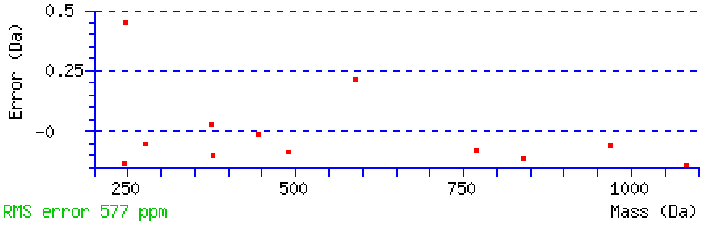


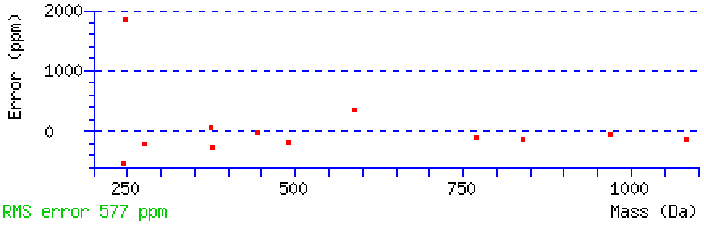


### All matches to this query

| Score | Mr(calc): | Delta | Sequence |
|---|---|---|---|
| 63.9 | 1211.5743 | -0.0000 | MLEAYNLTEK |
| 16.8 | 1211.5744 | -0.0001 | EIMFSDITEK |
| 13.1 | 1211.5686 | 0.0057 | NTELETLLAK |
| 10.0 | 1211.5798 | -0.0055 | AKETALAATEK |
| 8.9 | 1209.5754 | 1.9989 | ELQISQREK |
| 8.1 | 1209.5642 | 2.0101 | LEEEIQTLR |
| 6.5 | 1211.5798 | -0.0055 | NKNVTEAVKK |
| 5.3 | 1210.5764 | 0.9979 | ELSPEAICHR |
| 4.9 | 1211.5733 | 0.0010 | QKMTGLQAQK |
| 4.0 | 1209.5642 | 2.0101 | TAKQNPLTEK |

---

Spectrum No: 458; Query: 2287; Rank: 1

---

## Peptide View

MS/MS Fragmentation of **RIYYSDFSNQTINSMAEDGSNR**
Found in **IPI00205325**, Tax_Id=10116 Gene_Symbol=Lrp2 Low-density lipoprotein receptor-related protein 2 precursor

Match to Query 2287: 2568.126162 from(857.049330,3+)
Title: 100101RatKid_NS_deglyco_15.3390.3390.3.dta
Data file K:\NewmanPaper\Piliang\3SubProteomes\Piliang3SP\mgf5ppm\ERLIC_3SubProteomes5ppm.mgf

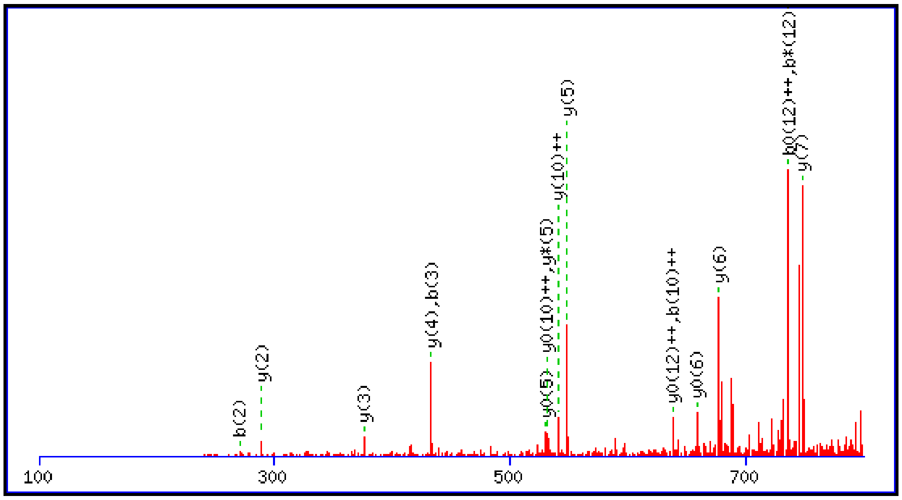

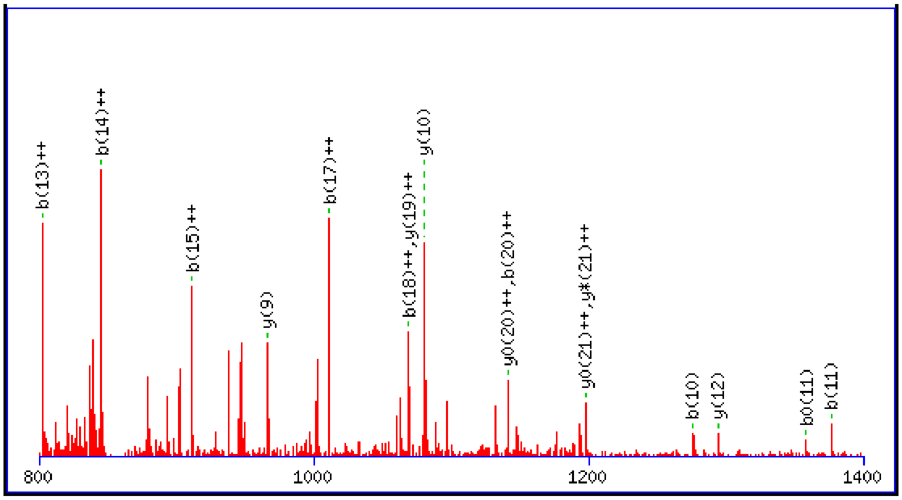


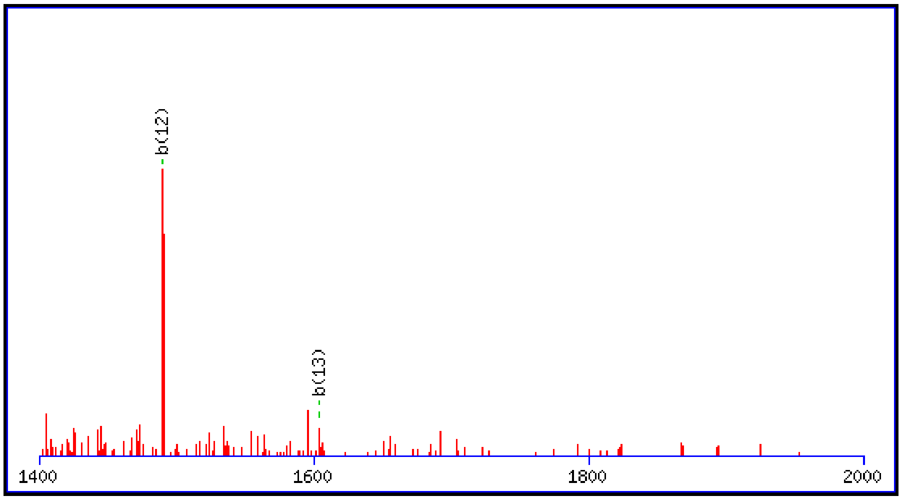


**Monoisotopic mass of neutral peptide Mr(calc):** 2568.1187
**Fixed modifications:** Carbamidomethyl (C)
**Variable modifications:**
**N9** : Deamidated_N (N)
**Ions Score:** 64 **Expect:** 0.00015
**Matches (Bold Red):** 36/240 fragment ions using 38 most intense peaks

| # | b | $b^{++}$ | b* | $b^{*++}$ | $b^0$ | $b^{0++}$ | Seq. | y | $y^{++}$ | y* | $y^{*++}$ | $y^0$ | $y^{0++}$ | # |
|---|---|---|---|---|---|---|---|---|---|---|---|---|---|---|
| 1 | 157.1084 | 79.0578 | 140.0818 | 70.5446 | | | R | | | | | | | 22 |
| 2 | **270.1925** | 135.5999 | 253.1659 | 127.0866 | | | I | 2413.0249 | 1207.0161 | 2395.9983 | **1198.5028** | 2395.0143 | **1198.0108** | 21 |
| 3 | **433.2558** | 217.1315 | 416.2292 | 208.6183 | | | Y | 2299.9408 | 1150.4740 | 2282.9142 | 1141.9608 | 2281.9302 | **1141.4687** | 20 |
| 4 | 596.3191 | 298.6632 | 579.2926 | 290.1499 | | | Y | 2136.8775 | **1068.9424** | 2119.8509 | 1060.4291 | 2118.8669 | 1059.9371 | 19 |
| 5 | 683.3511 | 342.1792 | 666.3246 | 333.6659 | 665.3406 | 333.1739 | S | 1973.8141 | 987.4107 | 1956.7876 | 978.8974 | 1955.8036 | 978.4054 | 18 |
| 6 | 798.3781 | 399.6927 | 781.3515 | 391.1794 | 780.3675 | 390.6874 | D | 1886.7821 | 943.8947 | 1869.7556 | 935.3814 | 1868.7715 | 934.8894 | 17 |
| 7 | 945.4465 | 473.2269 | 928.4199 | 464.7136 | 927.4359 | 464.2216 | F | 1771.7552 | 886.3812 | 1754.7286 | 877.8679 | 1753.7446 | 877.3759 | 16 |
| 8 | 1032.4785 | 516.7429 | 1015.4520 | 508.2296 | 1014.4680 | 507.7376 | S | 1624.6867 | 812.8470 | 1607.6602 | 804.3337 | 1606.6762 | 803.8417 | 15 |
| 9 | 1147.5055 | 574.2564 | 1130.4789 | 565.7431 | 1129.4949 | 565.2511 | N | 1537.6547 | 769.3310 | 1520.6282 | 760.8177 | 1519.6442 | 760.3257 | 14 |
| 10 | **1275.5640** | **638.2857** | 1258.5375 | 629.7724 | 1257.5535 | 629.2804 | Q | 1422.6278 | 711.8175 | 1405.6012 | 703.3043 | 1404.6172 | 702.8122 | 13 |
| 11 | **1376.6117** | 688.8095 | 1359.5852 | 680.2962 | **1358.6012** | 679.8042 | T | **1294.5692** | 647.7882 | 1277.5427 | 639.2750 | 1276.5586 | **638.7830** | 12 |
| 12 | **1489.6958** | 745.3515 | 1472.6692 | **736.8383** | 1471.6852 | **736.3462** | I | 1193.5215 | 597.2644 | 1176.4950 | 588.7511 | 1175.5110 | 588.2591 | 11 |
| 13 | **1603.7387** | **802.3730** | 1586.7122 | 793.8597 | 1585.7281 | 793.3677 | N | **1080.4375** | **540.7224** | 1063.4109 | 532.2091 | 1062.4269 | **531.7171** | 10 |
| 14 | 1690.7707 | **845.8890** | 1673.7442 | 837.3757 | 1672.7602 | 836.8837 | S | **966.3945** | 483.7009 | 949.3680 | 475.1876 | 948.3840 | 474.6956 | 9 |
| 15 | 1821.8112 | **911.4092** | 1804.7847 | 902.8960 | 1803.8007 | 902.4040 | M | 879.3625 | 440.1849 | 862.3360 | 431.6716 | 861.3519 | 431.1796 | 8 |
| 16 | 1892.8483 | 946.9278 | 1875.8218 | 938.4145 | 1874.8378 | 937.9225 | A | **748.3220** | 374.6646 | 731.2955 | 366.1514 | 730.3115 | 365.6594 | 7 |
| 17 | 2021.8909 | **1011.4491** | 2004.8644 | 1002.9358 | 2003.8804 | 1002.4438 | E | **677.2849** | 339.1461 | 660.2584 | 330.6328 | **659.2743** | 330.1408 | 6 |
| 18 | 2136.9179 | **1068.9626** | 2119.8913 | 1060.4493 | 2118.9073 | 1059.9573 | D | **548.2423** | 274.6248 | **531.2158** | 266.1115 | **530.2317** | 265.6195 | 5 |
| | | | | | | | | | | | | | | |

| | | | | | | | | | | | | | | |
|---|---|---|---|---|---|---|---|---|---|---|---|---|---|---|
| **19** | 2193.9393 | 1097.4733 | 2176.9128 | 1088.9600 | 2175.9288 | 1088.4680 | **G** | **433.2154** | 217.1113 | 416.1888 | 208.5980 | 415.2048 | 208.1060 | **4** |
| **20** | 2280.9714 | **1140.9893** | 2263.9448 | 1132.4760 | 2262.9608 | 1131.9840 | **S** | **376.1939** | 188.6006 | 359.1674 | 180.0873 | 358.1833 | 179.5953 | **3** |
| **21** | 2395.0143 | **1198.0108** | 2377.9877 | 1189.4975 | 2377.0037 | 1189.0055 | **N** | **289.1619** | 145.0846 | 272.1353 | 136.5713 | | | **2** |
| **22** | | | | | | | **R** | 175.1190 | 88.0631 | 158.0924 | 79.5498 | | | **1** |

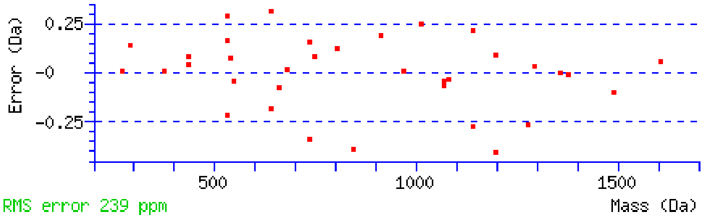


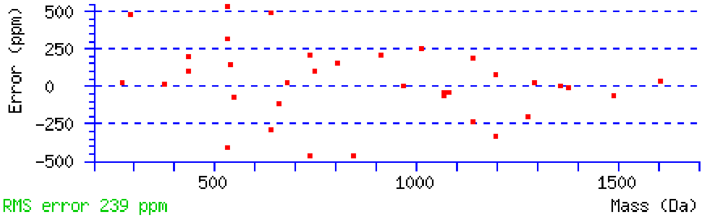


**All matches to this query**

| Score | Mr(calc): | Delta | Sequence |
|---|---|---|---|
| 63.8 | 2568.1187 | 0.0075 | RIYYSDFSNQTINSMAEDGSNR |
| 44.3 | 2567.1346 | 0.9915 | RIYYSDFSNQTINSMAEDGSNR |
| 39.3 | 2568.1187 | 0.0075 | RIYYSDFSNQTINSMAEDGSNR |
| 16.2 | 2568.1187 | 0.0075 | RIYYSDFSNQTINSMAEDGSNR |
| 6.9 | 2568.1455 | -0.0193 | IYNEFISVQATKEVNLDSCTR |
| 3.6 | 2568.1328 | -0.0067 | FLDPGFSSHQAVARTAGSVLLR |
| 1.1 | 2568.1387 | -0.0125 | KTSVISSPKPPATAARGETSTSR |
| 1.1 | 2568.1387 | -0.0125 | KTSVISSPKPPATAARGETSTSR |
| 0.3 | 2568.1328 | -0.0067 | FLDPGFSSHQAVARTAGSVLLR |

---

Spectrum No: 459; Query: 504; Rank: 1

---

## Peptide View

MS/MS Fragmentation of **LNFTGPGEPDSLR**
Found in **IPI00360541**, Tax_Id=10116 Gene_Symbol=Itgb2 integrin beta 2

Match to Query 504: 1402.677308 from(702.345930,2+)
Title: 100101RatKid_NS_deglyco_25.2526.2526.2.dta
Data file K:\NewmanPaper\Piliang\3SubProteomes\Piliang3SP\mgf5ppm\ERLIC_3SubProteomes5ppm.mgf

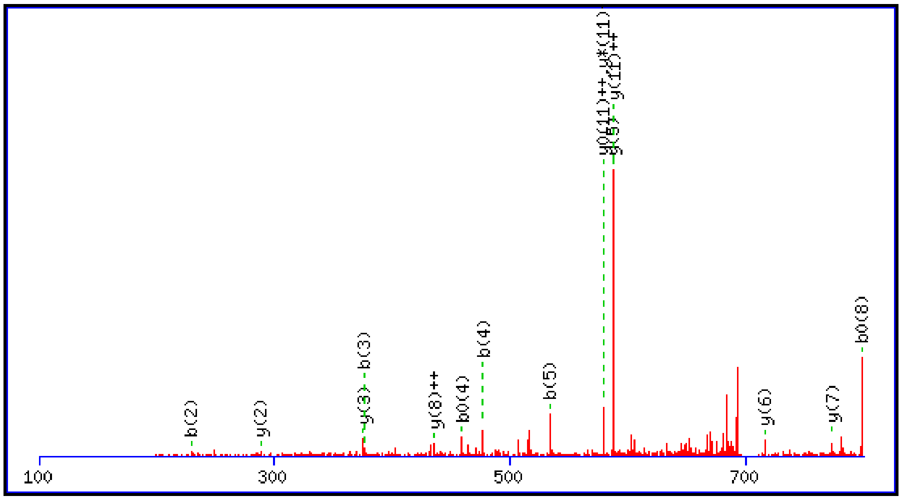

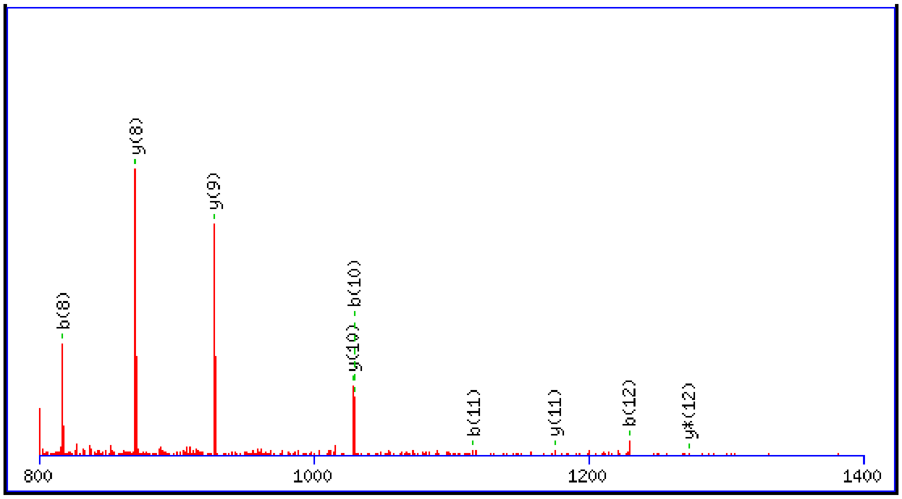


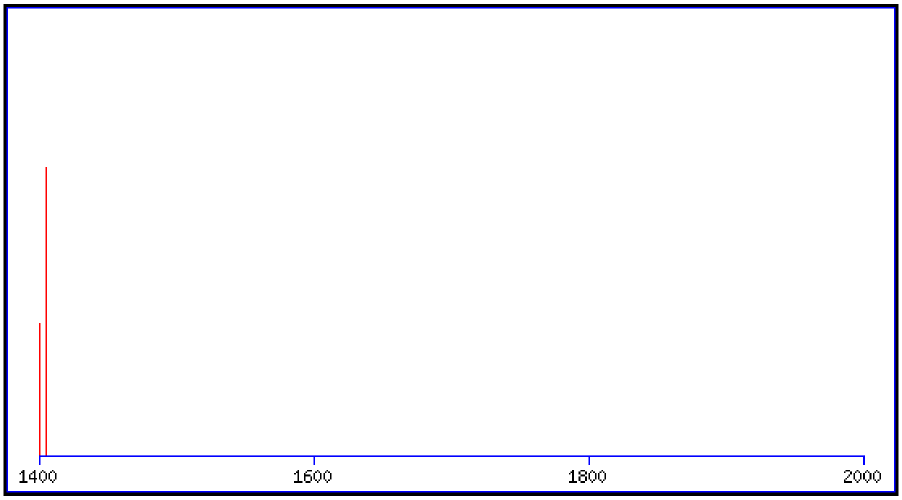


**Monoisotopic mass of neutral peptide Mr(calc):** 1402.6729
**Fixed modifications:** Carbamidomethyl (C)
**Variable modifications:**
**N2** : Deamidated_N (N)
**Ions Score:** 64 **Expect:** 6.1e-005
**Matches (Bold Red):** 24/132 fragment ions using 47 most intense peaks

| # | b | $b^{++}$ | b* | $b^{*++}$ | $b^0$ | $b^{0++}$ | Seq. | y | $y^{++}$ | y* | $y^{*++}$ | $y^0$ | $y^{0++}$ | # |
|---|---|---|---|---|---|---|---|---|---|---|---|---|---|---|
| 1 | 114.0913 | 57.5493 | | | | | L | | | | | | | 13 |
| 2 | **229.1183** | 115.0628 | 212.0917 | 106.5495 | | | N | 1290.5961 | 645.8017 | **1273.5695** | 637.2884 | 1272.5855 | 636.7964 | 12 |
| 3 | **376.1867** | 188.5970 | 359.1601 | 180.0837 | | | F | **1175.5691** | **588.2882** | 1158.5426 | **579.7749** | 1157.5586 | **579.2829** | 11 |
| 4 | **477.2344** | 239.1208 | 460.2078 | 230.6075 | **459.2238** | 230.1155 | T | **1028.5007** | 514.7540 | 1011.4742 | 506.2407 | 1010.4902 | 505.7487 | 10 |
| 5 | **534.2558** | 267.6316 | 517.2293 | 259.1183 | 516.2453 | 258.6263 | G | **927.4530** | 464.2302 | 910.4265 | 455.7169 | 909.4425 | 455.2249 | 9 |
| 6 | 631.3086 | 316.1579 | 614.2820 | 307.6447 | 613.2980 | 307.1527 | P | **870.4316** | **435.7194** | 853.4050 | 427.2061 | 852.4210 | 426.7141 | 8 |
| 7 | 688.3301 | 344.6687 | 671.3035 | 336.1554 | 670.3195 | 335.6634 | G | **773.3788** | 387.1930 | 756.3523 | 378.6798 | 755.3682 | 378.1878 | 7 |
| 8 | **817.3727** | 409.1900 | 800.3461 | 400.6767 | **799.3621** | 400.1847 | E | **716.3573** | 358.6823 | 699.3308 | 350.1690 | 698.3468 | 349.6770 | 6 |
| 9 | 914.4254 | 457.7163 | 897.3989 | 449.2031 | 896.4149 | 448.7111 | P | **587.3148** | 294.1610 | 570.2882 | 285.6477 | 569.3042 | 285.1557 | 5 |
| 10 | **1029.4524** | 515.2298 | 1012.4258 | 506.7165 | 1011.4418 | 506.2245 | D | 490.2620 | 245.6346 | 473.2354 | 237.1214 | 472.2514 | 236.6293 | 4 |
| 11 | **1116.4844** | 558.7458 | 1099.4578 | 550.2326 | 1098.4738 | 549.7406 | S | **375.2350** | 188.1212 | 358.2085 | 179.6079 | 357.2245 | 179.1159 | 3 |
| 12 | **1229.5685** | 615.2879 | 1212.5419 | 606.7746 | 1211.5579 | 606.2826 | L | **288.2030** | 144.6051 | 271.1765 | 136.0919 | | | 2 |
| 13 | | | | | | | R | 175.1190 | 88.0631 | 158.0924 | 79.5498 | | | 1 |

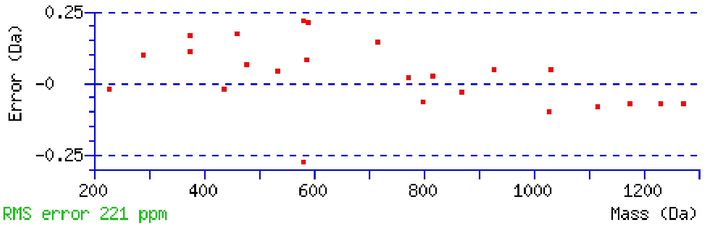


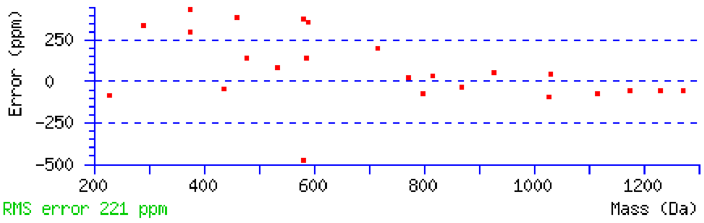


### All matches to this query

| Score | Mr(calc): | Delta | Sequence |
|---|---|---|---|
| 63.8 | 1402.6729 | 0.0045 | LNFTGPGEPDSLR |
| 13.5 | 1402.6745 | 0.0028 | LITYLDQTTQK |
| 13.5 | 1402.6745 | 0.0028 | LITYLDQTTQK |
| 8.4 | 1402.6731 | 0.0042 | MTMRMKTTSLR |
| 6.8 | 1402.6841 | -0.0067 | LNSSHEAFAASLR |
| 6.5 | 1402.6874 | -0.0101 | NPVMELNEKRR |
| 6.3 | 1402.6688 | 0.0085 | SSNLNVPSSDDIR |
| 5.4 | 1401.6615 | 1.0158 | VYNLVKETMPK |
| 5.0 | 1402.6792 | -0.0019 | VEARKTYMGLR |
| 4.4 | 1402.6646 | 0.0127 | FETVKLASWSR |

---

Spectrum No: 460; Query: 374; Rank: 1

---

## Peptide View

MS/MS Fragmentation of **DVNYTQIVVDR**
Found in **IPI00366011**, Tax_Id=10116 Gene_Symbol=Sema4d_predicted similar to semaphorin 4D

Match to Query 374: 1321.657108 from(661.835830,2+)
Title: 100101RatKid_NS_deglyco_19.2803.2803.2.dta
Data file K:\NewmanPaper\Piliang\3SubProteomes\Piliang3SP\mgf5ppm\ERLIC_3SubProteomes5ppm.mgf

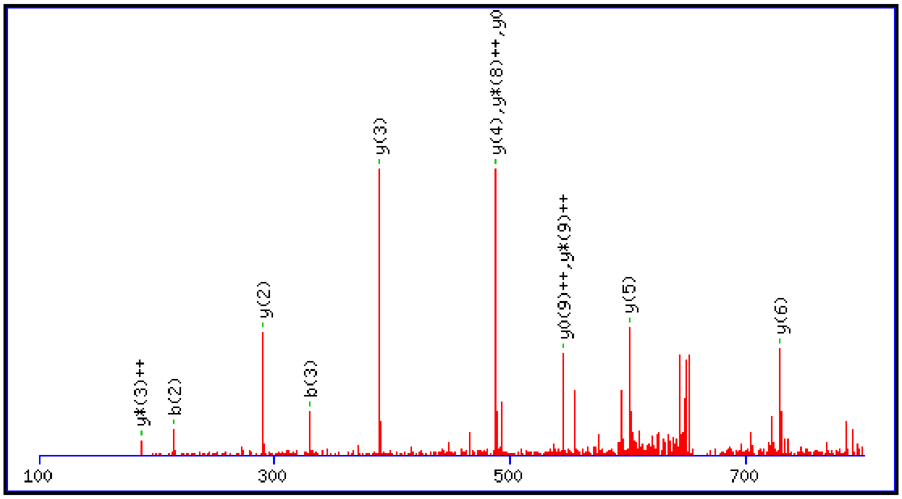

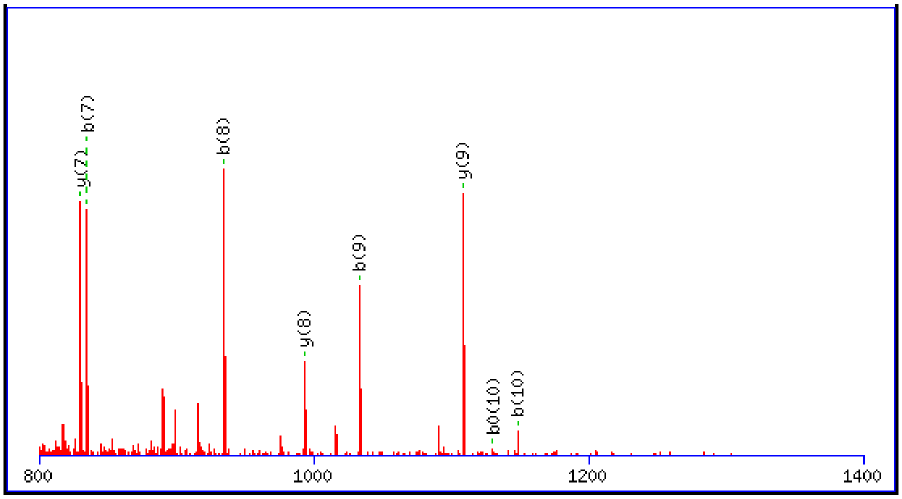


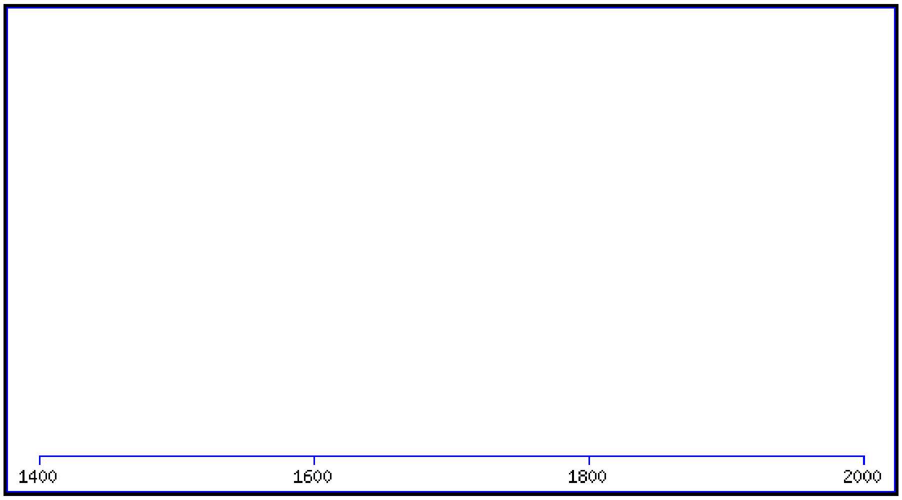


**Monoisotopic mass of neutral peptide Mr(calc):** 1321.6514
**Fixed modifications:** Carbamidomethyl (C)
**Variable modifications:**
**N3** : Deamidated_N (N)
**Ions Score:** 64 **Expect:** 5.3e-005
**Matches (Bold Red):** 20/114 fragment ions using 31 most intense peaks

| # | b | $b^{++}$ | b* | $b^{*++}$ | $b^0$ | $b^{0++}$ | Seq. | y | $y^{++}$ | y* | $y^{*++}$ | $y^0$ | $y^{0++}$ | # |
|---|---|---|---|---|---|---|---|---|---|---|---|---|---|---|
| 1 | 116.0342 | 58.5207 | | | 98.0237 | 49.5155 | D | | | | | | | 11 |
| 2 | **215.1026** | 108.0550 | | | 197.0921 | 99.0497 | V | 1207.6317 | 604.3195 | 1190.6052 | 595.8062 | 1189.6212 | 595.3142 | 10 |
| 3 | **330.1296** | 165.5684 | 313.1030 | 157.0551 | 312.1190 | 156.5631 | N | **1108.5633** | 554.7853 | 1091.5368 | **546.2720** | 1090.5527 | **545.7800** | 9 |
| 4 | 493.1929 | 247.1001 | 476.1663 | 238.5868 | 475.1823 | 238.0948 | Y | **993.5364** | 497.2718 | 976.5098 | **488.7586** | 975.5258 | **488.2665** | 8 |
| 5 | 594.2406 | 297.6239 | 577.2140 | 289.1107 | 576.2300 | 288.6186 | T | **830.4730** | 415.7402 | 813.4465 | 407.2269 | 812.4625 | 406.7349 | 7 |
| 6 | 722.2992 | 361.6532 | 705.2726 | 353.1399 | 704.2886 | 352.6479 | Q | **729.4254** | 365.2163 | 712.3988 | 356.7030 | 711.4148 | 356.2110 | 6 |
| 7 | **835.3832** | 418.1952 | 818.3567 | 409.6820 | 817.3727 | 409.1900 | I | **601.3668** | 301.1870 | 584.3402 | 292.6738 | 583.3562 | 292.1817 | 5 |
| 8 | **934.4516** | 467.7295 | 917.4251 | 459.2162 | 916.4411 | 458.7242 | V | **488.2827** | 244.6450 | 471.2562 | 236.1317 | 470.2722 | 235.6397 | 4 |
| 9 | **1033.5200** | 517.2637 | 1016.4935 | 508.7504 | 1015.5095 | 508.2584 | V | **389.2143** | 195.1108 | 372.1878 | **186.5975** | 371.2037 | 186.1055 | 3 |
| 10 | **1148.5470** | 574.7771 | 1131.5204 | 566.2639 | **1130.5364** | 565.7719 | D | **290.1459** | 145.5766 | 273.1193 | 137.0633 | 272.1353 | 136.5713 | 2 |
| 11 | | | | | | | R | 175.1190 | 88.0631 | 158.0924 | 79.5498 | | | 1 |

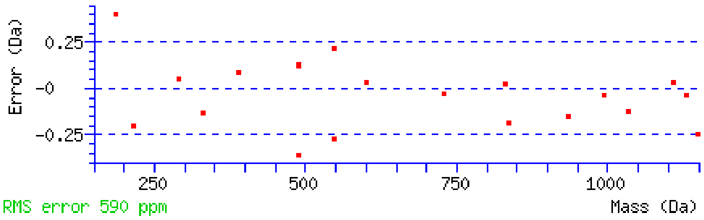


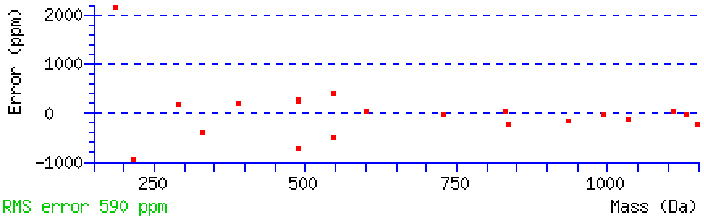

### All matches to this query

| Score | Mr(calc): | Delta | Sequence |
|---|---|---|---|
| 63.8 | 1321.6514 | 0.0057 | DVNYTQIVVDR |
| 14.0 | 1320.6551 | 1.0020 | TPRGTKTPINR |
| 4.7 | 1319.6411 | 2.0160 | WTTPNHHIWK |
| 4.5 | 1320.6551 | 1.0020 | TPRGTKTPINR |
| 2.1 | 1319.6374 | 2.0197 | VLDTPGPVSDLK |
| 0.0 | 1321.6473 | 0.0098 | TLSSKNNENTSK |

---

Spectrum No: 461; Query: 2268; Rank: 1

---

## Peptide View

MS/MS Fragmentation of **TGDFQLHTNVNNGTEFGGSIYQK**
Found in **IPI00198327**, Tax_Id=10116 Gene_Symbol=Vdac2 Voltage-dependent anion-selective channel protein 2

Match to Query 2268: 2527.157772 from(843.393200,3+)
Title: 091008RatKidney_NH4Format02_20.2989.2989.3.dta
Data file K:\NewmanPaper\Piliang\3SubProteomes\Piliang3SP\mgf5ppm\ERLIC_3SubProteomes5ppm.mgf

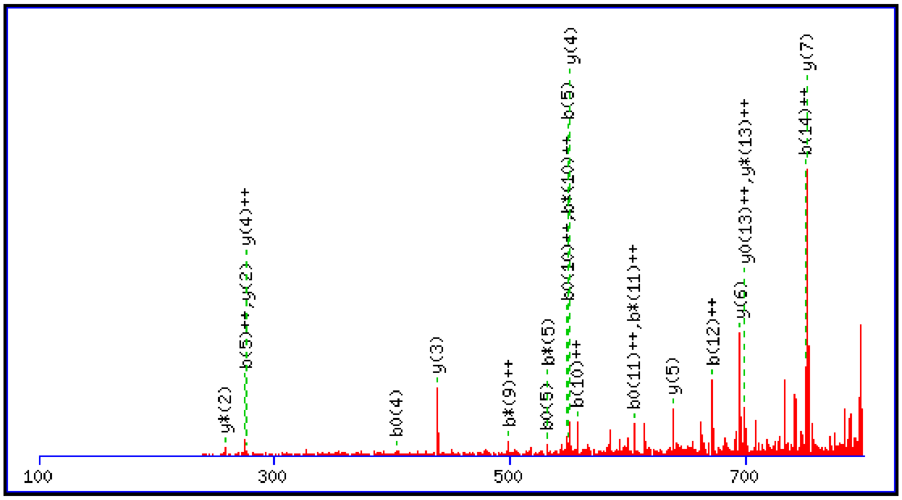


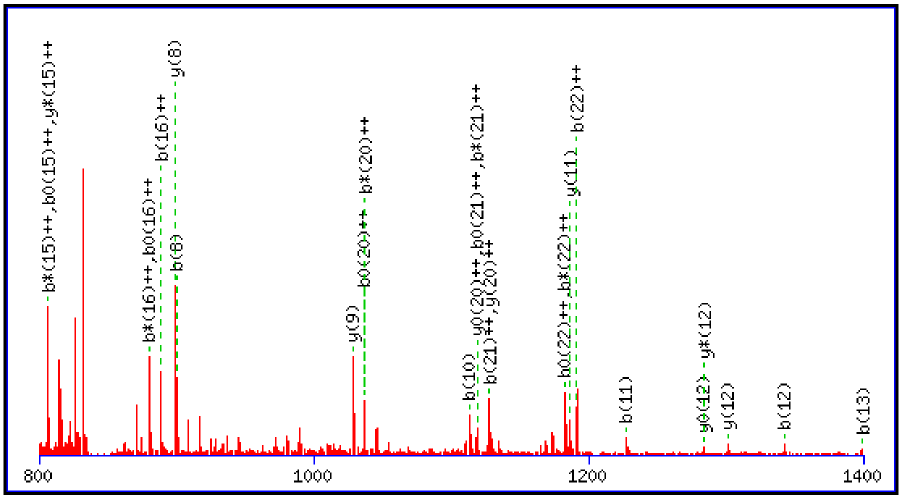

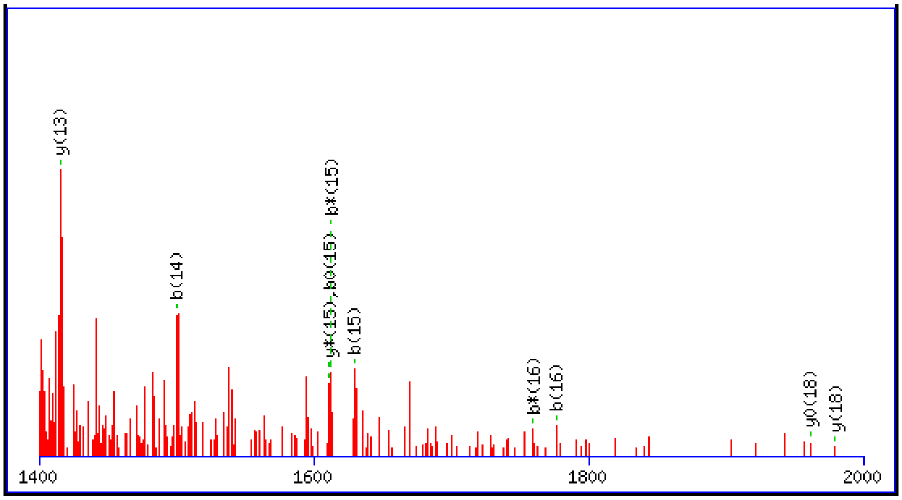


**Monoisotopic mass of neutral peptide Mr(calc):** 2527.1616
**Fixed modifications:** Carbamidomethyl (C)
**Variable modifications:**
**N12** : Deamidated_N (N)
**Ions Score:** 64 **Expect:** 0.00013
**Matches (Bold Red):** 60/248 fragment ions using 90 most intense peaks

| # | b | $b^{++}$ | b* | $b^{*++}$ | $b^{0}$ | $b^{0++}$ | Seq. | y | $y^{++}$ | y* | $y^{*++}$ | $y^{0}$ | $y^{0++}$ | # |
|---|---|---|---|---|---|---|---|---|---|---|---|---|---|---|
| 1 | 102.0550 | 51.5311 | | | 84.0444 | 42.5258 | T | | | | | | | 23 |
| 2 | 159.0764 | 80.0418 | | | 141.0659 | 71.0366 | G | 2427.1211 | 1214.0642 | 2410.0946 | 1205.5509 | 2409.1106 | 1205.0589 | 22 |
| 3 | 274.1034 | 137.5553 | | | 256.0928 | 128.5500 | D | 2370.0997 | 1185.5535 | 2353.0731 | 1177.0402 | 2352.0891 | 1176.5482 | 21 |
| 4 | 421.1718 | 211.0895 | | | **403.1612** | 202.0842 | F | 2255.0727 | **1128.0400** | 2238.0462 | 1119.5267 | 2237.0622 | **1119.0347** | 20 |
| 5 | **549.2304** | **275.1188** | **532.2038** | 266.6055 | **531.2198** | 266.1135 | Q | 2108.0043 | 1054.5058 | 2090.9778 | 1045.9925 | 2089.9938 | 1045.5005 | 19 |
| 6 | 662.3144 | 331.6608 | 645.2879 | 323.1476 | 644.3039 | 322.6556 | L | **1979.9457** | 990.4765 | 1962.9192 | 981.9632 | **1961.9352** | 981.4712 | 18 |
| 7 | 799.3733 | 400.1903 | 782.3468 | 391.6770 | 781.3628 | 391.1850 | H | 1866.8617 | 933.9345 | 1849.8351 | 925.4212 | 1848.8511 | 924.9292 | 17 |
| 8 | **900.4210** | 450.7141 | 883.3945 | 442.2009 | 882.4104 | 441.7089 | T | 1729.8028 | 865.4050 | 1712.7762 | 856.8917 | 1711.7922 | 856.3997 | 16 |
| 9 | 1014.4639 | 507.7356 | 997.4374 | **499.2223** | 996.4534 | 498.7303 | N | 1628.7551 | 814.8812 | **1611.7285** | **806.3679** | 1610.7445 | 805.8759 | 15 |
| 10 | **1113.5324** | **557.2698** | 1096.5058 | **548.7565** | 1095.5218 | **548.2645** | V | 1514.7122 | 757.8597 | 1497.6856 | 749.3464 | 1496.7016 | 748.8544 | 14 |
| 11 | **1227.5753** | 614.2913 | 1210.5487 | **605.7780** | 1209.5647 | **605.2860** | N | **1415.6437** | 708.3255 | 1398.6172 | **699.8122** | 1397.6332 | **699.3202** | 13 |
| 12 | **1342.6022** | **671.8047** | 1325.5757 | 663.2915 | 1324.5916 | 662.7995 | N | **1301.6008** | 651.3040 | **1284.5743** | 642.7908 | **1283.5903** | 642.2988 | 12 |
| 13 | **1399.6237** | 700.3155 | 1382.5971 | 691.8022 | 1381.6131 | 691.3102 | G | **1186.5739** | 593.7906 | 1169.5473 | 585.2773 | 1168.5633 | 584.7853 | 11 |
| 14 | **1500.6714** | **750.8393** | 1483.6448 | 742.3260 | 1482.6608 | 741.8340 | T | 1129.5524 | 565.2798 | 1112.5259 | 556.7666 | 1111.5419 | 556.2746 | 10 |
| 15 | **1629.7139** | 815.3606 | **1612.6874** | **806.8473** | **1611.7034** | **806.3553** | E | **1028.5047** | 514.7560 | 1011.4782 | 506.2427 | 1010.4942 | 505.7507 | 9 |
| 16 | **1776.7824** | **888.8948** | **1759.7558** | **880.3815** | 1758.7718 | **879.8895** | F | **899.4621** | 450.2347 | 882.4356 | 441.7214 | 881.4516 | 441.2294 | 8 |
| 17 | 1833.8038 | 917.4056 | 1816.7773 | 908.8923 | 1815.7933 | 908.4003 | G | **752.3937** | 376.7005 | 735.3672 | 368.1872 | 734.3832 | 367.6952 | 7 |
| 18 | 1890.8253 | 945.9163 | 1873.7987 | 937.4030 | 1872.8147 | 936.9110 | G | **695.3723** | 348.1898 | 678.3457 | 339.6765 | 677.3617 | 339.1845 | 6 |
| 19 | 1977.8573 | 989.4323 | 1960.8308 | 980.9190 | 1959.8468 | 980.4270 | S | **638.3508** | 319.6790 | 621.3243 | 311.1658 | 620.3402 | 310.6738 | 5 |
| 20 | 2090.9414 | 1045.9743 | 2073.9148 | **1037.4611** | 2072.9308 | **1036.9690** | I | **551.3188** | **276.1630** | 534.2922 | 267.6498 | | | 4 |
| 21 | 2254.0047 | **1127.5060** | 2236.9782 | **1118.9927** | 2235.9941 | **1118.5007** | Y | **438.2347** | 219.6210 | 421.2082 | 211.1077 | | | 3 |
| 22 | 2382.0633 | **1191.5353** | 2365.0367 | **1183.0220** | 2364.0527 | **1182.5300** | Q | **275.1714** | 138.0893 | **258.1448** | 129.5761 | | | 2 |
| 23 | | | | | | | K | 147.1128 | 74.0600 | 130.0863 | 65.5468 | | | 1 |

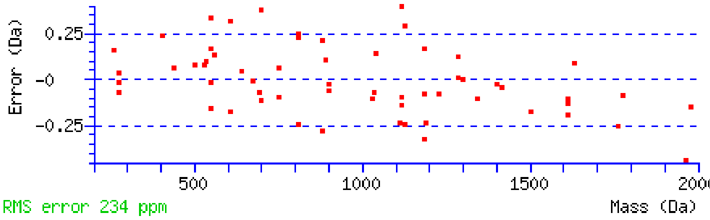


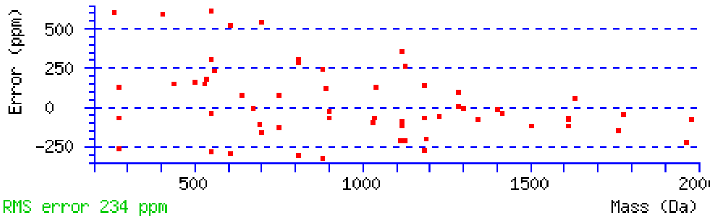


**All matches to this query**

| Score | Mr(calc): | Delta | Sequence |
|---|---|---|---|
| 63.7 | 2527.1616 | -0.0038 | TGDFQLHTNVNNGTEFGGSIYQK |
| 60.4 | 2527.1616 | -0.0038 | TGDFQLHTNVNNGTEFGGSIYQK |
| 54.2 | 2527.1616 | -0.0038 | TGDFQLHTNVNNGTEFGGSIYQK |
| 46.1 | 2526.1776 | 0.9802 | TGDFQLHTNVNNGTEFGGSIYQK |
| 3.5 | 2527.1348 | 0.0230 | NVSVANVCLLSVFQAITISPR |
| 3.1 | 2526.1590 | 0.9987 | SECVPAAAVTHVVEVLSLTAASR |
| 3.1 | 2526.1590 | 0.9987 | SECVPAAAVTHVVEVLSLTAASR |
| 3.1 | 2526.1590 | 0.9987 | SECVPAAAVTHVVEVLSLTAASR |
| 2.0 | 2526.1518 | 1.0059 | ILLLCVGEAGDTVQFAEYIQK |
| 1.6 | 2525.1702 | 1.9876 | CYGYYKNTPQLWSVPSELLK |

---

Spectrum No: 462; Query: 247; Rank: 1

---

## Peptide View

MS/MS Fragmentation of **ENISDPTSPVR**
Found in **IPI00363901**, Tax_Id=10116 Gene_Symbol=Igj Immunoglobulin joining chain

Match to Query 247: 1214.573748 from(608.294150,2+)
Title: 091008RatKidney_NH4Format01_31.584.584.2.dta
Data file K:\NewmanPaper\Piliang\3SubProteomes\Piliang3SP\mgf5ppm\ERLIC_3SubProteomes5ppm.mgf

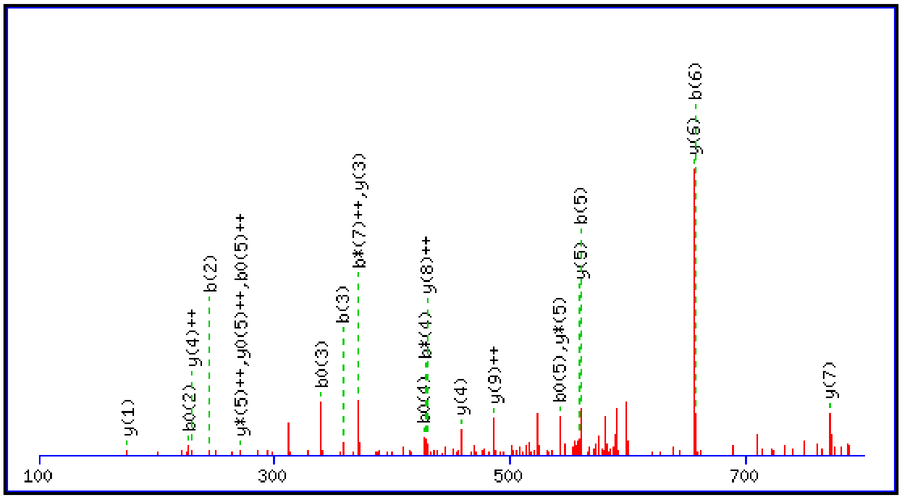


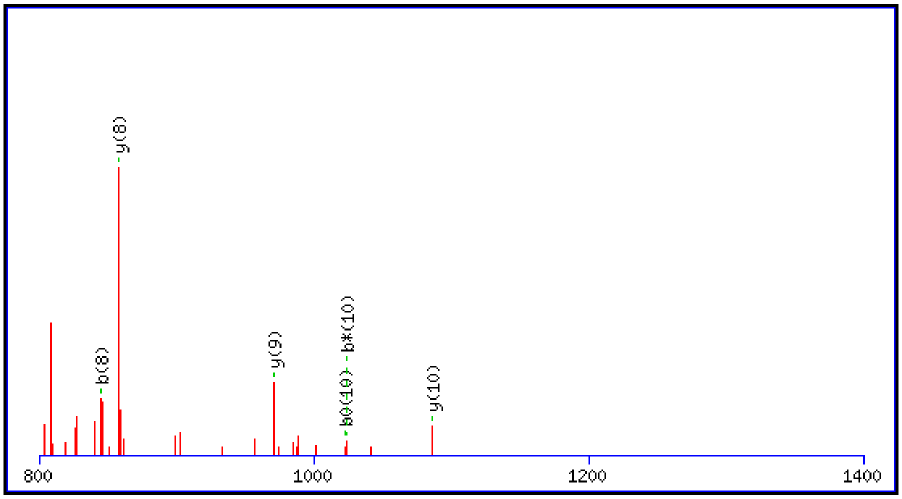

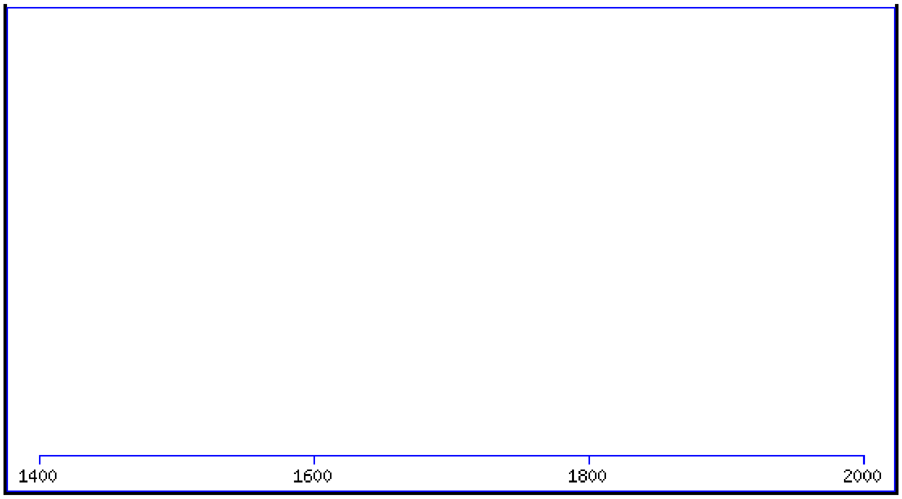


**Monoisotopic mass of neutral peptide Mr(calc):** 1214.5779
**Fixed modifications:** Carbamidomethyl (C)
**Variable modifications:**
**N2** : Deamidated_N (N)
**Ions Score:** 64 **Expect:** 5.8e-005
**Matches (Bold Red):** 29/112 fragment ions using 48 most intense peaks

| # | b | $b^{++}$ | b* | $b^{*++}$ | $b^{0}$ | $b^{0++}$ | Seq. | y | $y^{++}$ | y* | $y^{*++}$ | $y^{0}$ | $y^{0++}$ | # |
|---|---|---|---|---|---|---|---|---|---|---|---|---|---|---|
| 1 | 130.0499 | 65.5286 | | | 112.0393 | 56.5233 | E | | | | | | | 11 |
| 2 | **245.0768** | 123.0420 | 228.0503 | 114.5288 | **227.0662** | 114.0368 | N | **1086.5426** | 543.7749 | 1069.5160 | 535.2616 | 1068.5320 | 534.7696 | 10 |
| 3 | **358.1609** | 179.5841 | 341.1343 | 171.0708 | **340.1503** | 170.5788 | I | **971.5156** | **486.2615** | 954.4891 | 477.7482 | 953.5051 | 477.2562 | 9 |
| 4 | 445.1929 | 223.1001 | **428.1663** | 214.5868 | **427.1823** | 214.0948 | S | **858.4316** | **429.7194** | 841.4050 | 421.2061 | 840.4210 | 420.7141 | 8 |
| 5 | **560.2198** | 280.6136 | 543.1933 | 272.1003 | **542.2093** | **271.6083** | D | **771.3995** | 386.2034 | 754.3730 | 377.6901 | 753.3890 | 377.1981 | 7 |
| 6 | **657.2726** | 329.1399 | 640.2461 | 320.6267 | 639.2620 | 320.1347 | P | **656.3726** | 328.6899 | 639.3461 | 320.1767 | 638.3620 | 319.6847 | 6 |
| 7 | 758.3203 | 379.6638 | 741.2937 | **371.1505** | 740.3097 | 370.6585 | T | **559.3198** | 280.1636 | **542.2933** | **271.6503** | 541.3093 | **271.1583** | 5 |
| 8 | **845.3523** | 423.1798 | 828.3258 | 414.6665 | 827.3417 | 414.1745 | S | **458.2722** | **229.6397** | 441.2456 | 221.1264 | 440.2616 | 220.6344 | 4 |
| 9 | 942.4051 | 471.7062 | 925.3785 | 463.1929 | 924.3945 | 462.7009 | P | **371.2401** | 186.1237 | 354.2136 | 177.6104 | | | 3 |
| 10 | 1041.4735 | 521.2404 | **1024.4469** | 512.7271 | **1023.4629** | 512.2351 | V | 274.1874 | 137.5973 | 257.1608 | 129.0840 | | | 2 |
| 11 | | | | | | | R | **175.1190** | 88.0631 | 158.0924 | 79.5498 | | | 1 |

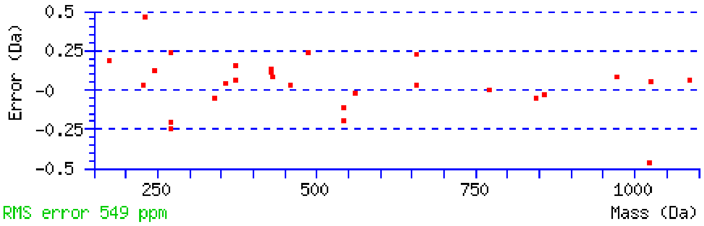


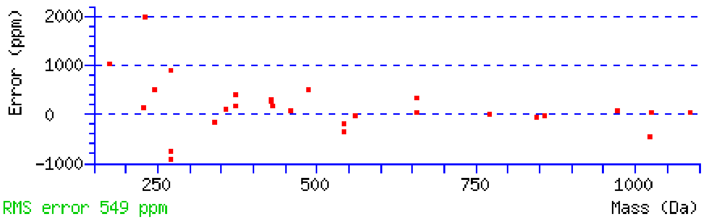


### All matches to this query

| Score | Mr(calc): | Delta | Sequence |
|---|---|---|---|
| 63.7 | 1214.5779 | -0.0041 | ENISDPTSPVR |
| 11.7 | 1213.5704 | 1.0034 | KTAEVGSSLSR |
| 8.3 | 1214.5809 | -0.0071 | AFKQNASLTR |
| 8.3 | 1213.5704 | 1.0034 | KTAEVGSSLSR |
| 8.3 | 1214.5779 | -0.0041 | QQTSDSNPPIK |
| 7.8 | 1214.5788 | -0.0050 | MITVNPAHMGK |
| 7.7 | 1212.5751 | 1.9986 | KAKDDATLSGK |
| 7.5 | 1212.5573 | 2.0164 | KNIMALSDGGK |
| 6.7 | 1214.5809 | -0.0071 | NKDVKAAFSR |
| 6.4 | 1213.5704 | 1.0034 | SLETQKTTAR |

Spectrum No: 463; Query: 214; Rank: 1

## Peptide View

MS/MS Fragmentation of **YNQSVSNPCK**
Found in **IPI00205325**, Tax_Id=10116 Gene_Symbol=Lrp2 Low-density lipoprotein receptor-related protein 2 precursor

Match to Query 214: 1196.509028 from(599.261790,2+)
Title: 091008RatKidney_NH4Format01_25.436.436.2.dta
Data file K:\NewmanPaper\Piliang\3SubProteomes\Piliang3SP\mgf5ppm\ERLIC_3SubProteomes5ppm.mgf

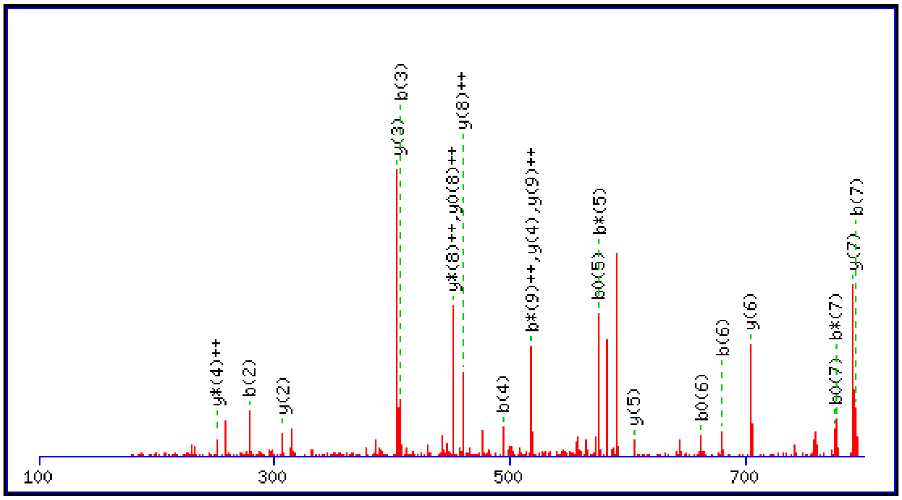


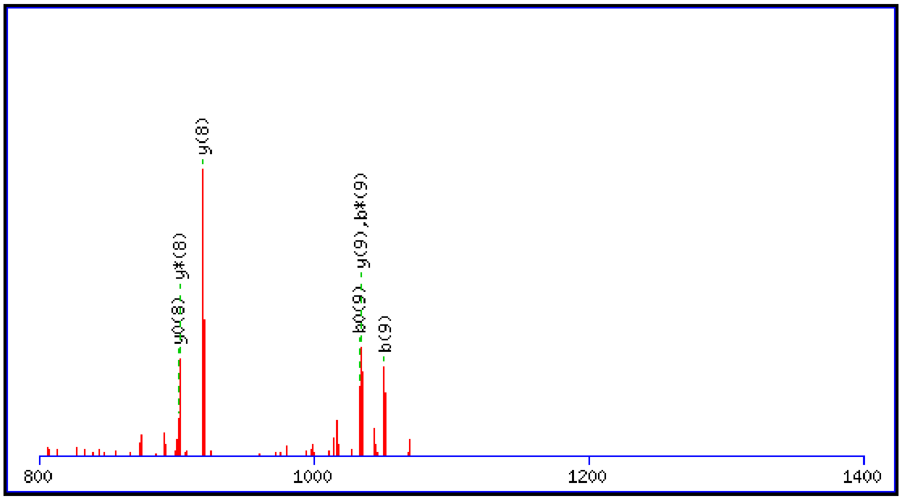


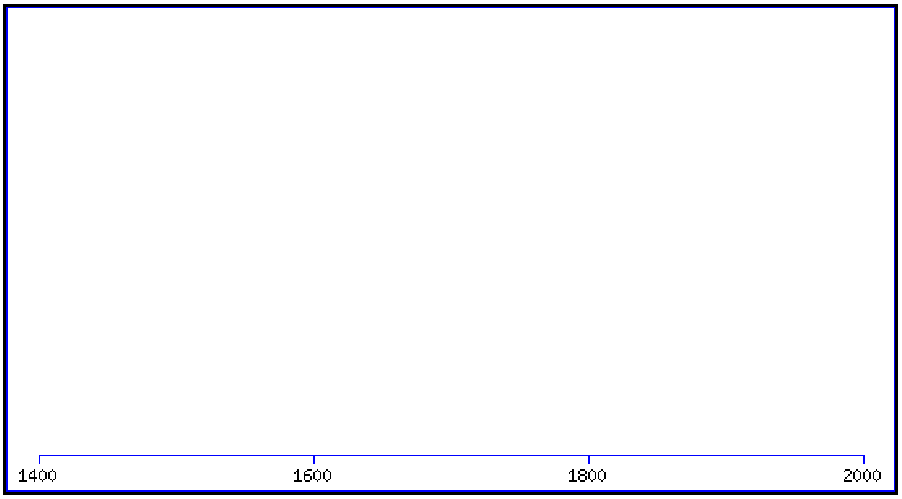

**Monoisotopic mass of neutral peptide Mr(calc):** 1196.5132
**Fixed modifications:** Carbamidomethyl (C)
**Variable modifications:**
**N2** : Deamidated_N (N)
**Ions Score:** 64 **Expect:** 4.2e-005
**Matches (Bold Red):** 29/92 fragment ions using 39 most intense peaks

| # | b | b$^{++}$ | b* | b*$^{++}$ | b$^{0}$ | b$^{0++}$ | Seq. | y | y$^{++}$ | y* | y*$^{++}$ | y$^{0}$ | y$^{0++}$ | # |
|---|---|---|---|---|---|---|---|---|---|---|---|---|---|---|
| 1 | 164.0706 | 82.5389 | | | | | Y | | | | | | | 10 |
| 2 | **279.0975** | 140.0524 | 262.0710 | 131.5391 | | | N | **1034.4571** | **517.7322** | 1017.4306 | 509.2189 | 1016.4466 | 508.7269 | 9 |
| 3 | **407.1561** | 204.0817 | 390.1296 | 195.5684 | | | Q | **919.4302** | **460.2187** | **902.4036** | **451.7055** | **901.4196** | **451.2135** | 8 |
| 4 | **494.1881** | 247.5977 | 477.1616 | 239.0844 | 476.1776 | 238.5924 | S | **791.3716** | 396.1894 | 774.3451 | 387.6762 | 773.3610 | 387.1842 | 7 |
| 5 | 593.2566 | 297.1319 | **576.2300** | 288.6186 | **575.2460** | 288.1266 | V | **704.3396** | 352.6734 | 687.3130 | 344.1602 | 686.3290 | 343.6681 | 6 |
| 6 | **680.2886** | 340.6479 | 663.2620 | 332.1347 | **662.2780** | 331.6427 | S | **605.2712** | 303.1392 | 588.2446 | 294.6259 | 587.2606 | 294.1339 | 5 |
| 7 | **794.3315** | 397.6694 | **777.3050** | 389.1561 | **776.3210** | 388.6641 | N | **518.2391** | 259.6232 | 501.2126 | **251.1099** | | | 4 |
| 8 | 891.3843 | 446.1958 | 874.3577 | 437.6825 | 873.3737 | 437.1905 | P | **404.1962** | 202.6017 | 387.1697 | 194.0885 | | | 3 |
| 9 | **1051.4149** | 526.2111 | **1034.3884** | **517.6978** | **1033.4044** | 517.2058 | C | **307.1435** | 154.0754 | 290.1169 | 145.5621 | | | 2 |
| 10 | | | | | | | K | 147.1128 | 74.0600 | 130.0863 | 65.5468 | | | 1 |

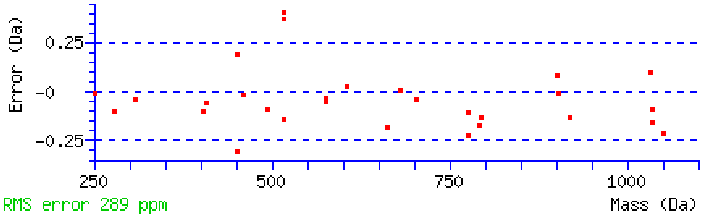


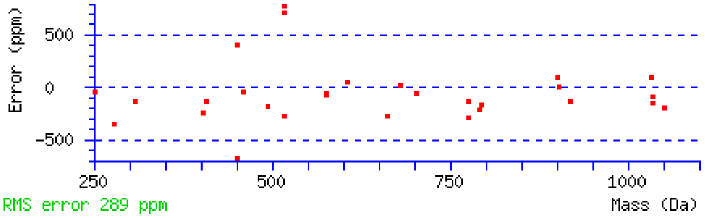


### All matches to this query

| Score | Mr(calc): | Delta | Sequence |
|---|---|---|---|
| 63.7 | 1196.5132 | -0.0041 | YNQSVSNPCK |
| 36.2 | 1196.5132 | -0.0041 | YNQSVSNPCK |
| 17.7 | 1196.5206 | -0.0115 | MWLEESQMK |
| 13.4 | 1196.5165 | -0.0075 | NSEMKNSMEK |
| 9.2 | 1196.5002 | 0.0088 | YYSLDELSK |
| 9.1 | 1196.5165 | -0.0075 | NAEMKNAMQK |
| 8.3 | 1196.5131 | -0.0041 | STLINTLFK |
| 7.3 | 1196.5131 | -0.0041 | RNSYNPGEMK |
| 7.2 | 1196.5121 | -0.0031 | HSRSRSMEK |
| 7.2 | 1196.5121 | -0.0031 | HSRSRSMEK |

---

Spectrum No: 464; Query: 2632; Rank: 1

---

## Peptide View

MS/MS Fragmentation of **ETIGPQQGCGGYLTEDNQSFVSPDSDSNGR**
Found in **IPI00196620**, Tax_Id=10116 Gene_Symbol=Cubn Cubilin precursor

Match to Query 2632: 3215.373402 from(1072.798410,3+)
Title: 100101RatKid_NS_deglyco_20.3067.3067.3.dta
Data file K:\NewmanPaper\Piliang\3SubProteomes\Piliang3SP\mgf5ppm\ERLIC_3SubProteomes5ppm.mgf

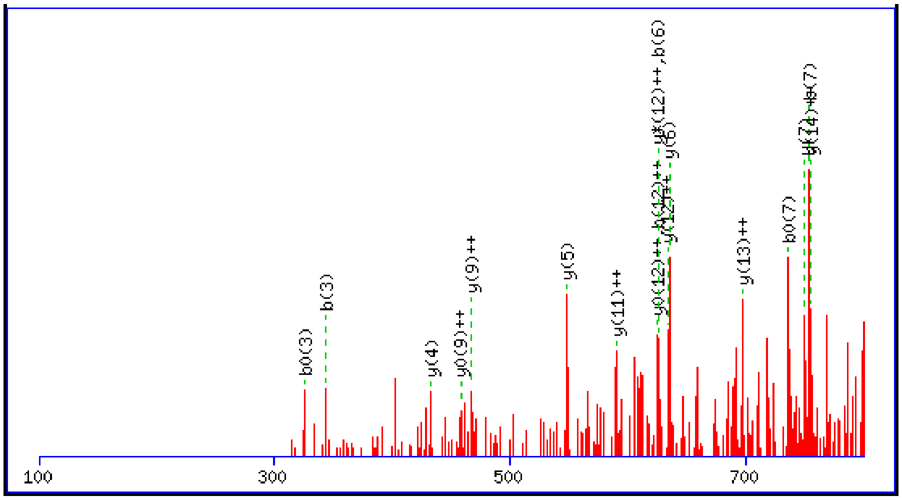


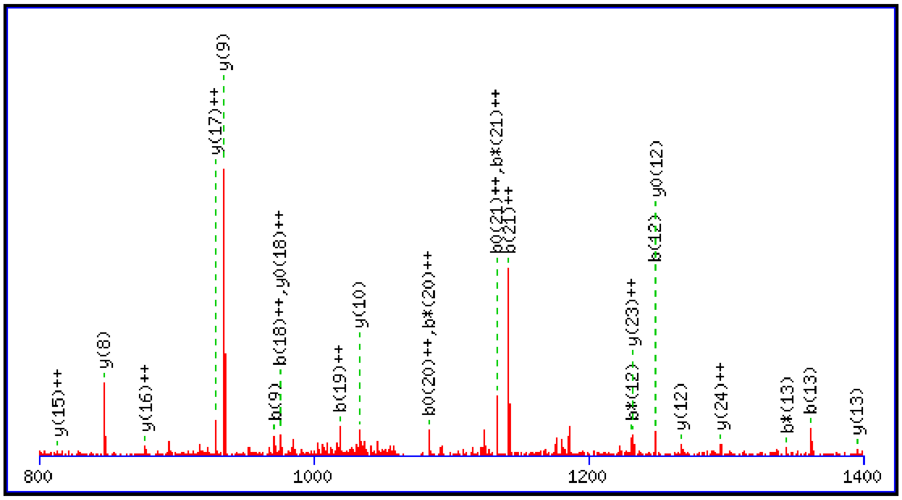


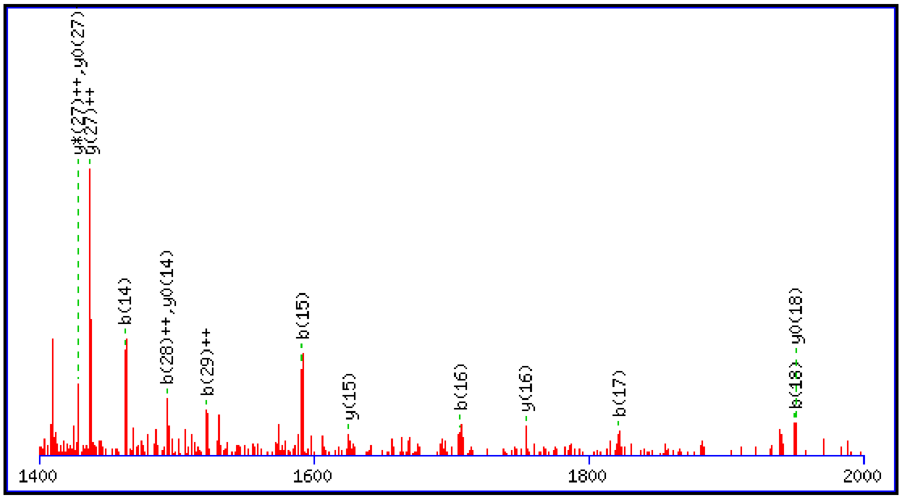


**Monoisotopic mass of neutral peptide Mr(calc):** 3215.3586
**Fixed modifications:** Carbamidomethyl (C)
**Variable modifications:**
**N17** : Deamidated_N (N)
**Ions Score:** 64 **Expect:** 0.00019
**Matches (Bold Red):** 56/332 fragment ions using 91 most intense peaks

| # | b | $b^{++}$ | b* | $b^{*++}$ | $b^0$ | $b^{0++}$ | Seq. | y | $y^{++}$ | y* | $y^{*++}$ | $y^0$ | $y^{0++}$ | # |
|---|---|---|---|---|---|---|---|---|---|---|---|---|---|---|
| 1 | 130.0499 | 65.5286 | | | 112.0393 | 56.5233 | E | | | | | | | 30 |
| 2 | 231.0975 | 116.0524 | | | 213.0870 | 107.0471 | T | 3087.3232 | 1544.1652 | 3070.2967 | 1535.6520 | 3069.3127 | 1535.1600 | 29 |
| 3 | **344.1816** | 172.5944 | | | **326.1710** | 163.5892 | I | 2986.2755 | 1493.6414 | 2969.2490 | 1485.1281 | 2968.2650 | 1484.6361 | 28 |
| 4 | 401.2031 | 201.1052 | | | 383.1925 | 192.0999 | G | 2873.1915 | **1437.0994** | 2856.1649 | **1428.5861** | 2855.1809 | **1428.0941** | 27 |
| 5 | 498.2558 | 249.6316 | | | 480.2453 | 240.6263 | P | 2816.1700 | 1408.5886 | 2799.1435 | 1400.0754 | 2798.1595 | 1399.5834 | 26 |

| | | | | | | | | | | | | | | |
|---|---|---|---|---|---|---|---|---|---|---|---|---|---|---|
| 6 | 626.3144 | 313.6608 | 609.2879 | 305.1476 | 608.3039 | 304.6556 | Q | 2719.1173 | 1360.0623 | 2702.0907 | 1351.5490 | 2701.1067 | 1351.0570 | 25 |
| 7 | 754.3730 | 377.6901 | 737.3464 | 369.1769 | 736.3624 | 368.6849 | Q | 2591.0587 | 1296.0330 | 2574.0321 | 1287.5197 | 2573.0481 | 1287.0277 | 24 |
| 8 | 811.3945 | 406.2009 | 794.3679 | 397.6876 | 793.3839 | 397.1956 | G | 2463.0001 | 1232.0037 | 2445.9735 | 1223.4904 | 2444.9895 | 1222.9984 | 23 |
| 9 | 971.4251 | 486.2162 | 954.3986 | 477.7029 | 953.4145 | 477.2109 | C | 2405.9786 | 1203.4930 | 2388.9521 | 1194.9797 | 2387.9681 | 1194.4877 | 22 |
| 10 | 1028.4466 | 514.7269 | 1011.4200 | 506.2137 | 1010.4360 | 505.7216 | G | 2245.9480 | 1123.4776 | 2228.9214 | 1114.9644 | 2227.9374 | 1114.4723 | 21 |
| 11 | 1085.4680 | 543.2377 | 1068.4415 | 534.7244 | 1067.4575 | 534.2324 | G | 2188.9265 | 1094.9669 | 2171.9000 | 1086.4536 | 2170.9160 | 1085.9616 | 20 |
| 12 | 1248.5314 | 624.7693 | 1231.5048 | 616.2560 | 1230.5208 | 615.7640 | Y | 2131.9051 | 1066.4562 | 2114.8785 | 1057.9429 | 2113.8945 | 1057.4509 | 19 |
| 13 | 1361.6154 | 681.3114 | 1344.5889 | 672.7981 | 1343.6049 | 672.3061 | L | 1968.8417 | 984.9245 | 1951.8152 | 976.4112 | 1950.8312 | 975.9192 | 18 |
| 14 | 1462.6631 | 731.8352 | 1445.6366 | 723.3219 | 1444.6525 | 722.8299 | T | 1855.7577 | 928.3825 | 1838.7311 | 919.8692 | 1837.7471 | 919.3772 | 17 |
| 15 | 1591.7057 | 796.3565 | 1574.6792 | 787.8432 | 1573.6951 | 787.3512 | E | 1754.7100 | 877.8586 | 1737.6834 | 869.3454 | 1736.6994 | 868.8533 | 16 |
| 16 | 1706.7326 | 853.8700 | 1689.7061 | 845.3567 | 1688.7221 | 844.8647 | D | 1625.6674 | 813.3373 | 1608.6408 | 804.8241 | 1607.6568 | 804.3321 | 15 |
| 17 | 1821.7596 | 911.3834 | 1804.7330 | 902.8702 | 1803.7490 | 902.3781 | N | 1510.6404 | 755.8239 | 1493.6139 | 747.3106 | 1492.6299 | 746.8186 | 14 |
| 18 | 1949.8182 | 975.4127 | 1932.7916 | 966.8994 | 1931.8076 | 966.4074 | Q | 1395.6135 | 698.3104 | 1378.5870 | 689.7971 | 1377.6029 | 689.3051 | 13 |
| 19 | 2036.8502 | 1018.9287 | 2019.8236 | 1010.4155 | 2018.8396 | 1009.9234 | S | 1267.5549 | 634.2811 | 1250.5284 | 625.7678 | 1249.5444 | 625.2758 | 12 |
| 20 | 2183.9186 | 1092.4629 | 2166.8921 | 1083.9497 | 2165.9080 | 1083.4577 | F | 1180.5229 | 590.7651 | 1163.4964 | 582.2518 | 1162.5123 | 581.7598 | 11 |
| 21 | 2282.9870 | 1141.9971 | 2265.9605 | 1133.4839 | 2264.9764 | 1132.9919 | V | 1033.4545 | 517.2309 | 1016.4279 | 508.7176 | 1015.4439 | 508.2256 | 10 |
| 22 | 2370.0190 | 1185.5132 | 2352.9925 | 1176.9999 | 2352.0085 | 1176.5079 | S | 934.3861 | 467.6967 | 917.3595 | 459.1834 | 916.3755 | 458.6914 | 9 |
| 23 | 2467.0718 | 1234.0395 | 2450.0453 | 1225.5263 | 2449.0612 | 1225.0343 | P | 847.3540 | 424.1807 | 830.3275 | 415.6674 | 829.3435 | 415.1754 | 8 |
| 24 | 2582.0987 | 1291.5530 | 2565.0722 | 1283.0397 | 2564.0882 | 1282.5477 | D | 750.3013 | 375.6543 | 733.2747 | 367.1410 | 732.2907 | 366.6490 | 7 |
| 25 | 2669.1308 | 1335.0690 | 2652.1042 | 1326.5558 | 2651.1202 | 1326.0637 | S | 635.2743 | 318.1408 | 618.2478 | 309.6275 | 617.2638 | 309.1355 | 6 |
| 26 | 2784.1577 | 1392.5825 | 2767.1312 | 1384.0692 | 2766.1472 | 1383.5772 | D | 548.2423 | 274.6248 | 531.2158 | 266.1115 | 530.2317 | 265.6195 | 5 |
| 27 | 2871.1897 | 1436.0985 | 2854.1632 | 1427.5852 | 2853.1792 | 1427.0932 | S | 433.2154 | 217.1113 | 416.1888 | 208.5980 | 415.2048 | 208.1060 | 4 |
| 28 | 2985.2327 | 1493.1200 | 2968.2061 | 1484.6067 | 2967.2221 | 1484.1147 | N | 346.1833 | 173.5953 | 329.1568 | 165.0820 | | | 3 |
| 29 | 3042.2541 | 1521.6307 | 3025.2276 | 1513.1174 | 3024.2436 | 1512.6254 | G | 232.1404 | 116.5738 | 215.1139 | 108.0606 | | | 2 |
| 30 | | | | | | | R | 175.1190 | 88.0631 | 158.0924 | 79.5498 | | | 1 |

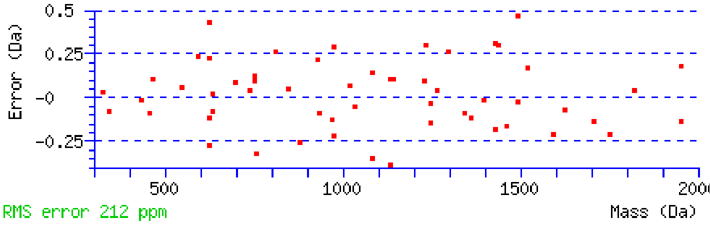


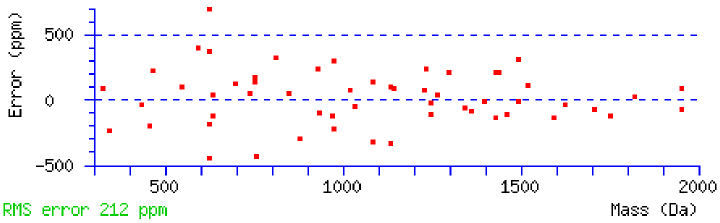


## All matches to this query

| Score | Mr(calc): | Delta | Sequence |
|---|---|---|---|
| 63.7 | 3215.3586 | 0.0148 | ETIGPQQGCGGYLTEDNQSFVSPDSDSNGR |
| 42.8 | 3214.3746 | 0.9988 | ETIGPQQGCGGYLTEDNQSFVSPDSDSNGR |
| 28.1 | 3215.3586 | 0.0148 | ETIGPQQGCGGYLTEDNQSFVSPDSDSNGR |
| 12.4 | 3215.4044 | -0.0310 | REALALQGSLEFDDAGGEGVTSHVDDEYR |
| 10.6 | 3215.3491 | 0.0243 | TWRGSSIHQDFLGFYVLDSFRMSAR |
| 10.6 | 3215.3491 | 0.0243 | TWRGSSIHQDFLGFYVLDSFRMSAR |
| 7.5 | 3215.3491 | 0.0243 | TWRGSSIHQDFLGFYVLDSFRMSAR |
| 5.7 | 3214.3573 | 1.0161 | FTEFQTQALQSFFETSAYPKDGEVER |
| 4.8 | 3214.3761 | 0.9973 | YNPSPDQSVSVTVTTNQAAPSSIALVQAK |
| 3.6 | 3215.3705 | 0.0029 | MASHEIEIGTIFTNINATDNHVHSMLK |

Spectrum No: 465; Query: 1145; Rank: 1

# Peptide View

MS/MS Fragmentation of **SDLNPANGSYPFQALR**
Found in **IPI00200195**, Tax_Id=10116 Gene_Symbol=LOC246120 Putative phospholipase B-like 2 precursor

Match to Query 1145: 1749.833768 from(875.924160,2+)
Title: 091008RatKidney_NH4Format01_24.3086.3086.2.dta
Data file K:\NewmanPaper\Piliang\3SubProteomes\Piliang3SP\mgf5ppm\ERLIC_3SubProteomes5ppm.mgf

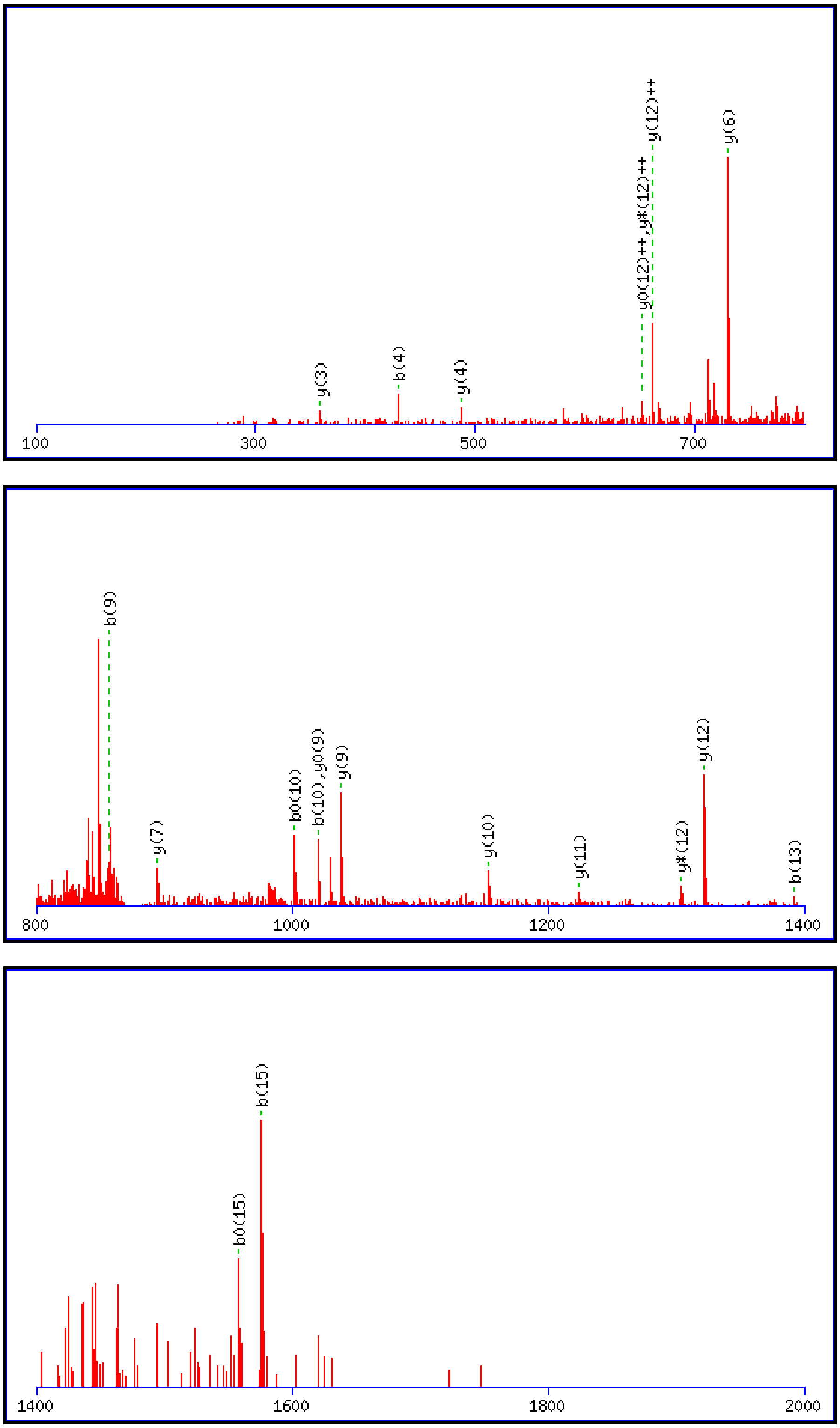


**Monoisotopic mass of neutral peptide Mr(calc):** 1749.8322
**Fixed modifications:** Carbamidomethyl (C)
**Variable modifications:**
**N7** : Deamidated_N (N)
**Ions Score:** 64 **Expect:** 8.6e-005
**Matches (Bold Red):** 20/160 fragment ions using 35 most intense peaks

| # | b | b++ | b* | b*++ | b0 | b0++ | Seq. | y | y++ | y* | y*++ | y0 | y0++ | # |
|---|---|---|---|---|---|---|---|---|---|---|---|---|---|---|
| 1 | 88.0393 | 44.5233 | | | 70.0287 | 35.5180 | S | | | | | | | 16 |
| 2 | 203.0662 | 102.0368 | | | 185.0557 | 93.0315 | D | 1663.8075 | 832.4074 | 1646.7809 | 823.8941 | 1645.7969 | 823.4021 | 15 |
| 3 | 316.1503 | 158.5788 | | | 298.1397 | 149.5735 | L | 1548.7805 | 774.8939 | 1531.7540 | 766.3806 | 1530.7699 | 765.8886 | 14 |
| 4 | **430.1932** | 215.6003 | 413.1667 | 207.0870 | 412.1827 | 206.5950 | N | 1435.6964 | 718.3519 | 1418.6699 | 709.8386 | 1417.6859 | 709.3466 | 13 |
| 5 | 527.2460 | 264.1266 | 510.2195 | 255.6134 | 509.2354 | 255.1214 | P | **1321.6535** | **661.3304** | **1304.6270** | **652.8171** | 1303.6430 | **652.3251** | 12 |
| 6 | 598.2831 | 299.6452 | 581.2566 | 291.1319 | 580.2726 | 290.6399 | A | **1224.6008** | 612.8040 | 1207.5742 | 604.2907 | 1206.5902 | 603.7987 | 11 |
| 7 | 713.3101 | 357.1587 | 696.2835 | 348.6454 | 695.2995 | 348.1534 | N | **1153.5636** | 577.2855 | 1136.5371 | 568.7722 | 1135.5531 | 568.2802 | 10 |
| 8 | 770.3315 | 385.6694 | 753.3050 | 377.1561 | 752.3210 | 376.6641 | G | **1038.5367** | 519.7720 | 1021.5102 | 511.2587 | **1020.5261** | 510.7667 | 9 |
| 9 | **857.3635** | 429.1854 | 840.3370 | 420.6721 | 839.3530 | 420.1801 | S | 981.5152 | 491.2613 | 964.4887 | 482.7480 | 963.5047 | 482.2560 | 8 |
| 10 | **1020.4269** | 510.7171 | 1003.4003 | 502.2038 | **1002.4163** | 501.7118 | Y | **894.4832** | 447.7452 | 877.4567 | 439.2320 | | | 7 |
| 11 | 1117.4796 | 559.2435 | 1100.4531 | 550.7302 | 1099.4691 | 550.2382 | P | **731.4199** | 366.2136 | 714.3933 | 357.7003 | | | 6 |
| 12 | 1264.5481 | 632.7777 | 1247.5215 | 624.2644 | 1246.5375 | 623.7724 | F | 634.3671 | 317.6872 | 617.3406 | 309.1739 | | | 5 |
| 13 | **1392.6066** | 696.8070 | 1375.5801 | 688.2937 | 1374.5961 | 687.8017 | Q | **487.2987** | 244.1530 | 470.2722 | 235.6397 | | | 4 |
| 14 | 1463.6437 | 732.3255 | 1446.6172 | 723.8122 | 1445.6332 | 723.3202 | A | **359.2401** | 180.1237 | 342.2136 | 171.6104 | | | 3 |
| 15 | **1576.7278** | 788.8675 | 1559.7013 | 780.3543 | **1558.7172** | 779.8623 | L | 288.2030 | 144.6051 | 271.1765 | 136.0919 | | | 2 |
| 16 | | | | | | | R | 175.1190 | 88.0631 | 158.0924 | 79.5498 | | | 1 |

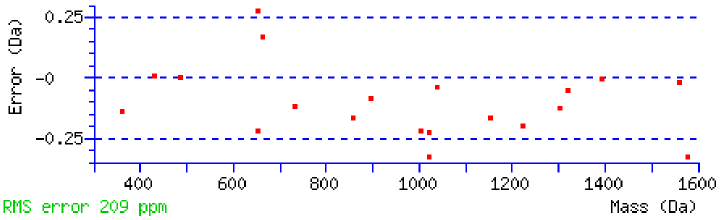


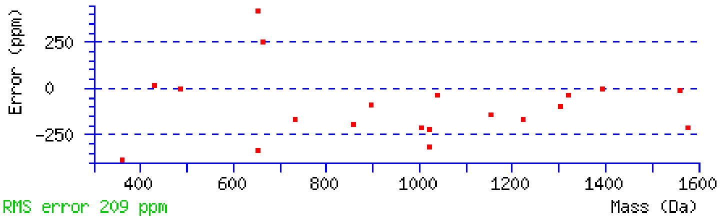


### All matches to this query

| Score | Mr(calc): | Delta | Sequence |
|---|---|---|---|
| 63.6 | 1749.8322 | 0.0016 | SDLNPANGSYPFQALR |
| 24.6 | 1749.8322 | 0.0016 | SDLNPANGSYPFQALR |
| 12.6 | 1749.8273 | 0.0065 | EKMTILAHDWAALR |
| 9.5 | 1749.8356 | -0.0018 | DSQVGTVNYMAPEAIR |
| 5.2 | 1749.8501 | -0.0163 | ETKTMAKVITTVLR |
| 5.1 | 1749.8506 | -0.0169 | SAEAGAAQAPGEXRGSVR |
| 1.5 | 1749.8355 | -0.0018 | FTVTPSTTQVVGILK |
| 0.9 | 1747.8294 | 2.0043 | KPSDYGYVSLGRAGAK |
| 0.9 | 1747.8294 | 2.0043 | KPSDYGYVSLGRAGAK |

---

Spectrum No: 466; Query: 2558; Rank: 1

---

## Peptide View

MS/MS Fragmentation of **MGAGEALGNASQLFEAWLKGNESIPVNLR**
Found in **IPI00327398**, Tax_Id=10116 Gene_Symbol=Enpep Isoform 1 of Glutamyl aminopeptidase

Match to Query 2558: 3073.533102 from(1025.518310,3+)
Title: 100101RatKid_NS_deglyco_14.7143.7143.3.dta
Data file K:\NewmanPaper\Piliang\3SubProteomes\Piliang3SP\mgf5ppm\ERLIC_3SubProteomes5ppm.mgf

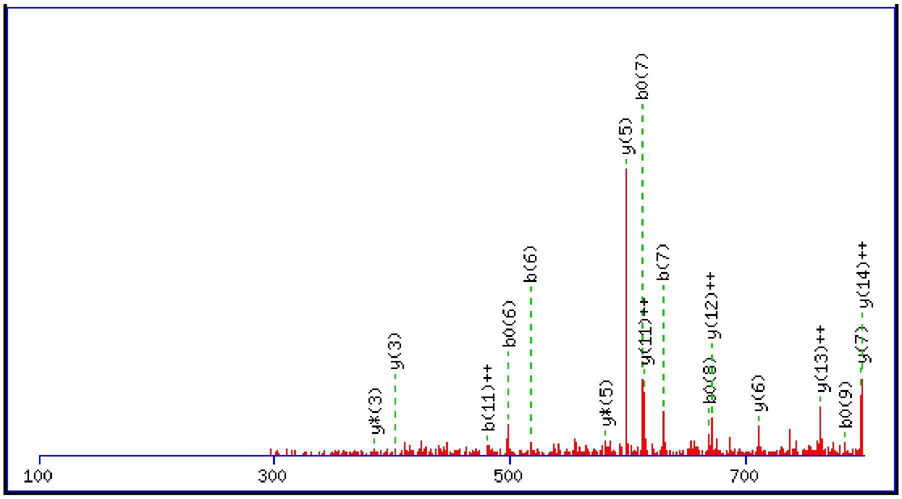


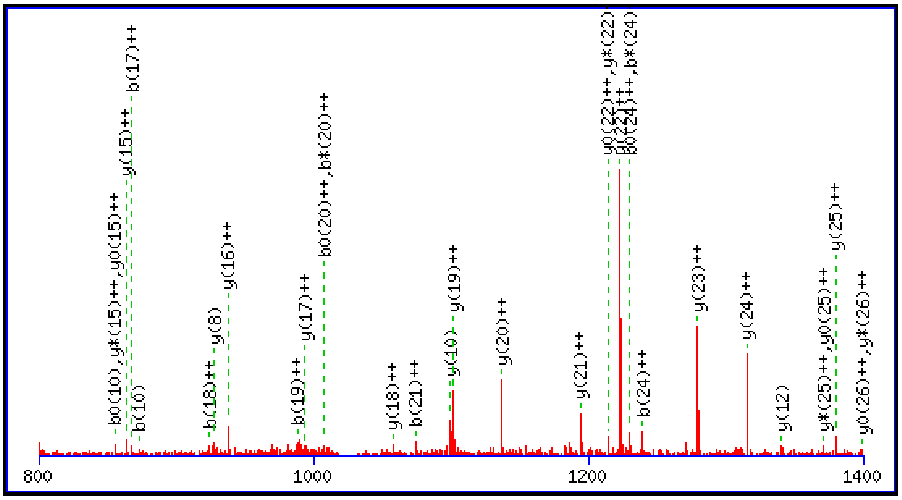


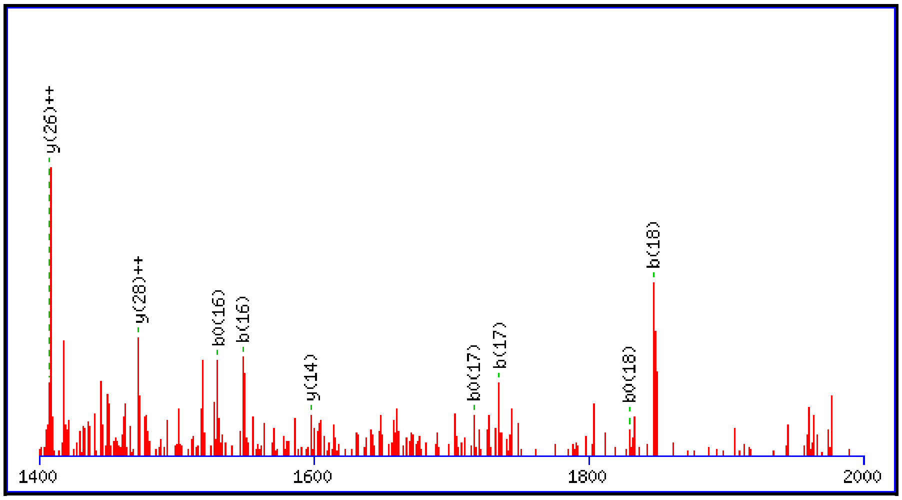


**Monoisotopic mass of neutral peptide Mr(calc):** 3073.5178
**Fixed modifications:** Carbamidomethyl (C)
**Variable modifications:**
**N9** : Deamidated_N (N)
**N21** : Deamidated_N (N)
**Ions Score:** 64 **Expect:** 0.0001
**Matches (Bold Red):** 59/300 fragment ions using 126 most intense peaks

| # | b | b++ | b* | b*++ | b0 | b0++ | Seq. | y | y++ | y* | y*++ | y0 | y0++ | # |
|---|---|---|---|---|---|---|---|---|---|---|---|---|---|---|
| 1 | 132.0478 | 66.5275 | | | | | M | | | | | | | 29 |
| 2 | 189.0692 | 95.0383 | | | | | G | 2943.4847 | **1472.2460** | 2926.4581 | 1463.7327 | 2925.4741 | 1463.2407 | 28 |
| 3 | 260.1063 | 130.5568 | | | | | A | 2886.4632 | 1443.7352 | 2869.4367 | 1435.2220 | 2868.4526 | 1434.7300 | 27 |
| 4 | 317.1278 | 159.0675 | | | | | G | 2815.4261 | **1408.2167** | 2798.3995 | **1399.7034** | 2797.4155 | **1399.2114** | 26 |
| | | | | | | | | | | | | | | |

| | | | | | | | | | | | | | | |
|---|---|---|---|---|---|---|---|---|---|---|---|---|---|---|
| 5 | 446.1704 | 223.5888 | | | 428.1598 | 214.5836 | E | 2758.4046 | **1379.7059** | 2741.3781 | **1371.1927** | 2740.3941 | **1370.7007** | 25 |
| 6 | **517.2075** | 259.1074 | | | **499.1969** | 250.1021 | A | 2629.3620 | **1315.1847** | 2612.3355 | 1306.6714 | 2611.3515 | 1306.1794 | 24 |
| 7 | **630.2916** | 315.6494 | | | **612.2810** | 306.6441 | L | 2558.3249 | **1279.6661** | 2541.2984 | 1271.1528 | 2540.3143 | 1270.6608 | 23 |
| 8 | 687.3130 | 344.1602 | | | **669.3025** | 335.1549 | G | 2445.2409 | **1223.1241** | 2428.2143 | **1214.6108** | 2427.2303 | **1214.1188** | 22 |
| 9 | 802.3400 | 401.6736 | 785.3134 | 393.1604 | **784.3294** | 392.6683 | N | 2388.2194 | **1194.6133** | 2371.1928 | 1186.1001 | 2370.2088 | 1185.6080 | 21 |
| 10 | **873.3771** | 437.1922 | 856.3505 | 428.6789 | **855.3665** | 428.1869 | A | 2273.1925 | **1137.0999** | 2256.1659 | 1128.5866 | 2255.1819 | 1128.0946 | 20 |
| 11 | 960.4091 | **480.7082** | 943.3826 | 472.1949 | 942.3986 | 471.7029 | S | 2202.1553 | **1101.5813** | 2185.1288 | 1093.0680 | 2184.1448 | 1092.5760 | 19 |
| 12 | 1088.4677 | 544.7375 | 1071.4411 | 536.2242 | 1070.4571 | 535.7322 | Q | 2115.1233 | **1058.0653** | 2098.0968 | 1049.5520 | 2097.1127 | 1049.0600 | 18 |
| 13 | 1201.5518 | 601.2795 | 1184.5252 | 592.7662 | 1183.5412 | 592.2742 | L | 1987.0647 | **994.0360** | 1970.0382 | 985.5227 | 1969.0542 | 985.0307 | 17 |
| 14 | 1348.6202 | 674.8137 | 1331.5936 | 666.3004 | 1330.6096 | 665.8084 | F | 1873.9807 | **937.4940** | 1856.9541 | 928.9807 | 1855.9701 | 928.4887 | 16 |
| 15 | 1477.6628 | 739.3350 | 1460.6362 | 730.8217 | 1459.6522 | 730.3297 | E | 1726.9123 | **863.9598** | 1709.8857 | **855.4465** | 1708.9017 | **854.9545** | 15 |
| 16 | **1548.6999** | 774.8536 | 1531.6733 | 766.3403 | **1530.6893** | 765.8483 | A | **1597.8697** | **799.4385** | 1580.8431 | 790.9252 | 1579.8591 | 790.4332 | 14 |
| 17 | **1734.7792** | **867.8932** | 1717.7526 | 859.3800 | **1716.7686** | 858.8880 | W | 1526.8325 | **763.9199** | 1509.8060 | 755.4066 | 1508.8220 | 754.9146 | 13 |
| 18 | **1847.8633** | **924.4353** | 1830.8367 | 915.9220 | **1829.8527** | 915.4300 | L | **1340.7532** | **670.8803** | 1323.7267 | 662.3670 | 1322.7427 | 661.8750 | 12 |
| 19 | 1975.9582 | **988.4827** | 1958.9317 | 979.9695 | 1957.9477 | 979.4775 | K | 1227.6692 | **614.3382** | 1210.6426 | 605.8249 | 1209.6586 | 605.3329 | 11 |
| 20 | 2032.9797 | 1016.9935 | 2015.9531 | **1008.4802** | 2014.9691 | **1007.9882** | G | **1099.5742** | 550.2907 | 1082.5477 | 541.7775 | 1081.5636 | 541.2855 | 10 |
| 21 | 2148.0066 | **1074.5069** | 2130.9801 | 1065.9937 | 2129.9961 | 1065.5017 | N | 1042.5527 | 521.7800 | 1025.5262 | 513.2667 | 1024.5422 | 512.7747 | 9 |
| 22 | 2277.0492 | 1139.0282 | 2260.0227 | 1130.5150 | 2259.0386 | 1130.0230 | E | **927.5258** | 464.2665 | 910.4993 | 455.7533 | 909.5152 | 455.2613 | 8 |
| 23 | 2364.0812 | 1182.5443 | 2347.0547 | 1174.0310 | 2346.0707 | 1173.5390 | S | **798.4832** | 399.7452 | 781.4567 | 391.2320 | 780.4726 | 390.7400 | 7 |
| 24 | 2477.1653 | **1239.0863** | 2460.1388 | **1230.5730** | 2459.1547 | **1230.0810** | I | **711.4512** | 356.2292 | 694.4246 | 347.7160 | | | 6 |
| 25 | 2574.2181 | 1287.6127 | 2557.1915 | 1279.0994 | 2556.2075 | 1278.6074 | P | **598.3671** | 299.6872 | **581.3406** | 291.1739 | | | 5 |
| 26 | 2673.2865 | 1337.1469 | 2656.2599 | 1328.6336 | 2655.2759 | 1328.1416 | V | 501.3144 | 251.1608 | 484.2878 | 242.6475 | | | 4 |
| 27 | 2787.3294 | 1394.1683 | 2770.3029 | 1385.6551 | 2769.3188 | 1385.1631 | N | **402.2459** | 201.6266 | **385.2194** | 193.1133 | | | 3 |
| 28 | 2900.4135 | 1450.7104 | 2883.3869 | 1442.1971 | 2882.4029 | 1441.7051 | L | 288.2030 | 144.6051 | 271.1765 | 136.0919 | | | 2 |
| 29 | | | | | | | R | 175.1190 | 88.0631 | 158.0924 | 79.5498 | | | 1 |

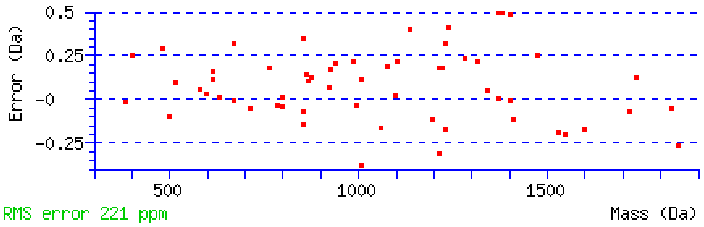


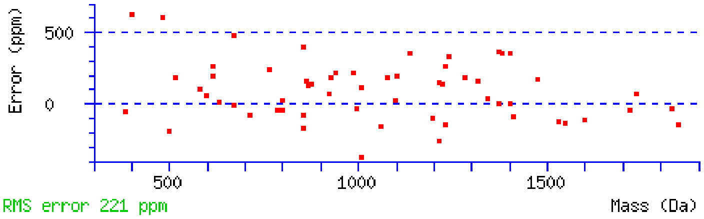


## All matches to this query

| Score | Mr(calc): | Delta | Sequence |
|---|---|---|---|
| 63.5 | 3073.5178 | 0.0153 | MGAGEALGNASQLFEAWLKGNESIPVNLR |
| 52.2 | 3073.5178 | 0.0153 | MGAGEALGNASQLFEAWLKGNESIPVNLR |
| 44.7 | 3073.5178 | 0.0153 | MGAGEALGNASQLFEAWLKGNESIPVNLR |
| 19.1 | 3072.5338 | 0.9993 | MGAGEALGNASQLFEAWLKGNESIPVNLR |
| 8.9 | 3072.5338 | 0.9993 | MGAGEALGNASQLFEAWLKGNESIPVNLR |
| 3.1 | 3072.5338 | 0.9993 | MGAGEALGNASQLFEAWLKGNESIPVNLR |
| 1.3 | 3071.5539 | 1.9792 | ALGTALIENNQLHVTLTQKQEEIASLR |
| 0.6 | 3073.5219 | 0.0112 | MQVRGREVSGLLISLTAVCLVVTPGSR |
| 0.6 | 3073.5219 | 0.0112 | MQVRGREVSGLLISLTAVCLVVTPGSR |
| 0.5 | 3072.5007 | 1.0324 | LSKNPYLNLASVLPSVCLSTAGKGTPPK |

Spectrum No: 467; Query: 539; Rank: 1

# Peptide View

MS/MS Fragmentation of **AIPTLVSDGNITVK**
Found in **IPI00212933**, Tax_Id=10116 Gene_Symbol=Slc5a1 Sodium/glucose cotransporter 1

Match to Query 539: 1427.790028 from(714.902290,2+)
Title: 100101RatKid_NS_deglyco_23.3148.3148.2.dta
Data file K:\NewmanPaper\Piliang\3SubProteomes\Piliang3SP\mgf5ppm\ERLIC_3SubProteomes5ppm.mgf

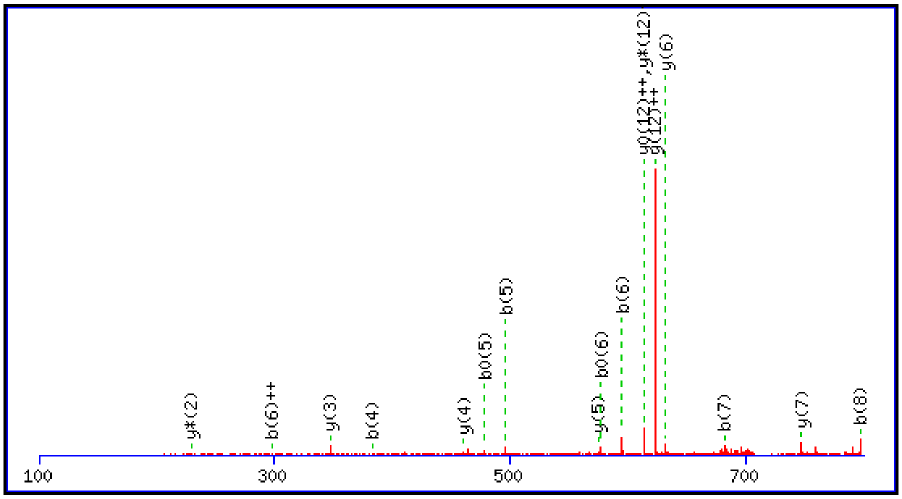


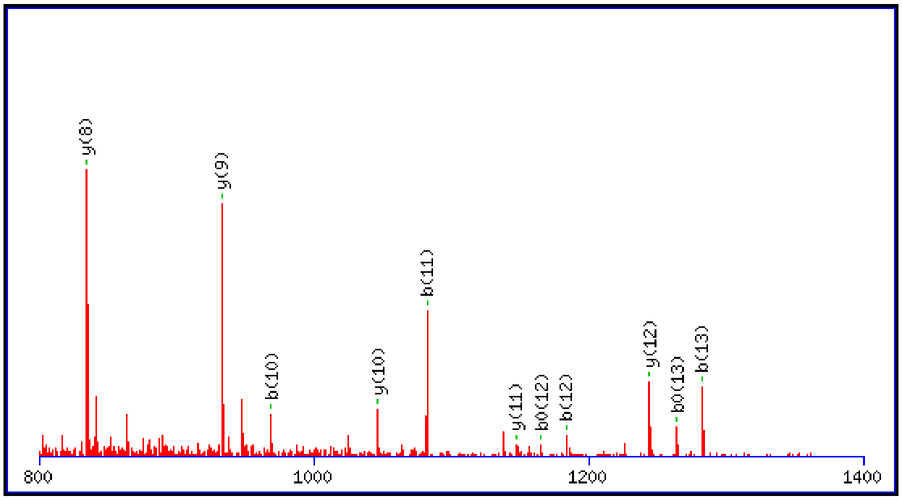


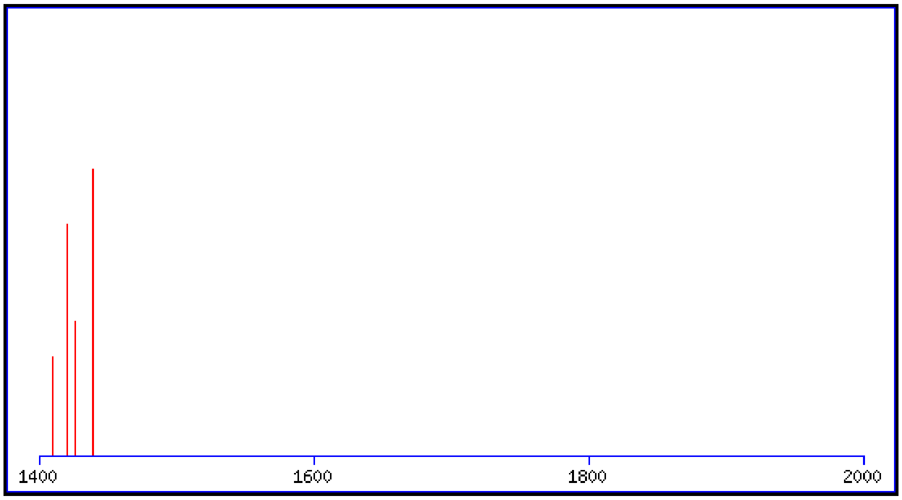


**Monoisotopic mass of neutral peptide Mr(calc):** 1427.7872
**Fixed modifications:** Carbamidomethyl (C)
**Variable modifications:**
**N10** : Deamidated_N (N)
**Ions Score:** 64 **Expect:** 3.1e-005
**Matches (Bold Red):** 29/128 fragment ions using 54 most intense peaks

| # | b | $b^{++}$ | b* | $b^{*++}$ | $b^0$ | $b^{0++}$ | Seq. | y | $y^{++}$ | y* | $y^{*++}$ | $y^0$ | $y^{0++}$ | # |
|---|---|---|---|---|---|---|---|---|---|---|---|---|---|---|
| 1 | 72.0444 | 36.5258 | | | | | A | | | | | | | 14 |
| 2 | 185.1285 | 93.0679 | | | | | I | 1357.7573 | 679.3823 | 1340.7308 | 670.8690 | 1339.7468 | 670.3770 | 13 |
| 3 | 282.1812 | 141.5942 | | | | | P | **1244.6733** | **622.8403** | 1227.6467 | **614.3270** | 1226.6627 | **613.8350** | 12 |
| 4 | **383.2289** | 192.1181 | | | 365.2183 | 183.1128 | T | **1147.6205** | 574.3139 | 1130.5939 | 565.8006 | 1129.6099 | 565.3086 | 11 |
| 5 | **496.3130** | 248.6601 | | | **478.3024** | 239.6548 | L | **1046.5728** | 523.7900 | 1029.5463 | 515.2768 | 1028.5622 | 514.7848 | 10 |
| 6 | **595.3814** | **298.1943** | | | **577.3708** | 289.1890 | V | **933.4887** | 467.2480 | 916.4622 | 458.7347 | 915.4782 | 458.2427 | 9 |
| 7 | **682.4134** | 341.7103 | | | 664.4028 | 332.7051 | S | **834.4203** | 417.7138 | 817.3938 | 409.2005 | 816.4098 | 408.7085 | 8 |
| 8 | **797.4403** | 399.2238 | | | 779.4298 | 390.2185 | D | **747.3883** | 374.1978 | 730.3618 | 365.6845 | 729.3777 | 365.1925 | 7 |
| 9 | 854.4618 | 427.7345 | | | 836.4512 | 418.7293 | G | **632.3614** | 316.6843 | 615.3348 | 308.1710 | **614.3508** | 307.6790 | 6 |
| 10 | **969.4887** | 485.2480 | 952.4622 | 476.7347 | 951.4782 | 476.2427 | N | **575.3399** | 288.1736 | 558.3133 | 279.6603 | 557.3293 | 279.1683 | 5 |
| 11 | **1082.5728** | 541.7900 | 1065.5463 | 533.2768 | 1064.5622 | 532.7848 | I | **460.3130** | 230.6601 | 443.2864 | 222.1468 | 442.3024 | 221.6548 | 4 |
| 12 | **1183.6205** | 592.3139 | 1166.5939 | 583.8006 | **1165.6099** | 583.3086 | T | **347.2289** | 174.1181 | 330.2023 | 165.6048 | 329.2183 | 165.1128 | 3 |
| 13 | **1282.6889** | 641.8481 | 1265.6624 | 633.3348 | **1264.6783** | 632.8428 | V | 246.1812 | 123.5942 | **229.1547** | 115.0810 | | | 2 |
| 14 | | | | | | | K | 147.1128 | 74.0600 | 130.0863 | 65.5468 | | | 1 |

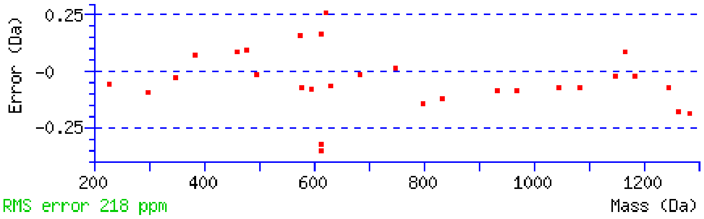


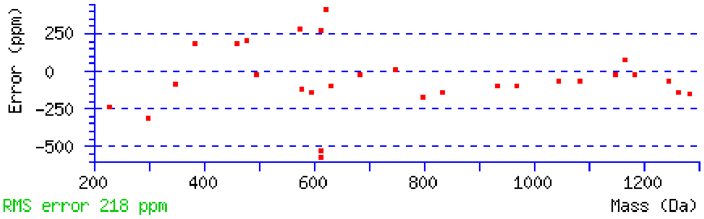


### All matches to this query

| Score | Mr(calc): | Delta | Sequence |
|---|---|---|---|
| 63.5 | 1427.7872 | 0.0029 | AIPTLVSDGNITVK |
| 5.0 | 1427.7871 | 0.0029 | DLPTLSNVQLISK |
| 4.8 | 1426.7780 | 1.0121 | NVIAPKGQEGSISK |
| 4.1 | 1427.7959 | -0.0059 | VLKELGFCVHVK |
| 3.2 | 1427.8024 | -0.0124 | ALLTVQEPPSFVK |
| 1.5 | 1425.7745 | 2.0156 | LQLGTPPPLLAAR |
| 1.2 | 1427.7901 | -0.0001 | SVLILPPRAGTPK |
| 0.9 | 1427.7844 | 0.0056 | ALRTENRNLLAR |
| 0.9 | 1427.7806 | 0.0094 | LAVLPEDRLQMK |
| 0.8 | 1425.7969 | 1.9931 | LARPPRLPVATR |

---

Spectrum No: 468; Query: 1766; Rank: 1

---

## Peptide View

MS/MS Fragmentation of **EGFSANYSVLQSSISEDFK**
Found in **IPI00212307**, Tax_Id=10116 Gene_Symbol=Nrp1 Neuropilin-1 precursor

Match to Query 1766: 2107.966568 from(1054.990560,2+)
Title: 100101RatKid_NS_deglyco_25.4615.4615.2.dta
Data file K:\NewmanPaper\Piliang\3SubProteomes\Piliang3SP\mgf5ppm\ERLIC_3SubProteomes5ppm.mgf

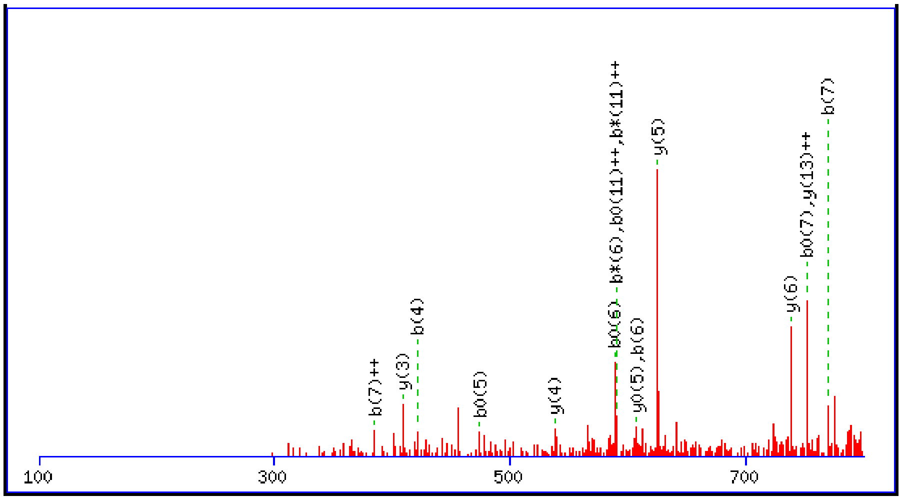


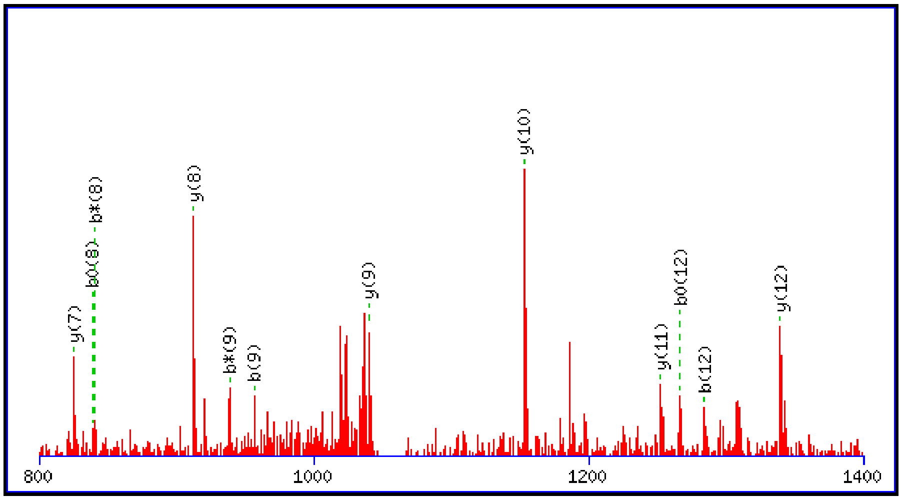


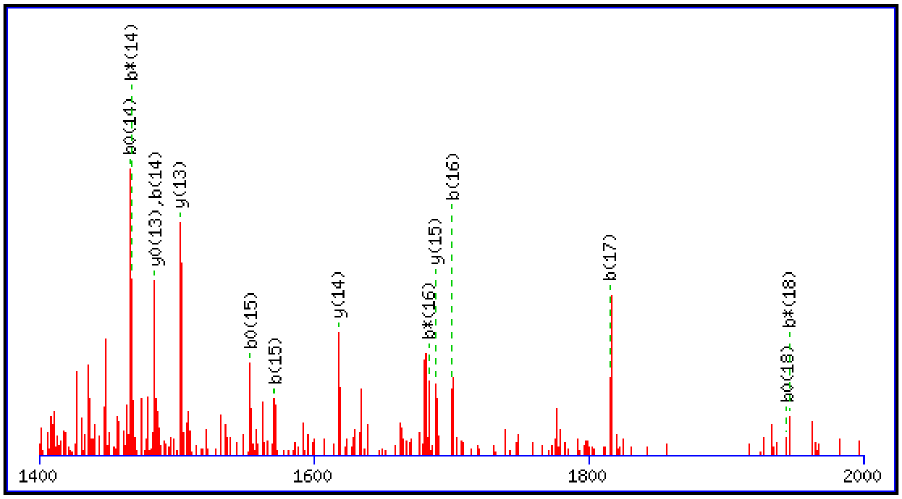


**Monoisotopic mass of neutral peptide Mr(calc):** 2107.9586
**Fixed modifications:** Carbamidomethyl (C)
**Variable modifications:**
**N6** : Deamidated_N (N)
**Ions Score:** 63 **Expect:** 0.00012
**Matches (Bold Red):** 42/202 fragment ions using 71 most intense peaks

| # | b | $b^{++}$ | b* | $b^{*++}$ | $b^{0}$ | $b^{0++}$ | Seq. | y | $y^{++}$ | y* | $y^{*++}$ | $y^{0}$ | $y^{0++}$ | # |
|---|---|---|---|---|---|---|---|---|---|---|---|---|---|---|
| 1 | 130.0499 | 65.5286 | | | 112.0393 | 56.5233 | E | | | | | | | 19 |
| 2 | 187.0713 | 94.0393 | | | 169.0608 | 85.0340 | G | 1979.9233 | 990.4653 | 1962.8967 | 981.9520 | 1961.9127 | 981.4600 | 18 |
| 3 | 334.1397 | 167.5735 | | | 316.1292 | 158.5682 | F | 1922.9018 | 961.9545 | 1905.8753 | 953.4413 | 1904.8912 | 952.9493 | 17 |
| 4 | **421.1718** | 211.0895 | | | 403.1612 | 202.0842 | S | 1775.8334 | 888.4203 | 1758.8068 | 879.9071 | 1757.8228 | 879.4151 | 16 |
| 5 | 492.2089 | 246.6081 | | | **474.1983** | 237.6028 | A | **1688.8014** | 844.9043 | 1671.7748 | 836.3910 | 1670.7908 | 835.8990 | 15 |

| | | | | | | | | | | | | | | |
|---|---|---|---|---|---|---|---|---|---|---|---|---|---|---|
| **6** | **607.2358** | 304.1216 | **590.2093** | 295.6083 | **589.2253** | 295.1163 | **N** | **1617.7643** | 809.3858 | 1600.7377 | 800.8725 | 1599.7537 | 800.3805 | **14** |
| **7** | **770.2992** | **385.6532** | 753.2726 | 377.1399 | **752.2886** | 376.6479 | **Y** | **1502.7373** | **751.8723** | 1485.7108 | 743.3590 | **1484.7268** | 742.8670 | **13** |
| **8** | 857.3312 | 429.1692 | **840.3046** | 420.6560 | **839.3206** | 420.1639 | **S** | **1339.6740** | 670.3406 | 1322.6474 | 661.8274 | 1321.6634 | 661.3353 | **12** |
| **9** | **956.3996** | 478.7034 | **939.3730** | 470.1902 | 938.3890 | 469.6982 | **V** | **1252.6420** | 626.8246 | 1235.6154 | 618.3113 | 1234.6314 | 617.8193 | **11** |
| **10** | 1069.4837 | 535.2455 | 1052.4571 | 526.7322 | 1051.4731 | 526.2402 | **L** | **1153.5735** | 577.2904 | 1136.5470 | 568.7771 | 1135.5630 | 568.2851 | **10** |
| **11** | 1197.5422 | 599.2748 | 1180.5157 | **590.7615** | 1179.5317 | **590.2695** | **Q** | **1040.4895** | 520.7484 | 1023.4629 | 512.2351 | 1022.4789 | 511.7431 | **9** |
| **12** | **1284.5743** | 642.7908 | 1267.5477 | 634.2775 | **1266.5637** | 633.7855 | **S** | **912.4309** | 456.7191 | 895.4044 | 448.2058 | 894.4203 | 447.7138 | **8** |
| **13** | 1371.6063 | 686.3068 | 1354.5797 | 677.7935 | 1353.5957 | 677.3015 | **S** | **825.3989** | 413.2031 | 808.3723 | 404.6898 | 807.3883 | 404.1978 | **7** |
| **14** | **1484.6904** | 742.8488 | **1467.6638** | 734.3355 | **1466.6798** | 733.8435 | **I** | **738.3668** | 369.6871 | 721.3403 | 361.1738 | 720.3563 | 360.6818 | **6** |
| **15** | **1571.7224** | 786.3648 | 1554.6958 | 777.8516 | **1553.7118** | 777.3595 | **S** | **625.2828** | 313.1450 | 608.2562 | 304.6318 | **607.2722** | 304.1397 | **5** |
| **16** | **1700.7650** | 850.8861 | **1683.7384** | 842.3729 | 1682.7544 | 841.8808 | **E** | **538.2508** | 269.6290 | 521.2242 | 261.1157 | 520.2402 | 260.6237 | **4** |
| **17** | **1815.7919** | 908.3996 | 1798.7654 | 899.8863 | 1797.7814 | 899.3943 | **D** | **409.2082** | 205.1077 | 392.1816 | 196.5944 | 391.1976 | 196.1024 | **3** |
| **18** | 1962.8603 | 981.9338 | **1945.8338** | 973.4205 | **1944.8498** | 972.9285 | **F** | 294.1812 | 147.5942 | 277.1547 | 139.0810 | | | **2** |
| **19** | | | | | | | **K** | 147.1128 | 74.0600 | 130.0863 | 65.5468 | | | **1** |

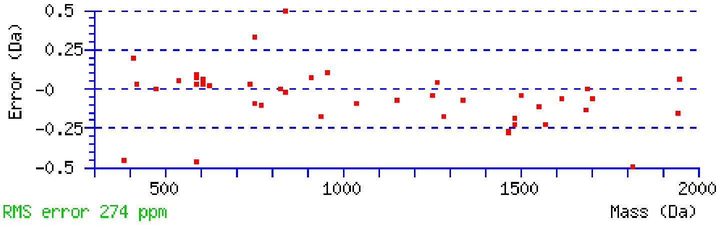


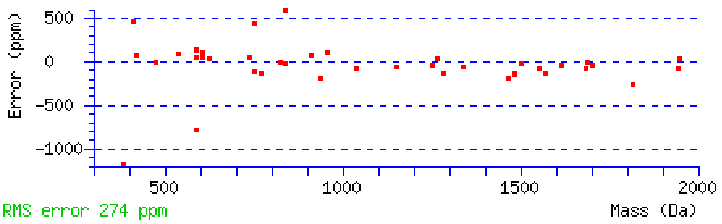


### All matches to this query

| Score | Mr(calc): | Delta | Sequence |
|---|---|---|---|
| 63.4 | 2107.9586 | 0.0080 | EGFSANYSVLQSSISEDFK |
| 40.9 | 2106.9746 | 0.9920 | EGFSANYSVLQSSISEDFK |
| 3.3 | 2107.9691 | -0.0025 | SIISETSNKIDTEIASLK |
| 3.0 | 2105.9499 | 2.0166 | RPMQQMQPTAPRNSNLR |
| 2.4 | 2107.9691 | -0.0025 | SIISETSNKIDTEIASLK |
| 1.8 | 2105.9656 | 2.0010 | LESQMMAMVERHETQVR |
| 1.8 | 2105.9656 | 2.0010 | LESQMMAMVERHETQVR |
| 1.1 | 2107.9510 | 0.0156 | KSNHLNHCDLSNILPHK |

---

Spectrum No: 469; Query: 1742; Rank: 1

---

## Peptide View

MS/MS Fragmentation of **IYHPNINSNGSICLDILR**
Found in **IPI00192159**, Tax_Id=10116 Gene_Symbol=Ube2d3 Ubiquitin-conjugating enzyme E2 D3

Match to Query 1742: 2099.048412 from(700.690080,3+)
Title: 091008RatKidney_NH4Format01_07.3658.3658.3.dta
Data file K:\NewmanPaper\Piliang\3SubProteomes\Piliang3SP\mgf5ppm\ERLIC_3SubProteomes5ppm.mgf

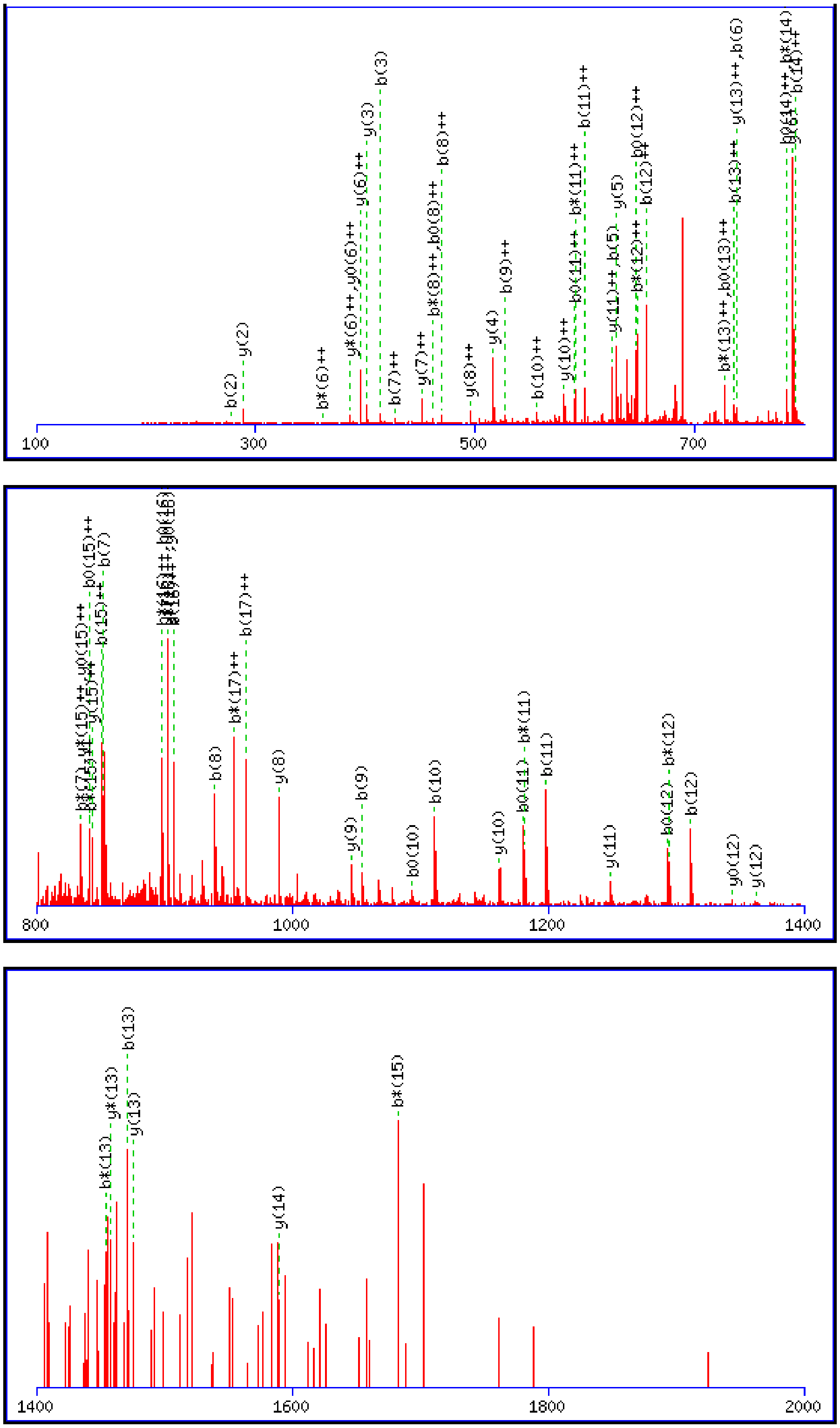


**Monoisotopic mass of neutral peptide Mr(calc):** 2099.0469
**Fixed modifications:** Carbamidomethyl (C)
**Variable modifications:**
**N9** : Deamidated_N (N)
**Ions Score:** 63 **Expect:** 8.8e-005
**Matches (Bold Red):** 74/176 fragment ions using 130 most intense peaks

| # | b | b++ | b* | b*++ | b0 | b0++ | Seq. | y | y++ | y* | y*++ | y0 | y0++ | # |
|---|---|---|---|---|---|---|---|---|---|---|---|---|---|---|
| 1 | 114.0913 | 57.5493 | | | | | I | | | | | | | 18 |
| 2 | **277.1547** | 139.0810 | | | | | Y | 1986.9702 | 993.9887 | 1969.9436 | 985.4755 | 1968.9596 | 984.9834 | 17 |
| 3 | **414.2136** | 207.6104 | | | | | H | 1823.9068 | 912.4571 | 1806.8803 | **903.9438** | 1805.8963 | **903.4518** | 16 |
| 4 | 511.2663 | 256.1368 | | | | | P | 1686.8479 | **843.9276** | 1669.8214 | **835.4143** | 1668.8374 | **834.9223** | 15 |
| 5 | **625.3093** | 313.1583 | 608.2827 | 304.6450 | | | N | **1589.7952** | 795.4012 | 1572.7686 | 786.8880 | 1571.7846 | 786.3959 | 14 |

| | | | | | | | | | | | | | | |
|---|---|---|---|---|---|---|---|---|---|---|---|---|---|---|
| 6 | **738.3933** | 369.7003 | 721.3668 | **361.1870** | | | I | **1475.7522** | **738.3798** | **1458.7257** | 729.8665 | 1457.7417 | 729.3745 | 13 |
| 7 | **852.4363** | **426.7218** | **835.4097** | 418.2085 | | | N | **1362.6682** | 681.8377 | 1345.6416 | 673.3245 | **1344.6576** | 672.8324 | 12 |
| 8 | **939.4683** | **470.2378** | 922.4417 | **461.7245** | 921.4577 | **461.2325** | S | **1248.6253** | **624.8163** | 1231.5987 | 616.3030 | 1230.6147 | 615.8110 | 11 |
| 9 | **1054.4952** | **527.7513** | 1037.4687 | 519.2380 | 1036.4847 | 518.7460 | N | **1161.5932** | **581.3003** | 1144.5667 | 572.7870 | 1143.5827 | 572.2950 | 10 |
| 10 | **1111.5167** | **556.2620** | 1094.4901 | 547.7487 | **1093.5061** | 547.2567 | G | **1046.5663** | 523.7868 | 1029.5397 | 515.2735 | 1028.5557 | 514.7815 | 9 |
| 11 | **1198.5487** | **599.7780** | **1181.5222** | **591.2647** | **1180.5382** | **590.7727** | S | **989.5448** | **495.2761** | 972.5183 | 486.7628 | 971.5343 | 486.2708 | 8 |
| 12 | **1311.6328** | **656.3200** | **1294.6062** | **647.8068** | **1293.6222** | **647.3147** | I | **902.5128** | **451.7600** | 885.4863 | 443.2468 | 884.5022 | 442.7548 | 7 |
| 13 | **1471.6634** | **736.3354** | **1454.6369** | **727.8221** | 1453.6529 | **727.3301** | C | **789.4287** | **395.2180** | 772.4022 | **386.7047** | 771.4182 | **386.2127** | 6 |
| 14 | 1584.7475 | **792.8774** | 1567.7209 | **784.3641** | 1566.7369 | **783.8721** | L | **629.3981** | 315.2027 | 612.3715 | 306.6894 | 611.3875 | 306.1974 | 5 |
| 15 | 1699.7744 | **850.3909** | **1682.7479** | **841.8776** | 1681.7639 | **841.3856** | D | **516.3140** | 258.6606 | 499.2875 | 250.1474 | 498.3035 | 249.6554 | 4 |
| 16 | 1812.8585 | **906.9329** | 1795.8320 | **898.4196** | 1794.8479 | **897.9276** | I | **401.2871** | 201.1472 | 384.2605 | 192.6339 | | | 3 |
| 17 | 1925.9426 | **963.4749** | 1908.9160 | **954.9616** | 1907.9320 | 954.4696 | L | **288.2030** | 144.6051 | 271.1765 | 136.0919 | | | 2 |
| 18 | | | | | | | R | 175.1190 | 88.0631 | 158.0924 | 79.5498 | | | 1 |

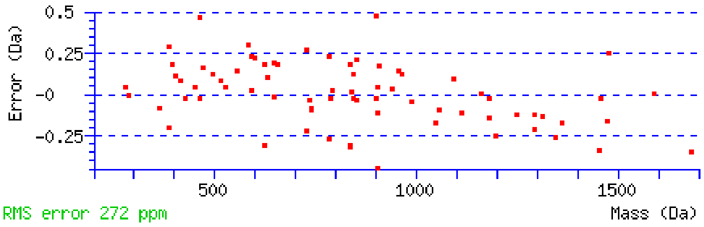


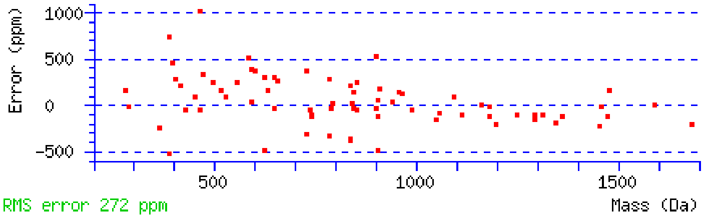


**All matches to this query**

| Score | Mr(calc): | Delta | Sequence |
|---|---|---|---|
| 63.4 | 2099.0469 | 0.0015 | IYHPNINSNGSICLDILR |
| 48.3 | 2099.0469 | 0.0015 | IYHPNINSNGSICLDILR |
| 41.0 | 2099.0469 | 0.0015 | IYHPNINSNGSICLDILR |
| 39.3 | 2098.0629 | 0.9855 | IYHPNINSNGSICLDILR |
| 14.6 | 2099.0663 | -0.0179 | RKINLNISYEINEVSVK |
| 8.7 | 2098.0360 | 1.0124 | FLEELRLSGNHLSHIPR |
| 8.3 | 2099.0663 | -0.0179 | RKINLNISYEINEVSVK |
| 5.1 | 2099.0485 | -0.0001 | LNKENNELYLELMKLR |
| 4.6 | 2098.0489 | 0.9995 | LSLKFRNGLTLSPVTHR |
| 2.9 | 2099.0486 | -0.0002 | RLMSSILTSIDASKPWSK |

---

Spectrum No: 470; Query: 157; Rank: 1

---

## Peptide View

MS/MS Fragmentation of **IYNASELPVR**
Found in **IPI00369937**, Tax_Id=10116 Gene_Symbol=Pigs GPI transamidase component PIG-S

Match to Query 157: 1161.601388 from(581.807970,2+)
Title: 091008RatKidney_NH4Format01_23.1693.1693.2.dta
Data file K:\NewmanPaper\Piliang\3SubProteomes\Piliang3SP\mgf5ppm\ERLIC_3SubProteomes5ppm.mgf

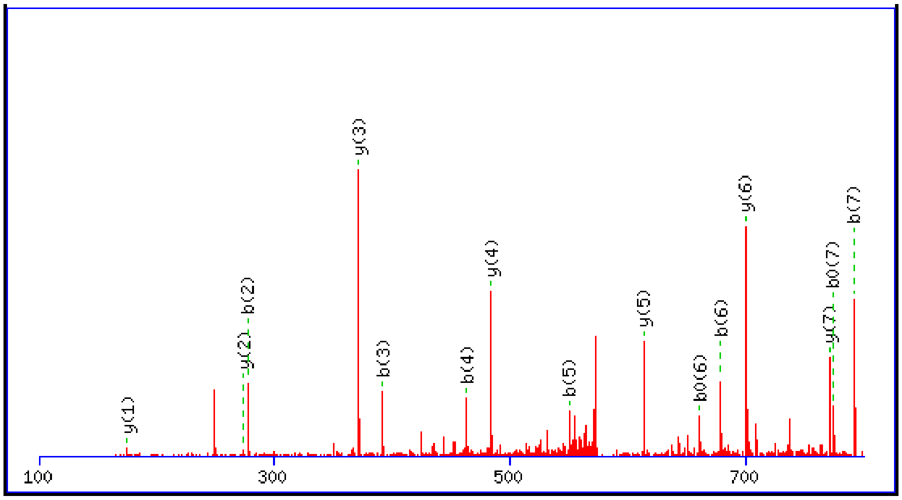


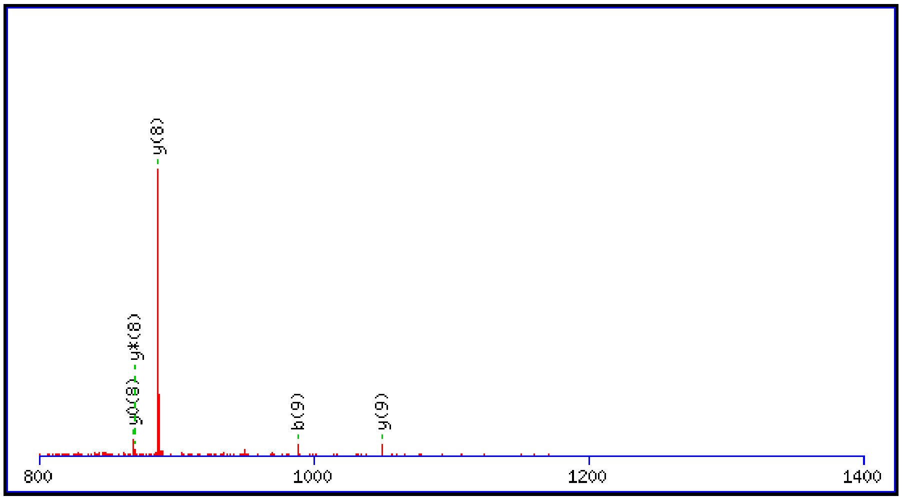


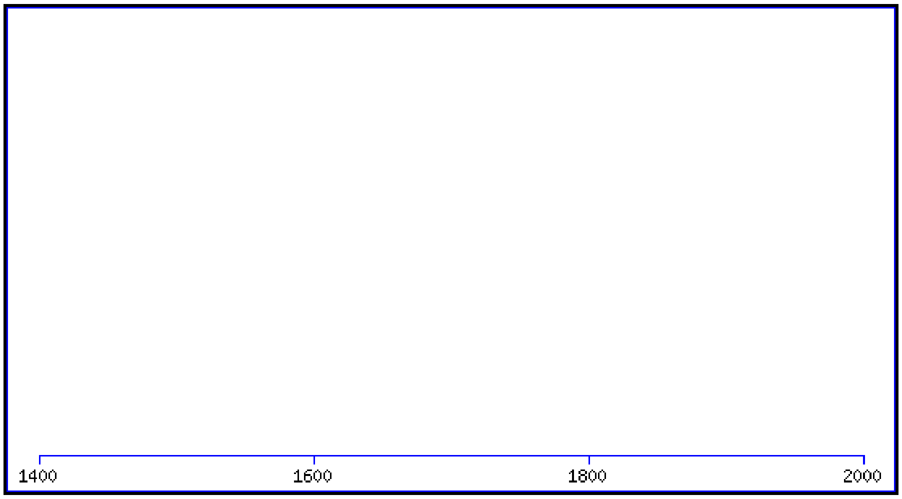


**Monoisotopic mass of neutral peptide Mr(calc):** 1161.6030
**Fixed modifications:** Carbamidomethyl (C)
**Variable modifications:**
**N3** : Deamidated_N (N)
**Ions Score:** 63 **Expect:** 5e-005
**Matches (Bold Red):** 20/88 fragment ions using 41 most intense peaks

| # | b | $b^{++}$ | b* | $b^{*++}$ | $b^0$ | $b^{0++}$ | Seq. | y | $y^{++}$ | y* | $y^{*++}$ | $y^0$ | $y^{0++}$ | # |
|---|---|---|---|---|---|---|---|---|---|---|---|---|---|---|
| 1 | 114.0913 | 57.5493 | | | | | I | | | | | | | 10 |
| 2 | **277.1547** | 139.0810 | | | | | Y | **1049.5262** | 525.2667 | 1032.4996 | 516.7535 | 1031.5156 | 516.2615 | 9 |
| 3 | **392.1816** | 196.5944 | 375.1551 | 188.0812 | | | N | **886.4629** | 443.7351 | **869.4363** | 435.2218 | **868.4523** | 434.7298 | 8 |
| 4 | **463.2187** | 232.1130 | 446.1922 | 223.5997 | | | A | **771.4359** | 386.2216 | 754.4094 | 377.7083 | 753.4254 | 377.2163 | 7 |
| 5 | **550.2507** | 275.6290 | 533.2242 | 267.1157 | 532.2402 | 266.6237 | S | **700.3988** | 350.7030 | 683.3723 | 342.1898 | 682.3883 | 341.6978 | 6 |

| | | | | | | | | | | | | | | |
|---|---|---|---|---|---|---|---|---|---|---|---|---|---|---|
| **6** | **679.2933** | 340.1503 | 662.2668 | 331.6370 | **661.2828** | 331.1450 | **E** | **613.3668** | 307.1870 | 596.3402 | 298.6738 | 595.3562 | 298.1817 | **5** |
| **7** | **792.3774** | 396.6923 | 775.3509 | 388.1791 | **774.3668** | 387.6871 | **L** | **484.3242** | 242.6657 | 467.2976 | 234.1525 | | | **4** |
| **8** | 889.4302 | 445.2187 | 872.4036 | 436.7054 | 871.4196 | 436.2134 | **P** | **371.2401** | 186.1237 | 354.2136 | 177.6104 | | | **3** |
| **9** | **988.4986** | 494.7529 | 971.4720 | 486.2397 | 970.4880 | 485.7476 | **V** | **274.1874** | 137.5973 | 257.1608 | 129.0840 | | | **2** |
| **10** | | | | | | | **R** | **175.1190** | 88.0631 | 158.0924 | 79.5498 | | | **1** |

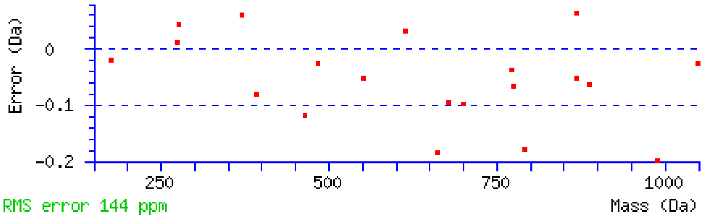


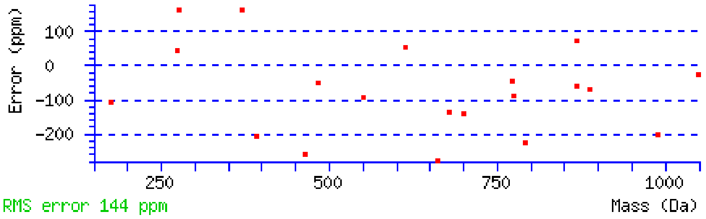


### All matches to this query

| Score | Mr(calc): | Delta | Sequence |
|---|---|---|---|
| 63.4 | 1161.6030 | -0.0016 | IYNASELPVR |
| 14.9 | 1161.5907 | 0.0107 | HLVLSSGTLR |
| 8.6 | 1160.5972 | 1.0042 | NKQIKCNEK |
| 6.4 | 1161.5907 | 0.0107 | HLVLSSGTLR |
| 6.2 | 1161.6030 | -0.0016 | LLLNGFDDVR |
| 5.9 | 1161.5998 | 0.0016 | ICXTQKLMR |
| 5.3 | 1161.5907 | 0.0107 | HLVLSSGTLR |
| 4.6 | 1161.5989 | 0.0024 | TRLDSSNLEK |
| 3.4 | 1161.6030 | -0.0016 | SSTLPELPYR |
| 2.6 | 1159.6019 | 1.9995 | EKELCQVVR |

---

Spectrum No: 471; Query: 584; Rank: 1

---

## Peptide View

MS/MS Fragmentation of **NSTLCDLCIGPAK**
Found in **IPI00196656**, Tax_Id=10116 Gene_Symbol=Srprb Ba1-667

Match to Query 584: 1448.667588 from(725.341070,2+)
Title: 100101RatKid_NS_deglyco_25.2621.2621.2.dta
Data file K:\NewmanPaper\Piliang\3SubProteomes\Piliang3SP\mgf5ppm\ERLIC_3SubProteomes5ppm.mgf

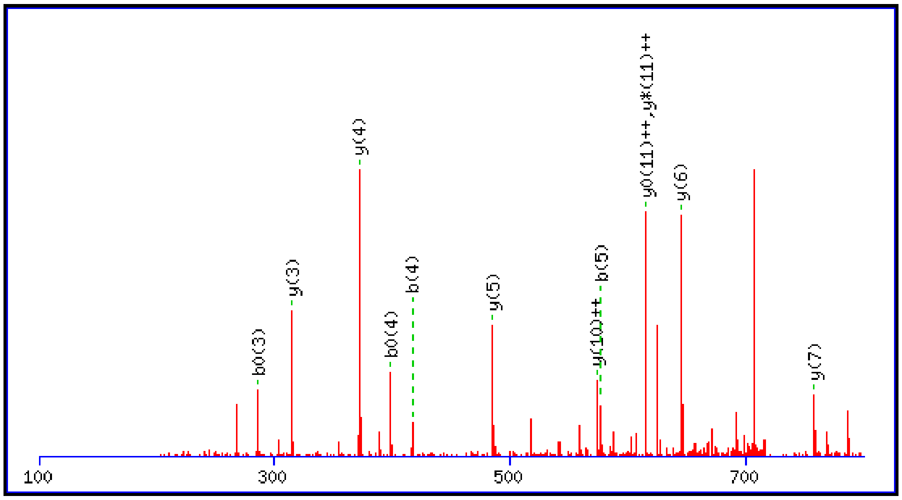

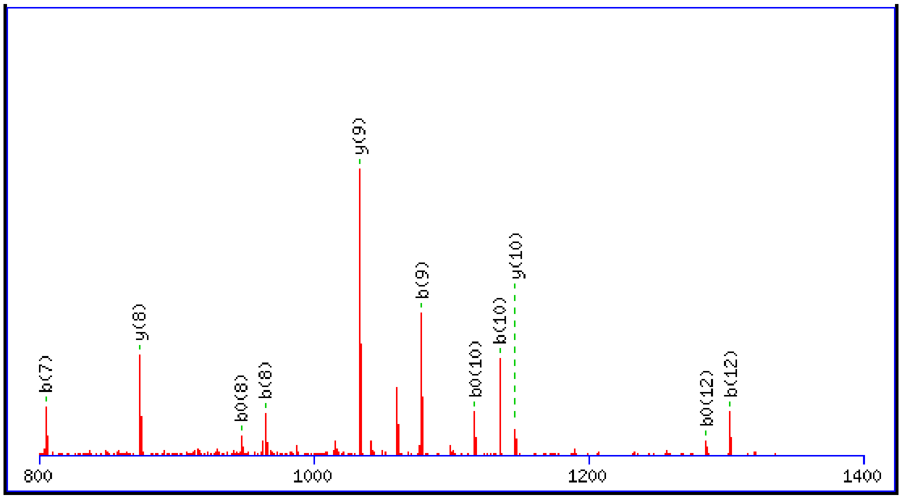


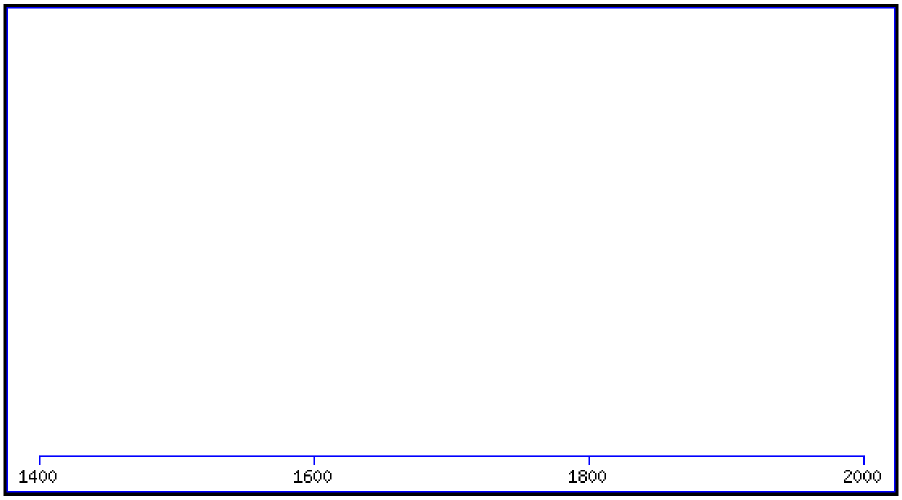


**Monoisotopic mass of neutral peptide Mr(calc):** 1448.6639
**Fixed modifications:** Carbamidomethyl (C)
**Variable modifications:**
**N1** : Deamidated_N (N)
**Ions Score:** 63 **Expect:** 6.9e-005
**Matches (Bold Red):** 23/128 fragment ions using 31 most intense peaks

| # | b | $b^{++}$ | b* | $b^{*++}$ | $b^0$ | $b^{0++}$ | Seq. | y | $y^{++}$ | y* | $y^{*++}$ | $y^0$ | $y^{0++}$ | # |
|---|---|---|---|---|---|---|---|---|---|---|---|---|---|---|
| 1 | 116.0342 | 58.5207 | 99.0077 | 50.0075 | | | N | | | | | | | 13 |
| 2 | 203.0662 | 102.0368 | 186.0397 | 93.5235 | 185.0557 | 93.0315 | S | 1334.6443 | 667.8258 | 1317.6177 | 659.3125 | 1316.6337 | 658.8205 | 12 |
| 3 | 304.1139 | 152.5606 | 287.0874 | 144.0473 | **286.1034** | 143.5553 | T | 1247.6123 | 624.3098 | 1230.5857 | **615.7965** | 1229.6017 | **615.3045** | 11 |
| 4 | **417.1980** | 209.1026 | 400.1714 | 200.5894 | **399.1874** | 200.0973 | L | **1146.5646** | **573.7859** | 1129.5380 | 565.2727 | 1128.5540 | 564.7806 | 10 |
| 5 | **577.2286** | 289.1180 | 560.2021 | 280.6047 | 559.2181 | 280.1127 | C | **1033.4805** | 517.2439 | 1016.4540 | 508.7306 | 1015.4699 | 508.2386 | 9 |
| 6 | 692.2556 | 346.6314 | 675.2290 | 338.1182 | 674.2450 | 337.6261 | D | **873.4499** | 437.2286 | 856.4233 | 428.7153 | 855.4393 | 428.2233 | 8 |
| 7 | **805.3396** | 403.1735 | 788.3131 | 394.6602 | 787.3291 | 394.1682 | L | **758.4229** | 379.7151 | 741.3964 | 371.2018 | | | 7 |
| 8 | **965.3703** | 483.1888 | 948.3437 | 474.6755 | **947.3597** | 474.1835 | C | **645.3389** | 323.1731 | 628.3123 | 314.6598 | | | 6 |
| 9 | **1078.4544** | 539.7308 | 1061.4278 | 531.2175 | 1060.4438 | 530.7255 | I | **485.3082** | 243.1577 | 468.2817 | 234.6445 | | | 5 |
| 10 | **1135.4758** | 568.2415 | 1118.4493 | 559.7283 | **1117.4653** | 559.2363 | G | **372.2241** | 186.6157 | 355.1976 | 178.1024 | | | 4 |
| 11 | 1232.5286 | 616.7679 | 1215.5020 | 608.2547 | 1214.5180 | 607.7626 | P | **315.2027** | 158.1050 | 298.1761 | 149.5917 | | | 3 |
| 12 | **1303.5657** | 652.2865 | 1286.5391 | 643.7732 | **1285.5551** | 643.2812 | A | 218.1499 | 109.5786 | 201.1234 | 101.0653 | | | 2 |
| 13 | | | | | | | K | 147.1128 | 74.0600 | 130.0863 | 65.5468 | | | 1 |

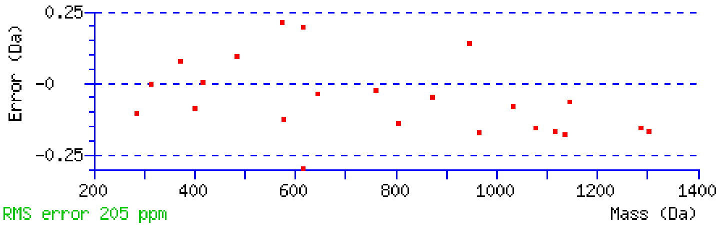


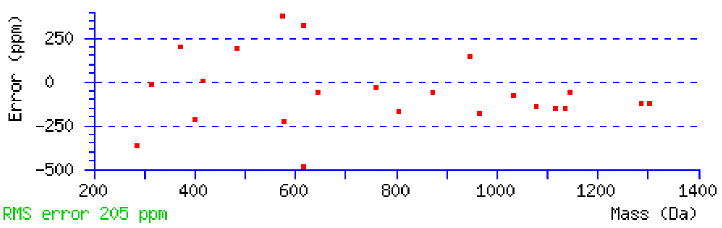


### All matches to this query

| Score | Mr(calc): | Delta | Sequence |
|---|---|---|---|
| 63.3 | 1448.6639 | 0.0036 | NSTLCDLCIGPAK |
| 11.6 | 1448.6538 | 0.0138 | ASRTTGGATALXK |
| 10.2 | 1448.6532 | 0.0144 | EGAGGGQDYPQTKAA |
| 8.5 | 1448.6639 | 0.0036 | QDGAVAMEMQPLK |
| 8.2 | 1448.6816 | -0.0141 | ELEEENMALRSK |
| 7.1 | 1448.6701 | -0.0025 | YLGQDFETLRK |
| 5.9 | 1448.6817 | -0.0141 | GKENTDSVVMQPK |
| 5.8 | 1448.6817 | -0.0141 | ERVMEIVDADEK |
| 5.8 | 1448.6622 | 0.0054 | NLGSLDVTIMYK |
| 5.8 | 1448.6557 | 0.0119 | EQMFRLLMASK |

---

Spectrum No: 472; Query: 646; Rank: 1

---

## Peptide View

MS/MS Fragmentation of **QSNGSIAIISSMAGK**
Found in **IPI00211096**, Tax_Id=10116 Gene_Symbol=Hsd11b1 Isoform 11-HSD1A of Corticosteroid 11-beta-dehydrogenase isozyme 1

Match to Query 646: 1479.719588 from(740.867070,2+)
Title: 091008RatKidney_NH4Format01_25.1481.1481.2.dta
Data file K:\NewmanPaper\Piliang\3SubProteomes\Piliang3SP\mgf5ppm\ERLIC_3SubProteomes5ppm.mgf

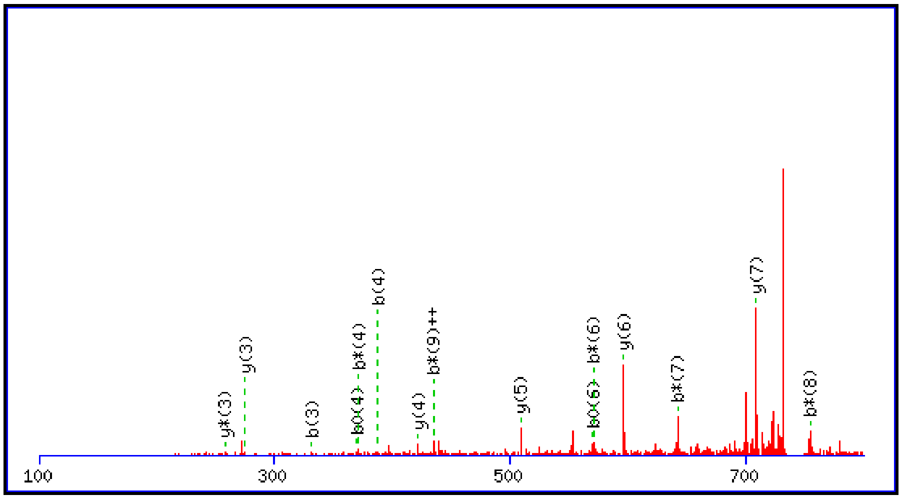

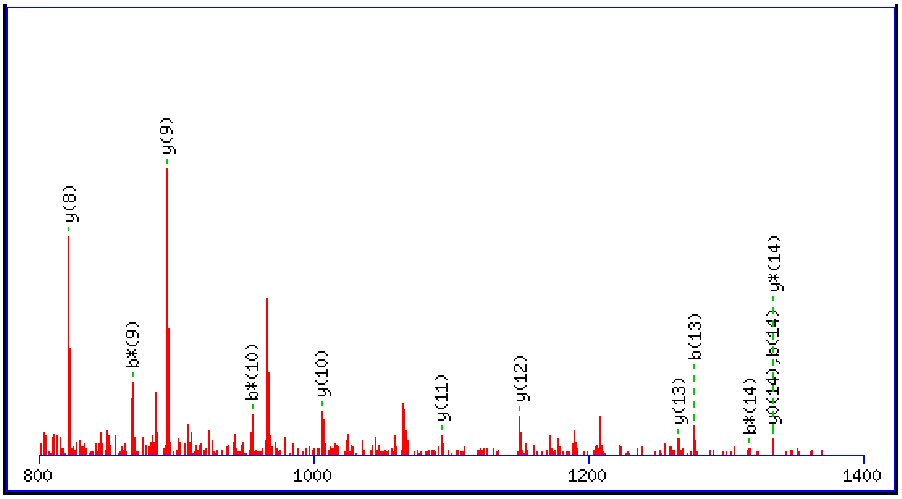


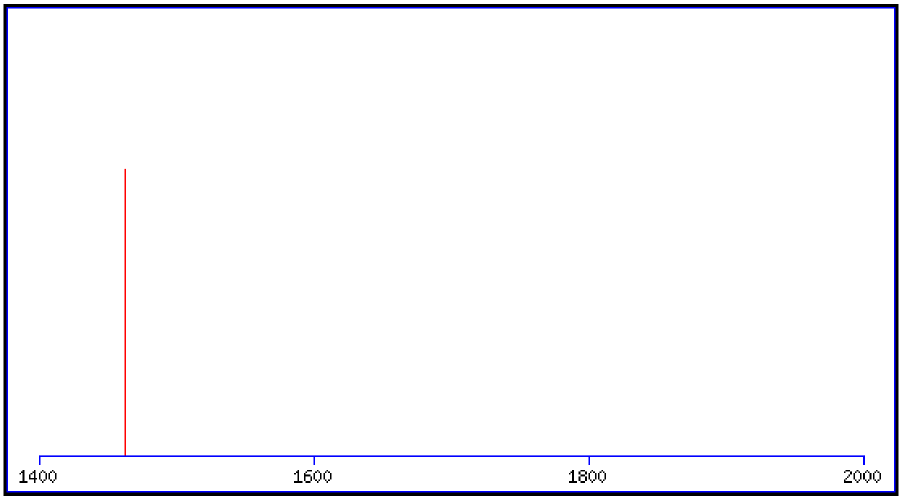


**Monoisotopic mass of neutral peptide Mr(calc):** 1479.7239
**Fixed modifications:** Carbamidomethyl (C)
**Variable modifications:**
**N3** : Deamidated_N (N)
**M12** : Oxidation (M)
**Ions Score:** 63 **Expect:** 7.1e-005
**Matches (Bold Red):** 28/158 fragment ions using 70 most intense peaks

| # | b | $b^{++}$ | b* | $b^{*++}$ | $b^{0}$ | $b^{0++}$ | Seq. | y | $y^{++}$ | y* | $y^{*++}$ | $y^{0}$ | $y^{0++}$ | # |
|---|---|---|---|---|---|---|---|---|---|---|---|---|---|---|
| 1 | 129.0659 | 65.0366 | 112.0393 | 56.5233 | | | Q | | | | | | | 15 |
| 2 | 216.0979 | 108.5526 | 199.0713 | 100.0393 | 198.0873 | 99.5473 | S | 1352.6726 | 676.8399 | **1335.6461** | 668.3267 | **1334.6620** | 667.8347 | 14 |
| 3 | **331.1248** | 166.0660 | 314.0983 | 157.5528 | 313.1143 | 157.0608 | N | **1265.6406** | 633.3239 | 1248.6140 | 624.8107 | 1247.6300 | 624.3186 | 13 |
| 4 | **388.1463** | 194.5768 | **371.1197** | 186.0635 | **370.1357** | 185.5715 | G | **1150.6136** | 575.8105 | 1133.5871 | 567.2972 | 1132.6031 | 566.8052 | 12 |
| 5 | 475.1783 | 238.0928 | 458.1518 | 229.5795 | 457.1677 | 229.0875 | S | **1093.5922** | 547.2997 | 1076.5656 | 538.7865 | 1075.5816 | 538.2944 | 11 |
| 6 | 588.2624 | 294.6348 | **571.2358** | 286.1216 | **570.2518** | 285.6295 | I | **1006.5601** | 503.7837 | 989.5336 | 495.2704 | 988.5496 | 494.7784 | 10 |
| 7 | 659.2995 | 330.1534 | **642.2729** | 321.6401 | 641.2889 | 321.1481 | A | **893.4761** | 447.2417 | 876.4495 | 438.7284 | 875.4655 | 438.2364 | 9 |
| 8 | 772.3836 | 386.6954 | **755.3570** | 378.1821 | 754.3730 | 377.6901 | I | **822.4390** | 411.7231 | 805.4124 | 403.2098 | 804.4284 | 402.7178 | 8 |
| 9 | 885.4676 | 443.2374 | **868.4411** | **434.7242** | 867.4571 | 434.2322 | I | **709.3549** | 355.1811 | 692.3284 | 346.6678 | 691.3443 | 346.1758 | 7 |
| 10 | 972.4996 | 486.7535 | **955.4731** | 478.2402 | 954.4891 | 477.7482 | S | **596.2708** | 298.6391 | 579.2443 | 290.1258 | 578.2603 | 289.6338 | 6 |
| 11 | 1059.5317 | 530.2695 | 1042.5051 | 521.7562 | 1041.5211 | 521.2642 | S | **509.2388** | 255.1230 | 492.2123 | 246.6098 | 491.2282 | 246.1178 | 5 |
| 12 | 1206.5671 | 603.7872 | 1189.5405 | 595.2739 | 1188.5565 | 594.7819 | M | **422.2068** | 211.6070 | 405.1802 | 203.0938 | | | 4 |
| 13 | **1277.6042** | 639.3057 | 1260.5776 | 630.7925 | 1259.5936 | 630.3005 | A | **275.1714** | 138.0893 | **258.1448** | 129.5761 | | | 3 |
| 14 | **1334.6257** | 667.8165 | **1317.5991** | 659.3032 | 1316.6151 | 658.8112 | G | 204.1343 | 102.5708 | 187.1077 | 94.0575 | | | 2 |
| 15 | | | | | | | K | 147.1128 | 74.0600 | 130.0863 | 65.5468 | | | 1 |

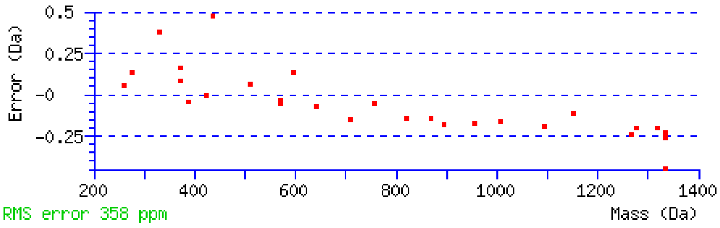


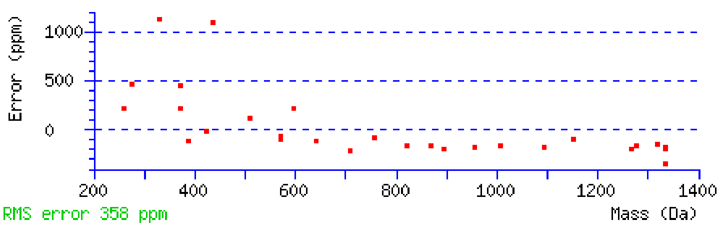


### All matches to this query

| Score | Mr(calc): | Delta | Sequence |
|---|---|---|---|
| 63.3 | 1479.7239 | -0.0043 | QSNGSIAIISSMAGK |
| 10.8 | 1479.7139 | 0.0057 | IKYKLSIVGDGK |
| 7.9 | 1478.7221 | 0.9975 | RMEEKPLSSMQK |
| 7.7 | 1479.7195 | 0.0001 | GSSQRLPVARNSK |
| 7.7 | 1479.7195 | 0.0001 | GSSQRLPVARNSK |
| 7.1 | 1479.7205 | -0.0009 | SNSSDTPPLPSPPGK |
| 7.0 | 1479.7334 | -0.0138 | VSLANLKPSLNSR |
| 6.9 | 1479.7334 | -0.0138 | LSSKVAAEVPQGSK |
| 6.5 | 1479.7334 | -0.0138 | SQGPSLDISALKGK |
| 6.4 | 1479.7235 | -0.0040 | KRVDPTVGWTGGK |

---

Spectrum No: 473; Query: 2599; Rank: 1

---

## Peptide View

MS/MS Fragmentation of **VNYEAETWDWIAETLSSNHMNFSSADR**
Found in **IPI00327398**, Tax_Id=10116 Gene_Symbol=Enpep Isoform 1 of Glutamyl aminopeptidase

Match to Query 2599: 3173.372922 from(1058.798250,3+)
Title: 100101RatKid_NS_deglyco_18.6588.6588.3.dta
Data file K:\NewmanPaper\Piliang\3SubProteomes\Piliang3SP\mgf5ppm\ERLIC_3SubProteomes5ppm.mgf

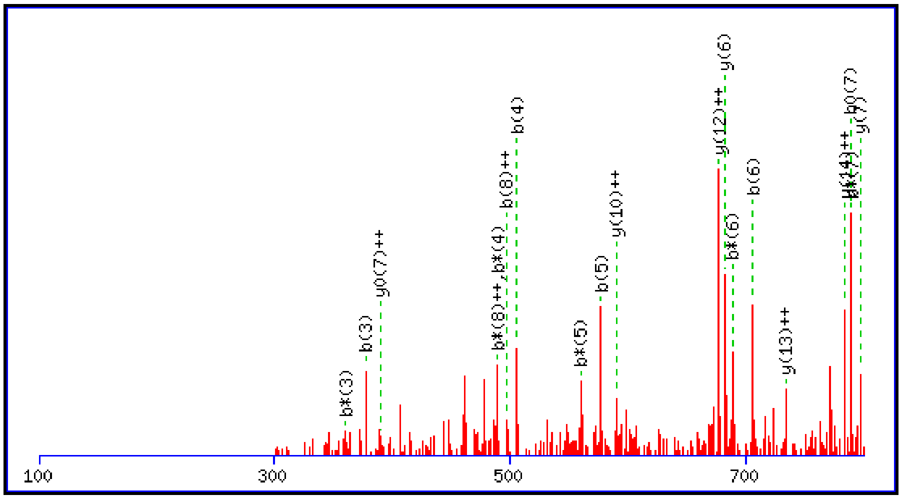

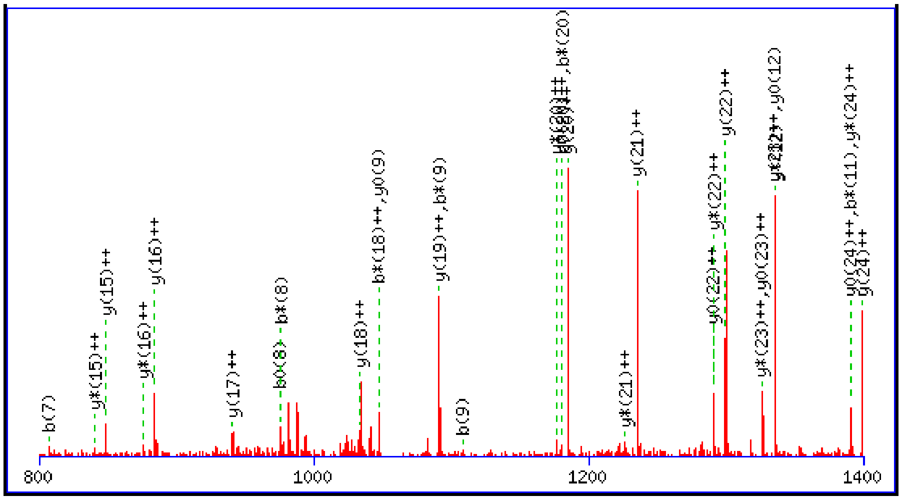


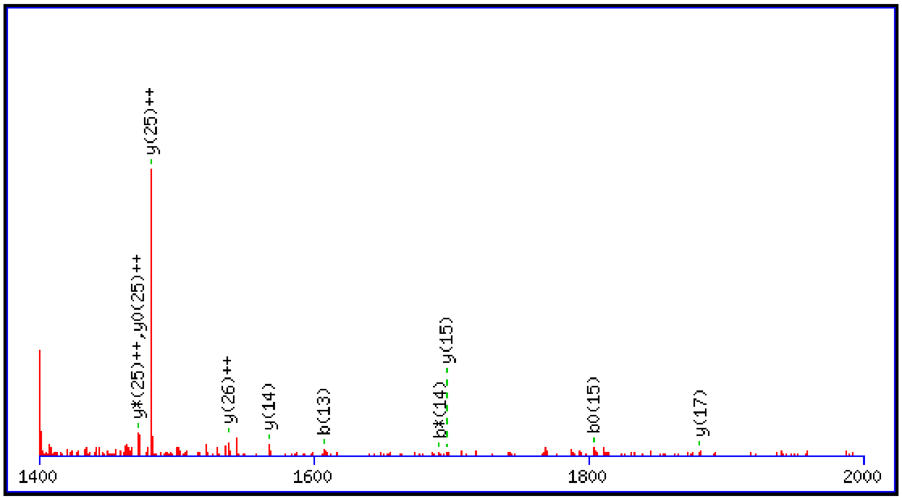


**Monoisotopic mass of neutral peptide Mr(calc):** 3173.3672
**Fixed modifications:** Carbamidomethyl (C)
**Variable modifications:**
**N21** : Deamidated_N (N)
**Ions Score:** 63 **Expect:** 0.00021
**Matches (Bold Red):** 61/302 fragment ions using 121 most intense peaks

| # | b | $b^{++}$ | b* | $b^{*++}$ | $b^0$ | $b^{0++}$ | Seq. | y | $y^{++}$ | y* | $y^{*++}$ | $y^0$ | $y^{0++}$ | # |
|---|---|---|---|---|---|---|---|---|---|---|---|---|---|---|
| 1 | 100.0757 | 50.5415 | | | | | V | | | | | | | 27 |
| 2 | 214.1186 | 107.5629 | 197.0921 | 99.0497 | | | N | 3075.3061 | **1538.1567** | 3058.2796 | 1529.6434 | 3057.2955 | 1529.1514 | 26 |
| 3 | **377.1819** | 189.0946 | **360.1554** | 180.5813 | | | Y | 2961.2632 | **1481.1352** | 2944.2366 | **1472.6220** | 2943.2526 | **1472.1299** | 25 |
| 4 | **506.2245** | 253.6159 | **489.1980** | 245.1026 | 488.2140 | 244.6106 | E | 2798.1999 | **1399.6036** | 2781.1733 | **1391.0903** | 2780.1893 | **1390.5983** | 24 |
| 5 | **577.2617** | 289.1345 | **560.2351** | 280.6212 | 559.2511 | 280.1292 | A | 2669.1573 | **1335.0823** | 2652.1307 | **1326.5690** | 2651.1467 | **1326.0770** | 23 |
| 6 | **706.3042** | 353.6558 | **689.2777** | 345.1425 | 688.2937 | 344.6505 | E | 2598.1201 | **1299.5637** | 2581.0936 | **1291.0504** | 2580.1096 | **1290.5584** | 22 |
| 7 | **807.3519** | 404.1796 | **790.3254** | 395.6663 | **789.3414** | 395.1743 | T | 2469.0776 | **1235.0424** | 2452.0510 | **1226.5291** | 2451.0670 | 1226.0371 | 21 |
| 8 | 993.4312 | **497.2193** | **976.4047** | **488.7060** | **975.4207** | 488.2140 | W | 2368.0299 | **1184.5186** | 2351.0033 | **1176.0053** | 2350.0193 | 1175.5133 | 20 |
| 9 | **1108.4582** | 554.7327 | **1091.4316** | 546.2195 | 1090.4476 | 545.7274 | D | 2181.9506 | **1091.4789** | 2164.9240 | 1082.9656 | 2163.9400 | 1082.4736 | 19 |
| 10 | 1294.5375 | 647.7724 | 1277.5109 | 639.2591 | 1276.5269 | 638.7671 | W | 2066.9236 | **1033.9654** | 2049.8971 | 1025.4522 | 2048.9131 | 1024.9602 | 18 |
| 11 | 1407.6216 | 704.3144 | **1390.5950** | 695.8011 | 1389.6110 | 695.3091 | I | **1880.8443** | **940.9258** | 1863.8178 | 932.4125 | 1862.8337 | 931.9205 | 17 |
| 12 | 1478.6587 | 739.8330 | 1461.6321 | 731.3197 | 1460.6481 | 730.8277 | A | 1767.7602 | **884.3838** | 1750.7337 | **875.8705** | 1749.7497 | 875.3785 | 16 |
| 13 | **1607.7013** | 804.3543 | 1590.6747 | 795.8410 | 1589.6907 | 795.3490 | E | **1696.7231** | **848.8652** | 1679.6966 | **840.3519** | 1678.7126 | 839.8599 | 15 |
| 14 | 1708.7489 | 854.8781 | **1691.7224** | 846.3648 | 1690.7384 | 845.8728 | T | **1567.6805** | **784.3439** | 1550.6540 | 775.8306 | 1549.6700 | 775.3386 | 14 |
| 15 | 1821.8330 | 911.4201 | 1804.8065 | 902.9069 | **1803.8224** | 902.4149 | L | 1466.6329 | **733.8201** | 1449.6063 | 725.3068 | 1448.6223 | 724.8148 | 13 |
| 16 | 1908.8650 | 954.9362 | 1891.8385 | 946.4229 | 1890.8545 | 945.9309 | S | 1353.5488 | **677.2780** | **1336.5222** | 668.7648 | **1335.5382** | 668.2728 | 12 |
| 17 | 1995.8971 | 998.4522 | 1978.8705 | 989.9389 | 1977.8865 | 989.4469 | S | 1266.5168 | 633.7620 | 1249.4902 | 625.2487 | 1248.5062 | 624.7567 | 11 |
| 18 | 2109.9400 | 1055.4736 | 2092.9134 | **1046.9604** | 2091.9294 | 1046.4684 | N | 1179.4847 | **590.2460** | 1162.4582 | 581.7327 | 1161.4742 | 581.2407 | 10 |

| | | | | | | | | | | | | | | |
|---|---|---|---|---|---|---|---|---|---|---|---|---|---|---|
| **19** | 2246.9989 | 1124.0031 | 2229.9724 | 1115.4898 | 2228.9883 | 1114.9978 | **H** | 1065.4418 | 533.2245 | 1048.4153 | 524.7113 | **1047.4312** | 524.2193 | **9** |
| **20** | 2378.0394 | 1189.5233 | 2361.0128 | **1181.0101** | 2360.0288 | **1180.5180** | **M** | 928.3829 | 464.6951 | 911.3564 | 456.1818 | 910.3723 | 455.6898 | **8** |
| **21** | 2493.0663 | 1247.0368 | 2476.0398 | 1238.5235 | 2475.0558 | 1238.0315 | **N** | **797.3424** | 399.1748 | 780.3159 | 390.6616 | 779.3318 | **390.1696** | **7** |
| **22** | 2640.1347 | 1320.5710 | 2623.1082 | 1312.0577 | 2622.1242 | 1311.5657 | **F** | **682.3155** | 341.6614 | 665.2889 | 333.1481 | 664.3049 | 332.6561 | **6** |
| **23** | 2727.1668 | 1364.0870 | 2710.1402 | 1355.5737 | 2709.1562 | 1355.0817 | **S** | 535.2471 | 268.1272 | 518.2205 | 259.6139 | 517.2365 | 259.1219 | **5** |
| **24** | 2814.1988 | 1407.6030 | 2797.1722 | 1399.0898 | 2796.1882 | 1398.5978 | **S** | 448.2150 | 224.6112 | 431.1885 | 216.0979 | 430.2045 | 215.6059 | **4** |
| **25** | 2885.2359 | 1443.1216 | 2868.2094 | 1434.6083 | 2867.2253 | 1434.1163 | **A** | 361.1830 | 181.0951 | 344.1565 | 172.5819 | 343.1724 | 172.0899 | **3** |
| **26** | 3000.2629 | 1500.6351 | 2983.2363 | 1492.1218 | 2982.2523 | 1491.6298 | **D** | 290.1459 | 145.5766 | 273.1193 | 137.0633 | 272.1353 | 136.5713 | **2** |
| **27** | | | | | | | **R** | 175.1190 | 88.0631 | 158.0924 | 79.5498 | | | **1** |

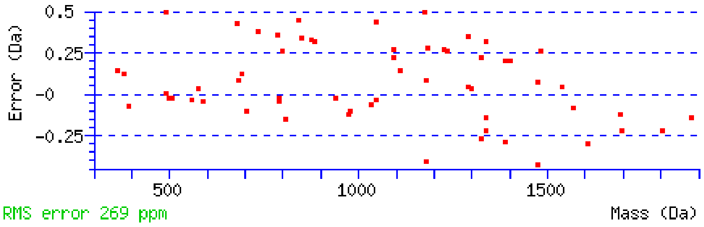


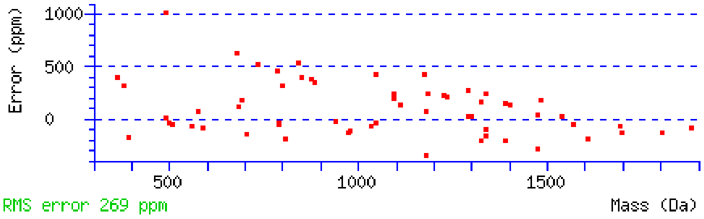


### All matches to this query

| Score | Mr(calc): | Delta | Sequence |
|---|---|---|---|
| 63.3 | 3173.3672 | 0.0057 | VNYEAETWDWIAETLSSNHMNFSSADR |
| 62.3 | 3173.3672 | 0.0057 | VNYEAETWDWIAETLSSNHMNFSSADR |
| 9.9 | 3173.3623 | 0.0106 | TKGSMLVSWTPPLDNGGSPITGYWLEK |
| 6.3 | 3173.3623 | 0.0106 | TKGSMLVSWTPPLDNGGSPITGYWLEK |
| 3.8 | 3171.3526 | 2.0204 | SALLYGTLSSEAPQDGESNQSGYCAMISR |
| 2.5 | 3173.3623 | 0.0106 | TKGSMLVSWTPPLDNGGSPITGYWLEK |
| 2.3 | 3171.3420 | 2.0310 | NRLQSVVVVPKNSTLPMEETSPCSSR |
| 1.2 | 3173.3811 | -0.0082 | NFSVGRQTTAPATMSTAASGTTMGLVEQAK |
| 1.0 | 3171.3354 | 2.0375 | EIEMYQGAAQYENPPHIYALADSMYR |
| 0.5 | 3173.3811 | -0.0082 | TLLNTQENMALSLANVSGRLSGMANAFR |

---

Spectrum No: 474; Query: 371; Rank: 1

---

## Peptide View

MS/MS Fragmentation of **ILCDNSDNITR**
Found in **IPI00197864**, Tax_Id=10116 Gene_Symbol=LOC314016 Melanoma-associated antigen MG50 (Fragment)

Match to Query 371: 1320.595788 from(661.305170,2+)
Title: 091008RatKidney_NH4Format01_24.997.997.2.dta
Data file K:\NewmanPaper\Piliang\3SubProteomes\Piliang3SP\mgf5ppm\ERLIC_3SubProteomes5ppm.mgf

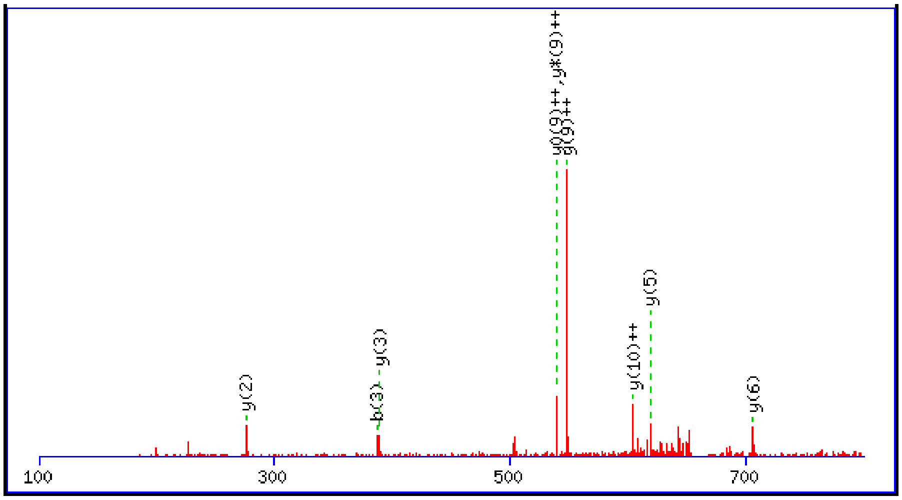


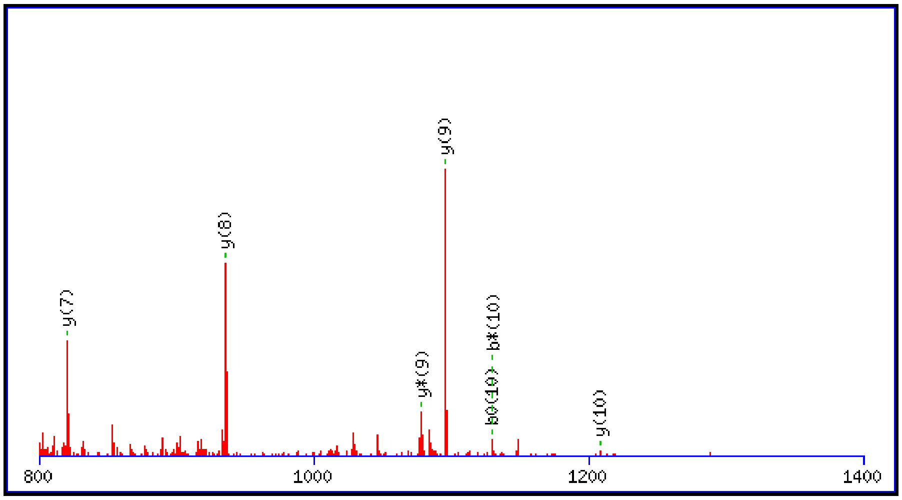


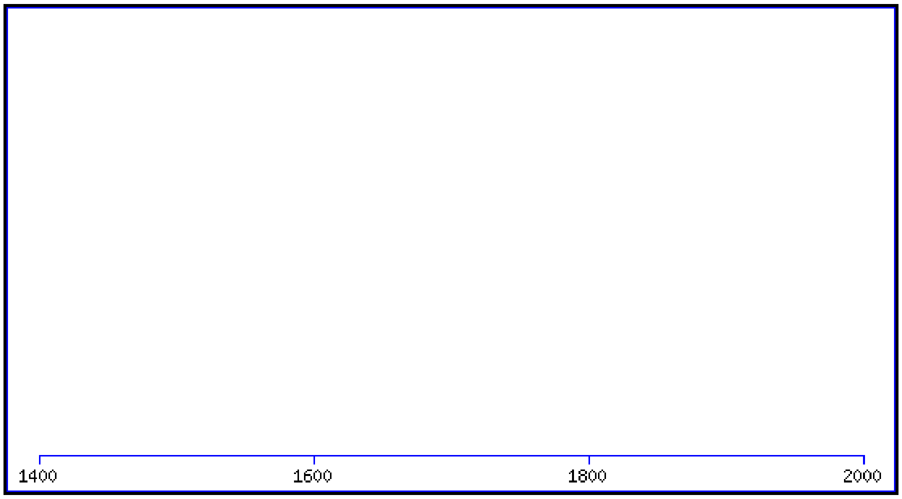


**Monoisotopic mass of neutral peptide Mr(calc):** 1320.5980
**Fixed modifications:** Carbamidomethyl (C)
**Variable modifications:**
**N8** : Deamidated_N (N)
**Ions Score:** 63 **Expect:** 6.4e-005
**Matches (Bold Red):** 16/104 fragment ions using 25 most intense peaks

| # | b | $b^{++}$ | b* | $b^{*++}$ | $b^0$ | $b^{0++}$ | Seq. | y | $y^{++}$ | y* | $y^{*++}$ | $y^0$ | $y^{0++}$ | # |
|---|---|---|---|---|---|---|---|---|---|---|---|---|---|---|
| 1 | 114.0913 | 57.5493 | | | | | I | | | | | | | 11 |
| 2 | 227.1754 | 114.0913 | | | | | L | **1208.5212** | **604.7642** | 1191.4946 | 596.2510 | 1190.5106 | 595.7589 | 10 |
| 3 | **387.2061** | 194.1067 | | | | | C | **1095.4371** | **548.2222** | **1078.4106** | **539.7089** | 1077.4266 | **539.2169** | 9 |
| 4 | 502.2330 | 251.6201 | | | 484.2224 | 242.6149 | D | **935.4065** | 468.2069 | 918.3799 | 459.6936 | 917.3959 | 459.2016 | 8 |
| 5 | 616.2759 | 308.6416 | 599.2494 | 300.1283 | 598.2654 | 299.6363 | N | **820.3795** | 410.6934 | 803.3530 | 402.1801 | 802.3690 | 401.6881 | 7 |

| | | | | | | | | | | | | | | |
|---|---|---|---|---|---|---|---|---|---|---|---|---|---|---|
| **6** | 703.3080 | 352.1576 | 686.2814 | 343.6443 | 685.2974 | 343.1523 | **S** | **706.3366** | 353.6719 | 689.3101 | 345.1587 | 688.3260 | 344.6667 | **6** |
| **7** | 818.3349 | 409.6711 | 801.3083 | 401.1578 | 800.3243 | 400.6658 | **D** | **619.3046** | 310.1559 | 602.2780 | 301.6427 | 601.2940 | 301.1506 | **5** |
| **8** | 933.3618 | 467.1846 | 916.3353 | 458.6713 | 915.3513 | 458.1793 | **N** | 504.2776 | 252.6425 | 487.2511 | 244.1292 | 486.2671 | 243.6372 | **4** |
| **9** | 1046.4459 | 523.7266 | 1029.4193 | 515.2133 | 1028.4353 | 514.7213 | **I** | **389.2507** | 195.1290 | 372.2241 | 186.6157 | 371.2401 | 186.1237 | **3** |
| **10** | 1147.4936 | 574.2504 | **1130.4670** | 565.7372 | **1129.4830** | 565.2451 | **T** | **276.1666** | 138.5870 | 259.1401 | 130.0737 | 258.1561 | 129.5817 | **2** |
| **11** | | | | | | | **R** | 175.1190 | 88.0631 | 158.0924 | 79.5498 | | | **1** |

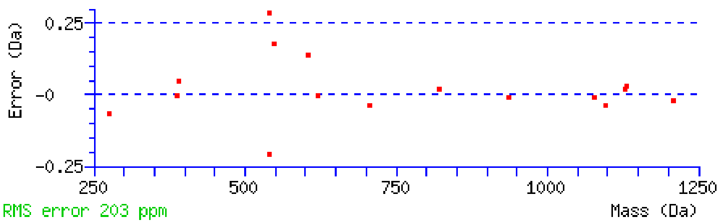


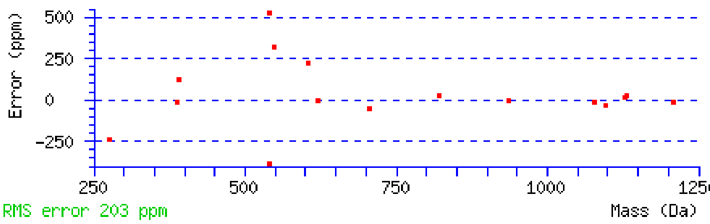


**All matches to this query**

| Score | Mr(calc): | Delta | Sequence |
|---|---|---|---|
| 63.2 | 1320.5980 | -0.0022 | ILCDNSDNITR |
| 47.2 | 1320.5980 | -0.0022 | ILCDNSDNITR |
| 13.7 | 1319.5983 | 0.9975 | RPAPSGGASGDLR |
| 8.7 | 1319.6027 | 0.9931 | KPCDEDLTSQK |
| 8.4 | 1320.6003 | -0.0045 | KDFINFISEK |
| 5.8 | 1320.5903 | 0.0054 | KPYIWEYSR |
| 5.1 | 1320.5971 | -0.0013 | MKPYSACIKK |
| 3.5 | 1320.6045 | -0.0087 | IVSSQSTAEGDVE |
| 3.1 | 1320.6083 | -0.0125 | IIYKNKCMR |
| 3.1 | 1320.5880 | 0.0078 | ILPDKVSFSR |

---

Spectrum No: 475; Query: 2059; Rank: 1

---

## Peptide View

MS/MS Fragmentation of **ELINALQINNTAVGHALVLPAR**
Found in **IPI00565677**, Tax_Id=10116 Gene_Symbol=Col6a3_predicted 289 kDa protein

Match to Query 2059: 2327.294952 from(776.772260,3+)
Title: 100101RatKid_NS_deglyco_14.4802.4802.3.dta
Data file K:\NewmanPaper\Piliang\3SubProteomes\Piliang3SP\mgf5ppm\ERLIC_3SubProteomes5ppm.mgf

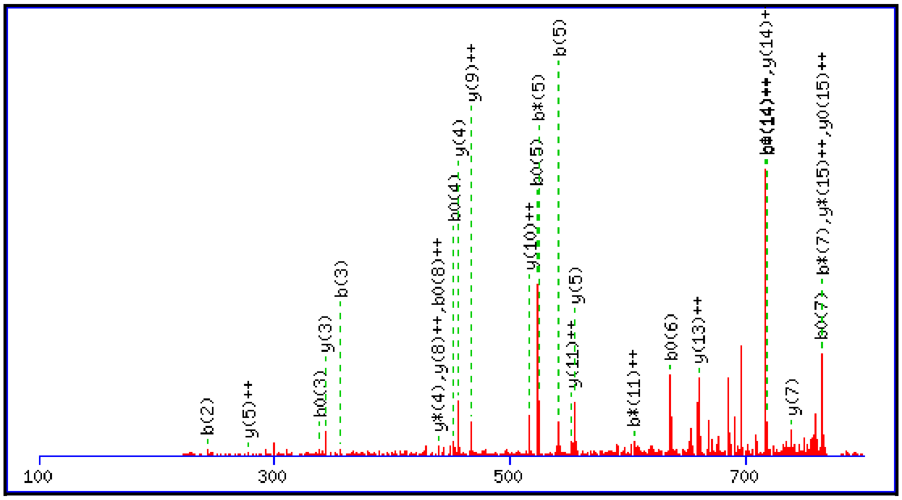

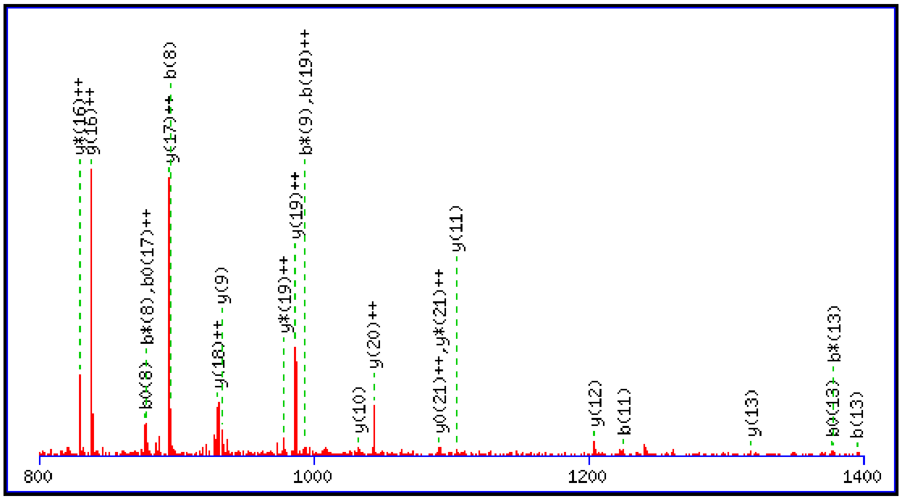


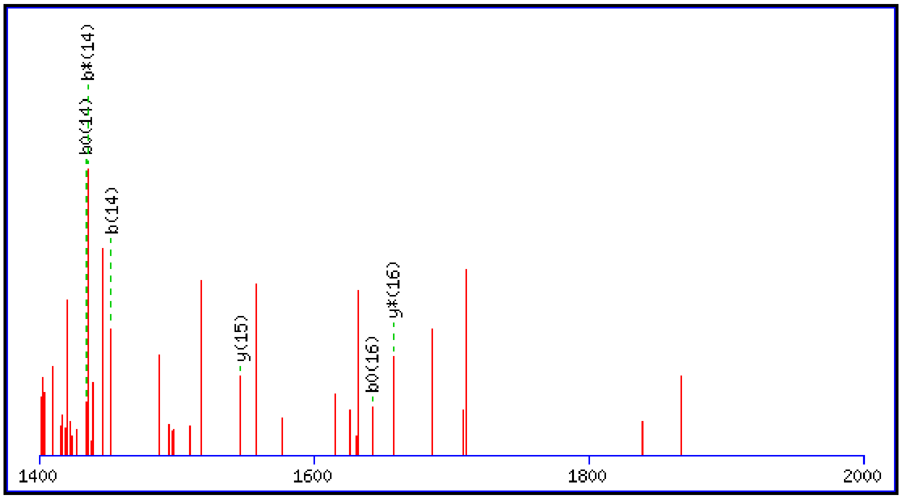


**Monoisotopic mass of neutral peptide Mr(calc):** 2327.2961
**Fixed modifications:** Carbamidomethyl (C)
**Variable modifications:**
**N9** : Deamidated_N (N)
**Ions Score:** 63 **Expect:** 1.5e-005
**Matches (Bold Red):** 58/224 fragment ions using 118 most intense peaks

| # | b | $b^{++}$ | b* | $b^{*++}$ | $b^0$ | $b^{0++}$ | Seq. | y | $y^{++}$ | y* | $y^{*++}$ | $y^0$ | $y^{0++}$ | # |
|---|---|---|---|---|---|---|---|---|---|---|---|---|---|---|
| 1 | 130.0499 | 65.5286 | | | 112.0393 | 56.5233 | E | | | | | | | 22 |
| 2 | **243.1339** | 122.0706 | | | 225.1234 | 113.0653 | L | 2199.2608 | 1100.1340 | 2182.2343 | **1091.6208** | 2181.2502 | **1091.1288** | 21 |
| 3 | **356.2180** | 178.6126 | | | **338.2074** | 169.6074 | I | 2086.1767 | **1043.5920** | 2069.1502 | 1035.0787 | 2068.1662 | 1034.5867 | 20 |
| 4 | 470.2609 | 235.6341 | 453.2344 | 227.1208 | **452.2504** | 226.6288 | N | 1973.0927 | **987.0500** | 1956.0661 | **978.5367** | 1955.0821 | 978.0447 | 19 |
| 5 | **541.2980** | 271.1527 | **524.2715** | 262.6394 | **523.2875** | 262.1474 | A | 1859.0498 | **930.0285** | 1842.0232 | 921.5152 | 1841.0392 | 921.0232 | 18 |
| 6 | 654.3821 | 327.6947 | 637.3556 | 319.1814 | **636.3715** | 318.6894 | L | 1788.0126 | **894.5100** | 1770.9861 | 885.9967 | 1770.0021 | 885.5047 | 17 |
| 7 | 782.4407 | 391.7240 | **765.4141** | 383.2107 | **764.4301** | 382.7187 | Q | 1674.9286 | **837.9679** | **1657.9020** | **829.4547** | 1656.9180 | 828.9626 | 16 |
| 8 | **895.5247** | 448.2660 | **878.4982** | 439.7527 | **877.5142** | **439.2607** | I | **1546.8700** | 773.9386 | 1529.8434 | **765.4254** | 1528.8594 | **764.9334** | 15 |
| 9 | 1010.5517 | 505.7795 | **993.5251** | 497.2662 | 992.5411 | 496.7742 | N | 1433.7859 | **717.3966** | 1416.7594 | 708.8833 | 1415.7754 | 708.3913 | 14 |
| 10 | 1124.5946 | 562.8009 | 1107.5681 | 554.2877 | 1106.5840 | 553.7957 | N | **1318.7590** | **659.8831** | 1301.7324 | 651.3699 | 1300.7484 | 650.8779 | 13 |
| 11 | **1225.6423** | 613.3248 | 1208.6157 | **604.8115** | 1207.6317 | 604.3195 | T | **1204.7161** | 602.8617 | 1187.6895 | 594.3484 | 1186.7055 | 593.8564 | 12 |
| 12 | 1296.6794 | 648.8433 | 1279.6529 | 640.3301 | 1278.6688 | 639.8381 | A | **1103.6684** | **552.3378** | 1086.6418 | 543.8246 | | | 11 |
| 13 | **1395.7478** | 698.3775 | **1378.7213** | 689.8643 | **1377.7372** | 689.3723 | V | **1032.6313** | **516.8193** | 1015.6047 | 508.3060 | | | 10 |
| 14 | **1452.7693** | 726.8883 | **1435.7427** | **718.3750** | **1434.7587** | **717.8830** | G | **933.5629** | **467.2851** | 916.5363 | 458.7718 | | | 9 |
| 15 | 1589.8282 | 795.4177 | 1572.8016 | 786.9045 | 1571.8176 | 786.4125 | H | 876.5414 | **438.7743** | 859.5148 | 430.2611 | | | 8 |
| 16 | 1660.8653 | 830.9363 | 1643.8388 | 822.4230 | **1642.8547** | 821.9310 | A | **739.4825** | 370.2449 | 722.4559 | 361.7316 | | | 7 |
| 17 | 1773.9494 | 887.4783 | 1756.9228 | 878.9650 | 1755.9388 | **878.4730** | L | 668.4454 | 334.7263 | 651.4188 | 326.2130 | | | 6 |
| 18 | 1873.0178 | 937.0125 | 1855.9912 | 928.4993 | 1855.0072 | 928.0072 | V | **555.3613** | **278.1843** | 538.3348 | 269.6710 | | | 5 |

| | | | | | | | | | | | | | | |
|---|---|---|---|---|---|---|---|---|---|---|---|---|---|---|
| **19** | 1986.1018 | **993.5546** | 1969.0753 | 985.0413 | 1968.0913 | 984.5493 | **L** | **456.2929** | 228.6501 | **439.2663** | 220.1368 | | | **4** |
| **20** | 2083.1546 | 1042.0809 | 2066.1281 | 1033.5677 | 2065.1440 | 1033.0757 | **P** | **343.2088** | 172.1081 | 326.1823 | 163.5948 | | | **3** |
| **21** | 2154.1917 | 1077.5995 | 2137.1652 | 1069.0862 | 2136.1812 | 1068.5942 | **A** | 246.1561 | 123.5817 | 229.1295 | 115.0684 | | | **2** |
| **22** | | | | | | | **R** | 175.1190 | 88.0631 | 158.0924 | 79.5498 | | | **1** |

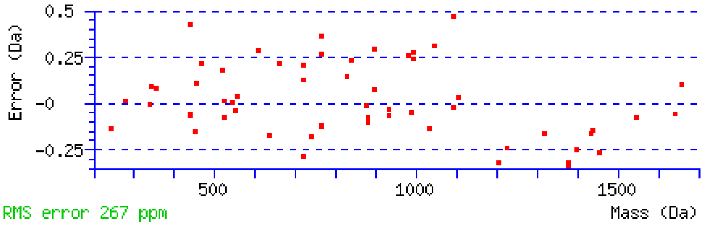


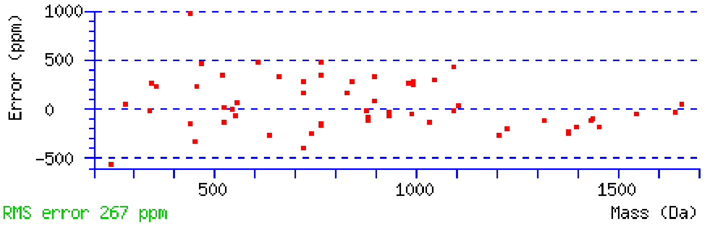


**All matches to this query**

| Score | Mr(calc): | Delta | Sequence |
|---|---|---|---|
| 63.2 | 2327.2961 | -0.0011 | ELINALQINNTAVGHALVLPAR |
| 60.7 | 2327.2961 | -0.0011 | ELINALQINNTAVGHALVLPAR |
| 31.0 | 2326.3121 | 0.9829 | ELINALQINNTAVGHALVLPAR |
| 30.4 | 2327.2961 | -0.0011 | ELINALQINNTAVGHALVLPAR |
| 1.8 | 2327.2974 | -0.0025 | EILQGLAHLHAHQVIHRDIK |

---

Spectrum No: 476; Query: 568; Rank: 1

---

## Peptide View

MS/MS Fragmentation of **LQYNSLLSNMSR**
Found in **IPI00324300**, Tax_Id=10116 Gene_Symbol=Ace Angiotensin-converting enzyme, somatic isoform precursor

Match to Query 568: 1442.689388 from(722.351970,2+)
Title: 091008RatKidney_NH4Format01_26.1899.1899.2.dta
Data file K:\NewmanPaper\Piliang\3SubProteomes\Piliang3SP\mgf5ppm\ERLIC_3SubProteomes5ppm.mgf

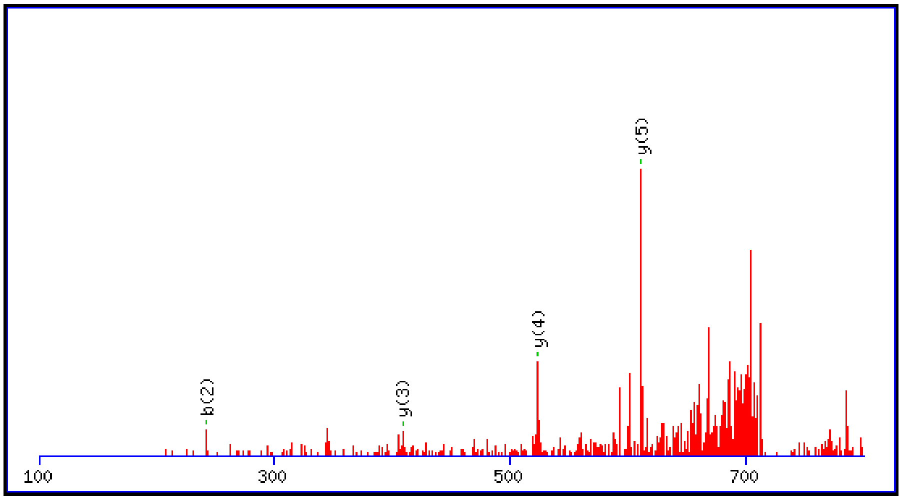

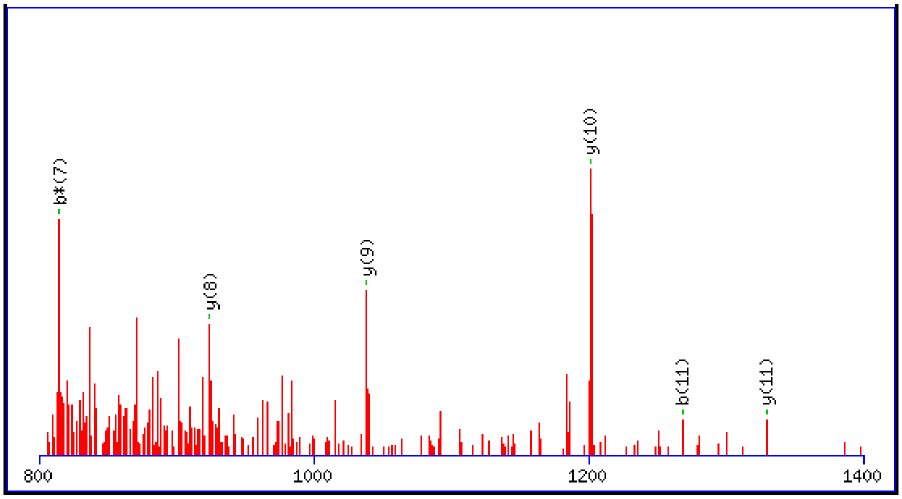


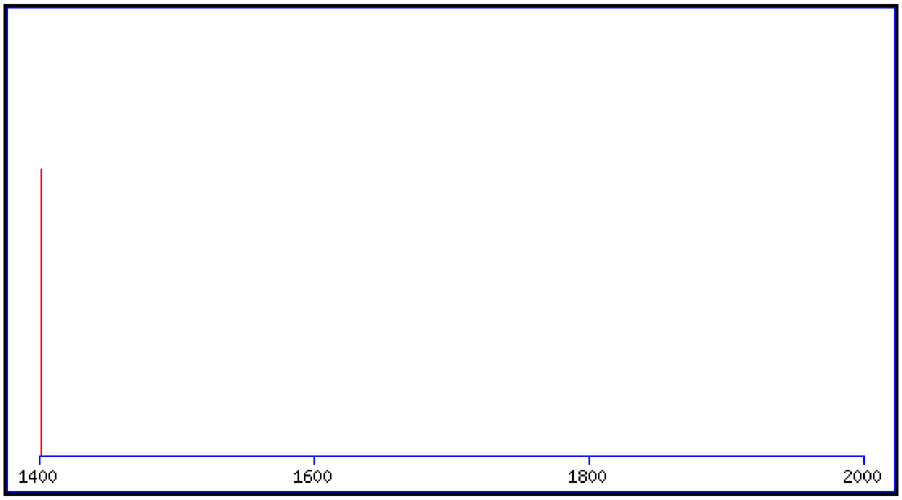


**Monoisotopic mass of neutral peptide Mr(calc):** 1441.6871
**Fixed modifications:** Carbamidomethyl (C)
**Variable modifications:**
**N9** : Deamidated_N (N)
**M10** : Oxidation (M)
**Ions Score:** 63 **Expect:** 7.5e-005
**Matches (Bold Red):** 10/120 fragment ions using 16 most intense peaks

| # | b | $b^{++}$ | b* | $b^{*++}$ | $b^{0}$ | $b^{0++}$ | Seq. | y | $y^{++}$ | y* | $y^{*++}$ | $y^{0}$ | $y^{0++}$ | # |
|---|---|---|---|---|---|---|---|---|---|---|---|---|---|---|
| 1 | 114.0913 | 57.5493 | | | | | L | | | | | | | 12 |
| 2 | **242.1499** | 121.5786 | 225.1234 | 113.0653 | | | Q | **1329.6103** | 665.3088 | 1312.5838 | 656.7955 | 1311.5998 | 656.3035 | 11 |
| 3 | 405.2132 | 203.1103 | 388.1867 | 194.5970 | | | Y | **1201.5518** | 601.2795 | 1184.5252 | 592.7662 | 1183.5412 | 592.2742 | 10 |
| 4 | 519.2562 | 260.1317 | 502.2296 | 251.6185 | | | N | **1038.4884** | 519.7479 | 1021.4619 | 511.2346 | 1020.4779 | 510.7426 | 9 |
| 5 | 606.2882 | 303.6477 | 589.2617 | 295.1345 | 588.2776 | 294.6425 | S | **924.4455** | 462.7264 | 907.4190 | 454.2131 | 906.4349 | 453.7211 | 8 |
| 6 | 719.3723 | 360.1898 | 702.3457 | 351.6765 | 701.3617 | 351.1845 | L | 837.4135 | 419.2104 | 820.3869 | 410.6971 | 819.4029 | 410.2051 | 7 |
| 7 | 832.4563 | 416.7318 | **815.4298** | 408.2185 | 814.4458 | 407.7265 | L | 724.3294 | 362.6683 | 707.3029 | 354.1551 | 706.3188 | 353.6631 | 6 |
| 8 | 919.4884 | 460.2478 | 902.4618 | 451.7345 | 901.4778 | 451.2425 | S | **611.2453** | 306.1263 | 594.2188 | 297.6130 | 593.2348 | 297.1210 | 5 |
| 9 | 1034.5153 | 517.7613 | 1017.4887 | 509.2480 | 1016.5047 | 508.7560 | N | **524.2133** | 262.6103 | 507.1868 | 254.0970 | 506.2028 | 253.6050 | 4 |
| 10 | 1181.5507 | 591.2790 | 1164.5241 | 582.7657 | 1163.5401 | 582.2737 | M | **409.1864** | 205.0968 | 392.1598 | 196.5836 | 391.1758 | 196.0915 | 3 |
| 11 | **1268.5827** | 634.7950 | 1251.5562 | 626.2817 | 1250.5722 | 625.7897 | S | 262.1510 | 131.5791 | 245.1244 | 123.0659 | 244.1404 | 122.5738 | 2 |
| 12 | | | | | | | R | 175.1190 | 88.0631 | 158.0924 | 79.5498 | | | 1 |

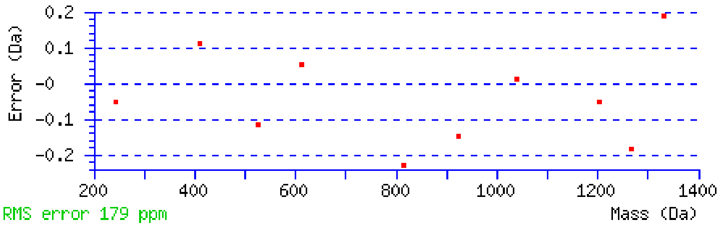


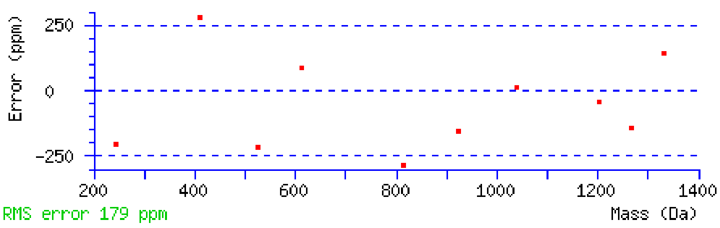


### All matches to this query

| Score | Mr(calc): | Delta | Sequence |
|---|---|---|---|
| 63.2 | 1441.6871 | 1.0023 | LQYNSLLSNMSR |
| 22.4 | 1441.6871 | 1.0023 | LQYNSLLSNMSR |
| 9.5 | 1441.6861 | 1.0033 | LRENRTLAMSR |
| 9.0 | 1441.6749 | 1.0145 | IKNAGGKVTMDGR |
| 5.3 | 1441.6748 | 1.0146 | LQMSERNILSR |
| 4.5 | 1441.6888 | 1.0006 | KIEVIEIMTDR |
| 4.0 | 1441.6888 | 1.0006 | LQSTAMTQELLK |
| 3.5 | 1441.6827 | 1.0067 | HNIATSQSVLHR |
| 3.0 | 1441.7000 | 0.9894 | LKLIASNSTMER |
| 2.8 | 1441.6827 | 1.0067 | HNIATSQSVLHR |

---

Spectrum No: 477; Query: 2177; Rank: 1

---

## Peptide View

MS/MS Fragmentation of **ASFNITLIHPNNLTALSNMLPK**
Found in **IPI00230862**, Tax_Id=10116 Gene_Symbol=Anpep Aminopeptidase N

Match to Query 2177: 2426.252022 from(809.757950,3+)
Title: 091008RatKidney_NH4Format01_24.4294.4294.3.dta
Data file K:\NewmanPaper\Piliang\3SubProteomes\Piliang3SP\mgf5ppm\ERLIC_3SubProteomes5ppm.mgf

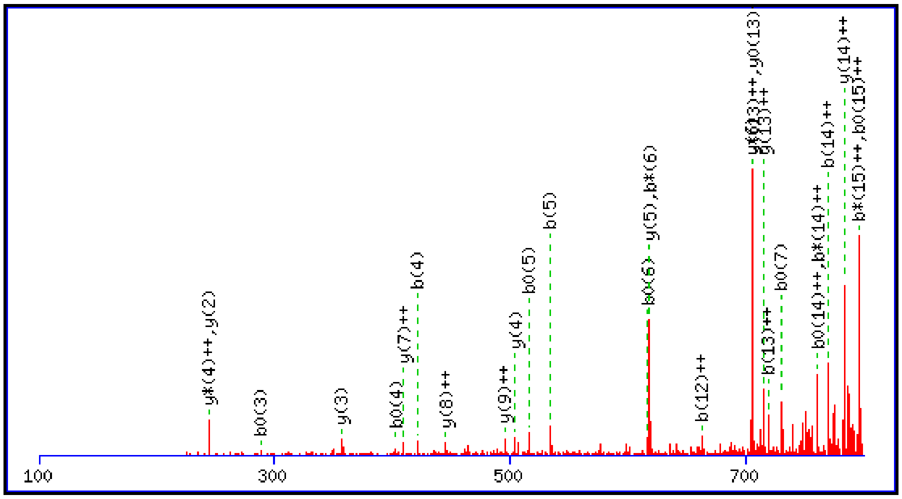

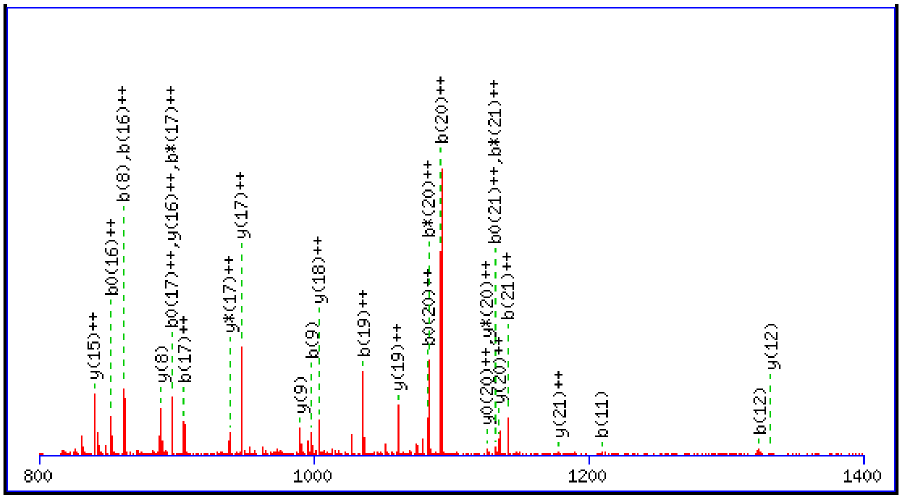


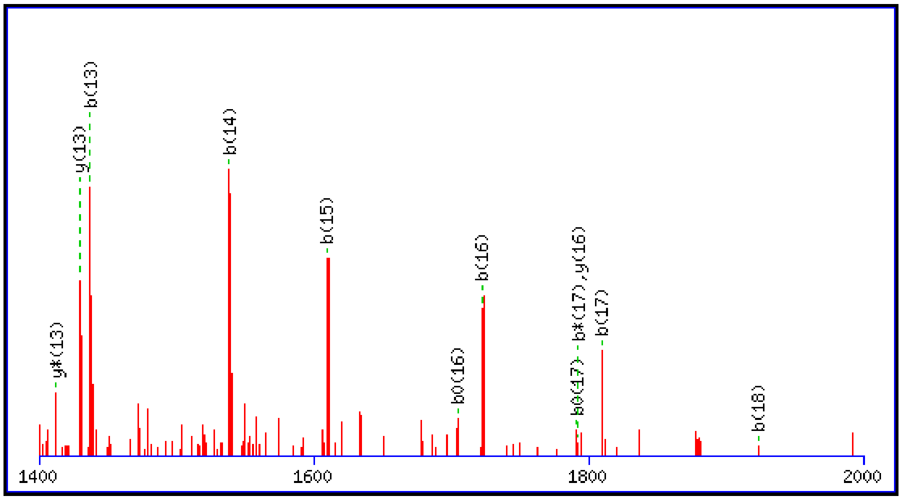


**Monoisotopic mass of neutral peptide Mr(calc):** 2426.2515
**Fixed modifications:** Carbamidomethyl (C)
**Variable modifications:**
**N4** : Deamidated_N (N)
**N12** : Deamidated_N (N)
**M19** : Oxidation (M)
**Ions Score:** 63 **Expect:** 6.8e-005
**Matches (Bold Red):** 69/234 fragment ions using 127 most intense peaks

| # | b | $b^{++}$ | b* | $b^{*++}$ | $b^{0}$ | $b^{0++}$ | Seq. | y | $y^{++}$ | y* | $y^{*++}$ | $y^{0}$ | $y^{0++}$ | # |
|---|---|---|---|---|---|---|---|---|---|---|---|---|---|---|
| 1 | 72.0444 | 36.5258 | | | | | A | | | | | | | 22 |
| 2 | 159.0764 | 80.0418 | | | 141.0659 | 71.0366 | S | 2356.2217 | **1178.6145** | 2339.1952 | 1170.1012 | 2338.2111 | 1169.6092 | 21 |
| 3 | 306.1448 | 153.5761 | | | **288.1343** | 144.5708 | F | 2269.1897 | **1135.0985** | 2252.1631 | **1126.5852** | 2251.1791 | **1126.0932** | 20 |
| 4 | **421.1718** | 211.0895 | 404.1452 | 202.5762 | **403.1612** | 202.0842 | N | 2122.1213 | **1061.5643** | 2105.0947 | 1053.0510 | 2104.1107 | 1052.5590 | 19 |
| 5 | **534.2558** | 267.6316 | 517.2293 | 259.1183 | **516.2453** | 258.6263 | I | 2007.0943 | **1004.0508** | 1990.0678 | 995.5375 | 1989.0838 | 995.0455 | 18 |
| 6 | 635.3035 | 318.1554 | **618.2770** | 309.6421 | **617.2929** | 309.1501 | T | 1894.0103 | **947.5088** | 1876.9837 | **938.9955** | 1875.9997 | 938.5035 | 17 |
| 7 | 748.3876 | 374.6974 | 731.3610 | 366.1842 | **730.3770** | 365.6921 | L | **1792.9626** | **896.9849** | 1775.9360 | 888.4717 | 1774.9520 | 887.9796 | 16 |
| 8 | **861.4716** | 431.2395 | 844.4451 | 422.7262 | 843.4611 | 422.2342 | I | 1679.8785 | **840.4429** | 1662.8520 | 831.9296 | 1661.8680 | 831.4376 | 15 |
| 9 | **998.5306** | 499.7689 | 981.5040 | 491.2556 | 980.5200 | 490.7636 | H | 1566.7945 | **783.9009** | 1549.7679 | 775.3876 | 1548.7839 | 774.8956 | 14 |
| 10 | 1095.5833 | 548.2953 | 1078.5568 | 539.7820 | 1077.5728 | 539.2900 | P | **1429.7355** | **715.3714** | **1412.7090** | **706.8581** | 1411.7250 | **706.3661** | 13 |
| 11 | **1209.6262** | 605.3168 | 1192.5997 | 596.8035 | 1191.6157 | 596.3115 | N | **1332.6828** | 666.8450 | 1315.6562 | 658.3318 | 1314.6722 | 657.8397 | 12 |
| 12 | **1324.6532** | **662.8302** | 1307.6266 | 654.3170 | 1306.6426 | 653.8249 | N | 1218.6398 | 609.8236 | 1201.6133 | 601.3103 | 1200.6293 | 600.8183 | 11 |
| 13 | **1437.7372** | **719.3723** | 1420.7107 | 710.8590 | 1419.7267 | 710.3670 | L | 1103.6129 | 552.3101 | 1086.5864 | 543.7968 | 1085.6023 | 543.3048 | 10 |
| 14 | **1538.7849** | **769.8961** | 1521.7584 | **761.3828** | 1520.7744 | **760.8908** | T | **990.5288** | **495.7681** | 973.5023 | 487.2548 | 972.5183 | 486.7628 | 9 |
| 15 | **1609.8220** | 805.4147 | 1592.7955 | **796.9014** | 1591.8115 | **796.4094** | A | **889.4812** | **445.2442** | 872.4546 | 436.7309 | 871.4706 | 436.2389 | 8 |
| 16 | **1722.9061** | **861.9567** | 1705.8795 | 853.4434 | **1704.8955** | **852.9514** | L | 818.4441 | **409.7257** | 801.4175 | 401.2124 | 800.4335 | 400.7204 | 7 |
| 17 | **1809.9381** | **905.4727** | **1792.9116** | **896.9594** | **1791.9276** | **896.4674** | S | **705.3600** | 353.1836 | 688.3334 | 344.6704 | 687.3494 | 344.1784 | 6 |

| | | | | | | | | | | | | | | |
|---|---|---|---|---|---|---|---|---|---|---|---|---|---|---|
| **18** | **1923.9811** | 962.4942 | 1906.9545 | 953.9809 | 1905.9705 | 953.4889 | **N** | **618.3280** | 309.6676 | 601.3014 | 301.1543 | | | **5** |
| **19** | 2071.0165 | **1036.0119** | 2053.9899 | 1027.4986 | 2053.0059 | 1027.0066 | **M** | **504.2850** | 252.6462 | 487.2585 | **244.1329** | | | **4** |
| **20** | 2184.1005 | **1092.5539** | 2167.0740 | **1084.0406** | 2166.0900 | **1083.5486** | **L** | **357.2496** | 179.1285 | 340.2231 | 170.6152 | | | **3** |
| **21** | 2281.1533 | **1141.0803** | 2264.1267 | **1132.5670** | 2263.1427 | **1132.0750** | **P** | **244.1656** | 122.5864 | 227.1390 | 114.0731 | | | **2** |
| **22** | | | | | | | **K** | 147.1128 | 74.0600 | 130.0863 | 65.5468 | | | **1** |

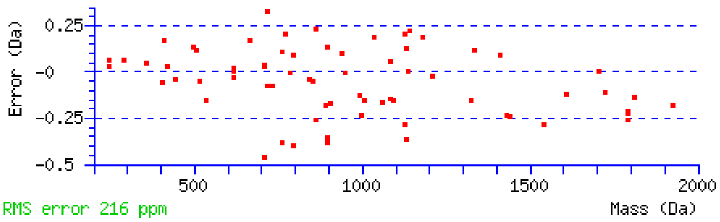


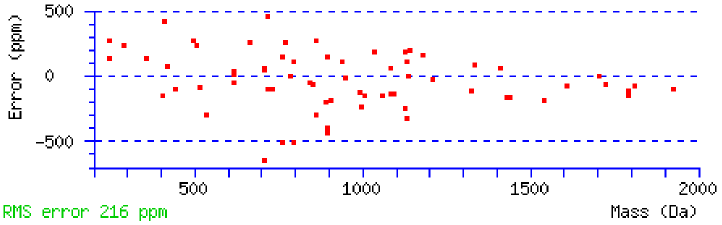


**All matches to this query**

| Score | Mr(calc): | Delta | Sequence |
|---|---|---|---|
| 63.1 | 2426.2515 | 0.0005 | ASFNITLIHPNNLTALSNMLPK |
| 63.1 | 2426.2515 | 0.0005 | ASFNITLIHPNNLTALSNMLPK |
| 55.2 | 2426.2515 | 0.0005 | ASFNITLIHPNNLTALSNMLPK |
| 48.5 | 2426.2515 | 0.0005 | ASFNITLIHPNNLTALSNMLPK |
| 32.8 | 2425.2675 | 0.9845 | ASFNITLIHPNNLTALSNMLPK |
| 32.8 | 2425.2675 | 0.9845 | ASFNITLIHPNNLTALSNMLPK |
| 31.7 | 2426.2515 | 0.0005 | ASFNITLIHPNNLTALSNMLPK |
| 31.7 | 2426.2515 | 0.0005 | ASFNITLIHPNNLTALSNMLPK |
| 31.3 | 2425.2675 | 0.9845 | ASFNITLIHPNNLTALSNMLPK |
| 23.9 | 2425.2675 | 0.9845 | ASFNITLIHPNNLTALSNMLPK |

---

Spectrum No: 478; Query: 51; Rank: 1

---

## Peptide View

MS/MS Fragmentation of **ANLSSQILK**
Found in **IPI00188338**, Tax_Id=10116 Gene_Symbol=Itih1_predicted inter-alpha trypsin inhibitor, heavy chain 1

Match to Query 51: 973.543048 from(487.778800,2+)
Title: 091008RatKidney_NH4Format01_29.1194.1194.2.dta
Data file K:\NewmanPaper\Piliang\3SubProteomes\Piliang3SP\mgf5ppm\ERLIC_3SubProteomes5ppm.mgf

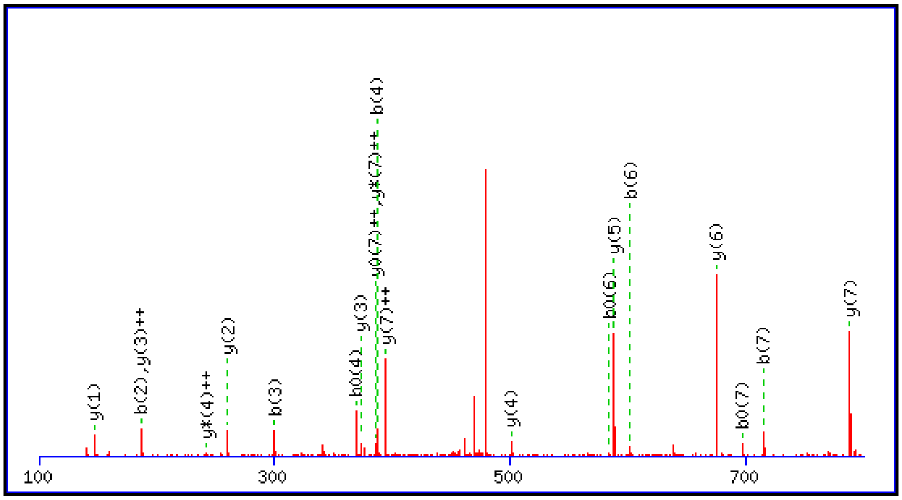

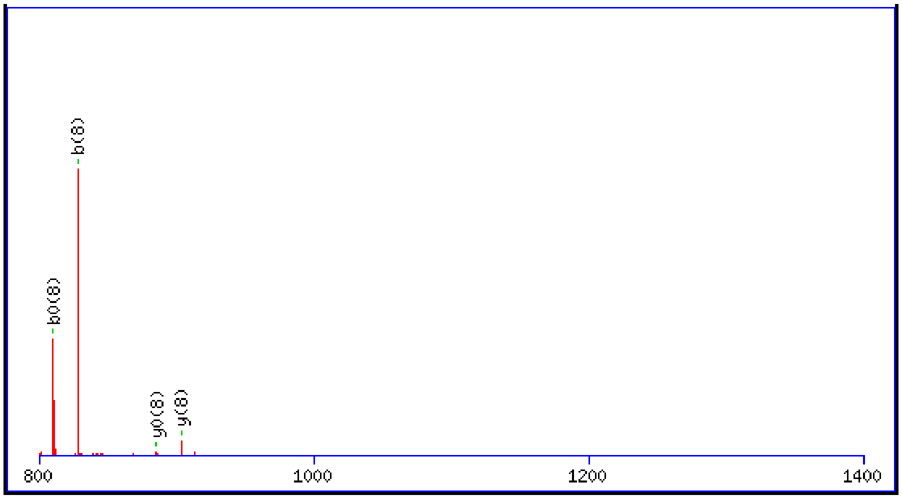


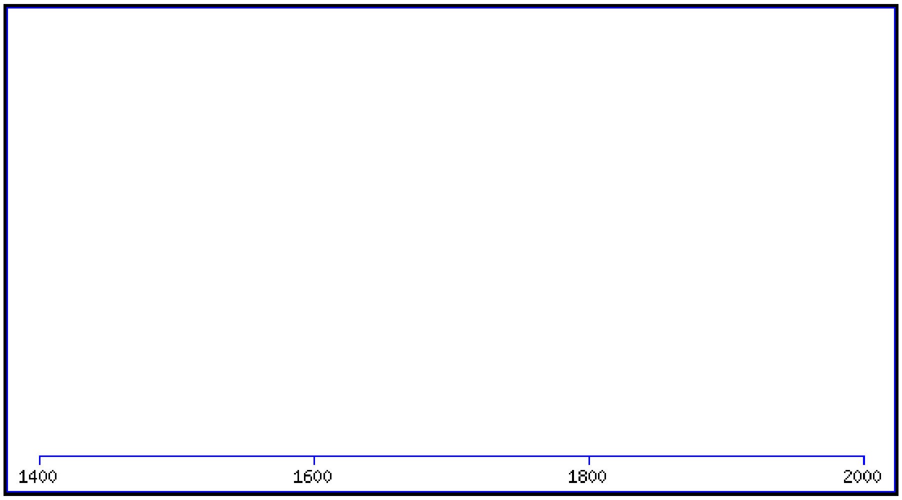


**Monoisotopic mass of neutral peptide Mr(calc):** 973.5444
**Fixed modifications:** Carbamidomethyl (C)
**Variable modifications:**
**N2** : Deamidated_N (N)
**Ions Score:** 63 **Expect:** 4.8e-005
**Matches (Bold Red):** 24/80 fragment ions using 43 most intense peaks

| # | b | $b^{++}$ | b* | $b^{*++}$ | $b^{0}$ | $b^{0++}$ | Seq. | y | $y^{++}$ | y* | $y^{*++}$ | $y^{0}$ | $y^{0++}$ | # |
|---|---|---|---|---|---|---|---|---|---|---|---|---|---|---|
| 1 | 72.0444 | 36.5258 | | | | | A | | | | | | | 9 |
| 2 | **187.0713** | 94.0393 | 170.0448 | 85.5260 | | | N | **903.5146** | 452.2609 | 886.4880 | 443.7476 | **885.5040** | 443.2556 | 8 |
| 3 | **300.1554** | 150.5813 | 283.1288 | 142.0681 | | | L | **788.4876** | **394.7475** | 771.4611 | **386.2342** | 770.4771 | **385.7422** | 7 |
| 4 | **387.1874** | 194.0973 | 370.1609 | 185.5841 | **369.1769** | 185.0921 | S | **675.4036** | 338.2054 | 658.3770 | 329.6921 | 657.3930 | 329.2001 | 6 |
| 5 | 474.2194 | 237.6134 | 457.1929 | 229.1001 | 456.2089 | 228.6081 | S | **588.3715** | 294.6894 | 571.3450 | 286.1761 | 570.3610 | 285.6841 | 5 |
| 6 | **602.2780** | 301.6427 | 585.2515 | 293.1294 | **584.2675** | 292.6374 | Q | **501.3395** | 251.1734 | 484.3130 | **242.6601** | | | 4 |
| 7 | **715.3621** | 358.1847 | 698.3355 | 349.6714 | **697.3515** | 349.1794 | I | **373.2809** | **187.1441** | 356.2544 | 178.6308 | | | 3 |
| 8 | **828.4462** | 414.7267 | 811.4196 | 406.2134 | **810.4356** | 405.7214 | L | **260.1969** | 130.6021 | 243.1703 | 122.0888 | | | 2 |
| 9 | | | | | | | K | **147.1128** | 74.0600 | 130.0863 | 65.5468 | | | 1 |

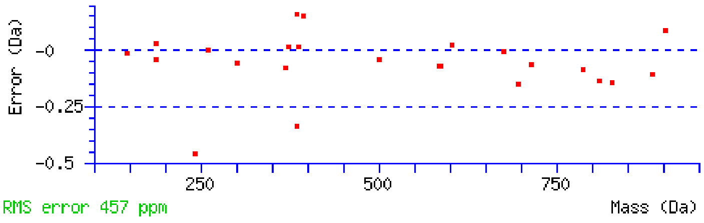


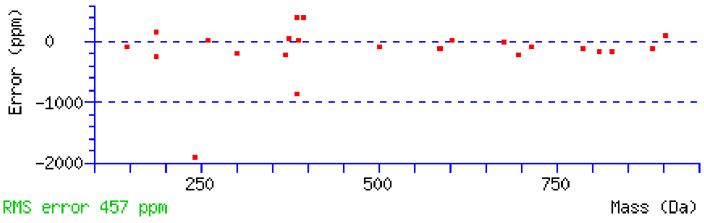


### All matches to this query

| Score | Mr(calc): | Delta | Sequence |
|---|---|---|---|
| 63.0 | 973.5444 | -0.0013 | ANLSSQILK |
| 22.7 | 973.5444 | -0.0013 | LVSSEQALK |
| 22.2 | 973.5444 | -0.0013 | SVLTAAGNLK |
| 18.1 | 973.5444 | -0.0013 | QENKTLLK |
| 17.0 | 973.5379 | 0.0052 | QGLEMRIK |
| 15.9 | 972.5465 | 0.9966 | QGLSRGLSR |
| 15.0 | 973.5444 | -0.0013 | SASQEVLIK |
| 12.4 | 973.5444 | -0.0013 | KATADNLIK |
| 11.3 | 973.5379 | 0.0052 | EGRKMPIK |
| 11.3 | 973.5345 | 0.0086 | WKDERLK |

---

Spectrum No: 479; Query: 148; Rank: 1

---

## Peptide View

MS/MS Fragmentation of **NPNGTVTVISR**
Found in **IPI00369591**, Tax_Id=10116 Gene_Symbol=Pvrl1 similar to poliovirus receptor-related 1

Match to Query 148: 1157.601088 from(579.807820,2+)
Title: 091008RatKidney_NoSalt_26.767.767.2.dta
Data file K:\NewmanPaper\Piliang\3SubProteomes\Piliang3SP\mgf5ppm\ERLIC_3SubProteomes5ppm.mgf

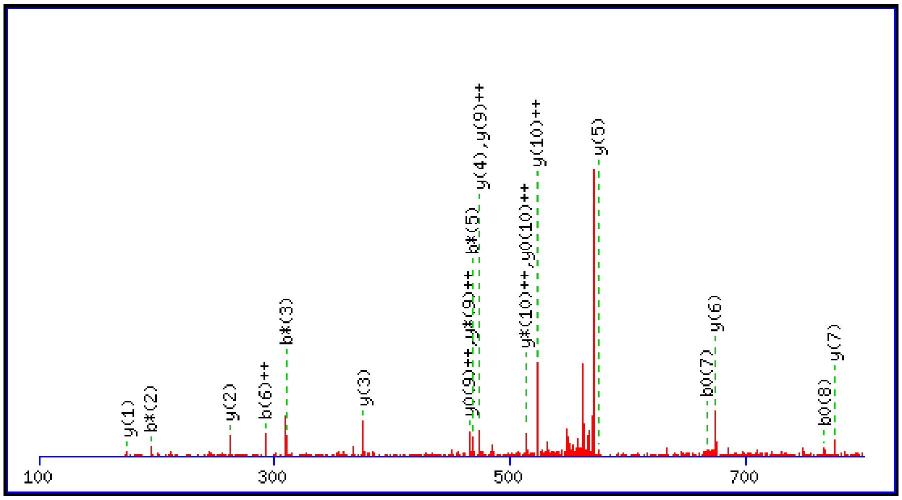


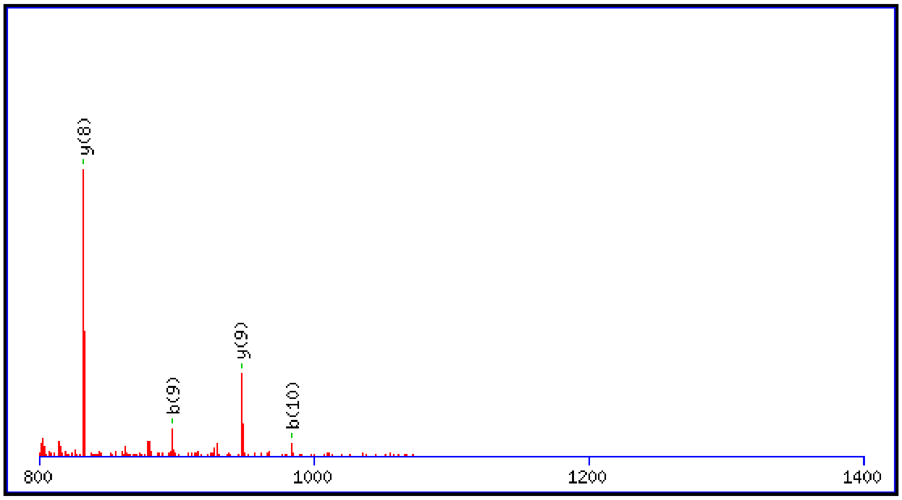

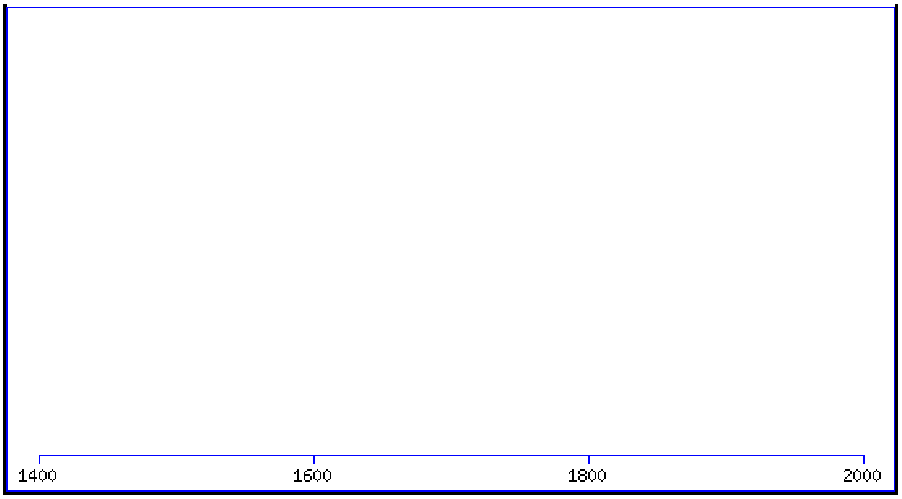


**Monoisotopic mass of neutral peptide Mr(calc):** 1157.6040
**Fixed modifications:** Carbamidomethyl (C)
**Variable modifications:**
**N3** : Deamidated_N (N)
**Ions Score:** 63 **Expect:** 5e-005
**Matches (Bold Red):** 23/110 fragment ions using 29 most intense peaks

| # | b | $b^{++}$ | b* | $b^{*++}$ | $b^0$ | $b^{0++}$ | Seq. | y | $y^{++}$ | y* | $y^{*++}$ | $y^0$ | $y^{0++}$ | # |
|---|---|---|---|---|---|---|---|---|---|---|---|---|---|---|
| 1 | 115.0502 | 58.0287 | 98.0237 | 49.5155 | | | N | | | | | | | 11 |
| 2 | 212.1030 | 106.5551 | **195.0764** | 98.0418 | | | P | 1044.5684 | **522.7878** | 1027.5418 | **514.2746** | 1026.5578 | **513.7826** | 10 |
| 3 | 327.1299 | 164.0686 | **310.1034** | 155.5553 | | | N | **947.5156** | **474.2615** | 930.4891 | **465.7482** | 929.5051 | **465.2562** | 9 |
| 4 | 384.1514 | 192.5793 | 367.1248 | 184.0660 | | | G | **832.4887** | 416.7480 | 815.4621 | 408.2347 | 814.4781 | 407.7427 | 8 |
| 5 | 485.1990 | 243.1032 | **468.1725** | 234.5899 | 467.1885 | 234.0979 | T | **775.4672** | 388.2373 | 758.4407 | 379.7240 | 757.4567 | 379.2320 | 7 |
| 6 | 584.2675 | **292.6374** | 567.2409 | 284.1241 | 566.2569 | 283.6321 | V | **674.4196** | 337.7134 | 657.3930 | 329.2001 | 656.4090 | 328.7081 | 6 |
| 7 | 685.3151 | 343.1612 | 668.2886 | 334.6479 | **667.3046** | 334.1559 | T | **575.3511** | 288.1792 | 558.3246 | 279.6659 | 557.3406 | 279.1739 | 5 |
| 8 | 784.3836 | 392.6954 | 767.3570 | 384.1821 | **766.3730** | 383.6901 | V | **474.3035** | 237.6554 | 457.2769 | 229.1421 | 456.2929 | 228.6501 | 4 |
| 9 | **897.4676** | 449.2374 | 880.4411 | 440.7242 | 879.4571 | 440.2322 | I | **375.2350** | 188.1212 | 358.2085 | 179.6079 | 357.2245 | 179.1159 | 3 |
| 10 | **984.4996** | 492.7535 | 967.4731 | 484.2402 | 966.4891 | 483.7482 | S | **262.1510** | 131.5791 | 245.1244 | 123.0659 | 244.1404 | 122.5738 | 2 |
| 11 | | | | | | | R | **175.1190** | 88.0631 | 158.0924 | 79.5498 | | | 1 |

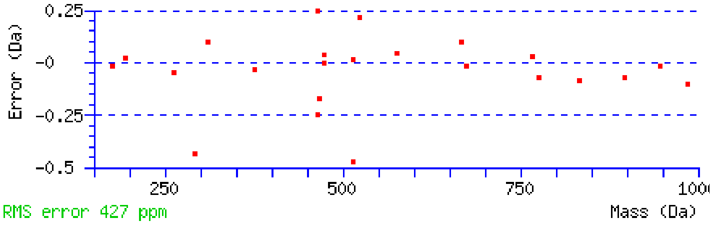


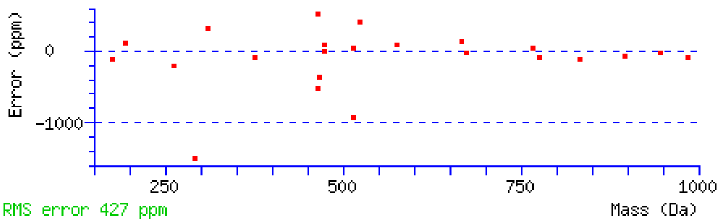


### All matches to this query

| Score | Mr(calc): | Delta | Sequence |
|---|---|---|---|
| 63.0 | 1157.6040 | -0.0030 | NPNGTVTVISR |
| 46.8 | 1157.6040 | -0.0030 | NPNGTVTVISR |
| 10.3 | 1157.5975 | 0.0036 | QVRPGMDLSR |
| 10.0 | 1156.6023 | 0.9988 | ATHIVQVMSR |
| 9.5 | 1157.6081 | -0.0070 | LVYTVSFSSR |
| 9.0 | 1157.5958 | 0.0053 | RFLTSVISR |
| 9.0 | 1157.5958 | 0.0053 | RFLTSVISR |
| 8.5 | 1157.6040 | -0.0029 | GKPTEASIEAR |
| 7.6 | 1156.5950 | 1.0060 | DILMFYLSR |
| 7.0 | 1156.6088 | 0.9923 | GVAQAALGDLDK |

Spectrum No: 480; Query: 2784; Rank: 1

# Peptide View

MS/MS Fragmentation of **MLVSNIDLGPTILDLAGYDLNKTQMDGTSLLPILK**
Found in **IPI00366226**, Tax_Id=10116 Gene_Symbol=Gns Glucosamine (N-acetyl)-6-sulfatase

Match to Query 2784: 3804.965922 from(1269.329250,3+)
Title: 091008RatKidney_NH4Format01_17.5595.5595.3.dta
Data file K:\NewmanPaper\Piliang\3SubProteomes\Piliang3SP\mgf5ppm\ERLIC_3SubProteomes5ppm.mgf

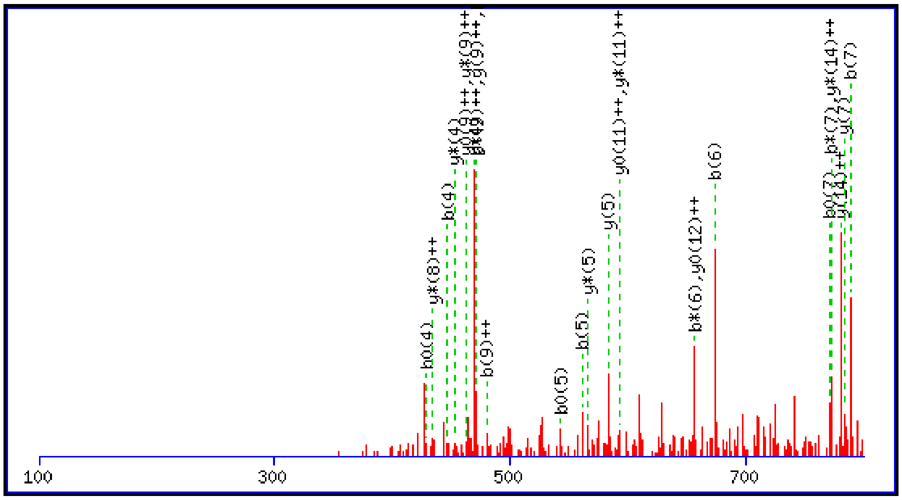


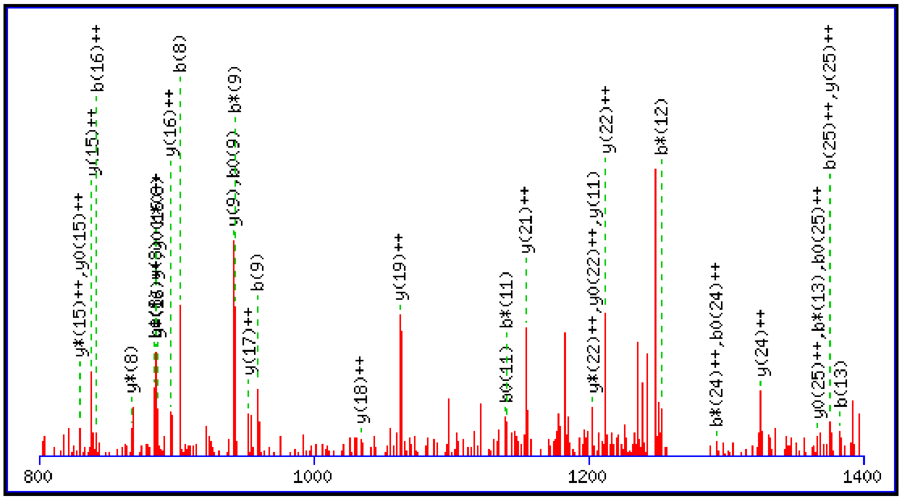


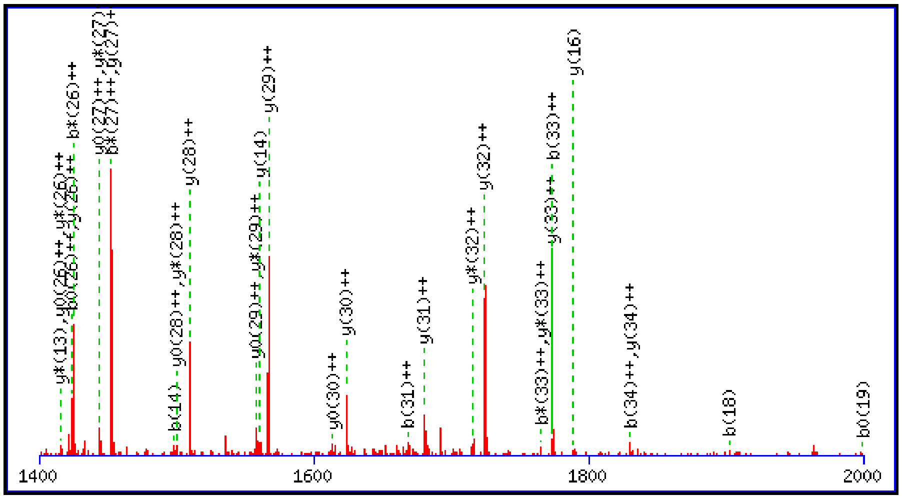

**Monoisotopic mass of neutral peptide Mr(calc):** 3804.9781
**Fixed modifications:** Carbamidomethyl (C)
**Variable modifications:**
**M1** : Oxidation (M)
**N21** : Deamidated_N (N)
**M25** : Oxidation (M)
**Ions Score:** 63 **Expect:** 5.7e-005
**Matches (Bold Red):** 96/382 fragment ions using 184 most intense peaks

| # | b | $b^{++}$ | b* | $b^{*++}$ | $b^0$ | $b^{0++}$ | Seq. | y | $y^{++}$ | y* | $y^{*++}$ | $y^0$ | $y^{0++}$ | # |
|---|---|---|---|---|---|---|---|---|---|---|---|---|---|---|
| 1 | 148.0427 | 74.5250 | | | | | M | | | | | | | 35 |
| 2 | 261.1267 | 131.0670 | | | | | L | 3658.9500 | **1829.9786** | 3641.9234 | 1821.4653 | 3640.9394 | 1820.9733 | 34 |
| 3 | 360.1952 | 180.6012 | | | | | V | 3545.8659 | **1773.4366** | 3528.8393 | **1764.9233** | 3527.8553 | 1764.4313 | 33 |
| 4 | **447.2272** | 224.1172 | | | **429.2166** | 215.1119 | S | 3446.7975 | **1723.9024** | 3429.7709 | **1715.3891** | 3428.7869 | 1714.8971 | 32 |
| 5 | **561.2701** | 281.1387 | 544.2436 | 272.6254 | **543.2595** | 272.1334 | N | 3359.7655 | **1680.3864** | 3342.7389 | 1671.8731 | 3341.7549 | 1671.3811 | 31 |
| 6 | **674.3542** | 337.6807 | **657.3276** | 329.1675 | 656.3436 | 328.6754 | I | 3245.7225 | **1623.3649** | 3228.6960 | 1614.8516 | 3227.7120 | **1614.3596** | 30 |
| 7 | **789.3811** | 395.1942 | **772.3546** | 386.6809 | **771.3706** | 386.1889 | D | 3132.6385 | **1566.8229** | 3115.6119 | **1558.3096** | 3114.6279 | **1557.8176** | 29 |
| 8 | **902.4652** | 451.7362 | **885.4386** | 443.2230 | **884.4546** | 442.7309 | L | 3017.6115 | **1509.3094** | 3000.5850 | **1500.7961** | 2999.6010 | **1500.3041** | 28 |
| 9 | **959.4866** | **480.2470** | **942.4601** | **471.7337** | **941.4761** | **471.2417** | G | 2904.5275 | **1452.7674** | 2887.5009 | **1444.2541** | 2886.5169 | **1443.7621** | 27 |
| 10 | 1056.5394 | 528.7733 | 1039.5129 | 520.2601 | 1038.5288 | 519.7681 | P | 2847.5060 | **1424.2566** | 2830.4794 | **1415.7434** | 2829.4954 | **1415.2514** | 26 |
| 11 | 1157.5871 | 579.2972 | **1140.5605** | 570.7839 | **1139.5765** | 570.2919 | T | 2750.4532 | **1375.7303** | 2733.4267 | 1367.2170 | 2732.4427 | **1366.7250** | 25 |
| 12 | 1270.6712 | 635.8392 | **1253.6446** | 627.3259 | 1252.6606 | 626.8339 | I | 2649.4055 | **1325.2064** | 2632.3790 | 1316.6931 | 2631.3950 | 1316.2011 | 24 |
| 13 | **1383.7552** | 692.3812 | **1366.7287** | 683.8680 | 1365.7447 | 683.3760 | L | 2536.3215 | 1268.6644 | 2519.2949 | 1260.1511 | 2518.3109 | 1259.6591 | 23 |
| 14 | **1498.7822** | 749.8947 | 1481.7556 | 741.3814 | 1480.7716 | 740.8894 | D | 2423.2374 | **1212.1223** | 2406.2109 | **1203.6091** | 2405.2269 | **1203.1171** | 22 |
| 15 | 1611.8662 | 806.4368 | 1594.8397 | 797.9235 | 1593.8557 | 797.4315 | L | 2308.2105 | **1154.6089** | 2291.1839 | 1146.0956 | 2290.1999 | 1145.6036 | 21 |
| 16 | 1682.9033 | **841.9553** | 1665.8768 | 833.4420 | 1664.8928 | 832.9500 | A | 2195.1264 | 1098.0668 | 2178.0999 | 1089.5536 | 2177.1158 | 1089.0616 | 20 |
| 17 | 1739.9248 | 870.4660 | 1722.8983 | 861.9528 | 1721.9142 | 861.4608 | G | 2124.0893 | **1062.5483** | 2107.0627 | 1054.0350 | 2106.0787 | 1053.5430 | 19 |
| 18 | **1902.9881** | 951.9977 | 1885.9616 | 943.4844 | 1884.9776 | 942.9924 | Y | 2067.0678 | **1034.0376** | 2050.0413 | 1025.5243 | 2049.0573 | 1025.0323 | 18 |
| 19 | 2018.0151 | 1009.5112 | 2000.9885 | 1000.9979 | **2000.0045** | 1000.5059 | D | 1904.0045 | **952.5059** | 1886.9780 | 943.9926 | 1885.9939 | 943.5006 | 17 |
| 20 | 2131.0991 | 1066.0532 | 2114.0726 | 1057.5399 | 2113.0886 | 1057.0479 | L | **1788.9776** | **894.9924** | 1771.9510 | **886.4791** | 1770.9670 | **885.9871** | 16 |
| 21 | 2246.1261 | 1123.5667 | 2229.0995 | 1115.0534 | 2228.1155 | 1114.5614 | N | 1675.8935 | **838.4504** | 1658.8669 | **829.9371** | 1657.8829 | **829.4451** | 15 |
| 22 | 2374.2210 | 1187.6142 | 2357.1945 | 1179.1009 | 2356.2105 | 1178.6089 | K | **1560.8666** | **780.9369** | 1543.8400 | **772.4236** | 1542.8560 | 771.9316 | 14 |
| 23 | 2475.2687 | 1238.1380 | 2458.2422 | 1229.6247 | 2457.2582 | 1229.1327 | T | 1432.7716 | 716.8894 | **1415.7451** | 708.3762 | 1414.7610 | 707.8842 | 13 |
| 24 | 2603.3273 | 1302.1673 | 2586.3007 | **1293.6540** | 2585.3167 | **1293.1620** | Q | 1331.7239 | 666.3656 | 1314.6974 | 657.8523 | 1313.7134 | **657.3603** | 12 |
| 25 | 2750.3627 | **1375.6850** | 2733.3362 | 1367.1717 | 2732.3521 | **1366.6797** | M | **1203.6653** | 602.3363 | 1186.6388 | **593.8230** | 1185.6548 | **593.3310** | 11 |
| 26 | 2865.3896 | 1433.1985 | 2848.3631 | **1424.6852** | 2847.3791 | **1424.1932** | D | 1056.6299 | 528.8186 | 1039.6034 | 520.3053 | 1038.6194 | 519.8133 | 10 |
| 27 | 2922.4111 | 1461.7092 | 2905.3846 | **1453.1959** | 2904.4005 | **1452.7039** | G | **941.6030** | **471.3051** | 924.5764 | **462.7919** | 923.5924 | **462.2999** | 9 |
| 28 | 3023.4588 | 1512.2330 | 3006.4322 | 1503.7198 | 3005.4482 | 1503.2277 | T | **884.5815** | 442.7944 | **867.5550** | **434.2811** | 866.5710 | 433.7891 | 8 |
| 29 | 3110.4908 | 1555.7490 | 3093.4643 | 1547.2358 | 3092.4802 | 1546.7438 | S | **783.5339** | 392.2706 | 766.5073 | 383.7573 | 765.5233 | 383.2653 | 7 |
| 30 | 3223.5749 | 1612.2911 | 3206.5483 | 1603.7778 | 3205.5643 | 1603.2858 | L | 696.5018 | 348.7546 | 679.4753 | 340.2413 | | | 6 |
| 31 | 3336.6589 | **1668.8331** | 3319.6324 | 1660.3198 | 3318.6484 | 1659.8278 | L | **583.4178** | 292.2125 | **566.3912** | 283.6992 | | | 5 |
| 32 | 3433.7117 | 1717.3595 | 3416.6852 | 1708.8462 | 3415.7011 | 1708.3542 | P | **470.3337** | 235.6705 | **453.3071** | 227.1572 | | | 4 |
| 33 | 3546.7958 | **1773.9015** | 3529.7692 | **1765.3882** | 3528.7852 | 1764.8962 | I | 373.2809 | 187.1441 | 356.2544 | 178.6308 | | | 3 |
| 34 | 3659.8798 | **1830.4436** | 3642.8533 | 1821.9303 | 3641.8693 | 1821.4383 | L | 260.1969 | 130.6021 | 243.1703 | 122.0888 | | | 2 |
| 35 | | | | | | | K | 147.1128 | 74.0600 | 130.0863 | 65.5468 | | | 1 |

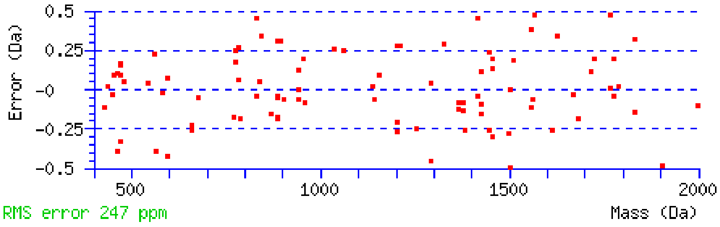


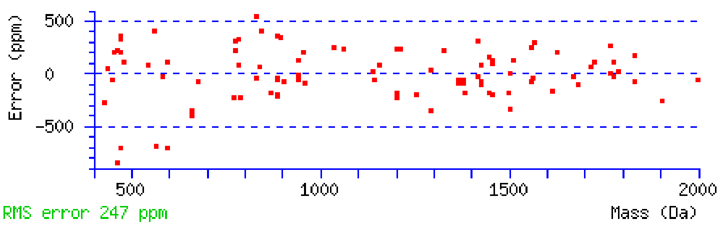


**All matches to this query**

| Score | Mr(calc): | Delta | Sequence |
|---|---|---|---|
| 62.9 | 3804.9781 | -0.0121 | MLVSNIDLGPTILDLAGYDLNKTQMDGTSLLPILK |
| 13.3 | 3803.9940 | 0.9719 | MLVSNIDLGPTILDLAGYDLNKTQMDGTSLLPILK |
| 6.8 | 3804.9781 | -0.0121 | MLVSNIDLGPTILDLAGYDLNKTQMDGTSLLPILK |
| 5.3 | 3803.9263 | 1.0396 | QNILSLPLGSQIITVPVNSSLSWHINKLRETGK |
| 5.2 | 3803.9263 | 1.0396 | QNILSLPLGSQIITVPVNSSLSWHINKLRETGK |
| 4.3 | 3803.9263 | 1.0396 | QNILSLPLGSQIITVPVNSSLSWHINKLRETGK |
| 3.4 | 3804.9282 | 0.0377 | AATDLERISNAEPEPRSLSLGGHVGFDSLPDQLVSK |
| 3.3 | 3803.9514 | 1.0145 | NLVSDLSPDVKMVLVNYIFFQGLWKKPFPSSR |
| 2.3 | 3803.9514 | 1.0145 | NLVSDLSPDVKMVLVNYIFFQGLWKKPFPSSR |
| 0.7 | 3803.9365 | 1.0294 | DIQYATSESVDLGEIMFSLCYLPTAGRLTLTVIK |

---

Spectrum No: 481; Query: 644; Rank: 1

---

## Peptide View

MS/MS Fragmentation of **VEAIGSLANTTMEK**
Found in **IPI00193397**, Tax_Id=10116 Gene_Symbol=Slc34a1 Sodium-dependent phosphate transport protein 2A

Match to Query 644: 1479.713788 from(740.864170,2+)
Title: 100101RatKid_NS_deglyco_26.1825.1825.2.dta
Data file K:\NewmanPaper\Piliang\3SubProteomes\Piliang3SP\mgf5ppm\ERLIC_3SubProteomes5ppm.mgf

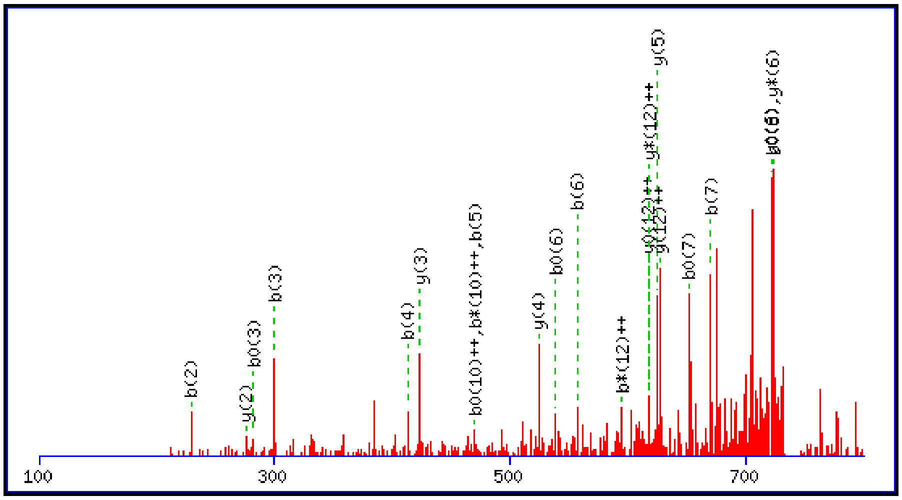


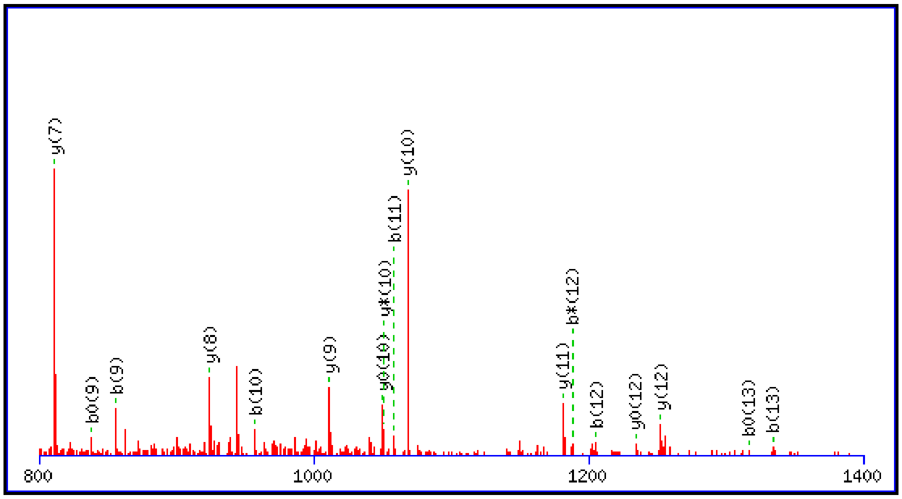

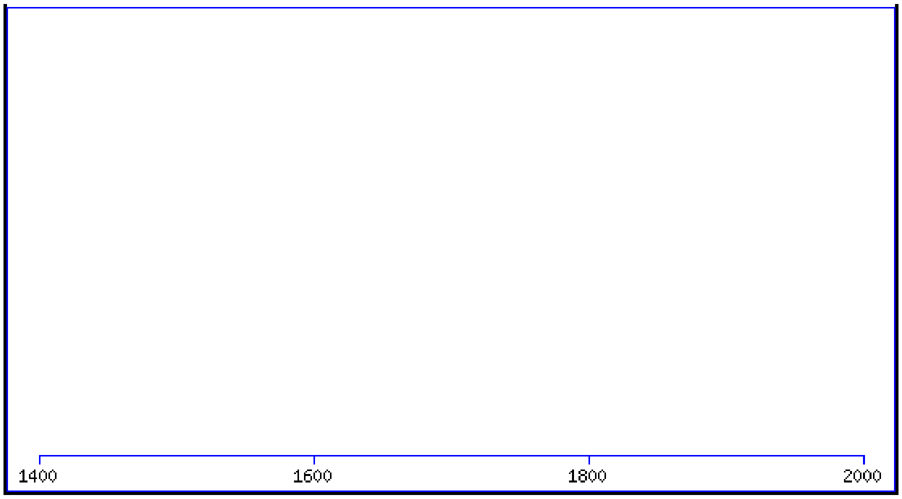


**Monoisotopic mass of neutral peptide Mr(calc):** 1479.7126
**Fixed modifications:** Carbamidomethyl (C)
**Variable modifications:**
**N9** : Deamidated_N (N)
**M12** : Oxidation (M)
**Ions Score:** 63 **Expect:** 7.9e-005
**Matches (Bold Red):** 39/136 fragment ions using 74 most intense peaks

| # | b | b++ | b* | b*++ | b0 | b0++ | Seq. | y | y++ | y* | y*++ | y0 | y0++ | # |
|---|---|---|---|---|---|---|---|---|---|---|---|---|---|---|
| 1 | 100.0757 | 50.5415 | | | | | V | | | | | | | 14 |
| 2 | **229.1183** | 115.0628 | | | 211.1077 | 106.0575 | E | 1381.6515 | 691.3294 | 1364.6250 | 682.8161 | 1363.6410 | 682.3241 | 13 |
| 3 | **300.1554** | 150.5813 | | | **282.1448** | 141.5761 | A | **1252.6089** | **626.8081** | 1235.5824 | **618.2948** | **1234.5984** | **617.8028** | 12 |
| 4 | **413.2395** | 207.1234 | | | 395.2289 | 198.1181 | I | **1181.5718** | 591.2896 | 1164.5453 | 582.7763 | 1163.5613 | 582.2843 | 11 |
| 5 | **470.2609** | 235.6341 | | | 452.2504 | 226.6288 | G | **1068.4878** | 534.7475 | **1051.4612** | 526.2342 | **1050.4772** | 525.7422 | 10 |
| 6 | **557.2930** | 279.1501 | | | **539.2824** | 270.1448 | S | **1011.4663** | 506.2368 | 994.4398 | 497.7235 | 993.4557 | 497.2315 | 9 |
| 7 | **670.3770** | 335.6921 | | | **652.3665** | 326.6869 | L | **924.4343** | 462.7208 | 907.4077 | 454.2075 | 906.4237 | 453.7155 | 8 |
| 8 | 741.4141 | 371.2107 | | | **723.4036** | 362.2054 | A | **811.3502** | 406.1787 | 794.3237 | 397.6655 | 793.3396 | 397.1735 | 7 |
| 9 | **856.4411** | 428.7242 | 839.4145 | 420.2109 | **838.4305** | 419.7189 | N | 740.3131 | 370.6602 | **723.2865** | 362.1469 | **722.3025** | 361.6549 | 6 |
| 10 | **957.4887** | 479.2480 | 940.4622 | **470.7347** | 939.4782 | **470.2427** | T | **625.2862** | 313.1467 | 608.2596 | 304.6334 | 607.2756 | 304.1414 | 5 |
| 11 | **1058.5364** | 529.7719 | 1041.5099 | 521.2586 | 1040.5259 | 520.7666 | T | **524.2385** | 262.6229 | 507.2119 | 254.1096 | 506.2279 | 253.6176 | 4 |
| 12 | **1205.5718** | 603.2896 | **1188.5453** | **594.7763** | 1187.5613 | 594.2843 | M | **423.1908** | 212.0990 | 406.1643 | 203.5858 | 405.1802 | 203.0938 | 3 |
| 13 | **1334.6144** | 667.8108 | 1317.5879 | 659.2976 | **1316.6039** | 658.8056 | E | **276.1554** | 138.5813 | 259.1288 | 130.0681 | 258.1448 | 129.5761 | 2 |
| 14 | | | | | | | K | 147.1128 | 74.0600 | 130.0863 | 65.5468 | | | 1 |

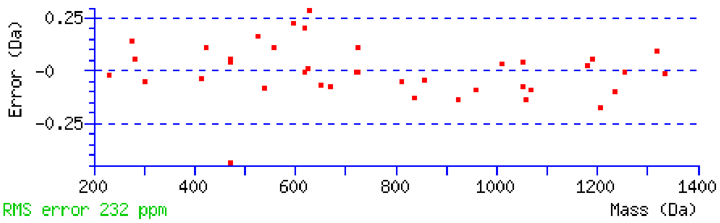


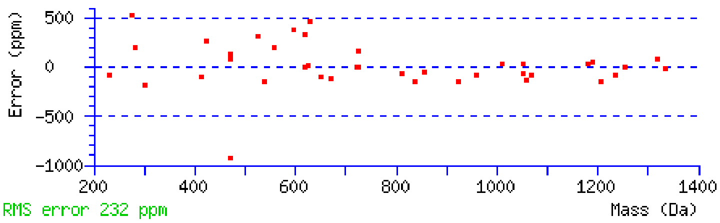


### All matches to this query

| Score | Mr(calc): | Delta | Sequence |
|---|---|---|---|
| 62.9 | 1479.7126 | 0.0011 | VEAIGSLANTTMEK |
| 2.9 | 1479.7222 | -0.0084 | DKSLADILDPSVK |
| 2.7 | 1479.7017 | 0.0121 | QVNQPMIRSATR |
| 0.9 | 1479.7083 | 0.0055 | VAGQNSPSGIQSKK |
| 0.2 | 1479.7221 | -0.0083 | ALNQSNTPLLSLK |

Spectrum No: 482; Query: 842; Rank: 1

## Peptide View

MS/MS Fragmentation of **NHPFNSSLEDPSSR**
Found in **IPI00373230**, Tax_Id=10116 Gene_Symbol=Muc1 similar to mucin 1, transmembrane

Match to Query 842: 1586.696232 from(529.906020,3+)
Title: 091008RatKidney_NH4Format01_24.1145.1145.3.dta
Data file K:\NewmanPaper\Piliang\3SubProteomes\Piliang3SP\mgf5ppm\ERLIC_3SubProteomes5ppm.mgf

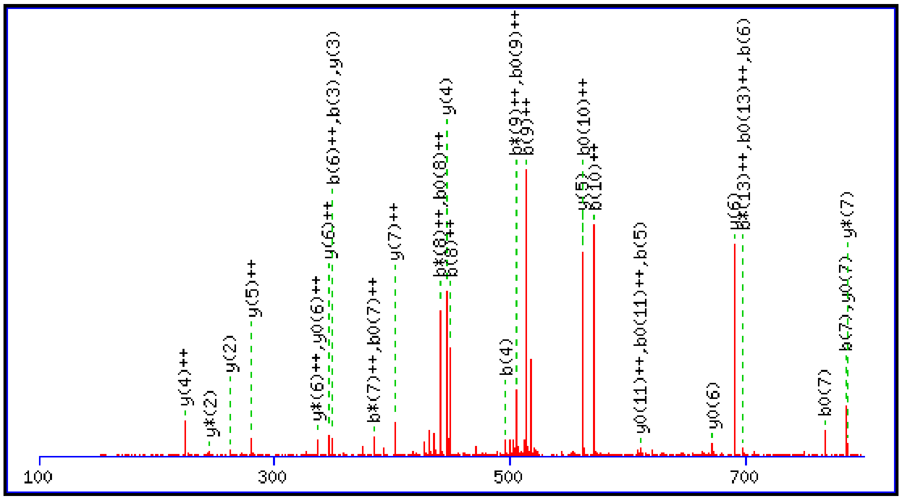


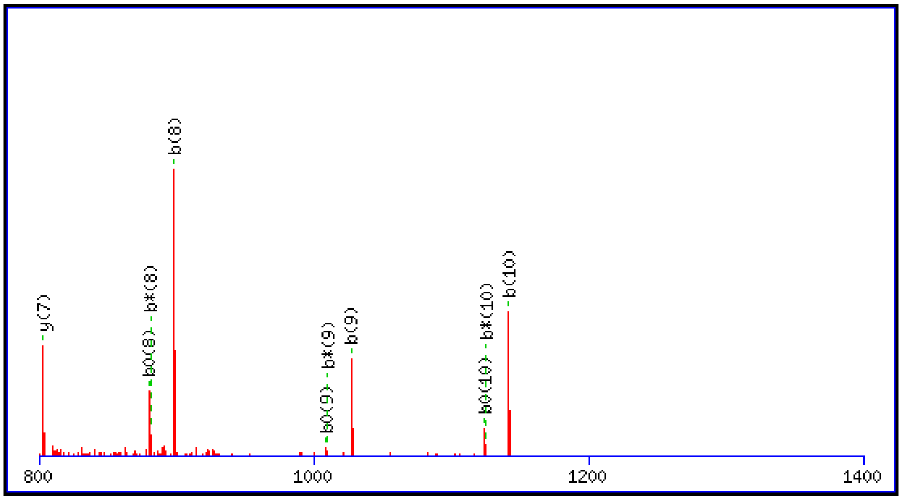


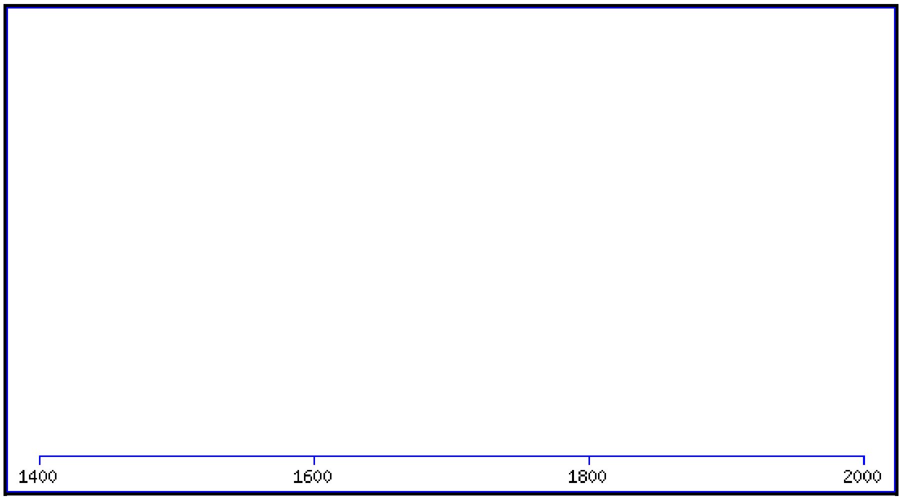


**Monoisotopic mass of neutral peptide Mr(calc):** 1586.6961
**Fixed modifications:** Carbamidomethyl (C)

**Variable modifications:**
**N5** : Deamidated_N (N)
**Ions Score:** 63 **Expect:** 8.3e-005
**Matches (Bold Red):** 46/144 fragment ions using 43 most intense peaks

| # | b | $b^{++}$ | b* | $b^{*++}$ | $b^0$ | $b^{0++}$ | Seq. | y | $y^{++}$ | y* | $y^{*++}$ | $y^0$ | $y^{0++}$ | # |
|---|---|---|---|---|---|---|---|---|---|---|---|---|---|---|
| 1 | 115.0502 | 58.0287 | 98.0237 | 49.5155 | | | N | | | | | | | 14 |
| 2 | 252.1091 | 126.5582 | 235.0826 | 118.0449 | | | H | 1473.6605 | 737.3339 | 1456.6339 | 728.8206 | 1455.6499 | 728.3286 | 13 |
| 3 | **349.1619** | 175.0846 | 332.1353 | 166.5713 | | | P | 1336.6015 | 668.8044 | 1319.5750 | 660.2911 | 1318.5910 | 659.7991 | 12 |
| 4 | **496.2303** | 248.6188 | 479.2037 | 240.1055 | | | F | 1239.5488 | 620.2780 | 1222.5222 | 611.7648 | 1221.5382 | **611.2727** | 11 |
| 5 | **611.2572** | 306.1323 | 594.2307 | 297.6190 | | | N | 1092.4804 | 546.7438 | 1075.4538 | 538.2305 | 1074.4698 | 537.7385 | 10 |
| 6 | **698.2893** | **349.6483** | 681.2627 | 341.1350 | 680.2787 | 340.6430 | S | 977.4534 | 489.2304 | 960.4269 | 480.7171 | 959.4429 | 480.2251 | 9 |
| 7 | **785.3213** | 393.1643 | 768.2947 | **384.6510** | **767.3107** | **384.1590** | S | 890.4214 | 445.7143 | 873.3949 | 437.2011 | 872.4108 | 436.7091 | 8 |
| 8 | **898.4053** | **449.7063** | **881.3788** | **441.1930** | **880.3948** | **440.7010** | L | **803.3894** | **402.1983** | **786.3628** | 393.6850 | **785.3788** | 393.1930 | 7 |
| 9 | **1027.4479** | **514.2276** | **1010.4214** | **505.7143** | **1009.4374** | **505.2223** | E | **690.3053** | **345.6563** | 673.2788 | **337.1430** | **672.2947** | **336.6510** | 6 |
| 10 | **1142.4749** | **571.7411** | **1125.4483** | 563.2278 | **1124.4643** | **562.7358** | D | **561.2627** | **281.1350** | 544.2362 | 272.6217 | 543.2522 | 272.1297 | 5 |
| 11 | 1239.5276 | 620.2675 | 1222.5011 | 611.7542 | 1221.5171 | **611.2622** | P | **446.2358** | **223.6215** | 429.2092 | 215.1082 | 428.2252 | 214.6162 | 4 |
| 12 | 1326.5597 | 663.7835 | 1309.5331 | 655.2702 | 1308.5491 | 654.7782 | S | **349.1830** | 175.0951 | 332.1565 | 166.5819 | 331.1724 | 166.0899 | 3 |
| 13 | 1413.5917 | 707.2995 | 1396.5652 | **698.7862** | 1395.5811 | **698.2942** | S | **262.1510** | 131.5791 | **245.1244** | 123.0659 | 244.1404 | 122.5738 | 2 |
| 14 | | | | | | | R | 175.1190 | 88.0631 | 158.0924 | 79.5498 | | | 1 |

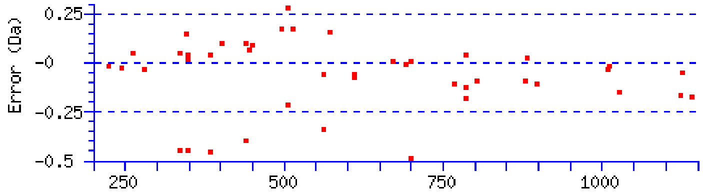


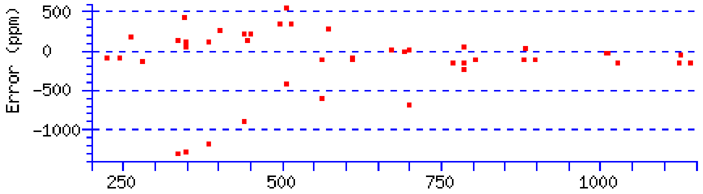


### All matches to this query

| Score | Mr(calc): | Delta | Sequence |
|---|---|---|---|
| 62.9 | 1586.6961 | 0.0001 | NHPFNSSLEDPSSR |
| 41.7 | 1586.6961 | 0.0001 | NHPFNSSLEDPSSR |
| 23.4 | 1585.7025 | 0.9937 | TPSPPEPEPAGTAQK |
| 23.1 | 1585.7025 | 0.9937 | TPSPPEPEPAGTAQK |
| 14.8 | 1586.6878 | 0.0084 | WTTNXRSLNSNK |
| 11.9 | 1585.6977 | 0.9986 | RCKDENYDLAMR |
| 10.8 | 1585.6798 | 1.0164 | QSRGMLLLYSMK |
| 10.0 | 1586.6912 | 0.0050 | SPIMNEFLGGRNR |
| 8.5 | 1586.6847 | 0.0116 | MAPMGWNRKASSR |
| 8.5 | 1586.6847 | 0.0116 | MAPMGWNRKASSR |

---

Spectrum No: 483; Query: 56; Rank: 1

---

## Peptide View

MS/MS Fragmentation of **LGACNNTLK**
Found in **IPI00372372**, Tax_Id=10116 Gene_Symbol=Serpinc1 Serine (Or cysteine) peptidase inhibitor, clade C (Antithrombin), member 1

Match to Query 56: 990.477968 from(496.246260,2+)
Title: 091008RatKidney_NH4Format01_29.350.350.2.dta
Data file K:\NewmanPaper\Piliang\3SubProteomes\Piliang3SP\mgf5ppm\ERLIC_3SubProteomes5ppm.mgf

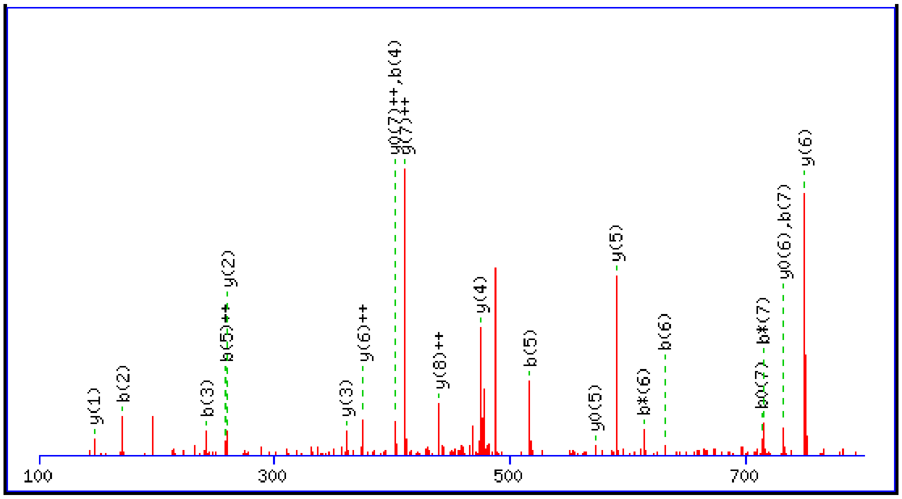


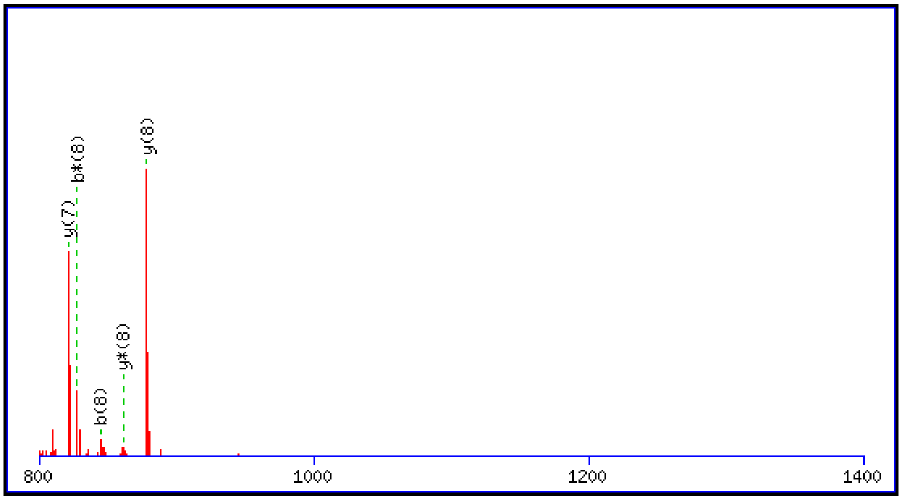


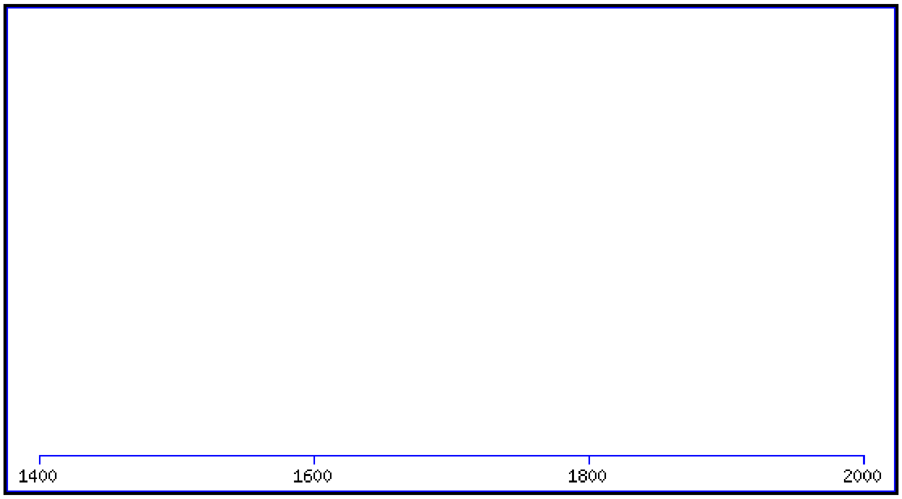


**Monoisotopic mass of neutral peptide Mr(calc):** 990.4804
**Fixed modifications:** Carbamidomethyl (C)
**Variable modifications:**
**N5** : Deamidated_N (N)
**Ions Score:** 63 **Expect:** 5.4e-005
**Matches (Bold Red):** 27/72 fragment ions using 45 most intense peaks

| # | b | b++ | b* | b*++ | b0 | b0++ | Seq. | y | y++ | y* | y*++ | y0 | y0++ | # |
|---|---|---|---|---|---|---|---|---|---|---|---|---|---|---|
| 1 | 114.0913 | 57.5493 | | | | | L | | | | | | | 9 |
| 2 | **171.1128** | 86.0600 | | | | | G | **878.4036** | **439.7055** | **861.3771** | 431.1922 | 860.3931 | 430.7002 | 8 |
| 3 | **242.1499** | 121.5786 | | | | | A | **821.3822** | **411.1947** | 804.3556 | 402.6815 | 803.3716 | **402.1894** | 7 |
| 4 | **402.1806** | 201.5939 | | | | | C | **750.3451** | **375.6762** | 733.3185 | 367.1629 | **732.3345** | 366.6709 | 6 |
| 5 | **517.2075** | **259.1074** | 500.1810 | 250.5941 | | | N | **590.3144** | 295.6608 | 573.2879 | 287.1476 | **572.3038** | 286.6556 | 5 |

| | | | | | | | | | | | | | | |
|---|---|---|---|---|---|---|---|---|---|---|---|---|---|---|
| 6 | 631.2504 | 316.1289 | 614.2239 | 307.6156 | | | N | 475.2875 | 238.1474 | 458.2609 | 229.6341 | 457.2769 | 229.1421 | 4 |
| 7 | 732.2981 | 366.6527 | 715.2716 | 358.1394 | 714.2875 | 357.6474 | T | 361.2445 | 181.1259 | 344.2180 | 172.6126 | 343.2340 | 172.1206 | 3 |
| 8 | 845.3822 | 423.1947 | 828.3556 | 414.6815 | 827.3716 | 414.1894 | L | 260.1969 | 130.6021 | 243.1703 | 122.0888 | | | 2 |
| 9 | | | | | | | K | 147.1128 | 74.0600 | 130.0863 | 65.5468 | | | 1 |

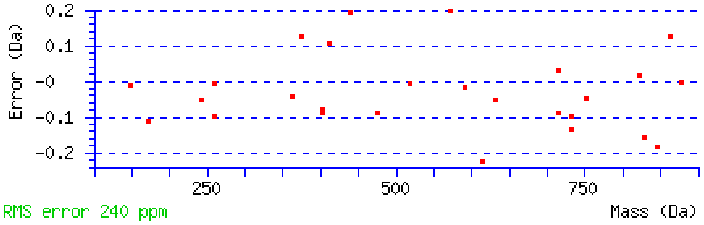


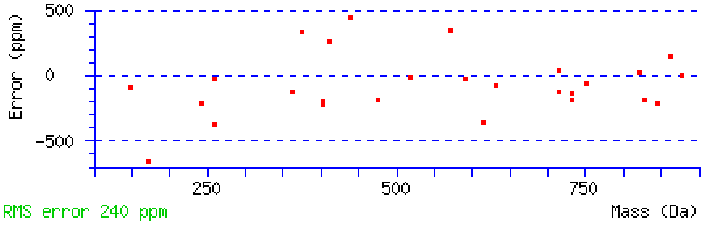


### All matches to this query

| Score | Mr(calc): | Delta | Sequence |
|---|---|---|---|
| 62.8 | 990.4804 | -0.0024 | LGACNNTLK |
| 61.0 | 990.4804 | -0.0024 | LGACNNTLK |
| 11.2 | 990.4787 | -0.0007 | LPDSPALAK |
| 10.4 | 990.4787 | -0.0007 | LQETVPPK |
| 8.8 | 990.4730 | 0.0050 | ASRSNNDVK |
| 8.4 | 990.4804 | -0.0025 | IGETMIDGR |
| 7.4 | 990.4770 | 0.0009 | THYNSELK |
| 7.2 | 990.4730 | 0.0050 | ASRSNNDVK |
| 6.1 | 990.4688 | 0.0092 | LQLHWSK |
| 5.0 | 990.4722 | 0.0058 | LKMSHPAK |

---

Spectrum No: 484; Query: 2299; Rank: 1

---

## Peptide View

MS/MS Fragmentation of **GTDPSLSPSPAAALPAPGTELSYLNR**
Found in **IPI00192504**, Tax_Id=10116 Gene_Symbol=Mrc2 Macrophage mannose receptor 2 precursor

Match to Query 2299: 2582.281568 from(1292.148060,2+)
Title: 091008RatKidney_NH4Format01_23.3513.3513.2.dta
Data file K:\NewmanPaper\Piliang\3SubProteomes\Piliang3SP\mgf5ppm\ERLIC_3SubProteomes5ppm.mgf

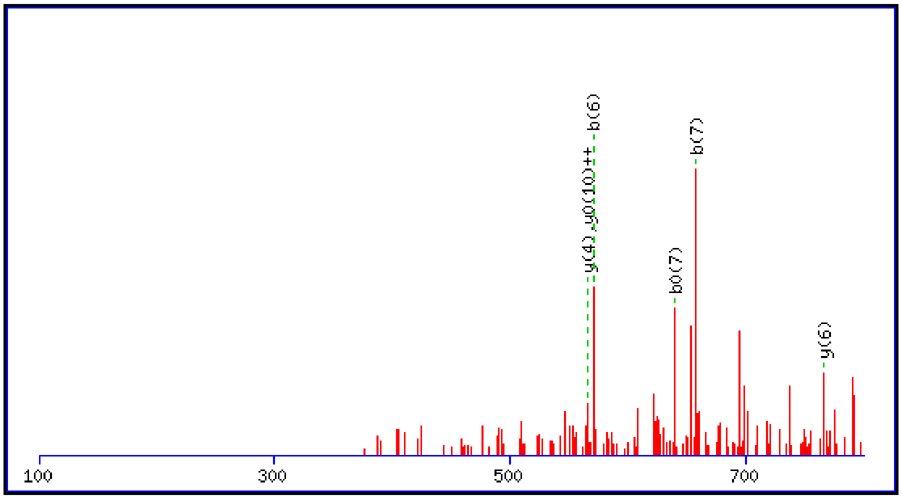

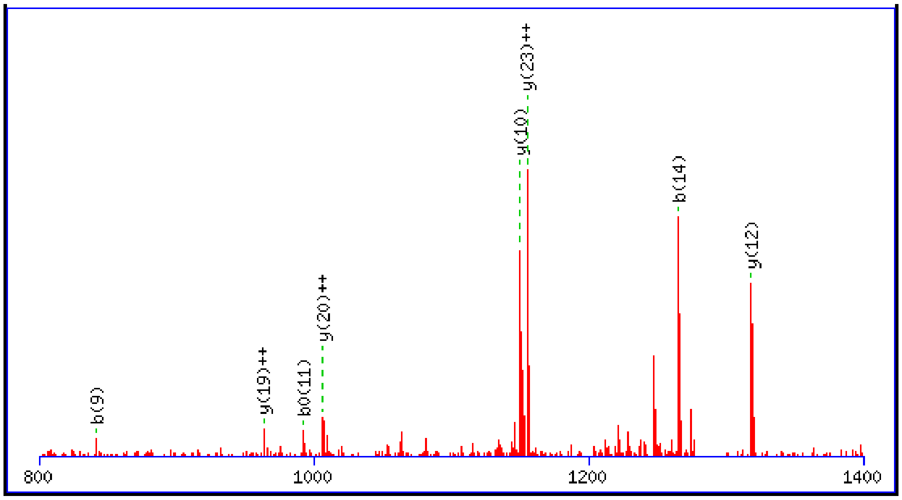


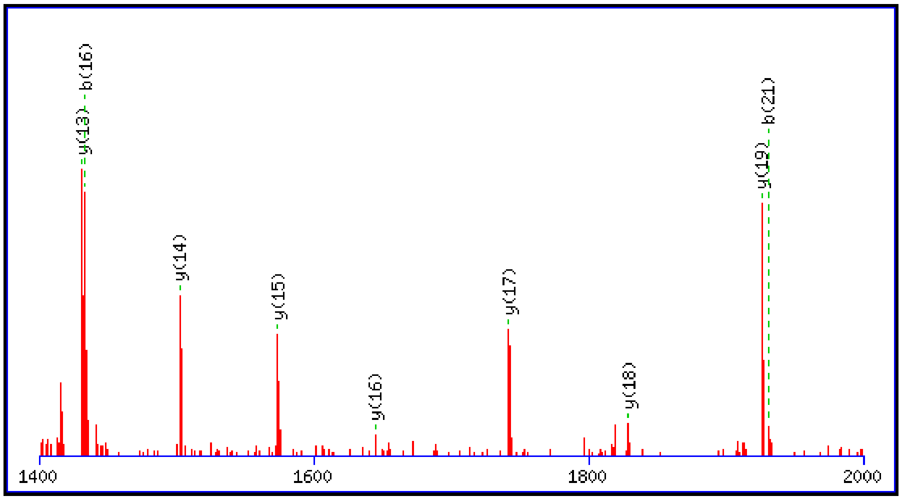


**Monoisotopic mass of neutral peptide Mr(calc):** 2582.2864
**Fixed modifications:** Carbamidomethyl (C)
**Variable modifications:**
**N25** : Deamidated_N (N)
**Ions Score:** 63 **Expect:** 0.00012
**Matches (Bold Red):** 23/242 fragment ions using 48 most intense peaks

| # | b | $b^{++}$ | b* | $b^{*++}$ | $b^{0}$ | $b^{0++}$ | Seq. | y | $y^{++}$ | y* | $y^{*++}$ | $y^{0}$ | $y^{0++}$ | # |
|---|---|---|---|---|---|---|---|---|---|---|---|---|---|---|
| 1 | 58.0287 | 29.5180 | | | | | G | | | | | | | 26 |
| 2 | 159.0764 | 80.0418 | | | 141.0659 | 71.0366 | T | 2526.2722 | 1263.6397 | 2509.2457 | 1255.1265 | 2508.2617 | 1254.6345 | 25 |
| 3 | 274.1034 | 137.5553 | | | 256.0928 | 128.5500 | D | 2425.2245 | 1213.1159 | 2408.1980 | 1204.6026 | 2407.2140 | 1204.1106 | 24 |
| 4 | 371.1561 | 186.0817 | | | 353.1456 | 177.0764 | P | 2310.1976 | **1155.6024** | 2293.1710 | 1147.0892 | 2292.1870 | 1146.5972 | 23 |
| 5 | 458.1882 | 229.5977 | | | 440.1776 | 220.5924 | S | 2213.1448 | 1107.0761 | 2196.1183 | 1098.5628 | 2195.1343 | 1098.0708 | 22 |
| 6 | **571.2722** | 286.1397 | | | 553.2617 | 277.1345 | L | 2126.1128 | 1063.5600 | 2109.0863 | 1055.0468 | 2108.1022 | 1054.5548 | 21 |
| 7 | **658.3042** | 329.6558 | | | **640.2937** | 320.6505 | S | 2013.0287 | **1007.0180** | 1996.0022 | 998.5047 | 1995.0182 | 998.0127 | 20 |
| 8 | 755.3570 | 378.1821 | | | 737.3464 | 369.1769 | P | **1925.9967** | **963.5020** | 1908.9702 | 954.9887 | 1907.9861 | 954.4967 | 19 |
| 9 | **842.3890** | 421.6982 | | | 824.3785 | 412.6929 | S | **1828.9439** | 914.9756 | 1811.9174 | 906.4623 | 1810.9334 | 905.9703 | 18 |
| 10 | 939.4418 | 470.2245 | | | 921.4312 | 461.2193 | P | **1741.9119** | 871.4596 | 1724.8854 | 862.9463 | 1723.9014 | 862.4543 | 17 |
| 11 | 1010.4789 | 505.7431 | | | **992.4684** | 496.7378 | A | **1644.8592** | 822.9332 | 1627.8326 | 814.4199 | 1626.8486 | 813.9279 | 16 |
| 12 | 1081.5160 | 541.2617 | | | 1063.5055 | 532.2564 | A | **1573.8220** | 787.4147 | 1556.7955 | 778.9014 | 1555.8115 | 778.4094 | 15 |
| 13 | 1152.5531 | 576.7802 | | | 1134.5426 | 567.7749 | A | **1502.7849** | 751.8961 | 1485.7584 | 743.3828 | 1484.7744 | 742.8908 | 14 |
| 14 | **1265.6372** | 633.3222 | | | 1247.6266 | 624.3170 | L | **1431.7478** | 716.3775 | 1414.7213 | 707.8643 | 1413.7372 | 707.3723 | 13 |
| 15 | 1362.6900 | 681.8486 | | | 1344.6794 | 672.8433 | P | **1318.6638** | 659.8355 | 1301.6372 | 651.3222 | 1300.6532 | 650.8302 | 12 |
| 16 | **1433.7271** | 717.3672 | | | 1415.7165 | 708.3619 | A | 1221.6110 | 611.3091 | 1204.5844 | 602.7959 | 1203.6004 | 602.3038 | 11 |
| 17 | 1530.7798 | 765.8936 | | | 1512.7693 | 756.8883 | P | **1150.5739** | 575.7906 | 1133.5473 | 567.2773 | 1132.5633 | **566.7853** | 10 |
| 18 | 1587.8013 | 794.4043 | | | 1569.7907 | 785.3990 | G | 1053.5211 | 527.2642 | 1036.4946 | 518.7509 | 1035.5105 | 518.2589 | 9 |

| | | | | | | | | | | | | | | |
|---|---|---|---|---|---|---|---|---|---|---|---|---|---|---|
| 19 | 1688.8490 | 844.9281 | | | 1670.8384 | 835.9229 | T | 996.4996 | 498.7535 | 979.4731 | 490.2402 | 978.4891 | 489.7482 | 8 |
| 20 | 1817.8916 | 909.4494 | | | 1799.8810 | 900.4441 | E | 895.4520 | 448.2296 | 878.4254 | 439.7163 | 877.4414 | 439.2243 | 7 |
| 21 | 1930.9756 | 965.9915 | | | 1912.9651 | 956.9862 | L | 766.4094 | 383.7083 | 749.3828 | 375.1950 | 748.3988 | 374.7030 | 6 |
| 22 | 2018.0077 | 1009.5075 | | | 1999.9971 | 1000.5022 | S | 653.3253 | 327.1663 | 636.2988 | 318.6530 | 635.3147 | 318.1610 | 5 |
| 23 | 2181.0710 | 1091.0391 | | | 2163.0604 | 1082.0339 | Y | 566.2933 | 283.6503 | 549.2667 | 275.1370 | | | 4 |
| 24 | 2294.1551 | 1147.5812 | | | 2276.1445 | 1138.5759 | L | 403.2300 | 202.1186 | 386.2034 | 193.6053 | | | 3 |
| 25 | 2409.1820 | 1205.0946 | 2392.1555 | 1196.5814 | 2391.1714 | 1196.0894 | N | 290.1459 | 145.5766 | 273.1193 | 137.0633 | | | 2 |
| 26 | | | | | | | R | 175.1190 | 88.0631 | 158.0924 | 79.5498 | | | 1 |

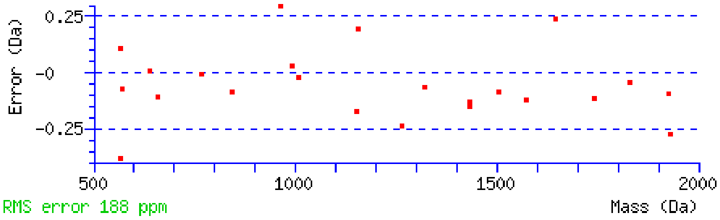


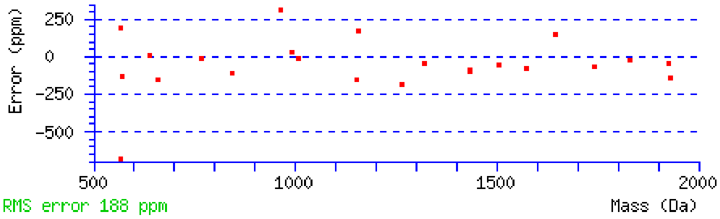


**All matches to this query**

| Score | Mr(calc): | Delta | Sequence |
|---|---|---|---|
| 62.8 | 2582.2864 | -0.0048 | GTDPSLSPSPAAALPAPGTELSYLNR |
| 9.8 | 2581.3024 | 0.9792 | GTDPSLSPSPAAALPAPGTELSYLNR |
| 0.7 | 2582.2733 | 0.0082 | EPTQPSDARGLIRWMLMVNPDR |
| 0.3 | 2582.2775 | 0.0041 | VVIQEMLKTVAQSQQLETSSQR |
| 0.3 | 2582.2775 | 0.0041 | VVIQEMLKTVAQSQQLETSSQR |
| 0.3 | 2582.2775 | 0.0041 | VVIQEMLKTVAQSQQLETSSQR |

---

Spectrum No: 485; Query: 2052; Rank: 1

---

## Peptide View

MS/MS Fragmentation of **GRQQSQEDGDYSPNGSAQPGDK**
Found in **IPI00364925**, Tax_Id=10116 Gene_Symbol=Dhtkd1 Probable 2-oxoglutarate dehydrogenase E1 component DHKTD1, mitochondrial precursor

Match to Query 2052: 2320.982022 from(774.667950,3+)
Title: 091008RatKidney_NH4Format01_23.376.376.3.dta
Data file K:\NewmanPaper\Piliang\3SubProteomes\Piliang3SP\mgf5ppm\ERLIC_3SubProteomes5ppm.mgf

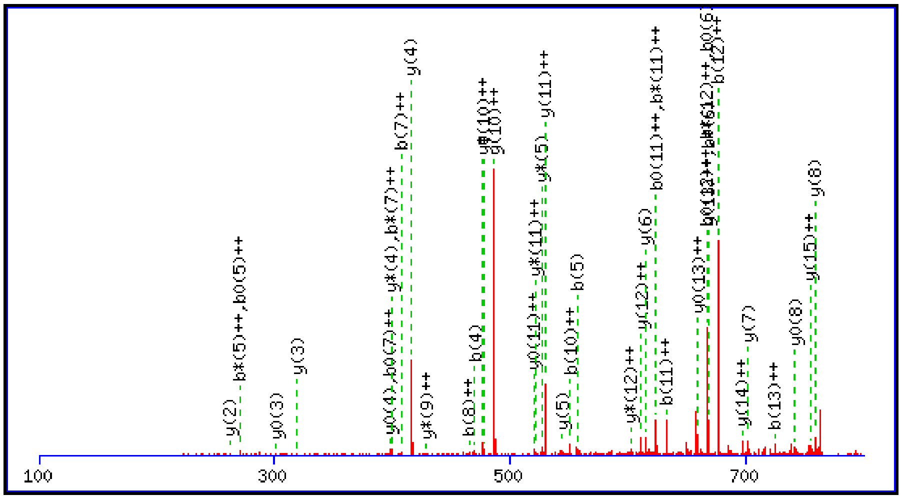

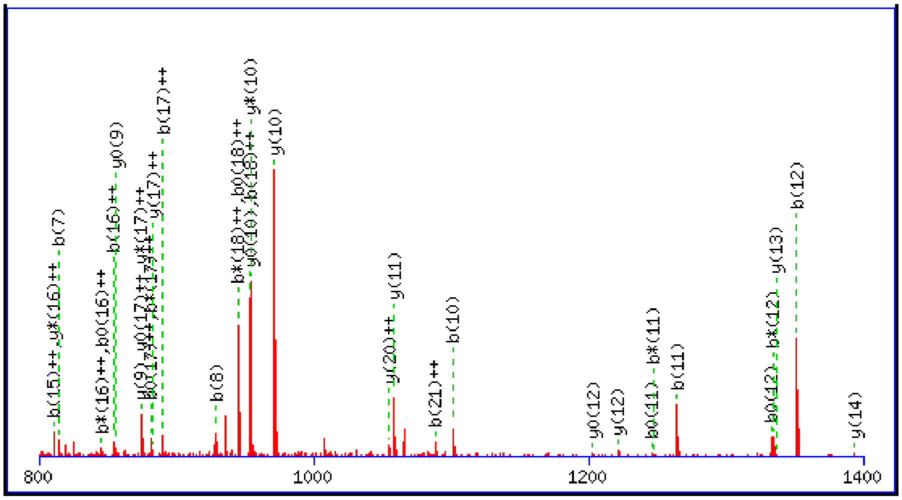


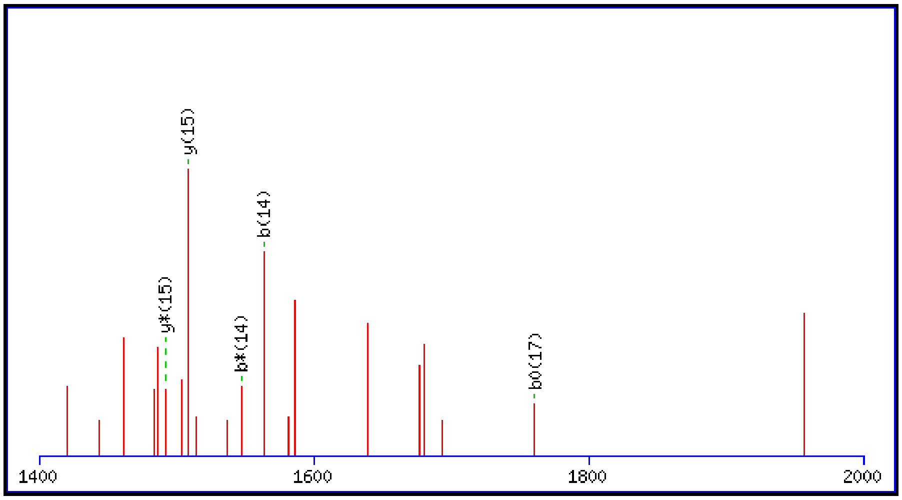


**Monoisotopic mass of neutral peptide Mr(calc):** 2320.9792
**Fixed modifications:** Carbamidomethyl (C)
**Variable modifications:**
**N14** : Deamidated_N (N)
**Ions Score:** 63 **Expect:** 0.00016
**Matches (Bold Red):** 83/240 fragment ions using 148 most intense peaks

| # | b | $b^{++}$ | b* | $b^{*++}$ | $b^{0}$ | $b^{0++}$ | Seq. | y | $y^{++}$ | y* | $y^{*++}$ | $y^{0}$ | $y^{0++}$ | # |
|---|---|---|---|---|---|---|---|---|---|---|---|---|---|---|
| 1 | 58.0287 | 29.5180 | | | | | G | | | | | | | 22 |
| 2 | 214.1299 | 107.5686 | 197.1033 | 99.0553 | | | R | 2264.9650 | 1132.9862 | 2247.9385 | 1124.4729 | 2246.9545 | 1123.9809 | 21 |
| 3 | 342.1884 | 171.5979 | 325.1619 | 163.0846 | | | Q | 2108.8639 | **1054.9356** | 2091.8374 | 1046.4223 | 2090.8534 | 1045.9303 | 20 |
| 4 | **470.2470** | 235.6271 | 453.2205 | 227.1139 | | | Q | 1980.8053 | 990.9063 | 1963.7788 | 982.3930 | 1962.7948 | 981.9010 | 19 |
| 5 | **557.2790** | 279.1432 | 540.2525 | **270.6299** | 539.2685 | **270.1379** | S | 1852.7468 | 926.8770 | 1835.7202 | 918.3637 | 1834.7362 | 917.8717 | 18 |
| 6 | 685.3376 | 343.1724 | **668.3111** | 334.6592 | **667.3270** | 334.1672 | Q | 1765.7147 | **883.3610** | 1748.6882 | **874.8477** | 1747.7042 | **874.3557** | 17 |
| 7 | **814.3802** | **407.6937** | 797.3537 | **399.1805** | 796.3696 | **398.6885** | E | 1637.6562 | 819.3317 | 1620.6296 | **810.8184** | 1619.6456 | 810.3264 | 16 |
| 8 | **929.4071** | **465.2072** | 912.3806 | 456.6939 | 911.3966 | 456.2019 | D | **1508.6136** | **754.8104** | **1491.5870** | 746.2971 | 1490.6030 | 745.8051 | 15 |
| 9 | 986.4286 | 493.7179 | 969.4021 | 485.2047 | 968.4180 | 484.7127 | G | **1393.5866** | **697.2969** | 1376.5601 | 688.7837 | 1375.5761 | 688.2917 | 14 |
| 10 | **1101.4556** | **551.2314** | 1084.4290 | 542.7181 | 1083.4450 | 542.2261 | D | **1336.5652** | **668.7862** | 1319.5386 | 660.2729 | 1318.5546 | **659.7809** | 13 |
| 11 | **1264.5189** | **632.7631** | **1247.4923** | **624.2498** | **1246.5083** | **623.7578** | Y | **1221.5382** | **611.2727** | 1204.5117 | **602.7595** | **1203.5277** | 602.2675 | 12 |
| 12 | **1351.5509** | **676.2791** | **1334.5244** | **667.7658** | **1333.5403** | **667.2738** | S | **1058.4749** | **529.7411** | 1041.4483 | **521.2278** | 1040.4643 | **520.7358** | 11 |
| 13 | 1448.6037 | **724.8055** | 1431.5771 | 716.2922 | 1430.5931 | 715.8002 | P | **971.4429** | **486.2251** | **954.4163** | **477.7118** | **953.4323** | **477.2198** | 10 |
| 14 | **1563.6306** | 782.3189 | **1546.6041** | 773.8057 | 1545.6200 | 773.3137 | N | **874.3901** | 437.6987 | 857.3635 | **429.1854** | **856.3795** | 428.6934 | 9 |
| 15 | 1620.6521 | **810.8297** | 1603.6255 | 802.3164 | 1602.6415 | 801.8244 | G | **759.3632** | 380.1852 | 742.3366 | 371.6719 | **741.3526** | 371.1799 | 8 |
| 16 | 1707.6841 | **854.3457** | 1690.6576 | **845.8324** | 1689.6735 | **845.3404** | S | **702.3417** | 351.6745 | 685.3151 | 343.1612 | 684.3311 | 342.6692 | 7 |
| 17 | 1778.7212 | **889.8642** | 1761.6947 | **881.3510** | **1760.7107** | **880.8590** | A | **615.3097** | 308.1585 | 598.2831 | 299.6452 | 597.2991 | 299.1532 | 6 |
| 18 | 1906.7798 | **953.8935** | 1889.7532 | **945.3803** | 1888.7692 | **944.8883** | Q | **544.2726** | 272.6399 | **527.2460** | 264.1266 | 526.2620 | 263.6346 | 5 |
| | | | | | | | | | | | | | | |

| | | | | | | | | | | | | | | |
|---|---|---|---|---|---|---|---|---|---|---|---|---|---|---|
| 19 | 2003.8326 | 1002.4199 | 1986.8060 | 993.9066 | 1985.8220 | 993.4146 | P | **416.2140** | 208.6106 | **399.1874** | 200.0974 | **398.2034** | 199.6053 | 4 |
| 20 | 2060.8540 | 1030.9307 | 2043.8275 | 1022.4174 | 2042.8435 | 1021.9254 | G | **319.1612** | 160.0842 | 302.1347 | 151.5710 | **301.1506** | 151.0790 | 3 |
| 21 | 2175.8810 | **1088.4441** | 2158.8544 | 1079.9308 | 2157.8704 | 1079.4388 | D | **262.1397** | 131.5735 | 245.1132 | 123.0602 | 244.1292 | 122.5682 | 2 |
| 22 | | | | | | | K | 147.1128 | 74.0600 | 130.0863 | 65.5468 | | | 1 |

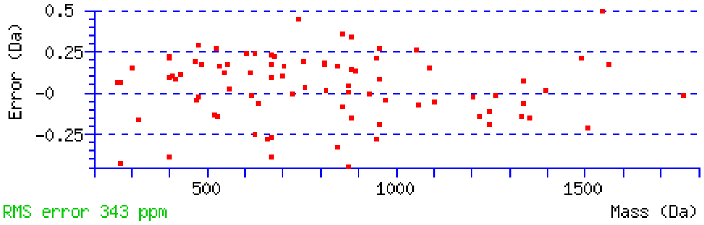


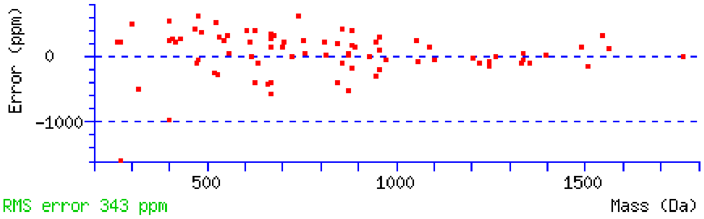


### All matches to this query

| Score | Mr(calc): | Delta | Sequence |
|---|---|---|---|
| 62.7 | 2320.9792 | 0.0028 | GRQQSQEDGDYSPNGSAQPGDK |
| 27.7 | 2319.9952 | 0.9868 | GRQQSQEDGDYSPNGSAQPGDK |
| 8.7 | 2321.0046 | -0.0225 | EQMMNSSMSSGSGSLRTSEKR |
| 8.7 | 2321.0046 | -0.0225 | EQMMNSSMSSGSGSLRTSEKR |
| 7.3 | 2318.9816 | 2.0004 | LSSSMCVTSDLLSDIYKPK |
| 5.1 | 2320.9879 | -0.0058 | TWKNNSHSKASETGSTWLK |
| 4.9 | 2320.9661 | 0.0160 | SRGPRGEGGAMSENIWSTLR |
| 4.9 | 2320.9661 | 0.0160 | SRGPRGEGGAMSENIWSTLR |
| 4.9 | 2320.9671 | 0.0150 | VTWTNGTIPPPVTEASSVPK |
| 4.7 | 2320.9743 | 0.0078 | TQAPQVSVSALQPSLPTNSR |

---

Spectrum No: 486; Query: 520; Rank: 1

---

## Peptide View

MS/MS Fragmentation of **LSNITNIGPLDMK**
Found in **IPI00421319**, Tax_Id=10116 Gene_Symbol=Tmem106b LRRGT00101

Match to Query 520: 1415.733888 from(708.874220,2+)
Title: 091008RatKidney_NH4Format02_25.3435.3435.2.dta
Data file K:\NewmanPaper\Piliang\3SubProteomes\Piliang3SP\mgf5ppm\ERLIC_3SubProteomes5ppm.mgf

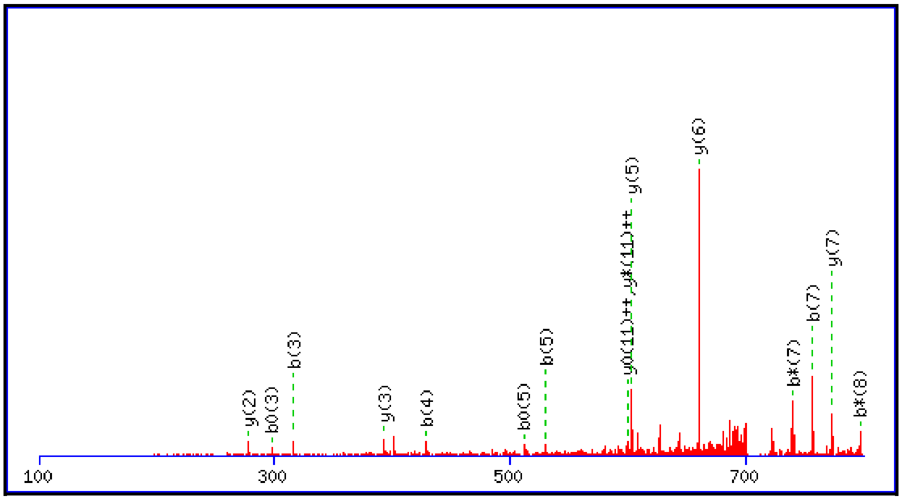

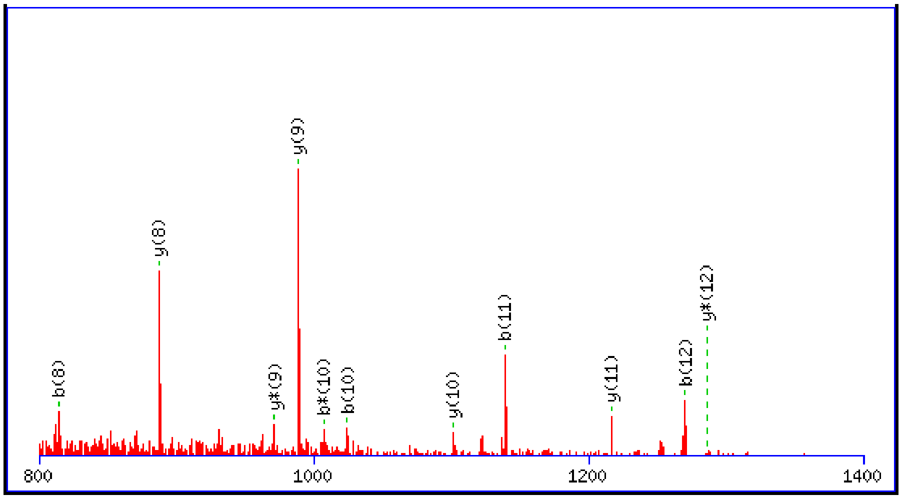


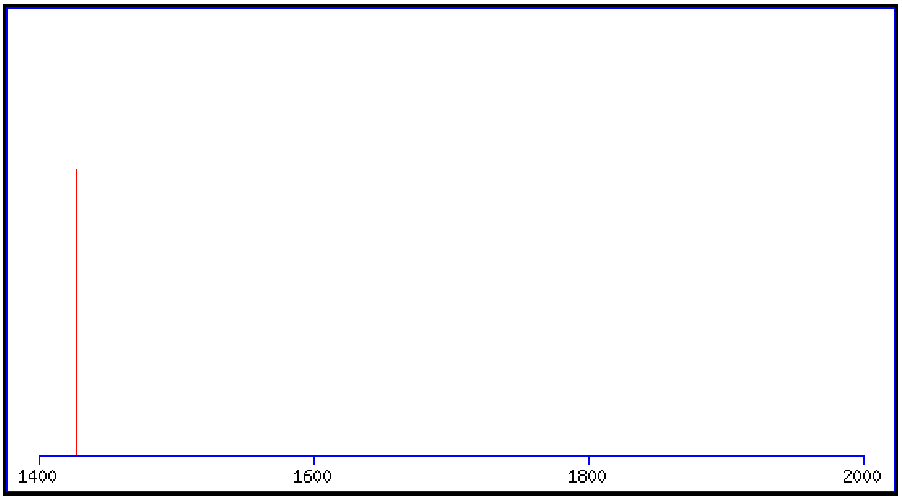


**Monoisotopic mass of neutral peptide Mr(calc):** 1415.7330
**Fixed modifications:** Carbamidomethyl (C)
**Variable modifications:**
**N3** : Deamidated_N (N)
**Ions Score:** 63 **Expect:** 7.2e-005
**Matches (Bold Red):** 26/134 fragment ions using 43 most intense peaks

| # | b | $b^{++}$ | b* | $b^{*++}$ | $b^{0}$ | $b^{0++}$ | Seq. | y | $y^{++}$ | y* | $y^{*++}$ | $y^{0}$ | $y^{0++}$ | # |
|---|---|---|---|---|---|---|---|---|---|---|---|---|---|---|
| 1 | 114.0913 | 57.5493 | | | | | L | | | | | | | 13 |
| 2 | 201.1234 | 101.0653 | | | 183.1128 | 92.0600 | S | 1303.6562 | 652.3317 | **1286.6297** | 643.8185 | 1285.6457 | 643.3265 | 12 |
| 3 | **316.1503** | 158.5788 | 299.1238 | 150.0655 | **298.1397** | 149.5735 | N | **1216.6242** | 608.8157 | 1199.5976 | **600.3025** | 1198.6136 | **599.8105** | 11 |
| 4 | **429.2344** | 215.1208 | 412.2078 | 206.6075 | 411.2238 | 206.1155 | I | **1101.5973** | 551.3023 | 1084.5707 | 542.7890 | 1083.5867 | 542.2970 | 10 |
| 5 | **530.2820** | 265.6447 | 513.2555 | 257.1314 | **512.2715** | 256.6394 | T | **988.5132** | 494.7602 | **971.4866** | 486.2470 | 970.5026 | 485.7550 | 9 |
| 6 | 644.3250 | 322.6661 | 627.2984 | 314.1529 | 626.3144 | 313.6608 | N | **887.4655** | 444.2364 | 870.4390 | 435.7231 | 869.4550 | 435.2311 | 8 |
| 7 | **757.4090** | 379.2082 | **740.3825** | 370.6949 | 739.3985 | 370.2029 | I | **773.4226** | 387.2149 | 756.3960 | 378.7017 | 755.4120 | 378.2096 | 7 |
| 8 | **814.4305** | 407.7189 | **797.4040** | 399.2056 | 796.4199 | 398.7136 | G | **660.3385** | 330.6729 | 643.3120 | 322.1596 | 642.3280 | 321.6676 | 6 |
| 9 | 911.4833 | 456.2453 | 894.4567 | 447.7320 | 893.4727 | 447.2400 | P | **603.3171** | 302.1622 | 586.2905 | 293.6489 | 585.3065 | 293.1569 | 5 |
| 10 | **1024.5673** | 512.7873 | **1007.5408** | 504.2740 | 1006.5568 | 503.7820 | L | 506.2643 | 253.6358 | 489.2377 | 245.1225 | 488.2537 | 244.6305 | 4 |
| 11 | **1139.5943** | 570.3008 | 1122.5677 | 561.7875 | 1121.5837 | 561.2955 | D | **393.1802** | 197.0938 | 376.1537 | 188.5805 | 375.1697 | 188.0885 | 3 |
| 12 | **1270.6348** | 635.8210 | 1253.6082 | 627.3077 | 1252.6242 | 626.8157 | M | **278.1533** | 139.5803 | 261.1267 | 131.0670 | | | 2 |
| 13 | | | | | | | K | 147.1128 | 74.0600 | 130.0863 | 65.5468 | | | 1 |

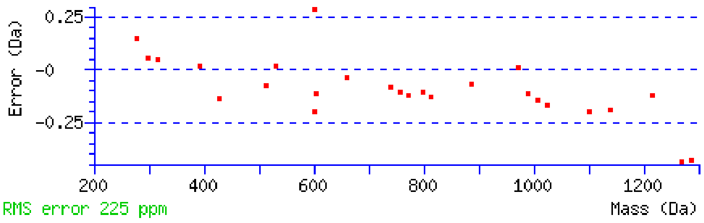


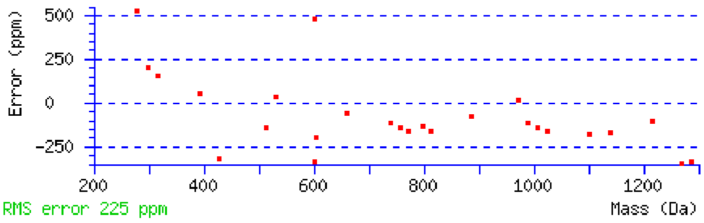


### All matches to this query

| Score | Mr(calc): | Delta | Sequence |
|---|---|---|---|
| 62.6 | 1415.7330 | 0.0009 | LSNITNIGPLDMK |
| 44.5 | 1415.7330 | 0.0009 | LSNITNIGPLDMK |
| 9.9 | 1415.7409 | -0.0070 | GLVNLYNLPGDNK |
| 7.2 | 1415.7286 | 0.0053 | TLNVLHNRGALK |
| 4.5 | 1413.7324 | 2.0015 | VTNPNARLRSER |
| 3.3 | 1413.7286 | 2.0053 | SGVLPGMPAVLSNR |
| 1.9 | 1413.7140 | 2.0199 | VWVASDPNLNAVK |
| 0.5 | 1415.7286 | 0.0053 | TLNVLHNRGALK |
| 0.2 | 1415.7256 | 0.0083 | SPESQNLIDGTKK |

---

Spectrum No: 487; Query: 1181; Rank: 1

---

## Peptide View

MS/MS Fragmentation of **LTSCATNASMCGDEAR**
Found in **IPI00369995**, Tax_Id=10116 Gene_Symbol=Lrp1 similar to low density lipoprotein receptor-related protein 1

Match to Query 1181: 1759.682968 from(880.848760,2+)
Title: 100101RatKid_NS_deglyco_23.527.527.2.dta
Data file K:\NewmanPaper\Piliang\3SubProteomes\Piliang3SP\mgf5ppm\ERLIC_3SubProteomes5ppm.mgf

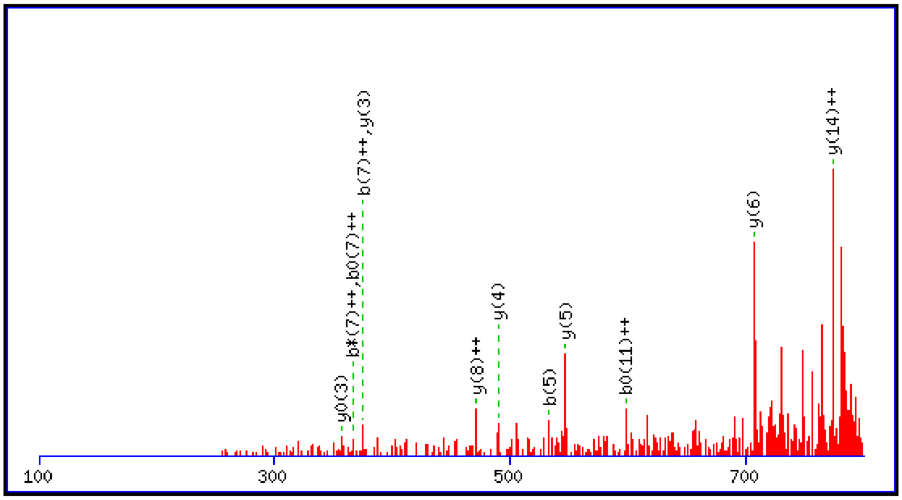

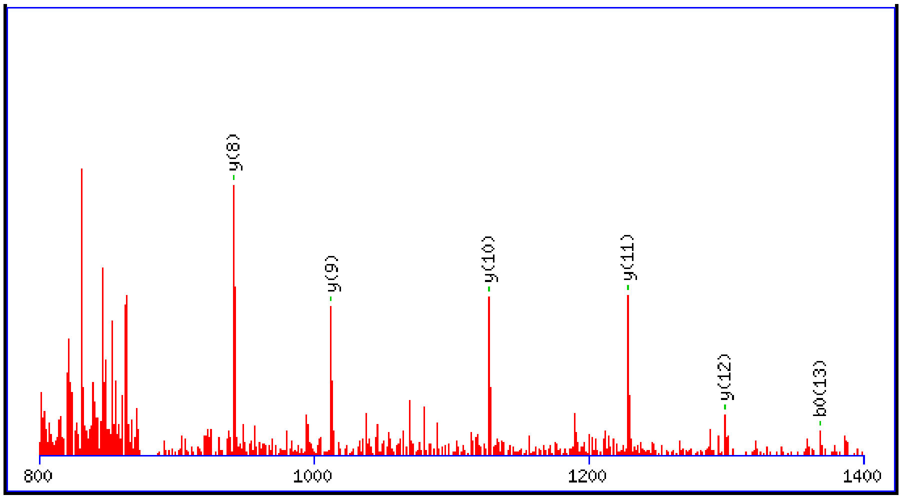


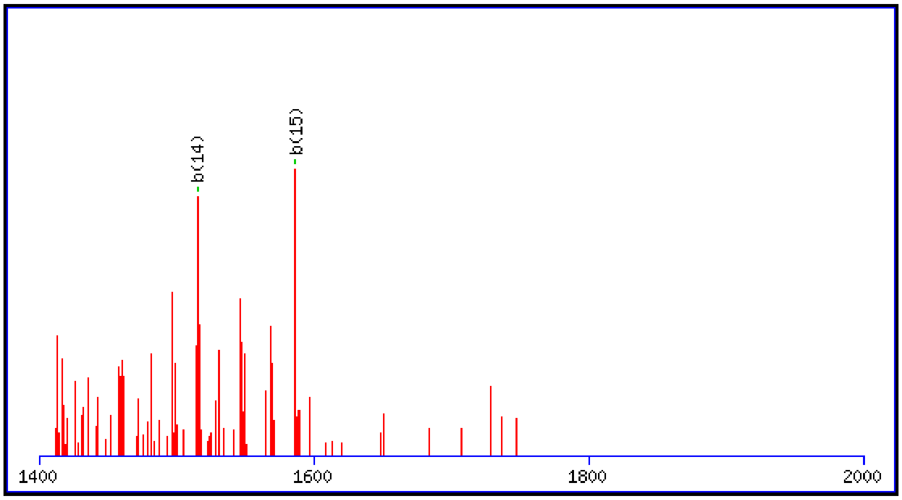


**Monoisotopic mass of neutral peptide Mr(calc):** 1759.6811
**Fixed modifications:** Carbamidomethyl (C)
**Variable modifications:**
**N7** : Deamidated_N (N)
**M10** : Oxidation (M)
**Ions Score:** 63 **Expect:** 6.5e-005
**Matches (Bold Red):** 20/162 fragment ions using 26 most intense peaks

| # | b | $b^{++}$ | b* | $b^{*++}$ | $b^0$ | $b^{0++}$ | Seq. | y | $y^{++}$ | y* | $y^{*++}$ | $y^0$ | $y^{0++}$ | # |
|---|---|---|---|---|---|---|---|---|---|---|---|---|---|---|
| 1 | 114.0913 | 57.5493 | | | | | L | | | | | | | 16 |
| 2 | 215.1390 | 108.0731 | | | 197.1285 | 99.0679 | T | 1647.6043 | 824.3058 | 1630.5778 | 815.7925 | 1629.5938 | 815.3005 | 15 |
| 3 | 302.1710 | 151.5892 | | | 284.1605 | 142.5839 | S | 1546.5567 | **773.7820** | 1529.5301 | 765.2687 | 1528.5461 | 764.7767 | 14 |
| 4 | 462.2017 | 231.6045 | | | 444.1911 | 222.5992 | C | 1459.5246 | 730.2660 | 1442.4981 | 721.7527 | 1441.5141 | 721.2607 | 13 |
| 5 | **533.2388** | 267.1230 | | | 515.2282 | 258.1178 | A | **1299.4940** | 650.2506 | 1282.4674 | 641.7374 | 1281.4834 | 641.2454 | 12 |
| 6 | 634.2865 | 317.6469 | | | 616.2759 | 308.6416 | T | **1228.4569** | 614.7321 | 1211.4303 | 606.2188 | 1210.4463 | 605.7268 | 11 |
| 7 | 749.3134 | **375.1604** | 732.2869 | **366.6471** | 731.3029 | **366.1551** | N | **1127.4092** | 564.2082 | 1110.3826 | 555.6950 | 1109.3986 | 555.2030 | 10 |
| 8 | 820.3505 | 410.6789 | 803.3240 | 402.1656 | 802.3400 | 401.6736 | A | **1012.3823** | 506.6948 | 995.3557 | 498.1815 | 994.3717 | 497.6895 | 9 |
| 9 | 907.3826 | 454.1949 | 890.3560 | 445.6816 | 889.3720 | 445.1896 | S | **941.3451** | **471.1762** | 924.3186 | 462.6629 | 923.3346 | 462.1709 | 8 |
| 10 | 1054.4180 | 527.7126 | 1037.3914 | 519.1993 | 1036.4074 | 518.7073 | M | 854.3131 | 427.6602 | 837.2866 | 419.1469 | 836.3026 | 418.6549 | 7 |
| 11 | 1214.4486 | 607.7279 | 1197.4221 | 599.2147 | 1196.4381 | **598.7227** | C | **707.2777** | 354.1425 | 690.2512 | 345.6292 | 689.2671 | 345.1372 | 6 |
| 12 | 1271.4701 | 636.2387 | 1254.4435 | 627.7254 | 1253.4595 | 627.2334 | G | **547.2471** | 274.1272 | 530.2205 | 265.6139 | 529.2365 | 265.1219 | 5 |
| 13 | 1386.4970 | 693.7522 | 1369.4705 | 685.2389 | **1368.4865** | 684.7469 | D | **490.2256** | 245.6164 | 473.1991 | 237.1032 | 472.2150 | 236.6112 | 4 |
[truncated: 486,775 more chars]
