# Supplementary material for: Simultaneous Analysis of Proteome, Phospho- and Glycoproteome of Rat Kidney Tissue with Electrostatic Repulsion Hydrophilic Interaction Chromatography
Source: PLoS One. 2011 Feb 23;6(2):e16884. doi: 10.1371/journal.pone.0016884 (PMC3044146; doi:10.1371/journal.pone.0016884)
Supplement: Data S2 — Tandem MS spectra_of phosphopeptides from ERLIC enrichment. (PDF) [file pone.0016884.s002.pdf]

Mascot Search Results

Results Generated by a Script Modified from Mascot Pepitde View
by Newman Sze, School of Biological Sciences, Nanyang Technological University

---

Spectrum No: 1; Query: 2759; Rank: 1

Peptide View

MS/MS Fragmentation of **MESEAGADDSAEEGDLLDDDDNEDRGDDQLELK**
Found in **IPI00187860**, Tax_Id=10116 Gene_Symbol=Hnrpc Heterogeneous nuclear ribonucleoprotein C

Match to Query 2759: 3691.409802 from(1231.477210,3+)
Title: 091008RatKidney_NoSalt_29.2636.2636.3.dta
Data file K:\NewmanPaper\Piliang\3SubProteomes\Piliang3SP\mgf5ppm\ERLIC_3SubProteomes5ppm.mgf

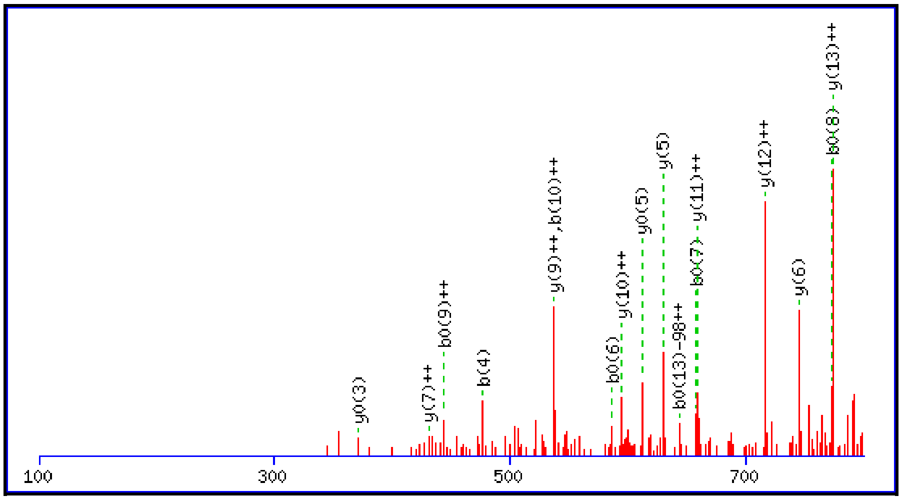

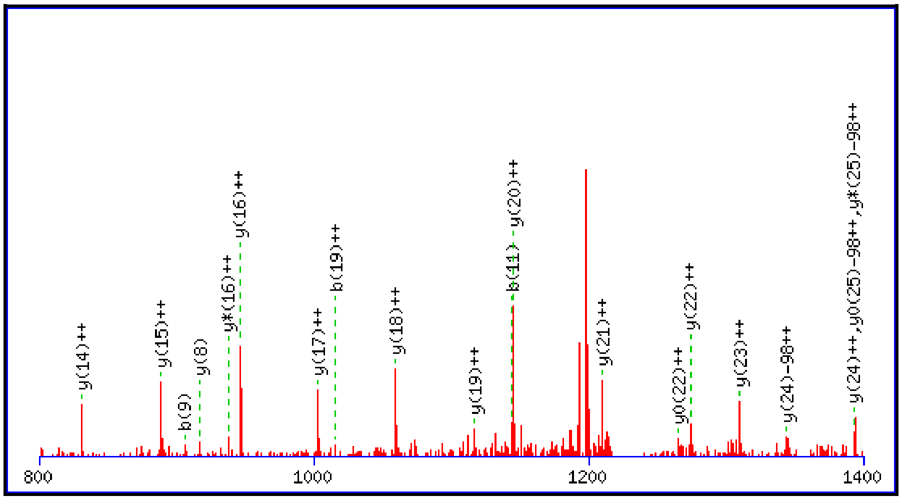

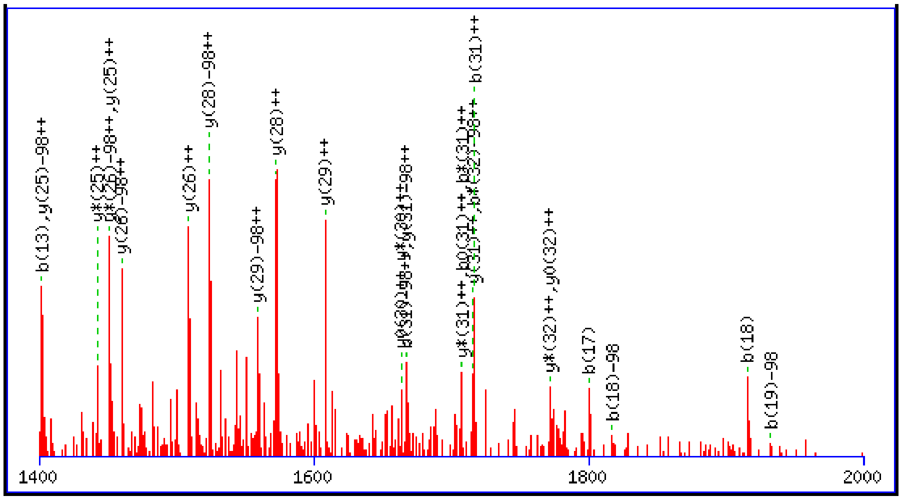

Monoisotopic mass of neutral peptide Mr(calc): 3690.4160
Fixed modifications: Carbamidomethyl (C)
Variable modifications:
S10 : Phospho (ST), with neutral losses 0.0000(shown in table), 97.9769
Ions Score: 131 **Expect:** 2.2e-011
Matches (Bold Red): 64/504 fragment ions using 96 most intense peaks

#	b	b^{++}	b*	b^{*++}	b^{0}	b^{0++}	Seq.	y	y^{++}	y*	y^{*++}	y^{0}	y^{0++}	#
1	132.0478	66.5275					M							33
2	261.0904	131.0488			243.0798	122.0435	E	3560.3828	1780.6950	3543.3562	**1772.1818**	3542.3722	**1771.6897**	32
3	348.1224	174.5648			330.1118	165.5595	S	3431.3402	**1716.1737**	3414.3136	**1707.6605**	3413.3296	1707.1684	31
4	**477.1650**	239.0861			459.1544	230.0808	E	3344.3082	1672.6577	3327.2816	**1664.1444**	3326.2976	**1663.6524**	30
5	548.2021	274.6047			530.1915	265.5994	A	3215.2656	**1608.1364**	3198.2390	1599.6231	3197.2550	1599.1311	29
6	605.2236	303.1154			**587.2130**	294.1101	G	3144.2284	**1572.6179**	3127.2019	1564.1046	3126.2179	1563.6126	28
7	676.2607	338.6340			**658.2501**	329.6287	A	3087.2070	1544.1071	3070.1804	1535.5939	3069.1964	1535.1018	27
8	791.2876	396.1474			**773.2770**	387.1422	D	3016.1699	**1508.5886**	2999.1433	1500.0753	2998.1593	1499.5833	26
9	**906.3146**	453.6609			888.3040	**444.6556**	D	2901.1429	**1451.0751**	2884.1164	**1442.5618**	2883.1324	1442.0698	25
10	1073.3129	**537.1601**			1055.3023	528.1548	S	2786.1160	**1393.5616**	2769.0894	1385.0484	2768.1054	1384.5563	24
11	**1144.3500**	572.6786			1126.3395	563.6734	A	2619.1176	**1310.0625**	2602.0911	1301.5492	2601.1071	1301.0572	23
12	1273.3926	637.1999			1255.3820	628.1947	E	2548.0805	**1274.5439**	2531.0540	1266.0306	2530.0700	**1265.5386**	22
13	**1402.4352**	701.7212			1384.4246	692.7160	E	2419.0379	**1210.0226**	2402.0114	1201.5093	2401.0274	1201.0173	21
14	1459.4567	730.2320			1441.4461	721.2267	G	2289.9953	**1145.5013**	2272.9688	1136.9880	2271.9848	1136.4960	20
15	1574.4836	787.7454			1556.4730	778.7402	D	2232.9739	**1116.9906**	2215.9473	1108.4773	2214.9633	1107.9853	19
16	1687.5677	844.2875			1669.5571	835.2822	L	2117.9469	**1059.4771**	2100.9204	1050.9638	2099.9364	1050.4718	18
17	**1800.6517**	900.8295			1782.6412	891.8242	L	2004.8629	**1002.9351**	1987.8363	994.4218	1986.8523	993.9298	17
18	**1915.6787**	958.3430			1897.6681	949.3377	D	1891.7788	**946.3930**	1874.7523	**937.8798**	1873.7682	937.3878	16
19	2030.7056	**1015.8565**			2012.6951	1006.8512	D	1776.7519	**888.8796**	1759.7253	880.3663	1758.7413	879.8743	15
20	2145.7326	1073.3699			2127.7220	1064.3646	D	1661.7249	**831.3661**	1644.6984	822.8528	1643.7143	822.3608	14
21	2260.7595	1130.8834			2242.7489	1121.8781	D	1546.6980	**773.8526**	1529.6714	765.3393	1528.6874	764.8473	13
22	2374.8024	1187.9049	2357.7759	1179.3916	2356.7919	1178.8996	N	1431.6710	**716.3392**	1414.6445	707.8259	1413.6605	707.3339	12
23	2503.8450	1252.4262	2486.8185	1243.9129	2485.8345	1243.4209	E	1317.6281	**659.3177**	1300.6016	650.8044	1299.6175	650.3124	11
24	2618.8720	1309.9396	2601.8454	1301.4264	2600.8614	1300.9343	D	1188.5855	**594.7964**	1171.5590	586.2831	1170.5749	585.7911	10
25	2774.9731	1387.9902	2757.9465	1379.4769	2756.9625	1378.9849	R	1073.5586	**537.2829**	1056.5320	528.7696	1055.5480	528.2776	9
26	2831.9945	1416.5009	2814.9680	1407.9876	2813.9840	1407.4956	G	**917.4575**	459.2324	900.4309	450.7191	899.4469	450.2271	8
27	2947.0215	1474.0144	2929.9949	1465.5011	2929.0109	1465.0091	D	860.4360	**430.7216**	843.4094	422.2084	842.4254	421.7164	7
28	3062.0484	1531.5279	3045.0219	1523.0146	3044.0379	1522.5226	D	**745.4090**	373.2082	728.3825	364.6949	727.3985	364.2029	6
29	3190.1070	1595.5571	3173.0805	1587.0439	3172.0964	1586.5519	Q	**630.3821**	315.6947	613.3556	307.1814	**612.3715**	306.6894	5
30	3303.1911	1652.0992	3286.1645	1643.5859	3285.1805	1643.0939	L	502.3235	251.6654	485.2970	243.1521	484.3130	242.6601	4
31	3432.2337	**1716.6205**	3415.2071	**1708.1072**	3414.2231	**1707.6152**	E	389.2395	195.1234	372.2129	186.6101	**371.2289**	186.1181	3
32	3545.3177	1773.1625	3528.2912	1764.6492	3527.3072	1764.1572	L	260.1969	130.6021	243.1703	122.0888			2

33							K	147.1128	74.0600	130.0863	65.5468			1

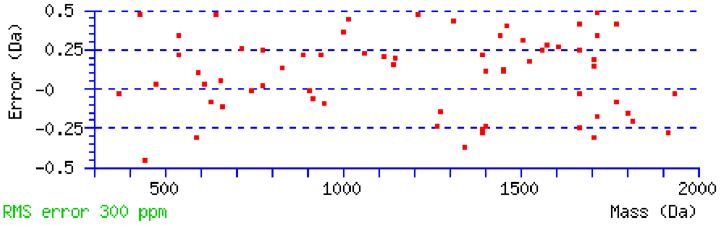

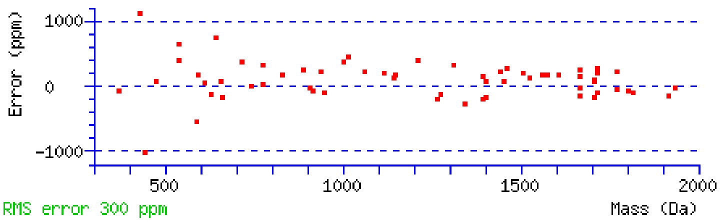

All matches to this query

Score	Mr(calc):	Delta	Sequence
131.3	3690.4160	0.9938	MESEAGADDSAEEGDLLDDDDNEDRGDDQLELK
131.0	3691.4000	0.0098	MESEAGADDSAEEGDLLDDDDNEDRGDDQLELK
76.2	3690.4160	0.9938	MESEAGADDSAEEGDLLDDDDNEDRGDDQLELK
74.6	3691.4000	0.0098	MESEAGADDSAEEGDLLDDDDNEDRGDDQLELK
13.7	3690.4268	0.9830	CIYTYGYYFSYFSNLSDQNEEPYVSITK
13.7	3690.4268	0.9830	CIYTYGYYFSYFSNLSDQNEEPYVSITK
13.7	3690.4268	0.9830	CIYTYGYYFSYFSNLSDQNEEPYVSITK
13.7	3690.4268	0.9830	CIYTYGYYFSYFSNLSDQNEEPYVSITK
13.7	3690.4268	0.9830	CIYTYGYYFSYFSNLSDQNEEPYVSITK
13.7	3690.4268	0.9830	CIYTYGYYFSYFSNLSDQNEEPYVSITK

Spectrum No: 2; Query: 2256; Rank: 1

Peptide View

MS/MS Fragmentation of **SASPDDDLGSSNWEAADLGNEER**
Found in **IPI00371185**, Tax_Id=10116 Gene_Symbol=RGD1311703 Similar to sid2057p

Match to Query 2256: 2513.994128 from(1258.004340,2+)
Title: 100101RatKid_NS_deglyco_24.3200.3200.2.dta
Data file K:\NewmanPaper\Piliang\3SubProteomes\Piliang3SP\mgf5ppm\ERLIC_3SubProteomes5ppm.mgf

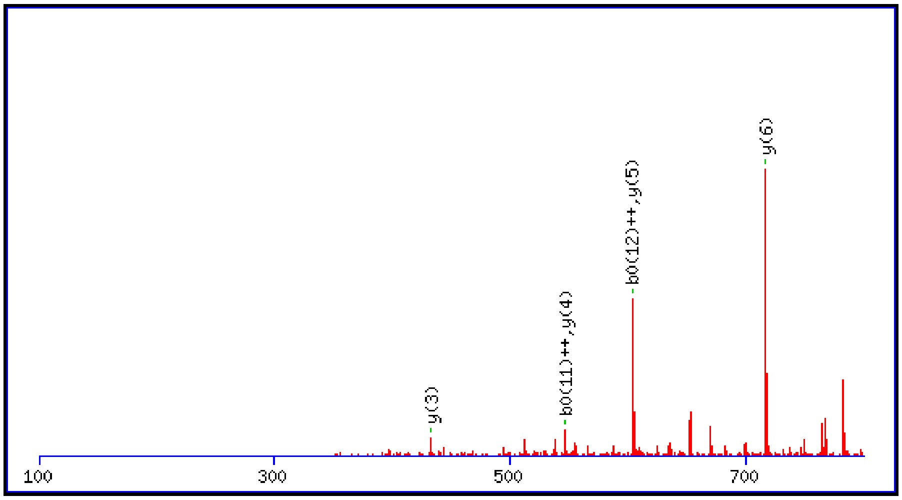

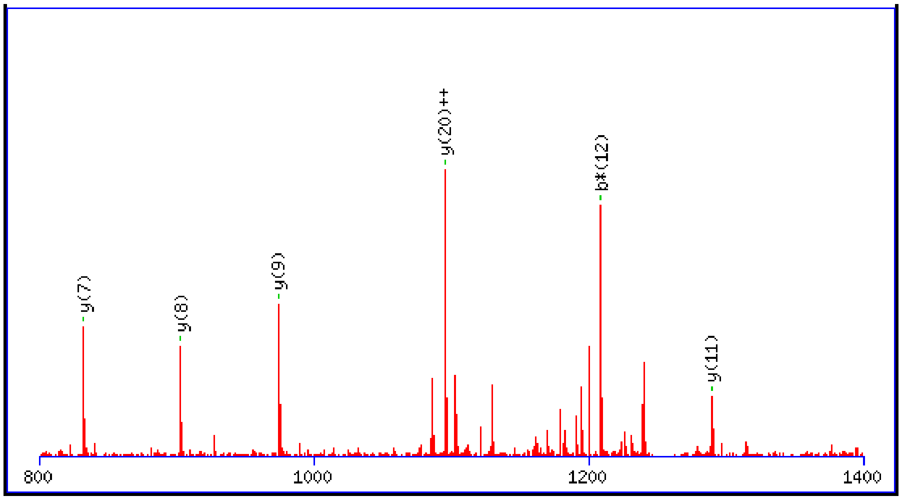

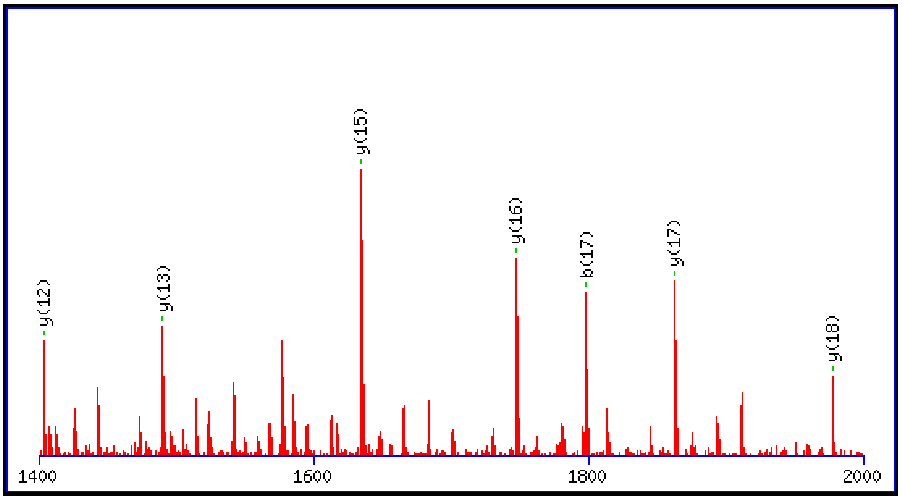

Monoisotopic mass of neutral peptide Mr(calc): 2513.9820
Fixed modifications: Carbamidomethyl (C)
Variable modifications:
S3 : Phospho (ST), with neutral losses 97.9769(shown in table), 0.0000
Ions Score: 129 **Expect:** 3.1e-011
Matches (Bold Red): 19/354 fragment ions using 20 most intense peaks

#	b	b^{++}	b*	b^{*++}	b^0	b^{0++}	Seq.	y	y^{++}	y*	y^{*++}	y^0	y^{0++}	#
1	88.0393	44.5233			70.0287	35.5180	S							23
2	159.0764	80.0418			141.0659	71.0366	A	2329.9803	1165.4938	2312.9538	1156.9805	2311.9698	1156.4885	22
3	228.0979	114.5526			210.0873	105.5473	S	2258.9432	1129.9753	2241.9167	1121.4620	2240.9327	1120.9700	21
4	325.1506	163.0790			307.1401	154.0737	P	2189.9218	**1095.4645**	2172.8952	1086.9513	2171.9112	1086.4592	20
5	440.1776	220.5924			422.1670	211.5871	D	2092.8690	1046.9381	2075.8425	1038.4249	2074.8584	1037.9329	19
6	555.2045	278.1059			537.1940	269.1006	D	**1977.8421**	989.4247	1960.8155	980.9114	1959.8315	980.4194	18
7	670.2315	335.6194			652.2209	326.6141	D	**1862.8151**	931.9112	1845.7886	923.3979	1844.8046	922.9059	17
8	783.3155	392.1614			765.3050	383.1561	L	**1747.7882**	874.3977	1730.7616	865.8845	1729.7776	865.3924	16
9	840.3370	420.6721			822.3264	411.6669	G	**1634.7041**	817.8557	1617.6776	809.3424	1616.6936	808.8504	15
10	927.3690	464.1881			909.3585	455.1829	S	1577.6827	789.3450	1560.6561	780.8317	1559.6721	780.3397	14
11	1014.4011	507.7042			996.3905	498.6989	S	**1490.6506**	745.8290	1473.6241	737.3157	1472.6401	736.8237	13
12	1128.4440	564.7256	1111.4174	556.2124	1110.4334	555.7203	N	**1403.6186**	702.3129	1386.5920	693.7997	1385.6080	693.3077	12
13	1314.5233	657.7653	1297.4967	649.2520	1296.5127	648.7600	W	**1289.5757**	645.2915	1272.5491	636.7782	1271.5651	636.2862	11
14	1443.5659	722.2866	1426.5393	713.7733	1425.5553	713.2813	E	1103.4964	552.2518	1086.4698	543.7385	1085.4858	543.2465	10
15	1514.6030	757.8051	1497.5764	749.2919	1496.5924	748.7999	A	**974.4538**	487.7305	957.4272	479.2172	956.4432	478.7252	9
16	1585.6401	793.3237	1568.6136	784.8104	1567.6295	784.3184	A	**903.4167**	452.2120	886.3901	443.6987	885.4061	443.2067	8
17	1700.6671	850.8372	1683.6405	842.3239	1682.6565	841.8319	D	**832.3795**	416.6934	815.3530	408.1801	814.3690	407.6881	7
18	1813.7511	907.3792	1796.7246	898.8659	1795.7406	898.3739	L	**717.3526**	359.1799	700.3260	350.6667	699.3420	350.1747	6

**19**	1870.7726	935.8899	1853.7460	927.3767	1852.7620	926.8846	**G**	**604.2685**	302.6379	587.2420	294.1246	586.2580	293.6326	**5**
**20**	1984.8155	992.9114	1967.7890	984.3981	1966.8049	983.9061	**N**	**547.2471**	274.1272	530.2205	265.6139	529.2365	265.1219	**4**
**21**	2113.8581	1057.4327	2096.8316	1048.9194	2095.8475	1048.4274	**E**	**433.2041**	217.1057	416.1776	208.5924	415.1936	208.1004	**3**
**22**	2242.9007	1121.9540	2225.8741	1113.4407	2224.8901	1112.9487	**E**	304.1615	152.5844	287.1350	144.0711	286.1510	143.5791	**2**
**23**							**R**	175.1190	88.0631	158.0924	79.5498			**1**

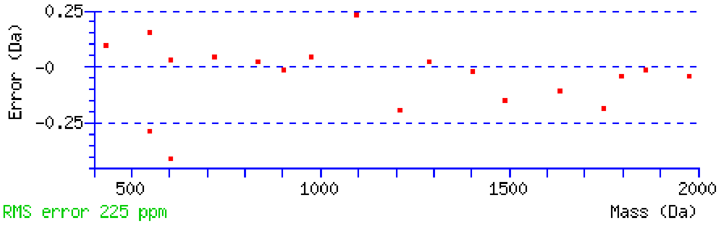

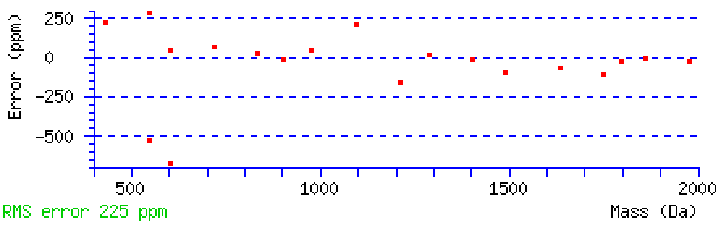

All matches to this query

Score	Mr(calc):	Delta	Sequence
129.0	2513.9820	0.0122	SASPDDDLGSSNWEAADLGNEER
129.0	2513.9820	0.0122	SASPDDDLGSSNWEAADLGNEER
63.5	2513.9820	0.0122	SASPDDDLGSSNWEAADLGNEER
50.4	2513.9820	0.0122	SASPDDDLGSSNWEAADLGNEER
4.2	2514.0144	-0.0202	TRLDHTDCINLYSCICGKK
3.7	2513.9737	0.0205	SHYSNIEANESEEERQFRK
2.8	2511.9808	2.0133	ERETSSIEPLPSDSPKEESR
2.5	2513.9718	0.0223	NXPCPARSSLRSALNVFSR
2.5	2513.9718	0.0223	NXPCPARSSLRSALNVFSR
0.7	2514.0122	-0.0181	MMQEQMTGAAMAMPADTNKAFK

Spectrum No: 3; Query: 2129; Rank: 1

Peptide View

MS/MS Fragmentation of **VWTSGQVEEYDLDADDINSR**
Found in **IPI00327202**, Tax_Id=10116 Gene_Symbol=Aqp1 Aquaporin-1

Match to Query 2129: 2390.987448 from(1196.501000,2+)
Title: 091008RatKidney_NoSalt_26.3115.3115.2.dta
Data file K:\NewmanPaper\Piliang\3SubProteomes\Piliang3SP\mgf5ppm\ERLIC_3SubProteomes5ppm.mgf

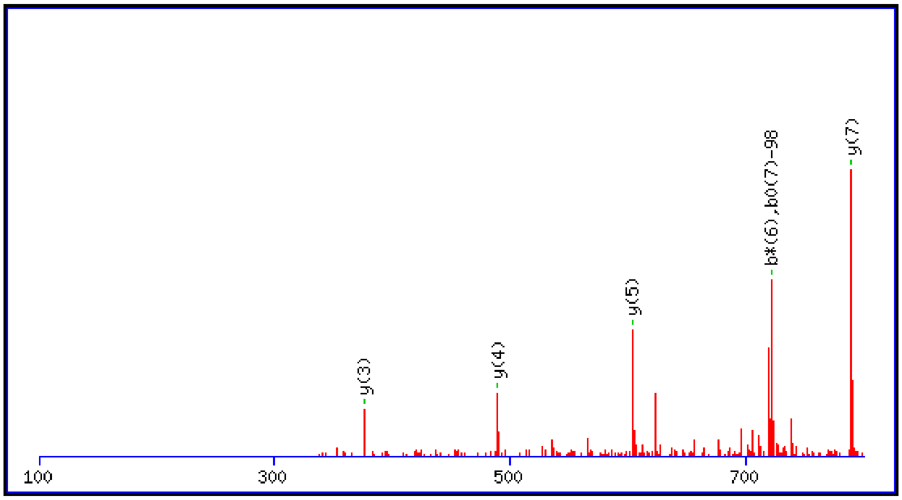

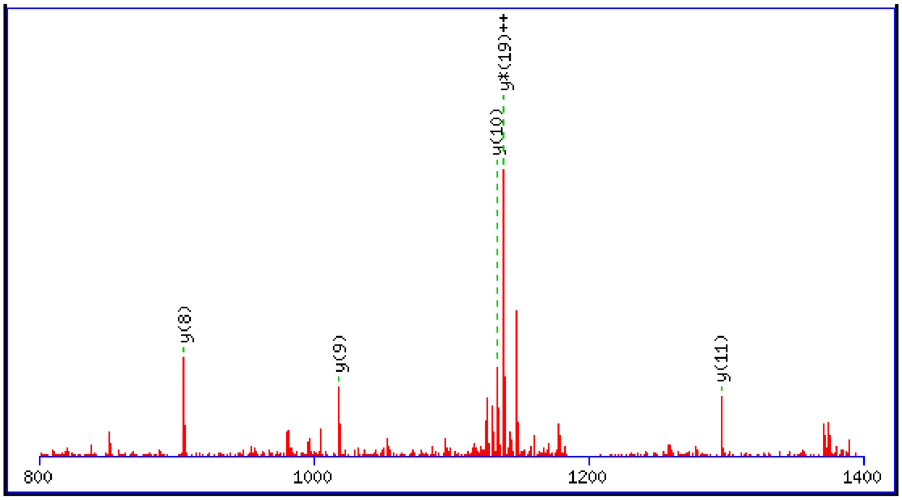

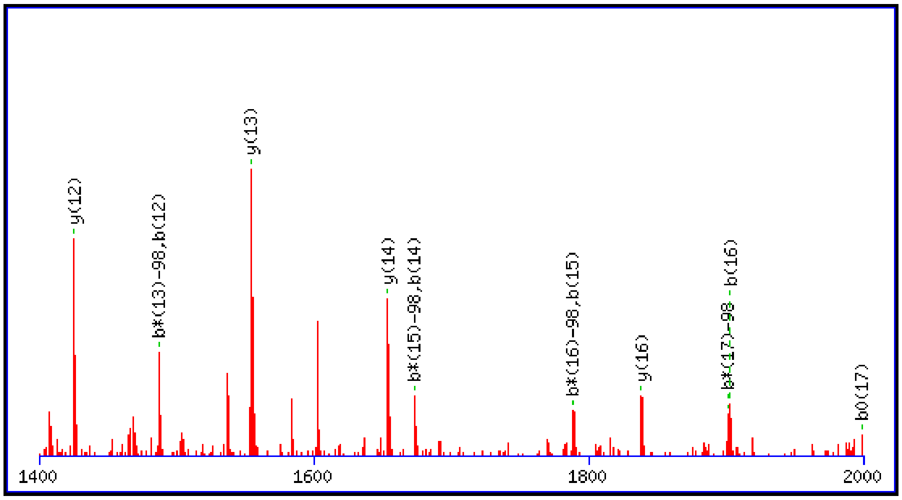

Monoisotopic mass of neutral peptide Mr(calc): 2390.9904
Fixed modifications: Carbamidomethyl (C)
Variable modifications:
S4 : Phospho (ST), with neutral losses 0.0000(shown in table), 97.9769
Ions Score: 123 **Expect:** 1.4e-010
Matches (Bold Red): 24/322 fragment ions using 26 most intense peaks

#	b	b++	b*	b*++	b0	b0++	Seq.	y	y++	y*	y*++	y0	y0++	#
1	100.0757	50.5415					V							20
2	286.1550	143.5811					W	2292.9292	1146.9683	2275.9027	**1138.4550**	2274.9187	1137.9630	19
3	387.2027	194.1050			369.1921	185.0997	T	2106.8499	1053.9286	2089.8234	1045.4153	2088.8394	1044.9233	18
4	554.2010	277.6042			536.1905	268.5989	S	2005.8022	1003.4048	1988.7757	994.8915	1987.7917	994.3995	17
5	611.2225	306.1149			593.2119	297.1096	G	**1838.8039**	919.9056	1821.7773	911.3923	1820.7933	910.9003	16
6	739.2811	370.1442	**722.2545**	361.6309	721.2705	361.1389	Q	1781.7824	891.3949	1764.7559	882.8816	1763.7719	882.3896	15
7	838.3495	419.6784	821.3229	411.1651	820.3389	410.6731	V	**1653.7239**	827.3656	1636.6973	818.8523	1635.7133	818.3603	14
8	967.3921	484.1997	950.3655	475.6864	949.3815	475.1944	E	**1554.6554**	777.8314	1537.6289	769.3181	1536.6449	768.8261	13
9	1096.4347	548.7210	1079.4081	540.2077	1078.4241	539.7157	E	**1425.6128**	713.3101	1408.5863	704.7968	1407.6023	704.3048	12
10	1259.4980	630.2526	1242.4715	621.7394	1241.4874	621.2474	Y	**1296.5703**	648.7888	1279.5437	640.2755	1278.5597	639.7835	11
11	1374.5249	687.7661	1357.4984	679.2528	1356.5144	678.7608	D	**1133.5069**	567.2571	1116.4804	558.7438	1115.4964	558.2518	10
12	**1487.6090**	744.3081	1470.5825	735.7949	1469.5984	735.3029	L	**1018.4800**	509.7436	1001.4534	501.2304	1000.4694	500.7383	9
13	1602.6360	801.8216	1585.6094	793.3083	1584.6254	792.8163	D	**905.3959**	453.2016	888.3694	444.6883	887.3853	444.1963	8
14	**1673.6731**	837.3402	1656.6465	828.8269	1655.6625	828.3349	A	**790.3690**	395.6881	773.3424	387.1748	772.3584	386.6828	7
15	**1788.7000**	894.8536	1771.6735	886.3404	1770.6894	885.8484	D	719.3319	360.1696	702.3053	351.6563	701.3213	351.1643	6
16	**1903.7270**	952.3671	1886.7004	943.8538	1885.7164	943.3618	D	**604.3049**	302.6561	587.2784	294.1428	586.2943	293.6508	5
17	2016.8110	1008.9091	1999.7845	1000.3959	**1998.8005**	999.9039	I	**489.2780**	245.1426	472.2514	236.6293	471.2674	236.1373	4
18	2130.8539	1065.9306	2113.8274	1057.4173	2112.8434	1056.9253	N	**376.1939**	188.6006	359.1674	180.0873	358.1833	179.5953	3

**19**	2217.8860	1109.4466	2200.8594	1100.9334	2199.8754	1100.4413	**S**	262.1510	131.5791	245.1244	123.0659	244.1404	122.5738	**2**
**20**							**R**	175.1190	88.0631	158.0924	79.5498			**1**

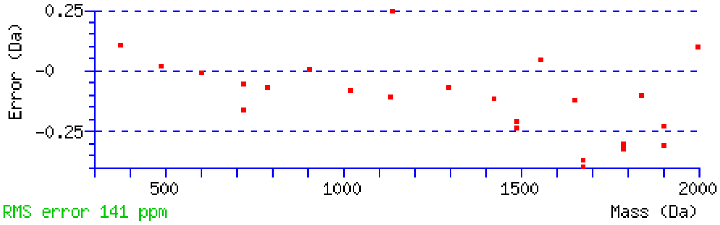

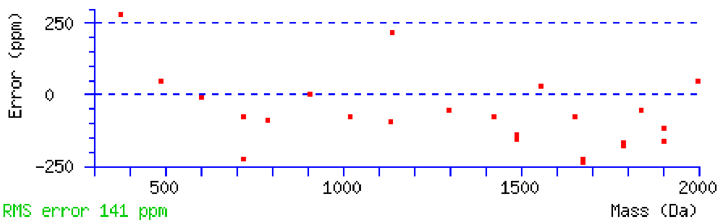

All matches to this query

Score	Mr(calc):	Delta	Sequence
122.9	2390.9904	-0.0029	VWTSGQVEEYDLDADDINSR
122.9	2390.9904	-0.0029	VWTSGQVEEYDLDADDINSR
39.0	2390.9904	-0.0029	VWTSGQVEEYDLDADDINSR
4.6	2390.9969	-0.0095	MEMWMENFAQRWDNLTQK
4.6	2390.9969	-0.0095	MEMWMENFAQRWDNLTQK
4.6	2390.9969	-0.0095	MEMWMENFAQRWDNLTQK
2.4	2390.9695	0.0180	NMESFLQMHGLSLDGCTAQR
0.5	2391.0041	-0.0166	DYYAHKVKSLMASLTNMSR
0.5	2391.0041	-0.0166	DYYAHKVKSLMASLTNMSR
0.5	2391.0041	-0.0166	DYYAHKVKSLMASLTNMSR

Spectrum No: 4; Query: 2394; Rank: 1

Peptide View

MS/MS Fragmentation of **SLAALDALNTDDENDEEEYEAWK**
Found in **IPI00209527**, Tax_Id=10116 Gene_Symbol=RGD1562232_predicted similar to microfibrillar-associated protein 1

Match to Query 2394: 2720.107268 from(1361.060910,2+)
Title: 100101RatKid_NS_deglyco_24.4376.4376.2.dta
Data file K:\NewmanPaper\Piliang\3SubProteomes\Piliang3SP\mgf5ppm\ERLIC_3SubProteomes5ppm.mgf

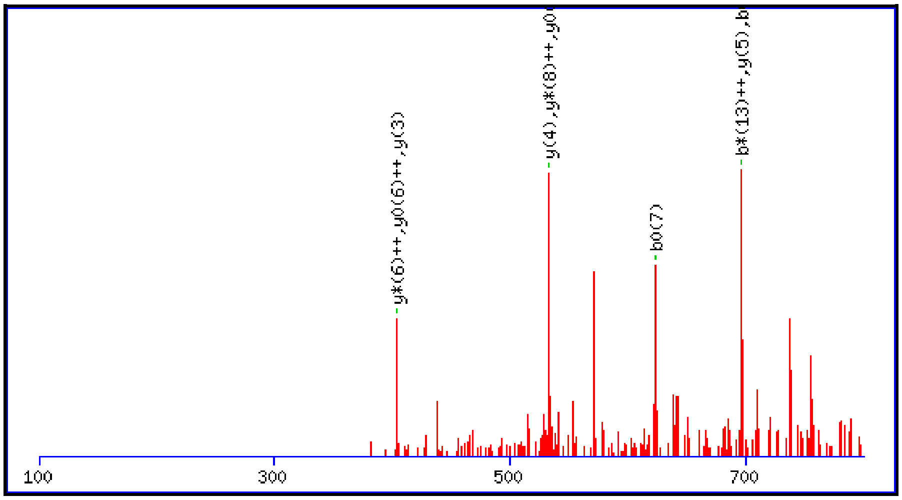

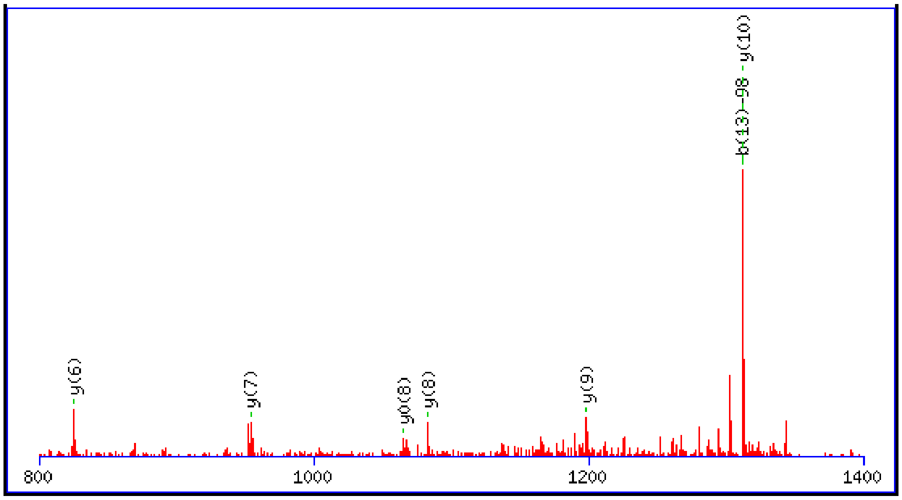

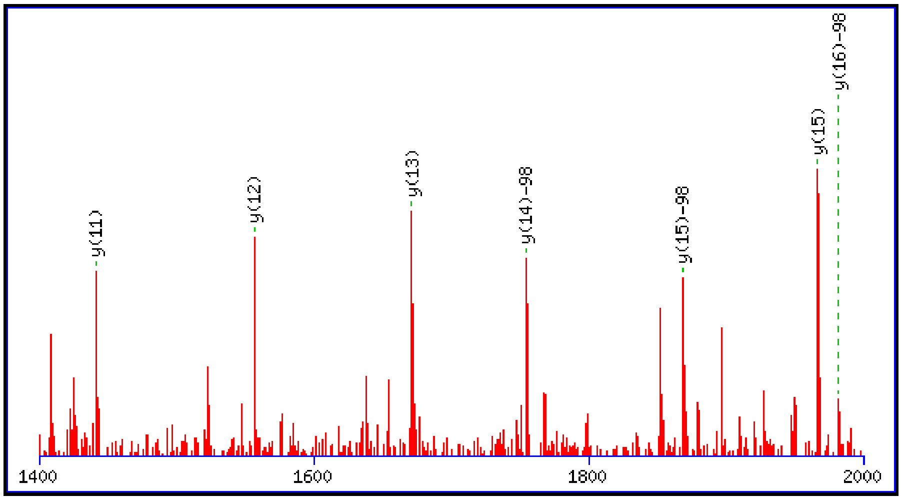

Monoisotopic mass of neutral peptide Mr(calc): 2720.1014
Fixed modifications: Carbamidomethyl (C)
Variable modifications:
T10 : Phospho (ST), with neutral losses 97.9769(shown in table), 0.0000
Ions Score: 118 **Expect:** 4.6e-010
Matches (Bold Red): 24/374 fragment ions using 23 most intense peaks

#	b	b^{++}	b*	b^{*++}	b^0	b^{0++}	Seq.	y	y^{++}	y*	y^{*++}	y^0	y^{0++}	#
1	88.0393	44.5233			70.0287	35.5180	S							23
2	201.1234	101.0653			183.1128	92.0600	L	2536.0998	1268.5535	2519.0732	1260.0403	2518.0892	1259.5483	22
3	272.1605	136.5839			254.1499	127.5786	A	2423.0157	1212.0115	2405.9892	1203.4982	2405.0052	1203.0062	21
4	343.1976	172.1024			325.1870	163.0972	A	2351.9786	1176.4929	2334.9521	1167.9797	2333.9680	1167.4877	20
5	456.2817	228.6445			438.2711	219.6392	L	2280.9415	1140.9744	2263.9150	1132.4611	2262.9309	1131.9691	19
6	571.3086	286.1579			553.2980	277.1527	D	2167.8574	1084.4324	2150.8309	1075.9191	2149.8469	1075.4271	18
7	642.3457	321.6765			**624.3352**	312.6712	A	2052.8305	1026.9189	2035.8039	1018.4056	2034.8199	1017.9136	17
8	755.4298	378.2185			737.4192	369.2132	L	**1981.7934**	991.4003	1964.7668	982.8871	1963.7828	982.3950	16
9	869.4727	435.2400	852.4462	426.7267	851.4621	426.2347	N	**1868.7093**	934.8583	1851.6828	926.3450	1850.6988	925.8530	15
10	952.5098	476.7585	935.4833	468.2453	934.4992	467.7533	T	**1754.6664**	877.8368	1737.6398	869.3236	1736.6558	868.8315	14
11	1067.5368	534.2720	1050.5102	525.7587	1049.5262	525.2667	D	**1671.6293**	836.3183	1654.6027	827.8050	1653.6187	827.3130	13
12	1182.5637	591.7855	1165.5372	583.2722	1164.5531	582.7802	D	**1556.6023**	778.8048	1539.5758	770.2915	1538.5918	769.7995	12
13	**1311.6063**	656.3068	1294.5797	647.7935	1293.5957	647.3015	E	**1441.5754**	721.2913	1424.5488	712.7781	1423.5648	712.2861	11
14	1425.6492	713.3282	1408.6227	704.8150	1407.6387	704.3230	N	**1312.5328**	656.7700	1295.5063	648.2568	1294.5222	647.7648	10
15	1540.6762	770.8417	1523.6496	762.3284	1522.6656	761.8364	D	**1198.4899**	599.7486	1181.4633	591.2353	1180.4793	590.7433	9
16	1669.7188	835.3630	1652.6922	826.8497	1651.7082	826.3577	E	**1083.4629**	542.2351	1066.4364	**533.7218**	**1065.4524**	**533.2298**	8
17	1798.7614	899.8843	1781.7348	891.3710	1780.7508	890.8790	E	**954.4203**	477.7138	937.3938	469.2005	936.4098	468.7085	7
18	1927.8039	964.4056	1910.7774	955.8923	1909.7934	955.4003	E	**825.3777**	413.1925	808.3512	**404.6792**	807.3672	**404.1872**	6

**19**	2090.8673	1045.9373	2073.8407	1037.4240	2072.8567	1036.9320	**Y**	**696.3352**	348.6712	679.3086	340.1579	678.3246	339.6659	**5**
**20**	2219.9099	1110.4586	2202.8833	1101.9453	2201.8993	1101.4533	**E**	**533.2718**	267.1396	516.2453	258.6263	515.2613	258.1343	**4**
**21**	2290.9470	1145.9771	2273.9204	1137.4639	2272.9364	1136.9718	**A**	**404.2292**	202.6183	387.2027	194.1050			**3**
**22**	2477.0263	1239.0168	2459.9997	1230.5035	2459.0157	1230.0115	**W**	333.1921	167.0997	316.1656	158.5864			**2**
**23**							**K**	147.1128	74.0600	130.0863	65.5468			**1**

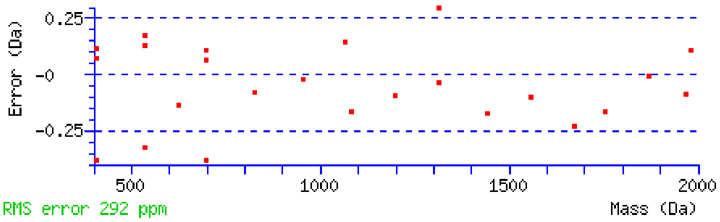

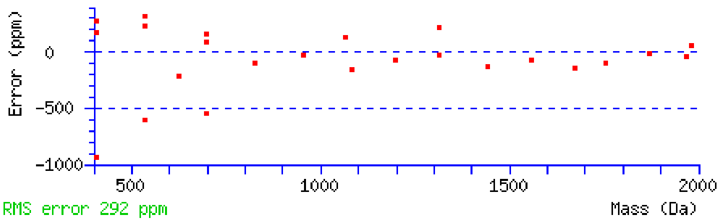

All matches to this query

Score	Mr(calc):	Delta	Sequence
117.6	2720.1014	0.0059	SLAALDALNTDDENDEEEYEAWK
93.3	2720.1014	0.0059	SLAALDALNTDDENDEEEYEAWK
7.5	2720.1014	0.0059	SLAALDALNTDDENDEEEYEAWK
2.1	2719.0900	1.0173	MNQCIKCLDDQYANPGGTHCLK
0.5	2720.1046	0.0027	NMGRFGSGMNMGRINEILSNALK
0.4	2718.0880	2.0192	AQSDSQEPIITVQPLESSLTQK
0.4	2718.0880	2.0192	AQSDSQEPIITVQPLESSLTQK
0.4	2719.0948	1.0125	VCSGEYPPKTDLMAFSPSLSDSR
0.4	2719.0948	1.0125	VCSGEYPPKTDLMAFSPSLSDSR
0.4	2719.0948	1.0125	VCSGEYPPKTDLMAFSPSLSDSR

Spectrum No: 5; Query: 2763; Rank: 1

Peptide View

MS/MS Fragmentation of **MESEAGADDSAEEGDLLDDDDNEDRGDDQLELK**
Found in **IPI00187860**, Tax_Id=10116 Gene_Symbol=Hnrpc Heterogeneous nuclear ribonucleoprotein C

Match to Query 2763: 3706.418292 from(1236.480040,3+)
Title: 091008RatKidney_NoSalt_30.2488.2488.3.dta
Data file K:\NewmanPaper\Piliang\3SubProteomes\Piliang3SP\mgf5ppm\ERLIC_3SubProteomes5ppm.mgf

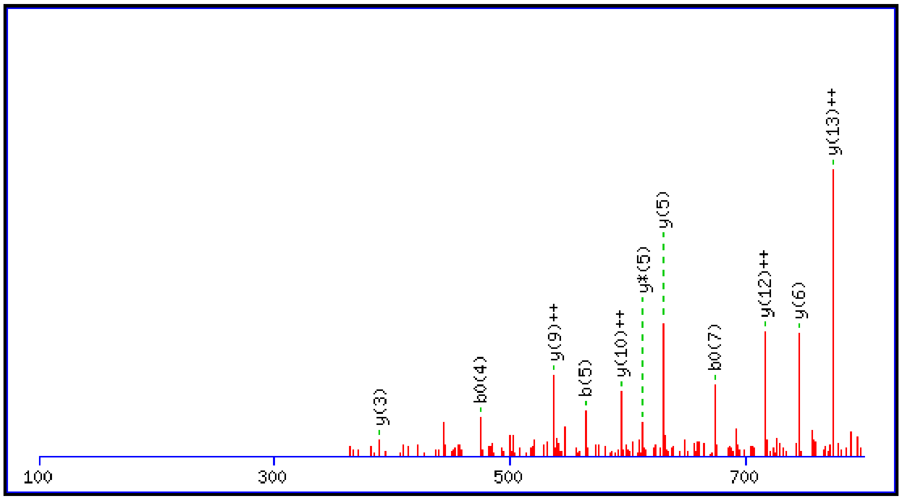

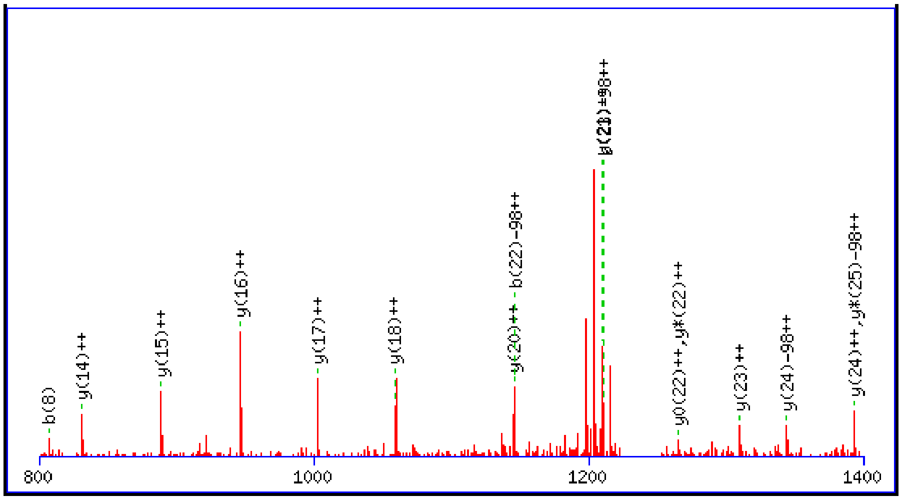

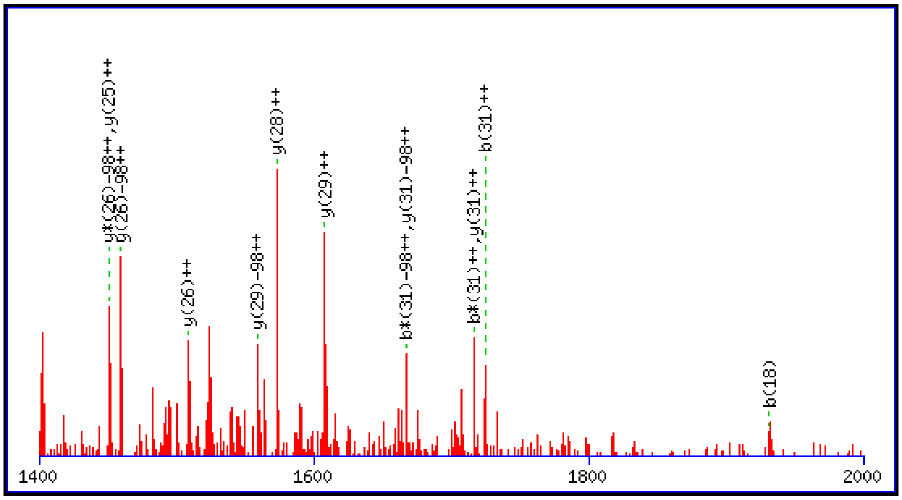

Monoisotopic mass of neutral peptide Mr(calc): 3706.4109
Fixed modifications: Carbamidomethyl (C)
Variable modifications:
M1 : Oxidation (M)
S10 : Phospho (ST), with neutral losses 0.0000(shown in table), 97.9769
Ions Score: 112 **Expect:** 1.8e-009
Matches (Bold Red): 40/504 fragment ions using 58 most intense peaks

#	b	b^{++}	b*	b^{*++}	b^{0}	b^{0++}	Seq.	y	y^{++}	y*	y^{*++}	y^{0}	y^{0++}	#
1	148.0427	74.5250					M							33
2	277.0853	139.0463			259.0747	130.0410	E	3560.3828	1780.6950	3543.3562	1772.1818	3542.3722	1771.6897	32
3	364.1173	182.5623			346.1067	173.5570	S	3431.3402	**1716.1737**	3414.3136	1707.6605	3413.3296	1707.1684	31
4	493.1599	247.0836			**475.1493**	238.0783	E	3344.3082	1672.6577	3327.2816	1664.1444	3326.2976	1663.6524	30
5	**564.1970**	282.6021			546.1864	273.5969	A	3215.2656	**1608.1364**	3198.2390	1599.6231	3197.2550	1599.1311	29
6	621.2185	311.1129			603.2079	302.1076	G	3144.2284	**1572.6179**	3127.2019	1564.1046	3126.2179	1563.6126	28
7	692.2556	346.6314			**674.2450**	337.6261	A	3087.2070	1544.1071	3070.1804	1535.5939	3069.1964	1535.1018	27
8	**807.2825**	404.1449			789.2720	395.1396	D	3016.1699	**1508.5886**	2999.1433	1500.0753	2998.1593	1499.5833	26
9	922.3095	461.6584			904.2989	452.6531	D	2901.1429	**1451.0751**	2884.1164	1442.5618	2883.1324	1442.0698	25
10	1089.3078	545.1576			1071.2973	536.1523	S	2786.1160	**1393.5616**	2769.0894	1385.0484	2768.1054	1384.5563	24
11	1160.3449	580.6761			1142.3344	571.6708	A	2619.1176	**1310.0625**	2602.0911	1301.5492	2601.1071	1301.0572	23
12	1289.3875	645.1974			1271.3770	636.1921	E	2548.0805	1274.5439	2531.0540	**1266.0306**	2530.0700	**1265.5386**	22
13	1418.4301	709.7187			1400.4196	700.7134	E	2419.0379	**1210.0226**	2402.0114	1201.5093	2401.0274	1201.0173	21
14	1475.4516	738.2294			1457.4410	729.2241	G	2289.9953	**1145.5013**	2272.9688	1136.9880	2271.9848	1136.4960	20
15	1590.4785	795.7429			1572.4680	786.7376	D	2232.9739	1116.9906	2215.9473	1108.4773	2214.9633	1107.9853	19
16	1703.5626	852.2849			1685.5520	843.2797	L	2117.9469	**1059.4771**	2100.9204	1050.9638	2099.9364	1050.4718	18
17	1816.6467	908.8270			1798.6361	899.8217	L	2004.8629	**1002.9351**	1987.8363	994.4218	1986.8523	993.9298	17
18	**1931.6736**	966.3404			1913.6630	957.3352	D	1891.7788	**946.3930**	1874.7523	937.8798	1873.7682	937.3878	16

19	2046.7005	1023.8539			2028.6900	1014.8486	D	1776.7519	888.8796	1759.7253	880.3663	1758.7413	879.8743	15
20	2161.7275	1081.3674			2143.7169	1072.3621	D	1661.7249	831.3661	1644.6984	822.8528	1643.7143	822.3608	14
21	2276.7544	1138.8809			2258.7439	1129.8756	D	1546.6980	773.8526	1529.6714	765.3393	1528.6874	764.8473	13
22	2390.7974	1195.9023	2373.7708	1187.3890	2372.7868	1186.8970	N	1431.6710	716.3392	1414.6445	707.8259	1413.6605	707.3339	12
23	2519.8400	1260.4236	2502.8134	1251.9103	2501.8294	1251.4183	E	1317.6281	659.3177	1300.6016	650.8044	1299.6175	650.3124	11
24	2634.8669	1317.9371	2617.8403	1309.4238	2616.8563	1308.9318	D	1188.5855	594.7964	1171.5590	586.2831	1170.5749	585.7911	10
25	2790.9680	1395.9876	2773.9415	1387.4744	2772.9574	1386.9824	R	1073.5586	537.2829	1056.5320	528.7696	1055.5480	528.2776	9
26	2847.9895	1424.4984	2830.9629	1415.9851	2829.9789	1415.4931	G	917.4575	459.2324	900.4309	450.7191	899.4469	450.2271	8
27	2963.0164	1482.0118	2945.9899	1473.4986	2945.0058	1473.0066	D	860.4360	430.7216	843.4094	422.2084	842.4254	421.7164	7
28	3078.0434	1539.5253	3061.0168	1531.0120	3060.0328	1530.5200	D	745.4090	373.2082	728.3825	364.6949	727.3985	364.2029	6
29	3206.1019	1603.5546	3189.0754	1595.0413	3188.0914	1594.5493	Q	630.3821	315.6947	613.3556	307.1814	612.3715	306.6894	5
30	3319.1860	1660.0966	3302.1594	1651.5834	3301.1754	1651.0914	L	502.3235	251.6654	485.2970	243.1521	484.3130	242.6601	4
31	3448.2286	1724.6179	3431.2020	1716.1047	3430.2180	1715.6127	E	389.2395	195.1234	372.2129	186.6101	371.2289	186.1181	3
32	3561.3127	1781.1600	3544.2861	1772.6467	3543.3021	1772.1547	L	260.1969	130.6021	243.1703	122.0888			2
33							K	147.1128	74.0600	130.0863	65.5468			1

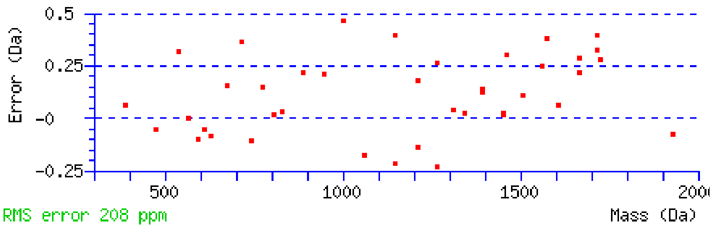

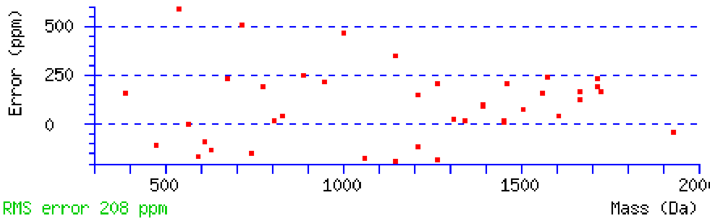

All matches to this query

Score	Mr(calc):	Delta	Sequence
112.4	3706.4109	0.0074	MESEAGADDSAEEGDLLDDDDNEDRGDDQLELK
55.0	3706.4109	0.0074	MESEAGADDSAEEGDLLDDDDNEDRGDDQLELK
6.2	3705.4462	0.9720	ASLNETPPMEKMPELIDNTGEICTNLGETAR
4.2	3706.4016	0.0167	GSCCVDQNVETGESMSSKPLFQKCQLIER
1.8	3705.4450	0.9733	NSNSNVIQVDQSGLGLPSRDYYLNKTENEK
1.8	3705.4450	0.9733	NSNSNVIQVDQSGLGLPSRDYYLNKTENEK
1.8	3705.4450	0.9733	NSNSNVIQVDQSGLGLPSRDYYLNKTENEK
0.9	3704.4052	2.0131	GLFTYSLSSKRSSPDDGNDVSPYSLSPVSNK
0.9	3704.4052	2.0131	GLFTYSLSSKRSSPDDGNDVSPYSLSPVSNK
0.7	3706.4016	0.0167	NGMEMTRPTPPHLTSAMAENILAAACESETR

Spectrum No: 6; Query: 2825; Rank: 1

Peptide View

MS/MS Fragmentation of **YGPVSVADTTGSGAADAKDDDDIDLFGSDDEEESEDAKR**
Found in **IPI00476899**, Tax_Id=10116 Gene_Symbol=Eef1b2_predicted eukaryotic translation elongation factor 1 beta 2

Match to Query 2825: 4142.701362 from(1381.907730,3+)
Title: 091008RatKidney_NoSalt_26.3185.3185.3.dta
Data file K:\NewmanPaper\Piliang\3SubProteomes\Piliang3SP\mgf5ppm\ERLIC_3SubProteomes5ppm.mgf

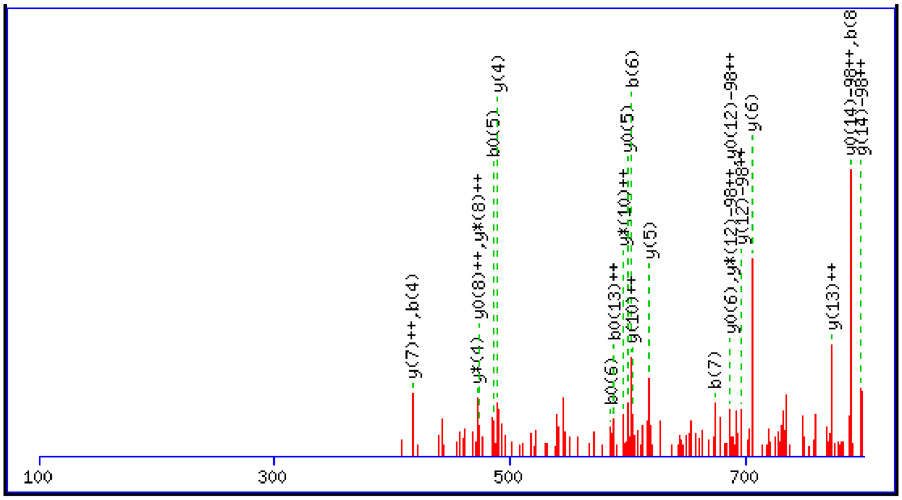

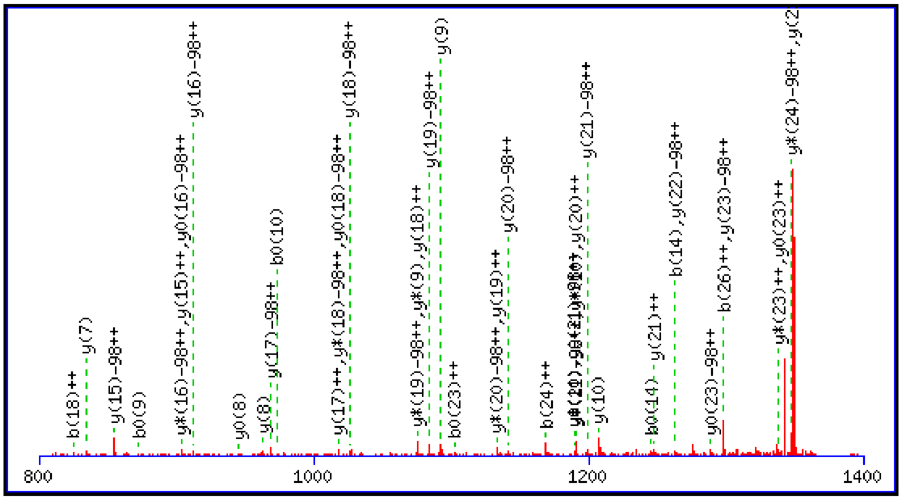

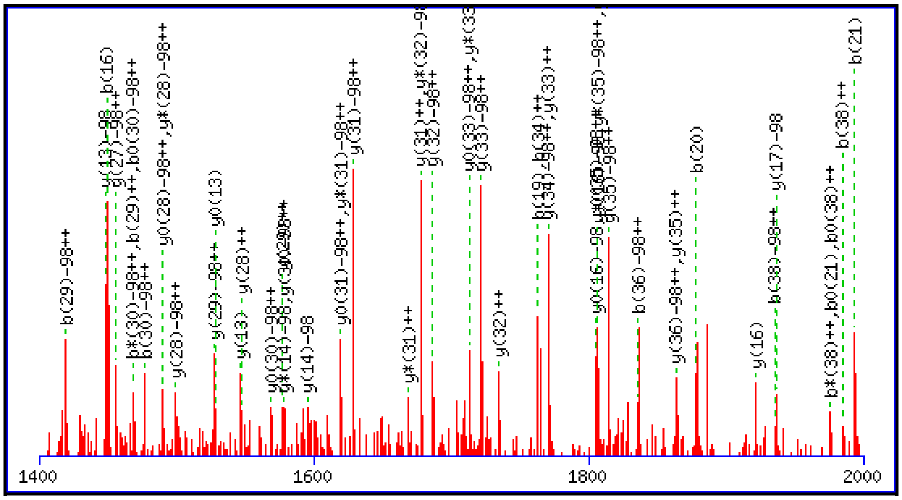

Monoisotopic mass of neutral peptide Mr(calc): 4141.6921
Fixed modifications: Carbamidomethyl (C)
Variable modifications:
S28 : Phospho (ST), with neutral losses 97.9769(shown in table), 0.0000
Ions Score: 111 **Expect:** 3.9e-009
Matches (Bold Red): 122/636 fragment ions using 161 most intense peaks

#	b	b++	b*	b*++	b0	b0++	Seq.	y	y++	y*	y*++	y0	y0++	#
1	164.0706	82.5389					Y							39
2	221.0921	111.0497					G	3881.6591	1941.3332	3864.6326	1932.8199	3863.6486	1932.3279	38
3	318.1448	159.5761					P	3824.6377	1912.8225	3807.6111	1904.3092	3806.6271	1903.8172	37
4	**417.2132**	209.1103					V	3727.5849	**1864.2961**	3710.5584	1855.7828	3709.5743	1855.2908	36
5	504.2453	252.6263			**486.2347**	243.6210	S	3628.5165	**1814.7619**	3611.4899	**1806.2486**	3610.5059	**1805.7566**	35

6	603.3137	302.1605			585.3031	293.1552	V	3541.4845	1771.2459	3524.4579	1762.7326	3523.4739	1762.2406	34
7	674.3508	337.6790			656.3402	328.6738	A	3442.4161	1721.7117	3425.3895	1713.1984	3424.4055	1712.7064	33
8	789.3777	395.1925			771.3672	386.1872	D	3371.3789	1686.1931	3354.3524	1677.6798	3353.3684	1677.1878	32
9	890.4254	445.7164			872.4149	436.7111	T	3256.3520	1628.6796	3239.3254	1620.1664	3238.3414	1619.6744	31
10	991.4731	496.2402			973.4625	487.2349	T	3155.3043	1578.1558	3138.2778	1569.6425	3137.2938	1569.1505	30
11	1048.4946	524.7509			1030.4840	515.7456	G	3054.2566	1527.6320	3037.2301	1519.1187	3036.2461	1518.6267	29
12	1135.5266	568.2669			1117.5160	559.2617	S	2997.2352	1499.1212	2980.2086	1490.6080	2979.2246	1490.1159	28
13	1192.5481	596.7777			1174.5375	587.7724	G	2910.2031	1455.6052	2893.1766	1447.0919	2892.1926	1446.5999	27
14	1263.5852	632.2962			1245.5746	623.2909	A	2853.1817	1427.0945	2836.1551	1418.5812	2835.1711	1418.0892	26
15	1334.6223	667.8148			1316.6117	658.8095	A	2782.1446	1391.5759	2765.1180	1383.0626	2764.1340	1382.5706	25
16	1449.6492	725.3283			1431.6387	716.3230	D	2711.1075	1356.0574	2694.0809	1347.5441	2693.0969	1347.0521	24
17	1520.6863	760.8468			1502.6758	751.8415	A	2596.0805	1298.5439	2579.0540	1290.0306	2578.0699	1289.5386	23
18	1648.7813	824.8943	1631.7548	816.3810	1630.7707	815.8890	K	2525.0434	1263.0253	2508.0168	1254.5121	2507.0328	1254.0201	22
19	1763.8083	882.4078	1746.7817	873.8945	1745.7977	873.4025	D	2396.9484	1198.9779	2379.9219	1190.4646	2378.9379	1189.9726	21
20	1878.8352	939.9212	1861.8086	931.4080	1860.8246	930.9160	D	2281.9215	1141.4644	2264.8949	1132.9511	2263.9109	1132.4591	20
21	1993.8621	997.4347	1976.8356	988.9214	1975.8516	988.4294	D	2166.8945	1083.9509	2149.8680	1075.4376	2148.8840	1074.9456	19
22	2108.8891	1054.9482	2091.8625	1046.4349	2090.8785	1045.9429	D	2051.8676	1026.4374	2034.8411	1017.9242	2033.8570	1017.4322	18
23	2221.9731	1111.4902	2204.9466	1102.9769	2203.9626	1102.4849	I	1936.8407	968.9240	1919.8141	960.4107	1918.8301	959.9187	17
24	2337.0001	1169.0037	2319.9735	1160.4904	2318.9895	1159.9984	D	1823.7566	912.3819	1806.7301	903.8687	1805.7460	903.3767	16
25	2450.0842	1225.5457	2433.0576	1217.0324	2432.0736	1216.5404	L	1708.7297	854.8685	1691.7031	846.3552	1690.7191	845.8632	15
26	2597.1526	1299.0799	2580.1260	1290.5666	2579.1420	1290.0746	F	1595.6456	798.3264	1578.6190	789.8132	1577.6350	789.3212	14
27	2654.1740	1327.5907	2637.1475	1319.0774	2636.1635	1318.5854	G	1448.5772	724.7922	1431.5506	716.2790	1430.5666	715.7869	13
28	2723.1955	1362.1014	2706.1689	1353.5881	2705.1849	1353.0961	S	1391.5557	696.2815	1374.5292	687.7682	1373.5451	687.2762	12
29	2838.2224	1419.6149	2821.1959	1411.1016	2820.2119	1410.6096	D	1322.5343	661.7708	1305.5077	653.2575	1304.5237	652.7655	11
30	2953.2494	1477.1283	2936.2228	1468.6150	2935.2388	1468.1230	D	1207.5073	604.2573	1190.4808	595.7440	1189.4968	595.2520	10
31	3082.2920	1541.6496	3065.2654	1533.1363	3064.2814	1532.6443	E	1092.4804	546.7438	1075.4538	538.2305	1074.4698	537.7385	9
32	3211.3346	1606.1709	3194.3080	1597.6576	3193.3240	1597.1656	E	963.4378	482.2225	946.4112	473.7093	945.4272	473.2172	8
33	3340.3771	1670.6922	3323.3506	1662.1789	3322.3666	1661.6869	E	834.3952	417.7012	817.3686	409.1880	816.3846	408.6959	7
34	3427.4092	1714.2082	3410.3826	1705.6950	3409.3986	1705.2029	S	705.3526	353.1799	688.3260	344.6667	687.3420	344.1747	6
35	3556.4518	1778.7295	3539.4252	1770.2162	3538.4412	1769.7242	E	618.3206	309.6639	601.2940	301.1506	600.3100	300.6586	5
36	3671.4787	1836.2430	3654.4522	1827.7297	3653.4681	1827.2377	D	489.2780	245.1426	472.2514	236.6293	471.2674	236.1373	4
37	3742.5158	1871.7616	3725.4893	1863.2483	3724.5053	1862.7563	A	374.2510	187.6292	357.2245	179.1159			3
38	3870.6108	1935.8090	3853.5842	1927.2958	3852.6002	1926.8038	K	303.2139	152.1106	286.1874	143.5973			2
39							R	175.1190	88.0631	158.0924	79.5498			1

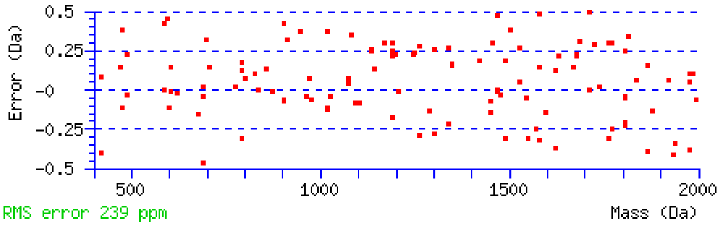

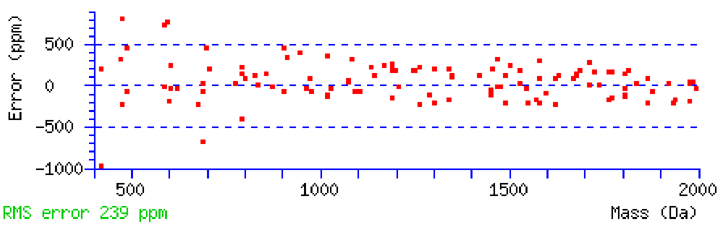

All matches to this query

Score	Mr(calc):	Delta	Sequence
111.1	4141.6921	1.0093	YGPVSVADTTGSGAADAKDDDDIDLFGSDDEEESEDAKR
96.1	4141.6921	1.0093	YGPVSVADTTGSGAADAKDDDDIDLFGSDDEEESEDAKR
51.2	4141.6921	1.0093	YGPVSVADTTGSGAADAKDDDDIDLFGSDDEEESEDAKR
45.5	4141.6921	1.0093	YGPVSVADTTGSGAADAKDDDDIDLFGSDDEEESEDAKR
40.0	4141.6921	1.0093	YGPVSVADTTGSGAADAKDDDDIDLFGSDDEEESEDAKR
24.2	4141.6921	1.0093	YGPVSVADTTGSGAADAKDDDDIDLFGSDDEEESEDAKR

19.5	4141.6921	1.0093	YGPVSVADTTGSGAADAKDDDDIDLFGSDDEEESEDAKR
9.2	4141.7389	0.9624	SNPGMDVSQMTFNPMNWRQMCLSSPSAVSVWTIER
8.5	4140.7302	1.9711	RLFTPKGPAAAPASASASALSAGSSSSCSSSSSLPASPASR
7.3	4140.6980	2.0033	SPHGGQGQXKEGQSPPSPHMKPMNWIQETSSTDTSGR

Spectrum No: 7; Query: 2082; Rank: 1

Peptide View

MS/MS Fragmentation of **SAEDLTEGSYDAILSAEQLEK**
Found in **IPI00372341**, Tax_Id=10116 Gene_Symbol=RGD1305915 armadillo repeat containing 10

Match to Query 2082: 2348.021208 from(1175.017880,2+)
Title: 091008RatKidney_NH4Format01_21.3976.3976.2.dta
Data file K:\NewmanPaper\Piliang\3SubProteomes\Piliang3SP\mgf5ppm\ERLIC_3SubProteomes5ppm.mgf

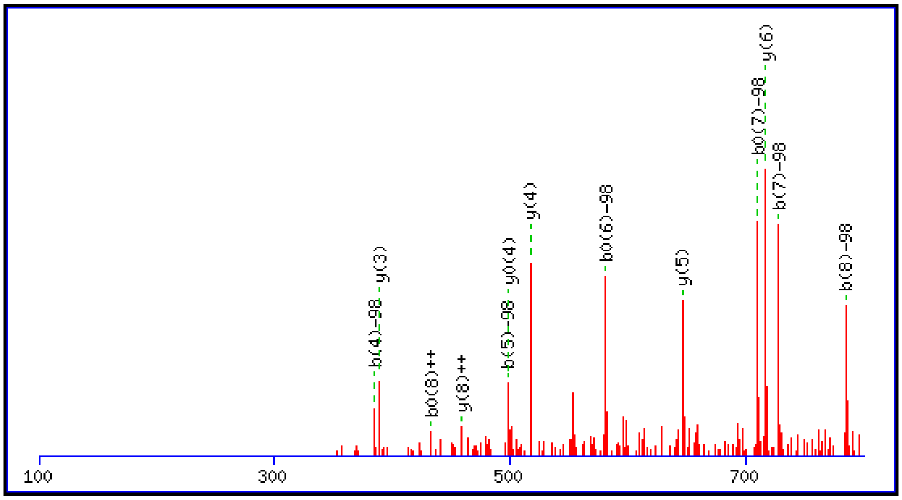

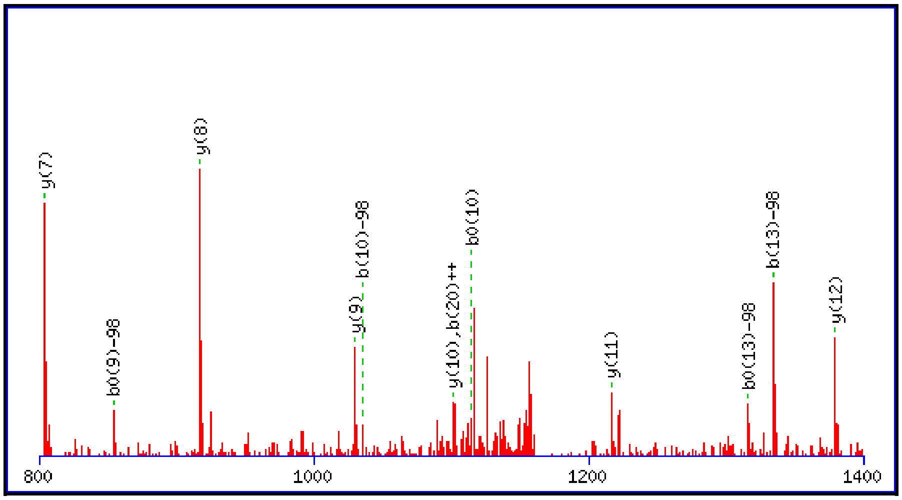

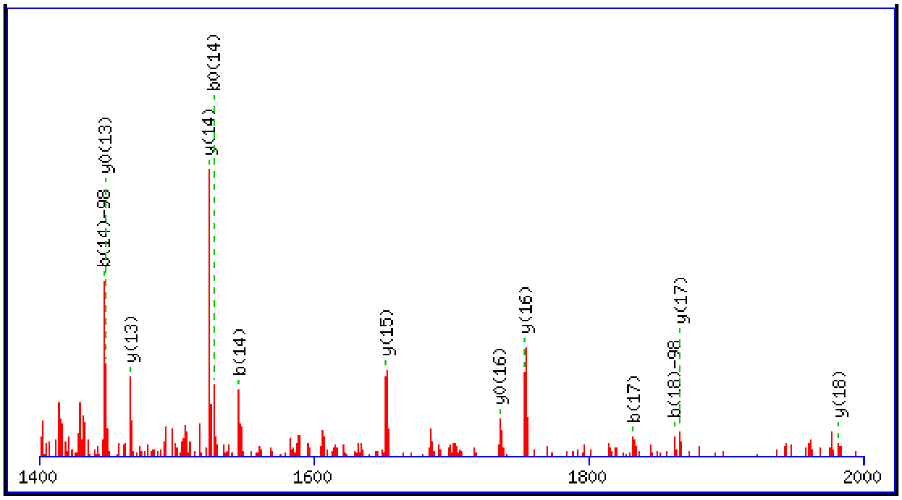

Monoisotopic mass of neutral peptide Mr(calc): 2348.0308
Fixed modifications: Carbamidomethyl (C)
Variable modifications:
S1 : Phospho (ST), with neutral losses 97.9769(shown in table), 0.0000
Ions Score: 104 **Expect:** 1.2e-008
Matches (Bold Red): 38/290 fragment ions using 64 most intense peaks

#	b	b++	b*	b*++	b0	b0++	Seq.	y	y++	y*	y*++	y0	y0++	#
1	70.0287	35.5180			52.0182	26.5127	S							21
2	141.0658	71.0366			123.0553	62.0313	A	2182.0398	1091.5235	2165.0132	1083.0102	2164.0292	1082.5182	20
3	270.1084	135.5579			252.0979	126.5526	E	2111.0027	1056.0050	2093.9761	1047.4917	2092.9921	1046.9997	19
4	**385.1354**	193.0713			367.1248	184.0660	D	**1981.9601**	991.4837	1964.9335	982.9704	1963.9495	982.4784	18
5	**498.2194**	249.6134			480.2089	240.6081	L	**1866.9331**	933.9702	1849.9066	925.4569	1848.9226	924.9649	17
6	599.2671	300.1372			**581.2566**	291.1319	T	**1753.8491**	877.4282	1736.8225	868.9149	**1735.8385**	868.4229	16
7	**728.3097**	364.6585			**710.2992**	355.6532	E	**1652.8014**	826.9043	1635.7748	818.3911	1634.7908	817.8990	15
8	**785.3312**	393.1692			767.3206	384.1639	G	**1523.7588**	762.3830	1506.7322	753.8698	1505.7482	753.3777	14
9	872.3632	436.6852			**854.3526**	427.6800	S	**1466.7373**	733.8723	1449.7108	725.3590	**1448.7268**	724.8670	13
10	**1035.4265**	518.2169			1017.4160	509.2116	Y	**1379.7053**	690.3563	1362.6787	681.8430	1361.6947	681.3510	12
11	1150.4535	575.7304			1132.4429	566.7251	D	**1216.6420**	608.8246	1199.6154	600.3113	1198.6314	599.8193	11
12	1221.4906	611.2489			1203.4800	602.2437	A	**1101.6150**	551.3111	1084.5885	542.7979	1083.6045	542.3059	10
13	**1334.5747**	667.7910			**1316.5641**	658.7857	I	**1030.5779**	515.7926	1013.5514	507.2793	1012.5673	506.7873	9
14	**1447.6587**	724.3330			1429.6482	715.3277	L	**917.4938**	**459.2506**	900.4673	450.7373	899.4833	450.2453	8
15	1534.6908	767.8490			1516.6802	758.8437	S	**804.4098**	402.7085	787.3832	394.1953	786.3992	393.7032	7
16	1605.7279	803.3676			1587.7173	794.3623	A	**717.3777**	359.1925	700.3512	350.6792	699.3672	350.1872	6
17	1734.7705	867.8889			1716.7599	858.8836	E	**646.3406**	323.6740	629.3141	315.1607	628.3301	314.6687	5
18	**1862.8290**	931.9182	1845.8025	923.4049	1844.8185	922.9129	Q	**517.2980**	259.1527	500.2715	250.6394	**499.2875**	250.1474	4
19	1975.9131	988.4602	1958.8866	979.9469	1957.9025	979.4549	L	**389.2395**	195.1234	372.2129	186.6101	371.2289	186.1181	3
20	2104.9557	1052.9815	2087.9291	1044.4682	2086.9451	1043.9762	E	276.1554	138.5813	259.1288	130.0681	258.1448	129.5761	2
21							K	147.1128	74.0600	130.0863	65.5468			1

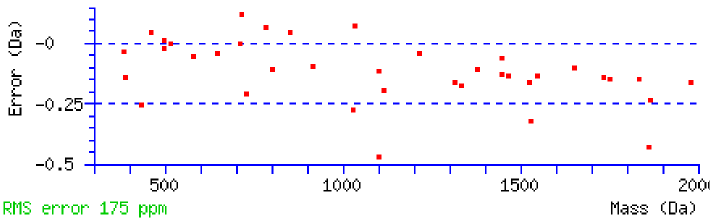

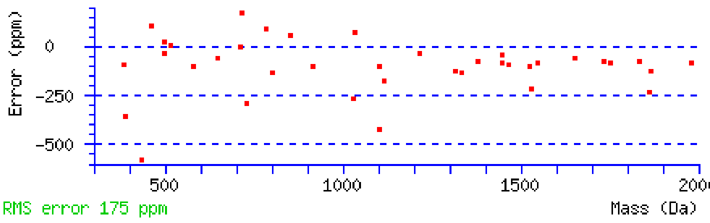

All matches to this query

Score	Mr(calc):	Delta	Sequence

104.3	2348.0308	-0.0096	SAEDLTEGSYDAILSAEQLEK
79.0	2348.0308	-0.0096	SAEDLTEGSYDAILSAEQLEK
51.1	2348.0308	-0.0096	SAEDLTEGSYDAILSAEQLEK
22.6	2348.0308	-0.0096	SAEDLTEGSYDAILSAEQLEK
4.8	2348.0308	-0.0096	SAEDLTEGSYDAILSAEQLEK

Spectrum No: 8; Query: 1938; Rank: 1

Peptide View

MS/MS Fragmentation of **TPEELDDSDFETEDFDVR**
Found in **IPI00358406**, Tax_Id=10116 Gene_Symbol=Catna1 Catenin (Cadherin-associated protein), alpha 1

Match to Query 1938: 2237.851528 from(1119.933040,2+)
Title: 091008RatKidney_NoSalt_30.3160.3160.2.dta
Data file K:\NewmanPaper\Piliang\3SubProteomes\Piliang3SP\mgf5ppm\ERLIC_3SubProteomes5ppm.mgf

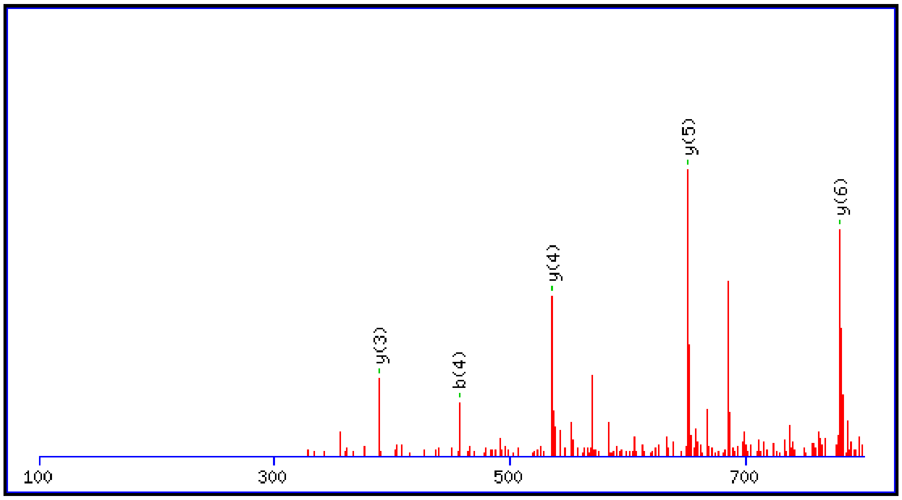

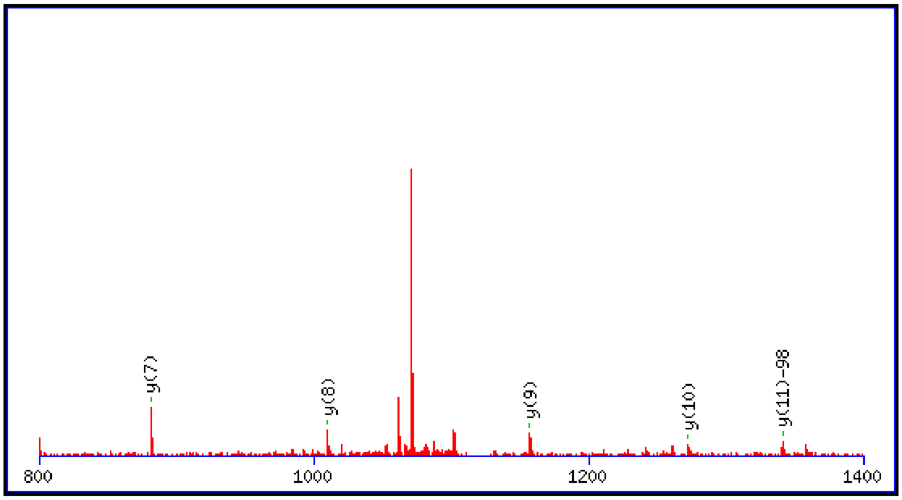

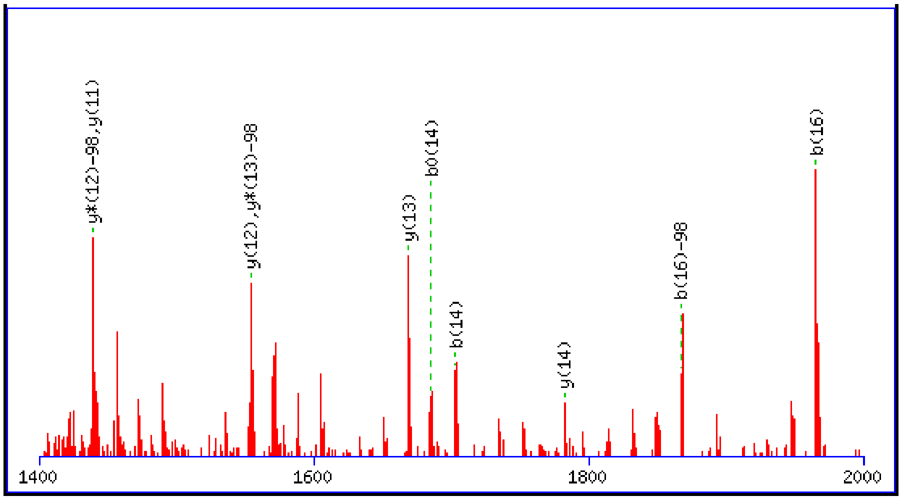

Monoisotopic mass of neutral peptide Mr(calc): 2237.8526
Fixed modifications: Carbamidomethyl (C)
Variable modifications:
S8 : Phospho (ST), with neutral losses 0.0000(shown in table), 97.9769
Ions Score: 104 **Expect:** 6.8e-009
Matches (Bold Red): 20/248 fragment ions using 28 most intense peaks

#	b	b^{++}	b^{0}	b^{0++}	Seq.	y	y^{++}	y*	y^{*++}	y^{0}	y^{0++}	#
1	102.0550	51.5311	84.0444	42.5258	T							18
2	199.1077	100.0575	181.0972	91.0522	P	2137.8121	1069.4097	2120.7856	1060.8964	2119.8016	1060.4044	17
3	328.1503	164.5788	310.1397	155.5735	E	2040.7594	1020.8833	2023.7328	1012.3701	2022.7488	1011.8780	16
4	**457.1929**	229.1001	439.1823	220.0948	E	1911.7168	956.3620	1894.6902	947.8488	1893.7062	947.3567	15
5	570.2770	285.6421	552.2664	276.6368	L	**1782.6742**	891.8407	1765.6476	883.3275	1764.6636	882.8355	14
6	685.3039	343.1556	667.2933	334.1503	D	**1669.5901**	835.2987	1652.5636	826.7854	1651.5796	826.2934	13
7	800.3309	400.6691	782.3203	391.6638	D	**1554.5632**	777.7852	1537.5366	769.2720	1536.5526	768.7799	12
8	967.3292	484.1682	949.3186	475.1630	S	**1439.5362**	720.2718	1422.5097	711.7585	1421.5257	711.2665	11
9	1082.3561	541.6817	1064.3456	532.6764	D	**1272.5379**	636.7726	1255.5113	628.2593	1254.5273	627.7673	10
10	1229.4246	615.2159	1211.4140	606.2106	F	**1157.5109**	579.2591	1140.4844	570.7458	1139.5004	570.2538	9
11	1358.4672	679.7372	1340.4566	670.7319	E	**1010.4425**	505.7249	993.4160	497.2116	992.4320	496.7196	8
12	1459.5148	730.2611	1441.5043	721.2558	T	**881.3999**	441.2036	864.3734	432.6903	863.3894	432.1983	7
13	1588.5574	794.7824	1570.5469	785.7771	E	**780.3523**	390.6798	763.3257	382.1665	762.3417	381.6745	6
14	**1703.5844**	852.2958	**1685.5738**	843.2905	D	**651.3097**	326.1585	634.2831	317.6452	633.2991	317.1532	5
15	1850.6528	925.8300	1832.6422	916.8247	F	**536.2827**	268.6450	519.2562	260.1317	518.2722	259.6397	4
16	**1965.6797**	983.3435	1947.6692	974.3382	D	**389.2143**	195.1108	372.1878	186.5975	371.2037	186.1055	3
17	2064.7481	1032.8777	2046.7376	1023.8724	V	274.1874	137.5973	257.1608	129.0840			2
18					R	175.1190	88.0631	158.0924	79.5498			1

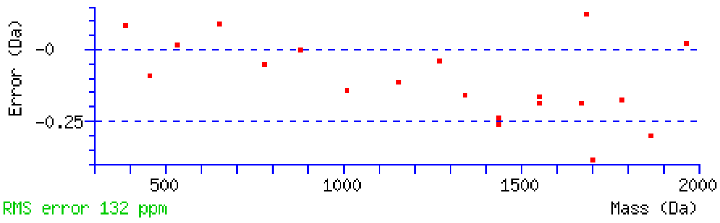

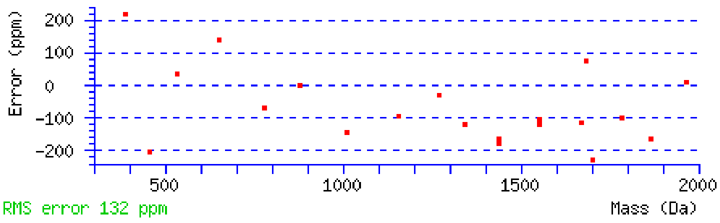

All matches to this query

Score	Mr(calc):	Delta	Sequence
103.6	2237.8526	-0.0010	TPEELDDSDFETEDFDVR
49.8	2237.8526	-0.0010	TPEELDDSDFETEDFDVR
42.1	2237.8526	-0.0010	TPEELDDSDFETEDFDVR

4.1	2236.8675	0.9841	EESQNETRDVLVWSNDR
2.0	2235.8512	2.0003	RSYVTMTATKIEITMR
2.0	2237.8451	0.0064	GIAAQPLFTGYCNYENKM
1.3	2236.8391	1.0124	TVSPRSDSVSPVMIRNR
0.5	2237.8378	0.0138	SYATGNWTYGDVLCISNR
0.1	2237.8661	-0.0146	DSSSGADACARPTEALQAGSK
0.0	2236.8374	1.0141	KSTAWNCSTASXRSSAVR

Spectrum No: 9; Query: 1706; Rank: 1

Peptide View

MS/MS Fragmentation of **KETESEAEDDNLDDLER**
Found in **IPI00779470**, Tax_Id=10116 Gene_Symbol=Srrm1_predicted 106 kDa protein

Match to Query 1706: 2086.830428 from(1044.422490,2+)
Title: 100101RatKid_NS_deglyco_23.1809.1809.2.dta
Data file K:\NewmanPaper\Piliang\3SubProteomes\Piliang3SP\mgf5ppm\ERLIC_3SubProteomes5ppm.mgf

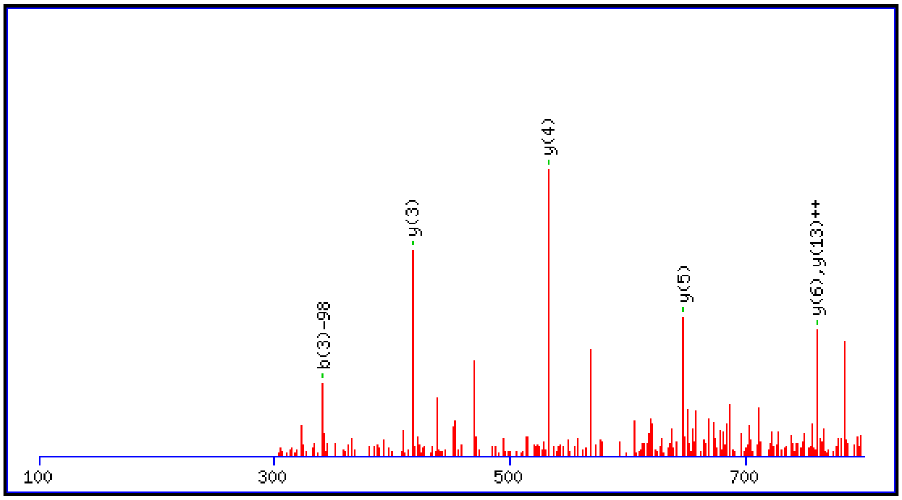

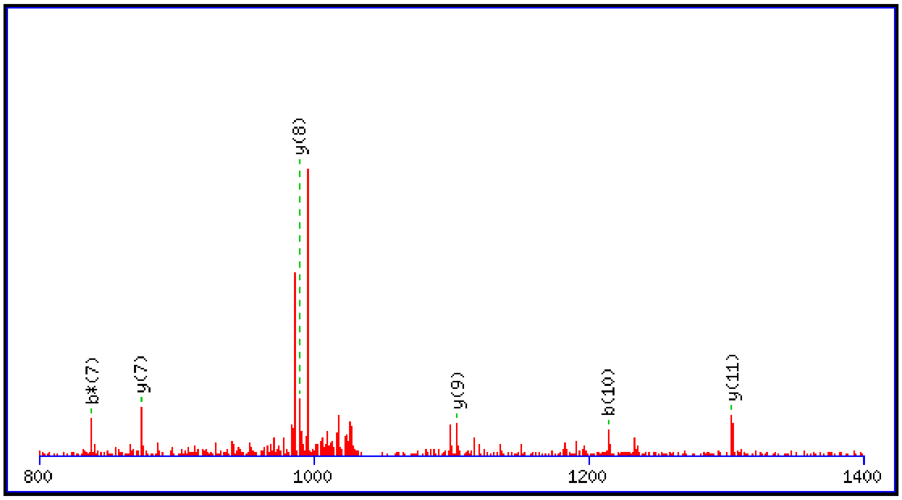

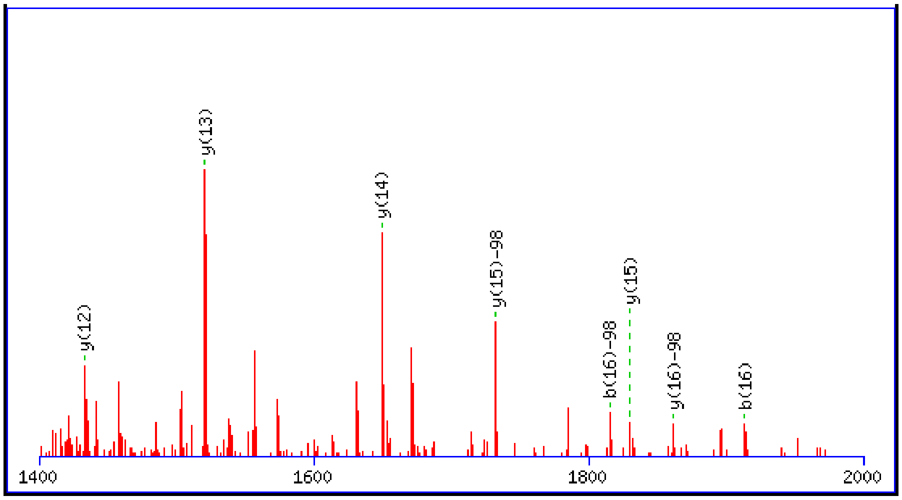

Monoisotopic mass of neutral peptide Mr(calc): 2086.8215
Fixed modifications: Carbamidomethyl (C)
Variable modifications:
T3 : Phospho (ST), with neutral losses 97.9769(shown in table), 0.0000
Ions Score: 101 **Expect:** 1.5e-008
Matches (Bold Red): 20/284 fragment ions using 27 most intense peaks

#	b	b++	b*	b*++	b0	b0++	Seq.	y	y++	y*	y*++	y0	y0++	#
1	129.1022	65.0548	112.0757	56.5415			K							17
2	258.1448	129.5761	241.1183	121.0628	240.1343	120.5708	E	**1861.7570**	931.3821	1844.7304	922.8689	1843.7464	922.3769	16
3	**341.1819**	171.0946	324.1554	162.5813	323.1714	162.0893	T	**1732.7144**	866.8608	1715.6879	858.3476	1714.7038	857.8556	15
4	470.2245	235.6159	453.1980	227.1026	452.2140	226.6106	E	**1649.6773**	825.3423	1632.6507	816.8290	1631.6667	816.3370	14
5	557.2566	279.1319	540.2300	270.6186	539.2460	270.1266	S	**1520.6347**	**760.8210**	1503.6082	752.3077	1502.6241	751.8157	13
6	686.2992	343.6532	669.2726	335.1399	668.2886	334.6479	E	**1433.6027**	717.3050	1416.5761	708.7917	1415.5921	708.2997	12
7	757.3363	379.1718	740.3097	370.6585	739.3257	370.1665	A	**1304.5601**	652.7837	1287.5335	644.2704	1286.5495	643.7784	11
8	886.3789	443.6931	869.3523	435.1798	868.3683	434.6878	E	1233.5230	617.2651	1216.4964	608.7518	1215.5124	608.2598	10
9	1001.4058	501.2065	984.3793	492.6933	983.3952	492.2013	D	**1104.4804**	552.7438	1087.4538	544.2305	1086.4698	543.7385	9
10	1116.4327	558.7200	1099.4062	550.2067	1098.4222	549.7147	D	**989.4534**	495.2304	972.4269	486.7171	971.4429	486.2251	8
11	1230.4757	615.7415	1213.4491	607.2282	1212.4651	606.7362	N	**874.4265**	437.7169	857.3999	429.2036	856.4159	428.7116	7
12	1343.5597	672.2835	1326.5332	663.7702	1325.5492	663.2782	L	**760.3836**	380.6954	743.3570	372.1821	742.3730	371.6901	6
13	1458.5867	729.7970	1441.5601	721.2837	1440.5761	720.7917	D	**647.2995**	324.1534	630.2729	315.6401	629.2889	315.1481	5
14	1573.6136	787.3104	1556.5871	778.7972	1555.6031	778.3052	D	**532.2726**	266.6399	515.2460	258.1266	514.2620	257.6346	4
15	1686.6977	843.8525	1669.6711	835.3392	1668.6871	834.8472	L	**417.2456**	209.1264	400.2191	200.6132	399.2350	200.1212	3
16	**1815.7403**	908.3738	1798.7137	899.8605	1797.7297	899.3685	E	304.1615	152.5844	287.1350	144.0711	286.1510	143.5791	2
17							R	175.1190	88.0631	158.0924	79.5498			1

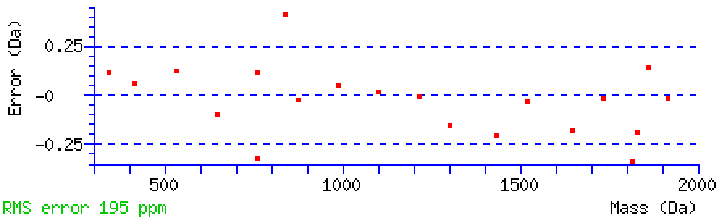

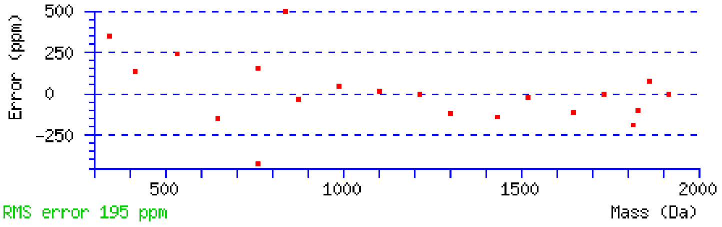

All matches to this query

Score	Mr(calc):	Delta	Sequence
101.2	2086.8215	0.0089	KETESEAEDDNLDDLER
73.3	2086.8215	0.0089	KETESEAEDDNLDDLER
5.0	2086.8360	-0.0056	QEYMEVTLTFVTEGHK
4.1	2086.8489	-0.0185	MNGTHFVLESPLNGCGTR

2.3	2086.8108	0.0196	MKYDGDTYYNSALKSR
2.3	2086.8108	0.0196	MKYDGDTYYNSALKSR
0.6	2086.8360	-0.0056	QEYMEVTLTFVTEGHK
0.2	2086.8108	0.0196	MKYDGDTYYNSALKSR

Spectrum No: 10; Query: 1331; Rank: 1

Peptide View

MS/MS Fragmentation of **SASSDTSEELNAQDSPK**
Found in **IPI00200898**, Tax_Id=10116 Gene_Symbol=Slc9a3r1 Ezrin-radixin-moesin-binding phosphoprotein 50

Match to Query 1331: 1844.740648 from(923.377600,2+)
Title: 091008RatKidney_NoSalt_28.701.701.2.dta
Data file K:\NewmanPaper\Piliang\3SubProteomes\Piliang3SP\mgf5ppm\ERLIC_3SubProteomes5ppm.mgf

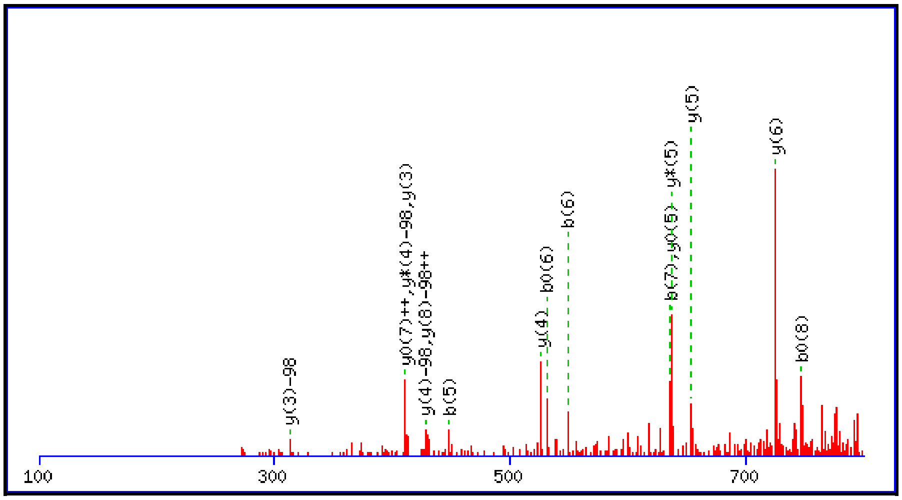

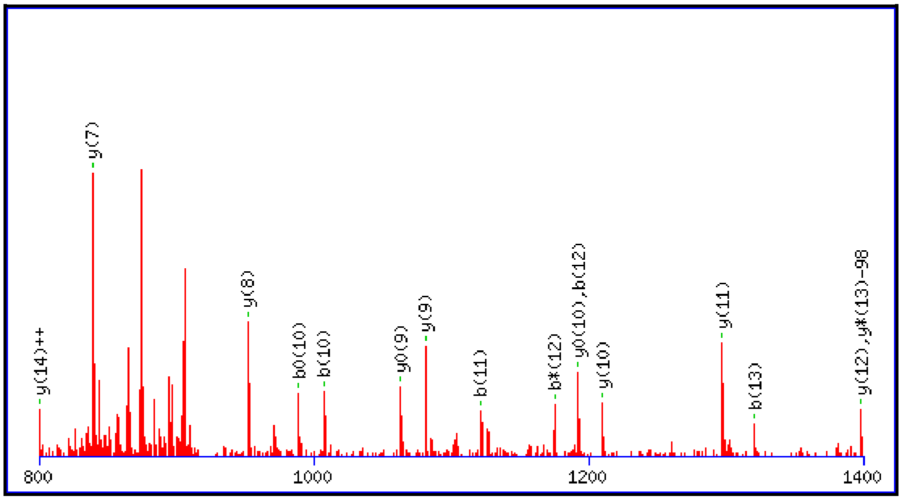

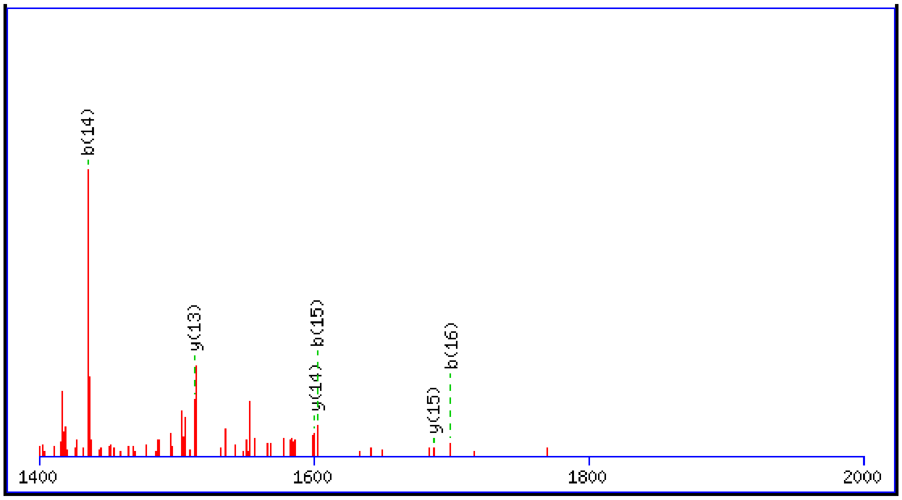

Monoisotopic mass of neutral peptide Mr(calc): 1844.7313
Fixed modifications: Carbamidomethyl (C)
Variable modifications:
S15 : Phospho (ST), with neutral losses 0.0000(shown in table), 97.9769
Ions Score: 101 **Expect:** 1.4e-008
Matches (Bold Red): 38/264 fragment ions using 49 most intense peaks

#	b	b^{++}	b*	b^{*++}	b^0	b^{0++}	Seq.	y	y^{++}	y*	y^{*++}	y^0	y^{0++}	#
1	88.0393	44.5233			70.0287	35.5180	S							17
2	159.0764	80.0418			141.0659	71.0366	A	1758.7066	879.8569	1741.6800	871.3436	1740.6960	870.8516	16
3	246.1084	123.5579			228.0979	114.5526	S	**1687.6694**	844.3384	1670.6429	835.8251	1669.6589	835.3331	15
4	333.1405	167.0739			315.1299	158.0686	S	**1600.6374**	**800.8223**	1583.6109	792.3091	1582.6268	791.8171	14
5	**448.1674**	224.5873			430.1569	215.5821	D	**1513.6054**	757.3063	1496.5788	748.7931	1495.5948	748.3010	13
6	**549.2151**	275.1112			**531.2045**	266.1059	T	**1398.5784**	699.7929	1381.5519	691.2796	1380.5679	690.7876	12
7	**636.2471**	318.6272			618.2366	309.6219	S	**1297.5308**	649.2690	1280.5042	640.7557	1279.5202	640.2637	11
8	765.2897	383.1485			**747.2792**	374.1432	E	**1210.4987**	605.7530	1193.4722	597.2397	**1192.4882**	596.7477	10
9	894.3323	447.6698			876.3217	438.6645	E	**1081.4561**	541.2317	1064.4296	532.7184	**1063.4456**	532.2264	9
10	**1007.4164**	504.2118			**989.4058**	495.2065	L	**952.4135**	476.7104	935.3870	468.1971	934.4030	467.7051	8
11	**1121.4593**	561.2333	1104.4328	552.7200	1103.4487	552.2280	N	**839.3295**	420.1684	822.3029	411.6551	821.3189	**411.1631**	7
12	**1192.4964**	596.7518	**1175.4699**	588.2386	1174.4859	587.7466	A	**725.2866**	363.1469	708.2600	354.6336	707.2760	354.1416	6
13	**1320.5550**	660.7811	1303.5284	652.2679	1302.5444	651.7759	Q	**654.2494**	327.6284	**637.2229**	319.1151	**636.2389**	318.6231	5
14	**1435.5819**	718.2946	1418.5554	709.7813	1417.5714	709.2893	D	**526.1909**	263.5991	509.1643	255.0858	508.1803	254.5938	4
15	**1602.5803**	801.7938	1585.5537	793.2805	1584.5697	792.7885	S	**411.1639**	206.0856	394.1374	197.5723	393.1534	197.0803	3
16	**1699.6331**	850.3202	1682.6065	841.8069	1681.6225	841.3149	P	244.1656	122.5864	227.1390	114.0731			2
17							K	147.1128	74.0600	130.0863	65.5468			1

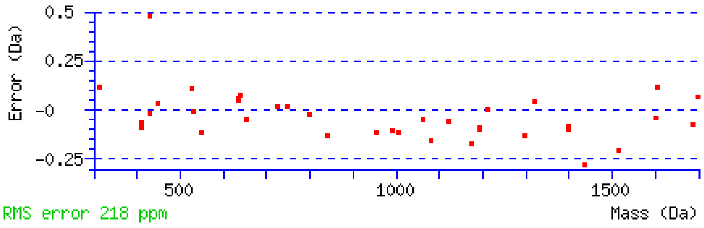

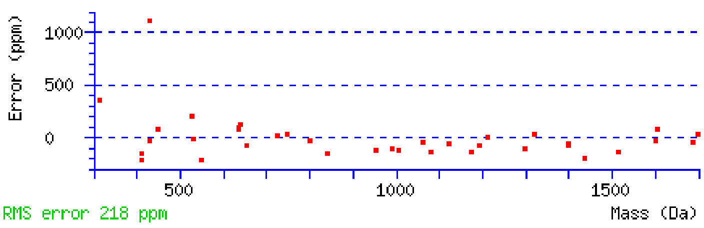

All matches to this query

Score	Mr(calc):	Delta	Sequence
100.7	1844.7313	0.0094	SASSDTSEELNAQDSPK
16.6	1844.7313	0.0094	SASSDTSEELNAQDSPK
16.2	1844.7313	0.0094	SASSDTSEELNAQDSPK
16.2	1844.7313	0.0094	SASSDTSEELNAQDSPK

14.6	1844.7313	0.0094	SASSDTSEELNAQDSPK
14.6	1844.7313	0.0094	SASSDTSEELNAQDSPK
9.5	1844.7417	-0.0010	LLLAHADCNSNLTDK
4.3	1844.7247	0.0159	DSQQDELDNLREMR
2.7	1844.7440	-0.0033	YDSEAIGLWLPLTK
1.4	1844.7248	0.0159	KTVEDADMDREDGER

Spectrum No: 11; Query: 2005; Rank: 1

Peptide View

MS/MS Fragmentation of **ASPALGSGPDGSGDSLEMSSLDR**
Found in **IPI00417750**, Tax_Id=10116 Gene_Symbol=Tomm70a Mitochondrial precursor proteins import receptor

Match to Query 2005: 2284.955688 from(1143.485120,2+)
Title: 091008RatKidney_NoSalt_26.2771.2771.2.dta
Data file K:\NewmanPaper\Piliang\3SubProteomes\Piliang3SP\mgf5ppm\ERLIC_3SubProteomes5ppm.mgf

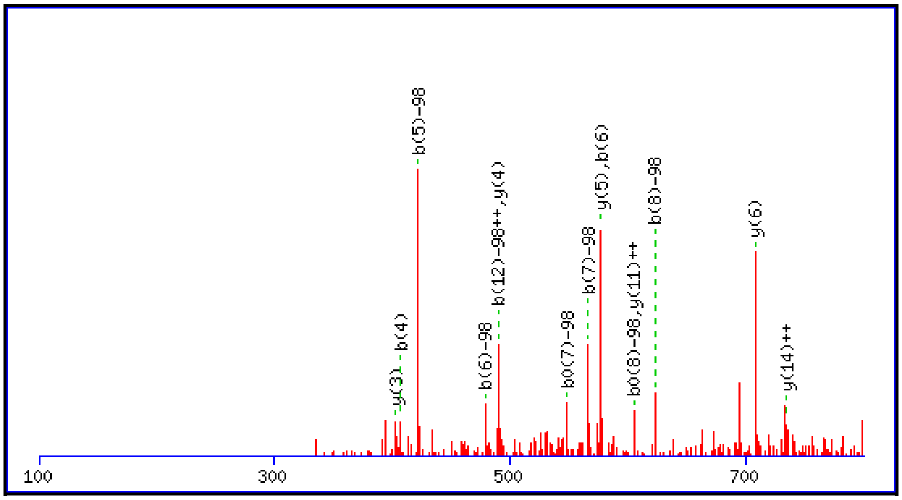

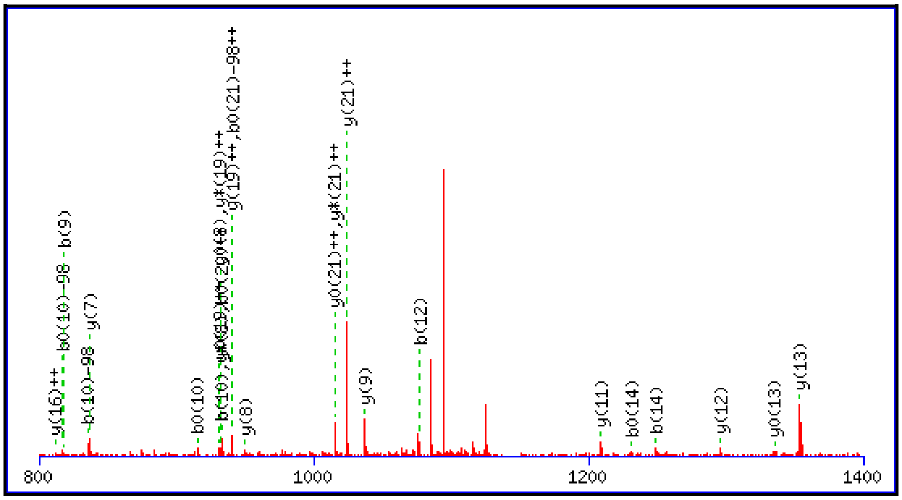

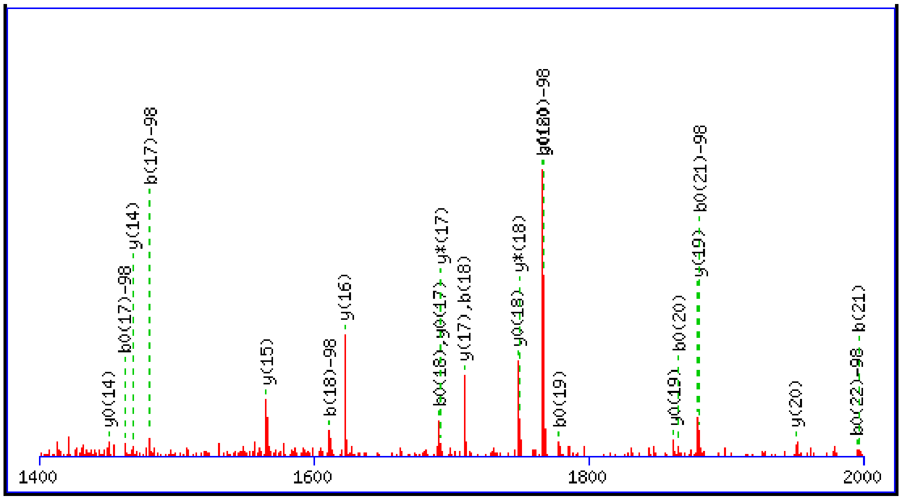

Monoisotopic mass of neutral peptide Mr(calc): 2284.9519
Fixed modifications: Carbamidomethyl (C)
Variable modifications:
S2 : Phospho (ST), with neutral losses 97.9769(shown in table), 0.0000
Ions Score: 100 **Expect:** 2.3e-008
Matches (Bold Red): 65/306 fragment ions using 106 most intense peaks

#	b	b++	b0	b0++	Seq.	y	y++	y*	y*++	y0	y0++	#
1	72.0444	36.5258			A							23
2	141.0658	71.0366	123.0553	62.0313	S	2116.9451	1058.9762	2099.9186	1050.4629	2098.9346	1049.9709	22
3	238.1186	119.5629	220.1080	110.5577	P	2047.9237	**1024.4655**	2030.8971	**1015.9522**	2029.9131	**1015.4602**	21
4	309.1557	155.0815	291.1452	146.0762	A	**1950.8709**	975.9391	1933.8444	967.4258	1932.8604	966.9338	20
5	**422.2398**	211.6235	404.2292	202.6182	L	**1879.8338**	**940.4205**	1862.8073	**931.9073**	**1861.8232**	**931.4153**	19
6	**479.2613**	240.1343	461.2507	231.1290	G	**1766.7497**	883.8785	**1749.7232**	875.3652	**1748.7392**	874.8732	18
7	**566.2933**	283.6503	**548.2827**	274.6450	S	**1709.7283**	855.3678	**1692.7017**	846.8545	**1691.7177**	846.3625	17
8	**623.3147**	312.1610	**605.3042**	303.1557	G	**1622.6963**	**811.8518**	1605.6697	803.3385	1604.6857	802.8465	16
9	720.3675	360.6874	702.3569	351.6821	P	**1565.6748**	783.3410	1548.6482	774.8278	1547.6642	774.3358	15
10	**835.3945**	418.2009	**817.3839**	409.1956	D	**1468.6220**	**734.8147**	1451.5955	726.3014	**1450.6115**	725.8094	14
11	892.4159	446.7116	874.4054	437.7063	G	**1353.5951**	677.3012	1336.5685	668.7879	**1335.5845**	668.2959	13
12	979.4479	**490.2276**	961.4374	481.2223	S	**1296.5736**	648.7904	1279.5471	640.2772	1278.5631	639.7852	12
13	1036.4694	518.7383	1018.4588	509.7331	G	**1209.5416**	**605.2744**	1192.5150	596.7612	1191.5310	596.2692	11
14	1151.4964	576.2518	1133.4858	567.2465	D	1152.5201	576.7637	1135.4936	568.2504	1134.5096	567.7584	10
15	1238.5284	619.7678	1220.5178	610.7625	S	**1037.4932**	519.2502	1020.4666	510.7370	1019.4826	510.2449	9
16	1351.6124	676.3099	1333.6019	667.3046	L	**950.4612**	475.7342	**933.4346**	467.2209	**932.4506**	466.7289	8
17	**1480.6550**	740.8312	**1462.6445**	731.8259	E	**837.3771**	419.1922	820.3505	410.6789	819.3665	410.1869	7
18	**1611.6955**	806.3514	1593.6850	797.3461	M	**708.3345**	354.6709	691.3080	346.1576	690.3239	345.6656	6
19	1698.7275	849.8674	1680.7170	840.8621	S	**577.2940**	289.1506	560.2675	280.6374	559.2835	280.1454	5
20	1785.7596	893.3834	**1767.7490**	884.3781	S	**490.2620**	245.6346	473.2354	237.1214	472.2514	236.6293	4
21	1898.8436	949.9255	**1880.8331**	**940.9202**	L	**403.2300**	202.1186	386.2034	193.6053	385.2194	193.1133	3
22	2013.8706	1007.4389	**1995.8600**	998.4336	D	290.1459	145.5766	273.1193	137.0633	272.1353	136.5713	2
23					R	175.1190	88.0631	158.0924	79.5498			1

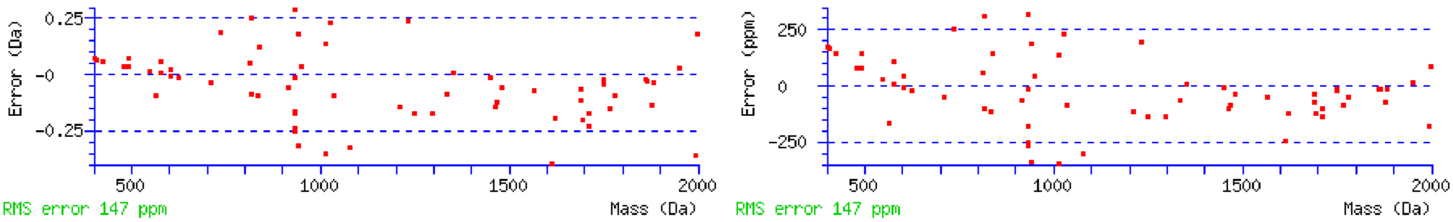

All matches to this query

Score	Mr(calc):	Delta	Sequence
100.4	2284.9519	0.0038	ASPALGSGPDGSGDSLEMSSLDR
81.3	2284.9519	0.0038	ASPALGSGPDGSGDSLEMSSLDR
38.1	2284.9519	0.0038	ASPALGSGPDGSGDSLEMSSLDR
24.5	2284.9519	0.0038	ASPALGSGPDGSGDSLEMSSLDR
15.7	2282.9457	2.0100	EKTYNALNNLSVNSENQGK
5.1	2282.9457	2.0100	EKTYNALNNLSVNSENQGK
5.0	2284.9519	0.0038	ASPALGSGPDGSGDSLEMSSLDR
1.6	2284.9671	-0.0114	VHSPSGAVEECHVSELEPDK
1.1	2283.9297	1.0260	EKTYNALNNLSVNSENQGK
1.1	2284.9519	0.0038	ASPALGSGPDGSGDSLEMSSLDR

Spectrum No: 12; Query: 2777; Rank: 1

Peptide View

MS/MS Fragmentation of **ELEQVDVSSEVNIVNPFALEPTILDNEDSDTKK**
Found in **IPI00339125**, Tax_Id=10116 Gene_Symbol=Slc1a1 Excitatory amino acid transporter 3

Match to Query 2777: 3766.776372 from(1256.599400,3+)
Title: 100101RatKid_NS_deglyco_19.5744.5744.3.dta
Data file K:\NewmanPaper\Piliang\3SubProteomes\Piliang3SP\mgf5ppm\ERLIC_3SubProteomes5ppm.mgf

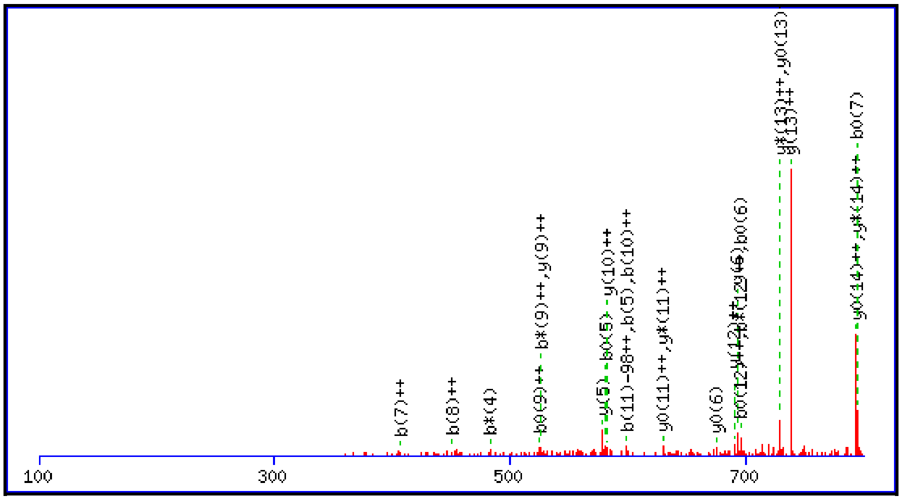

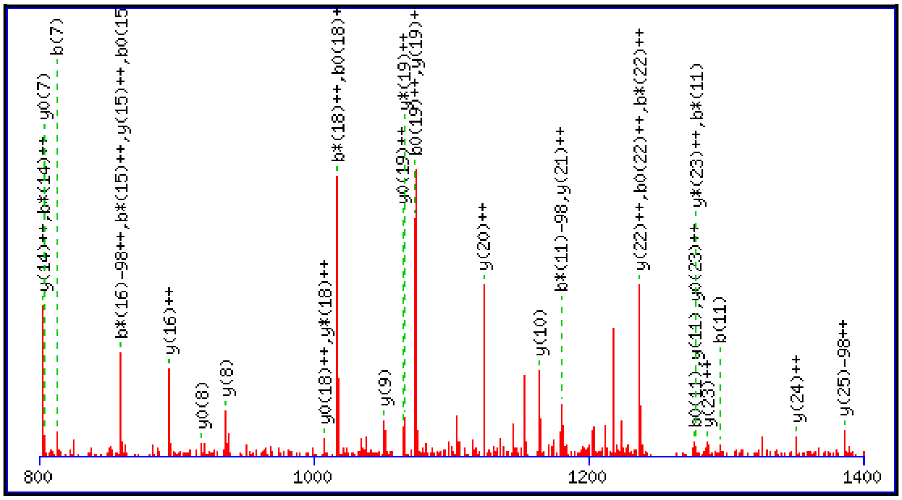

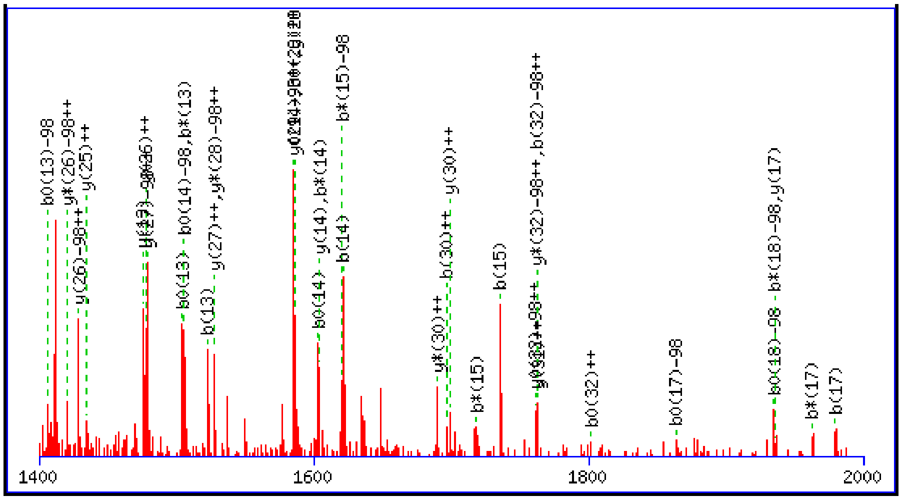

Monoisotopic mass of neutral peptide Mr(calc): 3766.7714
Fixed modifications: Carbamidomethyl (C)
Variable modifications:
S9 : Phospho (ST), with neutral losses 0.0000(shown in table), 97.9769
Ions Score: 99 **Expect:** 5.7e-008
Matches (Bold Red): 102/566 fragment ions using 123 most intense peaks

#	b	b^{++}	b^{*}	b^{*++}	b^{0}	b^{0++}	Seq.	y	y^{++}	y^{*}	y^{*++}	y^{0}	y^{0++}	#
1	130.0499	65.5286			112.0393	56.5233	E							33
2	243.1339	122.0706			225.1234	113.0653	L	3638.7361	1819.8717	3621.7095	1811.3584	3620.7255	1810.8664	32
3	372.1765	186.5919			354.1660	177.5866	E	3525.6520	**1763.3296**	3508.6255	1754.8164	3507.6414	1754.3244	31
4	500.2351	250.6212	**483.2086**	242.1079	482.2245	241.6159	Q	3396.6094	**1698.8083**	3379.5829	**1690.2951**	3378.5988	1689.8031	30
5	**599.3035**	300.1554	582.2770	291.6421	**581.2930**	291.1501	V	3268.5508	1634.7791	3251.5243	1626.2658	3250.5403	1625.7738	29
6	714.3305	357.6689	697.3039	349.1556	**696.3199**	348.6636	D	3169.4824	**1585.2448**	3152.4559	1576.7316	3151.4719	1576.2396	28
7	**813.3989**	**407.2031**	796.3723	398.6898	**795.3883**	398.1978	V	3054.4555	**1527.7314**	3037.4289	1519.2181	3036.4449	1518.7261	27
8	900.4309	**450.7191**	883.4044	442.2058	882.4203	441.7138	S	2955.3871	**1478.1972**	2938.3605	1469.6839	2937.3765	1469.1919	26
9	1067.4293	534.2183	1050.4027	**525.7050**	1049.4187	**525.2130**	S	2868.3550	**1434.6812**	2851.3285	1426.1679	2850.3445	1425.6759	25
10	1196.4718	**598.7396**	1179.4453	590.2263	1178.4613	589.7343	E	2701.3567	**1351.1820**	2684.3301	1342.6687	2683.3461	1342.1767	24
11	**1295.5403**	648.2738	**1278.5137**	639.7605	**1277.5297**	639.2685	V	2572.3141	**1286.6607**	2555.2875	**1278.1474**	2554.3035	**1277.6554**	23
12	1409.5832	705.2952	1392.5566	**696.7820**	1391.5726	**696.2899**	N	2473.2457	**1237.1265**	2456.2191	1228.6132	2455.2351	1228.1212	22
13	**1522.6673**	761.8373	**1505.6407**	753.3240	**1504.6567**	752.8320	I	2359.2027	**1180.1050**	2342.1762	1171.5917	2341.1922	1171.0997	21
14	**1621.7357**	811.3715	**1604.7091**	**802.8582**	**1603.7251**	802.3662	V	2246.1187	**1123.5630**	2229.0921	1115.0497	2228.1081	1114.5577	20
15	**1735.7786**	868.3929	**1718.7520**	**859.8797**	1717.7680	**859.3877**	N	2147.0503	**1074.0288**	2130.0237	**1065.5155**	2129.0397	**1065.0235**	19
16	1832.8314	916.9193	1815.8048	908.4060	1814.8208	907.9140	P	2033.0073	**1017.0073**	2015.9808	**1008.4940**	2014.9968	**1008.0020**	18
17	**1979.8998**	990.4535	**1962.8732**	981.9402	1961.8892	981.4482	F	**1935.9546**	968.4809	1918.9280	959.9677	1917.9440	959.4756	17
18	2050.9369	1025.9721	2033.9103	**1017.4588**	2032.9263	**1016.9668**	A	1788.8862	**894.9467**	1771.8596	886.4334	1770.8756	885.9414	16
19	2164.0210	1082.5141	2146.9944	**1074.0008**	2146.0104	**1073.5088**	L	1717.8491	**859.4282**	1700.8225	850.9149	1699.8385	850.4229	15
20	2293.0635	1147.0354	2276.0370	1138.5221	2275.0530	1138.0301	E	**1604.7650**	**802.8861**	1587.7384	**794.3729**	**1586.7544**	**793.8808**	14
21	2390.1163	1195.5618	2373.0898	1187.0485	2372.1057	1186.5565	P	**1475.7224**	**738.3648**	1458.6958	**729.8516**	1457.7118	**729.3596**	13
22	2491.1640	1246.0856	2474.1374	**1237.5724**	2473.1534	**1237.0803**	T	1378.6696	**689.8385**	1361.6431	681.3252	1360.6591	680.8332	12
23	2604.2481	1302.6277	2587.2215	1294.1144	2586.2375	1293.6224	I	**1277.6220**	639.3146	1260.5954	**630.8013**	1259.6114	**630.3093**	11
24	2717.3321	1359.1697	2700.3056	1350.6564	2699.3215	1350.1644	L	**1164.5379**	**582.7726**	1147.5113	574.2593	1146.5273	573.7673	10
25	2832.3591	1416.6832	2815.3325	1408.1699	2814.3485	1407.6779	D	**1051.4538**	**526.2305**	1034.4273	517.7173	1033.4433	517.2253	9
26	2946.4020	1473.7046	2929.3754	1465.1914	2928.3914	1464.6993	N	**936.4269**	468.7171	919.4003	460.2038	**918.4163**	459.7118	8
27	3075.4446	1538.2259	3058.4180	1529.7127	3057.4340	1529.2206	E	822.3840	411.6956	805.3574	403.1823	**804.3734**	402.6903	7
28	3190.4715	1595.7394	3173.4450	1587.2261	3172.4610	**1586.7341**	D	**693.3414**	347.1743	676.3148	338.6610	**675.3308**	338.1690	6
29	3277.5035	1639.2554	3260.4770	1630.7421	3259.4930	1630.2501	S	**578.3144**	289.6608	561.2879	281.1476	560.3039	280.6556	5
30	3392.5305	**1696.7689**	3375.5039	1688.2556	3374.5199	1687.7636	D	491.2824	246.1448	474.2558	237.6316	473.2718	237.1395	4
31	3493.5782	1747.2927	3476.5516	1738.7794	3475.5676	1738.2874	T	376.2554	188.6314	359.2289	180.1181	358.2449	179.6261	3
32	3621.6731	1811.3402	3604.6466	1802.8269	3603.6626	**1802.3349**	K	275.2078	138.1075	258.1812	129.5942			2

**33**							**K**	147.1128	74.0600	130.0863	65.5468			**1**

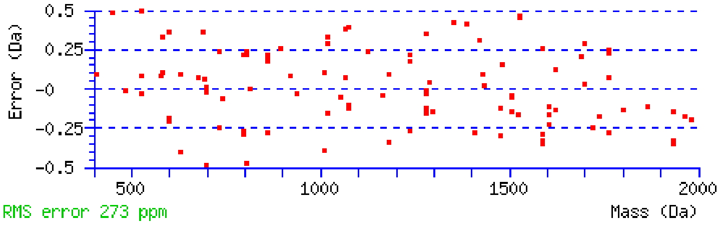

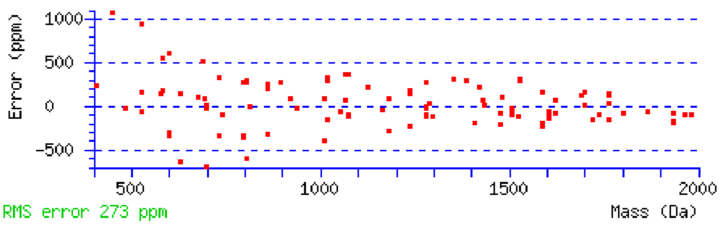

All matches to this query

Score	Mr(calc):	Delta	Sequence
98.6	3766.7714	0.0050	ELEQVDVSSEVNIVNPFALEPTILDNEDSDTKK
90.0	3766.7714	0.0050	ELEQVDVSSEVNIVNPFALEPTILDNEDSDTKK
13.1	3766.7714	0.0050	ELEQVDVSSEVNIVNPFALEPTILDNEDSDTKK
9.5	3766.7952	-0.0188	SGTVDPKARHLPESAGSPGQEGVSAVXAVENDSKPK
4.3	3765.7424	1.0340	GPNGYGFNLHSDKSKPGQFIRAVDPDSPAEASGLR
4.3	3765.7424	1.0340	GPNGYGFNLHSDKSKPGQFIRAVDPDSPAEASGLR
3.5	3766.7438	0.0325	VMCEDSLCSNLSVKNAIPTLILAALLSTVHLK
3.1	3764.7333	2.0431	SAPEPPTESPGHPALHPDDNTTFGRQSTPRVSFR
3.1	3764.7333	2.0431	SAPEPPTESPGHPALHPDDNTTFGRQSTPRVSFR
2.9	3764.7333	2.0431	SAPEPPTESPGHPALHPDDNTTFGRQSTPRVSFR

Spectrum No: 13; Query: 1323; Rank: 1

Peptide View

MS/MS Fragmentation of **SASSDTSEELNAQDSPK**
Found in **IPI00200898**, Tax_Id=10116 Gene_Symbol=Slc9a3r1 Ezrin-radixin-moesin-binding phosphoprotein 50

Match to Query 1323: 1844.731588 from(923.373070,2+)
Title: 091008RatKidney_NoSalt_27.1047.1047.2.dta
Data file K:\NewmanPaper\Piliang\3SubProteomes\Piliang3SP\mgf5ppm\ERLIC_3SubProteomes5ppm.mgf

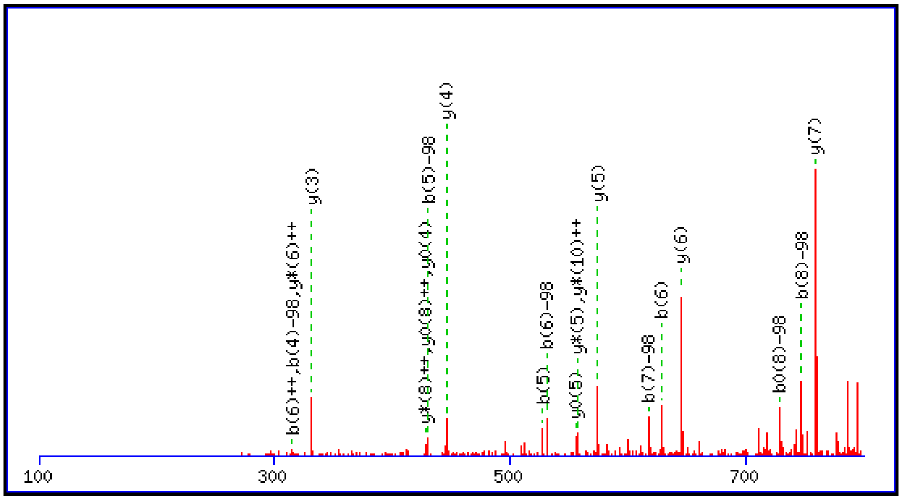

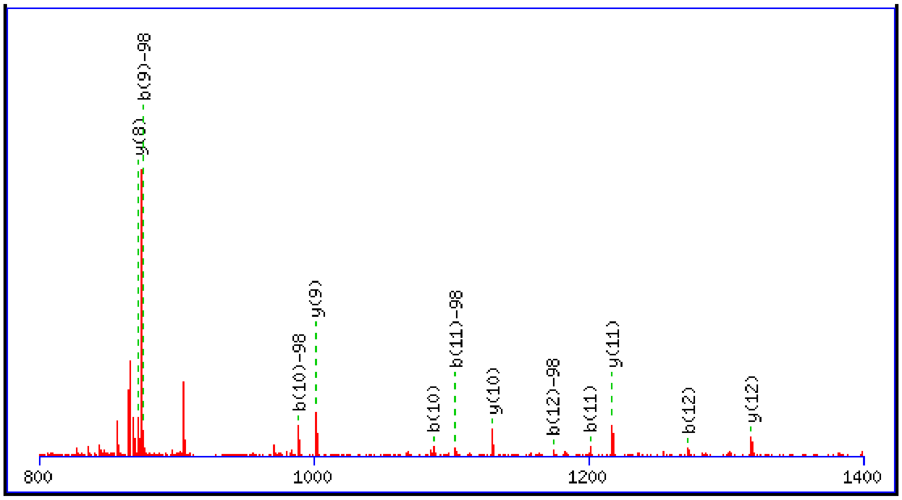

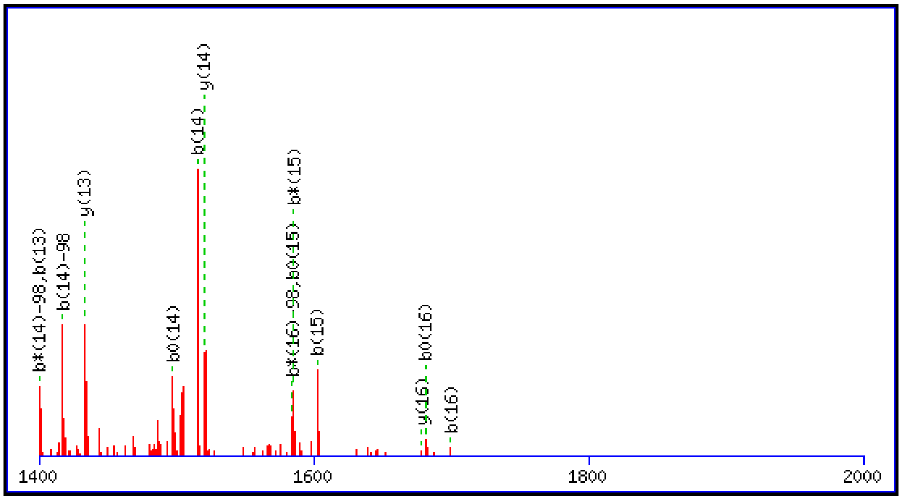

Monoisotopic mass of neutral peptide Mr(calc): 1844.7313
Fixed modifications: Carbamidomethyl (C)
Variable modifications:
S1 : Phospho (ST), with neutral losses 97.9769(shown in table), 0.0000
Ions Score: 97 **Expect:** 3e-008
Matches (Bold Red): 47/244 fragment ions using 65 most intense peaks

#	b	b^{++}	b*	b^{*++}	b^0	b^{0++}	Seq.	y	y^{++}	y*	y^{*++}	y^0	y^{0++}	#
1	70.0287	35.5180			52.0182	26.5127	S							17
2	141.0658	71.0366			123.0553	62.0313	A	**1678.7402**	839.8738	1661.7137	831.3605	1660.7297	830.8685	16
3	228.0979	114.5526			210.0873	105.5473	S	1607.7031	804.3552	1590.6766	795.8419	1589.6925	795.3499	15
4	**315.1299**	158.0686			297.1193	149.0633	S	**1520.6711**	760.8392	1503.6445	752.3259	1502.6605	751.8339	14
5	**430.1568**	215.5821			412.1463	206.5768	D	**1433.6391**	717.3232	1416.6125	708.8099	1415.6285	708.3179	13
6	**531.2045**	266.1059			513.1940	257.1006	T	**1318.6121**	659.8097	1301.5856	651.2964	1300.6016	650.8044	12
7	**618.2366**	309.6219			600.2260	300.6166	S	**1217.5644**	609.2859	1200.5379	600.7726	1199.5539	600.2806	11
8	**747.2791**	374.1432			**729.2686**	365.1379	E	**1130.5324**	565.7698	1113.5059	**557.2566**	1112.5218	556.7646	10
9	**876.3217**	438.6645			858.3112	429.6592	E	**1001.4898**	501.2485	984.4633	492.7353	983.4793	492.2433	9
10	**989.4058**	495.2065			971.3952	486.2013	L	**872.4472**	436.7272	855.4207	**428.2140**	854.4367	**427.7220**	8
11	**1103.4487**	552.2280	1086.4222	543.7147	1085.4382	543.2227	N	**759.3632**	380.1852	742.3366	371.6719	741.3526	371.1799	7
12	**1174.4858**	587.7466	1157.4593	579.2333	1156.4753	578.7413	A	**645.3202**	323.1638	628.2937	**314.6505**	627.3097	314.1585	6
13	1302.5444	651.7758	1285.5179	643.2626	1284.5339	642.7706	Q	**574.2831**	287.6452	**557.2566**	279.1319	**556.2726**	278.6399	5
14	**1417.5714**	709.2893	**1400.5448**	700.7760	1399.5608	700.2840	D	**446.2245**	223.6159	429.1980	215.1026	**428.2140**	214.6106	4
15	1504.6034	752.8053	1487.5768	744.2921	1486.5928	743.8001	S	**331.1976**	166.1024	314.1710	157.5892	313.1870	157.0972	3
16	1601.6562	801.3317	**1584.6296**	792.8184	1583.6456	792.3264	P	244.1656	122.5864	227.1390	114.0731			2
17							K	147.1128	74.0600	130.0863	65.5468			1

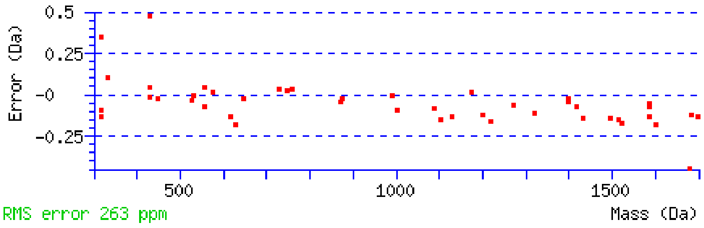

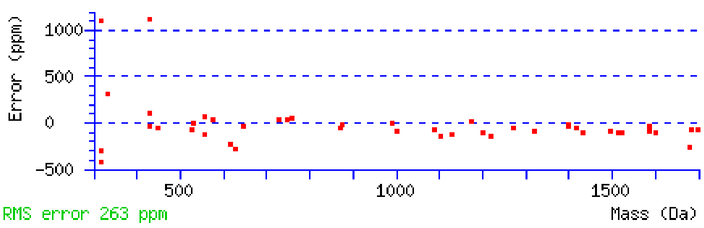

All matches to this query

Score	Mr(calc):	Delta	Sequence
97.0	1844.7313	0.0003	SASSDTSEELNAQDSPK
83.6	1844.7313	0.0003	SASSDTSEELNAQDSPK
78.0	1844.7313	0.0003	SASSDTSEELNAQDSPK
52.1	1844.7313	0.0003	SASSDTSEELNAQDSPK
40.2	1844.7313	0.0003	SASSDTSEELNAQDSPK
14.7	1842.7421	1.9895	ASGDFGSGLEESSNLHR
6.9	1842.7421	1.9895	ASGDFGSGLEESSNLHR
5.6	1843.7100	1.0216	NQSYLASSEPCSLTK
5.6	1843.7100	1.0216	NQSYLASSEPCSLTK
5.2	1843.7139	1.0177	ESNPNVSQNSTNHKK

Spectrum No: 14; Query: 1302; Rank: 1

Peptide View

MS/MS Fragmentation of **SSSVGSSSSYPISSAVPR**
Found in **IPI00209000**, Tax_Id=10116 Gene_Symbol=Plec1 Plectin 6

Match to Query 1302: 1833.814348 from(917.914450,2+)
Title: 091008RatKidney_NH4Format01_22.2154.2154.2.dta
Data file K:\NewmanPaper\Piliang\3SubProteomes\Piliang3SP\mgf5ppm\ERLIC_3SubProteomes5ppm.mgf

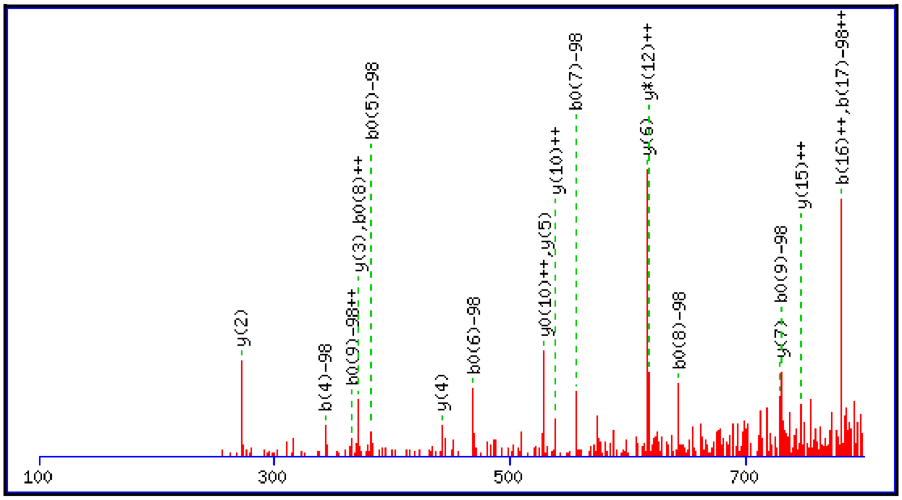

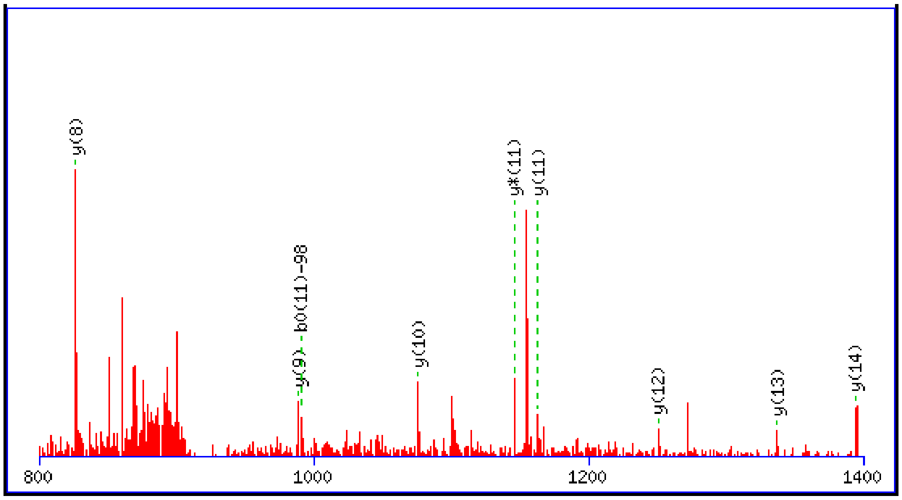

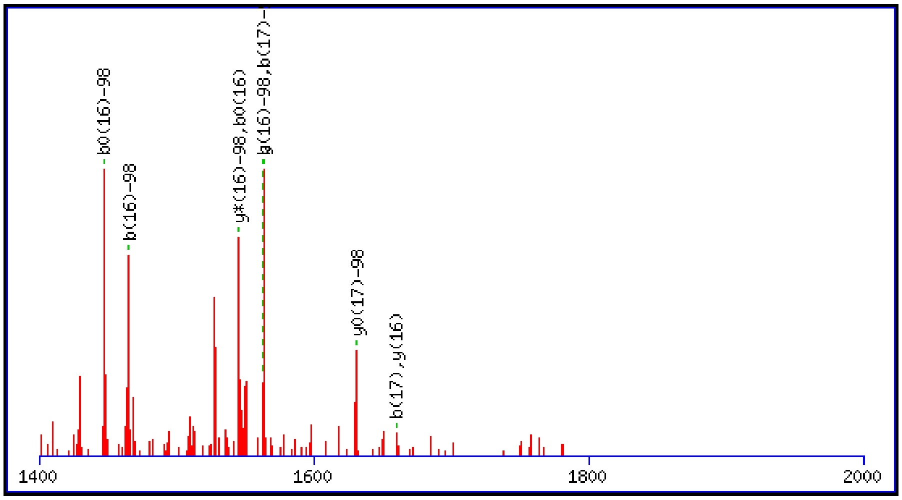

Monoisotopic mass of neutral peptide Mr(calc): 1833.8146
Fixed modifications: Carbamidomethyl (C)
Variable modifications:
S3 : Phospho (ST), with neutral losses 97.9769(shown in table), 0.0000
Ions Score: 96 **Expect:** 5.3e-008
Matches (Bold Red): 40/234 fragment ions using 70 most intense peaks

#	b	b^{++}	b^{0}	b^{0++}	Seq.	y	y^{++}	y^{*}	y^{*++}	y^{0}	y^{0++}	#
1	88.0393	44.5233	70.0287	35.5180	S							18
2	175.0713	88.0393	157.0608	79.0340	S	1649.8129	825.4101	1632.7864	816.8968	**1631.8024**	816.4048	17
3	244.0928	122.5500	226.0822	113.5447	S	**1562.7809**	**781.8941**	**1545.7544**	773.3808	1544.7703	772.8888	16
4	**343.1612**	172.0842	325.1506	163.0790	V	1493.7594	**747.3834**	1476.7329	738.8701	1475.7489	738.3781	15
5	400.1827	200.5950	**382.1721**	191.5897	G	**1394.6910**	697.8492	1377.6645	689.3359	1376.6805	688.8439	14
6	487.2147	244.1110	**469.2041**	235.1057	S	**1337.6696**	669.3384	1320.6430	660.8251	1319.6590	660.3331	13
7	574.2467	287.6270	**556.2362**	278.6217	S	**1250.6375**	625.8224	1233.6110	**617.3091**	1232.6270	616.8171	12
8	661.2787	331.1430	**643.2682**	322.1377	S	**1163.6055**	582.3064	**1146.5790**	573.7931	1145.5949	573.3011	11
9	748.3108	374.6590	**730.3002**	**365.6537**	S	**1076.5735**	**538.7904**	1059.5469	530.2771	1058.5629	**529.7851**	10
10	911.3741	456.1907	893.3635	447.1854	Y	**989.5415**	495.2744	972.5149	486.7611	971.5309	486.2691	9
11	1008.4269	504.7171	**990.4163**	495.7118	P	**826.4781**	413.7427	809.4516	405.2294	808.4676	404.7374	8
12	1121.5109	561.2591	1103.5004	552.2538	I	**729.4254**	365.2163	712.3988	356.7030	711.4148	356.2110	7
13	1208.5430	604.7751	1190.5324	595.7698	S	**616.3413**	308.6743	599.3148	300.1610	598.3307	299.6690	6
14	1295.5750	648.2911	1277.5644	639.2859	S	**529.3093**	265.1583	512.2827	256.6450	511.2987	256.1530	5
15	1366.6121	683.8097	1348.6015	674.8044	A	**442.2772**	221.6423	425.2507	213.1290			4
16	**1465.6805**	733.3439	**1447.6700**	724.3386	V	**371.2401**	186.1237	354.2136	177.6104			3
17	**1562.7333**	**781.8703**	1544.7227	772.8650	P	**272.1717**	136.5895	255.1452	128.0762			2
18					R	175.1190	88.0631	158.0924	79.5498			1

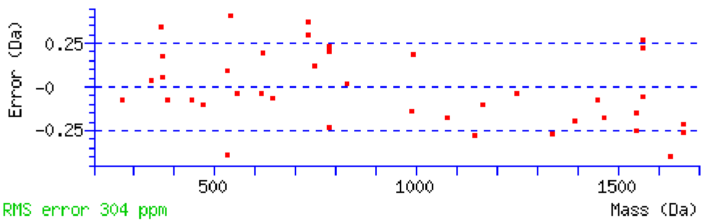

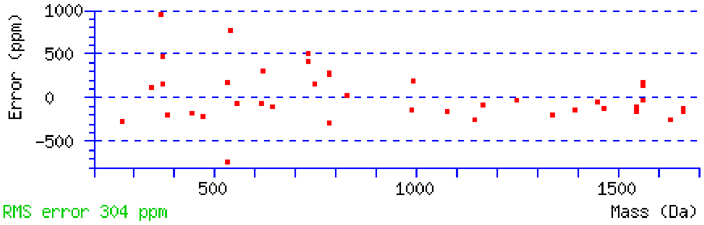

All matches to this query

Score	Mr(calc):	Delta	Sequence
95.9	1833.8146	-0.0002	SSSVGSSSSYPISSAVPR
77.3	1833.8146	-0.0002	SSSVGSSSSYPISSAVPR
77.3	1833.8146	-0.0002	SSSVGSSSSYPISSAVPR
54.6	1833.8146	-0.0002	SSSVGSSSSYPISSAVPR
52.4	1833.8146	-0.0002	SSSVGSSSSYPISSAVPR
38.6	1833.8146	-0.0002	SSSVGSSSSYPISSAVPR
38.6	1833.8146	-0.0002	SSSVGSSSSYPISSAVPR
28.6	1833.8146	-0.0002	SSSVGSSSSYPISSAVPR
6.7	1833.8006	0.0137	NRAGSPNPQSSSGELPR
5.0	1833.7977	0.0167	RDSDSDADEATPTTTPR

Spectrum No: 15; Query: 1327; Rank: 1

Peptide View

MS/MS Fragmentation of **SASSDTSEELNAQDSPK**
Found in **IPI00200898**, Tax_Id=10116 Gene_Symbol=Slc9a3r1 Ezrin-radixin-moesin-binding phosphoprotein 50

Match to Query 1327: 1844.736868 from(923.375710,2+)
Title: 100101RatKid_NS_deglyco_23.1163.1163.2.dta
Data file K:\NewmanPaper\Piliang\3SubProteomes\Piliang3SP\mgf5ppm\ERLIC_3SubProteomes5ppm.mgf

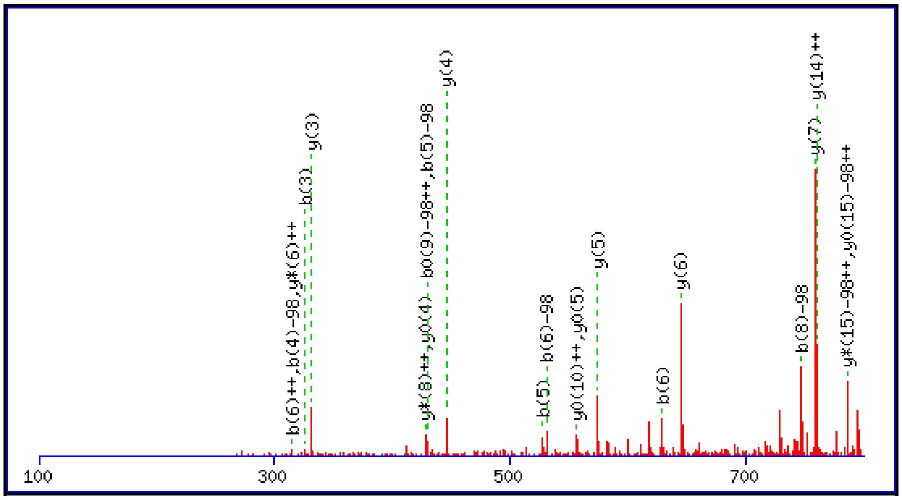

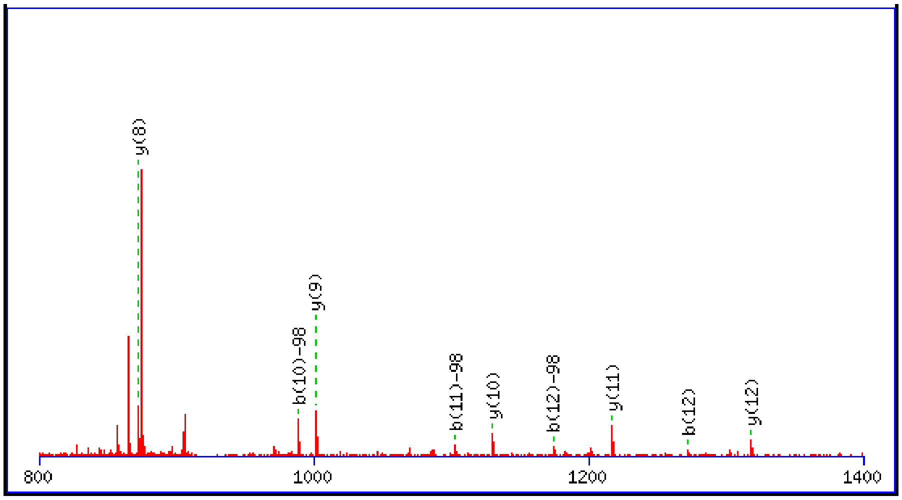

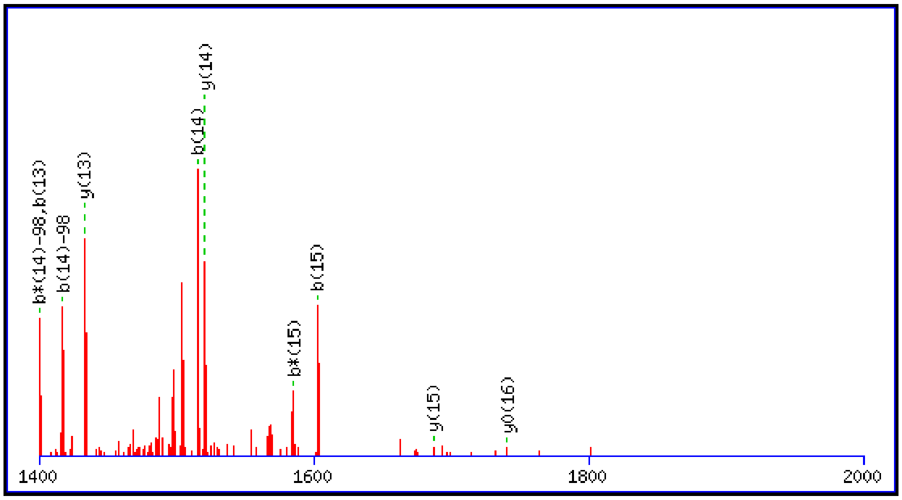

Monoisotopic mass of neutral peptide Mr(calc): 1844.7313
Fixed modifications: Carbamidomethyl (C)
Variable modifications:
S3 : Phospho (ST), with neutral losses 97.9769(shown in table), 0.0000
Ions Score: 94 **Expect:** 5.7e-008
Matches (Bold Red): 41/248 fragment ions using 48 most intense peaks

#	b	b^{++}	b*	b^{*++}	b^0	b^{0++}	Seq.	y	y^{++}	y*	y^{*++}	y^0	y^{0++}	#
1	88.0393	44.5233			70.0287	35.5180	S							17
2	159.0764	80.0418			141.0659	71.0366	A	1660.7297	830.8685	1643.7031	822.3552	1642.7191	821.8632	16
3	228.0979	114.5526			210.0873	105.5473	S	1589.6925	795.3499	1572.6660	**786.8366**	1571.6820	**786.3446**	15
4	**315.1299**	158.0686			297.1193	149.0633	S	**1520.6711**	**760.8392**	1503.6445	752.3259	1502.6605	751.8339	14
5	**430.1568**	215.5821			412.1463	206.5768	D	**1433.6391**	717.3232	1416.6125	708.8099	1415.6285	708.3179	13
6	**531.2045**	266.1059			513.1940	257.1006	T	**1318.6121**	659.8097	1301.5856	651.2964	1300.6016	650.8044	12
7	618.2366	309.6219			600.2260	300.6166	S	**1217.5644**	609.2859	1200.5379	600.7726	1199.5539	600.2806	11
8	**747.2791**	374.1432			729.2686	365.1379	E	**1130.5324**	565.7698	1113.5059	557.2566	1112.5218	**556.7646**	10
9	876.3217	438.6645			858.3112	**429.6592**	E	**1001.4898**	501.2485	984.4633	492.7353	983.4793	492.2433	9
10	**989.4058**	495.2065			971.3952	486.2013	L	**872.4472**	436.7272	855.4207	**428.2140**	854.4367	427.7220	8
11	**1103.4487**	552.2280	1086.4222	543.7147	1085.4382	543.2227	N	**759.3632**	380.1852	742.3366	371.6719	741.3526	371.1799	7
12	**1174.4858**	587.7466	1157.4593	579.2333	1156.4753	578.7413	A	**645.3202**	323.1638	628.2937	**314.6505**	627.3097	314.1585	6
13	1302.5444	651.7758	1285.5179	643.2626	1284.5339	642.7706	Q	**574.2831**	287.6452	557.2566	279.1319	**556.2726**	278.6399	5
14	**1417.5714**	709.2893	**1400.5448**	700.7760	1399.5608	700.2840	D	**446.2245**	223.6159	429.1980	215.1026	**428.2140**	214.6106	4
15	1504.6034	752.8053	1487.5768	744.2921	1486.5928	743.8001	S	**331.1976**	166.1024	314.1710	157.5892	313.1870	157.0972	3
16	1601.6562	801.3317	1584.6296	792.8184	1583.6456	792.3264	P	244.1656	122.5864	227.1390	114.0731			2
17							K	147.1128	74.0600	130.0863	65.5468			1

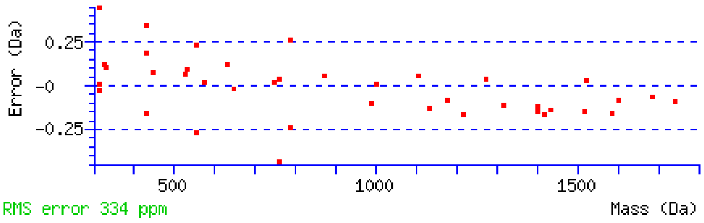

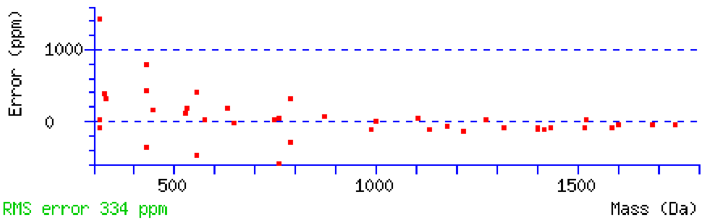

All matches to this query

Score	Mr(calc):	Delta	Sequence
94.4	1844.7313	0.0056	SASSDTSEELNAQDSPK
90.4	1844.7313	0.0056	SASSDTSEELNAQDSPK
84.5	1844.7313	0.0056	SASSDTSEELNAQDSPK
57.8	1844.7313	0.0056	SASSDTSEELNAQDSPK
46.0	1844.7313	0.0056	SASSDTSEELNAQDSPK
13.6	1842.7421	1.9947	ASGDFGSGLEESSNLHR
6.3	1842.7421	1.9947	ASGDFGSGLEESSNLHR
6.0	1842.7438	1.9931	FTLSSELEEERTSR
6.0	1842.7438	1.9931	FTLSSELEEERTSR
3.0	1844.7434	-0.0065	GDGDLSCINGDMEVRK

Spectrum No: 16; Query: 2293; Rank: 1

Peptide View

MS/MS Fragmentation of **LKGQEDNLASAVDATTGQEACDSD**
Found in **IPI00205868**, Tax_Id=10116 Gene_Symbol=Ccdc12_predicted coiled-coil domain containing 12

Match to Query 2293: 2574.050288 from(1288.032420,2+)
Title: 100101RatKid_NS_deglyco_26.2577.2577.2.dta
Data file K:\NewmanPaper\Piliang\3SubProteomes\Piliang3SP\mgf5ppm\ERLIC_3SubProteomes5ppm.mgf

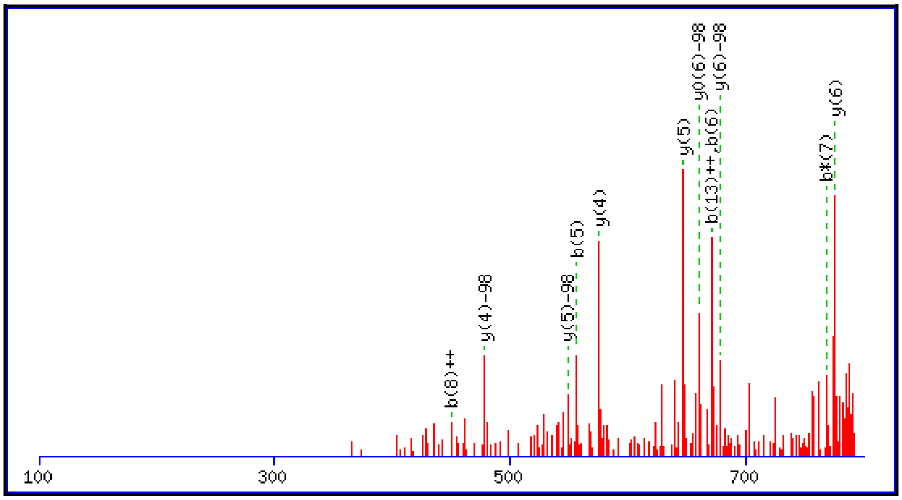

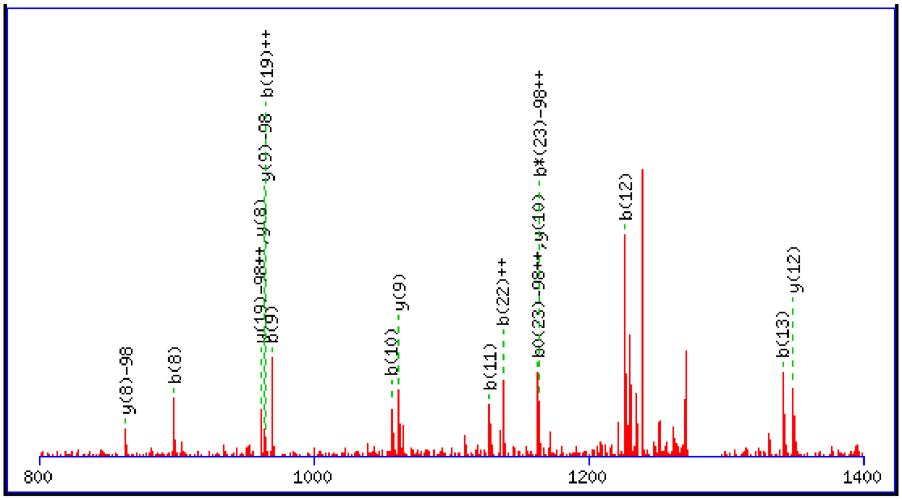

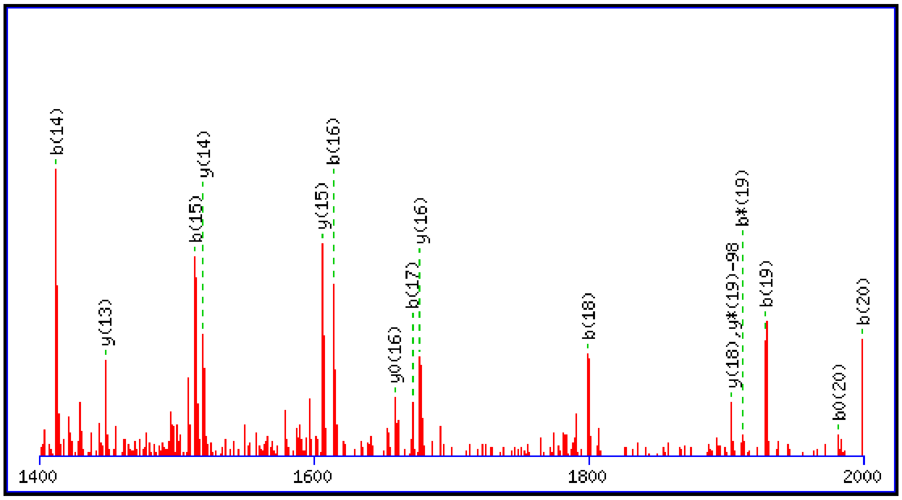

Monoisotopic mass of neutral peptide Mr(calc): 2574.0429
Fixed modifications: Carbamidomethyl (C)
Variable modifications:
S23 : Phospho (ST), with neutral losses 97.9769(shown in table), 0.0000
Ions Score: 94 **Expect:** 1.1e-007
Matches (Bold Red): 45/382 fragment ions using 72 most intense peaks

#	b	b^{++}	b*	b^{*++}	b^{0}	b^{0++}	Seq.	y	y^{++}	y*	y^{*++}	y^{0}	y^{0++}	#
1	114.0913	57.5493					L							24
2	242.1863	121.5968	225.1598	113.0835			K	2363.9892	1182.4982	2346.9626	1173.9850	2345.9786	1173.4930	23
3	299.2078	150.1075	282.1812	141.5942			G	2235.8942	1118.4508	2218.8677	1109.9375	2217.8837	1109.4455	22
4	427.2663	214.1368	410.2398	205.6235			Q	2178.8728	1089.9400	2161.8462	1081.4267	2160.8622	1080.9347	21
5	**556.3089**	278.6581	539.2824	270.1448	538.2984	269.6528	E	2050.8142	1025.9107	2033.7876	1017.3975	2032.8036	1016.9055	20
6	**671.3359**	336.1716	654.3093	327.6583	653.3253	327.1663	D	1921.7716	**961.3894**	**1904.7451**	952.8762	1903.7610	952.3842	19
7	785.3788	393.1930	**768.3523**	384.6798	767.3682	384.1878	N	1806.7447	903.8760	1789.7181	895.3627	1788.7341	894.8707	18
8	**898.4629**	**449.7351**	881.4363	441.2218	880.4523	440.7298	L	1692.7017	846.8545	1675.6752	838.3412	1674.6912	837.8492	17
9	**969.5000**	485.2536	952.4734	476.7404	951.4894	476.2483	A	1579.6177	790.3125	1562.5911	781.7992	1561.6071	781.3072	16
10	**1056.5320**	528.7696	1039.5055	520.2564	1038.5214	519.7644	S	1508.5806	754.7939	1491.5540	746.2806	1490.5700	745.7886	15
11	**1127.5691**	564.2882	1110.5426	555.7749	1109.5586	555.2829	A	1421.5485	711.2779	1404.5220	702.7646	1403.5380	702.2726	14
12	**1226.6375**	613.8224	1209.6110	605.3091	1208.6270	604.8171	V	1350.5114	675.7593	1333.4849	667.2461	1332.5008	666.7541	13
13	**1341.6645**	**671.3359**	1324.6379	662.8226	1323.6539	662.3306	D	1251.4430	626.2251	1234.4164	617.7119	1233.4324	617.2199	12
14	**1412.7016**	706.8544	1395.6751	698.3412	1394.6910	697.8492	A	1136.4161	568.7117	1119.3895	560.1984	1118.4055	559.7064	11
15	**1513.7493**	757.3783	1496.7227	748.8650	1495.7387	748.3730	T	1065.3789	533.1931	1048.3524	524.6798	1047.3684	524.1878	10
16	**1614.7970**	807.9021	1597.7704	799.3888	1596.7864	798.8968	T	**964.3313**	482.6693	947.3047	474.1560	946.3207	473.6640	9
17	**1671.8184**	836.4128	1654.7919	827.8996	1653.8079	827.4076	G	**863.2836**	432.1454	846.2570	423.6322	845.2730	423.1401	8
18	**1799.8770**	900.4421	1782.8505	891.9289	1781.8664	891.4369	Q	806.2621	403.6347	789.2356	395.1214	788.2516	394.6294	7

19	**1928.9196**	**964.9634**	**1911.8930**	956.4502	1910.9090	955.9582	E	**678.2035**	339.6054			**660.1930**	330.6001	6
20	**1999.9567**	1000.4820	1982.9302	991.9687	**1981.9461**	991.4767	A	**549.1609**	275.0841			531.1504	266.0788	5
21	2159.9874	1080.4973	2142.9608	1071.9840	2141.9768	1071.4920	C	**478.1238**	239.5656			460.1133	230.5603	4
22	2275.0143	**1138.0108**	2257.9877	1129.4975	2257.0037	1129.0055	D	318.0932	159.5502			300.0826	150.5449	3
23	2344.0358	1172.5215	2327.0092	**1164.0082**	2326.0252	**1163.5162**	S	203.0662	102.0368			185.0557	93.0315	2
24							D	134.0448	67.5260			116.0342	58.5207	1

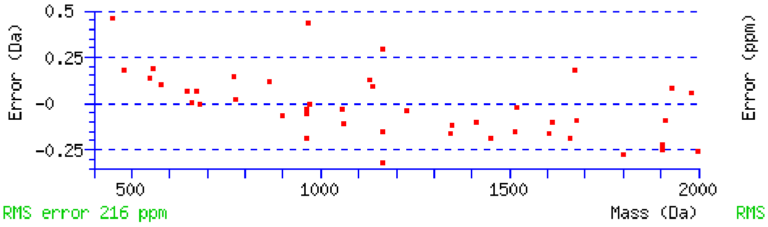

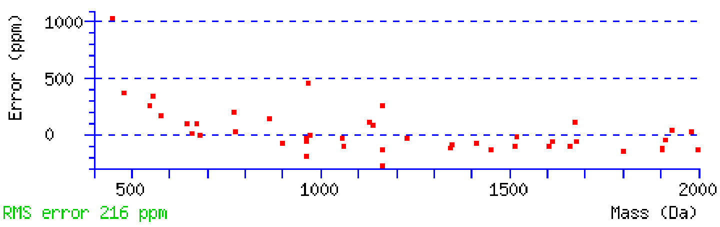

All matches to this query

Score	Mr(calc):	Delta	Sequence
93.7	2574.0429	0.0074	LKGQEDNLASAVDATTGQEACDSD
49.6	2574.0429	0.0074	LKGQEDNLASAVDATTGQEACDSD
49.5	2574.0429	0.0074	LKGQEDNLASAVDATTGQEACDSD
18.8	2574.0429	0.0074	LKGQEDNLASAVDATTGQEACDSD
3.9	2572.0407	2.0096	EEDSGEWKTTSEAATPNRYLK

Spectrum No: 17; Query: 2240; Rank: 1

Peptide View

MS/MS Fragmentation of **RASVCAEAYNPDEEEDDAESR**
Found in **IPI00365600**, Tax_Id=10116 Gene_Symbol=Prkar2b cAMP-dependent protein kinase type II-beta regulatory subunit

Match to Query 2240: 2492.956088 from(1247.485320,2+)
Title: 100101RatKid_NS_deglyco_22.1640.1640.2.dta
Data file K:\NewmanPaper\Piliang\3SubProteomes\Piliang3SP\mgf5ppm\ERLIC_3SubProteomes5ppm.mgf

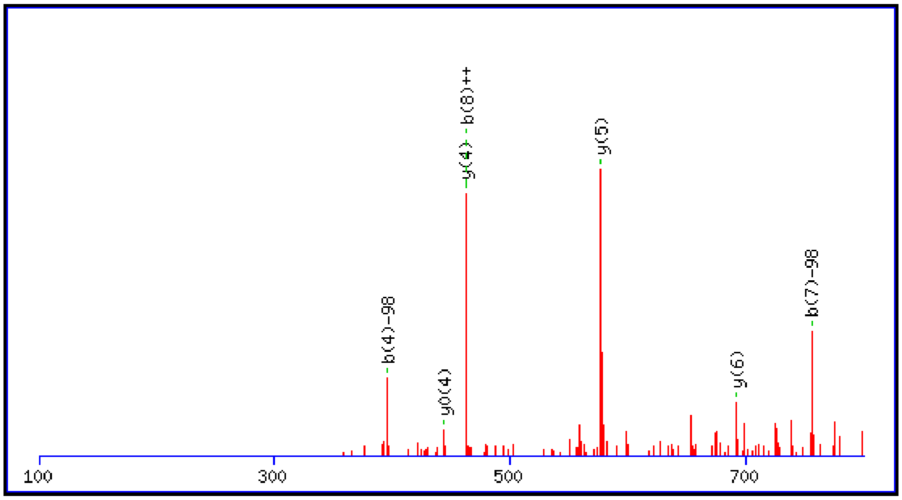

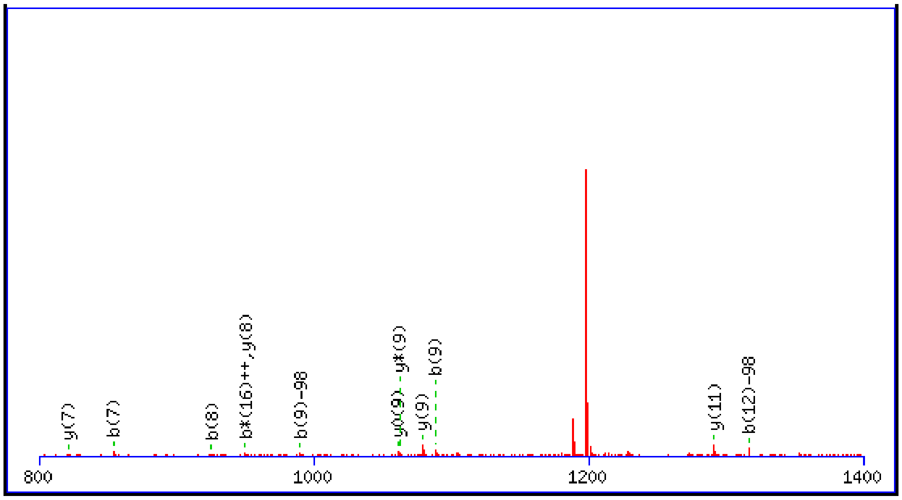

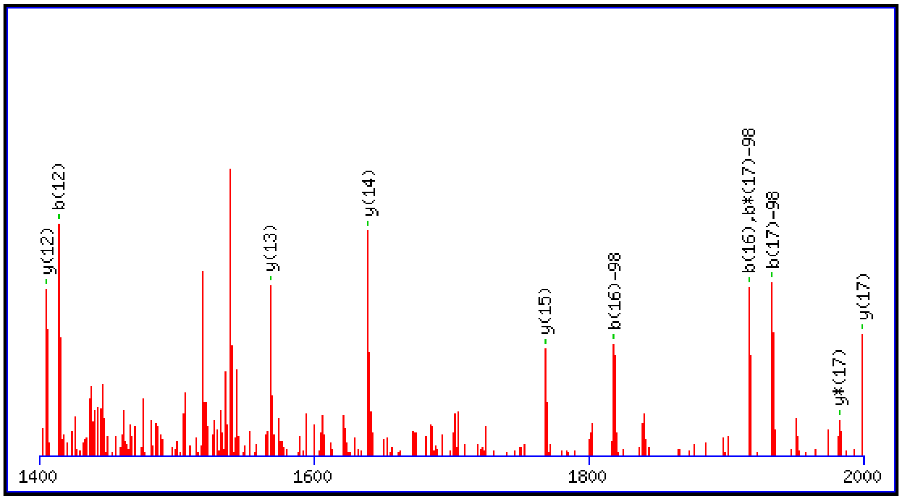

Monoisotopic mass of neutral peptide Mr(calc): 2491.9435
Fixed modifications: Carbamidomethyl (C)
Variable modifications:
S3 : Phospho (ST), with neutral losses 97.9769(shown in table), 0.0000
Ions Score: 92 **Expect:** 1.2e-007
Matches (Bold Red): 30/354 fragment ions using 42 most intense peaks

#	b	b^{++}	b*	b^{*++}	b^{0}	b^{0++}	Seq.	y	y^{++}	y*	y^{*++}	y^{0}	y^{0++}	#
1	157.1084	79.0578	140.0818	70.5446			R							21
2	228.1455	114.5764	211.1190	106.0631			A	2238.8728	1119.9400	2221.8462	1111.4267	2220.8622	1110.9347	20
3	297.1670	149.0871	280.1404	140.5738	279.1564	140.0818	S	2167.8357	1084.4215	2150.8091	1075.9082	2149.8251	1075.4162	19
4	**396.2354**	198.6213	379.2088	190.1080	378.2248	189.6160	V	2098.8142	1049.9107	2081.7876	1041.3975	2080.8036	1040.9055	18
5	556.2660	278.6366	539.2395	270.1234	538.2555	269.6314	C	**1999.7458**	1000.3765	**1982.7192**	991.8633	1981.7352	991.3712	17
6	627.3031	314.1552	610.2766	305.6419	609.2926	305.1499	A	1839.7151	920.3612	1822.6886	911.8479	1821.7046	911.3559	16
7	**756.3457**	378.6765	739.3192	370.1632	738.3352	369.6712	E	**1768.6780**	884.8426	1751.6515	876.3294	1750.6675	875.8374	15
8	827.3828	414.1951	810.3563	405.6818	809.3723	405.1898	A	**1639.6354**	820.3214	1622.6089	811.8081	1621.6249	811.3161	14
9	**990.4462**	495.7267	973.4196	487.2134	972.4356	486.7214	Y	**1568.5983**	784.8028	1551.5718	776.2895	1550.5878	775.7975	13
10	1104.4891	552.7482	1087.4625	544.2349	1086.4785	543.7429	N	**1405.5350**	703.2711	1388.5084	694.7579	1387.5244	694.2658	12
11	1201.5419	601.2746	1184.5153	592.7613	1183.5313	592.2693	P	**1291.4921**	646.2497	1274.4655	637.7364	1273.4815	637.2444	11
12	**1316.5688**	658.7880	1299.5423	650.2748	1298.5582	649.7828	D	1194.4393	597.7233	1177.4127	589.2100	1176.4287	588.7180	10
13	1445.6114	723.3093	1428.5848	714.7961	1427.6008	714.3041	E	**1079.4124**	540.2098	**1062.3858**	531.6965	**1061.4018**	531.2045	9
14	1574.6540	787.8306	1557.6274	779.3174	1556.6434	778.8254	E	**950.3698**	475.6885	933.3432	467.1752	932.3592	466.6832	8
15	1703.6966	852.3519	1686.6700	843.8387	1685.6860	843.3466	E	**821.3272**	411.1672	804.3006	402.6539	803.3166	402.1619	7
16	**1818.7235**	909.8654	1801.6970	901.3521	1800.7130	900.8601	D	**692.2846**	346.6459	675.2580	338.1327	674.2740	337.6406	6
17	**1933.7505**	967.3789	**1916.7239**	958.8656	1915.7399	958.3736	D	**577.2576**	289.1325	560.2311	280.6192	559.2471	280.1272	5
18	2004.7876	1002.8974	1987.7610	994.3842	1986.7770	993.8921	A	**462.2307**	231.6190	445.2041	223.1057	**444.2201**	222.6137	4

**19**	2133.8302	1067.4187	2116.8036	1058.9055	2115.8196	1058.4134	**E**	391.1936	196.1004	374.1670	187.5872	373.1830	187.0951	**3**
**20**	2220.8622	1110.9347	2203.8357	1102.4215	2202.8516	1101.9295	**S**	262.1510	131.5791	245.1244	123.0659	244.1404	122.5738	**2**
**21**							**R**	175.1190	88.0631	158.0924	79.5498			**1**

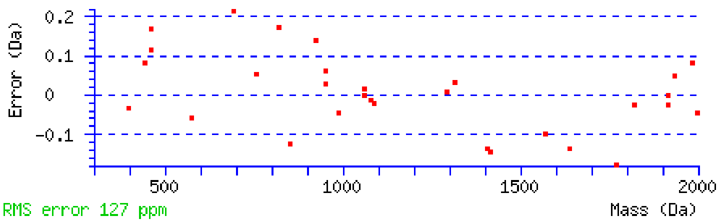

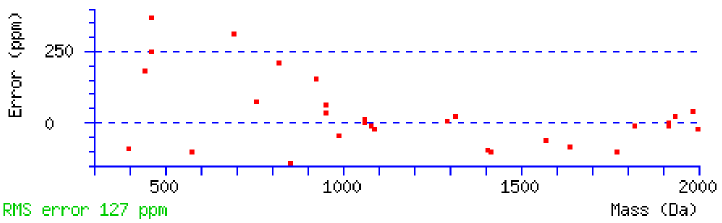

All matches to this query

Score	Mr(calc):	Delta	Sequence
92.3	2491.9435	1.0126	RASVCAEAYNPDEEEDDAESR
25.0	2491.9435	1.0126	RASVCAEAYNPDEEEDDAESR
5.5	2492.9501	0.0060	VESMGNGYGYLQVENMGNGYGR
3.9	2492.9781	-0.0220	TSSHLANDHRCPSQTYXSSTK
3.6	2492.9725	-0.0164	VKHNQENFSSVLYKENMGK
3.5	2491.9685	0.9876	VGGFNLPYTECYDPVTGEWK
3.1	2492.9781	-0.0220	TSSHLANDHRCPSQTYXSSTK
2.3	2492.9709	-0.0148	ESYDYQAACAPQPPGNLGAAPR
2.2	2491.9286	1.0275	LSTQPSCNSEHLIVFNGIR
2.2	2492.9709	-0.0148	ESYDYQAACAPQPPGNLGAAPR

Spectrum No: 18; Query: 2719; Rank: 1

Peptide View

MS/MS Fragmentation of **NEAAVPAAAAGSPVPVIAIPGILEDELEQTDAEAK**
Found in **IPI00204776**, Tax_Id=10116 Gene_Symbol=Akap8 A-kinase anchor protein 8

Match to Query 2719: 3535.743612 from(1179.588480,3+)
Title: 100101RatKid_NS_deglyco_18.6619.6619.3.dta
Data file K:\NewmanPaper\Piliang\3SubProteomes\Piliang3SP\mgf5ppm\ERLIC_3SubProteomes5ppm.mgf

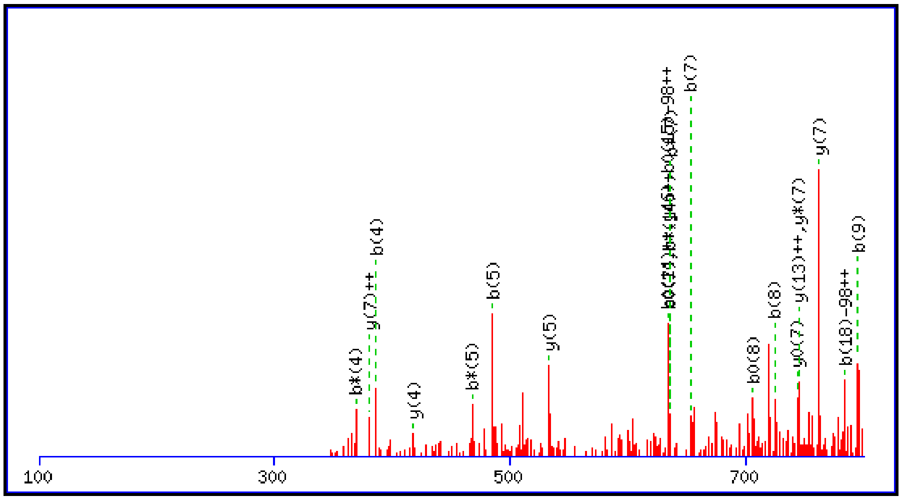

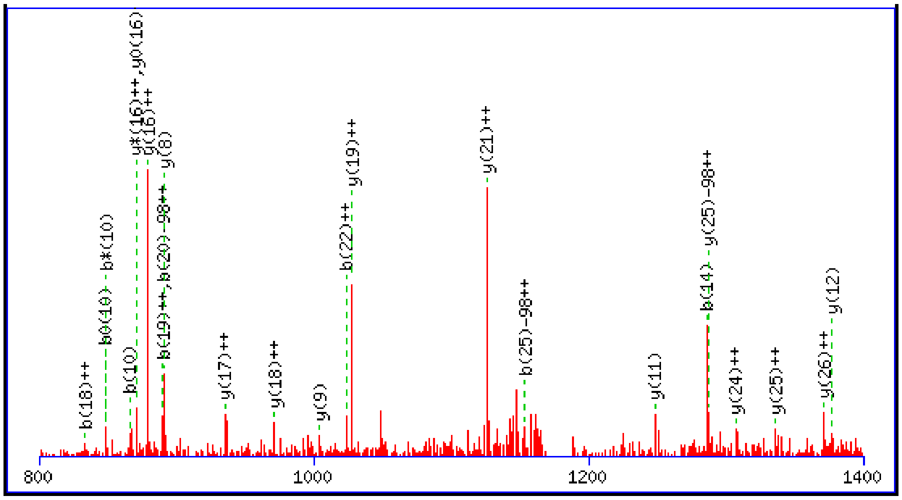

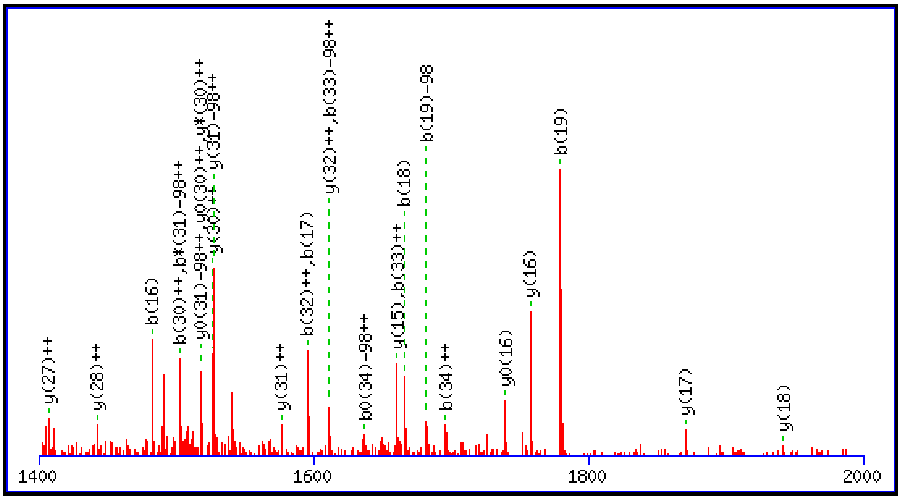

Monoisotopic mass of neutral peptide Mr(calc): 3535.7334
Fixed modifications: Carbamidomethyl (C)
Variable modifications:
S12 : Phospho (ST), with neutral losses 0.0000(shown in table), 97.9769
Ions Score: 91 **Expect:** 1.9e-007
Matches (Bold Red): 72/606 fragment ions using 99 most intense peaks

#	b	b^{++}	b*	b^{*++}	b^0	b^{0++}	Seq.	y	y^{++}	y*	y^{*++}	y^0	y^{0++}	#
1	115.0502	58.0287	98.0237	49.5155			N							35
2	244.0928	122.5500	227.0662	114.0368	226.0822	113.5448	E	3422.6978	1711.8526	3405.6713	1703.3393	3404.6873	1702.8473	34
3	315.1299	158.0686	298.1034	149.5553	297.1193	149.0633	A	3293.6552	1647.3313	3276.6287	1638.8180	3275.6447	1638.3260	33
4	**386.1670**	193.5872	**369.1405**	185.0739	368.1565	184.5819	A	3222.6181	**1611.8127**	3205.5916	1603.2994	3204.6076	1602.8074	32
5	**485.2354**	243.1214	**468.2089**	234.6081	467.2249	234.1161	V	3151.5810	**1576.2941**	3134.5545	1567.7809	3133.5704	1567.2889	31
6	582.2882	291.6477	565.2617	283.1345	564.2776	282.6425	P	3052.5126	**1526.7599**	3035.4861	**1518.2467**	3034.5020	**1517.7547**	30
7	**653.3253**	327.1663	**636.2988**	318.6530	**635.3148**	318.1610	A	2955.4598	1478.2336	2938.4333	1469.7203	2937.4493	1469.2283	29
8	**724.3624**	362.6849	707.3359	354.1716	**706.3519**	353.6796	A	2884.4227	**1442.7150**	2867.3962	1434.2017	2866.4122	1433.7097	28
9	**795.3995**	398.2034	778.3730	389.6901	777.3890	389.1981	A	2813.3856	**1407.1964**	2796.3591	1398.6832	2795.3750	1398.1912	27
10	**866.4367**	433.7220	**849.4101**	425.2087	**848.4261**	424.7167	A	2742.3485	**1371.6779**	2725.3219	1363.1646	2724.3379	1362.6726	26
11	923.4581	462.2327	906.4316	453.7194	905.4476	453.2274	G	2671.3114	**1336.1593**	2654.2848	1327.6461	2653.3008	1327.1540	25
12	1090.4565	545.7319	1073.4299	537.2186	1072.4459	536.7266	S	2614.2899	**1307.6486**	2597.2634	1299.1353	2596.2794	1298.6433	24
13	1187.5092	594.2583	1170.4827	585.7450	1169.4987	585.2530	P	2447.2916	1224.1494	2430.2650	1215.6361	2429.2810	1215.1441	23
14	**1286.5777**	643.7925	1269.5511	**635.2792**	1268.5671	**634.7872**	V	2350.2388	1175.6230	2333.2123	1167.1098	2332.2282	1166.6178	22
15	1383.6304	692.3188	1366.6039	683.8056	1365.6199	683.3136	P	2251.1704	**1126.0888**	2234.1438	1117.5756	2233.1598	1117.0835	21
16	**1482.6988**	741.8531	1465.6723	733.3398	1464.6883	732.8478	V	2154.1176	1077.5624	2137.0911	1069.0492	2136.1071	1068.5572	20
17	**1595.7829**	798.3951	1578.7563	789.8818	1577.7723	789.3898	I	2055.0492	**1028.0282**	2038.0227	1019.5150	2037.0386	1019.0230	19
18	**1666.8200**	**833.9136**	1649.7935	825.4004	1648.8094	824.9084	A	**1941.9651**	**971.4862**	1924.9386	962.9729	1923.9546	962.4809	18

19	1779.9041	890.4557	1762.8775	881.9424	1761.8935	881.4504	I	1870.9280	935.9677	1853.9015	927.4544	1852.9175	926.9624	17
20	1876.9568	938.9821	1859.9303	930.4688	1858.9463	929.9768	P	1757.8440	879.4256	1740.8174	870.9123	1739.8334	870.4203	16
21	1933.9783	967.4928	1916.9518	958.9795	1915.9677	958.4875	G	1660.7912	830.8992	1643.7647	822.3860	1642.7806	821.8940	15
22	2047.0624	1024.0348	2030.0358	1015.5215	2029.0518	1015.0295	I	1603.7697	802.3885	1586.7432	793.8752	1585.7592	793.3832	14
23	2160.1464	1080.5769	2143.1199	1072.0636	2142.1359	1071.5716	L	1490.6857	745.8465	1473.6591	737.3332	1472.6751	736.8412	13
24	2289.1890	1145.0981	2272.1625	1136.5849	2271.1785	1136.0929	E	1377.6016	689.3044	1360.5751	680.7912	1359.5910	680.2992	12
25	2404.2160	1202.6116	2387.1894	1194.0983	2386.2054	1193.6063	D	1248.5590	624.7831	1231.5325	616.2699	1230.5485	615.7779	11
26	2533.2586	1267.1329	2516.2320	1258.6196	2515.2480	1258.1276	E	1133.5321	567.2697	1116.5055	558.7564	1115.5215	558.2644	10
27	2646.3426	1323.6749	2629.3161	1315.1617	2628.3321	1314.6697	L	1004.4895	502.7484	987.4629	494.2351	986.4789	493.7431	9
28	2775.3852	1388.1962	2758.3587	1379.6830	2757.3746	1379.1910	E	891.4054	446.2063	874.3789	437.6931	873.3949	437.2011	8
29	2903.4438	1452.2255	2886.4172	1443.7123	2885.4332	1443.2203	Q	762.3628	381.6851	745.3363	373.1718	744.3523	372.6798	7
30	3004.4915	1502.7494	2987.4649	1494.2361	2986.4809	1493.7441	T	634.3042	317.6558	617.2777	309.1425	616.2937	308.6505	6
31	3119.5184	1560.2628	3102.4919	1551.7496	3101.5078	1551.2576	D	533.2566	267.1319	516.2300	258.6186	515.2460	258.1266	5
32	3190.5555	1595.7814	3173.5290	1587.2681	3172.5450	1586.7761	A	418.2296	209.6185	401.2031	201.1052	400.2191	200.6132	4
33	3319.5981	1660.3027	3302.5716	1651.7894	3301.5876	1651.2974	E	347.1925	174.0999	330.1660	165.5866	329.1819	165.0946	3
34	3390.6352	1695.8213	3373.6087	1687.3080	3372.6247	1686.8160	A	218.1499	109.5786	201.1234	101.0653			2
35							K	147.1128	74.0600	130.0863	65.5468			1

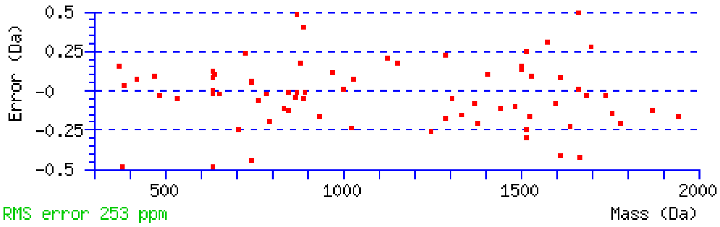

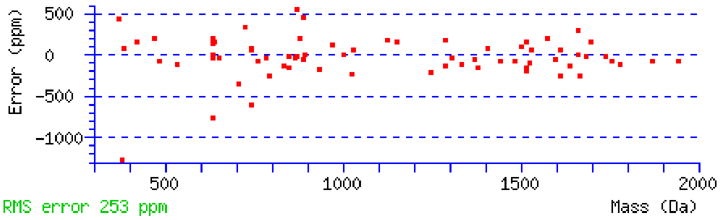

All matches to this query

Score	Mr(calc):	Delta	Sequence
91.3	3535.7334	0.0102	NEAAVPAAAAGSPVPVIAIPGILEDELEQTDAEAK
26.6	3535.7334	0.0102	NEAAVPAAAAGSPVPVIAIPGILEDELEQTDAEAK
5.8	3535.7088	0.0349	IREIWQITASLHGPPPGLDAYIAKMAAASGNK
4.7	3535.7323	0.0113	NGRIHFDMHGFLTNVILADLNLSASPFLLGR
4.5	3535.7323	0.0113	NGRIHFDMHGFLTNVILADLNLSASPFLLGR
2.2	3533.7248	2.0188	KVALWIAGLLGAGGTVSIVYIFGNNPVDENGTK
2.2	3533.7248	2.0188	KVALWIAGLLGAGGTVSIVYIFGNNPVDENGTK
0.2	3535.7088	0.0349	IREIWQITASLHGPPPGLDAYIAKMAAASGNK
0.2	3535.7088	0.0349	IREIWQITASLHGPPPGLDAYIAKMAAASGNK

Spectrum No: 19; Query: 1555; Rank: 1

Peptide View

MS/MS Fragmentation of **SSGSPYGGGYGSGGGSGGYGSR**
Found in **IPI00382376**, Tax_Id=10116 Gene_Symbol=Hnrpa3 Isoform 1 of Heterogeneous nuclear ribonucleoprotein A3

Match to Query 1555: 1989.748388 from(995.881470,2+)
Title: 091008RatKidney_NH4Format02_27.1026.1026.2.dta
Data file K:\NewmanPaper\Piliang\3SubProteomes\Piliang3SP\mgf5ppm\ERLIC_3SubProteomes5ppm.mgf

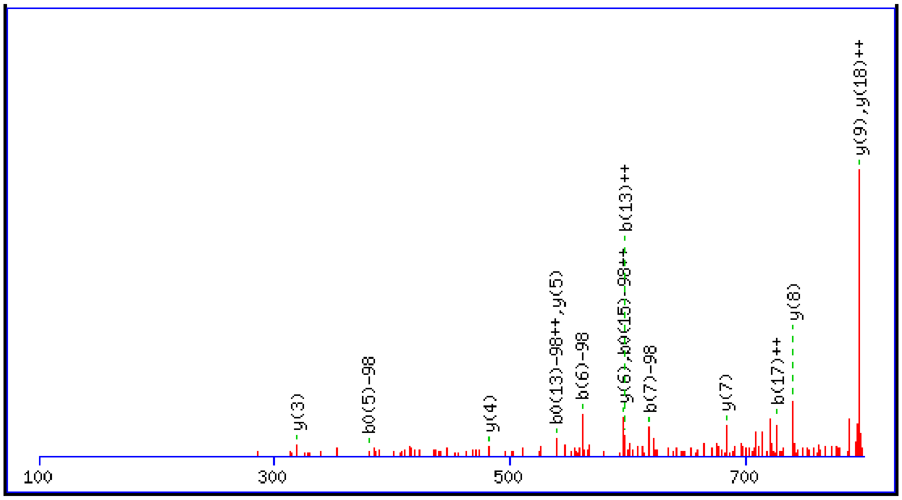

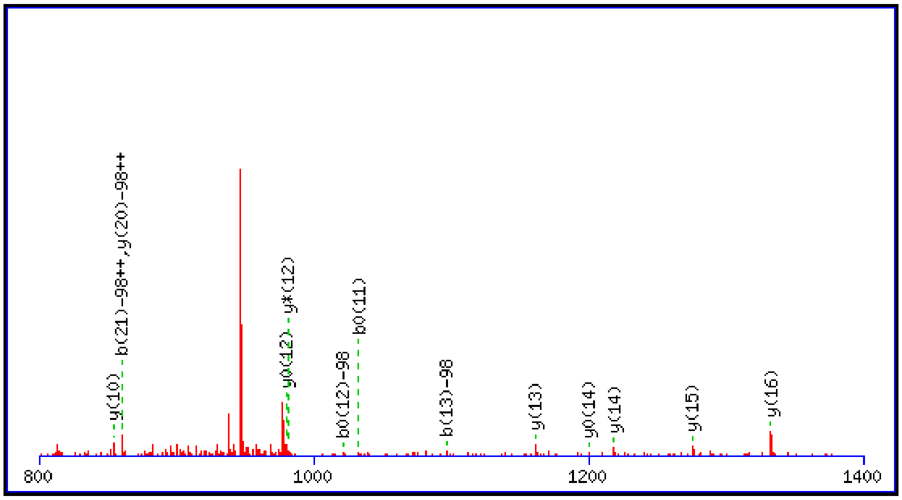

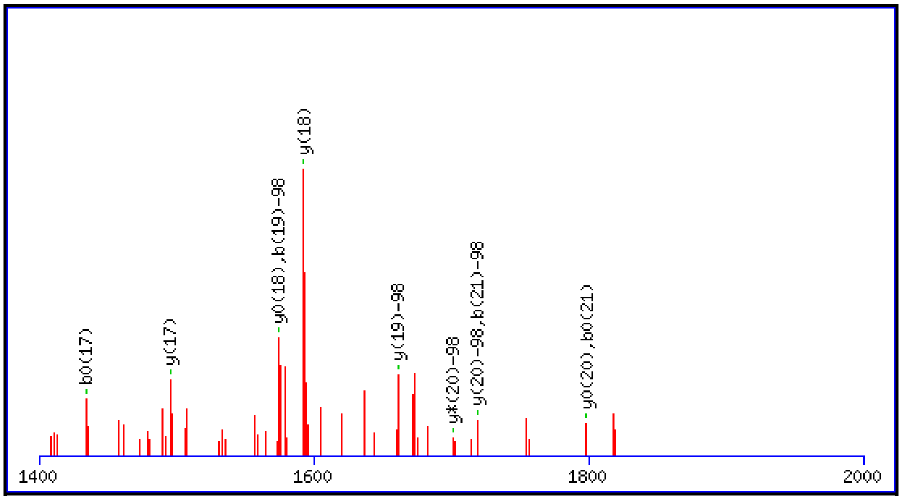

Monoisotopic mass of neutral peptide Mr(calc): 1989.7491
Fixed modifications: Carbamidomethyl (C)
Variable modifications:
S4 : Phospho (ST), with neutral losses 97.9769(shown in table), 0.0000
Ions Score: 91 **Expect:** 1.1e-007
Matches (Bold Red): 39/298 fragment ions using 70 most intense peaks

#	b	b++	b0	b0++	Seq.	y	y++	y*	y*++	y0	y0++	#
1	88.0393	44.5233	70.0287	35.5180	S							22
2	175.0713	88.0393	157.0608	79.0340	S	1805.7474	903.3773	1788.7208	894.8641	1787.7368	894.3720	21
3	232.0928	116.5500	214.0822	107.5448	G	**1718.7153**	**859.8613**	**1701.6888**	851.3480	1700.7048	850.8560	20
4	301.1143	151.0608	283.1037	142.0555	S	**1661.6939**	831.3506	1644.6673	822.8373	1643.6833	822.3453	19
5	398.1670	199.5871	**380.1565**	190.5819	P	**1592.6724**	**796.8399**	1575.6459	788.3266	**1574.6619**	787.8346	18

6	561.2303	281.1188	543.2198	272.1135	Y	1495.6197	748.3135	1478.5931	739.8002	1477.6091	739.3082	17
7	618.2518	309.6295	600.2412	300.6243	G	1332.5563	666.7818	1315.5298	658.2685	1314.5458	657.7765	16
8	675.2733	338.1403	657.2627	329.1350	G	1275.5349	638.2711	1258.5083	629.7578	1257.5243	629.2658	15
9	732.2947	366.6510	714.2842	357.6457	G	1218.5134	609.7603	1201.4869	601.2471	1200.5028	600.7551	14
10	895.3581	448.1827	877.3475	439.1774	Y	1161.4919	581.2496	1144.4654	572.7363	1143.4814	572.2443	13
11	952.3795	476.6934	934.3690	467.6881	G	998.4286	499.7179	981.4021	491.2047	980.4180	490.7127	12
12	1039.4116	520.2094	1021.4010	511.2041	S	941.4071	471.2072	924.3806	462.6939	923.3966	462.2019	11
13	1096.4330	548.7201	1078.4225	539.7149	G	854.3751	427.6912	837.3486	419.1779	836.3646	418.6859	10
14	1153.4545	577.2309	1135.4439	568.2256	G	797.3537	399.1805	780.3271	390.6672	779.3431	390.1752	9
15	1210.4760	605.7416	1192.4654	596.7363	G	740.3322	370.6697	723.3056	362.1565	722.3216	361.6645	8
16	1297.5080	649.2576	1279.4974	640.2523	S	683.3107	342.1590	666.2842	333.6457	665.3002	333.1537	7
17	1354.5294	677.7684	1336.5189	668.7631	G	596.2787	298.6430	579.2522	290.1297	578.2681	289.6377	6
18	1411.5509	706.2791	1393.5403	697.2738	G	539.2572	270.1323	522.2307	261.6190	521.2467	261.1270	5
19	1574.6142	787.8108	1556.6037	778.8055	Y	482.2358	241.6215	465.2092	233.1083	464.2252	232.6162	4
20	1631.6357	816.3215	1613.6251	807.3162	G	319.1724	160.0899	302.1459	151.5766	301.1619	151.0846	3
21	1718.6677	859.8375	1700.6572	850.8322	S	262.1510	131.5791	245.1244	123.0659	244.1404	122.5738	2
22					R	175.1190	88.0631	158.0924	79.5498			1

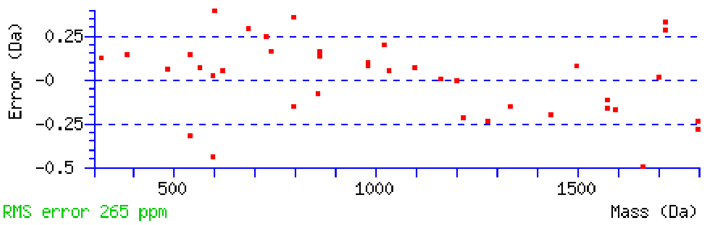

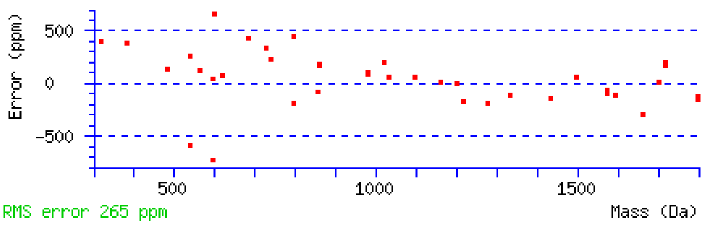

All matches to this query

Score	Mr(calc):	Delta	Sequence
90.9	1989.7491	-0.0007	SSGSPYGGGYGSGGGSGGYGSR
71.3	1989.7491	-0.0007	SSGSPYGGGYGSGGGSGGYGSR
71.3	1989.7491	-0.0007	SSGSPYGGGYGSGGGSGGYGSR
44.7	1989.7491	-0.0007	SSGSPYGGGYGSGGGSGGYGSR
38.5	1989.7491	-0.0007	SSGSPYGGGYGSGGGSGGYGSR
20.1	1989.7491	-0.0007	SSGSPYGGGYGSGGGSGGYGSR
8.1	1989.7491	-0.0007	SSGSPYGGGYGSGGGSGGYGSR
7.7	1989.7491	-0.0007	SSGSPYGGGYGSGGGSGGYGSR
6.9	1989.7659	-0.0175	LKFGNNWSQEYGSSGR
6.7	1989.7659	-0.0175	LKFGNNWSQEYGSSGR

Spectrum No: 20; Query: 1669; Rank: 1

Peptide View

MS/MS Fragmentation of **VLGPSSSENQEGTLTDSMK**
Found in **IPI00199060**, Tax_Id=10116 Gene_Symbol=Tgoln2 Trans golgi network (TGN) specific integral membrane protein TGN38 precursor

Match to Query 1669: 2058.890208 from(1030.452380,2+)
Title: 100101RatKid_NS_deglyco_19.2396.2396.2.dta
Data file K:\NewmanPaper\Piliang\3SubProteomes\Piliang3SP\mgf5ppm\ERLIC_3SubProteomes5ppm.mgf

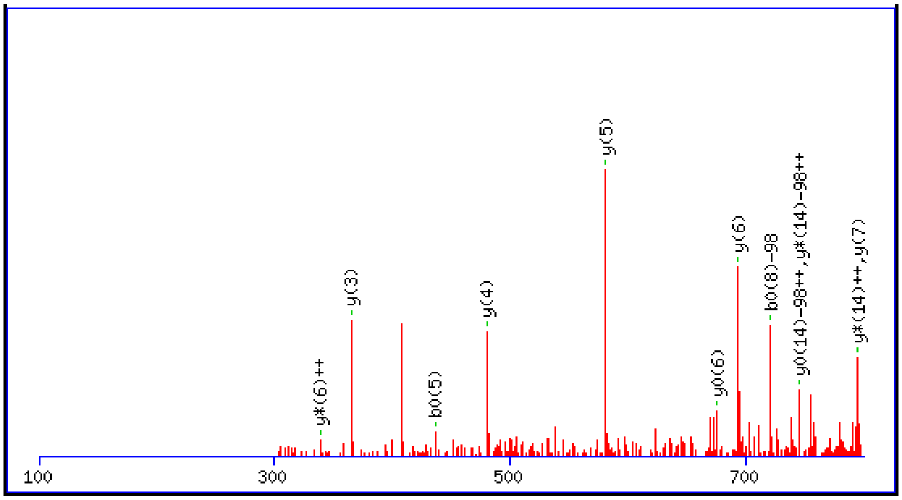

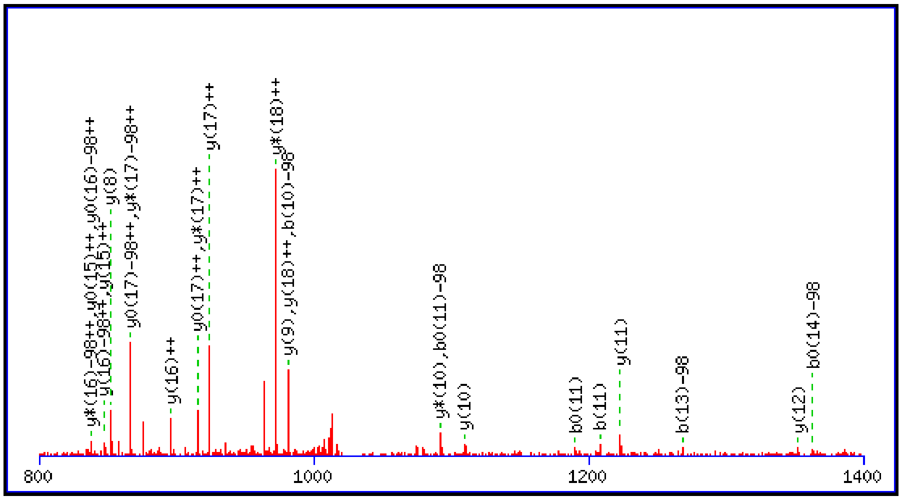

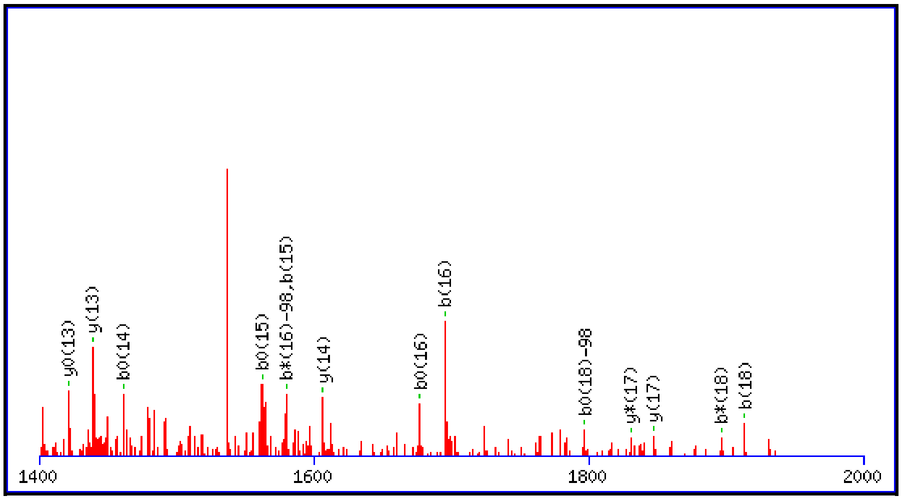

Monoisotopic mass of neutral peptide Mr(calc): 2058.8817
Fixed modifications: Carbamidomethyl (C)
Variable modifications:
S6 : Phospho (ST), with neutral losses 0.0000(shown in table), 97.9769
Ions Score: 90 **Expect:** 2.1e-007
Matches (Bold Red): 51/290 fragment ions using 61 most intense peaks

#	b	b++	b*	b*++	b0	b0++	Seq.	y	y++	y*	y*++	y0	y0++	#
1	100.0757	50.5415					V							19
2	213.1598	107.0835					L	1960.8205	**980.9139**	1943.7940	**972.4006**	1942.8100	971.9086	18
3	270.1812	135.5942					G	**1847.7365**	**924.3719**	**1830.7099**	**915.8586**	1829.7259	**915.3666**	17
4	367.2340	184.1206					P	1790.7150	**895.8611**	1773.6885	887.3479	1772.7044	886.8559	16
5	454.2660	227.6366			**436.2554**	218.6314	S	1693.6622	**847.3348**	1676.6357	838.8215	1675.6517	**838.3295**	15

6	621.2644	311.1358			603.2538	302.1305	S	**1606.6302**	803.8187	1589.6037	**795.3055**	1588.6197	794.8135	14
7	708.2964	354.6518			690.2858	345.6466	S	**1439.6319**	720.3196	1422.6053	711.8063	**1421.6213**	711.3143	13
8	837.3390	419.1731			819.3284	410.1678	E	**1352.5998**	676.8036	1335.5733	668.2903	1334.5893	667.7983	12
9	951.3819	476.1946	934.3554	467.6813	933.3713	467.1893	N	**1223.5572**	612.2823	1206.5307	603.7690	1205.5467	603.2770	11
10	1079.4405	540.2239	1062.4139	531.7106	1061.4299	531.2186	Q	**1109.5143**	555.2608	**1092.4878**	546.7475	1091.5038	546.2555	10
11	**1208.4831**	604.7452	1191.4565	596.2319	**1190.4725**	595.7399	E	**981.4557**	491.2315	964.4292	482.7182	963.4452	482.2262	9
12	1265.5045	633.2559	1248.4780	624.7426	1247.4940	624.2506	G	**852.4131**	426.7102	835.3866	418.1969	834.4026	417.7049	8
13	1366.5522	683.7798	1349.5257	675.2665	1348.5417	674.7745	T	**795.3917**	398.1995	778.3651	389.6862	777.3811	389.1942	7
14	1479.6363	740.3218	1462.6097	731.8085	**1461.6257**	731.3165	L	**694.3440**	347.6756	677.3175	**339.1624**	**676.3334**	338.6704	6
15	**1580.6840**	790.8456	1563.6574	782.3323	**1562.6734**	781.8403	T	**581.2599**	291.1336	564.2334	282.6203	563.2494	282.1283	5
16	**1695.7109**	848.3591	1678.6844	839.8458	**1677.7003**	839.3538	D	**480.2123**	240.6098	463.1857	232.0965	462.2017	231.6045	4
17	1782.7429	891.8751	1765.7164	883.3618	1764.7324	882.8698	S	**365.1853**	183.0963	348.1588	174.5830	347.1748	174.0910	3
18	**1913.7834**	957.3953	**1896.7569**	948.8821	1895.7729	948.3901	M	278.1533	139.5803	261.1267	131.0670			2
19							K	147.1128	74.0600	130.0863	65.5468			1

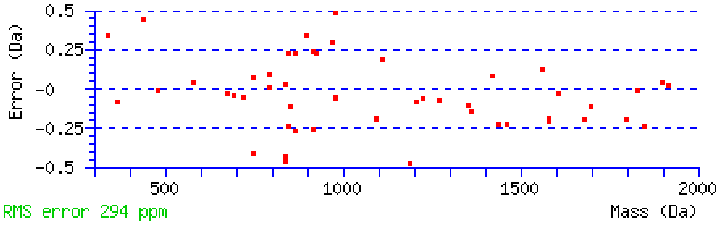

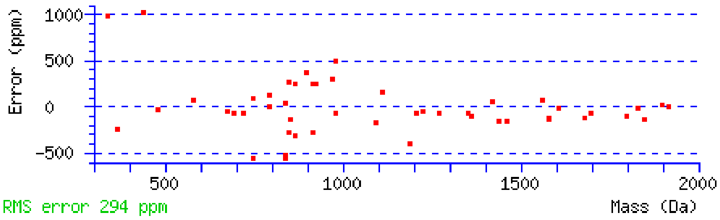

All matches to this query

Score	Mr(calc):	Delta	Sequence
90.4	2058.8817	0.0085	VLGPSSSENQEGTLTDSMK
75.9	2058.8817	0.0085	VLGPSSSENQEGTLTDSMK
75.8	2058.8817	0.0085	VLGPSSSENQEGTLTDSMK
20.5	2058.8817	0.0085	VLGPSSSENQEGTLTDSMK
6.9	2058.8817	0.0085	VLGPSSSENQEGTLTDSMK
3.8	2058.9016	-0.0114	TVREAPSECICPPGPPGR
0.7	2056.8999	1.9903	ILVGSFTGNMMTFKGNAR
0.7	2056.8999	1.9903	ILVGSFTGNMMTFKGNAR
0.5	2058.8813	0.0090	LVETIRTNYNDRYDK
0.3	2058.8952	-0.0049	SYNVSNLIDDLKNLYK

Spectrum No: 21; Query: 1322; Rank: 1

Peptide View

MS/MS Fragmentation of **SASSDTSEELNAQDSPK**
Found in **IPI00200898**, Tax_Id=10116 Gene_Symbol=Slc9a3r1 Ezrin-radixin-moesin-binding phosphoprotein 50

Match to Query 1322: 1844.729608 from(923.372080,2+)
Title: 091008RatKidney_NoSalt_27.1299.1299.2.dta
Data file K:\NewmanPaper\Piliang\3SubProteomes\Piliang3SP\mgf5ppm\ERLIC_3SubProteomes5ppm.mgf

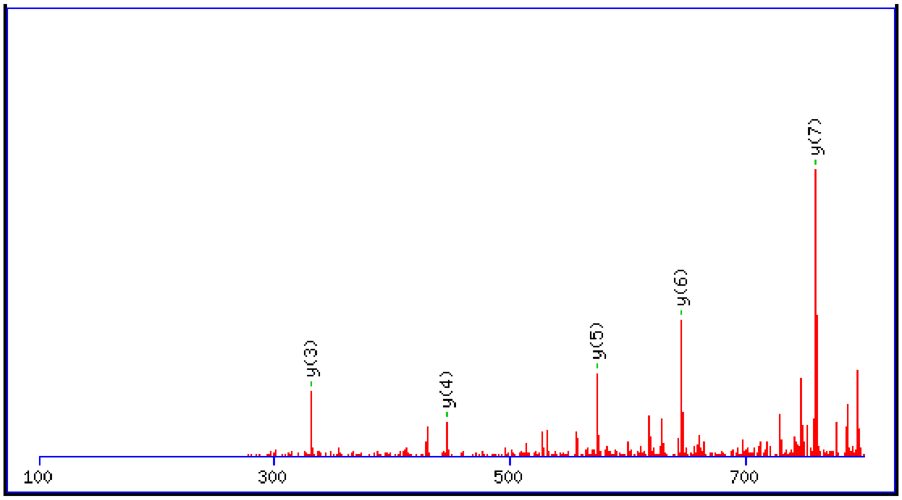

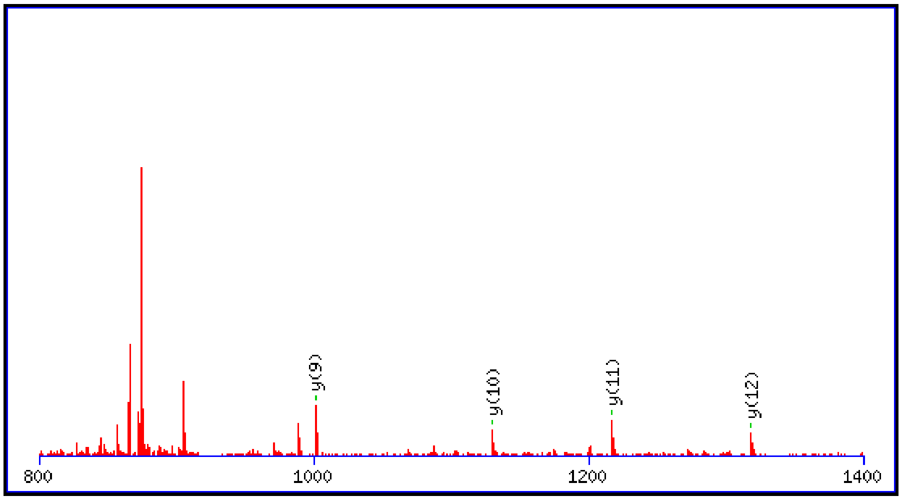

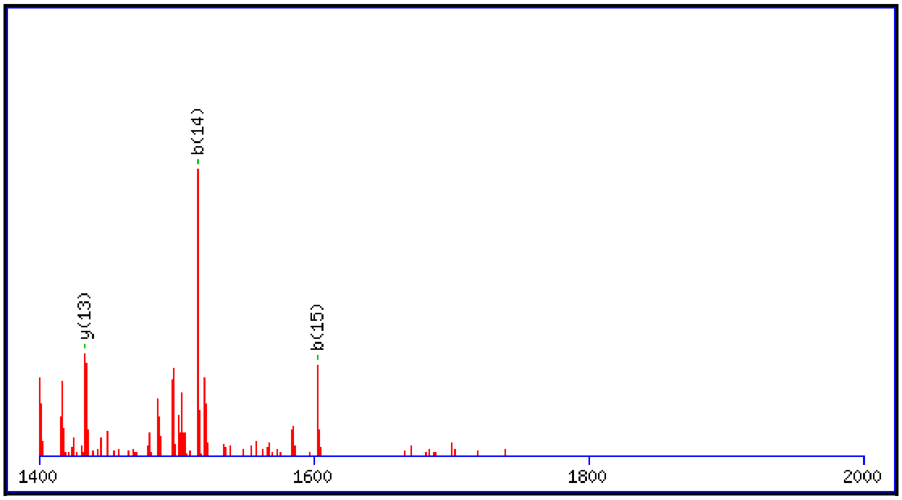

Monoisotopic mass of neutral peptide Mr(calc): 1844.7313
Fixed modifications: Carbamidomethyl (C)
Variable modifications:
S4 : Phospho (ST), with neutral losses 0.0000(shown in table), 97.9769
Ions Score: 90 **Expect:** 1.3e-007
Matches (Bold Red): 12/250 fragment ions using 19 most intense peaks

#	b	b++	b*	b*++	b0	b0++	Seq.	y	y++	y*	y*++	y0	y0++	#
1	88.0393	44.5233			70.0287	35.5180	S							17
2	159.0764	80.0418			141.0659	71.0366	A	1758.7066	879.8569	1741.6800	871.3436	1740.6960	870.8516	16
3	246.1084	123.5579			228.0979	114.5526	S	1687.6694	844.3384	1670.6429	835.8251	1669.6589	835.3331	15
4	413.1068	207.0570			395.0962	198.0518	S	1600.6374	800.8223	1583.6109	792.3091	1582.6268	791.8171	14
5	528.1337	264.5705			510.1232	255.5652	D	**1433.6391**	717.3232	1416.6125	708.8099	1415.6285	708.3179	13

**6**	629.1814	315.0943			611.1709	306.0891	**T**	**1318.6121**	659.8097	1301.5856	651.2964	1300.6016	650.8044	**12**
**7**	716.2134	358.6104			698.2029	349.6051	**S**	**1217.5644**	609.2859	1200.5379	600.7726	1199.5539	600.2806	**11**
**8**	845.2560	423.1317			827.2455	414.1264	**E**	**1130.5324**	565.7698	1113.5059	557.2566	1112.5218	556.7646	**10**
**9**	974.2986	487.6530			956.2881	478.6477	**E**	**1001.4898**	501.2485	984.4633	492.7353	983.4793	492.2433	**9**
**10**	1087.3827	544.1950			1069.3721	535.1897	**L**	872.4472	436.7272	855.4207	428.2140	854.4367	427.7220	**8**
**11**	1201.4256	601.2165	1184.3991	592.7032	1183.4151	592.2112	**N**	**759.3632**	380.1852	742.3366	371.6719	741.3526	371.1799	**7**
**12**	1272.4627	636.7350	1255.4362	628.2217	1254.4522	627.7297	**A**	**645.3202**	323.1638	628.2937	314.6505	627.3097	314.1585	**6**
**13**	1400.5213	700.7643	1383.4948	692.2510	1382.5108	691.7590	**Q**	**574.2831**	287.6452	557.2566	279.1319	556.2726	278.6399	**5**
**14**	**1515.5483**	758.2778	1498.5217	749.7645	1497.5377	749.2725	**D**	**446.2245**	223.6159	429.1980	215.1026	428.2140	214.6106	**4**
**15**	**1602.5803**	801.7938	1585.5537	793.2805	1584.5697	792.7885	**S**	**331.1976**	166.1024	314.1710	157.5892	313.1870	157.0972	**3**
**16**	1699.6331	850.3202	1682.6065	841.8069	1681.6225	841.3149	**P**	244.1656	122.5864	227.1390	114.0731			**2**
**17**							**K**	147.1128	74.0600	130.0863	65.5468			**1**

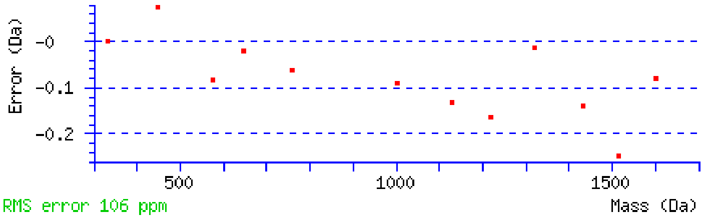

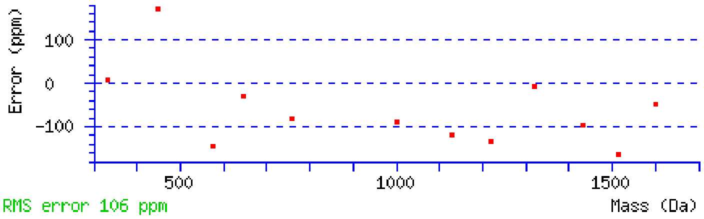

All matches to this query

Score	Mr(calc):	Delta	Sequence
90.4	1844.7313	-0.0017	SASSDTSEELNAQDSPK
90.4	1844.7313	-0.0017	SASSDTSEELNAQDSPK
90.4	1844.7313	-0.0017	SASSDTSEELNAQDSPK
57.7	1844.7313	-0.0017	SASSDTSEELNAQDSPK
43.5	1844.7313	-0.0017	SASSDTSEELNAQDSPK
9.7	1844.7313	-0.0017	SASSDTSEELNAQDSPK
6.0	1843.7139	1.0158	ESNPNVSQNSTNHKK
5.9	1843.7139	1.0158	ESNPNVSQNSTNHKK
5.8	1843.7261	1.0035	SYDEGPQNASSGEPGLR
5.8	1843.7261	1.0035	SYDEGPQNASSGEPGLR

Spectrum No: 22; Query: 549; Rank: 1

Peptide View

MS/MS Fragmentation of **TASGSSVTSLEGTR**
Found in **IPI00421389**, Tax_Id=10116 Gene_Symbol=Ndrg1 Protein NDRG1

Match to Query 549: 1431.619388 from(716.816970,2+)
Title: 091008RatKidney_NH4Format01_21.1301.1301.2.dta
Data file K:\NewmanPaper\Piliang\3SubProteomes\Piliang3SP\mgf5ppm\ERLIC_3SubProteomes5ppm.mgf

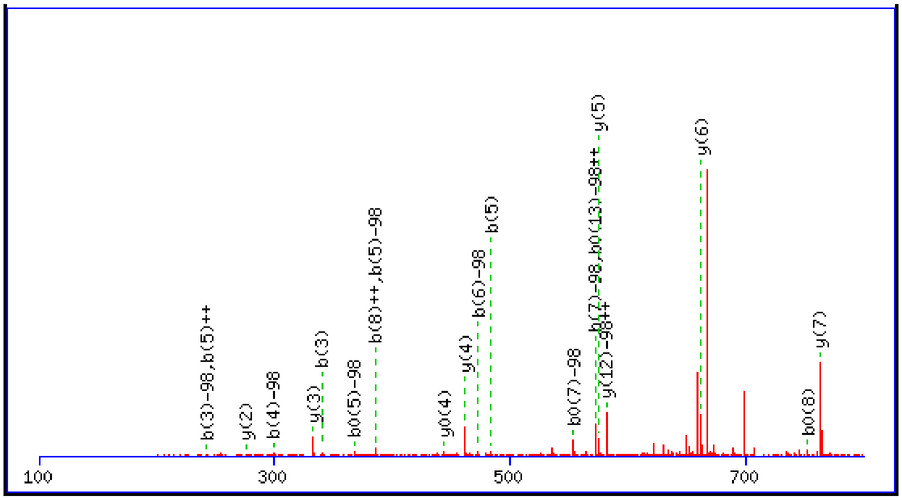

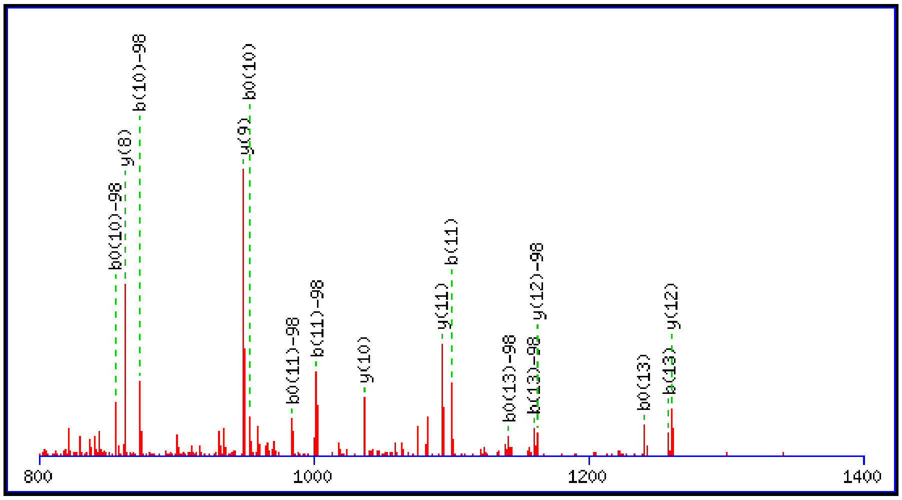

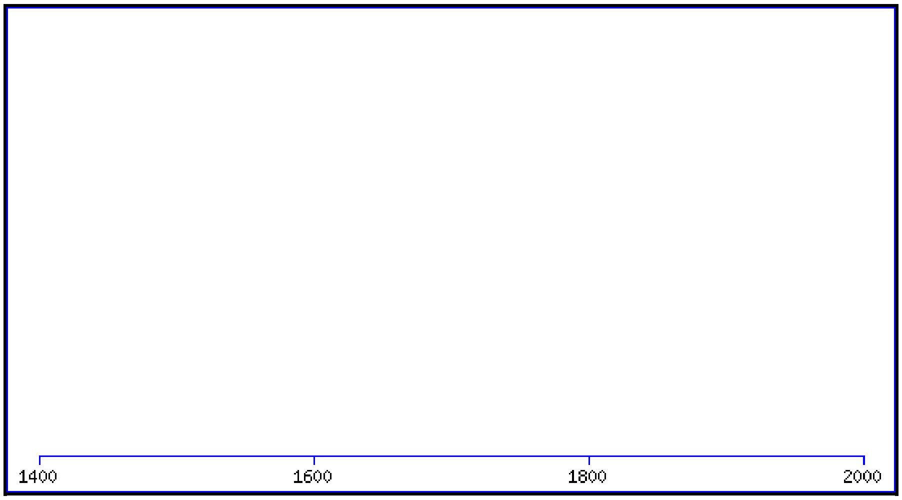

Monoisotopic mass of neutral peptide Mr(calc): 1431.6243
Fixed modifications: Carbamidomethyl (C)
Variable modifications:
S3 : Phospho (ST), with neutral losses 97.9769(shown in table), 0.0000
Ions Score: 90 **Expect:** 1.6e-007
Matches (Bold Red): 37/184 fragment ions using 47 most intense peaks

#	b	b^{++}	b^{0}	b^{0++}	Seq.	y	y^{++}	y*	y^{*++}	y^{0}	y^{0++}	#
1	102.0550	51.5311	84.0444	42.5258	T							14
2	173.0921	87.0497	155.0815	78.0444	A	1233.6070	617.3071	1216.5804	608.7938	1215.5964	608.3018	13
3	**242.1135**	121.5604	224.1030	112.5551	S	**1162.5698**	**581.7886**	1145.5433	573.2753	1144.5593	572.7833	12
4	**299.1350**	150.0711	281.1244	141.0658	G	**1093.5484**	547.2778	1076.5218	538.7646	1075.5378	538.2726	11
5	**386.1670**	193.5871	**368.1565**	184.5819	S	**1036.5269**	518.7671	1019.5004	510.2538	1018.5164	509.7618	10

6	473.1990	237.1032	455.1885	228.0979	S	949.4949	475.2511	932.4684	466.7378	931.4843	466.2458	9
7	572.2675	286.6374	554.2569	277.6321	V	862.4629	431.7351	845.4363	423.2218	844.4523	422.7298	8
8	673.3151	337.1612	655.3046	328.1559	T	763.3945	382.2009	746.3679	373.6876	745.3839	373.1956	7
9	760.3472	380.6772	742.3366	371.6719	S	662.3468	331.6770	645.3202	323.1638	644.3362	322.6717	6
10	873.4312	437.2193	855.4207	428.2140	L	575.3148	288.1610	558.2882	279.6477	557.3042	279.1557	5
11	1002.4738	501.7405	984.4633	492.7353	E	462.2307	231.6190	445.2041	223.1057	444.2201	222.6137	4
12	1059.4953	530.2513	1041.4847	521.2460	G	333.1881	167.0977	316.1615	158.5844	315.1775	158.0924	3
13	1160.5430	580.7751	1142.5324	571.7698	T	276.1666	138.5870	259.1401	130.0737	258.1561	129.5817	2
14					R	175.1190	88.0631	158.0924	79.5498			1

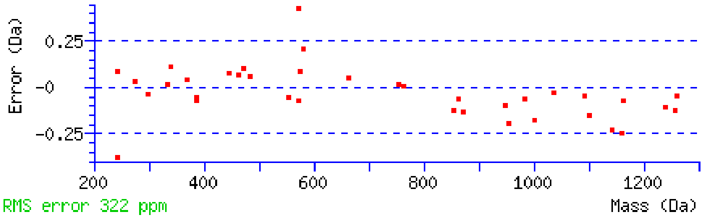

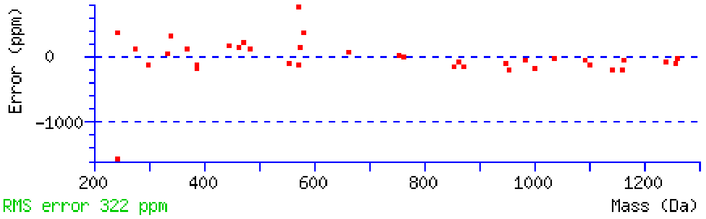

All matches to this query

Score	Mr(calc):	Delta	Sequence
89.9	1431.6243	-0.0049	TASGSSVTSLEGTR
79.1	1431.6243	-0.0049	TASGSSVTSLEGTR
60.9	1431.6243	-0.0049	TASGSSVTSLEGTR
47.4	1431.6243	-0.0049	TASGSSVTSLEGTR
43.4	1431.6243	-0.0049	TASGSSVTSLEGTR
33.5	1431.6243	-0.0049	TASGSSVTSLEGTR
10.7	1431.6243	-0.0049	TASGSSVTSLEGTR
7.1	1430.6068	1.0126	SRVLGGNSAASPR
6.4	1431.6330	-0.0136	RSCFPASLTASR
5.9	1431.6242	-0.0048	STKTETNQSSLR

Spectrum No: 23; Query: 1136; Rank: 1

Peptide View

MS/MS Fragmentation of **ATWGDGGDSSPSNVVSK**
Found in **IPI00197605**, Tax_Id=10116 Gene_Symbol=Snap23 Synaptosomal-associated protein 23

Match to Query 1136: 1742.714768 from(872.364660,2+)
Title: 091008RatKidney_NoSalt_26.1385.1385.2.dta
Data file K:\NewmanPaper\Piliang\3SubProteomes\Piliang3SP\mgf5ppm\ERLIC_3SubProteomes5ppm.mgf

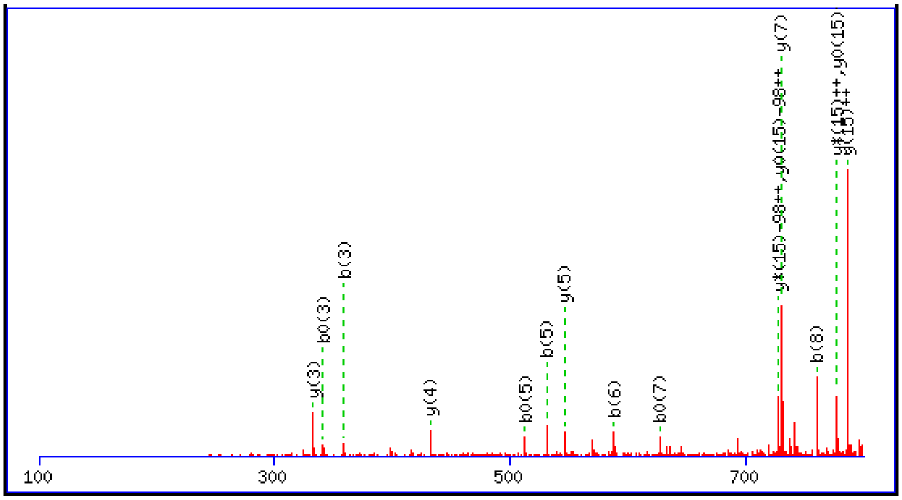

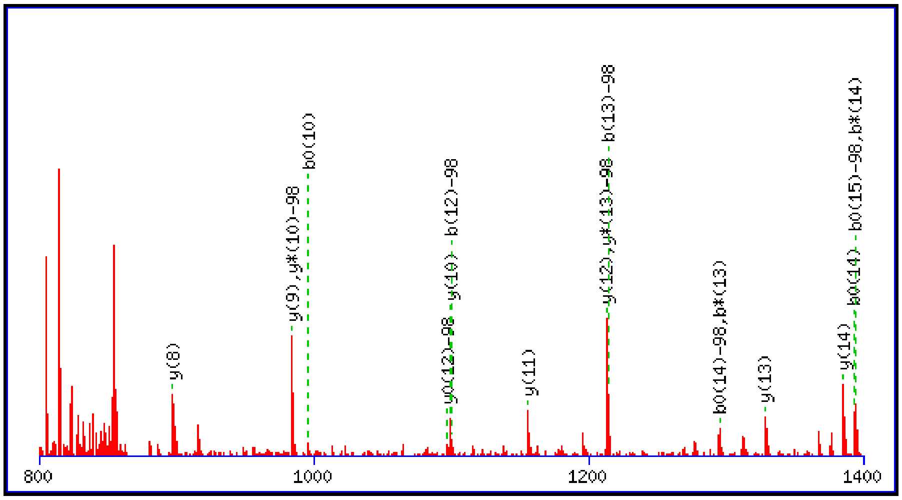

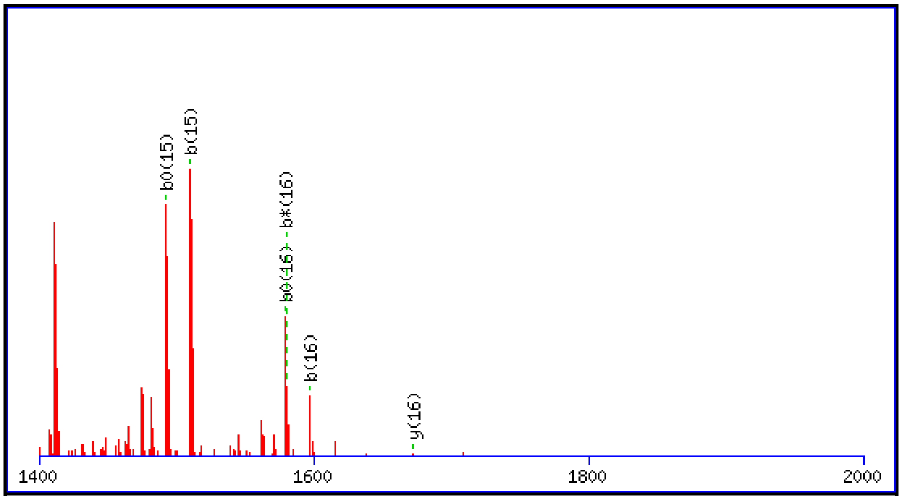

Monoisotopic mass of neutral peptide Mr(calc): 1742.7149
Fixed modifications: Carbamidomethyl (C)
Variable modifications:
S10 : Phospho (ST), with neutral losses 0.0000(shown in table), 97.9769
Ions Score: 90 **Expect:** 1.6e-007
Matches (Bold Red): 40/254 fragment ions using 49 most intense peaks

#	b	b^{++}	b*	b^{*++}	b^0	b^{0++}	Seq.	y	y^{++}	y*	y^{*++}	y^0	y^{0++}	#
1	72.0444	36.5258					A							17
2	173.0921	87.0497			155.0815	78.0444	T	**1672.6850**	836.8462	1655.6585	828.3329	1654.6745	827.8409	16
3	**359.1714**	180.0893			**341.1608**	171.0840	W	1571.6374	**786.3223**	1554.6108	**777.8090**	1553.6268	**777.3170**	15
4	416.1928	208.6001			398.1823	199.5948	G	**1385.5580**	693.2827	1368.5315	684.7694	1367.5475	684.2774	14
5	**531.2198**	266.1135			**513.2092**	257.1083	D	**1328.5366**	664.7719	1311.5100	656.2587	1310.5260	655.7666	13

**6**	**588.2413**	294.6243			570.2307	285.6190	**G**	**1213.5096**	607.2585	1196.4831	598.7452	1195.4991	598.2532	**12**
**7**	645.2627	323.1350			**627.2522**	314.1297	**G**	**1156.4882**	578.7477	1139.4616	570.2344	1138.4776	569.7424	**11**
**8**	**760.2897**	380.6485			742.2791	371.6432	**D**	**1099.4667**	550.2370	1082.4402	541.7237	1081.4561	541.2317	**10**
**9**	847.3217	424.1645			829.3111	415.1592	**S**	**984.4398**	492.7235	967.4132	484.2102	966.4292	483.7182	**9**
**10**	1014.3200	507.6637			**996.3095**	498.6584	**S**	**897.4077**	449.2075	880.3812	440.6942	879.3972	440.2022	**8**
**11**	1111.3728	556.1900			1093.3622	547.1848	**P**	**730.4094**	365.7083	713.3828	357.1951	712.3988	356.7030	**7**
**12**	1198.4048	599.7061			1180.3943	590.7008	**S**	633.3566	317.1819	616.3301	308.6687	615.3461	308.1767	**6**
**13**	1312.4478	656.7275	**1295.4212**	648.2142	1294.4372	647.7222	**N**	**546.3246**	273.6659	529.2980	265.1527	528.3140	264.6606	**5**
**14**	1411.5162	706.2617	**1394.4896**	697.7485	**1393.5056**	697.2564	**V**	**432.2817**	216.6445	415.2551	208.1312	414.2711	207.6392	**4**
**15**	**1510.5846**	755.7959	1493.5580	747.2827	**1492.5740**	746.7906	**V**	**333.2132**	167.1103	316.1867	158.5970	315.2027	158.1050	**3**
**16**	**1597.6166**	799.3119	**1580.5901**	790.7987	**1579.6061**	790.3067	**S**	234.1448	117.5761	217.1183	109.0628	216.1343	108.5708	**2**
**17**							**K**	147.1128	74.0600	130.0863	65.5468			**1**

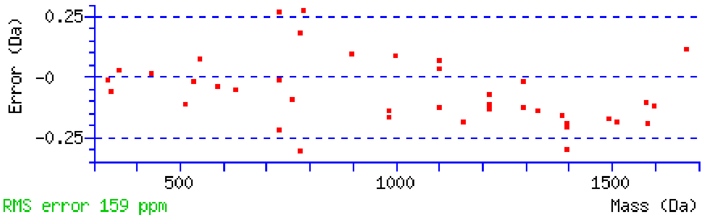

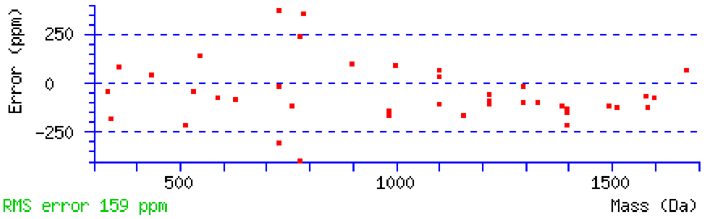

All matches to this query

Score	Mr(calc):	Delta	Sequence
89.7	1742.7149	-0.0001	ATWGDGGDSSPSNVVSK
80.6	1742.7149	-0.0001	ATWGDGGDSSPSNVVSK
73.8	1742.7149	-0.0001	ATWGDGGDSSPSNVVSK
34.9	1742.7149	-0.0001	ATWGDGGDSSPSNVVSK
34.4	1742.7149	-0.0001	ATWGDGGDSSPSNVVSK
8.3	1740.7233	1.9915	YSVKVRADGGTNSAR
4.3	1740.7233	1.9915	YSVKVRADGGTNSAR
3.6	1740.7233	1.9915	YSVKVRADGGTNSAR
3.2	1740.7233	1.9915	YSVKVRADGGTNSAR
2.7	1742.7229	-0.0081	MSTNNMSDPRRPNK

Spectrum No: 24; Query: 1632; Rank: 1

Peptide View

MS/MS Fragmentation of **ELGSLEEDANPDEEGVQK**
Found in **IPI00371736**, Tax_Id=10116 Gene_Symbol=LOC685174 similar to trinucleotide repeat containing 5

Match to Query 1632: 2037.838028 from(1019.926290,2+)
Title: 091008RatKidney_NoSalt_27.2220.2220.2.dta
Data file K:\NewmanPaper\Piliang\3SubProteomes\Piliang3SP\mgf5ppm\ERLIC_3SubProteomes5ppm.mgf

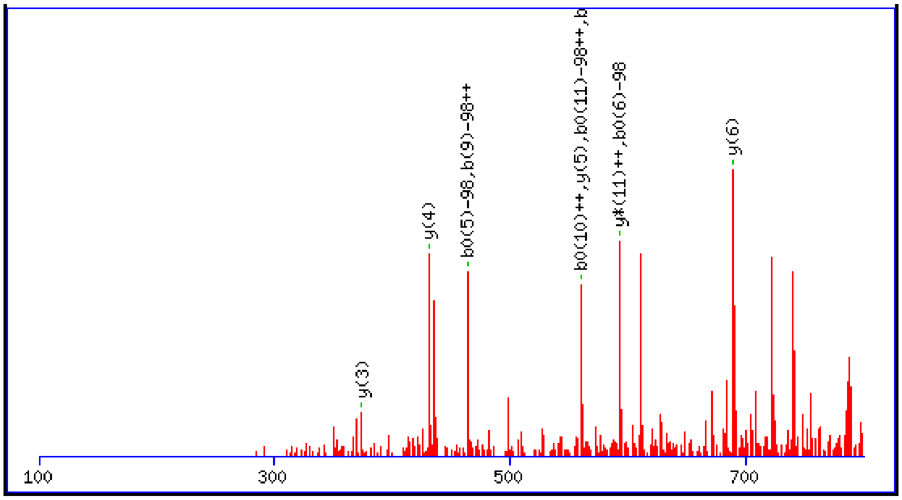

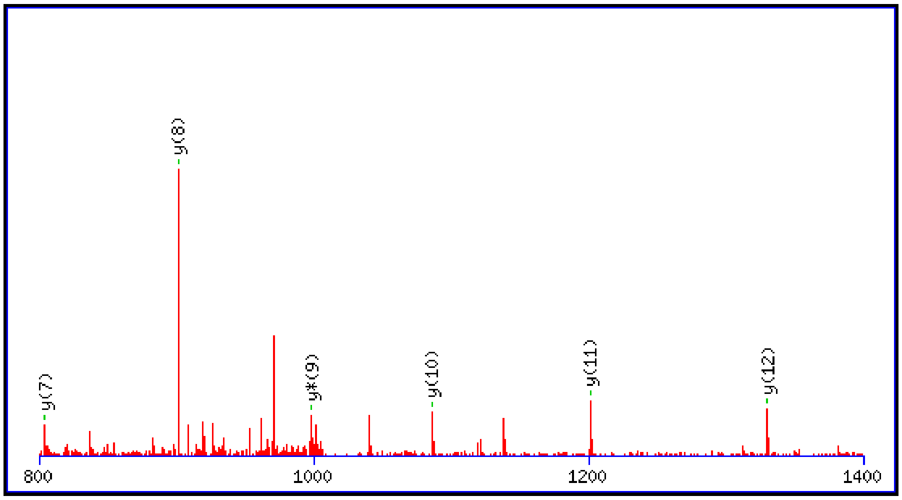

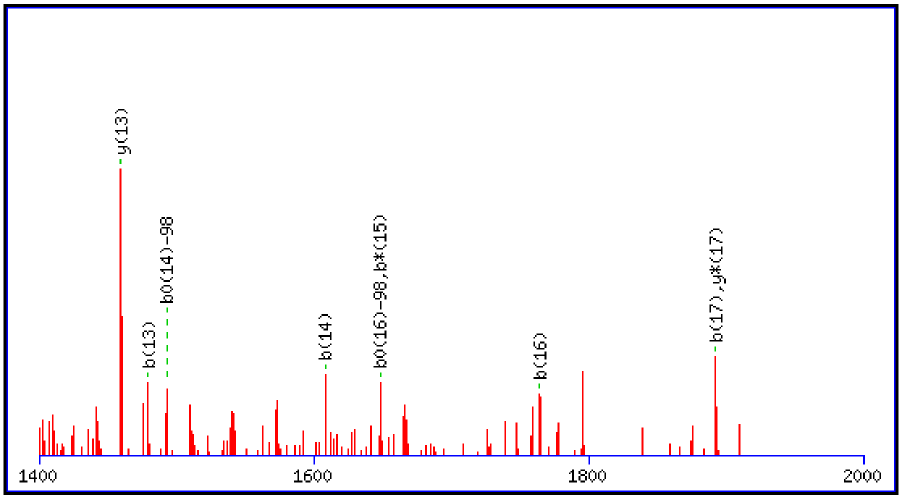

Monoisotopic mass of neutral peptide Mr(calc): 2037.8416
Fixed modifications: Carbamidomethyl (C)
Variable modifications:
S4 : Phospho (ST), with neutral losses 0.0000(shown in table), 97.9769
Ions Score: 89 **Expect:** 2.4e-007
Matches (Bold Red): 26/268 fragment ions using 24 most intense peaks

#	b	b^{++}	b*	b^{*++}	b^0	b^{0++}	Seq.	y	y^{++}	y*	y^{*++}	y^0	y^{0++}	#
1	130.0499	65.5286			112.0393	56.5233	E							18
2	243.1339	122.0706			225.1234	113.0653	L	1909.8063	955.4068	**1892.7797**	946.8935	1891.7957	946.4015	17
3	300.1554	150.5813			282.1448	141.5761	G	1796.7222	898.8647	1779.6957	890.3515	1778.7116	889.8595	16
4	467.1537	234.0805			449.1432	225.0752	S	1739.7007	870.3540	1722.6742	861.8407	1721.6902	861.3487	15
5	580.2378	290.6225			562.2272	281.6173	L	1572.7024	786.8548	1555.6758	778.3416	1554.6918	777.8495	14

6	709.2804	355.1438			691.2698	346.1386	E	1459.6183	730.3128	1442.5918	721.7995	1441.6078	721.3075	13
7	838.3230	419.6651			820.3124	410.6599	E	1330.5757	665.7915	1313.5492	657.2782	1312.5652	656.7862	12
8	953.3499	477.1786			935.3394	468.1733	D	1201.5331	601.2702	1184.5066	592.7569	1183.5226	592.2649	11
9	1024.3871	512.6972			1006.3765	503.6919	A	1086.5062	543.7567	1069.4796	535.2435	1068.4956	534.7515	10
10	1138.4300	569.7186	1121.4034	561.2054	1120.4194	560.7133	N	1015.4691	508.2382	998.4425	499.7249	997.4585	499.2329	9
11	1235.4827	618.2450	1218.4562	609.7317	1217.4722	609.2397	P	901.4262	451.2167	884.3996	442.7034	883.4156	442.2114	8
12	1350.5097	675.7585	1333.4831	667.2452	1332.4991	666.7532	D	804.3734	402.6903	787.3468	394.1771	786.3628	393.6851	7
13	1479.5523	740.2798	1462.5257	731.7665	1461.5417	731.2745	E	689.3464	345.1769	672.3199	336.6636	671.3359	336.1716	6
14	1608.5949	804.8011	1591.5683	796.2878	1590.5843	795.7958	E	560.3039	280.6556	543.2773	272.1423	542.2933	271.6503	5
15	1665.6163	833.3118	1648.5898	824.7985	1647.6058	824.3065	G	431.2613	216.1343	414.2347	207.6210			4
16	1764.6848	882.8460	1747.6582	874.3327	1746.6742	873.8407	V	374.2398	187.6235	357.2132	179.1103			3
17	1892.7433	946.8753	1875.7168	938.3620	1874.7328	937.8700	Q	275.1714	138.0893	258.1448	129.5761			2
18							K	147.1128	74.0600	130.0863	65.5468			1

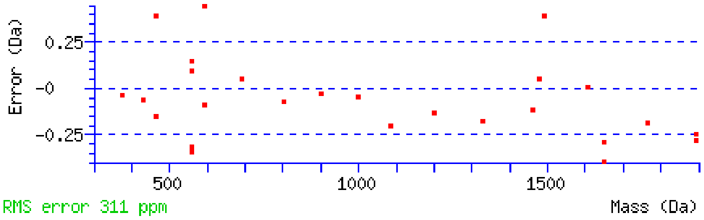

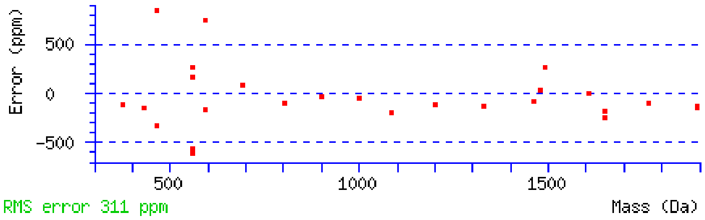

All matches to this query

Score	Mr(calc):	Delta	Sequence
89.4	2037.8416	-0.0035	ELGSLEEDANPDEEGVQK
8.5	2035.8289	2.0091	SKGTGDVQKPSQDEWSK
4.0	2037.8479	-0.0099	MGSSLGANILEDRSGGLGK
0.8	2037.8340	0.0040	MASRVNDQSQASRNGLK
0.6	2037.8350	0.0030	DSIMPGSKSSGNDSYELR
0.6	2036.8394	0.9986	ASSQREGNQTAFXGLWK
0.6	2036.8394	0.9986	ASSQREGNQTAFXGLWK
0.3	2037.8340	0.0040	MASRVNDQSQASRNGLK
0.2	2037.8537	-0.0156	DRQKEMDSFLAQMEAK
0.1	2037.8277	0.0103	GTTDPHDRTEDGNVSTEK

Spectrum No: 25; Query: 2203; Rank: 1

Peptide View

MS/MS Fragmentation of **FLENAAADFSDEDEDEDIDGR**
Found in **IPI00188880**, Tax_Id=10116 Gene_Symbol=Strn Striatin

Match to Query 2203: 2451.934028 from(1226.974290,2+)
Title: 100101RatKid_NS_deglyco_26.3044.3044.2.dta
Data file K:\NewmanPaper\Piliang\3SubProteomes\Piliang3SP\mgf5ppm\ERLIC_3SubProteomes5ppm.mgf

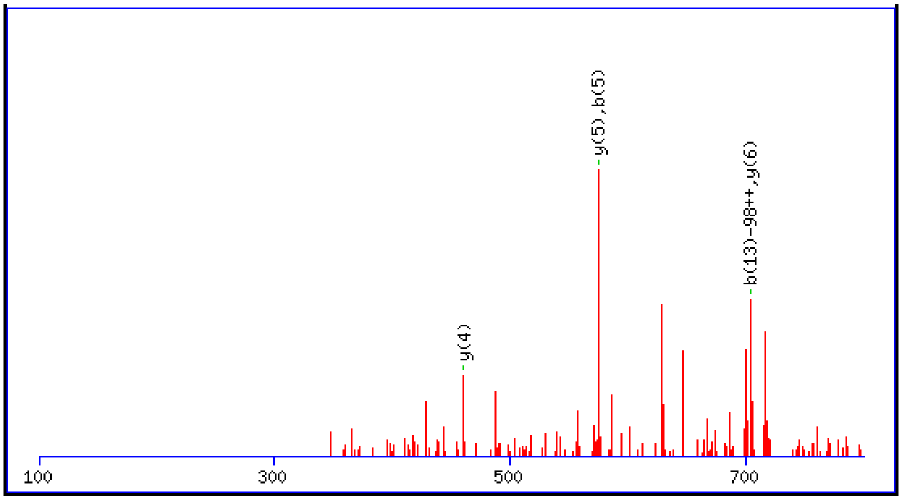

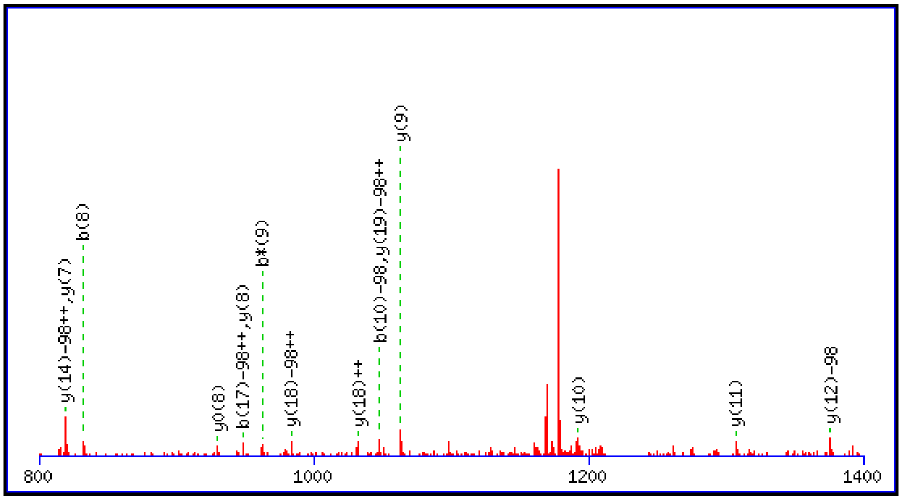

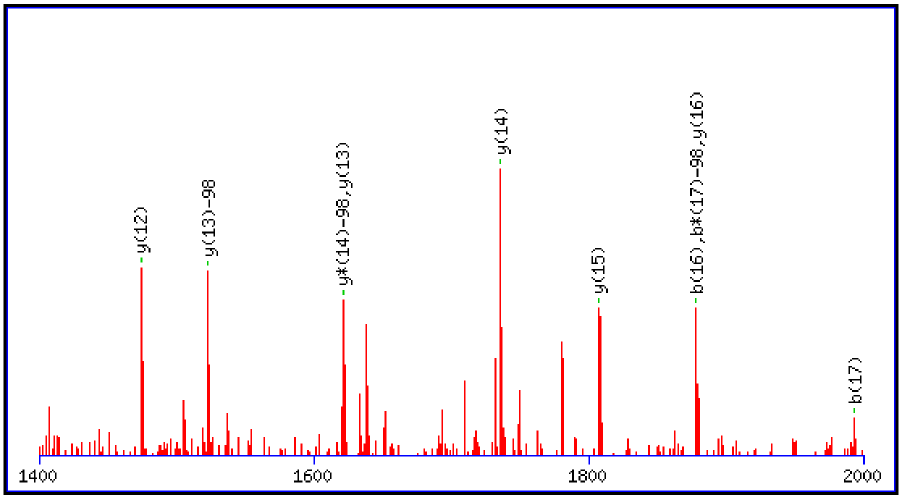

Monoisotopic mass of neutral peptide Mr(calc): 2451.9227
Fixed modifications: Carbamidomethyl (C)
Variable modifications:
S10 : Phospho (ST), with neutral losses 0.0000(shown in table), 97.9769
Ions Score: 89 **Expect:** 2.7e-007
Matches (Bold Red): 30/346 fragment ions using 31 most intense peaks

#	b	b++	b*	b*++	b0	b0++	Seq.	y	y++	y*	y*++	y0	y0++	#
1	148.0757	74.5415					F							21
2	261.1598	131.0835					L	2305.8616	1153.4344	2288.8351	1144.9212	2287.8510	1144.4292	20
3	390.2023	195.6048			372.1918	186.5995	E	2192.7775	1096.8924	2175.7510	1088.3791	2174.7670	1087.8871	19
4	504.2453	252.6263	487.2187	244.1130	486.2347	243.6210	N	2063.7350	**1032.3711**	2046.7084	1023.8578	2045.7244	1023.3658	18
5	**575.2824**	288.1448	558.2558	279.6316	557.2718	279.1395	A	1949.6920	975.3497	1932.6655	966.8364	1931.6815	966.3444	17

6	646.3195	323.6634	629.2930	315.1501	628.3089	314.6581	A	1878.6549	939.8311	1861.6284	931.3178	1860.6443	930.8258	16
7	717.3566	359.1819	700.3301	350.6687	699.3461	350.1767	A	1807.6178	904.3125	1790.5912	895.7993	1789.6072	895.3073	15
8	832.3836	416.6954	815.3570	408.1821	814.3730	407.6901	D	1736.5807	868.7940	1719.5541	860.2807	1718.5701	859.7887	14
9	979.4520	490.2296	962.4254	481.7163	961.4414	481.2243	F	1621.5537	811.2805	1604.5272	802.7672	1603.5432	802.2752	13
10	1146.4503	573.7288	1129.4238	565.2155	1128.4398	564.7235	S	1474.4853	737.7463	1457.4588	729.2330	1456.4748	728.7410	12
11	1261.4773	631.2423	1244.4507	622.7290	1243.4667	622.2370	D	1307.4870	654.2471	1290.4604	645.7339	1289.4764	645.2418	11
12	1390.5199	695.7636	1373.4933	687.2503	1372.5093	686.7583	E	1192.4600	596.7337	1175.4335	588.2204	1174.4495	587.7284	10
13	1505.5468	753.2770	1488.5203	744.7638	1487.5362	744.2718	D	1063.4174	532.2124	1046.3909	523.6991	1045.4069	523.2071	9
14	1634.5894	817.7983	1617.5628	809.2851	1616.5788	808.7931	E	948.3905	474.6989	931.3639	466.1856	930.3799	465.6936	8
15	1749.6163	875.3118	1732.5898	866.7985	1731.6058	866.3065	D	819.3479	410.1776	802.3214	401.6643	801.3373	401.1723	7
16	1878.6589	939.8331	1861.6324	931.3198	1860.6484	930.8278	E	704.3210	352.6641	687.2944	344.1508	686.3104	343.6588	6
17	1993.6859	997.3466	1976.6593	988.8333	1975.6753	988.3413	D	575.2784	288.1428	558.2518	279.6295	557.2678	279.1375	5
18	2106.7699	1053.8886	2089.7434	1045.3753	2088.7594	1044.8833	I	460.2514	230.6293	443.2249	222.1161	442.2409	221.6241	4
19	2221.7969	1111.4021	2204.7703	1102.8888	2203.7863	1102.3968	D	347.1674	174.0873	330.1408	165.5740	329.1568	165.0820	3
20	2278.8183	1139.9128	2261.7918	1131.3995	2260.8078	1130.9075	G	232.1404	116.5738	215.1139	108.0606			2
21							R	175.1190	88.0631	158.0924	79.5498			1

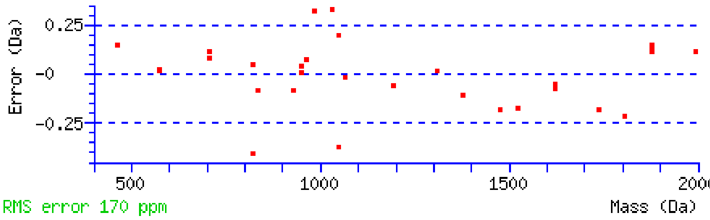

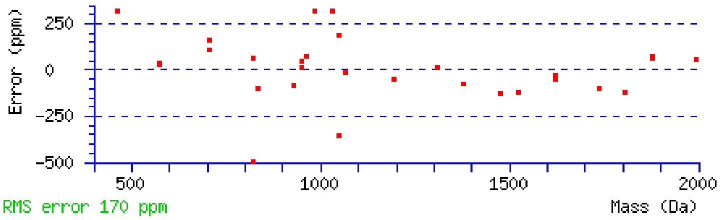

All matches to this query

Score	Mr(calc):	Delta	Sequence
88.9	2451.9227	0.0113	FLENAAADFSDEDEDEDIDGR
2.0	2451.9187	0.0153	IYCNASFLAGESCCNLSPTK
1.0	2451.9187	0.0153	IYCNASFLAGESCCNLSPTK
1.0	2449.9409	1.9931	FSDDAFNTTFISTIEIDFK
1.0	2449.9409	1.9931	FSDDAFNTTFISTIEIDFK
0.4	2449.9394	1.9946	IKAMMSQSGHEYDPINYMK
0.4	2450.9234	1.0106	IKAMMSQSGHEYDPINYMK
0.1	2449.9354	1.9986	MEMQEPKMNGTLSTGAAAGYR

Spectrum No: 26; Query: 1554; Rank: 1

Peptide View

MS/MS Fragmentation of **SSGSPYGGGYGSGGGSGGYGSR**
Found in **IPI00382376**, Tax_Id=10116 Gene_Symbol=Hnrpa3 Isoform 1 of Heterogeneous nuclear ribonucleoprotein A3

Match to Query 1554: 1989.743128 from(995.878840,2+)
Title: 091008RatKidney_NH4Format01_25.1053.1053.2.dta
Data file K:\NewmanPaper\Piliang\3SubProteomes\Piliang3SP\mgf5ppm\ERLIC_3SubProteomes5ppm.mgf

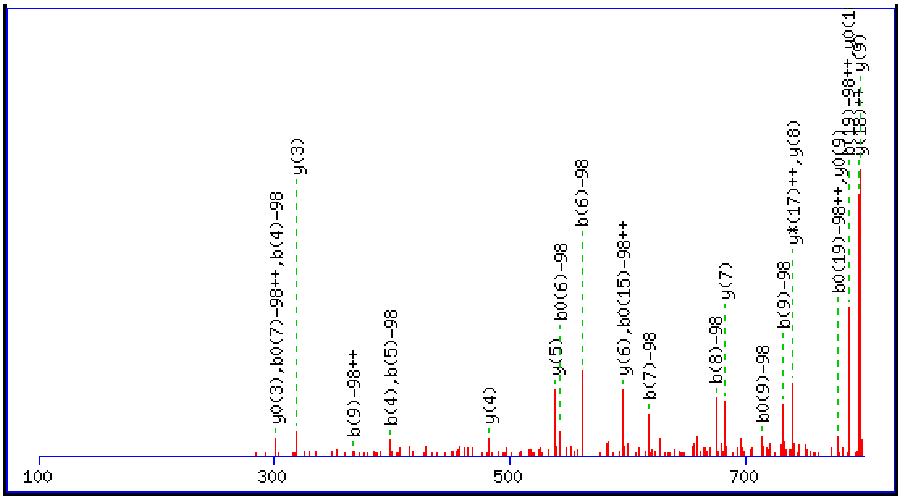

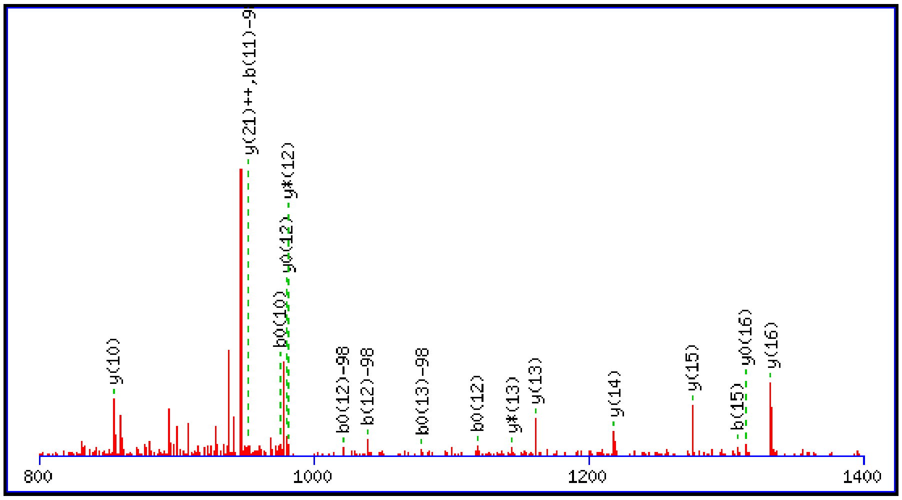

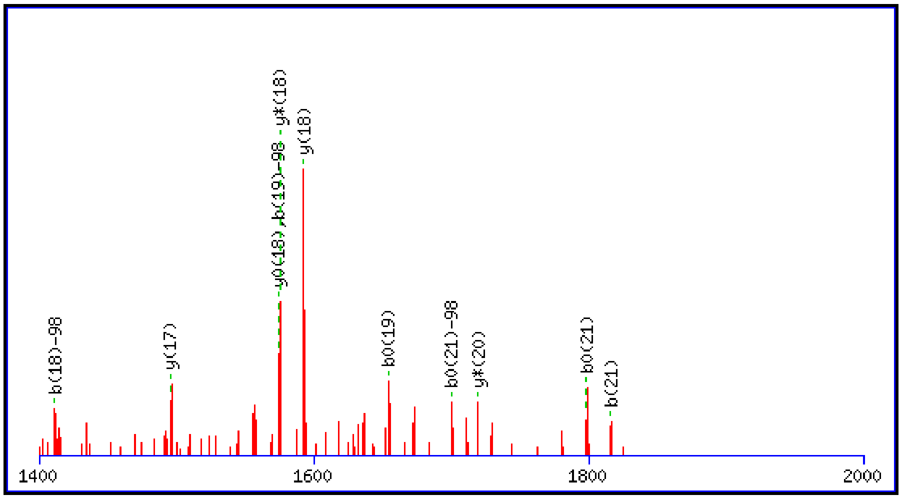

Monoisotopic mass of neutral peptide Mr(calc): 1989.7491
Fixed modifications: Carbamidomethyl (C)
Variable modifications:
S2 : Phospho (ST), with neutral losses 97.9769(shown in table), 0.0000
Ions Score: 88 **Expect:** 2.1e-007
Matches (Bold Red): 55/294 fragment ions using 81 most intense peaks

#	b	b++	b0	b0++	Seq.	y	y++	y*	y*++	y0	y0++	#
1	88.0393	44.5233	70.0287	35.5180	S							22
2	157.0608	79.0340	139.0502	70.0287	S	1805.7474	903.3773	1788.7208	894.8641	1787.7368	894.3720	21
3	214.0822	107.5447	196.0717	98.5395	G	1736.7259	868.8666	**1719.6994**	860.3533	1718.7154	859.8613	20
4	**301.1143**	151.0608	283.1037	142.0555	S	1679.7045	840.3559	1662.6779	831.8426	1661.6939	831.3506	19
5	**398.1670**	199.5871	380.1565	190.5819	P	**1592.6724**	**796.8399**	**1575.6459**	**788.3266**	**1574.6619**	**787.8346**	18

6	561.2303	281.1188	543.2198	272.1135	Y	1495.6197	748.3135	1478.5931	739.8002	1477.6091	739.3082	17
7	618.2518	309.6295	600.2412	300.6243	G	1332.5563	666.7818	1315.5298	658.2685	1314.5458	657.7765	16
8	675.2733	338.1403	657.2627	329.1350	G	1275.5349	638.2711	1258.5083	629.7578	1257.5243	629.2658	15
9	732.2947	366.6510	714.2842	357.6457	G	1218.5134	609.7603	1201.4869	601.2471	1200.5028	600.7551	14
10	895.3581	448.1827	877.3475	439.1774	Y	1161.4919	581.2496	1144.4654	572.7363	1143.4814	572.2443	13
11	952.3795	476.6934	934.3690	467.6881	G	998.4286	499.7179	981.4021	491.2047	980.4180	490.7127	12
12	1039.4116	520.2094	1021.4010	511.2041	S	941.4071	471.2072	924.3806	462.6939	923.3966	462.2019	11
13	1096.4330	548.7201	1078.4225	539.7149	G	854.3751	427.6912	837.3486	419.1779	836.3646	418.6859	10
14	1153.4545	577.2309	1135.4439	568.2256	G	797.3537	399.1805	780.3271	390.6672	779.3431	390.1752	9
15	1210.4760	605.7416	1192.4654	596.7363	G	740.3322	370.6697	723.3056	362.1565	722.3216	361.6645	8
16	1297.5080	649.2576	1279.4974	640.2523	S	683.3107	342.1590	666.2842	333.6457	665.3002	333.1537	7
17	1354.5294	677.7684	1336.5189	668.7631	G	596.2787	298.6430	579.2522	290.1297	578.2681	289.6377	6
18	1411.5509	706.2791	1393.5403	697.2738	G	539.2572	270.1323	522.2307	261.6190	521.2467	261.1270	5
19	1574.6142	787.8108	1556.6037	778.8055	Y	482.2358	241.6215	465.2092	233.1083	464.2252	232.6162	4
20	1631.6357	816.3215	1613.6251	807.3162	G	319.1724	160.0899	302.1459	151.5766	301.1619	151.0846	3
21	1718.6677	859.8375	1700.6572	850.8322	S	262.1510	131.5791	245.1244	123.0659	244.1404	122.5738	2
22					R	175.1190	88.0631	158.0924	79.5498			1

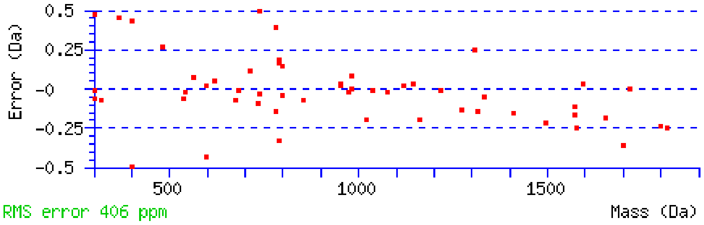

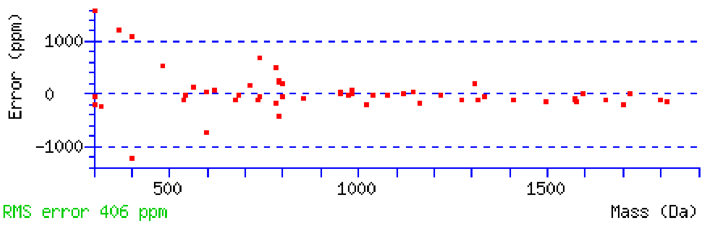

All matches to this query

Score	Mr(calc):	Delta	Sequence
87.7	1989.7491	-0.0059	SSGSPYGGGYGSGGGSGGYGSR
87.7	1989.7491	-0.0059	SSGSPYGGGYGSGGGSGGYGSR
86.1	1989.7491	-0.0059	SSGSPYGGGYGSGGGSGGYGSR
63.5	1989.7491	-0.0059	SSGSPYGGGYGSGGGSGGYGSR
58.3	1989.7491	-0.0059	SSGSPYGGGYGSGGGSGGYGSR
37.8	1989.7491	-0.0059	SSGSPYGGGYGSGGGSGGYGSR
19.3	1989.7491	-0.0059	SSGSPYGGGYGSGGGSGGYGSR
13.8	1989.7491	-0.0059	SSGSPYGGGYGSGGGSGGYGSR
4.0	1989.7491	-0.0059	SSGSPYGGGYGSGGGSGGYGSR
0.2	1987.7384	2.0048	QAPTSCSYNSTLTNQR

Spectrum No: 27; Query: 1028; Rank: 1

Peptide View

MS/MS Fragmentation of **DTEDTGSVGAPVSSPGK**
Found in **IPI00364728**, Tax_Id=10116 Gene_Symbol=RGD1304644 Similar to RIKEN cDNA 2310046K01

Match to Query 1028: 1682.703868 from(842.359210,2+)
Title: 091008RatKidney_NH4Format02_29.652.652.2.dta
Data file K:\NewmanPaper\Piliang\3SubProteomes\Piliang3SP\mgf5ppm\ERLIC_3SubProteomes5ppm.mgf

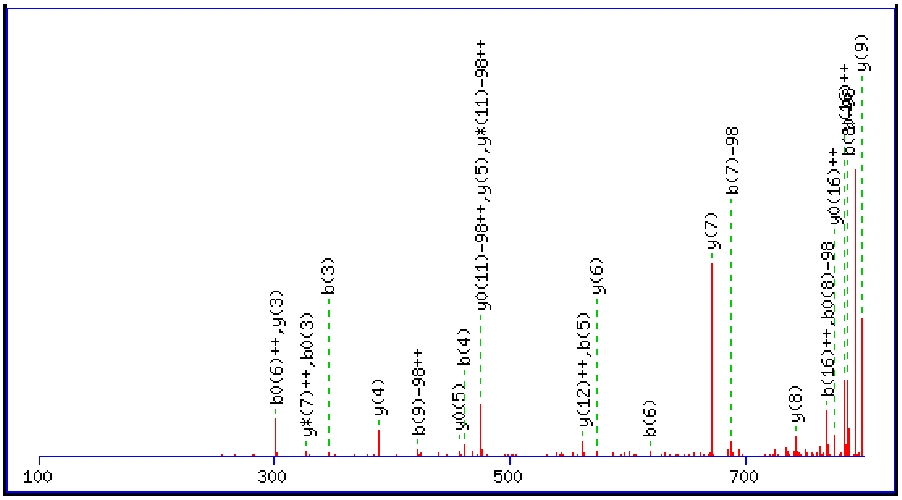

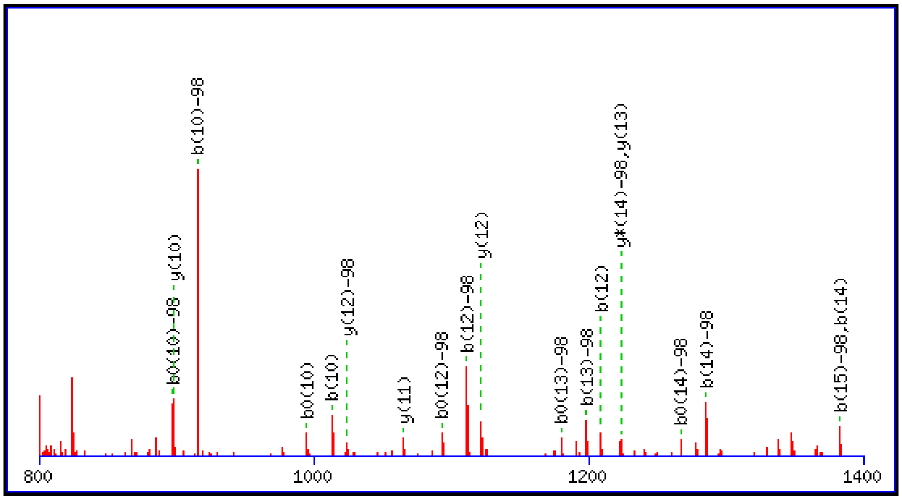

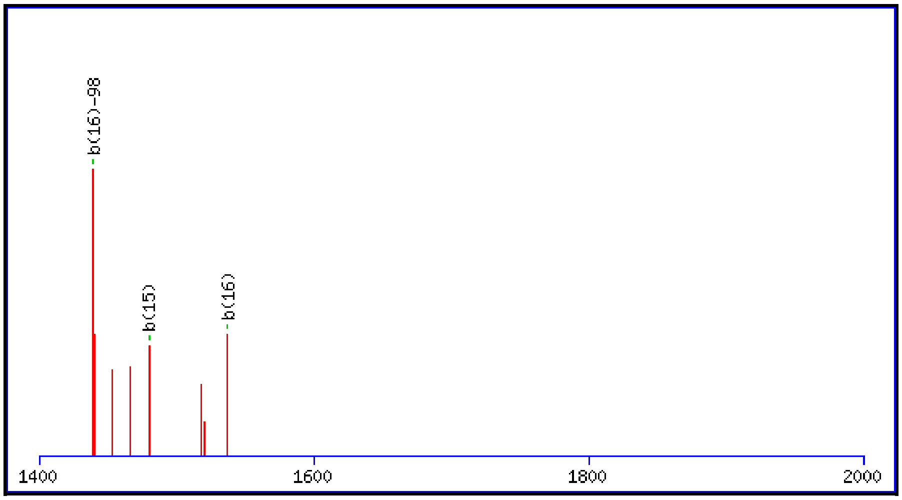

Monoisotopic mass of neutral peptide Mr(calc): 1682.7037
Fixed modifications: Carbamidomethyl (C)
Variable modifications:
S7 : Phospho (ST), with neutral losses 97.9769(shown in table), 0.0000
Ions Score: 87 **Expect:** 3.4e-007
Matches (Bold Red): 47/230 fragment ions using 59 most intense peaks

#	b	b^{++}	b^0	b^{0++}	Seq.	y	y^{++}	y*	y^{*++}	y^0	y^{0++}	#
1	116.0342	58.5207	98.0237	49.5155	D							17
2	217.0819	109.0446	199.0713	100.0393	T	1470.7071	735.8572	1453.6805	727.3439	1452.6965	726.8519	16
3	**346.1245**	173.5659	**328.1139**	164.5606	E	1369.6594	685.3333	1352.6328	676.8201	1351.6488	676.3281	15
4	**461.1514**	231.0794	443.1409	222.0741	D	1240.6168	620.8120	**1223.5903**	612.2988	1222.6062	611.8068	14
5	**562.1991**	281.6032	544.1885	272.5979	T	1125.5899	563.2986	1108.5633	554.7853	1107.5793	554.2933	13

6	619.2206	310.1139	601.2100	301.1086	G	1024.5422	512.7747	1007.5156	504.2615	1006.5316	503.7694	12
7	688.2420	344.6247	670.2315	335.6194	S	967.5207	484.2640	950.4942	475.7507	949.5101	475.2587	11
8	787.3104	394.1589	769.2999	385.1536	V	898.4993	449.7533	881.4727	441.2400	880.4887	440.7480	10
9	844.3319	422.6696	826.3213	413.6643	G	799.4308	400.2191	782.4043	391.7058	781.4203	391.2138	9
10	915.3690	458.1882	897.3585	449.1829	A	742.4094	371.7083	725.3828	363.1951	724.3988	362.7030	8
11	1012.4218	506.7145	994.4112	497.7092	P	671.3723	336.1898	654.3457	327.6765	653.3617	327.1845	7
12	1111.4902	556.2487	1093.4796	547.2435	V	574.3195	287.6634	557.2930	279.1501	556.3089	278.6581	6
13	1198.5222	599.7648	1180.5117	590.7595	S	475.2511	238.1292	458.2245	229.6159	457.2405	229.1239	5
14	1285.5543	643.2808	1267.5437	634.2755	S	388.2191	194.6132	371.1925	186.0999	370.2085	185.6079	4
15	1382.6070	691.8071	1364.5965	682.8019	P	301.1870	151.0972	284.1605	142.5839			3
16	1439.6285	720.3179	1421.6179	711.3126	G	204.1343	102.5708	187.1077	94.0575			2
17					K	147.1128	74.0600	130.0863	65.5468			1

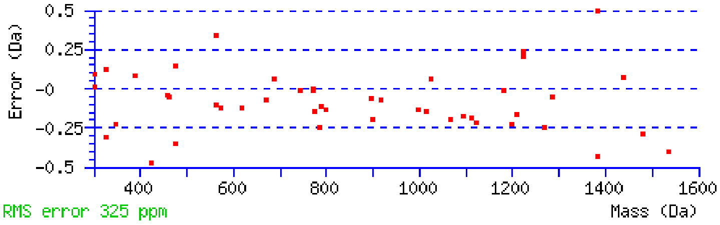

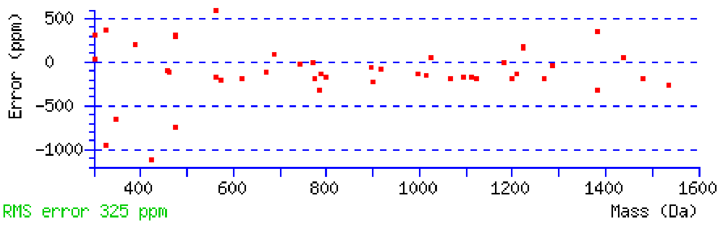

All matches to this query

Score	Mr(calc):	Delta	Sequence
86.9	1682.7037	0.0002	DTEDTGSVGAPVSSPGK
62.3	1682.7037	0.0002	DTEDTGSVGAPVSSPGK
52.6	1682.7037	0.0002	DTEDTGSVGAPVSSPGK
35.0	1682.7037	0.0002	DTEDTGSVGAPVSSPGK
27.7	1682.7037	0.0002	DTEDTGSVGAPVSSPGK
7.4	1682.6937	0.0101	GDSHSWGSDLSSLQK
7.2	1682.6937	0.0101	GDSHSWGSDLSSLQK
7.2	1682.6937	0.0101	GDSHSWGSDLSSLQK
6.4	1682.7124	-0.0085	DNSFLQVHNCIQK
5.9	1680.7131	1.9908	TDEKNPEDPLSEVK

Spectrum No: 28; Query: 1433; Rank: 1

Peptide View

MS/MS Fragmentation of **RGGGSGGGDESEGEEVDED**
Found in **IPI00189358**, Tax_Id=10116 Gene_Symbol=pur-beta Transcriptional activator protein Pur-beta

Match to Query 1433: 1916.654168 from(959.334360,2+)
Title: 091008RatKidney_NoSalt_33.302.302.2.dta
Data file K:\NewmanPaper\Piliang\3SubProteomes\Piliang3SP\mgf5ppm\ERLIC_3SubProteomes5ppm.mgf

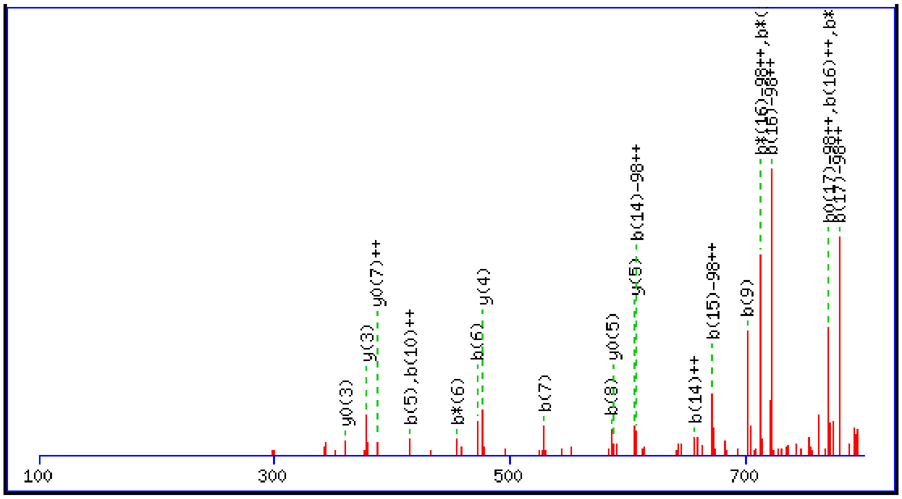

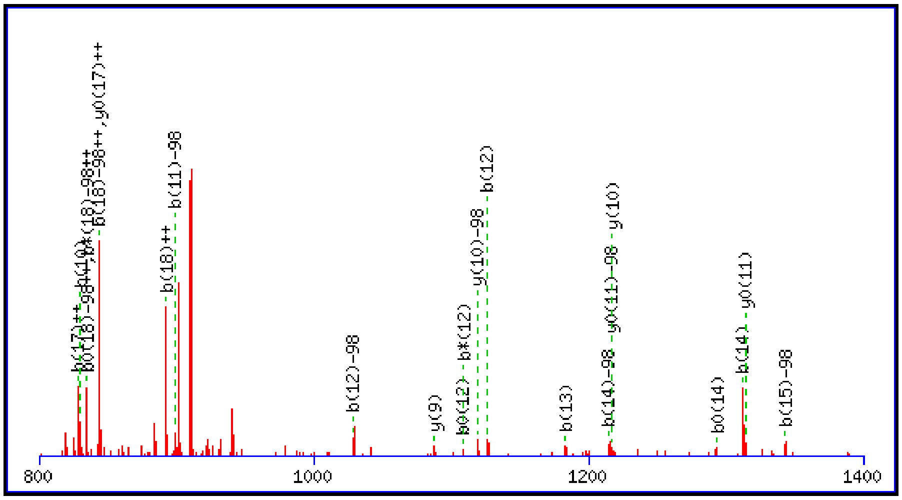

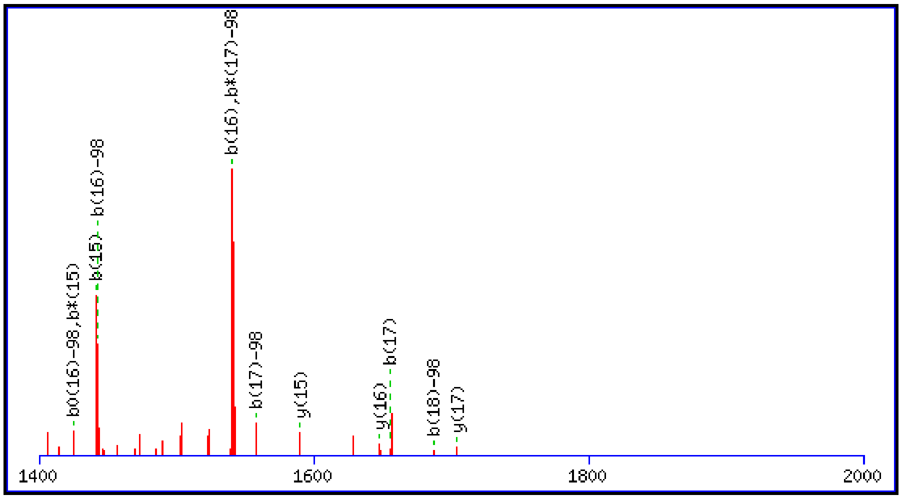

Monoisotopic mass of neutral peptide Mr(calc): 1916.6545
Fixed modifications: Carbamidomethyl (C)
Variable modifications:
S11 : Phospho (ST), with neutral losses 97.9769(shown in table), 0.0000
Ions Score: 86 **Expect:** 1.6e-007
Matches (Bold Red): 58/260 fragment ions using 75 most intense peaks

#	b	b^{++}	b*	b^{*++}	b^0	b^{0++}	Seq.	y	y^{++}	y^0	y^{0++}	#
1	157.1084	79.0578	140.0818	70.5446			R					19
2	214.1299	107.5686	197.1033	99.0553			G	1663.5838	832.2955	1645.5732	823.2902	18
3	271.1513	136.0793	254.1248	127.5660			G	1606.5623	803.7848	1588.5518	794.7795	17
4	328.1728	164.5900	311.1462	156.0768			G	1549.5409	775.2741	1531.5303	766.2688	16
5	**415.2048**	208.1060	398.1783	199.5928	397.1942	199.1008	S	1492.5194	746.7633	1474.5088	737.7581	15

6	**472.2263**	236.6168	**455.1997**	228.1035	454.2157	227.6115	G	1405.4874	703.2473	1387.4768	694.2420	14
7	**529.2477**	265.1275	512.2212	256.6142	511.2372	256.1222	G	1348.4659	674.7366	1330.4553	665.7313	13
8	**586.2692**	293.6382	569.2427	285.1250	568.2586	284.6330	G	1291.4444	646.2259	1273.4339	637.2206	12
9	**701.2961**	351.1517	684.2696	342.6384	683.2856	342.1464	D	1234.4230	617.7151	**1216.4124**	608.7098	11
10	**830.3387**	**415.6730**	813.3122	407.1597	812.3282	406.6677	E	**1119.3960**	560.2017	1101.3855	551.1964	10
11	**899.3602**	450.1837	882.3336	441.6705	881.3496	441.1785	S	990.3534	495.6804	972.3429	486.6751	9
12	**1028.4028**	514.7050	1011.3762	506.1918	1010.3922	505.6997	E	921.3320	461.1696	903.3214	452.1643	8
13	1085.4242	543.2158	1068.3977	534.7025	1067.4137	534.2105	G	792.2894	396.6483	774.2788	**387.6430**	7
14	**1214.4668**	**607.7371**	1197.4403	599.2238	1196.4563	598.7318	E	735.2679	368.1376	717.2574	359.1323	6
15	**1343.5094**	**672.2584**	1326.4829	663.7451	1325.4989	663.2531	E	**606.2253**	303.6163	**588.2148**	294.6110	5
16	**1442.5778**	**721.7926**	1425.5513	**713.2793**	**1424.5673**	**712.7873**	V	**477.1827**	239.0950	459.1722	230.0897	4
17	**1557.6048**	**779.3060**	**1540.5782**	**770.7928**	1539.5942	**770.3008**	D	**378.1143**	189.5608	**360.1038**	180.5555	3
18	**1686.6474**	**843.8273**	1669.6208	**835.3141**	1668.6368	**834.8220**	E	263.0874	132.0473	245.0768	123.0420	2
19							D	134.0448	67.5260	116.0342	58.5207	1

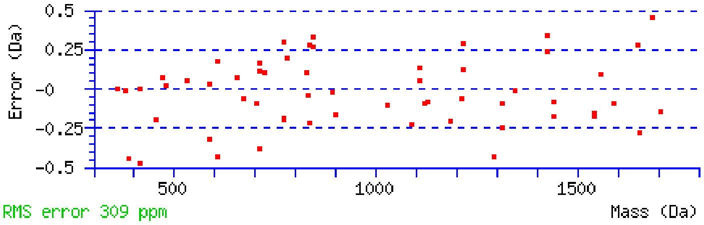

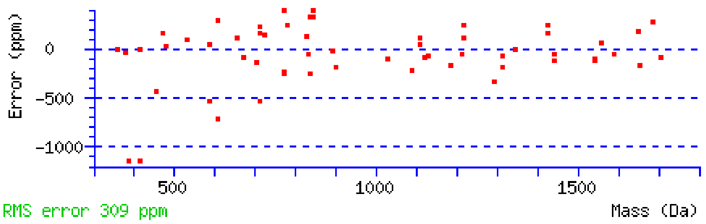

All matches to this query

Score	Mr(calc):	Delta	Sequence
85.6	1916.6545	-0.0004	RGGGSGGGDESEGEEVDED
25.2	1916.6545	-0.0004	RGGGSGGGDESEGEEVDED
2.0	1916.6639	-0.0097	XLRAGGAQGCGGDSGATR
0.6	1916.6649	-0.0107	MNNSTSAGVYANGNDNK
0.6	1916.6649	-0.0107	MNNSTSAGVYANGNDNK
0.6	1916.6649	-0.0107	MNNSTSAGVYANGNDNK
0.2	1914.6314	2.0228	MDNTTFGKLSSHLK

Spectrum No: 29; Query: 2288; Rank: 1

Peptide View

MS/MS Fragmentation of **SSPEIAPSDVTDESEVVTAADIEK**
Found in **IPI00358691**, Tax_Id=10116 Gene_Symbol=Eps15 Epidermal growth factor receptor pathway substrate 15

Match to Query 2288: 2568.143328 from(1285.078940,2+)
Title: 100101RatKid_NS_deglyco_23.3798.3798.2.dta
Data file K:\NewmanPaper\Piliang\3SubProteomes\Piliang3SP\mgf5ppm\ERLIC_3SubProteomes5ppm.mgf

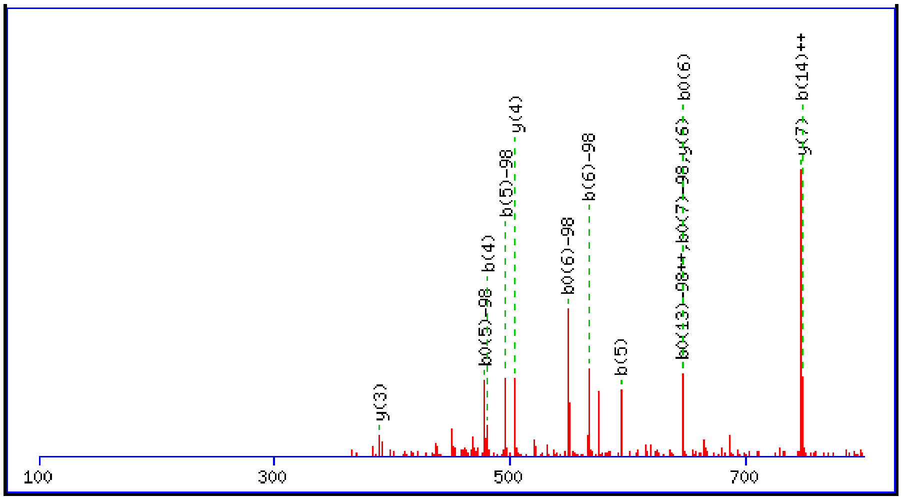

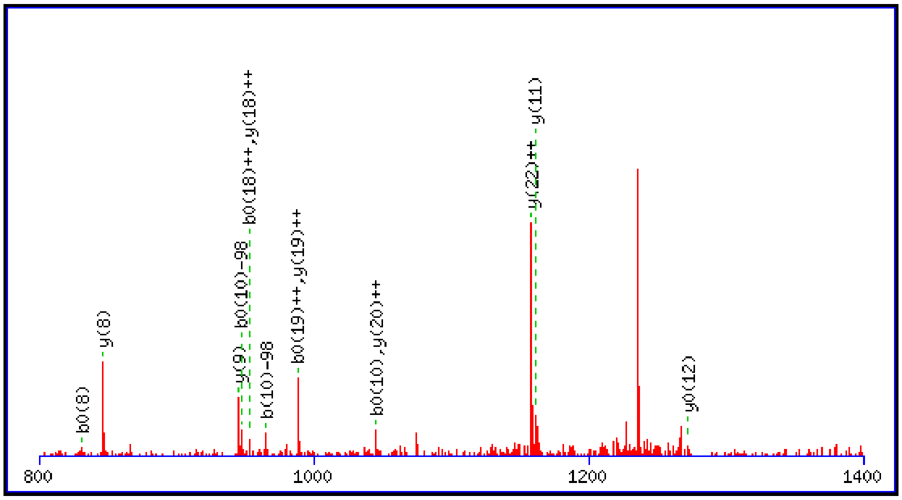

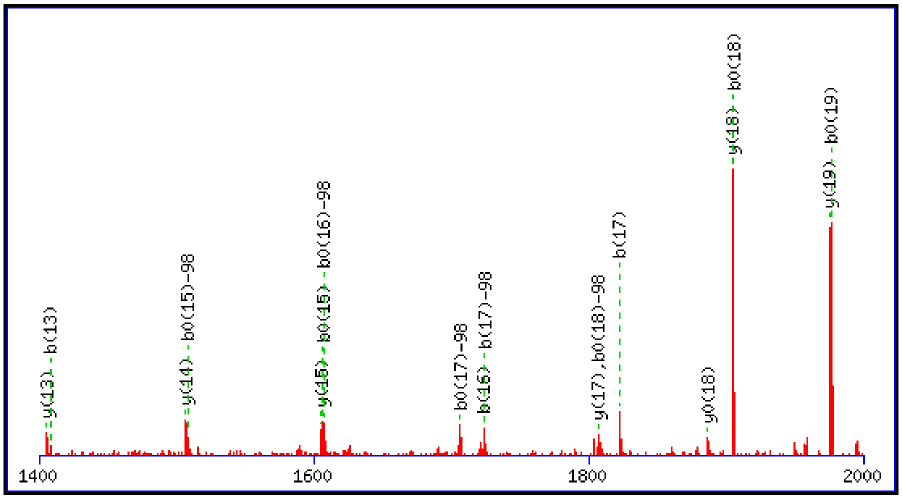

Monoisotopic mass of neutral peptide Mr(calc): 2568.1367
Fixed modifications: Carbamidomethyl (C)
Variable modifications:
S2 : Phospho (ST), with neutral losses 97.9769(shown in table), 0.0000
Ions Score: 85 **Expect:** 1.1e-006
Matches (Bold Red): 46/322 fragment ions using 56 most intense peaks

#	b	b^{++}	b^{0}	b^{0++}	Seq.	y	y^{++}	y^{*}	y^{*++}	y^{0}	y^{0++}	#
1	88.0393	44.5233	70.0287	35.5180	S							24
2	157.0608	79.0340	139.0502	70.0287	S	2384.1351	1192.5712	2367.1086	1184.0579	2366.1246	1183.5659	23
3	254.1135	127.5604	236.1030	118.5551	P	2315.1137	**1158.0605**	2298.0871	1149.5472	2297.1031	1149.0552	22
4	383.1561	192.0817	365.1456	183.0764	E	2218.0609	1109.5341	2201.0343	1101.0208	2200.0503	1100.5288	21
5	**496.2402**	248.6237	**478.2296**	239.6184	I	2089.0183	**1045.0128**	2071.9918	1036.4995	2071.0077	1036.0075	20

**6**	**567.2773**	284.1423	**549.2667**	275.1370	**A**	**1975.9342**	**988.4708**	1958.9077	979.9575	1957.9237	979.4655	**19**
**7**	664.3301	332.6687	**646.3195**	323.6634	**P**	**1904.8971**	**952.9522**	1887.8706	944.4389	**1886.8866**	943.9469	**18**
**8**	751.3621	376.1847	733.3515	367.1794	**S**	**1807.8444**	904.4258	1790.8178	895.9125	1789.8338	895.4205	**17**
**9**	866.3890	433.6982	848.3785	424.6929	**D**	1720.8123	860.9098	1703.7858	852.3965	1702.8018	851.9045	**16**
**10**	**965.4574**	483.2324	**947.4469**	474.2271	**V**	**1605.7854**	803.3963	1588.7588	794.8831	1587.7748	794.3911	**15**
**11**	1066.5051	533.7562	1048.4946	524.7509	**T**	**1506.7170**	753.8621	1489.6904	745.3489	1488.7064	744.8568	**14**
**12**	1181.5321	591.2697	1163.5215	582.2644	**D**	**1405.6693**	703.3383	1388.6427	694.8250	1387.6587	694.3330	**13**
**13**	1310.5747	655.7910	1292.5641	**646.7857**	**E**	1290.6424	645.8248	1273.6158	637.3115	**1272.6318**	636.8195	**12**
**14**	1397.6067	699.3070	1379.5961	690.3017	**S**	**1161.5998**	581.3035	1144.5732	572.7902	1143.5892	572.2982	**11**
**15**	1526.6493	763.8283	**1508.6387**	754.8230	**E**	1074.5677	537.7875	1057.5412	529.2742	1056.5572	528.7822	**10**
**16**	1625.7177	813.3625	**1607.7071**	804.3572	**V**	**945.5251**	473.2662	928.4986	464.7529	927.5146	464.2609	**9**
**17**	**1724.7861**	862.8967	**1706.7755**	853.8914	**V**	**846.4567**	423.7320	829.4302	415.2187	828.4462	414.7267	**8**
**18**	1825.8338	913.4205	**1807.8232**	904.4152	**T**	**747.3883**	374.1978	730.3618	365.6845	729.3777	365.1925	**7**
**19**	1896.8709	948.9391	1878.8603	939.9338	**A**	**646.3406**	323.6740	629.3141	315.1607	628.3301	314.6687	**6**
**20**	1967.9080	984.4576	1949.8975	975.4524	**A**	575.3035	288.1554	558.2770	279.6421	557.2930	279.1501	**5**
**21**	2082.9350	1041.9711	2064.9244	1032.9658	**D**	**504.2664**	252.6368	487.2399	244.1236	486.2558	243.6316	**4**
**22**	2196.0190	1098.5131	2178.0085	1089.5079	**I**	**389.2395**	195.1234	372.2129	186.6101	371.2289	186.1181	**3**
**23**	2325.0616	1163.0344	2307.0511	1154.0292	**E**	276.1554	138.5813	259.1288	130.0681	258.1448	129.5761	**2**
**24**					**K**	147.1128	74.0600	130.0863	65.5468			**1**

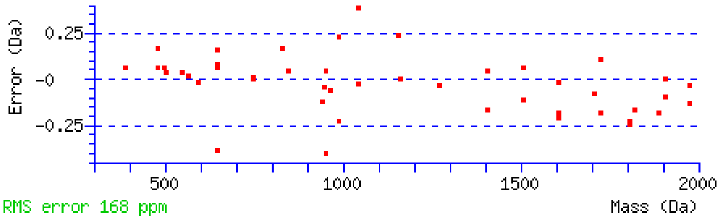

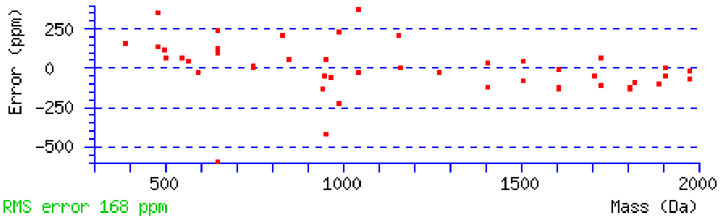

All matches to this query

Score	Mr(calc):	Delta	Sequence
85.3	2568.1367	0.0066	SSPEIAPSDVTDESEVVTAADIEK
85.3	2568.1367	0.0066	SSPEIAPSDVTDESEVVTAADIEK
49.1	2568.1367	0.0066	SSPEIAPSDVTDESEVVTAADIEK
28.6	2568.1367	0.0066	SSPEIAPSDVTDESEVVTAADIEK
20.1	2568.1367	0.0066	SSPEIAPSDVTDESEVVTAADIEK
5.5	2568.1351	0.0082	QMYSGIPIIAANMDTVGTFEMAK
2.0	2566.1173	2.0260	GGHMPPGGNSGELQGTHPPVLHAHS
1.3	2567.1209	1.0224	FFTGPMSDFKNVGLVFVNSKR
1.2	2568.1597	-0.0164	VISHMLSSHGGEIFLHVLSSSR
0.7	2567.1591	0.9843	GTPEKASESVKAETMTVSSLAIR

Spectrum No: 30; Query: 947; Rank: 1

Peptide View

MS/MS Fragmentation of **TLSPTPSAEGFQDGR**
Found in **IPI00198339**, Tax_Id=10116 Gene_Symbol=Ablim1_predicted 78 kDa protein

Match to Query 947: 1641.698248 from(821.856400,2+)
Title: 091008RatKidney_NoSalt_23.1542.1542.2.dta
Data file K:\NewmanPaper\Piliang\3SubProteomes\Piliang3SP\mgf5ppm\ERLIC_3SubProteomes5ppm.mgf

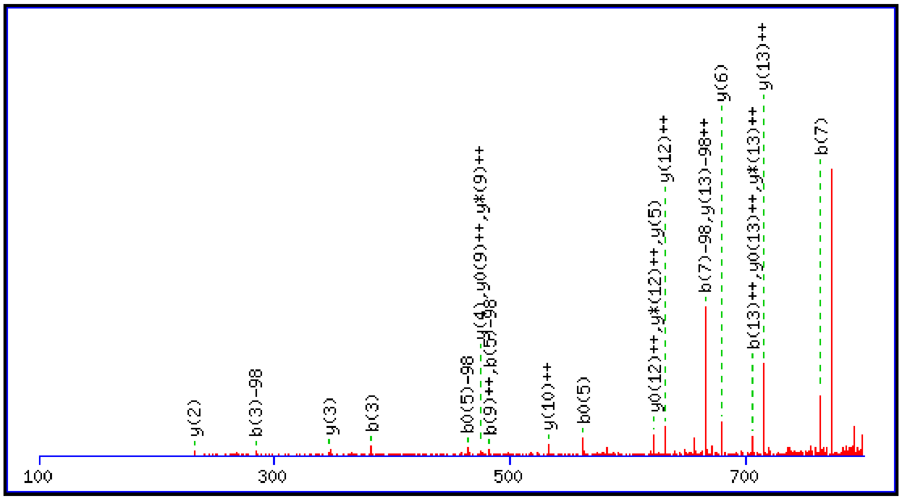

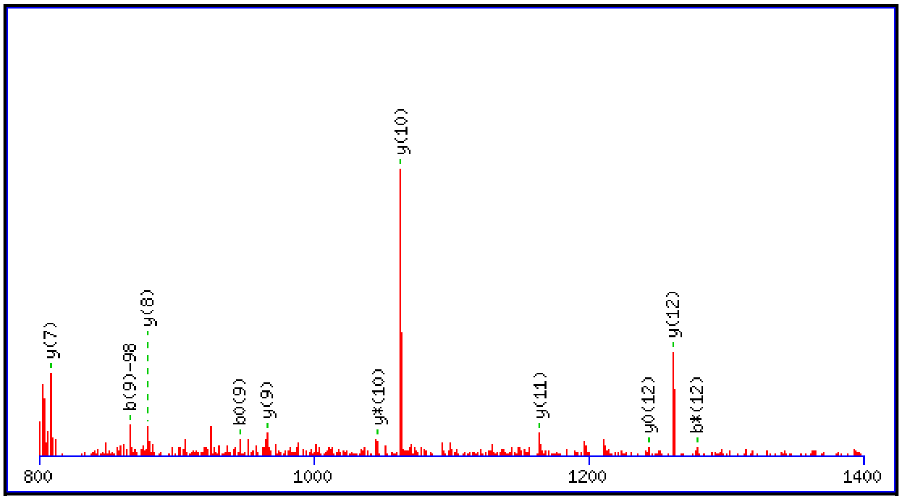

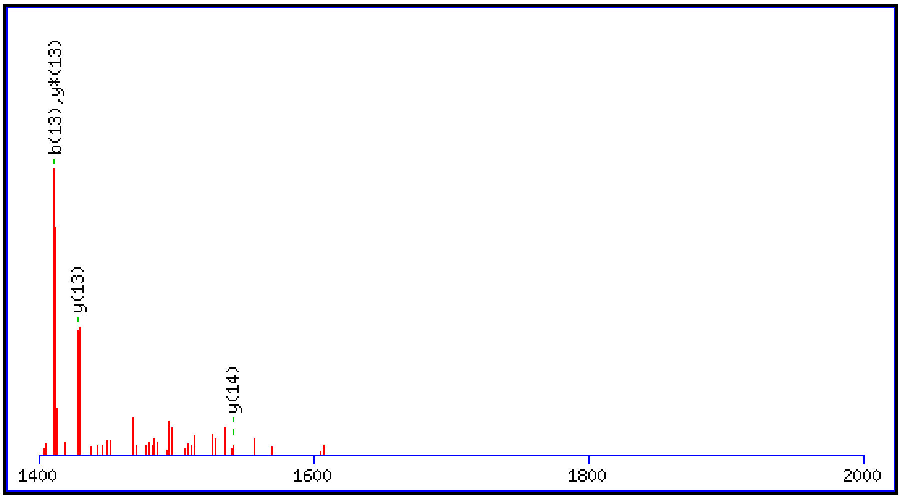

Monoisotopic mass of neutral peptide Mr(calc): 1641.7036
Fixed modifications: Carbamidomethyl (C)
Variable modifications:
S3 : Phospho (ST), with neutral losses 0.0000(shown in table), 97.9769
Ions Score: 85 **Expect:** 5.8e-007
Matches (Bold Red): 39/208 fragment ions using 57 most intense peaks

#	b	b^{++}	b*	b^{*++}	b^0	b^{0++}	Seq.	y	y^{++}	y*	y^{*++}	y^0	y^{0++}	#
1	102.0550	51.5311			84.0444	42.5258	T							15
2	215.1390	108.0731			197.1285	99.0679	L	**1541.6632**	771.3352	1524.6366	762.8220	1523.6526	762.3299	14
3	**382.1374**	191.5723			364.1268	182.5670	S	**1428.5791**	**714.7932**	**1411.5526**	**706.2799**	1410.5685	**705.7879**	13
4	479.1901	240.0987			461.1796	231.0934	P	**1261.5808**	**631.2940**	1244.5542	**622.7807**	**1243.5702**	**622.2887**	12
5	580.2378	290.6225			**562.2272**	281.6173	T	**1164.5280**	582.7676	1147.5014	574.2544	1146.5174	573.7624	11

**6**	677.2906	339.1489			659.2800	330.1436	**P**	**1063.4803**	**532.2438**	**1046.4538**	523.7305	1045.4697	523.2385	**10**
**7**	**764.3226**	382.6649			746.3120	373.6597	**S**	**966.4276**	483.7174	949.4010	**475.2041**	948.4170	**474.7121**	**9**
**8**	835.3597	418.1835			817.3492	409.1782	**A**	**879.3955**	440.2014	862.3690	431.6881	861.3850	431.1961	**8**
**9**	964.4023	**482.7048**			**946.3917**	473.6995	**E**	**808.3584**	404.6828	791.3319	396.1696	790.3478	395.6776	**7**
**10**	1021.4238	511.2155			1003.4132	502.2102	**G**	**679.3158**	340.1615	662.2893	331.6483	661.3053	331.1563	**6**
**11**	1168.4922	584.7497			1150.4816	575.7445	**F**	**622.2944**	311.6508	605.2678	303.1375	604.2838	302.6455	**5**
**12**	1296.5508	648.7790	**1279.5242**	640.2657	1278.5402	639.7737	**Q**	**475.2259**	238.1166	458.1994	229.6033	457.2154	229.1113	**4**
**13**	**1411.5777**	**706.2925**	1394.5512	697.7792	1393.5671	697.2872	**D**	**347.1674**	174.0873	330.1408	165.5740	329.1568	165.0820	**3**
**14**	1468.5992	734.8032	1451.5726	726.2900	1450.5886	725.7979	**G**	**232.1404**	116.5738	215.1139	108.0606			**2**
**15**							**R**	175.1190	88.0631	158.0924	79.5498			**1**

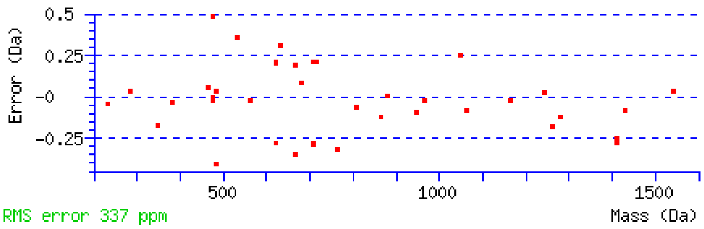

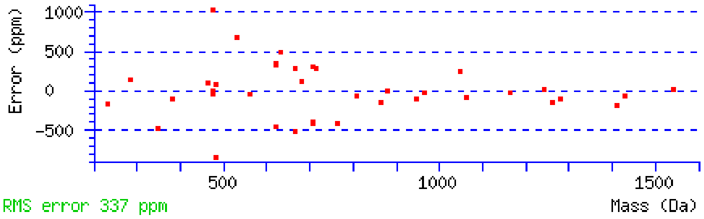

All matches to this query

Score	Mr(calc):	Delta	Sequence
84.8	1641.7036	-0.0053	TLSPTPSAEGFQDGR
68.5	1641.7036	-0.0053	TLSPTPSAEGFQDGR
58.2	1641.7036	-0.0053	TLSPTPSAEGFQDGR
34.9	1641.7036	-0.0053	TLSPTPSAEGFQDGR
4.6	1640.7004	0.9978	DGKTPNDELEIMKA
3.9	1641.7069	-0.0087	DNGIRPSSLEQMAK
3.4	1641.6913	0.0070	GASPASAAAQAPGASLR
1.7	1640.6888	1.0094	APKSSFIGDGDIFK
1.6	1641.6892	0.0091	DVPSTQKCPLMDR
1.2	1641.6892	0.0091	DVPSTQKCPLMDR

Spectrum No: 31; Query: 1509; Rank: 1

Peptide View

MS/MS Fragmentation of **VTGNPEDSCASEAPGSSPK**
Found in **IPI00191563**, Tax_Id=10116 Gene_Symbol=LOC690489 hypothetical protein LOC690489

Match to Query 1509: 1968.778608 from(985.396580,2+)
Title: 091008RatKidney_NoSalt_26.532.532.2.dta
Data file K:\NewmanPaper\Piliang\3SubProteomes\Piliang3SP\mgf5ppm\ERLIC_3SubProteomes5ppm.mgf

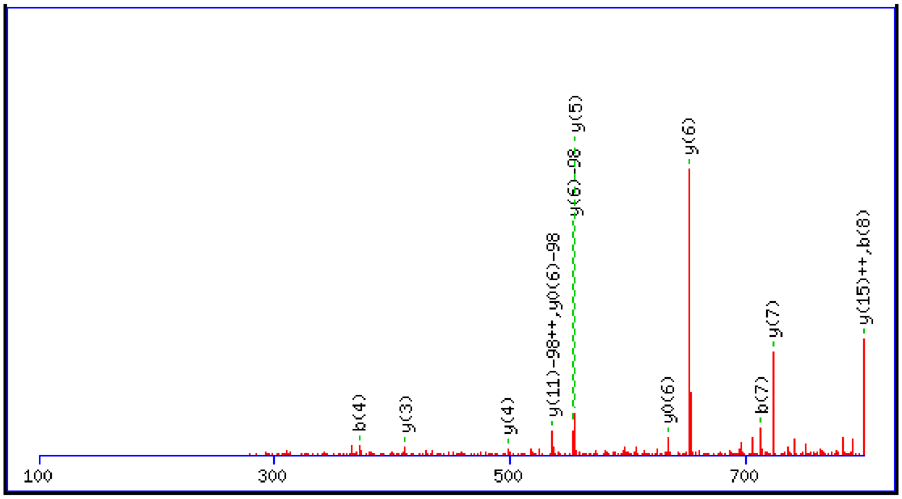

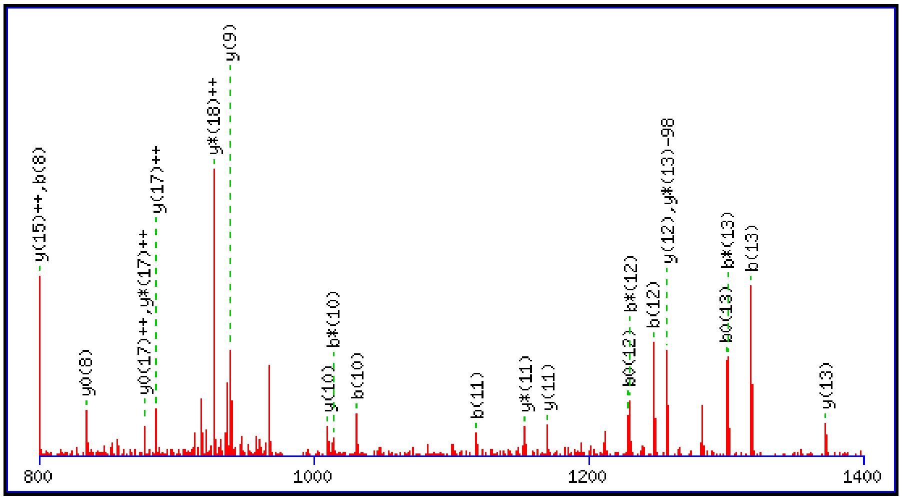

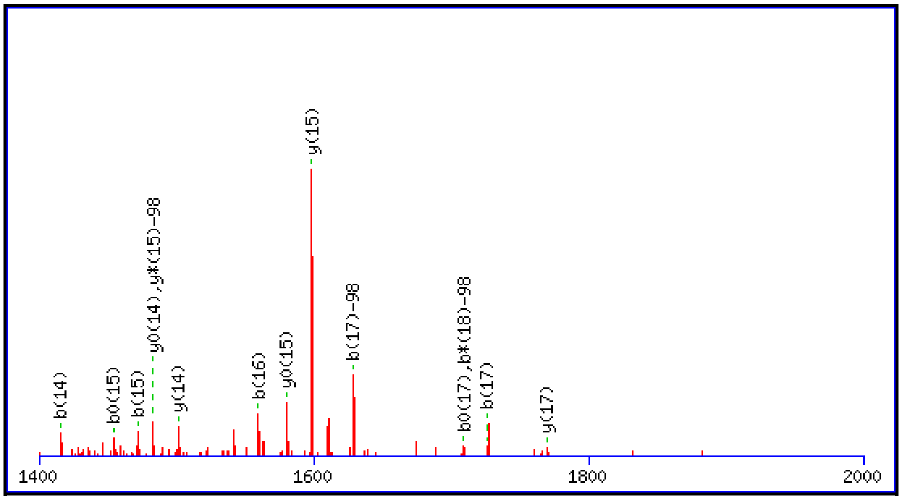

Monoisotopic mass of neutral peptide Mr(calc): 1968.7772
Fixed modifications: Carbamidomethyl (C)
Variable modifications:
S17 : Phospho (ST), with neutral losses 0.0000(shown in table), 97.9769
Ions Score: 85 **Expect:** 5.3e-007
Matches (Bold Red): 48/312 fragment ions using 61 most intense peaks

#	b	b^{++}	b*	b^{*++}	b^0	b^{0++}	Seq.	y	y^{++}	y*	y^{*++}	y^0	y^{0++}	#
1	100.0757	50.5415					V							19
2	201.1234	101.0653			183.1128	92.0600	T	1870.7161	935.8617	1853.6895	**927.3484**	1852.7055	926.8564	18
3	258.1448	129.5761			240.1343	120.5708	G	**1769.6684**	**885.3378**	1752.6418	**876.8246**	1751.6578	**876.3326**	17
4	**372.1878**	186.5975	355.1612	178.0842	354.1772	177.5922	N	1712.6469	856.8271	1695.6204	848.3138	1694.6364	847.8218	16
5	469.2405	235.1239	452.2140	226.6106	451.2300	226.1186	P	**1598.6040**	**799.8056**	1581.5775	791.2924	**1580.5934**	790.8004	15

**6**	598.2831	299.6452	581.2566	291.1319	580.2726	290.6399	**E**	**1501.5512**	751.2793	1484.5247	742.7660	**1483.5407**	742.2740	**14**
**7**	**713.3101**	357.1587	696.2835	348.6454	695.2995	348.1534	**D**	**1372.5086**	686.7580	1355.4821	678.2447	1354.4981	677.7527	**13**
**8**	**800.3421**	400.6747	783.3155	392.1614	782.3315	391.6694	**S**	**1257.4817**	629.2445	1240.4552	620.7312	1239.4711	620.2392	**12**
**9**	960.3727	480.6900	943.3462	472.1767	942.3622	471.6847	**C**	**1170.4497**	585.7285	**1153.4231**	577.2152	1152.4391	576.7232	**11**
**10**	**1031.4099**	516.2086	**1014.3833**	507.6953	1013.3993	507.2033	**A**	**1010.4190**	505.7132	993.3925	497.1999	992.4085	496.7079	**10**
**11**	**1118.4419**	559.7246	1101.4153	551.2113	1100.4313	550.7193	**S**	**939.3819**	470.1946	922.3554	461.6813	921.3713	461.1893	**9**
**12**	**1247.4845**	624.2459	**1230.4579**	615.7326	**1229.4739**	615.2406	**E**	852.3499	426.6786	835.3233	418.1653	**834.3393**	417.6733	**8**
**13**	**1318.5216**	659.7644	**1301.4950**	651.2512	**1300.5110**	650.7591	**A**	**723.3073**	362.1573	706.2807	353.6440	705.2967	353.1520	**7**
**14**	**1415.5744**	708.2908	1398.5478	699.7775	1397.5638	699.2855	**P**	**652.2702**	326.6387	635.2436	318.1255	**634.2596**	317.6334	**6**
**15**	**1472.5958**	736.8015	1455.5693	728.2883	**1454.5852**	727.7963	**G**	**555.2174**	278.1123	538.1909	269.5991	537.2068	269.1071	**5**
**16**	**1559.6278**	780.3176	1542.6013	771.8043	1541.6173	771.3123	**S**	**498.1959**	249.6016	481.1694	241.0883	480.1854	240.5963	**4**
**17**	**1726.6262**	863.8167	1709.5996	855.3035	**1708.6156**	854.8115	**S**	**411.1639**	206.0856	394.1374	197.5723	393.1534	197.0803	**3**
**18**	1823.6790	912.3431	1806.6524	903.8298	1805.6684	903.3378	**P**	244.1656	122.5864	227.1390	114.0731			**2**
**19**							**K**	147.1128	74.0600	130.0863	65.5468			**1**

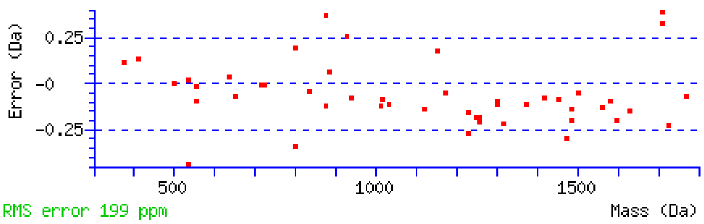

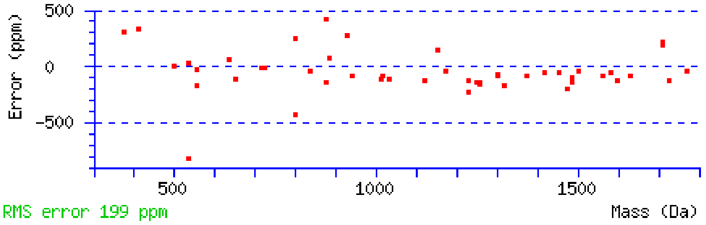

All matches to this query

Score	Mr(calc):	Delta	Sequence
84.6	1968.7772	0.0014	VTGNPEDSCASEAPGSSPK
70.0	1968.7772	0.0014	VTGNPEDSCASEAPGSSPK
35.8	1968.7772	0.0014	VTGNPEDSCASEAPGSSPK
31.5	1968.7772	0.0014	VTGNPEDSCASEAPGSSPK
13.0	1968.7647	0.0139	DKSLQMKYYVWPR
12.2	1968.7772	0.0014	VTGNPEDSCASEAPGSSPK
6.9	1967.7581	1.0205	LLYQGPSGPGAPASGGSGR
4.3	1968.7818	-0.0032	MISLQNTHLKATESR
2.8	1967.7792	0.9994	VTTERDSHTEVVISR
0.8	1967.7914	0.9872	EEDEASPPSALPPGLGSR

Spectrum No: 32; Query: 1199; Rank: 1

Peptide View

MS/MS Fragmentation of **SASPAPADVAPAQEDLR**
Found in **IPI00193648**, Tax_Id=10116 Gene_Symbol=G3bp similar to Ras-GTPase-activating protein binding protein 1

Match to Query 1199: 1773.795728 from(887.905140,2+)
Title: 091008RatKidney_NoSalt_25.1484.1484.2.dta
Data file K:\NewmanPaper\Piliang\3SubProteomes\Piliang3SP\mgf5ppm\ERLIC_3SubProteomes5ppm.mgf

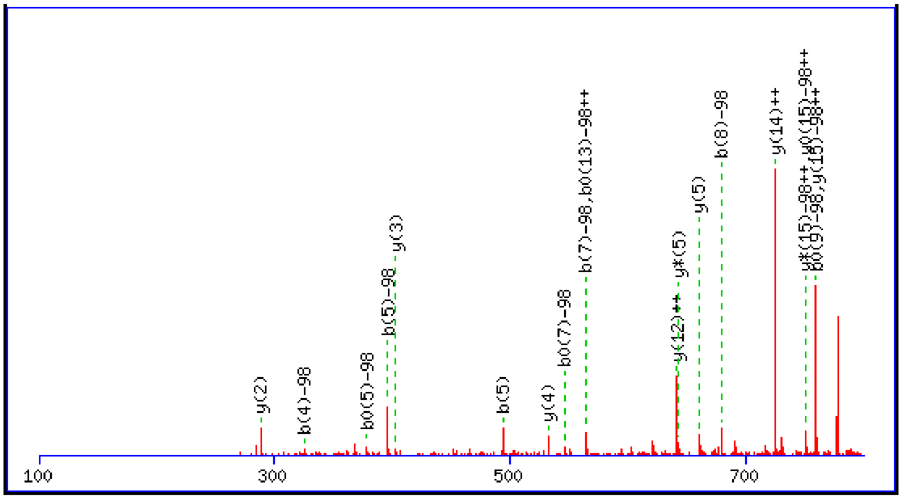

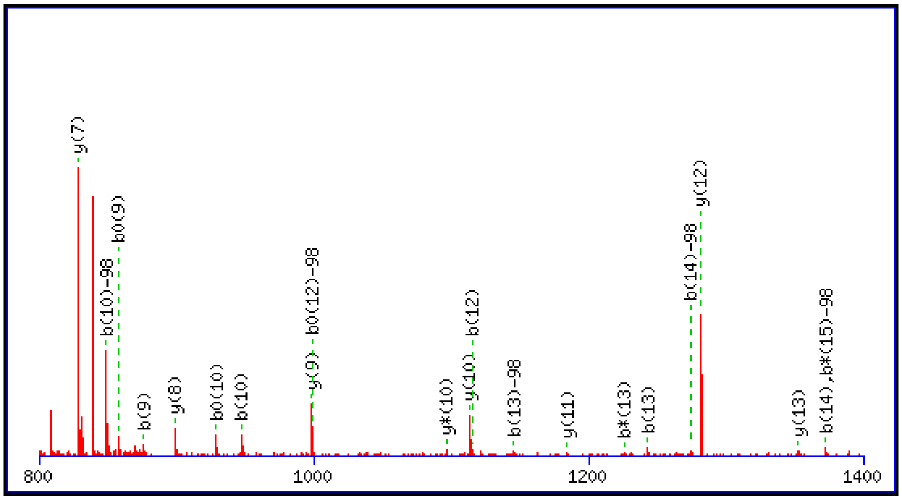

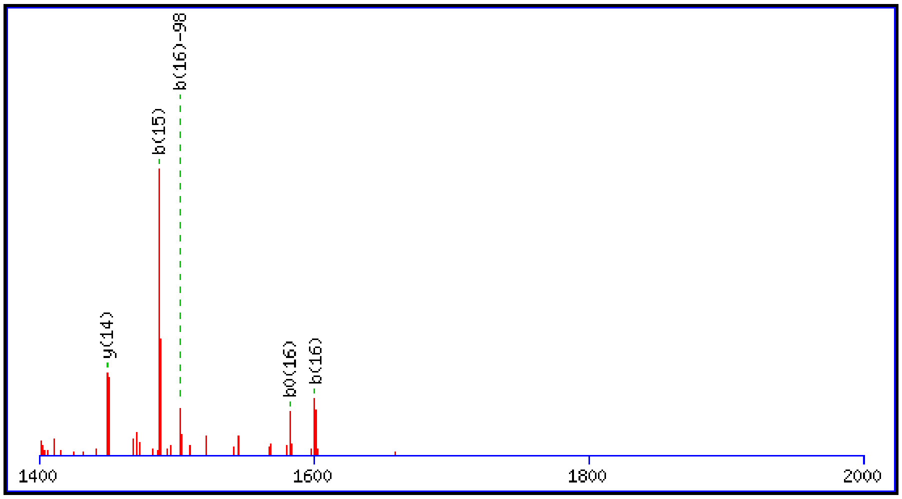

Monoisotopic mass of neutral peptide Mr(calc): 1773.7934
Fixed modifications: Carbamidomethyl (C)
Variable modifications:
S3 : Phospho (ST), with neutral losses 97.9769(shown in table), 0.0000
Ions Score: 84 **Expect:** 7.6e-007
Matches (Bold Red): 45/240 fragment ions using 66 most intense peaks

#	b	b^{++}	b*	b^{*++}	b^0	b^{0++}	Seq.	y	y^{++}	y*	y^{*++}	y^0	y^{0++}	#
1	88.0393	44.5233			70.0287	35.5180	S							17
2	159.0764	80.0418			141.0659	71.0366	A	1589.7918	795.3995	1572.7653	786.8863	1571.7812	786.3943	16
3	228.0979	114.5526			210.0873	105.5473	S	1518.7547	**759.8810**	1501.7281	**751.3677**	1500.7441	**750.8757**	15
4	**325.1506**	163.0790			307.1401	154.0737	P	**1449.7332**	**725.3703**	1432.7067	716.8570	1431.7227	716.3650	14
5	**396.1878**	198.5975			**378.1772**	189.5922	A	**1352.6805**	676.8439	1335.6539	668.3306	1334.6699	667.8386	13

6	493.2405	247.1239			475.2300	238.1186	P	**1281.6434**	**641.3253**	1264.6168	632.8120	1263.6328	632.3200	12
7	**564.2776**	282.6425			**546.2671**	273.6372	A	**1184.5906**	592.7989	1167.5640	584.2857	1166.5800	583.7937	11
8	**679.3046**	340.1559			661.2940	331.1506	D	**1113.5535**	557.2804	**1096.5269**	548.7671	1095.5429	548.2751	10
9	778.3730	389.6901			**760.3624**	380.6848	V	**998.5265**	499.7669	981.5000	491.2536	980.5160	490.7616	9
10	**849.4101**	425.2087			831.3995	416.2034	A	**899.4581**	450.2327	882.4316	441.7194	881.4476	441.2274	8
11	946.4629	473.7351			928.4523	464.7298	P	**828.4210**	414.7141	811.3945	406.2009	810.4104	405.7089	7
12	1017.5000	509.2536			**999.4894**	500.2483	A	731.3682	366.1878	714.3417	357.6745	713.3577	357.1825	6
13	**1145.5586**	573.2829	1128.5320	564.7696	1127.5480	**564.2776**	Q	**660.3311**	330.6692	**643.3046**	322.1559	642.3206	321.6639	5
14	**1274.6012**	637.8042	1257.5746	629.2909	1256.5906	628.7989	E	**532.2726**	266.6399	515.2460	258.1266	514.2620	257.6346	4
15	1389.6281	695.3177	**1372.6015**	686.8044	1371.6175	686.3124	D	**403.2300**	202.1186	386.2034	193.6053	385.2194	193.1133	3
16	**1502.7122**	751.8597	1485.6856	743.3464	1484.7016	742.8544	L	**288.2030**	144.6051	271.1765	136.0919			2
17							R	175.1190	88.0631	158.0924	79.5498			1

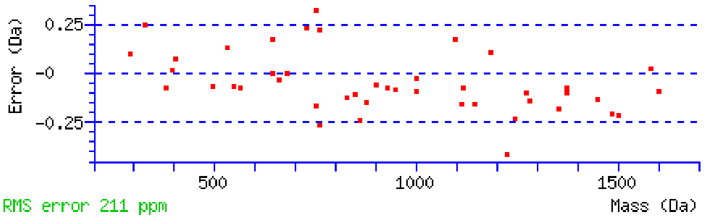

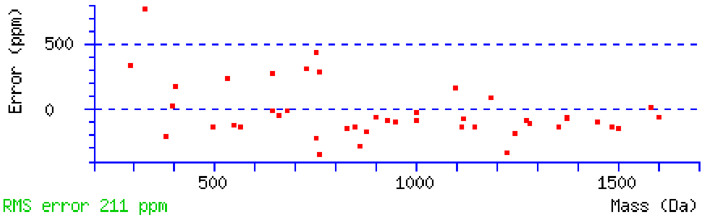

All matches to this query

Score	Mr(calc):	Delta	Sequence
84.4	1773.7934	0.0023	SASPAPADVAPAQEDLR
78.2	1773.7934	0.0023	SASPAPADVAPAQEDLR
9.3	1773.8042	-0.0085	ALEVTSMAMELNTLR
7.9	1773.8042	-0.0085	ALEVTSMAMELNTLR
5.5	1773.8026	-0.0068	ENLMASDHLDTPMLR
2.6	1772.8094	0.9863	IPSDPVEQAARPEER
1.1	1771.7818	2.0139	EVHFFVNASDVNSVK
0.4	1773.8080	-0.0123	SKVNQSEIGTRMSTR
0.4	1773.8080	-0.0123	SKVNQSEIGTRMSTR
0.3	1771.7818	2.0139	EVHFFVNASDVNSVK

Spectrum No: 33; Query: 1674; Rank: 1

Peptide View

MS/MS Fragmentation of **NLLEDDSDEEEDFFLR**
Found in **IPI00358945**, Tax_Id=10116 Gene_Symbol=Vamp4_predicted 16 kDa protein

Match to Query 1674: 2064.829048 from(1033.421800,2+)
Title: 100101RatKid_NS_deglyco_24.4951.4951.2.dta
Data file K:\NewmanPaper\Piliang\3SubProteomes\Piliang3SP\mgf5ppm\ERLIC_3SubProteomes5ppm.mgf

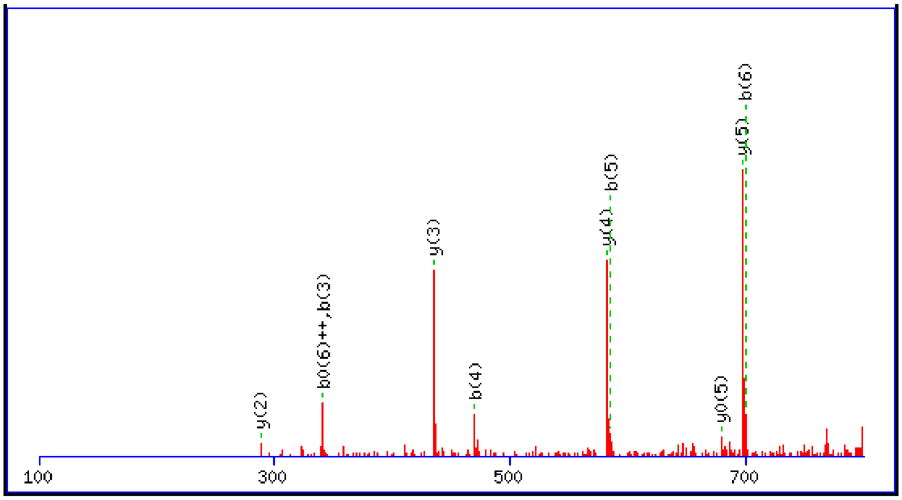

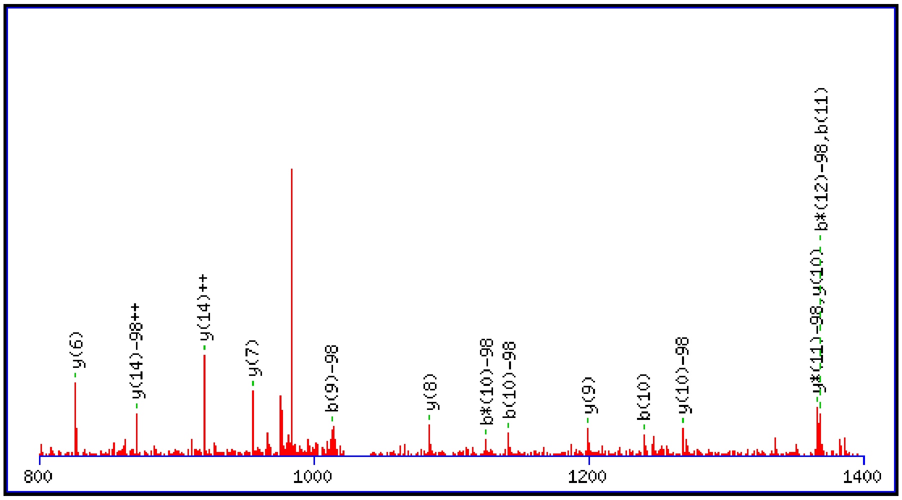

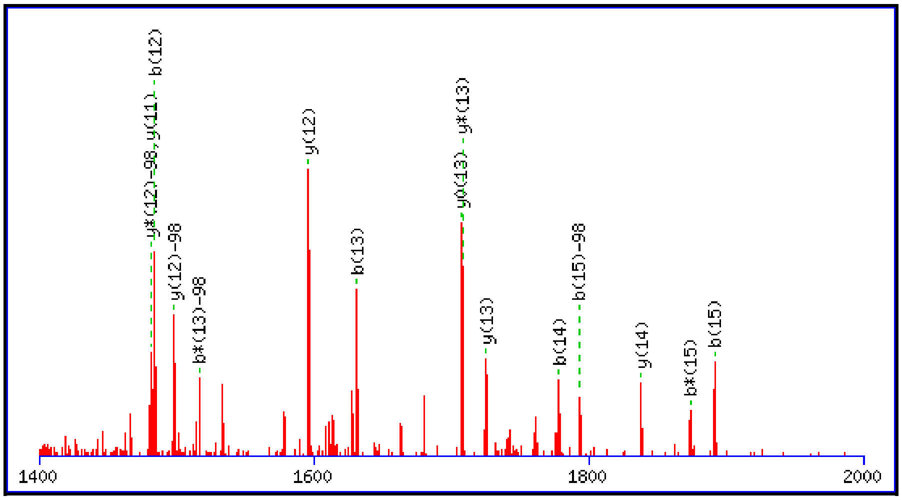

Monoisotopic mass of neutral peptide Mr(calc): 2064.8201
Fixed modifications: Carbamidomethyl (C)
Variable modifications:
S7 : Phospho (ST), with neutral losses 0.0000(shown in table), 97.9769
Ions Score: 84 **Expect:** 7.4e-007
Matches (Bold Red): 40/256 fragment ions using 54 most intense peaks

#	b	b^{++}	b*	b^{*++}	b^{0}	b^{0++}	Seq.	y	y^{++}	y*	y^{*++}	y^{0}	y^{0++}	#
1	115.0502	58.0287	98.0237	49.5155			N							16
2	228.1343	114.5708	211.1077	106.0575			L	1951.7845	976.3959	1934.7579	967.8826	1933.7739	967.3906	15
3	**341.2183**	171.1128	324.1918	162.5995			L	**1838.7004**	**919.8538**	1821.6739	911.3406	1820.6898	910.8486	14
4	**470.2609**	235.6341	453.2344	227.1208	452.2504	226.6288	E	**1725.6163**	863.3118	**1708.5898**	854.7985	**1707.6058**	854.3065	13
5	**585.2879**	293.1476	568.2613	284.6343	567.2773	284.1423	D	**1596.5737**	798.7905	1579.5472	790.2772	1578.5632	789.7852	12

6	700.3148	350.6610	683.2883	342.1478	682.3042	341.6558	D	1481.5468	741.2770	1464.5203	732.7638	1463.5362	732.2718	11
7	867.3132	434.1602	850.2866	425.6469	849.3026	425.1549	S	1366.5199	683.7636	1349.4933	675.2503	1348.5093	674.7583	10
8	982.3401	491.6737	965.3136	483.1604	964.3295	482.6684	D	1199.5215	600.2644	1182.4950	591.7511	1181.5109	591.2591	9
9	1111.3827	556.1950	1094.3561	547.6817	1093.3721	547.1897	E	1084.4946	542.7509	1067.4680	534.2376	1066.4840	533.7456	8
10	1240.4253	620.7163	1223.3987	612.2030	1222.4147	611.7110	E	955.4520	478.2296	938.4254	469.7164	937.4414	469.2243	7
11	1369.4679	685.2376	1352.4413	676.7243	1351.4573	676.2323	E	826.4094	413.7083	809.3828	405.1951	808.3988	404.7030	6
12	1484.4948	742.7511	1467.4683	734.2378	1466.4843	733.7458	D	697.3668	349.1870	680.3402	340.6738	679.3562	340.1817	5
13	1631.5632	816.2853	1614.5367	807.7720	1613.5527	807.2800	F	582.3398	291.6736	565.3133	283.1603			4
14	1778.6317	889.8195	1761.6051	881.3062	1760.6211	880.8142	F	435.2714	218.1394	418.2449	209.6261			3
15	1891.7157	946.3615	1874.6892	937.8482	1873.7052	937.3562	L	288.2030	144.6051	271.1765	136.0919			2
16							R	175.1190	88.0631	158.0924	79.5498			1

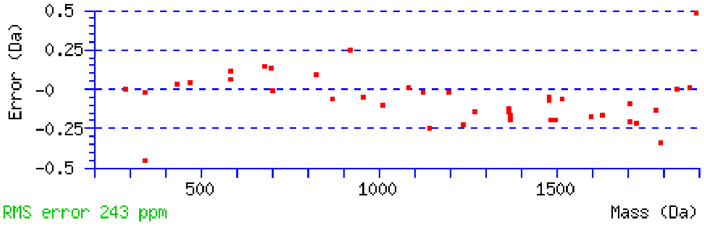

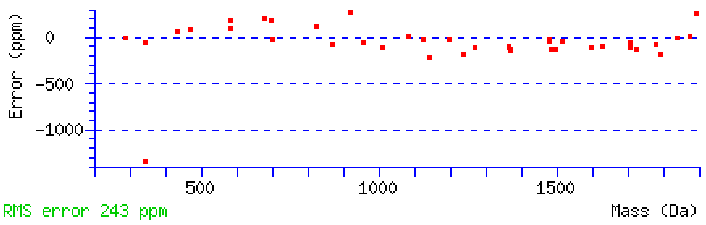

All matches to this query

Score	Mr(calc):	Delta	Sequence
83.8	2064.8201	0.0089	NLLEDDSDEEEDFFLR
14.9	2064.8161	0.0129	VFSFSSNPITMPMLMR
7.5	2064.8199	0.0091	SSMLFDTMQHHLALSR
6.8	2064.8161	0.0129	VFSFSSNPITMPMLMR
6.8	2064.8161	0.0129	VFSFSSNPITMPMLMR
6.5	2064.8472	-0.0181	YKELIQENNSYRIR
5.3	2064.8161	0.0129	VFSFSSNPITMPMLMR
5.3	2064.8161	0.0129	VFSFSSNPITMPMLMR
5.3	2064.8161	0.0129	VFSFSSNPITMPMLMR
5.0	2064.8264	0.0026	GEVITTYCPANNEPIAR

Spectrum No: 34; Query: 2124; Rank: 1

Peptide View

MS/MS Fragmentation of **GDSIEEILADSEDEDEEEER**
Found in **IPI00767073**, Tax_Id=10116 Gene_Symbol=LOC679127 similar to Protein KIAA0690

Match to Query 2124: 2387.903908 from(1194.959230,2+)
Title: 100101RatKid_NS_deglyco_26.3406.3406.2.dta
Data file K:\NewmanPaper\Piliang\3SubProteomes\Piliang3SP\mgf5ppm\ERLIC_3SubProteomes5ppm.mgf

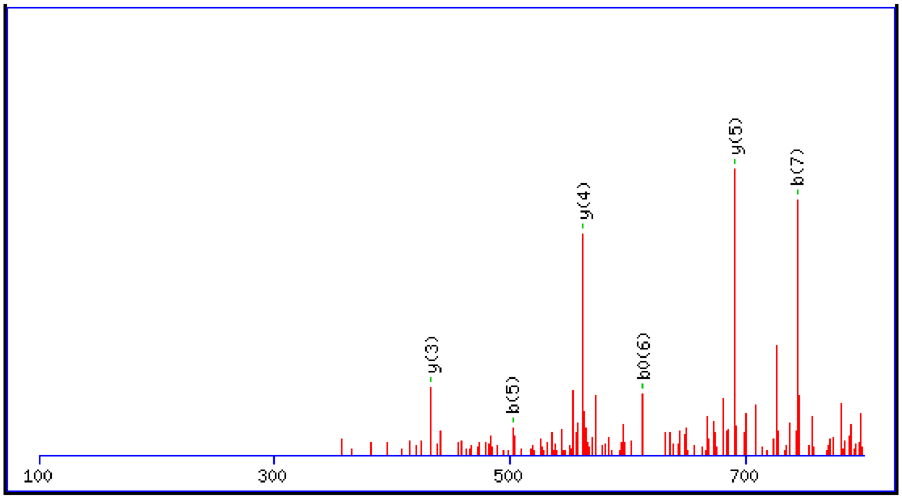

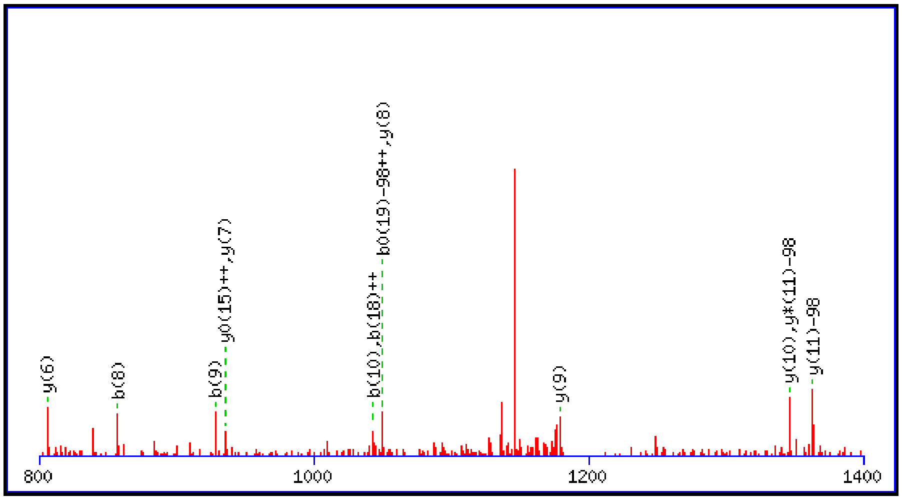

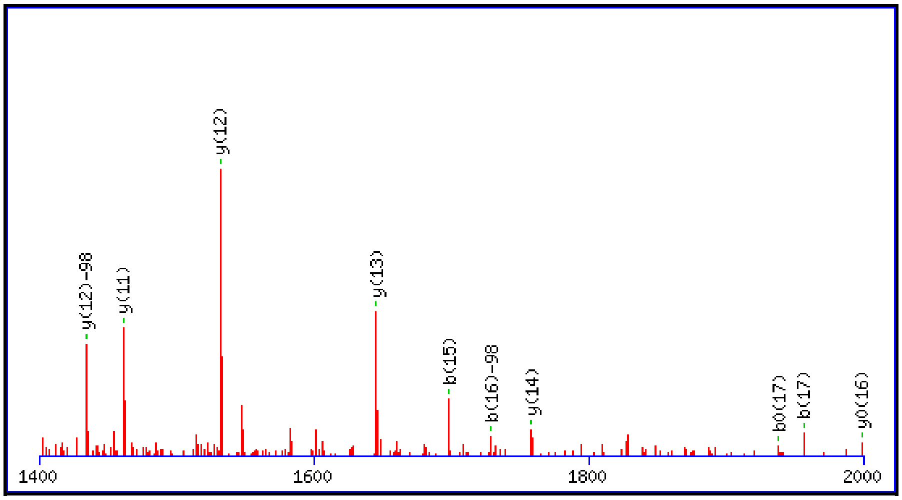

Monoisotopic mass of neutral peptide Mr(calc): 2387.9013
Fixed modifications: Carbamidomethyl (C)
Variable modifications:
S11 : Phospho (ST), with neutral losses 0.0000(shown in table), 97.9769
Ions Score: 84 **Expect:** 7.5e-007
Matches (Bold Red): 29/282 fragment ions using 36 most intense peaks

#	b	b^{++}	b^{0}	b^{0++}	Seq.	y	y^{++}	y*	y^{*++}	y^{0}	y^{0++}	#
1	58.0287	29.5180			G							20
2	173.0557	87.0315	155.0451	78.0262	D	2331.8872	1166.4472	2314.8606	1157.9339	2313.8766	1157.4419	19
3	260.0877	130.5475	242.0771	121.5422	S	2216.8602	1108.9337	2199.8337	1100.4205	2198.8496	1099.9285	18
4	373.1718	187.0895	355.1612	178.0842	I	2129.8282	1065.4177	2112.8016	1056.9045	2111.8176	1056.4124	17
5	**502.2144**	251.6108	484.2038	242.6055	E	2016.7441	1008.8757	1999.7176	1000.3624	**1998.7336**	999.8704	16

**6**	631.2570	316.1321	**613.2464**	307.1268	**E**	1887.7015	944.3544	1870.6750	935.8411	1869.6910	**935.3491**	**15**
**7**	**744.3410**	372.6742	726.3305	363.6689	**I**	**1758.6589**	879.8331	1741.6324	871.3198	1740.6484	870.8278	**14**
**8**	**857.4251**	429.2162	839.4145	420.2109	**L**	**1645.5749**	823.2911	1628.5483	814.7778	1627.5643	814.2858	**13**
**9**	**928.4622**	464.7347	910.4516	455.7295	**A**	**1532.4908**	766.7490	1515.4643	758.2358	1514.4802	757.7438	**12**
**10**	**1043.4891**	522.2482	1025.4786	513.2429	**D**	**1461.4537**	731.2305	1444.4271	722.7172	1443.4431	722.2252	**11**
**11**	1210.4875	605.7474	1192.4769	596.7421	**S**	**1346.4267**	673.7170	1329.4002	665.2037	1328.4162	664.7117	**10**
**12**	1339.5301	670.2687	1321.5195	661.2634	**E**	**1179.4284**	590.2178	1162.4018	581.7046	1161.4178	581.2126	**9**
**13**	1454.5570	727.7822	1436.5465	718.7769	**D**	**1050.3858**	525.6965	1033.3593	517.1833	1032.3752	516.6913	**8**
**14**	1583.5996	792.3035	1565.5891	783.2982	**E**	**935.3589**	468.1831	918.3323	459.6698	917.3483	459.1778	**7**
**15**	**1698.6266**	849.8169	1680.6160	840.8116	**D**	**806.3163**	403.6618	789.2897	395.1485	788.3057	394.6565	**6**
**16**	1827.6692	914.3382	1809.6586	905.3329	**E**	**691.2893**	346.1483	674.2628	337.6350	673.2788	337.1430	**5**
**17**	**1956.7118**	978.8595	**1938.7012**	969.8542	**E**	**562.2467**	281.6270	545.2202	273.1137	544.2362	272.6217	**4**
**18**	2085.7543	**1043.3808**	2067.7438	1034.3755	**E**	**433.2041**	217.1057	416.1776	208.5924	415.1936	208.1004	**3**
**19**	2214.7969	1107.9021	2196.7864	1098.8968	**E**	304.1615	152.5844	287.1350	144.0711	286.1510	143.5791	**2**
**20**					**R**	175.1190	88.0631	158.0924	79.5498			**1**

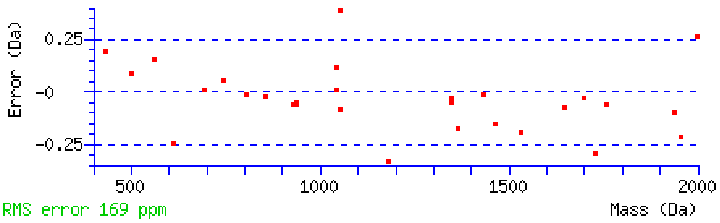

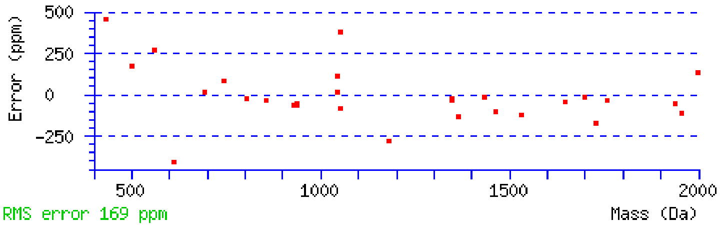

All matches to this query

Score	Mr(calc):	Delta	Sequence
83.7	2387.9013	0.0026	GDSIEEILADSEDEDEEEER
25.2	2387.9013	0.0026	GDSIEEILADSEDEDEEEER

Spectrum No: 35; Query: 1609; Rank: 1

Peptide View

MS/MS Fragmentation of **FHDSEGDDTEETEDYR**
Found in **IPI00382244**, Tax_Id=10116 Gene_Symbol=Bclaf1 Aa2-041

Match to Query 1609: 2023.691568 from(1012.853060,2+)
Title: 091008RatKidney_NoSalt_27.962.962.2.dta
Data file K:\NewmanPaper\Piliang\3SubProteomes\Piliang3SP\mgf5ppm\ERLIC_3SubProteomes5ppm.mgf

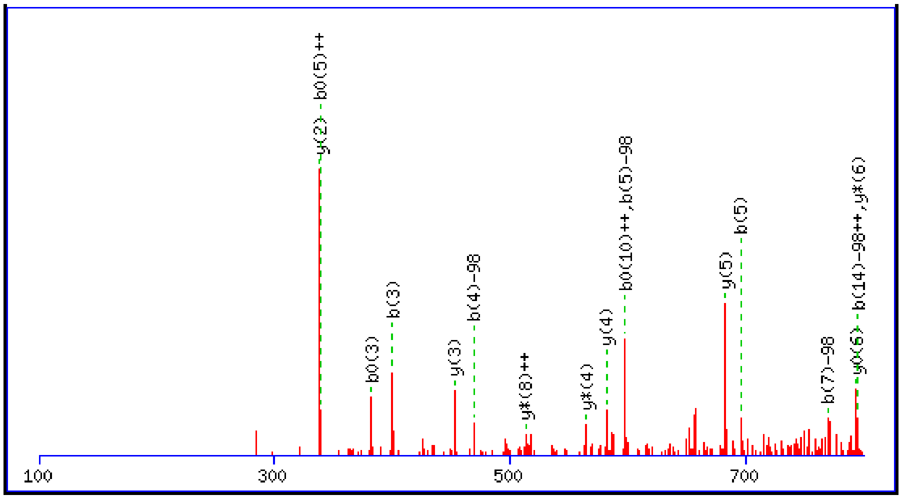

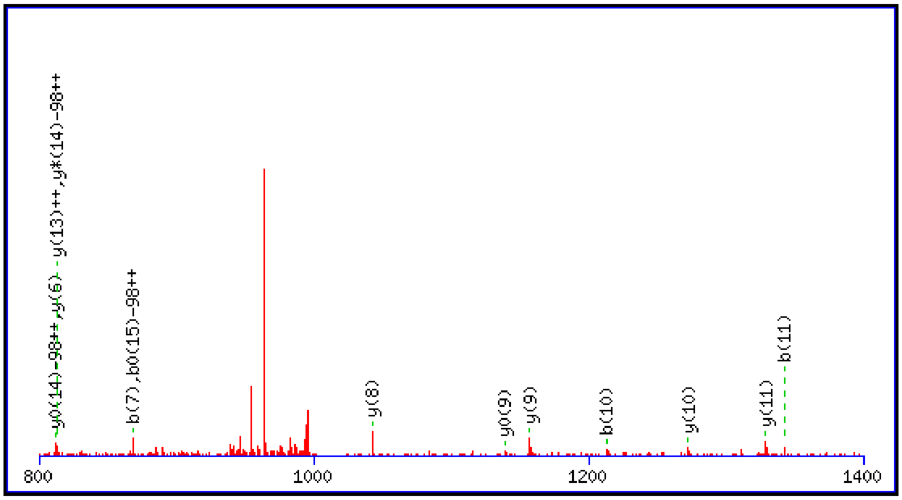

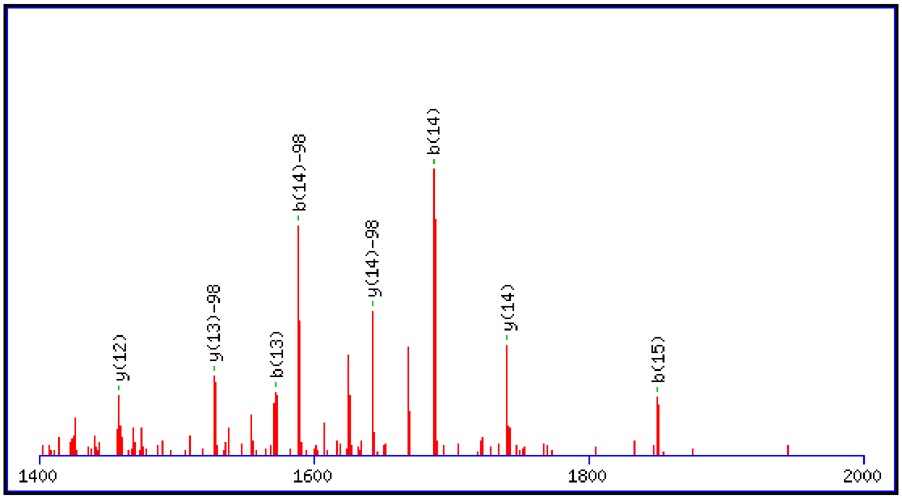

Monoisotopic mass of neutral peptide Mr(calc): 2023.6956
Fixed modifications: Carbamidomethyl (C)
Variable modifications:
S4 : Phospho (ST), with neutral losses 97.9769(shown in table), 0.0000
Ions Score: 83 **Expect:** 3.5e-007
Matches (Bold Red): 38/208 fragment ions using 55 most intense peaks

#	b	b^{++}	b^{0}	b^{0++}	Seq.	y	y^{++}	y*	y^{*++}	y^{0}	y^{0++}	#
1	148.0757	74.5415			F							16
2	285.1346	143.0709			H	1779.6576	890.3324	1762.6311	881.8192	1761.6471	881.3272	15
3	**400.1615**	200.5844	**382.1510**	191.5791	D	**1642.5987**	821.8030	1625.5722	**813.2897**	1624.5881	**812.7977**	14
4	**469.1830**	235.0951	451.1724	226.0899	S	**1527.5718**	764.2895	1510.5452	755.7762	1509.5612	755.2842	13
5	**598.2256**	299.6164	580.2150	290.6112	E	**1458.5503**	729.7788	1441.5238	721.2655	1440.5397	720.7735	12

**6**	655.2471	328.1272	637.2365	319.1219	**G**	**1329.5077**	665.2575	1312.4812	656.7442	1311.4971	656.2522	**11**
**7**	**770.2740**	385.6406	752.2634	376.6354	**D**	**1272.4862**	636.7468	1255.4597	628.2335	1254.4757	627.7415	**10**
**8**	885.3009	443.1541	867.2904	434.1488	**D**	**1157.4593**	579.2333	1140.4328	570.7200	**1139.4487**	570.2280	**9**
**9**	986.3486	493.6779	968.3381	484.6727	**T**	**1042.4324**	521.7198	1025.4058	**513.2065**	1024.4218	512.7145	**8**
**10**	1115.3912	558.1992	1097.3807	549.1940	**E**	941.3847	471.1960	924.3581	462.6827	923.3741	462.1907	**7**
**11**	1244.4338	622.7205	1226.4232	613.7153	**E**	**812.3421**	406.6747	**795.3155**	398.1614	**794.3315**	397.6694	**6**
**12**	1345.4815	673.2444	1327.4709	664.2391	**T**	**683.2995**	342.1534	666.2729	333.6401	665.2889	333.1481	**5**
**13**	1474.5241	737.7657	1456.5135	728.7604	**E**	**582.2518**	291.6295	**565.2253**	283.1163	564.2413	282.6243	**4**
**14**	**1589.5510**	**795.2792**	1571.5405	786.2739	**D**	**453.2092**	227.1083	436.1827	218.5950	435.1987	218.1030	**3**
**15**	1752.6144	876.8108	1734.6038	**867.8055**	**Y**	**338.1823**	169.5948	321.1557	161.0815			**2**
**16**					**R**	175.1190	88.0631	158.0924	79.5498			**1**

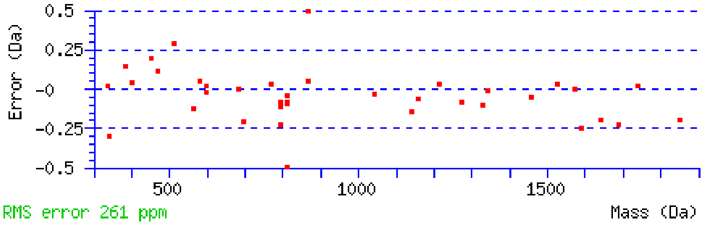

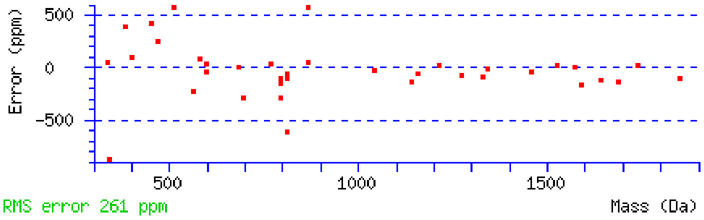

All matches to this query

Score	Mr(calc):	Delta	Sequence
82.9	2023.6956	-0.0041	FHDSEGDDTEETEDYR
35.1	2023.6956	-0.0041	FHDSEGDDTEETEDYR
25.5	2023.6956	-0.0041	FHDSEGDDTEETEDYR
3.6	2023.6882	0.0034	XGKSIXLFDRATTSXR
0.5	2021.6709	2.0207	ISSYTSYPMHDFYR

Spectrum No: 36; Query: 2649; Rank: 1

Peptide View

MS/MS Fragmentation of **GATPAEDDEDNDIDLFGSDEEEEDKEAAR**
Found in **IPI00197900**, Tax_Id=10116 Gene_Symbol=Eef1d Translation elongation factor 1-delta subunit

Match to Query 2649: 3261.274032 from(1088.098620,3+)
Title: 100101RatKid_NS_deglyco_24.2961.2961.3.dta
Data file K:\NewmanPaper\Piliang\3SubProteomes\Piliang3SP\mgf5ppm\ERLIC_3SubProteomes5ppm.mgf

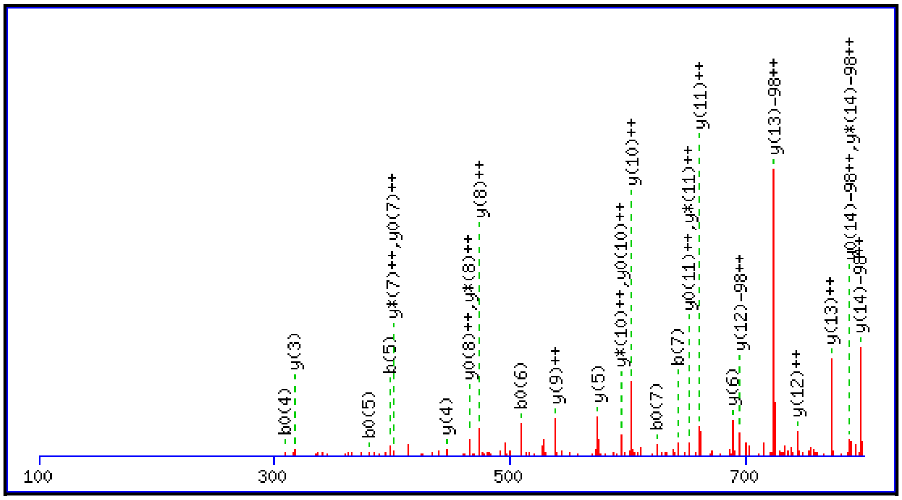

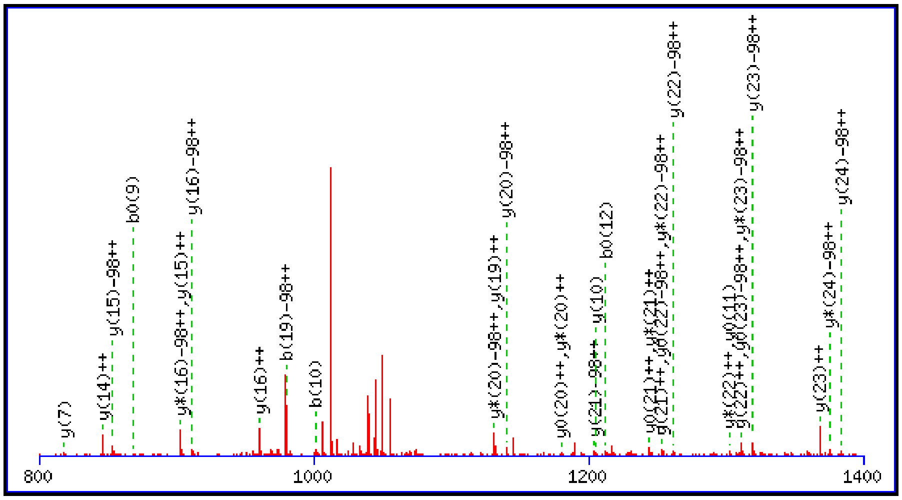

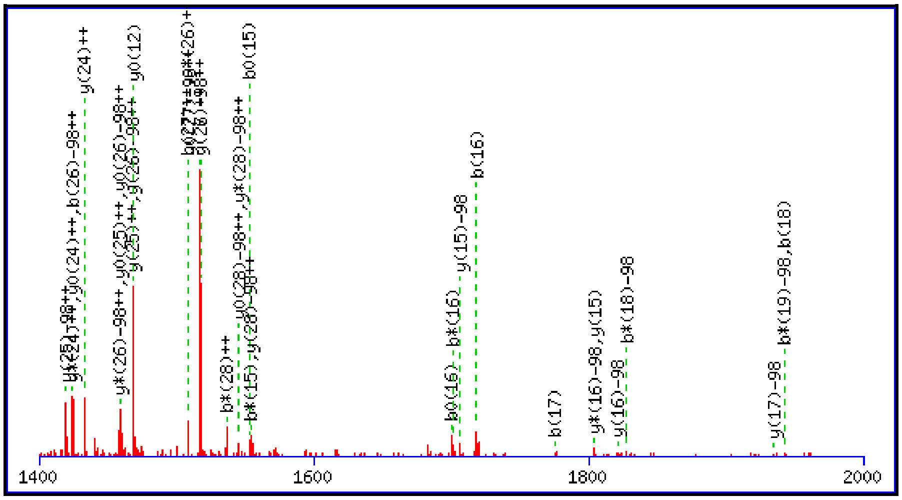

Monoisotopic mass of neutral peptide Mr(calc): 3261.2630
Fixed modifications: Carbamidomethyl (C)
Variable modifications:
S18 : Phospho (ST), with neutral losses 97.9769(shown in table), 0.0000
Ions Score: 83 **Expect:** 1.7e-006
Matches (Bold Red): 96/474 fragment ions using 137 most intense peaks

#	b	b^{++}	b*	b^{*++}	b^0	b^{0++}	Seq.	y	y^{++}	y*	y^{*++}	y^0	y^{0++}	#
1	58.0287	29.5180					G							29
2	129.0659	65.0366					A	3107.2720	**1554.1396**	3090.2454	**1545.6263**	3089.2614	**1545.1343**	28
3	230.1135	115.5604			212.1030	106.5551	T	3036.2348	**1518.6211**	3019.2083	1510.1078	3018.2243	**1509.6158**	27
4	327.1663	164.0868			**309.1557**	155.0815	P	2935.1872	**1468.0972**	2918.1606	**1459.5839**	2917.1766	**1459.0919**	26
5	**398.2034**	199.6053			**380.1928**	190.6001	A	2838.1344	**1419.5708**	2821.1078	1411.0576	2820.1238	1410.5656	25
6	527.2460	264.1266			**509.2354**	255.1214	E	2767.0973	**1384.0523**	2750.0707	**1375.5390**	2749.0867	1375.0470	24
7	**642.2729**	321.6401			**624.2624**	312.6348	D	2638.0547	**1319.5310**	2621.0281	**1311.0177**	2620.0441	**1310.5257**	23
8	757.2999	379.1536			739.2893	370.1483	D	2523.0277	**1262.0175**	2506.0012	**1253.5042**	2505.0172	**1253.0122**	22
9	886.3425	443.6749			**868.3319**	434.6696	E	2408.0008	**1204.5040**	2390.9743	1195.9908	2389.9902	1195.4988	21
10	**1001.3694**	501.1884			983.3589	492.1831	D	2278.9582	**1139.9827**	2261.9317	**1131.4695**	2260.9476	1130.9775	20
11	1115.4124	558.2098	1098.3858	549.6965	1097.4018	549.2045	N	2163.9313	1082.4693	2146.9047	1073.9560	2145.9207	1073.4640	19
12	1230.4393	615.7233	1213.4127	607.2100	**1212.4287**	606.7180	D	2049.8883	1025.4478	2032.8618	1016.9345	2031.8778	1016.4425	18
13	1343.5234	672.2653	1326.4968	663.7520	1325.5128	663.2600	I	**1934.8614**	967.9343	1917.8349	959.4211	1916.8508	958.9291	17
14	1458.5503	729.7788	1441.5238	721.2655	1440.5397	720.7735	D	**1821.7773**	**911.3923**	**1804.7508**	**902.8790**	1803.7668	902.3870	16
15	1571.6344	786.3208	**1554.6078**	777.8075	**1553.6238**	777.3155	L	**1706.7504**	**853.8788**	1689.7238	845.3656	1688.7398	844.8736	15
16	**1718.7028**	859.8550	**1701.6762**	851.3418	**1700.6922**	850.8497	F	1593.6663	**797.3368**	1576.6398	**788.8235**	1575.6558	**788.3315**	14
17	**1775.7242**	888.3658	1758.6977	879.8525	1757.7137	879.3605	G	1446.5979	**723.8026**	1429.5714	715.2893	1428.5873	714.7973	13
18	1844.7457	922.8765	**1827.7192**	914.3632	1826.7351	913.8712	S	1389.5765	**695.2919**	1372.5499	686.7786	1371.5659	686.2866	12

19	1959.7726	980.3900	1942.7461	971.8767	1941.7621	971.3847	D	1320.5550	660.7811	1303.5284	652.2679	1302.5444	651.7759	11
20	2088.8152	1044.9113	2071.7887	1036.3980	2070.8047	1035.9060	E	1205.5281	603.2677	1188.5015	594.7544	1187.5175	594.2624	10
21	2217.8578	1109.4326	2200.8313	1100.9193	2199.8473	1100.4273	E	1076.4855	538.7464	1059.4589	530.2331	1058.4749	529.7411	9
22	2346.9004	1173.9538	2329.8739	1165.4406	2328.8899	1164.9486	E	947.4429	474.2251	930.4163	465.7118	929.4323	465.2198	8
23	2475.9430	1238.4751	2458.9165	1229.9619	2457.9325	1229.4699	E	818.4003	409.7038	801.3737	401.1905	800.3897	400.6985	7
24	2590.9700	1295.9886	2573.9434	1287.4753	2572.9594	1286.9833	D	689.3577	345.1825	672.3311	336.6692	671.3471	336.1772	6
25	2719.0649	1360.0361	2702.0384	1351.5228	2701.0544	1351.0308	K	574.3307	287.6690	557.3042	279.1557	556.3202	278.6637	5
26	2848.1075	1424.5574	2831.0810	1416.0441	2830.0970	1415.5521	E	446.2358	223.6215	429.2092	215.1083	428.2252	214.6162	4
27	2919.1446	1460.0760	2902.1181	1451.5627	2901.1341	1451.0707	A	317.1932	159.1002	300.1666	150.5870			3
28	2990.1817	1495.5945	2973.1552	1487.0812	2972.1712	1486.5892	A	246.1561	123.5817	229.1295	115.0684			2
29							R	175.1190	88.0631	158.0924	79.5498			1

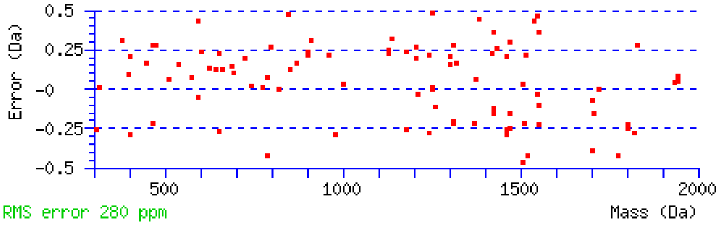

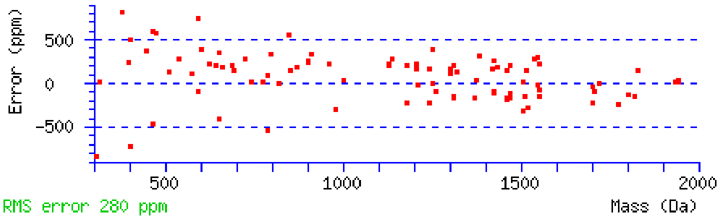

All matches to this query

Score	Mr(calc):	Delta	Sequence
82.8	3261.2630	0.0110	GATPAEDDEDNDIDLFGSDEEEEDKEAAR
22.6	3261.2630	0.0110	GATPAEDDEDNDIDLFGSDEEEEDKEAAR
3.6	3261.2927	-0.0186	MWGEELTSEESVFEVFEHYLSGEPNR
3.6	3261.2927	-0.0186	MWGEELTSEESVFEVFEHYLSGEPNR
1.5	3261.2984	-0.0244	LVLQQSDMASGNICEDLYATHGPMSLR
1.5	3260.2745	0.9995	ESAFVYAISSAGVVFAITRACSQGELK
1.1	3260.2814	0.9927	FITVYFDYSCEGDVPCTLDLSTKYK
1.0	3261.2916	-0.0176	QFANSVLPCYPGSNVENNLWQRPSNK
1.0	3261.2916	-0.0176	QFANSVLPCYPGSNVENNLWQRPSNK
0.8	3261.2876	-0.0136	CNNDTSWTVLASNVSNIITEFHQRGR

Spectrum No: 37; Query: 1365; Rank: 1

Peptide View

MS/MS Fragmentation of **EEASDDDMEGDEAVVR**
Found in **IPI00373118**, Tax_Id=10116 Gene_Symbol=Ascc3l1 79 kDa protein

Match to Query 1365: 1861.663148 from(931.838850,2+)
Title: 100101RatKid_NS_deglyco_25.962.962.2.dta
Data file K:\NewmanPaper\Piliang\3SubProteomes\Piliang3SP\mgf5ppm\ERLIC_3SubProteomes5ppm.mgf

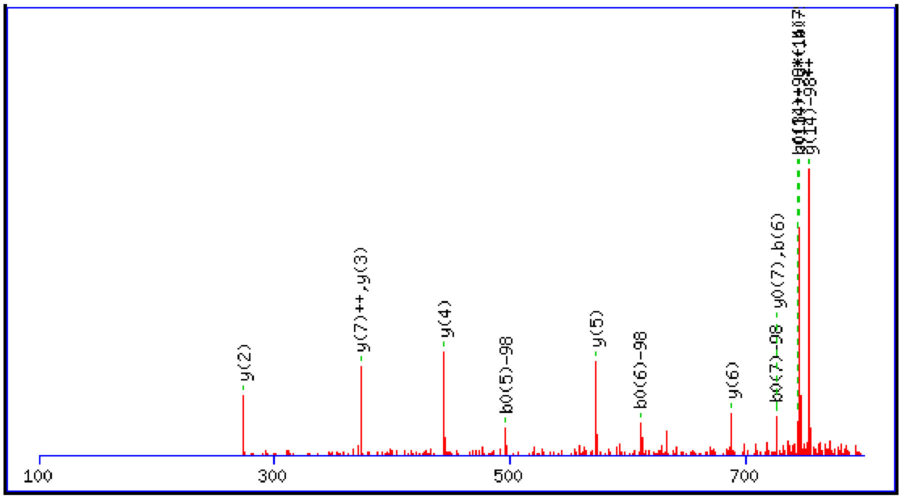

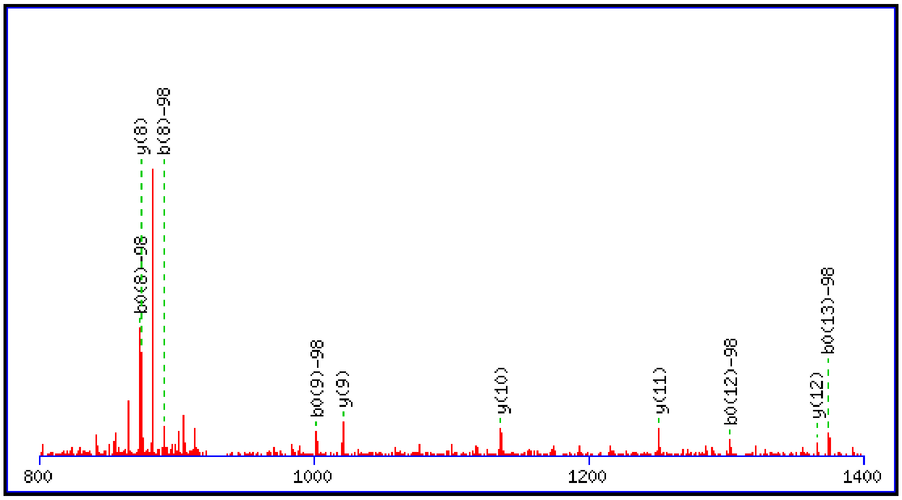

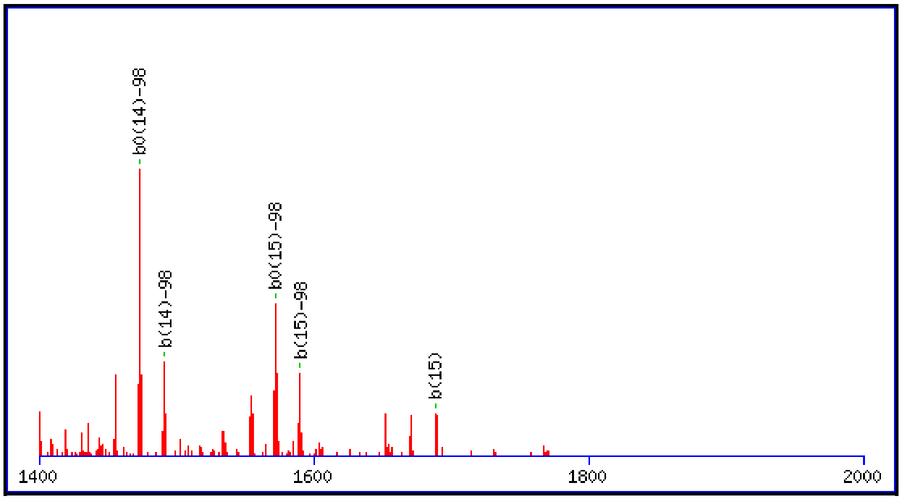

Monoisotopic mass of neutral peptide Mr(calc): 1861.6561
Fixed modifications: Carbamidomethyl (C)
Variable modifications:
S4 : Phospho (ST), with neutral losses 97.9769(shown in table), 0.0000
M8 : Oxidation (M)
Ions Score: 83 **Expect:** 4e-007
Matches (Bold Red): 32/208 fragment ions using 38 most intense peaks

#	b	b^{++}	b^0	b^{0++}	Seq.	y	y^{++}	y*	y^{*++}	y^0	y^{0++}	#
1	130.0499	65.5286	112.0393	56.5233	E							16
2	259.0925	130.0499	241.0819	121.0446	E	1635.6439	818.3256	1618.6173	809.8123	1617.6333	809.3203	15
3	330.1296	165.5684	312.1190	156.5631	A	1506.6013	**753.8043**	1489.5747	**745.2910**	1488.5907	**744.7990**	14
4	399.1510	200.0792	381.1405	191.0739	S	1435.5642	718.2857	1418.5376	709.7725	1417.5536	709.2804	13

5	514.1780	257.5926	**496.1674**	248.5873	D	**1366.5427**	683.7750	1349.5162	675.2617	1348.5322	674.7697	12
6	629.2049	315.1061	**611.1944**	306.1008	D	**1251.5158**	626.2615	1234.4892	617.7483	1233.5052	617.2562	11
7	**744.2319**	372.6196	**726.2213**	363.6143	D	**1136.4888**	568.7481	1119.4623	560.2348	1118.4783	559.7428	10
8	**891.2673**	446.1373	**873.2567**	437.1320	M	**1021.4619**	511.2346	1004.4353	502.7213	1003.4513	502.2293	9
9	1020.3099	510.6586	**1002.2993**	501.6533	E	**874.4265**	437.7169	857.3999	429.2036	856.4159	428.7116	8
10	1077.3313	539.1693	1059.3208	530.1640	G	**745.3839**	**373.1956**	728.3573	364.6823	**727.3733**	364.1903	7
11	1192.3583	596.6828	1174.3477	587.6775	D	**688.3624**	344.6849	671.3359	336.1716	670.3519	335.6796	6
12	1321.4009	661.2041	**1303.3903**	652.1988	E	**573.3355**	287.1714	556.3089	278.6581	555.3249	278.1661	5
13	1392.4380	696.7226	**1374.4274**	687.7173	A	**444.2929**	222.6501	427.2663	214.1368			4
14	**1491.5064**	746.2568	**1473.4958**	737.2515	V	**373.2558**	187.1315	356.2292	178.6183			3
15	**1590.5748**	795.7910	**1572.5642**	786.7858	V	**274.1874**	137.5973	257.1608	129.0840			2
16					R	175.1190	88.0631	158.0924	79.5498			1

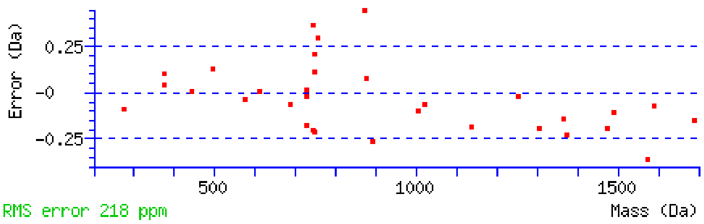

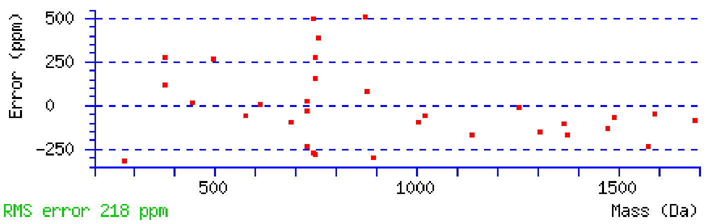

All matches to this query

Score	Mr(calc):	Delta	Sequence
82.8	1861.6561	0.0071	EEASDDDMEGDEAVVR
7.1	1861.6560	0.0071	MQNNSAENETAEGEEK
4.5	1861.6560	0.0071	MQNNSAENETAEGEEK
4.3	1861.6560	0.0071	MQNNSAENETAEGEEK
3.0	1860.6526	1.0105	NISCVVSDGSADDFSK
0.5	1861.6560	0.0071	MQNNSAENETAEGEEK
0.4	1861.6560	0.0071	MQNNSAENETAEGEEK
0.2	1861.6496	0.0136	DGMDNETGTESMVSHR

Spectrum No: 38; Query: 2737; Rank: 1

Peptide View

MS/MS Fragmentation of **EGQVEAAQPEQAAEAPAESSAQPNQLETGASSPER**
Found in **IPI00768543**, Tax_Id=10116 Gene_Symbol=Zbtb20_predicted zinc finger and BTB domain containing 20

Match to Query 2737: 3643.609092 from(1215.543640,3+)
Title: 100101RatKid_NS_deglyco_23.2054.2054.3.dta
Data file K:\NewmanPaper\Piliang\3SubProteomes\Piliang3SP\mgf5ppm\ERLIC_3SubProteomes5ppm.mgf

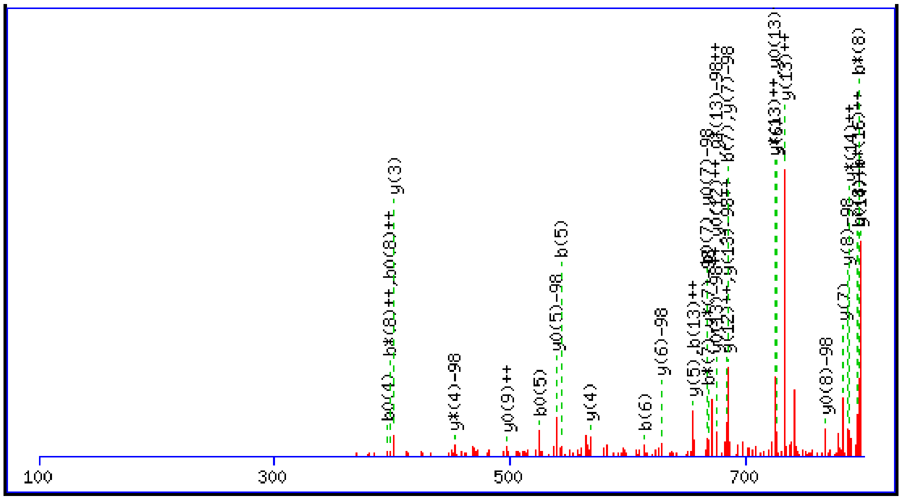

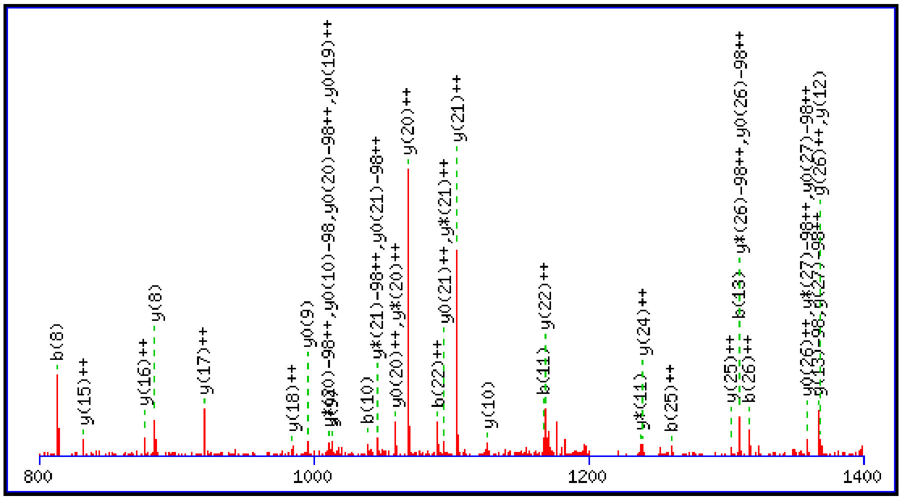

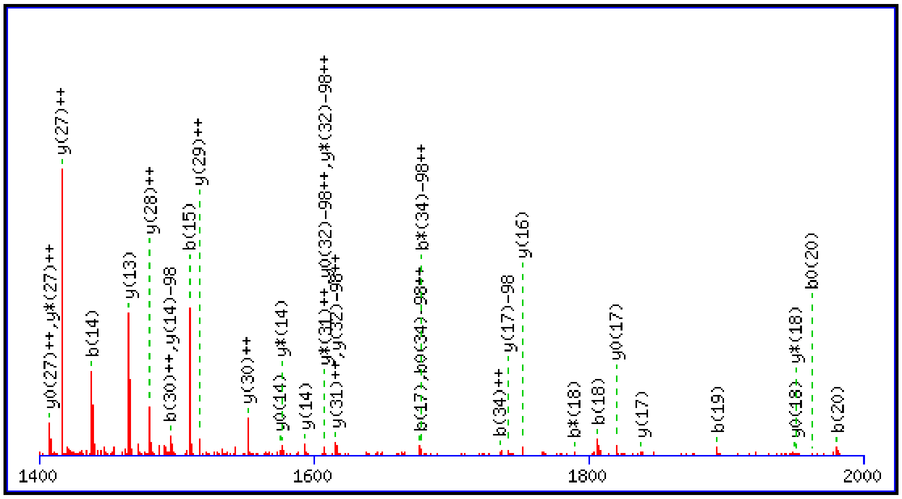

Monoisotopic mass of neutral peptide Mr(calc): 3643.5911
Fixed modifications: Carbamidomethyl (C)
Variable modifications:
S32 : Phospho (ST), with neutral losses 0.0000(shown in table), 97.9769
Ions Score: 83 **Expect:** 2.6e-006
Matches (Bold Red): 111/606 fragment ions using 153 most intense peaks

#	b	b^{++}	b*	b^{*++}	b^0	b^{0++}	Seq.	y	y^{++}	y*	y^{*++}	y^0	y^{0++}	#
1	130.0499	65.5286			112.0393	56.5233	E							35
2	187.0713	94.0393			169.0608	85.0340	G	3515.5558	1758.2815	3498.5293	1749.7683	3497.5453	1749.2763	34
3	315.1299	158.0686	298.1034	149.5553	297.1193	149.0633	Q	3458.5344	1729.7708	3441.5078	1721.2575	3440.5238	1720.7655	33
4	414.1983	207.6028	397.1718	199.0895	**396.1878**	198.5975	V	3330.4758	1665.7415	3313.4492	1657.2283	3312.4652	1656.7362	32
5	**543.2409**	272.1241	526.2144	263.6108	**525.2304**	263.1188	E	3231.4074	**1616.2073**	3214.3808	**1607.6940**	3213.3968	1607.2020	31

6	614.2780	307.6427	597.2515	299.1294	596.2675	298.6374	A	3102.3648	1551.6860	3085.3382	1543.1727	3084.3542	1542.6807	30
7	685.3151	343.1612	668.2886	334.6479	667.3046	334.1559	A	3031.3277	1516.1675	3014.3011	1507.6542	3013.3171	1507.1622	29
8	813.3737	407.1905	796.3472	398.6772	795.3632	398.1852	Q	2960.2905	1480.6489	2943.2640	1472.1356	2942.2800	1471.6436	28
9	910.4265	455.7169	893.3999	447.2036	892.4159	446.7116	P	2832.2320	1416.6196	2815.2054	1408.1063	2814.2214	1407.6143	27
10	1039.4691	520.2382	1022.4425	511.7249	1021.4585	511.2329	E	2735.1792	1368.0932	2718.1526	1359.5800	2717.1686	1359.0880	26
11	1167.5277	584.2675	1150.5011	575.7542	1149.5171	575.2622	Q	2606.1366	1303.5719	2589.1101	1295.0587	2588.1260	1294.5667	25
12	1238.5648	619.7860	1221.5382	611.2728	1220.5542	610.7807	A	2478.0780	1239.5427	2461.0515	1231.0294	2460.0675	1230.5374	24
13	1309.6019	655.3046	1292.5753	646.7913	1291.5913	646.2993	A	2407.0409	1204.0241	2390.0144	1195.5108	2389.0303	1195.0188	23
14	1438.6445	719.8259	1421.6179	711.3126	1420.6339	710.8206	E	2336.0038	1168.5055	2318.9772	1159.9923	2317.9932	1159.5003	22
15	1509.6816	755.3444	1492.6550	746.8312	1491.6710	746.3392	A	2206.9612	1103.9842	2189.9347	1095.4710	2188.9506	1094.9790	21
16	1606.7344	803.8708	1589.7078	795.3575	1588.7238	794.8655	P	2135.9241	1068.4657	2118.8975	1059.9524	2117.9135	1059.4604	20
17	1677.7715	839.3894	1660.7449	830.8761	1659.7609	830.3841	A	2038.8713	1019.9393	2021.8448	1011.4260	2020.8608	1010.9340	19
18	1806.8141	903.9107	1789.7875	895.3974	1788.8035	894.9054	E	1967.8342	984.4207	1950.8077	975.9075	1949.8236	975.4155	18
19	1893.8461	947.4267	1876.8195	938.9134	1875.8355	938.4214	S	1838.7916	919.8994	1821.7651	911.3862	1820.7811	910.8942	17
20	1980.8781	990.9427	1963.8516	982.4294	1962.8676	981.9374	S	1751.7596	876.3834	1734.7330	867.8702	1733.7490	867.3782	16
21	2051.9152	1026.4613	2034.8887	1017.9480	2033.9047	1017.4560	A	1664.7276	832.8674	1647.7010	824.3541	1646.7170	823.8621	15
22	2179.9738	1090.4905	2162.9473	1081.9773	2161.9632	1081.4853	Q	1593.6905	797.3489	1576.6639	788.8356	1575.6799	788.3436	14
23	2277.0266	1139.0169	2260.0000	1130.5037	2259.0160	1130.0116	P	1465.6319	733.3196	1448.6053	724.8063	1447.6213	724.3143	13
24	2391.0695	1196.0384	2374.0430	1187.5251	2373.0589	1187.0331	N	1368.5791	684.7932	1351.5526	676.2799	1350.5685	675.7879	12
25	2519.1281	1260.0677	2502.1015	1251.5544	2501.1175	1251.0624	Q	1254.5362	627.7717	1237.5096	619.2585	1236.5256	618.7664	11
26	2632.2121	1316.6097	2615.1856	1308.0964	2614.2016	1307.6044	L	1126.4776	563.7424	1109.4511	555.2292	1108.4670	554.7372	10
27	2761.2547	1381.1310	2744.2282	1372.6177	2743.2442	1372.1257	E	1013.3935	507.2004	996.3670	498.6871	995.3830	498.1951	9
28	2862.3024	1431.6548	2845.2759	1423.1416	2844.2919	1422.6496	T	884.3509	442.6791	867.3244	434.1658	866.3404	433.6738	8
29	2919.3239	1460.1656	2902.2973	1451.6523	2901.3133	1451.1603	G	783.3033	392.1553	766.2767	383.6420	765.2927	383.1500	7
30	2990.3610	1495.6841	2973.3344	1487.1709	2972.3504	1486.6789	A	726.2818	363.6445	709.2553	355.1313	708.2712	354.6393	6
31	3077.3930	1539.2002	3060.3665	1530.6869	3059.3825	1530.1949	S	655.2447	328.1260	638.2181	319.6127	637.2341	319.1207	5
32	3244.3914	1622.6993	3227.3648	1614.1861	3226.3808	1613.6940	S	568.2127	284.6100	551.1861	276.0967	550.2021	275.6047	4
33	3341.4441	1671.2257	3324.4176	1662.7124	3323.4336	1662.2204	P	401.2143	201.1108	384.1878	192.5975	383.2037	192.1055	3
34	3470.4867	1735.7470	3453.4602	1727.2337	3452.4762	1726.7417	E	304.1615	152.5844	287.1350	144.0711	286.1510	143.5791	2
35							R	175.1190	88.0631	158.0924	79.5498			1

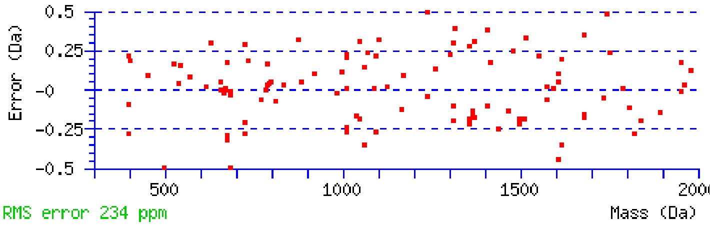

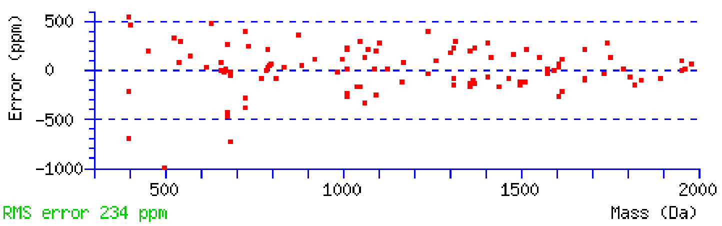

All matches to this query

Score	Mr(calc):	Delta	Sequence
82.7	3643.5911	0.0180	EGQVEAAQPEQAAEAPAESSAQPNQLETGASSPER
76.2	3643.5911	0.0180	EGQVEAAQPEQAAEAPAESSAQPNQLETGASSPER
65.6	3643.5911	0.0180	EGQVEAAQPEQAAEAPAESSAQPNQLETGASSPER
33.8	3643.5911	0.0180	EGQVEAAQPEQAAEAPAESSAQPNQLETGASSPER
20.0	3643.5911	0.0180	EGQVEAAQPEQAAEAPAESSAQPNQLETGASSPER
6.6	3642.5924	1.0167	EDRIIGMTVIQLQNIAEKGSYGAWYPLLK
4.6	3642.6198	0.9893	EIAEAYLGHPVTNAVITVPAYFNDSQRQATK
4.0	3642.5732	1.0359	FDDYNSDGSLTLGEFYTAFQVIQLSLAPEDK
3.1	3642.5699	1.0392	QEASTPERVDFPSSMEIASLQHTLDSQAGHSK
2.9	3642.6359	0.9732	KSTYGFRCLNVVLWLGYWAVQLNVCLSR

Spectrum No: 39; Query: 2842; Rank: 1

Peptide View

MS/MS Fragmentation of **TQNYPADWSDDESNNPFSSTDANGDSNPFDEDTTSGTEVR**
Found in **IPI00213498**, Tax_Id=10116 Gene_Symbol=Pacsin2 Isoform 1 of Protein kinase C and casein kinase substrate in neurons 2 protein

Match to Query 2842: 4463.750982 from(1488.924270,3+)
Title: 100101RatKid_NS_deglyco_25.4050.4050.3.dta
Data file K:\NewmanPaper\Piliang\3SubProteomes\Piliang3SP\mgf5ppm\ERLIC_3SubProteomes5ppm.mgf

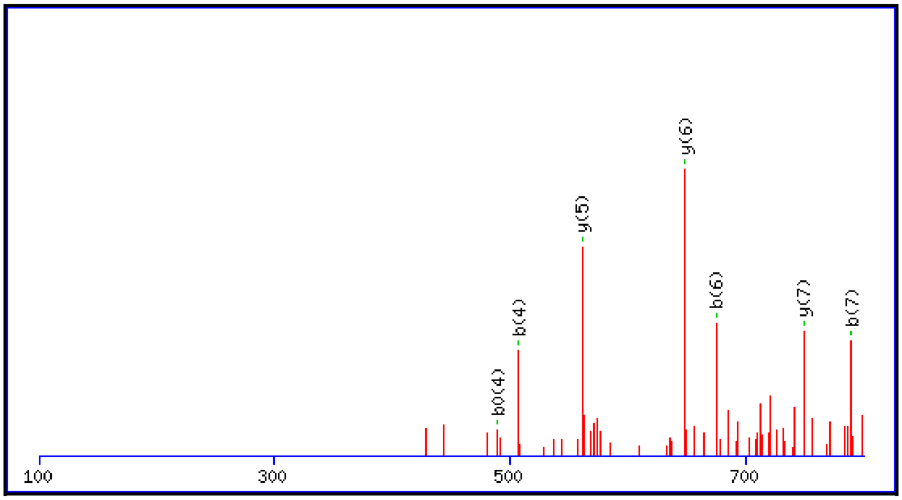

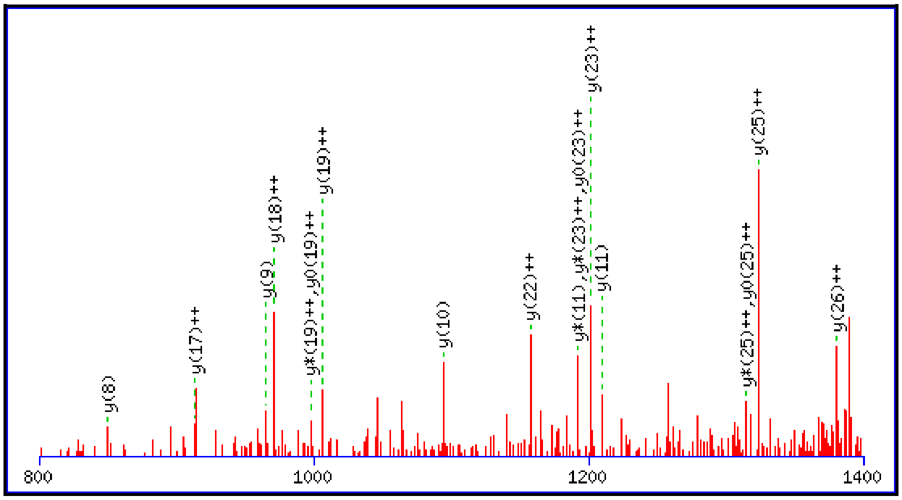

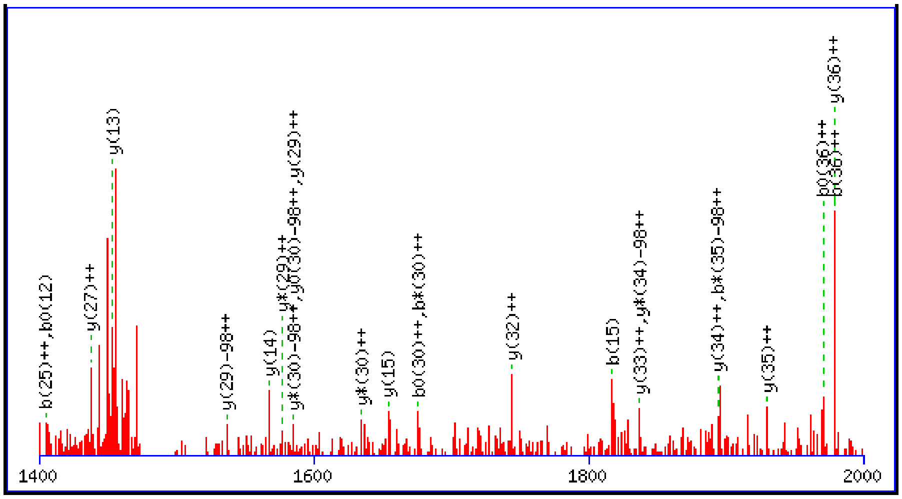

Monoisotopic mass of neutral peptide Mr(calc): 4461.7215
Fixed modifications: Carbamidomethyl (C)
Variable modifications:
S13 : Phospho (ST), with neutral losses 0.0000(shown in table), 97.9769
Ions Score: 83 **Expect:** 2.2e-006
Matches (Bold Red): 49/696 fragment ions using 68 most intense peaks

#	b	b^{++}	b*	b^{*++}	b^0	b^{0++}	Seq.	y	y^{++}	y*	y^{*++}	y^0	y^{0++}	#
1	102.0550	51.5311			84.0444	42.5258	T							40
2	230.1135	115.5604	213.0870	107.0471	212.1030	106.5551	Q	4361.6811	2181.3442	4344.6545	2172.8309	4343.6705	2172.3389	39
3	344.1565	172.5819	327.1299	164.0686	326.1459	163.5766	N	4233.6225	2117.3149	4216.5960	2108.8016	4215.6119	2108.3096	38
4	**507.2198**	254.1135	490.1932	245.6003	**489.2092**	245.1083	Y	4119.5796	2060.2934	4102.5530	2051.7802	4101.5690	2051.2881	37
5	604.2726	302.6399	587.2460	294.1266	586.2620	293.6346	P	3956.5163	**1978.7618**	3939.4897	1970.2485	3938.5057	1969.7565	36
6	**675.3097**	338.1585	658.2831	329.6452	657.2991	329.1532	A	3859.4635	**1930.2354**	3842.4369	1921.7221	3841.4529	1921.2301	35
7	**790.3366**	395.6719	773.3101	387.1587	772.3260	386.6667	D	3788.4264	**1894.7168**	3771.3998	1886.2035	3770.4158	1885.7115	34
8	976.4159	488.7116	959.3894	480.1983	958.4054	479.7063	W	3673.3994	**1837.2034**	3656.3729	1828.6901	3655.3889	1828.1981	33
9	1063.4480	532.2276	1046.4214	523.7143	1045.4374	523.2223	S	3487.3201	**1744.1637**	3470.2936	1735.6504	3469.3096	1735.1584	32
10	1178.4749	589.7411	1161.4483	581.2278	1160.4643	580.7358	D	3400.2881	1700.6477	3383.2615	1692.1344	3382.2775	1691.6424	31
11	1293.5018	647.2546	1276.4753	638.7413	1275.4913	638.2493	D	3285.2611	1643.1342	3268.2346	**1634.6209**	3267.2506	1634.1289	30
12	1422.5444	711.7759	1405.5179	703.2626	**1404.5339**	702.7706	E	3170.2342	**1585.6207**	3153.2077	**1577.1075**	3152.2236	1576.6155	29
13	1589.5428	795.2750	1572.5162	786.7618	1571.5322	786.2697	S	3041.1916	1521.0994	3024.1651	1512.5862	3023.1810	1512.0942	28
14	1703.5857	852.2965	1686.5592	843.7832	1685.5751	843.2912	N	2874.1933	**1437.6003**	2857.1667	1429.0870	2856.1827	1428.5950	27
15	**1817.6286**	909.3180	1800.6021	900.8047	1799.6181	900.3127	N	2760.1503	**1380.5788**	2743.1238	1372.0655	2742.1398	1371.5735	26
16	1914.6814	957.8443	1897.6549	949.3311	1896.6708	948.8391	P	2646.1074	**1323.5573**	2629.0809	**1315.0441**	2628.0968	**1314.5521**	25
17	2061.7498	1031.3785	2044.7233	1022.8653	2043.7392	1022.3733	F	2549.0546	1275.0310	2532.0281	1266.5177	2531.0441	1266.0257	24
18	2148.7818	1074.8946	2131.7553	1066.3813	2130.7713	1065.8893	S	2401.9862	**1201.4968**	2384.9597	**1192.9835**	2383.9757	**1192.4915**	23
19	2235.8139	1118.4106	2218.7873	1109.8973	2217.8033	1109.4053	S	2314.9542	**1157.9807**	2297.9276	1149.4675	2296.9436	1148.9755	22
20	2336.8615	1168.9344	2319.8350	1160.4211	2318.8510	1159.9291	T	2227.9222	1114.4647	2210.8956	1105.9514	2209.9116	1105.4594	21
21	2451.8885	1226.4479	2434.8619	1217.9346	2433.8779	1217.4426	D	2126.8745	1063.9409	2109.8479	1055.4276	2108.8639	1054.9356	20
22	2522.9256	1261.9664	2505.8991	1253.4532	2504.9150	1252.9612	A	2011.8475	**1006.4274**	1994.8210	**997.9141**	1993.8370	**997.4221**	19
23	2636.9685	1318.9879	2619.9420	1310.4746	2618.9580	1309.9826	N	1940.8104	**970.9089**	1923.7839	962.3956	1922.7999	961.9036	18
24	2693.9900	1347.4986	2676.9634	1338.9854	2675.9794	1338.4934	G	1826.7675	**913.8874**	1809.7410	905.3741	1808.7569	904.8821	17
25	2809.0169	**1405.0121**	2791.9904	1396.4988	2791.0064	1396.0068	D	1769.7460	885.3767	1752.7195	876.8634	1751.7355	876.3714	16
26	2896.0490	1448.5281	2879.0224	1440.0148	2878.0384	1439.5228	S	**1654.7191**	827.8632	1637.6926	819.3499	1636.7085	818.8579	15
27	3010.0919	1505.5496	2993.0653	1497.0363	2992.0813	1496.5443	N	**1567.6871**	784.3472	1550.6605	775.8339	1549.6765	775.3419	14
28	3107.1447	1554.0760	3090.1181	1545.5627	3089.1341	1545.0707	P	**1453.6441**	727.3257	1436.6176	718.8124	1435.6336	718.3204	13
29	3254.2131	1627.6102	3237.1865	1619.0969	3236.2025	1618.6049	F	1356.5914	678.7993	1339.5648	670.2861	1338.5808	669.7940	12
30	3369.2400	1685.1236	3352.2135	**1676.6104**	3351.2295	**1676.1184**	D	**1209.5230**	605.2651	**1192.4964**	596.7518	1191.5124	596.2598	11
31	3498.2826	1749.6449	3481.2561	1741.1317	3480.2720	1740.6397	E	**1094.4960**	547.7516	1077.4695	539.2384	1076.4855	538.7464	10
32	3613.3096	1807.1584	3596.2830	1798.6451	3595.2990	1798.1531	D	**965.4534**	483.2304	948.4269	474.7171	947.4429	474.2251	9

**33**	3714.3572	1857.6823	3697.3307	1849.1690	3696.3467	1848.6770	**T**	**850.4265**	425.7169	833.3999	417.2036	832.4159	416.7116	**8**
**34**	3815.4049	1908.2061	3798.3784	1899.6928	3797.3943	1899.2008	**T**	**749.3788**	375.1930	732.3523	366.6798	731.3682	366.1878	**7**
**35**	3902.4369	1951.7221	3885.4104	1943.2088	3884.4264	1942.7168	**S**	**648.3311**	324.6692	631.3046	316.1559	630.3206	315.6639	**6**
**36**	3959.4584	**1980.2328**	3942.4319	1971.7196	3941.4478	**1971.2276**	**G**	**561.2991**	281.1532	544.2726	272.6399	543.2885	272.1479	**5**
**37**	4060.5061	2030.7567	4043.4795	2022.2434	4042.4955	2021.7514	**T**	504.2776	252.6425	487.2511	244.1292	486.2671	243.6372	**4**
**38**	4189.5487	2095.2780	4172.5221	2086.7647	4171.5381	2086.2727	**E**	403.2300	202.1186	386.2034	193.6053	385.2194	193.1133	**3**
**39**	4288.6171	2144.8122	4271.5905	2136.2989	4270.6065	2135.8069	**V**	274.1874	137.5973	257.1608	129.0840			**2**
**40**							**R**	175.1190	88.0631	158.0924	79.5498			**1**

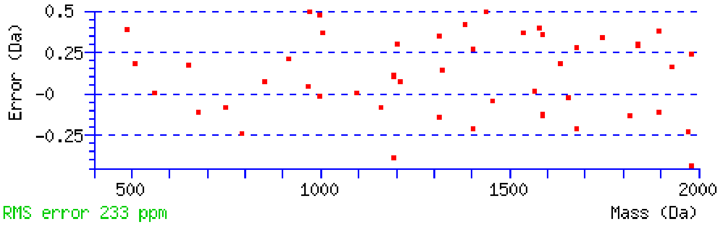

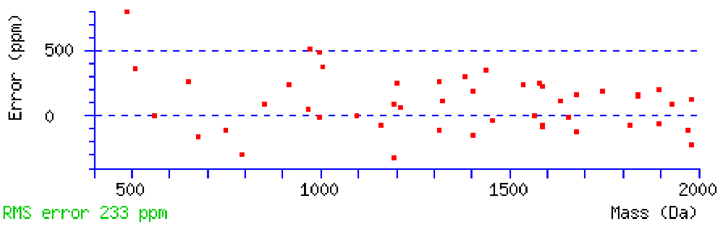

All matches to this query

Score	Mr(calc):	Delta	Sequence
82.7	4461.7215	2.0295	TQNYPADWSDDESNNPFSSTDANGDSNPFDEDTTSGTEVR
77.4	4462.7055	1.0455	TQNYPADWSDDESNNPFSSTDANGDSNPFDEDTTSGTEVR
76.3	4461.7215	2.0295	TQNYPADWSDDESNNPFSSTDANGDSNPFDEDTTSGTEVR
74.6	4462.7055	1.0455	TQNYPADWSDDESNNPFSSTDANGDSNPFDEDTTSGTEVR
74.6	4462.7055	1.0455	TQNYPADWSDDESNNPFSSTDANGDSNPFDEDTTSGTEVR
74.6	4462.7055	1.0455	TQNYPADWSDDESNNPFSSTDANGDSNPFDEDTTSGTEVR
74.6	4462.7055	1.0455	TQNYPADWSDDESNNPFSSTDANGDSNPFDEDTTSGTEVR
72.9	4462.7055	1.0455	TQNYPADWSDDESNNPFSSTDANGDSNPFDEDTTSGTEVR
72.9	4462.7055	1.0455	TQNYPADWSDDESNNPFSSTDANGDSNPFDEDTTSGTEVR
67.0	4462.7055	1.0455	TQNYPADWSDDESNNPFSSTDANGDSNPFDEDTTSGTEVR

Spectrum No: 40; Query: 2772; Rank: 1

Peptide View

MS/MS Fragmentation of **TATDENEDDLGSPPSPGDSSYYQDQVDEFHEAR**
Found in **IPI00204100**, Tax_Id=10116 Gene_Symbol=Crlz1 Something about silencing protein 10

Match to Query 2772: 3750.494112 from(1251.171980,3+)
Title: 100101RatKid_NS_deglyco_23.3334.3334.3.dta
Data file K:\NewmanPaper\Piliang\3SubProteomes\Piliang3SP\mgf5ppm\ERLIC_3SubProteomes5ppm.mgf

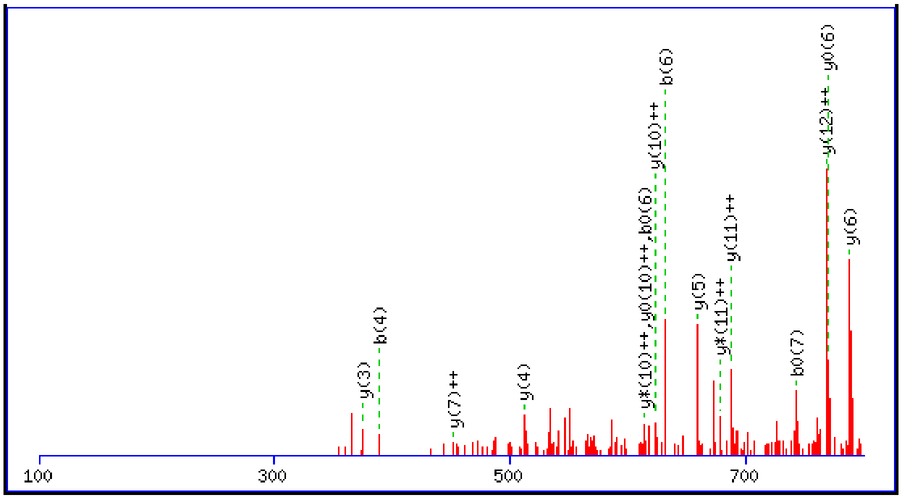

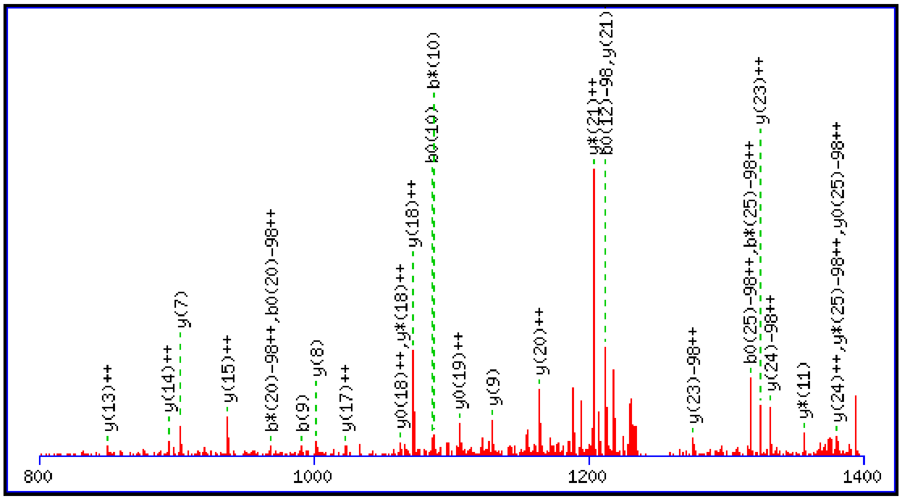

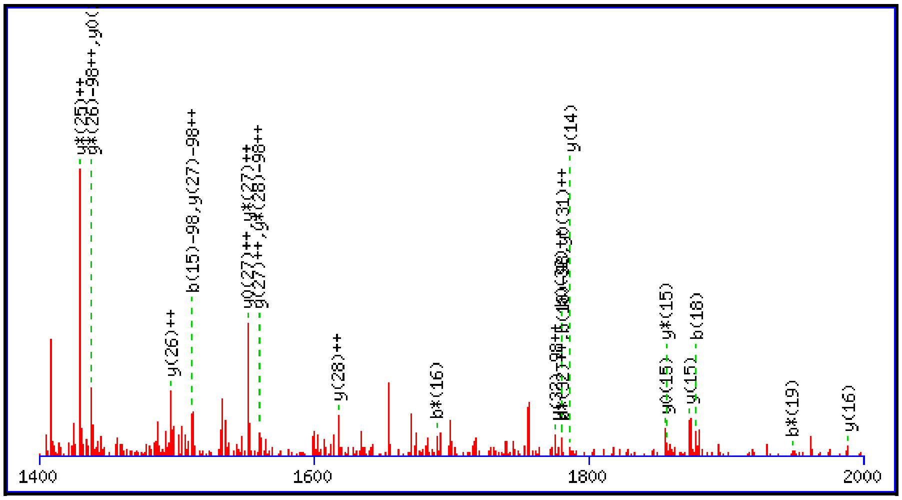

Monoisotopic mass of neutral peptide Mr(calc): 3750.4755
Fixed modifications: Carbamidomethyl (C)
Variable modifications:
S12 : Phospho (ST), with neutral losses 0.0000(shown in table), 97.9769
Ions Score: 83 **Expect:** 2.3e-006
Matches (Bold Red): 70/562 fragment ions using 121 most intense peaks

#	b	b++	b*	b*++	b0	b0++	Seq.	y	y++	y*	y*++	y0	y0++	#
1	102.0550	51.5311			84.0444	42.5258	T							33
2	173.0921	87.0497			155.0815	78.0444	A	3650.4351	1825.7212	3633.4085	1817.2079	3632.4245	1816.7159	32
3	274.1397	137.5735			256.1292	128.5682	T	3579.3980	1790.2026	3562.3714	1781.6894	3561.3874	**1781.1973**	31
4	**389.1667**	195.0870			371.1561	186.0817	D	3478.3503	1739.6788	3461.3237	1731.1655	3460.3397	1730.6735	30
5	518.2093	259.6083			500.1987	250.6030	E	3363.3234	1682.1653	3346.2968	1673.6520	3345.3128	1673.1600	29

6	**632.2522**	316.6297	615.2257	308.1165	**614.2416**	307.6245	N	3234.2808	**1617.6440**	3217.2542	1609.1307	3216.2702	1608.6387	28
7	761.2948	381.1510	744.2683	372.6378	**743.2842**	372.1458	E	3120.2378	**1560.6226**	3103.2113	**1552.1093**	3102.2273	**1551.6173**	27
8	876.3217	438.6645	859.2952	430.1512	858.3112	429.6592	D	2991.1952	**1496.1013**	2974.1687	1487.5880	2973.1847	1487.0960	26
9	**991.3487**	496.1780	974.3221	487.6647	973.3381	487.1727	D	2876.1683	**1438.5878**	2859.1417	**1430.0745**	2858.1577	1429.5825	25
10	1104.4328	552.7200	**1087.4062**	544.2067	**1086.4222**	543.7147	L	2761.1414	**1381.0743**	2744.1148	1372.5610	2743.1308	1372.0690	24
11	1161.4542	581.2307	1144.4277	572.7175	1143.4437	572.2255	G	2648.0573	**1324.5323**	2631.0307	1316.0190	2630.0467	1315.5270	23
12	1328.4526	664.7299	1311.4260	656.2166	1310.4420	655.7246	S	2591.0358	1296.0216	2574.0093	1287.5083	2573.0253	1287.0163	22
13	1425.5053	713.2563	1408.4788	704.7430	1407.4948	704.2510	P	2424.0375	**1212.5224**	2407.0109	**1204.0091**	2406.0269	1203.5171	21
14	1522.5581	761.7827	1505.5315	753.2694	1504.5475	752.7774	P	2326.9847	**1163.9960**	2309.9582	1155.4827	2308.9741	1154.9907	20
15	1609.5901	805.2987	1592.5636	796.7854	1591.5796	796.2934	S	2229.9319	1115.4696	2212.9054	1106.9563	2211.9214	**1106.4643**	19
16	1706.6429	853.8251	**1689.6163**	845.3118	1688.6323	844.8198	P	2142.8999	**1071.9536**	2125.8734	**1063.4403**	2124.8894	**1062.9483**	18
17	1763.6644	882.3358	1746.6378	873.8225	1745.6538	873.3305	G	2045.8472	**1023.4272**	2028.8206	1014.9139	2027.8366	1014.4219	17
18	**1878.6913**	939.8493	1861.6647	931.3360	1860.6807	930.8440	D	**1988.8257**	994.9165	1971.7991	986.4032	1970.8151	985.9112	16
19	1965.7233	983.3653	**1948.6968**	974.8520	1947.7128	974.3600	S	**1873.7987**	**937.4030**	**1856.7722**	928.8897	**1855.7882**	928.3977	15
20	2052.7554	1026.8813	2035.7288	1018.3680	2034.7448	1017.8760	S	**1786.7667**	**893.8870**	1769.7402	885.3737	1768.7562	884.8817	14
21	2215.8187	1108.4130	2198.7921	1099.8997	2197.8081	1099.4077	Y	1699.7347	**850.3710**	1682.7081	841.8577	1681.7241	841.3657	13
22	2378.8820	1189.9446	2361.8555	1181.4314	2360.8714	1180.9394	Y	1536.6714	**768.8393**	1519.6448	760.3260	1518.6608	759.8340	12
23	2506.9406	1253.9739	2489.9140	1245.4607	2488.9300	1244.9687	Q	1373.6080	**687.3077**	**1356.5815**	**678.7944**	1355.5975	678.3024	11
24	2621.9675	1311.4874	2604.9410	1302.9741	2603.9570	1302.4821	D	1245.5495	**623.2784**	1228.5229	**614.7651**	1227.5389	**614.2731**	10
25	2750.0261	1375.5167	2732.9996	1367.0034	2732.0155	1366.5114	Q	**1130.5225**	565.7649	1113.4960	557.2516	1112.5119	556.7596	9
26	2849.0945	1425.0509	2832.0680	1416.5376	2831.0840	1416.0456	V	**1002.4639**	501.7356	985.4374	493.2223	984.4534	492.7303	8
27	2964.1215	1482.5644	2947.0949	1474.0511	2946.1109	1473.5591	D	**903.3955**	**452.2014**	886.3690	443.6881	885.3850	443.1961	7
28	3093.1641	1547.0857	3076.1375	1538.5724	3075.1535	1538.0804	E	**788.3686**	394.6879	771.3420	386.1747	**770.3580**	385.6826	6
29	3240.2325	1620.6199	3223.2059	1612.1066	3222.2219	1611.6146	F	**659.3260**	330.1666	642.2994	321.6534	641.3154	321.1613	5
30	3377.2914	1689.1493	3360.2648	1680.6361	3359.2808	1680.1440	H	**512.2576**	256.6324	495.2310	248.1191	494.2470	247.6271	4
31	3506.3340	1753.6706	3489.3074	1745.1574	3488.3234	1744.6653	E	**375.1987**	188.1030	358.1721	179.5897	357.1881	179.0977	3
32	3577.3711	1789.1892	3560.3445	**1780.6759**	3559.3605	**1780.1839**	A	246.1561	123.5817	229.1295	115.0684			2
33							R	175.1190	88.0631	158.0924	79.5498			1

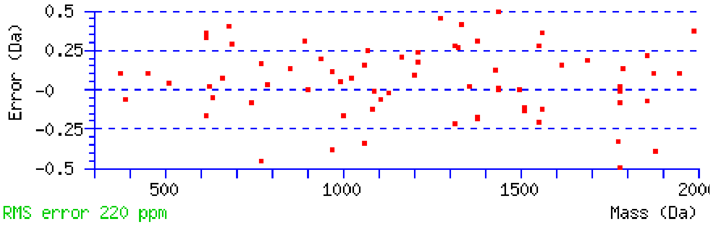

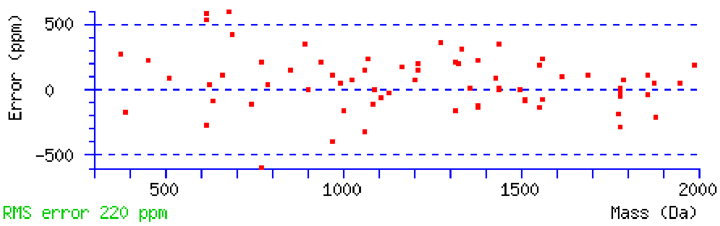

All matches to this query

Score	Mr(calc):	Delta	Sequence
82.6	3750.4755	0.0186	TATDENEDDLGSPPSPGDSSYYQDQVDEFHEAR
73.4	3750.4755	0.0186	TATDENEDDLGSPPSPGDSSYYQDQVDEFHEAR
57.6	3750.4755	0.0186	TATDENEDDLGSPPSPGDSSYYQDQVDEFHEAR
52.2	3750.4755	0.0186	TATDENEDDLGSPPSPGDSSYYQDQVDEFHEAR
43.4	3750.4755	0.0186	TATDENEDDLGSPPSPGDSSYYQDQVDEFHEAR
37.5	3750.4755	0.0186	TATDENEDDLGSPPSPGDSSYYQDQVDEFHEAR
33.5	3750.4755	0.0186	TATDENEDDLGSPPSPGDSSYYQDQVDEFHEAR
28.3	3750.4755	0.0186	TATDENEDDLGSPPSPGDSSYYQDQVDEFHEAR
7.6	3748.4619	2.0323	DVYRSQSYKAGIYSVELINDSLYDWHVK
6.0	3748.4619	2.0323	DVYRSQSYKAGIYSVELINDSLYDWHVK

Spectrum No: 41; Query: 1783; Rank: 1

Peptide View

MS/MS Fragmentation of **VFDDSDEKEDEEDTDVR**
Found in **IPI00765990**, Tax_Id=10116 Gene_Symbol=Htatsf1_predicted HIV TAT specific factor 1

Match to Query 1783: 2121.794908 from(1061.904730,2+)
Title: 100101RatKid_NS_deglyco_24.1330.1330.2.dta
Data file K:\NewmanPaper\Piliang\3SubProteomes\Piliang3SP\mgf5ppm\ERLIC_3SubProteomes5ppm.mgf

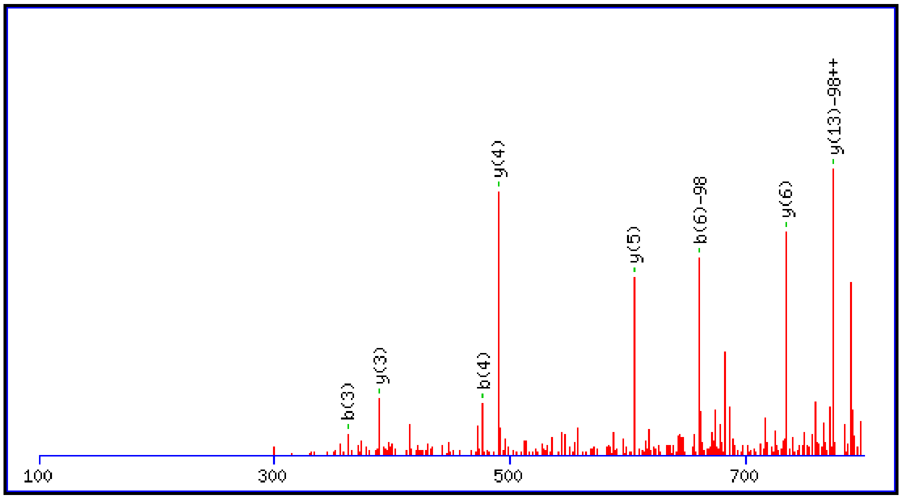

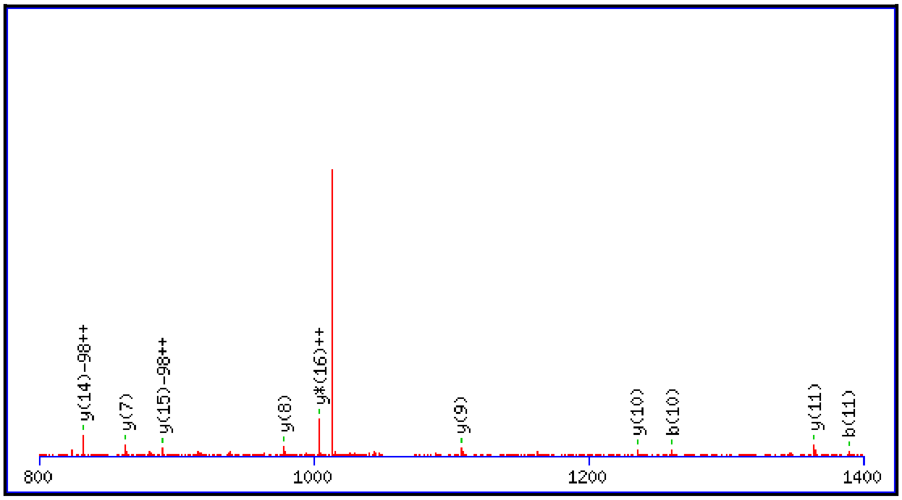

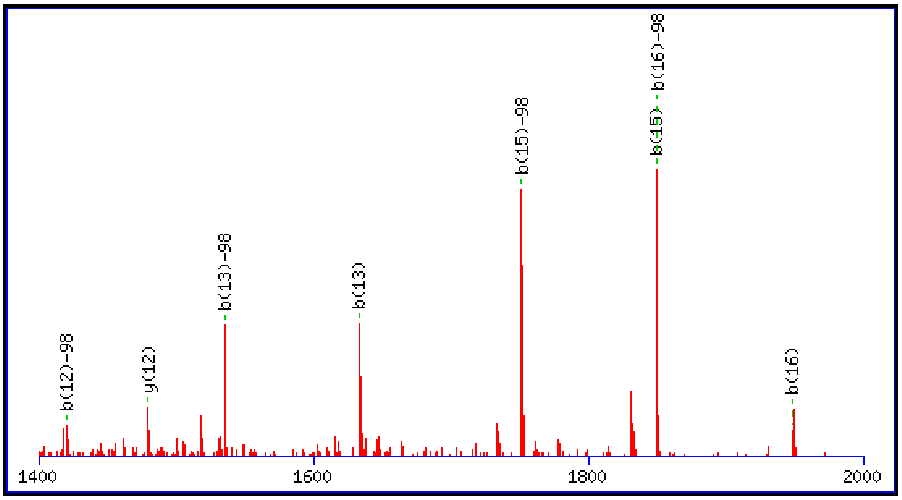

Monoisotopic mass of neutral peptide Mr(calc): 2121.7899
Fixed modifications: Carbamidomethyl (C)

Variable modifications:
S5 : Phospho (ST), with neutral losses 97.9769(shown in table), 0.0000
Ions Score: 82 **Expect:** 7.8e-007
Matches (Bold Red): 26/260 fragment ions using 40 most intense peaks

#	b	b^{++}	b^{*}	b^{*++}	b^{0}	b^{0++}	Seq.	y	y^{++}	y^{*}	y^{*++}	y^{0}	y^{0++}	#
1	100.0757	50.5415					V							17
2	247.1441	124.0757					F	1925.7519	963.3796	1908.7254	954.8663	1907.7413	954.3743	16
3	**362.1710**	181.5892			344.1605	172.5839	D	1778.6835	**889.8454**	1761.6569	881.3321	1760.6729	880.8401	15
4	**477.1980**	239.1026			459.1874	230.0974	D	1663.6566	**832.3319**	1646.6300	823.8186	1645.6460	823.3266	14
5	546.2194	273.6134			528.2089	264.6081	S	1548.6296	**774.8184**	1531.6031	766.3052	1530.6190	765.8132	13
6	**661.2464**	331.1268			643.2358	322.1216	D	**1479.6082**	740.3077	1462.5816	731.7944	1461.5976	731.3024	12
7	790.2890	395.6481			772.2784	386.6428	E	**1364.5812**	682.7942	1347.5547	674.2810	1346.5706	673.7890	11
8	918.3839	459.6956	901.3574	451.1823	900.3734	450.6903	K	**1235.5386**	618.2729	1218.5121	609.7597	1217.5281	609.2677	10
9	1047.4265	524.2169	1030.4000	515.7036	1029.4160	515.2116	E	**1107.4437**	554.2255	1090.4171	545.7122	1089.4331	545.2202	9
10	1162.4535	581.7304	1145.4269	573.2171	1144.4429	572.7251	D	**978.4011**	489.7042	961.3745	481.1909	960.3905	480.6989	8
11	1291.4961	646.2517	1274.4695	637.7384	1273.4855	637.2464	E	**863.3741**	432.1907	846.3476	423.6774	845.3636	423.1854	7
12	**1420.5387**	710.7730	1403.5121	702.2597	1402.5281	701.7677	E	**734.3315**	367.6694	717.3050	359.1561	716.3210	358.6641	6
13	**1535.5656**	768.2864	1518.5391	759.7732	1517.5550	759.2812	D	**605.2889**	303.1481	588.2624	294.6348	587.2784	294.1428	5
14	1636.6133	818.8103	1619.5867	810.2970	1618.6027	809.8050	T	**490.2620**	245.6346	473.2354	237.1214	472.2514	236.6293	4
15	**1751.6402**	876.3238	1734.6137	867.8105	1733.6297	867.3185	D	**389.2143**	195.1108	372.1878	186.5975	371.2037	186.1055	3
16	**1850.7086**	925.8580	1833.6821	917.3447	1832.6981	916.8527	V	274.1874	137.5973	257.1608	129.0840			2
17							R	175.1190	88.0631	158.0924	79.5498			1

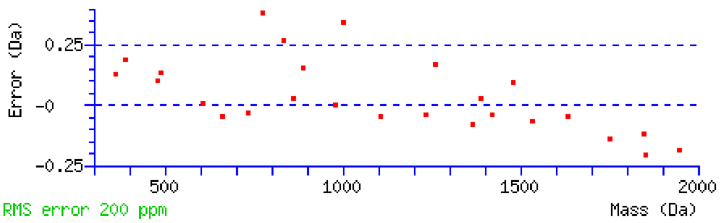

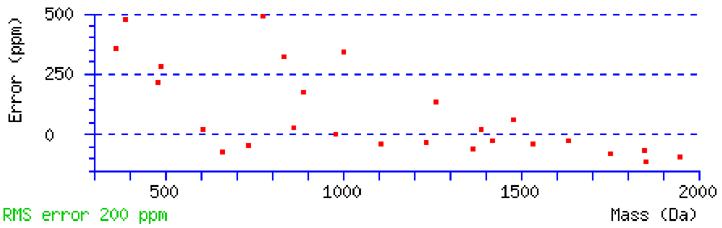

All matches to this query

Score	Mr(calc):	Delta	Sequence
82.4	2121.7899	0.0050	VFDDSDEKEDEEDTDVR
24.1	2121.7899	0.0050	VFDDSDEKEDEEDTDVR
8.7	2121.7872	0.0077	SHHKDHSDSESTSSDNSGK
4.0	2121.7872	0.0077	SHHKDHSDSESTSSDNSGK
4.0	2121.7872	0.0077	SHHKDHSDSESTSSDNSGK
3.7	2121.7741	0.0208	MGVQGNGTRQTNYGGRGK
3.5	2121.8003	-0.0054	EDSGTYVCTAENSVGFVK
3.4	2121.8003	-0.0054	EDSGTYVCTAENSVGFVK
3.4	2121.7908	0.0041	SHNLDMIEFSESCNVDK
3.3	2120.7901	1.0048	MGVQGNGTRQTNYGGRGK

Spectrum No: 42; Query: 2797; Rank: 1

Peptide View

MS/MS Fragmentation of **TFQQIQEEEDDDYPGSYSPQDPSAGPLLTEELIK**
Found in **IPI00364827**, Tax_Id=10116 Gene_Symbol=RGD1304782_predicted hypothetical protein LOC311591

Match to Query 2797: 3918.742332 from(1307.254720,3+)
Title: 091008RatKidney_NoSalt_26.4787.4787.3.dta
Data file K:\NewmanPaper\Piliang\3SubProteomes\Piliang3SP\mgf5ppm\ERLIC_3SubProteomes5ppm.mgf

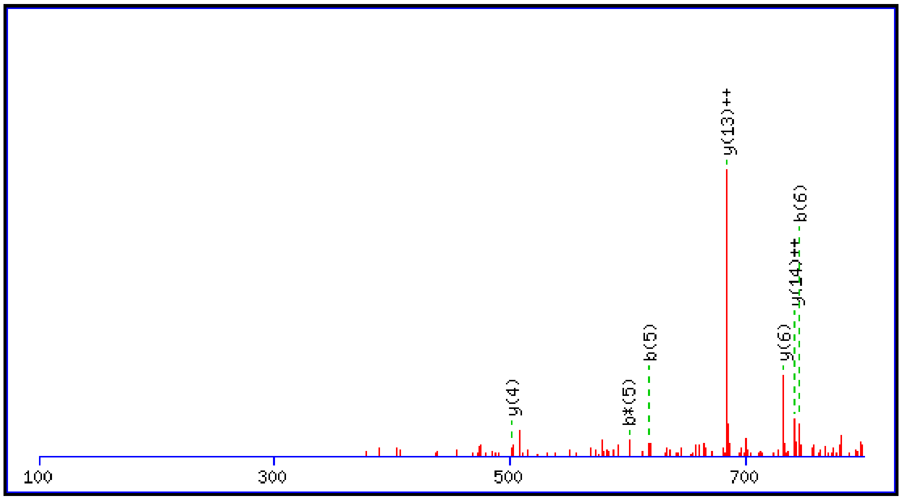

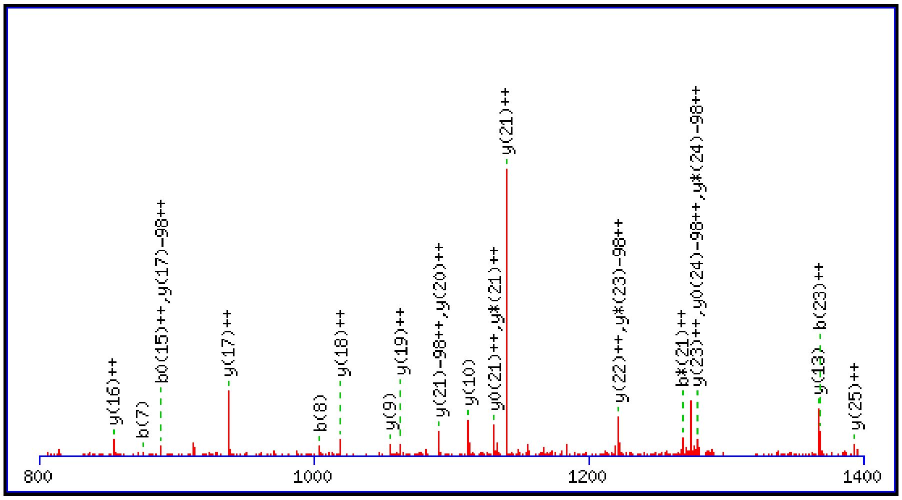

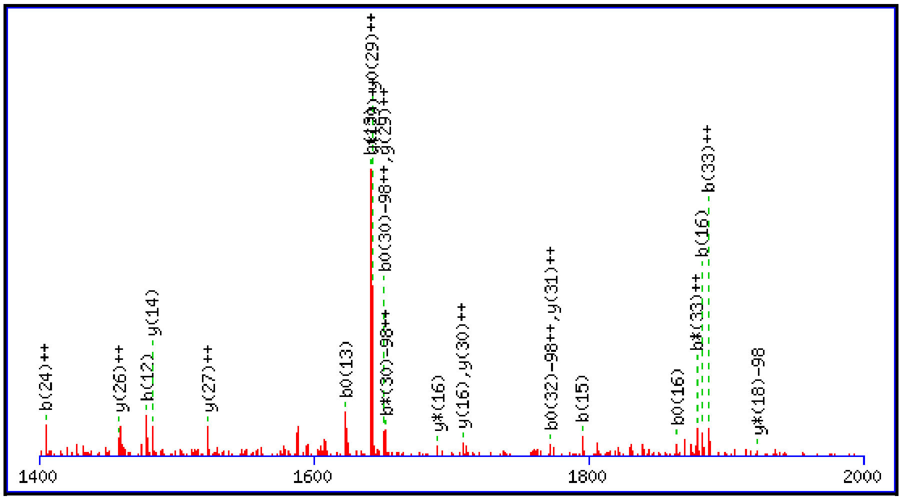

Monoisotopic mass of neutral peptide Mr(calc): 3918.7248
Fixed modifications: Carbamidomethyl (C)
Variable modifications:
S18 : Phospho (ST), with neutral losses 0.0000(shown in table), 97.9769
Ions Score: 82 **Expect:** 3.3e-006
Matches (Bold Red): 54/584 fragment ions using 85 most intense peaks

#	b	b^{++}	b*	b^{*++}	b^0	b^{0++}	Seq.	y	y^{++}	y*	y^{*++}	y^0	y^{0++}	#
1	102.0550	51.5311			84.0444	42.5258	T							34
2	249.1234	125.0653			231.1128	116.0600	F	3818.6844	1909.8459	3801.6579	1901.3326	3800.6739	1900.8406	33

3	377.1819	189.0946	360.1554	180.5813	359.1714	180.0893	Q	3671.6160	1836.3116	3654.5895	1827.7984	3653.6055	1827.3064	32
4	505.2405	253.1239	488.2140	244.6106	487.2300	244.1186	Q	3543.5574	**1772.2824**	3526.5309	1763.7691	3525.5469	1763.2771	31
5	**618.3246**	309.6659	**601.2980**	301.1527	600.3140	300.6607	I	3415.4989	**1708.2531**	3398.4723	1699.7398	3397.4883	1699.2478	30
6	**746.3832**	373.6952	729.3566	365.1819	728.3726	364.6899	Q	3302.4148	**1651.7110**	3285.3882	**1643.1978**	3284.4042	**1642.7058**	29
7	**875.4258**	438.2165	858.3992	429.7032	857.4152	429.2112	E	3174.3562	1587.6817	3157.3297	1579.1685	3156.3457	1578.6765	28
8	**1004.4684**	502.7378	987.4418	494.2245	986.4578	493.7325	E	3045.3136	**1523.1605**	3028.2871	1514.6472	3027.3031	1514.1552	27
9	1133.5109	567.2591	1116.4844	558.7458	1115.5004	558.2538	E	2916.2710	**1458.6392**	2899.2445	1450.1259	2898.2605	1449.6339	26
10	1248.5379	624.7726	1231.5113	616.2593	1230.5273	615.7673	D	2787.2284	**1394.1179**	2770.2019	1385.6046	2769.2179	1385.1126	25
11	1363.5648	682.2861	1346.5383	673.7728	1345.5543	673.2808	D	2672.2015	1336.6044	2655.1749	1328.0911	2654.1909	1327.5991	24
12	**1478.5918**	739.7995	1461.5652	731.2863	1460.5812	730.7942	D	2557.1746	**1279.0909**	2540.1480	1270.5776	2539.1640	1270.0856	23
13	**1641.6551**	821.3312	1624.6286	812.8179	**1623.6445**	812.3259	Y	2442.1476	**1221.5774**	2425.1211	1213.0642	2424.1370	1212.5722	22
14	1738.7079	869.8576	1721.6813	861.3443	1720.6973	860.8523	P	2279.0843	**1140.0458**	2262.0577	**1131.5325**	2261.0737	**1131.0405**	21
15	**1795.7293**	898.3683	1778.7028	889.8550	1777.7188	**889.3630**	G	2182.0315	**1091.5194**	2165.0050	1083.0061	2164.0210	1082.5141	20
16	**1882.7614**	941.8843	1865.7348	933.3710	**1864.7508**	932.8790	S	2125.0101	**1063.0087**	2107.9835	1054.4954	2106.9995	1054.0034	19
17	2045.8247	1023.4160	2028.7981	1014.9027	2027.8141	1014.4107	Y	2037.9780	**1019.4927**	2020.9515	1010.9794	2019.9675	1010.4874	18
18	2212.8230	1106.9152	2195.7965	1098.4019	2194.8125	1097.9099	S	1874.9147	**937.9610**	1857.8881	929.4477	1856.9041	928.9557	17
19	2309.8758	1155.4415	2292.8493	1146.9283	2291.8652	1146.4363	P	**1707.9163**	**854.4618**	**1690.8898**	845.9485	1689.9058	845.4565	16
20	2437.9344	1219.4708	2420.9078	1210.9576	2419.9238	1210.4655	Q	1610.8636	805.9354	1593.8370	797.4222	1592.8530	796.9301	15
21	2552.9613	1276.9843	2535.9348	**1268.4710**	2534.9508	1267.9790	D	**1482.8050**	**741.9061**	1465.7785	733.3929	1464.7944	732.9009	14
22	2650.0141	1325.5107	2632.9875	1316.9974	2632.0035	1316.5054	P	**1367.7781**	**684.3927**	1350.7515	675.8794	1349.7675	675.3874	13
23	2737.0461	**1369.0267**	2720.0196	1360.5134	2719.0356	1360.0214	S	1270.7253	635.8663	1253.6987	627.3530	1252.7147	626.8610	12
24	2808.0832	**1404.5453**	2791.0567	1396.0320	2790.0727	1395.5400	A	1183.6933	592.3503	1166.6667	583.8370	1165.6827	583.3450	11
25	2865.1047	1433.0560	2848.0781	1424.5427	2847.0941	1424.0507	G	**1112.6562**	556.8317	1095.6296	548.3184	1094.6456	547.8264	10
26	2962.1575	1481.5824	2945.1309	1473.0691	2944.1469	1472.5771	P	**1055.6347**	528.3210	1038.6081	519.8077	1037.6241	519.3157	9
27	3075.2415	1538.1244	3058.2150	1529.6111	3057.2310	1529.1191	L	958.5819	479.7946	941.5554	471.2813	940.5714	470.7893	8
28	3188.3256	1594.6664	3171.2990	1586.1532	3170.3150	1585.6611	L	845.4979	423.2526	828.4713	414.7393	827.4873	414.2473	7
29	3289.3733	1645.1903	3272.3467	1636.6770	3271.3627	1636.1850	T	**732.4138**	366.7105	715.3872	358.1973	714.4032	357.7053	6
30	3418.4159	1709.7116	3401.3893	1701.1983	3400.4053	1700.7063	E	631.3661	316.1867	614.3396	307.6734	613.3556	307.1814	5
31	3547.4585	1774.2329	3530.4319	1765.7196	3529.4479	1765.2276	E	**502.3235**	251.6654	485.2970	243.1521	484.3130	242.6601	4
32	3660.5425	1830.7749	3643.5160	1822.2616	3642.5320	1821.7696	L	373.2809	187.1441	356.2544	178.6308			3
33	3773.6266	**1887.3169**	3756.6000	**1878.8037**	3755.6160	1878.3116	I	260.1969	130.6021	243.1703	122.0888			2
34							K	147.1128	74.0600	130.0863	65.5468			1

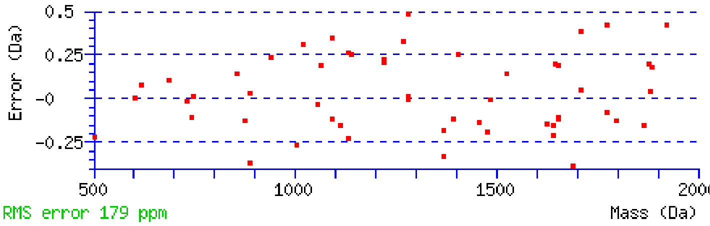

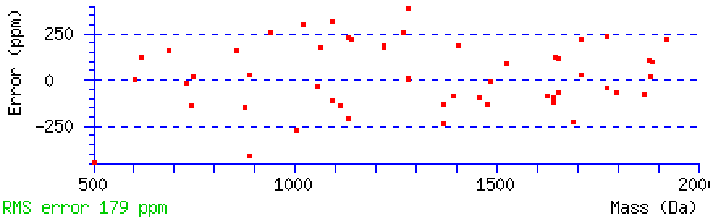

All matches to this query

Score	Mr(calc):	Delta	Sequence
82.1	3918.7248	0.0175	TFQQIQEEEDDDYPGSYSPQDPSAGPLLTEELIK
71.6	3918.7248	0.0175	TFQQIQEEEDDDYPGSYSPQDPSAGPLLTEELIK
59.2	3918.7248	0.0175	TFQQIQEEEDDDYPGSYSPQDPSAGPLLTEELIK
51.1	3918.7248	0.0175	TFQQIQEEEDDDYPGSYSPQDPSAGPLLTEELIK
43.0	3918.7248	0.0175	TFQQIQEEEDDDYPGSYSPQDPSAGPLLTEELIK
37.2	3918.7248	0.0175	TFQQIQEEEDDDYPGSYSPQDPSAGPLLTEELIK
13.7	3918.7248	0.0175	TFQQIQEEEDDDYPGSYSPQDPSAGPLLTEELIK
6.4	3917.7363	1.0060	GTTVGSAQANSTPSSVASVVASAESPASRQAAAKLALR

6.4	3918.7504	-0.0081	TFQNSLVHLGLNTMKSANICIGRPVLLTSLDGK
5.5	3917.7436	0.9987	RGDNLGSPALSIGFSVTLGHLLGIYFTGCSMNPAR

Spectrum No: 43; Query: 1381; Rank: 1

Peptide View

MS/MS Fragmentation of **SQSLPNSLDYAQTSER**
Found in **IPI00471759**, Tax_Id=10116 Gene_Symbol=RGD1308697 Protein FAM82C

Match to Query 1381: 1874.803168 from(938.408860,2+)
Title: 091008RatKidney_NoSalt_23.2049.2049.2.dta
Data file K:\NewmanPaper\Piliang\3SubProteomes\Piliang3SP\mgf5ppm\ERLIC_3SubProteomes5ppm.mgf

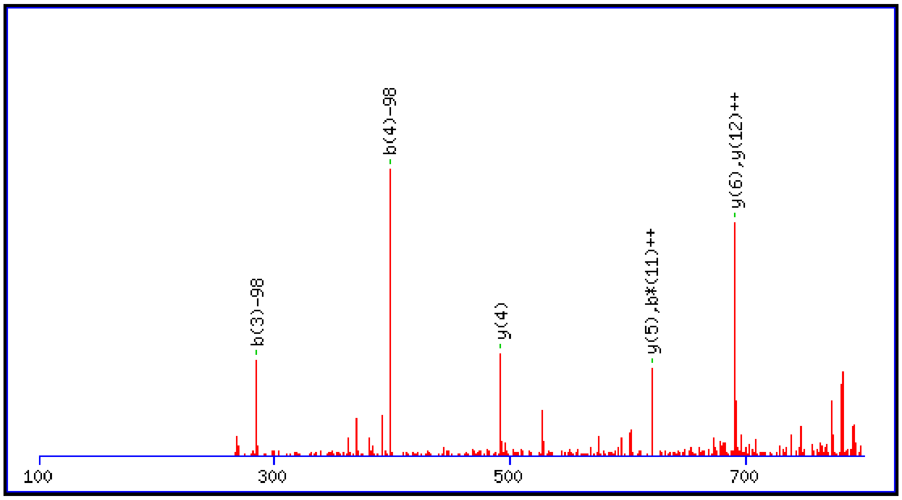

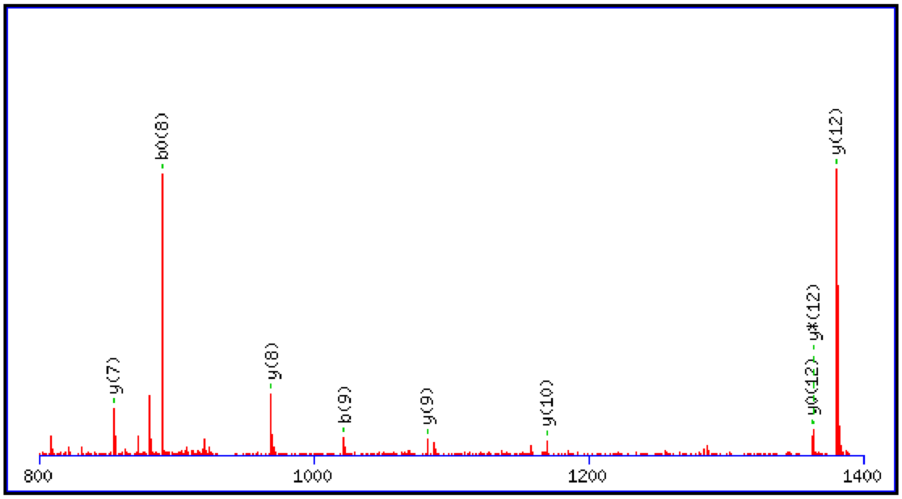

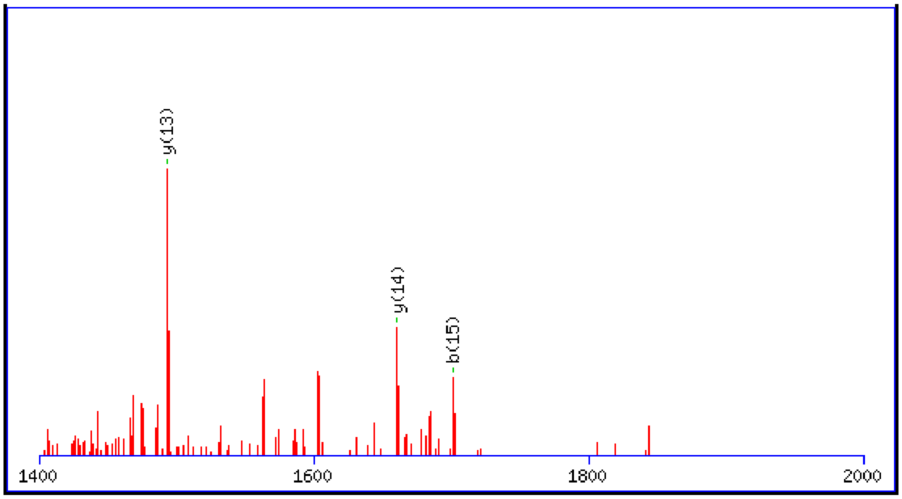

Monoisotopic mass of neutral peptide Mr(calc): 1874.8047
Fixed modifications: Carbamidomethyl (C)
Variable modifications:
S3 : Phospho (ST), with neutral losses 97.9769(shown in table), 0.0000
Ions Score: 82 **Expect:** 1.5e-006
Matches (Bold Red): 19/266 fragment ions using 24 most intense peaks

#	b	b++	b*	b*++	b0	b0++	Seq.	y	y++	y*	y*++	y0	y0++	#
1	88.0393	44.5233			70.0287	35.5180	S							16
2	216.0979	108.5526	199.0713	100.0393	198.0873	99.5473	Q	1690.8031	845.9052	1673.7765	837.3919	1672.7925	836.8999	15
3	**285.1193**	143.0633	268.0928	134.5500	267.1088	134.0580	S	1562.7445	781.8759	1545.7180	773.3626	1544.7340	772.8706	14
4	**398.2034**	199.6053	381.1769	191.0921	380.1928	190.6001	L	**1493.7231**	747.3652	1476.6965	738.8519	1475.7125	738.3599	13
5	495.2562	248.1317	478.2296	239.6184	477.2456	239.1264	P	**1380.6390**	**690.8231**	**1363.6125**	682.3099	**1362.6284**	681.8179	12
6	609.2991	305.1532	592.2725	296.6399	591.2885	296.1479	N	1283.5862	642.2968	1266.5597	633.7835	1265.5757	633.2915	11
7	696.3311	348.6692	679.3046	340.1559	678.3206	339.6639	S	**1169.5433**	585.2753	1152.5168	576.7620	1151.5327	576.2700	10
8	809.4152	405.2112	792.3886	396.6980	791.4046	396.2059	L	**1082.5113**	541.7593	1065.4847	533.2460	1064.5007	532.7540	9
9	924.4421	462.7247	907.4156	454.2114	906.4316	453.7194	D	**969.4272**	485.2172	952.4007	476.7040	951.4167	476.2120	8
10	1087.5055	544.2564	1070.4789	535.7431	1069.4949	535.2511	Y	**854.4003**	427.7038	837.3737	419.1905	836.3897	418.6985	7
11	1158.5426	579.7749	1141.5160	571.2616	1140.5320	570.7696	A	**691.3369**	346.1721	674.3104	337.6588	673.3264	337.1668	6
12	1286.6011	643.8042	1269.5746	635.2909	1268.5906	634.7989	Q	**620.2998**	310.6536	603.2733	302.1403	602.2893	301.6483	5
13	1387.6488	694.3281	1370.6223	685.8148	1369.6383	685.3228	T	**492.2413**	246.6243	475.2147	238.1110	474.2307	237.6190	4
14	1474.6809	737.8441	1457.6543	729.3308	1456.6703	728.8388	S	391.1936	196.1004	374.1670	187.5871	373.1830	187.0951	3
15	1603.7234	802.3654	1586.6969	793.8521	1585.7129	793.3601	E	304.1615	152.5844	287.1350	144.0711	286.1510	143.5791	2
16							R	175.1190	88.0631	158.0924	79.5498			1

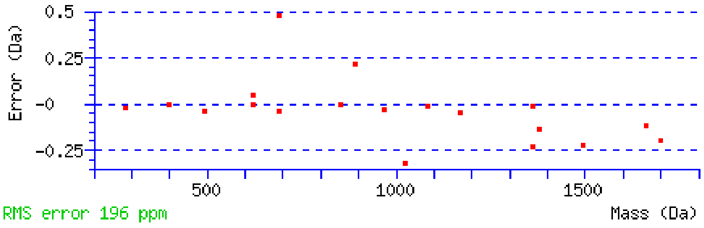

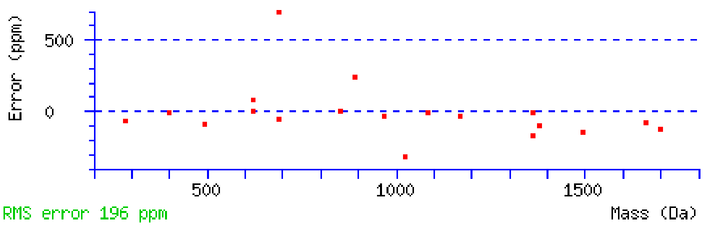

All matches to this query

Score	Mr(calc):	Delta	Sequence
81.6	1874.8047	-0.0015	SQSLPNSLDYAQTSER
77.3	1874.8047	-0.0015	SQSLPNSLDYAQTSER
47.3	1874.8047	-0.0015	SQSLPNSLDYAQTSER
11.3	1874.8047	-0.0015	SQSLPNSLDYAQTSER
5.0	1874.8216	-0.0185	DAYQVTDNLVLSIHK

3.3	1874.8118	-0.0086	GPYYTHLGAGPSVAAVR
2.8	1874.8077	-0.0045	QRLGSAALDSIHEYR
2.8	1874.8077	-0.0045	QRLGSAALDSIHEYR
2.6	1873.7900	1.0132	DSIENFTTDYKIIR
1.1	1874.8118	-0.0086	GPYYTHLGAGPSVAAVR

Spectrum No: 44; Query: 2458; Rank: 1

Peptide View

MS/MS Fragmentation of **VTLQDYHLPDSDEDEDEETAIRR**
Found in **IPI00193162**, Tax_Id=10116 Gene_Symbol=LOC499871 Similar to Zinc finger, FYVE domain containing 19

Match to Query 2458: 2825.216142 from(942.745990,3+)
Title: 100101RatKid_NS_deglyco_18.3129.3129.3.dta
Data file K:\NewmanPaper\Piliang\3SubProteomes\Piliang3SP\mgf5ppm\ERLIC_3SubProteomes5ppm.mgf

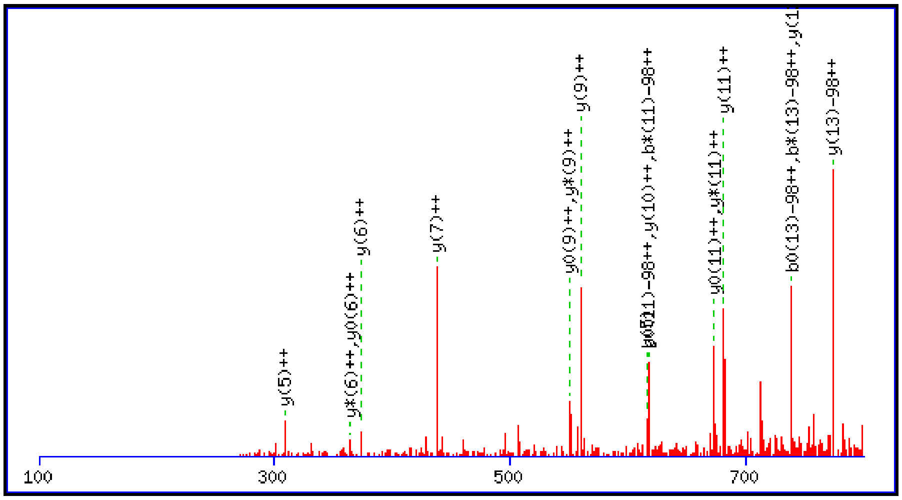

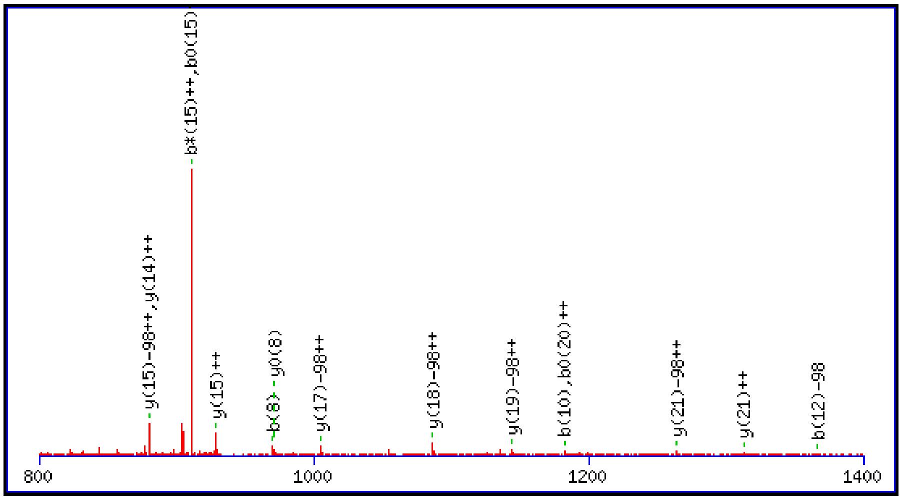

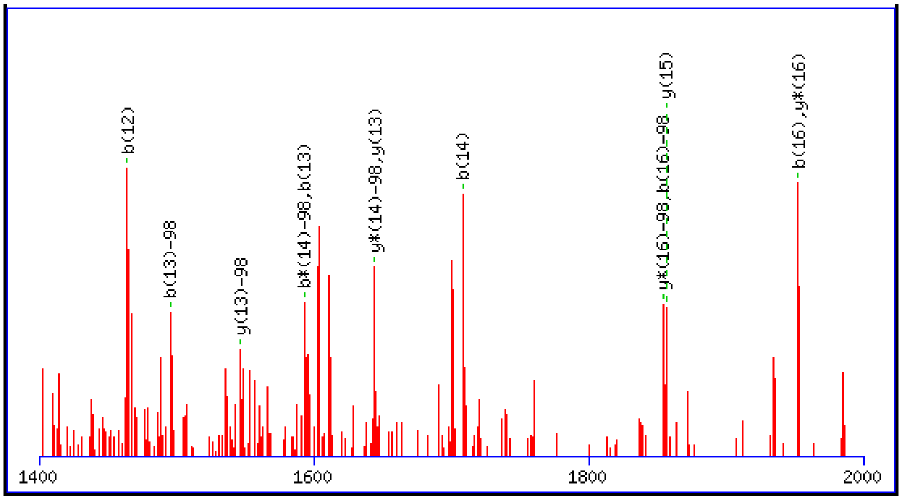

Monoisotopic mass of neutral peptide Mr(calc): 2825.2029
Fixed modifications: Carbamidomethyl (C)
Variable modifications:
S11 : Phospho (ST), with neutral losses 97.9769(shown in table), 0.0000
Ions Score: 81 **Expect:** 2.8e-006
Matches (Bold Red): 47/380 fragment ions using 43 most intense peaks

#	b	b^{++}	b*	b^{*++}	b^{0}	b^{0++}	Seq.	y	y^{++}	y*	y^{*++}	y^{0}	y^{0++}	#
1	100.0757	50.5415					V							23
2	201.1234	101.0653			183.1128	92.0600	T	2629.1649	1315.0861	2612.1383	1306.5728	2611.1543	1306.0808	22
3	314.2074	157.6074			296.1969	148.6021	L	2528.1172	**1264.5622**	2511.0906	1256.0490	2510.1066	1255.5569	21
4	442.2660	221.6366	425.2395	213.1234	424.2554	212.6314	Q	2415.0331	1208.0202	2398.0066	1199.5069	2397.0225	1199.0149	20
5	557.2930	279.1501	540.2664	270.6368	539.2824	270.1448	D	2286.9745	**1143.9909**	2269.9480	1135.4776	2268.9640	1134.9856	19
6	720.3563	360.6818	703.3297	352.1685	702.3457	351.6765	Y	2171.9476	**1086.4774**	2154.9210	1077.9642	2153.9370	1077.4721	18
7	857.4152	429.2112	840.3886	420.6980	839.4046	420.2060	H	2008.8843	**1004.9458**	1991.8577	996.4325	1990.8737	995.9405	17
8	**970.4993**	485.7533	953.4727	477.2400	952.4887	476.7480	L	1871.8253	936.4163	**1854.7988**	927.9030	1853.8148	927.4110	16
9	1067.5520	534.2796	1050.5255	525.7664	1049.5415	525.2744	P	1758.7413	**879.8743**	1741.7147	871.3610	1740.7307	870.8690	15
10	**1182.5790**	591.7931	1165.5524	583.2798	1164.5684	582.7878	D	1661.6885	831.3479	**1644.6620**	822.8346	1643.6780	822.3426	14
11	1251.6004	626.3038	1234.5739	**617.7906**	1233.5899	**617.2986**	S	**1546.6616**	**773.8344**	1529.6350	765.3212	1528.6510	764.8291	13
12	**1366.6274**	683.8173	1349.6008	675.3040	1348.6168	674.8120	D	1477.6401	**739.3237**	1460.6136	730.8104	1459.6296	730.3184	12
13	**1495.6700**	748.3386	1478.6434	**739.8253**	1477.6594	**739.3333**	E	1362.6132	**681.8102**	1345.5866	**673.2970**	1344.6026	**672.8049**	11
14	1610.6969	805.8521	**1593.6704**	797.3388	1592.6863	796.8468	D	1233.5706	**617.2889**	1216.5440	608.7757	1215.5600	608.2836	10
15	1739.7395	870.3734	1722.7129	861.8601	1721.7289	861.3681	E	1118.5436	**559.7755**	1101.5171	**551.2622**	1100.5331	**550.7702**	9
16	**1854.7664**	927.8869	1837.7399	919.3736	1836.7559	918.8816	D	989.5010	495.2542	972.4745	486.7409	**971.4905**	486.2489	8
17	1983.8090	992.4082	1966.7825	983.8949	1965.7985	983.4029	E	874.4741	**437.7407**	857.4476	429.2274	856.4635	428.7354	7
18	2112.8516	1056.9294	2095.8251	1048.4162	2094.8411	1047.9242	E	745.4315	**373.2194**	728.4050	**364.7061**	727.4209	**364.2141**	6
19	2213.8993	1107.4533	2196.8728	1098.9400	2195.8887	1098.4480	T	**616.3889**	**308.6981**	599.3624	300.1848	598.3784	299.6928	5
20	2284.9364	1142.9718	2267.9099	1134.4586	2266.9259	1133.9666	A	515.3412	258.1743	498.3147	249.6610			4
21	2398.0205	1199.5139	2380.9939	1191.0006	2380.0099	1190.5086	I	444.3041	222.6557	427.2776	214.1424			3
22	2554.1216	1277.5644	2537.0950	1269.0512	2536.1110	1268.5592	R	331.2201	166.1137	314.1935	157.6004			2
23							R	175.1190	88.0631	158.0924	79.5498			1

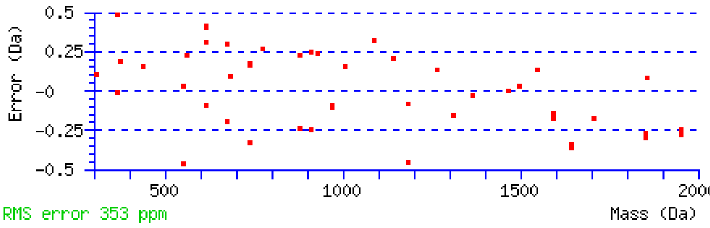

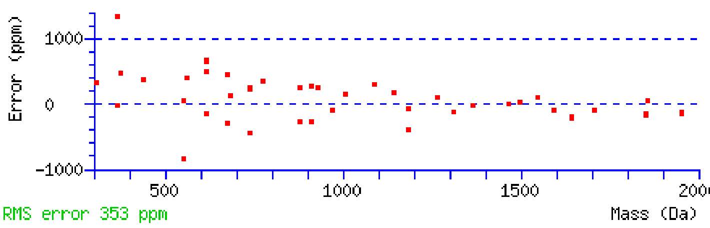

All matches to this query

Score	Mr(calc):	Delta	Sequence
81.1	2825.2029	0.0133	VTLQDYHLPDSDEDEDEETAIRR
31.4	2825.2029	0.0133	VTLQDYHLPDSDEDEDEETAIRR
27.9	2825.2029	0.0133	VTLQDYHLPDSDEDEDEETAIRR
20.0	2825.2029	0.0133	VTLQDYHLPDSDEDEDEETAIRR
10.5	2823.1848	2.0313	SNRDITQSSVVEINNETNLEDRK
7.0	2824.2383	0.9779	TQPXMELLLGDLIISLHSMTWR
7.0	2824.2383	0.9779	TQPXMELLLGDLIISLHSMTWR
4.4	2823.1848	2.0313	SNRDITQSSVVEINNETNLEDRK
4.4	2823.1848	2.0313	SNRDITQSSVVEINNETNLEDRK
4.3	2823.1848	2.0313	SNRDITQSSVVEINNETNLEDRK

Spectrum No: 45; Query: 2014; Rank: 1

Peptide View

MS/MS Fragmentation of **ASPALGSGPDGSGDSLEMSSLDR**
Found in **IPI00417750**, Tax_Id=10116 Gene_Symbol=Tomm70a Mitochondrial precursor proteins import receptor

Match to Query 2014: 2300.942588 from(1151.478570,2+)
Title: 091008RatKidney_NH4Format01_23.2003.2003.2.dta
Data file K:\NewmanPaper\Piliang\3SubProteomes\Piliang3SP\mgf5ppm\ERLIC_3SubProteomes5ppm.mgf

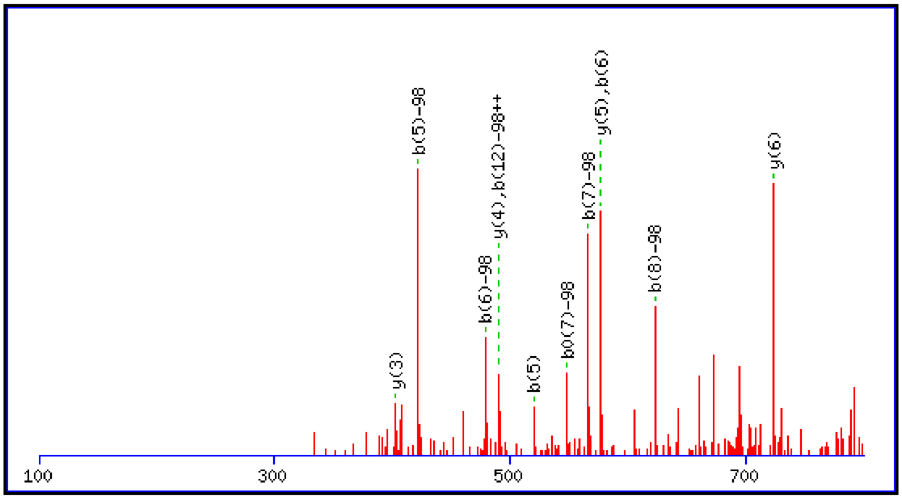

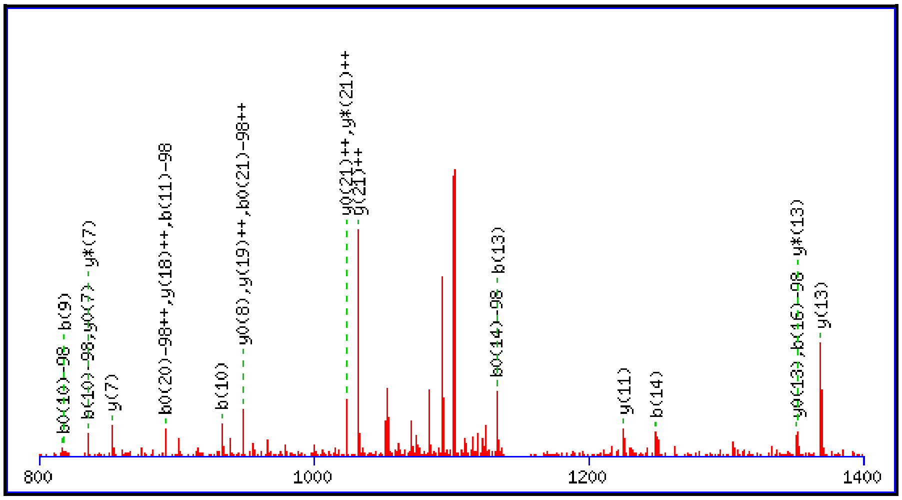

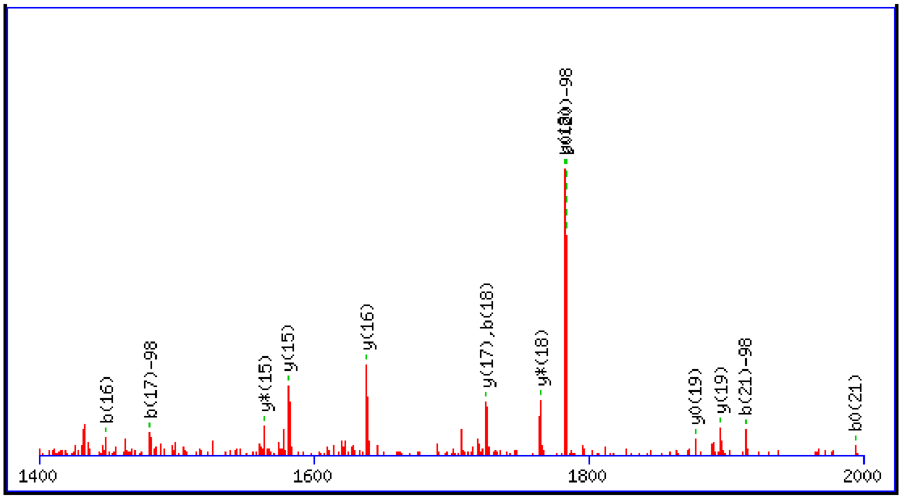

Monoisotopic mass of neutral peptide Mr(calc): 2300.9468
Fixed modifications: Carbamidomethyl (C)
Variable modifications:
S2 : Phospho (ST), with neutral losses 97.9769(shown in table), 0.0000
M18 : Oxidation (M)
Ions Score: 80 **Expect:** 2.6e-006
Matches (Bold Red): 50/306 fragment ions using 61 most intense peaks

#	b	b^{++}	b^{0}	b^{0++}	Seq.	y	y^{++}	y^{*}	y^{*++}	y^{0}	y^{0++}	#
1	72.0444	36.5258			A							23
2	141.0658	71.0366	123.0553	62.0313	S	2132.9401	1066.9737	2115.9135	1058.4604	2114.9295	1057.9684	22
3	238.1186	119.5629	220.1080	110.5577	P	2063.9186	**1032.4629**	2046.8921	**1023.9497**	2045.9080	**1023.4577**	21
4	309.1557	155.0815	291.1452	146.0762	A	1966.8658	983.9366	1949.8393	975.4233	1948.8553	974.9313	20
5	**422.2398**	211.6235	404.2292	202.6182	L	**1895.8287**	**948.4180**	1878.8022	939.9047	**1877.8182**	939.4127	19
6	**479.2613**	240.1343	461.2507	231.1290	G	**1782.7447**	**891.8760**	**1765.7181**	883.3627	1764.7341	882.8707	18
7	**566.2933**	283.6503	**548.2827**	274.6450	S	**1725.7232**	863.3652	1708.6967	854.8520	1707.7126	854.3600	17
8	**623.3147**	312.1610	605.3042	303.1557	G	**1638.6912**	819.8492	1621.6646	811.3360	1620.6806	810.8439	16
9	720.3675	360.6874	702.3569	351.6821	P	**1581.6697**	791.3385	**1564.6432**	782.8252	1563.6591	782.3332	15
10	**835.3945**	418.2009	**817.3839**	409.1956	D	1484.6169	742.8121	1467.5904	734.2988	1466.6064	733.8068	14
11	**892.4159**	446.7116	874.4054	437.7063	G	**1369.5900**	685.2986	**1352.5635**	676.7854	**1351.5794**	676.2934	13
12	979.4479	**490.2276**	961.4374	481.2223	S	1312.5685	656.7879	1295.5420	648.2746	1294.5580	647.7826	12
13	1036.4694	518.7383	1018.4588	509.7331	G	**1225.5365**	613.2719	1208.5100	604.7586	1207.5259	604.2666	11
14	1151.4964	576.2518	**1133.4858**	567.2465	D	1168.5150	584.7612	1151.4885	576.2479	1150.5045	575.7559	10
15	1238.5284	619.7678	1220.5178	610.7625	S	1053.4881	527.2477	1036.4616	518.7344	1035.4775	518.2424	9
16	**1351.6124**	676.3099	1333.6019	667.3046	L	966.4561	483.7317	949.4295	475.2184	**948.4455**	474.7264	8
17	**1480.6550**	740.8312	1462.6445	731.8259	E	**853.3720**	427.1896	**836.3455**	418.6764	**835.3614**	418.1844	7
18	1627.6904	814.3489	1609.6799	805.3436	M	**724.3294**	362.6683	707.3029	354.1551	706.3189	353.6631	6
19	1714.7225	857.8649	1696.7119	848.8596	S	**577.2940**	289.1506	560.2675	280.6374	559.2834	280.1454	5
20	1801.7545	901.3809	**1783.7439**	**892.3756**	S	**490.2620**	245.6346	473.2354	237.1214	472.2514	236.6293	4
21	**1914.8386**	957.9229	1896.8280	**948.9176**	L	**403.2300**	202.1186	386.2034	193.6053	385.2194	193.1133	3
22	2029.8655	1015.4364	2011.8549	1006.4311	D	290.1459	145.5766	273.1193	137.0633	272.1353	136.5713	2
23					R	175.1190	88.0631	158.0924	79.5498			1

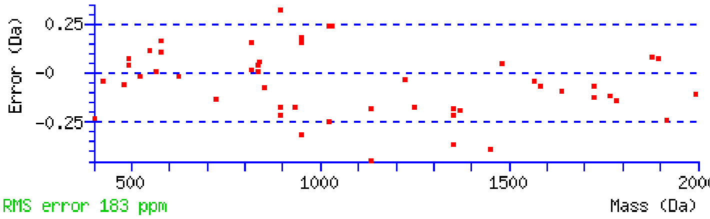

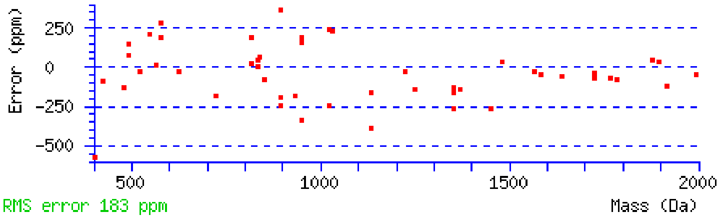

All matches to this query

Score	Mr(calc):	Delta	Sequence
80.0	2300.9468	-0.0042	ASPALGSGPDGSGDSLEMSSLDR
63.9	2300.9468	-0.0042	ASPALGSGPDGSGDSLEMSSLDR
46.5	2300.9468	-0.0042	ASPALGSGPDGSGDSLEMSSLDR
30.1	2300.9468	-0.0042	ASPALGSGPDGSGDSLEMSSLDR
16.1	2300.9468	-0.0042	ASPALGSGPDGSGDSLEMSSLDR
11.7	2300.9468	-0.0042	ASPALGSGPDGSGDSLEMSSLDR

Spectrum No: 46; Query: 803; Rank: 1

Peptide View

MS/MS Fragmentation of **IEDVGSDEEDDSGK**
Found in **IPI00471584**, Tax_Id=10116 Gene_Symbol=Hspcb Heat shock protein HSP 90-beta

Match to Query 803: 1573.564408 from(787.789480,2+)
Title: 091008RatKidney_NoSalt_31.519.519.2.dta
Data file K:\NewmanPaper\Piliang\3SubProteomes\Piliang3SP\mgf5ppm\ERLIC_3SubProteomes5ppm.mgf

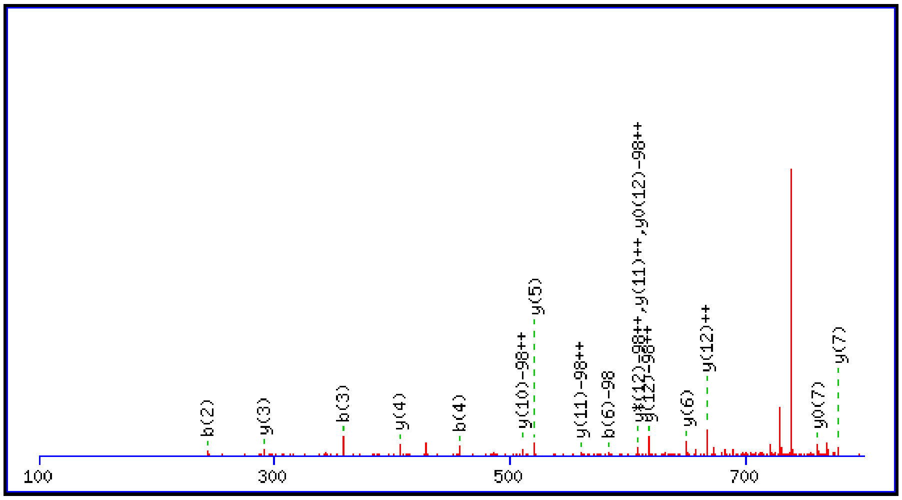

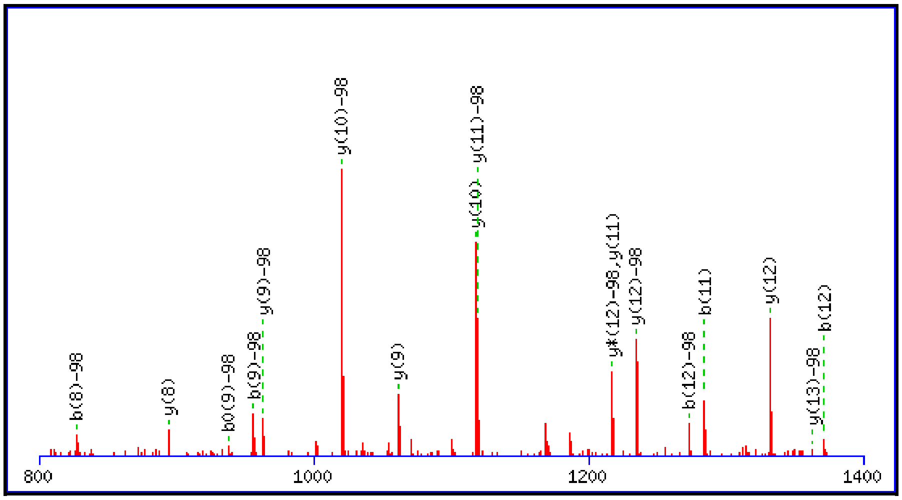

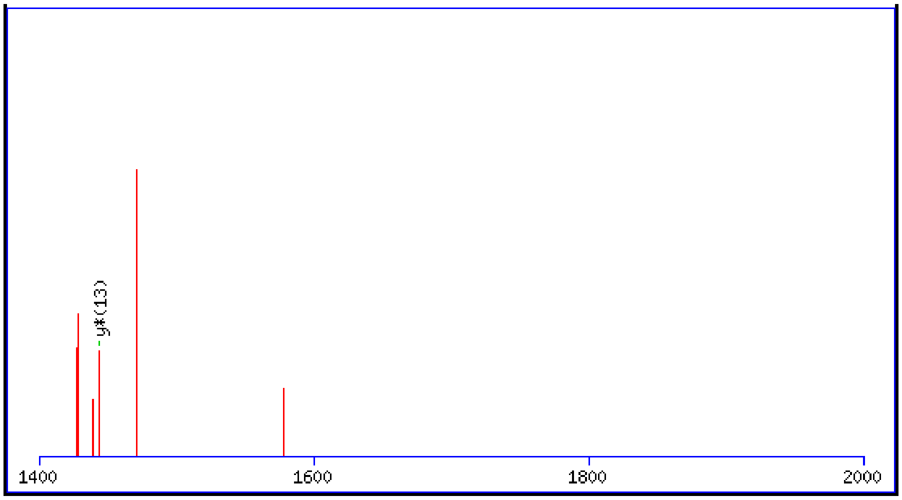

Monoisotopic mass of neutral peptide Mr(calc): 1573.5669
Fixed modifications: Carbamidomethyl (C)
Variable modifications:
S6 : Phospho (ST), with neutral losses 97.9769(shown in table), 0.0000
Ions Score: 80 **Expect:** 6.8e-007
Matches (Bold Red): 35/186 fragment ions using 56 most intense peaks

#	b	b^{++}	b^{0}	b^{0++}	Seq.	y	y^{++}	y*	y^{*++}	y^{0}	y^{0++}	#
1	114.0913	57.5493			I							14
2	**243.1339**	122.0706	225.1234	113.0653	E	**1363.5132**	682.2602	1346.4866	673.7470	1345.5026	673.2549	13
3	**358.1609**	179.5841	340.1503	170.5788	D	**1234.4706**	**617.7389**	**1217.4440**	**609.2257**	1216.4600	**608.7336**	12
4	**457.2293**	229.1183	439.2187	220.1130	V	**1119.4436**	**560.2255**	1102.4171	551.7122	1101.4331	551.2202	11
5	514.2508	257.6290	496.2402	248.6237	G	**1020.3752**	**510.6913**	1003.3487	502.1780	1002.3647	501.6860	10
6	**583.2722**	292.1397	565.2616	283.1345	S	**963.3538**	482.1805	946.3272	473.6672	945.3432	473.1752	9
7	698.2992	349.6532	680.2886	340.6479	D	**894.3323**	447.6698	877.3058	439.1565	876.3217	438.6645	8
8	**827.3417**	414.1745	809.3312	405.1692	E	**779.3054**	390.1563	762.2788	381.6430	**761.2948**	381.1510	7
9	**956.3843**	478.6958	**938.3738**	469.6905	E	**650.2628**	325.6350	633.2362	317.1218	632.2522	316.6297	6
10	1071.4113	536.2093	1053.4007	527.2040	D	**521.2202**	261.1137	504.1936	252.6005	503.2096	252.1084	5
11	1186.4382	593.7228	1168.4277	584.7175	D	**406.1932**	203.6003	389.1667	195.0870	388.1827	194.5950	4
12	**1273.4703**	637.2388	1255.4597	628.2335	S	**291.1663**	146.0868	274.1397	137.5735	273.1557	137.0815	3
13	1330.4917	665.7495	1312.4812	656.7442	G	204.1343	102.5708	187.1077	94.0575			2
14					K	147.1128	74.0600	130.0863	65.5468			1

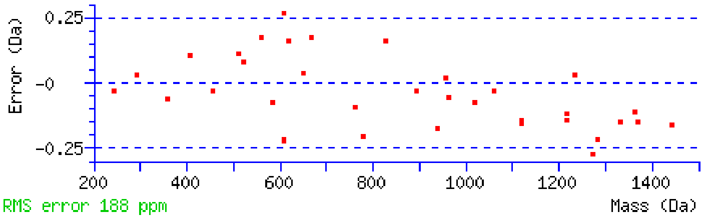

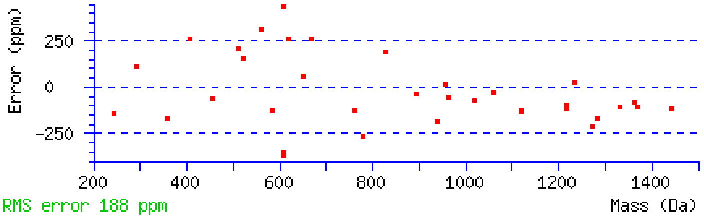

All matches to this query

Score	Mr(calc):	Delta	Sequence
79.7	1573.5669	-0.0025	IEDVGSDEEDDSGK
28.6	1573.5669	-0.0025	IEDVGSDEEDDSGK
0.7	1573.5633	0.0011	SGSYRPNNMSRK
0.1	1571.5493	2.0151	MTKDSPTPLGGGR
0.1	1571.5571	2.0073	SAYASNPPKQNR

Spectrum No: 47; Query: 2525; Rank: 1

Peptide View

MS/MS Fragmentation of **TRSPDVISSASTALSQDIPEIASEALSR**
Found in **IPI00207643**, Tax_Id=10116 Gene_Symbol=LOC361399;RGD1309136_predicted LRRG00115

Match to Query 2525: 2980.442892 from(994.488240,3+)
Title: 100101RatKid_NS_deglyco_13.6756.6756.3.dta
Data file K:\NewmanPaper\Piliang\3SubProteomes\Piliang3SP\mgf5ppm\ERLIC_3SubProteomes5ppm.mgf

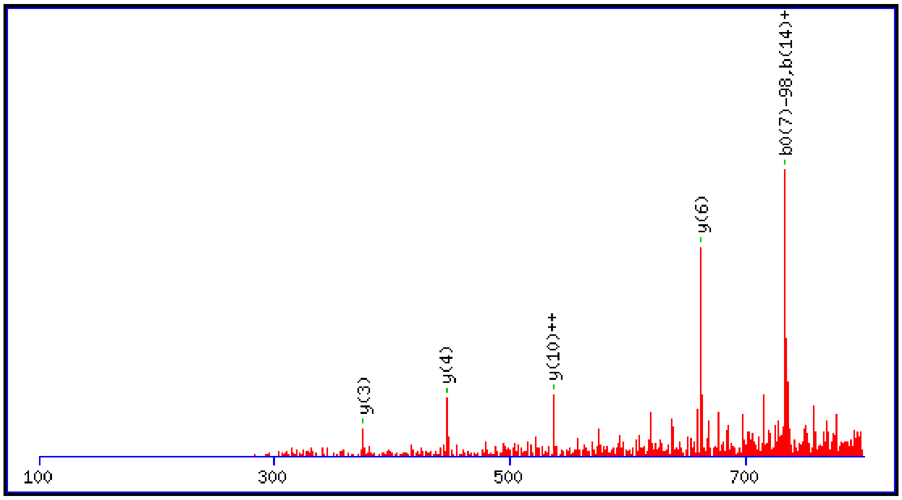

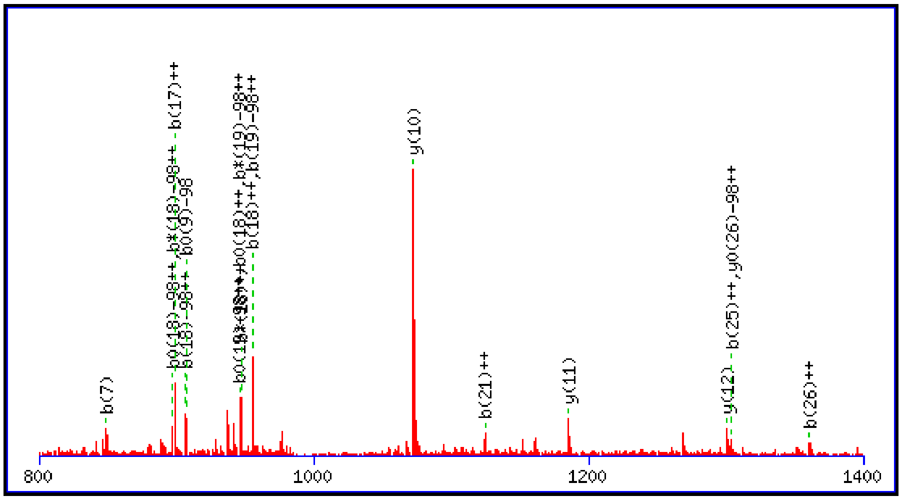

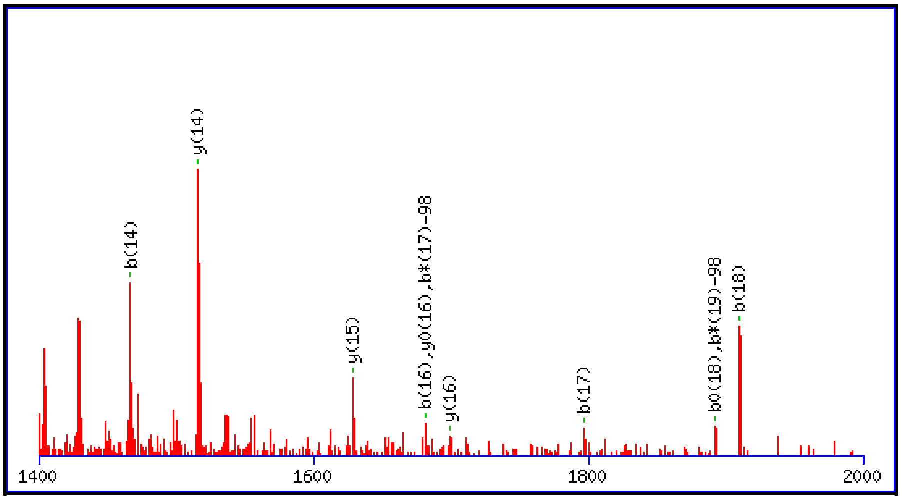

Monoisotopic mass of neutral peptide Mr(calc): 2980.4390
Fixed modifications: Carbamidomethyl (C)
Variable modifications:

S3 : Phospho (ST), with neutral losses 0.0000(shown in table), 97.9769
Ions Score: 80 **Expect:** 3.3e-006
Matches (Bold Red): 37/482 fragment ions using 45 most intense peaks

#	b	b^{++}	b^{*}	b^{*++}	b^{0}	b^{0++}	Seq.	y	y^{++}	y^{*}	y^{*++}	y^{0}	y^{0++}	#
1	102.0550	51.5311			84.0444	42.5258	T							28
2	258.1561	129.5817	241.1295	121.0684	240.1455	120.5764	R	2880.3986	1440.7030	2863.3721	1432.1897	2862.3881	1431.6977	27
3	425.1544	213.0808	408.1279	204.5676	407.1439	204.0756	S	2724.2975	1362.6524	2707.2710	1354.1391	2706.2870	1353.6471	26
4	522.2072	261.6072	505.1806	253.0940	504.1966	252.6019	P	2557.2992	1279.1532	2540.2726	1270.6399	2539.2886	1270.1479	25
5	637.2341	319.1207	620.2076	310.6074	619.2236	310.1154	D	2460.2464	1230.6268	2443.2199	1222.1136	2442.2358	1221.6216	24
6	736.3025	368.6549	719.2760	360.1416	718.2920	359.6496	V	2345.2195	1173.1134	2328.1929	1164.6001	2327.2089	1164.1081	23
7	**849.3866**	425.1969	832.3601	416.6837	831.3760	416.1917	I	2246.1510	1123.5792	2229.1245	1115.0659	2228.1405	1114.5739	22
8	936.4186	468.7130	919.3921	460.1997	918.4081	459.7077	S	2133.0670	1067.0371	2116.0404	1058.5239	2115.0564	1058.0318	21
9	1023.4507	512.2290	1006.4241	503.7157	1005.4401	503.2237	S	2046.0350	1023.5211	2029.0084	1015.0078	2028.0244	1014.5158	20
10	1094.4878	547.7475	1077.4612	539.2343	1076.4772	538.7422	A	1959.0029	980.0051	1941.9764	971.4918	1940.9924	970.9998	19
11	1181.5198	591.2635	1164.4933	582.7503	1163.5092	582.2583	S	1887.9658	944.4865	1870.9393	935.9733	1869.9552	935.4813	18
12	1282.5675	641.7874	1265.5409	633.2741	1264.5569	632.7821	T	1800.9338	900.9705	1783.9072	892.4573	1782.9232	891.9652	17
13	1353.6046	677.3059	1336.5780	668.7927	1335.5940	668.3007	A	**1699.8861**	850.4467	1682.8596	841.9334	**1681.8755**	841.4414	16
14	**1466.6887**	**733.8480**	1449.6621	725.3347	1448.6781	724.8427	L	**1628.8490**	814.9281	1611.8224	806.4149	1610.8384	805.9229	15
15	1553.7207	777.3640	1536.6941	768.8507	1535.7101	768.3587	S	**1515.7649**	758.3861	1498.7384	749.8728	1497.7544	749.3808	14
16	**1681.7793**	841.3933	1664.7527	832.8800	1663.7687	832.3880	Q	1428.7329	714.8701	1411.7064	706.3568	1410.7223	705.8648	13
17	**1796.8062**	**898.9067**	1779.7797	890.3935	1778.7956	889.9015	D	**1300.6743**	650.8408	1283.6478	642.3275	1282.6638	641.8355	12
18	**1909.8903**	**955.4488**	1892.8637	**946.9355**	**1891.8797**	**946.4435**	I	**1185.6474**	593.3273	1168.6208	584.8141	1167.6368	584.3220	11
19	2006.9430	1003.9752	1989.9165	995.4619	1988.9325	994.9699	P	**1072.5633**	**536.7853**	1055.5368	528.2720	1054.5527	527.7800	10
20	2135.9856	1068.4965	2118.9591	1059.9832	2117.9751	1059.4912	E	975.5106	488.2589	958.4840	479.7456	957.5000	479.2536	9
21	2249.0697	**1125.0385**	2232.0431	1116.5252	2231.0591	1116.0332	I	846.4680	423.7376	829.4414	415.2243	828.4574	414.7323	8
22	2320.1068	1160.5570	2303.0803	1152.0438	2302.0962	1151.5518	A	**733.3839**	367.1956	716.3573	358.6823	715.3733	358.1903	7
23	2407.1388	1204.0731	2390.1123	1195.5598	2389.1283	1195.0678	S	**662.3468**	331.6770	645.3202	323.1638	644.3362	322.6717	6
24	2536.1814	1268.5944	2519.1549	1260.0811	2518.1709	1259.5891	E	575.3148	288.1610	558.2882	279.6477	557.3042	279.1557	5
25	2607.2185	**1304.1129**	2590.1920	1295.5996	2589.2080	1295.1076	A	**446.2722**	223.6397	429.2456	215.1264	428.2616	214.6344	4
26	2720.3026	**1360.6549**	2703.2761	1352.1417	2702.2920	1351.6497	L	**375.2350**	188.1212	358.2085	179.6079	357.2245	179.1159	3
27	2807.3346	1404.1710	2790.3081	1395.6577	2789.3241	1395.1657	S	262.1510	131.5791	245.1244	123.0659	244.1404	122.5738	2
28							R	175.1190	88.0631	158.0924	79.5498			1

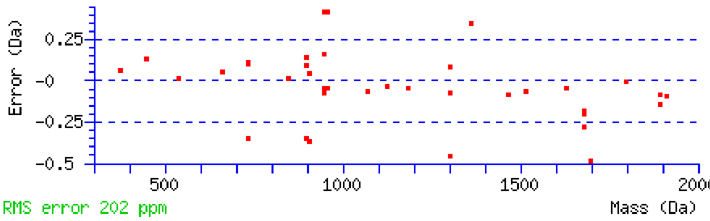

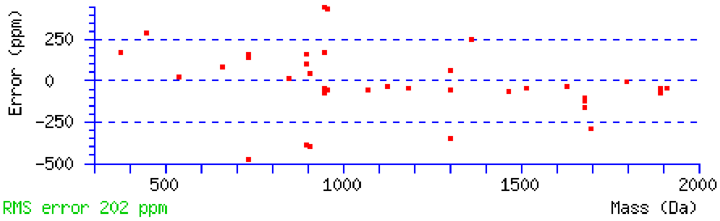

All matches to this query

Score	Mr(calc):	Delta	Sequence
79.6	2980.4390	0.0039	TRSPDVISSASTALSQDIPEIASEALSR
79.6	2980.4390	0.0039	TRSPDVISSASTALSQDIPEIASEALSR
75.1	2980.4390	0.0039	TRSPDVISSASTALSQDIPEIASEALSR
75.1	2980.4390	0.0039	TRSPDVISSASTALSQDIPEIASEALSR
75.1	2980.4390	0.0039	TRSPDVISSASTALSQDIPEIASEALSR
75.1	2980.4390	0.0039	TRSPDVISSASTALSQDIPEIASEALSR
34.3	2980.4390	0.0039	TRSPDVISSASTALSQDIPEIASEALSR
5.2	2980.4188	0.0241	TVLAEYNMSCDDTGKLILKPRPHVQ

5.0	2979.4257	1.0172	MALWSGAGGQPPASAAAAPAEPRSEQQATR
4.9	2980.4690	-0.0261	NDSYTLHKILPNKVDPLVSLMMVEK

Spectrum No: 48; Query: 2078; Rank: 1

Peptide View

MS/MS Fragmentation of **ETQSPAPLQNSSGDPAEEESPV**
Found in **IPI00214787**, Tax_Id=10116 Gene_Symbol=Slc16a1 Monocarboxylate transporter 1

Match to Query 2078: 2347.967948 from(1174.991250,2+)
Title: 091008RatKidney_NoSalt_35.1361.1361.2.dta
Data file K:\NewmanPaper\Piliang\3SubProteomes\Piliang3SP\mgf5ppm\ERLIC_3SubProteomes5ppm.mgf

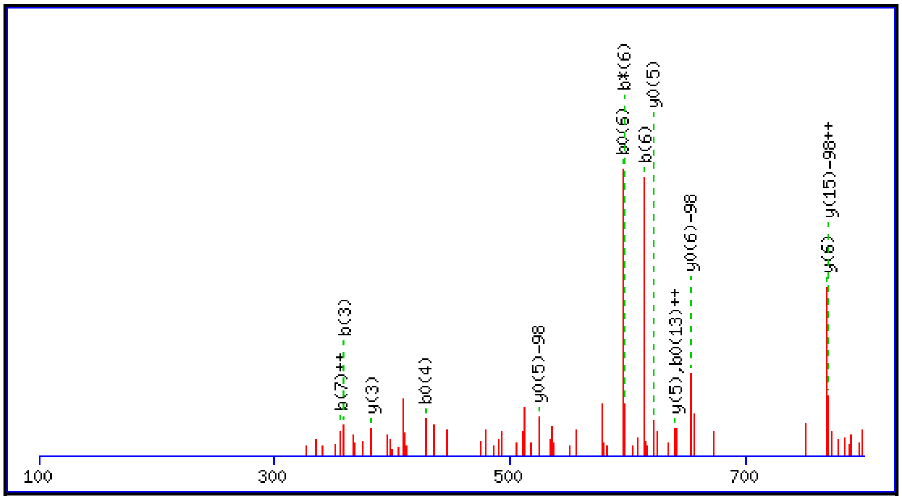

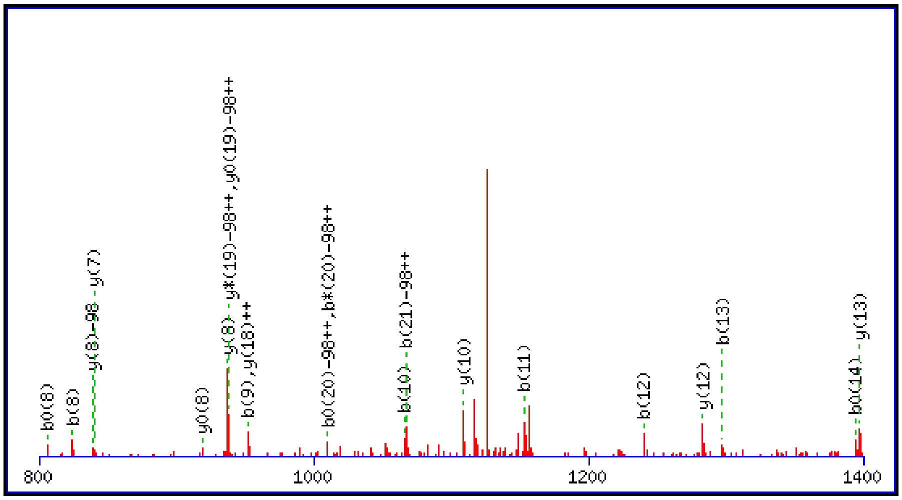

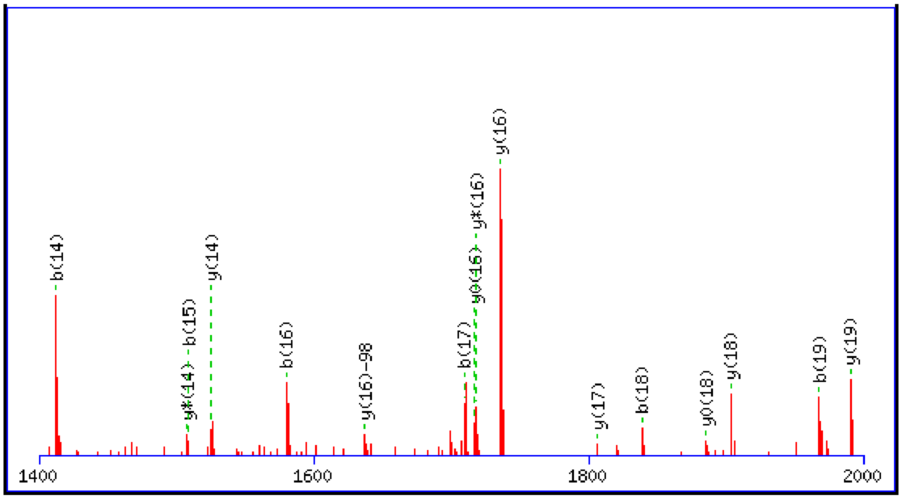

Monoisotopic mass of neutral peptide Mr(calc): 2347.9693
Fixed modifications: Carbamidomethyl (C)
Variable modifications:
S20 : Phospho (ST), with neutral losses 0.0000(shown in table), 97.9769
Ions Score: 79 **Expect:** 2.9e-006
Matches (Bold Red): 51/326 fragment ions using 75 most intense peaks

#	b	b^{++}	b^{*}	b^{*++}	b^{0}	b^{0++}	Seq.	y	y^{++}	y^{*}	y^{*++}	y^{0}	y^{0++}	#
1	130.0499	65.5286			112.0393	56.5233	E							22
2	231.0975	116.0524			213.0870	107.0471	T	2219.9340	1110.4706	2202.9074	1101.9574	2201.9234	1101.4653	21
3	**359.1561**	180.0817	342.1296	171.5684	341.1456	171.0764	Q	2118.8863	1059.9468	2101.8598	1051.4335	2100.8757	1050.9415	20
4	446.1882	223.5977	429.1616	215.0844	**428.1776**	214.5924	S	**1990.8277**	995.9175	1973.8012	987.4042	1972.8172	986.9122	19
5	543.2409	272.1241	526.2144	263.6108	525.2304	263.1188	P	**1903.7957**	**952.4015**	1886.7692	943.8882	**1885.7851**	943.3962	18
6	**614.2780**	307.6427	**597.2515**	299.1294	**596.2675**	298.6374	A	**1806.7429**	903.8751	1789.7164	895.3618	1788.7324	894.8698	17
7	711.3308	**356.1690**	694.3042	347.6558	693.3202	347.1638	P	**1735.7058**	868.3566	**1718.6793**	859.8433	**1717.6953**	859.3513	16
8	**824.4149**	412.7111	807.3883	404.1978	**806.4043**	403.7058	L	1638.6531	819.8302	1621.6265	811.3169	1620.6425	810.8249	15
9	**952.4734**	476.7404	935.4469	468.2271	934.4629	467.7351	Q	**1525.5690**	763.2881	**1508.5424**	754.7749	1507.5584	754.2829	14
10	**1066.5164**	533.7618	1049.4898	525.2485	1048.5058	524.7565	N	**1397.5104**	699.2588	1380.4839	690.7456	1379.4999	690.2536	13
11	**1153.5484**	577.2778	1136.5218	568.7646	1135.5378	568.2726	S	**1283.4675**	642.2374			1265.4569	633.2321	12
12	**1240.5804**	620.7938	1223.5539	612.2806	1222.5699	611.7886	S	1196.4355	598.7214			1178.4249	589.7161	11
13	**1297.6019**	649.3046	1280.5753	640.7913	1279.5913	**640.2993**	G	**1109.4034**	555.2054			1091.3929	546.2001	10
14	**1412.6288**	706.8181	1395.6023	698.3048	**1394.6183**	697.8128	D	1052.3820	526.6946			1034.3714	517.6893	9
15	**1509.6816**	755.3444	1492.6550	746.8312	1491.6710	746.3392	P	**937.3550**	469.1812			**919.3445**	460.1759	8
16	**1580.7187**	790.8630	1563.6922	782.3497	1562.7081	781.8577	A	**840.3023**	420.6548			822.2917	411.6495	7
17	**1709.7613**	855.3843	1692.7348	846.8710	1691.7507	846.3790	E	**769.2652**	385.1362			751.2546	376.1309	6
18	**1838.8039**	919.9056	1821.7773	911.3923	1820.7933	910.9003	E	**640.2226**	320.6149			**622.2120**	311.6096	5
19	**1967.8465**	984.4269	1950.8199	975.9136	1949.8359	975.4216	E	511.1800	256.0936			493.1694	247.0883	4
20	2134.8448	1067.9261	2117.8183	1059.4128	2116.8343	1058.9208	S	**382.1374**	191.5723			364.1268	182.5670	3
21	2231.8976	1116.4524	2214.8711	1107.9392	2213.8870	1107.4472	P	215.1390	108.0731					2
22							V	118.0863	59.5468					1

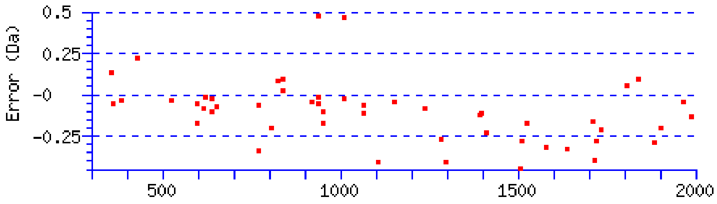

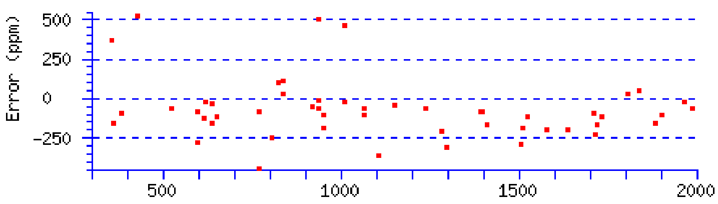

All matches to this query

Score	Mr(calc):	Delta	Sequence
79.5	2347.9693	-0.0013	ETQSPAPLQNSSGDPAEEESPV
10.8	2347.9693	-0.0013	ETQSPAPLQNSSGDPAEEESPV
9.9	2347.9693	-0.0013	ETQSPAPLQNSSGDPAEEESPV
3.9	2347.9693	-0.0013	ETQSPAPLQNSSGDPAEEESPV
2.9	2347.9491	0.0189	HRTTSQRAAIHSSAPRPR
2.9	2347.9491	0.0189	HRTTSQRAAIHSSAPRPR
2.4	2346.9596	1.0083	TLNANNMDTLIECQSEGDIK
1.9	2346.9743	0.9936	NRADHRSSPNVANQPPSPGGK
1.1	2345.9500	2.0179	DKVGQYSLYIGNSKVTVR
0.2	2347.9732	-0.0052	RVGLPSMVPSPQTCWGDSSK

Spectrum No: 49; Query: 1411; Rank: 1

Peptide View

MS/MS Fragmentation of **SVPTIDSGNEDDDSSFK**
Found in **IPI00366370**, Tax_Id=10116 Gene_Symbol=Eif5b eukaryotic translation initiation factor 5B

Match to Query 1411: 1891.735608 from(946.875080,2+)
Title: 091008RatKidney_NoSalt_29.1647.1647.2.dta
Data file K:\NewmanPaper\Piliang\3SubProteomes\Piliang3SP\mgf5ppm\ERLIC_3SubProteomes5ppm.mgf

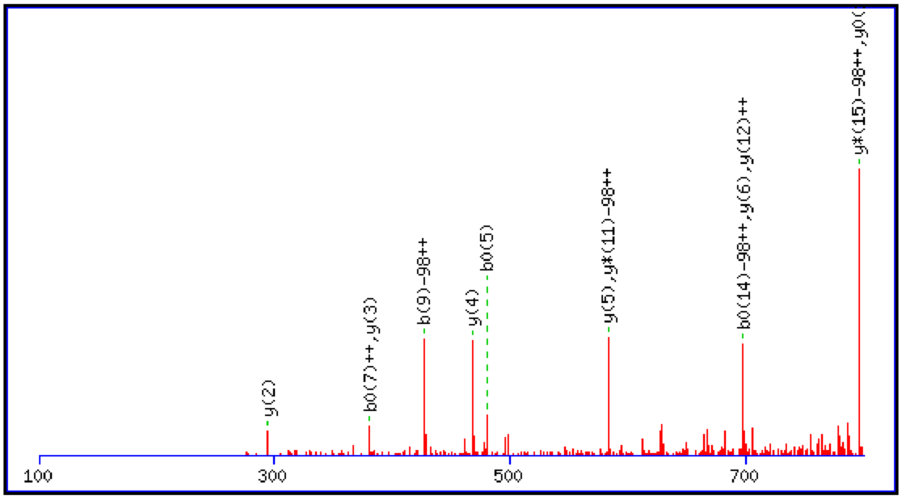

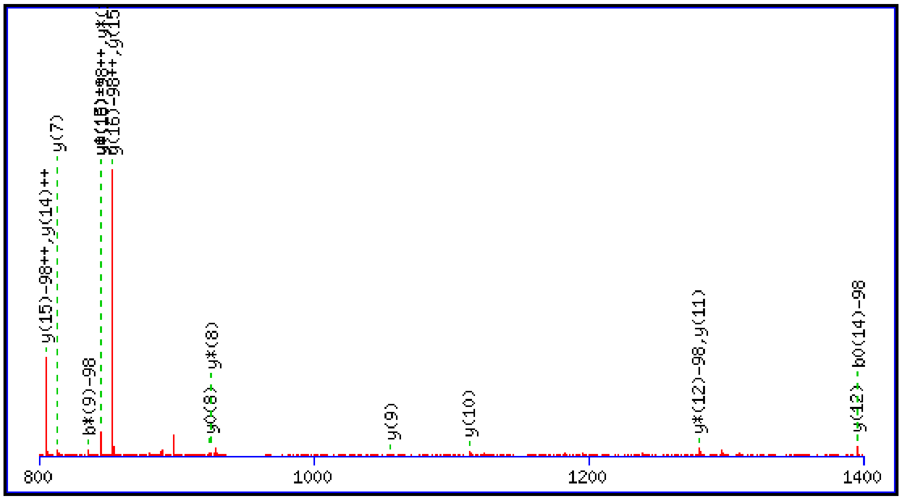

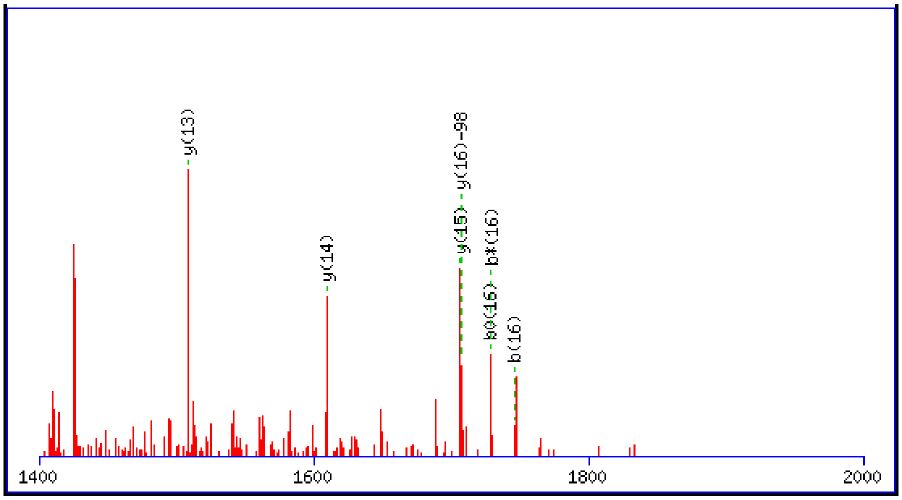

Monoisotopic mass of neutral peptide Mr(calc): 1891.7361
Fixed modifications: Carbamidomethyl (C)
Variable modifications:
S7 : Phospho (ST), with neutral losses 0.0000(shown in table), 97.9769
Ions Score: 79 **Expect:** 2.4e-006
Matches (Bold Red): 40/264 fragment ions using 43 most intense peaks

#	b	b^{++}	b*	b^{*++}	b^0	b^{0++}	Seq.	y	y^{++}	y*	y^{*++}	y^0	y^{0++}	#
1	88.0393	44.5233			70.0287	35.5180	S							17
2	187.1077	94.0575			169.0972	85.0522	V	1805.7113	903.3593	1788.6848	894.8460	1787.7007	894.3540	16
3	284.1605	142.5839			266.1499	133.5786	P	**1706.6429**	**853.8251**	1689.6163	**845.3118**	1688.6323	**844.8198**	15
4	385.2082	193.1077			367.1976	184.1024	T	**1609.5901**	**805.2987**	1592.5636	796.7854	1591.5796	**796.2934**	14
5	498.2922	249.6498			**480.2817**	240.6445	I	**1508.5424**	754.7749	1491.5159	746.2616	1490.5319	745.7696	13
6	613.3192	307.1632			595.3086	298.1579	D	**1395.4584**	**698.2328**	1378.4318	689.7196	1377.4478	689.2275	12
7	780.3175	390.6624			762.3070	**381.6571**	S	**1280.4314**	640.7194	1263.4049	632.2061	1262.4209	631.7141	11
8	837.3390	419.1731			819.3284	410.1678	G	**1113.4331**	557.2202	1096.4065	548.7069	1095.4225	548.2149	10
9	951.3819	476.1946	934.3554	467.6813	933.3713	467.1893	N	**1056.4116**	528.7094	1039.3851	520.1962	1038.4011	519.7042	9
10	1080.4245	540.7159	1063.3980	532.2026	1062.4139	531.7106	E	942.3687	471.6880	**925.3421**	463.1747	**924.3581**	462.6827	8
11	1195.4514	598.2294	1178.4249	589.7161	1177.4409	589.2241	D	**813.3261**	407.1667	**796.2996**	398.6534	795.3155	398.1614	7
12	1310.4784	655.7428	1293.4518	647.2296	1292.4678	646.7376	D	**698.2992**	349.6532	681.2726	341.1399	680.2886	340.6479	6
13	1425.5053	713.2563	1408.4788	704.7430	1407.4948	704.2510	D	**583.2722**	292.1397	566.2457	283.6265	565.2617	283.1345	5
14	1512.5374	756.7723	1495.5108	748.2590	1494.5268	747.7670	S	**468.2453**	234.6263	451.2187	226.1130	450.2347	225.6210	4
15	1599.5694	800.2883	1582.5428	791.7751	1581.5588	791.2831	S	**381.2132**	191.1103	364.1867	182.5970	363.2027	182.1050	3
16	**1746.6378**	873.8225	**1729.6113**	865.3093	**1728.6272**	864.8173	F	**294.1812**	147.5942	277.1547	139.0810			2
17							K	147.1128	74.0600	130.0863	65.5468			1

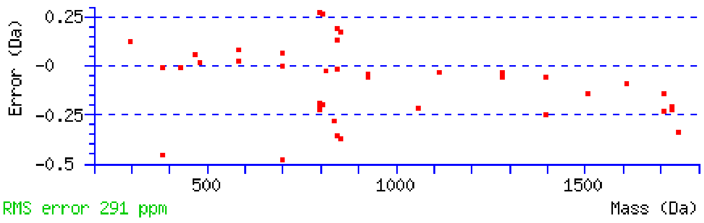

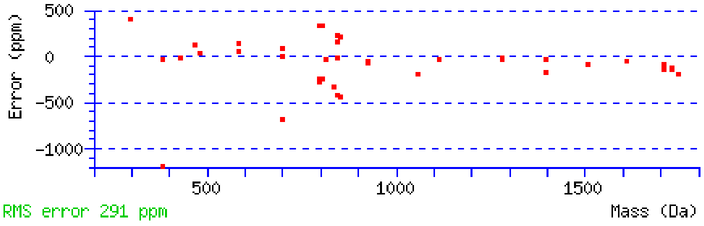

All matches to this query

Score	Mr(calc):	Delta	Sequence
79.4	1891.7361	-0.0005	SVPTIDSGNEDDDSSFK
42.4	1891.7361	-0.0005	SVPTIDSGNEDDDSSFK
30.3	1891.7361	-0.0005	SVPTIDSGNEDDDSSFK
30.3	1891.7361	-0.0005	SVPTIDSGNEDDDSSFK

30.0	1891.7361	-0.0005	SVPTIDSGNEDDDSSFK
7.9	1890.7455	0.9902	LASIFSGTGTDPAVSTK
5.5	1891.7390	-0.0034	WNDDPEARELSKSGK
4.3	1890.7146	1.0210	SNSXGEGARSERSPGNK
4.3	1889.7306	2.0050	SNSXGEGARSERSPGNK
3.9	1890.7455	0.9902	LASIFSGTGTDPAVSTK

Spectrum No: 50; Query: 1966; Rank: 1

Peptide View

MS/MS Fragmentation of **GETPQGSSEECDLSGSCTER**
Found in **IPI00203529**, Tax_Id=10116 Gene_Symbol=Slc34a3 Sodium-dependent phosphate transport protein 2C

Match to Query 1966: 2264.827368 from(1133.420960,2+)
Title: 100101RatKid_NS_deglyco_24.1093.1093.2.dta
Data file K:\NewmanPaper\Piliang\3SubProteomes\Piliang3SP\mgf5ppm\ERLIC_3SubProteomes5ppm.mgf

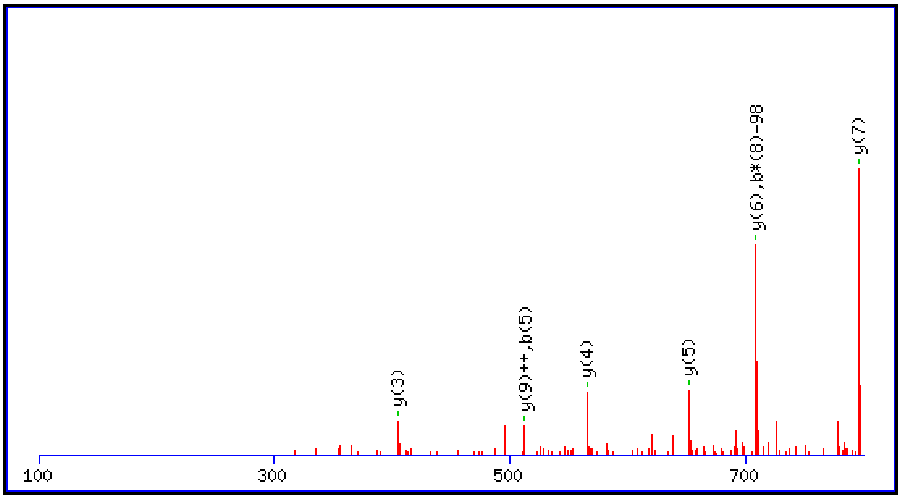

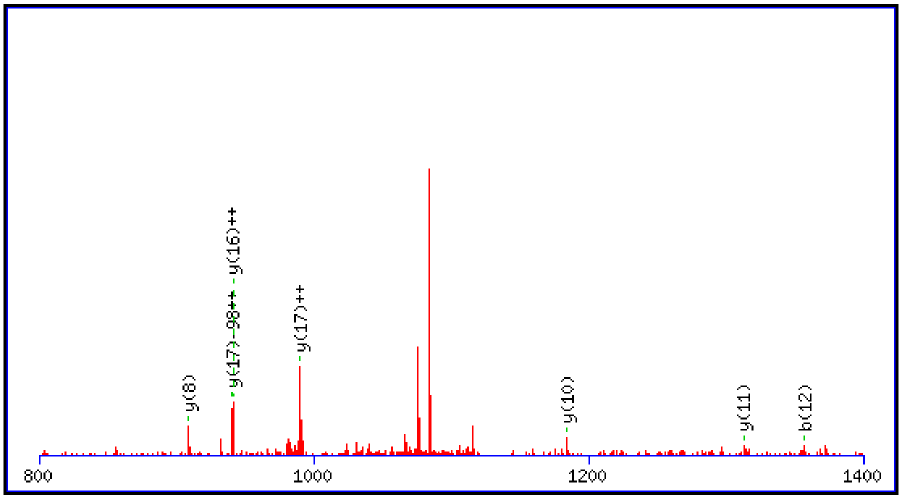

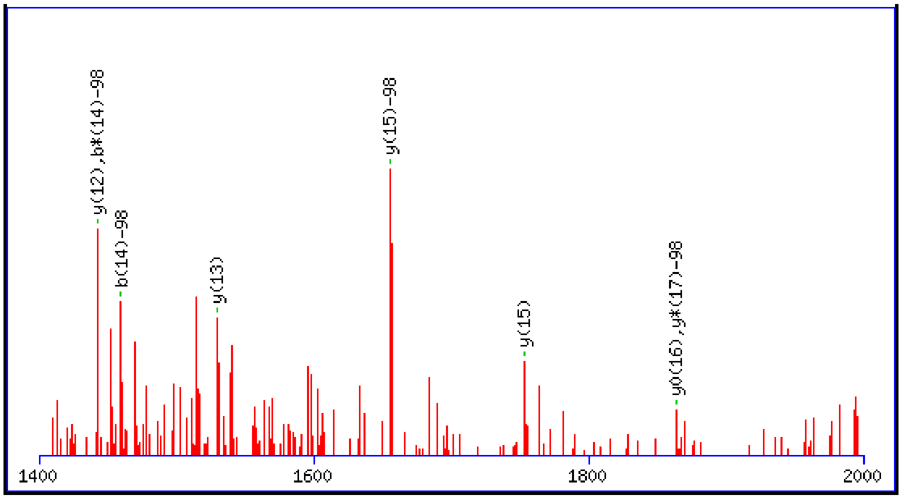

Monoisotopic mass of neutral peptide Mr(calc): 2264.8199
Fixed modifications: Carbamidomethyl (C)
Variable modifications:
S7 : Phospho (ST), with neutral losses 0.0000(shown in table), 97.9769
Ions Score: 79 **Expect:** 1.3e-006
Matches (Bold Red): 23/330 fragment ions using 29 most intense peaks

#	b	b++	b*	b*++	b0	b0++	Seq.	y	y++	y*	y*++	y0	y0++	#
1	58.0287	29.5180					G							20
2	187.0713	94.0393			169.0608	85.0340	E	2208.8057	1104.9065	2191.7791	1096.3932	2190.7951	1095.9012	19
3	288.1190	144.5631			270.1084	135.5579	T	2079.7631	1040.3852	2062.7366	1031.8719	2061.7525	1031.3799	18
4	385.1718	193.0895			367.1612	184.0842	P	1978.7154	**989.8613**	1961.6889	981.3481	1960.7049	980.8561	17
5	**513.2304**	257.1188	496.2038	248.6055	495.2198	248.1135	Q	1881.6627	**941.3350**	1864.6361	932.8217	**1863.6521**	932.3297	16
6	570.2518	285.6295	553.2253	277.1163	552.2413	276.6243	G	**1753.6041**	877.3057	1736.5775	868.7924	1735.5935	868.3004	15
7	737.2502	369.1287	720.2236	360.6154	719.2396	360.1234	S	1696.5826	848.7949	1679.5561	840.2817	1678.5721	839.7897	14
8	824.2822	412.6447	807.2556	404.1315	806.2716	403.6395	S	**1529.5843**	765.2958	1512.5577	756.7825	1511.5737	756.2905	13
9	953.3248	477.1660	936.2982	468.6528	935.3142	468.1608	E	**1442.5522**	721.7798	1425.5257	713.2665	1424.5417	712.7745	12
10	1082.3674	541.6873	1065.3408	533.1741	1064.3568	532.6820	E	**1313.5096**	657.2585	1296.4831	648.7452	1295.4991	648.2532	11
11	1242.3980	621.7027	1225.3715	613.1894	1224.3875	612.6974	C	**1184.4670**	592.7372	1167.4405	584.2239	1166.4565	583.7319	10
12	**1357.4250**	679.2161	1340.3984	670.7029	1339.4144	670.2108	D	1024.4364	**512.7218**	1007.4099	504.2086	1006.4258	503.7166	9
13	1470.5090	735.7582	1453.4825	727.2449	1452.4985	726.7529	L	**909.4095**	455.2084	892.3829	446.6951	891.3989	446.2031	8
14	1557.5411	779.2742	1540.5145	770.7609	1539.5305	770.2689	S	**796.3254**	398.6663	779.2988	390.1531	778.3148	389.6611	7
15	1614.5625	807.7849	1597.5360	799.2716	1596.5520	798.7796	G	**709.2934**	355.1503	692.2668	346.6370	691.2828	346.1450	6
16	1701.5946	851.3009	1684.5680	842.7876	1683.5840	842.2956	S	**652.2719**	326.6396	635.2454	318.1263	634.2613	317.6343	5
17	1861.6252	931.3162	1844.5987	922.8030	1843.6146	922.3110	C	**565.2399**	283.1236	548.2133	274.6103	547.2293	274.1183	4
18	1962.6729	981.8401	1945.6463	973.3268	1944.6623	972.8348	T	**405.2092**	203.1082	388.1827	194.5950	387.1987	194.1030	3
19	2091.7155	1046.3614	2074.6889	1037.8481	2073.7049	1037.3561	E	304.1615	152.5844	287.1350	144.0711	286.1510	143.5791	2
20							R	175.1190	88.0631	158.0924	79.5498			1

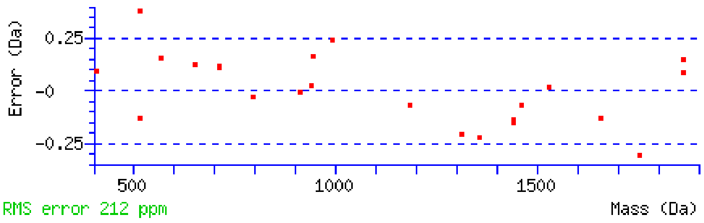

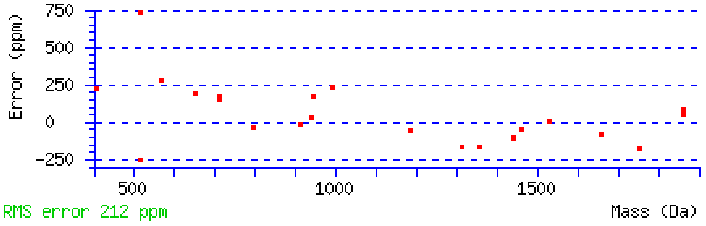

All matches to this query

Score	Mr(calc):	Delta	Sequence
79.4	2264.8199	0.0075	GETPQGSSEECDLSGSCTER

64.1	2264.8199	0.0075	GETPQGSSEECDLSGSCTER
56.6	2264.8199	0.0075	GETPQGSSEECDLSGSCTER
11.6	2264.8199	0.0075	GETPQGSSEECDLSGSCTER
4.0	2262.8347	1.9927	TLSQCGAGVFEYFNSKSK
4.0	2262.8347	1.9927	TLSQCGAGVFEYFNSKSK
2.4	2264.8199	0.0075	GETPQGSSEECDLSGSCTER
0.6	2263.8381	0.9892	SSQQTLCSEWKYDSEEK
0.6	2263.8020	1.0254	KGGCMGESVQMENSMGTLR
0.4	2263.8381	0.9892	SSQQTLCSEWKYDSEEK

Spectrum No: 51; Query: 983; Rank: 1

Peptide View

MS/MS Fragmentation of **VLSSTSEEDEPGVVK**
Found in **IPI00200370**, Tax_Id=10116 Gene_Symbol=RGD1566426_predicted similar to nuclear receptor coactivator 7

Match to Query 983: 1654.735488 from(828.375020,2+)
Title: 091008RatKidney_NH4Format02_26.1326.1326.2.dta
Data file K:\NewmanPaper\Piliang\3SubProteomes\Piliang3SP\mgf5ppm\ERLIC_3SubProteomes5ppm.mgf

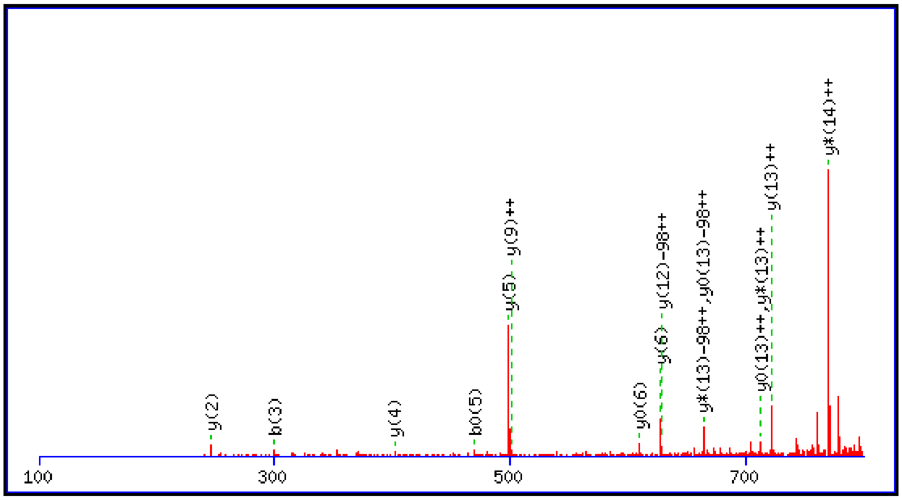

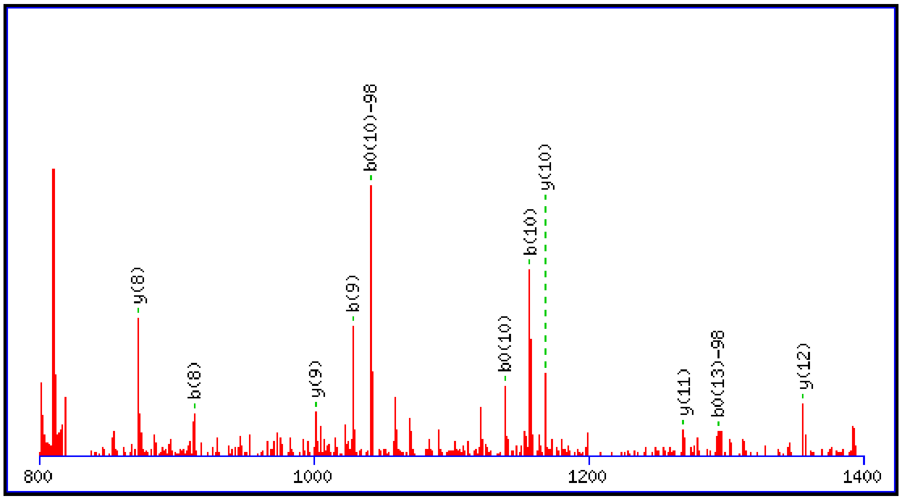

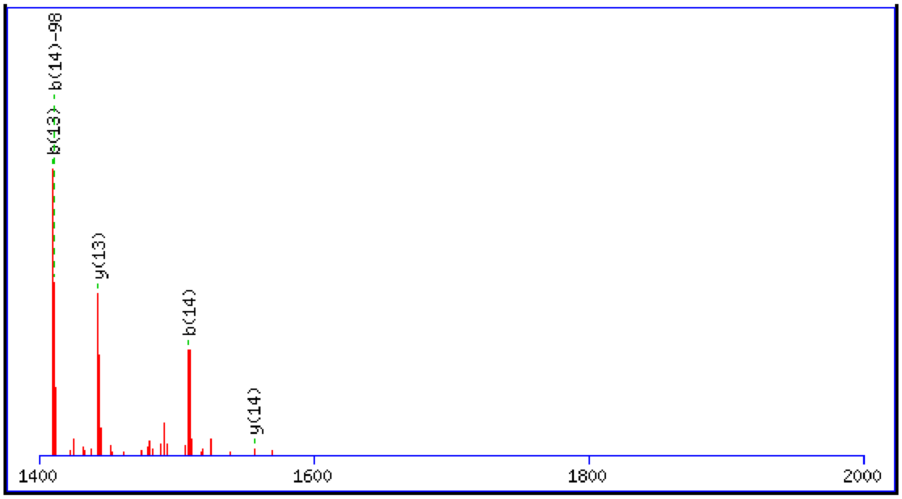

Monoisotopic mass of neutral peptide Mr(calc): 1654.7339
Fixed modifications: Carbamidomethyl (C)
Variable modifications:
S6 : Phospho (ST), with neutral losses 0.0000(shown in table), 97.9769
Ions Score: 79 **Expect:** 2.3e-006
Matches (Bold Red): 31/192 fragment ions using 47 most intense peaks

#	b	b^{++}	b^0	b^{0++}	Seq.	y	y^{++}	y^*	y^{*++}	y^0	y^{0++}	#
1	100.0757	50.5415			V							15
2	213.1598	107.0835			L	**1556.6727**	778.8400	1539.6462	**770.3267**	1538.6622	769.8347	14
3	**300.1918**	150.5995	282.1812	141.5942	S	**1443.5887**	**722.2980**	1426.5621	**713.7847**	1425.5781	**713.2927**	13
4	387.2238	194.1155	369.2132	185.1103	S	**1356.5566**	678.7820	1339.5301	670.2687	1338.5461	669.7767	12
5	488.2715	244.6394	**470.2609**	235.6341	T	**1269.5246**	635.2659	1252.4981	626.7527	1251.5140	626.2607	11
6	655.2698	328.1386	637.2593	319.1333	S	**1168.4769**	584.7421	1151.4504	576.2288	1150.4664	575.7368	10
7	784.3124	392.6599	766.3019	383.6546	E	**1001.4786**	**501.2429**	984.4520	492.7297	983.4680	492.2376	9
8	**913.3550**	457.1812	895.3445	448.1759	E	**872.4360**	436.7216	855.4094	428.2084	854.4254	427.7164	8
9	**1028.3820**	514.6946	1010.3714	505.6893	D	743.3934	372.2003	726.3668	363.6871	725.3828	363.1951	7
10	**1157.4246**	579.2159	**1139.4140**	570.2106	E	**628.3665**	314.6869	611.3399	306.1736	**610.3559**	305.6816	6
11	1254.4773	627.7423	1236.4668	618.7370	P	**499.3239**	250.1656	482.2973	241.6523			5
12	1311.4988	656.2530	1293.4882	647.2478	G	**402.2711**	201.6392	385.2445	193.1259			4
13	**1410.5672**	705.7872	1392.5566	696.7820	V	345.2496	173.1285	328.2231	164.6152			3
14	**1509.6356**	755.3214	1491.6251	746.3162	V	**246.1812**	123.5942	229.1547	115.0810			2
15					K	147.1128	74.0600	130.0863	65.5468			1

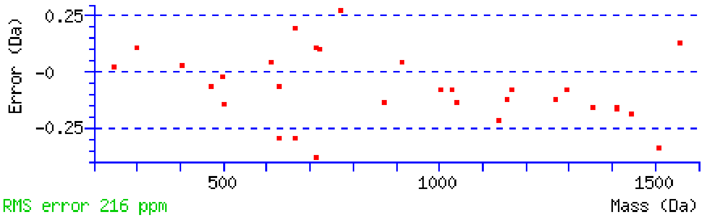

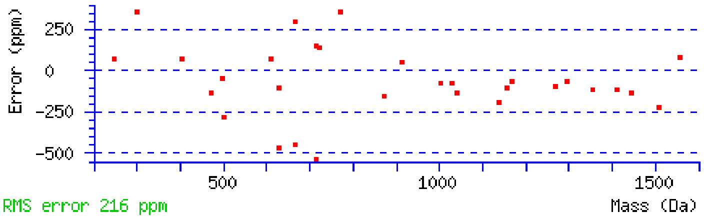

All matches to this query

Score	Mr(calc):	Delta	Sequence
79.1	1654.7339	0.0016	VLSSTSEEDEPGVVK
65.8	1654.7339	0.0016	VLSSTSEEDEPGVVK
53.7	1654.7339	0.0016	VLSSTSEEDEPGVVK
46.5	1654.7339	0.0016	VLSSTSEEDEPGVVK
7.8	1654.7509	-0.0154	LPNGVSDSMEASGFTK
7.5	1653.7416	0.9939	SVYVVGSATASLVNK

6.8	1654.7368	-0.0014	VLAVNGVSLEGATHK
6.4	1653.7416	0.9939	SVYVVGSATASLVNK
6.4	1653.7416	0.9939	SVYVVGSATASLVNK
5.5	1653.7415	0.9939	ISYASKVKENLNK

Spectrum No: 52; Query: 1495; Rank: 1

Peptide View

MS/MS Fragmentation of **DWEDDSDEDMSNFDR**
Found in **IPI00365935**, Tax_Id=10116 Gene_Symbol=Ptges3_predicted Prostaglandin E synthase 3

Match to Query 1495: 1954.625908 from(978.320230,2+)
Title: 100101RatKid_NS_deglyco_27.2610.2610.2.dta
Data file K:\NewmanPaper\Piliang\3SubProteomes\Piliang3SP\mgf5ppm\ERLIC_3SubProteomes5ppm.mgf

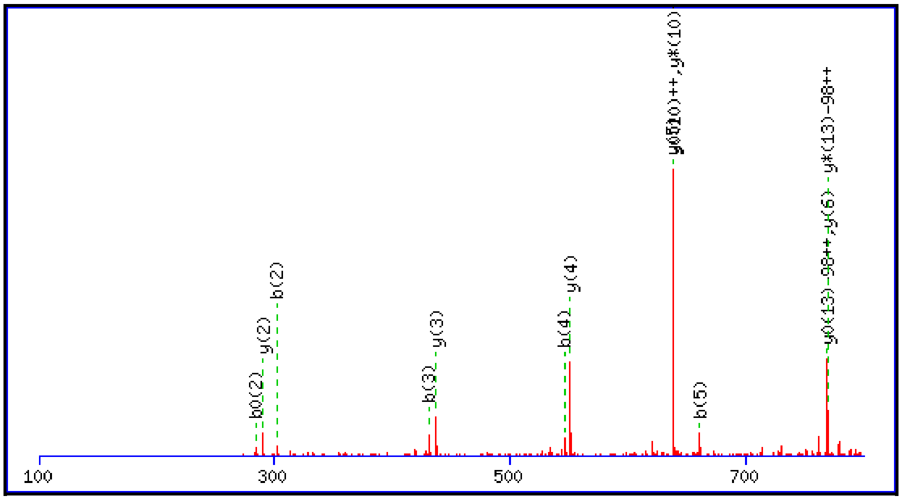

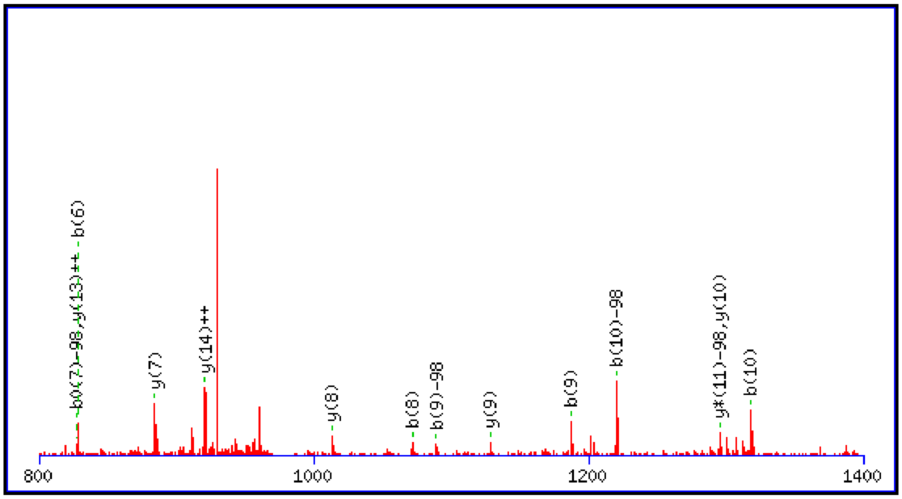

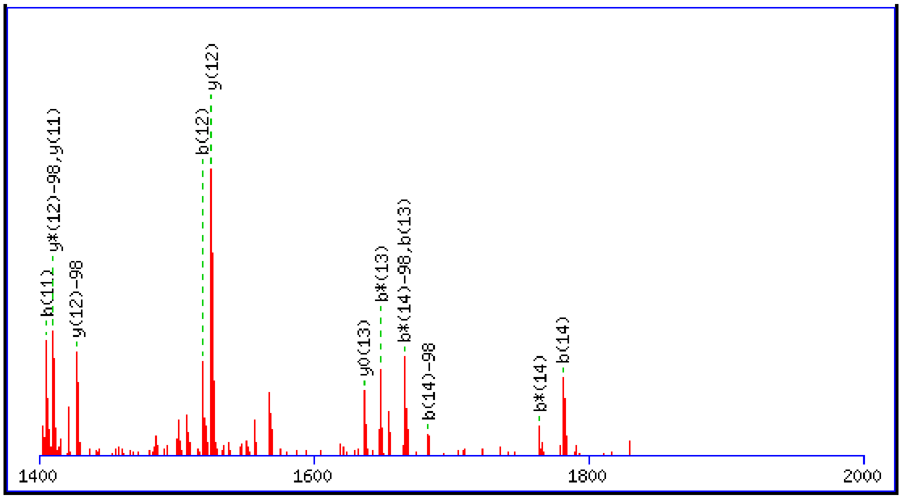

Monoisotopic mass of neutral peptide Mr(calc): 1954.6200
Fixed modifications: Carbamidomethyl (C)
Variable modifications:
S6 : Phospho (ST), with neutral losses 0.0000(shown in table), 97.9769
Ions Score: 79 **Expect:** 4.4e-007
Matches (Bold Red): 41/216 fragment ions using 55 most intense peaks

#	b	b^{++}	b*	b^{*++}	b^0	b^{0++}	Seq.	y	y^{++}	y*	y^{*++}	y^0	y^{0++}	#
1	116.0342	58.5207			98.0237	49.5155	D							15
2	**302.1135**	151.5604			**284.1030**	142.5551	W	1840.6004	**920.8038**	1823.5738	912.2905	1822.5898	911.7985	14
3	**431.1561**	216.0817			413.1456	207.0764	E	1654.5211	**827.7642**	1637.4945	819.2509	**1636.5105**	818.7589	13
4	**546.1831**	273.5952			528.1725	264.5899	D	**1525.4785**	763.2429	1508.4519	754.7296	1507.4679	754.2376	12
5	**661.2100**	331.1086			643.1994	322.1034	D	**1410.4515**	705.7294	1393.4250	697.2161	1392.4410	696.7241	11
6	**828.2084**	414.6078			810.1978	405.6025	S	**1295.4246**	648.2159	1278.3980	**639.7027**	1277.4140	**639.2106**	10
7	943.2353	472.1213			925.2247	463.1160	D	**1128.4262**	564.7168	1111.3997	556.2035	1110.4157	555.7115	9
8	**1072.2779**	536.6426			1054.2673	527.6373	E	**1013.3993**	507.2033	996.3727	498.6900	995.3887	498.1980	8
9	**1187.3048**	594.1561			1169.2943	585.1508	D	**884.3567**	442.6820	867.3301	434.1687	866.3461	433.6767	7
10	**1318.3453**	659.6763			1300.3348	650.6710	M	**769.3297**	385.1685	752.3032	376.6552	751.3192	376.1632	6
11	**1405.3774**	703.1923			1387.3668	694.1870	S	**638.2893**	319.6483	621.2627	311.1350	620.2787	310.6430	5
12	**1519.4203**	760.2138	1502.3937	751.7005	1501.4097	751.2085	N	**551.2572**	276.1323	534.2307	267.6190	533.2467	267.1270	4
13	**1666.4887**	833.7480	**1649.4621**	825.2347	1648.4781	824.7427	F	**437.2143**	219.1108	420.1878	210.5975	419.2037	210.1055	3
14	**1781.5156**	891.2615	**1764.4891**	882.7482	1763.5051	882.2562	D	**290.1459**	145.5766	273.1193	137.0633	272.1353	136.5713	2
15							R	175.1190	88.0631	158.0924	79.5498			1

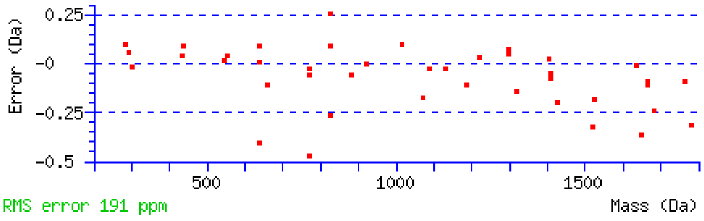

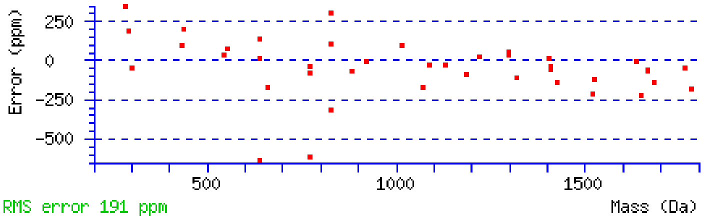

All matches to this query

Score	Mr(calc):	Delta	Sequence
79.0	1954.6200	0.0059	DWEDDSDEDMSNFDR
30.7	1954.6200	0.0059	DWEDDSDEDMSNFDR
1.6	1954.6433	-0.0174	ATGMKYNLPSWCDR

Spectrum No: 53; Query: 2736; Rank: 1

Peptide View

MS/MS Fragmentation of **EGQVEAAQPEQAAEAPAESSAQPNQLETGASSPER**
Found in **IPI00768543**, Tax_Id=10116 Gene_Symbol=Zbtb20_predicted zinc finger and BTB domain containing 20

Match to Query 2736: 3643.596342 from(1215.539390,3+)
Title: 091008RatKidney_NoSalt_27.1896.1896.3.dta
Data file K:\NewmanPaper\Piliang\3SubProteomes\Piliang3SP\mgf5ppm\ERLIC_3SubProteomes5ppm.mgf

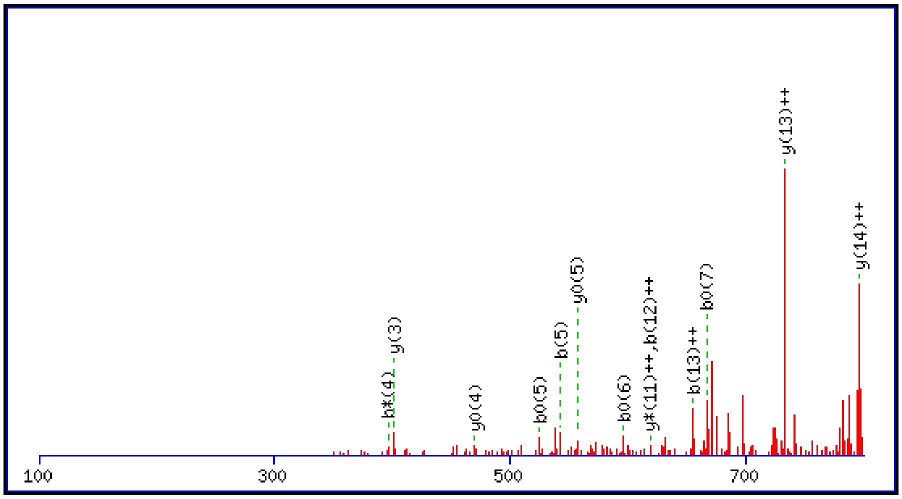

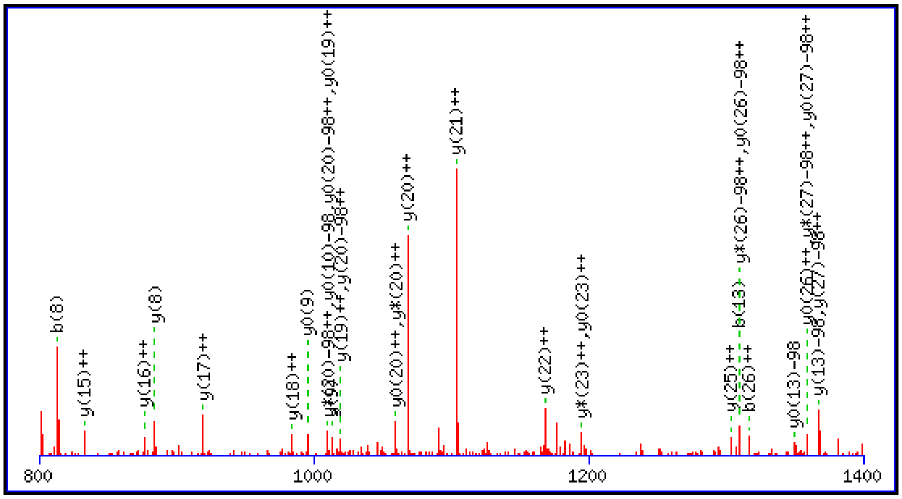

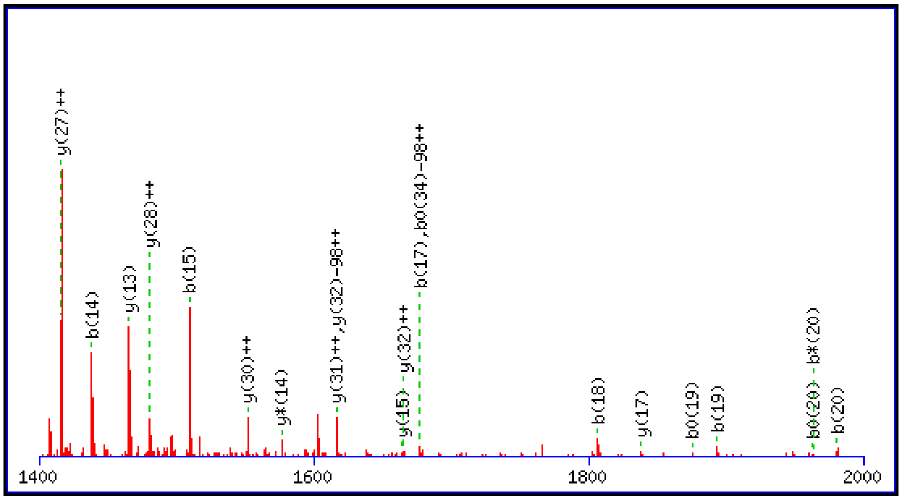

Monoisotopic mass of neutral peptide Mr(calc): 3643.5911
Fixed modifications: Carbamidomethyl (C)
Variable modifications:
T28 : Phospho (ST), with neutral losses 0.0000(shown in table), 97.9769
Ions Score: 78 **Expect:** 7e-006

Matches (Bold Red): 65/606 fragment ions using 92 most intense peaks

#	b	b^{++}	b*	b^{*++}	b^0	b^{0++}	Seq.	y	y^{++}	y*	y^{*++}	y^0	y^{0++}	#
1	130.0499	65.5286			112.0393	56.5233	E							35
2	187.0713	94.0393			169.0608	85.0340	G	3515.5558	1758.2815	3498.5293	1749.7683	3497.5453	1749.2763	34
3	315.1299	158.0686	298.1034	149.5553	297.1193	149.0633	Q	3458.5344	1729.7708	3441.5078	1721.2575	3440.5238	1720.7655	33
4	414.1983	207.6028	**397.1718**	199.0895	396.1878	198.5975	V	3330.4758	**1665.7415**	3313.4492	1657.2283	3312.4652	1656.7362	32
5	**543.2409**	272.1241	526.2144	263.6108	**525.2304**	263.1188	E	3231.4074	**1616.2073**	3214.3808	1607.6940	3213.3968	1607.2020	31
6	614.2780	307.6427	597.2515	299.1294	**596.2675**	298.6374	A	3102.3648	**1551.6860**	3085.3382	1543.1727	3084.3542	1542.6807	30
7	685.3151	343.1612	668.2886	334.6479	**667.3046**	334.1559	A	3031.3277	1516.1675	3014.3011	1507.6542	3013.3171	1507.1622	29
8	**813.3737**	407.1905	796.3472	398.6772	795.3632	398.1852	Q	2960.2905	**1480.6489**	2943.2640	1472.1356	2942.2800	1471.6436	28
9	910.4265	455.7169	893.3999	447.2036	892.4159	446.7116	P	2832.2320	**1416.6196**	2815.2054	1408.1063	2814.2214	1407.6143	27
10	1039.4691	520.2382	1022.4425	511.7249	1021.4585	511.2329	E	2735.1792	1368.0932	2718.1526	1359.5800	2717.1686	**1359.0880**	26
11	1167.5277	584.2675	1150.5011	575.7542	1149.5171	575.2622	Q	2606.1366	**1303.5719**	2589.1101	1295.0587	2588.1260	1294.5667	25
12	1238.5648	**619.7860**	1221.5382	611.2728	1220.5542	610.7807	A	2478.0780	1239.5427	2461.0515	1231.0294	2460.0675	1230.5374	24
13	**1309.6019**	**655.3046**	1292.5753	646.7913	1291.5913	646.2993	A	2407.0409	1204.0241	2390.0144	**1195.5108**	2389.0303	**1195.0188**	23
14	**1438.6445**	719.8259	1421.6179	711.3126	1420.6339	710.8206	E	2336.0038	**1168.5055**	2318.9772	1159.9923	2317.9932	1159.5003	22
15	**1509.6816**	755.3444	1492.6550	746.8312	1491.6710	746.3392	A	2206.9612	**1103.9842**	2189.9347	1095.4710	2188.9506	1094.9790	21
16	1606.7344	803.8708	1589.7078	795.3575	1588.7238	794.8655	P	2135.9241	**1068.4657**	2118.8975	**1059.9524**	2117.9135	**1059.4604**	20
17	**1677.7715**	839.3894	1660.7449	830.8761	1659.7609	830.3841	A	2038.8713	**1019.9393**	2021.8448	1011.4260	2020.8608	**1010.9340**	19
18	**1806.8141**	903.9107	1789.7875	895.3974	1788.8035	894.9054	E	1967.8342	**984.4207**	1950.8077	975.9075	1949.8236	975.4155	18
19	**1893.8461**	947.4267	1876.8195	938.9134	**1875.8355**	938.4214	S	**1838.7916**	**919.8994**	1821.7651	911.3862	1820.7811	910.8942	17
20	**1980.8781**	990.9427	**1963.8516**	982.4294	**1962.8676**	981.9374	S	1751.7596	**876.3834**	1734.7330	867.8702	1733.7490	867.3782	16
21	2051.9152	1026.4613	2034.8887	1017.9480	2033.9047	1017.4560	A	**1664.7276**	**832.8674**	1647.7010	824.3541	1646.7170	823.8621	15
22	2179.9738	1090.4905	2162.9473	1081.9773	2161.9632	1081.4853	Q	1593.6905	**797.3489**	**1576.6639**	788.8356	1575.6799	788.3436	14
23	2277.0266	1139.0169	2260.0000	1130.5037	2259.0160	1130.0116	P	**1465.6319**	**733.3196**	1448.6053	724.8063	1447.6213	724.3143	13
24	2391.0695	1196.0384	2374.0430	1187.5251	2373.0589	1187.0331	N	1368.5791	684.7932	1351.5526	676.2799	1350.5685	675.7879	12
25	2519.1281	1260.0677	2502.1015	1251.5544	2501.1175	1251.0624	Q	1254.5362	627.7717	1237.5096	**619.2585**	1236.5256	618.7664	11
26	2632.2121	**1316.6097**	2615.1856	1308.0964	2614.2016	1307.6044	L	1126.4776	563.7424	1109.4511	555.2292	1108.4670	554.7372	10
27	2761.2547	1381.1310	2744.2282	1372.6177	2743.2442	1372.1257	E	**1013.3935**	507.2004	996.3670	498.6871	**995.3830**	498.1951	9
28	2942.2687	1471.6380	2925.2422	1463.1247	2924.2582	1462.6327	T	**884.3509**	442.6791	867.3244	434.1658	866.3404	433.6738	8
29	2999.2902	1500.1487	2982.2637	1491.6355	2981.2796	1491.1435	G	703.3369	352.1721	686.3104	343.6588	685.3264	343.1668	7
30	3070.3273	1535.6673	3053.3008	1527.1540	3052.3168	1526.6620	A	646.3155	323.6614	629.2889	315.1481	628.3049	314.6561	6
31	3157.3593	1579.1833	3140.3328	1570.6700	3139.3488	1570.1780	S	575.2784	288.1428	558.2518	279.6295	**557.2678**	279.1375	5
32	3244.3914	1622.6993	3227.3648	1614.1861	3226.3808	1613.6940	S	488.2463	244.6268	471.2198	236.1135	**470.2358**	235.6215	4
33	3341.4441	1671.2257	3324.4176	1662.7124	3323.4336	1662.2204	P	**401.2143**	201.1108	384.1878	192.5975	383.2037	192.1055	3
34	3470.4867	1735.7470	3453.4602	1727.2337	3452.4762	1726.7417	E	304.1615	152.5844	287.1350	144.0711	286.1510	143.5791	2
35							R	175.1190	88.0631	158.0924	79.5498			1

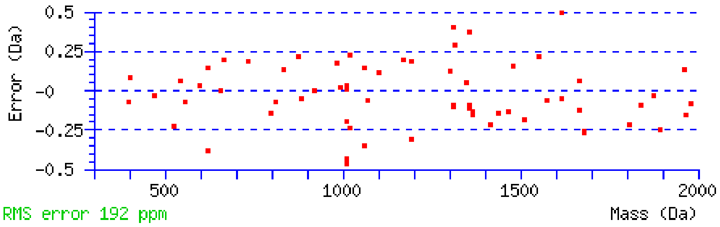

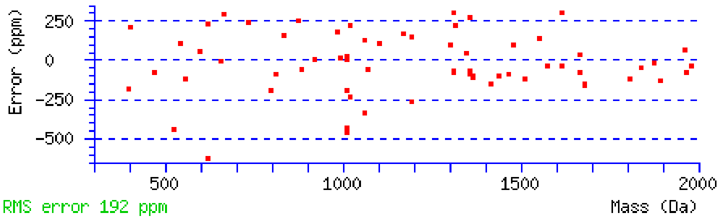

All matches to this query

Score	Mr(calc):	Delta	Sequence
78.5	3643.5911	0.0053	EGQVEAAQPEQAAEAPAESSAQPNQLETGASSPER
77.2	3643.5911	0.0053	EGQVEAAQPEQAAEAPAESSAQPNQLETGASSPER

75.9	3643.5911	0.0053	EGQVEAAQPEQAAEAPAESSAQPNQLETGASSPER
42.3	3643.5911	0.0053	EGQVEAAQPEQAAEAPAESSAQPNQLETGASSPER
28.6	3643.5911	0.0053	EGQVEAAQPEQAAEAPAESSAQPNQLETGASSPER
8.2	3642.5885	1.0078	RSVTFIEAQPEPTAAPTDMLPATGQPQSCGPGR
7.2	3642.5885	1.0078	RSVTFIEAQPEPTAAPTDMLPATGQPQSCGPGR
5.8	3642.5885	1.0078	RSVTFIEAQPEPTAAPTDMLPATGQPQSCGPGR
5.8	3641.5607	2.0357	YHDSQARPAERGSATVSFGDLARLNDTMSQGK
5.6	3641.6221	1.9742	TTSETLKHKINPSAGETAPQAIEVLLYTPGR

Spectrum No: 54; Query: 1208; Rank: 1

Peptide View

MS/MS Fragmentation of **TLSNAEDYLDDEDSD**
Found in **IPI00202703**, Tax_Id=10116 Gene_Symbol=Ostf1 Osteoclast-stimulating factor 1

Match to Query 1208: 1780.623368 from(891.318960,2+)
Title: 100101RatKid_NS_deglyco_27.2442.2442.2.dta
Data file K:\NewmanPaper\Piliang\3SubProteomes\Piliang3SP\mgf5ppm\ERLIC_3SubProteomes5ppm.mgf

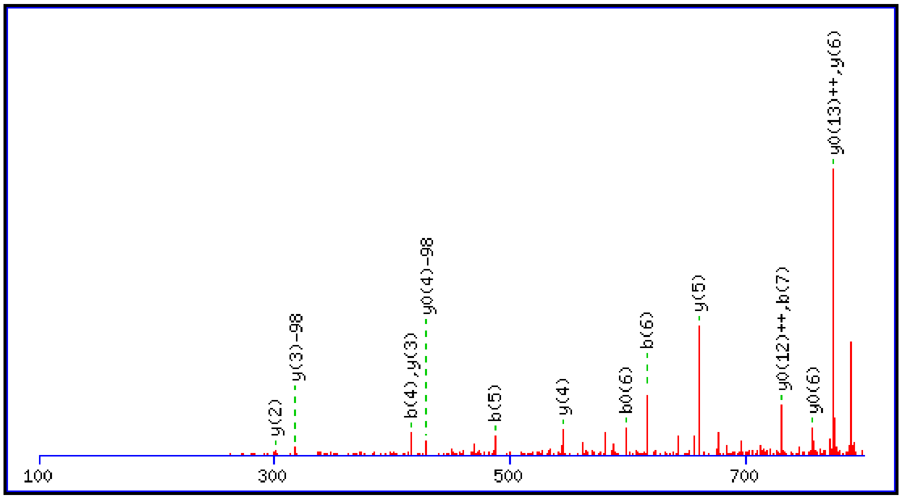

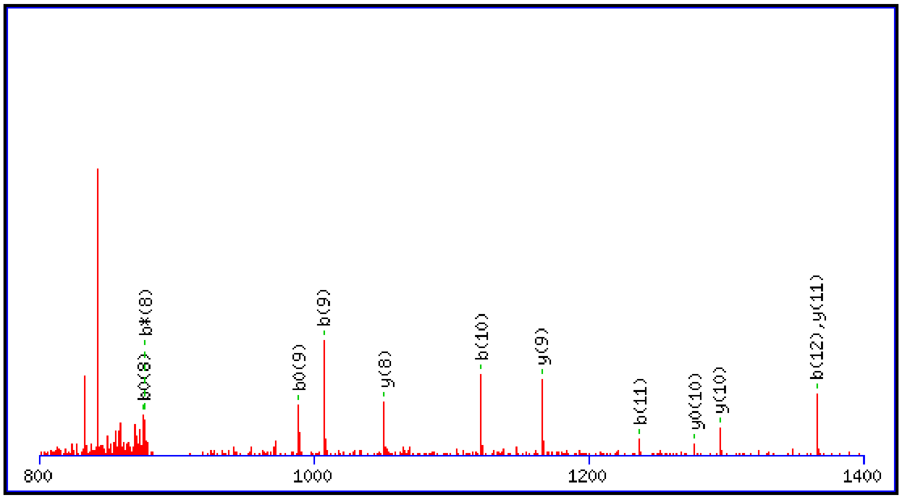

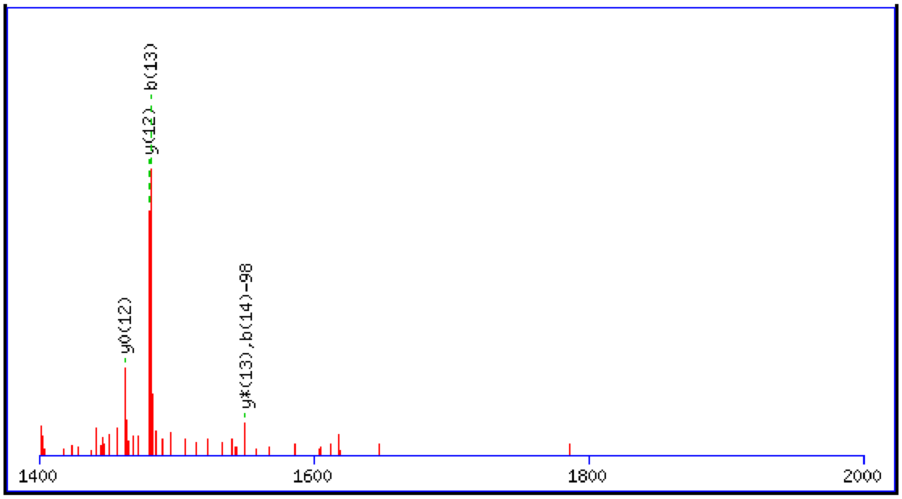

Monoisotopic mass of neutral peptide Mr(calc): 1780.6200
Fixed modifications: Carbamidomethyl (C)
Variable modifications:
S14 : Phospho (ST), with neutral losses 0.0000(shown in table), 97.9769
Ions Score: 79 **Expect:** 9.6e-007
Matches (Bold Red): 32/204 fragment ions using 35 most intense peaks

#	b	b^{++}	b*	b^{*++}	b^{0}	b^{0++}	Seq.	y	y^{++}	y*	y^{*++}	y^{0}	y^{0++}	#
1	102.0550	51.5311			84.0444	42.5258	T							15
2	215.1390	108.0731			197.1285	99.0679	L	1680.5796	840.7934	1663.5531	832.2802	1662.5691	831.7882	14
3	302.1710	151.5892			284.1605	142.5839	S	1567.4956	784.2514	**1550.4690**	775.7381	1549.4850	**775.2461**	13
4	**416.2140**	208.6106	399.1874	200.0974	398.2034	199.6053	N	**1480.4635**	740.7354	1463.4370	732.2221	**1462.4530**	**731.7301**	12
5	**487.2511**	244.1292	470.2245	235.6159	469.2405	235.1239	A	**1366.4206**	683.7139			1348.4100	674.7087	11
6	**616.2937**	308.6505	599.2671	300.1372	**598.2831**	299.6452	E	**1295.3835**	648.1954			**1277.3729**	639.1901	10
7	**731.3206**	366.1640	714.2941	357.6507	713.3101	357.1587	D	**1166.3409**	583.6741			1148.3303	574.6688	9
8	894.3840	447.6956	**877.3574**	439.1823	**876.3734**	438.6903	Y	**1051.3140**	526.1606			1033.3034	517.1553	8
9	**1007.4680**	504.2376	990.4415	495.7244	**989.4575**	495.2324	L	888.2506	444.6289			870.2401	435.6237	7
10	**1122.4950**	561.7511	1105.4684	553.2378	1104.4844	552.7458	D	**775.1666**	388.0869			**757.1560**	379.0816	6
11	**1237.5219**	619.2646	1220.4954	610.7513	1219.5113	610.2593	D	**660.1396**	330.5734			642.1291	321.5682	5
12	**1366.5645**	683.7859	1349.5379	675.2726	1348.5539	674.7806	E	**545.1127**	273.0600			527.1021	264.0547	4
13	**1481.5914**	741.2994	1464.5649	732.7861	1463.5809	732.2941	D	**416.0701**	208.5387			398.0595	199.5334	3
14	1648.5898	824.7985	1631.5632	816.2853	1630.5792	815.7933	S	**301.0431**	151.0252			283.0326	142.0199	2
15							D	134.0448	67.5260			116.0342	58.5207	1

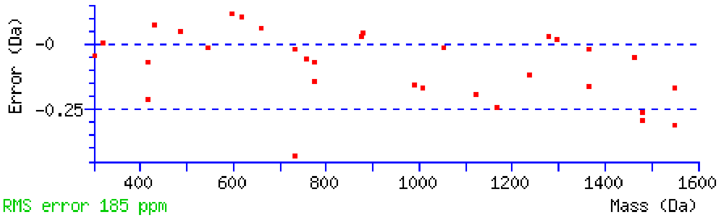

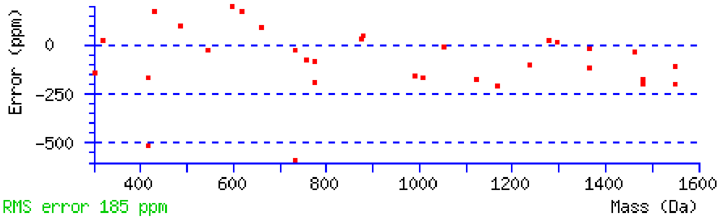

All matches to this query

Score	Mr(calc):	Delta	Sequence
78.5	1780.6200	0.0034	TLSNAEDYLDDEDSD
13.9	1780.6200	0.0034	TLSNAEDYLDDEDSD
13.4	1780.6200	0.0034	TLSNAEDYLDDEDSD
13.4	1780.6200	0.0034	TLSNAEDYLDDEDSD
3.8	1780.6239	-0.0005	MAVYQTYANDMNGK
0.9	1780.6239	-0.0005	MAVYQTYANDMNGK

0.0	1780.6239	-0.0005	MAVYQTYANDMNGK

Spectrum No: 55; Query: 1273; Rank: 1

Peptide View

MS/MS Fragmentation of **IEDVGSDEEDDSGKDK**
Found in **IPI00471584**, Tax_Id=10116 Gene_Symbol=Hspcb Heat shock protein HSP 90-beta

Match to Query 1273: 1816.684088 from(909.349320,2+)
Title: 091008RatKidney_NoSalt_28.469.469.2.dta
Data file K:\NewmanPaper\Piliang\3SubProteomes\Piliang3SP\mgf5ppm\ERLIC_3SubProteomes5ppm.mgf

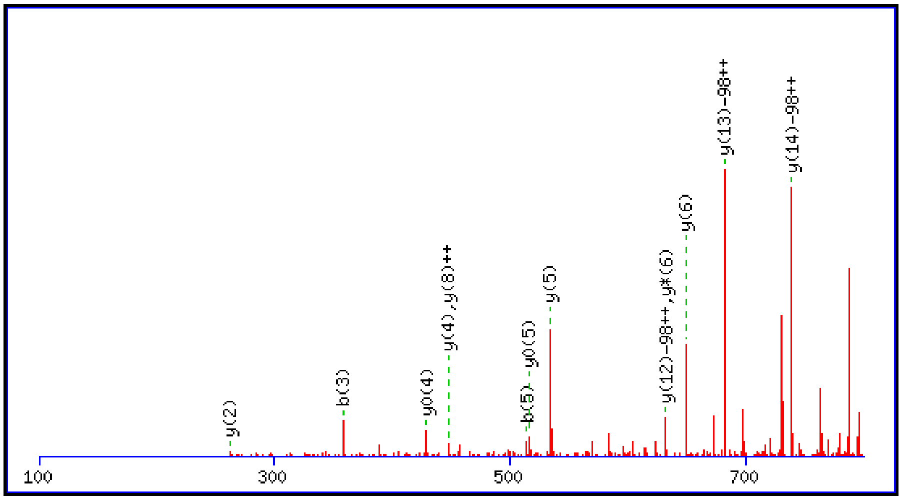

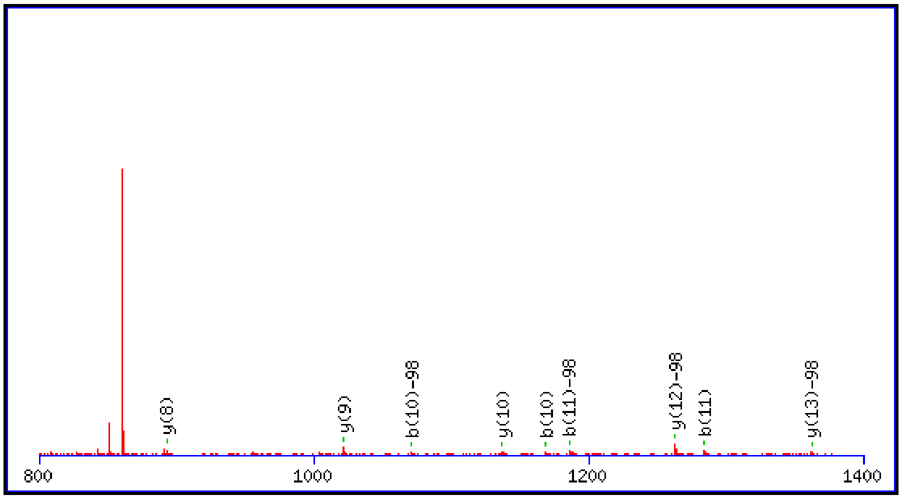

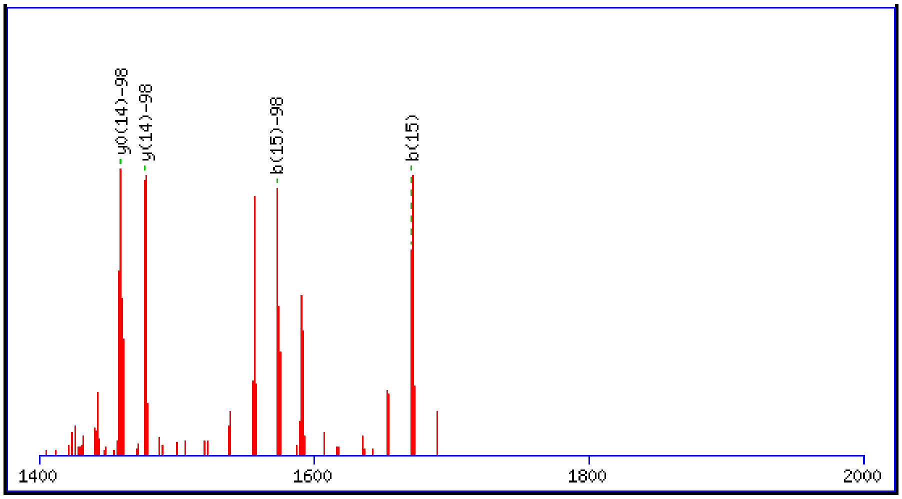

Monoisotopic mass of neutral peptide Mr(calc): 1816.6888
Fixed modifications: Carbamidomethyl (C)
Variable modifications:
S6 : Phospho (ST), with neutral losses 97.9769(shown in table), 0.0000
Ions Score: 79 **Expect:** 1.6e-006
Matches (Bold Red): 26/224 fragment ions using 35 most intense peaks

#	b	b^{++}	b*	b^{*++}	b^0	b^{0++}	Seq.	y	y^{++}	y*	y^{*++}	y^0	y^{0++}	#
1	114.0913	57.5493					I							16
2	243.1339	122.0706			225.1234	113.0653	E	1606.6351	803.8212	1589.6085	795.3079	1588.6245	794.8159	15
3	**358.1609**	179.5841			340.1503	170.5788	D	**1477.5925**	**739.2999**	1460.5659	730.7866	**1459.5819**	730.2946	14
4	457.2293	229.1183			439.2187	220.1130	V	**1362.5656**	**681.7864**	1345.5390	673.2731	1344.5550	672.7811	13
5	**514.2508**	257.6290			496.2402	248.6237	G	**1263.4971**	**632.2522**	1246.4706	623.7389	1245.4866	623.2469	12
6	583.2722	292.1397			565.2616	283.1345	S	1206.4757	603.7415	1189.4491	595.2282	1188.4651	594.7362	11
7	698.2992	349.6532			680.2886	340.6479	D	**1137.4542**	569.2307	1120.4277	560.7175	1119.4437	560.2255	10
8	827.3417	414.1745			809.3312	405.1692	E	**1022.4273**	511.7173	1005.4007	503.2040	1004.4167	502.7120	9
9	956.3843	478.6958			938.3738	469.6905	E	**893.3847**	**447.1960**	876.3581	438.6827	875.3741	438.1907	8
10	**1071.4113**	536.2093			1053.4007	527.2040	D	764.3421	382.6747	747.3155	374.1614	746.3315	373.6694	7
11	**1186.4382**	593.7228			1168.4277	584.7175	D	**649.3151**	325.1612	**632.2886**	316.6479	631.3046	316.1559	6
12	1273.4703	637.2388			1255.4597	628.2335	S	**534.2882**	267.6477	517.2617	259.1345	**516.2776**	258.6425	5
13	1330.4917	665.7495			1312.4812	656.7442	G	**447.2562**	224.1317	430.2296	215.6185	**429.2456**	215.1264	4
14	1458.5867	729.7970	1441.5601	721.2837	1440.5761	720.7917	K	390.2347	195.6210	373.2082	187.1077	372.2241	186.6157	3
15	**1573.6136**	787.3104	1556.5871	778.7972	1555.6031	778.3052	D	**262.1397**	131.5735	245.1132	123.0602	244.1292	122.5682	2
16							K	147.1128	74.0600	130.0863	65.5468			1

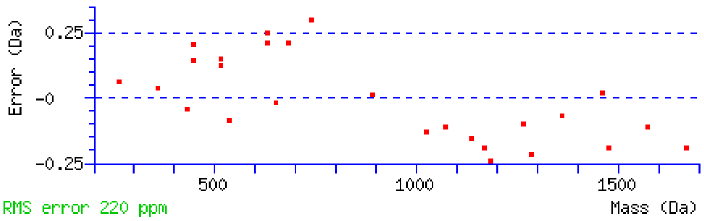

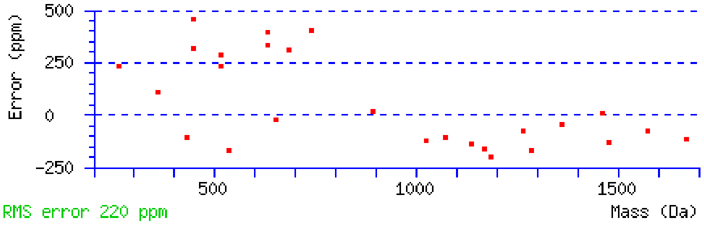

All matches to this query

Score	Mr(calc):	Delta	Sequence
78.5	1816.6888	-0.0047	IEDVGSDEEDDSGKDK
49.1	1816.6888	-0.0047	IEDVGSDEEDDSGKDK
4.7	1816.6926	-0.0085	NRPXASPTMTMGTAK
2.7	1815.6895	0.9946	LQQELMTLMMSGDK
2.4	1816.6935	-0.0094	CQVLQSEDEQSNSGR

2.3	1816.6869	-0.0028	QEPKQSNCESCQSR
2.3	1816.6811	0.0030	SNDGHRTWSCGMWK
2.2	1816.6892	-0.0051	MAAGTSNYWEDLRK
2.2	1816.6957	-0.0117	YNFNADTNVELSLR
1.9	1816.7017	-0.0176	ECEPLSMLSCVCPR

Spectrum No: 56; Query: 1129; Rank: 1

Peptide View

MS/MS Fragmentation of **AEEEGGSDGSAAEAEPR**
Found in **IPI00363771**, Tax_Id=10116 Gene_Symbol=Eif3s9 108 kDa protein

Match to Query 1129: 1740.644048 from(871.329300,2+)
Title: 091008RatKidney_NoSalt_28.437.437.2.dta
Data file K:\NewmanPaper\Piliang\3SubProteomes\Piliang3SP\mgf5ppm\ERLIC_3SubProteomes5ppm.mgf

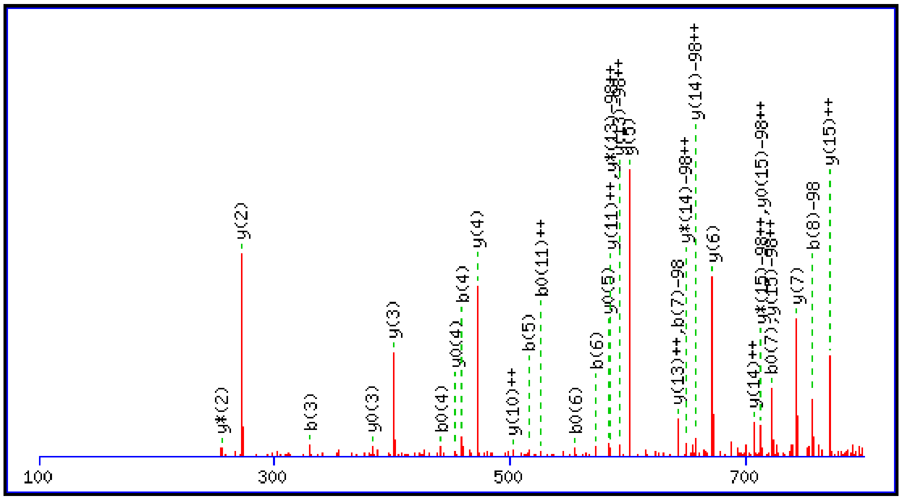

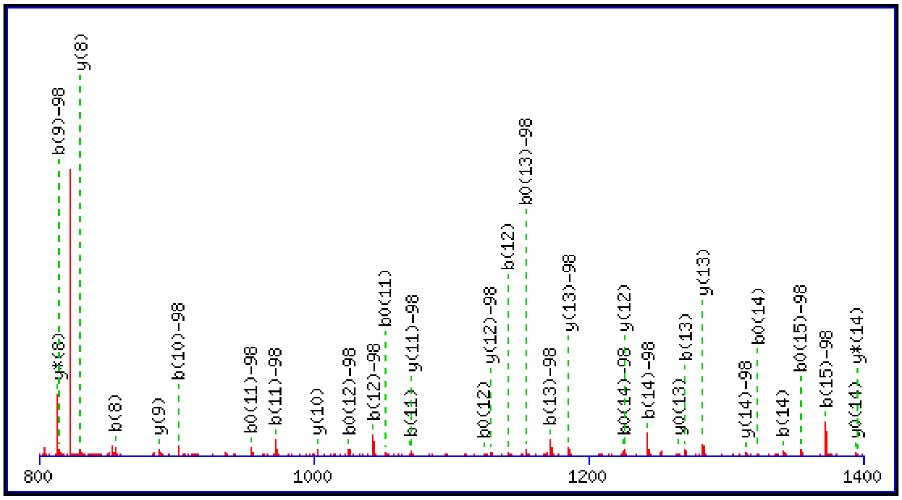

Mascot Search Results: Peptide View

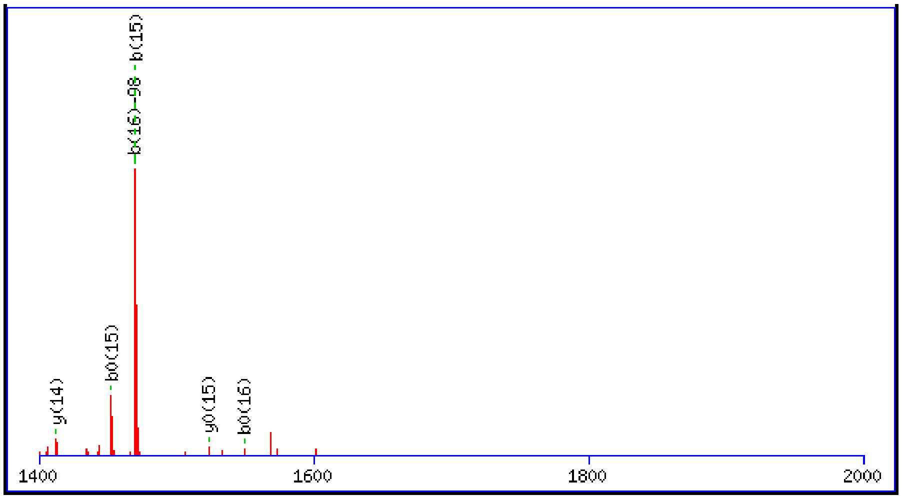

Monoisotopic mass of neutral peptide Mr(calc): 1740.6475
Fixed modifications: Carbamidomethyl (C)
Variable modifications:
S7 : Phospho (ST), with neutral losses 97.9769(shown in table), 0.0000
Ions Score: 78 **Expect:** 1.4e-006
Matches (Bold Red): 71/230 fragment ions using 120 most intense peaks

#	b	b^{++}	b^{0}	b^{0++}	Seq.	y	y^{++}	y*	y^{*++}	y^{0}	y^{0++}	#
1	72.0444	36.5258			A							17
2	201.0870	101.0471	183.0764	92.0418	E	1572.6408	786.8241	1555.6143	778.3108	1554.6303	777.8188	16
3	**330.1296**	165.5684	312.1190	156.5631	E	1443.5982	**722.3028**	1426.5717	**713.7895**	1425.5877	**713.2975**	15
4	**459.1722**	230.0897	**441.1616**	221.0844	E	**1314.5557**	**657.7815**	1297.5291	**649.2682**	1296.5451	648.7762	14
5	**516.1936**	258.6005	498.1831	249.5952	G	**1185.5131**	**593.2602**	1168.4865	**584.7469**	1167.5025	584.2549	13
6	**573.2151**	287.1112	**555.2045**	278.1059	G	**1128.4916**	564.7494	1111.4651	556.2362	1110.4810	555.7442	12
7	**642.2366**	321.6219	624.2260	312.6166	S	**1071.4701**	536.2387	1054.4436	527.7254	1053.4596	527.2334	11
8	**757.2635**	379.1354	739.2529	370.1301	D	**1002.4487**	**501.7280**	985.4221	493.2147	984.4381	492.7227	10
9	**814.2850**	407.6461	796.2744	398.6408	G	**887.4217**	444.2145	870.3952	435.7012	869.4112	435.2092	9
10	**901.3170**	451.1621	883.3064	442.1568	S	**830.4003**	415.7038	**813.3737**	407.1905	812.3897	406.6985	8
11	**972.3541**	486.6807	**954.3435**	477.6754	A	**743.3682**	372.1878	726.3417	363.6745	725.3577	363.1825	7
12	**1043.3912**	522.1992	**1025.3807**	513.1940	A	**672.3311**	336.6692	655.3046	328.1559	654.3206	327.6639	6
13	**1172.4338**	586.7205	**1154.4232**	577.7153	E	**601.2940**	301.1506	584.2675	292.6374	**583.2835**	292.1454	5
14	**1243.4709**	622.2391	**1225.4604**	613.2338	A	**472.2514**	236.6293	455.2249	228.1161	**454.2409**	227.6241	4
15	**1372.5135**	686.7604	**1354.5030**	677.7551	E	**401.2143**	201.1108	384.1878	192.5975	**383.2037**	192.1055	3
16	**1469.5663**	735.2868	1451.5557	726.2815	P	**272.1717**	136.5895	**255.1452**	128.0762			2
17					R	175.1190	88.0631	158.0924	79.5498			1

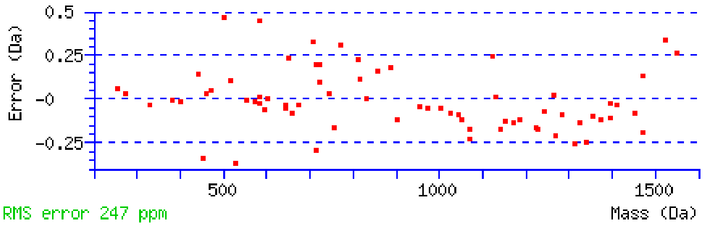

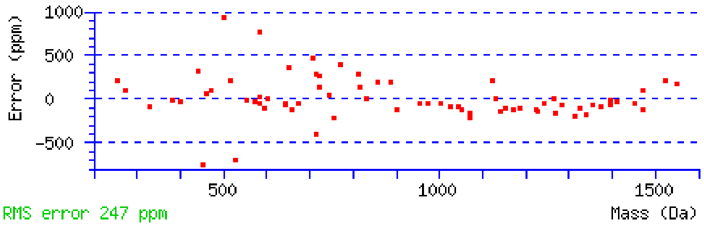

All matches to this query

Score	Mr(calc):	Delta	Sequence
78.3	1740.6475	-0.0035	AEEEGGSDGSAAEAEPR
65.0	1740.6475	-0.0035	AEEEGGSDGSAAEAEPR
11.7	1740.6410	0.0031	LGTEATSNGLVHSSK
11.3	1740.6281	0.0160	DKYGINDNGTTEPR

9.3	1740.6491	-0.0051	VTADDDGSYHCFFK
8.4	1740.6281	0.0160	DKYGINDNGTTEPR
6.8	1740.6410	0.0031	LGTEATSNGLVHSSK
0.9	1740.6427	0.0013	MGSTPATTEGSTAAQR
0.9	1740.6427	0.0013	MGSTPATTEGSTAAQR
0.6	1740.6491	-0.0051	VTADDDGSYHCFFK

Spectrum No: 57; Query: 1772; Rank: 1

Peptide View

MS/MS Fragmentation of **KGSLDSDNDDSDCPYSEK**
Found in **IPI00199985**, Tax_Id=10116 Gene_Symbol=Slc4a4 Isoform 1 of Electrogenic sodium bicarbonate cotransporter 1

Match to Query 1772: 2110.771348 from(1056.392950,2+)
Title: 100101RatKid_NS_deglyco_23.723.723.2.dta
Data file K:\NewmanPaper\Piliang\3SubProteomes\Piliang3SP\mgf5ppm\ERLIC_3SubProteomes5ppm.mgf

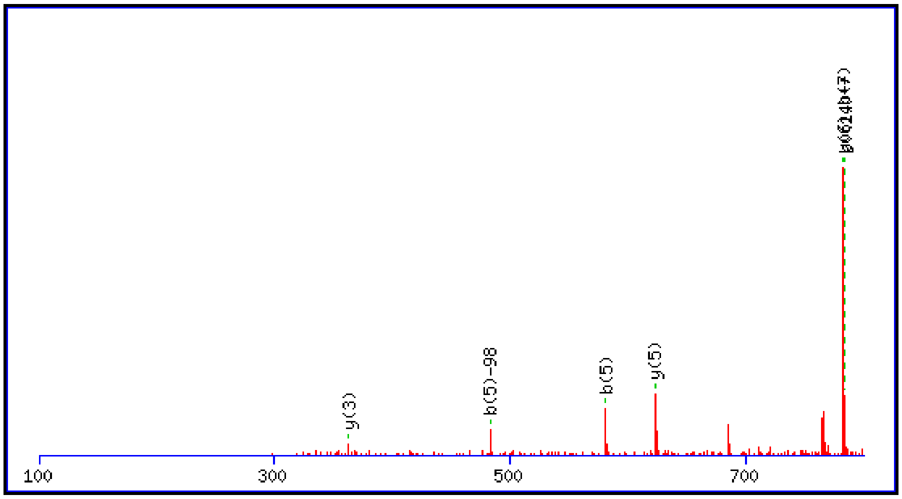

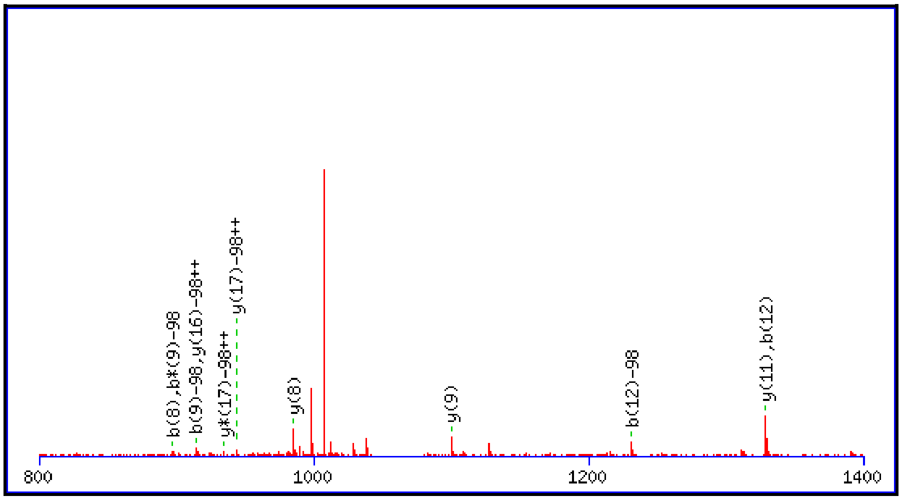

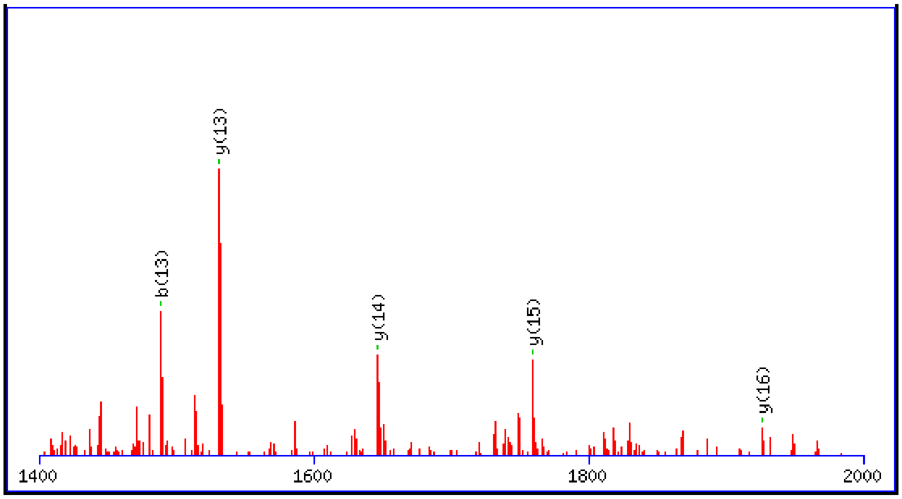

Monoisotopic mass of neutral peptide Mr(calc): 2110.7674
Fixed modifications: Carbamidomethyl (C)
Variable modifications:
S3 : Phospho (ST), with neutral losses 97.9769(shown in table), 0.0000
Ions Score: 78 **Expect:** 1.9e-006
Matches (Bold Red): 23/300 fragment ions using 26 most intense peaks

#	b	b^{++}	b*	b^{*++}	b^0	b^{0++}	Seq.	y	y^{++}	y*	y^{*++}	y^0	y^{0++}	#
1	129.1022	65.0548	112.0757	56.5415			K							18
2	186.1237	93.5655	169.0972	85.0522			G	1885.7028	**943.3551**	1868.6763	**934.8418**	1867.6923	934.3498	17
3	255.1452	128.0762	238.1186	119.5629	237.1346	119.0709	S	1828.6814	**914.8443**	1811.6548	906.3311	1810.6708	905.8390	16
4	368.2292	184.6182	351.2027	176.1050	350.2187	175.6130	L	**1759.6599**	880.3336	1742.6334	871.8203	1741.6494	871.3283	15
5	**483.2562**	242.1317	466.2296	233.6184	465.2456	233.1264	D	**1646.5759**	823.7916	1629.5493	815.2783	1628.5653	814.7863	14
6	570.2882	285.6477	553.2616	277.1345	552.2776	276.6425	S	**1531.5489**	766.2781	1514.5224	757.7648	1513.5384	757.2728	13
7	685.3151	343.1612	668.2886	334.6479	667.3046	334.1559	D	1444.5169	722.7621	1427.4903	714.2488	1426.5063	713.7568	12
8	799.3581	400.1827	782.3315	391.6694	781.3475	391.1774	N	**1329.4900**	665.2486	1312.4634	656.7353	1311.4794	656.2433	11
9	**914.3850**	457.6961	**897.3585**	449.1829	896.3744	448.6909	D	1215.4470	608.2272	1198.4205	599.7139	1197.4365	599.2219	10
10	1029.4119	515.2096	1012.3854	506.6963	1011.4014	506.2043	D	**1100.4201**	550.7137	1083.3935	542.2004	1082.4095	541.7084	9
11	1116.4440	558.7256	1099.4174	550.2124	1098.4334	549.7203	S	**985.3931**	493.2002	968.3666	484.6869	967.3826	484.1949	8
12	**1231.4709**	616.2391	1214.4444	607.7258	1213.4604	607.2338	D	898.3611	449.6842	881.3346	441.1709	880.3505	440.6789	7
13	1391.5016	696.2544	1374.4750	687.7411	1373.4910	687.2491	C	**783.3342**	392.1707	766.3076	383.6574	765.3236	383.1654	6
14	1488.5543	744.7808	1471.5278	736.2675	1470.5438	735.7755	P	**623.3035**	312.1554	606.2770	303.6421	605.2930	303.1501	5
15	1651.6177	826.3125	1634.5911	817.7992	1633.6071	817.3072	Y	526.2508	263.6290	509.2242	255.1157	508.2402	254.6237	4
16	1738.6497	869.8285	1721.6231	861.3152	1720.6391	860.8232	S	**363.1874**	182.0974	346.1609	173.5841	345.1769	173.0921	3
17	1867.6923	934.3498	1850.6657	925.8365	1849.6817	925.3445	E	276.1554	138.5813	259.1288	130.0681	258.1448	129.5761	2
18							K	147.1128	74.0600	130.0863	65.5468			1

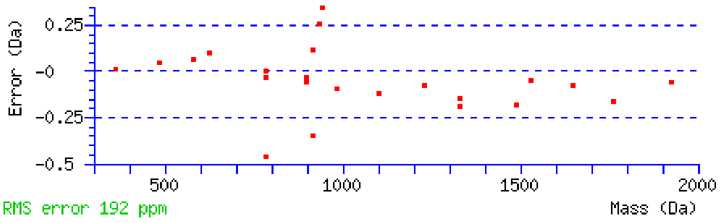

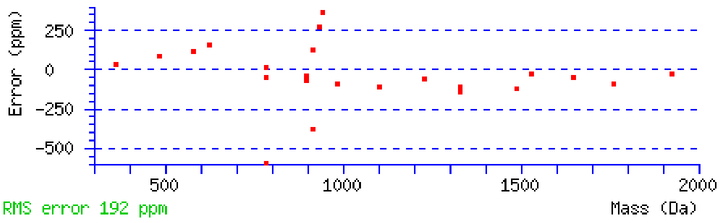

All matches to this query

Score	Mr(calc):	Delta	Sequence
78.1	2110.7674	0.0039	KGSLDSDNDDSDCPYSEK
44.9	2110.7674	0.0039	KGSLDSDNDDSDCPYSEK
19.3	2110.7674	0.0039	KGSLDSDNDDSDCPYSEK

11.6	2110.7674	0.0039	KGSLDSDNDDSDCPYSEK
3.8	2110.7907	-0.0193	DVQVTCPNEMHLLSTK
0.0	2109.7881	0.9833	GMGVFSTGTDASGIQSLSR

Spectrum No: 58; Query: 1080; Rank: 1

Peptide View

MS/MS Fragmentation of **SSTPLPTVSSSAENTR**
Found in **IPI00208114**, Tax_Id=10116 Gene_Symbol=Tmpo Lamina-associated polypeptide 2 isoform beta

Match to Query 1080: 1712.768808 from(857.391680,2+)
Title: 100101RatKid_NS_deglyco_16.1467.1467.2.dta
Data file K:\NewmanPaper\Piliang\3SubProteomes\Piliang3SP\mgf5ppm\ERLIC_3SubProteomes5ppm.mgf

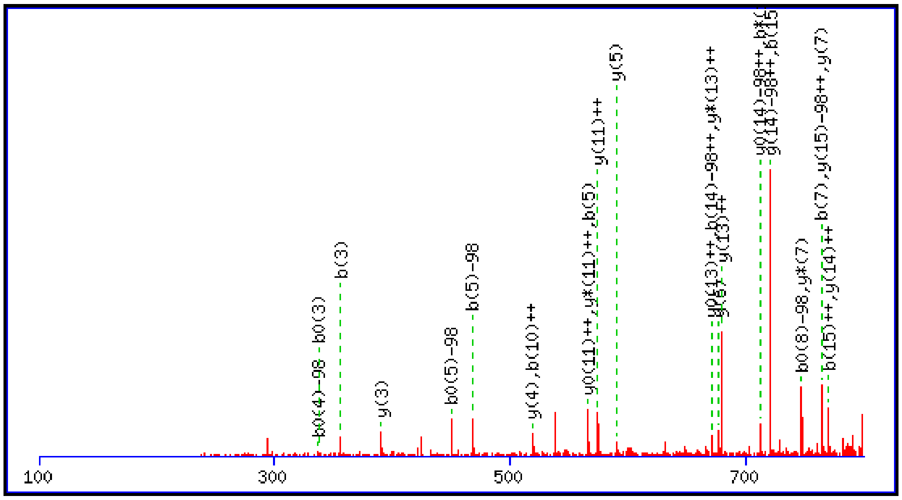

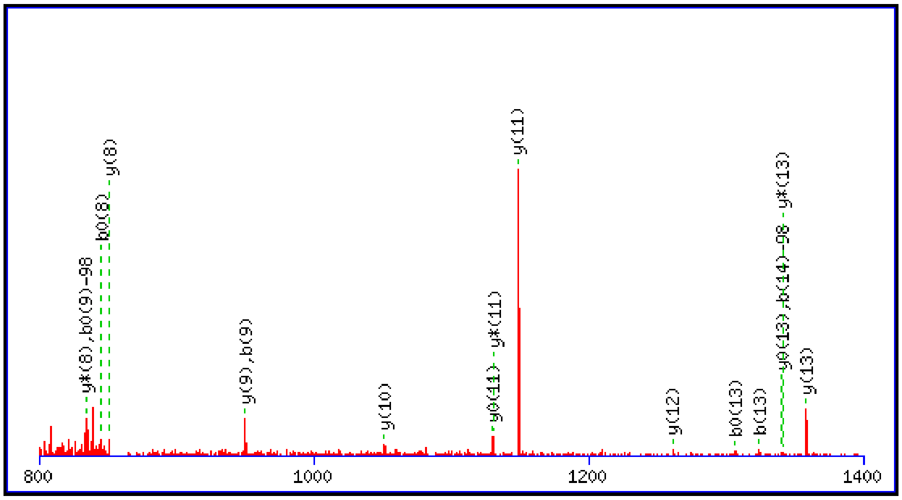

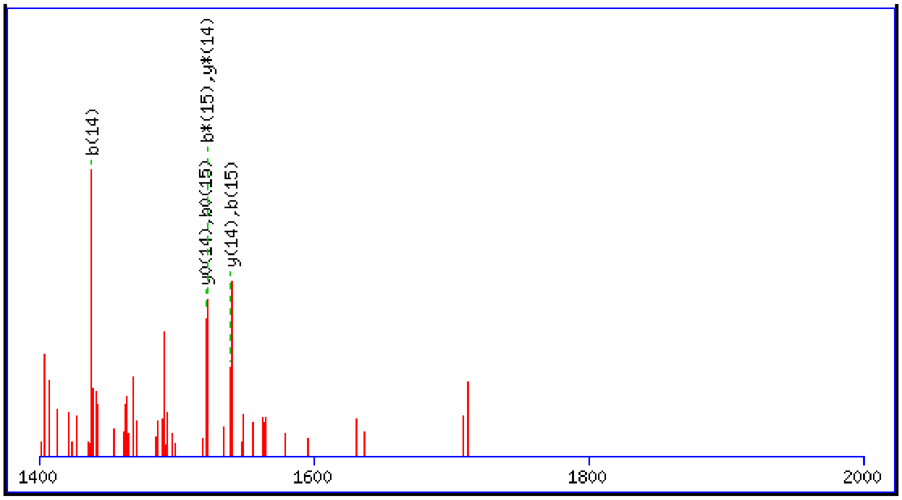

Monoisotopic mass of neutral peptide Mr(calc): 1712.7618
Fixed modifications: Carbamidomethyl (C)
Variable modifications:
T3 : Phospho (ST), with neutral losses 0.0000(shown in table), 97.9769
Ions Score: 78 **Expect:** 3.7e-006
Matches (Bold Red): 55/220 fragment ions using 62 most intense peaks

#	b	b^{++}	b*	b^{*++}	b^0	b^{0++}	Seq.	y	y^{++}	y*	y^{*++}	y^0	y^{0++}	#
1	88.0393	44.5233			70.0287	35.5180	S							16
2	175.0713	88.0393			157.0608	79.0340	S	1626.7371	813.8722	1609.7105	805.3589	1608.7265	804.8669	15
3	**356.0853**	178.5463			**338.0748**	169.5410	T	**1539.7050**	**770.3562**	**1522.6785**	761.8429	**1521.6945**	761.3509	14
4	453.1381	227.0727			435.1275	218.0674	P	**1358.6910**	**679.8492**	**1341.6645**	**671.3359**	**1340.6805**	**670.8439**	13
5	**566.2222**	283.6147			548.2116	274.6094	L	**1261.6383**	631.3228	1244.6117	622.8095	1243.6277	622.3175	12
6	663.2749	332.1411			645.2644	323.1358	P	**1148.5542**	**574.7807**	**1131.5277**	**566.2675**	**1130.5436**	**565.7755**	11
7	**764.3226**	382.6649			746.3120	373.6597	T	**1051.5014**	526.2544	1034.4749	517.7411	1033.4909	517.2491	10
8	863.3910	432.1991			**845.3805**	423.1939	V	**950.4538**	475.7305	933.4272	467.2172	932.4432	466.7252	9
9	**950.4230**	475.7152			932.4125	466.7099	S	**851.3853**	426.1963	**834.3588**	417.6830	833.3748	417.1910	8
10	1037.4551	**519.2312**			1019.4445	510.2259	S	**764.3533**	382.6803	**747.3268**	374.1670	746.3428	373.6750	7
11	1124.4871	562.7472			1106.4765	553.7419	S	**677.3213**	339.1643	660.2947	330.6510	659.3107	330.1590	6
12	1195.5242	598.2657			1177.5137	589.2605	A	**590.2893**	295.6483	573.2627	287.1350	572.2787	286.6430	5
13	**1324.5668**	662.7870			**1306.5562**	653.7818	E	**519.2522**	260.1297	502.2256	251.6164	501.2416	251.1244	4
14	**1438.6097**	719.8085	1421.5832	711.2952	1420.5992	710.8032	N	**390.2096**	195.6084	373.1830	187.0951	372.1990	186.6031	3
15	**1539.6574**	**770.3323**	**1522.6309**	761.8191	**1521.6469**	761.3271	T	276.1666	138.5870	259.1401	130.0737	258.1561	129.5817	2
16							R	175.1190	88.0631	158.0924	79.5498			1

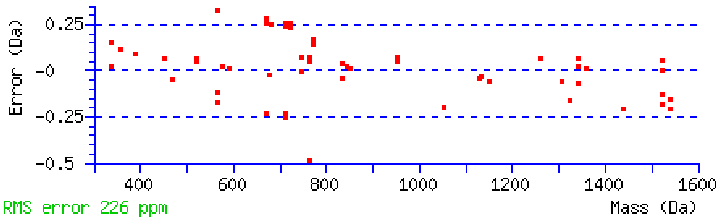

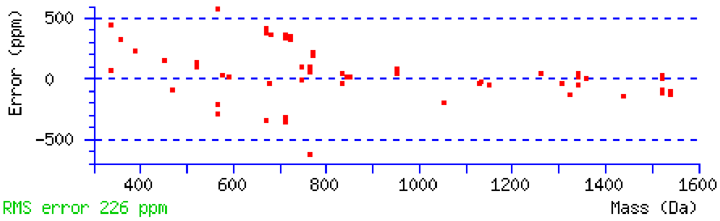

All matches to this query

Score	Mr(calc):	Delta	Sequence
78.0	1712.7618	0.0070	SSTPLPTVSSSAENTR
66.5	1712.7618	0.0070	SSTPLPTVSSSAENTR
66.5	1712.7618	0.0070	SSTPLPTVSSSAENTR
51.4	1712.7618	0.0070	SSTPLPTVSSSAENTR
29.9	1712.7618	0.0070	SSTPLPTVSSSAENTR

17.9	1712.7618	0.0070	SSTPLPTVSSSAENTR
10.9	1712.7618	0.0070	SSTPLPTVSSSAENTR
8.9	1712.7576	0.0112	STSKLSYGFLTPPR
8.9	1712.7576	0.0112	STSKLSYGFLTPPR
8.9	1712.7576	0.0112	STSKLSYGFLTPPR

Spectrum No: 59; Query: 2218; Rank: 1

Peptide View

MS/MS Fragmentation of **EGEEPTVYSDDEEPKDEAARK**
Found in **IPI00480820**, Tax_Id=10116 Gene_Symbol=Pgrmc1 Membrane-associated progesterone receptor component 1

Match to Query 2218: 2473.011162 from(825.344330,3+)
Title: 091008RatKidney_NH4Format01_21.1001.1001.3.dta
Data file K:\NewmanPaper\Piliang\3SubProteomes\Piliang3SP\mgf5ppm\ERLIC_3SubProteomes5ppm.mgf

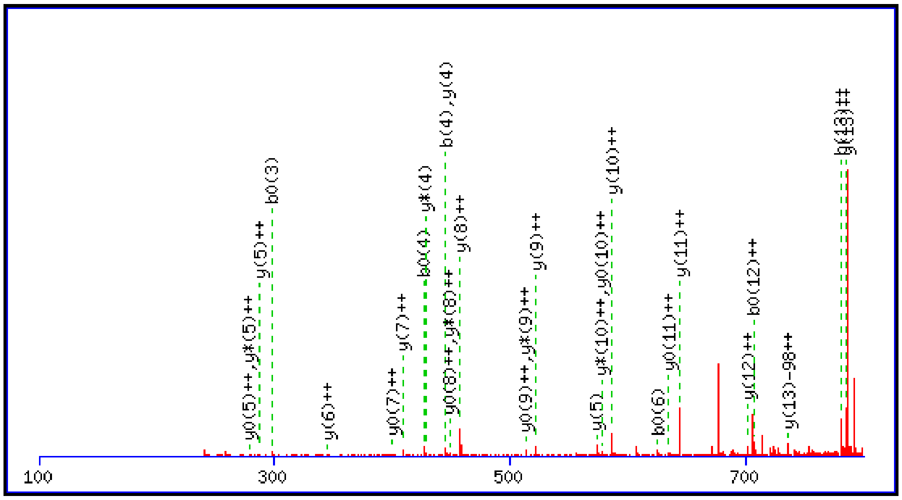

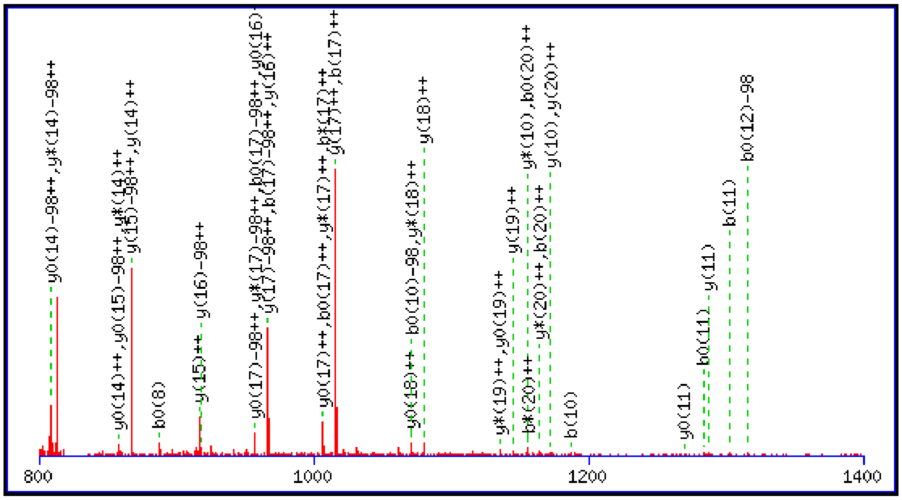

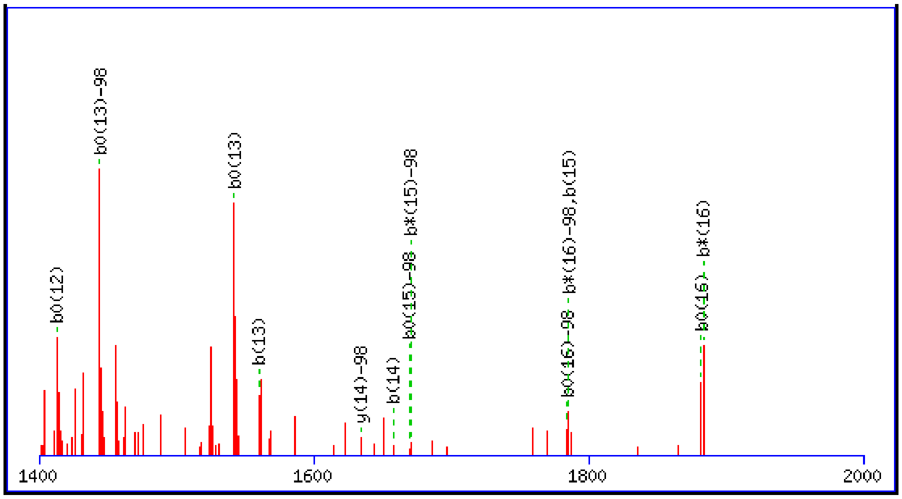

Monoisotopic mass of neutral peptide Mr(calc): 2473.0169
Fixed modifications: Carbamidomethyl (C)
Variable modifications:
S9 : Phospho (ST), with neutral losses 0.0000(shown in table), 97.9769
Ions Score: 77 **Expect:** 5.2e-006
Matches (Bold Red): 86/312 fragment ions using 117 most intense peaks

#	b	b^{++}	b*	b^{*++}	b^0	b^{0++}	Seq.	y	y^{++}	y*	y^{*++}	y^0	y^{0++}	#
1	130.0499	65.5286			112.0393	56.5233	E							21
2	187.0713	94.0393			169.0608	85.0340	G	2344.9817	**1172.9945**	2327.9551	**1164.4812**	2326.9711	1163.9892	20
3	316.1139	158.5606			**298.1034**	149.5553	E	2287.9602	**1144.4837**	2270.9337	**1135.9705**	2269.9496	**1135.4785**	19
4	**445.1565**	223.0819			**427.1460**	214.0766	E	2158.9176	**1079.9624**	2141.8911	**1071.4492**	2140.9070	**1070.9572**	18
5	542.2093	271.6083			524.1987	262.6030	P	2029.8750	**1015.4411**	2012.8485	**1006.9279**	2011.8645	**1006.4359**	17
6	643.2570	322.1321			**625.2464**	313.1268	T	1932.8223	**966.9148**	1915.7957	958.4015	1914.8117	**957.9095**	16
7	742.3254	371.6663			724.3148	362.6610	V	1831.7746	**916.3909**	1814.7480	907.8777	1813.7640	907.3856	15
8	905.3887	453.1980			**887.3781**	444.1927	Y	1732.7062	**866.8567**	1715.6796	**858.3434**	1714.6956	**857.8514**	14
9	1072.3871	536.6972			1054.3765	527.6919	S	1569.6428	**785.3251**	1552.6163	776.8118	1551.6323	776.3198	13
10	**1187.4140**	594.2106			1169.4034	585.2054	D	1402.6445	**701.8259**	1385.6179	693.3126	1384.6339	692.8206	12
11	**1302.4409**	651.7241			**1284.4304**	642.7188	D	**1287.6175**	**644.3124**	1270.5910	635.7991	**1269.6070**	**635.3071**	11
12	1431.4835	716.2454			**1413.4730**	**707.2401**	E	**1172.5906**	**586.7989**	**1155.5640**	**578.2857**	1154.5800	**577.7937**	10
13	**1560.5261**	**780.7667**			**1542.5156**	771.7614	E	1043.5480	**522.2776**	1026.5215	**513.7644**	1025.5374	**513.2724**	9
14	**1657.5789**	829.2931			1639.5683	820.2878	P	914.5054	**457.7563**	897.4789	**449.2431**	896.4948	**448.7511**	8
15	**1785.6739**	893.3406	1768.6473	884.8273	1767.6633	884.3353	K	817.4526	**409.2300**	800.4261	400.7167	799.4421	**400.2247**	7
16	1900.7008	950.8540	**1883.6743**	942.3408	**1882.6902**	941.8488	D	689.3577	**345.1825**	672.3311	336.6692	671.3471	336.1772	6
17	2029.7434	**1015.3753**	2012.7168	**1006.8621**	2011.7328	**1006.3701**	E	**574.3307**	**287.6690**	557.3042	**279.1557**	556.3202	**278.6637**	5
18	2100.7805	1050.8939	2083.7540	1042.3806	2082.7699	1041.8886	A	**445.2881**	223.1477	**428.2616**	214.6344			4
19	2171.8176	1086.4124	2154.7911	1077.8992	2153.8071	1077.4072	A	374.2510	187.6292	357.2245	179.1159			3
20	2327.9187	**1164.4630**	2310.8922	**1155.9497**	2309.9082	**1155.4577**	R	303.2139	152.1106	286.1874	143.5973			2
21							K	147.1128	74.0600	130.0863	65.5468			1

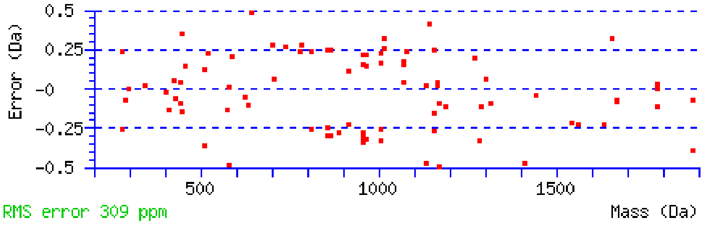

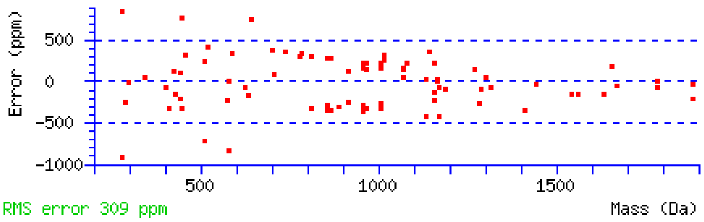

All matches to this query

Score	Mr(calc):	Delta	Sequence

77.1	2473.0169	-0.0058	EGEEPTVYSDDEEPKDEAARK
67.0	2473.0169	-0.0058	EGEEPTVYSDDEEPKDEAARK
49.7	2473.0169	-0.0058	EGEEPTVYSDDEEPKDEAARK
3.3	2472.9995	0.0117	MAAAGSGGGGGRYYRGSNQGGXAPK
1.6	2471.0043	2.0069	ADNFEYSDPVDGSISKNQGLR
1.6	2471.9883	1.0229	ADNFEYSDPVDGSISKNQGLR
1.4	2470.9839	2.0272	FKSLMELVEYYKHHSLK
1.4	2471.9843	1.0269	VSRDKISDNNEQETSNYDAK
1.4	2471.9843	1.0269	VSRDKISDNNEQETSNYDAK
1.4	2471.9843	1.0269	VSRDKISDNNEQETSNYDAK

Spectrum No: 60; Query: 2573; Rank: 1

Peptide View

MS/MS Fragmentation of **RSSLDAEIDSLTSILADLECSSPYKPR**
Found in **IPI00362080**, Tax_Id=10116 Gene_Symbol=Lpp 66 kDa protein

Match to Query 2573: 3102.465776 from(776.623720,4+)
Title: 091008RatKidney_NH4Format02_21.6092.6092.4.dta
Data file K:\NewmanPaper\Piliang\3SubProteomes\Piliang3SP\mgf5ppm\ERLIC_3SubProteomes5ppm.mgf

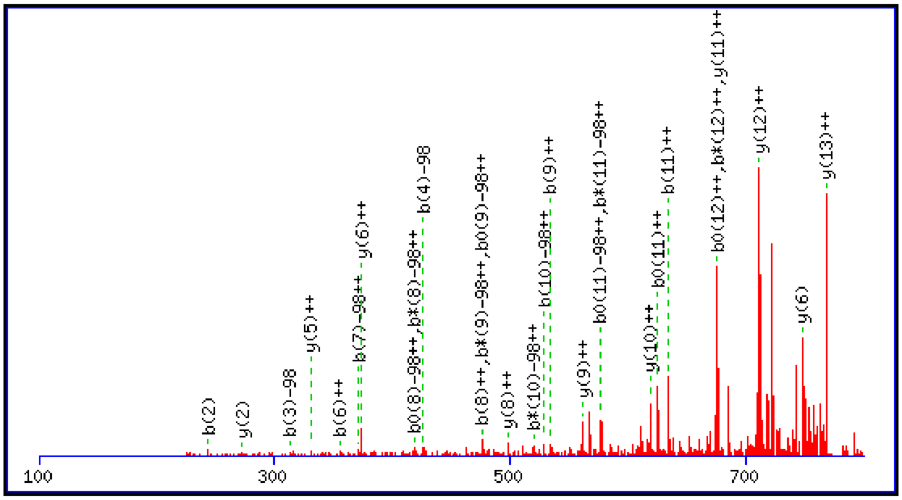

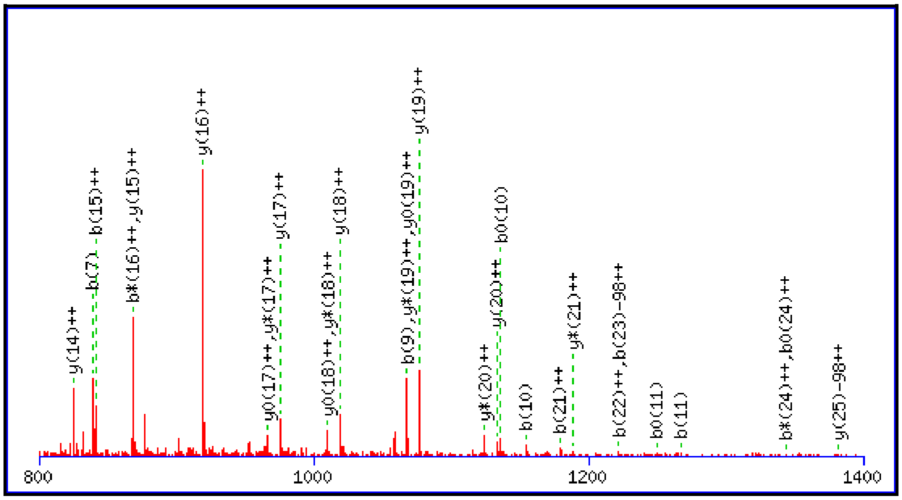

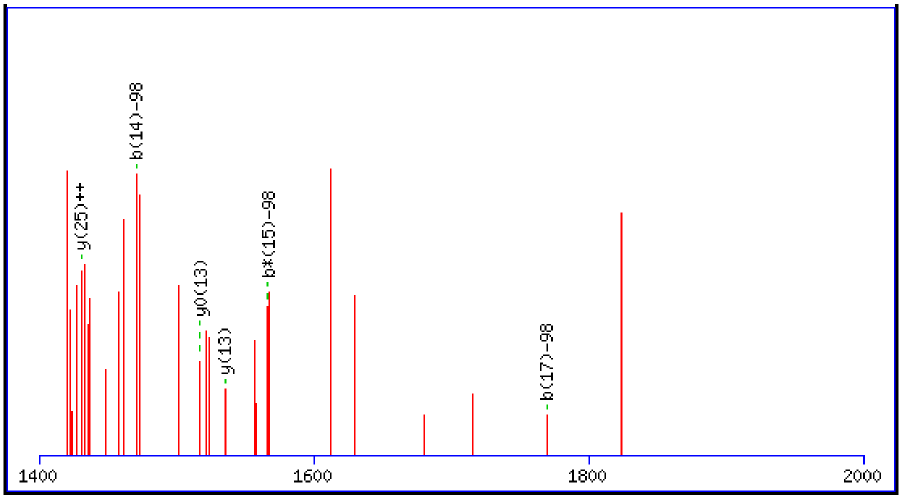

Monoisotopic mass of neutral peptide Mr(calc): 3102.4580
Fixed modifications: Carbamidomethyl (C)
Variable modifications:
S3 : Phospho (ST), with neutral losses 97.9769(shown in table), 0.0000
Ions Score: 77 **Expect:** 7.1e-006
Matches (Bold Red): 64/456 fragment ions using 90 most intense peaks

#	b	b^{++}	b*	b^{*++}	b^0	b^{0++}	Seq.	y	y^{++}	y*	y^{*++}	y^0	y^{0++}	#
1	157.1084	79.0578	140.0818	70.5446			R							27
2	**244.1404**	122.5738	227.1139	114.0606	226.1298	113.5686	S	2849.3873	1425.1973	2832.3608	1416.6840	2831.3768	1416.1920	26
3	**313.1619**	157.0846	296.1353	148.5713	295.1513	148.0793	S	2762.3553	**1381.6813**	2745.3287	1373.1680	2744.3447	1372.6760	25
4	**426.2459**	213.6266	409.2194	205.1133	408.2354	204.6213	L	2693.3338	1347.1706	2676.3073	1338.6573	2675.3233	1338.1653	24
5	541.2729	271.1401	524.2463	262.6268	523.2623	262.1348	D	2580.2498	1290.6285	2563.2232	1282.1153	2562.2392	1281.6232	23
6	612.3100	306.6586	595.2834	298.1454	594.2994	297.6534	A	2465.2228	1233.1151	2448.1963	1224.6018	2447.2123	1224.1098	22
7	741.3526	**371.1799**	724.3260	362.6667	723.3420	362.1746	E	2394.1857	1197.5965	2377.1592	**1189.0832**	2376.1752	1188.5912	21
8	854.4366	427.7220	837.4101	**419.2087**	836.4261	**418.7167**	I	2265.1431	**1133.0752**	2248.1166	**1124.5619**	2247.1326	1124.0699	20
9	969.4636	485.2354	952.4370	**476.7222**	951.4530	**476.2302**	D	2152.0591	**1076.5332**	2135.0325	**1068.0199**	2134.0485	**1067.5279**	19
10	1056.4956	**528.7514**	1039.4691	**520.2382**	1038.4851	519.7462	S	2037.0321	**1019.0197**	2020.0056	**1010.5064**	2019.0216	**1010.0144**	18
11	1169.5797	585.2935	1152.5531	**576.7802**	1151.5691	**576.2882**	L	1950.0001	**975.5037**	1932.9735	**966.9904**	1931.9895	**966.4984**	17
12	1270.6274	635.8173	1253.6008	627.3040	1252.6168	626.8120	T	1836.9160	**918.9617**	1819.8895	910.4484	1818.9055	909.9564	16
13	1357.6594	679.3333	1340.6328	670.8201	1339.6488	670.3281	S	1735.8683	**868.4378**	1718.8418	859.9245	1717.8578	859.4325	15
14	**1470.7435**	735.8754	1453.7169	727.3621	1452.7329	726.8701	I	1648.8363	**824.9218**	1631.8098	816.4085	1630.8258	815.9165	14
15	1583.8275	792.4174	**1566.8010**	783.9041	1565.8170	783.4121	L	**1535.7523**	**768.3798**	1518.7257	759.8665	**1517.7417**	759.3745	13
16	1654.8646	827.9360	1637.8381	819.4227	1636.8541	818.9307	A	1422.6682	**711.8377**	1405.6416	703.3245	1404.6576	702.8325	12
17	**1769.8916**	885.4494	1752.8650	876.9362	1751.8810	876.4441	D	1351.6311	**676.3192**	1334.6045	667.8059	1333.6205	667.3139	11
18	1882.9756	941.9915	1865.9491	933.4782	1864.9651	932.9862	L	1236.6041	**618.8057**	1219.5776	610.2924	1218.5936	609.8004	10
19	2012.0182	1006.5128	1994.9917	997.9995	1994.0077	997.5075	E	1123.5201	**562.2637**	1106.4935	553.7504	1105.5095	553.2584	9
20	2172.0489	1086.5281	2155.0223	1078.0148	2154.0383	1077.5228	C	994.4775	**497.7424**	977.4509	489.2291	976.4669	488.7371	8
21	2259.0809	1130.0441	2242.0544	1121.5308	2241.0703	1121.0388	S	834.4468	417.7271	817.4203	409.2138	816.4363	408.7218	7
22	2346.1129	1173.5601	2329.0864	1165.0468	2328.1024	1164.5548	S	**747.4148**	**374.2110**	730.3883	365.6978	729.4042	365.2058	6
23	2443.1657	**1222.0865**	2426.1392	1213.5732	2425.1551	1213.0812	P	660.3828	**330.6950**	643.3562	322.1817			5
24	2606.2290	1303.6182	2589.2025	1295.1049	2588.2185	1294.6129	Y	563.3300	282.1686	546.3035	273.6554			4
25	2734.3240	1367.6656	2717.2974	1359.1524	2716.3134	1358.6604	K	400.2667	200.6370	383.2401	192.1237			3
26	2831.3768	1416.1920	2814.3502	1407.6787	2813.3662	1407.1867	P	**272.1717**	136.5895	255.1452	128.0762			2
27							R	175.1190	88.0631	158.0924	79.5498			1

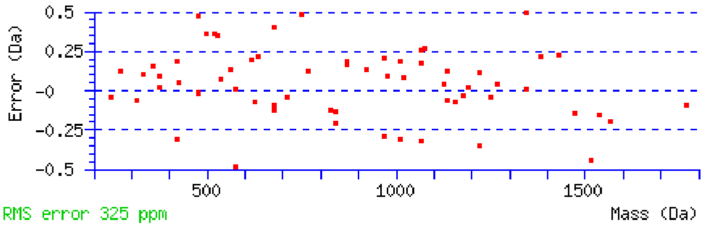

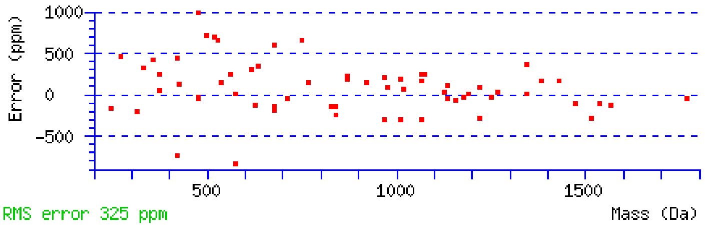

All matches to this query

Score	Mr(calc):	Delta	Sequence
76.8	3102.4580	0.0078	RSSLDAEIDSLTSILADLECSSPYKPR
74.1	3102.4580	0.0078	RSSLDAEIDSLTSILADLECSSPYKPR
66.4	3102.4580	0.0078	RSSLDAEIDSLTSILADLECSSPYKPR
55.5	3102.4580	0.0078	RSSLDAEIDSLTSILADLECSSPYKPR
45.9	3102.4580	0.0078	RSSLDAEIDSLTSILADLECSSPYKPR
5.8	3100.4723	1.9935	STTPKAHLPLGQSNSCGSVLPHSLATMTK
5.6	3100.4301	2.0357	TPLRTSTGGISTLGLQELKEQSPCPNR
5.0	3101.4335	1.0323	EASQEGSVCHSGHSGVMLISLAPGEPYFR
4.3	3100.4852	1.9806	KALATSVLTGEAGALPSTHVVVAGMTKCR
4.2	3102.4514	0.0144	LLLYSNRLMSLDSGLLANLGALTELR

Spectrum No: 61; Query: 2501; Rank: 1

Peptide View

MS/MS Fragmentation of **YEVAAQNEADEADGSAQGDGAGPAAEQVK**
Found in **IPI00470251**, Tax_Id=10116 Gene_Symbol=Slc7a7 Y+L amino acid transporter 1

Match to Query 2501: 2927.211642 from(976.744490,3+)
Title: 091008RatKidney_NoSalt_28.1570.1570.3.dta
Data file K:\NewmanPaper\Piliang\3SubProteomes\Piliang3SP\mgf5ppm\ERLIC_3SubProteomes5ppm.mgf

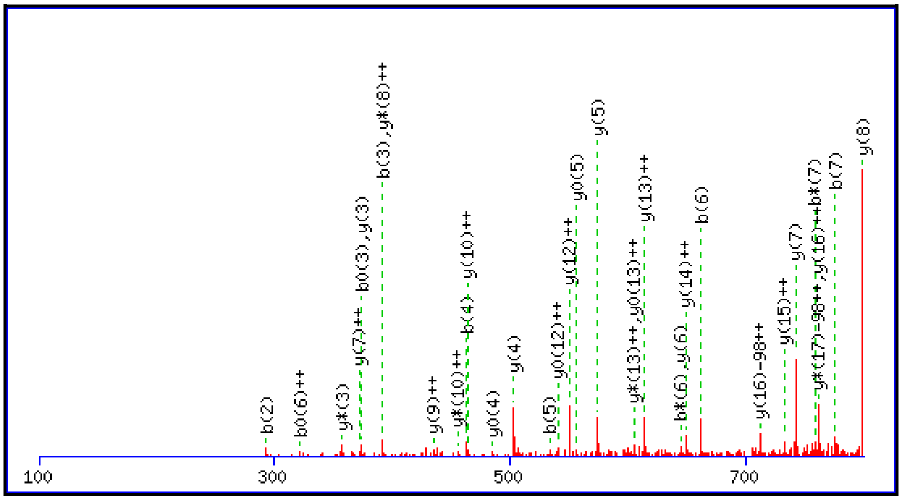

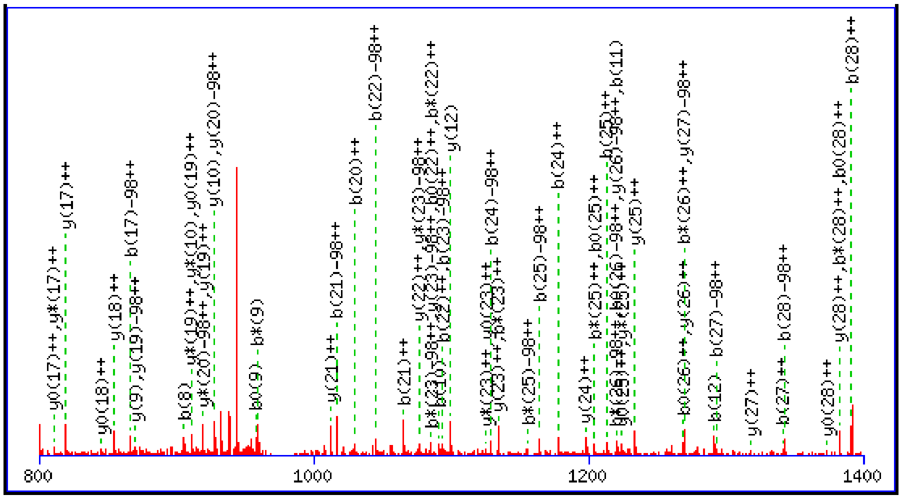

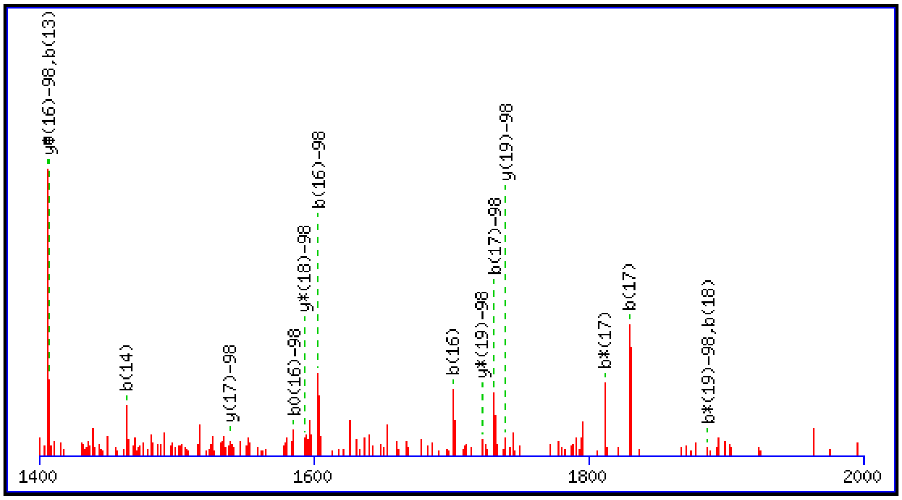

Monoisotopic mass of neutral peptide Mr(calc): 2927.2094
Fixed modifications: Carbamidomethyl (C)
Variable modifications:
S15 : Phospho (ST), with neutral losses 0.0000(shown in table), 97.9769
Ions Score: 77 **Expect:** 7.2e-006
Matches (Bold Red): 116/486 fragment ions using 173 most intense peaks

#	b	b^{++}	b*	b^{*++}	b^0	b^{0++}	Seq.	y	y^{++}	y*	y^{*++}	y^0	y^{0++}	#
1	164.0706	82.5389					Y							29
2	**293.1132**	147.0602			275.1026	138.0550	E	2765.1534	**1383.0803**	2748.1268	1374.5671	2747.1428	**1374.0750**	28
3	**392.1816**	196.5944			**374.1710**	187.5892	V	2636.1108	**1318.5590**	2619.0842	1310.0458	2618.1002	1309.5537	27
4	**463.2187**	232.1130			445.2082	223.1077	A	2537.0424	**1269.0248**	2520.0158	1260.5115	2519.0318	1260.0195	26
5	**534.2558**	267.6316			516.2453	258.6263	A	2466.0053	**1233.5063**	2448.9787	**1224.9930**	2447.9947	**1224.5010**	25
6	**662.3144**	331.6608	**645.2879**	323.1476	644.3039	**322.6556**	Q	2394.9681	**1197.9877**	2377.9416	1189.4744	2376.9576	1188.9824	24
7	**776.3573**	388.6823	**759.3308**	380.1690	758.3468	379.6770	N	2266.9096	**1133.9584**	2249.8830	**1125.4451**	2248.8990	**1124.9531**	23
8	**905.3999**	453.2036	888.3734	444.6903	887.3894	444.1983	E	2152.8666	**1076.9370**	2135.8401	1068.4237	2134.8561	1067.9317	22
9	976.4371	488.7222	**959.4105**	480.2089	**958.4265**	479.7169	A	2023.8240	**1012.4157**	2006.7975	1003.9024	2005.8135	1003.4104	21
10	**1091.4640**	546.2356	1074.4374	537.7224	1073.4534	537.2304	D	1952.7869	976.8971	1935.7604	968.3838	1934.7764	967.8918	20
11	**1220.5066**	610.7569	1203.4800	602.2437	1202.4960	601.7516	E	1837.7600	**919.3836**	1820.7334	**910.8704**	1819.7494	**910.3783**	19
12	**1291.5437**	646.2755	1274.5172	637.7622	1273.5331	637.2702	A	1708.7174	**854.8623**	1691.6908	846.3491	1690.7068	**845.8571**	18
13	**1406.5706**	703.7890	1389.5441	695.2757	1388.5601	694.7837	D	1637.6803	**819.3438**	1620.6537	**810.8305**	1619.6697	**810.3385**	17
14	**1463.5921**	732.2997	1446.5656	723.7864	1445.5815	723.2944	G	1522.6533	**761.8303**	1505.6268	753.3170	1504.6428	752.8250	16
15	1630.5905	815.7989	1613.5639	807.2856	1612.5799	806.7936	S	1465.6319	**733.3196**	1448.6053	724.8063	1447.6213	724.3143	15
16	**1701.6276**	851.3174	1684.6010	842.8042	1683.6170	842.3121	A	1298.6335	**649.8204**	1281.6070	641.3071	1280.6230	640.8151	14
17	**1829.6862**	915.3467	**1812.6596**	906.8334	1811.6756	906.3414	Q	1227.5964	**614.3018**	1210.5699	**605.7886**	1209.5858	**605.2966**	13
18	**1886.7076**	943.8574	1869.6811	935.3442	1868.6971	934.8522	G	**1099.5378**	**550.2726**	1082.5113	541.7593	1081.5273	**541.2673**	12

19	2001.7346	1001.3709	1984.7080	992.8576	1983.7240	992.3656	D	1042.5164	521.7618	1025.4898	513.2485	1024.5058	512.7565	11
20	2058.7560	**1029.8816**	2041.7295	1021.3684	2040.7455	1020.8764	G	**927.4894**	**464.2483**	**910.4629**	**455.7351**	909.4789	455.2431	10
21	2129.7931	**1065.4002**	2112.7666	1056.8869	2111.7826	1056.3949	A	**870.4680**	**435.7376**	853.4414	427.2243	852.4574	426.7323	9
22	2186.8146	**1093.9109**	2169.7881	**1085.3977**	2168.8040	**1084.9057**	G	**799.4308**	400.2191	782.4043	**391.7058**	781.4203	391.2138	8
23	2283.8674	1142.4373	2266.8408	**1133.9240**	2265.8568	1133.4320	P	**742.4094**	**371.7083**	725.3828	363.1951	724.3988	362.7030	7
24	2354.9045	**1177.9559**	2337.8779	1169.4426	2336.8939	1168.9506	A	**645.3566**	323.1819	628.3301	314.6687	627.3461	314.1767	6
25	2425.9416	**1213.4744**	2408.9150	**1204.9612**	2407.9310	**1204.4692**	A	**574.3195**	287.6634	557.2930	279.1501	**556.3089**	278.6581	5
26	2554.9842	1277.9957	2537.9576	**1269.4825**	2536.9736	**1268.9904**	E	**503.2824**	252.1448	486.2558	243.6316	**485.2718**	243.1396	4
27	2683.0428	**1342.0250**	2666.0162	1333.5117	2665.0322	1333.0197	Q	**374.2398**	187.6235	**357.2132**	179.1103			3
28	2782.1112	**1391.5592**	2765.0846	**1383.0460**	2764.1006	**1382.5539**	V	246.1812	123.5942	229.1547	115.0810			2
29							K	147.1128	74.0600	130.0863	65.5468			1

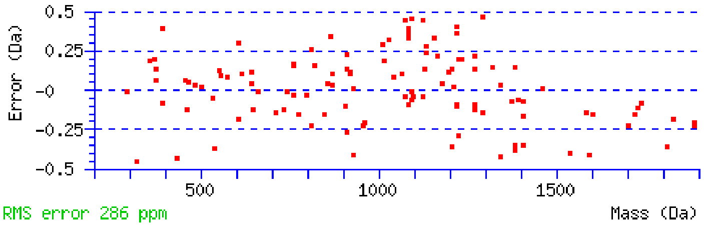

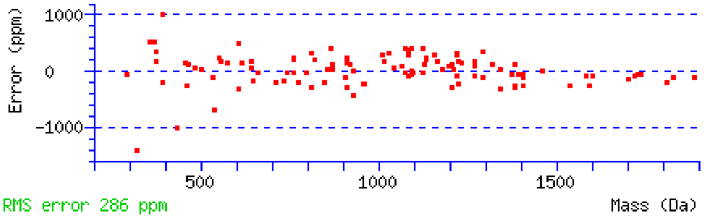

All matches to this query

Score	Mr(calc):	Delta	Sequence
76.6	2927.2094	0.0022	YEVAAQNEADEADGSAQGDGAGPAAEQVK
19.0	2927.2094	0.0022	YEVAAQNEADEADGSAQGDGAGPAAEQVK
5.5	2927.2224	-0.0108	DLCLVENSISFYDHLANLEGSLEK
2.6	2926.1994	1.0123	MAEAEATQLKEEGNQHFQRQDYK
2.2	2927.1834	0.0283	MAEAEATQLKEEGNQHFQRQDYK
1.4	2927.2349	-0.0233	SGDGGPLSAGAAGSSPGAGGGWAAARPGSQTR
1.4	2927.2349	-0.0233	SGDGGPLSAGAAGSSPGAGGGWAAARPGSQTR
1.4	2927.2349	-0.0233	SGDGGPLSAGAAGSSPGAGGGWAAARPGSQTR
1.4	2927.2349	-0.0233	SGDGGPLSAGAAGSSPGAGGGWAAARPGSQTR
1.1	2926.2050	1.0066	SEACGYPVIIQKYKPYSNGAANQK

Spectrum No: 62; Query: 1352; Rank: 1

Peptide View

MS/MS Fragmentation of **SSQDSDTPAQASPPEEK**
Found in **IPI00400617**, Tax_Id=10116 Gene_Symbol=Arhgap27 Rho GTPase activating protein 27

Match to Query 1352: 1852.732888 from(927.373720,2+)
Title: 091008RatKidney_NoSalt_28.551.551.2.dta
Data file K:\NewmanPaper\Piliang\3SubProteomes\Piliang3SP\mgf5ppm\ERLIC_3SubProteomes5ppm.mgf

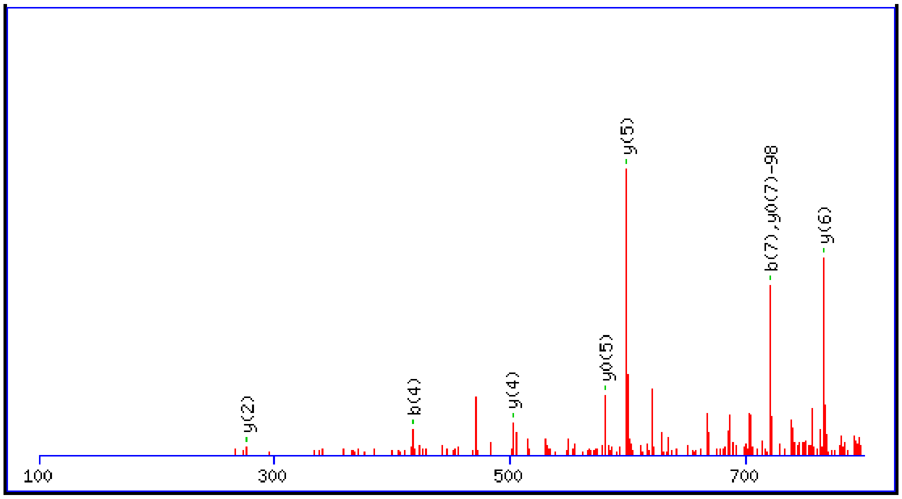

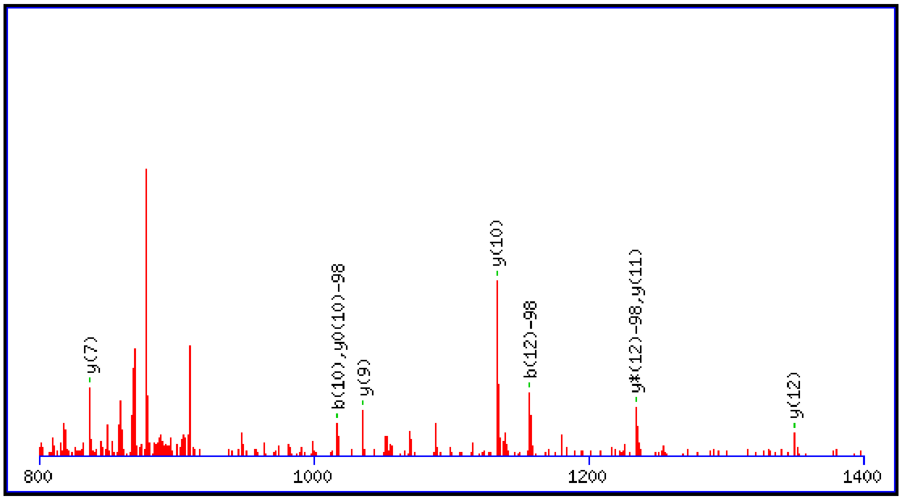

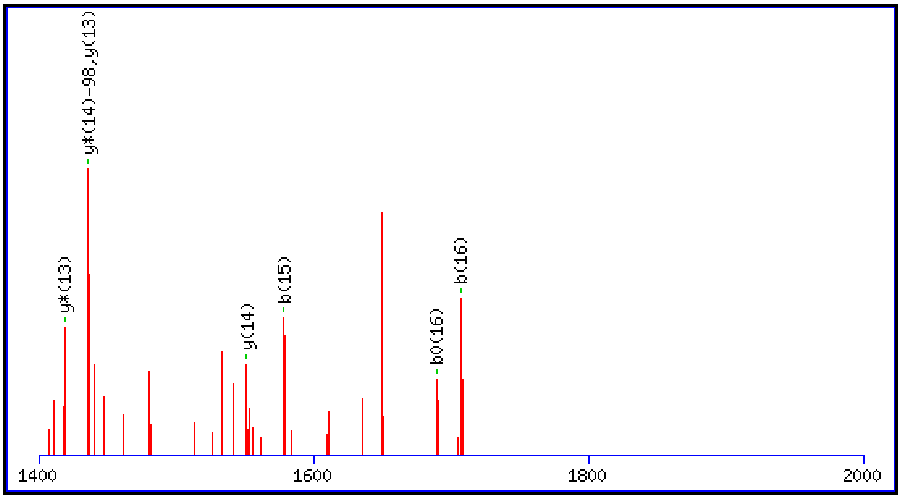

Monoisotopic mass of neutral peptide Mr(calc): 1852.7364
Fixed modifications: Carbamidomethyl (C)
Variable modifications:
S12 : Phospho (ST), with neutral losses 0.0000(shown in table), 97.9769
Ions Score: 76 **Expect:** 3.9e-006
Matches (Bold Red): 24/282 fragment ions using 36 most intense peaks

#	b	b^{++}	b*	b^{*++}	b^0	b^{0++}	Seq.	y	y^{++}	y*	y^{*++}	y^0	y^{0++}	#
1	88.0393	44.5233			70.0287	35.5180	S							17
2	175.0713	88.0393			157.0608	79.0340	S	1766.7116	883.8595	1749.6851	875.3462	1748.7011	874.8542	16
3	303.1299	152.0686	286.1034	143.5553	285.1193	143.0633	Q	1679.6796	840.3434	1662.6531	831.8302	1661.6690	831.3382	15
4	**418.1569**	209.5821	401.1303	201.0688	400.1463	200.5768	D	**1551.6210**	776.3142	1534.5945	767.8009	1533.6105	767.3089	14
5	505.1889	253.0981	488.1623	244.5848	487.1783	244.0928	S	**1436.5941**	718.8007	**1419.5675**	710.2874	1418.5835	709.7954	13

6	620.2158	310.6116	603.1893	302.0983	602.2053	301.6063	D	**1349.5621**	675.2847	1332.5355	666.7714	1331.5515	666.2794	12
7	**721.2635**	361.1354	704.2370	352.6221	703.2529	352.1301	T	**1234.5351**	617.7712	1217.5086	609.2579	1216.5246	608.7659	11
8	818.3163	409.6618	801.2897	401.1485	800.3057	400.6565	P	**1133.4874**	567.2474	1116.4609	558.7341	1115.4769	558.2421	10
9	889.3534	445.1803	872.3268	436.6671	871.3428	436.1750	A	**1036.4347**	518.7210	1019.4081	510.2077	1018.4241	509.7157	9
10	**1017.4120**	509.2096	1000.3854	500.6963	999.4014	500.2043	Q	965.3976	483.2024	948.3710	474.6891	947.3870	474.1971	8
11	1088.4491	544.7282	1071.4225	536.2149	1070.4385	535.7229	A	**837.3390**	419.1731	820.3124	410.6599	819.3284	410.1678	7
12	1255.4474	628.2274	1238.4209	619.7141	1237.4369	619.2221	S	**766.3019**	383.6546	749.2753	375.1413	748.2913	374.6493	6
13	1352.5002	676.7537	1335.4736	668.2405	1334.4896	667.7485	P	**599.3035**	300.1554	582.2770	291.6421	**581.2930**	291.1501	5
14	1449.5530	725.2801	1432.5264	716.7668	1431.5424	716.2748	P	**502.2508**	251.6290	485.2242	243.1157	484.2402	242.6237	4
15	**1578.5955**	789.8014	1561.5690	781.2881	1560.5850	780.7961	E	405.1980	203.1026	388.1714	194.5894	387.1874	194.0974	3
16	**1707.6381**	854.3227	1690.6116	845.8094	**1689.6276**	845.3174	E	**276.1554**	138.5813	259.1288	130.0681	258.1448	129.5761	2
17							K	147.1128	74.0600	130.0863	65.5468			1

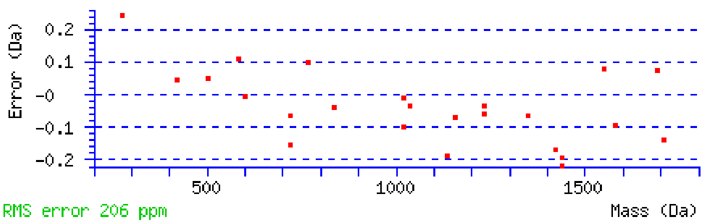

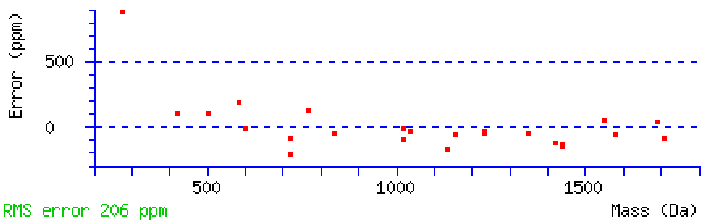

All matches to this query

Score	Mr(calc):	Delta	Sequence
76.3	1852.7364	-0.0035	SSQDSDTPAQASPPEEK
36.2	1852.7364	-0.0035	SSQDSDTPAQASPPEEK
17.0	1852.7364	-0.0035	SSQDSDTPAQASPPEEK
9.9	1852.7364	-0.0035	SSQDSDTPAQASPPEEK
9.3	1852.7364	-0.0035	SSQDSDTPAQASPPEEK
2.4	1852.7182	0.0147	YGEKYASNYSGGNKR
2.2	1852.7491	-0.0162	GMAYTGKYFDQASYR
0.9	1852.7491	-0.0162	GMAYTGKYFDQASYR

Spectrum No: 63; Query: 1286; Rank: 1

Peptide View

MS/MS Fragmentation of **SGSGSPPPTTEEVAATTR**
Found in **IPI00201120**, Tax_Id=10116 Gene_Symbol=Slc5a2 Low affinity Na-dependent glucose transporter SGLT2 delta e trans

Match to Query 1286: 1823.789568 from(912.902060,2+)
Title: 091008RatKidney_NoSalt_23.905.905.2.dta
Data file K:\NewmanPaper\Piliang\3SubProteomes\Piliang3SP\mgf5ppm\ERLIC_3SubProteomes5ppm.mgf

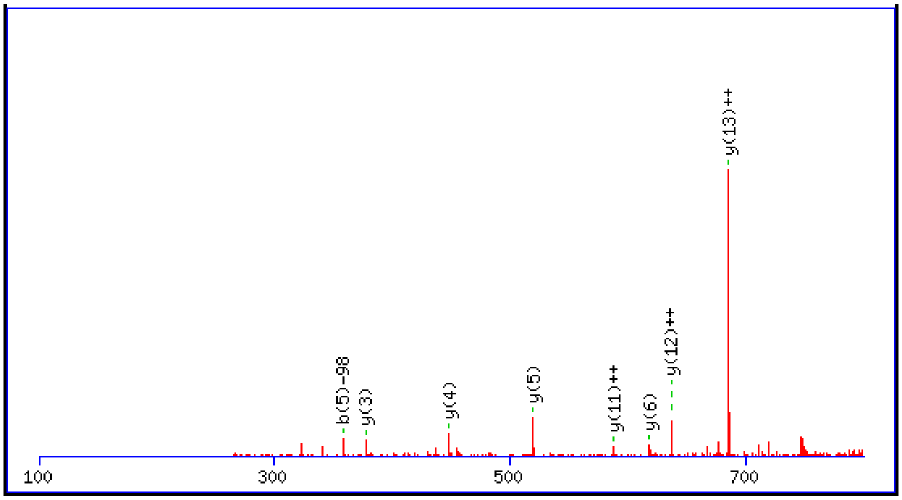

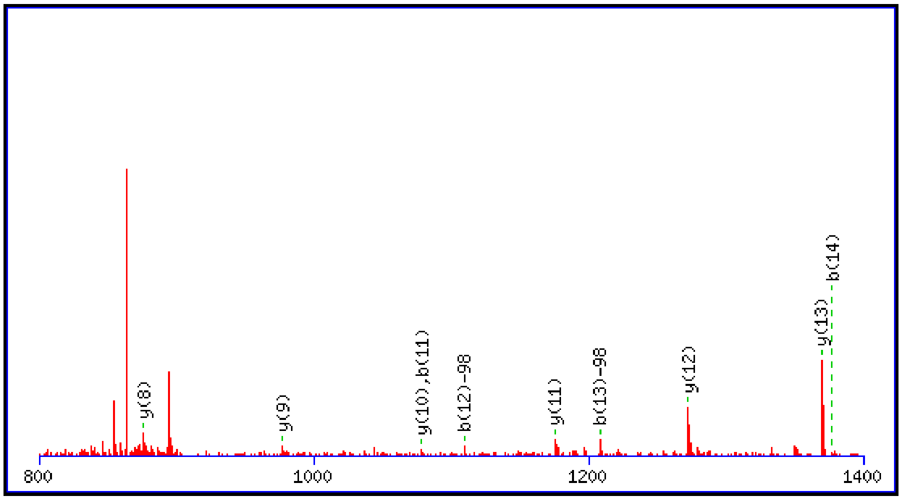

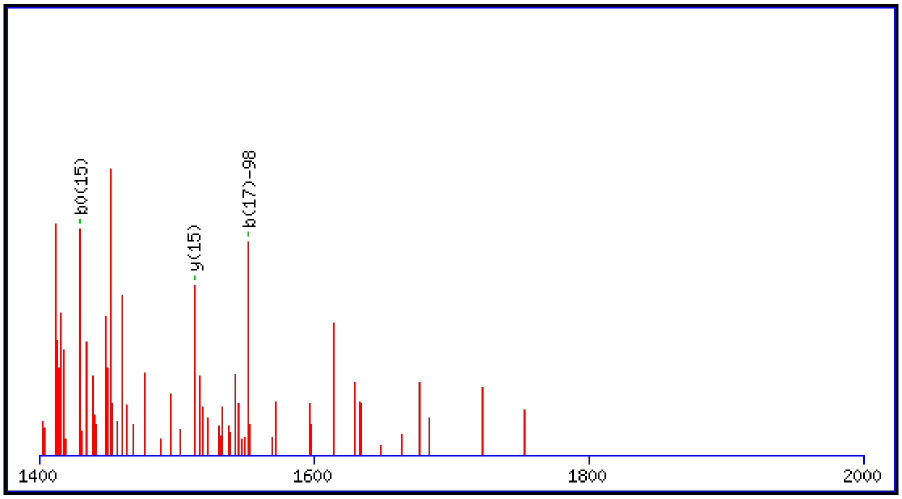

Monoisotopic mass of neutral peptide Mr(calc): 1823.7939
Fixed modifications: Carbamidomethyl (C)
Variable modifications:
S3 : Phospho (ST), with neutral losses 97.9769(shown in table), 0.0000
Ions Score: 76 **Expect:** 5.2e-006
Matches (Bold Red): 21/240 fragment ions using 40 most intense peaks

#	b	b^{++}	b^{0}	b^{0++}	Seq.	y	y^{++}	y*	y^{*++}	y^{0}	y^{0++}	#
1	88.0393	44.5233	70.0287	35.5180	S							18
2	145.0608	73.0340	127.0502	64.0287	G	1639.7922	820.3997	1622.7657	811.8865	1621.7816	811.3945	17
3	214.0822	107.5448	196.0717	98.5395	S	1582.7707	791.8890	1565.7442	783.3757	1564.7602	782.8837	16
4	271.1037	136.0555	253.0931	127.0502	G	**1513.7493**	757.3783	1496.7227	748.8650	1495.7387	748.3730	15
5	**358.1357**	179.5715	340.1252	170.5662	S	1456.7278	728.8675	1439.7013	720.3543	1438.7173	719.8623	14

6	455.1885	228.0979	437.1779	219.0926	P	**1369.6958**	**685.3515**	1352.6692	676.8383	1351.6852	676.3462	13
7	552.2412	276.6243	534.2307	267.6190	P	**1272.6430**	**636.8252**	1255.6165	628.3119	1254.6325	627.8199	12
8	649.2940	325.1506	631.2834	316.1454	P	**1175.5903**	**588.2988**	1158.5637	579.7855	1157.5797	579.2935	11
9	750.3417	375.6745	732.3311	366.6692	T	**1078.5375**	539.7724	1061.5109	531.2591	1060.5269	530.7671	10
10	851.3894	426.1983	833.3788	417.1930	T	**977.4898**	489.2485	960.4633	480.7353	959.4793	480.2433	9
11	980.4320	490.7196	962.4214	481.7143	E	**876.4421**	438.7247	859.4156	430.2114	858.4316	429.7194	8
12	**1109.4746**	555.2409	1091.4640	546.2356	E	747.3995	374.2034	730.3730	365.6901	729.3890	365.1981	7
13	**1208.5430**	604.7751	1190.5324	595.7698	V	**618.3570**	309.6821	601.3304	301.1688	600.3464	300.6768	6
14	1279.5801	640.2937	1261.5695	631.2884	A	**519.2885**	260.1479	502.2620	251.6346	501.2780	251.1426	5
15	1350.6172	675.8122	1332.6066	666.8070	A	**448.2514**	224.6293	431.2249	216.1161	430.2409	215.6241	4
16	1451.6649	726.3361	1433.6543	717.3308	T	**377.2143**	189.1108	360.1878	180.5975	359.2037	180.1055	3
17	**1552.7126**	776.8599	1534.7020	767.8546	T	276.1666	138.5870	259.1401	130.0737	258.1561	129.5817	2
18					R	175.1190	88.0631	158.0924	79.5498			1

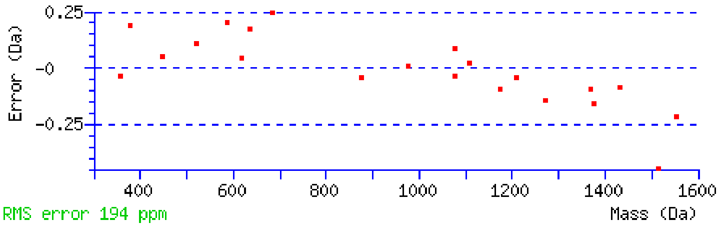

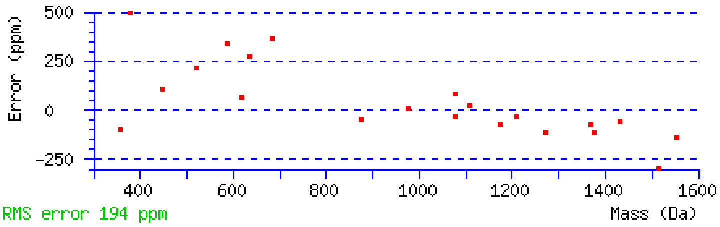

All matches to this query

Score	Mr(calc):	Delta	Sequence
76.0	1823.7939	-0.0043	SGSGSPPPTTEEVAATTR
76.0	1823.7939	-0.0043	SGSGSPPPTTEEVAATTR
63.2	1823.7939	-0.0043	SGSGSPPPTTEEVAATTR
23.2	1823.7939	-0.0043	SGSGSPPPTTEEVAATTR
17.5	1823.7939	-0.0043	SGSGSPPPTTEEVAATTR
13.5	1823.7896	-0.0000	NVLDTSWPTPPPALR
11.6	1823.7882	0.0014	MIISAHPNLMSCNTR
11.6	1823.7882	0.0014	MIISAHPNLMSCNTR
7.6	1823.7896	-0.0000	NVLDTSWPTPPPALR
7.6	1823.7896	-0.0000	NVLDTSWPTPPPALR

Spectrum No: 64; Query: 1922; Rank: 1

Peptide View

MS/MS Fragmentation of **IQQFDDGGSDEEDIWEEK**
Found in **IPI00766645**, Tax_Id=10116 Gene_Symbol=Saps3_predicted similar to SAPS domain family, member 3

Match to Query 1922: 2218.868448 from(1110.441500,2+)
Title: 100101RatKid_NS_deglyco_25.3292.3292.2.dta
Data file K:\NewmanPaper\Piliang\3SubProteomes\Piliang3SP\mgf5ppm\ERLIC_3SubProteomes5ppm.mgf

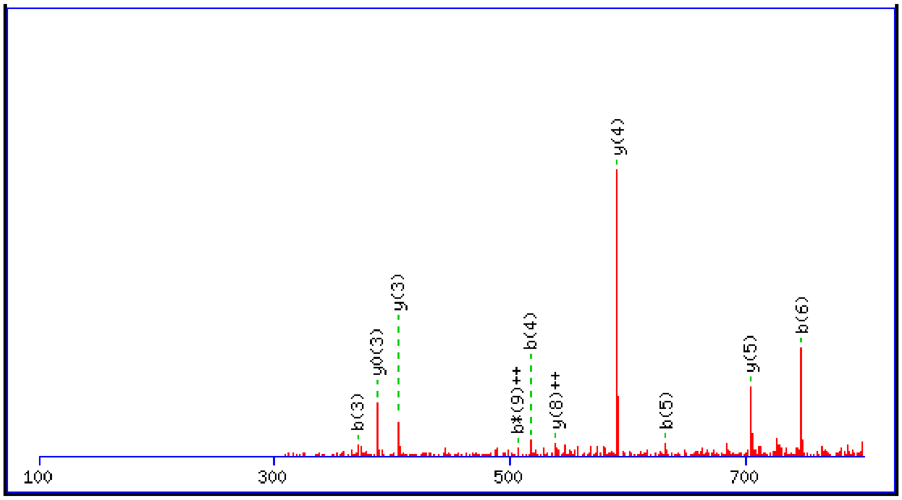

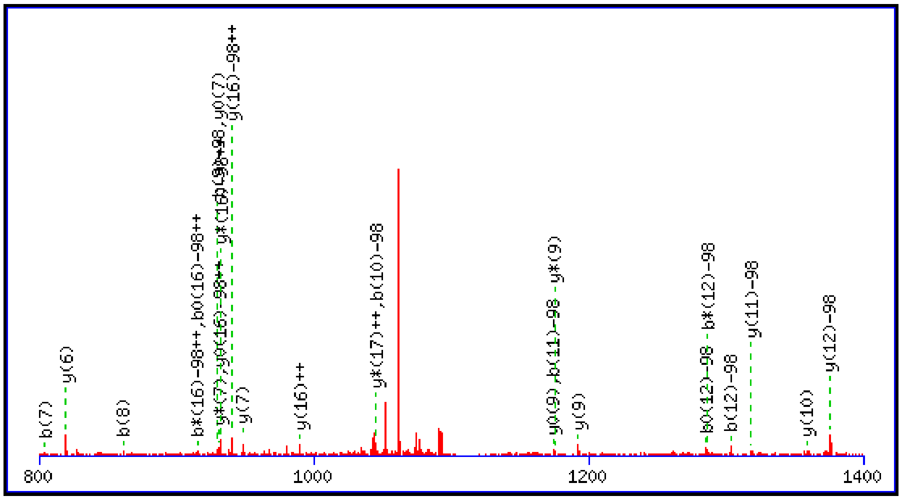

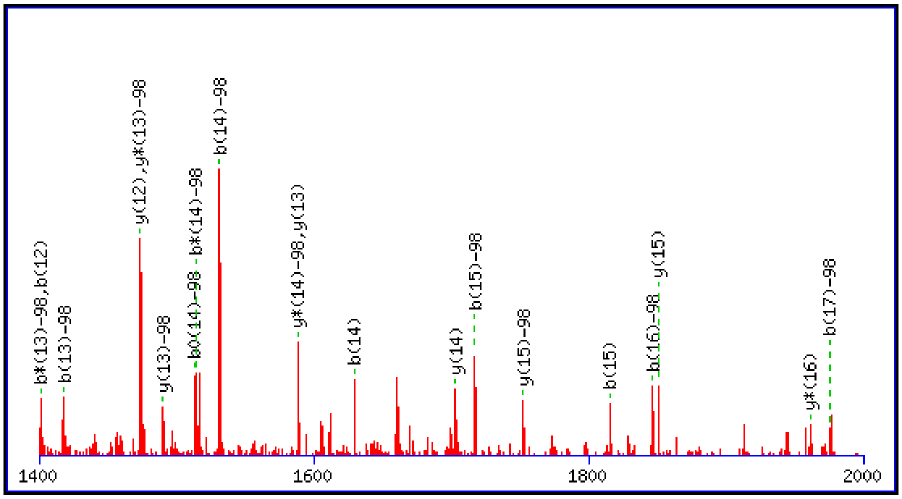

Monoisotopic mass of neutral peptide Mr(calc): 2218.8580
Fixed modifications: Carbamidomethyl (C)
Variable modifications:
S9 : Phospho (ST), with neutral losses 97.9769(shown in table), 0.0000
Ions Score: 76 **Expect:** 5e-006
Matches (Bold Red): 55/294 fragment ions using 80 most intense peaks

#	b	b^{++}	b*	b^{*++}	b^0	b^{0++}	Seq.	y	y^{++}	y*	y^{*++}	y^0	y^{0++}	#
1	114.0913	57.5493					I							18
2	242.1499	121.5786	225.1234	113.0653			Q	2008.8043	1004.9058	1991.7777	996.3925	1990.7937	995.9005	17
3	**370.2085**	185.6079	353.1819	177.0946			Q	1880.7457	**940.8765**	1863.7192	**932.3632**	1862.7351	**931.8712**	16
4	**517.2769**	259.1421	500.2504	250.6288			F	**1752.6871**	876.8472	1735.6606	868.3339	1734.6766	867.8419	15
5	**632.3039**	316.6556	615.2773	308.1423	614.2933	307.6503	D	1605.6187	803.3130	**1588.5922**	794.7997	1587.6081	794.3077	14

6	747.3308	374.1690	730.3042	365.6558	729.3202	365.1638	D	1490.5918	745.7995	1473.5652	737.2862	1472.5812	736.7942	13
7	804.3523	402.6798	787.3257	394.1665	786.3417	393.6745	G	1375.5648	688.2860	1358.5383	679.7728	1357.5543	679.2808	12
8	861.3737	431.1905	844.3472	422.6772	843.3632	422.1852	G	1318.5434	659.7753	1301.5168	651.2620	1300.5328	650.7700	11
9	930.3952	465.7012	913.3686	457.1880	912.3846	456.6959	S	1261.5219	631.2646	1244.4953	622.7513	1243.5113	622.2593	10
10	1045.4221	523.2147	1028.3956	514.7014	1027.4116	514.2094	D	1192.5004	596.7539	1175.4739	588.2406	1174.4899	587.7486	9
11	1174.4647	587.7360	1157.4382	579.2227	1156.4542	578.7307	E	1077.4735	539.2404	1060.4469	530.7271	1059.4629	530.2351	8
12	1303.5073	652.2573	1286.4808	643.7440	1285.4967	643.2520	E	948.4309	474.7191	931.4044	466.2058	930.4203	465.7138	7
13	1418.5343	709.7708	1401.5077	701.2575	1400.5237	700.7655	D	819.3883	410.1978	802.3618	401.6845	801.3777	401.1925	6
14	1531.6183	766.3128	1514.5918	757.7995	1513.6078	757.3075	I	704.3614	352.6843	687.3348	344.1710	686.3508	343.6790	5
15	1717.6976	859.3525	1700.6711	850.8392	1699.6871	850.3472	W	591.2773	296.1423	574.2508	287.6290	573.2667	287.1370	4
16	1846.7402	923.8737	1829.7137	915.3605	1828.7297	914.8685	E	405.1980	203.1026	388.1714	194.5894	387.1874	194.0974	3
17	1975.7828	988.3950	1958.7563	979.8818	1957.7723	979.3898	E	276.1554	138.5813	259.1288	130.0681	258.1448	129.5761	2
18							K	147.1128	74.0600	130.0863	65.5468			1

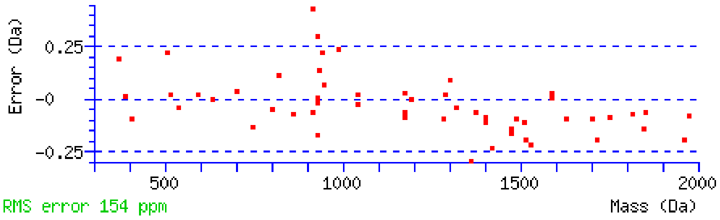

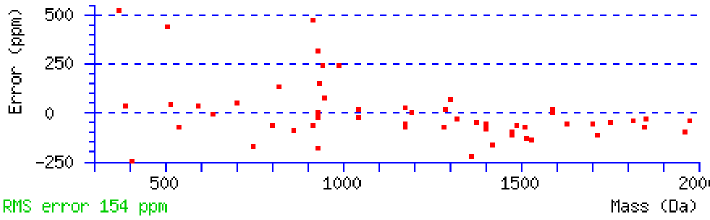

All matches to this query

Score	Mr(calc):	Delta	Sequence
75.5	2218.8580	0.0105	IQQFDDGGSDEEDIWEEK
1.6	2218.8697	-0.0012	IWTAHYDPNHCFIETR
1.5	2218.8749	-0.0064	AEFDEASDVYSVVWDTVK
1.5	2218.8749	-0.0064	AEFDEASDVYSVVWDTVK
0.8	2217.8762	0.9923	KSSGISEIKTEALSPHSK
0.8	2217.8762	0.9923	KSSGISEIKTEALSPHSK
0.5	2217.8762	0.9923	KSSGISEIKTEALSPHSK
0.5	2217.8762	0.9923	KSSGISEIKTEALSPHSK
0.3	2218.8797	-0.0112	SLPNLKKTLSGDSSSDSSR
0.2	2218.8878	-0.0193	SIDEMNNAWENLNRTWK

Spectrum No: 65; Query: 1626; Rank: 1

Peptide View

MS/MS Fragmentation of **GDSETDLEALFNAVMNPK**
Found in **IPI00188053**, Tax_Id=10116 Gene_Symbol=Yap1 Yes-associated protein

Match to Query 1626: 2029.873128 from(1015.943840,2+)
Title: 091008RatKidney_NoSalt_22.5986.5986.2.dta
Data file K:\NewmanPaper\Piliang\3SubProteomes\Piliang3SP\mgf5ppm\ERLIC_3SubProteomes5ppm.mgf

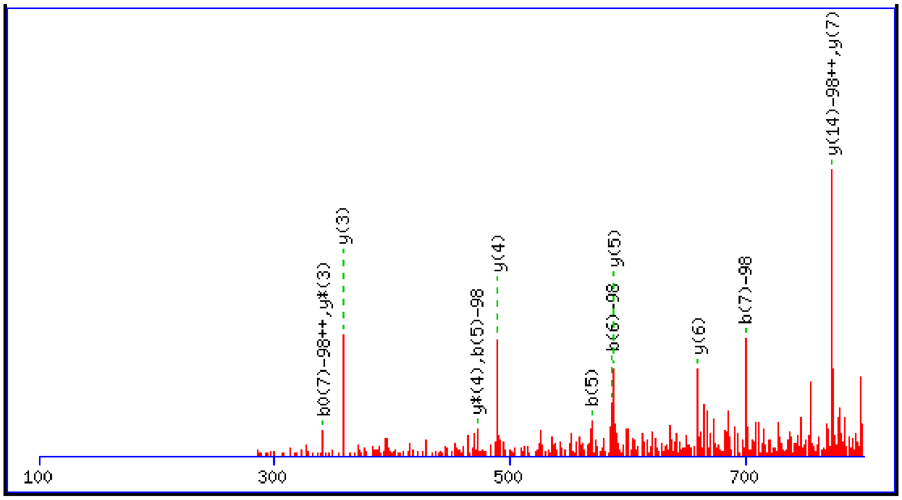

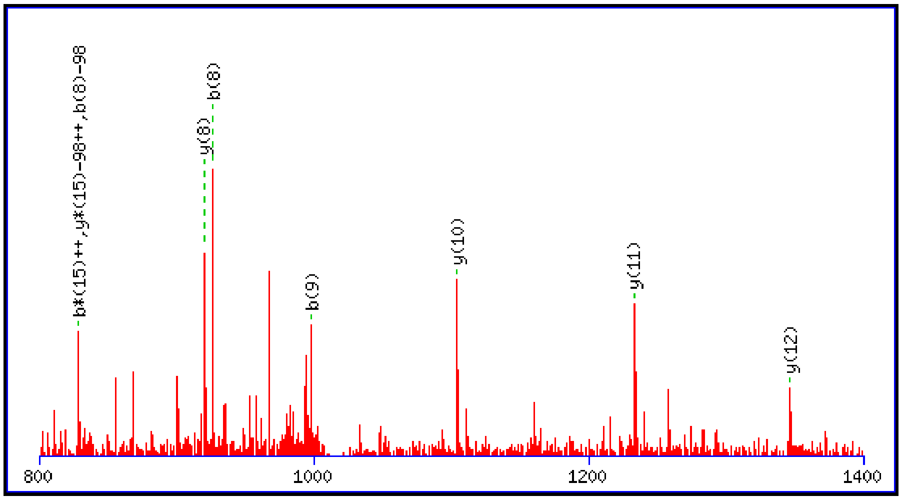

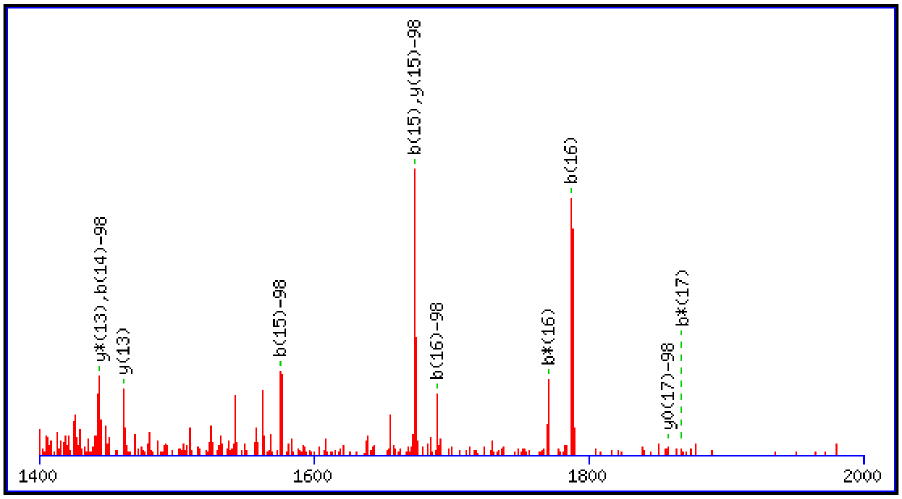

Monoisotopic mass of neutral peptide Mr(calc): 2029.8704
Fixed modifications: Carbamidomethyl (C)
Variable modifications:
T5 : Phospho (ST), with neutral losses 97.9769(shown in table), 0.0000
Ions Score: 75 **Expect:** 7.1e-006
Matches (Bold Red): 33/248 fragment ions using 47 most intense peaks

#	b	b++	b*	b*++	b0	b0++	Seq.	y	y++	y*	y*++	y0	y0++	#
1	58.0287	29.5180					G							18
2	173.0557	87.0315			155.0451	78.0262	D	1875.8793	938.4433	1858.8527	929.9300	**1857.8687**	929.4380	17
3	260.0877	130.5475			242.0771	121.5422	S	1760.8524	880.9298	1743.8258	872.4165	1742.8418	871.9245	16
4	389.1303	195.0688			371.1197	186.0635	E	**1673.8203**	837.4138	1656.7938	**828.9005**	1655.8098	828.4085	15
5	**472.1674**	236.5873			454.1568	227.5821	T	1544.7777	**772.8925**	1527.7512	764.3792	1526.7672	763.8872	14

**6**	**587.1944**	294.1008			569.1838	285.0955	**D**	**1461.7406**	731.3740	**1444.7141**	722.8607	1443.7301	722.3687	**13**
**7**	**700.2784**	350.6428			682.2679	**341.6376**	**L**	**1346.7137**	673.8605	1329.6871	665.3472	1328.7031	664.8552	**12**
**8**	**829.3210**	415.1641			811.3104	406.1589	**E**	**1233.6296**	617.3184	1216.6031	608.8052	1215.6191	608.3132	**11**
**9**	900.3581	450.6827			882.3476	441.6774	**A**	**1104.5870**	552.7972	1087.5605	544.2839			**10**
**10**	1013.4422	507.2247			995.4316	498.2194	**L**	1033.5499	517.2786	1016.5234	508.7653			**9**
**11**	1160.5106	580.7589			1142.5000	571.7537	**F**	**920.4658**	460.7366	903.4393	452.2233			**8**
**12**	1274.5535	637.7804	1257.5270	629.2671	1256.5430	628.7751	**N**	**773.3974**	387.2024	756.3709	378.6891			**7**
**13**	1345.5906	673.2990	1328.5641	664.7857	1327.5801	664.2937	**A**	**659.3545**	330.1809	642.3280	321.6676			**6**
**14**	**1444.6591**	722.8332	1427.6325	714.3199	1426.6485	713.8279	**V**	**588.3174**	294.6623	571.2908	286.1491			**5**
**15**	**1575.6995**	788.3534	1558.6730	779.8401	1557.6890	779.3481	**M**	**489.2490**	245.1281	**472.2224**	236.6149			**4**
**16**	**1689.7425**	845.3749	1672.7159	836.8616	1671.7319	836.3696	**N**	**358.2085**	179.6079	**341.1819**	171.0946			**3**
**17**	1786.7952	893.9013	1769.7687	885.3880	1768.7847	884.8960	**P**	244.1656	122.5864	227.1390	114.0731			**2**
**18**							**K**	147.1128	74.0600	130.0863	65.5468			**1**

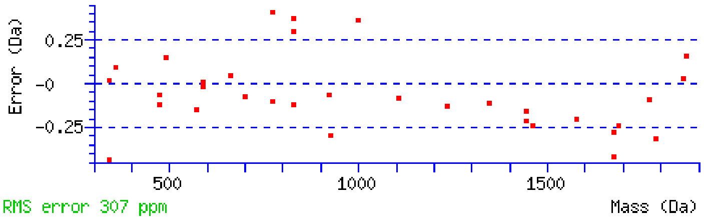

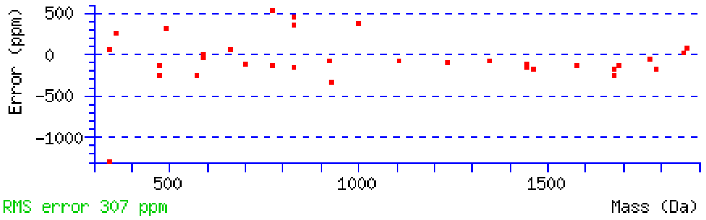

All matches to this query

Score	Mr(calc):	Delta	Sequence
75.3	2029.8704	0.0028	GDSETDLEALFNAVMNPK
69.0	2029.8704	0.0028	GDSETDLEALFNAVMNPK
5.4	2029.8703	0.0028	ELELTNGSNGGLEFNPMK
3.3	2029.8686	0.0045	FLDINLYKDNNITTGK
3.3	2029.8686	0.0045	FLDINLYKDNNITTGK
0.3	2027.8642	2.0089	ASSYLLEEFLQGNLER

Spectrum No: 66; Query: 1334; Rank: 1

Peptide View

MS/MS Fragmentation of **EEASDDDMEGDEAVVR**
Found in **IPI00373118**, Tax_Id=10116 Gene_Symbol=Ascc3l1 79 kDa protein

Match to Query 1334: 1845.668888 from(923.841720,2+)
Title: 100101RatKid_NS_deglyco_25.1568.1568.2.dta
Data file K:\NewmanPaper\Piliang\3SubProteomes\Piliang3SP\mgf5ppm\ERLIC_3SubProteomes5ppm.mgf

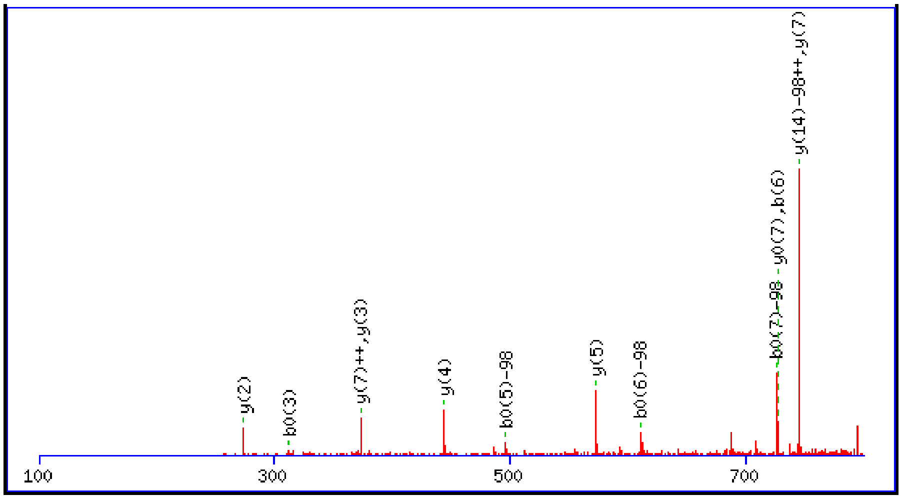

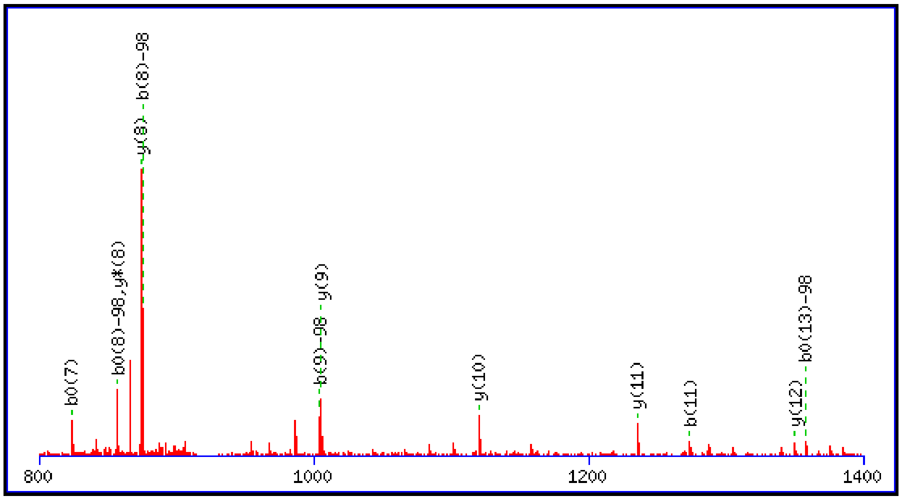

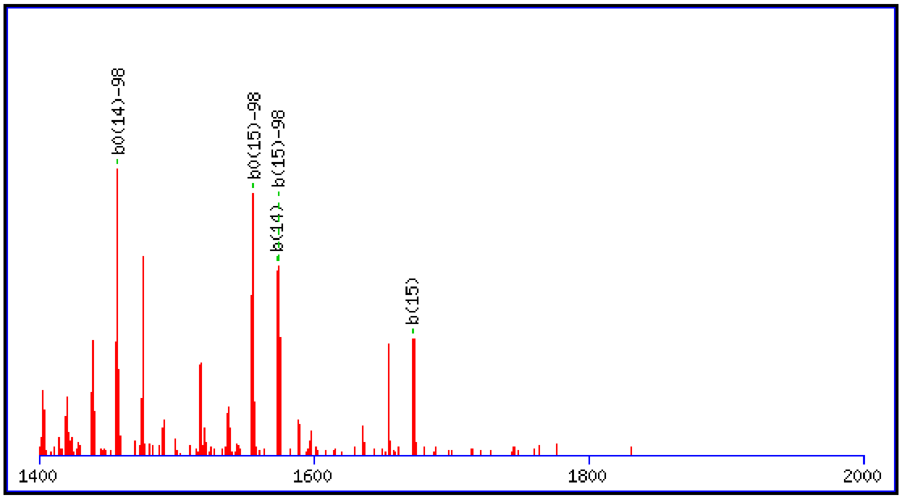

Monoisotopic mass of neutral peptide Mr(calc): 1845.6612
Fixed modifications: Carbamidomethyl (C)
Variable modifications:
S4 : Phospho (ST), with neutral losses 97.9769(shown in table), 0.0000
Ions Score: 75 **Expect:** 2.4e-006
Matches (Bold Red): 30/208 fragment ions using 38 most intense peaks

#	b	b++	b0	b0++	Seq.	y	y++	y*	y*++	y0	y0++	#
1	130.0499	65.5286	112.0393	56.5233	E							16
2	259.0925	130.0499	241.0819	121.0446	E	1619.6490	810.3281	1602.6224	801.8148	1601.6384	801.3228	15
3	330.1296	165.5684	**312.1190**	156.5631	A	1490.6064	**745.8068**	1473.5798	737.2935	1472.5958	736.8015	14
4	399.1510	200.0792	381.1405	191.0739	S	1419.5693	710.2883	1402.5427	701.7750	1401.5587	701.2830	13
5	514.1780	257.5926	**496.1674**	248.5873	D	**1350.5478**	675.7775	1333.5213	667.2643	1332.5372	666.7723	12

6	629.2049	315.1061	611.1944	306.1008	D	1235.5209	618.2641	1218.4943	609.7508	1217.5103	609.2588	11
7	744.2319	372.6196	726.2213	363.6143	D	1120.4939	560.7506	1103.4674	552.2373	1102.4833	551.7453	10
8	875.2723	438.1398	857.2618	429.1345	M	1005.4670	503.2371	988.4404	494.7238	987.4564	494.2318	9
9	1004.3149	502.6611	986.3044	493.6558	E	874.4265	437.7169	857.3999	429.2036	856.4159	428.7116	8
10	1061.3364	531.1718	1043.3258	522.1666	G	745.3839	373.1956	728.3573	364.6823	727.3733	364.1903	7
11	1176.3633	588.6853	1158.3528	579.6800	D	688.3624	344.6849	671.3359	336.1716	670.3519	335.6796	6
12	1305.4059	653.2066	1287.3954	644.2013	E	573.3355	287.1714	556.3089	278.6581	555.3249	278.1661	5
13	1376.4431	688.7252	1358.4325	679.7199	A	444.2929	222.6501	427.2663	214.1368			4
14	1475.5115	738.2594	1457.5009	729.2541	V	373.2558	187.1315	356.2292	178.6183			3
15	1574.5799	787.7936	1556.5693	778.7883	V	274.1874	137.5973	257.1608	129.0840			2
16					R	175.1190	88.0631	158.0924	79.5498			1

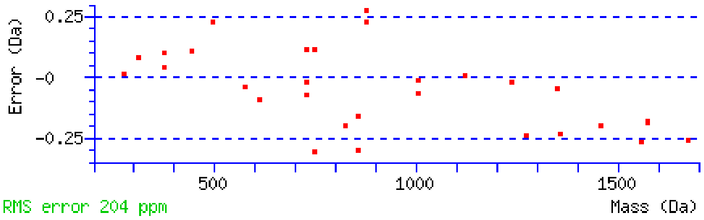

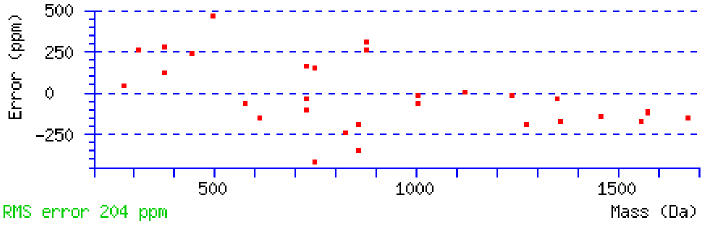

All matches to this query

Score	Mr(calc):	Delta	Sequence
75.3	1845.6612	0.0077	EEASDDDMEGDEAVVR
8.3	1843.6650	2.0039	ESGQEGSTDNDDSCSEK
1.4	1845.6546	0.0142	DGMDNETGTESMVSHR
1.0	1845.6624	0.0065	ESFSENTKAPAAPTR
0.8	1845.6789	-0.0100	EEEDDEKETSTNPSR
0.8	1845.6789	-0.0100	EEEDDEKETSTNPSR
0.8	1845.6789	-0.0100	EEEDDEKETSTNPSR
0.8	1845.6789	-0.0100	EEEDDEKETSTNPSR

Spectrum No: 67; Query: 2684; Rank: 1

Peptide View

MS/MS Fragmentation of **KGATPAEDDEDNDIDLFGSDEEEEDKEAAR**
Found in **IPI00197900**, Tax_Id=10116 Gene_Symbol=Eef1d Translation elongation factor 1-delta subunit

Match to Query 2684: 3389.361942 from(1130.794590,3+)
Title: 091008RatKidney_NoSalt_26.2506.2506.3.dta
Data file K:\NewmanPaper\Piliang\3SubProteomes\Piliang3SP\mgf5ppm\ERLIC_3SubProteomes5ppm.mgf

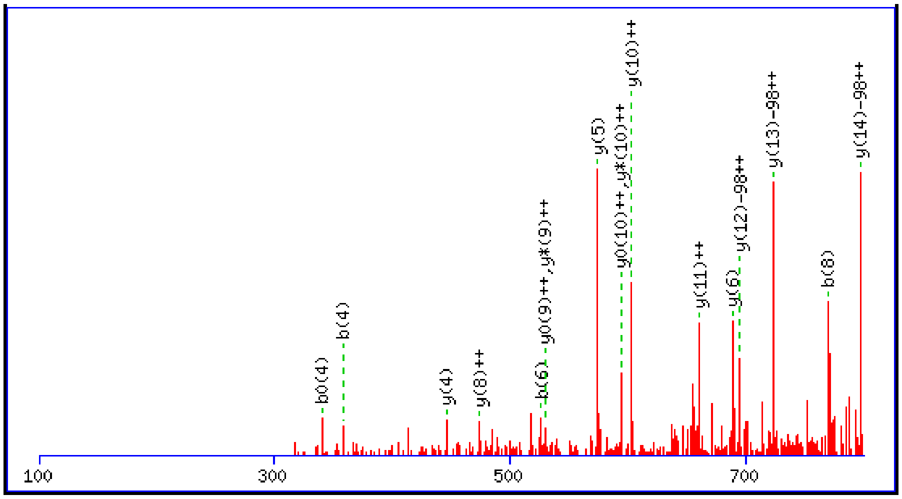

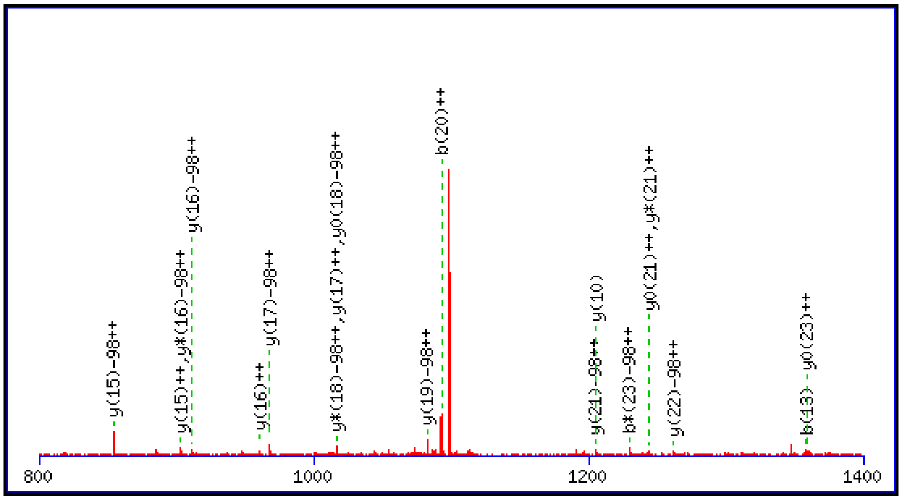

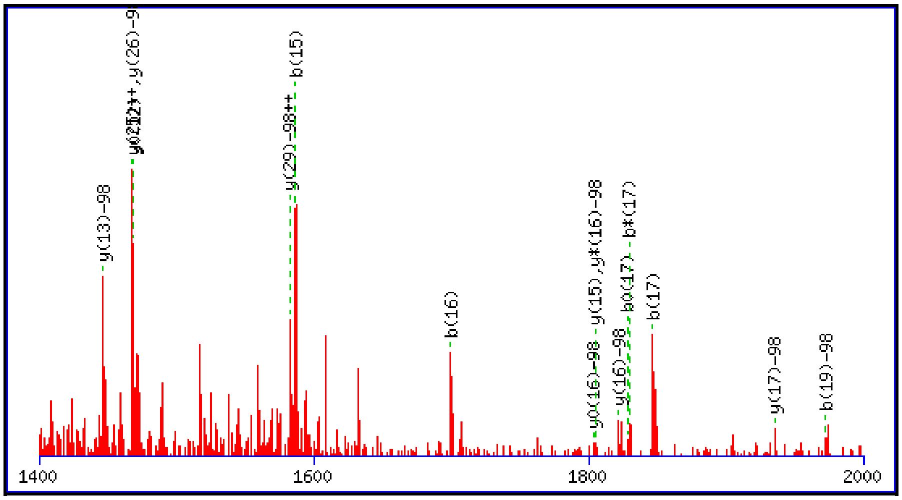

Monoisotopic mass of neutral peptide Mr(calc): 3389.3580
Fixed modifications: Carbamidomethyl (C)
Variable modifications:
S19 : Phospho (ST), with neutral losses 97.9769(shown in table), 0.0000
Ions Score: 75 **Expect:** 1.2e-005
Matches (Bold Red): 52/510 fragment ions using 65 most intense peaks

#	b	b^{++}	b*	b^{*++}	b^0	b^{0++}	Seq.	y	y^{++}	y*	y^{*++}	y^0	y^{0++}	#
**1**	129.1022	65.0548	112.0757	56.5415			**K**							**30**
**2**	186.1237	93.5655	169.0972	85.0522			**G**	3164.2934	**1582.6503**	3147.2669	1574.1371	3146.2829	1573.6451	**29**
**3**	257.1608	129.0840	240.1343	120.5708			**A**	3107.2720	1554.1396	3090.2454	1545.6263	3089.2614	1545.1343	**28**
**4**	**358.2085**	179.6079	341.1819	171.0946	**340.1979**	170.6026	**T**	3036.2348	1518.6211	3019.2083	1510.1078	3018.2243	1509.6158	**27**
**5**	455.2613	228.1343	438.2347	219.6210	437.2507	219.1290	**P**	2935.1872	**1468.0972**	2918.1606	1459.5839	2917.1766	1459.0919	**26**

6	526.2984	263.6528	509.2718	255.1396	508.2878	254.6475	A	2838.1344	1419.5708	2821.1078	1411.0576	2820.1238	1410.5656	25
7	655.3410	328.1741	638.3144	319.6608	637.3304	319.1688	E	2767.0973	1384.0523	2750.0707	1375.5390	2749.0867	1375.0470	24
8	770.3679	385.6876	753.3414	377.1743	752.3573	376.6823	D	2638.0547	1319.5310	2621.0281	1311.0177	2620.0441	1310.5257	23
9	885.3949	443.2011	868.3683	434.6878	867.3843	434.1958	D	2523.0277	1262.0175	2506.0012	1253.5042	2505.0172	1253.0122	22
10	1014.4374	507.7224	997.4109	499.2091	996.4269	498.7171	E	2408.0008	1204.5040	2390.9743	1195.9908	2389.9902	1195.4988	21
11	1129.4644	565.2358	1112.4378	556.7226	1111.4538	556.2305	D	2278.9582	1139.9827	2261.9317	1131.4695	2260.9476	1130.9775	20
12	1243.5073	622.2573	1226.4808	613.7440	1225.4968	613.2520	N	2163.9313	1082.4693	2146.9047	1073.9560	2145.9207	1073.4640	19
13	1358.5343	679.7708	1341.5077	671.2575	1340.5237	670.7655	D	2049.8883	1025.4478	2032.8618	1016.9345	2031.8778	1016.4425	18
14	1471.6183	736.3128	1454.5918	727.7995	1453.6078	727.3075	I	1934.8614	967.9343	1917.8349	959.4211	1916.8508	958.9291	17
15	1586.6453	793.8263	1569.6187	785.3130	1568.6347	784.8210	D	1821.7773	911.3923	1804.7508	902.8790	1803.7668	902.3870	16
16	1699.7293	850.3683	1682.7028	841.8550	1681.7188	841.3630	L	1706.7504	853.8788	1689.7238	845.3656	1688.7398	844.8736	15
17	1846.7977	923.9025	1829.7712	915.3892	1828.7872	914.8972	F	1593.6663	797.3368	1576.6398	788.8235	1575.6558	788.3315	14
18	1903.8192	952.4132	1886.7927	943.9000	1885.8086	943.4080	G	1446.5979	723.8026	1429.5714	715.2893	1428.5873	714.7973	13
19	1972.8407	986.9240	1955.8141	978.4107	1954.8301	977.9187	S	1389.5765	695.2919	1372.5499	686.7786	1371.5659	686.2866	12
20	2087.8676	1044.4374	2070.8411	1035.9242	2069.8570	1035.4322	D	1320.5550	660.7811	1303.5284	652.2679	1302.5444	651.7759	11
21	2216.9102	1108.9587	2199.8837	1100.4455	2198.8996	1099.9535	E	1205.5281	603.2677	1188.5015	594.7544	1187.5175	594.2624	10
22	2345.9528	1173.4800	2328.9262	1164.9668	2327.9422	1164.4748	E	1076.4855	538.7464	1059.4589	530.2331	1058.4749	529.7411	9
23	2474.9954	1238.0013	2457.9688	1229.4881	2456.9848	1228.9960	E	947.4429	474.2251	930.4163	465.7118	929.4323	465.2198	8
24	2604.0380	1302.5226	2587.0114	1294.0094	2586.0274	1293.5173	E	818.4003	409.7038	801.3737	401.1905	800.3897	400.6985	7
25	2719.0649	1360.0361	2702.0384	1351.5228	2701.0544	1351.0308	D	689.3577	345.1825	672.3311	336.6692	671.3471	336.1772	6
26	2847.1599	1424.0836	2830.1333	1415.5703	2829.1493	1415.0783	K	574.3307	287.6690	557.3042	279.1557	556.3202	278.6637	5
27	2976.2025	1488.6049	2959.1759	1480.0916	2958.1919	1479.5996	E	446.2358	223.6215	429.2092	215.1083	428.2252	214.6162	4
28	3047.2396	1524.1234	3030.2130	1515.6102	3029.2290	1515.1182	A	317.1932	159.1002	300.1666	150.5870			3
29	3118.2767	1559.6420	3101.2502	1551.1287	3100.2661	1550.6367	A	246.1561	123.5817	229.1295	115.0684			2
30							R	175.1190	88.0631	158.0924	79.5498			1

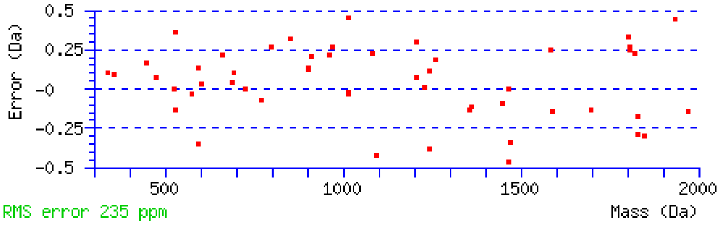

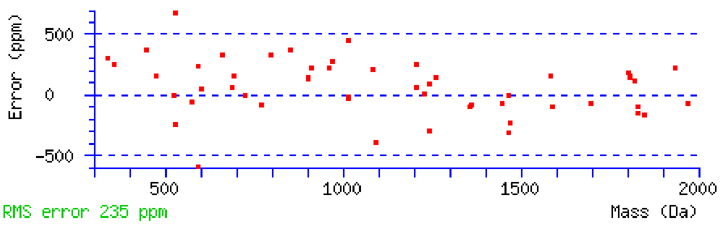

All matches to this query

Score	Mr(calc):	Delta	Sequence
75.1	3389.3580	0.0040	KGATPAEDDEDNDIDLFGSDEEEEDKEAAR
8.6	3388.3752	0.9867	RLLYGSLVCMSKDNFETFLFATVSNR
6.1	3389.3580	0.0040	KGATPAEDDEDNDIDLFGSDEEEEDKEAAR
5.6	3389.3560	0.0060	SPDKPGKGSYWTLHPDSGNMFENGCYLR
5.4	3389.3699	-0.0079	DYIQILSSPNFSLDQYCEQMWYREK
4.3	3389.3560	0.0060	SPDKPGKGSYWTLHPDSGNMFENGCYLR
3.3	3389.3522	0.0097	DMQSMVDSIYADLSHSNLYQSLTKDKK
3.2	3389.3560	0.0060	SPDKPGKGSYWTLHPDSGNMFENGCYLR
3.2	3389.3560	0.0060	SPDKPGKGSYWTLHPDSGNMFENGCYLR
3.2	3389.3560	0.0060	SPDKPGKGSYWTLHPDSGNMFENGCYLR

Spectrum No: 68; Query: 2722; Rank: 1

Peptide View

MS/MS Fragmentation of **AALLAQYADVTDEEDEADEKADPGASTANIGSDK**
Found in **IPI00372936**, Tax_Id=10116 Gene_Symbol=Ccdc43 similar to CG9586-PB

Match to Query 2722: 3560.553012 from(1187.858280,3+)
Title: 100101RatKid_NS_deglyco_23.3490.3490.3.dta
Data file K:\NewmanPaper\Piliang\3SubProteomes\Piliang3SP\mgf5ppm\ERLIC_3SubProteomes5ppm.mgf

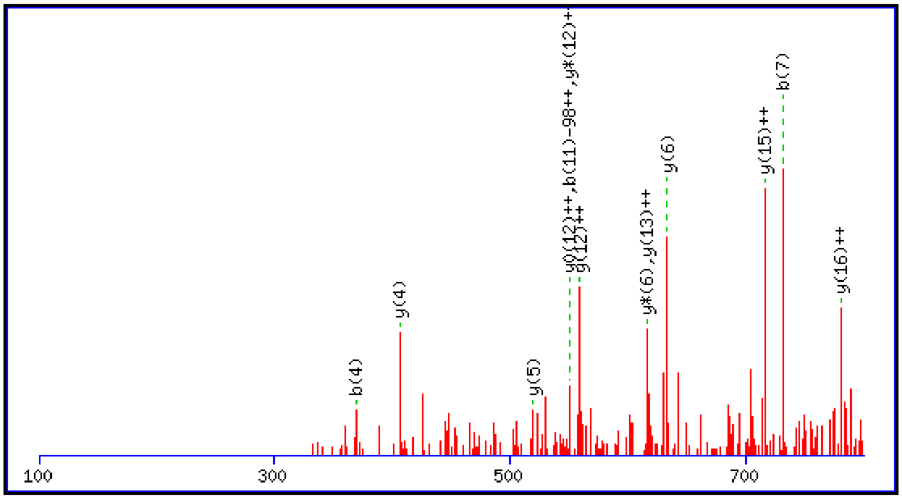

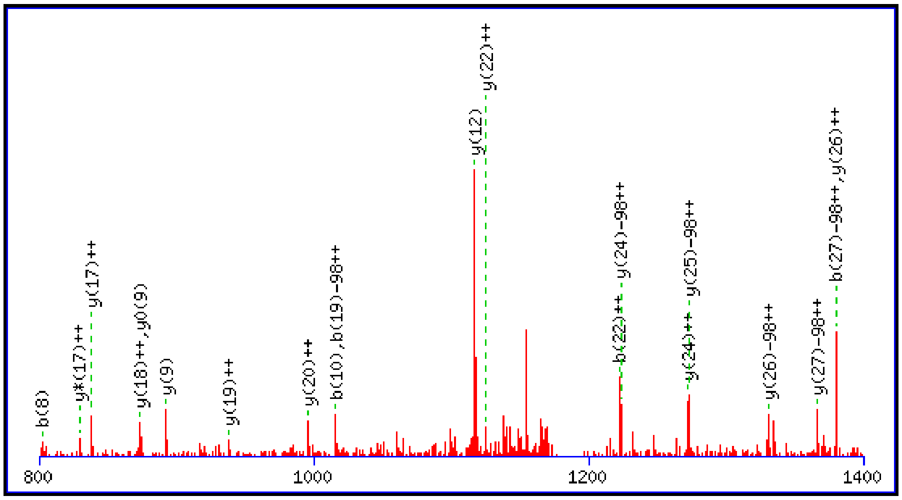

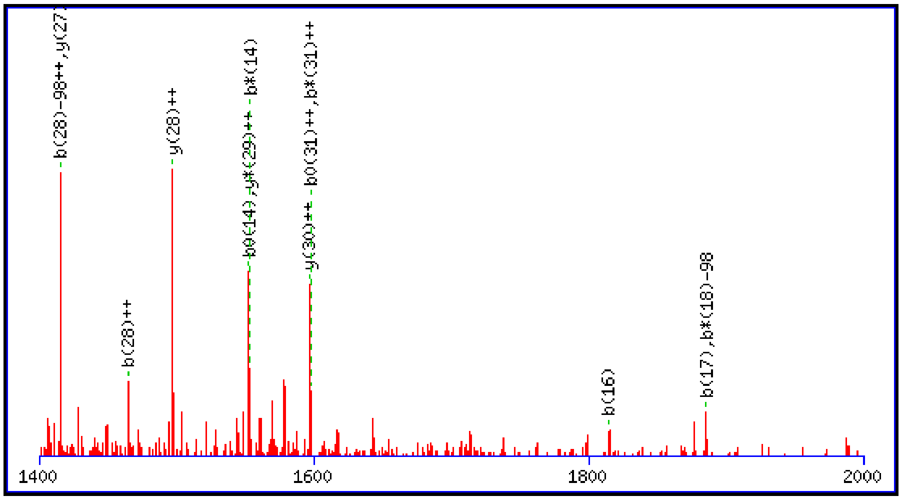

Monoisotopic mass of neutral peptide Mr(calc): 3559.5363
Fixed modifications: Carbamidomethyl (C)
Variable modifications:
T11 : Phospho (ST), with neutral losses 0.0000(shown in table), 97.9769
Ions Score: 75 **Expect:** 1.5e-005
Matches (Bold Red): 46/566 fragment ions using 58 most intense peaks

#	b	b++	b*	b*++	b0	b0++	Seq.	y	y++	y*	y*++	y0	y0++	#
1	72.0444	36.5258					A							34
2	143.0815	72.0444					A	3489.5065	1745.2569	3472.4799	1736.7436	3471.4959	1736.2516	33
3	256.1656	128.5864					L	3418.4694	1709.7383	3401.4428	1701.2250	3400.4588	1700.7330	32
4	**369.2496**	185.1285					L	3305.3853	1653.1963	3288.3587	1644.6830	3287.3747	1644.1910	31
5	440.2867	220.6470					A	3192.3012	**1596.6542**	3175.2747	1588.1410	3174.2907	1587.6490	30
6	568.3453	284.6763	551.3188	276.1630			Q	3121.2641	1561.1357	3104.2376	**1552.6224**	3103.2535	1552.1304	29
7	**731.4087**	366.2080	714.3821	357.6947			Y	2993.2055	**1497.1064**	2976.1790	1488.5931	2975.1950	1488.1011	28
8	**802.4458**	401.7265	785.4192	393.2132			A	2830.1422	**1415.5747**	2813.1157	1407.0615	2812.1316	1406.5695	27
9	917.4727	459.2400	900.4462	450.7267	899.4621	450.2347	D	2759.1051	**1380.0562**	2742.0785	1371.5429	2741.0945	1371.0509	26
10	**1016.5411**	508.7742	999.5146	500.2609	998.5306	499.7689	V	2644.0781	1322.5427	2627.0516	1314.0294	2626.0676	1313.5374	25
11	1197.5551	599.2812	1180.5286	590.7679	1179.5446	590.2759	T	2545.0097	**1273.0085**	2527.9832	1264.4952	2526.9992	1264.0032	24
12	1312.5821	656.7947	1295.5555	648.2814	1294.5715	647.7894	D	2363.9957	1182.5015	2346.9692	1173.9882	2345.9852	1173.4962	23
13	1441.6247	721.3160	1424.5981	712.8027	1423.6141	712.3107	E	2248.9688	**1124.9880**	2231.9422	1116.4748	2230.9582	1115.9827	22
14	1570.6673	785.8373	**1553.6407**	777.3240	**1552.6567**	776.8320	E	2119.9262	1060.4667	2102.8996	1051.9535	2101.9156	1051.4615	21
15	1685.6942	843.3507	1668.6677	834.8375	1667.6836	834.3455	D	1990.8836	**995.9454**	1973.8571	987.4322	1972.8730	986.9402	20
16	**1814.7368**	907.8720	1797.7102	899.3588	1796.7262	898.8668	E	1875.8567	**938.4320**	1858.8301	929.9187	1857.8461	929.4267	19
17	**1885.7739**	943.3906	1868.7474	934.8773	1867.7633	934.3853	A	1746.8141	**873.9107**	1729.7875	865.3974	1728.8035	864.9054	18
18	2000.8008	1000.9041	1983.7743	992.3908	1982.7903	991.8988	D	1675.7769	**838.3921**	1658.7504	**829.8788**	1657.7664	829.3868	17
19	2129.8434	1065.4254	2112.8169	1056.9121	2111.8329	1056.4201	E	1560.7500	**780.8786**	1543.7235	772.3654	1542.7394	771.8734	16
20	2257.9384	1129.4728	2240.9119	1120.9596	2239.9278	1120.4676	K	1431.7074	**716.3573**	1414.6809	707.8441	1413.6968	707.3521	15
21	2328.9755	1164.9914	2311.9490	1156.4781	2310.9650	1155.9861	A	1303.6125	652.3099	1286.5859	643.7966	1285.6019	643.3046	14
22	2444.0025	**1222.5049**	2426.9759	1213.9916	2425.9919	1213.4996	D	1232.5753	**616.7913**	1215.5488	608.2780	1214.5648	607.7860	13
23	2541.0552	1271.0313	2524.0287	1262.5180	2523.0447	1262.0260	P	**1117.5484**	**559.2778**	1100.5218	**550.7646**	1099.5378	**550.2726**	12
24	2598.0767	1299.5420	2581.0501	1291.0287	2580.0661	1290.5367	G	1020.4956	510.7515	1003.4691	502.2382	1002.4851	501.7462	11
25	2669.1138	1335.0605	2652.0873	1326.5473	2651.1032	1326.0553	A	963.4742	482.2407	946.4476	473.7274	945.4636	473.2354	10
26	2756.1458	1378.5766	2739.1193	1370.0633	2738.1353	1369.5713	S	**892.4371**	446.7222	875.4105	438.2089	**874.4265**	437.7169	9
27	2857.1935	1429.1004	2840.1670	1420.5871	2839.1829	1420.0951	T	805.4050	403.2061	788.3785	394.6929	787.3945	394.2009	8
28	2928.2306	**1464.6190**	2911.2041	1456.1057	2910.2201	1455.6137	A	704.3573	352.6823	687.3308	344.1690	686.3468	343.6770	7
29	3042.2736	1521.6404	3025.2470	1513.1271	3024.2630	1512.6351	N	**633.3202**	317.1638	**616.2937**	308.6505	615.3097	308.1585	6
30	3155.3576	1578.1824	3138.3311	1569.6692	3137.3471	1569.1772	I	**519.2773**	260.1423	502.2508	251.6290	501.2667	251.1370	5
31	3212.3791	1606.6932	3195.3525	**1598.1799**	3194.3685	**1597.6879**	G	**406.1932**	203.6003	389.1667	195.0870	388.1827	194.5950	4
32	3299.4111	1650.2092	3282.3846	1641.6959	3281.4005	1641.2039	S	349.1718	175.0895	332.1452	166.5763	331.1612	166.0842	3
33	3414.4381	1707.7227	3397.4115	1699.2094	3396.4275	1698.7174	D	262.1397	131.5735	245.1132	123.0602	244.1292	122.5682	2
34							K	147.1128	74.0600	130.0863	65.5468			1

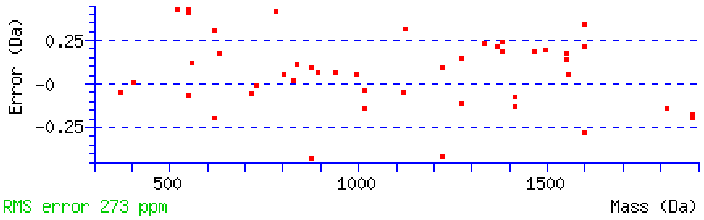

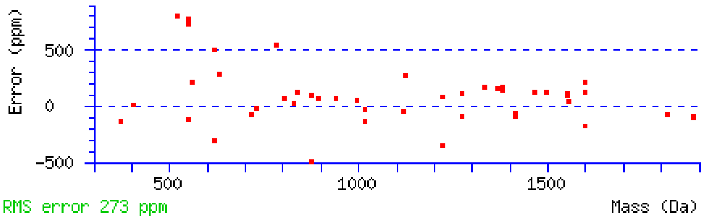

All matches to this query

Score	Mr(calc):	Delta	Sequence
74.9	3559.5363	1.0168	AALLAQYADVTDEEDEADEKADPGASTANIGSDK
57.4	3560.5203	0.0327	AALLAQYADVTDEEDEADEKADPGASTANIGSDK
49.5	3559.5363	1.0168	AALLAQYADVTDEEDEADEKADPGASTANIGSDK
31.6	3559.5363	1.0168	AALLAQYADVTDEEDEADEKADPGASTANIGSDK

30.5	3560.5203	0.0327	AALLAQYADVTDEEDEADEKADPGASTANIGSDK
27.4	3559.5363	1.0168	AALLAQYADVTDEEDEADEKADPGASTANIGSDK
20.8	3560.5203	0.0327	AALLAQYADVTDEEDEADEKADPGASTANIGSDK
20.8	3560.5203	0.0327	AALLAQYADVTDEEDEADEKADPGASTANIGSDK
13.9	3559.5363	1.0168	AALLAQYADVTDEEDEADEKADPGASTANIGSDK
12.2	3560.5203	0.0327	AALLAQYADVTDEEDEADEKADPGASTANIGSDK

Spectrum No: 69; Query: 1705; Rank: 1

Peptide View

MS/MS Fragmentation of **KETESEAEDDNLDDLER**
Found in **IPI00779470**, Tax_Id=10116 Gene_Symbol=Srrm1_predicted 106 kDa protein

Match to Query 1705: 2086.829808 from(1044.422180,2+)
Title: 100101RatKid_NS_deglyco_23.1873.1873.2.dta
Data file K:\NewmanPaper\Piliang\3SubProteomes\Piliang3SP\mgf5ppm\ERLIC_3SubProteomes5ppm.mgf

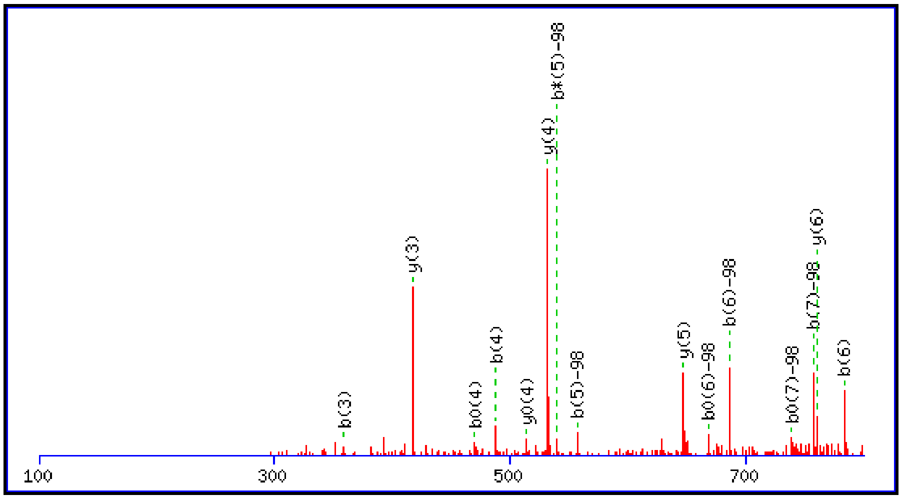

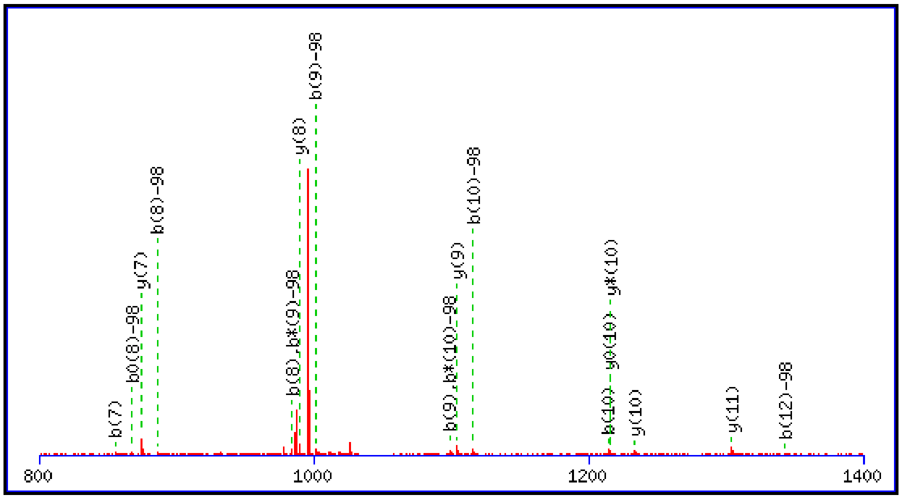

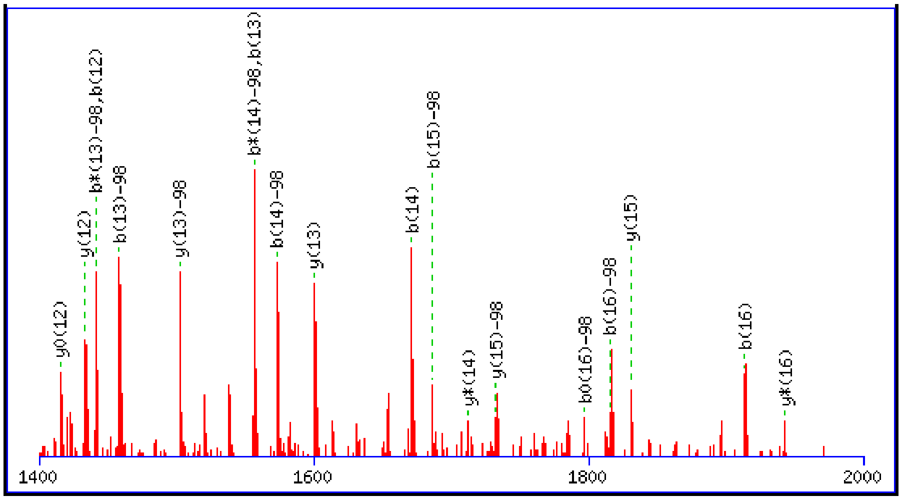

Monoisotopic mass of neutral peptide Mr(calc): 2086.8215
Fixed modifications: Carbamidomethyl (C)
Variable modifications:
S5 : Phospho (ST), with neutral losses 97.9769(shown in table), 0.0000
Ions Score: 74 **Expect:** 6.9e-006
Matches (Bold Red): 52/284 fragment ions using 90 most intense peaks

#	b	b^{++}	b*	b^{*++}	b^0	b^{0++}	Seq.	y	y^{++}	y*	y^{*++}	y^0	y^{0++}	#
1	129.1022	65.0548	112.0757	56.5415			K							17
2	258.1448	129.5761	241.1183	121.0628	240.1343	120.5708	E	1861.7570	931.3821	1844.7304	922.8689	1843.7464	922.3769	16
3	**359.1925**	180.0999	342.1660	171.5866	341.1819	171.0946	T	**1732.7144**	866.8608	1715.6879	858.3476	1714.7038	857.8556	15
4	**488.2351**	244.6212	471.2086	236.1079	**470.2245**	235.6159	E	1631.6667	816.3370	1614.6402	807.8237	1613.6562	807.3317	14
5	**557.2566**	279.1319	**540.2300**	270.6186	539.2460	270.1266	S	**1502.6241**	751.8157	1485.5976	743.3024	1484.6136	742.8104	13
6	**686.2992**	343.6532	669.2726	335.1399	**668.2886**	334.6479	E	**1433.6027**	717.3050	1416.5761	708.7917	**1415.5921**	708.2997	12
7	**757.3363**	379.1718	740.3097	370.6585	**739.3257**	370.1665	A	**1304.5601**	652.7837	1287.5335	644.2704	1286.5495	643.7784	11
8	**886.3789**	443.6931	869.3523	435.1798	**868.3683**	434.6878	E	**1233.5230**	617.2651	**1216.4964**	608.7518	**1215.5124**	608.2598	10
9	**1001.4058**	501.2065	**984.3793**	492.6933	983.3952	492.2013	D	**1104.4804**	552.7438	1087.4538	544.2305	1086.4698	543.7385	9
10	**1116.4327**	558.7200	**1099.4062**	550.2067	1098.4222	549.7147	D	**989.4534**	495.2304	972.4269	486.7171	971.4429	486.2251	8
11	1230.4757	615.7415	1213.4491	607.2282	1212.4651	606.7362	N	**874.4265**	437.7169	857.3999	429.2036	856.4159	428.7116	7
12	**1343.5597**	672.2835	1326.5332	663.7702	1325.5492	663.2782	L	**760.3836**	380.6954	743.3570	372.1821	742.3730	371.6901	6
13	**1458.5867**	729.7970	**1441.5601**	721.2837	1440.5761	720.7917	D	**647.2995**	324.1534	630.2729	315.6401	629.2889	315.1481	5
14	**1573.6136**	787.3104	**1556.5871**	778.7972	1555.6031	778.3052	D	**532.2726**	266.6399	515.2460	258.1266	**514.2620**	257.6346	4
15	**1686.6977**	843.8525	1669.6711	835.3392	1668.6871	834.8472	L	**417.2456**	209.1264	400.2191	200.6132	399.2350	200.1212	3
16	**1815.7403**	908.3738	1798.7137	899.8605	**1797.7297**	899.3685	E	304.1615	152.5844	287.1350	144.0711	286.1510	143.5791	2
17							R	175.1190	88.0631	158.0924	79.5498			1

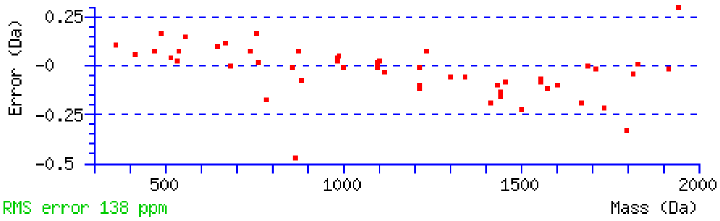

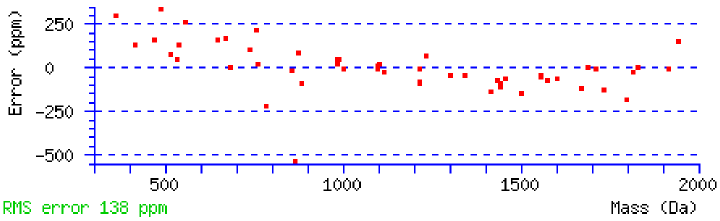

All matches to this query

Score	Mr(calc):	Delta	Sequence
74.4	2086.8215	0.0083	KETESEAEDDNLDDLER
62.0	2086.8215	0.0083	KETESEAEDDNLDDLER
1.4	2086.8449	-0.0151	ASEESRDMDSKCHQTVK
1.3	2086.8438	-0.0140	QFISWLQDVDDKYDR

1.1	2086.8377	-0.0079	YMADMNELFSQVDEKR
1.0	2086.8410	-0.0112	NNMTASMFDLSMKDKTR
0.7	2086.8410	-0.0112	NNMTASMFDLSMKDKTR
0.7	2086.8410	-0.0112	NNMTASMFDLSMKDKTR
0.7	2086.8410	-0.0112	NNMTASMFDLSMKDKTR
0.6	2086.8220	0.0078	SMKNRPNPYYEDLER

Spectrum No: 70; Query: 2753; Rank: 1

Peptide View

MS/MS Fragmentation of **ALENGEADEPSFSDPEDFVDDVSEEELLGDVLK**
Found in **IPI00363771**, Tax_Id=10116 Gene_Symbol=Eif3s9 108 kDa protein

Match to Query 2753: 3688.579182 from(1230.533670,3+)
Title: 100101RatKid_NS_deglyco_24.6330.6330.3.dta
Data file K:\NewmanPaper\Piliang\3SubProteomes\Piliang3SP\mgf5ppm\ERLIC_3SubProteomes5ppm.mgf

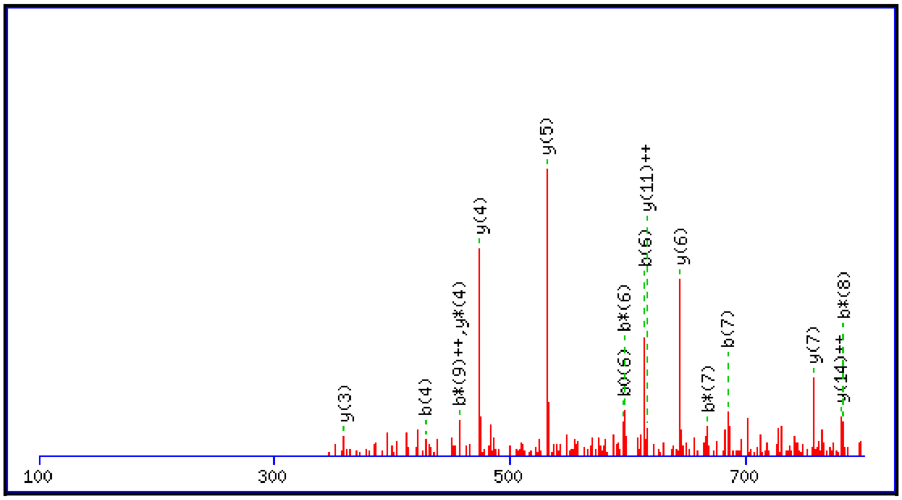

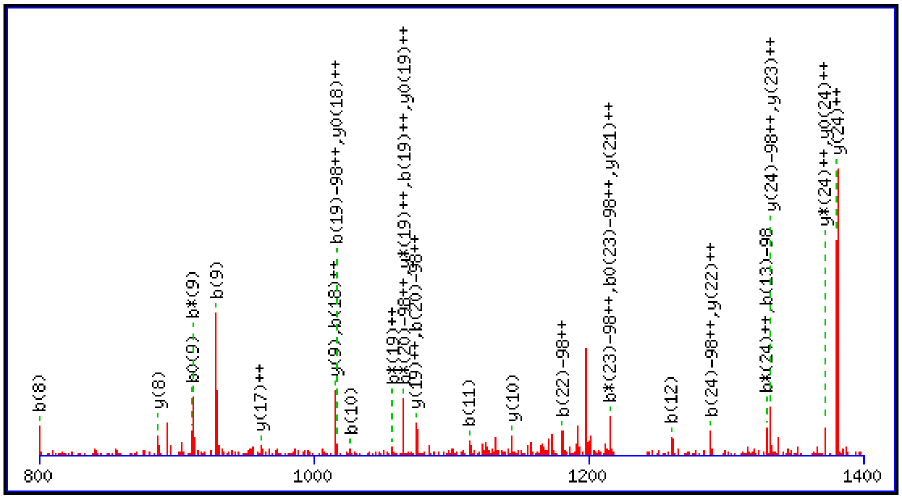

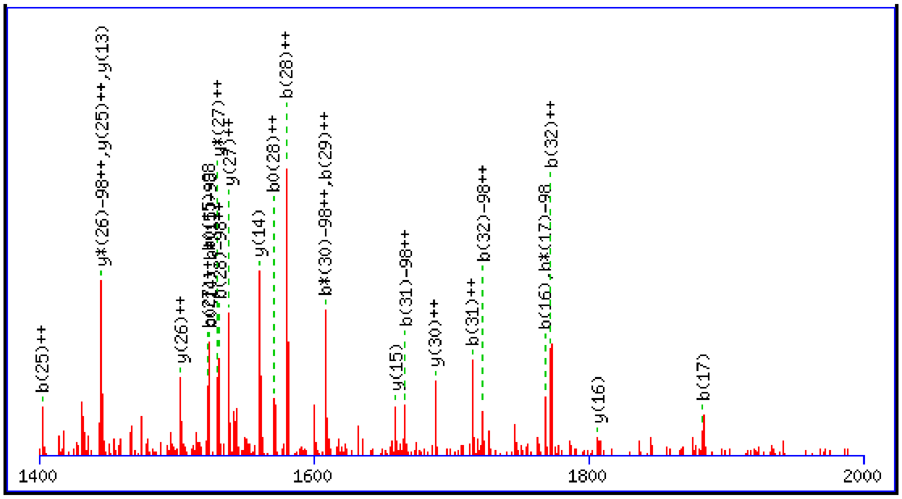

Monoisotopic mass of neutral peptide Mr(calc): 3688.5717
Fixed modifications: Carbamidomethyl (C)
Variable modifications:
S13 : Phospho (ST), with neutral losses 0.0000(shown in table), 97.9769
Ions Score: 74 **Expect:** 1.7e-005
Matches (Bold Red): 77/560 fragment ions using 110 most intense peaks

#	b	b^{++}	b*	b^{*++}	b^{0}	b^{0++}	Seq.	y	y^{++}	y*	y^{*++}	y^{0}	y^{0++}	#
1	72.0444	36.5258					A							33
2	185.1285	93.0679					L	3618.5418	1809.7746	3601.5153	1801.2613	3600.5313	1800.7693	32
3	314.1710	157.5892			296.1605	148.5839	E	3505.4578	1753.2325	3488.4312	1744.7193	3487.4472	1744.2272	31
4	**428.2140**	214.6106	411.1874	206.0974	410.2034	205.6053	N	3376.4152	**1688.7112**	3359.3886	1680.1980	3358.4046	1679.7059	30
5	485.2354	243.1214	468.2089	234.6081	467.2249	234.1161	G	3262.3723	1631.6898	3245.3457	1623.1765	3244.3617	1622.6845	29
6	**614.2780**	307.6427	**597.2515**	299.1294	**596.2675**	298.6374	E	3205.3508	1603.1790	3188.3242	1594.6658	3187.3402	1594.1738	28
7	**685.3151**	343.1612	**668.2886**	334.6479	667.3046	334.1559	A	3076.3082	**1538.6577**	3059.2817	**1530.1445**	3058.2976	1529.6525	27
8	**800.3421**	400.6747	**783.3155**	392.1614	782.3315	391.6694	D	3005.2711	**1503.1392**	2988.2445	1494.6259	2987.2605	1494.1339	26
9	**929.3847**	465.1960	**912.3581**	**456.6827**	**911.3741**	456.1907	E	2890.2441	**1445.6257**	2873.2176	1437.1124	2872.2336	1436.6204	25
10	**1026.4374**	513.7224	1009.4109	505.2091	1008.4269	504.7171	P	2761.2016	**1381.1044**	2744.1750	**1372.5911**	2743.1910	**1372.0991**	24
11	**1113.4695**	557.2384	1096.4429	548.7251	1095.4589	548.2331	S	2664.1488	**1332.5780**	2647.1222	1324.0648	2646.1382	1323.5727	23
12	**1260.5379**	630.7726	1243.5113	622.2593	1242.5273	621.7673	F	2577.1168	**1289.0620**	2560.0902	1280.5487	2559.1062	1280.0567	22
13	1427.5362	714.2718	1410.5097	705.7585	1409.5257	705.2665	S	2430.0483	**1215.5278**	2413.0218	1207.0145	2412.0378	1206.5225	21
14	1542.5632	771.7852	1525.5366	763.2720	**1524.5526**	762.7799	D	2263.0500	1132.0286	2246.0234	1123.5154	2245.0394	1123.0234	20
15	1639.6159	820.3116	1622.5894	811.7983	1621.6054	811.3063	P	2148.0231	**1074.5152**	2130.9965	**1066.0019**	2130.0125	**1065.5099**	19
16	**1768.6585**	884.8329	1751.6320	876.3196	1750.6480	875.8276	E	2050.9703	1025.9888	2033.9437	1017.4755	2032.9597	**1016.9835**	18
17	**1883.6855**	942.3464	1866.6589	933.8331	1865.6749	933.3411	D	1921.9277	**961.4675**	1904.9011	952.9542	1903.9171	952.4622	17
18	2030.7539	**1015.8806**	2013.7273	1007.3673	2012.7433	1006.8753	F	**1806.9008**	903.9540	1789.8742	895.4407	1788.8902	894.9487	16
19	2129.8223	**1065.4148**	2112.7958	**1056.9015**	2111.8117	1056.4095	V	**1659.8323**	830.4198	1642.8058	821.9065	1641.8218	821.4145	15
20	2244.8493	1122.9283	2227.8227	1114.4150	2226.8387	1113.9230	D	**1560.7639**	**780.8856**	1543.7374	772.3723	1542.7534	771.8803	14
21	2359.8762	1180.4417	2342.8496	1171.9285	2341.8656	1171.4365	D	**1445.7370**	723.3721	1428.7104	714.8589	1427.7264	714.3668	13
22	2458.9446	1229.9759	2441.9181	1221.4627	2440.9340	1220.9707	V	1330.7100	665.8587	1313.6835	657.3454	1312.6995	656.8534	12
23	2545.9766	1273.4920	2528.9501	1264.9787	2527.9661	1264.4867	S	1231.6416	**616.3245**	1214.6151	607.8112	1213.6311	607.3192	11
24	2675.0192	1338.0133	2657.9927	**1329.5000**	2657.0087	1329.0080	E	**1144.6096**	572.8084	1127.5830	564.2952	1126.5990	563.8032	10
25	2804.0618	**1402.5345**	2787.0353	1394.0213	2786.0513	1393.5293	E	**1015.5670**	508.2871	998.5405	499.7739	997.5564	499.2819	9
26	2933.1044	1467.0558	2916.0779	1458.5426	2915.0939	1458.0506	E	**886.5244**	443.7658	869.4979	435.2526	868.5138	434.7606	8
27	3046.1885	**1523.5979**	3029.1619	1515.0846	3028.1779	1514.5926	L	**757.4818**	379.2445	740.4553	370.7313	739.4713	370.2393	7
28	3159.2725	**1580.1399**	3142.2460	1571.6266	3141.2620	**1571.1346**	L	**644.3978**	322.7025	627.3712	314.1892	626.3872	313.6972	6
29	3216.2940	**1608.6506**	3199.2675	1600.1374	3198.2834	1599.6454	G	**531.3137**	266.1605	514.2871	257.6472	513.3031	257.1552	5
30	3331.3210	1666.1641	3314.2944	1657.6508	3313.3104	1657.1588	D	**474.2922**	237.6498	**457.2657**	229.1365	456.2817	228.6445	4
31	3430.3894	**1715.6983**	3413.3628	1707.1850	3412.3788	1706.6930	V	**359.2653**	180.1363	342.2387	171.6230			3
32	3543.4734	**1772.2404**	3526.4469	1763.7271	3525.4629	1763.2351	L	260.1969	130.6021	243.1703	122.0888			2

33							K	147.1128	74.0600	130.0863	65.5468		1

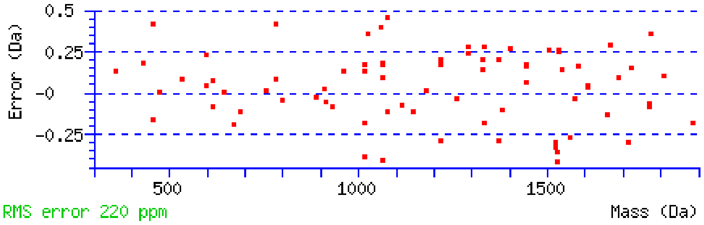

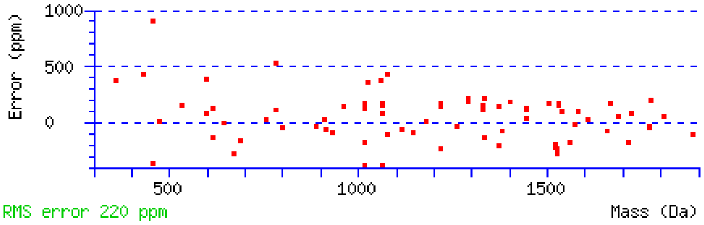

All matches to this query

Score	Mr(calc):	Delta	Sequence
74.3	3688.5717	0.0075	ALENGEADEPSFSDPEDFVDDVSEEELLGDVLK
58.6	3688.5717	0.0075	ALENGEADEPSFSDPEDFVDDVSEEELLGDVLK
40.6	3688.5717	0.0075	ALENGEADEPSFSDPEDFVDDVSEEELLGDVLK
9.9	3687.5995	0.9797	AASNSTAPPVSPTQQPVNTAELALTSPNAAPTSEK
8.8	3687.5995	0.9797	AASNSTAPPVSPTQQPVNTAELALTSPNAAPTSEK
8.0	3686.5808	1.9984	QTSITSFVTSQPGMANGGNQKNASSLKENQINR
6.8	3686.5808	1.9984	QTSITSFVTSQPGMANGGNQKNASSLKENQINR
6.6	3688.5835	-0.0043	AASNSTAPPVSPTQQPVNTAELALTSPNAAPTSEK
5.6	3687.5933	0.9859	GFNLPNGQDTIICTESGWTPQPICIPSETCSK
5.6	3688.6058	-0.0266	SGQKQLFYVGHSQGTTIAFIAFSTNPELAKK

Spectrum No: 71; Query: 552; Rank: 1

Peptide View

MS/MS Fragmentation of **TASGSSVTSLEGTR**
Found in **IPI00421389**, Tax_Id=10116 Gene_Symbol=Ndrg1 Protein NDRG1

Match to Query 552: 1431.620448 from(716.817500,2+)
Title: 091008RatKidney_NoSalt_21.1137.1137.2.dta
Data file K:\NewmanPaper\Piliang\3SubProteomes\Piliang3SP\mgf5ppm\ERLIC_3SubProteomes5ppm.mgf

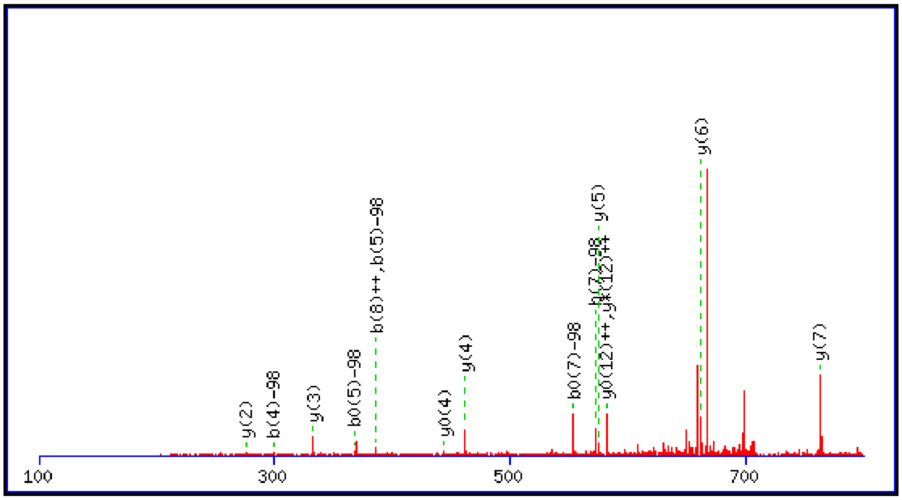

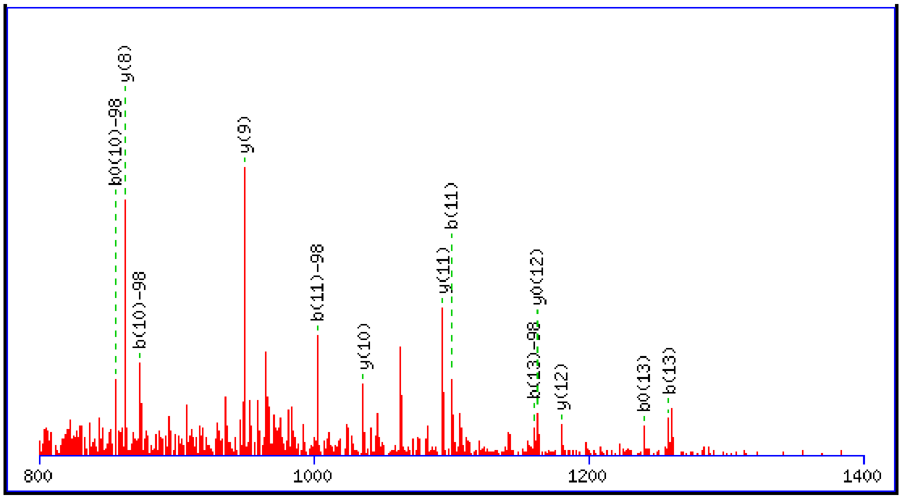

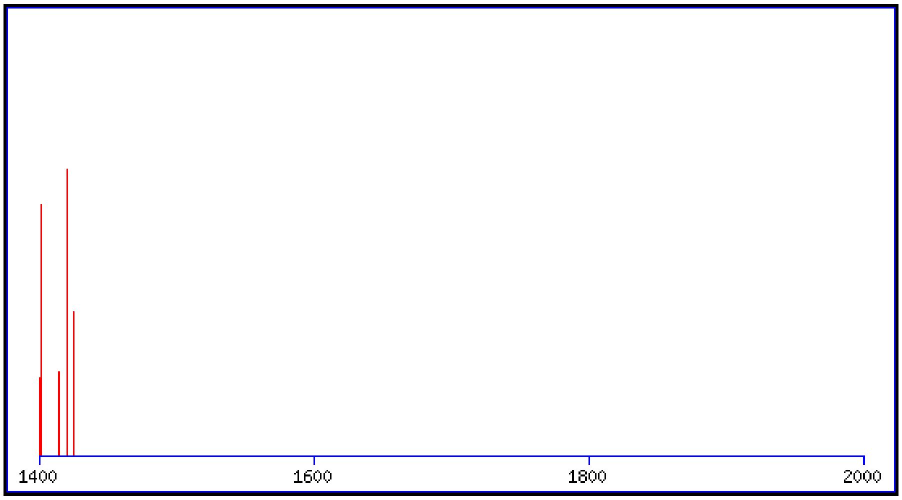

Monoisotopic mass of neutral peptide Mr(calc): 1431.6243
Fixed modifications: Carbamidomethyl (C)
Variable modifications:
T1 : Phospho (ST), with neutral losses 97.9769(shown in table), 0.0000
Ions Score: 74 **Expect:** 6.5e-006
Matches (Bold Red): 28/180 fragment ions using 56 most intense peaks

#	b	b^{++}	b^{0}	b^{0++}	Seq.	y	y^{++}	y*	y^{*++}	y^{0}	y^{0++}	#
1	84.0444	42.5258	66.0338	33.5205	T							14
2	155.0815	78.0444	137.0709	69.0391	A	1251.6175	626.3124	1234.5910	617.7991	1233.6070	617.3071	13
3	242.1135	121.5604	224.1030	112.5551	S	**1180.5804**	590.7938	1163.5539	**582.2806**	**1162.5699**	**581.7886**	12
4	**299.1350**	150.0711	281.1244	141.0658	G	**1093.5484**	547.2778	1076.5218	538.7646	1075.5378	538.2726	11
5	**386.1670**	193.5871	**368.1565**	184.5819	S	**1036.5269**	518.7671	1019.5004	510.2538	1018.5164	509.7618	10
6	473.1990	237.1032	455.1885	228.0979	S	**949.4949**	475.2511	932.4684	466.7378	931.4843	466.2458	9
7	**572.2675**	286.6374	**554.2569**	277.6321	V	**862.4629**	431.7351	845.4363	423.2218	844.4523	422.7298	8
8	673.3151	337.1612	655.3046	328.1559	T	**763.3945**	382.2009	746.3679	373.6876	745.3839	373.1956	7
9	760.3472	380.6772	742.3366	371.6719	S	**662.3468**	331.6770	645.3202	323.1638	644.3362	322.6717	6
10	**873.4312**	437.2193	**855.4207**	428.2140	L	**575.3148**	288.1610	558.2882	279.6477	557.3042	279.1557	5
11	**1002.4738**	501.7405	984.4633	492.7353	E	**462.2307**	231.6190	445.2041	223.1057	**444.2201**	222.6137	4
12	1059.4953	530.2513	1041.4847	521.2460	G	**333.1881**	167.0977	316.1615	158.5844	315.1775	158.0924	3
13	**1160.5430**	580.7751	1142.5324	571.7698	T	**276.1666**	138.5870	259.1401	130.0737	258.1561	129.5817	2
14					R	175.1190	88.0631	158.0924	79.5498			1

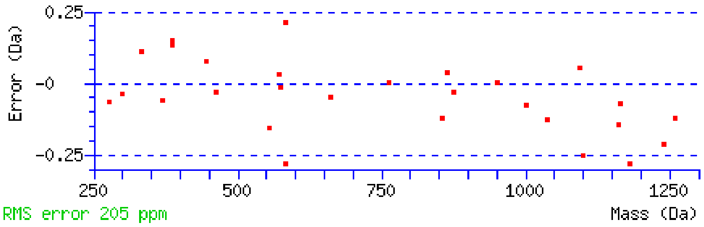

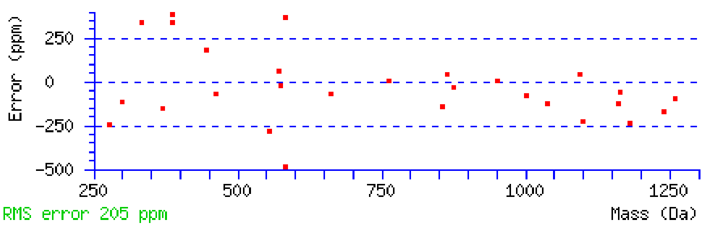

All matches to this query

Score	Mr(calc):	Delta	Sequence
74.2	1431.6243	-0.0038	TASGSSVTSLEGTR
72.7	1431.6243	-0.0038	TASGSSVTSLEGTR
48.9	1431.6243	-0.0038	TASGSSVTSLEGTR
37.4	1431.6243	-0.0038	TASGSSVTSLEGTR
20.0	1431.6243	-0.0038	TASGSSVTSLEGTR
14.2	1431.6243	-0.0038	TASGSSVTSLEGTR
7.4	1430.6191	1.0013	GADAASPPPATGSPR
7.4	1430.6191	1.0013	GADAASPPPATGSPR
0.9	1431.6200	0.0004	YLSQEGLPXPR
0.9	1431.6200	0.0004	YLSQEGLPXPR

Spectrum No: 72; Query: 628; Rank: 1

Peptide View

MS/MS Fragmentation of **SGYSSPGSPGTPGSR**
Found in **IPI00230972**, Tax_Id=10116 Gene_Symbol=Mapt Isoform Tau-E of Microtubule-associated protein tau

Match to Query 628: 1472.591268 from(737.302910,2+)
Title: 091008RatKidney_NH4Format02_26.584.584.2.dta
Data file K:\NewmanPaper\Piliang\3SubProteomes\Piliang3SP\mgf5ppm\ERLIC_3SubProteomes5ppm.mgf

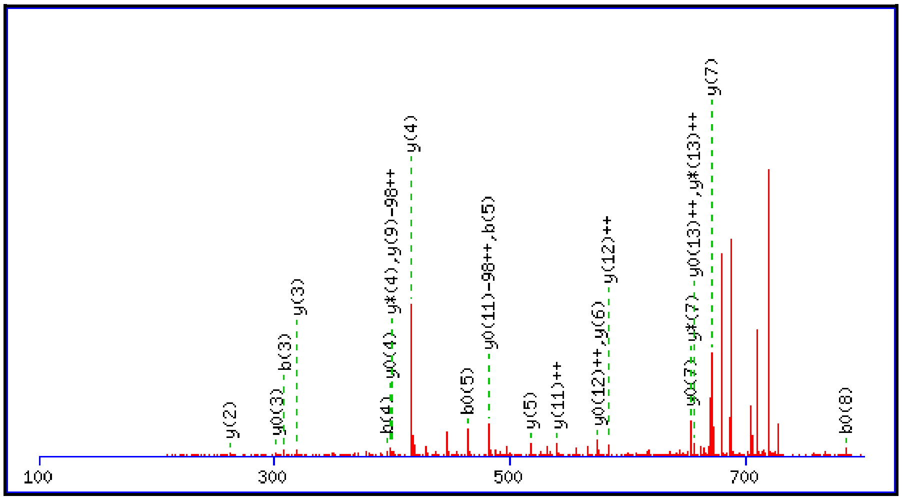

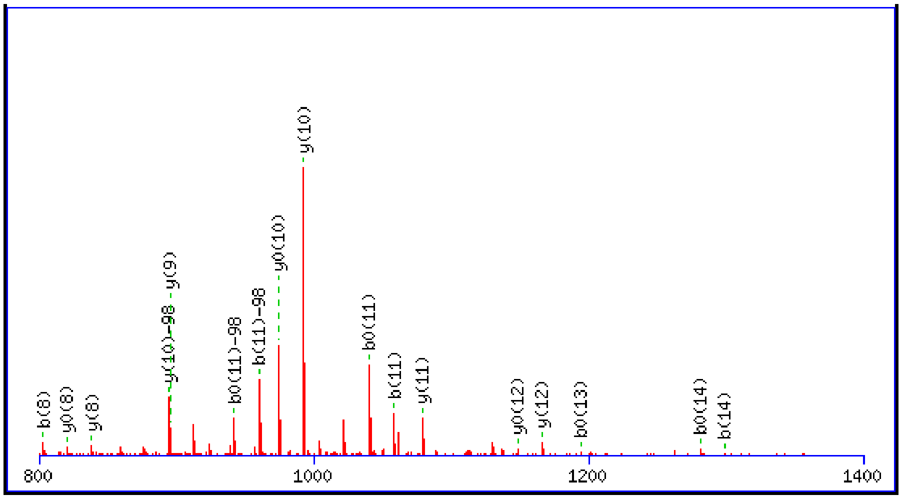

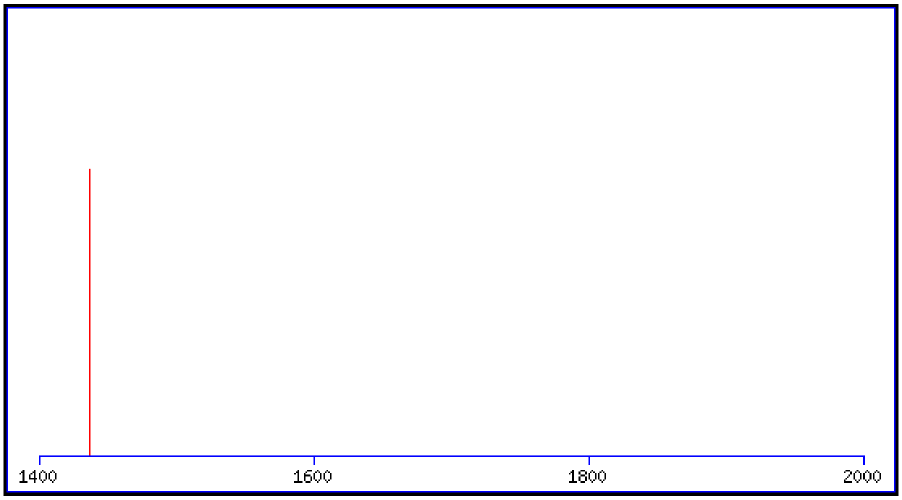

Monoisotopic mass of neutral peptide Mr(calc): 1472.5933
Fixed modifications: Carbamidomethyl (C)
Variable modifications:
S8 : Phospho (ST), with neutral losses 0.0000(shown in table), 97.9769
Ions Score: 74 **Expect:** 4.6e-006
Matches (Bold Red): 40/208 fragment ions using 61 most intense peaks

#	b	b^{++}	b^{0}	b^{0++}	Seq.	y	y^{++}	y^{*}	y^{*++}	y^{0}	y^{0++}	#
1	88.0393	44.5233	70.0287	35.5180	S							15
2	145.0608	73.0340	127.0502	64.0287	G	1386.5685	693.7879	1369.5420	685.2746	1368.5580	684.7826	14
3	**308.1241**	154.5657	290.1135	145.5604	Y	1329.5471	665.2772	1312.5205	**656.7639**	1311.5365	**656.2719**	13
4	**395.1561**	198.0817	377.1456	189.0764	S	**1166.4838**	**583.7455**	1149.4572	575.2322	**1148.4732**	**574.7402**	12
5	**482.1882**	241.5977	**464.1776**	232.5924	S	**1079.4517**	**540.2295**	1062.4252	531.7162	1061.4412	531.2242	11
6	579.2409	290.1241	561.2304	281.1188	P	**992.4197**	496.7135	975.3931	488.2002	**974.4091**	487.7082	10
7	636.2624	318.6348	618.2518	309.6295	G	**895.3669**	448.1871	878.3404	439.6738	877.3564	439.1818	9
8	**803.2607**	402.1340	**785.2502**	393.1287	S	**838.3455**	419.6764	821.3189	411.1631	**820.3349**	410.6711	8
9	900.3135	450.6604	882.3029	441.6551	P	**671.3471**	336.1772	**654.3206**	327.6639	**653.3365**	327.1719	7
10	957.3350	479.1711	939.3244	470.1658	G	**574.2944**	287.6508	557.2678	279.1375	556.2838	278.6455	6
11	**1058.3826**	529.6950	**1040.3721**	520.6897	T	**517.2729**	259.1401	500.2463	250.6268	499.2623	250.1348	5
12	1155.4354	578.2213	1137.4248	569.2161	P	**416.2252**	208.6162	**399.1987**	200.1030	**398.2146**	199.6110	4
13	1212.4569	606.7321	**1194.4463**	597.7268	G	**319.1724**	160.0899	302.1459	151.5766	**301.1619**	151.0846	3
14	**1299.4889**	650.2481	**1281.4783**	641.2428	S	**262.1510**	131.5791	245.1244	123.0659	244.1404	122.5738	2
15					R	175.1190	88.0631	158.0924	79.5498			1

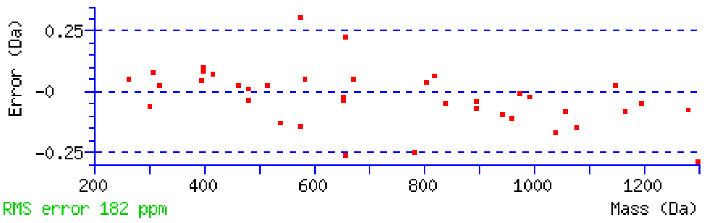

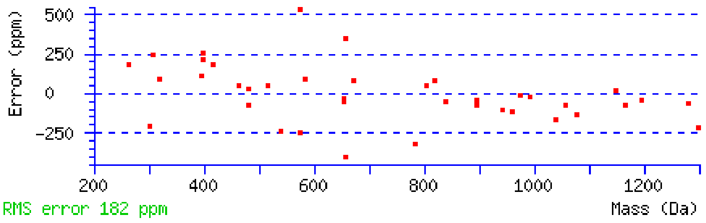

All matches to this query

Score	Mr(calc):	Delta	Sequence
74.1	1472.5933	-0.0020	SGYSSPGSPGTPGSR
39.1	1472.5933	-0.0020	SGYSSPGSPGTPGSR
36.2	1472.5933	-0.0020	SGYSSPGSPGTPGSR
29.3	1472.5933	-0.0020	SGYSSPGSPGTPGSR
23.4	1472.5933	-0.0020	SGYSSPGSPGTPGSR
19.7	1472.5933	-0.0020	SGYSSPGSPGTPGSR
15.1	1472.5933	-0.0020	SGYSSPGSPGTPGSR
6.0	1472.5901	0.0011	MPTGTSSCRPGSR
6.0	1472.5901	0.0011	MPTGTSSCRPGSR
6.0	1472.5901	0.0011	MPTGTSSCRPGSR

Spectrum No: 73; Query: 1229; Rank: 1

Peptide View

MS/MS Fragmentation of **STSPPPSPEVWAESR**
Found in **IPI00778976**, Tax_Id=10116 Gene_Symbol=Epb4.9_predicted 45 kDa protein

Match to Query 1229: 1785.699848 from(893.857200,2+)
Title: 091008RatKidney_NoSalt_32.1846.1846.2.dta
Data file K:\NewmanPaper\Piliang\3SubProteomes\Piliang3SP\mgf5ppm\ERLIC_3SubProteomes5ppm.mgf

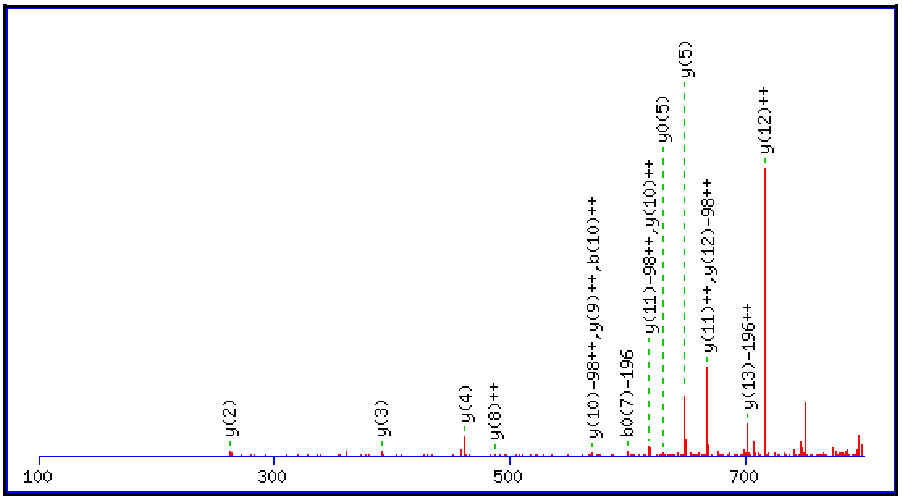

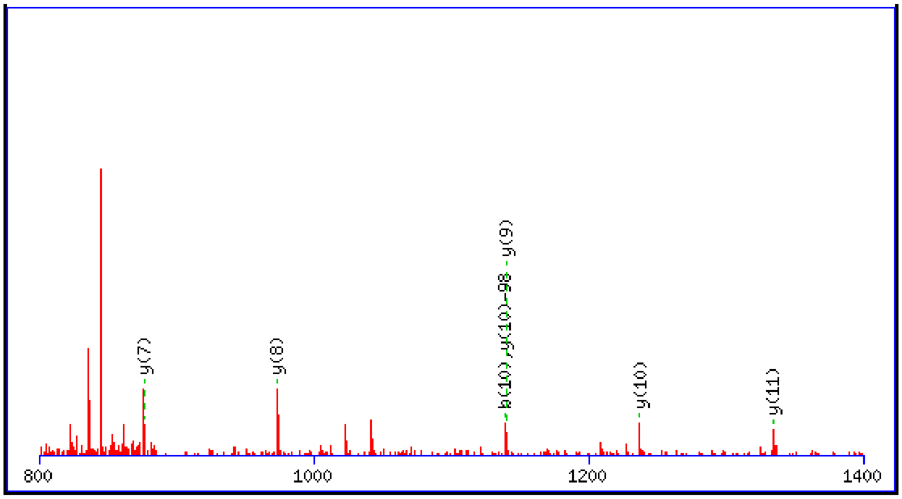

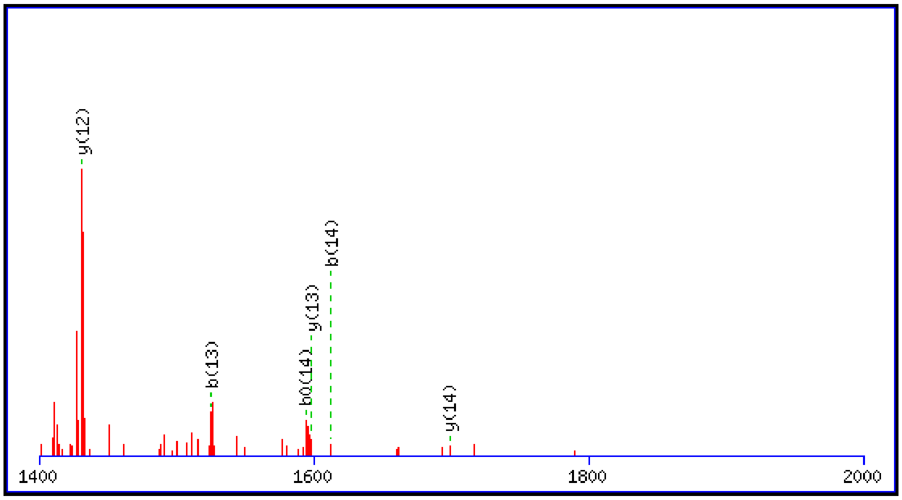

Monoisotopic mass of neutral peptide Mr(calc): 1785.7012
Fixed modifications: Carbamidomethyl (C)
Variable modifications:
S3 : Phospho (ST), with neutral losses 0.0000(shown in table), 97.9769
S7 : Phospho (ST), with neutral losses 0.0000(shown in table), 97.9769
Ions Score: 74 **Expect:** 5.5e-006
Matches (Bold Red): 29/222 fragment ions using 44 most intense peaks

#	b	b^{++}	b^{0}	b^{0++}	Seq.	y	y^{++}	y*	y^{*++}	y^{0}	y^{0++}	#
1	88.0393	44.5233	70.0287	35.5180	S							15
2	189.0870	95.0471	171.0764	86.0418	T	**1699.6764**	850.3419	1682.6499	841.8286	1681.6659	841.3366	14
3	356.0853	178.5463	338.0748	169.5410	S	**1598.6288**	799.8180	1581.6022	791.3047	1580.6182	790.8127	13
4	453.1381	227.0727	435.1275	218.0674	P	**1431.6304**	**716.3188**	1414.6039	707.8056	1413.6199	707.3136	12
5	550.1909	275.5991	532.1803	266.5938	P	**1334.5777**	**667.7925**	1317.5511	659.2792	1316.5671	658.7872	11
6	647.2436	324.1255	629.2331	315.1202	P	**1237.5249**	**619.2661**	1220.4983	610.7528	1219.5143	610.2608	10
7	814.2420	407.6246	796.2314	398.6193	S	**1140.4721**	**570.7397**	1123.4456	562.2264	1122.4616	561.7344	9
8	911.2947	456.1510	893.2842	447.1457	P	**973.4738**	**487.2405**	956.4472	478.7272	955.4632	478.2352	8
9	1040.3373	520.6723	1022.3268	511.6670	E	**876.4210**	438.7141	859.3945	430.2009	858.4104	429.7089	7
10	**1139.4058**	**570.2065**	1121.3952	561.2012	V	747.3784	374.1928	730.3519	365.6796	729.3678	365.1876	6
11	1325.4851	663.2462	1307.4745	654.2409	W	**648.3100**	324.6586	631.2835	316.1454	**630.2994**	315.6534	5
12	1396.5222	698.7647	1378.5116	689.7594	A	**462.2307**	231.6190	445.2041	223.1057	444.2201	222.6137	4
13	**1525.5648**	763.2860	1507.5542	754.2807	E	**391.1936**	196.1004	374.1670	187.5872	373.1830	187.0951	3
14	**1612.5968**	806.8020	**1594.5862**	797.7968	S	**262.1510**	131.5791	245.1244	123.0659	244.1404	122.5738	2
15					R	175.1190	88.0631	158.0924	79.5498			1

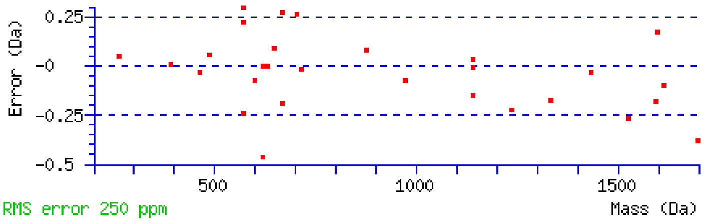

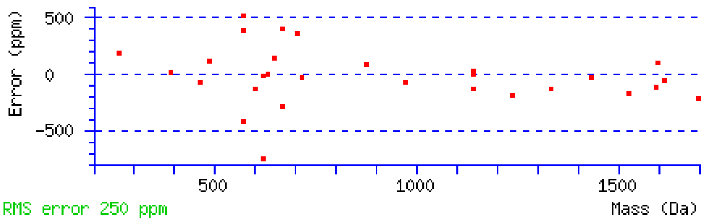

All matches to this query

Score	Mr(calc):	Delta	Sequence
73.9	1785.7012	-0.0013	STSPPPSPEVWAESR
73.9	1785.7012	-0.0013	STSPPPSPEVWAESR
60.9	1785.7012	-0.0013	STSPPPSPEVWAESR
26.0	1785.7012	-0.0013	STSPPPSPEVWAESR
17.2	1785.7012	-0.0013	STSPPPSPEVWAESR
17.2	1785.7012	-0.0013	STSPPPSPEVWAESR
7.1	1785.7012	-0.0013	STSPPPSPEVWAESR
7.1	1785.7012	-0.0013	STSPPPSPEVWAESR
0.9	1783.6957	2.0041	RPVSHGPSRDPSPR
0.9	1784.6978	1.0021	NHTTYNQSCKWHK

Spectrum No: 74; Query: 1506; Rank: 1

Peptide View

MS/MS Fragmentation of **LLHEDLDESDDDVDEK**
Found in **IPI00365595**, Tax_Id=10116 Gene_Symbol=Ddef2_predicted development and differentiation enhancing factor 2

Match to Query 1506: 1965.777108 from(983.895830,2+)
Title: 100101RatKid_NS_deglyco_24.1596.1596.2.dta
Data file K:\NewmanPaper\Piliang\3SubProteomes\Piliang3SP\mgf5ppm\ERLIC_3SubProteomes5ppm.mgf

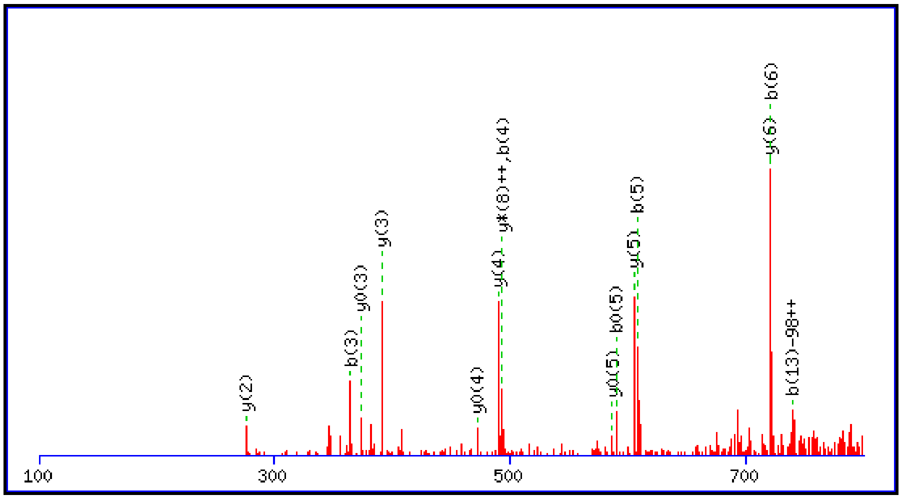

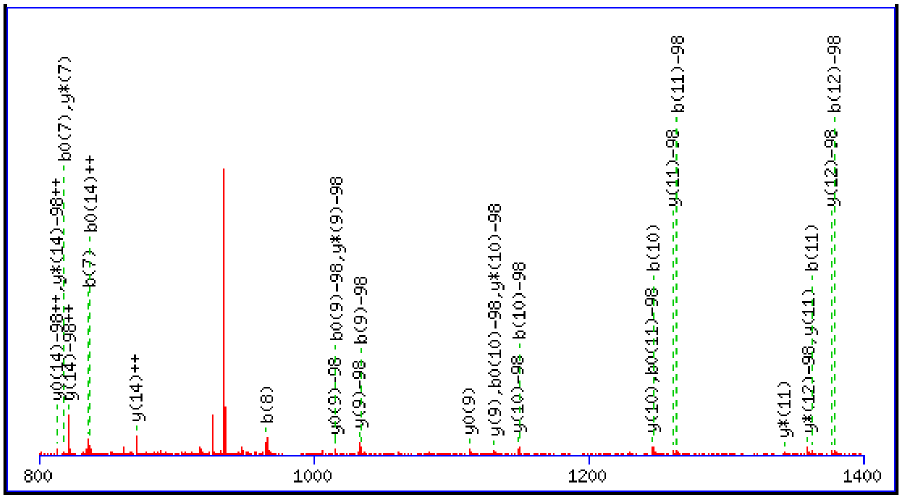

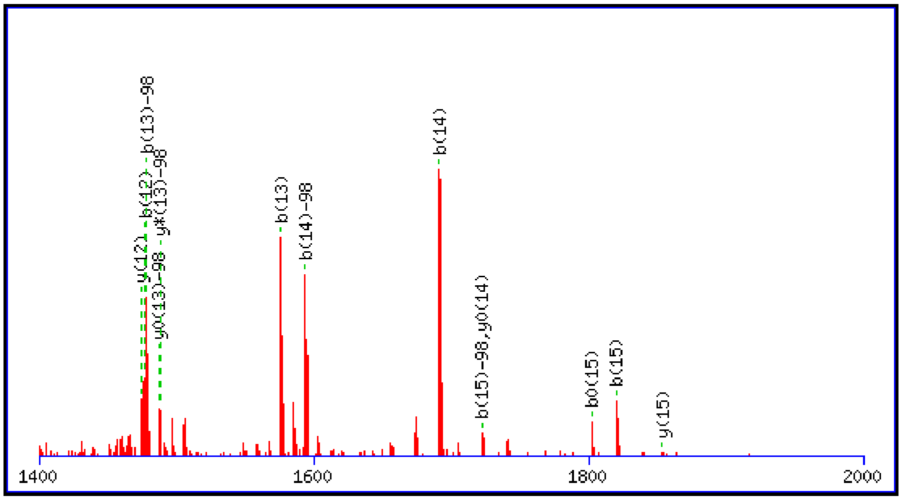

Monoisotopic mass of neutral peptide Mr(calc): 1965.7728
Fixed modifications: Carbamidomethyl (C)
Variable modifications:
S9 : Phospho (ST), with neutral losses 97.9769(shown in table), 0.0000
Ions Score: 74 **Expect:** 7e-006
Matches (Bold Red): 59/218 fragment ions using 92 most intense peaks

#	b	b^{++}	b^0	b^{0++}	Seq.	y	y^{++}	y*	y^{*++}	y^0	y^{0++}	#
1	114.0913	57.5493			L							16
2	227.1754	114.0913			L	1755.7192	878.3632	1738.6926	869.8499	1737.7086	869.3579	15
3	**364.2343**	182.6208			H	1642.6351	**821.8212**	1625.6085	**813.3079**	1624.6245	**812.8159**	14
4	**493.2769**	247.1421	475.2663	238.1368	E	1505.5762	753.2917	**1488.5496**	744.7785	**1487.5656**	744.2864	13
5	**608.3039**	304.6556	**590.2933**	295.6503	D	**1376.5336**	688.7704	**1359.5070**	680.2572	1358.5230	679.7651	12
6	**721.3879**	361.1976	703.3774	352.1923	L	**1261.5066**	631.2570	1244.4801	622.7437	1243.4961	622.2517	11
7	**836.4149**	418.7111	**818.4043**	409.7058	D	**1148.4226**	574.7149	**1131.3960**	566.2017	1130.4120	565.7096	10
8	**965.4575**	483.2324	947.4469	474.2271	E	**1033.3956**	517.2015	**1016.3691**	508.6882	**1015.3851**	508.1962	9
9	**1034.4789**	517.7431	**1016.4683**	508.7378	S	904.3530	452.6802	887.3265	444.1669	886.3425	443.6749	8
10	**1149.5059**	575.2566	**1131.4953**	566.2513	D	835.3316	418.1694	**818.3050**	409.6562	817.3210	409.1641	7
11	**1264.5328**	632.7700	**1246.5222**	623.7648	D	**720.3046**	360.6560	703.2781	352.1427	702.2941	351.6507	6
12	**1379.5597**	690.2835	1361.5492	681.2782	D	**605.2777**	303.1425	588.2511	294.6292	**587.2671**	294.1372	5
13	**1478.6282**	**739.8177**	1460.6176	730.8124	V	**490.2508**	245.6290	473.2242	237.1157	**472.2402**	236.6237	4
14	**1593.6551**	797.3312	1575.6445	788.3259	D	**391.1823**	196.0948	374.1558	187.5815	**373.1718**	187.0895	3
15	**1722.6977**	861.8525	1704.6871	852.8472	E	**276.1554**	138.5813	259.1288	130.0681	258.1448	129.5761	2
16					K	147.1128	74.0600	130.0863	65.5468			1

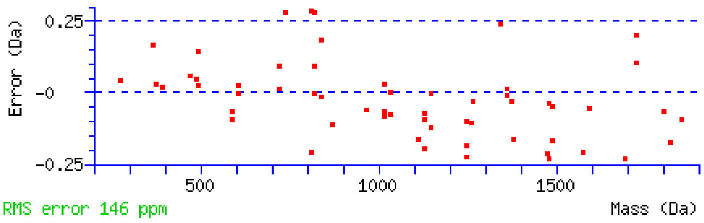

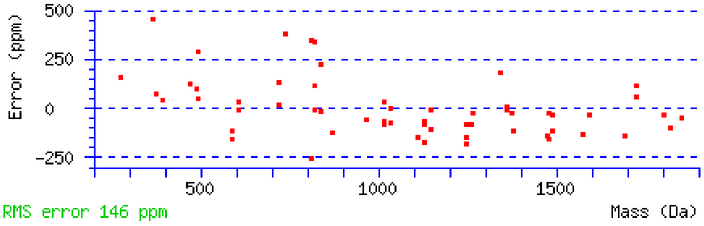

All matches to this query

Score	Mr(calc):	Delta	Sequence
73.6	1965.7728	0.0043	LLHEDLDESDDDVDEK
5.3	1965.7948	-0.0177	EMEAELNSTMKTVEDK
5.3	1965.7948	-0.0177	EMEAELNSTMKTVEDK
3.3	1963.7754	2.0017	TSNSYTAGGLFKESWR
2.4	1965.7744	0.0027	SCQGSCAALSGLTGCTTR
2.4	1965.7744	0.0027	SCQGSCAALSGLTGCTTR
2.4	1965.7744	0.0027	SCQGSCAALSGLTGCTTR
2.1	1963.7635	2.0137	VISQTNLITTVTPEK
1.8	1965.7849	-0.0078	VMNMEESNNGSLSAEFK
1.8	1965.7849	-0.0078	VMNMEESNNGSLSAEFK

Spectrum No: 75; Query: 1992; Rank: 1

Peptide View

MS/MS Fragmentation of **RFSHDGEEEEEDEDYGTR**
Found in **IPI00198768**, Tax_Id=10116 Gene_Symbol=Npuk68 Nuclear protein UKp68

Match to Query 1992: 2278.833942 from(760.618590,3+)
Title: 100101RatKid_NS_deglyco_19.1015.1015.3.dta
Data file K:\NewmanPaper\Piliang\3SubProteomes\Piliang3SP\mgf5ppm\ERLIC_3SubProteomes5ppm.mgf

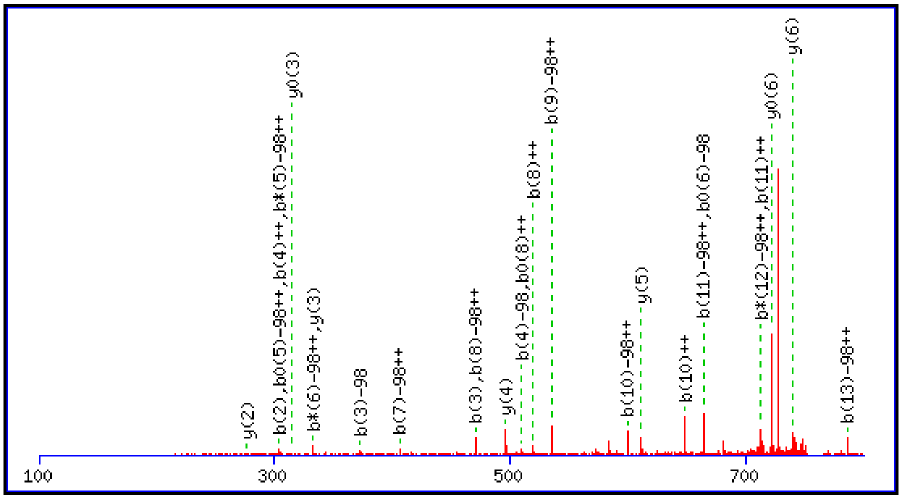

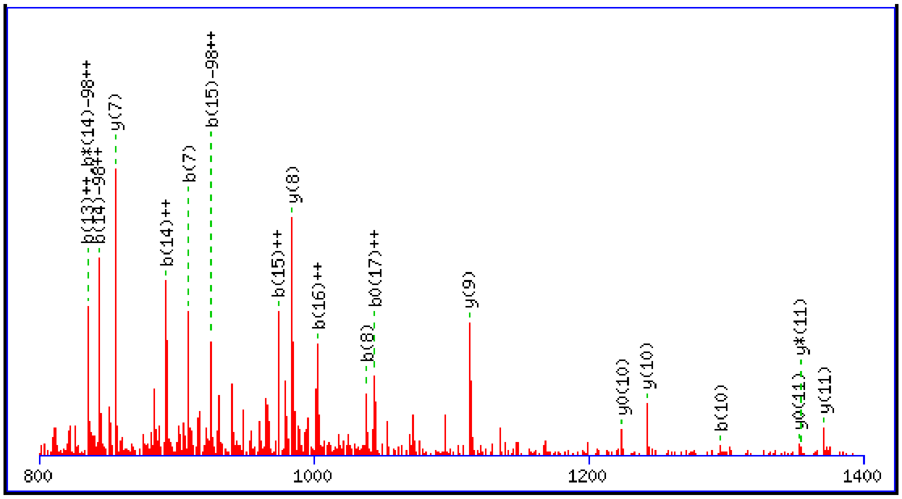

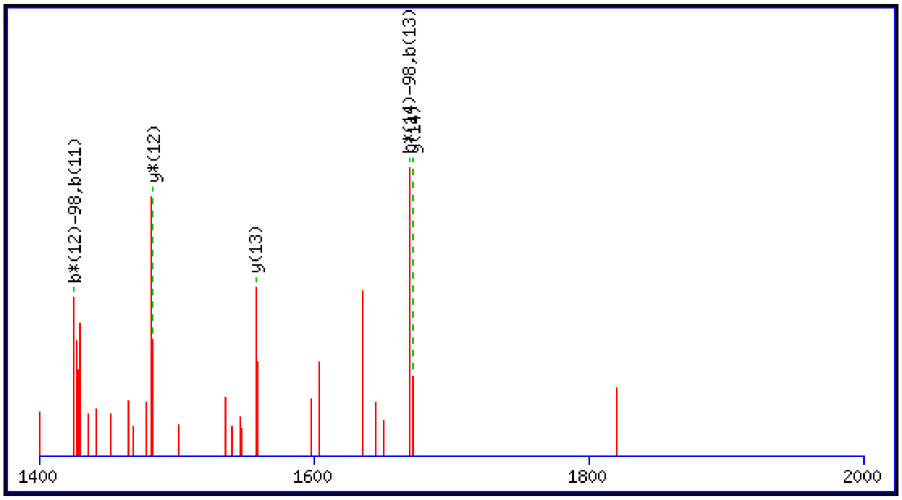

Monoisotopic mass of neutral peptide Mr(calc): 2278.8288
Fixed modifications: Carbamidomethyl (C)
Variable modifications:
S3 : Phospho (ST), with neutral losses 97.9769(shown in table), 0.0000
Ions Score: 73 **Expect:** 6.1e-006
Matches (Bold Red): 53/300 fragment ions using 67 most intense peaks

#	b	b^{++}	b*	b^{*++}	b^{0}	b^{0++}	Seq.	y	y^{++}	y*	y^{*++}	y^{0}	y^{0++}	#
1	157.1084	79.0578	140.0818	70.5446			R							18
2	**304.1768**	152.5920	287.1503	144.0788			F	2025.7581	1013.3827	2008.7315	1004.8694	2007.7475	1004.3774	17
3	**373.1983**	187.1028	356.1717	178.5895	355.1877	178.0975	S	1878.6896	939.8485	1861.6631	931.3352	1860.6791	930.8432	16
4	**510.2572**	255.6322	493.2306	247.1189	492.2466	246.6269	H	1809.6682	905.3377	1792.6416	896.8245	1791.6576	896.3324	15
5	625.2841	313.1457	608.2576	**304.6324**	607.2735	**304.1404**	D	**1672.6093**	836.8083	1655.5827	828.2950	1654.5987	827.8030	14
6	682.3056	341.6564	665.2790	**333.1432**	**664.2950**	332.6511	G	**1557.5823**	779.2948	1540.5558	770.7815	1539.5718	770.2895	13
7	811.3482	**406.1777**	794.3216	397.6644	793.3376	397.1724	E	1500.5609	750.7841	**1483.5343**	742.2708	1482.5503	741.7788	12
8	940.3908	**470.6990**	923.3642	462.1857	922.3802	461.6937	E	**1371.5183**	686.2628	**1354.4917**	677.7495	**1353.5077**	677.2575	11
9	1069.4334	**535.2203**	1052.4068	526.7070	1051.4228	526.2150	E	**1242.4757**	621.7415	1225.4491	613.2282	**1224.4651**	612.7362	10
10	1198.4759	**599.7416**	1181.4494	591.2283	1180.4654	590.7363	E	**1113.4331**	557.2202	1096.4065	548.7069	1095.4225	548.2149	9
11	1327.5185	**664.2629**	1310.4920	655.7496	1309.5080	655.2576	E	**984.3905**	492.6989	967.3639	484.1856	966.3799	483.6936	8
12	1442.5455	721.7764	**1425.5189**	**713.2631**	1424.5349	712.7711	D	**855.3479**	428.1776	838.3214	419.6643	837.3373	419.1723	7
13	1571.5881	**786.2977**	1554.5615	777.7844	1553.5775	777.2924	E	**740.3210**	370.6641	723.2944	362.1508	**722.3104**	361.6588	6
14	1686.6150	**843.8111**	**1669.5885**	**835.2979**	1668.6045	834.8059	D	**611.2784**	306.1428	594.2518	297.6295	593.2678	297.1375	5
15	1849.6783	**925.3428**	1832.6518	916.8295	1831.6678	916.3375	Y	**496.2514**	248.6293	479.2249	240.1161	478.2409	239.6241	4
16	1906.6998	953.8535	1889.6733	945.3403	1888.6892	944.8483	G	**333.1881**	167.0977	316.1615	158.5844	**315.1775**	158.0924	3
17	2007.7475	1004.3774	1990.7209	995.8641	1989.7369	995.3721	T	**276.1666**	138.5870	259.1401	130.0737	258.1561	129.5817	2
18							R	175.1190	88.0631	158.0924	79.5498			1

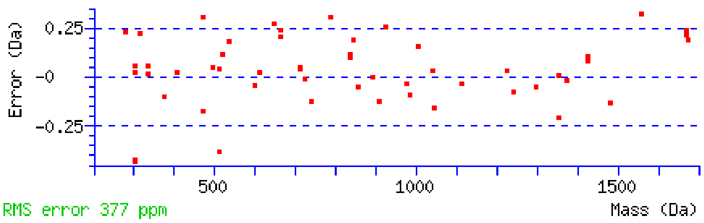

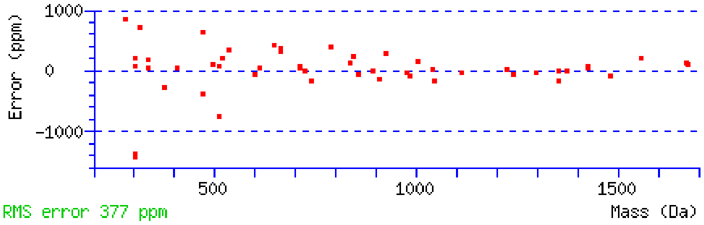

All matches to this query

Score	Mr(calc):	Delta	Sequence
73.3	2278.8288	0.0052	RFSHDGEEEEEDEDYGTR
15.8	2276.8487	1.9852	TGSSTNNNEEEKSRLLEK
15.8	2276.8487	1.9852	TGSSTNNNEEEKSRLLEK
15.8	2276.8487	1.9852	TGSSTNNNEEEKSRLLEK
15.8	2276.8487	1.9852	TGSSTNNNEEEKSRLLEK
15.8	2276.8487	1.9852	TGSSTNNNEEEKSRLLEK
15.8	2276.8487	1.9852	TGSSTNNNEEEKSRLLEK
11.1	2277.8327	1.0012	TGSSTNNNEEEKSRLLEK
11.1	2277.8327	1.0012	TGSSTNNNEEEKSRLLEK
11.1	2277.8327	1.0012	TGSSTNNNEEEKSRLLEK

Spectrum No: 76; Query: 1513; Rank: 1

Peptide View

MS/MS Fragmentation of **DWEDDSDEDMSNFDR**
Found in **IPI00365935**, Tax_Id=10116 Gene_Symbol=Ptges3_predicted Prostaglandin E synthase 3

Match to Query 1513: 1970.617328 from(986.315940,2+)
Title: 100101RatKid_NS_deglyco_27.1860.1860.2.dta
Data file K:\NewmanPaper\Piliang\3SubProteomes\Piliang3SP\mgf5ppm\ERLIC_3SubProteomes5ppm.mgf

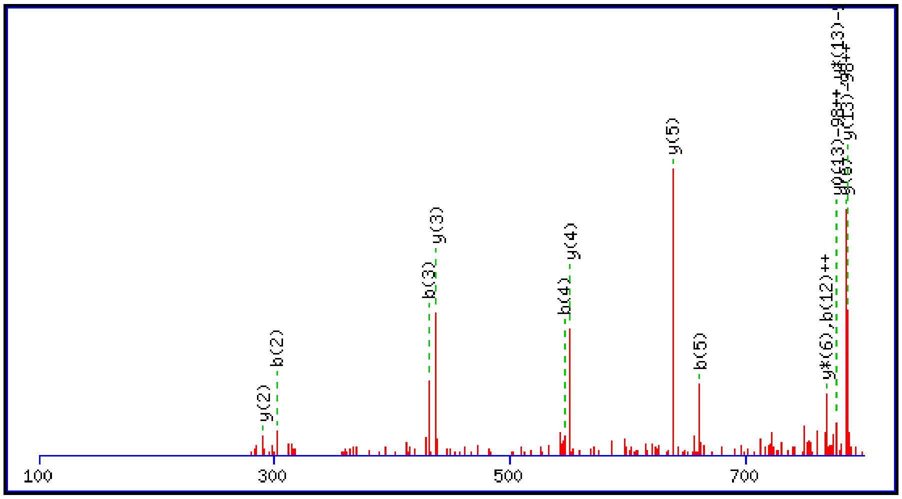

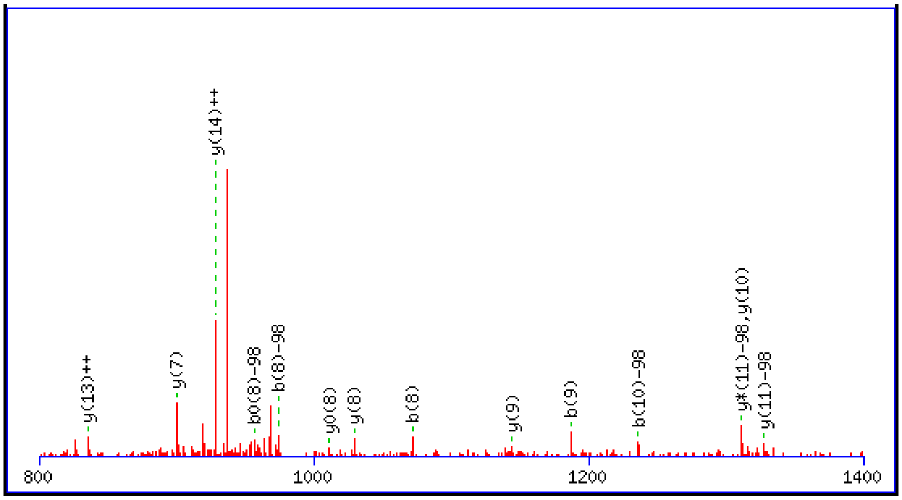

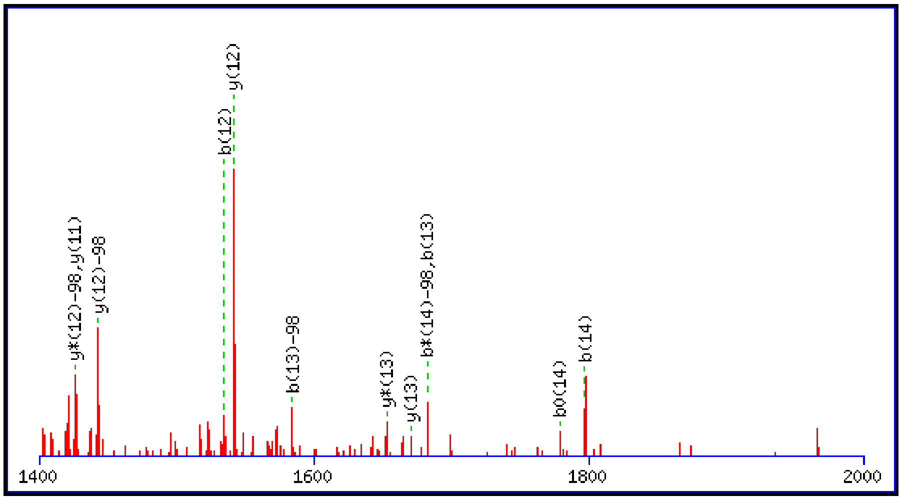

Monoisotopic mass of neutral peptide Mr(calc): 1970.6150
Fixed modifications: Carbamidomethyl (C)
Variable modifications:
S6 : Phospho (ST), with neutral losses 0.0000(shown in table), 97.9769
M10 : Oxidation (M)
Ions Score: 73 **Expect:** 1.5e-006
Matches (Bold Red): 40/216 fragment ions using 67 most intense peaks

#	b	b^{++}	b*	b^{*++}	b^0	b^{0++}	Seq.	y	y^{++}	y*	y^{*++}	y^0	y^{0++}	#
1	116.0342	58.5207			98.0237	49.5155	D							15
2	**302.1135**	151.5604			284.1030	142.5551	W	1856.5953	**928.8013**	1839.5687	920.2880	1838.5847	919.7960	14
3	**431.1561**	216.0817			413.1456	207.0764	E	**1670.5160**	**835.7616**	**1653.4894**	827.2484	1652.5054	826.7563	13
4	**546.1831**	273.5952			528.1725	264.5899	D	**1541.4734**	771.2403	1524.4468	762.7271	1523.4628	762.2350	12
5	**661.2100**	331.1086			643.1994	322.1034	D	**1426.4464**	713.7269	1409.4199	705.2136	1408.4359	704.7216	11
6	828.2084	414.6078			810.1978	405.6025	S	**1311.4195**	656.2134	1294.3930	647.7001	1293.4089	647.2081	10
7	943.2353	472.1213			925.2247	463.1160	D	**1144.4211**	572.7142	1127.3946	564.2009	1126.4106	563.7089	9
8	**1072.2779**	536.6426			1054.2673	527.6373	E	**1029.3942**	515.2007	1012.3677	506.6875	**1011.3836**	506.1955	8
9	**1187.3048**	594.1561			1169.2943	585.1508	D	**900.3516**	450.6794	883.3251	442.1662	882.3410	441.6742	7
10	1334.3402	667.6738			1316.3297	658.6685	M	**785.3247**	393.1660	**768.2981**	384.6527	767.3141	384.1607	6
11	1421.3723	711.1898			1403.3617	702.1845	S	**638.2893**	319.6483	621.2627	311.1350	620.2787	310.6430	5
12	**1535.4152**	**768.2112**	1518.3887	759.6980	1517.4046	759.2060	N	**551.2572**	276.1323	534.2307	267.6190	533.2467	267.1270	4
13	**1682.4836**	841.7454	1665.4571	833.2322	1664.4731	832.7402	F	**437.2143**	219.1108	420.1878	210.5975	419.2037	210.1055	3
14	**1797.5106**	899.2589	1780.4840	890.7456	**1779.5000**	890.2536	D	**290.1459**	145.5766	273.1193	137.0633	272.1353	136.5713	2
15							R	175.1190	88.0631	158.0924	79.5498			1

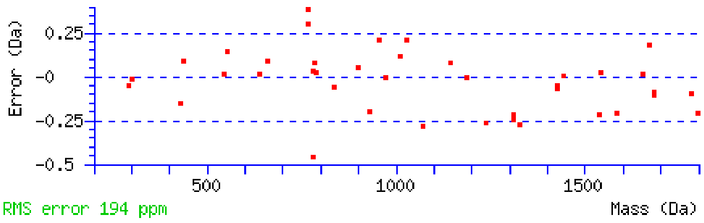

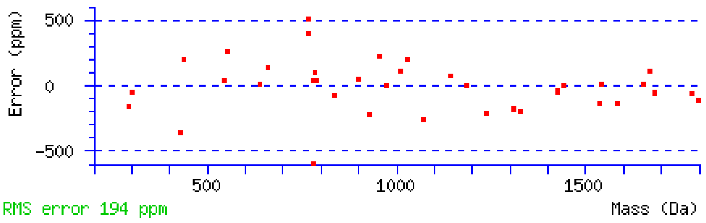

All matches to this query

Score	Mr(calc):	Delta	Sequence
73.2	1970.6150	0.0024	DWEDDSDEDMSNFDR
24.5	1970.6150	0.0024	DWEDDSDEDMSNFDR

Spectrum No: 77; Query: 2310; Rank: 1

Peptide View

MS/MS Fragmentation of **KVEEEQEADEEDVSEEETENR**
Found in **IPI00365626**, Tax_Id=10116 Gene_Symbol=Txndc1 Thioredoxin domain containing 1

Match to Query 2310: 2602.017468 from(1302.016010,2+)
Title: 100101RatKid_NS_deglyco_24.1022.1022.2.dta
Data file K:\NewmanPaper\Piliang\3SubProteomes\Piliang3SP\mgf5ppm\ERLIC_3SubProteomes5ppm.mgf

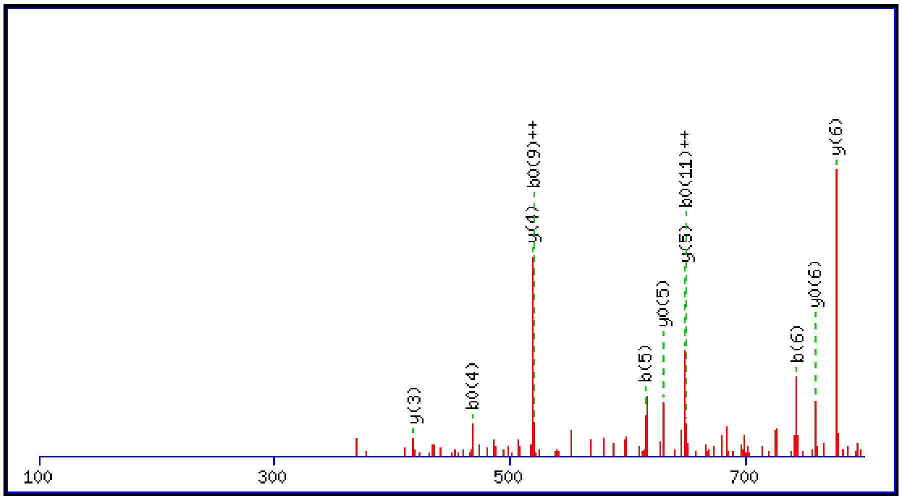

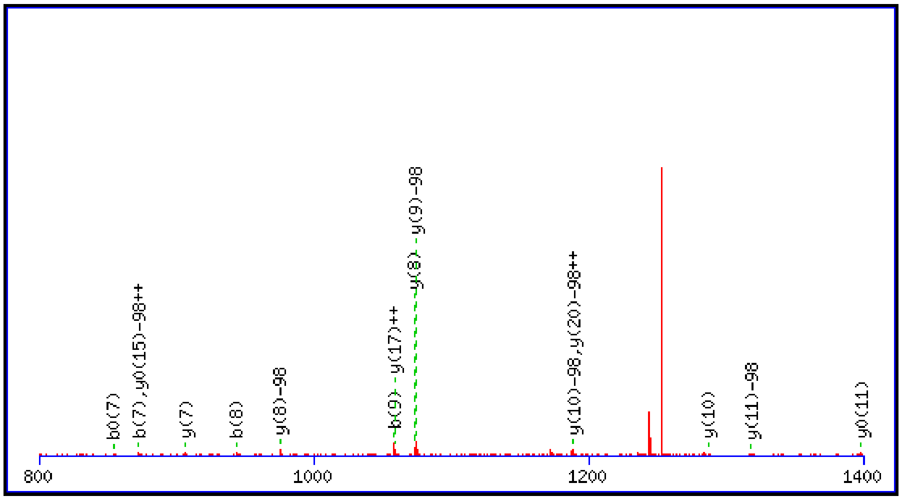

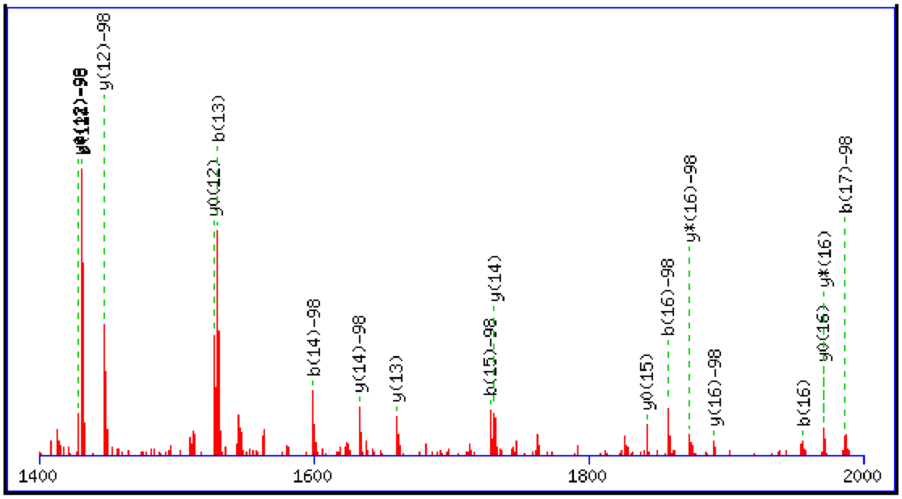

Monoisotopic mass of neutral peptide Mr(calc): 2602.0079
Fixed modifications: Carbamidomethyl (C)
Variable modifications:
S14 : Phospho (ST), with neutral losses 97.9769(shown in table), 0.0000
Ions Score: 72 **Expect:** 1.3e-005
Matches (Bold Red): 45/352 fragment ions using 58 most intense peaks

#	b	b^{++}	b^{*}	b^{*++}	b^{0}	b^{0++}	Seq.	y	y^{++}	y^{*}	y^{*++}	y^{0}	y^{0++}	#
1	129.1022	65.0548	112.0757	56.5415			K							21
2	228.1707	114.5890	211.1441	106.0757			V	2376.9434	**1188.9753**	2359.9168	1180.4620	2358.9328	1179.9700	20
3	357.2132	179.1103	340.1867	170.5970	339.2027	170.1050	E	2277.8749	1139.4411	2260.8484	1130.9278	2259.8644	1130.4358	19
4	486.2558	243.6316	469.2293	235.1183	**468.2453**	234.6263	E	2148.8323	1074.9198	2131.8058	1066.4065	2130.8218	1065.9145	18
5	**615.2984**	308.1529	598.2719	299.6396	597.2879	299.1476	E	2019.7898	1010.3985	2002.7632	1001.8852	2001.7792	1001.3932	17
6	**743.3570**	372.1821	726.3305	363.6689	725.3464	363.1769	Q	**1890.7472**	945.8772	**1873.7206**	937.3639	1872.7366	936.8719	16
7	**872.3996**	436.7034	855.3731	428.1902	**854.3890**	427.6982	E	1762.6886	881.8479	1745.6620	873.3347	1744.6780	**872.8426**	15
8	**943.4367**	472.2220	926.4102	463.7087	925.4262	463.2167	A	**1633.6460**	817.3266	1616.6194	808.8134	1615.6354	808.3213	14
9	**1058.4637**	529.7355	1041.4371	521.2222	1040.4531	**520.7302**	D	1562.6089	781.8081	1545.5823	773.2948	1544.5983	772.8028	13
10	1187.5063	594.2568	1170.4797	585.7435	1169.4957	585.2515	E	**1447.5819**	724.2946	**1430.5554**	715.7813	**1429.5714**	715.2893	12
11	1316.5488	658.7781	1299.5223	650.2648	1298.5383	**649.7728**	E	**1318.5393**	659.7733	1301.5128	651.2600	1300.5288	650.7680	11
12	**1431.5758**	716.2915	1414.5492	707.7783	1413.5652	707.2863	D	**1189.4967**	595.2520	1172.4702	586.7387	1171.4862	586.2467	10
13	**1530.6442**	765.8257	1513.6177	757.3125	1512.6336	756.8205	V	**1074.4698**	537.7385	1057.4433	529.2253	1056.4592	528.7333	9
14	**1599.6657**	800.3365	1582.6391	791.8232	1581.6551	791.3312	S	**975.4014**	488.2043	958.3748	479.6911	957.3908	479.1990	8
15	**1728.7083**	864.8578	1711.6817	856.3445	1710.6977	855.8525	E	**906.3799**	453.6936	889.3534	445.1803	888.3694	444.6883	7
16	**1857.7508**	929.3791	1840.7243	920.8658	1839.7403	920.3738	E	**777.3373**	389.1723	760.3108	380.6590	**759.3268**	380.1670	6
17	**1986.7934**	993.9004	1969.7669	985.3871	1968.7829	984.8951	E	**648.2947**	324.6510	631.2682	316.1377	**630.2842**	315.6457	5
18	2087.8411	1044.4242	2070.8146	1035.9109	2069.8306	1035.4189	T	**519.2522**	260.1297	502.2256	251.6164	501.2416	251.1244	4
19	2216.8837	1108.9455	2199.8572	1100.4322	2198.8731	1099.9402	E	**418.2045**	209.6059	401.1779	201.0926	400.1939	200.6006	3
20	2330.9266	1165.9670	2313.9001	1157.4537	2312.9161	1156.9617	N	289.1619	145.0846	272.1353	136.5713			2
21							R	175.1190	88.0631	158.0924	79.5498			1

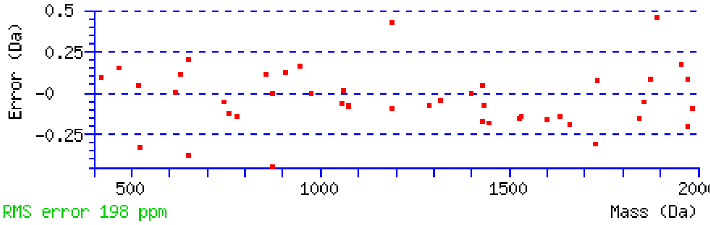

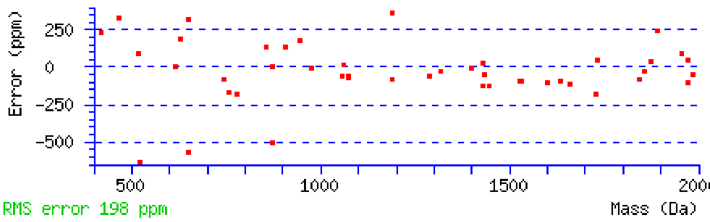

All matches to this query

Score	Mr(calc):	Delta	Sequence

72.3	2602.0079	0.0096	KVEEEQEADEEDVSEEETENR
55.5	2602.0079	0.0096	KVEEEQEADEEDVSEEETENR
7.5	2602.0132	0.0042	XFDDDEGEEEDEAAAAAAAAIGYR
6.0	2601.0309	0.9866	RQQFSPLSDYEGQEEELNGSK
3.6	2602.0374	-0.0199	CRYMDSKMKPLWLVYSNR
3.0	2602.0382	-0.0207	SWLPQSLLSLTKCTTQTYR
2.8	2602.0382	-0.0207	SWLPQSLLSLTKCTTQTYR
2.7	2601.0206	0.9969	DAASPSVDMSKTVGMTAEKWWE
2.4	2602.0353	-0.0178	RHLANMMGEDPETFTEEDIDR
2.4	2602.0353	-0.0178	RHLANMMGEDPETFTEEDIDR

Spectrum No: 78; Query: 888; Rank: 1

Peptide View

MS/MS Fragmentation of **EDEISPPPPNPVVK**
Found in **IPI00231770**, Tax_Id=10116 Gene_Symbol=Prkar1a cAMP-dependent protein kinase type I-alpha regulatory subunit

Match to Query 888: 1596.750548 from(799.382550,2+)
Title: 100101RatKid_NS_deglyco_20.1982.1982.2.dta
Data file K:\NewmanPaper\Piliang\3SubProteomes\Piliang3SP\mgf5ppm\ERLIC_3SubProteomes5ppm.mgf

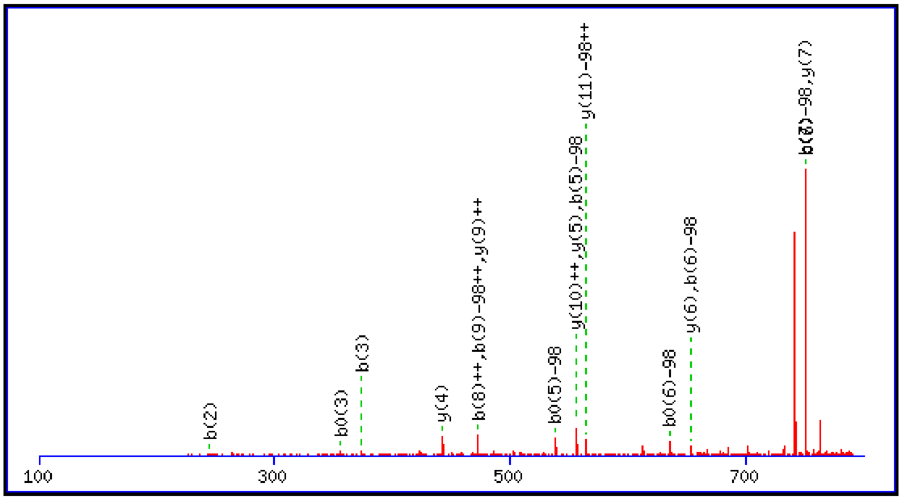

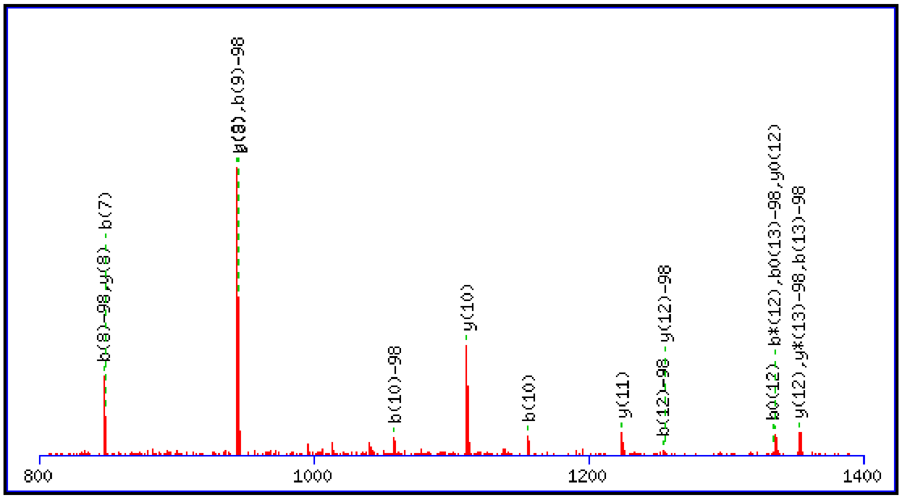

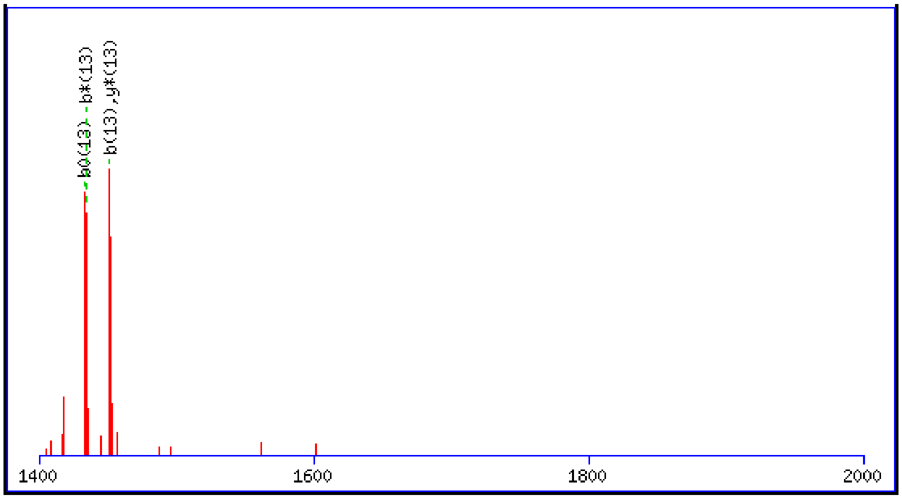

Monoisotopic mass of neutral peptide Mr(calc): 1596.7436
Fixed modifications: Carbamidomethyl (C)
Variable modifications:
S5 : Phospho (ST), with neutral losses 97.9769(shown in table), 0.0000
Ions Score: 72 **Expect:** 1.1e-005
Matches (Bold Red): 41/188 fragment ions using 46 most intense peaks

#	b	b^{++}	b*	b^{*++}	b^0	b^{0++}	Seq.	y	y^{++}	y*	y^{*++}	y^0	y^{0++}	#
1	130.0499	65.5286			112.0393	56.5233	E							14
2	**245.0768**	123.0420			227.0662	114.0368	D	1370.7314	685.8694	**1353.7049**	677.3561	1352.7209	676.8641	13
3	**374.1194**	187.5633			**356.1088**	178.5581	E	**1255.7045**	628.3559	1238.6779	619.8426	1237.6939	619.3506	12
4	487.2035	244.1054			469.1929	235.1001	I	1126.6619	**563.8346**	1109.6354	555.3213	1108.6513	554.8293	11
5	**556.2249**	278.6161			**538.2144**	269.6108	S	1013.5778	507.2926	996.5513	498.7793	995.5673	498.2873	10
6	**653.2777**	327.1425			**635.2671**	318.1372	P	**944.5564**	**472.7818**	927.5298	464.2686			9
7	**750.3305**	375.6689			732.3199	366.6636	P	**847.5036**	424.2554	830.4771	415.7422			8
8	**847.3832**	424.1952			829.3727	415.1900	P	**750.4509**	375.7291	733.4243	367.2158			7
9	**944.4360**	**472.7216**			926.4254	463.7163	P	**653.3981**	327.2027	636.3715	318.6894			6
10	**1058.4789**	529.7431	1041.4524	521.2298	1040.4683	520.7378	N	**556.3453**	278.6763	539.3188	270.1630			5
11	1155.5317	578.2695	1138.5051	569.7562	1137.5211	569.2642	P	**442.3024**	221.6548	425.2758	213.1416			4
12	**1254.6001**	627.8037	1237.5735	619.2904	1236.5895	618.7984	V	345.2496	173.1285	328.2231	164.6152			3
13	**1353.6685**	677.3379	1336.6420	668.8246	**1335.6579**	668.3326	V	246.1812	123.5942	229.1547	115.0810			2
14							K	147.1128	74.0600	130.0863	65.5468			1

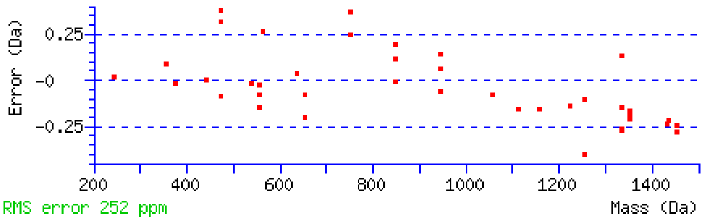

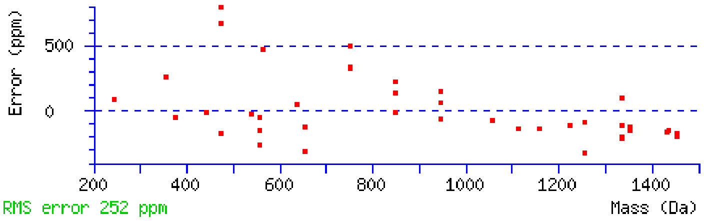

All matches to this query

Score	Mr(calc):	Delta	Sequence
72.2	1596.7436	0.0069	EDEISPPPPNPVVK
16.5	1596.7548	-0.0043	NYEPLQTPSARIK
10.3	1596.7623	-0.0117	MQYILLSPPSPQK
10.3	1596.7623	-0.0117	MQYILLSPPSPQK
7.3	1596.7565	-0.0060	NPTKLPLTLNPTK
6.4	1596.7565	-0.0060	NPTKLPLTLNPTK
5.5	1595.7457	1.0049	NEPTLPREFTRR

5.5	1596.7559	-0.0053	NIEPTEYIDDLFK
4.5	1595.7613	0.9892	ENRGIFEDMTKAGK
4.4	1596.7647	-0.0142	SSLKEQIVENLEK

Spectrum No: 79; Query: 2420; Rank: 1

Peptide View

MS/MS Fragmentation of **SSSLSDPLEESSPIAAIFDTENLEK**
Found in **IPI00366081**, Tax_Id=10116 Gene_Symbol=Dsp similar to desmoplakin isoform I isoform 2

Match to Query 2420: 2759.261388 from(1380.637970,2+)
Title: 091008RatKidney_NoSalt_23.5627.5627.2.dta
Data file K:\NewmanPaper\Piliang\3SubProteomes\Piliang3SP\mgf5ppm\ERLIC_3SubProteomes5ppm.mgf

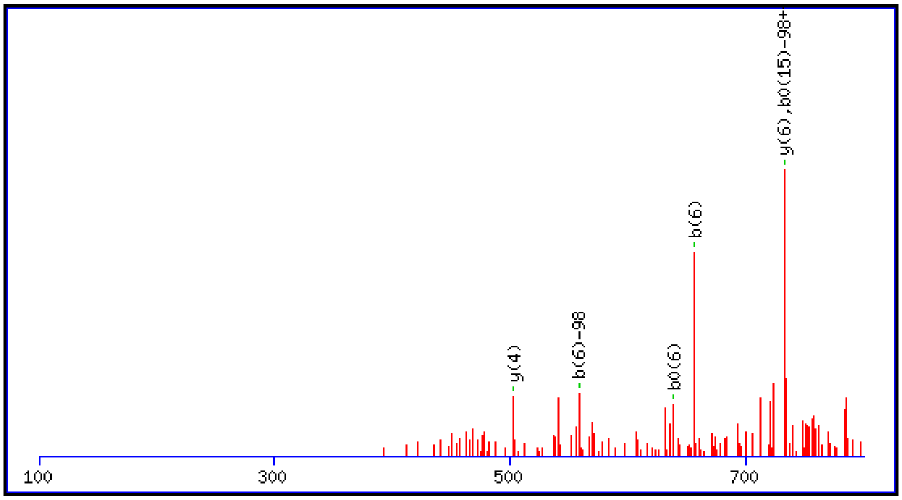

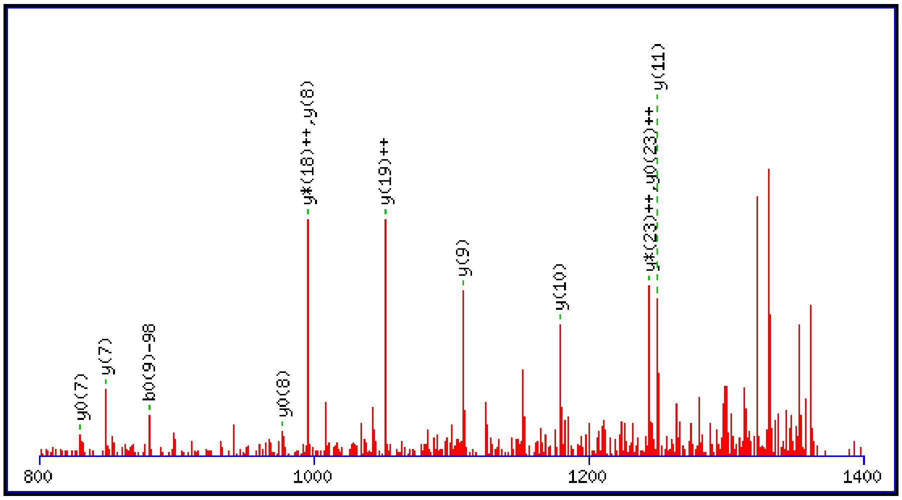

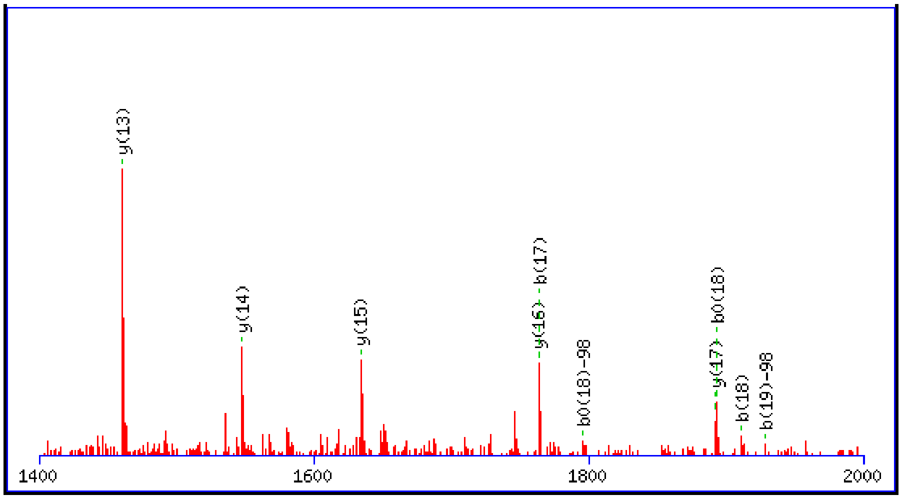

Monoisotopic mass of neutral peptide Mr(calc): 2758.2473
Fixed modifications: Carbamidomethyl (C)
Variable modifications:
S2 : Phospho (ST), with neutral losses 0.0000(shown in table), 97.9769
Ions Score: 72 **Expect:** 2.3e-005
Matches (Bold Red): 28/348 fragment ions using 39 most intense peaks

#	b	b^{++}	b*	b^{*++}	b^0	b^{0++}	Seq.	y	y^{++}	y*	y^{*++}	y^0	y^{0++}	#
1	88.0393	44.5233			70.0287	35.5180	S							25
2	255.0377	128.0225			237.0271	119.0172	S	2672.2226	1336.6149	2655.1961	1328.1017	2654.2121	1327.6097	24
3	342.0697	171.5385			324.0591	162.5332	S	2505.2243	1253.1158	2488.1977	**1244.6025**	2487.2137	**1244.1105**	23
4	455.1537	228.0805			437.1432	219.0752	L	2418.1922	1209.5998	2401.1657	1201.0865	2400.1817	1200.5945	22
5	542.1858	271.5965			524.1752	262.5912	S	2305.1082	1153.0577	2288.0816	1144.5445	2287.0976	1144.0524	21
6	**657.2127**	329.1100			**639.2022**	320.1047	D	2218.0761	1109.5417	2201.0496	1101.0284	2200.0656	1100.5364	20
7	754.2655	377.6364			736.2549	368.6311	P	2103.0492	**1052.0282**	2086.0227	1043.5150	2085.0386	1043.0230	19
8	867.3495	434.1784			849.3390	425.1731	L	2005.9964	1003.5019	1988.9699	**994.9886**	1987.9859	994.4966	18
9	996.3921	498.6997			978.3816	489.6944	E	**1892.9124**	946.9598	1875.8858	938.4466	1874.9018	937.9545	17
10	1125.4347	563.2210			1107.4242	554.2157	E	**1763.8698**	882.4385	1746.8432	873.9253	1745.8592	873.4332	16
11	1212.4668	606.7370			1194.4562	597.7317	S	**1634.8272**	817.9172	1617.8006	809.4040	1616.8166	808.9120	15
12	1299.4988	650.2530			1281.4882	641.2478	S	**1547.7952**	774.4012	1530.7686	765.8879	1529.7846	765.3959	14
13	1396.5516	698.7794			1378.5410	689.7741	P	**1460.7631**	730.8852	1443.7366	722.3719	1442.7526	721.8799	13
14	1509.6356	755.3214			1491.6251	746.3162	I	1363.7104	682.3588	1346.6838	673.8456	1345.6998	673.3535	12
15	1580.6727	790.8400			1562.6622	781.8347	A	**1250.6263**	625.8168	1233.5998	617.3035	1232.6157	616.8115	11
16	1651.7098	826.3586			1633.6993	817.3533	A	**1179.5892**	590.2982	1162.5626	581.7850	1161.5786	581.2930	10
17	**1764.7939**	882.9006			1746.7833	873.8953	I	**1108.5521**	554.7797	1091.5255	546.2664	1090.5415	545.7744	9
18	**1911.8623**	956.4348			**1893.8518**	947.4295	F	**995.4680**	498.2376	978.4415	489.7244	**977.4575**	489.2324	8
19	2026.8893	1013.9483			2008.8787	1004.9430	D	**848.3996**	424.7034	831.3731	416.1902	**830.3890**	415.6982	7
20	2127.9369	1064.4721			2109.9264	1055.4668	T	**733.3727**	367.1900	716.3461	358.6767	715.3621	358.1847	6
21	2256.9795	1128.9934			2238.9690	1119.9881	E	632.3250	316.6661	615.2984	308.1529	614.3144	307.6608	5
22	2371.0225	1186.0149	2353.9959	1177.5016	2353.0119	1177.0096	N	**503.2824**	252.1448	486.2558	243.6316	485.2718	243.1395	4
23	2484.1065	1242.5569	2467.0800	1234.0436	2466.0960	1233.5516	L	389.2395	195.1234	372.2129	186.6101	371.2289	186.1181	3
24	2613.1491	1307.0782	2596.1226	1298.5649	2595.1386	1298.0729	E	276.1554	138.5813	259.1288	130.0681	258.1448	129.5761	2
25							K	147.1128	74.0600	130.0863	65.5468			1

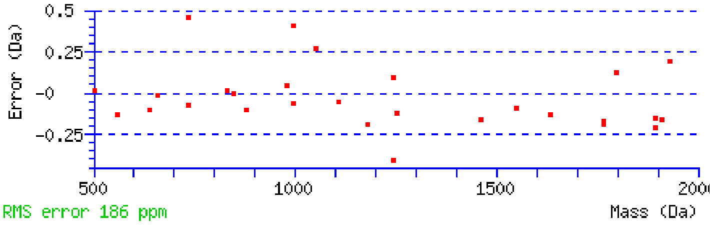

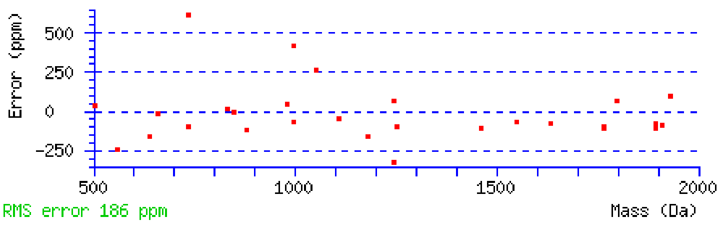

All matches to this query

Score	Mr(calc):	Delta	Sequence
72.0	2758.2473	1.0141	SSSLSDPLEESSPIAAIFDTENLEK
72.0	2758.2473	1.0141	SSSLSDPLEESSPIAAIFDTENLEK
69.2	2758.2473	1.0141	SSSLSDPLEESSPIAAIFDTENLEK
69.2	2758.2473	1.0141	SSSLSDPLEESSPIAAIFDTENLEK
34.3	2758.2473	1.0141	SSSLSDPLEESSPIAAIFDTENLEK
25.8	2758.2473	1.0141	SSSLSDPLEESSPIAAIFDTENLEK
3.7	2758.2377	1.0237	VDSRTALSVGGAPCTVLNVMLDCDK
3.5	2759.2877	-0.0263	IHVGYKDLMENYQIVVSNLAAER
3.4	2757.2560	2.0054	VKNGHVLESCAFYKEMDALVNCR
3.2	2759.2793	-0.0179	SYLLSVRPEASLSSNRLSHPSSGR

Spectrum No: 80; Query: 2750; Rank: 1

Peptide View

MS/MS Fragmentation of **EGSLGDELLPLGYAEPEPQEGASAGAPSPTLDLASR**
Found in **IPI00359734**, Tax_Id=10116 Gene_Symbol=Kctd12_predicted similar to Potassium channel tetramerisation domain containing protein 12

Match to Query 2750: 3673.705242 from(1225.575690,3+)
Title: 091008RatKidney_NoSalt_25.5170.5170.3.dta
Data file K:\NewmanPaper\Piliang\3SubProteomes\Piliang3SP\mgf5ppm\ERLIC_3SubProteomes5ppm.mgf

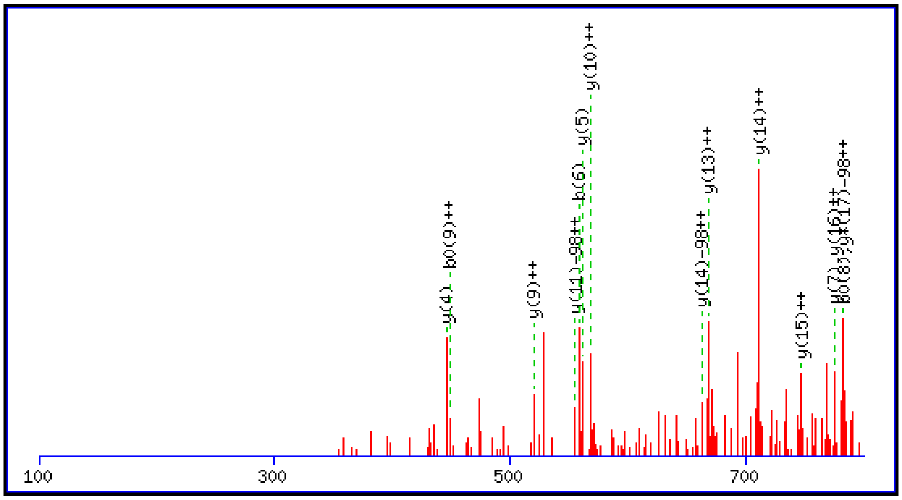

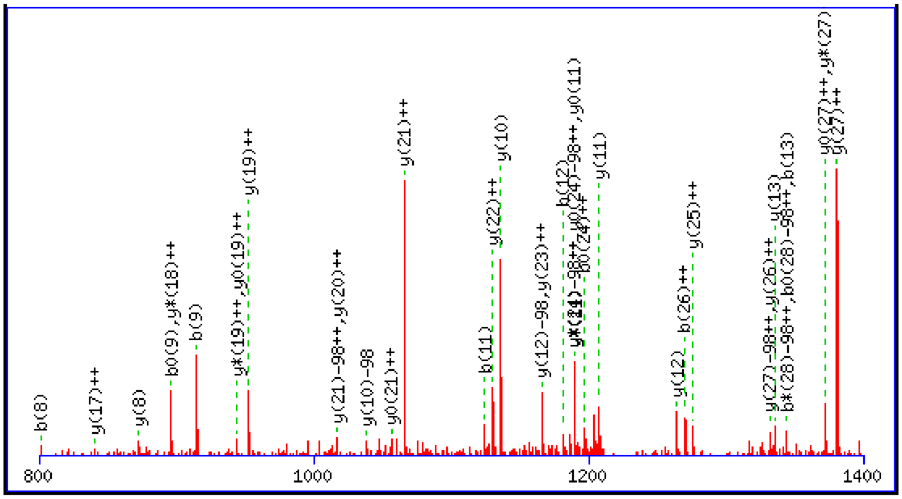

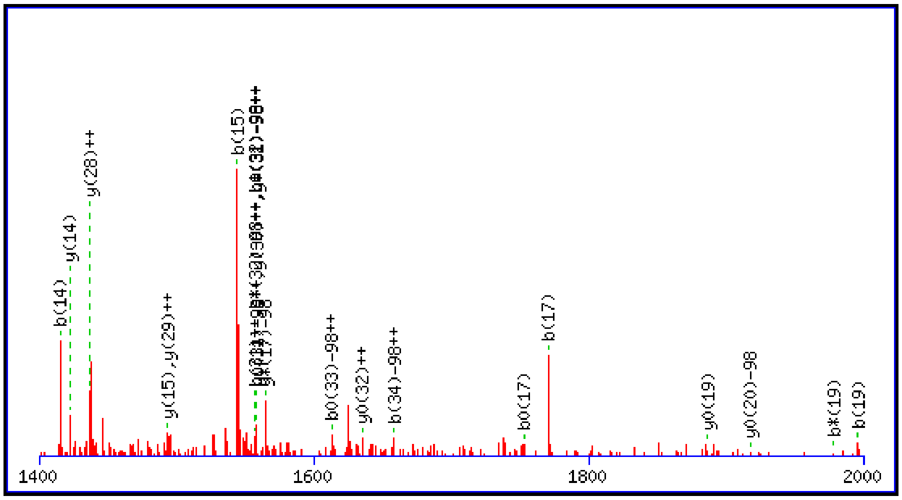

Monoisotopic mass of neutral peptide Mr(calc): 3673.7036
Fixed modifications: Carbamidomethyl (C)
Variable modifications:
S28 : Phospho (ST), with neutral losses 0.0000(shown in table), 97.9769
Ions Score: 72 **Expect:** 2.7e-005
Matches (Bold Red): 75/592 fragment ions using 123 most intense peaks

#	b	b^{++}	b*	b^{*++}	b^0	b^{0++}	Seq.	y	y^{++}	y*	y^{*++}	y^0	y^{0++}	#
1	130.0499	65.5286			112.0393	56.5233	E							36
2	187.0713	94.0393			169.0608	85.0340	G	3545.6683	1773.3378	3528.6418	1764.8245	3527.6578	1764.3325	35
3	274.1034	137.5553			256.0928	128.5500	S	3488.6469	1744.8271	3471.6203	1736.3138	3470.6363	1735.8218	34
4	387.1874	194.0974			369.1769	185.0921	L	3401.6148	1701.3111	3384.5883	1692.7978	3383.6043	1692.3058	33
5	444.2089	222.6081			426.1983	213.6028	G	3288.5308	1644.7690	3271.5042	1636.2557	3270.5202	**1635.7637**	32
6	**559.2358**	280.1216			541.2253	271.1163	D	3231.5093	1616.2583	3214.4828	1607.7450	3213.4987	1607.2530	31
7	688.2784	344.6429			670.2679	335.6376	E	3116.4824	**1558.7448**	3099.4558	1550.2315	3098.4718	1549.7395	30
8	**801.3625**	401.1849			**783.3519**	392.1796	L	2987.4398	**1494.2235**	2970.4132	1485.7102	2969.4292	1485.2182	29
9	**914.4466**	457.7269			**896.4360**	**448.7216**	L	2874.3557	**1437.6815**	2857.3292	1429.1682	2856.3451	1428.6762	28
10	1011.4993	506.2533			993.4888	497.2480	P	2761.2716	**1381.1395**	2744.2451	**1372.6262**	2743.2611	**1372.1342**	27
11	**1124.5834**	562.7953			1106.5728	553.7900	L	2664.2189	**1332.6131**	2647.1923	1324.0998	2646.2083	1323.6078	26
12	**1181.6048**	591.3061			1163.5943	582.3008	G	2551.1348	**1276.0710**	2534.1083	1267.5578	2533.1242	1267.0658	25
13	**1344.6682**	672.8377			1326.6576	663.8324	Y	2494.1134	1247.5603	2477.0868	1239.0470	2476.1028	1238.5550	24
14	**1415.7053**	708.3563			1397.6947	699.3510	A	2331.0500	**1166.0286**	2314.0235	1157.5154	2313.0395	1157.0234	23
15	**1544.7479**	772.8776			1526.7373	763.8723	E	2260.0129	**1130.5101**	2242.9864	1121.9968	2242.0023	1121.5048	22
16	1641.8006	821.4040			1623.7901	812.3987	P	2130.9703	**1065.9888**	2113.9438	1057.4755	2112.9597	**1056.9835**	21
17	**1770.8432**	885.9253			**1752.8327**	876.9200	E	2033.9176	**1017.4624**	2016.8910	1008.9491	2015.9070	1008.4571	20
18	1867.8960	934.4516			1849.8854	925.4464	P	1904.8750	**952.9411**	1887.8484	**944.4278**	**1886.8644**	**943.9358**	19

**19**	**1995.9546**	998.4809	**1978.9280**	989.9677	1977.9440	989.4756	**Q**	1807.8222	904.4147	1790.7956	**895.9015**	1789.8116	895.4095	**18**
**20**	2124.9972	1063.0022	2107.9706	1054.4889	2106.9866	1053.9969	**E**	1679.7636	**840.3854**	1662.7371	831.8722	1661.7531	831.3802	**17**
**21**	2182.0186	1091.5130	2164.9921	1082.9997	2164.0081	1082.5077	**G**	1550.7210	**775.8641**	1533.6945	767.3509	1532.7105	766.8589	**16**
**22**	2253.0558	1127.0315	2236.0292	1118.5182	2235.0452	1118.0262	**A**	**1493.6996**	**747.3534**	1476.6730	738.8401	1475.6890	738.3481	**15**
**23**	2340.0878	1170.5475	2323.0612	1162.0343	2322.0772	1161.5422	**S**	**1422.6624**	**711.8349**	1405.6359	703.3216	1404.6519	702.8296	**14**
**24**	2411.1249	1206.0661	2394.0983	1197.5528	2393.1143	**1197.0608**	**A**	**1335.6304**	**668.3188**	1318.6039	659.8056	1317.6199	659.3136	**13**
**25**	2468.1464	1234.5768	2451.1198	1226.0635	2450.1358	1225.5715	**G**	**1264.5933**	632.8003	1247.5668	624.2870	1246.5827	623.7950	**12**
**26**	2539.1835	**1270.0954**	2522.1569	1261.5821	2521.1729	1261.0901	**A**	**1207.5718**	604.2896	**1190.5453**	595.7763	**1189.5613**	595.2843	**11**
**27**	2636.2362	1318.6218	2619.2097	1310.1085	2618.2257	1309.6165	**P**	**1136.5347**	**568.7710**	1119.5082	560.2577	1118.5242	559.7657	**10**
**28**	2803.2346	1402.1209	2786.2080	1393.6077	2785.2240	1393.1156	**S**	1039.4820	**520.2446**	1022.4554	511.7313	1021.4714	511.2393	**9**
**29**	2900.2874	1450.6473	2883.2608	1442.1340	2882.2768	1441.6420	**P**	**872.4836**	436.7454	855.4571	428.2322	854.4730	427.7402	**8**
**30**	3001.3350	1501.1712	2984.3085	1492.6579	2983.3245	1492.1659	**T**	**775.4308**	388.2191	758.4043	379.7058	757.4203	379.2138	**7**
**31**	3114.4191	**1557.7132**	3097.3925	1549.1999	3096.4085	1548.7079	**L**	674.3832	337.6952	657.3566	329.1819	656.3726	328.6899	**6**
**32**	3229.4460	1615.2267	3212.4195	1606.7134	3211.4355	1606.2214	**D**	**561.2991**	281.1532	544.2726	272.6399	543.2885	272.1479	**5**
**33**	3342.5301	1671.7687	3325.5036	1663.2554	3324.5195	1662.7634	**L**	**446.2722**	223.6397	429.2456	215.1264	428.2616	214.6344	**4**
**34**	3413.5672	1707.2872	3396.5407	1698.7740	3395.5567	1698.2820	**A**	333.1881	167.0977	316.1615	158.5844	315.1775	158.0924	**3**
**35**	3500.5992	1750.8033	3483.5727	1742.2900	3482.5887	1741.7980	**S**	262.1510	131.5791	245.1244	123.0659	244.1404	122.5738	**2**
**36**							**R**	175.1190	88.0631	158.0924	79.5498			**1**

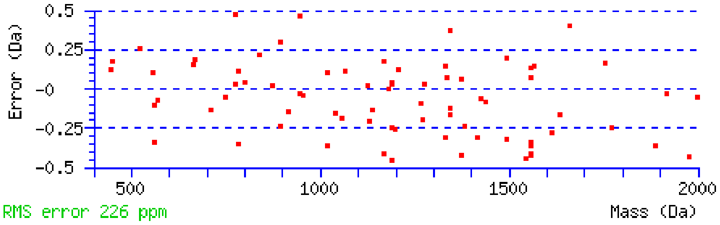

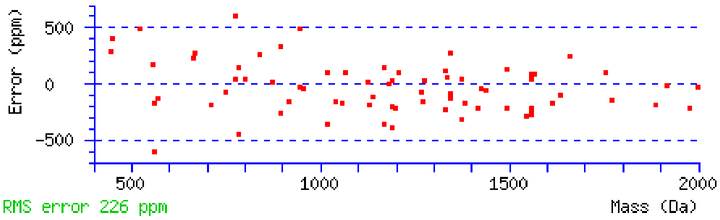

All matches to this query

Score	Mr(calc):	Delta	Sequence
71.9	3673.7036	0.0016	EGSLGDELLPLGYAEPEPQEGASAGAPSPTLDLASR
70.8	3673.7036	0.0016	EGSLGDELLPLGYAEPEPQEGASAGAPSPTLDLASR
69.7	3673.7036	0.0016	EGSLGDELLPLGYAEPEPQEGASAGAPSPTLDLASR
50.8	3673.7036	0.0016	EGSLGDELLPLGYAEPEPQEGASAGAPSPTLDLASR
8.5	3673.6962	0.0090	DNNNKDVIVAVMYTVVTPMLNPFIYSLRNR
7.6	3673.6962	0.0090	DNNNKDVIVAVMYTVVTPMLNPFIYSLRNR
7.6	3673.6962	0.0090	DNNNKDVIVAVMYTVVTPMLNPFIYSLRNR
7.0	3673.6962	0.0090	DNNNKDVIVAVMYTVVTPMLNPFIYSLRNR
6.2	3673.6962	0.0090	DNNNKDVIVAVMYTVVTPMLNPFIYSLRNR
6.2	3673.6962	0.0090	DNNNKDVIVAVMYTVVTPMLNPFIYSLRNR

Spectrum No: 81; Query: 1551; Rank: 1

Peptide View

MS/MS Fragmentation of **AASDGQYENQSPEATSPR**
Found in **IPI00362755**, Tax_Id=10116 Gene_Symbol=Tns similar to tensin

Match to Query 1551: 1986.802248 from(994.408400,2+)
Title: 100101RatKid_NS_deglyco_23.808.808.2.dta
Data file K:\NewmanPaper\Piliang\3SubProteomes\Piliang3SP\mgf5ppm\ERLIC_3SubProteomes5ppm.mgf

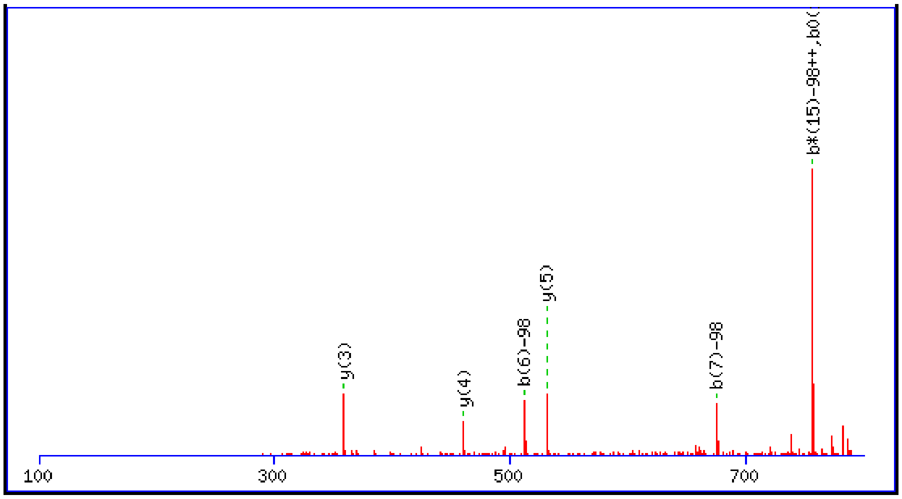

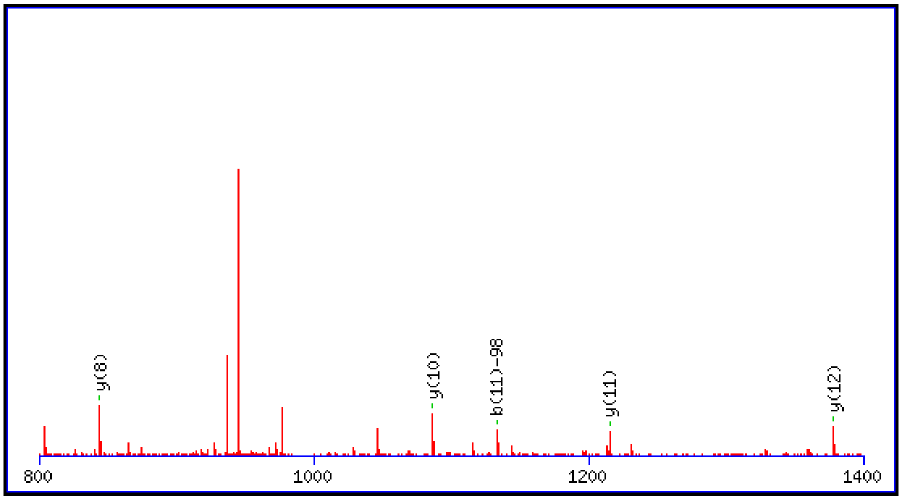

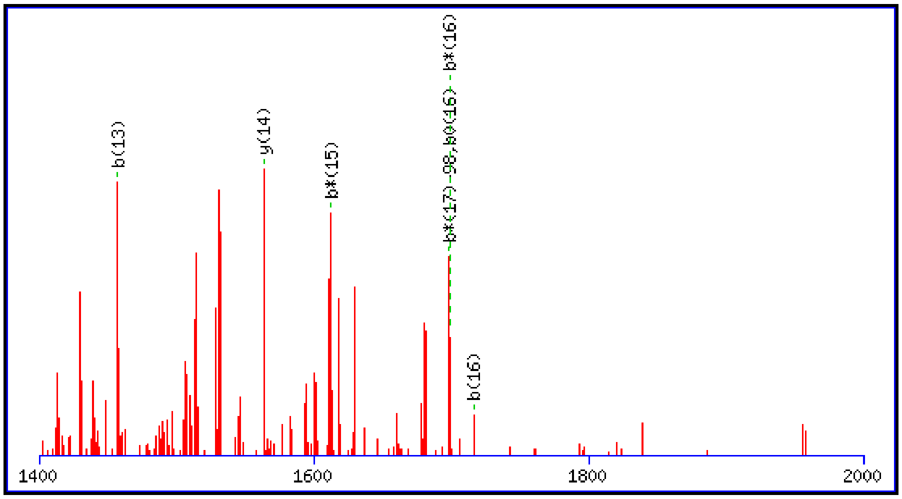

Monoisotopic mass of neutral peptide Mr(calc): 1986.7956
Fixed modifications: Carbamidomethyl (C)
Variable modifications:
S3 : Phospho (ST), with neutral losses 97.9769(shown in table), 0.0000
Ions Score: 72 **Expect:** 1.2e-005
Matches (Bold Red): 20/282 fragment ions using 23 most intense peaks

#	b	b^{++}	b*	b^{*++}	b^0	b^{0++}	Seq.	y	y^{++}	y*	y^{*++}	y^0	y^{0++}	#
1	72.0444	36.5258					A							18
2	143.0815	72.0444					A	1818.7889	909.8981	1801.7624	901.3848	1800.7783	900.8928	17
3	212.1030	106.5551			194.0924	97.5498	S	1747.7518	874.3795	1730.7252	865.8663	1729.7412	865.3743	16
4	327.1299	164.0686			309.1193	155.0633	D	1678.7303	839.8688	1661.7038	831.3555	1660.7198	830.8635	15
5	384.1514	192.5793			366.1408	183.5740	G	**1563.7034**	782.3553	1546.6768	773.8421	1545.6928	773.3501	14

6	**512.2099**	256.6086	495.1834	248.0953	494.1994	247.6033	Q	1506.6819	753.8446	1489.6554	745.3313	1488.6714	744.8393	13
7	**675.2733**	338.1403	658.2467	329.6270	657.2627	329.1350	Y	**1378.6233**	689.8153	1361.5968	681.3020	1360.6128	680.8100	12
8	804.3159	402.6616	787.2893	394.1483	786.3053	393.6563	E	**1215.5600**	608.2836	1198.5335	599.7704	1197.5495	599.2784	11
9	918.3588	459.6830	901.3322	451.1698	900.3482	450.6778	N	**1086.5174**	543.7624	1069.4909	535.2491	1068.5069	534.7571	10
10	1046.4174	523.7123	1029.3908	515.1990	1028.4068	514.7070	Q	972.4745	486.7409	955.4480	478.2276	954.4639	477.7356	9
11	**1133.4494**	567.2283	1116.4229	558.7151	1115.4388	558.2231	S	**844.4159**	422.7116	827.3894	414.1983	826.4054	413.7063	8
12	1230.5022	615.7547	1213.4756	607.2414	1212.4916	606.7494	P	**757.3839**	379.1956	740.3573	370.6823	739.3733	370.1903	7
13	1359.5448	680.2760	1342.5182	671.7627	1341.5342	671.2707	E	660.3311	330.6692	643.3046	322.1559	642.3206	321.6639	6
14	1430.5819	715.7946	1413.5553	707.2813	1412.5713	706.7893	A	**531.2885**	266.1479	514.2620	257.6346	513.2780	257.1426	5
15	1531.6296	766.3184	1514.6030	**757.8051**	1513.6190	**757.3131**	T	**460.2514**	230.6293	443.2249	222.1161	442.2409	221.6241	4
16	1618.6616	809.8344	1601.6350	801.3212	1600.6510	800.8291	S	**359.2037**	180.1055	342.1772	171.5922	341.1932	171.1002	3
17	1715.7143	858.3608	**1698.6878**	849.8475	1697.7038	849.3555	P	272.1717	136.5895	255.1452	128.0762			2
18							R	175.1190	88.0631	158.0924	79.5498			1

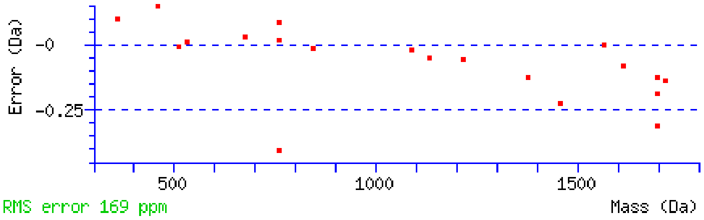

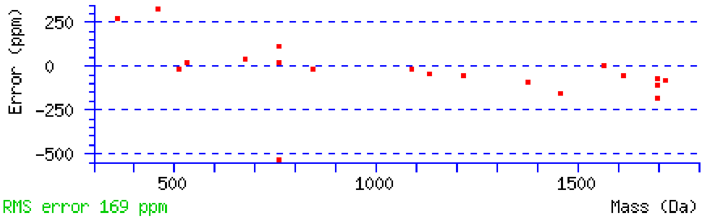

All matches to this query

Score	Mr(calc):	Delta	Sequence
71.9	1986.7956	0.0066	AASDGQYENQSPEATSPR
30.1	1986.7956	0.0066	AASDGQYENQSPEATSPR
21.2	1986.7956	0.0066	AASDGQYENQSPEATSPR
14.3	1986.7956	0.0066	AASDGQYENQSPEATSPR
5.9	1985.8116	0.9906	QSPFASSSEHSSENGSLR
2.1	1984.7784	2.0238	LNKDEYEFFTYLDK
1.5	1986.7860	0.0162	DENYLDKESKQTTEK
1.3	1984.7784	2.0238	LNKDEYEFFTYLDK
1.0	1986.7956	0.0066	QSPFASSSEHSSENGSLR
1.0	1986.7956	0.0066	QSPFASSSEHSSENGSLR

Spectrum No: 82; Query: 1869; Rank: 1

Peptide View

MS/MS Fragmentation of **RGSGDTSSLIDPDTSLSELR**
Found in **IPI00373564**, Tax_Id=10116 Gene_Symbol=Lrrfip2 Leucine-rich repeat flightless-interacting protein 2

Match to Query 1869: 2184.998228 from(1093.506390,2+)
Title: 091008RatKidney_NoSalt_21.3372.3372.2.dta
Data file K:\NewmanPaper\Piliang\3SubProteomes\Piliang3SP\mgf5ppm\ERLIC_3SubProteomes5ppm.mgf

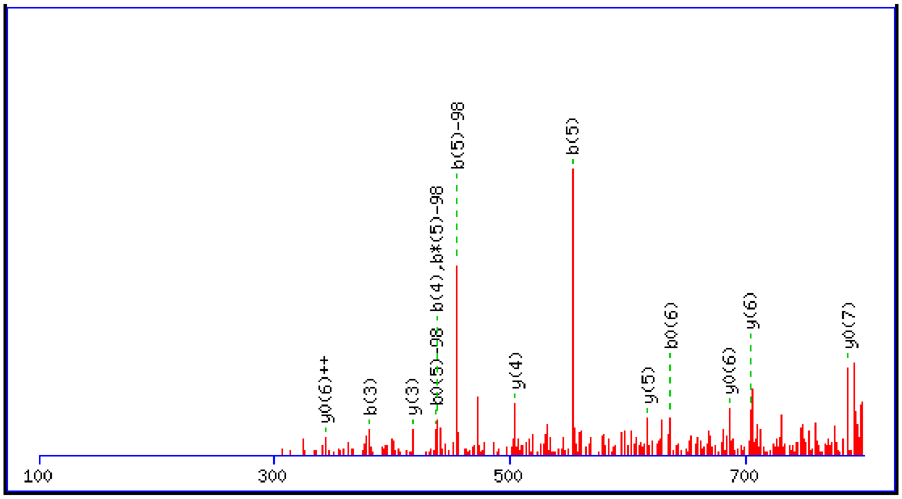

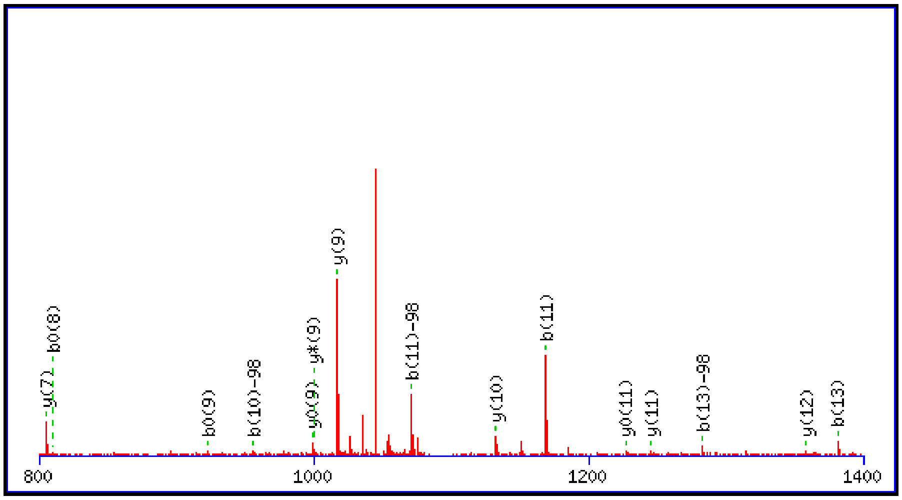

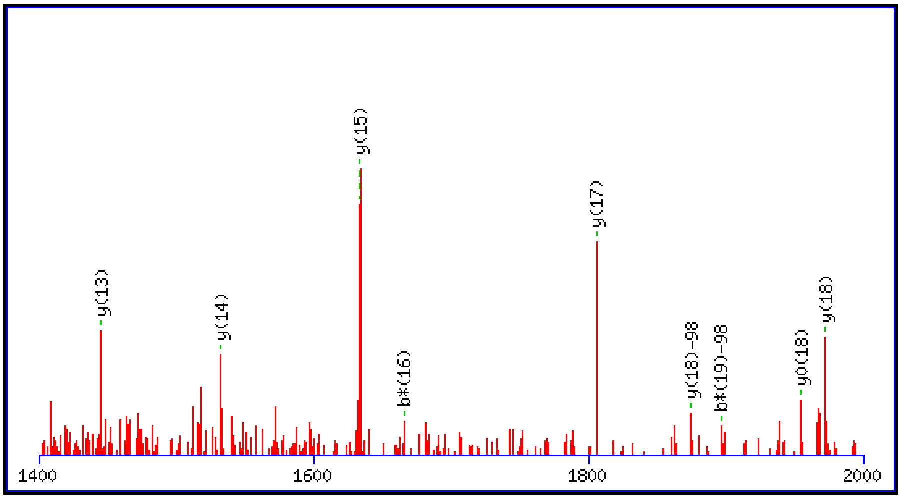

Monoisotopic mass of neutral peptide Mr(calc): 2184.9900
Fixed modifications: Carbamidomethyl (C)
Variable modifications:
S3 : Phospho (ST), with neutral losses 97.9769(shown in table), 0.0000
Ions Score: 72 **Expect:** 1.9e-005
Matches (Bold Red): 38/334 fragment ions using 68 most intense peaks

#	b	b^{++}	b*	b^{*++}	b^{0}	b^{0++}	Seq.	y	y^{++}	y*	y^{*++}	y^{0}	y^{0++}	#
1	157.1084	79.0578	140.0818	70.5446			R							20
2	214.1299	107.5686	197.1033	99.0553			G	1931.9192	966.4633	1914.8927	957.9500	1913.9087	957.4580	19
3	283.1513	142.0793	266.1248	133.5660	265.1407	133.0740	S	**1874.8978**	937.9525	1857.8712	929.4393	1856.8872	928.9472	18
4	340.1728	170.5900	323.1462	162.0767	322.1622	161.5847	G	**1805.8763**	903.4418	1788.8498	894.9285	1787.8658	894.4365	17
5	**455.1997**	228.1035	**438.1732**	219.5902	**437.1891**	219.0982	D	1748.8549	874.9311	1731.8283	866.4178	1730.8443	865.9258	16

**6**	556.2474	278.6273	539.2208	270.1141	538.2368	269.6221	**T**	**1633.8279**	817.4176	1616.8014	808.9043	1615.8174	808.4123	**15**
**7**	643.2794	322.1433	626.2529	313.6301	625.2689	313.1381	**S**	**1532.7802**	766.8938	1515.7537	758.3805	1514.7697	757.8885	**14**
**8**	730.3114	365.6594	713.2849	357.1461	712.3009	356.6541	**S**	**1445.7482**	723.3777	1428.7217	714.8645	1427.7376	714.3725	**13**
**9**	843.3955	422.2014	826.3690	413.6881	825.3849	413.1961	**L**	**1358.7162**	679.8617	1341.6896	671.3485	1340.7056	670.8564	**12**
**10**	**956.4796**	478.7434	939.4530	470.2302	938.4690	469.7381	**I**	**1245.6321**	623.3197	1228.6056	614.8064	**1227.6216**	614.3144	**11**
**11**	**1071.5065**	536.2569	1054.4800	527.7436	1053.4960	527.2516	**D**	**1132.5481**	566.7777	1115.5215	558.2644	1114.5375	557.7724	**10**
**12**	1168.5593	584.7833	1151.5327	576.2700	1150.5487	575.7780	**P**	**1017.5211**	509.2642	**1000.4946**	500.7509	**999.5106**	500.2589	**9**
**13**	**1283.5862**	642.2968	1266.5597	633.7835	1265.5757	633.2915	**D**	920.4684	460.7378	903.4418	452.2245	902.4578	451.7325	**8**
**14**	1384.6339	692.8206	1367.6074	684.3073	1366.6233	683.8153	**T**	**805.4414**	403.2243	788.4149	394.7111	**787.4308**	394.2191	**7**
**15**	1471.6659	736.3366	1454.6394	727.8233	1453.6554	727.3313	**S**	**704.3937**	352.7005	687.3672	344.1872	**686.3832**	**343.6952**	**6**
**16**	1584.7500	792.8786	1567.7234	784.3654	1566.7394	783.8734	**L**	**617.3617**	309.1845	600.3352	300.6712	599.3511	300.1792	**5**
**17**	1671.7820	836.3947	1654.7555	827.8814	1653.7715	827.3894	**S**	**504.2776**	252.6425	487.2511	244.1292	486.2671	243.6372	**4**
**18**	1800.8246	900.9159	1783.7981	892.4027	1782.8141	891.9107	**E**	**417.2456**	209.1264	400.2191	200.6132	399.2350	200.1212	**3**
**19**	1913.9087	957.4580	**1896.8821**	948.9447	1895.8981	948.4527	**L**	288.2030	144.6051	271.1765	136.0919			**2**
**20**							**R**	175.1190	88.0631	158.0924	79.5498			**1**

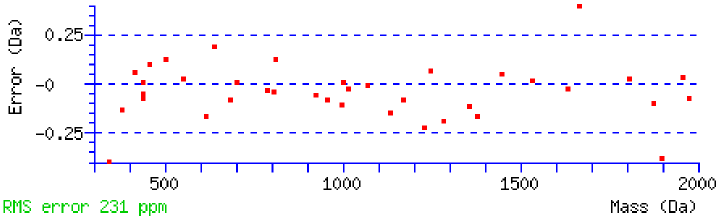

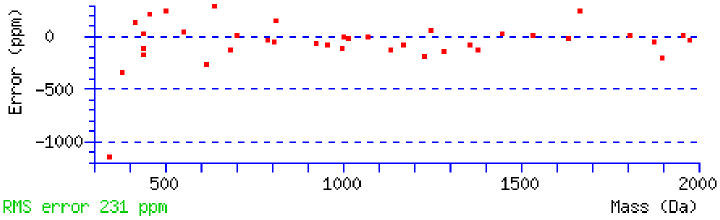

All matches to this query

Score	Mr(calc):	Delta	Sequence
71.8	2184.9900	0.0083	RGSGDTSSLIDPDTSLSELR
48.7	2184.9900	0.0083	RGSGDTSSLIDPDTSLSELR
40.6	2184.9900	0.0083	RGSGDTSSLIDPDTSLSELR
33.0	2184.9900	0.0083	RGSGDTSSLIDPDTSLSELR
8.0	2184.9900	0.0083	RGSGDTSSLIDPDTSLSELR
6.8	2184.9900	0.0083	RGSGDTSSLIDPDTSLSELR
3.5	2185.0166	-0.0184	MSLIINTFYSNQEIFLR
1.6	2185.0166	-0.0184	MSLIINTFYSNQEIFLR
0.5	2184.9900	0.0083	RGSGDTSSLIDPDTSLSELR

Spectrum No: 83; Query: 2042; Rank: 1

Peptide View

MS/MS Fragmentation of **VVDYSQFQESDDADEDYGR**
Found in **IPI00189138**, Tax_Id=10116 Gene_Symbol=Nucks Nuclear ubiquitous casein and cyclin-dependent kinases substrate

Match to Query 2042: 2316.876672 from(773.299500,3+)
Title: 100101RatKid_NS_deglyco_25.2377.2377.3.dta
Data file K:\NewmanPaper\Piliang\3SubProteomes\Piliang3SP\mgf5ppm\ERLIC_3SubProteomes5ppm.mgf

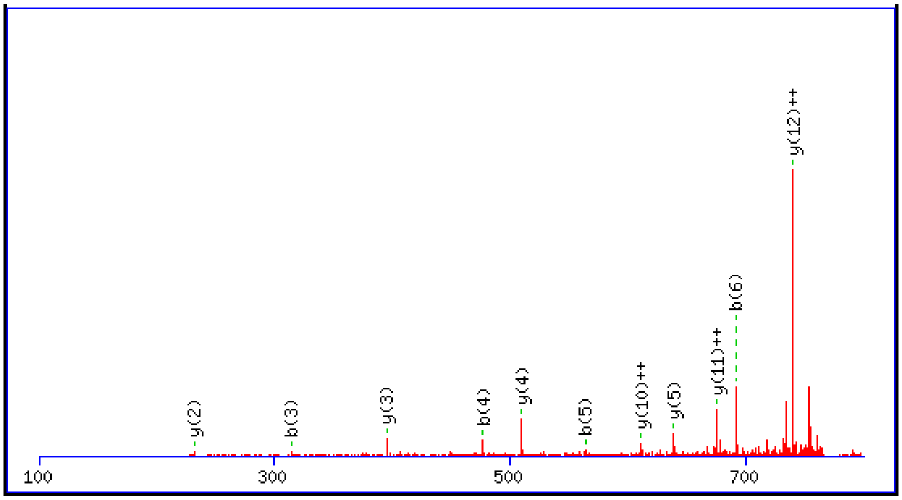

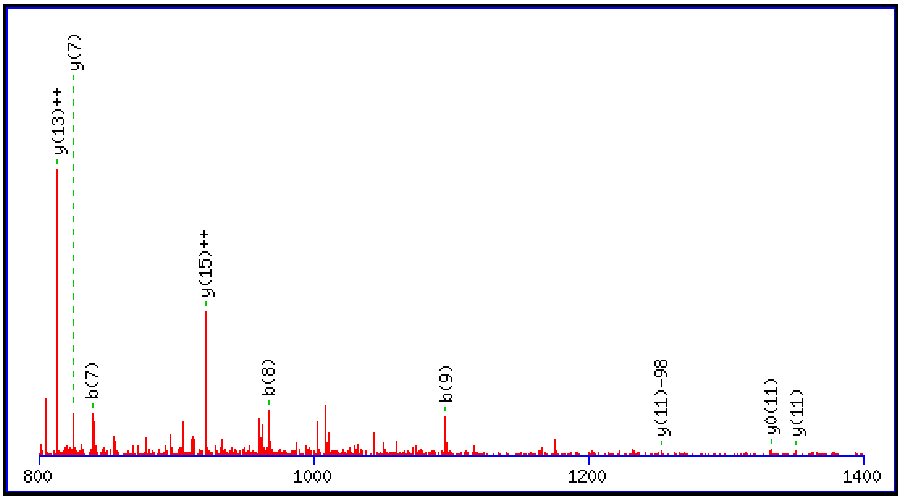

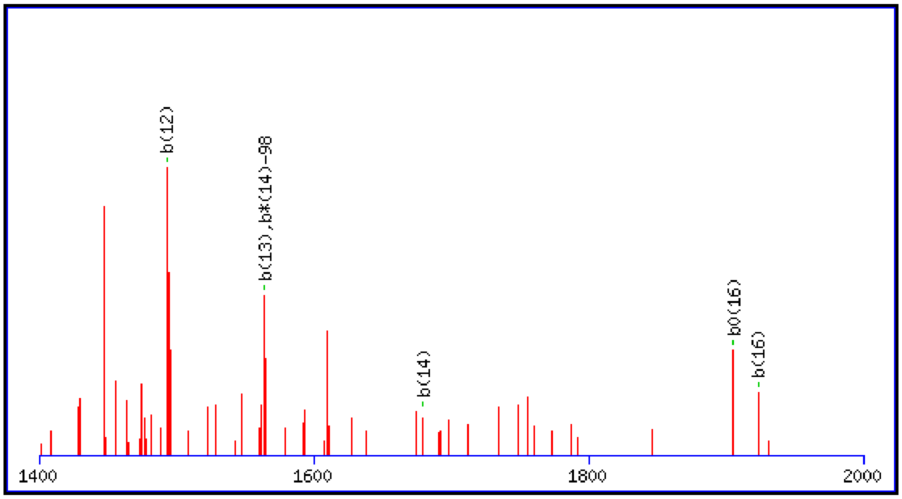

Monoisotopic mass of neutral peptide Mr(calc): 2316.8696
Fixed modifications: Carbamidomethyl (C)
Variable modifications:
S10 : Phospho (ST), with neutral losses 0.0000(shown in table), 97.9769
Ions Score: 72 **Expect:** 1e-005
Matches (Bold Red): 26/304 fragment ions using 44 most intense peaks

#	b	b++	b*	b*++	b0	b0++	Seq.	y	y++	y*	y*++	y0	y0++	#
1	100.0757	50.5415					V							19
2	199.1441	100.0757					V	2218.8085	1109.9079	2201.7819	1101.3946	2200.7979	1100.9026	18
3	**314.1710**	157.5892			296.1605	148.5839	D	2119.7400	1060.3737	2102.7135	1051.8604	2101.7295	1051.3684	17
4	**477.2344**	239.1208			459.2238	230.1155	Y	2004.7131	1002.8602	1987.6865	994.3469	1986.7025	993.8549	16
5	**564.2664**	282.6368			546.2558	273.6316	S	1841.6498	**921.3285**	1824.6232	912.8152	1823.6392	912.3232	15

6	**692.3250**	346.6661	675.2984	338.1529	674.3144	337.6608	Q	1754.6177	877.8125	1737.5912	869.2992	1736.6072	868.8072	14
7	**839.3934**	420.2003	822.3668	411.6871	821.3828	411.1951	F	1626.5592	**813.7832**	1609.5326	805.2699	1608.5486	804.7779	13
8	**967.4520**	484.2296	950.4254	475.7164	949.4414	475.2243	Q	1479.4907	**740.2490**	1462.4642	731.7357	1461.4802	731.2437	12
9	**1096.4946**	548.7509	1079.4680	540.2376	1078.4840	539.7456	E	**1351.4322**	**676.2197**	1334.4056	667.7064	**1333.4216**	667.2144	11
10	1263.4929	632.2501	1246.4664	623.7368	1245.4824	623.2448	S	1222.3896	**611.6984**	1205.3630	603.1852	1204.3790	602.6931	10
11	1378.5199	689.7636	1361.4933	681.2503	1360.5093	680.7583	D	1055.3912	528.1993	1038.3647	519.6860	1037.3807	519.1940	9
12	**1493.5468**	747.2770	1476.5203	738.7638	1475.5362	738.2718	D	940.3643	470.6858	923.3377	462.1725	922.3537	461.6805	8
13	**1564.5839**	782.7956	1547.5574	774.2823	1546.5734	773.7903	A	**825.3373**	413.1723	808.3108	404.6590	807.3268	404.1670	7
14	**1679.6109**	840.3091	1662.5843	831.7958	1661.6003	831.3038	D	754.3002	377.6538	737.2737	369.1405	736.2897	368.6485	6
15	1808.6535	904.8304	1791.6269	896.3171	1790.6429	895.8251	E	**639.2733**	320.1403	622.2467	311.6270	621.2627	311.1350	5
16	**1923.6804**	962.3438	1906.6538	953.8306	**1905.6698**	953.3386	D	**510.2307**	255.6190	493.2041	247.1057	492.2201	246.6137	4
17	2086.7437	1043.8755	2069.7172	1035.3622	2068.7332	1034.8702	Y	**395.2037**	198.1055	378.1772	189.5922			3
18	2143.7652	1072.3862	2126.7386	1063.8730	2125.7546	1063.3810	G	**232.1404**	116.5738	215.1139	108.0606			2
19							R	175.1190	88.0631	158.0924	79.5498			1

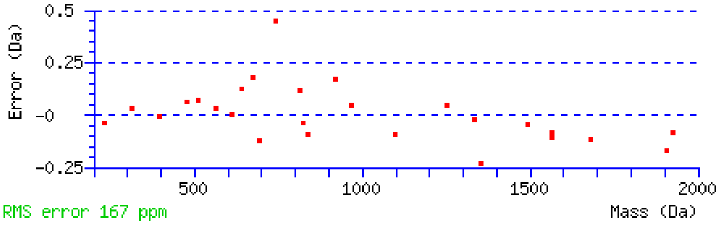

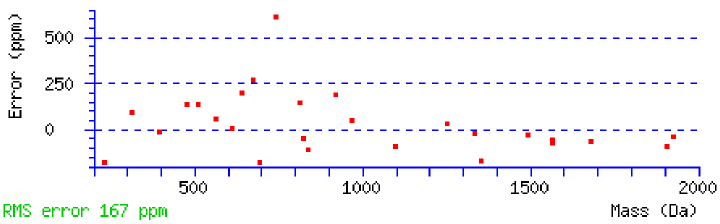

All matches to this query

Score	Mr(calc):	Delta	Sequence
71.8	2316.8696	0.0071	VVDYSQFQESDDADEDYGR
30.2	2316.8696	0.0071	VVDYSQFQESDDADEDYGR
23.4	2316.8696	0.0071	VVDYSQFQESDDADEDYGR
16.0	2316.8696	0.0071	VVDYSQFQESDDADEDYGR
4.2	2314.8757	2.0010	NTGGDSKTAPSSPLTSPSDTR
0.9	2314.8757	2.0010	NTGGDSKTAPSSPLTSPSDTR
0.5	2316.8623	0.0144	TKAADASLTAEPTENSSMPR
0.4	2316.8967	-0.0200	RGTGNIQLPGFSGGTWGSER
0.3	2316.8945	-0.0179	MGINFHHPGTDNIMALNTR
0.2	2314.8757	2.0010	NTGGDSKTAPSSPLTSPSDTR

Spectrum No: 84; Query: 1484; Rank: 1

Peptide View

MS/MS Fragmentation of **IEDVGSDEEDDSGKDKK**
Found in **IPI00471584**, Tax_Id=10116 Gene_Symbol=Hspcb Heat shock protein HSP 90-beta

Match to Query 1484: 1944.785228 from(973.399890,2+)
Title: 091008RatKidney_NH4Format01_23.285.285.2.dta
Data file K:\NewmanPaper\Piliang\3SubProteomes\Piliang3SP\mgf5ppm\ERLIC_3SubProteomes5ppm.mgf

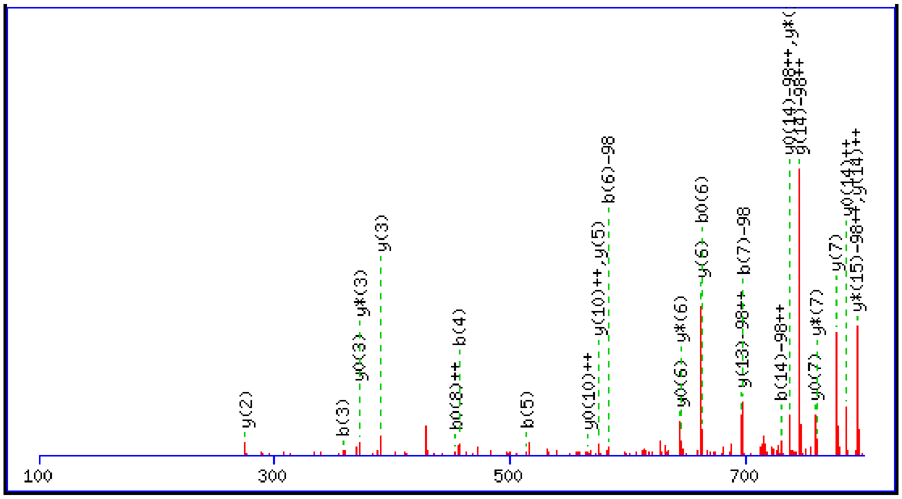

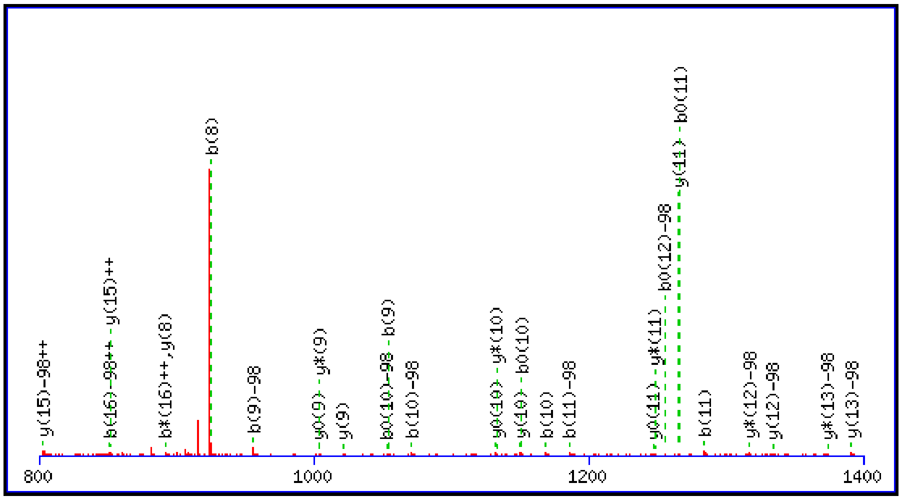

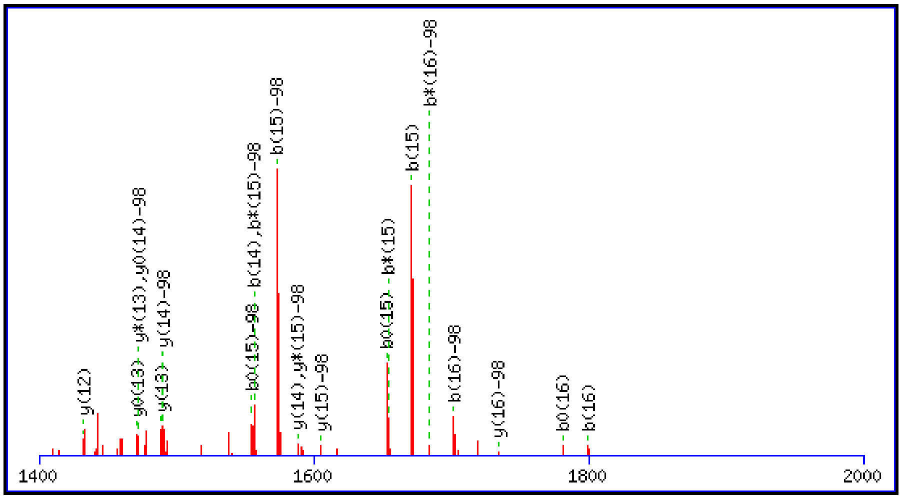

Monoisotopic mass of neutral peptide Mr(calc): 1944.7837
Fixed modifications: Carbamidomethyl (C)
Variable modifications:
S6 : Phospho (ST), with neutral losses 97.9769(shown in table), 0.0000
Ions Score: 72 **Expect:** 1.2e-005
Matches (Bold Red): 79/240 fragment ions using 136 most intense peaks

#	b	b^{++}	b*	b^{*++}	b^0	b^{0++}	Seq.	y	y^{++}	y*	y^{*++}	y^0	y^{0++}	#
1	114.0913	57.5493					I							17
2	243.1339	122.0706			225.1234	113.0653	E	**1734.7301**	867.8687	1717.7035	859.3554	1716.7195	858.8634	16
3	**358.1609**	179.5841			340.1503	170.5788	D	**1605.6875**	**803.3474**	**1588.6609**	**794.8341**	1587.6769	794.3421	15
4	**457.2293**	229.1183			439.2187	220.1130	V	**1490.6605**	**745.8339**	1473.6340	**737.3206**	**1472.6499**	**736.8286**	14
5	**514.2508**	257.6290			496.2402	248.6237	G	**1391.5921**	**696.2997**	**1374.5656**	687.7864	1373.5815	687.2944	13

**6**	**583.2722**	292.1397			565.2616	283.1345	**S**	**1334.5706**	667.7890	**1317.5441**	659.2757	1316.5601	658.7837	**12**
**7**	**698.2992**	349.6532			680.2886	340.6479	**D**	**1265.5492**	633.2782	**1248.5226**	624.7650	**1247.5386**	624.2729	**11**
**8**	827.3417	414.1745			809.3312	405.1692	**E**	**1150.5222**	**575.7648**	**1133.4957**	567.2515	**1132.5117**	**566.7595**	**10**
**9**	**956.3843**	478.6958			938.3738	469.6905	**E**	**1021.4796**	511.2435	**1004.4531**	502.7302	**1003.4691**	502.2382	**9**
**10**	**1071.4113**	536.2093			**1053.4007**	527.2040	**D**	**892.4371**	446.7222	875.4105	438.2089	874.4265	437.7169	**8**
**11**	**1186.4382**	593.7228			1168.4277	584.7175	**D**	**777.4101**	389.2087	**760.3836**	380.6954	**759.3995**	380.2034	**7**
**12**	1273.4703	637.2388			**1255.4597**	628.2335	**S**	**662.3832**	331.6952	**645.3566**	323.1819	**644.3726**	322.6899	**6**
**13**	1330.4917	665.7495			1312.4812	656.7442	**G**	**575.3511**	288.1792	558.3246	279.6659	557.3406	279.1739	**5**
**14**	1458.5867	**729.7970**	1441.5601	721.2837	1440.5761	720.7917	**K**	518.3297	259.6685	501.3031	251.1552	500.3191	250.6632	**4**
**15**	**1573.6136**	787.3104	**1556.5871**	778.7972	**1555.6031**	778.3052	**D**	**390.2347**	195.6210	**373.2082**	187.1077	**372.2241**	186.6157	**3**
**16**	**1701.7086**	**851.3579**	**1684.6820**	842.8447	1683.6980	842.3526	**K**	**275.2078**	138.1075	258.1812	129.5942			**2**
**17**							**K**	147.1128	74.0600	130.0863	65.5468			**1**

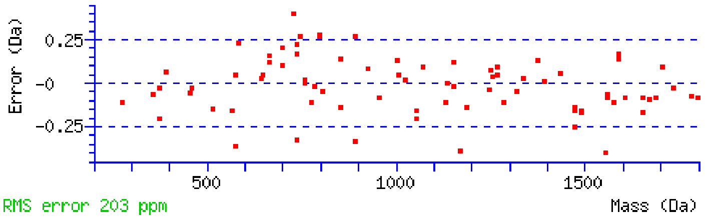

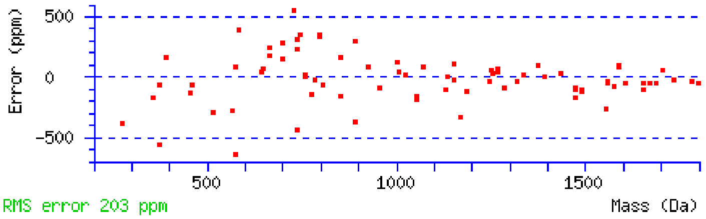

All matches to this query

Score	Mr(calc):	Delta	Sequence
71.5	1944.7837	0.0015	IEDVGSDEEDDSGKDKK
55.7	1944.7837	0.0015	IEDVGSDEEDDSGKDKK
13.9	1942.7784	2.0068	DSPESVTQMLAAYLSR
10.4	1944.7975	-0.0123	NISNMSNMNSSRMDSPK
9.2	1944.7808	0.0044	YTNRYDEGIYHIGGK
9.1	1944.7846	0.0007	MQEIEEMEKESGPGQK
8.3	1944.8006	-0.0154	TPEAVEEPAEDTSGIIK
8.2	1942.7784	2.0068	DSPESVTQMLAAYLSR
7.9	1944.7729	0.0123	ENIRDSPKFEVYCK
7.5	1944.7846	0.0007	MQEIEEMEKESGPGQK

Spectrum No: 85; Query: 2499; Rank: 1

Peptide View

MS/MS Fragmentation of **DFSPQDSFDDDELDAAVADPDEFER**
Found in **IPI00197559**, Tax_Id=10116 Gene_Symbol=Pla2g4a Cytosolic phospholipase A2

Match to Query 2499: 2924.129928 from(1463.072240,2+)
Title: 100101RatKid_NS_deglyco_26.4345.4345.2.dta
Data file K:\NewmanPaper\Piliang\3SubProteomes\Piliang3SP\mgf5ppm\ERLIC_3SubProteomes5ppm.mgf

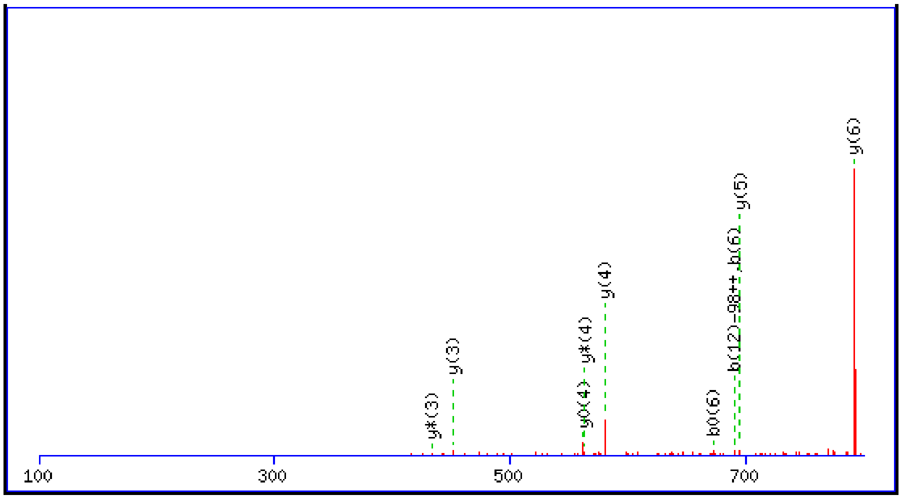

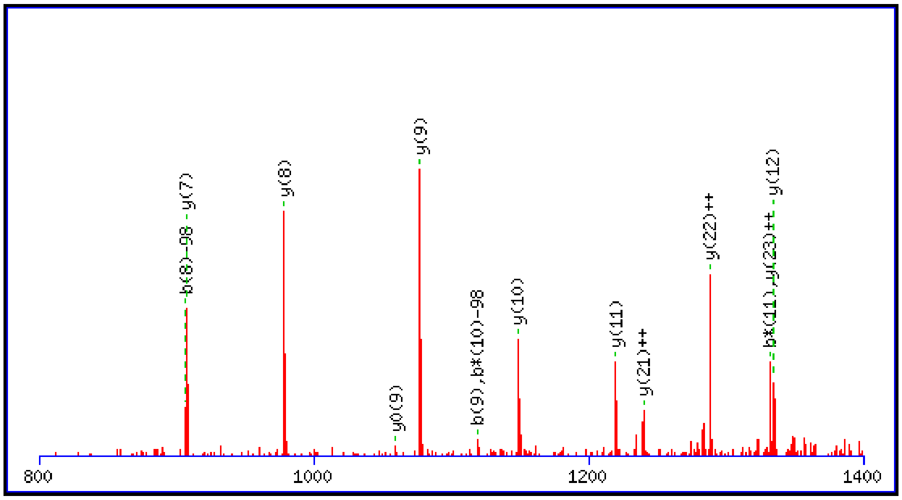

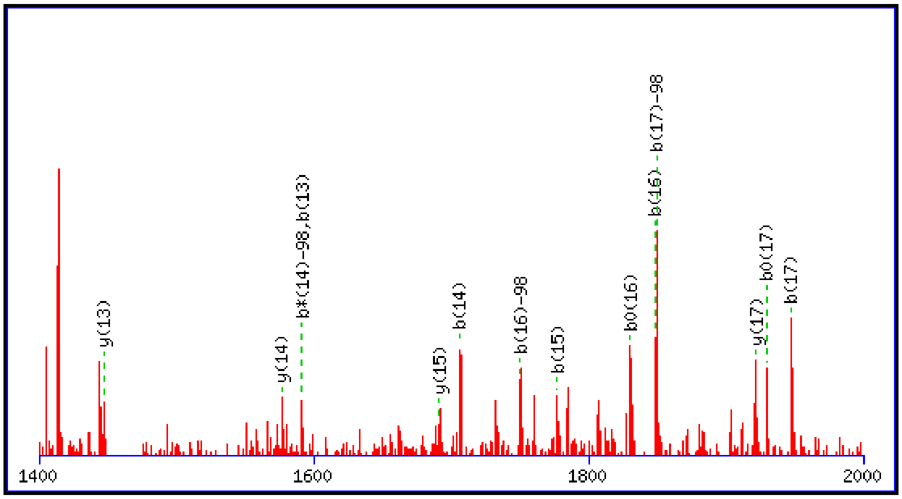

Monoisotopic mass of neutral peptide Mr(calc): 2924.1186
Fixed modifications: Carbamidomethyl (C)
Variable modifications:
S7 : Phospho (ST), with neutral losses 0.0000(shown in table), 97.9769
Ions Score: 72 **Expect:** 1.7e-005
Matches (Bold Red): 38/422 fragment ions using 64 most intense peaks

#	b	b^{++}	b*	b^{*++}	b^0	b^{0++}	Seq.	y	y^{++}	y*	y^{*++}	y^0	y^{0++}	#
1	116.0342	58.5207			98.0237	49.5155	D							25
2	263.1026	132.0550			245.0921	123.0497	F	2810.0989	1405.5531	2793.0723	1397.0398	2792.0883	1396.5478	24
3	350.1347	175.5710			332.1241	166.5657	S	2663.0305	**1332.0189**	2646.0039	1323.5056	2645.0199	1323.0136	23
4	447.1874	224.0974			429.1769	215.0921	P	2575.9984	**1288.5029**	2558.9719	1279.9896	2557.9879	1279.4976	22
5	575.2460	288.1266	558.2195	279.6134	557.2354	279.1214	Q	2478.9457	**1239.9765**	2461.9191	1231.4632	2460.9351	1230.9712	21

6	690.2729	345.6401	673.2464	337.1268	672.2624	336.6348	D	2350.8871	1175.9472	2333.8605	1167.4339	2332.8765	1166.9419	20
7	857.2713	429.1393	840.2447	420.6260	839.2607	420.1340	S	2235.8602	1118.4337	2218.8336	1109.9204	2217.8496	1109.4284	19
8	1004.3397	502.6735	987.3132	494.1602	986.3291	493.6682	F	2068.8618	1034.9345	2051.8353	1026.4213	2050.8512	1025.9293	18
9	1119.3667	560.1870	1102.3401	551.6737	1101.3561	551.1817	D	1921.7934	961.4003	1904.7668	952.8871	1903.7828	952.3950	17
10	1234.3936	617.7004	1217.3670	609.1872	1216.3830	608.6952	D	1806.7664	903.8869	1789.7399	895.3736	1788.7559	894.8816	16
11	1349.4205	675.2139	1332.3940	666.7006	1331.4100	666.2086	D	1691.7395	846.3734	1674.7130	837.8601	1673.7289	837.3681	15
12	1478.4631	739.7352	1461.4366	731.2219	1460.4526	730.7299	E	1576.7126	788.8599	1559.6860	780.3466	1558.7020	779.8546	14
13	1591.5472	796.2772	1574.5206	787.7640	1573.5366	787.2720	L	1447.6700	724.3386	1430.6434	715.8253	1429.6594	715.3333	13
14	1706.5741	853.7907	1689.5476	845.2774	1688.5636	844.7854	D	1334.5859	667.7966	1317.5594	659.2833	1316.5753	658.7913	12
15	1777.6113	889.3093	1760.5847	880.7960	1759.6007	880.3040	A	1219.5590	610.2831	1202.5324	601.7698	1201.5484	601.2778	11
16	1848.6484	924.8278	1831.6218	916.3145	1830.6378	915.8225	A	1148.5218	574.7646	1131.4953	566.2513	1130.5113	565.7593	10
17	1947.7168	974.3620	1930.6902	965.8488	1929.7062	965.3567	V	1077.4847	539.2460	1060.4582	530.7327	1059.4742	530.2407	9
18	2018.7539	1009.8806	2001.7273	1001.3673	2000.7433	1000.8753	A	978.4163	489.7118	961.3898	481.1985	960.4058	480.7065	8
19	2133.7808	1067.3941	2116.7543	1058.8808	2115.7703	1058.3888	D	907.3792	454.1932	890.3527	445.6800	889.3686	445.1880	7
20	2230.8336	1115.9204	2213.8071	1107.4072	2212.8230	1106.9152	P	792.3523	396.6798	775.3257	388.1665	774.3417	387.6745	6
21	2345.8605	1173.4339	2328.8340	1164.9206	2327.8500	1164.4286	D	695.2995	348.1534	678.2729	339.6401	677.2889	339.1481	5
22	2474.9031	1237.9552	2457.8766	1229.4419	2456.8926	1228.9499	E	580.2726	290.6399	563.2460	282.1266	562.2620	281.6346	4
23	2621.9716	1311.4894	2604.9450	1302.9761	2603.9610	1302.4841	F	451.2300	226.1186	434.2034	217.6053	433.2194	217.1133	3
24	2751.0141	1376.0107	2733.9876	1367.4974	2733.0036	1367.0054	E	304.1615	152.5844	287.1350	144.0711	286.1510	143.5791	2
25							R	175.1190	88.0631	158.0924	79.5498			1

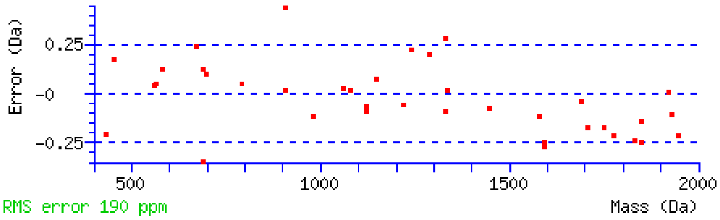

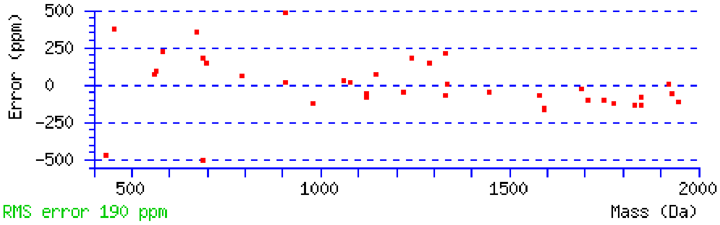

All matches to this query

Score	Mr(calc):	Delta	Sequence
71.6	2924.1186	0.0114	DFSPQDSFDDDELDAAVADPDEFER
70.0	2924.1186	0.0114	DFSPQDSFDDDELDAAVADPDEFER
4.5	2922.1001	2.0299	AVSVRASNTAAATRSTDTNSGSALSK
1.6	2922.1462	1.9837	IHAENLYGISDPLVSDSMKARDR
0.4	2923.1336	0.9963	LDIDSAPITARNTDIMCTIGPASR
0.0	2924.1217	0.0083	YSFMESLLQNQVVLVSGDSKCGK

Spectrum No: 86; Query: 1288; Rank: 1

Peptide View

MS/MS Fragmentation of **SGSGSPPPTTEEVAATTR**
Found in **IPI00201120**, Tax_Id=10116 Gene_Symbol=Slc5a2 Low affinity Na-dependent glucose transporter SGLT2 delta e trans

Match to Query 1288: 1823.800328 from(912.907440,2+)
Title: 091008RatKidney_NH4Format01_22.1087.1087.2.dta
Data file K:\NewmanPaper\Piliang\3SubProteomes\Piliang3SP\mgf5ppm\ERLIC_3SubProteomes5ppm.mgf

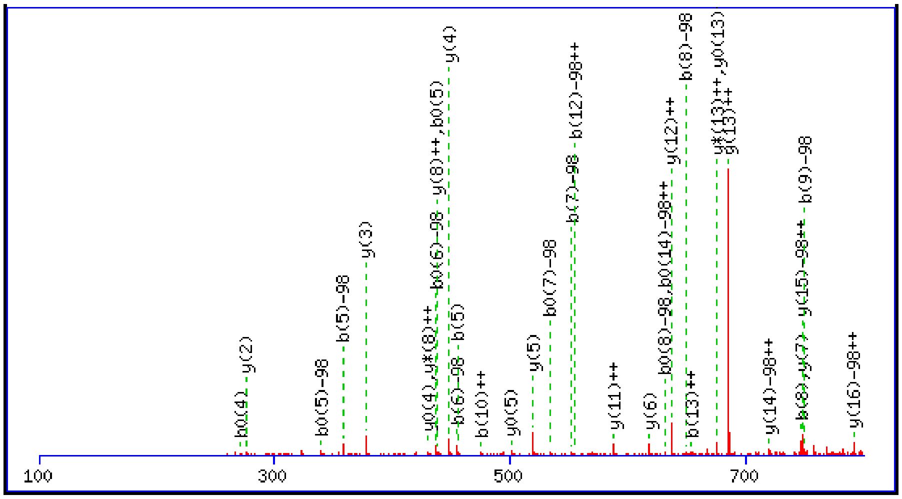

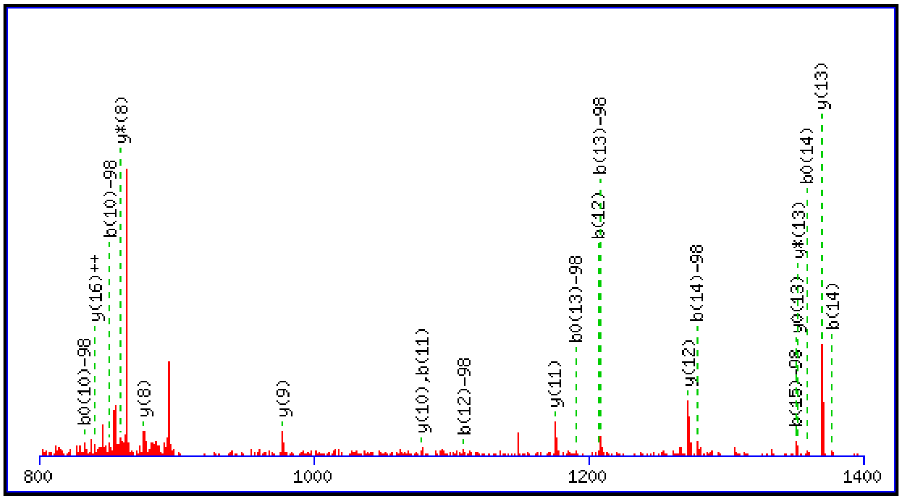

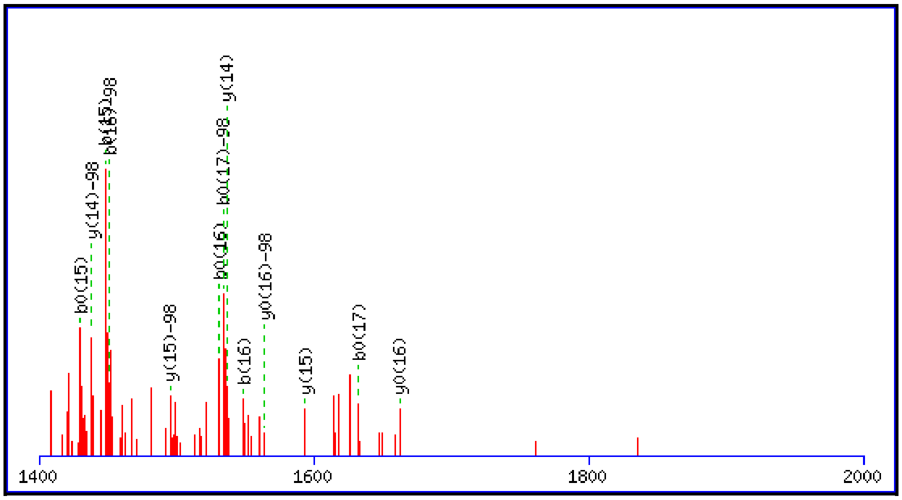

Monoisotopic mass of neutral peptide Mr(calc): 1823.7939
Fixed modifications: Carbamidomethyl (C)
Variable modifications:
S5 : Phospho (ST), with neutral losses 97.9769(shown in table), 0.0000
Ions Score: 71 **Expect:** 1.5e-005
Matches (Bold Red): 69/244 fragment ions using 137 most intense peaks

#	b	b^{++}	b^0	b^{0++}	Seq.	y	y^{++}	y*	y^{*++}	y^0	y^{0++}	#
1	88.0393	44.5233	70.0287	35.5180	S							18
2	145.0608	73.0340	127.0502	64.0287	G	1639.7922	820.3997	1622.7657	811.8865	1621.7816	811.3945	17
3	232.0928	116.5500	214.0822	107.5448	S	1582.7707	**791.8890**	1565.7442	783.3757	**1564.7602**	782.8837	16
4	289.1143	145.0608	**271.1037**	136.0555	G	**1495.7387**	**748.3730**	1478.7122	739.8597	1477.7281	739.3677	15
5	**358.1357**	179.5715	**340.1252**	170.5662	S	**1438.7172**	**719.8623**	1421.6907	711.3490	1420.7067	710.8570	14

6	**455.1885**	228.0979	**437.1779**	219.0926	P	**1369.6958**	**685.3515**	**1352.6692**	**676.8383**	**1351.6852**	**676.3462**	13
7	**552.2412**	276.6243	**534.2307**	267.6190	P	**1272.6430**	**636.8252**	1255.6165	628.3119	1254.6325	627.8199	12
8	**649.2940**	325.1506	**631.2834**	316.1454	P	**1175.5903**	**588.2988**	1158.5637	579.7855	1157.5797	579.2935	11
9	**750.3417**	375.6745	732.3311	366.6692	T	**1078.5375**	539.7724	1061.5109	531.2591	1060.5269	530.7671	10
10	**851.3894**	426.1983	**833.3788**	417.1930	T	**977.4898**	489.2485	960.4633	480.7353	959.4793	480.2433	9
11	980.4320	490.7196	962.4214	481.7143	E	**876.4421**	**438.7247**	**859.4156**	**430.2114**	858.4316	429.7194	8
12	**1109.4746**	**555.2409**	1091.4640	546.2356	E	**747.3995**	374.2034	730.3730	365.6901	729.3890	365.1981	7
13	**1208.5430**	604.7751	**1190.5324**	595.7698	V	**618.3570**	309.6821	601.3304	301.1688	600.3464	300.6768	6
14	**1279.5801**	640.2937	1261.5695	**631.2884**	A	**519.2885**	260.1479	502.2620	251.6346	**501.2780**	251.1426	5
15	**1350.6172**	675.8122	1332.6066	666.8070	A	**448.2514**	224.6293	431.2249	216.1161	**430.2409**	215.6241	4
16	**1451.6649**	726.3361	1433.6543	717.3308	T	**377.2143**	189.1108	360.1878	180.5975	359.2037	180.1055	3
17	1552.7126	776.8599	**1534.7020**	767.8546	T	**276.1666**	138.5870	259.1401	130.0737	258.1561	129.5817	2
18					R	175.1190	88.0631	158.0924	79.5498			1

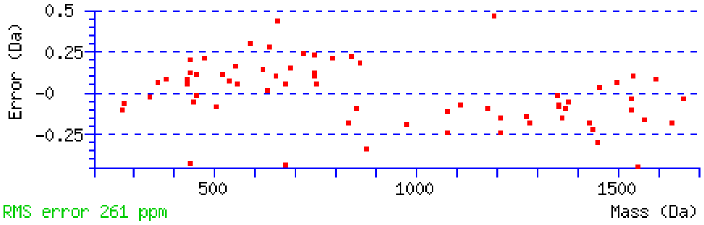

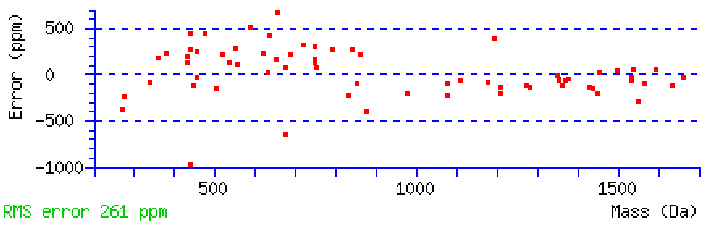

All matches to this query

Score	Mr(calc):	Delta	Sequence
71.5	1823.7939	0.0065	SGSGSPPPTTEEVAATTR
58.2	1823.7939	0.0065	SGSGSPPPTTEEVAATTR
58.2	1823.7939	0.0065	SGSGSPPPTTEEVAATTR
44.3	1823.7939	0.0065	SGSGSPPPTTEEVAATTR
36.2	1823.7939	0.0065	SGSGSPPPTTEEVAATTR
11.0	1823.7896	0.0107	NVLDTSWPTPPPALR
7.4	1822.7838	1.0165	IPMPPSSPQPRSTPR
4.4	1823.7939	0.0065	SGSGSPPPTTEEVAATTR
4.4	1822.8032	0.9971	TSSVKSPKPLSPSLR
4.4	1822.7804	1.0199	KEVPFREAPAYSNR

Spectrum No: 87; Query: 1518; Rank: 1

Peptide View

MS/MS Fragmentation of **AADPPAENSSAPEAEQGGAE**
Found in **IPI00551815**, Tax_Id=10116 Gene_Symbol=Ybx1-ps3 Ybx1 protein

Match to Query 1518: 1976.760768 from(989.387660,2+)
Title: 091008RatKidney_NoSalt_34.549.549.2.dta
Data file K:\NewmanPaper\Piliang\3SubProteomes\Piliang3SP\mgf5ppm\ERLIC_3SubProteomes5ppm.mgf

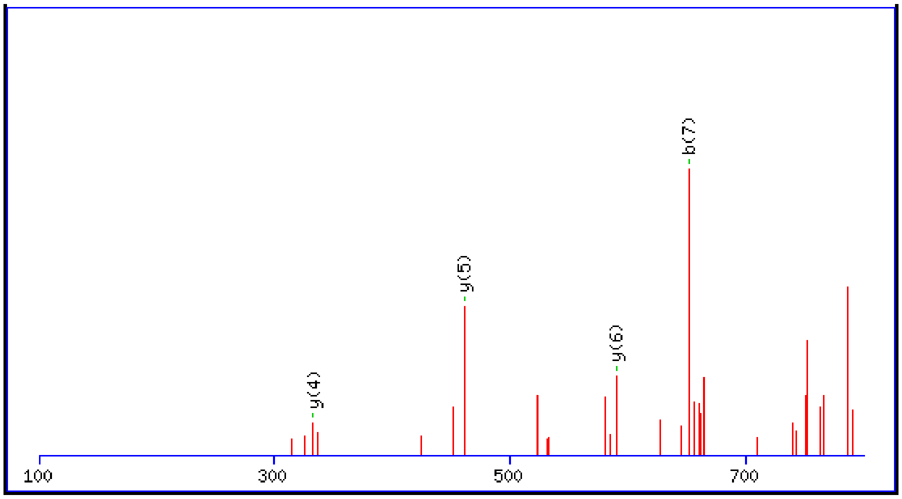

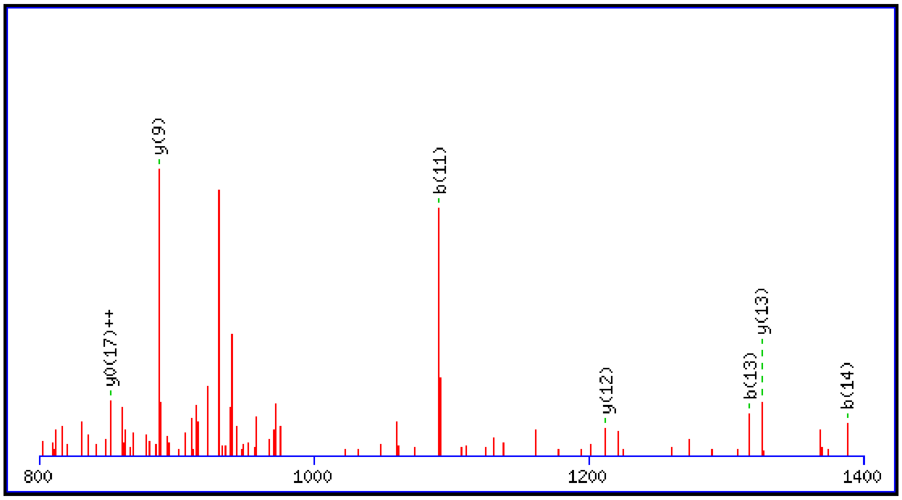

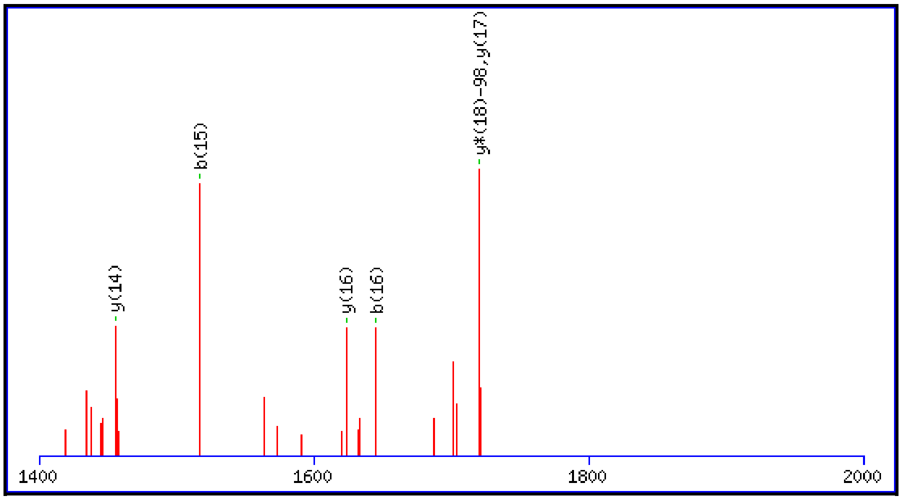

Monoisotopic mass of neutral peptide Mr(calc): 1976.7636
Fixed modifications: Carbamidomethyl (C)
Variable modifications:
S10 : Phospho (ST), with neutral losses 0.0000(shown in table), 97.9769
Ions Score: 71 **Expect:** 1e-005
Matches (Bold Red): 18/316 fragment ions using 21 most intense peaks

#	b	b^{++}	b*	b^{*++}	b^{0}	b^{0++}	Seq.	y	y^{++}	y*	y^{*++}	y^{0}	y^{0++}	#
1	72.0444	36.5258					A							20
2	143.0815	72.0444					A	1906.7338	953.8706	1889.7073	945.3573	1888.7233	944.8653	19
3	258.1084	129.5579			240.0979	120.5526	D	1835.6967	918.3520	1818.6702	909.8387	1817.6862	909.3467	18
4	355.1612	178.0842			337.1506	169.0790	P	**1720.6698**	860.8385	1703.6432	852.3253	1702.6592	**851.8332**	17
5	452.2140	226.6106			434.2034	217.6053	P	**1623.6170**	812.3121	1606.5905	803.7989	1605.6064	803.3069	16

**6**	523.2511	262.1292			505.2405	253.1239	**A**	1526.5642	763.7858	1509.5377	755.2725	1508.5537	754.7805	**15**
**7**	**652.2937**	326.6505			634.2831	317.6452	**E**	**1455.5271**	728.2672	1438.5006	719.7539	1437.5166	719.2619	**14**
**8**	766.3366	383.6719	749.3101	375.1587	748.3260	374.6667	**N**	**1326.4845**	663.7459	1309.4580	655.2326	1308.4740	654.7406	**13**
**9**	853.3686	427.1880	836.3421	418.6747	835.3581	418.1827	**S**	**1212.4416**	606.7244	1195.4151	598.2112	1194.4310	597.7192	**12**
**10**	1020.3670	510.6871	1003.3404	502.1739	1002.3564	501.6819	**S**	1125.4096	563.2084	1108.3830	554.6952	1107.3990	554.2031	**11**
**11**	**1091.4041**	546.2057	1074.3776	537.6924	1073.3935	537.2004	**A**	958.4112	479.7093	941.3847	471.1960	940.4007	470.7040	**10**
**12**	1188.4569	594.7321	1171.4303	586.2188	1170.4463	585.7268	**P**	**887.3741**	444.1907	870.3476	435.6774	869.3636	435.1854	**9**
**13**	**1317.4995**	659.2534	1300.4729	650.7401	1299.4889	650.2481	**E**	790.3214	395.6643	773.2948	387.1510	772.3108	386.6590	**8**
**14**	**1388.5366**	694.7719	1371.5100	686.2587	1370.5260	685.7666	**A**	661.2788	331.1430	644.2522	322.6297	643.2682	322.1377	**7**
**15**	**1517.5792**	759.2932	1500.5526	750.7799	1499.5686	750.2879	**E**	**590.2416**	295.6245	573.2151	287.1112	572.2311	286.6192	**6**
**16**	**1645.6377**	823.3225	1628.6112	814.8092	1627.6272	814.3172	**Q**	**461.1991**	231.1032	444.1725	222.5899	443.1885	222.0979	**5**
**17**	1702.6592	**851.8332**	1685.6327	843.3200	1684.6486	842.8280	**G**	**333.1405**	167.0739			315.1299	158.0686	**4**
**18**	1759.6807	880.3440	1742.6541	871.8307	1741.6701	871.3387	**G**	276.1190	138.5631			258.1084	129.5579	**3**
**19**	1830.7178	915.8625	1813.6912	907.3493	1812.7072	906.8572	**A**	219.0975	110.0524			201.0870	101.0471	**2**
**20**							**E**	148.0604	74.5339			130.0499	65.5286	**1**

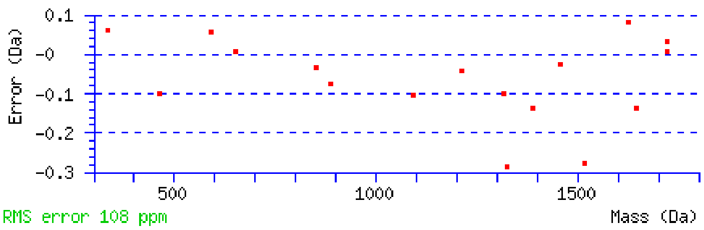

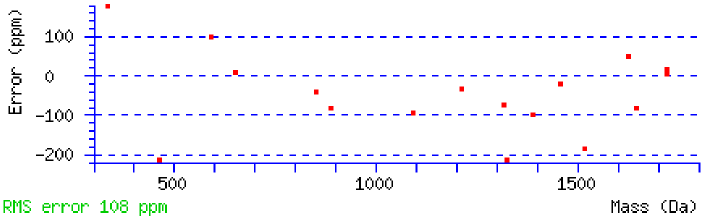

All matches to this query

Score	Mr(calc):	Delta	Sequence
71.2	1976.7636	-0.0029	AADPPAENSSAPEAEQGGAE
71.2	1976.7636	-0.0029	AADPPAENSSAPEAEQGGAE

Spectrum No: 88; Query: 1095; Rank: 1

Peptide View

MS/MS Fragmentation of **LLDAEDVDVPSPDEK**
Found in **IPI00359003**, Tax_Id=10116 Gene_Symbol=LOC362587 831 kDa protein

Match to Query 1095: 1720.747988 from(861.381270,2+)
Title: 100101RatKid_NS_deglyco_24.2601.2601.2.dta
Data file K:\NewmanPaper\Piliang\3SubProteomes\Piliang3SP\mgf5ppm\ERLIC_3SubProteomes5ppm.mgf

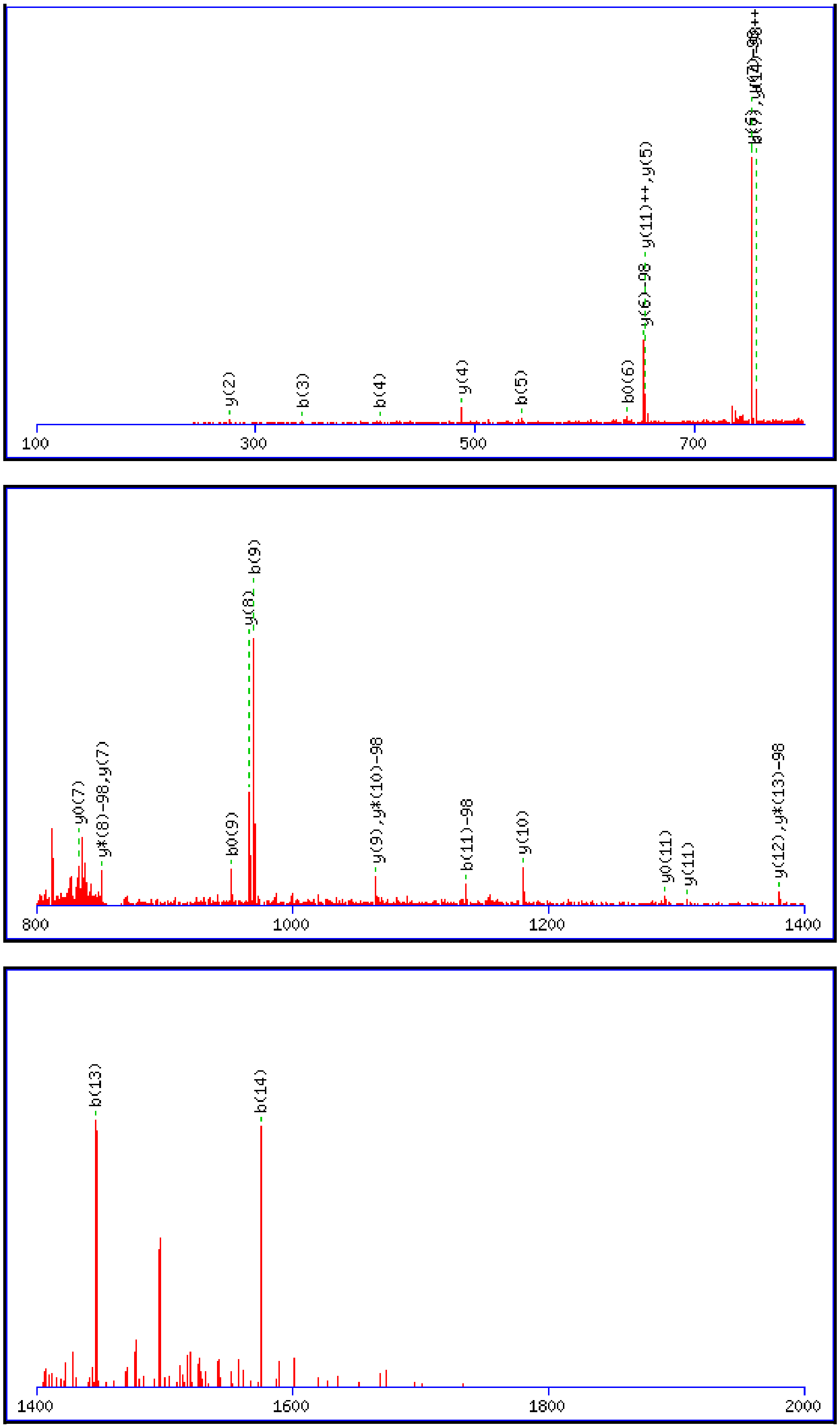

Monoisotopic mass of neutral peptide Mr(calc): 1720.7444
Fixed modifications: Carbamidomethyl (C)
Variable modifications:
S11 : Phospho (ST), with neutral losses 0.0000(shown in table), 97.9769
Ions Score: 71 **Expect:** 1.4e-005
Matches (Bold Red): 29/210 fragment ions using 48 most intense peaks

#	b	b++	b0	b0++	Seq.	y	y++	y*	y*++	y0	y0++	#
1	114.0913	57.5493			L							15
2	227.1754	114.0913			L	1608.6676	804.8375	1591.6411	796.3242	1590.6571	795.8322	14
3	**342.2023**	171.6048	324.1918	162.5995	D	1495.5836	748.2954	1478.5570	739.7822	1477.5730	739.2901	13
4	**413.2395**	207.1234	395.2289	198.1181	A	**1380.5566**	690.7820	1363.5301	682.2687	1362.5461	681.7767	12
5	**542.2821**	271.6447	524.2715	262.6394	E	**1309.5195**	**655.2634**	1292.4930	646.7501	**1291.5090**	646.2581	11

6	657.3090	329.1581	**639.2984**	320.1529	D	**1180.4769**	590.7421	1163.4504	582.2288	1162.4664	581.7368	10
7	**756.3774**	378.6923	738.3668	369.6871	V	**1065.4500**	533.2286	1048.4234	524.7154	1047.4394	524.2234	9
8	871.4044	436.2058	853.3938	427.2005	D	**966.3816**	483.6944	949.3550	475.1812	948.3710	474.6891	8
9	**970.4728**	485.7400	**952.4622**	476.7347	V	**851.3546**	426.1810	834.3281	417.6677	**833.3441**	417.1757	7
10	1067.5255	534.2664	1049.5150	525.2611	P	**752.2862**	376.6467	735.2597	368.1335	734.2757	367.6415	6
11	1234.5239	617.7656	1216.5133	608.7603	S	**655.2335**	328.1204	638.2069	319.6071	637.2229	319.1151	5
12	1331.5766	666.2920	1313.5661	657.2867	P	**488.2351**	244.6212	471.2086	236.1079	470.2245	235.6159	4
13	**1446.6036**	723.8054	1428.5930	714.8002	D	391.1823	196.0948	374.1558	187.5815	373.1718	187.0895	3
14	**1575.6462**	788.3267	1557.6356	779.3214	E	**276.1554**	138.5813	259.1288	130.0681	258.1448	129.5761	2
15					K	147.1128	74.0600	130.0863	65.5468			1

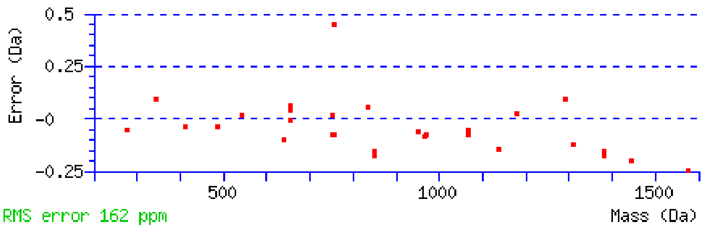

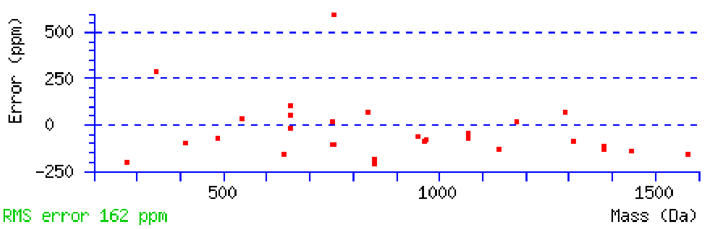

All matches to this query

Score	Mr(calc):	Delta	Sequence
71.0	1720.7444	0.0036	LLDAEDVDVPSPDEK
10.0	1720.7486	-0.0007	GSMLALAMVSLEMEK
10.0	1720.7486	-0.0007	GSMLALAMVSLEMEK
9.3	1720.7586	-0.0106	RKKPNEYSVSPEK
8.6	1720.7586	-0.0106	RKKPNEYSVSPEK
8.0	1720.7491	-0.0011	KMTLFNQEKNESR
7.3	1719.7535	0.9945	GWPTXLPSARSSFR
6.2	1718.7529	1.9951	ATISSPLELEGTVSR
6.2	1718.7529	1.9951	ATISSPLELEGTVSR
6.2	1718.7529	1.9951	ATISSPLELEGTVSR

Spectrum No: 89; Query: 1230; Rank: 1

Peptide View

MS/MS Fragmentation of **STSPPPSPEVWAESR**
Found in **IPI00778976**, Tax_Id=10116 Gene_Symbol=Epb4.9_predicted 45 kDa protein

Match to Query 1230: 1785.703468 from(893.859010,2+)
Title: 100101RatKid_NS_deglyco_26.2432.2432.2.dta
Data file K:\NewmanPaper\Piliang\3SubProteomes\Piliang3SP\mgf5ppm\ERLIC_3SubProteomes5ppm.mgf

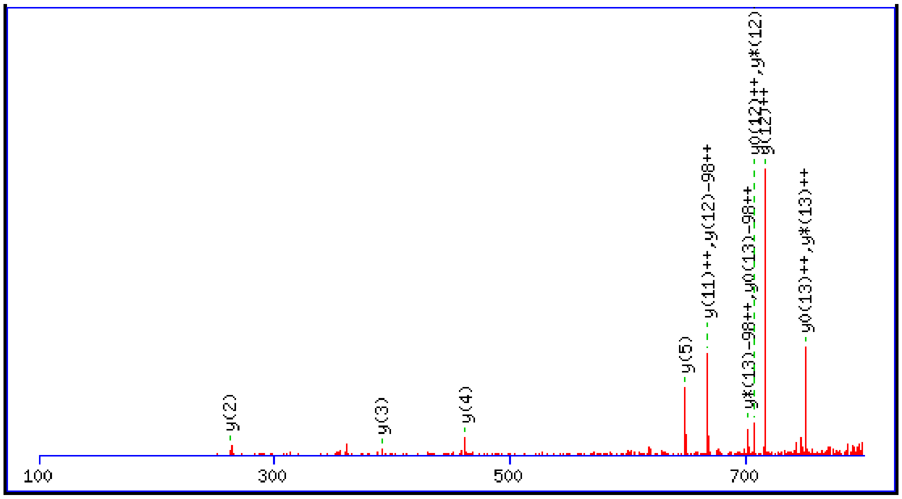

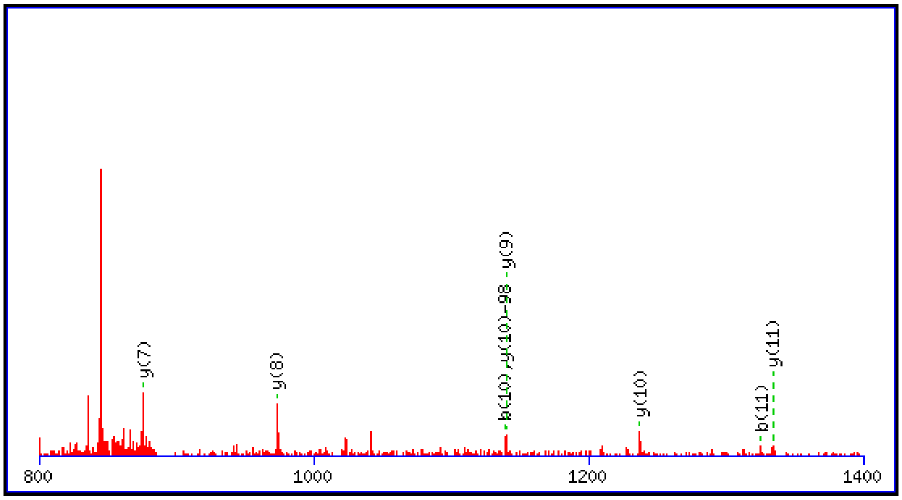

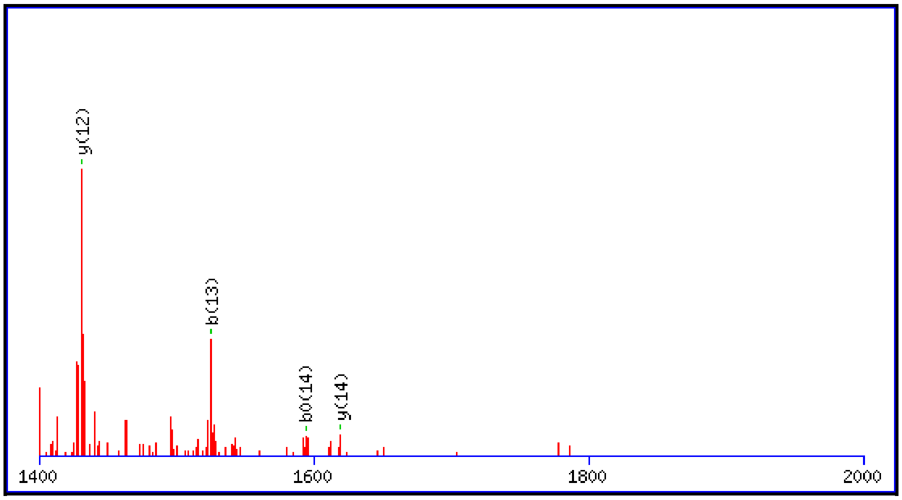

Monoisotopic mass of neutral peptide Mr(calc): 1785.7012
Fixed modifications: Carbamidomethyl (C)
Variable modifications:
S1 : Phospho (ST), with neutral losses 0.0000(shown in table), 97.9769
S7 : Phospho (ST), with neutral losses 0.0000(shown in table), 97.9769
Ions Score: 71 **Expect:** 1.2e-005
Matches (Bold Red): 25/230 fragment ions using 43 most intense peaks

#	b	b^{++}	b^{0}	b^{0++}	Seq.	y	y^{++}	y*	y^{*++}	y^{0}	y^{0++}	#
1	168.0056	84.5065	149.9951	75.5012	S							15
2	269.0533	135.0303	251.0427	126.0250	T	**1619.7101**	810.3587	1602.6836	801.8454	1601.6996	801.3534	14
3	356.0853	178.5463	338.0748	169.5410	S	1518.6624	759.8349	1501.6359	**751.3216**	1500.6519	**750.8296**	13
4	453.1381	227.0727	435.1275	218.0674	P	**1431.6304**	**716.3188**	1414.6039	**707.8056**	1413.6199	**707.3136**	12

**5**	550.1909	275.5991	532.1803	266.5938	**P**	**1334.5777**	**667.7925**	1317.5511	659.2792	1316.5671	658.7872	**11**
**6**	647.2436	324.1255	629.2331	315.1202	**P**	**1237.5249**	619.2661	1220.4983	610.7528	1219.5143	610.2608	**10**
**7**	814.2420	407.6246	796.2314	398.6193	**S**	**1140.4721**	570.7397	1123.4456	562.2264	1122.4616	561.7344	**9**
**8**	911.2947	456.1510	893.2842	447.1457	**P**	**973.4738**	487.2405	956.4472	478.7272	955.4632	478.2352	**8**
**9**	1040.3373	520.6723	1022.3268	511.6670	**E**	**876.4210**	438.7141	859.3945	430.2009	858.4104	429.7089	**7**
**10**	**1139.4058**	570.2065	1121.3952	561.2012	**V**	747.3784	374.1928	730.3519	365.6796	729.3678	365.1876	**6**
**11**	**1325.4851**	663.2462	1307.4745	654.2409	**W**	**648.3100**	324.6586	631.2835	316.1454	630.2994	315.6534	**5**
**12**	1396.5222	698.7647	1378.5116	689.7594	**A**	**462.2307**	231.6190	445.2041	223.1057	444.2201	222.6137	**4**
**13**	**1525.5648**	763.2860	1507.5542	754.2807	**E**	**391.1936**	196.1004	374.1670	187.5871	373.1830	187.0951	**3**
**14**	1612.5968	806.8020	**1594.5862**	797.7968	**S**	**262.1510**	131.5791	245.1244	123.0659	244.1404	122.5738	**2**
**15**					**R**	175.1190	88.0631	158.0924	79.5498			**1**

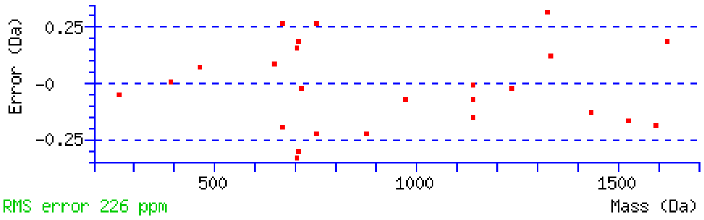

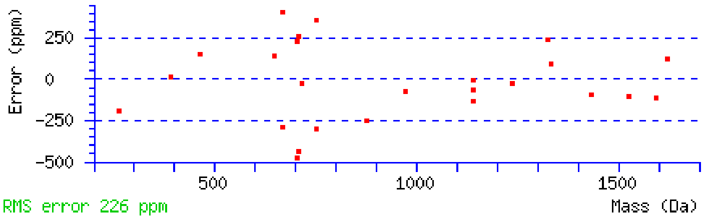

All matches to this query

Score	Mr(calc):	Delta	Sequence
70.9	1785.7012	0.0023	STSPPPSPEVWAESR
58.0	1785.7012	0.0023	STSPPPSPEVWAESR
58.0	1785.7012	0.0023	STSPPPSPEVWAESR
25.9	1785.7012	0.0023	STSPPPSPEVWAESR
25.9	1785.7012	0.0023	STSPPPSPEVWAESR
17.2	1785.7012	0.0023	STSPPPSPEVWAESR
14.9	1785.7012	0.0023	STSPPPSPEVWAESR
7.6	1785.7012	0.0023	STSPPPSPEVWAESR
7.6	1785.7012	0.0023	STSPPPSPEVWAESR
1.9	1783.7137	1.9897	NHTTYNQSCKWHK

Spectrum No: 90; Query: 1856; Rank: 1

Peptide View

MS/MS Fragmentation of **SPDEATAADQESEDDLSASR**
Found in **IPI00766690**, Tax_Id=10116 Gene_Symbol=Farp1_predicted FERM, RhoGEF (Arhgef) and pleckstrin domain protein 1

Match to Query 1856: 2172.843288 from(1087.428920,2+)
Title: 100101RatKid_NS_deglyco_25.1198.1198.2.dta
Data file K:\NewmanPaper\Piliang\3SubProteomes\Piliang3SP\mgf5ppm\ERLIC_3SubProteomes5ppm.mgf

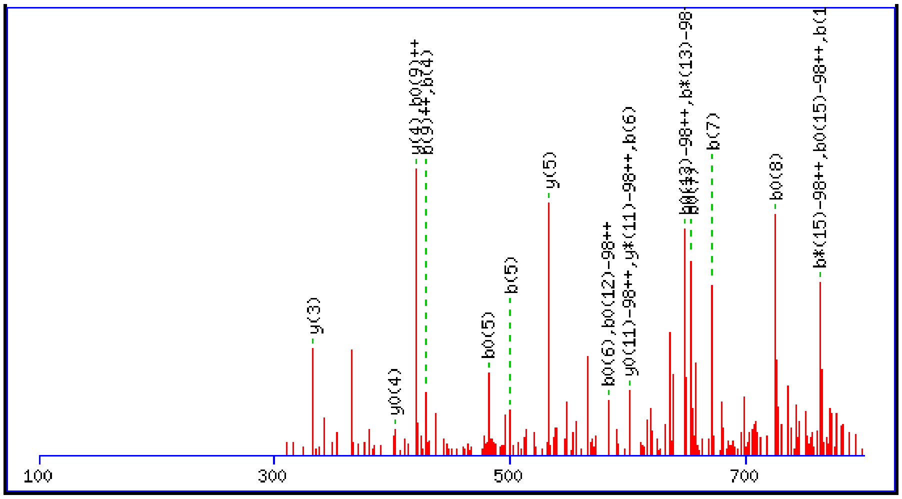

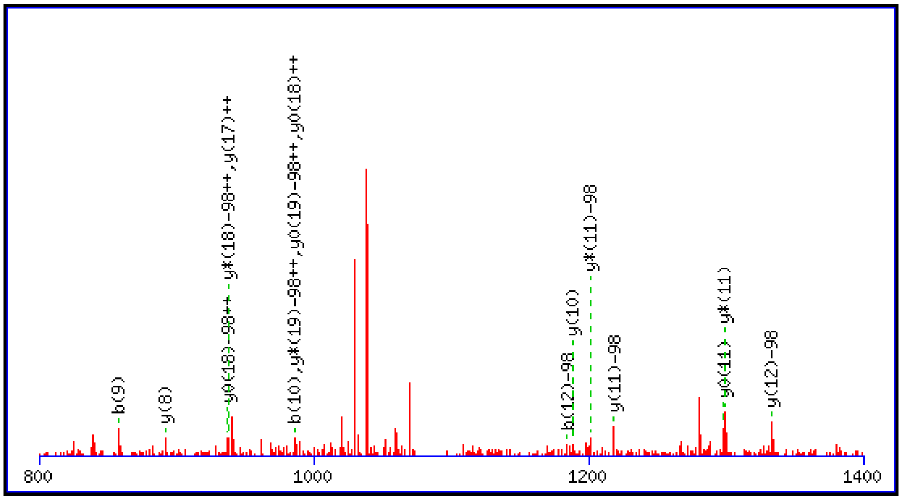

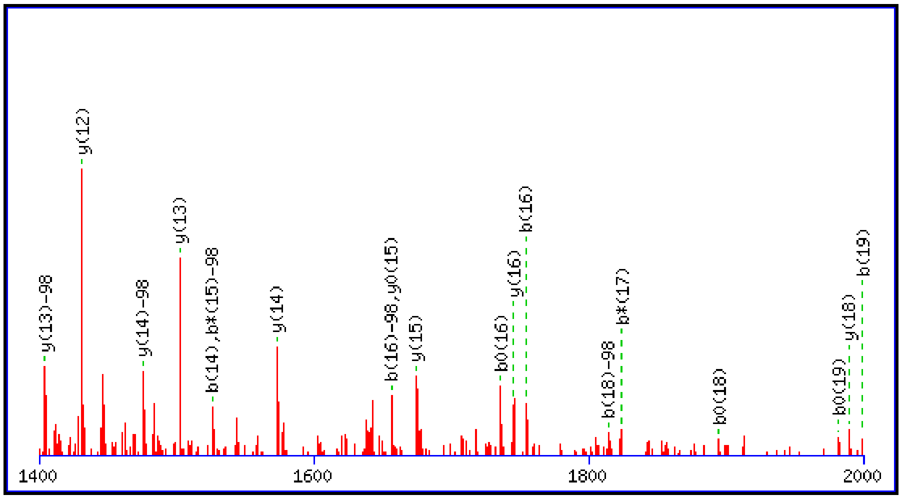

Monoisotopic mass of neutral peptide Mr(calc): 2172.8332
Fixed modifications: Carbamidomethyl (C)
Variable modifications:
S12 : Phospho (ST), with neutral losses 0.0000(shown in table), 97.9769
Ions Score: 71 **Expect:** 1.6e-005
Matches (Bold Red): 59/322 fragment ions using 72 most intense peaks

#	b	b^{++}	b*	b^{*++}	b^0	b^{0++}	Seq.	y	y^{++}	y*	y^{*++}	y^0	y^{0++}	#
1	88.0393	44.5233			70.0287	35.5180	S							20
2	185.0921	93.0497			167.0815	84.0444	P	2086.8085	1043.9079	2069.7819	1035.3946	2068.7979	1034.9026	19
3	300.1190	150.5631			282.1084	141.5579	D	**1989.7557**	995.3815	1972.7291	986.8682	1971.7451	**986.3762**	18
4	**429.1616**	215.0844			411.1510	206.0792	E	1874.7287	**937.8680**	1857.7022	929.3547	1856.7182	928.8627	17
5	**500.1987**	250.6030			**482.1882**	241.5977	A	**1745.6862**	873.3467	1728.6596	864.8334	1727.6756	864.3414	16

6	601.2464	301.1268			583.2358	292.1216	T	1674.6490	837.8282	1657.6225	829.3149	1656.6385	828.8229	15
7	672.2835	336.6454			654.2729	327.6401	A	1573.6014	787.3043	1556.5748	778.7910	1555.5908	778.2990	14
8	743.3206	372.1640			725.3101	363.1587	A	1502.5642	751.7858	1485.5377	743.2725	1484.5537	742.7805	13
9	858.3476	429.6774			840.3370	420.6721	D	1431.5271	716.2672	1414.5006	707.7539	1413.5166	707.2619	12
10	986.4061	493.7067	969.3796	485.1934	968.3956	484.7014	Q	1316.5002	658.7537	1299.4736	650.2405	1298.4896	649.7484	11
11	1115.4487	558.2280	1098.4222	549.7147	1097.4382	549.2227	E	1188.4416	594.7244	1171.4151	586.2112	1170.4310	585.7192	10
12	1282.4471	641.7272	1265.4205	633.2139	1264.4365	632.7219	S	1059.3990	530.2031	1042.3725	521.6899	1041.3885	521.1979	9
13	1411.4897	706.2485	1394.4631	697.7352	1393.4791	697.2432	E	892.4007	446.7040	875.3741	438.1907	874.3901	437.6987	8
14	1526.5166	763.7620	1509.4901	755.2487	1508.5061	754.7567	D	763.3581	382.1827	746.3315	373.6694	745.3475	373.1774	7
15	1641.5436	821.2754	1624.5170	812.7621	1623.5330	812.2701	D	648.3311	324.6692	631.3046	316.1559	630.3206	315.6639	6
16	1754.6276	877.8175	1737.6011	869.3042	1736.6171	868.8122	L	533.3042	267.1557	516.2776	258.6425	515.2936	258.1504	5
17	1841.6597	921.3335	1824.6331	912.8202	1823.6491	912.3282	S	420.2201	210.6137	403.1936	202.1004	402.2096	201.6084	4
18	1912.6968	956.8520	1895.6702	948.3388	1894.6862	947.8467	A	333.1881	167.0977	316.1615	158.5844	315.1775	158.0924	3
19	1999.7288	1000.3680	1982.7023	991.8548	1981.7182	991.3628	S	262.1510	131.5791	245.1244	123.0659	244.1404	122.5738	2
20							R	175.1190	88.0631	158.0924	79.5498			1

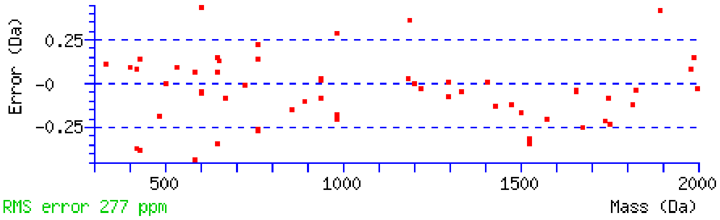

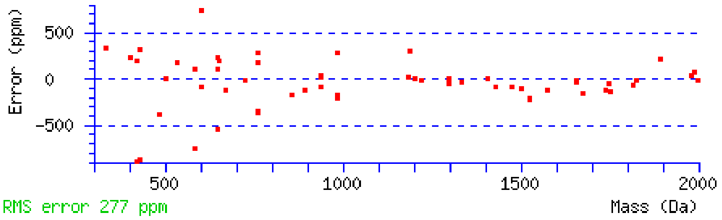

All matches to this query

Score	Mr(calc):	Delta	Sequence
70.8	2172.8332	0.0101	SPDEATAADQESEDDLSASR
51.1	2172.8332	0.0101	SPDEATAADQESEDDLSASR
41.4	2172.8332	0.0101	SPDEATAADQESEDDLSASR
36.2	2172.8332	0.0101	SPDEATAADQESEDDLSASR
27.9	2172.8332	0.0101	SPDEATAADQESEDDLSASR
2.8	2172.8606	-0.0173	TTGCYGASCHVPDISSPER
1.4	2170.8571	1.9862	GITWGEDTLMEYLENPK

Spectrum No: 91; Query: 1854; Rank: 1

Peptide View

MS/MS Fragmentation of **AAFDDAIAELDTLSEESYK**
Found in **IPI00325135**, Tax_Id=10116 Gene_Symbol=Ywhae 14-3-3 protein epsilon

Match to Query 1854: 2166.935268 from(1084.474910,2+)
Title: 100101RatKid_NS_deglyco_22.4748.4748.2.dta
Data file K:\NewmanPaper\Piliang\3SubProteomes\Piliang3SP\mgf5ppm\ERLIC_3SubProteomes5ppm.mgf

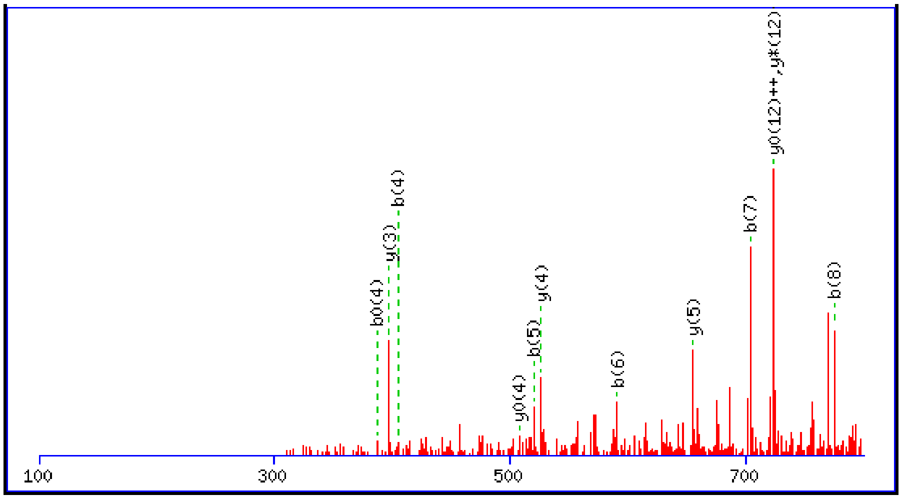

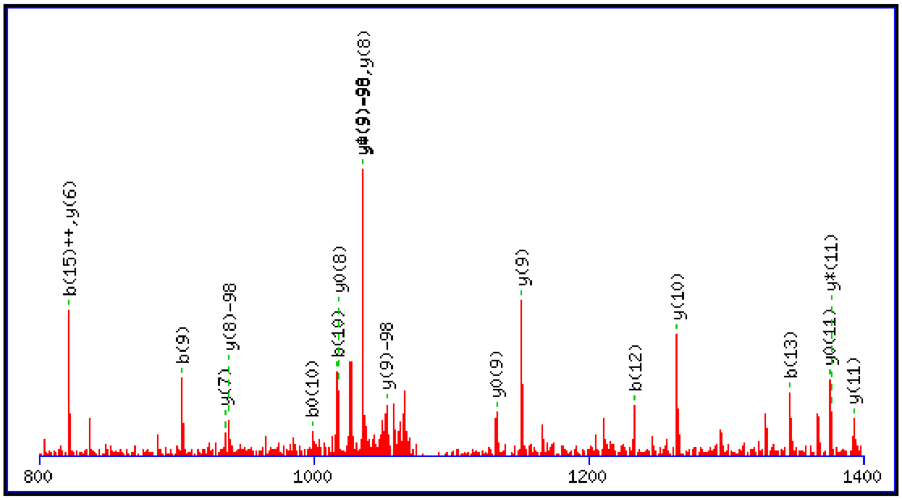

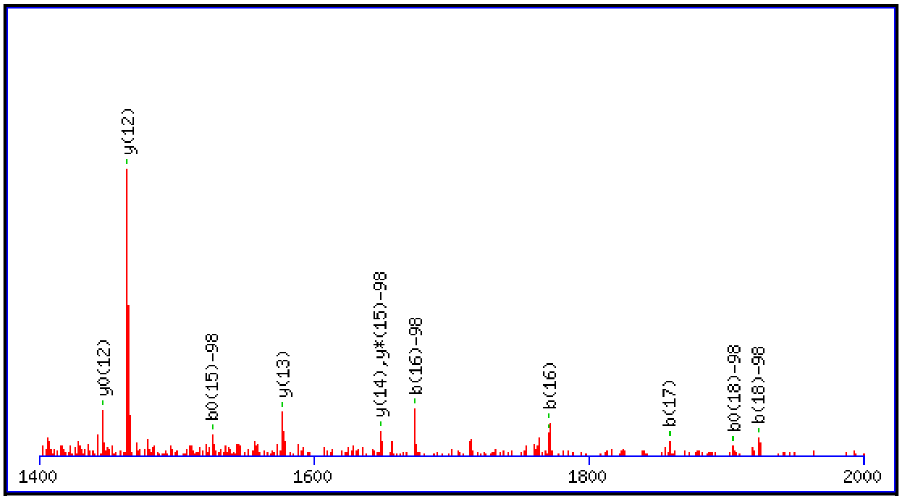

Monoisotopic mass of neutral peptide Mr(calc): 2166.9245
Fixed modifications: Carbamidomethyl (C)
Variable modifications:
S14 : Phospho (ST), with neutral losses 0.0000(shown in table), 97.9769
Ions Score: 71 **Expect:** 2.2e-005
Matches (Bold Red): 44/268 fragment ions using 64 most intense peaks

#	b	b^{++}	b^0	b^{0++}	Seq.	y	y^{++}	y*	y^{*++}	y^0	y^{0++}	#
1	72.0444	36.5258			A							19
2	143.0815	72.0444			A	2096.8947	1048.9510	2079.8682	1040.4377	2078.8842	1039.9457	18
3	290.1499	145.5786			F	2025.8576	1013.4325	2008.8311	1004.9192	2007.8471	1004.4272	17
4	**405.1769**	203.0921	**387.1663**	194.0868	D	1878.7892	939.8982	1861.7627	931.3850	1860.7787	930.8930	16
5	**520.2038**	260.6055	502.1932	251.6003	D	1763.7623	882.3848	1746.7357	873.8715	1745.7517	873.3795	15

6	591.2409	296.1241	573.2304	287.1188	A	1648.7353	824.8713	1631.7088	816.3580	1630.7248	815.8660	14
7	704.3250	352.6661	686.3144	343.6608	I	1577.6982	789.3527	1560.6717	780.8395	1559.6877	780.3475	13
8	775.3621	388.1847	757.3515	379.1794	A	1464.6142	732.8107	1447.5876	724.2974	1446.6036	723.8054	12
9	904.4047	452.7060	886.3941	443.7007	E	1393.5770	697.2922	1376.5505	688.7789	1375.5665	688.2869	11
10	1017.4888	509.2480	999.4782	500.2427	L	1264.5344	632.7709	1247.5079	624.2576	1246.5239	623.7656	10
11	1132.5157	566.7615	1114.5051	557.7562	D	1151.4504	576.2288	1134.4238	567.7156	1133.4398	567.2235	9
12	1233.5634	617.2853	1215.5528	608.2800	T	1036.4234	518.7154	1019.3969	510.2021	1018.4129	509.7101	8
13	1346.6474	673.8274	1328.6369	664.8221	L	935.3758	468.1915	918.3492	459.6782	917.3652	459.1862	7
14	1513.6458	757.3265	1495.6352	748.3213	S	822.2917	411.6495	805.2652	403.1362	804.2811	402.6442	6
15	1642.6884	821.8478	1624.6778	812.8425	E	655.2933	328.1503	638.2668	319.6370	637.2828	319.1450	5
16	1771.7310	886.3691	1753.7204	877.3638	E	526.2508	263.6290	509.2242	255.1157	508.2402	254.6237	4
17	1858.7630	929.8851	1840.7524	920.8799	S	397.2082	199.1077	380.1816	190.5944	379.1976	190.1024	3
18	2021.8263	1011.4168	2003.8158	1002.4115	Y	310.1761	155.5917	293.1496	147.0784			2
19					K	147.1128	74.0600	130.0863	65.5468			1

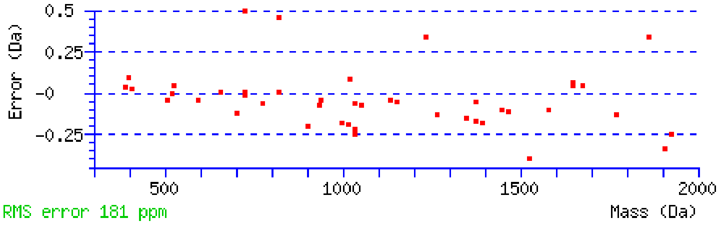

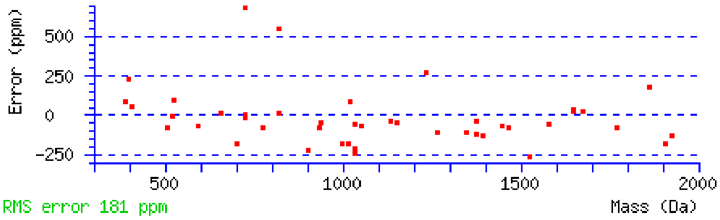

All matches to this query

Score	Mr(calc):	Delta	Sequence
70.8	2166.9245	0.0107	AAFDDAIAELDTLSEESYK
49.3	2166.9245	0.0107	AAFDDAIAELDTLSEESYK
45.3	2166.9245	0.0107	AAFDDAIAELDTLSEESYK
36.1	2166.9245	0.0107	AAFDDAIAELDTLSEESYK
4.9	2166.9430	-0.0077	TKSESRLEFTSSGSANTER
3.0	2166.9430	-0.0077	TKSESRLEFTSSGSANTER
0.8	2166.9316	0.0037	TYYWKSLFIDTVASDAK
0.8	2166.9316	0.0037	TYYWKSLFIDTVASDAK

Spectrum No: 92; Query: 2610; Rank: 1

Peptide View

MS/MS Fragmentation of **GPAGSPCEEGDDAEEDGTSDLRDEASSVTR**
Found in **IPI00400666**, Tax_Id=10116 Gene_Symbol=Nhn1 Isoform A of Zinc finger CCCH domain-containing protein 18

Match to Query 2610: 3188.246322 from(1063.756050,3+)
Title: 100101RatKid_NS_deglyco_24.2637.2637.3.dta
Data file K:\NewmanPaper\Piliang\3SubProteomes\Piliang3SP\mgf5ppm\ERLIC_3SubProteomes5ppm.mgf

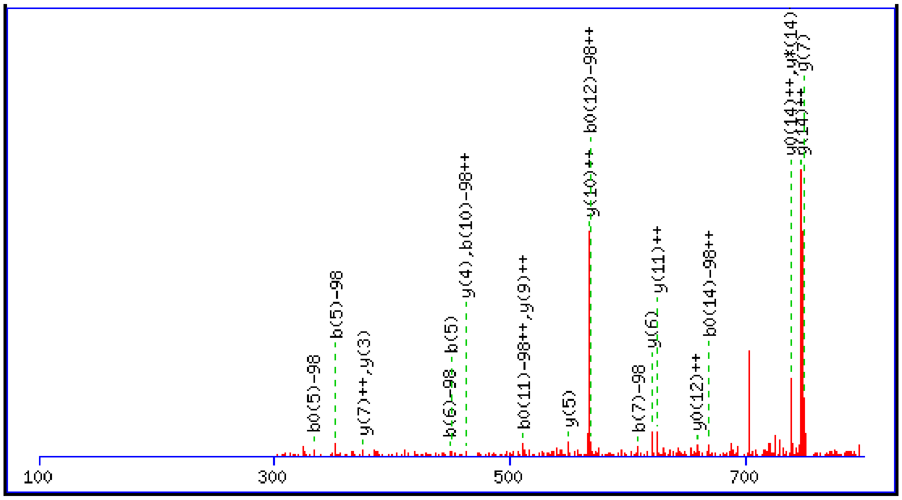

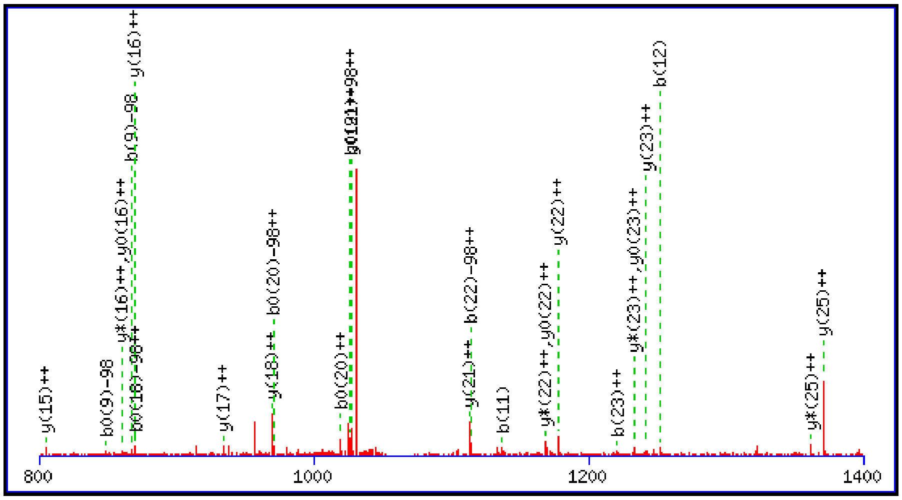

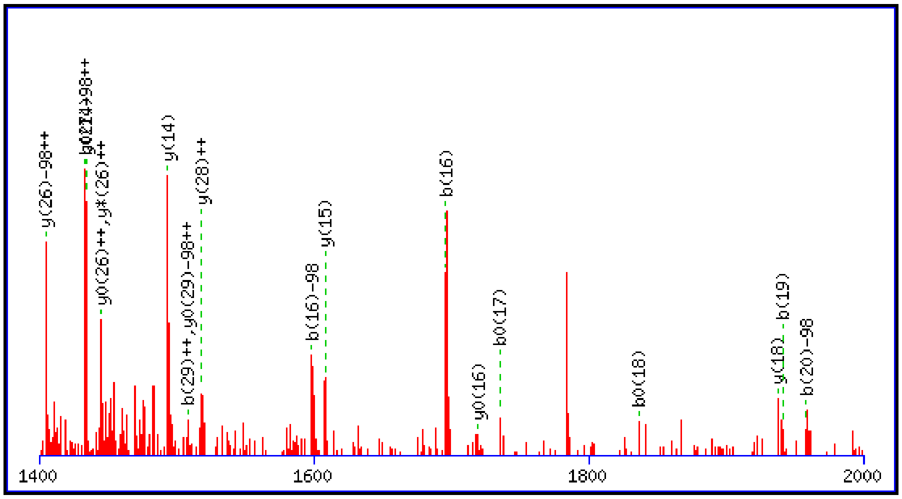

Monoisotopic mass of neutral peptide Mr(calc): 3188.2361
Fixed modifications: Carbamidomethyl (C)
Variable modifications:
S5 : Phospho (ST), with neutral losses 97.9769(shown in table), 0.0000
Ions Score: 71 **Expect:** 2.7e-005
Matches (Bold Red): 67/436 fragment ions using 109 most intense peaks

#	b	b^{++}	b*	b^{*++}	b^0	b^{0++}	Seq.	y	y^{++}	y*	y^{*++}	y^0	y^{0++}	#
1	58.0287	29.5180					G							30
2	155.0815	78.0444					P	3034.2450	1517.6262	3017.2185	1509.1129	3016.2345	**1508.6209**	29
3	226.1186	113.5629					A	2937.1923	1469.0998	2920.1657	1460.5865	2919.1817	1460.0945	28
4	283.1401	142.0737					G	2866.1552	**1433.5812**	2849.1286	1425.0679	2848.1446	1424.5759	27
5	**352.1615**	176.5844			**334.1510**	167.5791	S	2809.1337	**1405.0705**	2792.1071	1396.5572	2791.1231	1396.0652	26

6	**449.2143**	225.1108			431.2037	216.1055	P	2740.1122	**1370.5598**	2723.0857	**1362.0465**	2722.1017	1361.5545	25
7	**609.2450**	305.1261			591.2344	296.1208	C	2643.0595	1322.0334	2626.0329	1313.5201	2625.0489	1313.0281	24
8	**738.2875**	369.6474			720.2770	360.6421	E	2483.0288	**1242.0180**	2466.0023	**1233.5048**	2465.0183	**1233.0128**	23
9	**867.3301**	434.1687			**849.3196**	425.1634	E	2353.9862	**1177.4968**	2336.9597	**1168.9835**	2335.9757	**1168.4915**	22
10	924.3516	**462.6794**			906.3410	453.6742	G	2224.9436	**1112.9755**	2207.9171	1104.4622	2206.9331	1103.9702	21
11	1039.3785	520.1929			1021.3680	**511.1876**	D	2167.9222	1084.4647	2150.8956	1075.9514	2149.9116	1075.4594	20
12	1154.4055	577.7064			1136.3949	**568.7011**	D	2052.8952	**1026.9513**	2035.8687	1018.4380	2034.8847	1017.9460	19
13	1225.4426	613.2249			1207.4320	604.2197	A	**1937.8683**	**969.4378**	1920.8417	960.9245	1919.8577	960.4325	18
14	1354.4852	677.7462			1336.4746	**668.7410**	E	1866.8312	**933.9192**	1849.8046	925.4059	1848.8206	924.9139	17
15	1483.5278	742.2675			1465.5172	733.2622	E	1737.7886	**869.3979**	1720.7620	**860.8847**	**1719.7780**	**860.3926**	16
16	**1598.5547**	799.7810			1580.5442	790.7757	D	**1608.7460**	**804.8766**	1591.7194	796.3634	1590.7354	795.8713	15
17	1655.5762	828.2917			1637.5656	819.2865	G	**1493.7190**	**747.3632**	1476.6925	**738.8499**	1475.7085	**738.3579**	14
18	1756.6239	878.8156			1738.6133	**869.8103**	T	1436.6976	718.8524	1419.6710	710.3392	1418.6870	709.8471	13
19	1843.6559	922.3316			1825.6453	913.3263	S	1335.6499	668.3286	1318.6233	659.8153	1317.6393	**659.3233**	12
20	**1958.6828**	979.8451			1940.6723	**970.8398**	D	1248.6179	**624.8126**	1231.5913	616.2993	1230.6073	615.8073	11
21	2071.7669	1036.3871			2053.7563	**1027.3818**	L	1133.5909	**567.2991**	1116.5644	558.7858	1115.5804	558.2938	10
22	2227.8680	**1114.4376**	2210.8415	1105.9244	2209.8575	1105.4324	R	1020.5069	**510.7571**	1003.4803	502.2438	1002.4963	501.7518	9
23	2342.8950	1171.9511	2325.8684	1163.4378	2324.8844	1162.9458	D	864.4058	432.7065	847.3792	424.1932	846.3952	423.7012	8
24	2471.9376	1236.4724	2454.9110	1227.9591	2453.9270	1227.4671	E	**749.3788**	**375.1930**	732.3523	366.6798	731.3682	366.1878	7
25	2542.9747	1271.9910	2525.9481	1263.4777	2524.9641	1262.9857	A	**620.3362**	310.6717	603.3097	302.1585	602.3257	301.6665	6
26	2630.0067	1315.5070	2612.9801	1306.9937	2611.9961	1306.5017	S	**549.2991**	275.1532	532.2726	266.6399	531.2885	266.1479	5
27	2717.0387	1359.0230	2700.0122	1350.5097	2699.0282	1350.0177	S	**462.2671**	231.6372	445.2405	223.1239	444.2565	222.6319	4
28	2816.1071	1408.5572	2799.0806	1400.0439	2798.0966	1399.5519	V	**375.2350**	188.1212	358.2085	179.6079	357.2245	179.1159	3
29	2917.1548	1459.0810	2900.1283	1450.5678	2899.1443	1450.0758	T	276.1666	138.5870	259.1401	130.0737	258.1561	129.5817	2
30							R	175.1190	88.0631	158.0924	79.5498			1

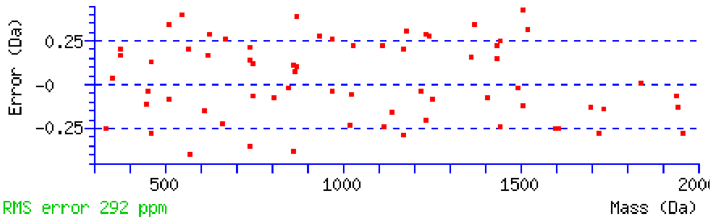

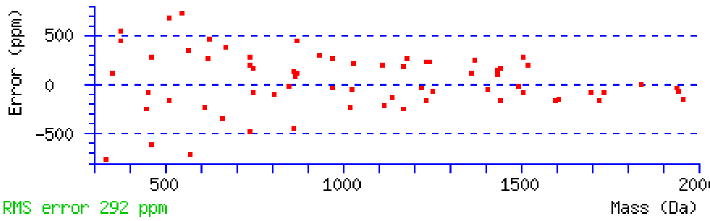

All matches to this query

Score	Mr(calc):	Delta	Sequence
70.7	3188.2361	0.0102	GPAGSPCEEGDDAEEDGTSDLRDEASSVTR
13.8	3188.2361	0.0102	GPAGSPCEEGDDAEEDGTSDLRDEASSVTR
10.4	3188.2361	0.0102	GPAGSPCEEGDDAEEDGTSDLRDEASSVTR
10.1	3187.2334	1.0130	TITMRDTMSDLPEIQNGASAPEISYK
5.8	3187.2210	1.0254	NQATAGASHSEDEDEDYVPYVPLRQR
5.8	3187.2210	1.0254	NQATAGASHSEDEDEDYVPYVPLRQR
5.1	3187.2115	1.0348	YAEGSSFPNTTSGGGADKSQGSEDVTCYR
3.4	3187.2334	1.0130	TITMRDTMSDLPEIQNGASAPEISYK
2.6	3187.2334	1.0130	TITMRDTMSDLPEIQNGASAPEISYK
2.6	3187.2334	1.0130	TITMRDTMSDLPEIQNGASAPEISYK

Spectrum No: 93; Query: 1336; Rank: 1

Peptide View

MS/MS Fragmentation of **LADDEGDSDSESVGQSR**
Found in **IPI00370333**, Tax_Id=10116 Gene_Symbol=Baz1b similar to Bromodomain adjacent to zinc finger domain protein 1B

Match to Query 1336: 1845.689208 from(923.851880,2+)
Title: 091008RatKidney_NoSalt_31.551.551.2.dta
Data file K:\NewmanPaper\Piliang\3SubProteomes\Piliang3SP\mgf5ppm\ERLIC_3SubProteomes5ppm.mgf

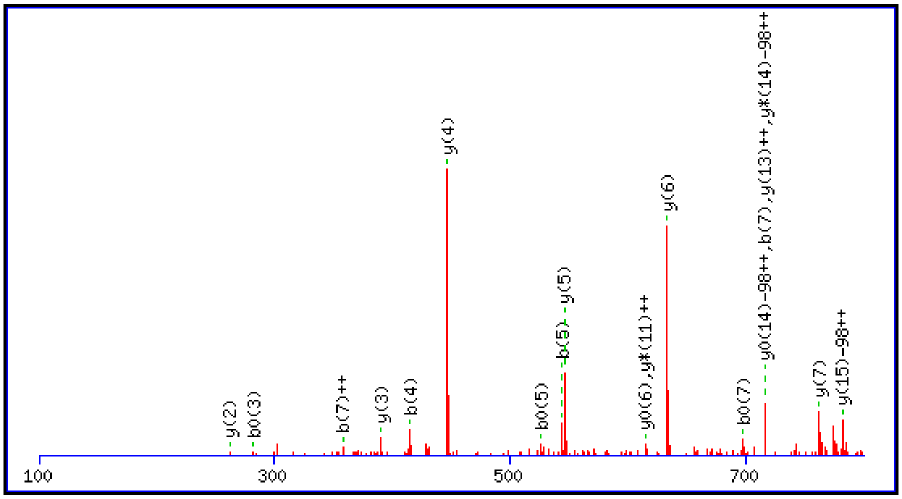

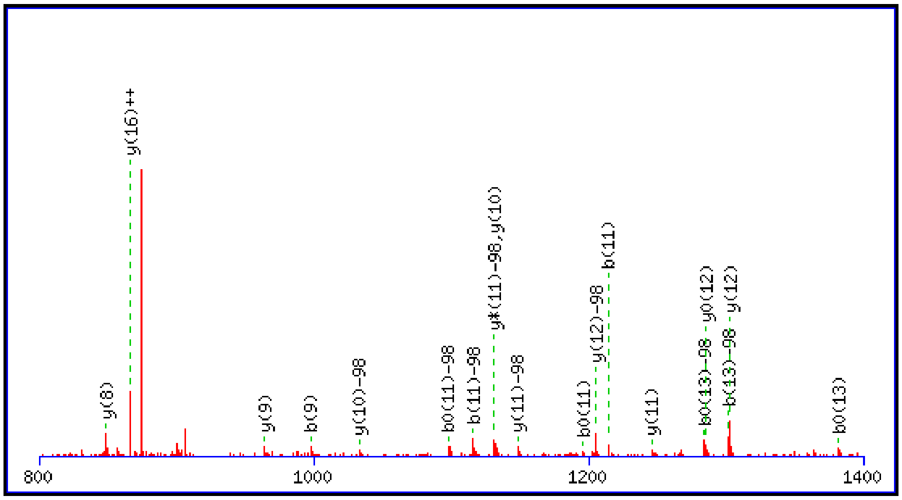

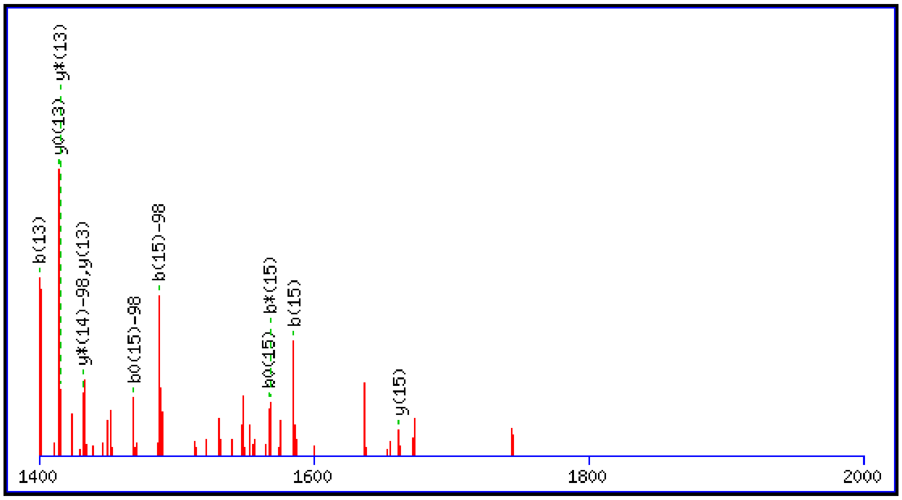

Monoisotopic mass of neutral peptide Mr(calc): 1845.6902
Fixed modifications: Carbamidomethyl (C)
Variable modifications:
S8 : Phospho (ST), with neutral losses 0.0000(shown in table), 97.9769
Ions Score: 71 **Expect:** 8.3e-006
Matches (Bold Red): 49/240 fragment ions using 67 most intense peaks

#	b	b++	b*	b*++	b0	b0++	Seq.	y	y++	y*	y*++	y0	y0++	#
1	114.0913	57.5493					L							17
2	185.1285	93.0679					A	1733.6134	**867.3103**	1716.5868	858.7971	1715.6028	858.3050	16
3	300.1554	150.5813			**282.1448**	141.5761	D	**1662.5763**	831.7918	1645.5497	823.2785	1644.5657	822.7865	15
4	**415.1823**	208.0948			397.1718	199.0895	D	1547.5493	774.2783	1530.5228	765.7650	1529.5388	765.2730	14
5	**544.2249**	272.6161			**526.2144**	263.6108	E	**1432.5224**	**716.7648**	**1415.4958**	708.2516	**1414.5118**	707.7595	13
6	601.2464	301.1268			583.2358	292.1216	G	**1303.4798**	652.2435	1286.4532	643.7303	**1285.4692**	643.2382	12
7	**716.2733**	**358.6403**			**698.2628**	349.6350	D	**1246.4583**	623.7328	1229.4318	**615.2195**	1228.4478	614.7275	11
8	883.2717	442.1395			865.2611	433.1342	S	**1131.4314**	566.2193	1114.4048	557.7061	1113.4208	557.2140	10
9	**998.2986**	499.6530			980.2881	490.6477	D	**964.4330**	482.7202	947.4065	474.2069	946.4225	473.7149	9
10	1085.3307	543.1690			1067.3201	534.1637	S	**849.4061**	425.2067	832.3795	416.6934	831.3955	416.2014	8
11	**1214.3733**	607.6903			**1196.3627**	598.6850	E	**762.3741**	381.6907	745.3475	373.1774	744.3635	372.6854	7
12	1301.4053	651.2063			1283.3947	642.2010	S	**633.3315**	317.1694	616.3049	308.6561	**615.3209**	308.1641	6
13	**1400.4737**	700.7405			**1382.4631**	691.7352	V	**546.2994**	273.6534	529.2729	265.1401	528.2889	264.6481	5
14	1457.4952	729.2512			1439.4846	720.2459	G	**447.2310**	224.1191	430.2045	215.6059	429.2205	215.1139	4
15	**1585.5537**	793.2805	**1568.5272**	784.7672	**1567.5432**	784.2752	Q	**390.2096**	195.6084	373.1830	187.0951	372.1990	186.6031	3
16	1672.5858	836.7965	1655.5592	828.2832	1654.5752	827.7912	S	**262.1510**	131.5791	245.1244	123.0659	244.1404	122.5738	2
17							R	175.1190	88.0631	158.0924	79.5498			1

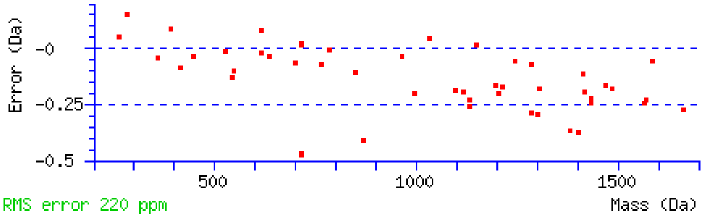

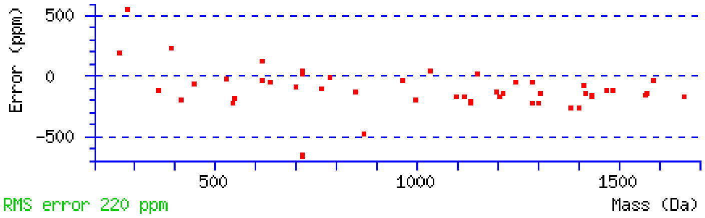

All matches to this query

Score	Mr(calc):	Delta	Sequence
70.7	1845.6902	-0.0010	LADDEGDSDSESVGQSR
46.9	1845.6902	-0.0010	LADDEGDSDSESVGQSR
29.3	1845.6902	-0.0010	LADDEGDSDSESVGQSR
12.8	1845.6902	-0.0010	LADDEGDSDSESVGQSR
5.2	1844.6867	1.0025	DDAPASVHSEITDSNK
3.2	1845.6951	-0.0058	KEMNVSCWDIEPGEG
2.7	1843.6978	1.9914	ANSTPKIDTLCTQR
2.7	1844.6818	1.0074	ANSTPKIDTLCTQR
1.5	1843.6914	1.9978	NSGELEATSSFLESGR
1.5	1843.6914	1.9978	NSGELEATSSFLESGR

Spectrum No: 94; Query: 1328; Rank: 1

Peptide View

MS/MS Fragmentation of **SASSDTSEELNAQDSPK**
Found in **IPI00200898**, Tax_Id=10116 Gene_Symbol=Slc9a3r1 Ezrin-radixin-moesin-binding phosphoprotein 50

Match to Query 1328: 1844.737128 from(923.375840,2+)
Title: 100101RatKid_NS_deglyco_24.866.866.2.dta
Data file K:\NewmanPaper\Piliang\3SubProteomes\Piliang3SP\mgf5ppm\ERLIC_3SubProteomes5ppm.mgf

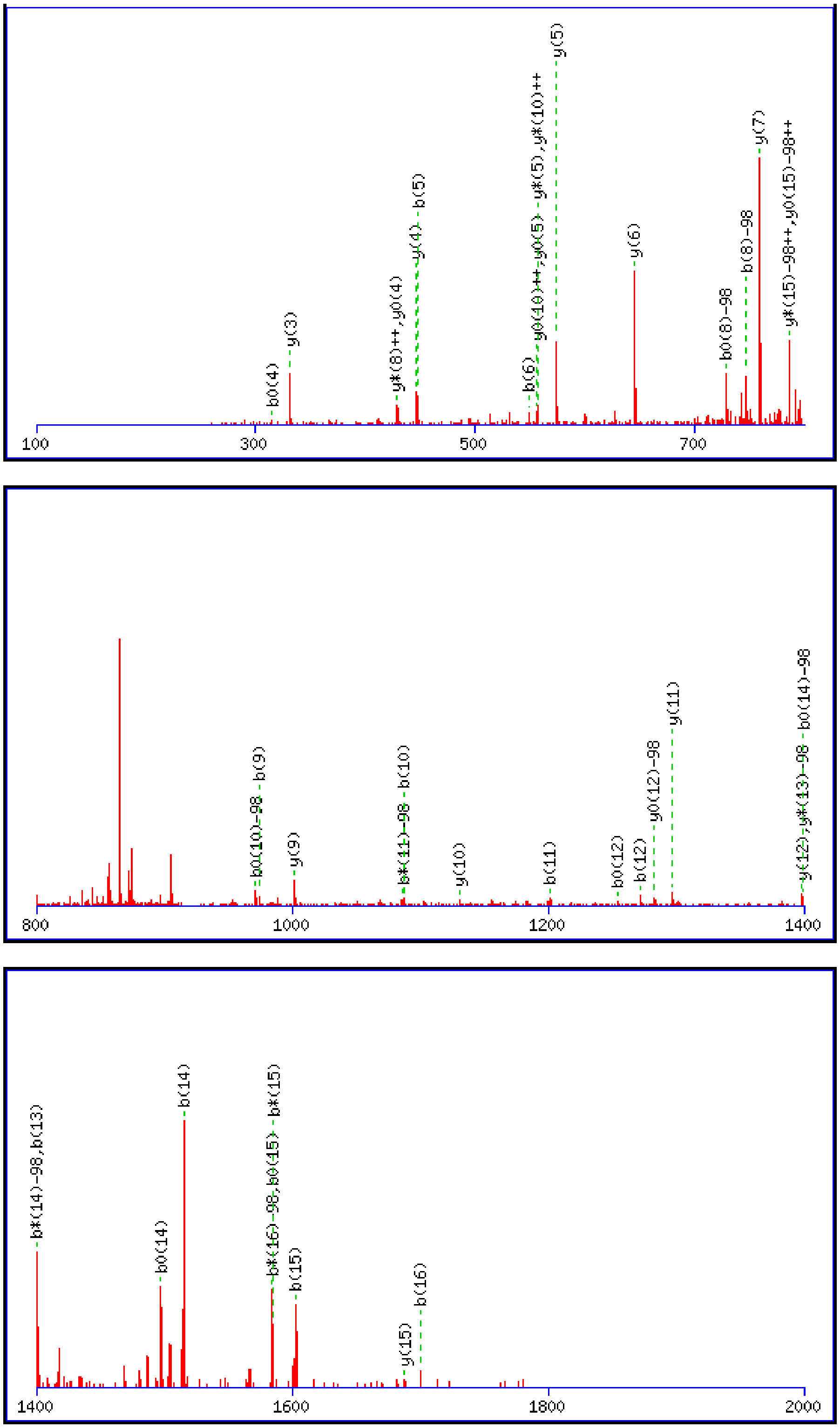

Monoisotopic mass of neutral peptide Mr(calc): 1844.7313
Fixed modifications: Carbamidomethyl (C)
Variable modifications:
S7 : Phospho (ST), with neutral losses 0.0000(shown in table), 97.9769
Ions Score: 71 **Expect:** 1.4e-005
Matches (Bold Red): 42/256 fragment ions using 52 most intense peaks

#	b	b^{++}	b*	b^{*++}	b^0	b^{0++}	Seq.	y	y^{++}	y*	y^{*++}	y^0	y^{0++}	#
1	88.0393	44.5233			70.0287	35.5180	S							17
2	159.0764	80.0418			141.0659	71.0366	A	1758.7066	879.8569	1741.6800	871.3436	1740.6960	870.8516	16
3	246.1084	123.5579			228.0979	114.5526	S	**1687.6694**	844.3384	1670.6429	835.8251	1669.6589	835.3331	15
4	333.1405	167.0739			**315.1299**	158.0686	S	1600.6374	800.8223	1583.6109	792.3091	1582.6268	791.8171	14
5	**448.1674**	224.5873			430.1569	215.5821	D	1513.6054	757.3063	1496.5788	748.7931	1495.5948	748.3010	13

6	549.2151	275.1112			531.2045	266.1059	T	1398.5784	699.7929	1381.5519	691.2796	1380.5679	690.7876	12
7	716.2134	358.6104			698.2029	349.6051	S	1297.5308	649.2690	1280.5042	640.7557	1279.5202	640.2637	11
8	845.2560	423.1317			827.2455	414.1264	E	1130.5324	565.7698	1113.5059	557.2566	1112.5218	556.7646	10
9	974.2986	487.6530			956.2881	478.6477	E	1001.4898	501.2485	984.4633	492.7353	983.4793	492.2433	9
10	1087.3827	544.1950			1069.3721	535.1897	L	872.4472	436.7272	855.4207	428.2140	854.4367	427.7220	8
11	1201.4256	601.2165	1184.3991	592.7032	1183.4151	592.2112	N	759.3632	380.1852	742.3366	371.6719	741.3526	371.1799	7
12	1272.4627	636.7350	1255.4362	628.2217	1254.4522	627.7297	A	645.3202	323.1638	628.2937	314.6505	627.3097	314.1585	6
13	1400.5213	700.7643	1383.4948	692.2510	1382.5108	691.7590	Q	574.2831	287.6452	557.2566	279.1319	556.2726	278.6399	5
14	1515.5483	758.2778	1498.5217	749.7645	1497.5377	749.2725	D	446.2245	223.6159	429.1980	215.1026	428.2140	214.6106	4
15	1602.5803	801.7938	1585.5537	793.2805	1584.5697	792.7885	S	331.1976	166.1024	314.1710	157.5892	313.1870	157.0972	3
16	1699.6331	850.3202	1682.6065	841.8069	1681.6225	841.3149	P	244.1656	122.5864	227.1390	114.0731			2
17							K	147.1128	74.0600	130.0863	65.5468			1

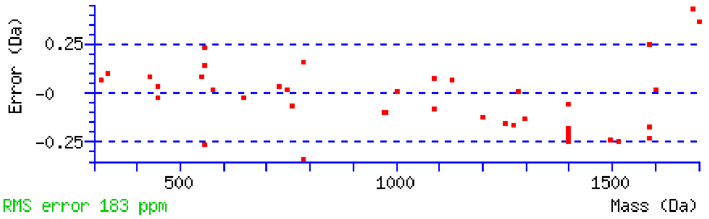

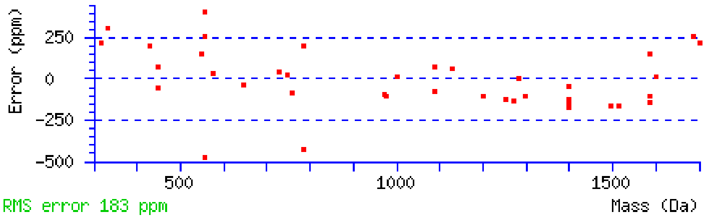

All matches to this query

Score	Mr(calc):	Delta	Sequence
70.6	1844.7313	0.0059	SASSDTSEELNAQDSPK
57.0	1844.7313	0.0059	SASSDTSEELNAQDSPK
53.1	1844.7313	0.0059	SASSDTSEELNAQDSPK
52.6	1844.7313	0.0059	SASSDTSEELNAQDSPK
51.3	1844.7313	0.0059	SASSDTSEELNAQDSPK
10.3	1843.7261	1.0110	SYDEGPQNASSGEPGLR
10.3	1843.7261	1.0110	SYDEGPQNASSGEPGLR
5.1	1844.7423	-0.0052	NVVYAIAFNNPYGDK
4.8	1844.7254	0.0117	TGNGNTDFIWVGPDNR
4.6	1843.7430	0.9941	DGAAVYSFINALATNR

Spectrum No: 95; Query: 1851; Rank: 1

Peptide View

MS/MS Fragmentation of **KETESEAEDDNLDDLER**
Found in **IPI00779470**, Tax_Id=10116 Gene_Symbol=Srrm1_predicted 106 kDa protein

Match to Query 1851: 2166.794388 from(1084.404470,2+)
Title: 100101RatKid_NS_deglyco_26.2052.2052.2.dta
Data file K:\NewmanPaper\Piliang\3SubProteomes\Piliang3SP\mgf5ppm\ERLIC_3SubProteomes5ppm.mgf

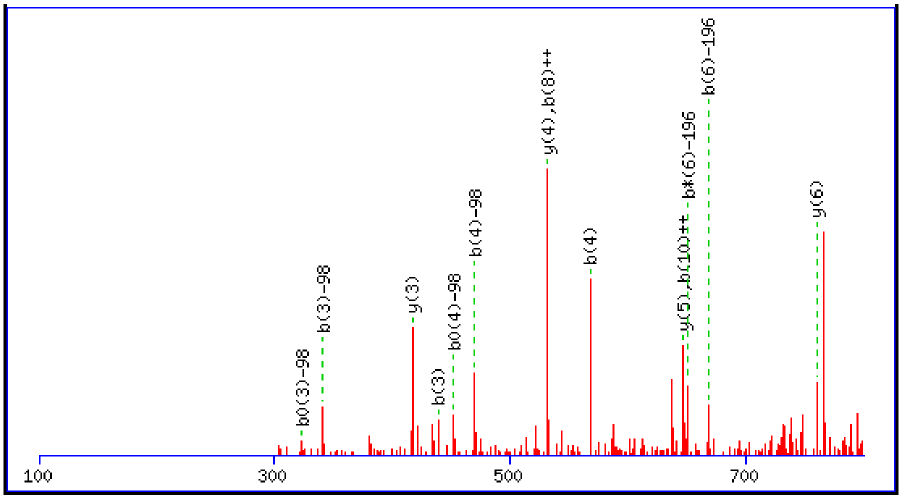

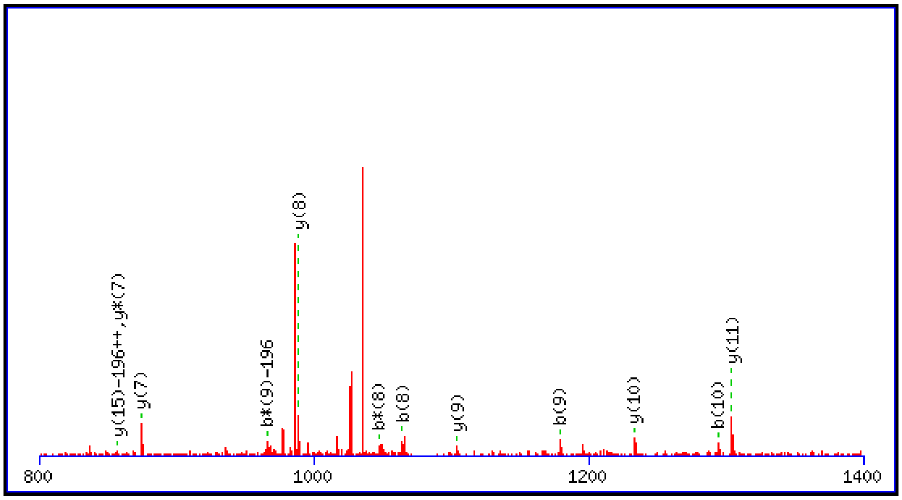

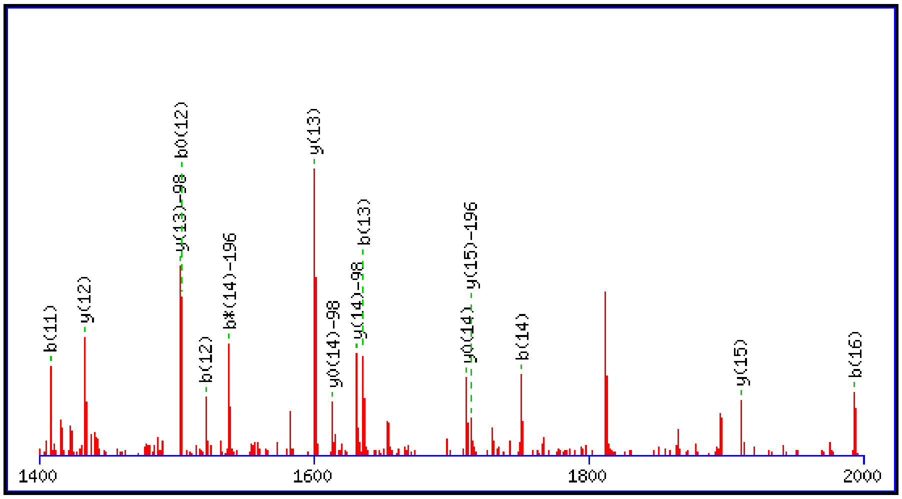

Monoisotopic mass of neutral peptide Mr(calc): 2166.7879
Fixed modifications: Carbamidomethyl (C)
Variable modifications:
T3 : Phospho (ST), with neutral losses 97.9769(shown in table), 0.0000
S5 : Phospho (ST), with neutral losses 97.9769(shown in table), 0.0000
Ions Score: 71 **Expect:** 1.1e-005
Matches (Bold Red): 41/296 fragment ions using 76 most intense peaks

#	b	b^{++}	b*	b^{*++}	b^{0}	b^{0++}	Seq.	y	y^{++}	y*	y^{*++}	y^{0}	y^{0++}	#
1	129.1022	65.0548	112.0757	56.5415			K							17
2	258.1448	129.5761	241.1183	121.0628	240.1343	120.5708	E	1843.7464	922.3768	1826.7199	913.8636	1825.7359	913.3716	16
3	**341.1819**	171.0946	324.1554	162.5813	**323.1714**	162.0893	T	**1714.7038**	**857.8556**	1697.6773	849.3423	1696.6933	848.8503	15
4	**470.2245**	235.6159	453.1980	227.1026	**452.2140**	226.6106	E	**1631.6667**	816.3370	1614.6402	807.8237	**1613.6562**	807.3317	14

**5**	539.2460	270.1266	522.2194	261.6134	521.2354	261.1213	**S**	**1502.6241**	751.8157	1485.5976	743.3024	1484.6136	742.8104	**13**
**6**	**668.2886**	334.6479	**651.2620**	326.1347	650.2780	325.6426	**E**	**1433.6027**	717.3050	1416.5761	708.7917	1415.5921	708.2997	**12**
**7**	739.3257	370.1665	722.2991	361.6532	721.3151	361.1612	**A**	**1304.5601**	652.7837	1287.5335	644.2704	1286.5495	643.7784	**11**
**8**	868.3683	434.6878	851.3417	426.1745	850.3577	425.6825	**E**	**1233.5230**	617.2651	1216.4964	608.7518	1215.5124	608.2598	**10**
**9**	983.3952	492.2013	**966.3687**	483.6880	965.3847	483.1960	**D**	**1104.4804**	552.7438	1087.4538	544.2305	1086.4698	543.7385	**9**
**10**	1098.4222	549.7147	1081.3956	541.2015	1080.4116	540.7094	**D**	**989.4534**	495.2304	972.4269	486.7171	971.4429	486.2251	**8**
**11**	1212.4651	606.7362	1195.4386	598.2229	1194.4545	597.7309	**N**	**874.4265**	437.7169	**857.3999**	429.2036	856.4159	428.7116	**7**
**12**	1325.5492	663.2782	1308.5226	654.7649	1307.5386	654.2729	**L**	**760.3836**	380.6954	743.3570	372.1821	742.3730	371.6901	**6**
**13**	1440.5761	720.7917	1423.5496	712.2784	1422.5655	711.7864	**D**	**647.2995**	324.1534	630.2729	315.6401	629.2889	315.1481	**5**
**14**	1555.6031	778.3052	**1538.5765**	769.7919	1537.5925	769.2999	**D**	**532.2726**	266.6399	515.2460	258.1266	514.2620	257.6346	**4**
**15**	1668.6871	834.8472	1651.6606	826.3339	1650.6766	825.8419	**L**	**417.2456**	209.1264	400.2191	200.6132	399.2350	200.1212	**3**
**16**	1797.7297	899.3685	1780.7032	890.8552	1779.7191	890.3632	**E**	304.1615	152.5844	287.1350	144.0711	286.1510	143.5791	**2**
**17**							**R**	175.1190	88.0631	158.0924	79.5498			**1**

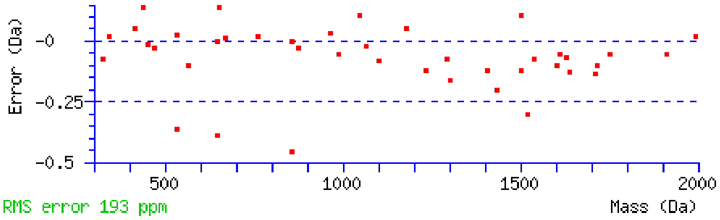

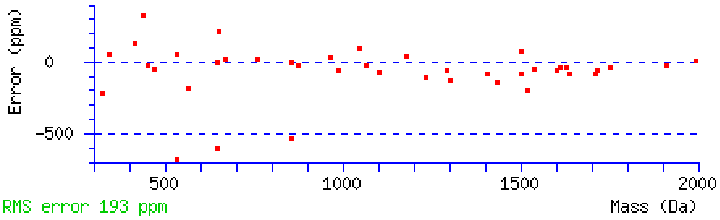

All matches to this query

Score	Mr(calc):	Delta	Sequence
70.6	2166.7879	0.0065	KETESEAEDDNLDDLER
1.7	2166.8150	-0.0207	HYYVMVICYIYFTR
1.7	2166.8150	-0.0207	HYYVMVICYIYFTR
1.0	2166.8078	-0.0134	VPSTSPTADPAAALHPSTK
1.0	2166.8078	-0.0134	VPSTSPTADPAAALHPSTK
1.0	2166.8078	-0.0134	VPSTSPTADPAAALHPSTK
1.0	2166.8078	-0.0134	VPSTSPTADPAAALHPSTK
1.0	2166.8078	-0.0134	VPSTSPTADPAAALHPSTK
1.0	2166.8078	-0.0134	VPSTSPTADPAAALHPSTK
1.0	2166.8078	-0.0134	VPSTSPTADPAAALHPSTK

Spectrum No: 96; Query: 1580; Rank: 1

Peptide View

MS/MS Fragmentation of **TQPDGTSVPGEPASPISQR**
Found in **IPI00368473**, Tax_Id=10116 Gene_Symbol=Numa1 similar to nuclear mitotic apparatus protein 1

Match to Query 1580: 2002.895748 from(1002.455150,2+)
Title: 091008RatKidney_NH4Format02_26.1474.1474.2.dta
Data file K:\NewmanPaper\Piliang\3SubProteomes\Piliang3SP\mgf5ppm\ERLIC_3SubProteomes5ppm.mgf

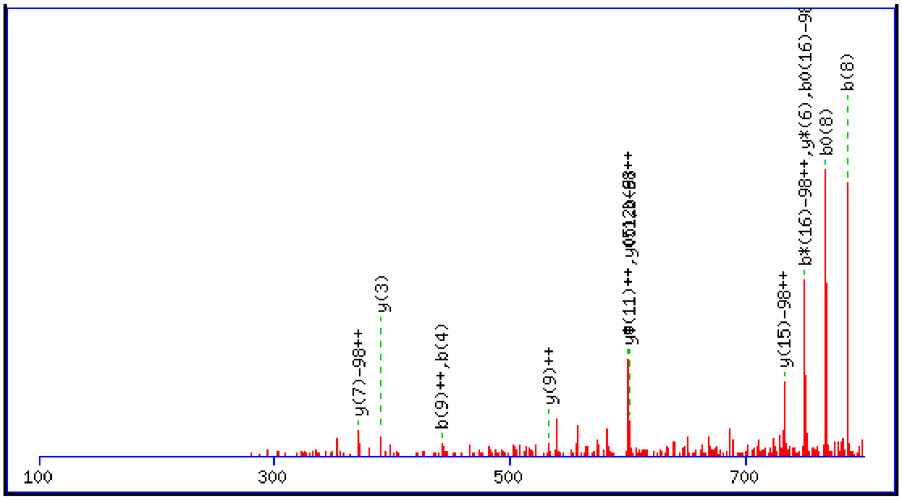

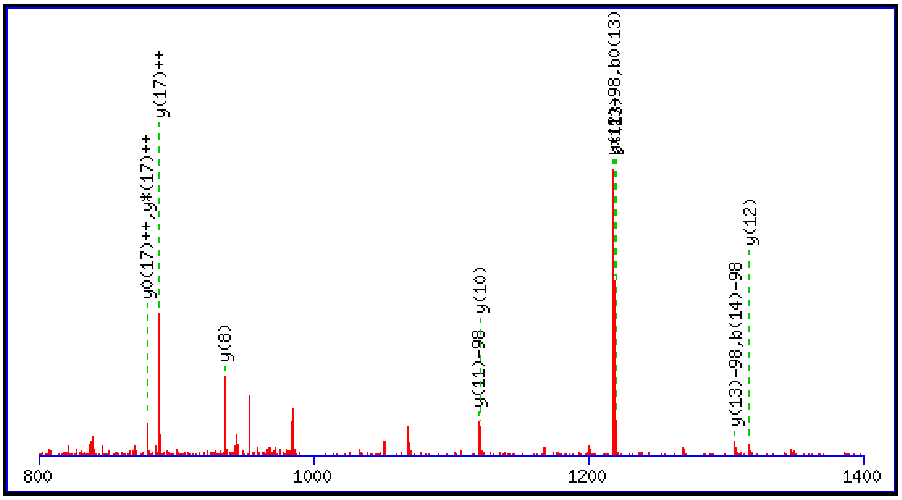

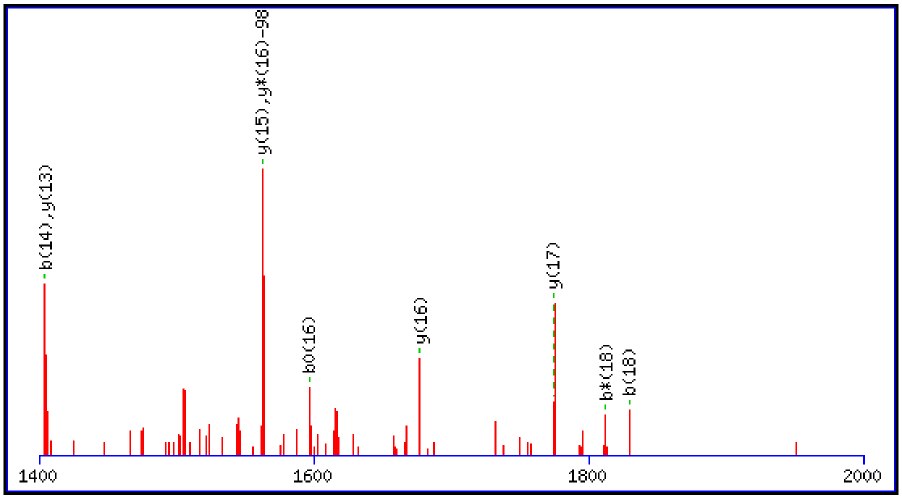

Monoisotopic mass of neutral peptide Mr(calc): 2002.8997
Fixed modifications: Carbamidomethyl (C)
Variable modifications:
S14 : Phospho (ST), with neutral losses 0.0000(shown in table), 97.9769
Ions Score: 70 **Expect:** 2.1e-005
Matches (Bold Red): 38/318 fragment ions using 39 most intense peaks

#	b	b^{++}	b*	b^{*++}	b^{0}	b^{0++}	Seq.	y	y^{++}	y*	y^{*++}	y^{0}	y^{0++}	#
1	102.0550	51.5311			84.0444	42.5258	T							19
2	230.1135	115.5604	213.0870	107.0471	212.1030	106.5551	Q	1902.8593	951.9333	1885.8328	943.4200	1884.8487	942.9280	18
3	327.1663	164.0868	310.1397	155.5735	309.1557	155.0815	P	**1774.8007**	**887.9040**	1757.7742	**879.3907**	1756.7902	**878.8987**	17
4	**442.1932**	221.6003	425.1667	213.0870	424.1827	212.5950	D	**1677.7480**	839.3776	1660.7214	830.8643	1659.7374	830.3723	16
5	499.2147	250.1110	482.1882	241.5977	481.2041	241.1057	G	**1562.7210**	781.8641	1545.6945	773.3509	1544.7105	772.8589	15

**6**	**600.2624**	300.6348	583.2358	292.1216	582.2518	291.6295	**T**	1505.6996	753.3534	1488.6730	744.8401	1487.6890	744.3481	**14**
**7**	687.2944	344.1508	670.2679	335.6376	669.2838	335.1456	**S**	**1404.6519**	702.8296	1387.6253	694.3163	1386.6413	693.8243	**13**
**8**	**786.3628**	393.6851	769.3363	385.1718	**768.3523**	384.6798	**V**	**1317.6199**	659.3136	1300.5933	650.8003	1299.6093	650.3083	**12**
**9**	883.4156	**442.2114**	866.3890	433.6982	865.4050	433.2062	**P**	**1218.5514**	609.7794	1201.5249	**601.2661**	1200.5409	**600.7741**	**11**
**10**	940.4371	470.7222	923.4105	462.2089	922.4265	461.7169	**G**	**1121.4987**	561.2530	1104.4721	552.7397	1103.4881	552.2477	**10**
**11**	1069.4796	535.2435	1052.4531	526.7302	1051.4691	526.2382	**E**	1064.4772	**532.7422**	1047.4507	524.2290	1046.4666	523.7370	**9**
**12**	1166.5324	583.7698	1149.5059	575.2566	1148.5218	574.7646	**P**	**935.4346**	468.2209	918.4081	459.7077	917.4241	459.2157	**8**
**13**	1237.5695	619.2884	**1220.5430**	610.7751	**1219.5590**	610.2831	**A**	838.3819	419.6946	821.3553	411.1813	820.3713	410.6893	**7**
**14**	**1404.5679**	702.7876	1387.5413	694.2743	1386.5573	693.7823	**S**	767.3447	384.1760	**750.3182**	375.6627	749.3342	375.1707	**6**
**15**	1501.6206	751.3140	1484.5941	742.8007	1483.6101	742.3087	**P**	**600.3464**	300.6768	583.3198	292.1636	582.3358	291.6715	**5**
**16**	1614.7047	807.8560	1597.6782	799.3427	**1596.6941**	798.8507	**I**	503.2936	252.1504	486.2671	243.6372	485.2831	243.1452	**4**
**17**	1701.7367	851.3720	1684.7102	842.8587	1683.7262	842.3667	**S**	**390.2096**	195.6084	373.1830	187.0951	372.1990	186.6031	**3**
**18**	**1829.7953**	915.4013	**1812.7688**	906.8880	1811.7847	906.3960	**Q**	303.1775	152.0924	286.1510	143.5791			**2**
**19**							**R**	175.1190	88.0631	158.0924	79.5498			**1**

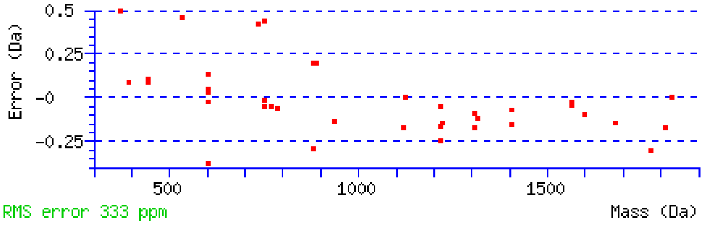

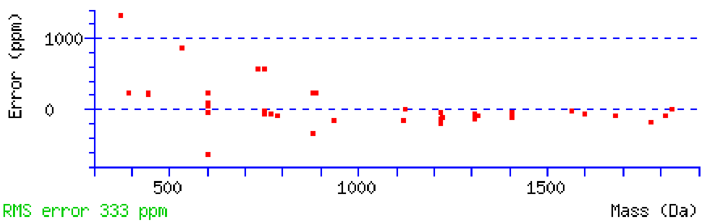

All matches to this query

Score	Mr(calc):	Delta	Sequence
70.4	2002.8997	-0.0040	TQPDGTSVPGEPASPISQR
49.3	2002.8997	-0.0040	TQPDGTSVPGEPASPISQR
36.3	2002.8997	-0.0040	TQPDGTSVPGEPASPISQR
29.4	2002.8997	-0.0040	TQPDGTSVPGEPASPISQR
11.9	2002.8997	-0.0040	TQPDGTSVPGEPASPISQR
6.7	2002.8919	0.0039	TTVTVTMEVPSGGKTEDR
5.2	2002.8955	0.0003	VEFTTGAYPRLSLSFR
5.2	2002.8955	0.0003	VEFTTGAYPRLSLSFR
5.2	2002.8955	0.0003	VEFTTGAYPRLSLSFR
4.9	2002.8874	0.0083	SALNVVNSSQNRTTVPR

Spectrum No: 97; Query: 2044; Rank: 1

Peptide View

MS/MS Fragmentation of **KGAEEEEEEEDDDSEEEIK**
Found in **IPI00778976**, Tax_Id=10116 Gene_Symbol=Epb4.9_predicted 45 kDa protein

Match to Query 2044: 2317.848468 from(1159.931510,2+)
Title: 091008RatKidney_NoSalt_29.678.678.2.dta
Data file K:\NewmanPaper\Piliang\3SubProteomes\Piliang3SP\mgf5ppm\ERLIC_3SubProteomes5ppm.mgf

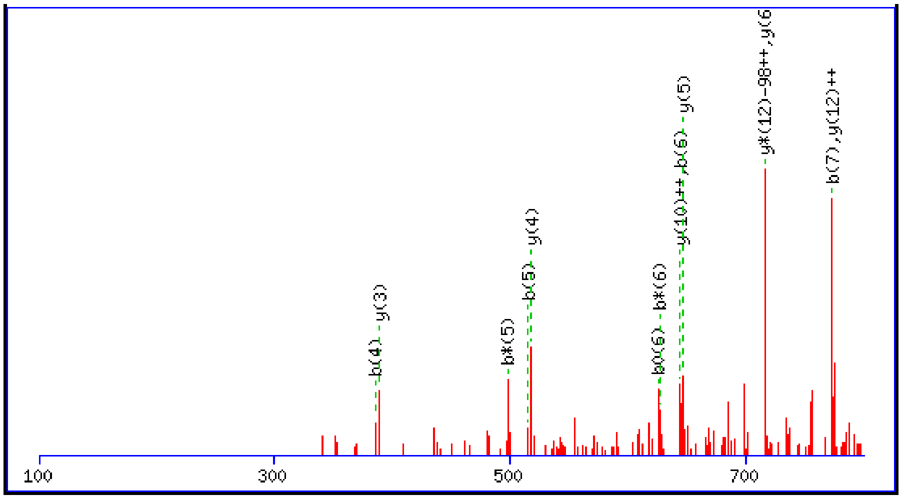

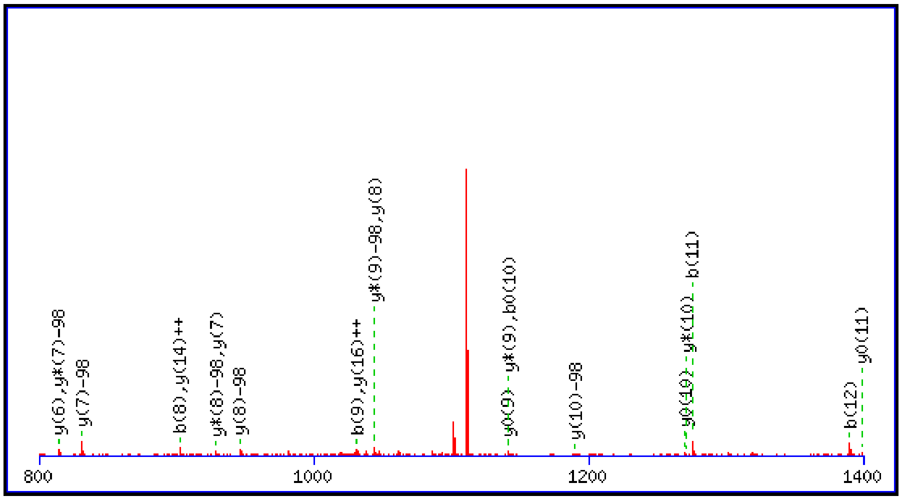

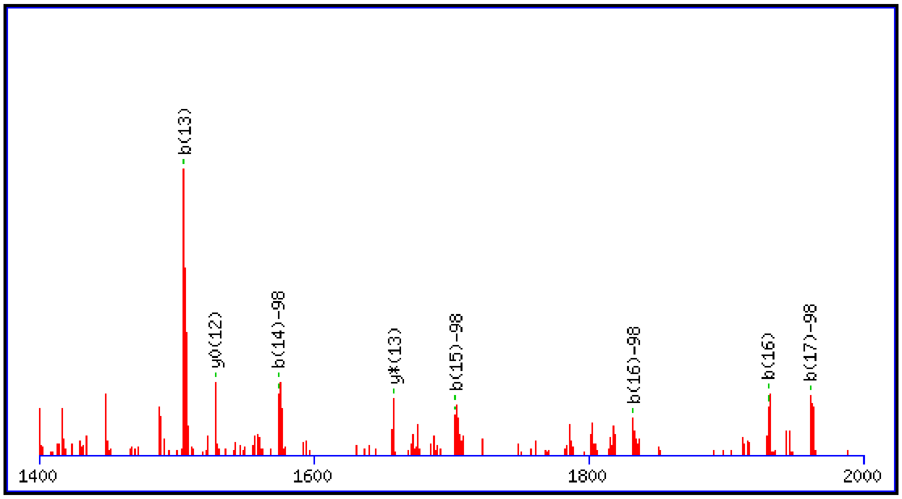

Monoisotopic mass of neutral peptide Mr(calc): 2317.8482
Fixed modifications: Carbamidomethyl (C)
Variable modifications:
S14 : Phospho (ST), with neutral losses 97.9769(shown in table), 0.0000
Ions Score: 70 **Expect:** 1.4e-005
Matches (Bold Red): 43/314 fragment ions using 59 most intense peaks

#	b	b^{++}	b*	b^{*++}	b^0	b^{0++}	Seq.	y	y^{++}	y*	y^{*++}	y^0	y^{0++}	#
1	129.1022	65.0548	112.0757	56.5415			K							19
2	186.1237	93.5655	169.0972	85.0522			G	2092.7837	1046.8955	2075.7571	1038.3822	2074.7731	1037.8902	18
3	257.1608	129.0840	240.1343	120.5708			A	2035.7622	1018.3847	2018.7356	1009.8715	2017.7516	1009.3795	17
4	**386.2034**	193.6053	369.1769	185.0921	368.1928	184.6001	E	1964.7251	982.8662	1947.6985	974.3529	1946.7145	973.8609	16
5	**515.2460**	258.1266	**498.2195**	249.6134	497.2354	249.1214	E	1835.6825	918.3449	1818.6559	909.8316	1817.6719	909.3396	15

6	**644.2886**	322.6479	**627.2620**	314.1347	**626.2780**	313.6427	E	1706.6399	853.8236	1689.6133	845.3103	1688.6293	844.8183	14
7	**773.3312**	387.1692	756.3046	378.6560	755.3206	378.1640	E	1577.5973	789.3023	1560.5708	780.7890	1559.5867	780.2970	13
8	**902.3738**	451.6905	885.3472	443.1773	884.3632	442.6852	E	1448.5547	724.7810	1431.5282	**716.2677**	1430.5441	715.7757	12
9	**1031.4164**	516.2118	1014.3898	507.6986	1013.4058	507.2065	E	1319.5121	660.2597	1302.4856	651.7464	1301.5016	651.2544	11
10	1160.4590	580.7331	1143.4324	572.2198	**1142.4484**	571.7278	E	**1190.4695**	595.7384	1173.4430	587.2251	1172.4590	586.7331	10
11	**1275.4859**	638.2466	1258.4594	629.7333	1257.4753	629.2413	D	1061.4269	531.2171	**1044.4004**	522.7038	1043.4164	522.2118	9
12	**1390.5129**	695.7601	1373.4863	687.2468	1372.5023	686.7548	D	**946.4000**	473.7036	**929.3734**	465.1904	928.3894	464.6984	8
13	**1505.5398**	753.2735	1488.5132	744.7603	1487.5292	744.2683	D	**831.3730**	416.1902	**814.3465**	407.6769	813.3625	407.1849	7
14	**1574.5613**	787.7843	1557.5347	779.2710	1556.5507	778.7790	S	**716.3461**	358.6767	699.3196	350.1634	698.3355	349.6714	6
15	**1703.6038**	852.3056	1686.5773	843.7923	1685.5933	843.3003	E	**647.3246**	324.1660	630.2981	315.6527	629.3141	315.1607	5
16	**1832.6464**	916.8269	1815.6199	908.3136	1814.6359	907.8216	E	**518.2821**	259.6447	501.2555	251.1314	500.2715	250.6394	4
17	**1961.6890**	981.3482	1944.6625	972.8349	1943.6785	972.3429	E	**389.2395**	195.1234	372.2129	186.6101	371.2289	186.1181	3
18	2074.7731	1037.8902	2057.7465	1029.3769	2056.7625	1028.8849	I	260.1969	130.6021	243.1703	122.0888			2
19							K	147.1128	74.0600	130.0863	65.5468			1

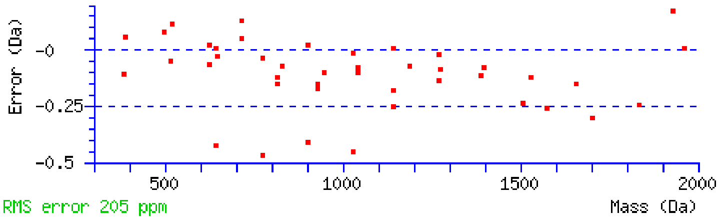

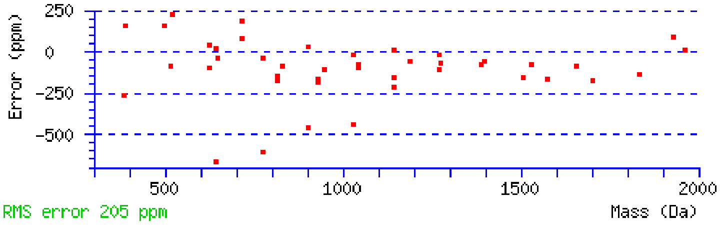

All matches to this query

Score	Mr(calc):	Delta	Sequence
69.8	2317.8482	0.0003	KGAEEEEEEEEDDDSEEEIK
4.9	2317.8595	-0.0110	NNPCTNYSMLPETMIDLK
4.9	2317.8595	-0.0110	NNPCTNYSMLPETMIDLK
4.5	2317.8595	-0.0110	NNPCTNYSMLPETMIDLK
4.3	2317.8595	-0.0110	NNPCTNYSMLPETMIDLK
4.3	2317.8595	-0.0110	NNPCTNYSMLPETMIDLK
4.0	2317.8595	-0.0110	NNPCTNYSMLPETMIDLK
3.7	2316.8606	0.9878	NPSNRNQDDMAPSGPNSPIK
3.7	2317.8447	0.0038	NPSNRNQDDMAPSGPNSPIK
3.5	2316.8606	0.9878	NPSNRNQDDMAPSGPNSPIK

Spectrum No: 98; Query: 2680; Rank: 1

Peptide View

MS/MS Fragmentation of **RTDPDHYETDYTTGGESCDELEEDWLR**
Found in **IPI00421524**, Tax_Id=10116 Gene_Symbol=Ocln;Tpmt Occludin

Match to Query 2680: 3369.321612 from(1124.114480,3+)
Title: 100101RatKid_NS_deglyco_20.3566.3566.3.dta
Data file K:\NewmanPaper\Piliang\3SubProteomes\Piliang3SP\mgf5ppm\ERLIC_3SubProteomes5ppm.mgf

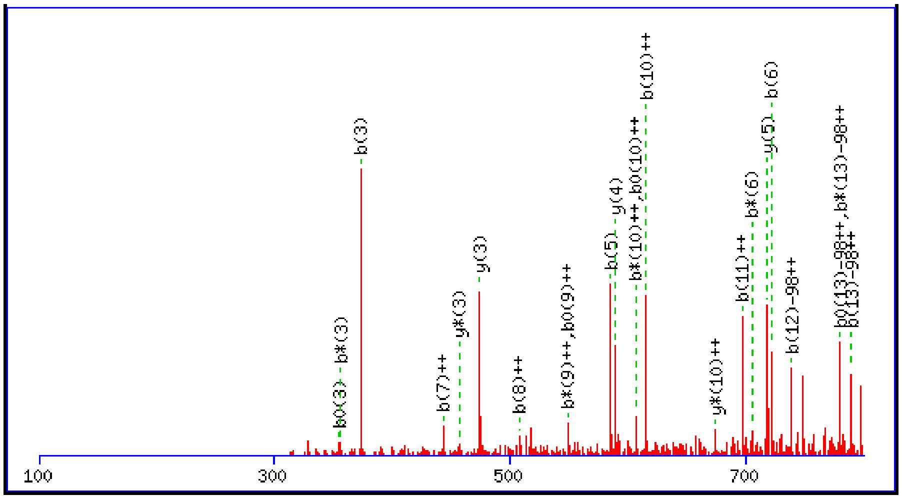

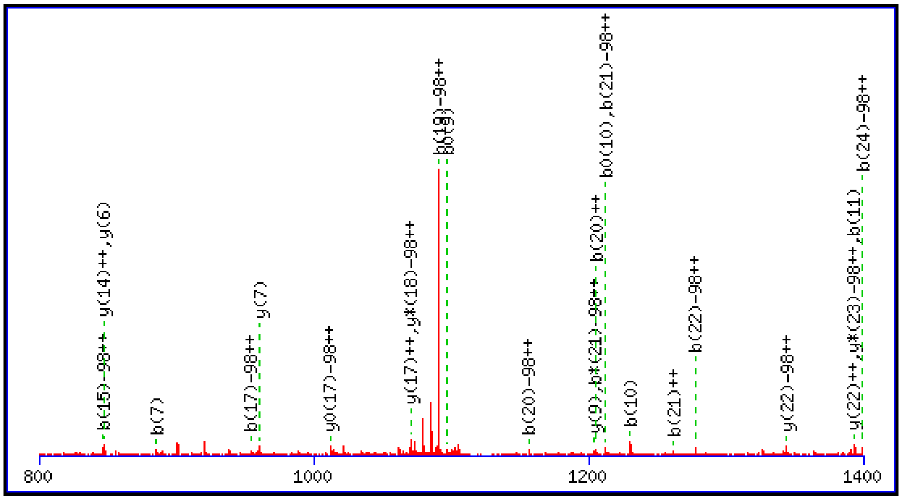

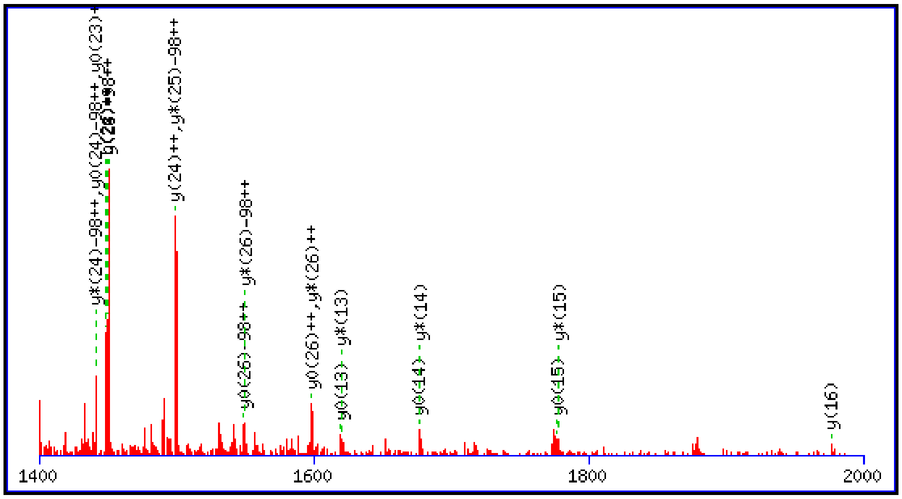

Monoisotopic mass of neutral peptide Mr(calc): 3368.3089
Fixed modifications: Carbamidomethyl (C)
Variable modifications:
T12 : Phospho (ST), with neutral losses 97.9769(shown in table), 0.0000
Ions Score: 70 **Expect:** 3.7e-005
Matches (Bold Red): 69/460 fragment ions using 92 most intense peaks

#	b	b^{++}	b*	b^{*++}	b^0	b^{0++}	Seq.	y	y^{++}	y*	y^{*++}	y^0	y^{0++}	#
1	157.1084	79.0578	140.0818	70.5446			R							27
2	258.1561	129.5817	241.1295	121.0684	240.1455	120.5764	T	3115.2382	1558.1227	3098.2116	**1549.6094**	3097.2276	**1549.1174**	26
3	**373.1830**	187.0951	**356.1565**	178.5819	**355.1724**	178.0899	D	3014.1905	1507.5989	2997.1639	**1499.0856**	2996.1799	1498.5936	25
4	470.2358	235.6215	453.2092	227.1083	452.2252	226.6162	P	2899.1635	**1450.0854**	2882.1370	**1441.5721**	2881.1530	**1441.0801**	24
5	**585.2627**	293.1350	568.2362	284.6217	567.2522	284.1297	D	2802.1108	1401.5590	2785.0842	**1393.0457**	2784.1002	1392.5537	23

6	**722.3216**	361.6645	**705.2951**	353.1512	704.3111	352.6592	H	2687.0838	**1344.0456**	2670.0573	1335.5323	2669.0733	1335.0403	22
7	**885.3850**	**443.1961**	868.3584	434.6828	867.3744	434.1908	Y	2550.0249	1275.5161	2532.9984	1267.0028	2532.0143	1266.5108	21
8	1014.4276	**507.7174**	997.4010	499.2041	996.4170	498.7121	E	2386.9616	1193.9844	2369.9350	1185.4712	2368.9510	1184.9791	20
9	1115.4752	558.2413	1098.4487	**549.7280**	**1097.4647**	**549.2360**	T	2257.9190	1129.4631	2240.8924	1120.9499	2239.9084	1120.4579	19
10	**1230.5022**	**615.7547**	1213.4756	**607.2414**	**1212.4916**	**606.7494**	D	2156.8713	1078.9393	2139.8448	**1070.4260**	2138.8607	1069.9340	18
11	**1393.5655**	**697.2864**	1376.5390	688.7731	1375.5549	688.2811	Y	2041.8444	1021.4258	2024.8178	1012.9125	2023.8338	**1012.4205**	17
12	1476.6026	**738.8049**	1459.5761	730.2917	1458.5920	729.7997	T	1878.7810	939.8942	1861.7545	931.3809	1860.7705	930.8889	16
13	1577.6503	**789.3288**	1560.6237	**780.8155**	1559.6397	**780.3235**	T	1795.7439	898.3756	**1778.7174**	889.8623	**1777.7334**	889.3703	15
14	1634.6718	817.8395	1617.6452	809.3262	1616.6612	808.8342	G	1694.6963	**847.8518**	**1677.6697**	839.3385	**1676.6857**	838.8465	14
15	1691.6932	**846.3502**	1674.6667	837.8370	1673.6827	837.3450	G	1637.6748	819.3410	**1620.6482**	810.8278	**1619.6642**	810.3358	13
16	1820.7358	910.8715	1803.7093	902.3583	1802.7252	901.8663	E	1580.6533	790.8303	1563.6268	782.3170	1562.6428	781.8250	12
17	1907.7678	**954.3876**	1890.7413	945.8743	1889.7573	945.3823	S	1451.6107	726.3090	1434.5842	717.7957	1433.6002	717.3037	11
18	2067.7985	1034.4029	2050.7719	1025.8896	2049.7879	1025.3976	C	1364.5787	682.7930	1347.5522	**674.2797**	1346.5681	673.7877	10
19	2182.8254	**1091.9164**	2165.7989	1083.4031	2164.8149	1082.9111	D	**1204.5481**	602.7777	1187.5215	594.2644	1186.5375	593.7724	9
20	2311.8680	**1156.4376**	2294.8415	1147.9244	2293.8575	1147.4324	E	1089.5211	545.2642	1072.4946	536.7509	1071.5106	536.2589	8
21	2424.9521	**1212.9797**	2407.9255	**1204.4664**	2406.9415	1203.9744	L	**960.4785**	480.7429	943.4520	472.2296	942.4680	471.7376	7
22	2553.9947	**1277.5010**	2536.9681	1268.9877	2535.9841	1268.4957	E	**847.3945**	424.2009	830.3679	415.6876	829.3839	415.1956	6
23	2683.0373	1342.0223	2666.0107	1333.5090	2665.0267	1333.0170	E	**718.3519**	359.6796	701.3253	351.1663	700.3413	350.6743	5
24	2798.0642	**1399.5357**	2781.0377	1391.0225	2780.0536	1390.5305	D	**589.3093**	295.1583	572.2827	286.6450	571.2987	286.1530	4
25	2984.1435	1492.5754	2967.1170	1484.0621	2966.1330	1483.5701	W	**474.2823**	237.6448	**457.2558**	229.1315			3
26	3097.2276	**1549.1174**	3080.2010	1540.6042	3079.2170	1540.1122	L	288.2030	144.6051	271.1765	136.0919			2
27							R	175.1190	88.0631	158.0924	79.5498			1

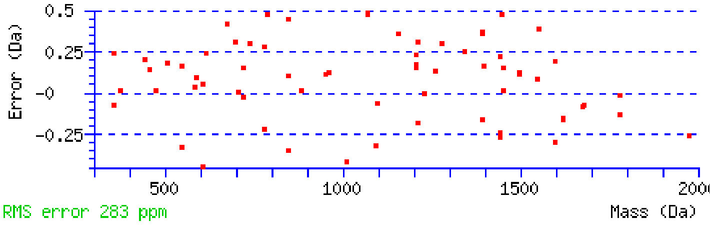

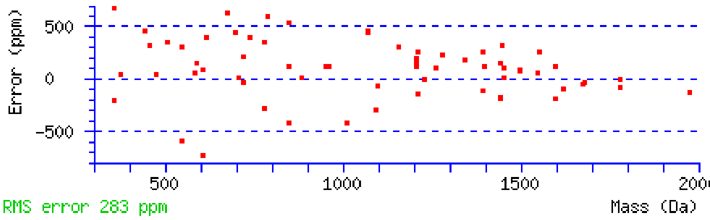

All matches to this query

Score	Mr(calc):	Delta	Sequence
69.7	3368.3089	1.0127	RTDPDHYETDYTTGGESCDELEEDWLR
69.3	3368.3089	1.0127	RTDPDHYETDYTTGGESCDELEEDWLR
67.4	3368.3089	1.0127	RTDPDHYETDYTTGGESCDELEEDWLR
66.0	3368.3089	1.0127	RTDPDHYETDYTTGGESCDELEEDWLR
34.6	3368.3089	1.0127	RTDPDHYETDYTTGGESCDELEEDWLR
20.5	3368.3089	1.0127	RTDPDHYETDYTTGGESCDELEEDWLR
14.7	3369.3363	-0.0147	GNLKGDCEFSLGGIVGTCNPSTGRLSTPR
10.4	3368.2930	1.0286	CGPHHKFSTTFEPGLSSAQKQPSSVVR
10.0	3369.3363	-0.0147	GNLKGDCEFSLGGIVGTCNPSTGRLSTPR
10.0	3369.3363	-0.0147	GNLKGDCEFSLGGIVGTCNPSTGRLSTPR

Spectrum No: 99; Query: 1814; Rank: 1

Peptide View

MS/MS Fragmentation of **LDGPIENVSEDEAQSSSQR**
Found in **IPI00471864**, Tax_Id=10116 Gene_Symbol=RGD1307509 Similar to RIKEN cDNA 1700108L22

Match to Query 1814: 2139.896948 from(1070.955750,2+)
Title: 091008RatKidney_NoSalt_27.1621.1621.2.dta
Data file K:\NewmanPaper\Piliang\3SubProteomes\Piliang3SP\mgf5ppm\ERLIC_3SubProteomes5ppm.mgf

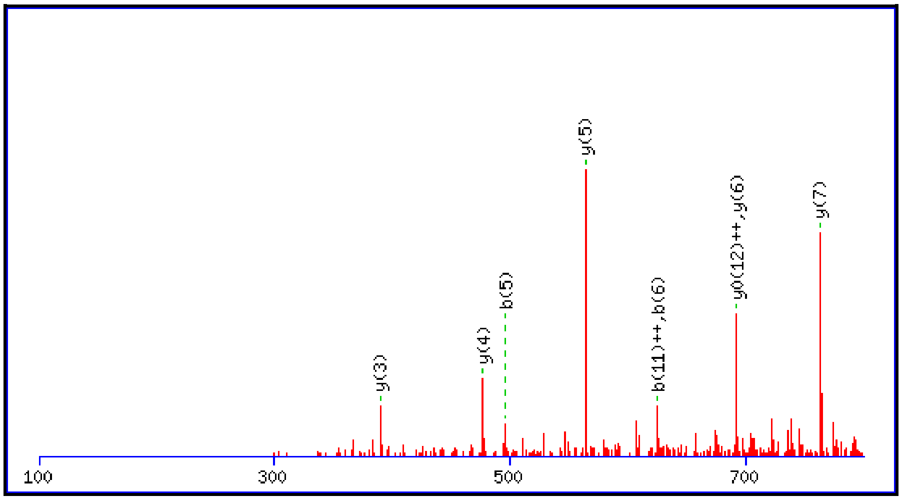

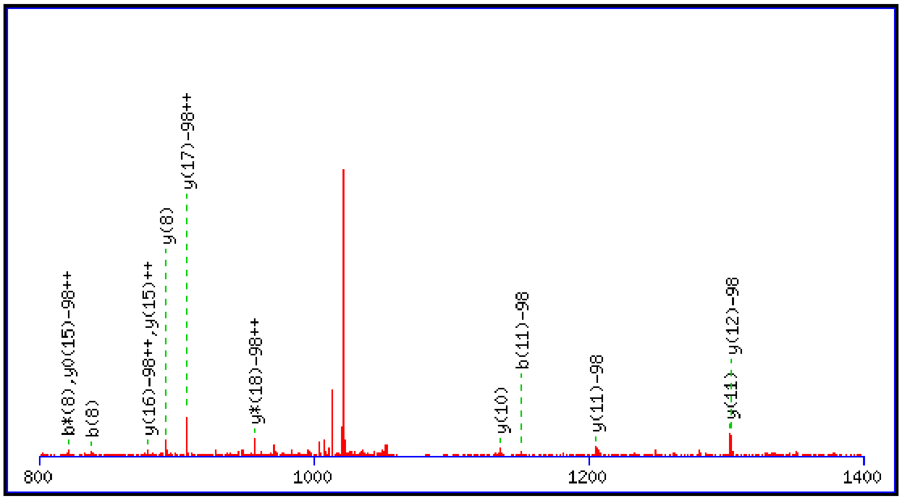

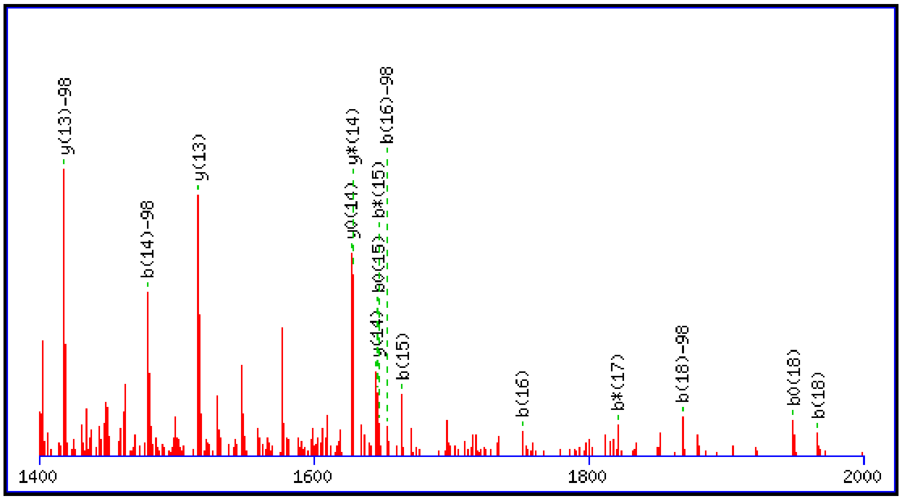

Monoisotopic mass of neutral peptide Mr(calc): 2139.8957
Fixed modifications: Carbamidomethyl (C)
Variable modifications:
S9 : Phospho (ST), with neutral losses 97.9769(shown in table), 0.0000
Ions Score: 70 **Expect:** 2.4e-005
Matches (Bold Red): 37/306 fragment ions using 49 most intense peaks

#	b	b^{++}	b*	b^{*++}	b^0	b^{0++}	Seq.	y	y^{++}	y*	y^{*++}	y^0	y^{0++}	#
1	114.0913	57.5493					L							19
2	229.1183	115.0628			211.1077	106.0575	D	1929.8421	965.4247	1912.8155	**956.9114**	1911.8315	956.4194	18

**3**	286.1397	143.5735			268.1292	134.5682	**G**	1814.8151	**907.9112**	1797.7886	899.3979	1796.8046	898.9059	**17**
**4**	383.1925	192.0999			365.1819	183.0946	**P**	1757.7937	**879.4005**	1740.7671	870.8872	1739.7831	870.3952	**16**
**5**	**496.2766**	248.6419			478.2660	239.6366	**I**	1660.7409	830.8741	1643.7143	822.3608	1642.7303	**821.8688**	**15**
**6**	**625.3192**	313.1632			607.3086	304.1579	**E**	1547.6568	774.3321	1530.6303	765.8188	1529.6463	765.3268	**14**
**7**	739.3621	370.1847	722.3355	361.6714	721.3515	361.1794	**N**	**1418.6142**	709.8108	1401.5877	701.2975	1400.6037	700.8055	**13**
**8**	**838.4305**	419.7189	**821.4040**	411.2056	820.4199	410.7136	**V**	**1304.5713**	652.7893	1287.5448	644.2760	1286.5607	643.7840	**12**
**9**	907.4520	454.2296	890.4254	445.7163	889.4414	445.2243	**S**	**1205.5029**	603.2551	1188.4763	594.7418	1187.4923	594.2498	**11**
**10**	1036.4946	518.7509	1019.4680	510.2376	1018.4840	509.7456	**E**	**1136.4814**	568.7444	1119.4549	560.2311	1118.4709	559.7391	**10**
**11**	**1151.5215**	576.2644	1134.4950	567.7511	1133.5109	567.2591	**D**	1007.4388	504.2231	990.4123	495.7098	989.4283	495.2178	**9**
**12**	1280.5641	640.7857	1263.5375	632.2724	1262.5535	631.7804	**E**	**892.4119**	446.7096	875.3854	438.1963	874.4013	437.7043	**8**
**13**	1351.6012	676.3042	1334.5747	667.7910	1333.5906	667.2990	**A**	**763.3693**	382.1883	746.3428	373.6750	745.3587	373.1830	**7**
**14**	**1479.6598**	740.3335	1462.6332	731.8203	1461.6492	731.3282	**Q**	**692.3322**	346.6697	675.3056	338.1565	674.3216	337.6645	**6**
**15**	1566.6918	783.8495	1549.6653	775.3363	1548.6812	774.8443	**S**	**564.2736**	282.6404	547.2471	274.1272	546.2630	273.6352	**5**
**16**	**1653.7238**	827.3656	1636.6973	818.8523	1635.7133	818.3603	**S**	**477.2416**	239.1244	460.2150	230.6112	459.2310	230.1191	**4**
**17**	1740.7559	870.8816	1723.7293	862.3683	1722.7453	861.8763	**S**	**390.2096**	195.6084	373.1830	187.0951	372.1990	186.6031	**3**
**18**	**1868.8144**	934.9109	1851.7879	926.3976	1850.8039	925.9056	**Q**	303.1775	152.0924	286.1510	143.5791			**2**
**19**							**R**	175.1190	88.0631	158.0924	79.5498			**1**

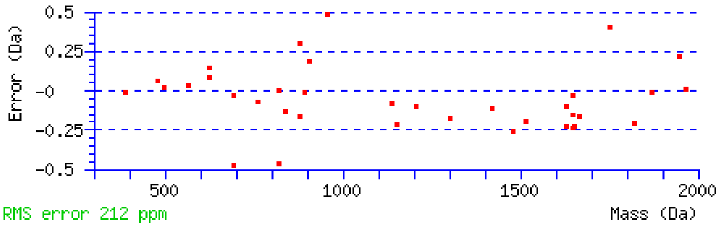

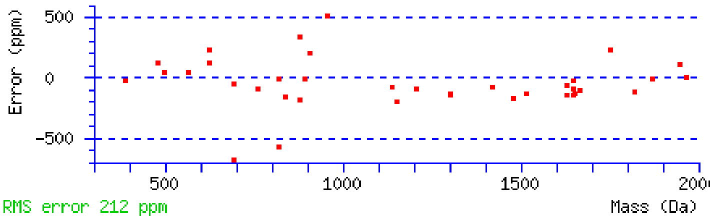

All matches to this query

Score	Mr(calc):	Delta	Sequence
69.5	2139.8957	0.0012	LDGPIENVSEDEAQSSSQR
31.5	2139.8957	0.0012	LDGPIENVSEDEAQSSSQR
25.6	2139.8957	0.0012	LDGPIENVSEDEAQSSSQR
19.9	2139.8957	0.0012	LDGPIENVSEDEAQSSSQR
5.5	2139.9011	-0.0041	NNNSNQNFFKEGSYIQR
5.5	2139.9011	-0.0041	NNNSNQNFFKEGSYIQR
5.4	2139.9011	-0.0041	NNNSNQNFFKEGSYIQR
3.2	2139.8845	0.0124	HTEETQAIPESSTKETGTD
2.7	2139.9055	-0.0085	FLGSNDEEMSYENNPHIK
2.6	2139.8955	0.0014	LNKDEYEFFTYLDKR

Spectrum No: 100; Query: 2120; Rank: 1

Peptide View

MS/MS Fragmentation of **KSLDSDESEDEDDDYQQKR**
Found in **IPI00208277**, Tax_Id=10116 Gene_Symbol=Pdap1 28 kDa heat- and acid-stable phosphoprotein

Match to Query 2120: 2380.924092 from(794.648640,3+)
Title: 100101RatKid_NS_deglyco_22.888.888.3.dta
Data file K:\NewmanPaper\Piliang\3SubProteomes\Piliang3SP\mgf5ppm\ERLIC_3SubProteomes5ppm.mgf

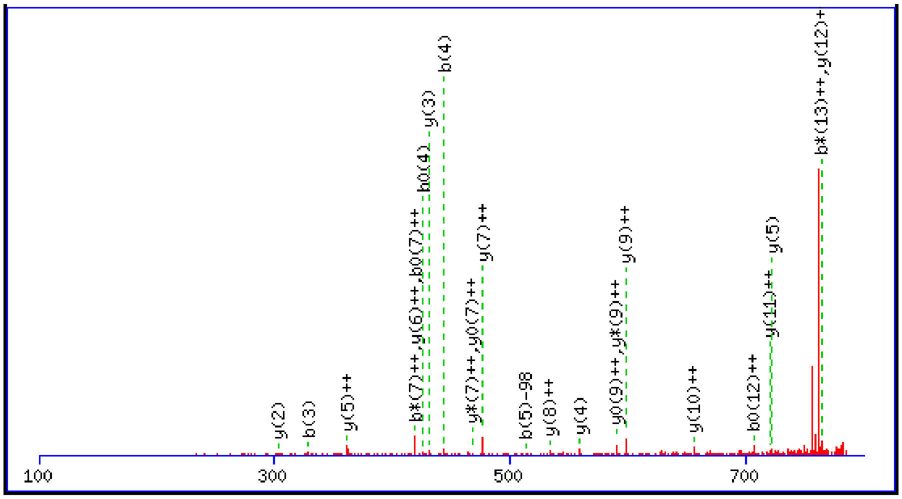

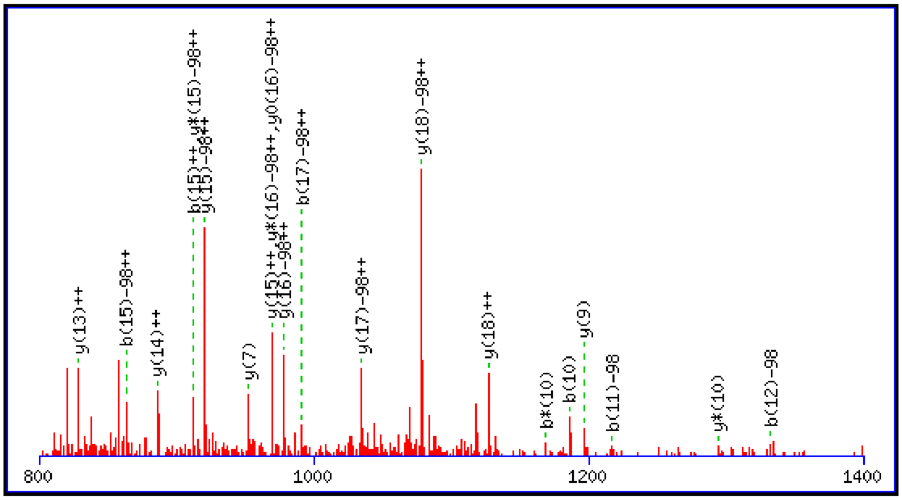

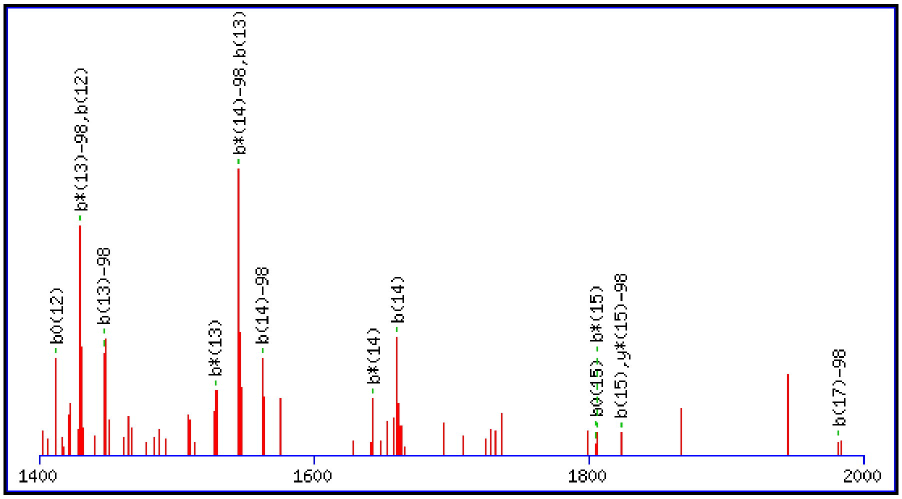

Monoisotopic mass of neutral peptide Mr(calc): 2380.9180
Fixed modifications: Carbamidomethyl (C)
Variable modifications:
S5 : Phospho (ST), with neutral losses 97.9769(shown in table), 0.0000
Ions Score: 69 **Expect:** 2.4e-005
Matches (Bold Red): 61/312 fragment ions using 92 most intense peaks

#	b	b⁺⁺	b*	b*⁺⁺	b⁰	b⁰⁺⁺	Seq.	y	y⁺⁺	y*	y*⁺⁺	y⁰	y⁰⁺⁺	#
1	129.1022	65.0548	112.0757	56.5415			K							19
2	216.1343	108.5708	199.1077	100.0575	198.1237	99.5655	S	2155.8534	**1078.4303**	2138.8269	1069.9171	2137.8428	1069.4251	18
3	**329.2183**	165.1128	312.1918	156.5995	311.2078	156.1075	L	2068.8214	**1034.9143**	2051.7948	1026.4011	2050.8108	1025.9090	17
4	**444.2453**	222.6263	427.2187	214.1130	**426.2347**	213.6210	D	1955.7373	**978.3723**	1938.7108	**969.8590**	1937.7268	**969.3670**	16
5	**513.2667**	257.1370	496.2402	248.6237	495.2562	248.1317	S	1840.7104	**920.8588**	**1823.6838**	**912.3456**	1822.6998	911.8535	15

**6**	628.2937	314.6505	611.2671	306.1372	610.2831	305.6452	**D**	1771.6889	**886.3481**	1754.6624	877.8348	1753.6784	877.3428	**14**
**7**	757.3363	379.1718	740.3097	370.6585	739.3257	370.1665	**E**	1656.6620	**828.8346**	1639.6354	820.3214	1638.6514	819.8293	**13**
**8**	844.3683	422.6878	827.3417	414.1745	826.3577	413.6825	**S**	1527.6194	**764.3133**	1510.5928	755.8001	1509.6088	755.3080	**12**
**9**	973.4109	487.2091	956.3843	478.6958	955.4003	478.2038	**E**	1440.5874	**720.7973**	1423.5608	712.2840	1422.5768	711.7920	**11**
**10**	1088.4378	544.7226	1071.4113	536.2093	1070.4273	535.7173	**D**	1311.5448	**656.2760**	**1294.5182**	647.7627	1293.5342	647.2707	**10**
**11**	**1217.4804**	609.2438	1200.4539	600.7306	1199.4699	600.2386	**E**	**1196.5178**	**598.7625**	1179.4913	**590.2493**	1178.5073	**589.7573**	**9**
**12**	**1332.5074**	666.7573	1315.4808	658.2440	1314.4968	657.7520	**D**	1067.4752	**534.2413**	1050.4487	525.7280	1049.4647	525.2360	**8**
**13**	**1447.5343**	724.2708	**1430.5078**	715.7575	1429.5237	715.2655	**D**	**952.4483**	**476.7278**	935.4217	**468.2145**	934.4377	**467.7225**	**7**
**14**	**1562.5613**	781.7843	**1545.5347**	773.2710	1544.5507	772.7790	**D**	837.4213	**419.2143**	820.3948	410.7010	819.4108	410.2090	**6**
**15**	1725.6246	**863.3159**	1708.5980	854.8027	1707.6140	854.3106	**Y**	**722.3944**	**361.7008**	705.3679	353.1876			**5**
**16**	1853.6832	927.3452	1836.6566	918.8319	1835.6726	918.3399	**Q**	**559.3311**	280.1692	542.3045	271.6559			**4**
**17**	**1981.7417**	**991.3745**	1964.7152	982.8612	1963.7312	982.3692	**Q**	**431.2725**	216.1399	414.2459	207.6266			**3**
**18**	2109.8367	1055.4220	2092.8102	1046.9087	2091.8261	1046.4167	**K**	**303.2139**	152.1106	286.1874	143.5973			**2**
**19**							**R**	175.1190	88.0631	158.0924	79.5498			**1**

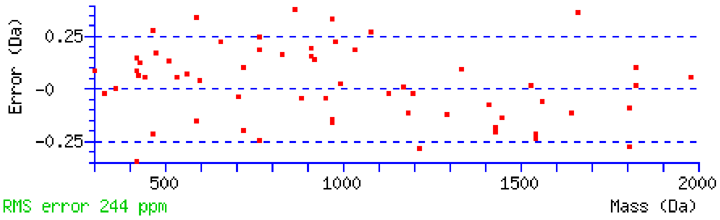

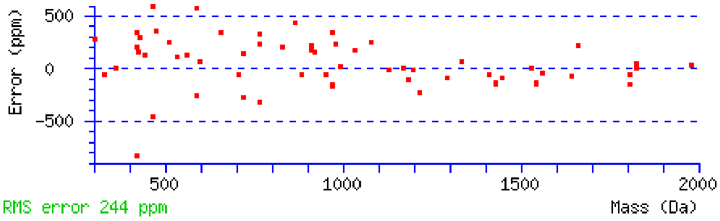

All matches to this query

Score	Mr(calc):	Delta	Sequence
69.3	2380.9180	0.0061	KSLDSDESEDEDDDYQQKR
45.4	2380.9180	0.0061	KSLDSDESEDEDDDYQQKR
40.7	2380.9180	0.0061	KSLDSDESEDEDDDYQQKR
1.8	2378.8946	2.0295	CRYYHDRYTTTLYTAR
1.8	2378.8957	2.0284	DSESSPNDFSVSLKAQGKNK
1.7	2379.9054	1.0187	YGSGSIYTLLPPMSFGSKR
1.3	2380.9065	0.0176	QMALPGSLTSASLKENNLTS
1.3	2380.9259	-0.0018	VSLWTLCTATCGNYGFQSR
1.3	2380.9259	-0.0018	VSLWTLCTATCGNYGFQSR
1.3	2380.9259	-0.0018	VSLWTLCTATCGNYGFQSR

Spectrum No: 101; Query: 1476; Rank: 1

Peptide View

MS/MS Fragmentation of **HSSLPTESDEDIAPAQR**
Found in **IPI00198486**, Tax_Id=10116 Gene_Symbol=Ap3d1 similar to adaptor-related protein complex 3, delta 1 subunit

Match to Query 1476: 1931.828548 from(966.921550,2+)
Title: 091008RatKidney_NoSalt_22.1337.1337.2.dta
Data file K:\NewmanPaper\Piliang\3SubProteomes\Piliang3SP\mgf5ppm\ERLIC_3SubProteomes5ppm.mgf

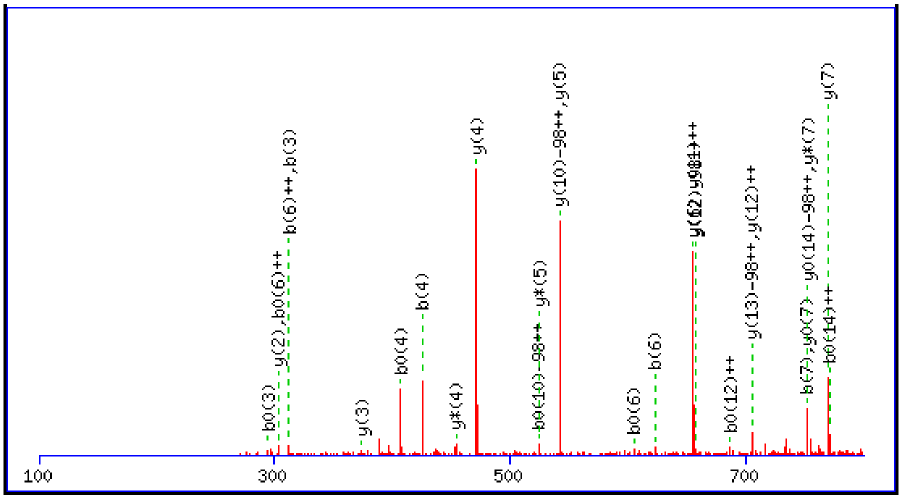

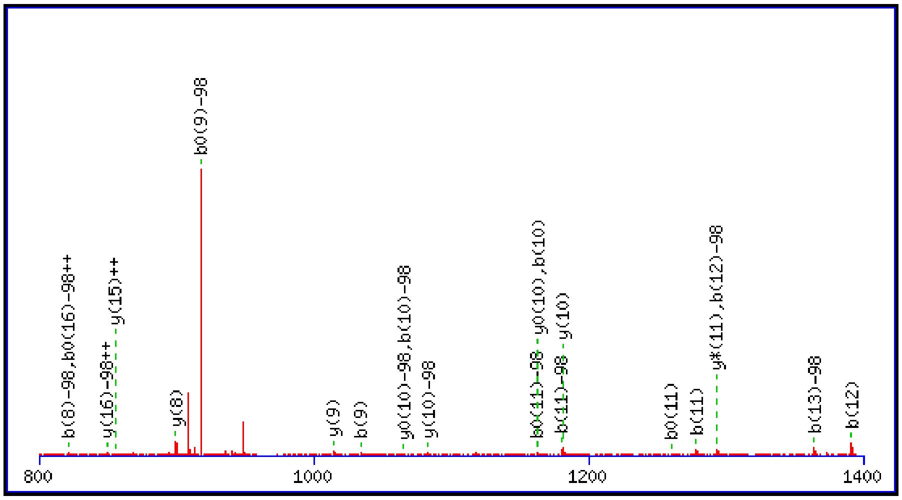

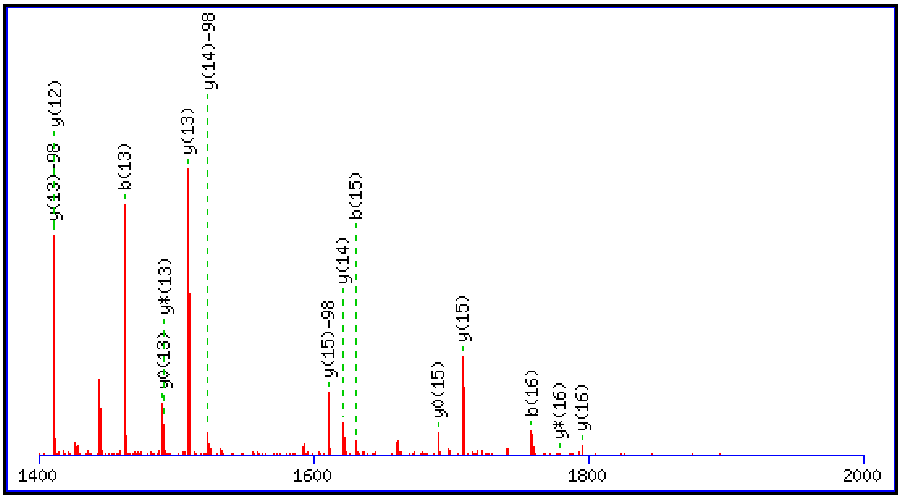

Monoisotopic mass of neutral peptide Mr(calc): 1931.8262
Fixed modifications: Carbamidomethyl (C)
Variable modifications:
S8 : Phospho (ST), with neutral losses 0.0000(shown in table), 97.9769
Ions Score: 69 **Expect:** 2.5e-005
Matches (Bold Red): 65/228 fragment ions using 92 most intense peaks

#	b	b^{++}	b*	b^{*++}	b^0	b^{0++}	Seq.	y	y^{++}	y*	y^{*++}	y^0	y^{0++}	#
1	138.0662	69.5367					H							17
2	225.0982	113.0527			207.0877	104.0475	S	**1795.7746**	898.3909	**1778.7480**	889.8777	1777.7640	889.3856	16
3	**312.1302**	156.5688			**294.1197**	147.5635	S	**1708.7425**	**854.8749**	1691.7160	846.3616	**1690.7320**	845.8696	15
4	**425.2143**	213.1108			**407.2037**	204.1055	L	**1621.7105**	811.3589	1604.6840	802.8456	1603.7000	802.3536	14
5	522.2671	261.6372			504.2565	252.6319	P	**1508.6265**	754.8169	**1491.5999**	746.3036	**1490.6159**	745.8116	13

6	623.3148	312.1610			605.3042	303.1557	T	1411.5737	706.2905	1394.5471	697.7772	1393.5631	697.2852	12
7	752.3573	376.6823			734.3468	367.6770	E	1310.5260	655.7666	1293.4995	647.2534	1292.5154	646.7614	11
8	919.3557	460.1815			901.3451	451.1762	S	1181.4834	591.2453	1164.4569	582.7321	1163.4729	582.2401	10
9	1034.3826	517.6950			1016.3721	508.6897	D	1014.4851	507.7462	997.4585	499.2329	996.4745	498.7409	9
10	1163.4252	582.2163			1145.4147	573.2110	E	899.4581	450.2327	882.4316	441.7194	881.4476	441.2274	8
11	1278.4522	639.7297			1260.4416	630.7244	D	770.4155	385.7114	753.3890	377.1981	752.4050	376.7061	7
12	1391.5362	696.2718			1373.5257	687.2665	I	655.3886	328.1979	638.3620	319.6847			6
13	1462.5734	731.7903			1444.5628	722.7850	A	542.3045	271.6559	525.2780	263.1426			5
14	1559.6261	780.3167			1541.6156	771.3114	P	471.2674	236.1373	454.2409	227.6241			4
15	1630.6632	815.8353			1612.6527	806.8300	A	374.2146	187.6110	357.1881	179.0977			3
16	1758.7218	879.8645	1741.6953	871.3513	1740.7112	870.8593	Q	303.1775	152.0924	286.1510	143.5791			2
17							R	175.1190	88.0631	158.0924	79.5498			1

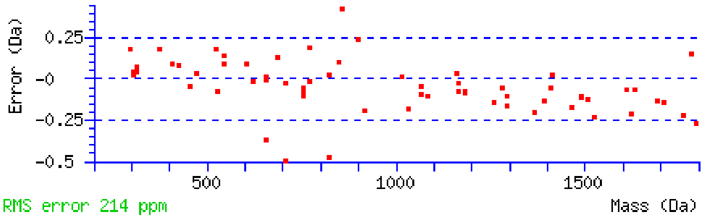

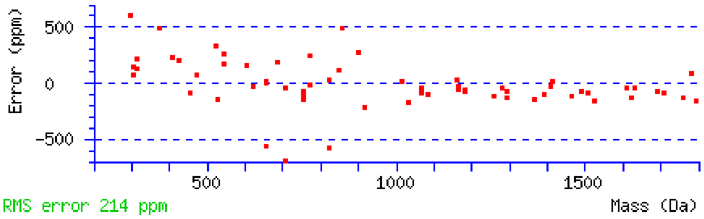

All matches to this query

Score	Mr(calc):	Delta	Sequence
69.3	1931.8262	0.0024	HSSLPTESDEDIAPAQR
58.3	1931.8262	0.0024	HSSLPTESDEDIAPAQR
49.7	1931.8262	0.0024	HSSLPTESDEDIAPAQR
48.4	1931.8262	0.0024	HSSLPTESDEDIAPAQR
8.6	1931.8179	0.0106	KASGNYAGPPSSALPAQR
7.0	1931.8271	0.0015	QDILPGKSQACGCFSKG
5.1	1931.8179	0.0106	KASGNYAGPPSSALPAQR
3.4	1929.8403	1.9882	KIFTNPTTDRGLISK
3.4	1931.8179	0.0106	KASGNYAGPPSSALPAQR
3.4	1931.8179	0.0106	KASGNYAGPPSSALPAQR

Spectrum No: 102; Query: 1369; Rank: 1

Peptide View

MS/MS Fragmentation of **ESTESSNTTIEDEDVK**
Found in **IPI00212226**, Tax_Id=10116 Gene_Symbol=Camk2d Isoform Delta 1 of Calcium/calmodulin-dependent protein kinase type II delta chain

Match to Query 1369: 1862.728268 from(932.371410,2+)
Title: 091008RatKidney_NoSalt_28.810.810.2.dta
Data file K:\NewmanPaper\Piliang\3SubProteomes\Piliang3SP\mgf5ppm\ERLIC_3SubProteomes5ppm.mgf

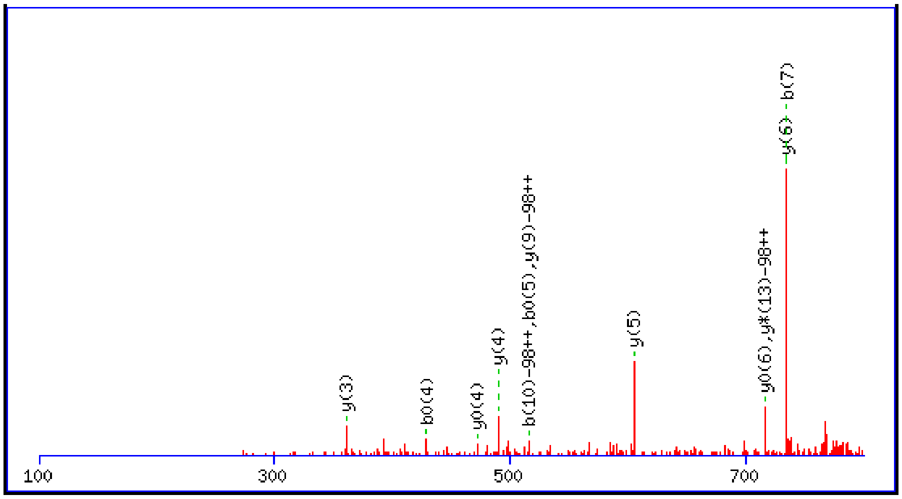

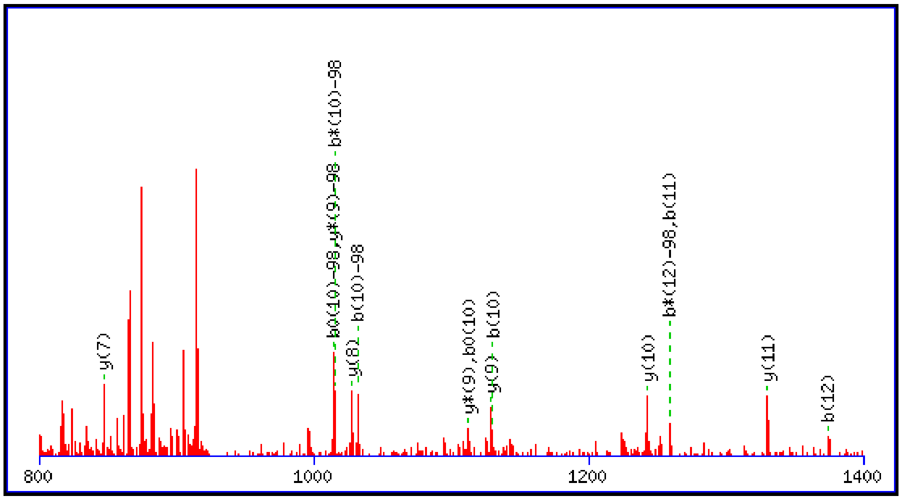

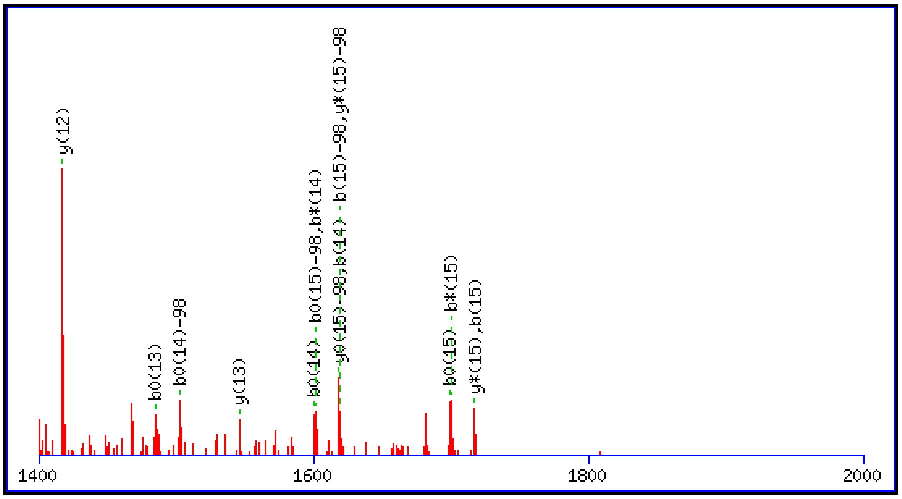

Monoisotopic mass of neutral peptide Mr(calc): 1862.7306
Fixed modifications: Carbamidomethyl (C)
Variable modifications:
T9 : Phospho (ST), with neutral losses 0.0000(shown in table), 97.9769
Ions Score: 69 **Expect:** 1.8e-005
Matches (Bold Red): 42/254 fragment ions using 54 most intense peaks

#	b	b^{++}	b*	b^{*++}	b^0	b^{0++}	Seq.	y	y^{++}	y*	y^{*++}	y^0	y^{0++}	#
1	130.0499	65.5286			112.0393	56.5233	E							16
2	217.0819	109.0446			199.0713	100.0393	S	1734.6953	867.8513	**1717.6688**	859.3380	1716.6848	858.8460	15
3	318.1296	159.5684			300.1190	150.5631	T	1647.6633	824.3353	1630.6367	815.8220	1629.6527	815.3300	14
4	447.1722	224.0897			**429.1616**	215.0844	E	**1546.6156**	773.8114	1529.5891	765.2982	1528.6050	764.8062	13
5	534.2042	267.6057			**516.1936**	258.6005	S	**1417.5730**	709.2901	1400.5465	700.7769	1399.5625	700.2849	12

**6**	621.2362	311.1218			603.2257	302.1165	**S**	**1330.5410**	665.7741	1313.5144	657.2609	1312.5304	656.7689	**11**
**7**	**735.2792**	368.1432	718.2526	359.6299	717.2686	359.1379	**N**	**1243.5090**	622.2581	1226.4824	613.7448	1225.4984	613.2528	**10**
**8**	836.3268	418.6671	819.3003	410.1538	818.3163	409.6618	**T**	**1129.4660**	565.2367	**1112.4395**	556.7234	1111.4555	556.2314	**9**
**9**	1017.3408	509.1741	1000.3143	500.6608	999.3303	500.1688	**T**	**1028.4184**	514.7128	1011.3918	506.1995	1010.4078	505.7075	**8**
**10**	**1130.4249**	565.7161	1113.3983	557.2028	**1112.4143**	556.7108	**I**	**847.4044**	424.2058	830.3778	415.6925	829.3938	415.2005	**7**
**11**	**1259.4675**	630.2374	1242.4409	621.7241	1241.4569	621.2321	**E**	**734.3203**	367.6638	717.2937	359.1505	**716.3097**	358.6585	**6**
**12**	**1374.4944**	687.7509	1357.4679	679.2376	1356.4839	678.7456	**D**	**605.2777**	303.1425	588.2511	294.6292	587.2671	294.1372	**5**
**13**	1503.5370	752.2722	1486.5105	743.7589	**1485.5265**	743.2669	**E**	**490.2508**	245.6290	473.2242	237.1157	**472.2402**	236.6237	**4**
**14**	**1618.5640**	809.7856	**1601.5374**	801.2723	**1600.5534**	800.7803	**D**	**361.2082**	181.1077	344.1816	172.5944	343.1976	172.1024	**3**
**15**	**1717.6324**	859.3198	**1700.6058**	850.8066	**1699.6218**	850.3145	**V**	246.1812	123.5942	229.1547	115.0810			**2**
**16**							**K**	147.1128	74.0600	130.0863	65.5468			**1**

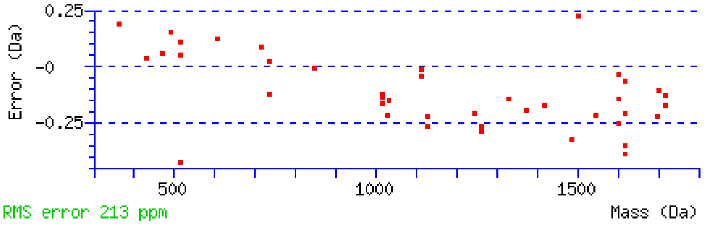

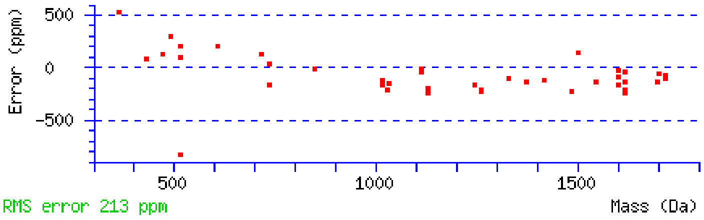

All matches to this query

Score	Mr(calc):	Delta	Sequence
69.2	1862.7306	-0.0024	ESTESSNTTIEDEDVK
57.1	1862.7306	-0.0024	ESTESSNTTIEDEDVK
34.5	1862.7306	-0.0024	ESTESSNTTIEDEDVK
27.4	1862.7306	-0.0024	ESTESSNTTIEDEDVK
26.0	1862.7306	-0.0024	ESTESSNTTIEDEDVK
24.8	1862.7306	-0.0024	ESTESSNTTIEDEDVK
6.3	1862.7434	-0.0151	VCNYVDWIEDTIAAN
2.7	1862.7279	0.0003	NARSYCTMIRGMVK
2.4	1862.7279	0.0003	NARSYCTMIRGMVK
0.8	1862.7309	-0.0026	SNSXGEGARSERSPGNK

Spectrum No: 103; Query: 1916; Rank: 1

Peptide View

MS/MS Fragmentation of **TAFDEAIAELDTLSEESYK**
Found in **IPI00324893**, Tax_Id=10116 Gene_Symbol=Ywhaz 14-3-3 protein zeta/delta

Match to Query 1916: 2210.949348 from(1106.481950,2+)
Title: 091008RatKidney_NoSalt_25.5183.5183.2.dta
Data file K:\NewmanPaper\Piliang\3SubProteomes\Piliang3SP\mgf5ppm\ERLIC_3SubProteomes5ppm.mgf

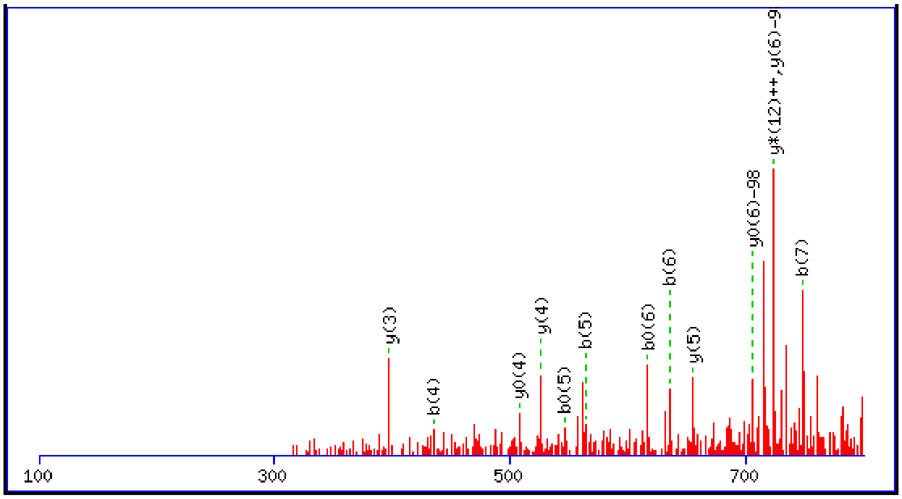

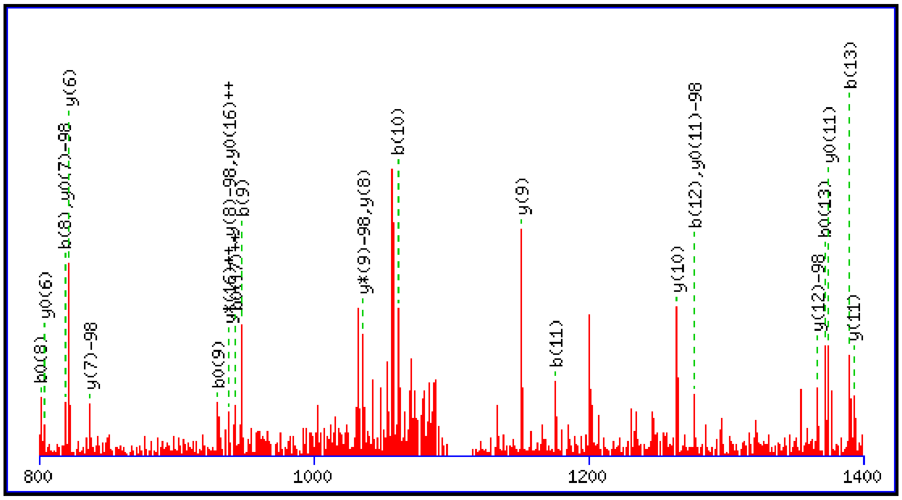

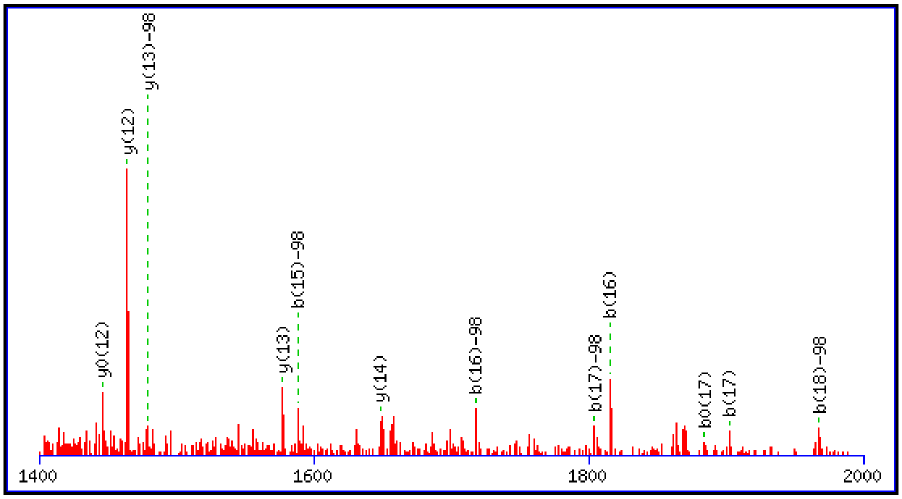

Monoisotopic mass of neutral peptide Mr(calc): 2210.9508
Fixed modifications: Carbamidomethyl (C)
Variable modifications:
S14 : Phospho (ST), with neutral losses 97.9769(shown in table), 0.0000
Ions Score: 69 **Expect:** 3.4e-005
Matches (Bold Red): 50/274 fragment ions using 94 most intense peaks

#	b	b^{++}	b^{0}	b^{0++}	Seq.	y	y^{++}	y*	y^{*++}	y^{0}	y^{0++}	#
1	102.0550	51.5311	84.0444	42.5258	T							19
2	173.0921	87.0497	155.0815	78.0444	A	2012.9335	1006.9704	1995.9070	998.4571	1994.9229	997.9651	18
3	320.1605	160.5839	302.1499	151.5786	F	1941.8964	971.4518	1924.8698	962.9386	1923.8858	962.4465	17
4	**435.1874**	218.0974	417.1769	209.0921	D	1794.8280	897.9176	1777.8014	889.4044	1776.8174	888.9123	16
5	**564.2300**	282.6186	**546.2195**	273.6134	E	1679.8010	840.4042	1662.7745	831.8909	1661.7905	831.3989	15

6	**635.2671**	318.1372	**617.2566**	309.1319	A	1550.7584	775.8829	1533.7319	767.3696	1532.7479	766.8776	14
7	**748.3512**	374.6792	730.3406	365.6740	I	**1479.7213**	740.3643	1462.6948	731.8510	1461.7108	731.3590	13
8	**819.3883**	410.1978	**801.3777**	401.1925	A	**1366.6373**	683.8223	1349.6107	675.3090	1348.6267	674.8170	12
9	**948.4309**	474.7191	**930.4203**	465.7138	E	1295.6001	648.3037	1278.5736	639.7904	**1277.5896**	639.2984	11
10	**1061.5150**	531.2611	1043.5044	522.2558	L	1166.5576	583.7824	1149.5310	575.2691	1148.5470	574.7771	10
11	**1176.5419**	588.7746	1158.5313	579.7693	D	1053.4735	527.2404	**1036.4469**	518.7271	1035.4629	518.2351	9
12	**1277.5896**	639.2984	1259.5790	630.2932	T	**938.4465**	469.7269	921.4200	461.2136	920.4360	460.7216	8
13	**1390.6737**	695.8405	**1372.6631**	686.8352	L	**837.3989**	419.2031	820.3723	410.6898	**819.3883**	410.1978	7
14	1459.6951	730.3512	1441.6845	721.3459	S	**724.3148**	362.6610	707.2883	354.1478	**706.3042**	353.6558	6
15	**1588.7377**	794.8725	1570.7271	785.8672	E	**655.2933**	328.1503	638.2668	319.6370	637.2828	319.1450	5
16	**1717.7803**	859.3938	1699.7697	850.3885	E	**526.2508**	263.6290	509.2242	255.1157	**508.2402**	254.6237	4
17	**1804.8123**	902.9098	1786.8018	893.9045	S	**397.2082**	199.1077	380.1816	190.5944	379.1976	190.1024	3
18	**1967.8757**	984.4415	1949.8651	975.4362	Y	310.1761	155.5917	293.1496	147.0784			2
19					K	147.1128	74.0600	130.0863	65.5468			1

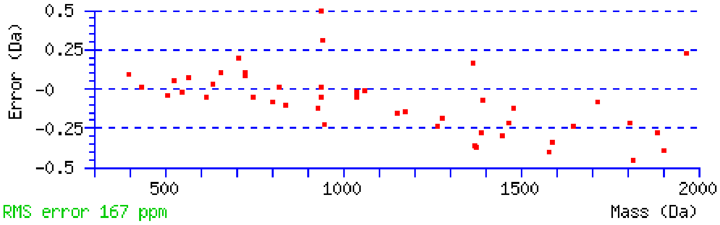

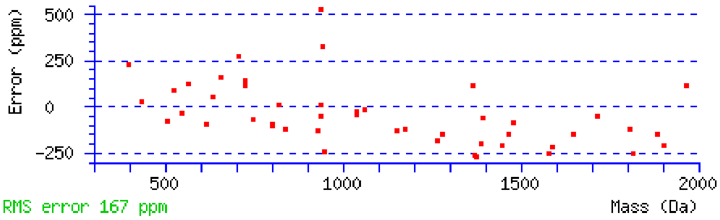

All matches to this query

Score	Mr(calc):	Delta	Sequence
68.9	2210.9508	-0.0014	TAFDEAIAELDTLSEESYK
57.1	2210.9508	-0.0014	TAFDEAIAELDTLSEESYK
46.9	2210.9508	-0.0014	TAFDEAIAELDTLSEESYK
29.2	2210.9508	-0.0014	TAFDEAIAELDTLSEESYK
20.3	2210.9508	-0.0014	TAFDEAIAELDTLSEESYK
2.8	2210.9386	0.0108	MYLSNNPNSHTDNSAKSSDK
2.8	2210.9386	0.0108	MYLSNNPNSHTDNSAKSSDK
2.1	2210.9376	0.0117	LFNMKISLLISDSSFQK
1.4	2209.9536	0.9957	LFNMKISLLISDSSFQK
0.5	2210.9386	0.0108	MYLSNNPNSHTDNSAKSSDK

Spectrum No: 104; Query: 1444; Rank: 1

Peptide View

MS/MS Fragmentation of **GPDRTPPSEEDSAEAER**
Found in **IPI00325260**, Tax_Id=10116 Gene_Symbol=Sgta Small glutamine-rich tetratricopeptide repeat-containing protein A

Match to Query 1444: 1921.771752 from(641.597860,3+)
Title: 100101RatKid_NS_deglyco_19.527.527.3.dta
Data file K:\NewmanPaper\Piliang\3SubProteomes\Piliang3SP\mgf5ppm\ERLIC_3SubProteomes5ppm.mgf

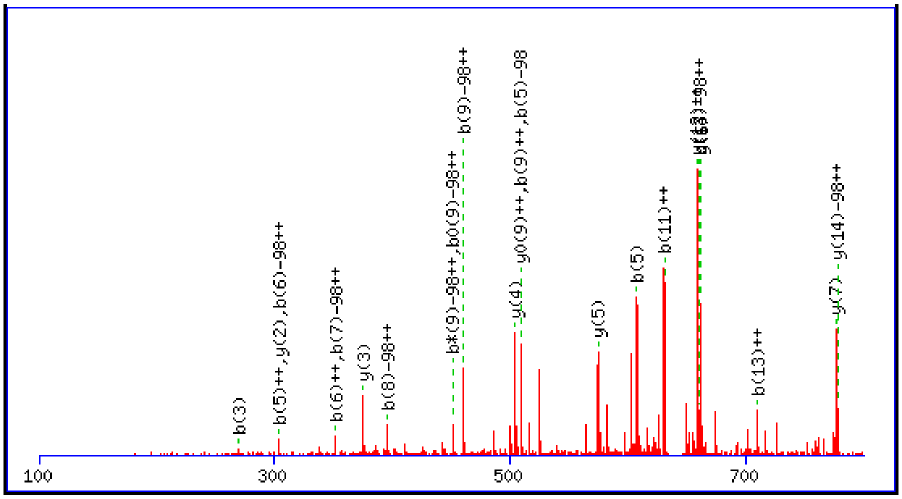

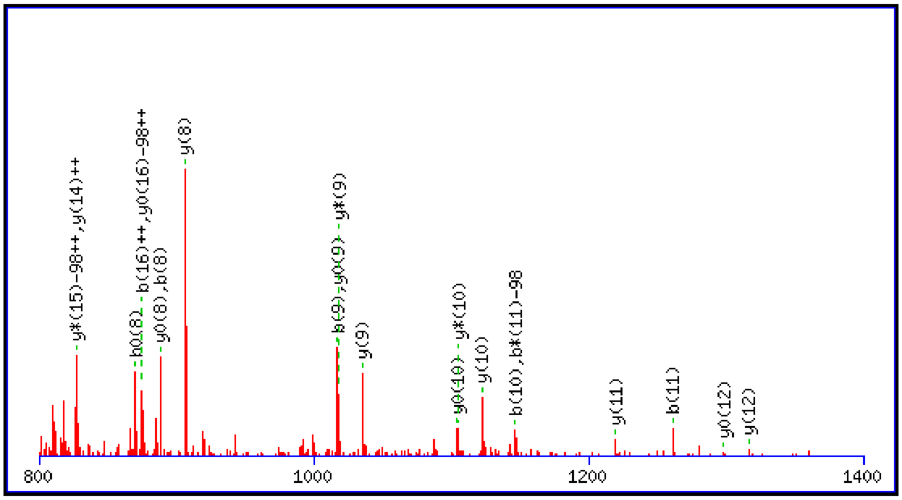

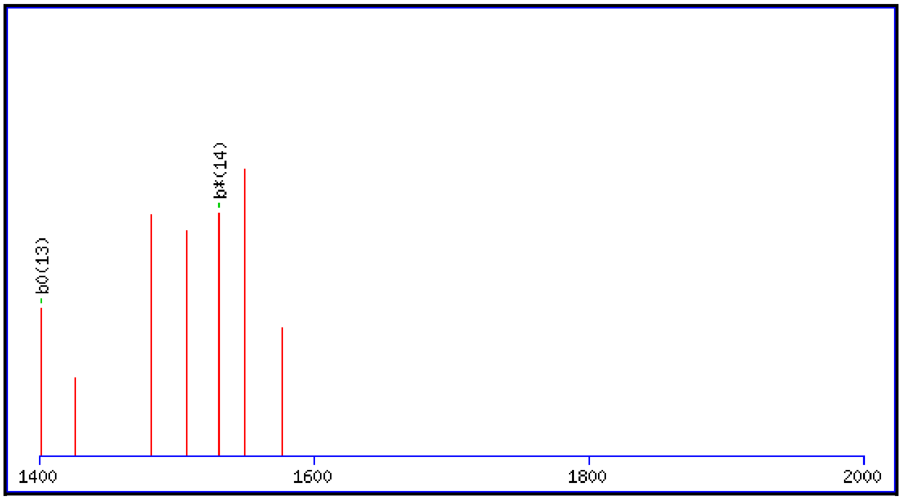

Monoisotopic mass of neutral peptide Mr(calc): 1921.7691
Fixed modifications: Carbamidomethyl (C)
Variable modifications:
T5 : Phospho (ST), with neutral losses 97.9769(shown in table), 0.0000
Ions Score: 69 **Expect:** 2.2e-005
Matches (Bold Red): 47/276 fragment ions using 51 most intense peaks

#	b	b^{++}	b*	b^{*++}	b^0	b^{0++}	Seq.	y	y^{++}	y*	y^{*++}	y^0	y^{0++}	#
1	58.0287	29.5180					G							17
2	155.0815	78.0444					P	1767.7780	884.3926	1750.7515	875.8794	1749.7674	**875.3874**	16
3	**270.1084**	135.5579			252.0979	126.5526	D	1670.7252	835.8663	1653.6987	**827.3530**	1652.7147	826.8610	15
4	426.2096	213.6084	409.1830	205.0951	408.1990	204.6031	R	1555.6983	**778.3528**	1538.6717	769.8395	1537.6877	769.3475	14
5	**509.2467**	255.1270	492.2201	246.6137	491.2361	246.1217	T	1399.5972	700.3022	1382.5706	691.7890	1381.5866	691.2969	13

6	606.2994	303.6534	589.2729	295.1401	588.2889	294.6481	P	1316.5601	658.7837	1299.5335	650.2704	1298.5495	649.7784	12
7	703.3522	352.1797	686.3256	343.6665	685.3416	343.1745	P	1219.5073	610.2573	1202.4808	601.7440	1201.4968	601.2520	11
8	790.3842	395.6957	773.3577	387.1825	772.3737	386.6905	S	1122.4546	561.7309	1105.4280	553.2176	1104.4440	552.7256	10
9	919.4268	460.2170	902.4003	451.7038	901.4162	451.2118	E	1035.4225	518.2149	1018.3960	509.7016	1017.4120	509.2096	9
10	1048.4694	524.7383	1031.4429	516.2251	1030.4588	515.7331	E	906.3799	453.6936	889.3534	445.1803	888.3694	444.6883	8
11	1163.4964	582.2518	1146.4698	573.7385	1145.4858	573.2465	D	777.3373	389.1723	760.3108	380.6590	759.3268	380.1670	7
12	1250.5284	625.7678	1233.5018	617.2546	1232.5178	616.7625	S	662.3104	331.6588	645.2838	323.1456	644.2998	322.6536	6
13	1321.5655	661.2864	1304.5389	652.7731	1303.5549	652.2811	A	575.2784	288.1428	558.2518	279.6295	557.2678	279.1375	5
14	1450.6081	725.8077	1433.5815	717.2944	1432.5975	716.8024	E	504.2413	252.6243	487.2147	244.1110	486.2307	243.6190	4
15	1521.6452	761.3262	1504.6187	752.8130	1503.6346	752.3210	A	375.1987	188.1030	358.1721	179.5897	357.1881	179.0977	3
16	1650.6878	825.8475	1633.6612	817.3343	1632.6772	816.8423	E	304.1615	152.5844	287.1350	144.0711	286.1510	143.5791	2
17							R	175.1190	88.0631	158.0924	79.5498			1

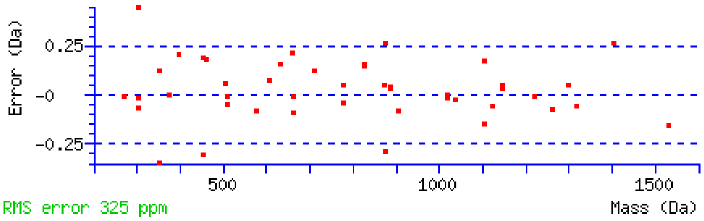

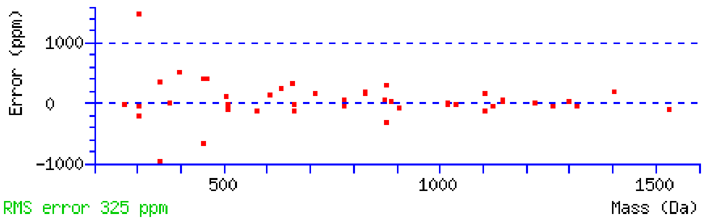

All matches to this query

Score	Mr(calc):	Delta	Sequence
68.9	1921.7691	0.0027	GPDRTPPSEEDSAEAER
39.2	1921.7691	0.0027	GPDRTPPSEEDSAEAER
23.9	1921.7691	0.0027	GPDRTPPSEEDSAEAER
5.5	1920.7859	0.9858	RYTCIENQNCQIDK
5.5	1920.7859	0.9858	RYTCIENQNCQIDK
5.3	1921.7872	-0.0155	MTMTTMPESLNSPVSGK
4.4	1919.7631	2.0086	QFDIANYELFSSTPK
4.2	1921.7642	0.0075	TEVXTHLCSNSKTSGK
3.8	1921.7872	-0.0155	MTMTTMPESLNSPVSGK
3.7	1921.7642	0.0075	TEVXTHLCSNSKTSGK

Spectrum No: 105; Query: 2157; Rank: 1

Peptide View

MS/MS Fragmentation of **TVQKDADEEDSDEETSHLER**
Found in **IPI00656420**, Tax_Id=10116 Gene_Symbol=LOC683897 Similar to Protein C6orf203

Match to Query 2157: 2411.955162 from(804.992330,3+)
Title: 091008RatKidney_NoSalt_25.511.511.3.dta
Data file K:\NewmanPaper\Piliang\3SubProteomes\Piliang3SP\mgf5ppm\ERLIC_3SubProteomes5ppm.mgf

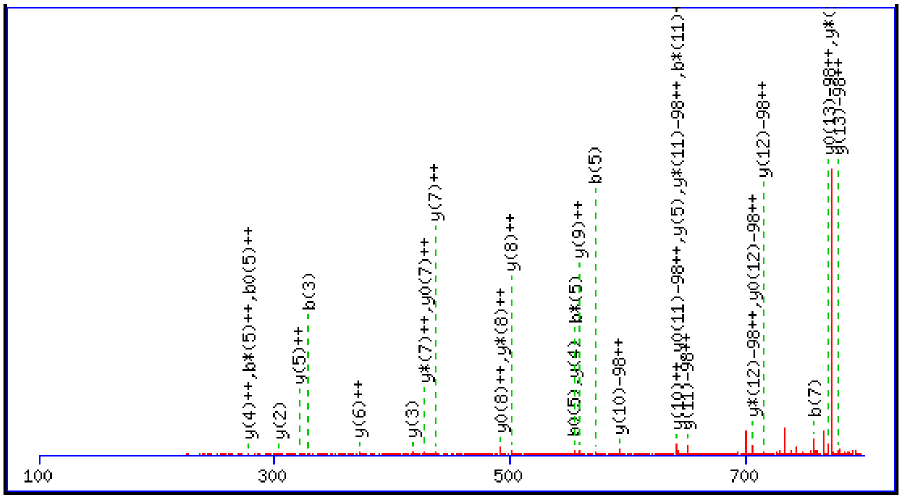

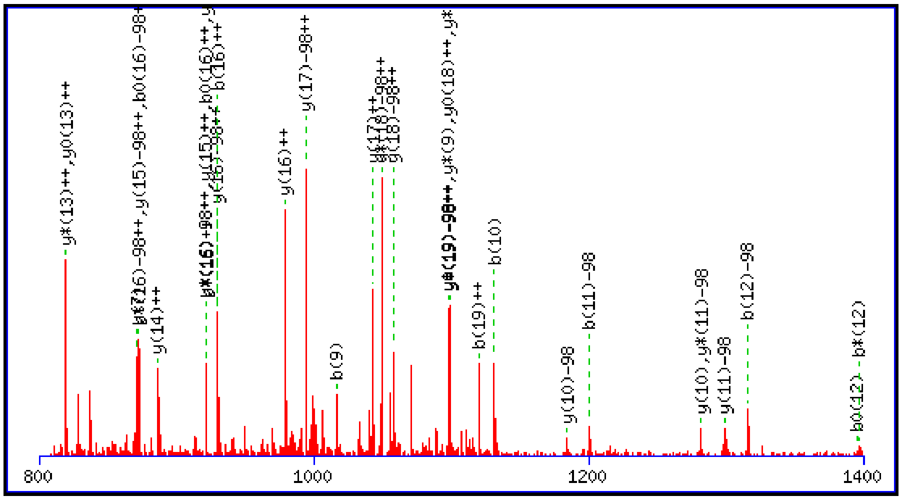

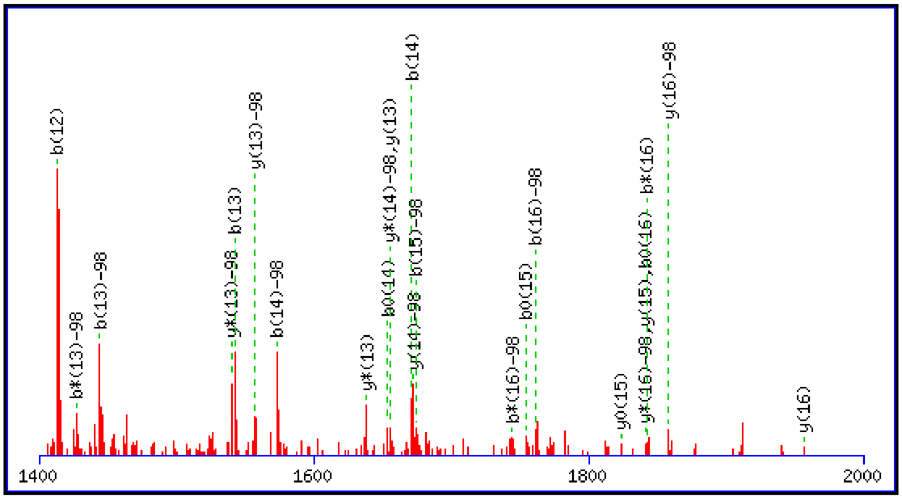

Monoisotopic mass of neutral peptide Mr(calc): 2411.9602
Fixed modifications: Carbamidomethyl (C)
Variable modifications:
S11 : Phospho (ST), with neutral losses 97.9769(shown in table), 0.0000
Ions Score: 69 **Expect:** 2.9e-005
Matches (Bold Red): 92/336 fragment ions using 114 most intense peaks

#	b	b++	b*	b*++	b0	b0++	Seq.	y	y++	y*	y*++	y0	y0++	#
1	102.0550	51.5311			84.0444	42.5258	T							20
2	201.1234	101.0653			183.1128	92.0600	V	2213.9429	1107.4751	2196.9163	**1098.9618**	2195.9323	**1098.4698**	19
3	**329.1819**	165.0946	312.1554	156.5813	311.1714	156.0893	Q	2114.8745	**1057.9409**	2097.8479	**1049.4276**	2096.8639	1048.9356	18
4	457.2769	229.1421	440.2504	220.6288	439.2663	220.1368	K	1986.8159	**993.9116**	1969.7894	985.3983	1968.8053	984.9063	17
5	**572.3039**	286.6556	**555.2773**	**278.1423**	**554.2933**	**277.6503**	D	**1858.7209**	**929.8641**	**1841.6944**	**921.3508**	1840.7104	**920.8588**	16

6	643.3410	322.1741	626.3144	313.6608	625.3304	313.1688	A	1743.6940	**872.3506**	1726.6675	863.8374	1725.6834	863.3454	15
7	**758.3679**	379.6876	741.3414	371.1743	740.3573	370.6823	D	**1672.6569**	836.8321	**1655.6303**	828.3188	1654.6463	827.8268	14
8	887.4105	444.2089	870.3840	435.6956	869.3999	435.2036	E	**1557.6299**	**779.3186**	**1540.6034**	**770.8053**	1539.6194	**770.3133**	13
9	**1016.4531**	508.7302	999.4265	500.2169	998.4425	499.7249	E	1428.5873	**714.7973**	1411.5608	**706.2840**	1410.5768	**705.7920**	12
10	**1131.4800**	566.2437	1114.4535	557.7304	1113.4695	557.2384	D	**1299.5448**	**650.2760**	**1282.5182**	**641.7627**	1281.5342	**641.2707**	11
11	**1200.5015**	600.7544	1183.4749	592.2411	1182.4909	591.7491	S	**1184.5178**	**592.7625**	1167.4913	584.2493	1166.5072	583.7573	10
12	**1315.5284**	658.2679	1298.5019	649.7546	1297.5179	649.2626	D	1115.4964	**558.2518**	**1098.4698**	549.7385	1097.4858	549.2465	9
13	**1444.5710**	722.7892	**1427.5445**	714.2759	1426.5605	713.7839	E	1000.4694	**500.7383**	983.4429	**492.2251**	982.4588	**491.7331**	8
14	**1573.6136**	787.3105	1556.5871	778.7972	1555.6031	778.3052	E	**871.4268**	**436.2170**	854.4003	**427.7038**	853.4163	**427.2118**	7
15	**1674.6613**	837.8343	1657.6348	829.3210	1656.6507	828.8290	T	742.3842	**371.6958**	725.3577	363.1825	724.3737	362.6905	6
16	**1761.6933**	881.3503	**1744.6668**	**872.8370**	1743.6828	**872.3450**	S	**641.3365**	**321.1719**	624.3100	312.6586	623.3260	312.1666	5
17	1898.7522	949.8798	1881.7257	941.3665	1880.7417	940.8745	H	**554.3045**	**277.6559**	537.2780	269.1426	536.2940	268.6506	4
18	2011.8363	1006.4218	1994.8098	997.9085	1993.8257	997.4165	L	**417.2456**	209.1264	400.2191	200.6132	399.2350	200.1212	3
19	2140.8789	1070.9431	2123.8524	1062.4298	2122.8683	1061.9378	E	**304.1615**	152.5844	287.1350	144.0711	286.1510	143.5791	2
20							R	175.1190	88.0631	158.0924	79.5498			1

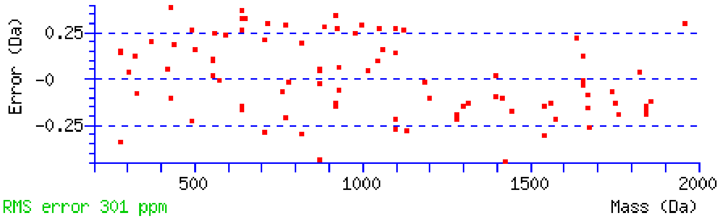

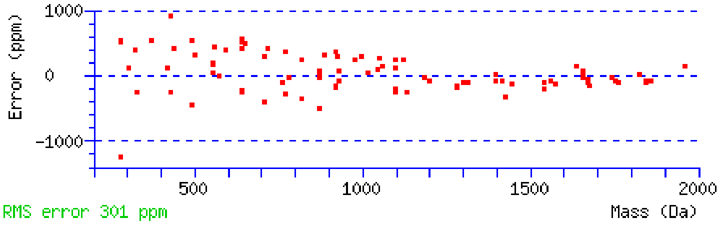

All matches to this query

Score	Mr(calc):	Delta	Sequence
68.9	2411.9602	-0.0050	TVQKDADEEDSDEETSHLER
52.3	2411.9602	-0.0050	TVQKDADEEDSDEETSHLER
46.3	2411.9602	-0.0050	TVQKDADEEDSDEETSHLER
24.6	2411.9602	-0.0050	TVQKDADEEDSDEETSHLER
8.4	2410.9405	1.0146	KSYTLDMVSNKSPEASAEAK
8.2	2410.9405	1.0146	KSYTLDMVSNKSPEASAEAK
8.2	2410.9405	1.0146	KSYTLDMVSNKSPEASAEAK
8.0	2410.9405	1.0146	KSYTLDMVSNKSPEASAEAK
7.3	2410.9405	1.0146	KSYTLDMVSNKSPEASAEAK
6.4	2409.9486	2.0066	AYGVVSMCIGTGMGAAAVFEYP

Spectrum No: 106; Query: 884; Rank: 1

Peptide View

MS/MS Fragmentation of **DAVAVAPPPSPSLPAK**
Found in **IPI00205325**, Tax_Id=10116 Gene_Symbol=Lrp2 Low-density lipoprotein receptor-related protein 2 precursor

Match to Query 884: 1595.803268 from(798.908910,2+)
Title: 091008RatKidney_NH4Format01_13.2138.2138.2.dta
Data file K:\NewmanPaper\Piliang\3SubProteomes\Piliang3SP\mgf5ppm\ERLIC_3SubProteomes5ppm.mgf

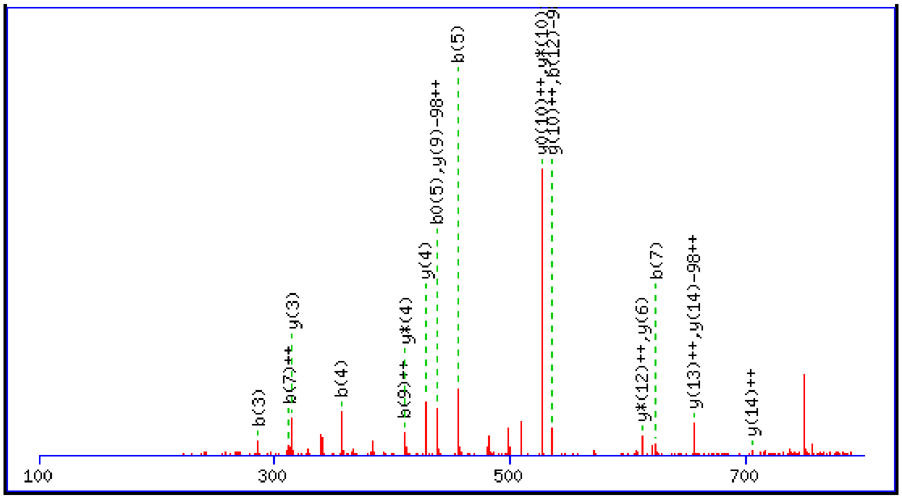

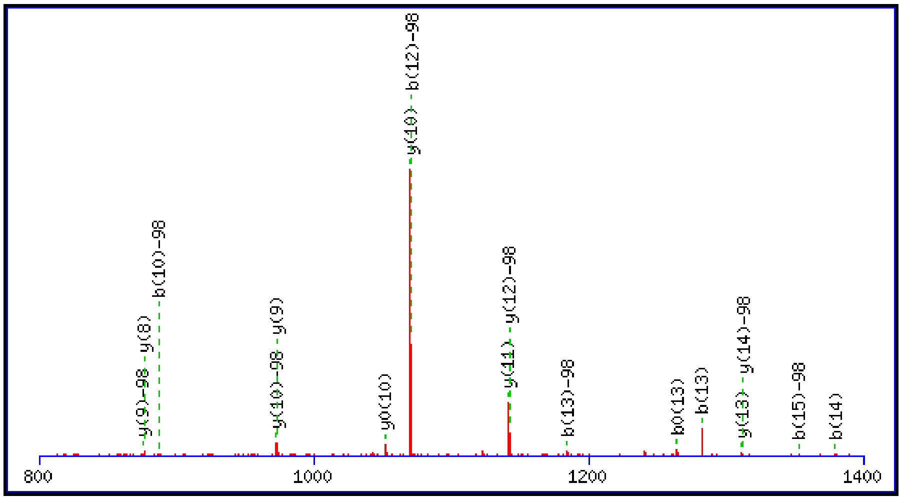

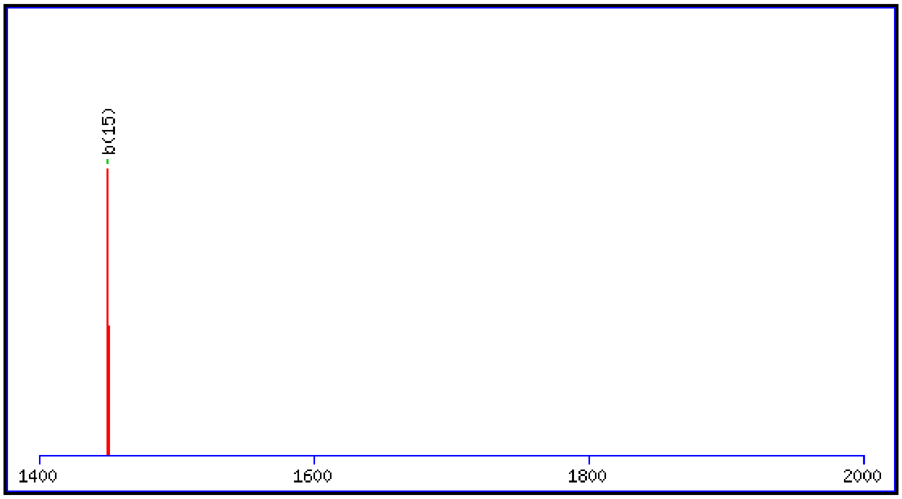

Monoisotopic mass of neutral peptide Mr(calc): 1595.7960
Fixed modifications: Carbamidomethyl (C)
Variable modifications:
S10 : Phospho (ST), with neutral losses 97.9769(shown in table), 0.0000
Ions Score: 69 **Expect:** 2e-005
Matches (Bold Red): 40/220 fragment ions using 42 most intense peaks

#	b	b^{++}	b^{0}	b^{0++}	Seq.	y	y^{++}	y^{*}	y^{*++}	y^{0}	y^{0++}	#
1	116.0342	58.5207	98.0237	49.5155	D							16
2	187.0713	94.0393	169.0608	85.0340	A	1383.7995	692.4034	1366.7729	683.8901	1365.7889	683.3981	15
3	**286.1397**	143.5735	268.1292	134.5682	V	**1312.7623**	**656.8848**	1295.7358	648.3715	1294.7518	647.8795	14
4	**357.1769**	179.0921	339.1663	170.0868	A	1213.6939	607.3506	1196.6674	598.8373	1195.6834	598.3453	13
5	**456.2453**	228.6263	**438.2347**	219.6210	V	**1142.6568**	571.8320	1125.6303	563.3188	1124.6462	562.8268	12

6	**527.2824**	264.1448	509.2718	255.1396	A	1043.5884	522.2978	1026.5619	513.7846	1025.5778	513.2926	11
7	**624.3352**	**312.6712**	606.3246	303.6659	P	**972.5513**	486.7793	955.5247	478.2660	954.5407	477.7740	10
8	721.3879	361.1976	703.3774	352.1923	P	**875.4985**	**438.2529**	858.4720	429.7396	857.4880	429.2476	9
9	818.4407	**409.7240**	800.4301	400.7187	P	778.4458	389.7265	761.4192	381.2132	760.4352	380.7212	8
10	**887.4621**	444.2347	869.4516	435.2294	S	681.3930	341.2001	664.3664	332.6869	663.3824	332.1949	7
11	984.5149	492.7611	966.5043	483.7558	P	**612.3715**	306.6894	595.3450	298.1761	594.3610	297.6841	6
12	**1071.5469**	**536.2771**	1053.5364	**527.2718**	S	515.3188	258.1630	498.2922	249.6498	497.3082	249.1577	5
13	**1184.6310**	592.8191	1166.6204	583.8139	L	**428.2867**	214.6470	**411.2602**	206.1337			4
14	1281.6838	641.3455	1263.6732	632.3402	P	**315.2027**	158.1050	298.1761	149.5917			3
15	**1352.7209**	676.8641	1334.7103	667.8588	A	218.1499	109.5786	201.1234	101.0653			2
16					K	147.1128	74.0600	130.0863	65.5468			1

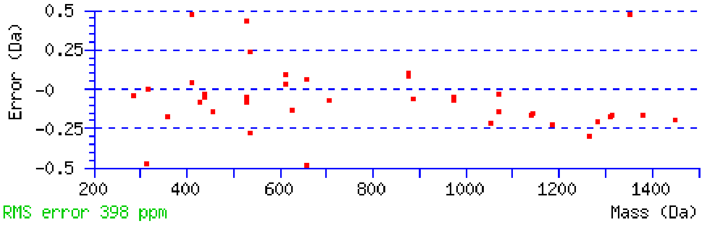

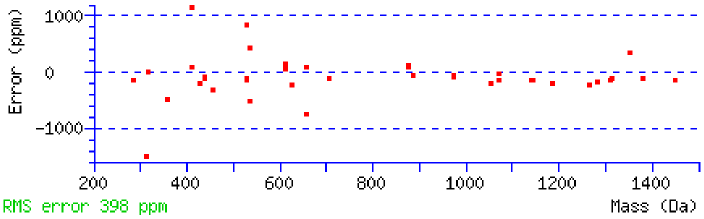

All matches to this query

Score	Mr(calc):	Delta	Sequence
68.6	1595.7960	0.0073	DAVAVAPPPSPSLPAK
57.7	1595.7960	0.0073	DAVAVAPPPSPSLPAK
10.9	1595.8128	-0.0095	SAPEAAPGSTRPGRSR
10.0	1595.7928	0.0104	LVLSLPVNMRCSK
9.6	1595.8072	-0.0040	SAPVTALEPGRKYK
7.5	1594.8120	0.9913	QLNLGAPFLSSLVR
5.3	1595.8072	-0.0040	IKNPEGGLYVAVTR
4.3	1595.7977	0.0056	ELMSPPDATQAAPLR
3.9	1595.8171	-0.0138	DKISTKIEVLNEK
3.9	1595.8171	-0.0138	DKISTKIEVLNEK

Spectrum No: 107; Query: 771; Rank: 1

Peptide View

MS/MS Fragmentation of **ASSLGEIDDSSELR**
Found in **IPI00214079**, Tax_Id=10116 Gene_Symbol=Pkn2 Serine/threonine-protein kinase N2

Match to Query 771: 1557.659848 from(779.837200,2+)
Title: 100101RatKid_NS_deglyco_22.2440.2440.2.dta
Data file K:\NewmanPaper\Piliang\3SubProteomes\Piliang3SP\mgf5ppm\ERLIC_3SubProteomes5ppm.mgf

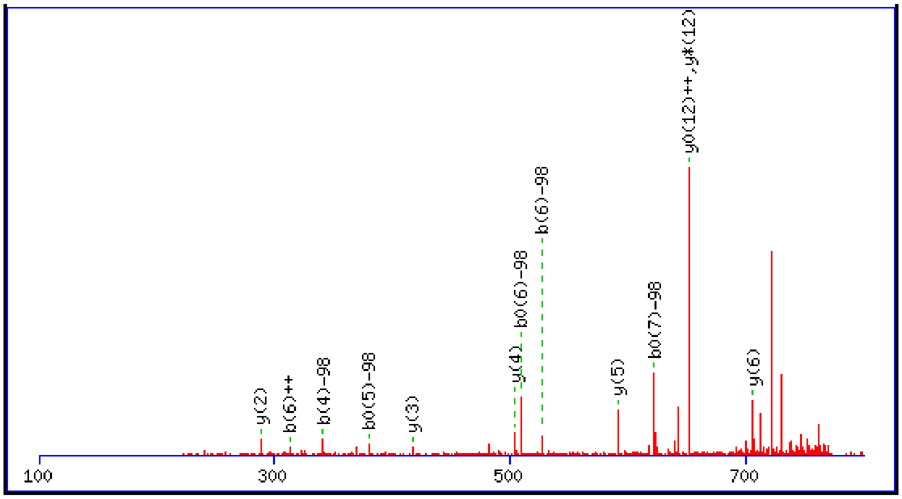

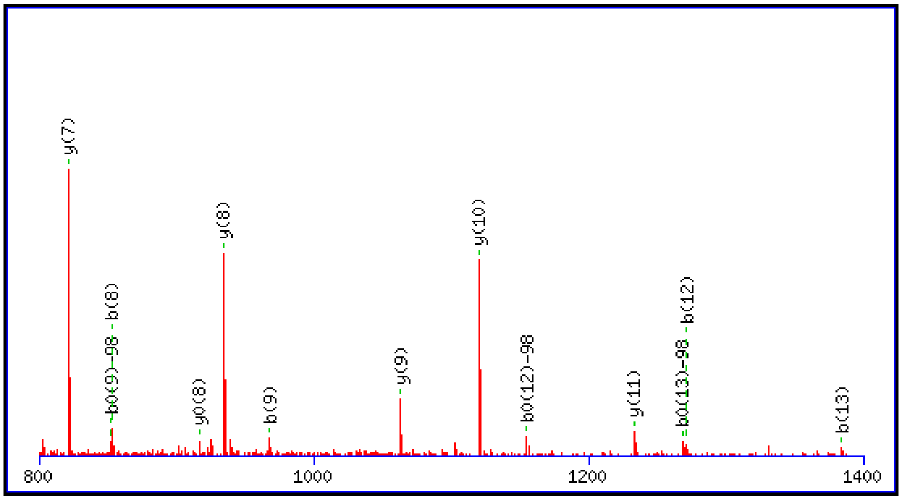

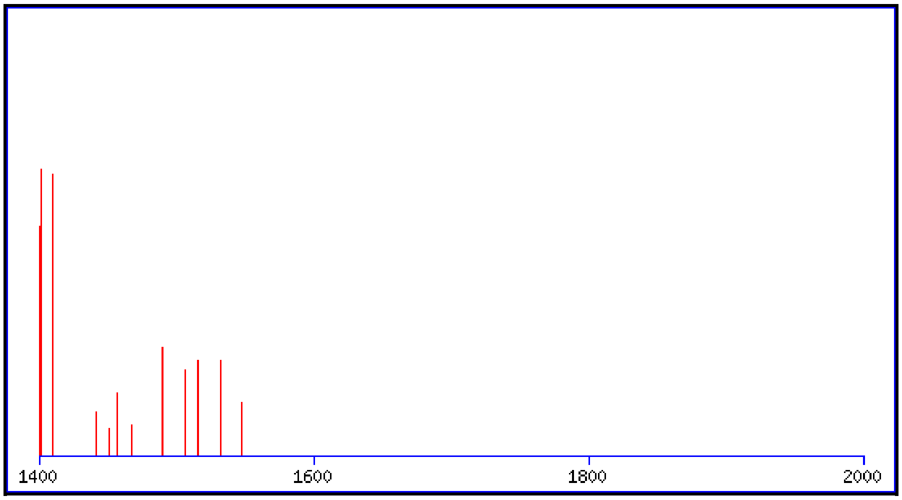

Monoisotopic mass of neutral peptide Mr(calc): 1557.6559
Fixed modifications: Carbamidomethyl (C)
Variable modifications:
S2 : Phospho (ST), with neutral losses 97.9769(shown in table), 0.0000
Ions Score: 69 **Expect:** 2.1e-005
Matches (Bold Red): 26/178 fragment ions using 47 most intense peaks

#	b	b^{++}	b^0	b^{0++}	Seq.	y	y^{++}	y*	y^{*++}	y^0	y^{0++}	#
1	72.0444	36.5258			A							14
2	141.0658	71.0366	123.0553	62.0313	S	1389.6492	695.3282	1372.6227	686.8150	1371.6387	686.3230	13
3	228.0979	114.5526	210.0873	105.5473	S	1320.6278	660.8175	1303.6012	**652.3042**	1302.6172	**651.8122**	12
4	**341.1819**	171.0946	323.1714	162.0893	L	**1233.5957**	617.3015	1216.5692	608.7882	1215.5852	608.2962	11
5	398.2034	199.6053	**380.1928**	190.6001	G	**1120.5117**	560.7595	1103.4851	552.2462	1102.5011	551.7542	10

**6**	**527.2460**	264.1266	**509.2354**	255.1214	**E**	**1063.4902**	532.2487	1046.4637	523.7355	1045.4796	523.2435	**9**
**7**	640.3301	320.6687	**622.3195**	311.6634	**I**	**934.4476**	467.7274	917.4211	459.2142	**916.4371**	458.7222	**8**
**8**	755.3570	378.1821	737.3464	369.1769	**D**	**821.3636**	411.1854	804.3370	402.6721	803.3530	402.1801	**7**
**9**	870.3839	435.6956	**852.3734**	426.6903	**D**	**706.3366**	353.6719	689.3101	345.1587	688.3260	344.6667	**6**
**10**	957.4160	479.2116	939.4054	470.2063	**S**	**591.3097**	296.1585	574.2831	287.6452	573.2991	287.1532	**5**
**11**	1044.4480	522.7276	1026.4374	513.7224	**S**	**504.2776**	252.6425	487.2511	244.1292	486.2671	243.6372	**4**
**12**	1173.4906	587.2489	**1155.4800**	578.2437	**E**	**417.2456**	209.1264	400.2191	200.6132	399.2350	200.1212	**3**
**13**	1286.5747	643.7910	**1268.5641**	634.7857	**L**	**288.2030**	144.6051	271.1765	136.0919			**2**
**14**					**R**	175.1190	88.0631	158.0924	79.5498			**1**

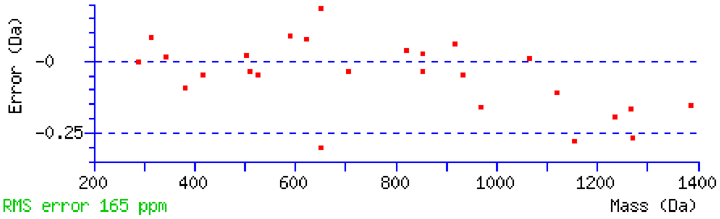

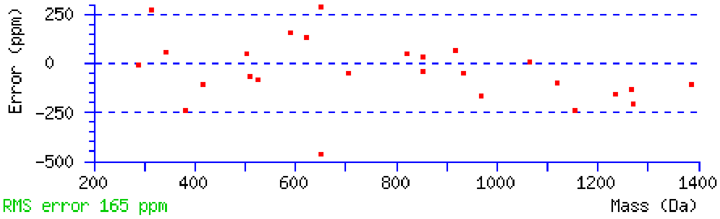

All matches to this query

Score	Mr(calc):	Delta	Sequence
68.6	1557.6559	0.0039	ASSLGEIDDSSELR
66.9	1557.6559	0.0039	ASSLGEIDDSSELR
5.2	1555.6428	2.0170	GSLGVLTMSQLMK
4.3	1556.6711	0.9888	IKEGLVSVMSYR
0.5	1555.6589	2.0010	VISLDTEVLTVK

Spectrum No: 108; Query: 2245; Rank: 1

Peptide View

MS/MS Fragmentation of **GSLDSDNDDSDCPYSEKVPSIK**
Found in **IPI00199985**, Tax_Id=10116 Gene_Symbol=Slc4a4 Isoform 1 of Electrogenic sodium bicarbonate cotransporter 1

Match to Query 2245: 2506.993048 from(1254.503800,2+)
Title: 091008RatKidney_NH4Format02_26.2169.2169.2.dta
Data file K:\NewmanPaper\Piliang\3SubProteomes\Piliang3SP\mgf5ppm\ERLIC_3SubProteomes5ppm.mgf

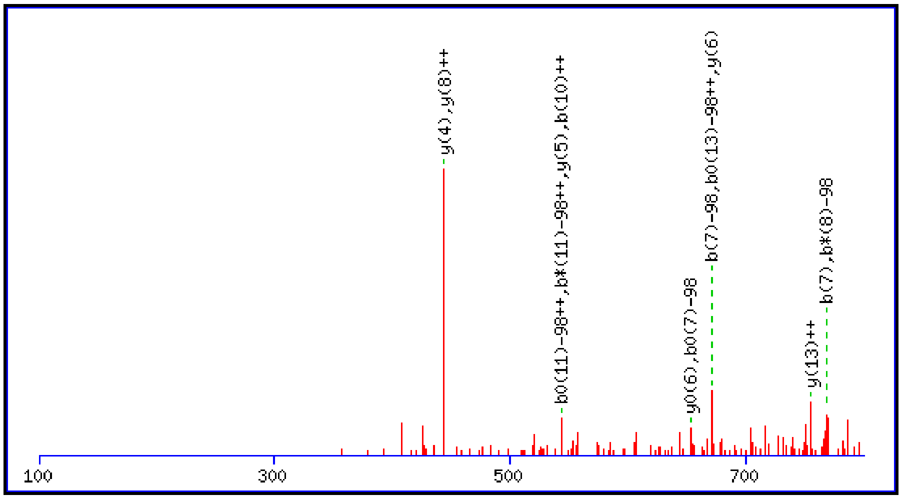

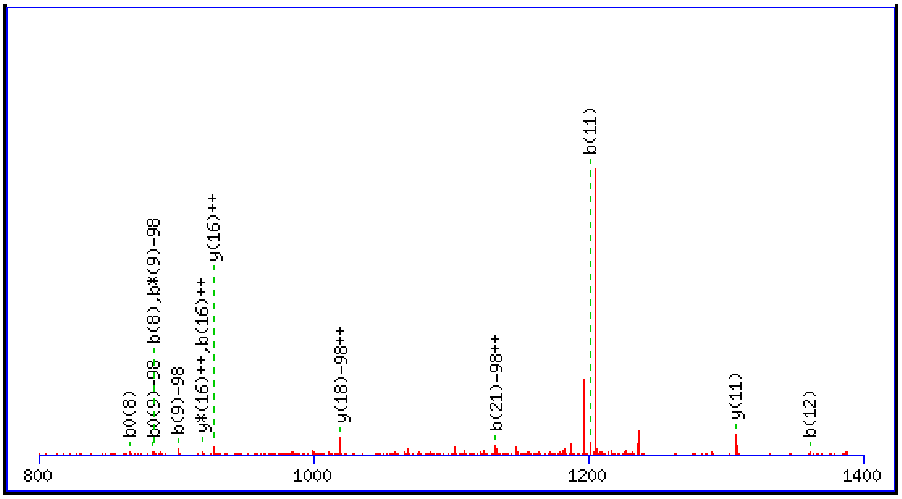

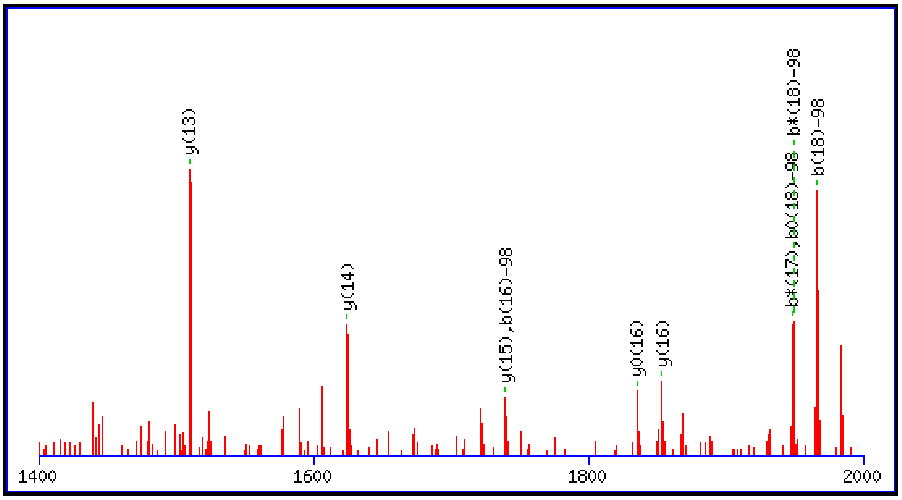

Monoisotopic mass of neutral peptide Mr(calc): 2507.0047
Fixed modifications: Carbamidomethyl (C)
Variable modifications:
S5 : Phospho (ST), with neutral losses 97.9769(shown in table), 0.0000
Ions Score: 68 **Expect:** 3.8e-005
Matches (Bold Red): 37/356 fragment ions using 33 most intense peaks

#	b	b^{++}	b*	b^{*++}	b^0	b^{0++}	Seq.	y	y^{++}	y*	y^{*++}	y^0	y^{0++}	#
1	58.0287	29.5180					G							22
2	145.0608	73.0340			127.0502	64.0287	S	2353.0136	1177.0104	2335.9871	1168.4972	2335.0031	1168.0052	21
3	258.1448	129.5761			240.1343	120.5708	L	2265.9816	1133.4944	2248.9550	1124.9812	2247.9710	1124.4892	20
4	373.1718	187.0895			355.1612	178.0842	D	2152.8975	1076.9524	2135.8710	1068.4391	2134.8870	1067.9471	19
5	442.1932	221.6003			424.1827	212.5950	S	2037.8706	**1019.4389**	2020.8440	1010.9257	2019.8600	1010.4336	18
6	557.2202	279.1137			539.2096	270.1084	D	1968.8491	984.9282	1951.8226	976.4149	1950.8386	975.9229	17
7	**671.2631**	336.1352	654.2366	327.6219	**653.2525**	327.1299	N	**1853.8222**	**927.4147**	1836.7956	**918.9015**	**1835.8116**	918.4094	16
8	786.2900	393.6487	**769.2635**	385.1354	768.2795	384.6434	D	**1739.7793**	870.3933	1722.7527	861.8800	1721.7687	861.3880	15
9	**901.3170**	451.1621	**884.2904**	442.6489	**883.3064**	442.1568	D	**1624.7523**	812.8798	1607.7258	804.3665	1606.7417	803.8745	14
10	988.3490	494.6781	971.3225	486.1649	970.3384	485.6729	S	**1509.7254**	**755.3663**	1492.6988	746.8530	1491.7148	746.3610	13
11	1103.3760	552.1916	1086.3494	**543.6783**	1085.3654	**543.1863**	D	1422.6933	711.8503	1405.6668	703.3370	1404.6828	702.8450	12
12	1263.4066	632.2069	1246.3801	623.6937	1245.3960	623.2017	C	**1307.6664**	654.3368	1290.6399	645.8236	1289.6558	645.3316	11
13	1360.4594	680.7333	1343.4328	672.2200	1342.4488	**671.7280**	P	1147.6358	574.3215	1130.6092	565.8082	1129.6252	565.3162	10
14	1523.5227	762.2650	1506.4962	753.7517	1505.5121	753.2597	Y	1050.5830	525.7951	1033.5564	517.2819	1032.5724	516.7898	9
15	1610.5547	805.7810	1593.5282	797.2677	1592.5442	796.7757	S	887.5197	**444.2635**	870.4931	435.7502	869.5091	435.2582	8
16	**1739.5973**	870.3023	1722.5708	861.7890	1721.5868	861.2970	E	800.4876	400.7475	783.4611	392.2342	782.4771	391.7422	7
17	1867.6923	934.3498	1850.6657	925.8365	1849.6817	925.3445	K	**671.4450**	336.2262	654.4185	327.7129	**653.4345**	327.2209	6
18	**1966.7607**	983.8840	**1949.7341**	975.3707	**1948.7501**	974.8787	V	**543.3501**	272.1787	526.3235	263.6654	525.3395	263.1734	5

**19**	2063.8135	1032.4104	2046.7869	1023.8971	2045.8029	1023.4051	**P**	**444.2817**	222.6445	427.2551	214.1312	426.2711	213.6392	**4**
**20**	2150.8455	1075.9264	2133.8189	1067.4131	2132.8349	1066.9211	**S**	347.2289	174.1181	330.2023	165.6048	329.2183	165.1128	**3**
**21**	2263.9296	**1132.4684**	2246.9030	1123.9551	2245.9190	1123.4631	**I**	260.1969	130.6021	243.1703	122.0888			**2**
**22**							**K**	147.1128	74.0600	130.0863	65.5468			**1**

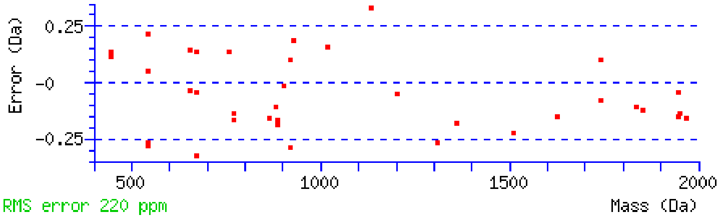

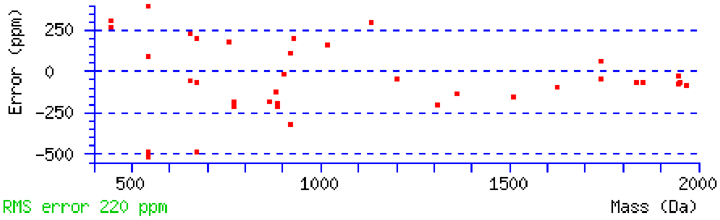

All matches to this query

Score	Mr(calc):	Delta	Sequence
68.3	2507.0047	-0.0117	GSLDSDNDDSDCPYSEKVPSIK
68.3	2507.0047	-0.0117	GSLDSDNDDSDCPYSEKVPSIK
27.9	2507.0047	-0.0117	GSLDSDNDDSDCPYSEKVPSIK
15.6	2507.0047	-0.0117	GSLDSDNDDSDCPYSEKVPSIK
8.6	2506.9848	0.0082	QYGITLYSHPYGGALLNEDR
8.6	2506.9848	0.0082	QYGITLYSHPYGGALLNEDR
7.9	2506.9982	-0.0051	QGDKKTFDECVAEGGSDCAPEK
7.5	2506.9982	-0.0051	QGDKKTFDECVAEGGSDCAPEK
6.4	2507.0124	-0.0193	VTQNAQRQYDRTMGLSCFR
5.4	2505.9816	1.0114	LAYNENDNTYYAMKVLSKK

Spectrum No: 109; Query: 2349; Rank: 1

Peptide View

MS/MS Fragmentation of **TENQPAVLEDAPDNTETGSVCTKV**
Found in **IPI00203446**, Tax_Id=10116 Gene_Symbol=Slc23a1 Solute carrier family 23 member 1

Match to Query 2349: 2654.139148 from(1328.076850,2+)
Title: 091008RatKidney_NoSalt_26.2541.2541.2.dta
Data file K:\NewmanPaper\Piliang\3SubProteomes\Piliang3SP\mgf5ppm\ERLIC_3SubProteomes5ppm.mgf

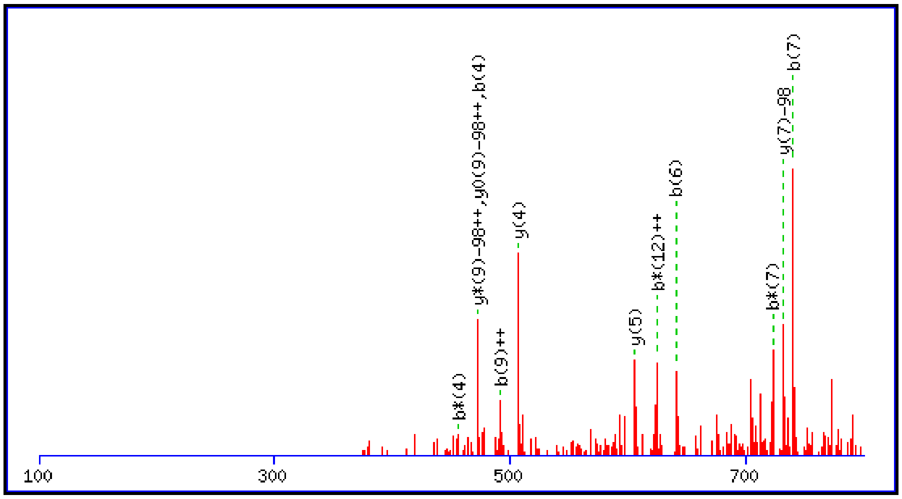

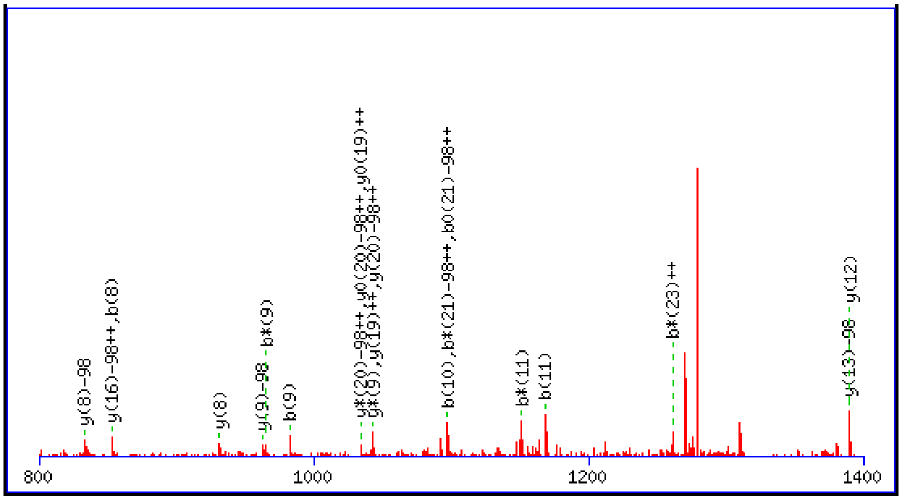

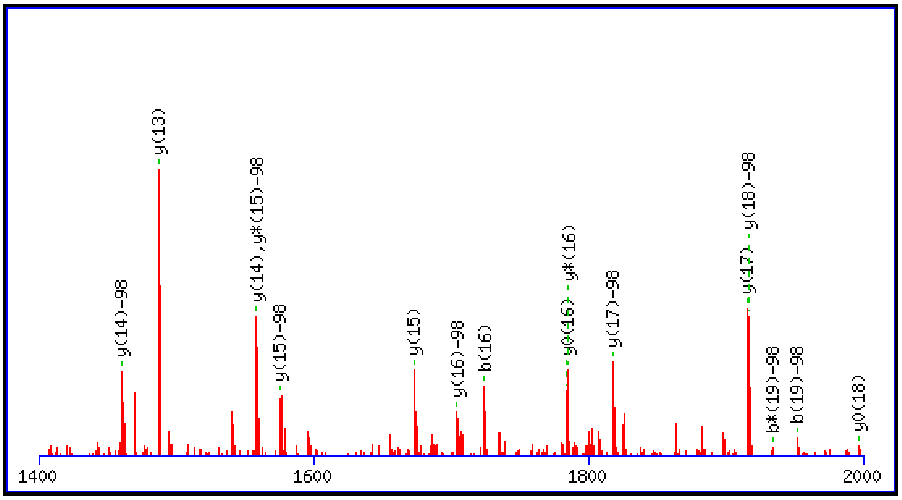

Monoisotopic mass of neutral peptide Mr(calc): 2654.1419
Fixed modifications: Carbamidomethyl (C)
Variable modifications:
S19 : Phospho (ST), with neutral losses 97.9769(shown in table), 0.0000
Ions Score: 68 **Expect:** 5.4e-005
Matches (Bold Red): 49/404 fragment ions using 58 most intense peaks

#	b	b^{++}	b*	b^{*++}	b^0	b^{0++}	Seq.	y	y^{++}	y*	y^{*++}	y^0	y^{0++}	#
1	102.0550	51.5311			84.0444	42.5258	T							24
2	231.0975	116.0524			213.0870	107.0471	E	2456.1246	1228.5659	2439.0980	1220.0526	2438.1140	1219.5606	23
3	345.1405	173.0739	328.1139	164.5606	327.1299	164.0686	N	2327.0820	1164.0446	2310.0554	1155.5314	2309.0714	1155.0393	22
4	**473.1991**	237.1032	**456.1725**	228.5899	455.1885	228.0979	Q	2213.0390	1107.0232	2196.0125	1098.5099	2195.0285	1098.0179	21
5	570.2518	285.6295	553.2253	277.1163	552.2413	276.6243	P	2084.9805	**1042.9939**	2067.9539	**1034.4806**	2066.9699	**1033.9886**	20
6	**641.2889**	321.1481	624.2624	312.6348	623.2784	312.1428	A	1987.9277	994.4675	1970.9012	985.9542	1969.9171	985.4622	19
7	**740.3573**	370.6823	**723.3308**	362.1690	722.3468	361.6770	V	**1916.8906**	958.9489	1899.8640	950.4357	1898.8800	949.9437	18
8	**853.4414**	427.2243	836.4149	418.7111	835.4308	418.2191	L	**1817.8222**	909.4147	1800.7956	900.9015	1799.8116	900.4094	17
9	**982.4840**	**491.7456**	**965.4575**	483.2324	964.4734	482.7404	E	**1704.7381**	**852.8727**	1687.7116	844.3594	1686.7275	843.8674	16
10	**1097.5109**	549.2591	1080.4844	540.7458	1079.5004	540.2538	D	**1575.6955**	788.3514	**1558.6690**	779.8381	1557.6850	779.3461	15
11	**1168.5481**	584.7777	**1151.5215**	576.2644	1150.5375	575.7724	A	**1460.6686**	730.8379	1443.6420	722.3247	1442.6580	721.8326	14
12	1265.6008	633.3040	1248.5743	**624.7908**	1247.5903	624.2988	P	**1389.6315**	695.3194	1372.6049	686.8061	1371.6209	686.3141	13
13	1380.6278	690.8175	1363.6012	682.3042	1362.6172	681.8122	D	1292.5787	646.7930	1275.5522	638.2797	1274.5681	637.7877	12
14	1494.6707	747.8390	1477.6441	739.3257	1476.6601	738.8337	N	1177.5518	589.2795	1160.5252	580.7662	1159.5412	580.2742	11
15	1595.7184	798.3628	1578.6918	789.8495	1577.7078	789.3575	T	1063.5088	532.2581	1046.4823	523.7448	1045.4983	523.2528	10
16	**1724.7610**	862.8841	1707.7344	854.3708	1706.7504	853.8788	E	**962.4612**	481.7342	945.4346	**473.2209**	944.4506	**472.7289**	9
17	1825.8086	913.4080	1808.7821	904.8947	1807.7981	904.4027	T	**833.4186**	417.2129	816.3920	408.6996	815.4080	408.2076	8
18	1882.8301	941.9187	1865.8036	933.4054	1864.8195	932.9134	G	**732.3709**	366.6891	715.3443	358.1758	714.3603	357.6838	7

**19**	**1951.8516**	976.4294	**1934.8250**	967.9161	1933.8410	967.4241	**S**	675.3494	338.1783	658.3229	329.6651	657.3389	329.1731	**6**
**20**	2050.9200	1025.9636	2033.8934	1017.4504	2032.9094	1016.9583	**V**	**606.3280**	303.6676	589.3014	295.1543	588.3174	294.6623	**5**
**21**	2210.9506	1105.9790	2193.9241	**1097.4657**	2192.9401	**1096.9737**	**C**	**507.2595**	254.1334	490.2330	245.6201	489.2490	245.1281	**4**
**22**	2311.9983	1156.5028	2294.9718	1147.9895	2293.9877	1147.4975	**T**	347.2289	174.1181	330.2023	165.6048	329.2183	165.1128	**3**
**23**	2440.0933	1220.5503	2423.0667	1212.0370	2422.0827	1211.5450	**K**	246.1812	123.5942	229.1547	115.0810			**2**
**24**							**V**	118.0863	59.5468					**1**

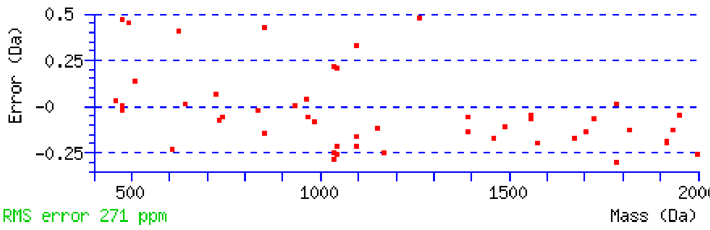

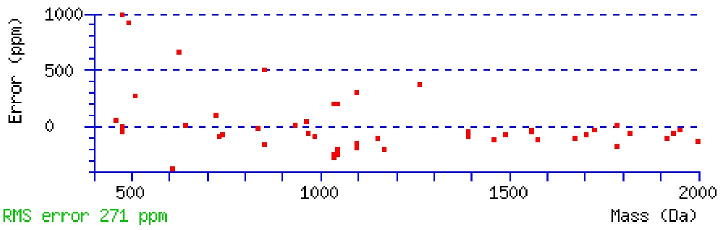

All matches to this query

Score	Mr(calc):	Delta	Sequence
68.0	2654.1419	-0.0027	TENQPAVLEDAPDNTETGSVCTKV
58.4	2654.1419	-0.0027	TENQPAVLEDAPDNTETGSVCTKV
48.6	2654.1419	-0.0027	TENQPAVLEDAPDNTETGSVCTKV
42.5	2654.1419	-0.0027	TENQPAVLEDAPDNTETGSVCTKV
6.7	2654.1151	0.0240	YMYSDEIDLEADTVLATLYAAK
6.7	2654.1151	0.0240	YMYSDEIDLEADTVLATLYAAK
6.7	2654.1419	-0.0027	TENQPAVLEDAPDNTETGSVCTKV
3.3	2654.1367	0.0025	SQHAHVIEDLHCNLCDVDVSAR
3.0	2654.1626	-0.0234	EVNPPNEGKNPTEASSKSQQKPK
2.0	2652.1451	1.9941	THAAYNLLFFASGGGKFNYQGTK

Spectrum No: 110; Query: 757; Rank: 1

Peptide View

MS/MS Fragmentation of **DEILPTTPISEQK**
Found in **IPI00195999**, Tax_Id=10116 Gene_Symbol=RGD1560831_predicted similar to 40S ribosomal protein S3

Match to Query 757: 1549.727108 from(775.870830,2+)
Title: 091008RatKidney_NoSalt_25.2092.2092.2.dta
Data file K:\NewmanPaper\Piliang\3SubProteomes\Piliang3SP\mgf5ppm\ERLIC_3SubProteomes5ppm.mgf

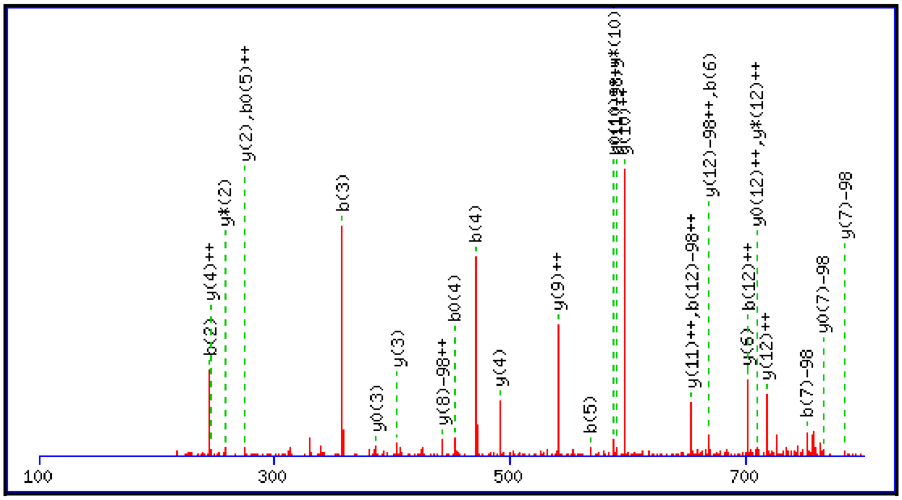

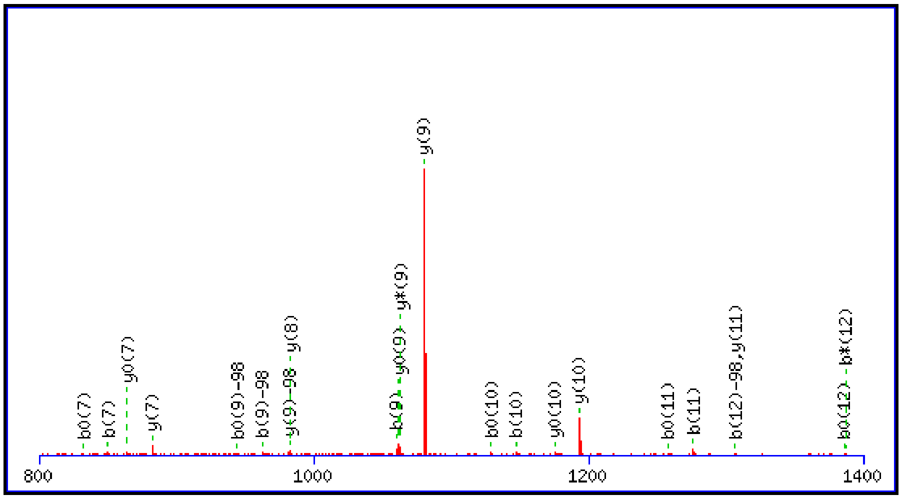

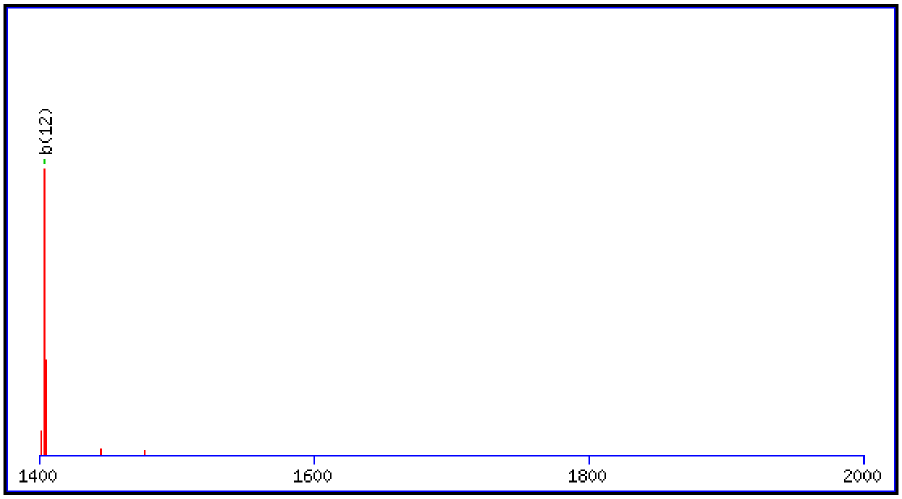

Monoisotopic mass of neutral peptide Mr(calc): 1549.7276
Fixed modifications: Carbamidomethyl (C)
Variable modifications:
T7 : Phospho (ST), with neutral losses 0.0000(shown in table), 97.9769
Ions Score: 68 **Expect:** 2.9e-005
Matches (Bold Red): 53/180 fragment ions using 67 most intense peaks

#	b	b++	b*	b*++	b0	b0++	Seq.	y	y++	y*	y*++	y0	y0++	#
1	116.0342	58.5207			98.0237	49.5155	D							13
2	**245.0768**	123.0420			227.0662	114.0368	E	1435.7080	**718.3576**	1418.6814	**709.8444**	1417.6974	**709.3524**	12
3	**358.1609**	179.5841			340.1503	170.5788	I	**1306.6654**	**653.8363**	1289.6389	645.3231	1288.6548	644.8311	11
4	**471.2449**	236.1261			**453.2344**	227.1208	L	**1193.5813**	**597.2943**	1176.5548	**588.7810**	**1175.5708**	**588.2890**	10
5	**568.2977**	284.6525			550.2871	**275.6472**	P	**1080.4973**	**540.7523**	**1063.4707**	532.2390	**1062.4867**	531.7470	9
6	**669.3454**	335.1763			651.3348	326.1710	T	**983.4445**	492.2259	966.4180	483.7126	965.4339	483.2206	8
7	**850.3594**	425.6833			**832.3488**	416.6780	T	**882.3968**	441.7021	865.3703	433.1888	**864.3863**	432.6968	7
8	947.4122	474.2097			929.4016	465.2044	P	**701.3828**	351.1951	684.3563	342.6818	683.3723	342.1898	6
9	**1060.4962**	530.7517			1042.4856	521.7465	I	604.3301	302.6687	587.3035	294.1554	586.3195	293.6634	5
10	**1147.5282**	574.2678			**1129.5177**	565.2625	S	**491.2460**	**246.1266**	474.2195	237.6134	473.2354	237.1214	4
11	**1276.5708**	638.7891			**1258.5603**	629.7838	E	**404.2140**	202.6106	387.1874	194.0974	**386.2034**	193.6053	3
12	**1404.6294**	**702.8183**	**1387.6029**	694.3051	**1386.6188**	693.8131	Q	**275.1714**	138.0893	**258.1448**	129.5761			2
13							K	147.1128	74.0600	130.0863	65.5468			1

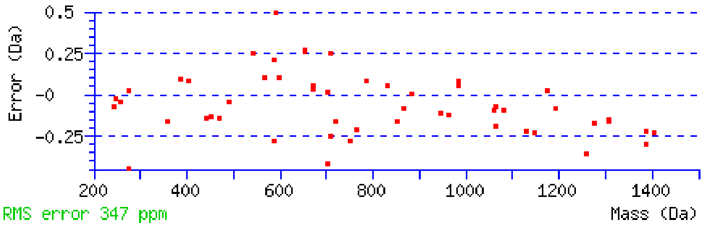

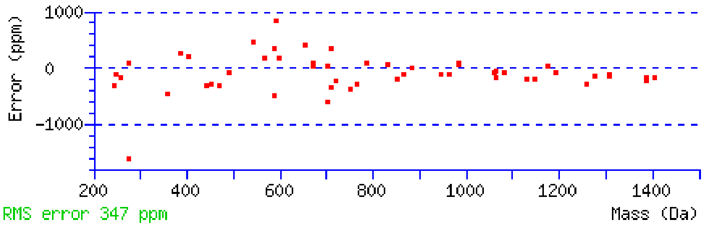

All matches to this query

Score	Mr(calc):	Delta	Sequence
67.8	1549.7276	-0.0005	DEILPTTPISEQK
53.7	1549.7276	-0.0005	DEILPTTPISEQK
45.9	1549.7276	-0.0005	DEILPTTPISEQK
12.9	1549.7406	-0.0135	GAQTLSDLRSEMDK
12.4	1549.7177	0.0094	NLEPVSWSSLNPK
12.4	1549.7177	0.0094	NLEPVSWSSLNPK
12.4	1549.7177	0.0094	NLEPVSWSSLNPK
12.4	1547.7071	2.0200	IIKNPMDLSTIK
12.1	1549.7372	-0.0101	DLQNYQSNQLAQK
10.3	1549.7372	-0.0101	ENNLELQGYQTNK

Spectrum No: 111; Query: 1303; Rank: 1

Peptide View

MS/MS Fragmentation of **SSSVGSSSSYPISSAVPR**
Found in **IPI00209000**, Tax_Id=10116 Gene_Symbol=Plec1 Plectin 6

Match to Query 1303: 1833.817008 from(917.915780,2+)
Title: 091008RatKidney_NH4Format01_22.2119.2119.2.dta
Data file K:\NewmanPaper\Piliang\3SubProteomes\Piliang3SP\mgf5ppm\ERLIC_3SubProteomes5ppm.mgf

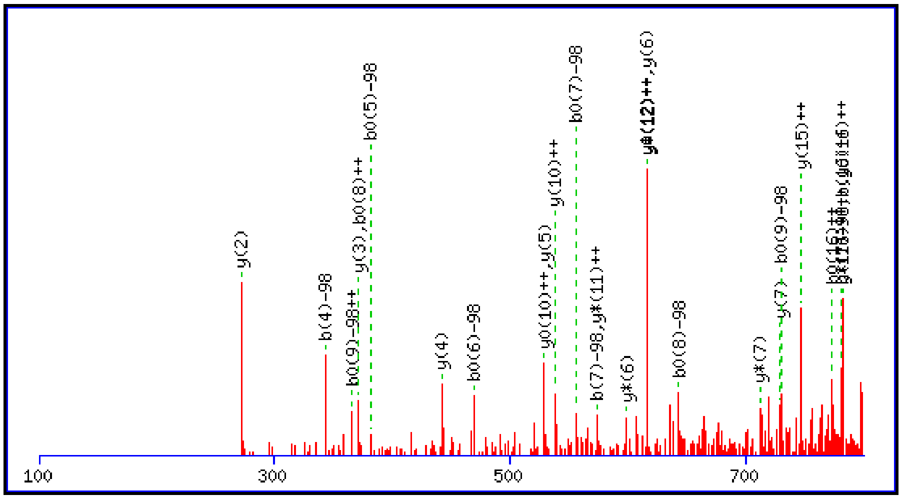

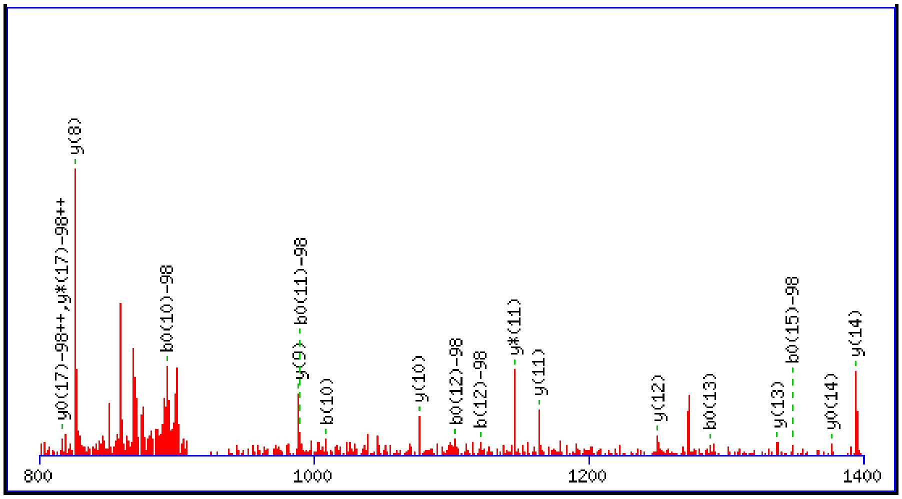

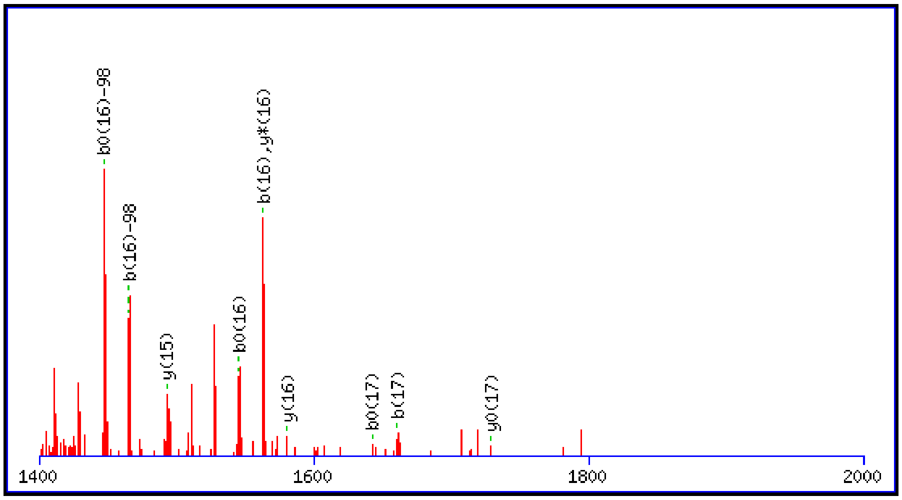

Monoisotopic mass of neutral peptide Mr(calc): 1833.8146
Fixed modifications: Carbamidomethyl (C)
Variable modifications:
S2 : Phospho (ST), with neutral losses 97.9769(shown in table), 0.0000
Ions Score: 68 **Expect:** 3.5e-005
Matches (Bold Red): 56/232 fragment ions using 131 most intense peaks

#	b	b^{++}	b^0	b^{0++}	Seq.	y	y^{++}	y*	y^{*++}	y^0	y^{0++}	#
1	88.0393	44.5233	70.0287	35.5180	S							18
2	157.0608	79.0340	139.0502	70.0287	S	1649.8129	825.4101	1632.7864	**816.8968**	1631.8024	**816.4048**	17
3	244.0928	122.5500	226.0822	113.5447	S	**1580.7915**	790.8994	**1563.7649**	**782.3861**	1562.7809	**781.8941**	16
4	**343.1612**	172.0842	325.1506	163.0790	V	**1493.7594**	**747.3834**	1476.7329	738.8701	1475.7489	738.3781	15
5	400.1827	200.5950	**382.1721**	191.5897	G	**1394.6910**	697.8492	1377.6645	689.3359	**1376.6805**	688.8439	14
6	487.2147	244.1110	**469.2041**	235.1057	S	**1337.6696**	669.3384	1320.6430	660.8251	1319.6590	660.3331	13
7	**574.2467**	287.6270	**556.2362**	278.6217	S	**1250.6375**	625.8224	1233.6110	**617.3091**	1232.6270	**616.8171**	12
8	661.2787	331.1430	**643.2682**	322.1377	S	**1163.6055**	582.3064	**1146.5790**	**573.7931**	1145.5949	573.3011	11
9	748.3108	374.6590	**730.3002**	**365.6537**	S	**1076.5735**	**538.7904**	1059.5469	530.2771	1058.5629	**529.7851**	10
10	911.3741	456.1907	**893.3635**	447.1854	Y	**989.5415**	495.2744	972.5149	486.7611	971.5309	486.2691	9
11	1008.4269	504.7171	**990.4163**	495.7118	P	**826.4781**	413.7427	809.4516	405.2294	808.4676	404.7374	8
12	**1121.5109**	561.2591	**1103.5004**	552.2538	I	**729.4254**	365.2163	**712.3988**	356.7030	711.4148	356.2110	7
13	1208.5430	604.7751	1190.5324	595.7698	S	**616.3413**	308.6743	**599.3148**	300.1610	598.3307	299.6690	6
14	1295.5750	648.2911	1277.5644	639.2859	S	**529.3093**	265.1583	512.2827	256.6450	511.2987	256.1530	5
15	1366.6121	683.8097	**1348.6015**	674.8044	A	**442.2772**	221.6423	425.2507	213.1290			4
16	**1465.6805**	733.3439	**1447.6700**	724.3386	V	**371.2401**	186.1237	354.2136	177.6104			3
17	1562.7333	**781.8703**	1544.7227	772.8650	P	**272.1717**	136.5895	255.1452	128.0762			2
18					R	175.1190	88.0631	158.0924	79.5498			1

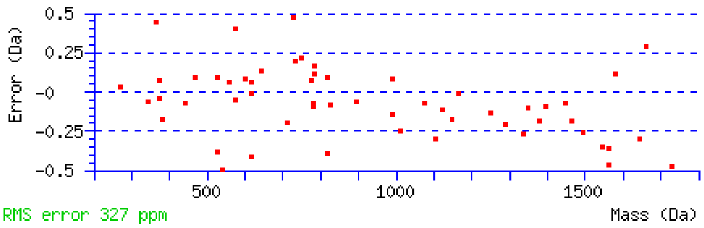

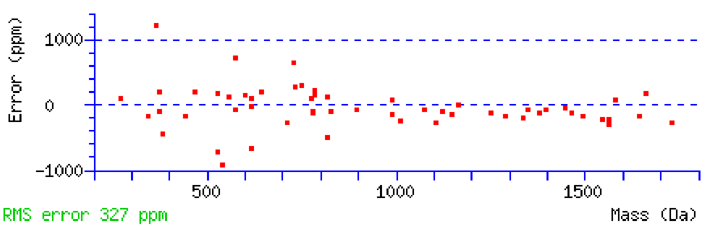

All matches to this query

Score	Mr(calc):	Delta	Sequence
67.7	1833.8146	0.0024	SSSVGSSSSYPISSAVPR
67.7	1833.8146	0.0024	SSSVGSSSSYPISSAVPR
58.5	1833.8146	0.0024	SSSVGSSSSYPISSAVPR
40.6	1833.8146	0.0024	SSSVGSSSSYPISSAVPR
31.3	1833.8146	0.0024	SSSVGSSSSYPISSAVPR
21.0	1833.8146	0.0024	SSSVGSSSSYPISSAVPR
21.0	1833.8146	0.0024	SSSVGSSSSYPISSAVPR
12.0	1833.8146	0.0024	SSSVGSSSSYPISSAVPR
2.8	1833.8006	0.0164	NRAGSPNPQSSSGELPR
2.8	1833.8006	0.0164	NRAGSPNPQSSSGELPR

Spectrum No: 112; Query: 2728; Rank: 1

Peptide View

MS/MS Fragmentation of **DLGHPVEEEDESGDQEDDDDELDDGDRDQDI**
Found in **IPI00324618**, Tax_Id=10116 Gene_Symbol=Vdp General vesicular transport factor p115

Match to Query 2728: 3595.315182 from(1199.445670,3+)
Title: 100101RatKid_NS_deglyco_27.2267.2267.3.dta
Data file K:\NewmanPaper\Piliang\3SubProteomes\Piliang3SP\mgf5ppm\ERLIC_3SubProteomes5ppm.mgf

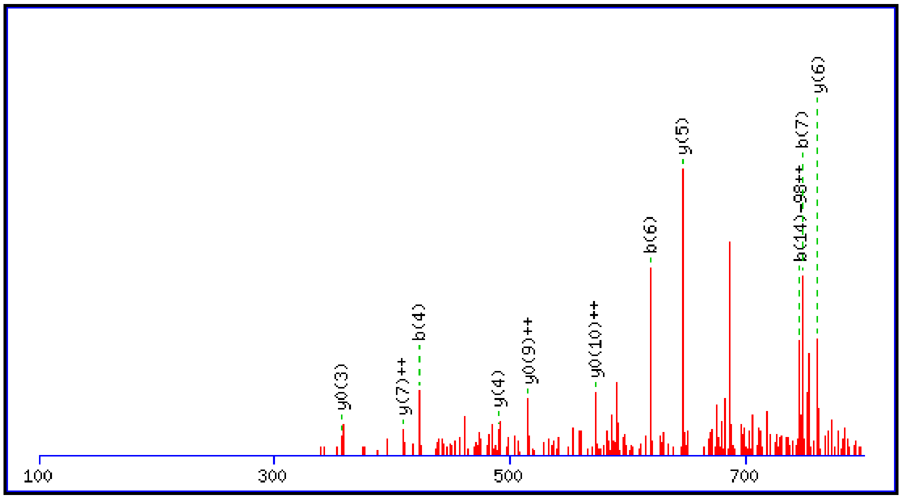

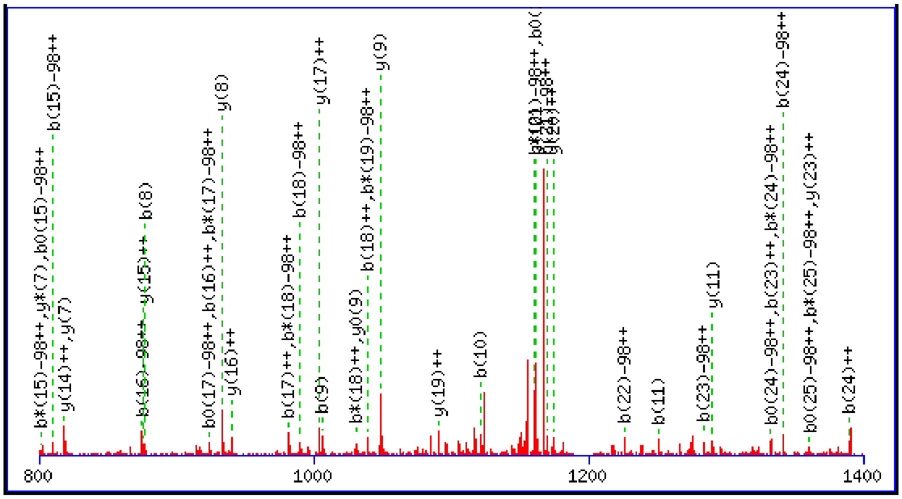

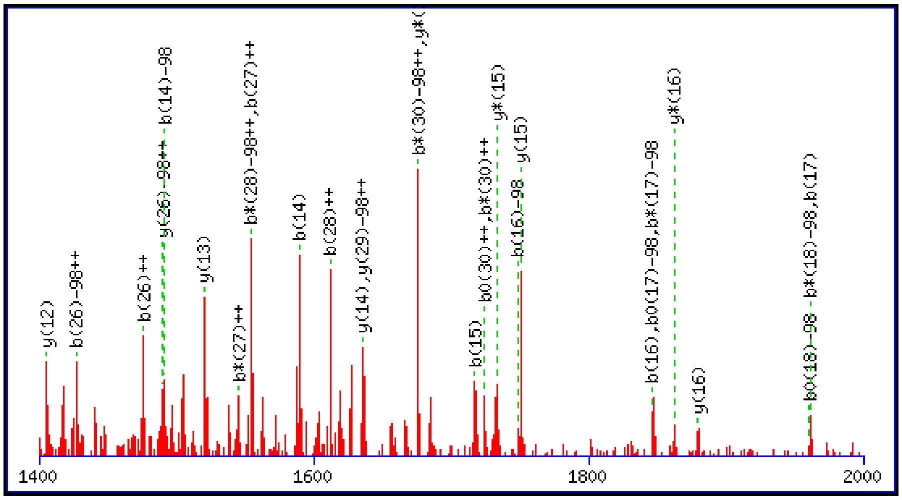

Monoisotopic mass of neutral peptide Mr(calc): 3595.3027
Fixed modifications: Carbamidomethyl (C)
Variable modifications:
S12 : Phospho (ST), with neutral losses 97.9769(shown in table), 0.0000
Ions Score: 68 **Expect:** 3.8e-005
Matches (Bold Red): 83/500 fragment ions using 113 most intense peaks

#	b	b++	b*	b*++	b0	b0++	Seq.	y	y++	y*	y*++	y0	y0++	#
1	116.0342	58.5207			98.0237	49.5155	D							31
2	229.1183	115.0628			211.1077	106.0575	L	3383.3062	1692.1567	3366.2796	1683.6434	3365.2956	1683.1514	30
3	286.1397	143.5735			268.1292	134.5682	G	3270.2221	**1635.6147**	3253.1956	1627.1014	3252.2115	1626.6094	29
4	**423.1987**	212.1030			405.1881	203.0977	H	3213.2006	1607.1040	3196.1741	1598.5907	3195.1901	1598.0987	28
5	520.2514	260.6293			502.2409	251.6241	P	3076.1417	1538.5745	3059.1152	1530.0612	3058.1312	1529.5692	27
6	**619.3198**	310.1636			601.3093	301.1583	V	2979.0890	**1490.0481**	2962.0624	1481.5348	2961.0784	1481.0428	26
7	**748.3624**	374.6849			730.3519	365.6796	E	2880.0206	1440.5139	2862.9940	1432.0006	2862.0100	1431.5086	25
8	**877.4050**	439.2061			859.3945	430.2009	E	2750.9780	1375.9926	2733.9514	1367.4793	2732.9674	1366.9873	24
9	**1006.4476**	503.7274			988.4371	494.7222	E	2621.9354	1311.4713	2604.9088	1302.9580	2603.9248	1302.4660	23
10	**1121.4746**	561.2409			1103.4640	552.2356	D	2492.8928	1246.9500	2475.8662	1238.4367	2474.8822	1237.9447	22
11	**1250.5172**	625.7622			1232.5066	616.7569	E	2377.8658	1189.4366	2360.8393	1180.9233	2359.8553	1180.4313	21
12	1319.5386	660.2729			1301.5280	651.2677	S	2248.8232	1124.9153	2231.7967	1116.4020	2230.8127	1115.9100	20
13	1376.5601	688.7837			1358.5495	679.7784	G	2179.8018	**1090.4045**	2162.7752	1081.8913	2161.7912	1081.3992	19
14	**1491.5870**	**746.2971**			1473.5765	737.2919	D	2122.7803	1061.8938	2105.7538	1053.3805	2104.7698	1052.8885	18
15	1619.6456	**810.3264**	1602.6190	**801.8132**	1601.6350	**801.3212**	Q	2007.7534	**1004.3803**	1990.7268	995.8670	1989.7428	995.3750	17
16	**1748.6882**	**874.8477**	1731.6616	866.3345	1730.6776	865.8424	E	**1879.6948**	**940.3510**	**1862.6682**	931.8378	1861.6842	931.3458	16
17	1863.7151	932.3612	**1846.6886**	**923.8479**	**1845.7046**	**923.3559**	D	**1750.6522**	**875.8297**	**1733.6257**	867.3165	1732.6416	866.8245	15
18	1978.7421	**989.8747**	**1961.7155**	**981.3614**	**1960.7315**	980.8694	D	**1635.6253**	**818.3163**	1618.5987	809.8030	1617.6147	809.3110	14

**19**	2093.7690	1047.3881	2076.7425	**1038.8749**	2075.7584	1038.3829	**D**	**1520.5983**	760.8028	1503.5718	752.2895	1502.5878	751.7975	**13**
**20**	2208.7960	1104.9016	2191.7694	1096.3883	2190.7854	1095.8963	**D**	**1405.5714**	703.2893	1388.5448	694.7760	1387.5608	694.2840	**12**
**21**	2337.8386	**1169.4229**	2320.8120	**1160.9096**	2319.8280	**1160.4176**	**E**	**1290.5444**	645.7759	1273.5179	637.2626	1272.5339	636.7706	**11**
**22**	2450.9226	**1225.9649**	2433.8961	1217.4517	2432.9120	1216.9597	**L**	**1161.5018**	581.2546	1144.4753	572.7413	1143.4913	**572.2493**	**10**
**23**	2565.9496	**1283.4784**	2548.9230	1274.9651	2547.9390	1274.4731	**D**	**1048.4178**	524.7125	1031.3912	516.1992	**1030.4072**	**515.7072**	**9**
**24**	2680.9765	**1340.9919**	2663.9500	**1332.4786**	2662.9659	**1331.9866**	**D**	**933.3908**	467.1991	916.3643	458.6858	915.3803	458.1938	**8**
**25**	2737.9980	1369.5026	2720.9714	**1360.9893**	2719.9874	**1360.4973**	**G**	**818.3639**	**409.6856**	**801.3373**	401.1723	800.3533	400.6803	**7**
**26**	2853.0249	**1427.0161**	2835.9984	1418.5028	2835.0143	1418.0108	**D**	**761.3424**	381.1748	744.3159	372.6616	743.3319	372.1696	**6**
**27**	3009.1260	1505.0666	2992.0995	1496.5534	2991.1155	1496.0614	**R**	**646.3155**	323.6614	629.2889	315.1481	628.3049	314.6561	**5**
**28**	3124.1530	1562.5801	3107.1264	**1554.0668**	3106.1424	1553.5748	**D**	**490.2144**	245.6108	473.1878	237.0975	472.2038	236.6055	**4**
**29**	3252.2115	1626.6094	3235.1850	1618.0961	3234.2010	1617.6041	**Q**	375.1874	188.0974	358.1609	179.5841	**357.1769**	179.0921	**3**
**30**	3367.2385	1684.1229	3350.2119	**1675.6096**	3349.2279	1675.1176	**D**	247.1288	124.0681			229.1183	115.0628	**2**
**31**							**I**	132.1019	66.5546					**1**

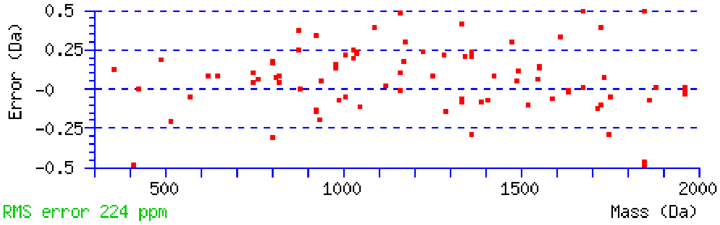

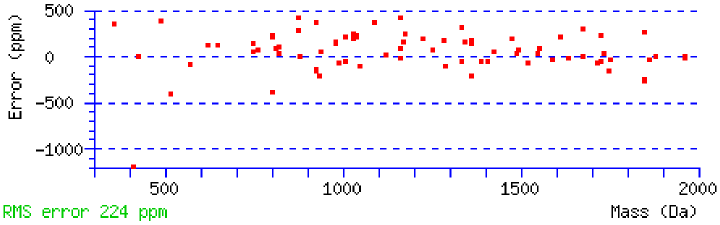

All matches to this query

Score	Mr(calc):	Delta	Sequence
67.5	3595.3027	0.0124	DLGHPVEEEDESGDQEDDDDELDDGDRDQDI
4.4	3593.2926	2.0226	QQYNFLRSSRIWVHPTPFTSTMPPK
3.5	3595.3274	-0.0122	YELVLSKNSTLFWNTESSELPDSFCTK
3.5	3594.3434	0.9718	YELVLSKNSTLFWNTESSELPDSFCTK
3.5	3595.3274	-0.0122	YELVLSKNSTLFWNTESSELPDSFCTK
2.1	3593.3261	1.9891	CSSGNIGSYSVHWYQQHEGRSPTTMIYK
1.6	3595.3420	-0.0268	MYTARWVTNNDTFAPEDPCFFCDVCFR
1.6	3595.3420	-0.0268	MYTARWVTNNDTFAPEDPCFFCDVCFR
1.6	3595.3420	-0.0268	MYTARWVTNNDTFAPEDPCFFCDVCFR
1.6	3595.3420	-0.0268	MYTARWVTNNDTFAPEDPCFFCDVCFR

Spectrum No: 113; Query: 1014; Rank: 1

Peptide View

MS/MS Fragmentation of **GSDEVTASSAATGTSPR**
Found in **IPI00195926**, Tax_Id=10116 Gene_Symbol=Cast Isoform 5 of Calpastatin

Match to Query 1014: 1672.692548 from(837.353550,2+)
Title: 091008RatKidney_NoSalt_26.498.498.2.dta
Data file K:\NewmanPaper\Piliang\3SubProteomes\Piliang3SP\mgf5ppm\ERLIC_3SubProteomes5ppm.mgf

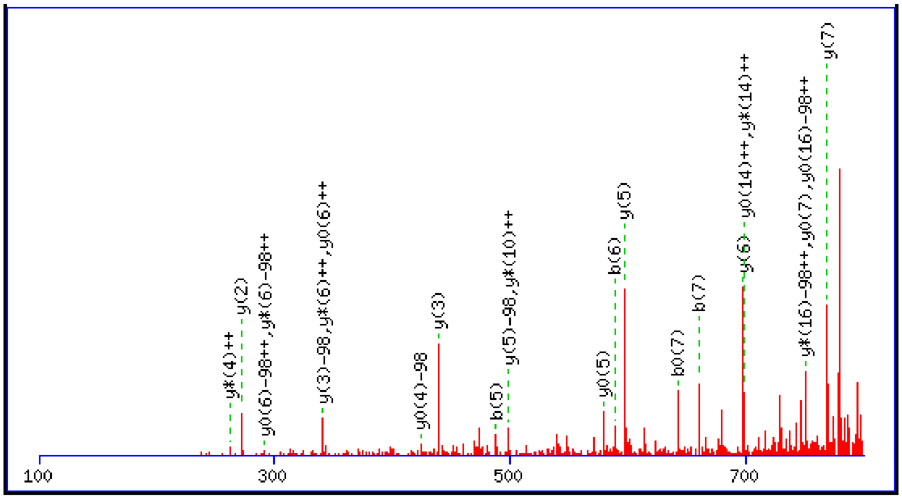

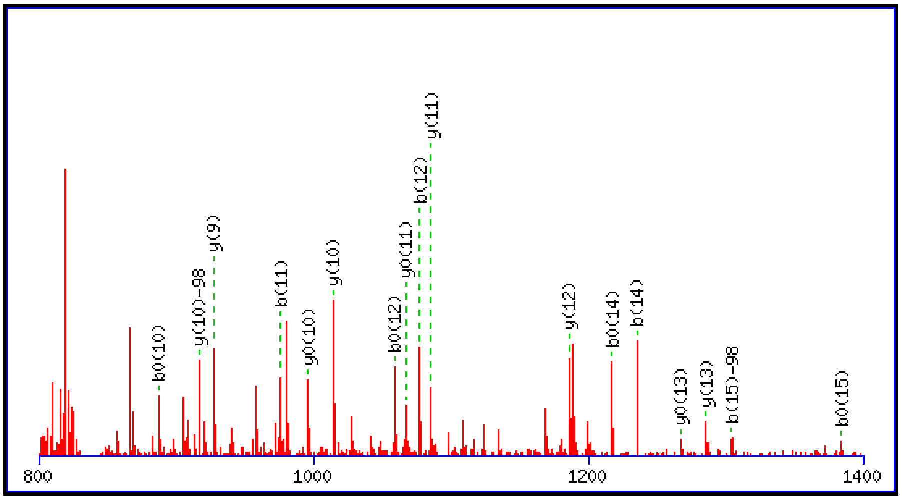

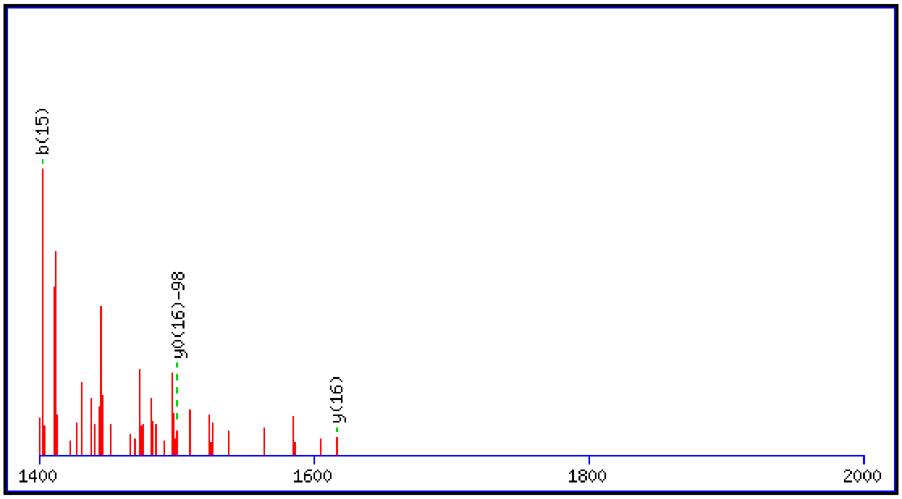

Monoisotopic mass of neutral peptide Mr(calc): 1672.6941
Fixed modifications: Carbamidomethyl (C)
Variable modifications:
S15 : Phospho (ST), with neutral losses 0.0000(shown in table), 97.9769
Ions Score: 68 **Expect:** 2.6e-005
Matches (Bold Red): 44/246 fragment ions using 64 most intense peaks

#	b	b^{++}	b^{0}	b^{0++}	Seq.	y	y^{++}	y*	y^{*++}	y^{0}	y^{0++}	#
1	58.0287	29.5180			G							17
2	145.0608	73.0340	127.0502	64.0287	S	**1616.6799**	808.8436	1599.6534	800.3303	1598.6694	799.8383	16
3	260.0877	130.5475	242.0771	121.5422	D	1529.6479	765.3276	1512.6214	756.8143	1511.6374	756.3223	15
4	389.1303	195.0688	371.1197	186.0635	E	1414.6210	707.8141	1397.5944	**699.3009**	1396.6104	**698.8088**	14
5	**488.1987**	244.6030	470.1882	235.5977	V	**1285.5784**	643.2928	1268.5518	634.7796	**1267.5678**	634.2875	13

6	**589.2464**	295.1268	571.2358	286.1216	T	**1186.5100**	593.7586	1169.4834	585.2453	1168.4994	584.7533	12
7	**660.2835**	330.6454	**642.2729**	321.6401	A	**1085.4623**	543.2348	1068.4357	534.7215	**1067.4517**	534.2295	11
8	747.3155	374.1614	729.3050	365.1561	S	**1014.4252**	507.7162	997.3986	**499.2030**	**996.4146**	498.7109	10
9	834.3476	417.6774	816.3370	408.6721	S	**927.3931**	464.2002	910.3666	455.6869	909.3826	455.1949	9
10	905.3847	453.1960	**887.3741**	444.1907	A	840.3611	420.6842	823.3346	412.1709	822.3506	411.6789	8
11	**976.4218**	488.7145	958.4112	479.7093	A	**769.3240**	385.1656	752.2975	376.6524	**751.3134**	376.1604	7
12	**1077.4695**	539.2384	**1059.4589**	530.2331	T	**698.2869**	349.6471	681.2603	**341.1338**	680.2763	**340.6418**	6
13	1134.4909	567.7491	1116.4804	558.7438	G	**597.2392**	299.1232	580.2127	290.6100	**579.2286**	290.1180	5
14	**1235.5386**	618.2729	**1217.5281**	609.2677	T	540.2177	270.6125	523.1912	**262.0992**	522.2072	261.6072	4
15	**1402.5370**	701.7721	**1384.5264**	692.7668	S	**439.1701**	220.0887	422.1435	211.5754	421.1595	211.0834	3
16	1499.5897	750.2985	1481.5792	741.2932	P	**272.1717**	136.5895	255.1452	128.0762			2
17					R	175.1190	88.0631	158.0924	79.5498			1

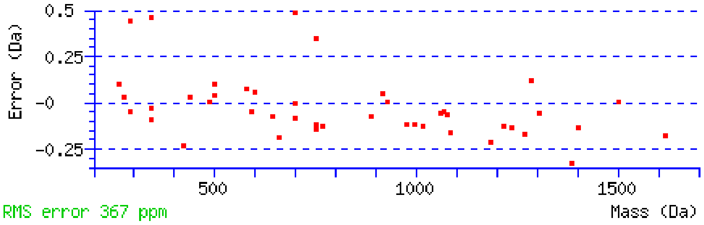

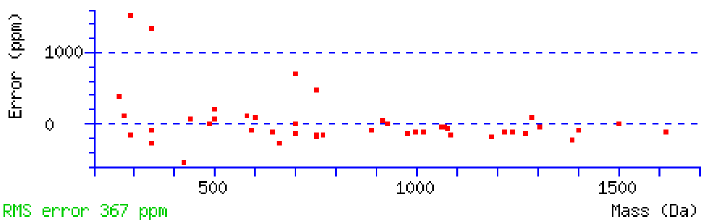

All matches to this query

Score	Mr(calc):	Delta	Sequence
67.6	1672.6941	-0.0016	GSDEVTASSAATGTSPR
53.1	1672.6941	-0.0016	GSDEVTASSAATGTSPR
38.8	1672.6941	-0.0016	GSDEVTASSAATGTSPR
27.8	1672.6941	-0.0016	GSDEVTASSAATGTSPR
22.9	1672.6941	-0.0016	GSDEVTASSAATGTSPR
14.4	1672.6941	-0.0016	GSDEVTASSAATGTSPR
13.3	1672.6941	-0.0016	GSDEVTASSAATGTSPR
5.8	1670.6776	2.0150	SLASFCNNVTKIR
4.5	1670.6855	2.0070	SAHWDTVTPQTLR
1.1	1672.6950	-0.0024	AASESAQAQVCMNVK

Spectrum No: 114; Query: 2803; Rank: 1

Peptide View

MS/MS Fragmentation of **VQGEAVSNIQENTQTPTVQEESEEEEVDETGVEVK**
Found in **IPI00371266**, Tax_Id=10116 Gene_Symbol=Naca_predicted nascent-polypeptide-associated complex alpha polypeptide

Match to Query 2803: 3940.739172 from(1314.587000,3+)
Title: 100101RatKid_NS_deglyco_24.2976.2976.3.dta
Data file K:\NewmanPaper\Piliang\3SubProteomes\Piliang3SP\mgf5ppm\ERLIC_3SubProteomes5ppm.mgf

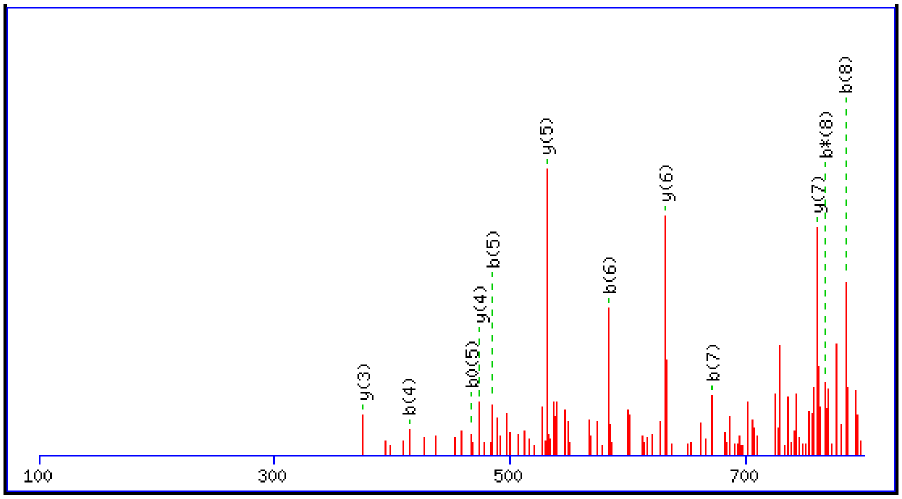

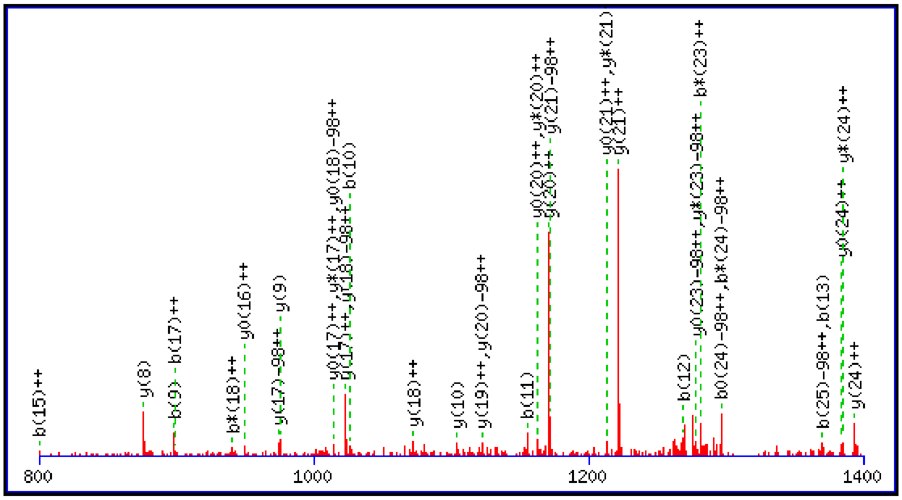

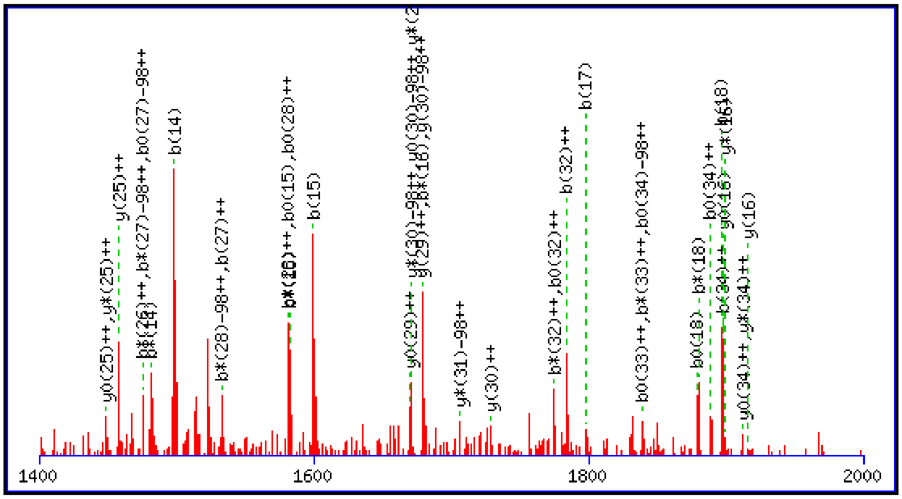

Monoisotopic mass of neutral peptide Mr(calc): 3939.7270
Fixed modifications: Carbamidomethyl (C)
Variable modifications:
S22 : Phospho (ST), with neutral losses 0.0000(shown in table), 97.9769
Ions Score: 67 **Expect:** 8.9e-005
Matches (Bold Red): 90/600 fragment ions using 126 most intense peaks

#	b	b^{++}	b*	b^{*++}	b^0	b^{0++}	Seq.	y	y^{++}	y*	y^{*++}	y^0	y^{0++}	#
1	100.0757	50.5415					V							35
2	228.1343	114.5708	211.1077	106.0575			Q	3841.6659	1921.3366	3824.6393	**1912.8233**	3823.6553	**1912.3313**	34
3	285.1557	143.0815	268.1292	134.5682			G	3713.6073	1857.3073	3696.5808	1848.7940	3695.5967	1848.3020	33
4	**414.1983**	207.6028	397.1718	199.0895	396.1878	198.5975	E	3656.5858	1828.7966	3639.5593	1820.2833	3638.5753	1819.7913	32
5	**485.2354**	243.1214	468.2089	234.6081	**467.2249**	234.1161	A	3527.5432	1764.2753	3510.5167	1755.7620	3509.5327	1755.2700	31

6	584.3039	292.6556	567.2773	284.1423	566.2933	283.6503	V	3456.5061	1728.7567	3439.4796	1720.2434	3438.4956	1719.7514	30
7	671.3359	336.1716	654.3093	327.6583	653.3253	327.1663	S	3357.4377	1679.2225	3340.4112	1670.7092	3339.4272	1670.2172	29
8	785.3788	393.1930	768.3523	384.6798	767.3682	384.1878	N	3270.4057	1635.7065	3253.3791	1627.1932	3252.3951	1626.7012	28
9	898.4629	449.7351	881.4363	441.2218	880.4523	440.7298	I	3156.3628	1578.6850	3139.3362	1570.1717	3138.3522	1569.6797	27
10	1026.5215	513.7644	1009.4949	505.2511	1008.5109	504.7591	Q	3043.2787	1522.1430	3026.2521	1513.6297	3025.2681	1513.1377	26
11	1155.5640	578.2857	1138.5375	569.7724	1137.5535	569.2804	E	2915.2201	1458.1137	2898.1936	1449.6004	2897.2096	1449.1084	25
12	1269.6070	635.3071	1252.5804	626.7938	1251.5964	626.3018	N	2786.1775	1393.5924	2769.1510	1385.0791	2768.1670	1384.5871	24
13	1370.6546	685.8310	1353.6281	677.3177	1352.6441	676.8257	T	2672.1346	1336.5709	2655.1080	1328.0577	2654.1240	1327.5657	23
14	1498.7132	749.8603	1481.6867	741.3470	1480.7027	740.8550	Q	2571.0869	1286.0471	2554.0604	1277.5338	2553.0764	1277.0418	22
15	1599.7609	800.3841	1582.7344	791.8708	1581.7503	791.3788	T	2443.0283	1222.0178	2426.0018	1213.5045	2425.0178	1213.0125	21
16	1696.8137	848.9105	1679.7871	840.3972	1678.8031	839.9052	P	2341.9807	1171.4940	2324.9541	1162.9807	2323.9701	1162.4887	20
17	1797.8613	899.4343	1780.8348	890.9210	1779.8508	890.4290	T	2244.9279	1122.9676	2227.9013	1114.4543	2226.9173	1113.9623	19
18	1896.9298	948.9685	1879.9032	940.4552	1878.9192	939.9632	V	2143.8802	1072.4437	2126.8537	1063.9305	2125.8697	1063.4385	18
19	2024.9883	1012.9978	2007.9618	1004.4845	2006.9778	1003.9925	Q	2044.8118	1022.9095	2027.7853	1014.3963	2026.8012	1013.9043	17
20	2154.0309	1077.5191	2137.0044	1069.0058	2136.0204	1068.5138	E	1916.7532	958.8803	1899.7267	950.3670	1898.7427	949.8750	16
21	2283.0735	1142.0404	2266.0470	1133.5271	2265.0630	1133.0351	E	1787.7106	894.3590	1770.6841	885.8457	1769.7001	885.3537	15
22	2450.0719	1225.5396	2433.0453	1217.0263	2432.0613	1216.5343	S	1658.6680	829.8377	1641.6415	821.3244	1640.6575	820.8324	14
23	2579.1145	1290.0609	2562.0879	1281.5476	2561.1039	1281.0556	E	1491.6697	746.3385	1474.6431	737.8252	1473.6591	737.3332	13
24	2708.1571	1354.5822	2691.1305	1346.0689	2690.1465	1345.5769	E	1362.6271	681.8172	1345.6005	673.3039	1344.6165	672.8119	12
25	2837.1997	1419.1035	2820.1731	1410.5902	2819.1891	1410.0982	E	1233.5845	617.2959	1216.5580	608.7826	1215.5739	608.2906	11
26	2966.2423	1483.6248	2949.2157	1475.1115	2948.2317	1474.6195	E	1104.5419	552.7746	1087.5154	544.2613	1086.5313	543.7693	10
27	3065.3107	1533.1590	3048.2841	1524.6457	3047.3001	1524.1537	V	975.4993	488.2533	958.4728	479.7400	957.4888	479.2480	9
28	3180.3376	1590.6724	3163.3111	1582.1592	3162.3270	1581.6672	D	876.4309	438.7191	859.4044	430.2058	858.4203	429.7138	8
29	3309.3802	1655.1937	3292.3537	1646.6805	3291.3696	1646.1885	E	761.4040	381.2056	744.3774	372.6923	743.3934	372.2003	7
30	3410.4279	1705.7176	3393.4013	1697.2043	3392.4173	1696.7123	T	632.3614	316.6843	615.3348	308.1710	614.3508	307.6790	6
31	3467.4493	1734.2283	3450.4228	1725.7150	3449.4388	1725.2230	G	531.3137	266.1605	514.2871	257.6472	513.3031	257.1552	5
32	3566.5178	1783.7625	3549.4912	1775.2492	3548.5072	1774.7572	V	474.2922	237.6498	457.2657	229.1365	456.2817	228.6445	4
33	3695.5604	1848.2838	3678.5338	1839.7705	3677.5498	1839.2785	E	375.2238	188.1155	358.1973	179.6023	357.2132	179.1103	3
34	3794.6288	1897.8180	3777.6022	1889.3047	3776.6182	1888.8127	V	246.1812	123.5942	229.1547	115.0810			2
35							K	147.1128	74.0600	130.0863	65.5468			1

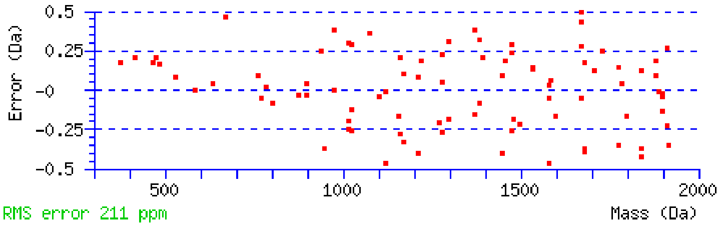

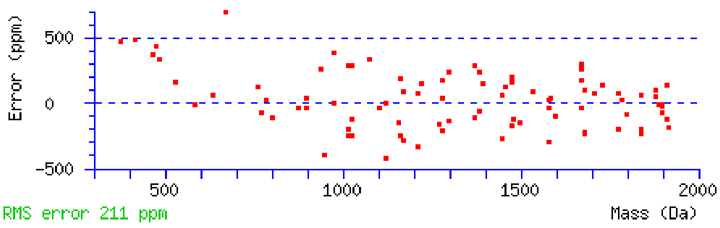

All matches to this query

Score	Mr(calc):	Delta	Sequence
67.5	3939.7270	1.0122	VQGEAVSNIQENTQTPTVQEESEEEEVDETGVEVK
60.2	3940.7110	0.0282	VQGEAVSNIQENTQTPTVQEESEEEEVDETGVEVK
55.8	3940.7110	0.0282	VQGEAVSNIQENTQTPTVQEESEEEEVDETGVEVK
54.7	3940.7110	0.0282	VQGEAVSNIQENTQTPTVQEESEEEEVDETGVEVK
53.1	3939.7270	1.0122	VQGEAVSNIQENTQTPTVQEESEEEEVDETGVEVK
52.6	3940.7110	0.0282	VQGEAVSNIQENTQTPTVQEESEEEEVDETGVEVK
46.1	3939.7270	1.0122	VQGEAVSNIQENTQTPTVQEESEEEEVDETGVEVK
43.0	3939.7270	1.0122	VQGEAVSNIQENTQTPTVQEESEEEEVDETGVEVK
42.5	3940.7110	0.0282	VQGEAVSNIQENTQTPTVQEESEEEEVDETGVEVK
42.0	3940.7110	0.0282	VQGEAVSNIQENTQTPTVQEESEEEEVDETGVEVK

Spectrum No: 115; Query: 2155; Rank: 1

Peptide View

MS/MS Fragmentation of **SKESLQEAGKSDANTDLIGGSPK**
Found in **IPI00214787**, Tax_Id=10116 Gene_Symbol=Slc16a1 Monocarboxylate transporter 1

Match to Query 2155: 2411.120052 from(804.713960,3+)
Title: 091008RatKidney_NH4Format01_19.1390.1390.3.dta
Data file K:\NewmanPaper\Piliang\3SubProteomes\Piliang3SP\mgf5ppm\ERLIC_3SubProteomes5ppm.mgf

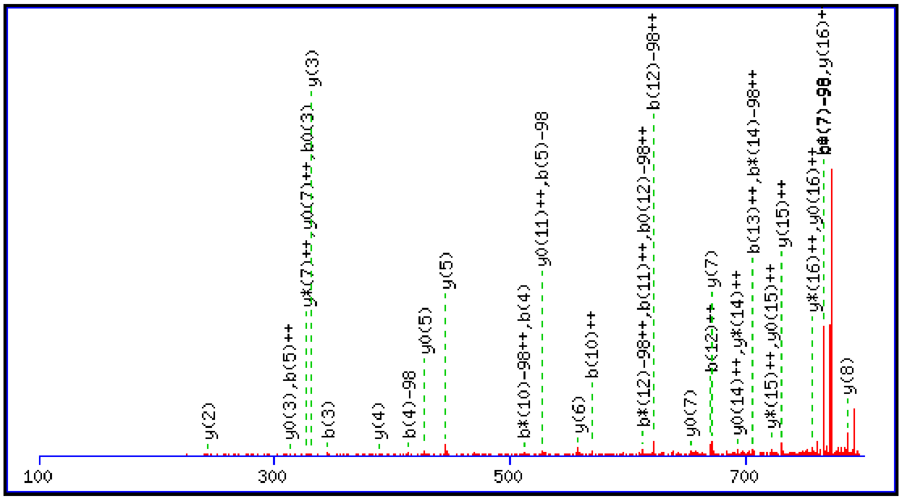

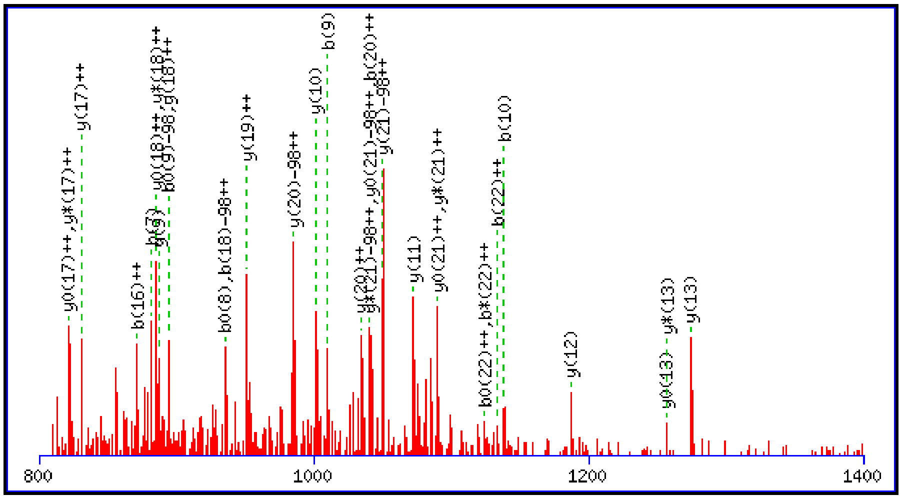

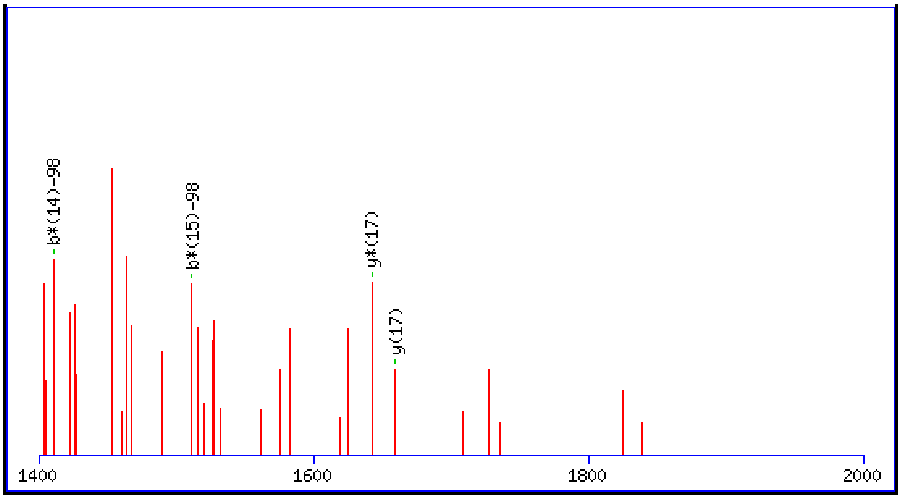

Monoisotopic mass of neutral peptide Mr(calc): 2411.1217
Fixed modifications: Carbamidomethyl (C)
Variable modifications:
S4 : Phospho (ST), with neutral losses 97.9769(shown in table), 0.0000
Ions Score: 67 **Expect:** 5.4e-005
Matches (Bold Red): 74/390 fragment ions using 104 most intense peaks

#	b	b^{++}	b*	b^{*++}	b^{0}	b^{0++}	Seq.	y	y^{++}	y*	y^{*++}	y^{0}	y^{0++}	#
1	88.0393	44.5233			70.0287	35.5180	S							23
2	216.1343	108.5708	199.1077	100.0575	198.1237	99.5655	K	2227.1201	1114.0637	2210.0935	1105.5504	2209.1095	1105.0584	22
3	**345.1769**	173.0921	328.1503	164.5788	**327.1663**	164.0868	E	2099.0251	**1050.0162**	2081.9986	**1041.5029**	2081.0145	**1041.0109**	21
4	**414.1983**	207.6028	397.1718	199.0895	396.1878	198.5975	S	1969.9825	**985.4949**	1952.9560	976.9816	1951.9720	976.4896	20
5	**527.2824**	264.1448	510.2558	255.6316	509.2718	255.1395	L	1900.9611	**950.9842**	1883.9345	942.4709	1882.9505	941.9789	19
6	655.3410	328.1741	638.3144	319.6608	637.3304	319.1688	Q	1787.8770	**894.4421**	1770.8504	**885.9289**	1769.8664	**885.4369**	18
7	784.3836	392.6954	**767.3570**	384.1821	**766.3730**	383.6901	E	**1659.8184**	**830.4128**	**1642.7919**	**821.8996**	1641.8079	**821.4076**	17
8	855.4207	428.2140	838.3941	419.7007	837.4101	419.2087	A	1530.7758	**765.8916**	1513.7493	**757.3783**	1512.7653	**756.8863**	16
9	912.4421	456.7247	895.4156	448.2114	**894.4316**	447.7194	G	1459.7387	**730.3730**	1442.7122	**721.8597**	1441.7281	**721.3677**	15
10	1040.5371	520.7722	1023.5105	**512.2589**	1022.5265	511.7669	K	1402.7172	701.8623	1385.6907	**693.3490**	1384.7067	**692.8570**	14
11	1127.5691	564.2882	1110.5426	555.7749	1109.5586	555.2829	S	**1274.6223**	637.8148	**1257.5957**	629.3015	**1256.6117**	628.8095	13
12	1242.5961	**621.8017**	1225.5695	**613.2884**	1224.5855	**612.7964**	D	**1187.5903**	594.2988	1170.5637	585.7855	1169.5797	585.2935	12
13	1313.6332	657.3202	1296.6066	648.8070	1295.6226	648.3149	A	**1072.5633**	536.7853	1055.5368	528.2720	1054.5527	**527.7800**	11
14	1427.6761	714.3417	**1410.6496**	**705.8284**	1409.6655	705.3364	N	**1001.5262**	501.2667	984.4997	492.7535	983.5156	492.2615	10
15	1528.7238	764.8655	**1511.6972**	756.3523	1510.7132	755.8602	T	**887.4833**	444.2453	870.4567	435.7320	869.4727	435.2400	9
16	1643.7507	822.3790	1626.7242	813.8657	1625.7402	813.3737	D	**786.4356**	393.7214	769.4090	385.2082	768.4250	384.7162	8
17	1756.8348	878.9210	1739.8082	870.4078	1738.8242	869.9158	L	**671.4087**	336.2080	654.3821	**327.6947**	**653.3981**	**327.2027**	7
18	1869.9189	**935.4631**	1852.8923	926.9498	1851.9083	926.4578	I	**558.3246**	279.6659	541.2980	271.1527	540.3140	270.6606	6
19	1926.9403	963.9738	1909.9138	955.4605	1908.9298	954.9685	G	**445.2405**	223.1239	428.2140	214.6106	**427.2300**	214.1186	5
20	1983.9618	992.4845	1966.9352	983.9713	1965.9512	983.4792	G	**388.2191**	194.6132	371.1925	186.0999	370.2085	185.6079	4
21	2070.9938	1036.0005	2053.9673	1027.4873	2052.9832	1026.9953	S	**331.1976**	166.1024	314.1710	157.5892	**313.1870**	157.0972	3
22	2168.0466	1084.5269	2151.0200	1076.0137	2150.0360	1075.5216	P	**244.1656**	122.5864	227.1390	114.0731			2
23							K	147.1128	74.0600	130.0863	65.5468			1

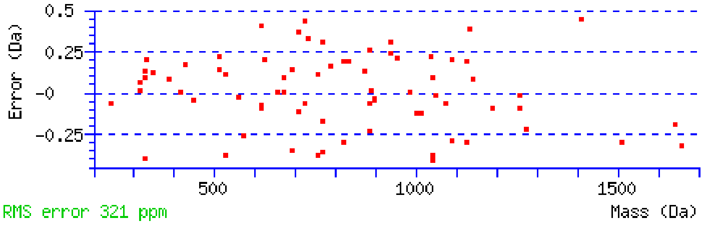

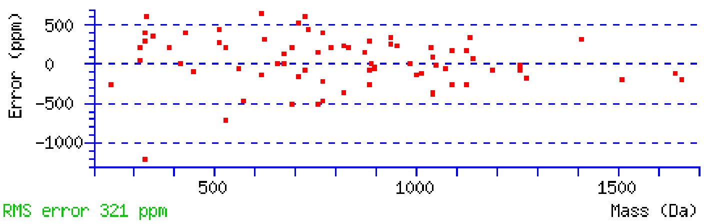

All matches to this query

Score	Mr(calc):	Delta	Sequence
67.4	2411.1217	-0.0016	SKESLQEAGKSDANTDLIGGSPK
64.9	2411.1217	-0.0016	SKESLQEAGKSDANTDLIGGSPK
34.2	2411.1217	-0.0016	SKESLQEAGKSDANTDLIGGSPK
16.1	2411.1217	-0.0016	SKESLQEAGKSDANTDLIGGSPK
11.8	2410.0948	1.0253	SSVDSDTGQTEIRAMKAEIHR
11.8	2410.0948	1.0253	SSVDSDTGQTEIRAMKAEIHR
11.8	2410.0948	1.0253	SSVDSDTGQTEIRAMKAEIHR
9.7	2411.1217	-0.0016	SKESLQEAGKSDANTDLIGGSPK
9.2	2411.1198	0.0002	MAAAAAAAAAAMTAAGGGGAGAARSLSR
5.3	2411.0966	0.0235	LSSKMRLHPLPAMTSCPTPK

Spectrum No: 116; Query: 1503; Rank: 1

Peptide View

MS/MS Fragmentation of **AESPETSAVESTQSTPQK**
Found in **IPI00764370**, Tax_Id=10116 Gene_Symbol=Aprin_predicted androgen-induced proliferation inhibitor isoform 1

Match to Query 1503: 1955.838628 from(978.926590,2+)
Title: 100101RatKid_NS_deglyco_21.1085.1085.2.dta
Data file K:\NewmanPaper\Piliang\3SubProteomes\Piliang3SP\mgf5ppm\ERLIC_3SubProteomes5ppm.mgf

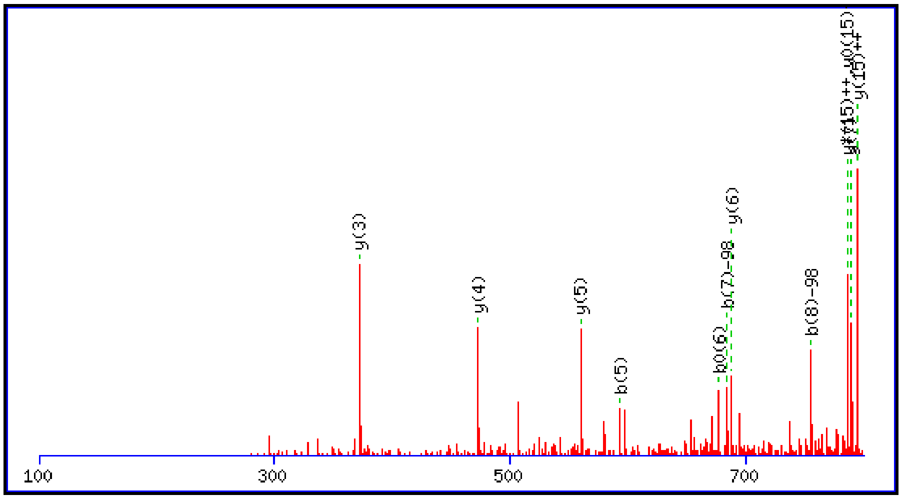

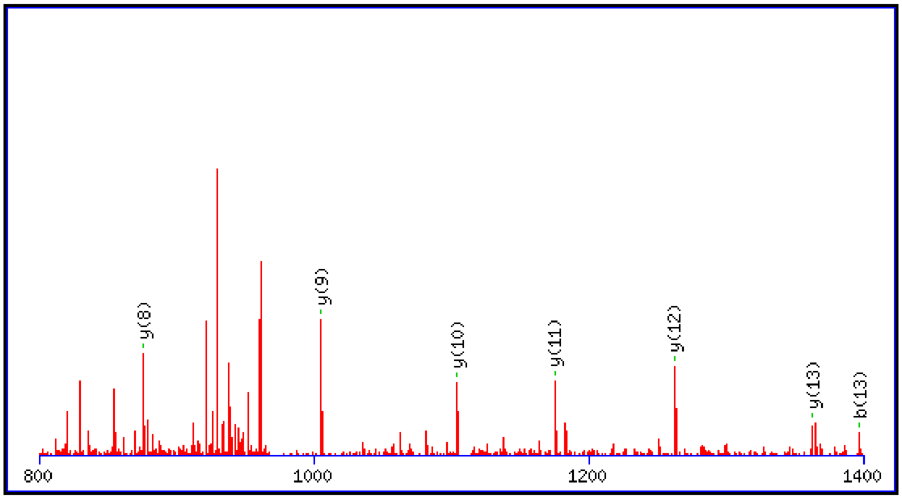

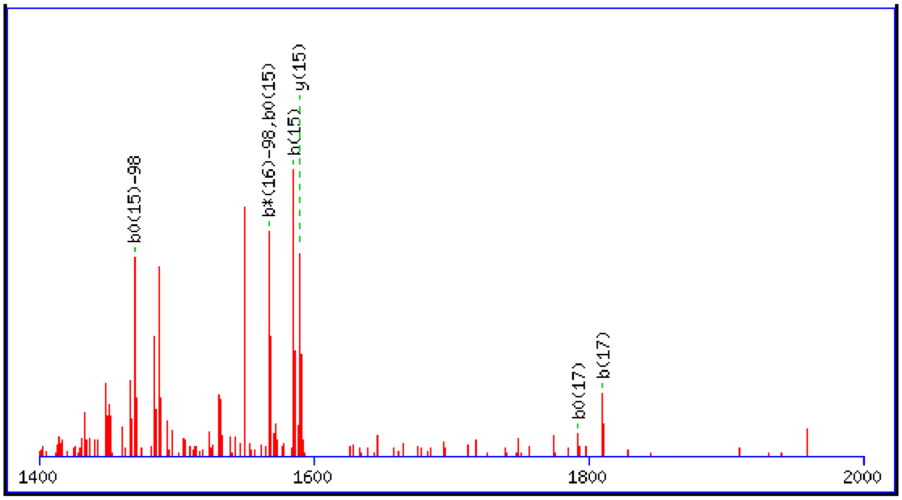

Monoisotopic mass of neutral peptide Mr(calc): 1955.8361
Fixed modifications: Carbamidomethyl (C)
Variable modifications:
S3 : Phospho (ST), with neutral losses 0.0000(shown in table), 97.9769
Ions Score: 67 **Expect:** 3.9e-005
Matches (Bold Red): 26/254 fragment ions using 42 most intense peaks

#	b	b^{++}	b*	b^{*++}	b^{0}	b^{0++}	Seq.	y	y^{++}	y*	y^{*++}	y^{0}	y^{0++}	#
1	72.0444	36.5258					A							18
2	201.0870	101.0471			183.0764	92.0418	E	1885.8063	943.4068	1868.7797	934.8935	1867.7957	934.4015	17
3	368.0853	184.5463			350.0748	175.5410	S	1756.7637	878.8855	1739.7371	870.3722	1738.7531	869.8802	16
4	465.1381	233.0727			447.1275	224.0674	P	**1589.7653**	**795.3863**	1572.7388	**786.8730**	1571.7548	**786.3810**	15
5	**594.1807**	297.5940			576.1701	288.5887	E	1492.7126	746.8599	1475.6860	738.3466	1474.7020	737.8546	14
6	695.2284	348.1178			**677.2178**	339.1125	T	**1363.6700**	682.3386	1346.6434	673.8253	1345.6594	673.3333	13
7	782.2604	391.6338			764.2498	382.6286	S	**1262.6223**	631.8148	1245.5957	623.3015	1244.6117	622.8095	12
8	853.2975	427.1524			835.2869	418.1471	A	**1175.5903**	588.2988	1158.5637	579.7855	1157.5797	579.2935	11
9	952.3659	476.6866			934.3554	467.6813	V	**1104.5531**	552.7802	1087.5266	544.2669	1086.5426	543.7749	10
10	1081.4085	541.2079			1063.3980	532.2026	E	**1005.4847**	503.2460	988.4582	494.7327	987.4742	494.2407	9
11	1168.4405	584.7239			1150.4300	575.7186	S	**876.4421**	438.7247	859.4156	430.2114	858.4316	429.7194	8
12	1269.4882	635.2478			1251.4777	626.2425	T	**789.4101**	395.2087	772.3836	386.6954	771.3995	386.2034	7
13	**1397.5468**	699.2770	1380.5203	690.7638	1379.5362	690.2718	Q	**688.3624**	344.6849	671.3359	336.1716	670.3519	335.6796	6
14	1484.5788	742.7931	1467.5523	734.2798	1466.5683	733.7878	S	**560.3039**	280.6556	543.2773	272.1423	542.2933	271.6503	5
15	**1585.6265**	793.3169	1568.6000	784.8036	**1567.6159**	784.3116	T	**473.2718**	237.1396	456.2453	228.6263	455.2613	228.1343	4
16	1682.6793	841.8433	1665.6527	833.3300	1664.6687	832.8380	P	**372.2241**	186.6157	355.1976	178.1024			3
17	**1810.7379**	905.8726	1793.7113	897.3593	**1792.7273**	896.8673	Q	275.1714	138.0893	258.1448	129.5761			2
18							K	147.1128	74.0600	130.0863	65.5468			1

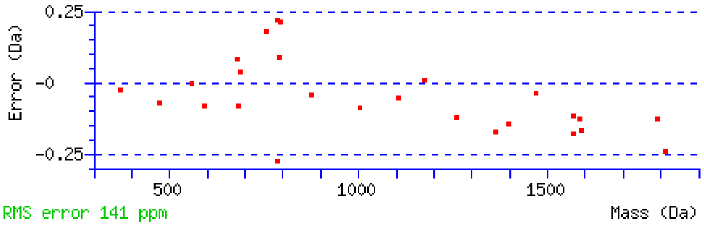

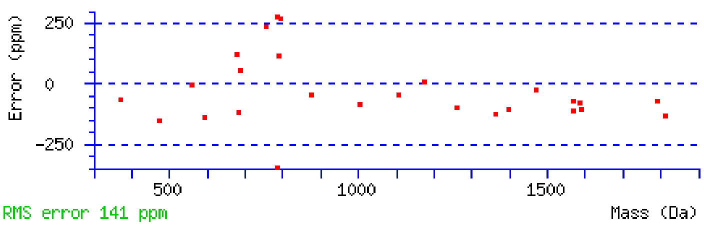

All matches to this query

Score	Mr(calc):	Delta	Sequence
67.4	1955.8361	0.0025	AESPETSAVESTQSTPQK
41.7	1955.8361	0.0025	AESPETSAVESTQSTPQK
29.7	1955.8361	0.0025	AESPETSAVESTQSTPQK

4.8	1955.8361	0.0025	AESPETSAVESTQSTPQK
4.0	1954.8187	1.0200	EYITSSVAQQSSSPRR
3.0	1955.8361	0.0025	AESPETSAVESTQSTPQK

Spectrum No: 117; Query: 1498; Rank: 1

Peptide View

MS/MS Fragmentation of **DADEEDSDEETSHLER**
Found in **IPI00656420**, Tax_Id=10116 Gene_Symbol=LOC683897 Similar to Protein C6orf203

Match to Query 1498: 1955.692048 from(978.853300,2+)
Title: 091008RatKidney_NH4Format01_26.519.519.2.dta
Data file K:\NewmanPaper\Piliang\3SubProteomes\Piliang3SP\mgf5ppm\ERLIC_3SubProteomes5ppm.mgf

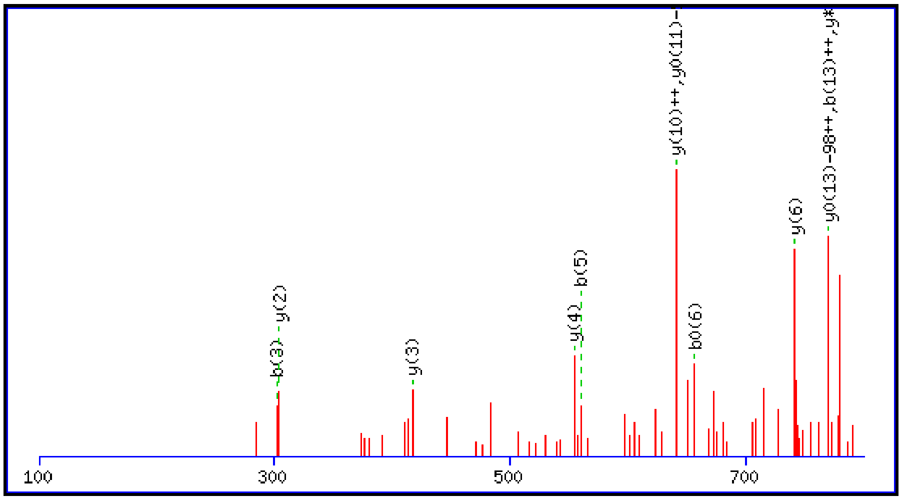

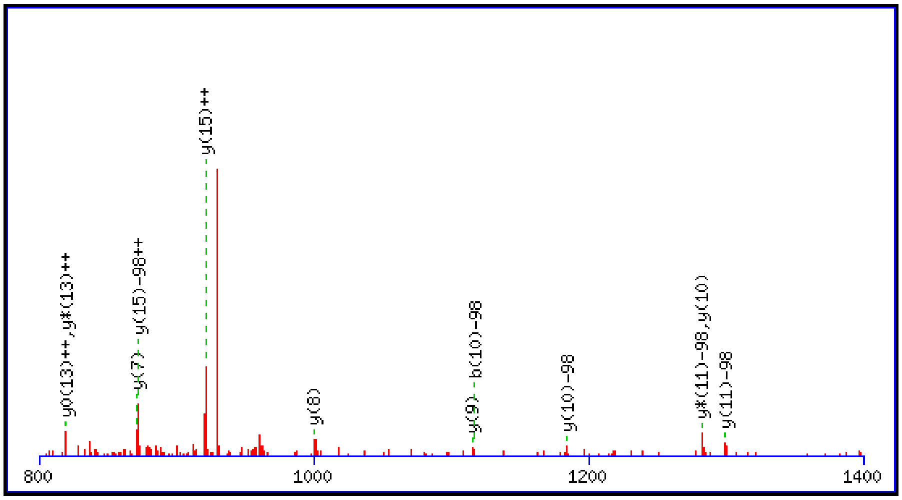

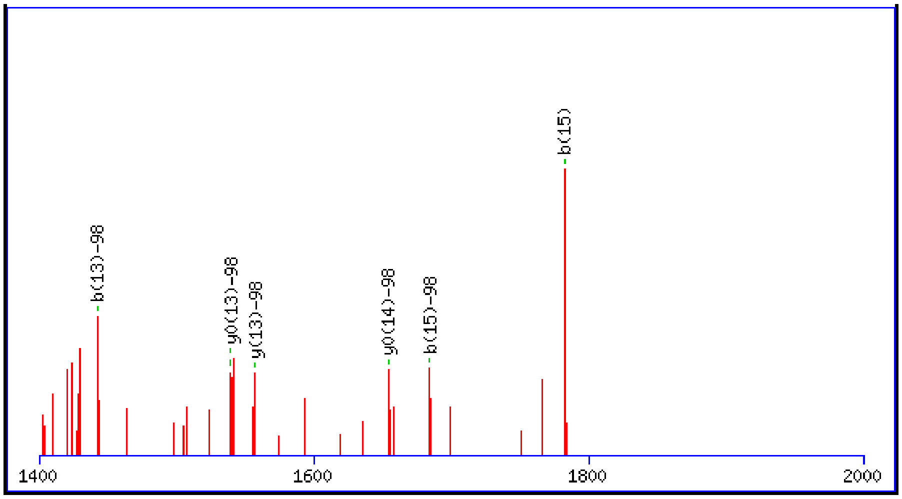

Monoisotopic mass of neutral peptide Mr(calc): 1955.6905
Fixed modifications: Carbamidomethyl (C)
Variable modifications:
S7 : Phospho (ST), with neutral losses 97.9769(shown in table), 0.0000
Ions Score: 67 **Expect:** 1.6e-005
Matches (Bold Red): 32/220 fragment ions using 37 most intense peaks

#	b	b^{++}	b^0	b^{0++}	Seq.	y	y^{++}	y*	y^{*++}	y^0	y^{0++}	#
1	116.0342	58.5207	98.0237	49.5155	D							16
2	187.0713	94.0393	169.0608	85.0340	A	1743.6940	**872.3506**	1726.6675	863.8374	1725.6834	863.3454	15
3	**302.0983**	151.5528	284.0877	142.5475	D	1672.6569	836.8321	1655.6303	828.3188	**1654.6463**	827.8268	14
4	431.1409	216.0741	413.1303	207.0688	E	**1557.6299**	779.3186	1540.6034	**770.8053**	**1539.6194**	**770.3133**	13
5	**560.1835**	280.5954	542.1729	271.5901	E	1428.5873	714.7973	1411.5608	706.2840	1410.5768	705.7920	12
6	675.2104	338.1088	**657.1998**	329.1036	D	**1299.5448**	650.2760	**1282.5182**	**641.7627**	1281.5342	**641.2707**	11
7	744.2319	372.6196	726.2213	363.6143	S	**1184.5178**	592.7625	1167.4913	584.2493	1166.5072	583.7573	10
8	859.2588	430.1330	841.2482	421.1278	D	**1115.4964**	558.2518	1098.4698	549.7385	1097.4858	549.2465	9
9	988.3014	494.6543	970.2908	485.6491	E	**1000.4694**	500.7383	983.4429	492.2251	982.4588	491.7331	8
10	**1117.3440**	559.1756	1099.3334	550.1704	E	**871.4268**	436.2170	854.4003	427.7038	853.4163	427.2118	7
11	1218.3917	609.6995	1200.3811	600.6942	T	**742.3842**	371.6958	725.3577	363.1825	724.3737	362.6905	6
12	1305.4237	653.2155	1287.4131	644.2102	S	**641.3365**	321.1719	624.3100	312.6586	623.3260	312.1666	5
13	**1442.4826**	721.7449	1424.4720	712.7397	H	**554.3045**	277.6559	537.2780	269.1426	536.2940	268.6506	4
14	1555.5667	778.2870	1537.5561	769.2817	L	**417.2456**	209.1264	400.2191	200.6132	399.2350	200.1212	3
15	**1684.6093**	842.8083	1666.5987	833.8030	E	**304.1615**	152.5844	287.1350	144.0711	286.1510	143.5791	2
16					R	175.1190	88.0631	158.0924	79.5498			1

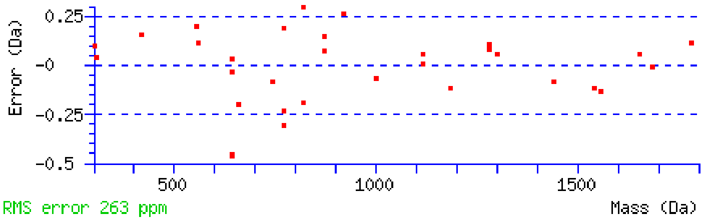

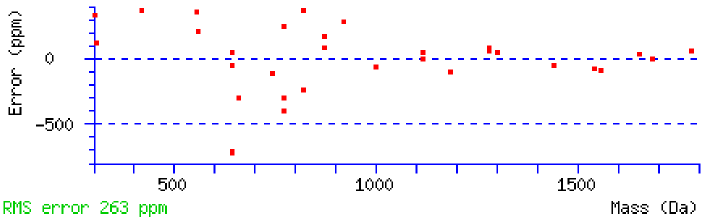

All matches to this query

Score	Mr(calc):	Delta	Sequence
67.3	1955.6905	0.0015	DADEEDSDEETSHLER
26.3	1955.6905	0.0015	DADEEDSDEETSHLER
19.6	1955.6905	0.0015	DADEEDSDEETSHLER
1.2	1955.7078	-0.0157	EDHHRSQGGSPPVTGR
0.7	1955.6969	-0.0048	MDNISRNVGNASESQR

0.7	1955.6969	-0.0048	MDNISRNVGNASESQR
0.6	1955.6905	0.0015	NFSDMKMMLSEYQR
0.1	1955.6905	0.0015	NFSDMKMMLSEYQR
0.1	1954.6822	1.0099	STNISFTNMVSVDER

Spectrum No: 118; Query: 2640; Rank: 1

Peptide View

MS/MS Fragmentation of **GDGGSTPTPGDSLQNPDTSSEALSEPESQRR**
Found in **IPI00372081**, Tax_Id=10116 Gene_Symbol=RGD1310887_predicted hypothetical protein LOC296623

Match to Query 2640: 3251.380572 from(1084.800800,3+)
Title: 091008RatKidney_NH4Format01_24.1807.1807.3.dta
Data file K:\NewmanPaper\Piliang\3SubProteomes\Piliang3SP\mgf5ppm\ERLIC_3SubProteomes5ppm.mgf

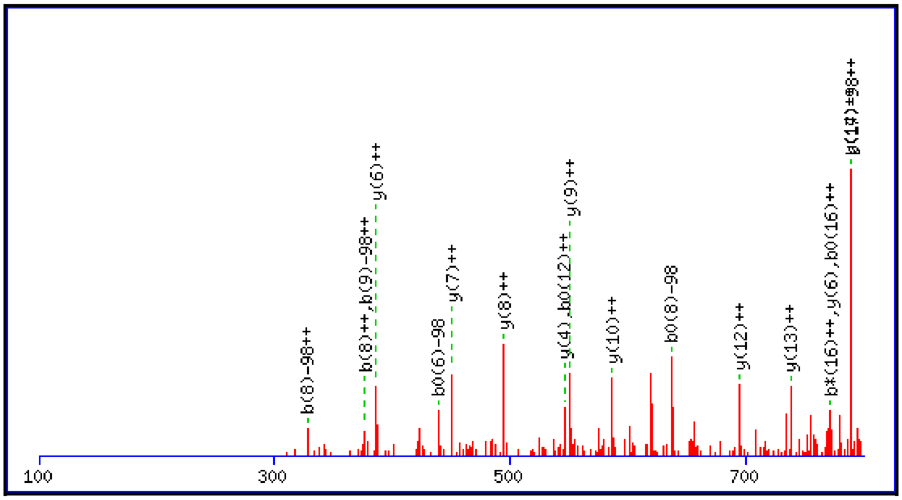

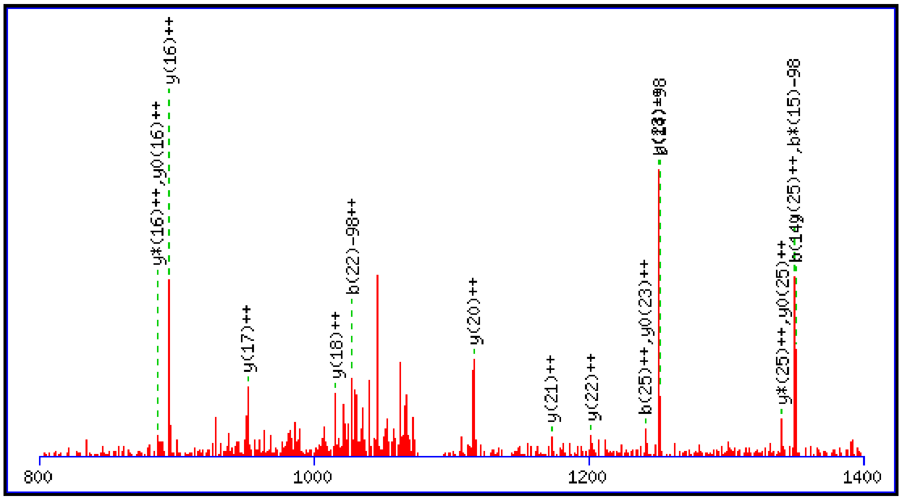

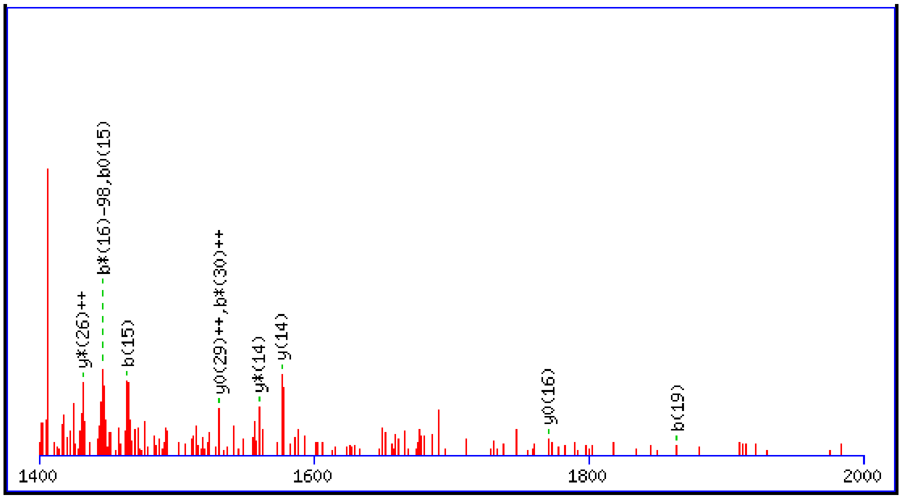

Monoisotopic mass of neutral peptide Mr(calc): 3251.3852
Fixed modifications: Carbamidomethyl (C)
Variable modifications:
T6 : Phospho (ST), with neutral losses 0.0000(shown in table), 97.9769
Ions Score: 67 **Expect:** 7.8e-005
Matches (Bold Red): 47/490 fragment ions using 70 most intense peaks

#	b	b^{++}	b^{*}	b^{*++}	b^{0}	b^{0++}	Seq.	y	y^{++}	y^{*}	y^{*++}	y^{0}	y^{0++}	#
1	58.0287	29.5180					G							31
2	173.0557	87.0315			155.0451	78.0262	D	3195.3710	1598.1891	3178.3444	1589.6759	3177.3604	1589.1838	30
3	230.0771	115.5422			212.0666	106.5369	G	3080.3440	1540.6757	3063.3175	1532.1624	3062.3335	**1531.6704**	29
4	287.0986	144.0529			269.0880	135.0477	G	3023.3226	1512.1649	3006.2960	1503.6516	3005.3120	1503.1596	28
5	374.1306	187.5690			356.1201	178.5637	S	2966.3011	1483.6542	2949.2746	1475.1409	2948.2905	1474.6489	27
6	555.1446	278.0760			537.1341	269.0707	T	2879.2691	1440.1382	2862.2425	**1431.6249**	2861.2585	1431.1329	26
7	652.1974	326.6023			634.1868	317.5971	P	2698.2551	**1349.6312**	2681.2285	**1341.1179**	2680.2445	**1340.6259**	25
8	753.2451	**377.1262**			735.2345	368.1209	T	2601.2023	1301.1048	2584.1758	1292.5915	2583.1917	1292.0995	24
9	850.2978	425.6526			832.2873	416.6473	P	2500.1546	**1250.5810**	2483.1281	1242.0677	2482.1441	**1241.5757**	23
10	907.3193	454.1633			889.3087	445.1580	G	2403.1019	**1202.0546**	2386.0753	1193.5413	2385.0913	1193.0493	22
11	1022.3463	511.6768			1004.3357	502.6715	D	2346.0804	**1173.5438**	2329.0539	1165.0306	2328.0698	1164.5386	21
12	1109.3783	555.1928			1091.3677	**546.1875**	S	2231.0535	**1116.0304**	2214.0269	1107.5171	2213.0429	1107.0251	20
13	1222.4623	611.7348			1204.4518	602.7295	L	2144.0214	1072.5144	2126.9949	1064.0011	2126.0109	1063.5091	19
14	**1350.5209**	675.7641	1333.4944	667.2508	1332.5104	666.7588	Q	2030.9374	**1015.9723**	2013.9108	1007.4590	2012.9268	1006.9670	18
15	**1464.5639**	732.7856	1447.5373	724.2723	**1446.5533**	723.7803	N	1902.8788	**951.9430**	1885.8522	943.4298	1884.8682	942.9377	17
16	1561.6166	781.3119	1544.5901	**772.7987**	1543.6061	**772.3067**	P	1788.8359	**894.9216**	1771.8093	**886.4083**	**1770.8253**	**885.9163**	16
17	1676.6436	838.8254	1659.6170	830.3121	1658.6330	829.8201	D	1691.7831	846.3952	1674.7565	837.8819	1673.7725	837.3899	15
18	1777.6912	889.3493	1760.6647	880.8360	1759.6807	880.3440	T	**1576.7562**	**788.8817**	**1559.7296**	780.3684	1558.7456	779.8764	14
19	**1864.7233**	932.8653	1847.6967	924.3520	1846.7127	923.8600	S	1475.7085	**738.3579**	1458.6819	729.8446	1457.6979	729.3526	13
20	1951.7553	976.3813	1934.7287	967.8680	1933.7447	967.3760	S	1388.6764	**694.8419**	1371.6499	686.3286	1370.6659	685.8366	12
21	2080.7979	1040.9026	2063.7713	1032.3893	2062.7873	1031.8973	E	1301.6444	651.3258	1284.6179	642.8126	1283.6339	642.3206	11
22	2151.8350	1076.4211	2134.8085	1067.9079	2133.8244	1067.4159	A	1172.6018	**586.8046**	1155.5753	578.2913	1154.5913	577.7993	10
23	2264.9191	1132.9632	2247.8925	1124.4499	2246.9085	1123.9579	L	1101.5647	**551.2860**	1084.5382	542.7727	1083.5541	542.2807	9
24	2351.9511	1176.4792	2334.9245	1167.9659	2333.9405	1167.4739	S	988.4806	**494.7440**	971.4541	486.2307	970.4701	485.7387	8
25	2480.9937	**1241.0005**	2463.9671	1232.4872	2462.9831	1231.9952	E	901.4486	**451.2279**	884.4221	442.7147	883.4381	442.2227	7
26	2578.0465	1289.5269	2561.0199	1281.0136	2560.0359	1280.5216	P	**772.4060**	**386.7067**	755.3795	378.1934	754.3955	377.7014	6
27	2707.0890	1354.0482	2690.0625	1345.5349	2689.0785	1345.0429	E	675.3533	338.1803	658.3267	329.6670	657.3427	329.1750	5
28	2794.1211	1397.5642	2777.0945	1389.0509	2776.1105	1388.5589	S	**546.3107**	273.6590	529.2841	265.1457	528.3001	264.6537	4
29	2922.1797	1461.5935	2905.1531	1453.0802	2904.1691	1452.5882	Q	459.2786	230.1430	442.2521	221.6297			3
30	3078.2808	1539.6440	3061.2542	**1531.1307**	3060.2702	1530.6387	R	331.2201	166.1137	314.1935	157.6004			2
31							R	175.1190	88.0631	158.0924	79.5498			1

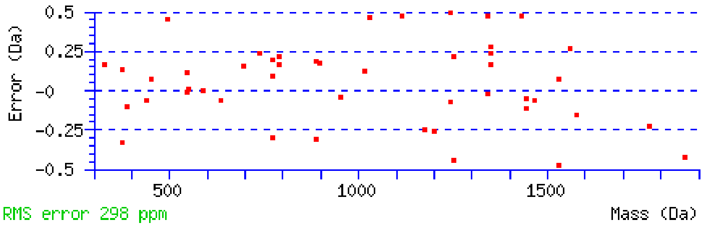

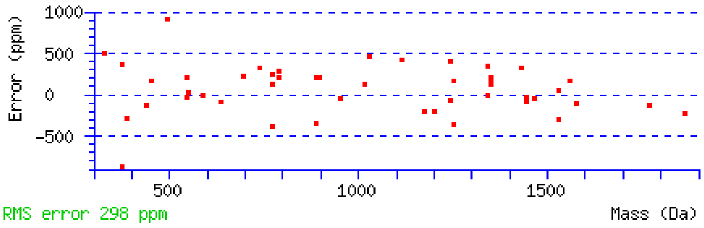

All matches to this query

Score	Mr(calc):	Delta	Sequence
67.2	3251.3852	-0.0046	GDGGSTPTPGDSLQNPDTSSEALSEPESQRR
65.6	3251.3852	-0.0046	GDGGSTPTPGDSLQNPDTSSEALSEPESQRR
65.1	3251.3852	-0.0046	GDGGSTPTPGDSLQNPDTSSEALSEPESQRR
32.7	3251.3852	-0.0046	GDGGSTPTPGDSLQNPDTSSEALSEPESQRR
9.6	3251.3852	-0.0046	GDGGSTPTPGDSLQNPDTSSEALSEPESQRR
9.6	3249.4001	1.9804	SAVMSISSVTENGGSPQGIKSPMKPPGPNR
7.0	3251.3883	-0.0077	NSFVMEYVGEIITSEEAERRGQIYDR
5.3	3251.3852	-0.0046	GDGGSTPTPGDSLQNPDTSSEALSEPESQRR
4.6	3250.3842	0.9964	SAVMSISSVTENGGSPQGIKSPMKPPGPNR
3.7	3250.3842	0.9964	SAVMSISSVTENGGSPQGIKSPMKPPGPNR

Spectrum No: 119; Query: 1164; Rank: 1

Peptide View

MS/MS Fragmentation of **SRTASGSSVTSLEGTR**
Found in **IPI00421389**, Tax_Id=10116 Gene_Symbol=Ndrg1 Protein NDRG1

Match to Query 1164: 1754.715668 from(878.365110,2+)
Title: 091008RatKidney_NoSalt_25.998.998.2.dta
Data file K:\NewmanPaper\Piliang\3SubProteomes\Piliang3SP\mgf5ppm\ERLIC_3SubProteomes5ppm.mgf

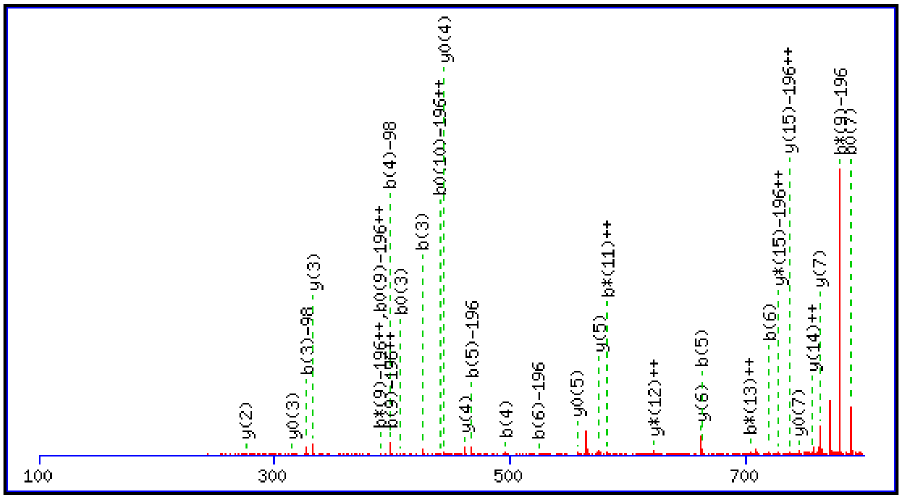

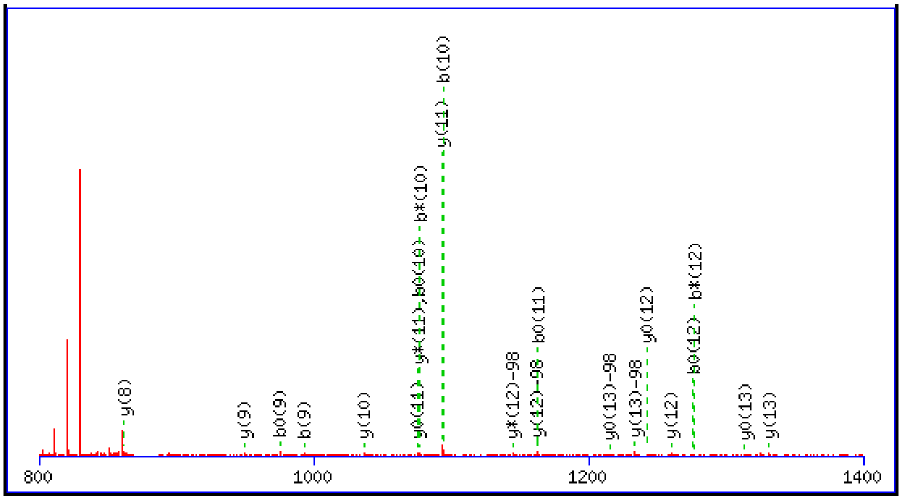

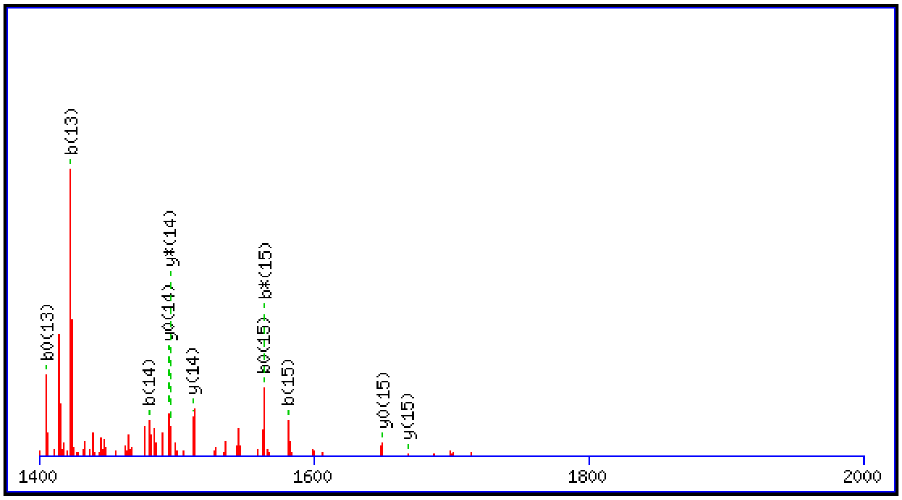

Monoisotopic mass of neutral peptide Mr(calc): 1754.7237
Fixed modifications: Carbamidomethyl (C)
Variable modifications:
T3 : Phospho (ST), with neutral losses 0.0000(shown in table), 97.9769
S5 : Phospho (ST), with neutral losses 0.0000(shown in table), 97.9769
Ions Score: 67 **Expect:** 3e-005
Matches (Bold Red): 64/278 fragment ions using 131 most intense peaks

#	b	b^{++}	b*	b^{*++}	b^0	b^{0++}	Seq.	y	y^{++}	y*	y^{*++}	y^0	y^{0++}	#
1	88.0393	44.5233			70.0287	35.5180	S							16
2	244.1404	122.5738	227.1139	114.0606	226.1298	113.5686	R	**1668.6990**	834.8531	1651.6724	826.3398	**1650.6884**	825.8478	15
3	**425.1544**	213.0808	408.1279	204.5676	**407.1439**	204.0756	T	**1512.5979**	**756.8026**	**1495.5713**	748.2893	**1494.5873**	747.7973	14
4	**496.1915**	248.5994	479.1650	240.0861	478.1810	239.5941	A	**1331.5839**	666.2956	1314.5573	657.7823	**1313.5733**	657.2903	13
5	**663.1899**	332.0986	646.1633	323.5853	645.1793	323.0933	S	**1260.5467**	630.7770	1243.5202	**622.2637**	**1242.5362**	621.7717	12
6	**720.2113**	360.6093	703.1848	352.0960	702.2008	351.6040	G	**1093.5484**	547.2778	**1076.5218**	538.7646	**1075.5378**	538.2726	11
7	807.2434	404.1253	790.2168	395.6121	**789.2328**	395.1200	S	**1036.5269**	518.7671	1019.5004	510.2538	1018.5164	509.7618	10
8	894.2754	447.6413	877.2489	439.1281	876.2648	438.6361	S	**949.4949**	475.2511	932.4684	466.7378	931.4843	466.2458	9
9	**993.3438**	497.1755	976.3173	488.6623	**975.3333**	488.1703	V	**862.4629**	431.7351	845.4363	423.2218	844.4523	422.7298	8
10	**1094.3915**	547.6994	**1077.3649**	539.1861	**1076.3809**	538.6941	T	**763.3945**	382.2009	746.3679	373.6876	**745.3839**	373.1956	7
11	1181.4235	591.2154	1164.3970	**582.7021**	**1163.4130**	582.2101	S	**662.3468**	331.6770	645.3202	323.1638	644.3362	322.6717	6
12	1294.5076	647.7574	**1277.4810**	639.2442	**1276.4970**	638.7521	L	**575.3148**	288.1610	558.2882	279.6477	**557.3042**	279.1557	5
13	**1423.5502**	712.2787	1406.5236	**703.7655**	**1405.5396**	703.2734	E	**462.2307**	231.6190	445.2041	223.1057	**444.2201**	222.6137	4
14	**1480.5716**	740.7895	1463.5451	732.2762	1462.5611	731.7842	G	**333.1881**	167.0977	316.1615	158.5844	**315.1775**	158.0924	3
15	**1581.6193**	791.3133	**1564.5928**	782.8000	**1563.6088**	782.3080	T	**276.1666**	138.5870	259.1401	130.0737	258.1561	129.5817	2
16							R	175.1190	88.0631	158.0924	79.5498			1

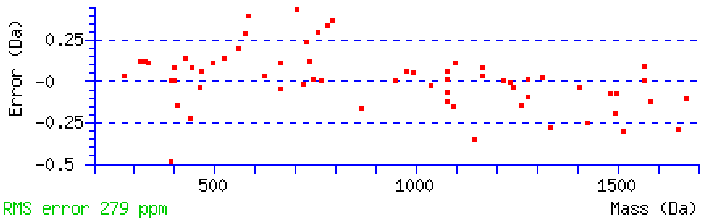

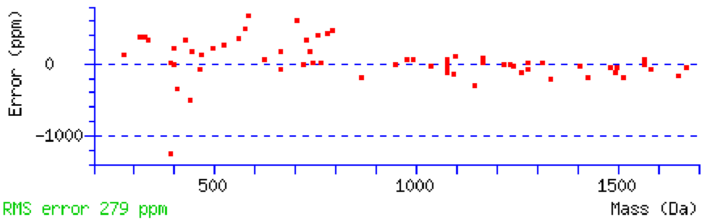

All matches to this query

Score	Mr(calc):	Delta	Sequence
67.0	1754.7237	-0.0081	SRTASGSSVTSLEGTR
31.9	1754.7237	-0.0081	SRTASGSSVTSLEGTR
27.8	1754.7237	-0.0081	SRTASGSSVTSLEGTR
20.6	1754.7237	-0.0081	SRTASGSSVTSLEGTR
9.6	1754.7237	-0.0081	SRTASGSSVTSLEGTR
4.8	1754.6987	0.0169	AVNISDSNMTLTWK
3.8	1752.7089	2.0067	GMCLSPSSLVSGRAR
3.8	1752.7089	2.0067	GMCLSPSSLVSGRAR
3.8	1754.7165	-0.0008	KYGTDPSTGEKLDGK
3.8	1754.7165	-0.0008	KYGTNPSTGEKLDGK

Spectrum No: 120; Query: 1371; Rank: 1

Peptide View

MS/MS Fragmentation of **VAGEAAETDSEPEPEPR**
Found in **IPI00367023**, Tax_Id=10116 Gene_Symbol=LOC686812;LOC680476 similar to reduced pigmentation protein

Match to Query 1371: 1862.757428 from(932.385990,2+)
Title: 091008RatKidney_NoSalt_27.721.721.2.dta
Data file K:\NewmanPaper\Piliang\3SubProteomes\Piliang3SP\mgf5ppm\ERLIC_3SubProteomes5ppm.mgf

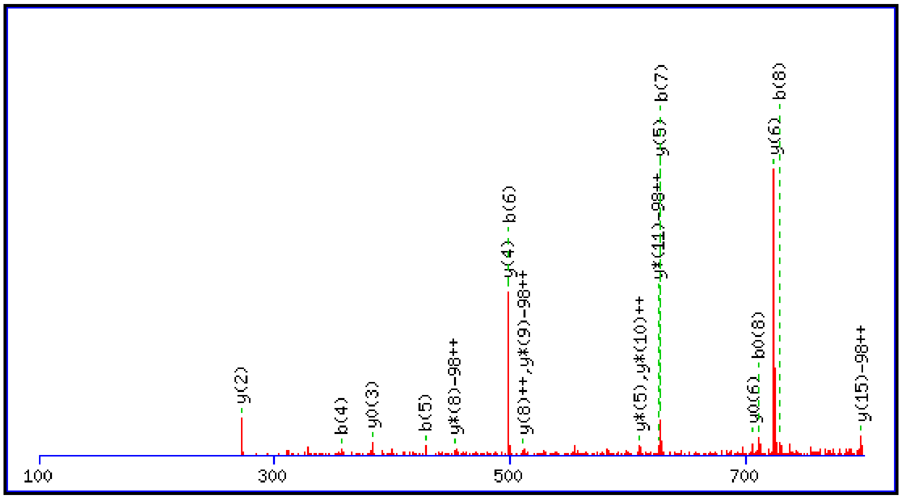

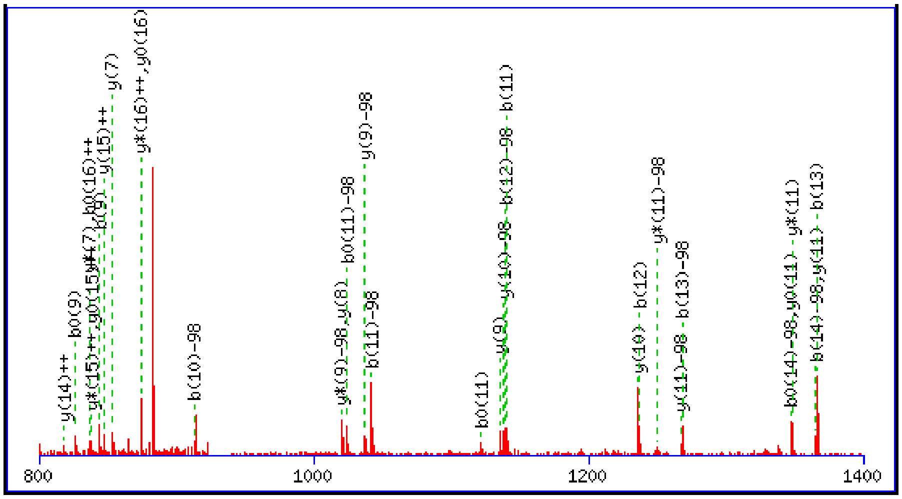

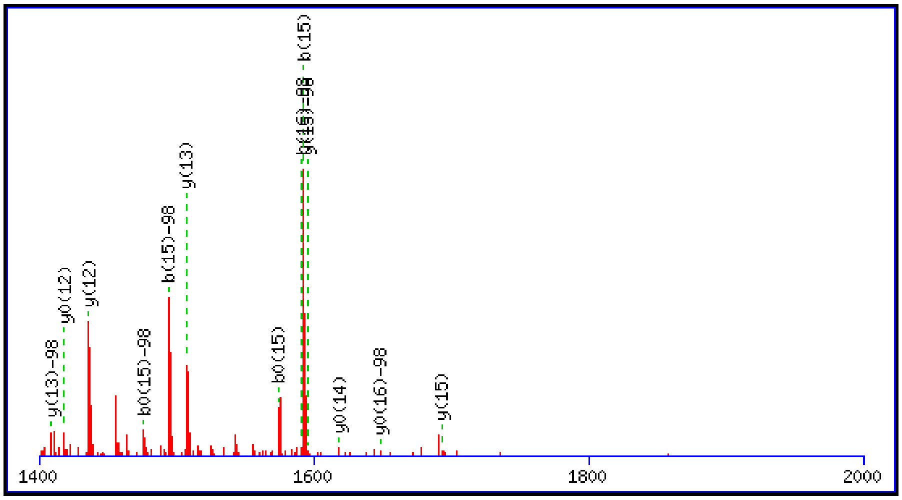

Monoisotopic mass of neutral peptide Mr(calc): 1862.7571
Fixed modifications: Carbamidomethyl (C)
Variable modifications:
S10 : Phospho (ST), with neutral losses 97.9769(shown in table), 0.0000
Ions Score: 67 **Expect:** 3.5e-005
Matches (Bold Red): 70/232 fragment ions using 115 most intense peaks

#	b	b^{++}	b^0	b^{0++}	Seq.	y	y^{++}	y^*	y^{*++}	y^0	y^{0++}	#
1	100.0757	50.5415			V							17
2	171.1128	86.0600			A	1666.7191	833.8632	1649.6925	825.3499	**1648.7085**	824.8579	16
3	228.1343	114.5708			G	**1595.6820**	**798.3446**	1578.6554	789.8314	1577.6714	789.3393	15
4	**357.1769**	179.0921	339.1663	170.0868	E	1538.6605	769.8339	1521.6340	761.3206	1520.6500	760.8286	14
5	**428.2140**	214.6106	410.2034	205.6053	A	**1409.6179**	705.3126	1392.5914	696.7993	1391.6074	696.3073	13
6	**499.2511**	250.1292	481.2405	241.1239	A	1338.5808	669.7940	1321.5543	661.2808	1320.5702	660.7888	12
7	**628.2937**	314.6505	**610.2831**	305.6452	E	**1267.5437**	634.2755	**1250.5171**	**625.7622**	1249.5331	625.2702	11
8	**729.3414**	365.1743	**711.3308**	356.1690	T	**1138.5011**	569.7542	1121.4746	561.2409	1120.4905	560.7489	10
9	**844.3683**	422.6878	**826.3577**	413.6825	D	**1037.4534**	519.2303	**1020.4269**	**510.7171**	1019.4429	510.2251	9
10	**913.3898**	457.1985	895.3792	448.1932	S	922.4265	461.7169	905.3999	**453.2036**	904.4159	452.7116	8
11	**1042.4324**	521.7198	**1024.4218**	512.7145	E	**853.4050**	427.2061	**836.3785**	418.6929	835.3945	418.2009	7
12	**1139.4851**	570.2462	1121.4746	561.2409	P	**724.3624**	362.6849	707.3359	354.1716	**706.3519**	353.6796	6
13	**1268.5277**	634.7675	**1250.5171**	**625.7622**	E	**627.3097**	314.1585	**610.2831**	305.6452	609.2991	305.1532	5
14	**1365.5805**	683.2939	**1347.5699**	674.2886	P	**498.2671**	249.6372	481.2405	241.1239	480.2565	240.6319	4
15	**1494.6231**	747.8152	**1476.6125**	738.8099	E	401.2143	201.1108	384.1878	192.5975	**383.2037**	192.1055	3
16	**1591.6758**	796.3416	1573.6653	787.3363	P	**272.1717**	136.5895	255.1452	128.0762			2
17					R	175.1190	88.0631	158.0924	79.5498			1

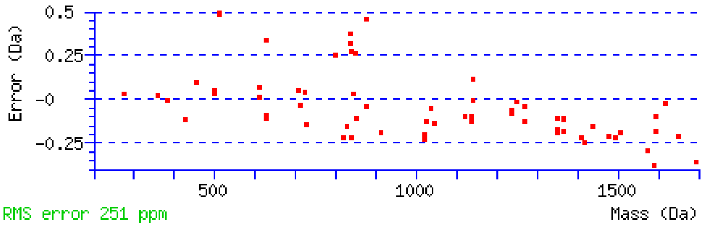

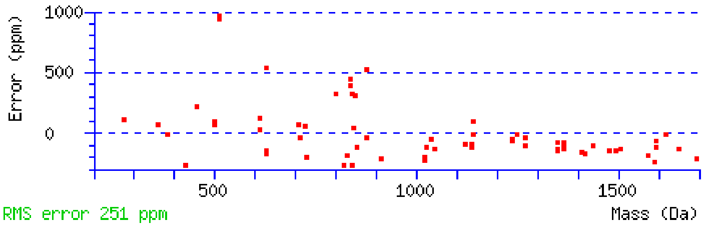

All matches to this query

Score	Mr(calc):	Delta	Sequence
66.8	1862.7571	0.0003	VAGEAAETDSEPEPEPR
62.1	1862.7571	0.0003	VAGEAAETDSEPEPEPR
8.6	1862.7749	-0.0174	MDYAMKSLSLLYPR
8.6	1862.7749	-0.0174	MDYAMKSLSLLYPR
8.4	1862.7462	0.0112	RRNTGGTGGDYAPAPGR
7.8	1862.7462	0.0112	RRNTGGTGGDYAPAPGR
5.6	1862.7749	-0.0174	MDYAMKSLSLLYPR
4.7	1862.7535	0.0039	AEAEQSILYAHCRR
3.8	1862.7749	-0.0174	MDYAMKSLSLLYPR
0.9	1862.7601	-0.0027	YVSVHVDNSRDLGNK

Spectrum No: 121; Query: 959; Rank: 1

Peptide View

MS/MS Fragmentation of **SASSGAEGDVSSEREP**
Found in **IPI00357936**, Tax_Id=10116 Gene_Symbol=Hars2_predicted histidyl tRNA synthetase 2

Match to Query 959: 1643.636648 from(822.825600,2+)
Title: 100101RatKid_NS_deglyco_23.832.832.2.dta
Data file K:\NewmanPaper\Piliang\3SubProteomes\Piliang3SP\mgf5ppm\ERLIC_3SubProteomes5ppm.mgf

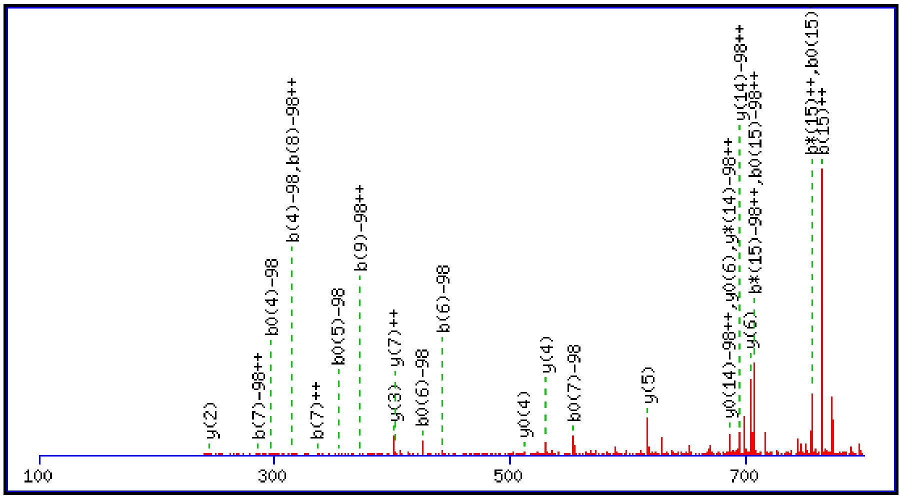

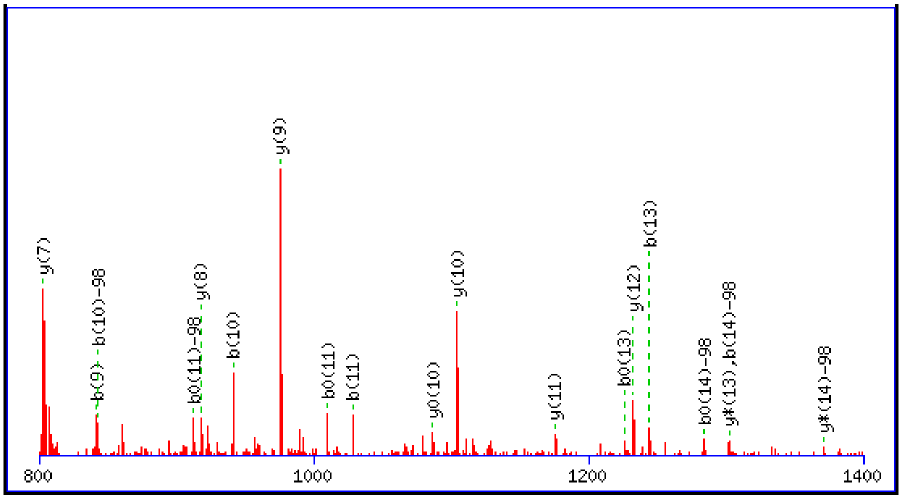

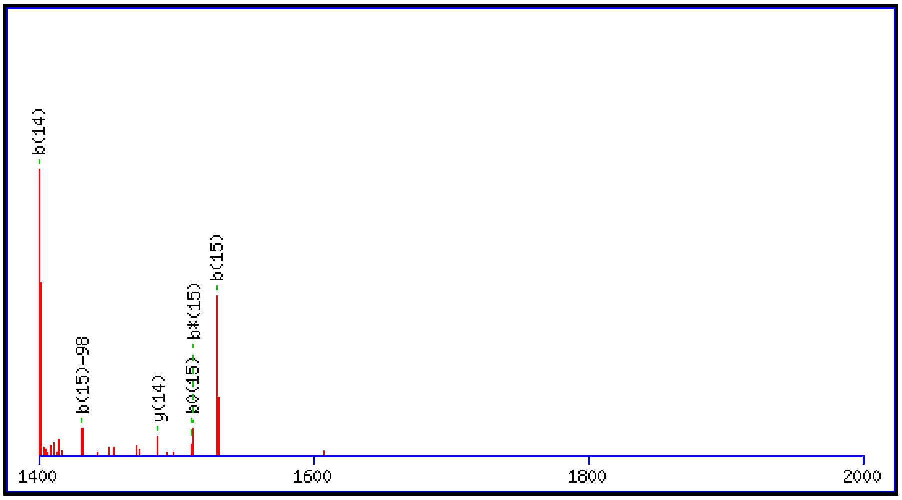

Monoisotopic mass of neutral peptide Mr(calc): 1643.6312
Fixed modifications: Carbamidomethyl (C)
Variable modifications:
S3 : Phospho (ST), with neutral losses 0.0000(shown in table), 97.9769
Ions Score: 67 **Expect:** 2.4e-005
Matches (Bold Red): 51/216 fragment ions using 75 most intense peaks

#	b	b^{++}	b*	b^{*++}	b^0	b^{0++}	Seq.	y	y^{++}	y*	y^{*++}	y^0	y^{0++}	#
1	88.0393	44.5233			70.0287	35.5180	S							16
2	159.0764	80.0418			141.0659	71.0366	A	1557.6064	779.3069	1540.5799	770.7936	1539.5959	770.3016	15
3	326.0748	163.5410			308.0642	154.5357	S	**1486.5693**	743.7883	1469.5428	735.2750	1468.5588	734.7830	14
4	413.1068	207.0570			395.0962	198.0518	S	1319.5710	660.2891	**1302.5444**	651.7759	1301.5604	651.2838	13
5	470.1283	235.5678			452.1177	226.5625	G	**1232.5390**	616.7731	1215.5124	608.2598	1214.5284	607.7678	12
6	541.1654	271.0863			523.1548	262.0810	A	**1175.5175**	588.2624	1158.4909	579.7491	1157.5069	579.2571	11
7	670.2080	**335.6076**			652.1974	326.6023	E	**1104.4804**	552.7438	1087.4538	544.2305	**1086.4698**	543.7385	10
8	727.2294	364.1184			709.2189	355.1131	G	**975.4378**	488.2225	958.4112	479.7093	957.4272	479.2172	9
9	**842.2564**	421.6318			824.2458	412.6265	D	**918.4163**	459.7118	901.3898	451.1985	900.4058	450.7065	8
10	**941.3248**	471.1660			923.3142	462.1608	V	**803.3894**	**402.1983**	786.3628	393.6850	785.3788	393.1930	7
11	**1028.3568**	514.6820			**1010.3463**	505.6768	S	**704.3210**	352.6641	687.2944	344.1508	**686.3104**	343.6588	6
12	1115.3888	558.1981			1097.3783	549.1928	S	**617.2889**	309.1481	600.2624	300.6348	599.2784	300.1428	5
13	**1244.4314**	622.7194			**1226.4209**	613.7141	E	**530.2569**	265.6321	513.2304	257.1188	**512.2463**	256.6268	4
14	**1400.5326**	700.7699	1383.5060	692.2566	1382.5220	691.7646	R	**401.2143**	201.1108	384.1878	192.5975	383.2037	192.1055	3
15	**1529.5751**	**765.2912**	**1512.5486**	**756.7779**	**1511.5646**	**756.2859**	E	**245.1132**	123.0602			227.1026	114.0550	2
16							P	116.0706	58.5389					1

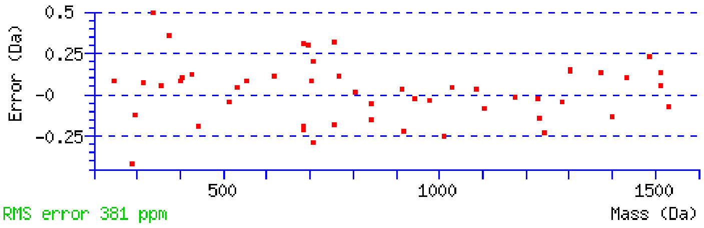

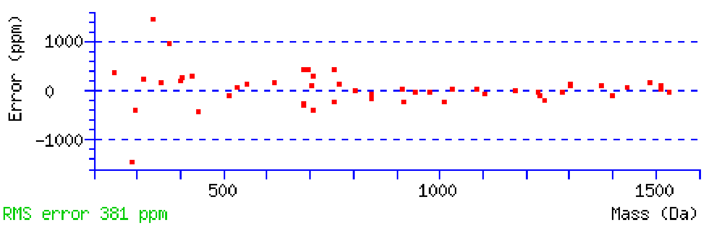

All matches to this query

Score	Mr(calc):	Delta	Sequence
66.7	1643.6312	0.0055	SASSGAEGDVSSEREP
66.2	1643.6312	0.0055	SASSGAEGDVSSEREP
65.0	1643.6312	0.0055	SASSGAEGDVSSEREP
16.4	1643.6312	0.0055	SASSGAEGDVSSEREP
9.4	1643.6312	0.0055	SASSGAEGDVSSEREP
8.4	1641.6398	1.9968	IEMFTEEESLVR
7.2	1643.6440	-0.0073	DDTEFQHPCFLR
7.0	1643.6225	0.0141	DEERGGMEAMESCK
7.0	1643.6303	0.0063	RPTESEPMGSSIPP
4.1	1642.6405	0.9961	LQKLGEGNSSITR

Spectrum No: 122; Query: 547; Rank: 1

Peptide View

MS/MS Fragmentation of **AGGAGEGSDDDTSLT**
Found in **IPI00555188**, Tax_Id=10116 Gene_Symbol=Stard10 START domain containing 10

Match to Query 547: 1431.500948 from(716.757750,2+)
Title: 091008RatKidney_NoSalt_39.427.427.2.dta
Data file K:\NewmanPaper\Piliang\3SubProteomes\Piliang3SP\mgf5ppm\ERLIC_3SubProteomes5ppm.mgf

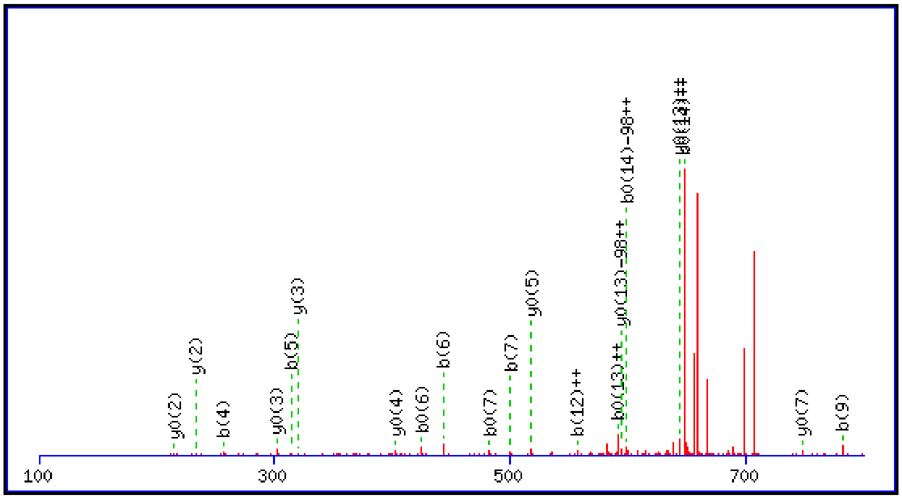

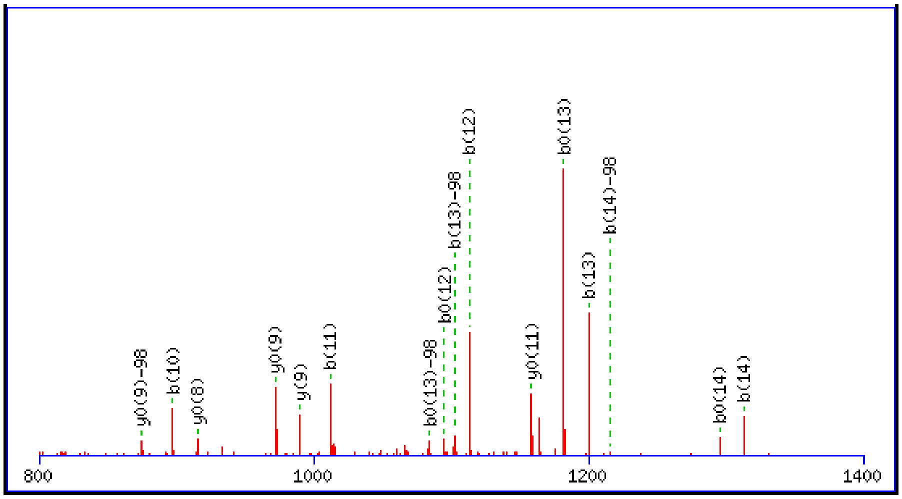

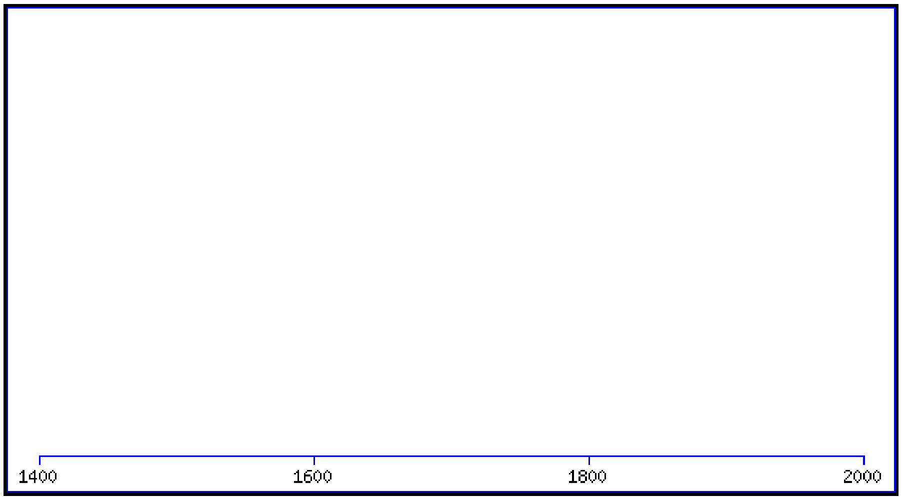

Monoisotopic mass of neutral peptide Mr(calc): 1431.5039
Fixed modifications: Carbamidomethyl (C)
Variable modifications:
S8 : Phospho (ST), with neutral losses 0.0000(shown in table), 97.9769
Ions Score: 67 **Expect:** 1.2e-005
Matches (Bold Red): 36/158 fragment ions using 53 most intense peaks

#	b	b++	b0	b0++	Seq.	y	y++	y0	y0++	#
1	72.0444	36.5258			A					15
2	129.0659	65.0366			G	1361.4740	681.2407	1343.4635	672.2354	14
3	186.0873	93.5473			G	1304.4526	652.7299	1286.4420	**643.7246**	13
4	**257.1244**	129.0659			A	1247.4311	624.2192	1229.4205	615.2139	12
5	**314.1459**	157.5766			G	1176.3940	588.7006	**1158.3834**	579.6954	11
6	**443.1885**	222.0979	**425.1779**	213.0926	E	1119.3725	560.1899	1101.3620	551.1846	10
7	**500.2100**	250.6086	**482.1994**	241.6033	G	**990.3299**	495.6686	**972.3194**	486.6633	9
8	667.2083	334.1078	649.1977	325.1025	S	933.3085	467.1579	**915.2979**	458.1526	8
9	**782.2352**	391.6213	764.2247	382.6160	D	766.3101	383.6587	**748.2996**	374.6534	7
10	**897.2622**	449.1347	879.2516	440.1295	D	651.2832	326.1452	633.2726	317.1399	6
11	**1012.2891**	506.6482	994.2786	497.6429	D	536.2562	268.6318	**518.2457**	259.6265	5
12	**1113.3368**	**557.1720**	**1095.3262**	548.1668	T	421.2293	211.1183	**403.2187**	202.1130	4
13	**1200.3688**	600.6881	**1182.3583**	**591.6828**	S	**320.1816**	160.5944	**302.1710**	151.5892	3
14	**1313.4529**	657.2301	**1295.4423**	**648.2248**	L	**233.1496**	117.0784	**215.1390**	108.0731	2
15					T	120.0655	60.5364	102.0550	51.5311	1

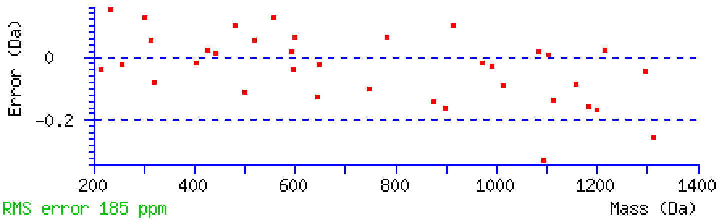

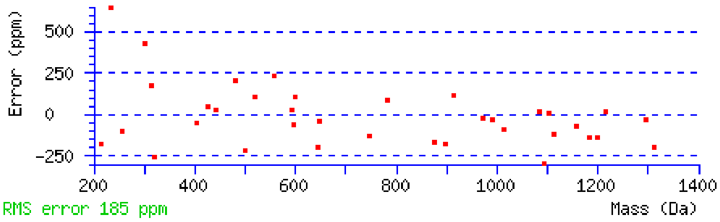

All matches to this query

Score	Mr(calc):	Delta	Sequence
66.7	1431.5039	-0.0029	AGGAGEGSDDDTSLT
36.3	1431.5039	-0.0029	AGGAGEGSDDDTSLT
32.1	1431.5039	-0.0029	AGGAGEGSDDDTSLT
19.3	1431.5039	-0.0029	AGGAGEGSDDDTSLT
5.5	1431.5030	-0.0021	YSSTGLGICNSL
1.9	1431.4973	0.0037	DSSLVQLSESK
1.9	1431.4973	0.0037	DSSLVQLSESK
1.3	1430.5051	0.9959	HPGSQMSDVASR
0.9	1430.5051	0.9959	HPGSQMSDVASR

Spectrum No: 123; Query: 1243; Rank: 1

Peptide View

MS/MS Fragmentation of **NLPDLTAQETETSLSP**
Found in **IPI00391873**, Tax_Id=10116 Gene_Symbol=Htf9c HpaII tinys locus 9c

Match to Query 1243: 1794.793748 from(898.404150,2+)
Title: 091008RatKidney_NoSalt_34.2401.2401.2.dta
Data file K:\NewmanPaper\Piliang\3SubProteomes\Piliang3SP\mgf5ppm\ERLIC_3SubProteomes5ppm.mgf

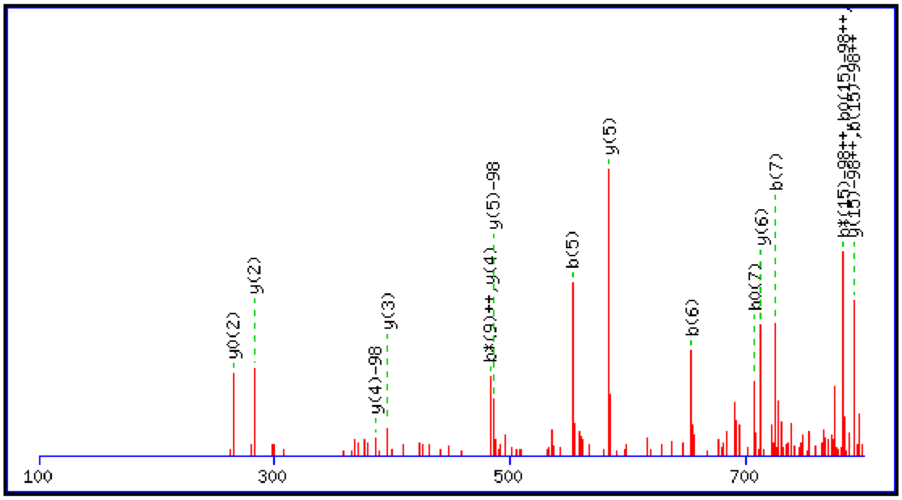

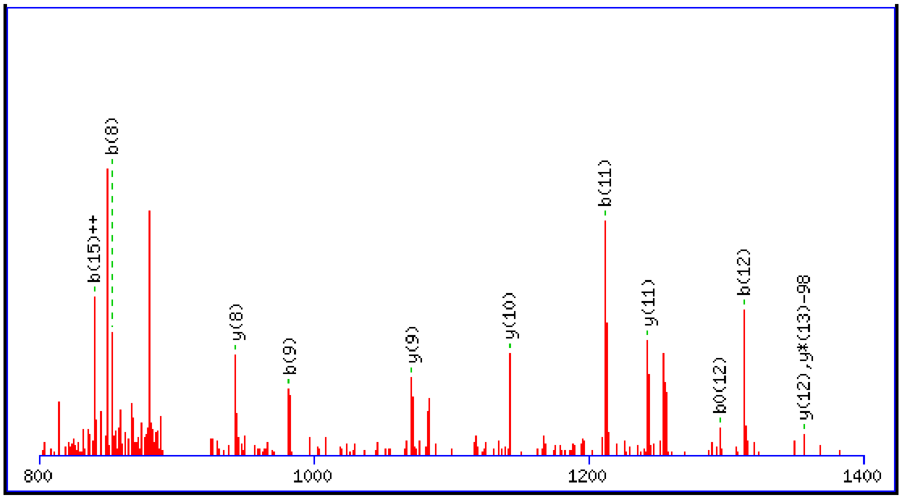

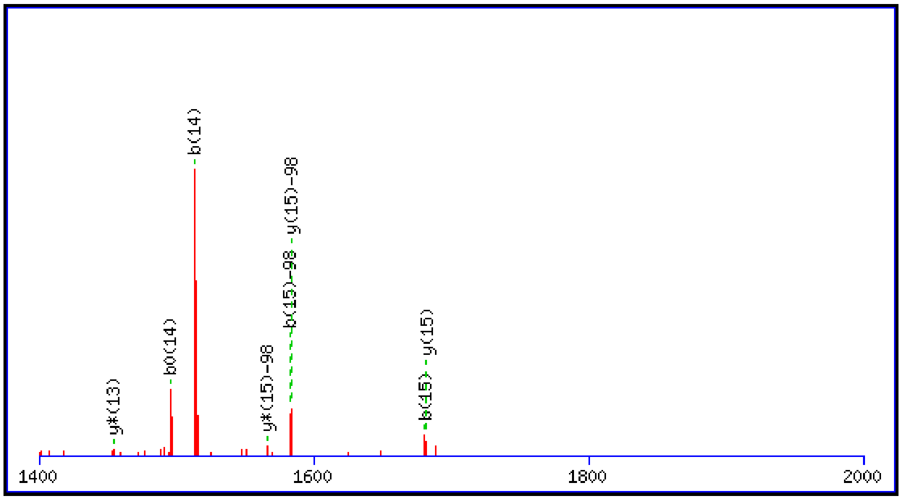

Monoisotopic mass of neutral peptide Mr(calc): 1794.7924
Fixed modifications: Carbamidomethyl (C)
Variable modifications:
S15 : Phospho (ST), with neutral losses 0.0000(shown in table), 97.9769
Ions Score: 66 **Expect:** 5.5e-005
Matches (Bold Red): 38/232 fragment ions using 57 most intense peaks

#	b	b++	b*	b*++	b0	b0++	Seq.	y	y++	y*	y*++	y0	y0++	#
1	115.0502	58.0287	98.0237	49.5155			N							16
2	228.1343	114.5708	211.1077	106.0575			L	**1681.7568**	841.3820	1664.7303	832.8688	1663.7462	832.3768	15
3	325.1870	163.0972	308.1605	154.5839			P	1568.6727	784.8400	1551.6462	776.3267	1550.6622	775.8347	14
4	440.2140	220.6106	423.1874	212.0974	422.2034	211.6053	D	1471.6200	736.3136	**1454.5934**	727.8003	1453.6094	727.3083	13
5	**553.2980**	277.1527	536.2715	268.6394	535.2875	268.1474	L	**1356.5930**	678.8002	1339.5665	670.2869	1338.5825	669.7949	12
6	**654.3457**	327.6765	637.3192	319.1632	636.3352	318.6712	T	**1243.5090**	622.2581	1226.4824	613.7448	1225.4984	613.2528	11
7	**725.3828**	363.1951	708.3563	354.6818	**707.3723**	354.1898	A	**1142.4613**	571.7343	1125.4347	563.2210	1124.4507	562.7290	10
8	**853.4414**	427.2243	836.4149	418.7111	835.4308	418.2191	Q	**1071.4242**	536.2157	1054.3976	527.7024	1053.4136	527.2104	9
9	**982.4840**	491.7456	965.4575	**483.2324**	964.4734	482.7404	E	**943.3656**	472.1864			925.3550	463.1812	8
10	1083.5317	542.2695	1066.5051	533.7562	1065.5211	533.2642	T	814.3230	407.6651			796.3124	398.6599	7
11	**1212.5743**	606.7908	1195.5477	598.2775	1194.5637	597.7855	E	**713.2753**	357.1413			695.2648	348.1360	6
12	**1313.6220**	657.3146	1296.5954	648.8013	**1295.6114**	648.3093	T	**584.2327**	292.6200			566.2222	283.6147	5
13	1400.6540	700.8306	1383.6274	692.3174	1382.6434	691.8253	S	**483.1850**	242.0962			465.1745	233.0909	4
14	**1513.7380**	757.3727	1496.7115	748.8594	**1495.7275**	748.3674	L	**396.1530**	198.5801			378.1425	189.5749	3
15	**1680.7364**	**840.8718**	1663.7098	832.3586	1662.7258	831.8666	S	**283.0690**	142.0381			**265.0584**	133.0328	2
16							P	116.0706	58.5389					1

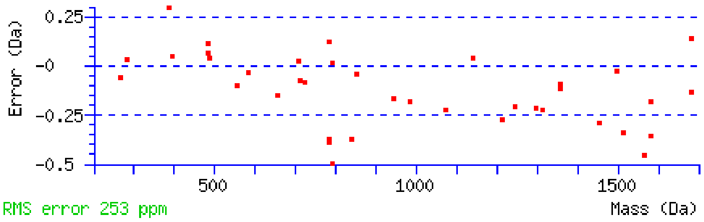

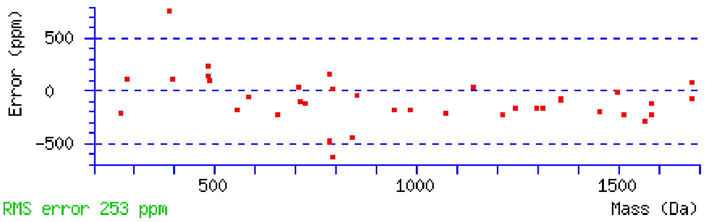

All matches to this query

Score	Mr(calc):	Delta	Sequence
66.3	1794.7924	0.0013	NLPDLTAQETETSLSP
47.9	1794.7924	0.0013	NLPDLTAQETETSLSP
32.1	1794.7924	0.0013	NLPDLTAQETETSLSP
19.8	1794.7924	0.0013	NLPDLTAQETETSLSP
4.3	1794.7897	0.0041	RDAAAAAAAANANANATAK
2.1	1794.7937	0.0000	NSENGIHFLLNRDGK

Spectrum No: 124; Query: 2628; Rank: 1

Peptide View

MS/MS Fragmentation of **VDIITEEMPENALPSDEDDKDPNDPYR**
Found in **IPI00198486**, Tax_Id=10116 Gene_Symbol=Ap3d1 similar to adaptor-related protein complex 3, delta 1 subunit

Match to Query 2628: 3212.346732 from(1071.789520,3+)
Title: 091008RatKidney_NH4Format01_23.2463.2463.3.dta
Data file K:\NewmanPaper\Piliang\3SubProteomes\Piliang3SP\mgf5ppm\ERLIC_3SubProteomes5ppm.mgf

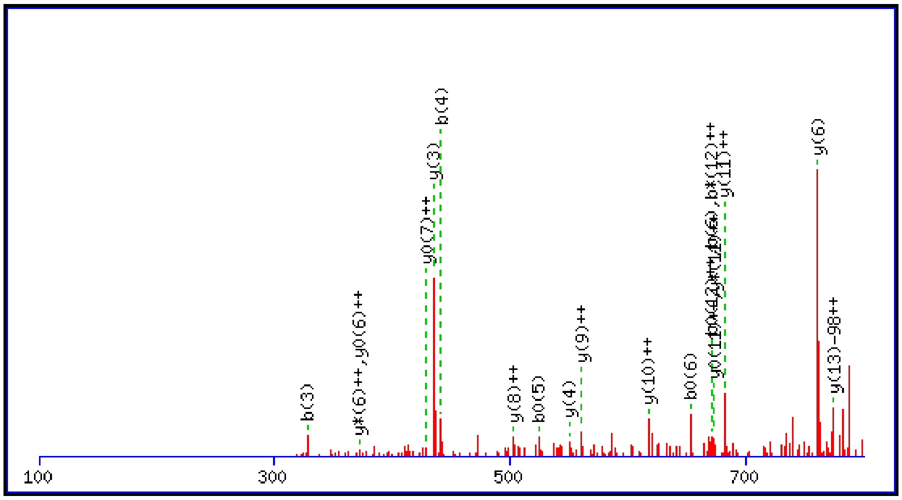

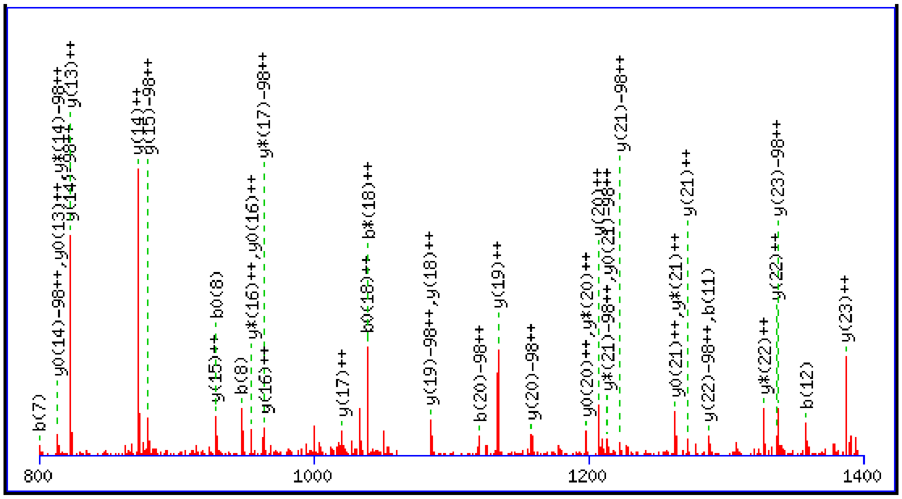

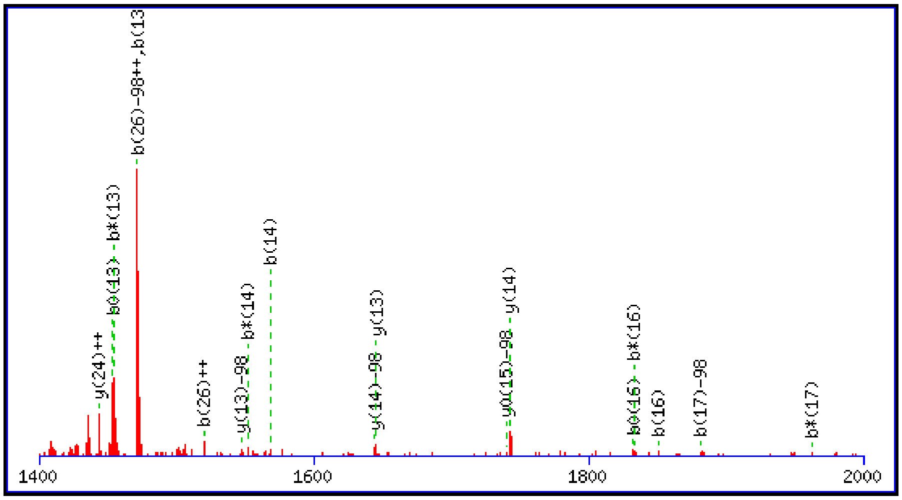

Monoisotopic mass of neutral peptide Mr(calc): 3212.3380
Fixed modifications: Carbamidomethyl (C)
Variable modifications:
M8 : Oxidation (M)
S15 : Phospho (ST), with neutral losses 0.0000(shown in table), 97.9769
Ions Score: 66 **Expect:** 9.3e-005
Matches (Bold Red): 77/440 fragment ions using 127 most intense peaks

#	b	b^{++}	b*	b^{*++}	b^0	b^{0++}	Seq.	y	y^{++}	y*	y^{*++}	y^0	y^{0++}	#
1	100.0757	50.5415					V							27
2	215.1026	108.0550			197.0921	99.0497	D	3114.2769	1557.6421	3097.2504	1549.1288	3096.2664	1548.6368	26
3	**328.1867**	164.5970			310.1761	155.5917	I	2999.2500	1500.1286	2982.2234	1491.6154	2981.2394	1491.1233	25
4	**441.2708**	221.1390			423.2602	212.1337	I	2886.1659	**1443.5866**	2869.1394	1435.0733	2868.1554	1434.5813	24
5	542.3184	271.6629			**524.3079**	262.6576	T	2773.0819	**1387.0446**	2756.0553	1378.5313	2755.0713	1378.0393	23
6	**671.3610**	336.1842			**653.3505**	327.1789	E	2672.0342	**1336.5207**	2655.0076	**1328.0075**	2654.0236	1327.5154	22
7	**800.4036**	400.7055			782.3931	391.7002	E	2542.9916	**1271.9994**	2525.9650	**1263.4862**	2524.9810	**1262.9941**	21
8	**947.4390**	474.2232			**929.4285**	465.2179	M	2413.9490	**1207.4781**	2396.9224	**1198.9649**	2395.9384	**1198.4728**	20
9	1044.4918	522.7495			1026.4812	513.7443	P	2266.9136	**1133.9604**	2249.8870	1125.4472	2248.9030	1124.9551	19
10	1173.5344	587.2708			1155.5238	578.2655	E	2169.8608	**1085.4340**	2152.8343	1076.9208	2151.8503	1076.4288	18
11	**1287.5773**	644.2923	1270.5508	635.7790	1269.5667	635.2870	N	2040.8182	**1020.9128**	2023.7917	1012.3995	2022.8077	1011.9075	17
12	**1358.6144**	679.8109	1341.5879	**671.2976**	1340.6039	**670.8056**	A	1926.7753	**963.8913**	1909.7488	**955.3780**	1908.7647	**954.8860**	16
13	**1471.6985**	736.3529	**1454.6719**	727.8396	**1453.6879**	727.3476	L	1855.7382	**928.3727**	1838.7116	919.8595	1837.7276	919.3674	15
14	**1568.7513**	784.8793	**1551.7247**	776.3660	1550.7407	775.8740	P	**1742.6541**	**871.8307**	1725.6276	863.3174	1724.6436	862.8254	14
15	1735.7496	868.3784	1718.7231	859.8652	1717.7390	859.3732	S	**1645.6014**	**823.3043**	1628.5748	814.7910	1627.5908	**814.2990**	13
16	**1850.7766**	925.8919	**1833.7500**	917.3786	**1832.7660**	916.8866	D	1478.6030	739.8051	1461.5765	731.2919	1460.5924	730.7999	12
17	1979.8191	990.4132	**1962.7926**	981.8999	1961.8086	981.4079	E	1363.5761	**682.2917**	1346.5495	**673.7784**	1345.5655	**673.2864**	11
18	2094.8461	1047.9267	2077.8195	**1039.4134**	2076.8355	**1038.9214**	D	1234.5335	**617.7704**	1217.5069	609.2571	1216.5229	608.7651	10

**19**	2209.8730	1105.4402	2192.8465	1096.9269	2191.8625	1096.4349	**D**	1119.5065	**560.2569**	1102.4800	551.7436	1101.4960	551.2516	**9**
**20**	2337.9680	1169.4876	2320.9414	1160.9744	2319.9574	1160.4824	**K**	1004.4796	**502.7434**	987.4530	494.2302	986.4690	493.7381	**8**
**21**	2452.9949	1227.0011	2435.9684	1218.4878	2434.9844	1217.9958	**D**	876.3846	438.6959	859.3581	430.1827	858.3741	**429.6907**	**7**
**22**	2550.0477	1275.5275	2533.0212	1267.0142	2532.0371	1266.5222	**P**	**761.3577**	381.1825	744.3311	**372.6692**	743.3471	**372.1772**	**6**
**23**	2664.0906	1332.5490	2647.0641	1324.0357	2646.0801	1323.5437	**N**	664.3049	332.6561	647.2784	324.1428	646.2943	323.6508	**5**
**24**	2779.1176	1390.0624	2762.0910	1381.5491	2761.1070	1381.0571	**D**	**550.2620**	275.6346	533.2354	267.1214	532.2514	266.6293	**4**
**25**	2876.1703	1438.5888	2859.1438	1430.0755	2858.1598	1429.5835	**P**	**435.2350**	218.1212	418.2085	209.6079			**3**
**26**	3039.2337	**1520.1205**	3022.2071	1511.6072	3021.2231	1511.1152	**Y**	338.1823	169.5948	321.1557	161.0815			**2**
**27**							**R**	175.1190	88.0631	158.0924	79.5498			**1**

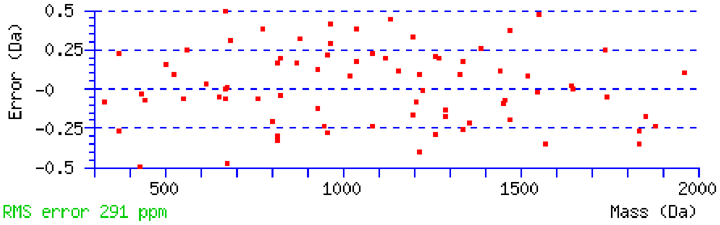

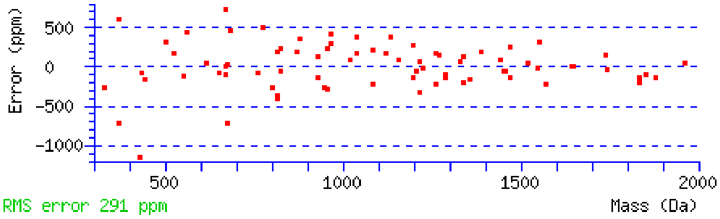

All matches to this query

Score	Mr(calc):	Delta	Sequence
66.2	3212.3380	0.0087	VDIITEEMPENALPSDEDDKDPNDPYR
27.6	3212.3380	0.0087	VDIITEEMPENALPSDEDDKDPNDPYR
10.2	3212.3380	0.0087	VDIITEEMPENALPSDEDDKDPNDPYR
7.3	3212.3509	-0.0042	VNLGSGEITNGQALNTSLEDFEPMETKR
7.3	3212.3509	-0.0042	VNLGSGEITNGQALNTSLEDFEPMETKR
4.3	3212.3699	-0.0231	EVGIGSAGARRPLCSAPSPVPGQCEAPPR
2.6	3212.3509	-0.0042	VNLGSGEITNGQALNTSLEDFEPMETKR
2.5	3212.3644	-0.0176	SQEQDSSFHETDGNTVRNTSRFSSSTTK
1.8	3212.3402	0.0066	GASLDNIYMIGVSLGAHIAGFVGEMYSGK
1.0	3212.3447	0.0021	FQAFWGNSPTVHVPREPGENPTLTYK

Spectrum No: 125; Query: 1707; Rank: 1

Peptide View

MS/MS Fragmentation of **SLDDSEEEDDEDSGHSSR**
Found in **IPI00198146**, Tax_Id=10116 Gene_Symbol=Pkd2_predicted similar to polycystin 2

Match to Query 1707: 2087.699808 from(1044.857180,2+)
Title: 091008RatKidney_NoSalt_29.320.320.2.dta
Data file K:\NewmanPaper\Piliang\3SubProteomes\Piliang3SP\mgf5ppm\ERLIC_3SubProteomes5ppm.mgf

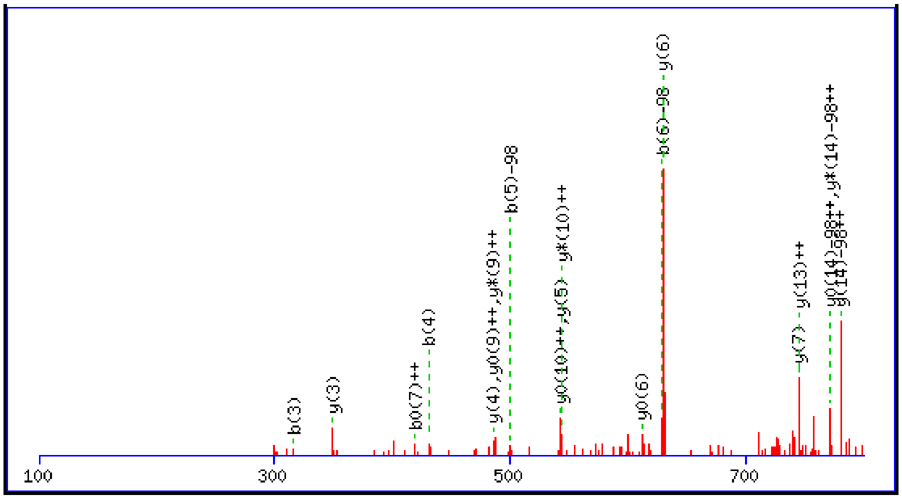

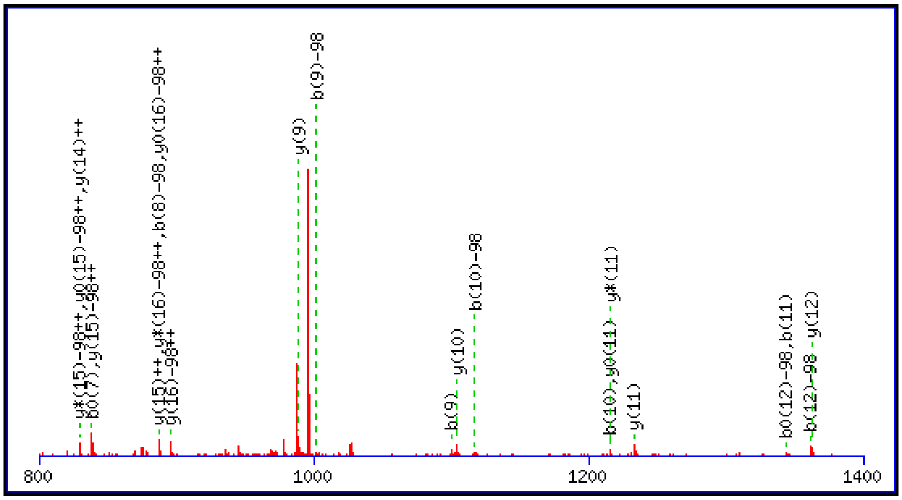

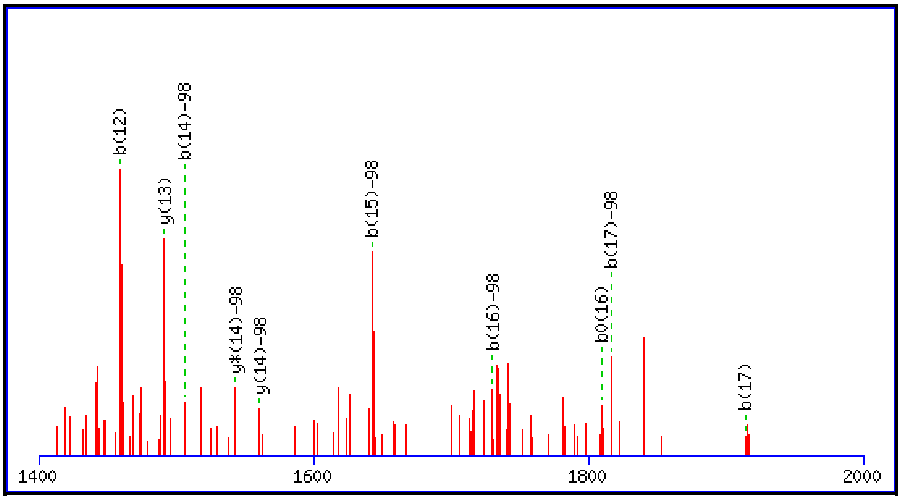

Monoisotopic mass of neutral peptide Mr(calc): 2087.7077
Fixed modifications: Carbamidomethyl (C)
Variable modifications:
S5 : Phospho (ST), with neutral losses 97.9769(shown in table), 0.0000
Ions Score: 66 **Expect:** 1.5e-005
Matches (Bold Red): 52/244 fragment ions using 87 most intense peaks

#	b	b^{++}	b^{0}	b^{0++}	Seq.	y	y^{++}	y^{*}	y^{*++}	y^{0}	y^{0++}	#
1	88.0393	44.5233	70.0287	35.5180	S							18
2	201.1234	101.0653	183.1128	92.0600	L	1903.7060	952.3566	1886.6795	943.8434	1885.6955	943.3514	17
3	**316.1503**	158.5788	298.1397	149.5735	D	1790.6220	**895.8146**	1773.5954	**887.3013**	1772.6114	**886.8093**	16
4	**431.1773**	216.0923	413.1667	207.0870	D	1675.5950	**838.3011**	1658.5685	**829.7879**	1657.5844	**829.2959**	15
5	**500.1987**	250.6030	482.1881	241.5977	S	**1560.5681**	**780.7877**	**1543.5415**	**772.2744**	1542.5575	**771.7824**	14

6	**629.2413**	315.1243	611.2307	306.1190	E	**1491.5466**	**746.2769**	1474.5201	737.7637	1473.5360	737.2717	13
7	758.2839	379.6456	740.2733	370.6403	E	**1362.5040**	681.7556	1345.4775	673.2424	1344.4935	672.7504	12
8	**887.3265**	444.1669	869.3159	435.1616	E	**1233.4614**	617.2344	**1216.4349**	608.7211	**1215.4509**	608.2291	11
9	**1002.3534**	501.6804	984.3429	492.6751	D	**1104.4188**	552.7131	1087.3923	**544.1998**	1086.4083	**543.7078**	10
10	**1117.3804**	559.1938	1099.3698	550.1885	D	**989.3919**	495.1996	972.3653	**486.6863**	971.3813	**486.1943**	9
11	1246.4230	623.7151	1228.4124	614.7098	E	874.3649	437.6861	857.3384	429.1728	856.3544	428.6808	8
12	**1361.4499**	681.2286	**1343.4393**	672.2233	D	**745.3224**	373.1648	728.2958	364.6515	727.3118	364.1595	7
13	1448.4819	724.7446	1430.4714	715.7393	S	**630.2954**	315.6513	613.2689	307.1381	**612.2848**	306.6461	6
14	**1505.5034**	753.2553	1487.4928	744.2501	G	**543.2634**	272.1353	526.2368	263.6221	525.2528	263.1300	5
15	**1642.5623**	821.7848	1624.5518	812.7795	H	**486.2419**	243.6246	469.2154	235.1113	468.2314	234.6193	4
16	**1729.5943**	865.3008	1711.5838	856.2955	S	**349.1830**	175.0951	332.1565	166.5819	331.1724	166.0899	3
17	**1816.6264**	908.8168	1798.6158	899.8115	S	262.1510	131.5791	245.1244	123.0659	244.1404	122.5738	2
18					R	175.1190	88.0631	158.0924	79.5498			1

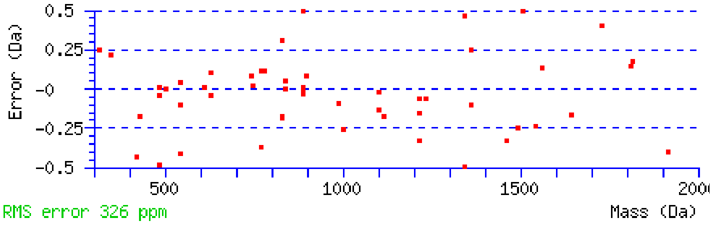

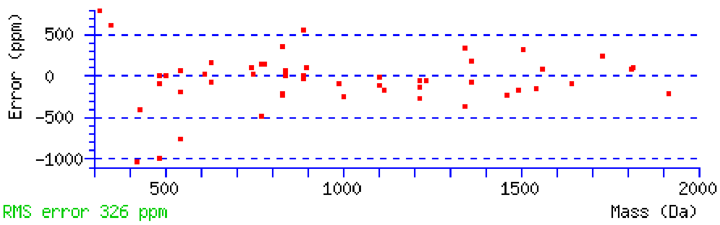

All matches to this query

Score	Mr(calc):	Delta	Sequence
66.0	2087.7077	-0.0078	SLDDSEEEDDEDSGHSSR
46.8	2087.7077	-0.0078	SLDDSEEEDDEDSGHSSR
22.0	2087.7077	-0.0078	SLDDSEEEDDEDSGHSSR
10.3	2087.7077	-0.0078	SLDDSEEEDDEDSGHSSR
8.1	2087.7077	-0.0078	SLDDSEEEDDEDSGHSSR
0.2	2087.6985	0.0013	NYVSETMASPGKDNYR
0.1	2087.6985	0.0013	NYVSETMASPGKDNYR

Spectrum No: 126; Query: 2027; Rank: 1

Peptide View

MS/MS Fragmentation of **SSTLEPEDEDDEDEDTMTR**
Found in **IPI00359020**, Tax_Id=10116 Gene_Symbol=RGD1308876_predicted hypothetical protein LOC362597

Match to Query 2027: 2308.815488 from(1155.415020,2+)
Title: 100101RatKid_NS_deglyco_26.1276.1276.2.dta
Data file K:\NewmanPaper\Piliang\3SubProteomes\Piliang3SP\mgf5ppm\ERLIC_3SubProteomes5ppm.mgf

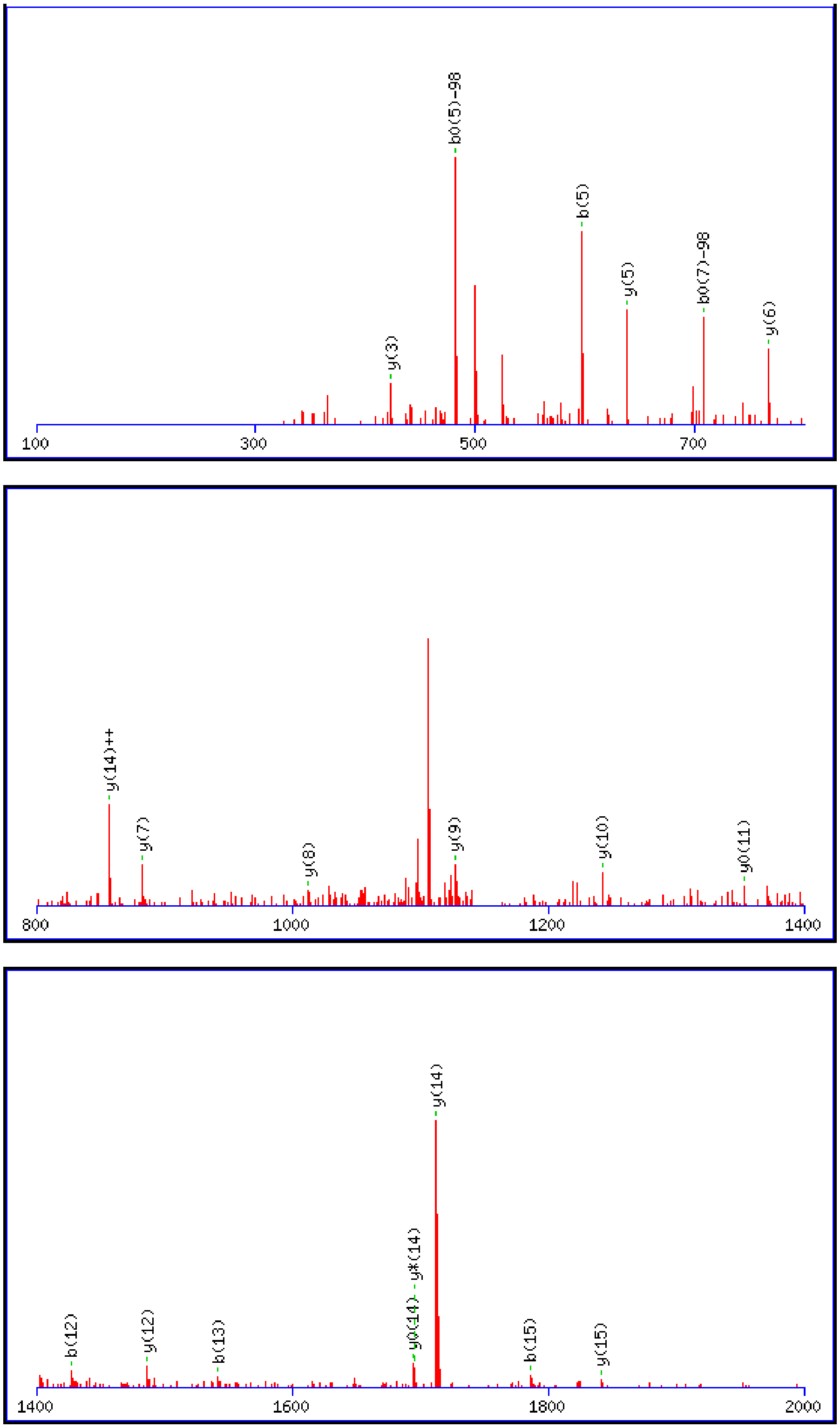

Monoisotopic mass of neutral peptide Mr(calc): 2308.8050
Fixed modifications: Carbamidomethyl (C)
Variable modifications:
T3 : Phospho (ST), with neutral losses 0.0000(shown in table), 97.9769
M17 : Oxidation (M)
Ions Score: 66 **Expect:** 2.8e-005
Matches (Bold Red): 20/254 fragment ions using 37 most intense peaks

#	b	b++	b0	b0++	Seq.	y	y++	y*	y*++	y0	y0++	#
1	88.0393	44.5233	70.0287	35.5180	S							19
2	175.0713	88.0393	157.0608	79.0340	S	2222.7803	1111.8938	2205.7537	1103.3805	2204.7697	1102.8885	18
3	356.0853	178.5463	338.0748	169.5410	T	2135.7482	1068.3778	2118.7217	1059.8645	2117.7377	1059.3725	17
4	469.1694	235.0883	451.1588	226.0831	L	1954.7342	977.8707	1937.7077	969.3575	1936.7237	968.8655	16

**5**	**598.2120**	299.6096	580.2014	290.6044	**E**	**1841.6502**	921.3287	1824.6236	912.8154	1823.6396	912.3234	**15**
**6**	695.2648	348.1360	677.2542	339.1307	**P**	**1712.6076**	**856.8074**	**1695.5810**	848.2941	**1694.5970**	847.8021	**14**
**7**	824.3073	412.6573	806.2968	403.6520	**E**	1615.5548	808.2810	1598.5283	799.7678	1597.5442	799.2758	**13**
**8**	939.3343	470.1708	921.3237	461.1655	**D**	**1486.5122**	743.7597	1469.4857	735.2465	1468.5016	734.7545	**12**
**9**	1068.3769	534.6921	1050.3663	525.6868	**E**	1371.4853	686.2463	1354.4587	677.7330	**1353.4747**	677.2410	**11**
**10**	1183.4038	592.2056	1165.3933	583.2003	**D**	**1242.4427**	621.7250	1225.4161	613.2117	1224.4321	612.7197	**10**
**11**	1298.4308	649.7190	1280.4202	640.7137	**D**	**1127.4157**	564.2115	1110.3892	555.6982	1109.4052	555.2062	**9**
**12**	**1427.4734**	714.2403	1409.4628	705.2350	**E**	**1012.3888**	506.6980	995.3622	498.1848	994.3782	497.6927	**8**
**13**	**1542.5003**	771.7538	1524.4897	762.7485	**D**	**883.3462**	442.1767	866.3196	433.6635	865.3356	433.1715	**7**
**14**	1671.5429	836.2751	1653.5323	827.2698	**E**	**768.3193**	384.6633	751.2927	376.1500	750.3087	375.6580	**6**
**15**	**1786.5698**	893.7886	1768.5593	884.7833	**D**	**639.2767**	320.1420	622.2501	311.6287	621.2661	311.1367	**5**
**16**	1887.6175	944.3124	1869.6070	935.3071	**T**	524.2497	262.6285	507.2232	254.1152	506.2391	253.6232	**4**
**17**	2034.6529	1017.8301	2016.6424	1008.8248	**M**	**423.2020**	212.1047	406.1755	203.5914	405.1915	203.0994	**3**
**18**	2135.7006	1068.3539	2117.6900	1059.3487	**T**	276.1666	138.5870	259.1401	130.0737	258.1561	129.5817	**2**
**19**					**R**	175.1190	88.0631	158.0924	79.5498			**1**

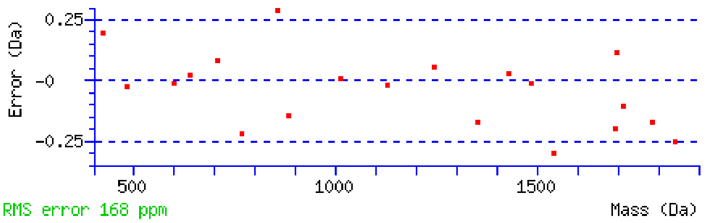

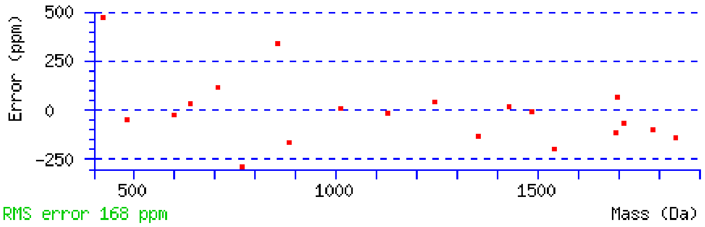

All matches to this query

Score	Mr(calc):	Delta	Sequence
65.9	2308.8050	0.0105	SSTLEPEDEDDEDEDTMTR
65.9	2308.8050	0.0105	SSTLEPEDEDDEDEDTMTR
65.9	2308.8050	0.0105	SSTLEPEDEDDEDEDTMTR
3.2	2308.8221	-0.0066	AYGLLVATTSRDSADTLR
1.8	2308.8221	-0.0066	AYGLLVATTSRDSADTLR
1.8	2308.8221	-0.0066	AYGLLVATTSRDSADTLR
1.7	2308.8221	-0.0066	AYGLLVATTSRDSADTLR
1.1	2308.8221	-0.0066	AYGLLVATTSRDSADTLR
0.1	2308.7956	0.0199	TPSSPKSSSSSTTGEKITK

Spectrum No: 127; Query: 1837; Rank: 1

Peptide View

MS/MS Fragmentation of **SPLEPESPGWPGFGDTTTAK**
Found in **IPI00373011**, Tax_Id=10116 Gene_Symbol=eplin similar to Epithelial protein lost in neoplasm

Match to Query 1837: 2152.943428 from(1077.478990,2+)
Title: 100101RatKid_NS_deglyco_08.3963.3963.2.dta
Data file K:\NewmanPaper\Piliang\3SubProteomes\Piliang3SP\mgf5ppm\ERLIC_3SubProteomes5ppm.mgf

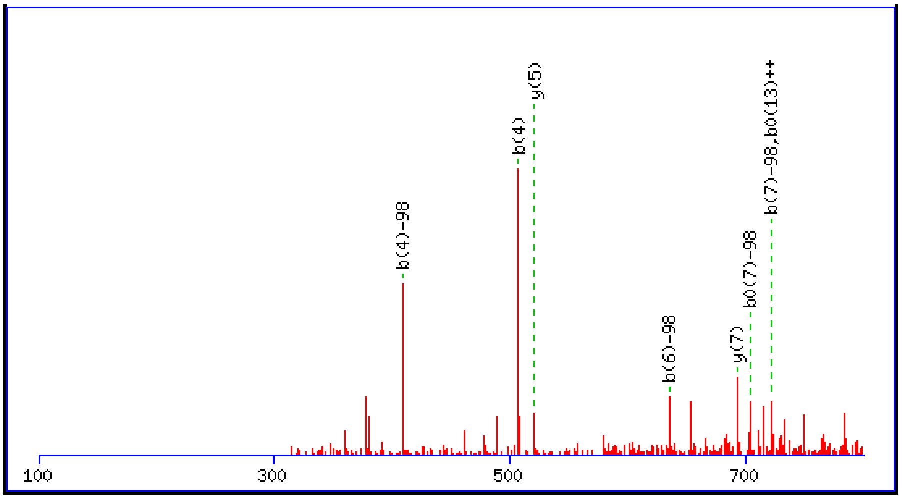

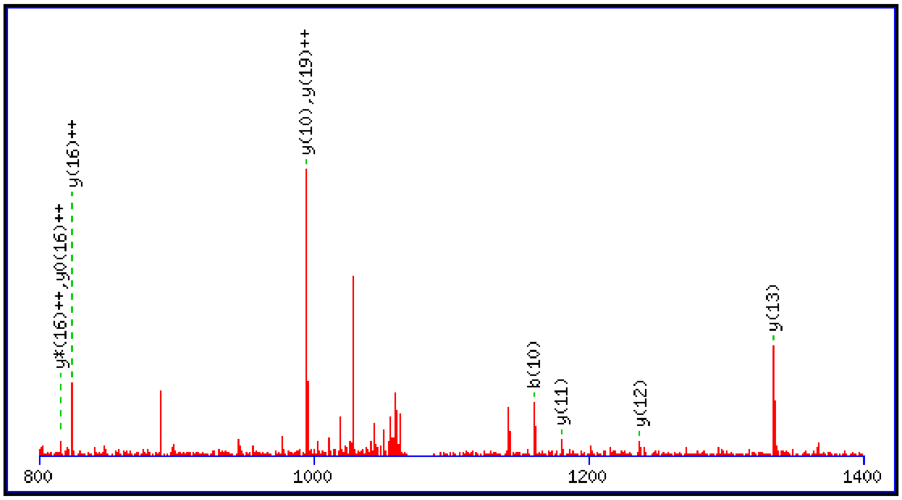

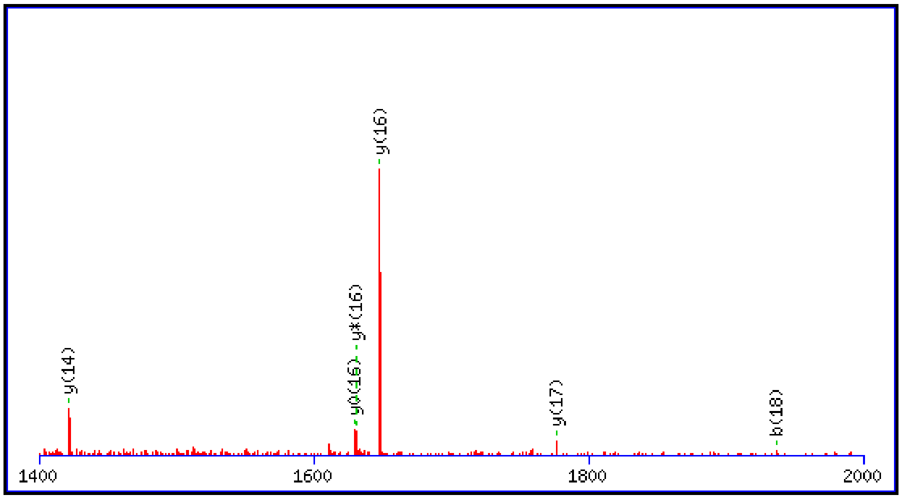

Monoisotopic mass of neutral peptide Mr(calc): 2152.9354
Fixed modifications: Carbamidomethyl (C)
Variable modifications:
S1 : Phospho (ST), with neutral losses 97.9769(shown in table), 0.0000
Ions Score: 66 **Expect:** 6.6e-005
Matches (Bold Red): 23/262 fragment ions using 30 most intense peaks

#	b	b^{++}	b^0	b^{0++}	Seq.	y	y^{++}	y^*	y^{*++}	y^0	y^{0++}	#
1	70.0287	35.5180	52.0182	26.5127	S							20
2	167.0815	84.0444	149.0709	75.0391	P	1986.9444	**993.9758**	1969.9178	985.4625	1968.9338	984.9705	19
3	280.1656	140.5864	262.1550	131.5811	L	1889.8916	945.4494	1872.8650	936.9362	1871.8810	936.4441	18
4	**409.2082**	205.1077	391.1976	196.1024	E	**1776.8075**	888.9074	1759.7810	880.3941	1758.7970	879.9021	17
5	506.2609	253.6341	488.2504	244.6288	P	**1647.7649**	**824.3861**	**1630.7384**	**815.8728**	**1629.7544**	**815.3808**	16

6	635.3035	318.1554	617.2929	309.1501	E	1550.7122	775.8597	1533.6856	767.3464	1532.7016	766.8544	15
7	722.3355	361.6714	704.3250	352.6661	S	1421.6696	711.3384	1404.6430	702.8252	1403.6590	702.3331	14
8	819.3883	410.1978	801.3777	401.1925	P	1334.6375	667.8224	1317.6110	659.3091	1316.6270	658.8171	13
9	876.4098	438.7085	858.3992	429.7032	G	1237.5848	619.2960	1220.5582	610.7828	1219.5742	610.2907	12
10	1062.4891	531.7482	1044.4785	522.7429	W	1180.5633	590.7853	1163.5368	582.2720	1162.5528	581.7800	11
11	1159.5418	580.2746	1141.5313	571.2693	P	994.4840	497.7456	977.4575	489.2324	976.4734	488.7404	10
12	1216.5633	608.7853	1198.5527	599.7800	G	897.4312	449.2193	880.4047	440.7060	879.4207	440.2140	9
13	1363.6317	682.3195	1345.6212	673.3142	F	840.4098	420.7085	823.3832	412.1953	822.3992	411.7032	8
14	1420.6532	710.8302	1402.6426	701.8249	G	693.3414	347.1743	676.3148	338.6610	675.3308	338.1690	7
15	1535.6801	768.3437	1517.6696	759.3384	D	636.3199	318.6636	619.2933	310.1503	618.3093	309.6583	6
16	1636.7278	818.8675	1618.7172	809.8623	T	521.2930	261.1501	504.2664	252.6368	503.2824	252.1448	5
17	1737.7755	869.3914	1719.7649	860.3861	T	420.2453	210.6263	403.2187	202.1130	402.2347	201.6210	4
18	1838.8232	919.9152	1820.8126	910.9099	T	319.1976	160.1024	302.1710	151.5892	301.1870	151.0972	3
19	1909.8603	955.4338	1891.8497	946.4285	A	218.1499	109.5786	201.1234	101.0653			2
20					K	147.1128	74.0600	130.0863	65.5468			1

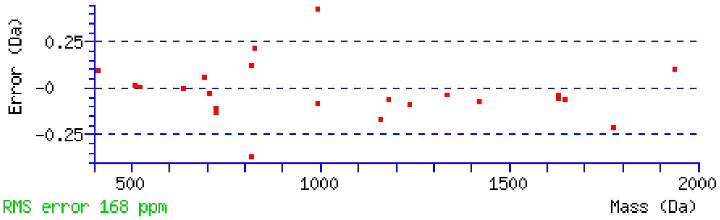

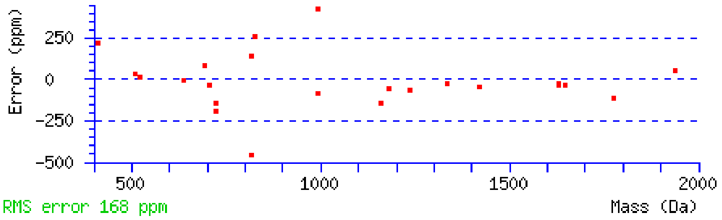

All matches to this query

Score	Mr(calc):	Delta	Sequence
65.7	2152.9354	0.0080	SPLEPESPGWPGFGDTTTAK
20.7	2152.9354	0.0080	SPLEPESPGWPGFGDTTTAK
9.9	2151.9378	1.0057	IAIGVIFSLSKDEDENNK
8.9	2150.9538	1.9897	IAIGVIFSLSKDEDENNK
5.8	2151.9378	1.0057	IAIGVIFSLSKDEDENNK
4.4	2152.9476	-0.0042	LTNALVGMSPSSSLSALSSR
4.0	2152.9476	-0.0042	LTNALVGMSPSSSLSALSSR
1.2	2150.9304	2.0131	EPTSVKQQDSGCHLVNSGK
1.2	2150.9304	2.0131	EPTSVKQQDSGCHLVNSGK
1.0	2152.9613	-0.0178	KVSVTINDDGTLHMHFSR

Spectrum No: 128; Query: 930; Rank: 1

Peptide View

MS/MS Fragmentation of **SSPPATDPGPVPSSPR**
Found in **IPI00371410**, Tax_Id=10116 Gene_Symbol=RGD1309471 Isoform 1 of SAPK substrate protein 1

Match to Query 930: 1627.718088 from(814.866320,2+)
Title: 091008RatKidney_NoSalt_23.834.834.2.dta
Data file K:\NewmanPaper\Piliang\3SubProteomes\Piliang3SP\mgf5ppm\ERLIC_3SubProteomes5ppm.mgf

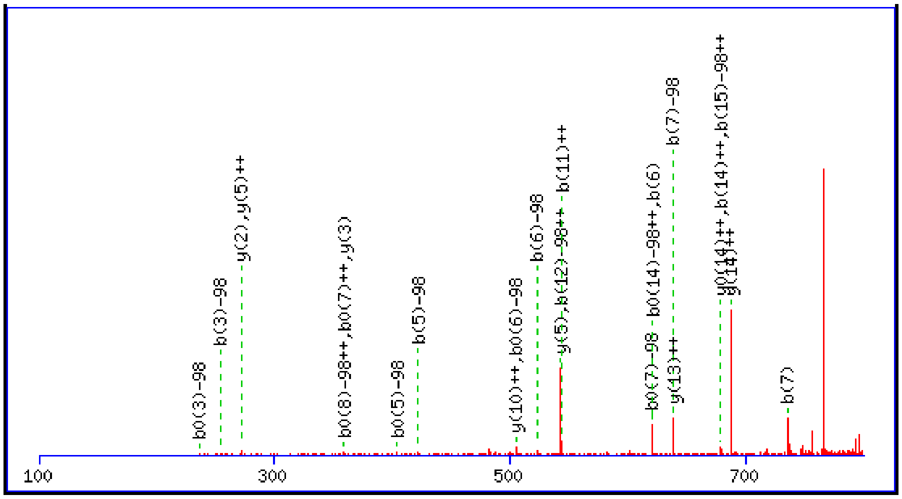

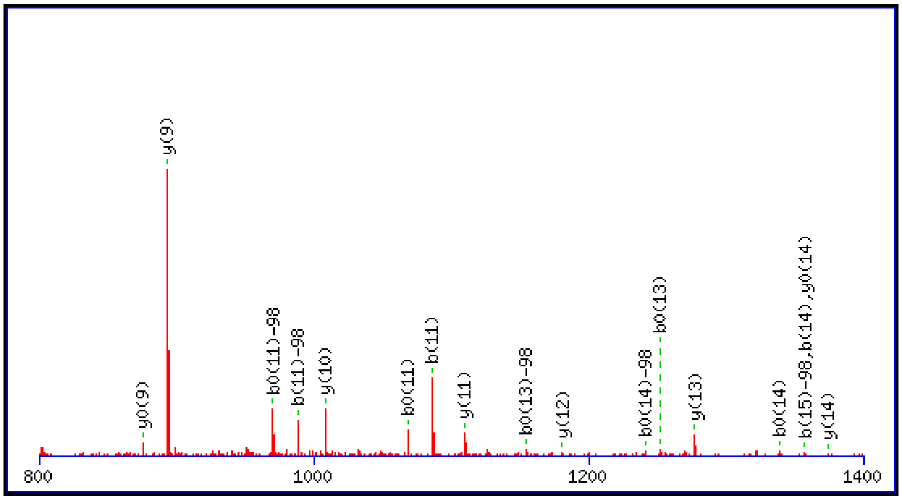

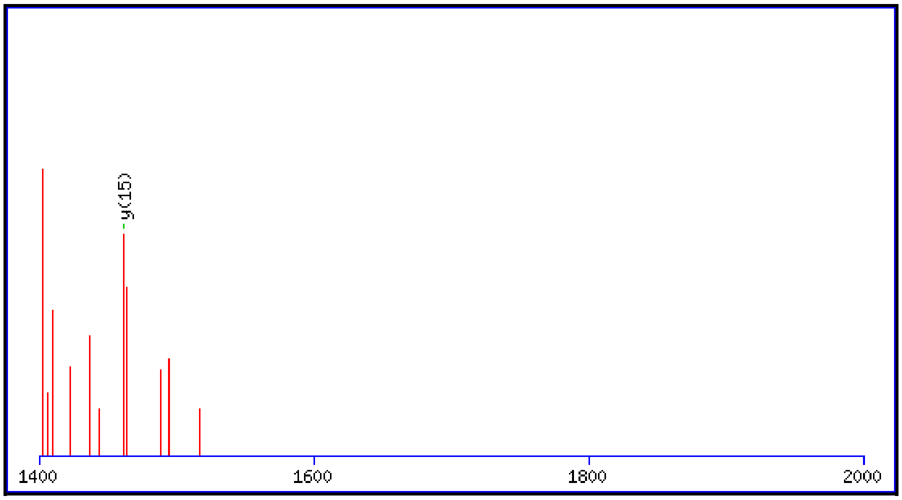

Monoisotopic mass of neutral peptide Mr(calc): 1627.7243
Fixed modifications: Carbamidomethyl (C)
Variable modifications:
S1 : Phospho (ST), with neutral losses 97.9769(shown in table), 0.0000
Ions Score: 66 **Expect:** 4.9e-005
Matches (Bold Red): 44/206 fragment ions using 55 most intense peaks

#	b	b^{++}	b^0	b^{0++}	Seq.	y	y^{++}	y*	y^{*++}	y^0	y^{0++}	#
1	70.0287	35.5180	52.0182	26.5127	S							16
2	157.0608	79.0340	139.0502	70.0287	S	**1461.7332**	731.3703	1444.7067	722.8570	1443.7227	722.3650	15
3	**254.1135**	127.5604	**236.1030**	118.5551	P	**1374.7012**	**687.8542**	1357.6747	679.3410	**1356.6906**	**678.8490**	14
4	351.1663	176.0868	333.1557	167.0815	P	**1277.6484**	**639.3279**	1260.6219	630.8146	1259.6379	630.3226	13
5	**422.2034**	211.6053	**404.1928**	202.6001	A	**1180.5957**	590.8015	1163.5691	582.2882	1162.5851	581.7962	12

6	**523.2511**	262.1292	**505.2405**	253.1239	T	**1109.5586**	555.2829	1092.5320	546.7696	1091.5480	546.2776	11
7	**638.2780**	319.6427	**620.2675**	310.6374	D	**1008.5109**	**504.7591**	991.4843	496.2458	990.5003	495.7538	10
8	735.3308	368.1690	717.3202	**359.1637**	P	**893.4839**	447.2456	876.4574	438.7323	**875.4734**	438.2403	9
9	792.3523	396.6798	774.3417	387.6745	G	796.4312	398.7192	779.4046	390.2060	778.4206	389.7139	8
10	889.4050	445.2061	871.3945	436.2009	P	739.4097	370.2085	722.3832	361.6952	721.3991	361.2032	7
11	**988.4734**	494.7404	**970.4629**	485.7351	V	642.3570	321.6821	625.3304	313.1688	624.3464	312.6768	6
12	1085.5262	**543.2667**	1067.5156	534.2615	P	**543.2885**	**272.1479**	526.2620	263.6346	525.2780	263.1426	5
13	1172.5582	586.7827	**1154.5477**	577.7775	S	446.2358	223.6215	429.2092	215.1082	428.2252	214.6162	4
14	1259.5903	630.2988	**1241.5797**	**621.2935**	S	**359.2037**	180.1055	342.1772	171.5922	341.1932	171.1002	3
15	**1356.6430**	**678.8251**	1338.6324	669.8199	P	**272.1717**	136.5895	255.1452	128.0762			2
16					R	175.1190	88.0631	158.0924	79.5498			1

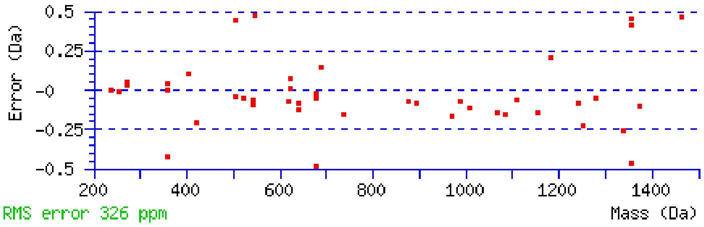

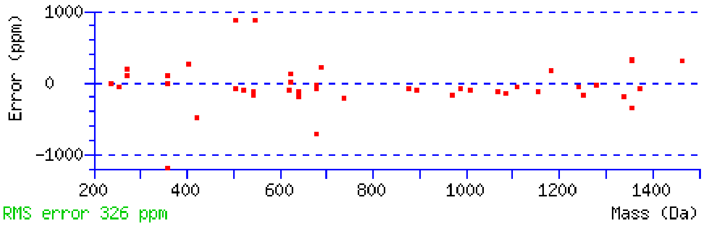

All matches to this query

Score	Mr(calc):	Delta	Sequence
65.6	1627.7243	-0.0062	SSPPATDPGPVPSSPR
54.1	1627.7243	-0.0062	SSPPATDPGPVPSSPR
34.4	1627.7243	-0.0062	SSPPATDPGPVPSSPR
6.9	1627.7243	-0.0062	SSPPATDPGPVPSSPR
5.1	1627.7104	0.0077	RKLSGNGTDSXNGR
3.4	1627.7315	-0.0134	PRTTENSLRGSSSR
3.4	1627.7315	-0.0134	PRTTENSLRGSSSR
3.4	1627.7315	-0.0134	PRTTENSLRGSSSR
2.7	1627.7104	0.0077	SGHSAESSKHKEHK
2.0	1627.7144	0.0037	IQFGNGFSHSDALR

Spectrum No: 129; Query: 2639; Rank: 1

Peptide View

MS/MS Fragmentation of **AKPAAQSEEETAASPAASPTPQSAQEPSAPGK**
Found in **IPI00363771**, Tax_Id=10116 Gene_Symbol=Eif3s9 108 kDa protein

Match to Query 2639: 3250.419222 from(1084.480350,3+)
Title: 100101RatKid_NS_deglyco_24.989.989.3.dta
Data file K:\NewmanPaper\Piliang\3SubProteomes\Piliang3SP\mgf5ppm\ERLIC_3SubProteomes5ppm.mgf

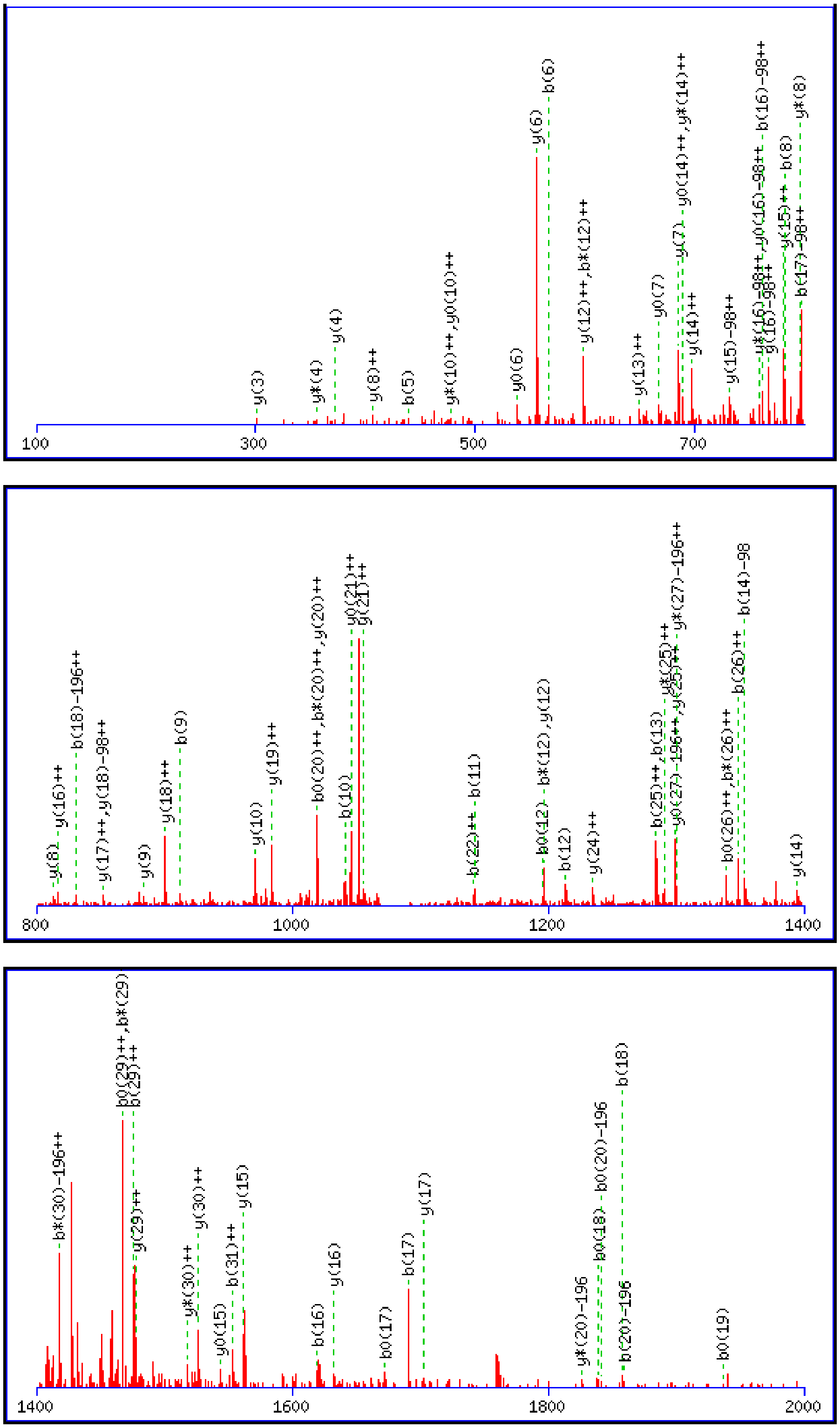

Monoisotopic mass of neutral peptide Mr(calc): 3250.4068
Fixed modifications: Carbamidomethyl (C)
Variable modifications:
S14 : Phospho (ST), with neutral losses 0.0000(shown in table), 97.9769
S18 : Phospho (ST), with neutral losses 0.0000(shown in table), 97.9769
Ions Score: 65 **Expect:** 0.00012
Matches (Bold Red): 82/560 fragment ions using 130 most intense peaks

#	b	b^{++}	b*	b^{*++}	b^0	b^{0++}	Seq.	y	y^{++}	y*	y^{*++}	y^0	y^{0++}	#
1	72.0444	36.5258					A							32
2	200.1394	100.5733	183.1128	92.0600			K	3180.3770	1590.6921	3163.3504	1582.1789	3162.3664	1581.6868	31
3	297.1921	149.0997	280.1656	140.5864			P	3052.2820	**1526.6446**	3035.2555	**1518.1314**	3034.2715	1517.6394	30
4	368.2292	184.6183	351.2027	176.1050			A	2955.2293	**1478.1183**	2938.2027	1469.6050	2937.2187	1469.1130	29

5	**439.2663**	220.1368	422.2398	211.6235			A	2884.1921	1442.5997	2867.1656	1434.0864	2866.1816	1433.5944	28
6	**567.3249**	284.1661	550.2984	275.6528			Q	2813.1550	1407.0812	2796.1285	1398.5679	2795.1445	1398.0759	27
7	654.3570	327.6821	637.3304	319.1688	636.3464	318.6768	S	2685.0964	1343.0519	2668.0699	1334.5386	2667.0859	1334.0466	26
8	**783.3995**	392.2034	766.3730	383.6901	765.3890	383.1981	E	2598.0644	**1299.5358**	2581.0379	**1291.0226**	2580.0539	1290.5306	25
9	**912.4421**	456.7247	895.4156	448.2114	894.4316	447.7194	E	2469.0218	**1235.0146**	2451.9953	1226.5013	2451.0113	1226.0093	24
10	**1041.4847**	521.2460	1024.4582	512.7327	1023.4742	512.2407	E	2339.9792	1170.4933	2322.9527	1161.9800	2321.9687	1161.4880	23
11	**1142.5324**	571.7698	1125.5059	563.2566	1124.5218	562.7646	T	2210.9366	1105.9720	2193.9101	1097.4587	2192.9261	1096.9667	22
12	**1213.5695**	607.2884	**1196.5430**	**598.7751**	**1195.5590**	598.2831	A	2109.8890	**1055.4481**	2092.8624	1046.9348	2091.8784	**1046.4428**	21
13	**1284.6066**	642.8070	1267.5801	634.2937	1266.5961	633.8017	A	2038.8518	**1019.9296**	2021.8253	1011.4163	2020.8413	1010.9243	20
14	1451.6050	726.3061	1434.5784	717.7929	1433.5944	717.3008	S	1967.8147	**984.4110**	1950.7882	975.8977	1949.8042	975.4057	19
15	1548.6578	774.8325	1531.6312	766.3192	1530.6472	765.8272	P	1800.8164	**900.9118**	1783.7898	892.3986	1782.8058	891.9065	18
16	**1619.6949**	810.3511	1602.6683	801.8378	1601.6843	801.3458	A	**1703.7636**	**852.3854**	1686.7371	843.8722	1685.7531	843.3802	17
17	**1690.7320**	845.8696	1673.7054	837.3564	**1672.7214**	836.8643	A	**1632.7265**	**816.8669**	1615.7000	808.3536	1614.7159	807.8616	16
18	**1857.7303**	929.3688	1840.7038	920.8555	**1839.7198**	920.3635	S	**1561.6894**	**781.3483**	1544.6628	772.8351	**1543.6788**	772.3430	15
19	1954.7831	977.8952	1937.7565	969.3819	**1936.7725**	968.8899	P	**1394.6910**	**697.8492**	1377.6645	**689.3359**	1376.6805	**688.8439**	14
20	2055.8308	1028.4190	2038.8042	**1019.9058**	2037.8202	**1019.4137**	T	1297.6383	**649.3228**	1280.6117	640.8095	1279.6277	640.3175	13
21	2152.8835	1076.9454	2135.8570	1068.4321	2134.8730	1067.9401	P	**1196.5906**	**598.7989**	1179.5640	590.2857	1178.5800	589.7937	12
22	2280.9421	**1140.9747**	2263.9156	1132.4614	2262.9316	1131.9694	Q	1099.5378	550.2726	1082.5113	541.7593	1081.5273	541.2673	11
23	2367.9741	1184.4907	2350.9476	1175.9774	2349.9636	1175.4854	S	**971.4793**	486.2433	954.4527	**477.7300**	953.4687	**477.2380**	10
24	2439.0113	1220.0093	2421.9847	1211.4960	2421.0007	1211.0040	A	**884.4472**	442.7272	867.4207	434.2140	866.4367	433.7220	9
25	2567.0698	**1284.0386**	2550.0433	1275.5253	2549.0593	1275.0333	Q	**813.4101**	**407.2087**	**796.3836**	398.6954	795.3995	398.2034	8
26	2696.1124	**1348.5599**	2679.0859	**1340.0466**	2678.1019	**1339.5546**	E	**685.3515**	343.1794	668.3250	334.6661	**667.3410**	334.1741	7
27	2793.1652	1397.0862	2776.1386	1388.5730	2775.1546	1388.0810	P	**556.3089**	278.6581	539.2824	270.1448	**538.2984**	269.6528	6
28	2880.1972	1440.6022	2863.1707	1432.0890	2862.1867	1431.5970	S	459.2562	230.1317	442.2296	221.6185	441.2456	221.1264	5
29	2951.2343	**1476.1208**	2934.2078	**1467.6075**	2933.2238	**1467.1155**	A	**372.2241**	186.6157	**355.1976**	178.1024			4
30	3048.2871	1524.6472	3031.2606	1516.1339	3030.2765	1515.6419	P	**301.1870**	151.0972	284.1605	142.5839			3
31	3105.3086	**1553.1579**	3088.2820	1544.6446	3087.2980	1544.1526	G	204.1343	102.5708	187.1077	94.0575			2
32							K	147.1128	74.0600	130.0863	65.5468			1

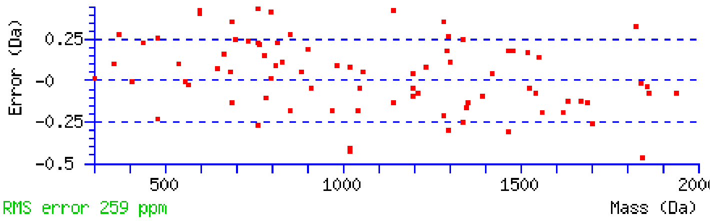

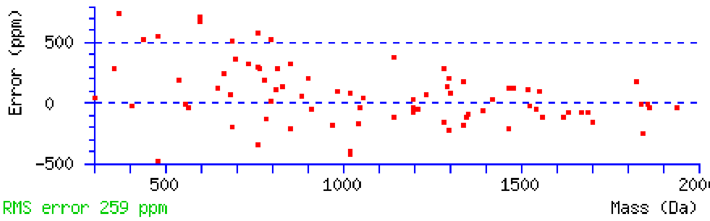

All matches to this query

Score	Mr(calc):	Delta	Sequence
65.3	3250.4068	0.0125	AKPAAQSEEETAASPAASPTPQSAQEPSAPGK
36.3	3250.4068	0.0125	AKPAAQSEEETAASPAASPTPQSAQEPSAPGK
26.6	3250.4068	0.0125	AKPAAQSEEETAASPAASPTPQSAQEPSAPGK
22.3	3250.4068	0.0125	AKPAAQSEEETAASPAASPTPQSAQEPSAPGK
16.4	3250.4068	0.0125	AKPAAQSEEETAASPAASPTPQSAQEPSAPGK
7.0	3249.3862	1.0330	NQNLQVTERMTLSPSNCQLRIHDVR
7.0	3249.3862	1.0330	NQNLQVTERMTLSPSNCQLRIHDVR
7.0	3249.3862	1.0330	NQNLQVTERMTLSPSNCQLRIHDVR
7.0	3249.3862	1.0330	NQNLQVTERMTLSPSNCQLRIHDVR
7.0	3249.3862	1.0330	NQNLQVTERMTLSPSNCQLRIHDVR

Spectrum No: 130; Query: 1736; Rank: 1

Peptide View

MS/MS Fragmentation of **SLDSDESEDEDDDYQQK**
Found in **IPI00208277**, Tax_Id=10116 Gene_Symbol=Pdap1 28 kDa heat- and acid-stable phosphoprotein

Match to Query 1736: 2096.718268 from(1049.366410,2+)
Title: 091008RatKidney_NoSalt_33.792.792.2.dta
Data file K:\NewmanPaper\Piliang\3SubProteomes\Piliang3SP\mgf5ppm\ERLIC_3SubProteomes5ppm.mgf

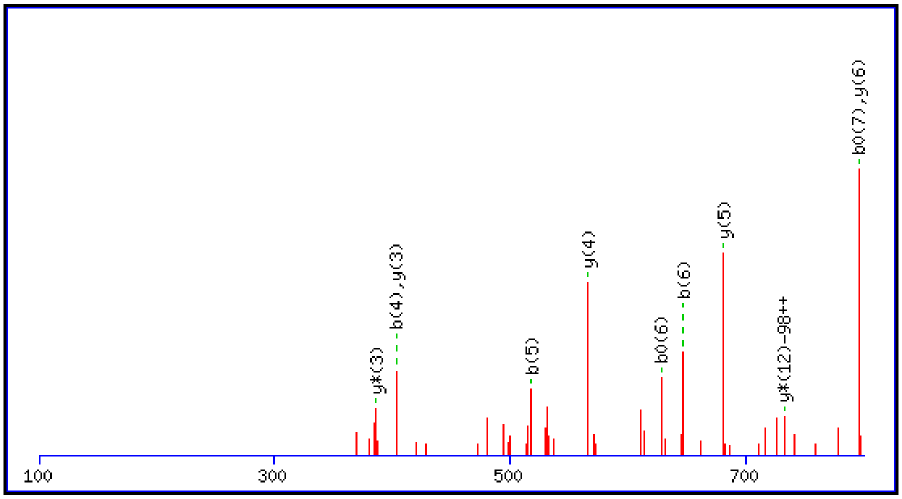

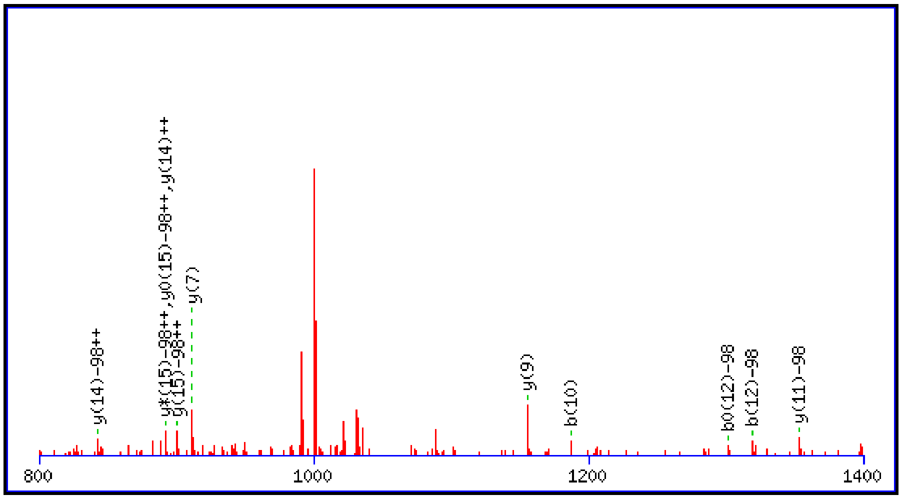

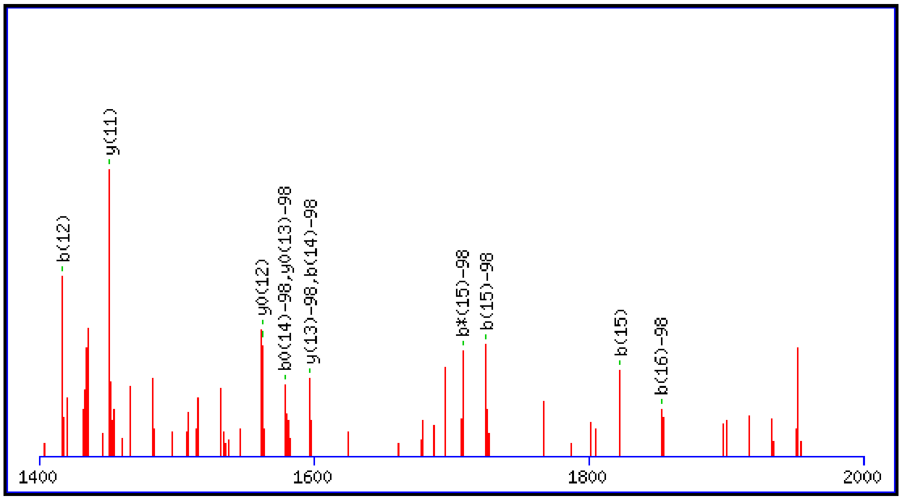

Monoisotopic mass of neutral peptide Mr(calc): 2096.7219
Fixed modifications: Carbamidomethyl (C)

Variable modifications:
S7 : Phospho (ST), with neutral losses 97.9769(shown in table), 0.0000
Ions Score: 65 **Expect:** 2e-005
Matches (Bold Red): 33/236 fragment ions using 36 most intense peaks

#	b	b^{++}	b*	b^{*++}	b^0	b^{0++}	Seq.	y	y^{++}	y*	y^{*++}	y^0	y^{0++}	#
1	88.0393	44.5233			70.0287	35.5180	S							17
2	201.1234	101.0653			183.1128	92.0600	L	1912.7203	956.8638	1895.6937	948.3505	1894.7097	947.8585	16
3	316.1503	158.5788			298.1397	149.5735	D	1799.6362	**900.3217**	1782.6097	**891.8085**	1781.6256	**891.3165**	15
4	**403.1823**	202.0948			385.1718	193.0895	S	1684.6093	**842.8083**	1667.5827	834.2950	1666.5987	833.8030	14
5	**518.2093**	259.6083			500.1987	250.6030	D	**1597.5772**	799.2923	1580.5507	790.7790	**1579.5667**	790.2870	13
6	**647.2519**	324.1296			**629.2413**	315.1243	E	1482.5503	741.7788	1465.5237	**733.2655**	1464.5397	732.7735	12
7	716.2733	358.6403			698.2628	349.6350	S	**1353.5077**	677.2575	1336.4812	668.7442	1335.4971	668.2522	11
8	845.3159	423.1616			827.3054	414.1563	E	1284.4862	642.7468	1267.4597	634.2335	1266.4757	633.7415	10
9	960.3429	480.6751			942.3323	471.6698	D	**1155.4437**	578.2255	1138.4171	569.7122	1137.4331	569.2202	9
10	1089.3855	545.1964			1071.3749	536.1911	E	1040.4167	520.7120	1023.3902	512.1987	1022.4061	511.7067	8
11	1204.4124	602.7098			1186.4018	593.7046	D	**911.3741**	456.1907	894.3476	447.6774	893.3636	447.1854	7
12	**1319.4393**	660.2233			**1301.4288**	651.2180	D	**796.3472**	398.6772	779.3206	390.1640	778.3366	389.6719	6
13	1434.4663	717.7368			1416.4557	708.7315	D	**681.3202**	341.1638	664.2937	332.6505	663.3097	332.1585	5
14	**1597.5296**	799.2684			**1579.5191**	790.2632	Y	**566.2933**	283.6503	549.2667	275.1370			4
15	**1725.5882**	863.2977	**1708.5616**	854.7845	1707.5776	854.2925	Q	**403.2300**	202.1186	**386.2034**	193.6053			3
16	**1853.6468**	927.3270	1836.6202	918.8138	1835.6362	918.3217	Q	275.1714	138.0893	258.1448	129.5761			2
17							K	147.1128	74.0600	130.0863	65.5468			1

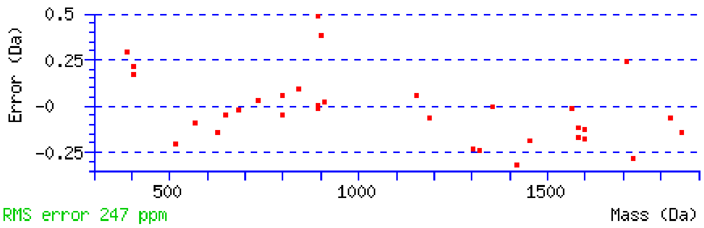

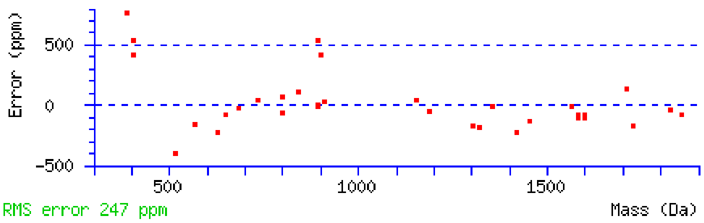

All matches to this query

Score	Mr(calc):	Delta	Sequence
65.2	2096.7219	-0.0036	SLDSDESEDEDDDYQQK
36.8	2096.7219	-0.0036	SLDSDESEDEDDDYQQK
35.6	2096.7219	-0.0036	SLDSDESEDEDDDYQQK
7.0	2096.7219	-0.0036	SLDSDESEDEDDDYQQK
0.3	2094.7126	2.0057	MADDVDPQQTNNTAEER

Spectrum No: 131; Query: 1435; Rank: 1

Peptide View

MS/MS Fragmentation of **EIQGDGPPSSPTNDPTVK**
Found in **IPI00369880**, Tax_Id=10116 Gene_Symbol=Chd3 similar to chromodomain helicase DNA binding protein 3 isoform 3

Match to Query 1435: 1917.844888 from(959.929720,2+)
Title: 100101RatKid_NS_deglyco_23.1044.1044.2.dta
Data file K:\NewmanPaper\Piliang\3SubProteomes\Piliang3SP\mgf5ppm\ERLIC_3SubProteomes5ppm.mgf

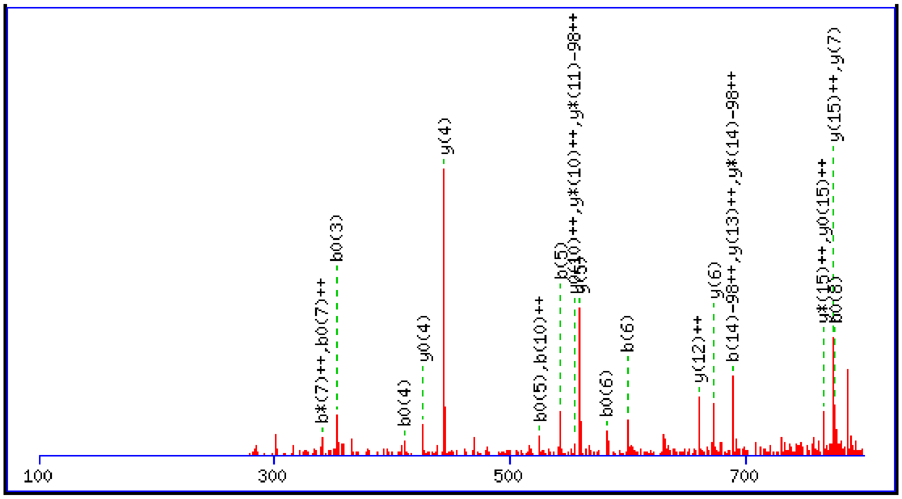

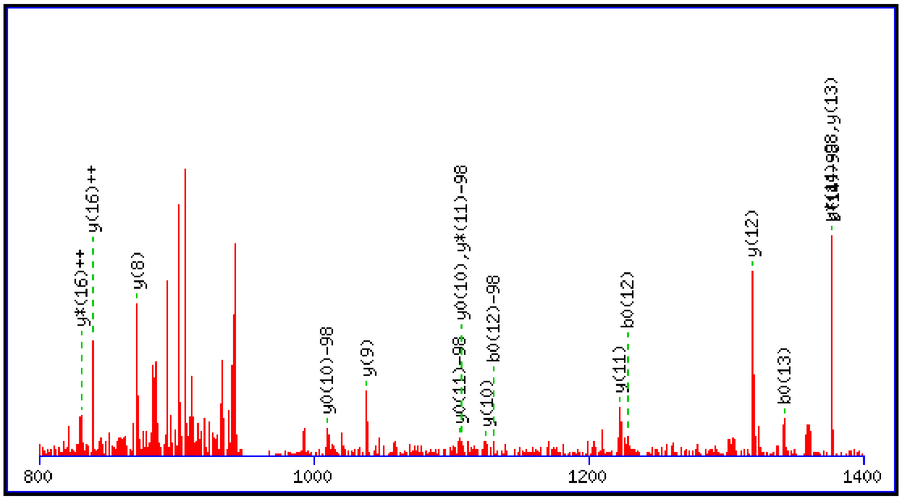

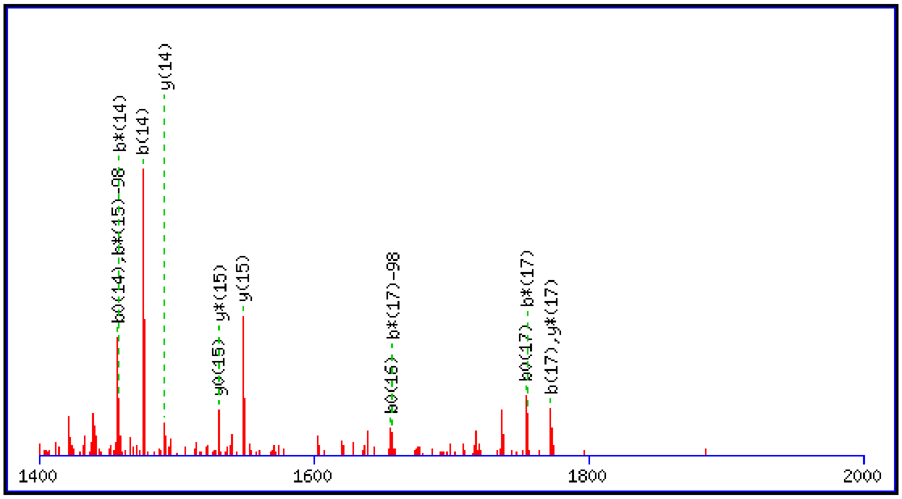

Monoisotopic mass of neutral peptide Mr(calc): 1917.8357
Fixed modifications: Carbamidomethyl (C)
Variable modifications:
S10 : Phospho (ST), with neutral losses 0.0000(shown in table), 97.9769
Ions Score: 65 **Expect:** 7e-005
Matches (Bold Red): 56/298 fragment ions using 85 most intense peaks

#	b	b^{++}	b*	b^{*++}	b^0	b^{0++}	Seq.	y	y^{++}	y*	y^{*++}	y^0	y^{0++}	#
1	130.0499	65.5286			112.0393	56.5233	E							18
2	243.1339	122.0706			225.1234	113.0653	I	1789.8004	895.4038	**1772.7738**	886.8906	1771.7898	886.3986	17
3	371.1925	186.0999	354.1660	177.5866	**353.1819**	177.0946	Q	1676.7163	**838.8618**	1659.6898	**830.3485**	1658.7058	829.8565	16
4	428.2140	214.6106	411.1874	206.0974	**410.2034**	205.6053	G	**1548.6578**	**774.8325**	**1531.6312**	**766.3192**	**1530.6472**	**765.8272**	15
5	**543.2409**	272.1241	526.2144	263.6108	**525.2304**	263.1188	D	**1491.6363**	746.3218	1474.6097	737.8085	1473.6257	737.3165	14

**6**	**600.2624**	300.6348	583.2358	292.1216	**582.2518**	291.6295	**G**	**1376.6093**	**688.8083**	1359.5828	680.2950	1358.5988	679.8030	**13**
**7**	697.3151	349.1612	680.2886	**340.6479**	679.3046	**340.1559**	**P**	**1319.5879**	**660.2976**	1302.5613	651.7843	1301.5773	651.2923	**12**
**8**	794.3679	397.6876	777.3414	389.1743	**776.3573**	388.6823	**P**	**1222.5351**	611.7712	1205.5086	603.2579	1204.5246	602.7659	**11**
**9**	881.3999	441.2036	864.3734	432.6903	863.3894	432.1983	**S**	**1125.4824**	563.2448	1108.4558	**554.7315**	**1107.4718**	**554.2395**	**10**
**10**	1048.3983	**524.7028**	1031.3717	516.1895	1030.3877	515.6975	**S**	**1038.4503**	519.7288	1021.4238	511.2155	1020.4398	510.7235	**9**
**11**	1145.4511	573.2292	1128.4245	564.7159	1127.4405	564.2239	**P**	**871.4520**	436.2296	854.4254	427.7164	853.4414	427.2243	**8**
**12**	1246.4987	623.7530	1229.4722	615.2397	**1228.4882**	614.7477	**T**	**774.3992**	387.7032	757.3727	379.1900	756.3886	378.6980	**7**
**13**	1360.5417	680.7745	1343.5151	672.2612	**1342.5311**	671.7692	**N**	**673.3515**	337.1794	656.3250	328.6661	655.3410	328.1741	**6**
**14**	**1475.5686**	738.2879	**1458.5421**	729.7747	**1457.5580**	729.2827	**D**	**559.3086**	280.1579	542.2821	271.6447	541.2980	271.1527	**5**
**15**	1572.6214	786.8143	1555.5948	778.3010	1554.6108	777.8090	**P**	**444.2817**	222.6445	427.2551	214.1312	**426.2711**	213.6392	**4**
**16**	1673.6690	837.3382	1656.6425	828.8249	**1655.6585**	828.3329	**T**	347.2289	174.1181	330.2023	165.6048	329.2183	165.1128	**3**
**17**	**1772.7375**	886.8724	**1755.7109**	878.3591	**1754.7269**	877.8671	**V**	246.1812	123.5942	229.1547	115.0810			**2**
**18**							**K**	147.1128	74.0600	130.0863	65.5468			**1**

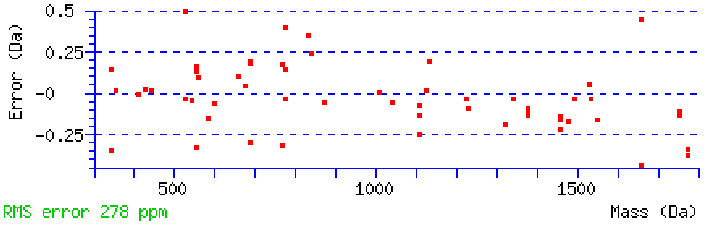

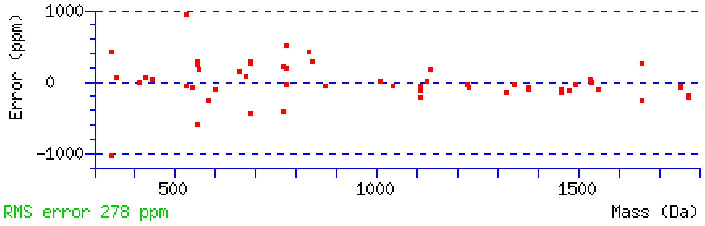

All matches to this query

Score	Mr(calc):	Delta	Sequence
65.2	1917.8357	0.0092	EIQGDGPPSSPTNDPTVK
54.5	1917.8357	0.0092	EIQGDGPPSSPTNDPTVK
45.6	1917.8357	0.0092	EIQGDGPPSSPTNDPTVK
18.3	1917.8357	0.0092	EIQGDGPPSSPTNDPTVK
3.6	1916.8564	0.9885	MVNSSRVQPQPPGDPGR
3.6	1916.8564	0.9885	MVNSSRVQPQPPGDPGR
3.5	1915.8313	2.0136	SGDLAPPESPSSPGQASPR
3.0	1915.8533	1.9916	MQANNTKAVSTCAEAIK
2.0	1917.8431	0.0018	GVISSVDGMSLEGFPSEK
1.5	1915.8533	1.9916	MQANNTKAVSTCAEAIK

Spectrum No: 132; Query: 2601; Rank: 1

Peptide View

MS/MS Fragmentation of **TNTNVNCPIECFMPLDVQADRDDSRE**
Found in **IPI00200757**, Tax_Id=10116 Gene_Symbol=Fn1 Isoform 1 of Fibronectin precursor

Match to Query 2601: 3175.316232 from(1059.446020,3+)
Title: 100101RatKid_NS_deglyco_19.4368.4368.3.dta
Data file K:\NewmanPaper\Piliang\3SubProteomes\Piliang3SP\mgf5ppm\ERLIC_3SubProteomes5ppm.mgf

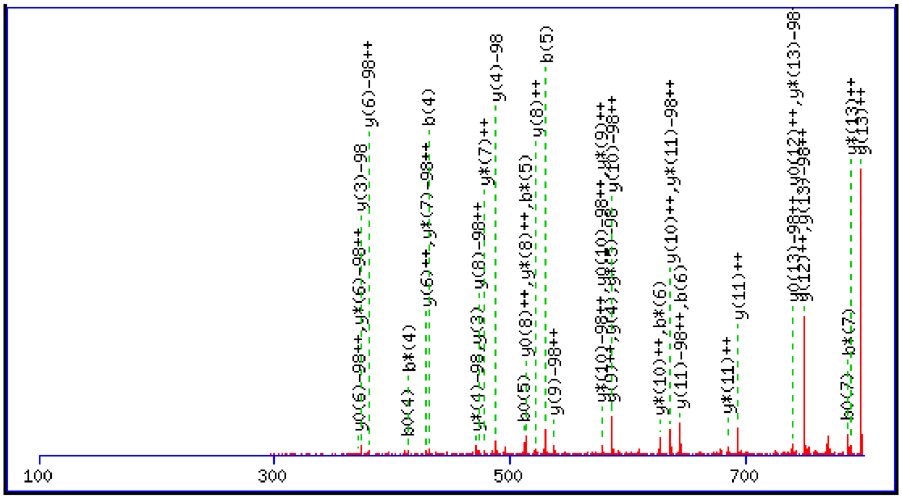

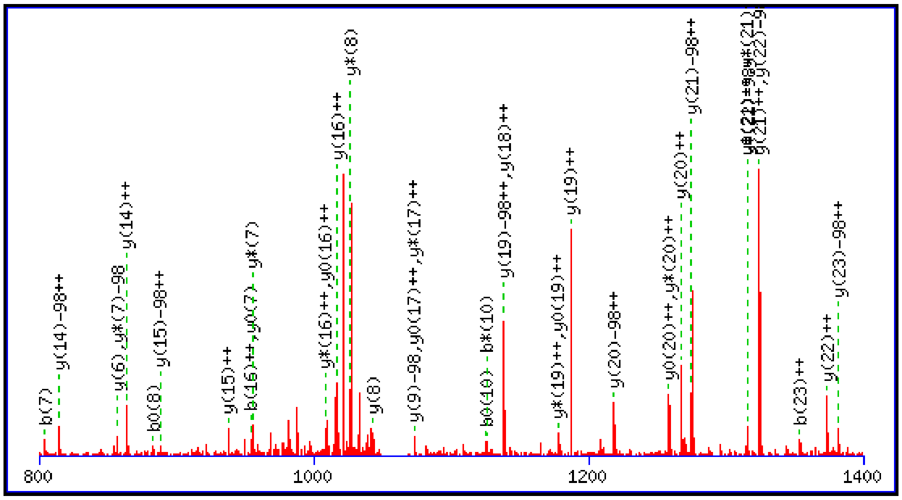

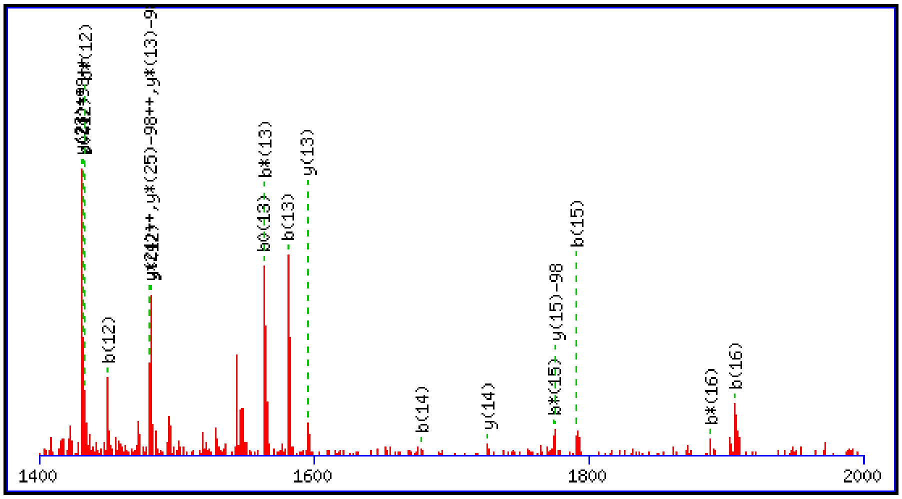

Monoisotopic mass of neutral peptide Mr(calc): 3175.3046
Fixed modifications: Carbamidomethyl (C)
Variable modifications:
S24 : Phospho (ST), with neutral losses 0.0000(shown in table), 97.9769
Ions Score: 65 **Expect:** 0.00013
Matches (Bold Red): 106/446 fragment ions using 151 most intense peaks

#	b	b^{++}	b*	b^{*++}	b^{0}	b^{0++}	Seq.	y	y^{++}	y*	y^{*++}	y^{0}	y^{0++}	#
1	102.0550	51.5311			84.0444	42.5258	T							26
2	216.0979	108.5526	199.0713	100.0393	198.0873	99.5473	N	3075.2642	1538.1357	3058.2377	1529.6225	3057.2536	1529.1305	25
3	317.1456	159.0764	300.1190	150.5631	299.1350	150.0711	T	2961.2213	**1481.1143**	2944.1947	1472.6010	2943.2107	1472.1090	24
4	**431.1885**	216.0979	**414.1619**	207.5846	**413.1779**	207.0926	N	2860.1736	**1430.5904**	2843.1471	1422.0772	2842.1630	1421.5852	23
5	**530.2569**	265.6321	**513.2304**	257.1188	**512.2463**	256.6268	V	2746.1307	**1373.5690**	2729.1041	1365.0557	2728.1201	1364.5637	22

6	644.2998	322.6536	627.2733	314.1403	626.2893	313.6483	N	2647.0623	1324.0348	2630.0357	1315.5215	2629.0517	1315.0295	21
7	804.3305	402.6689	787.3039	394.1556	786.3199	393.6636	C	2533.0193	1267.0133	2515.9928	1258.5000	2515.0088	1258.0080	20
8	901.3832	451.1953	884.3567	442.6820	883.3727	442.1900	P	2372.9887	1186.9980	2355.9621	1178.4847	2354.9781	1177.9927	19
9	1014.4673	507.7373	997.4408	499.2240	996.4567	498.7320	I	2275.9359	1138.4716	2258.9094	1129.9583	2257.9254	1129.4663	18
10	1143.5099	572.2586	1126.4834	563.7453	1125.4993	563.2533	E	2162.8519	1081.9296	2145.8253	1073.4163	2144.8413	1072.9243	17
11	1303.5405	652.2739	1286.5140	643.7606	1285.5300	643.2686	C	2033.8093	1017.4083	2016.7827	1008.8950	2015.7987	1008.4030	16
12	1450.6090	725.8081	1433.5824	717.2948	1432.5984	716.8028	F	1873.7786	937.3929	1856.7521	928.8797	1855.7680	928.3877	15
13	1581.6494	791.3284	1564.6229	782.8151	1563.6389	782.3231	M	1726.7102	863.8587	1709.6837	855.3455	1708.6996	854.8535	14
14	1678.7022	839.8547	1661.6757	831.3415	1660.6916	830.8495	P	1595.6697	798.3385	1578.6432	789.8252	1577.6591	789.3332	13
15	1791.7863	896.3968	1774.7597	887.8835	1773.7757	887.3915	L	1498.6170	749.8121	1481.5904	741.2988	1480.6064	740.8068	12
16	1906.8132	953.9102	1889.7867	945.3970	1888.8027	944.9050	D	1385.5329	693.2701	1368.5063	684.7568	1367.5223	684.2648	11
17	2005.8816	1003.4445	1988.8551	994.9312	1987.8711	994.4392	V	1270.5059	635.7566	1253.4794	627.2433	1252.4954	626.7513	10
18	2133.9402	1067.4737	2116.9137	1058.9605	2115.9296	1058.4685	Q	1171.4375	586.2224	1154.4110	577.7091	1153.4270	577.2171	9
19	2204.9773	1102.9923	2187.9508	1094.4790	2186.9668	1093.9870	A	1043.3790	522.1931	1026.3524	513.6798	1025.3684	513.1878	8
20	2320.0043	1160.5058	2302.9777	1151.9925	2301.9937	1151.5005	D	972.3418	486.6746	955.3153	478.1613	954.3313	477.6693	7
21	2476.1054	1238.5563	2459.0788	1230.0431	2458.0948	1229.5510	R	857.3149	429.1611	840.2883	420.6478	839.3043	420.1558	6
22	2591.1323	1296.0698	2574.1058	1287.5565	2573.1218	1287.0645	D	701.2138	351.1105	684.1872	342.5973	683.2032	342.1052	5
23	2706.1593	1353.5833	2689.1327	1345.0700	2688.1487	1344.5780	D	586.1868	293.5971	569.1603	285.0838	568.1763	284.5918	4
24	2873.1576	1437.0824	2856.1311	1428.5692	2855.1471	1428.0772	S	471.1599	236.0836	454.1333	227.5703	453.1493	227.0783	3
25	3029.2587	1515.1330	3012.2322	1506.6197	3011.2482	1506.1277	R	304.1615	152.5844	287.1350	144.0711	286.1510	143.5791	2
26							E	148.0604	74.5339			130.0499	65.5286	1

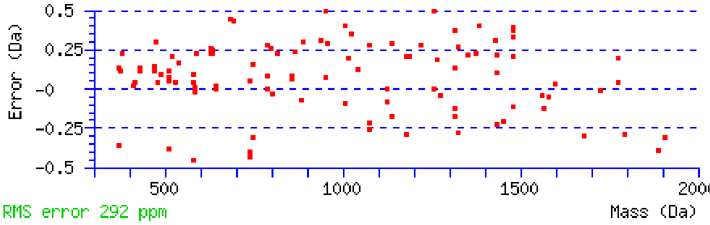

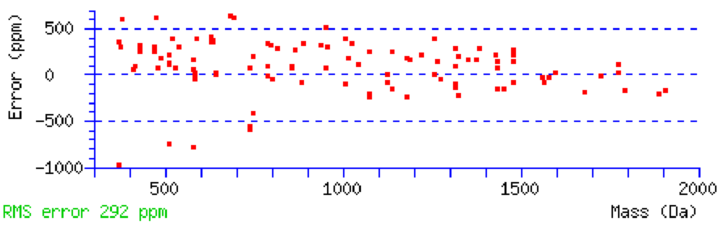

All matches to this query

Score	Mr(calc):	Delta	Sequence
65.2	3175.3046	0.0116	TNTNVNCPIECFMPLDVQADRDDSRE
4.4	3173.3252	1.9910	TVNTEALMTVFHPQNLENLDSKMRK
3.2	3174.3051	1.0112	FGPESKNSPFTCSLPTLPAKVYMGVK
2.9	3174.3035	1.0127	MNSNNRDSSALNHSSKPLPMAPEEKNK
2.5	3173.3158	2.0005	TGFSMSVCELPLPAQSCNSIGSSSPSAVK
2.5	3174.2998	1.0165	TGFSMSVCELPLPAQSCNSIGSSSPSAVK
2.5	3173.3158	2.0005	TGFSMSVCELPLPAQSCNSIGSSSPSAVK
2.5	3174.2998	1.0165	TGFSMSVCELPLPAQSCNSIGSSSPSAVK
2.3	3173.3195	1.9967	MNSNNRDSSALNHSSKPLPMAPEEKNK
2.3	3174.3035	1.0127	MNSNNRDSSALNHSSKPLPMAPEEKNK

Spectrum No: 133; Query: 1576; Rank: 1

Peptide View

MS/MS Fragmentation of **VLHAQCHSTPDSAEDVR**
Found in **IPI00327469**, Tax_Id=10116 Gene_Symbol=Ahsg Alpha-2-HS-glycoprotein precursor

Match to Query 1576: 2000.839048 from(1001.426800,2+)

Title: 091008RatKidney_NH4Format02_24.513.513.2.dta
Data file K:\NewmanPaper\Piliang\3SubProteomes\Piliang3SP\mgf5ppm\ERLIC_3SubProteomes5ppm.mgf

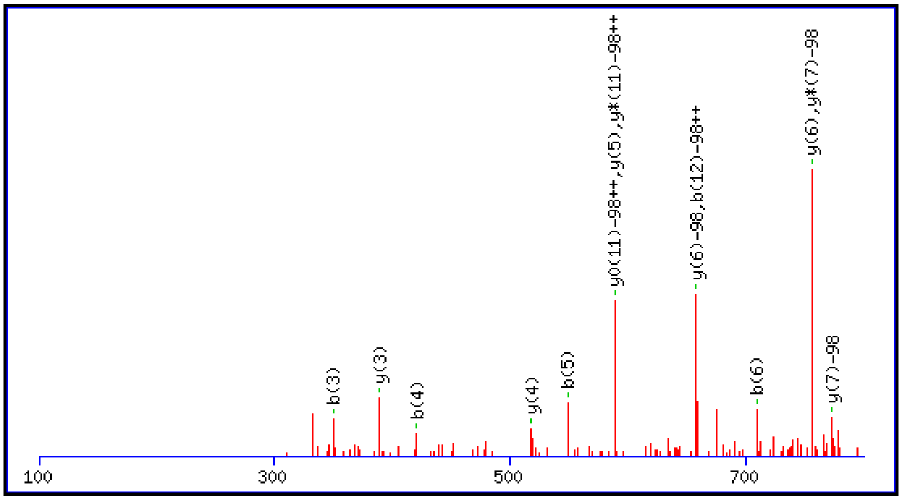

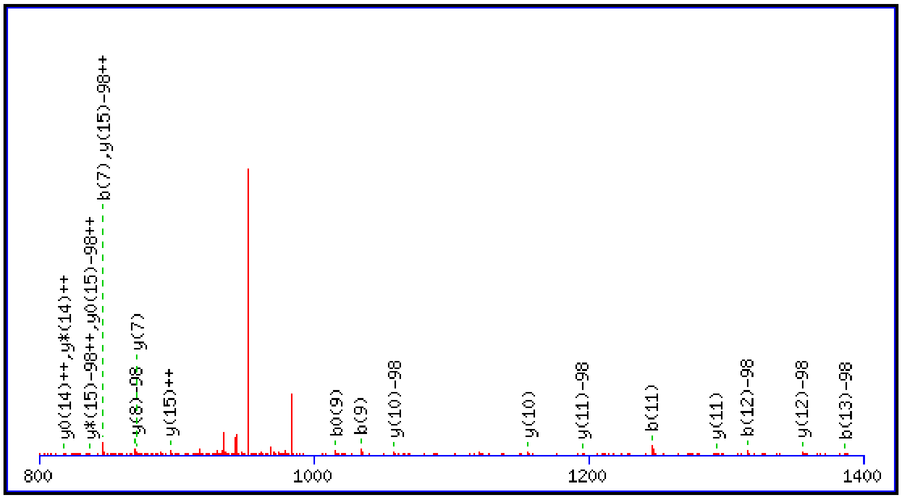

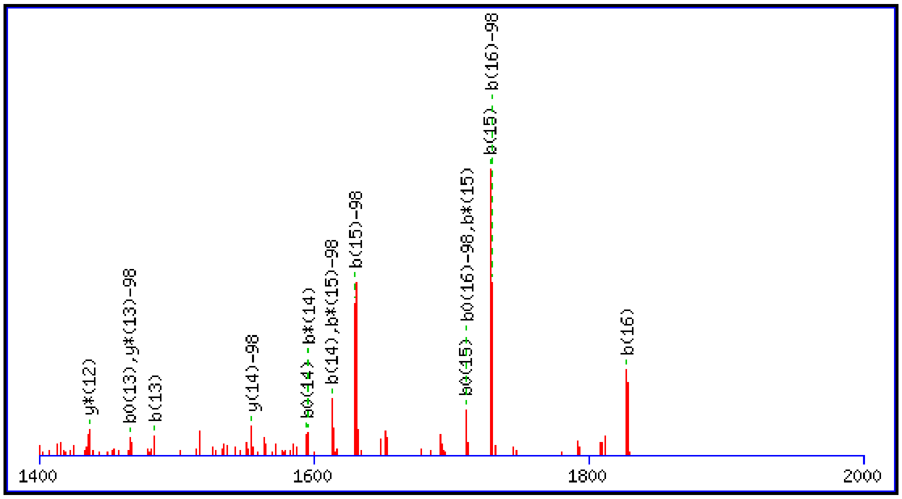

Monoisotopic mass of neutral peptide Mr(calc): 2000.8412
Fixed modifications: Carbamidomethyl (C)
Variable modifications:
S12 : Phospho (ST), with neutral losses 97.9769(shown in table), 0.0000
Ions Score: 65 **Expect:** 6.8e-005
Matches (Bold Red): 49/262 fragment ions using 68 most intense peaks

#	b	b^{++}	b*	b^{*++}	b^{0}	b^{0++}	Seq.	y	y^{++}	y*	y^{*++}	y^{0}	y^{0++}	#
1	100.0757	50.5415					V							17
2	213.1598	107.0835					L	1804.8031	902.9052	1787.7766	894.3919	1786.7926	893.8999	16
3	**350.2187**	175.6130					H	1691.7191	**846.3632**	1674.6925	**837.8499**	1673.7085	**837.3579**	15

4	**421.2558**	211.1315					A	**1554.6601**	777.8337	1537.6336	769.3204	1536.6496	768.8284	14
5	**549.3144**	275.1608	532.2878	266.6475			Q	1483.6230	742.3152	**1466.5965**	733.8019	1465.6125	733.3099	13
6	**709.3450**	355.1761	692.3185	346.6629			C	**1355.5644**	678.2859	1338.5379	669.7726	1337.5539	669.2806	12
7	**846.4039**	423.7056	829.3774	415.1923			H	**1195.5338**	598.2705	1178.5072	**589.7573**	1177.5232	**589.2653**	11
8	933.4359	467.2216	916.4094	458.7083	915.4254	458.2163	S	**1058.4749**	529.7411	1041.4483	521.2278	1040.4643	520.7358	10
9	**1034.4836**	517.7455	1017.4571	509.2322	**1016.4731**	508.7402	T	971.4429	486.2251	954.4163	477.7118	953.4323	477.2198	9
10	1131.5364	566.2718	1114.5098	557.7586	1113.5258	557.2666	P	**870.3952**	435.7012	853.3686	427.1880	852.3846	426.6959	8
11	**1246.5633**	623.7853	1229.5368	615.2720	1228.5528	614.7800	D	**773.3424**	387.1748	**756.3159**	378.6616	755.3319	378.1696	7
12	**1315.5848**	**658.2960**	1298.5582	649.7828	1297.5742	649.2908	S	**658.3155**	329.6614	641.2889	321.1481	640.3049	320.6561	6
13	**1386.6219**	693.8146	1369.5954	685.3013	1368.6113	684.8093	A	**589.2940**	295.1506	572.2675	286.6374	571.2835	286.1454	5
14	1515.6645	758.3359	1498.6379	749.8226	1497.6539	749.3306	E	**518.2569**	259.6321	501.2304	251.1188	500.2463	250.6268	4
15	**1630.6914**	815.8494	**1613.6649**	807.3361	1612.6809	806.8441	D	**389.2143**	195.1108	372.1878	186.5975	371.2037	186.1055	3
16	**1729.7599**	865.3836	1712.7333	856.8703	**1711.7493**	856.3783	V	274.1874	137.5973	257.1608	129.0840			2
17							R	175.1190	88.0631	158.0924	79.5498			1

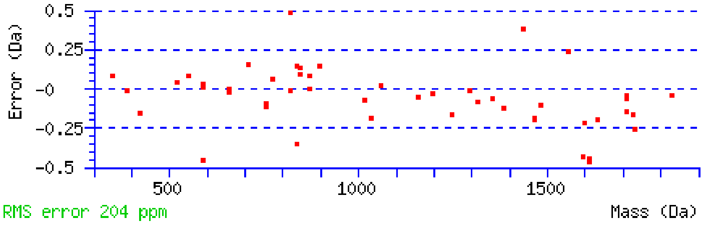

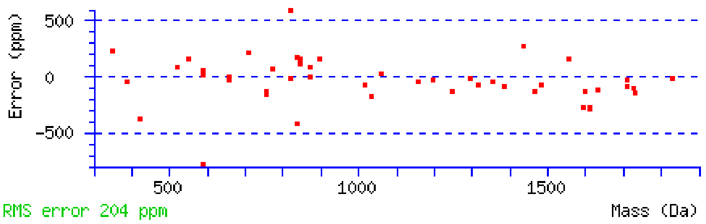

All matches to this query

Score	Mr(calc):	Delta	Sequence
64.9	2000.8412	-0.0021	VLHAQCHSTPDSAEDVR
53.4	2000.8412	-0.0021	VLHAQCHSTPDSAEDVR
53.4	2000.8412	-0.0021	VLHAQCHSTPDSAEDVR
7.0	2000.8576	-0.0185	TDDVSEKTSLADQEEVR
6.8	2000.8299	0.0091	KSCGSHEVEENGSVFVR
4.5	2000.8576	-0.0185	TDDVSEKTSLADQEEVR
4.3	2000.8550	-0.0160	IPGMYPLASANQEAESSR
4.2	1999.8343	1.0048	AFSTHTFSENPRPPPR
4.2	1999.8343	1.0048	AFSTHTFSENPRPPPR
3.9	1999.8185	1.0205	VNAVYMKMPVSLDGER

Spectrum No: 134; Query: 2581; Rank: 1

Peptide View

MS/MS Fragmentation of **RESLTSFGNGPLSPGGPSKPGGGGGGPGSGSTSR**
Found in **IPI00210027**, Tax_Id=10116 Gene_Symbol=Itpr1 Isoform 1 of Inositol 1,4,5-trisphosphate receptor type 1

Match to Query 2581: 3135.430376 from(784.864870,4+)
Title: 091008RatKidney_NH4Format01_19.2153.2153.4.dta
Data file K:\NewmanPaper\Piliang\3SubProteomes\Piliang3SP\mgf5ppm\ERLIC_3SubProteomes5ppm.mgf

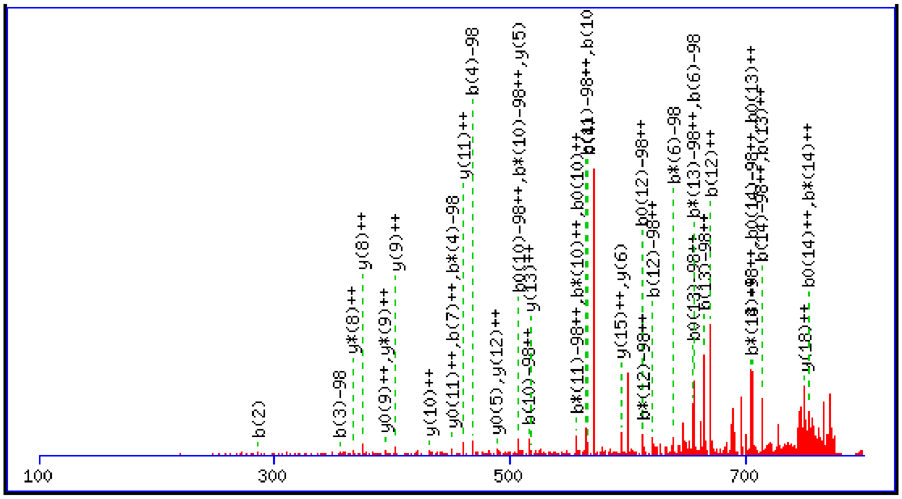

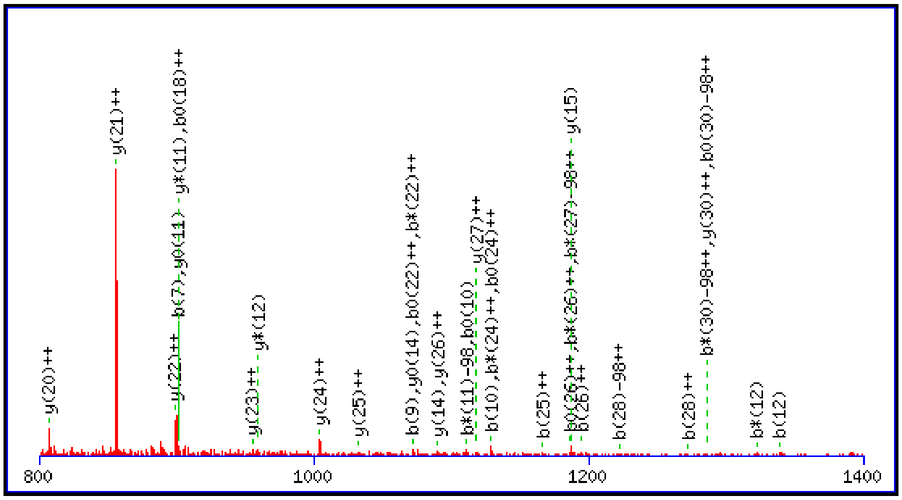

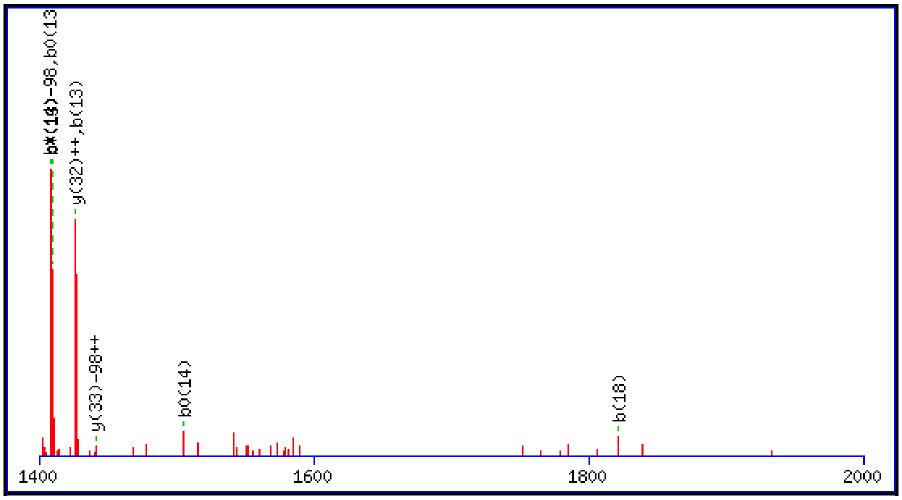

Monoisotopic mass of neutral peptide Mr(calc): 3135.4371
Fixed modifications: Carbamidomethyl (C)
Variable modifications:
S3 : Phospho (ST), with neutral losses 97.9769(shown in table), 0.0000
Ions Score: 65 **Expect:** 0.00014
Matches (Bold Red): 90/590 fragment ions using 140 most intense peaks

#	b	b^{++}	b*	b^{*++}	b^0	b^{0++}	Seq.	y	y^{++}	y*	y^{*++}	y^0	y^{0++}	#
1	157.1084	79.0578	140.0818	70.5446			R							34
2	**286.1510**	143.5791	269.1244	135.0659	268.1404	134.5738	E	2882.3663	**1441.6868**	2865.3398	1433.1735	2864.3558	1432.6815	33
3	**355.1724**	178.0899	338.1459	169.5766	337.1619	169.0846	S	2753.3238	1377.1655	2736.2972	1368.6522	2735.3132	1368.1602	32
4	**468.2565**	234.6319	**451.2300**	226.1186	450.2459	225.6266	L	2684.3023	1342.6548	2667.2757	1334.1415	2666.2917	1333.6495	31
5	569.3042	285.1557	552.2776	276.6425	551.2936	276.1504	T	2571.2182	**1286.1128**	2554.1917	1277.5995	2553.2077	1277.1075	30

6	656.3362	328.6717	639.3097	320.1585	638.3256	319.6665	S	2470.1706	1235.5889	2453.1440	1227.0756	2452.1600	1226.5836	29
7	803.4046	402.2059	786.3781	393.6927	785.3941	393.2007	F	2383.1385	1192.0729	2366.1120	1183.5596	2365.1280	1183.0676	28
8	860.4261	430.7167	843.3995	422.2034	842.4155	421.7114	G	2236.0701	1118.5387	2219.0436	1110.0254	2218.0595	1109.5334	27
9	974.4690	487.7381	957.4425	479.2249	956.4584	478.7329	N	2179.0486	1090.0280	2162.0221	1081.5147	2161.0381	1081.0227	26
10	1031.4905	516.2489	1014.4639	507.7356	1013.4799	507.2436	G	2065.0057	1033.0065	2047.9792	1024.4932	2046.9952	1024.0012	25
11	1128.5432	564.7753	1111.5167	556.2620	1110.5327	555.7700	P	2007.9843	1004.4958	1990.9577	995.9825	1989.9737	995.4905	24
12	1241.6273	621.3173	1224.6008	612.8040	1223.6167	612.3120	L	1910.9315	955.9694	1893.9049	947.4561	1892.9209	946.9641	23
13	1328.6593	664.8333	1311.6328	656.3200	1310.6488	655.8280	S	1797.8474	899.4274	1780.8209	890.9141	1779.8369	890.4221	22
14	1425.7121	713.3597	1408.6855	704.8464	1407.7015	704.3544	P	1710.8154	855.9113	1693.7889	847.3981	1692.8048	846.9061	21
15	1482.7336	741.8704	1465.7070	733.3571	1464.7230	732.8651	G	1613.7626	807.3850	1596.7361	798.8717	1595.7521	798.3797	20
16	1539.7550	770.3812	1522.7285	761.8679	1521.7445	761.3759	G	1556.7412	778.8742	1539.7146	770.3610	1538.7306	769.8689	19
17	1636.8078	818.9075	1619.7812	810.3943	1618.7972	809.9022	P	1499.7197	750.3635	1482.6932	741.8502	1481.7091	741.3582	18
18	1723.8398	862.4235	1706.8133	853.9103	1705.8293	853.4183	S	1402.6669	701.8371	1385.6404	693.3238	1384.6564	692.8318	17
19	1851.9348	926.4710	1834.9082	917.9578	1833.9242	917.4657	K	1315.6349	658.3211	1298.6084	649.8078	1297.6244	649.3158	16
20	1948.9875	974.9974	1931.9610	966.4841	1930.9770	965.9921	P	1187.5400	594.2736	1170.5134	585.7603	1169.5294	585.2683	15
21	2006.0090	1003.5081	1988.9825	994.9949	1987.9984	994.5029	G	1090.4872	545.7472	1073.4606	537.2340	1072.4766	536.7420	14
22	2063.0305	1032.0189	2046.0039	1023.5056	2045.0199	1023.0136	G	1033.4657	517.2365	1016.4392	508.7232	1015.4552	508.2312	13
23	2120.0519	1060.5296	2103.0254	1052.0163	2102.0414	1051.5243	G	976.4443	488.7258	959.4177	480.2125	958.4337	479.7205	12
24	2177.0734	1089.0403	2160.0468	1080.5271	2159.0628	1080.0351	G	919.4228	460.2150	902.3962	451.7018	901.4122	451.2098	11
25	2234.0949	1117.5511	2217.0683	1109.0378	2216.0843	1108.5458	G	862.4013	431.7043	845.3748	423.1910	844.3908	422.6990	10
26	2291.1163	1146.0618	2274.0898	1137.5485	2273.1058	1137.0565	G	805.3799	403.1936	788.3533	394.6803	787.3693	394.1883	9
27	2388.1691	1194.5882	2371.1425	1186.0749	2370.1585	1185.5829	P	748.3584	374.6828	731.3319	366.1696	730.3478	365.6776	8
28	2445.1906	1223.0989	2428.1640	1214.5856	2427.1800	1214.0936	G	651.3056	326.1565	634.2791	317.6432	633.2951	317.1512	7
29	2532.2226	1266.6149	2515.1960	1258.1017	2514.2120	1257.6096	S	594.2842	297.6457	577.2576	289.1325	576.2736	288.6404	6
30	2589.2440	1295.1257	2572.2175	1286.6124	2571.2335	1286.1204	G	507.2522	254.1297	490.2256	245.6164	489.2416	245.1244	5
31	2676.2761	1338.6417	2659.2495	1330.1284	2658.2655	1329.6364	S	450.2307	225.6190	433.2041	217.1057	432.2201	216.6137	4
32	2777.3238	1389.1655	2760.2972	1380.6522	2759.3132	1380.1602	T	363.1987	182.1030	346.1721	173.5897	345.1881	173.0977	3
33	2864.3558	1432.6815	2847.3292	1424.1683	2846.3452	1423.6762	S	262.1510	131.5791	245.1244	123.0659	244.1404	122.5738	2
34							R	175.1190	88.0631	158.0924	79.5498			1

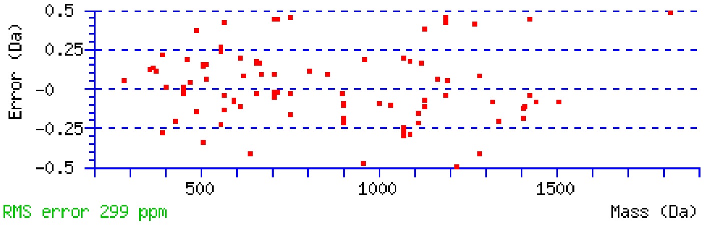

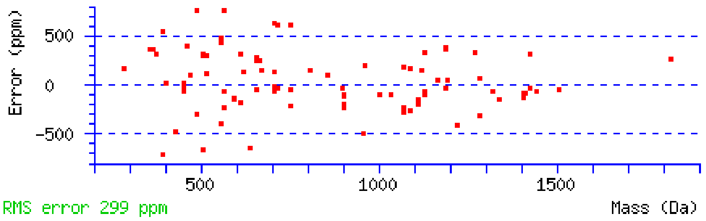

All matches to this query

Score	Mr(calc):	Delta	Sequence
64.8	3135.4371	-0.0067	RESLTSFGNGPLSPGGPSKPGGGGGGGPGSGSTSR
53.7	3135.4371	-0.0067	RESLTSFGNGPLSPGGPSKPGGGGGGGPGSGSTSR
53.7	3135.4371	-0.0067	RESLTSFGNGPLSPGGPSKPGGGGGGGPGSGSTSR
29.1	3135.4371	-0.0067	RESLTSFGNGPLSPGGPSKPGGGGGGGPGSGSTSR
5.8	3135.4371	-0.0067	RESLTSFGNGPLSPGGPSKPGGGGGGGPGSGSTSR
5.6	3133.4145	2.0158	LLQVTCTQMPPFICGALYLVSEILK
5.1	3134.4218	1.0085	EITARCPLAMTEELLQDLAQYKTHK
5.1	3134.4218	1.0085	EITARCPLAMTEELLQDLAQYKTHK
3.4	3135.4552	-0.0248	DGTSHPAMCLQNIDGLNLENLKIKMSR
3.4	3135.4552	-0.0248	DGTSHPAMCLQNIDGLNLENLKIKMSR

Spectrum No: 135; Query: 1088; Rank: 1

Peptide View

MS/MS Fragmentation of **SSLSSSSPSAGDTVTEK**
Found in **IPI00360075**, Tax_Id=10116 Gene_Symbol=Lrpprc Leucine-rich PPR motif-containing protein, mitochondrial precursor

Match to Query 1088: 1718.724688 from(860.369620,2+)
Title: 091008RatKidney_NoSalt_25.608.608.2.dta
Data file K:\NewmanPaper\Piliang\3SubProteomes\Piliang3SP\mgf5ppm\ERLIC_3SubProteomes5ppm.mgf

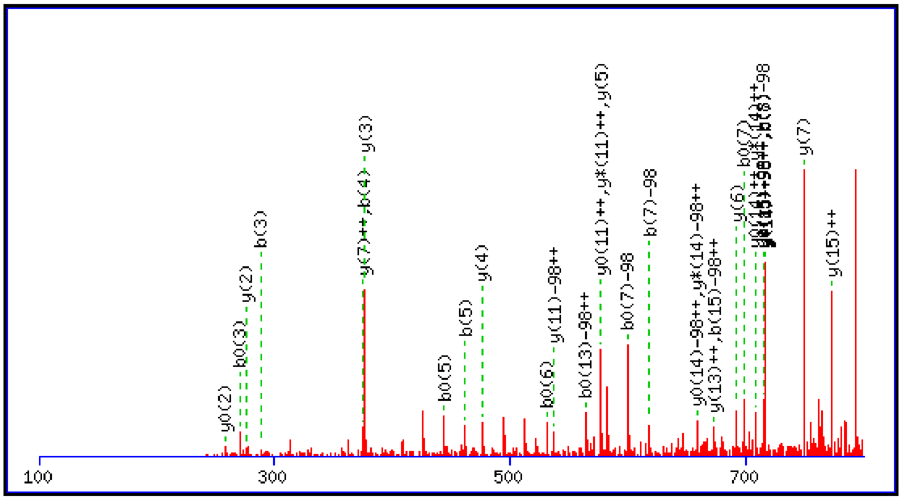

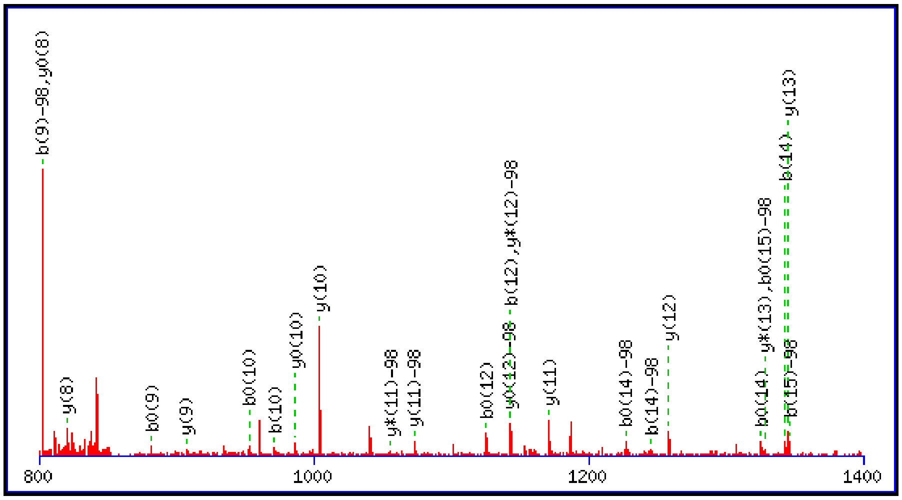

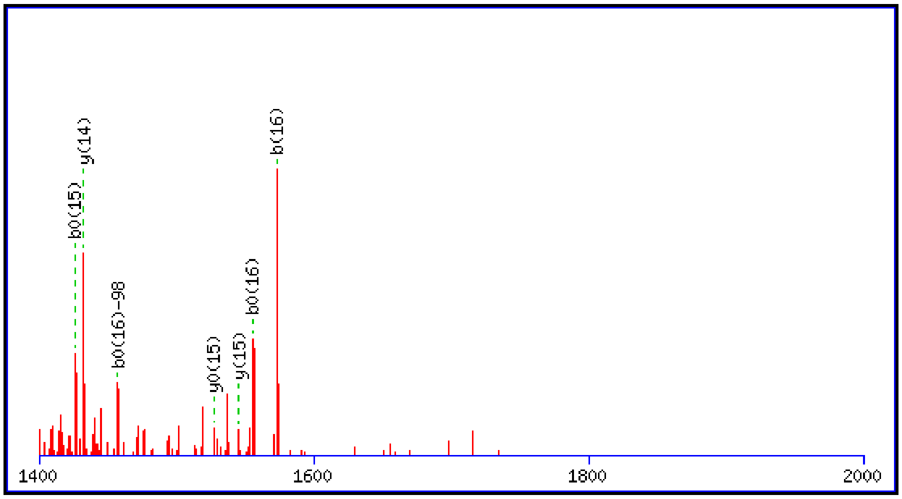

Monoisotopic mass of neutral peptide Mr(calc): 1718.7248
Fixed modifications: Carbamidomethyl (C)
Variable modifications:
S7 : Phospho (ST), with neutral losses 0.0000(shown in table), 97.9769
Ions Score: 65 **Expect:** 5.6e-005
Matches (Bold Red): 65/234 fragment ions using 121 most intense peaks

#	b	b^{++}	b^{0}	b^{0++}	Seq.	y	y^{++}	y^{*}	y^{*++}	y^{0}	y^{0++}	#
1	88.0393	44.5233	70.0287	35.5180	S							17
2	175.0713	88.0393	157.0608	79.0340	S	1632.7000	816.8536	1615.6735	808.3404	1614.6894	807.8484	16
3	**288.1554**	144.5813	**270.1448**	135.5761	L	**1545.6680**	**773.3376**	1528.6414	764.8244	**1527.6574**	764.3323	15
4	**375.1874**	188.0974	357.1769	179.0921	S	**1432.5839**	**716.7956**	1415.5574	**708.2823**	1414.5734	**707.7903**	14
5	**462.2195**	231.6134	**444.2089**	222.6081	S	**1345.5519**	**673.2796**	**1328.5253**	664.7663	1327.5413	664.2743	13
6	549.2515	275.1294	**531.2409**	266.1241	S	**1258.5199**	629.7636	1241.4933	621.2503	1240.5093	620.7583	12
7	**716.2498**	358.6286	**698.2393**	349.6233	S	**1171.4878**	586.2476	1154.4613	**577.7343**	1153.4773	**577.2423**	11
8	813.3026	407.1549	795.2920	398.1497	P	**1004.4895**	502.7484	987.4629	494.2351	**986.4789**	493.7431	10
9	900.3346	450.6710	**882.3241**	441.6657	S	**907.4367**	454.2220	890.4102	445.7087	889.4262	445.2167	9
10	**971.3717**	486.1895	**953.3612**	477.1842	A	**820.4047**	410.7060	803.3781	402.1927	**802.3941**	401.7007	8
11	1028.3932	514.7002	1010.3826	505.6950	G	**749.3676**	**375.1874**	732.3410	366.6742	731.3570	366.1821	7
12	**1143.4201**	572.2137	**1125.4096**	563.2084	D	**692.3461**	346.6767	675.3196	338.1634	674.3355	337.6714	6
13	1244.4678	622.7376	1226.4573	613.7323	T	**577.3192**	289.1632	560.2926	280.6499	559.3086	280.1579	5
14	**1343.5362**	672.2718	**1325.5257**	663.2665	V	**476.2715**	238.6394	459.2449	230.1261	458.2609	229.6341	4
15	1444.5839	722.7956	**1426.5734**	713.7903	T	**377.2031**	189.1052	360.1765	180.5919	359.1925	180.0999	3
16	**1573.6265**	787.3169	**1555.6159**	778.3116	E	**276.1554**	138.5813	259.1288	130.0681	**258.1448**	129.5761	2
17					K	147.1128	74.0600	130.0863	65.5468			1

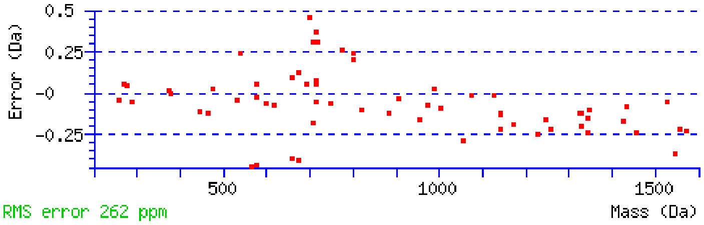

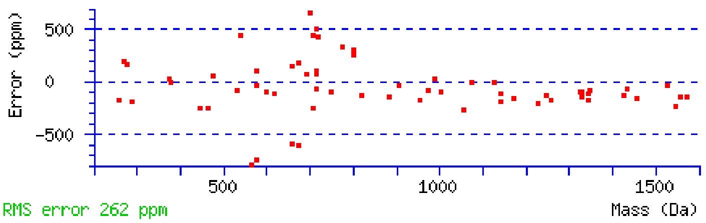

All matches to this query

Score	Mr(calc):	Delta	Sequence
64.8	1718.7248	-0.0001	SSLSSSSPSAGDTVTEK
55.5	1718.7248	-0.0001	SSLSSSSPSAGDTVTEK
51.3	1718.7248	-0.0001	SSLSSSSPSAGDTVTEK
44.6	1718.7248	-0.0001	SSLSSSSPSAGDTVTEK
34.2	1718.7248	-0.0001	SSLSSSSPSAGDTVTEK
31.1	1718.7248	-0.0001	SSLSSSSPSAGDTVTEK
27.2	1718.7248	-0.0001	SSLSSSSPSAGDTVTEK
24.4	1718.7248	-0.0001	SSLSSSSPSAGDTVTEK
18.4	1717.7164	1.0083	QNMSSSMAQQSKSPK
14.1	1718.7248	-0.0001	SSLSSSSPSAGDTVTEK

Spectrum No: 136; Query: 2373; Rank: 1

Peptide View

MS/MS Fragmentation of **RSASPDDDLGSSNWEAADLGNEER**
Found in **IPI00371185**, Tax_Id=10116 Gene_Symbol=RGD1311703 Similar to sid2057p

Match to Query 2373: 2670.089922 from(891.037250,3+)
Title: 091008RatKidney_NH4Format01_23.2372.2372.3.dta
Data file K:\NewmanPaper\Piliang\3SubProteomes\Piliang3SP\mgf5ppm\ERLIC_3SubProteomes5ppm.mgf

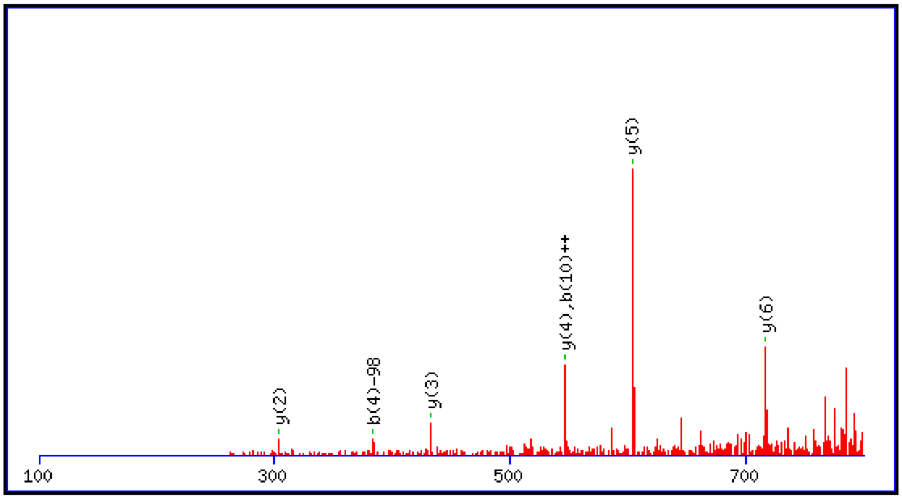

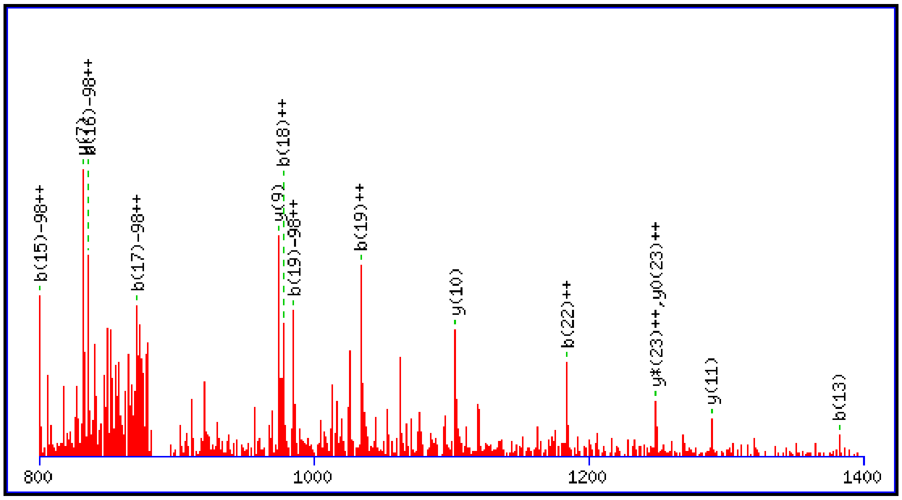

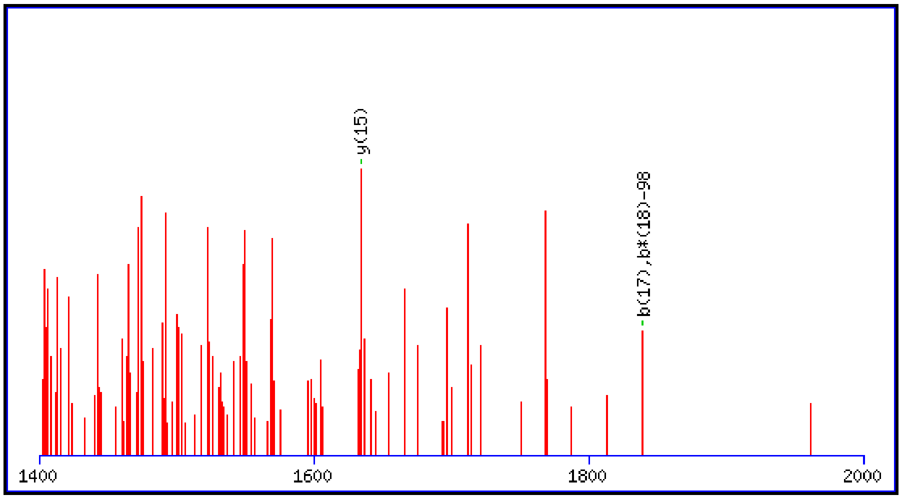

Monoisotopic mass of neutral peptide Mr(calc): 2670.0831
Fixed modifications: Carbamidomethyl (C)
Variable modifications:
S4 : Phospho (ST), with neutral losses 0.0000(shown in table), 97.9769
Ions Score: 65 **Expect:** 9.3e-005
Matches (Bold Red): 25/410 fragment ions using 28 most intense peaks

#	b	b^{++}	b*	b^{*++}	b^0	b^{0++}	Seq.	y	y^{++}	y*	y^{*++}	y^0	y^{0++}	#
1	157.1084	79.0578	140.0818	70.5446			R							24
2	244.1404	122.5738	227.1139	114.0606	226.1298	113.5686	S	2514.9893	1257.9983	2497.9627	**1249.4850**	2496.9787	**1248.9930**	23

**3**	315.1775	158.0924	298.1510	149.5791	297.1670	149.0871	**A**	2427.9572	1214.4823	2410.9307	1205.9690	2409.9467	1205.4770	**22**
**4**	482.1759	241.5916	465.1493	233.0783	464.1653	232.5863	**S**	2356.9201	1178.9637	2339.8936	1170.4504	2338.9096	1169.9584	**21**
**5**	579.2286	290.1180	562.2021	281.6047	561.2181	281.1127	**P**	2189.9218	1095.4645	2172.8952	1086.9513	2171.9112	1086.4592	**20**
**6**	694.2556	347.6314	677.2290	339.1182	676.2450	338.6261	**D**	2092.8690	1046.9381	2075.8425	1038.4249	2074.8584	1037.9329	**19**
**7**	809.2825	405.1449	792.2560	396.6316	791.2720	396.1396	**D**	1977.8421	989.4247	1960.8155	980.9114	1959.8315	980.4194	**18**
**8**	924.3095	462.6584	907.2829	454.1451	906.2989	453.6531	**D**	1862.8151	931.9112	1845.7886	923.3979	1844.8046	922.9059	**17**
**9**	1037.3935	519.2004	1020.3670	510.6871	1019.3830	510.1951	**L**	1747.7882	874.3977	1730.7616	865.8845	1729.7776	865.3924	**16**
**10**	1094.4150	**547.7111**	1077.3885	539.1979	1076.4044	538.7059	**G**	**1634.7041**	817.8557	1617.6776	809.3424	1616.6936	808.8504	**15**
**11**	1181.4470	591.2272	1164.4205	582.7139	1163.4365	582.2219	**S**	1577.6827	789.3450	1560.6561	780.8317	1559.6721	780.3397	**14**
**12**	1268.4791	634.7432	1251.4525	626.2299	1250.4685	625.7379	**S**	1490.6506	745.8290	1473.6241	737.3157	1472.6401	736.8237	**13**
**13**	**1382.5220**	691.7646	1365.4954	683.2514	1364.5114	682.7593	**N**	1403.6186	702.3129	1386.5920	693.7997	1385.6080	693.3077	**12**
**14**	1568.6013	784.8043	1551.5747	776.2910	1550.5907	775.7990	**W**	**1289.5757**	645.2915	1272.5491	636.7782	1271.5651	636.2862	**11**
**15**	1697.6439	849.3256	1680.6173	840.8123	1679.6333	840.3203	**E**	**1103.4964**	552.2518	1086.4698	543.7385	1085.4858	543.2465	**10**
**16**	1768.6810	884.8441	1751.6545	876.3309	1750.6704	875.8389	**A**	**974.4538**	487.7305	957.4272	479.2172	956.4432	478.7252	**9**
**17**	**1839.7181**	920.3627	1822.6916	911.8494	1821.7076	911.3574	**A**	903.4167	452.2120	886.3901	443.6987	885.4061	443.2067	**8**
**18**	1954.7451	**977.8762**	1937.7185	969.3629	1936.7345	968.8709	**D**	**832.3795**	416.6934	815.3530	408.1801	814.3690	407.6881	**7**
**19**	2067.8291	**1034.4182**	2050.8026	1025.9049	2049.8186	1025.4129	**L**	**717.3526**	359.1799	700.3260	350.6667	699.3420	350.1747	**6**
**20**	2124.8506	1062.9289	2107.8240	1054.4157	2106.8400	1053.9237	**G**	**604.2685**	302.6379	587.2420	294.1246	586.2580	293.6326	**5**
**21**	2238.8935	1119.9504	2221.8670	1111.4371	2220.8830	1110.9451	**N**	**547.2471**	274.1272	530.2205	265.6139	529.2365	265.1219	**4**
**22**	2367.9361	**1184.4717**	2350.9096	1175.9584	2349.9255	1175.4664	**E**	**433.2041**	217.1057	416.1776	208.5924	415.1936	208.1004	**3**
**23**	2496.9787	**1248.9930**	2479.9522	1240.4797	2478.9681	1239.9877	**E**	**304.1615**	152.5844	287.1350	144.0711	286.1510	143.5791	**2**
**24**							**R**	175.1190	88.0631	158.0924	79.5498			**1**

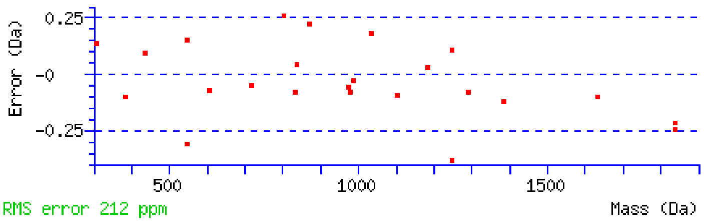

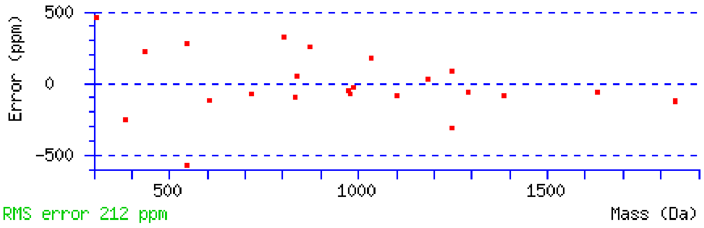

All matches to this query

Score	Mr(calc):	Delta	Sequence
64.8	2670.0831	0.0069	RSASPDDDLGSSNWEAADLGNEER
64.8	2670.0831	0.0069	RSASPDDDLGSSNWEAADLGNEER
51.8	2670.0831	0.0069	RSASPDDDLGSSNWEAADLGNEER
51.8	2670.0831	0.0069	RSASPDDDLGSSNWEAADLGNEER
4.2	2670.1116	-0.0217	SYISANVTGFKCVTGMSCLMRK
4.2	2670.1116	-0.0217	SYISANVTGFKCVTGMSCLMRK
4.2	2670.1116	-0.0217	SYISANVTGFKCVTGMSCLMRK
3.6	2670.1116	-0.0217	SYISANVTGFKCVTGMSCLMRK
2.5	2670.1130	-0.0231	LVLICSVANTTENITYLWYR
2.3	2668.0659	2.0241	LTATTQKQEQIINTMTQDLR

Spectrum No: 137; Query: 623; Rank: 1

Peptide View

MS/MS Fragmentation of **SSTSFANIQENST**
Found in **IPI00564119**, Tax_Id=10116 Gene_Symbol=LOC499474 Similar to osteopetrosis associated transmembrane protein 1

Match to Query 623: 1464.577828 from(733.296190,2+)
Title: 091008RatKidney_NoSalt_34.1143.1143.2.dta
Data file K:\NewmanPaper\Piliang\3SubProteomes\Piliang3SP\mgf5ppm\ERLIC_3SubProteomes5ppm.mgf

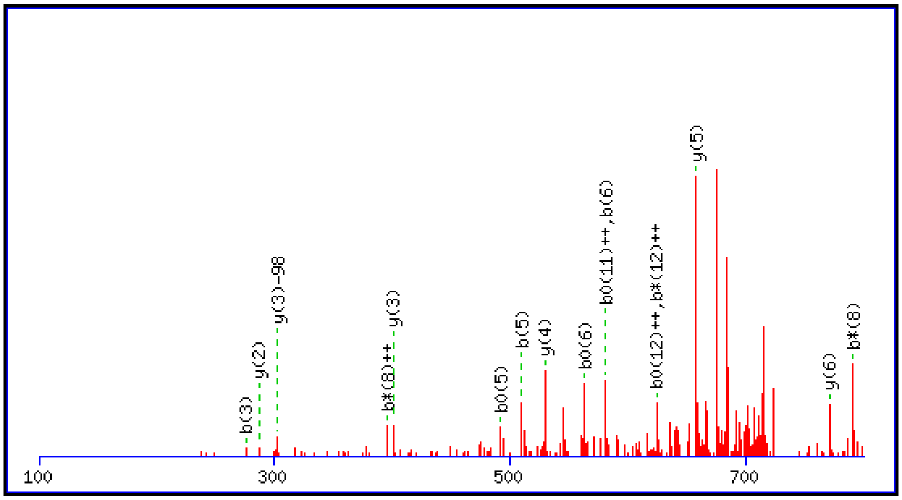

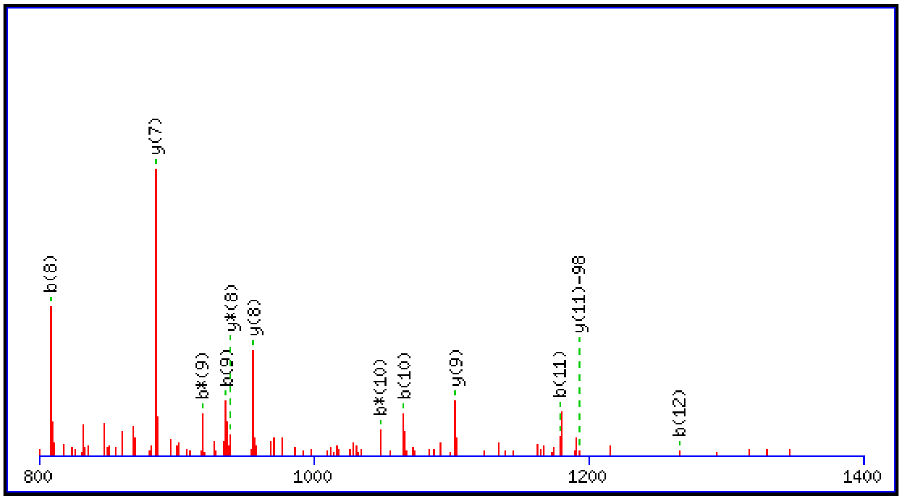

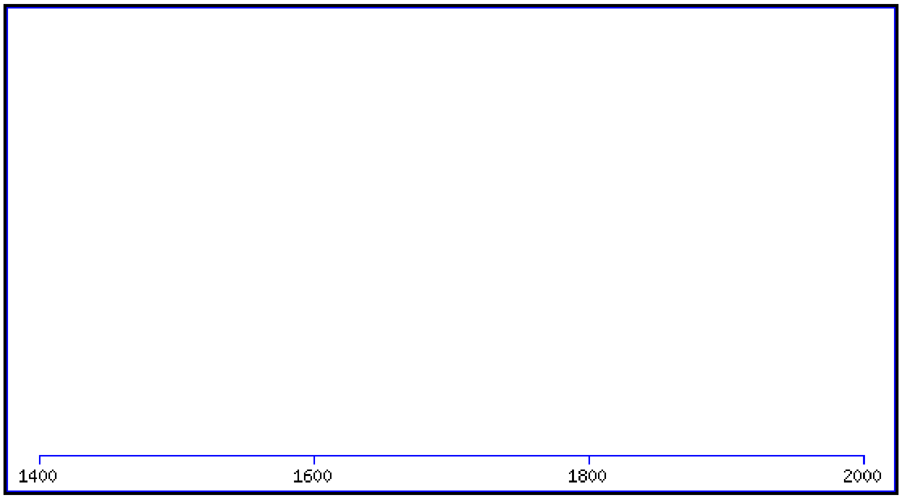

Monoisotopic mass of neutral peptide Mr(calc): 1464.5770
Fixed modifications: Carbamidomethyl (C)
Variable modifications:
T13 : Phospho (ST), with neutral losses 0.0000(shown in table), 97.9769
Ions Score: 65 **Expect:** 3.1e-005
Matches (Bold Red): 28/196 fragment ions using 42 most intense peaks

#	b	b^{++}	b*	b^{*++}	b^0	b^{0++}	Seq.	y	y^{++}	y*	y^{*++}	y^0	y^{0++}	#
1	88.0393	44.5233			70.0287	35.5180	S							13
2	175.0713	88.0393			157.0608	79.0340	S	1378.5522	689.7798	1361.5257	681.2665	1360.5417	680.7745	12

**3**	**276.1190**	138.5631			258.1084	129.5579	**T**	1291.5202	646.2637	1274.4936	637.7505	1273.5096	637.2585	**11**
**4**	363.1510	182.0792			345.1405	173.0739	**S**	1190.4725	595.7399	1173.4460	587.2266	1172.4620	586.7346	**10**
**5**	**510.2195**	255.6134			**492.2089**	246.6081	**F**	**1103.4405**	552.2239	1086.4139	543.7106	1085.4299	543.2186	**9**
**6**	**581.2566**	291.1319			**563.2460**	282.1266	**A**	**956.3721**	478.6897	**939.3455**	470.1764	938.3615	469.6844	**8**
**7**	695.2995	348.1534	678.2729	339.6401	677.2889	339.1481	**N**	**885.3350**	443.1711	868.3084	434.6578	867.3244	434.1658	**7**
**8**	**808.3836**	404.6954	**791.3570**	**396.1821**	790.3730	395.6901	**I**	**771.2920**	386.1497	754.2655	377.6364	753.2815	377.1444	**6**
**9**	**936.4421**	468.7247	**919.4156**	460.2114	918.4316	459.7194	**Q**	**658.2080**	329.6076	641.1814	321.0943	640.1974	320.6023	**5**
**10**	**1065.4847**	533.2460	**1048.4582**	524.7327	1047.4742	524.2407	**E**	**530.1494**	265.5783	513.1228	257.0651	512.1388	256.5731	**4**
**11**	**1179.5277**	590.2675	1162.5011	581.7542	1161.5171	**581.2622**	**N**	**401.1068**	201.0570	384.0803	192.5438	383.0962	192.0518	**3**
**12**	**1266.5597**	633.7835	1249.5331	**625.2702**	1248.5491	**624.7782**	**S**	**287.0639**	144.0356			269.0533	135.0303	**2**
**13**							**T**	200.0318	100.5196			182.0213	91.5143	**1**

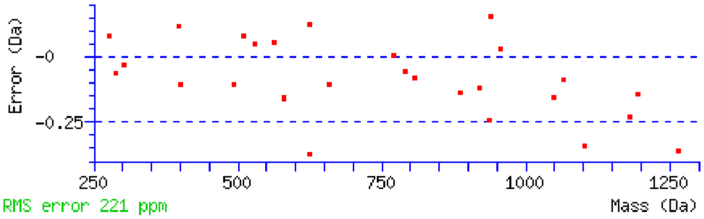

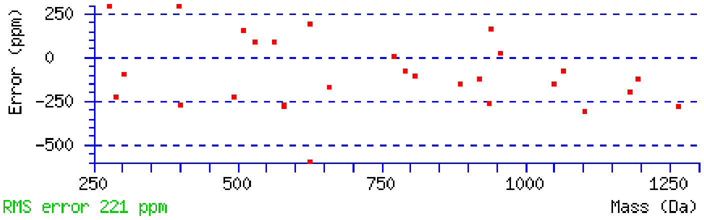

All matches to this query

Score	Mr(calc):	Delta	Sequence
64.6	1464.5770	0.0009	SSTSFANIQENST
62.4	1464.5770	0.0009	SSTSFANIQENST
7.0	1464.5816	-0.0037	VYSAISSSKRK
2.4	1464.5816	-0.0037	VYSAISSSKRK
2.4	1464.5816	-0.0037	VYSAISSSKRK
0.2	1464.5870	-0.0091	MGGTFAMCSALCK

Spectrum No: 138; Query: 2561; Rank: 1

Peptide View

MS/MS Fragmentation of **EREESEDELEEPNGSNPVDIDIDPNR**
Found in **IPI00368364**, Tax_Id=10116 Gene_Symbol=- 42 kDa protein

Match to Query 2561: 3076.292712 from(1026.438180,3+)
Title: 100101RatKid_NS_deglyco_23.3232.3232.3.dta
Data file K:\NewmanPaper\Piliang\3SubProteomes\Piliang3SP\mgf5ppm\ERLIC_3SubProteomes5ppm.mgf

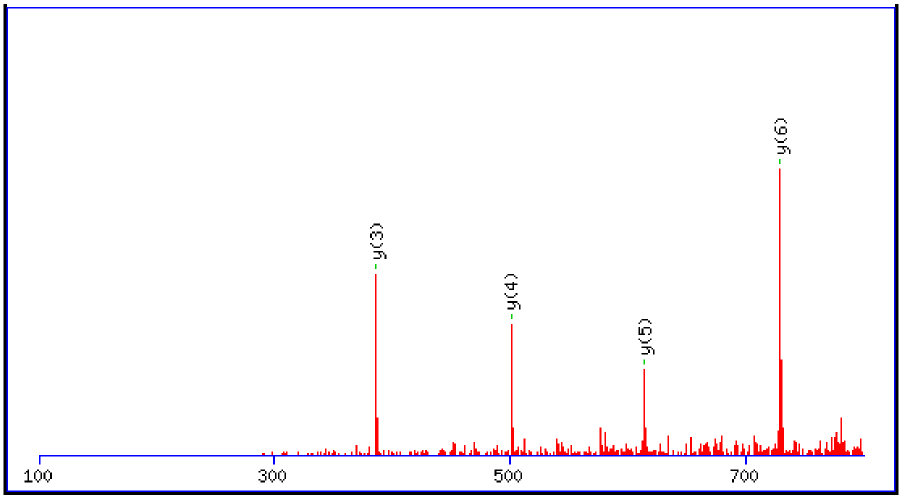

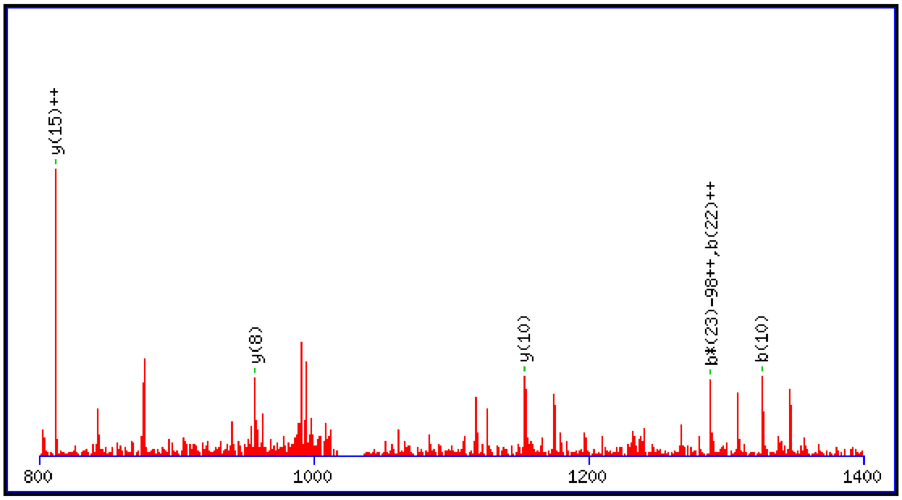

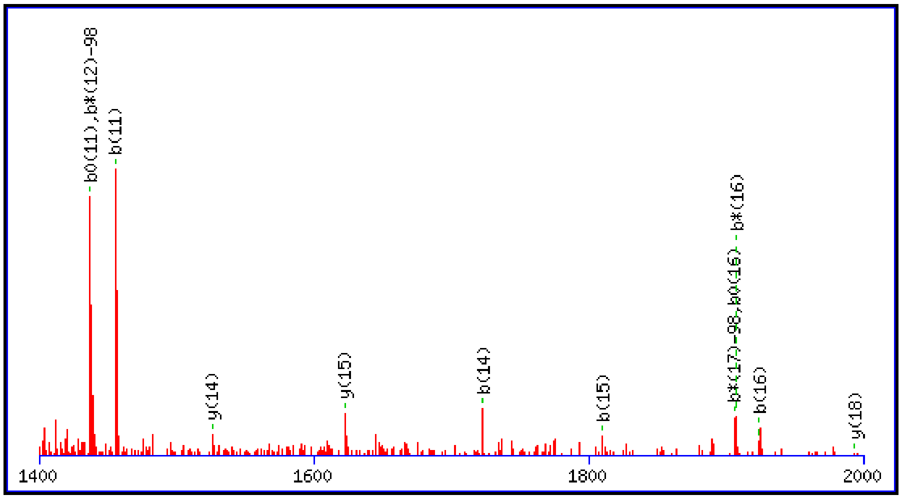

Monoisotopic mass of neutral peptide Mr(calc): 3076.2782
Fixed modifications: Carbamidomethyl (C)
Variable modifications:
S5 : Phospho (ST), with neutral losses 0.0000(shown in table), 97.9769
Ions Score: 64 **Expect:** 0.00015
Matches (Bold Red): 22/442 fragment ions using 40 most intense peaks

#	b	b^{++}	b*	b^{*++}	b^0	b^{0++}	Seq.	y	y^{++}	y*	y^{*++}	y^0	y^{0++}	#
1	130.0499	65.5286			112.0393	56.5233	E							26
2	286.1510	143.5791	269.1244	135.0659	268.1404	134.5738	R	2948.2429	1474.6251	2931.2164	1466.1118	2930.2324	1465.6198	25
3	415.1936	208.1004	398.1670	199.5872	397.1830	199.0951	E	2792.1418	1396.5745	2775.1153	1388.0613	2774.1312	1387.5693	24
4	544.2362	272.6217	527.2096	264.1084	526.2256	263.6164	E	2663.0992	1332.0532	2646.0727	1323.5400	2645.0886	1323.0480	23
5	711.2345	356.1209	694.2080	347.6076	693.2240	347.1156	S	2534.0566	1267.5319	2517.0301	1259.0187	2516.0461	1258.5267	22

6	840.2771	420.6422	823.2506	412.1289	822.2665	411.6369	E	2367.0583	1184.0328	2350.0317	1175.5195	2349.0477	1175.0275	21
7	955.3041	478.1557	938.2775	469.6424	937.2935	469.1504	D	2238.0157	1119.5115	2220.9891	1110.9982	2220.0051	1110.5062	20
8	1084.3466	542.6770	1067.3201	534.1637	1066.3361	533.6717	E	2122.9887	1061.9980	2105.9622	1053.4847	2104.9782	1052.9927	19
9	1197.4307	599.2190	1180.4042	590.7057	1179.4201	590.2137	L	**1993.9461**	997.4767	1976.9196	988.9634	1975.9356	988.4714	18
10	**1326.4733**	663.7403	1309.4468	655.2270	1308.4627	654.7350	E	1880.8621	940.9347	1863.8355	932.4214	1862.8515	931.9294	17
11	**1455.5159**	728.2616	1438.4893	719.7483	**1437.5053**	719.2563	E	1751.8195	876.4134	1734.7929	867.9001	1733.8089	867.4081	16
12	1552.5687	776.7880	1535.5421	768.2747	1534.5581	767.7827	P	**1622.7769**	**811.8921**	1605.7503	803.3788	1604.7663	802.8868	15
13	1666.6116	833.8094	1649.5850	825.2962	1648.6010	824.8041	N	**1525.7241**	763.3657	1508.6976	754.8524	1507.7136	754.3604	14
14	**1723.6331**	862.3202	1706.6065	853.8069	1705.6225	853.3149	G	1411.6812	706.3442	1394.6546	697.8310	1393.6706	697.3390	13
15	**1810.6651**	905.8362	1793.6385	897.3229	1792.6545	896.8309	S	1354.6597	677.8335	1337.6332	669.3202	1336.6492	668.8282	12
16	**1924.7080**	962.8576	**1907.6815**	954.3444	**1906.6974**	953.8524	N	1267.6277	634.3175	1250.6012	625.8042	1249.6171	625.3122	11
17	2021.7608	1011.3840	2004.7342	1002.8707	2003.7502	1002.3787	P	**1153.5848**	577.2960	1136.5582	568.7828	1135.5742	568.2907	10
18	2120.8292	1060.9182	2103.8026	1052.4050	2102.8186	1051.9129	V	1056.5320	528.7696	1039.5055	520.2564	1038.5214	519.7644	9
19	2235.8561	1118.4317	2218.8296	1109.9184	2217.8456	1109.4264	D	**957.4636**	479.2354	940.4371	470.7222	939.4530	470.2302	8
20	2348.9402	1174.9737	2331.9136	1166.4605	2330.9296	1165.9685	I	842.4367	421.7220	825.4101	413.2087	824.4261	412.7167	7
21	2463.9671	1232.4872	2446.9406	1223.9739	2445.9566	1223.4819	D	**729.3526**	365.1799	712.3260	356.6667	711.3420	356.1747	6
22	2577.0512	**1289.0292**	2560.0246	1280.5160	2559.0406	1280.0240	I	**614.3257**	307.6665	597.2991	299.1532	596.3151	298.6612	5
23	2692.0781	1346.5427	2675.0516	1338.0294	2674.0676	1337.5374	D	**501.2416**	251.1244	484.2150	242.6112	483.2310	242.1191	4
24	2789.1309	1395.0691	2772.1044	1386.5558	2771.1203	1386.0638	P	**386.2146**	193.6110	369.1881	185.0977			3
25	2903.1738	1452.0906	2886.1473	1443.5773	2885.1633	1443.0853	N	289.1619	145.0846	272.1353	136.5713			2
26							R	175.1190	88.0631	158.0924	79.5498			1

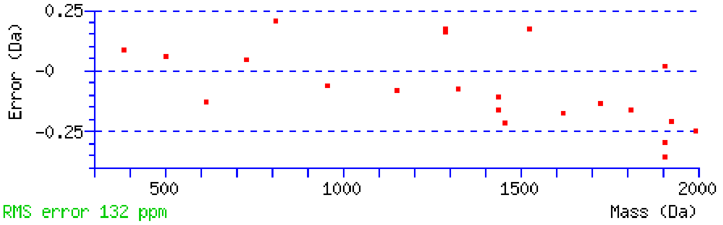

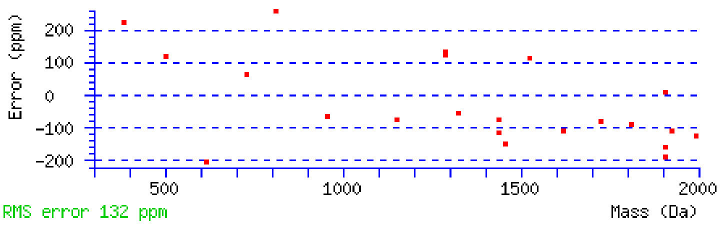

All matches to this query

Score	Mr(calc):	Delta	Sequence
64.3	3076.2782	0.0145	EREESEDELEEPNGSNPVDIDIDPNR
14.7	3076.2782	0.0145	EREESEDELEEPNGSNPVDIDIDPNR
6.2	3076.3138	-0.0210	VQFSTDSRVQTMFEILLNENSEADK
6.2	3076.3138	-0.0210	VQFSTDSRVQTMFEILLNENSEADK
6.2	3076.3138	-0.0210	VQFSTDSRVQTMFEILLNENSEADK
3.1	3075.2916	1.0011	KSAGGGGGAGGGGGGGGSSSGGGSPQSYEELQTQR
2.2	3076.2852	0.0075	TFHNQLGEAEDGGPPELKYLGSDAQSR
2.2	3076.2852	0.0075	TFHNQLGEAEDGGPPELKYLGSDAQSR
1.9	3076.2886	0.0041	DIDKVNVXTGIYHGGEPLCDNVNTQR
1.9	3074.3094	1.9834	DIDKVNVXTGIYHGGEPLCDNVNTQR

Spectrum No: 139; Query: 2812; Rank: 1

Peptide View

MS/MS Fragmentation of **YGPVSVADTTGSGAADAKDDDDIDLFGSDDEEESEDAK**
Found in **IPI00476899**, Tax_Id=10116 Gene_Symbol=Eef1b2_predicted eukaryotic translation elongation factor 1 beta 2

Match to Query 2812: 3985.602552 from(1329.541460,3+)

Title: 100101RatKid_NS_deglyco_25.3629.3629.3.dta
Data file K:\NewmanPaper\Piliang\3SubProteomes\Piliang3SP\mgf5ppm\ERLIC_3SubProteomes5ppm.mgf

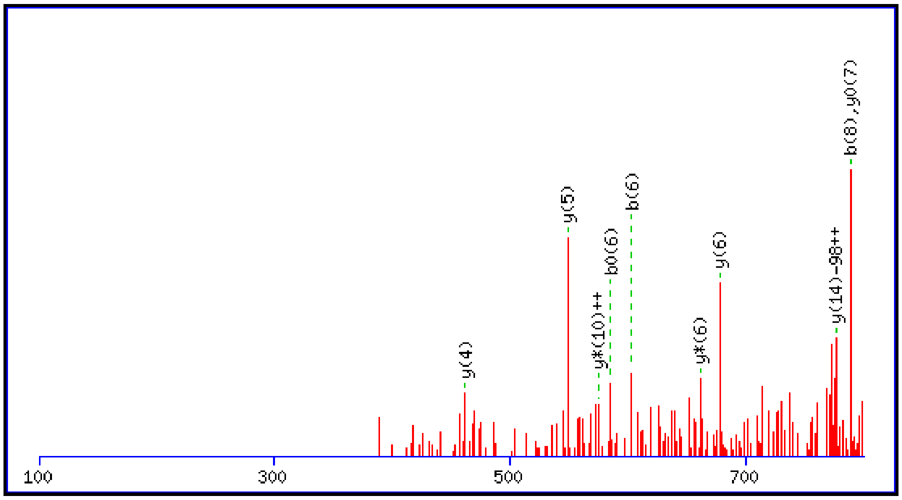

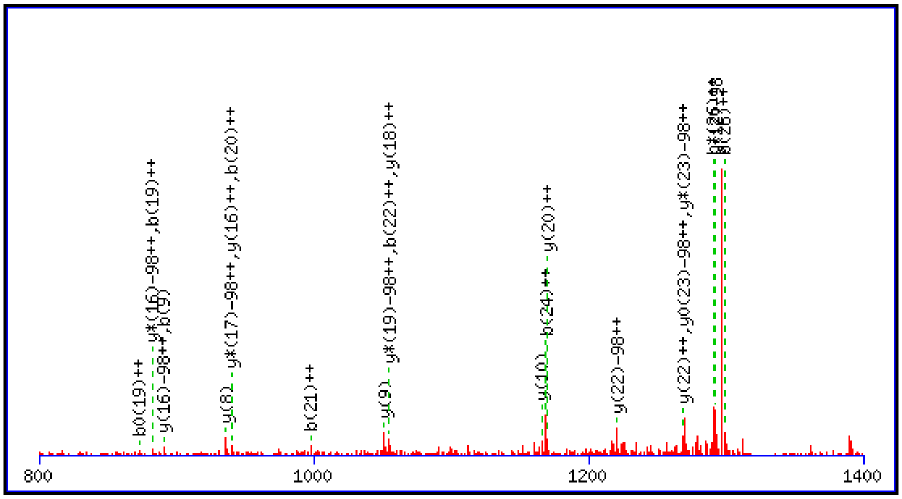

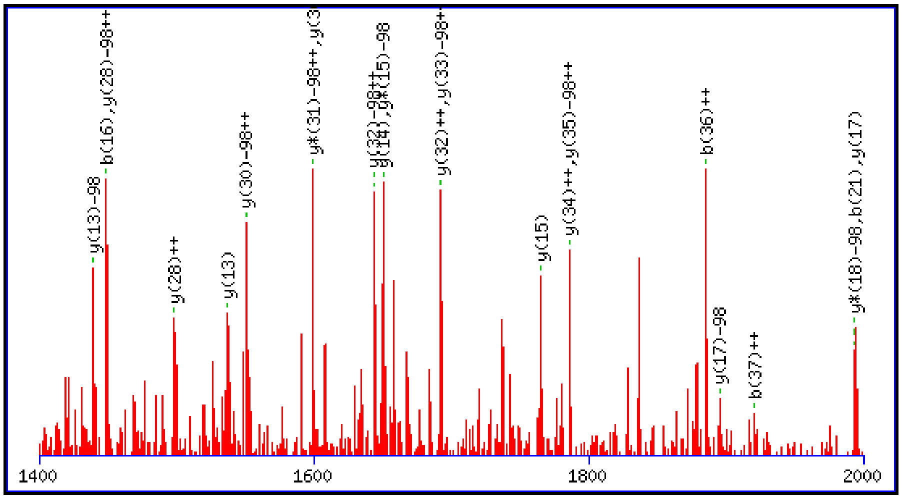

Monoisotopic mass of neutral peptide Mr(calc): 3985.5910
Fixed modifications: Carbamidomethyl (C)
Variable modifications:
S28 : Phospho (ST), with neutral losses 97.9769(shown in table), 0.0000
Ions Score: 64 **Expect:** 0.00018
Matches (Bold Red): 56/620 fragment ions using 54 most intense peaks

#	b	b^{++}	b*	b^{*++}	b^0	b^{0++}	Seq.	y	y^{++}	y*	y^{*++}	y^0	y^{0++}	#
1	164.0706	82.5389					Y							38
2	221.0921	111.0497					G	3725.5580	1863.2827	3708.5315	1854.7694	3707.5475	1854.2774	37
3	318.1448	159.5761					P	3668.5366	1834.7719	3651.5100	1826.2586	3650.5260	1825.7666	36

4	417.2132	209.1103					V	3571.4838	**1786.2455**	3554.4572	1777.7323	3553.4732	1777.2403	35
5	504.2453	252.6263			486.2347	243.6210	S	3472.4154	1736.7113	3455.3888	1728.1981	3454.4048	1727.7060	34
6	**603.3137**	302.1605			**585.3031**	293.1552	V	3385.3834	**1693.1953**	3368.3568	1684.6820	3367.3728	1684.1900	33
7	674.3508	337.6790			656.3402	328.6738	A	3286.3149	**1643.6611**	3269.2884	1635.1478	3268.3044	1634.6558	32
8	**789.3777**	395.1925			771.3672	386.1872	D	3215.2778	1608.1426	3198.2513	**1599.6293**	3197.2673	1599.1373	31
9	**890.4254**	445.7164			872.4149	436.7111	T	3100.2509	**1550.6291**	3083.2243	1542.1158	3082.2403	1541.6238	30
10	991.4731	496.2402			973.4625	487.2349	T	2999.2032	1500.1052	2982.1767	1491.5920	2981.1926	1491.1000	29
11	1048.4946	524.7509			1030.4840	515.7456	G	2898.1555	**1449.5814**	2881.1290	1441.0681	2880.1450	1440.5761	28
12	1135.5266	568.2669			1117.5160	559.2617	S	2841.1341	1421.0707	2824.1075	1412.5574	2823.1235	1412.0654	27
13	1192.5481	596.7777			1174.5375	587.7724	G	2754.1020	1377.5547	2737.0755	1369.0414	2736.0915	1368.5494	26
14	1263.5852	632.2962			1245.5746	623.2909	A	2697.0806	1349.0439	2680.0540	1340.5306	2679.0700	1340.0386	25
15	1334.6223	667.8148			1316.6117	658.8095	A	2626.0435	1313.5254	2609.0169	1305.0121	2608.0329	1304.5201	24
16	**1449.6492**	725.3283			1431.6387	716.3230	D	2555.0063	1278.0068	2537.9798	**1269.4935**	2536.9958	**1269.0015**	23
17	1520.6863	760.8468			1502.6758	751.8415	A	2439.9794	**1220.4933**	2422.9529	1211.9801	2421.9688	1211.4881	22
18	1648.7813	824.8943	1631.7548	816.3810	1630.7707	815.8890	K	2368.9423	1184.9748	2351.9157	1176.4615	2350.9317	1175.9695	21
19	1763.8083	**882.4078**	1746.7817	873.8945	1745.7977	**873.4025**	D	2240.8473	1120.9273	2223.8208	1112.4140	2222.8368	1111.9220	20
20	1878.8352	**939.9212**	1861.8086	931.4080	1860.8246	930.9160	D	2125.8204	1063.4138	2108.7938	**1054.9006**	2107.8098	1054.4085	19
21	**1993.8621**	**997.4347**	1976.8356	988.9214	1975.8516	988.4294	D	2010.7934	1005.9004	**1993.7669**	997.3871	1992.7829	996.8951	18
22	2108.8891	**1054.9482**	2091.8625	1046.4349	2090.8785	1045.9429	D	**1895.7665**	948.3869	1878.7399	**939.8736**	1877.7559	939.3816	17
23	2221.9731	1111.4902	2204.9466	1102.9769	2203.9626	1102.4849	I	1780.7396	**890.8734**	1763.7130	**882.3601**	1762.7290	881.8681	16
24	2337.0001	**1169.0037**	2319.9735	1160.4904	2318.9895	1159.9984	D	1667.6555	834.3314	**1650.6289**	825.8181	1649.6449	825.3261	15
25	2450.0842	1225.5457	2433.0576	1217.0324	2432.0736	1216.5404	L	1552.6285	**776.8179**	1535.6020	768.3046	1534.6180	767.8126	14
26	2597.1526	**1299.0799**	2580.1260	**1290.5666**	2579.1420	1290.0746	F	**1439.5445**	720.2759	1422.5179	711.7626	1421.5339	711.2706	13
27	2654.1740	1327.5907	2637.1475	1319.0774	2636.1635	1318.5854	G	**1292.4761**	646.7417	1275.4495	638.2284	1274.4655	637.7364	12
28	2723.1955	1362.1014	2706.1689	1353.5881	2705.1849	1353.0961	S	1235.4546	618.2309	1218.4281	609.7177	1217.4440	609.2257	11
29	2838.2224	1419.6149	2821.1959	1411.1016	2820.2119	1410.6096	D	**1166.4331**	583.7202	1149.4066	**575.2069**	1148.4226	574.7149	10
30	2953.2494	1477.1283	2936.2228	1468.6150	2935.2388	1468.1230	D	**1051.4062**	526.2067	1034.3797	517.6935	1033.3956	517.2015	9
31	3082.2920	1541.6496	3065.2654	1533.1363	3064.2814	1532.6443	E	**936.3793**	468.6933	919.3527	460.1800	918.3687	459.6880	8
32	3211.3346	1606.1709	3194.3080	1597.6576	3193.3240	1597.1656	E	807.3367	404.1720	790.3101	395.6587	**789.3261**	395.1667	7
33	3340.3771	1670.6922	3323.3506	1662.1789	3322.3666	1661.6869	E	**678.2941**	339.6507	**661.2675**	331.1374	660.2835	330.6454	6
34	3427.4092	1714.2082	3410.3826	1705.6950	3409.3986	1705.2029	S	**549.2515**	275.1294	532.2249	266.6161	531.2409	266.1241	5
35	3556.4518	1778.7295	3539.4252	1770.2162	3538.4412	1769.7242	E	**462.2195**	231.6134	445.1929	223.1001	444.2089	222.6081	4
36	3671.4787	1836.2430	3654.4522	1827.7297	3653.4681	1827.2377	D	333.1769	167.0921	316.1503	158.5788	315.1663	158.0868	3
37	3742.5158	1871.7616	3725.4893	1863.2483	3724.5053	1862.7563	A	218.1499	109.5786	201.1234	101.0653			2
38							K	147.1128	74.0600	130.0863	65.5468			1

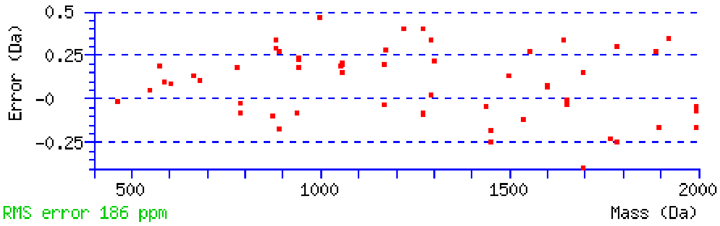

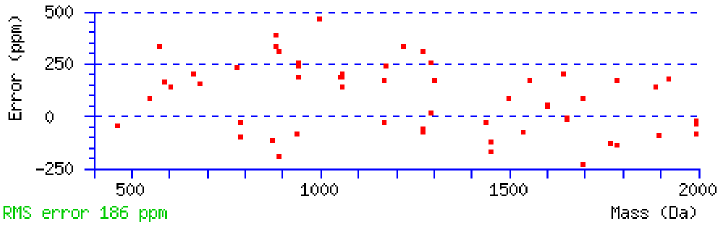

All matches to this query

Score	Mr(calc):	Delta	Sequence
63.9	3985.5910	0.0116	YGPVSVADTTGSGAADAKDDDDIDLFGSDDEEESEDAK
38.4	3985.5910	0.0116	YGPVSVADTTGSGAADAKDDDDIDLFGSDDEEESEDAK
25.1	3985.5910	0.0116	YGPVSVADTTGSGAADAKDDDDIDLFGSDDEEESEDAK
24.7	3985.5910	0.0116	YGPVSVADTTGSGAADAKDDDDIDLFGSDDEEESEDAK
20.0	3985.5910	0.0116	YGPVSVADTTGSGAADAKDDDDIDLFGSDDEEESEDAK

19.3	3985.5910	0.0116	YGPVSVADTTGSGAADAKDDDDIDLFGSDDEEESEDAK
14.7	3985.5910	0.0116	YGPVSVADTTGSGAADAKDDDDIDLFGSDDEEESEDAK
8.7	3985.6384	-0.0359	VNLEPMHTEIKMSGDVADSTDARSTFGQVESGNDR
8.1	3985.6384	-0.0359	VNLEPMHTEIKMSGDVADSTDARSTFGQVESGNDR
7.5	3985.6384	-0.0359	VNLEPMHTEIKMSGDVADSTDARSTFGQVESGNDR

Spectrum No: 140; Query: 1079; Rank: 1

Peptide View

MS/MS Fragmentation of **SSTPLPTVSSSAENTR**
Found in **IPI00208114**, Tax_Id=10116 Gene_Symbol=Tmpo Lamina-associated polypeptide 2 isoform beta

Match to Query 1079: 1712.761988 from(857.388270,2+)
Title: 091008RatKidney_NoSalt_22.1118.1118.2.dta
Data file K:\NewmanPaper\Piliang\3SubProteomes\Piliang3SP\mgf5ppm\ERLIC_3SubProteomes5ppm.mgf

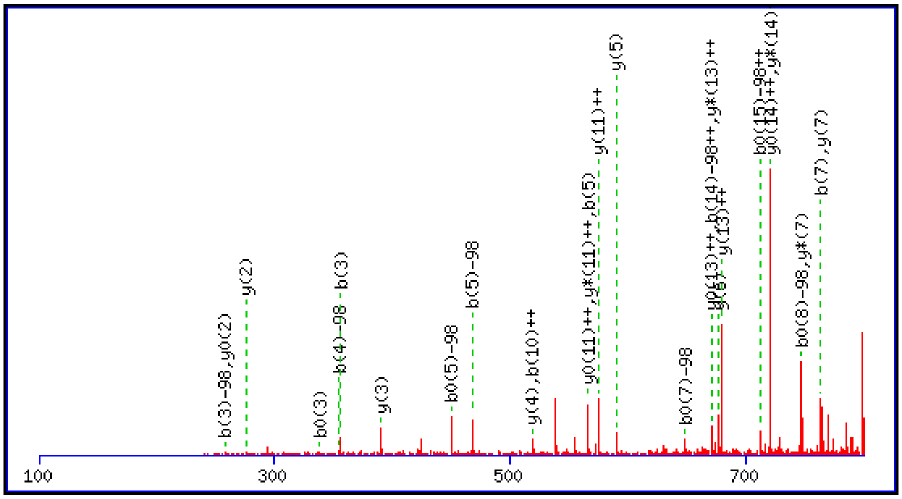

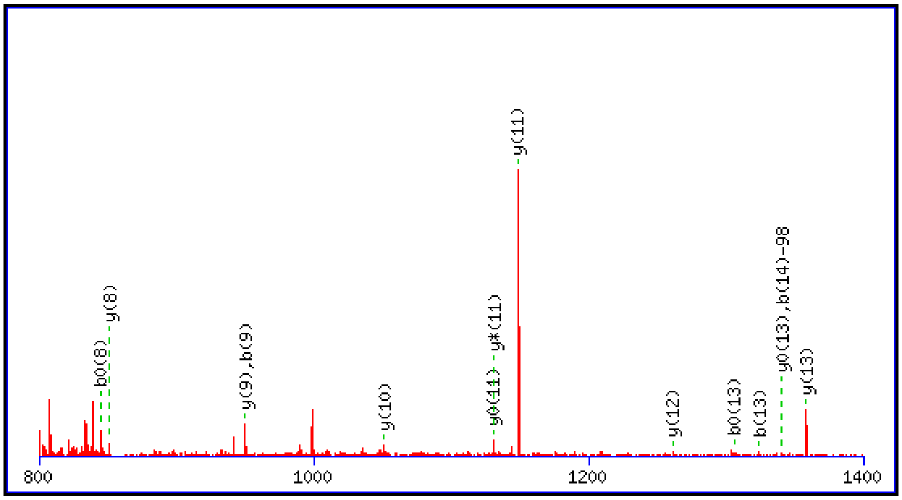

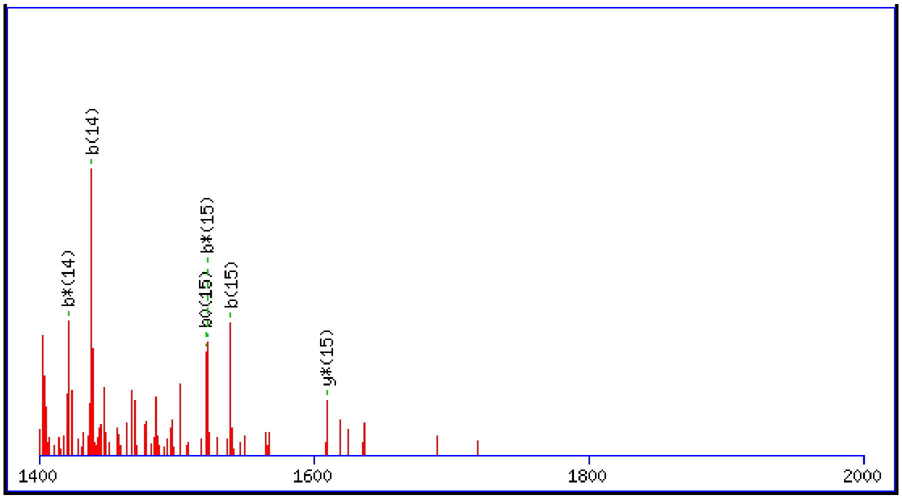

Monoisotopic mass of neutral peptide Mr(calc): 1712.7618
Fixed modifications: Carbamidomethyl (C)
Variable modifications:
S2 : Phospho (ST), with neutral losses 0.0000(shown in table), 97.9769
Ions Score: 64 **Expect:** 9.9e-005
Matches (Bold Red): 50/218 fragment ions using 78 most intense peaks

#	b	b^{++}	b*	b^{*++}	b^0	b^{0++}	Seq.	y	y^{++}	y*	y^{*++}	y^0	y^{0++}	#
1	88.0393	44.5233			70.0287	35.5180	S							16
2	255.0377	128.0225			237.0271	119.0172	S	1626.7371	813.8722	**1609.7105**	805.3589	1608.7265	804.8669	15
3	**356.0853**	178.5463			**338.0748**	169.5410	T	1459.7387	730.3730	1442.7122	**721.8597**	1441.7281	**721.3677**	14
4	453.1381	227.0727			435.1275	218.0674	P	**1358.6910**	**679.8492**	1341.6645	**671.3359**	**1340.6805**	**670.8439**	13
5	**566.2222**	283.6147			548.2116	274.6094	L	**1261.6383**	631.3228	1244.6117	622.8095	1243.6277	622.3175	12
6	663.2749	332.1411			645.2644	323.1358	P	**1148.5542**	**574.7807**	**1131.5277**	**566.2675**	**1130.5436**	**565.7755**	11
7	**764.3226**	382.6649			746.3120	373.6597	T	**1051.5014**	526.2544	1034.4749	517.7411	1033.4909	517.2491	10
8	863.3910	432.1991			**845.3805**	423.1939	V	**950.4538**	475.7305	933.4272	467.2172	932.4432	466.7252	9
9	**950.4230**	475.7152			932.4125	466.7099	S	**851.3853**	426.1963	834.3588	417.6830	833.3748	417.1910	8
10	1037.4551	**519.2312**			1019.4445	510.2259	S	**764.3533**	382.6803	**747.3268**	374.1670	746.3428	373.6750	7
11	1124.4871	562.7472			1106.4765	553.7419	S	**677.3213**	339.1643	660.2947	330.6510	659.3107	330.1590	6
12	1195.5242	598.2657			1177.5137	589.2605	A	**590.2893**	295.6483	573.2627	287.1350	572.2787	286.6430	5
13	**1324.5668**	662.7870			**1306.5562**	653.7818	E	**519.2522**	260.1297	502.2256	251.6164	501.2416	251.1244	4
14	**1438.6097**	719.8085	**1421.5832**	711.2952	1420.5992	710.8032	N	**390.2096**	195.6084	373.1830	187.0951	372.1990	186.6031	3
15	**1539.6574**	770.3323	**1522.6309**	761.8191	**1521.6469**	761.3271	T	**276.1666**	138.5870	259.1401	130.0737	**258.1561**	129.5817	2
16							R	175.1190	88.0631	158.0924	79.5498			1

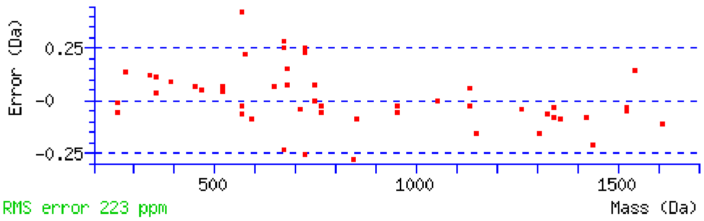

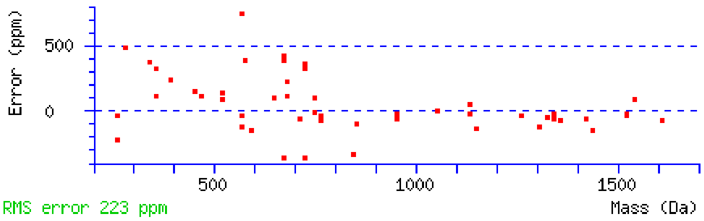

All matches to this query

Score	Mr(calc):	Delta	Sequence
63.6	1712.7618	0.0002	SSTPLPTVSSSAENTR
62.8	1712.7618	0.0002	SSTPLPTVSSSAENTR
62.3	1712.7618	0.0002	SSTPLPTVSSSAENTR
38.0	1712.7618	0.0002	SSTPLPTVSSSAENTR
19.8	1712.7618	0.0002	SSTPLPTVSSSAENTR

14.1	1712.7618	0.0002	SSTPLPTVSSSAENTR
8.3	1712.7618	0.0002	SSTPLPTVSSSAENTR
3.0	1710.7620	2.0000	LRQSTSAKTKPVR
2.7	1712.7601	0.0018	AADPPENSSAPGTEQSR
1.4	1712.7706	-0.0086	HPSLGTCPSASAPVPR

Spectrum No: 141; Query: 2566; Rank: 1

Peptide View

MS/MS Fragmentation of **MLAESDESGDEESVSQTDKTELQNTLR**
Found in **IPI00870224**, Tax_Id=10116 Gene_Symbol=Osbp_predicted oxysterol binding protein

Match to Query 2566: 3091.324542 from(1031.448790,3+)
Title: 100101RatKid_NS_deglyco_20.2900.2900.3.dta
Data file K:\NewmanPaper\Piliang\3SubProteomes\Piliang3SP\mgf5ppm\ERLIC_3SubProteomes5ppm.mgf

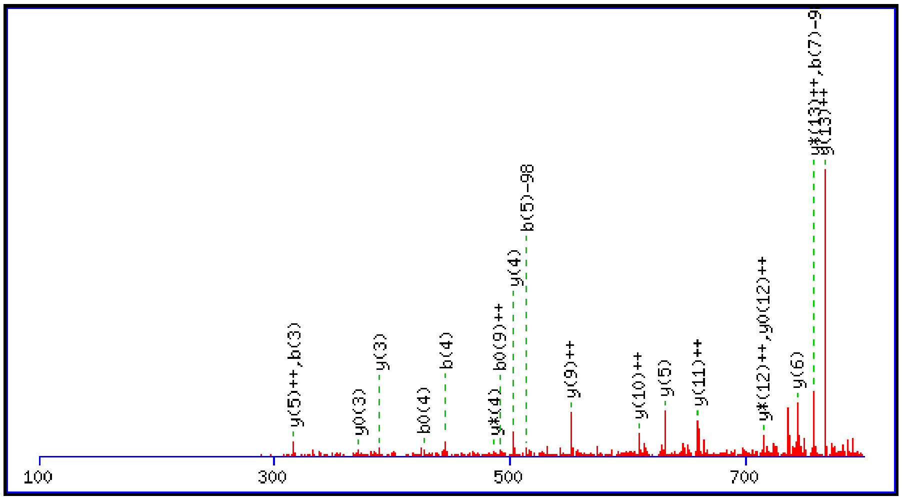

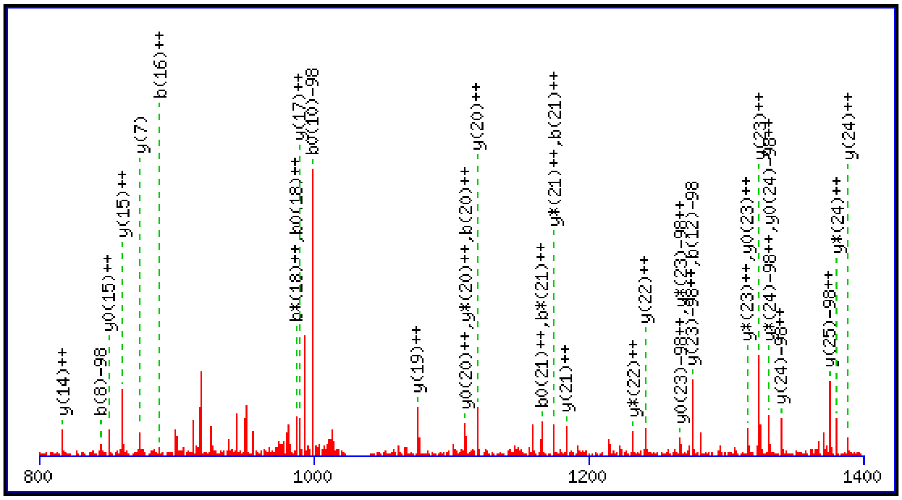

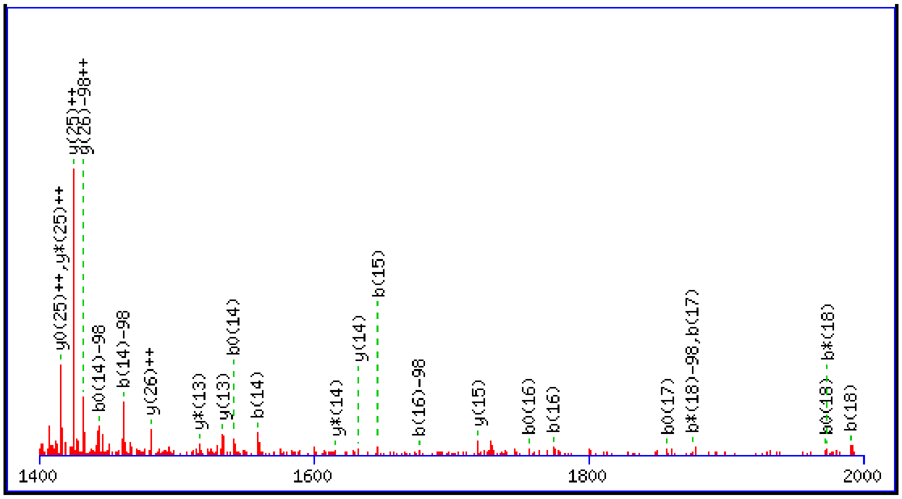

Monoisotopic mass of neutral peptide Mr(calc): 3091.3176
Fixed modifications: Carbamidomethyl (C)
Variable modifications:
S5 : Phospho (ST), with neutral losses 97.9769(shown in table), 0.0000
Ions Score: 64 **Expect:** 0.00018
Matches (Bold Red): 80/406 fragment ions using 135 most intense peaks

#	b	b^{++}	b^{*}	b^{*++}	b^{0}	b^{0++}	Seq.	y	y^{++}	y^{*}	y^{*++}	y^{0}	y^{0++}	#
1	132.0478	66.5275					M							27
2	245.1318	123.0696					L	2863.3076	**1432.1574**	2846.2810	1423.6441	2845.2970	1423.1521	26
3	**316.1689**	158.5881					A	2750.2235	**1375.6154**	2733.1969	1367.1021	2732.2129	1366.6101	25
4	**445.2115**	223.1094			**427.2010**	214.1041	E	2679.1864	**1340.0968**	2662.1598	**1331.5836**	2661.1758	**1331.0915**	24
5	**514.2330**	257.6201			496.2224	248.6148	S	2550.1438	**1275.5755**	2533.1172	**1267.0623**	2532.1332	**1266.5702**	23
6	629.2599	315.1336			611.2494	306.1283	D	2481.1223	**1241.0648**	2464.0958	**1232.5515**	2463.1118	1232.0595	22
7	**758.3025**	379.6549			740.2920	370.6496	E	2366.0954	**1183.5513**	2349.0688	**1175.0381**	2348.0848	1174.5460	21
8	**845.3346**	423.1709			827.3240	414.1656	S	2237.0528	**1119.0300**	2220.0262	**1110.5168**	2219.0422	**1110.0248**	20
9	902.3560	451.6816			884.3455	442.6764	G	2150.0208	**1075.5140**	2132.9942	1067.0007	2132.0102	1066.5087	19
10	1017.3830	509.1951			**999.3724**	500.1898	D	2092.9993	1047.0033	2075.9727	1038.4900	2074.9887	1037.9980	18
11	1146.4256	573.7164			1128.4150	564.7111	E	1977.9724	**989.4898**	1960.9458	980.9765	1959.9618	980.4845	17
12	**1275.4681**	638.2377			1257.4576	629.2324	E	1848.9298	924.9685	1831.9032	916.4552	1830.9192	915.9632	16
13	1362.5002	681.7537			1344.4896	672.7484	S	**1719.8872**	**860.4472**	1702.8606	851.9339	1701.8766	**851.4419**	15
14	**1461.5686**	731.2879			**1443.5580**	722.2826	V	**1632.8551**	**816.9312**	**1615.8286**	808.4179	1614.8446	807.9259	14
15	1548.6006	774.8039			1530.5901	765.7987	S	**1533.7867**	**767.3970**	**1516.7602**	**758.8837**	1515.7762	**758.3917**	13
16	**1676.6592**	838.8332	1659.6326	830.3200	1658.6486	829.8280	Q	1446.7547	723.8810	1429.7282	**715.3677**	1428.7441	**714.8757**	12
17	1777.7069	889.3571	1760.6803	880.8438	1759.6963	880.3518	T	1318.6961	**659.8517**	1301.6696	651.3384	1300.6856	650.8464	11
18	1892.7338	946.8705	**1875.7073**	938.3573	1874.7233	937.8653	D	1217.6484	**609.3279**	1200.6219	600.8146	1199.6379	600.3226	10
19	2020.8288	1010.9180	2003.8022	1002.4048	2002.8182	1001.9127	K	1102.6215	**551.8144**	1085.5950	543.3011	1084.6109	542.8091	9
20	2121.8765	1061.4419	2104.8499	1052.9286	2103.8659	1052.4366	T	974.5265	487.7669	957.5000	479.2536	956.5160	478.7616	8
21	2250.9191	1125.9632	2233.8925	1117.4499	2232.9085	1116.9579	E	**873.4789**	437.2431	856.4523	428.7298	855.4683	428.2378	7
22	2364.0031	1182.5052	2346.9766	1173.9919	2345.9925	1173.4999	L	**744.4363**	372.7218	727.4097	364.2085	726.4257	363.7165	6
23	2492.0617	1246.5345	2475.0351	1238.0212	2474.0511	1237.5292	Q	**631.3522**	**316.1797**	614.3257	307.6665	613.3416	307.1745	5
24	2606.1046	1303.5559	2589.0781	1295.0427	2588.0941	1294.5507	N	**503.2936**	252.1504	**486.2671**	243.6372	485.2831	243.1452	4
25	2707.1523	1354.0798	2690.1257	1345.5665	2689.1417	1345.0745	T	**389.2507**	195.1290	372.2241	186.6157	**371.2401**	186.1237	3
26	2820.2364	1410.6218	2803.2098	1402.1085	2802.2258	1401.6165	L	288.2030	144.6051	271.1765	136.0919			2
27							R	175.1190	88.0631	158.0924	79.5498			1

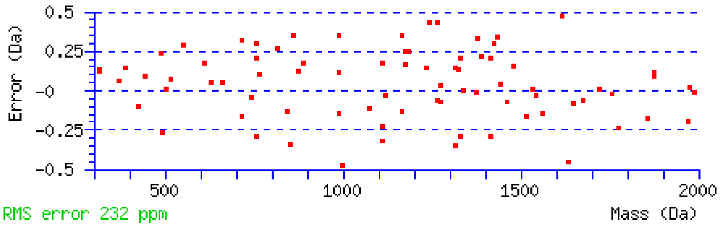

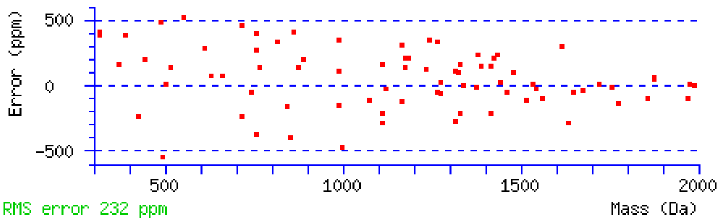

All matches to this query

Score	Mr(calc):	Delta	Sequence
63.6	3091.3176	0.0069	MLAESDESGDEESVSQTDKTELQNTLR
62.4	3091.3176	0.0069	MLAESDESGDEESVSQTDKTELQNTLR
41.5	3091.3176	0.0069	MLAESDESGDEESVSQTDKTELQNTLR
29.4	3091.3176	0.0069	MLAESDESGDEESVSQTDKTELQNTLR
26.0	3091.3176	0.0069	MLAESDESGDEESVSQTDKTELQNTLR
19.5	3091.3176	0.0069	MLAESDESGDEESVSQTDKTELQNTLR
7.0	3089.3252	1.9993	AEDEYYVNEPLYLNTFTNALGNAEYMK
7.0	3089.3252	1.9993	AEDEYYVNEPLYLNTFTNALGNAEYMK
3.4	3091.3298	-0.0053	KYHNSANKVNGVSSGGGGVWSGAGSPSSQR
3.4	3089.3252	1.9993	AEDEYYVNEPLYLNTFTNALGNAEYMK

Spectrum No: 142; Query: 2198; Rank: 1

Peptide View

MS/MS Fragmentation of **KVVDYSQFQESDDADEDYGR**
Found in **IPI00189138**, Tax_Id=10116 Gene_Symbol=Nucks Nuclear ubiquitous casein and cyclin-dependent kinases substrate

Match to Query 2198: 2444.969668 from(1223.492110,2+)
Title: 100101RatKid_NS_deglyco_24.2081.2081.2.dta
Data file K:\NewmanPaper\Piliang\3SubProteomes\Piliang3SP\mgf5ppm\ERLIC_3SubProteomes5ppm.mgf

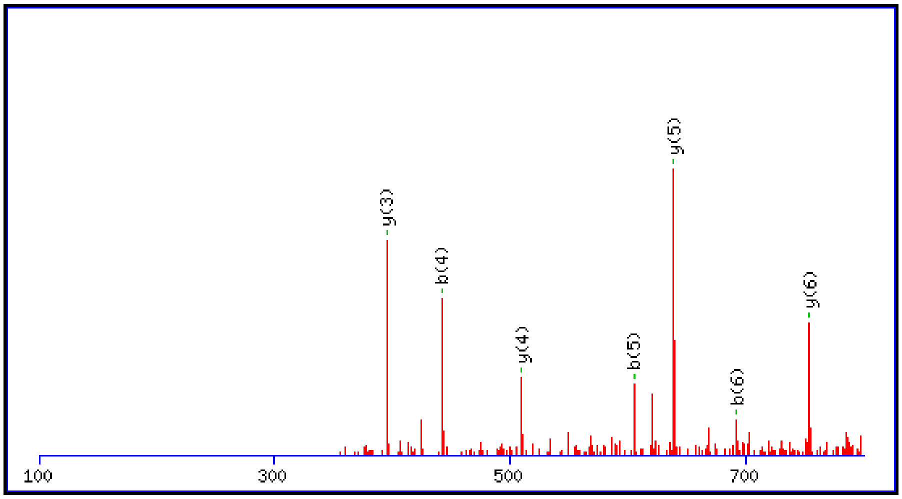

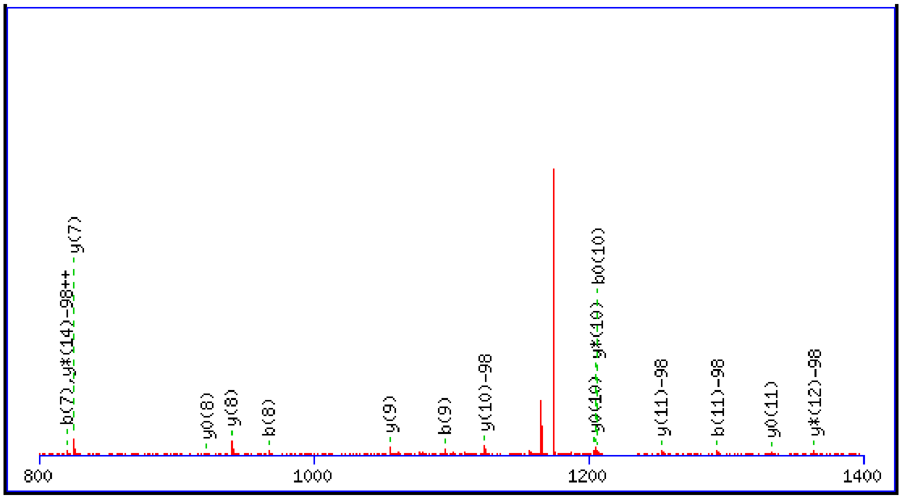

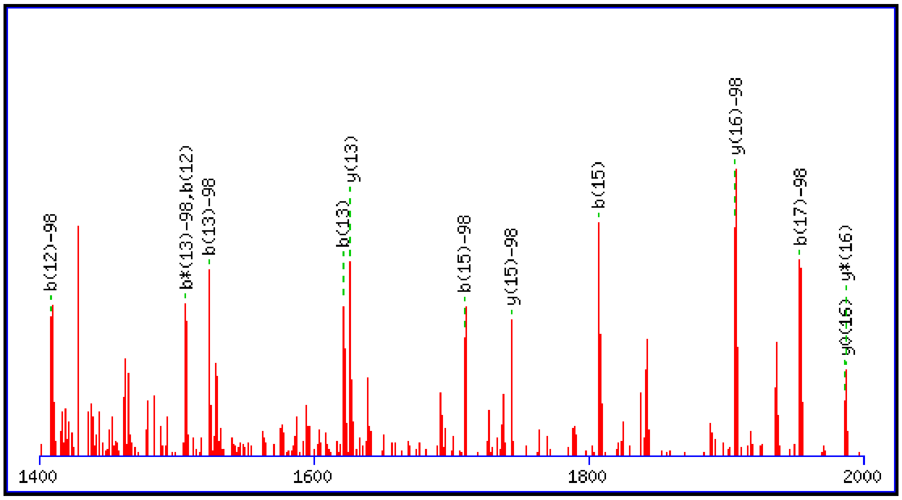

Monoisotopic mass of neutral peptide Mr(calc): 2444.9646
Fixed modifications: Carbamidomethyl (C)
Variable modifications:
S11 : Phospho (ST), with neutral losses 97.9769(shown in table), 0.0000
Ions Score: 64 **Expect:** 0.0001
Matches (Bold Red): 36/330 fragment ions using 57 most intense peaks

#	b	b^{++}	b*	b^{*++}	b^0	b^{0++}	Seq.	y	y^{++}	y*	y^{*++}	y^0	y^{0++}	#
1	129.1022	65.0548	112.0757	56.5415			K							20
2	228.1707	114.5890	211.1441	106.0757			V	2219.9000	1110.4536	2202.8734	1101.9403	2201.8894	1101.4483	19
3	327.2391	164.1232	310.2125	155.6099			V	2120.8316	1060.9194	2103.8050	1052.4061	2102.8210	1051.9141	18
4	**442.2660**	221.6366	425.2395	213.1234	424.2554	212.6314	D	2021.7631	1011.3852	2004.7366	1002.8719	2003.7526	1002.3799	17
5	**605.3293**	303.1683	588.3028	294.6550	587.3188	294.1630	Y	**1906.7362**	953.8717	1889.7097	945.3585	1888.7256	944.8665	16
6	**692.3614**	346.6843	675.3348	338.1710	674.3508	337.6790	S	**1743.6729**	872.3401	1726.6463	863.8268	1725.6623	863.3348	15
7	**820.4199**	410.7136	803.3934	402.2003	802.4094	401.7083	Q	1656.6408	828.8241	1639.6143	**820.3108**	1638.6303	819.8188	14
8	**967.4884**	484.2478	950.4618	475.7345	949.4778	475.2425	F	1528.5823	764.7948	1511.5557	756.2815	1510.5717	755.7895	13
9	**1095.5469**	548.2771	1078.5204	539.7638	1077.5364	539.2718	Q	1381.5139	691.2606	**1364.4873**	682.7473	1363.5033	682.2553	12
10	1224.5895	612.7984	1207.5630	604.2851	**1206.5790**	603.7931	E	**1253.4553**	627.2313	1236.4287	618.7180	1235.4447	618.2260	11
11	**1293.6110**	647.3091	1276.5844	638.7959	1275.6004	638.3038	S	**1124.4127**	562.7100	1107.3861	554.1967	1106.4021	553.7047	10
12	**1408.6379**	704.8226	1391.6114	696.3093	1390.6274	695.8173	D	**1055.3912**	528.1993	1038.3647	519.6860	1037.3807	519.1940	9
13	**1523.6649**	762.3361	**1506.6383**	753.8228	1505.6543	753.3308	D	**940.3643**	470.6858	923.3377	462.1725	**922.3537**	461.6805	8
14	1594.7020	797.8546	1577.6754	789.3414	1576.6914	788.8493	A	**825.3373**	413.1723	808.3108	404.6590	807.3268	404.1670	7
15	**1709.7289**	855.3681	1692.7024	846.8548	1691.7184	846.3628	D	**754.3002**	377.6538	737.2737	369.1405	736.2897	368.6485	6
16	1838.7715	919.8894	1821.7450	911.3761	1820.7610	910.8841	E	**639.2733**	320.1403	622.2467	311.6270	621.2627	311.1350	5
17	**1953.7985**	977.4029	1936.7719	968.8896	1935.7879	968.3976	D	**510.2307**	255.6190	493.2041	247.1057	492.2201	246.6137	4
18	2116.8618	1058.9345	2099.8352	1050.4213	2098.8512	1049.9293	Y	**395.2037**	198.1055	378.1772	189.5922			3

19	2173.8833	1087.4453	2156.8567	1078.9320	2155.8727	1078.4400	G	232.1404	116.5738	215.1139	108.0606			2
20							R	175.1190	88.0631	158.0924	79.5498			1

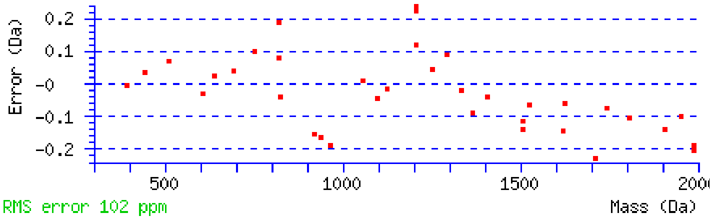

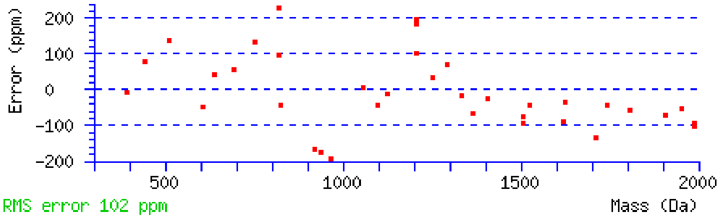

All matches to this query

Score	Mr(calc):	Delta	Sequence
63.6	2444.9646	0.0051	KVVDYSQFQESDDADEDYGR
43.9	2444.9646	0.0051	KVVDYSQFQESDDADEDYGR
29.4	2444.9646	0.0051	KVVDYSQFQESDDADEDYGR
6.0	2444.9535	0.0161	LTQYMDGNGMPKTMQSEETR
2.6	2444.9646	0.0051	KVVDYSQFQESDDADEDYGR
1.8	2442.9486	2.0210	SGRYRDTVLLPQTNFPMK
1.3	2443.9598	1.0099	IYPELYSFHLRFPTWR
1.2	2444.9500	0.0196	ELYRNVMESNYETLVSLK

Spectrum No: 143; Query: 1965; Rank: 1

Peptide View

MS/MS Fragmentation of **GETPQGSSEECDLSGSCTER**
Found in **IPI00203529**, Tax_Id=10116 Gene_Symbol=Slc34a3 Sodium-dependent phosphate transport protein 2C

Match to Query 1965: 2264.827108 from(1133.420830,2+)
Title: 100101RatKid_NS_deglyco_24.1194.1194.2.dta
Data file K:\NewmanPaper\Piliang\3SubProteomes\Piliang3SP\mgf5ppm\ERLIC_3SubProteomes5ppm.mgf

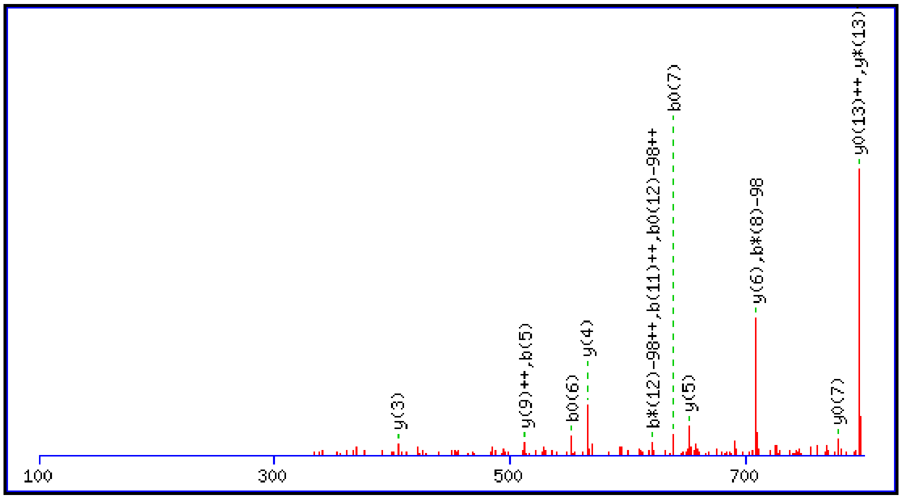

Mascot Search Results: Peptide View

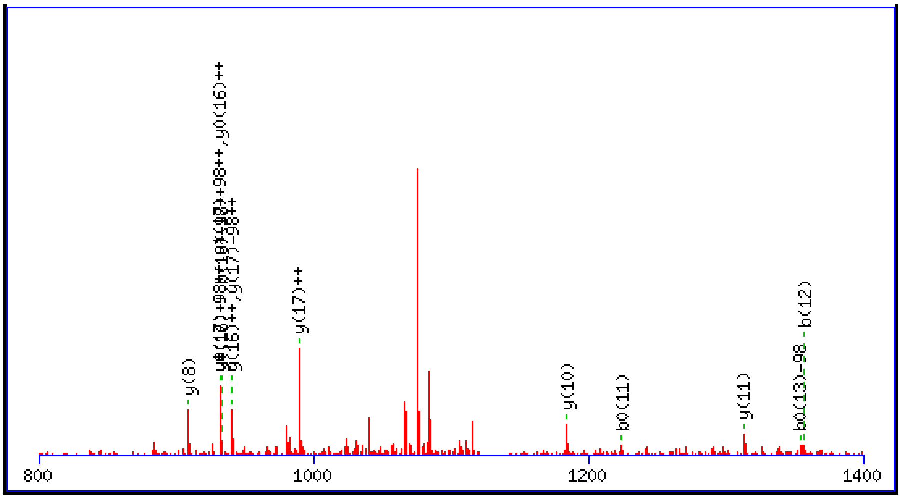

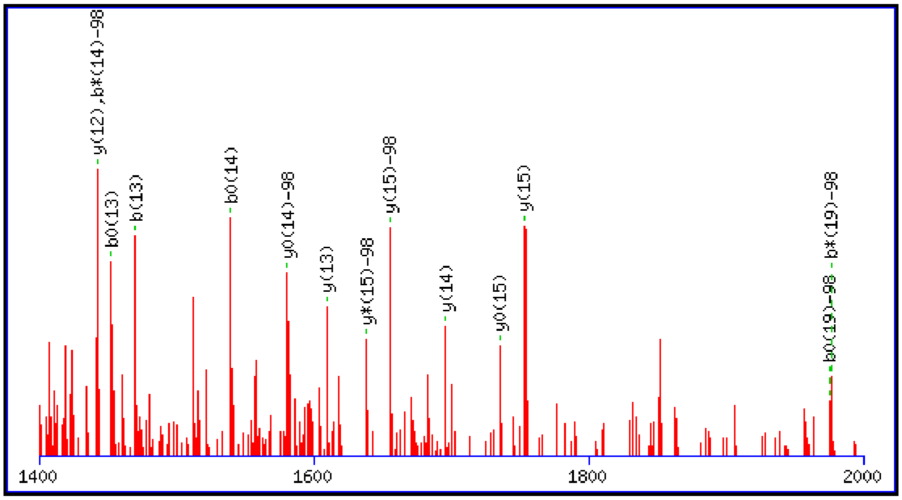

Monoisotopic mass of neutral peptide Mr(calc): 2264.8199
Fixed modifications: Carbamidomethyl (C)
Variable modifications:
S8 : Phospho (ST), with neutral losses 0.0000(shown in table), 97.9769
Ions Score: 64 **Expect:** 5.1e-005
Matches (Bold Red): 44/330 fragment ions using 55 most intense peaks

#	b	b^{++}	b*	b^{*++}	b^0	b^{0++}	Seq.	y	y^{++}	y*	y^{*++}	y^0	y^{0++}	#
1	58.0287	29.5180					G							20
2	187.0713	94.0393			169.0608	85.0340	E	2208.8057	1104.9065	2191.7791	1096.3932	2190.7951	1095.9012	19
3	288.1190	144.5631			270.1084	135.5579	T	2079.7631	1040.3852	2062.7366	1031.8719	2061.7525	1031.3799	18
4	385.1718	193.0895			367.1612	184.0842	P	1978.7154	**989.8613**	1961.6889	981.3481	1960.7049	980.8561	17
5	**513.2304**	257.1188	496.2038	248.6055	495.2198	248.1135	Q	1881.6627	**941.3350**	1864.6361	**932.8217**	1863.6521	**932.3297**	16
6	570.2518	285.6295	553.2253	277.1163	**552.2413**	276.6243	G	**1753.6041**	877.3057	1736.5775	868.7924	**1735.5935**	868.3004	15
7	657.2838	329.1456	640.2573	320.6323	**639.2733**	320.1403	S	**1696.5826**	848.7949	1679.5561	840.2817	1678.5721	839.7897	14
8	824.2822	412.6447	807.2556	404.1315	806.2716	403.6395	S	**1609.5506**	805.2789	1592.5240	**796.7657**	1591.5400	**796.2736**	13
9	953.3248	477.1660	936.2982	468.6528	935.3142	468.1608	E	**1442.5522**	721.7798	1425.5257	713.2665	1424.5417	712.7745	12
10	1082.3674	541.6873	1065.3408	533.1741	1064.3568	532.6820	E	**1313.5096**	657.2585	1296.4831	648.7452	1295.4991	648.2532	11
11	1242.3980	**621.7027**	1225.3715	613.1894	**1224.3875**	612.6974	C	**1184.4670**	592.7372	1167.4405	584.2239	1166.4565	583.7319	10
12	**1357.4250**	679.2161	1340.3984	670.7029	1339.4144	670.2108	D	1024.4364	**512.7218**	1007.4099	504.2086	1006.4258	503.7166	9
13	**1470.5090**	735.7582	1453.4825	727.2449	**1452.4985**	726.7529	L	**909.4095**	455.2084	892.3829	446.6951	891.3989	446.2031	8
14	1557.5411	779.2742	1540.5145	770.7609	**1539.5305**	770.2689	S	**796.3254**	398.6663	779.2988	390.1531	**778.3148**	389.6611	7
15	1614.5625	807.7849	1597.5360	799.2716	1596.5520	798.7796	G	**709.2934**	355.1503	692.2668	346.6370	691.2828	346.1450	6
16	1701.5946	851.3009	1684.5680	842.7876	1683.5840	842.2956	S	**652.2719**	326.6396	635.2454	318.1263	634.2613	317.6343	5
17	1861.6252	931.3162	1844.5987	922.8030	1843.6146	922.3110	C	**565.2399**	283.1236	548.2133	274.6103	547.2293	274.1183	4
18	1962.6729	981.8401	1945.6463	973.3268	1944.6623	972.8348	T	**405.2092**	203.1082	388.1827	194.5950	387.1987	194.1030	3

**19**	2091.7155	1046.3614	2074.6889	1037.8481	2073.7049	1037.3561	**E**	304.1615	152.5844	287.1350	144.0711	286.1510	143.5791	**2**
**20**							**R**	175.1190	88.0631	158.0924	79.5498			**1**

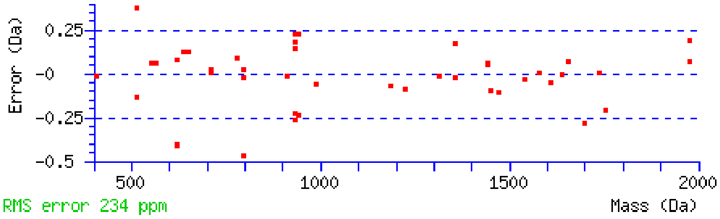

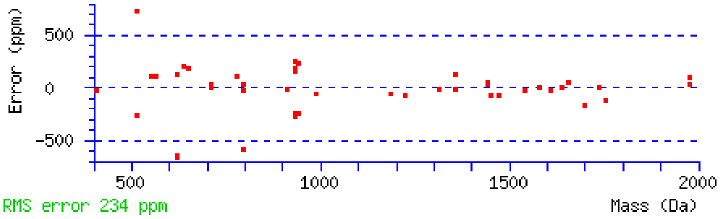

All matches to this query

Score	Mr(calc):	Delta	Sequence
63.6	2264.8199	0.0072	GETPQGSSEECDLSGSCTER
59.4	2264.8199	0.0072	GETPQGSSEECDLSGSCTER
34.6	2264.8199	0.0072	GETPQGSSEECDLSGSCTER
16.1	2264.8199	0.0072	GETPQGSSEECDLSGSCTER
7.6	2264.8199	0.0072	GETPQGSSEECDLSGSCTER
1.6	2264.8199	0.0072	GETPQGSSEECDLSGSCTER
0.7	2263.8164	1.0107	MCVSSSSNNHDEAPVLNDK
0.7	2263.8164	1.0107	MCVSSSSNNHDEAPVLNDK
0.0	2263.8460	0.9811	DNGHIQSPNYPDDYRPSK

Spectrum No: 144; Query: 428; Rank: 1

Peptide View

MS/MS Fragmentation of **LCLSTVDLEVK**
Found in **IPI00369678**, Tax_Id=10116 Gene_Symbol=Slc43a2_predicted solute carrier family 43, member 2

Match to Query 428: 1355.641508 from(678.828030,2+)
Title: 100101RatKid_NS_deglyco_13.4042.4042.2.dta
Data file K:\NewmanPaper\Piliang\3SubProteomes\Piliang3SP\mgf5ppm\ERLIC_3SubProteomes5ppm.mgf

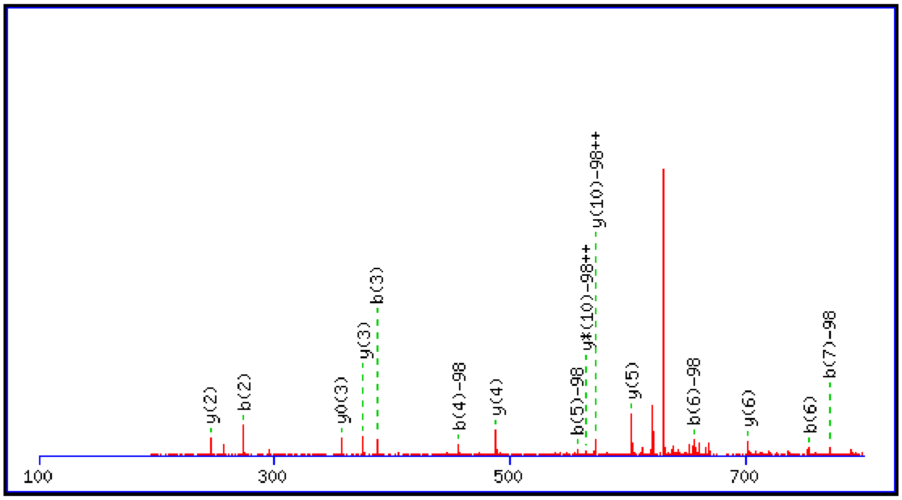

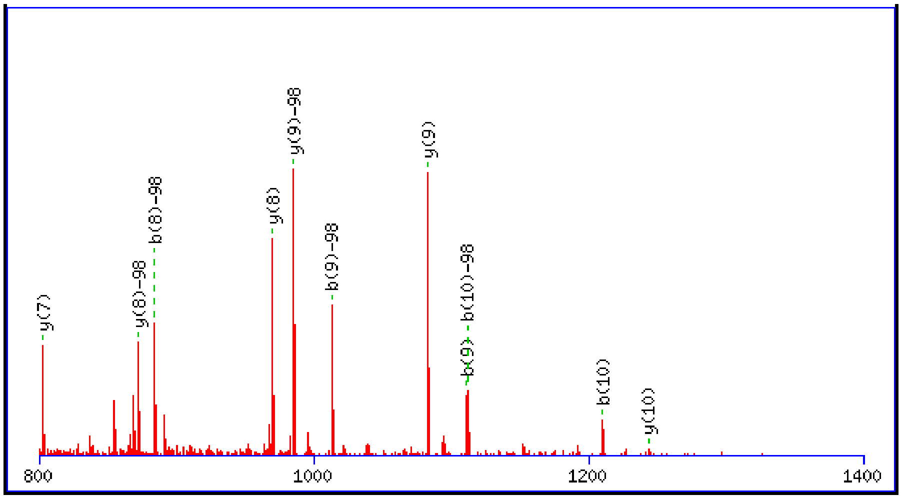

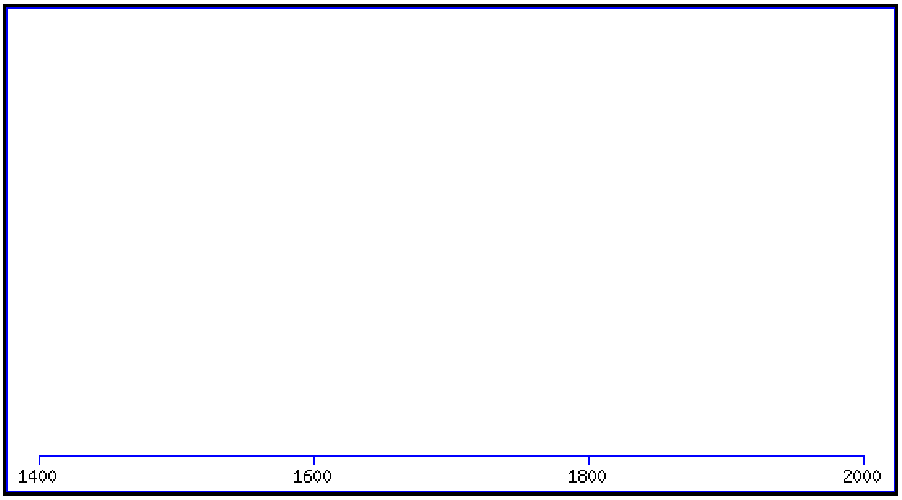

Monoisotopic mass of neutral peptide Mr(calc): 1355.6408
Fixed modifications: Carbamidomethyl (C)
Variable modifications:
S4 : Phospho (ST), with neutral losses 97.9769(shown in table), 0.0000
Ions Score: 63 **Expect:** 6.9e-005
Matches (Bold Red): 26/136 fragment ions using 37 most intense peaks

#	b	b^{++}	b^{0}	b^{0++}	Seq.	y	y^{++}	y*	y^{*++}	y^{0}	y^{0++}	#
1	114.0913	57.5493			L							11
2	**274.1220**	137.5646			C	1145.5871	**573.2972**	1128.5605	**564.7839**	1127.5765	564.2919	10
3	**387.2061**	194.1067			L	**985.5564**	493.2819	968.5299	484.7686	967.5459	484.2766	9
4	**456.2275**	228.6174	438.2169	219.6121	S	**872.4724**	436.7398	855.4458	428.2265	854.4618	427.7345	8
5	**557.2752**	279.1412	539.2646	270.1359	T	**803.4509**	402.2291	786.4244	393.7158	785.4403	393.2238	7
6	**656.3436**	328.6754	638.3330	319.6702	V	**702.4032**	351.7053	685.3767	343.1920	684.3927	342.7000	6
7	**771.3705**	386.1889	753.3600	377.1836	D	**603.3348**	302.1710	586.3083	293.6578	585.3243	293.1658	5
8	**884.4546**	442.7309	866.4440	433.7257	L	**488.3079**	244.6576	471.2813	236.1443	470.2973	235.6523	4
9	**1013.4972**	507.2522	995.4866	498.2470	E	**375.2238**	188.1155	358.1973	179.6023	**357.2132**	179.1103	3
10	**1112.5656**	556.7864	1094.5551	547.7812	V	**246.1812**	123.5942	229.1547	115.0810			2
11					K	147.1128	74.0600	130.0863	65.5468			1

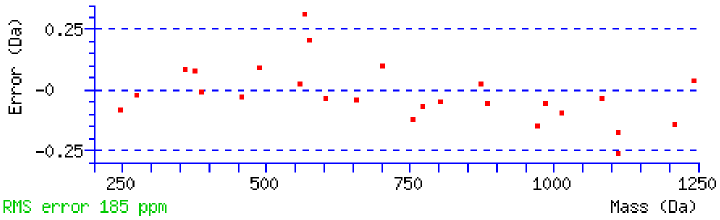

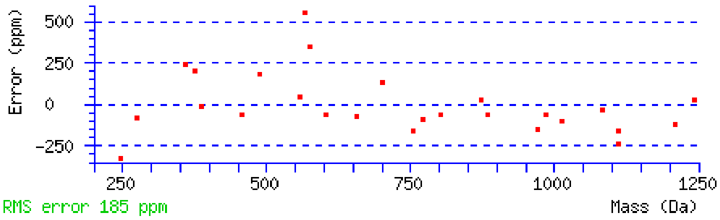

All matches to this query

Score	Mr(calc):	Delta	Sequence
63.3	1355.6408	0.0007	LCLSTVDLEVK
54.6	1355.6408	0.0007	LCLSTVDLEVK
14.3	1355.6421	-0.0006	MNIPFRIGNSK
10.3	1353.6429	1.9986	LEVEAVSVSDVK
9.0	1355.6486	-0.0071	LASSDTFPVIAR
8.7	1355.6520	-0.0105	MDKSELVQKAK
8.6	1355.6486	-0.0071	EKEGFPITSLR
7.9	1355.6486	-0.0071	LASSDTFPVIAR
7.6	1355.6407	0.0008	KMPNILTSNLK
7.6	1355.6407	0.0008	KMPNILTSNLK

Spectrum No: 145; Query: 1734; Rank: 1

Peptide View

MS/MS Fragmentation of **SLDSDESEDEDDDYQQK**
Found in **IPI00208277**, Tax_Id=10116 Gene_Symbol=Pdap1 28 kDa heat- and acid-stable phosphoprotein

Match to Query 1734: 2096.714668 from(1049.364610,2+)
Title: 091008RatKidney_NH4Format01_29.791.791.2.dta
Data file K:\NewmanPaper\Piliang\3SubProteomes\Piliang3SP\mgf5ppm\ERLIC_3SubProteomes5ppm.mgf

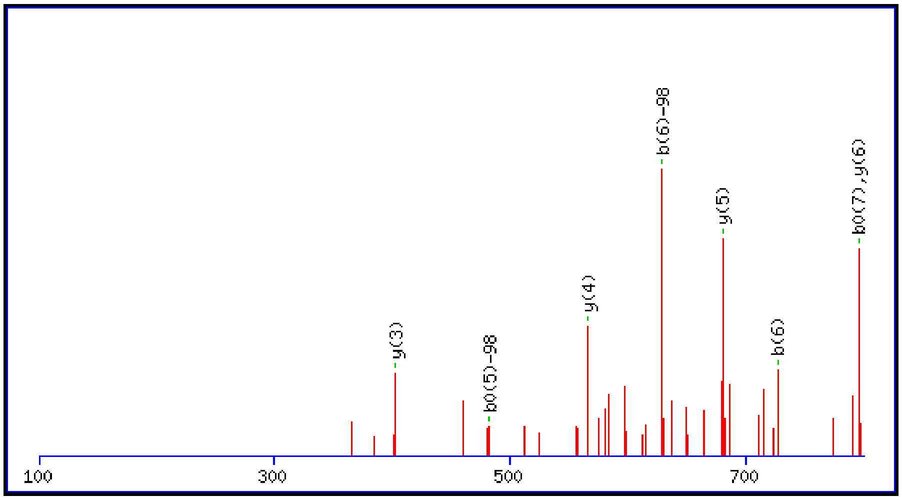

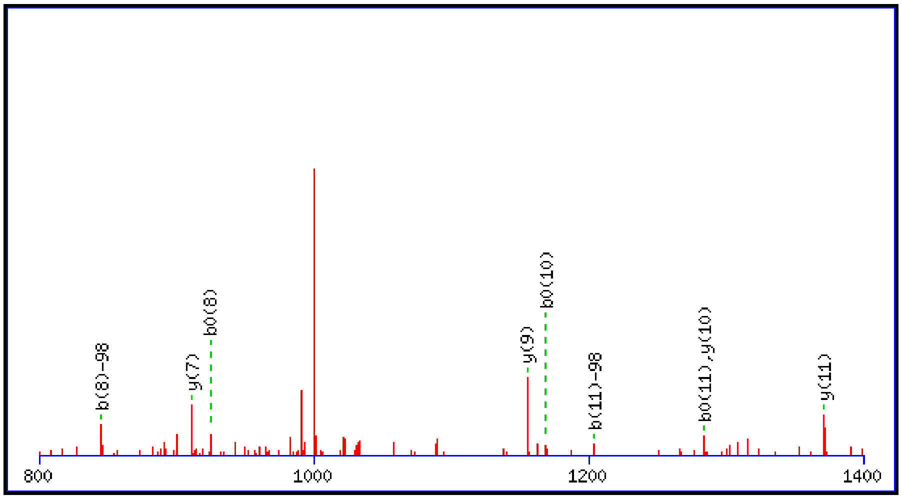

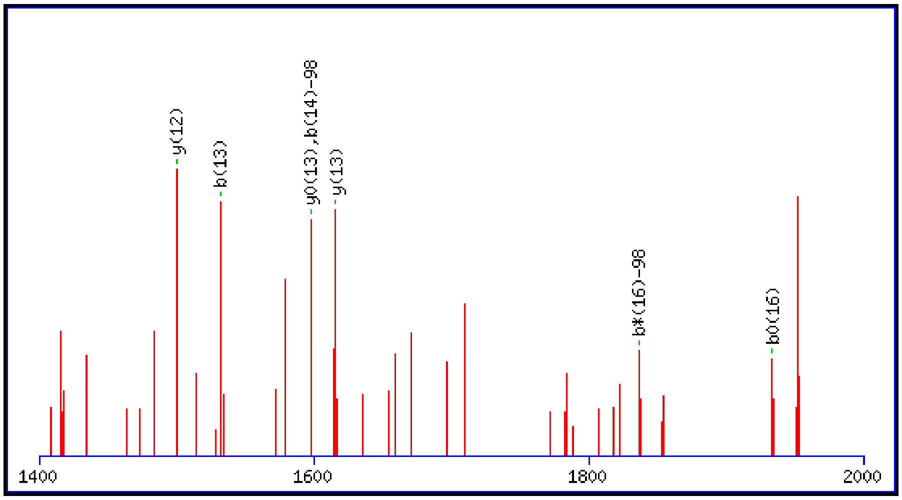

Monoisotopic mass of neutral peptide Mr(calc): 2096.7219
Fixed modifications: Carbamidomethyl (C)
Variable modifications:
S4 : Phospho (ST), with neutral losses 97.9769(shown in table), 0.0000
Ions Score: 63 **Expect:** 3.1e-005
Matches (Bold Red): 24/230 fragment ions using 34 most intense peaks

#	b	b^{++}	b*	b^{*++}	b^{0}	b^{0++}	Seq.	y	y^{++}	y*	y^{*++}	y^{0}	y^{0++}	#
1	88.0393	44.5233			70.0287	35.5180	S							17
2	201.1234	101.0653			183.1128	92.0600	L	1912.7203	956.8638	1895.6937	948.3505	1894.7097	947.8585	16
3	316.1503	158.5788			298.1397	149.5735	D	1799.6362	900.3217	1782.6097	891.8085	1781.6256	891.3165	15
4	385.1718	193.0895			367.1612	184.0842	S	1684.6093	842.8083	1667.5827	834.2950	1666.5987	833.8030	14
5	500.1987	250.6030			**482.1881**	241.5977	D	**1615.5878**	808.2975	1598.5613	799.7843	**1597.5772**	799.2923	13
6	**629.2413**	315.1243			611.2307	306.1190	E	**1500.5609**	750.7841	1483.5343	742.2708	1482.5503	741.7788	12
7	716.2733	358.6403			698.2628	349.6350	S	**1371.5183**	686.2628	1354.4917	677.7495	1353.5077	677.2575	11
8	**845.3159**	423.1616			827.3054	414.1563	E	**1284.4862**	642.7468	1267.4597	634.2335	1266.4757	633.7415	10
9	960.3429	480.6751			942.3323	471.6698	D	**1155.4437**	578.2255	1138.4171	569.7122	1137.4331	569.2202	9
10	1089.3855	545.1964			1071.3749	536.1911	E	1040.4167	520.7120	1023.3902	512.1987	1022.4061	511.7067	8
11	**1204.4124**	602.7098			1186.4018	593.7046	D	**911.3741**	456.1907	894.3476	447.6774	893.3636	447.1854	7
12	1319.4393	660.2233			1301.4288	651.2180	D	**796.3472**	398.6772	779.3206	390.1640	778.3366	389.6719	6
13	1434.4663	717.7368			1416.4557	708.7315	D	**681.3202**	341.1638	664.2937	332.6505	663.3097	332.1585	5
14	**1597.5296**	799.2684			1579.5191	790.2632	Y	**566.2933**	283.6503	549.2667	275.1370			4
15	1725.5882	863.2977	1708.5616	854.7845	1707.5776	854.2925	Q	**403.2300**	202.1186	386.2034	193.6053			3
16	1853.6468	927.3270	**1836.6202**	918.8138	1835.6362	918.3217	Q	275.1714	138.0893	258.1448	129.5761			2
17							K	147.1128	74.0600	130.0863	65.5468			1

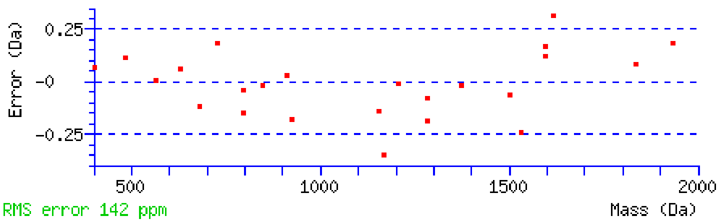

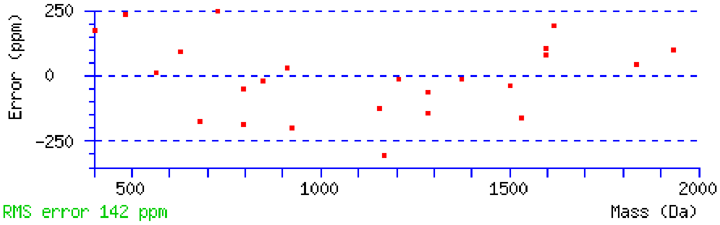

All matches to this query

Score	Mr(calc):	Delta	Sequence
63.2	2096.7219	-0.0072	SLDSDESEDEDDDYQQK
63.2	2096.7219	-0.0072	SLDSDESEDEDDDYQQK
35.3	2096.7219	-0.0072	SLDSDESEDEDDDYQQK
2.3	2096.7010	0.0137	EQELESCACADTDNSCK

1.8	2096.7010	0.0137	EQELESCACADTDNSCK
1.8	2096.7010	0.0137	EQELESCACADTDNSCK
0.2	2096.7041	0.0105	HYSDMMVLMTYMASEE

Spectrum No: 146; Query: 2236; Rank: 1

Peptide View

MS/MS Fragmentation of **KFPDRLADDEGDSDSESVGQSR**
Found in **IPI00370333**, Tax_Id=10116 Gene_Symbol=Baz1b similar to Bromodomain adjacent to zinc finger domain protein 1B

Match to Query 2236: 2489.041512 from(830.687780,3+)
Title: 100101RatKid_NS_deglyco_20.1604.1604.3.dta
Data file K:\NewmanPaper\Piliang\3SubProteomes\Piliang3SP\mgf5ppm\ERLIC_3SubProteomes5ppm.mgf

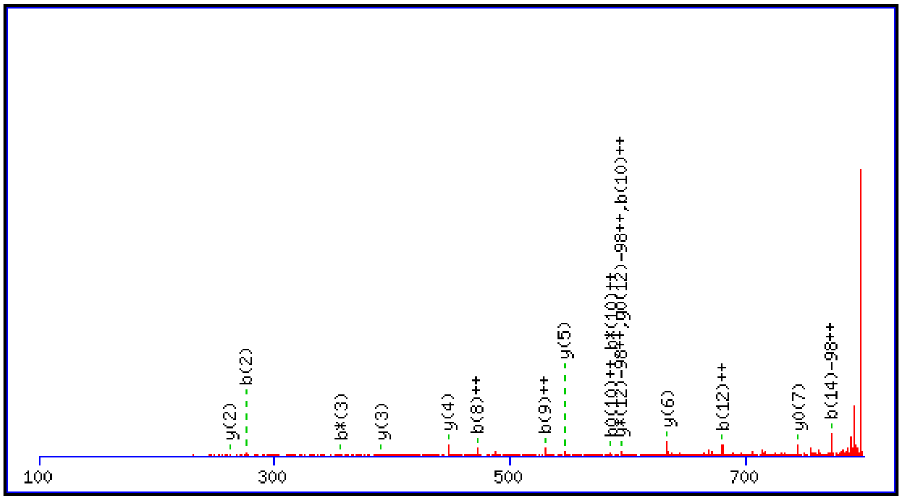

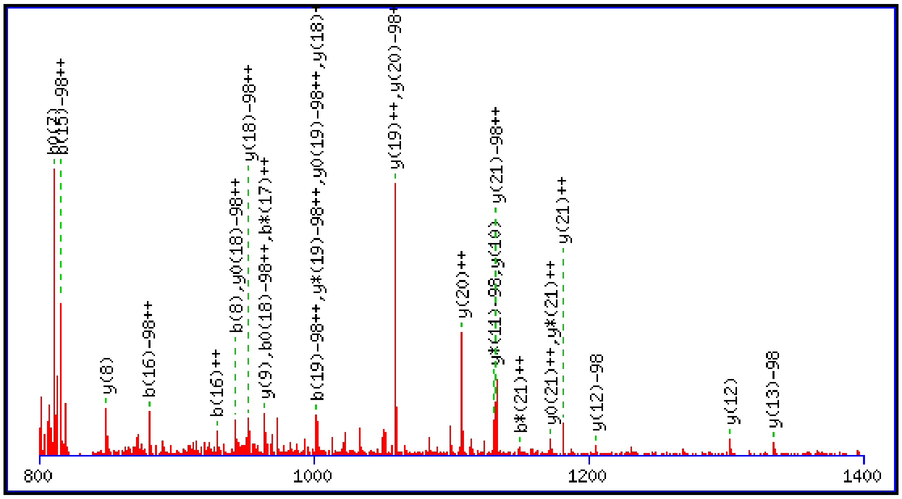

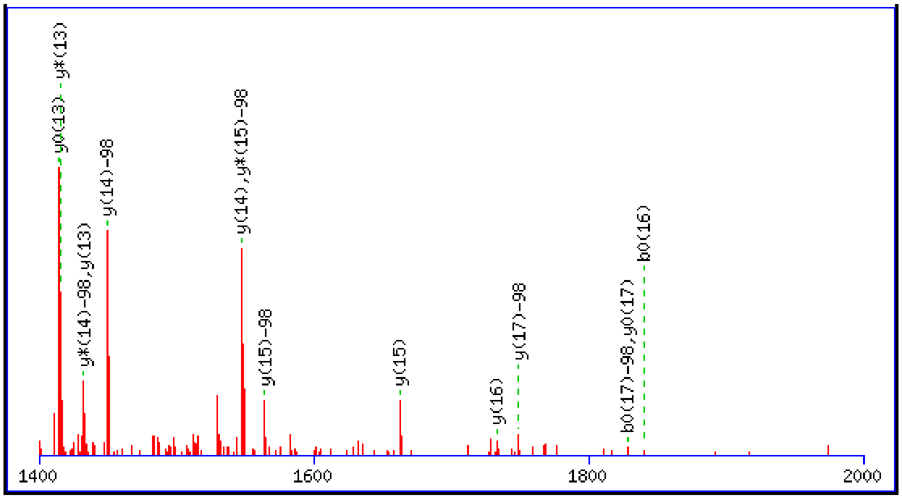

Monoisotopic mass of neutral peptide Mr(calc): 2489.0344
Fixed modifications: Carbamidomethyl (C)
Variable modifications:
S13 : Phospho (ST), with neutral losses 0.0000(shown in table), 97.9769
Ions Score: 63 **Expect:** 0.00015
Matches (Bold Red): 59/370 fragment ions using 73 most intense peaks

#	b	b^{++}	b*	b^{*++}	b^{0}	b^{0++}	Seq.	y	y^{++}	y*	y^{*++}	y^{0}	y^{0++}	#
1	129.1022	65.0548	112.0757	56.5415			K							22
2	**276.1707**	138.5890	259.1441	130.0757			F	2361.9467	**1181.4770**	2344.9201	**1172.9637**	2343.9361	**1172.4717**	21
3	373.2234	187.1153	**356.1969**	178.6021			P	2214.8783	**1107.9428**	2197.8517	1099.4295	2196.8677	1098.9375	20
4	488.2504	244.6288	471.2238	236.1155	470.2398	235.6235	D	2117.8255	**1059.4164**	2100.7989	1050.9031	2099.8149	1050.4111	19
5	644.3515	322.6794	627.3249	314.1661	626.3409	313.6741	R	2002.7986	**1001.9029**	1985.7720	993.3896	1984.7880	992.8976	18
6	757.4355	379.2214	740.4090	370.7081	739.4250	370.2161	L	1846.6974	923.8524	1829.6709	915.3391	**1828.6869**	914.8471	17
7	828.4726	414.7400	811.4461	406.2267	**810.4621**	405.7347	A	**1733.6134**	867.3103	1716.5868	858.7971	1715.6028	858.3050	16
8	**943.4996**	**472.2534**	926.4730	463.7402	925.4890	463.2482	D	**1662.5763**	831.7918	1645.5497	823.2785	1644.5657	822.7865	15
9	1058.5265	**529.7669**	1041.5000	521.2536	1040.5160	520.7616	D	**1547.5493**	774.2783	1530.5228	765.7650	1529.5388	765.2730	14
10	1187.5691	**594.2882**	1170.5426	**585.7749**	1169.5586	**585.2829**	E	**1432.5224**	716.7648	**1415.4958**	708.2516	**1414.5118**	707.7595	13
11	1244.5906	622.7989	1227.5640	614.2857	1226.5800	613.7937	G	**1303.4798**	652.2435	1286.4532	643.7303	1285.4692	643.2382	12
12	1359.6175	**680.3124**	1342.5910	671.7991	1341.6070	671.3071	D	1246.4583	623.7328	1229.4318	615.2195	1228.4478	614.7275	11
13	1526.6159	763.8116	1509.5893	755.2983	1508.6053	754.8063	S	**1131.4314**	566.2193	1114.4048	557.7061	1113.4208	557.2140	10
14	1641.6428	821.3251	1624.6163	812.8118	1623.6323	812.3198	D	**964.4330**	482.7202	947.4065	474.2069	946.4225	473.7149	9
15	1728.6749	864.8411	1711.6483	856.3278	1710.6643	855.8358	S	**849.4061**	425.2067	832.3795	416.6934	831.3955	416.2014	8
16	1857.7175	**929.3624**	1840.6909	920.8491	**1839.7069**	920.3571	E	762.3741	381.6907	745.3475	373.1774	**744.3635**	372.6854	7
17	1944.7495	972.8784	1927.7229	**964.3651**	1926.7389	963.8731	S	**633.3315**	317.1694	616.3049	308.6561	615.3209	308.1641	6
18	2043.8179	1022.4126	2026.7913	1013.8993	2025.8073	1013.4073	V	**546.2994**	273.6534	529.2729	265.1401	528.2889	264.6481	5
19	2100.8394	1050.9233	2083.8128	1042.4100	2082.8288	1041.9180	G	**447.2310**	224.1191	430.2045	215.6059	429.2205	215.1139	4
20	2228.8979	1114.9526	2211.8714	1106.4393	2210.8874	1105.9473	Q	**390.2096**	195.6084	373.1830	187.0951	372.1990	186.6031	3
21	2315.9300	1158.4686	2298.9034	**1149.9553**	2297.9194	1149.4633	S	**262.1510**	131.5791	245.1244	123.0659	244.1404	122.5738	2
22							R	175.1190	88.0631	158.0924	79.5498			1

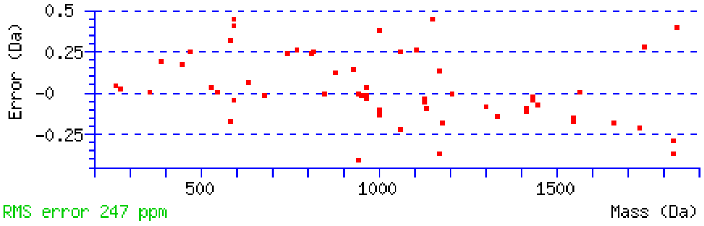

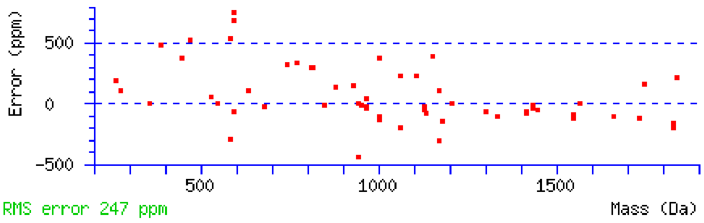

All matches to this query

Score	Mr(calc):	Delta	Sequence
63.0	2489.0344	0.0071	KFPDRLADDEGDSDSESVGQSR
50.8	2489.0344	0.0071	KFPDRLADDEGDSDSESVGQSR
43.0	2489.0344	0.0071	KFPDRLADDEGDSDSESVGQSR
17.5	2489.0344	0.0071	KFPDRLADDEGDSDSESVGQSR
6.6	2489.0582	-0.0167	SGGNQGAKIIQSFLWYLNPR
5.6	2488.0381	1.0034	THRQGSSSVSGSPAPSTSGTSTPR
5.6	2488.0381	1.0034	THRQGSSSVSGSPAPSTSGTSTPR
5.6	2488.0381	1.0034	THRQGSSSVSGSPAPSTSGTSTPR
5.6	2488.0381	1.0034	THRQGSSSVSGSPAPSTSGTSTPR
4.4	2489.0570	-0.0155	GKDEAMAIGGHWSPSLDGPNPEK

Spectrum No: 147; Query: 1863; Rank: 1

Peptide View

MS/MS Fragmentation of **VAQSDGEESPAEEGQLLGER**
Found in **IPI00210147**, Tax_Id=10116 Gene_Symbol=Sec31l1 Isoform 1 of Protein transport protein Sec31A

Match to Query 1863: 2179.934108 from(1090.974330,2+)
Title: 100101RatKid_NS_deglyco_23.2400.2400.2.dta
Data file K:\NewmanPaper\Piliang\3SubProteomes\Piliang3SP\mgf5ppm\ERLIC_3SubProteomes5ppm.mgf

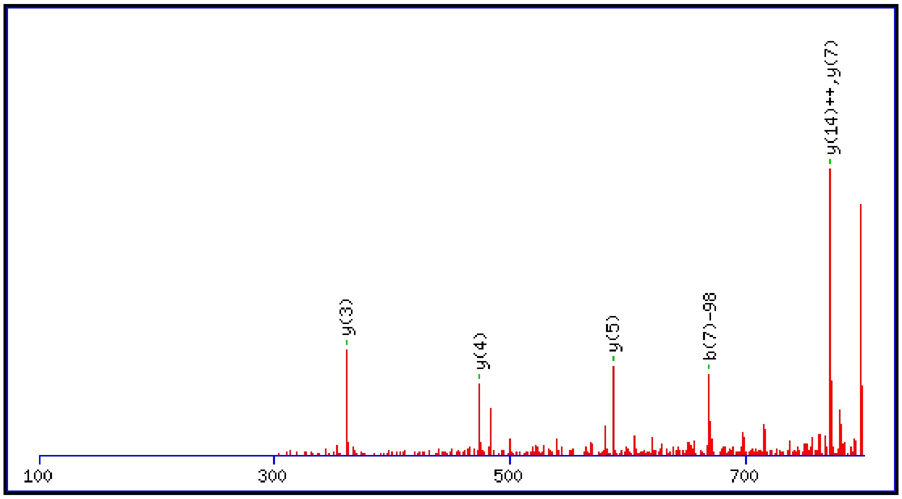

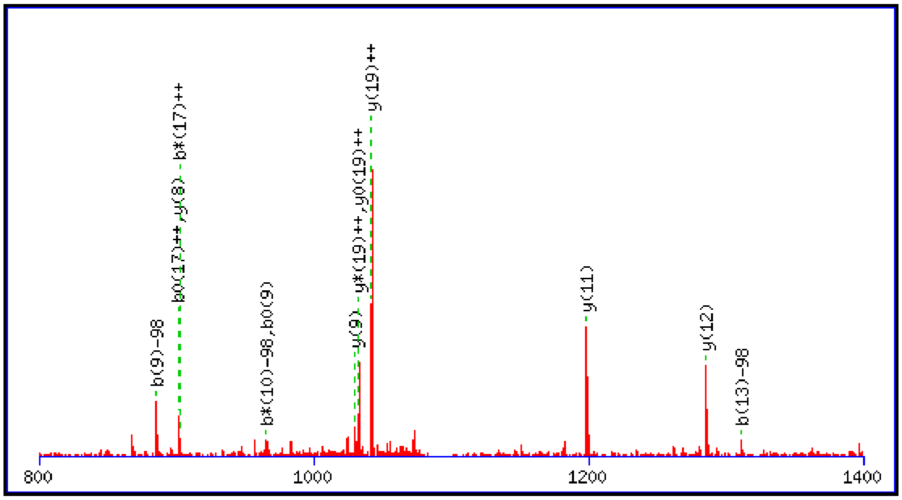

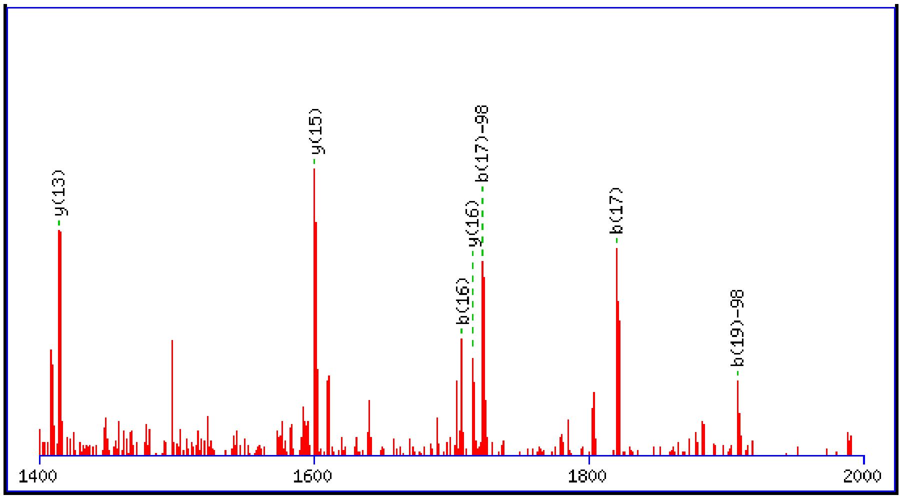

Monoisotopic mass of neutral peptide Mr(calc): 2179.9270
Fixed modifications: Carbamidomethyl (C)
Variable modifications:
S4 : Phospho (ST), with neutral losses 97.9769(shown in table), 0.0000
Ions Score: 63 **Expect:** 0.00012
Matches (Bold Red): 26/330 fragment ions using 34 most intense peaks

#	b	b^{++}	b*	b^{*++}	b^{0}	b^{0++}	Seq.	y	y^{++}	y*	y^{*++}	y^{0}	y^{0++}	#
1	100.0757	50.5415					V							20
2	171.1128	86.0600					A	1983.8890	992.4481	1966.8625	983.9349	1965.8784	983.4429	19
3	299.1714	150.0893	282.1448	141.5761			Q	1912.8519	956.9296	1895.8253	948.4163	1894.8413	947.9243	18
4	368.1928	184.6001	351.1663	176.0868	350.1823	175.5948	S	1784.7933	892.9003	1767.7668	884.3870	1766.7828	883.8950	17
5	483.2198	242.1135	466.1932	233.6003	465.2092	233.1082	D	**1715.7719**	858.3896	1698.7453	849.8763	1697.7613	849.3843	16
6	540.2412	270.6243	523.2147	262.1110	522.2307	261.6190	G	**1600.7449**	800.8761	1583.7184	792.3628	1582.7344	791.8708	15
7	**669.2838**	335.1456	652.2573	326.6323	651.2733	326.1403	E	1543.7235	**772.3654**	1526.6969	763.8521	1525.7129	763.3601	14
8	798.3264	399.6669	781.2999	391.1536	780.3159	390.6616	E	**1414.6809**	707.8441	1397.6543	699.3308	1396.6703	698.8388	13
9	**885.3585**	443.1829	868.3319	434.6696	867.3479	434.1776	S	**1285.6383**	643.3228	1268.6117	634.8095	1267.6277	634.3175	12
10	982.4112	491.7092	**965.3847**	483.1960	964.4007	482.7040	P	**1198.6062**	599.8068	1181.5797	591.2935	1180.5957	590.8015	11
11	1053.4483	527.2278	1036.4218	518.7145	1035.4378	518.2225	A	1101.5535	551.2804	1084.5269	542.7671	1083.5429	542.2751	10
12	1182.4909	591.7491	1165.4644	583.2358	1164.4804	582.7438	E	**1030.5164**	515.7618	1013.4898	507.2485	1012.5058	506.7565	9
13	**1311.5335**	656.2704	1294.5070	647.7571	1293.5230	647.2651	E	**901.4738**	451.2405	884.4472	442.7272	883.4632	442.2352	8
14	1368.5550	684.7811	1351.5284	676.2679	1350.5444	675.7758	G	**772.4312**	386.7192	755.4046	378.2060	754.4206	377.7139	7
15	1496.6136	748.8104	1479.5870	740.2971	1478.6030	739.8051	Q	715.4097	358.2085	698.3832	349.6952	697.3991	349.2032	6
16	1609.6976	805.3525	1592.6711	796.8392	1591.6871	796.3472	L	**587.3511**	294.1792	570.3246	285.6659	569.3406	285.1739	5
17	**1722.7817**	861.8945	1705.7551	853.3812	1704.7711	852.8892	L	**474.2671**	237.6372	457.2405	229.1239	456.2565	228.6319	4
18	1779.8032	890.4052	1762.7766	881.8919	1761.7926	881.3999	G	**361.1830**	181.0951	344.1565	172.5819	343.1724	172.0899	3
19	**1908.8458**	954.9265	1891.8192	946.4132	1890.8352	945.9212	E	304.1615	152.5844	287.1350	144.0711	286.1510	143.5791	2
20							R	175.1190	88.0631	158.0924	79.5498			1

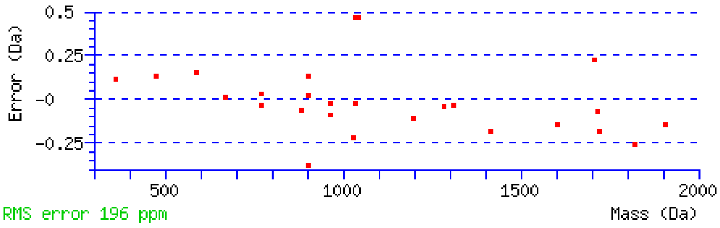

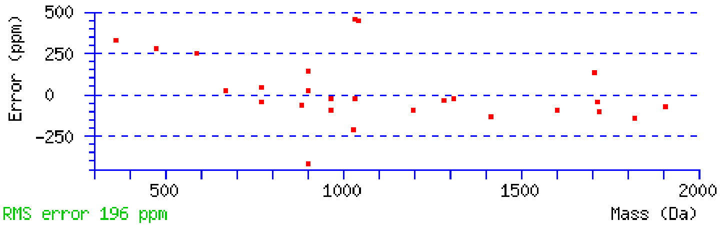

All matches to this query

Score	Mr(calc):	Delta	Sequence
63.0	2179.9270	0.0071	VAQSDGEESPAEEGQLLGER

26.3	2179.9270	0.0071	VAQSDGEESPAEEGQLLGER
11.1	2177.9309	2.0032	TVCIISCQNEQCGLIFR
7.2	2179.9445	-0.0104	MMKYWTNFARQGNPNSR
4.6	2179.9357	-0.0016	GAYTLVTFSAKNNTIVNK
1.0	2179.9275	0.0066	AFAPESGRLMYTINSAHR
1.0	2179.9453	-0.0111	NPESFAAGERRVTPAYVR
0.7	2178.9496	0.9846	VMNLIIFHSQMLDSVEK
0.4	2177.9300	2.0041	NVMLLPVGSADDGAHSQNEK

Spectrum No: 148; Query: 716; Rank: 1

Peptide View

MS/MS Fragmentation of **AEDGAAPSPSSETPK**
Found in **IPI00371946**, Tax_Id=10116 Gene_Symbol=LOC681252 similar to Myristoylated alanine-rich C-kinase substrate

Match to Query 716: 1522.615828 from(762.315190,2+)
Title: 091008RatKidney_NoSalt_25.430.430.2.dta
Data file K:\NewmanPaper\Piliang\3SubProteomes\Piliang3SP\mgf5ppm\ERLIC_3SubProteomes5ppm.mgf

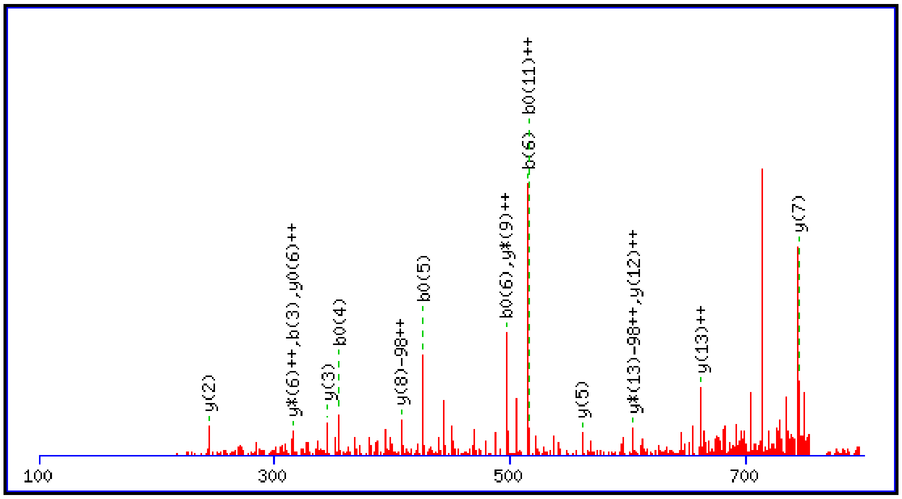

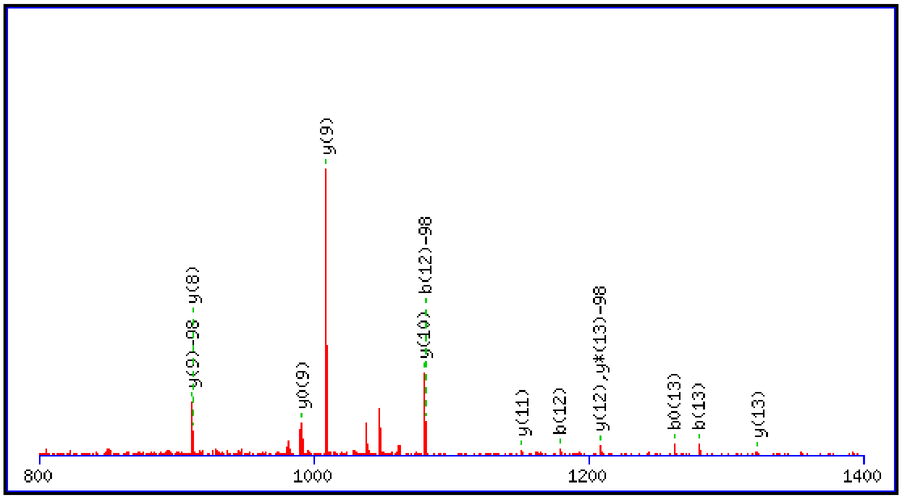

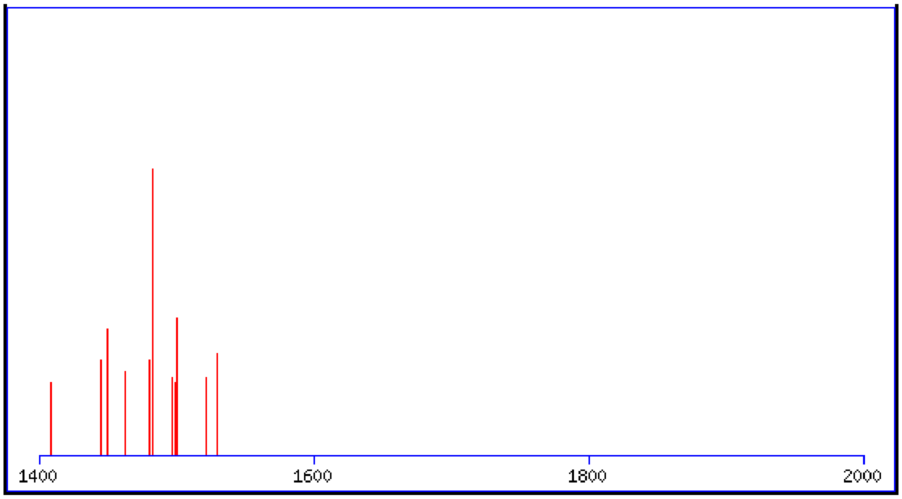

Monoisotopic mass of neutral peptide Mr(calc): 1522.6188
Fixed modifications: Carbamidomethyl (C)
Variable modifications:
S8 : Phospho (ST), with neutral losses 0.0000(shown in table), 97.9769
Ions Score: 63 **Expect:** 6.1e-005
Matches (Bold Red): 30/204 fragment ions using 48 most intense peaks

#	b	b++	b0	b0++	Seq.	y	y++	y*	y*++	y0	y0++	#
1	72.0444	36.5258			A							15
2	201.0870	101.0471	183.0764	92.0418	E	1452.5890	726.7981	1435.5625	718.2849	1434.5784	717.7929	14
3	**316.1139**	158.5606	298.1034	149.5553	D	**1323.5464**	**662.2768**	1306.5199	653.7636	1305.5358	653.2716	13
4	373.1354	187.0713	**355.1248**	178.0661	G	**1208.5195**	**604.7634**	1191.4929	596.2501	1190.5089	595.7581	12
5	444.1725	222.5899	**426.1619**	213.5846	A	**1151.4980**	576.2526	1134.4715	567.7394	1133.4874	567.2474	11
6	**515.2096**	258.1084	**497.1991**	249.1032	A	**1080.4609**	540.7341	1063.4343	532.2208	1062.4503	531.7288	10
7	612.2624	306.6348	594.2518	297.6295	P	**1009.4238**	505.2155	992.3972	**496.7023**	**991.4132**	496.2102	9
8	779.2607	390.1340	761.2502	381.1287	S	**912.3710**	456.6891	895.3445	448.1759	894.3604	447.6839	8
9	876.3135	438.6604	858.3029	429.6551	P	**745.3727**	373.1900	728.3461	364.6767	727.3621	364.1847	7
10	963.3455	482.1764	945.3350	473.1711	S	648.3199	324.6636	631.2933	**316.1503**	630.3093	**315.6583**	6
11	1050.3776	525.6924	1032.3670	**516.6871**	S	**561.2879**	281.1476	544.2613	272.6343	543.2773	272.1423	5
12	**1179.4201**	590.2137	1161.4096	581.2084	E	474.2558	237.6316	457.2293	229.1183	456.2453	228.6263	4
13	**1280.4678**	640.7376	**1262.4573**	631.7323	T	**345.2132**	173.1103	328.1867	164.5970	327.2027	164.1050	3
14	1377.5206	689.2639	1359.5100	680.2587	P	**244.1656**	122.5864	227.1390	114.0731			2
15					K	147.1128	74.0600	130.0863	65.5468			1

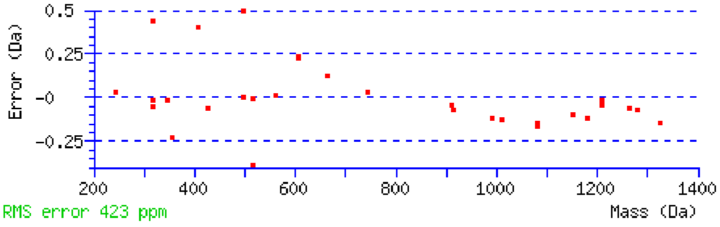

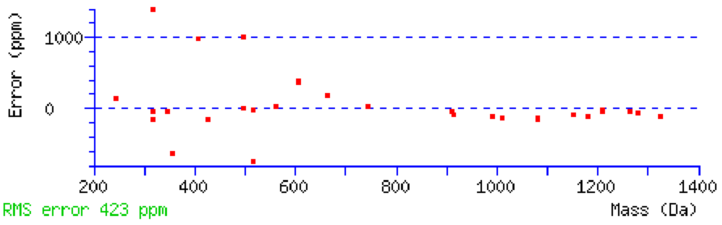

All matches to this query

Score	Mr(calc):	Delta	Sequence
63.0	1522.6188	-0.0030	AEDGAAPSPSSETPK
51.0	1522.6188	-0.0030	AEDGAAPSPSSETPK
40.2	1522.6188	-0.0030	AEDGAAPSPSSETPK
30.7	1522.6188	-0.0030	AEDGAAPSPSSETPK
10.0	1522.6146	0.0012	TFSYTPSYAKAK
10.0	1522.6146	0.0012	TFSYTPSYAKAK

8.2	1522.6218	-0.0059	YNRKPAETNLR
3.1	1522.6301	-0.0142	NTVAQDTETPPNR
2.5	1521.6200	0.9958	TYMLRRYTSR
2.5	1522.6146	0.0012	TFSYTPSYAKAK

Spectrum No: 149; Query: 1215; Rank: 1

Peptide View

MS/MS Fragmentation of **TPSQEGSQDLTQEQR**
Found in **IPI00766157**, Tax_Id=10116 Gene_Symbol=RGD1559939_predicted hypothetical protein LOC500006

Match to Query 1215: 1782.748608 from(892.381580,2+)
Title: 100101RatKid_NS_deglyco_22.1032.1032.2.dta
Data file K:\NewmanPaper\Piliang\3SubProteomes\Piliang3SP\mgf5ppm\ERLIC_3SubProteomes5ppm.mgf

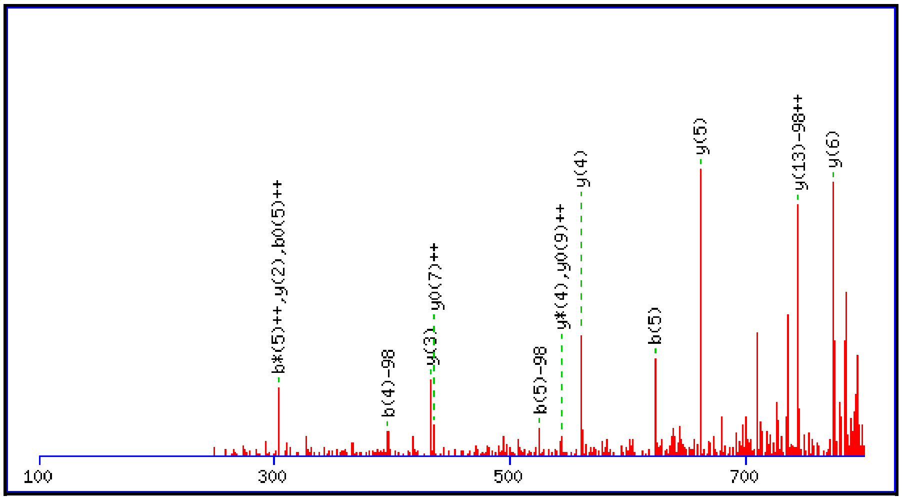

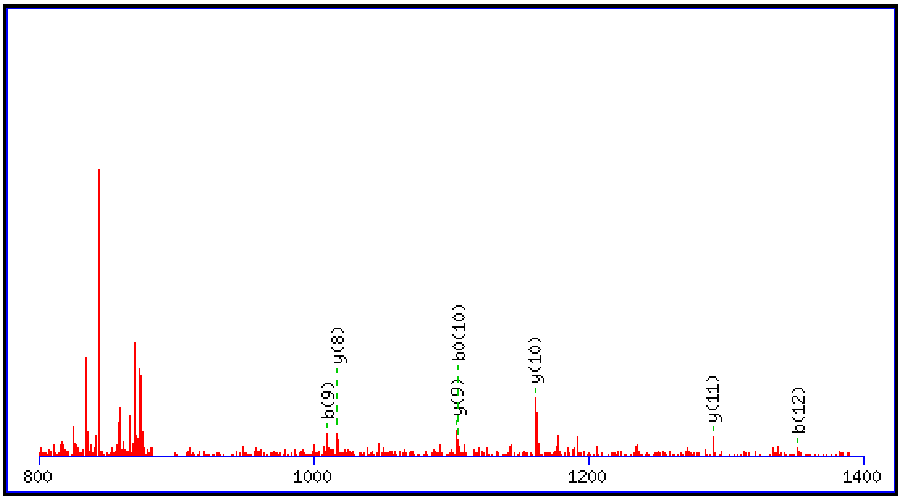

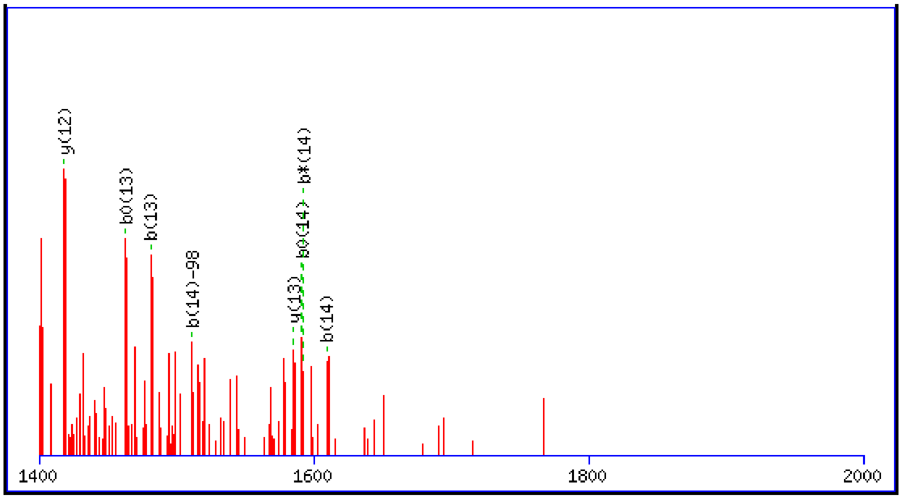

Monoisotopic mass of neutral peptide Mr(calc): 1782.7421
Fixed modifications: Carbamidomethyl (C)
Variable modifications:
S3 : Phospho (ST), with neutral losses 0.0000(shown in table), 97.9769
Ions Score: 63 **Expect:** 9e-005
Matches (Bold Red): 29/240 fragment ions using 46 most intense peaks

#	b	b^{++}	b*	b^{*++}	b^0	b^{0++}	Seq.	y	y^{++}	y*	y^{*++}	y^0	y^{0++}	#
1	102.0550	51.5311			84.0444	42.5258	T							15
2	199.1077	100.0575			181.0972	91.0522	P	1682.7017	841.8545	1665.6752	833.3412	1664.6912	832.8492	14
3	366.1061	183.5567			348.0955	174.5514	S	**1585.6490**	793.3281	1568.6224	784.8149	1567.6384	784.3228	13
4	494.1646	247.5860	477.1381	239.0727	476.1541	238.5807	Q	**1418.6506**	709.8290	1401.6241	701.3157	1400.6401	700.8237	12
5	**623.2072**	312.1073	606.1807	**303.5940**	605.1967	**303.1020**	E	**1290.5921**	645.7997	1273.5655	637.2864	1272.5815	636.7944	11
6	680.2287	340.6180	663.2022	332.1047	662.2181	331.6127	G	**1161.5495**	581.2784	1144.5229	572.7651	1143.5389	572.2731	10
7	767.2607	384.1340	750.2342	375.6207	749.2502	375.1287	S	**1104.5280**	552.7676	1087.5014	544.2544	1086.5174	**543.7624**	9
8	895.3193	448.1633	878.2928	439.6500	877.3087	439.1580	Q	**1017.4960**	509.2516	1000.4694	500.7383	999.4854	500.2463	8
9	**1010.3463**	505.6768	993.3197	497.1635	992.3357	496.6715	D	889.4374	445.2223	872.4108	436.7091	871.4268	**436.2170**	7
10	1123.4303	562.2188	1106.4038	553.7055	**1105.4198**	553.2135	L	**774.4104**	387.7089	757.3839	379.1956	756.3999	378.7036	6
11	1224.4780	612.7426	1207.4514	604.2294	1206.4674	603.7374	T	**661.3264**	331.1668	644.2998	322.6536	643.3158	322.1615	5
12	**1352.5366**	676.7719	1335.5100	668.2587	1334.5260	667.7666	Q	**560.2787**	280.6430	**543.2522**	272.1297	542.2681	271.6377	4
13	**1481.5792**	741.2932	1464.5526	732.7799	**1463.5686**	732.2879	E	**432.2201**	216.6137	415.1936	208.1004	414.2096	207.6084	3
14	**1609.6377**	805.3225	**1592.6112**	796.8092	**1591.6272**	796.3172	Q	**303.1775**	152.0924	286.1510	143.5791			2
15							R	175.1190	88.0631	158.0924	79.5498			1

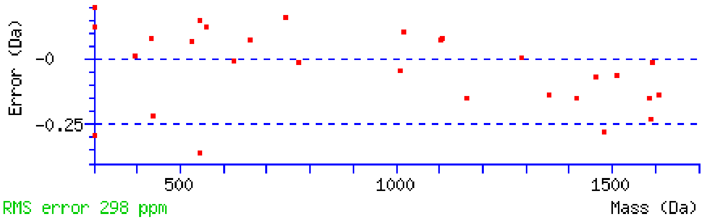

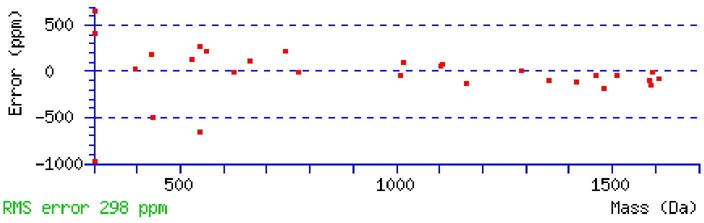

All matches to this query

Score	Mr(calc):	Delta	Sequence
62.9	1782.7421	0.0065	TPSQEGSQDLTQEQR
55.2	1782.7421	0.0065	TPSQEGSQDLTQEQR
22.5	1782.7421	0.0065	TPSQEGSQDLTQEQR
7.7	1782.7421	0.0065	TPSQEGSQDLTQEQR
2.1	1781.7461	1.0025	AHTGEKTYECNQCGK
1.4	1781.7477	1.0009	MFSSSRSMPALTSER

Spectrum No: 150; Query: 1651; Rank: 1

Peptide View

MS/MS Fragmentation of **KEESEESDDDMGFGLFD**
Found in **IPI00188804**, Tax_Id=10116 Gene_Symbol=Rplp2 60S acidic ribosomal protein P2

Match to Query 1651: 2044.718088 from(1023.366320,2+)
Title: 100101RatKid_NS_deglyco_25.3651.3651.2.dta
Data file K:\NewmanPaper\Piliang\3SubProteomes\Piliang3SP\mgf5ppm\ERLIC_3SubProteomes5ppm.mgf

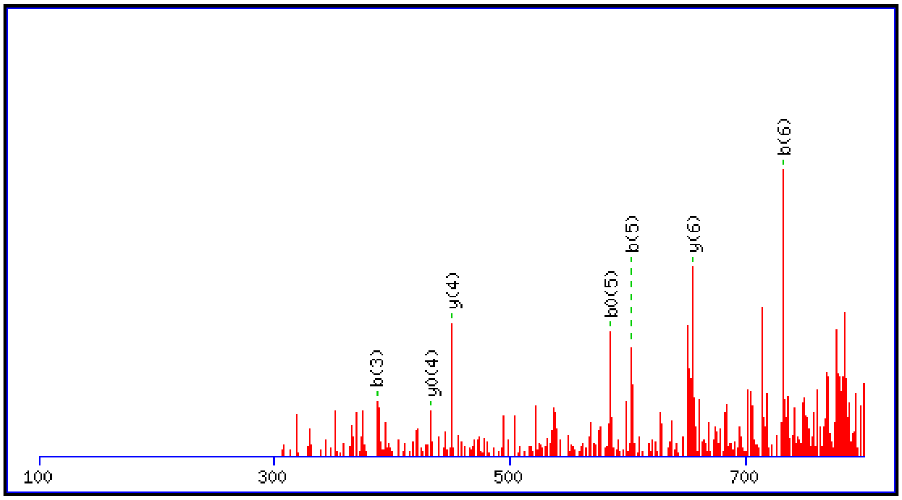

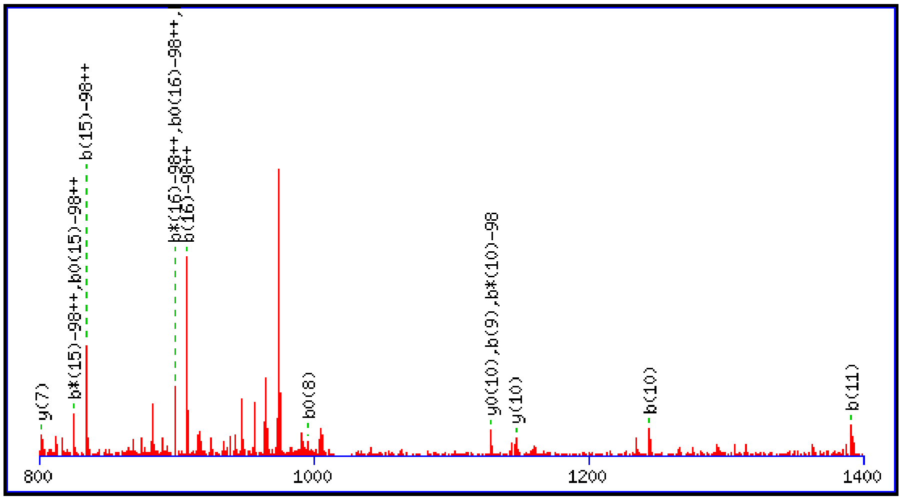

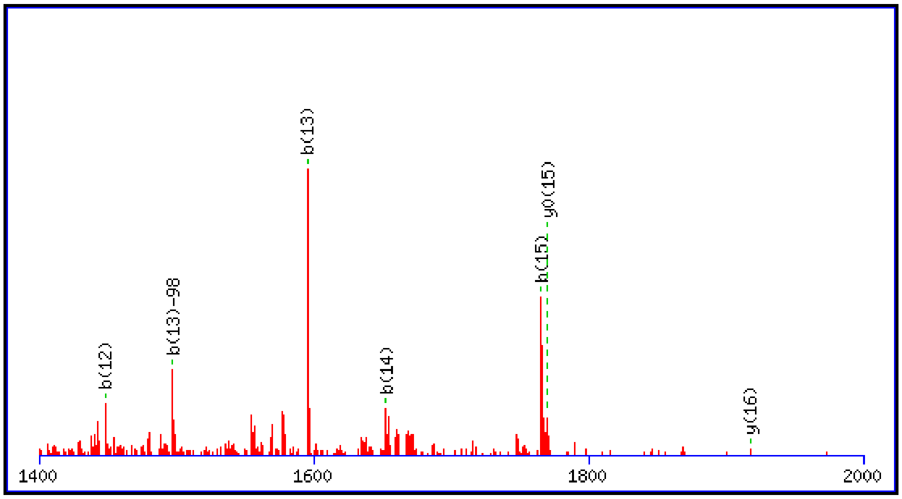

Monoisotopic mass of neutral peptide Mr(calc): 2044.7133
Fixed modifications: Carbamidomethyl (C)
Variable modifications:
S7 : Phospho (ST), with neutral losses 0.0000(shown in table), 97.9769
M11 : Oxidation (M)
Ions Score: 63 **Expect:** 4.5e-005
Matches (Bold Red): 31/242 fragment ions using 53 most intense peaks

#	b	b^{++}	b*	b^{*++}	b^0	b^{0++}	Seq.	y	y^{++}	y^0	y^{0++}	#
1	129.1022	65.0548	112.0757	56.5415			K					17
2	258.1448	129.5761	241.1183	121.0628	240.1343	120.5708	E	**1917.6256**	959.3164	1899.6150	950.3112	16
3	**387.1874**	194.0974	370.1609	185.5841	369.1769	185.0921	E	1788.5830	894.7951	**1770.5724**	885.7899	15
4	474.2195	237.6134	457.1929	229.1001	456.2089	228.6081	S	1659.5404	830.2738	1641.5298	821.2686	14
5	**603.2620**	302.1347	586.2355	293.6214	**585.2515**	293.1294	E	1572.5084	786.7578	1554.4978	777.7525	13
6	**732.3046**	366.6560	715.2781	358.1427	714.2941	357.6507	E	1443.4658	722.2365	1425.4552	713.2312	12
7	**899.3030**	450.1551	882.2764	441.6419	881.2924	441.1499	S	1314.4232	657.7152	1296.4126	648.7099	11
8	1014.3299	507.6686	997.3034	499.1553	**996.3194**	498.6633	D	**1147.4248**	574.2161	**1129.4143**	565.2108	10
9	**1129.3569**	565.1821	1112.3303	556.6688	1111.3463	556.1768	D	1032.3979	516.7026	1014.3873	507.6973	9
10	**1244.3838**	622.6955	1227.3573	614.1823	1226.3733	613.6903	D	917.3710	459.1891	**899.3604**	450.1838	8
11	**1391.4192**	696.2133	1374.3927	687.7000	1373.4087	687.2080	M	**802.3440**	401.6756	784.3334	392.6704	7
12	**1448.4407**	724.7240	1431.4141	716.2107	1430.4301	715.7187	G	**655.3086**	328.1579	637.2980	319.1527	6
13	**1595.5091**	798.2582	1578.4826	789.7449	1577.4985	789.2529	F	598.2871	299.6472	580.2766	290.6419	5
14	**1652.5306**	826.7689	1635.5040	818.2556	1634.5200	817.7636	G	**451.2187**	226.1130	**433.2082**	217.1077	4
15	**1765.6146**	883.3110	1748.5881	874.7977	1747.6041	874.3057	L	394.1973	197.6023	376.1867	188.5970	3
16	1912.6830	956.8452	1895.6565	948.3319	1894.6725	947.8399	F	281.1132	141.0602	263.1026	132.0550	2
17							D	134.0448	67.5260	116.0342	58.5207	1

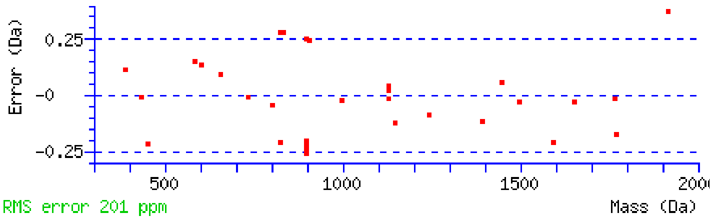

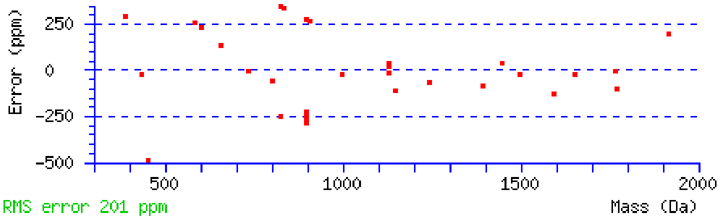

All matches to this query

Score	Mr(calc):	Delta	Sequence
62.8	2044.7133	0.0048	KEESEESDDDMGFGLFD
40.4	2044.7133	0.0048	KEESEESDDDMGFGLFD

Spectrum No: 151; Query: 2790; Rank: 1

Peptide View

MS/MS Fragmentation of **KEDSDEDEDEEDEDDSDEDEDEEDEFEPPVVK**
Found in **IPI00231827**, Tax_Id=10116 Gene_Symbol=Ncl Nucleolin

Match to Query 2790: 3881.368182 from(1294.796670,3+)
Title: 100101RatKid_NS_deglyco_28.2430.2430.3.dta
Data file K:\NewmanPaper\Piliang\3SubProteomes\Piliang3SP\mgf5ppm\ERLIC_3SubProteomes5ppm.mgf

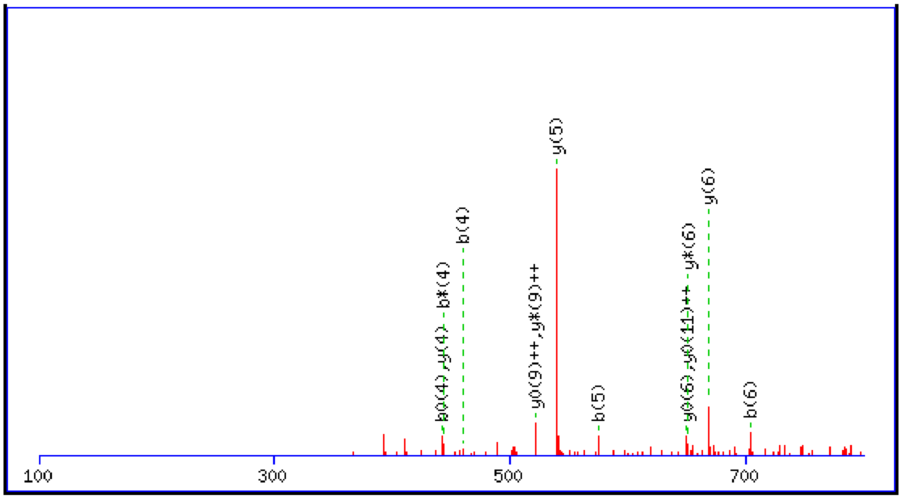

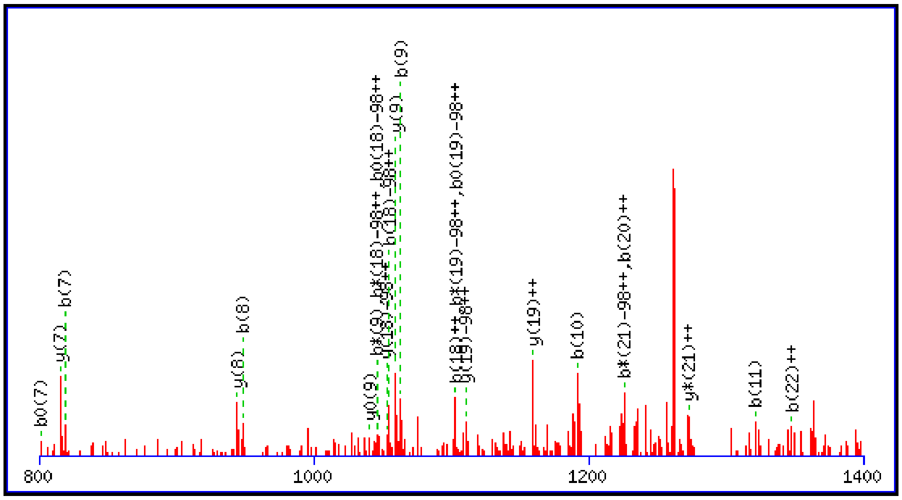

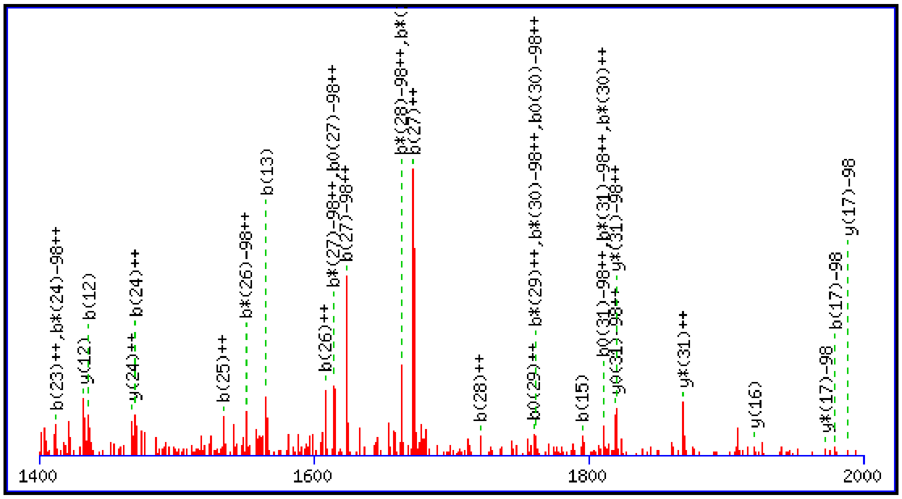

Monoisotopic mass of neutral peptide Mr(calc): 3881.3491
Fixed modifications: Carbamidomethyl (C)
Variable modifications:
S16 : Phospho (ST), with neutral losses 97.9769(shown in table), 0.0000
Ions Score: 63 **Expect:** 9.4e-005
Matches (Bold Red): 72/546 fragment ions using 104 most intense peaks

#	b	b^{++}	b*	b^{*++}	b^0	b^{0++}	Seq.	y	y^{++}	y*	y^{*++}	y^0	y^{0++}	#
1	129.1022	65.0548	112.0757	56.5415			K							32
2	258.1448	129.5761	241.1183	121.0628	240.1343	120.5708	E	3656.2845	1828.6459	3639.2580	**1820.1326**	3638.2740	**1819.6406**	31
3	373.1718	187.0895	356.1452	178.5763	355.1612	178.0842	D	3527.2420	1764.1246	3510.2154	1755.6113	3509.2314	1755.1193	30
4	**460.2038**	230.6055	**443.1773**	222.0923	**442.1932**	221.6003	S	3412.2150	1706.6111	3395.1885	1698.0979	3394.2044	1697.6059	29
5	**575.2307**	288.1190	558.2042	279.6057	557.2202	279.1137	D	3325.1830	1663.0951	3308.1564	1654.5819	3307.1724	1654.0898	28

6	**704.2733**	352.6403	687.2468	344.1270	686.2628	343.6350	E	3210.1560	1605.5817	3193.1295	1597.0684	3192.1455	1596.5764	27
7	**819.3003**	410.1538	802.2737	401.6405	**801.2897**	401.1485	D	3081.1134	1541.0604	3064.0869	1532.5471	3063.1029	1532.0551	26
8	**948.3429**	474.6751	931.3163	466.1618	930.3323	465.6698	E	2966.0865	1483.5469	2949.0600	1475.0336	2948.0759	1474.5416	25
9	**1063.3698**	532.1885	**1046.3433**	523.6753	1045.3593	523.1833	D	2837.0439	1419.0256	2820.0174	1410.5123	2819.0333	1410.0203	24
10	**1192.4124**	596.7098	1175.3859	588.1966	1174.4018	587.7046	E	2722.0170	1361.5121	2704.9904	1352.9988	2704.0064	1352.5068	23
11	**1321.4550**	661.2311	1304.4285	652.7179	1303.4444	652.2259	E	2592.9744	1296.9908	2575.9478	1288.4776	2574.9638	1287.9855	22
12	**1436.4819**	718.7446	1419.4554	710.2313	1418.4714	709.7393	D	2463.9318	1232.4695	2446.9052	1223.9563	2445.9212	1223.4642	21
13	**1565.5245**	783.2659	1548.4980	774.7526	1547.5140	774.2606	E	2348.9048	1174.9561	2331.8783	1166.4428	2330.8943	1165.9508	20
14	1680.5515	840.7794	**1663.5249**	832.2661	1662.5409	831.7741	D	2219.8622	**1110.4348**	2202.8357	1101.9215	2201.8517	1101.4295	19
15	**1795.5784**	898.2929	1778.5519	889.7796	1777.5679	889.2876	D	2104.8353	**1052.9213**	2087.8088	1044.4080	2086.8247	1043.9160	18
16	1864.5999	932.8036	1847.5733	924.2903	1846.5893	923.7983	S	**1989.8084**	995.4078	**1972.7818**	986.8945	1971.7978	986.4025	17
17	**1979.6268**	990.3171	1962.6003	981.8038	1961.6163	981.3118	D	**1920.7869**	960.8971	1903.7604	952.3838	1902.7763	951.8918	16
18	2108.6694	**1054.8383**	2091.6429	**1046.3251**	2090.6589	**1045.8331**	E	1805.7600	903.3836	1788.7334	894.8703	1787.7494	894.3783	15
19	2223.6964	1112.3518	2206.6698	**1103.8385**	2205.6858	**1103.3465**	D	1676.7174	838.8623	1659.6908	830.3490	1658.7068	829.8570	14
20	2352.7390	1176.8731	2335.7124	1168.3598	2334.7284	1167.8678	E	1561.6904	781.3489	1544.6639	772.8356	1543.6799	772.3436	13
21	2467.7659	1234.3866	2450.7393	**1225.8733**	2449.7553	1225.3813	D	**1432.6478**	716.8276	1415.6213	708.3143	1414.6373	707.8223	12
22	2596.8085	1298.9079	2579.7819	1290.3946	2578.7979	1289.9026	E	1317.6209	659.3141	1300.5943	650.8008	1299.6103	**650.3088**	11
23	2725.8511	1363.4292	2708.8245	1354.9159	2707.8405	1354.4239	E	1188.5783	594.7928	1171.5517	586.2795	1170.5677	585.7875	10
24	2840.8780	1420.9427	2823.8515	**1412.4294**	2822.8675	1411.9374	D	**1059.5357**	530.2715	1042.5092	**521.7582**	**1041.5251**	**521.2662**	9
25	2969.9206	1485.4639	2952.8941	1476.9507	2951.9101	1476.4587	E	**944.5088**	472.7580	927.4822	464.2447	926.4982	463.7527	8
26	3116.9890	1558.9982	3099.9625	**1550.4849**	3098.9785	1549.9929	F	**815.4662**	408.2367	798.4396	399.7234	797.4556	399.2314	7
27	3246.0316	**1623.5195**	3229.0051	**1615.0062**	3228.0211	**1614.5142**	E	**668.3978**	334.7025	**651.3712**	326.1892	**650.3872**	325.6972	6
28	3343.0844	1672.0458	3326.0578	**1663.5326**	3325.0738	1663.0406	P	**539.3552**	270.1812	522.3286	261.6679			5
29	3440.1372	1720.5722	3423.1106	1712.0589	3422.1266	1711.5669	P	**442.3024**	221.6548	425.2758	213.1416			4
30	3539.2056	1770.1064	3522.1790	**1761.5931**	3521.1950	**1761.1011**	V	345.2496	173.1285	328.2231	164.6152			3
31	3638.2740	**1819.6406**	3621.2474	**1811.1274**	3620.2634	**1810.6353**	V	246.1812	123.5942	229.1547	115.0810			2
32							K	147.1128	74.0600	130.0863	65.5468			1

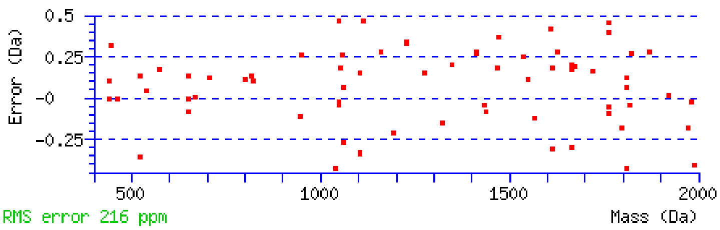

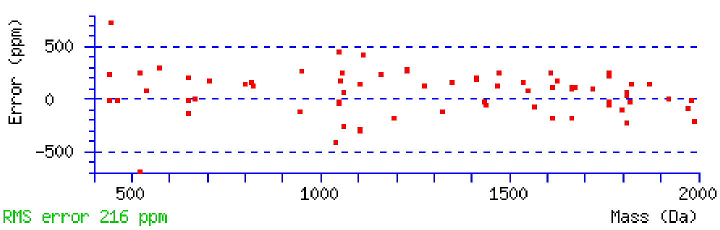

All matches to this query

Score	Mr(calc):	Delta	Sequence
62.7	3881.3491	0.0191	KEDSDEDEDEEDEDDSDEDEDEEDEFEPPVVK
21.0	3881.3491	0.0191	KEDSDEDEDEEDEDDSDEDEDEEDEFEPPVVK
6.2	3881.3703	-0.0021	YMNNYTSSYGNEWSTPDTMKRYSMYLTPK
6.2	3881.3703	-0.0021	YMNNYTSSYGNEWSTPDTMKRYSMYLTPK
5.8	3881.3703	-0.0021	YMNNYTSSYGNEWSTPDTMKRYSMYLTPK
3.0	3881.3703	-0.0021	YMNNYTSSYGNEWSTPDTMKRYSMYLTPK
2.8	3880.3298	1.0384	QSCIMAPYKSNSPKFSNCSYEEMYSVVNK
2.5	3881.3703	-0.0021	YMNNYTSSYGNEWSTPDTMKRYSMYLTPK
2.1	3880.3863	0.9819	YMNNYTSSYGNEWSTPDTMKRYSMYLTPK
1.8	3880.3863	0.9819	YMNNYTSSYGNEWSTPDTMKRYSMYLTPK

Spectrum No: 152; Query: 1535; Rank: 1

Peptide View

MS/MS Fragmentation of **HIVSNDSSDSDDEAQGPK**
Found in **IPI00197559**, Tax_Id=10116 Gene_Symbol=Pla2g4a Cytosolic phospholipase A2

Match to Query 1535: 1979.781928 from(990.898240,2+)
Title: 100101RatKid_NS_deglyco_23.363.363.2.dta
Data file K:\NewmanPaper\Piliang\3SubProteomes\Piliang3SP\mgf5ppm\ERLIC_3SubProteomes5ppm.mgf

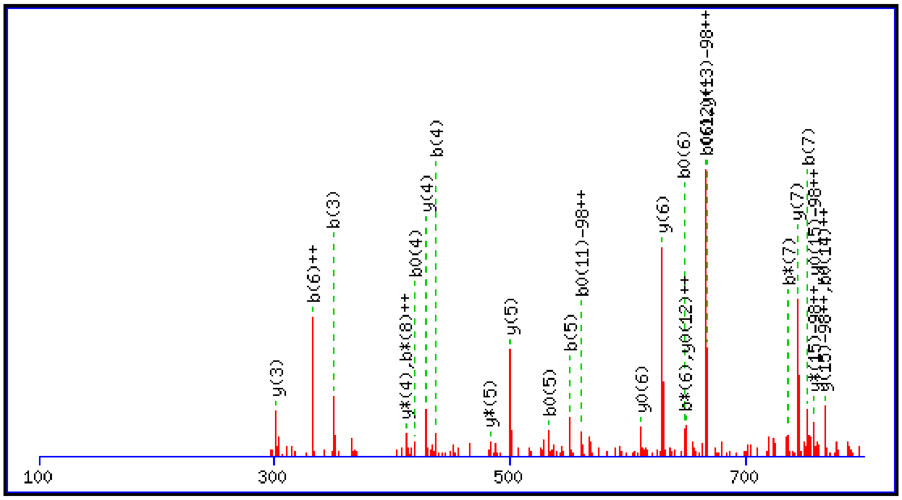

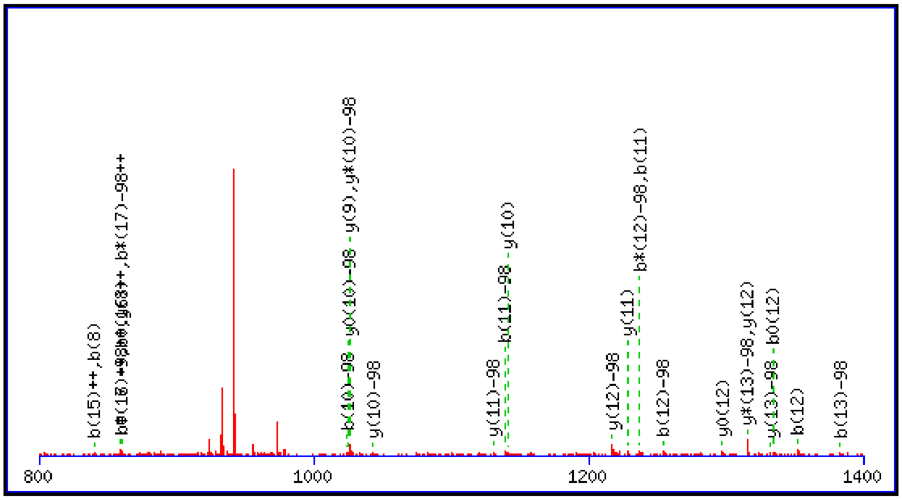

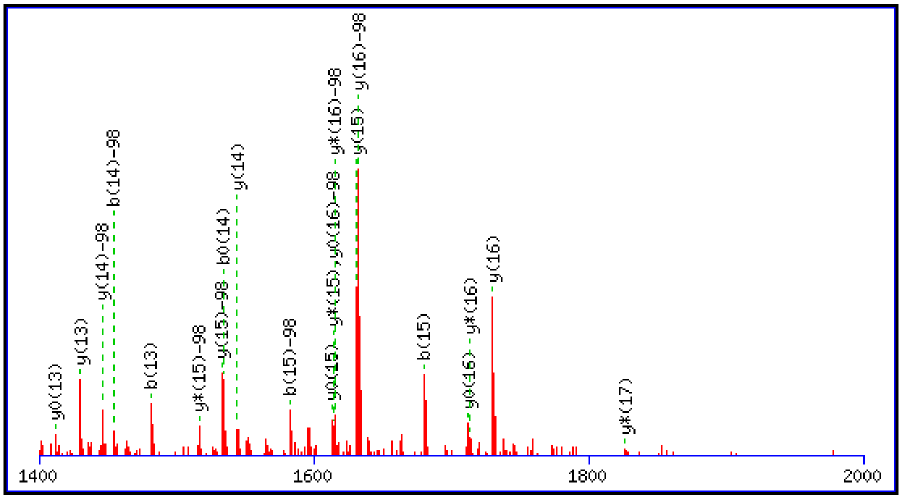

Monoisotopic mass of neutral peptide Mr(calc): 1979.7746
Fixed modifications: Carbamidomethyl (C)
Variable modifications:

S10 : Phospho (ST), with neutral losses 97.9769(shown in table), 0.0000
Ions Score: 63 **Expect:** 8.4e-005
Matches (Bold Red): 76/282 fragment ions using 129 most intense peaks

#	b	b^{++}	b*	b^{*++}	b^{0}	b^{0++}	Seq.	y	y^{++}	y*	y^{*++}	y^{0}	y^{0++}	#
1	138.0662	69.5367					H							18
2	251.1503	126.0788					I	1745.7460	873.3767	1728.7195	864.8634	1727.7355	864.3714	17
3	**350.2187**	175.6130					V	**1632.6620**	816.8346	**1615.6354**	808.3213	**1614.6514**	807.8293	16
4	**437.2507**	219.1290			**419.2401**	210.1237	S	**1533.5936**	**767.3004**	**1516.5670**	**758.7871**	1515.5830	**758.2951**	15
5	**551.2936**	276.1504	534.2671	267.6372	**533.2831**	267.1452	N	**1446.5615**	723.7844	1429.5350	715.2711	1428.5510	714.7791	14
6	**666.3206**	**333.6639**	**649.2940**	325.1506	**648.3100**	324.6586	D	**1332.5186**	**666.7629**	**1315.4921**	658.2497	1314.5080	657.7577	13
7	**753.3526**	377.1799	**736.3260**	368.6667	735.3420	368.1747	S	**1217.4917**	609.2495	1200.4651	600.7362	1199.4811	600.2442	12
8	**840.3846**	420.6959	823.3581	**412.1827**	822.3741	411.6907	S	**1130.4596**	565.7335	1113.4331	557.2202	1112.4491	556.7282	11
9	955.4116	478.2094	938.3850	469.6961	937.4010	469.2041	D	**1043.4276**	522.2174	**1026.4011**	513.7042	**1025.4170**	513.2122	10
10	**1024.4330**	512.7201	1007.4065	504.2069	1006.4225	503.7149	S	928.4007	464.7040	911.3741	456.1907	910.3901	455.6987	9
11	**1139.4600**	570.2336	1122.4334	561.7203	1121.4494	**561.2283**	D	**859.3792**	430.1932	842.3527	421.6800	841.3686	421.1880	8
12	**1254.4869**	627.7471	**1237.4604**	619.2338	1236.4763	618.7418	D	**744.3523**	372.6798	727.3257	364.1665	726.3417	363.6745	7
13	**1383.5295**	692.2684	1366.5029	683.7551	1365.5189	683.2631	E	**629.3253**	315.1663	612.2988	306.6530	**611.3148**	306.1610	6
14	**1454.5666**	727.7869	1437.5401	719.2737	1436.5560	718.7817	A	**500.2827**	250.6450	**483.2562**	242.1317			5
15	**1582.6252**	791.8162	1565.5986	783.3030	1564.6146	782.8110	Q	**429.2456**	215.1264	**412.2191**	206.6132			4
16	1639.6467	820.3270	1622.6201	811.8137	1621.6361	811.3217	G	**301.1870**	151.0972	284.1605	142.5839			3
17	1736.6994	868.8533	1719.6729	**860.3401**	1718.6889	**859.8481**	P	244.1656	122.5864	227.1390	114.0731			2
18							K	147.1128	74.0600	130.0863	65.5468			1

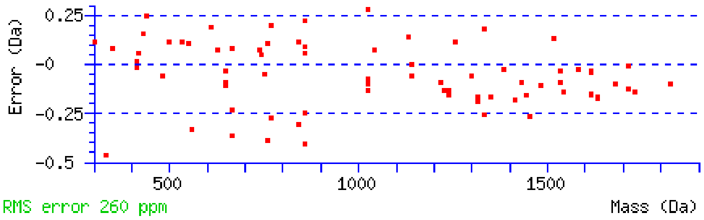

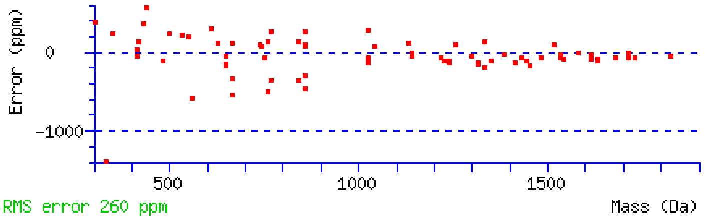

All matches to this query

Score	Mr(calc):	Delta	Sequence
62.7	1979.7746	0.0074	HIVSNDSSDSDDEAQGPK
56.0	1979.7746	0.0074	HIVSNDSSDSDDEAQGPK
53.8	1979.7746	0.0074	HIVSNDSSDSDDEAQGPK
34.4	1979.7746	0.0074	HIVSNDSSDSDDEAQGPK
0.8	1979.7735	0.0084	RGRSPRPSSSSDSDSDK
0.5	1979.7672	0.0147	IHTGEKPYQCNVCSK
0.4	1979.7890	-0.0070	CFYRAIITEVDDYR
0.0	1979.7944	-0.0125	LSFGTFKGSEQLNRR

Spectrum No: 153; Query: 2448; Rank: 1

Peptide View

MS/MS Fragmentation of **MESEAGADDSAEEGDLLDDDDNEDR**
Found in **IPI00187860**, Tax_Id=10116 Gene_Symbol=Hnrpc Heterogeneous nuclear ribonucleoprotein C

Match to Query 2448: 2807.964972 from(936.995600,3+)
Title: 091008RatKidney_NH4Format01_32.1366.1366.3.dta
Data file K:\NewmanPaper\Piliang\3SubProteomes\Piliang3SP\mgf5ppm\ERLIC_3SubProteomes5ppm.mgf

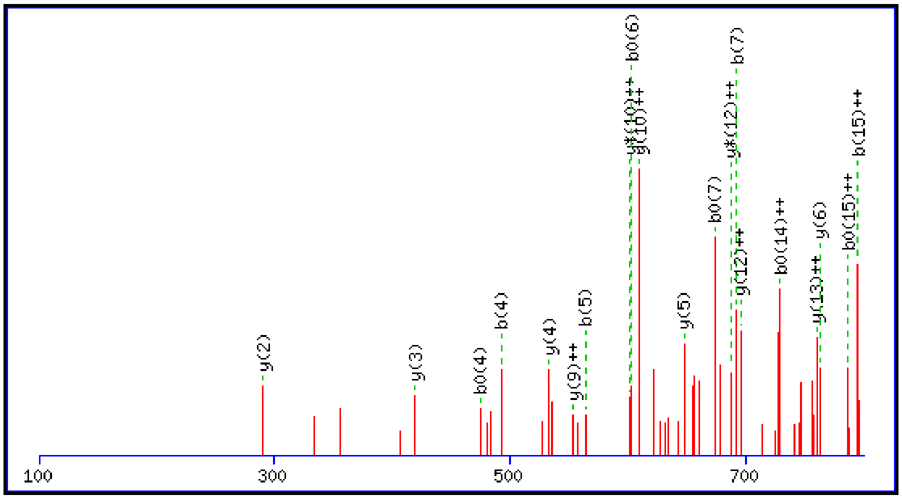

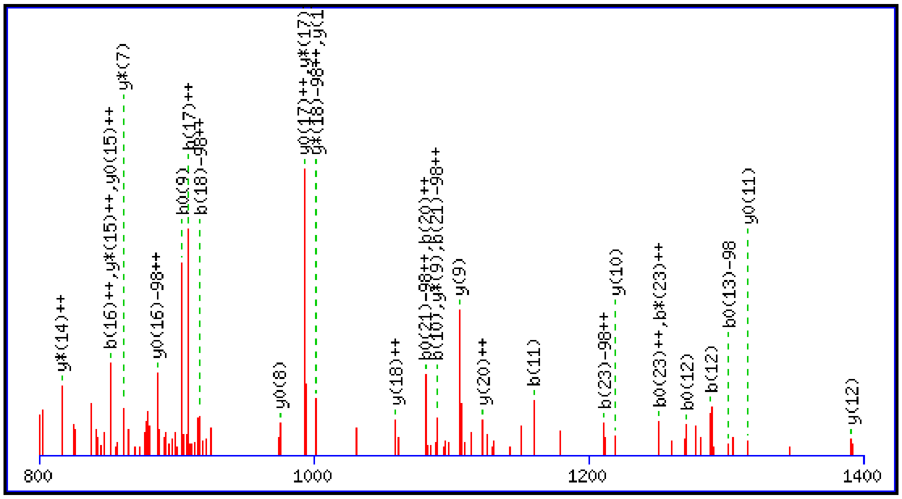

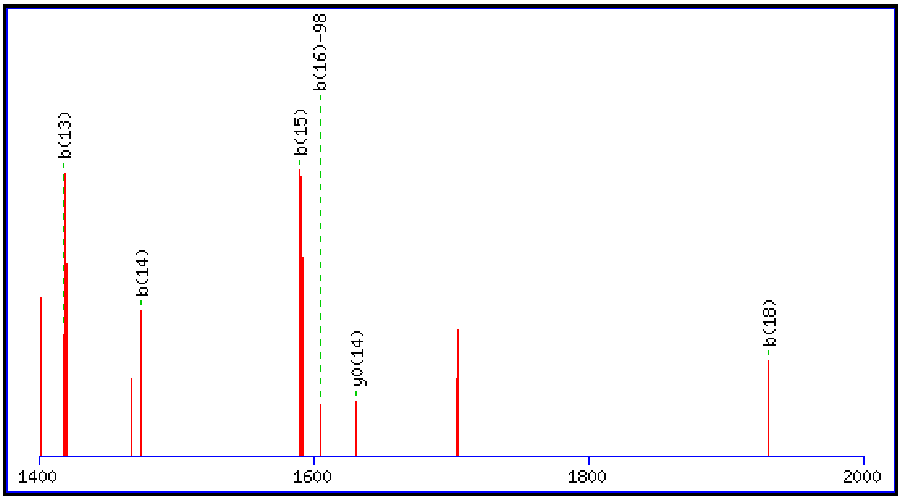

Monoisotopic mass of neutral peptide Mr(calc): 2807.9713
Fixed modifications: Carbamidomethyl (C)
Variable modifications:
M1 : Oxidation (M)
S10 : Phospho (ST), with neutral losses 0.0000(shown in table), 97.9769
Ions Score: 63 **Expect:** 5.9e-005
Matches (Bold Red): 59/362 fragment ions using 79 most intense peaks

#	b	b^{++}	b*	b^{*++}	b^0	b^{0++}	Seq.	y	y^{++}	y*	y^{*++}	y^0	y^{0++}	#
1	148.0427	74.5250					M							25
2	277.0853	139.0463			259.0747	130.0410	E	2661.9432	1331.4752	2644.9166	1322.9619	2643.9326	1322.4699	24
3	364.1173	182.5623			346.1067	173.5570	S	2532.9006	1266.9539	2515.8740	1258.4406	2514.8900	1257.9486	23
4	**493.1599**	247.0836			**475.1493**	238.0783	E	2445.8685	1223.4379	2428.8420	1214.9246	2427.8580	1214.4326	22

5	**564.1970**	282.6021			546.1864	273.5969	A	2316.8260	1158.9166	2299.7994	1150.4033	2298.8154	1149.9113	21
6	621.2185	311.1129			**603.2079**	302.1076	G	2245.7888	**1123.3981**	2228.7623	1114.8848	2227.7783	1114.3928	20
7	**692.2556**	346.6314			**674.2450**	337.6261	A	2188.7674	1094.8873	2171.7408	1086.3741	2170.7568	1085.8820	19
8	807.2825	404.1449			789.2720	395.1396	D	2117.7303	**1059.3688**	2100.7037	1050.8555	2099.7197	1050.3635	18
9	922.3095	461.6584			**904.2989**	452.6531	D	2002.7033	**1001.8553**	1985.6768	**993.3420**	1984.6928	**992.8500**	17
10	**1089.3078**	545.1576			1071.2973	536.1523	S	1887.6764	944.3418	1870.6498	935.8286	1869.6658	935.3365	16
11	**1160.3449**	580.6761			1142.3344	571.6708	A	1720.6780	860.8426	1703.6515	**852.3294**	1702.6675	**851.8374**	15
12	**1289.3875**	645.1974			**1271.3770**	636.1921	E	1649.6409	825.3241	1632.6144	**816.8108**	**1631.6303**	816.3188	14
13	**1418.4301**	709.7187			1400.4196	700.7134	E	1520.5983	**760.8028**	1503.5718	752.2895	1502.5877	751.7975	13
14	**1475.4516**	738.2294			1457.4410	**729.2241**	G	**1391.5557**	**696.2815**	1374.5292	**687.7682**	1373.5452	687.2762	12
15	**1590.4785**	**795.7429**			1572.4680	**786.7376**	D	1334.5343	667.7708	1317.5077	659.2575	**1316.5237**	658.7655	11
16	1703.5626	**852.2849**			1685.5520	843.2797	L	**1219.5073**	**610.2573**	1202.4808	**601.7440**	1201.4967	601.2520	10
17	1816.6467	**908.8270**			1798.6361	899.8217	L	**1106.4233**	**553.7153**	**1089.3967**	545.2020	1088.4127	544.7100	9
18	**1931.6736**	966.3404			1913.6630	957.3352	D	**993.3392**	497.1732	976.3126	488.6600	**975.3286**	488.1679	8
19	2046.7005	1023.8539			2028.6900	1014.8486	D	878.3122	439.6598	**861.2857**	431.1465	860.3017	430.6545	7
20	2161.7275	**1081.3674**			2143.7169	1072.3621	D	**763.2853**	382.1463	746.2588	373.6330	745.2747	373.1410	6
21	2276.7544	1138.8809			2258.7439	1129.8756	D	**648.2584**	324.6328	631.2318	316.1195	630.2478	315.6275	5
22	2390.7974	1195.9023	2373.7708	1187.3890	2372.7868	1186.8970	N	**533.2314**	267.1193	516.2049	258.6061	515.2208	258.1141	4
23	2519.8400	1260.4236	2502.8134	**1251.9103**	2501.8294	**1251.4183**	E	**419.1885**	210.0979	402.1619	201.5846	401.1779	200.0926	3
24	2634.8669	1317.9371	2617.8403	1309.4238	2616.8563	1308.9318	D	**290.1459**	145.5766	273.1193	137.0633	272.1353	136.5713	2
25							R	175.1190	88.0631	158.0924	79.5498			1

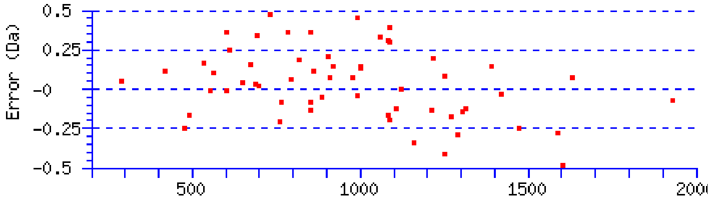

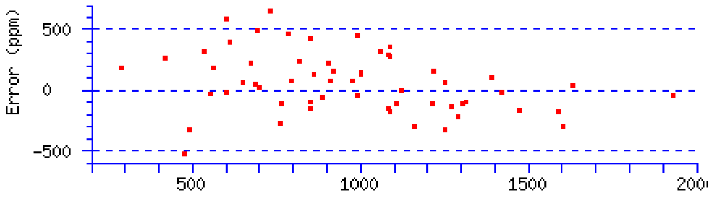

All matches to this query

Score	Mr(calc):	Delta	Sequence
62.6	2807.9713	-0.0063	MESEAGADDSAEEGDLLDDDDNEDR
44.3	2807.9713	-0.0063	MESEAGADDSAEEGDLLDDDDNEDR
1.3	2807.9757	-0.0107	YDNEYGYSNRVVDLMVYTASK
1.3	2807.9757	-0.0107	YDNEYGYSNRVVDLMVYTASK
1.3	2807.9757	-0.0107	YDNEYGYSNRVVDLMVYTASK
1.3	2807.9757	-0.0107	YDNEYGYSNRVVDLMVYTASK
0.9	2807.9757	-0.0107	YDNEYGYSNRVVDLMVYTASK
0.9	2807.9757	-0.0107	YDNEYGYSNRVVDLMVYTASK
0.9	2807.9757	-0.0107	YDNEYGYSNRVVDLMVYTASK

Spectrum No: 154; Query: 2206; Rank: 1

Peptide View

MS/MS Fragmentation of **RVSVCAETFNPDEEEDNDPR**
Found in **IPI00196684**, Tax_Id=10116 Gene_Symbol=Prkar2a cAMP-dependent protein kinase type II-alpha regulatory subunit

Match to Query 2206: 2457.983128 from(1229.998840,2+)
Title: 091008RatKidney_NoSalt_24.1850.1850.2.dta

Data file K:\NewmanPaper\Piliang\3SubProteomes\Piliang3SP\mgf5ppm\ERLIC_3SubProteomes5ppm.mgf

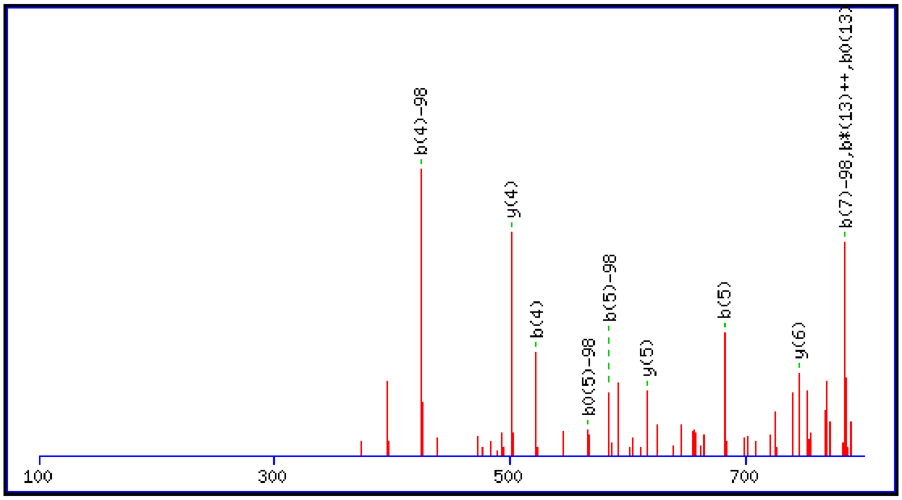

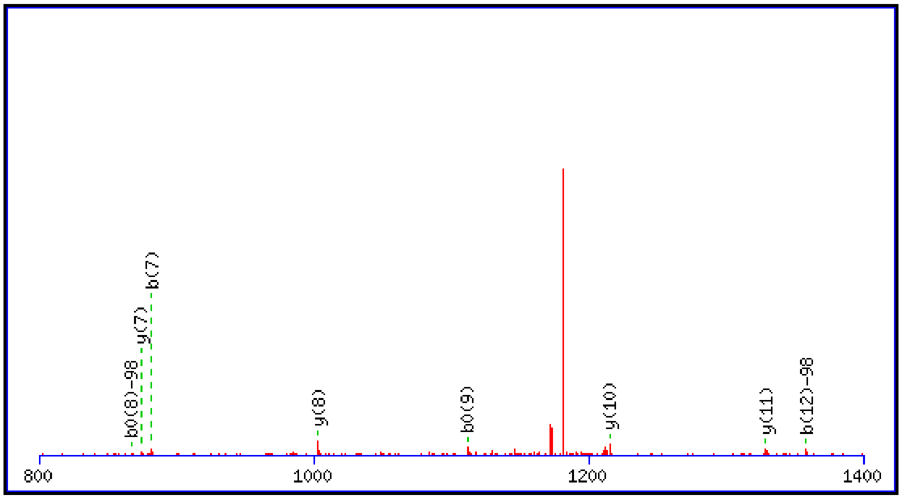

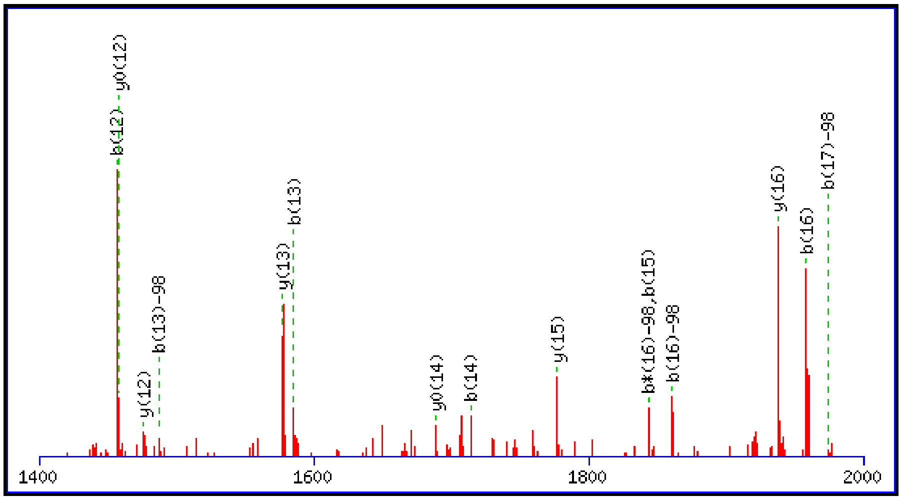

Monoisotopic mass of neutral peptide Mr(calc): 2457.9744
Fixed modifications: Carbamidomethyl (C)
Variable modifications:
S3 : Phospho (ST), with neutral losses 97.9769(shown in table), 0.0000
Ions Score: 63 **Expect:** 0.00012
Matches (Bold Red): 34/334 fragment ions using 55 most intense peaks

#	b	b^{++}	b*	b^{*++}	b^0	b^{0++}	Seq.	y	y^{++}	y*	y^{*++}	y^0	y^{0++}	#
1	157.1084	79.0578	140.0818	70.5446			R							20
2	256.1768	128.5920	239.1503	120.0788			V	2204.9037	1102.9555	2187.8771	1094.4422	2186.8931	1093.9502	19
3	325.1983	163.1028	308.1717	154.5895	307.1877	154.0975	S	2105.8353	1053.4213	2088.8087	1044.9080	2087.8247	1044.4160	18
4	**424.2667**	212.6370	407.2401	204.1237	406.2561	203.6317	V	2036.8138	1018.9105	2019.7873	1010.3973	2018.8032	1009.9053	17

5	**584.2973**	292.6523	567.2708	284.1390	**566.2868**	283.6470	C	**1937.7454**	969.3763	1920.7188	960.8631	1919.7348	960.3711	16
6	655.3344	328.1709	638.3079	319.6576	637.3239	319.1656	A	**1777.7147**	889.3610	1760.6882	880.8477	1759.7042	880.3557	15
7	**784.3770**	392.6922	767.3505	384.1789	766.3665	383.6869	E	1706.6776	853.8425	1689.6511	845.3292	**1688.6671**	844.8372	14
8	885.4247	443.2160	868.3982	434.7027	**867.4141**	434.2107	T	**1577.6350**	789.3212	1560.6085	780.8079	1559.6245	780.3159	13
9	1032.4931	516.7502	1015.4666	508.2369	1014.4826	507.7449	F	**1476.5874**	738.7973	1459.5608	730.2840	**1458.5768**	729.7920	12
10	1146.5360	573.7717	1129.5095	565.2584	1128.5255	564.7664	N	**1329.5189**	665.2631	1312.4924	656.7498	1311.5084	656.2578	11
11	1243.5888	622.2980	1226.5623	613.7848	1225.5782	613.2928	P	**1215.4760**	608.2416	1198.4495	599.7284	1197.4655	599.2364	10
12	**1358.6158**	679.8115	1341.5892	671.2982	1340.6052	670.8062	D	1118.4233	559.7153	1101.3967	551.2020	1100.4127	550.7100	9
13	**1487.6583**	744.3328	1470.6318	735.8195	1469.6478	735.3275	E	**1003.3963**	502.2018	986.3698	493.6885	985.3857	493.1965	8
14	1616.7009	808.8541	1599.6744	800.3408	1598.6904	799.8488	E	**874.3537**	437.6805	857.3272	429.1672	856.3432	428.6752	7
15	1745.7435	873.3754	1728.7170	864.8621	1727.7330	864.3701	E	**745.3111**	373.1592	728.2846	364.6459	727.3006	364.1539	6
16	**1860.7705**	930.8889	**1843.7439**	922.3756	1842.7599	921.8836	D	**616.2685**	308.6379	599.2420	300.1246	598.2580	299.6326	5
17	**1974.8134**	987.9103	1957.7869	979.3971	1956.8028	978.9051	N	**501.2416**	251.1244	484.2150	242.6112	483.2310	242.1191	4
18	2089.8403	1045.4238	2072.8138	1036.9105	2071.8298	1036.4185	D	387.1987	194.1030	370.1721	185.5897	369.1881	185.0977	3
19	2186.8931	1093.9502	2169.8666	1085.4369	2168.8825	1084.9449	P	272.1717	136.5895	255.1452	128.0762			2
20							R	175.1190	88.0631	158.0924	79.5498			1

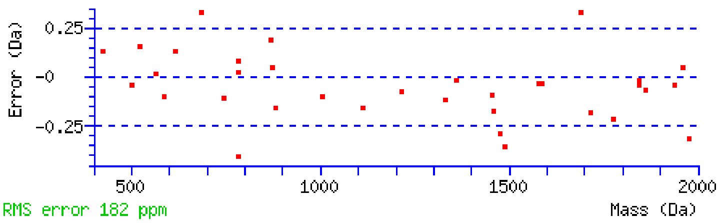

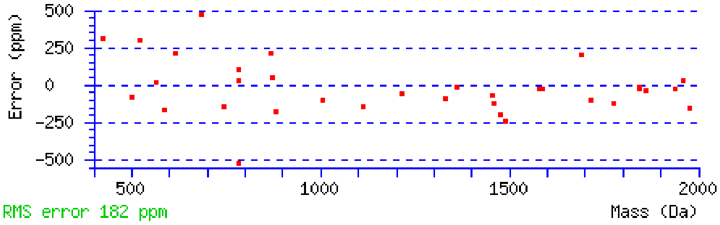

All matches to this query

Score	Mr(calc):	Delta	Sequence
62.6	2457.9744	0.0087	RVSVCAETFNPDEEEDNDPR
39.5	2457.9744	0.0087	RVSVCAETFNPDEEEDNDPR
3.9	2457.9784	0.0047	FYSKEETGPVQSSHDMNSYR
2.6	2457.0003	0.9829	NQVVSSANGELNMDDPTVGRSNA
2.6	2457.9843	-0.0011	NQVVSSANGELNMDDPTVGRSNA
2.6	2457.0003	0.9829	NQVVSSANGELNMDDPTVGRSNA
2.6	2457.9843	-0.0011	NQVVSSANGELNMDDPTVGRSNA
2.2	2457.0003	0.9829	NQVVSSANGELNMDDPTVGRSNA
2.2	2457.0003	0.9829	NQVVSSANGELNMDDPTVGRSNA
2.0	2457.9790	0.0041	GQAAAVTPEQMAYLSPEQRR

Spectrum No: 155; Query: 1420; Rank: 1

Peptide View

MS/MS Fragmentation of **SPPDQSAVPNTPPSTPVK**
Found in **IPI00231810**, Tax_Id=10116 Gene_Symbol=Add1 Isoform 2 of Alpha-adducin

Match to Query 1420: 1897.884568 from(949.949560,2+)
Title: 100101RatKid_NS_deglyco_18.1974.1974.2.dta
Data file K:\NewmanPaper\Piliang\3SubProteomes\Piliang3SP\mgf5ppm\ERLIC_3SubProteomes5ppm.mgf

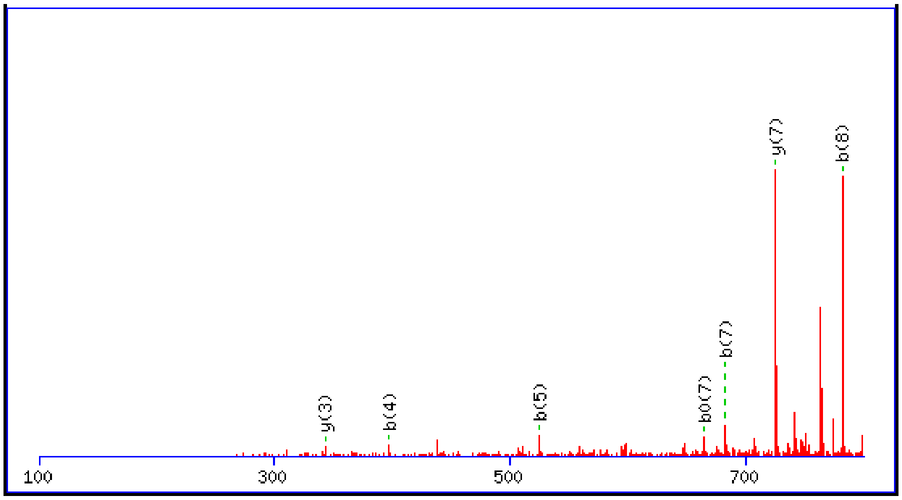

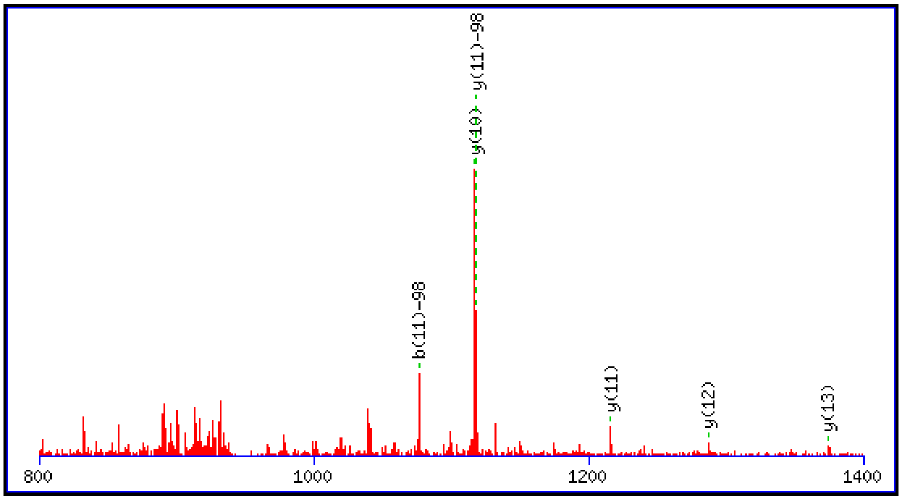

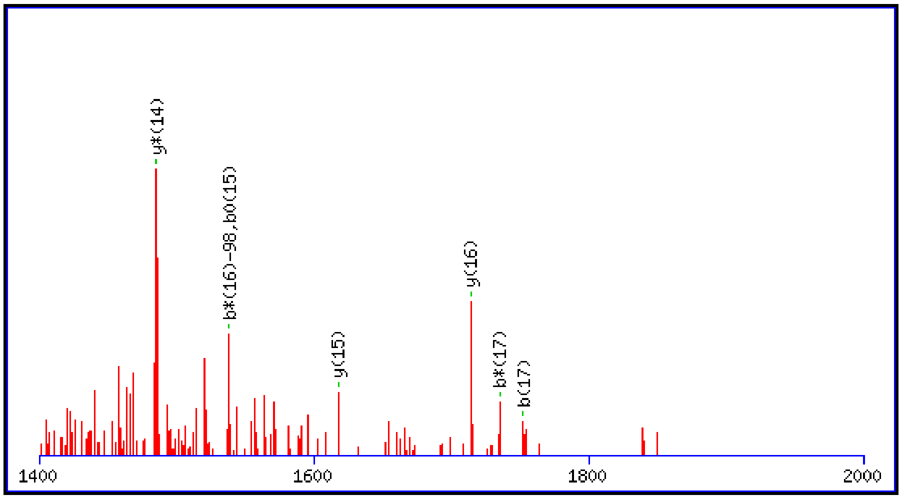

Monoisotopic mass of neutral peptide Mr(calc): 1897.8823
Fixed modifications: Carbamidomethyl (C)
Variable modifications:
T11 : Phospho (ST), with neutral losses 0.0000(shown in table), 97.9769
Ions Score: 62 **Expect:** 0.00014
Matches (Bold Red): 20/292 fragment ions using 28 most intense peaks

#	b	b^{++}	b*	b^{*++}	b^0	b^{0++}	Seq.	y	y^{++}	y*	y^{*++}	y^0	y^{0++}	#
1	88.0393	44.5233			70.0287	35.5180	S							18
2	185.0921	93.0497			167.0815	84.0444	P	1811.8575	906.4324	1794.8310	897.9191	1793.8470	897.4271	17
3	282.1448	141.5761			264.1343	132.5708	P	**1714.8048**	857.9060	1697.7782	849.3927	1696.7942	848.9007	16
4	**397.1718**	199.0895			379.1612	190.0842	D	**1617.7520**	809.3796	1600.7254	800.8664	1599.7414	800.3743	15
5	**525.2304**	263.1188	508.2038	254.6055	507.2198	254.1135	Q	1502.7250	751.8662	**1485.6985**	743.3529	1484.7145	742.8609	14

**6**	612.2624	306.6348	595.2358	298.1216	594.2518	297.6295	**S**	**1374.6665**	687.8369	1357.6399	679.3236	1356.6559	678.8316	**13**
**7**	**683.2995**	342.1534	666.2729	333.6401	**665.2889**	333.1481	**A**	**1287.6344**	644.3209	1270.6079	635.8076	1269.6239	635.3156	**12**
**8**	**782.3679**	391.6876	765.3414	383.1743	764.3573	382.6823	**V**	**1216.5973**	608.8023	1199.5708	600.2890	1198.5868	599.7970	**11**
**9**	879.4207	440.2140	862.3941	431.7007	861.4101	431.2087	**P**	**1117.5289**	559.2681	1100.5024	550.7548	1099.5183	550.2628	**10**
**10**	993.4636	497.2354	976.4371	488.7222	975.4530	488.2302	**N**	1020.4761	510.7417	1003.4496	502.2284	1002.4656	501.7364	**9**
**11**	1174.4776	587.7424	1157.4511	579.2292	1156.4670	578.7372	**T**	906.4332	453.7202	889.4067	445.2070	888.4227	444.7150	**8**
**12**	1271.5304	636.2688	1254.5038	627.7555	1253.5198	627.2635	**P**	**725.4192**	363.2132	708.3927	354.7000	707.4087	354.2080	**7**
**13**	1368.5831	684.7952	1351.5566	676.2819	1350.5726	675.7899	**P**	628.3665	314.6869	611.3399	306.1736	610.3559	305.6816	**6**
**14**	1455.6152	728.3112	1438.5886	719.7979	1437.6046	719.3059	**S**	531.3137	266.1605	514.2871	257.6472	513.3031	257.1552	**5**
**15**	1556.6628	778.8351	1539.6363	770.3218	**1538.6523**	769.8298	**T**	444.2817	222.6445	427.2551	214.1312	426.2711	213.6392	**4**
**16**	1653.7156	827.3614	1636.6891	818.8482	1635.7050	818.3562	**P**	**343.2340**	172.1206	326.2074	163.6074			**3**
**17**	**1752.7840**	876.8956	**1735.7575**	868.3824	1734.7735	867.8904	**V**	246.1812	123.5942	229.1547	115.0810			**2**
**18**							**K**	147.1128	74.0600	130.0863	65.5468			**1**

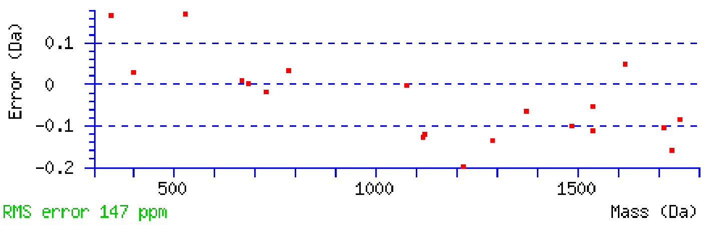

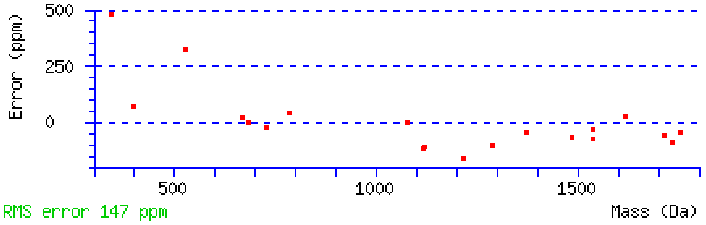

All matches to this query

Score	Mr(calc):	Delta	Sequence
62.3	1897.8823	0.0023	SPPDQSAVPNTPPSTPVK
47.0	1897.8823	0.0023	SPPDQSAVPNTPPSTPVK
47.0	1897.8823	0.0023	SPPDQSAVPNTPPSTPVK
16.6	1897.8823	0.0023	SPPDQSAVPNTPPSTPVK
10.4	1897.8823	0.0023	SPPDQSAVPNTPPSTPVK
9.7	1897.8740	0.0106	WKLPSPVTQVNAPSSK
9.7	1897.8740	0.0106	WKLPSPVTQVNAPSSK
4.4	1897.8700	0.0146	RTVSEPSLSGLHLNTK
4.4	1897.8700	0.0146	RTVSEPSLSGLHLNTK
4.4	1897.8700	0.0146	RTVSEPSLSGLHLNTK

Spectrum No: 156; Query: 1149; Rank: 1

Peptide View

MS/MS Fragmentation of **ESLKEEDESDDDNM**
Found in **IPI00476178**, Tax_Id=10116 Gene_Symbol=Psma3;Psma3l Proteasome subunit alpha type-3

Match to Query 1149: 1750.573028 from(876.293790,2+)
Title: 091008RatKidney_NoSalt_33.277.277.2.dta
Data file K:\NewmanPaper\Piliang\3SubProteomes\Piliang3SP\mgf5ppm\ERLIC_3SubProteomes5ppm.mgf

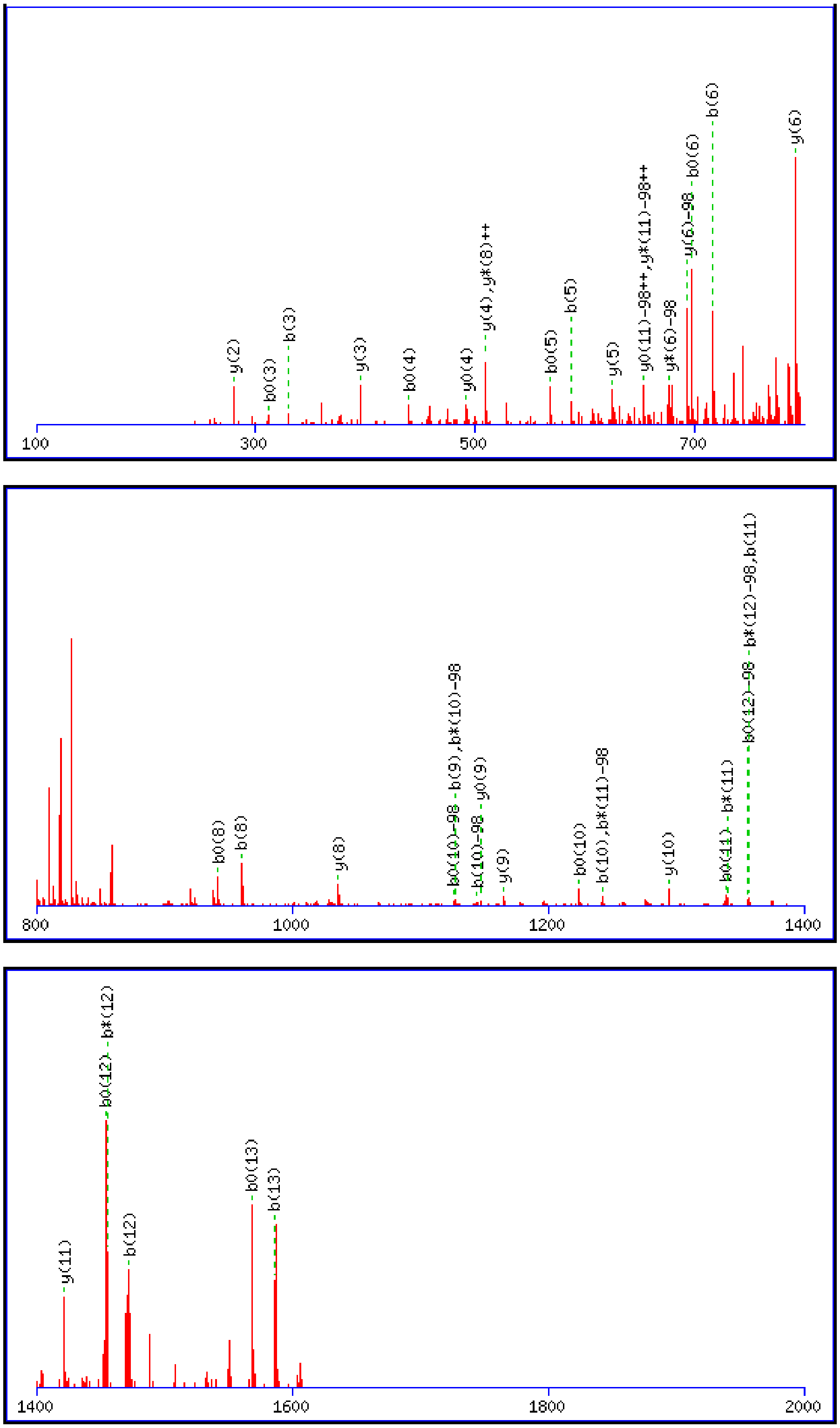

Monoisotopic mass of neutral peptide Mr(calc): 1750.5764
Fixed modifications: Carbamidomethyl (C)
Variable modifications:
S9 : Phospho (ST), with neutral losses 0.0000(shown in table), 97.9769
M14 : Oxidation (M)
Ions Score: 62 **Expect:** 2.5e-005
Matches (Bold Red): 42/222 fragment ions using 50 most intense peaks

#	b	b^{++}	b*	b^{*++}	b^0	b^{0++}	Seq.	y	y^{++}	y*	y^{*++}	y^0	y^{0++}	#
1	130.0499	65.5286			112.0393	56.5233	E							14
2	217.0819	109.0446			199.0713	100.0393	S	1622.5411	811.7742	1605.5146	803.2609	1604.5306	802.7689	13
3	**330.1660**	165.5866			**312.1554**	156.5813	L	1535.5091	768.2582	1518.4826	759.7449	1517.4985	759.2529	12
4	458.2609	229.6341	441.2344	221.1208	**440.2504**	220.6288	K	**1422.4250**	711.7162	1405.3985	703.2029	1404.4145	702.7109	11

5	**587.3035**	294.1554	570.2770	285.6421	**569.2930**	285.1501	E	**1294.3301**	647.6687	1277.3035	639.1554	1276.3195	638.6634	10
6	**716.3461**	358.6767	699.3196	350.1634	**698.3355**	349.6714	E	**1165.2875**	583.1474	1148.2609	574.6341	**1147.2769**	574.1421	9
7	831.3731	416.1902	814.3465	407.6769	813.3625	407.1849	D	**1036.2449**	518.6261	1019.2183	**510.1128**	1018.2343	509.6208	8
8	**960.4156**	480.7115	943.3891	472.1982	**942.4051**	471.7062	E	921.2179	461.1126	904.1914	452.5993	903.2074	452.1073	7
9	**1127.4140**	564.2106	1110.3874	555.6974	1109.4034	555.2054	S	**792.1754**	396.5913	775.1488	388.0780	774.1648	387.5860	6
10	**1242.4409**	621.7241	1225.4144	613.2108	**1224.4304**	612.7188	D	**625.1770**	313.0921	608.1505	304.5789	607.1664	304.0869	5
11	**1357.4679**	679.2376	**1340.4413**	670.7243	**1339.4573**	670.2323	D	**510.1501**	255.5787	493.1235	247.0654	**492.1395**	246.5734	4
12	**1472.4948**	736.7511	**1455.4683**	728.2378	**1454.4843**	727.7458	D	**395.1231**	198.0652	378.0966	189.5519	377.1126	189.0599	3
13	**1586.5378**	793.7725	1569.5112	785.2592	**1568.5272**	784.7672	N	**280.0962**	140.5517	263.0696	132.0384			2
14							M	166.0532	83.5303					1

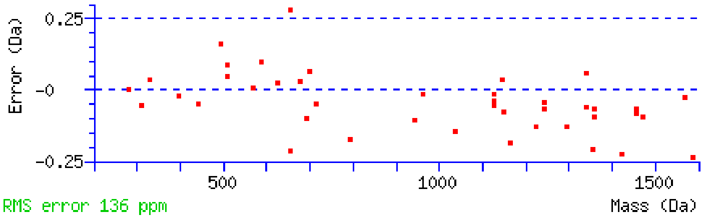

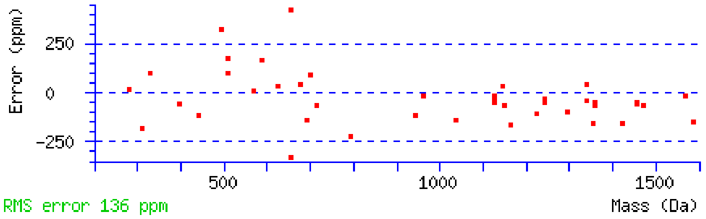

All matches to this query

Score	Mr(calc):	Delta	Sequence
61.8	1750.5764	-0.0034	ESLKEEDESDDDNM
40.1	1750.5764	-0.0034	ESLKEEDESDDDNM
0.3	1749.5645	1.0085	TNSTGGSSGNSVGGGSGK

Spectrum No: 157; Query: 2364; Rank: 1

Peptide View

MS/MS Fragmentation of **TCDSPQNPMDLISDPVPDSPFPR**
Found in **IPI00359825**, Tax_Id=10116 Gene_Symbol=Usp10 Ubiquitin carboxyl-terminal hydrolase

Match to Query 2364: 2664.136068 from(1333.075310,2+)
Title: 100101RatKid_NS_deglyco_19.5231.5231.2.dta
Data file K:\NewmanPaper\Piliang\3SubProteomes\Piliang3SP\mgf5ppm\ERLIC_3SubProteomes5ppm.mgf

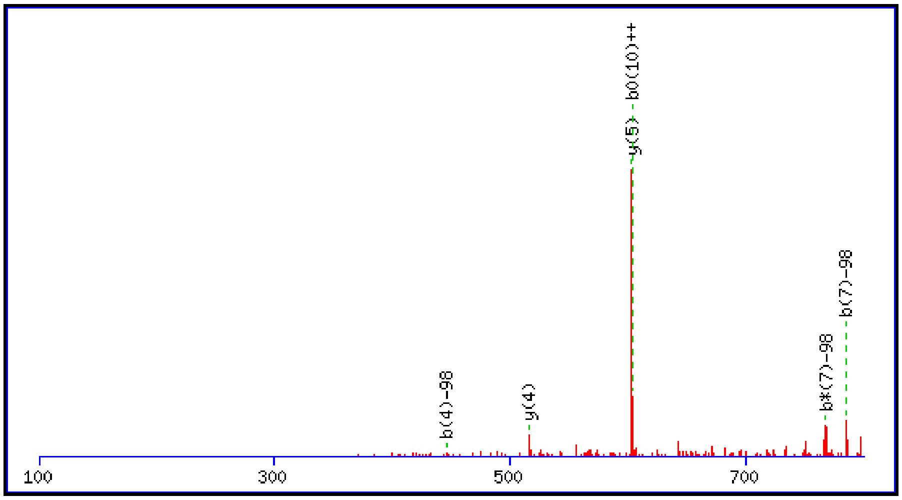

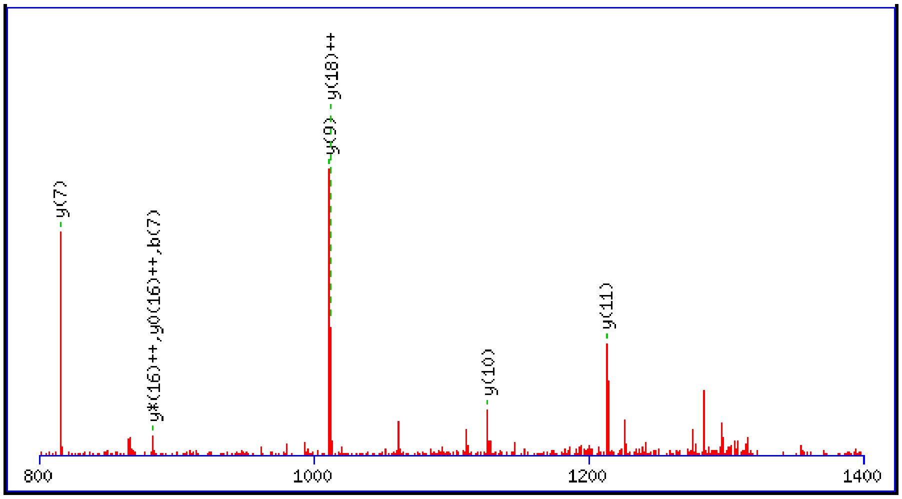

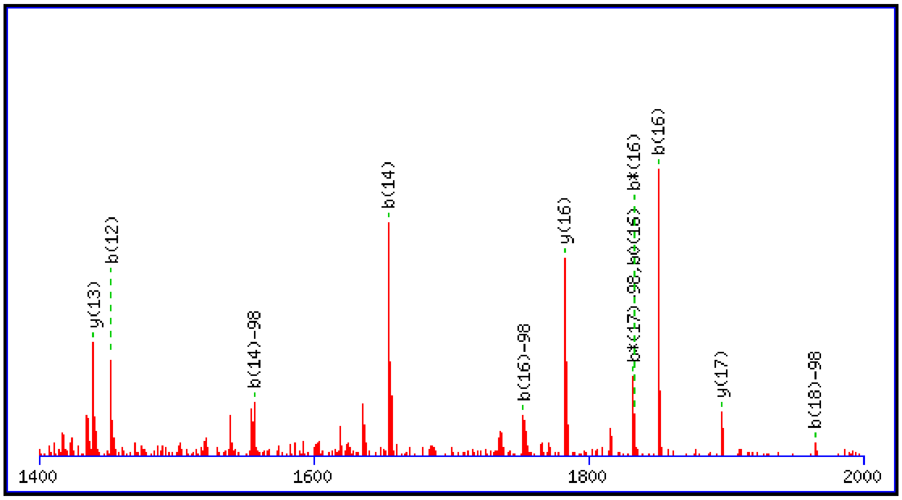

Monoisotopic mass of neutral peptide Mr(calc): 2664.1237
Fixed modifications: Carbamidomethyl (C)
Variable modifications:
S4 : Phospho (ST), with neutral losses 97.9769(shown in table), 0.0000
Ions Score: 61 **Expect:** 0.00027
Matches (Bold Red): 26/374 fragment ions using 38 most intense peaks

#	b	b^{++}	b*	b^{*++}	b^0	b^{0++}	Seq.	y	y^{++}	y*	y^{*++}	y^0	y^{0++}	#
1	102.0550	51.5311			84.0444	42.5258	T							23
2	262.0856	131.5464			244.0750	122.5412	C	2466.1064	1233.5568	2449.0799	1225.0436	2448.0958	1224.5516	22
3	377.1125	189.0599			359.1020	180.0546	D	2306.0758	1153.5415	2289.0492	1145.0282	2288.0652	1144.5362	21
4	**446.1340**	223.5706			428.1234	214.5654	S	2191.0488	1096.0280	2174.0223	1087.5148	2173.0383	1087.0228	20
5	543.1868	272.0970			525.1762	263.0917	P	2122.0274	1061.5173	2105.0008	1053.0040	2104.0168	1052.5120	19
6	671.2453	336.1263	654.2188	327.6130	653.2348	327.1210	Q	2024.9746	**1012.9909**	2007.9481	1004.4777	2006.9640	1003.9857	18
7	**785.2883**	393.1478	**768.2617**	384.6345	767.2777	384.1425	N	**1896.9160**	948.9617	1879.8895	940.4484	1878.9055	939.9564	17
8	882.3410	441.6742	865.3145	433.1609	864.3305	432.6689	P	**1782.8731**	891.9402	1765.8465	**883.4269**	1764.8625	**882.9349**	16
9	1013.3815	507.1944	996.3550	498.6811	995.3710	498.1891	M	1685.8203	843.4138	1668.7938	834.9005	1667.8098	834.4085	15
10	1128.4085	564.7079	1111.3819	556.1946	1110.3979	555.7026	D	1554.7798	777.8936	1537.7533	769.3803	1536.7693	768.8883	14
11	1241.4925	621.2499	1224.4660	612.7366	1223.4820	612.2446	L	**1439.7529**	720.3801	1422.7264	711.8668	1421.7423	711.3748	13
12	1354.5766	677.7919	1337.5500	669.2787	1336.5660	668.7867	I	1326.6688	663.8381	1309.6423	655.3248	1308.6583	654.8328	12
13	1441.6086	721.3079	1424.5821	712.7947	1423.5981	712.3027	S	**1213.5848**	607.2960	1196.5582	598.7828	1195.5742	598.2907	11
14	**1556.6356**	778.8214	1539.6090	770.3081	1538.6250	769.8161	D	**1126.5528**	563.7800	1109.5262	555.2667	1108.5422	554.7747	10
15	1653.6883	827.3478	1636.6618	818.8345	1635.6778	818.3425	P	**1011.5258**	506.2665	994.4993	497.7533	993.5152	497.2613	9
16	**1752.7567**	876.8820	1735.7302	868.3687	1734.7462	867.8767	V	914.4730	457.7402	897.4465	449.2269	896.4625	448.7349	8
17	1849.8095	925.4084	**1832.7830**	916.8951	1831.7989	916.4031	P	**815.4046**	408.2060	798.3781	399.6927	797.3941	399.2007	7
18	**1964.8364**	982.9219	1947.8099	974.4086	1946.8259	973.9166	D	718.3519	359.6796	701.3253	351.1663	700.3413	350.6743	6

**19**	2051.8685	1026.4379	2034.8419	1017.9246	2033.8579	1017.4326	**S**	**603.3249**	302.1661	586.2984	293.6528	585.3144	293.1608	**5**
**20**	2148.9212	1074.9643	2131.8947	1066.4510	2130.9107	1065.9590	**P**	**516.2929**	258.6501	499.2663	250.1368			**4**
**21**	2295.9897	1148.4985	2278.9631	1139.9852	2277.9791	1139.4932	**F**	419.2401	210.1237	402.2136	201.6104			**3**
**22**	2393.0424	1197.0248	2376.0159	1188.5116	2375.0319	1188.0196	**P**	272.1717	136.5895	255.1452	128.0762			**2**
**23**							**R**	175.1190	88.0631	158.0924	79.5498			**1**

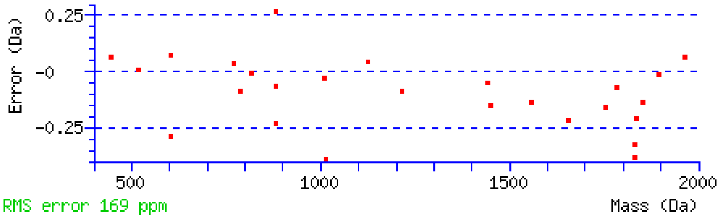

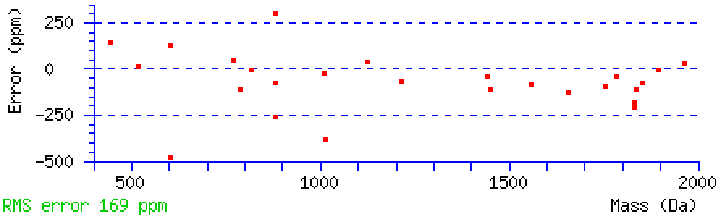

All matches to this query

Score	Mr(calc):	Delta	Sequence
61.2	2664.1237	0.0123	TCDSPQNPMDLISDPVPDSPFPR
61.2	2664.1237	0.0123	TCDSPQNPMDLISDPVPDSPFPR
22.3	2664.1237	0.0123	TCDSPQNPMDLISDPVPDSPFPR
7.0	2663.1339	1.0021	CIYRQAGTPLAEDQDQSLVPDK
6.0	2663.1339	1.0021	CIYRQAGTPLAEDQDQSLVPDK
4.5	2664.1568	-0.0207	RENKNLQEEISDLTDQVSLSGK
4.5	2664.1568	-0.0207	RENKNLQEEISDLTDQVSLSGK
4.0	2664.1146	0.0215	GLWSENQLASSSPTPGSFPQGTEK
3.1	2664.1438	-0.0077	NYIVEDGDIIFFKFNTPQQSK
3.1	2664.1438	-0.0077	NYIVEDGDIIFFKFNTPQQSK

Spectrum No: 158; Query: 1021; Rank: 1

Peptide View

MS/MS Fragmentation of **KYVISDEEEEEDD**
Found in **IPI00471640**, Tax_Id=10116 Gene_Symbol=Leo1 RNA polymerase-associated protein LEO1

Match to Query 1021: 1678.611668 from(840.313110,2+)
Title: 091008RatKidney_NoSalt_32.1254.1254.2.dta
Data file K:\NewmanPaper\Piliang\3SubProteomes\Piliang3SP\mgf5ppm\ERLIC_3SubProteomes5ppm.mgf

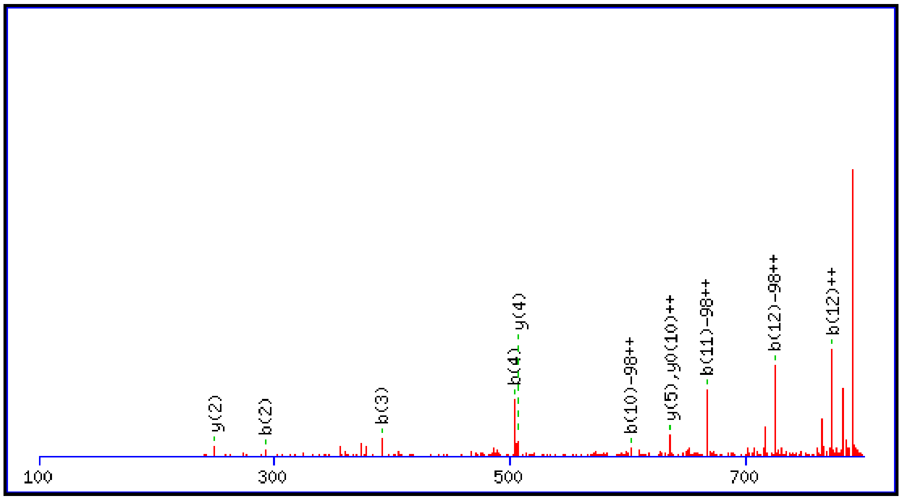

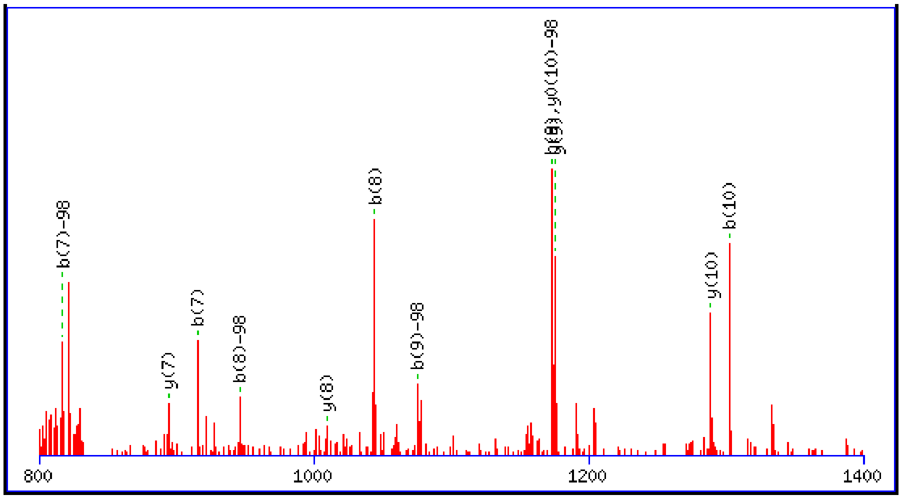

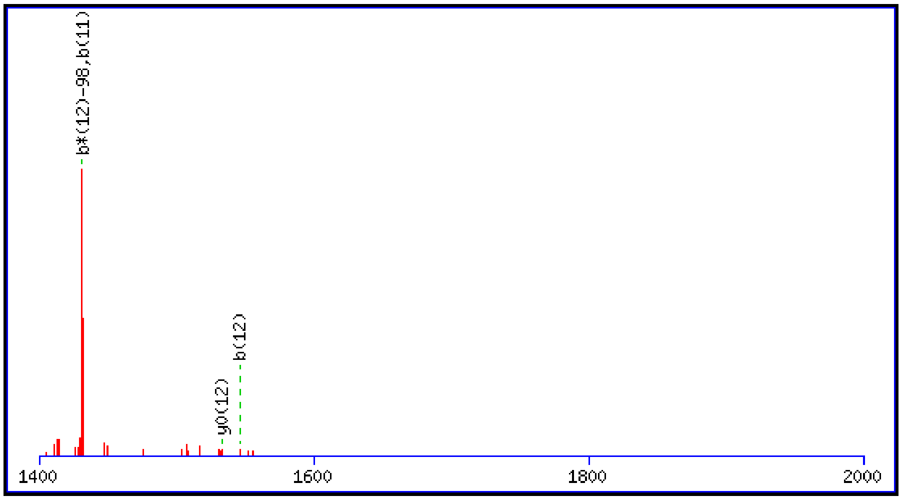

Monoisotopic mass of neutral peptide Mr(calc): 1678.6134
Fixed modifications: Carbamidomethyl (C)
Variable modifications:
S5 : Phospho (ST), with neutral losses 0.0000(shown in table), 97.9769
Ions Score: 61 **Expect:** 5.8e-005
Matches (Bold Red): 27/176 fragment ions using 34 most intense peaks

#	b	b++	b*	b*++	b0	b0++	Seq.	y	y++	y0	y0++	#
1	129.1022	65.0548	112.0757	56.5415			K					13
2	**292.1656**	146.5864	275.1390	138.0731			Y	1551.5258	776.2665	**1533.5152**	767.2613	12
3	**391.2340**	196.1206	374.2074	187.6074			V	1388.4625	694.7349	1370.4519	685.7296	11
4	**504.3180**	252.6627	487.2915	244.1494			I	**1289.3941**	645.2007	1271.3835	**636.1954**	10
5	671.3164	336.1618	654.2898	327.6486	653.3058	327.1566	S	**1176.3100**	588.6586	1158.2994	579.6533	9
6	786.3433	393.6753	769.3168	385.1620	768.3328	384.6700	D	**1009.3116**	505.1595	991.3011	496.1542	8
7	**915.3859**	458.1966	898.3594	449.6833	897.3754	449.1913	E	**894.2847**	447.6460	876.2741	438.6407	7
8	**1044.4285**	522.7179	1027.4020	514.2046	1026.4180	513.7126	E	765.2421	383.1247	747.2315	374.1194	6
9	**1173.4711**	587.2392	1156.4446	578.7259	1155.4606	578.2339	E	**636.1995**	318.6034	618.1889	309.5981	5
10	**1302.5137**	651.7605	1285.4872	643.2472	1284.5031	642.7552	E	**507.1569**	254.0821	489.1463	245.0768	4
11	**1431.5563**	716.2818	1414.5298	707.7685	1413.5457	707.2765	E	378.1143	189.5608	360.1038	180.5555	3
12	**1546.5832**	**773.7953**	1529.5567	765.2820	1528.5727	764.7900	D	**249.0717**	125.0395	231.0612	116.0342	2
13							D	134.0448	67.5260	116.0342	58.5207	1

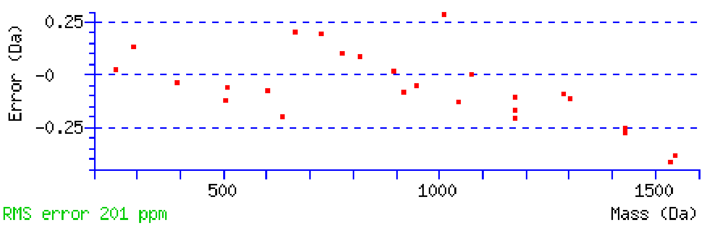

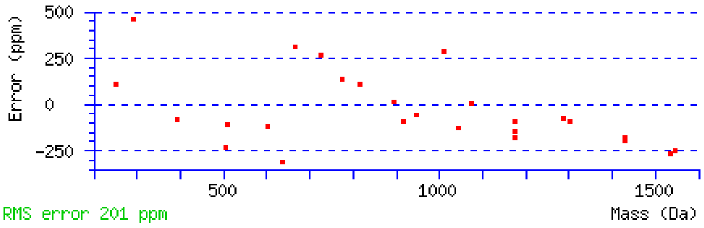

All matches to this query

Score	Mr(calc):	Delta	Sequence
61.1	1678.6134	-0.0018	KYVISDEEEEEDD
30.4	1678.6134	-0.0018	KYVISDEEEEEDD
1.6	1676.5885	2.0232	YKTENSLNQPSR
0.2	1677.5946	1.0170	DMENRQNTGDCMK

Spectrum No: 159; Query: 1969; Rank: 1

Peptide View

MS/MS Fragmentation of **SDSSTEESGQDVLENTFSQK**
Found in **IPI00211776**, Tax_Id=10116 Gene_Symbol=Golgb1 Rat GCP360

Match to Query 1969: 2266.908908 from(1134.461730,2+)
Title: 091008RatKidney_NoSalt_27.2951.2951.2.dta
Data file K:\NewmanPaper\Piliang\3SubProteomes\Piliang3SP\mgf5ppm\ERLIC_3SubProteomes5ppm.mgf

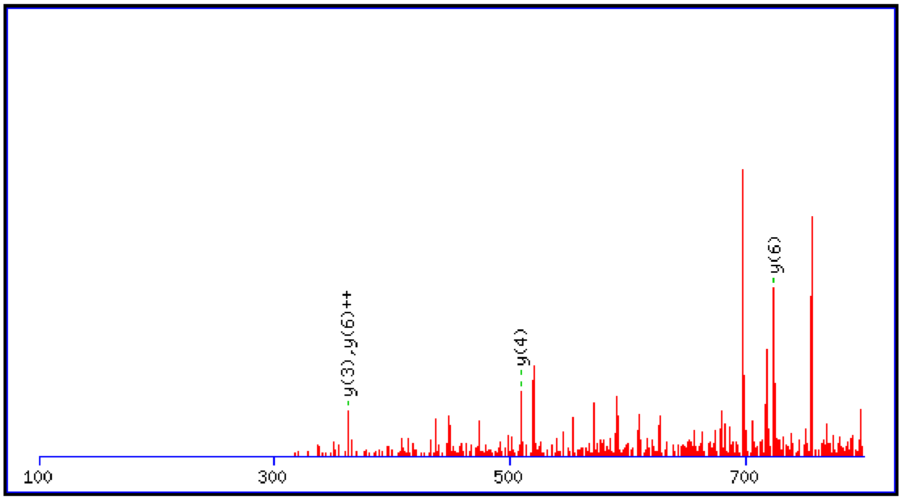

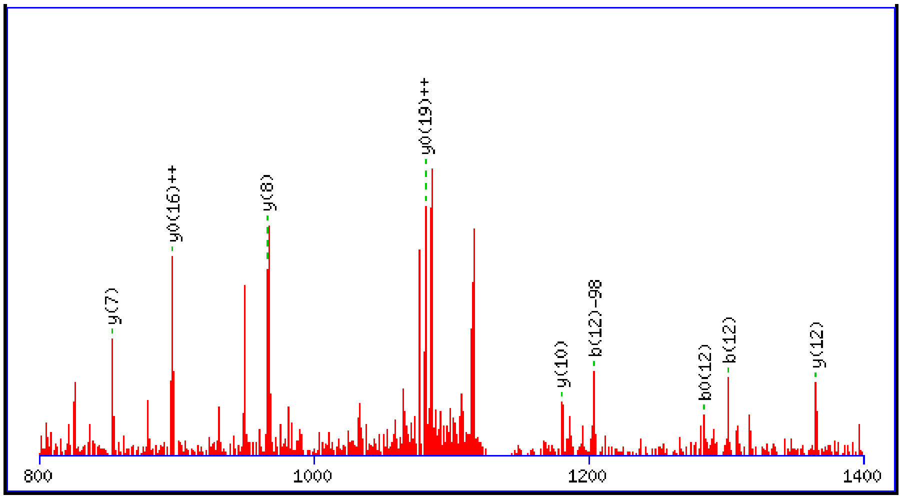

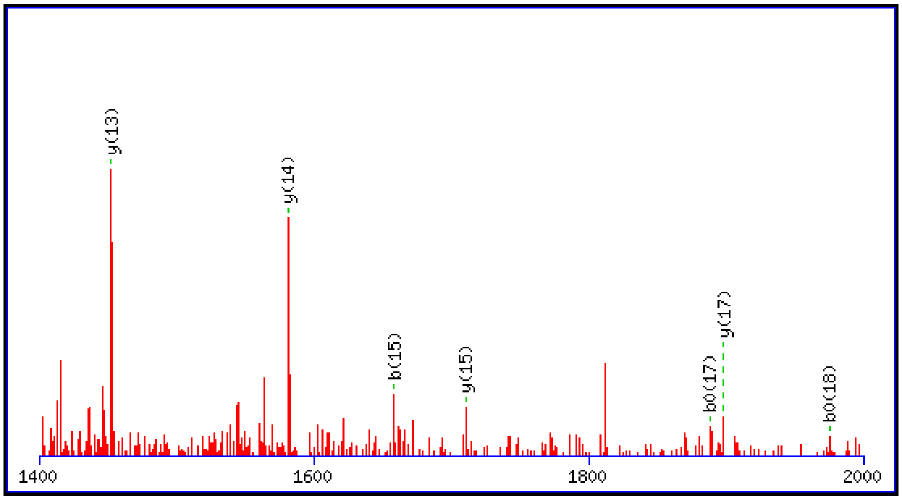

Monoisotopic mass of neutral peptide Mr(calc): 2266.9115
Fixed modifications: Carbamidomethyl (C)
Variable modifications:
S3 : Phospho (ST), with neutral losses 0.0000(shown in table), 97.9769
Ions Score: 61 **Expect:** 0.00016
Matches (Bold Red): 20/306 fragment ions using 40 most intense peaks

#	b	b^{++}	b*	b^{*++}	b^0	b^{0++}	Seq.	y	y^{++}	y*	y^{*++}	y^0	y^{0++}	#
1	88.0393	44.5233			70.0287	35.5180	S							20
2	203.0662	102.0368			185.0557	93.0315	D	2180.8867	1090.9470	2163.8602	1082.4337	2162.8761	**1081.9417**	19
3	370.0646	185.5359			352.0540	176.5307	S	2065.8598	1033.4335	2048.8332	1024.9202	2047.8492	1024.4282	18
4	457.0966	229.0520			439.0861	220.0467	S	**1898.8614**	949.9343	1881.8349	941.4211	1880.8508	940.9291	17
5	558.1443	279.5758			540.1337	270.5705	T	1811.8294	906.4183	1794.8028	897.9051	1793.8188	**897.4130**	16
6	687.1869	344.0971			669.1763	335.0918	E	**1710.7817**	855.8945	1693.7552	847.3812	1692.7711	846.8892	15
7	816.2295	408.6184			798.2189	399.6131	E	**1581.7391**	791.3732	1564.7126	782.8599	1563.7285	782.3679	14
8	903.2615	452.1344			885.2510	443.1291	S	**1452.6965**	726.8519	1435.6700	718.3386	1434.6859	717.8466	13
9	960.2830	480.6451			942.2724	471.6398	G	**1365.6645**	683.3359	1348.6379	674.8226	1347.6539	674.3306	12
10	1088.3416	544.6744	1071.3150	536.1611	1070.3310	535.6691	Q	1308.6430	654.8251	1291.6165	646.3119	1290.6325	645.8199	11
11	1203.3685	602.1879	1186.3420	593.6746	1185.3579	593.1826	D	**1180.5844**	590.7959	1163.5579	582.2826	1162.5739	581.7906	10
12	**1302.4369**	651.7221	1285.4104	643.2088	**1284.4264**	642.7168	V	1065.5575	533.2824	1048.5310	524.7691	1047.5469	524.2771	9
13	1415.5210	708.2641	1398.4944	699.7509	1397.5104	699.2588	L	**966.4891**	483.7482	949.4625	475.2349	948.4785	474.7429	8
14	1544.5636	772.7854	1527.5370	764.2722	1526.5530	763.7801	E	**853.4050**	427.2061	836.3785	418.6929	835.3945	418.2009	7
15	**1658.6065**	829.8069	1641.5800	821.2936	1640.5959	820.8016	N	**724.3624**	**362.6849**	707.3359	354.1716	706.3519	353.6796	6
16	1759.6542	880.3307	1742.6276	871.8175	1741.6436	871.3254	T	610.3195	305.6634	593.2930	297.1501	592.3089	296.6581	5
17	1906.7226	953.8649	1889.6960	945.3517	**1888.7120**	944.8597	F	**509.2718**	255.1396	492.2453	246.6263	491.2613	246.1343	4
18	1993.7546	997.3810	1976.7281	988.8677	**1975.7441**	988.3757	S	**362.2034**	181.6053	345.1769	173.0921	344.1928	172.6001	3

19	2121.8132	1061.4102	2104.7867	1052.8970	2103.8026	1052.4050	Q	275.1714	138.0893	258.1448	129.5761		2
20							K	147.1128	74.0600	130.0863	65.5468		1

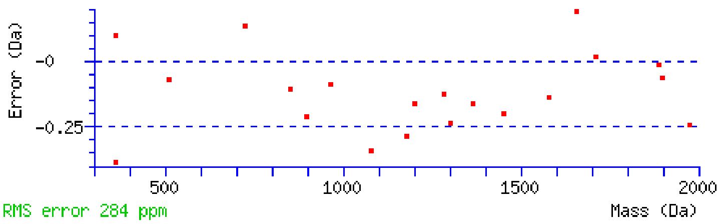

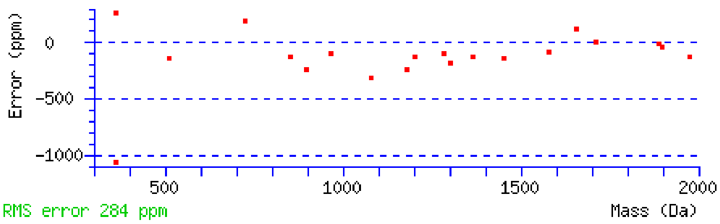

All matches to this query

Score	Mr(calc):	Delta	Sequence
61.1	2266.9115	-0.0025	SDSSTEESGQDVLENTFSQK
59.5	2266.9115	-0.0025	SDSSTEESGQDVLENTFSQK
55.7	2266.9115	-0.0025	SDSSTEESGQDVLENTFSQK
54.7	2266.9115	-0.0025	SDSSTEESGQDVLENTFSQK
33.7	2266.9115	-0.0025	SDSSTEESGQDVLENTFSQK
6.5	2265.9009	1.0080	VTEEENTSSGADASSASDTTVK
6.5	2265.9009	1.0080	VTEEENTSSGADASSASDTTVK
1.1	2265.9266	0.9823	EDFHNLMTVSPSLVGTTNK
0.5	2265.9149	0.9940	KNLYSPIAGYWLTASGER
0.1	2265.9009	1.0080	VTEEENTSSGADASSASDTTVK

Spectrum No: 160; Query: 1390; Rank: 1

Peptide View

MS/MS Fragmentation of **DVEDMELSDVEDDGSK**
Found in **IPI00368287**, Tax_Id=10116 Gene_Symbol=- 157 kDa protein

Match to Query 1390: 1877.679348 from(939.846950,2+)
Title: 091008RatKidney_NH4Format01_28.1631.1631.2.dta
Data file K:\NewmanPaper\Piliang\3SubProteomes\Piliang3SP\mgf5ppm\ERLIC_3SubProteomes5ppm.mgf

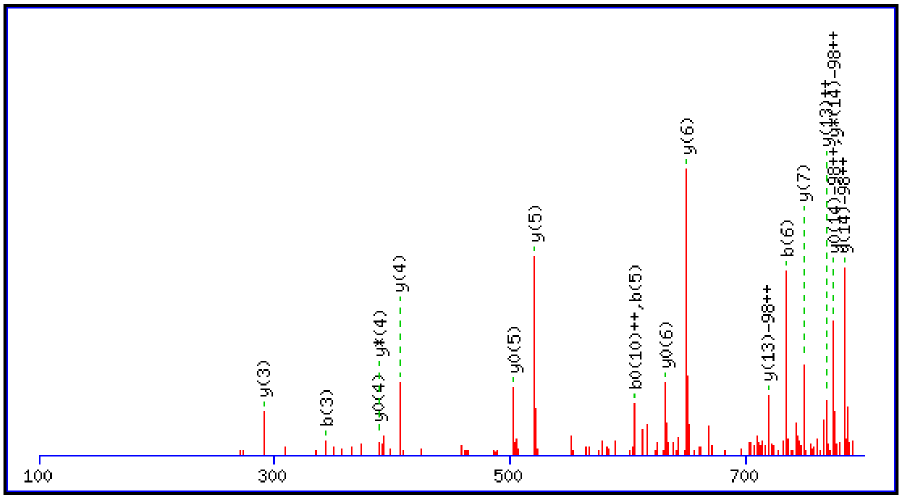

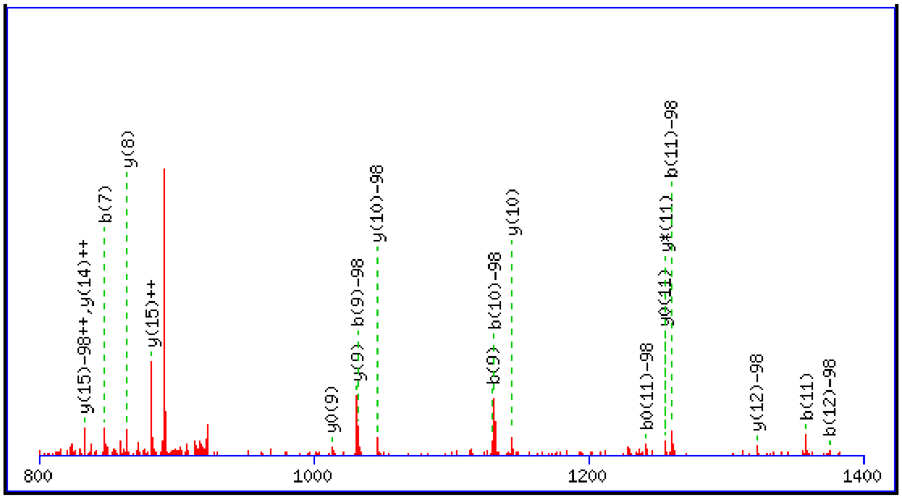

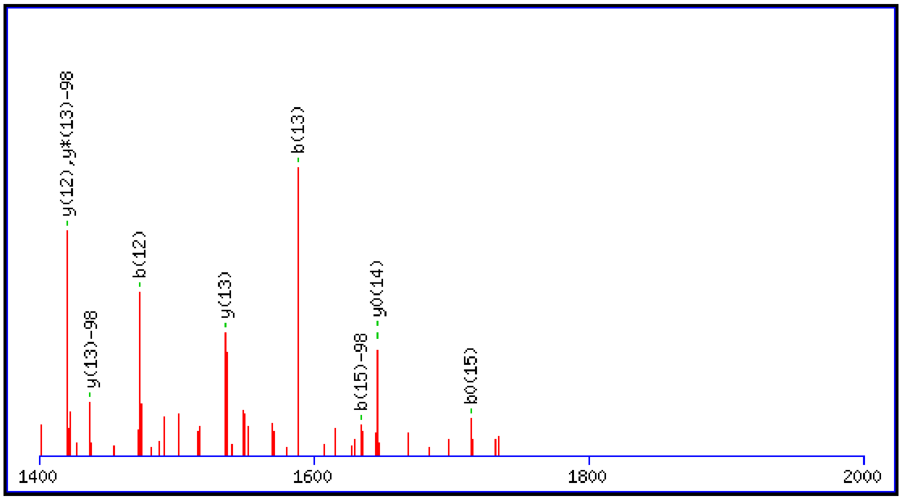

Monoisotopic mass of neutral peptide Mr(calc): 1877.6762
Fixed modifications: Carbamidomethyl (C)
Variable modifications:
M5 : Oxidation (M)
S8 : Phospho (ST), with neutral losses 97.9769(shown in table), 0.0000
Ions Score: 61 **Expect:** 7.5e-005
Matches (Bold Red): 46/222 fragment ions using 64 most intense peaks

#	b	b^{++}	b^{0}	b^{0++}	Seq.	y	y^{++}	y*	y^{*++}	y^{0}	y^{0++}	#
1	116.0342	58.5207	98.0237	49.5155	D							16
2	215.1026	108.0550	197.0921	99.0497	V	1665.6796	**833.3434**	1648.6531	824.8302	1647.6690	824.3382	15
3	**344.1452**	172.5763	326.1347	163.5710	E	1566.6112	**783.8092**	1549.5846	**775.2960**	1548.6006	**774.8039**	14
4	459.1722	230.0897	441.1616	221.0844	D	**1437.5686**	**719.2879**	**1420.5420**	710.7747	1419.5580	710.2827	13
5	**606.2076**	303.6074	588.1970	294.6021	M	**1322.5416**	661.7745	1305.5151	653.2612	1304.5311	652.7692	12
6	**735.2502**	368.1287	717.2396	359.1234	E	1175.5062	588.2568	1158.4797	579.7435	1157.4957	579.2515	11
7	**848.3342**	424.6708	830.3237	415.6655	L	**1046.4637**	523.7355	1029.4371	515.2222	1028.4531	514.7302	10
8	917.3557	459.1815	899.3451	450.1762	S	933.3796	467.1934	916.3530	458.6802	915.3690	458.1881	9
9	**1032.3826**	516.6950	1014.3721	507.6897	D	**864.3581**	432.6827	847.3316	424.1694	846.3476	423.6774	8
10	**1131.4510**	566.2292	1113.4405	557.2239	V	**749.3312**	375.1692	732.3046	366.6560	731.3206	366.1640	7
11	**1260.4936**	630.7505	**1242.4831**	621.7452	E	**650.2628**	325.6350	633.2362	317.1218	**632.2522**	316.6297	6
12	**1375.5206**	688.2639	1357.5100	679.2586	D	**521.2202**	261.1137	504.1936	252.6005	**503.2096**	252.1084	5
13	1490.5475	745.7774	1472.5370	736.7721	D	**406.1932**	203.6003	**389.1667**	195.0870	**388.1827**	194.5950	4
14	1547.5690	774.2881	1529.5584	765.2828	G	**291.1663**	146.0868	274.1397	137.5735	273.1557	137.0815	3
15	**1634.6010**	817.8041	1616.5904	808.7989	S	234.1448	117.5761	217.1183	109.0628	216.1343	108.5708	2
16					K	147.1128	74.0600	130.0863	65.5468			1

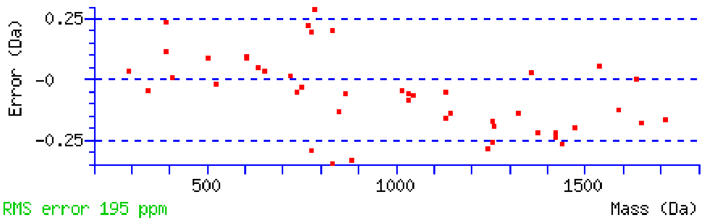

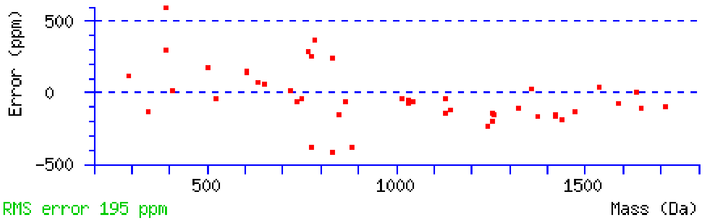

All matches to this query

Score	Mr(calc):	Delta	Sequence
60.8	1877.6762	0.0032	DVEDMELSDVEDDGSK
23.5	1877.6762	0.0032	DVEDMELSDVEDDGSK
6.1	1877.6696	0.0097	ECSSGLDSNEMSPQEK
4.5	1876.6868	0.9925	SRYSLHENMDKIK

Spectrum No: 161; Query: 1033; Rank: 1

Peptide View

MS/MS Fragmentation of **DLLSDLQDISDSER**
Found in **IPI00563558**, Tax_Id=10116 Gene_Symbol=Cdc2l1 cell division cycle 2 homolog (S.pombe)-like 1

Match to Query 1033: 1684.716748 from(843.365650,2+)
Title: 091008RatKidney_NH4Format01_25.3500.3500.2.dta
Data file K:\NewmanPaper\Piliang\3SubProteomes\Piliang3SP\mgf5ppm\ERLIC_3SubProteomes5ppm.mgf

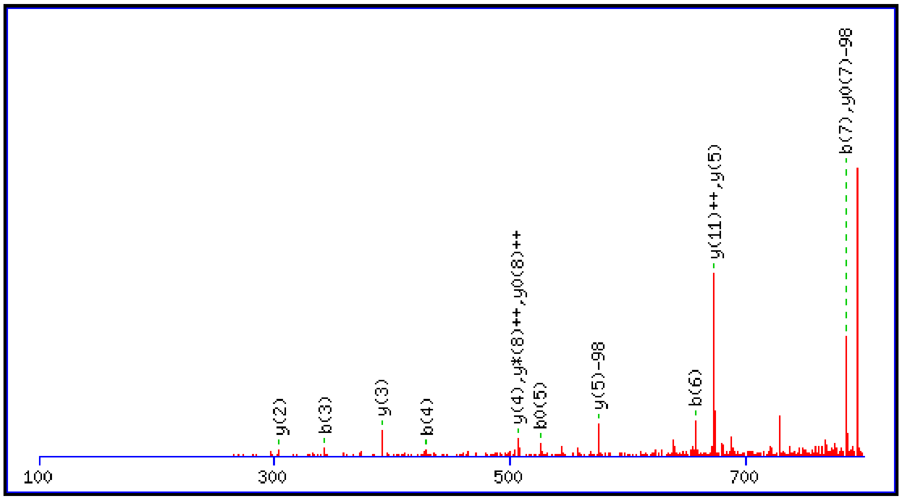

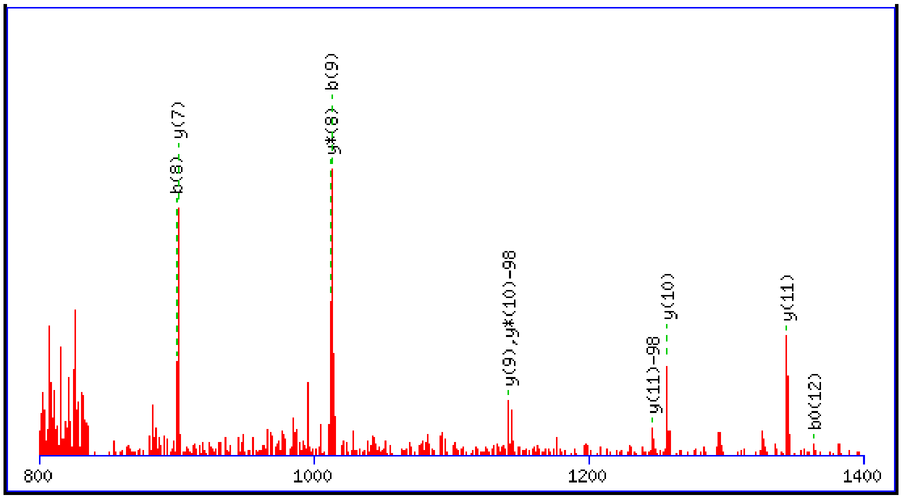

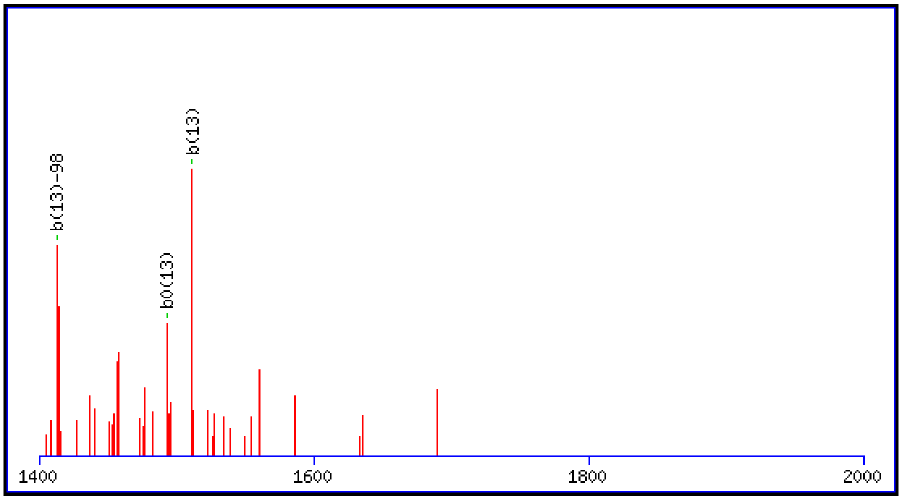

Monoisotopic mass of neutral peptide Mr(calc): 1684.7193
Fixed modifications: Carbamidomethyl (C)
Variable modifications:
S10 : Phospho (ST), with neutral losses 0.0000(shown in table), 97.9769
Ions Score: 61 **Expect:** 0.00014
Matches (Bold Red): 27/220 fragment ions using 39 most intense peaks

#	b	b^{++}	b*	b^{*++}	b^0	b^{0++}	Seq.	y	y^{++}	y*	y^{*++}	y^0	y^{0++}	#
1	116.0342	58.5207			98.0237	49.5155	D							14
2	229.1183	115.0628			211.1077	106.0575	L	1570.6996	785.8534	1553.6731	777.3402	1552.6891	776.8482	13
3	**342.2023**	171.6048			324.1918	162.5995	L	1457.6156	729.3114	1440.5890	720.7981	1439.6050	720.3061	12
4	**429.2344**	215.1208			411.2238	206.1155	S	**1344.5315**	**672.7694**	1327.5049	664.2561	1326.5209	663.7641	11
5	544.2613	272.6343			**526.2508**	263.6290	D	**1257.4995**	629.2534	1240.4729	620.7401	1239.4889	620.2481	10
6	**657.3454**	329.1763			639.3348	320.1710	L	**1142.4725**	571.7399	1125.4460	563.2266	1124.4620	562.7346	9
7	**785.4040**	393.2056	768.3774	384.6923	767.3934	384.2003	Q	1029.3885	515.1979	**1012.3619**	**506.6846**	1011.3779	**506.1926**	8
8	**900.4309**	450.7191	883.4044	442.2058	882.4203	441.7138	D	**901.3299**	451.1686	884.3033	442.6553	883.3193	442.1633	7
9	**1013.5150**	507.2611	996.4884	498.7478	995.5044	498.2558	I	786.3029	393.6551	769.2764	385.1418	768.2924	384.6498	6
10	1180.5133	590.7603	1163.4868	582.2470	1162.5028	581.7550	S	**673.2189**	337.1131	656.1923	328.5998	655.2083	328.1078	5
11	1295.5403	648.2738	1278.5137	639.7605	1277.5297	639.2685	D	**506.2205**	253.6139	489.1940	245.1006	488.2100	244.6086	4
12	1382.5723	691.7898	1365.5457	683.2765	**1364.5617**	682.7845	S	**391.1936**	196.1004	374.1670	187.5871	373.1830	187.0951	3
13	**1511.6149**	756.3111	1494.5883	747.7978	**1493.6043**	747.3058	E	**304.1615**	152.5844	287.1350	144.0711	286.1510	143.5791	2
14							R	175.1190	88.0631	158.0924	79.5498			1

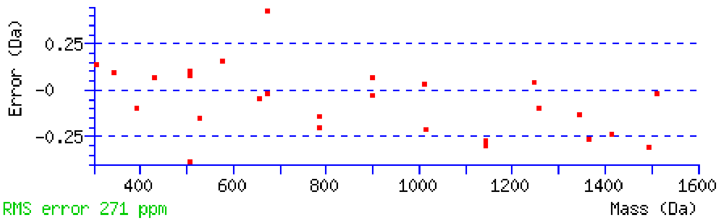

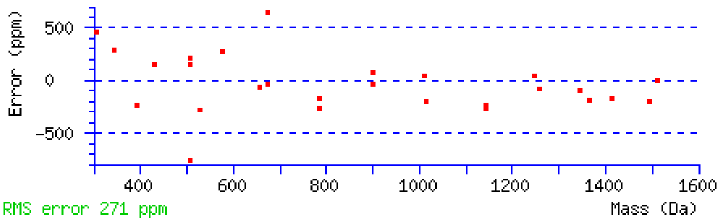

All matches to this query

Score	Mr(calc):	Delta	Sequence
60.8	1684.7193	-0.0025	DLLSDLQDISDSER
50.0	1684.7193	-0.0025	DLLSDLQDISDSER
19.6	1684.7193	-0.0025	DLLSDLQDISDSER
11.8	1684.7239	-0.0072	SNSIQQMEQLNRR
11.8	1684.7239	-0.0072	SNSIQQMEQLNRR
7.1	1684.7222	-0.0055	NLISGGHLSLQTER
6.8	1682.6954	2.0214	AAGKXFTQVNSEDK
6.4	1684.7094	0.0074	AVSTWXQTESQPSR
5.6	1684.7028	0.0139	WLCNDQIDAGTRR
4.3	1682.7124	2.0044	DSGFFMLRKDSER

Spectrum No: 162; Query: 2262; Rank: 1

Peptide View

MS/MS Fragmentation of **IYHLPDAESDEDEDFKEQTR**
Found in **IPI00208304**, Tax_Id=10116 Gene_Symbol=Sept2 Septin-2

Match to Query 2262: 2516.029348 from(1259.021950,2+)
Title: 091008RatKidney_NH4Format02_25.2531.2531.2.dta
Data file K:\NewmanPaper\Piliang\3SubProteomes\Piliang3SP\mgf5ppm\ERLIC_3SubProteomes5ppm.mgf

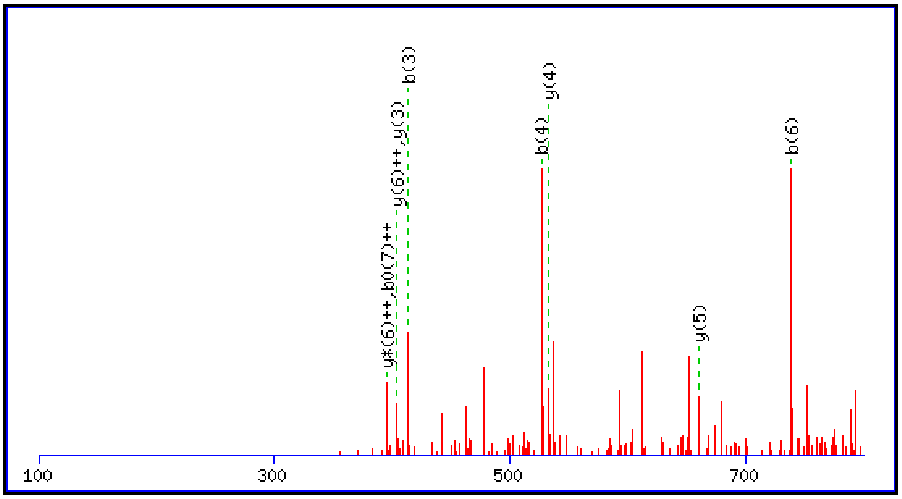

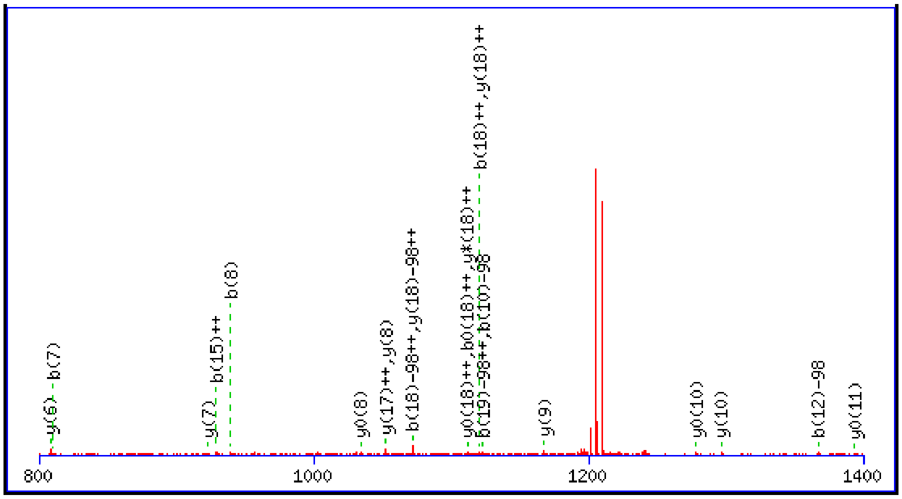

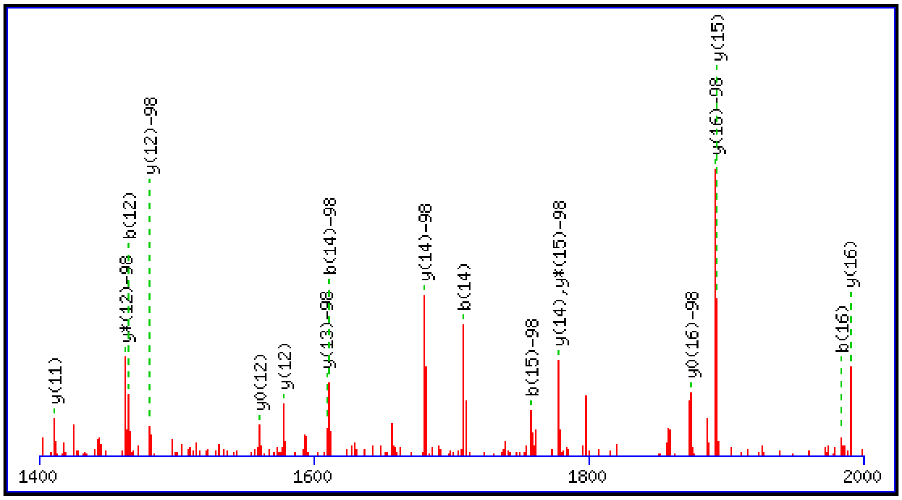

Monoisotopic mass of neutral peptide Mr(calc): 2516.0380
Fixed modifications: Carbamidomethyl (C)
Variable modifications:
S9 : Phospho (ST), with neutral losses 97.9769(shown in table), 0.0000
Ions Score: 60 **Expect:** 0.00027
Matches (Bold Red): 49/286 fragment ions using 78 most intense peaks

#	b	b^{++}	b*	b^{*++}	b^{0}	b^{0++}	Seq.	y	y^{++}	y*	y^{*++}	y^{0}	y^{0++}	#
1	114.0913	57.5493					I							20
2	277.1547	139.0810					Y	2305.9844	1153.4958	2288.9578	1144.9825	2287.9738	1144.4905	19
3	**414.2136**	207.6104					H	2142.9210	**1071.9642**	2125.8945	1063.4509	2124.9105	1062.9589	18
4	**527.2976**	264.1525					L	2005.8621	1003.4347	1988.8356	994.9214	1987.8516	994.4294	17
5	624.3504	312.6788					P	**1892.7781**	946.8927	1875.7515	938.3794	**1874.7675**	937.8874	16
6	**739.3774**	370.1923			721.3668	361.1870	D	1795.7253	898.3663	**1778.6988**	889.8530	1777.7147	889.3610	15
7	**810.4145**	405.7109			792.4039	**396.7056**	A	**1680.6984**	840.8528	1663.6718	832.3395	1662.6878	831.8475	14
8	**939.4571**	470.2322			921.4465	461.2269	E	**1609.6612**	805.3343	1592.6347	796.8210	1591.6507	796.3290	13
9	1008.4785	504.7429			990.4679	495.7376	S	**1480.6187**	740.8130	**1463.5921**	732.2997	1462.6081	731.8077	12
10	**1123.5055**	562.2564			1105.4949	553.2511	D	**1411.5972**	706.3022	1394.5706	697.7890	**1393.5866**	697.2970	11
11	1252.5481	626.7777			1234.5375	617.7724	E	**1296.5703**	648.7888	1279.5437	640.2755	**1278.5597**	639.7835	10
12	**1367.5750**	684.2911			1349.5644	675.2859	D	**1167.5277**	584.2675	1150.5011	575.7542	1149.5171	575.2622	9
13	1496.6176	748.8124			1478.6070	739.8071	E	**1052.5007**	526.7540	1035.4742	518.2407	**1034.4902**	517.7487	8
14	**1611.6445**	806.3259			1593.6340	797.3206	D	**923.4581**	462.2327	906.4316	453.7194	905.4476	453.2274	7
15	**1758.7129**	879.8601			1740.7024	870.8548	F	**808.4312**	**404.7192**	791.4046	**396.2060**	790.4206	395.7139	6
16	1886.8079	943.9076	1869.7814	935.3943	1868.7973	934.9023	K	**661.3628**	331.1850	644.3362	322.6717	643.3522	322.1797	5
17	2015.8505	1008.4289	1998.8240	999.9156	1997.8399	999.4236	E	**533.2678**	267.1375	516.2413	258.6243	515.2572	258.1323	4
18	2143.9091	**1072.4582**	2126.8825	1063.9449	2125.8985	1063.4529	Q	**404.2252**	202.6162	387.1987	194.1030	386.2146	193.6110	3

**19**	2244.9568	1122.9820	2227.9302	1114.4687	2226.9462	1113.9767	**T**	276.1666	138.5870	259.1401	130.0737	258.1561	129.5817	**2**
**20**							**R**	175.1190	88.0631	158.0924	79.5498			**1**

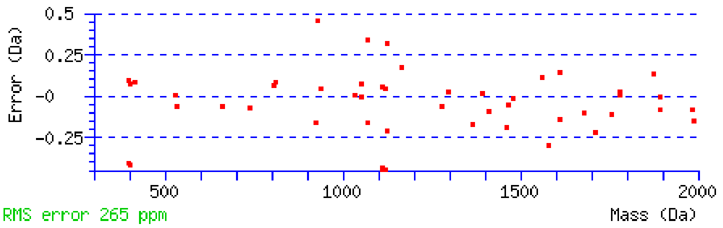

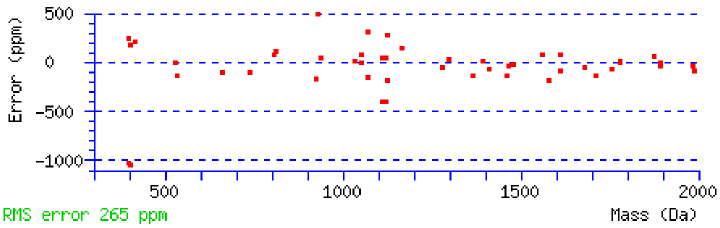

All matches to this query

Score	Mr(calc):	Delta	Sequence
60.4	2516.0380	-0.0087	IYHLPDAESDEDEDFKEQTR
20.7	2516.0380	-0.0087	IYHLPDAESDEDEDFKEQTR
11.5	2516.0380	-0.0087	IYHLPDAESDEDEDFKEQTR
5.5	2516.0285	0.0009	XFDDDEGEEEDEAAAAAAAAIGYR
4.1	2515.0379	0.9914	SPAASLPYEGLIGTYTCSTPRN
4.1	2515.0379	0.9914	SPAASLPYEGLIGTYTCSTPRN
2.8	2516.0150	0.0143	SPVYCRYQFHKTPVHETK
2.8	2516.0150	0.0143	SPVYCRYQFHKTPVHETK
2.7	2514.0266	2.0028	SMDFRNYMMRDENLEPLPK
2.5	2515.0508	0.9786	FSINTDDIMGTSLTVRAKYK

Spectrum No: 163; Query: 1457; Rank: 1

Peptide View

MS/MS Fragmentation of **SASSDTSEELNAQDSPK**
Found in **IPI00200898**, Tax_Id=10116 Gene_Symbol=Slc9a3r1 Ezrin-radixin-moesin-binding phosphoprotein 50

Match to Query 1457: 1924.694348 from(963.354450,2+)
Title: 091008RatKidney_NoSalt_37.586.586.2.dta
Data file K:\NewmanPaper\Piliang\3SubProteomes\Piliang3SP\mgf5ppm\ERLIC_3SubProteomes5ppm.mgf

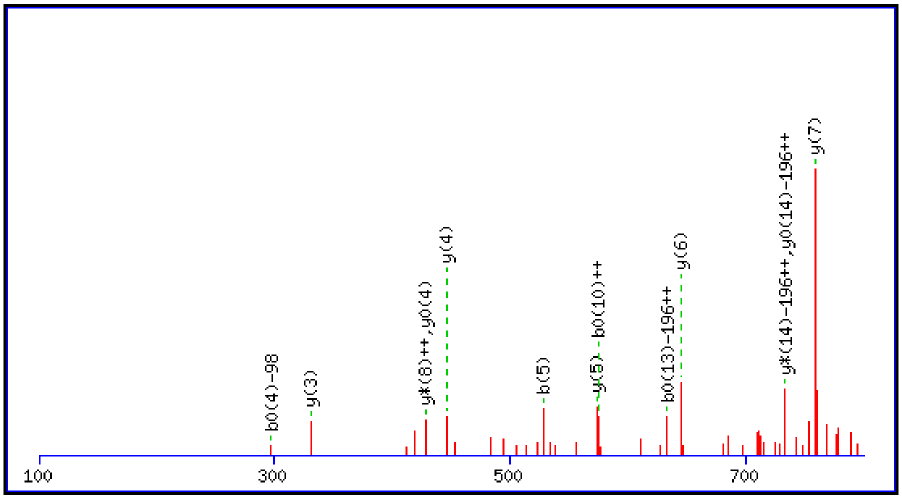

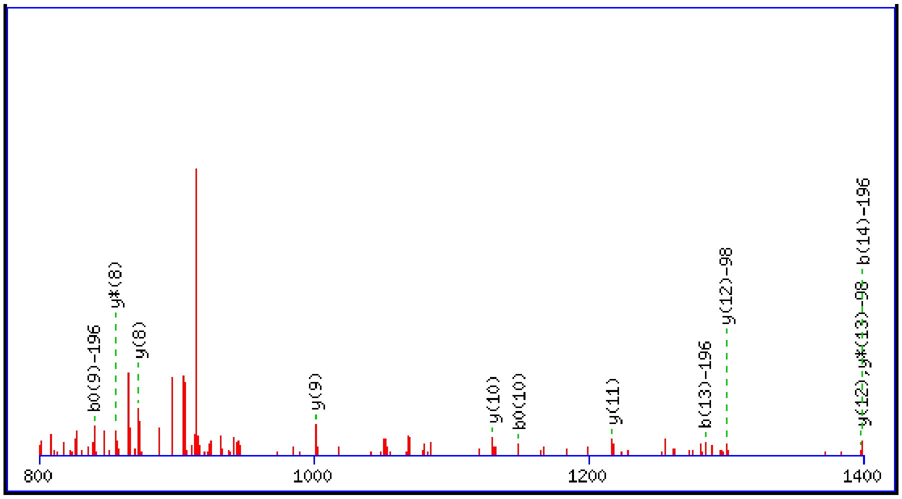

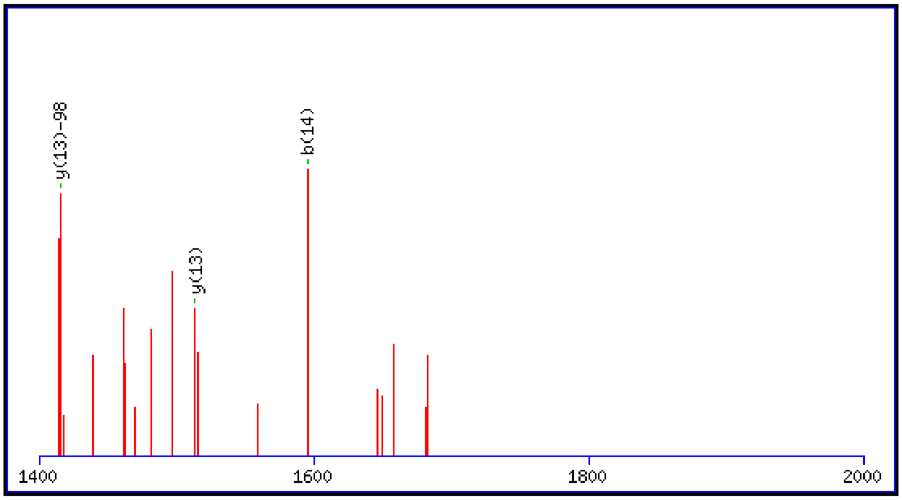

Monoisotopic mass of neutral peptide Mr(calc): 1924.6976
Fixed modifications: Carbamidomethyl (C)
Variable modifications:
S4 : Phospho (ST), with neutral losses 97.9769(shown in table), 0.0000
T6 : Phospho (ST), with neutral losses 97.9769(shown in table), 0.0000
Ions Score: 60 **Expect:** 8.2e-005
Matches (Bold Red): 28/262 fragment ions using 49 most intense peaks

#	b	b^{++}	b*	b^{*++}	b^{0}	b^{0++}	Seq.	y	y^{++}	y*	y^{*++}	y^{0}	y^{0++}	#
1	88.0393	44.5233			70.0287	35.5180	S							17
2	159.0764	80.0418			141.0659	71.0366	A	1642.7191	821.8632	1625.6925	813.3499	1624.7085	812.8579	16
3	246.1084	123.5579			228.0979	114.5526	S	1571.6820	786.3446	1554.6554	777.8313	1553.6714	777.3393	15
4	315.1299	158.0686			**297.1193**	149.0633	S	1484.6499	742.8286	1467.6234	**734.3153**	1466.6394	**733.8233**	14
5	430.1568	215.5821			412.1463	206.5768	D	**1415.6285**	708.3179	**1398.6019**	699.8046	1397.6179	699.3126	13
6	513.1940	257.1006			495.1834	248.0953	T	**1300.6015**	650.8044	1283.5750	642.2911	1282.5910	641.7991	12
7	600.2260	300.6166			582.2154	291.6113	S	**1217.5644**	609.2859	1200.5379	600.7726	1199.5539	600.2806	11
8	729.2686	365.1379			711.2580	356.1326	E	**1130.5324**	565.7698	1113.5059	557.2566	1112.5218	556.7646	10
9	858.3112	429.6592			**840.3006**	420.6539	E	**1001.4898**	501.2485	984.4633	492.7353	983.4793	492.2433	9
10	971.3952	486.2013			953.3847	477.1960	L	**872.4472**	436.7272	**855.4207**	**428.2140**	854.4367	427.7220	8
11	1085.4382	543.2227	1068.4116	534.7094	1067.4276	534.2174	N	**759.3632**	380.1852	742.3366	371.6719	741.3526	371.1799	7
12	1156.4753	578.7413	1139.4487	570.2280	1138.4647	569.7360	A	**645.3202**	323.1638	628.2937	314.6505	627.3097	314.1585	6
13	**1284.5338**	642.7706	1267.5073	634.2573	1266.5233	**633.7653**	Q	**574.2831**	287.6452	557.2566	279.1319	556.2726	278.6399	5
14	**1399.5608**	700.2840	1382.5342	691.7708	1381.5502	691.2788	D	**446.2245**	223.6159	429.1980	215.1026	**428.2140**	214.6106	4
15	1486.5928	743.8000	1469.5663	735.2868	1468.5823	734.7948	S	**331.1976**	166.1024	314.1710	157.5892	313.1870	157.0972	3
16	1583.6456	792.3264	1566.6190	783.8132	1565.6350	783.3211	P	244.1656	122.5864	227.1390	114.0731			2
17							K	147.1128	74.0600	130.0863	65.5468			1

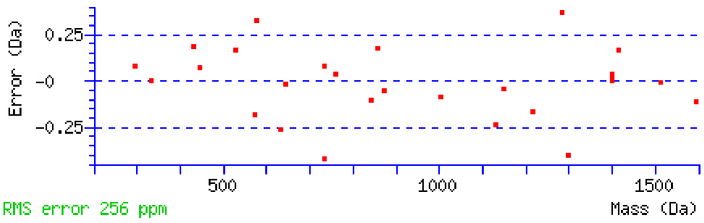

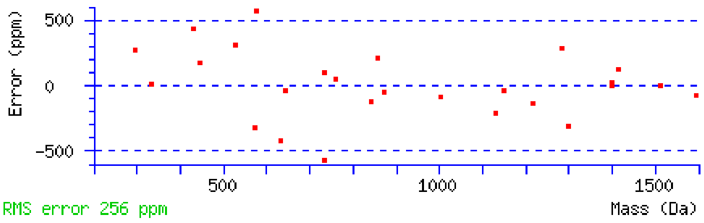

All matches to this query

Score	Mr(calc):	Delta	Sequence
60.3	1924.6976	-0.0033	SASSDTSEELNAQDSPK
58.3	1924.6976	-0.0033	SASSDTSEELNAQDSPK
58.3	1924.6976	-0.0033	SASSDTSEELNAQDSPK
50.7	1924.6976	-0.0033	SASSDTSEELNAQDSPK
48.8	1924.6976	-0.0033	SASSDTSEELNAQDSPK
48.8	1924.6976	-0.0033	SASSDTSEELNAQDSPK
38.7	1924.6976	-0.0033	SASSDTSEELNAQDSPK
38.7	1924.6976	-0.0033	SASSDTSEELNAQDSPK
38.7	1924.6976	-0.0033	SASSDTSEELNAQDSPK
35.5	1924.6976	-0.0033	SASSDTSEELNAQDSPK

Spectrum No: 164; Query: 1773; Rank: 1

Peptide View

MS/MS Fragmentation of **KGSLDSDNDDSDCPYSEK**
Found in **IPI00199985**, Tax_Id=10116 Gene_Symbol=Slc4a4 Isoform 1 of Electrogenic sodium bicarbonate cotransporter 1

Match to Query 1773: 2110.772592 from(704.598140,3+)
Title: 100101RatKid_NS_deglyco_23.810.810.3.dta
Data file K:\NewmanPaper\Piliang\3SubProteomes\Piliang3SP\mgf5ppm\ERLIC_3SubProteomes5ppm.mgf

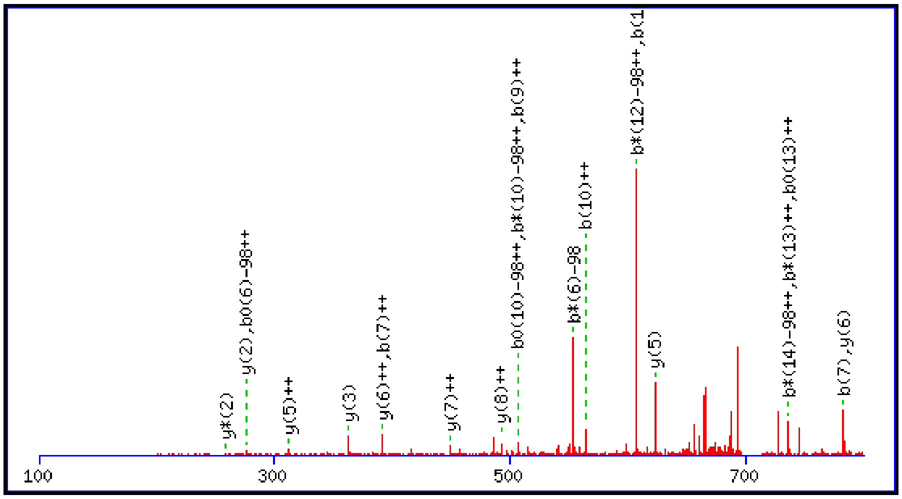

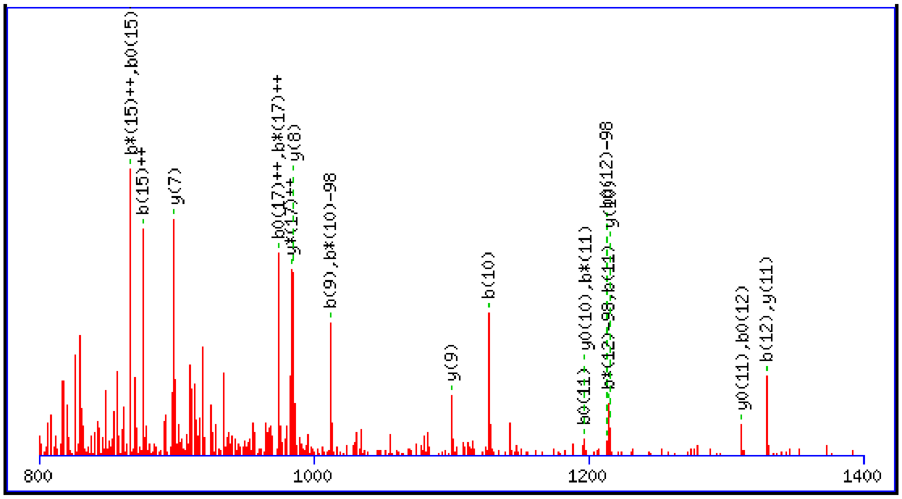

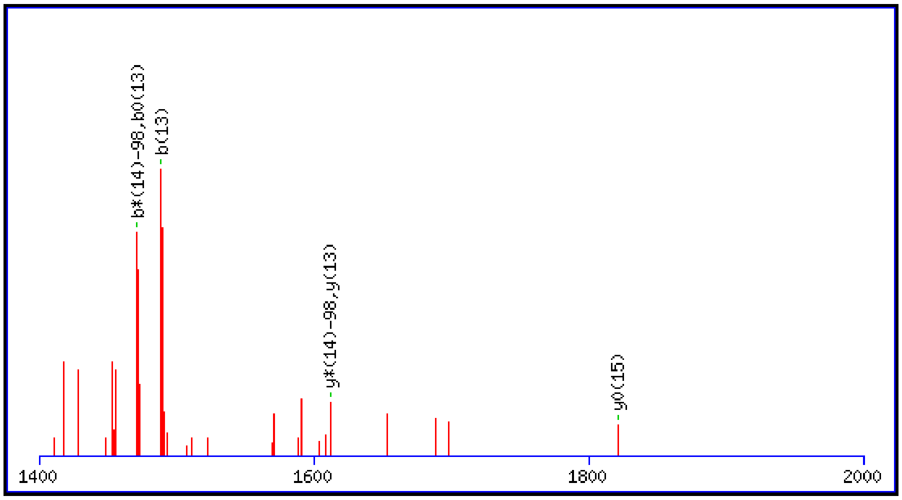

Monoisotopic mass of neutral peptide Mr(calc): 2110.7674
Fixed modifications: Carbamidomethyl (C)
Variable modifications:
S6 : Phospho (ST), with neutral losses 0.0000(shown in table), 97.9769
Ions Score: 60 **Expect:** 0.00011
Matches (Bold Red): 52/300 fragment ions using 50 most intense peaks

#	b	b^{++}	b*	b^{*++}	b^0	b^{0++}	Seq.	y	y^{++}	y*	y^{*++}	y^0	y^{0++}	#
1	129.1022	65.0548	112.0757	56.5415			K							18
2	186.1237	93.5655	169.0972	85.0522			G	1983.6797	992.3435	1966.6532	**983.8302**	1965.6692	983.3382	17
3	273.1557	137.0815	256.1292	128.5682	255.1452	128.0762	S	1926.6583	963.8328	1909.6317	955.3195	1908.6477	954.8275	16
4	386.2398	193.6235	369.2132	185.1103	368.2292	184.6183	L	1839.6263	920.3168	1822.5997	911.8035	**1821.6157**	911.3115	15
5	501.2667	251.1370	484.2402	242.6237	483.2562	242.1317	D	1726.5422	863.7747	1709.5156	855.2615	1708.5316	854.7695	14
6	668.2651	334.6362	651.2385	326.1229	650.2545	325.6309	S	**1611.5152**	806.2613	1594.4887	797.7480	1593.5047	797.2560	13
7	**783.2920**	**392.1497**	766.2655	383.6364	765.2815	383.1444	D	1444.5169	722.7621	1427.4903	714.2488	1426.5063	713.7568	12
8	897.3350	449.1711	880.3084	440.6578	879.3244	440.1658	N	**1329.4900**	665.2486	1312.4634	656.7353	**1311.4794**	656.2433	11
9	**1012.3619**	**506.6846**	995.3354	498.1713	994.3513	497.6793	D	**1215.4470**	**608.2272**	1198.4205	599.7139	**1197.4365**	599.2219	10
10	**1127.3888**	**564.1981**	1110.3623	555.6848	1109.3783	555.1928	D	**1100.4201**	550.7137	1083.3935	542.2004	1082.4095	541.7084	9
11	**1214.4209**	**607.7141**	**1197.3943**	599.2008	**1196.4103**	598.7088	S	**985.3931**	**493.2002**	968.3666	484.6869	967.3826	484.1949	8
12	**1329.4478**	665.2275	1312.4213	656.7143	**1311.4373**	656.2223	D	**898.3611**	**449.6842**	881.3346	441.1709	880.3505	440.6789	7
13	**1489.4785**	745.2429	1472.4519	**736.7296**	**1471.4679**	**736.2376**	C	**783.3342**	**392.1707**	766.3076	383.6574	765.3236	383.1654	6
14	1586.5312	793.7693	1569.5047	785.2560	1568.5207	784.7640	P	**623.3035**	**312.1554**	606.2770	303.6421	605.2930	303.1501	5
15	1749.5946	**875.3009**	1732.5680	**866.7876**	1731.5840	**866.2956**	Y	526.2508	263.6290	509.2242	255.1157	508.2402	254.6237	4
16	1836.6266	918.8169	1819.6000	910.3037	1818.6160	909.8116	S	**363.1874**	182.0974	346.1609	173.5841	345.1769	173.0921	3
17	1965.6692	983.3382	1948.6426	**974.8250**	1947.6586	**974.3329**	E	**276.1554**	138.5813	**259.1288**	130.0681	258.1448	129.5761	2
18							K	147.1128	74.0600	130.0863	65.5468			1

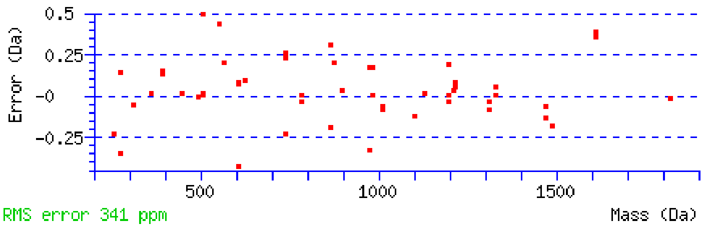

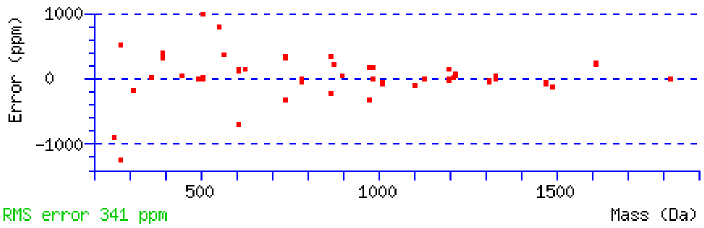

All matches to this query

Score	Mr(calc):	Delta	Sequence
60.3	2110.7674	0.0052	KGSLDSDNDDSDCPYSEK
47.9	2110.7674	0.0052	KGSLDSDNDDSDCPYSEK
18.2	2110.7674	0.0052	KGSLDSDNDDSDCPYSEK
10.3	2108.7848	1.9878	SADGVSEDGNDSDFPQFNK
9.5	2108.7848	1.9878	SADGVSEDGNDSDFPQFNK
7.9	2109.7688	1.0038	SADGVSEDGNDSDFPQFNK
7.7	2109.7688	1.0038	SADGVSEDGNDSDFPQFNK
7.2	2108.7848	1.9878	SADGVSEDGNDSDFPQFNK
7.0	2108.7848	1.9878	SADGVSEDGNDSDFPQFNK
5.7	2108.7495	2.0231	WRTVSTXPCRNPPR

Spectrum No: 165; Query: 1862; Rank: 1

Peptide View

MS/MS Fragmentation of **VAQSDGEESPAEEGQLLGER**
Found in **IPI00210147**, Tax_Id=10116 Gene_Symbol=Sec31l1 Isoform 1 of Protein transport protein Sec31A

Match to Query 1862: 2179.933748 from(1090.974150,2+)
Title: 100101RatKid_NS_deglyco_23.2202.2202.2.dta
Data file K:\NewmanPaper\Piliang\3SubProteomes\Piliang3SP\mgf5ppm\ERLIC_3SubProteomes5ppm.mgf

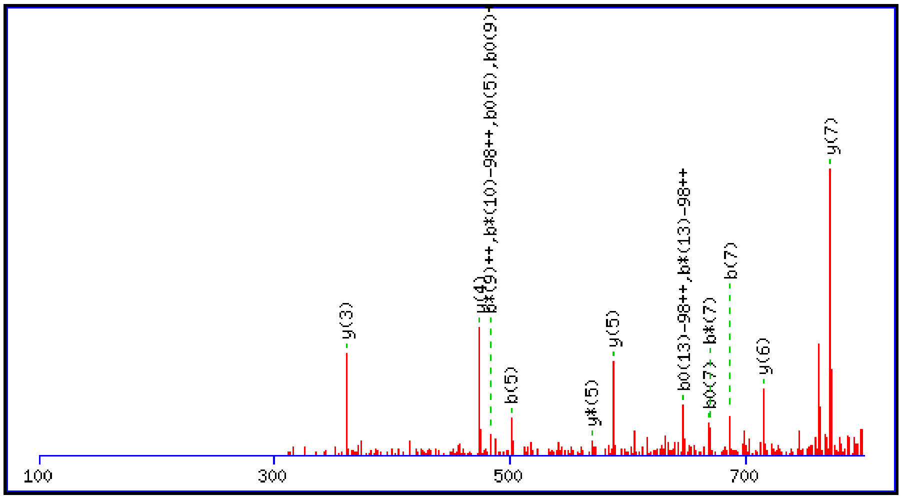

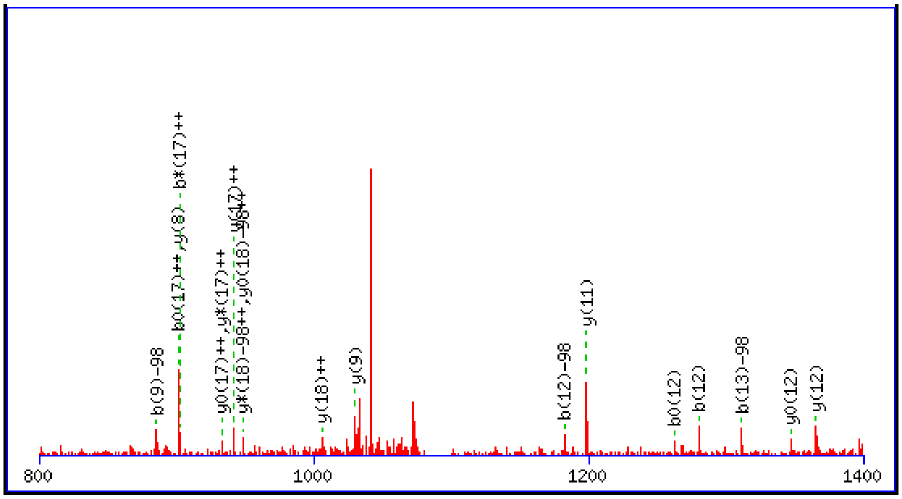

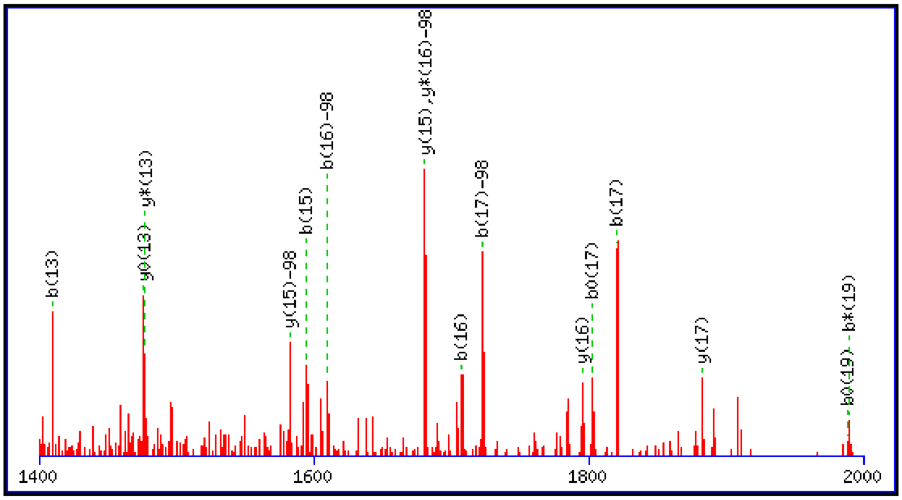

Monoisotopic mass of neutral peptide Mr(calc): 2179.9270
Fixed modifications: Carbamidomethyl (C)
Variable modifications:
S9 : Phospho (ST), with neutral losses 0.0000(shown in table), 97.9769
Ions Score: 60 **Expect:** 0.00026
Matches (Bold Red): 50/330 fragment ions using 72 most intense peaks

#	b	b^{++}	b*	b^{*++}	b^0	b^{0++}	Seq.	y	y^{++}	y*	y^{*++}	y^0	y^{0++}	#
1	100.0757	50.5415					V							20
2	171.1128	86.0600					A	2081.8659	1041.4366	2064.8394	1032.9233	2063.8553	1032.4313	19
3	299.1714	150.0893	282.1448	141.5761			Q	2010.8288	**1005.9180**	1993.8022	997.4048	1992.8182	996.9128	18
4	386.2034	193.6053	369.1769	185.0921	368.1928	184.6001	S	**1882.7702**	**941.8887**	1865.7437	**933.3755**	1864.7597	**932.8835**	17
5	**501.2304**	251.1188	484.2038	242.6055	**483.2198**	242.1135	D	**1795.7382**	898.3727	1778.7116	889.8595	1777.7276	889.3674	16
6	558.2518	279.6295	541.2253	271.1163	540.2413	270.6243	G	**1680.7112**	840.8593	1663.6847	832.3460	1662.7007	831.8540	15
7	**687.2944**	344.1508	**670.2679**	335.6376	**669.2838**	335.1456	E	1623.6898	812.3485	1606.6632	803.8353	1605.6792	803.3432	14
8	816.3370	408.6721	799.3105	400.1589	798.3264	399.6669	E	1494.6472	747.8272	**1477.6206**	739.3140	**1476.6366**	738.8219	13
9	983.3354	492.1713	966.3088	**483.6580**	965.3248	**483.1660**	S	**1365.6046**	683.3059	1348.5780	674.7927	**1347.5940**	674.3007	12
10	1080.3881	540.6977	1063.3616	532.1844	1062.3776	531.6924	P	**1198.6062**	599.8068	1181.5797	591.2935	1180.5957	590.8015	11
11	1151.4252	576.2163	1134.3987	567.7030	1133.4147	567.2110	A	1101.5535	551.2804	1084.5269	542.7671	1083.5429	542.2751	10
12	**1280.4678**	640.7376	1263.4413	632.2243	**1262.4573**	631.7323	E	**1030.5164**	515.7618	1013.4898	507.2485	1012.5058	506.7565	9
13	**1409.5104**	705.2588	1392.4839	696.7456	1391.4999	696.2536	E	**901.4738**	451.2405	884.4472	442.7272	883.4632	442.2352	8
14	1466.5319	733.7696	1449.5053	725.2563	1448.5213	724.7643	G	**772.4312**	386.7192	755.4046	378.2060	754.4206	377.7139	7
15	**1594.5905**	797.7989	1577.5639	789.2856	1576.5799	788.7936	Q	**715.4097**	358.2085	698.3832	349.6952	697.3991	349.2032	6
16	**1707.6745**	854.3409	1690.6480	845.8276	1689.6640	845.3356	L	**587.3511**	294.1792	**570.3246**	285.6659	569.3406	285.1739	5
17	**1820.7586**	910.8829	1803.7320	**902.3697**	**1802.7480**	**901.8776**	L	**474.2671**	237.6372	457.2405	229.1239	456.2565	228.6319	4
18	1877.7801	939.3937	1860.7535	930.8804	1859.7695	930.3884	G	**361.1830**	181.0951	344.1565	172.5819	343.1724	172.0899	3

**19**	2006.8226	1003.9150	**1989.7961**	995.4017	**1988.8121**	994.9097	**E**	304.1615	152.5844	287.1350	144.0711	286.1510	143.5791	**2**
**20**							**R**	175.1190	88.0631	158.0924	79.5498			**1**

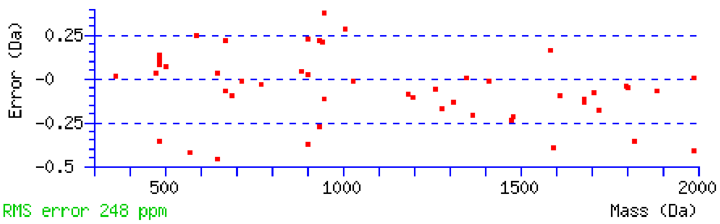

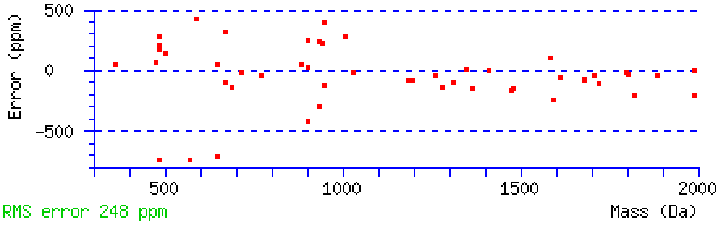

All matches to this query

Score	Mr(calc):	Delta	Sequence
59.8	2179.9270	0.0067	VAQSDGEESPAEEGQLLGER
28.4	2179.9270	0.0067	VAQSDGEESPAEEGQLLGER
3.5	2177.9309	2.0029	TVCIISCQNEQCGLIFR
3.0	2179.9445	-0.0107	MMKYWTNFARQGNPNSR
1.1	2179.9414	-0.0077	DQMTPSYLLYRYAKQK

Spectrum No: 166; Query: 772; Rank: 1

Peptide View

MS/MS Fragmentation of **ASSLGEIDDSSELR**
Found in **IPI00214079**, Tax_Id=10116 Gene_Symbol=Pkn2 Serine/threonine-protein kinase N2

Match to Query 772: 1557.663408 from(779.838980,2+)
Title: 091008RatKidney_NoSalt_26.2080.2080.2.dta
Data file K:\NewmanPaper\Piliang\3SubProteomes\Piliang3SP\mgf5ppm\ERLIC_3SubProteomes5ppm.mgf

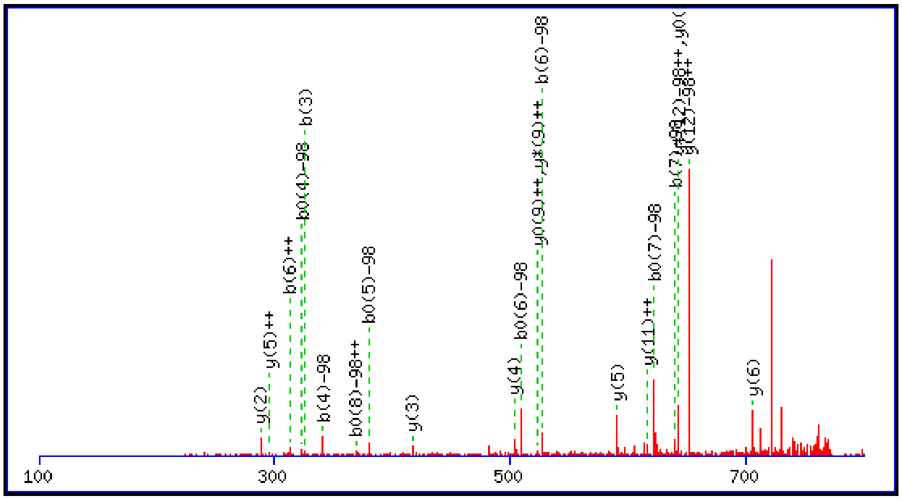

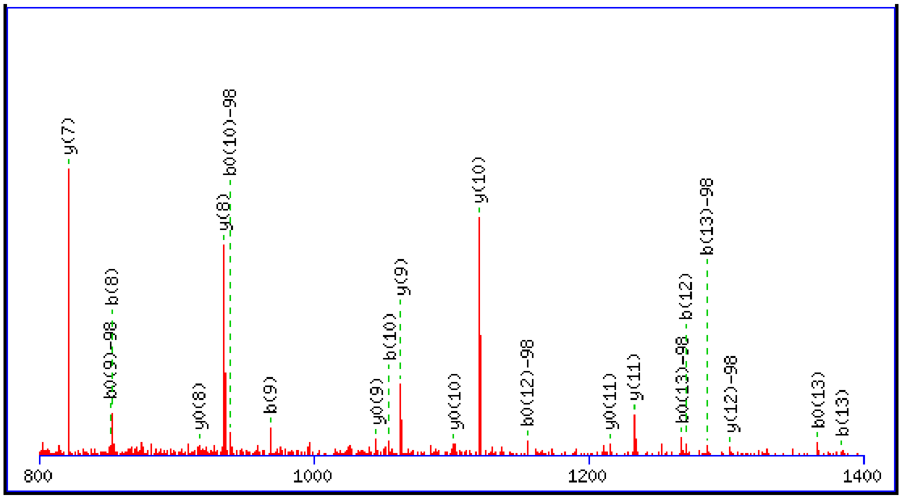

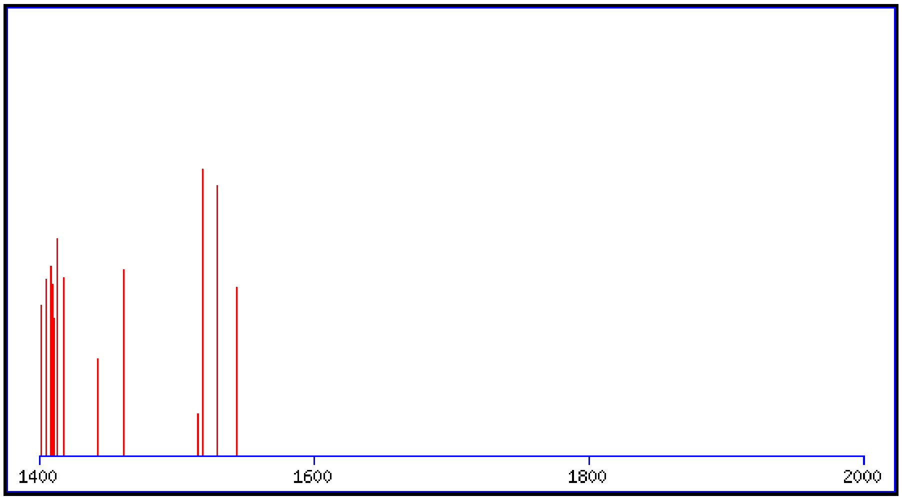

Monoisotopic mass of neutral peptide Mr(calc): 1557.6559
Fixed modifications: Carbamidomethyl (C)
Variable modifications:
S3 : Phospho (ST), with neutral losses 97.9769(shown in table), 0.0000
Ions Score: 60 **Expect:** 0.00016
Matches (Bold Red): 43/180 fragment ions using 100 most intense peaks

#	b	b^{++}	b^0	b^{0++}	Seq.	y	y^{++}	y*	y^{*++}	y^0	y^{0++}	#
1	72.0444	36.5258			A							14
2	159.0764	80.0418	141.0659	71.0366	S	1389.6492	695.3282	1372.6227	686.8150	1371.6387	686.3230	13
3	228.0979	114.5526	210.0873	105.5473	S	**1302.6172**	**651.8122**	1285.5906	**643.2990**	1284.6066	**642.8070**	12
4	**341.1819**	171.0946	**323.1714**	162.0893	L	**1233.5957**	**617.3015**	1216.5692	608.7882	**1215.5852**	608.2962	11
5	398.2034	199.6053	**380.1928**	190.6001	G	**1120.5117**	560.7595	1103.4851	552.2462	**1102.5011**	551.7542	10
6	**527.2460**	264.1266	**509.2354**	255.1214	E	**1063.4902**	532.2487	1046.4637	**523.7355**	**1045.4796**	**523.2435**	9
7	**640.3301**	320.6687	**622.3195**	311.6634	I	**934.4476**	467.7274	917.4211	459.2142	**916.4371**	458.7222	8
8	755.3570	378.1821	737.3464	**369.1769**	D	**821.3636**	411.1854	804.3370	402.6721	803.3530	402.1801	7
9	870.3839	435.6956	**852.3734**	426.6903	D	**706.3366**	353.6719	689.3101	345.1587	688.3260	344.6667	6
10	957.4160	479.2116	**939.4054**	470.2063	S	**591.3097**	**296.1585**	574.2831	287.6452	573.2991	287.1532	5
11	1044.4480	522.7276	1026.4374	513.7224	S	**504.2776**	252.6425	487.2511	244.1292	486.2671	243.6372	4
12	1173.4906	587.2489	**1155.4800**	578.2437	E	**417.2456**	209.1264	400.2191	200.6132	399.2350	200.1212	3
13	**1286.5747**	643.7910	**1268.5641**	634.7857	L	**288.2030**	144.6051	271.1765	136.0919			2
14					R	175.1190	88.0631	158.0924	79.5498			1

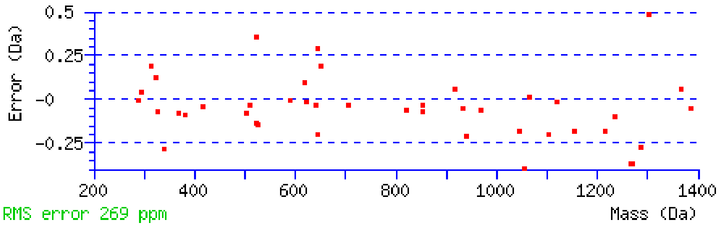

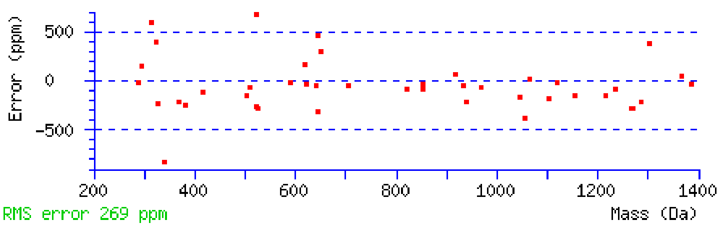

All matches to this query

Score	Mr(calc):	Delta	Sequence
59.8	1557.6559	0.0075	ASSLGEIDDSSELR
56.1	1557.6559	0.0075	ASSLGEIDDSSELR
6.4	1557.6559	0.0075	ASSLGEIDDSSELR
4.0	1556.6711	0.9923	IKEGLVSVMSYR
0.6	1557.6559	0.0075	ASSLGEIDDSSELR

Spectrum No: 167; Query: 2383; Rank: 1

Peptide View

MS/MS Fragmentation of **DTSATFQSVDGSPQAEQPPLESTSK**
Found in **IPI00364213**, Tax_Id=10116 Gene_Symbol=Zc3hc1_predicted similar to Nuclear-interacting partner of ALK

Match to Query 2383: 2686.159152 from(896.393660,3+)
Title: 091008RatKidney_NoSalt_28.2084.2084.3.dta
Data file K:\NewmanPaper\Piliang\3SubProteomes\Piliang3SP\mgf5ppm\ERLIC_3SubProteomes5ppm.mgf

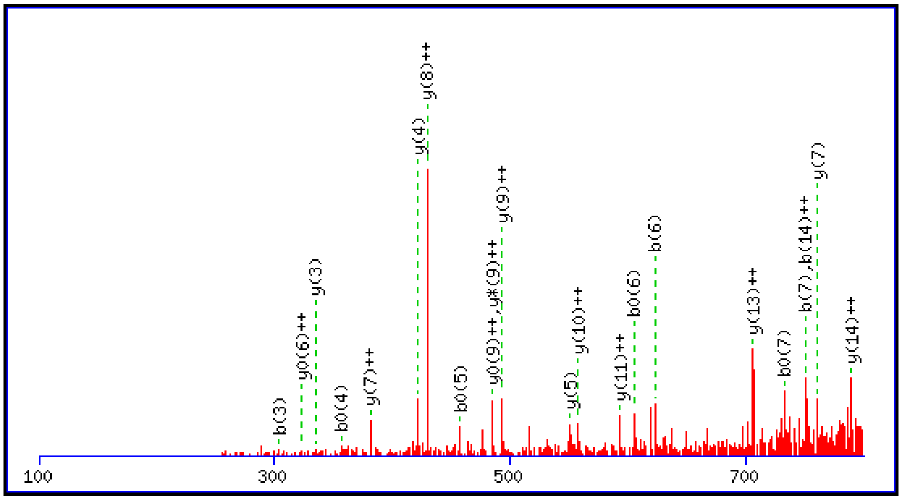

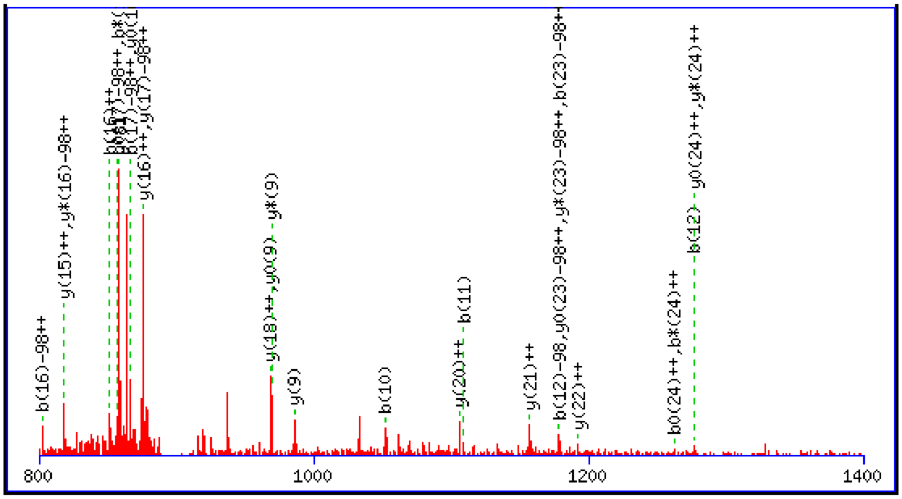

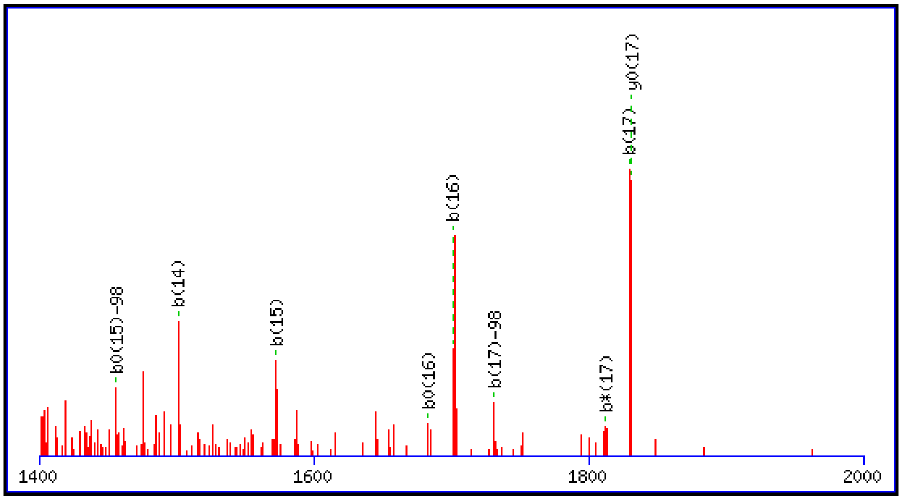

Monoisotopic mass of neutral peptide Mr(calc): 2686.1647
Fixed modifications: Carbamidomethyl (C)
Variable modifications:
S12 : Phospho (ST), with neutral losses 0.0000(shown in table), 97.9769
Ions Score: 59 **Expect:** 0.00041
Matches (Bold Red): 60/418 fragment ions using 90 most intense peaks

#	b	b^{++}	b*	b^{*++}	b^0	b^{0++}	Seq.	y	y^{++}	y*	y^{*++}	y^0	y^{0++}	#
1	116.0342	58.5207			98.0237	49.5155	D							25
2	217.0819	109.0446			199.0713	100.0393	T	2572.1450	1286.5762	2555.1185	**1278.0629**	2554.1345	**1277.5709**	24
3	**304.1139**	152.5606			286.1034	143.5553	S	2471.0974	1236.0523	2454.0708	1227.5390	2453.0868	1227.0470	23
4	375.1510	188.0792			**357.1405**	179.0739	A	2384.0653	**1192.5363**	2367.0388	1184.0230	2366.0548	1183.5310	22
5	476.1987	238.6030			**458.1882**	229.5977	T	2313.0282	**1157.0177**	2296.0017	1148.5045	2295.0177	1148.0125	21
6	**623.2671**	312.1372			**605.2566**	303.1319	F	2211.9805	**1106.4939**	2194.9540	1097.9806	2193.9700	1097.4886	20
7	**751.3257**	376.1665	734.2992	367.6532	**733.3151**	367.1612	Q	2064.9121	1032.9597	2047.8856	1024.4464	2046.9016	1023.9544	19
8	838.3577	419.6825	821.3312	411.1692	820.3472	410.6772	S	1936.8536	**968.9304**	1919.8270	960.4171	1918.8430	959.9251	18
9	937.4262	469.2167	920.3996	460.7034	919.4156	460.2114	V	1849.8215	925.4144	1832.7950	916.9011	**1831.8110**	916.4091	17
10	**1052.4531**	526.7302	1035.4265	518.2169	1034.4425	517.7249	D	1750.7531	**875.8802**	1733.7266	867.3669	1732.7425	**866.8749**	16
11	**1109.4746**	555.2409	1092.4480	546.7276	1091.4640	546.2356	G	1635.7262	**818.3667**	1618.6996	809.8534	1617.7156	809.3614	15
12	**1276.4729**	638.7401	1259.4464	630.2268	1258.4623	629.7348	S	1578.7047	**789.8560**	1561.6782	781.3427	1560.6941	780.8507	14
13	1373.5257	687.2665	1356.4991	678.7532	1355.5151	678.2612	P	1411.7064	**706.3568**	1394.6798	697.8435	1393.6958	697.3515	13
14	**1501.5843**	**751.2958**	1484.5577	742.7825	1483.5737	742.2905	Q	1314.6536	657.8304	1297.6270	649.3172	1296.6430	648.8251	12
15	**1572.6214**	786.8143	1555.5948	778.3010	1554.6108	777.8090	A	1186.5950	**593.8011**	1169.5685	585.2879	1168.5844	584.7959	11
16	**1701.6640**	**851.3356**	1684.6374	842.8223	**1683.6534**	842.3303	E	1115.5579	**558.2826**	1098.5313	549.7693	1097.5473	549.2773	10
17	**1829.7225**	915.3649	**1812.6960**	906.8516	1811.7120	906.3596	Q	**986.5153**	**493.7613**	**969.4888**	**485.2480**	**968.5047**	**484.7560**	9
18	1926.7753	963.8913	1909.7488	955.3780	1908.7647	954.8860	P	**858.4567**	**429.7320**	841.4302	421.2187	840.4462	420.7267	8

**19**	2023.8281	1012.4177	2006.8015	1003.9044	2005.8175	1003.4124	**P**	**761.4040**	**381.2056**	744.3774	372.6923	743.3934	372.2003	**7**
**20**	2136.9121	1068.9597	2119.8856	1060.4464	2118.9016	1059.9544	**L**	664.3512	332.6792	647.3246	324.1660	646.3406	**323.6740**	**6**
**21**	2265.9547	1133.4810	2248.9282	1124.9677	2247.9442	1124.4757	**E**	**551.2671**	276.1372	534.2406	267.6239	533.2566	267.1319	**5**
**22**	2352.9868	1176.9970	2335.9602	1168.4837	2334.9762	1167.9917	**S**	**422.2245**	211.6159	405.1980	203.1026	404.2140	202.6106	**4**
**23**	2454.0344	1227.5209	2437.0079	1219.0076	2436.0239	1218.5156	**T**	**335.1925**	168.0999	318.1660	159.5866	317.1819	159.0946	**3**
**24**	2541.0665	1271.0369	2524.0399	**1262.5236**	2523.0559	**1262.0316**	**S**	234.1448	117.5761	217.1183	109.0628	216.1343	108.5708	**2**
**25**							**K**	147.1128	74.0600	130.0863	65.5468			**1**

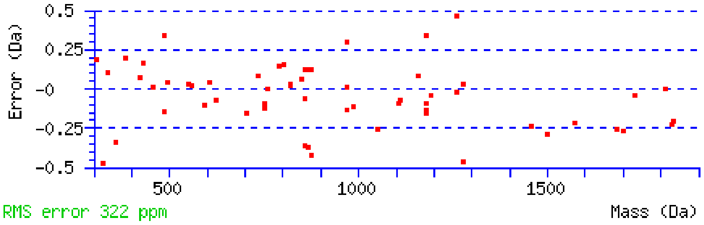

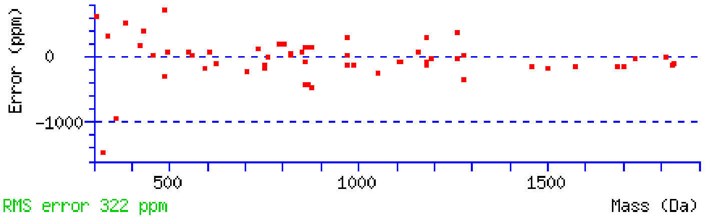

All matches to this query

Score	Mr(calc):	Delta	Sequence
59.3	2686.1647	-0.0056	DTSATFQSVDGSPQAEQPPLESTSK
35.5	2686.1647	-0.0056	DTSATFQSVDGSPQAEQPPLESTSK
28.4	2686.1647	-0.0056	DTSATFQSVDGSPQAEQPPLESTSK
27.1	2686.1647	-0.0056	DTSATFQSVDGSPQAEQPPLESTSK
27.1	2686.1647	-0.0056	DTSATFQSVDGSPQAEQPPLESTSK
13.8	2686.1647	-0.0056	DTSATFQSVDGSPQAEQPPLESTSK
13.8	2686.1647	-0.0056	DTSATFQSVDGSPQAEQPPLESTSK
13.8	2686.1647	-0.0056	DTSATFQSVDGSPQAEQPPLESTSK
2.5	2684.1708	1.9883	DIFKFEQGMFLKNMSTIIAEY
2.5	2684.1708	1.9883	DIFKFEQGMFLKNMSTIIAEY

Spectrum No: 168; Query: 474; Rank: 1

Peptide View

MS/MS Fragmentation of **GVQVETISSGDGR**
Found in **IPI00231434**, Tax_Id=10116 Gene_Symbol=Fkbp1a FK506-binding protein 1A

Match to Query 474: 1383.606388 from(692.810470,2+)
Title: 100101RatKid_NS_deglyco_21.1256.1256.2.dta
Data file K:\NewmanPaper\Piliang\3SubProteomes\Piliang3SP\mgf5ppm\ERLIC_3SubProteomes5ppm.mgf

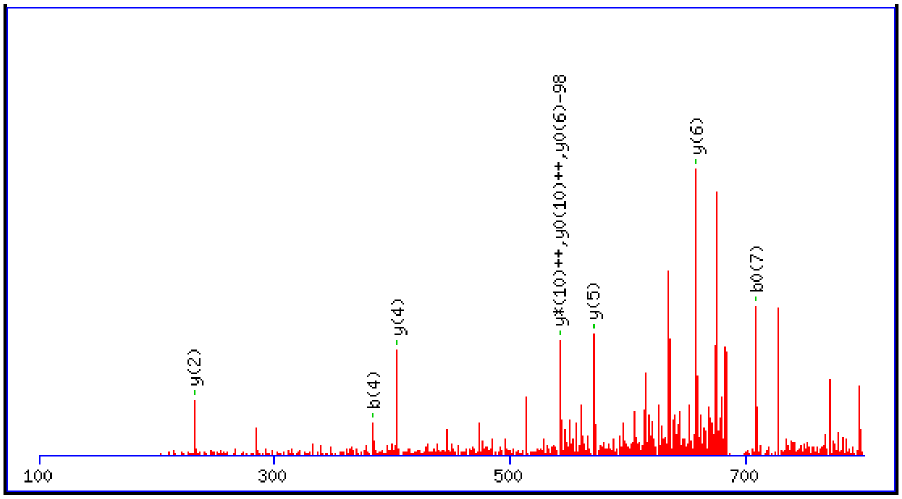

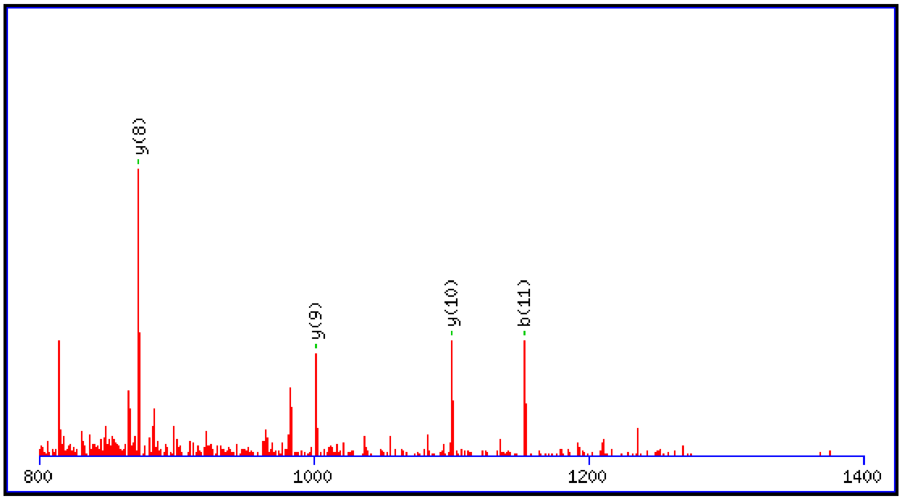

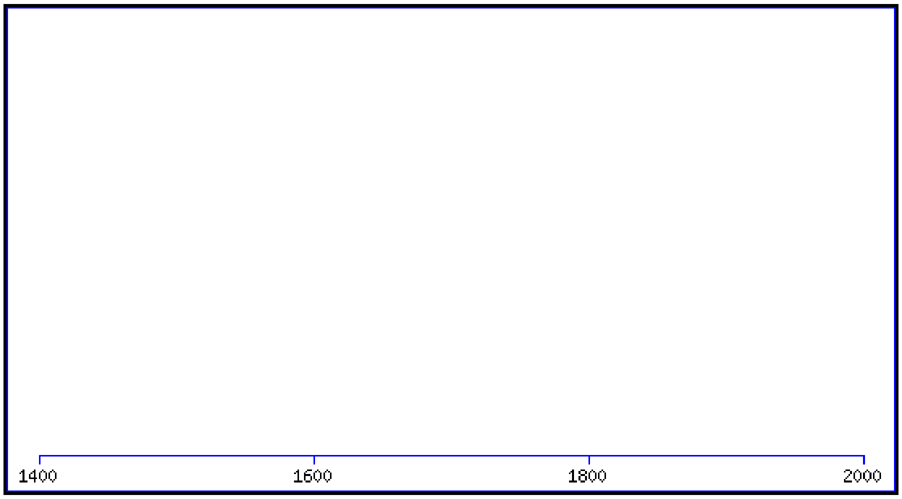

Monoisotopic mass of neutral peptide Mr(calc): 1383.6031
Fixed modifications: Carbamidomethyl (C)
Variable modifications:
S9 : Phospho (ST), with neutral losses 0.0000(shown in table), 97.9769
Ions Score: 59 **Expect:** 0.00015
Matches (Bold Red): 13/200 fragment ions using 23 most intense peaks

#	b	b++	b*	b*++	b0	b0++	Seq.	y	y++	y*	y*++	y0	y0++	#
1	58.0287	29.5180					G							13
2	157.0972	79.0522					V	1327.5889	664.2981	1310.5624	655.7848	1309.5784	655.2928	12
3	285.1557	143.0815	268.1292	134.5682			Q	1228.5205	614.7639	1211.4940	606.2506	1210.5100	605.7586	11
4	**384.2241**	192.6157	367.1976	184.1024			V	**1100.4620**	550.7346	1083.4354	**542.2213**	1082.4514	**541.7293**	10
5	513.2667	257.1370	496.2402	248.6237	495.2562	248.1317	E	**1001.3935**	501.2004	984.3670	492.6871	983.3830	492.1951	9

6	614.3144	307.6608	597.2879	299.1476	596.3039	298.6556	T	**872.3509**	436.6791	855.3244	428.1658	854.3404	427.6738	8
7	727.3985	364.2029	710.3719	355.6896	**709.3879**	355.1976	I	771.3033	386.1553	754.2767	377.6420	753.2927	377.1500	7
8	814.4305	407.7189	797.4040	399.2056	796.4199	398.7136	S	**658.2192**	329.6132	641.1927	321.1000	640.2086	320.6080	6
9	981.4289	491.2181	964.4023	482.7048	963.4183	482.2128	S	**571.1872**	286.0972	554.1606	277.5840	553.1766	277.0919	5
10	1038.4503	519.7288	1021.4238	511.2155	1020.4398	510.7235	G	**404.1888**	202.5980	387.1623	194.0848	386.1783	193.5928	4
11	**1153.4773**	577.2423	1136.4507	568.7290	1135.4667	568.2370	D	347.1674	174.0873	330.1408	165.5740	329.1568	165.0820	3
12	1210.4987	605.7530	1193.4722	597.2397	1192.4882	596.7477	G	**232.1404**	116.5738	215.1139	108.0606			2
13							R	175.1190	88.0631	158.0924	79.5498			1

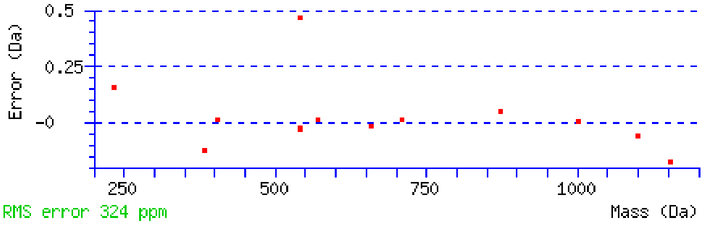

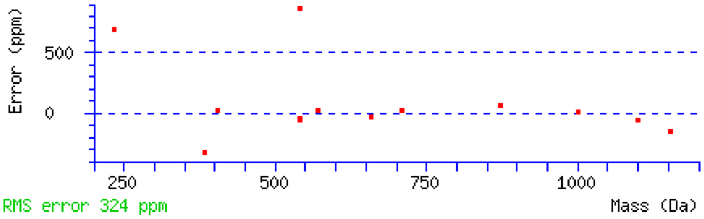

All matches to this query

Score	Mr(calc):	Delta	Sequence
59.3	1383.6031	0.0032	GVQVETISSGDGR
43.5	1383.6031	0.0032	GVQVETISSGDGR
26.3	1383.6031	0.0032	GVQVETISSGDGR
13.7	1382.6062	1.0001	AVNDSTSDFNTGR
3.9	1383.5965	0.0098	VLATVTKTVGR
3.0	1383.6105	-0.0041	LMAENSTTALPR
1.4	1383.6006	0.0058	WQSLPRCTEK
0.1	1383.6144	-0.0080	KLSGNGTDSXNGR

Spectrum No: 169; Query: 2681; Rank: 1

Peptide View

MS/MS Fragmentation of **AVEGPSPSPEPEETPAESSATDPTAGTGEASGER**
Found in **IPI00366074**, Tax_Id=10116 Gene_Symbol=Rreb1_predicted 188 kDa protein

Match to Query 2681: 3377.426472 from(1126.816100,3+)
Title: 100101RatKid_NS_deglyco_24.1735.1735.3.dta
Data file K:\NewmanPaper\Piliang\3SubProteomes\Piliang3SP\mgf5ppm\ERLIC_3SubProteomes5ppm.mgf

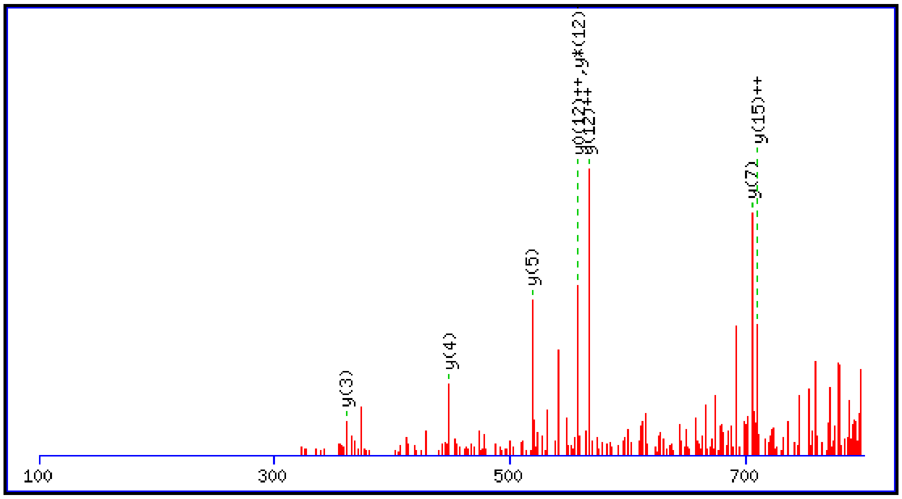

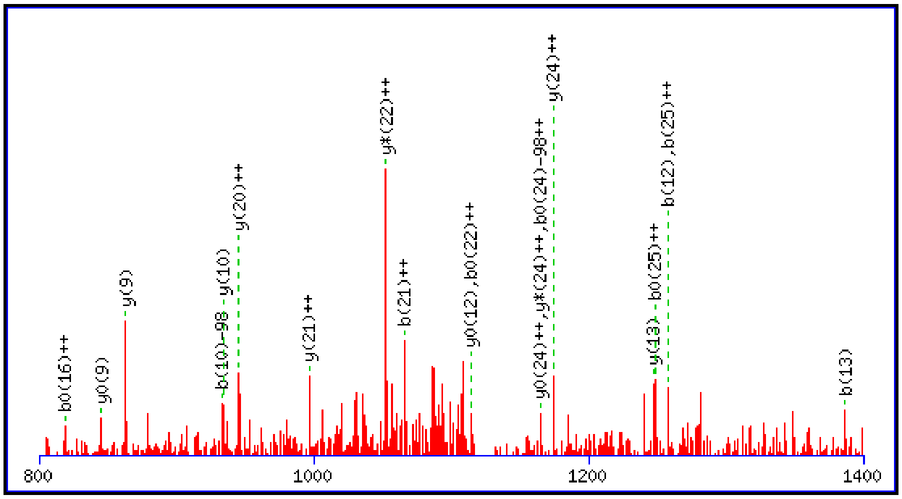

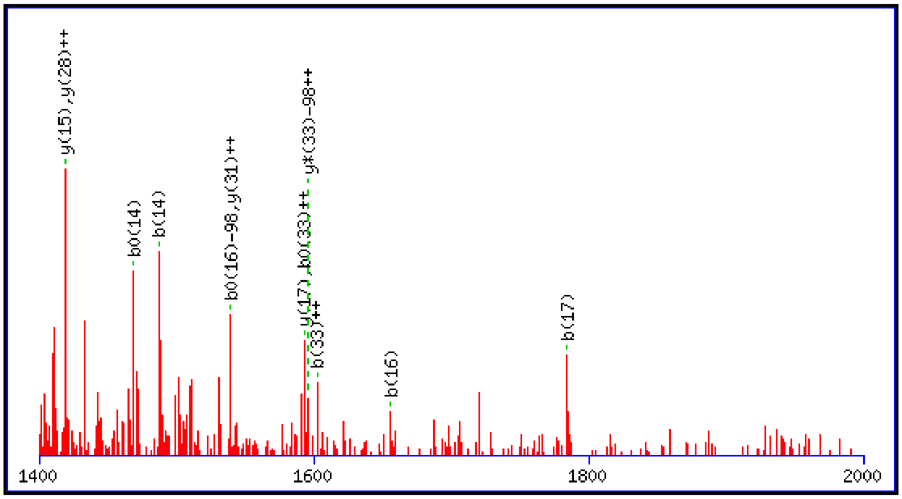

Monoisotopic mass of neutral peptide Mr(calc): 3376.4104
Fixed modifications: Carbamidomethyl (C)
Variable modifications:
S8 : Phospho (ST), with neutral losses 0.0000(shown in table), 97.9769
Ions Score: 59 **Expect:** 0.00052
Matches (Bold Red): 40/470 fragment ions using 53 most intense peaks

#	b	b^{++}	b^0	b^{0++}	Seq.	y	y^{++}	y*	y^{*++}	y^0	y^{0++}	#
1	72.0444	36.5258			A							34
2	171.1128	86.0600			V	3306.3805	1653.6939	3289.3540	1645.1806	3288.3700	1644.6886	33
3	300.1554	150.5813	282.1448	141.5761	E	3207.3121	1604.1597	3190.2856	1595.6464	3189.3016	1595.1544	32
4	357.1769	179.0921	339.1663	170.0868	G	3078.2695	**1539.6384**	3061.2430	1531.1251	3060.2590	1530.6331	31
5	454.2296	227.6185	436.2191	218.6132	P	3021.2481	1511.1277	3004.2215	1502.6144	3003.2375	1502.1224	30
6	541.2617	271.1345	523.2511	262.1292	S	2924.1953	1462.6013	2907.1688	1454.0880	2906.1847	1453.5960	29
7	638.3144	319.6608	620.3039	310.6556	P	2837.1633	**1419.0853**	2820.1367	1410.5720	2819.1527	1410.0800	28
8	805.3128	403.1600	787.3022	394.1547	S	2740.1105	1370.5589	2723.0840	1362.0456	2722.0999	1361.5536	27
9	902.3655	451.6864	884.3550	442.6811	P	2573.1122	1287.0597	2556.0856	1278.5464	2555.1016	1278.0544	26
10	1031.4081	516.2077	1013.3976	507.2024	E	2476.0594	1238.5333	2459.0328	1230.0201	2458.0488	1229.5281	25
11	1128.4609	564.7341	1110.4503	555.7288	P	2347.0168	**1174.0120**	2329.9903	**1165.4988**	2329.0062	**1165.0068**	24
12	**1257.5035**	629.2554	1239.4929	620.2501	E	2249.9640	1125.4857	2232.9375	1116.9724	2231.9535	1116.4804	23
13	**1386.5461**	693.7767	1368.5355	684.7714	E	2120.9214	1060.9644	2103.8949	**1052.4511**	2102.9109	1051.9591	22
14	**1487.5938**	744.3005	**1469.5832**	735.2952	T	1991.8789	**996.4431**	1974.8523	987.9298	1973.8683	987.4378	21
15	1584.6465	792.8269	1566.6360	783.8216	P	1890.8312	**945.9192**	1873.8046	937.4059	1872.8206	936.9139	20
16	**1655.6836**	828.3455	1637.6731	**819.3402**	A	1793.7784	897.3928	1776.7519	888.8796	1775.7678	888.3876	19
17	**1784.7262**	892.8668	1766.7157	883.8615	E	1722.7413	861.8743	1705.7147	853.3610	1704.7307	852.8690	18
18	1871.7583	936.3828	1853.7477	927.3775	S	**1593.6987**	797.3530	1576.6722	788.8397	1575.6881	788.3477	17

**19**	1958.7903	979.8988	1940.7797	970.8935	**S**	1506.6667	753.8370	1489.6401	745.3237	1488.6561	744.8317	**16**
**20**	2029.8274	1015.4173	2011.8168	1006.4121	**A**	**1419.6346**	**710.3210**	1402.6081	701.8077	1401.6241	701.3157	**15**
**21**	2130.8751	**1065.9412**	2112.8645	1056.9359	**T**	1348.5975	674.8024	1331.5710	666.2891	1330.5870	665.7971	**14**
**22**	2245.9020	1123.4546	2227.8915	**1114.4494**	**D**	**1247.5499**	624.2786	1230.5233	615.7653	1229.5393	615.2733	**13**
**23**	2342.9548	1171.9810	2324.9442	1162.9757	**P**	1132.5229	**566.7651**	1115.4964	**558.2518**	**1114.5123**	**557.7598**	**12**
**24**	2444.0025	1222.5049	2425.9919	1213.4996	**T**	1035.4701	518.2387	1018.4436	509.7254	1017.4596	509.2334	**11**
**25**	2515.0396	**1258.0234**	2497.0290	**1249.0181**	**A**	**934.4225**	467.7149	917.3959	459.2016	916.4119	458.7096	**10**
**26**	2572.0610	1286.5342	2554.0505	1277.5289	**G**	**863.3854**	432.1963	846.3588	423.6830	**845.3748**	423.1910	**9**
**27**	2673.1087	1337.0580	2655.0982	1328.0527	**T**	806.3639	403.6856	789.3373	395.1723	788.3533	394.6803	**8**
**28**	2730.1302	1365.5687	2712.1196	1356.5634	**G**	**705.3162**	353.1617	688.2897	344.6485	687.3056	344.1565	**7**
**29**	2859.1728	1430.0900	2841.1622	1421.0847	**E**	648.2947	324.6510	631.2682	316.1377	630.2842	315.6457	**6**
**30**	2930.2099	1465.6086	2912.1993	1456.6033	**A**	**519.2522**	260.1297	502.2256	251.6164	501.2416	251.1244	**5**
**31**	3017.2419	1509.1246	2999.2314	1500.1193	**S**	**448.2150**	224.6112	431.1885	216.0979	430.2045	215.6059	**4**
**32**	3074.2634	1537.6353	3056.2528	1528.6300	**G**	**361.1830**	181.0951	344.1565	172.5819	343.1724	172.0899	**3**
**33**	3203.3060	**1602.1566**	3185.2954	**1593.1513**	**E**	304.1615	152.5844	287.1350	144.0711	286.1510	143.5791	**2**
**34**					**R**	175.1190	88.0631	158.0924	79.5498			**1**

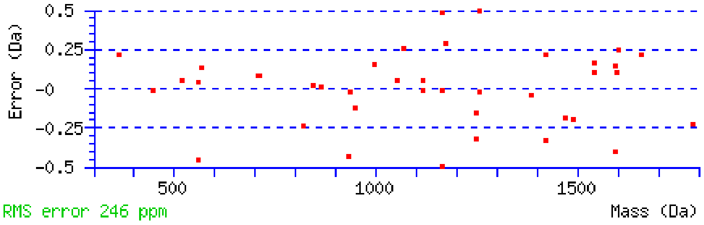

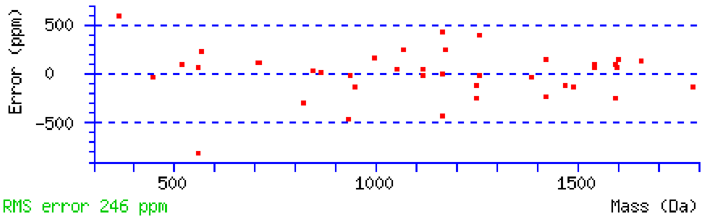

All matches to this query

Score	Mr(calc):	Delta	Sequence
59.1	3376.4104	1.0161	AVEGPSPSPEPEETPAESSATDPTAGTGEASGER
52.8	3376.4104	1.0161	AVEGPSPSPEPEETPAESSATDPTAGTGEASGER
43.5	3376.4104	1.0161	AVEGPSPSPEPEETPAESSATDPTAGTGEASGER
26.7	3376.4104	1.0161	AVEGPSPSPEPEETPAESSATDPTAGTGEASGER
22.5	3376.4104	1.0161	AVEGPSPSPEPEETPAESSATDPTAGTGEASGER
14.9	3376.4104	1.0161	AVEGPSPSPEPEETPAESSATDPTAGTGEASGER
7.5	3376.4104	1.0161	AVEGPSPSPEPEETPAESSATDPTAGTGEASGER
4.2	3376.4104	1.0161	AVEGPSPSPEPEETPAESSATDPTAGTGEASGER
1.7	3375.3937	2.0328	TAMVAALSPADINYDETLSTLRYADRAK
1.5	3375.4463	1.9802	SSDGLQNPPSLPGGPDXPQLNGDPGVTGPPGAK

Spectrum No: 170; Query: 2697; Rank: 1

Peptide View

MS/MS Fragmentation of **VSEEAESQQWDTSKGDQVSQNGLPAEQGSPR**
Found in **IPI00373419**, Tax_Id=10116 Gene_Symbol=Spnb2 Non-erythrocyte beta-spectrin

Match to Query 2697: 3423.496302 from(1142.172710,3+)
Title: 100101RatKid_NS_deglyco_20.2123.2123.3.dta
Data file K:\NewmanPaper\Piliang\3SubProteomes\Piliang3SP\mgf5ppm\ERLIC_3SubProteomes5ppm.mgf

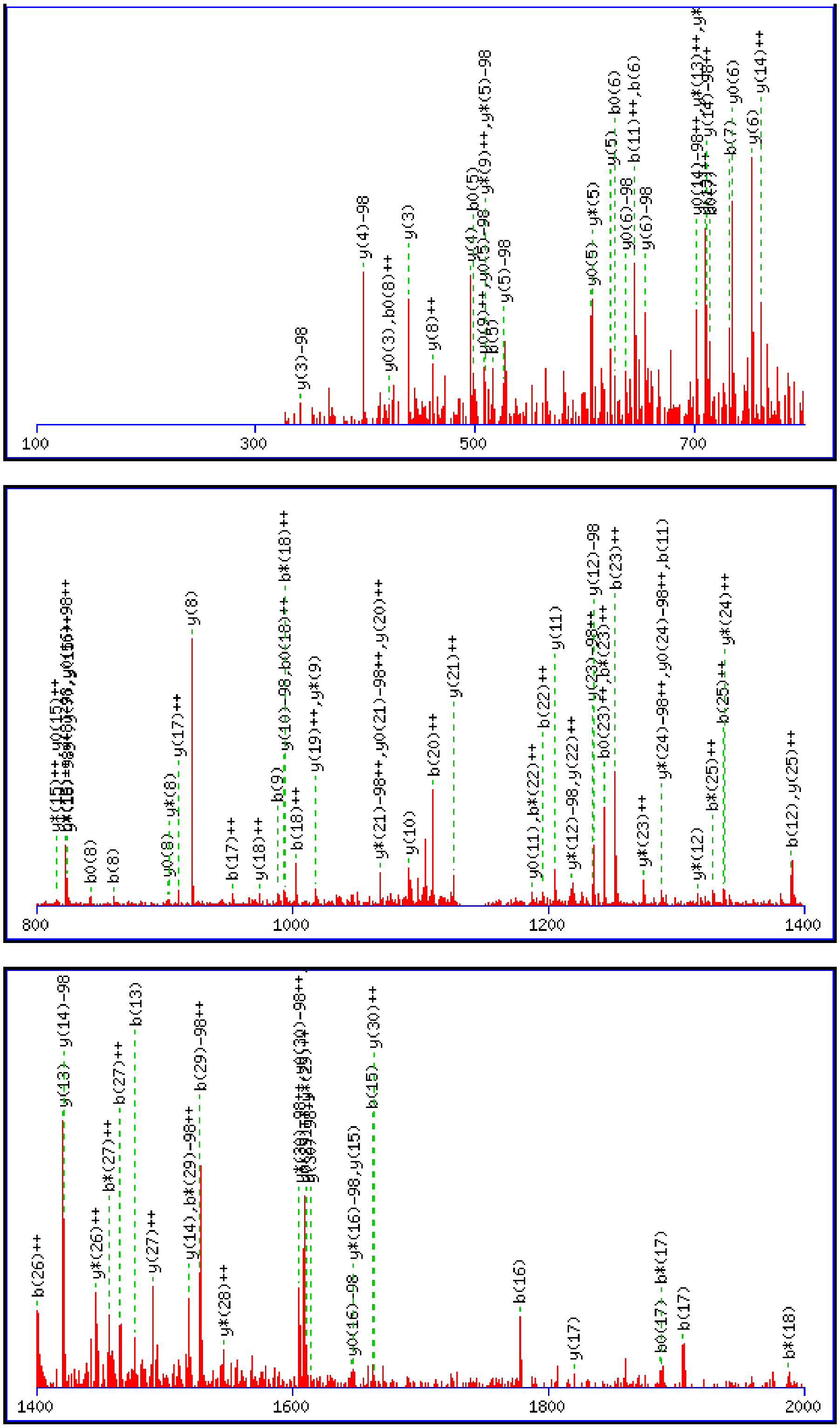

Monoisotopic mass of neutral peptide Mr(calc): 3423.4852
Fixed modifications: Carbamidomethyl (C)
Variable modifications:
S29 : Phospho (ST), with neutral losses 0.0000(shown in table), 97.9769
Ions Score: 59 **Expect:** 0.00059
Matches (Bold Red): 111/520 fragment ions using 158 most intense peaks

#	b	b^{++}	b*	b^{*++}	b^0	b^{0++}	Seq.	y	y^{++}	y*	y^{*++}	y^0	y^{0++}	#
1	100.0757	50.5415					V							31
2	187.1077	94.0575			169.0972	85.0522	S	3325.4241	**1663.2157**	3308.3975	1654.7024	3307.4135	1654.2104	30
3	316.1503	158.5788			298.1397	149.5735	E	3238.3920	1619.6997	3221.3655	**1611.1864**	3220.3815	**1610.6944**	29
4	445.1929	223.1001			427.1823	214.0948	E	3109.3495	1555.1784	3092.3229	**1546.6651**	3091.3389	1546.1731	28
5	**516.2300**	258.6186			**498.2195**	249.6134	A	2980.3069	**1490.6571**	2963.2803	1482.1438	2962.2963	1481.6518	27

6	**645.2726**	323.1399			**627.2620**	314.1347	E	2909.2697	1455.1385	2892.2432	**1446.6252**	2891.2592	1446.1332	26
7	**732.3046**	366.6560			**714.2941**	357.6507	S	2780.2272	**1390.6172**	2763.2006	1382.1039	2762.2166	1381.6119	25
8	**860.3632**	430.6852	843.3367	422.1720	**842.3527**	**421.6800**	Q	2693.1951	1347.1012	2676.1686	**1338.5879**	2675.1846	1338.0959	24
9	**988.4218**	494.7145	971.3952	486.2013	970.4112	485.7093	Q	2565.1365	1283.0719	2548.1100	**1274.5586**	2547.1260	1274.0666	23
10	1174.5011	587.7542	1157.4746	579.2409	1156.4905	578.7489	W	2437.0780	**1219.0426**	2420.0514	1210.5293	2419.0674	1210.0373	22
11	**1289.5281**	**645.2677**	1272.5015	636.7544	1271.5175	636.2624	D	2250.9987	**1126.0030**	2233.9721	1117.4897	2232.9881	1116.9977	21
12	**1390.5757**	695.7915	1373.5492	687.2782	1372.5652	686.7862	T	2135.9717	**1068.4895**	2118.9452	1059.9762	2117.9611	1059.4842	20
13	**1477.6078**	739.3075	1460.5812	730.7942	1459.5972	730.3022	S	2034.9240	**1017.9657**	2017.8975	1009.4524	2016.9135	1008.9604	19
14	**1605.7027**	803.3550	1588.6762	794.8417	1587.6922	794.3497	K	1947.8920	**974.4496**	1930.8655	965.9364	1929.8814	965.4444	18
15	**1662.7242**	831.8657	1645.6976	**823.3525**	1644.7136	822.8604	G	**1819.7970**	**910.4022**	1802.7705	901.8889	1801.7865	901.3969	17
16	**1777.7511**	889.3792	1760.7246	880.8659	1759.7406	880.3739	D	1762.7756	881.8914	1745.7490	873.3782	1744.7650	872.8861	16
17	**1905.8097**	**953.4085**	**1888.7832**	944.8952	**1887.7991**	944.4032	Q	**1647.7486**	**824.3780**	1630.7221	**815.8647**	1629.7381	**815.3727**	15
18	2004.8781	**1002.9427**	**1987.8516**	**994.4294**	1986.8676	**993.9374**	V	**1519.6901**	**760.3487**	1502.6635	751.8354	1501.6795	751.3434	14
19	2091.9101	1046.4587	2074.8836	1037.9454	2073.8996	1037.4534	S	**1420.6216**	**710.8145**	1403.5951	**702.3012**	1402.6111	701.8092	13
20	2219.9687	**1110.4880**	2202.9422	1101.9747	2201.9582	1101.4827	Q	1333.5896	667.2984	**1316.5631**	658.7852	1315.5790	658.2932	12
21	2334.0117	1167.5095	2316.9851	1158.9962	2316.0011	1158.5042	N	**1205.5310**	603.2692	1188.5045	594.7559	**1187.5205**	594.2639	11
22	2391.0331	**1196.0202**	2374.0066	**1187.5069**	2373.0226	1187.0149	G	**1091.4881**	546.2477	1074.4616	537.7344	1073.4775	537.2424	10
23	2504.1172	**1252.5622**	2487.0906	**1244.0490**	2486.1066	**1243.5569**	L	1034.4666	517.7370	**1017.4401**	**509.2237**	1016.4561	**508.7317**	9
24	2601.1699	1301.0886	2584.1434	1292.5753	2583.1594	1292.0833	P	**921.3826**	**461.1949**	**904.3560**	452.6817	**903.3720**	452.1896	8
25	2672.2071	**1336.6072**	2655.1805	**1328.0939**	2654.1965	1327.6019	A	**824.3298**	412.6685	807.3033	404.1553	806.3193	403.6633	7
26	2801.2497	**1401.1285**	2784.2231	1392.6152	2783.2391	1392.1232	E	**753.2927**	377.1500	736.2662	368.6367	**735.2821**	368.1447	6
27	2929.3082	**1465.1578**	2912.2817	**1456.6445**	2911.2977	1456.1525	Q	**624.2501**	312.6287	**607.2236**	304.1154	**606.2395**	303.6234	5
28	2986.3297	1493.6685	2969.3031	1485.1552	2968.3191	1484.6632	G	**496.1915**	248.5994	479.1650	240.0861	478.1810	239.5941	4
29	3153.3280	1577.1677	3136.3015	1568.6544	3135.3175	1568.1624	S	**439.1701**	220.0887	422.1435	211.5754	**421.1595**	211.0834	3
30	3250.3808	1625.6940	3233.3543	1617.1808	3232.3702	1616.6888	P	272.1717	136.5895	255.1452	128.0762			2
31							R	175.1190	88.0631	158.0924	79.5498			1

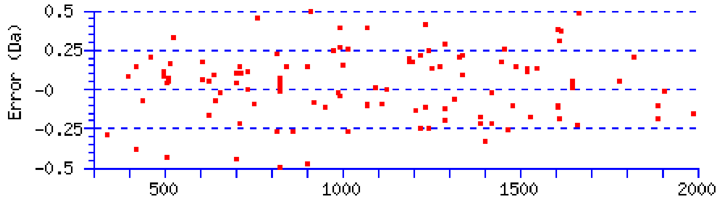

RMS error 258 ppm

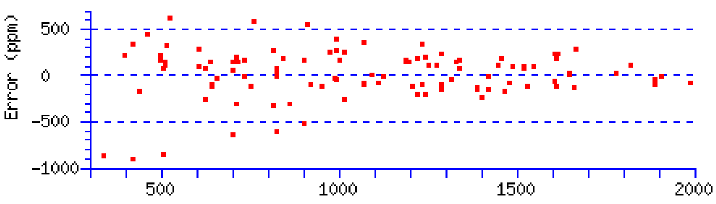

RMS error 258 ppm

All matches to this query

Score	Mr(calc):	Delta	Sequence
59.0	3423.4852	0.0111	VSEEAESQQWDTSKGDQVSQNGLPAEQGSPR
31.6	3423.4852	0.0111	VSEEAESQQWDTSKGDQVSQNGLPAEQGSPR
6.1	3423.4852	0.0111	VSEEAESQQWDTSKGDQVSQNGLPAEQGSPR
4.2	3423.4852	0.0111	VSEEAESQQWDTSKGDQVSQNGLPAEQGSPR
2.1	3423.5000	-0.0037	TLEAEFNNPCPLTPEPGEGPRKLEGCTNSK
2.0	3422.4974	0.9989	EPPDGEPACPGALEDTGDPVKEPAVSPGGPSASR
2.0	3422.4974	0.9989	EPPDGEPACPGALEDTGDPVKEPAVSPGGPSASR
1.8	3421.4801	2.0162	EMGNIRNQAVSMANLGHLTLKSCMQQSAR
1.8	3421.4801	2.0162	EMGNIRNQAVSMANLGHLTLKSCMQQSAR
1.8	3421.4801	2.0162	EMGNIRNQAVSMANLGHLTLKSCMQQSAR

Spectrum No: 171; Query: 2697; Rank: 1

Peptide View

MS/MS Fragmentation of **VSEEAESQQWDTSKGDQVSQNGLPAEQGSPR**
Found in **IPI00373419**, Tax_Id=10116 Gene_Symbol=Spnb2 Non-erythrocyte beta-spectrin

Match to Query 2697: 3423.496302 from(1142.172710,3+)
Title: 100101RatKid_NS_deglyco_20.2123.2123.3.dta
Data file K:\NewmanPaper\Piliang\3SubProteomes\Piliang3SP\mgf5ppm\ERLIC_3SubProteomes5ppm.mgf

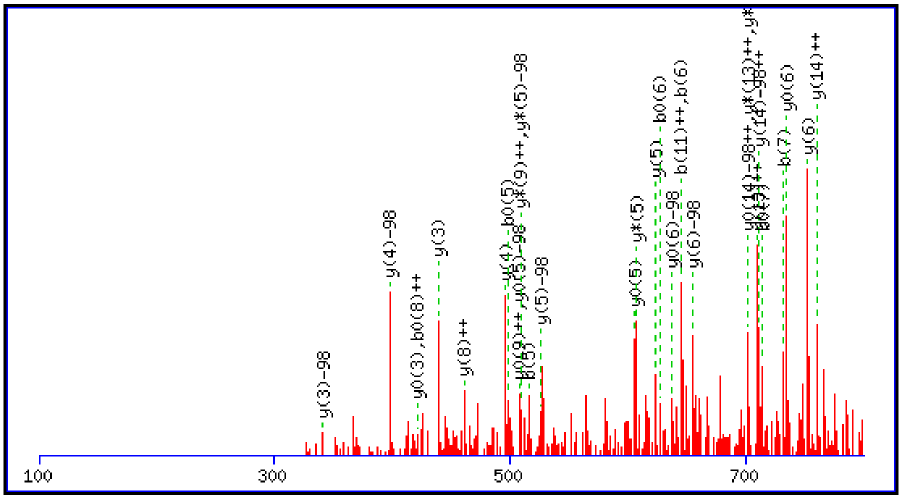

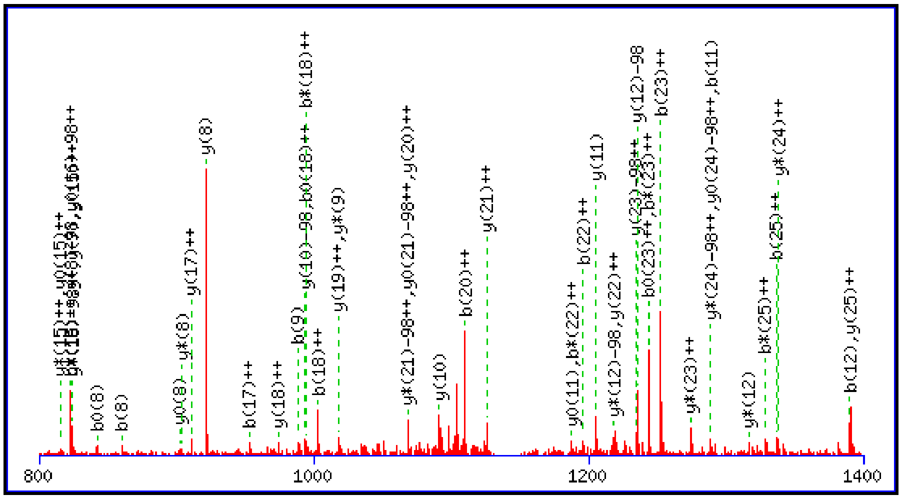

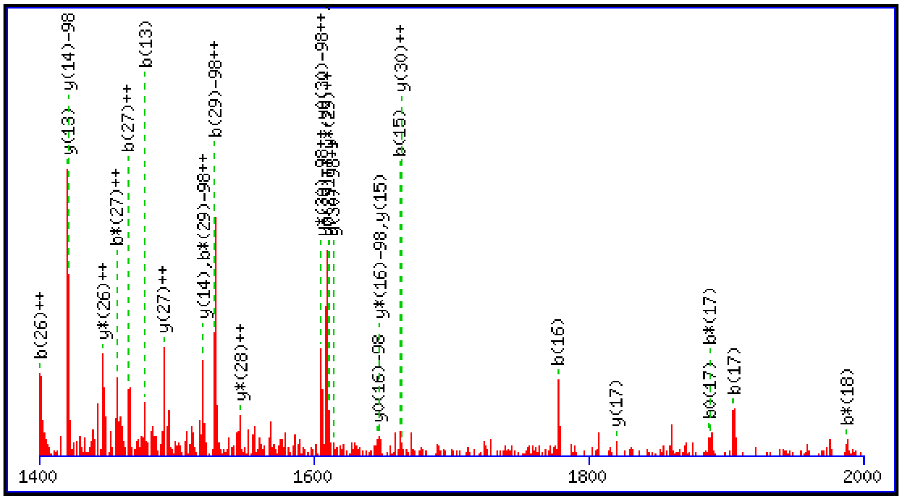

Monoisotopic mass of neutral peptide Mr(calc): 3423.4852
Fixed modifications: Carbamidomethyl (C)
Variable modifications:
S29 : Phospho (ST), with neutral losses 0.0000(shown in table), 97.9769
Ions Score: 59 **Expect:** 0.00059

Matches (Bold Red): 111/520 fragment ions using 158 most intense peaks

#	b	b^{++}	b*	b^{*++}	b^0	b^{0++}	Seq.	y	y^{++}	y*	y^{*++}	y^0	y^{0++}	#
1	100.0757	50.5415					V							31
2	187.1077	94.0575			169.0972	85.0522	S	3325.4241	**1663.2157**	3308.3975	1654.7024	3307.4135	1654.2104	30
3	316.1503	158.5788			298.1397	149.5735	E	3238.3920	1619.6997	3221.3655	**1611.1864**	3220.3815	**1610.6944**	29
4	445.1929	223.1001			427.1823	214.0948	E	3109.3495	1555.1784	3092.3229	**1546.6651**	3091.3389	1546.1731	28
5	**516.2300**	258.6186			**498.2195**	249.6134	A	2980.3069	**1490.6571**	2963.2803	1482.1438	2962.2963	1481.6518	27
6	**645.2726**	323.1399			**627.2620**	314.1347	E	2909.2697	1455.1385	2892.2432	**1446.6252**	2891.2592	1446.1332	26
7	**732.3046**	366.6560			**714.2941**	357.6507	S	2780.2272	**1390.6172**	2763.2006	1382.1039	2762.2166	1381.6119	25
8	**860.3632**	430.6852	843.3367	422.1720	**842.3527**	**421.6800**	Q	2693.1951	1347.1012	2676.1686	**1338.5879**	2675.1846	1338.0959	24
9	**988.4218**	494.7145	971.3952	486.2013	970.4112	485.7093	Q	2565.1365	1283.0719	2548.1100	**1274.5586**	2547.1260	1274.0666	23
10	1174.5011	587.7542	1157.4746	579.2409	1156.4905	578.7489	W	2437.0780	**1219.0426**	2420.0514	1210.5293	2419.0674	1210.0373	22
11	**1289.5281**	**645.2677**	1272.5015	636.7544	1271.5175	636.2624	D	2250.9987	**1126.0030**	2233.9721	1117.4897	2232.9881	1116.9977	21
12	**1390.5757**	695.7915	1373.5492	687.2782	1372.5652	686.7862	T	2135.9717	**1068.4895**	2118.9452	1059.9762	2117.9611	1059.4842	20
13	**1477.6078**	739.3075	1460.5812	730.7942	1459.5972	730.3022	S	2034.9240	**1017.9657**	2017.8975	1009.4524	2016.9135	1008.9604	19
14	**1605.7027**	803.3550	1588.6762	794.8417	1587.6922	794.3497	K	1947.8920	**974.4496**	1930.8655	965.9364	1929.8814	965.4444	18
15	**1662.7242**	831.8657	1645.6976	**823.3525**	1644.7136	822.8604	G	**1819.7970**	**910.4022**	1802.7705	901.8889	1801.7865	901.3969	17
16	**1777.7511**	889.3792	1760.7246	880.8659	1759.7406	880.3739	D	1762.7756	881.8914	1745.7490	873.3782	1744.7650	872.8861	16
17	**1905.8097**	**953.4085**	**1888.7832**	944.8952	**1887.7991**	944.4032	Q	**1647.7486**	**824.3780**	1630.7221	**815.8647**	1629.7381	**815.3727**	15
18	2004.8781	**1002.9427**	**1987.8516**	**994.4294**	1986.8676	**993.9374**	V	**1519.6901**	**760.3487**	1502.6635	751.8354	1501.6795	751.3434	14
19	2091.9101	1046.4587	2074.8836	1037.9454	2073.8996	1037.4534	S	**1420.6216**	**710.8145**	1403.5951	**702.3012**	1402.6111	701.8092	13
20	2219.9687	**1110.4880**	2202.9422	1101.9747	2201.9582	1101.4827	Q	1333.5896	667.2984	**1316.5631**	658.7852	1315.5790	658.2932	12
21	2334.0117	1167.5095	2316.9851	1158.9962	2316.0011	1158.5042	N	**1205.5310**	603.2692	1188.5045	594.7559	**1187.5205**	594.2639	11
22	2391.0331	**1196.0202**	2374.0066	**1187.5069**	2373.0226	1187.0149	G	**1091.4881**	546.2477	1074.4616	537.7344	1073.4775	537.2424	10
23	2504.1172	**1252.5622**	2487.0906	**1244.0490**	2486.1066	**1243.5569**	L	1034.4666	517.7370	**1017.4401**	**509.2237**	1016.4561	**508.7317**	9
24	2601.1699	1301.0886	2584.1434	1292.5753	2583.1594	1292.0833	P	**921.3826**	**461.1949**	**904.3560**	452.6817	**903.3720**	452.1896	8
25	2672.2071	**1336.6072**	2655.1805	**1328.0939**	2654.1965	1327.6019	A	**824.3298**	412.6685	807.3033	404.1553	806.3193	403.6633	7
26	2801.2497	**1401.1285**	2784.2231	1392.6152	2783.2391	1392.1232	E	**753.2927**	377.1500	736.2662	368.6367	**735.2821**	368.1447	6
27	2929.3082	**1465.1578**	2912.2817	**1456.6445**	2911.2977	1456.1525	Q	**624.2501**	312.6287	**607.2236**	304.1154	**606.2395**	303.6234	5
28	2986.3297	1493.6685	2969.3031	1485.1552	2968.3191	1484.6632	G	**496.1915**	248.5994	479.1650	240.0861	478.1810	239.5941	4
29	3153.3280	1577.1677	3136.3015	1568.6544	3135.3175	1568.1624	S	**439.1701**	220.0887	422.1435	211.5754	**421.1595**	211.0834	3
30	3250.3808	1625.6940	3233.3543	1617.1808	3232.3702	1616.6888	P	272.1717	136.5895	255.1452	128.0762			2
31							R	175.1190	88.0631	158.0924	79.5498			1

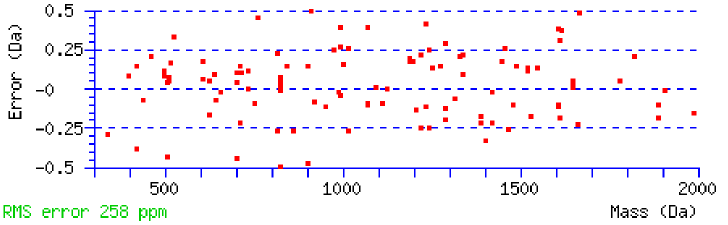

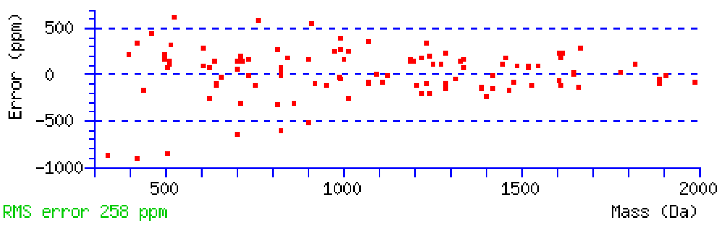

All matches to this query

Score	Mr(calc):	Delta	Sequence
59.0	3423.4852	0.0111	VSEEAESQQWDTSKGDQVSQNGLPAEQGSPR
31.6	3423.4852	0.0111	VSEEAESQQWDTSKGDQVSQNGLPAEQGSPR
6.1	3423.4852	0.0111	VSEEAESQQWDTSKGDQVSQNGLPAEQGSPR
4.2	3423.4852	0.0111	VSEEAESQQWDTSKGDQVSQNGLPAEQGSPR
2.1	3423.5000	-0.0037	TLEAEFNNPCPLTPEPGEGPRKLEGCTNSK
2.0	3422.4974	0.9989	EPPDGEPACPGALEDTGDPVKEPAVSPGGPSASR

2.0	3422.4974	0.9989	EPPDGEPACPGALEDTGDPVKEPAVSPGGPSASR
1.8	3421.4801	2.0162	EMGNIRNQAVSMANLGHLTLKSCMQQSAR
1.8	3421.4801	2.0162	EMGNIRNQAVSMANLGHLTLKSCMQQSAR
1.8	3421.4801	2.0162	EMGNIRNQAVSMANLGHLTLKSCMQQSAR

Spectrum No: 172; Query: 2708; Rank: 1

Peptide View

MS/MS Fragmentation of **AENDVDNELLDYEDDEVETAAGADGTEAPAKK**
Found in **IPI00215291**, Tax_Id=10116 Gene_Symbol=Bat1a;Atp6v1g2 Spliceosome RNA helicase Bat1

Match to Query 2708: 3473.447136 from(869.369060,4+)
Title: 100101RatKid_NS_deglyco_21.4147.4147.4.dta
Data file K:\NewmanPaper\Piliang\3SubProteomes\Piliang3SP\mgf5ppm\ERLIC_3SubProteomes5ppm.mgf

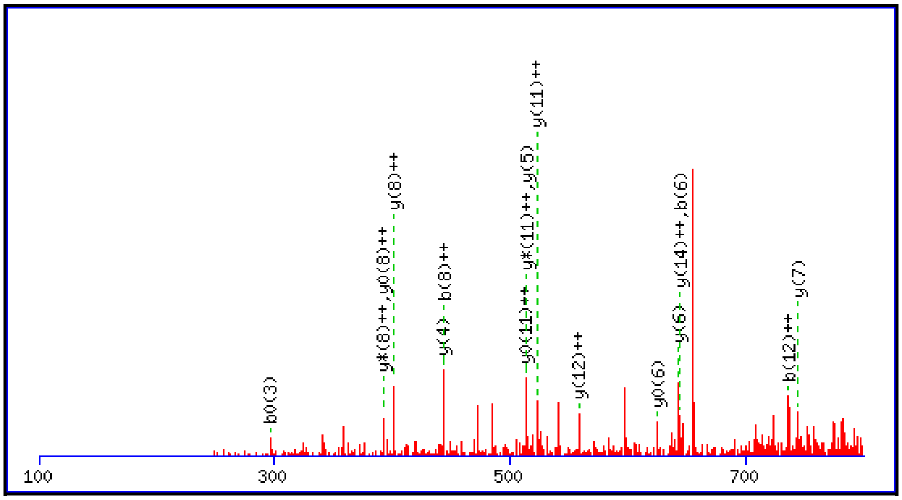

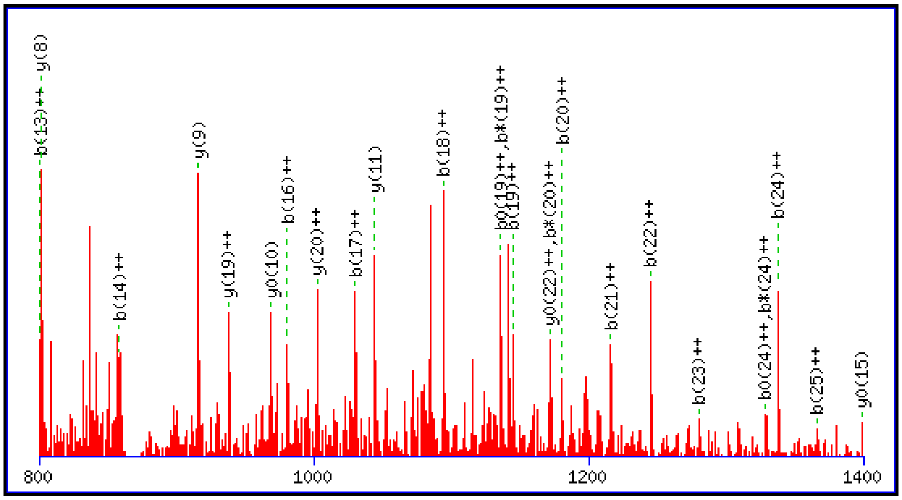

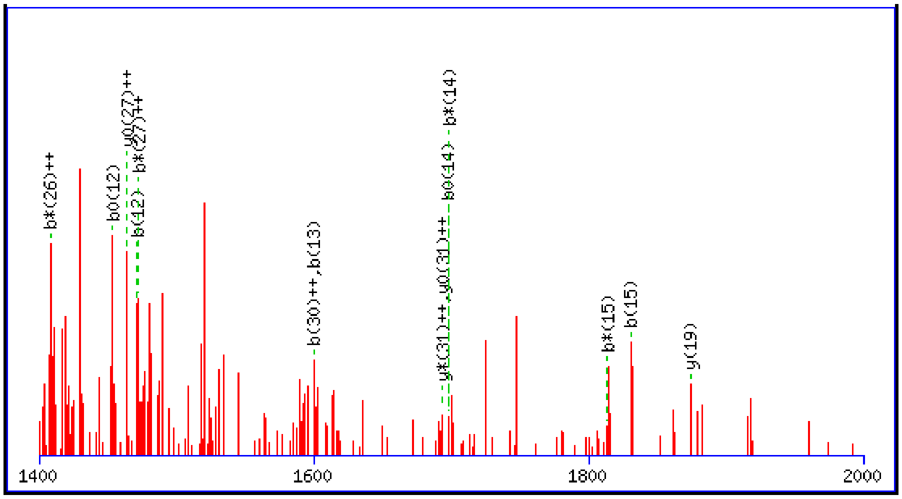

Monoisotopic mass of neutral peptide Mr(calc): 3473.4519
Fixed modifications: Carbamidomethyl (C)
Variable modifications:
**Y12 : ** Phospho (Y)
Ions Score: 59 **Expect:** 0.00057
Matches (Bold Red): 56/356 fragment ions using 110 most intense peaks

#	b	b^{++}	b*	b^{*++}	b^0	b^{0++}	Seq.	y	y^{++}	y*	y^{*++}	y^0	y^{0++}	#
1	72.0444	36.5258					A							32
2	201.0870	101.0471			183.0764	92.0418	E	3403.4221	1702.2147	3386.3955	**1693.7014**	3385.4115	**1693.2094**	31
3	315.1299	158.0686	298.1034	149.5553	**297.1193**	149.0633	N	3274.3795	1637.6934	3257.3529	1629.1801	3256.3689	1628.6881	30
4	430.1569	215.5821	413.1303	207.0688	412.1463	206.5768	D	3160.3365	1580.6719	3143.3100	1572.1586	3142.3260	1571.6666	29
5	529.2253	265.1163	512.1987	256.6030	511.2147	256.1110	V	3045.3096	1523.1584	3028.2831	1514.6452	3027.2990	1514.1532	28
6	**644.2522**	322.6297	627.2257	314.1165	626.2416	313.6245	D	2946.2412	1473.6242	2929.2146	1465.1110	2928.2306	**1464.6190**	27
7	758.2951	379.6512	741.2686	371.1379	740.2846	370.6459	N	2831.2142	1416.1108	2814.1877	1407.5975	2813.2037	1407.1055	26
8	887.3377	**444.1725**	870.3112	435.6592	869.3272	435.1672	E	2717.1713	1359.0893	2700.1448	1350.5760	2699.1608	1350.0840	25
9	1000.4218	500.7145	983.3952	492.2013	982.4112	491.7093	L	2588.1287	1294.5680	2571.1022	1286.0547	2570.1182	1285.5627	24
10	1113.5059	557.2566	1096.4793	548.7433	1095.4953	548.2513	L	2475.0447	1238.0260	2458.0181	1229.5127	2457.0341	1229.0207	23
11	1228.5328	614.7700	1211.5063	606.2568	1210.5222	605.7648	D	2361.9606	1181.4839	2344.9340	1172.9707	2343.9500	**1172.4787**	22
12	**1471.5625**	**736.2849**	1454.5359	727.7716	**1453.5519**	727.2796	Y	2246.9337	1123.9705	2229.9071	1115.4572	2228.9231	1114.9652	21
13	**1600.6050**	**800.8062**	1583.5785	792.2929	1582.5945	791.8009	E	2003.9040	**1002.4556**	1986.8775	993.9424	1985.8934	993.4504	20
14	1715.6320	**858.3196**	**1698.6054**	849.8064	**1697.6214**	849.3144	D	**1874.8614**	**937.9343**	1857.8349	929.4211	1856.8508	928.9291	19
15	**1830.6589**	915.8331	**1813.6324**	907.3198	1812.6484	906.8278	D	1759.8345	880.4209	1742.8079	871.9076	1741.8239	871.4156	18
16	1959.7015	**980.3544**	1942.6750	971.8411	1941.6910	971.3491	E	1644.8075	822.9074	1627.7810	814.3941	1626.7970	813.9021	17
17	2058.7699	**1029.8886**	2041.7434	1021.3753	2040.7594	1020.8833	V	1515.7649	758.3861	1498.7384	749.8728	1497.7544	749.3808	16
18	2187.8125	**1094.4099**	2170.7860	1085.8966	2169.8020	1085.4046	E	1416.6965	708.8519	1399.6700	700.3386	**1398.6860**	699.8466	15
19	2288.8602	**1144.9337**	2271.8337	**1136.4205**	2270.8496	**1135.9285**	T	1287.6539	**644.3306**	1270.6274	635.8173	1269.6434	635.3253	14
20	2359.8973	**1180.4523**	2342.8708	**1171.9390**	2341.8868	1171.4470	A	1186.6062	593.8068	1169.5797	585.2935	1168.5957	584.8015	13
21	2430.9344	**1215.9709**	2413.9079	1207.4576	2412.9239	1206.9656	A	1115.5691	**558.2882**	1098.5426	549.7749	1097.5586	549.2829	12
22	2487.9559	**1244.4816**	2470.9294	1235.9683	2469.9453	1235.4763	G	**1044.5320**	**522.7696**	1027.5055	**514.2564**	1026.5215	**513.7644**	11
23	2558.9930	**1280.0001**	2541.9665	1271.4869	2540.9825	1270.9949	A	987.5106	494.2589	970.4840	485.7456	**969.5000**	485.2536	10
24	2674.0200	**1337.5136**	2656.9934	**1329.0003**	2656.0094	**1328.5083**	D	**916.4734**	458.7404	899.4469	450.2271	898.4629	449.7351	9
25	2731.0414	**1366.0244**	2714.0149	1357.5111	2713.0309	1357.0191	G	**801.4465**	**401.2269**	784.4199	**392.7136**	783.4359	**392.2216**	8
26	2832.0891	1416.5482	2815.0626	**1408.0349**	2814.0785	1407.5429	T	**744.4250**	372.7162	727.3985	364.2029	726.4145	363.7109	7
27	2961.1317	1481.0695	2944.1051	**1472.5562**	2943.1211	1472.0642	E	**643.3774**	322.1923	626.3508	313.6790	**625.3668**	313.1870	6
28	3032.1688	1516.5880	3015.1423	1508.0748	3014.1582	1507.5828	A	**514.3348**	257.6710	497.3082	249.1577			5
29	3129.2216	1565.1144	3112.1950	1556.6012	3111.2110	1556.1091	P	**443.2976**	222.1525	426.2711	213.6392			4
30	3200.2587	**1600.6330**	3183.2321	1592.1197	3182.2481	1591.6277	A	346.2449	173.6261	329.2183	165.1128			3
31	3328.3537	1664.6805	3311.3271	1656.1672	3310.3431	1655.6752	K	275.2078	138.1075	258.1812	129.5942			2
32							K	147.1128	74.0600	130.0863	65.5468			1

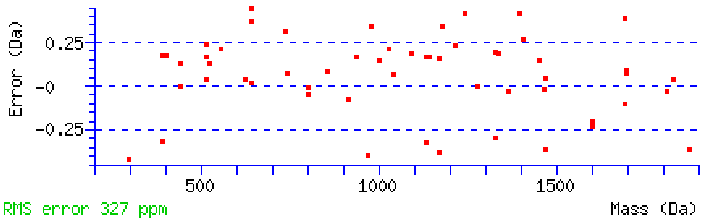

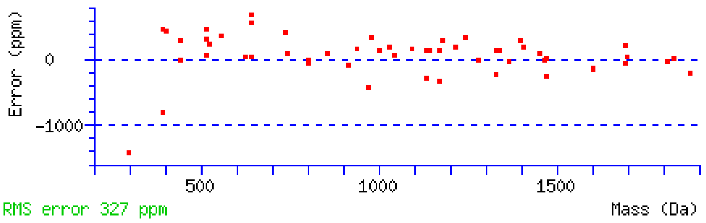

All matches to this query

Score	Mr(calc):	Delta	Sequence
58.8	3473.4519	-0.0047	AENDVDNELLDYEDDEVETAAGADGTEAPAKK
24.4	3473.4519	-0.0047	AENDVDNELLDYEDDEVETAAGADGTEAPAKK
7.6	3472.4687	0.9784	DMAAAMMYTVVTPMLNPFIYSLRNRDMK
5.3	3473.4700	-0.0228	VTFQFSYGTKETSYGLSFFKPQPGNFEK
3.5	3473.4664	-0.0192	VNYHCQELQNPEETSGTAEASAMEPAEFSTF
3.0	3473.4234	0.0238	LTFVEMDIESQPECAYDHLEVFDGRDAK
3.0	3473.4628	-0.0156	DGVYSCPICAKNFNSKESFVPHVTLHVK
3.0	3473.4700	-0.0228	VTFQFSYGTKETSYGLSFFKPQPGNFEK
2.0	3472.4189	1.0282	SYVPGSVNCGVMNSNGPECPRSGRATFFHK
2.0	3473.4700	-0.0228	VTFQFSYGTKETSYGLSFFKPQPGNFEK

Spectrum No: 173; Query: 1936; Rank: 1

Peptide View

MS/MS Fragmentation of **TPEELDDSDFETEDFDVR**
Found in **IPI00358406**, Tax_Id=10116 Gene_Symbol=Catna1 Catenin (Cadherin-associated protein), alpha 1

Match to Query 1936: 2237.849292 from(746.957040,3+)
Title: 091008RatKidney_NoSalt_30.3036.3036.3.dta
Data file K:\NewmanPaper\Piliang\3SubProteomes\Piliang3SP\mgf5ppm\ERLIC_3SubProteomes5ppm.mgf

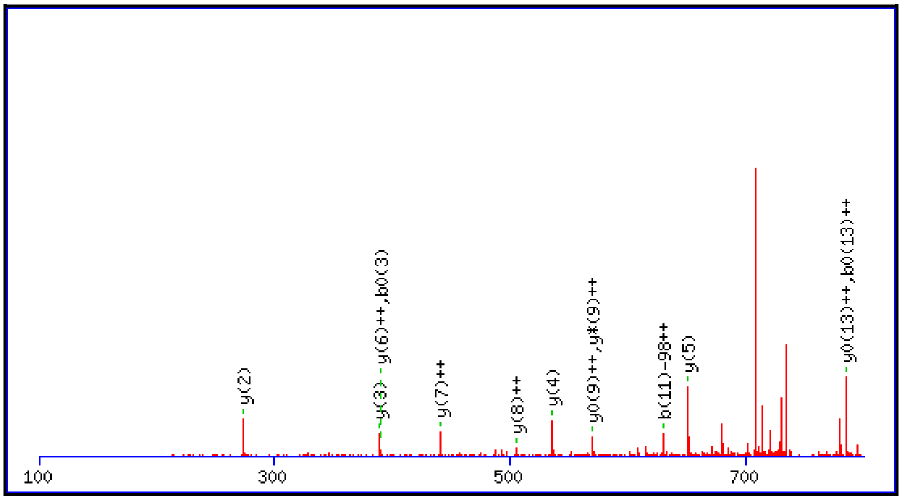

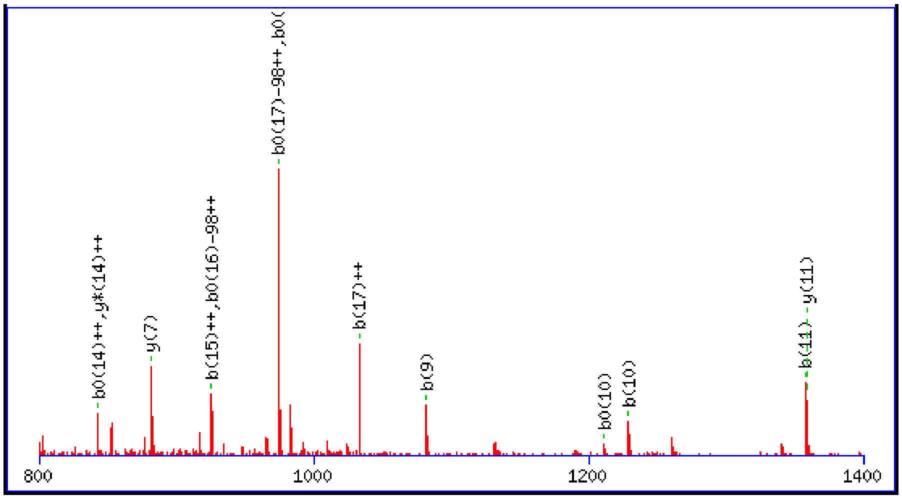

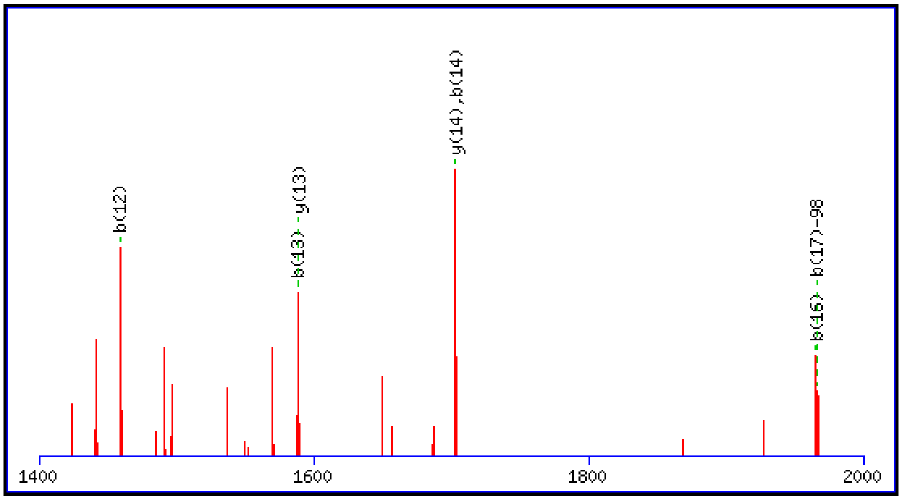

Monoisotopic mass of neutral peptide Mr(calc): 2237.8526
Fixed modifications: Carbamidomethyl (C)
Variable modifications:
T1 : Phospho (ST), with neutral losses 0.0000(shown in table), 97.9769
Ions Score: 59 **Expect:** 0.00021
Matches (Bold Red): 33/234 fragment ions using 38 most intense peaks

#	b	b^{++}	b^{0}	b^{0++}	Seq.	y	y^{++}	y*	y^{*++}	y^{0}	y^{0++}	#
1	182.0213	91.5143	164.0107	82.5090	T							18
2	279.0740	140.0407	261.0635	131.0354	P	2057.8458	1029.4265	2040.8193	1020.9133	2039.8353	1020.4213	17
3	408.1166	204.5620	**390.1061**	195.5567	E	1960.7931	980.9002	1943.7665	972.3869	1942.7825	971.8949	16
4	537.1592	269.0833	519.1487	260.0780	E	1831.7505	916.3789	1814.7239	907.8656	1813.7399	907.3736	15
5	650.2433	325.6253	632.2327	316.6200	L	**1702.7079**	851.8576	1685.6813	**843.3443**	1684.6973	842.8523	14
6	765.2702	383.1388	747.2597	374.1335	D	**1589.6238**	795.3155	1572.5973	786.8023	1571.6132	**786.3103**	13
7	880.2972	440.6522	862.2866	431.6469	D	1474.5969	737.8021	1457.5703	729.2888	1456.5863	728.7968	12
8	967.3292	484.1682	949.3186	475.1630	S	**1359.5699**	680.2886	1342.5434	671.7753	1341.5594	671.2833	11
9	**1082.3562**	541.6817	1064.3456	532.6764	D	1272.5379	636.7726	1255.5113	628.2593	1254.5273	627.7673	10
10	**1229.4246**	615.2159	**1211.4140**	606.2106	F	1157.5109	579.2591	1140.4844	**570.7458**	1139.5004	**570.2538**	9
11	**1358.4672**	679.7372	1340.4566	670.7319	E	1010.4425	**505.7249**	993.4160	497.2116	992.4320	496.7196	8
12	**1459.5148**	730.2611	1441.5043	721.2558	T	**881.3999**	**441.2036**	864.3734	432.6903	863.3894	432.1983	7
13	**1588.5574**	794.7824	1570.5469	**785.7771**	E	780.3523	**390.6798**	763.3257	382.1665	762.3417	381.6745	6
14	**1703.5844**	852.2958	1685.5738	**843.2905**	D	**651.3097**	326.1585	634.2831	317.6452	633.2991	317.1532	5
15	1850.6528	**925.8300**	1832.6422	916.8247	F	**536.2827**	268.6450	519.2562	260.1317	518.2722	259.6397	4
16	**1965.6797**	983.3435	1947.6692	**974.3382**	D	**389.2143**	195.1108	372.1878	186.5975	371.2037	186.1055	3
17	2064.7481	**1032.8777**	2046.7376	1023.8724	V	**274.1874**	137.5973	257.1608	129.0840			2
18					R	175.1190	88.0631	158.0924	79.5498			1

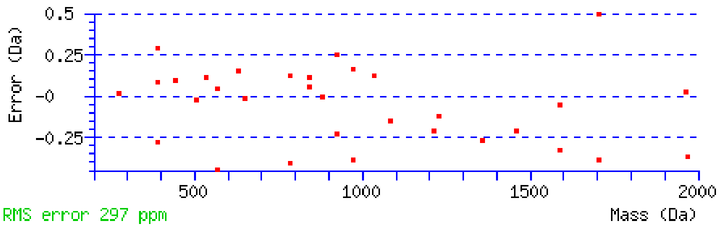

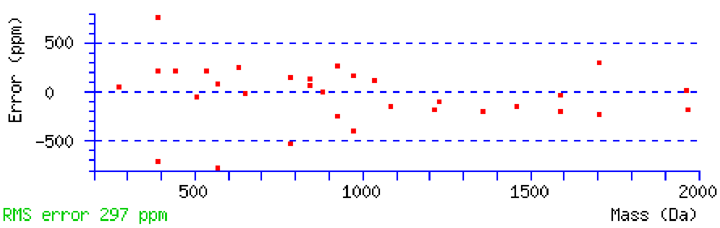

All matches to this query

Score	Mr(calc):	Delta	Sequence
58.6	2237.8526	-0.0033	TPEELDDSDFETEDFDVR
46.2	2237.8526	-0.0033	TPEELDDSDFETEDFDVR
17.3	2237.8526	-0.0033	TPEELDDSDFETEDFDVR
8.0	2237.8549	-0.0056	NMAEATSGEAPENGVTTGSVR
7.4	2237.8549	-0.0056	NMAEATSGEAPENGVTTGSVR
6.3	2236.8552	0.9941	GLAVSTGNNRGHSSGAAQPSK
6.2	2237.8658	-0.0165	GGSLRVGGGSFGGGSLYGGGGSR
3.7	2237.8684	-0.0191	LTAESPNPSPFGDDVPTVR
3.7	2237.8684	-0.0191	LTAESPNPSPFGDDVPTVR
3.7	2237.8684	-0.0191	LTAESPNPSPFGDDVPTVR

Spectrum No: 174; Query: 2342; Rank: 1

Peptide View

MS/MS Fragmentation of **DMPGGFLFDGLSDDEDDFHPSTR**
Found in **IPI00191651**, Tax_Id=10116 Gene_Symbol=Bmsc-UbP Bone marrow stromal cell-derived ubiquitin-like protein

Match to Query 2342: 2649.043482 from(884.021770,3+)
Title: 100101RatKid_NS_deglyco_21.5074.5074.3.dta
Data file K:\NewmanPaper\Piliang\3SubProteomes\Piliang3SP\mgf5ppm\ERLIC_3SubProteomes5ppm.mgf

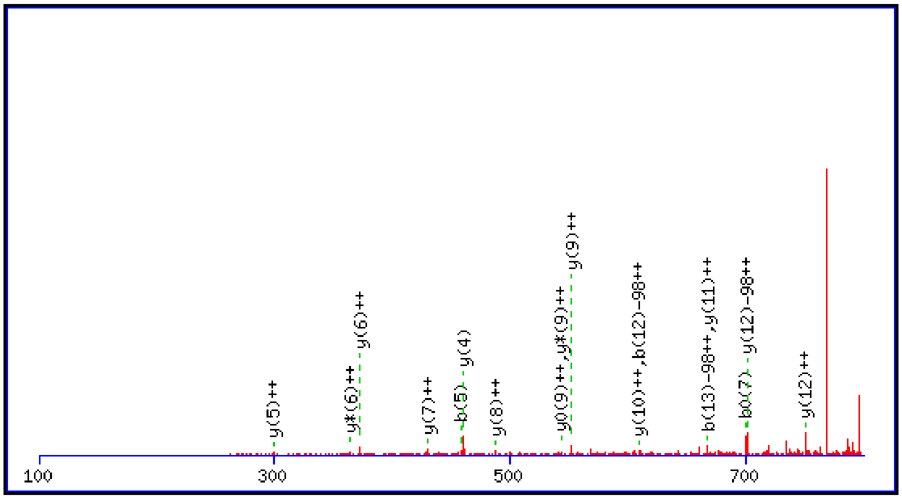

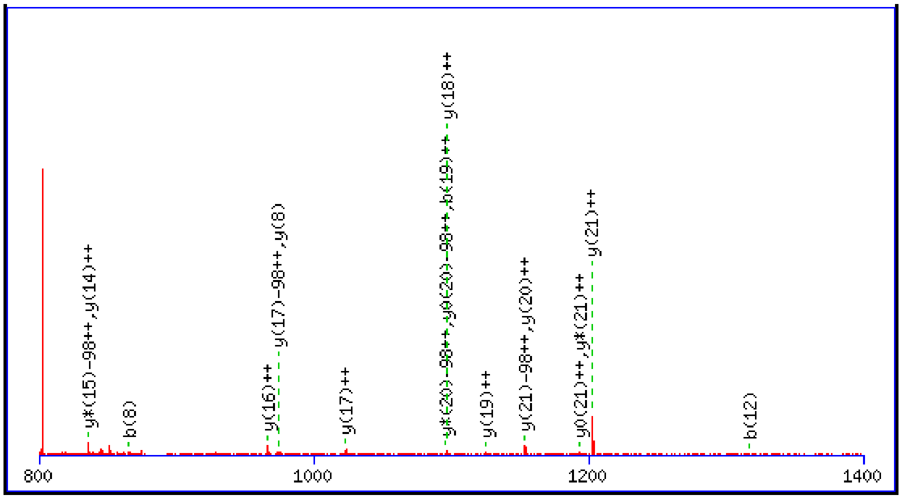

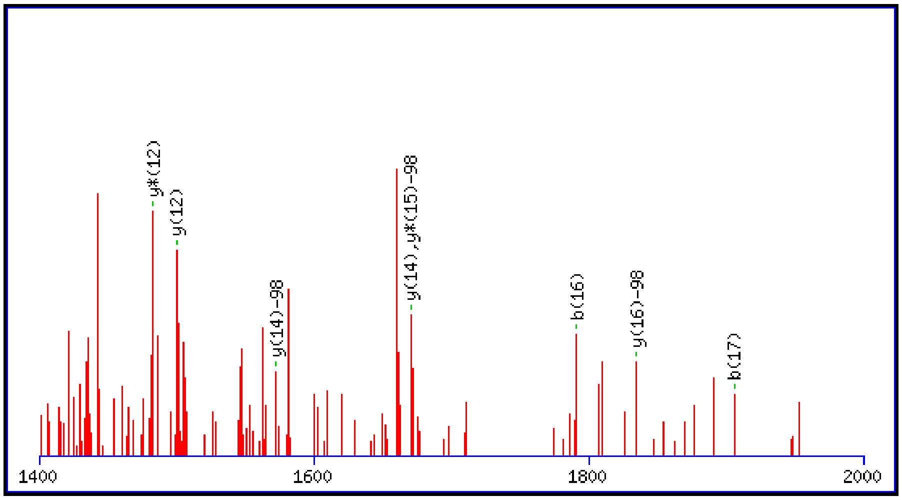

Monoisotopic mass of neutral peptide Mr(calc): 2649.0367
Fixed modifications: Carbamidomethyl (C)
Variable modifications:
S12 : Phospho (ST), with neutral losses 0.0000(shown in table), 97.9769
Ions Score: 59 **Expect:** 0.00033
Matches (Bold Red): 43/328 fragment ions using 90 most intense peaks

#	b	b^{++}	b^{0}	b^{0++}	Seq.	y	y^{++}	y*	y^{*++}	y^{0}	y^{0++}	#
1	116.0342	58.5207	98.0237	49.5155	D							23
2	247.0747	124.0410	229.0641	115.0357	M	2535.0170	1268.0121	2517.9905	1259.4989	2517.0064	1259.0069	22
3	344.1275	172.5674	326.1169	163.5621	P	2403.9765	**1202.4919**	2386.9500	**1193.9786**	2385.9660	**1193.4866**	21
4	401.1489	201.0781	383.1384	192.0728	G	2306.9238	**1153.9655**	2289.8972	1145.4522	2288.9132	1144.9602	20
5	**458.1704**	229.5888	440.1598	220.5836	G	2249.9023	**1125.4548**	2232.8757	1116.9415	2231.8917	1116.4495	19
6	605.2388	303.1230	587.2282	294.1178	F	2192.8808	**1096.9441**	2175.8543	1088.4308	2174.8703	1087.9388	18
7	718.3229	359.6651	**700.3123**	350.6598	L	2045.8124	**1023.4098**	2028.7859	1014.8966	2027.8018	1014.4046	17
8	**865.3913**	433.1993	847.3807	424.1940	F	1932.7284	**966.8678**	1915.7018	958.3545	1914.7178	957.8625	16
9	980.4182	490.7128	962.4077	481.7075	D	1785.6599	893.3336	1768.6334	884.8203	1767.6494	884.3283	15
10	1037.4397	519.2235	1019.4291	510.2182	G	**1670.6330**	**835.8201**	1653.6064	827.3069	1652.6224	826.8149	14
11	1150.5238	575.7655	1132.5132	566.7602	L	1613.6115	807.3094	1596.5850	798.7961	1595.6010	798.3041	13
12	**1317.5221**	659.2647	1299.5115	650.2594	S	**1500.5275**	**750.7674**	**1483.5009**	742.2541	1482.5169	741.7621	12
13	1432.5491	716.7782	1414.5385	707.7729	D	1333.5291	**667.2682**	1316.5026	658.7549	1315.5185	658.2629	11
14	1547.5760	774.2916	1529.5654	765.2864	D	1218.5022	**609.7547**	1201.4756	601.2414	1200.4916	600.7494	10
15	1676.6186	838.8129	1658.6080	829.8077	E	1103.4752	**552.2413**	1086.4487	**543.7280**	1085.4647	**543.2360**	9
16	**1791.6455**	896.3264	1773.6350	887.3211	D	**974.4326**	**487.7200**	957.4061	479.2067	956.4221	478.7147	8
17	**1906.6725**	953.8399	1888.6619	944.8346	D	859.4057	**430.2065**	842.3791	421.6932	841.3951	421.2012	7
18	2053.7409	1027.3741	2035.7303	1018.3688	F	744.3787	**372.6930**	727.3522	**364.1797**	726.3682	363.6877	6

19	2190.7998	1095.9035	2172.7892	1086.8983	H	597.3103	299.1588	580.2838	290.6455	579.2998	290.1535	5
20	2287.8526	1144.4299	2269.8420	1135.4246	P	460.2514	230.6293	443.2249	222.1161	442.2409	221.6241	4
21	2374.8846	1187.9459	2356.8740	1178.9407	S	363.1987	182.1030	346.1721	173.5897	345.1881	173.0977	3
22	2475.9323	1238.4698	2457.9217	1229.4645	T	276.1666	138.5870	259.1401	130.0737	258.1561	129.5817	2
23					R	175.1190	88.0631	158.0924	79.5498			1

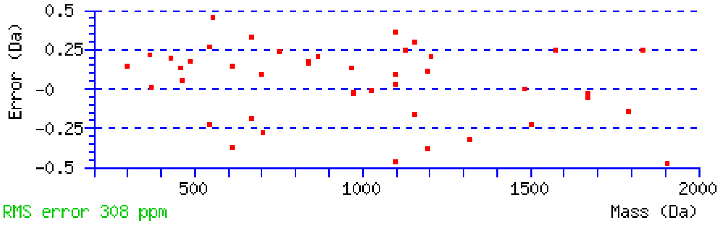

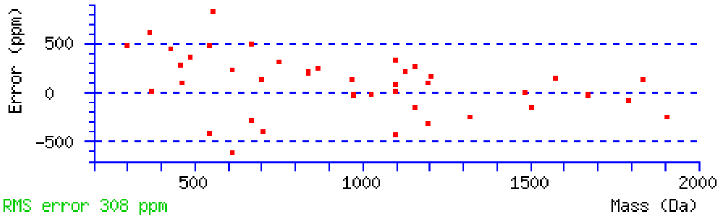

All matches to this query

Score	Mr(calc):	Delta	Sequence
58.7	2649.0367	0.0068	DMPGGFLFDGLSDDEDDFHPSTR
8.9	2647.0211	2.0223	TDDQENVSVDVPSPAQENEEASK
8.3	2648.0139	1.0296	QTSYSNNRSPTNSTGMWKDSPK
8.3	2648.0139	1.0296	QTSYSNNRSPTNSTGMWKDSPK
7.6	2649.0316	0.0119	MVSGNRGTSLNDSYHSRDSSFR
5.3	2647.0171	2.0264	QNQNVDTSSSSTVSATSSKGGDSNK
5.3	2647.0171	2.0264	QNQNVDTSSSSTVSATSSKGGDSNK
5.3	2647.0171	2.0264	QNQNVDTSSSSTVSATSSKGGDSNK
5.3	2647.0171	2.0264	QNQNVDTSSSSTVSATSSKGGDSNK
5.3	2647.0171	2.0264	QNQNVDTSSSSTVSATSSKGGDSNK

Spectrum No: 175; Query: 2099; Rank: 1

Peptide View

MS/MS Fragmentation of **TEEVGENTQVEDTEPSSPVTK**
Found in **IPI00373107**, Tax_Id=10116 Gene_Symbol=Trp53bp1_predicted similar to transformation related protein 53 binding protein 1

Match to Query 2099: 2355.011928 from(1178.513240,2+)
Title: 100101RatKid_NS_deglyco_24.1351.1351.2.dta
Data file K:\NewmanPaper\Piliang\3SubProteomes\Piliang3SP\mgf5ppm\ERLIC_3SubProteomes5ppm.mgf

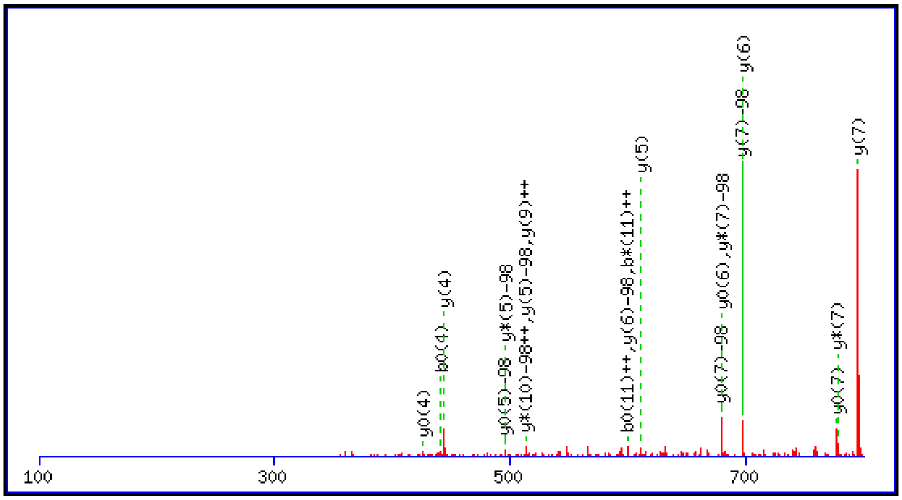

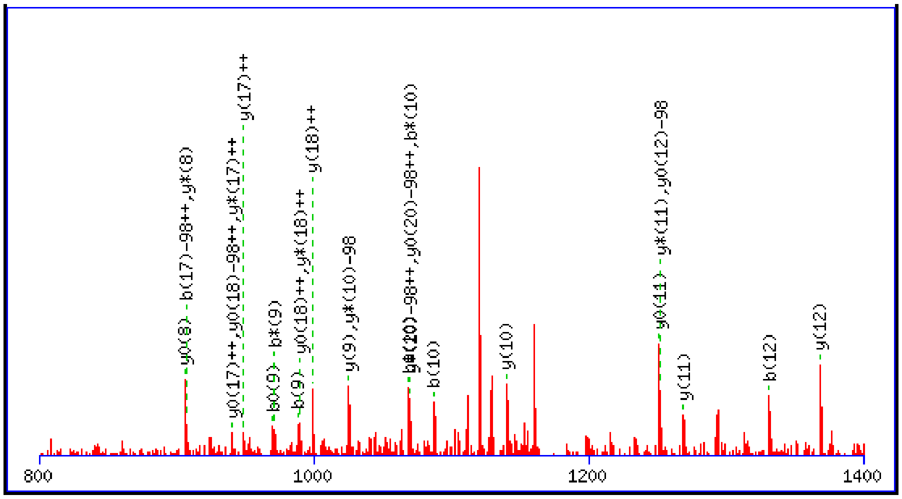

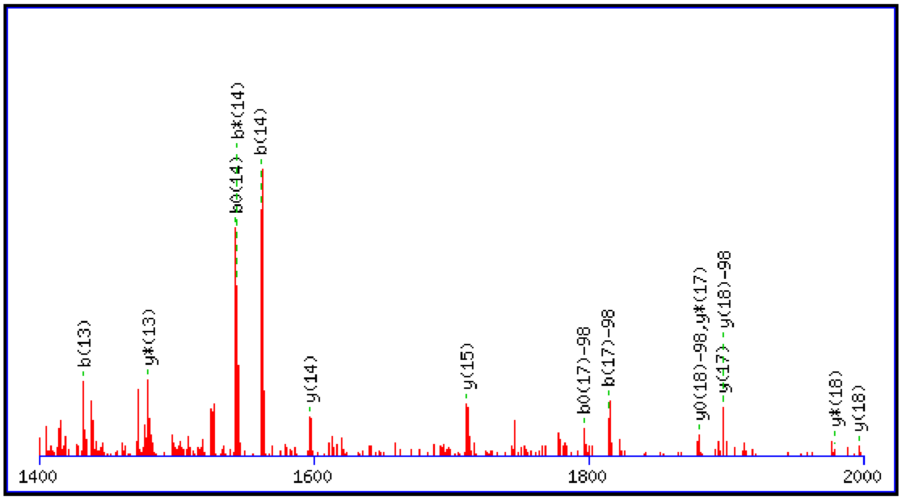

Monoisotopic mass of neutral peptide Mr(calc): 2355.0003
Fixed modifications: Carbamidomethyl (C)
Variable modifications:
S17 : Phospho (ST), with neutral losses 0.0000(shown in table), 97.9769
Ions Score: 59 **Expect:** 0.00041
Matches (Bold Red): 62/346 fragment ions using 78 most intense peaks

#	b	b^{++}	b*	b^{*++}	b^0	b^{0++}	Seq.	y	y^{++}	y*	y^{*++}	y^0	y^{0++}	#
1	102.0550	51.5311			84.0444	42.5258	T							21
2	231.0975	116.0524			213.0870	107.0471	E	2254.9599	1127.9836	2237.9333	1119.4703	2236.9493	1118.9783	20
3	360.1401	180.5737			342.1296	171.5684	E	2125.9173	1063.4623	2108.8907	1054.9490	2107.9067	1054.4570	19
4	459.2086	230.1079			**441.1980**	221.1026	V	**1996.8747**	**998.9410**	**1979.8481**	**990.4277**	1978.8641	**989.9357**	18
5	516.2300	258.6186			498.2195	249.6134	G	**1897.8063**	**949.4068**	**1880.7797**	**940.8935**	1879.7957	**940.4015**	17
6	645.2726	323.1399			627.2620	314.1347	E	1840.7848	920.8960	1823.7583	912.3828	1822.7742	911.8908	16
7	759.3155	380.1614	742.2890	371.6481	741.3050	371.1561	N	**1711.7422**	856.3747	1694.7157	847.8615	1693.7316	847.3695	15
8	860.3632	430.6852	843.3367	422.1720	842.3527	421.6800	T	**1597.6993**	799.3533	1580.6727	790.8400	1579.6887	790.3480	14
9	**988.4218**	494.7145	**971.3952**	486.2013	**970.4112**	485.7093	Q	1496.6516	748.8294	**1479.6251**	740.3162	1478.6410	739.8242	13
10	**1087.4902**	544.2487	**1070.4637**	535.7355	**1069.4796**	535.2435	V	**1368.5930**	684.8002	1351.5665	676.2869	1350.5825	675.7949	12
11	1216.5328	608.7700	1199.5063	**600.2568**	1198.5222	**599.7648**	E	**1269.5246**	635.2659	**1252.4981**	626.7527	**1251.5140**	626.2607	11
12	**1331.5597**	666.2835	1314.5332	657.7702	1313.5492	657.2782	D	**1140.4820**	570.7446	1123.4555	562.2314	1122.4715	561.7394	10
13	**1432.6074**	716.8074	1415.5809	708.2941	1414.5969	707.8021	T	**1025.4551**	**513.2312**	1008.4285	504.7179	1007.4445	504.2259	9
14	**1561.6500**	781.3286	**1544.6235**	772.8154	**1543.6395**	772.3234	E	924.4074	462.7073	**907.3808**	454.1941	**906.3968**	453.7021	8
15	1658.7028	829.8550	1641.6762	821.3418	1640.6922	820.8497	P	**795.3648**	398.1860	**778.3383**	389.6728	**777.3542**	389.1808	7
16	1745.7348	873.3710	1728.7083	864.8578	1727.7242	864.3658	S	**698.3120**	349.6597	681.2855	341.1464	**680.3015**	340.6544	6
17	1912.7332	956.8702	1895.7066	948.3569	1894.7226	947.8649	S	**611.2800**	306.1436	594.2535	297.6304	593.2694	297.1384	5
18	2009.7859	1005.3966	1992.7594	996.8833	1991.7754	996.3913	P	**444.2817**	222.6445	427.2551	214.1312	**426.2711**	213.6392	4

**19**	2108.8543	1054.9308	2091.8278	1046.4175	2090.8438	1045.9255	**V**	347.2289	174.1181	330.2023	165.6048	329.2183	165.1128	**3**
**20**	2209.9020	1105.4546	2192.8755	1096.9414	2191.8915	1096.4494	**T**	248.1605	124.5839	231.1339	116.0706	230.1499	115.5786	**2**
**21**							**K**	147.1128	74.0600	130.0863	65.5468			**1**

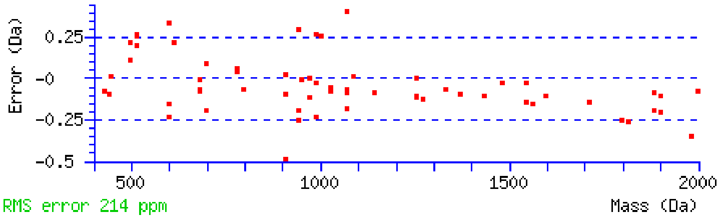

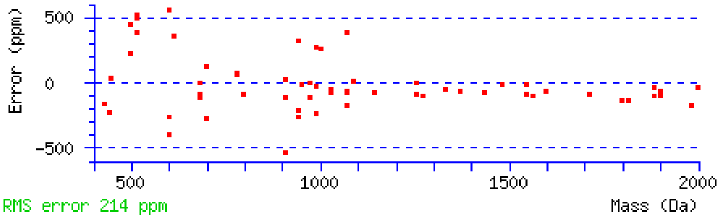

All matches to this query

Score	Mr(calc):	Delta	Sequence
58.7	2355.0003	0.0117	TEEVGENTQVEDTEPSSPVTK
50.1	2355.0003	0.0117	TEEVGENTQVEDTEPSSPVTK
48.9	2355.0003	0.0117	TEEVGENTQVEDTEPSSPVTK
30.3	2355.0003	0.0117	TEEVGENTQVEDTEPSSPVTK
10.8	2355.0003	0.0117	TEEVGENTQVEDTEPSSPVTK
4.7	2355.0003	0.0117	TEEVGENTQVEDTEPSSPVTK
2.4	2355.0124	-0.0004	ENLQPNSPVGMVTVMDADKGR
2.2	2354.0284	0.9836	ENLQPNSPVGMVTVMDADKGR

Spectrum No: 176; Query: 2147; Rank: 1

Peptide View

MS/MS Fragmentation of **ASVDSETESSPGVNETAAASGQR**
Found in **IPI00211999**, Tax_Id=10116 Gene_Symbol=Rnmt 46 kDa protein

Match to Query 2147: 2408.944628 from(1205.479590,2+)
Title: 100101RatKid_NS_deglyco_27.1173.1173.2.dta
Data file K:\NewmanPaper\Piliang\3SubProteomes\Piliang3SP\mgf5ppm\ERLIC_3SubProteomes5ppm.mgf

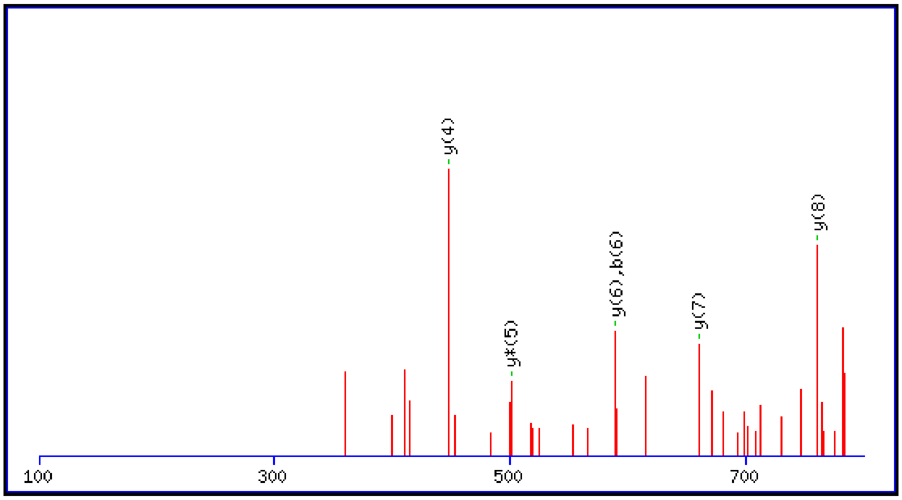

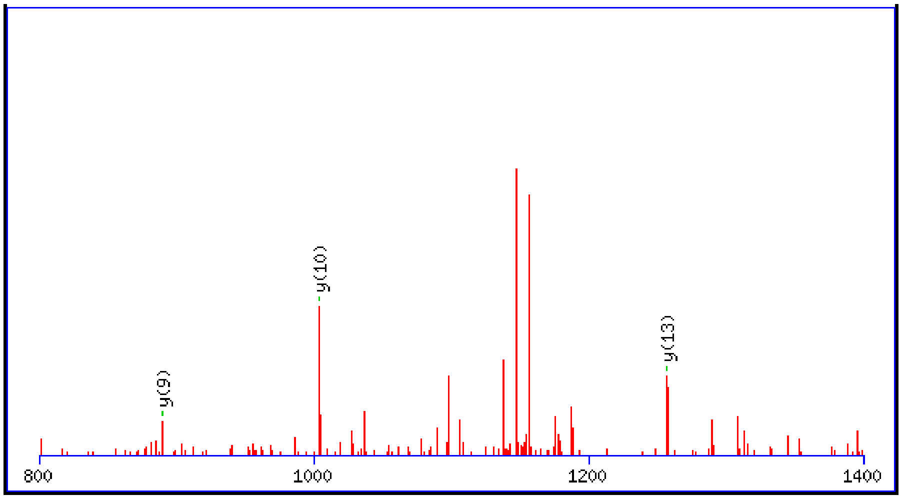

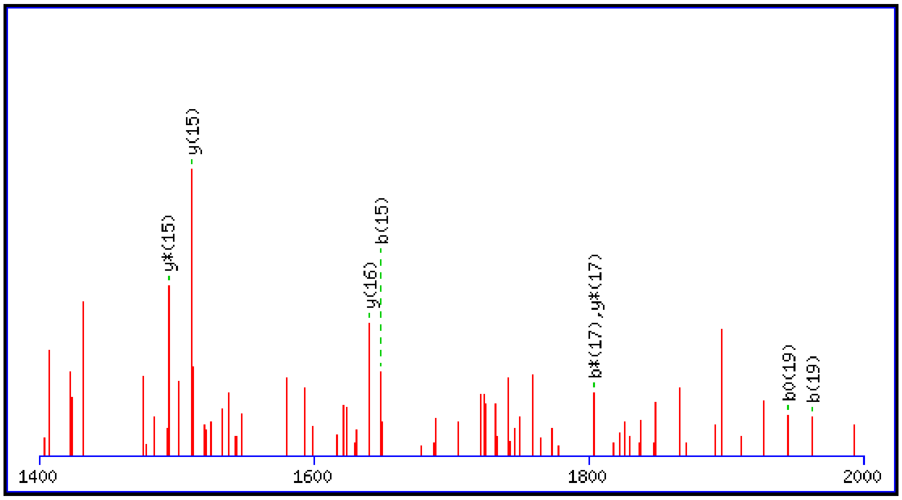

Monoisotopic mass of neutral peptide Mr(calc): 2408.9370
Fixed modifications: Carbamidomethyl (C)
Variable modifications:
T7 : Phospho (ST), with neutral losses 0.0000(shown in table), 97.9769
S9 : Phospho (ST), with neutral losses 0.0000(shown in table), 97.9769
Ions Score: 59 **Expect:** 0.0003
Matches (Bold Red): 17/360 fragment ions using 24 most intense peaks

#	b	b++	b*	b*++	b0	b0++	Seq.	y	y++	y*	y*++	y0	y0++	#
1	72.0444	36.5258					A							23
2	159.0764	80.0418			141.0659	71.0366	S	2338.9072	1169.9572	2321.8806	1161.4440	2320.8966	1160.9519	22
3	258.1448	129.5761			240.1343	120.5708	V	2251.8752	1126.4412	2234.8486	1117.9279	2233.8646	1117.4359	21
4	373.1718	187.0895			355.1612	178.0842	D	2152.8067	1076.9070	2135.7802	1068.3937	2134.7962	1067.9017	20
5	460.2038	230.6055			442.1932	221.6003	S	2037.7798	1019.3935	2020.7533	1010.8803	2019.7692	1010.3883	19
6	**589.2464**	295.1268			571.2358	286.1216	E	1950.7478	975.8775	1933.7212	967.3643	1932.7372	966.8722	18
7	770.2604	385.6338			752.2498	376.6286	T	1821.7052	911.3562	**1804.6786**	902.8430	1803.6946	902.3509	17
8	899.3030	450.1551			881.2924	441.1499	E	**1640.6912**	820.8492	1623.6646	812.3360	1622.6806	811.8439	16
9	1066.3013	533.6543			1048.2908	524.6490	S	**1511.6486**	756.3279	**1494.6220**	747.8147	1493.6380	747.3226	15
10	1153.3334	577.1703			1135.3228	568.1650	S	1344.6502	672.8288	1327.6237	664.3155	1326.6397	663.8235	14
11	1250.3861	625.6967			1232.3756	616.6914	P	**1257.6182**	629.3127	1240.5917	620.7995	1239.6076	620.3075	13
12	1307.4076	654.2074			1289.3970	645.2022	G	1160.5654	580.7864	1143.5389	572.2731	1142.5549	571.7811	12
13	1406.4760	703.7416			1388.4654	694.7364	V	1103.5440	552.2756	1086.5174	543.7624	1085.5334	543.2703	11
14	1520.5189	760.7631	1503.4924	752.2498	1502.5084	751.7578	N	**1004.4756**	502.7414	987.4490	494.2281	986.4650	493.7361	10
15	**1649.5615**	825.2844	1632.5350	816.7711	1631.5510	816.2791	E	**890.4326**	445.7200	873.4061	437.2067	872.4221	436.7147	9
16	1750.6092	875.8082	1733.5827	867.2950	1732.5986	866.8030	T	**761.3900**	381.1987	744.3635	372.6854	743.3795	372.1934	8
17	1821.6463	911.3268	**1804.6198**	902.8135	1803.6358	902.3215	A	**660.3424**	330.6748	643.3158	322.1615	642.3318	321.6695	7
18	1892.6834	946.8454	1875.6569	938.3321	1874.6729	937.8401	A	**589.3053**	295.1563	572.2787	286.6430	571.2947	286.1510	6

19	1963.7206	982.3639	1946.6940	973.8506	1945.7100	973.3586	A	518.2681	259.6377	501.2416	251.1244	500.2576	250.6324	5
20	2050.7526	1025.8799	2033.7260	1017.3667	2032.7420	1016.8746	S	447.2310	224.1191	430.2045	215.6059	429.2205	215.1139	4
21	2107.7740	1054.3907	2090.7475	1045.8774	2089.7635	1045.3854	G	360.1990	180.6031	343.1724	172.0899			3
22	2235.8326	1118.4200	2218.8061	1109.9067	2217.8221	1109.4147	Q	303.1775	152.0924	286.1510	143.5791			2
23							R	175.1190	88.0631	158.0924	79.5498			1

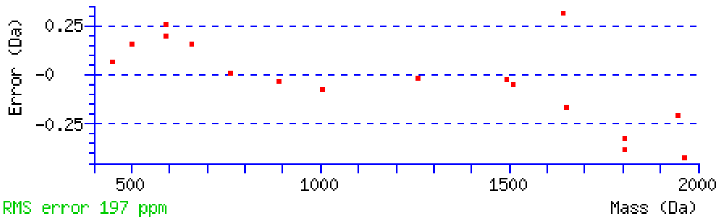

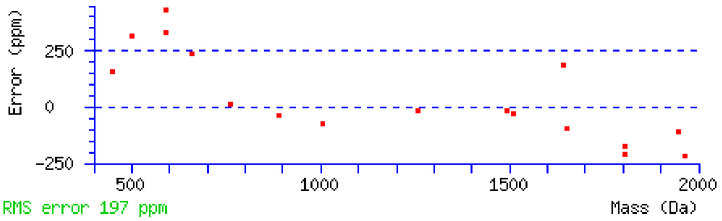

All matches to this query

Score	Mr(calc):	Delta	Sequence
58.6	2408.9370	0.0076	ASVDSETESSPGVNETAAASGQR
33.1	2408.9370	0.0076	ASVDSETESSPGVNETAAASGQR
28.2	2408.9370	0.0076	ASVDSETESSPGVNETAAASGQR
28.2	2408.9370	0.0076	ASVDSETESSPGVNETAAASGQR
3.7	2408.9380	0.0066	ASIMFMQNLNSVVEFCTEK
3.6	2408.9267	0.0180	MDPEISEQDEEQKTVCVGR
3.2	2408.9370	0.0076	ASVDSETESSPGVNETAAASGQR
0.3	2407.9540	0.9906	ASIMFMQNLNSVVEFCTEK
0.3	2407.9487	0.9959	QGPDAEYTEKLQQYASGRK
0.2	2408.9580	-0.0133	NPSNRNQDYMASSEPNYPTK

Spectrum No: 177; Query: 531; Rank: 1

Peptide View

MS/MS Fragmentation of **SGDETPGSEAPGDK**
Found in **IPI00360386**, Tax_Id=10116 Gene_Symbol=RGD1562784_predicted similar to heterogeneous nuclear ribonucleoprotein U isoform a

Match to Query 531: 1425.527248 from(713.770900,2+)
Title: 091008RatKidney_NH4Format02_28.254.254.2.dta
Data file K:\NewmanPaper\Piliang\3SubProteomes\Piliang3SP\mgf5ppm\ERLIC_3SubProteomes5ppm.mgf

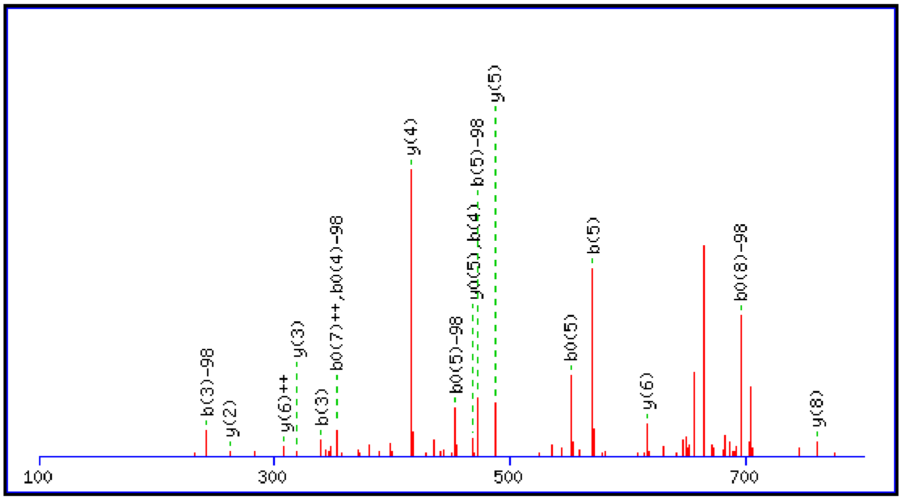

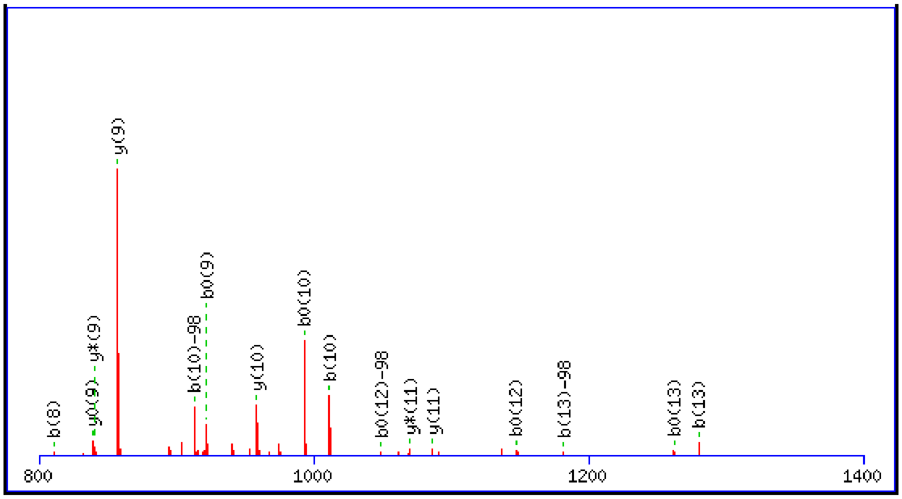

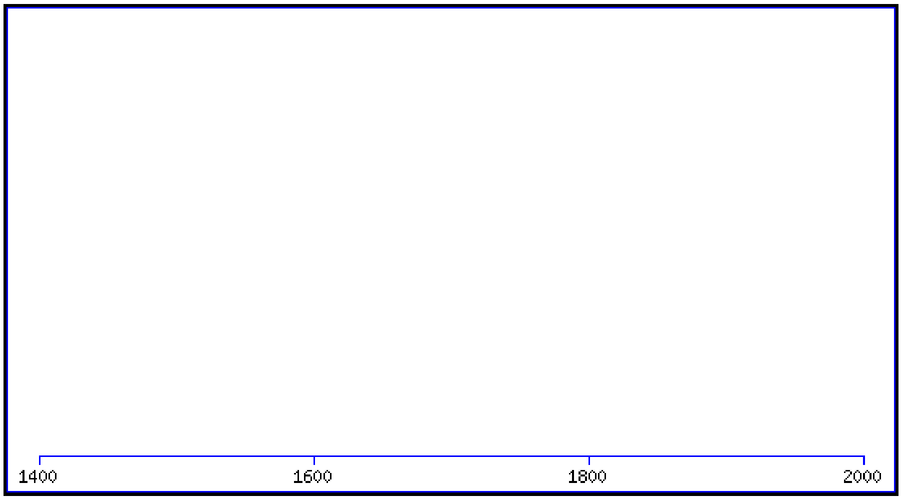

Monoisotopic mass of neutral peptide Mr(calc): 1425.5297
Fixed modifications: Carbamidomethyl (C)
Variable modifications:
S1 : Phospho (ST), with neutral losses 97.9769(shown in table), 0.0000
Ions Score: 59 **Expect:** 0.00011
Matches (Bold Red): 34/180 fragment ions using 47 most intense peaks

#	b	b^{++}	b^0	b^{0++}	Seq.	y	y^{++}	y*	y^{*++}	y^0	y^{0++}	#
1	70.0287	35.5180	52.0182	26.5127	S							14
2	127.0502	64.0287	109.0396	55.0235	G	1259.5386	630.2729	1242.5121	621.7597	1241.5281	621.2677	13
3	**242.0771**	121.5422	224.0666	112.5369	D	1202.5172	601.7622	1185.4906	593.2489	1184.5066	592.7569	12
4	371.1197	186.0635	**353.1092**	177.0582	E	**1087.4902**	544.2487	**1070.4637**	535.7355	1069.4796	535.2435	11
5	**472.1674**	236.5873	**454.1568**	227.5821	T	**958.4476**	479.7274	941.4211	471.2142	940.4371	470.7222	10
6	569.2202	285.1137	551.2096	276.1084	P	**857.3999**	429.2036	**840.3734**	420.6903	**839.3894**	420.1983	9
7	626.2416	313.6245	608.2311	304.6192	G	**760.3472**	380.6772	743.3206	372.1640	742.3366	371.6719	8
8	713.2737	357.1405	**695.2631**	348.1352	S	703.3257	352.1665	686.2992	343.6532	685.3151	343.1612	7
9	842.3163	421.6618	824.3057	412.6565	E	**616.2937**	**308.6505**	599.2671	300.1372	598.2831	299.6452	6
10	**913.3534**	457.1803	895.3428	448.1750	A	**487.2511**	244.1292	470.2245	235.6159	**469.2405**	235.1239	5
11	1010.4061	505.7067	992.3956	496.7014	P	**416.2140**	208.6106	399.1874	200.0974	398.2034	199.6053	4
12	1067.4276	534.2174	**1049.4170**	525.2122	G	**319.1612**	160.0842	302.1347	151.5710	301.1506	151.0790	3
13	**1182.4545**	591.7309	1164.4440	582.7256	D	**262.1397**	131.5735	245.1132	123.0602	244.1292	122.5682	2
14					K	147.1128	74.0600	130.0863	65.5468			1

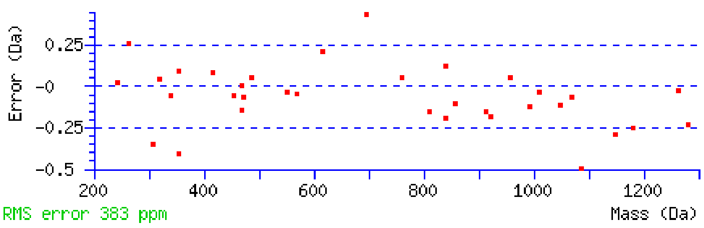

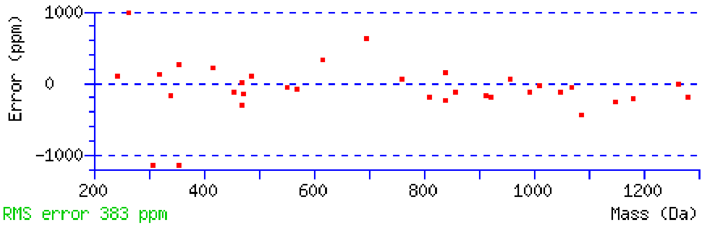

All matches to this query

Score	Mr(calc):	Delta	Sequence
58.6	1425.5297	-0.0024	SGDETPGSEAPGDK
34.2	1425.5297	-0.0024	SGDETPGSEAPGDK
12.8	1425.5297	-0.0024	SGDETPGSEAPGDK
7.7	1425.5384	-0.0111	SGNEGREFYMR
0.3	1425.5248	0.0025	GKTNSQCLETK
0.3	1425.5248	0.0025	GKTNSQCLETK

Spectrum No: 178; Query: 2033; Rank: 1

Peptide View

MS/MS Fragmentation of **NLTSSSLNDISDKPEKDQLK**
Found in **IPI00199985**, Tax_Id=10116 Gene_Symbol=Slc4a4 Isoform 1 of Electrogenic sodium bicarbonate cotransporter 1

Match to Query 2033: 2311.097308 from(1156.555930,2+)
Title: 091008RatKidney_NH4Format01_17.1697.1697.2.dta
Data file K:\NewmanPaper\Piliang\3SubProteomes\Piliang3SP\mgf5ppm\ERLIC_3SubProteomes5ppm.mgf

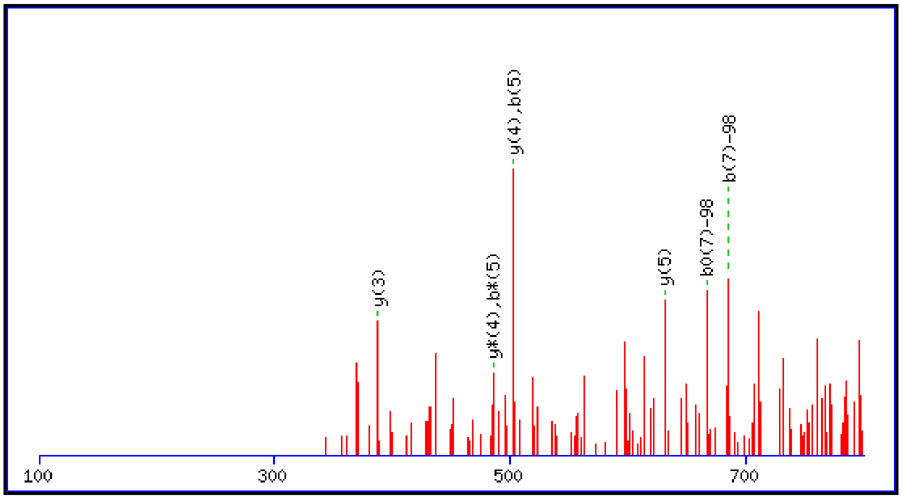

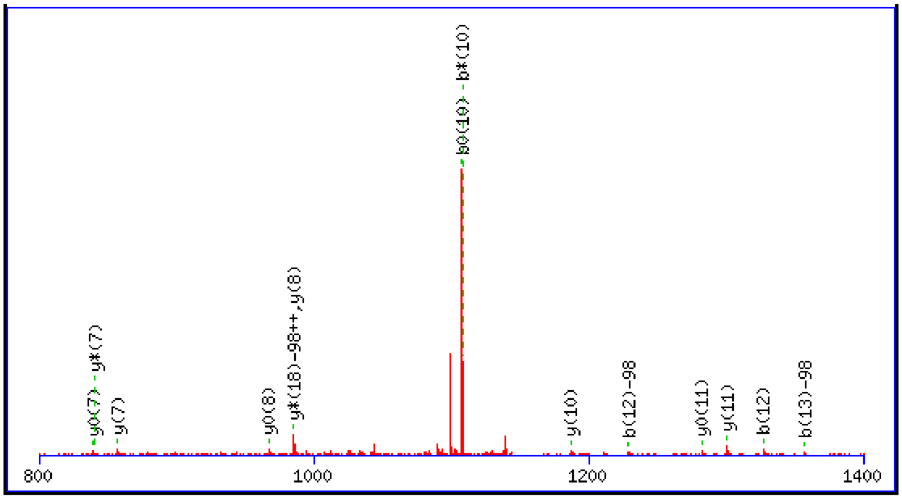

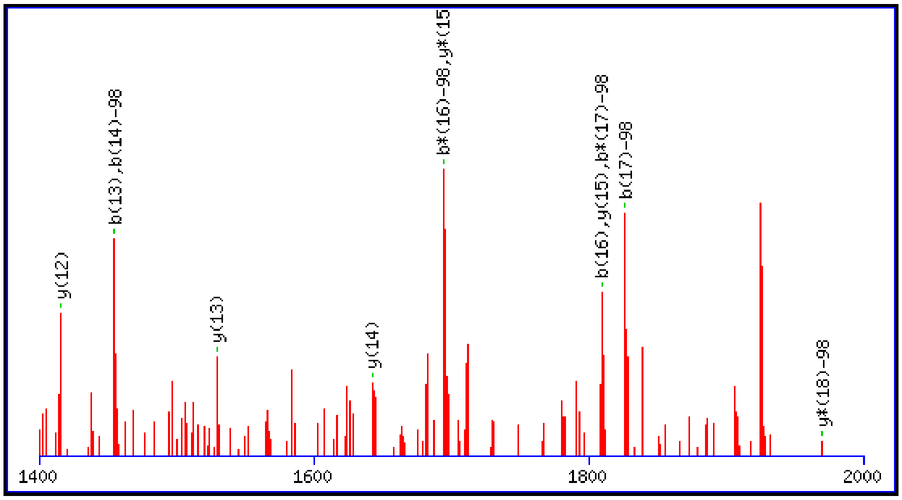

Monoisotopic mass of neutral peptide Mr(calc): 2311.0944
Fixed modifications: Carbamidomethyl (C)
Variable modifications:
S6 : Phospho (ST), with neutral losses 97.9769(shown in table), 0.0000
Ions Score: 58 **Expect:** 0.00039
Matches (Bold Red): 34/332 fragment ions using 46 most intense peaks

#	b	b++	b*	b*++	b0	b0++	Seq.	y	y++	y*	y*++	y0	y0++	#
1	115.0502	58.0287	98.0237	49.5155			N							20
2	228.1343	114.5708	211.1077	106.0575			L	2100.0819	1050.5446	2083.0553	1042.0313	2082.0713	1041.5393	19
3	329.1819	165.0946	312.1554	156.5813	311.1714	156.0893	T	1986.9978	994.0026	**1969.9713**	**985.4893**	1968.9873	984.9973	18
4	416.2140	208.6106	399.1874	200.0974	398.2034	199.6053	S	1885.9502	943.4787	1868.9236	934.9654	1867.9396	934.4734	17
5	**503.2460**	252.1266	**486.2195**	243.6134	485.2354	243.1214	S	1798.9181	899.9627	1781.8916	891.4494	1780.9076	890.9574	16
6	572.2675	286.6374	555.2409	278.1241	554.2569	277.6321	S	1711.8861	856.4467	**1694.8595**	847.9334	1693.8755	847.4414	15
7	**685.3515**	343.1794	668.3250	334.6661	**667.3410**	334.1741	L	**1642.8646**	821.9360	1625.8381	813.4227	1624.8541	812.9307	14
8	799.3944	400.2009	782.3679	391.6876	781.3839	391.1956	N	**1529.7806**	765.3939	1512.7540	756.8807	1511.7700	756.3886	13
9	914.4214	457.7143	897.3948	449.2011	896.4108	448.7091	D	**1415.7377**	708.3725	1398.7111	699.8592	1397.7271	699.3672	12
10	1027.5055	514.2564	1010.4789	505.7431	1009.4949	505.2511	I	**1300.7107**	650.8590	1283.6842	642.3457	**1282.7001**	641.8537	11
11	1114.5375	557.7724	1097.5109	549.2591	1096.5269	548.7671	S	**1187.6266**	594.3170	1170.6001	585.8037	1169.6161	585.3117	10
12	**1229.5644**	615.2859	1212.5379	606.7726	1211.5539	606.2806	D	1100.5946	550.8009	1083.5681	542.2877	1082.5841	541.7957	9
13	**1357.6594**	679.3333	1340.6328	670.8201	1339.6488	670.3281	K	**985.5677**	493.2875	968.5411	484.7742	**967.5571**	484.2822	8
14	**1454.7122**	727.8597	1437.6856	719.3464	1436.7016	718.8544	P	**857.4727**	429.2400	**840.4462**	420.7267	**839.4621**	420.2347	7
15	1583.7547	792.3810	1566.7282	783.8677	1565.7442	783.3757	E	760.4199	380.7136	743.3934	372.2003	742.4094	371.7083	6
16	1711.8497	856.4285	**1694.8232**	847.9152	1693.8391	847.4232	K	**631.3774**	316.1923	614.3508	307.6790	613.3668	307.1870	5
17	**1826.8767**	913.9420	**1809.8501**	905.4287	1808.8661	904.9367	D	**503.2824**	252.1448	**486.2558**	243.6316	485.2718	243.1396	4
18	1954.9352	977.9713	1937.9087	969.4580	1936.9247	968.9660	Q	**388.2554**	194.6314	371.2289	186.1181			3

19	2068.0193	1034.5133	2050.9927	1026.0000	2050.0087	1025.5080	L	260.1969	130.6021	243.1703	122.0888			2
20							K	147.1128	74.0600	130.0863	65.5468			1

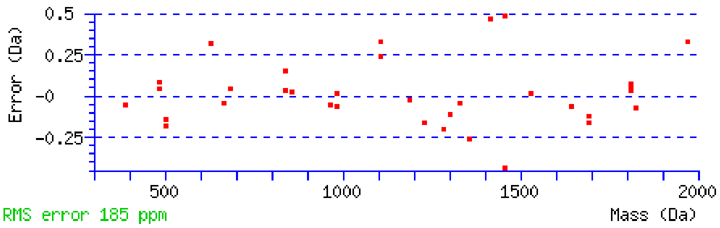

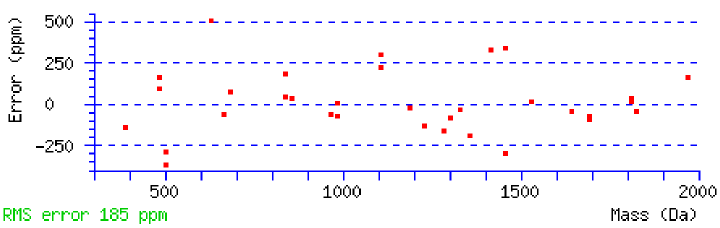

All matches to this query

Score	Mr(calc):	Delta	Sequence
58.5	2311.0944	0.0029	NLTSSSLNDISDKPEKDQLK
57.9	2311.0944	0.0029	NLTSSSLNDISDKPEKDQLK
57.9	2311.0944	0.0029	NLTSSSLNDISDKPEKDQLK
57.9	2311.0944	0.0029	NLTSSSLNDISDKPEKDQLK
32.8	2311.1097	-0.0124	GKSNNVTEFILLGLTQDPAGR
23.8	2311.0944	0.0029	NLTSSSLNDISDKPEKDQLK
6.8	2311.1097	-0.0124	GKSNNVTEFILLGLTQDPAGR
6.5	2310.0810	1.0163	EIYNTYKNNRVASLLGPAK
6.4	2311.1097	-0.0124	GKSNNVTEFILLGLTQDPAGR
6.2	2311.0919	0.0054	CQQPAVIFIDEIDSLLSQR

Spectrum No: 179; Query: 812; Rank: 1

Peptide View

MS/MS Fragmentation of **GSGTASDDEFENLR**
Found in **IPI00201213**, Tax_Id=10116 Gene_Symbol=LOC501546 LOC501546 protein

Match to Query 812: 1576.607288 from(789.310920,2+)
Title: 100101RatKid_NS_deglyco_22.2273.2273.2.dta
Data file K:\NewmanPaper\Piliang\3SubProteomes\Piliang3SP\mgf5ppm\ERLIC_3SubProteomes5ppm.mgf

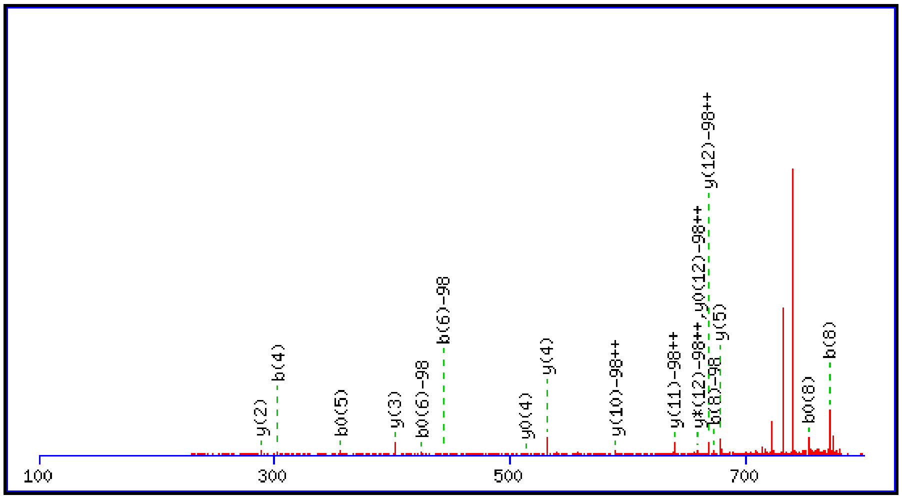

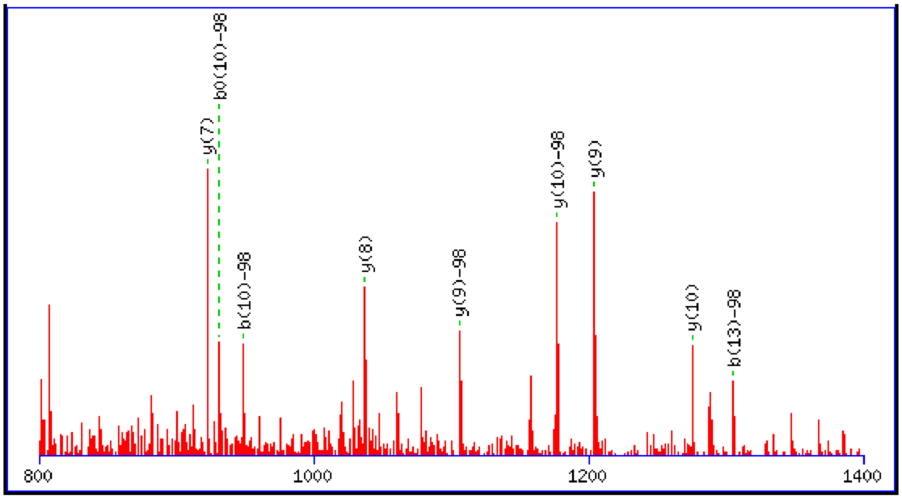

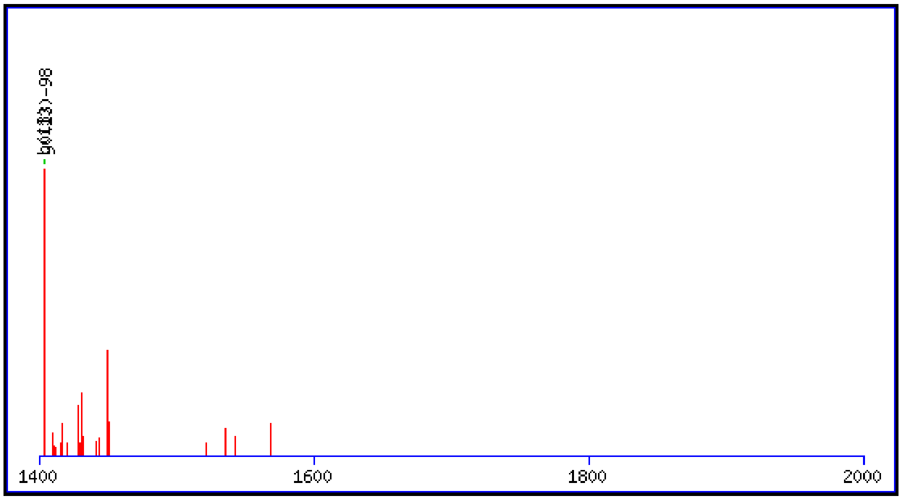

Monoisotopic mass of neutral peptide Mr(calc): 1576.6042
Fixed modifications: Carbamidomethyl (C)
Variable modifications:
S6 : Phospho (ST), with neutral losses 97.9769(shown in table), 0.0000
Ions Score: 58 **Expect:** 0.00015
Matches (Bold Red): 28/192 fragment ions using 40 most intense peaks

#	b	b++	b*	b*++	b0	b0++	Seq.	y	y++	y*	y*++	y0	y0++	#
1	58.0287	29.5180					G							14
2	145.0608	73.0340			127.0502	64.0287	S	1422.6132	711.8102	1405.5866	703.2969	**1404.6026**	702.8049	13
3	202.0822	101.5448			184.0717	92.5395	G	1335.5811	**668.2942**	1318.5546	**659.7809**	1317.5706	**659.2889**	12
4	**303.1299**	152.0686			285.1193	143.0633	T	1278.5597	**639.7835**	1261.5331	631.2702	1260.5491	630.7782	11
5	374.1670	187.5872			**356.1565**	178.5819	A	**1177.5120**	**589.2596**	1160.4854	580.7464	1159.5014	580.2544	10
6	**443.1885**	222.0979			**425.1779**	213.0926	S	**1106.4749**	553.7411	1089.4483	545.2278	1088.4643	544.7358	9
7	558.2154	279.6114			540.2049	270.6061	D	**1037.4534**	519.2304	1020.4269	510.7171	1019.4429	510.2251	8
8	**673.2424**	337.1248			655.2318	328.1195	D	**922.4265**	461.7169	905.3999	453.2036	904.4159	452.7116	7
9	802.2850	401.6461			784.2744	392.6408	E	807.3995	404.2034	790.3730	395.6901	789.3890	395.1981	6
10	**949.3534**	475.1803			**931.3428**	466.1750	F	**678.3569**	339.6821	661.3304	331.1688	660.3464	330.6768	5
11	1078.3960	539.7016			1060.3854	530.6963	E	**531.2885**	266.1479	514.2620	257.6346	**513.2780**	257.1426	4
12	1192.4389	596.7231	1175.4123	588.2098	1174.4283	587.7178	N	**402.2459**	201.6266	385.2194	193.1133			3
13	**1305.5230**	653.2651	1288.4964	644.7518	1287.5124	644.2598	L	**288.2030**	144.6051	271.1765	136.0919			2
14							R	175.1190	88.0631	158.0924	79.5498			1

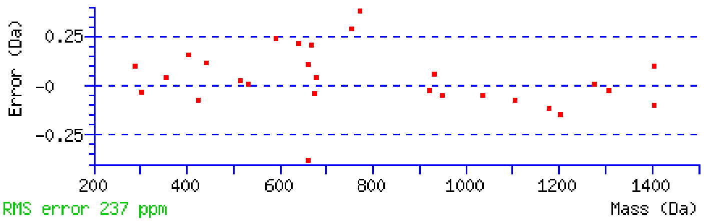

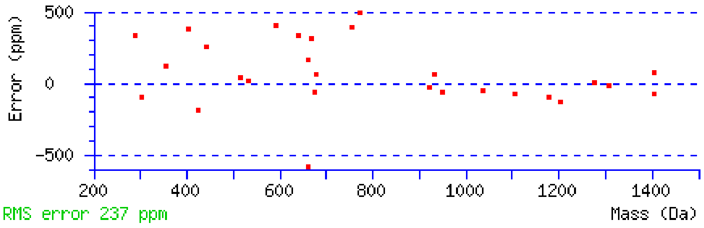

All matches to this query

Score	Mr(calc):	Delta	Sequence
58.4	1576.6042	0.0030	GSGTASDDEFENLR
37.3	1576.6042	0.0030	GSGTASDDEFENLR
37.3	1576.6042	0.0030	GSGTASDDEFENLR
7.7	1576.6150	-0.0077	NEESMEGTMVTLR
4.8	1576.6171	-0.0098	DSEQSKDNLPRK
3.5	1576.6150	-0.0077	TMTELEIDMNQR
3.5	1576.6150	-0.0077	TMTELEIDMNQR
3.0	1576.5994	0.0079	VTQMSPQEDVAGR
2.6	1576.6146	-0.0073	ANYNSAKMALYR
2.3	1576.6150	-0.0077	TMTELEIDMNQR

Spectrum No: 180; Query: 2775; Rank: 1

Peptide View

MS/MS Fragmentation of **LASPSGSTSSGLEVVAPEVTSAPVSGPGILDDSATICR**
Found in **IPI00194567**, Tax_Id=10116 Gene_Symbol=Trim28 similar to Transcription intermediary factor 1-beta

Match to Query 2775: 3763.801362 from(1255.607730,3+)
Title: 100101RatKid_NS_deglyco_18.4950.4950.3.dta
Data file K:\NewmanPaper\Piliang\3SubProteomes\Piliang3SP\mgf5ppm\ERLIC_3SubProteomes5ppm.mgf

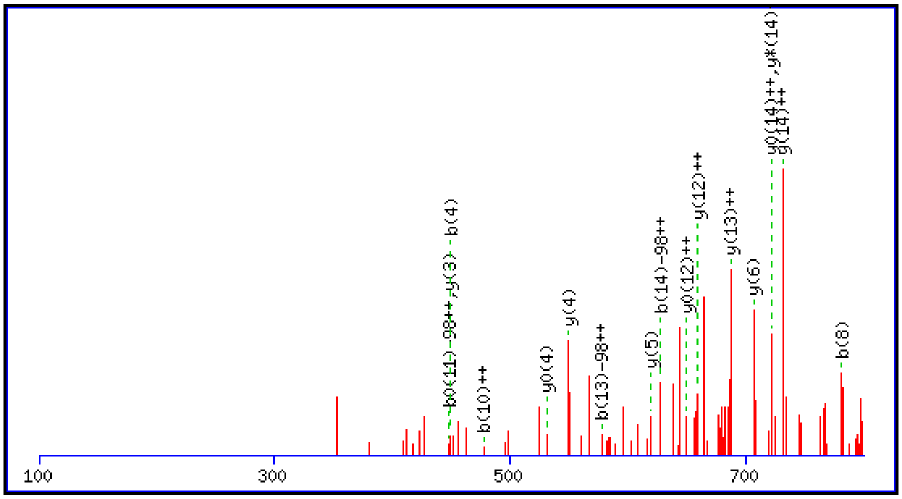

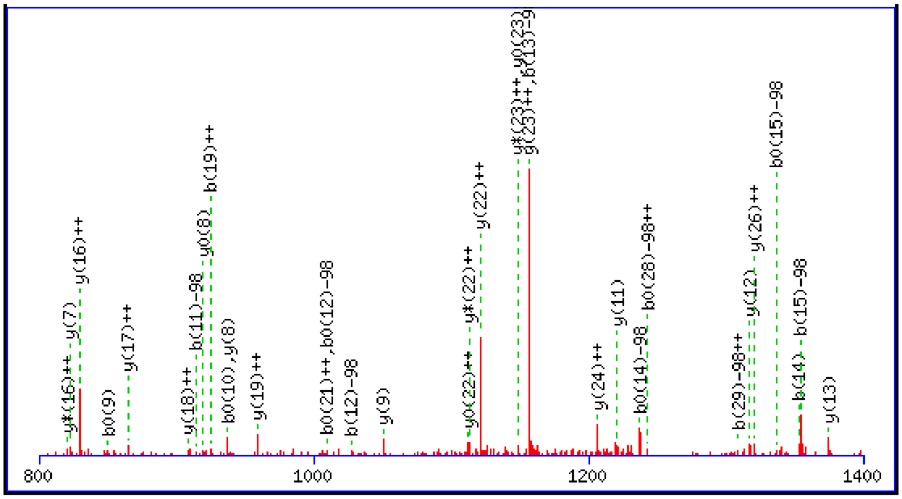

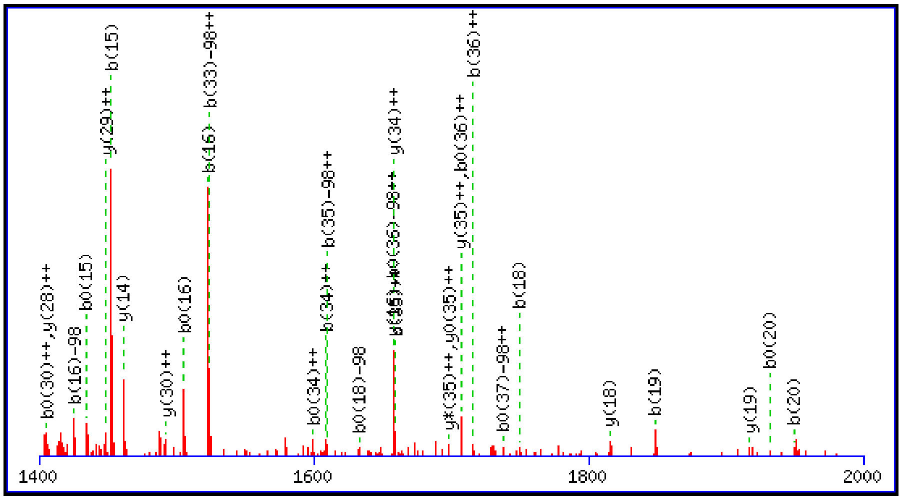

Monoisotopic mass of neutral peptide Mr(calc): 3763.7863
Fixed modifications: Carbamidomethyl (C)
Variable modifications:
S3 : Phospho (ST), with neutral losses 97.9769(shown in table), 0.0000
Ions Score: 58 **Expect:** 0.00051
Matches (Bold Red): 82/512 fragment ions using 168 most intense peaks

#	b	b^{++}	b^{0}	b^{0++}	Seq.	y	y^{++}	y*	y^{*++}	y^{0}	y^{0++}	#
1	114.0913	57.5493			L							38
2	185.1285	93.0679			A	3553.7326	1777.3700	3536.7061	1768.8567	3535.7221	1768.3647	37
3	254.1499	127.5786	236.1393	118.5733	S	3482.6955	1741.8514	3465.6690	1733.3381	3464.6850	1732.8461	36
4	351.2027	176.1050	333.1921	167.0997	P	3413.6741	**1707.3407**	3396.6475	**1698.8274**	3395.6635	**1698.3354**	35
5	438.2347	219.6210	420.2241	210.6157	S	3316.6213	**1658.8143**	3299.5948	1650.3010	3298.6107	1649.8090	34
6	495.2562	248.1317	477.2456	239.1264	G	3229.5893	1615.2983	3212.5627	1606.7850	3211.5787	1606.2930	33
7	582.2882	291.6477	564.2776	282.6425	S	3172.5678	1586.7875	3155.5413	1578.2743	3154.5573	1577.7823	32
8	683.3359	342.1716	665.3253	333.1663	T	3085.5358	1543.2715	3068.5092	1534.7583	3067.5252	1534.2662	31
9	770.3679	385.6876	752.3573	376.6823	S	2984.4881	**1492.7477**	2967.4616	1484.2344	2966.4775	1483.7424	30
10	857.3999	429.2036	839.3894	420.1983	S	2897.4561	**1449.2317**	2880.4295	1440.7184	2879.4455	1440.2264	29
11	**914.4214**	457.7143	896.4108	**448.7091**	G	2810.4241	**1405.7157**	2793.3975	1397.2024	2792.4135	1396.7104	28
12	**1027.5055**	514.2564	**1009.4949**	505.2511	L	2753.4026	1377.2049	2736.3760	1368.6917	2735.3920	1368.1996	27
13	**1156.5480**	**578.7777**	1138.5375	569.7724	E	2640.3185	**1320.6629**	2623.2920	1312.1496	2622.3080	1311.6576	26
14	1255.6165	**628.3119**	**1237.6059**	619.3066	V	2511.2759	1256.1416	2494.2494	1247.6283	2493.2654	1247.1363	25
15	**1354.6849**	677.8461	**1336.6743**	668.8408	V	2412.2075	**1206.6074**	2395.1810	1198.0941	2394.1970	1197.6021	24
16	**1425.7220**	713.3646	1407.7114	704.3594	A	2313.1391	**1157.0732**	2296.1126	**1148.5599**	2295.1285	**1148.0679**	23
17	1522.7748	761.8910	1504.7642	752.8857	P	2242.1020	**1121.5546**	2225.0754	**1113.0414**	2224.0914	**1112.5494**	22
18	1651.8173	826.4123	**1633.8068**	817.4070	E	2145.0492	1073.0283	2128.0227	1064.5150	2127.0387	1064.0230	21

19	1750.8858	875.9465	1732.8752	866.9412	V	2016.0066	1008.5070	1998.9801	999.9937	1997.9961	999.5017	20
20	1851.9334	926.4704	1833.9229	917.4651	T	**1916.9382**	**958.9727**	1899.9117	950.4595	1898.9277	949.9675	19
21	1938.9655	969.9864	1920.9549	960.9811	S	**1815.8905**	**908.4489**	1798.8640	899.9356	1797.8800	899.4436	18
22	2010.0026	1005.5049	1991.9920	996.4996	A	1728.8585	**864.9329**	1711.8320	856.4196	1710.8479	855.9276	17
23	2107.0553	1054.0313	2089.0448	1045.0260	P	**1657.8214**	**829.4143**	1640.7948	**820.9011**	1639.8108	820.4091	16
24	2206.1238	1103.5655	2188.1132	1094.5602	V	1560.7686	780.8880	1543.7421	772.3747	1542.7581	771.8827	15
25	2293.1558	1147.0815	2275.1452	1138.0763	S	**1461.7002**	**731.3537**	1444.6737	**722.8405**	1443.6897	**722.3485**	14
26	2350.1773	1175.5923	2332.1667	1166.5870	G	**1374.6682**	**687.8377**	1357.6416	679.3245	1356.6576	678.8325	13
27	2447.2300	1224.1186	2429.2195	1215.1134	P	**1317.6467**	**659.3270**	1300.6202	650.8137	1299.6362	**650.3217**	12
28	2504.2515	1252.6294	2486.2409	**1243.6241**	G	**1220.5940**	610.8006	1203.5674	602.2873	1202.5834	601.7953	11
29	2617.3355	**1309.1714**	2599.3250	1300.1661	I	1163.5725	582.2899	1146.5460	573.7766	1145.5619	573.2846	10
30	2730.4196	1365.7134	2712.4090	1356.7082	L	**1050.4884**	525.7479	1033.4619	517.2346	1032.4779	516.7426	9
31	2845.4466	1423.2269	2827.4360	1414.2216	D	**937.4044**	469.2058	920.3778	460.6925	**919.3938**	460.2005	8
32	2960.4735	1480.7404	2942.4629	1471.7351	D	**822.3774**	411.6924	805.3509	403.1791	804.3669	402.6871	7
33	3047.5055	**1524.2564**	3029.4950	1515.2511	S	**707.3505**	354.1789	690.3239	345.6656	689.3399	345.1736	6
34	3118.5426	1559.7750	3100.5321	1550.7697	A	**620.3185**	310.6629	603.2919	302.1496	602.3079	301.6576	5
35	3219.5903	**1610.2988**	3201.5798	1601.2935	T	**549.2813**	275.1443	532.2548	266.6310	**531.2708**	266.1390	4
36	3332.6744	1666.8408	3314.6638	**1657.8355**	I	**448.2337**	224.6205	431.2071	216.1072			3
37	3492.7050	1746.8562	3474.6945	**1737.8509**	C	335.1496	168.0784	318.1231	159.5652			2
38					R	175.1190	88.0631	158.0924	79.5498			1

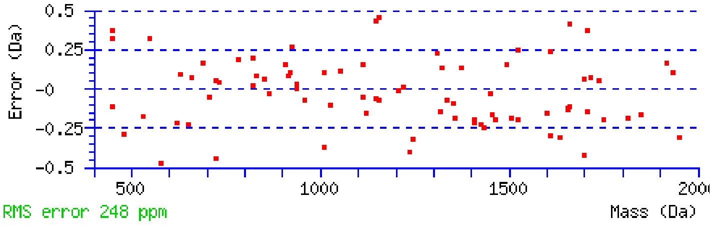

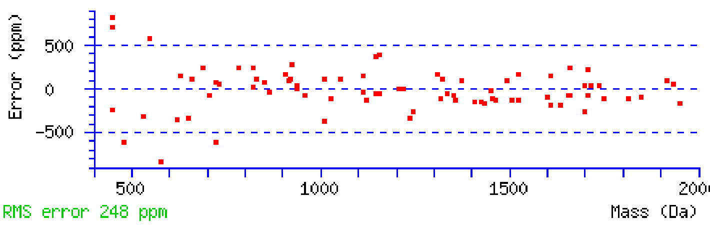

All matches to this query

Score	Mr(calc):	Delta	Sequence
58.4	3763.7863	0.0150	LASPSGSTSSGLEVVAPEVTSAPVSGPGILDDSATICR
53.5	3763.7863	0.0150	LASPSGSTSSGLEVVAPEVTSAPVSGPGILDDSATICR
52.7	3763.7863	0.0150	LASPSGSTSSGLEVVAPEVTSAPVSGPGILDDSATICR
52.0	3763.7863	0.0150	LASPSGSTSSGLEVVAPEVTSAPVSGPGILDDSATICR
48.0	3763.7863	0.0150	LASPSGSTSSGLEVVAPEVTSAPVSGPGILDDSATICR
43.5	3763.7863	0.0150	LASPSGSTSSGLEVVAPEVTSAPVSGPGILDDSATICR
17.6	3763.7863	0.0150	LASPSGSTSSGLEVVAPEVTSAPVSGPGILDDSATICR
12.8	3763.7863	0.0150	LASPSGSTSSGLEVVAPEVTSAPVSGPGILDDSATICR
7.4	3763.8090	-0.0076	TLKCSTLLVVGDNSPAVEAVVSAADXLFEFGGTKK
4.3	3763.7863	0.0150	LASPSGSTSSGLEVVAPEVTSAPVSGPGILDDSATICR

Spectrum No: 181; Query: 2047; Rank: 1

Peptide View

MS/MS Fragmentation of **AKPSPAPSPTISAPDASGPQKR**
Found in **IPI00371516**, Tax_Id=10116 Gene_Symbol=LOC289233 similar to Peroxisomal biogenesis factor 19

Match to Query 2047: 2319.060042 from(774.027290,3+)
Title: 091008RatKidney_NoSalt_21.763.763.3.dta

Data file K:\NewmanPaper\Piliang\3SubProteomes\Piliang3SP\mgf5ppm\ERLIC_3SubProteomes5ppm.mgf

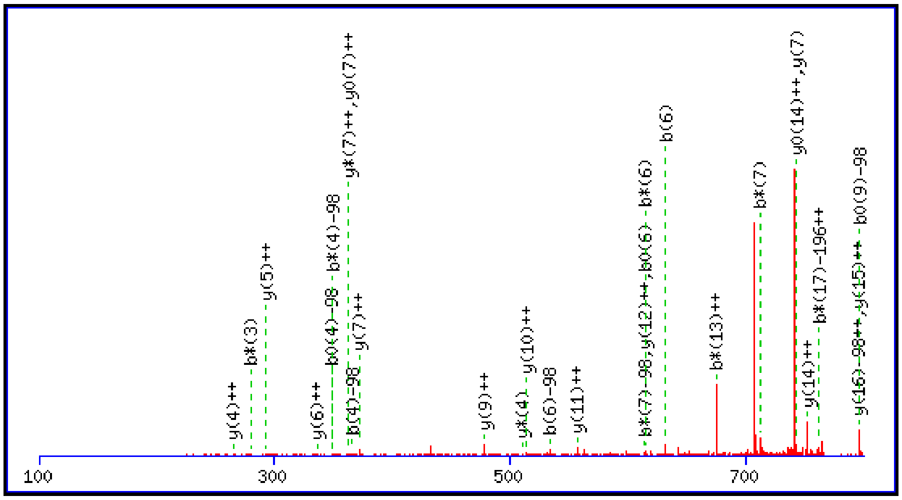

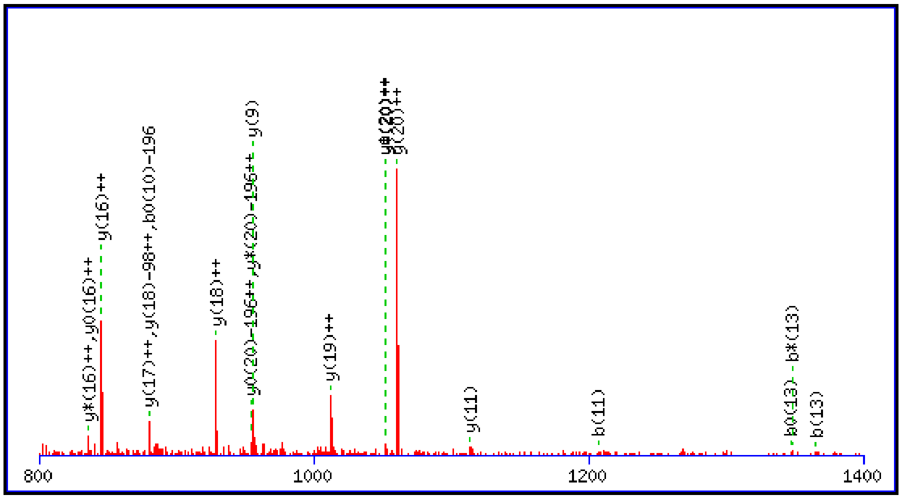

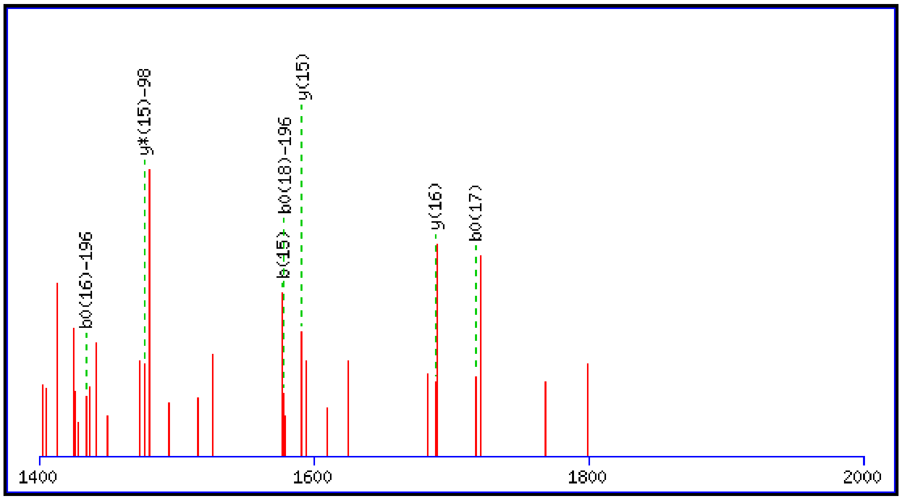

Monoisotopic mass of neutral peptide Mr(calc): 2319.0661
Fixed modifications: Carbamidomethyl (C)
Variable modifications:
S4 : Phospho (ST), with neutral losses 0.0000(shown in table), 97.9769
T10 : Phospho (ST), with neutral losses 0.0000(shown in table), 97.9769
Ions Score: 58 **Expect:** 0.00052
Matches (Bold Red): 55/396 fragment ions using 115 most intense peaks

#	b	b^{++}	b*	b^{*++}	b^0	b^{0++}	Seq.	y	y^{++}	y*	y^{*++}	y^0	y^{0++}	#
1	72.0444	36.5258					A							22
2	200.1394	100.5733	183.1128	92.0600			K	2249.0363	1125.0218	2232.0097	1116.5085	2231.0257	1116.0165	21
3	297.1921	149.0997	**280.1656**	140.5864			P	2120.9413	**1060.9743**	2103.9148	**1052.4610**	2102.9308	**1051.9690**	20

4	464.1905	232.5989	447.1639	224.0856	446.1799	223.5936	S	2023.8886	**1012.4479**	2006.8620	1003.9346	2005.8780	1003.4426	19
5	561.2432	281.1253	544.2167	272.6120	543.2327	272.1200	P	1856.8902	**928.9487**	1839.8637	920.4355	1838.8796	919.9435	18
6	**632.2803**	316.6438	**615.2538**	308.1305	**614.2698**	307.6385	A	1759.8374	**880.4224**	1742.8109	871.9091	1741.8269	871.4171	17
7	729.3331	365.1702	**712.3066**	356.6569	711.3225	356.1649	P	**1688.8003**	**844.9038**	1671.7738	**836.3905**	1670.7898	**835.8985**	16
8	816.3651	408.6862	799.3386	400.1729	798.3546	399.6809	S	**1591.7476**	**796.3774**	1574.7210	787.8641	1573.7370	787.3721	15
9	913.4179	457.2126	896.3914	448.6993	895.4073	448.2073	P	1504.7155	**752.8614**	1487.6890	744.3481	1486.7050	**743.8561**	14
10	1094.4319	547.7196	1077.4054	539.2063	1076.4213	538.7143	T	1407.6628	704.3350	1390.6362	695.8218	1389.6522	695.3297	13
11	**1207.5160**	604.2616	1190.4894	595.7483	1189.5054	595.2563	I	1226.6488	**613.8280**	1209.6222	605.3148	1208.6382	604.8227	12
12	1294.5480	647.7776	1277.5214	639.2644	1276.5374	638.7724	S	**1113.5647**	**557.2860**	1096.5382	548.7727	1095.5541	548.2807	11
13	**1365.5851**	683.2962	**1348.5586**	**674.7829**	**1347.5745**	674.2909	A	1026.5327	**513.7700**	1009.5061	505.2567	1008.5221	504.7647	10
14	1462.6379	731.8226	1445.6113	723.3093	1444.6273	722.8173	P	**955.4956**	**478.2514**	938.4690	469.7381	937.4850	469.2461	9
15	**1577.6648**	789.3360	1560.6383	780.8228	1559.6543	780.3308	D	858.4428	429.7250	841.4163	421.2118	840.4322	420.7198	8
16	1648.7019	824.8546	1631.6754	816.3413	1630.6914	815.8493	A	**743.4159**	**372.2116**	726.3893	**363.6983**	725.4053	**363.2063**	7
17	1735.7340	868.3706	1718.7074	859.8573	**1717.7234**	859.3653	S	672.3787	**336.6930**	655.3522	328.1797	654.3682	327.6877	6
18	1792.7554	896.8814	1775.7289	888.3681	1774.7449	887.8761	G	585.3467	**293.1770**	568.3202	284.6637			5
19	1889.8082	945.4077	1872.7816	936.8945	1871.7976	936.4025	P	528.3253	**264.6663**	**511.2987**	256.1530			4
20	2017.8668	1009.4370	2000.8402	1000.9237	1999.8562	1000.4317	Q	431.2725	216.1399	414.2459	207.6266			3
21	2145.9617	1073.4845	2128.9352	1064.9712	2127.9512	1064.4792	K	303.2139	152.1106	286.1874	143.5973			2
22							R	175.1190	88.0631	158.0924	79.5498			1

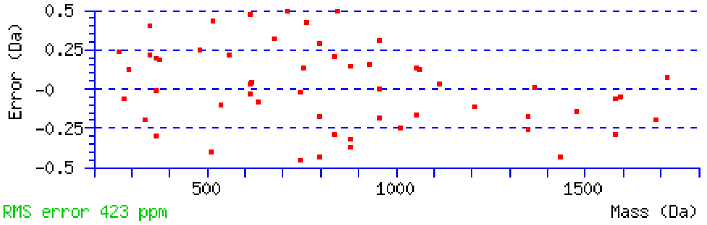

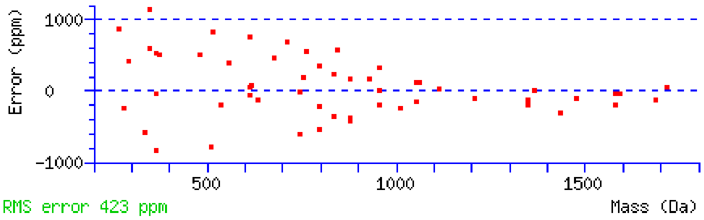

All matches to this query

Score	Mr(calc):	Delta	Sequence
58.2	2319.0661	-0.0061	AKPSPAPSPTISAPDASGPQKR
56.7	2319.0661	-0.0061	AKPSPAPSPTISAPDASGPQKR
38.8	2319.0661	-0.0061	AKPSPAPSPTISAPDASGPQKR
26.1	2319.0661	-0.0061	AKPSPAPSPTISAPDASGPQKR
15.7	2319.0661	-0.0061	AKPSPAPSPTISAPDASGPQKR
12.7	2319.0661	-0.0061	AKPSPAPSPTISAPDASGPQKR
7.1	2319.0661	-0.0061	AKPSPAPSPTISAPDASGPQKR
6.5	2319.0527	0.0073	VFTGLQKLPSQSNMMADINK
4.3	2318.0684	0.9917	GHGLSITPLPAGHMIGGTIWK
2.5	2317.0518	2.0082	VSFHFGGPARSLRSTNPTAR

Spectrum No: 182; Query: 866; Rank: 1

Peptide View

MS/MS Fragmentation of **VTNDISPESSPGVGR**
Found in **IPI00359962**, Tax_Id=10116 Gene_Symbol=Pcm1 similar to pericentriolar material 1

Match to Query 866: 1593.706188 from(797.860370,2+)
Title: 091008RatKidney_NoSalt_24.762.762.2.dta
Data file K:\NewmanPaper\Piliang\3SubProteomes\Piliang3SP\mgf5ppm\ERLIC_3SubProteomes5ppm.mgf

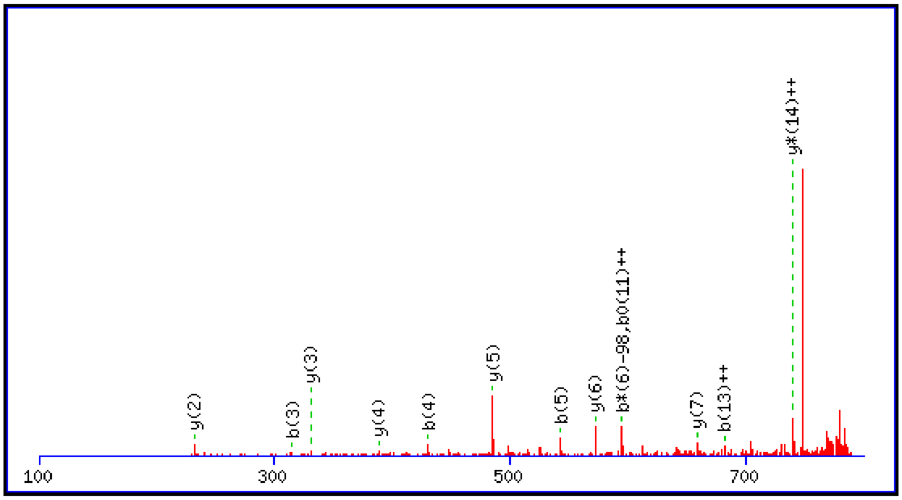

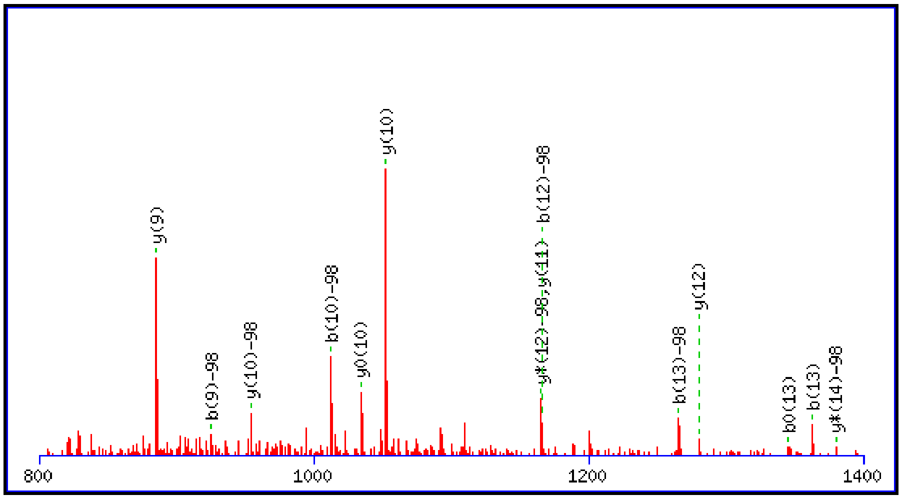

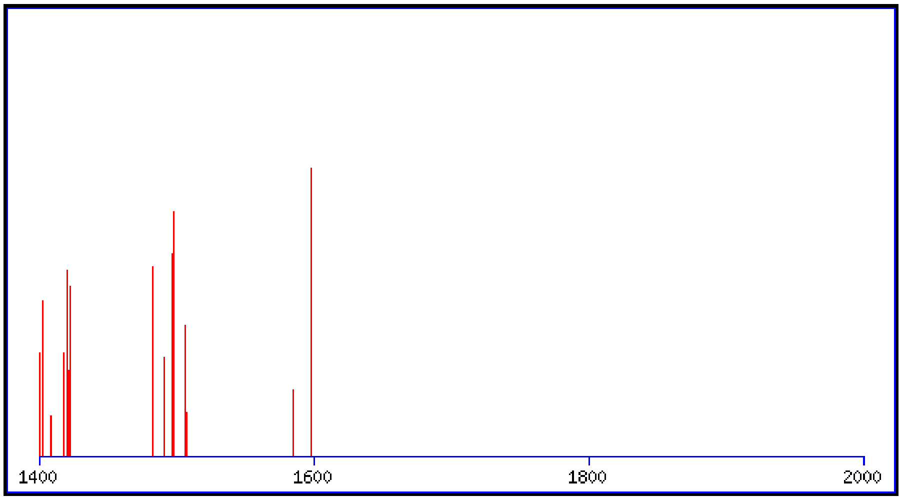

Monoisotopic mass of neutral peptide Mr(calc): 1593.7036
Fixed modifications: Carbamidomethyl (C)
Variable modifications:
S6 : Phospho (ST), with neutral losses 0.0000(shown in table), 97.9769
Ions Score: 58 **Expect:** 0.00025
Matches (Bold Red): 27/236 fragment ions using 45 most intense peaks

#	b	b^{++}	b*	b^{*++}	b^0	b^{0++}	Seq.	y	y^{++}	y*	y^{*++}	y^0	y^{0++}	#
1	100.0757	50.5415					V							15
2	201.1234	101.0653			183.1128	92.0600	T	1495.6424	748.3249	1478.6159	**739.8116**	1477.6319	739.3196	14
3	**315.1663**	158.0868	298.1397	149.5735	297.1557	149.0815	N	1394.5948	697.8010	1377.5682	689.2877	1376.5842	688.7957	13
4	**430.1932**	215.6003	413.1667	207.0870	412.1827	206.5950	D	**1280.5518**	640.7796	1263.5253	632.2663	1262.5413	631.7743	12
5	**543.2773**	272.1423	526.2508	263.6290	525.2667	263.1370	I	**1165.5249**	583.2661	1148.4983	574.7528	1147.5143	574.2608	11

**6**	710.2757	355.6415	693.2491	347.1282	692.2651	346.6362	**S**	**1052.4408**	526.7240	1035.4143	518.2108	**1034.4303**	517.7188	**10**
**7**	807.3284	404.1678	790.3019	395.6546	789.3179	395.1626	**P**	**885.4425**	443.2249	868.4159	434.7116	867.4319	434.2196	**9**
**8**	936.3710	468.6891	919.3445	460.1759	918.3604	459.6839	**E**	788.3897	394.6985	771.3632	386.1852	770.3791	385.6932	**8**
**9**	1023.4030	512.2052	1006.3765	503.6919	1005.3925	503.1999	**S**	**659.3471**	330.1772	642.3206	321.6639	641.3365	321.1719	**7**
**10**	1110.4351	555.7212	1093.4085	547.2079	1092.4245	546.7159	**S**	**572.3151**	286.6612	555.2885	278.1479	554.3045	277.6559	**6**
**11**	1207.4878	604.2476	1190.4613	595.7343	1189.4773	**595.2423**	**P**	**485.2831**	243.1452	468.2565	234.6319			**5**
**12**	1264.5093	632.7583	1247.4827	624.2450	1246.4987	623.7530	**G**	**388.2303**	194.6188	371.2037	186.1055			**4**
**13**	**1363.5777**	**682.2925**	1346.5512	673.7792	**1345.5671**	673.2872	**V**	**331.2088**	166.1081	314.1823	157.5948			**3**
**14**	1420.5992	710.8032	1403.5726	702.2900	1402.5886	701.7979	**G**	**232.1404**	116.5738	215.1139	108.0606			**2**
**15**							**R**	175.1190	88.0631	158.0924	79.5498			**1**

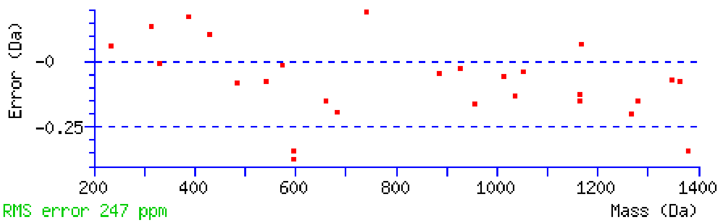

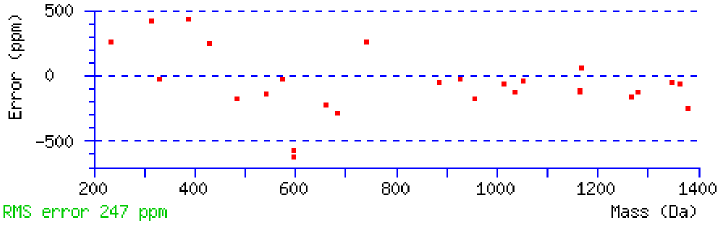

All matches to this query

Score	Mr(calc):	Delta	Sequence
58.2	1593.7036	0.0026	VTNDISPESSPGVGR
40.3	1593.7036	0.0026	VTNDISPESSPGVGR
35.5	1593.7036	0.0026	VTNDISPESSPGVGR
27.2	1593.7036	0.0026	VTNDISPESSPGVGR
8.6	1593.6937	0.0125	VSQNGGTGHANPTFK
3.8	1593.6937	0.0125	VSQNGGTGHANPTFK
3.1	1591.6879	2.0183	NNDVLTPTPDSSPR
3.1	1591.6879	2.0183	NNDVLTPTPDSSPR
2.1	1593.6937	0.0125	GTQGPPGATGFPGSAGR
1.1	1593.7106	-0.0044	FSDKFSLHLKGR

Spectrum No: 183; Query: 2053; Rank: 1

Peptide View

MS/MS Fragmentation of **RQLHIEGASLELSDDDTESK**
Found in **IPI00211813**, Tax_Id=10116 Gene_Symbol=Myh10 Myosin-10

Match to Query 2053: 2322.047712 from(775.023180,3+)
Title: 100101RatKid_NS_deglyco_08.2688.2688.3.dta
Data file K:\NewmanPaper\Piliang\3SubProteomes\Piliang3SP\mgf5ppm\ERLIC_3SubProteomes5ppm.mgf

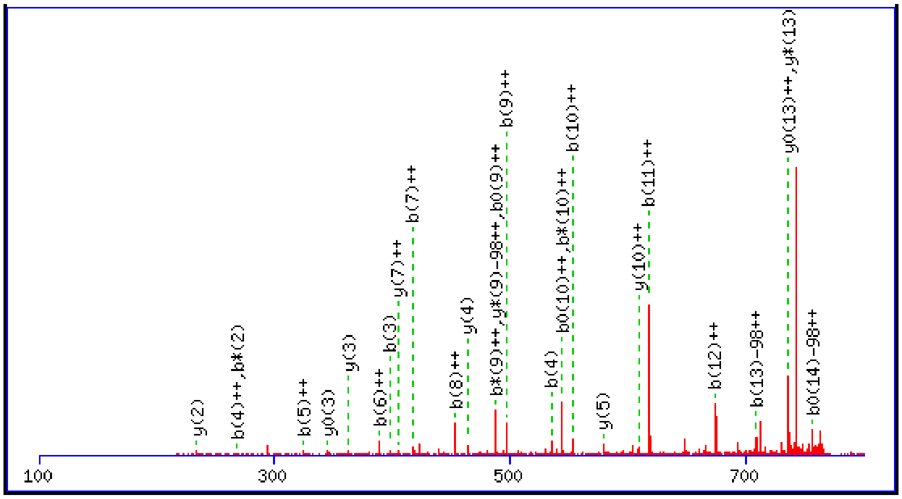

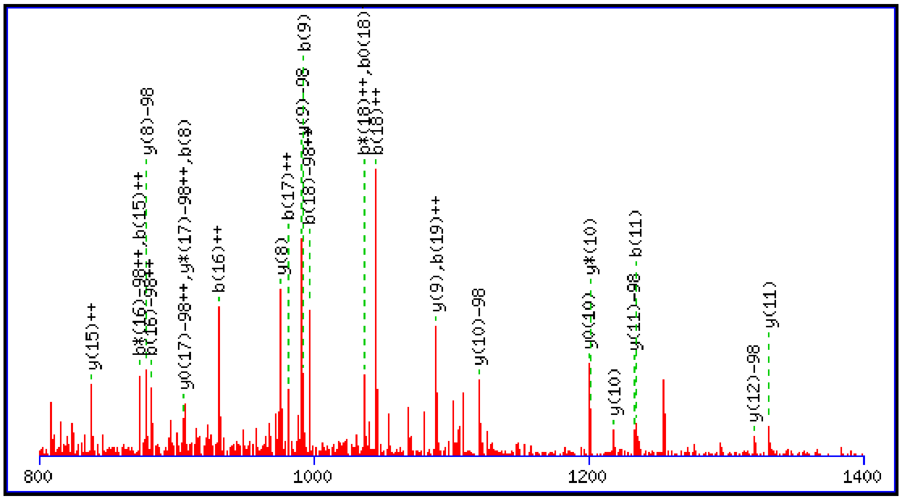

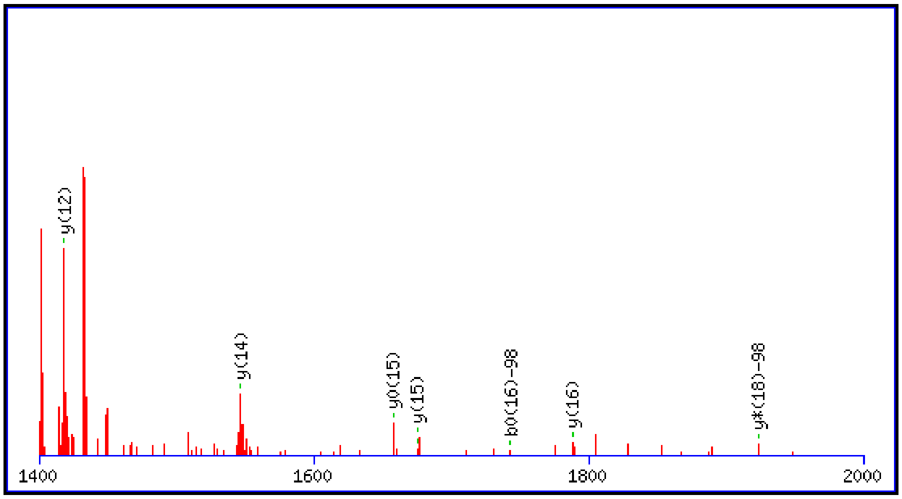

Monoisotopic mass of neutral peptide Mr(calc): 2322.0376
Fixed modifications: Carbamidomethyl (C)
Variable modifications:
S13 : Phospho (ST), with neutral losses 0.0000(shown in table), 97.9769
Ions Score: 58 **Expect:** 0.00044
Matches (Bold Red): 62/330 fragment ions using 106 most intense peaks

#	b	b^{++}	b*	b*$^{++}$	b^{0}	b^{0++}	Seq.	y	y^{++}	y*	y*$^{++}$	y^{0}	y^{0++}	#
1	157.1084	79.0578	140.0818	70.5446			R							20
2	285.1670	143.0871	**268.1404**	134.5738			Q	2166.9438	1083.9755	2149.9173	1075.4623	2148.9333	1074.9703	19
3	**398.2510**	199.6292	381.2245	191.1159			L	2038.8852	1019.9463	2021.8587	1011.4330	2020.8747	1010.9410	18
4	**535.3099**	**268.1586**	518.2834	259.6453			H	1925.8012	963.4042	1908.7746	954.8910	1907.7906	954.3989	17
5	648.3940	**324.7006**	631.3675	316.1874			I	**1788.7423**	894.8748	1771.7157	886.3615	1770.7317	885.8695	16

**6**	777.4366	**389.2219**	760.4100	380.7087	759.4260	380.2167	**E**	**1675.6582**	**838.3327**	1658.6317	829.8195	**1657.6476**	829.3275	**15**
**7**	834.4581	**417.7327**	817.4315	409.2194	816.4475	408.7274	**G**	**1546.6156**	773.8114	1529.5891	765.2982	1528.6050	764.8062	**14**
**8**	**905.4952**	**453.2512**	888.4686	444.7380	887.4846	444.2459	**A**	1489.5941	745.3007	1472.5676	**736.7874**	1471.5836	**736.2954**	**13**
**9**	**992.5272**	**496.7672**	975.5007	**488.2540**	974.5166	**487.7620**	**S**	**1418.5570**	709.7822	1401.5305	701.2689	1400.5465	700.7769	**12**
**10**	1105.6113	**553.3093**	1088.5847	**544.7960**	1087.6007	**544.3040**	**L**	**1331.5250**	666.2661	1314.4985	657.7529	1313.5144	657.2609	**11**
**11**	**1234.6539**	**617.8306**	1217.6273	609.3173	1216.6433	608.8253	**E**	**1218.4409**	**609.7241**	**1201.4144**	601.2108	**1200.4304**	600.7188	**10**
**12**	1347.7379	**674.3726**	1330.7114	665.8593	1329.7274	665.3673	**L**	**1089.3983**	545.2028	1072.3718	536.6895	1071.3878	536.1975	**9**
**13**	1514.7363	757.8718	1497.7097	749.3585	1496.7257	748.8665	**S**	**976.3143**	488.6608	959.2877	480.1475	958.3037	479.6555	**8**
**14**	1629.7632	815.3852	1612.7367	806.8720	1611.7527	806.3800	**D**	809.3159	**405.1616**	792.2894	396.6483	791.3054	396.1563	**7**
**15**	1744.7902	**872.8987**	1727.7636	864.3854	1726.7796	863.8934	**D**	694.2890	347.6481	677.2624	339.1349	676.2784	338.6429	**6**
**16**	1859.8171	**930.4122**	1842.7906	921.8989	1841.8065	921.4069	**D**	**579.2620**	290.1347	562.2355	281.6214	561.2515	281.1294	**5**
**17**	1960.8648	**980.9360**	1943.8382	972.4228	1942.8542	971.9307	**T**	**464.2351**	232.6212	447.2086	224.1079	446.2245	223.6159	**4**
**18**	2089.9074	**1045.4573**	2072.8808	**1036.9441**	2071.8968	**1036.4520**	**E**	**363.1874**	182.0974	346.1609	173.5841	**345.1769**	173.0921	**3**
**19**	2176.9394	**1088.9733**	2159.9129	1080.4601	2158.9288	1079.9681	**S**	**234.1448**	117.5761	217.1183	109.0628	216.1343	108.5708	**2**
**20**							**K**	147.1128	74.0600	130.0863	65.5468			**1**

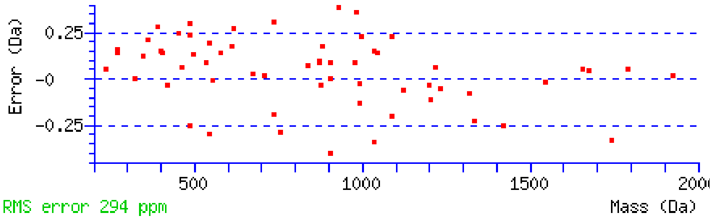

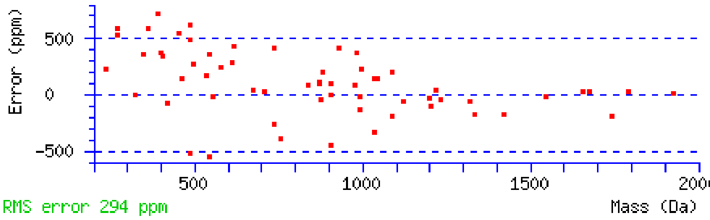

All matches to this query

Score	Mr(calc):	Delta	Sequence
58.2	2322.0376	0.0101	RQLHIEGASLELSDDDTESK
43.3	2322.0376	0.0101	RQLHIEGASLELSDDDTESK
35.1	2322.0376	0.0101	RQLHIEGASLELSDDDTESK
26.0	2322.0376	0.0101	RQLHIEGASLELSDDDTESK
13.5	2322.0524	-0.0047	ELPPELLAEIESTMPLCER
13.2	2322.0637	-0.0159	TAMAKAGYTNQVVIGMDVAASK
12.6	2322.0524	-0.0047	ELPPELLAEIESTMPLCER
12.0	2322.0637	-0.0159	TAMAKAGYTNQVVIGMDVAASK
8.2	2322.0637	-0.0159	TAMAKAGYTNQVVIGMDVAASK
7.4	2322.0352	0.0125	YNGAIQVVMNQDGTGFTVANK

Spectrum No: 184; Query: 2117; Rank: 1

Peptide View

MS/MS Fragmentation of **NWEDEDFYDSDDDTFLDR**
Found in **IPI00562860**, Tax_Id=10116 Gene_Symbol=- 57 kDa protein

Match to Query 2117: 2375.844228 from(1188.929390,2+)
Title: 100101RatKid_NS_deglyco_26.4276.4276.2.dta
Data file K:\NewmanPaper\Piliang\3SubProteomes\Piliang3SP\mgf5ppm\ERLIC_3SubProteomes5ppm.mgf

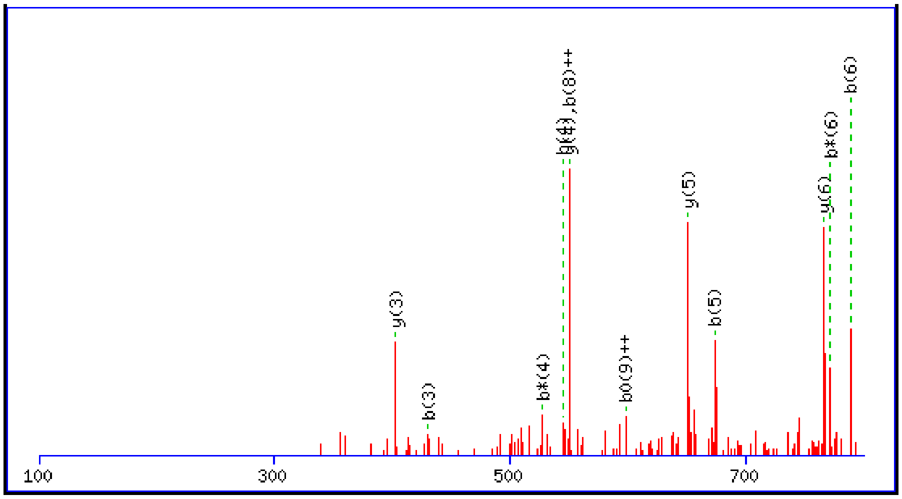

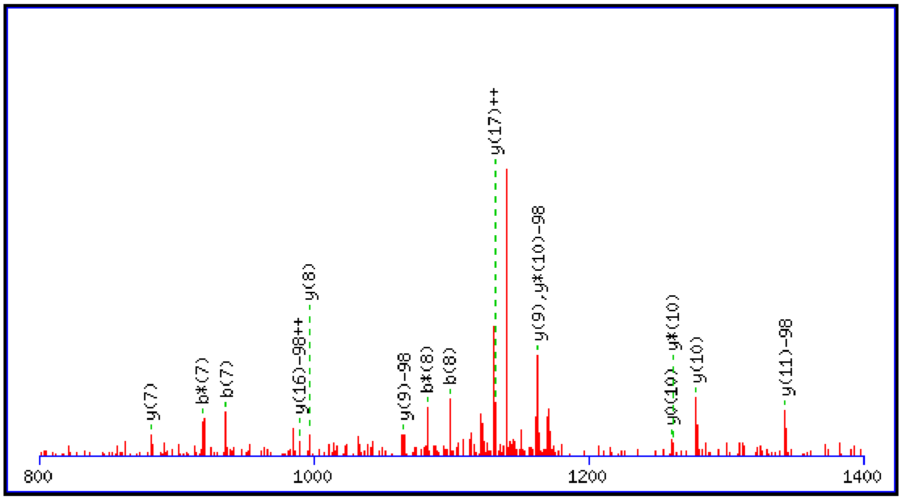

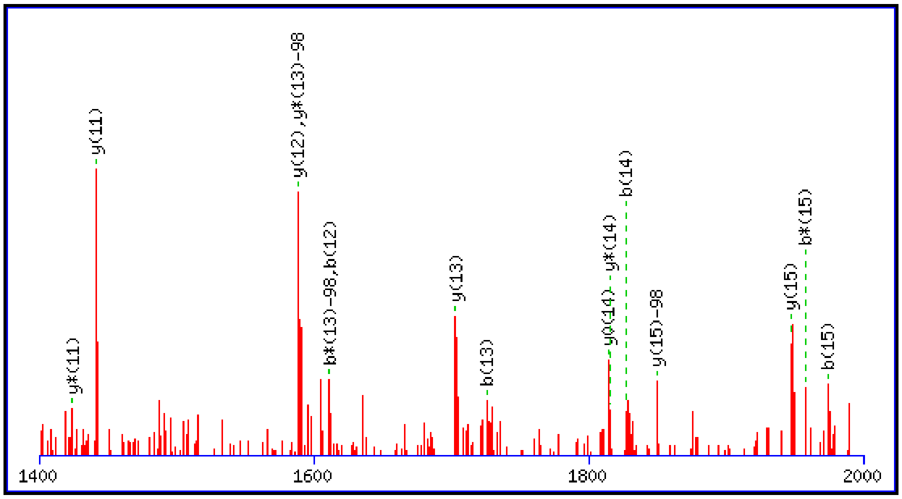

Monoisotopic mass of neutral peptide Mr(calc): 2375.8380
Fixed modifications: Carbamidomethyl (C)
Variable modifications:
S10 : Phospho (ST), with neutral losses 0.0000(shown in table), 97.9769
Ions Score: 58 **Expect:** 0.00016
Matches (Bold Red): 42/300 fragment ions using 72 most intense peaks

#	b	b^{++}	b*	b^{*++}	b^0	b^{0++}	Seq.	y	y^{++}	y*	y^{*++}	y^0	y^{0++}	#
1	115.0502	58.0287	98.0237	49.5155			N							18
2	301.1295	151.0684	284.1030	142.5551			W	2262.8023	**1131.9048**	2245.7758	1123.3915	2244.7917	1122.8995	17
3	**430.1721**	215.5897	413.1456	207.0764	412.1615	206.5844	E	2076.7230	1038.8651	2059.6964	1030.3519	2058.7124	1029.8599	16
4	**545.1991**	273.1032	**528.1725**	264.5899	527.1885	264.0979	D	**1947.6804**	974.3438	1930.6538	965.8306	1929.6698	965.3386	15
5	**674.2416**	337.6245	657.2151	329.1112	656.2311	328.6192	E	1832.6535	916.8304	**1815.6269**	908.3171	**1814.6429**	907.8251	14

6	789.2686	395.1379	772.2420	386.6247	771.2580	386.1326	D	1703.6109	852.3091	1686.5843	843.7958	1685.6003	843.3038	13
7	936.3370	468.6721	919.3105	460.1589	918.3264	459.6669	F	1588.5839	794.7956	1571.5574	786.2823	1570.5734	785.7903	12
8	1099.4003	550.2038	1082.3738	541.6905	1081.3898	541.1985	Y	1441.5155	721.2614	1424.4890	712.7481	1423.5049	712.2561	11
9	1214.4273	607.7173	1197.4007	599.2040	1196.4167	598.7120	D	1278.4522	639.7297	1261.4256	631.2165	1260.4416	630.7244	10
10	1381.4256	691.2165	1364.3991	682.7032	1363.4151	682.2112	S	1163.4252	582.2163	1146.3987	573.7030	1145.4147	573.2110	9
11	1496.4526	748.7299	1479.4260	740.2166	1478.4420	739.7246	D	996.4269	498.7171	979.4003	490.2038	978.4163	489.7118	8
12	1611.4795	806.2434	1594.4530	797.7301	1593.4689	797.2381	D	881.3999	441.2036	864.3734	432.6903	863.3894	432.1983	7
13	1726.5065	863.7569	1709.4799	855.2436	1708.4959	854.7516	D	766.3730	383.6901	749.3464	375.1769	748.3624	374.6849	6
14	1827.5541	914.2807	1810.5276	905.7674	1809.5436	905.2754	T	651.3461	326.1767	634.3195	317.6634	633.3355	317.1714	5
15	1974.6225	987.8149	1957.5960	979.3016	1956.6120	978.8096	F	550.2984	275.6528	533.2718	267.1396	532.2878	266.6475	4
16	2087.7066	1044.3569	2070.6801	1035.8437	2069.6960	1035.3517	L	403.2300	202.1186	386.2034	193.6053	385.2194	193.1133	3
17	2202.7336	1101.8704	2185.7070	1093.3571	2184.7230	1092.8651	D	290.1459	145.5766	273.1193	137.0633	272.1353	136.5713	2
18							R	175.1190	88.0631	158.0924	79.5498			1

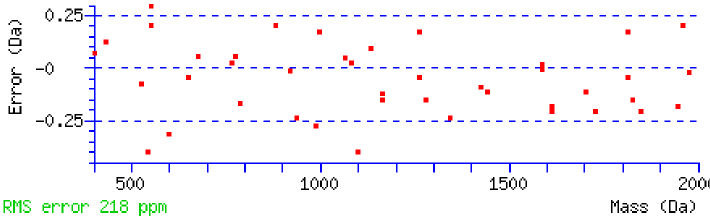

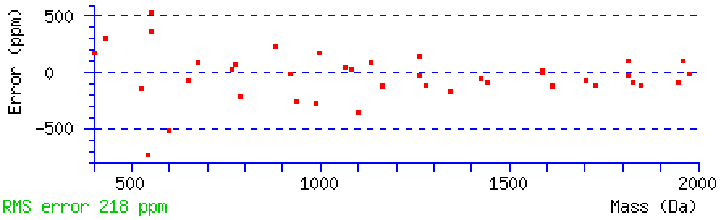

All matches to this query

Score	Mr(calc):	Delta	Sequence
58.0	2375.8380	0.0063	NWEDEDFYDSDDDTFLDR
33.8	2375.8380	0.0063	NWEDEDFYDSDDDTFLDR
31.0	2375.8380	0.0063	NWEDEDFYDSDDDTFLDR
2.9	2375.8379	0.0063	DSAKANPSCSVRPGTGDSSDK

Spectrum No: 185; Query: 441; Rank: 1

Peptide View

MS/MS Fragmentation of **GEPNVSYICSR**
Found in **IPI00189904**, Tax_Id=10116 Gene_Symbol=Gsk3a Glycogen synthase kinase-3 alpha

Match to Query 441: 1360.544988 from(681.279770,2+)
Title: 091008RatKidney_NoSalt_22.1237.1237.2.dta
Data file K:\NewmanPaper\Piliang\3SubProteomes\Piliang3SP\mgf5ppm\ERLIC_3SubProteomes5ppm.mgf

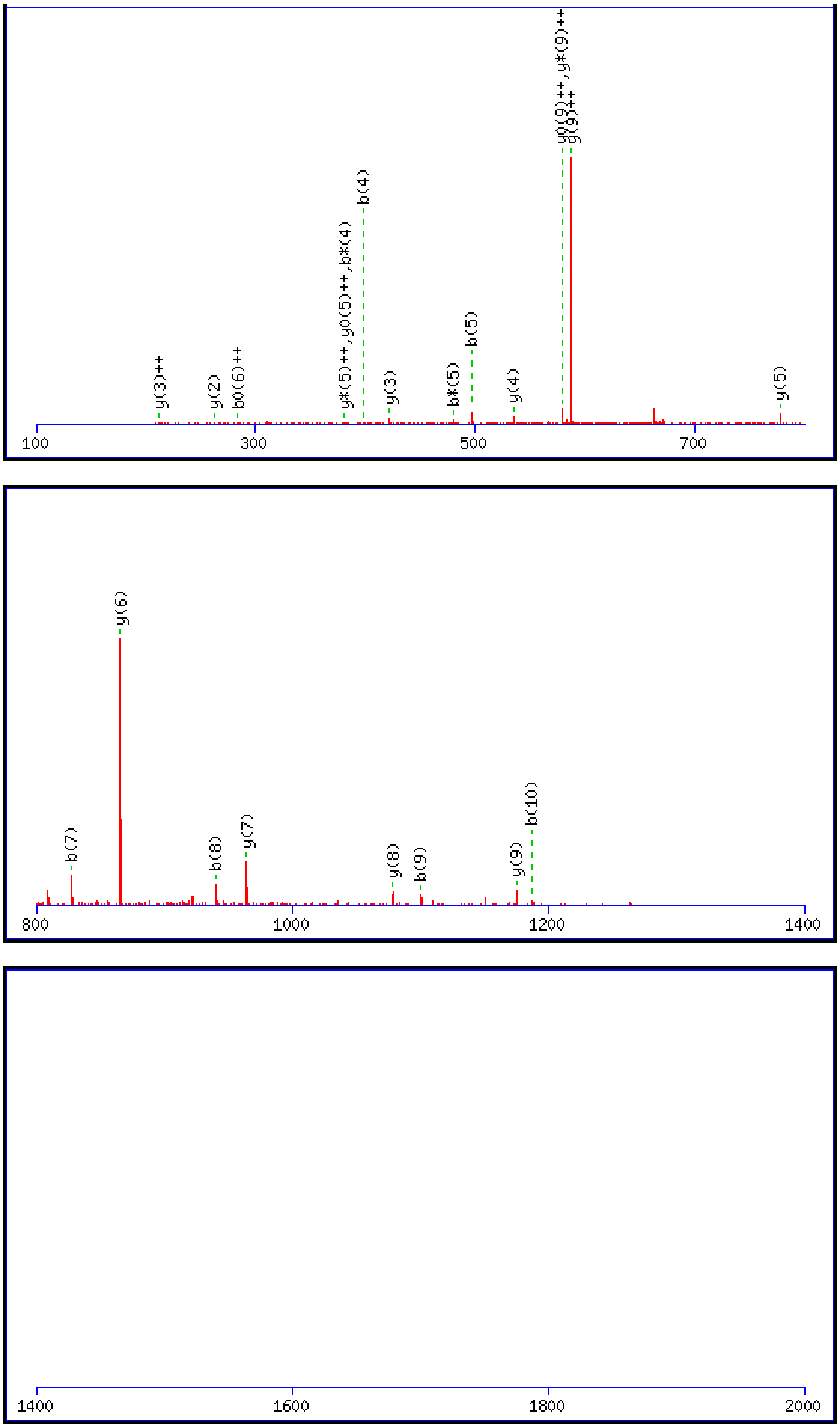

Monoisotopic mass of neutral peptide Mr(calc): 1360.5482
Fixed modifications: Carbamidomethyl (C)
Variable modifications:
Y7 : Phospho (Y)
Ions Score: 58 **Expect:** 0.00014
Matches (Bold Red): 23/110 fragment ions using 35 most intense peaks

#	b	b^{++}	b*	b^{*++}	b^{0}	b^{0++}	Seq.	y	y^{++}	y*	y^{*++}	y^{0}	y^{0++}	#
1	58.0287	29.5180					G							11
2	187.0713	94.0393			169.0608	85.0340	E	1304.5341	652.7707	1287.5075	644.2574	1286.5235	643.7654	10
3	284.1241	142.5657			266.1135	133.5604	P	**1175.4915**	**588.2494**	1158.4649	**579.7361**	1157.4809	**579.2441**	9
4	**398.1670**	199.5872	**381.1405**	191.0739	380.1565	190.5819	N	**1078.4387**	539.7230	1061.4122	531.2097	1060.4282	530.7177	8
5	**497.2354**	249.1214	**480.2089**	240.6081	479.2249	240.1161	V	**964.3958**	482.7015	947.3692	474.1883	946.3852	473.6962	7

**6**	584.2675	292.6374	567.2409	284.1241	566.2569	**283.6321**	**S**	**865.3274**	433.1673	848.3008	424.6541	847.3168	424.1620	**6**
**7**	**827.2971**	414.1522	810.2706	405.6389	809.2866	405.1469	**Y**	**778.2953**	389.6513	761.2688	**381.1380**	760.2848	**380.6460**	**5**
**8**	**940.3812**	470.6942	923.3546	462.1810	922.3706	461.6889	**I**	**535.2657**	268.1365	518.2391	259.6232	517.2551	259.1312	**4**
**9**	**1100.4118**	550.7096	1083.3853	542.1963	1082.4013	541.7043	**C**	**422.1816**	**211.5945**	405.1551	203.0812	404.1711	202.5892	**3**
**10**	**1187.4439**	594.2256	1170.4173	585.7123	1169.4333	585.2203	**S**	**262.1510**	131.5791	245.1244	123.0659	244.1404	122.5738	**2**
**11**							**R**	175.1190	88.0631	158.0924	79.5498			**1**

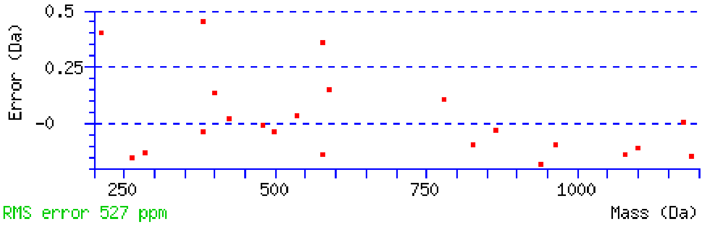

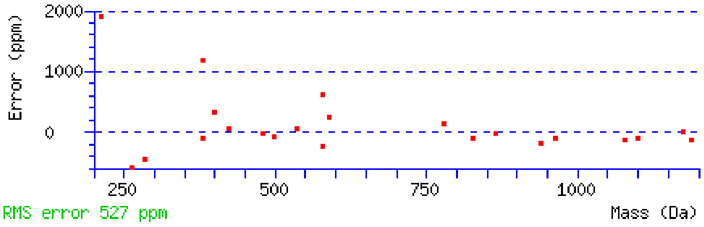

All matches to this query

Score	Mr(calc):	Delta	Sequence
57.9	1360.5482	-0.0033	GEPNVSYICSR
46.1	1360.5482	-0.0033	GEPNVSYICSR
26.9	1360.5482	-0.0033	GEPNVSYICSR
12.7	1358.5286	2.0164	SLDXRNRDDK
8.0	1359.5528	0.9922	SRRSNGGESSSR
4.1	1360.5516	-0.0066	IDTSCVCALSR
4.1	1360.5516	-0.0066	IDTSCVCALSR
3.8	1360.5330	0.0120	GEETKAMESQR
3.8	1358.5288	2.0162	CSFLYDVTMK
3.6	1358.5268	2.0181	SNILTDRHDK

Spectrum No: 186; Query: 1471; Rank: 1

Peptide View

MS/MS Fragmentation of **EESEESDEDMGFGLFD**
Found in **IPI00200147**, Tax_Id=10116 Gene_Symbol=Arbp 60S acidic ribosomal protein P0

Match to Query 1471: 1930.639628 from(966.327090,2+)
Title: 100101RatKid_NS_deglyco_29.4061.4061.2.dta
Data file K:\NewmanPaper\Piliang\3SubProteomes\Piliang3SP\mgf5ppm\ERLIC_3SubProteomes5ppm.mgf

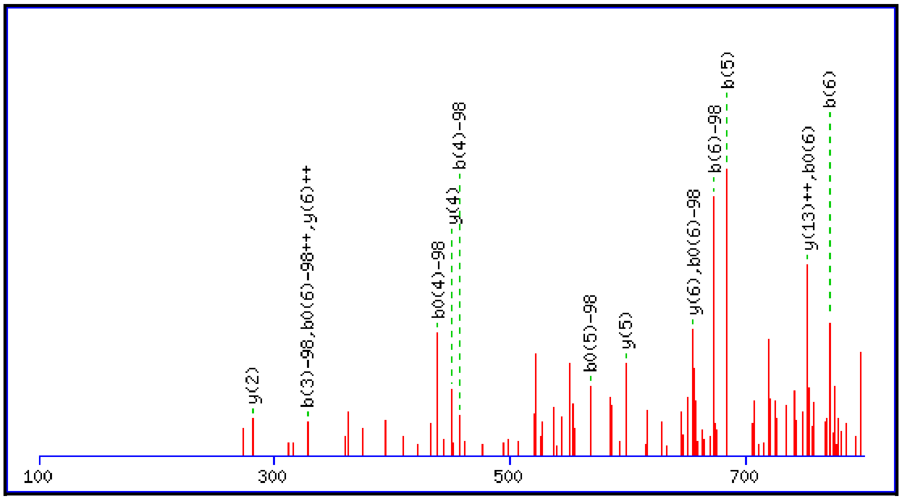

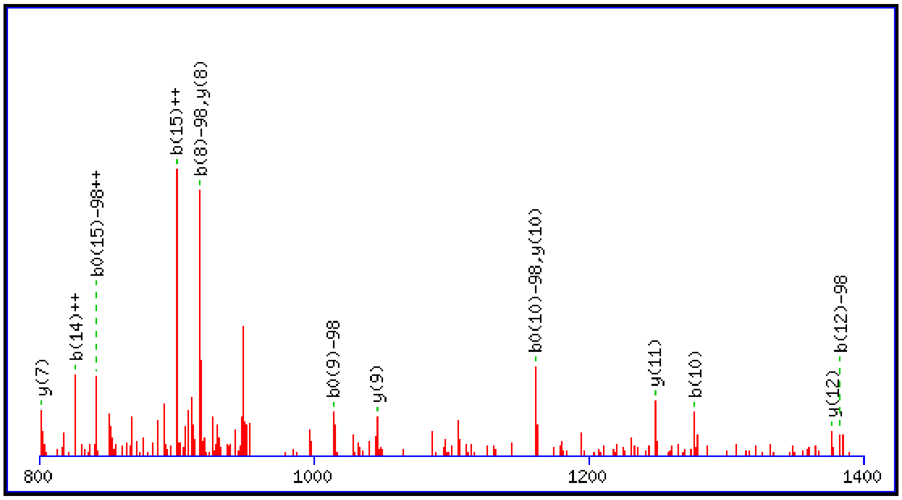

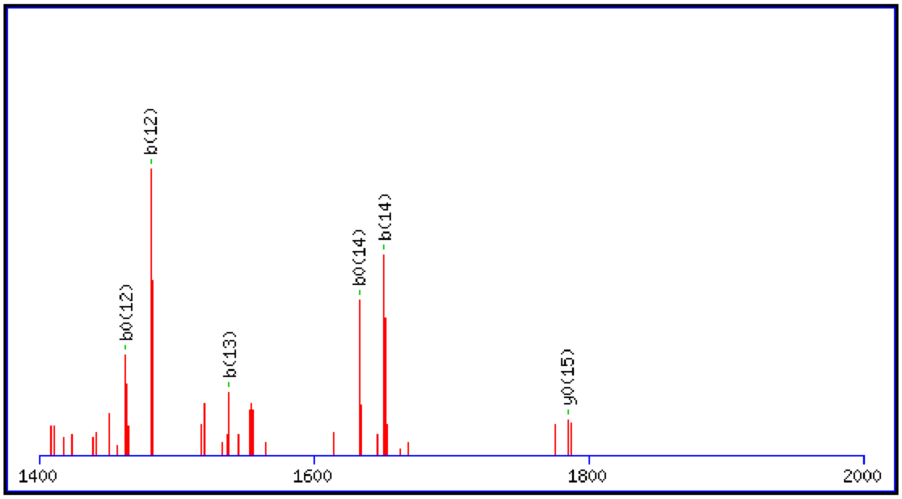

Monoisotopic mass of neutral peptide Mr(calc): 1930.6340
Fixed modifications: Carbamidomethyl (C)
Variable modifications:
S3 : Phospho (ST), with neutral losses 97.9769(shown in table), 0.0000
M10 : Oxidation (M)
Ions Score: 58 **Expect:** 8.1e-005
Matches (Bold Red): 36/180 fragment ions using 47 most intense peaks

#	b	b^{++}	b^0	b^{0++}	Seq.	y	y^{++}	y^0	y^{0++}	#
1	130.0499	65.5286	112.0393	56.5233	E					16
2	259.0925	130.0499	241.0819	121.0446	E	1704.6218	852.8145	1686.6112	843.8092	15
3	**328.1139**	164.5606	310.1034	155.5553	S	1575.5792	788.2932	1557.5686	779.2879	14
4	**457.1565**	229.0819	**439.1459**	220.0766	E	1506.5577	**753.7825**	1488.5471	744.7772	13
5	586.1991	293.6032	**568.1885**	284.5979	E	**1377.5151**	689.2612	1359.5045	680.2559	12
6	**673.2311**	337.1192	**655.2206**	**328.1139**	S	**1248.4725**	624.7399	1230.4620	615.7346	11
7	788.2581	394.6327	770.2475	385.6274	D	**1161.4405**	581.2239	1143.4299	572.2186	10
8	**917.3007**	459.1540	899.2901	450.1487	E	**1046.4135**	523.7104	1028.4030	514.7051	9
9	1032.3276	516.6674	**1014.3170**	507.6622	D	**917.3710**	459.1891	899.3604	450.1838	8
10	1179.3630	590.1851	**1161.3525**	581.1799	M	**802.3440**	401.6756	784.3334	392.6704	7
11	1236.3845	618.6959	1218.3739	609.6906	G	**655.3086**	**328.1579**	637.2980	319.1527	6
12	**1383.4529**	692.2301	1365.4423	683.2248	F	**598.2871**	299.6472	580.2766	290.6419	5
13	1440.4744	720.7408	1422.4638	711.7355	G	**451.2187**	226.1130	433.2082	217.1077	4
14	1553.5584	777.2828	1535.5479	768.2776	L	394.1973	197.6023	376.1867	188.5970	3
15	1700.6268	850.8171	1682.6163	**841.8118**	F	**281.1132**	141.0602	263.1026	132.0550	2
16					D	134.0448	67.5260	116.0342	58.5207	1

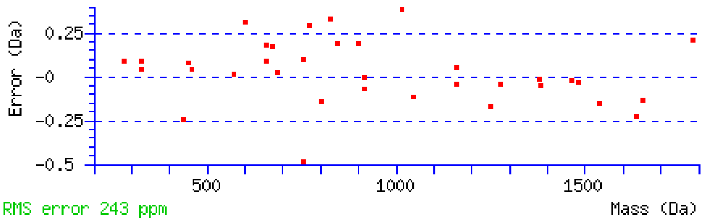

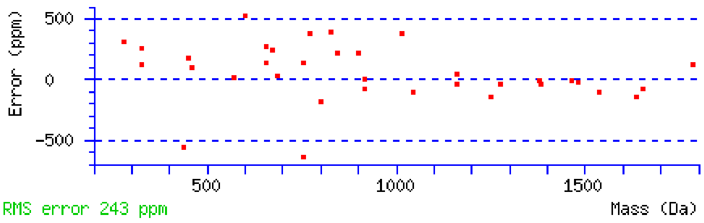

All matches to this query

Score	Mr(calc):	Delta	Sequence
57.9	1930.6340	0.0057	EESEESDEDMGFGLFD
46.9	1930.6340	0.0057	EESEESEDDMGFGLFD
36.0	1930.6340	0.0057	EESEESDEDMGFGLFD
28.7	1930.6340	0.0057	EESEESEDDMGFGLFD
2.9	1929.6196	1.0200	LSGLDGQRDSSSSSSK
0.8	1928.6489	1.9907	EESEESEDDVGIGLFD

Spectrum No: 187; Query: 596; Rank: 1

Peptide View

MS/MS Fragmentation of **IVSAQSLAEDDVE**
Found in **IPI00208207**, Tax_Id=10116 Gene_Symbol=Tomm20 Mitochondrial import receptor subunit TOM20 homolog

Match to Query 596: 1454.620268 from(728.317410,2+)
Title: 091008RatKidney_NoSalt_33.1993.1993.2.dta
Data file K:\NewmanPaper\Piliang\3SubProteomes\Piliang3SP\mgf5ppm\ERLIC_3SubProteomes5ppm.mgf

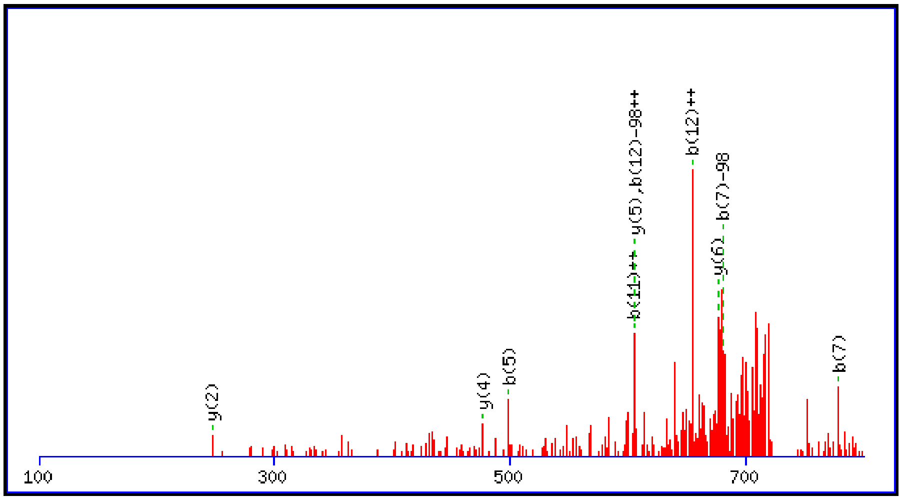

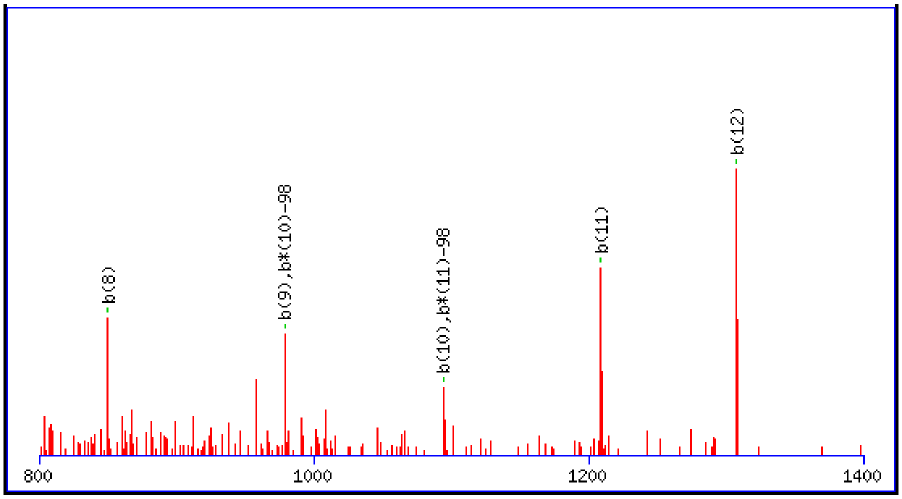

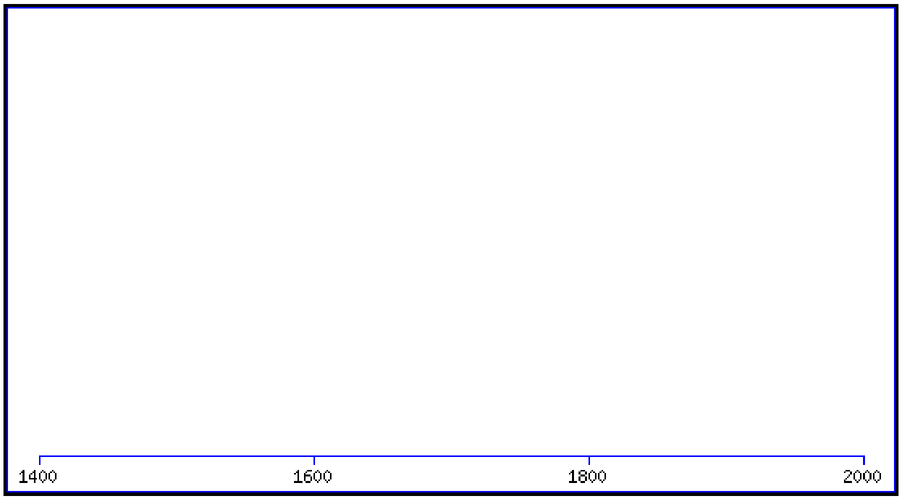

Monoisotopic mass of neutral peptide Mr(calc): 1454.6178
Fixed modifications: Carbamidomethyl (C)
Variable modifications:
S6 : Phospho (ST), with neutral losses 0.0000(shown in table), 97.9769
Ions Score: 58 **Expect:** 0.00021
Matches (Bold Red): 17/186 fragment ions using 35 most intense peaks

#	b	b^{++}	b*	b^{*++}	b^0	b^{0++}	Seq.	y	y^{++}	y*	y^{*++}	y^0	y^{0++}	#
1	114.0913	57.5493					I							13
2	213.1598	107.0835					V	1342.5410	671.7741	1325.5144	663.2609	1324.5304	662.7689	12
3	300.1918	150.5995			282.1812	141.5942	S	1243.4726	622.2399	1226.4460	613.7267	1225.4620	613.2346	11
4	371.2289	186.1181			353.2183	177.1128	A	1156.4405	578.7239	1139.4140	570.2106	1138.4300	569.7186	10
5	**499.2875**	250.1474	482.2609	241.6341	481.2769	241.1421	Q	1085.4034	543.2054	1068.3769	534.6921	1067.3929	534.2001	9
6	666.2858	333.6466	649.2593	325.1333	648.2753	324.6413	S	957.3449	479.1761			939.3343	470.1708	8
7	**779.3699**	390.1886	762.3433	381.6753	761.3593	381.1833	L	790.3465	395.6769			772.3359	386.6716	7
8	**850.4070**	425.7071	833.3805	417.1939	832.3964	416.7019	A	**677.2624**	339.1349			659.2519	330.1296	6
9	**979.4496**	490.2284	962.4230	481.7152	961.4390	481.2232	E	**606.2253**	303.6163			588.2148	294.6110	5
10	**1094.4765**	547.7419	1077.4500	539.2286	1076.4660	538.7366	D	**477.1827**	239.0950			459.1722	230.0897	4
11	**1209.5035**	**605.2554**	1192.4769	596.7421	1191.4929	596.2501	D	362.1558	181.5815			344.1452	172.5763	3
12	**1308.5719**	**654.7896**	1291.5453	646.2763	1290.5613	645.7843	V	**247.1288**	124.0681			229.1183	115.0628	2
13							E	148.0604	74.5339			130.0499	65.5286	1

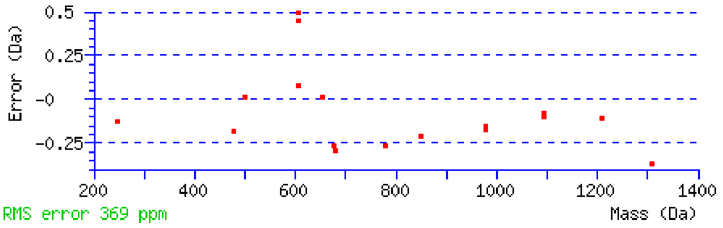

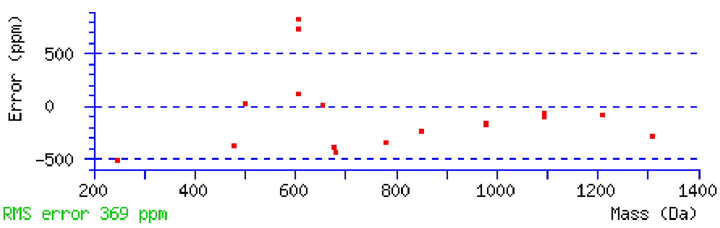

All matches to this query

Score	Mr(calc):	Delta	Sequence
57.9	1454.6178	0.0025	IVSAQSLAEDDVE
42.6	1454.6178	0.0025	IVSAQSLAEDDVE
5.2	1454.6191	0.0012	SNHRFENETIK
4.7	1454.6330	-0.0127	FPNVASNSAEPIK
4.3	1453.6126	1.0076	WLPVQDSGTEDK
3.5	1454.6177	0.0026	MVNRGNCSKGCR
3.4	1453.6272	0.9930	MSANDAITTLHGK
3.4	1453.6272	0.9930	MSANDAITTLHGK
2.8	1454.6281	-0.0079	LSSMKFKDPVK
2.7	1453.6234	0.9969	EKMSMYNITLK

Spectrum No: 188; Query: 1470; Rank: 1

Peptide View

MS/MS Fragmentation of **RVIENTDGSEEEMDAR**
Found in **IPI00464464**, Tax_Id=10116 Gene_Symbol=Myh11 Isoform 1 of Myosin-11 (Fragment)

Match to Query 1470: 1929.782982 from(644.268270,3+)
Title: 100101RatKid_NS_deglyco_20.911.911.3.dta
Data file K:\NewmanPaper\Piliang\3SubProteomes\Piliang3SP\mgf5ppm\ERLIC_3SubProteomes5ppm.mgf

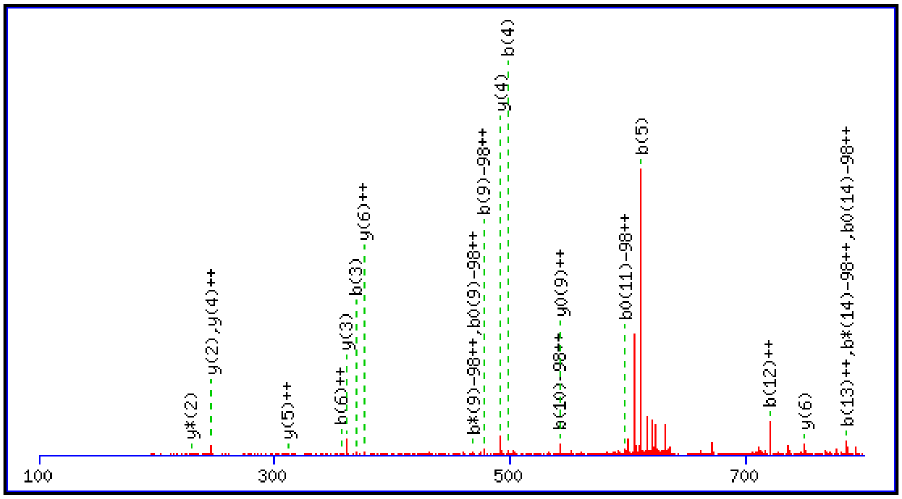

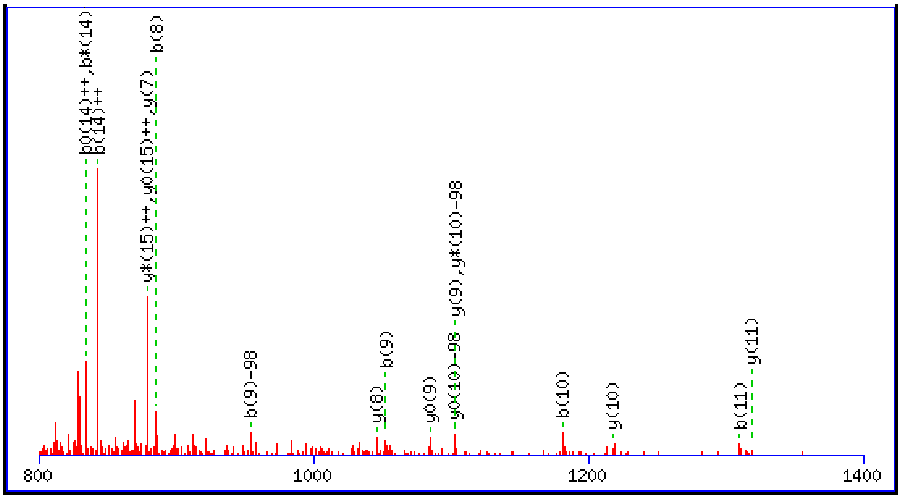

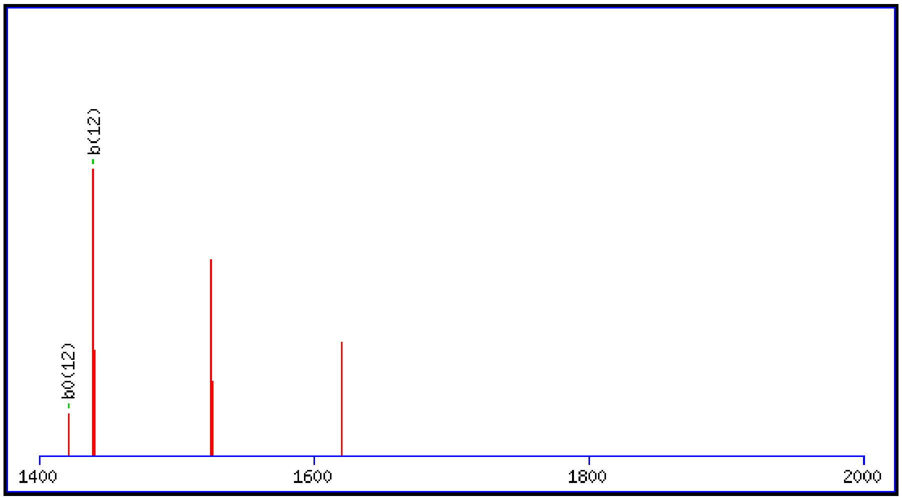

Monoisotopic mass of neutral peptide Mr(calc): 1929.7775
Fixed modifications: Carbamidomethyl (C)
Variable modifications:
S9 : Phospho (ST), with neutral losses 0.0000(shown in table), 97.9769
Ions Score: 58 **Expect:** 0.0003
Matches (Bold Red): 43/260 fragment ions using 52 most intense peaks

#	b	b^{++}	b*	b^{*++}	b^0	b^{0++}	Seq.	y	y^{++}	y*	y^{*++}	y^0	y^{0++}	#
1	157.1084	79.0578	140.0818	70.5446			R							16
2	256.1768	128.5920	239.1503	120.0788			V	1774.6837	887.8455	1757.6572	**879.3322**	1756.6731	**878.8402**	15
3	**369.2609**	185.1341	352.2343	176.6208			I	1675.6153	838.3113	1658.5887	829.7980	1657.6047	829.3060	14
4	**498.3035**	249.6554	481.2769	241.1421	480.2929	240.6501	E	1562.5312	781.7693	1545.5047	773.2560	1544.5207	772.7640	13
5	**612.3464**	306.6768	595.3198	298.1636	594.3358	297.6715	N	1433.4886	717.2480	1416.4621	708.7347	1415.4781	708.2427	12
6	713.3941	**357.2007**	696.3675	348.6874	695.3835	348.1954	T	**1319.4457**	660.2265	1302.4192	651.7132	1301.4351	651.2212	11
7	828.4210	414.7141	811.3945	406.2009	810.4104	405.7089	D	**1218.3980**	609.7027	1201.3715	601.1894	1200.3875	600.6974	10
8	**885.4425**	443.2249	868.4159	434.7116	867.4319	434.2196	G	**1103.3711**	552.1892	1086.3445	543.6759	**1085.3605**	**543.1839**	9
9	**1052.4408**	526.7240	1035.4143	518.2108	1034.4303	517.7188	S	**1046.3496**	523.6785	1029.3231	515.1652	1028.3391	514.6732	8
10	**1181.4834**	591.2453	1164.4569	582.7321	1163.4729	582.2401	E	**879.3513**	440.1793	862.3247	431.6660	861.3407	431.1740	7
11	**1310.5260**	655.7666	1293.4995	647.2534	1292.5154	646.7614	E	**750.3087**	**375.6580**	733.2821	367.1447	732.2981	366.6527	6
12	**1439.5686**	**720.2879**	1422.5421	711.7747	**1421.5580**	711.2827	E	621.2661	**311.1367**	604.2395	302.6234	603.2555	302.1314	5
13	1570.6091	**785.8082**	1553.5825	777.2949	1552.5985	776.8029	M	**492.2235**	**246.6154**	475.1969	238.1021	474.2129	237.6101	4
14	1685.6360	**843.3217**	1668.6095	**834.8084**	1667.6255	**834.3164**	D	**361.1830**	181.0951	344.1565	172.5819	343.1724	172.0899	3
15	1756.6731	**878.8402**	1739.6466	870.3269	1738.6626	869.8349	A	**246.1561**	123.5817	**229.1295**	115.0684			2
16							R	175.1190	88.0631	158.0924	79.5498			1

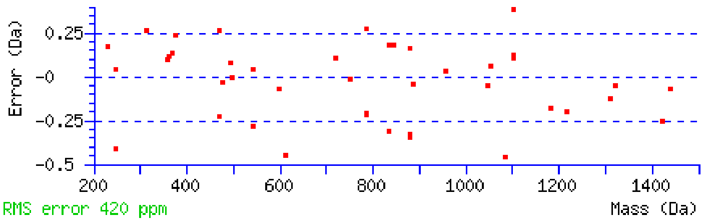

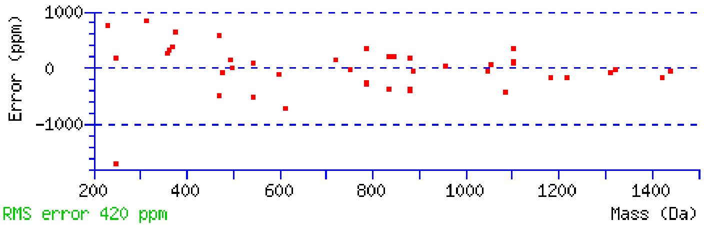

All matches to this query

Score	Mr(calc):	Delta	Sequence
57.6	1929.7775	0.0055	RVIENTDGSEEEMDAR
31.9	1929.7775	0.0055	RVIENTDGSEEEMDAR
13.4	1929.7767	0.0063	CVTMSSAVVQLYAADR
3.4	1928.7988	0.9842	TLESLSKRWDLWR
2.7	1929.7983	-0.0153	GRGSSSHAPVTKENSQK
2.7	1929.7983	-0.0153	GRGSSSHAPVTKENSQK
2.7	1929.7983	-0.0153	GRGSSSHAPVTKENSQK
2.6	1929.7767	0.0063	CVTMSSAVVQLYAADR
2.6	1929.7767	0.0063	CVTMSSAVVQLYAADR
2.3	1929.7798	0.0032	DYVANEVASLFISADR

Spectrum No: 189; Query: 897; Rank: 1

Peptide View

MS/MS Fragmentation of **VDSTTCLFPVEEK**
Found in **IPI00190908**, Tax_Id=10116 Gene_Symbol=Gfpt1 79 kDa protein

Match to Query 897: 1603.684048 from(802.849300,2+)
Title: 091008RatKidney_NoSalt_22.2669.2669.2.dta
Data file K:\NewmanPaper\Piliang\3SubProteomes\Piliang3SP\mgf5ppm\ERLIC_3SubProteomes5ppm.mgf

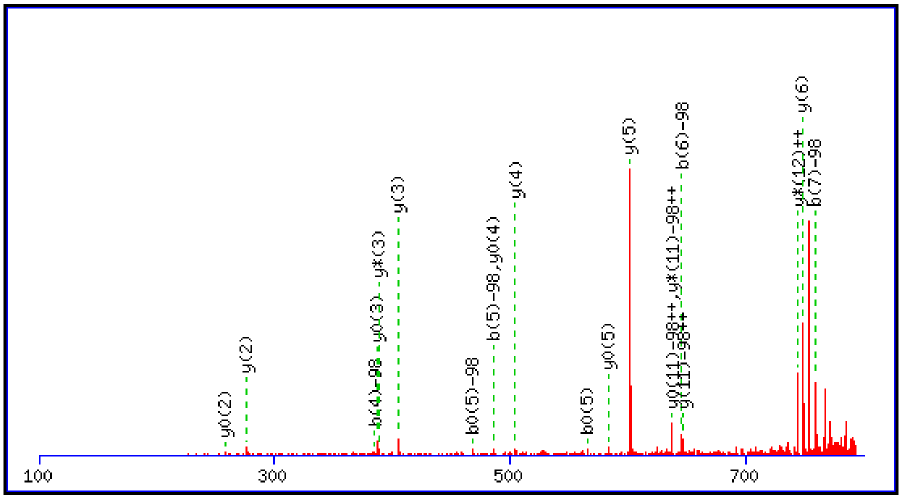

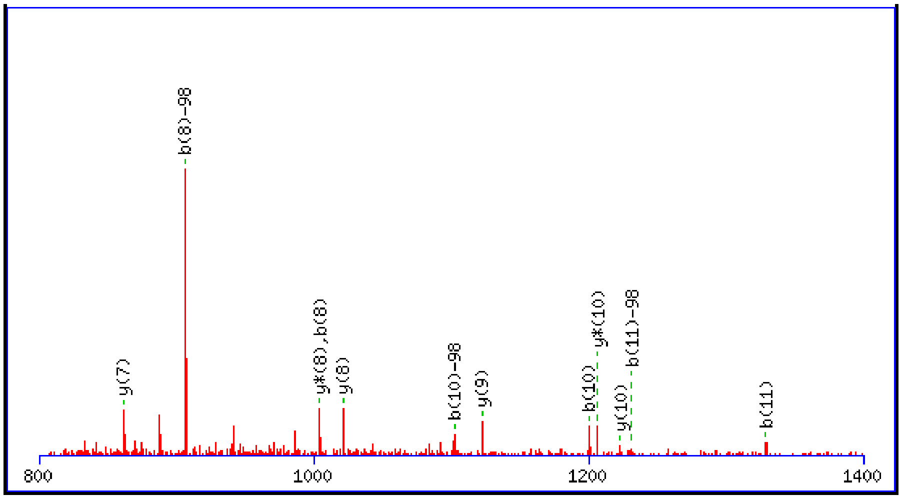

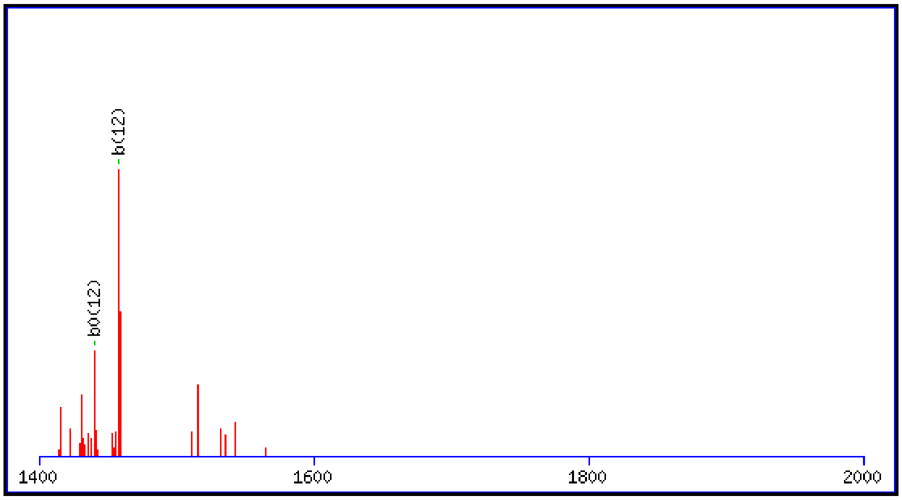

Monoisotopic mass of neutral peptide Mr(calc): 1603.6841
Fixed modifications: Carbamidomethyl (C)
Variable modifications:
S3 : Phospho (ST), with neutral losses 97.9769(shown in table), 0.0000
Ions Score: 58 **Expect:** 0.00026
Matches (Bold Red): 34/168 fragment ions using 50 most intense peaks

#	b	b^{++}	b^0	b^{0++}	Seq.	y	y^{++}	y*	y^{*++}	y^0	y^{0++}	#
1	100.0757	50.5415			V							13
2	215.1026	108.0550	197.0921	99.0497	D	1407.6461	704.3267	1390.6195	695.8134	1389.6355	695.3214	12
3	284.1241	142.5657	266.1135	133.5604	S	1292.6191	**646.8132**	1275.5926	**638.2999**	1274.6085	**637.8079**	11
4	**385.1718**	193.0895	367.1612	184.0842	T	**1223.5977**	612.3025	**1206.5711**	603.7892	1205.5871	603.2972	10
5	**486.2194**	243.6134	**468.2089**	234.6081	T	**1122.5500**	561.7786	1105.5234	553.2654	1104.5394	552.7733	9
6	**646.2501**	323.6287	628.2395	314.6234	C	**1021.5023**	511.2548	**1004.4757**	502.7415	1003.4917	502.2495	8
7	**759.3342**	380.1707	741.3236	371.1654	L	**861.4716**	431.2395	844.4451	422.7262	843.4611	422.2342	7
8	**906.4026**	453.7049	888.3920	444.6996	F	**748.3876**	374.6974	731.3610	366.1842	730.3770	365.6921	6
9	1003.4553	502.2313	985.4448	493.2260	P	**601.3192**	301.1632	584.2926	292.6499	**583.3086**	292.1579	5
10	**1102.5238**	551.7655	1084.5132	542.7602	V	**504.2664**	252.6368	487.2399	244.1236	**486.2558**	243.6316	4
11	**1231.5663**	616.2868	1213.5558	607.2815	E	**405.1980**	203.1026	**388.1714**	194.5894	**387.1874**	194.0974	3
12	1360.6089	680.8081	1342.5984	671.8028	E	**276.1554**	138.5813	259.1288	130.0681	**258.1448**	129.5761	2
13					K	147.1128	74.0600	130.0863	65.5468			1

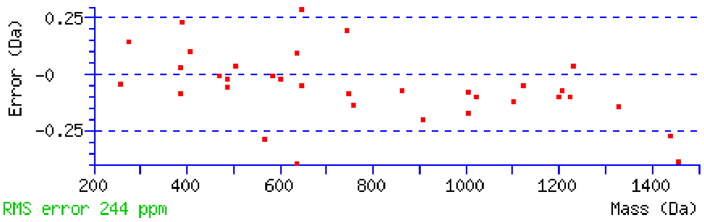

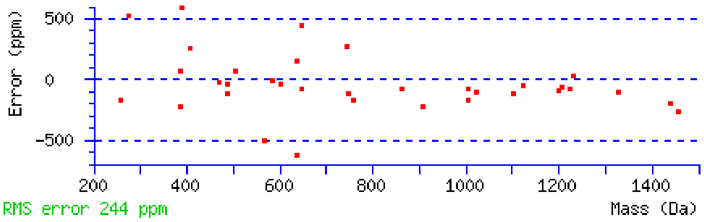

All matches to this query

Score	Mr(calc):	Delta	Sequence
57.7	1603.6841	-0.0000	VDSTTCLFPVEEK
44.9	1603.6841	-0.0000	VDSTTCLFPVEEK
35.4	1603.6841	-0.0000	VDSTTCLFPVEEK
7.8	1603.6953	-0.0112	CQKSLNEFLEEK
7.4	1603.6888	-0.0047	NCFSHLMKSEKK
7.2	1603.6987	-0.0146	DAKMAETCVVKEK
7.1	1603.6991	-0.0151	THSPVRDQAEGAEK
7.1	1603.6991	-0.0151	THSPVRDQAEGAEK
3.4	1601.6756	2.0084	LLQQSMGETNTER
3.4	1601.6756	2.0084	LLQQSMGETNTER

Spectrum No: 190; Query: 2153; Rank: 1

Peptide View

MS/MS Fragmentation of **YEKEFGDGSDENEVEEQEAK**
Found in **IPI00324841**, Tax_Id=10116 Gene_Symbol=Gs3 47 kDa protein

Match to Query 2153: 2410.940748 from(1206.477650,2+)
Title: 100101RatKid_NS_deglyco_22.1693.1693.2.dta
Data file K:\NewmanPaper\Piliang\3SubProteomes\Piliang3SP\mgf5ppm\ERLIC_3SubProteomes5ppm.mgf

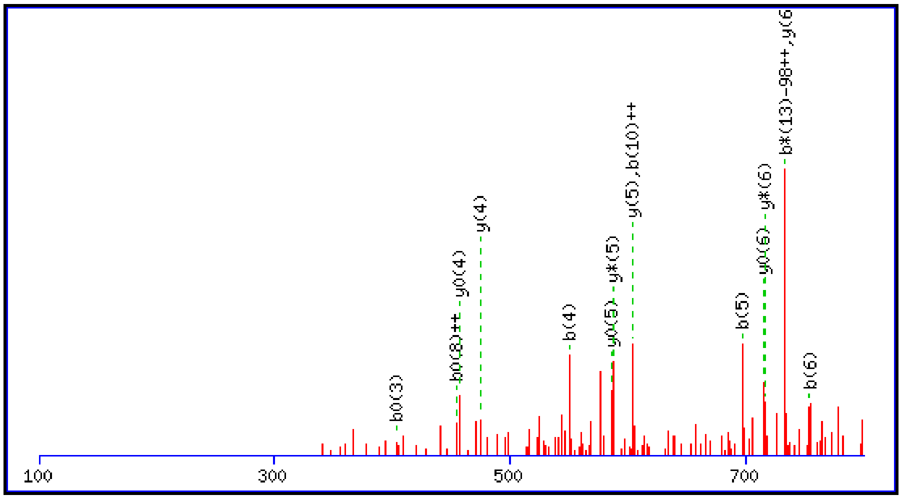

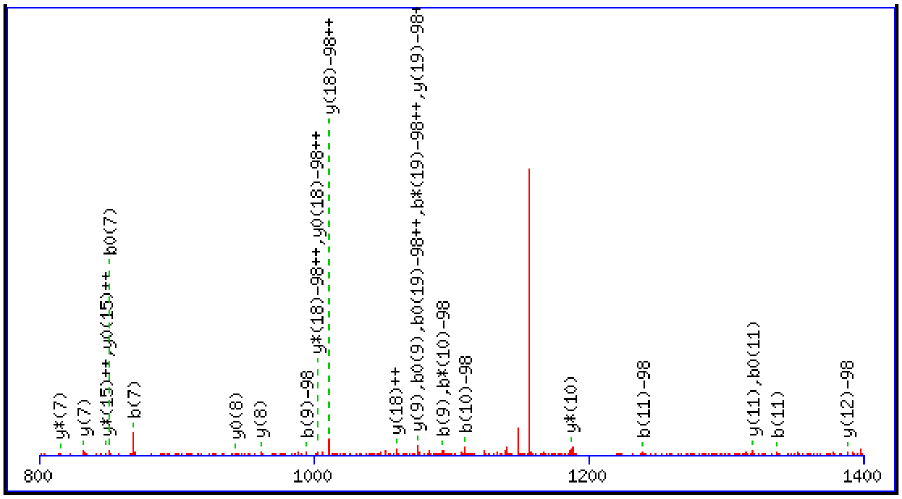

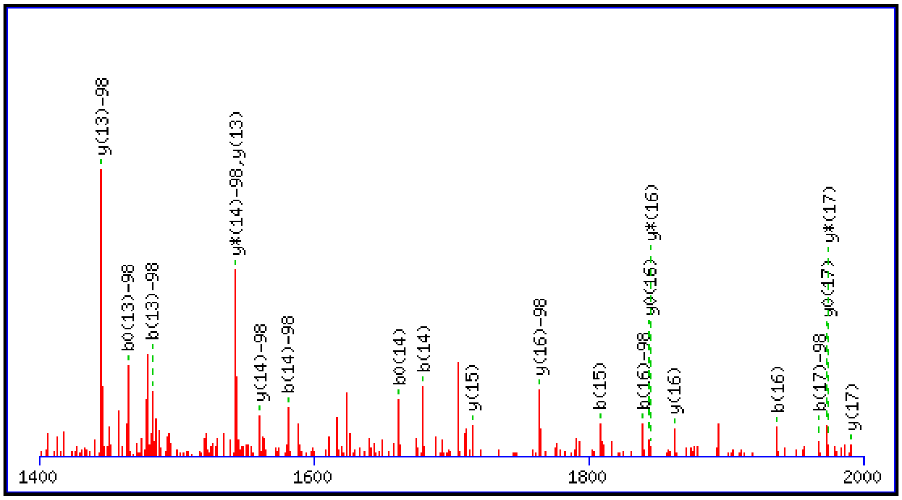

Monoisotopic mass of neutral peptide Mr(calc): 2410.9325
Fixed modifications: Carbamidomethyl (C)
Variable modifications:
S9 : Phospho (ST), with neutral losses 97.9769(shown in table), 0.0000
Ions Score: 58 **Expect:** 0.00038
Matches (Bold Red): 63/332 fragment ions using 89 most intense peaks

#	b	b^{++}	b*	b^{*++}	b^0	b^{0++}	Seq.	y	y^{++}	y*	y^{*++}	y^0	y^{0++}	#
1	164.0706	82.5389					Y							20
2	293.1132	147.0602			275.1026	138.0550	E	2150.8996	**1075.9535**	2133.8731	1067.4402	2132.8891	1066.9482	19
3	421.2082	211.1077	404.1816	202.5944	**403.1976**	202.1024	K	2021.8570	**1011.4322**	2004.8305	**1002.9189**	2003.8465	**1002.4269**	18
4	**550.2508**	275.6290	533.2242	267.1157	532.2402	266.6237	E	1893.7621	947.3847	1876.7355	938.8714	1875.7515	938.3794	17
5	**697.3192**	349.1632	680.2926	340.6499	679.3086	340.1579	F	**1764.7195**	882.8634	1747.6929	874.3501	1746.7089	873.8581	16
6	**754.3406**	377.6740	737.3141	369.1607	736.3301	368.6687	G	1617.6511	809.3292	1600.6245	800.8159	1599.6405	800.3239	15
7	**869.3676**	435.1874	852.3410	426.6742	**851.3570**	426.1821	D	**1560.6296**	780.8184	**1543.6031**	772.3052	1542.6190	771.8132	14
8	926.3890	463.6982	909.3625	455.1849	908.3785	**454.6929**	G	**1445.6027**	723.3050	1428.5761	714.7917	1427.5921	714.2997	13
9	**995.4105**	498.2089	978.3839	489.6956	977.3999	489.2036	S	**1388.5812**	694.7942	1371.5547	686.2810	1370.5706	685.7890	12
10	**1110.4374**	555.7224	**1093.4109**	547.2091	1092.4269	546.7171	D	**1319.5597**	660.2835	1302.5332	651.7702	1301.5492	651.2782	11
11	**1239.4800**	620.2437	1222.4535	611.7304	1221.4695	611.2384	E	1204.5328	602.7700	**1187.5063**	594.2568	1186.5222	593.7648	10
12	1353.5230	677.2651	1336.4964	668.7518	1335.5124	668.2598	N	**1075.4902**	538.2487	1058.4637	529.7355	1057.4796	529.2435	9
13	**1482.5656**	741.7864	1465.5390	**733.2731**	**1464.5550**	732.7811	E	**961.4473**	481.2273	944.4207	472.7140	**943.4367**	472.2220	8
14	**1581.6340**	791.3206	1564.6074	782.8073	1563.6234	782.3153	V	**832.4047**	416.7060	**815.3781**	408.1927	814.3941	407.7007	7
15	1710.6766	855.8419	1693.6500	847.3286	1692.6660	846.8366	E	**733.3363**	367.1718	**716.3097**	358.6585	**715.3257**	358.1665	6
16	**1839.7192**	920.3632	1822.6926	911.8499	1821.7086	911.3579	E	**604.2937**	302.6505	**587.2671**	294.1372	**586.2831**	293.6452	5
17	**1967.7777**	984.3925	1950.7512	975.8792	1949.7672	975.3872	Q	**475.2511**	238.1292	458.2245	229.6159	**457.2405**	229.1239	4
18	2096.8203	1048.9138	2079.7938	1040.4005	2078.8098	1039.9085	E	347.1925	174.0999	330.1660	165.5866	329.1819	165.0946	3

19	2167.8574	1084.4324	2150.8309	1075.9191	2149.8469	1075.4271	A	218.1499	109.5786	201.1234	101.0653			2
20							K	147.1128	74.0600	130.0863	65.5468			1

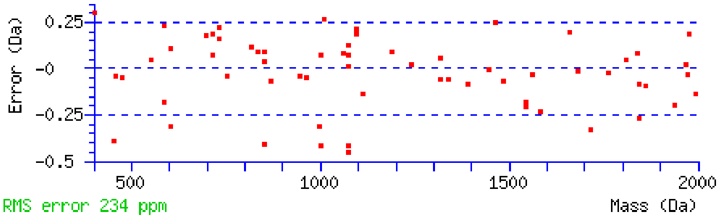

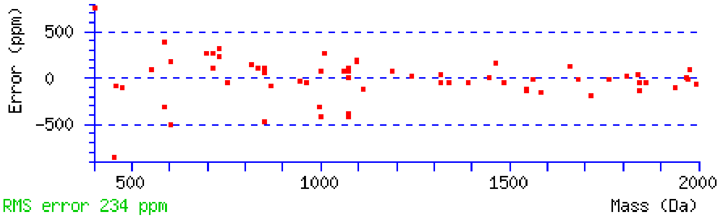

All matches to this query

Score	Mr(calc):	Delta	Sequence
57.6	2410.9325	0.0082	YEKEFGDGSDENEVEEQEAK
16.2	2410.9325	0.0082	YEKEFGDGSDENEVEEQEAK
7.1	2410.9488	-0.0080	NNGGKMENTQLTLNLQTENK
7.1	2410.9488	-0.0080	NNGGKMENTQLTLNLQTENK
4.0	2410.9639	-0.0231	MDPPSELYYLKLPFEWK
3.6	2409.9240	1.0167	SSPSSGHLRSDAVISSPDDTR
3.3	2410.9348	0.0059	IEVNESGTVASSSTAILVSAR
3.3	2410.9348	0.0059	IEVNESGTVASSSTAILVSAR
2.5	2408.9289	2.0118	MIDPDEEKAKQDPSYYLK
2.0	2408.9224	2.0183	FAAMSYGRPPPDVEGMTSLK

Spectrum No: 191; Query: 2674; Rank: 1

Peptide View

MS/MS Fragmentation of **AENDVDNELLDYEDDEVETAAGADGTEAPAK**
Found in **IPI00215291**, Tax_Id=10116 Gene_Symbol=Bat1a;Atp6v1g2 Spliceosome RNA helicase Bat1

Match to Query 2674: 3345.348132 from(1116.123320,3+)
Title: 091008RatKidney_NH4Format01_22.3879.3879.3.dta
Data file K:\NewmanPaper\Piliang\3SubProteomes\Piliang3SP\mgf5ppm\ERLIC_3SubProteomes5ppm.mgf

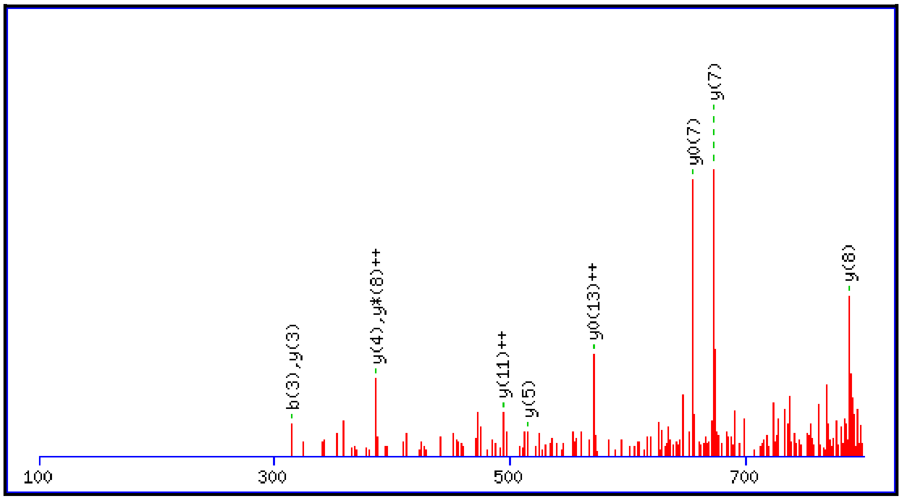

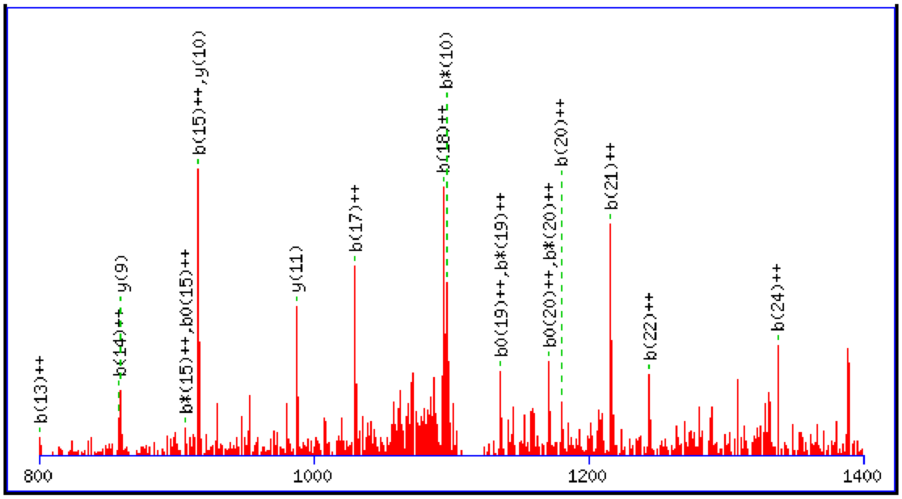

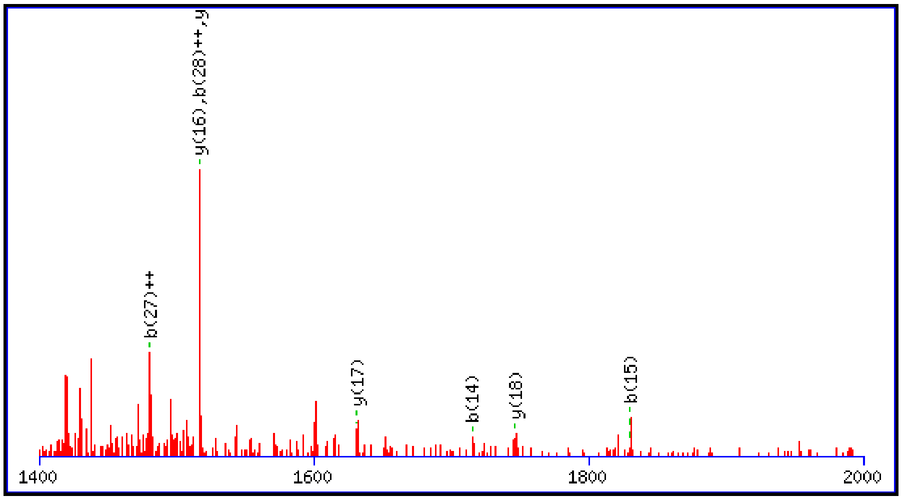

Monoisotopic mass of neutral peptide Mr(calc): 3345.3569
Fixed modifications: Carbamidomethyl (C)
Variable modifications:
Y12 : Phospho (Y)
Ions Score: 58 **Expect:** 0.00062
Matches (Bold Red): 37/346 fragment ions using 53 most intense peaks

#	b	b^{++}	b*	b^{*++}	b^0	b^{0++}	Seq.	y	y^{++}	y*	y^{*++}	y^0	y^{0++}	#
1	72.0444	36.5258					A							31
2	201.0870	101.0471			183.0764	92.0418	E	3275.3271	1638.1672	3258.3006	1629.6539	3257.3165	1629.1619	30
3	**315.1299**	158.0686	298.1034	149.5553	297.1193	149.0633	N	3146.2845	1573.6459	3129.2580	1565.1326	3128.2739	1564.6406	29
4	430.1569	215.5821	413.1303	207.0688	412.1463	206.5768	D	3032.2416	**1516.6244**	3015.2150	1508.1112	3014.2310	1507.6191	28
5	529.2253	265.1163	512.1987	256.6030	511.2147	256.1110	V	2917.2146	1459.1110	2900.1881	1450.5977	2899.2041	1450.1057	27
6	644.2522	322.6297	627.2257	314.1165	626.2416	313.6245	D	2818.1462	1409.5768	2801.1197	1401.0635	2800.1357	1400.5715	26
7	758.2951	379.6512	741.2686	371.1379	740.2846	370.6459	N	2703.1193	1352.0633	2686.0927	1343.5500	2685.1087	1343.0580	25
8	887.3377	444.1725	870.3112	435.6592	869.3272	435.1672	E	2589.0764	1295.0418	2572.0498	1286.5285	2571.0658	1286.0365	24
9	1000.4218	500.7145	983.3952	492.2013	982.4112	491.7093	L	2460.0338	1230.5205	2443.0072	1222.0072	2442.0232	1221.5152	23
10	1113.5059	557.2566	**1096.4793**	548.7433	1095.4953	548.2513	L	2346.9497	1173.9785	2329.9232	1165.4652	2328.9391	1164.9732	22
11	1228.5328	614.7700	1211.5063	606.2568	1210.5222	605.7648	D	2233.8656	1117.4365	2216.8391	1108.9232	2215.8551	1108.4312	21
12	1471.5625	736.2849	1454.5359	727.7716	1453.5519	727.2796	Y	2118.8387	1059.9230	2101.8121	1051.4097	2100.8281	1050.9177	20
13	1600.6050	**800.8062**	1583.5785	792.2929	1582.5945	791.8009	E	1875.8090	938.4082	1858.7825	929.8949	1857.7985	929.4029	19
14	**1715.6320**	**858.3196**	1698.6054	849.8064	1697.6214	849.3144	D	**1746.7664**	873.8869	1729.7399	865.3736	1728.7559	864.8816	18
15	**1830.6589**	**915.8331**	1813.6324	**907.3198**	1812.6484	**906.8278**	D	**1631.7395**	816.3734	1614.7130	807.8601	1613.7289	807.3681	17
16	1959.7015	980.3544	1942.6750	971.8411	1941.6910	971.3491	E	**1516.7126**	758.8599	1499.6860	750.3466	1498.7020	749.8546	16
17	2058.7699	**1029.8886**	2041.7434	1021.3753	2040.7594	1020.8833	V	1387.6700	694.3386	1370.6434	685.8253	1369.6594	685.3333	15
18	2187.8125	**1094.4099**	2170.7860	1085.8966	2169.8020	1085.4046	E	1288.6016	644.8044	1271.5750	636.2911	1270.5910	635.7991	14

**19**	2288.8602	1144.9337	2271.8337	**1136.4205**	2270.8496	**1135.9285**	**T**	1159.5590	580.2831	1142.5324	571.7698	1141.5484	**571.2778**	**13**
**20**	2359.8973	**1180.4523**	2342.8708	**1171.9390**	2341.8868	**1171.4470**	**A**	1058.5113	529.7593	1041.4847	521.2460	1040.5007	520.7540	**12**
**21**	2430.9344	**1215.9709**	2413.9079	1207.4576	2412.9239	1206.9656	**A**	**987.4742**	**494.2407**	970.4476	485.7274	969.4636	485.2354	**11**
**22**	2487.9559	**1244.4816**	2470.9294	1235.9683	2469.9453	1235.4763	**G**	**916.4371**	458.7222	899.4105	450.2089	898.4265	449.7169	**10**
**23**	2558.9930	1280.0001	2541.9665	1271.4869	2540.9825	1270.9949	**A**	**859.4156**	430.2114	842.3890	421.6982	841.4050	421.2062	**9**
**24**	2674.0200	**1337.5136**	2656.9934	1329.0003	2656.0094	1328.5083	**D**	**788.3785**	394.6929	771.3519	**386.1796**	770.3679	385.6876	**8**
**25**	2731.0414	1366.0244	2714.0149	1357.5111	2713.0309	1357.0191	**G**	**673.3515**	337.1794	656.3250	328.6661	**655.3410**	328.1741	**7**
**26**	2832.0891	1416.5482	2815.0626	1408.0349	2814.0785	1407.5429	**T**	616.3301	308.6687	599.3035	300.1554	598.3195	299.6634	**6**
**27**	2961.1317	**1481.0695**	2944.1051	1472.5562	2943.1211	1472.0642	**E**	**515.2824**	258.1448	498.2558	249.6316	497.2718	249.1396	**5**
**28**	3032.1688	**1516.5880**	3015.1423	1508.0748	3014.1582	1507.5828	**A**	**386.2398**	193.6235	369.2132	185.1103			**4**
**29**	3129.2216	1565.1144	3112.1950	1556.6012	3111.2110	1556.1091	**P**	**315.2027**	158.1050	298.1761	149.5917			**3**
**30**	3200.2587	1600.6330	3183.2321	1592.1197	3182.2481	1591.6277	**A**	218.1499	109.5786	201.1234	101.0653			**2**
**31**							**K**	147.1128	74.0600	130.0863	65.5468			**1**

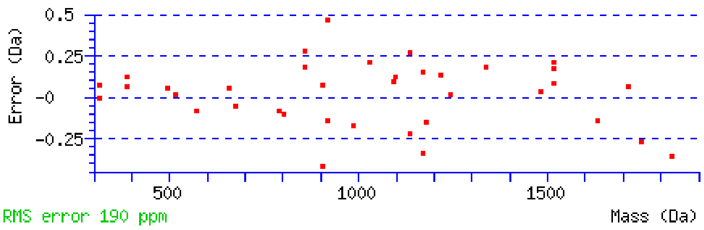

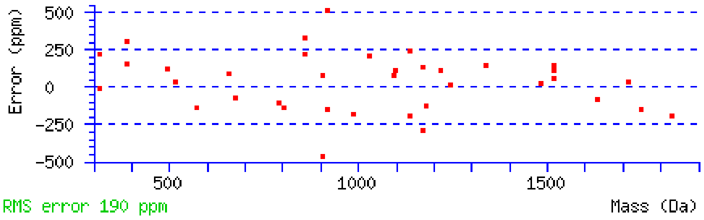

All matches to this query

Score	Mr(calc):	Delta	Sequence
57.6	3345.3569	-0.0088	AENDVDNELLDYEDDEVETAAGADGTEAPAK
17.5	3345.3569	-0.0088	AENDVDNELLDYEDDEVETAAGADGTEAPAK
9.0	3343.3311	2.0170	DHIKSLNRLDFITNESDLMYDNVVR
8.1	3343.3546	1.9935	YVAVNLATSHPHYDEAGNVLNMGTSIADK
7.1	3343.3546	1.9935	YVAVNLATSHPHYDEAGNVLNMGTSIADK
7.1	3343.3546	1.9935	YVAVNLATSHPHYDEAGNVLNMGTSIADK
6.8	3343.3546	1.9935	YVAVNLATSHPHYDEAGNVLNMGTSIADK
6.7	3343.3167	2.0315	GKCTMLVIGSVIGGTINSLTHTLSLYK
6.7	3343.3167	2.0315	GKCTMLVIGSVIGGTINSLTHTLSLYK
6.7	3343.3167	2.0315	GKCTMLVIGSVIGGTINSLTHTLSLYK

Spectrum No: 192; Query: 480; Rank: 1

Peptide View

MS/MS Fragmentation of **DTANLVKEDSDV**
Found in **IPI00205325**, Tax_Id=10116 Gene_Symbol=Lrp2 Low-density lipoprotein receptor-related protein 2 precursor

Match to Query 480: 1384.577028 from(693.295790,2+)
Title: 100101RatKid_NS_deglyco_25.1598.1598.2.dta
Data file K:\NewmanPaper\Piliang\3SubProteomes\Piliang3SP\mgf5ppm\ERLIC_3SubProteomes5ppm.mgf

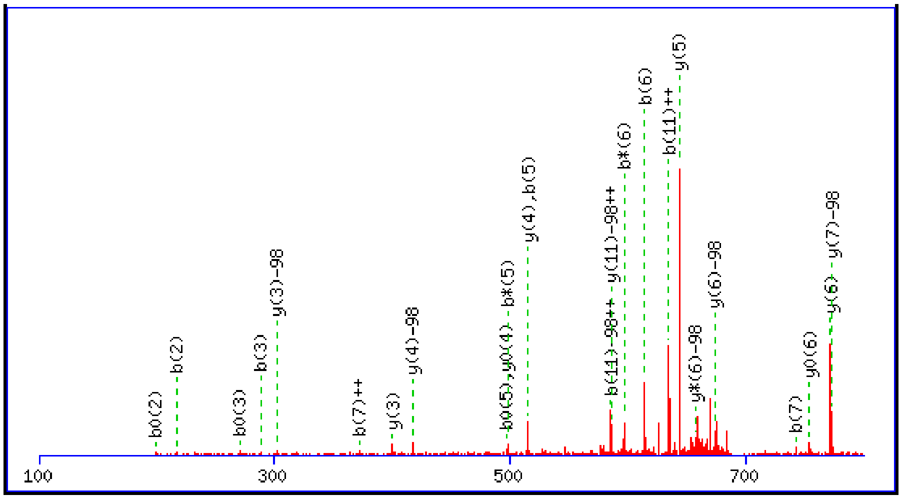

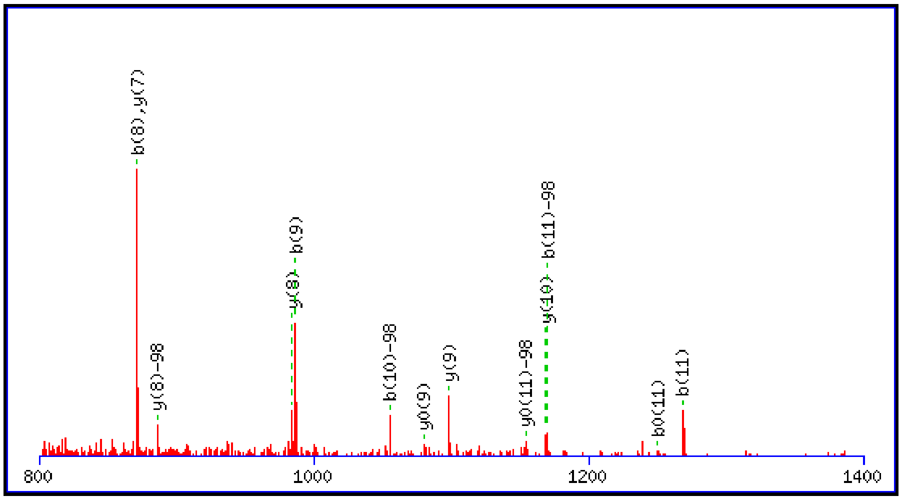

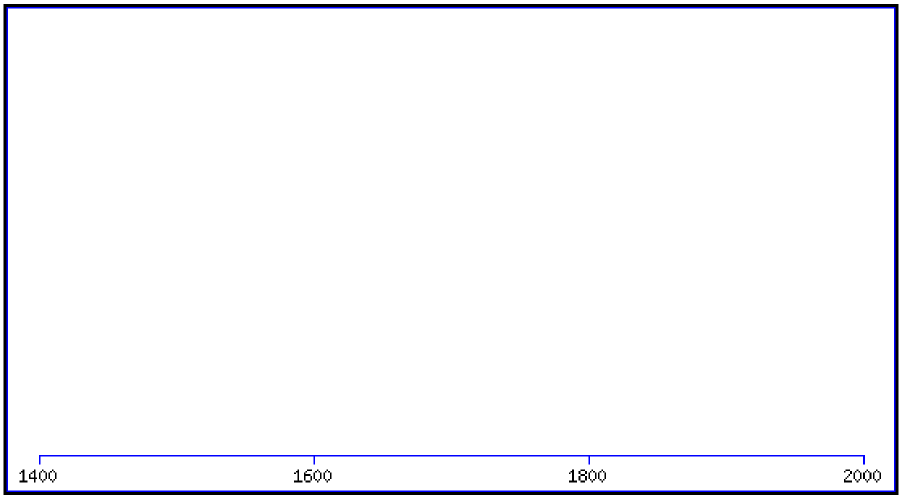

Monoisotopic mass of neutral peptide Mr(calc): 1384.5759
Fixed modifications: Carbamidomethyl (C)
Variable modifications:
S10 : Phospho (ST), with neutral losses 97.9769(shown in table), 0.0000
Ions Score: 58 **Expect:** 0.00019
Matches (Bold Red): 38/174 fragment ions using 70 most intense peaks

#	b	b^{++}	b*	b^{*++}	b^0	b^{0++}	Seq.	y	y^{++}	y*	y^{*++}	y^0	y^{0++}	#
1	116.0342	58.5207			98.0237	49.5155	D							12
2	**217.0819**	109.0446			**199.0713**	100.0393	T	1172.5794	**586.7933**	1155.5528	578.2800	**1154.5688**	577.7880	11
3	**288.1190**	144.5631			**270.1084**	135.5579	A	1071.5317	536.2695	1054.5051	527.7562	1053.5211	527.2642	10
4	402.1619	201.5846	385.1354	193.0713	384.1514	192.5793	N	1000.4946	500.7509	983.4680	492.2376	982.4840	491.7456	9
5	**515.2460**	258.1266	**498.2195**	249.6134	**497.2354**	249.1214	L	**886.4516**	443.7295	869.4251	435.2162	868.4411	434.7242	8

**6**	**614.3144**	307.6608	**597.2879**	299.1476	596.3039	298.6556	**V**	**773.3676**	387.1874	756.3410	378.6741	755.3570	378.1821	**7**
**7**	**742.4094**	**371.7083**	725.3828	363.1951	724.3988	362.7030	**K**	**674.2992**	337.6532	**657.2726**	329.1399	656.2886	328.6479	**6**
**8**	**871.4520**	436.2296	854.4254	427.7164	853.4414	427.2243	**E**	546.2042	273.6057			528.1936	264.6005	**5**
**9**	**986.4789**	493.7431	969.4524	485.2298	968.4684	484.7378	**D**	**417.1616**	209.0844			399.1510	200.0792	**4**
**10**	**1055.5004**	528.2538	1038.4738	519.7405	1037.4898	519.2485	**S**	**302.1347**	151.5710			284.1241	142.5657	**3**
**11**	**1170.5273**	**585.7673**	1153.5008	577.2540	1152.5168	576.7620	**D**	233.1132	117.0602			215.1026	108.0550	**2**
**12**							**V**	118.0863	59.5468					**1**

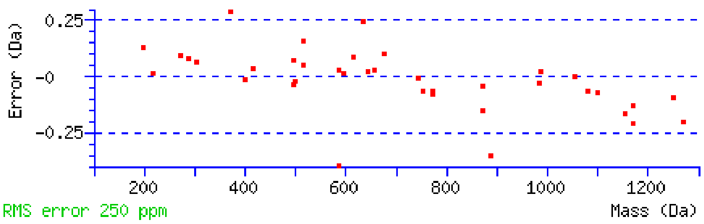

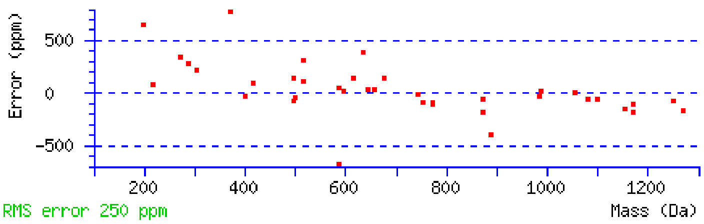

All matches to this query

Score	Mr(calc):	Delta	Sequence
57.6	1384.5759	0.0011	DTANLVKEDSDV
12.9	1384.5759	0.0011	DTANLVKEDSDV
8.1	1384.5694	0.0076	EDGTVCELLXR
3.1	1384.5901	-0.0131	QSPSPSTRPIR
0.6	1384.5751	0.0020	MSTLYVTAVPK
0.6	1383.5643	1.0128	CSPGWLADGSVR
0.3	1383.5659	1.0112	YGVTMEQIKR
0.0	1384.5676	0.0094	SLTKYSELER

Spectrum No: 193; Query: 2617; Rank: 1

Peptide View

MS/MS Fragmentation of **VDIITEEMPENALPSDEDDKDPNDPYR**
Found in **IPI00198486**, Tax_Id=10116 Gene_Symbol=Ap3d1 similar to adaptor-related protein complex 3, delta 1 subunit

Match to Query 2617: 3196.342602 from(1066.454810,3+)
Title: 091008RatKidney_NoSalt_26.3221.3221.3.dta
Data file K:\NewmanPaper\Piliang\3SubProteomes\Piliang3SP\mgf5ppm\ERLIC_3SubProteomes5ppm.mgf

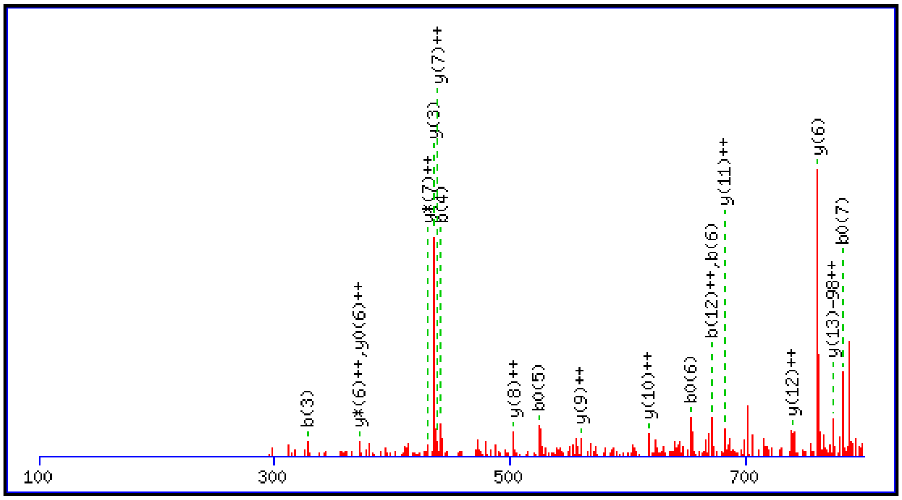

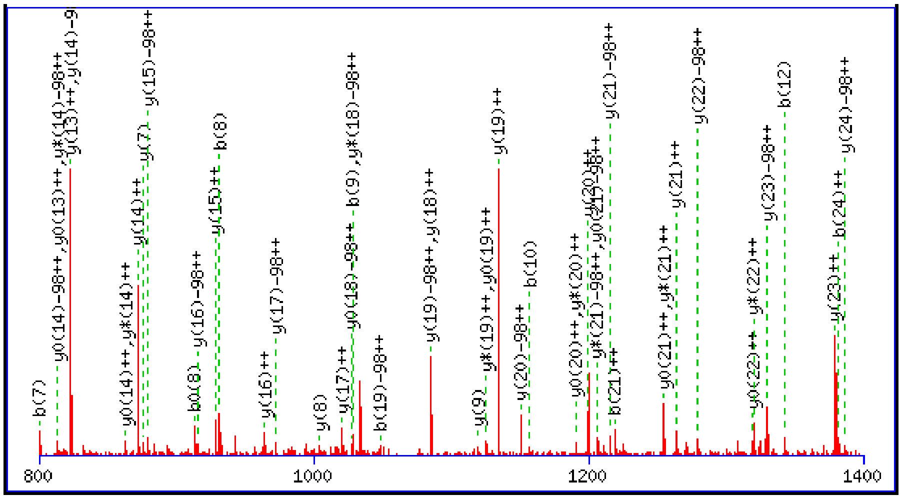

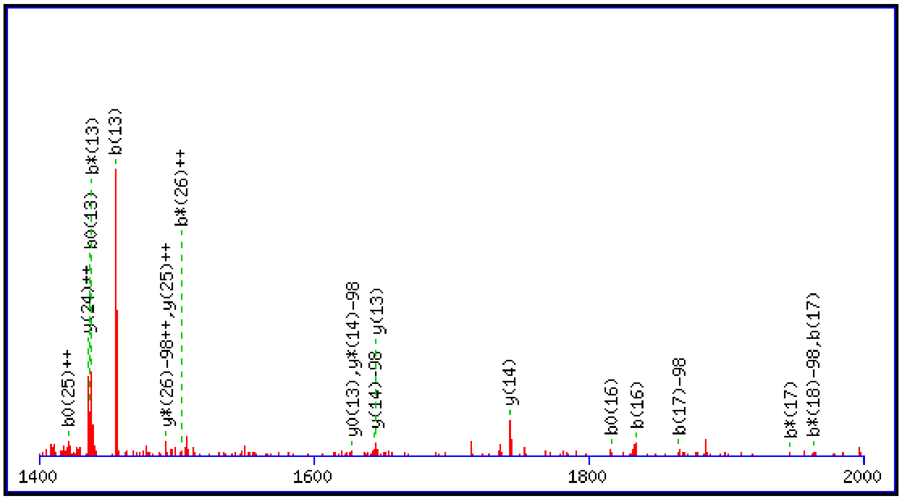

Monoisotopic mass of neutral peptide Mr(calc): 3196.3431
Fixed modifications: Carbamidomethyl (C)
Variable modifications:
S15 : Phospho (ST), with neutral losses 0.0000(shown in table), 97.9769
Ions Score: 58 **Expect:** 0.00069
Matches (Bold Red): 87/440 fragment ions using 180 most intense peaks

#	b	b++	b*	b*++	b0	b0++	Seq.	y	y++	y*	y*++	y0	y0++	#
1	100.0757	50.5415					V							27
2	215.1026	108.0550			197.0921	99.0497	D	3098.2820	1549.6446	3081.2555	1541.1314	3080.2714	1540.6394	26
3	**328.1867**	164.5970			310.1761	155.5917	I	2983.2551	**1492.1312**	2966.2285	1483.6179	2965.2445	1483.1259	25
4	**441.2708**	221.1390			423.2602	212.1337	I	2870.1710	**1435.5891**	2853.1444	1427.0759	2852.1604	1426.5839	24
5	542.3184	271.6629			**524.3079**	262.6576	T	2757.0869	**1379.0471**	2740.0604	1370.5338	2739.0764	1370.0418	23
6	**671.3610**	336.1842			**653.3505**	327.1789	E	2656.0393	1328.5233	2639.0127	**1320.0100**	2638.0287	**1319.5180**	22
7	**800.4036**	400.7055			**782.3931**	391.7002	E	2526.9967	**1264.0020**	2509.9701	**1255.4887**	2508.9861	**1254.9967**	21
8	**931.4441**	466.2257			**913.4335**	457.2204	M	2397.9541	**1199.4807**	2380.9275	**1190.9674**	2379.9435	**1190.4754**	20
9	**1028.4969**	514.7521			1010.4863	505.7468	P	2266.9136	**1133.9604**	2249.8870	**1125.4472**	2248.9030	**1124.9551**	19
10	**1157.5395**	579.2734			1139.5289	570.2681	E	2169.8608	**1085.4340**	2152.8343	1076.9208	2151.8503	1076.4288	18
11	1271.5824	636.2948	1254.5558	627.7816	1253.5718	627.2896	N	2040.8182	**1020.9128**	2023.7917	1012.3995	2022.8077	1011.9075	17
12	**1342.6195**	**671.8134**	1325.5930	663.3001	1324.6089	662.8081	A	1926.7753	**963.8913**	1909.7488	955.3780	1908.7647	954.8860	16
13	**1455.7036**	728.3554	**1438.6770**	719.8422	**1437.6930**	719.3501	L	1855.7382	**928.3727**	1838.7116	919.8595	1837.7276	919.3674	15
14	1552.7563	776.8818	1535.7298	768.3685	1534.7458	767.8765	P	**1742.6541**	**871.8307**	1725.6276	**863.3174**	1724.6436	**862.8254**	14
15	1719.7547	860.3810	1702.7281	851.8677	1701.7441	851.3757	S	**1645.6014**	**823.3043**	1628.5748	814.7910	**1627.5908**	**814.2990**	13
16	**1834.7816**	917.8945	1817.7551	909.3812	**1816.7711**	908.8892	D	1478.6030	**739.8051**	1461.5765	731.2919	1460.5924	730.7999	12
17	**1963.8242**	982.4158	**1946.7977**	973.9025	1945.8137	973.4105	E	1363.5761	**682.2917**	1346.5495	673.7784	1345.5655	673.2864	11
18	2078.8512	1039.9292	2061.8246	1031.4159	2060.8406	1030.9239	D	1234.5335	**617.7704**	1217.5069	609.2571	1216.5229	608.7651	10

19	2193.8781	1097.4427	2176.8516	1088.9294	2175.8675	1088.4374	D	1119.5065	560.2569	1102.4800	551.7436	1101.4960	551.2516	9
20	2321.9731	1161.4902	2304.9465	1152.9769	2303.9625	1152.4849	K	1004.4796	502.7434	987.4530	494.2302	986.4690	493.7381	8
21	2437.0000	1219.0036	2419.9735	1210.4904	2418.9895	1209.9984	D	876.3846	438.6959	859.3581	430.1827	858.3741	429.6907	7
22	2534.0528	1267.5300	2517.0262	1259.0168	2516.0422	1258.5247	P	761.3577	381.1825	744.3311	372.6692	743.3471	372.1772	6
23	2648.0957	1324.5515	2631.0692	1316.0382	2630.0851	1315.5462	N	664.3049	332.6561	647.2784	324.1428	646.2943	323.6508	5
24	2763.1227	1382.0650	2746.0961	1373.5517	2745.1121	1373.0597	D	550.2620	275.6346	533.2354	267.1214	532.2514	266.6293	4
25	2860.1754	1430.5913	2843.1489	1422.0781	2842.1648	1421.5861	P	435.2350	218.1212	418.2085	209.6079			3
26	3023.2387	1512.1230	3006.2122	1503.6097	3005.2282	1503.1177	Y	338.1823	169.5948	321.1557	161.0815			2
27							R	175.1190	88.0631	158.0924	79.5498			1

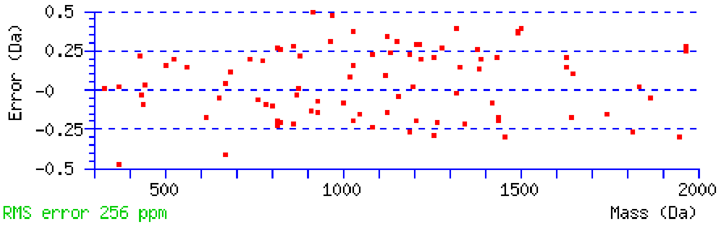

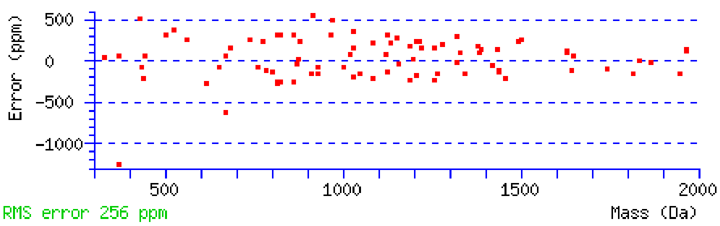

All matches to this query

Score	Mr(calc):	Delta	Sequence
57.5	3196.3431	-0.0005	VDIITEEMPENALPSDEDDKDPNDPYR
28.3	3196.3431	-0.0005	VDIITEEMPENALPSDEDDKDPNDPYR
6.5	3194.3328	2.0098	CDLKANLIRNGCGGEIESPASSTHVLR
6.5	3194.3328	2.0098	CDLKANLIRNGCGGEIESPASSTHVLR
6.5	3194.3328	2.0098	CDLKANLIRNGCGGEIESPASSTHVLR
6.0	3195.3522	0.9904	RNFTSAFTGTSWTTSAQIGTLLQLFK
5.3	3194.3529	1.9897	NKVDLFSVHPTSGVISLSGRLNYDEK
4.7	3196.3431	-0.0005	VDIITEEMPENALPSDEDDKDPNDPYR
3.0	3195.3216	1.0210	TQESLTAEDSPGLKSAFTGPGILVSTNR
1.4	3196.3322	0.0104	HGTKTVSALGLDPSGARLVTGGYDYDVK

Spectrum No: 194; Query: 2079; Rank: 1

Peptide View

MS/MS Fragmentation of **ETQSPAPLQNSSGDPAEEESPV**
Found in **IPI00214787**, Tax_Id=10116 Gene_Symbol=Slc16a1 Monocarboxylate transporter 1

Match to Query 2079: 2347.968828 from(1174.991690,2+)
Title: 091008RatKidney_NoSalt_33.1613.1613.2.dta
Data file K:\NewmanPaper\Piliang\3SubProteomes\Piliang3SP\mgf5ppm\ERLIC_3SubProteomes5ppm.mgf

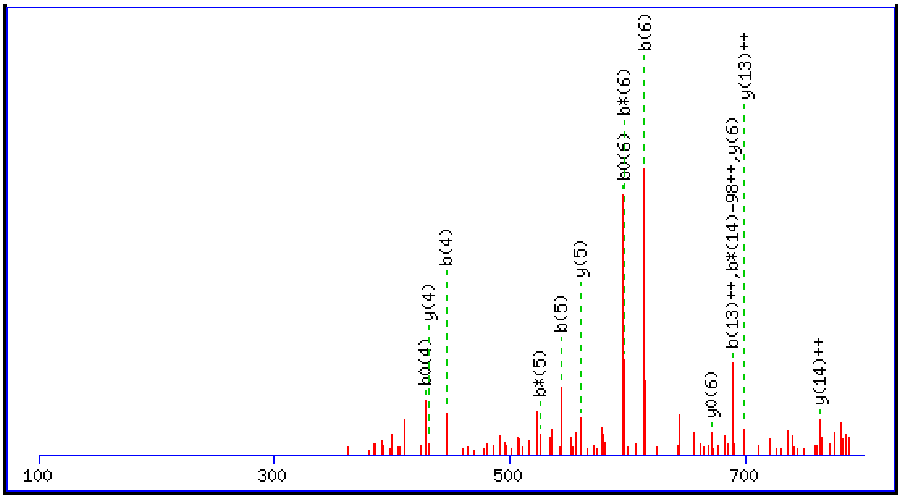

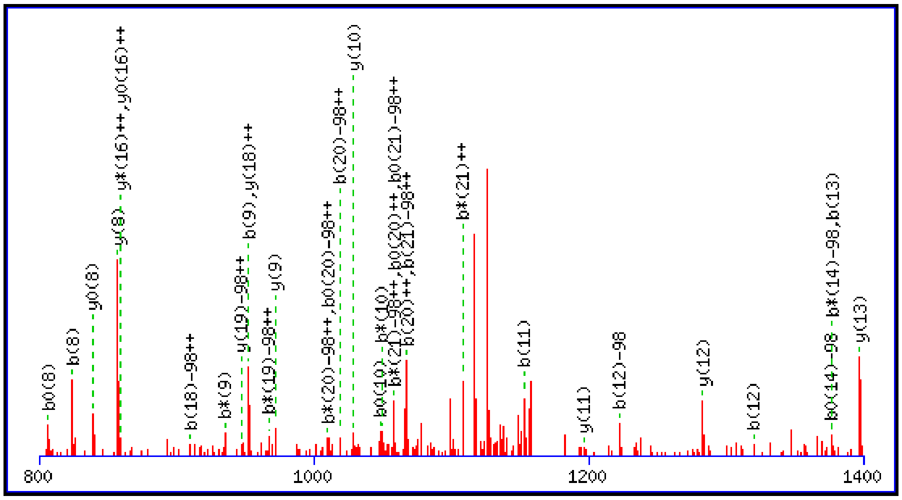

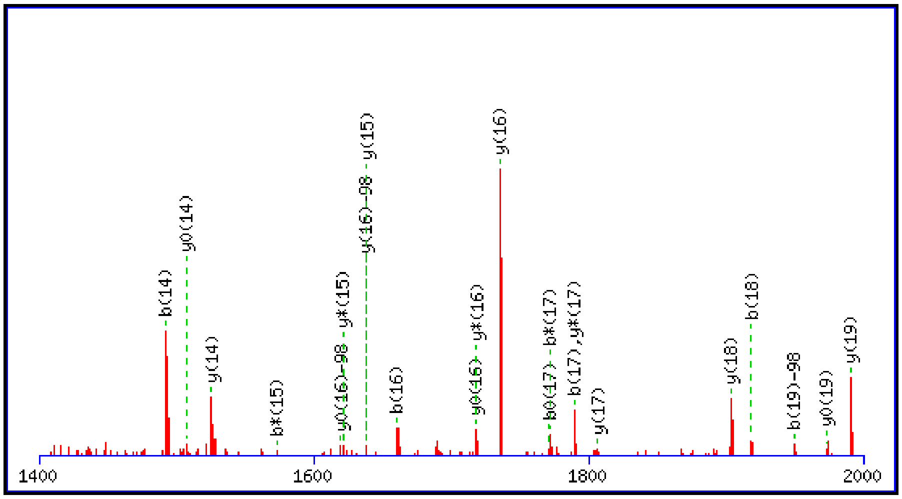

Monoisotopic mass of neutral peptide Mr(calc): 2347.9693
Fixed modifications: Carbamidomethyl (C)
Variable modifications:
S12 : Phospho (ST), with neutral losses 0.0000(shown in table), 97.9769
Ions Score: 57 **Expect:** 0.00046
Matches (Bold Red): 71/342 fragment ions using 141 most intense peaks

#	b	b^{++}	b*	b^{*++}	b^{0}	b^{0++}	Seq.	y	y^{++}	y*	y^{*++}	y^{0}	y^{0++}	#
1	130.0499	65.5286			112.0393	56.5233	E							22
2	231.0975	116.0524			213.0870	107.0471	T	2219.9340	1110.4706	2202.9074	1101.9574	2201.9234	1101.4653	21
3	359.1561	180.0817	342.1296	171.5684	341.1456	171.0764	Q	2118.8863	1059.9468	2101.8598	1051.4335	2100.8757	1050.9415	20
4	**446.1882**	223.5977	429.1616	215.0844	**428.1776**	214.5924	S	**1990.8277**	995.9175	1973.8012	987.4042	**1972.8172**	986.9122	19
5	**543.2409**	272.1241	**526.2144**	263.6108	525.2304	263.1188	P	**1903.7957**	**952.4015**	1886.7692	943.8882	1885.7851	943.3962	18

6	614.2780	307.6427	597.2515	299.1294	596.2675	298.6374	A	1806.7429	903.8751	1789.7164	895.3618	1788.7324	894.8698	17
7	711.3308	356.1690	694.3042	347.6558	693.3202	347.1638	P	1735.7058	868.3566	1718.6793	859.8433	1717.6953	859.3513	16
8	824.4149	412.7111	807.3883	404.1978	806.4043	403.7058	L	1638.6531	819.8302	1621.6265	811.3169	1620.6425	810.8249	15
9	952.4734	476.7404	935.4469	468.2271	934.4629	467.7351	Q	1525.5690	763.2881	1508.5424	754.7749	1507.5584	754.2829	14
10	1066.5164	533.7618	1049.4898	525.2485	1048.5058	524.7565	N	1397.5104	699.2588	1380.4839	690.7456	1379.4999	690.2536	13
11	1153.5484	577.2778	1136.5218	568.7646	1135.5378	568.2726	S	1283.4675	642.2374			1265.4569	633.2321	12
12	1320.5467	660.7770	1303.5202	652.2637	1302.5362	651.7717	S	1196.4355	598.7214			1178.4249	589.7161	11
13	1377.5682	689.2877	1360.5417	680.7745	1359.5576	680.2825	G	1029.4371	515.2222			1011.4265	506.2169	10
14	1492.5952	746.8012	1475.5686	738.2879	1474.5846	737.7959	D	972.4156	486.7115			954.4051	477.7062	9
15	1589.6479	795.3276	1572.6214	786.8143	1571.6374	786.3223	P	857.3887	429.1980			839.3781	420.1927	8
16	1660.6850	830.8462	1643.6585	822.3329	1642.6745	821.8409	A	760.3359	380.6716			742.3254	371.6663	7
17	1789.7276	895.3674	1772.7011	886.8542	1771.7171	886.3622	E	689.2988	345.1531			671.2883	336.1478	6
18	1918.7702	959.8887	1901.7437	951.3755	1900.7597	950.8835	E	560.2562	280.6318			542.2457	271.6265	5
19	2047.8128	1024.4100	2030.7863	1015.8968	2029.8022	1015.4048	E	431.2136	216.1105			413.2031	207.1052	4
20	2134.8448	1067.9261	2117.8183	1059.4128	2116.8343	1058.9208	S	302.1710	151.5892			284.1605	142.5839	3
21	2231.8976	1116.4524	2214.8711	1107.9392	2213.8870	1107.4472	P	215.1390	108.0731					2
22							V	118.0863	59.5468					1

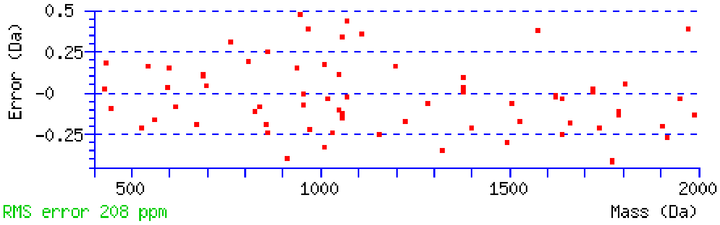

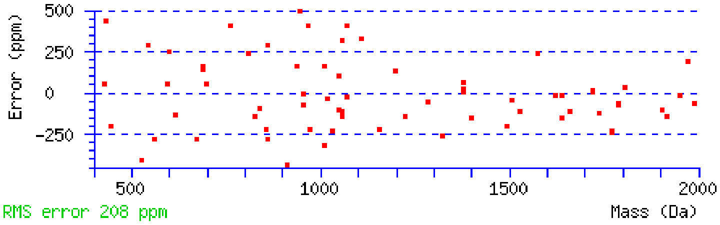

All matches to this query

Score	Mr(calc):	Delta	Sequence
57.5	2347.9693	-0.0005	ETQSPAPLQNSSGDPAEEESPV
50.5	2347.9693	-0.0005	ETQSPAPLQNSSGDPAEEESPV
20.6	2347.9693	-0.0005	ETQSPAPLQNSSGDPAEEESPV
10.4	2347.9693	-0.0005	ETQSPAPLQNSSGDPAEEESPV
9.6	2347.9693	-0.0005	ETQSPAPLQNSSGDPAEEESPV
3.1	2346.9535	1.0154	TYNALNNLSVNSENQGKIK
1.4	2346.9741	0.9948	VTNIVSFDDDDEEQGAGDTLK
1.1	2347.9474	0.0215	MTTTFTXEWTLPDVTTRS
0.9	2347.9837	-0.0149	LYWADAGMKTIEFAGLDGSK
0.8	2347.9837	-0.0149	LYWADAGMKTIEFAGLDGSK

Spectrum No: 195; Query: 2424; Rank: 1

Peptide View

MS/MS Fragmentation of **GLMAGGRPEGQYSEDEDTDTEEYR**
Found in **IPI00196562**, Tax_Id=10116 Gene_Symbol=Ric8a similar to synembryn

Match to Query 2424: 2784.089622 from(929.037150,3+)
Title: 100101RatKid_NS_deglyco_21.1977.1977.3.dta
Data file K:\NewmanPaper\Piliang\3SubProteomes\Piliang3SP\mgf5ppm\ERLIC_3SubProteomes5ppm.mgf

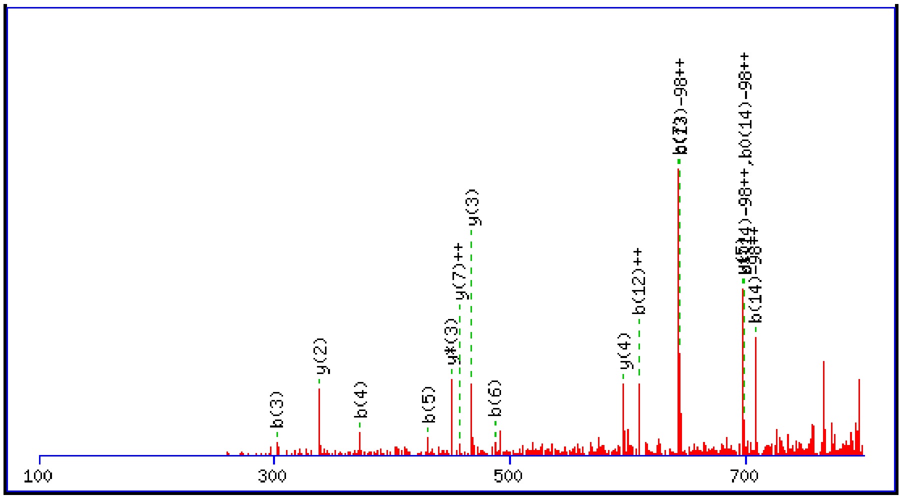

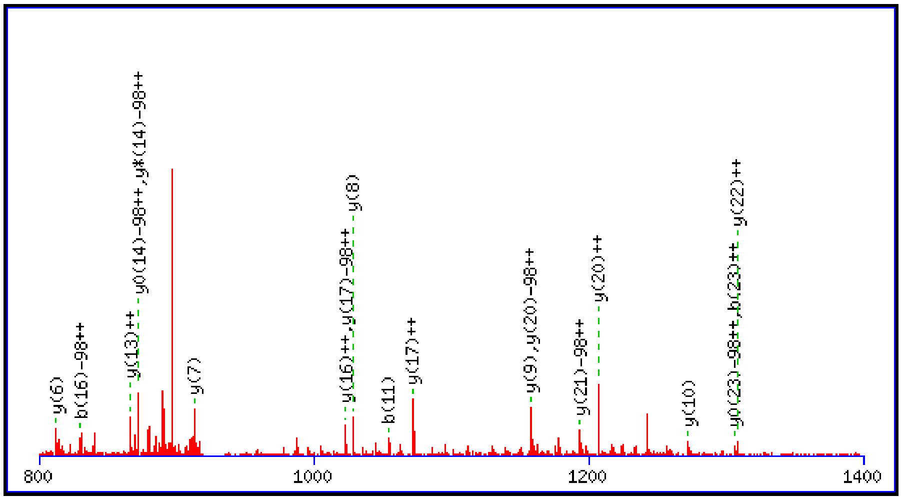

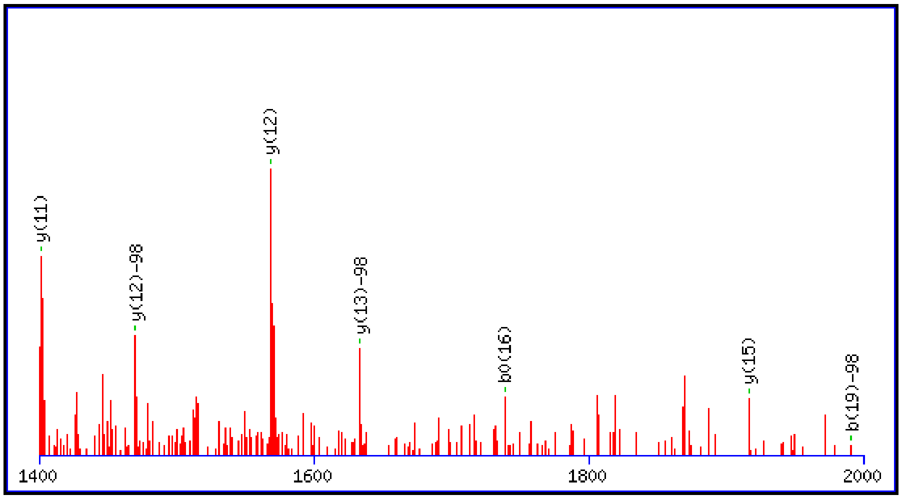

Monoisotopic mass of neutral peptide Mr(calc): 2784.0858
Fixed modifications: Carbamidomethyl (C)
Variable modifications:
S13 : Phospho (ST), with neutral losses 97.9769(shown in table), 0.0000
Ions Score: 57 **Expect:** 0.00044
Matches (Bold Red): 42/382 fragment ions using 74 most intense peaks

#	b	b^{++}	b*	b^{*++}	b^0	b^{0++}	Seq.	y	y^{++}	y*	y^{*++}	y^0	y^{0++}	#
1	58.0287	29.5180					G							24
2	171.1128	86.0600					L	2630.0947	1315.5510	2613.0682	1307.0377	2612.0842	**1306.5457**	23
3	**302.1533**	151.5803					M	2517.0107	1259.0090	2499.9841	1250.4957	2499.0001	1250.0037	22
4	**373.1904**	187.0988					A	2385.9702	**1193.4887**	2368.9436	1184.9755	2367.9596	1184.4834	21
5	**430.2119**	215.6096					G	2314.9331	**1157.9702**	2297.9065	1149.4569	2296.9225	1148.9649	20

6	**487.2333**	244.1203					G	2257.9116	1129.4594	2240.8850	1120.9462	2239.9010	1120.4542	19
7	**643.3344**	322.1709	626.3079	313.6576			R	2200.8901	1100.9487	2183.8636	1092.4354	2182.8796	1091.9434	18
8	740.3872	370.6972	723.3607	362.1840			P	2044.7890	**1022.8982**	2027.7625	1014.3849	2026.7785	1013.8929	17
9	869.4298	435.2185	852.4033	426.7053	851.4192	426.2133	E	1947.7363	974.3718	1930.7097	965.8585	1929.7257	965.3665	16
10	926.4513	463.7293	909.4247	455.2160	908.4407	454.7240	G	1818.6937	909.8505	1801.6671	901.3372	1800.6831	900.8452	15
11	**1054.5098**	527.7586	1037.4833	519.2453	1036.4993	518.7533	Q	1761.6722	881.3397	1744.6457	**872.8265**	1743.6616	**872.3345**	14
12	1217.5732	**609.2902**	1200.5466	600.7769	1199.5626	600.2849	Y	**1633.6136**	817.3105	1616.5871	808.7972	1615.6031	808.3052	13
13	1286.5946	**643.8010**	1269.5681	635.2877	1268.5841	634.7957	S	**1470.5503**	735.7788	1453.5237	727.2655	1452.5397	726.7735	12
14	1415.6372	**708.3222**	1398.6107	**699.8090**	1397.6267	**699.3170**	E	**1401.5288**	701.2681	1384.5023	692.7548	1383.5183	692.2628	11
15	1530.6642	765.8357	1513.6376	757.3224	1512.6536	756.8304	D	**1272.4862**	636.7468	1255.4597	628.2335	1254.4757	627.7415	10
16	1659.7068	**830.3570**	1642.6802	821.8437	1641.6962	821.3517	E	**1157.4593**	579.2333	1140.4328	570.7200	1139.4487	570.2280	9
17	1774.7337	887.8705	1757.7071	879.3572	1756.7231	878.8652	D	**1028.4167**	514.7120	1011.3902	506.1987	1010.4061	505.7067	8
18	1875.7814	938.3943	1858.7548	929.8811	1857.7708	929.3890	T	**913.3898**	**457.1985**	896.3632	448.6852	895.3792	448.1932	7
19	**1990.8083**	995.9078	1973.7818	987.3945	1972.7978	986.9025	D	**812.3421**	406.6747	795.3155	398.1614	794.3315	397.6694	6
20	2091.8560	1046.4316	2074.8294	1037.9184	2073.8454	1037.4264	T	**697.3151**	349.1612	680.2886	340.6479	679.3046	340.1559	5
21	2220.8986	1110.9529	2203.8720	1102.4397	2202.8880	1101.9477	E	**596.2675**	298.6374	579.2409	290.1241	578.2569	289.6321	4
22	2349.9412	1175.4742	2332.9146	1166.9610	2331.9306	1166.4689	E	**467.2249**	234.1161	**450.1983**	225.6028	449.2143	225.1108	3
23	2513.0045	1257.0059	2495.9780	1248.4926	2494.9939	1248.0006	Y	**338.1823**	169.5948	321.1557	161.0815			2
24							R	175.1190	88.0631	158.0924	79.5498			1

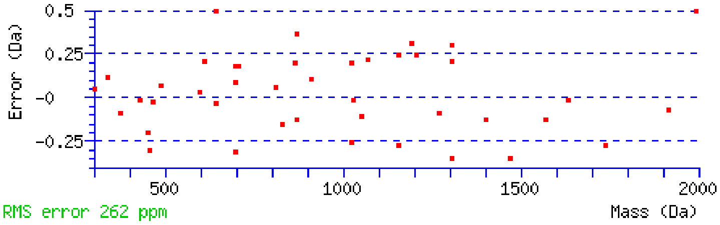

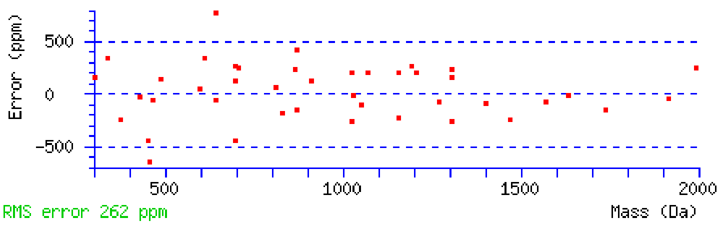

All matches to this query

Score	Mr(calc):	Delta	Sequence
57.4	2784.0858	0.0038	GLMAGGRPEGQYSEDEDTDTEEYR
42.7	2784.0858	0.0038	GLMAGGRPEGQYSEDEDTDTEEYR
26.1	2784.0858	0.0038	GLMAGGRPEGQYSEDEDTDTEEYR
18.1	2784.0858	0.0038	GLMAGGRPEGQYSEDEDTDTEEYR
8.9	2784.0858	0.0038	GLMAGGRPEGQYSEDEDTDTEEYR
4.2	2784.1023	-0.0126	SGSGNSGGGRGDGFGGNDNFGRGGNFSGR
1.1	2782.0700	2.0196	SIQGHNDYMCPATNQCTIDKNR
1.1	2782.0700	2.0196	SIQGHNDYMCPATNQCTIDKNR
0.6	2784.1066	-0.0170	REAYNDSSDEESSSHHGPGVQCAHQ
0.5	2784.1130	-0.0234	VSSVFYTTVVPMLNPLVYSLR

Spectrum No: 196; Query: 2780; Rank: 1

Peptide View

MS/MS Fragmentation of **SSVLPSTLTASAPSNAVSVVSSVDSTQVPDAGGESPGSR**
Found in **IPI00870284**, Tax_Id=10116 Gene_Symbol=Lrba_predicted LPS-responsive beige-like anchor

Match to Query 2780: 3780.779892 from(1261.267240,3+)
Title: 091008RatKidney_NoSalt_26.4206.4206.3.dta
Data file K:\NewmanPaper\Piliang\3SubProteomes\Piliang3SP\mgf5ppm\ERLIC_3SubProteomes5ppm.mgf

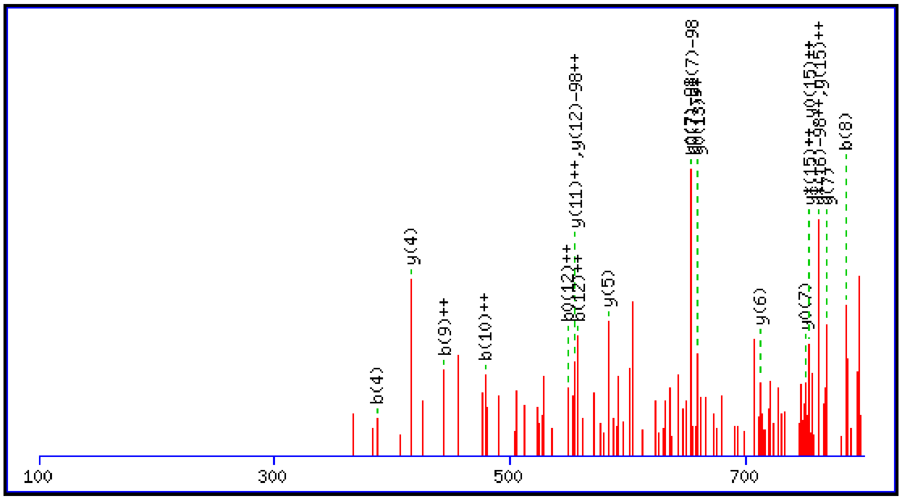

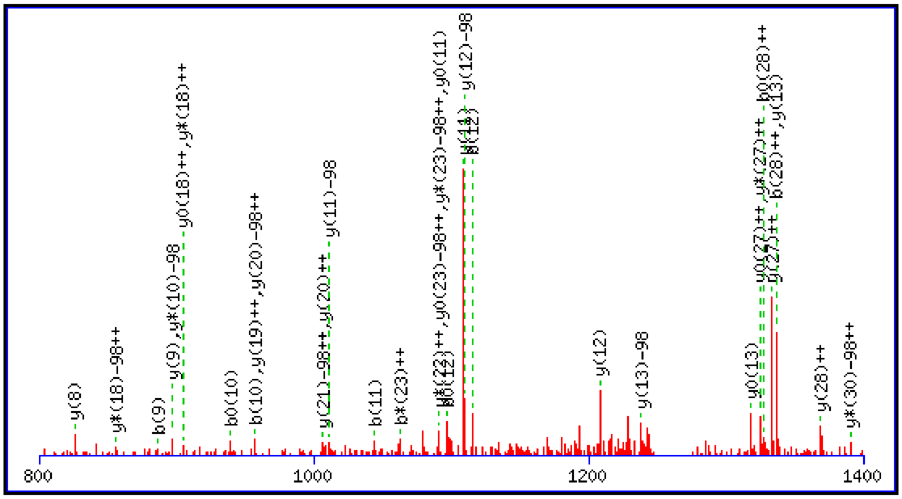

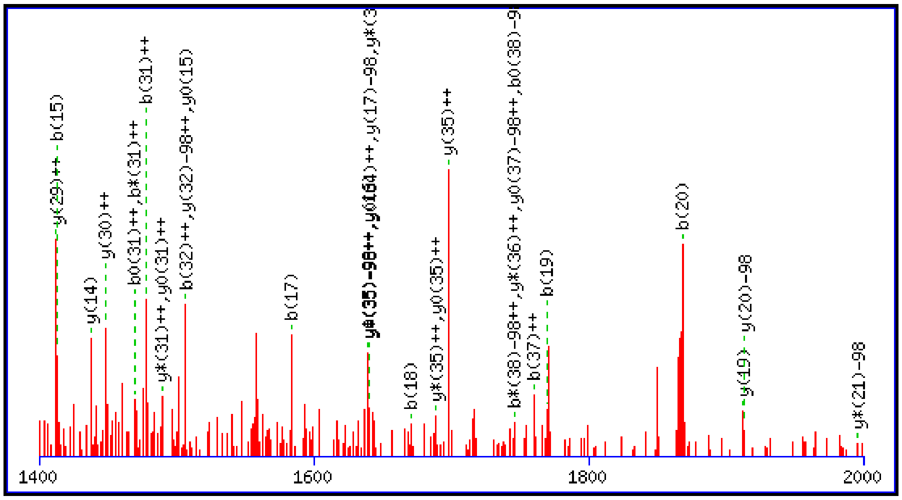

Monoisotopic mass of neutral peptide Mr(calc): 3779.7739
Fixed modifications: Carbamidomethyl (C)
Variable modifications:
S35 : Phospho (ST), with neutral losses 0.0000(shown in table), 97.9769
Ions Score: 57 **Expect:** 0.00071
Matches (Bold Red): 90/654 fragment ions using 123 most intense peaks

#	b	b++	b*	b*++	b0	b0++	Seq.	y	y++	y*	y*++	y0	y0++	#
1	88.0393	44.5233			70.0287	35.5180	S							39
2	175.0713	88.0393			157.0608	79.0340	S	3693.7491	1847.3782	3676.7225	1838.8649	3675.7385	1838.3729	38
3	274.1397	137.5735			256.1292	128.5682	V	3606.7171	1803.8622	3589.6905	1795.3489	3588.7065	1794.8569	37
4	**387.2238**	194.1155			369.2132	185.1103	L	3507.6487	1754.3280	3490.6221	**1745.8147**	3489.6381	1745.3227	36
5	484.2766	242.6419			466.2660	233.6366	P	3394.5646	**1697.7859**	3377.5380	**1689.2727**	3376.5540	**1688.7806**	35

6	571.3086	286.1579			553.2980	277.1527	S	3297.5118	1649.2595	3280.4853	**1640.7463**	3279.5013	**1640.2543**	34
7	672.3563	336.6818			**654.3457**	327.6765	T	3210.4798	1605.7435	3193.4532	1597.2303	3192.4692	1596.7383	33
8	**785.4403**	393.2238			767.4298	384.2185	L	3109.4321	1555.2197	3092.4056	1546.7064	3091.4216	1546.2144	32
9	**886.4880**	**443.7477**			868.4775	434.7424	T	2996.3481	1498.6777	2979.3215	**1490.1644**	2978.3375	**1489.6724**	31
10	**957.5251**	**479.2662**			**939.5146**	470.2609	A	2895.3004	**1448.1538**	2878.2738	1439.6405	2877.2898	1439.1485	30
11	**1044.5572**	522.7822			1026.5466	513.7769	S	2824.2633	**1412.6353**	2807.2367	1404.1220	2806.2527	1403.6300	29
12	**1115.5943**	**558.3008**			**1097.5837**	**549.2955**	A	2737.2312	**1369.1193**	2720.2047	1360.6060	2719.2207	1360.1140	28
13	1212.6470	606.8272			1194.6365	597.8219	P	2666.1941	**1333.6007**	2649.1676	**1325.0874**	2648.1836	**1324.5954**	27
14	1299.6791	650.3432			1281.6685	641.3379	S	2569.1414	1285.0743	2552.1148	1276.5610	2551.1308	1276.0690	26
15	**1413.7220**	707.3646	1396.6955	698.8514	1395.7114	698.3594	N	2482.1093	1241.5583	2465.0828	1233.0450	2464.0988	1232.5530	25
16	1484.7591	742.8832	1467.7326	734.3699	1466.7485	733.8779	A	2368.0664	1184.5368	2351.0398	1176.0236	2350.0558	1175.5316	24
17	**1583.8275**	792.4174	1566.8010	783.9041	1565.8170	783.4121	V	2297.0293	1149.0183	2280.0027	1140.5050	2279.0187	1140.0130	23
18	**1670.8596**	835.9334	1653.8330	827.4201	1652.8490	826.9281	S	2197.9609	1099.4841	2180.9343	**1090.9708**	2179.9503	1090.4788	22
19	**1769.9280**	885.4676	1752.9014	876.9543	1751.9174	876.4623	V	2110.9288	1055.9681	2093.9023	1047.4548	2092.9183	1046.9628	21
20	**1868.9964**	935.0018	1851.9698	926.4886	1850.9858	925.9965	V	2011.8604	**1006.4339**	1994.8339	997.9206	1993.8499	997.4286	20
21	1956.0284	978.5178	1939.0019	970.0046	1938.0178	969.5126	S	**1912.7920**	**956.8996**	1895.7655	948.3864	1894.7814	947.8944	19
22	2043.0604	1022.0339	2026.0339	1013.5206	2025.0499	1013.0286	S	1825.7600	913.3836	1808.7334	**904.8704**	1807.7494	**904.3783**	18
23	2142.1289	1071.5681	2125.1023	**1063.0548**	2124.1183	1062.5628	V	1738.7280	869.8676	1721.7014	861.3543	1720.7174	860.8623	17
24	2257.1558	1129.0815	2240.1292	1120.5683	2239.1452	1120.0763	D	**1639.6595**	820.3334	1622.6330	811.8201	1621.6490	811.3281	16
25	2344.1878	1172.5976	2327.1613	1164.0843	2326.1773	1163.5923	S	1524.6326	**762.8199**	1507.6061	**754.3067**	**1506.6220**	**753.8147**	15
26	2445.2355	1223.1214	2428.2090	1214.6081	2427.2249	1214.1161	T	**1437.6006**	719.3039	1420.5740	710.7907	1419.5900	710.2986	14
27	2573.2941	1287.1507	2556.2675	1278.6374	2555.2835	1278.1454	Q	**1336.5529**	668.7801	1319.5263	660.2668	**1318.5423**	**659.7748**	13
28	2672.3625	**1336.6849**	2655.3359	1328.1716	2654.3519	**1327.6796**	V	**1208.4943**	604.7508	1191.4678	596.2375	1190.4838	595.7455	12
29	2769.4153	1385.2113	2752.3887	1376.6980	2751.4047	1376.2060	P	**1109.4259**	**555.2166**	1092.3994	546.7033	**1091.4153**	546.2113	11
30	2884.4422	1442.7247	2867.4157	1434.2115	2866.4316	1433.7195	D	1012.3731	506.6902	995.3466	498.1769	994.3626	497.6849	10
31	2955.4793	**1478.2433**	2938.4528	**1469.7300**	2937.4688	**1469.2380**	A	**897.3462**	449.1767	880.3196	440.6635	879.3356	440.1715	9
32	3012.5008	**1506.7540**	2995.4742	1498.2408	2994.4902	1497.7487	G	**826.3091**	413.6582	809.2825	405.1449	808.2985	404.6529	8
33	3069.5222	1535.2648	3052.4957	1526.7515	3051.5117	1526.2595	G	**769.2876**	385.1474	752.2611	376.6342	**751.2771**	376.1422	7
34	3198.5648	1599.7861	3181.5383	1591.2728	3180.5543	1590.7808	E	**712.2662**	356.6367	695.2396	348.1234	694.2556	347.6314	6
35	3365.5632	1683.2852	3348.5366	1674.7720	3347.5526	1674.2799	S	**583.2236**	292.1154	566.1970	283.6021	565.2130	283.1101	5
36	3462.6160	1731.8116	3445.5894	1723.2983	3444.6054	1722.8063	P	**416.2252**	208.6162	399.1987	200.1030	398.2146	199.6110	4
37	3519.6374	**1760.3223**	3502.6109	1751.8091	3501.6269	1751.3171	G	319.1724	160.0899	302.1459	151.5766	301.1619	151.0846	3
38	3606.6694	1803.8384	3589.6429	1795.3251	3588.6589	1794.8331	S	262.1510	131.5791	245.1244	123.0659	244.1404	122.5738	2
39							R	175.1190	88.0631	158.0924	79.5498			1

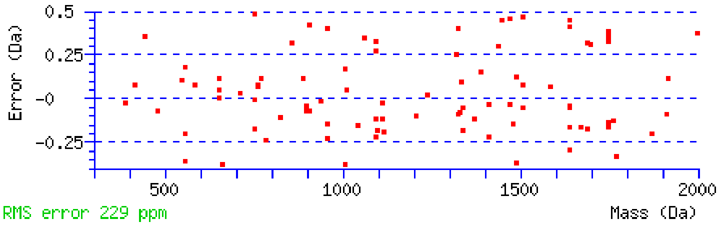

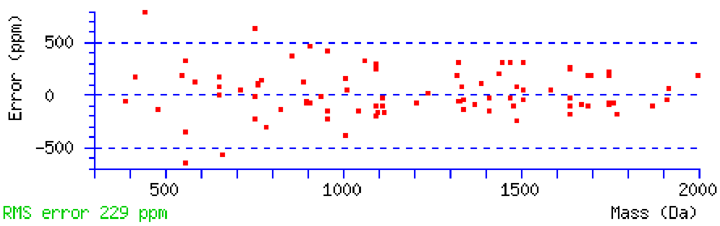

All matches to this query

Score	Mr(calc):	Delta	Sequence
57.4	3779.7739	1.0060	SSVLPSTLTASAPSNAVSVVSSVDSTQVPDAGGESPGSR
50.8	3779.7739	1.0060	SSVLPSTLTASAPSNAVSVVSSVDSTQVPDAGGESPGSR
46.4	3780.7579	0.0220	SSVLPSTLTASAPSNAVSVVSSVDSTQVPDAGGESPGSR
41.1	3780.7579	0.0220	SSVLPSTLTASAPSNAVSVVSSVDSTQVPDAGGESPGSR
24.7	3779.7739	1.0060	SSVLPSTLTASAPSNAVSVVSSVDSTQVPDAGGESPGSR
24.1	3779.7739	1.0060	SSVLPSTLTASAPSNAVSVVSSVDSTQVPDAGGESPGSR

20.9	3780.7579	0.0220	SSVLPSTLTASAPSNAVSVVSSVDSTQVPDAGGESPGSR
20.4	3780.7579	0.0220	SSVLPSTLTASAPSNAVSVVSSVDSTQVPDAGGESPGSR
19.4	3779.7739	1.0060	SSVLPSTLTASAPSNAVSVVSSVDSTQVPDAGGESPGSR
18.1	3780.7579	0.0220	SSVLPSTLTASAPSNAVSVVSSVDSTQVPDAGGESPGSR

Spectrum No: 197; Query: 931; Rank: 1

Peptide View

MS/MS Fragmentation of **SSPPATDPGPVPSSPR**
Found in **IPI00371410**, Tax_Id=10116 Gene_Symbol=RGD1309471 Isoform 1 of SAPK substrate protein 1

Match to Query 931: 1627.719548 from(814.867050,2+)
Title: 091008RatKidney_NH4Format02_26.1036.1036.2.dta
Data file K:\NewmanPaper\Piliang\3SubProteomes\Piliang3SP\mgf5ppm\ERLIC_3SubProteomes5ppm.mgf

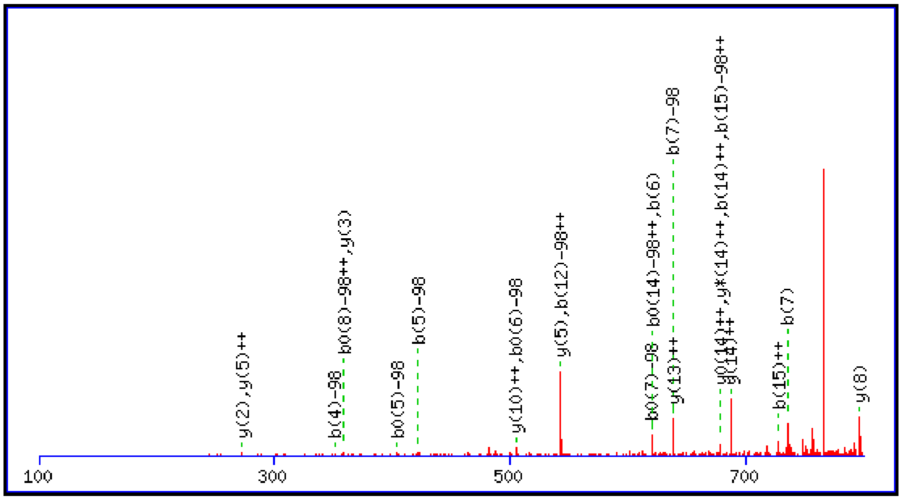

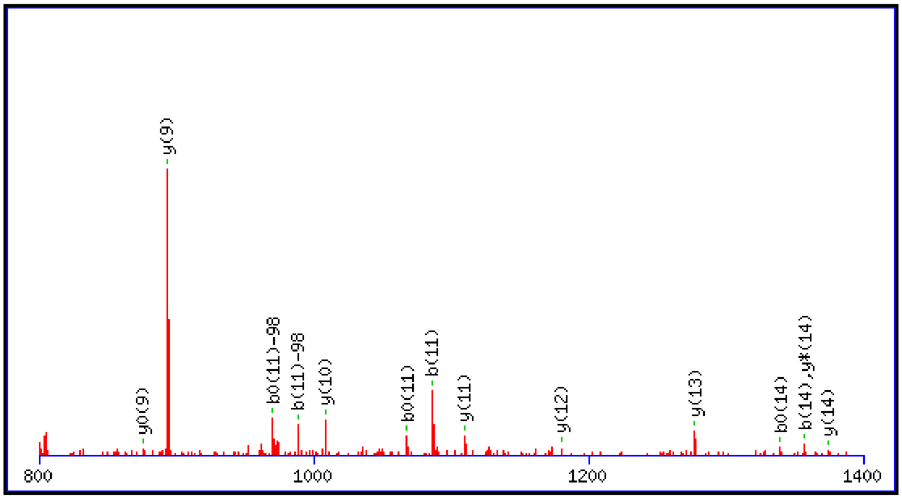

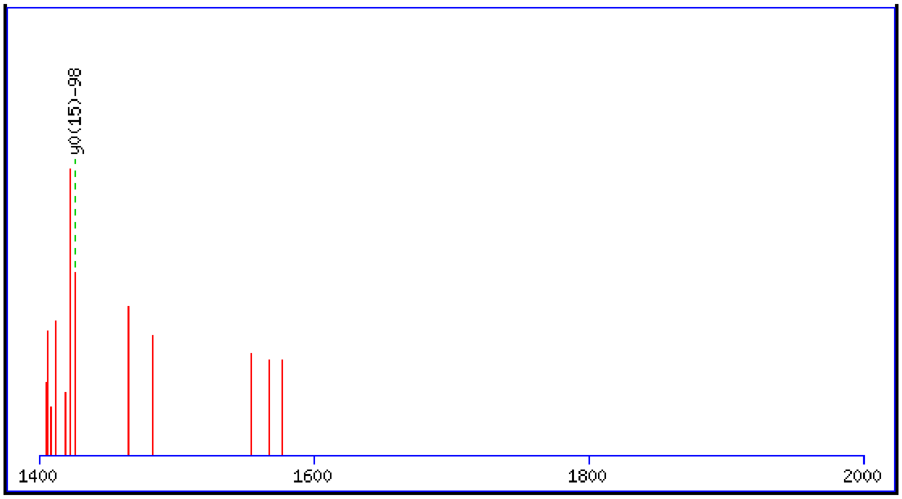

Monoisotopic mass of neutral peptide Mr(calc): 1627.7243
Fixed modifications: Carbamidomethyl (C)
Variable modifications:
S2 : Phospho (ST), with neutral losses 97.9769(shown in table), 0.0000
Ions Score: 57 **Expect:** 0.00035
Matches (Bold Red): 39/208 fragment ions using 63 most intense peaks

#	b	b^{++}	b^0	b^{0++}	Seq.	y	y^{++}	y*	y^{*++}	y^0	y^{0++}	#
1	88.0393	44.5233	70.0287	35.5180	S							16
2	157.0608	79.0340	139.0502	70.0287	S	1443.7227	722.3650	1426.6961	713.8517	**1425.7121**	713.3597	15
3	254.1135	127.5604	236.1030	118.5551	P	**1374.7012**	**687.8542**	**1357.6747**	**679.3410**	1356.6906	**678.8490**	14
4	**351.1663**	176.0868	333.1557	167.0815	P	**1277.6484**	**639.3279**	1260.6219	630.8146	1259.6379	630.3226	13
5	**422.2034**	211.6053	**404.1928**	202.6001	A	**1180.5957**	590.8015	1163.5691	582.2882	1162.5851	581.7962	12
6	523.2511	262.1292	**505.2405**	253.1239	T	**1109.5586**	555.2829	1092.5320	546.7696	1091.5480	546.2776	11
7	**638.2780**	319.6427	**620.2675**	310.6374	D	**1008.5109**	**504.7591**	991.4843	496.2458	990.5003	495.7538	10
8	735.3308	368.1690	717.3202	**359.1637**	P	**893.4839**	447.2456	876.4574	438.7323	**875.4734**	438.2403	9
9	792.3523	396.6798	774.3417	387.6745	G	**796.4312**	398.7192	779.4046	390.2060	778.4206	389.7139	8
10	889.4050	445.2061	871.3945	436.2009	P	739.4097	370.2085	722.3832	361.6952	721.3991	361.2032	7
11	**988.4734**	494.7404	**970.4629**	485.7351	V	642.3570	321.6821	625.3304	313.1688	624.3464	312.6768	6
12	1085.5262	**543.2667**	1067.5156	534.2615	P	**543.2885**	**272.1479**	526.2620	263.6346	525.2780	263.1426	5
13	1172.5582	586.7827	1154.5477	577.7775	S	446.2358	223.6215	429.2092	215.1082	428.2252	214.6162	4
14	1259.5903	630.2988	1241.5797	**621.2935**	S	**359.2037**	180.1055	342.1772	171.5922	341.1932	171.1002	3
15	1356.6430	**678.8251**	1338.6324	669.8199	P	**272.1717**	136.5895	255.1452	128.0762			2
16					R	175.1190	88.0631	158.0924	79.5498			1

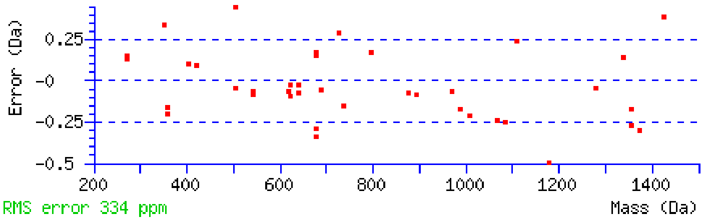

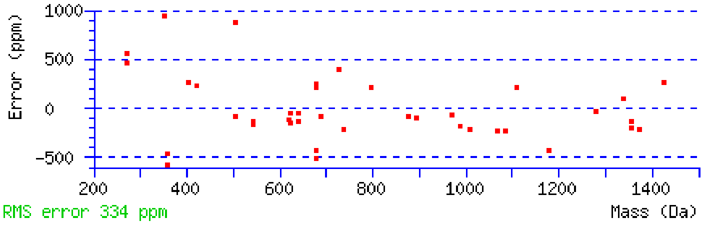

All matches to this query

Score	Mr(calc):	Delta	Sequence
57.1	1627.7243	-0.0048	SSPPATDPGPVPSSPR
55.7	1627.7243	-0.0048	SSPPATDPGPVPSSPR
22.7	1627.7243	-0.0048	SSPPATDPGPVPSSPR
5.2	1627.7090	0.0105	ISNLQTQQNVAGTSS
4.6	1627.7243	-0.0048	SSPPATDPGPVPSSPR

4.4	1626.7250	0.9945	GPVSTSELSKGTDNR
3.5	1627.7090	0.0105	ISNLQTQQNVAGTSS
3.1	1626.7250	0.9945	GPVSTSELSKGTDNR
2.6	1627.7315	-0.0119	PRTTENSLRGSSSR
2.6	1627.7315	-0.0119	PRTTENSLRGSSSR

Spectrum No: 198; Query: 2643; Rank: 1

Peptide View

MS/MS Fragmentation of **FGEESKEPFSDEDEDDNDDVEPISEFR**
Found in **IPI00231294**, Tax_Id=10116 Gene_Symbol=Clns1a Clns1a protein

Match to Query 2643: 3255.258462 from(1086.093430,3+)
Title: 091008RatKidney_NH4Format01_24.3267.3267.3.dta
Data file K:\NewmanPaper\Piliang\3SubProteomes\Piliang3SP\mgf5ppm\ERLIC_3SubProteomes5ppm.mgf

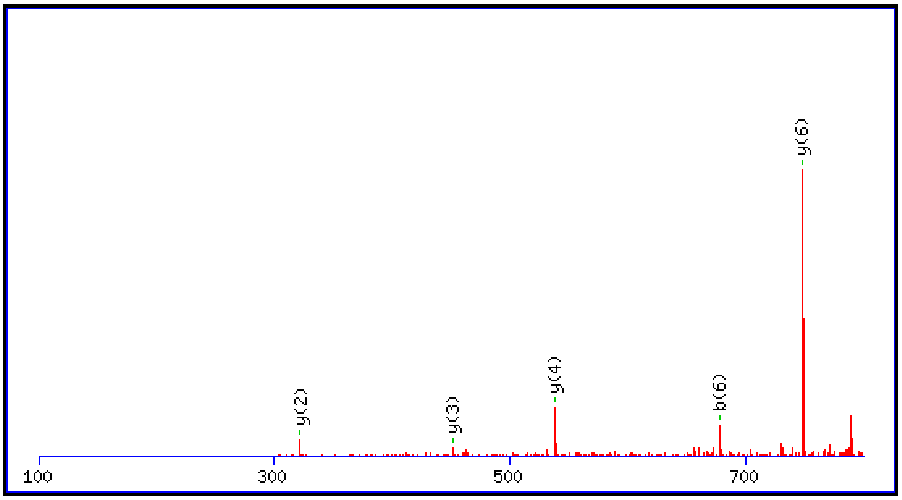

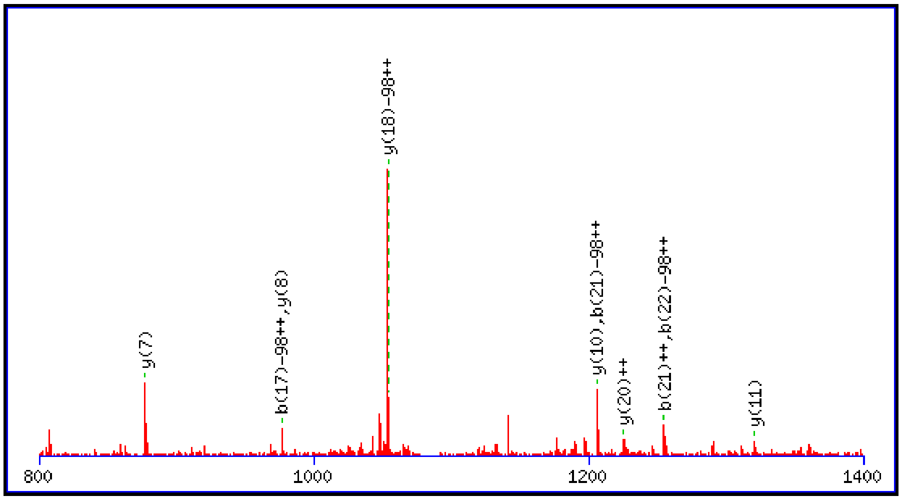

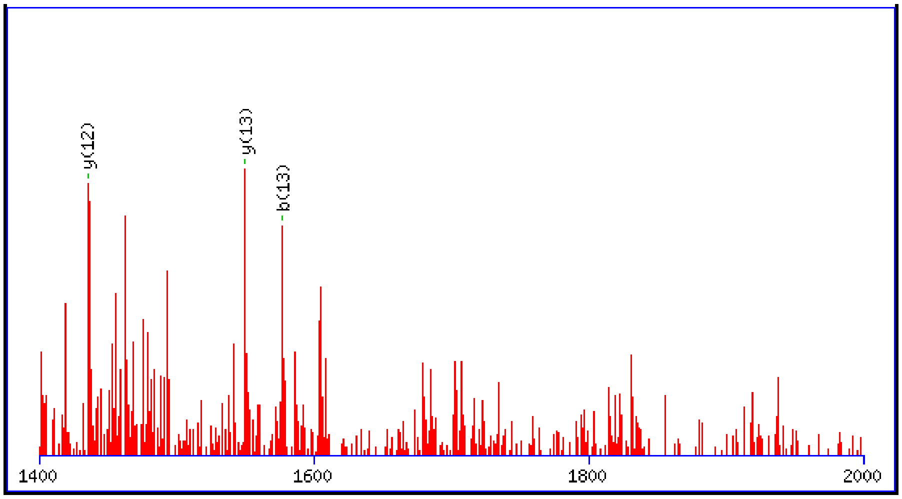

Monoisotopic mass of neutral peptide Mr(calc): 3255.2565
Fixed modifications: Carbamidomethyl (C)
Variable modifications:
S10 : Phospho (ST), with neutral losses 97.9769(shown in table), 0.0000
Ions Score: 57 **Expect:** 0.00061
Matches (Bold Red): 18/450 fragment ions using 31 most intense peaks

#	b	b++	b*	b*++	b0	b0++	Seq.	y	y++	y*	y*++	y0	y0++	#
1	148.0757	74.5415					F							27
2	205.0972	103.0522					G	3011.2185	1506.1129	2994.1919	1497.5996	2993.2079	1497.1076	26
3	334.1397	167.5735			316.1292	158.5682	E	2954.1970	1477.6021	2937.1704	1469.0889	2936.1864	1468.5969	25
4	463.1823	232.0948			445.1718	223.0895	E	2825.1544	1413.0808	2808.1279	1404.5676	2807.1438	1404.0756	24
5	550.2144	275.6108			532.2038	266.6055	S	2696.1118	1348.5595	2679.0853	1340.0463	2678.1012	1339.5543	23
6	**678.3093**	339.6583	661.2828	331.1450	660.2988	330.6530	K	2609.0798	1305.0435	2592.0532	1296.5303	2591.0692	1296.0382	22
7	807.3519	404.1796	790.3254	395.6663	789.3414	395.1743	E	2480.9848	1240.9960	2463.9583	1232.4828	2462.9743	1231.9908	21
8	904.4047	452.7060	887.3781	444.1927	886.3941	443.7007	P	2351.9422	1176.4748	2334.9157	1167.9615	2333.9317	1167.4695	20
9	1051.4731	526.2402	1034.4466	517.7269	1033.4625	517.2349	F	2254.8895	1127.9484	2237.8629	1119.4351	2236.8789	1118.9431	19
10	1120.4946	560.7509	1103.4680	552.2376	1102.4840	551.7456	S	2107.8210	**1054.4142**	2090.7945	1045.9009	2089.8105	1045.4089	18
11	1235.5215	618.2644	1218.4950	609.7511	1217.5109	609.2591	D	2038.7996	1019.9034	2021.7730	1011.3902	2020.7890	1010.8982	17
12	1364.5641	682.7857	1347.5375	674.2724	1346.5535	673.7804	E	1923.7726	962.3900	1906.7461	953.8767	1905.7621	953.3847	16
13	1479.5910	740.2992	1462.5645	731.7859	1461.5805	731.2939	D	1794.7301	897.8687	1777.7035	889.3554	1776.7195	888.8634	15
14	1608.6336	804.8205	1591.6071	796.3072	1590.6231	795.8152	E	1679.7031	840.3552	1662.6766	831.8419	1661.6925	831.3499	14
15	1723.6606	862.3339	1706.6340	853.8207	1705.6500	853.3286	D	**1550.6605**	775.8339	1533.6340	767.3206	1532.6500	766.8286	13
16	1838.6875	919.8474	1821.6610	911.3341	1820.6770	910.8421	D	**1435.6336**	718.3204	1418.6070	709.8072	1417.6230	709.3151	12
17	1952.7304	**976.8689**	1935.7039	968.3556	1934.7199	967.8636	N	**1320.6066**	660.8070	1303.5801	652.2937	1302.5961	651.8017	11
18	2067.7574	1034.3823	2050.7308	1025.8691	2049.7468	1025.3770	D	**1206.5637**	603.7855	1189.5372	595.2722	1188.5531	594.7802	10
19	2182.7843	1091.8958	2165.7578	1083.3825	2164.7738	1082.8905	D	1091.5368	546.2720	1074.5102	537.7587	1073.5262	537.2667	9
20	2281.8527	1141.4300	2264.8262	1132.9167	2263.8422	1132.4247	V	**976.5098**	488.7585	959.4833	480.2453	958.4993	479.7533	8
21	2410.8953	**1205.9513**	2393.8688	1197.4380	2392.8848	1196.9460	E	**877.4414**	439.2243	860.4149	430.7111	859.4308	430.2191	7
22	2507.9481	**1254.4777**	2490.9216	1245.9644	2489.9375	1245.4724	P	**748.3988**	374.7030	731.3723	366.1898	730.3883	365.6978	6
23	2621.0322	1311.0197	2604.0056	1302.5064	2603.0216	1302.0144	I	651.3461	326.1767	634.3195	317.6634	633.3355	317.1714	5
24	2708.0642	1354.5357	2691.0376	1346.0225	2690.0536	1345.5305	S	**538.2620**	269.6346	521.2354	261.1214	520.2514	260.6293	4
25	2837.1068	1419.0570	2820.0802	1410.5438	2819.0962	1410.0517	E	**451.2300**	226.1186	434.2034	217.6053	433.2194	217.1133	3
26	2984.1752	1492.5912	2967.1486	1484.0780	2966.1646	1483.5860	F	**322.1874**	161.5973	305.1608	153.0840			2
27							R	175.1190	88.0631	158.0924	79.5498			1

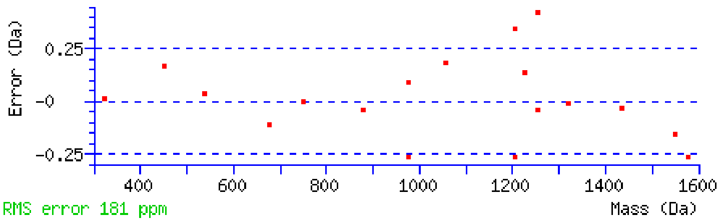

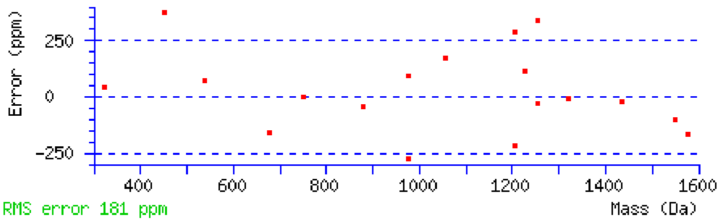

All matches to this query

Score	Mr(calc):	Delta	Sequence
57.0	3255.2565	0.0020	FGEESKEPFSDEDEDDNDDVEPISEFR
56.8	3255.2565	0.0020	FGEESKEPFSDEDEDDNDDVEPISEFR
2.8	3253.2406	2.0178	VDSFNDSTFAIAYTPVTPTTQEIMNR
2.0	3253.2406	2.0178	VDSFNDSTFAIAYTPVTPTTQEIMNR
1.5	3254.2246	1.0338	VDSFNDSTFAIAYTPVTPTTQEIMNR
1.5	3254.2856	0.9729	MSSTYCGNSSAKMSVHEVSESSLSLEQK
1.3	3253.2648	1.9937	TGTWSPERGPPIDMPNSQPNSQSVEMR
1.3	3254.2246	1.0338	VDSFNDSTFAIAYTPVTPTTQEIMNR
0.9	3254.2488	1.0097	TGTWSPERGPPIDMPNSQPNSQSVEMR
0.9	3254.2488	1.0097	TGTWSPERGPPIDMPNSQPNSQSVEMR

Spectrum No: 199; Query: 1455; Rank: 1

Peptide View

MS/MS Fragmentation of **SASSDTSEELNAQDSPK**
Found in **IPI00200898**, Tax_Id=10116 Gene_Symbol=Slc9a3r1 Ezrin-radixin-moesin-binding phosphoprotein 50

Match to Query 1455: 1924.691348 from(963.352950,2+)
Title: 091008RatKidney_NoSalt_36.753.753.2.dta
Data file K:\NewmanPaper\Piliang\3SubProteomes\Piliang3SP\mgf5ppm\ERLIC_3SubProteomes5ppm.mgf

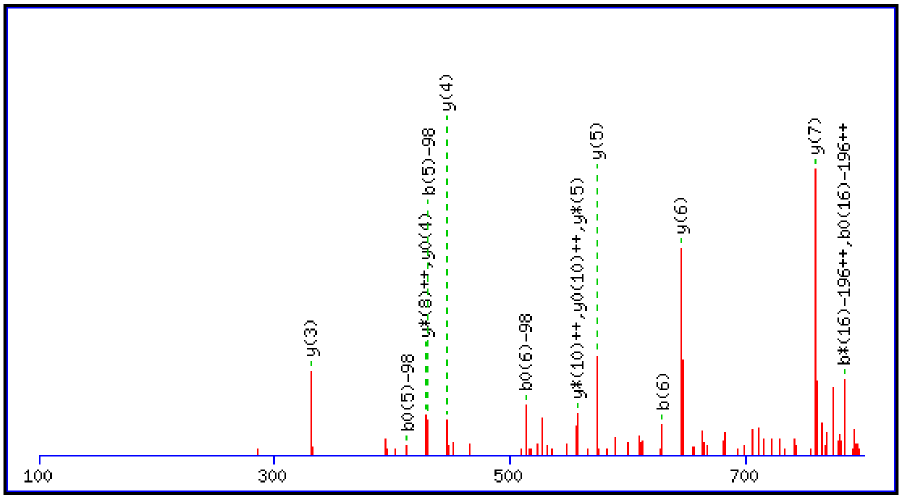

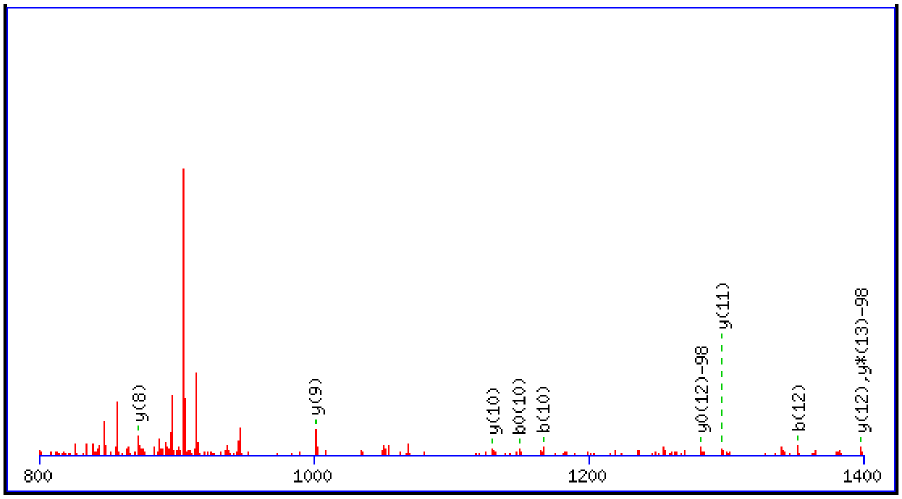

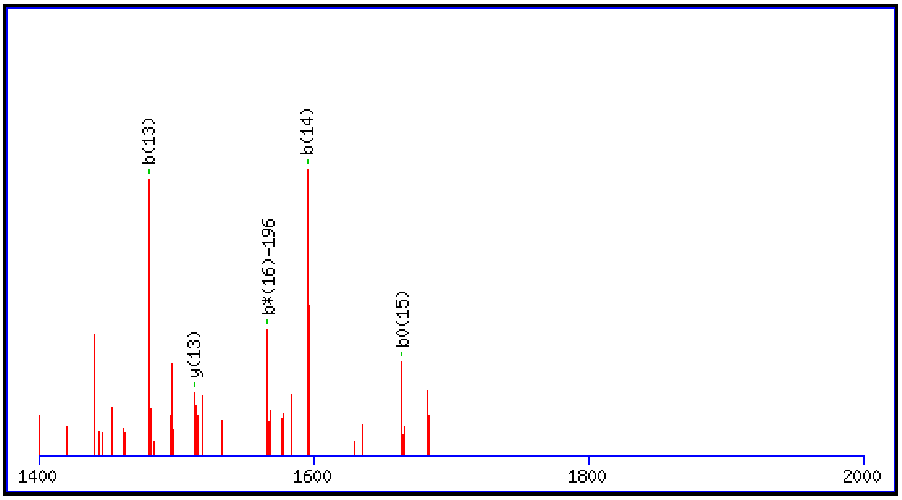

Monoisotopic mass of neutral peptide Mr(calc): 1924.6976
Fixed modifications: Carbamidomethyl (C)
Variable modifications:
S4 : Phospho (ST), with neutral losses 0.0000(shown in table), 97.9769
S7 : Phospho (ST), with neutral losses 0.0000(shown in table), 97.9769
Ions Score: 57 **Expect:** 0.00017
Matches (Bold Red): 31/268 fragment ions using 51 most intense peaks

#	b	b^{++}	b*	b^{*++}	b^0	b^{0++}	Seq.	y	y^{++}	y*	y^{*++}	y^0	y^{0++}	#
1	88.0393	44.5233			70.0287	35.5180	S							17
2	159.0764	80.0418			141.0659	71.0366	A	1838.6729	919.8401	1821.6463	911.3268	1820.6623	910.8348	16
3	246.1084	123.5579			228.0979	114.5526	S	1767.6358	884.3215	1750.6092	875.8082	1749.6252	875.3162	15
4	413.1068	207.0570			395.0962	198.0518	S	1680.6037	840.8055	1663.5772	832.2922	1662.5932	831.8002	14
5	528.1337	264.5705			510.1232	255.5652	D	**1513.6054**	757.3063	1496.5788	748.7931	1495.5948	748.3010	13
6	**629.1814**	315.0943			611.1709	306.0891	T	**1398.5784**	699.7929	1381.5519	691.2796	1380.5679	690.7876	12
7	796.1798	398.5935			778.1692	389.5882	S	**1297.5308**	649.2690	1280.5042	640.7557	1279.5202	640.2637	11
8	925.2224	463.1148			907.2118	454.1095	E	**1130.5324**	565.7698	1113.5059	**557.2566**	1112.5218	**556.7646**	10
9	1054.2650	527.6361			1036.2544	518.6308	E	**1001.4898**	501.2485	984.4633	492.7353	983.4793	492.2433	9
10	**1167.3490**	584.1781			**1149.3385**	575.1729	L	**872.4472**	436.7272	855.4207	**428.2140**	854.4367	427.7220	8
11	1281.3919	641.1996	1264.3654	632.6863	1263.3814	632.1943	N	**759.3632**	380.1852	742.3366	371.6719	741.3526	371.1799	7
12	**1352.4291**	676.7182	1335.4025	668.2049	1334.4185	667.7129	A	**645.3202**	323.1638	628.2937	314.6505	627.3097	314.1585	6
13	**1480.4876**	740.7475	1463.4611	732.2342	1462.4771	731.7422	Q	**574.2831**	287.6452	**557.2566**	279.1319	556.2726	278.6399	5
14	**1595.5146**	798.2609	1578.4880	789.7477	1577.5040	789.2556	D	**446.2245**	223.6159	429.1980	215.1026	**428.2140**	214.6106	4
15	1682.5466	841.7769	1665.5201	833.2637	**1664.5360**	832.7717	S	**331.1976**	166.1024	314.1710	157.5892	313.1870	157.0972	3
16	1779.5994	890.3033	1762.5728	881.7901	1761.5888	881.2980	P	244.1656	122.5864	227.1390	114.0731			2
17							K	147.1128	74.0600	130.0863	65.5468			1

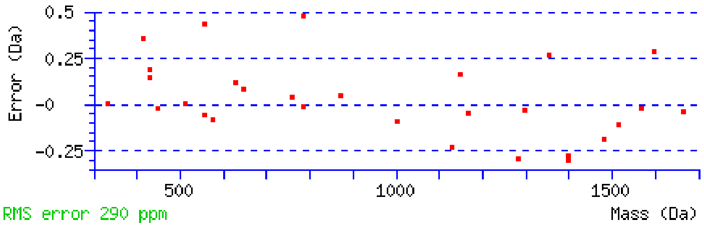

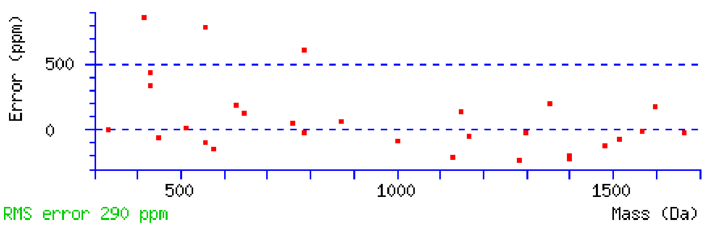

All matches to this query

Score	Mr(calc):	Delta	Sequence
57.0	1924.6976	-0.0063	SASSDTSEELNAQDSPK
57.0	1924.6976	-0.0063	SASSDTSEELNAQDSPK
57.0	1924.6976	-0.0063	SASSDTSEELNAQDSPK
44.0	1924.6976	-0.0063	SASSDTSEELNAQDSPK
44.0	1924.6976	-0.0063	SASSDTSEELNAQDSPK
44.0	1924.6976	-0.0063	SASSDTSEELNAQDSPK
37.2	1924.6976	-0.0063	SASSDTSEELNAQDSPK
26.8	1924.6976	-0.0063	SASSDTSEELNAQDSPK
26.8	1924.6976	-0.0063	SASSDTSEELNAQDSPK
26.8	1924.6976	-0.0063	SASSDTSEELNAQDSPK

Spectrum No: 200; Query: 1849; Rank: 1

Peptide View

MS/MS Fragmentation of **KELGSLEEDANPDEEGVQK**
Found in **IPI00371736**, Tax_Id=10116 Gene_Symbol=LOC685174 similar to trinucleotide repeat containing 5

Match to Query 1849: 2165.947128 from(1083.980840,2+)
Title: 100101RatKid_NS_deglyco_19.2028.2028.2.dta
Data file K:\NewmanPaper\Piliang\3SubProteomes\Piliang3SP\mgf5ppm\ERLIC_3SubProteomes5ppm.mgf

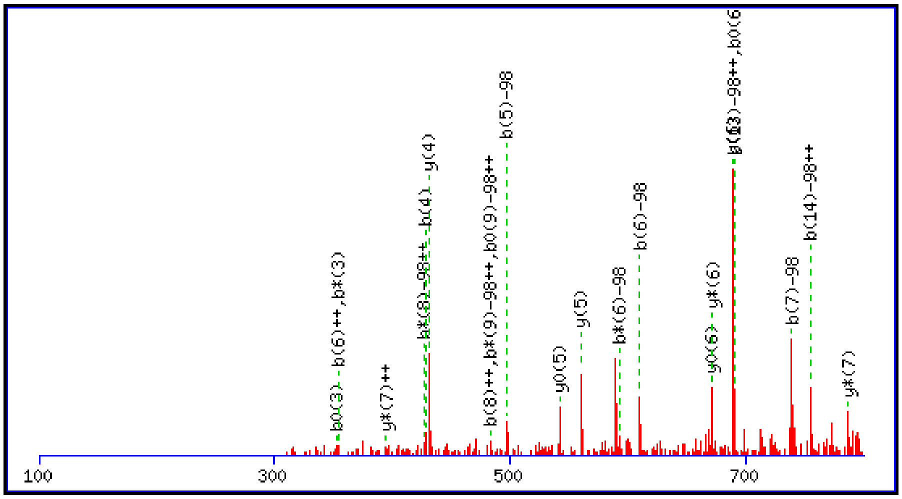

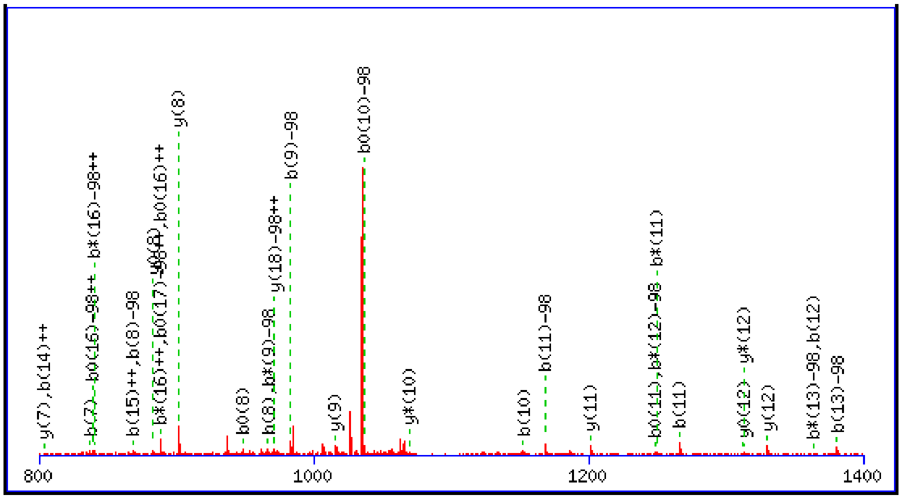

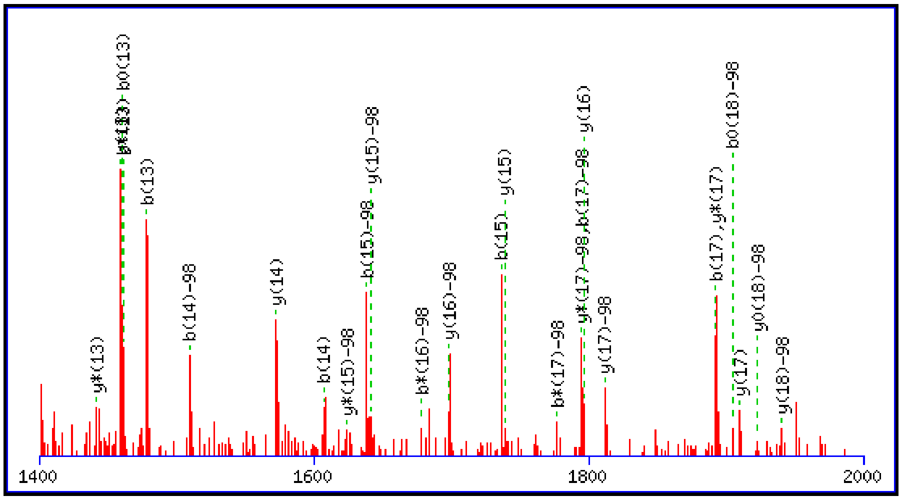

Monoisotopic mass of neutral peptide Mr(calc): 2165.9365
Fixed modifications: Carbamidomethyl (C)
Variable modifications:
S5 : Phospho (ST), with neutral losses 97.9769(shown in table), 0.0000
Ions Score: 57 **Expect:** 0.00052
Matches (Bold Red): 83/314 fragment ions using 158 most intense peaks

#	b	b^{++}	b*	b^{*++}	b^0	b^{0++}	Seq.	y	y^{++}	y*	y^{*++}	y^0	y^{0++}	#
1	129.1022	65.0548	112.0757	56.5415			K							19
2	258.1448	129.5761	241.1183	121.0628	240.1343	120.5708	E	**1940.8720**	**970.9396**	1923.8454	962.4263	**1922.8614**	961.9343	18
3	371.2289	186.1181	**354.2023**	177.6048	**353.2183**	177.1128	L	**1811.8294**	906.4183	**1794.8028**	897.9050	1793.8188	897.4130	17
4	**428.2504**	214.6288	411.2238	206.1155	410.2398	205.6235	G	**1698.7453**	849.8763	1681.7188	841.3630	1680.7347	840.8710	16
5	**497.2718**	249.1395	480.2453	240.6263	479.2613	240.1343	S	**1641.7238**	821.3656	**1624.6973**	812.8523	1623.7133	812.3603	15
6	**610.3559**	305.6816	**593.3293**	297.1683	592.3453	296.6763	L	**1572.7024**	786.8548	1555.6758	778.3416	1554.6918	777.8495	14
7	**739.3985**	370.2029	722.3719	361.6896	721.3879	361.1976	E	**1459.6183**	730.3128	**1442.5918**	721.7995	1441.6078	721.3075	13
8	**868.4411**	434.7242	851.4145	**426.2109**	850.4305	425.7189	E	**1330.5757**	665.7915	**1313.5492**	657.2782	**1312.5652**	656.7862	12
9	**983.4680**	492.2376	**966.4415**	**483.7244**	965.4574	**483.2324**	D	**1201.5331**	601.2702	1184.5066	592.7569	1183.5226	592.2649	11
10	1054.5051	527.7562	1037.4786	519.2429	**1036.4946**	518.7509	A	1086.5062	543.7567	**1069.4796**	535.2435	1068.4956	534.7515	10
11	**1168.5481**	584.7777	1151.5215	576.2644	1150.5375	575.7724	N	**1015.4691**	508.2382	998.4425	499.7249	997.4585	499.2329	9
12	1265.6008	633.3040	**1248.5743**	624.7908	1247.5902	624.2988	P	**901.4262**	451.2167	884.3996	442.7034	**883.4156**	442.2114	8
13	**1380.6278**	**690.8175**	**1363.6012**	682.3042	1362.6172	681.8122	D	**804.3734**	402.6903	**787.3468**	**394.1771**	786.3628	393.6851	7
14	**1509.6704**	**755.3388**	1492.6438	746.8255	1491.6598	746.3335	E	**689.3464**	345.1769	**672.3199**	336.6636	**671.3359**	336.1716	6
15	**1638.7129**	819.8601	1621.6864	811.3468	1620.7024	810.8548	E	**560.3039**	280.6556	543.2773	272.1423	**542.2933**	271.6503	5
16	1695.7344	848.3708	**1678.7079**	**839.8576**	1677.7238	**839.3656**	G	**431.2613**	216.1343	414.2347	207.6210			4
17	**1794.8028**	897.9050	**1777.7763**	889.3918	1776.7923	**888.8998**	V	374.2398	187.6235	357.2132	179.1103			3
18	**1922.8614**	961.9343	1905.8349	953.4211	**1904.8508**	952.9291	Q	275.1714	138.0893	258.1448	129.5761			2

19							K	147.1128	74.0600	130.0863	65.5468			1

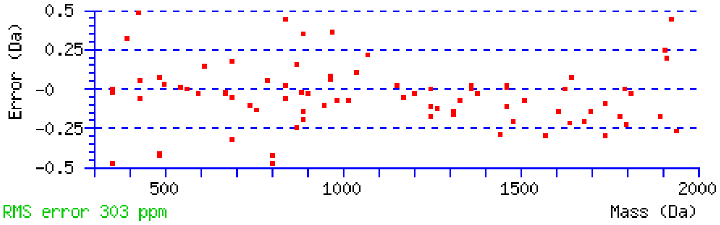

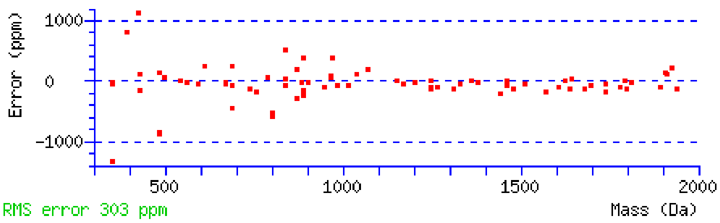

All matches to this query

Score	Mr(calc):	Delta	Sequence
56.9	2165.9365	0.0106	KELGSLEEDANPDEEGVQK
4.2	2165.9670	-0.0199	ESPGGSLYLNEAGFNFVRK
3.6	2165.9590	-0.0119	TKSESRLEFTSSGSANTER
2.9	2165.9258	0.0213	YRCFTFGIGEGASTSLIK
2.2	2164.9569	0.9903	GQGVNHVPTQNPYSPRVR
1.9	2165.9664	-0.0193	VKDMAAPGTSSAPAPTAGNAEK
1.5	2163.9425	2.0046	FSDTRLSQTMLGHVGLNK
1.5	2163.9425	2.0046	FSDTRLSQTMLGHVGLNK
1.5	2163.9435	2.0036	NVPVLFNDTETNLAGMYGK
1.4	2164.9390	1.0081	DRVIGLMMTACDLCSVTK

Mascot: http://www.matrixscience.com/

{MATRIX SCIENCE} Mascot Search Results

Results Generated by a Script Modified from Mascot Pepitde View
by Newman Sze, School of Biological Sciences, Nanyang Technological University

---

Spectrum No: 201; Query: 1684; Rank: 1

Peptide View

MS/MS Fragmentation of **LKFSDDEDEEDVVKDGR**
Found in **IPI00363963**, Tax_Id=10116 Gene_Symbol=LOC296637 similar to HLA-B associated transcript-2 isoform a

Match to Query 1684: 2074.878462 from(692.633430,3+)
Title: 100101RatKid_NS_deglyco_18.2307.2307.3.dta
Data file K:\NewmanPaper\Piliang\3SubProteomes\Piliang3SP\mgf5ppm\ERLIC_3SubProteomes5ppm.mgf

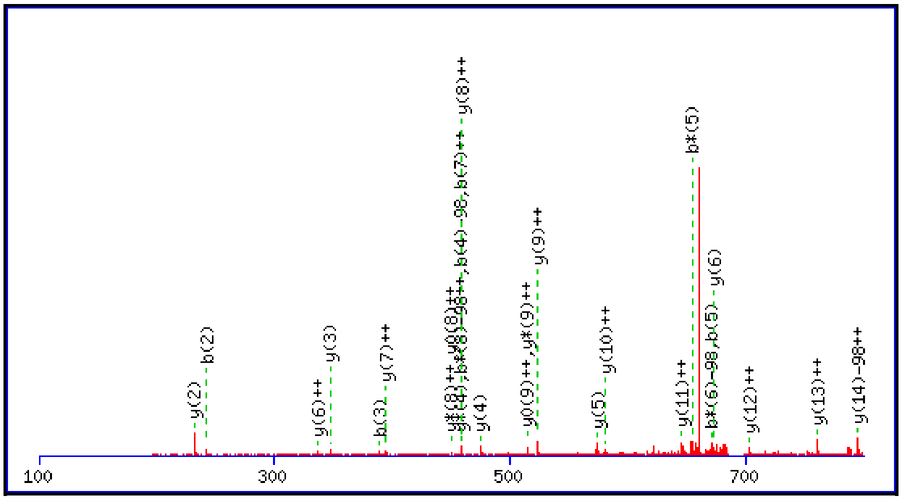

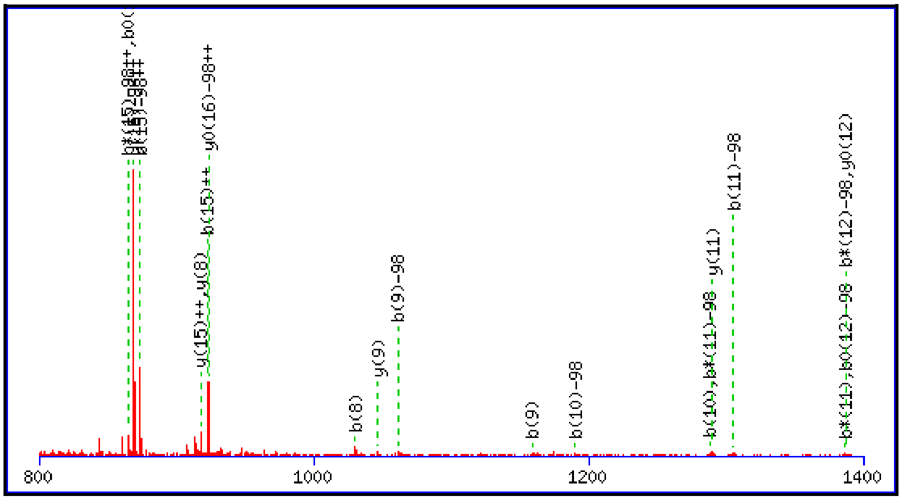

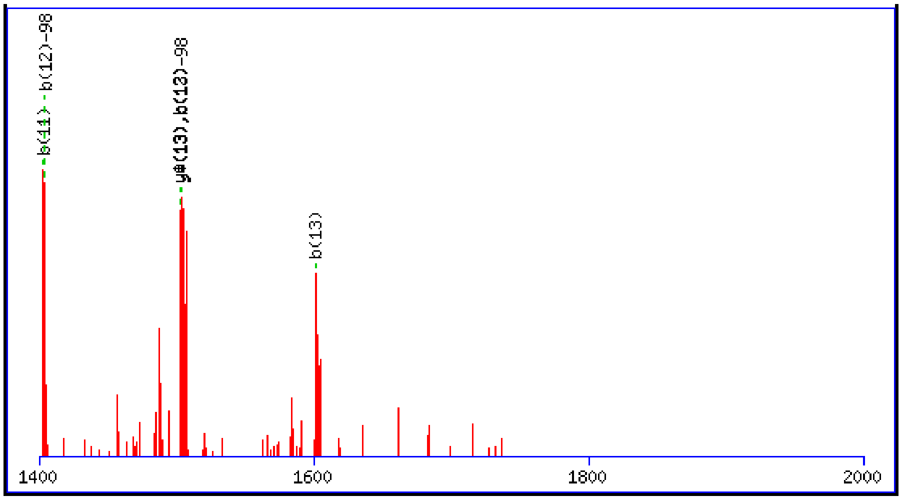

Monoisotopic mass of neutral peptide Mr(calc): 2074.8732
Fixed modifications: Carbamidomethyl (C)
Variable modifications:
S4 : Phospho (ST), with neutral losses 97.9769(shown in table), 0.0000
Ions Score: 57 **Expect:** 0.00054
Matches (Bold Red): 56/276 fragment ions using 79 most intense peaks

#	b	b++	b*	b*++	b0	b0++	Seq.	y	y++	y*	y*++	y0	y0++	#
1	114.0913	57.5493					L							17
2	**242.1863**	121.5968	225.1598	113.0835			K	1864.8195	932.9134	1847.7930	924.4001	1846.8090	**923.9081**	16
3	**389.2547**	195.1310	372.2282	186.6177			F	1736.7246	**868.8659**	1719.6980	860.3526	1718.7140	859.8606	15
4	**458.2762**	229.6417	441.2496	221.1284	440.2656	220.6364	S	1589.6562	**795.3317**	1572.6296	786.8184	1571.6456	786.3264	14
5	573.3031	287.1552	556.2766	278.6419	555.2926	278.1499	D	1520.6347	**760.8210**	**1503.6082**	752.3077	**1502.6241**	751.8157	13
6	688.3301	344.6687	**671.3035**	336.1554	670.3195	335.6634	D	1405.6078	**703.3075**	1388.5812	694.7942	**1387.5972**	694.3022	12
7	817.3727	409.1900	800.3461	400.6767	799.3621	400.1847	E	**1290.5808**	**645.7940**	1273.5543	637.2808	1272.5703	636.7888	11
8	932.3996	466.7034	915.3730	**458.1902**	914.3890	457.6982	D	1161.5382	**581.2727**	1144.5117	572.7595	1143.5277	572.2675	10
9	**1061.4422**	531.2247	1044.4156	522.7115	1043.4316	522.2194	E	**1046.5113**	**523.7593**	1029.4847	**515.2460**	1028.5007	**514.7540**	9
10	**1190.4848**	595.7460	1173.4582	587.2328	1172.4742	586.7407	E	**917.4687**	**459.2380**	900.4421	**450.7247**	899.4581	**450.2327**	8
11	**1305.5117**	653.2595	**1288.4852**	644.7462	1287.5012	644.2542	D	788.4261	**394.7167**	771.3995	386.2034	770.4155	385.7114	7
12	**1404.5801**	702.7937	**1387.5536**	694.2804	**1386.5696**	693.7884	V	**673.3992**	**337.2032**	656.3726	328.6899	655.3886	328.1979	6
13	**1503.6486**	752.3279	1486.6220	743.8146	1485.6380	743.3226	V	**574.3307**	287.6690	557.3042	279.1557	556.3202	278.6637	5
14	1631.7435	816.3754	1614.7170	807.8621	1613.7330	807.3701	K	**475.2623**	238.1348	**458.2358**	229.6215	457.2518	229.1295	4
15	1746.7705	**873.8889**	1729.7439	**865.3756**	1728.7599	**864.8836**	D	**347.1674**	174.0873	330.1408	165.5740	329.1568	165.0820	3
16	1803.7919	902.3996	1786.7654	893.8863	1785.7814	893.3943	G	**232.1404**	116.5738	215.1139	108.0606			2
17							R	175.1190	88.0631	158.0924	79.5498			1

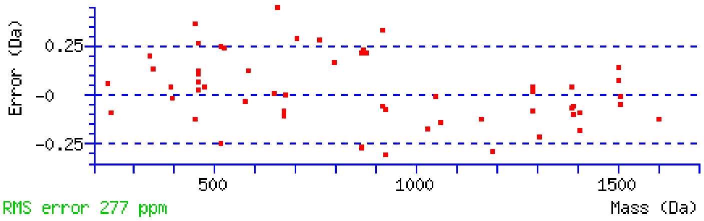

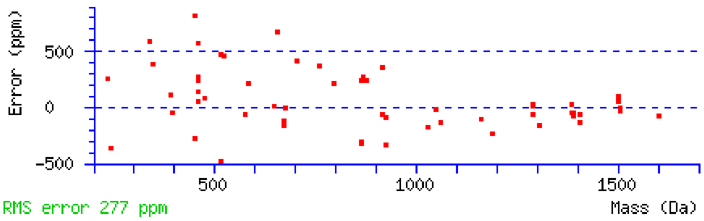

All matches to this query

Score	Mr(calc):	Delta	Sequence
56.8	2074.8732	0.0052	LKFSDDEDEEDVVKDGR
4.6	2073.8632	1.0153	LSYPHPGTDNLLMLNGR
2.8	2074.8943	-0.0158	SEEEQSSASVKKDETNVK
2.7	2074.8692	0.0093	DDEDSVPATNTAKSRSSSK

2.7	2074.8692	0.0093	DDEDSVPATNTAKSRSSSK
2.7	2074.8692	0.0093	DDEDSVPATNTAKSRSSSK
2.7	2074.8692	0.0093	DDEDSVPATNTAKSRSSSK
2.7	2073.8730	1.0054	ASSKLALMKAQEENSYK
2.5	2074.8893	-0.0109	NPFLGMIQNWTETAMDK
2.5	2072.8560	2.0224	STSITQTQLSKMMSNQK

Spectrum No: 202; Query: 2785; Rank: 1

Peptide View

MS/MS Fragmentation of **GSAPHSESDIPEQEEEILGSDDDEQEDPNDYCK**
Found in **IPI00372823**, Tax_Id=10116 Gene_Symbol=Srpk1 Serine/arginine-rich protein specific kinase 1

Match to Query 2785: 3813.474492 from(1272.165440,3+)
Title: 100101RatKid_NS_deglyco_24.2818.2818.3.dta
Data file K:\NewmanPaper\Piliang\3SubProteomes\Piliang3SP\mgf5ppm\ERLIC_3SubProteomes5ppm.mgf

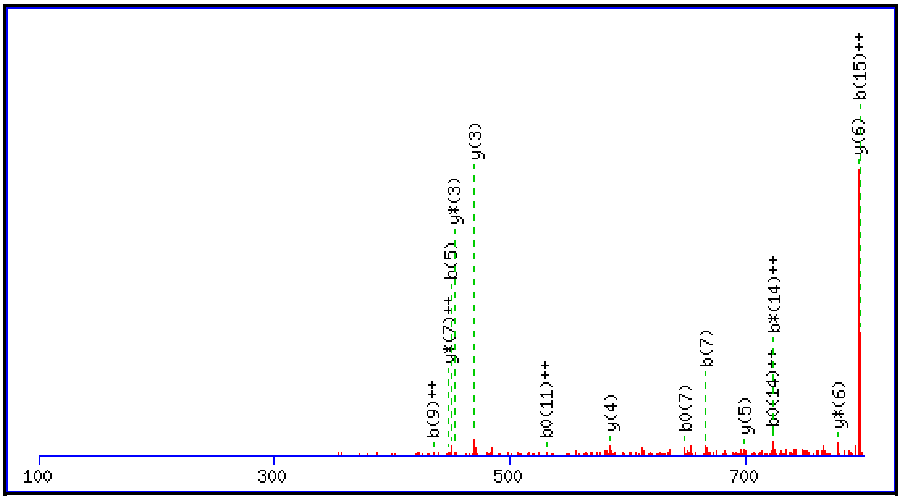

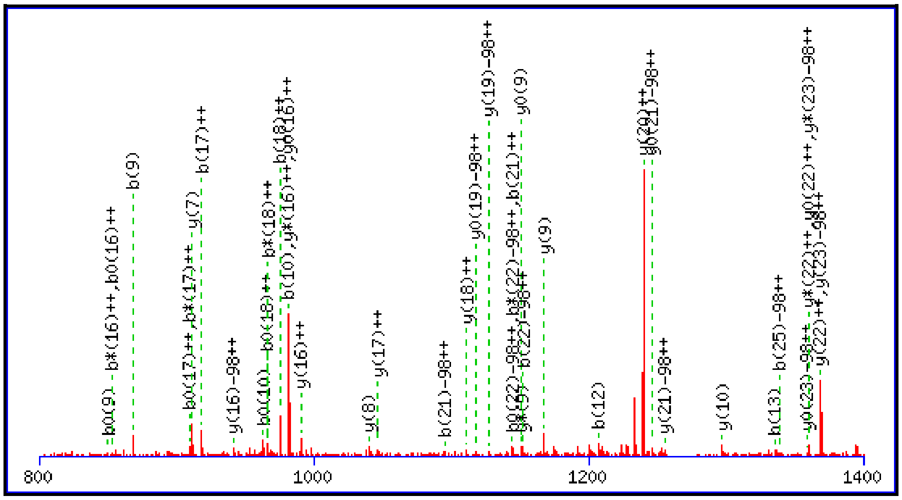

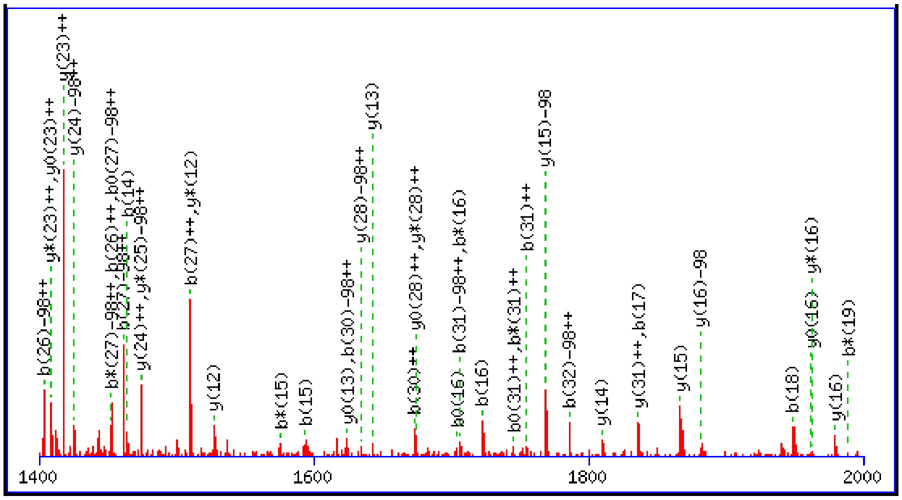

Monoisotopic mass of neutral peptide Mr(calc): 3813.4632
Fixed modifications: Carbamidomethyl (C)
Variable modifications:
S20 : Phospho (ST), with neutral losses 97.9769(shown in table), 0.0000
Ions Score: 57 **Expect:** 0.00071
Matches (Bold Red): 101/544 fragment ions using 165 most intense peaks

#	b	b++	b*	b*++	b0	b0++	Seq.	y	y++	y*	y*++	y0	y0++	#
1	58.0287	29.5180					G							33
2	145.0608	73.0340			127.0502	64.0287	S	3659.4722	1830.2397	3642.4456	1821.7265	3641.4616	1821.2344	32
3	216.0979	108.5526			198.0873	99.5473	A	3572.4402	1786.7237	3555.4136	1778.2104	3554.4296	1777.7184	31
4	313.1506	157.0790			295.1401	148.0737	P	3501.4030	1751.2052	3484.3765	1742.6919	3483.3925	1742.1999	30
5	**450.2096**	225.6084			432.1990	216.6031	H	3404.3503	1702.6788	3387.3237	1694.1655	3386.3397	1693.6735	29
6	537.2416	269.1244			519.2310	260.1191	S	3267.2914	**1634.1493**	3250.2648	1625.6360	3249.2808	1625.1440	28
7	**666.2842**	333.6457			**648.2736**	324.6404	E	3180.2593	1590.6333	3163.2328	1582.1200	3162.2488	1581.6280	27
8	753.3162	377.1617			735.3056	368.1565	S	3051.2167	1526.1120	3034.1902	1517.5987	3033.2062	1517.1067	26
9	**868.3431**	**434.6752**			**850.3326**	425.6699	D	2964.1847	1482.5960	2947.1582	**1474.0827**	2946.1742	1473.5907	25
10	**981.4272**	491.2172			**963.4166**	482.2120	I	2849.1578	**1425.0825**	2832.1312	1416.5693	2831.1472	1416.0772	24
11	1078.4800	539.7436			1060.4694	**530.7383**	P	2736.0737	**1368.5405**	2719.0472	**1360.0272**	2718.0631	**1359.5352**	23
12	**1207.5226**	604.2649			1189.5120	595.2596	E	2639.0209	1320.0141	2621.9944	1311.5008	2621.0104	1311.0088	22
13	**1335.5811**	668.2942	1318.5546	659.7809	1317.5706	659.2889	Q	2509.9784	**1255.4928**	2492.9518	1246.9795	2491.9678	**1246.4875**	21
14	**1464.6237**	732.8155	1447.5972	**724.3022**	1446.6132	**723.8102**	E	2381.9198	1191.4635	2364.8932	1182.9503	2363.9092	1182.4582	20
15	**1593.6663**	**797.3368**	**1576.6398**	788.8235	1575.6558	788.3315	E	2252.8772	**1126.9422**	2235.8506	1118.4290	2234.8666	**1117.9369**	19
16	**1722.7089**	861.8581	**1705.6824**	**853.3448**	**1704.6984**	**852.8528**	E	2123.8346	1062.4209	2106.8080	1053.9077	2105.8240	1053.4157	18
17	**1835.7930**	**918.4001**	1818.7664	**909.8869**	1817.7824	**909.3949**	I	1994.7920	997.8996	1977.7655	989.3864	1976.7814	988.8944	17
18	**1948.8771**	**974.9422**	1931.8505	**966.4289**	1930.8665	**965.9369**	L	**1881.7079**	**941.3576**	1864.6814	932.8443	1863.6974	932.3523	16
19	2005.8985	1003.4529	**1988.8720**	994.9396	1987.8880	994.4476	G	**1768.6239**	884.8156	1751.5973	876.3023	1750.6133	875.8103	15
20	2074.9200	1037.9636	2057.8934	1029.4504	2056.9094	1028.9583	S	1711.6024	856.3048	1694.5759	847.7916	1693.5918	847.2996	14
21	2189.9469	**1095.4771**	2172.9204	1086.9638	2171.9364	1086.4718	D	**1642.5810**	821.7941	1625.5544	813.2808	**1624.5704**	812.7888	13
22	2304.9739	**1152.9906**	2287.9473	**1144.4773**	2286.9633	**1143.9853**	D	**1527.5540**	764.2806	**1510.5275**	755.7674	1509.5434	755.2754	12
23	2420.0008	1210.5040	2402.9743	1201.9908	2401.9902	1201.4988	D	1412.5271	706.7672	1395.5005	698.2539	1394.5165	697.7619	11
24	2549.0434	1275.0253	2532.0168	1266.5121	2531.0328	1266.0201	E	**1297.5001**	649.2537	1280.4736	640.7404	1279.4896	640.2484	10
25	2677.1020	**1339.0546**	2660.0754	1330.5414	2659.0914	1330.0493	Q	**1168.4575**	584.7324	**1151.4310**	576.2191	**1150.4470**	575.7271	9
26	2806.1446	**1403.5759**	2789.1180	1395.0626	2788.1340	1394.5706	E	**1040.3990**	520.7031	1023.3724	512.1898	1022.3884	511.6978	8
27	2921.1715	**1461.0894**	2904.1450	**1452.5761**	2903.1609	**1452.0841**	D	**911.3564**	456.1818	894.3298	**447.6685**	893.3458	447.1765	7
28	3018.2243	1509.6158	3001.1977	1501.1025	3000.2137	1500.6105	P	**796.3294**	398.6683	**779.3029**	390.1551	778.3189	389.6631	6
29	3132.2672	1566.6372	3115.2407	1558.1240	3114.2566	1557.6320	N	**699.2767**	350.1420	682.2501	341.6287	681.2661	341.1367	5
30	3247.2941	**1624.1507**	3230.2676	1615.6374	3229.2836	1615.1454	D	**585.2337**	293.1205	568.2072	284.6072	567.2232	284.1152	4
31	3410.3575	**1705.6824**	3393.3309	1697.1691	3392.3469	1696.6771	Y	**470.2068**	235.6070	**453.1802**	227.0938			3
32	3570.3881	**1785.6977**	3553.3616	1777.1844	3552.3776	1776.6924	C	307.1435	154.0754	290.1169	145.5621			2

33							K	147.1128	74.0600	130.0863	65.5468			1

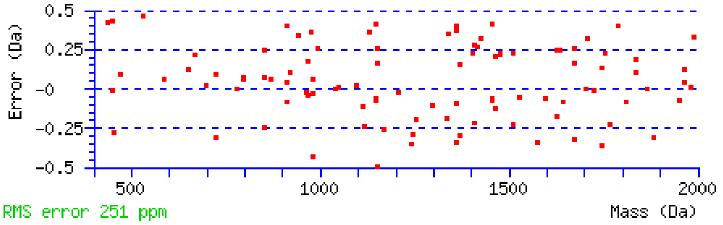

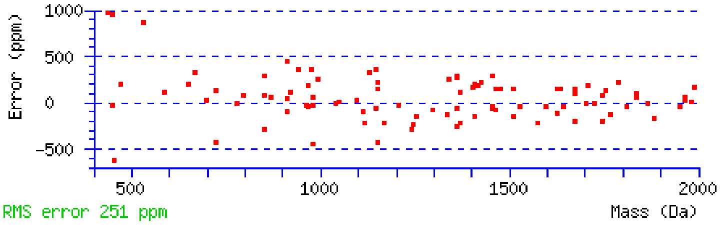

All matches to this query

Score	Mr(calc):	Delta	Sequence
56.8	3813.4632	0.0113	GSAPHSESDIPEQEEEILGSDDDEQEDPNDYCK
30.0	3813.4632	0.0113	GSAPHSESDIPEQEEEILGSDDDEQEDPNDYCK
29.4	3813.4632	0.0113	GSAPHSESDIPEQEEEILGSDDDEQEDPNDYCK
28.1	3813.4632	0.0113	GSAPHSESDIPEQEEEILGSDDDEQEDPNDYCK
24.6	3813.4632	0.0113	GSAPHSESDIPEQEEEILGSDDDEQEDPNDYCK
5.3	3813.5016	-0.0271	ECDWSGSLLQYSEALNIASYAKSEEILIPK
5.1	3813.5016	-0.0271	ECDWSGSLLQYSEALNIASYAKSEEILIPK
4.5	3813.4992	-0.0247	MYTIPLYEDLCTGALKSFALEVFYQTQGR
4.5	3813.4992	-0.0247	MYTIPLYEDLCTGALKSFALEVFYQTQGR
4.2	3813.5016	-0.0271	ECDWSGSLLQYSEALNIASYAKSEEILIPK

Spectrum No: 203; Query: 2391; Rank: 1

Peptide View

MS/MS Fragmentation of **SDISERPSVDDVESETGSTGALETR**
Found in **IPI00373280**, Tax_Id=10116 Gene_Symbol=Tmem24 72 kDa protein

Match to Query 2391: 2716.179642 from(906.400490,3+)
Title: 100101RatKid_NS_deglyco_20.2417.2417.3.dta
Data file K:\NewmanPaper\Piliang\3SubProteomes\Piliang3SP\mgf5ppm\ERLIC_3SubProteomes5ppm.mgf

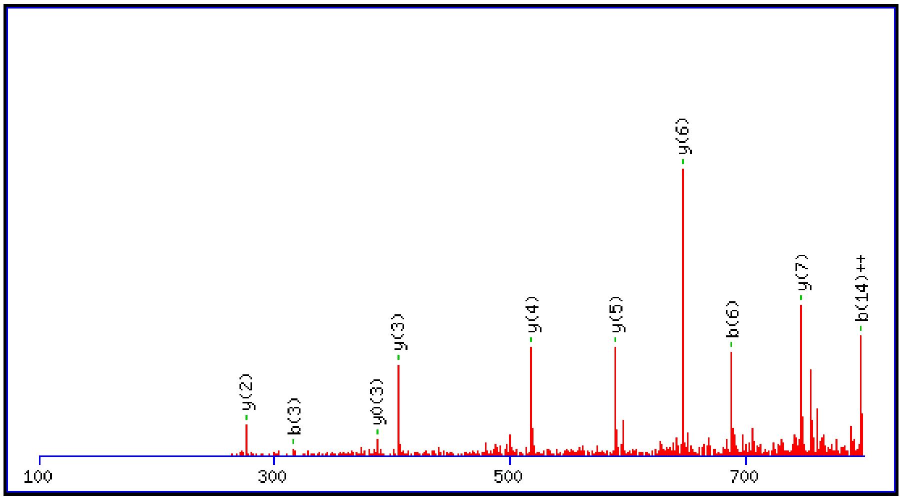

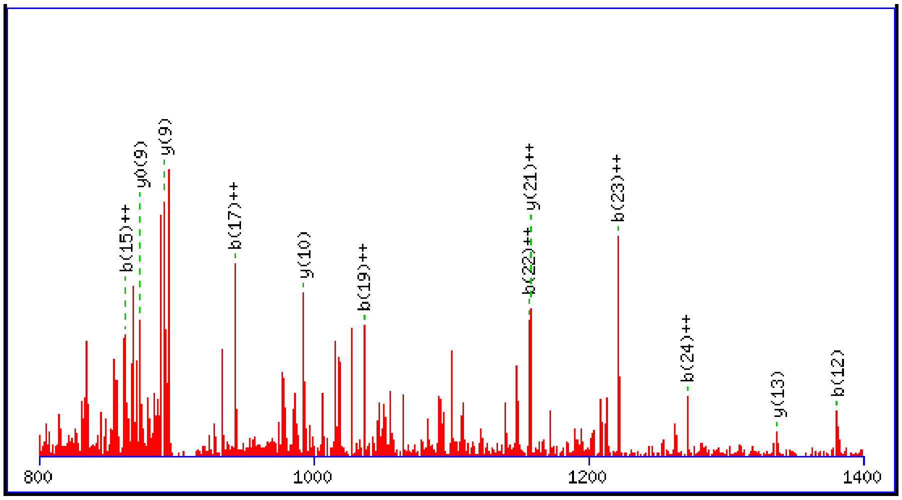

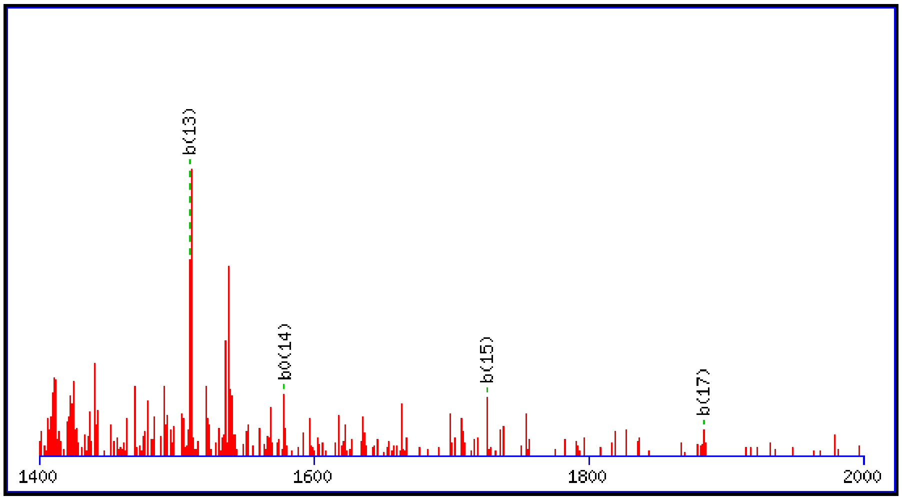

Monoisotopic mass of neutral peptide Mr(calc): 2716.1712
Fixed modifications: Carbamidomethyl (C)
Variable modifications:
S8 : Phospho (ST), with neutral losses 0.0000(shown in table), 97.9769
Ions Score: 57 **Expect:** 0.00081
Matches (Bold Red): 26/420 fragment ions using 52 most intense peaks

#	b	b^{++}	b*	b^{*++}	b^0	b^{0++}	Seq.	y	y^{++}	y*	y^{*++}	y^0	y^{0++}	#
1	88.0393	44.5233			70.0287	35.5180	S							25
2	203.0662	102.0368			185.0557	93.0315	D	2630.1465	1315.5769	2613.1199	1307.0636	2612.1359	1306.5716	24
3	**316.1503**	158.5788			298.1397	149.5735	I	2515.1196	1258.0634	2498.0930	1249.5501	2497.1090	1249.0581	23
4	403.1823	202.0948			385.1718	193.0895	S	2402.0355	1201.5214	2385.0089	1193.0081	2384.0249	1192.5161	22
5	532.2249	266.6161			514.2144	257.6108	E	2315.0035	**1158.0054**	2297.9769	1149.4921	2296.9929	1149.0001	21
6	**688.3260**	344.6667	671.2995	336.1534	670.3155	335.6614	R	2185.9609	1093.4841	2168.9343	1084.9708	2167.9503	1084.4788	20
7	785.3788	393.1930	768.3523	384.6798	767.3682	384.1878	P	2029.8598	1015.4335	2012.8332	1006.9202	2011.8492	1006.4282	19
8	952.3772	476.6922	935.3506	468.1789	934.3666	467.6869	S	1932.8070	966.9071	1915.7804	958.3939	1914.7964	957.9019	18
9	1051.4456	526.2264	1034.4190	517.7132	1033.4350	517.2211	V	1765.8086	883.4080	1748.7821	874.8947	1747.7981	874.4027	17
10	1166.4725	583.7399	1149.4460	575.2266	1148.4620	574.7346	D	1666.7402	833.8738	1649.7137	825.3605	1648.7297	824.8685	16
11	1281.4995	641.2534	1264.4729	632.7401	1263.4889	632.2481	D	1551.7133	776.3603	1534.6867	767.8470	1533.7027	767.3550	15
12	**1380.5679**	690.7876	1363.5413	682.2743	1362.5573	681.7823	V	1436.6863	718.8468	1419.6598	710.3335	1418.6758	709.8415	14
13	**1509.6105**	755.3089	1492.5839	746.7956	1491.5999	746.3036	E	**1337.6179**	669.3126	1320.5914	660.7993	1319.6074	660.3073	13
14	1596.6425	**798.8249**	1579.6159	790.3116	**1578.6319**	789.8196	S	1208.5753	604.7913	1191.5488	596.2780	1190.5648	595.7860	12
15	**1725.6851**	**863.3462**	1708.6585	854.8329	1707.6745	854.3409	E	1121.5433	561.2753	1104.5168	552.7620	1103.5327	552.2700	11
16	1826.7328	913.8700	1809.7062	905.3567	1808.7222	904.8647	T	**992.5007**	496.7540	975.4742	488.2407	974.4902	487.7487	10
17	**1883.7542**	**942.3808**	1866.7277	933.8675	1865.7437	933.3755	G	**891.4530**	446.2302	874.4265	437.7169	**873.4425**	437.2249	9
18	1970.7863	985.8968	1953.7597	977.3835	1952.7757	976.8915	S	834.4316	417.7194	817.4050	409.2062	816.4210	408.7141	8

**19**	2071.8339	**1036.4206**	2054.8074	1027.9073	2053.8234	1027.4153	**T**	**747.3995**	374.2034	730.3730	365.6901	729.3890	365.1981	**7**
**20**	2128.8554	1064.9313	2111.8289	1056.4181	2110.8448	1055.9261	**G**	**646.3519**	323.6796	629.3253	315.1663	628.3413	314.6743	**6**
**21**	2199.8925	1100.4499	2182.8660	1091.9366	2181.8820	1091.4446	**A**	**589.3304**	295.1688	572.3039	286.6556	571.3198	286.1636	**5**
**22**	2312.9766	**1156.9919**	2295.9500	1148.4787	2294.9660	1147.9866	**L**	**518.2933**	259.6503	501.2667	251.1370	500.2827	250.6450	**4**
**23**	2442.0192	**1221.5132**	2424.9926	1212.9999	2424.0086	1212.5079	**E**	**405.2092**	203.1082	388.1827	194.5950	**387.1987**	194.1030	**3**
**24**	2543.0669	**1272.0371**	2526.0403	1263.5238	2525.0563	1263.0318	**T**	**276.1666**	138.5870	259.1401	130.0737	258.1561	129.5817	**2**
**25**							**R**	175.1190	88.0631	158.0924	79.5498			**1**

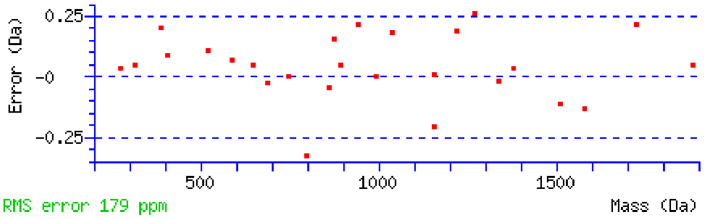

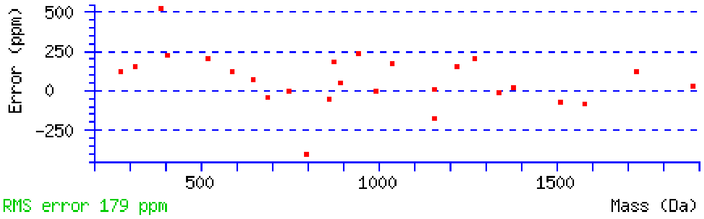

All matches to this query

Score	Mr(calc):	Delta	Sequence
56.6	2716.1712	0.0084	SDISERPSVDDVESETGSTGALETR
47.5	2716.1712	0.0084	SDISERPSVDDVESETGSTGALETR
43.9	2716.1712	0.0084	SDISERPSVDDVESETGSTGALETR
41.0	2716.1712	0.0084	SDISERPSVDDVESETGSTGALETR
23.0	2716.1712	0.0084	SDISERPSVDDVESETGSTGALETR
14.4	2716.1712	0.0084	SDISERPSVDDVESETGSTGALETR
8.8	2716.1712	0.0084	SDISERPSVDDVESETGSTGALETR
5.5	2715.1837	0.9959	AAPVTRQVSGCAGRVPSPTGSDTER
3.8	2714.1531	2.0266	GFCSVPDESSALDDRSTALPPEER
3.4	2715.1636	1.0160	SPVTGEWHTFNDSSVTPMSSSQVR

Spectrum No: 204; Query: 2678; Rank: 1

Peptide View

MS/MS Fragmentation of **DDGDEEGLLTHSEEELEHSQDTDAEDGALQ**
Found in **IPI00373147**, Tax_Id=10116 Gene_Symbol=Raly HnRNP-associated with lethal yellow

Match to Query 2678: 3363.318942 from(1122.113590,3+)
Title: 100101RatKid_NS_deglyco_25.2910.2910.3.dta
Data file K:\NewmanPaper\Piliang\3SubProteomes\Piliang3SP\mgf5ppm\ERLIC_3SubProteomes5ppm.mgf

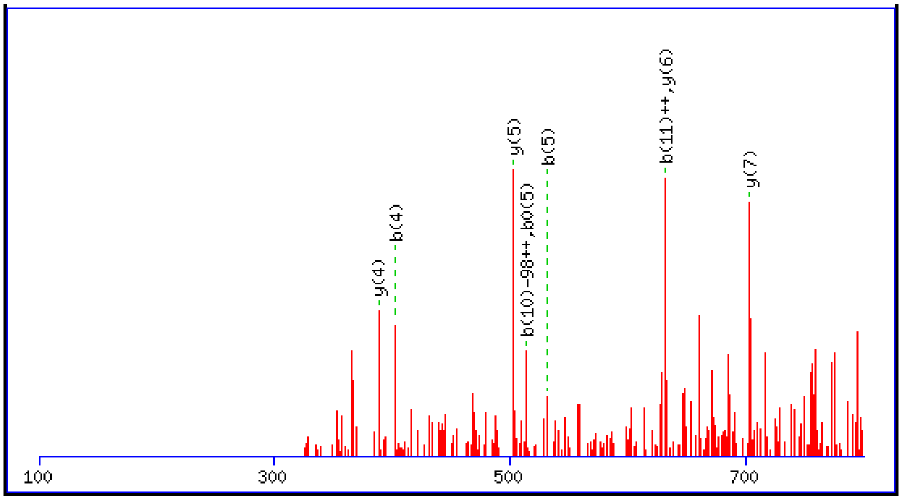

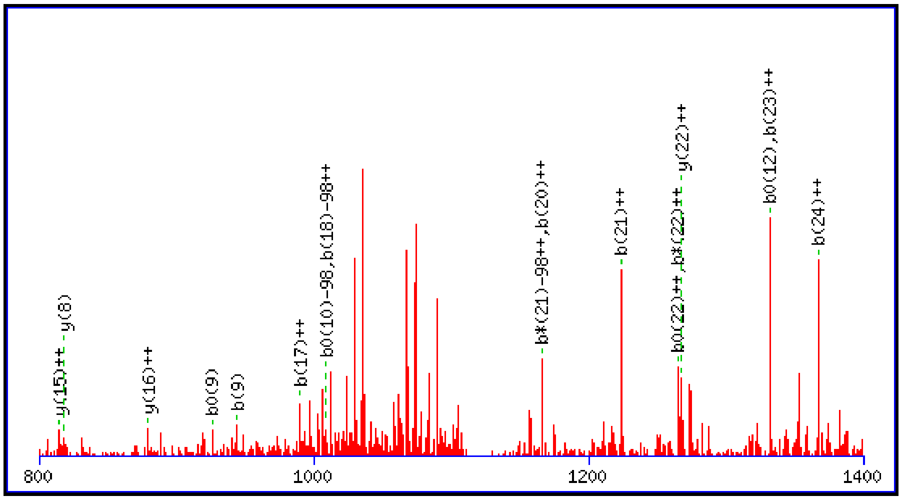

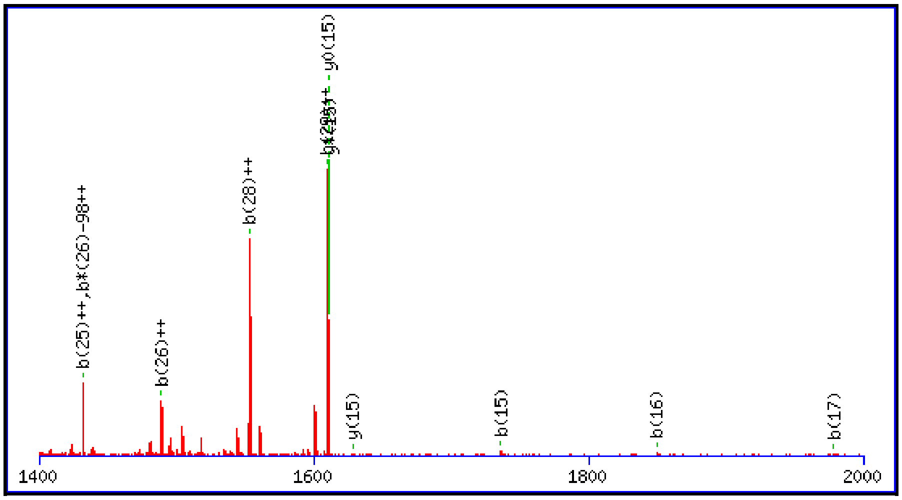

Monoisotopic mass of neutral peptide Mr(calc): 3363.3060
Fixed modifications: Carbamidomethyl (C)
Variable modifications:
T10 : Phospho (ST), with neutral losses 0.0000(shown in table), 97.9769
Ions Score: 57 **Expect:** 0.0007
Matches (Bold Red): 37/456 fragment ions using 52 most intense peaks

#	b	b++	b*	b*++	b0	b0++	Seq.	y	y++	y*	y*++	y0	y0++	#
1	116.0342	58.5207			98.0237	49.5155	D							30
2	231.0612	116.0342			213.0506	107.0289	D	3249.2863	1625.1468	3232.2598	1616.6335	3231.2757	1616.1415	29
3	288.0826	144.5450			270.0721	135.5397	G	3134.2594	1567.6333	3117.2328	1559.1200	3116.2488	1558.6280	28
4	**403.1096**	202.0584			385.0990	193.0531	D	3077.2379	1539.1226	3060.2113	1530.6093	3059.2273	1530.1173	27
5	**532.1522**	266.5797			**514.1416**	257.5744	E	2962.2110	1481.6091	2945.1844	1473.0958	2944.2004	1472.6038	26

**6**	661.1948	331.1010			643.1842	322.0957	**E**	2833.1684	1417.0878	2816.1418	1408.5745	2815.1578	1408.0825	**25**
**7**	718.2162	359.6117			700.2057	350.6065	**G**	2704.1258	1352.5665	2687.0992	1344.0532	2686.1152	1343.5612	**24**
**8**	831.3003	416.1538			813.2897	407.1485	**L**	2647.1043	1324.0558	2630.0778	1315.5425	2629.0937	1315.0505	**23**
**9**	**944.3843**	472.6958			**926.3738**	463.6905	**L**	2534.0202	**1267.5138**	2516.9937	1259.0005	2516.0097	1258.5085	**22**
**10**	1125.3984	563.2028			1107.3878	554.1975	**T**	2420.9362	1210.9717	2403.9096	1202.4584	2402.9256	1201.9664	**21**
**11**	1262.4573	**631.7323**			1244.4467	622.7270	**H**	2239.9222	1120.4647	2222.8956	1111.9514	2221.9116	1111.4594	**20**
**12**	1349.4893	675.2483			**1331.4787**	666.2430	**S**	2102.8633	1051.9353	2085.8367	1043.4220	2084.8527	1042.9300	**19**
**13**	1478.5319	739.7696			1460.5213	730.7643	**E**	2015.8312	1008.4193	1998.8047	999.9060	1997.8207	999.4140	**18**
**14**	1607.5745	804.2909			1589.5639	795.2856	**E**	1886.7886	943.8980	1869.7621	935.3847	1868.7781	934.8927	**17**
**15**	**1736.6171**	868.8122			1718.6065	859.8069	**E**	1757.7460	**879.3767**	1740.7195	870.8634	1739.7355	870.3714	**16**
**16**	**1849.7011**	925.3542			1831.6906	916.3489	**L**	**1628.7035**	**814.8554**	**1611.6769**	806.3421	**1610.6929**	805.8501	**15**
**17**	**1978.7437**	**989.8755**			1960.7332	980.8702	**E**	1515.6194	758.3133	1498.5928	749.8001	1497.6088	749.3080	**14**
**18**	2115.8026	1058.4050			2097.7921	1049.3997	**H**	1386.5768	693.7920	1369.5502	685.2788	1368.5662	684.7868	**13**
**19**	2202.8347	1101.9210			2184.8241	1092.9157	**S**	1249.5179	625.2626	1232.4913	616.7493	1231.5073	616.2573	**12**
**20**	2330.8932	**1165.9503**	2313.8667	1157.4370	2312.8827	1156.9450	**Q**	1162.4859	581.7466	1145.4593	573.2333	1144.4753	572.7413	**11**
**21**	2445.9202	**1223.4637**	2428.8936	1214.9505	2427.9096	1214.4584	**D**	1034.4273	517.7173	1017.4007	509.2040	1016.4167	508.7120	**10**
**22**	2546.9679	1273.9876	2529.9413	**1265.4743**	2528.9573	**1264.9823**	**T**	919.4003	460.2038	902.3738	451.6905	901.3898	451.1985	**9**
**23**	2661.9948	**1331.5010**	2644.9683	1322.9878	2643.9842	1322.4958	**D**	**818.3527**	409.6800	801.3261	401.1667	800.3421	400.6747	**8**
**24**	2733.0319	**1367.0196**	2716.0054	1358.5063	2715.0214	1358.0143	**A**	**703.3257**	352.1665	686.2992	343.6532	685.3151	343.1612	**7**
**25**	2862.0745	**1431.5409**	2845.0480	1423.0276	2844.0640	1422.5356	**E**	**632.2886**	316.6479	615.2620	308.1347	614.2780	307.6427	**6**
**26**	2977.1015	**1489.0544**	2960.0749	1480.5411	2959.0909	1480.0491	**D**	**503.2460**	252.1266	486.2195	243.6134	485.2354	243.1214	**5**
**27**	3034.1229	1517.5651	3017.0964	1509.0518	3016.1124	1508.5598	**G**	**388.2191**	194.6132	371.1925	186.0999			**4**
**28**	3105.1600	**1553.0837**	3088.1335	1544.5704	3087.1495	1544.0784	**A**	331.1976	166.1024	314.1710	157.5892			**3**
**29**	3218.2441	**1609.6257**	3201.2176	1601.1124	3200.2335	1600.6204	**L**	260.1605	130.5839	243.1339	122.0706			**2**
**30**							**Q**	147.0764	74.0418	130.0499	65.5286			**1**

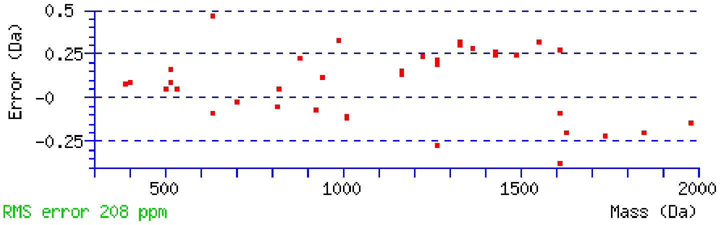

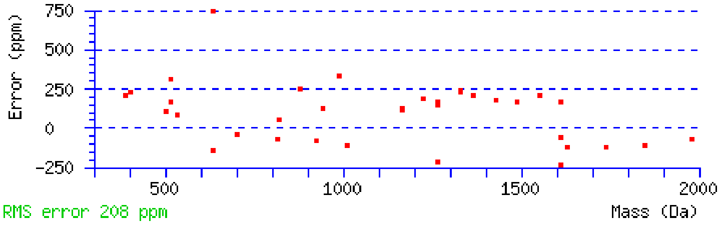

All matches to this query

Score	Mr(calc):	Delta	Sequence
56.6	3363.3060	0.0130	DDGDEEGLLTHSEEELEHSQDTDAEDGALQ
50.0	3363.3060	0.0130	DDGDEEGLLTHSEEELEHSQDTDAEDGALQ
31.1	3363.3060	0.0130	DDGDEEGLLTHSEEELEHSQDTDAEDGALQ
22.1	3363.3060	0.0130	DDGDEEGLLTHSEEELEHSQDTDAEDGALQ
16.5	3363.3390	-0.0201	ASTVEEANKAEENGSEALMTLDDQANKLK
16.5	3363.3390	-0.0201	ASTVEEANKAEENGSEALMTLDDQANKLK
6.4	3363.3237	-0.0047	IEIPLTSNGGDVGAEYVVESTGVFTTMEK
6.4	3363.3237	-0.0047	IEIPLTSNGGDVGAEYVVESTGVFTTMEK
6.4	3363.3237	-0.0047	IEIPLTSNGGDVGAEYVVESTGVFTTMEK
6.4	3363.3237	-0.0047	IEIPLTSNGGDVGAEYVVESTGVFTTMEK

Spectrum No: 205; Query: 1447; Rank: 1

Peptide View

MS/MS Fragmentation of **SATPPPTEPASLPQEPPK**
Found in **IPI00560565**, Tax_Id=10116 Gene_Symbol=RGD1309571 54 kDa protein

Match to Query 1447: 1922.904628 from(962.459590,2+)
Title: 091008RatKidney_NoSalt_21.1457.1457.2.dta
Data file K:\NewmanPaper\Piliang\3SubProteomes\Piliang3SP\mgf5ppm\ERLIC_3SubProteomes5ppm.mgf

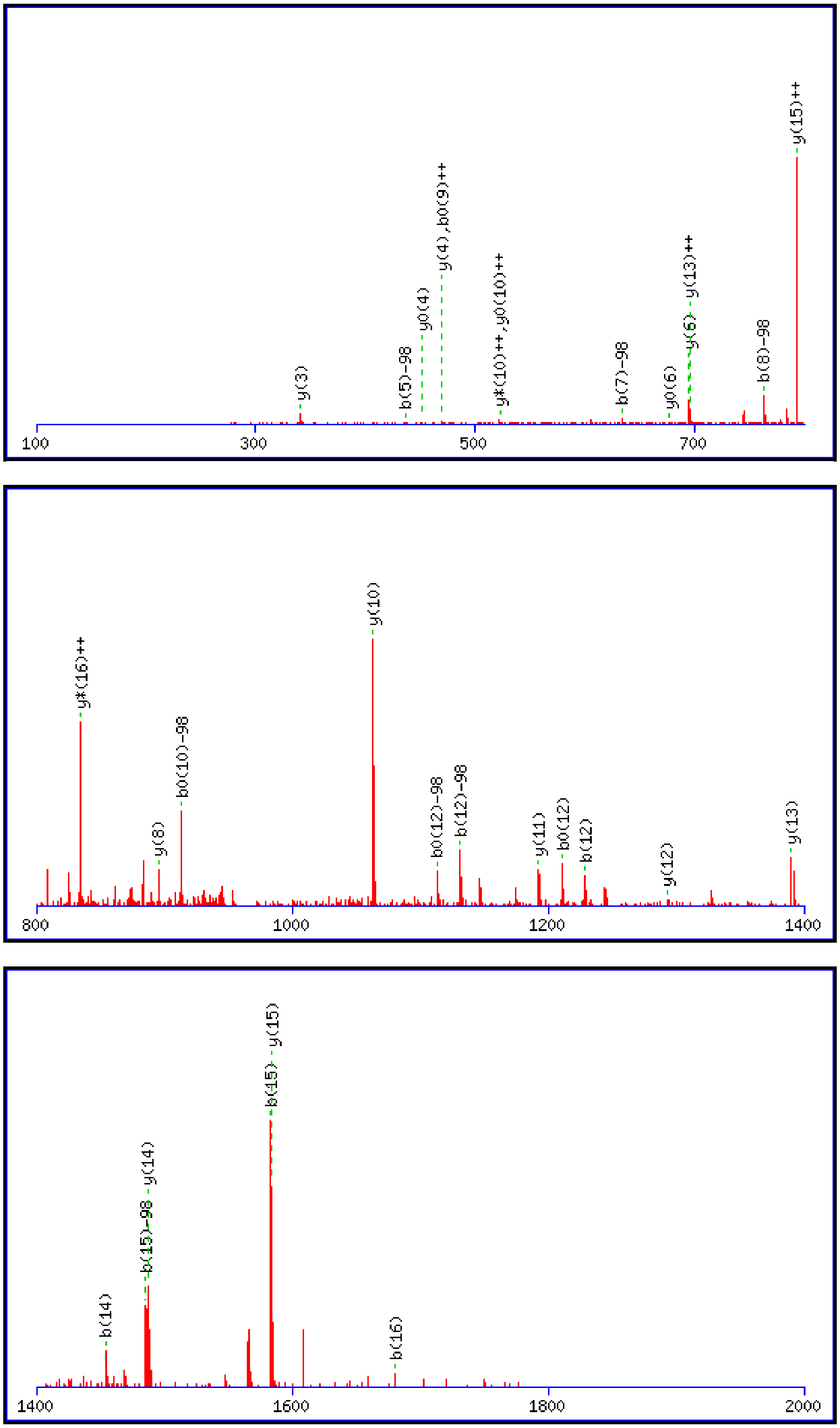

Monoisotopic mass of neutral peptide Mr(calc): 1922.9026
Fixed modifications: Carbamidomethyl (C)
Variable modifications:
S1 : Phospho (ST), with neutral losses 97.9769(shown in table), 0.0000
Ions Score: 56 **Expect:** 0.00054
Matches (Bold Red): 30/248 fragment ions using 52 most intense peaks

#	b	b^{++}	b*	b^{*++}	b^0	b^{0++}	Seq.	y	y^{++}	y*	y^{*++}	y^0	y^{0++}	#
1	70.0287	35.5180			52.0182	26.5127	S							18
2	141.0658	71.0366			123.0553	62.0313	A	1756.9116	878.9594	1739.8850	870.4462	1738.9010	869.9542	17
3	242.1135	121.5604			224.1030	112.5551	T	1685.8745	843.4409	1668.8479	**834.9276**	1667.8639	834.4356	16
4	339.1663	170.0868			321.1557	161.0815	P	**1584.8268**	**792.9170**	1567.8003	784.4038	1566.8162	783.9118	15
5	**436.2191**	218.6132			418.2085	209.6079	P	**1487.7740**	744.3907	1470.7475	735.8774	1469.7635	735.3854	14
6	533.2718	267.1395			515.2613	258.1343	P	**1390.7213**	**695.8643**	1373.6947	687.3510	1372.7107	686.8590	13
7	**634.3195**	317.6634			616.3089	308.6581	T	**1293.6685**	647.3379	1276.6420	638.8246	1275.6579	638.3326	12
8	**763.3621**	382.1847			745.3515	373.1794	E	**1192.6208**	596.8141	1175.5943	588.3008	1174.6103	587.8088	11
9	860.4149	430.7111			842.4043	421.7058	P	**1063.5782**	532.2928	1046.5517	**523.7795**	1045.5677	**523.2875**	10
10	931.4520	466.2296			**913.4414**	457.2243	A	966.5255	483.7664	949.4989	475.2531	948.5149	474.7611	9
11	1018.4840	509.7456			1000.4734	500.7404	S	**895.4884**	448.2478	878.4618	439.7345	877.4778	439.2425	8
12	**1131.5681**	566.2877			**1113.5575**	557.2824	L	808.4563	404.7318	791.4298	396.2185	790.4458	395.7265	7
13	1228.6208	614.8140			1210.6103	605.8088	P	**695.3723**	348.1898	678.3457	339.6765	**677.3617**	339.1845	6
14	1356.6794	678.8433	1339.6529	670.3301	1338.6688	669.8381	Q	598.3195	299.6634	581.2930	291.1501	580.3089	290.6581	5
15	**1485.7220**	743.3646	1468.6954	734.8514	1467.7114	734.3594	E	**470.2609**	235.6341	453.2344	227.1208	**452.2504**	226.6288	4
16	1582.7748	791.8910	1565.7482	783.3777	1564.7642	782.8857	P	**341.2183**	171.1128	324.1918	162.5995			3
17	1679.8275	840.4174	1662.8010	831.9041	1661.8170	831.4121	P	244.1656	122.5864	227.1390	114.0731			2
18							K	147.1128	74.0600	130.0863	65.5468			1

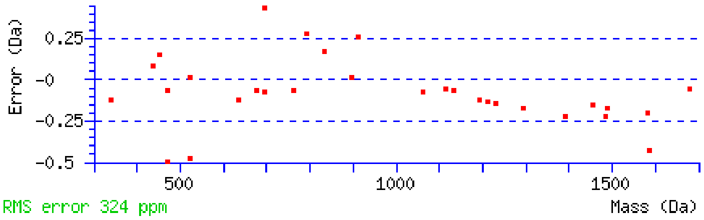

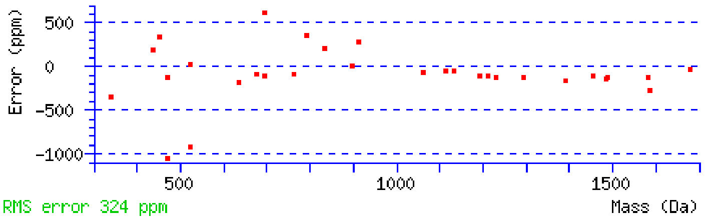

All matches to this query

Score	Mr(calc):	Delta	Sequence
56.4	1922.9026	0.0020	SATPPPTEPASLPQEPPK
54.6	1922.9026	0.0020	SATPPPTEPASLPQEPPK
25.9	1922.9026	0.0020	SATPPPTEPASLPQEPPK
9.9	1922.9172	-0.0126	VGTTLLLLALLPGTSSK
9.9	1922.9172	-0.0126	VGTTLLLLALLPGTSSK
9.3	1920.9056	1.9990	SGLPLYCLEKYTEIR
7.3	1922.9026	0.0020	SATPPPTEPASLPQEPPK
6.3	1922.9172	-0.0126	VGTTLLLLALLPGTSSK
6.0	1922.9098	-0.0051	DRLWSSPAFSPTCPFR
3.5	1922.9172	-0.0126	VGTTLLLLALLPGTSSK

Spectrum No: 206; Query: 2070; Rank: 1

Peptide View

MS/MS Fragmentation of **EGEEPTVYSDDEEPKDEAAR**
Found in **IPI00480820**, Tax_Id=10116 Gene_Symbol=Pgrmc1 Membrane-associated progesterone receptor component 1

Match to Query 2070: 2344.918048 from(1173.466300,2+)
Title: 091008RatKidney_NH4Format02_27.1087.1087.2.dta

Data file K:\NewmanPaper\Piliang\3SubProteomes\Piliang3SP\mgf5ppm\ERLIC_3SubProteomes5ppm.mgf

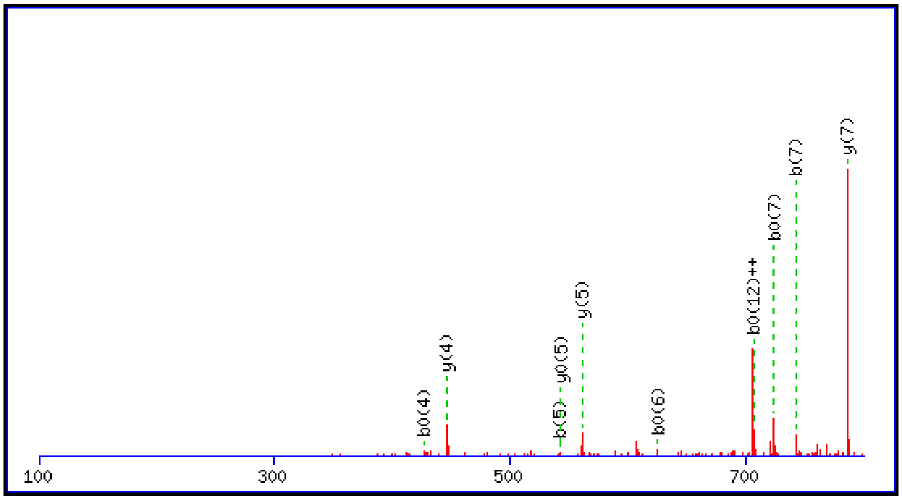

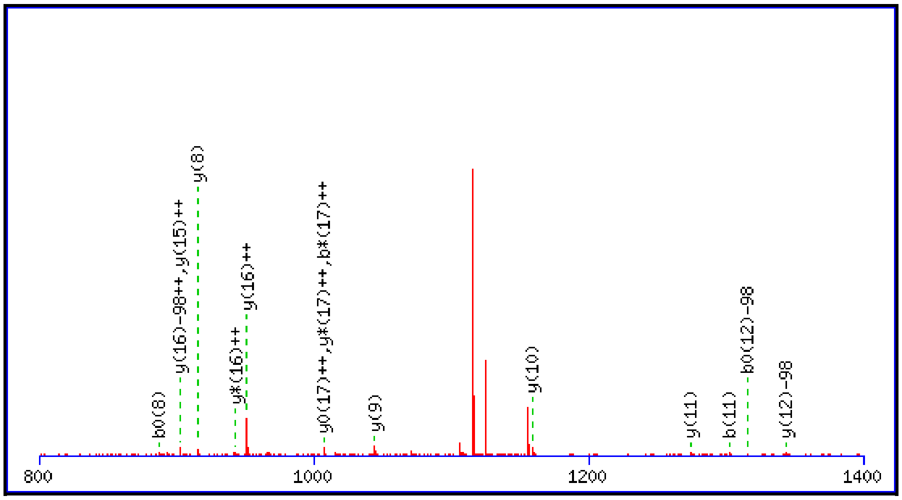

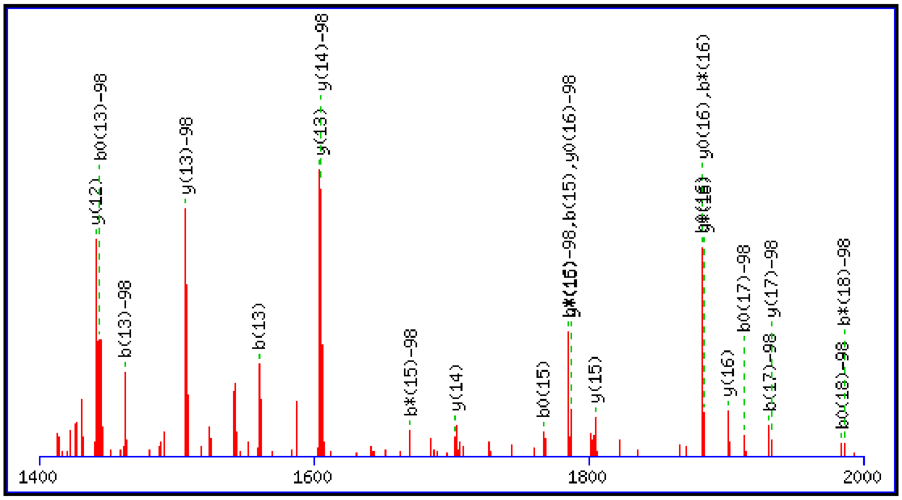

Monoisotopic mass of neutral peptide Mr(calc): 2344.9220
Fixed modifications: Carbamidomethyl (C)
Variable modifications:
S9 : Phospho (ST), with neutral losses 0.0000(shown in table), 97.9769
Ions Score: 56 **Expect:** 0.00051
Matches (Bold Red): 50/296 fragment ions using 73 most intense peaks

#	b	b++	b*	b*++	b0	b0++	Seq.	y	y++	y*	y*++	y0	y0++	#
1	130.0499	65.5286			112.0393	56.5233	E							20
2	187.0713	94.0393			169.0608	85.0340	G	2216.8867	1108.9470	2199.8602	1100.4337	2198.8761	1099.9417	19
3	316.1139	158.5606			298.1034	149.5553	E	2159.8652	1080.4363	2142.8387	1071.9230	2141.8547	1071.4310	18
4	445.1565	223.0819			**427.1460**	214.0766	E	2030.8226	1015.9150	2013.7961	**1007.4017**	2012.8121	**1006.9097**	17

5	**542.2093**	271.6083			524.1987	262.6030	P	**1901.7801**	**951.3937**	**1884.7535**	**942.8804**	**1883.7695**	942.3884	16
6	643.2570	322.1321			**625.2464**	313.1268	T	**1804.7273**	**902.8673**	**1787.7007**	894.3540	1786.7167	893.8620	15
7	**742.3254**	371.6663			**724.3148**	362.6610	V	**1703.6796**	852.3434	1686.6531	843.8302	1685.6690	843.3382	14
8	905.3887	453.1980			**887.3781**	444.1927	Y	**1604.6112**	802.8092	1587.5846	794.2960	1586.6006	793.8040	13
9	1072.3871	536.6972			1054.3765	527.6919	S	**1441.5479**	721.2776	1424.5213	712.7643	1423.5373	712.2723	12
10	1187.4140	594.2106			1169.4034	585.2054	D	**1274.5495**	637.7784	1257.5230	629.2651	1256.5390	628.7731	11
11	**1302.4409**	651.7241			1284.4304	642.7188	D	**1159.5226**	580.2649	1142.4960	571.7516	1141.5120	571.2596	10
12	1431.4835	716.2454			1413.4730	**707.2401**	E	**1044.4956**	522.7515	1027.4691	514.2382	1026.4851	513.7462	9
13	**1560.5261**	780.7667			1542.5156	771.7614	E	**915.4530**	458.2302	898.4265	449.7169	897.4425	449.2249	8
14	1657.5789	829.2931			1639.5683	820.2878	P	**786.4104**	393.7089	769.3839	385.1956	768.3999	384.7036	7
15	**1785.6739**	893.3406	1768.6473	884.8273	**1767.6633**	884.3353	K	689.3577	345.1825	672.3311	336.6692	671.3471	336.1772	6
16	1900.7008	950.8540	**1883.6743**	942.3408	**1882.6902**	941.8488	D	**561.2627**	281.1350	544.2362	272.6217	**543.2522**	272.1297	5
17	2029.7434	1015.3753	2012.7168	**1006.8621**	2011.7328	1006.3701	E	**446.2358**	223.6215	429.2092	215.1083	428.2252	214.6162	4
18	2100.7805	1050.8939	2083.7540	1042.3806	2082.7699	1041.8886	A	317.1932	159.1002	300.1666	150.5870			3
19	2171.8176	1086.4124	2154.7911	1077.8992	2153.8071	1077.4072	A	246.1561	123.5817	229.1295	115.0684			2
20							R	175.1190	88.0631	158.0924	79.5498			1

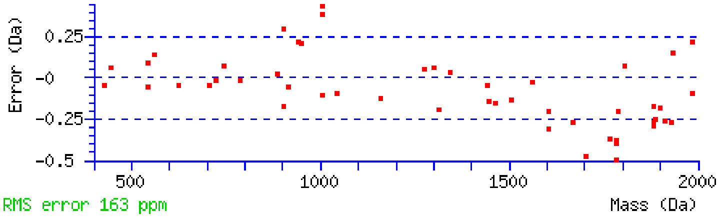

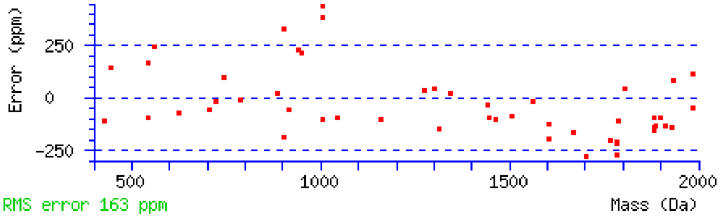

All matches to this query

Score	Mr(calc):	Delta	Sequence
56.4	2344.9220	-0.0039	EGEEPTVYSDDEEPKDEAAR
44.4	2344.9220	-0.0039	EGEEPTVYSDDEEPKDEAAR
37.7	2344.9220	-0.0039	EGEEPTVYSDDEEPKDEAAR
7.2	2344.9407	-0.0226	DWAAVLGDDTADDGNLDLDMK
4.9	2343.9014	1.0167	TVLEKTSDRGVPFSVSMR
4.2	2343.9014	1.0167	TVLEKTSDRGVPFSVSMR
4.2	2343.9014	1.0167	TVLEKTSDRGVPFSVSMR
3.8	2343.9202	0.9979	NPSNRNQDYMAPSEPNTPTK
3.8	2344.9371	-0.0190	NPDINMTFEQVYRDCKR
3.5	2344.9106	0.0075	SENEGLMRSGQLQCYLGDK

Spectrum No: 207; Query: 1600; Rank: 1

Peptide View

MS/MS Fragmentation of **INENSDKEAEVEESSEK**
Found in **IPI00363433**, Tax_Id=10116 Gene_Symbol=Fnbp4 similar to formin binding protein 4

Match to Query 1600: 2015.826552 from(672.949460,3+)
Title: 100101RatKid_NS_deglyco_19.708.708.3.dta
Data file K:\NewmanPaper\Piliang\3SubProteomes\Piliang3SP\mgf5ppm\ERLIC_3SubProteomes5ppm.mgf

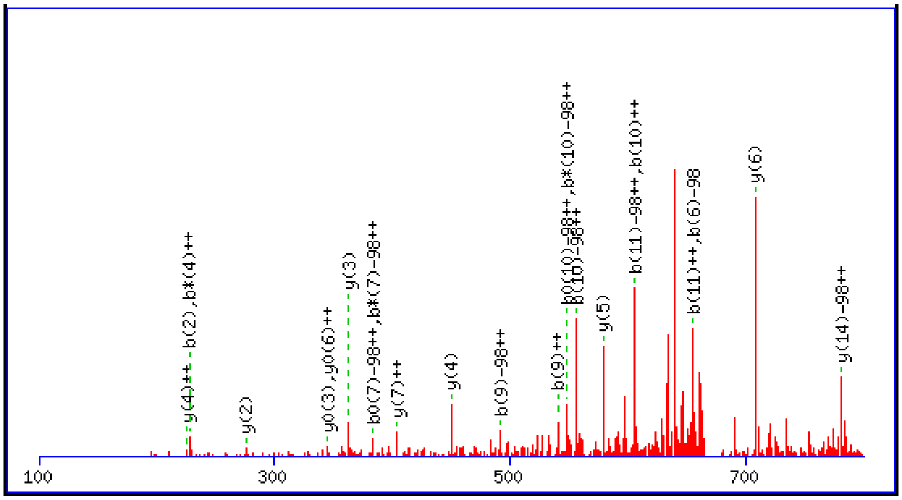

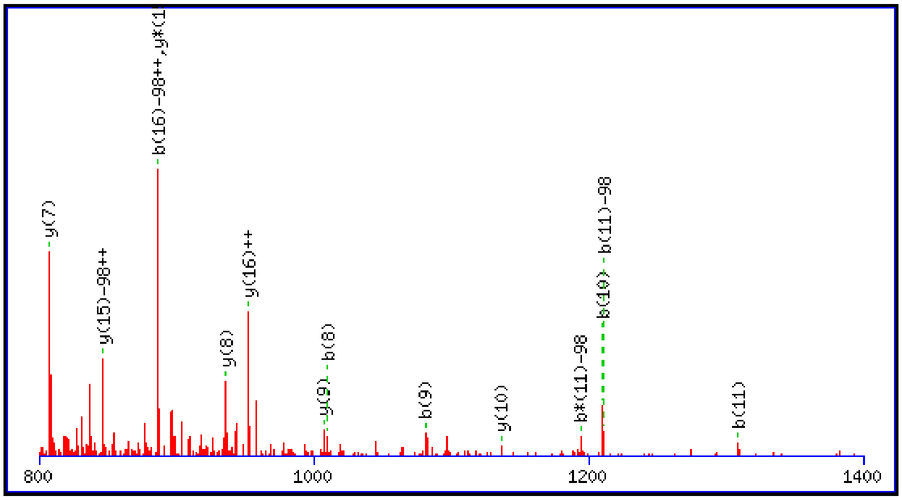

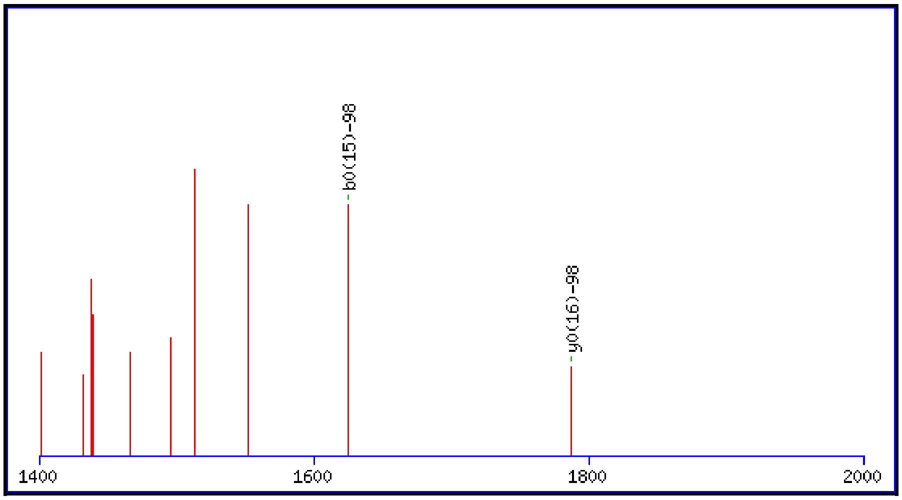

Monoisotopic mass of neutral peptide Mr(calc): 2015.8208
Fixed modifications: Carbamidomethyl (C)
Variable modifications:
S5 : Phospho (ST), with neutral losses 97.9769(shown in table), 0.0000
Ions Score: 56 **Expect:** 0.00043
Matches (Bold Red): 40/280 fragment ions using 47 most intense peaks

#	b	b^{++}	b*	b^{*++}	b^0	b^{0++}	Seq.	y	y^{++}	y*	y^{*++}	y^0	y^{0++}	#
1	114.0913	57.5493					I							17
2	**228.1343**	114.5708	211.1077	106.0575			N	1805.7672	903.3872	1788.7406	894.8739	**1787.7566**	894.3819	16
3	357.1769	179.0921	340.1503	170.5788	339.1663	170.0868	E	1691.7242	**846.3658**	1674.6977	837.8525	1673.7137	837.3605	15
4	471.2198	236.1135	454.1932	**227.6003**	453.2092	227.1082	N	1562.6816	**781.8445**	1545.6551	773.3312	1544.6711	772.8392	14
5	540.2412	270.6243	523.2147	262.1110	522.2307	261.6190	S	1448.6387	724.8230	1431.6122	716.3097	1430.6282	715.8177	13

6	655.2682	328.1377	638.2416	319.6245	637.2576	319.1324	D	1379.6173	690.3123	1362.5907	681.7990	1361.6067	681.3070	12
7	783.3631	392.1852	766.3366	383.6719	765.3526	383.1799	K	1264.5903	632.7988	1247.5638	624.2855	1246.5798	623.7935	11
8	912.4057	456.7065	895.3792	448.1932	894.3952	447.7012	E	1136.4954	568.7513	1119.4688	560.2380	1118.4848	559.7460	10
9	983.4429	492.2251	966.4163	483.7118	965.4323	483.2198	A	1007.4528	504.2300	990.4262	495.7167	989.4422	495.2247	9
10	1112.4854	556.7464	1095.4589	548.2331	1094.4749	547.7411	E	936.4156	468.7115	919.3891	460.1982	918.4051	459.7062	8
11	1211.5539	606.2806	1194.5273	597.7673	1193.5433	597.2753	V	807.3731	404.1902	790.3465	395.6769	789.3625	395.1849	7
12	1340.5965	670.8019	1323.5699	662.2886	1322.5859	661.7966	E	708.3046	354.6560	691.2781	346.1427	690.2941	345.6507	6
13	1469.6390	735.3232	1452.6125	726.8099	1451.6285	726.3179	E	579.2620	290.1347	562.2355	281.6214	561.2515	281.1294	5
14	1556.6711	778.8392	1539.6445	770.3259	1538.6605	769.8339	S	450.2195	225.6134	433.1929	217.1001	432.2089	216.6081	4
15	1643.7031	822.3552	1626.6766	813.8419	1625.6925	813.3499	S	363.1874	182.0974	346.1609	173.5841	345.1769	173.0921	3
16	1772.7457	886.8765	1755.7191	878.3632	1754.7351	877.8712	E	276.1554	138.5813	259.1288	130.0681	258.1448	129.5761	2
17							K	147.1128	74.0600	130.0863	65.5468			1

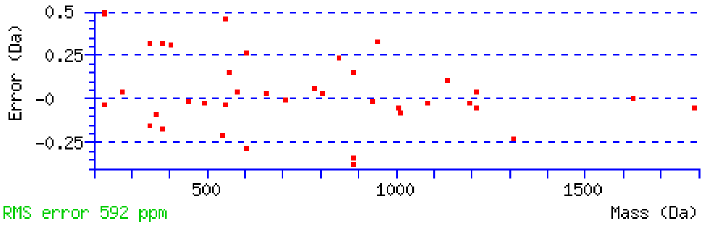

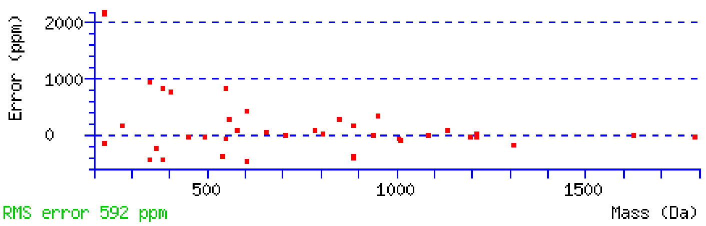

All matches to this query

Score	Mr(calc):	Delta	Sequence
56.3	2015.8208	0.0058	INENSDKEAEVEESSEK
11.1	2015.8443	-0.0178	SFALDSEGEEASQESSSEK
6.3	2015.8230	0.0035	CNECDKAFSVHSNLQK
4.6	2015.8268	-0.0003	RKIDTHPSPSHSSTVK
3.3	2015.8279	-0.0013	YNGDGNSDIFNLTVTVK
1.1	2013.8039	2.0226	YIAIVSYEQRQNYK
0.6	2013.8155	2.0110	YEEMREEASNILLNK
0.1	2015.8268	-0.0003	RKIDTHPSPSHSSTVK
0.1	2013.8039	2.0226	YIAIVSYEQRQNYK

Spectrum No: 208; Query: 1366; Rank: 1

Peptide View

MS/MS Fragmentation of **DVEDMELSDVEDDGSK**
Found in **IPI00368287**, Tax_Id=10116 Gene_Symbol=- 157 kDa protein

Match to Query 1366: 1861.680588 from(931.847570,2+)
Title: 091008RatKidney_NH4Format02_28.2250.2250.2.dta
Data file K:\NewmanPaper\Piliang\3SubProteomes\Piliang3SP\mgf5ppm\ERLIC_3SubProteomes5ppm.mgf

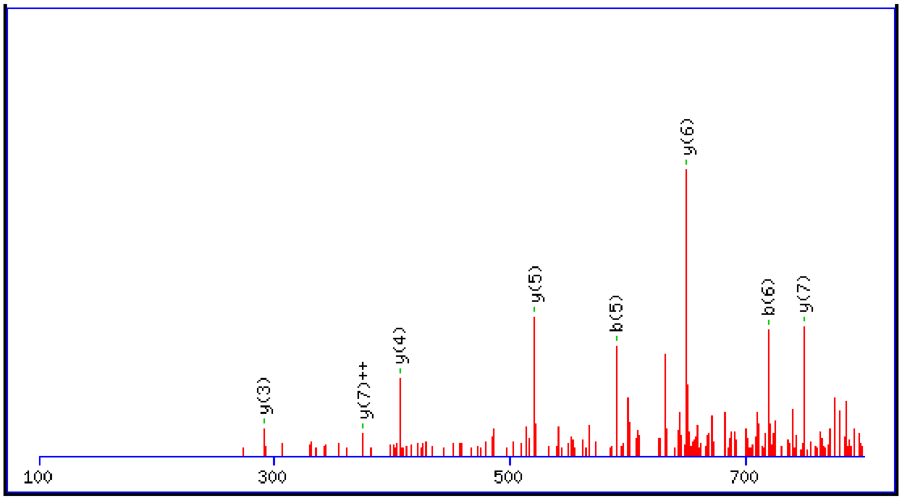

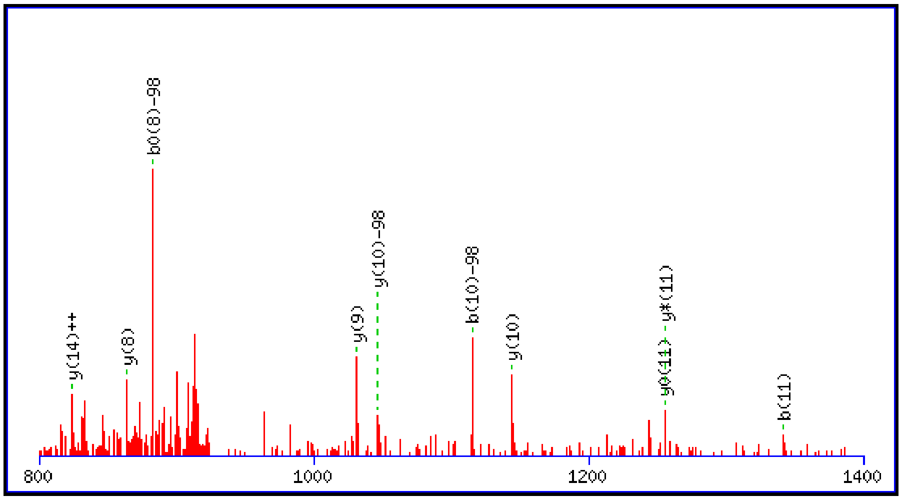

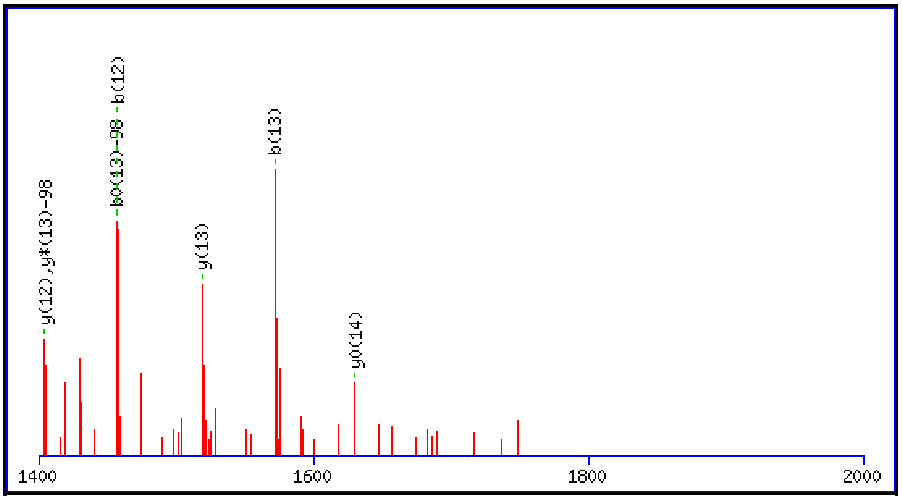

Monoisotopic mass of neutral peptide Mr(calc): 1861.6812
Fixed modifications: Carbamidomethyl (C)
Variable modifications:
S8 : Phospho (ST), with neutral losses 0.0000(shown in table), 97.9769
Ions Score: 56 **Expect:** 0.00023
Matches (Bold Red): 25/222 fragment ions using 38 most intense peaks

#	b	b^{++}	b^0	b^{0++}	Seq.	y	y^{++}	y*	y^{*++}	y^0	y^{0++}	#
1	116.0342	58.5207	98.0237	49.5155	D							16
2	215.1026	108.0550	197.0921	99.0497	V	1747.6616	874.3344	1730.6350	865.8212	1729.6510	865.3291	15
3	344.1452	172.5763	326.1347	163.5710	E	1648.5932	**824.8002**	1631.5666	816.2869	**1630.5826**	815.7949	14
4	459.1722	230.0897	441.1616	221.0844	D	**1519.5506**	760.2789	1502.5240	751.7656	1501.5400	751.2736	13
5	**590.2127**	295.6100	572.2021	286.6047	M	**1404.5236**	702.7655	1387.4971	694.2522	1386.5131	693.7602	12

6	**719.2552**	360.1313	701.2447	351.1260	E	1273.4831	637.2452	**1256.4566**	628.7319	**1255.4726**	628.2399	11
7	832.3393	416.6733	814.3287	407.6680	L	**1144.4405**	572.7239	1127.4140	564.2106	1126.4300	563.7186	10
8	999.3377	500.1725	981.3271	491.1672	S	**1031.3565**	516.1819	1014.3299	507.6686	1013.3459	507.1766	9
9	1114.3646	557.6859	1096.3540	548.6807	D	**864.3581**	432.6827	847.3316	424.1694	846.3476	423.6774	8
10	1213.4330	607.2201	1195.4225	598.2149	V	**749.3312**	**375.1692**	732.3046	366.6560	731.3206	366.1640	7
11	**1342.4756**	671.7414	1324.4650	662.7362	E	**650.2628**	325.6350	633.2362	317.1218	632.2522	316.6297	6
12	**1457.5026**	729.2549	1439.4920	720.2496	D	**521.2202**	261.1137	504.1936	252.6005	503.2096	252.1084	5
13	**1572.5295**	786.7684	1554.5189	777.7631	D	**406.1932**	203.6003	389.1667	195.0870	388.1827	194.5950	4
14	1629.5510	815.2791	1611.5404	806.2738	G	**291.1663**	146.0868	274.1397	137.5735	273.1557	137.0815	3
15	1716.5830	858.7951	1698.5724	849.7899	S	234.1448	117.5761	217.1183	109.0628	216.1343	108.5708	2
16					K	147.1128	74.0600	130.0863	65.5468			1

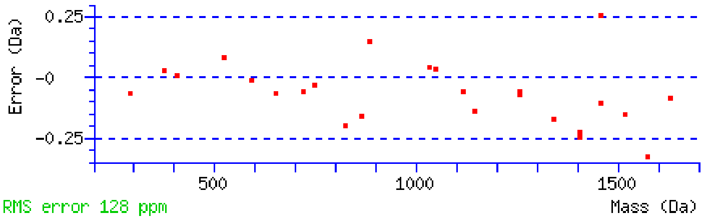

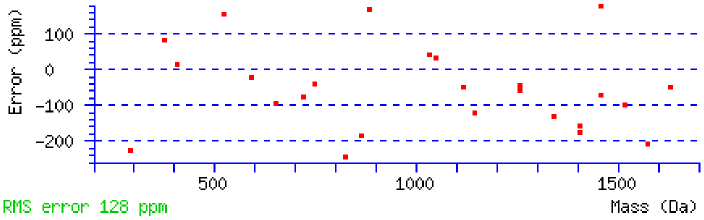

All matches to this query

Score	Mr(calc):	Delta	Sequence
56.0	1861.6812	-0.0007	DVEDMELSDVEDDGSK
6.1	1861.6812	-0.0007	DVEDMELSDVEDDGSK

Spectrum No: 209; Query: 281; Rank: 1

Peptide View

MS/MS Fragmentation of **GVAPADSPDAPR**
Found in **IPI00370649**, Tax_Id=10116 Gene_Symbol=LOC368062 similar to Ankyrin repeat and IBR domain-containing protein 1

Match to Query 281: 1231.519768 from(616.767160,2+)
Title: 091008RatKidney_NH4Format02_26.524.524.2.dta
Data file K:\NewmanPaper\Piliang\3SubProteomes\Piliang3SP\mgf5ppm\ERLIC_3SubProteomes5ppm.mgf

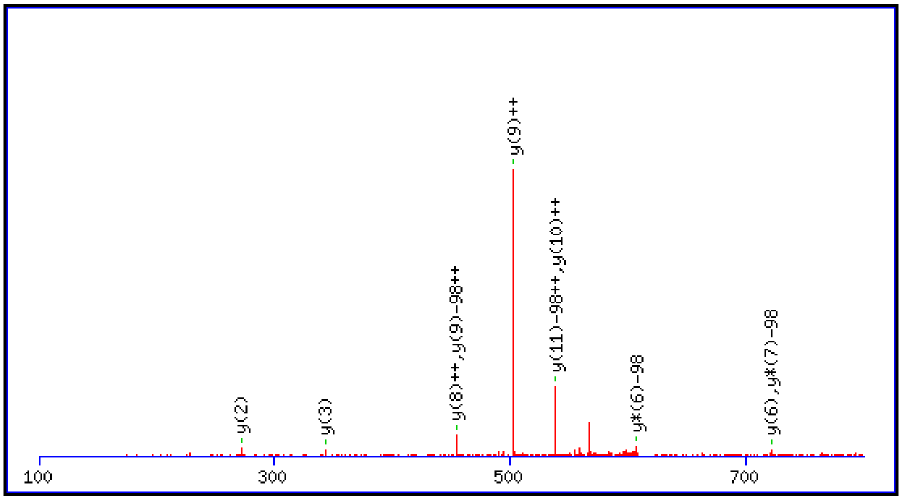

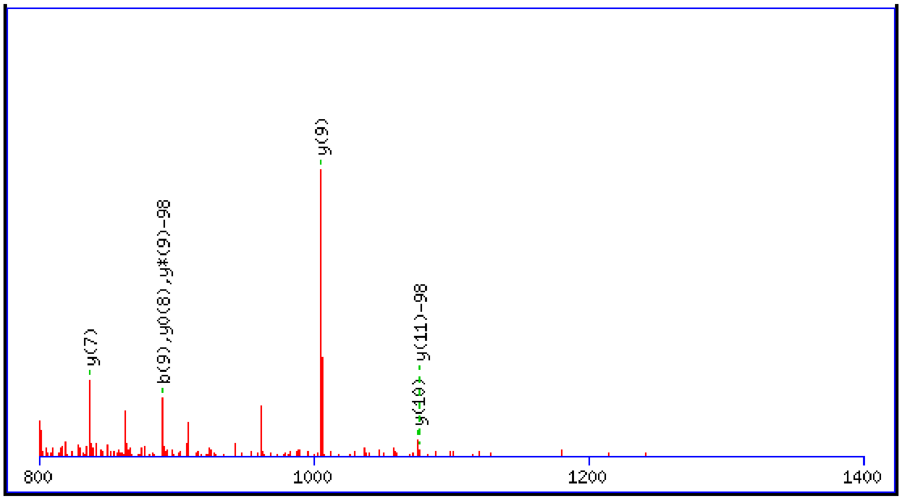

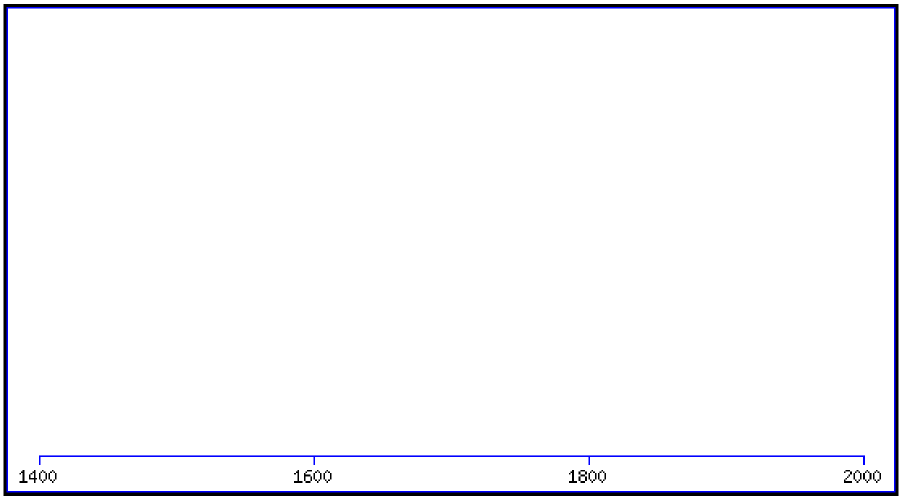

Monoisotopic mass of neutral peptide Mr(calc): 1231.5234
Fixed modifications: Carbamidomethyl (C)
Variable modifications:
S7 : Phospho (ST), with neutral losses 0.0000(shown in table), 97.9769
Ions Score: 56 **Expect:** 0.00024
Matches (Bold Red): 17/150 fragment ions using 15 most intense peaks

#	b	b^{++}	b^0	b^{0++}	Seq.	y	y^{++}	y*	y^{*++}	y^0	y^{0++}	#
1	58.0287	29.5180			G							12
2	157.0972	79.0522			V	1175.5092	588.2583	1158.4827	579.7450	1157.4987	579.2530	11
3	228.1343	114.5708			A	**1076.4408**	**538.7241**	1059.4143	530.2108	1058.4303	529.7188	10
4	325.1870	163.0972			P	**1005.4037**	**503.2055**	988.3772	494.6922	987.3931	494.2002	9
5	396.2241	198.6157			A	908.3509	**454.6791**	891.3244	446.1658	**890.3404**	445.6738	8
6	511.2511	256.1292	493.2405	247.1239	D	**837.3138**	419.1606	820.2873	410.6473	819.3033	410.1553	7
7	678.2494	339.6284	660.2389	330.6231	S	**722.2869**	361.6471	705.2603	353.1338	704.2763	352.6418	6
8	775.3022	388.1547	757.2916	379.1495	P	555.2885	278.1479	538.2620	269.6346	537.2780	269.1426	5
9	**890.3291**	445.6682	872.3186	436.6629	D	458.2358	229.6215	441.2092	221.1082	440.2252	220.6162	4
10	961.3663	481.1868	943.3557	472.1815	A	**343.2088**	172.1081	326.1823	163.5948			3
11	1058.4190	529.7132	1040.4085	520.7079	P	**272.1717**	136.5895	255.1452	128.0762			2
12					R	175.1190	88.0631	158.0924	79.5498			1

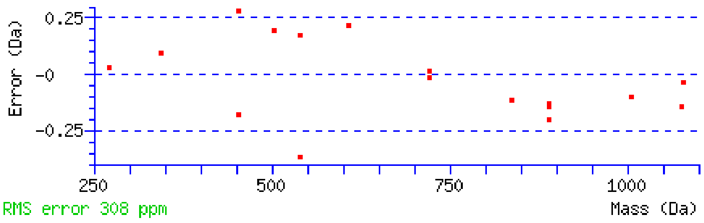

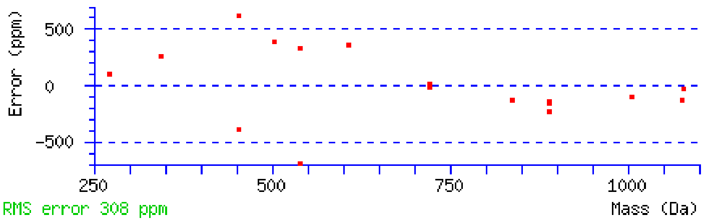

All matches to this query

Score	Mr(calc):	Delta	Sequence
56.0	1231.5234	-0.0037	GVAPADSPDAPR
7.9	1231.5316	-0.0118	NLENNNLEDR
6.4	1230.5282	0.9916	GASFITSTNPR
6.4	1230.5282	0.9916	GASFITSTNPR
6.4	1230.5282	0.9916	GASFITSTNPR
6.4	1230.5282	0.9916	GASFITSTNPR
5.8	1231.5203	-0.0005	GASKRCSVCK
5.6	1231.5274	-0.0077	EDWIAYISR
3.2	1231.5250	-0.0053	NLNPGLKTSK
3.2	1231.5234	-0.0036	VQDNHLSPNK

Spectrum No: 210; Query: 2267; Rank: 1

Peptide View

MS/MS Fragmentation of **DSGSDEDFLMEDDDDSDYGSSK**
Found in **IPI00189138**, Tax_Id=10116 Gene_Symbol=Nucks Nuclear ubiquitous casein and cyclin-dependent kinases substrate

Match to Query 2267: 2523.837668 from(1262.926110,2+)
Title: 100101RatKid_NS_deglyco_28.2267.2267.2.dta
Data file K:\NewmanPaper\Piliang\3SubProteomes\Piliang3SP\mgf5ppm\ERLIC_3SubProteomes5ppm.mgf

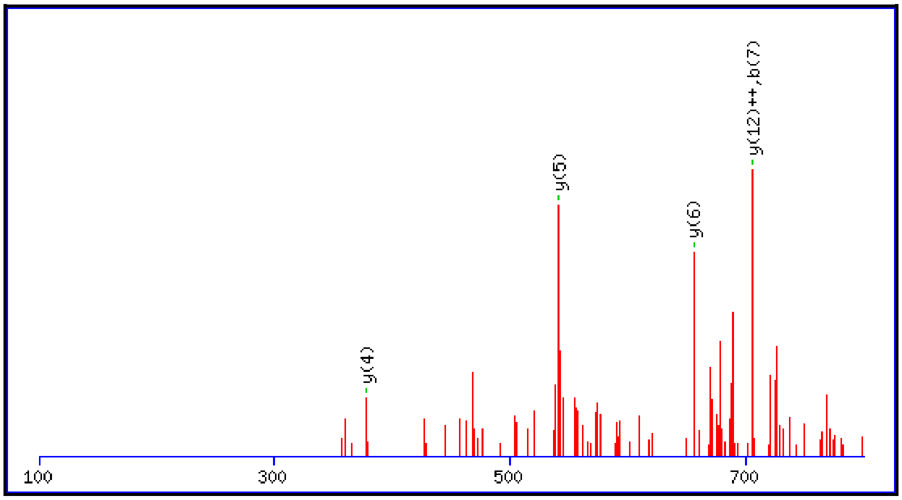

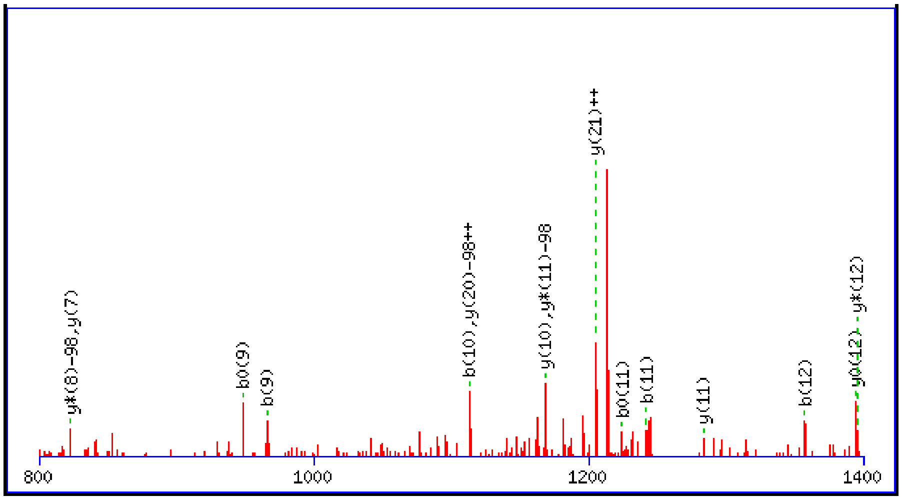

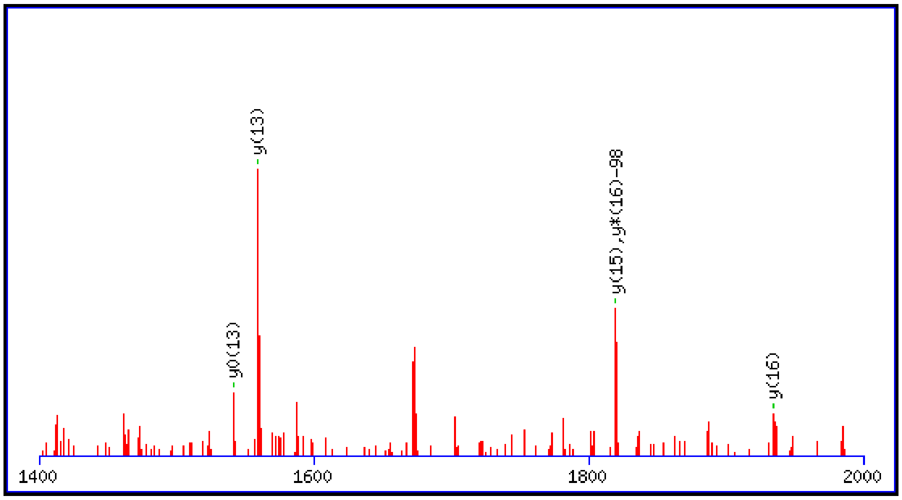

Monoisotopic mass of neutral peptide Mr(calc): 2523.8269
Fixed modifications: Carbamidomethyl (C)
Variable modifications:
M10 : Oxidation (M)
S16 : Phospho (ST), with neutral losses 0.0000(shown in table), 97.9769
Ions Score: 56 **Expect:** 0.00023
Matches (Bold Red): 25/322 fragment ions using 35 most intense peaks

#	b	b^{++}	b^{0}	b^{0++}	Seq.	y	y^{++}	y*	y^{*++}	y^{0}	y^{0++}	#
1	116.0342	58.5207	98.0237	49.5155	D							22
2	203.0662	102.0368	185.0557	93.0315	S	2409.8072	**1205.4072**	2392.7806	1196.8940	2391.7966	1196.4020	21
3	260.0877	130.5475	242.0771	121.5422	G	2322.7752	1161.8912	2305.7486	1153.3779	2304.7646	1152.8859	20
4	347.1197	174.0635	329.1092	165.0582	S	2265.7537	1133.3805	2248.7272	1124.8672	2247.7431	1124.3752	19
5	462.1467	231.5770	444.1361	222.5717	D	2178.7217	1089.8645	2161.6951	1081.3512	2160.7111	1080.8592	18
6	591.1893	296.0983	573.1787	287.0930	E	2063.6947	1032.3510	2046.6682	1023.8377	2045.6842	1023.3457	17
7	**706.2162**	353.6117	688.2057	344.6065	D	**1934.6521**	967.8297	1917.6256	959.3164	1916.6416	958.8244	16
8	853.2846	427.1460	835.2741	418.1407	F	**1819.6252**	910.3162	1802.5986	901.8030	1801.6146	901.3110	15
9	**966.3687**	483.6880	**948.3581**	474.6827	L	1672.5568	836.7820	1655.5302	828.2688	1654.5462	827.7767	14
10	**1113.4041**	557.2057	1095.3935	548.2004	M	**1559.4727**	780.2400	1542.4462	771.7267	**1541.4622**	771.2347	13
11	**1242.4467**	621.7270	**1224.4361**	612.7217	E	1412.4373	**706.7223**	**1395.4108**	698.2090	**1394.4267**	697.7170	12
12	**1357.4736**	679.2405	1339.4631	670.2352	D	**1283.3947**	642.2010	1266.3682	633.6877	1265.3842	633.1957	11
13	1472.5006	736.7539	1454.4900	727.7486	D	**1168.3678**	584.6875	1151.3412	576.1743	1150.3572	575.6822	10
14	1587.5275	794.2674	1569.5170	785.2621	D	1053.3408	527.1741	1036.3143	518.6608	1035.3303	518.1688	9
15	1702.5545	851.7809	1684.5439	842.7756	D	938.3139	469.6606	921.2873	461.1473	920.3033	460.6553	8
16	1869.5528	935.2800	1851.5423	926.2748	S	**823.2869**	412.1471	806.2604	403.6338	805.2764	403.1418	7
17	1984.5798	992.7935	1966.5692	983.7882	D	**656.2886**	328.6479	639.2620	320.1347	638.2780	319.6427	6
18	2147.6431	1074.3252	2129.6325	1065.3199	Y	**541.2617**	271.1345	524.2351	262.6212	523.2511	262.1292	5

**19**	2204.6646	1102.8359	2186.6540	1093.8306	**G**	**378.1983**	189.6028	361.1718	181.0895	360.1878	180.5975	**4**
**20**	2291.6966	1146.3519	2273.6860	1137.3466	**S**	321.1769	161.0921	304.1503	152.5788	303.1663	152.0868	**3**
**21**	2378.7286	1189.8679	2360.7180	1180.8627	**S**	234.1448	117.5761	217.1183	109.0628	216.1343	108.5708	**2**
**22**					**K**	147.1128	74.0600	130.0863	65.5468			**1**

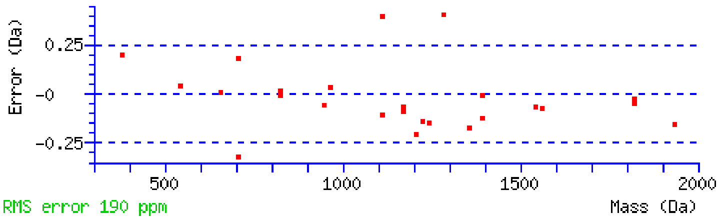

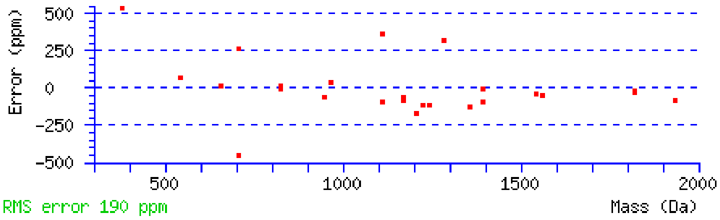

All matches to this query

Score	Mr(calc):	Delta	Sequence
55.9	2523.8269	0.0108	DSGSDEDFLMEDDDDSDYGSSK
30.6	2523.8269	0.0108	DSGSDEDFLMEDDDDSDYGSSK
21.3	2523.8269	0.0108	DSGSDEDFLMEDDDDSDYGSSK
21.3	2523.8269	0.0108	DSGSDEDFLMEDDDDSDYGSSK
4.0	2523.8269	0.0108	DSGSDEDFLMEDDDDSDYGSSK
3.8	2523.8269	0.0108	DSGSDEDFLMEDDDDSDYGSSK

Spectrum No: 211; Query: 2771; Rank: 1

Peptide View

MS/MS Fragmentation of **NSTPSEPDSGQGPPPEEEEGEEEAAKEEAEAQGVR**
Found in **IPI00326566**, Tax_Id=10116 Gene_Symbol=Hdgf Hepatoma-derived growth factor

Match to Query 2771: 3745.543332 from(1249.521720,3+)
Title: 091008RatKidney_NoSalt_25.2693.2693.3.dta
Data file K:\NewmanPaper\Piliang\3SubProteomes\Piliang3SP\mgf5ppm\ERLIC_3SubProteomes5ppm.mgf

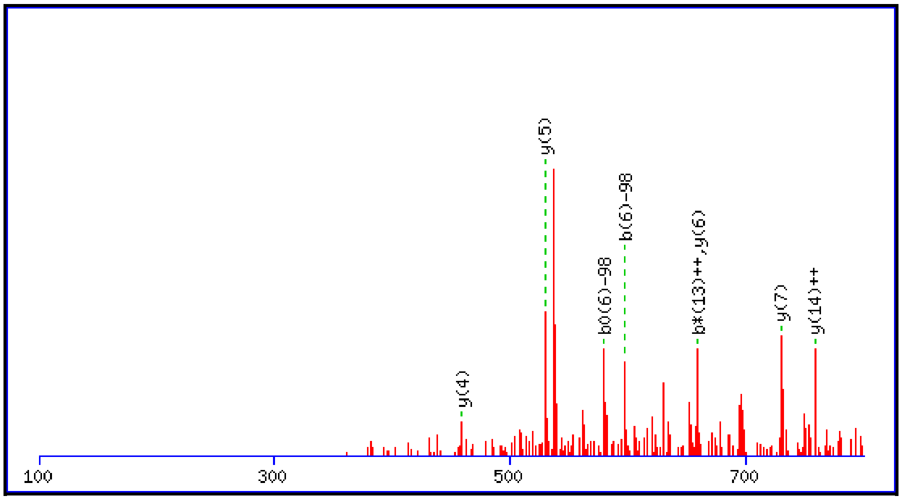

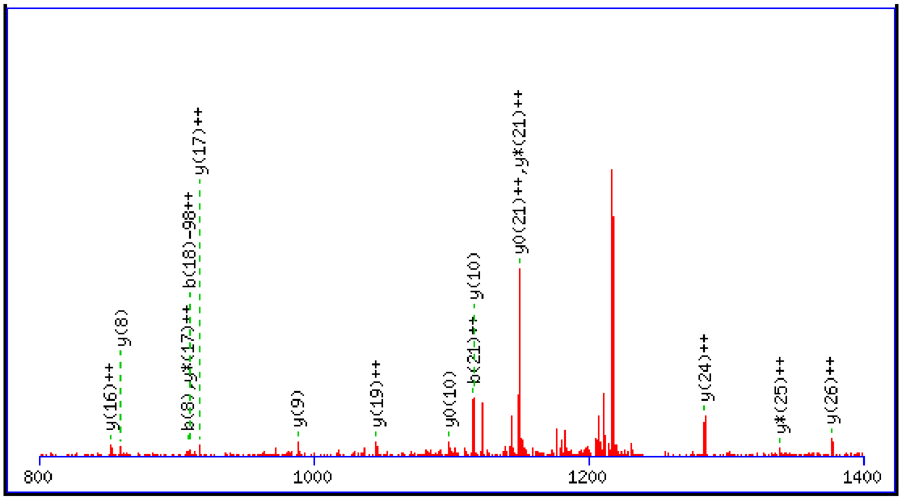

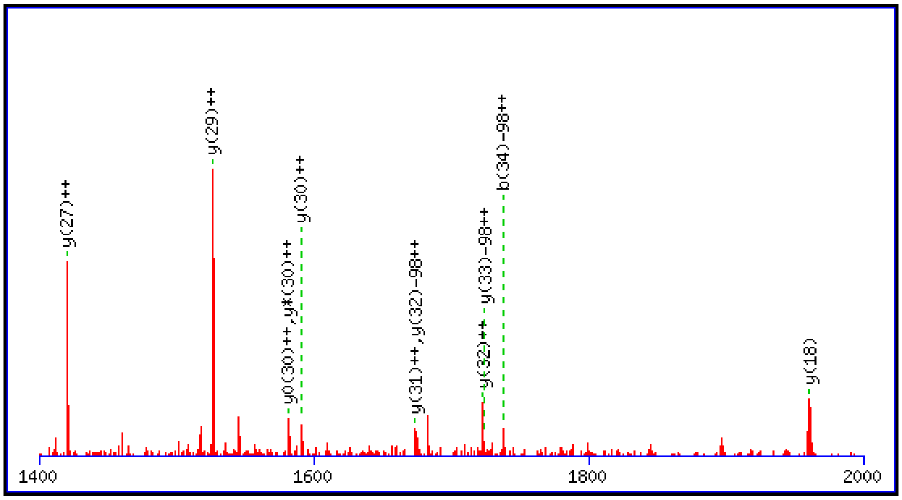

Monoisotopic mass of neutral peptide Mr(calc): 3745.5388
Fixed modifications: Carbamidomethyl (C)
Variable modifications:
S5 : Phospho (ST), with neutral losses 0.0000(shown in table), 97.9769
Ions Score: 56 **Expect:** 0.0012
Matches (Bold Red): 35/600 fragment ions using 57 most intense peaks

#	b	b^{++}	b*	b^{*++}	b^0	b^{0++}	Seq.	y	y^{++}	y*	y^{*++}	y^0	y^{0++}	#
1	115.0502	58.0287	98.0237	49.5155			N							35
2	202.0822	101.5448	185.0557	93.0315	184.0717	92.5395	S	3632.5032	1816.7552	3615.4766	1808.2419	3614.4926	1807.7499	34
3	303.1299	152.0686	286.1034	143.5553	285.1193	143.0633	T	3545.4711	1773.2392	3528.4446	1764.7259	3527.4606	1764.2339	33
4	400.1827	200.5950	383.1561	192.0817	382.1721	191.5897	P	3444.4235	**1722.7154**	3427.3969	1714.2021	3426.4129	1713.7101	32
5	567.1810	284.0942	550.1545	275.5809	549.1705	275.0889	S	3347.3707	**1674.1890**	3330.3442	1665.6757	3329.3601	1665.1837	31
6	696.2236	348.6154	679.1971	340.1022	678.2131	339.6102	E	3180.3723	**1590.6898**	3163.3458	**1582.1765**	3162.3618	**1581.6845**	30
7	793.2764	397.1418	776.2498	388.6286	775.2658	388.1365	P	3051.3298	**1526.1685**	3034.3032	1517.6552	3033.3192	1517.1632	29
8	**908.3033**	454.6553	891.2768	446.1420	890.2928	445.6500	D	2954.2770	1477.6421	2937.2504	1469.1289	2936.2664	1468.6369	28
9	995.3354	498.1713	978.3088	489.6580	977.3248	489.1660	S	2839.2500	**1420.1287**	2822.2235	1411.6154	2821.2395	1411.1234	27
10	1052.3568	526.6820	1035.3303	518.1688	1034.3463	517.6768	G	2752.2180	**1376.6126**	2735.1915	1368.0994	2734.2075	1367.6074	26
11	1180.4154	590.7113	1163.3888	582.1981	1162.4048	581.7061	Q	2695.1966	1348.1019	2678.1700	**1339.5886**	2677.1860	1339.0966	25
12	1237.4369	619.2221	1220.4103	610.7088	1219.4263	610.2168	G	2567.1380	**1284.0726**	2550.1114	1275.5594	2549.1274	1275.0673	24
13	1334.4896	667.7485	1317.4631	**659.2352**	1316.4791	658.7432	P	2510.1165	1255.5619	2493.0900	1247.0486	2492.1059	1246.5566	23
14	1431.5424	716.2748	1414.5158	707.7616	1413.5318	707.2695	P	2413.0637	1207.0355	2396.0372	1198.5222	2395.0532	1198.0302	22
15	1528.5952	764.8012	1511.5686	756.2879	1510.5846	755.7959	P	2316.0110	1158.5091	2298.9844	**1149.9959**	2298.0004	**1149.5038**	21
16	1657.6377	829.3225	1640.6112	820.8092	1639.6272	820.3172	E	2218.9582	1109.9827	2201.9317	1101.4695	2200.9477	1100.9775	20
17	1786.6803	893.8438	1769.6538	885.3305	1768.6698	884.8385	E	2089.9156	**1045.4615**	2072.8891	1036.9482	2071.9051	1036.4562	19
18	1915.7229	958.3651	1898.6964	949.8518	1897.7124	949.3598	E	**1960.8730**	980.9402	1943.8465	972.4269	1942.8625	971.9349	18

19	2044.7655	1022.8864	2027.7390	1014.3731	2026.7550	1013.8811	E	1831.8304	916.4189	1814.8039	907.9056	1813.8199	907.4136	17
20	2101.7870	1051.3971	2084.7604	1042.8839	2083.7764	1042.3918	G	1702.7878	851.8976	1685.7613	843.3843	1684.7773	842.8923	16
21	2230.8296	1115.9184	2213.8030	1107.4052	2212.8190	1106.9131	E	1645.7664	823.3868	1628.7398	814.8736	1627.7558	814.3815	15
22	2359.8722	1180.4397	2342.8456	1171.9265	2341.8616	1171.4344	E	1516.7238	758.8655	1499.6972	750.3523	1498.7132	749.8603	14
23	2488.9148	1244.9610	2471.8882	1236.4477	2470.9042	1235.9557	E	1387.6812	694.3442	1370.6547	685.8310	1369.6706	685.3390	13
24	2559.9519	1280.4796	2542.9253	1271.9663	2541.9413	1271.4743	A	1258.6386	629.8229	1241.6121	621.3097	1240.6280	620.8177	12
25	2630.9890	1315.9981	2613.9624	1307.4849	2612.9784	1306.9929	A	1187.6015	594.3044	1170.5749	585.7911	1169.5909	585.2991	11
26	2759.0840	1380.0456	2742.0574	1371.5323	2741.0734	1371.0403	K	1116.5644	558.7858	1099.5378	550.2726	1098.5538	549.7805	10
27	2888.1266	1444.5669	2871.1000	1436.0536	2870.1160	1435.5616	E	988.4694	494.7383	971.4429	486.2251	970.4589	485.7331	9
28	3017.1691	1509.0882	3000.1426	1500.5749	2999.1586	1500.0829	E	859.4268	430.2170	842.4003	421.7038	841.4163	421.2118	8
29	3088.2063	1544.6068	3071.1797	1536.0935	3070.1957	1535.6015	A	730.3842	365.6958	713.3577	357.1825	712.3737	356.6905	7
30	3217.2489	1609.1281	3200.2223	1600.6148	3199.2383	1600.1228	E	659.3471	330.1772	642.3206	321.6639	641.3366	321.1719	6
31	3288.2860	1644.6466	3271.2594	1636.1333	3270.2754	1635.6413	A	530.3045	265.6559	513.2780	257.1426			5
32	3416.3445	1708.6759	3399.3180	1700.1626	3398.3340	1699.6706	Q	459.2674	230.1373	442.2409	221.6241			4
33	3473.3660	1737.1866	3456.3395	1728.6734	3455.3554	1728.1814	G	331.2088	166.1081	314.1823	157.5948			3
34	3572.4344	1786.7208	3555.4079	1778.2076	3554.4239	1777.7156	V	274.1874	137.5973	257.1608	129.0840			2
35							R	175.1190	88.0631	158.0924	79.5498			1

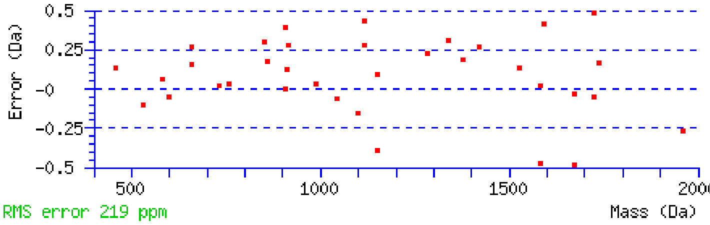

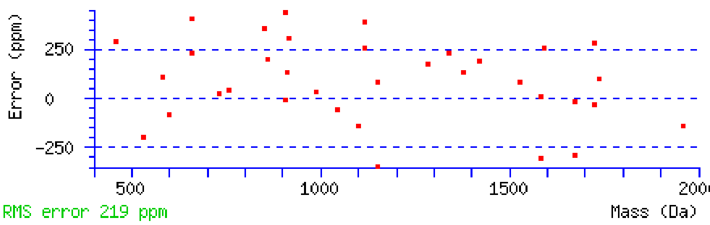

All matches to this query

Score	Mr(calc):	Delta	Sequence
55.9	3745.5388	0.0046	NSTPSEPDSGQGPPPEEEEGEEEAAKEEAEAQGVR
51.4	3745.5388	0.0046	NSTPSEPDSGQGPPPEEEEGEEEAAKEEAEAQGVR
51.4	3745.5388	0.0046	NSTPSEPDSGQGPPPEEEEGEEEAAKEEAEAQGVR
35.7	3745.5388	0.0046	NSTPSEPDSGQGPPPEEEEGEEEAAKEEAEAQGVR
4.2	3745.5663	-0.0230	ESANTKASLXLSDRTMPIMGEGLGLIPGMMER
3.8	3744.5205	1.0228	AKTSPVEGHLPSPMPHLSQDHASSAGEQYSDTK
3.5	3743.5368	2.0065	DTLQRPDSSAPNSGTAGMVETTAYALLTSLNLK
3.5	3743.5368	2.0065	DTLQRPDSSAPNSGTAGMVETTAYALLTSLNLK
3.2	3745.5105	0.0328	YDDMASAMKAVTELNEPLSNEDRNLLSVAYK
3.2	3745.5105	0.0328	YDDMASAMKAVTELNEPLSNEDRNLLSVAYK

Spectrum No: 212; Query: 2620; Rank: 1

Peptide View

MS/MS Fragmentation of **DLPAETLETQNTPNVVEAHSIQLENDEI**
Found in **IPI00205637**, Tax_Id=10116 Gene_Symbol=Cgref1 Cell growth regulator with EF hand domain protein 1

Match to Query 2620: 3198.465462 from(1067.162430,3+)
Title: 100101RatKid_NS_deglyco_23.4469.4469.3.dta
Data file K:\NewmanPaper\Piliang\3SubProteomes\Piliang3SP\mgf5ppm\ERLIC_3SubProteomes5ppm.mgf

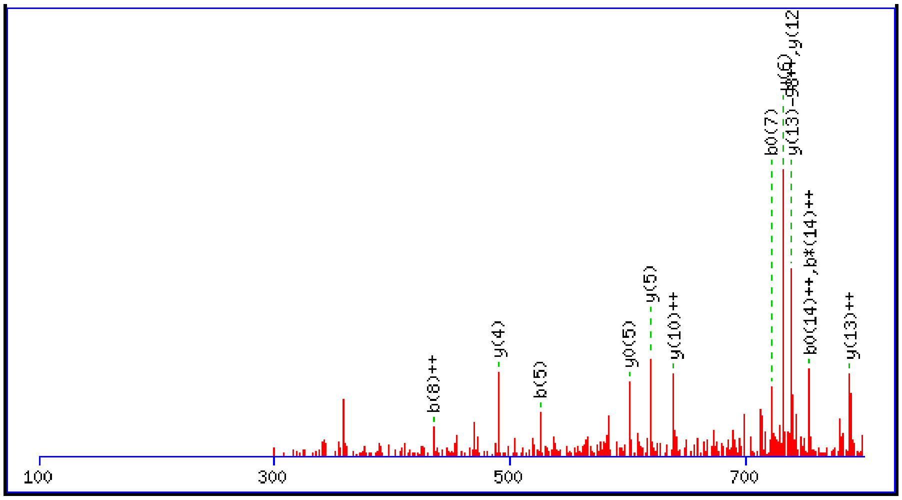

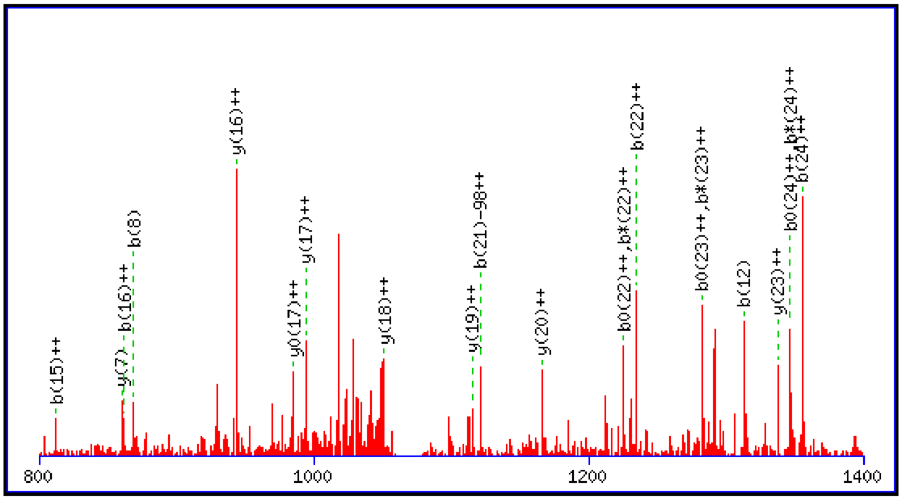

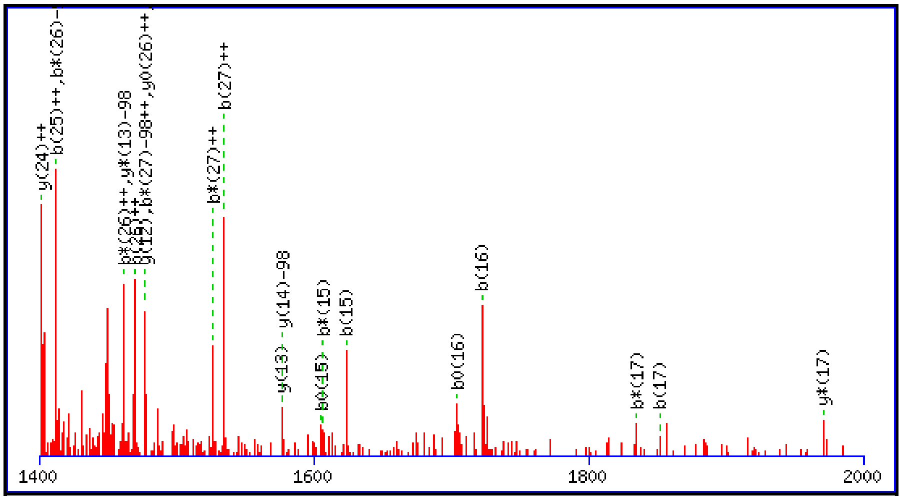

Monoisotopic mass of neutral peptide Mr(calc): 3198.4605
Fixed modifications: Carbamidomethyl (C)
Variable modifications:
S20 : Phospho (ST), with neutral losses 0.0000(shown in table), 97.9769
Ions Score: 56 **Expect:** 0.0011
Matches (Bold Red): 56/460 fragment ions using 72 most intense peaks

#	b	b++	b*	b*++	b0	b0++	Seq.	y	y++	y*	y*++	y0	y0++	#
1	116.0342	58.5207			98.0237	49.5155	D							28
2	229.1183	115.0628			211.1077	106.0575	L	3084.4409	1542.7241	3067.4143	1534.2108	3066.4303	1533.7188	27
3	326.1710	163.5892			308.1605	154.5839	P	2971.3568	1486.1821	2954.3303	**1477.6688**	2953.3463	**1477.1768**	26
4	397.2082	199.1077			379.1976	190.1024	A	2874.3041	1437.6557	2857.2775	1429.1424	2856.2935	1428.6504	25
5	**526.2508**	263.6290			508.2402	254.6237	E	2803.2669	**1402.1371**	2786.2404	1393.6238	2785.2564	1393.1318	24

6	627.2984	314.1529			609.2879	305.1476	T	2674.2244	**1337.6158**	2657.1978	1329.1025	2656.2138	1328.6105	23
7	740.3825	370.6949			**722.3719**	361.6896	L	2573.1767	1287.0920	2556.1501	1278.5787	2555.1661	1278.0867	22
8	**869.4251**	**435.2162**			851.4145	426.2109	E	2460.0926	1230.5499	2443.0661	1222.0367	2442.0820	1221.5447	21
9	970.4728	485.7400			952.4622	476.7347	T	2331.0500	**1166.0286**	2314.0235	1157.5154	2313.0395	1157.0234	20
10	1098.5313	549.7693	1081.5048	541.2560	1080.5208	540.7640	Q	2230.0023	**1115.5048**	2212.9758	1106.9915	2211.9918	1106.4995	19
11	1212.5743	606.7908	1195.5477	598.2775	1194.5637	597.7855	N	2101.9438	**1051.4755**	2084.9172	1042.9622	2083.9332	1042.4702	18
12	**1313.6220**	657.3146	1296.5954	648.8013	1295.6114	648.3093	T	1987.9008	**994.4541**	**1970.8743**	985.9408	1969.8903	**985.4488**	17
13	1410.6747	705.8410	1393.6482	697.3277	1392.6642	696.8357	P	1886.8532	**943.9302**	1869.8266	935.4169	1868.8426	934.9249	16
14	1524.7176	762.8625	1507.6911	**754.3492**	1506.7071	**753.8572**	N	1789.8004	895.4038	1772.7738	886.8906	1771.7898	886.3986	15
15	**1623.7861**	**812.3967**	**1606.7595**	803.8834	**1605.7755**	803.3914	V	1675.7575	838.3824	1658.7309	829.8691	1657.7469	829.3771	14
16	**1722.8545**	**861.9309**	1705.8279	853.4176	**1704.8439**	852.9256	V	**1576.6891**	**788.8482**	1559.6625	780.3349	1558.6785	779.8429	13
17	**1851.8971**	926.4522	**1834.8705**	917.9389	1833.8865	917.4469	E	**1477.6206**	**739.3140**	1460.5941	730.8007	1459.6101	730.3087	12
18	1922.9342	961.9707	1905.9076	953.4575	1904.9236	952.9654	A	1348.5780	674.7927	1331.5515	666.2794	1330.5675	665.7874	11
19	2059.9931	1030.5002	2042.9665	1021.9869	2041.9825	1021.4949	H	1277.5409	**639.2741**	1260.5144	630.7608	1259.5304	630.2688	10
20	2226.9914	1113.9994	2209.9649	1105.4861	2208.9809	1104.9941	S	1140.4820	570.7446	1123.4555	562.2314	1122.4715	561.7394	9
21	2340.0755	1170.5414	2323.0490	1162.0281	2322.0649	1161.5361	I	973.4837	487.2455	956.4571	478.7322	955.4731	478.2402	8
22	2468.1341	**1234.5707**	2451.1075	**1226.0574**	2450.1235	**1225.5654**	Q	**860.3996**	430.7034	843.3731	422.1902	842.3890	421.6982	7
23	2581.2181	1291.1127	2564.1916	**1282.5994**	2563.2076	**1282.1074**	L	**732.3410**	366.6742	715.3145	358.1609	714.3305	357.6689	6
24	2710.2607	**1355.6340**	2693.2342	**1347.1207**	2692.2502	**1346.6287**	E	**619.2570**	310.1321	602.2304	301.6188	**601.2464**	301.1268	5
25	2824.3037	**1412.6555**	2807.2771	1404.1422	2806.2931	1403.6502	N	**490.2144**	245.6108	473.1878	237.0975	472.2038	236.6055	4
26	2939.3306	**1470.1689**	2922.3041	**1461.6557**	2921.3200	1461.1637	D	376.1714	188.5894			358.1609	179.5841	3
27	3068.3732	**1534.6902**	3051.3467	**1526.1770**	3050.3626	1525.6850	E	261.1445	131.0759			243.1339	122.0706	2
28							I	132.1019	66.5546					1

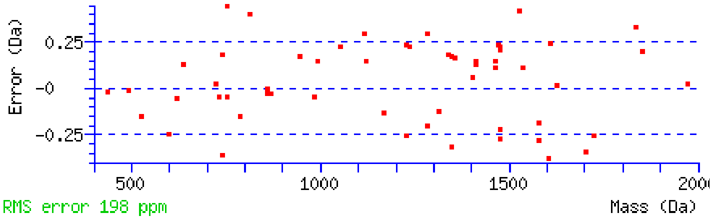

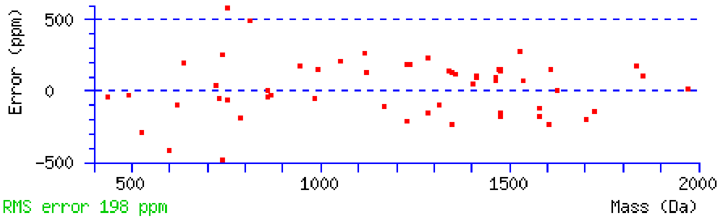

All matches to this query

Score	Mr(calc):	Delta	Sequence
55.9	3198.4605	0.0049	DLPAETLETQNTPNVVEAHSIQLENDEI
19.9	3198.4605	0.0049	DLPAETLETQNTPNVVEAHSIQLENDEI
18.4	3198.4605	0.0049	DLPAETLETQNTPNVVEAHSIQLENDEI
18.4	3198.4605	0.0049	DLPAETLETQNTPNVVEAHSIQLENDEI
15.1	3196.4756	1.9899	MYKLEGQEAQGDLMGSNLSLYLMRXVR
15.1	3196.4756	1.9899	MYKLEGQEAQGDLMGSNLSLYLMRXVR
7.4	3198.4470	0.0185	LQCMMRAGTTLVECKSGYGLNLETELK
7.4	3198.4470	0.0185	LQCMMRAGTTLVECKSGYGLNLETELK
5.2	3196.4756	1.9899	MYKLEGQEAQGDLMGSNLSLYLMRXVR
5.2	3196.4756	1.9899	MYKLEGQEAQGDLMGSNLSLYLMRXVR

Spectrum No: 213; Query: 1575; Rank: 1

Peptide View

MS/MS Fragmentation of **SASSDTSEELNAQDSPKR**

Found in **IPI00200898**, Tax_Id=10116 Gene_Symbol=Slc9a3r1 Ezrin-radixin-moesin-binding phosphoprotein 50

Match to Query 1575: 2000.837322 from(667.953050,3+)
Title: 100101RatKid_NS_deglyco_18.1141.1141.3.dta
Data file K:\NewmanPaper\Piliang\3SubProteomes\Piliang3SP\mgf5ppm\ERLIC_3SubProteomes5ppm.mgf

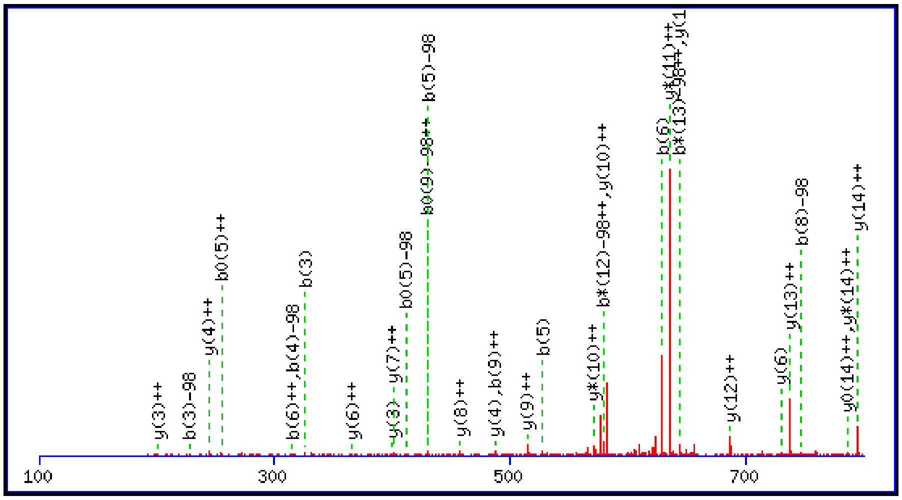

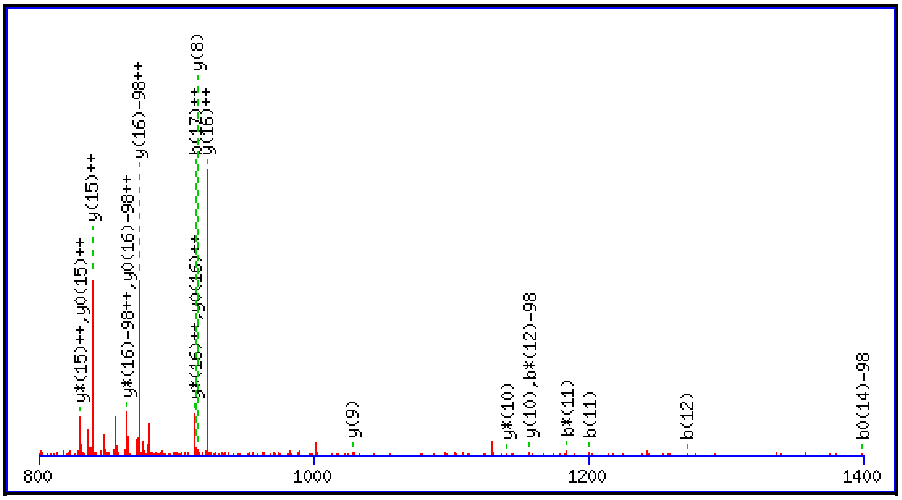

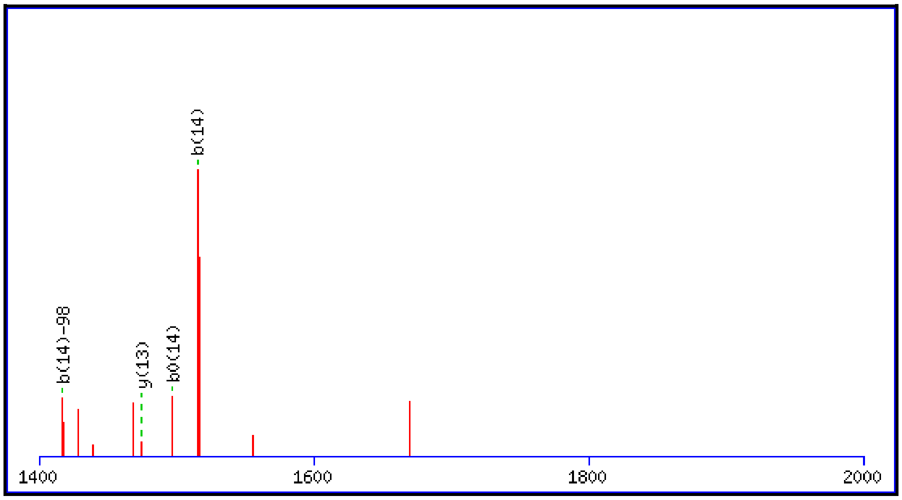

Monoisotopic mass of neutral peptide Mr(calc): 2000.8324
Fixed modifications: Carbamidomethyl (C)
Variable modifications:
S3 : Phospho (ST), with neutral losses 97.9769(shown in table), 0.0000
Ions Score: 56 **Expect:** 0.00054
Matches (Bold Red): 55/264 fragment ions using 111 most intense peaks

#	b	b^{++}	b*	b^{*++}	b^0	b^{0++}	Seq.	y	y^{++}	y*	y^{*++}	y^0	y^{0++}	#
1	88.0393	44.5233			70.0287	35.5180	S							18

**2**	159.0764	80.0418			141.0659	71.0366	**A**	1816.8308	908.9190	1799.8042	900.4057	1798.8202	899.9137	**17**
**3**	**228.0979**	114.5526			210.0873	105.5473	**S**	1745.7937	**873.4005**	1728.7671	**864.8872**	1727.7831	**864.3952**	**16**
**4**	**315.1299**	158.0686			297.1193	149.0633	**S**	1676.7722	**838.8897**	1659.7456	**830.3765**	1658.7616	**829.8845**	**15**
**5**	**430.1568**	215.5821			**412.1463**	206.5768	**D**	1589.7402	**795.3737**	1572.7136	**786.8604**	1571.7296	**786.3684**	**14**
**6**	531.2045	266.1059			513.1940	257.1006	**T**	**1474.7132**	**737.8603**	1457.6867	729.3470	1456.7027	728.8550	**13**
**7**	618.2366	309.6219			600.2260	300.6166	**S**	1373.6655	**687.3364**	1356.6390	678.8231	1355.6550	678.3311	**12**
**8**	**747.2791**	374.1432			729.2686	365.1379	**E**	1286.6335	**643.8204**	1269.6070	**635.3071**	1268.6230	634.8151	**11**
**9**	876.3217	438.6645			858.3112	**429.6592**	**E**	**1157.5909**	**579.2991**	**1140.5644**	**570.7858**	1139.5804	570.2938	**10**
**10**	989.4058	495.2065			971.3952	486.2013	**L**	**1028.5483**	**514.7778**	1011.5218	506.2645	1010.5378	505.7725	**9**
**11**	1103.4487	552.2280	1086.4222	543.7147	1085.4382	543.2227	**N**	**915.4643**	**458.2358**	898.4377	449.7225	897.4537	449.2305	**8**
**12**	1174.4858	587.7466	**1157.4593**	**579.2333**	1156.4753	578.7413	**A**	801.4213	**401.2143**	784.3948	392.7010	783.4108	392.2090	**7**
**13**	1302.5444	651.7758	1285.5179	**643.2626**	1284.5339	642.7706	**Q**	**730.3842**	**365.6958**	713.3577	357.1825	712.3737	356.6905	**6**
**14**	**1417.5714**	709.2893	1400.5448	700.7760	**1399.5608**	700.2840	**D**	602.3257	301.6665	585.2991	293.1532	584.3151	292.6612	**5**
**15**	1504.6034	752.8053	1487.5768	744.2921	1486.5928	743.8001	**S**	**487.2987**	**244.1530**	470.2722	235.6397	469.2881	235.1477	**4**
**16**	1601.6562	801.3317	1584.6296	792.8184	1583.6456	792.3264	**P**	**400.2667**	**200.6370**	383.2401	192.1237			**3**
**17**	1729.7511	865.3792	1712.7246	856.8659	1711.7406	856.3739	**K**	303.2139	152.1106	286.1874	143.5973			**2**
**18**							**R**	175.1190	88.0631	158.0924	79.5498			**1**

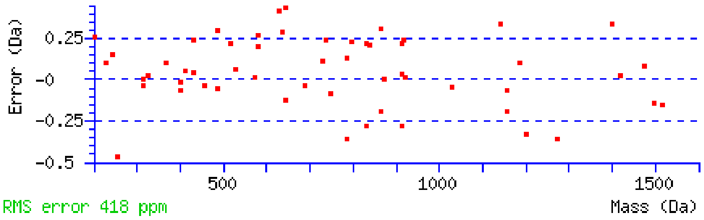

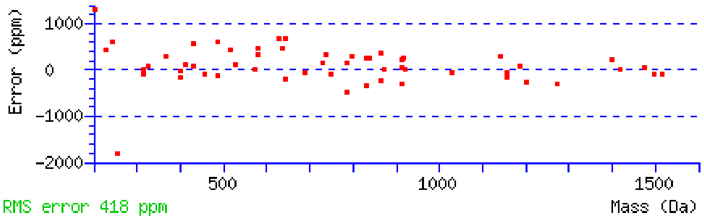

All matches to this query

Score	Mr(calc):	Delta	Sequence
55.8	2000.8324	0.0049	SASSDTSEELNAQDSPKR
53.7	2000.8324	0.0049	SASSDTSEELNAQDSPKR
46.6	2000.8324	0.0049	SASSDTSEELNAQDSPKR
30.7	2000.8324	0.0049	SASSDTSEELNAQDSPKR
23.9	2000.8324	0.0049	SASSDTSEELNAQDSPKR
4.0	1999.8176	1.0197	LNFTASSLNDNISGTKR
3.9	1999.8299	1.0074	VKTSAYSEDSDSFEEVK
3.8	1998.8496	1.9878	SRPTSAPSRHAASNMLR
3.6	2000.8337	0.0036	RSSSKTYGNGYSSNSNGR
3.4	2000.8429	-0.0056	SSIYPMPKSSIYPMPK

Spectrum No: 214; Query: 2757; Rank: 1

Peptide View

MS/MS Fragmentation of **SVERLPSVEEAEVSKPSPPASKDEGEEFQAILR**
Found in **IPI00358106**, Tax_Id=10116 Gene_Symbol=- 57 kDa protein

Match to Query 2757: 3689.799136 from(923.457060,4+)
Title: 100101RatKid_NS_deglyco_10.3702.3702.4.dta
Data file K:\NewmanPaper\Piliang\3SubProteomes\Piliang3SP\mgf5ppm\ERLIC_3SubProteomes5ppm.mgf

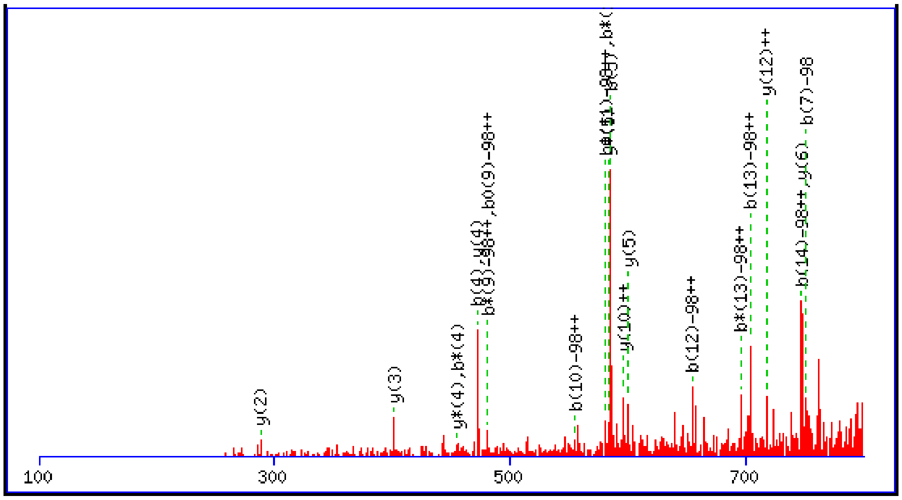

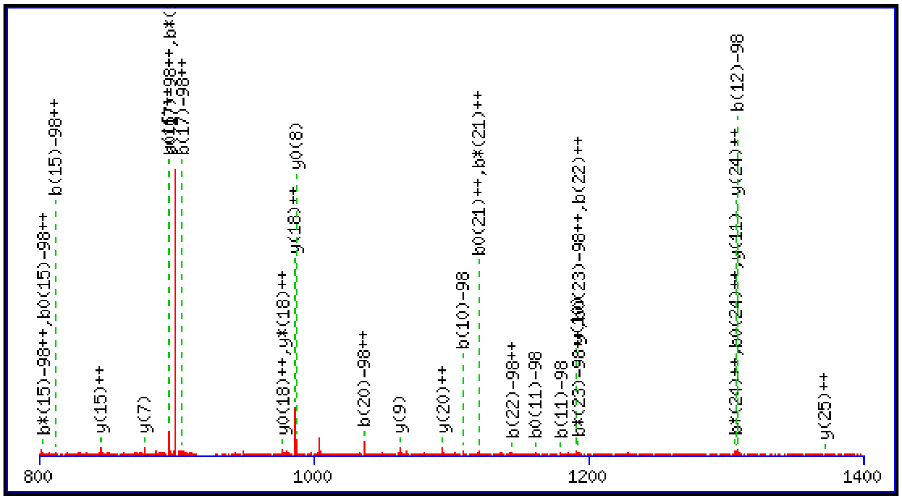

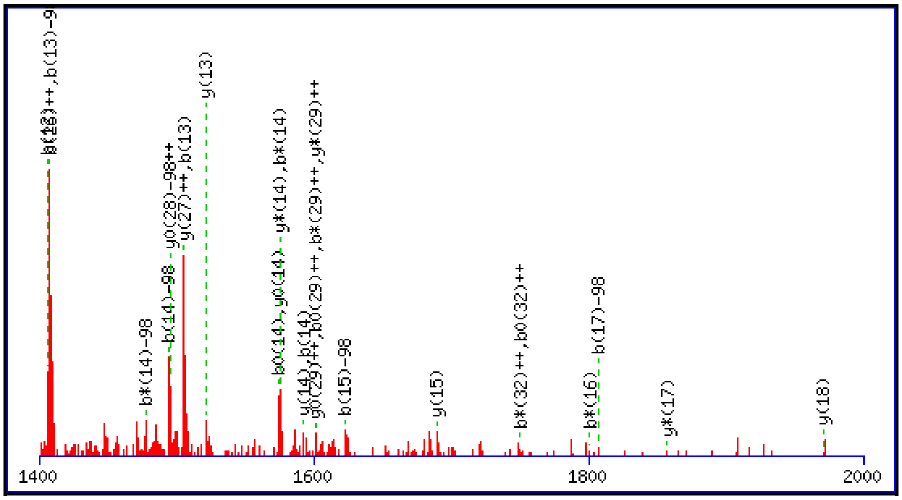

Monoisotopic mass of neutral peptide Mr(calc): 3689.7825
Fixed modifications: Carbamidomethyl (C)
Variable modifications:
S7 : Phospho (ST), with neutral losses 97.9769(shown in table), 0.0000
Ions Score: 56 **Expect:** 0.00083
Matches (Bold Red): 81/558 fragment ions using 129 most intense peaks

#	b	b^{++}	b*	b^{*++}	b^0	b^{0++}	Seq.	y	y^{++}	y*	y^{*++}	y^0	y^{0++}	#
1	88.0393	44.5233			70.0287	35.5180	S							33
2	187.1077	94.0575			169.0972	85.0522	V	3505.7809	1753.3941	3488.7544	1744.8808	3487.7703	1744.3888	32
3	316.1503	158.5788			298.1397	149.5735	E	3406.7125	1703.8599	3389.6859	1695.3466	3388.7019	1694.8546	31
4	**472.2514**	236.6293	**455.2249**	228.1161	454.2409	227.6241	R	3277.6699	1639.3386	3260.6434	1630.8253	3259.6593	1630.3333	30
5	**585.3355**	293.1714	568.3089	284.6581	567.3249	284.1661	L	3121.5688	1561.2880	3104.5422	1552.7748	3103.5582	1552.2828	29

6	682.3883	341.6978	665.3617	333.1845	664.3777	332.6925	P	3008.4847	1504.7460	2991.4582	1496.2327	2990.4742	**1495.7407**	28
7	**751.4097**	376.2085	734.3832	367.6952	733.3991	367.2032	S	2911.4320	1456.2196	2894.4054	1447.7063	2893.4214	1447.2143	27
8	850.4781	425.7427	833.4516	417.2294	832.4676	416.7374	V	2842.4105	1421.7089	2825.3840	1413.1956	2824.3999	1412.7036	26
9	979.5207	490.2640	962.4942	**481.7507**	961.5101	**481.2587**	E	2743.3421	**1372.1747**	2726.3155	1363.6614	2725.3315	1363.1694	25
10	**1108.5633**	**554.7853**	1091.5368	546.2720	1090.5527	545.7800	E	2614.2995	**1307.6534**	2597.2730	1299.1401	2596.2889	1298.6481	24
11	**1179.6004**	590.3038	1162.5739	**581.7906**	**1161.5899**	**581.2986**	A	2485.2569	1243.1321	2468.2304	1234.6188	2467.2463	1234.1268	23
12	**1308.6430**	**654.8251**	1291.6165	646.3119	1290.6324	645.8199	E	2414.2198	1207.6135	2397.1932	1199.1003	2396.2092	1198.6083	22
13	**1407.7114**	**704.3594**	1390.6849	**695.8461**	1389.7009	695.3541	V	2285.1772	1143.0922	2268.1507	1134.5790	2267.1666	1134.0870	21
14	**1494.7435**	**747.8754**	**1477.7169**	739.3621	1476.7329	738.8701	S	2186.1088	**1093.5580**	2169.0822	1085.0448	2168.0982	1084.5527	20
15	**1622.8384**	**811.9228**	1605.8119	**803.4096**	1604.8279	**802.9176**	K	2099.0768	1050.0420	2082.0502	1041.5287	2081.0662	1041.0367	19
16	1719.8912	860.4492	1702.8646	851.9360	1701.8806	851.4439	P	**1970.9818**	**985.9945**	1953.9552	**977.4813**	1952.9712	**976.9893**	18
17	**1806.9232**	**903.9652**	1789.8967	**895.4520**	1788.9126	**894.9600**	S	1873.9290	937.4682	**1856.9025**	928.9549	1855.9185	928.4629	17
18	1903.9760	952.4916	1886.9494	943.9784	1885.9654	943.4863	P	1786.8970	**893.9521**	1769.8705	885.4389	1768.8864	884.9469	16
19	2001.0287	1001.0180	1984.0022	992.5047	1983.0182	992.0127	P	**1689.8442**	**845.4258**	1672.8177	836.9125	1671.8337	836.4205	15
20	2072.0659	**1036.5366**	2055.0393	1028.0233	2054.0553	1027.5313	A	**1592.7915**	796.8994	**1575.7649**	788.3861	**1574.7809**	787.8941	14
21	2159.0979	1080.0526	2142.0713	1071.5393	2141.0873	1071.0473	S	**1521.7544**	761.3808	1504.7278	752.8675	1503.7438	752.3755	13
22	2287.1928	**1144.1001**	2270.1663	1135.5868	2269.1823	1135.0948	K	1434.7223	**717.8648**	1417.6958	709.3515	1416.7118	708.8595	12
23	2402.2198	1201.6135	2385.1932	**1193.1003**	2384.2092	**1192.6082**	D	**1306.6274**	653.8173	1289.6008	645.3040	1288.6168	644.8120	11
24	2531.2624	1266.1348	2514.2358	1257.6216	2513.2518	1257.1295	E	**1191.6004**	**596.3039**	1174.5739	587.7906	1173.5899	587.2986	10
25	2588.2838	1294.6456	2571.2573	1286.1323	2570.2733	1285.6403	G	**1062.5578**	531.7826	1045.5313	523.2693	1044.5473	522.7773	9
26	2717.3264	1359.1669	2700.2999	1350.6536	2699.3159	1350.1616	E	1005.5364	503.2718	988.5098	494.7585	**987.5258**	494.2665	8
27	2846.3690	1423.6882	2829.3425	1415.1749	2828.3585	1414.6829	E	**876.4938**	438.7505	859.4672	430.2373	858.4832	429.7452	7
28	2993.4374	1497.2224	2976.4109	1488.7091	2975.4269	1488.2171	F	**747.4512**	374.2292	730.4246	365.7160			6
29	3121.4960	1561.2516	3104.4695	1552.7384	3103.4855	1552.2464	Q	**600.3828**	300.6950	**583.3562**	292.1817			5
30	3192.5331	1596.7702	3175.5066	1588.2569	3174.5226	1587.7649	A	**472.3242**	236.6657	**455.2976**	228.1525			4
31	3305.6172	1653.3122	3288.5907	1644.7990	3287.6066	1644.3070	I	**401.2871**	201.1472	384.2605	192.6339			3
32	3418.7013	1709.8543	3401.6747	1701.3410	3400.6907	1700.8490	L	**288.2030**	144.6051	271.1765	136.0919			2
33							R	175.1190	88.0631	158.0924	79.5498			1

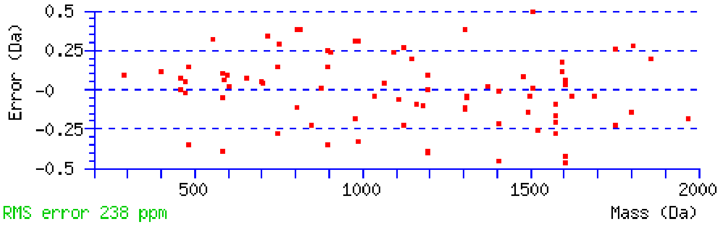

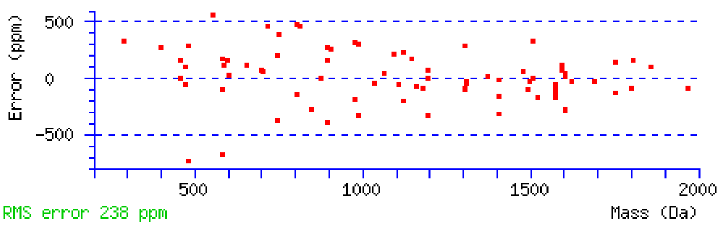

All matches to this query

Score	Mr(calc):	Delta	Sequence
55.7	3689.7825	0.0166	SVERLPSVEEAEVSKPSPPASKDEGEEFQAILR
48.6	3689.7825	0.0166	SVERLPSVEEAEVSKPSPPASKDEGEEFQAILR
35.2	3689.7825	0.0166	SVERLPSVEEAEVSKPSPPASKDEGEEFQAILR
24.8	3689.7825	0.0166	SVERLPSVEEAEVSKPSPPASKDEGEEFQAILR
19.6	3689.7825	0.0166	SVERLPSVEEAEVSKPSPPASKDEGEEFQAILR
9.5	3687.7910	2.0081	GPQVTVDGEAVALPVATAHVSVTAEGRNVVLQTNK
8.3	3688.7751	1.0241	GPQVTVDGEAVALPVATAHVSVTAEGRNVVLQTNK
7.9	3689.7986	0.0006	EHAMLISAGFNLSSSLTIILVSYAFIIAAILR
6.5	3689.7654	0.0337	FTATDRDIADTVYYEFIGTQKEFAINEAIIR
6.5	3689.7654	0.0337	FTATDRDIADTVYYEFIGTQKEFAINEAIIR

Spectrum No: 215; Query: 1595; Rank: 1

Peptide View

MS/MS Fragmentation of **HSSLPTESDEDIAPAQR**
Found in **IPI00198486**, Tax_Id=10116 Gene_Symbol=Ap3d1 similar to adaptor-related protein complex 3, delta 1 subunit

Match to Query 1595: 2011.797908 from(1006.906230,2+)
Title: 100101RatKid_NS_deglyco_24.1663.1663.2.dta
Data file K:\NewmanPaper\Piliang\3SubProteomes\Piliang3SP\mgf5ppm\ERLIC_3SubProteomes5ppm.mgf

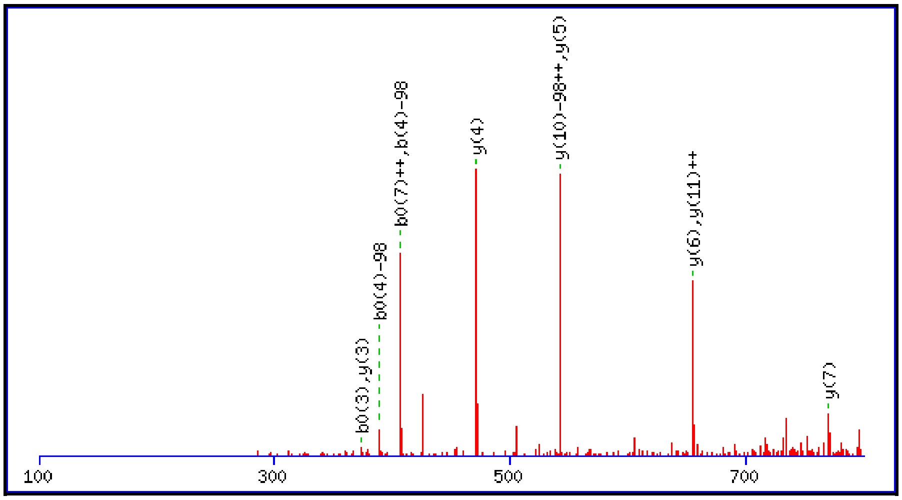

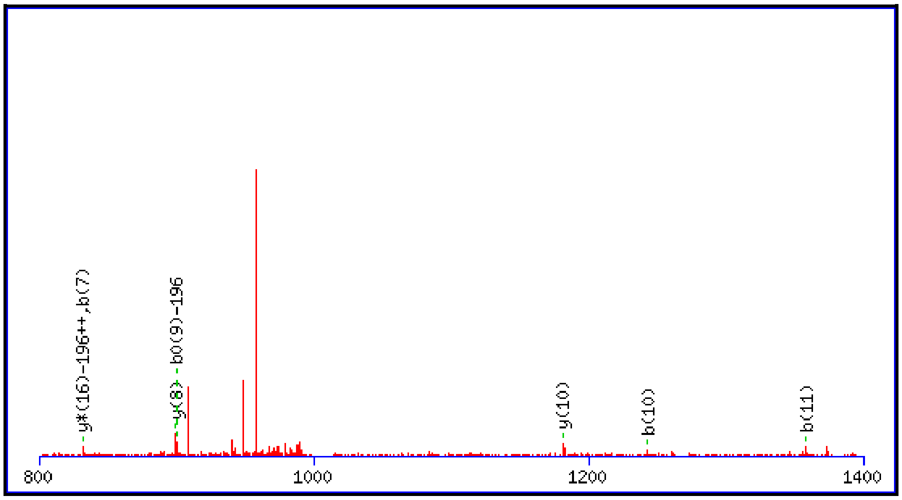

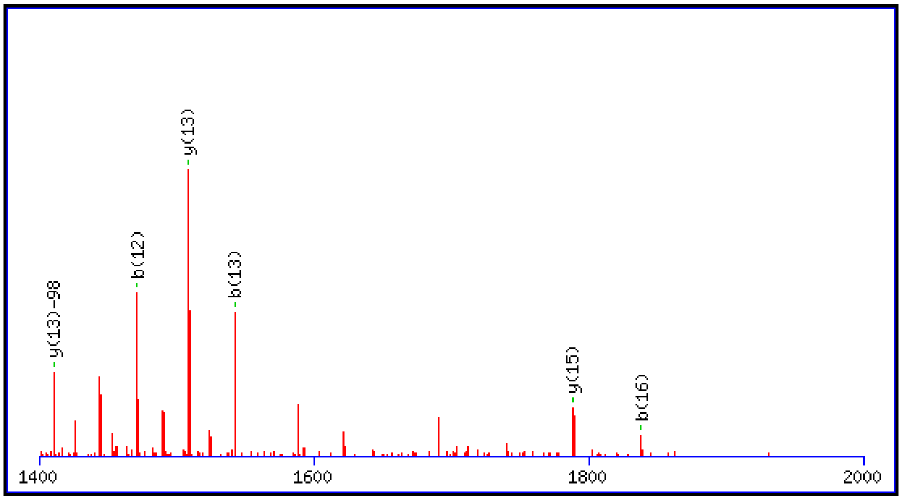

Monoisotopic mass of neutral peptide Mr(calc): 2011.7925
Fixed modifications: Carbamidomethyl (C)

```
Variable modifications:
S3     : Phospho (ST), with neutral losses 0.0000(shown in table), 97.9769
S8     : Phospho (ST), with neutral losses 0.0000(shown in table), 97.9769
Ions Score: 56  Expect: 0.00047
Matches (Bold Red): 24/248 fragment ions using 37 most intense peaks
```

#	b	b^{++}	b*	b^{*++}	b^0	b^{0++}	Seq.	y	y^{++}	y*	y^{*++}	y^0	y^{0++}	#
1	138.0662	69.5367					H							17
2	225.0982	113.0527			207.0877	104.0475	S	1875.7409	938.3741	1858.7143	929.8608	1857.7303	929.3688	16
3	392.0966	196.5519			**374.0860**	187.5466	S	**1788.7089**	894.8581	1771.6823	886.3448	1770.6983	885.8528	15
4	505.1806	253.0940			487.1701	244.0887	L	1621.7105	811.3589	1604.6840	802.8456	1603.7000	802.3536	14
5	602.2334	301.6203			584.2228	292.6151	P	**1508.6265**	754.8169	1491.5999	746.3036	1490.6159	745.8116	13
6	703.2811	352.1442			685.2705	343.1389	T	1411.5737	706.2905	1394.5471	697.7772	1393.5631	697.2852	12
7	**832.3237**	416.6655			814.3131	**407.6602**	E	1310.5260	**655.7666**	1293.4995	647.2534	1292.5154	646.7614	11
8	999.3220	500.1646			981.3115	491.1594	S	**1181.4834**	591.2453	1164.4569	582.7321	1163.4729	582.2401	10
9	1114.3490	557.6781			1096.3384	548.6728	D	1014.4851	507.7462	997.4585	499.2329	996.4745	498.7409	9
10	**1243.3916**	622.1994			1225.3810	613.1941	E	**899.4581**	450.2327	882.4316	441.7194	881.4476	441.2274	8
11	**1358.4185**	679.7129			1340.4079	670.7076	D	**770.4155**	385.7114	753.3890	377.1981	752.4050	376.7061	7
12	**1471.5026**	736.2549			1453.4920	727.2496	I	**655.3886**	328.1979	638.3620	319.6847			6
13	**1542.5397**	771.7735			1524.5291	762.7682	A	**542.3045**	271.6559	525.2780	263.1426			5
14	1639.5924	820.2999			1621.5819	811.2946	P	**471.2674**	236.1373	454.2409	227.6241			4
15	1710.6296	855.8184			1692.6190	846.8131	A	**374.2146**	187.6110	357.1881	179.0977			3
16	**1838.6881**	919.8477	1821.6616	911.3344	1820.6776	910.8424	Q	303.1775	152.0924	286.1510	143.5791			2
17							R	175.1190	88.0631	158.0924	79.5498			1

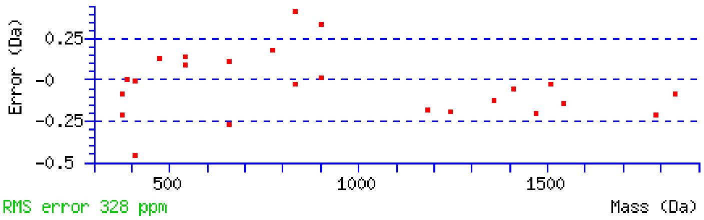

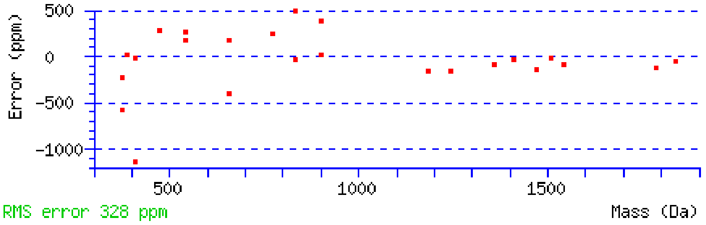

All matches to this query

Score	Mr(calc):	Delta	Sequence
55.6	2011.7925	0.0054	HSSLPTESDEDIAPAQR
54.8	2011.7925	0.0054	HSSLPTESDEDIAPAQR
45.1	2011.7925	0.0054	HSSLPTESDEDIAPAQR
39.1	2011.7925	0.0054	HSSLPTESDEDIAPAQR
30.0	2011.7925	0.0054	HSSLPTESDEDIAPAQR
18.5	2011.7925	0.0054	HSSLPTESDEDIAPAQR
7.1	2010.8002	0.9977	KASGNYAGPPSSALPAQR
2.7	2010.7755	1.0224	SDCTLPDSASSGNKGRAK
2.7	2010.7755	1.0224	SDCTLPDSASSGNKGRAK
2.4	2011.7918	0.0061	THTDERPYTCDICHK

Spectrum No: 216; Query: 2783; Rank: 1

Peptide View

MS/MS Fragmentation of **TVSETPAVPPVSEDEDDDDDATPPPVIAPRPEHTK**
Found in **IPI00198685**, Tax_Id=10116 Gene_Symbol=Pak1 Serine/threonine-protein kinase PAK 1

Match to Query 2783: 3803.729292 from(1268.917040,3+)
Title: 100101RatKid_NS_deglyco_21.2486.2486.3.dta
Data file K:\NewmanPaper\Piliang\3SubProteomes\Piliang3SP\mgf5ppm\ERLIC_3SubProteomes5ppm.mgf

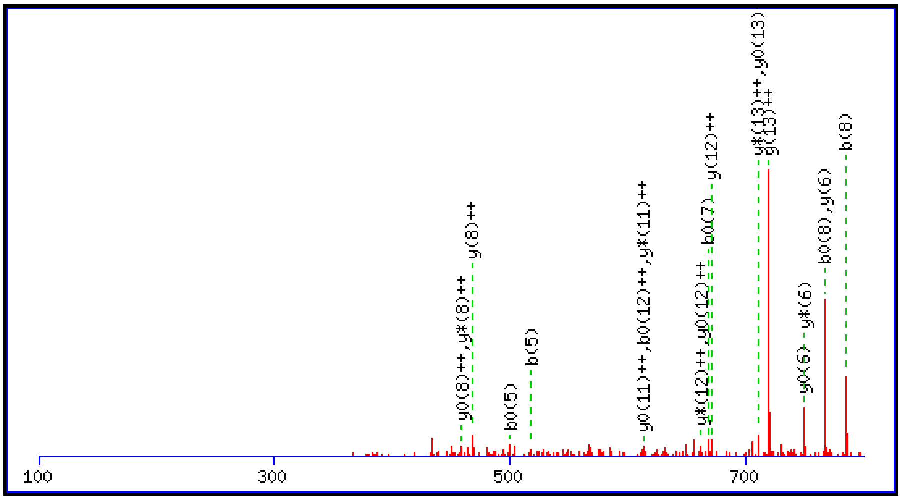

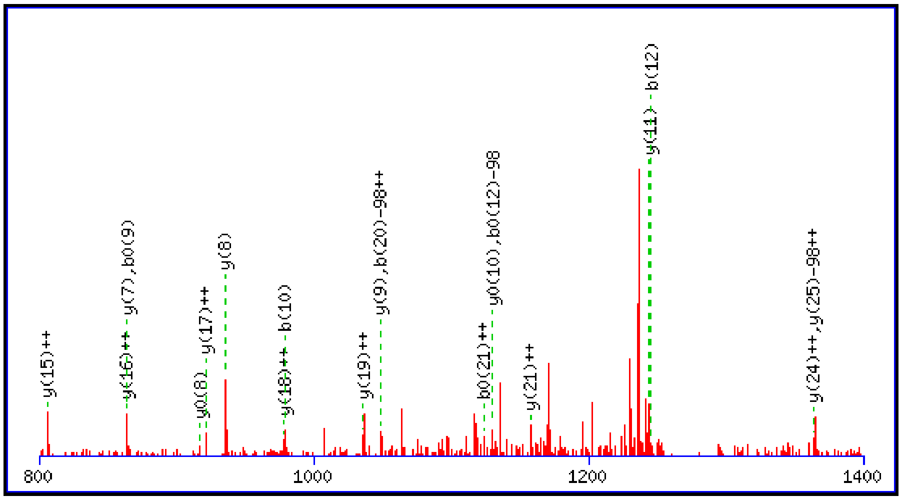

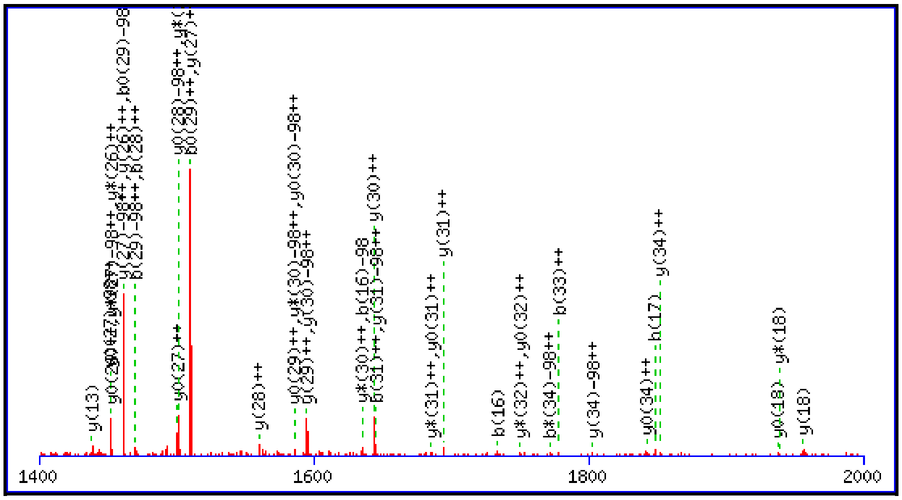

Monoisotopic mass of neutral peptide Mr(calc): 3802.7099
Fixed modifications: Carbamidomethyl (C)
Variable modifications:
S12 : Phospho (ST), with neutral losses 0.0000(shown in table), 97.9769
Ions Score: 56 **Expect:** 0.0014
Matches (Bold Red): 84/516 fragment ions using 140 most intense peaks

#	b	b^{++}	b*	b^{*++}	b^0	b^{0++}	Seq.	y	y^{++}	y*	y^{*++}	y^0	y^{0++}	#
1	102.0550	51.5311			84.0444	42.5258	T							35
2	201.1234	101.0653			183.1128	92.0600	V	3702.6694	**1851.8384**	3685.6429	1843.3251	3684.6589	**1842.8331**	34

3	288.1554	144.5813			270.1448	135.5761	S	3603.6010	1802.3042	3586.5745	1793.7909	3585.5905	1793.2989	33
4	417.1980	209.1026			399.1874	200.0974	E	3516.5690	1758.7881	3499.5425	**1750.2749**	3498.5584	**1749.7829**	32
5	**518.2457**	259.6265			**500.2351**	250.6212	T	3387.5264	**1694.2668**	3370.4999	**1685.7536**	3369.5158	**1685.2616**	31
6	615.2984	308.1529			597.2879	299.1476	P	3286.4787	**1643.7430**	3269.4522	**1635.2297**	3268.4682	1634.7377	30
7	686.3355	343.6714			**668.3250**	334.6661	A	3189.4260	**1595.2166**	3172.3994	1586.7033	3171.4154	**1586.2113**	29
8	**785.4040**	393.2056			**767.3934**	384.2003	V	3118.3889	**1559.6981**	3101.3623	1551.1848	3100.3783	1550.6928	28
9	882.4567	441.7320			**864.4462**	432.7267	P	3019.3204	**1510.1639**	3002.2939	**1501.6506**	3001.3099	**1501.1586**	27
10	**979.5095**	490.2584			961.4989	481.2531	P	2922.2677	**1461.6375**	2905.2411	**1453.1242**	2904.2571	**1452.6322**	26
11	1078.5779	539.7926			1060.5673	530.7873	V	2825.2149	1413.1111	2808.1884	1404.5978	2807.2043	1404.1058	25
12	**1245.5763**	623.2918			1227.5657	**614.2865**	S	2726.1465	**1363.5769**	2709.1200	1355.0636	2708.1359	1354.5716	24
13	1374.6188	687.8131			1356.6083	678.8078	E	2559.1481	1280.0777	2542.1216	1271.5644	2541.1376	1271.0724	23
14	1489.6458	745.3265			1471.6352	736.3213	D	2430.1056	1215.5564	2413.0790	1207.0431	2412.0950	1206.5511	22
15	1618.6884	809.8478			1600.6778	800.8425	E	2315.0786	**1158.0429**	2298.0521	1149.5297	2297.0680	1149.0377	21
16	**1733.7153**	867.3613			1715.7048	858.3560	D	2186.0360	1093.5216	2169.0095	1085.0084	2168.0255	1084.5164	20
17	**1848.7423**	924.8748			1830.7317	915.8695	D	2071.0091	**1036.0082**	2053.9825	1027.4949	2052.9985	1027.0029	19
18	1963.7692	982.3882			1945.7586	973.3830	D	**1955.9821**	**978.4947**	**1938.9556**	969.9814	**1937.9716**	969.4894	18
19	2078.7962	1039.9017			2060.7856	1030.8964	D	1840.9552	**920.9812**	1823.9286	912.4680	1822.9446	911.9760	17
20	2193.8231	1097.4152			2175.8125	1088.4099	D	1725.9282	**863.4678**	1708.9017	854.9545	1707.9177	854.4625	16
21	2264.8602	1132.9337			2246.8496	**1123.9285**	A	1610.9013	**805.9543**	1593.8748	797.4410	1592.8907	796.9490	15
22	2365.9079	1183.4576			2347.8973	1174.4523	T	1539.8642	770.4357	1522.8376	761.9225	1521.8536	761.4305	14
23	2462.9607	1231.9840			2444.9501	1222.9787	P	**1438.8165**	**719.9119**	1421.7900	**711.3986**	1420.8059	**710.9066**	13
24	2560.0134	1280.5103			2542.0029	1271.5051	P	1341.7637	**671.3855**	1324.7372	**662.8722**	1323.7532	**662.3802**	12
25	2657.0662	1329.0367			2639.0556	1320.0314	P	**1244.7110**	622.8591	1227.6844	**614.3459**	1226.7004	**613.8538**	11
26	2756.1346	1378.5709			2738.1240	1369.5657	V	1147.6582	574.3327	1130.6317	565.8195	**1129.6477**	565.3275	10
27	2869.2187	1435.1130			2851.2081	1426.1077	I	**1048.5898**	524.7985	1031.5633	516.2853	1030.5792	515.7933	9
28	2940.2558	**1470.6315**			2922.2452	**1461.6262**	A	**935.5057**	**468.2565**	918.4792	**459.7432**	**917.4952**	**459.2512**	8
29	3037.3085	1519.1579			3019.2980	**1510.1526**	P	**864.4686**	432.7380	847.4421	424.2247	846.4581	423.7327	7
30	3193.4097	1597.2085	3176.3831	1588.6952	3175.3991	1588.2032	R	**767.4159**	384.2116	**750.3893**	375.6983	**749.4053**	375.2063	6
31	3290.4624	**1645.7348**	3273.4359	1637.2216	3272.4519	1636.7296	P	611.3148	306.1610	594.2882	297.6477	593.3042	297.1557	5
32	3419.5050	1710.2561	3402.4785	1701.7429	3401.4944	1701.2509	E	514.2620	257.6346	497.2354	249.1214	496.2514	248.6293	4
33	3556.5639	**1778.7856**	3539.5374	1770.2723	3538.5534	1769.7803	H	385.2194	193.1133	368.1928	184.6001	367.2088	184.1081	3
34	3657.6116	1829.3094	3640.5851	1820.7962	3639.6010	1820.3042	T	248.1605	124.5839	231.1339	116.0706	230.1499	115.5786	2
35							K	147.1128	74.0600	130.0863	65.5468			1

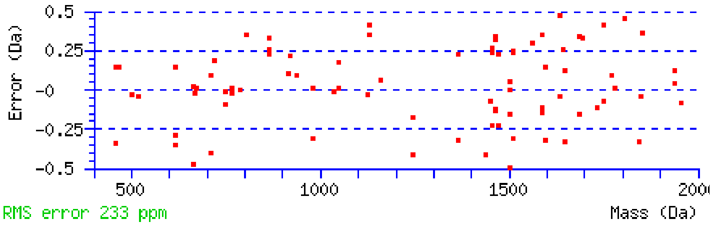

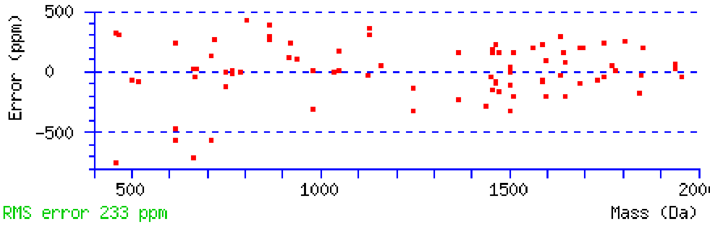

All matches to this query

Score	Mr(calc):	Delta	Sequence
55.6	3802.7099	1.0194	TVSETPAVPPVSEDEDDDDDATPPPVIAPRPEHTK
27.1	3802.7099	1.0194	TVSETPAVPPVSEDEDDDDDATPPPVIAPRPEHTK
21.9	3802.7099	1.0194	TVSETPAVPPVSEDEDDDDDATPPPVIAPRPEHTK
21.9	3802.7099	1.0194	TVSETPAVPPVSEDEDDDDDATPPPVIAPRPEHTK
19.6	3802.7099	1.0194	TVSETPAVPPVSEDEDDDDDATPPPVIAPRPEHTK
18.4	3802.7099	1.0194	TVSETPAVPPVSEDEDDDDDATPPPVIAPRPEHTK
7.0	3801.6892	2.0401	ADTTTPTPTAILAPGSPASPPGSLEPKAARLPPMR

3.5	3802.7557	0.9736	DIDKVNVXTGIYHGGEPLCDNVNTQRVPCSNPR
3.3	3801.7557	1.9736	VTGLQEGNTYEFRVSAENRAGIGPPSDASNPVLMK
2.4	3802.7567	0.9726	EGGFAHALVLPSDPGSEQDPACEDPCPSVGPALVATR

Spectrum No: 217; Query: 2562; Rank: 1

Peptide View

MS/MS Fragmentation of **VLSSTINNQVAEADEAAEEEAPDSPGVEK**
Found in **IPI00366572**, Tax_Id=10116 Gene_Symbol=Arhgap12_predicted Rho GTPase activating protein 12

Match to Query 2562: 3078.365142 from(1027.128990,3+)
Title: 100101RatKid_NS_deglyco_24.3205.3205.3.dta
Data file K:\NewmanPaper\Piliang\3SubProteomes\Piliang3SP\mgf5ppm\ERLIC_3SubProteomes5ppm.mgf

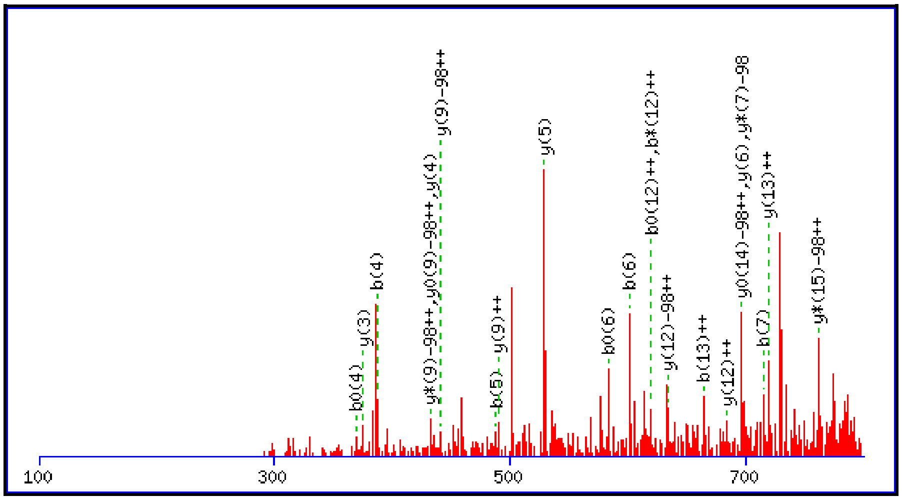

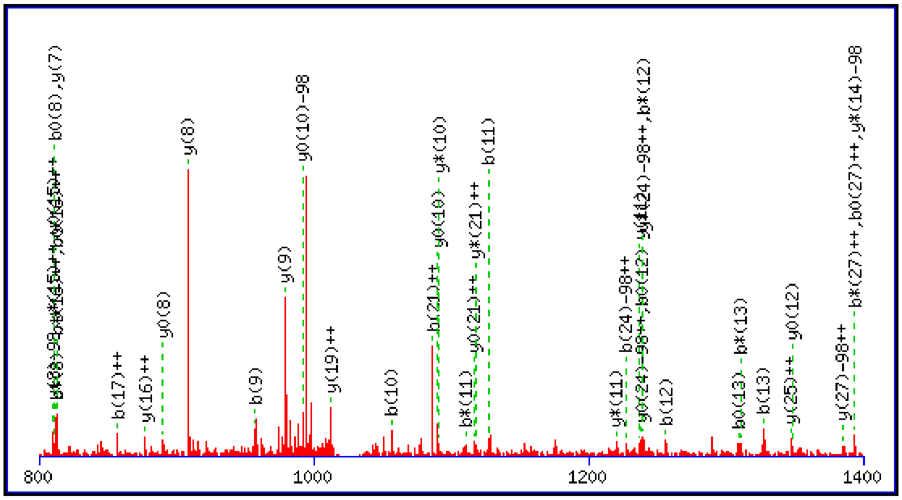

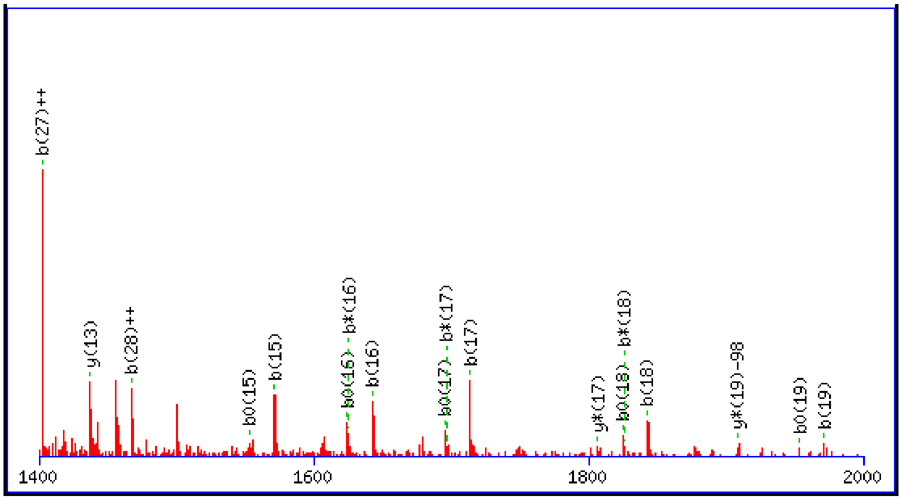

Monoisotopic mass of neutral peptide Mr(calc): 3078.3554
Fixed modifications: Carbamidomethyl (C)
Variable modifications:
S24 : Phospho (ST), with neutral losses 0.0000(shown in table), 97.9769
Ions Score: 56 **Expect:** 0.0012
Matches (Bold Red): 82/486 fragment ions using 155 most intense peaks

#	b	b^{++}	b*	b^{*++}	b^0	b^{0++}	Seq.	y	y^{++}	y*	y^{*++}	y^0	y^{0++}	#
1	100.0757	50.5415					V							29
2	213.1598	107.0835					L	2980.2943	1490.6508	2963.2677	1482.1375	2962.2837	1481.6455	28
3	300.1918	150.5995			282.1812	141.5942	S	2867.2102	1434.1087	2850.1837	1425.5955	2849.1997	1425.1035	27
4	**387.2238**	194.1155			**369.2132**	185.1103	S	2780.1782	1390.5927	2763.1516	1382.0795	2762.1676	1381.5875	26
5	**488.2715**	244.6394			470.2609	235.6341	T	2693.1462	**1347.0767**	2676.1196	1338.5634	2675.1356	1338.0714	25
6	**601.3556**	301.1814			**583.3450**	292.1761	I	2592.0985	1296.5529	2575.0719	1288.0396	2574.0879	1287.5476	24
7	**715.3985**	358.2029	698.3719	349.6896	697.3879	349.1976	N	2479.0144	1240.0108	2461.9879	1231.4976	2461.0039	1231.0056	23
8	829.4414	415.2243	**812.4149**	406.7111	**811.4308**	406.2191	N	2364.9715	1182.9894	2347.9449	1174.4761	2346.9609	1173.9841	22
9	**957.5000**	479.2536	940.4734	470.7404	939.4894	470.2483	Q	2250.9286	1125.9679	2233.9020	**1117.4546**	2232.9180	**1116.9626**	21
10	**1056.5684**	528.7878	1039.5419	520.2746	1038.5578	519.7826	V	2122.8700	1061.9386	2105.8434	1053.4254	2104.8594	1052.9334	20
11	**1127.6055**	564.3064	**1110.5790**	555.7931	1109.5949	555.3011	A	2023.8016	**1012.4044**	2006.7750	1003.8912	2005.7910	1003.3991	19
12	**1256.6481**	628.8277	**1239.6216**	**620.3144**	**1238.6375**	**619.8224**	E	1952.7645	976.8859	1935.7379	968.3726	1934.7539	967.8806	18
13	**1327.6852**	**664.3462**	**1310.6587**	655.8330	**1309.6747**	655.3410	A	1823.7219	912.3646	**1806.6953**	903.8513	1805.7113	903.3593	17
14	1442.7122	721.8597	1425.6856	713.3464	1424.7016	712.8544	D	1752.6848	**876.8460**	1735.6582	868.3327	1734.6742	867.8407	16
15	**1571.7548**	786.3810	1554.7282	777.8677	**1553.7442**	777.3757	E	1637.6578	819.3325	1620.6313	**810.8193**	1619.6472	**810.3273**	15
16	**1642.7919**	821.8996	**1625.7653**	**813.3863**	**1624.7813**	**812.8943**	A	1508.6152	754.8112	1491.5887	746.2980	1490.6047	745.8060	14
17	**1713.8290**	**857.4181**	**1696.8024**	848.9049	**1695.8184**	848.4128	A	**1437.5781**	**719.2927**	1420.5516	710.7794	1419.5675	710.2874	13
18	**1842.8716**	921.9394	**1825.8450**	913.4262	**1824.8610**	912.9341	E	1366.5410	**683.7741**	1349.5144	675.2609	**1348.5304**	674.7689	12
19	**1971.9142**	986.4607	1954.8876	977.9474	**1953.9036**	977.4554	E	**1237.4984**	619.2528	**1220.4718**	610.7396	1219.4878	610.2476	11
20	2100.9568	1050.9820	2083.9302	1042.4687	2082.9462	1041.9767	E	1108.4558	554.7315	**1091.4293**	546.2183	**1090.4452**	545.7263	10
21	2171.9939	**1086.5006**	2154.9673	1077.9873	2153.9833	1077.4953	A	**979.4132**	**490.2102**	962.3867	481.6970	961.4026	481.2050	9
22	2269.0466	1135.0270	2252.0201	1126.5137	2251.0361	1126.0217	P	**908.3761**	454.6917	891.3495	446.1784	**890.3655**	445.6864	8
23	2384.0736	1192.5404	2367.0470	1184.0272	2366.0630	1183.5351	D	**811.3233**	406.1653	794.2968	397.6520	793.3128	397.1600	7
24	2551.0719	1276.0396	2534.0454	1267.5263	2533.0614	1267.0343	S	**696.2964**	348.6518	679.2698	340.1386	678.2858	339.6466	6
25	2648.1247	1324.5660	2631.0982	1316.0527	2630.1141	1315.5607	P	**529.2980**	265.1527	512.2715	256.6394	511.2875	256.1474	5
26	2705.1462	1353.0767	2688.1196	1344.5634	2687.1356	1344.0714	G	**432.2453**	216.6263	415.2187	208.1130	414.2347	207.6210	4
27	2804.2146	**1402.6109**	2787.1880	**1394.0977**	2786.2040	**1393.6056**	V	**375.2238**	188.1155	358.1973	179.6023	357.2132	179.1103	3
28	2933.2572	**1467.1322**	2916.2306	1458.6189	2915.2466	1458.1269	E	276.1554	138.5813	259.1288	130.0681	258.1448	129.5761	2
29							K	147.1128	74.0600	130.0863	65.5468			1

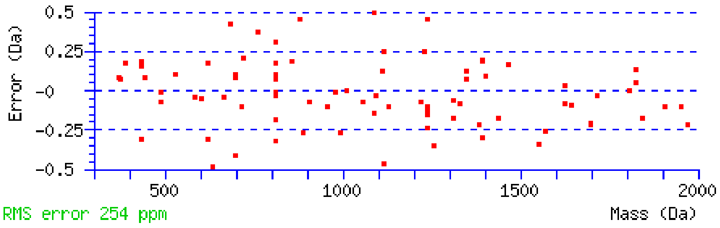

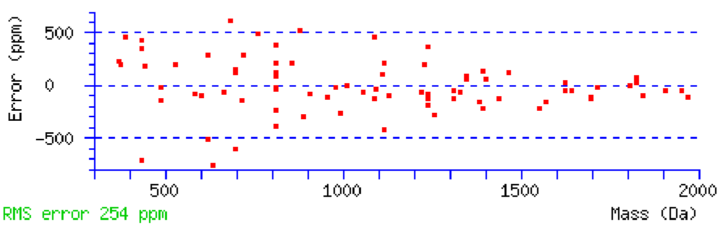

All matches to this query

Score	Mr(calc):	Delta	Sequence
55.5	3078.3554	0.0098	VLSSTINNQVAEADEAAEEEAPDSPGVEK
12.3	3077.3361	1.0291	LPSARGAAAAPVAPMSPRPVTATPAASAR
12.3	3077.3361	1.0291	LPSARGAAAAPVAPMSPRPVTATPAASAR
6.0	3078.3554	0.0098	VLSSTINNQVAEADEAAEEEAPDSPGVEK
5.5	3078.3554	0.0098	VLSSTINNQVAEADEAAEEEAPDSPGVEK
5.2	3078.3554	0.0098	VLSSTINNQVAEADEAAEEEAPDSPGVEK
3.8	3076.3671	1.9980	MIRAFAQPSTPPYQPLSSCLSEDTER
3.8	3077.3714	0.9938	ESTEVFARDSPITVSDSSESNEATPSKK
3.8	3078.3554	0.0098	ESTEVFARDSPITVSDSSESNEATPSKK
3.8	3077.3714	0.9938	ESTEVFARDSPITVSDSSESNEATPSKK

Spectrum No: 218; Query: 1572; Rank: 1

Peptide View

MS/MS Fragmentation of **VDPSLMEDSDDGPSLPTK**
Found in **IPI00202231**, Tax_Id=10116 Gene_Symbol=RGD1306324 Protein RER1

Match to Query 1572: 1997.823408 from(999.918980,2+)
Title: 091008RatKidney_NH4Format01_25.1857.1857.2.dta
Data file K:\NewmanPaper\Piliang\3SubProteomes\Piliang3SP\mgf5ppm\ERLIC_3SubProteomes5ppm.mgf

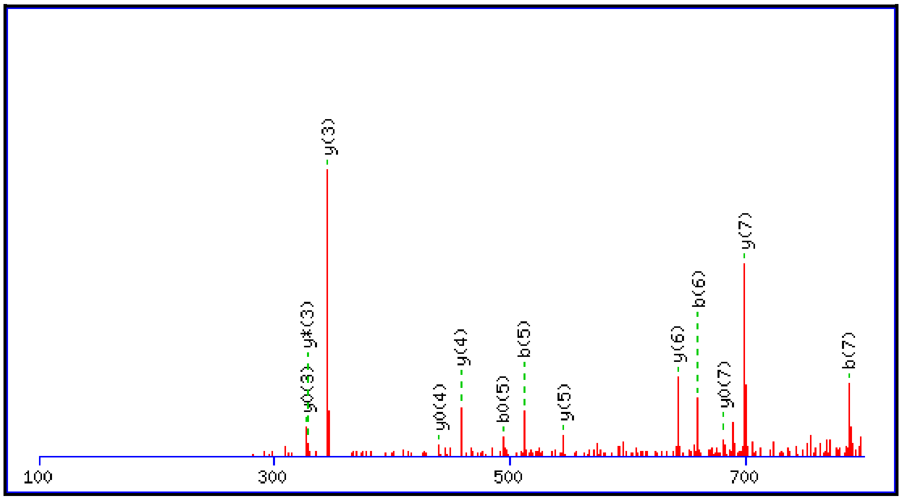

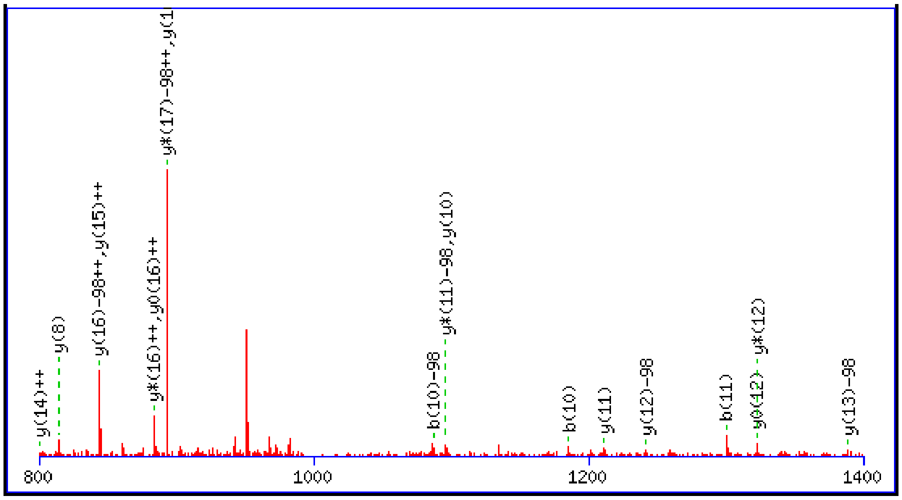

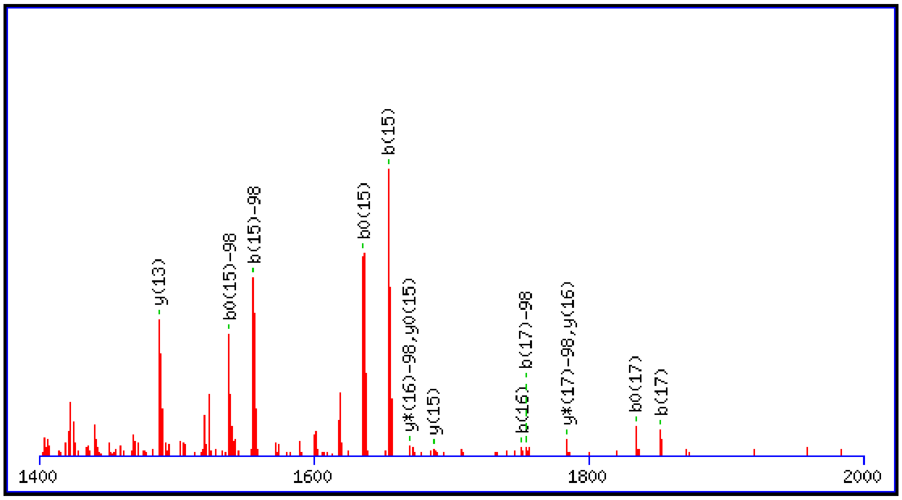

Monoisotopic mass of neutral peptide Mr(calc): 1997.8177
Fixed modifications: Carbamidomethyl (C)
Variable modifications:
M6 : Oxidation (M)
S9 : Phospho (ST), with neutral losses 0.0000(shown in table), 97.9769
Ions Score: 56 **Expect:** 0.00056
Matches (Bold Red): 45/250 fragment ions using 78 most intense peaks

#	b	b^{++}	b^{0}	b^{0++}	Seq.	y	y^{++}	y*	y^{*++}	y^{0}	y^{0++}	#
1	100.0757	50.5415			V							18
2	215.1026	108.0550	197.0921	99.0497	D	1899.7565	950.3819	1882.7300	941.8686	1881.7460	941.3766	17
3	312.1554	156.5813	294.1448	147.5761	P	**1784.7296**	**892.8684**	1767.7031	**884.3552**	1766.7190	**883.8632**	16
4	399.1874	200.0974	381.1769	191.0921	S	**1687.6768**	**844.3421**	1670.6503	835.8288	**1669.6663**	835.3368	15
5	**512.2715**	256.6394	**494.2609**	247.6341	L	1600.6448	**800.8260**	1583.6183	792.3128	1582.6342	791.8208	14
6	**659.3069**	330.1571	641.2963	321.1518	M	**1487.5607**	744.2840	1470.5342	735.7707	1469.5502	735.2787	13
7	**788.3495**	394.6784	770.3389	385.6731	E	1340.5253	670.7663	**1323.4988**	662.2530	**1322.5148**	661.7610	12
8	903.3764	452.1919	885.3659	443.1866	D	**1211.4827**	606.2450	1194.4562	597.7317	1193.4722	597.2397	11
9	1070.3748	535.6910	1052.3642	526.6857	S	**1096.4558**	548.7315	1079.4293	540.2183	1078.4452	539.7263	10
10	**1185.4017**	593.2045	1167.3912	584.1992	D	929.4575	465.2324	912.4309	456.7191	911.4469	456.2271	9
11	**1300.4287**	650.7180	1282.4181	641.7127	D	**814.4305**	407.7189	797.4040	399.2056	796.4199	398.7136	8
12	1357.4501	679.2287	1339.4396	670.2234	G	**699.4036**	350.2054	682.3770	341.6921	**681.3930**	341.2001	7
13	1454.5029	727.7551	1436.4923	718.7498	P	**642.3821**	321.6947	625.3556	313.1814	624.3715	312.6894	6
14	1541.5349	771.2711	1523.5244	762.2658	S	**545.3293**	273.1683	528.3028	264.6550	527.3188	264.1630	5
15	**1654.6190**	827.8131	**1636.6084**	818.8078	L	**458.2973**	229.6523	441.2708	221.1390	**440.2867**	220.6470	4
16	**1751.6718**	876.3395	1733.6612	867.3342	P	**345.2132**	173.1103	**328.1867**	164.5970	**327.2027**	164.1050	3
17	**1852.7194**	926.8634	**1834.7089**	917.8581	T	248.1605	124.5839	231.1339	116.0706	230.1499	115.5786	2
18					K	147.1128	74.0600	130.0863	65.5468			1

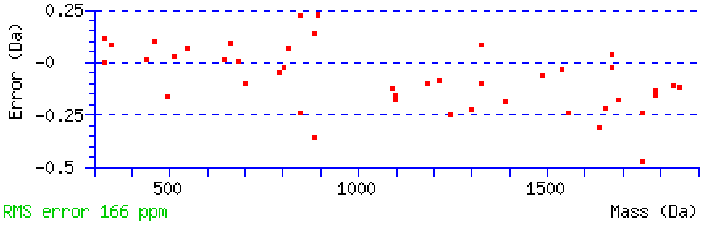

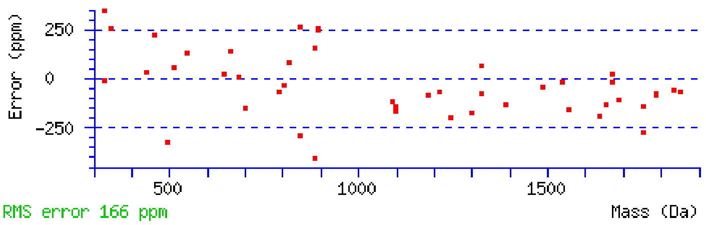

All matches to this query

Score	Mr(calc):	Delta	Sequence
55.5	1997.8177	0.0057	VDPSLMEDSDDGPSLPTK
31.1	1997.8177	0.0057	VDPSLMEDSDDGPSLPTK
29.1	1997.8177	0.0057	VDPSLMEDSDDGPSLPTK
14.8	1997.8177	0.0057	VDPSLMEDSDDGPSLPTK
4.6	1995.8261	1.9973	IETTESTSAVHKASTMK
4.4	1997.8226	0.0008	KSELGLGWWWGNSYR
2.9	1997.8325	-0.0091	LVINTQSFPNNYWDK
2.5	1997.8067	0.0167	MVTSKNRTTNNQYHK
2.5	1997.8067	0.0167	MVTSKNRTTNNQYHK
2.2	1995.8227	2.0007	EDSDFKRLPDSALSEK

Spectrum No: 219; Query: 1430; Rank: 1

Peptide View

MS/MS Fragmentation of **ADSEGDLSENDDGAGDLR**
Found in **IPI00204776**, Tax_Id=10116 Gene_Symbol=Akap8 A-kinase anchor protein 8

Match to Query 1430: 1914.711288 from(958.362920,2+)
Title: 091008RatKidney_NoSalt_30.1225.1225.2.dta
Data file K:\NewmanPaper\Piliang\3SubProteomes\Piliang3SP\mgf5ppm\ERLIC_3SubProteomes5ppm.mgf

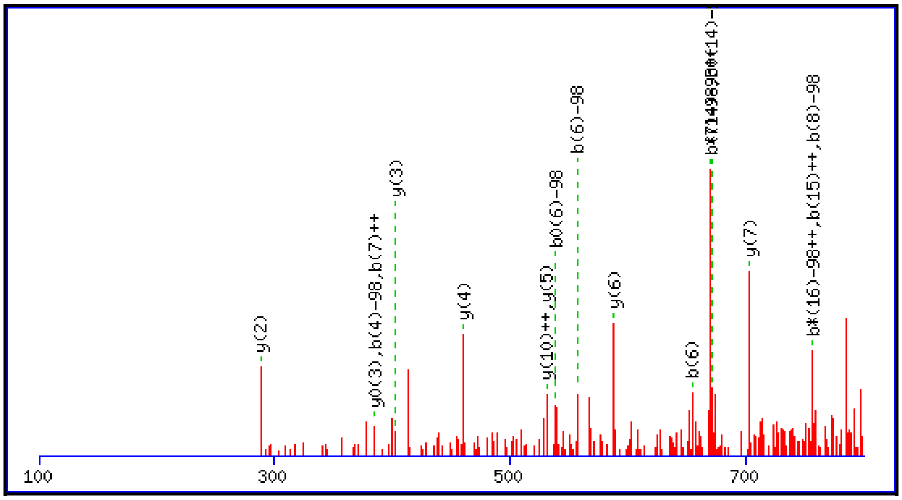

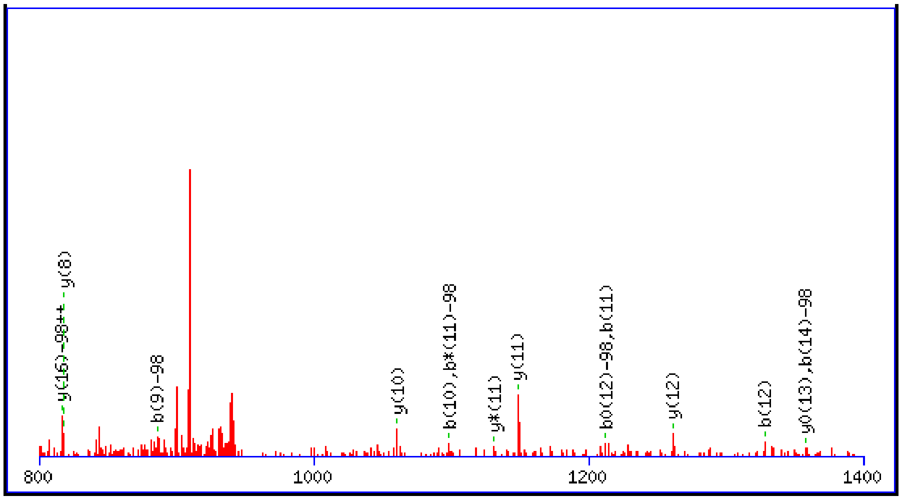

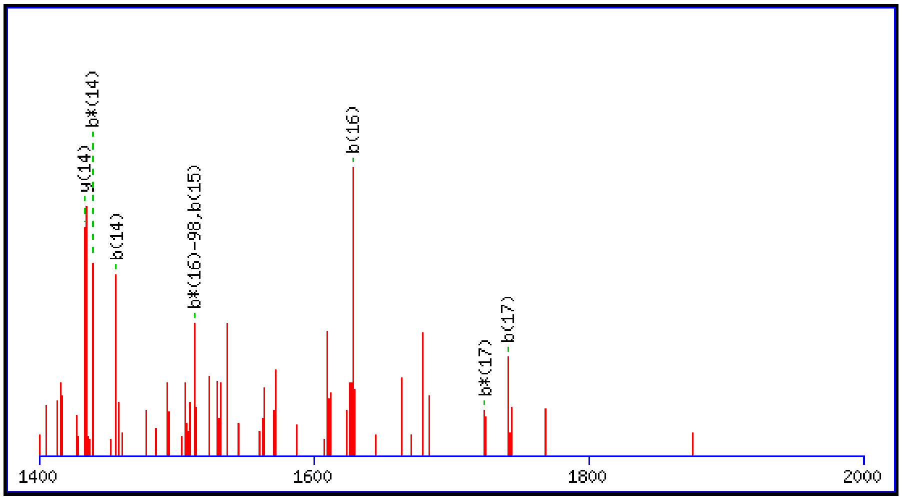

Monoisotopic mass of neutral peptide Mr(calc): 1914.7116
Fixed modifications: Carbamidomethyl (C)
Variable modifications:
S3 : Phospho (ST), with neutral losses 97.9769(shown in table), 0.0000
Ions Score: 55 **Expect:** 0.00031
Matches (Bold Red): 41/268 fragment ions using 66 most intense peaks

#	b	b^{++}	b*	b^{*++}	b^0	b^{0++}	Seq.	y	y^{++}	y*	y^{*++}	y^0	y^{0++}	#
1	72.0444	36.5258					A							18
2	187.0713	94.0393			169.0608	85.0340	D	1746.7049	873.8561	1729.6783	865.3428	1728.6943	864.8508	17
3	256.0928	128.5500			238.0822	119.5448	S	1631.6780	**816.3426**	1614.6514	807.8293	1613.6674	807.3373	16
4	**385.1354**	193.0713			367.1248	184.0660	E	1562.6565	781.8319	1545.6300	773.3186	1544.6459	772.8266	15
5	442.1568	221.5821			424.1463	212.5768	G	**1433.6139**	717.3106	1416.5874	708.7973	1415.6033	708.3053	14
6	**557.1838**	279.0955			**539.1732**	270.0903	D	1376.5924	688.7999	1359.5659	680.2866	**1358.5819**	679.7946	13
7	**670.2679**	335.6376			652.2573	326.6323	L	**1261.5655**	631.2864	1244.5390	622.7731	1243.5549	622.2811	12
8	**757.2999**	379.1536			739.2893	370.1483	S	**1148.4814**	574.7444	**1131.4549**	566.2311	1130.4709	565.7391	11
9	**886.3425**	443.6749			868.3319	434.6696	E	**1061.4494**	**531.2283**	1044.4229	522.7151	1043.4388	522.2231	10
10	1000.3854	500.6963	983.3589	492.1831	982.3748	491.6911	N	932.4068	466.7070	915.3803	458.1938	914.3962	457.7018	9
11	1115.4123	558.2098	**1098.3858**	549.6965	1097.4018	549.2045	D	**818.3639**	409.6856	801.3373	401.1723	800.3533	400.6803	8
12	1230.4393	615.7233	1213.4127	607.2100	**1212.4287**	606.7180	D	**703.3369**	352.1721	686.3104	343.6588	685.3264	343.1668	7
13	1287.4608	644.2340	1270.4342	635.7207	1269.4502	635.2287	G	**588.3100**	294.6586	571.2835	286.1454	570.2994	285.6534	6
14	**1358.4979**	679.7526	1341.4713	**671.2393**	1340.4873	**670.7473**	A	**531.2885**	266.1479	514.2620	257.6346	513.2780	257.1426	5
15	1415.5193	708.2633	1398.4928	699.7500	1397.5088	699.2580	G	**460.2514**	230.6293	443.2249	222.1161	442.2409	221.6241	4
16	1530.5463	765.7768	**1513.5197**	**757.2635**	1512.5357	756.7715	D	**403.2300**	202.1186	386.2034	193.6053	**385.2194**	193.1133	3
17	1643.6303	822.3188	1626.6038	813.8055	1625.6198	813.3135	L	**288.2030**	144.6051	271.1765	136.0919			2
18							R	175.1190	88.0631	158.0924	79.5498			1

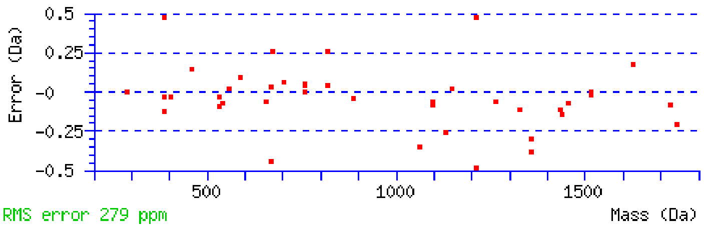

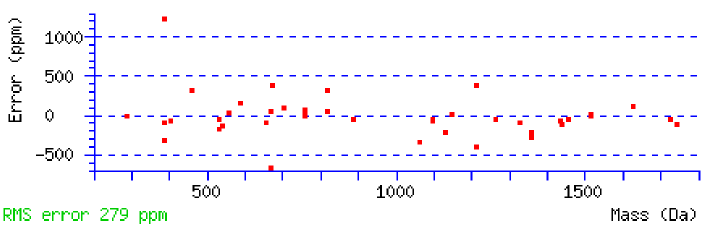

All matches to this query

Score	Mr(calc):	Delta	Sequence
55.4	1914.7116	-0.0003	ADSEGDLSENDDGAGDLR
36.3	1914.7116	-0.0003	ADSEGDLSENDDGAGDLR

Spectrum No: 220; Query: 2259; Rank: 1

Peptide View

MS/MS Fragmentation of **ESDDKPEIEDVGSDEEEEEKK**
Found in **IPI00210566**, Tax_Id=10116 Gene_Symbol=Hspca Heat shock protein HSP 90-alpha

Match to Query 2259: 2515.010172 from(839.344000,3+)
Title: 091008RatKidney_NH4Format01_24.1220.1220.3.dta
Data file K:\NewmanPaper\Piliang\3SubProteomes\Piliang3SP\mgf5ppm\ERLIC_3SubProteomes5ppm.mgf

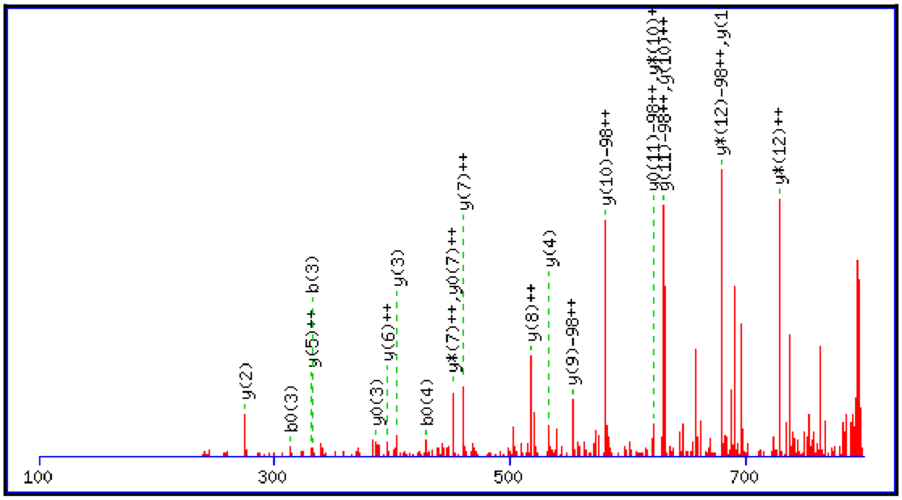

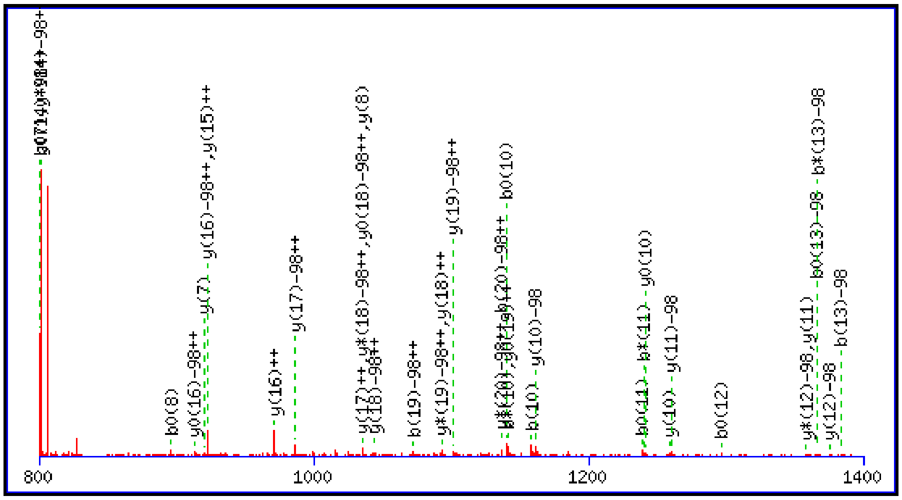

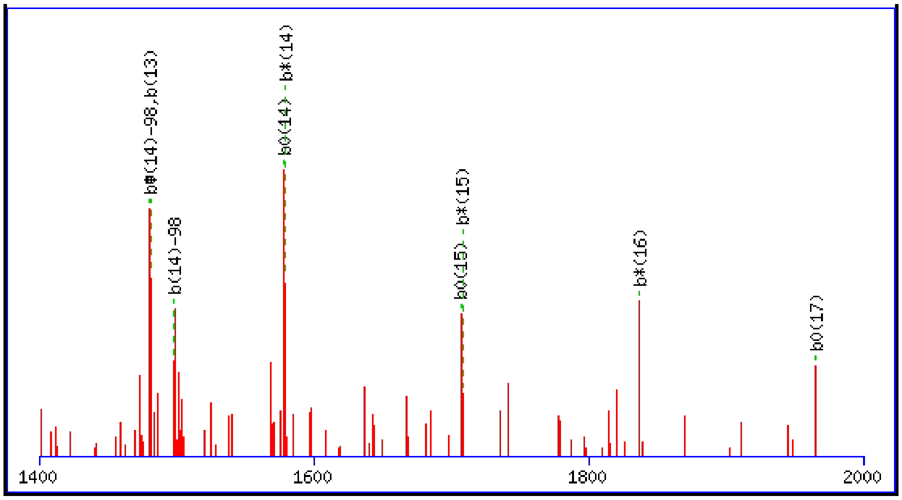

Monoisotopic mass of neutral peptide Mr(calc): 2515.0010
Fixed modifications: Carbamidomethyl (C)
Variable modifications:
S13 : Phospho (ST), with neutral losses 97.9769(shown in table), 0.0000
Ions Score: 55 **Expect:** 0.00075
Matches (Bold Red): 72/348 fragment ions using 87 most intense peaks

#	b	b^{++}	b^{*}	b^{*++}	b^{0}	b^{0++}	Seq.	y	y^{++}	y^{*}	y^{*++}	y^{0}	y^{0++}	#
1	130.0499	65.5286			112.0393	56.5233	E							21
2	217.0819	109.0446			199.0713	100.0393	S	2288.9888	1144.9981	2271.9623	**1136.4848**	2270.9783	1135.9928	20
3	**332.1088**	166.5581			**314.0983**	157.5528	D	2201.9568	**1101.4820**	2184.9303	**1092.9688**	2183.9463	1092.4768	19
4	447.1358	224.0715			**429.1252**	215.0662	D	2086.9299	**1043.9686**	2069.9033	**1035.4553**	2068.9193	**1034.9633**	18
5	575.2307	288.1190	558.2042	279.6057	557.2202	279.1137	K	1971.9029	**986.4551**	1954.8764	977.9418	1953.8924	977.4498	17
6	672.2835	336.6454	655.2570	328.1321	654.2729	327.6401	P	1843.8080	**922.4076**	1826.7814	913.8943	1825.7974	**913.4023**	16
7	**801.3261**	401.1667	784.2996	392.6534	783.3155	392.1614	E	1746.7552	873.8812	1729.7287	865.3680	1728.7446	864.8760	15
8	914.4102	457.7087	897.3836	449.1954	**896.3996**	448.7034	I	1617.7126	809.3599	1600.6861	**800.8467**	1599.7020	**800.3547**	14
9	1043.4528	522.2300	1026.4262	513.7167	1025.4422	513.2247	E	1504.6285	752.8179	1487.6020	744.3046	1486.6180	743.8126	13
10	**1158.4797**	579.7435	**1141.4532**	571.2302	**1140.4691**	570.7382	D	**1375.5860**	688.2966	**1358.5594**	**679.7833**	1357.5754	**679.2913**	12
11	1257.5481	629.2777	**1240.5216**	620.7644	**1239.5376**	620.2724	V	**1260.5590**	**630.7831**	1243.5325	**622.2699**	1242.5484	**621.7779**	11
12	1314.5696	657.7884	1297.5430	649.2752	**1296.5590**	648.7831	G	**1161.4906**	**581.2489**	1144.4640	572.7357	1143.4800	572.2437	10
13	**1383.5910**	692.2992	**1366.5645**	683.7859	**1365.5805**	683.2939	S	1104.4691	**552.7382**	1087.4426	544.2249	1086.4586	543.7329	9
14	**1498.6180**	749.8126	**1481.5914**	741.2994	**1480.6074**	740.8073	D	**1035.4477**	**518.2275**	1018.4211	509.7142	1017.4371	509.2222	8
15	1627.6606	814.3339	1610.6340	805.8207	1609.6500	805.3286	E	**920.4207**	**460.7140**	903.3942	**452.2007**	902.4102	**451.7087**	7
16	1756.7032	878.8552	1739.6766	870.3419	1738.6926	869.8499	E	791.3781	**396.1927**	774.3516	387.6794	773.3676	387.1874	6
17	1885.7458	943.3765	1868.7192	934.8632	1867.7352	934.3712	E	662.3355	**331.6714**	645.3090	323.1581	644.3250	322.6661	5
18	2014.7884	1007.8978	1997.7618	999.3845	1996.7778	998.8925	E	**533.2930**	267.1501	516.2664	258.6368	515.2824	258.1448	4
19	2143.8309	**1072.4191**	2126.8044	1063.9058	2125.8204	1063.4138	E	**404.2504**	202.6288	387.2238	194.1155	**386.2398**	193.6235	3
20	2271.9259	**1136.4666**	2254.8994	1127.9533	2253.9153	1127.4613	K	**275.2078**	138.1075	258.1812	129.5942			2
21							K	147.1128	74.0600	130.0863	65.5468			1

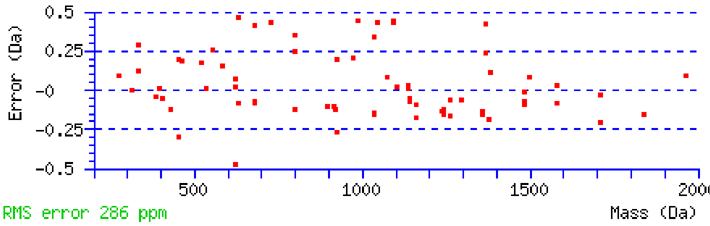

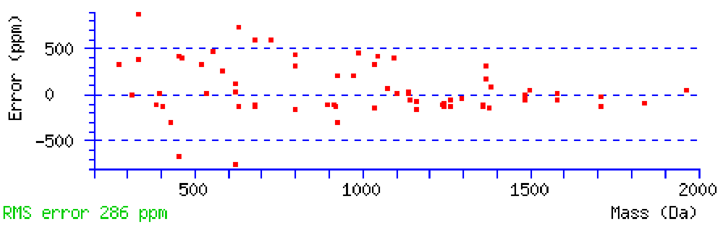

All matches to this query

Score	Mr(calc):	Delta	Sequence

55.4	2515.0010	0.0092	ESDDKPEIEDVGSDEEEEEKK
19.2	2515.0010	0.0092	ESDDKPEIEDVGSDEEEEEKK
5.6	2515.0225	-0.0123	YMNNWDNIFSFGEKMIHQK
4.0	2513.9919	1.0183	LPDTLEDSKSSDSHSDSDDEQK
2.6	2512.9788	2.0314	LEPIPEELEEDTSATDEKTK
2.4	2514.9892	0.0209	FITTATPENRDHTMENLIR
1.4	2512.9988	2.0114	LLSGPGTAASDTPEYGMKTPHK
1.2	2514.9926	0.0175	NGTVMAPDLPEMLDLAGTRSR
1.0	2513.9902	1.0200	YVTTCAFAPNTLLLATGSMDK
1.0	2513.0259	1.9843	YTEVFPPAPVRPAYSFYNR

Spectrum No: 221; Query: 2038; Rank: 1

Peptide View

MS/MS Fragmentation of **KLEKEEEEGISQESSEEEQ**
Found in **IPI00203725**, Tax_Id=10116 Gene_Symbol=Hmga1 Isoform HMG-I of High mobility group protein HMG-I/HMG-Y

Match to Query 2038: 2315.955702 from(772.992510,3+)
Title: 091008RatKidney_NoSalt_27.731.731.3.dta
Data file K:\NewmanPaper\Piliang\3SubProteomes\Piliang3SP\mgf5ppm\ERLIC_3SubProteomes5ppm.mgf

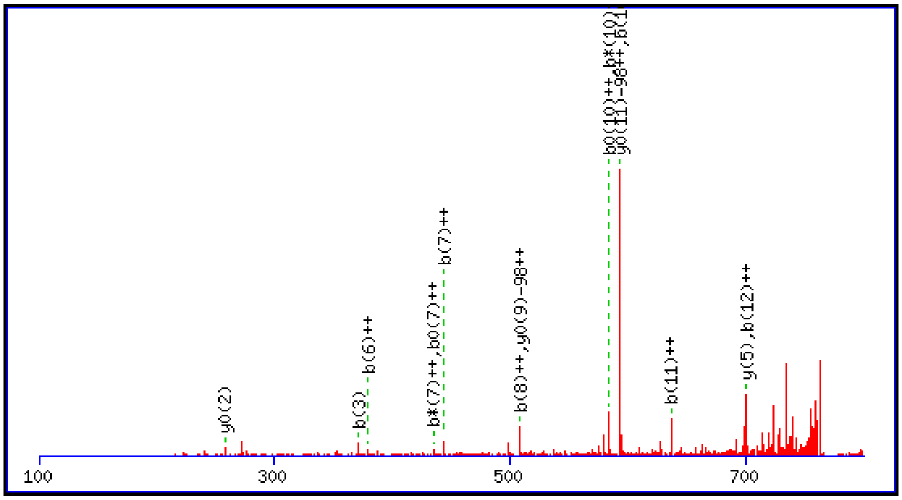

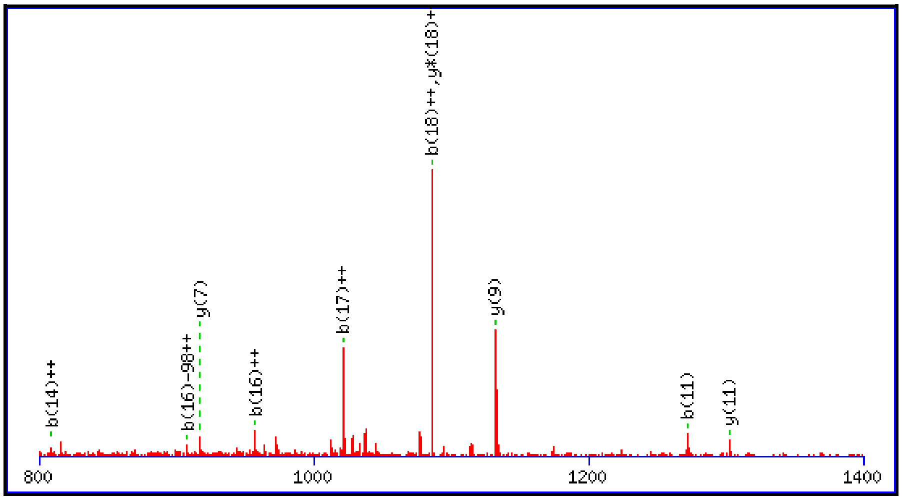

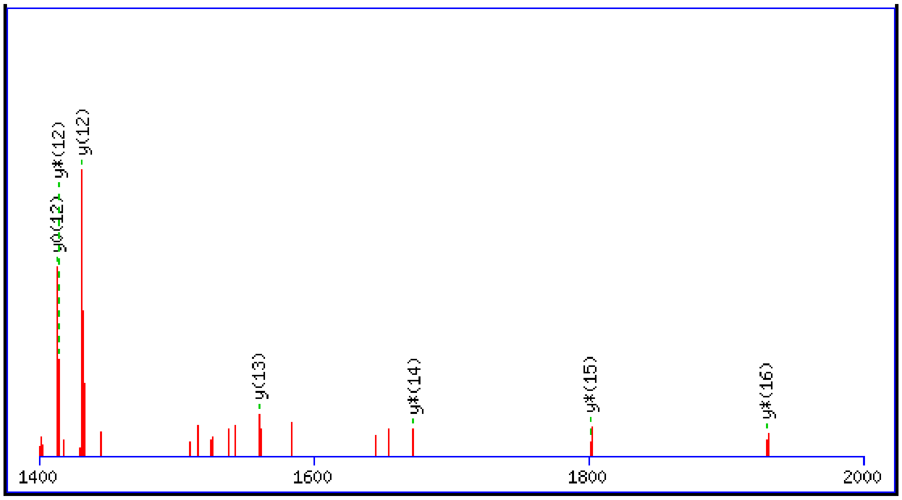

Monoisotopic mass of neutral peptide Mr(calc): 2315.9529
Fixed modifications: Carbamidomethyl (C)
Variable modifications:
S15 : Phospho (ST), with neutral losses 0.0000(shown in table), 97.9769
Ions Score: 55 **Expect:** 0.00082
Matches (Bold Red): 33/318 fragment ions using 39 most intense peaks

#	b	b^{++}	b*	b^{*++}	b^0	b^{0++}	Seq.	y	y^{++}	y*	y^{*++}	y^0	y^{0++}	#
1	129.1022	65.0548	112.0757	56.5415			K							19
2	242.1863	121.5968	225.1598	113.0835			L	2188.8653	1094.9363	2171.8387	**1086.4230**	2170.8547	**1085.9310**	18
3	**371.2289**	186.1181	354.2023	177.6048	353.2183	177.1128	E	2075.7812	1038.3943	2058.7547	1029.8810	2057.7707	1029.3890	17
4	499.3239	250.1656	482.2973	241.6523	481.3133	241.1603	K	1946.7386	973.8730	**1929.7121**	965.3597	1928.7281	964.8677	16
5	628.3665	314.6869	611.3399	306.1736	610.3559	305.6816	E	1818.6437	909.8255	**1801.6171**	901.3122	1800.6331	900.8202	15
6	757.4090	**379.2082**	740.3825	370.6949	739.3985	370.2029	E	1689.6011	845.3042	**1672.5745**	836.7909	1671.5905	836.2989	14
7	886.4516	**443.7295**	869.4251	**435.2162**	868.4411	**434.7242**	E	**1560.5585**	780.7829	1543.5319	772.2696	1542.5479	771.7776	13
8	1015.4942	**508.2508**	998.4677	499.7375	997.4837	499.2455	E	**1431.5159**	716.2616	**1414.4893**	707.7483	**1413.5053**	707.2563	12
9	1072.5157	536.7615	1055.4891	528.2482	1054.5051	527.7562	G	**1302.4733**	651.7403	1285.4468	643.2270	1284.4627	642.7350	11
10	1185.5998	**593.3035**	1168.5732	**584.7902**	1167.5892	**584.2982**	I	1245.4518	623.2296	1228.4253	614.7163	1227.4413	614.2243	10
11	**1272.6318**	**636.8195**	1255.6052	628.3063	1254.6212	627.8142	S	**1132.3678**	566.6875	1115.3412	558.1743	1114.3572	557.6822	9
12	1400.6904	**700.8488**	1383.6638	692.3355	1382.6798	691.8435	Q	1045.3357	523.1715	1028.3092	514.6582	1027.3252	514.1662	8
13	1529.7330	765.3701	1512.7064	756.8568	1511.7224	756.3648	E	**917.2772**	459.1422	900.2506	450.6289	899.2666	450.1369	7
14	1616.7650	**808.8861**	1599.7384	800.3729	1598.7544	799.8808	S	788.2346	394.6209	771.2080	386.1077	770.2240	385.6156	6
15	1783.7633	892.3853	1766.7368	883.8720	1765.7528	883.3800	S	**701.2026**	351.1049	684.1760	342.5916	683.1920	342.0996	5
16	1912.8059	**956.9066**	1895.7794	948.3933	1894.7954	947.9013	E	534.2042	267.6057	517.1776	259.0925	516.1936	258.6005	4
17	2041.8485	**1021.4279**	2024.8220	1012.9146	2023.8380	1012.4226	E	405.1616	203.0844	388.1351	194.5712	387.1510	194.0792	3
18	2170.8911	**1085.9492**	2153.8646	1077.4359	2152.8806	1076.9439	E	276.1190	138.5631	259.0925	130.0499	**258.1084**	129.5579	2
19							Q	147.0764	74.0418	130.0499	65.5286			1

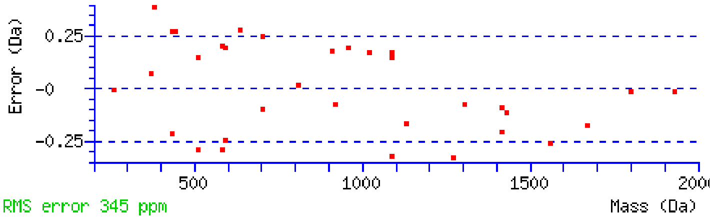

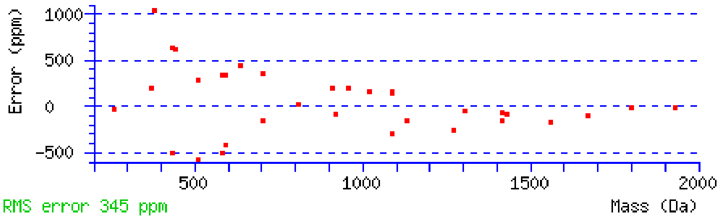

All matches to this query

Score	Mr(calc):	Delta	Sequence
55.3	2315.9529	0.0028	KLEKEEEEGISQESSEEEQ
55.3	2315.9529	0.0028	KLEKEEEEGISQESSEEEQ

28.3	2315.9529	0.0028	KLEKEEEEGISQESSEEEQ
12.1	2315.9388	0.0169	EKYPYHSFIGEESVASGEK
12.1	2315.9388	0.0169	EKYPYHSFIGEESVASGEK
11.8	2315.9388	0.0169	EKYPYHSFIGEESVASGEK
11.6	2315.9388	0.0169	EKYPYHSFIGEESVASGEK
11.6	2315.9388	0.0169	EKYPYHSFIGEESVASGEK
11.6	2315.9388	0.0169	EKYPYHSFIGEESVASGEK
9.5	2314.9694	0.9863	SYCAEIAPNVSFENGKTIR

Spectrum No: 222; Query: 2696; Rank: 1

Peptide View

MS/MS Fragmentation of **VASTKYEVAAQNEADEADGSAQGDGAGPAAEQVK**
Found in **IPI00470251**, Tax_Id=10116 Gene_Symbol=Slc7a7 Y+L amino acid transporter 1

Match to Query 2696: 3413.497752 from(1138.839860,3+)
Title: 100101RatKid_NS_deglyco_21.1920.1920.3.dta
Data file K:\NewmanPaper\Piliang\3SubProteomes\Piliang3SP\mgf5ppm\ERLIC_3SubProteomes5ppm.mgf

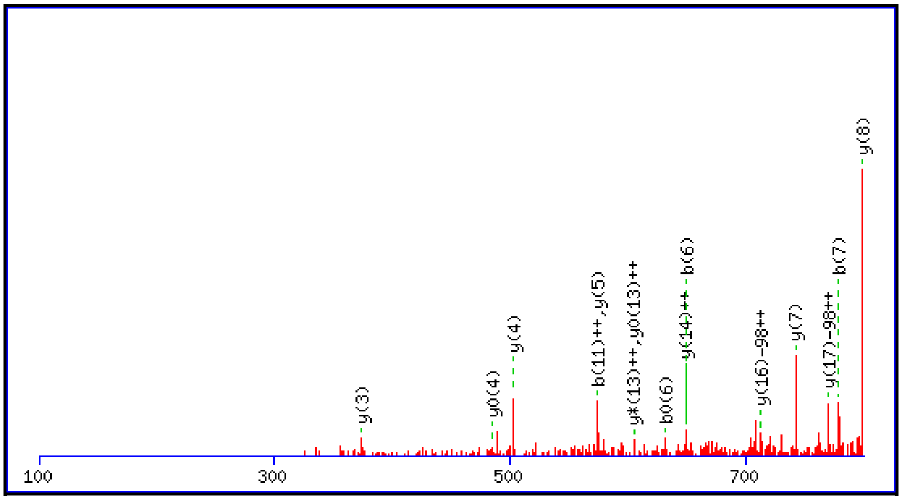

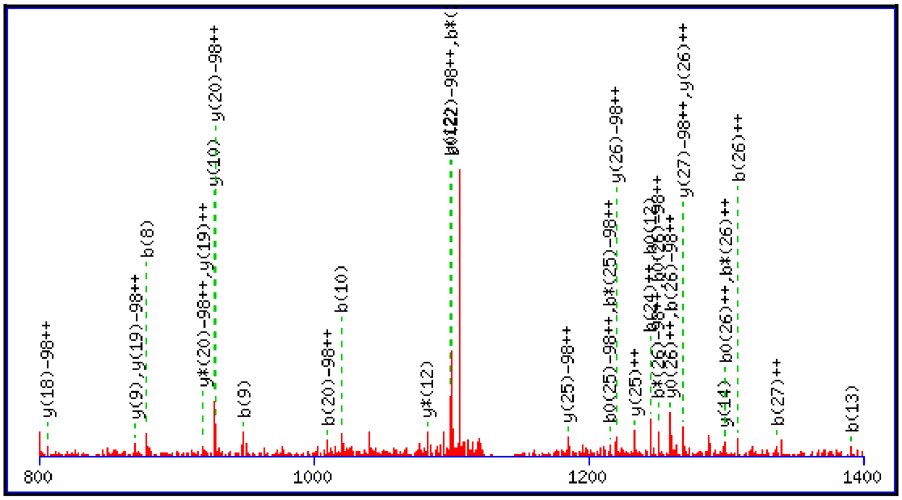

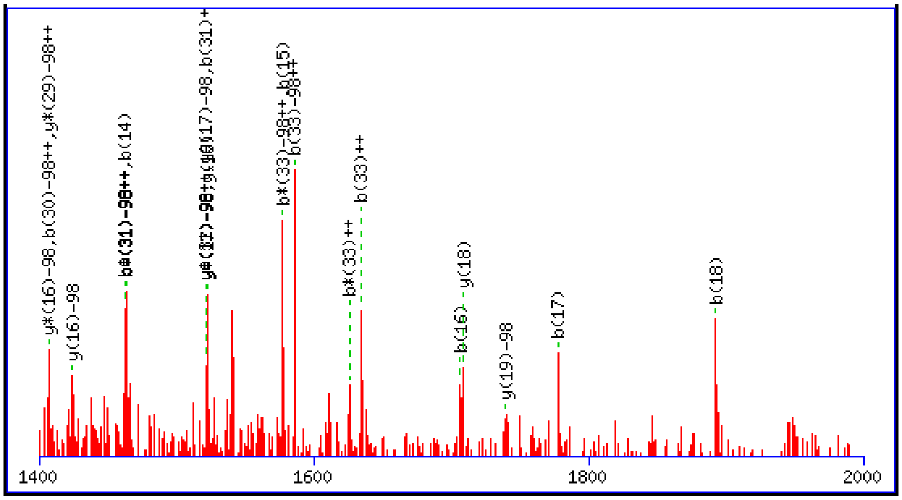

Monoisotopic mass of neutral peptide Mr(calc): 3413.4896
Fixed modifications: Carbamidomethyl (C)
Variable modifications:
S20 : Phospho (ST), with neutral losses 97.9769(shown in table), 0.0000
Ions Score: 55 **Expect:** 0.0014
Matches (Bold Red): 72/576 fragment ions using 99 most intense peaks

#	b	b^{++}	b*	b^{*++}	b^{0}	b^{0++}	Seq.	y	y^{++}	y*	y^{*++}	y^{0}	y^{0++}	#
1	100.0757	50.5415					V							34
2	171.1128	86.0600					A	3217.4516	1609.2294	3200.4250	1600.7162	3199.4410	1600.2242	33
3	258.1448	129.5761			240.1343	120.5708	S	3146.4145	1573.7109	3129.3879	1565.1976	3128.4039	1564.7056	32
4	359.1925	180.0999			341.1819	171.0946	T	3059.3825	1530.1949	3042.3559	**1521.6816**	3041.3719	**1521.1896**	31
5	487.2875	244.1474	470.2609	235.6341	469.2769	235.1421	K	2958.3348	1479.6710	2941.3082	1471.1578	2940.3242	1470.6657	30
6	**650.3508**	325.6790	633.3243	317.1658	**632.3402**	316.6738	Y	2830.2398	1415.6235	2813.2133	**1407.1103**	2812.2292	1406.6183	29
7	**779.3934**	390.2003	762.3668	381.6871	761.3828	381.1951	E	2667.1765	1334.0919	2650.1499	1325.5786	2649.1659	1325.0866	28
8	**878.4618**	439.7345	861.4353	431.2213	860.4512	430.7293	V	2538.1339	**1269.5706**	2521.1073	1261.0573	2520.1233	1260.5653	27
9	**949.4989**	475.2531	932.4724	466.7398	931.4884	466.2478	A	2439.0655	**1220.0364**	2422.0389	1211.5231	2421.0549	1211.0311	26
10	**1020.5360**	510.7717	1003.5095	502.2584	1002.5255	501.7664	A	2368.0284	**1184.5178**	2351.0018	1176.0045	2350.0178	1175.5125	25
11	1148.5946	**574.8009**	1131.5681	566.2877	1130.5841	565.7957	Q	2296.9912	1148.9993	2279.9647	1140.4860	2278.9807	1139.9940	24
12	1262.6375	631.8224	1245.6110	623.3091	**1244.6270**	622.8171	N	2168.9327	1084.9700	2151.9061	1076.4567	2150.9221	1075.9647	23
13	**1391.6801**	696.3437	1374.6536	687.8304	1373.6696	687.3384	E	2054.8897	1027.9485	2037.8632	1019.4352	2036.8792	1018.9432	22
14	**1462.7173**	731.8623	1445.6907	723.3490	1444.7067	722.8570	A	1925.8471	963.4272	1908.8206	954.9139	1907.8366	954.4219	21
15	**1577.7442**	789.3757	1560.7176	780.8625	1559.7336	780.3705	D	1854.8100	**927.9087**	1837.7835	**919.3954**	1836.7995	918.9034	20
16	**1706.7868**	853.8970	1689.7602	845.3838	1688.7762	844.8917	E	**1739.7831**	**870.3952**	1722.7565	861.8819	1721.7725	861.3899	19
17	**1777.8239**	889.4156	1760.7974	880.9023	1759.8133	880.4103	A	1610.7405	**805.8739**	1593.7140	797.3606	1592.7299	796.8686	18
18	**1892.8508**	946.9291	1875.8243	938.4158	1874.8403	937.9238	D	1539.7034	**770.3553**	**1522.6768**	761.8421	**1521.6928**	761.3500	17
19	1949.8723	975.4398	1932.8458	966.9265	1931.8617	966.4345	G	**1424.6764**	**712.8419**	**1407.6499**	704.3286	1406.6659	703.8366	16
20	2018.8938	**1009.9505**	2001.8672	1001.4372	2000.8832	1000.9452	S	1367.6550	684.3311	1350.6284	675.8179	1349.6444	675.3258	15
21	2089.9309	1045.4691	2072.9043	1036.9558	2071.9203	1036.4638	A	**1298.6335**	**649.8204**	1281.6070	641.3071	1280.6230	640.8151	14
22	2217.9895	1109.4984	2200.9629	**1100.9851**	2199.9789	**1100.4931**	Q	1227.5964	614.3018	1210.5699	**605.7886**	1209.5858	**605.2966**	13
23	2275.0109	1138.0091	2257.9844	1129.4958	2257.0004	1129.0038	G	**1099.5378**	550.2726	**1082.5113**	541.7593	1081.5273	541.2673	12
24	2390.0379	1195.5226	2373.0113	1187.0093	2372.0273	1186.5173	D	1042.5164	521.7618	1025.4898	513.2485	1024.5058	512.7565	11
25	2447.0593	1224.0333	2430.0328	**1215.5200**	2429.0488	**1215.0280**	G	**927.4894**	464.2483	910.4629	455.7351	909.4789	455.2431	10
26	2518.0964	**1259.5519**	2501.0699	**1251.0386**	2500.0859	**1250.5466**	A	**870.4680**	435.7376	853.4414	427.2243	852.4574	426.7323	9
27	2575.1179	1288.0626	2558.0914	1279.5493	2557.1073	1279.0573	G	**799.4308**	400.2191	782.4043	391.7058	781.4203	391.2138	8
28	2672.1707	1336.5890	2655.1441	1328.0757	2654.1601	1327.5837	P	**742.4094**	371.7083	725.3828	363.1951	724.3988	362.7030	7
29	2743.2078	1372.1075	2726.1812	1363.5943	2725.1972	1363.1022	A	645.3566	323.1819	628.3301	314.6687	627.3461	314.1767	6
30	2814.2449	**1407.6261**	2797.2183	1399.1128	2796.2343	1398.6208	A	**574.3195**	287.6634	557.2930	279.1501	556.3089	278.6581	5
31	2943.2875	1472.1474	2926.2609	**1463.6341**	2925.2769	**1463.1421**	E	**503.2824**	252.1448	486.2558	243.6316	**485.2718**	243.1396	4
32	3071.3461	1536.1767	3054.3195	1527.6634	3053.3355	1527.1714	Q	**374.2398**	187.6235	357.2132	179.1103			3

33	3170.4145	1585.7109	3153.3879	1577.1976	3152.4039	1576.7056	V	246.1812	123.5942	229.1547	115.0810			2
34							K	147.1128	74.0600	130.0863	65.5468			1

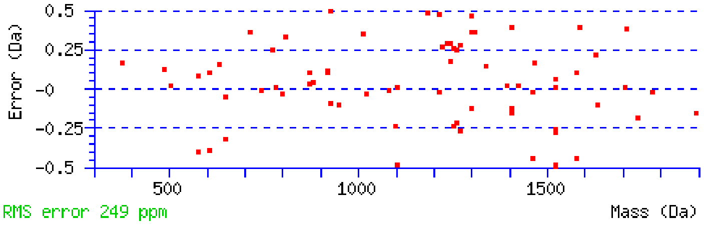

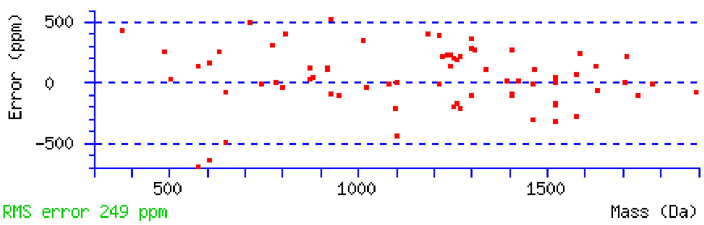

All matches to this query

Score	Mr(calc):	Delta	Sequence
55.3	3413.4896	0.0082	VASTKYEVAAQNEADEADGSAQGDGAGPAAEQVK
17.8	3413.4896	0.0082	VASTKYEVAAQNEADEADGSAQGDGAGPAAEQVK
17.8	3413.4896	0.0082	VASTKYEVAAQNEADEADGSAQGDGAGPAAEQVK
14.5	3413.4896	0.0082	VASTKYEVAAQNEADEADGSAQGDGAGPAAEQVK
11.5	3412.5083	0.9894	SRNFIYFITLSSFLTSFHNQRNPRPK
11.5	3412.5083	0.9894	SRNFIYFITLSSFLTSFHNQRNPRPK
7.3	3413.5091	-0.0113	MLEDCVQETIHEHNKLAANSDHLMQIQK
6.9	3412.5083	0.9894	SRNFIYFITLSSFLTSFHNQRNPRPK
6.6	3412.5083	0.9894	SRNFIYFITLSSFLTSFHNQRNPRPK
6.6	3412.5083	0.9894	SRNFIYFITLSSFLTSFHNQRNPRPK

Spectrum No: 223; Query: 942; Rank: 1

Peptide View

MS/MS Fragmentation of **SQDSYPGSPSLSPR**
Found in **IPI00365184**, Tax_Id=10116 Gene_Symbol=Ranbp10_predicted similar to Ran-binding protein 10

Match to Query 942: 1636.619548 from(819.317050,2+)
Title: 100101RatKid_NS_deglyco_27.1426.1426.2.dta
Data file K:\NewmanPaper\Piliang\3SubProteomes\Piliang3SP\mgf5ppm\ERLIC_3SubProteomes5ppm.mgf

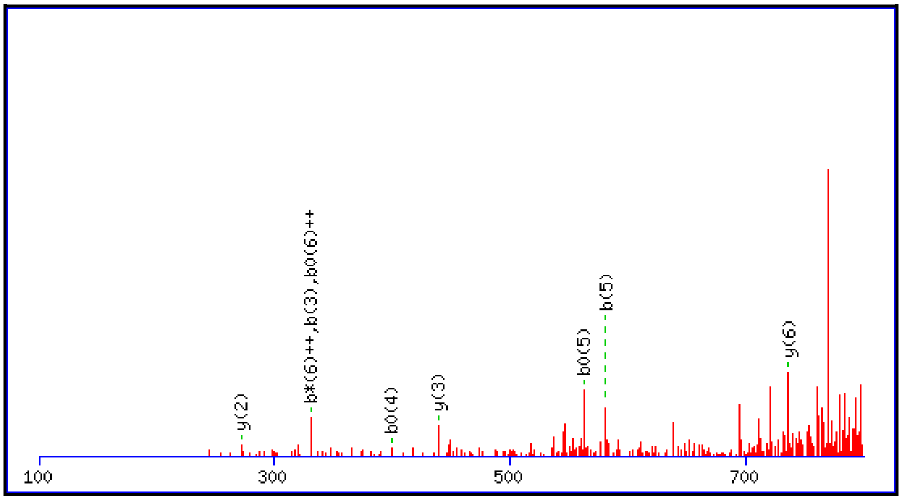

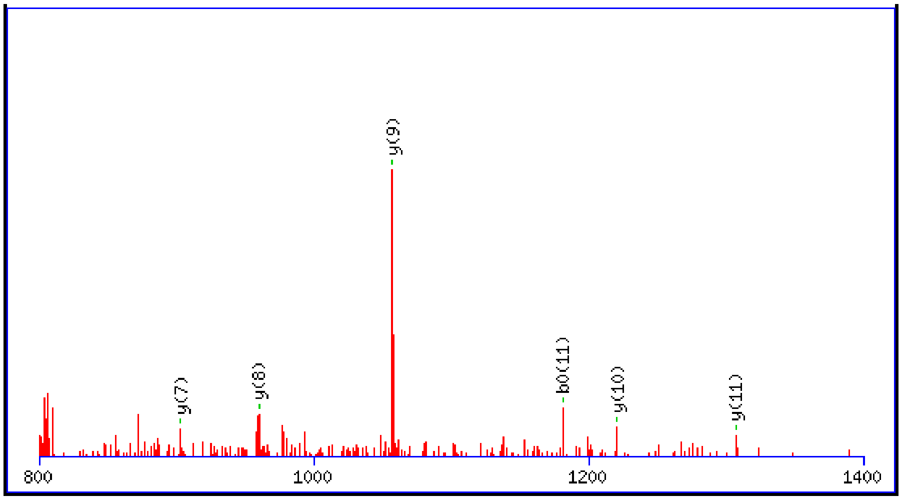

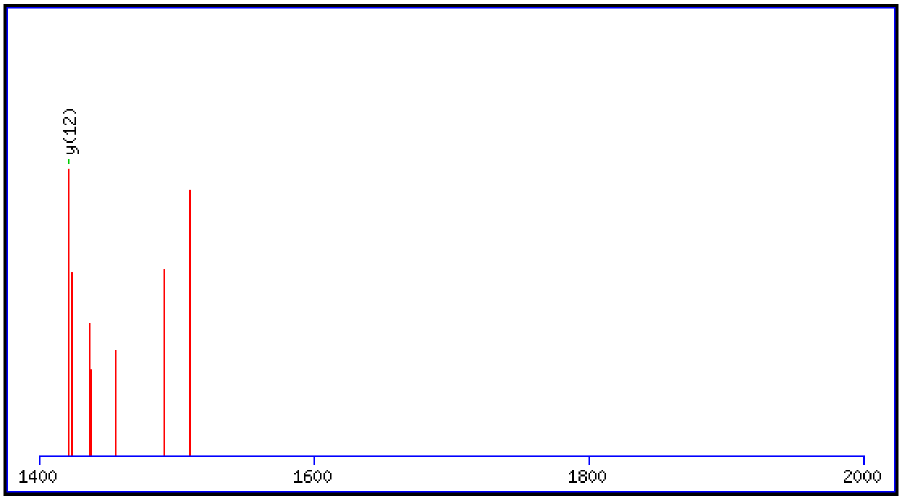

Monoisotopic mass of neutral peptide Mr(calc): 1636.6171
Fixed modifications: Carbamidomethyl (C)
Variable modifications:
S8 : Phospho (ST), with neutral losses 0.0000(shown in table), 97.9769
S12 : Phospho (ST), with neutral losses 0.0000(shown in table), 97.9769
Ions Score: 55 **Expect:** 0.00029
Matches (Bold Red): 16/252 fragment ions using 30 most intense peaks

#	b	b^{++}	b*	b^{*++}	b^{0}	b^{0++}	Seq.	y	y^{++}	y*	y^{*++}	y^{0}	y^{0++}	#
1	88.0393	44.5233			70.0287	35.5180	S							14
2	216.0979	108.5526	199.0713	100.0393	198.0873	99.5473	Q	1550.5924	775.7998	1533.5658	767.2866	1532.5818	766.7945	13
3	**331.1248**	166.0661	314.0983	157.5528	313.1143	157.0608	D	**1422.5338**	711.7705	1405.5073	703.2573	1404.5232	702.7653	12
4	418.1569	209.5821	401.1303	201.0688	**400.1463**	200.5768	S	**1307.5069**	654.2571	1290.4803	645.7438	1289.4963	645.2518	11
5	**581.2202**	291.1137	564.1936	282.6005	**563.2096**	282.1084	Y	**1220.4748**	610.7411	1203.4483	602.2278	1202.4643	601.7358	10
6	678.2729	339.6401	661.2464	**331.1268**	660.2624	**330.6348**	P	**1057.4115**	529.2094	1040.3850	520.6961	1039.4009	520.2041	9
7	735.2944	368.1508	718.2679	359.6376	717.2838	359.1456	G	**960.3587**	480.6830	943.3322	472.1697	942.3482	471.6777	8
8	902.2928	451.6500	885.2662	443.1367	884.2822	442.6447	S	**903.3373**	452.1723	886.3107	443.6590	885.3267	443.1670	7
9	999.3455	500.1764	982.3190	491.6631	981.3350	491.1711	P	**736.3389**	368.6731	719.3124	360.1598	718.3284	359.6678	6
10	1086.3776	543.6924	1069.3510	535.1791	1068.3670	534.6871	S	639.2862	320.1467	622.2596	311.6334	621.2756	311.1414	5
11	1199.4616	600.2344	1182.4351	591.7212	**1181.4511**	591.2292	L	552.2541	276.6307	535.2276	268.1174	534.2436	267.6254	4
12	1366.4600	683.7336	1349.4334	675.2203	1348.4494	674.7283	S	**439.1701**	220.0887	422.1435	211.5754	421.1595	211.0834	3
13	1463.5127	732.2600	1446.4862	723.7467	1445.5022	723.2547	P	**272.1717**	136.5895	255.1452	128.0762			2
14							R	175.1190	88.0631	158.0924	79.5498			1

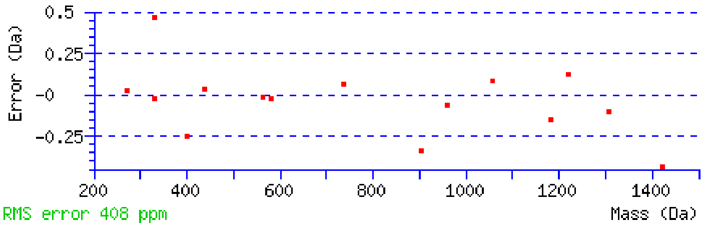

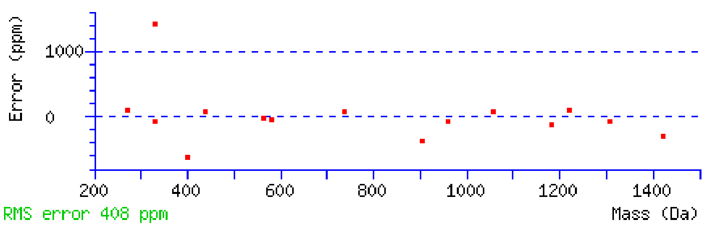

All matches to this query

Score	Mr(calc):	Delta	Sequence
55.2	1636.6171	0.0024	SQDSYPGSPSLSPR
42.6	1636.6171	0.0024	SQDSYPGSPSLSPR
40.5	1636.6171	0.0024	SQDSYPGSPSLSPR
24.1	1636.6171	0.0024	SQDSYPGSPSLSPR
15.8	1636.6171	0.0024	SQDSYPGSPSLSPR
9.0	1636.6171	0.0024	SQDSYPGSPSLSPR
3.9	1636.6171	0.0024	SQDSYPGSPSLSPR
3.3	1636.6171	0.0024	SQDSYPGSPSLSPR
3.0	1636.6171	0.0024	SQDSYPGSPSLSPR
1.8	1636.6131	0.0065	NSSIDSGEAEVGRR

Spectrum No: 224; Query: 2583; Rank: 1

Peptide View

MS/MS Fragmentation of **VKPPPSPTTEGPSLQPDLAPEEAAGAQRPK**
Found in **IPI00364858**, Tax_Id=10116 Gene_Symbol=Akap2 A kinase (PRKA) anchor protein 2

Match to Query 2583: 3145.552332 from(1049.524720,3+)
Title: 091008RatKidney_NH4Format02_24.2514.2514.3.dta
Data file K:\NewmanPaper\Piliang\3SubProteomes\Piliang3SP\mgf5ppm\ERLIC_3SubProteomes5ppm.mgf

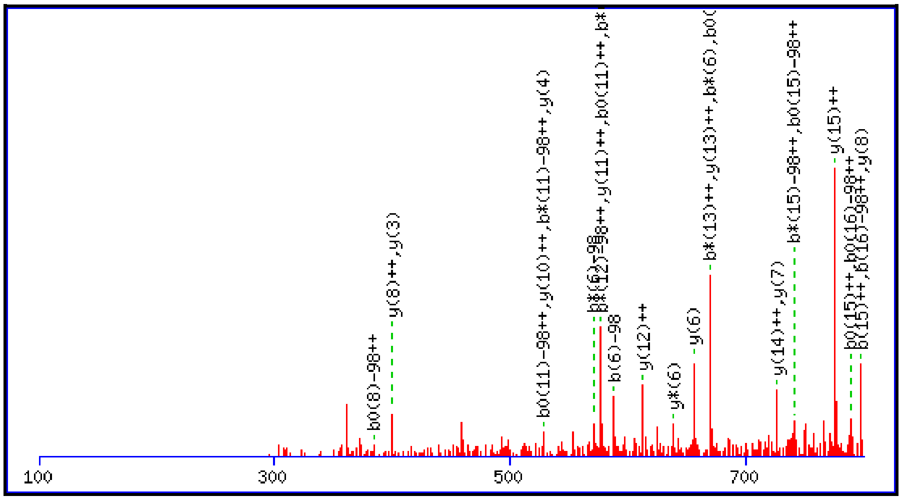

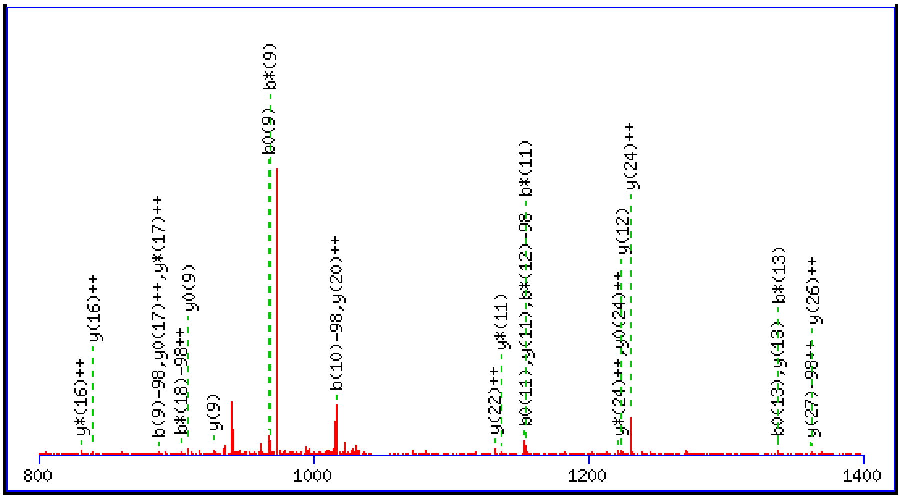

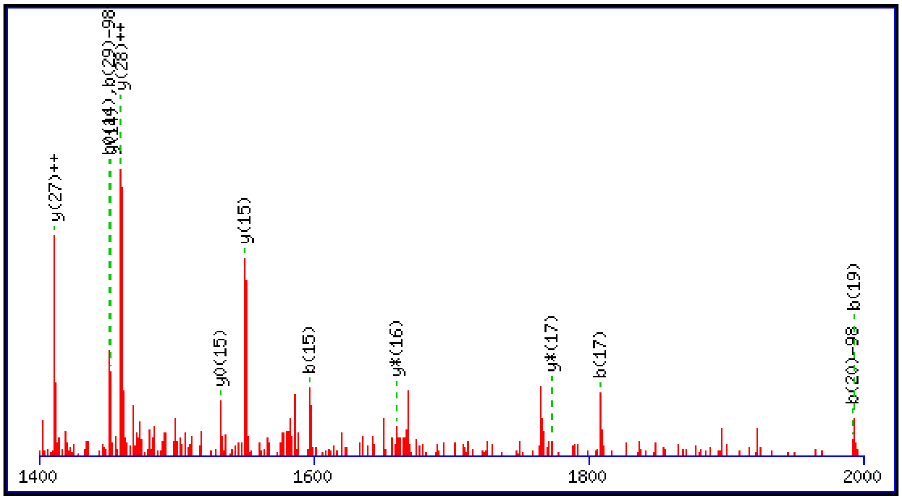

Monoisotopic mass of neutral peptide Mr(calc): 3144.5492
Fixed modifications: Carbamidomethyl (C)
Variable modifications:
S6 : Phospho (ST), with neutral losses 0.0000(shown in table), 97.9769
Ions Score: 55 **Expect:** 0.00079
Matches (Bold Red): 72/494 fragment ions using 98 most intense peaks

#	b	b^{++}	b*	b^{*++}	b^0	b^{0++}	Seq.	y	y^{++}	y*	y^{*++}	y^0	y^{0++}	#
1	100.0757	50.5415					V							30
2	228.1707	114.5890	211.1441	106.0757			K	3046.4881	1523.7477	3029.4616	1515.2344	3028.4776	1514.7424	29
3	325.2234	163.1153	308.1969	154.6021			P	2918.3932	**1459.7002**	2901.3666	**1451.1869**	2900.3826	**1450.6949**	28
4	422.2762	211.6417	405.2496	203.1285			P	2821.3404	**1411.1738**	2804.3138	1402.6606	2803.3298	1402.1686	27
5	519.3289	260.1681	502.3024	251.6548			P	2724.2876	**1362.6475**	2707.2611	1354.1342	2706.2771	1353.6422	26
6	686.3273	343.6673	**669.3007**	335.1540	668.3167	334.6620	S	2627.2349	1314.1211	2610.2083	1305.6078	2609.2243	1305.1158	25
7	783.3801	392.1937	766.3535	383.6804	765.3695	383.1884	P	2460.2365	**1230.6219**	2443.2100	**1222.1086**	2442.2259	**1221.6166**	24
8	884.4277	442.7175	867.4012	434.2042	866.4172	433.7122	T	2363.1837	1182.0955	2346.1572	1173.5822	2345.1732	1173.0902	23
9	985.4754	493.2413	**968.4489**	484.7281	**967.4649**	484.2361	T	2262.1361	**1131.5717**	2245.1095	1123.0584	2244.1255	1122.5664	22
10	1114.5180	557.7626	1097.4915	549.2494	1096.5074	548.7574	E	2161.0884	1081.0478	2144.0618	1072.5346	2143.0778	1072.0425	21
11	1171.5395	586.2734	**1154.5129**	**577.7601**	**1153.5289**	**577.2681**	G	2032.0458	**1016.5265**	2015.0192	1008.0133	2014.0352	1007.5213	20
12	1268.5922	634.7998	1251.5657	626.2865	1250.5817	625.7945	P	1975.0243	988.0158	1957.9978	979.5025	1957.0138	979.0105	19
13	1355.6243	678.3158	**1338.5977**	**669.8025**	**1337.6137**	**669.3105**	S	1877.9716	939.4894	1860.9450	930.9761	1859.9610	930.4841	18
14	1468.7083	734.8578	1451.6818	726.3445	**1450.6978**	725.8525	L	1790.9395	895.9734	**1773.9130**	**887.4601**	1772.9290	**886.9681**	17
15	**1596.7669**	**798.8871**	1579.7404	790.3738	1578.7563	**789.8818**	Q	1677.8555	**839.4314**	**1660.8289**	**830.9181**	1659.8449	830.4261	16
16	1693.8197	847.4135	1676.7931	838.9002	1675.8091	838.4082	P	**1549.7969**	**775.4021**	1532.7703	766.8888	**1531.7863**	766.3968	15
17	**1808.8466**	904.9269	1791.8201	896.4137	1790.8361	895.9217	D	**1452.7441**	**726.8757**	1435.7176	718.3624	1434.7336	717.8704	14
18	1921.9307	961.4690	1904.9041	952.9557	1903.9201	952.4637	L	**1337.7172**	**669.3622**	1320.6906	660.8490	1319.7066	660.3570	13

19	1992.9678	996.9875	1975.9412	988.4743	1974.9572	987.9823	A	1224.6331	612.8202	1207.6066	604.3069	1206.6226	603.8149	12
20	2090.0206	1045.5139	2072.9940	1037.0006	2072.0100	1036.5086	P	1153.5960	577.3016	1136.5695	568.7884	1135.5854	568.2964	11
21	2219.0632	1110.0352	2202.0366	1101.5219	2201.0526	1101.0299	E	1056.5432	528.7753	1039.5167	520.2620	1038.5327	519.7700	10
22	2348.1057	1174.5565	2331.0792	1166.0432	2330.0952	1165.5512	E	927.5007	464.2540	910.4741	455.7407	909.4901	455.2487	9
23	2419.1429	1210.0751	2402.1163	1201.5618	2401.1323	1201.0698	A	798.4581	399.7327	781.4315	391.2194			8
24	2490.1800	1245.5936	2473.1534	1237.0804	2472.1694	1236.5883	A	727.4209	364.2141	710.3944	355.7008			7
25	2547.2014	1274.1044	2530.1749	1265.5911	2529.1909	1265.0991	G	656.3838	328.6956	639.3573	320.1823			6
26	2618.2386	1309.6229	2601.2120	1301.1096	2600.2280	1300.6176	A	599.3624	300.1848	582.3358	291.6715			5
27	2746.2971	1373.6522	2729.2706	1365.1389	2728.2866	1364.6469	Q	528.3253	264.6663	511.2987	256.1530			4
28	2902.3982	1451.7028	2885.3717	1443.1895	2884.3877	1442.6975	R	400.2667	200.6370	383.2401	192.1237			3
29	2999.4510	1500.2291	2982.4245	1491.7159	2981.4404	1491.2239	P	244.1656	122.5864	227.1390	114.0731			2
30							K	147.1128	74.0600	130.0863	65.5468			1

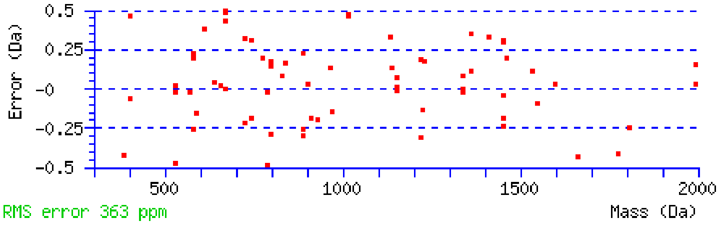

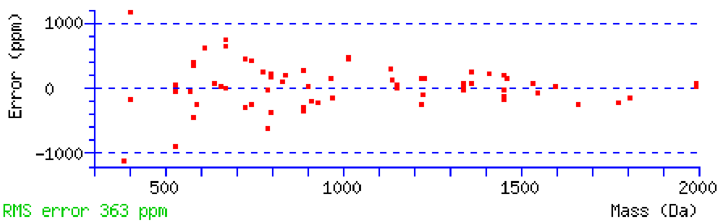

All matches to this query

Score	Mr(calc):	Delta	Sequence
55.1	3144.5492	1.0031	VKPPPSPTTEGPSLQPDLAPEEAAGAQRPK
54.6	3144.5492	1.0031	VKPPPSPTTEGPSLQPDLAPEEAAGAQRPK
46.0	3144.5492	1.0031	VKPPPSPTTEGPSLQPDLAPEEAAGAQRPK
37.0	3144.5492	1.0031	VKPPPSPTTEGPSLQPDLAPEEAAGAQRPK
3.9	3144.5314	1.0209	ATSVLYSVLSPTLNPLIYTLRNKDVK
3.3	3144.5314	1.0209	ATSVLYSVLSPTLNPLIYTLRNKDVK
3.1	3144.5178	1.0345	EQLAKQTVAIAELTEELQSKCIQLNK
2.8	3144.5314	1.0209	ATSVLYSVLSPTLNPLIYTLRNKDVK
2.8	3144.5314	1.0209	ATSVLYSVLSPTLNPLIYTLRNKDVK
2.8	3144.5314	1.0209	ATSVLYSVLSPTLNPLIYTLRNKDVK

Spectrum No: 225; Query: 2283; Rank: 1

Peptide View

MS/MS Fragmentation of **TEDGGWEWSDDEFDEESEEGK**
Found in **IPI00371139**, Tax_Id=10116 Gene_Symbol=Oxsr1_predicted oxidative-stress responsive 1

Match to Query 2283: 2555.891008 from(1278.952780,2+)
Title: 100101RatKid_NS_deglyco_26.3689.3689.2.dta
Data file K:\NewmanPaper\Piliang\3SubProteomes\Piliang3SP\mgf5ppm\ERLIC_3SubProteomes5ppm.mgf

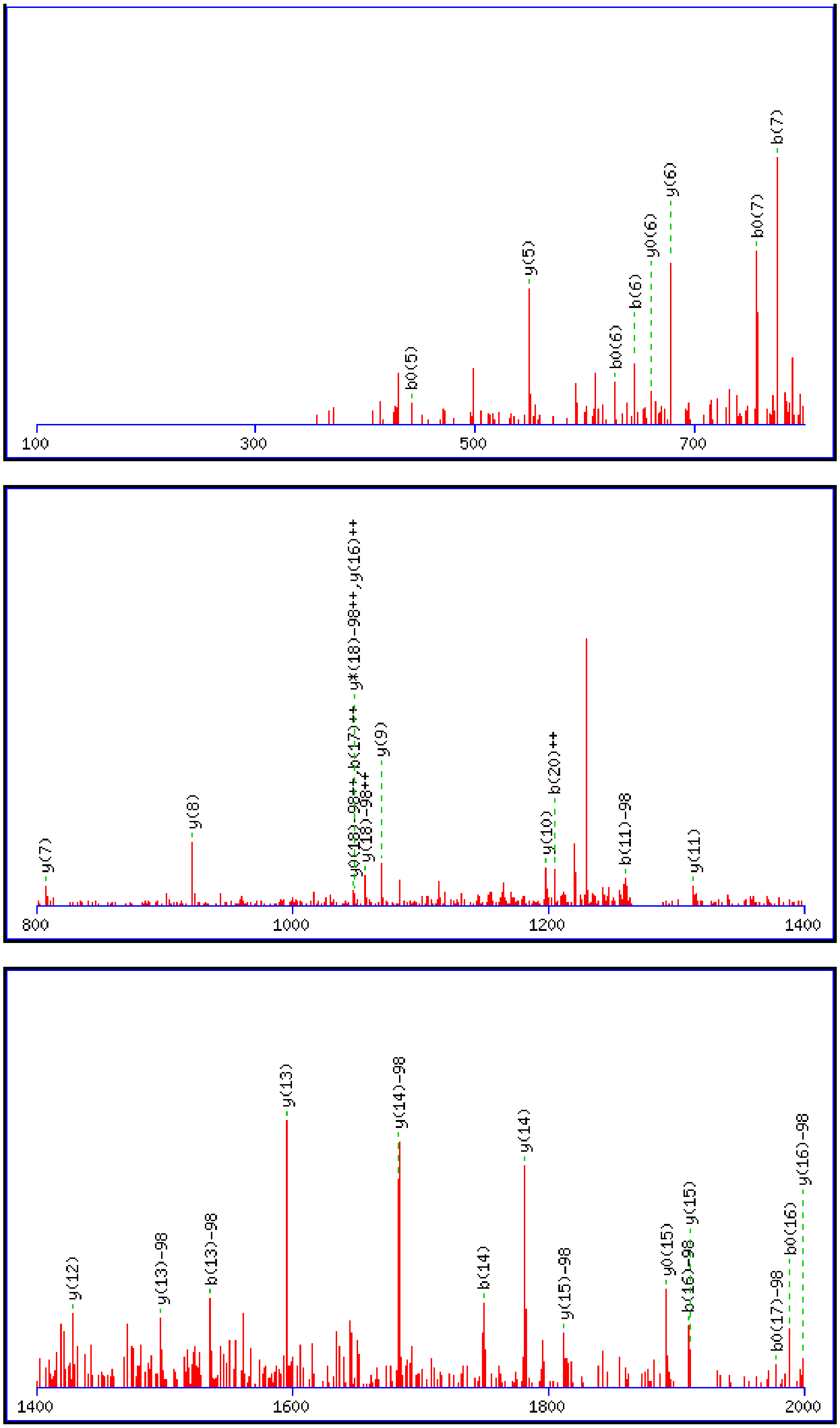

Monoisotopic mass of neutral peptide Mr(calc): 2554.8809
Fixed modifications: Carbamidomethyl (C)
Variable modifications:
S9 : Phospho (ST), with neutral losses 97.9769(shown in table), 0.0000
Ions Score: 55 **Expect:** 0.00033
Matches (Bold Red): 34/292 fragment ions using 55 most intense peaks

#	b	b^{++}	b^{0}	b^{0++}	Seq.	y	y^{++}	y^{*}	y^{*++}	y^{0}	y^{0++}	#
1	102.0550	51.5311	84.0444	42.5258	T							21
2	231.0975	116.0524	213.0870	107.0471	E	2356.8636	1178.9355	2339.8371	1170.4222	2338.8531	1169.9302	20
3	346.1245	173.5659	328.1139	164.5606	D	2227.8211	1114.4142	2210.7945	1105.9009	2209.8105	1105.4089	19
4	403.1460	202.0766	385.1354	193.0713	G	2112.7941	**1056.9007**	2095.7676	**1048.3874**	2094.7835	**1047.8954**	18
5	460.1674	230.5873	**442.1569**	221.5821	G	2055.7726	1028.3900	2038.7461	1019.8767	2037.7621	1019.3847	17

6	646.2467	323.6270	628.2362	314.6217	W	1998.7512	999.8792	1981.7246	991.3660	1980.7406	990.8739	16
7	775.2893	388.1483	757.2788	379.1430	E	1812.6719	906.8396	1795.6453	898.3263	1794.6613	897.8343	15
8	961.3686	481.1880	943.3581	472.1827	W	1683.6293	842.3183	1666.6027	833.8050	1665.6187	833.3130	14
9	1030.3901	515.6987	1012.3795	506.6934	S	1497.5500	749.2786	1480.5234	740.7653	1479.5394	740.2733	13
10	1145.4170	573.2122	1127.4065	564.2069	D	1428.5285	714.7679	1411.5020	706.2546	1410.5179	705.7626	12
11	1260.4440	630.7256	1242.4334	621.7203	D	1313.5016	657.2544	1296.4750	648.7411	1295.4910	648.2491	11
12	1389.4866	695.2469	1371.4760	686.2416	E	1198.4746	599.7409	1181.4481	591.2277	1180.4641	590.7357	10
13	1536.5550	768.7811	1518.5444	759.7758	F	1069.4320	535.2197	1052.4055	526.7064	1051.4215	526.2144	9
14	1651.5819	826.2946	1633.5714	817.2893	D	922.3636	461.6854	905.3371	453.1722	904.3530	452.6802	8
15	1780.6245	890.8159	1762.6140	881.8106	E	807.3367	404.1720	790.3101	395.6587	789.3261	395.1667	7
16	1909.6671	955.3372	1891.6566	946.3319	E	678.2941	339.6507	661.2675	331.1374	660.2835	330.6454	6
17	1996.6991	998.8532	1978.6886	989.8479	S	549.2515	275.1294	532.2249	266.6161	531.2409	266.1241	5
18	2125.7417	1063.3745	2107.7312	1054.3692	E	462.2195	231.6134	445.1929	223.1001	444.2089	222.6081	4
19	2254.7843	1127.8958	2236.7738	1118.8905	E	333.1769	167.0921	316.1503	158.5788	315.1663	158.0868	3
20	2311.8058	1156.4065	2293.7952	1147.4013	G	204.1343	102.5708	187.1077	94.0575			2
21					K	147.1128	74.0600	130.0863	65.5468			1

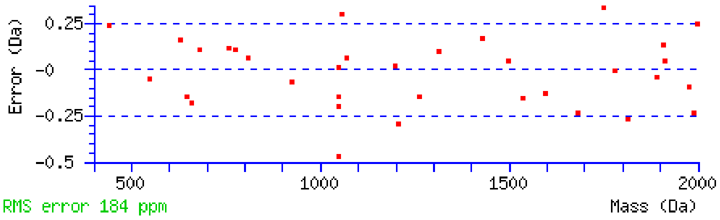

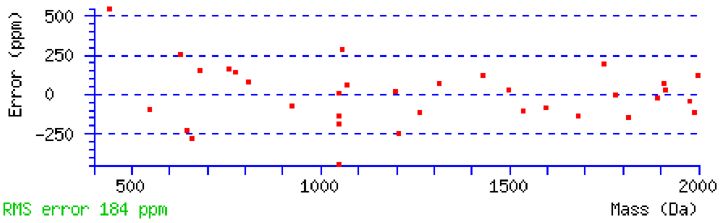

All matches to this query

Score	Mr(calc):	Delta	Sequence
55.0	2554.8809	1.0101	TEDGGWEWSDDEFDEESEEGK
30.2	2554.8809	1.0101	TEDGGWEWSDDEFDEESEEGK
7.6	2554.8809	1.0101	TEDGGWEWSDDEFDEESEEGK
1.8	2554.9027	0.9883	ALDKVTSDYHSGSSSSDEDTSK
1.8	2555.9073	-0.0163	WINDVEDSYGQQWTYEQR
1.5	2555.8848	0.0062	KSPMKSESSPADLSHSISPR
0.3	2555.8848	0.0062	KSPMKSESSPADLSHSISPR

Spectrum No: 226; Query: 1617; Rank: 1

Peptide View

MS/MS Fragmentation of **GLSASLPDLDSESWIEVK**
Found in **IPI00369227**, Tax_Id=10116 Gene_Symbol=Larp1_predicted similar to la related protein isoform 2

Match to Query 1617: 2025.939508 from(1013.977030,2+)
Title: 091008RatKidney_NoSalt_22.4986.4986.2.dta
Data file K:\NewmanPaper\Piliang\3SubProteomes\Piliang3SP\mgf5ppm\ERLIC_3SubProteomes5ppm.mgf

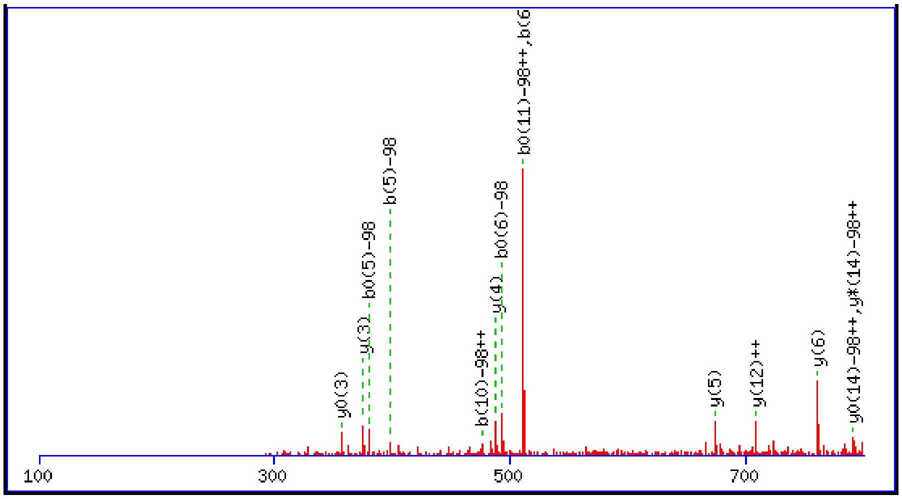

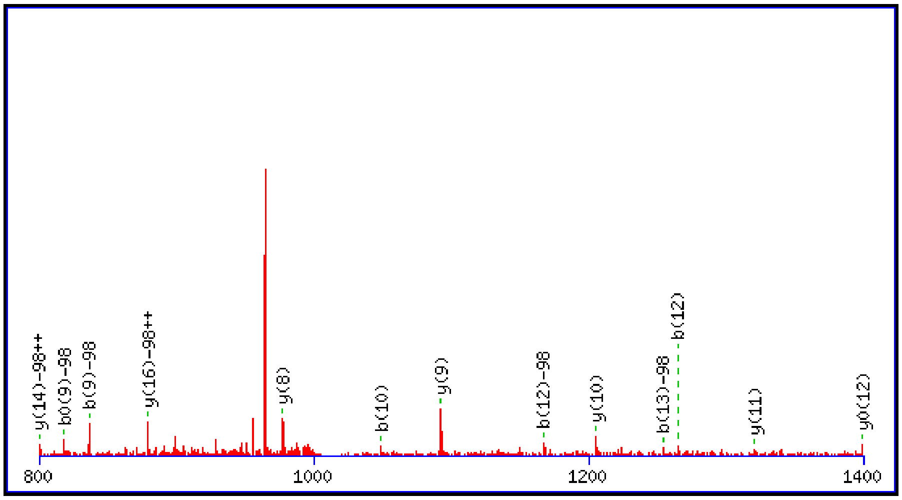

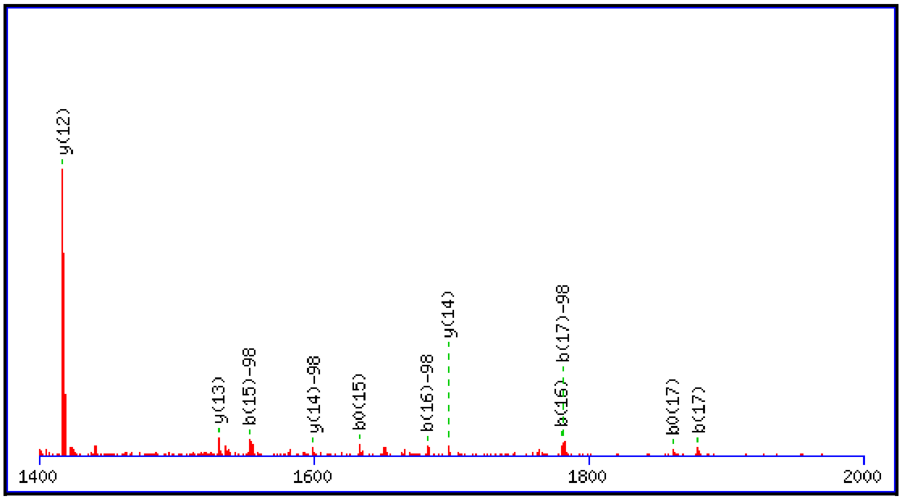

Monoisotopic mass of neutral peptide Mr(calc): 2024.9343
Fixed modifications: Carbamidomethyl (C)
Variable modifications:
S5 : Phospho (ST), with neutral losses 97.9769(shown in table), 0.0000
Ions Score: 55 **Expect:** 0.00082
Matches (Bold Red): 38/238 fragment ions using 74 most intense peaks

#	b	b^{++}	b^0	b^{0++}	Seq.	y	y^{++}	y*	y^{*++}	y^0	y^{0++}	#
1	58.0287	29.5180			G							18
2	171.1128	86.0600			L	1870.9433	935.9753	1853.9167	927.4620	1852.9327	926.9700	17
3	258.1448	129.5761	240.1343	120.5708	S	1757.8592	**879.4332**	1740.8327	870.9200	1739.8486	870.4280	16
4	329.1819	165.0946	311.1714	156.0893	A	1670.8272	835.9172	1653.8006	827.4040	1652.8166	826.9119	15
5	**398.2034**	199.6053	**380.1928**	190.6001	S	**1599.7901**	**800.3987**	1582.7635	**791.8854**	1581.7795	**791.3934**	14

6	**511.2875**	256.1474	**493.2769**	247.1421	L	**1530.7686**	765.8879	1513.7421	757.3747	1512.7581	756.8827	13
7	608.3402	304.6738	590.3297	295.6685	P	**1417.6846**	**709.3459**	1400.6580	700.8326	**1399.6740**	700.3406	12
8	723.3672	362.1872	705.3566	353.1819	D	**1320.6318**	660.8195	1303.6052	652.3063	1302.6212	651.8142	11
9	**836.4512**	418.7293	**818.4407**	409.7240	L	**1205.6048**	603.3061	1188.5783	594.7928	1187.5943	594.3008	10
10	951.4782	**476.2427**	933.4676	467.2374	D	**1092.5208**	546.7640	1075.4942	538.2508	1074.5102	537.7587	9
11	1038.5102	519.7587	1020.4996	**510.7535**	S	**977.4938**	489.2506	960.4673	480.7373	959.4833	480.2453	8
12	**1167.5528**	584.2800	1149.5422	575.2748	E	890.4618	445.7345	873.4353	437.2213	872.4512	436.7293	7
13	**1254.5848**	627.7961	1236.5743	618.7908	S	**761.4192**	381.2132	744.3927	372.7000	743.4087	372.2080	6
14	1440.6641	720.8357	1422.6536	711.8304	W	**674.3872**	337.6972	657.3606	329.1840	656.3766	328.6919	5
15	**1553.7482**	777.3777	1535.7376	768.3725	I	**488.3079**	244.6576	471.2813	236.1443	470.2973	235.6523	4
16	**1682.7908**	841.8990	1664.7802	832.8938	E	**375.2238**	188.1155	358.1973	179.6023	**357.2132**	179.1103	3
17	**1781.8592**	891.4332	1763.8486	882.4280	V	246.1812	123.5942	229.1547	115.0810			2
18					K	147.1128	74.0600	130.0863	65.5468			1

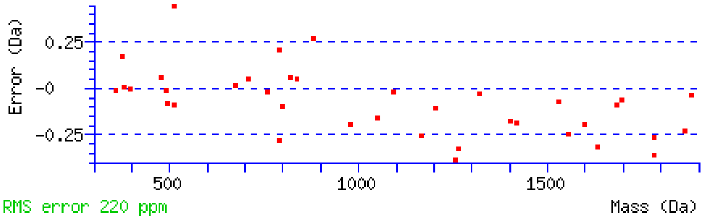

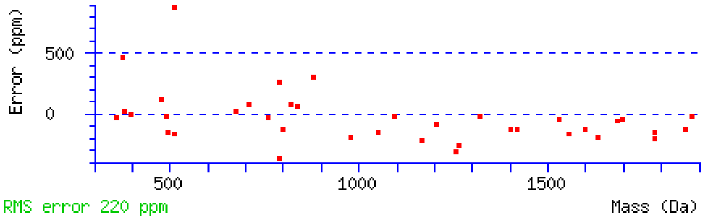

All matches to this query

Score	Mr(calc):	Delta	Sequence
54.9	2024.9343	1.0052	GLSASLPDLDSESWIEVK
54.3	2024.9343	1.0052	GLSASLPDLDSESWIEVK
12.3	2024.9343	1.0052	GLSASLPDLDSESWIEVK
7.8	2024.9343	1.0052	GLSASLPDLDSESWIEVK
6.7	2024.9469	0.9926	NKINHLNEHAFTHLQK
6.1	2024.9469	0.9926	NKINHLNEHAFTHLQK
5.5	2024.9543	0.9852	MAKGYGAISVATSWPPGPR
2.4	2024.9469	0.9926	NKINHLNEHAFTHLQK
1.4	2025.9595	-0.0199	TEAVCLSAPHLASPPATPK
1.4	2024.9303	1.0092	SNLRIVGIEENEDSQLK

Spectrum No: 227; Query: 1685; Rank: 1

Peptide View

MS/MS Fragmentation of **HGSPTAPIGAGSPEFTEQGR**
Found in **IPI00371183**, Tax_Id=10116 Gene_Symbol=LOC499249 similar to pleckstrin homology domain containing, family A member 2

Match to Query 1685: 2074.907232 from(692.643020,3+)
Title: 091008RatKidney_NH4Format01_18.1687.1687.3.dta
Data file K:\NewmanPaper\Piliang\3SubProteomes\Piliang3SP\mgf5ppm\ERLIC_3SubProteomes5ppm.mgf

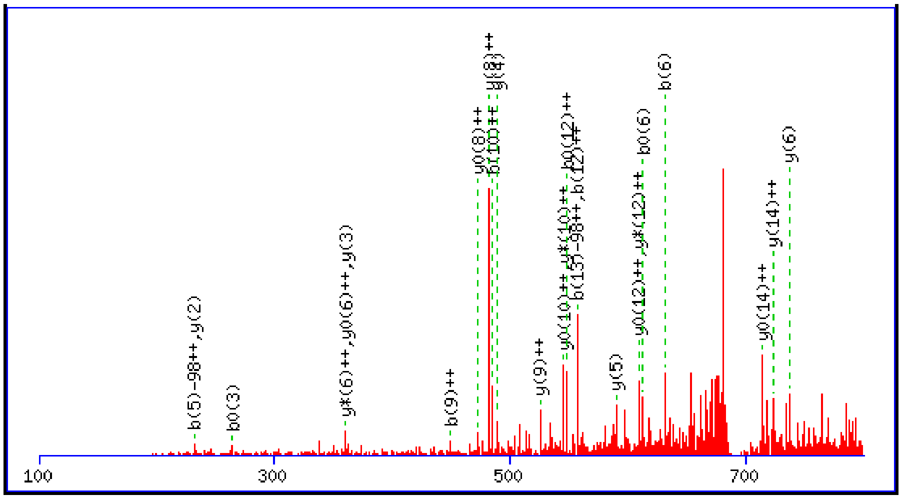

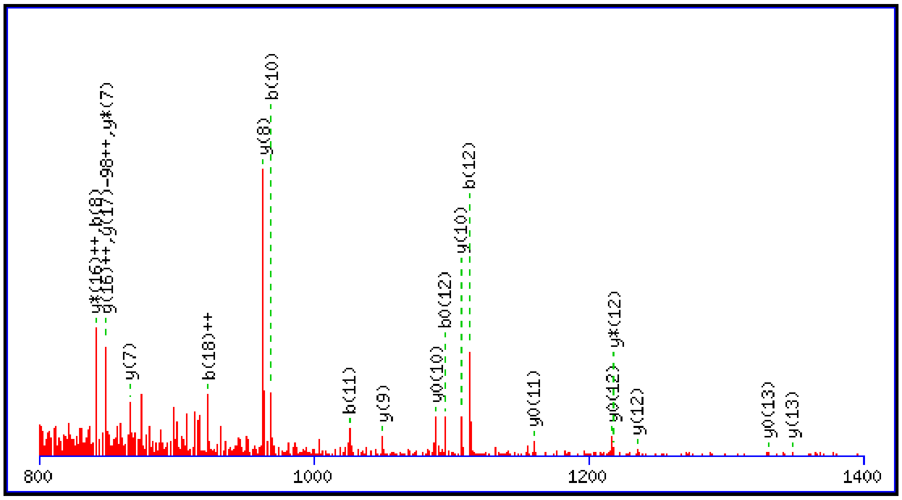

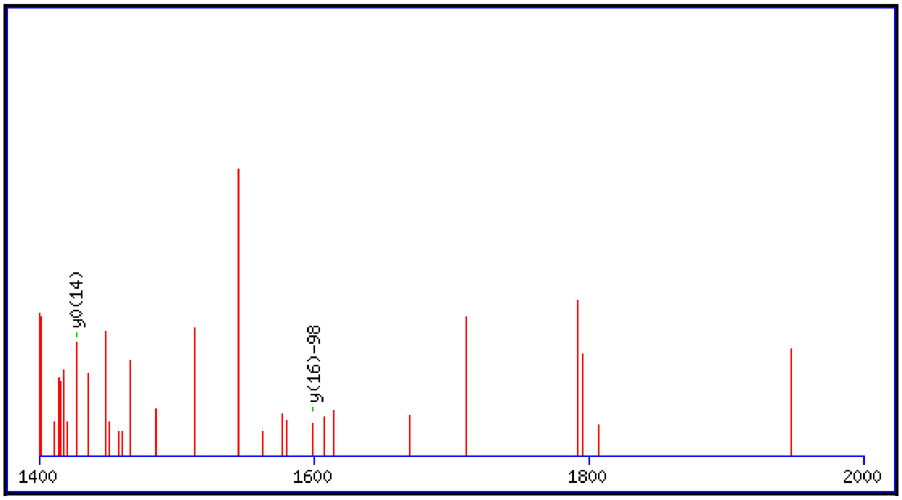

Monoisotopic mass of neutral peptide Mr(calc): 2074.9110
Fixed modifications: Carbamidomethyl (C)
Variable modifications:
T5 : Phospho (ST), with neutral losses 0.0000(shown in table), 97.9769
Ions Score: 55 **Expect:** 0.00099
Matches (Bold Red): 48/272 fragment ions using 75 most intense peaks

#	b	b^{++}	b*	b^{*++}	b^0	b^{0++}	Seq.	y	y^{++}	y*	y^{*++}	y^0	y^{0++}	#
1	138.0662	69.5367					H							20
2	195.0877	98.0475					G	1938.8593	969.9333	1921.8328	961.4200	1920.8487	960.9280	19
3	282.1197	141.5635			**264.1091**	132.5582	S	1881.8378	941.4226	1864.8113	932.9093	1863.8273	932.4173	18
4	379.1724	190.0899			361.1619	181.0846	P	1794.8058	897.9065	1777.7793	889.3933	1776.7953	888.9013	17
5	560.1864	280.5969			542.1759	271.5916	T	1697.7531	**849.3802**	1680.7265	**840.8669**	1679.7425	840.3749	16

**6**	**631.2236**	316.1154			**613.2130**	307.1101	**A**	1516.7390	758.8732	1499.7125	750.3599	1498.7285	749.8679	**15**
**7**	728.2763	364.6418			710.2658	355.6365	**P**	1445.7019	**723.3546**	1428.6754	714.8413	**1427.6914**	**714.3493**	**14**
**8**	**841.3604**	421.1838			823.3498	412.1786	**I**	**1348.6492**	674.8282	1331.6226	666.3149	**1330.6386**	665.8229	**13**
**9**	898.3819	**449.6946**			880.3713	440.6893	**G**	**1235.5651**	618.2862	**1218.5386**	**609.7729**	**1217.5545**	**609.2809**	**12**
**10**	**969.4190**	**485.2131**			951.4084	476.2078	**A**	1178.5436	589.7755	1161.5171	581.2622	**1160.5331**	580.7702	**11**
**11**	**1026.4404**	513.7239			1008.4299	504.7186	**G**	**1107.5065**	554.2569	1090.4800	**545.7436**	**1089.4960**	**545.2516**	**10**
**12**	**1113.4725**	**557.2399**			**1095.4619**	**548.2346**	**S**	**1050.4851**	**525.7462**	1033.4585	517.2329	1032.4745	516.7409	**9**
**13**	1210.5252	605.7662			1192.5147	596.7610	**P**	**963.4530**	**482.2302**	946.4265	473.7169	945.4425	**473.2249**	**8**
**14**	1339.5678	670.2875			1321.5573	661.2823	**E**	**866.4003**	433.7038	**849.3737**	425.1905	848.3897	424.6985	**7**
**15**	1486.6362	743.8218			1468.6257	734.8165	**F**	**737.3577**	369.1825	720.3311	**360.6692**	719.3471	**360.1772**	**6**
**16**	1587.6839	794.3456			1569.6733	785.3403	**T**	**590.2893**	295.6483	573.2627	287.1350	572.2787	286.6430	**5**
**17**	1716.7265	858.8669			1698.7159	849.8616	**E**	**489.2416**	245.1244	472.2150	236.6112	471.2310	236.1191	**4**
**18**	1844.7851	**922.8962**	1827.7585	914.3829	1826.7745	913.8909	**Q**	**360.1990**	180.6031	343.1724	172.0899			**3**
**19**	1901.8065	951.4069	1884.7800	942.8936	1883.7960	942.4016	**G**	**232.1404**	116.5738	215.1139	108.0606			**2**
**20**							**R**	175.1190	88.0631	158.0924	79.5498			**1**

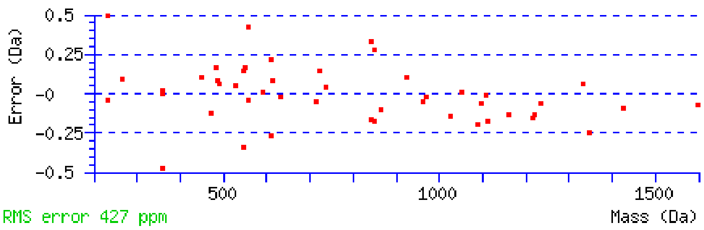

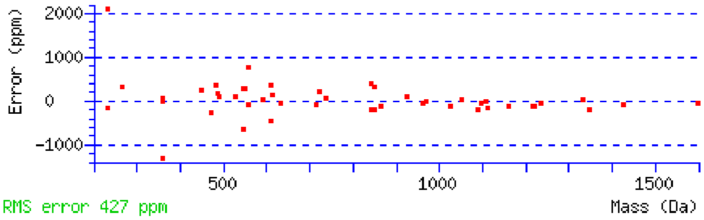

All matches to this query

Score	Mr(calc):	Delta	Sequence
54.8	2074.9110	-0.0037	HGSPTAPIGAGSPEFTEQGR
53.6	2074.9110	-0.0037	HGSPTAPIGAGSPEFTEQGR
47.1	2074.9110	-0.0037	HGSPTAPIGAGSPEFTEQGR
10.7	2074.9110	-0.0037	HGSPTAPIGAGSPEFTEQGR

Spectrum No: 228; Query: 2488; Rank: 1

Peptide View

MS/MS Fragmentation of **DGELPVEDDIDLSDVELDDLEKDEL**
Found in **IPI00365929**, Tax_Id=10116 Gene_Symbol=Pdia6 protein disulfide isomerase associated 6

Match to Query 2488: 2909.251408 from(1455.632980,2+)
Title: 091008RatKidney_NH4Format01_25.4385.4385.2.dta
Data file K:\NewmanPaper\Piliang\3SubProteomes\Piliang3SP\mgf5ppm\ERLIC_3SubProteomes5ppm.mgf

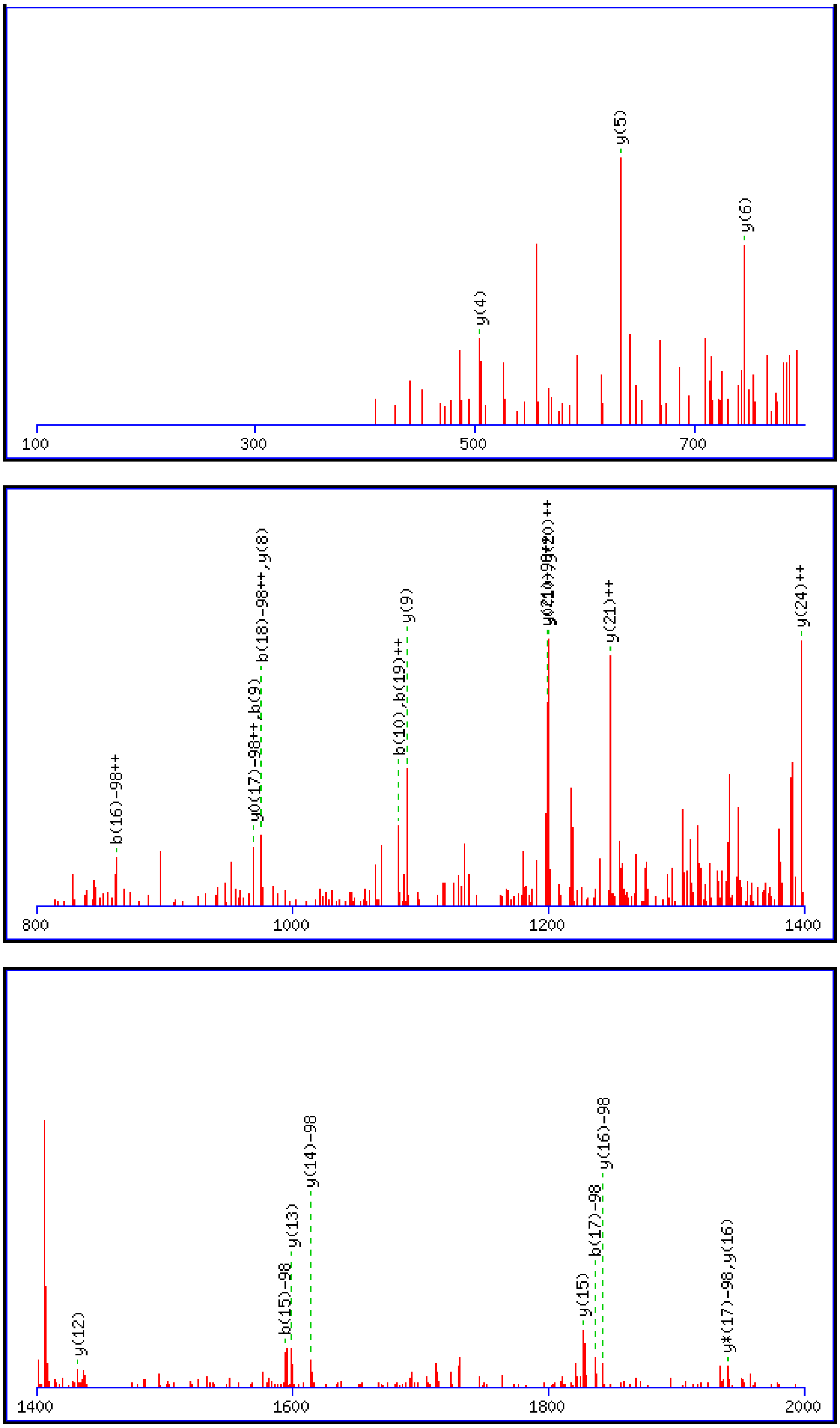

Monoisotopic mass of neutral peptide Mr(calc): 2909.2478
Fixed modifications: Carbamidomethyl (C)
Variable modifications:
S13 : Phospho (ST), with neutral losses 0.0000(shown in table), 97.9769
Ions Score: 55 **Expect:** 0.0012
Matches (Bold Red): 25/364 fragment ions using 28 most intense peaks

#	b	b++	b*	b*++	b0	b0++	Seq.	y	y++	y*	y*++	y0	y0++	#
1	116.0342	58.5207			98.0237	49.5155	D							25
2	173.0557	87.0315			155.0451	78.0262	G	2795.2282	**1398.1177**	2778.2016	1389.6044	2777.2176	1389.1124	24
3	302.0983	151.5528			284.0877	142.5475	E	2738.2067	1369.6070	2721.1801	1361.0937	2720.1961	1360.6017	23
4	415.1823	208.0948			397.1718	199.0895	L	2609.1641	1305.0857	2592.1376	1296.5724	2591.1535	1296.0804	22
5	512.2351	256.6212			494.2245	247.6159	P	2496.0800	**1248.5437**	2479.0535	1240.0304	2478.0695	1239.5384	21

**6**	611.3035	306.1554			593.2930	297.1501	**V**	2399.0273	**1200.0173**	2382.0007	1191.5040	2381.0167	1191.0120	**20**
**7**	740.3461	370.6767			722.3355	361.6714	**E**	2299.9589	1150.4831	2282.9323	1141.9698	2281.9483	1141.4778	**19**
**8**	855.3731	428.1902			837.3625	419.1849	**D**	2170.9163	1085.9618	2153.8897	1077.4485	2152.9057	1076.9565	**18**
**9**	**970.4000**	485.7036			952.3894	476.6984	**D**	2055.8893	1028.4483	2038.8628	1019.9350	2037.8788	1019.4430	**17**
**10**	**1083.4841**	542.2457			1065.4735	533.2404	**I**	**1940.8624**	970.9348	1923.8358	962.4216	1922.8518	961.9295	**16**
**11**	1198.5110	599.7591			1180.5004	590.7539	**D**	**1827.7783**	914.3928	1810.7518	905.8795	1809.7678	905.3875	**15**
**12**	1311.5951	656.3012			1293.5845	647.2959	**L**	1712.7514	856.8793	1695.7248	848.3661	1694.7408	847.8740	**14**
**13**	1478.5934	739.8003			1460.5829	730.7951	**S**	**1599.6673**	800.3373	1582.6408	791.8240	1581.6567	791.3320	**13**
**14**	1593.6204	797.3138			1575.6098	788.3085	**D**	**1432.6690**	716.8381	1415.6424	708.3248	1414.6584	707.8328	**12**
**15**	1692.6888	846.8480			1674.6782	837.8427	**V**	1317.6420	659.3246	1300.6155	650.8114	1299.6315	650.3194	**11**
**16**	1821.7314	911.3693			1803.7208	902.3640	**E**	1218.5736	609.7904	1201.5471	601.2772	**1200.5630**	600.7852	**10**
**17**	1934.8154	967.9114			1916.8049	958.9061	**L**	**1089.5310**	545.2691	1072.5045	536.7559	1071.5204	536.2639	**9**
**18**	2049.8424	1025.4248			2031.8318	1016.4195	**D**	**976.4469**	488.7271	959.4204	480.2138	958.4364	479.7218	**8**
**19**	2164.8693	**1082.9383**			2146.8588	1073.9330	**D**	861.4200	431.2136	844.3935	422.7004	843.4094	422.2084	**7**
**20**	2277.9534	1139.4803			2259.9428	1130.4750	**L**	**746.3931**	373.7002	729.3665	365.1869	728.3825	364.6949	**6**
**21**	2406.9960	1204.0016			2388.9854	1194.9963	**E**	**633.3090**	317.1581	616.2824	308.6449	615.2984	308.1529	**5**
**22**	2535.0909	1268.0491	2518.0644	1259.5358	2517.0804	1259.0438	**K**	**504.2664**	252.6368	487.2399	244.1236	486.2558	243.6316	**4**
**23**	2650.1179	1325.5626	2633.0913	1317.0493	2632.1073	1316.5573	**D**	376.1714	188.5894			358.1609	179.5841	**3**
**24**	2779.1605	1390.0839	2762.1339	1381.5706	2761.1499	1381.0786	**E**	261.1445	131.0759			243.1339	122.0706	**2**
**25**							**L**	132.1019	66.5546					**1**

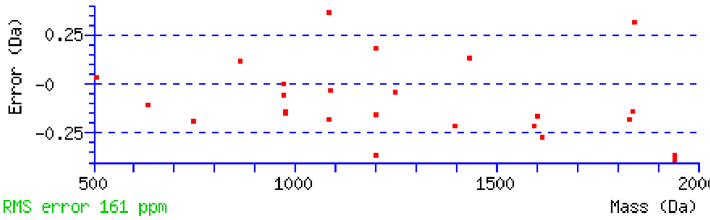

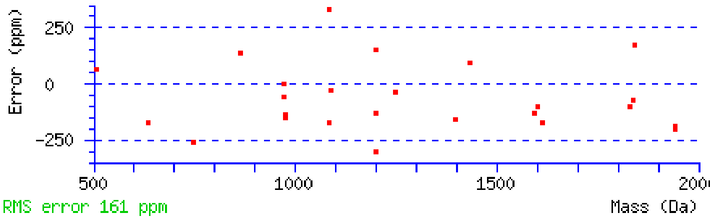

All matches to this query

Score	Mr(calc):	Delta	Sequence
54.8	2909.2478	0.0036	DGELPVEDDIDLSDVELDDLEKDEL
8.8	2909.2604	-0.0090	EKEYSENLDSSFRHDIDLQSTTSK
8.8	2909.2604	-0.0090	EKEYSENLDSSFRHDIDLQSTTSK
5.8	2909.2604	-0.0090	EKEYSENLDSSFRHDIDLQSTTSK
3.7	2909.2604	-0.0090	EKEYSENLDSSFRHDIDLQSTTSK
1.5	2907.2537	1.9977	TQESPNMPVPPLSMASSPRMGNRPSC
1.5	2908.2377	1.0137	TQESPNMPVPPLSMASSPRMGNRPSC
1.1	2907.2589	1.9926	AGTGMDNVNLEAAMTKSILVMNTPNGK
1.1	2907.2589	1.9926	AGTGMDNVNLEAAMTKSILVMNTPNGK
0.9	2908.2764	0.9751	EKEYSENLDSSFRHDIDLQSTTSK

Spectrum No: 229; Query: 581; Rank: 1

Peptide View

MS/MS Fragmentation of **SYEDLTELEDR**
Found in **IPI00195732**, Tax_Id=10116 Gene_Symbol=Mon1a_predicted similar to MON1 homolog A

Match to Query 581: 1448.574148 from(725.294350,2+)
Title: 091008RatKidney_NoSalt_26.2617.2617.2.dta

Data file K:\NewmanPaper\Piliang\3SubProteomes\Piliang3SP\mgf5ppm\ERLIC_3SubProteomes5ppm.mgf

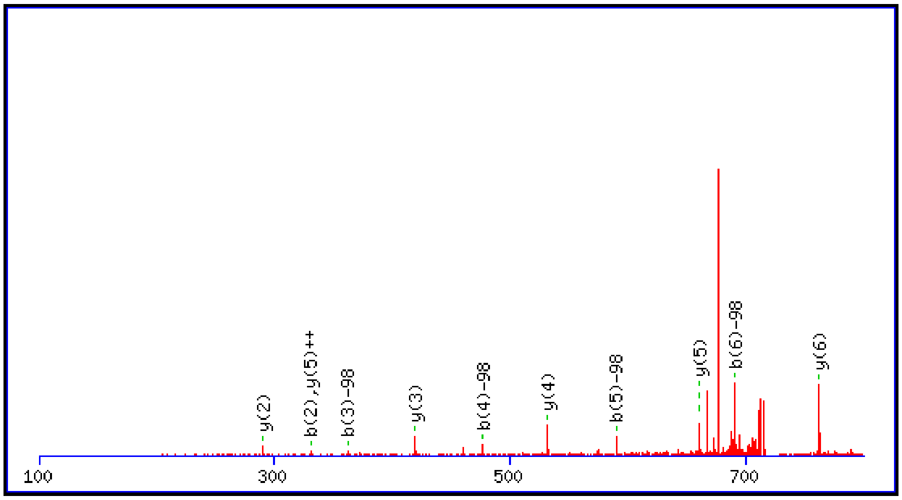

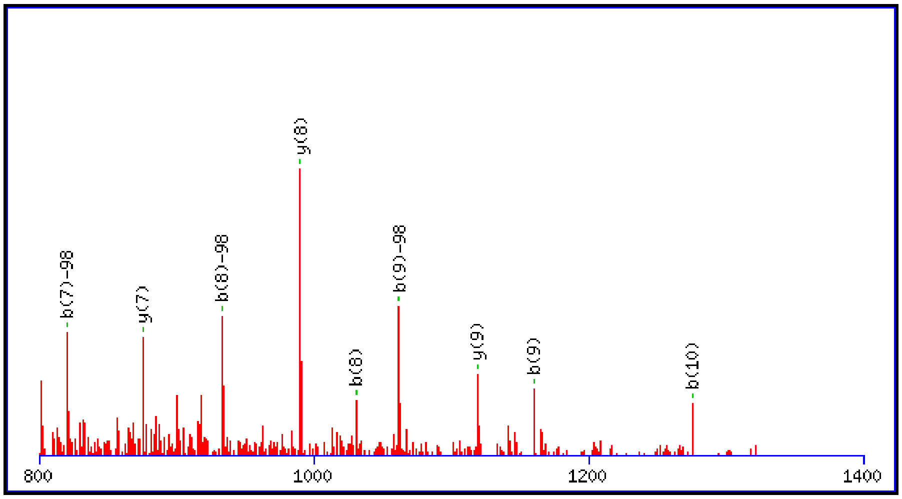

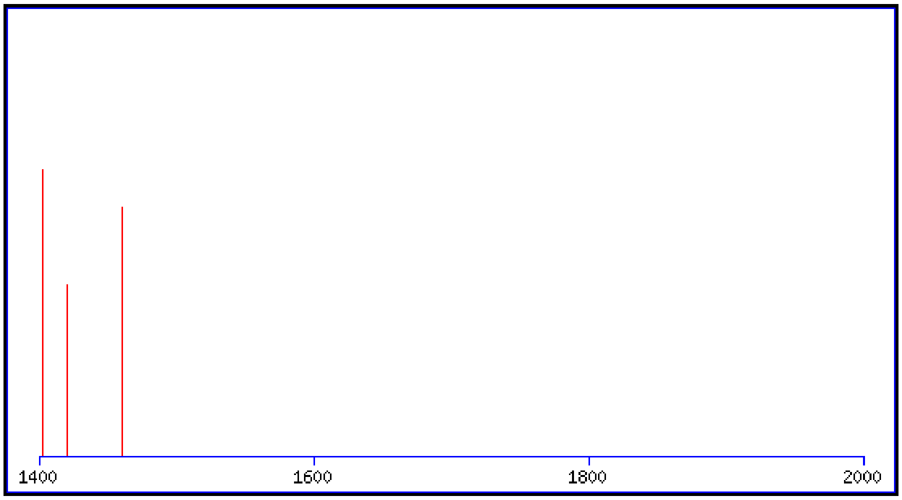

Monoisotopic mass of neutral peptide Mr(calc): 1448.5708
Fixed modifications: Carbamidomethyl (C)
Variable modifications:
S1 : Phospho (ST), with neutral losses 97.9769(shown in table), 0.0000
Ions Score: 55 **Expect:** 0.00035
Matches (Bold Red): 20/138 fragment ions using 35 most intense peaks

#	b	b^{++}	b^0	b^{0++}	Seq.	y	y^{++}	y*	y^{*++}	y^0	y^{0++}	#
1	70.0287	35.5180	52.0182	26.5127	S							11
2	233.0921	117.0497	215.0815	108.0444	Y	1282.5798	641.7935	1265.5532	633.2802	1264.5692	632.7882	10
3	**362.1347**	181.5710	344.1241	172.5657	E	**1119.5164**	560.2619	1102.4899	551.7486	1101.5059	551.2566	9
4	**477.1616**	239.0844	459.1510	230.0792	D	**990.4738**	495.7406	973.4473	487.2273	972.4633	486.7353	8

**5**	**590.2457**	295.6265	572.2351	286.6212	**L**	**875.4469**	438.2271	858.4203	429.7138	857.4363	429.2218	**7**
**6**	**691.2933**	346.1503	673.2828	337.1450	**T**	**762.3628**	381.6851	745.3363	373.1718	744.3523	372.6798	**6**
**7**	**820.3359**	410.6716	802.3254	401.6663	**E**	**661.3151**	**331.1612**	644.2886	322.6479	643.3046	322.1559	**5**
**8**	**933.4200**	467.2136	915.4094	458.2084	**L**	**532.2726**	266.6399	515.2460	258.1266	514.2620	257.6346	**4**
**9**	**1062.4626**	531.7349	1044.4520	522.7297	**E**	**419.1885**	210.0979	402.1619	201.5846	401.1779	201.0926	**3**
**10**	1177.4895	589.2484	1159.4790	580.2431	**D**	**290.1459**	145.5766	273.1193	137.0633	272.1353	136.5713	**2**
**11**					**R**	175.1190	88.0631	158.0924	79.5498			**1**

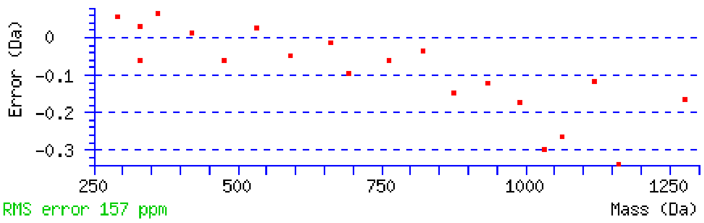

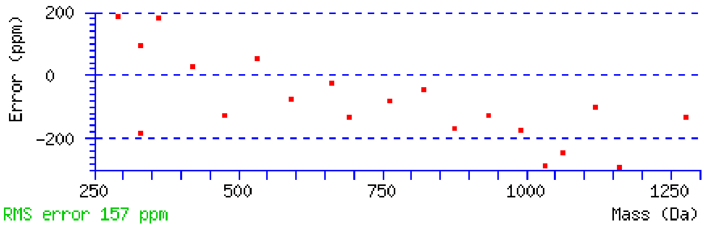

All matches to this query

Score	Mr(calc):	Delta	Sequence
54.6	1448.5708	0.0033	SYEDLTELEDR
34.0	1448.5708	0.0033	SYEDLTELEDR
14.5	1448.5708	0.0033	SYEDLTELEDR
6.8	1448.5793	-0.0052	HNQATENNERR
5.9	1448.5820	-0.0079	QSDDTSYLLGNR
5.9	1448.5820	-0.0079	QSDDTSYLLGNR
5.9	1448.5820	-0.0079	QSDDTSYLLGNR
2.3	1447.5715	1.0026	SDSEEKSDSEKK
2.3	1447.5715	1.0026	SDSEEKSDSEKK
2.1	1448.5711	0.0031	ASGHYSAERRR

Spectrum No: 230; Query: 1605; Rank: 1

Peptide View

MS/MS Fragmentation of **EGPEPPEEVPAPTTPPAPK**
Found in **IPI00417730**, Tax_Id=10116 Gene_Symbol=Bat2 Large proline-rich protein BAT2

Match to Query 1605: 2018.927388 from(1010.470970,2+)
Title: 100101RatKid_NS_deglyco_19.2120.2120.2.dta
Data file K:\NewmanPaper\Piliang\3SubProteomes\Piliang3SP\mgf5ppm\ERLIC_3SubProteomes5ppm.mgf

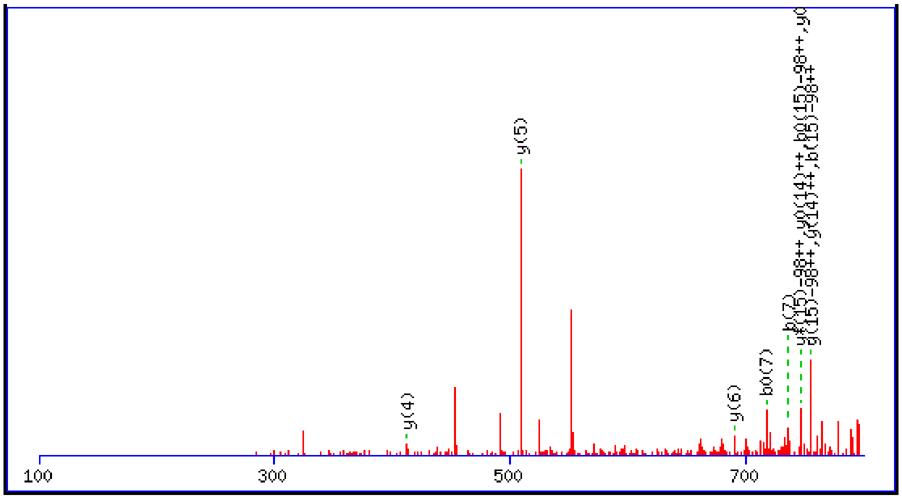

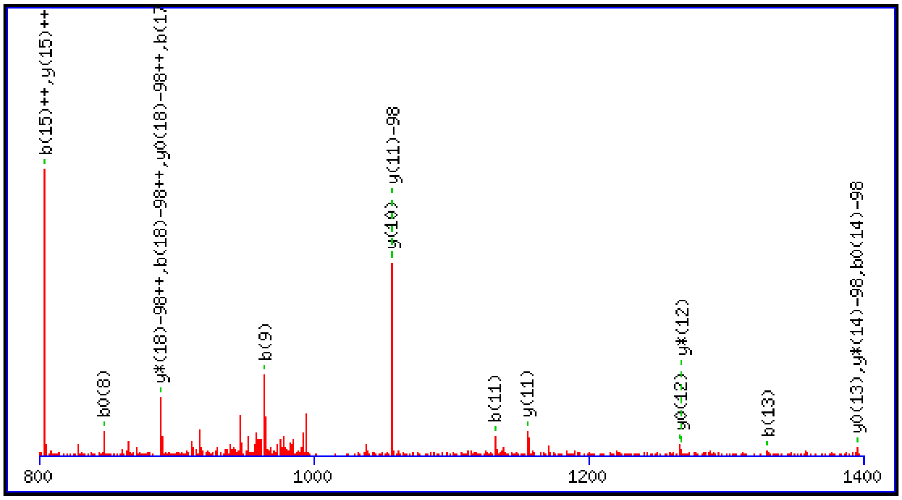

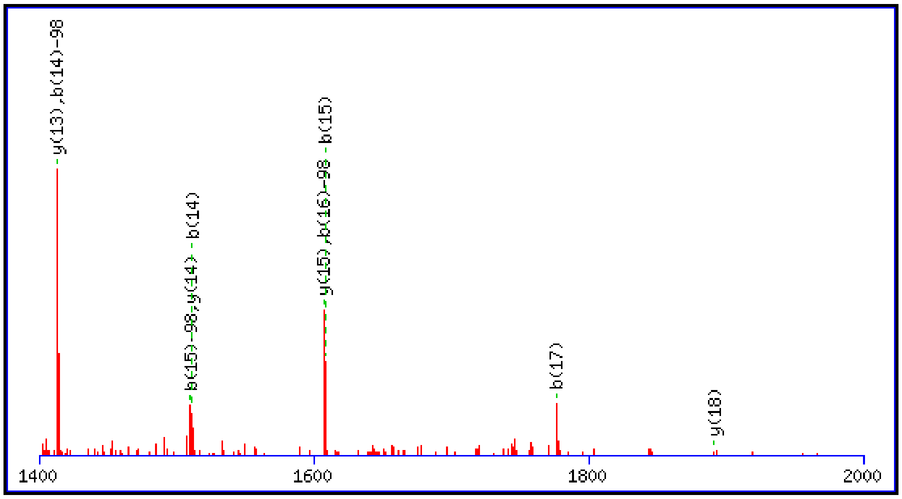

Monoisotopic mass of neutral peptide Mr(calc): 2018.9238
Fixed modifications: Carbamidomethyl (C)
Variable modifications:
T14 : Phospho (ST), with neutral losses 0.0000(shown in table), 97.9769
Ions Score: 55 **Expect:** 0.00088
Matches (Bold Red): 42/268 fragment ions using 44 most intense peaks

#	b	b^{++}	b^0	b^{0++}	Seq.	y	y^{++}	y^*	y^{*++}	y^0	y^{0++}	#
1	130.0499	65.5286	112.0393	56.5233	E							19
2	187.0713	94.0393	169.0608	85.0340	G	**1890.8885**	945.9479	1873.8619	937.4346	1872.8779	936.9426	18
3	284.1241	142.5657	266.1135	133.5604	P	1833.8670	917.4371	1816.8405	908.9239	1815.8565	908.4319	17
4	413.1667	207.0870	395.1561	198.0817	E	1736.8143	868.9108	1719.7877	860.3975	1718.8037	859.9055	16
5	510.2195	255.6134	492.2089	246.6081	P	**1607.7717**	**804.3895**	1590.7451	795.8762	1589.7611	795.3842	15

6	607.2722	304.1397	589.2617	295.1345	P	1510.7189	755.8631	1493.6923	747.3498	1492.7083	746.8578	14
7	736.3148	368.6610	718.3042	359.6558	E	1413.6661	707.3367	1396.6396	698.8234	1395.6556	698.3314	13
8	865.3574	433.1823	847.3468	424.1771	E	1284.6235	642.8154	1267.5970	634.3021	1266.6130	633.8101	12
9	964.4258	482.7165	946.4153	473.7113	V	1155.5809	578.2941	1138.5544	569.7808	1137.5704	569.2888	11
10	1061.4786	531.2429	1043.4680	522.2376	P	1056.5125	528.7599	1039.4860	520.2466	1038.5020	519.7546	10
11	1132.5157	566.7615	1114.5051	557.7562	A	959.4598	480.2335	942.4332	471.7202	941.4492	471.2282	9
12	1229.5685	615.2879	1211.5579	606.2826	P	888.4227	444.7150	871.3961	436.2017	870.4121	435.7097	8
13	1330.6161	665.8117	1312.6056	656.8064	T	791.3699	396.1886	774.3433	387.6753	773.3593	387.1833	7
14	1511.6301	756.3187	1493.6196	747.3134	T	690.3222	345.6647	673.2957	337.1515	672.3116	336.6595	6
15	1608.6829	804.8451	1590.6723	795.8398	P	509.3082	255.1577	492.2817	246.6445			5
16	1705.7357	853.3715	1687.7251	844.3662	P	412.2554	206.6314	395.2289	198.1181			4
17	1776.7728	888.8900	1758.7622	879.8847	A	315.2027	158.1050	298.1761	149.5917			3
18	1873.8255	937.4164	1855.8150	928.4111	P	244.1656	122.5864	227.1390	114.0731			2
19					K	147.1128	74.0600	130.0863	65.5468			1

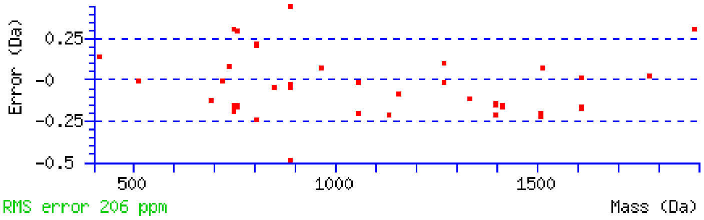

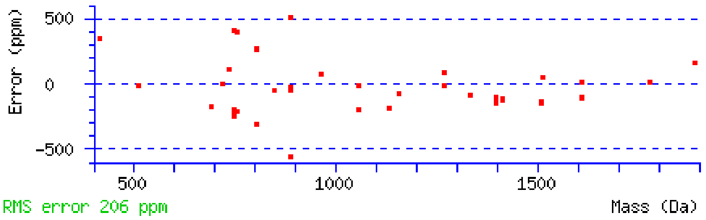

All matches to this query

Score	Mr(calc):	Delta	Sequence
54.6	2018.9238	0.0036	EGPEPPEEVPAPTTPPAPK
52.4	2018.9238	0.0036	EGPEPPEEVPAPTTPPAPK
7.0	2017.9048	1.0226	GTGPVQIPPGEWHSSFXR
7.0	2017.9048	1.0226	GTGPVQIPPGEWHSSFXR
7.0	2017.9048	1.0226	GTGPVQIPPGEWHSSFXR
3.0	2018.9075	0.0199	SRELTGGVDGIASIESIR
1.7	2017.9179	1.0095	EISVAIKTLKVGYTEK
1.3	2018.9091	0.0183	EHNTPDKAMWGFACTVR
0.9	2018.9334	-0.0060	GLGGFEENNHLFLNGGSQK
0.9	2017.9202	1.0072	GPXAGPSGPSATQEQQQGHR

Spectrum No: 231; Query: 2715; Rank: 1

Peptide View

MS/MS Fragmentation of **EAGVVDRESEGISDPELEQTDYCADPLPEGR**
Found in **IPI00564481**, Tax_Id=10116 Gene_Symbol=RGD1565449_predicted 72 kDa protein

Match to Query 2715: 3512.508702 from(1171.843510,3+)
Title: 100101RatKid_NS_deglyco_22.3294.3294.3.dta
Data file K:\NewmanPaper\Piliang\3SubProteomes\Piliang3SP\mgf5ppm\ERLIC_3SubProteomes5ppm.mgf

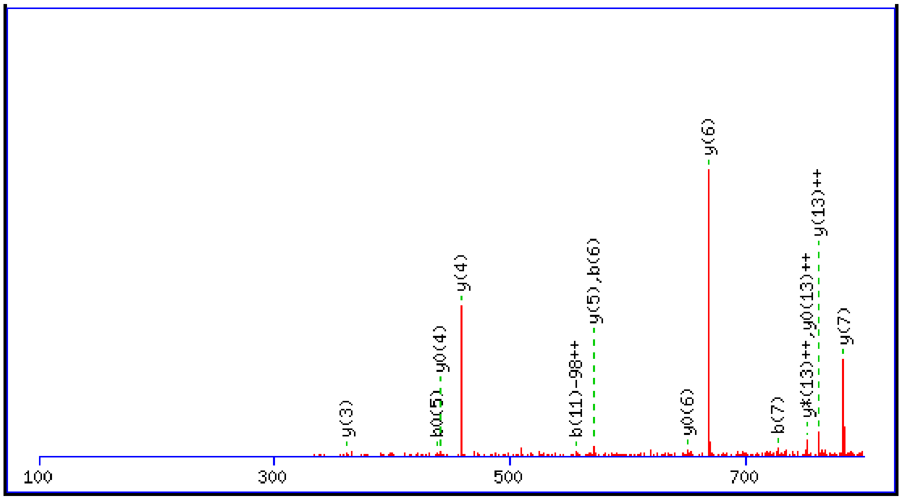

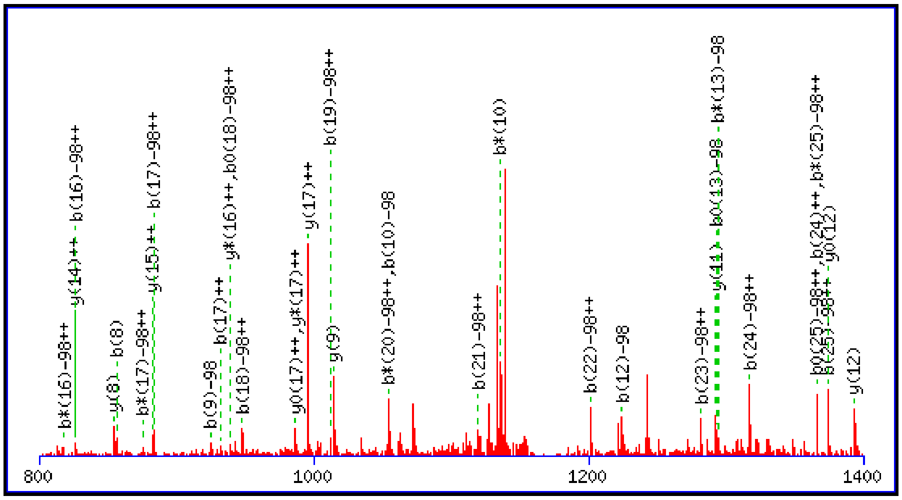

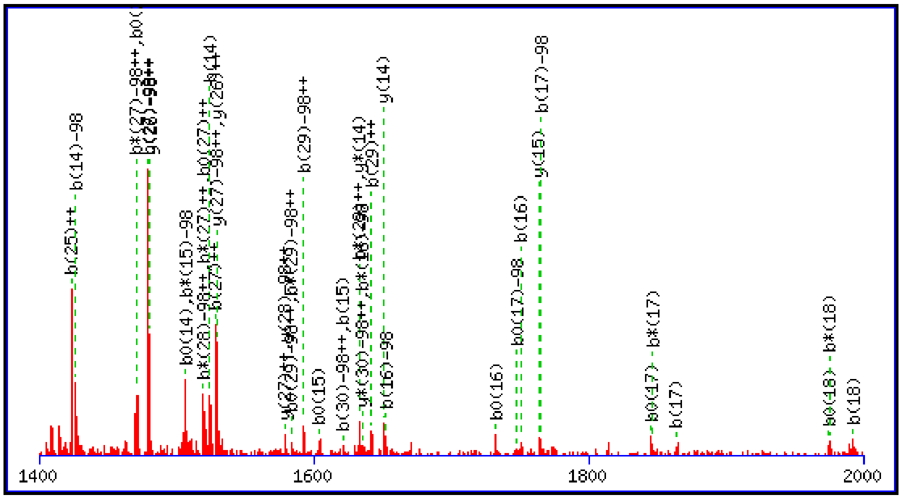

Monoisotopic mass of neutral peptide Mr(calc): 3512.4926
Fixed modifications: Carbamidomethyl (C)
Variable modifications:
S9 : Phospho (ST), with neutral losses 97.9769(shown in table), 0.0000
Ions Score: 55 **Expect:** 0.0017
Matches (Bold Red): 90/524 fragment ions using 141 most intense peaks

#	b	b^{++}	b*	b^{*++}	b^0	b^{0++}	Seq.	y	y^{++}	y*	y^{*++}	y^0	y^{0++}	#
1	130.0499	65.5286			112.0393	56.5233	E							31
2	201.0870	101.0471			183.0764	92.0418	A	3286.4805	1643.7439	3269.4539	**1635.2306**	3268.4699	1634.7386	30
3	258.1084	129.5579			240.0979	120.5526	G	3215.4433	1608.2253	3198.4168	1599.7120	3197.4328	1599.2200	29
4	357.1769	179.0921			339.1663	170.0868	V	3158.4219	**1579.7146**	3141.3953	1571.2013	3140.4113	1570.7093	28
5	456.2453	228.6263			**438.2347**	219.6210	V	3059.3535	**1530.1804**	3042.3269	1521.6671	3041.3429	1521.1751	27

6	**571.2722**	286.1397			553.2617	277.1345	D	2960.2850	**1480.6462**	2943.2585	1472.1329	2942.2745	1471.6409	26
7	**727.3733**	364.1903	710.3468	355.6770	709.3628	355.1850	R	2845.2581	1423.1327	2828.2316	1414.6194	2827.2475	1414.1274	25
8	**856.4159**	428.7116	839.3894	420.1983	838.4054	419.7063	E	2689.1570	1345.0821	2672.1304	1336.5689	2671.1464	1336.0769	24
9	**925.4374**	463.2223	908.4108	454.7091	907.4268	454.2170	S	2560.1144	1280.5608	2543.0878	1272.0476	2542.1038	1271.5556	23
10	**1054.4800**	527.7436	1037.4534	519.2303	1036.4694	518.7383	E	2491.0929	1246.0501	2474.0664	1237.5368	2473.0824	1237.0448	22
11	1111.5014	**556.2544**	1094.4749	547.7411	1093.4909	547.2491	G	2362.0503	1181.5288	2345.0238	1173.0155	2344.0398	1172.5235	21
12	**1224.5855**	612.7964	1207.5590	604.2831	1206.5749	603.7911	I	2305.0289	1153.0181	2288.0023	1144.5048	2287.0183	1144.0128	20
13	1311.6175	656.3124	**1294.5910**	647.7991	**1293.6070**	647.3071	S	2191.9448	1096.4760	2174.9183	1087.9628	2173.9343	1087.4708	19
14	**1426.6445**	713.8259	1409.6179	705.3126	1408.6339	704.8206	D	2104.9128	1052.9600	2087.8862	1044.4468	2086.9022	1043.9548	18
15	1523.6972	762.3523	**1506.6707**	753.8390	1505.6867	753.3470	P	1989.8858	**995.4466**	1972.8593	**986.9333**	1971.8753	**986.4413**	17
16	**1652.7398**	**826.8736**	**1635.7133**	**818.3603**	1634.7293	817.8683	E	1892.8331	946.9202	1875.8065	**938.4069**	1874.8225	937.9149	16
17	**1765.8239**	**883.4156**	1748.7973	**874.9023**	**1747.8133**	874.4103	L	**1763.7905**	**882.3989**	1746.7639	873.8856	1745.7799	873.3936	15
18	1894.8665	**947.9369**	1877.8399	939.4236	1876.8559	**938.9316**	E	**1650.7064**	**825.8569**	**1633.6799**	817.3436	1632.6959	816.8516	14
19	2022.9251	**1011.9662**	2005.8985	1003.4529	2004.9145	1002.9609	Q	1521.6638	**761.3356**	1504.6373	**752.8223**	1503.6533	**752.3303**	13
20	2123.9727	1062.4900	2106.9462	**1053.9767**	2105.9622	1053.4847	T	**1393.6053**	697.3063	1376.5787	688.7930	**1375.5947**	688.3010	12
21	2238.9997	**1120.0035**	2221.9731	1111.4902	2220.9891	1110.9982	D	**1292.5576**	646.7824	1275.5310	638.2692	1274.5470	637.7771	11
22	2402.0630	**1201.5351**	2385.0365	1193.0219	2384.0524	1192.5299	Y	1177.5306	589.2690	1160.5041	580.7557	1159.5201	580.2637	10
23	2562.0937	**1281.5505**	2545.0671	1273.0372	2544.0831	1272.5452	C	**1014.4673**	507.7373	997.4408	499.2240	996.4567	498.7320	9
24	2633.1308	**1317.0690**	2616.1042	1308.5558	2615.1202	1308.0637	A	**854.4367**	427.7220	837.4101	419.2087	836.4261	418.7167	8
25	2748.1577	**1374.5825**	2731.1312	**1366.0692**	2730.1472	**1365.5772**	D	**783.3995**	392.2034	766.3730	383.6901	765.3890	383.1981	7
26	2845.2105	1423.1089	2828.1839	1414.5956	2827.1999	1414.1036	P	**668.3726**	334.6899	651.3461	326.1767	**650.3620**	325.6847	6
27	2958.2945	**1479.6509**	2941.2680	**1471.1376**	2940.2840	**1470.6456**	L	**571.3198**	286.1636	554.2933	277.6503	553.3093	277.1583	5
28	3055.3473	1528.1773	3038.3208	**1519.6640**	3037.3367	1519.1720	P	**458.2358**	229.6215	441.2092	221.1083	**440.2252**	220.6162	4
29	3184.3899	**1592.6986**	3167.3634	**1584.1853**	3166.3793	**1583.6933**	E	**361.1830**	181.0951	344.1565	172.5819	343.1724	172.0899	3
30	3241.4114	**1621.2093**	3224.3848	1612.6960	3223.4008	1612.2040	G	232.1404	116.5738	215.1139	108.0606			2
31							R	175.1190	88.0631	158.0924	79.5498			1

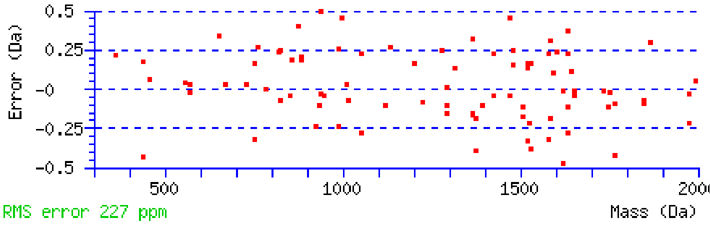

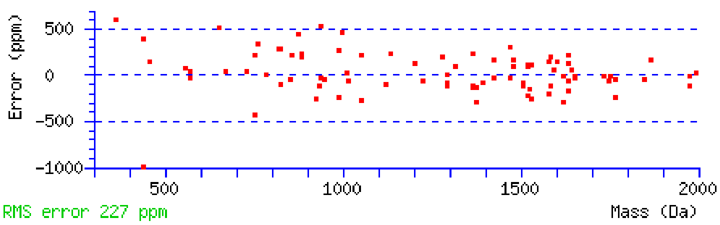

All matches to this query

Score	Mr(calc):	Delta	Sequence
54.6	3512.4926	0.0161	EAGVVDRESEGISDPELEQTDYCADPLPEGR
52.4	3512.4926	0.0161	EAGVVDRESEGISDPELEQTDYCADPLPEGR
30.4	3512.4926	0.0161	EAGVVDRESEGISDPELEQTDYCADPLPEGR
13.4	3512.4926	0.0161	EAGVVDRESEGISDPELEQTDYCADPLPEGR
10.5	3511.5224	0.9863	NLSANSFTLMLMSKNLQGNYTCSATNVLSR
10.5	3511.5224	0.9863	NLSANSFTLMLMSKNLQGNYTCSATNVLSR
10.2	3512.4959	0.0128	LDLTSVTEGSDNYKKNMNALSSSDNVVLLK
10.2	3512.4959	0.0128	LDLTSVTEGSDNYKKNMNALSSSDNVVLLK
9.0	3511.5119	0.9968	LDLTSVTEGSDNYKKNMNALSSSDNVVLLK
8.5	3511.4795	1.0292	YFRSLTAMTYTWLLTATTSVFNNDFCLD

Spectrum No: 232; Query: 881; Rank: 1

Peptide View

MS/MS Fragmentation of **DAVAVAPPPSPSLPAK**
Found in **IPI00205325**, Tax_Id=10116 Gene_Symbol=Lrp2 Low-density lipoprotein receptor-related protein 2 precursor

Match to Query 881: 1595.796028 from(798.905290,2+)
Title: 091008RatKidney_NH4Format02_25.2181.2181.2.dta
Data file K:\NewmanPaper\Piliang\3SubProteomes\Piliang3SP\mgf5ppm\ERLIC_3SubProteomes5ppm.mgf

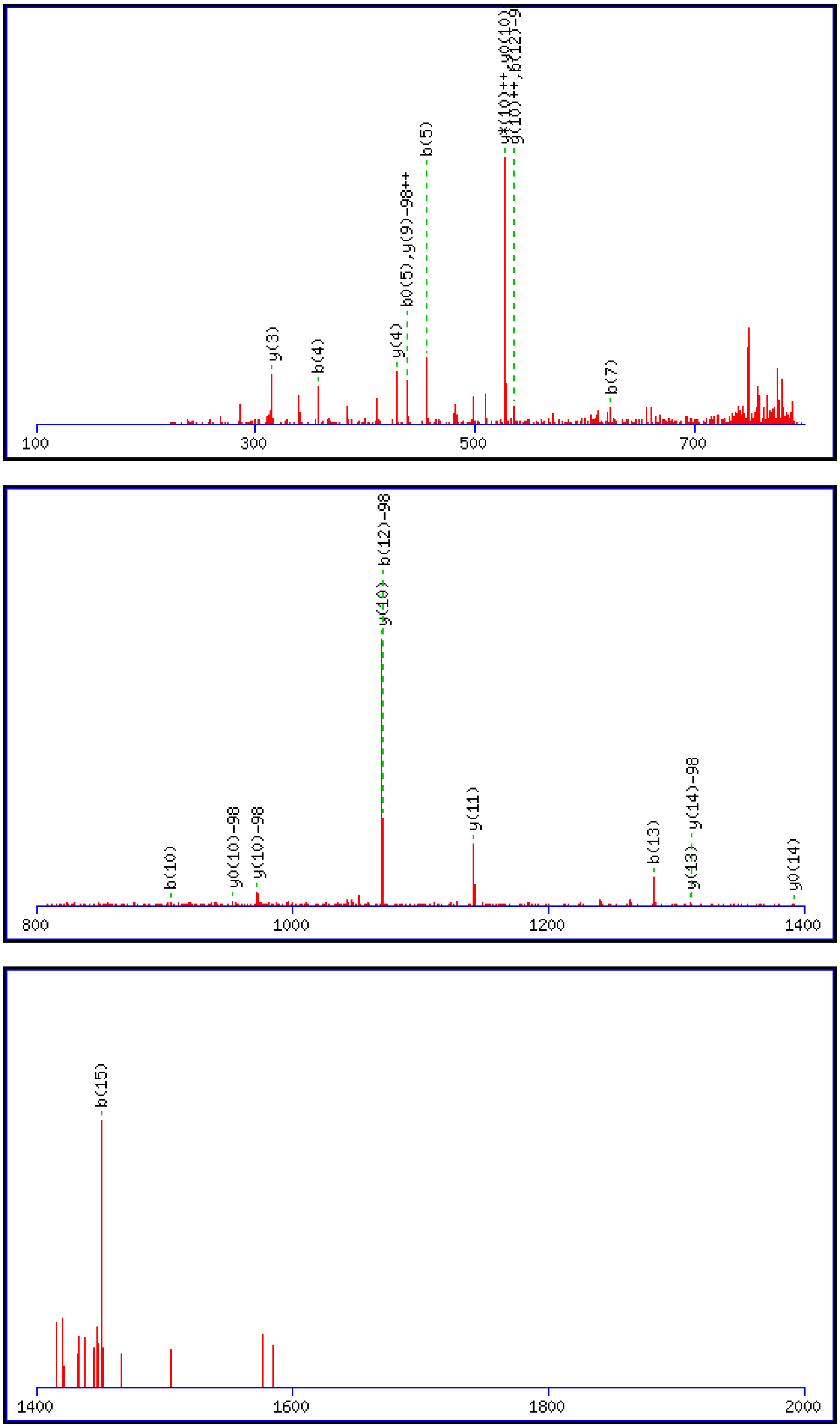

Monoisotopic mass of neutral peptide Mr(calc): 1595.7960
Fixed modifications: Carbamidomethyl (C)
Variable modifications:
S12 : Phospho (ST), with neutral losses 0.0000(shown in table), 97.9769
Ions Score: 55 **Expect:** 0.00055

Matches (Bold Red): 24/224 fragment ions using 46 most intense peaks

#	b	b^{++}	b^0	b^{0++}	Seq.	y	y^{++}	y*	y^{*++}	y^0	y^{0++}	#
1	116.0342	58.5207	98.0237	49.5155	D							16
2	187.0713	94.0393	169.0608	85.0340	A	1481.7764	741.3918	1464.7498	732.8785	1463.7658	732.3865	15
3	286.1397	143.5735	268.1292	134.5682	V	1410.7392	705.8733	1393.7127	697.3600	**1392.7287**	696.8680	14
4	**357.1769**	179.0921	339.1663	170.0868	A	**1311.6708**	656.3391	1294.6443	647.8258	1293.6603	647.3338	13
5	**456.2453**	228.6263	**438.2347**	219.6210	V	1240.6337	620.8205	1223.6072	612.3072	1222.6231	611.8152	12
6	**527.2824**	264.1448	509.2718	255.1396	A	**1141.5653**	571.2863	1124.5387	562.7730	1123.5547	562.2810	11
7	**624.3352**	312.6712	606.3246	303.6659	P	**1070.5282**	**535.7677**	1053.5016	**527.2545**	1052.5176	**526.7624**	10
8	721.3879	361.1976	703.3774	352.1923	P	973.4754	487.2413	956.4489	478.7281	955.4649	478.2361	9
9	818.4407	409.7240	800.4301	400.7187	P	876.4227	438.7150	859.3961	430.2017	858.4121	429.7097	8
10	**905.4727**	453.2400	887.4621	444.2347	S	779.3699	390.1886	762.3433	381.6753	761.3593	381.1833	7
11	1002.5255	501.7664	984.5149	492.7611	P	692.3379	346.6726	675.3113	338.1593	674.3273	337.6673	6
12	1169.5238	585.2656	1151.5133	576.2603	S	595.2851	298.1462	578.2585	289.6329	577.2745	289.1409	5
13	**1282.6079**	641.8076	1264.5973	632.8023	L	**428.2867**	214.6470	411.2602	206.1337			4
14	1379.6607	690.3340	1361.6501	681.3287	P	**315.2027**	158.1050	298.1761	149.5917			3
15	**1450.6978**	725.8525	1432.6872	716.8472	A	218.1499	109.5786	201.1234	101.0653			2
16					K	147.1128	74.0600	130.0863	65.5468			1

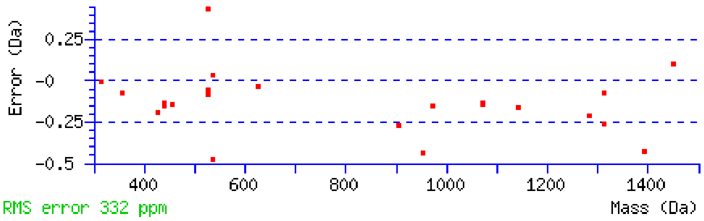

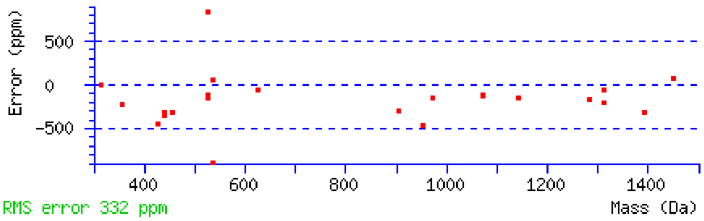

All matches to this query

Score	Mr(calc):	Delta	Sequence
54.5	1595.7960	0.0000	DAVAVAPPPSPSLPAK
40.8	1595.7960	0.0000	DAVAVAPPPSPSLPAK
12.5	1595.7823	0.0138	SFLVFINLYCIK
9.4	1595.7848	0.0113	IEQEFLTEALPVK
9.2	1595.8072	-0.0112	SAPVTALEPGRKYK
4.0	1595.8072	-0.0112	IKNPEGGLYVAVTR
3.1	1595.7977	-0.0017	ELMSPPDATQAAPLR
1.2	1594.7773	1.0188	KKVVAFSSVTELK
1.2	1594.7773	1.0188	KKVVAFSSVTELK
1.2	1594.7773	1.0188	KKVVAFSSVTELK

Spectrum No: 233; Query: 1548; Rank: 1

Peptide View

MS/MS Fragmentation of **AASDGQYENQSPEATSPR**
Found in **IPI00362755**, Tax_Id=10116 Gene_Symbol=Tns similar to tensin

Match to Query 1548: 1986.795068 from(994.404810,2+)
Title: 091008RatKidney_NoSalt_26.545.545.2.dta
Data file K:\NewmanPaper\Piliang\3SubProteomes\Piliang3SP\mgf5ppm\ERLIC_3SubProteomes5ppm.mgf

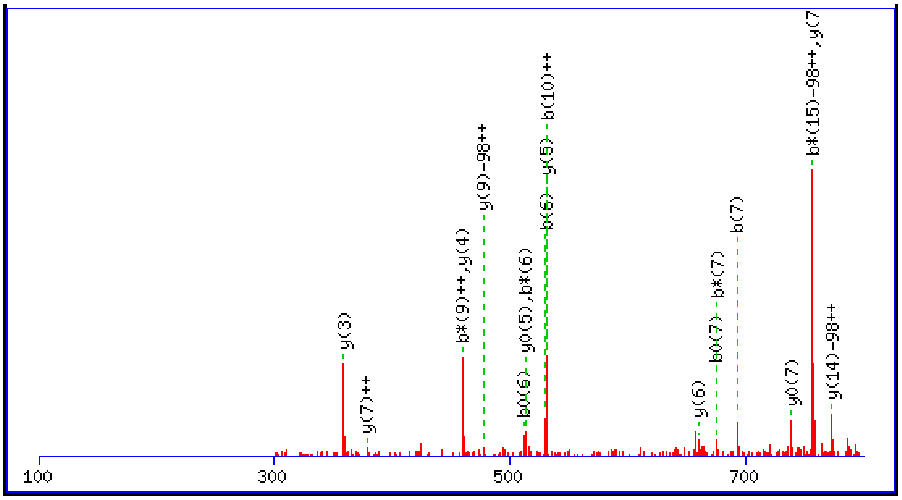

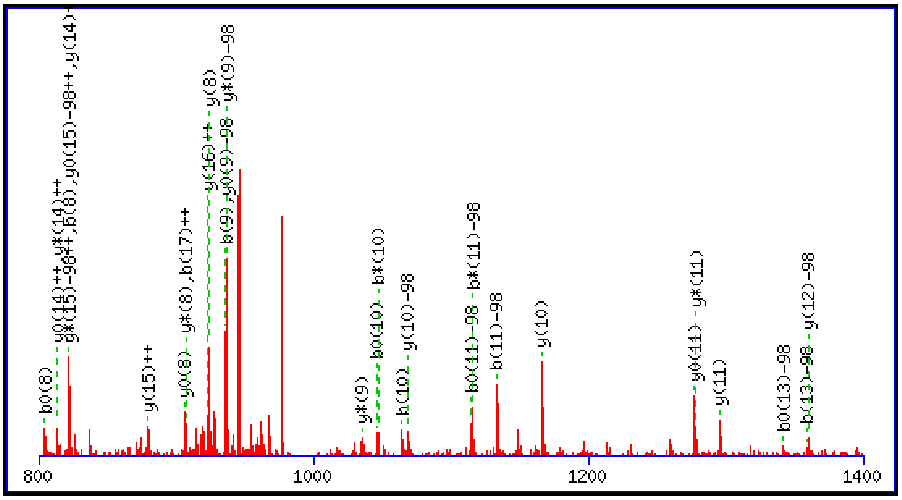

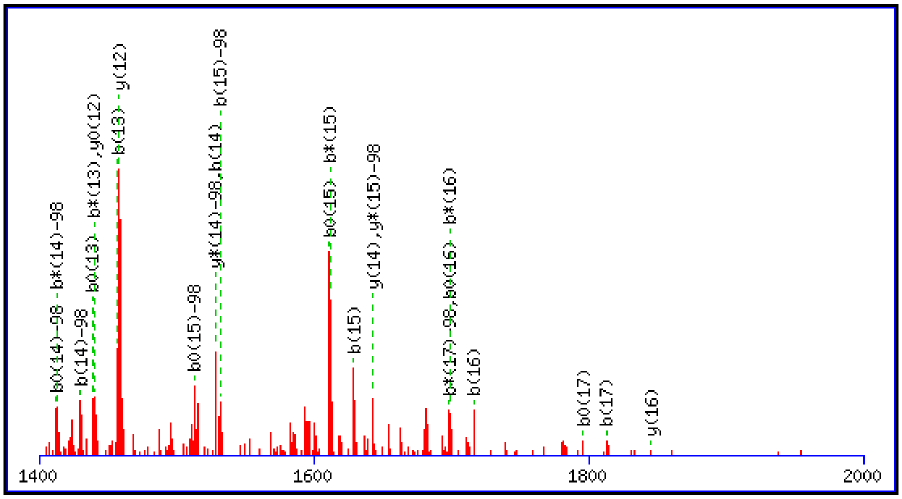

Monoisotopic mass of neutral peptide Mr(calc): 1986.7956
Fixed modifications: Carbamidomethyl (C)
Variable modifications:
S11 : Phospho (ST), with neutral losses 0.0000(shown in table), 97.9769
Ions Score: 54 **Expect:** 0.00063
Matches (Bold Red): 75/288 fragment ions using 106 most intense peaks

#	b	b^{++}	b*	b^{*++}	b^0	b^{0++}	Seq.	y	y^{++}	y*	y^{*++}	y^0	y^{0++}	#
1	72.0444	36.5258					A							18
2	143.0815	72.0444					A	1916.7658	958.8865	1899.7393	950.3733	1898.7552	949.8813	17
3	230.1135	115.5604			212.1030	106.5551	S	**1845.7287**	**923.3680**	1828.7021	914.8547	1827.7181	914.3627	16
4	345.1405	173.0739			327.1299	164.0686	D	1758.6967	**879.8520**	1741.6701	871.3387	1740.6861	870.8467	15
5	402.1619	201.5846			384.1514	192.5793	G	**1643.6697**	**822.3385**	1626.6432	**813.8252**	1625.6592	**813.3332**	14

6	**530.2205**	265.6139	**513.1940**	257.1006	**512.2100**	256.6086	Q	1586.6483	793.8278	1569.6217	785.3145	1568.6377	784.8225	13
7	**693.2838**	347.1456	**676.2573**	338.6323	**675.2733**	338.1403	Y	**1458.5897**	729.7985	1441.5631	721.2852	**1440.5791**	720.7932	12
8	**822.3264**	411.6669	805.2999	403.1536	**804.3159**	402.6616	E	**1295.5263**	648.2668	**1278.4998**	639.7535	**1277.5158**	639.2615	11
9	**936.3694**	468.6883	919.3428	**460.1750**	918.3588	459.6830	N	**1166.4838**	583.7455	1149.4572	575.2322	1148.4732	574.7402	10
10	**1064.4279**	**532.7176**	**1047.4014**	524.2043	**1046.4174**	523.7123	Q	1052.4408	526.7241	**1035.4143**	518.2108	1034.4303	517.7188	9
11	1231.4263	616.2168	1214.3997	607.7035	1213.4157	607.2115	S	**924.3822**	462.6948	**907.3557**	454.1815	**906.3717**	453.6895	8
12	1328.4791	664.7432	1311.4525	656.2299	1310.4685	655.7379	P	**757.3839**	**379.1956**	740.3573	370.6823	**739.3733**	370.1903	7
13	**1457.5217**	729.2645	**1440.4951**	720.7512	**1439.5111**	720.2592	E	**660.3311**	330.6692	643.3046	322.1559	642.3206	321.6639	6
14	**1528.5588**	764.7830	1511.5322	756.2697	1510.5482	755.7777	A	**531.2885**	266.1479	514.2620	257.6346	**513.2780**	257.1426	5
15	**1629.6064**	815.3069	**1612.5799**	806.7936	**1611.5959**	806.3016	T	**460.2514**	230.6293	443.2249	222.1161	442.2409	221.6241	4
16	**1716.6385**	858.8229	**1699.6119**	850.3096	**1698.6279**	849.8176	S	**359.2037**	180.1055	342.1772	171.5922	341.1932	171.1002	3
17	**1813.6912**	**907.3493**	1796.6647	898.8360	**1795.6807**	898.3440	P	272.1717	136.5895	255.1452	128.0762			2
18							R	175.1190	88.0631	158.0924	79.5498			1

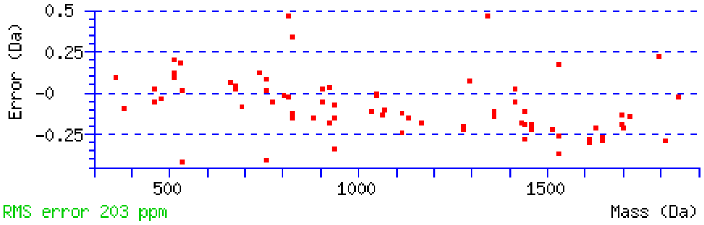

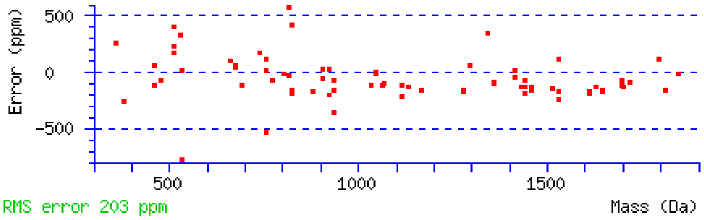

All matches to this query

Score	Mr(calc):	Delta	Sequence
54.5	1986.7956	-0.0005	AASDGQYENQSPEATSPR
25.6	1986.7956	-0.0005	AASDGQYENQSPEATSPR
24.5	1986.7956	-0.0005	AASDGQYENQSPEATSPR
18.6	1986.7956	-0.0005	AASDGQYENQSPEATSPR
17.6	1986.7956	-0.0005	AASDGQYENQSPEATSPR
2.3	1985.8004	0.9947	ENTEGEYTNLEHESVR
1.9	1986.7812	0.0139	EATLTMDQVSSLPALR
1.8	1986.7812	0.0139	EATLTMDQVSSLPALR
0.1	1985.7744	1.0207	DHLYGVHNPVMTSPSR

Spectrum No: 234; Query: 2030; Rank: 1

Peptide View

MS/MS Fragmentation of **SSTLEPEDEDDEDEDTMTR**
Found in **IPI00359020**, Tax_Id=10116 Gene_Symbol=RGD1308876_predicted hypothetical protein LOC362597

Match to Query 2030: 2309.802528 from(1155.908540,2+)
Title: 091008RatKidney_NH4Format02_31.612.612.2.dta
Data file K:\NewmanPaper\Piliang\3SubProteomes\Piliang3SP\mgf5ppm\ERLIC_3SubProteomes5ppm.mgf

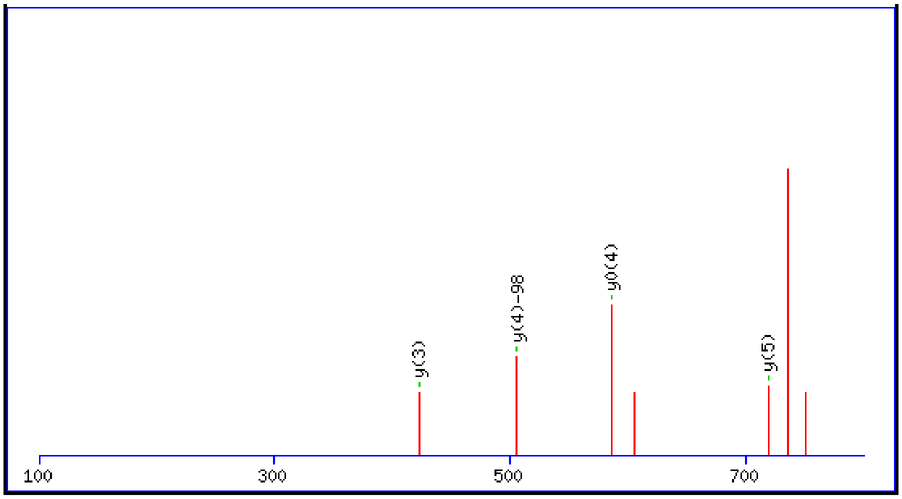

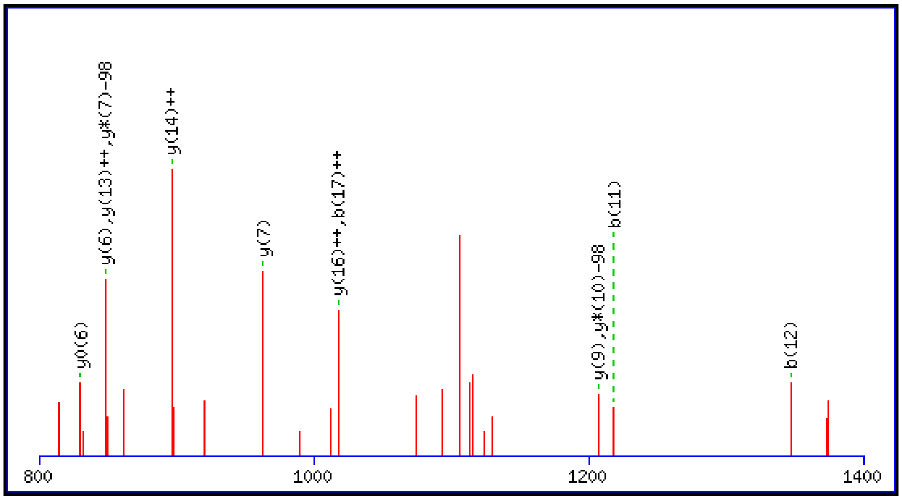

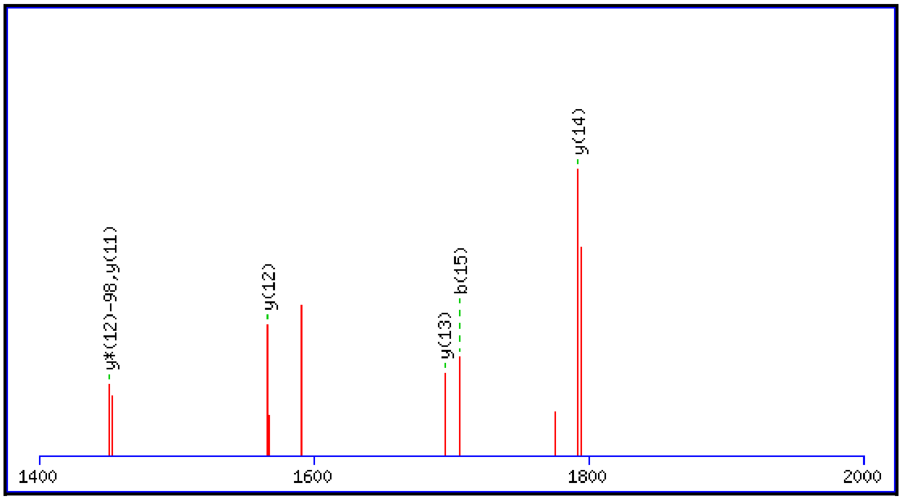

Monoisotopic mass of neutral peptide Mr(calc): 2308.8050
Fixed modifications: Carbamidomethyl (C)
Variable modifications:
T16 : Phospho (ST), with neutral losses 0.0000(shown in table), 97.9769
M17 : Oxidation (M)
Ions Score: 54 **Expect:** 0.00034
Matches (Bold Red): 22/280 fragment ions using 28 most intense peaks

#	b	b^{++}	b^0	b^{0++}	Seq.	y	y^{++}	y*	y^{*++}	y^0	y^{0++}	#
1	88.0393	44.5233	70.0287	35.5180	S							19
2	175.0713	88.0393	157.0608	79.0340	S	2222.7803	1111.8938	2205.7537	1103.3805	2204.7697	1102.8885	18
3	276.1190	138.5631	258.1084	129.5579	T	2135.7482	1068.3778	2118.7217	1059.8645	2117.7377	1059.3725	17
4	389.2031	195.1052	371.1925	186.0999	L	2034.7005	**1017.8539**	2017.6740	1009.3406	2016.6900	1008.8486	16

**5**	518.2457	259.6265	500.2351	250.6212	**E**	1921.6165	961.3119	1904.5899	952.7986	1903.6059	952.3066	**15**
**6**	615.2984	308.1529	597.2879	299.1476	**P**	**1792.5739**	**896.7906**	1775.5473	888.2773	1774.5633	887.7853	**14**
**7**	744.3410	372.6742	726.3305	363.6689	**E**	**1695.5211**	**848.2642**	1678.4946	839.7509	1677.5106	839.2589	**13**
**8**	859.3680	430.1876	841.3574	421.1823	**D**	**1566.4785**	783.7429	1549.4520	775.2296	1548.4680	774.7376	**12**
**9**	988.4106	494.7089	970.4000	485.7036	**E**	**1451.4516**	726.2294	1434.4250	717.7162	1433.4410	717.2242	**11**
**10**	1103.4375	552.2224	1085.4269	543.2171	**D**	1322.4090	661.7081	1305.3824	653.1949	1304.3984	652.7029	**10**
**11**	**1218.4644**	609.7359	1200.4539	600.7306	**D**	**1207.3821**	604.1947	1190.3555	595.6814	1189.3715	595.1894	**9**
**12**	**1347.5070**	674.2572	1329.4965	665.2519	**E**	1092.3551	546.6812	1075.3286	538.1679	1074.3445	537.6759	**8**
**13**	1462.5340	731.7706	1444.5234	722.7653	**D**	**963.3125**	482.1599	946.2860	473.6466	945.3020	473.1546	**7**
**14**	1591.5766	796.2919	1573.5660	787.2866	**E**	**848.2856**	424.6464	831.2590	416.1332	**830.2750**	415.6411	**6**
**15**	**1706.6035**	853.8054	1688.5930	844.8001	**D**	**719.2430**	360.1251	702.2164	351.6119	701.2324	351.1198	**5**
**16**	1887.6175	944.3124	1869.6070	935.3071	**T**	604.2160	302.6117	587.1895	294.0984	**586.2055**	293.6064	**4**
**17**	2034.6529	**1017.8301**	2016.6424	1008.8248	**M**	**423.2020**	212.1047	406.1755	203.5914	405.1915	203.0994	**3**
**18**	2135.7006	1068.3539	2117.6900	1059.3487	**T**	276.1666	138.5870	259.1401	130.0737	258.1561	129.5817	**2**
**19**					**R**	175.1190	88.0631	158.0924	79.5498			**1**

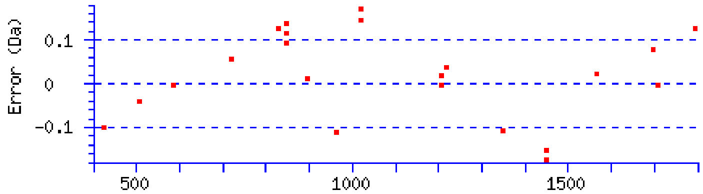

RMS error 106 ppm

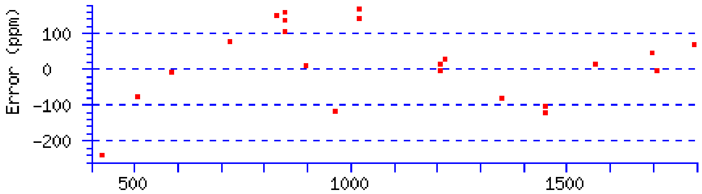

RMS error 106 ppm

All matches to this query

Score	Mr(calc):	Delta	Sequence
54.4	2308.8050	0.9975	SSTLEPEDEDDEDEDTMTR
43.1	2308.8050	0.9975	SSTLEPEDEDDEDEDTMTR
7.2	2309.8241	-0.0216	DCYPEGAIYPQDELSVSK
6.9	2309.8241	-0.0216	DCYPEGAIYPQDELSVSK
6.9	2309.8241	-0.0216	DCYPEGAIYPQDELSVSK
0.2	2308.8207	0.9818	CPEDWYADTEQTHCLPK
0.2	2308.8207	0.9818	CPEDWYANTEQTHCLPK

Spectrum No: 235; Query: 2524; Rank: 1

Peptide View

MS/MS Fragmentation of **TRSPDVISSASTALSQDIPEIASEALSR**
Found in **IPI00207643**, Tax_Id=10116 Gene_Symbol=LOC361399;RGD1309136_predicted LRRG00115

Match to Query 2524: 2980.438422 from(994.486750,3+)
Title: 091008RatKidney_NH4Format02_24.5718.5718.3.dta
Data file K:\NewmanPaper\Piliang\3SubProteomes\Piliang3SP\mgf5ppm\ERLIC_3SubProteomes5ppm.mgf

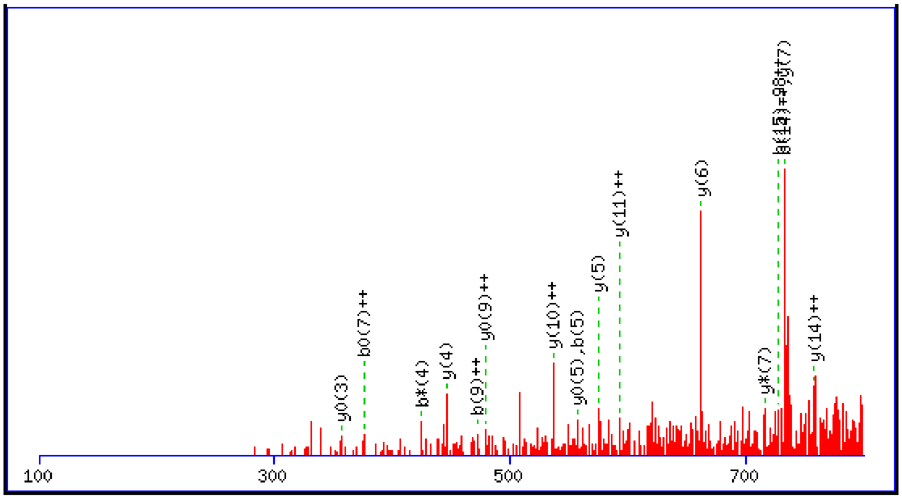

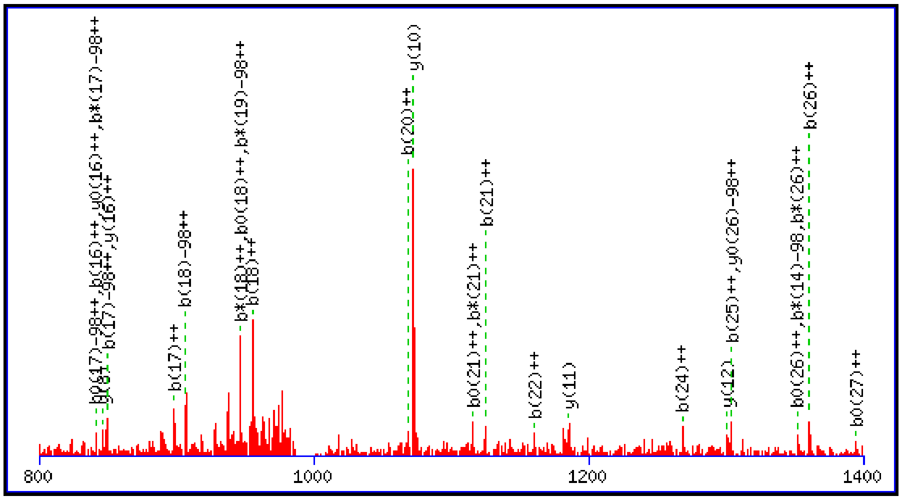

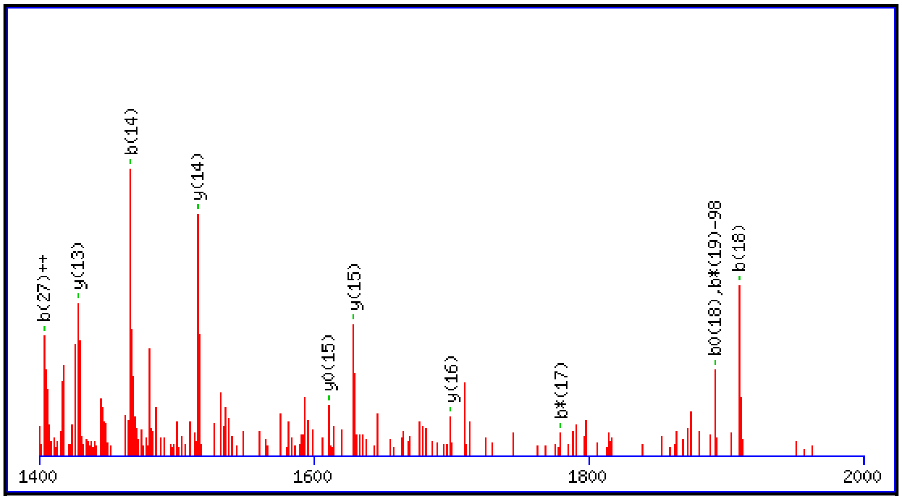

Monoisotopic mass of neutral peptide Mr(calc): 2980.4390
Fixed modifications: Carbamidomethyl (C)
Variable modifications:
T12 : Phospho (ST), with neutral losses 0.0000(shown in table), 97.9769
Ions Score: 54 **Expect:** 0.0011
Matches (Bold Red): 57/482 fragment ions using 97 most intense peaks

#	b	b++	b*	b*++	b0	b0++	Seq.	y	y++	y*	y*++	y0	y0++	#
1	102.0550	51.5311			84.0444	42.5258	T							28
2	258.1561	129.5817	241.1295	121.0684	240.1455	120.5764	R	2880.3986	1440.7030	2863.3721	1432.1897	2862.3881	1431.6977	27
3	345.1881	173.0977	328.1615	164.5844	327.1775	164.0924	S	2724.2975	1362.6524	2707.2710	1354.1391	2706.2870	1353.6471	26
4	442.2409	221.6241	**425.2143**	213.1108	424.2303	212.6188	P	2637.2655	1319.1364	2620.2389	1310.6231	2619.2549	1310.1311	25
5	**557.2678**	279.1375	540.2413	270.6243	539.2572	270.1323	D	2540.2127	1270.6100	2523.1862	1262.0967	2522.2022	1261.6047	24

**6**	656.3362	328.6717	639.3097	320.1585	638.3257	319.6665	**V**	2425.1858	1213.0965	2408.1592	1204.5833	2407.1752	1204.0912	**23**
**7**	769.4203	385.2138	752.3937	376.7005	751.4097	**376.2085**	**I**	2326.1174	1163.5623	2309.0908	1155.0490	2308.1068	1154.5570	**22**
**8**	856.4523	428.7298	839.4258	420.2165	838.4417	419.7245	**S**	2213.0333	1107.0203	2196.0068	1098.5070	2195.0227	1098.0150	**21**
**9**	943.4843	**472.2458**	926.4578	463.7325	925.4738	463.2405	**S**	2126.0013	1063.5043	2108.9747	1054.9910	2107.9907	1054.4990	**20**
**10**	1014.5214	507.7644	997.4949	499.2511	996.5109	498.7591	**A**	2038.9693	1019.9883	2021.9427	1011.4750	2020.9587	1010.9830	**19**
**11**	1101.5535	551.2804	1084.5269	542.7671	1083.5429	542.2751	**S**	1967.9321	984.4697	1950.9056	975.9564	1949.9216	975.4644	**18**
**12**	1282.5675	641.7874	1265.5409	633.2741	1264.5569	632.7821	**T**	1880.9001	940.9537	1863.8736	932.4404	1862.8895	931.9484	**17**
**13**	1353.6046	677.3059	1336.5780	668.7927	1335.5940	668.3007	**A**	**1699.8861**	**850.4467**	1682.8596	841.9334	1681.8755	**841.4414**	**16**
**14**	**1466.6887**	**733.8480**	1449.6621	725.3347	1448.6781	724.8427	**L**	**1628.8490**	814.9281	1611.8224	806.4149	**1610.8384**	805.9229	**15**
**15**	1553.7207	777.3640	1536.6941	768.8507	1535.7101	768.3587	**S**	**1515.7649**	**758.3861**	1498.7384	749.8728	1497.7544	749.3808	**14**
**16**	1681.7793	**841.3933**	1664.7527	832.8800	1663.7687	832.3880	**Q**	**1428.7329**	714.8701	1411.7064	706.3568	1410.7223	705.8648	**13**
**17**	1796.8062	**898.9067**	**1779.7797**	890.3935	1778.7956	889.9015	**D**	**1300.6743**	650.8408	1283.6478	642.3275	1282.6638	641.8355	**12**
**18**	**1909.8903**	**955.4488**	1892.8637	**946.9355**	**1891.8797**	**946.4435**	**I**	**1185.6474**	**593.3273**	1168.6208	584.8141	1167.6368	584.3220	**11**
**19**	2006.9430	1003.9752	1989.9165	995.4619	1988.9325	994.9699	**P**	**1072.5633**	**536.7853**	1055.5368	528.2720	1054.5527	527.7800	**10**
**20**	2135.9856	**1068.4965**	2118.9591	1059.9832	2117.9751	1059.4912	**E**	975.5106	488.2589	958.4840	479.7456	957.5000	**479.2536**	**9**
**21**	2249.0697	**1125.0385**	2232.0431	**1116.5252**	2231.0591	**1116.0332**	**I**	**846.4680**	423.7376	829.4414	415.2243	828.4574	414.7323	**8**
**22**	2320.1068	**1160.5570**	2303.0803	1152.0438	2302.0962	1151.5518	**A**	**733.3839**	367.1956	**716.3573**	358.6823	715.3733	358.1903	**7**
**23**	2407.1388	1204.0731	2390.1123	1195.5598	2389.1283	1195.0678	**S**	**662.3468**	331.6770	645.3202	323.1638	644.3362	322.6717	**6**
**24**	2536.1814	**1268.5944**	2519.1549	1260.0811	2518.1709	1259.5891	**E**	**575.3148**	288.1610	558.2882	279.6477	**557.3042**	279.1557	**5**
**25**	2607.2185	**1304.1129**	2590.1920	1295.5996	2589.2080	1295.1076	**A**	**446.2722**	223.6397	429.2456	215.1264	428.2616	214.6344	**4**
**26**	2720.3026	**1360.6549**	2703.2761	**1352.1417**	2702.2920	**1351.6497**	**L**	375.2350	188.1212	358.2085	179.6079	**357.2245**	179.1159	**3**
**27**	2807.3346	**1404.1710**	2790.3081	1395.6577	2789.3241	**1395.1657**	**S**	262.1510	131.5791	245.1244	123.0659	244.1404	122.5738	**2**
**28**							**R**	175.1190	88.0631	158.0924	79.5498			**1**

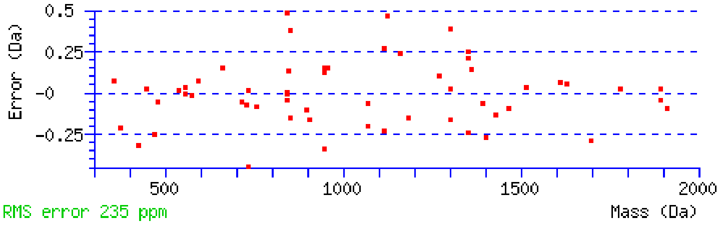

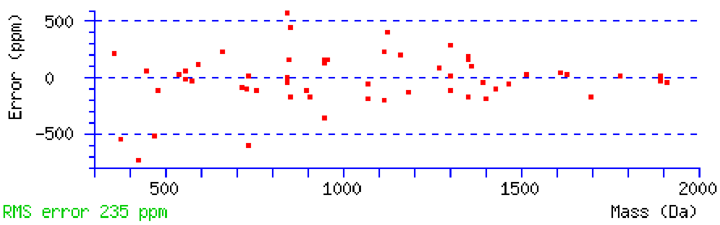

All matches to this query

Score	Mr(calc):	Delta	Sequence
54.3	2980.4390	-0.0006	TRSPDVISSASTALSQDIPEIASEALSR
54.3	2980.4390	-0.0006	TRSPDVISSASTALSQDIPEIASEALSR
52.1	2980.4390	-0.0006	TRSPDVISSASTALSQDIPEIASEALSR
52.1	2980.4390	-0.0006	TRSPDVISSASTALSQDIPEIASEALSR
48.0	2980.4390	-0.0006	TRSPDVISSASTALSQDIPEIASEALSR
48.0	2980.4390	-0.0006	TRSPDVISSASTALSQDIPEIASEALSR
28.0	2980.4390	-0.0006	TRSPDVISSASTALSQDIPEIASEALSR
6.0	2979.4313	1.0071	QAWNKLSYVNHAEDLASKLLQCSPK
2.1	2980.4347	0.0037	MIISAHPNLMSCNTRKHTPLHLAAR
1.4	2978.4056	2.0328	QLTPEEMENDLVTVVTQKKNDNPTR

Spectrum No: 236; Query: 2690; Rank: 1

Peptide View

MS/MS Fragmentation of **LSDGEYISDGEEGEDAITGDTDKYLGPQDLK**

Found in **IPI00199445**, Tax_Id=10116 Gene_Symbol=Ank3 ankyrin 3, epithelial isoform 2

Match to Query 2690: 3409.475442 from(1137.499090,3+)
Title: 100101RatKid_NS_deglyco_22.4006.4006.3.dta
Data file K:\NewmanPaper\Piliang\3SubProteomes\Piliang3SP\mgf5ppm\ERLIC_3SubProteomes5ppm.mgf

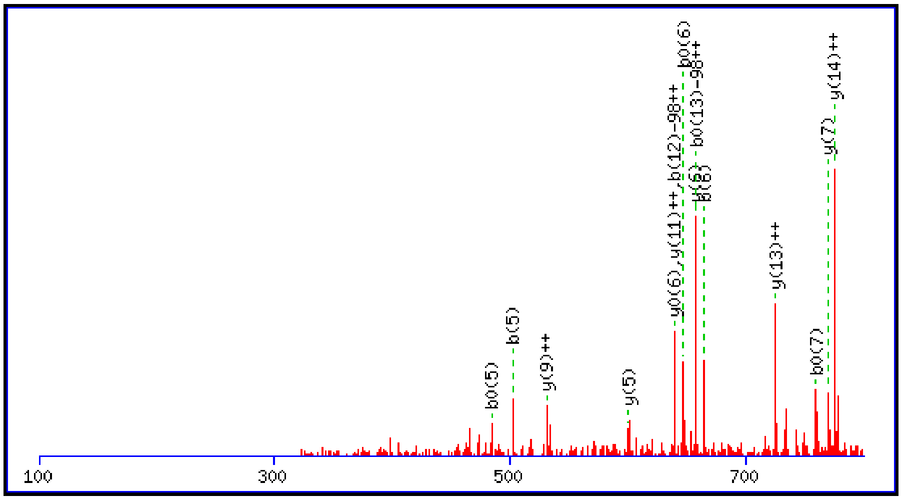

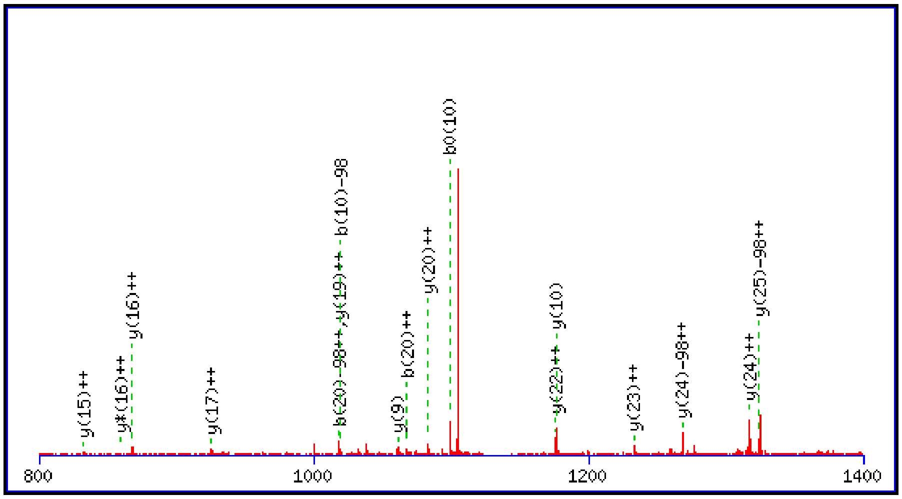

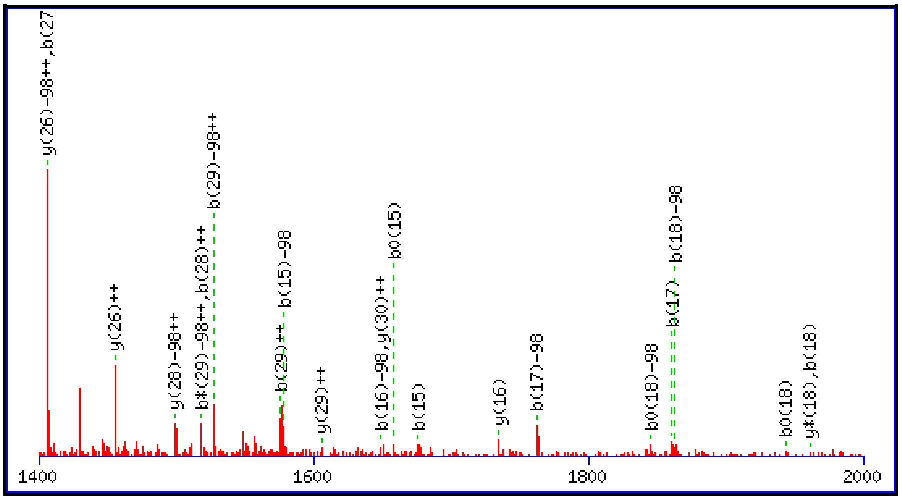

Monoisotopic mass of neutral peptide Mr(calc): 3409.4610
Fixed modifications: Carbamidomethyl (C)
Variable modifications:
S8 : Phospho (ST), with neutral losses 97.9769(shown in table), 0.0000
Ions Score: 54 **Expect:** 0.0017
Matches (Bold Red): 55/460 fragment ions using 97 most intense peaks

#	b	b++	b*	b*++	b0	b0++	Seq.	y	y++	y*	y*++	y0	y0++	#
1	114.0913	57.5493					L							31

2	201.1234	101.0653			183.1128	92.0600	S	3199.4073	1600.2073	3182.3808	1591.6940	3181.3968	1591.2020	30
3	316.1503	158.5788			298.1397	149.5735	D	3112.3753	1556.6913	3095.3488	1548.1780	3094.3647	1547.6860	29
4	373.1718	187.0895			355.1612	178.0842	G	2997.3484	**1499.1778**	2980.3218	1490.6645	2979.3378	1490.1725	28
5	**502.2144**	251.6108			**484.2038**	242.6055	E	2940.3269	1470.6671	2923.3003	1462.1538	2922.3163	1461.6618	27
6	**665.2777**	333.1425			**647.2671**	324.1372	Y	2811.2843	**1406.1458**	2794.2578	1397.6325	2793.2737	1397.1405	26
7	778.3618	389.6845			**760.3512**	380.6792	I	2648.2210	**1324.6141**	2631.1944	1316.1008	2630.2104	1315.6088	25
8	847.3832	424.1952			829.3727	415.1900	S	2535.1369	**1268.0721**	2518.1104	1259.5588	2517.1263	1259.0668	24
9	962.4102	481.7087			944.3996	472.7034	D	2466.1155	**1233.5614**	2449.0889	1225.0481	2448.1049	1224.5561	23
10	**1019.4316**	510.2194			1001.4211	501.2142	G	2351.0885	**1176.0479**	2334.0620	1167.5346	2333.0779	1167.0426	22
11	1148.4742	574.7407			1130.4637	565.7355	E	2294.0670	1147.5372	2277.0405	1139.0239	2276.0565	1138.5319	21
12	1277.5168	**639.2620**			1259.5062	630.2568	E	2165.0245	**1083.0159**	2147.9979	1074.5026	2147.0139	1074.0106	20
13	1334.5383	667.7728			1316.5277	**658.7675**	G	2035.9819	**1018.4946**	2018.9553	1009.9813	2017.9713	1009.4893	19
14	1463.5809	732.2941			1445.5703	723.2888	E	1978.9604	989.9838	**1961.9338**	981.4706	1960.9498	980.9786	18
15	**1578.6078**	789.8075			1560.5972	780.8023	D	1849.9178	**925.4625**	1832.8913	916.9493	1831.9072	916.4573	17
16	**1649.6449**	825.3261			1631.6344	816.3208	A	**1734.8909**	**867.9491**	1717.8643	**859.4358**	1716.8803	858.9438	16
17	**1762.7290**	881.8681			1744.7184	872.8628	I	1663.8537	**832.4305**	1646.8272	823.9172	1645.8432	823.4252	15
18	**1863.7767**	932.3920			**1845.7661**	923.3867	T	1550.7697	**775.8885**	1533.7431	767.3752	1532.7591	766.8832	14
19	1920.7981	960.9027			1902.7876	951.8974	G	1449.7220	**725.3646**	1432.6955	716.8514	1431.7114	716.3594	13
20	2035.8251	**1018.4162**			2017.8145	1009.4109	D	1392.7005	696.8539	1375.6740	688.3406	1374.6900	687.8486	12
21	2136.8728	1068.9400			2118.8622	1059.9347	T	1277.6736	**639.3404**	1260.6470	630.8272	1259.6630	630.3352	11
22	2251.8997	1126.4535			2233.8891	1117.4482	D	**1176.6259**	588.8166	1159.5994	580.3033	1158.6154	579.8113	10
23	2379.9947	1190.5010	2362.9681	1181.9877	2361.9841	1181.4957	K	**1061.5990**	**531.3031**	1044.5724	522.7899	1043.5884	522.2978	9
24	2543.0580	1272.0326	2526.0314	1263.5194	2525.0474	1263.0273	Y	933.5040	467.2556	916.4775	458.7424	915.4934	458.2504	8
25	2656.1421	1328.5747	2639.1155	1320.0614	2638.1315	1319.5694	L	**770.4407**	385.7240	753.4141	377.2107	752.4301	376.7187	7
26	2713.1635	1357.0854	2696.1370	1348.5721	2695.1530	1348.0801	G	**657.3566**	329.1819	640.3301	320.6687	**639.3461**	320.1767	6
27	2810.2163	**1405.6118**	2793.1897	1397.0985	2792.2057	1396.6065	P	**600.3352**	300.6712	583.3086	292.1579	582.3246	291.6659	5
28	2938.2749	1469.6411	2921.2483	1461.1278	2920.2643	1460.6358	Q	503.2824	252.1448	486.2558	243.6316	485.2718	243.1396	4
29	3053.3018	**1527.1545**	3036.2753	**1518.6413**	3035.2912	1518.1493	D	375.2238	188.1155	358.1973	179.6023	357.2132	179.1103	3
30	3166.3859	1583.6966	3149.3593	1575.1833	3148.3753	1574.6913	L	260.1969	130.6021	243.1703	122.0888			2
31							K	147.1128	74.0600	130.0863	65.5468			1

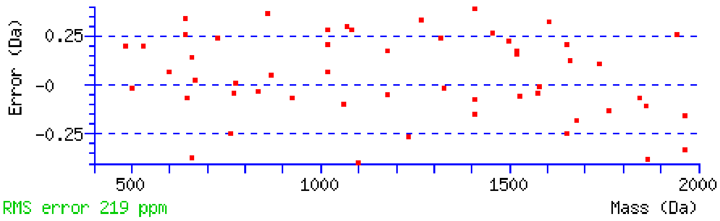

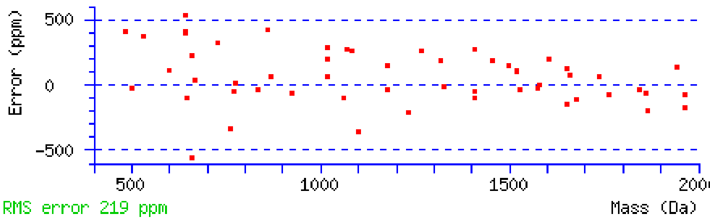

All matches to this query

Score	Mr(calc):	Delta	Sequence
54.2	3409.4610	0.0144	LSDGEYISDGEEGEDAITGDTDKYLGPQDLK
43.5	3409.4610	0.0144	LSDGEYISDGEEGEDAITGDTDKYLGPQDLK
41.2	3409.4610	0.0144	LSDGEYISDGEEGEDAITGDTDKYLGPQDLK
13.5	3409.4610	0.0144	LSDGEYISDGEEGEDAITGDTDKYLGPQDLK
5.4	3408.4451	1.0303	VLQLKNVHVSDTGRYVCVAVNVAGMTDK
3.8	3409.4889	-0.0135	YCMFNDDIQGTASVAVAGILAALRITKNR
3.6	3408.5052	0.9702	GLGKEITLLMQTLNTLSTPEEKLAALCK
3.5	3408.4482	1.0273	LANFLVRSSNNLGPVLPPTNVGSNTYGKR
3.4	3409.5043	-0.0289	ANFSNILENEFTVSGLTEDAAYEFRVIAK
3.4	3409.5043	-0.0289	ANFSNILENEFTVSGLTEDAAYEFRVIAK

Spectrum No: 237; Query: 1055; Rank: 1

Peptide View

MS/MS Fragmentation of **VDNSSLTGESEPQTR**
Found in **IPI00205693**, Tax_Id=10116 Gene_Symbol=Atp1a2 Sodium/potassium-transporting ATPase subunit alpha-2 precursor

Match to Query 1055: 1698.708168 from(850.361360,2+)
Title: 091008RatKidney_NoSalt_26.656.656.2.dta
Data file K:\NewmanPaper\Piliang\3SubProteomes\Piliang3SP\mgf5ppm\ERLIC_3SubProteomes5ppm.mgf

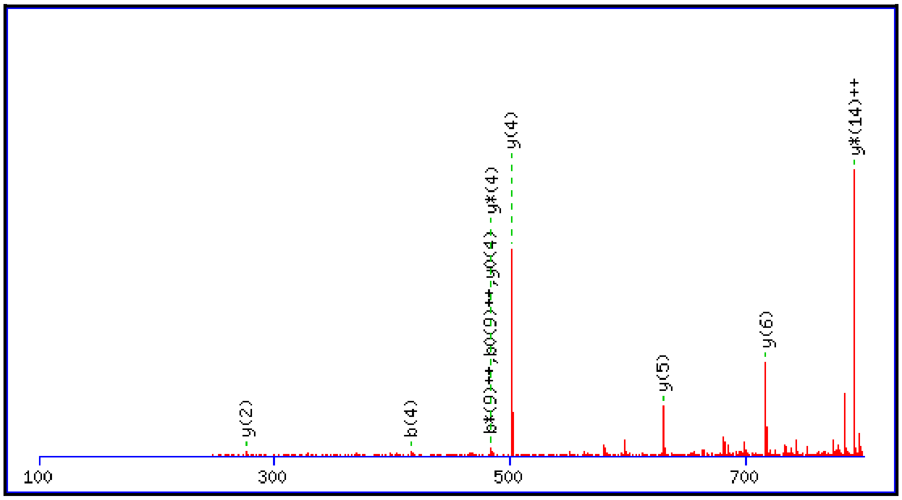

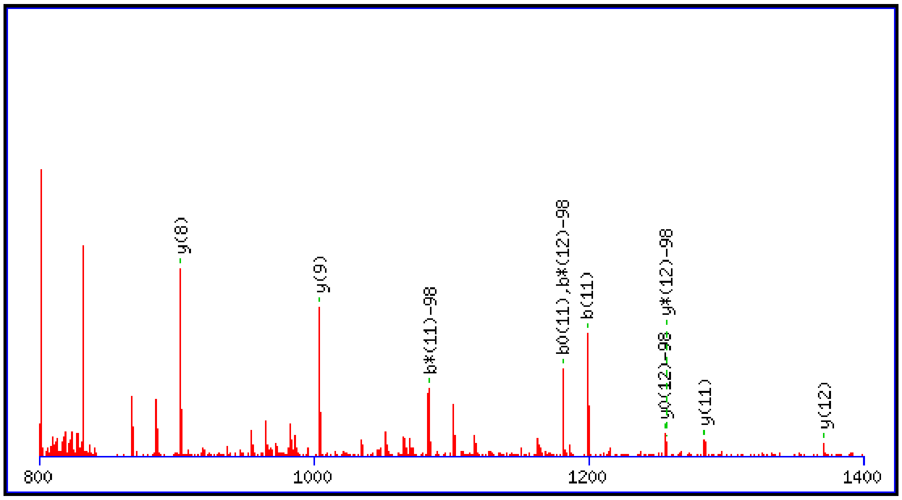

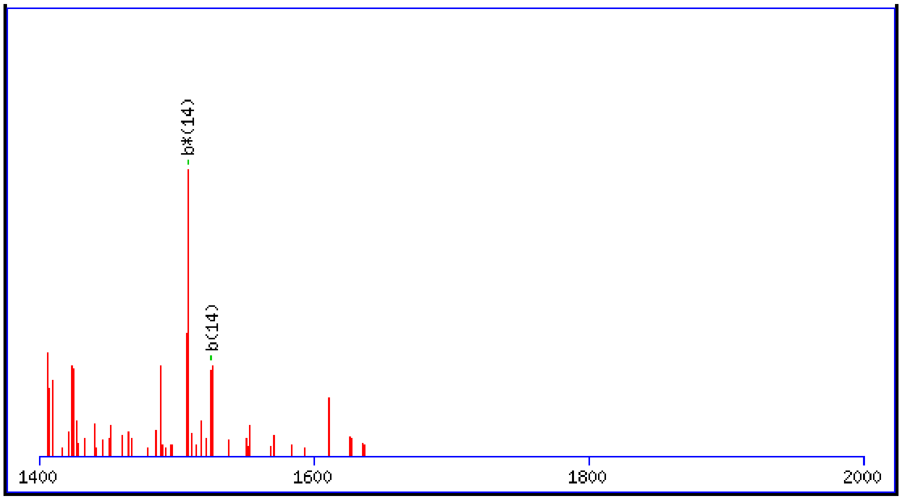

Monoisotopic mass of neutral peptide Mr(calc): 1698.7098
Fixed modifications: Carbamidomethyl (C)
Variable modifications:
S5 : Phospho (ST), with neutral losses 0.0000(shown in table), 97.9769
Ions Score: 54 **Expect:** 0.00084
Matches (Bold Red): 22/244 fragment ions using 25 most intense peaks

#	b	b++	b*	b*++	b0	b0++	Seq.	y	y++	y*	y*++	y0	y0++	#
1	100.0757	50.5415					V							15
2	215.1026	108.0550			197.0921	99.0497	D	1600.6486	800.8280	1583.6221	**792.3147**	1582.6381	791.8227	14
3	329.1456	165.0764	312.1190	156.5631	311.1350	156.0711	N	1485.6217	743.3145	1468.5952	734.8012	1467.6111	734.3092	13
4	**416.1776**	208.5924	399.1510	200.0792	398.1670	199.5871	S	**1371.5788**	686.2930	1354.5522	677.7798	1353.5682	677.2877	12
5	583.1759	292.0916	566.1494	283.5783	565.1654	283.0863	S	**1284.5467**	642.7770	1267.5202	634.2637	1266.5362	633.7717	11
6	696.2600	348.6336	679.2335	340.1204	678.2494	339.6284	L	1117.5484	559.2778	1100.5218	550.7646	1099.5378	550.2726	10
7	797.3077	399.1575	780.2811	390.6442	779.2971	390.1522	T	**1004.4643**	502.7358	987.4378	494.2225	986.4538	493.7305	9
8	854.3291	427.6682	837.3026	419.1549	836.3186	418.6629	G	**903.4167**	452.2120	886.3901	443.6987	885.4061	443.2067	8
9	983.3717	492.1895	966.3452	**483.6762**	965.3612	**483.1842**	E	846.3952	423.7012	829.3686	415.1880	828.3846	414.6959	7
10	1070.4038	535.7055	1053.3772	527.1922	1052.3932	526.7002	S	**717.3526**	359.1799	700.3260	350.6667	699.3420	350.1747	6
11	**1199.4464**	600.2268	1182.4198	591.7135	**1181.4358**	591.2215	E	**630.3206**	315.6639	613.2940	307.1506	612.3100	306.6586	5
12	1296.4991	648.7532	1279.4726	640.2399	1278.4886	639.7479	P	**501.2780**	251.1426	**484.2514**	242.6294	**483.2674**	242.1373	4
13	1424.5577	712.7825	1407.5312	704.2692	1406.5471	703.7772	Q	404.2252	202.6162	387.1987	194.1030	386.2146	193.6110	3
14	**1525.6054**	763.3063	**1508.5788**	754.7931	1507.5948	754.3010	T	**276.1666**	138.5870	259.1401	130.0737	258.1561	129.5817	2
15							R	175.1190	88.0631	158.0924	79.5498			1

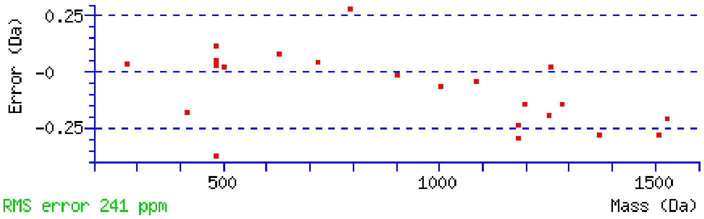

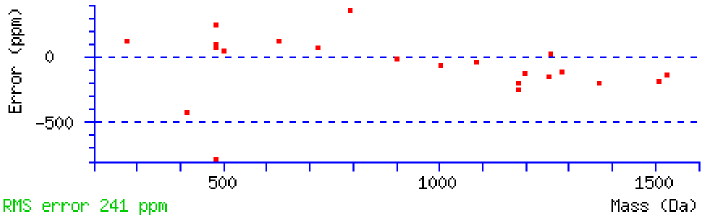

All matches to this query

Score	Mr(calc):	Delta	Sequence
54.2	1698.7098	-0.0016	VDNSSLTGESEPQTR
54.0	1698.7098	-0.0016	VDNSSLTGESEPQTR
44.4	1698.7098	-0.0016	VDNSSLTGESEPQTR
43.2	1698.7098	-0.0016	VDNSSLTGESEPQTR
21.5	1698.7098	-0.0016	VDNSSLTGESEPQTR
8.9	1698.7113	-0.0031	YCPATFYSSPGLTR

8.9	1698.7113	-0.0032	YCPSTFFSSPGLTR
5.6	1698.7155	-0.0073	GRDDEGSLNNAYMEK
4.9	1698.7155	-0.0073	GRDDEGSLNNAYMEK
4.3	1698.6984	0.0098	KMRVSGVMTQGASR

Spectrum No: 238; Query: 1190; Rank: 1

Peptide View

MS/MS Fragmentation of **SSSPILSEEEAEGSLR**
Found in **IPI00764230**, Tax_Id=10116 Gene_Symbol=LOC682920 similar to phosphatase and actin regulator 4

Match to Query 1190: 1769.777228 from(885.895890,2+)
Title: 091008RatKidney_NoSalt_22.2283.2283.2.dta
Data file K:\NewmanPaper\Piliang\3SubProteomes\Piliang3SP\mgf5ppm\ERLIC_3SubProteomes5ppm.mgf

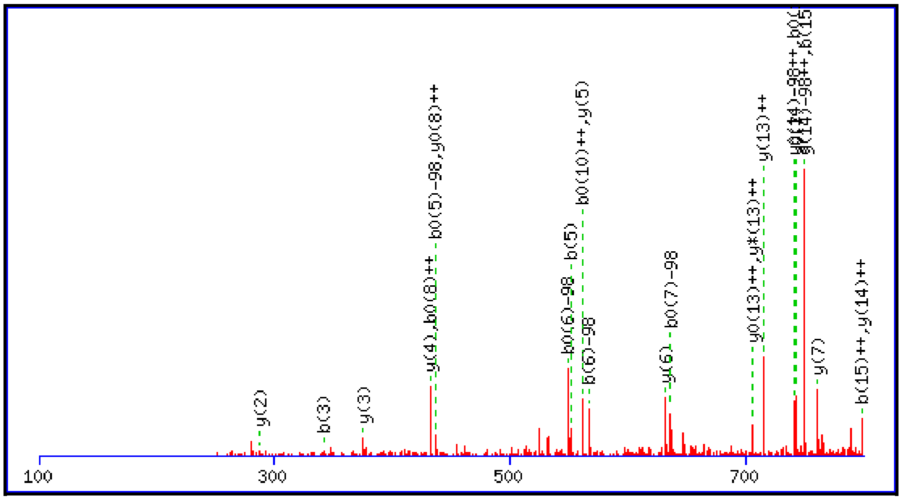

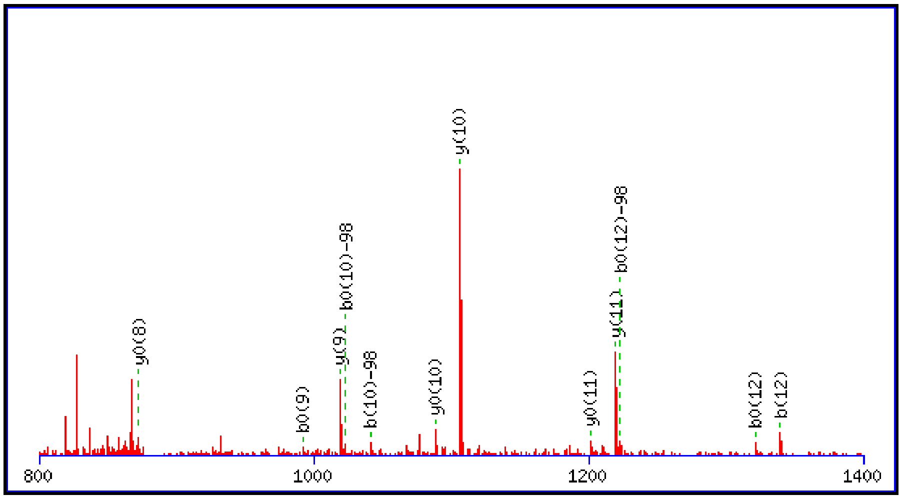

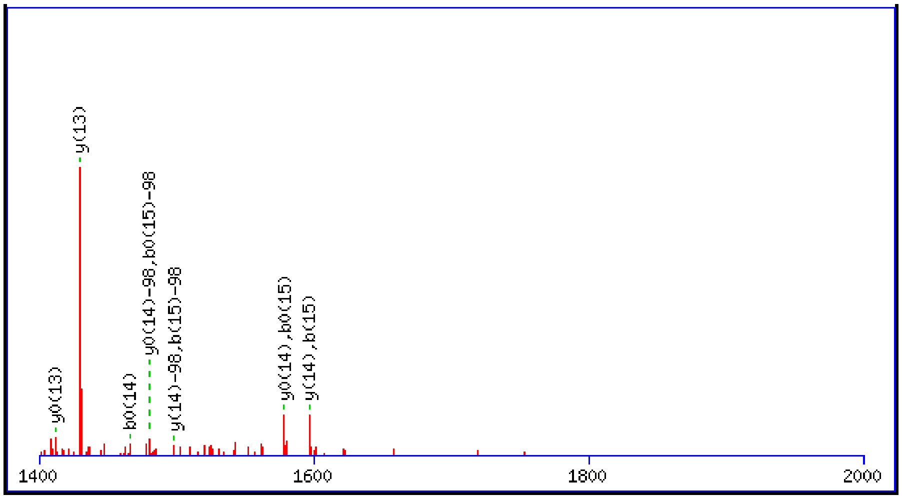

Monoisotopic mass of neutral peptide Mr(calc): 1769.7720
Fixed modifications: Carbamidomethyl (C)
Variable modifications:
S3 : Phospho (ST), with neutral losses 97.9769(shown in table), 0.0000
Ions Score: 54 **Expect:** 0.00078
Matches (Bold Red): 49/210 fragment ions using 81 most intense peaks

#	b	b++	b0	b0++	Seq.	y	y++	y*	y*++	y0	y0++	#
1	88.0393	44.5233	70.0287	35.5180	S							16
2	175.0713	88.0393	157.0608	79.0340	S	1585.7704	793.3888	1568.7438	784.8756	1567.7598	784.3836	15
3	244.0928	122.5500	226.0822	113.5447	S	**1498.7384**	**749.8728**	1481.7118	**741.3595**	**1480.7278**	**740.8675**	14
4	341.1456	171.0764	323.1350	162.0711	P	**1429.7169**	**715.3621**	1412.6904	**706.8488**	**1411.7063**	**706.3568**	13
5	454.2296	227.6184	**436.2191**	218.6132	I	1332.6641	666.8357	1315.6376	658.3224	1314.6536	657.8304	12
6	**567.3137**	284.1605	**549.3031**	275.1552	L	**1219.5801**	610.2937	1202.5535	601.7804	**1201.5695**	601.2884	11
7	654.3457	327.6765	**636.3351**	318.6712	S	**1106.4960**	553.7516	1089.4695	545.2384	**1088.4855**	544.7464	10
8	783.3883	392.1978	765.3777	383.1925	E	**1019.4640**	510.2356	1002.4374	501.7224	1001.4534	501.2304	9
9	912.4309	456.7191	894.4203	447.7138	E	890.4214	445.7143	873.3949	437.2011	**872.4108**	**436.7091**	8
10	**1041.4735**	521.2404	**1023.4629**	512.2351	E	**761.3788**	381.1930	744.3523	372.6798	**743.3682**	372.1878	7
11	1112.5106	556.7589	1094.5000	547.7537	A	**632.3362**	316.6717	615.3097	308.1585	614.3256	307.6665	6
12	1241.5532	621.2802	**1223.5426**	612.2750	E	**561.2991**	281.1532	544.2726	272.6399	543.2885	272.1479	5
13	1298.5747	649.7910	1280.5641	640.7857	G	**432.2565**	216.6319	415.2300	208.1186	414.2459	207.6266	4
14	1385.6067	693.3070	1367.5961	684.3017	S	**375.2350**	188.1212	358.2085	179.6079	357.2245	179.1159	3
15	**1498.6908**	**749.8490**	**1480.6802**	**740.8437**	L	**288.2030**	144.6051	271.1765	136.0919			2
16					R	175.1190	88.0631	158.0924	79.5498			1

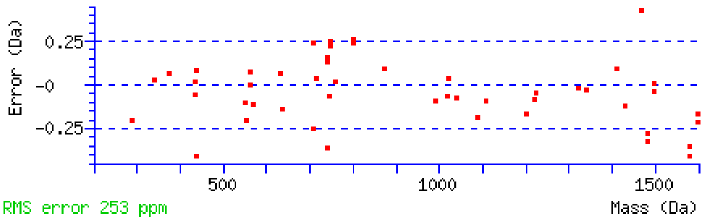

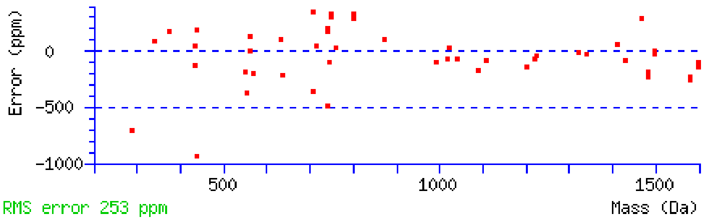

All matches to this query

Score	Mr(calc):	Delta	Sequence
54.1	1769.7720	0.0052	SSSPILSEEEAEGSLR
46.3	1769.7720	0.0052	SSSPILSEEEAEGSLR
46.3	1769.7720	0.0052	SSSPILSEEEAEGSLR
35.5	1769.7720	0.0052	SSSPILSEEEAEGSLR
8.5	1769.7862	-0.0090	ASAAKYSGSRSLGLSR

7.3	1769.7790	-0.0018	LELGHNPLTYTVPR
7.0	1769.7720	0.0052	SSSPILSEEEAEGSLR
4.8	1769.7790	-0.0018	LELGHNPLTYTVPR
4.6	1769.7862	-0.0090	ASAAKYSGSRSLGLSR
2.6	1769.7903	-0.0130	YPRPASVPPSPSLSR

Spectrum No: 239; Query: 2146; Rank: 1

Peptide View

MS/MS Fragmentation of **IVSQNVGDVFPGIQDSGQDTPR**
Found in **IPI00768543**, Tax_Id=10116 Gene_Symbol=Zbtb20_predicted zinc finger and BTB domain containing 20

Match to Query 2146: 2408.097582 from(803.706470,3+)
Title: 091008RatKidney_NH4Format01_22.3016.3016.3.dta
Data file K:\NewmanPaper\Piliang\3SubProteomes\Piliang3SP\mgf5ppm\ERLIC_3SubProteomes5ppm.mgf

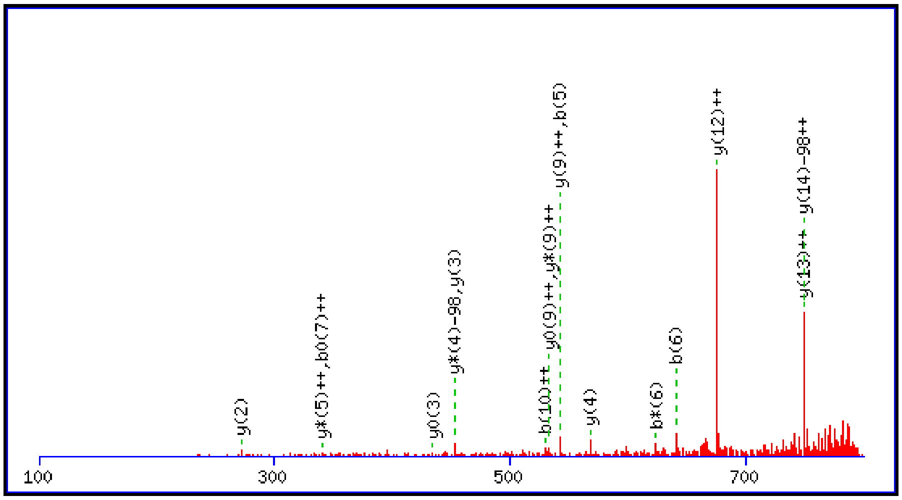

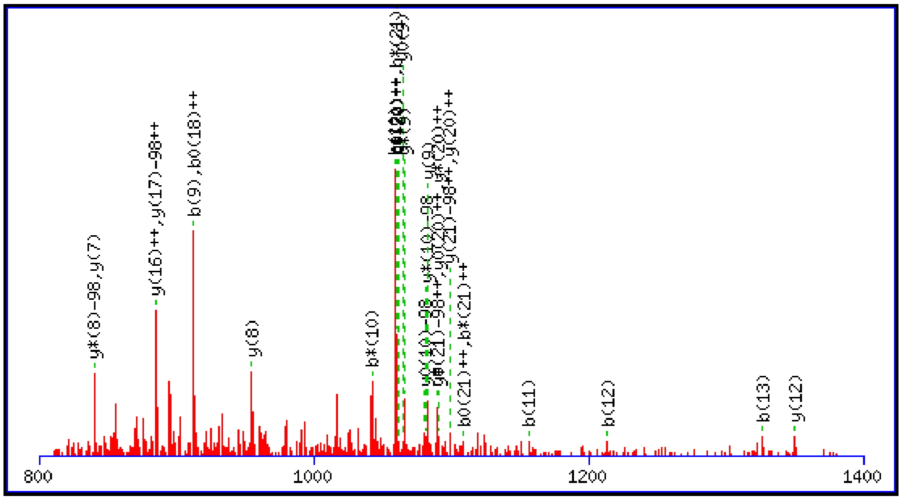

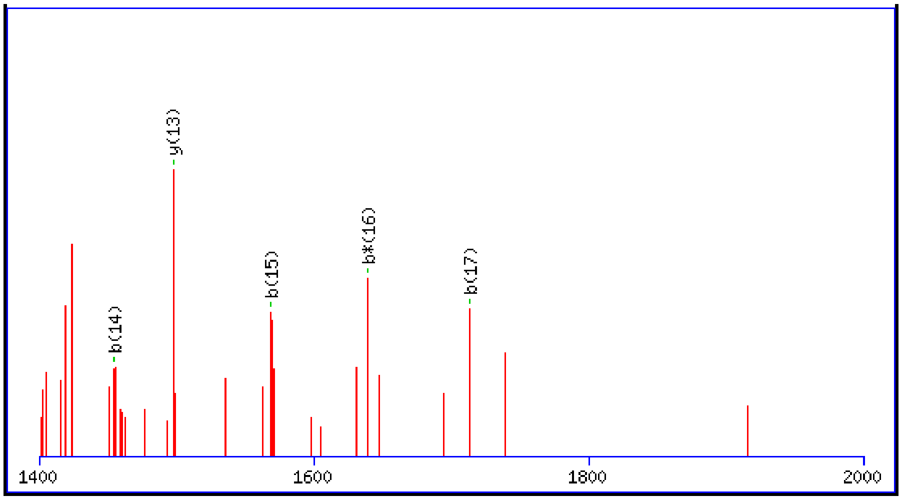

Monoisotopic mass of neutral peptide Mr(calc): 2408.1010
Fixed modifications: Carbamidomethyl (C)
Variable modifications:
T20 : Phospho (ST), with neutral losses 0.0000(shown in table), 97.9769
Ions Score: 54 **Expect:** 0.0013
Matches (Bold Red): 52/364 fragment ions using 62 most intense peaks

#	b	b^{++}	b*	b^{*++}	b^0	b^{0++}	Seq.	y	y^{++}	y*	y^{*++}	y^0	y^{0++}	#
1	114.0913	57.5493					I							22
2	213.1598	107.0835					V	2296.0241	1148.5157	2278.9976	1140.0024	2278.0136	1139.5104	21
3	300.1918	150.5995			282.1812	141.5942	S	2196.9557	**1098.9815**	2179.9292	**1090.4682**	2178.9452	**1089.9762**	20
4	428.2504	214.6288	411.2238	206.1155	410.2398	205.6235	Q	2109.9237	1055.4655	2092.8971	1046.9522	2091.9131	1046.4602	19
5	**542.2933**	271.6503	525.2667	263.1370	524.2827	262.6450	N	1981.8651	991.4362	1964.8386	982.9229	1963.8546	982.4309	18
6	**641.3617**	321.1845	**624.3352**	312.6712	623.3511	312.1792	V	1867.8222	934.4147	1850.7956	925.9015	1849.8116	925.4095	17
7	698.3832	349.6952	681.3566	341.1819	680.3726	**340.6899**	G	1768.7538	**884.8805**	1751.7272	876.3673	1750.7432	875.8752	16
8	813.4101	407.2087	796.3836	398.6954	795.3995	398.2034	D	1711.7323	856.3698	1694.7058	847.8565	1693.7218	847.3645	15
9	**912.4785**	456.7429	895.4520	448.2296	894.4680	447.7376	V	1596.7054	798.8563	1579.6788	790.3430	1578.6948	789.8510	14
10	**1059.5469**	**530.2771**	**1042.5204**	521.7638	1041.5364	521.2718	F	**1497.6370**	**749.3221**	1480.6104	740.8088	1479.6264	740.3168	13
11	**1156.5997**	578.8035	1139.5732	570.2902	1138.5891	569.7982	P	**1350.5685**	**675.7879**	1333.5420	667.2746	1332.5580	666.7826	12
12	**1213.6212**	607.3142	1196.5946	598.8009	1195.6106	598.3089	G	1253.5158	627.2615	1236.4892	618.7483	1235.5052	618.2562	11
13	**1326.7052**	663.8563	1309.6787	655.3430	1308.6947	654.8510	I	1196.4943	598.7508	1179.4678	590.2375	1178.4838	589.7455	10
14	**1454.7638**	727.8855	1437.7373	719.3723	1436.7532	718.8803	Q	**1083.4103**	**542.2088**	**1066.3837**	**533.6955**	**1065.3997**	**533.2035**	9
15	**1569.7907**	785.3990	1552.7642	776.8857	1551.7802	776.3937	D	**955.3517**	478.1795	938.3251	469.6662	937.3411	469.1742	8
16	1656.8228	828.9150	**1639.7962**	820.4018	1638.8122	819.9097	S	**840.3247**	420.6660	823.2982	412.1527	822.3142	411.6607	7
17	**1713.8442**	857.4258	1696.8177	848.9125	1695.8337	848.4205	G	753.2927	377.1500	736.2662	368.6367	735.2821	368.1447	6
18	1841.9028	921.4550	1824.8763	912.9418	1823.8923	**912.4498**	Q	696.2712	348.6393	679.2447	**340.1260**	678.2607	339.6340	5
19	1956.9298	978.9685	1939.9032	970.4552	1938.9192	969.9632	D	**568.2127**	284.6100	551.1861	276.0967	550.2021	275.6047	4
20	2137.9438	1069.4755	2120.9172	**1060.9622**	2119.9332	**1060.4702**	T	**453.1857**	227.0965	436.1592	218.5832	**435.1752**	218.0912	3
21	2234.9965	1118.0019	2217.9700	**1109.4886**	2216.9860	**1108.9966**	P	**272.1717**	136.5895	255.1452	128.0762			2
22							R	175.1190	88.0631	158.0924	79.5498			1

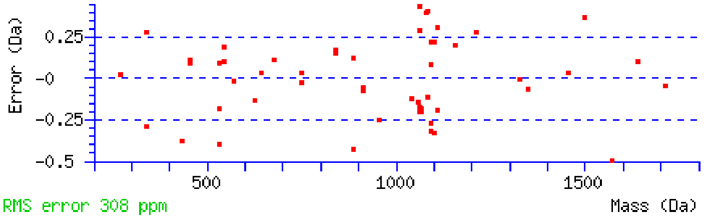

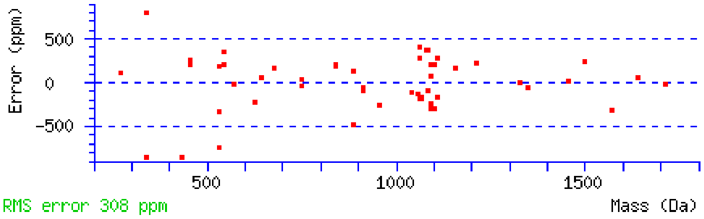

All matches to this query

Score	Mr(calc):	Delta	Sequence
54.0	2408.1010	-0.0034	IVSQNVGDVFPGIQDSGQDTPR
38.6	2408.1010	-0.0034	IVSQNVGDVFPGIQDSGQDTPR
10.5	2408.0855	0.0121	YTVPGYKDSLDFFTKLAQR
4.9	2408.1204	-0.0228	KLTHAAPCMLFMKGTPQEPR
3.1	2408.0880	0.0096	VAFSRLHTFGDENEDDSELAR
2.6	2408.0764	0.0212	VASTTLGRLSTVAHRQSTQR
1.4	2406.0749	2.0227	EELLCAVCYDPFRDAVTLR
0.8	2407.0701	1.0274	SSQGQRMSVYEQMIQWPIK
0.7	2407.0943	1.0033	VLCRWMAATVLRAPTXTTR
0.7	2408.0821	0.0155	AKSALAHALQSSRHDCDLLR

Spectrum No: 240; Query: 715; Rank: 1

Peptide View

MS/MS Fragmentation of **AEDGAAPSPSSETPK**
Found in **IPI00371946**, Tax_Id=10116 Gene_Symbol=LOC681252 similar to Myristoylated alanine-rich C-kinase substrate

Match to Query 715: 1522.613008 from(762.313780,2+)
Title: 091008RatKidney_NH4Format02_27.395.395.2.dta
Data file K:\NewmanPaper\Piliang\3SubProteomes\Piliang3SP\mgf5ppm\ERLIC_3SubProteomes5ppm.mgf

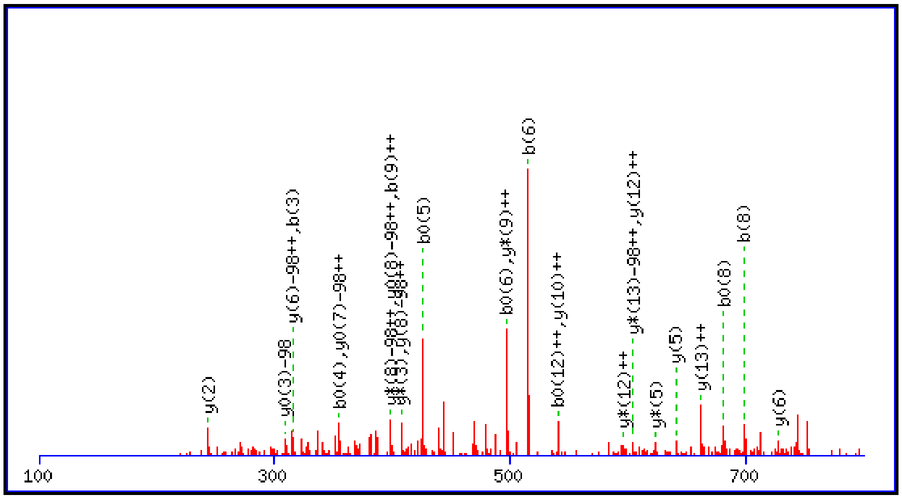

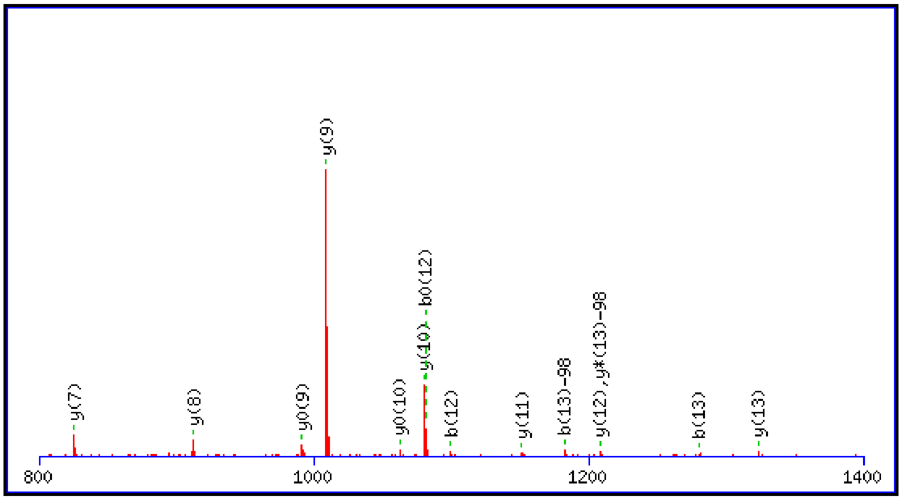

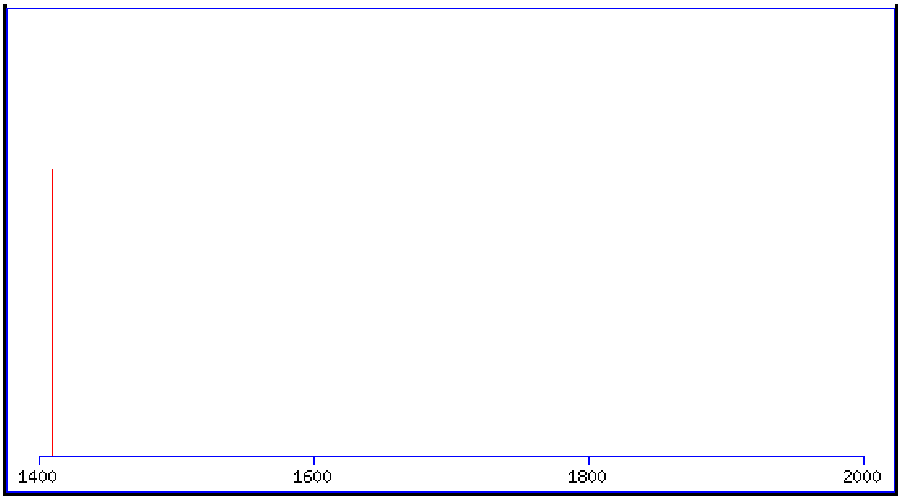

Monoisotopic mass of neutral peptide Mr(calc): 1522.6188
Fixed modifications: Carbamidomethyl (C)
Variable modifications:
T13 : Phospho (ST), with neutral losses 0.0000(shown in table), 97.9769
Ions Score: 54 **Expect:** 0.00046
Matches (Bold Red): 40/214 fragment ions using 52 most intense peaks

#	b	b++	b0	b0++	Seq.	y	y++	y*	y*++	y0	y0++	#
1	72.0444	36.5258			A							15
2	201.0870	101.0471	183.0764	92.0418	E	1452.5890	726.7981	1435.5625	718.2849	1434.5784	717.7929	14
3	**316.1139**	158.5606	298.1034	149.5553	D	**1323.5464**	**662.2768**	1306.5199	653.7636	1305.5358	653.2716	13
4	373.1354	187.0713	**355.1248**	178.0661	G	**1208.5195**	**604.7634**	1191.4929	**596.2501**	1190.5089	595.7581	12
5	444.1725	222.5899	**426.1619**	213.5846	A	**1151.4980**	576.2526	1134.4715	567.7394	1133.4874	567.2474	11
6	**515.2096**	258.1084	**497.1991**	249.1032	A	**1080.4609**	**540.7341**	1063.4343	532.2208	**1062.4503**	531.7288	10
7	612.2624	306.6348	594.2518	297.6295	P	**1009.4238**	505.2155	992.3972	**496.7023**	**991.4132**	496.2102	9
8	**699.2944**	350.1508	**681.2838**	341.1456	S	**912.3710**	456.6891	895.3445	448.1759	894.3604	447.6839	8
9	796.3472	**398.6772**	778.3366	389.6719	P	**825.3390**	413.1731	808.3124	404.6599	807.3284	404.1678	7
10	883.3792	442.1932	865.3686	433.1880	S	**728.2862**	364.6467	711.2597	356.1335	710.2757	355.6415	6
11	970.4112	485.7093	952.4007	476.7040	S	**641.2542**	321.1307	**624.2276**	312.6175	623.2436	312.1255	5
12	**1099.4538**	550.2305	**1081.4433**	**541.2253**	E	554.2222	277.6147	537.1956	269.1014	536.2116	268.6094	4
13	**1280.4678**	640.7376	1262.4573	631.7323	T	425.1796	213.0934	**408.1530**	204.5801	407.1690	204.0881	3
14	1377.5206	689.2639	1359.5100	680.2587	P	**244.1656**	122.5864	227.1390	114.0731			2
15					K	147.1128	74.0600	130.0863	65.5468			1

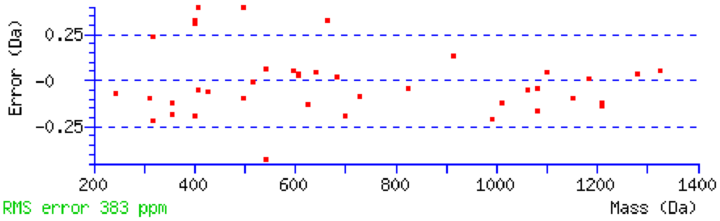

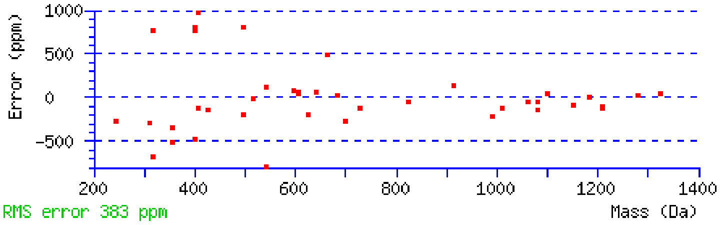

All matches to this query

Score	Mr(calc):	Delta	Sequence
54.0	1522.6188	-0.0058	AEDGAAPSPSSETPK
51.1	1522.6188	-0.0058	AEDGAAPSPSSETPK
51.1	1522.6188	-0.0058	AEDGAAPSPSSETPK
38.1	1522.6188	-0.0058	AEDGAAPSPSSETPK
9.4	1522.6146	-0.0016	TFSYTPSYAKAK
9.4	1522.6146	-0.0016	TFSYTPSYAKAK

9.3	1522.6146	-0.0016	TFSYTPSYAKAK
7.7	1522.6201	-0.0071	ENPRYSSYQGSR
7.0	1521.6187	0.9943	TISLSGCLDTAPK
6.4	1520.6208	1.9922	CRQSVLEGLGSR

Spectrum No: 241; Query: 2657; Rank: 1

Peptide View

MS/MS Fragmentation of **YQDEVFGGFVTEPQEESEEEVEEPEER**
Found in **IPI00193648**, Tax_Id=10116 Gene_Symbol=G3bp similar to Ras-GTPase-activating protein binding protein 1

Match to Query 2657: 3295.319142 from(1099.446990,3+)
Title: 091008RatKidney_NoSalt_28.3535.3535.3.dta
Data file K:\NewmanPaper\Piliang\3SubProteomes\Piliang3SP\mgf5ppm\ERLIC_3SubProteomes5ppm.mgf

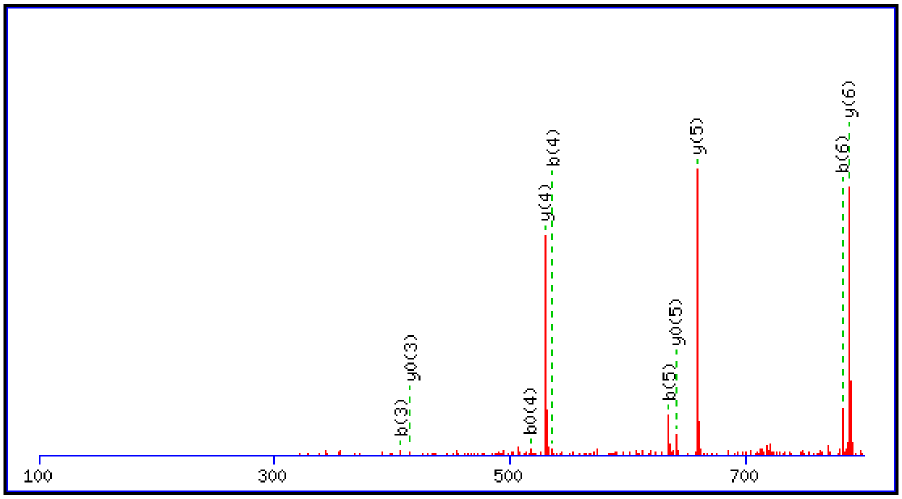

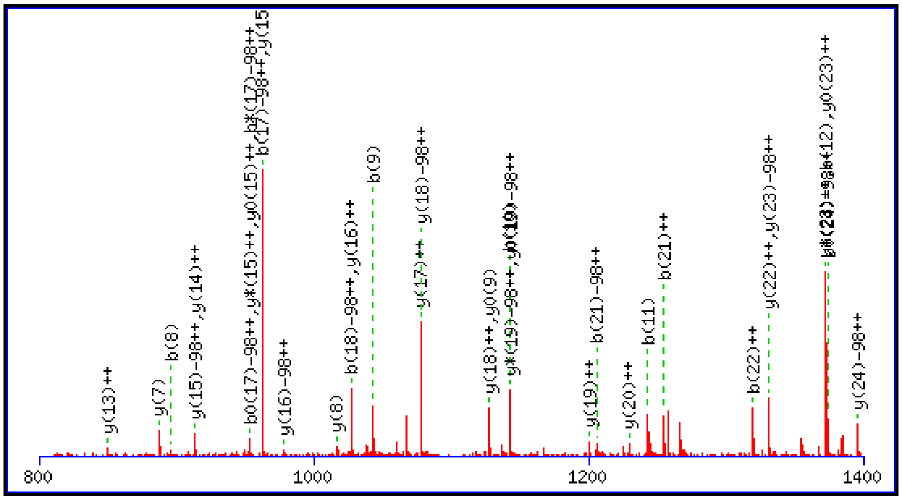

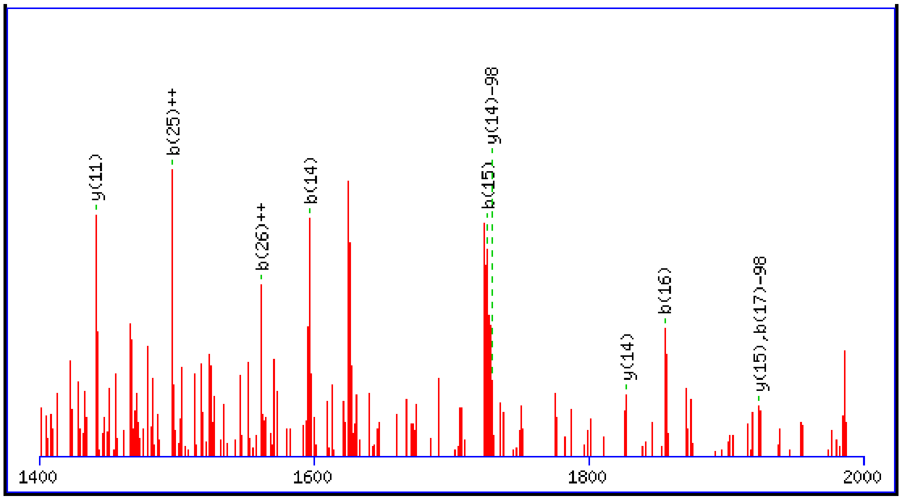

Monoisotopic mass of neutral peptide Mr(calc): 3295.3242
Fixed modifications: Carbamidomethyl (C)
Variable modifications:
S17 : Phospho (ST), with neutral losses 97.9769(shown in table), 0.0000
Ions Score: 54 **Expect:** 0.0015
Matches (Bold Red): 56/460 fragment ions using 90 most intense peaks

#	b	b^{++}	b*	b^{*++}	b^0	b^{0++}	Seq.	y	y^{++}	y*	y^{*++}	y^0	y^{0++}	#
1	164.0706	82.5389					Y							27
2	292.1292	146.5682	275.1026	138.0550			Q	3035.2912	1518.1493	3018.2647	1509.6360	3017.2807	1509.1440	26
3	**407.1561**	204.0817	390.1296	195.5684	389.1456	195.0764	D	2907.2327	1454.1200	2890.2061	1445.6067	2889.2221	1445.1147	25
4	**536.1987**	268.6030	519.1722	260.0897	**518.1882**	259.5977	E	2792.2057	**1396.6065**	2775.1792	1388.0932	2774.1951	1387.6012	24
5	**635.2671**	318.1372	618.2406	309.6239	617.2566	309.1319	V	2663.1631	**1332.0852**	2646.1366	1323.5719	2645.1526	1323.0799	23
6	**782.3355**	391.6714	765.3090	383.1581	764.3250	382.6661	F	2564.0947	1282.5510	2547.0682	1274.0377	2546.0841	1273.5457	22
7	839.3570	420.1821	822.3305	411.6689	821.3464	411.1769	G	2417.0263	1209.0168	2399.9997	1200.5035	2399.0157	1200.0115	21
8	**896.3785**	448.6929	879.3519	440.1796	878.3679	439.6876	G	2360.0048	1180.5061	2342.9783	1171.9928	2341.9943	1171.5008	20
9	**1043.4469**	522.2271	1026.4203	513.7138	1025.4363	513.2218	F	2302.9834	1151.9953	2285.9568	**1143.4820**	2284.9728	**1142.9900**	19
10	**1142.5153**	571.7613	1125.4888	563.2480	1124.5047	562.7560	V	2155.9150	**1078.4611**	2138.8884	1069.9478	2137.9044	1069.4558	18
11	**1243.5630**	622.2851	1226.5364	613.7719	1225.5524	613.2798	T	2056.8465	1028.9269	2039.8200	1020.4136	2038.8360	1019.9216	17
12	**1372.6056**	686.8064	1355.5790	678.2932	1354.5950	677.8011	E	1955.7989	**978.4031**	1938.7723	969.8898	1937.7883	969.3978	16
13	1469.6583	735.3328	1452.6318	726.8195	1451.6478	726.3275	P	1826.7563	**913.8818**	1809.7297	905.3685	1808.7457	904.8765	15
14	**1597.7169**	799.3621	1580.6904	790.8488	1579.7064	790.3568	Q	**1729.7035**	865.3554	1712.6770	856.8421	1711.6929	856.3501	14
15	**1726.7595**	863.8834	1709.7330	855.3701	1708.7489	854.8781	E	1601.6449	801.3261	1584.6184	792.8128	1583.6344	792.3208	13
16	**1855.8021**	928.4047	1838.7756	919.8914	1837.7915	919.3994	E	1472.6023	736.8048	1455.5758	728.2915	1454.5918	727.7995	12
17	**1924.8236**	**962.9154**	1907.7970	**954.4021**	1906.8130	**953.9101**	S	1343.5597	672.2835	1326.5332	663.7702	1325.5492	663.2782	11
18	2053.8662	**1027.4367**	2036.8396	1018.9234	2035.8556	1018.4314	E	1274.5383	637.7728	1257.5117	629.2595	1256.5277	628.7675	10
19	2182.9087	1091.9580	2165.8822	1083.4447	2164.8982	1082.9527	E	1145.4957	573.2515	1128.4691	564.7382	**1127.4851**	564.2462	9
20	2311.9513	1156.4793	2294.9248	1147.9660	2293.9408	1147.4740	E	**1016.4531**	508.7302	999.4265	500.2169	998.4425	499.7249	8
21	2411.0198	**1206.0135**	2393.9932	1197.5002	2393.0092	1197.0082	V	**887.4105**	444.2089	870.3840	435.6956	869.3999	435.2036	7
22	2540.0623	1270.5348	2523.0358	1262.0215	2522.0518	1261.5295	E	**788.3421**	394.6747	771.3155	386.1614	770.3315	385.6694	6
23	2669.1049	1335.0561	2652.0784	1326.5428	2651.0944	1326.0508	E	**659.2995**	330.1534	642.2729	321.6401	**641.2889**	321.1481	5
24	2766.1577	1383.5825	2749.1312	1375.0692	2748.1471	**1374.5772**	P	**530.2569**	265.6321	513.2304	257.1188	512.2463	256.6268	4
25	2895.2003	1448.1038	2878.1737	1439.5905	2877.1897	1439.0985	E	433.2041	217.1057	416.1776	208.5924	**415.1936**	208.1004	3
26	3024.2429	1512.6251	3007.2163	1504.1118	3006.2323	1503.6198	E	304.1615	152.5844	287.1350	144.0711	286.1510	143.5791	2
27							R	175.1190	88.0631	158.0924	79.5498			1

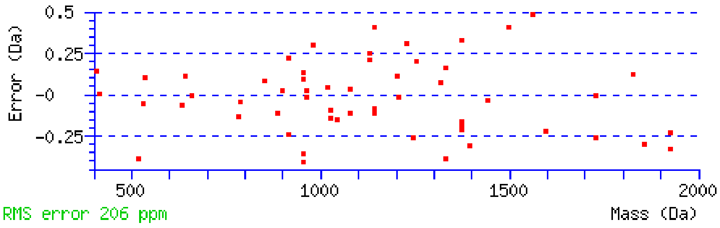

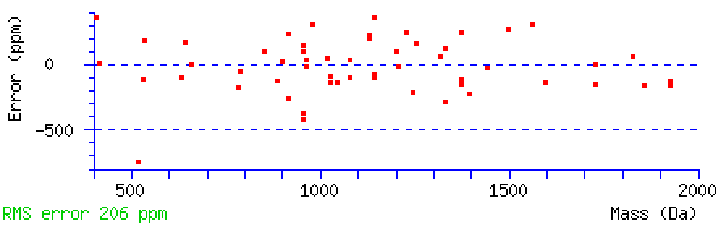

All matches to this query

Score	Mr(calc):	Delta	Sequence
54.0	3295.3242	-0.0050	YQDEVFGGFVTEPQEESEEEVEEPEER
22.9	3295.3242	-0.0050	YQDEVFGGFVTEPQEESEEEVEEPEER
6.1	3294.2895	1.0296	LYSPVPSVSRELSSPVTFPDGRSIPK
5.5	3295.2942	0.0249	TNQSFLELLNMLIEMTTKDLSSMER
3.8	3295.3369	-0.0177	MASHEIEIGTIFTNINATDNHVHSMLK
3.5	3295.3369	-0.0177	MASHEIEIGTIFTNINATDNHVHSMLK
3.1	3294.2978	1.0213	NGSSQNGRNERSHMPGTYLTTVIPYEK
3.0	3294.2978	1.0213	NGSSQNGRNERSHMPGTYLTTVIPYEK
3.0	3294.2978	1.0213	NGSSQNGRNERSHMPGTYLTTVIPYEK
2.8	3295.3242	-0.0050	YQDEVFGGFVTEPQEESEEEVEEPEER

Spectrum No: 242; Query: 958; Rank: 1

Peptide View

MS/MS Fragmentation of **SASSGAEGDVSSEREP**
Found in **IPI00357936**, Tax_Id=10116 Gene_Symbol=Hars2_predicted histidyl tRNA synthetase 2

Match to Query 958: 1643.629908 from(822.822230,2+)
Title: 091008RatKidney_NoSalt_26.710.710.2.dta
Data file K:\NewmanPaper\Piliang\3SubProteomes\Piliang3SP\mgf5ppm\ERLIC_3SubProteomes5ppm.mgf

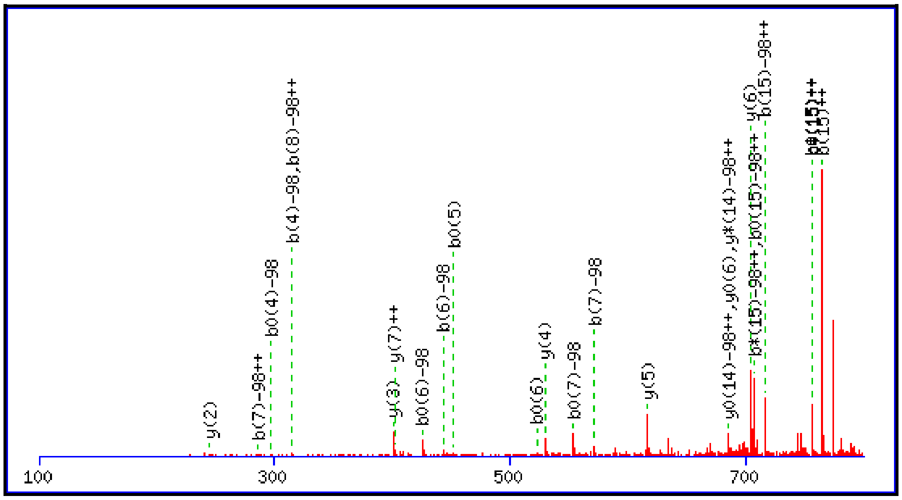

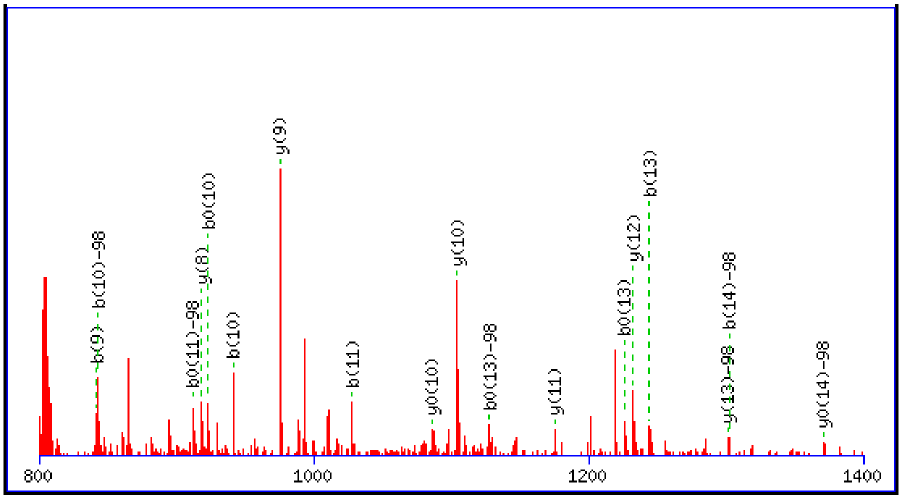

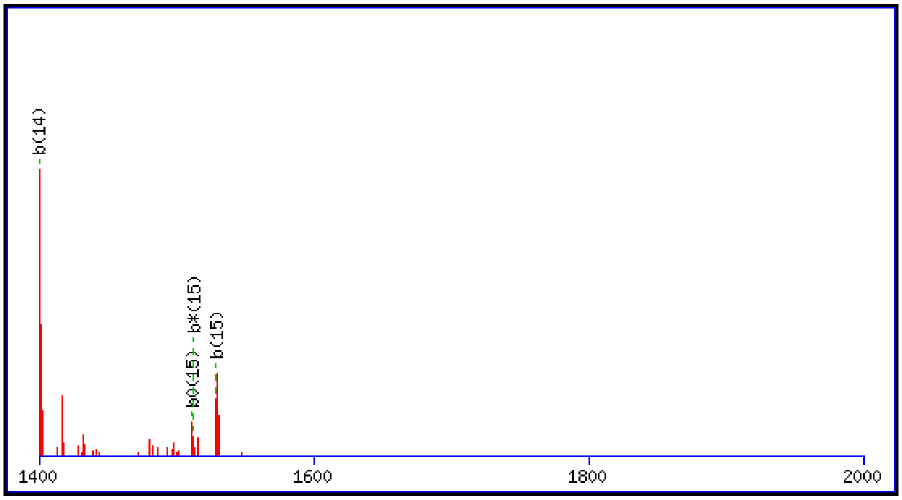

Monoisotopic mass of neutral peptide Mr(calc): 1643.6312
Fixed modifications: Carbamidomethyl (C)
Variable modifications:
S4 : Phospho (ST), with neutral losses 97.9769(shown in table), 0.0000
Ions Score: 54 **Expect:** 0.00043
Matches (Bold Red): 47/218 fragment ions using 86 most intense peaks

#	b	b^{++}	b*	b^{*++}	b^{0}	b^{0++}	Seq.	y	y^{++}	y*	y^{*++}	y^{0}	y^{0++}	#
1	88.0393	44.5233			70.0287	35.5180	S							16
2	159.0764	80.0418			141.0659	71.0366	A	1459.6295	730.3184	1442.6030	721.8051	1441.6190	721.3131	15
3	246.1084	123.5579			228.0979	114.5526	S	1388.5924	694.7999	1371.5659	**686.2866**	**1370.5819**	**685.7946**	14
4	**315.1299**	158.0686			**297.1193**	149.0633	S	**1301.5604**	651.2838	1284.5339	642.7706	1283.5498	642.2786	13
5	372.1514	186.5793			354.1408	177.5740	G	**1232.5390**	616.7731	1215.5124	608.2598	1214.5284	607.7678	12
6	**443.1885**	222.0979			**425.1779**	213.0926	A	**1175.5175**	588.2624	1158.4909	579.7491	1157.5069	579.2571	11
7	**572.2311**	**286.6192**			**554.2205**	277.6139	E	**1104.4804**	552.7438	1087.4538	544.2305	**1086.4698**	543.7385	10
8	629.2525	**315.1299**			611.2420	306.1246	G	**975.4378**	488.2225	958.4112	479.7093	957.4272	479.2172	9
9	744.2795	372.6434			726.2689	363.6381	D	**918.4163**	459.7118	901.3898	451.1985	900.4058	450.7065	8
10	**843.3479**	422.1776			825.3373	413.1723	V	803.3894	**402.1983**	786.3628	393.6850	785.3788	393.1930	7
11	930.3799	465.6936			**912.3694**	456.6883	S	**704.3210**	352.6641	687.2944	344.1508	**686.3104**	343.6588	6
12	1017.4119	509.2096			999.4014	500.2043	S	**617.2889**	309.1481	600.2624	300.6348	599.2784	300.1428	5
13	1146.4545	573.7309			**1128.4440**	564.7256	E	**530.2569**	265.6321	513.2304	257.1188	512.2463	256.6268	4
14	**1302.5557**	651.7815	1285.5291	643.2682	1284.5451	642.7762	R	**401.2143**	201.1108	384.1878	192.5975	383.2037	192.1055	3
15	1431.5982	**716.3028**	1414.5717	**707.7895**	1413.5877	**707.2975**	E	**245.1132**	123.0602			227.1026	114.0550	2
16							P	116.0706	58.5389					1

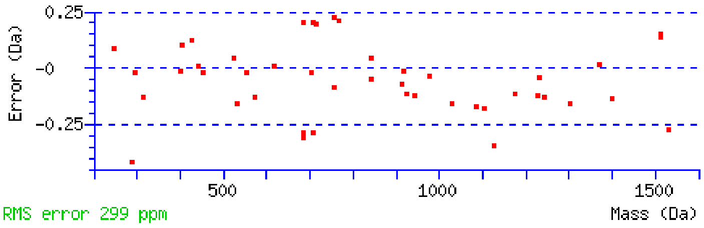

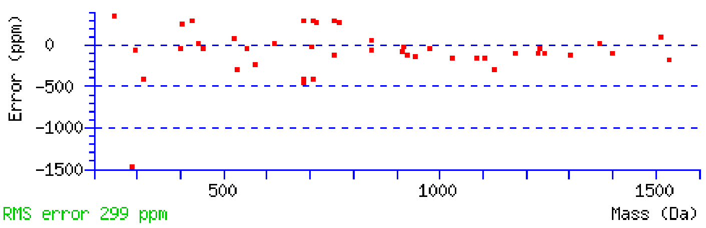

All matches to this query

Score	Mr(calc):	Delta	Sequence
53.8	1643.6312	-0.0013	SASSGAEGDVSSEREP
45.0	1643.6312	-0.0013	SASSGAEGDVSSEREP
44.2	1643.6312	-0.0013	SASSGAEGDVSSEREP
7.5	1643.6416	-0.0117	INTYTTCLGASQR
6.6	1641.6394	1.9905	LPLSWTLGSWSR
5.8	1643.6312	-0.0013	EDQNTGSTLDQEAR
5.3	1643.6312	-0.0013	SASSGAEGDVSSEREP
4.9	1643.6440	-0.0141	DDTEFQHPCFLR
4.2	1643.6382	-0.0083	HYLSQYRSSDTK
3.2	1642.6154	1.0145	NESIQNRQSSLK

Spectrum No: 243; Query: 1448; Rank: 1

Peptide View

MS/MS Fragmentation of **SATPPPTEPASLPQEPPK**
Found in **IPI00560565**, Tax_Id=10116 Gene_Symbol=RGD1309571 54 kDa protein

Match to Query 1448: 1922.906208 from(962.460380,2+)
Title: 091008RatKidney_NoSalt_21.1544.1544.2.dta
Data file K:\NewmanPaper\Piliang\3SubProteomes\Piliang3SP\mgf5ppm\ERLIC_3SubProteomes5ppm.mgf

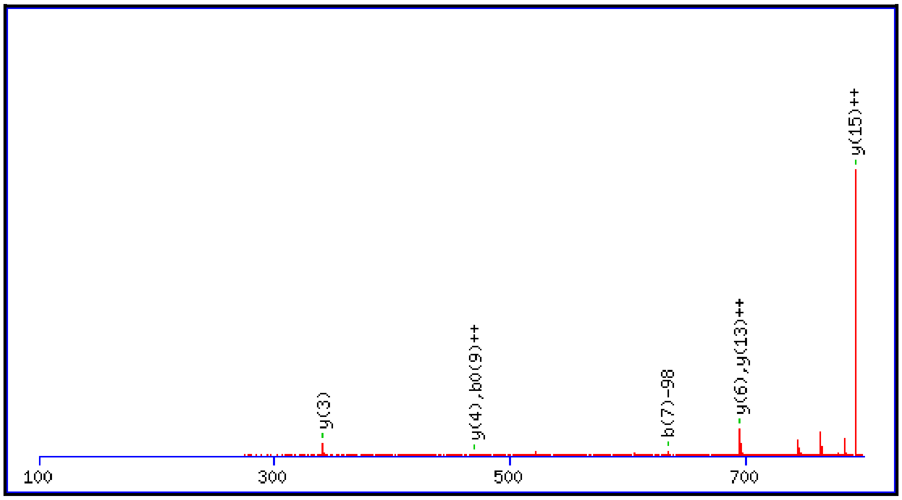

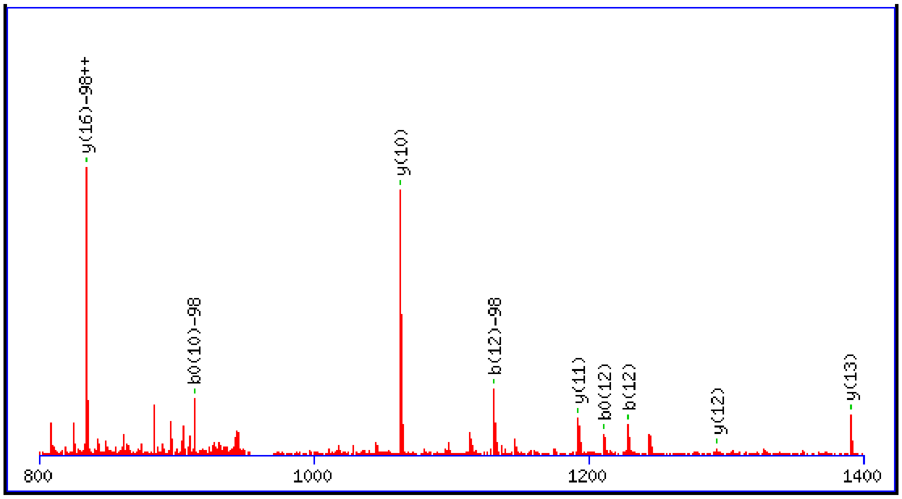

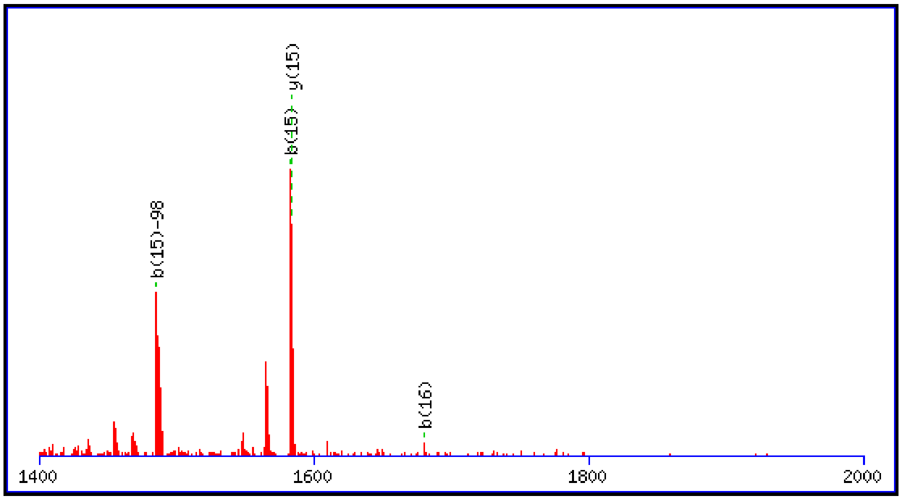

Monoisotopic mass of neutral peptide Mr(calc): 1922.9026
Fixed modifications: Carbamidomethyl (C)
Variable modifications:
T3 : Phospho (ST), with neutral losses 97.9769(shown in table), 0.0000
Ions Score: 54 **Expect:** 0.001
Matches (Bold Red): 20/252 fragment ions using 38 most intense peaks

#	b	b^{++}	b*	b^{*++}	b^0	b^{0++}	Seq.	y	y^{++}	y*	y^{*++}	y^0	y^{0++}	#
1	88.0393	44.5233			70.0287	35.5180	S							18
2	159.0764	80.0418			141.0659	71.0366	A	1738.9010	869.9541	1721.8745	861.4409	1720.8905	860.9489	17
3	242.1135	121.5604			224.1030	112.5551	T	1667.8639	**834.4356**	1650.8374	825.9223	1649.8533	825.4303	16
4	339.1663	170.0868			321.1557	161.0815	P	**1584.8268**	**792.9170**	1567.8003	784.4038	1566.8162	783.9118	15
5	436.2191	218.6132			418.2085	209.6079	P	1487.7740	744.3907	1470.7475	735.8774	1469.7635	735.3854	14
6	533.2718	267.1395			515.2613	258.1343	P	**1390.7213**	**695.8643**	1373.6947	687.3510	1372.7107	686.8590	13
7	**634.3195**	317.6634			616.3089	308.6581	T	**1293.6685**	647.3379	1276.6420	638.8246	1275.6579	638.3326	12
8	763.3621	382.1847			745.3515	373.1794	E	**1192.6208**	596.8141	1175.5943	588.3008	1174.6103	587.8088	11
9	860.4149	430.7111			842.4043	421.7058	P	**1063.5782**	532.2928	1046.5517	523.7795	1045.5677	523.2875	10
10	931.4520	466.2296			**913.4414**	457.2243	A	966.5255	483.7664	949.4989	475.2531	948.5149	474.7611	9
11	1018.4840	509.7456			1000.4734	500.7404	S	895.4884	448.2478	878.4618	439.7345	877.4778	439.2425	8
12	**1131.5681**	566.2877			1113.5575	557.2824	L	808.4563	404.7318	791.4298	396.2185	790.4458	395.7265	7
13	1228.6208	614.8140			1210.6103	605.8088	P	**695.3723**	348.1898	678.3457	339.6765	677.3617	339.1845	6
14	1356.6794	678.8433	1339.6529	670.3301	1338.6688	669.8381	Q	598.3195	299.6634	581.2930	291.1501	580.3089	290.6581	5
15	**1485.7220**	743.3646	1468.6954	734.8514	1467.7114	734.3594	E	**470.2609**	235.6341	453.2344	227.1208	452.2504	226.6288	4
16	1582.7748	791.8910	1565.7482	783.3777	1564.7642	782.8857	P	**341.2183**	171.1128	324.1918	162.5995			3
17	1679.8275	840.4174	1662.8010	831.9041	1661.8170	831.4121	P	244.1656	122.5864	227.1390	114.0731			2
18							K	147.1128	74.0600	130.0863	65.5468			1

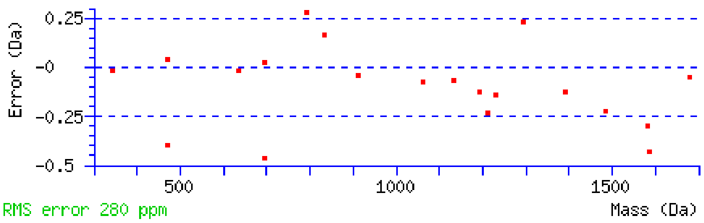

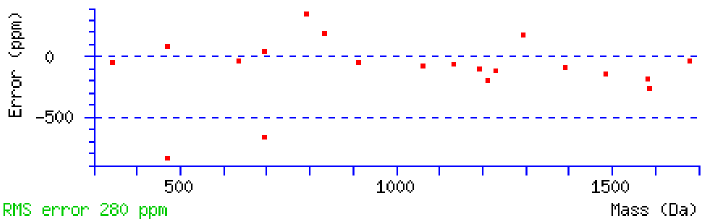

All matches to this query

Score	Mr(calc):	Delta	Sequence
53.7	1922.9026	0.0036	SATPPPTEPASLPQEPPK
53.7	1922.9026	0.0036	SATPPPTEPASLPQEPPK
22.1	1922.9026	0.0036	SATPPPTEPASLPQEPPK
10.7	1922.9026	0.0036	SATPPPTEPASLPQEPPK
3.6	1922.9155	-0.0093	QVKKTQEAVSELIYK
3.1	1922.9172	-0.0110	VGTTLLLLALLPGTSSK
3.1	1922.9172	-0.0110	VGTTLLLLALLPGTSSK
3.1	1922.9172	-0.0110	VGTTLLLLALLPGTSSK
2.0	1920.8940	2.0122	GLSLYPWIPTSPKFR
1.7	1920.8972	2.0090	RTAPGSTRPASPLTPPR

Spectrum No: 244; Query: 1144; Rank: 1

Peptide View

MS/MS Fragmentation of **GVENPAFVPSSPDTPR**
Found in **IPI00393407**, Tax_Id=10116 Gene_Symbol=Slco4c1 Kidney-specific organic anion transporter

Match to Query 1144: 1748.782948 from(875.398750,2+)
Title: 100101RatKid_NS_deglyco_08.2404.2404.2.dta
Data file K:\NewmanPaper\Piliang\3SubProteomes\Piliang3SP\mgf5ppm\ERLIC_3SubProteomes5ppm.mgf

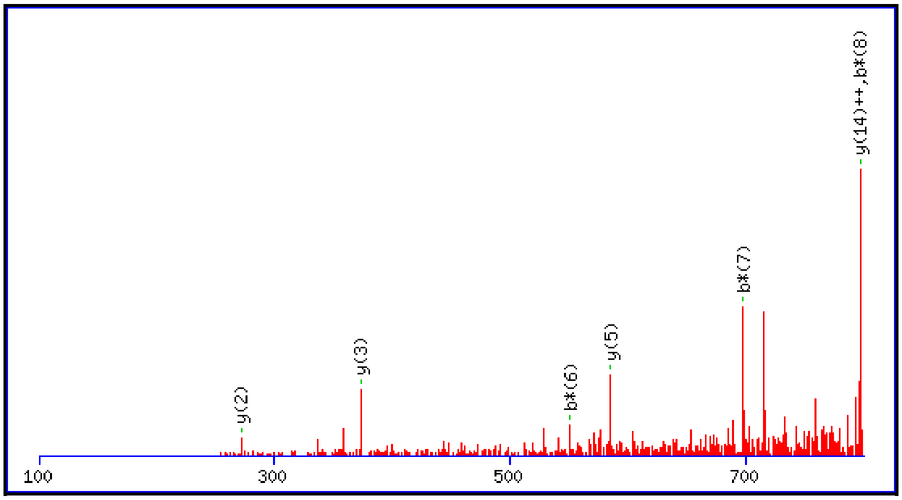

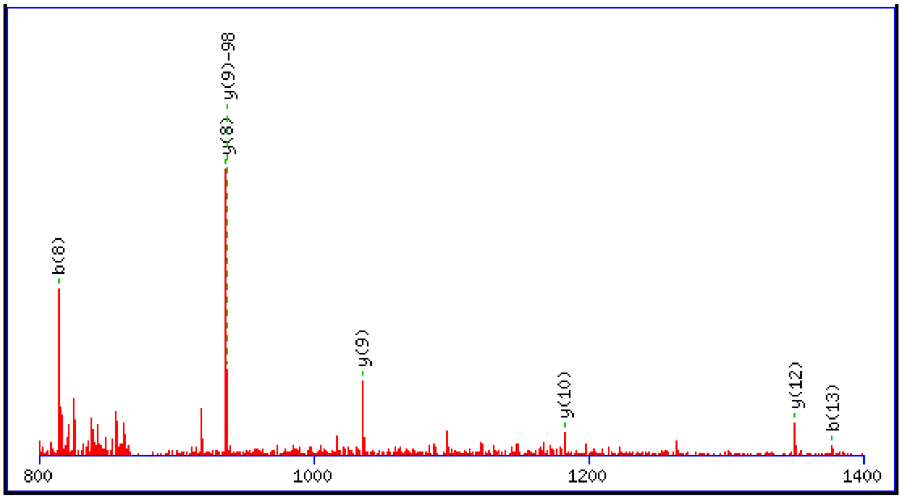

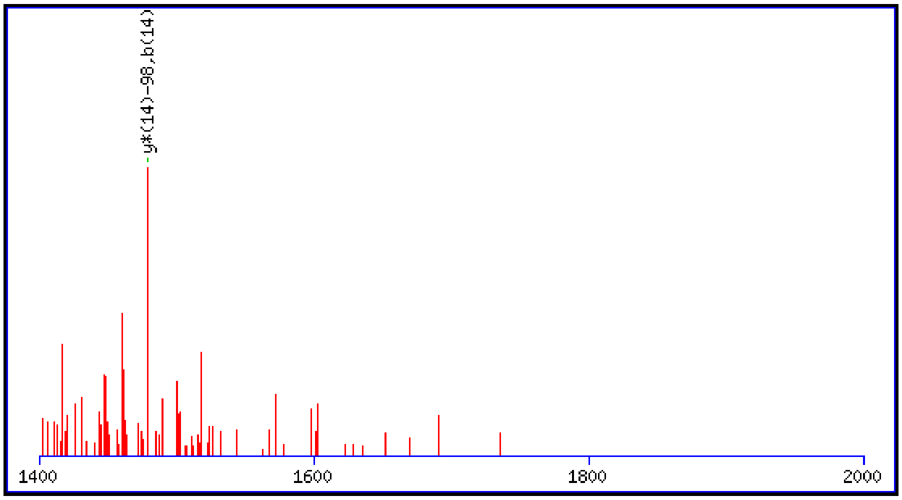

Monoisotopic mass of neutral peptide Mr(calc): 1748.7771
Fixed modifications: Carbamidomethyl (C)
Variable modifications:
S11 : Phospho (ST), with neutral losses 0.0000(shown in table), 97.9769
Ions Score: 54 **Expect:** 0.00093
Matches (Bold Red): 16/256 fragment ions using 23 most intense peaks

#	b	b^{++}	b*	b^{*++}	b^0	b^{0++}	Seq.	y	y^{++}	y*	y^{*++}	y^0	y^{0++}	#
1	58.0287	29.5180					G							16
2	157.0972	79.0522					V	1692.7629	846.8851	1675.7363	838.3718	1674.7523	837.8798	15
3	286.1397	143.5735			268.1292	134.5682	E	1593.6945	**797.3509**	1576.6679	788.8376	1575.6839	788.3456	14
4	400.1827	200.5950	383.1561	192.0817	382.1721	191.5897	N	1464.6519	732.8296	1447.6253	724.3163	1446.6413	723.8243	13
5	497.2354	249.1214	480.2089	240.6081	479.2249	240.1161	P	**1350.6090**	675.8081	1333.5824	667.2948	1332.5984	666.8028	12
6	568.2726	284.6399	**551.2460**	276.1266	550.2620	275.6346	A	1253.5562	627.2817	1236.5296	618.7685	1235.5456	618.2764	11
7	715.3410	358.1741	**698.3144**	349.6608	697.3304	349.1688	F	**1182.5191**	591.7632	1165.4925	583.2499	1164.5085	582.7579	10
8	**814.4094**	407.7083	**797.3828**	399.1951	796.3988	398.7030	V	**1035.4507**	518.2290	1018.4241	509.7157	1017.4401	509.2237	9
9	911.4621	456.2347	894.4356	447.7214	893.4516	447.2294	P	**936.3822**	468.6948	919.3557	460.1815	918.3717	459.6895	8
10	998.4942	499.7507	981.4676	491.2374	980.4836	490.7454	S	839.3295	420.1684	822.3029	411.6551	821.3189	411.1631	7
11	1165.4925	583.2499	1148.4660	574.7366	1147.4820	574.2446	S	752.2975	376.6524	735.2709	368.1391	734.2869	367.6471	6
12	1262.5453	631.7763	1245.5187	623.2630	1244.5347	622.7710	P	**585.2991**	293.1532	568.2726	284.6399	567.2885	284.1479	5
13	**1377.5722**	689.2898	1360.5457	680.7765	1359.5617	680.2845	D	488.2463	244.6268	471.2198	236.1135	470.2358	235.6215	4
14	**1478.6199**	739.8136	1461.5934	731.3003	1460.6093	730.8083	T	**373.2194**	187.1133	356.1928	178.6001	355.2088	178.1081	3
15	1575.6727	788.3400	1558.6461	779.8267	1557.6621	779.3347	P	**272.1717**	136.5895	255.1452	128.0762			2
16							R	175.1190	88.0631	158.0924	79.5498			1

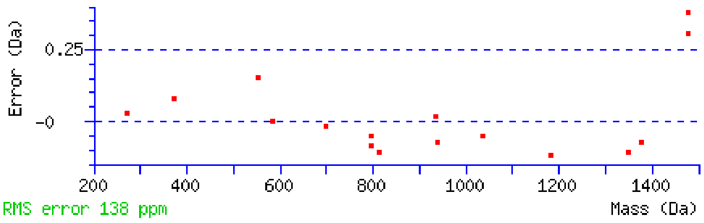

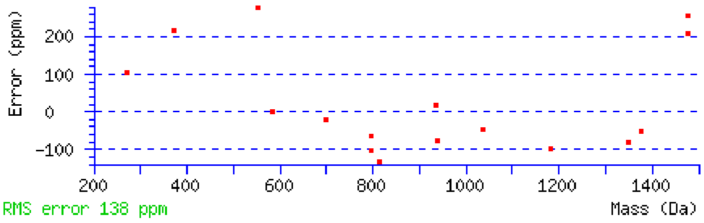

All matches to this query

Score	Mr(calc):	Delta	Sequence
53.6	1748.7771	0.0059	GVENPAFVPSSPDTPR
53.6	1748.7771	0.0059	GVENPAFVPSSPDTPR
25.6	1748.7771	0.0059	GVENPAFVPSSPDTPR
2.1	1746.7800	2.0029	KMPRYYPTEDVPR
1.3	1747.7818	1.0011	SAGVATFVIQEEFDR
1.3	1747.7818	1.0011	SAGVATFVLQEEFDR
1.1	1748.7982	-0.0152	ANDPSLQEVNLNNIK
0.9	1748.7899	-0.0070	SSPDPAVNPVPKPKR
0.8	1747.7818	1.0011	SAGVATFVIQEEFDR
0.8	1747.7818	1.0011	SAGVATFVLQEEFDR

Spectrum No: 245; Query: 2585; Rank: 1

Peptide View

MS/MS Fragmentation of **SAEDGSPDGETEPSPSPSPGLASDDPNPGSSQ**
Found in **IPI00393341**, Tax_Id=10116 Gene_Symbol=Sult2b1_predicted Isoform 2 of Sulfotransferase family cytosolic 2B member 1

Match to Query 2585: 3147.243552 from(1050.088460,3+)
Title: 100101RatKid_NS_deglyco_27.1687.1687.3.dta
Data file K:\NewmanPaper\Piliang\3SubProteomes\Piliang3SP\mgf5ppm\ERLIC_3SubProteomes5ppm.mgf

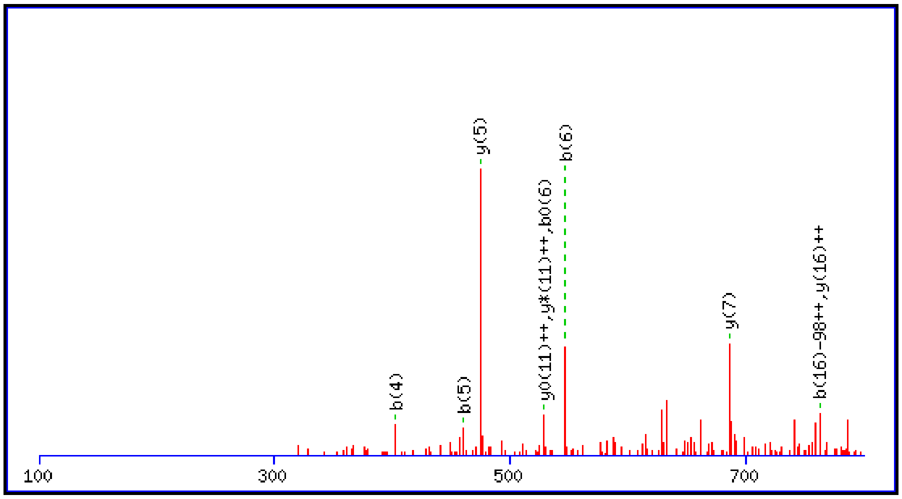

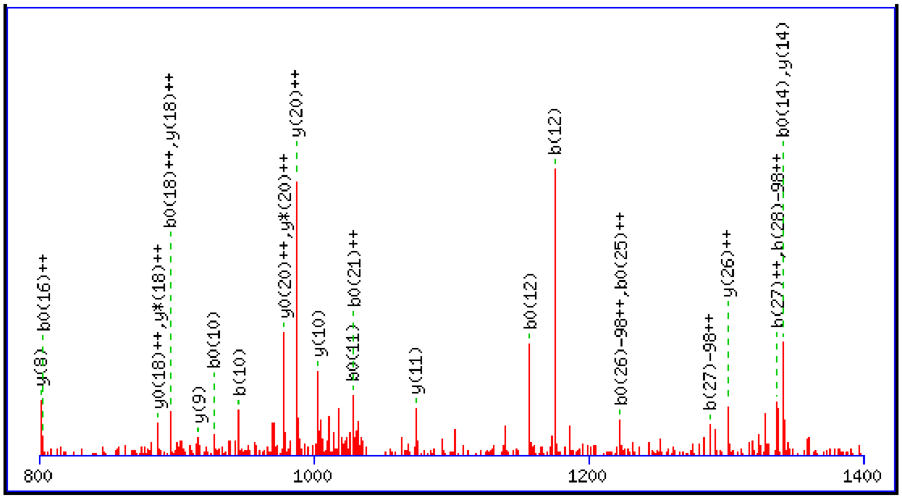

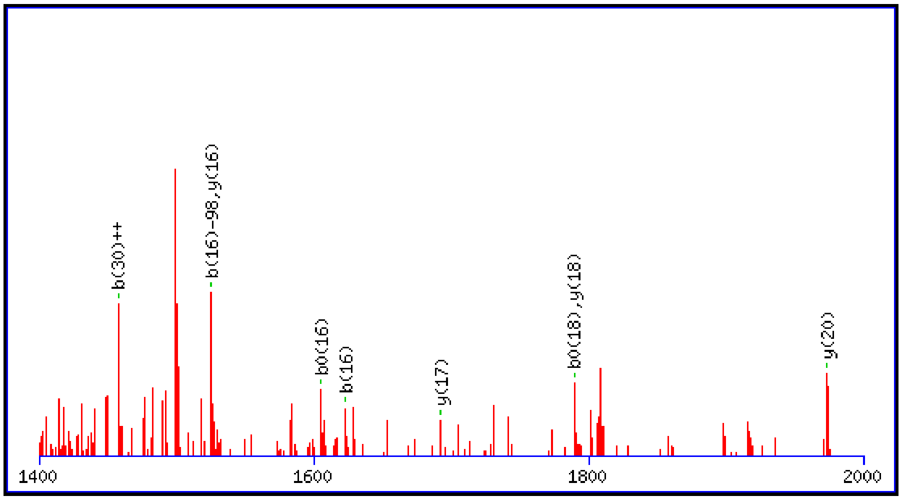

Monoisotopic mass of neutral peptide Mr(calc): 3147.2314
Fixed modifications: Carbamidomethyl (C)
Variable modifications:
S16 : Phospho (ST), with neutral losses 0.0000(shown in table), 97.9769
Ions Score: 54 **Expect:** 0.0014
Matches (Bold Red): 45/482 fragment ions using 56 most intense peaks

#	b	b^{++}	b*	b^{*++}	b^0	b^{0++}	Seq.	y	y^{++}	y*	y^{*++}	y^0	y^{0++}	#
1	88.0393	44.5233			70.0287	35.5180	S							32
2	159.0764	80.0418			141.0659	71.0366	A	3061.2066	1531.1069	3044.1800	1522.5937	3043.1960	1522.1017	31
3	288.1190	144.5631			270.1084	135.5579	E	2990.1695	1495.5884	2973.1429	1487.0751	2972.1589	1486.5831	30
4	**403.1460**	202.0766			385.1354	193.0713	D	2861.1269	1431.0671	2844.1003	1422.5538	2843.1163	1422.0618	29
5	**460.1674**	230.5873			442.1569	221.5821	G	2746.0999	1373.5536	2729.0734	1365.0403	2728.0894	1364.5483	28
6	**547.1994**	274.1034			**529.1889**	265.0981	S	2689.0785	1345.0429	2672.0519	1336.5296	2671.0679	1336.0376	27
7	644.2522	322.6297			626.2416	313.6245	P	2602.0465	**1301.5269**	2585.0199	1293.0136	2584.0359	1292.5216	26
8	759.2792	380.1432			741.2686	371.1379	D	2504.9937	1253.0005	2487.9671	1244.4872	2486.9831	1243.9952	25
9	816.3006	408.6539			798.2901	399.6487	G	2389.9667	1195.4870	2372.9402	1186.9737	2371.9562	1186.4817	24
10	**945.3432**	473.1752			**927.3326**	464.1700	E	2332.9453	1166.9763	2315.9187	1158.4630	2314.9347	1157.9710	23
11	1046.3909	523.6991			**1028.3803**	514.6938	T	2203.9027	1102.4550	2186.8761	1093.9417	2185.8921	1093.4497	22
12	**1175.4335**	588.2204			**1157.4229**	579.2151	E	2102.8550	1051.9311	2085.8285	1043.4179	2084.8444	1042.9259	21
13	1272.4862	636.7468			1254.4757	627.7415	P	**1973.8124**	**987.4098**	1956.7859	**978.8966**	1955.8018	**978.4046**	20
14	1359.5183	680.2628			**1341.5077**	671.2575	S	1876.7597	938.8835	1859.7331	930.3702	1858.7491	929.8782	19
15	1456.5710	728.7892			1438.5605	719.7839	P	**1789.7276**	**895.3674**	1772.7011	**886.8542**	1771.7171	**886.3622**	18
16	**1623.5694**	812.2883			**1605.5588**	**803.2831**	S	**1692.6749**	846.8411	1675.6483	838.3278	1674.6643	837.8358	17
17	1720.6222	860.8147			1702.6116	851.8094	P	**1525.6765**	**763.3419**	1508.6500	754.8286	1507.6659	754.3366	16
18	1807.6542	904.3307			**1789.6436**	**895.3254**	S	1428.6237	714.8155	1411.5972	706.3022	1410.6132	705.8102	15

19	1904.7069	952.8571			1886.6964	943.8518	P	1341.5917	671.2995	1324.5652	662.7862	1323.5811	662.2942	14
20	1961.7284	981.3678			1943.7178	972.3626	G	1244.5390	622.7731	1227.5124	614.2598	1226.5284	613.7678	13
21	2074.8125	1037.9099			2056.8019	1028.9046	L	1187.5175	594.2624	1170.4909	585.7491	1169.5069	585.2571	12
22	2145.8496	1073.4284			2127.8390	1064.4231	A	1074.4334	537.7203	1057.4069	529.2071	1056.4229	528.7151	11
23	2232.8816	1116.9444			2214.8711	1107.9392	S	1003.3963	502.2018	986.3698	493.6885	985.3857	493.1965	10
24	2347.9086	1174.4579			2329.8980	1165.4526	D	916.3643	458.6858	899.3377	450.1725	898.3537	449.6805	9
25	2462.9355	1231.9714			2444.9249	1222.9661	D	801.3373	401.1723	784.3108	392.6590	783.3268	392.1670	8
26	2559.9883	1280.4978			2541.9777	1271.4925	P	686.3104	343.6588	669.2838	335.1456	668.2998	334.6536	7
27	2674.0312	1337.5192	2657.0046	1329.0060	2656.0206	1328.5140	N	589.2576	295.1325	572.2311	286.6192	571.2471	286.1272	6
28	2771.0840	1386.0456	2754.0574	1377.5323	2753.0734	1377.0403	P	475.2147	238.1110	458.1882	229.5977	457.2041	229.1057	5
29	2828.1054	1414.5563	2811.0789	1406.0431	2810.0949	1405.5511	G	378.1619	189.5846	361.1354	181.0713	360.1514	180.5793	4
30	2915.1374	1458.0724	2898.1109	1449.5591	2897.1269	1449.0671	S	321.1405	161.0739	304.1139	152.5606	303.1299	152.0686	3
31	3002.1695	1501.5884	2985.1429	1493.0751	2984.1589	1492.5831	S	234.1084	117.5579	217.0819	109.0446	216.0979	108.5526	2
32							Q	147.0764	74.0418	130.0499	65.5286			1

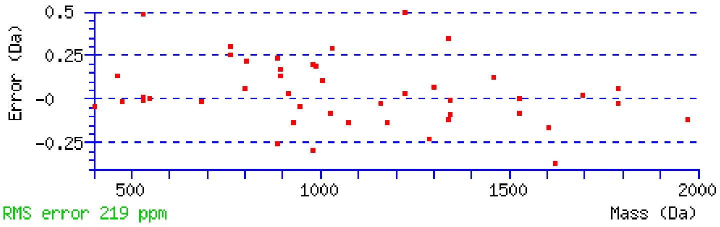

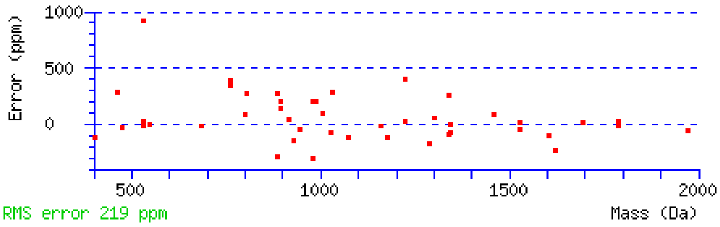

All matches to this query

Score	Mr(calc):	Delta	Sequence
53.5	3147.2314	0.0122	SAEDGSPDGETEPSPSPSPGLASDDPNPGSSQ
52.1	3147.2314	0.0122	SAEDGSPDGETEPSPSPSPGLASDDPNPGSSQ
38.3	3147.2314	0.0122	SAEDGSPDGETEPSPSPSPGLASDDPNPGSSQ
36.4	3147.2314	0.0122	SAEDGSPDGETEPSPSPSPGLASDDPNPGSSQ
33.8	3147.2314	0.0122	SAEDGSPDGETEPSPSPSPGLASDDPNPGSSQ
27.4	3147.2314	0.0122	SAEDGSPDGETEPSPSPSPGLASDDPNPGSSQ
18.7	3147.2314	0.0122	SAEDGSPDGETEPSPSPSPGLASDDPNPGSSQ
17.0	3147.2314	0.0122	SAEDGSPDGETEPSPSPSPGLASDDPNPGSSQ
16.1	3147.2314	0.0122	SAEDGSPDGETEPSPSPSPGLASDDPNPGSSQ
7.7	3145.2182	2.0254	MNNGDVDLTNDRYSMVGGNLVINNPDK

Spectrum No: 246; Query: 2742; Rank: 1

Peptide View

MS/MS Fragmentation of **DGDDEDGSDEDVVHNEDIHFEPIVSLPEVEVK**
Found in **IPI00371944**, Tax_Id=10116 Gene_Symbol=RGD1560047_predicted similar to RAN binding protein 2

Match to Query 2742: 3657.569772 from(1220.197200,3+)
Title: 100101RatKid_NS_deglyco_23.4754.4754.3.dta
Data file K:\NewmanPaper\Piliang\3SubProteomes\Piliang3SP\mgf5ppm\ERLIC_3SubProteomes5ppm.mgf

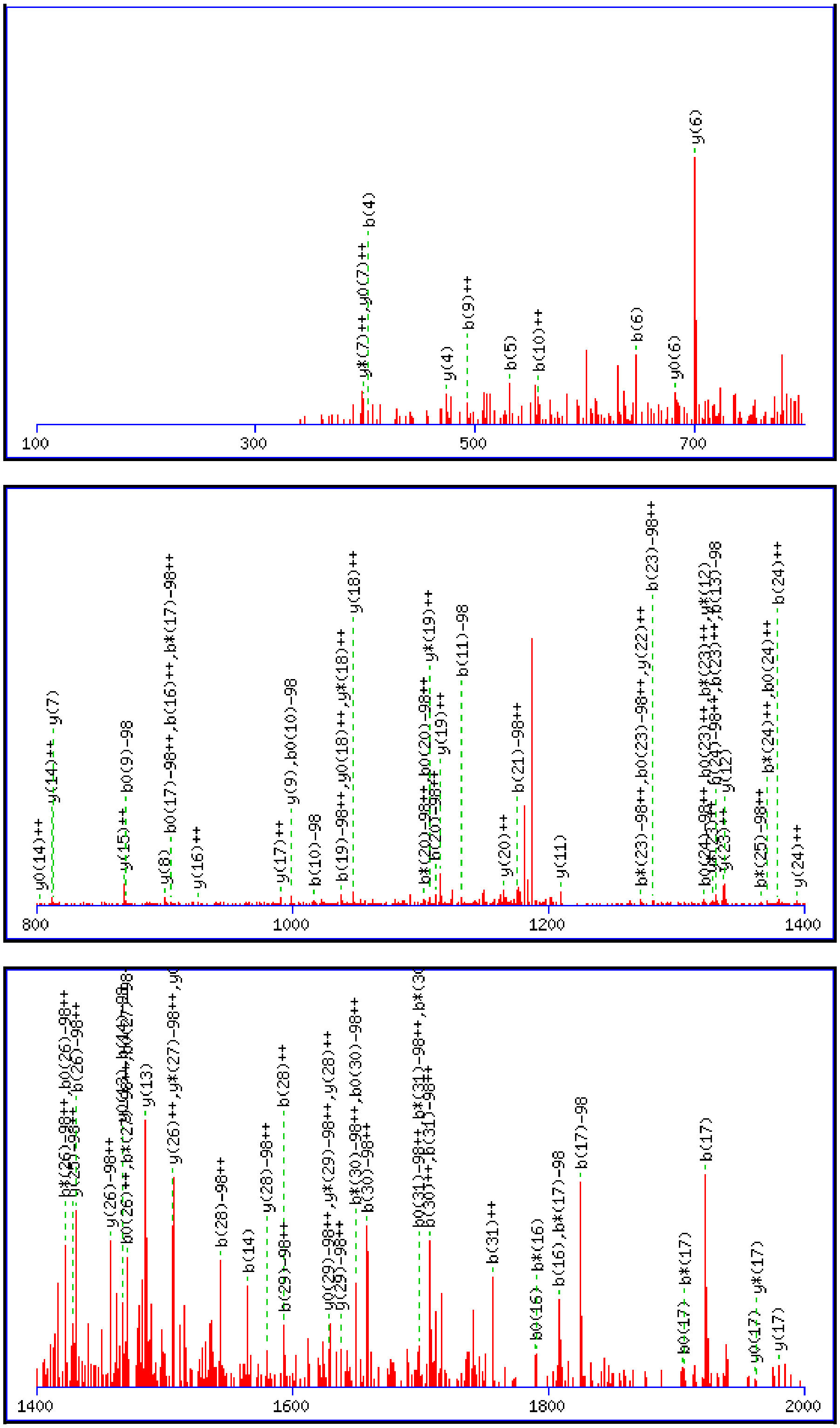

Monoisotopic mass of neutral peptide Mr(calc): 3657.5520
Fixed modifications: Carbamidomethyl (C)
Variable modifications:
S8 : Phospho (ST), with neutral losses 97.9769(shown in table), 0.0000
Ions Score: 54 **Expect:** 0.0022
Matches (Bold Red): 99/512 fragment ions using 171 most intense peaks

#	b	b++	b*	b*++	b0	b0++	Seq.	y	y++	y*	y*++	y0	y0++	#
1	116.0342	58.5207			98.0237	49.5155	D							32
2	173.0557	87.0315			155.0451	78.0262	G	3445.5554	1723.2813	3428.5288	1714.7681	3427.5448	1714.2760	31
3	288.0826	144.5450			270.0721	135.5397	D	3388.5339	1694.7706	3371.5074	1686.2573	3370.5234	1685.7653	30
4	**403.1096**	202.0584			385.0990	193.0531	D	3273.5070	**1637.2571**	3256.4804	**1628.7439**	3255.4964	**1628.2518**	29
5	**532.1522**	266.5797			514.1416	257.5744	E	3158.4800	**1579.7437**	3141.4535	1571.2304	3140.4695	1570.7384	28

**6**	**647.1791**	324.0932			629.1685	315.0879	**D**	3029.4374	1515.2224	3012.4109	**1506.7091**	3011.4269	**1506.2171**	**27**
**7**	704.2006	352.6039			686.1900	343.5986	**G**	2914.4105	**1457.7089**	2897.3840	1449.1956	2896.3999	1448.7036	**26**
**8**	773.2220	387.1147			755.2115	378.1094	**S**	2857.3890	**1429.1982**	2840.3625	1420.6849	2839.3785	1420.1929	**25**
**9**	888.2490	444.6281			**870.2384**	435.6228	**D**	2788.3676	**1394.6874**	2771.3410	1386.1742	2770.3570	1385.6821	**24**
**10**	**1017.2916**	509.1494			**999.2810**	500.1441	**E**	2673.3406	**1337.1740**	2656.3141	**1328.6607**	2655.3301	1328.1687	**23**
**11**	**1132.3185**	566.6629			1114.3079	557.6576	**D**	2544.2980	**1272.6527**	2527.2715	1264.1394	2526.2875	1263.6474	**22**
**12**	1231.3869	616.1971			1213.3764	607.1918	**V**	2429.2711	1215.1392	2412.2446	1206.6259	2411.2605	1206.1339	**21**
**13**	**1330.4553**	665.7313			1312.4448	656.7260	**V**	2330.2027	**1165.6050**	2313.1761	1157.0917	2312.1921	1156.5997	**20**
**14**	**1467.5142**	734.2608			1449.5037	725.2555	**H**	2231.1343	**1116.0708**	2214.1077	**1107.5575**	2213.1237	1107.0655	**19**
**15**	1581.5572	791.2822	1564.5306	782.7689	1563.5466	782.2769	**N**	2094.0754	**1047.5413**	2077.0488	**1039.0280**	2076.0648	**1038.5360**	**18**
**16**	1710.5998	855.8035	1693.5732	847.2902	1692.5892	846.7982	**E**	**1980.0324**	**990.5199**	**1963.0059**	982.0066	**1962.0219**	981.5146	**17**
**17**	**1825.6267**	913.3170	**1808.6002**	**904.8037**	1807.6161	**904.3117**	**D**	1850.9898	**925.9986**	1833.9633	917.4853	1832.9793	916.9933	**16**
**18**	1938.7108	969.8590	1921.6842	961.3457	1920.7002	960.8537	**I**	1735.9629	**868.4851**	1718.9364	859.9718	1717.9523	859.4798	**15**
**19**	2075.7697	**1038.3885**	2058.7431	1029.8752	2057.7591	1029.3832	**H**	1622.8788	**811.9431**	1605.8523	803.4298	1604.8683	**802.9378**	**14**
**20**	2222.8381	**1111.9227**	2205.8115	**1103.4094**	2204.8275	**1102.9174**	**F**	**1485.8199**	743.4136	1468.7934	734.9003	**1467.8094**	734.4083	**13**
**21**	2351.8807	**1176.4440**	2334.8541	1167.9307	2333.8701	1167.4387	**E**	**1338.7515**	669.8794	**1321.7250**	661.3661	1320.7409	660.8741	**12**
**22**	2448.9335	1224.9704	2431.9069	1216.4571	2430.9229	1215.9651	**P**	**1209.7089**	605.3581	1192.6824	596.8448	1191.6984	596.3528	**11**
**23**	2562.0175	**1281.5124**	2544.9910	**1272.9991**	2544.0070	**1272.5071**	**I**	1112.6562	556.8317	1095.6296	548.3184	1094.6456	547.8264	**10**
**24**	2661.0859	**1331.0466**	2644.0594	1322.5333	2643.0754	**1322.0413**	**V**	**999.5721**	500.2897	982.5455	491.7764	981.5615	491.2844	**9**
**25**	2748.1180	1374.5626	2731.0914	**1366.0493**	2730.1074	1365.5573	**S**	**900.5037**	450.7555	883.4771	442.2422	882.4931	441.7502	**8**
**26**	2861.2020	**1431.1046**	2844.1755	**1422.5914**	2843.1915	**1422.0994**	**L**	**813.4716**	407.2395	796.4451	**398.7262**	795.4611	**398.2342**	**7**
**27**	2958.2548	1479.6310	2941.2282	**1471.1178**	2940.2442	**1470.6257**	**P**	**700.3876**	350.6974	683.3610	342.1842	**682.3770**	341.6921	**6**
**28**	3087.2974	**1544.1523**	3070.2708	1535.6391	3069.2868	1535.1470	**E**	603.3348	302.1710	586.3083	293.6578	585.3243	293.1658	**5**
**29**	3186.3658	**1593.6865**	3169.3392	1585.1733	3168.3552	1584.6813	**V**	**474.2922**	237.6498	457.2657	229.1365	456.2817	228.6445	**4**
**30**	3315.4084	**1658.2078**	3298.3818	**1649.6946**	3297.3978	**1649.2025**	**E**	375.2238	188.1155	358.1973	179.6023	357.2132	179.1103	**3**
**31**	3414.4768	**1707.7420**	3397.4503	**1699.2288**	3396.4662	**1698.7368**	**V**	246.1812	123.5942	229.1547	115.0810			**2**
**32**							**K**	147.1128	74.0600	130.0863	65.5468			**1**

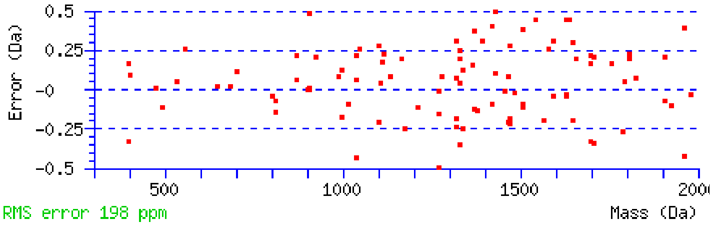

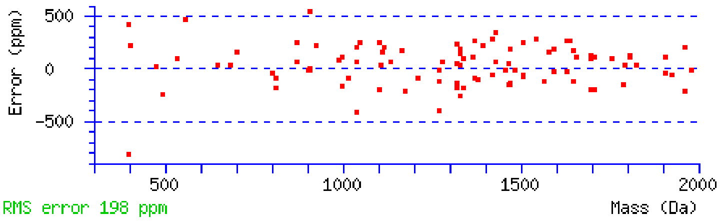

All matches to this query

Score	Mr(calc):	Delta	Sequence
53.5	3657.5520	0.0178	DGDDEDGSDEDVVHNEDIHFEPIVSLPEVEVK
7.1	3656.5449	1.0249	QYASVSNLFNTKKNALQDHFATFLNEQCK
5.3	3657.5849	-0.0151	VAMIDETKNSLYQYFLSYLQSLLLFPPK
5.3	3657.5849	-0.0151	VAMIDETKNSLYQYFLSYLQSLLLFPPK
5.2	3655.5456	2.0241	FQLSSVVSPYERQLNLDGYAQCLEDGSGKR
5.2	3655.5456	2.0241	FQLSSVVSPYERQLNLDGYAQCLEDGSGKR
5.2	3655.5456	2.0241	FQLSSVVSPYERQLNLDGYAQCLEDGSGKR
5.0	3656.5425	1.0273	QVSQIKYATSNGVHIRSFDSEIELMQVTR
4.9	3657.5849	-0.0151	VAMIDETKNSLYQYFLSYLQSLLLFPPK
4.9	3657.5849	-0.0151	VAMIDETKNSLYQYFLSYLQSLLLFPPK

Spectrum No: 247; Query: 677; Rank: 1

Peptide View

MS/MS Fragmentation of **ASAAEGSEASPPSLR**
Found in **IPI00358407**, Tax_Id=10116 Gene_Symbol=RGD1563576_predicted hypothetical protein LOC306617

Match to Query 677: 1508.649328 from(755.331940,2+)
Title: 091008RatKidney_NoSalt_23.861.861.2.dta
Data file K:\NewmanPaper\Piliang\3SubProteomes\Piliang3SP\mgf5ppm\ERLIC_3SubProteomes5ppm.mgf

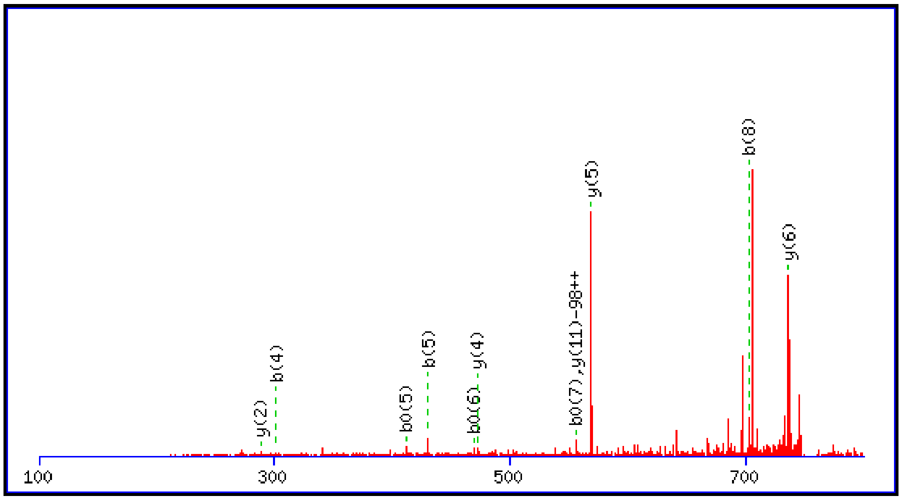

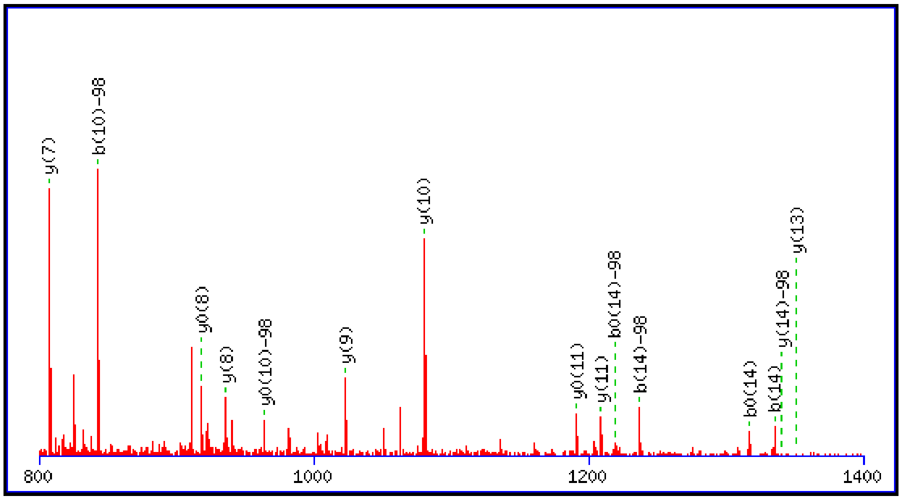

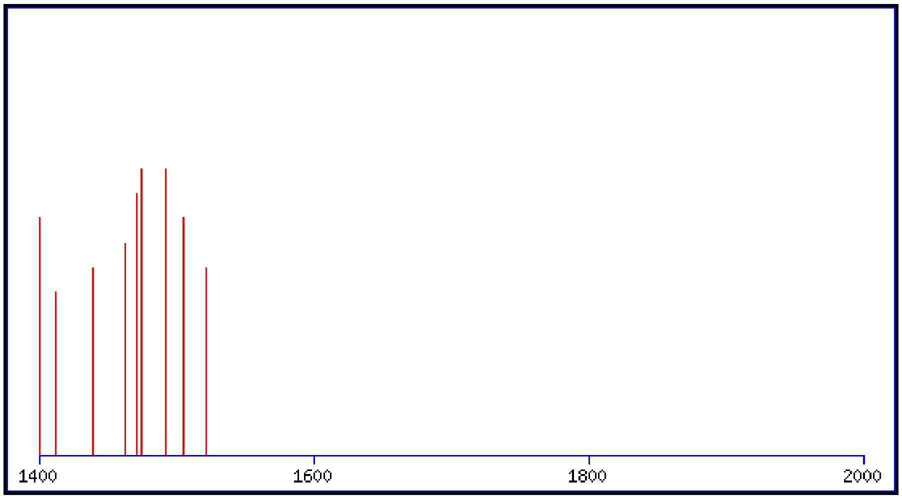

Monoisotopic mass of neutral peptide Mr(calc): 1508.6508
Fixed modifications: Carbamidomethyl (C)
Variable modifications:

S10 : Phospho (ST), with neutral losses 0.0000(shown in table), 97.9769
Ions Score: 53 **Expect:** 0.00062
Matches (Bold Red): 26/208 fragment ions using 54 most intense peaks

#	b	b^{++}	b^{0}	b^{0++}	Seq.	y	y^{++}	y*	y^{*++}	y^{0}	y^{0++}	#
1	72.0444	36.5258			A							15
2	159.0764	80.0418	141.0659	71.0366	S	1438.6210	719.8141	1421.5944	711.3008	1420.6104	710.8088	14
3	230.1135	115.5604	212.1030	106.5551	A	**1351.5889**	676.2981	1334.5624	667.7848	1333.5784	667.2928	13
4	**301.1506**	151.0790	283.1401	142.0737	A	1280.5518	640.7796	1263.5253	632.2663	1262.5413	631.7743	12
5	**430.1932**	215.6003	**412.1827**	206.5950	E	**1209.5147**	605.2610	1192.4882	596.7477	**1191.5042**	596.2557	11
6	487.2147	244.1110	**469.2041**	235.1057	G	**1080.4721**	540.7397	1063.4456	532.2264	1062.4616	531.7344	10
7	574.2467	287.6270	**556.2362**	278.6217	S	**1023.4507**	512.2290	1006.4241	503.7157	1005.4401	503.2237	9
8	**703.2893**	352.1483	685.2788	343.1430	E	**936.4186**	468.7130	919.3921	460.1997	**918.4081**	459.7077	8
9	774.3264	387.6669	756.3159	378.6616	A	**807.3760**	404.1917	790.3495	395.6784	789.3655	395.1864	7
10	941.3248	471.1660	923.3142	462.1608	S	**736.3389**	368.6731	719.3124	360.1598	718.3284	359.6678	6
11	1038.3776	519.6924	1020.3670	510.6871	P	**569.3406**	285.1739	552.3140	276.6606	551.3300	276.1686	5
12	1135.4303	568.2188	1117.4198	559.2135	P	**472.2878**	236.6475	455.2613	228.1343	454.2772	227.6423	4
13	1222.4623	611.7348	1204.4518	602.7295	S	375.2350	188.1212	358.2085	179.6079	357.2245	179.1159	3
14	**1335.5464**	668.2768	**1317.5358**	659.2716	L	**288.2030**	144.6051	271.1765	136.0919			2
15					R	175.1190	88.0631	158.0924	79.5498			1

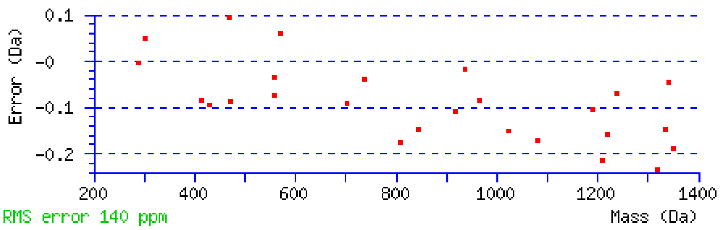

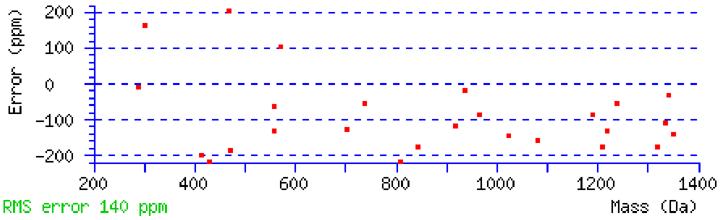

All matches to this query

Score	Mr(calc):	Delta	Sequence
53.4	1508.6508	-0.0014	ASAAEGSEASPPSLR
32.7	1508.6508	-0.0014	ASAAEGSEASPPSLR
19.2	1508.6508	-0.0014	ASAAEGSEASPPSLR
14.7	1508.6508	-0.0014	ASAAEGSEASPPSLR
4.5	1508.6384	0.0109	TFQAYLPHCHR
3.7	1508.6561	-0.0068	EINFYHNLHSR
1.8	1508.6633	-0.0140	REQRYQEQHR
1.6	1508.6517	-0.0023	ICRQDTSFMKK
1.4	1508.6578	-0.0085	QINWTVLYRR
0.9	1508.6443	0.0051	NTHDSEMLGLRR

Spectrum No: 248; Query: 695; Rank: 1

Peptide View

MS/MS Fragmentation of **TASGSSVTSLEGTR**
Found in **IPI00421389**, Tax_Id=10116 Gene_Symbol=Ndrg1 Protein NDRG1

Match to Query 695: 1511.593368 from(756.803960,2+)
Title: 100101RatKid_NS_deglyco_26.1661.1661.2.dta
Data file K:\NewmanPaper\Piliang\3SubProteomes\Piliang3SP\mgf5ppm\ERLIC_3SubProteomes5ppm.mgf

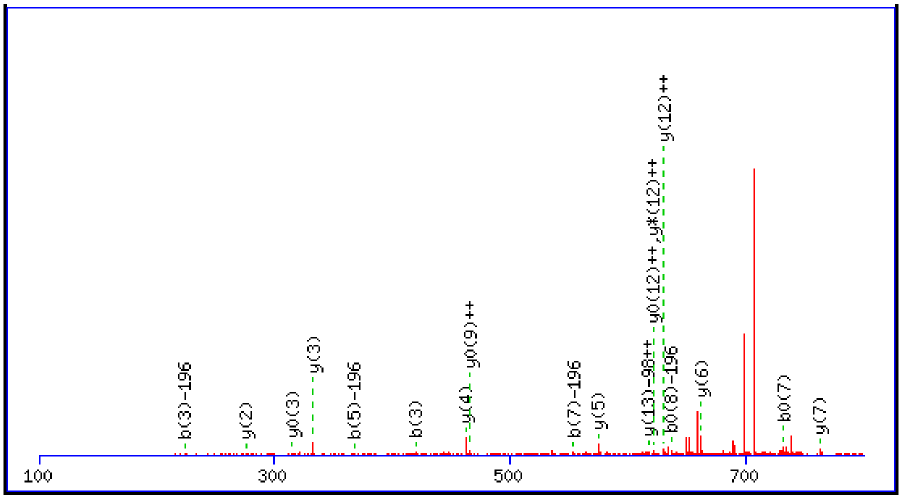

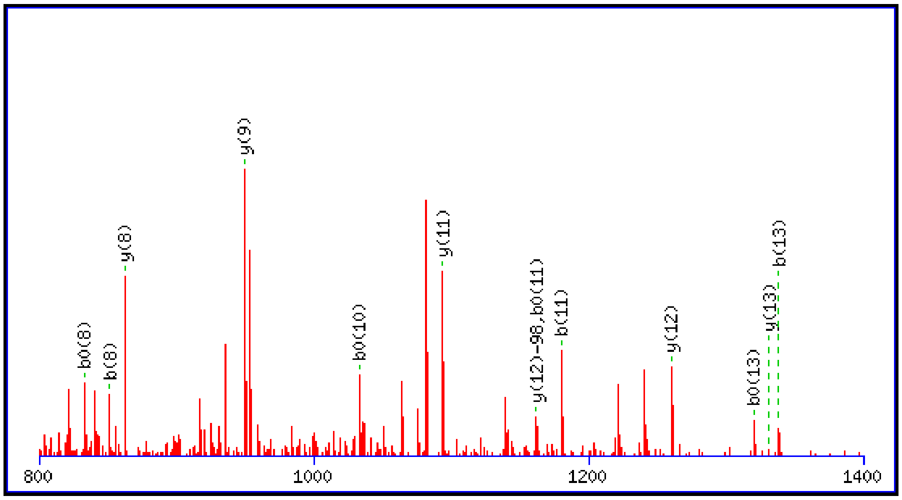

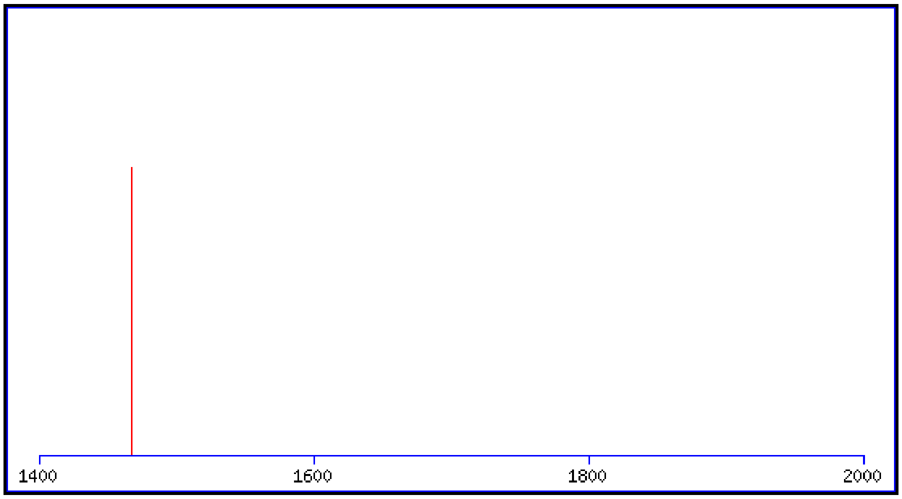

Monoisotopic mass of neutral peptide Mr(calc): 1511.5906
Fixed modifications: Carbamidomethyl (C)
Variable modifications:
T1 : Phospho (ST), with neutral losses 0.0000(shown in table), 97.9769
S3 : Phospho (ST), with neutral losses 0.0000(shown in table), 97.9769
Ions Score: 53 **Expect:** 0.00066
Matches (Bold Red): 31/192 fragment ions using 70 most intense peaks

#	b	b^{++}	b^{0}	b^{0++}	Seq.	y	y^{++}	y*	y^{*++}	y^{0}	y^{0++}	#
1	182.0213	91.5143	164.0107	82.5090	T							14
2	253.0584	127.0328	235.0478	118.0276	A	**1331.5839**	666.2956	1314.5573	657.7823	1313.5733	657.2903	13
3	**420.0567**	210.5320	402.0462	201.5267	S	**1260.5467**	**630.7770**	1243.5202	**622.2637**	1242.5362	**621.7717**	12
4	477.0782	239.0427	459.0676	230.0375	G	**1093.5484**	547.2778	1076.5218	538.7646	1075.5378	538.2726	11

5	564.1102	282.5588	546.0997	273.5535	S	1036.5269	518.7671	1019.5004	510.2538	1018.5164	509.7618	10
6	651.1423	326.0748	633.1317	317.0695	S	**949.4949**	475.2511	932.4684	466.7378	931.4843	**466.2458**	9
7	750.2107	375.6090	**732.2001**	366.6037	V	**862.4629**	431.7351	845.4363	423.2218	844.4523	422.7298	8
8	**851.2584**	426.1328	**833.2478**	417.1275	T	**763.3945**	382.2009	746.3679	373.6876	745.3839	373.1956	7
9	938.2904	469.6488	920.2798	460.6435	S	**662.3468**	331.6770	645.3202	323.1638	644.3362	322.6717	6
10	1051.3744	526.1909	**1033.3639**	517.1856	L	**575.3148**	288.1610	558.2882	279.6477	557.3042	279.1557	5
11	**1180.4170**	590.7122	**1162.4065**	581.7069	E	**462.2307**	231.6190	445.2041	223.1057	444.2201	222.6137	4
12	1237.4385	619.2229	1219.4279	610.2176	G	**333.1881**	167.0977	316.1615	158.5844	**315.1775**	158.0924	3
13	**1338.4862**	669.7467	**1320.4756**	660.7414	T	**276.1666**	138.5870	259.1401	130.0737	258.1561	129.5817	2
14					R	175.1190	88.0631	158.0924	79.5498			1

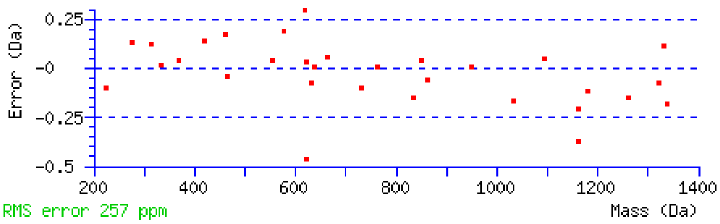

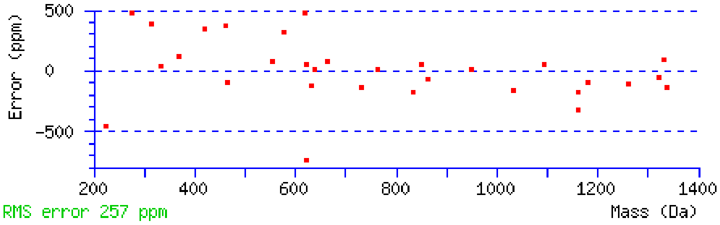

All matches to this query

Score	Mr(calc):	Delta	Sequence
53.3	1511.5906	0.0028	TASGSSVTSLEGTR
27.8	1511.5906	0.0028	TASGSSVTSLEGTR
15.9	1511.5906	0.0028	TASGSSVTSLEGTR
9.1	1509.5959	1.9975	SITFVVFKNSK
8.7	1511.5824	0.0110	MSSYSSDRDRGR
7.2	1511.6042	-0.0108	YDPTNENKHGTR
7.2	1511.6042	-0.0108	YDPTNENKHGTR
6.3	1511.5906	0.0028	TASGSSVTSLEGTR
6.2	1511.5906	0.0028	TASGSSVTSLEGTR
3.3	1511.5921	0.0013	YAMRYFATVSK

Spectrum No: 249; Query: 1154; Rank: 1

Peptide View

MS/MS Fragmentation of **AELGMNDSPSQSPPVK**
Found in **IPI00869599**, Tax_Id=10116 Gene_Symbol=Acss2_predicted acyl-CoA synthetase short-chain family member 2

Match to Query 1154: 1751.740528 from(876.877540,2+)
Title: 091008RatKidney_NH4Format01_20.962.962.2.dta
Data file K:\NewmanPaper\Piliang\3SubProteomes\Piliang3SP\mgf5ppm\ERLIC_3SubProteomes5ppm.mgf

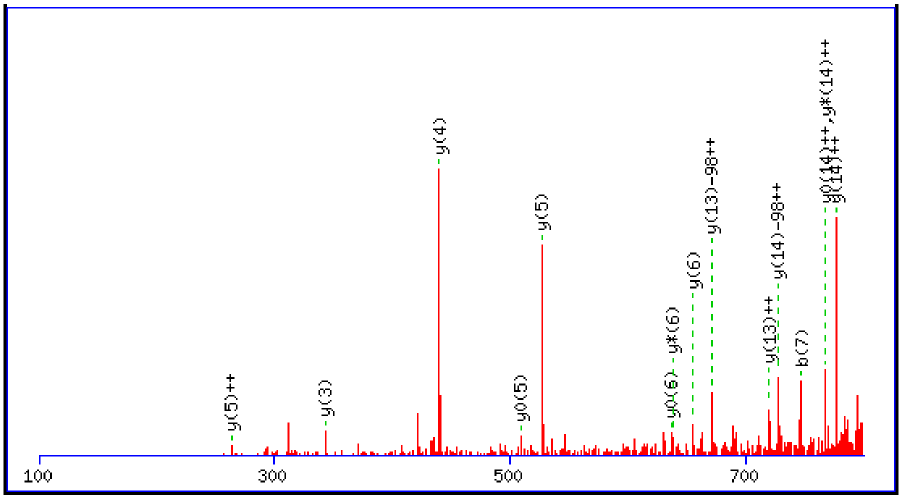

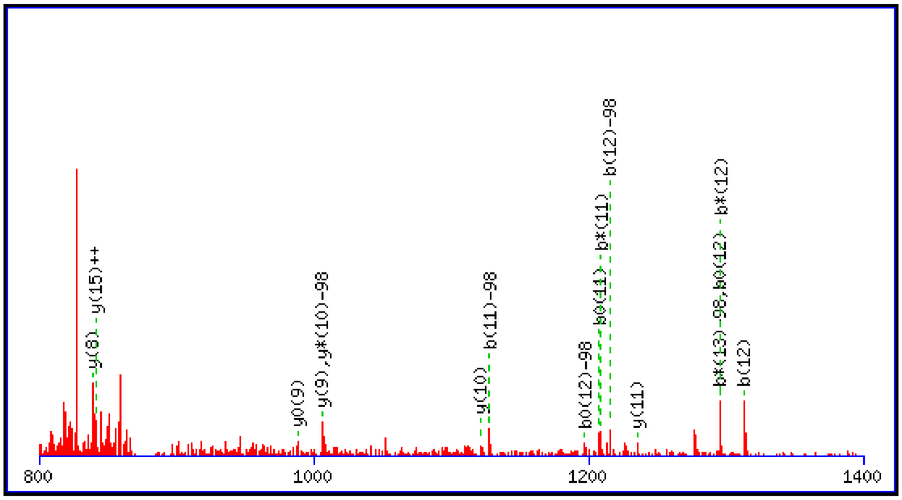

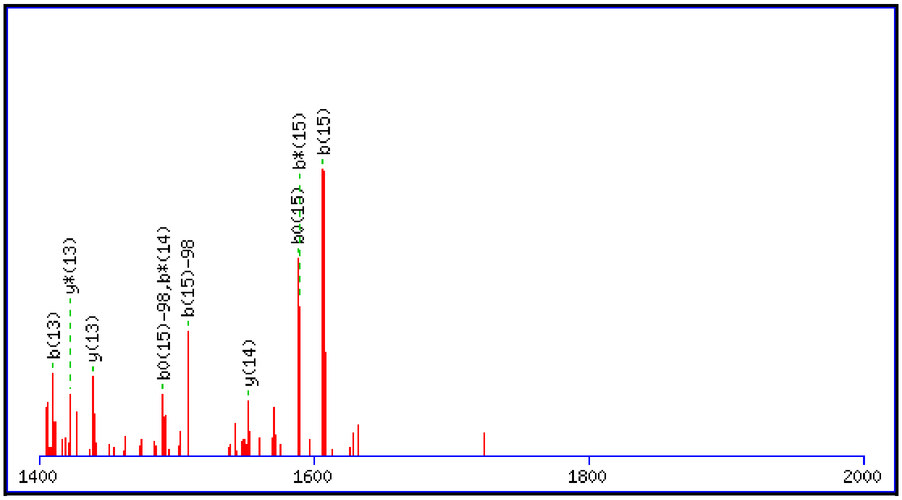

Monoisotopic mass of neutral peptide Mr(calc): 1751.7437
Fixed modifications: Carbamidomethyl (C)
Variable modifications:
M5 : Oxidation (M)
S8 : Phospho (ST), with neutral losses 0.0000(shown in table), 97.9769
Ions Score: 53 **Expect:** 0.0008
Matches (Bold Red): 41/250 fragment ions using 72 most intense peaks

#	b	b^{++}	b*	b^{*++}	b^0	b^{0++}	Seq.	y	y^{++}	y*	y^{*++}	y^0	y^{0++}	#
1	72.0444	36.5258					A							16
2	201.0870	101.0471			183.0764	92.0418	E	1681.7139	**841.3606**	1664.6873	832.8473	1663.7033	832.3553	15
3	314.1710	157.5892			296.1605	148.5839	L	**1552.6713**	**776.8393**	1535.6447	**768.3260**	1534.6607	**767.8340**	14
4	371.1925	186.0999			353.1819	177.0946	G	**1439.5872**	**720.2973**	**1422.5607**	711.7840	1421.5767	711.2920	13

**5**	518.2279	259.6176			500.2174	250.6123	**M**	1382.5658	691.7865	1365.5392	683.2732	1364.5552	682.7812	**12**
**6**	632.2708	316.6391	615.2443	308.1258	614.2603	307.6338	**N**	**1235.5304**	618.2688	1218.5038	609.7555	1217.5198	609.2635	**11**
**7**	**747.2978**	374.1525	730.2712	365.6393	729.2872	365.1472	**D**	**1121.4874**	561.2474	1104.4609	552.7341	1103.4769	552.2421	**10**
**8**	914.2961	457.6517	897.2696	449.1384	896.2856	448.6464	**S**	**1006.4605**	503.7339	989.4339	495.2206	**988.4499**	494.7286	**9**
**9**	1011.3489	506.1781	994.3224	497.6648	993.3383	497.1728	**P**	**839.4621**	420.2347	822.4356	411.7214	821.4516	411.2294	**8**
**10**	1098.3809	549.6941	1081.3544	541.1808	1080.3704	540.6888	**S**	742.4094	371.7083	725.3828	363.1951	724.3988	362.7030	**7**
**11**	1226.4395	613.7234	**1209.4130**	605.2101	**1208.4289**	604.7181	**Q**	**655.3774**	328.1923	**638.3508**	319.6790	**637.3668**	319.1870	**6**
**12**	**1313.4715**	657.2394	**1296.4450**	648.7261	**1295.4610**	648.2341	**S**	**527.3188**	**264.1630**	510.2922	255.6498	**509.3082**	255.1577	**5**
**13**	**1410.5243**	705.7658	1393.4978	697.2525	1392.5137	696.7605	**P**	**440.2867**	220.6470	423.2602	212.1337			**4**
**14**	1507.5771	754.2922	**1490.5505**	745.7789	1489.5665	745.2869	**P**	**343.2340**	172.1206	326.2074	163.6074			**3**
**15**	**1606.6455**	803.8264	**1589.6189**	795.3131	**1588.6349**	794.8211	**V**	246.1812	123.5942	229.1547	115.0810			**2**
**16**							**K**	147.1128	74.0600	130.0863	65.5468			**1**

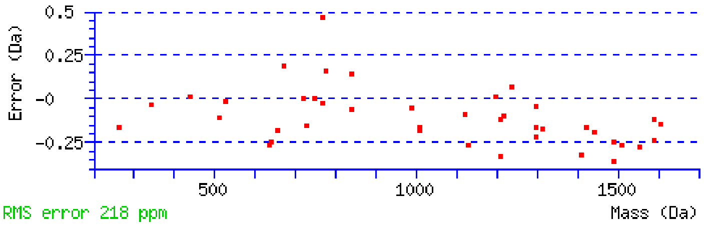

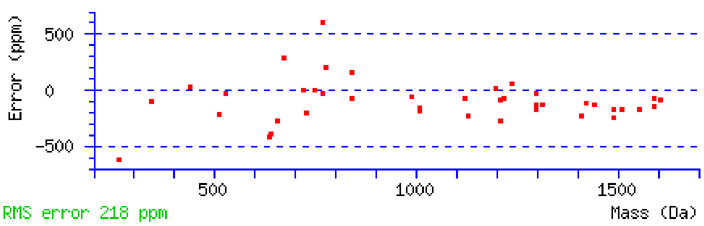

All matches to this query

Score	Mr(calc):	Delta	Sequence
53.2	1751.7437	-0.0032	AELGMNDSPSQSPPVK
42.4	1751.7437	-0.0032	AELGMNDSPSQSPPVK
20.6	1751.7437	-0.0032	AELGMNDSPSQSPPVK
6.1	1749.7223	2.0182	GSPSSAIDISLDSSVR
4.5	1751.7532	-0.0127	TNKNLEQPSSFTVK
4.5	1751.7532	-0.0127	TNKNLEQPSSFTVK
3.3	1751.7420	-0.0014	ASYQEEVIKNSPVK
1.6	1751.7453	-0.0048	QLESMGISLETSGIK
1.6	1751.7453	-0.0048	QLESMGISLETSGIK
1.4	1751.7532	-0.0127	TNKNLEQPSSFTVK

Spectrum No: 250; Query: 2506; Rank: 1

Peptide View

MS/MS Fragmentation of **LHKTEDGGWEWSDDEFDEESEEGK**
Found in **IPI00371139**, Tax_Id=10116 Gene_Symbol=Oxsr1_predicted oxidative-stress responsive 1

Match to Query 2506: 2933.124372 from(978.715400,3+)
Title: 100101RatKid_NS_deglyco_21.3156.3156.3.dta
Data file K:\NewmanPaper\Piliang\3SubProteomes\Piliang3SP\mgf5ppm\ERLIC_3SubProteomes5ppm.mgf

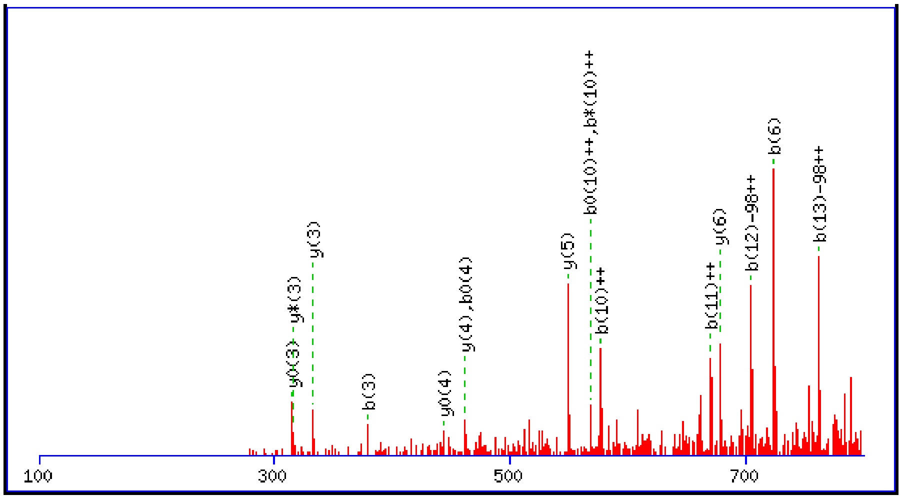

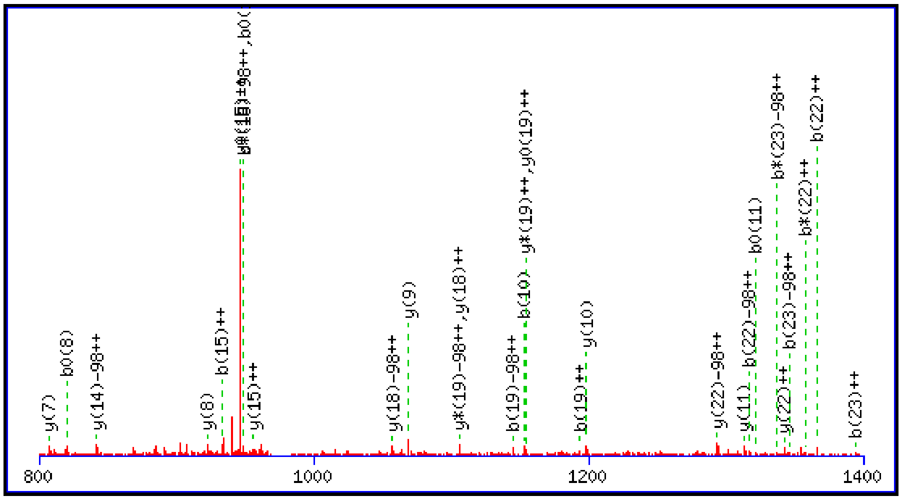

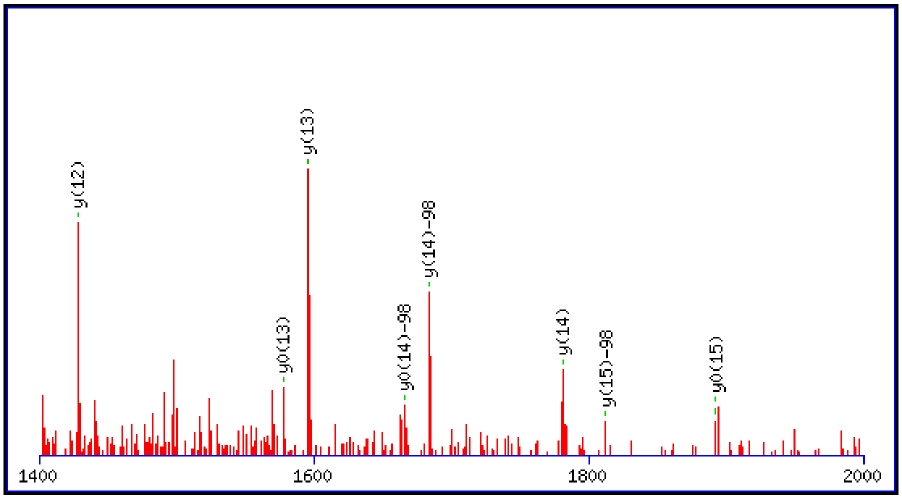

Monoisotopic mass of neutral peptide Mr(calc): 2933.1189
Fixed modifications: Carbamidomethyl (C)
Variable modifications:
S12 : Phospho (ST), with neutral losses 97.9769(shown in table), 0.0000
Ions Score: 53 **Expect:** 0.0011
Matches (Bold Red): 53/400 fragment ions using 76 most intense peaks

#	b	b++	b*	b*++	b0	b0++	Seq.	y	y++	y*	y*++	y0	y0++	#
1	114.0913	57.5493					L							24
2	251.1503	126.0788					H	2723.0652	1362.0362	2706.0386	1353.5230	2705.0546	1353.0310	23
3	**379.2452**	190.1262	362.2187	181.6130			K	2586.0063	**1293.5068**	2568.9797	1284.9935	2567.9957	1284.5015	22
4	480.2929	240.6501	463.2663	232.1368	**462.2823**	231.6448	T	2457.9113	1229.4593	2440.8848	1220.9460	2439.9008	1220.4540	21
5	609.3355	305.1714	592.3089	296.6581	591.3249	296.1661	E	2356.8636	1178.9355	2339.8371	1170.4222	2338.8531	1169.9302	20

**6**	**724.3624**	362.6849	707.3359	354.1716	706.3519	353.6796	**D**	2227.8211	1114.4142	2210.7945	**1105.9009**	2209.8105	1105.4089	**19**
**7**	781.3839	391.1956	764.3573	382.6823	763.3733	382.1903	**G**	2112.7941	**1056.9007**	2095.7676	1048.3874	2094.7835	1047.8954	**18**
**8**	838.4054	419.7063	821.3788	411.1930	**820.3948**	410.7010	**G**	2055.7726	1028.3900	2038.7461	1019.8767	2037.7621	1019.3847	**17**
**9**	1024.4847	512.7460	1007.4581	504.2327	1006.4741	503.7407	**W**	1998.7512	999.8792	1981.7246	991.3660	1980.7406	990.8739	**16**
**10**	**1153.5273**	**577.2673**	1136.5007	**568.7540**	1135.5167	**568.2620**	**E**	**1812.6719**	906.8396	1795.6453	898.3263	1794.6613	897.8343	**15**
**11**	1339.6066	**670.3069**	1322.5800	661.7937	**1321.5960**	661.3016	**W**	**1683.6293**	**842.3183**	1666.6027	833.8050	**1665.6187**	833.3130	**14**
**12**	1408.6280	**704.8177**	1391.6015	696.3044	1390.6175	695.8124	**S**	1497.5500	749.2786	1480.5234	740.7653	1479.5394	740.2733	**13**
**13**	1523.6550	**762.3311**	1506.6284	753.8179	1505.6444	753.3258	**D**	**1428.5285**	714.7679	1411.5020	706.2546	1410.5179	705.7626	**12**
**14**	1638.6819	819.8446	1621.6554	811.3313	1620.6714	810.8393	**D**	**1313.5016**	657.2544	1296.4750	648.7411	1295.4910	648.2491	**11**
**15**	1767.7245	884.3659	1750.6980	875.8526	1749.7139	875.3606	**E**	**1198.4746**	599.7409	1181.4481	591.2277	1180.4641	590.7357	**10**
**16**	1914.7929	957.9001	1897.7664	**949.3868**	1896.7824	**948.8948**	**F**	**1069.4320**	535.2197	1052.4055	526.7064	1051.4215	526.2144	**9**
**17**	2029.8199	1015.4136	2012.7933	1006.9003	2011.8093	1006.4083	**D**	**922.3636**	461.6854	905.3371	453.1722	904.3530	452.6802	**8**
**18**	2158.8625	1079.9349	2141.8359	1071.4216	2140.8519	1070.9296	**E**	**807.3367**	404.1720	790.3101	395.6587	789.3261	395.1667	**7**
**19**	2287.9051	**1144.4562**	2270.8785	1135.9429	2269.8945	1135.4509	**E**	**678.2941**	339.6507	661.2675	331.1374	660.2835	330.6454	**6**
**20**	2374.9371	1187.9722	2357.9105	1179.4589	2356.9265	1178.9669	**S**	**549.2515**	275.1294	532.2249	266.6161	531.2409	266.1241	**5**
**21**	2503.9797	1252.4935	2486.9531	1243.9802	2485.9691	1243.4882	**E**	**462.2195**	231.6134	445.1929	223.1001	**444.2089**	222.6081	**4**
**22**	2633.0223	**1317.0148**	2615.9957	1308.5015	2615.0117	1308.0095	**E**	**333.1769**	167.0921	**316.1503**	158.5788	**315.1663**	158.0868	**3**
**23**	2690.0437	**1345.5255**	2673.0172	**1337.0122**	2672.0332	1336.5202	**G**	204.1343	102.5708	187.1077	94.0575			**2**
**24**							**K**	147.1128	74.0600	130.0863	65.5468			**1**

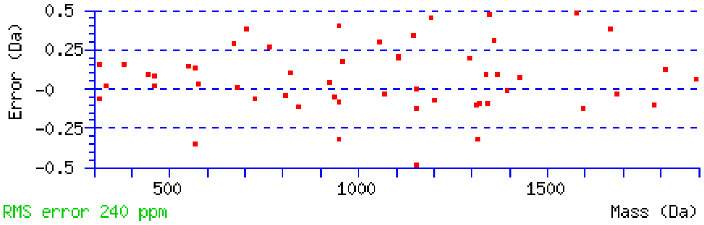

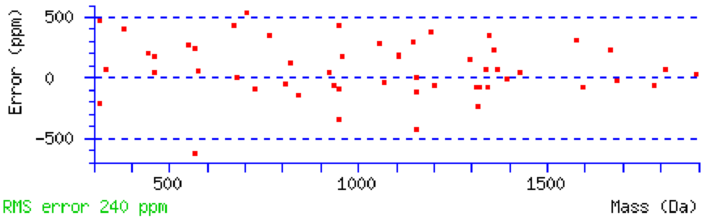

All matches to this query

Score	Mr(calc):	Delta	Sequence
53.1	2933.1189	0.0055	LHKTEDGGWEWSDDEFDEESEEGK
29.9	2933.1189	0.0055	LHKTEDGGWEWSDDEFDEESEEGK
16.2	2933.1189	0.0055	LHKTEDGGWEWSDDEFDEESEEGK
5.6	2932.0937	1.0307	ASEENLLSSNSISNSDQDSSPTANPK
5.6	2932.0937	1.0307	ASEENLLSSNSISNSDQDSSPTANPK
5.6	2932.0937	1.0307	ASEENLLSSNSISNSDQDSSPTANPK
5.4	2931.1102	2.0141	MGTQHDAACRLSNNHNTEREHFK
5.3	2932.1235	1.0008	ASTTTITPSPTTTLPVSTAGTSTSPR
3.4	2931.1283	1.9961	QDNEQLARSGTNCLENLVISNGEK
3.3	2932.1123	1.0121	QDNEQLARSGTNCLENLVISNGEK

Spectrum No: 251; Query: 1245; Rank: 1

Peptide View

MS/MS Fragmentation of **DHSPTPSVFNSDEER**
Found in **IPI00193470**, Tax_Id=10116 Gene_Symbol=Fip1l1 65 kDa protein

Match to Query 1245: 1795.702212 from(599.574680,3+)
Title: 091008RatKidney_NH4Format01_21.1386.1386.3.dta
Data file K:\NewmanPaper\Piliang\3SubProteomes\Piliang3SP\mgf5ppm\ERLIC_3SubProteomes5ppm.mgf

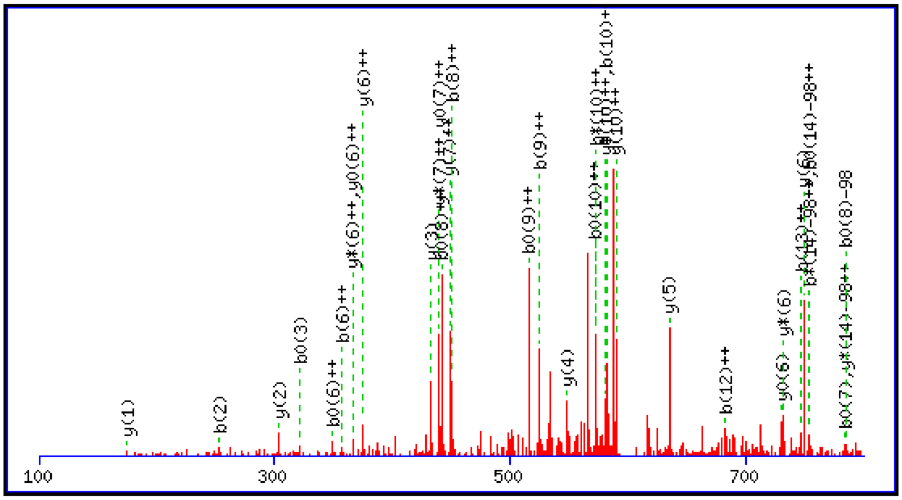

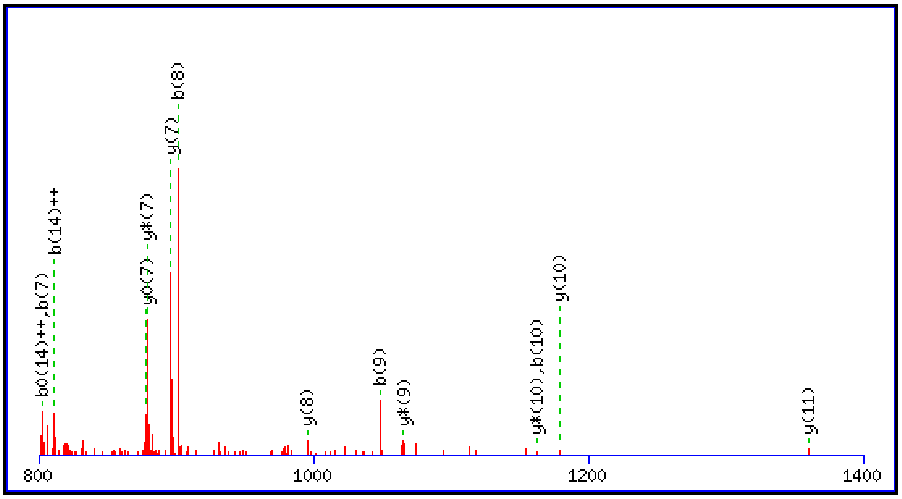

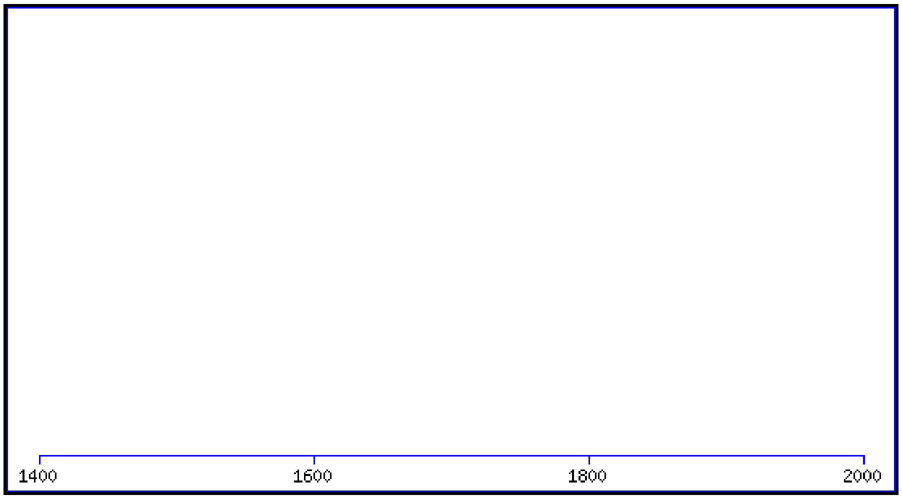

Monoisotopic mass of neutral peptide Mr(calc): 1795.7050
Fixed modifications: Carbamidomethyl (C)
Variable modifications:
T5 : Phospho (ST), with neutral losses 0.0000(shown in table), 97.9769
Ions Score: 53 **Expect:** 0.00069
Matches (Bold Red): 49/222 fragment ions using 76 most intense peaks

#	b	b^{++}	b*	b^{*++}	b^0	b^{0++}	Seq.	y	y^{++}	y*	y^{*++}	y^0	y^{0++}	#
1	116.0342	58.5207			98.0237	49.5155	D							15
2	**253.0931**	127.0502			235.0826	118.0449	H	1681.6854	841.3463	1664.6588	832.8330	1663.6748	832.3410	14
3	340.1252	170.5662			**322.1146**	161.5609	S	1544.6265	772.8169	1527.5999	764.3036	1526.6159	763.8116	13
4	437.1779	219.0926			419.1674	210.0873	P	1457.5944	729.3008	1440.5679	720.7876	1439.5839	720.2956	12
5	618.1919	309.5996			600.1814	300.5943	T	**1360.5417**	680.7745	1343.5151	672.2612	1342.5311	671.7692	11

**6**	715.2447	**358.1260**			697.2341	**349.1207**	**P**	**1179.5277**	**590.2675**	**1162.5011**	**581.7542**	1161.5171	**581.2622**	**10**
**7**	**802.2767**	401.6420			**784.2662**	392.6367	**S**	1082.4749	541.7411	**1065.4483**	533.2278	1064.4643	532.7358	**9**
**8**	**901.3451**	**451.1762**			883.3346	**442.1709**	**V**	**995.4429**	498.2251	978.4163	489.7118	977.4323	489.2198	**8**
**9**	**1048.4135**	**524.7104**			1030.4030	**515.7051**	**F**	**896.3745**	**448.6909**	**879.3479**	**440.1776**	**878.3639**	**439.6856**	**7**
**10**	**1162.4565**	**581.7319**	1145.4299	**573.2186**	1144.4459	**572.7266**	**N**	**749.3060**	**375.1567**	**732.2795**	**366.6434**	**731.2955**	**366.1514**	**6**
**11**	1249.4885	625.2479	1232.4620	616.7346	1231.4779	616.2426	**S**	**635.2631**	318.1352	618.2366	309.6219	617.2525	309.1299	**5**
**12**	1364.5154	**682.7614**	1347.4889	674.2481	1346.5049	673.7561	**D**	**548.2311**	274.6192	531.2045	266.1059	530.2205	265.6139	**4**
**13**	1493.5580	**747.2827**	1476.5315	738.7694	1475.5475	738.2774	**E**	**433.2041**	217.1057	416.1776	208.5924	415.1936	208.1004	**3**
**14**	1622.6006	**811.8040**	1605.5741	803.2907	1604.5901	**802.7987**	**E**	**304.1615**	152.5844	287.1350	144.0711	286.1510	143.5791	**2**
**15**							**R**	**175.1190**	88.0631	158.0924	79.5498			**1**

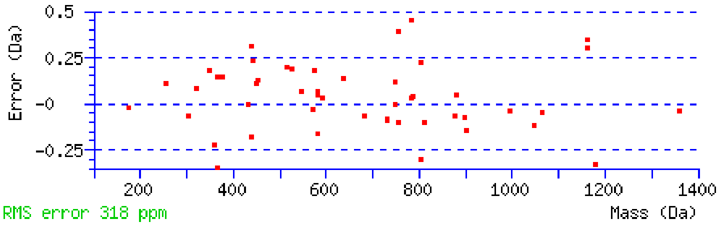

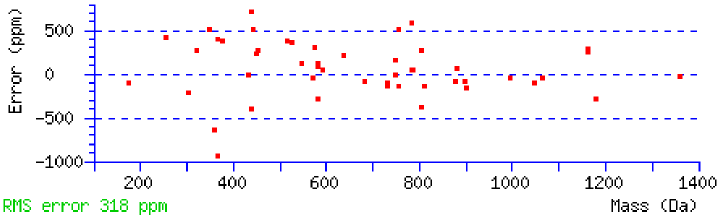

All matches to this query

Score	Mr(calc):	Delta	Sequence
53.0	1795.7050	-0.0028	DHSPTPSVFNSDEER
46.7	1795.7050	-0.0028	DHSPTPSVFNSDEER
43.2	1795.7050	-0.0028	DHSPTPSVFNSDEER
15.7	1795.6985	0.0037	EGGSQNCSFPRASYR
10.3	1795.6985	0.0037	EGGSQNCSFPRASYR
9.8	1795.7131	-0.0109	VHTGEKPCRDNSCR
9.5	1795.7193	-0.0170	RFSQTSNFTQHQR
9.3	1795.6929	0.0093	TLFSLMQYSDEFR
8.6	1795.7193	-0.0170	RFSQTSNFTQHQR
8.6	1795.7193	-0.0170	RFSQTSNFTQHQR

Spectrum No: 252; Query: 2833; Rank: 1

Peptide View

MS/MS Fragmentation of **HTSDYEDTDTEGGAYTDQELDETLNDEVGTPPESAITR**
Found in **IPI00870042**, Tax_Id=10116 Gene_Symbol=Tjp1_predicted tight junction protein 1

Match to Query 2833: 4251.739872 from(1418.253900,3+)
Title: 091008RatKidney_NoSalt_27.3375.3375.3.dta
Data file K:\NewmanPaper\Piliang\3SubProteomes\Piliang3SP\mgf5ppm\ERLIC_3SubProteomes5ppm.mgf

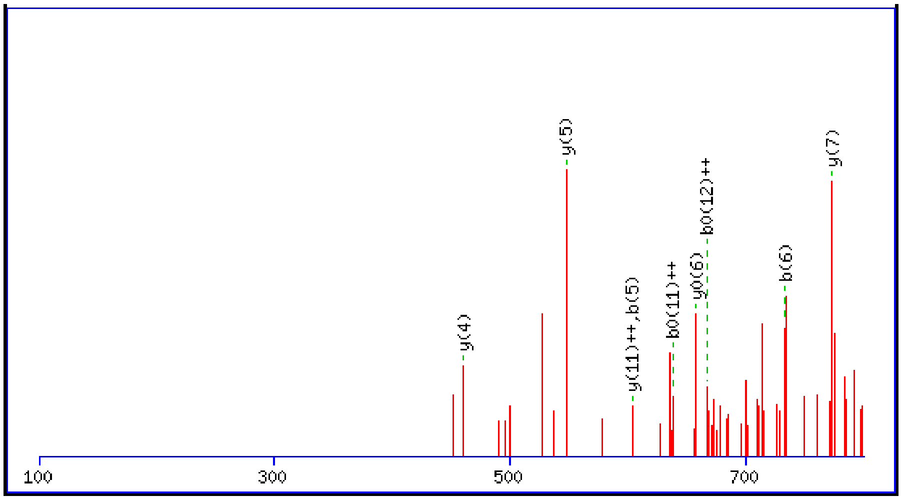

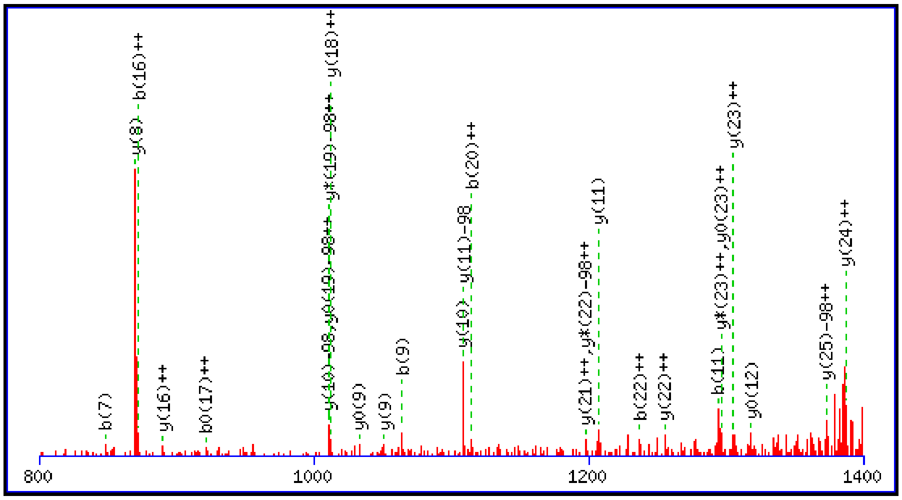

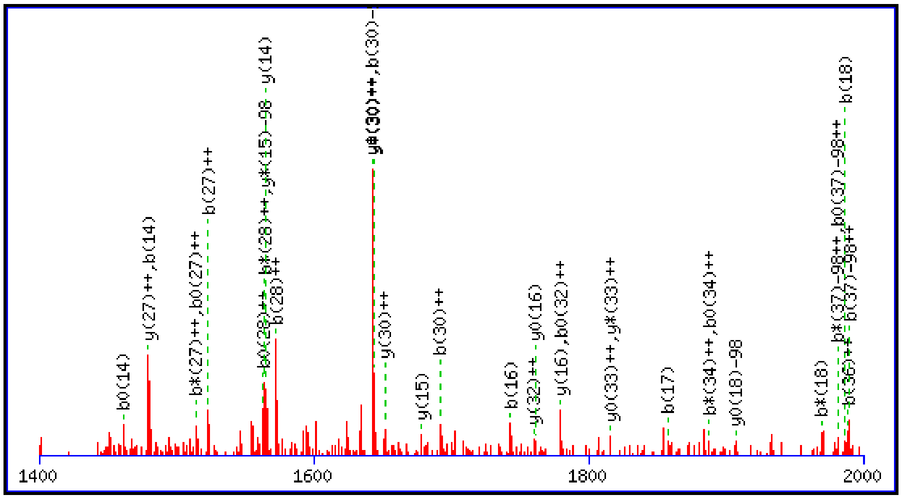

Monoisotopic mass of neutral peptide Mr(calc): 4250.7449
Fixed modifications: Carbamidomethyl (C)
Variable modifications:
T30 : Phospho (ST), with neutral losses 0.0000(shown in table), 97.9769
Ions Score: 53 **Expect:** 0.0024
Matches (Bold Red): 71/628 fragment ions using 101 most intense peaks

#	b	b++	b*	b*++	b0	b0++	Seq.	y	y++	y*	y*++	y0	y0++	#
1	138.0662	69.5367					H							38
2	239.1139	120.0606			221.1033	111.0553	T	4114.6932	2057.8502	4097.6667	2049.3370	4096.6827	2048.8450	37
3	326.1459	163.5766			308.1353	154.5713	S	4013.6455	2007.3264	3996.6190	1998.8131	3995.6350	1998.3211	36
4	441.1728	221.0901			423.1623	212.0848	D	3926.6135	1963.8104	3909.5870	1955.2971	3908.6029	1954.8051	35
5	**604.2362**	302.6217			586.2256	293.6164	Y	3811.5866	1906.2969	3794.5600	1897.7836	3793.5760	1897.2916	34

6	**733.2788**	367.1430			715.2682	358.1377	E	3648.5232	1824.7653	3631.4967	**1816.2520**	3630.5127	**1815.7600**	33
7	**848.3057**	424.6565			830.2951	415.6512	D	3519.4806	**1760.2440**	3502.4541	1751.7307	3501.4701	1751.2387	32
8	949.3534	475.1803			931.3428	466.1750	T	3404.4537	1702.7305	3387.4272	1694.2172	3386.4431	1693.7252	31
9	**1064.3803**	532.6938			1046.3698	523.6885	D	3303.4060	**1652.2066**	3286.3795	**1643.6934**	3285.3955	**1643.2014**	30
10	1165.4280	583.2176			1147.4174	574.2124	T	3188.3791	1594.6932	3171.3525	1586.1799	3170.3685	1585.6879	29
11	**1294.4706**	647.7389			1276.4600	**638.7337**	E	3087.3314	1544.1693	3070.3049	1535.6561	3069.3208	1535.1641	28
12	1351.4921	676.2497			1333.4815	**667.2444**	G	2958.2888	**1479.6480**	2941.2623	1471.1348	2940.2782	1470.6428	27
13	1408.5135	704.7604			1390.5030	695.7551	G	2901.2673	1451.1373	2884.2408	1442.6240	2883.2568	1442.1320	26
14	**1479.5506**	740.2790			**1461.5401**	731.2737	A	2844.2459	1422.6266	2827.2193	1414.1133	2826.2353	1413.6213	25
15	**1642.6140**	821.8106			1624.6034	812.8053	Y	2773.2088	**1387.1080**	2756.1822	1378.5947	2755.1982	1378.1027	24
16	**1743.6616**	**872.3345**			1725.6511	863.3292	T	2610.1454	**1305.5764**	2593.1189	**1297.0631**	2592.1349	**1296.5711**	23
17	**1858.6886**	929.8479			1840.6780	**920.8427**	D	2509.0978	**1255.0525**	2492.0712	1246.5392	2491.0872	1246.0472	22
18	**1986.7472**	993.8772	**1969.7206**	985.3639	1968.7366	984.8719	Q	2394.0708	**1197.5390**	2377.0443	1189.0258	2376.0603	1188.5338	21
19	2115.7898	1058.3985	2098.7632	1049.8852	2097.7792	1049.3932	E	2266.0122	1133.5098	2248.9857	1124.9965	2248.0017	1124.5045	20
20	2228.8738	**1114.9406**	2211.8473	1106.4273	2210.8633	1105.9353	L	2136.9696	1068.9885	2119.9431	1060.4752	2118.9591	1059.9832	19
21	2343.9008	1172.4540	2326.8742	1163.9407	2325.8902	1163.4487	D	2023.8856	**1012.4464**	2006.8590	1003.9332	2005.8750	1003.4411	18
22	2472.9434	**1236.9753**	2455.9168	1228.4620	2454.9328	1227.9700	E	1908.8586	954.9330	1891.8321	946.4197	1890.8481	945.9277	17
23	2573.9910	1287.4992	2556.9645	1278.9859	2555.9805	1278.4939	T	**1779.8160**	**890.4117**	1762.7895	881.8984	**1761.8055**	881.4064	16
24	2687.0751	1344.0412	2670.0486	1335.5279	2669.0645	1335.0359	L	**1678.7684**	839.8878	1661.7418	831.3745	1660.7578	830.8825	15
25	2801.1180	1401.0627	2784.0915	1392.5494	2783.1075	1392.0574	N	**1565.6843**	783.3458	1548.6578	774.8325	1547.6737	774.3405	14
26	2916.1450	1458.5761	2899.1184	1450.0629	2898.1344	1449.5708	D	1451.6414	726.3243	1434.6148	717.8111	1433.6308	717.3190	13
27	3045.1876	**1523.0974**	3028.1610	**1514.5841**	3027.1770	**1514.0921**	E	1336.6144	668.8109	1319.5879	660.2976	**1318.6039**	659.8056	12
28	3144.2560	**1572.6316**	3127.2294	**1564.1184**	3126.2454	**1563.6263**	V	**1207.5718**	**604.2896**	1190.5453	595.7763	1189.5613	595.2843	11
29	3201.2774	1601.1424	3184.2509	1592.6291	3183.2669	1592.1371	G	**1108.5034**	554.7554	1091.4769	546.2421	1090.4929	545.7501	10
30	3382.2914	**1691.6494**	3365.2649	1683.1361	3364.2809	1682.6441	T	**1051.4820**	526.2446	1034.4554	517.7313	**1033.4714**	517.2393	9
31	3479.3442	1740.1757	3462.3177	1731.6625	3461.3336	1731.1705	P	**870.4680**	435.7376	853.4414	427.2243	852.4574	426.7323	8
32	3576.3970	1788.7021	3559.3704	1780.1889	3558.3864	**1779.6968**	P	**773.4152**	387.2112	756.3886	378.6980	755.4046	378.2060	7
33	3705.4396	1853.2234	3688.4130	1844.7101	3687.4290	1844.2181	E	676.3624	338.6849	659.3359	330.1716	**658.3519**	329.6796	6
34	3792.4716	1896.7394	3775.4450	**1888.2262**	3774.4610	**1887.7342**	S	**547.3198**	274.1636	530.2933	265.6503	529.3093	265.1583	5
35	3863.5087	1932.2580	3846.4822	1923.7447	3845.4981	1923.2527	A	**460.2878**	230.6475	443.2613	222.1343	442.2772	221.6423	4
36	3976.5928	**1988.8000**	3959.5662	1980.2868	3958.5822	1979.7947	I	389.2507	195.1290	372.2241	186.6157	371.2401	186.1237	3
37	4077.6405	2039.3239	4060.6139	2030.8106	4059.6299	2030.3186	T	276.1666	138.5870	259.1401	130.0737	258.1561	129.5817	2
38							R	175.1190	88.0631	158.0924	79.5498			1

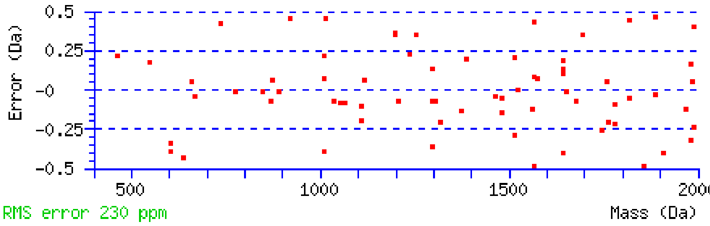

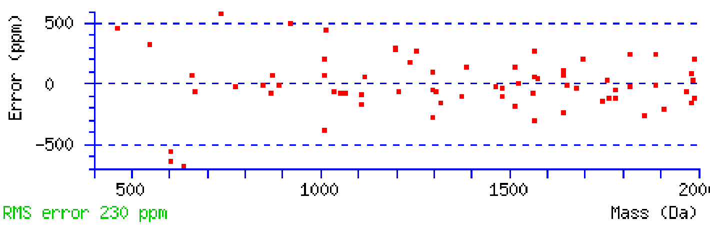

All matches to this query

Score	Mr(calc):	Delta	Sequence
52.9	4250.7449	0.9950	HTSDYEDTDTEGGAYTDQELDETLNDEVGTPPESAITR
52.2	4251.7289	0.0110	HTSDYEDTDTEGGAYTDQELDETLNDEVGTPPESAITR
44.7	4251.7289	0.0110	HTSDYEDTDTEGGAYTDQELDETLNDEVGTPPESAITR
42.9	4250.7449	0.9950	HTSDYEDTDTEGGAYTDQELDETLNDEVGTPPESAITR
39.7	4250.7449	0.9950	HTSDYEDTDTEGGAYTDQELDETLNDEVGTPPESAITR
39.7	4251.7289	0.0110	HTSDYEDTDTEGGAYTDQELDETLNDEVGTPPESAITR
38.5	4251.7289	0.0110	HTSDYEDTDTEGGAYTDQELDETLNDEVGTPPESAITR

32.8	4250.7449	0.9950	HTSDYEDTDTEGGAYTDQELDETLNDEVGTPPESAITR
27.7	4251.7289	0.0110	HTSDYEDTDTEGGAYTDQELDETLNDEVGTPPESAITR
22.3	4251.7289	0.0110	HTSDYEDTDTEGGAYTDQELDETLNDEVGTPPESAITR

Spectrum No: 253; Query: 1210; Rank: 1

Peptide View

MS/MS Fragmentation of **FQEQECPPSPEPTR**
Found in **IPI00190290**, Tax_Id=10116 Gene_Symbol=Rras2 Related RAS viral (R-ras) oncogene homolog 2

Match to Query 1210: 1780.717668 from(891.366110,2+)
Title: 100101RatKid_NS_deglyco_20.1100.1100.2.dta
Data file K:\NewmanPaper\Piliang\3SubProteomes\Piliang3SP\mgf5ppm\ERLIC_3SubProteomes5ppm.mgf

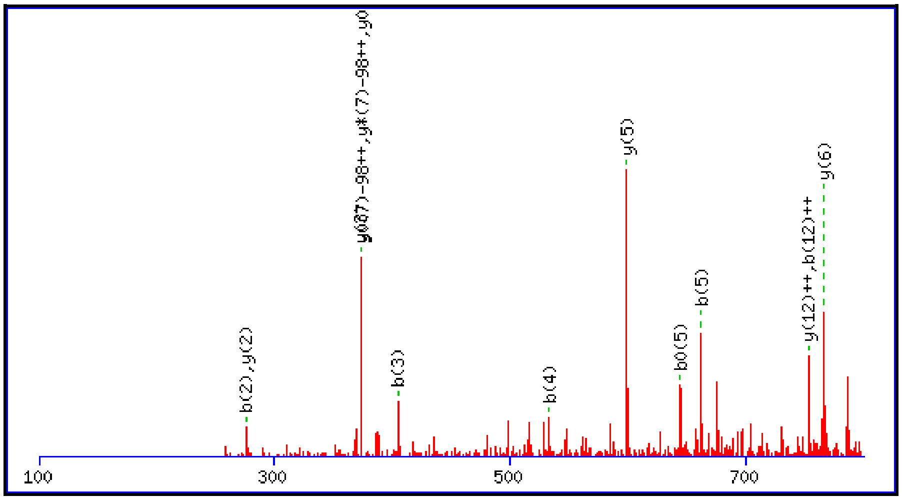

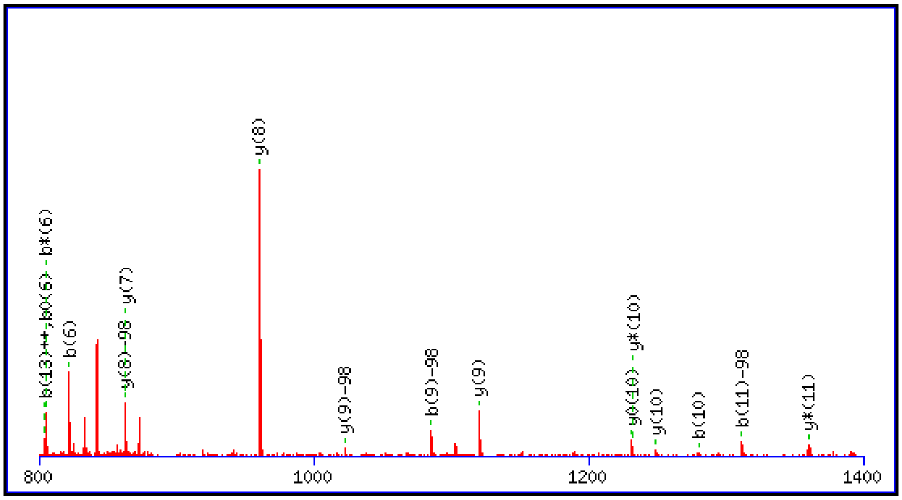

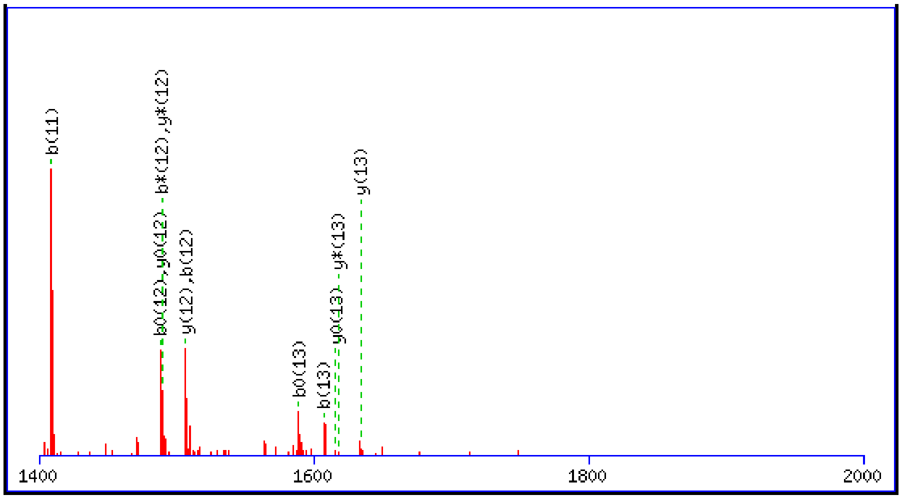

Monoisotopic mass of neutral peptide Mr(calc): 1780.7128
Fixed modifications: Carbamidomethyl (C)
Variable modifications:
S9 : Phospho (ST), with neutral losses 0.0000(shown in table), 97.9769
Ions Score: 53 **Expect:** 0.00078
Matches (Bold Red): 42/226 fragment ions using 58 most intense peaks

#	b	b^{++}	b*	b^{*++}	b^0	b^{0++}	Seq.	y	y^{++}	y*	y^{*++}	y^0	y^{0++}	#
1	148.0757	74.5415					F							14
2	**276.1343**	138.5708	259.1077	130.0575			Q	**1634.6516**	817.8294	**1617.6251**	809.3162	**1616.6411**	808.8242	13
3	**405.1769**	203.0921	388.1503	194.5788	387.1663	194.0868	E	**1506.5930**	**753.8002**	**1489.5665**	745.2869	**1488.5825**	744.7949	12
4	**533.2354**	267.1214	516.2089	258.6081	515.2249	258.1161	Q	1377.5505	689.2789	**1360.5239**	680.7656	1359.5399	680.2736	11
5	**662.2780**	331.6427	645.2515	323.1294	**644.2675**	322.6374	E	**1249.4919**	625.2496	**1232.4653**	616.7363	**1231.4813**	616.2443	10
6	**822.3087**	411.6580	**805.2821**	403.1447	**804.2981**	402.6527	C	**1120.4493**	560.7283	1103.4227	552.2150	1102.4387	551.7230	9
7	919.3614	460.1844	902.3349	451.6711	901.3509	451.1791	P	**960.4186**	480.7130	943.3921	472.1997	942.4081	471.7077	8
8	1016.4142	508.7107	999.3877	500.1975	998.4036	499.7055	P	**863.3659**	432.1866	846.3393	423.6733	845.3553	423.1813	7
9	1183.4126	592.2099	1166.3860	583.6966	1165.4020	583.2046	S	**766.3131**	383.6602	749.2866	375.1469	748.3025	**374.6549**	6
10	**1280.4653**	640.7363	1263.4388	632.2230	1262.4548	631.7310	P	**599.3148**	300.1610	582.2882	291.6477	581.3042	291.1557	5
11	**1409.5079**	705.2576	1392.4814	696.7443	1391.4974	696.2523	E	502.2620	251.6346	485.2354	243.1214	484.2514	242.6293	4
12	**1506.5607**	**753.7840**	**1489.5341**	745.2707	**1488.5501**	744.7787	P	**373.2194**	187.1133	356.1928	178.6001	355.2088	178.1081	3
13	**1607.6084**	**804.3078**	1590.5818	795.7945	**1589.5978**	795.3025	T	**276.1666**	138.5870	259.1401	130.0737	258.1561	129.5817	2
14							R	175.1190	88.0631	158.0924	79.5498			1

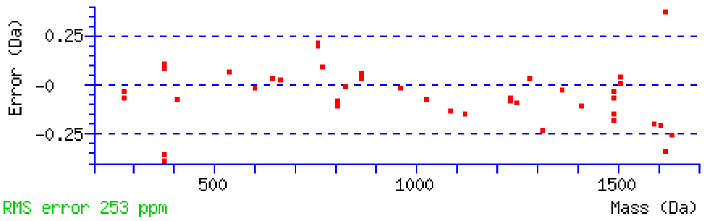

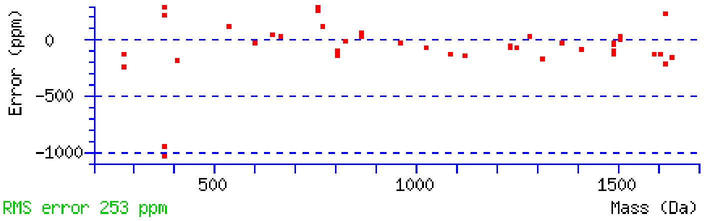

All matches to this query

Score	Mr(calc):	Delta	Sequence
52.9	1780.7128	0.0049	FQEQECPPSPEPTR
25.4	1780.7128	0.0049	FQEQECPPSPEPTR
7.3	1780.7096	0.0081	ILCCNCVSDFRTR
4.9	1780.7005	0.0172	FQHVMSITRNNR
4.7	1780.7057	0.0120	MQNNSAENETAEGEEK
3.1	1780.7136	0.0040	MNCYMNFPTAVPTR
1.9	1779.7256	0.9921	ILCCNCVSDFRTR

1.5	1779.7135	1.0042	AAEPPPCDVSTGQESR
0.7	1779.7287	0.9890	FKTSVGSAKYVPEK
0.4	1780.7281	-0.0104	SNQLTKKINSTDDR

Spectrum No: 254; Query: 536; Rank: 1

Peptide View

MS/MS Fragmentation of **KVMDSDEDDDY**
Found in **IPI00193547**, Tax_Id=10116 Gene_Symbol=Pdcd5_predicted programmed cell death 5

Match to Query 536: 1426.444908 from(714.229730,2+)
Title: 091008RatKidney_NoSalt_33.410.410.2.dta
Data file K:\NewmanPaper\Piliang\3SubProteomes\Piliang3SP\mgf5ppm\ERLIC_3SubProteomes5ppm.mgf

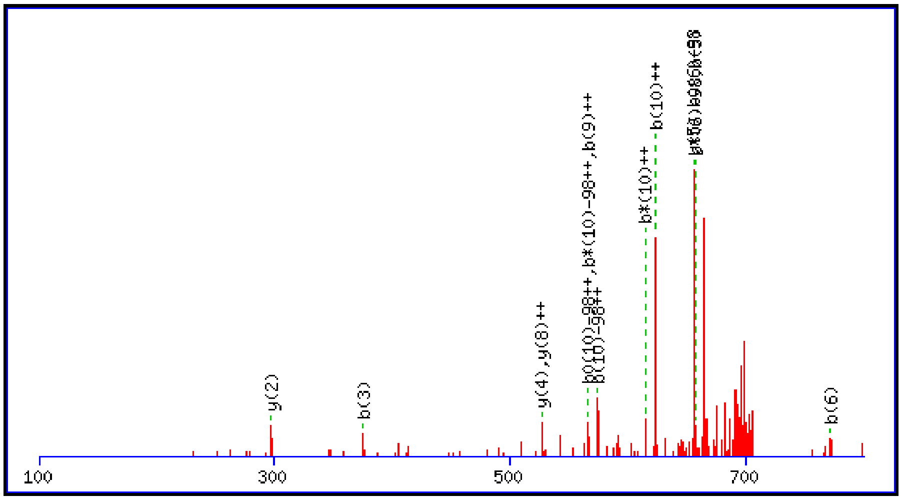

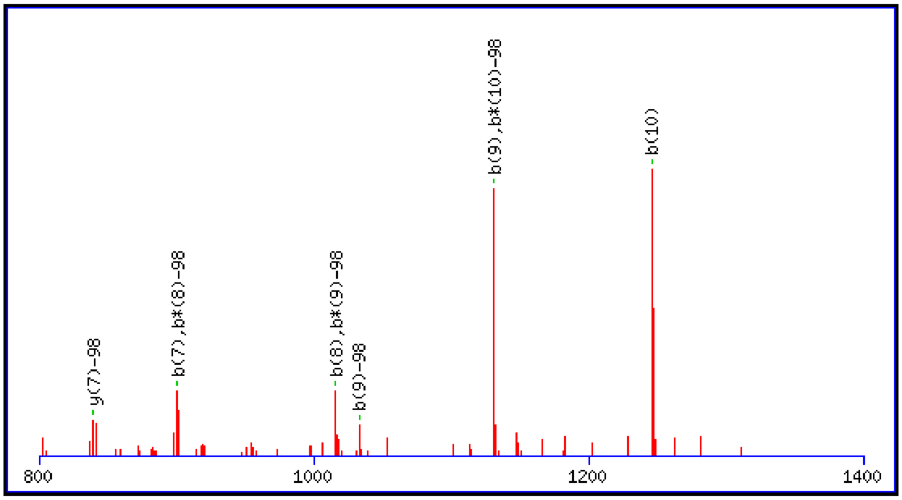

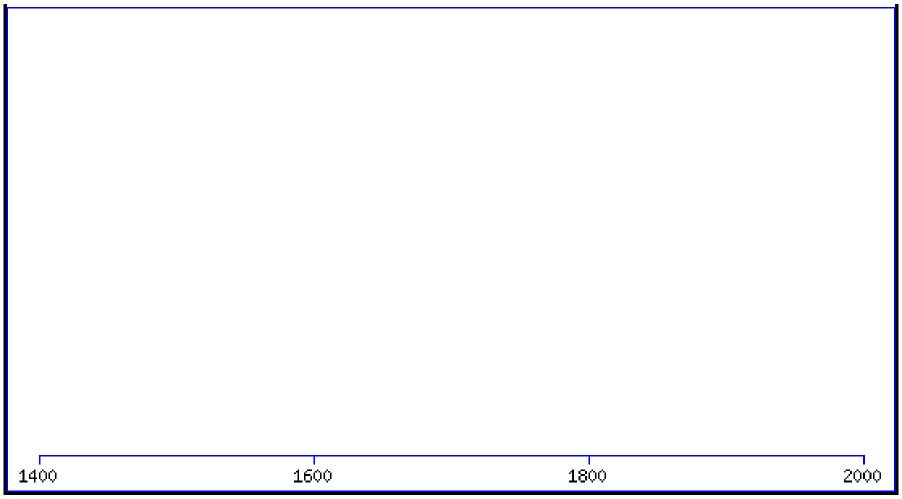

Monoisotopic mass of neutral peptide Mr(calc): 1426.4483
Fixed modifications: Carbamidomethyl (C)
Variable modifications:
M3 : Oxidation (M)
S5 : Phospho (ST), with neutral losses 0.0000(shown in table), 97.9769
Ions Score: 53 **Expect:** 0.00013
Matches (Bold Red): 24/144 fragment ions using 40 most intense peaks

#	b	b^{++}	b*	b^{*++}	b^{0}	b^{0++}	Seq.	y	y^{++}	y^{0}	y^{0++}	#
1	129.1022	65.0548	112.0757	56.5415			K					11
2	228.1707	114.5890	211.1441	106.0757			V	1299.3606	650.1840	1281.3501	641.1787	10
3	**375.2061**	188.1067	358.1795	179.5934			M	1200.2922	600.6498	1182.2817	591.6445	9
4	490.2330	245.6201	473.2065	237.1069	472.2224	236.6149	D	1053.2568	**527.1321**	1035.2463	518.1268	8
5	**657.2314**	329.1193	640.2048	320.6060	639.2208	320.1140	S	938.2299	469.6186	920.2193	460.6133	7
6	**772.2583**	386.6328	755.2317	378.1195	754.2477	377.6275	D	771.2315	386.1194	753.2210	377.1141	6
7	**901.3009**	451.1541	884.2743	442.6408	883.2903	442.1488	E	**656.2046**	328.6059	638.1940	319.6007	5
8	**1016.3278**	508.6676	999.3013	500.1543	998.3173	499.6623	D	**527.1620**	264.0846	509.1514	255.0794	4
9	**1131.3548**	**566.1810**	1114.3282	557.6678	1113.3442	557.1757	D	412.1351	206.5712	394.1245	197.5659	3
10	**1246.3817**	**623.6945**	1229.3552	**615.1812**	1228.3712	614.6892	D	**297.1081**	149.0577	279.0975	140.0524	2
11							Y	182.0812	91.5442			1

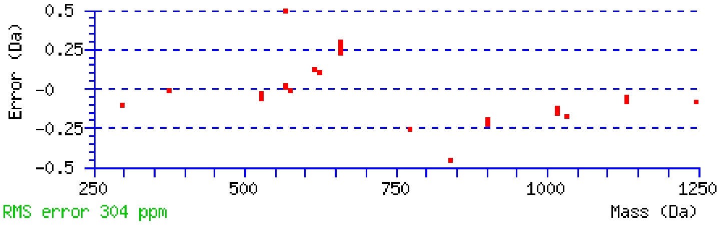

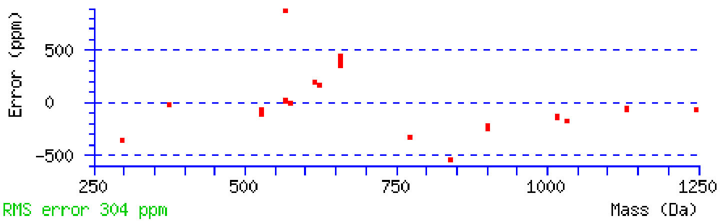

All matches to this query

Score	Mr(calc):	Delta	Sequence
52.9	1426.4483	-0.0034	KVMDSDEDDDY

Spectrum No: 255; Query: 403; Rank: 1

Peptide View

MS/MS Fragmentation of **DTTDLSVEASPK**
Found in **IPI00368799**, Tax_Id=10116 Gene_Symbol=RGD1306067 ESF1 homolog

Match to Query 403: 1341.569588 from(671.792070,2+)

Title: 091008RatKidney_NoSalt_28.943.943.2.dta
Data file K:\NewmanPaper\Piliang\3SubProteomes\Piliang3SP\mgf5ppm\ERLIC_3SubProteomes5ppm.mgf

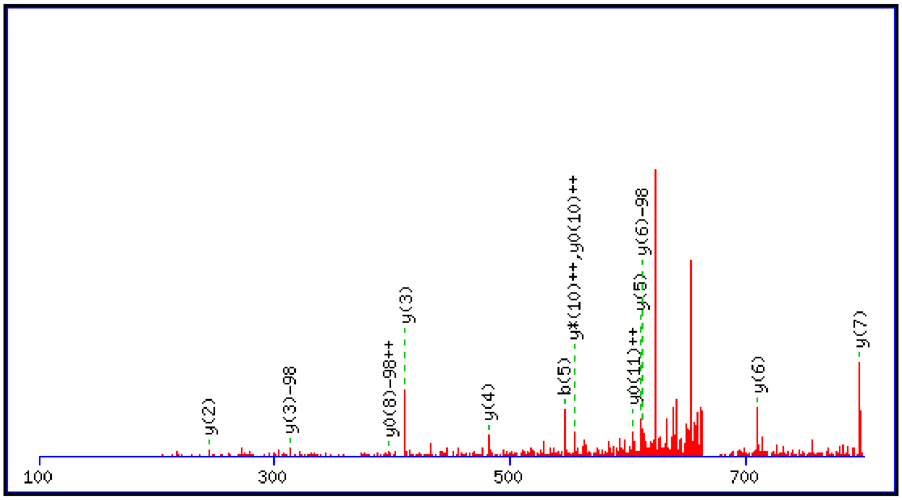

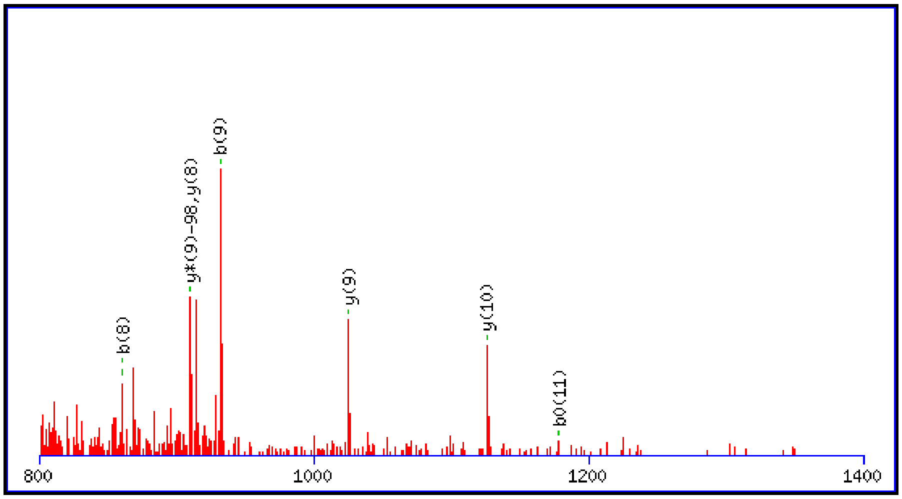

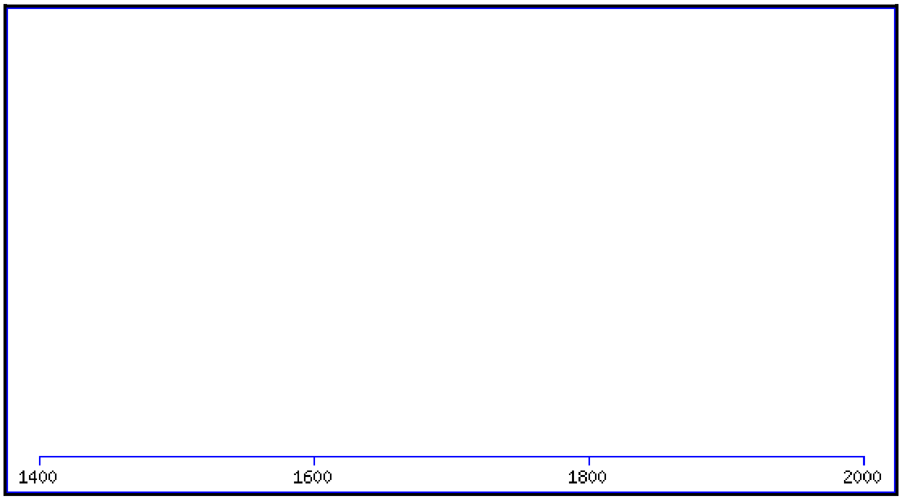

Monoisotopic mass of neutral peptide Mr(calc): 1341.5701
Fixed modifications: Carbamidomethyl (C)
Variable modifications:
S10 : Phospho (ST), with neutral losses 0.0000(shown in table), 97.9769
Ions Score: 53 **Expect:** 0.00066
Matches (Bold Red): 20/168 fragment ions using 33 most intense peaks

#	b	b^{++}	b^{0}	b^{0++}	Seq.	y	y^{++}	y*	y*$^{++}$	y^{0}	y^{0++}	#
1	116.0342	58.5207	98.0237	49.5155	D							12
2	217.0819	109.0446	199.0713	100.0393	T	1227.5504	614.2789	1210.5239	605.7656	1209.5399	**605.2736**	11
3	318.1296	159.5684	300.1190	150.5631	T	**1126.5028**	563.7550	1109.4762	**555.2417**	1108.4922	**554.7497**	10

**4**	433.1565	217.0819	415.1460	208.0766	**D**	**1025.4551**	513.2312	1008.4285	504.7179	1007.4445	504.2259	**9**
**5**	**546.2406**	273.6239	528.2300	264.6186	**L**	**910.4281**	455.7177	893.4016	447.2044	892.4176	446.7124	**8**
**6**	633.2726	317.1399	615.2620	308.1347	**S**	**797.3441**	399.1757	780.3175	390.6624	779.3335	390.1704	**7**
**7**	732.3410	366.6742	714.3305	357.6689	**V**	**710.3120**	355.6597	693.2855	347.1464	692.3015	346.6544	**6**
**8**	**861.3836**	431.1954	843.3731	422.1902	**E**	**611.2436**	306.1255	594.2171	297.6122	593.2331	297.1202	**5**
**9**	**932.4207**	466.7140	914.4102	457.7087	**A**	**482.2010**	241.6042	465.1745	233.0909	464.1905	232.5989	**4**
**10**	1099.4191	550.2132	1081.4085	541.2079	**S**	**411.1639**	206.0856	394.1374	197.5723	393.1534	197.0803	**3**
**11**	1196.4718	598.7396	**1178.4613**	589.7343	**P**	**244.1656**	122.5864	227.1390	114.0731			**2**
**12**					**K**	147.1128	74.0600	130.0863	65.5468			**1**

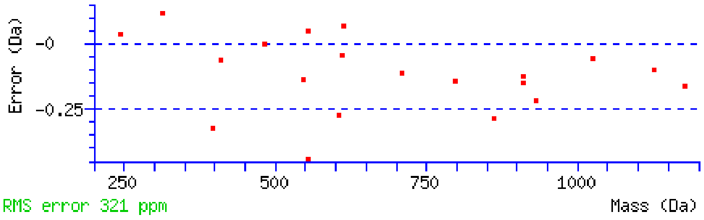

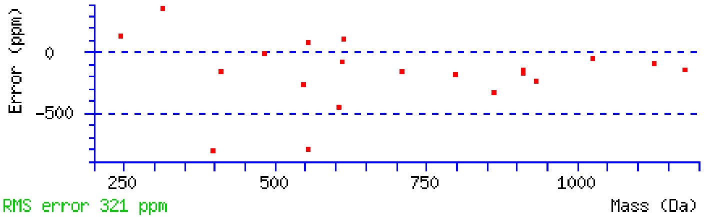

All matches to this query

Score	Mr(calc):	Delta	Sequence
52.8	1341.5701	-0.0005	DTTDLSVEASPK
18.5	1341.5701	-0.0005	DTTDLSVEASPK
3.3	1341.5635	0.0060	AMNVLTEAEER
2.4	1339.5509	2.0187	VGRTMRGYPK
2.3	1341.5701	-0.0005	DTTDLSVEASPK
2.2	1340.5579	1.0117	ESSEDKNTSESK
1.4	1340.5537	1.0159	DTSSDPEILWV
1.3	1340.5705	0.9991	NTDRDHSHSGSK
1.1	1340.5778	0.9918	ALQKLSSGSYK
1.1	1340.5778	0.9918	ALQKLSSGSYK

Spectrum No: 256; Query: 1664; Rank: 1

Peptide View

MS/MS Fragmentation of **KEESEEESEDDMGFGLFD**
Found in **IPI00200145**, Tax_Id=10116 Gene_Symbol=Rplp1 60S acidic ribosomal protein P1

Match to Query 1664: 2058.729188 from(1030.371870,2+)
Title: 091008RatKidney_NoSalt_29.2987.2987.2.dta
Data file K:\NewmanPaper\Piliang\3SubProteomes\Piliang3SP\mgf5ppm\ERLIC_3SubProteomes5ppm.mgf

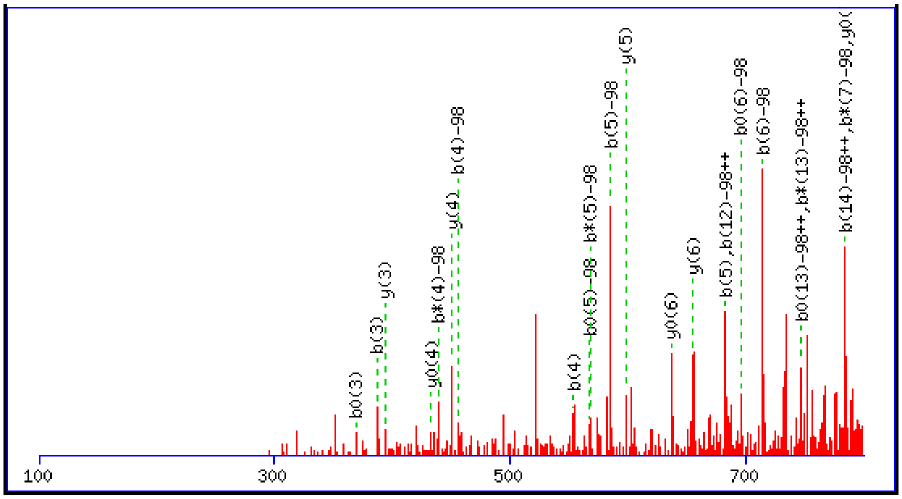

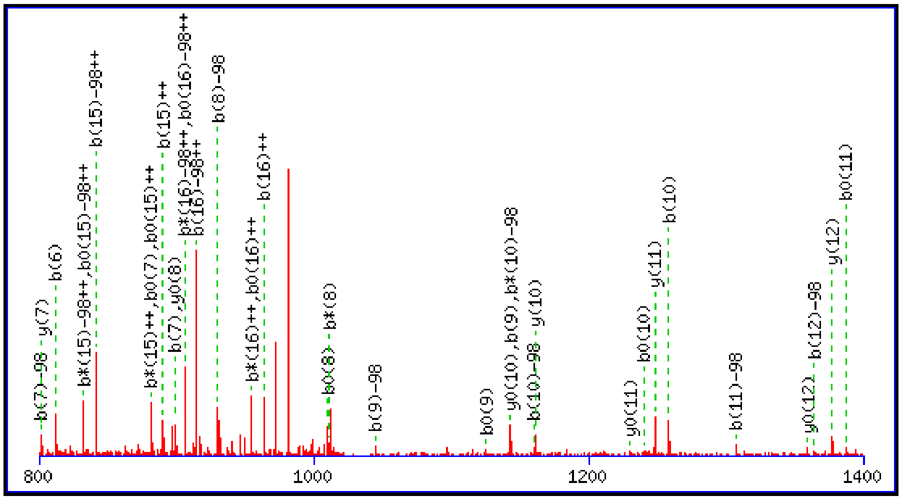

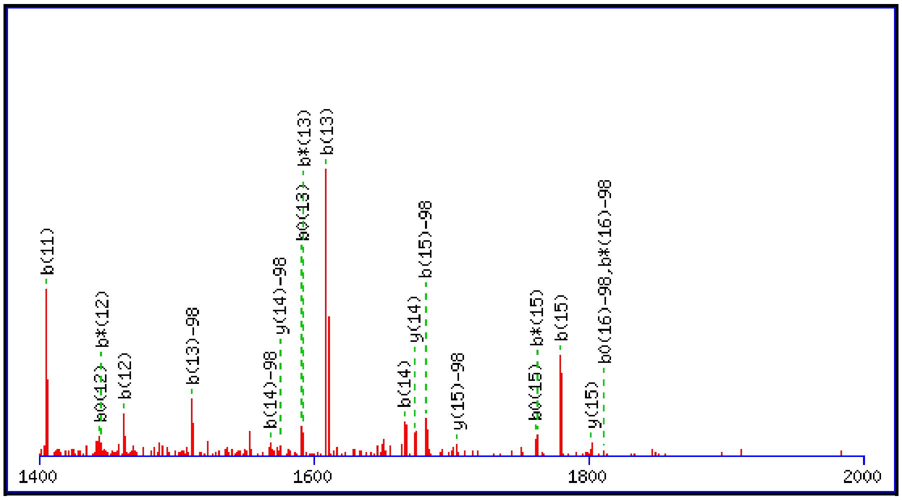

Monoisotopic mass of neutral peptide Mr(calc): 2058.7289
Fixed modifications: Carbamidomethyl (C)
Variable modifications:
S4 : Phospho (ST), with neutral losses 97.9769(shown in table), 0.0000
M11 : Oxidation (M)
Ions Score: 53 **Expect:** 0.00046
Matches (Bold Red): 80/248 fragment ions using 134 most intense peaks

#	b	b^{++}	b*	b^{*++}	b^{0}	b^{0++}	Seq.	y	y^{++}	y^{0}	y^{0++}	#
1	129.1022	65.0548	112.0757	56.5415			K					17
2	258.1448	129.5761	241.1183	121.0628	240.1343	120.5708	E	1833.6643	917.3358	1815.6538	908.3305	16
3	**387.1874**	194.0974	370.1609	185.5841	**369.1769**	185.0921	E	**1704.6218**	852.8145	1686.6112	843.8092	15
4	**456.2089**	228.6081	**439.1823**	220.0948	438.1983	219.6028	S	**1575.5792**	788.2932	1557.5686	779.2879	14

5	585.2515	293.1294	568.2249	284.6161	567.2409	284.1241	E	1506.5577	753.7825	1488.5471	744.7772	13
6	714.2941	357.6507	697.2675	349.1374	696.2835	348.6454	E	1377.5151	689.2612	1359.5045	680.2559	12
7	801.3261	401.1667	784.2995	392.6534	783.3155	392.1614	S	1248.4725	624.7399	1230.4620	615.7346	11
8	930.3687	465.6880	913.3421	457.1747	912.3581	456.6827	E	1161.4405	581.2239	1143.4299	572.2186	10
9	1045.3956	523.2015	1028.3691	514.6882	1027.3851	514.1962	D	1032.3979	516.7026	1014.3873	507.6973	9
10	1160.4226	580.7149	1143.3960	572.2017	1142.4120	571.7096	D	917.3710	459.1891	899.3604	450.1838	8
11	1307.4580	654.2326	1290.4314	645.7194	1289.4474	645.2273	M	802.3440	401.6756	784.3334	392.6704	7
12	1364.4794	682.7434	1347.4529	674.2301	1346.4689	673.7381	G	655.3086	328.1579	637.2980	319.1527	6
13	1511.5479	756.2776	1494.5213	747.7643	1493.5373	747.2723	F	598.2871	299.6472	580.2766	290.6419	5
14	1568.5693	784.7883	1551.5428	776.2750	1550.5588	775.7830	G	451.2187	226.1130	433.2082	217.1077	4
15	1681.6534	841.3303	1664.6268	832.8171	1663.6428	832.3250	L	394.1973	197.6023	376.1867	188.5970	3
16	1828.7218	914.8645	1811.6952	906.3513	1810.7112	905.8593	F	281.1132	141.0602	263.1026	132.0550	2
17							D	134.0448	67.5260	116.0342	58.5207	1

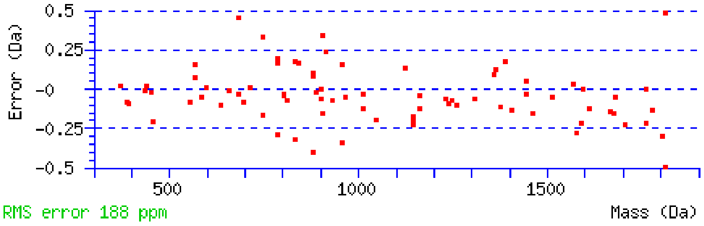

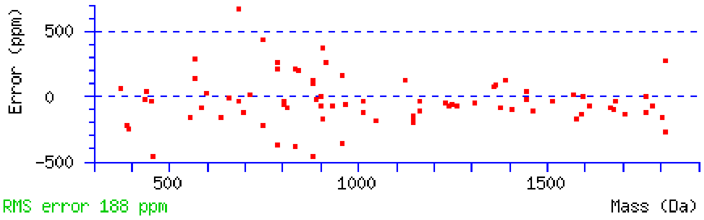

All matches to this query

Score	Mr(calc):	Delta	Sequence
52.9	2058.7289	0.0003	KEESEEESEDDMGFGLFD
44.9	2058.7289	0.0003	KEESEEESDEDMGFGLFD
37.8	2058.7289	0.0003	KEESEEESEDDMGFGLFD
32.8	2058.7289	0.0003	KEESEEESDEDMGFGLFD
0.1	2057.7284	1.0008	DWSSFFMNSLTLGGQK

Spectrum No: 257; Query: 2584; Rank: 1

Peptide View

MS/MS Fragmentation of **VKPPPSPTTEGPSLQPDLAPEEAAGAQRPK**
Found in **IPI00364858**, Tax_Id=10116 Gene_Symbol=Akap2 A kinase (PRKA) anchor protein 2

Match to Query 2584: 3145.563192 from(1049.528340,3+)
Title: 091008RatKidney_NH4Format02_24.2503.2503.3.dta
Data file K:\NewmanPaper\Piliang\3SubProteomes\Piliang3SP\mgf5ppm\ERLIC_3SubProteomes5ppm.mgf

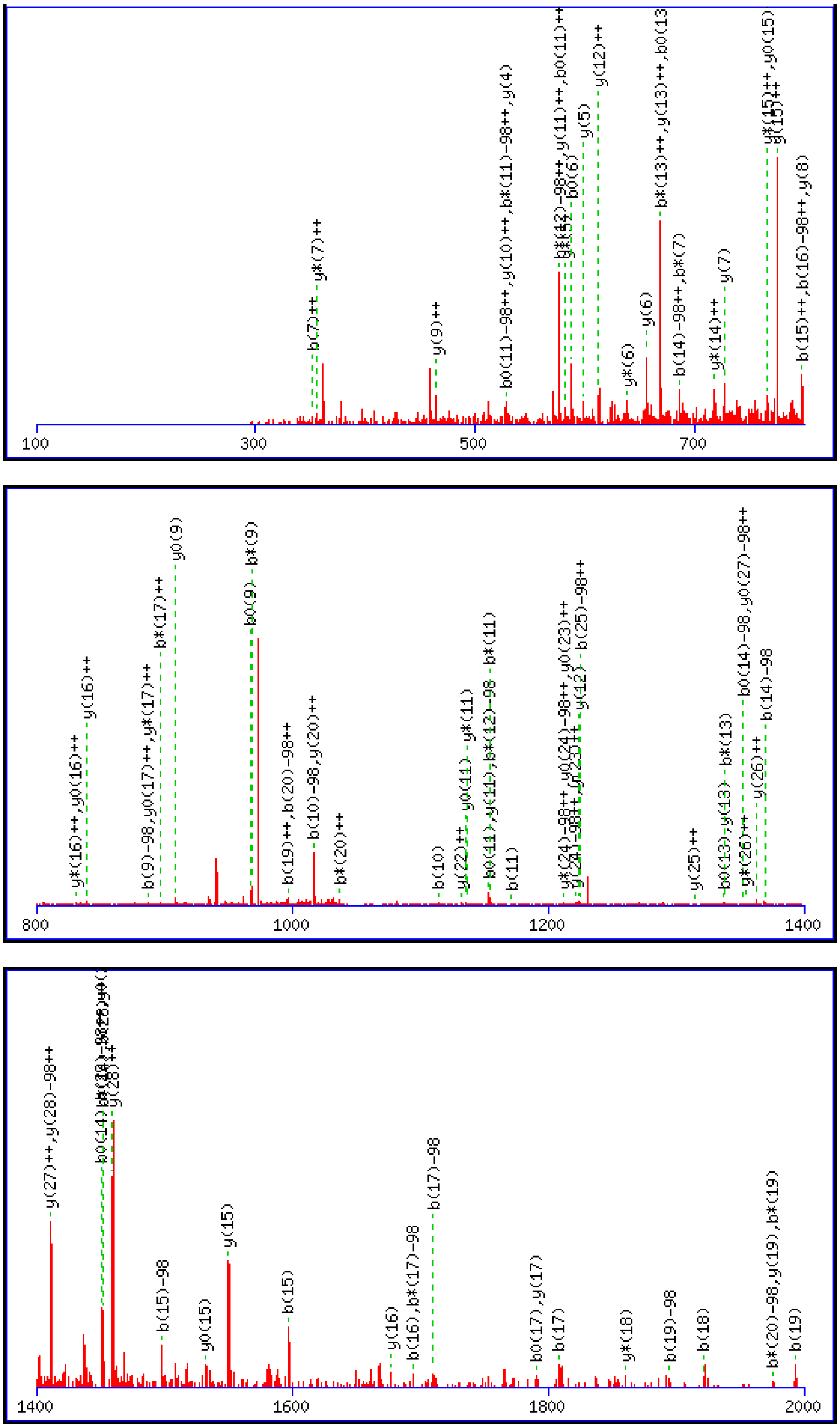

Monoisotopic mass of neutral peptide Mr(calc): 3144.5492
Fixed modifications: Carbamidomethyl (C)
Variable modifications:
T8 : Phospho (ST), with neutral losses 0.0000(shown in table), 97.9769
Ions Score: 53 **Expect:** 0.0013
Matches (Bold Red): 97/494 fragment ions using 144 most intense peaks

#	b	b^{++}	b*	b*$^{++}$	b^{0}	b^{0++}	Seq.	y	y^{++}	y*	y*$^{++}$	y^{0}	y^{0++}	#
1	100.0757	50.5415					V							30
2	228.1707	114.5890	211.1441	106.0757			K	3046.4881	1523.7477	3029.4616	1515.2344	3028.4776	1514.7424	29
3	325.2234	163.1153	308.1969	154.6021			P	2918.3932	**1459.7002**	2901.3666	**1451.1869**	2900.3826	**1450.6949**	28
4	422.2762	211.6417	405.2496	203.1285			P	2821.3404	**1411.1738**	2804.3138	1402.6606	2803.3298	1402.1686	27
5	519.3289	260.1681	502.3024	251.6548			P	2724.2876	**1362.6475**	2707.2611	**1354.1342**	2706.2771	1353.6422	26

6	606.3610	303.6841	589.3344	295.1709	**588.3504**	294.6788	S	2627.2349	**1314.1211**	2610.2083	1305.6078	2609.2243	1305.1158	25
7	703.4137	**352.2105**	**686.3872**	343.6972	685.4032	343.2052	P	2540.2028	1270.6051	2523.1763	1262.0918	2522.1923	1261.5998	24
8	884.4277	442.7175	867.4012	434.2042	866.4172	433.7122	T	2443.1501	**1222.0787**	2426.1235	1213.5654	2425.1395	**1213.0734**	23
9	985.4754	493.2413	**968.4489**	484.7281	**967.4649**	484.2361	T	2262.1361	**1131.5717**	2245.1095	1123.0584	2244.1255	1122.5664	22
10	**1114.5180**	557.7626	1097.4915	549.2494	1096.5074	548.7574	E	2161.0884	1081.0478	2144.0618	1072.5346	2143.0778	1072.0425	21
11	**1171.5395**	586.2734	**1154.5129**	**577.7601**	**1153.5289**	**577.2681**	G	2032.0458	**1016.5265**	2015.0192	1008.0133	2014.0352	1007.5213	20
12	1268.5922	634.7998	1251.5657	626.2865	1250.5817	625.7945	P	**1975.0243**	988.0158	1957.9978	979.5025	1957.0138	979.0105	19
13	1355.6243	678.3158	**1338.5977**	**669.8025**	**1337.6137**	**669.3105**	S	1877.9716	939.4894	**1860.9450**	930.9761	1859.9610	930.4841	18
14	1468.7083	734.8578	**1451.6818**	726.3445	**1450.6978**	725.8525	L	**1790.9395**	895.9734	1773.9130	**887.4601**	1772.9290	**886.9681**	17
15	**1596.7669**	**798.8871**	1579.7404	790.3738	1578.7563	789.8818	Q	**1677.8555**	**839.4314**	1660.8289	**830.9181**	1659.8449	**830.4261**	16
16	**1693.8197**	847.4135	1676.7931	838.9002	1675.8091	838.4082	P	**1549.7969**	**775.4021**	1532.7703	**766.8888**	**1531.7863**	**766.3968**	15
17	**1808.8466**	904.9269	1791.8201	**896.4137**	**1790.8361**	895.9217	D	1452.7441	726.8757	1435.7176	**718.3624**	1434.7336	717.8704	14
18	**1921.9307**	961.4690	1904.9041	952.9557	1903.9201	952.4637	L	**1337.7172**	**669.3622**	1320.6906	660.8490	1319.7066	660.3570	13
19	**1992.9678**	**996.9875**	**1975.9412**	988.4743	1974.9572	987.9823	A	**1224.6331**	**612.8202**	1207.6066	604.3069	1206.6226	603.8149	12
20	2090.0206	1045.5139	2072.9940	**1037.0006**	2072.0100	1036.5086	P	**1153.5960**	**577.3016**	**1136.5695**	568.7884	**1135.5854**	568.2964	11
21	2219.0632	1110.0352	2202.0366	1101.5219	2201.0526	1101.0299	E	1056.5432	**528.7753**	1039.5167	520.2620	1038.5327	519.7700	10
22	2348.1057	1174.5565	2331.0792	1166.0432	2330.0952	1165.5512	E	927.5007	**464.2540**	910.4741	455.7407	**909.4901**	455.2487	9
23	2419.1429	1210.0751	2402.1163	1201.5618	2401.1323	1201.0698	A	**798.4581**	399.7327	781.4315	391.2194			8
24	2490.1800	1245.5936	2473.1534	1237.0804	2472.1694	1236.5883	A	**727.4209**	364.2141	710.3944	**355.7008**			7
25	2547.2014	1274.1044	2530.1749	1265.5911	2529.1909	1265.0991	G	**656.3838**	328.6956	**639.3573**	320.1823			6
26	2618.2386	1309.6229	2601.2120	1301.1096	2600.2280	1300.6176	A	**599.3624**	300.1848	**582.3358**	291.6715			5
27	2746.2971	1373.6522	2729.2706	1365.1389	2728.2866	1364.6469	Q	**528.3253**	264.6663	511.2987	256.1530			4
28	2902.3982	**1451.7028**	2885.3717	1443.1895	2884.3877	1442.6975	R	400.2667	200.6370	383.2401	192.1237			3
29	2999.4510	1500.2291	2982.4245	1491.7159	2981.4404	1491.2239	P	244.1656	122.5864	227.1390	114.0731			2
30							K	147.1128	74.0600	130.0863	65.5468			1

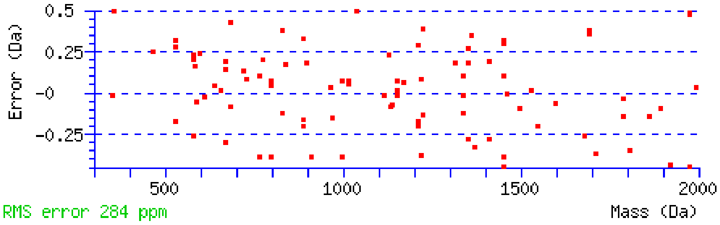

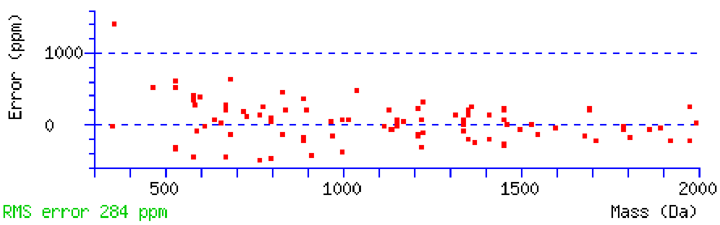

All matches to this query

Score	Mr(calc):	Delta	Sequence
52.6	3144.5492	1.0140	VKPPPSPTTEGPSLQPDLAPEEAAGAQRPK
51.5	3144.5492	1.0140	VKPPPSPTTEGPSLQPDLAPEEAAGAQRPK
48.2	3144.5492	1.0140	VKPPPSPTTEGPSLQPDLAPEEAAGAQRPK
39.2	3144.5492	1.0140	VKPPPSPTTEGPSLQPDLAPEEAAGAQRPK
3.8	3143.5531	2.0101	YEVPKWLSPSSILLLQQMLQVDPKR
3.8	3144.5314	1.0318	ATSVLYSVLSPTLNPLIYTLRNKDVK
3.5	3144.5314	1.0318	ATSVLYSVLSPTLNPLIYTLRNKDVK
3.1	3144.5314	1.0318	ATSVLYSVLSPTLNPLIYTLRNKDVK
3.1	3144.5314	1.0318	ATSVLYSVLSPTLNPLIYTLRNKDVK
3.0	3144.5604	1.0028	DLDLLNPAARQNLEALQSSGLEKIHYR

Spectrum No: 258; Query: 1662; Rank: 1

Peptide View

MS/MS Fragmentation of **TDSREDEISPPPPNPVVK**
Found in **IPI00231770**, Tax_Id=10116 Gene_Symbol=Prkar1a cAMP-dependent protein kinase type I-alpha regulatory subunit

Match to Query 1662: 2055.948928 from(1028.981740,2+)
Title: 091008RatKidney_NoSalt_22.1374.1374.2.dta
Data file K:\NewmanPaper\Piliang\3SubProteomes\Piliang3SP\mgf5ppm\ERLIC_3SubProteomes5ppm.mgf

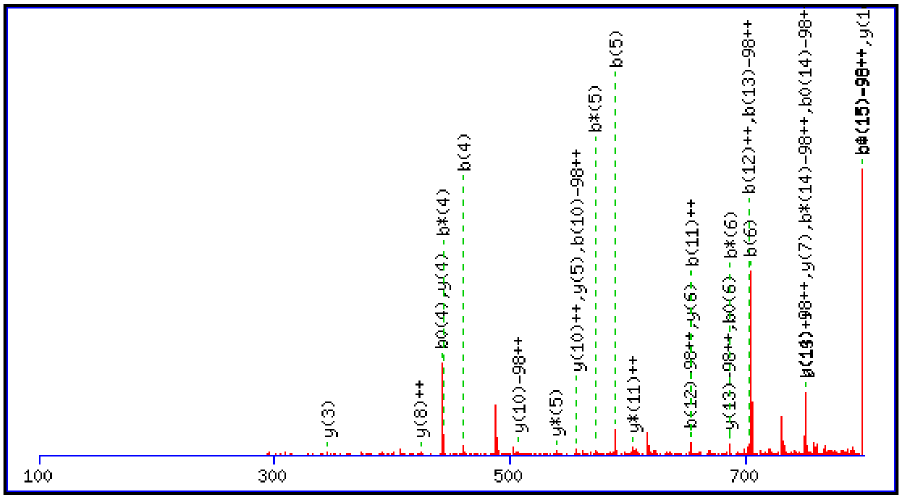

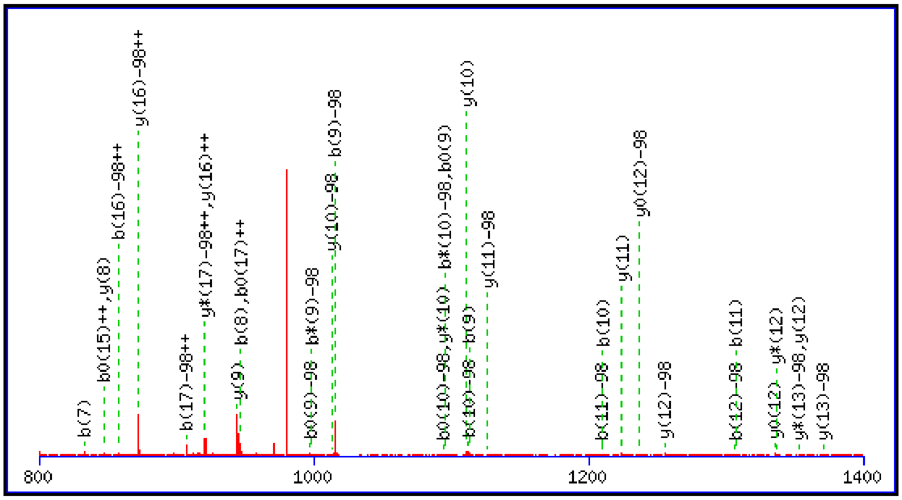

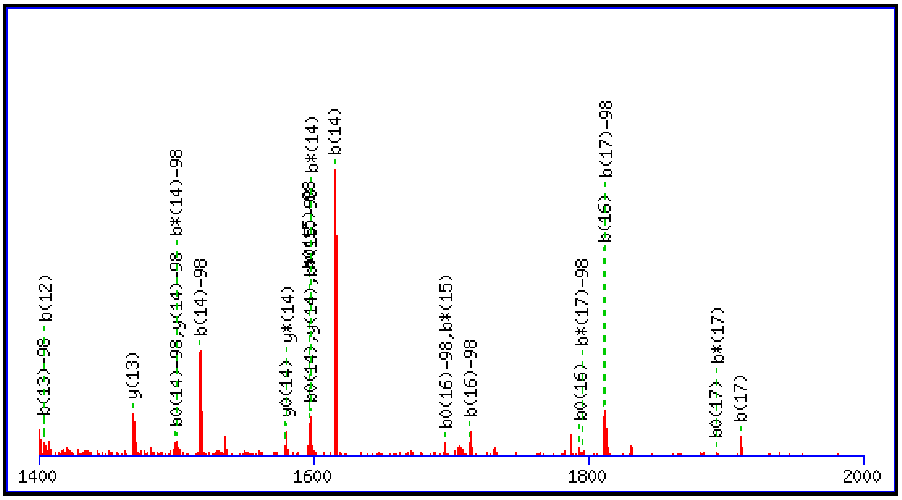

Monoisotopic mass of neutral peptide Mr(calc): 2055.9514
Fixed modifications: Carbamidomethyl (C)
Variable modifications:
S9 : Phospho (ST), with neutral losses 97.9769(shown in table), 0.0000
Ions Score: 52 **Expect:** 0.0014
Matches (Bold Red): 93/282 fragment ions using 158 most intense peaks

#	b	b^{++}	b*	b*$^{++}$	b^{0}	b^{0++}	Seq.	y	y^{++}	y*	y*$^{++}$	y^{0}	y^{0++}	#
1	102.0550	51.5311			84.0444	42.5258	T							18
2	217.0819	109.0446			199.0713	100.0393	D	1857.9341	929.4707	1840.9076	920.9574	1839.9235	920.4654	17
3	304.1139	152.5606			286.1034	143.5553	S	1742.9072	871.9572	1725.8806	863.4439	1724.8966	862.9519	16
4	460.2150	230.6112	443.1885	222.0979	442.2045	221.6059	R	1655.8751	828.4412	1638.8486	819.9279	1637.8646	819.4359	15
5	589.2576	295.1325	572.2311	286.6192	571.2471	286.1272	E	1499.7740	750.3907	1482.7475	741.8774	1481.7635	741.3854	14
6	704.2846	352.6459	687.2580	344.1327	686.2740	343.6406	D	1370.7314	685.8694	1353.7049	677.3561	1352.7209	676.8641	13
7	833.3272	417.1672	816.3006	408.6539	815.3166	408.1619	E	1255.7045	628.3559	1238.6779	619.8426	1237.6939	619.3506	12
8	946.4112	473.7093	929.3847	465.1960	928.4007	464.7040	I	1126.6619	563.8346	1109.6354	555.3213	1108.6513	554.8293	11
9	1015.4327	508.2200	998.4061	499.7067	997.4221	499.2147	S	1013.5778	507.2926	996.5513	498.7793	995.5673	498.2873	10
10	1112.4855	556.7464	1095.4589	548.2331	1094.4749	547.7411	P	944.5564	472.7818	927.5298	464.2686			9
11	1209.5382	605.2727	1192.5117	596.7595	1191.5276	596.2675	P	847.5036	424.2554	830.4771	415.7422			8
12	1306.5910	653.7991	1289.5644	645.2859	1288.5804	644.7938	P	750.4509	375.7291	733.4243	367.2158			7
13	1403.6437	702.3255	1386.6172	693.8122	1385.6332	693.3202	P	653.3981	327.2027	636.3715	318.6894			6
14	1517.6867	759.3470	1500.6601	750.8337	1499.6761	750.3417	N	556.3453	278.6763	539.3188	270.1630			5
15	1614.7394	807.8734	1597.7129	799.3601	1596.7289	798.8681	P	442.3024	221.6548	425.2758	213.1416			4
16	1713.8078	857.4076	1696.7813	848.8943	1695.7973	848.4023	V	345.2496	173.1285	328.2231	164.6152			3
17	1812.8763	906.9418	1795.8497	898.4285	1794.8657	897.9365	V	246.1812	123.5942	229.1547	115.0810			2
18							K	147.1128	74.0600	130.0863	65.5468			1

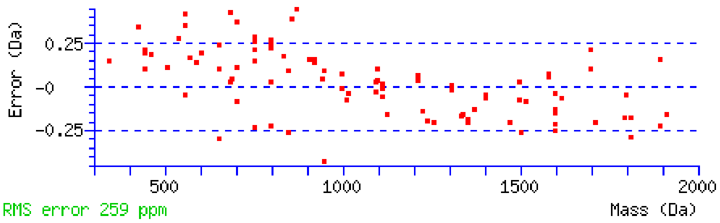

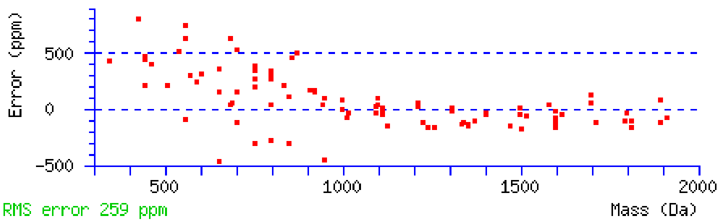

All matches to this query

Score	Mr(calc):	Delta	Sequence
52.5	2055.9514	-0.0025	TDSREDEISPPPPNPVVK
29.0	2055.9514	-0.0025	TDSREDEISPPPPNPVVK
28.1	2055.9514	-0.0025	TDSREDEISPPPPNPVVK
12.1	2054.9483	1.0007	KMEEVSNTPLLLTVEASD
7.1	2055.9401	0.0088	LDGRPITEDEYLENALK
7.0	2054.9561	0.9928	DSRDVGEEKELLDFVPK
6.2	2053.9252	2.0238	ITTTVGLNIGTVDVGKAR
6.2	2053.9252	2.0238	ITTTVGLNIGTVDVGKAR
6.2	2053.9252	2.0238	ITTTVGLNIGTVDVGKAR
5.7	2054.9595	0.9895	LEAELQAASTNKEEALMK

Spectrum No: 259; Query: 1639; Rank: 1

Peptide View

MS/MS Fragmentation of **KEESEESDEDMGFGLFD**
Found in **IPI00734740**, Tax_Id=10116 Gene_Symbol=LOC498555 similar to 60S acidic ribosomal protein P2

Match to Query 1639: 2042.730008 from(1022.372280,2+)
Title: 091008RatKidney_NH4Format02_26.3987.3987.2.dta

Data file K:\NewmanPaper\Piliang\3SubProteomes\Piliang3SP\mgf5ppm\ERLIC_3SubProteomes5ppm.mgf

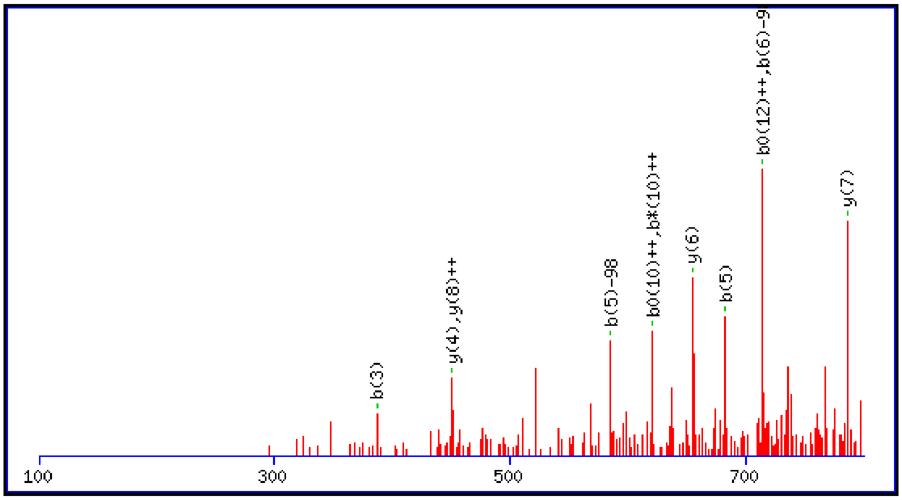

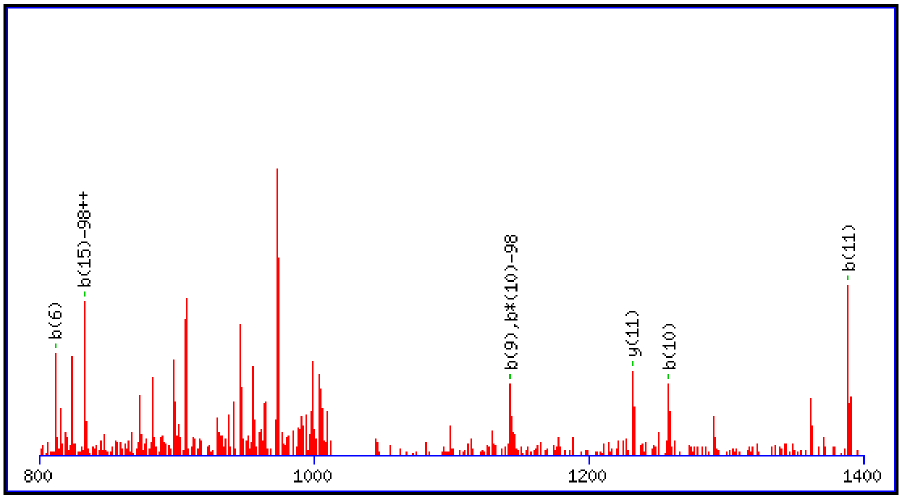

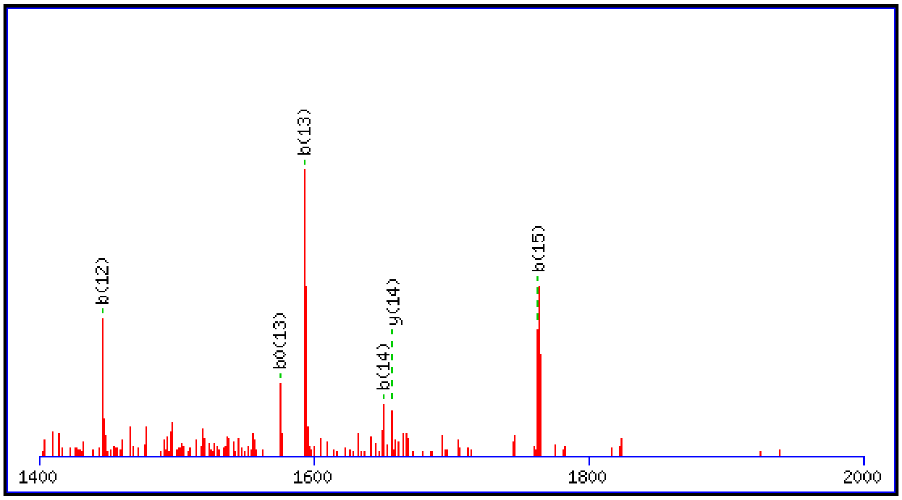

Monoisotopic mass of neutral peptide Mr(calc): 2042.7340
Fixed modifications: Carbamidomethyl (C)
Variable modifications:
S4 : Phospho (ST), with neutral losses 0.0000(shown in table), 97.9769
Ions Score: 52 **Expect:** 0.00054
Matches (Bold Red): 24/248 fragment ions using 39 most intense peaks

#	b	b^{++}	b*	b^{*++}	b^0	b^{0++}	Seq.	y	y^{++}	y^0	y^{0++}	#
1	129.1022	65.0548	112.0757	56.5415			K					17
2	258.1448	129.5761	241.1183	121.0628	240.1343	120.5708	E	1915.6463	958.3268	1897.6358	949.3215	16
3	**387.1874**	194.0974	370.1609	185.5841	369.1769	185.0921	E	1786.6037	893.8055	1768.5932	884.8002	15
4	554.1858	277.5965	537.1592	269.0833	536.1752	268.5912	S	**1657.5611**	829.2842	1639.5506	820.2789	14

**5**	**683.2284**	342.1178	666.2018	333.6045	665.2178	333.1125	**E**	1490.5628	745.7850	1472.5522	736.7797	**13**
**6**	**812.2710**	406.6391	795.2444	398.1258	794.2604	397.6338	**E**	1361.5202	681.2637	1343.5096	672.2585	**12**
**7**	899.3030	450.1551	882.2764	441.6419	881.2924	441.1499	**S**	**1232.4776**	616.7424	1214.4670	607.7372	**11**
**8**	1014.3299	507.6686	997.3034	499.1553	996.3194	498.6633	**D**	1145.4456	573.2264	1127.4350	564.2211	**10**
**9**	**1143.3725**	572.1899	1126.3460	563.6766	1125.3620	563.1846	**E**	1030.4186	515.7130	1012.4081	506.7077	**9**
**10**	**1258.3995**	629.7034	1241.3729	**621.1901**	1240.3889	**620.6981**	**D**	901.3760	**451.1917**	883.3655	442.1864	**8**
**11**	**1389.4400**	695.2236	1372.4134	686.7103	1371.4294	686.2183	**M**	**786.3491**	393.6782	768.3385	384.6729	**7**
**12**	**1446.4614**	723.7343	1429.4349	715.2211	1428.4509	**714.7291**	**G**	**655.3086**	328.1579	637.2980	319.1527	**6**
**13**	**1593.5298**	797.2686	1576.5033	788.7553	**1575.5193**	788.2633	**F**	598.2871	299.6472	580.2766	290.6419	**5**
**14**	**1650.5513**	825.7793	1633.5247	817.2660	1632.5407	816.7740	**G**	**451.2187**	226.1130	433.2082	217.1077	**4**
**15**	**1763.6354**	882.3213	1746.6088	873.8080	1745.6248	873.3160	**L**	394.1973	197.6023	376.1867	188.5970	**3**
**16**	1910.7038	955.8555	1893.6772	947.3423	1892.6932	946.8502	**F**	281.1132	141.0602	263.1026	132.0550	**2**
**17**							**D**	134.0448	67.5260	116.0342	58.5207	**1**

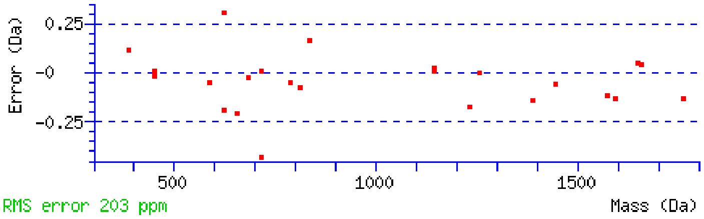

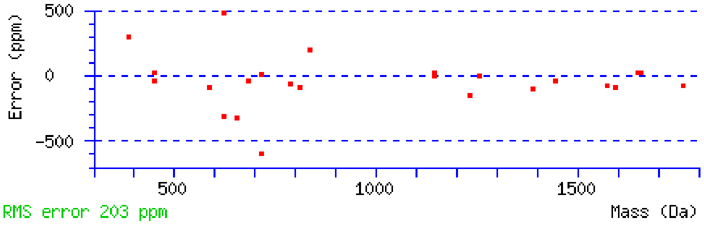

All matches to this query

Score	Mr(calc):	Delta	Sequence
52.4	2042.7340	-0.0040	KEESEESDEDMGFGLFD
52.4	2042.7340	-0.0040	KEESEESEDDMGFGLFD
30.9	2042.7340	-0.0040	KEESEESDEDMGFGLFD
30.9	2042.7340	-0.0040	KEESEESEDDMGFGLFD

Spectrum No: 260; Query: 2369; Rank: 1

Peptide View

MS/MS Fragmentation of **VTLQDYHLPDSDEDEDEETAIR**
Found in **IPI00193162**, Tax_Id=10116 Gene_Symbol=LOC499871 Similar to Zinc finger, FYVE domain containing 19

Match to Query 2369: 2669.113182 from(890.711670,3+)
Title: 100101RatKid_NS_deglyco_22.3258.3258.3.dta
Data file K:\NewmanPaper\Piliang\3SubProteomes\Piliang3SP\mgf5ppm\ERLIC_3SubProteomes5ppm.mgf

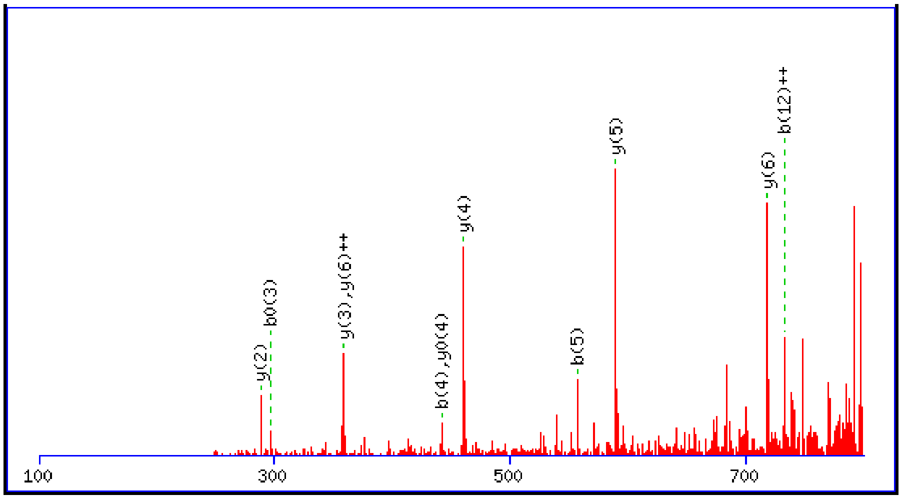

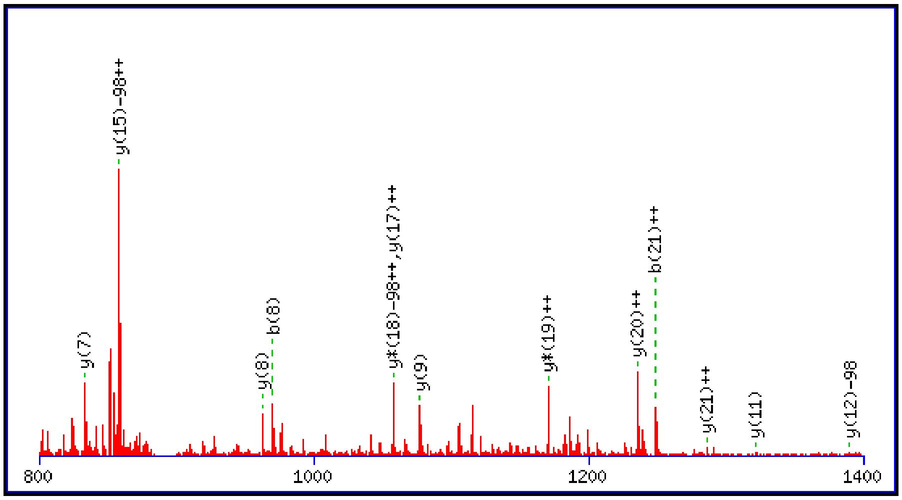

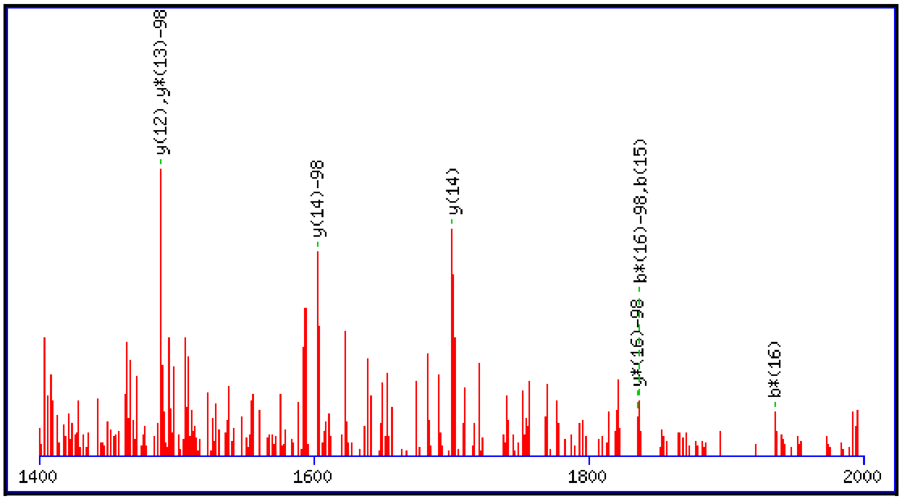

Monoisotopic mass of neutral peptide Mr(calc): 2669.1018
Fixed modifications: Carbamidomethyl (C)
Variable modifications:
S11 : Phospho (ST), with neutral losses 97.9769(shown in table), 0.0000
Ions Score: 52 **Expect:** 0.0018
Matches (Bold Red): 32/364 fragment ions using 47 most intense peaks

#	b	b++	b*	b*++	b0	b0++	Seq.	y	y++	y*	y*++	y0	y0++	#
1	100.0757	50.5415					V							22
2	201.1234	101.0653			183.1128	92.0600	T	2473.0637	1237.0355	2456.0372	1228.5222	2455.0532	1228.0302	21
3	314.2074	157.6074			**296.1969**	148.6021	L	2372.0161	1186.5117	2354.9895	1177.9984	2354.0055	1177.5064	20
4	**442.2660**	221.6366	425.2395	213.1234	424.2554	212.6314	Q	2258.9320	1129.9696	2241.9054	1121.4564	2240.9214	1120.9644	19
5	**557.2930**	279.1501	540.2664	270.6368	539.2824	270.1448	D	2130.8734	1065.9403	2113.8469	**1057.4271**	2112.8629	1056.9351	18

**6**	720.3563	360.6818	703.3297	352.1685	702.3457	351.6765	**Y**	2015.8465	1008.4269	1998.8199	999.9136	1997.8359	999.4216	**17**
**7**	857.4152	429.2112	840.3886	420.6980	839.4046	420.2060	**H**	1852.7831	926.8952	**1835.7566**	918.3819	1834.7726	917.8899	**16**
**8**	**970.4993**	485.7533	953.4727	477.2400	952.4887	476.7480	**L**	1715.7242	**858.3658**	1698.6977	849.8525	1697.7137	849.3605	**15**
**9**	1067.5520	534.2796	1050.5255	525.7664	1049.5415	525.2744	**P**	**1602.6402**	801.8237	1585.6136	793.3105	1584.6296	792.8184	**14**
**10**	1182.5790	591.7931	1165.5524	583.2798	1164.5684	582.7878	**D**	1505.5874	753.2973	**1488.5609**	744.7841	1487.5768	744.2921	**13**
**11**	1251.6004	626.3038	1234.5739	617.7906	1233.5899	617.2986	**S**	**1390.5605**	695.7839	1373.5339	687.2706	1372.5499	686.7786	**12**
**12**	1366.6274	683.8173	1349.6008	675.3040	1348.6168	674.8120	**D**	**1321.5390**	661.2731	1304.5125	652.7599	1303.5284	652.2679	**11**
**13**	1495.6700	748.3386	1478.6434	739.8253	1477.6594	739.3333	**E**	1206.5121	603.7597	1189.4855	595.2464	1188.5015	594.7544	**10**
**14**	1610.6969	805.8521	1593.6704	797.3388	1592.6863	796.8468	**D**	**1077.4695**	539.2384	1060.4429	530.7251	1059.4589	530.2331	**9**
**15**	1739.7395	870.3734	1722.7129	861.8601	1721.7289	861.3681	**E**	**962.4425**	481.7249	945.4160	473.2116	944.4320	472.7196	**8**
**16**	1854.7664	927.8869	**1837.7399**	919.3736	1836.7559	918.8816	**D**	**833.3999**	417.2036	816.3734	408.6903	815.3894	408.1983	**7**
**17**	1983.8090	992.4082	1966.7825	983.8949	1965.7985	983.4029	**E**	**718.3730**	**359.6901**	701.3464	351.1769	700.3624	350.6849	**6**
**18**	2112.8516	1056.9294	2095.8251	1048.4162	2094.8411	1047.9242	**E**	**589.3304**	295.1688	572.3039	286.6556	571.3198	286.1636	**5**
**19**	2213.8993	1107.4533	2196.8728	1098.9400	2195.8887	1098.4480	**T**	**460.2878**	230.6475	443.2613	222.1343	**442.2772**	221.6423	**4**
**20**	2284.9364	1142.9718	2267.9099	1134.4586	2266.9259	1133.9666	**A**	**359.2401**	180.1237	342.2136	171.6104			**3**
**21**	2398.0205	1199.5139	2380.9939	1191.0006	2380.0099	1190.5086	**I**	**288.2030**	144.6051	271.1765	136.0919			**2**
**22**							**R**	175.1190	88.0631	158.0924	79.5498			**1**

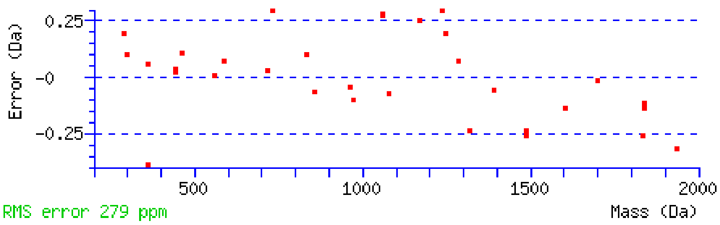

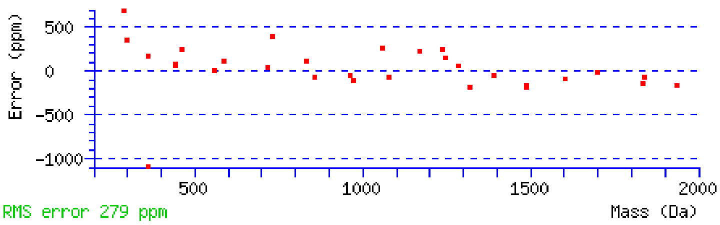

All matches to this query

Score	Mr(calc):	Delta	Sequence
52.3	2669.1018	0.0114	VTLQDYHLPDSDEDEDEETAIR
37.4	2669.1018	0.0114	VTLQDYHLPDSDEDEDEETAIR
26.7	2669.1018	0.0114	VTLQDYHLPDSDEDEDEETAIR
8.6	2669.1018	0.0114	VTLQDYHLPDSDEDEDEETAIR
1.3	2667.0876	2.0256	TEAGCLIRYNVSSTPRLQSMK
1.3	2667.0876	2.0256	TEAGCLIRYNVSSTPRLQSMK
1.3	2667.0876	2.0256	TEAGCLIRYNVSSTPRLQSMK

Spectrum No: 261; Query: 1639; Rank: 1

Peptide View

MS/MS Fragmentation of **KEESEESDEDMGFGLFD**
Found in **IPI00734740**, Tax_Id=10116 Gene_Symbol=LOC498555 similar to 60S acidic ribosomal protein P2

Match to Query 1639: 2042.730008 from(1022.372280,2+)
Title: 091008RatKidney_NH4Format02_26.3987.3987.2.dta
Data file K:\NewmanPaper\Piliang\3SubProteomes\Piliang3SP\mgf5ppm\ERLIC_3SubProteomes5ppm.mgf

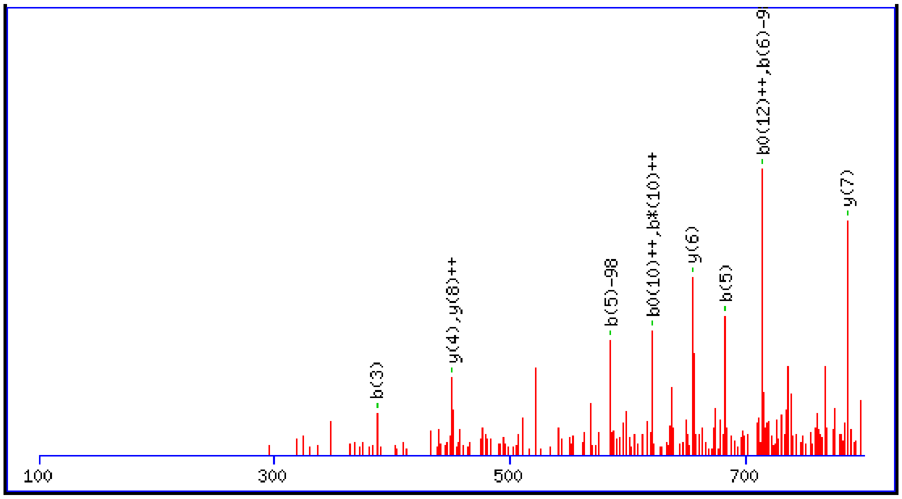

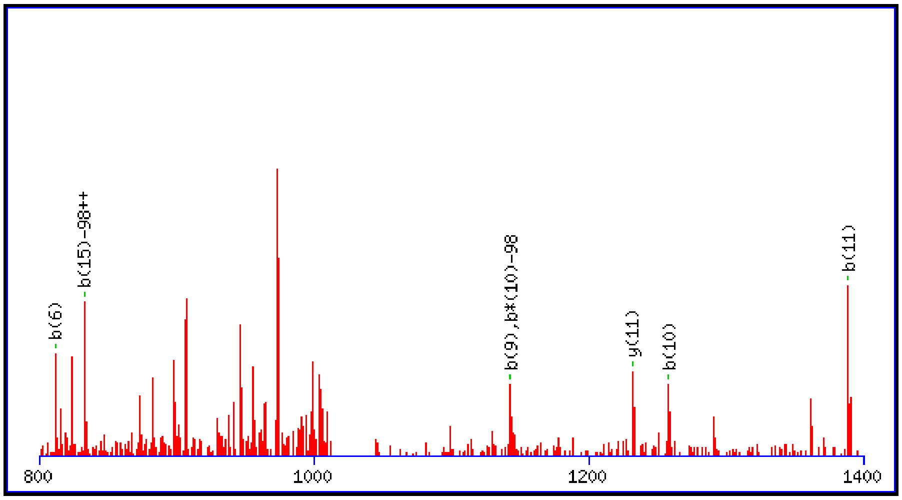

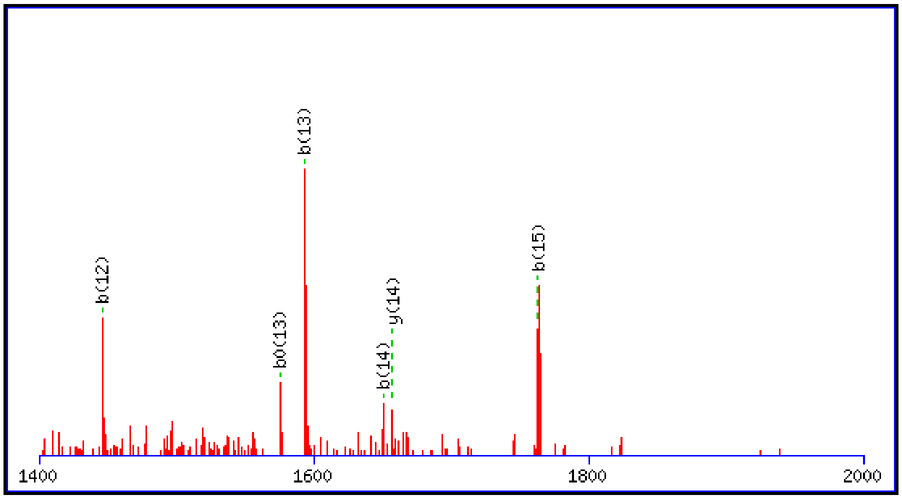

Monoisotopic mass of neutral peptide Mr(calc): 2042.7340
Fixed modifications: Carbamidomethyl (C)
Variable modifications:
S4 : Phospho (ST), with neutral losses 0.0000(shown in table), 97.9769
Ions Score: 52 **Expect:** 0.00054
Matches (Bold Red): 24/248 fragment ions using 39 most intense peaks

#	b	b^{++}	b*	b^{*++}	b^0	b^{0++}	Seq.	y	y^{++}	y^0	y^{0++}	#
1	129.1022	65.0548	112.0757	56.5415			K					17
2	258.1448	129.5761	241.1183	121.0628	240.1343	120.5708	E	1915.6463	958.3268	1897.6358	949.3215	16
3	**387.1874**	194.0974	370.1609	185.5841	369.1769	185.0921	E	1786.6037	893.8055	1768.5932	884.8002	15
4	554.1858	277.5965	537.1592	269.0833	536.1752	268.5912	S	**1657.5611**	829.2842	1639.5506	820.2789	14
5	**683.2284**	342.1178	666.2018	333.6045	665.2178	333.1125	E	1490.5628	745.7850	1472.5522	736.7797	13

6	812.2710	406.6391	795.2444	398.1258	794.2604	397.6338	E	1361.5202	681.2637	1343.5096	672.2585	12
7	899.3030	450.1551	882.2764	441.6419	881.2924	441.1499	S	1232.4776	616.7424	1214.4670	607.7372	11
8	1014.3299	507.6686	997.3034	499.1553	996.3194	498.6633	D	1145.4456	573.2264	1127.4350	564.2211	10
9	1143.3725	572.1899	1126.3460	563.6766	1125.3620	563.1846	E	1030.4186	515.7130	1012.4081	506.7077	9
10	1258.3995	629.7034	1241.3729	621.1901	1240.3889	620.6981	D	901.3760	451.1917	883.3655	442.1864	8
11	1389.4400	695.2236	1372.4134	686.7103	1371.4294	686.2183	M	786.3491	393.6782	768.3385	384.6729	7
12	1446.4614	723.7343	1429.4349	715.2211	1428.4509	714.7291	G	655.3086	328.1579	637.2980	319.1527	6
13	1593.5298	797.2686	1576.5033	788.7553	1575.5193	788.2633	F	598.2871	299.6472	580.2766	290.6419	5
14	1650.5513	825.7793	1633.5247	817.2660	1632.5407	816.7740	G	451.2187	226.1130	433.2082	217.1077	4
15	1763.6354	882.3213	1746.6088	873.8080	1745.6248	873.3160	L	394.1973	197.6023	376.1867	188.5970	3
16	1910.7038	955.8555	1893.6772	947.3423	1892.6932	946.8502	F	281.1132	141.0602	263.1026	132.0550	2
17							D	134.0448	67.5260	116.0342	58.5207	1

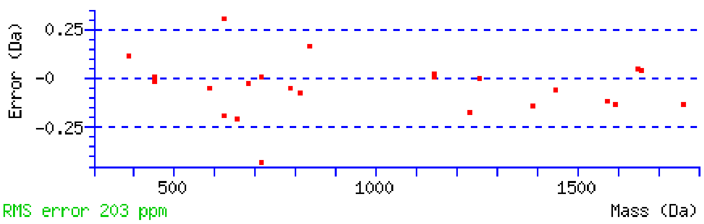

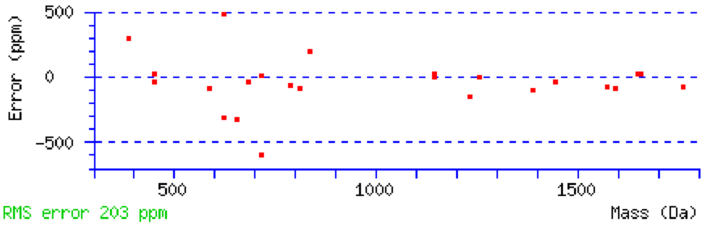

All matches to this query

Score	Mr(calc):	Delta	Sequence
52.4	2042.7340	-0.0040	KEESEESDEDMGFGLFD
52.4	2042.7340	-0.0040	KEESEESEDDMGFGLFD
30.9	2042.7340	-0.0040	KEESEESDEDMGFGLFD
30.9	2042.7340	-0.0040	KEESEESEDDMGFGLFD

Spectrum No: 262; Query: 2132; Rank: 1

Peptide View

MS/MS Fragmentation of **NLTSSSLNDISDKPEKDQLK**
Found in **IPI00199985**, Tax_Id=10116 Gene_Symbol=Slc4a4 Isoform 1 of Electrogenic sodium bicarbonate cotransporter 1

Match to Query 2132: 2391.058242 from(798.026690,3+)
Title: 091008RatKidney_NH4Format01_23.1639.1639.3.dta
Data file K:\NewmanPaper\Piliang\3SubProteomes\Piliang3SP\mgf5ppm\ERLIC_3SubProteomes5ppm.mgf

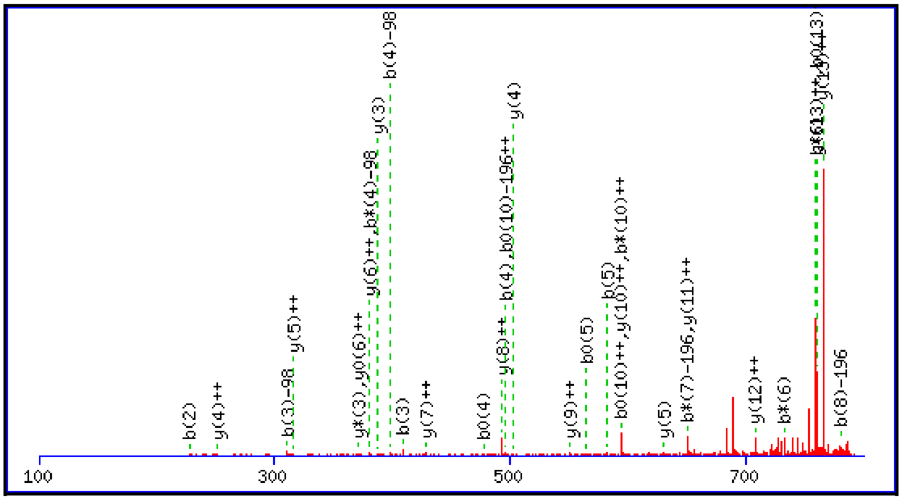

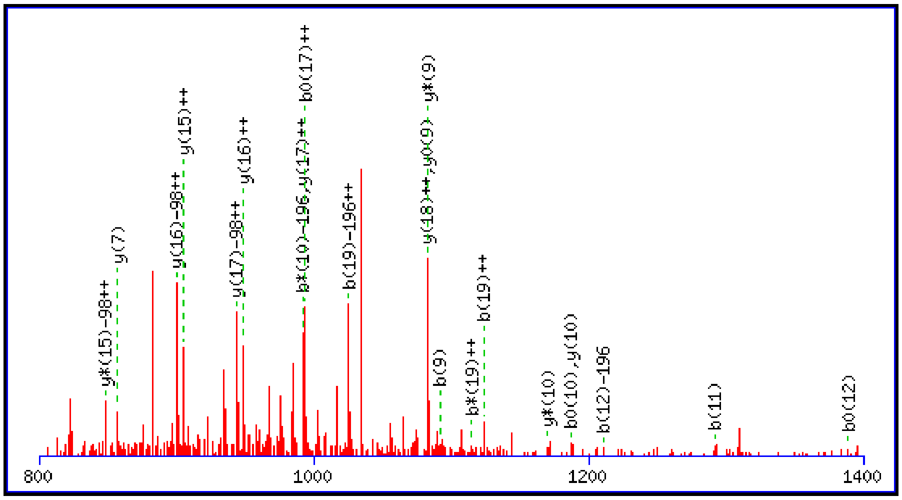

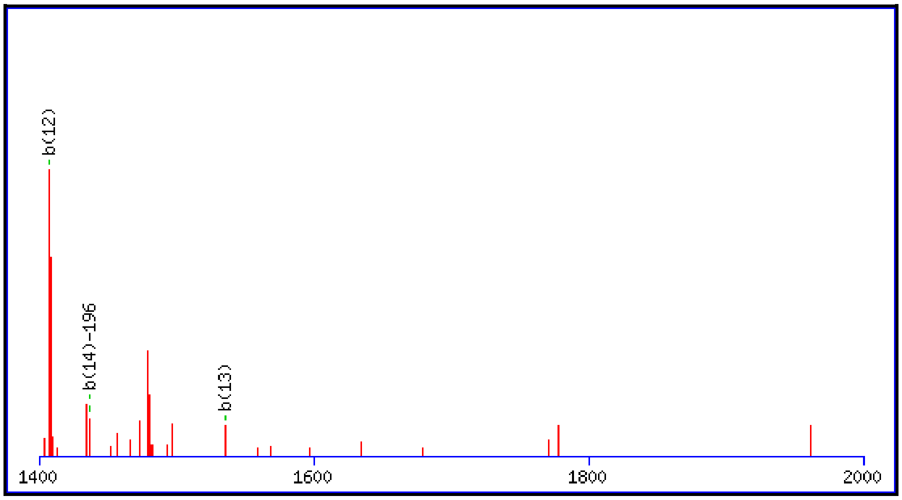

Monoisotopic mass of neutral peptide Mr(calc): 2391.0607
Fixed modifications: Carbamidomethyl (C)
Variable modifications:
T3 : Phospho (ST), with neutral losses 0.0000(shown in table), 97.9769
S6 : Phospho (ST), with neutral losses 0.0000(shown in table), 97.9769
Ions Score: 52 **Expect:** 0.0018
Matches (Bold Red): 58/350 fragment ions using 113 most intense peaks

#	b	b^{++}	b*	b^{*++}	b^0	b^{0++}	Seq.	y	y^{++}	y*	y^{*++}	y^0	y^{0++}	#
1	115.0502	58.0287	98.0237	49.5155			N							20
2	**228.1343**	114.5708	211.1077	106.0575			L	2278.0251	1139.5162	2260.9986	1131.0029	2260.0146	1130.5109	19
3	**409.1483**	205.0778	392.1217	196.5645	391.1377	196.0725	T	2164.9411	**1082.9742**	2147.9145	1074.4609	2146.9305	1073.9689	18
4	**496.1803**	248.5938	479.1537	240.0805	**478.1697**	239.5885	S	1983.9270	**992.4672**	1966.9005	983.9539	1965.9165	983.4619	17
5	**583.2123**	292.1098	566.1858	283.5965	**565.2018**	283.1045	S	1896.8950	**948.9511**	1879.8685	940.4379	1878.8845	939.9459	16
6	750.2107	375.6090	**733.1841**	367.0957	732.2001	366.6037	S	1809.8630	**905.4351**	1792.8364	896.9219	1791.8524	896.4299	15
7	863.2947	432.1510	846.2682	423.6377	845.2842	423.1457	L	1642.8646	821.9360	1625.8381	813.4227	1624.8541	812.9307	14
8	977.3377	489.1725	960.3111	480.6592	959.3271	480.1672	N	1529.7806	**765.3939**	1512.7540	756.8807	1511.7700	756.3886	13
9	**1092.3646**	546.6859	1075.3381	538.1727	1074.3540	537.6807	D	1415.7377	**708.3725**	1398.7111	699.8592	1397.7271	699.3672	12
10	1205.4487	603.2280	1188.4221	**594.7147**	**1187.4381**	**594.2227**	I	1300.7107	**650.8590**	1283.6842	642.3457	1282.7001	641.8537	11
11	**1292.4807**	646.7440	1275.4542	638.2307	1274.4701	637.7387	S	**1187.6266**	**594.3170**	**1170.6001**	585.8037	1169.6161	585.3117	10
12	**1407.5076**	704.2575	1390.4811	695.7442	**1389.4971**	695.2522	D	1100.5946	**550.8009**	**1083.5681**	542.2877	**1082.5841**	541.7957	9
13	**1535.6026**	768.3049	1518.5761	**759.7917**	1517.5920	**759.2997**	K	985.5677	**493.2875**	968.5411	484.7742	967.5571	484.2822	8
14	1632.6554	816.8313	1615.6288	808.3181	1614.6448	807.8260	P	**857.4727**	**429.2400**	840.4462	420.7267	839.4621	420.2347	7
15	1761.6980	881.3526	1744.6714	872.8393	1743.6874	872.3473	E	**760.4199**	**380.7136**	743.3934	372.2003	742.4094	**371.7083**	6
16	1889.7929	945.4001	1872.7664	936.8868	1871.7824	936.3948	K	**631.3774**	**316.1923**	614.3508	307.6790	613.3668	307.1870	5
17	2004.8199	1002.9136	1987.7933	994.4003	1986.8093	**993.9083**	D	**503.2824**	**252.1448**	486.2558	243.6316	485.2718	243.1396	4

18	2132.8785	1066.9429	2115.8519	1058.4296	2114.8679	1057.9376	Q	388.2554	194.6314	371.2289	186.1181			3
19	2245.9625	1123.4849	2228.9360	1114.9716	2227.9519	1114.4796	L	260.1969	130.6021	243.1703	122.0888			2
20							K	147.1128	74.0600	130.0863	65.5468			1

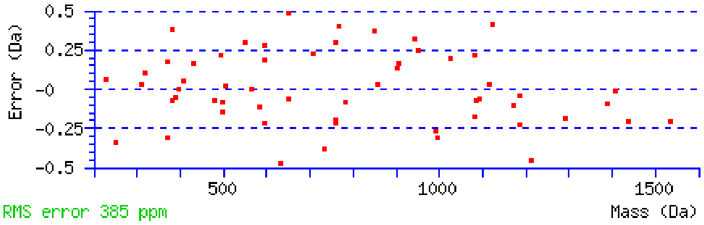

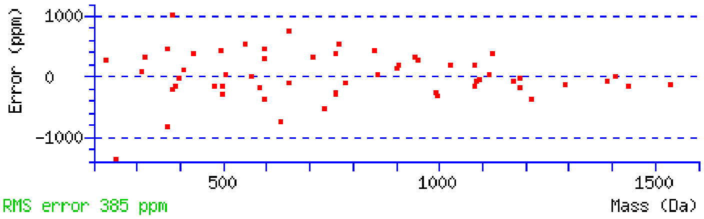

All matches to this query

Score	Mr(calc):	Delta	Sequence
52.2	2391.0607	-0.0025	NLTSSSLNDISDKPEKDQLK
46.0	2391.0607	-0.0025	NLTSSSLNDISDKPEKDQLK
45.1	2391.0607	-0.0025	NLTSSSLNDISDKPEKDQLK
43.3	2391.0607	-0.0025	NLTSSSLNDISDKPEKDQLK
37.9	2391.0607	-0.0025	NLTSSSLNDISDKPEKDQLK
36.6	2391.0607	-0.0025	NLTSSSLNDISDKPEKDQLK
28.3	2391.0607	-0.0025	NLTSSSLNDISDKPEKDQLK
22.6	2391.0607	-0.0025	NLTSSSLNDISDKPEKDQLK
16.3	2391.0607	-0.0025	NLTSSSLNDISDKPEKDQLK
10.7	2391.0607	-0.0025	NLTSSSLNDISDKPEKDQLK

Spectrum No: 263; Query: 2517; Rank: 1

Peptide View

MS/MS Fragmentation of **LHGGFDSDCSEDGEALNGEPELDLTSK**
Found in **IPI00366437**, Tax_Id=10116 Gene_Symbol=Gtpbp1_predicted 76 kDa protein

Match to Query 2517: 2971.216572 from(991.412800,3+)
Title: 100101RatKid_NS_deglyco_21.3259.3259.3.dta
Data file K:\NewmanPaper\Piliang\3SubProteomes\Piliang3SP\mgf5ppm\ERLIC_3SubProteomes5ppm.mgf

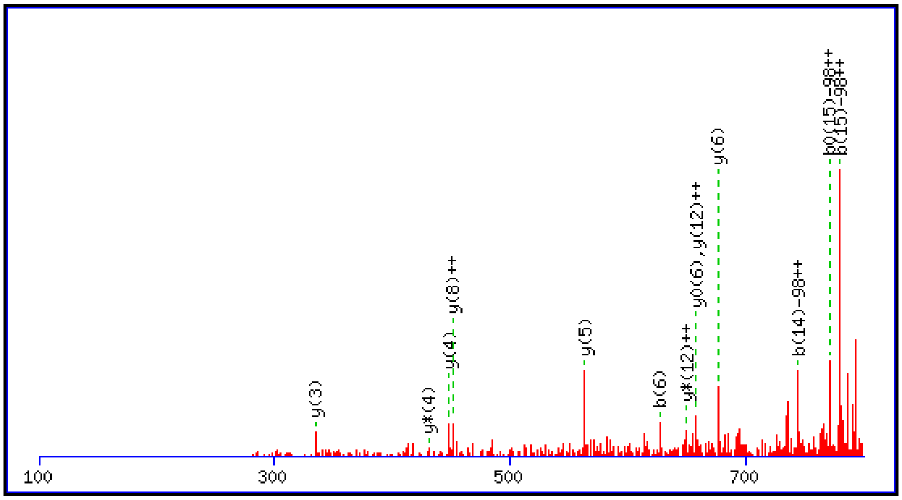

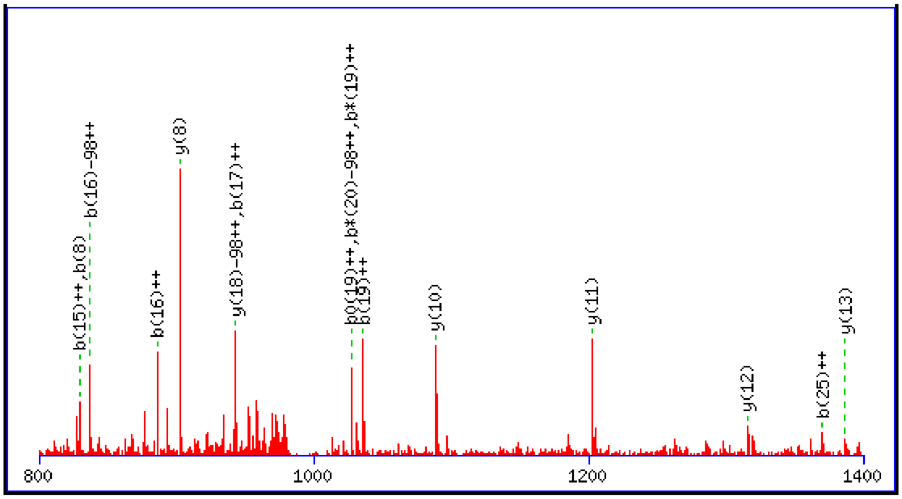

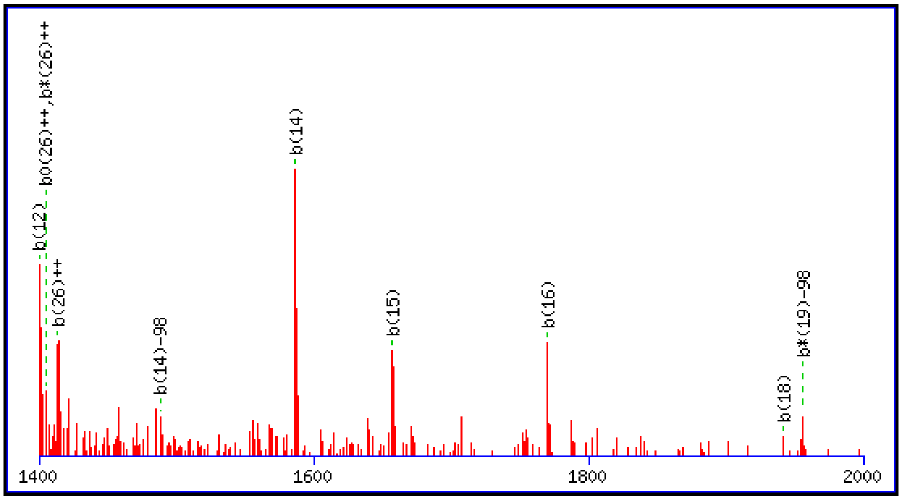

Monoisotopic mass of neutral peptide Mr(calc): 2971.2067
Fixed modifications: Carbamidomethyl (C)
Variable modifications:
S10 : Phospho (ST), with neutral losses 97.9769(shown in table), 0.0000
Ions Score: 52 **Expect:** 0.0022
Matches (Bold Red): 39/410 fragment ions using 61 most intense peaks

#	b	b^{++}	b^{*}	b^{*++}	b^{0}	b^{0++}	Seq.	y	y^{++}	y^{*}	y^{*++}	y^{0}	y^{0++}	#
1	114.0913	57.5493					L							27
2	251.1503	126.0788					H	2761.1530	1381.0801	2744.1264	1372.5668	2743.1424	1372.0748	26
3	308.1717	154.5895					G	2624.0941	1312.5507	2607.0675	1304.0374	2606.0835	1303.5454	25
4	365.1932	183.1002					G	2567.0726	1284.0399	2550.0460	1275.5267	2549.0620	1275.0347	24
5	512.2616	256.6344					F	2510.0511	1255.5292	2493.0246	1247.0159	2492.0406	1246.5239	23
6	**627.2885**	314.1479			609.2780	305.1426	D	2362.9827	1181.9950	2345.9562	1173.4817	2344.9721	1172.9897	22
7	714.3206	357.6639			696.3100	348.6586	S	2247.9558	1124.4815	2230.9292	1115.9682	2229.9452	1115.4762	21
8	**829.3475**	415.1774			811.3369	406.1721	D	2160.9237	1080.9655	2143.8972	1072.4522	2142.9132	1071.9602	20
9	989.3782	495.1927			971.3676	486.1874	C	2045.8968	1023.4520	2028.8703	1014.9388	2027.8862	1014.4468	19
10	1058.3996	529.7034			1040.3890	520.6982	S	1885.8662	**943.4367**	1868.8396	934.9234	1867.8556	934.4314	18
11	1187.4422	594.2247			1169.4316	585.2195	E	1816.8447	908.9260	1799.8181	900.4127	1798.8341	899.9207	17
12	1302.4691	651.7382			1284.4586	642.7329	D	1687.8021	844.4047	1670.7756	835.8914	1669.7915	835.3994	16
13	1359.4906	680.2489			1341.4800	671.2437	G	1572.7752	786.8912	1555.7486	778.3779	1554.7646	777.8859	15
14	**1488.5332**	**744.7702**			1470.5226	735.7650	E	1515.7537	758.3805	1498.7271	749.8672	1497.7431	749.3752	14
15	1559.5703	**780.2888**			1541.5598	**771.2835**	A	**1386.7111**	693.8592	1369.6846	685.3459	1368.7005	684.8539	13
16	1672.6544	**836.8308**			1654.6438	827.8255	L	**1315.6740**	**658.3406**	1298.6474	**649.8274**	1297.6634	649.3353	12
17	1786.6973	893.8523	1769.6708	885.3390	1768.6867	884.8470	N	**1202.5899**	601.7986	1185.5634	593.2853	1184.5794	592.7933	11
18	1843.7188	922.3630	1826.6922	913.8498	1825.7082	913.3577	G	**1088.5470**	544.7771	1071.5204	536.2639	1070.5364	535.7719	10

**19**	1972.7614	986.8843	**1955.7348**	978.3710	1954.7508	977.8790	**E**	1031.5255	516.2664	1014.4990	507.7531	1013.5150	507.2611	**9**
**20**	2069.8141	1035.4107	2052.7876	**1026.8974**	2051.8036	1026.4054	**P**	**902.4829**	**451.7451**	885.4564	443.2318	884.4724	442.7398	**8**
**21**	2198.8567	1099.9320	2181.8302	1091.4187	2180.8462	1090.9267	**E**	805.4302	403.2187	788.4036	394.7055	787.4196	394.2134	**7**
**22**	2311.9408	1156.4740	2294.9142	1147.9608	2293.9302	1147.4687	**L**	**676.3876**	338.6974	659.3610	330.1842	**658.3770**	329.6921	**6**
**23**	2426.9677	1213.9875	2409.9412	1205.4742	2408.9572	1204.9822	**D**	**563.3035**	282.1554	546.2770	273.6421	545.2930	273.1501	**5**
**24**	2540.0518	1270.5295	2523.0252	1262.0163	2522.0412	1261.5243	**L**	**448.2766**	224.6419	**431.2500**	216.1287	430.2660	215.6366	**4**
**25**	2641.0995	1321.0534	2624.0729	1312.5401	2623.0889	1312.0481	**T**	**335.1925**	168.0999	318.1660	159.5866	317.1819	159.0946	**3**
**26**	2728.1315	1364.5694	2711.1050	1356.0561	2710.1209	1355.5641	**S**	234.1448	117.5761	217.1183	109.0628	216.1343	108.5708	**2**
**27**							**K**	147.1128	74.0600	130.0863	65.5468			**1**

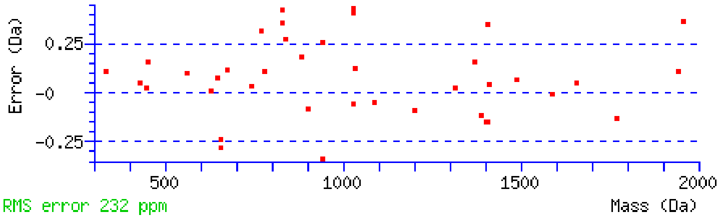

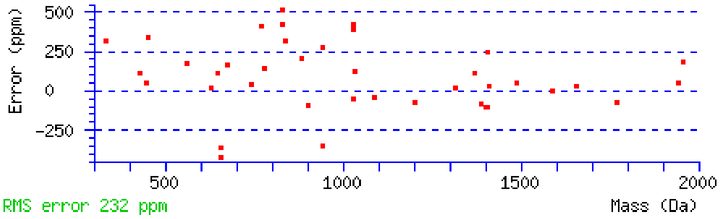

All matches to this query

Score	Mr(calc):	Delta	Sequence
52.1	2971.2067	0.0099	LHGGFDSDCSEDGEALNGEPELDLTSK
45.5	2971.2067	0.0099	LHGGFDSDCSEDGEALNGEPELDLTSK
6.7	2971.2360	-0.0195	DGTSQPDMCLQTDIMKEDFIKEMK
6.7	2971.2360	-0.0195	DGTSQPDMCLQTDIMKEDFIKEMK
6.6	2971.2266	-0.0100	TNSSHITCDENGSWVYTTFCARKR
4.4	2971.2381	-0.0216	MAIGEFDQKASSQPSTTPVEEERMK
4.1	2971.2381	-0.0216	MAIGEFDQKASSQPSTTPVEEERMK
2.8	2971.2456	-0.0290	FYGDNPQTSPNLGRIINQKHFER
2.5	2971.2081	0.0085	DRPDLQSIIALCEEKMQPVSSCR
1.9	2969.2276	1.9890	LGLSVFTHHRVDSEGPSTAPSATRK

Spectrum No: 264; Query: 624; Rank: 1

Peptide View

MS/MS Fragmentation of **TLSDESIYSSQR**
Found in **IPI00209335**, Tax_Id=10116 Gene_Symbol=Sipa1l1 Signal-induced proliferation-associated 1-like protein 1

Match to Query 624: 1464.616308 from(733.315430,2+)
Title: 100101RatKid_NS_deglyco_19.1797.1797.2.dta
Data file K:\NewmanPaper\Piliang\3SubProteomes\Piliang3SP\mgf5ppm\ERLIC_3SubProteomes5ppm.mgf

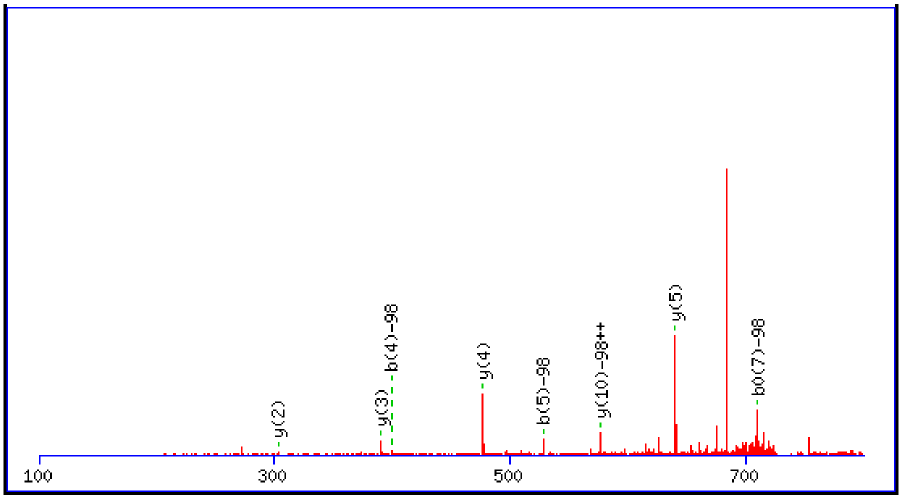

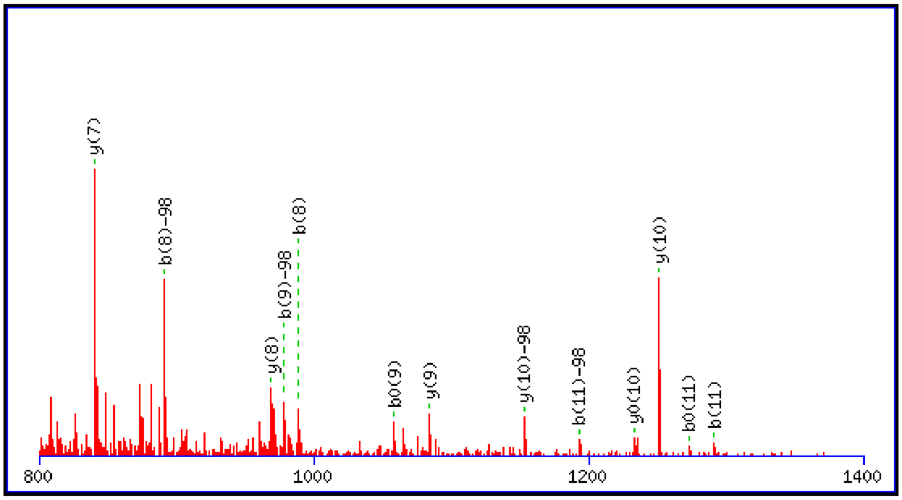

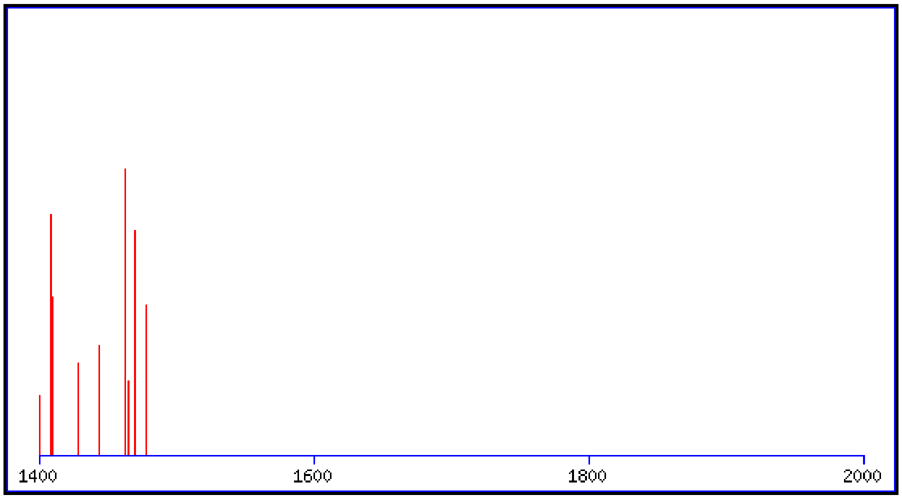

Monoisotopic mass of neutral peptide Mr(calc): 1464.6133
Fixed modifications: Carbamidomethyl (C)
Variable modifications:
S3 : Phospho (ST), with neutral losses 97.9769(shown in table), 0.0000
Ions Score: 52 **Expect:** 0.00087
Matches (Bold Red): 21/158 fragment ions using 33 most intense peaks

#	b	b^{++}	b*	b^{*++}	b^0	b^{0++}	Seq.	y	y^{++}	y*	y^{*++}	y^0	y^{0++}	#
1	102.0550	51.5311			84.0444	42.5258	T							12
2	215.1390	108.0731			197.1285	99.0679	L	1266.5961	633.8017	1249.5695	625.2884	1248.5855	624.7964	11
3	284.1605	142.5839			266.1499	133.5786	S	**1153.5120**	**577.2596**	1136.4855	568.7464	1135.5014	568.2544	10
4	**399.1874**	200.0973			381.1769	191.0921	D	**1084.4905**	542.7489	1067.4640	534.2356	1066.4800	533.7436	9
5	**528.2300**	264.6186			510.2194	255.6134	E	**969.4636**	485.2354	952.4371	476.7222	951.4530	476.2302	8

**6**	615.2620	308.1347			597.2515	299.1294	**S**	**840.4210**	420.7141	823.3945	412.2009	822.4104	411.7089	**7**
**7**	728.3461	364.6767			**710.3355**	355.6714	**I**	753.3890	377.1981	736.3624	368.6849	735.3784	368.1928	**6**
**8**	**891.4094**	446.2084			873.3989	437.2031	**Y**	**640.3049**	320.6561	623.2784	312.1428	622.2944	311.6508	**5**
**9**	**978.4415**	489.7244			960.4309	480.7191	**S**	**477.2416**	239.1244	460.2150	230.6112	459.2310	230.1191	**4**
**10**	1065.4735	533.2404			1047.4629	524.2351	**S**	**390.2096**	195.6084	373.1830	187.0951	372.1990	186.6031	**3**
**11**	**1193.5321**	597.2697	1176.5055	588.7564	1175.5215	588.2644	**Q**	**303.1775**	152.0924	286.1510	143.5791			**2**
**12**							**R**	175.1190	88.0631	158.0924	79.5498			**1**

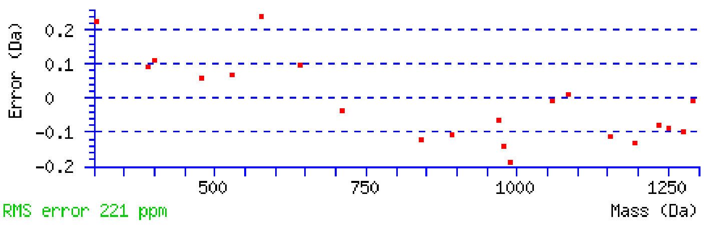

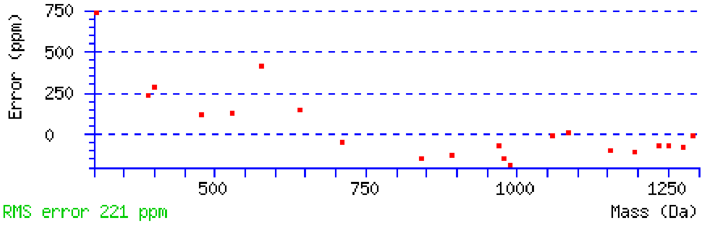

All matches to this query

Score	Mr(calc):	Delta	Sequence
52.1	1464.6133	0.0030	TLSDESIYSSQR
42.0	1464.6133	0.0030	TLSDESIYSSQR
20.5	1464.6133	0.0030	TLSDESIYSSQR
6.4	1463.6058	1.0105	DISIQLSSAQSR
5.1	1464.6133	0.0030	TLSDESIYSSQR
4.5	1464.6068	0.0095	DHQDTMLEIQR
3.4	1464.6163	-0.0000	YSTKSNLTVHR
3.4	1464.6163	-0.0000	YSTKSNLTVHR
3.4	1464.6163	-0.0000	YSTKSNLTVHR
2.9	1464.6133	0.0030	TLSDESIYSSQR

Spectrum No: 265; Query: 1172; Rank: 1

Peptide View

MS/MS Fragmentation of **SRTASGSSVTSLEGTR**
Found in **IPI00421389**, Tax_Id=10116 Gene_Symbol=Ndrg1 Protein NDRG1

Match to Query 1172: 1754.726502 from(585.916110,3+)
Title: 100101RatKid_NS_deglyco_21.1376.1376.3.dta
Data file K:\NewmanPaper\Piliang\3SubProteomes\Piliang3SP\mgf5ppm\ERLIC_3SubProteomes5ppm.mgf

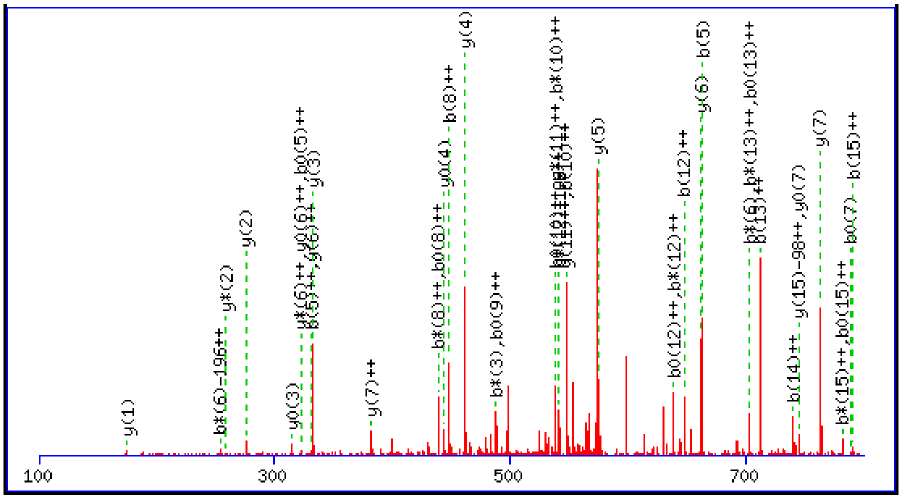

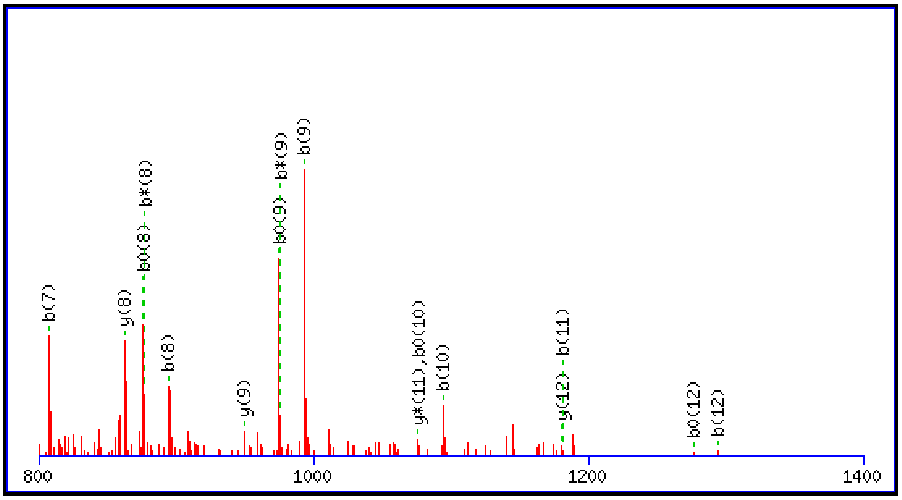

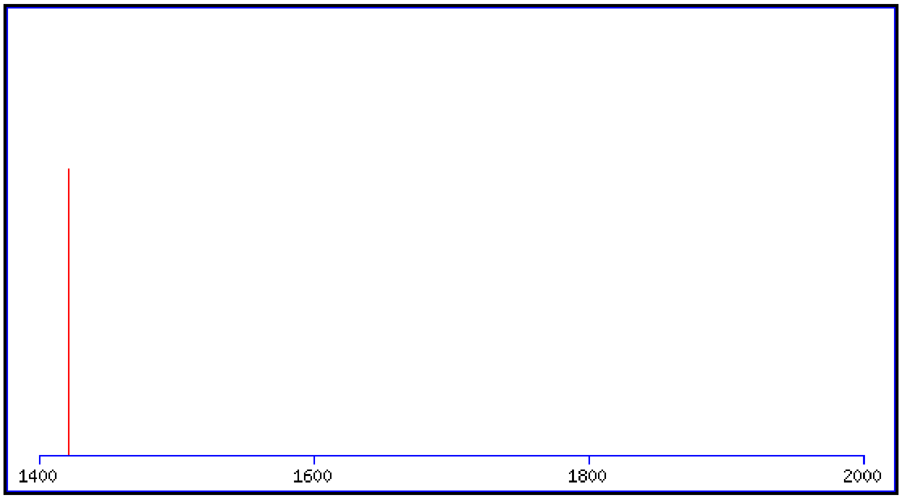

Monoisotopic mass of neutral peptide Mr(calc): 1754.7237
Fixed modifications: Carbamidomethyl (C)
Variable modifications:
S1 : Phospho (ST), with neutral losses 0.0000(shown in table), 97.9769
T3 : Phospho (ST), with neutral losses 0.0000(shown in table), 97.9769
Ions Score: 52 **Expect:** 0.001
Matches (Bold Red): 59/276 fragment ions using 79 most intense peaks

#	b	b^{++}	b*	b^{*++}	b^0	b^{0++}	Seq.	y	y^{++}	y*	y^{*++}	y^0	y^{0++}	#
1	168.0056	84.5065			149.9951	75.5012	S							16
2	324.1067	162.5570	307.0802	154.0437	306.0962	153.5517	R	1588.7326	794.8700	1571.7061	786.3567	1570.7221	785.8647	15
3	505.1207	253.0640	**488.0942**	244.5507	487.1102	244.0587	T	1432.6315	716.8194	1415.6050	708.3061	1414.6210	707.8141	14
4	576.1579	288.5826	559.1313	280.0693	558.1473	279.5773	A	1251.6175	626.3124	1234.5910	617.7991	1233.6070	617.3071	13

**5**	**663.1899**	**332.0986**	646.1633	323.5853	645.1793	**323.0933**	**S**	**1180.5804**	590.7938	1163.5539	582.2806	1162.5699	581.7886	**12**
**6**	720.2113	360.6093	**703.1848**	352.0960	702.2008	351.6040	**G**	1093.5484	**547.2778**	**1076.5218**	**538.7646**	1075.5378	538.2726	**11**
**7**	**807.2434**	404.1253	790.2168	395.6121	**789.2328**	395.1200	**S**	1036.5269	518.7671	1019.5004	510.2538	1018.5164	509.7618	**10**
**8**	**894.2754**	**447.6413**	**877.2489**	**439.1281**	**876.2648**	**438.6361**	**S**	**949.4949**	475.2511	932.4684	466.7378	931.4843	466.2458	**9**
**9**	**993.3438**	497.1755	**976.3173**	488.6623	**975.3333**	**488.1703**	**V**	**862.4629**	431.7351	845.4363	423.2218	844.4523	422.7298	**8**
**10**	**1094.3915**	**547.6994**	1077.3649	**539.1861**	**1076.3809**	**538.6941**	**T**	**763.3945**	**382.2009**	746.3679	373.6876	**745.3839**	373.1956	**7**
**11**	**1181.4235**	591.2154	1164.3970	582.7021	1163.4130	582.2101	**S**	**662.3468**	**331.6770**	645.3202	**323.1638**	644.3362	**322.6717**	**6**
**12**	**1294.5076**	**647.7574**	1277.4810	**639.2442**	**1276.4970**	**638.7521**	**L**	**575.3148**	288.1610	558.2882	279.6477	557.3042	279.1557	**5**
**13**	1423.5502	**712.2787**	1406.5236	**703.7655**	1405.5396	**703.2734**	**E**	**462.2307**	231.6190	445.2041	223.1057	**444.2201**	222.6137	**4**
**14**	1480.5716	**740.7895**	1463.5451	732.2762	1462.5611	731.7842	**G**	**333.1881**	167.0977	316.1615	158.5844	**315.1775**	158.0924	**3**
**15**	1581.6193	**791.3133**	1564.5928	**782.8000**	1563.6088	**782.3080**	**T**	**276.1666**	138.5870	**259.1401**	130.0737	258.1561	129.5817	**2**
**16**							**R**	**175.1190**	88.0631	158.0924	79.5498			**1**

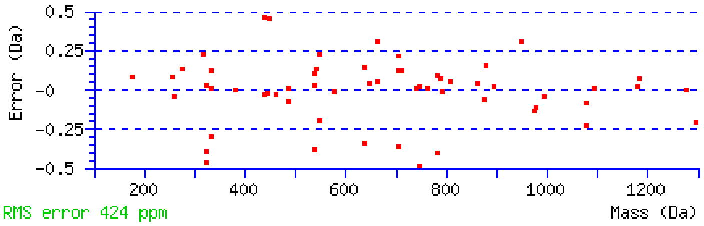

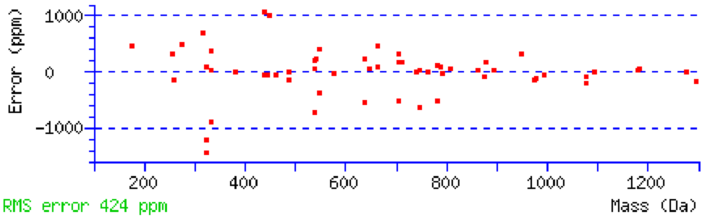

All matches to this query

Score	Mr(calc):	Delta	Sequence
52.0	1754.7237	0.0028	SRTASGSSVTSLEGTR
42.4	1754.7237	0.0028	SRTASGSSVTSLEGTR
40.3	1754.7237	0.0028	SRTASGSSVTSLEGTR
39.7	1754.7237	0.0028	SRTASGSSVTSLEGTR
17.8	1754.7352	-0.0087	DVSCSLTNTHFCASR
16.9	1754.7237	0.0028	SRTASGSSVTSLEGTR
8.1	1753.7359	0.9906	DNSCDQLCTKQETR
8.0	1754.7237	0.0028	SRTASGSSVTSLEGTR
6.1	1752.7178	2.0087	YEMLQDNVEGYRR
5.5	1754.7158	0.0107	SMGGEFCVAAVFGTSR

Spectrum No: 266; Query: 2069; Rank: 1

Peptide View

MS/MS Fragmentation of **LVREDENDASDDEDDDEKR**
Found in **IPI00197076**, Tax_Id=10116 Gene_Symbol=LOC681004;LOC683880 similar to GC-rich sequence DNA-binding factor homolog isoform 2

Match to Query 2069: 2343.895152 from(782.305660,3+)
Title: 091008RatKidney_NoSalt_27.319.319.3.dta
Data file K:\NewmanPaper\Piliang\3SubProteomes\Piliang3SP\mgf5ppm\ERLIC_3SubProteomes5ppm.mgf

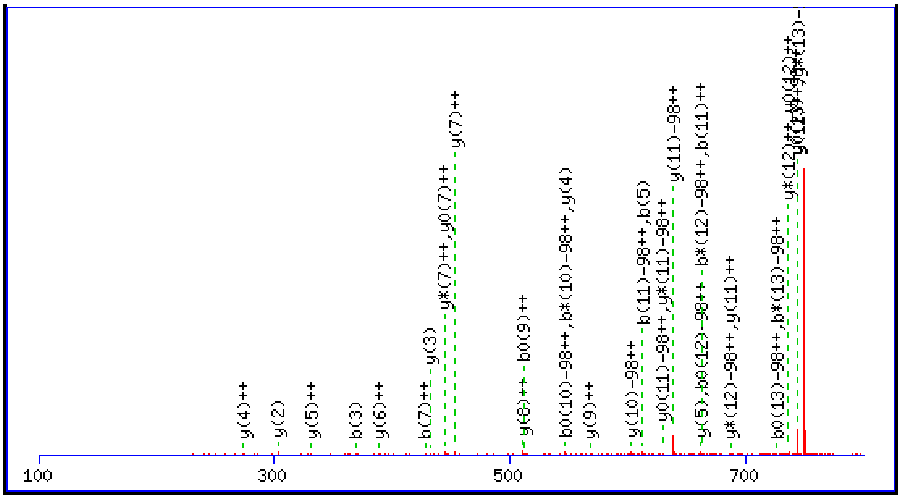

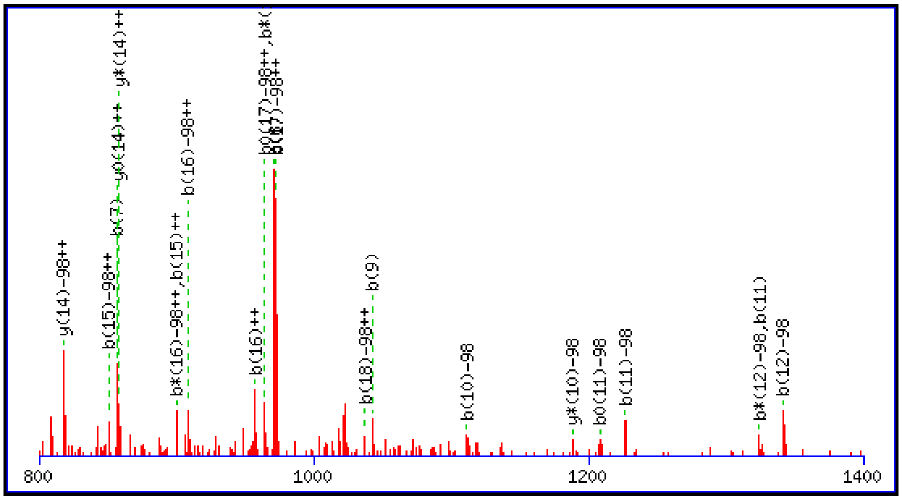

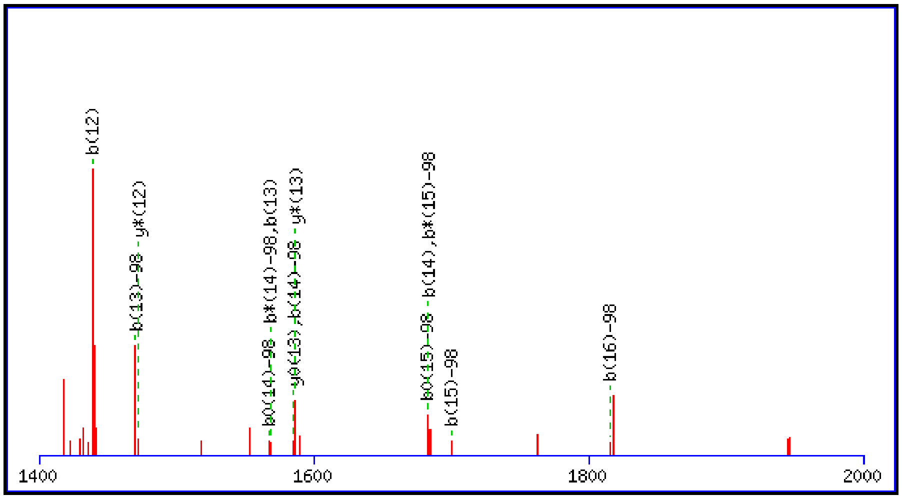

Monoisotopic mass of neutral peptide Mr(calc): 2343.8976
Fixed modifications: Carbamidomethyl (C)
Variable modifications:
S10 : Phospho (ST), with neutral losses 97.9769(shown in table), 0.0000
Ions Score: 52 **Expect:** 0.0011
Matches (Bold Red): 71/310 fragment ions using 97 most intense peaks

#	b	b++	b*	b*++	b0	b0++	Seq.	y	y++	y*	y*++	y0	y0++	#
1	114.0913	57.5493					L							19
2	213.1598	107.0835					V	2133.8439	1067.4256	2116.8174	1058.9123	2115.8333	1058.4203	18
3	**369.2609**	185.1341	352.2343	176.6208			R	2034.7755	1017.8914	2017.7489	1009.3781	2016.7649	1008.8861	17
4	498.3035	249.6554	481.2769	241.1421	480.2929	240.6501	E	1878.6744	939.8408	1861.6478	931.3276	1860.6638	930.8355	16
5	**613.3304**	307.1688	596.3039	298.6556	595.3198	298.1636	D	1749.6318	875.3195	1732.6052	866.8063	1731.6212	866.3143	15

**6**	742.3730	371.6901	725.3464	363.1769	724.3624	362.6849	**E**	1634.6048	**817.8061**	1617.5783	809.2928	1616.5943	808.8008	**14**
**7**	**856.4159**	**428.7116**	839.3894	420.1983	838.4054	419.7063	**N**	1505.5623	753.2848	1488.5357	**744.7715**	1487.5517	**744.2795**	**13**
**8**	**971.4429**	486.2251	954.4163	477.7118	953.4323	477.2198	**D**	1391.5193	696.2633	1374.4928	**687.7500**	1373.5088	687.2580	**12**
**9**	**1042.4800**	521.7436	1025.4534	513.2304	1024.4694	**512.7383**	**A**	1276.4924	**638.7498**	1259.4658	**630.2366**	1258.4818	**629.7445**	**11**
**10**	**1111.5014**	556.2544	1094.4749	**547.7411**	1093.4909	**547.2491**	**S**	1205.4553	**603.2313**	**1188.4287**	594.7180	1187.4447	594.2260	**10**
**11**	**1226.5284**	**613.7678**	1209.5018	605.2546	**1208.5178**	604.7625	**D**	1136.4338	**568.7205**	1119.4073	560.2073	1118.4233	559.7153	**9**
**12**	**1341.5553**	671.2813	**1324.5288**	**662.7680**	1323.5448	**662.2760**	**D**	1021.4069	**511.2071**	1004.3803	502.6938	1003.3963	502.2018	**8**
**13**	**1470.5979**	735.8026	1453.5714	**727.2893**	1452.5873	**726.7973**	**E**	906.3799	**453.6936**	889.3534	**445.1803**	888.3694	**444.6883**	**7**
**14**	**1585.6249**	793.3161	**1568.5983**	784.8028	**1567.6143**	784.3108	**D**	777.3373	**389.1723**	760.3108	380.6590	759.3268	380.1670	**6**
**15**	**1700.6518**	**850.8295**	**1683.6253**	842.3163	**1682.6412**	841.8243	**D**	**662.3104**	**331.6588**	645.2838	323.1456	644.2998	322.6536	**5**
**16**	**1815.6787**	**908.3430**	1798.6522	**899.8297**	1797.6682	899.3377	**D**	**547.2835**	**274.1454**	530.2569	265.6321	529.2729	265.1401	**4**
**17**	1944.7213	**972.8643**	1927.6948	**964.3510**	1926.7108	**963.8590**	**E**	**432.2565**	216.6319	415.2300	208.1186	414.2459	207.6266	**3**
**18**	2072.8163	**1036.9118**	2055.7897	1028.3985	2054.8057	1027.9065	**K**	**303.2139**	152.1106	286.1874	143.5973			**2**
**19**							**R**	175.1190	88.0631	158.0924	79.5498			**1**

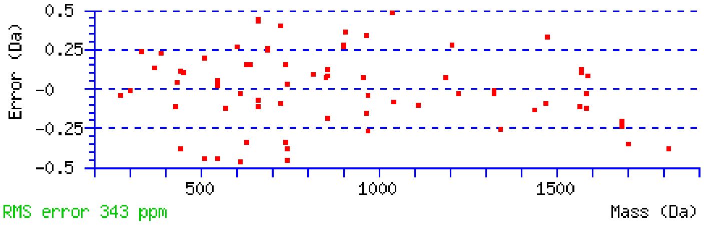

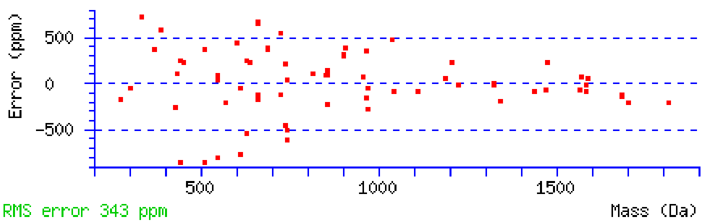

All matches to this query

Score	Mr(calc):	Delta	Sequence
52.0	2343.8976	-0.0024	LVREDENDASDDEDDDEKR
5.5	2342.9006	0.9945	KSNVIVMCGTPETFYNVK
4.9	2341.8800	2.0152	SKGASSSRALMNLHNNEAGR
4.8	2342.8704	1.0248	AFTDLTRPYSIFSYNTK
4.8	2342.8799	1.0153	MSESGEMSEFGYIMELLAK
4.8	2342.8799	1.0153	MSESGEMSEFGYIMELLAK
4.8	2341.8745	2.0207	TDTQHKIMDDFTTKTLK
4.8	2341.8745	2.0207	TDTQHKIMDDFTTKTLK
4.6	2341.8800	2.0152	SKGASSSRALMNLHNNEAGR
4.6	2341.8800	2.0152	SKGASSSRALMNLHNNEAGR

Spectrum No: 267; Query: 1165; Rank: 1

Peptide View

MS/MS Fragmentation of **SRTASGSSVTSLEGTR**
Found in **IPI00421389**, Tax_Id=10116 Gene_Symbol=Ndrg1 Protein NDRG1

Match to Query 1165: 1754.719332 from(585.913720,3+)
Title: 091008RatKidney_NH4Format01_26.994.994.3.dta
Data file K:\NewmanPaper\Piliang\3SubProteomes\Piliang3SP\mgf5ppm\ERLIC_3SubProteomes5ppm.mgf

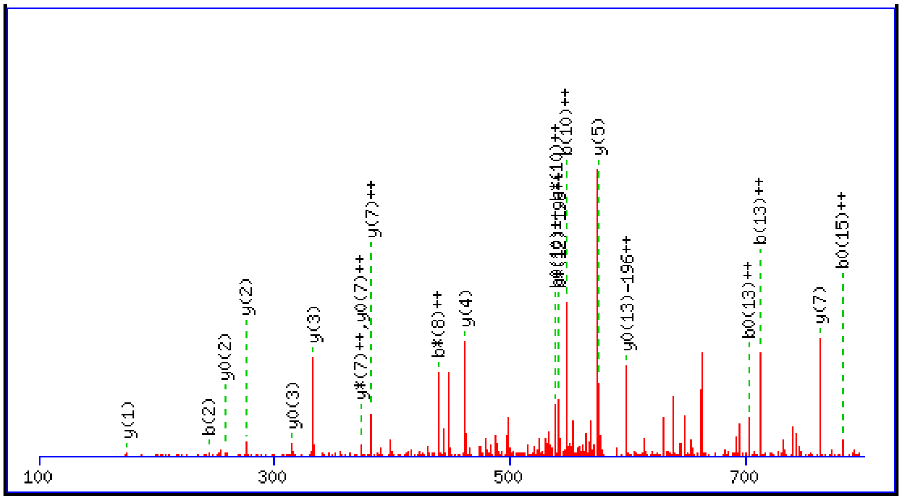

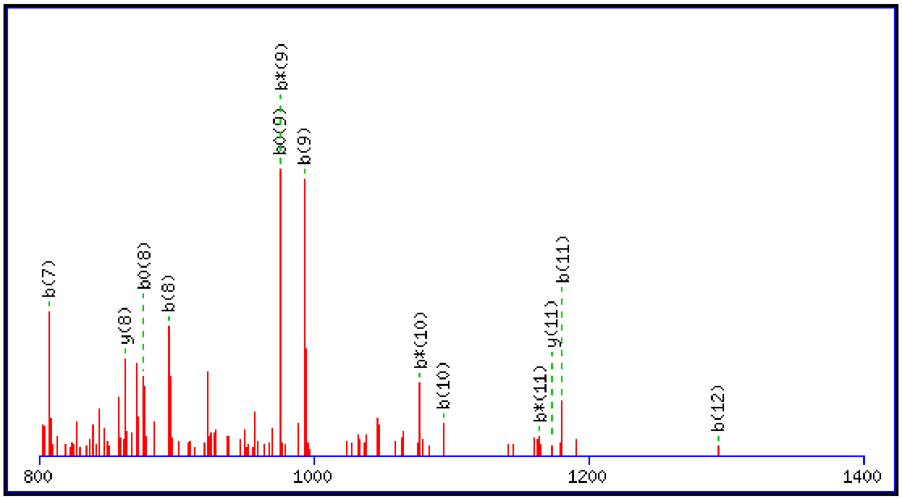

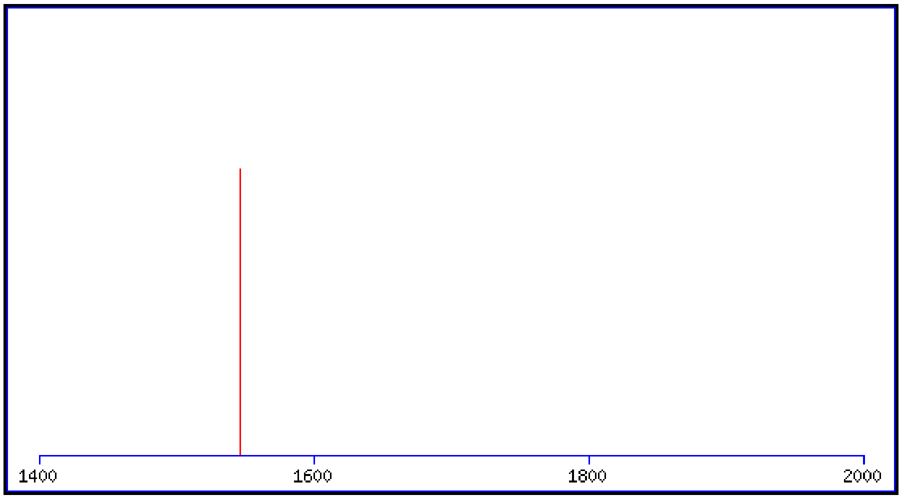

Monoisotopic mass of neutral peptide Mr(calc): 1754.7237
Fixed modifications: Carbamidomethyl (C)
Variable modifications:
S5 : Phospho (ST), with neutral losses 0.0000(shown in table), 97.9769
S7 : Phospho (ST), with neutral losses 0.0000(shown in table), 97.9769
Ions Score: 52 **Expect:** 0.00099
Matches (Bold Red): 34/278 fragment ions using 50 most intense peaks

#	b	b^{++}	b*	b^{*++}	b^0	b^{0++}	Seq.	y	y^{++}	y*	y^{*++}	y^0	y^{0++}	#
1	88.0393	44.5233			70.0287	35.5180	S							16
2	**244.1404**	122.5738	227.1139	114.0606	226.1298	113.5686	R	1668.6990	834.8531	1651.6724	826.3398	1650.6884	825.8478	15
3	345.1881	173.0977	328.1615	164.5844	327.1775	164.0924	T	1512.5979	756.8026	1495.5713	748.2893	1494.5873	747.7973	14
4	416.2252	208.6162	399.1987	200.1030	398.2146	199.6110	A	1411.5502	706.2787	1394.5236	697.7655	1393.5396	697.2734	13

**5**	583.2236	292.1154	566.1970	283.6021	565.2130	283.1101	**S**	1340.5131	670.7602	1323.4865	662.2469	1322.5025	661.7549	**12**
**6**	640.2450	320.6262	623.2185	312.1129	622.2345	311.6209	**G**	**1173.5147**	587.2610	1156.4882	578.7477	1155.5042	578.2557	**11**
**7**	**807.2434**	404.1253	790.2168	395.6121	789.2328	395.1200	**S**	1116.4933	558.7503	1099.4667	550.2370	1098.4827	549.7450	**10**
**8**	**894.2754**	447.6413	877.2489	**439.1281**	**876.2648**	438.6361	**S**	949.4949	475.2511	932.4684	466.7378	931.4843	466.2458	**9**
**9**	**993.3438**	497.1755	**976.3173**	488.6623	**975.3333**	488.1703	**V**	**862.4629**	431.7351	845.4363	423.2218	844.4523	422.7298	**8**
**10**	**1094.3915**	**547.6994**	**1077.3649**	**539.1861**	1076.3809	**538.6941**	**T**	**763.3945**	**382.2009**	746.3679	**373.6876**	745.3839	**373.1956**	**7**
**11**	**1181.4235**	591.2154	**1164.3970**	582.7021	1163.4130	582.2101	**S**	662.3468	331.6770	645.3202	323.1638	644.3362	322.6717	**6**
**12**	**1294.5076**	647.7574	1277.4810	639.2442	1276.4970	638.7521	**L**	**575.3148**	288.1610	558.2882	279.6477	557.3042	279.1557	**5**
**13**	1423.5502	**712.2787**	1406.5236	703.7655	1405.5396	**703.2734**	**E**	**462.2307**	231.6190	445.2041	223.1057	444.2201	222.6137	**4**
**14**	1480.5716	740.7895	1463.5451	732.2762	1462.5611	731.7842	**G**	**333.1881**	167.0977	316.1615	158.5844	**315.1775**	158.0924	**3**
**15**	1581.6193	791.3133	1564.5928	782.8000	1563.6088	**782.3080**	**T**	**276.1666**	138.5870	259.1401	130.0737	**258.1561**	129.5817	**2**
**16**							**R**	**175.1190**	88.0631	158.0924	79.5498			**1**

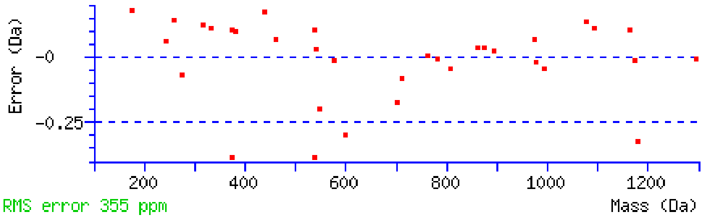

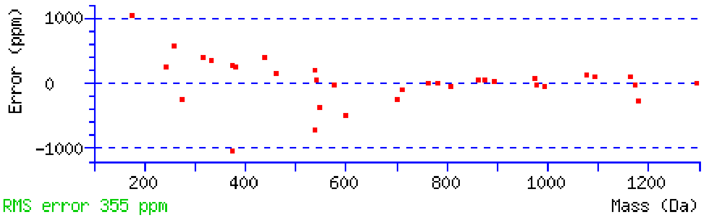

All matches to this query

Score	Mr(calc):	Delta	Sequence
51.9	1754.7237	-0.0044	SRTASGSSVTSLEGTR
48.7	1754.7237	-0.0044	SRTASGSSVTSLEGTR
44.6	1754.7237	-0.0044	SRTASGSSVTSLEGTR
40.3	1754.7237	-0.0044	SRTASGSSVTSLEGTR
17.3	1754.7237	-0.0044	SRTASGSSVTSLEGTR
10.8	1754.7352	-0.0159	DVSCSLTNTHFCASR
9.2	1754.7237	-0.0044	SRTASGSSVTSLEGTR
5.5	1754.7252	-0.0059	IPMNASWKTAFGTR
3.8	1753.7090	1.0104	LLASPNPTISSTSAR
3.8	1752.6962	2.0231	NQAIYAAVDDEDANDE

Spectrum No: 268; Query: 2665; Rank: 1

Peptide View

MS/MS Fragmentation of **NYQQNYQNSESGEKNEGSESAPEGQAQQR**
Found in **IPI00551815**, Tax_Id=10116 Gene_Symbol=Ybx1-ps3 Ybx1 protein

Match to Query 2665: 3336.366012 from(1113.129280,3+)
Title: 100101RatKid_NS_deglyco_19.804.804.3.dta
Data file K:\NewmanPaper\Piliang\3SubProteomes\Piliang3SP\mgf5ppm\ERLIC_3SubProteomes5ppm.mgf

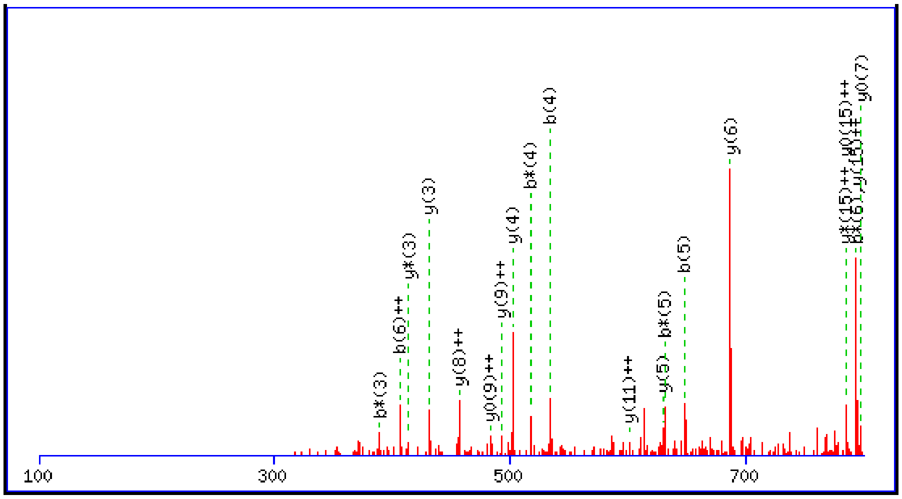

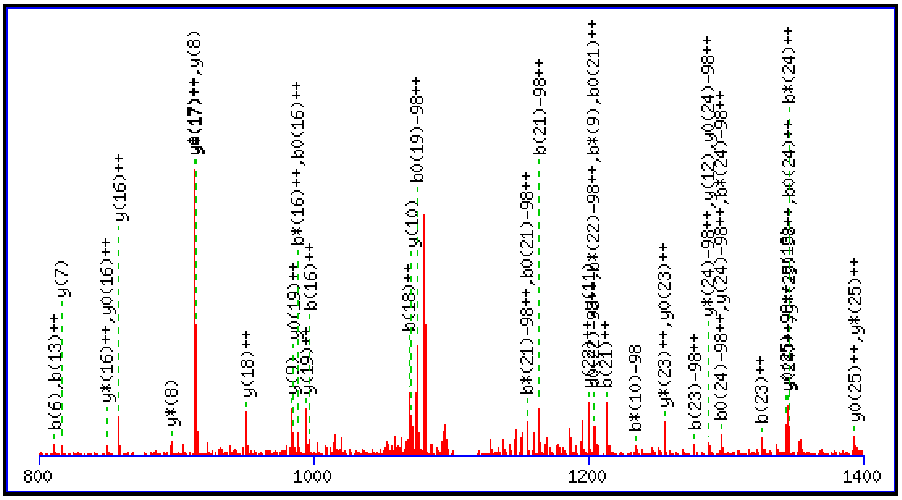

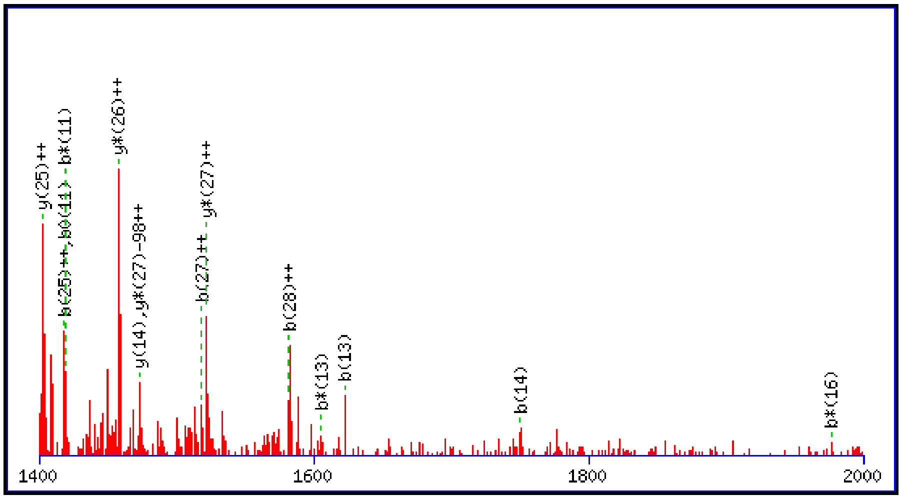

Monoisotopic mass of neutral peptide Mr(calc): 3336.3552
Fixed modifications: Carbamidomethyl (C)
Variable modifications:
S9 : Phospho (ST), with neutral losses 0.0000(shown in table), 97.9769
Ions Score: 52 **Expect:** 0.0025
Matches (Bold Red): 84/476 fragment ions using 124 most intense peaks

#	b	b^{++}	b*	b^{*++}	b^{0}	b^{0++}	Seq.	y	y^{++}	y*	y^{*++}	y^{0}	y^{0++}	#
1	115.0502	58.0287	98.0237	49.5155			N							29
2	278.1135	139.5604	261.0870	131.0471			Y	3223.3196	1612.1634	3206.2931	1603.6502	3205.3090	1603.1582	28
3	**406.1721**	203.5897	**389.1456**	195.0764			Q	3060.2563	1530.6318	3043.2297	**1522.1185**	3042.2457	1521.6265	27
4	**534.2307**	267.6190	**517.2041**	259.1057			Q	2932.1977	1466.6025	2915.1712	**1458.0892**	2914.1871	1457.5972	26
5	**648.2736**	324.6404	**631.2471**	316.1272			N	2804.1391	**1402.5732**	2787.1126	**1394.0599**	2786.1286	**1393.5679**	25

6	811.3369	406.1721	794.3104	397.6588			Y	2690.0962	1345.5517	2673.0696	1337.0385	2672.0856	1336.5465	24
7	939.3955	470.2014	922.3690	461.6881			Q	2527.0329	1264.0201	2510.0063	1255.5068	2509.0223	1255.0148	23
8	1053.4384	527.2229	1036.4119	518.7096			N	2398.9743	1199.9908	2381.9477	1191.4775	2380.9637	1190.9855	22
9	1220.4368	610.7220	1203.4103	602.2088	1202.4262	601.7168	S	2284.9314	1142.9693	2267.9048	1134.4560	2266.9208	1133.9640	21
10	1349.4794	675.2433	1332.4528	666.7301	1331.4688	666.2381	E	2117.9330	1059.4701	2100.9065	1050.9569	2099.9224	1050.4649	20
11	1436.5114	718.7593	1419.4849	710.2461	1418.5009	709.7541	S	1988.8904	994.9488	1971.8639	986.4356	1970.8799	985.9436	19
12	1493.5329	747.2701	1476.5063	738.7568	1475.5223	738.2648	G	1901.8584	951.4328	1884.8318	942.9196	1883.8478	942.4275	18
13	1622.5755	811.7914	1605.5489	803.2781	1604.5649	802.7861	E	1844.8369	922.9221	1827.8104	914.4088	1826.8264	913.9168	17
14	1750.6704	875.8389	1733.6439	867.3256	1732.6599	866.8336	K	1715.7943	858.4008	1698.7678	849.8875	1697.7838	849.3955	16
15	1864.7134	932.8603	1847.6868	924.3470	1846.7028	923.8550	N	1587.6994	794.3533	1570.6728	785.8400	1569.6888	785.3480	15
16	1993.7560	997.3816	1976.7294	988.8683	1975.7454	988.3763	E	1473.6564	737.3319	1456.6299	728.8186	1455.6459	728.3266	14
17	2050.7774	1025.8924	2033.7509	1017.3791	2032.7669	1016.8871	G	1344.6138	672.8106	1327.5873	664.2973	1326.6033	663.8053	13
18	2137.8095	1069.4084	2120.7829	1060.8951	2119.7989	1060.4031	S	1287.5924	644.2998	1270.5658	635.7866	1269.5818	635.2945	12
19	2266.8520	1133.9297	2249.8255	1125.4164	2248.8415	1124.9244	E	1200.5604	600.7838	1183.5338	592.2705	1182.5498	591.7785	11
20	2353.8841	1177.4457	2336.8575	1168.9324	2335.8735	1168.4404	S	1071.5178	536.2625	1054.4912	527.7492	1053.5072	527.2572	10
21	2424.9212	1212.9642	2407.8946	1204.4510	2406.9106	1203.9590	A	984.4857	492.7465	967.4592	484.2332	966.4752	483.7412	9
22	2521.9740	1261.4906	2504.9474	1252.9773	2503.9634	1252.4853	P	913.4486	457.2279	896.4221	448.7147	895.4381	448.2227	8
23	2651.0165	1326.0119	2633.9900	1317.4986	2633.0060	1317.0066	E	816.3959	408.7016	799.3693	400.1883	798.3853	399.6963	7
24	2708.0380	1354.5226	2691.0115	1346.0094	2690.0274	1345.5174	G	687.3533	344.1803	670.3267	335.6670			6
25	2836.0966	1418.5519	2819.0700	1410.0387	2818.0860	1409.5466	Q	630.3318	315.6695	613.3053	307.1563			5
26	2907.1337	1454.0705	2890.1072	1445.5572	2889.1231	1445.0652	A	502.2732	251.6402	485.2467	243.1270			4
27	3035.1923	1518.0998	3018.1657	1509.5865	3017.1817	1509.0945	Q	431.2361	216.1217	414.2096	207.6084			3
28	3163.2509	1582.1291	3146.2243	1573.6158	3145.2403	1573.1238	Q	303.1775	152.0924	286.1510	143.5791			2
29							R	175.1190	88.0631	158.0924	79.5498			1

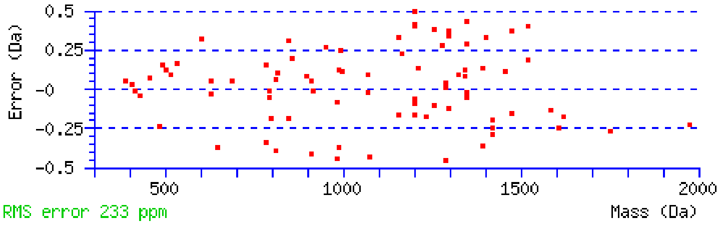

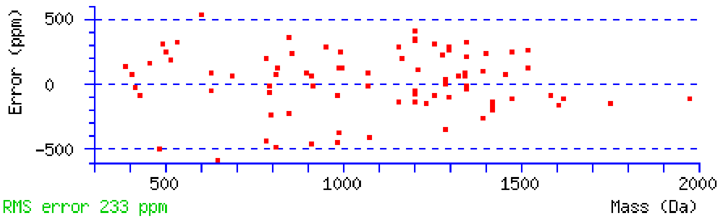

All matches to this query

Score	Mr(calc):	Delta	Sequence
51.9	3336.3552	0.0108	NYQQNYQNSESGEKNEGSESAPEGQAQQR
47.3	3336.3552	0.0108	NYQQNYQNSESGEKNEGSESAPEGQAQQR
45.3	3336.3552	0.0108	NYQQNYQNSESGEKNEGSESAPEGQAQQR
26.6	3336.3552	0.0108	NYQQNYQNSESGEKNEGSESAPEGQAQQR
23.4	3336.3552	0.0108	NYQQNYQNSESGEKNEGSESAPEGQAQQR
16.5	3336.3552	0.0108	NYQQNYQNSESGEKNEGSESAPEGQAQQR
8.2	3336.3845	-0.0184	TALLFLSSSLGTERLSSGAGMLDIQELK
4.3	3336.3845	-0.0184	TALLFLSSSLGTERLSSGAGMLDIQELK
4.1	3336.3968	-0.0308	EMTPSSFTDLGPGSYAMASTLLASSGQVEAR
2.1	3336.3448	0.0212	TGSVGSDLQFLGTANGHQDKVCASLNFGSK

Spectrum No: 269; Query: 1237; Rank: 1

Peptide View

MS/MS Fragmentation of **VIENTDGSEEEMDAR**
Found in **IPI00464464**, Tax_Id=10116 Gene_Symbol=Myh11 Isoform 1 of Myosin-11 (Fragment)

Match to Query 1237: 1789.678288 from(895.846420,2+)
Title: 091008RatKidney_NH4Format01_25.671.671.2.dta
Data file K:\NewmanPaper\Piliang\3SubProteomes\Piliang3SP\mgf5ppm\ERLIC_3SubProteomes5ppm.mgf

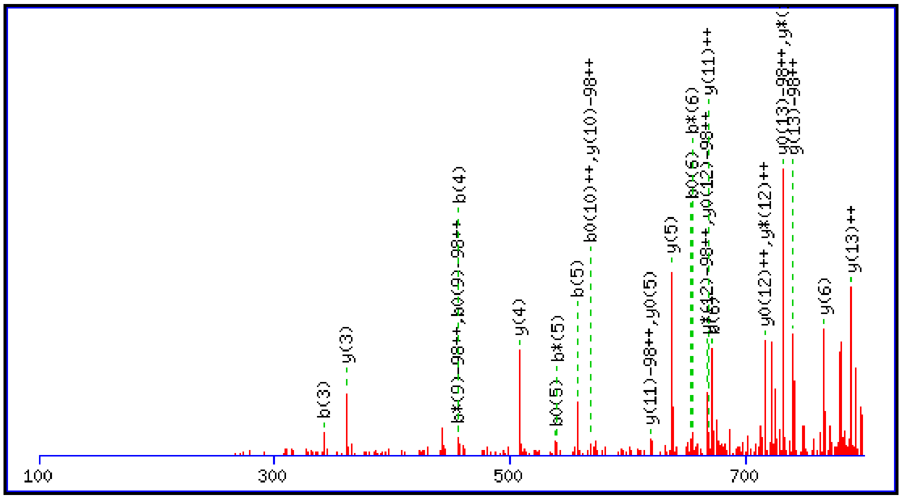

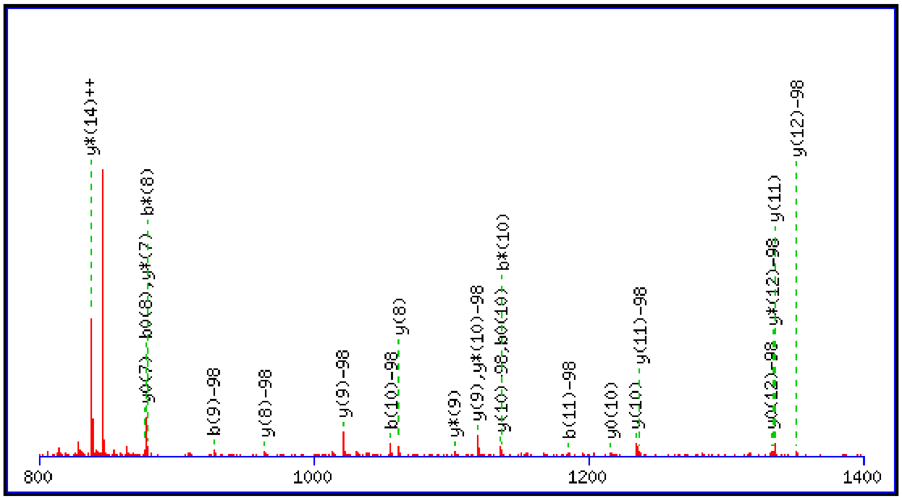

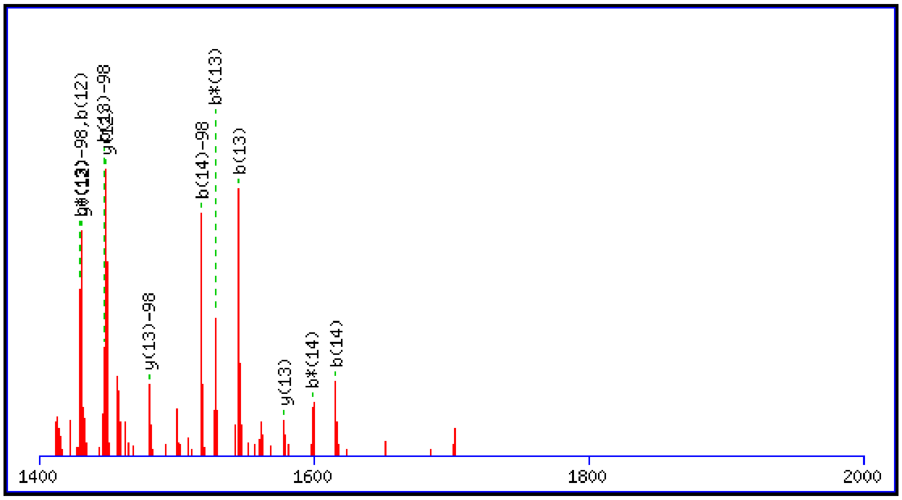

Monoisotopic mass of neutral peptide Mr(calc): 1789.6713
Fixed modifications: Carbamidomethyl (C)
Variable modifications:
S8 : Phospho (ST), with neutral losses 97.9769(shown in table), 0.0000
M12 : Oxidation (M)
Ions Score: 52 **Expect:** 0.00085
Matches (Bold Red): 63/238 fragment ions using 85 most intense peaks

		++		++	0	0++			++		++	0	0++	

#	b	b	b*	b*	b	b	Seq.	y	y	y*	y*	y	y	#
1	100.0757	50.5415					V							15
2	213.1598	107.0835					I	1593.6333	797.3203	1576.6068	788.8070	1575.6228	788.3150	14
3	**342.2023**	171.6048			324.1918	162.5995	E	**1480.5493**	**740.7783**	1463.5227	**732.2650**	1462.5387	**731.7730**	13
4	**456.2453**	228.6263	439.2187	220.1130	438.2347	219.6210	N	**1351.5067**	676.2570	**1334.4801**	**667.7437**	**1333.4961**	**667.2517**	12
5	**557.2930**	279.1501	**540.2664**	270.6368	**539.2824**	270.1448	T	**1237.4637**	**619.2355**	1220.4372	610.7222	1219.4532	610.2302	11
6	**672.3199**	336.6636	**655.2933**	328.1503	**654.3093**	327.6583	D	**1136.4161**	**568.7117**	**1119.3895**	560.1984	1118.4055	559.7064	10
7	729.3414	365.1743	712.3148	356.6610	711.3308	356.1690	G	**1021.3891**	511.1982	1004.3626	502.6849	1003.3785	502.1929	9
8	798.3628	399.6850	781.3363	391.1718	780.3523	390.6798	S	**964.3676**	482.6875	947.3411	474.1742	946.3571	473.6822	8
9	**927.4054**	464.2063	910.3789	**455.6931**	909.3948	**455.2011**	E	895.3462	448.1767	**878.3196**	439.6635	**877.3356**	439.1715	7
10	**1056.4480**	528.7276	1039.4215	520.2144	1038.4374	519.7224	E	**766.3036**	383.6554	749.2771	375.1422	748.2930	374.6502	6
11	**1185.4906**	593.2489	1168.4640	584.7357	1167.4800	584.2437	E	**637.2610**	319.1341	620.2345	310.6209	**619.2504**	310.1289	5
12	1332.5260	666.7666	1315.4995	658.2534	1314.5154	657.7614	M	**508.2184**	254.6128	491.1919	246.0996	490.2078	245.6076	4
13	**1447.5529**	724.2801	**1430.5264**	715.7668	1429.5424	715.2748	D	**361.1830**	181.0951	344.1565	172.5819	343.1724	172.0899	3
14	**1518.5901**	759.7987	1501.5635	751.2854	1500.5795	750.7934	A	246.1561	123.5817	229.1295	115.0684			2
15							R	175.1190	88.0631	158.0924	79.5498			1

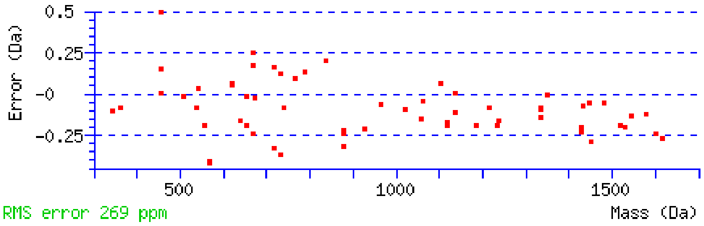

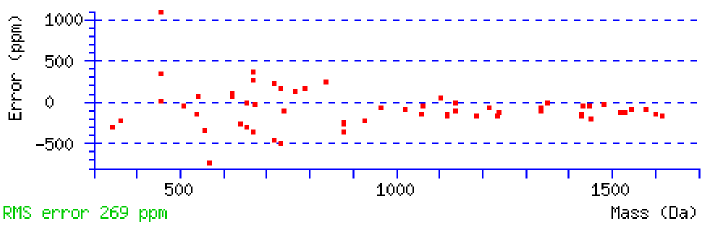

All matches to this query

Score	Mr(calc):	Delta	Sequence
51.9	1789.6713	0.0070	VIENTDGSEEEMDAR
38.1	1789.6713	0.0070	VIENTDGSEEEMDAR
12.4	1789.6801	-0.0018	CLANGSWTDMDTPSR
10.8	1789.6866	-0.0083	CAEPLFESNNSVNAR
10.8	1789.6866	-0.0083	CAEPLFESNNSVNAR
10.4	1789.6801	-0.0018	CLANGSWTDMDTPSR
2.4	1789.6817	-0.0034	MPNGLDSMSLSPPGAR
2.3	1789.6900	-0.0117	TAMINGETGSHSMDVK
2.3	1789.6900	-0.0117	TAMINGETGSHSMDVK
2.3	1789.6900	-0.0117	TAMINGETGSHSMDVK

Spectrum No: 270; Query: 1143; Rank: 1

Peptide View

MS/MS Fragmentation of **ISVPALPGLTPSSTQTP**
Found in **IPI00188142**, Tax_Id=10116 Gene_Symbol=Acy3 Aspartoacylase (Aminoacylase) 3

Match to Query 1143: 1744.866988 from(873.440770,2+)
Title: 091008RatKidney_NH4Format01_25.3664.3664.2.dta
Data file K:\NewmanPaper\Piliang\3SubProteomes\Piliang3SP\mgf5ppm\ERLIC_3SubProteomes5ppm.mgf

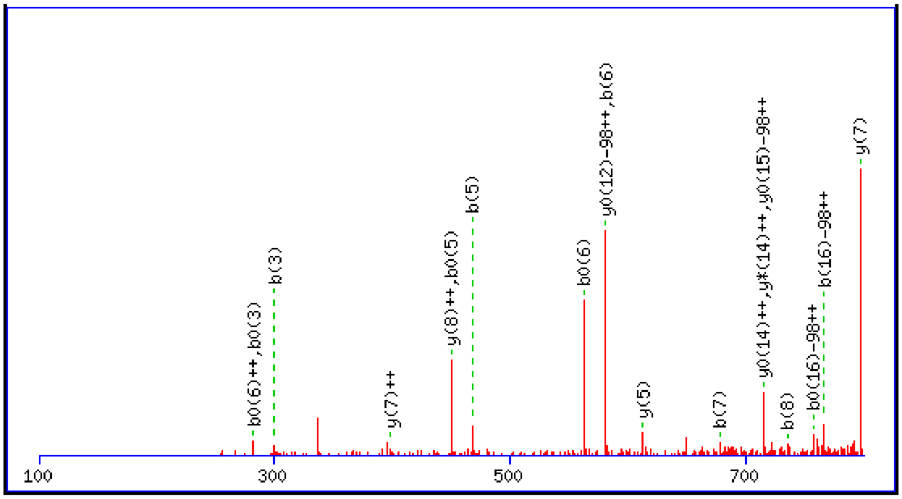

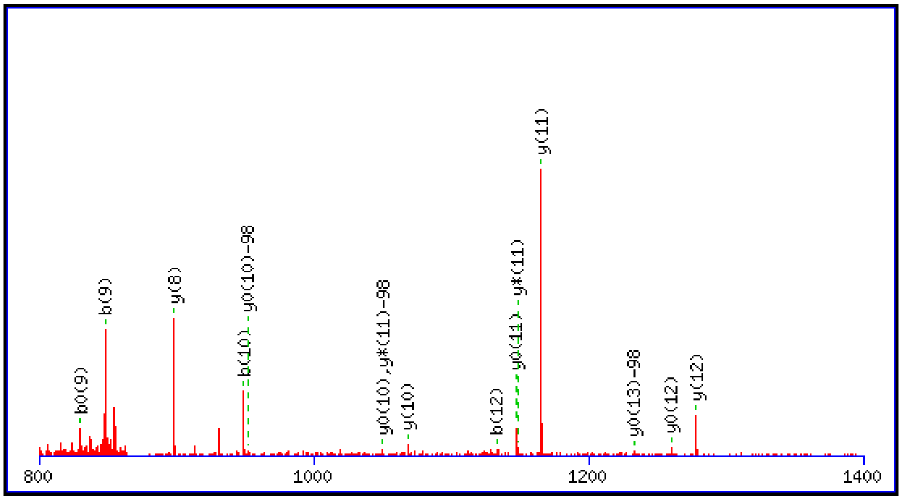

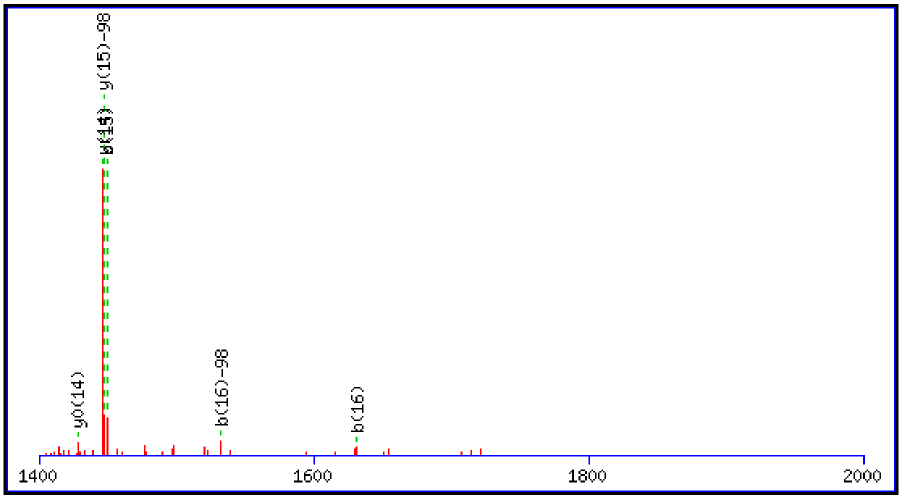

Monoisotopic mass of neutral peptide Mr(calc): 1744.8648
Fixed modifications: Carbamidomethyl (C)
Variable modifications:
T16 : Phospho (ST), with neutral losses 0.0000(shown in table), 97.9769
Ions Score: 52 **Expect:** 0.0011
Matches (Bold Red): 40/250 fragment ions using 67 most intense peaks

#	b	b^{++}	b*	b^{*++}	b^0	b^{0++}	Seq.	y	y^{++}	y*	y^{*++}	y^0	y^{0++}	#
1	114.0913	57.5493					I							17
2	201.1234	101.0653			183.1128	92.0600	S	1632.7880	816.8977	1615.7615	808.3844	1614.7775	807.8924	16
3	**300.1918**	150.5995			**282.1812**	141.5942	V	1545.7560	773.3816	1528.7295	764.8684	1527.7454	764.3764	15
4	397.2445	199.1259			379.2340	190.1206	P	**1446.6876**	723.8474	1429.6610	**715.3342**	**1428.6770**	**714.8422**	14
5	**468.2817**	234.6445			**450.2711**	225.6392	A	1349.6348	675.3211	1332.6083	666.8078	1331.6243	666.3158	13

**6**	**581.3657**	291.1865			**563.3552**	**282.1812**	**L**	**1278.5977**	639.8025	1261.5712	631.2892	**1260.5872**	630.7972	**12**
**7**	**678.4185**	339.7129			660.4079	330.7076	**P**	**1165.5137**	583.2605	**1148.4871**	574.7472	**1147.5031**	574.2552	**11**
**8**	**735.4400**	368.2236			717.4294	359.2183	**G**	**1068.4609**	534.7341	1051.4343	526.2208	**1050.4503**	525.7288	**10**
**9**	**848.5240**	424.7656			**830.5135**	415.7604	**L**	1011.4394	506.2234	994.4129	497.7101	993.4289	497.2181	**9**
**10**	**949.5717**	475.2895			931.5611	466.2842	**T**	**898.3554**	**449.6813**	881.3288	441.1680	880.3448	440.6760	**8**
**11**	1046.6245	523.8159			1028.6139	514.8106	**P**	**797.3077**	**399.1575**	780.2811	390.6442	779.2971	390.1522	**7**
**12**	**1133.6565**	567.3319			1115.6459	558.3266	**S**	700.2549	350.6311	683.2284	342.1178	682.2444	341.6258	**6**
**13**	1220.6885	610.8479			1202.6780	601.8426	**S**	**613.2229**	307.1151	596.1963	298.6018	595.2123	298.1098	**5**
**14**	1321.7362	661.3717			1303.7256	652.3665	**T**	526.1909	263.5991	509.1643	255.0858	508.1803	254.5938	**4**
**15**	**1449.7948**	725.4010	1432.7682	716.8877	1431.7842	716.3957	**Q**	425.1432	213.0752	408.1166	204.5620	407.1326	204.0699	**3**
**16**	**1630.8088**	815.9080	1613.7822	807.3948	1612.7982	806.9027	**T**	297.0846	149.0459			279.0740	140.0407	**2**
**17**							**P**	116.0706	58.5389					**1**

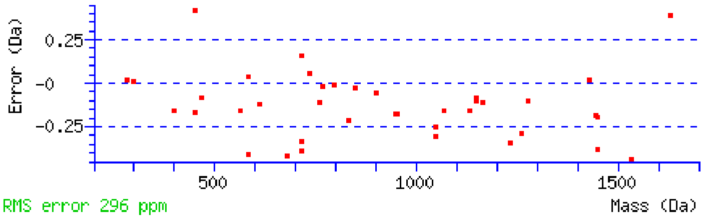

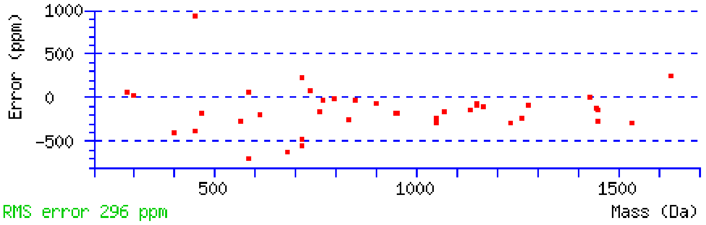

All matches to this query

Score	Mr(calc):	Delta	Sequence
51.9	1744.8648	0.0022	ISVPALPGLTPSSTQTP
42.2	1744.8648	0.0022	ISVPALPGLTPSSTQTP
42.2	1744.8648	0.0022	ISVPALPGLTPSSTQTP
32.5	1744.8648	0.0022	ISVPALPGLTPSSTQTP
30.1	1744.8648	0.0022	ISVPALPGLTPSSTQTP
6.7	1744.8549	0.0121	NFTSINSLPQLFKR
6.0	1743.8709	0.9961	NFTSINSLPQLFKR
4.3	1744.8549	0.0121	NFTSINSLPQLFKR
1.7	1742.8716	1.9954	QLRTDSLLREFXSK
1.3	1744.8632	0.0038	DSFKHLIGGLDDVSNK

Spectrum No: 271; Query: 1658; Rank: 1

Peptide View

MS/MS Fragmentation of **TSEPEAETNAGNSPVTTNR**
Found in **IPI00367930**, Tax_Id=10116 Gene_Symbol=RGD1562952_predicted similar to Erbb2 interacting protein isoform 1

Match to Query 1658: 2053.859562 from(685.627130,3+)
Title: 091008RatKidney_NoSalt_26.560.560.3.dta
Data file K:\NewmanPaper\Piliang\3SubProteomes\Piliang3SP\mgf5ppm\ERLIC_3SubProteomes5ppm.mgf

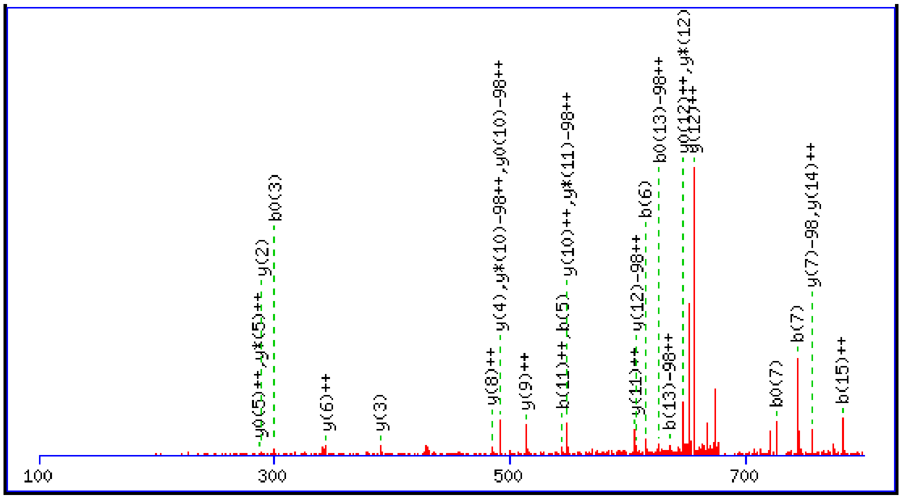

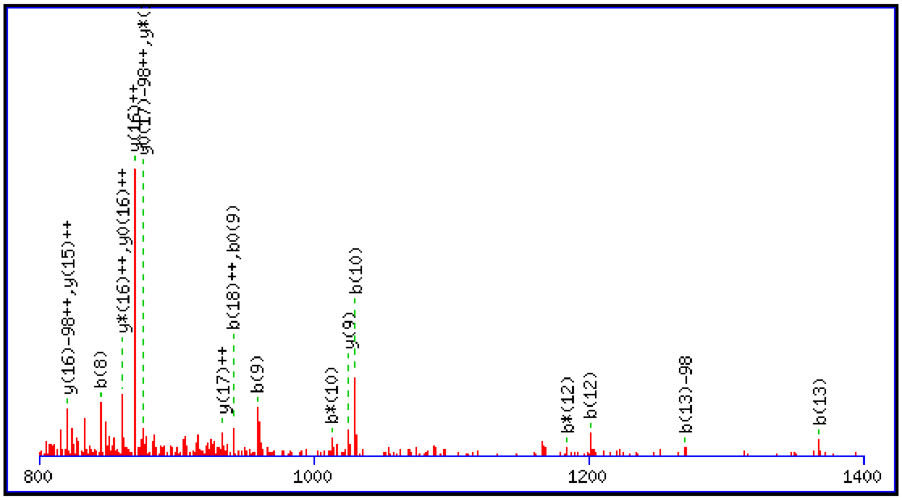

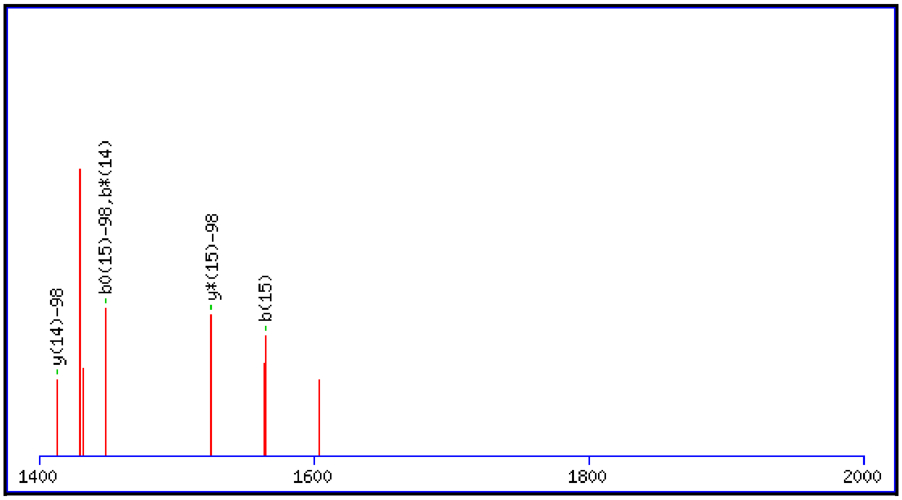

Monoisotopic mass of neutral peptide Mr(calc): 2053.8589
Fixed modifications: Carbamidomethyl (C)
Variable modifications:
S13 : Phospho (ST), with neutral losses 0.0000(shown in table), 97.9769
Ions Score: 52 **Expect:** 0.0015
Matches (Bold Red): 52/304 fragment ions using 68 most intense peaks

#	b	b++	b*	b*++	b0	b0++	Seq.	y	y++	y*	y*++	y0	y0++	#
1	102.0550	51.5311			84.0444	42.5258	T							19
2	189.0870	95.0471			171.0764	86.0418	S	1953.8186	977.4129	1936.7920	968.8996	1935.8080	968.4076	18
3	318.1296	159.5684			**300.1190**	150.5631	E	1866.7865	**933.8969**	1849.7600	925.3836	1848.7760	924.8916	17
4	415.1823	208.0948			397.1718	199.0895	P	1737.7439	**869.3756**	1720.7174	**860.8623**	1719.7334	**860.3703**	16
5	**544.2249**	272.6161			526.2144	263.6108	E	1640.6912	**820.8492**	1623.6646	812.3360	1622.6806	811.8439	15

6	**615.2620**	308.1347			597.2515	299.1294	A	1511.6486	**756.3279**	1494.6220	747.8147	1493.6380	747.3226	14
7	**744.3046**	372.6560			**726.2941**	363.6507	E	1440.6115	720.8094	1423.5849	712.2961	1422.6009	711.8041	13
8	**845.3523**	423.1798			827.3418	414.1745	T	1311.5689	**656.2881**	1294.5423	**647.7748**	1293.5583	**647.2828**	12
9	**959.3952**	480.2013	942.3687	471.6880	**941.3847**	471.1960	N	1210.5212	**605.7642**	1193.4947	597.2510	1192.5106	596.7590	11
10	**1030.4324**	515.7198	**1013.4058**	507.2065	1012.4218	506.7145	A	1096.4783	**548.7428**	1079.4517	540.2295	1078.4677	539.7375	10
11	1087.4538	**544.2305**	1070.4273	535.7173	1069.4433	535.2253	G	**1025.4412**	**513.2242**	1008.4146	504.7109	1007.4306	504.2189	9
12	**1201.4968**	601.2520	**1184.4702**	592.7387	1183.4862	592.2467	N	968.4197	**484.7135**	951.3931	476.2002	950.4091	475.7082	8
13	**1368.4951**	684.7512	1351.4686	676.2379	1350.4845	675.7459	S	854.3768	427.6920	837.3502	419.1787	836.3662	418.6867	7
14	1465.5479	733.2776	**1448.5213**	724.7643	1447.5373	724.2723	P	687.3784	**344.1928**	670.3519	335.6796	669.3679	335.1876	6
15	**1564.6163**	**782.8118**	1547.5897	774.2985	1546.6057	773.8065	V	590.3257	295.6665	573.2991	**287.1532**	572.3151	**286.6612**	5
16	1665.6640	833.3356	1648.6374	824.8223	1647.6534	824.3303	T	**491.2572**	246.1323	474.2307	237.6190	473.2467	237.1270	4
17	1766.7116	883.8595	1749.6851	875.3462	1748.7011	874.8542	T	**390.2096**	195.6084	373.1830	187.0951	372.1990	186.6031	3
18	1880.7546	**940.8809**	1863.7280	932.3676	1862.7440	931.8756	N	**289.1619**	145.0846	272.1353	136.5713			2
19							R	175.1190	88.0631	158.0924	79.5498			1

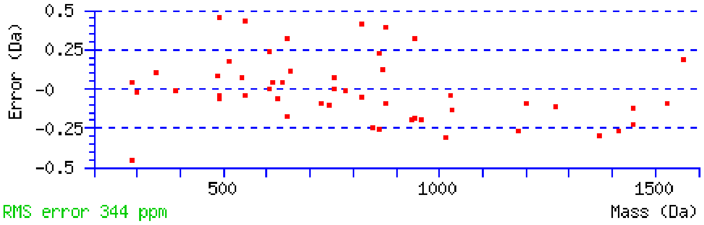

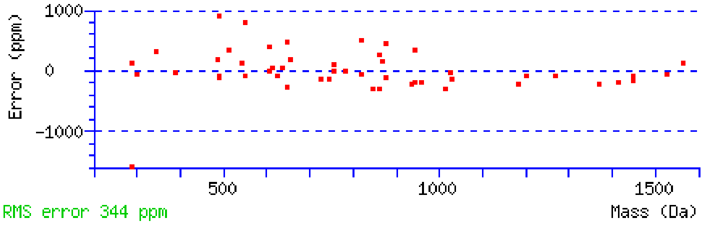

All matches to this query

Score	Mr(calc):	Delta	Sequence
51.9	2053.8589	0.0006	TSEPEAETNAGNSPVTTNR
38.3	2053.8589	0.0006	TSEPEAETNAGNSPVTTNR
31.8	2053.8589	0.0006	TSEPEAETNAGNSPVTTNR
24.8	2053.8589	0.0006	TSEPEAETNAGNSPVTTNR
4.7	2053.8425	0.0170	DRNNPMHSGPQXSSGATR
3.4	2053.8589	0.0006	TSEPEAETNAGNSPVTTNR
3.4	2053.8589	0.0006	TSEPEAETNAGNSPVTTNR
3.3	2051.8674	1.9921	TRSQTSVSSSAGLSSAPGGR
3.2	2051.8659	1.9937	FKFTSVTDSLEQVLAK
3.2	2051.8659	1.9937	FKFTSVTDSLEQVLAK

Spectrum No: 272; Query: 2494; Rank: 1

Peptide View

MS/MS Fragmentation of **EELEQQTDGDCDEEDDDKDGEMPK**
Found in **IPI00382218**, Tax_Id=10116 Gene_Symbol=Tloc1 Ab2-292

Match to Query 2494: 2922.032472 from(975.018100,3+)
Title: 100101RatKid_NS_deglyco_25.767.767.3.dta
Data file K:\NewmanPaper\Piliang\3SubProteomes\Piliang3SP\mgf5ppm\ERLIC_3SubProteomes5ppm.mgf

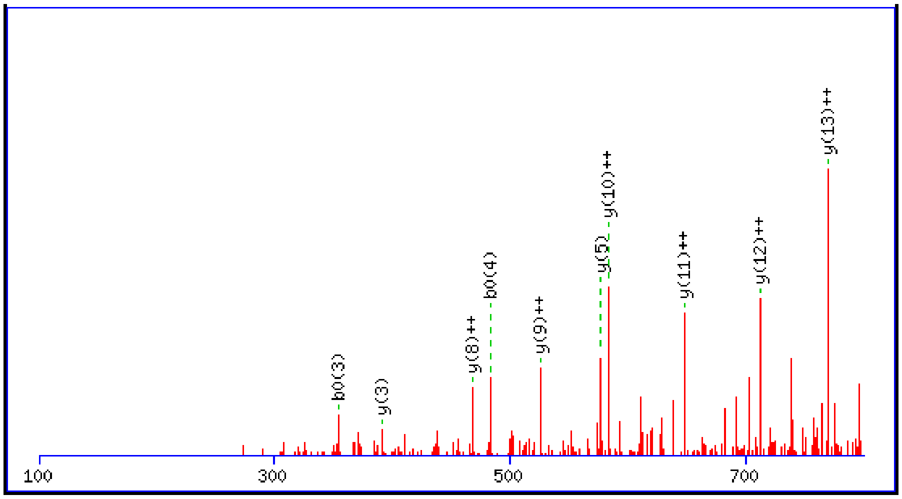

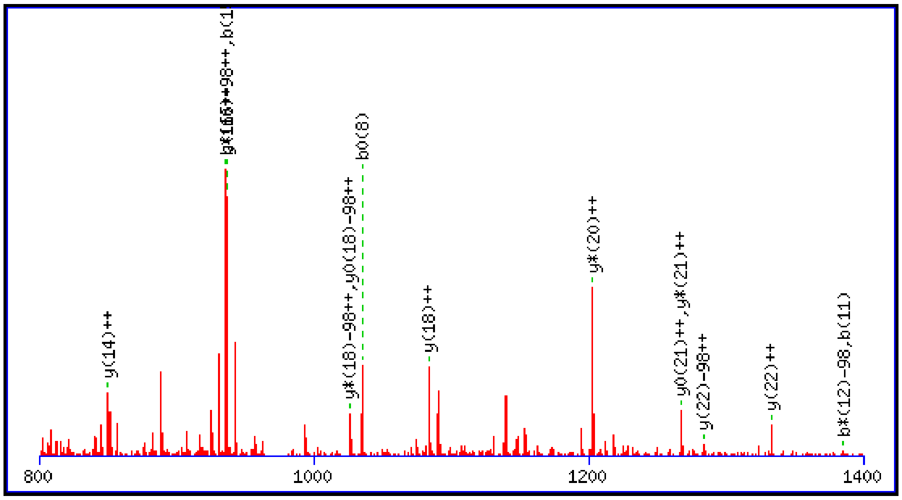

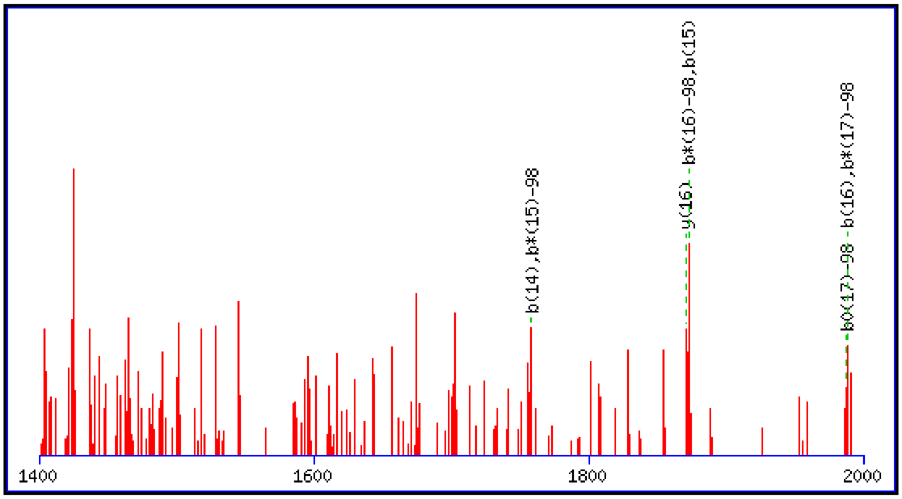

Monoisotopic mass of neutral peptide Mr(calc): 2922.0216
Fixed modifications: Carbamidomethyl (C)
Variable modifications:
T7 : Phospho (ST), with neutral losses 0.0000(shown in table), 97.9769
M22 : Oxidation (M)
Ions Score: 52 **Expect:** 0.00089
Matches (Bold Red): 35/400 fragment ions using 47 most intense peaks

#	b	b++	b*	b*++	b0	b0++	Seq.	y	y++	y*	y*++	y0	y0++	#
1	130.0499	65.5286			112.0393	56.5233	E							24
2	259.0925	130.0499			241.0819	121.0446	E	2793.9863	1397.4968	2776.9598	1388.9835	2775.9757	1388.4915	23
3	372.1765	186.5919			**354.1660**	177.5866	L	2664.9437	**1332.9755**	2647.9172	1324.4622	2646.9331	1323.9702	22
4	501.2191	251.1132			**483.2086**	242.1079	E	2551.8596	1276.4335	2534.8331	**1267.9202**	2533.8491	**1267.4282**	21

**5**	629.2777	315.1425	612.2511	306.6292	611.2671	306.1372	**Q**	2422.8171	1211.9122	2405.7905	**1203.3989**	2404.8065	1202.9069	**20**
**6**	757.3363	379.1718	740.3097	370.6585	739.3257	370.1665	**Q**	2294.7585	1147.8829	2277.7319	1139.3696	2276.7479	1138.8776	**19**
**7**	938.3503	469.6788	921.3237	461.1655	920.3397	460.6735	**T**	2166.6999	**1083.8536**	2149.6733	1075.3403	2148.6893	1074.8483	**18**
**8**	1053.3772	527.1922	1036.3507	518.6790	**1035.3667**	518.1870	**D**	1985.6859	993.3466	1968.6593	984.8333	1967.6753	984.3413	**17**
**9**	1110.3987	555.7030	1093.3721	547.1897	1092.3881	546.6977	**G**	**1870.6589**	**935.8331**	1853.6324	927.3198	1852.6484	926.8278	**16**
**10**	1225.4256	613.2165	1208.3991	604.7032	1207.4151	604.2112	**D**	1813.6375	907.3224	1796.6109	898.8091	1795.6269	898.3171	**15**
**11**	**1385.4563**	693.2318	1368.4297	684.7185	1367.4457	684.2265	**C**	1698.6105	**849.8089**	1681.5840	841.2956	1680.6000	840.8036	**14**
**12**	1500.4832	750.7452	1483.4567	742.2320	1482.4727	741.7400	**D**	1538.5799	**769.7936**	1521.5533	761.2803	1520.5693	760.7883	**13**
**13**	1629.5258	815.2665	1612.4993	806.7533	1611.5152	806.2613	**E**	1423.5530	**712.2801**	1406.5264	703.7668	1405.5424	703.2748	**12**
**14**	**1758.5684**	879.7878	1741.5419	871.2746	1740.5578	870.7826	**E**	1294.5104	**647.7588**	1277.4838	639.2455	1276.4998	638.7535	**11**
**15**	**1873.5953**	**937.3013**	1856.5688	928.7880	1855.5848	928.2960	**D**	1165.4678	**583.2375**	1148.4412	574.7242	1147.4572	574.2322	**10**
**16**	**1988.6223**	994.8148	1971.5957	986.3015	1970.6117	985.8095	**D**	1050.4408	**525.7240**	1033.4143	517.2108	1032.4303	516.7188	**9**
**17**	2103.6492	1052.3283	2086.6227	1043.8150	2085.6387	1043.3230	**D**	935.4139	**468.2106**	918.3873	459.6973	917.4033	459.2053	**8**
**18**	2231.7442	1116.3757	2214.7176	1107.8625	2213.7336	1107.3705	**K**	820.3869	410.6971	803.3604	402.1838	802.3764	401.6918	**7**
**19**	2346.7711	1173.8892	2329.7446	1165.3759	2328.7606	1164.8839	**D**	692.2920	346.6496	675.2654	338.1363	674.2814	337.6443	**6**
**20**	2403.7926	1202.3999	2386.7661	1193.8867	2385.7820	1193.3947	**G**	**577.2650**	289.1362	560.2385	280.6229	559.2545	280.1309	**5**
**21**	2532.8352	1266.9212	2515.8086	1258.4080	2514.8246	1257.9160	**E**	520.2436	260.6254	503.2170	252.1121	502.2330	251.6201	**4**
**22**	2679.8706	1340.4389	2662.8441	1331.9257	2661.8600	1331.4337	**M**	**391.2010**	196.1041	374.1744	187.5908			**3**
**23**	2776.9234	1388.9653	2759.8968	1380.4520	2758.9128	1379.9600	**P**	244.1656	122.5864	227.1390	114.0731			**2**
**24**							**K**	147.1128	74.0600	130.0863	65.5468			**1**

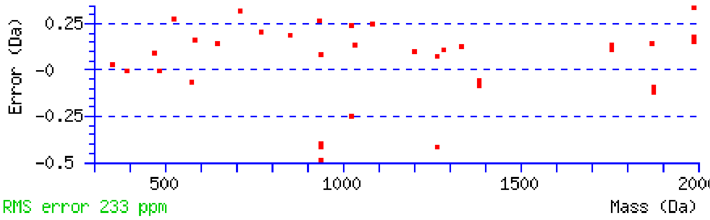

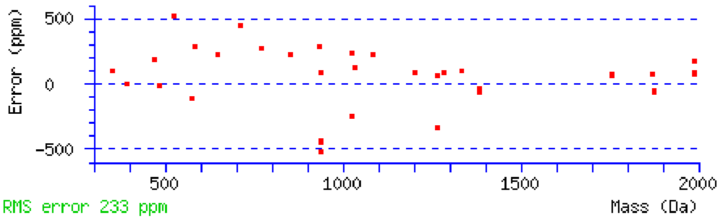

All matches to this query

Score	Mr(calc):	Delta	Sequence
51.8	2922.0216	0.0109	EELEQQTDGDCDEEDDDKDGEMPK
4.7	2920.0311	2.0014	GNGTGAQDPSTDHTCXPHLSNYHTR
4.1	2920.0311	2.0014	GNGTGAQDPSTDHTCXPHLSNYHTR
2.7	2920.0101	2.0224	EANYSVPLNVSEVVVYDSTISR
2.7	2921.0501	0.9824	DLDAYLVQNITCGRDGSEPQDSND
2.7	2922.0341	-0.0016	DLDAYLVQNITCGRDGSEPQDSND
2.2	2921.0106	1.0219	DGGEPCSPPGPFDGCEDDYNYYSRS
2.2	2921.0106	1.0219	DGGEPCSPPGPFDGCEDDYNYYSRS
1.0	2921.0160	1.0165	TDEFQLHTNVNDGTEFGGSIYQK
0.9	2920.0352	1.9972	AVYCYDPVEDYWMHVQNTFSR

Spectrum No: 273; Query: 2519; Rank: 1

Peptide View

MS/MS Fragmentation of **NTSPHSEPCSSDSVSEPECTTDSSSSK**
Found in **IPI00204814**, Tax_Id=10116 Gene_Symbol=Vps54 Vacuolar protein sorting-associated protein 54

Match to Query 2519: 2978.119272 from(993.713700,3+)
Title: 100101RatKid_NS_deglyco_20.777.777.3.dta

Data file K:\NewmanPaper\Piliang\3SubProteomes\Piliang3SP\mgf5ppm\ERLIC_3SubProteomes5ppm.mgf

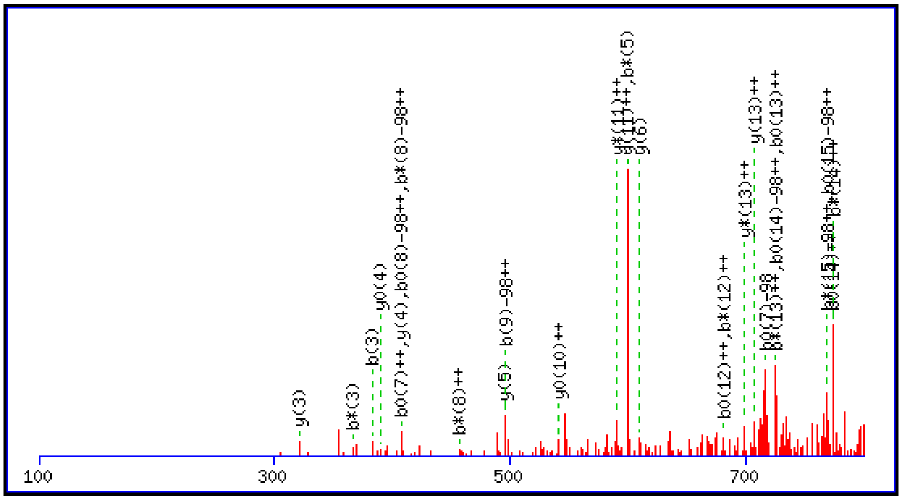

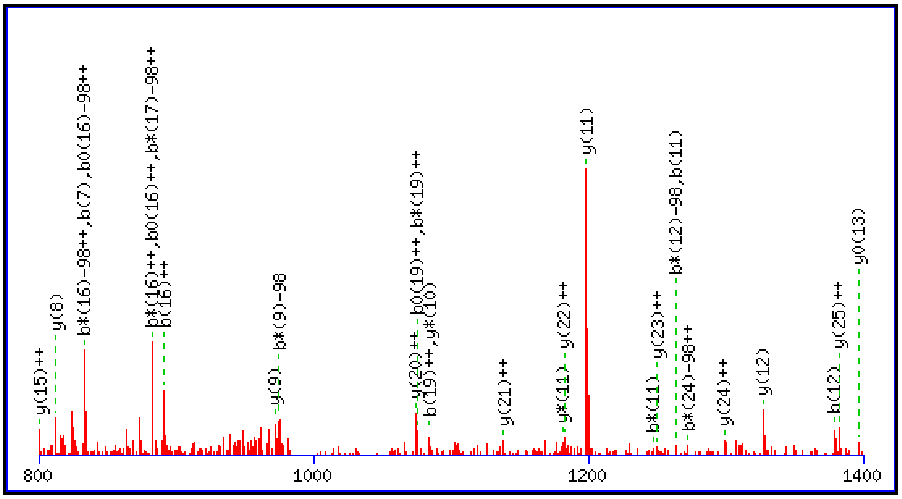

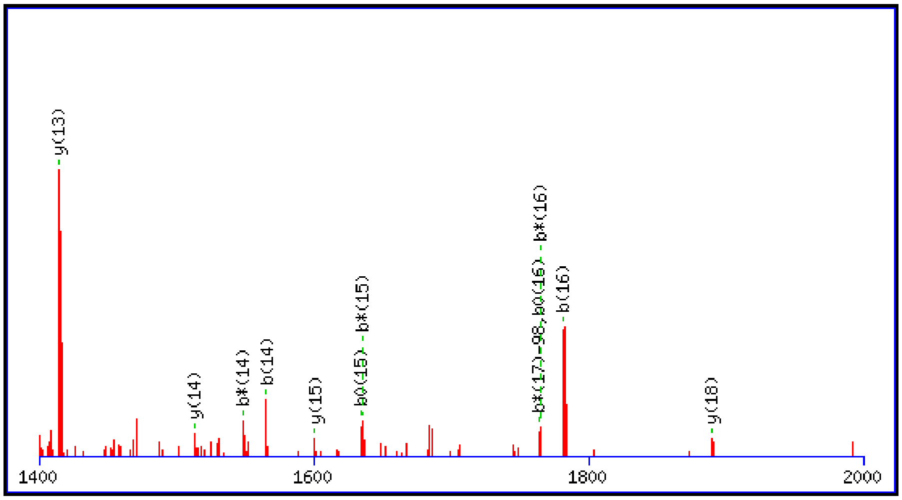

Monoisotopic mass of neutral peptide Mr(calc): 2978.1067
Fixed modifications: Carbamidomethyl (C)
Variable modifications:
S3 : Phospho (ST), with neutral losses 0.0000(shown in table), 97.9769
Ions Score: 52 **Expect:** 0.0014
Matches (Bold Red): 70/464 fragment ions using 114 most intense peaks

#	b	b^{++}	b*	b^{*++}	b^0	b^{0++}	Seq.	y	y^{++}	y*	y^{*++}	y^0	y^{0++}	#
1	115.0502	58.0287	98.0237	49.5155			N							27
2	216.0979	108.5526	199.0713	100.0393	198.0873	99.5473	T	2865.0710	1433.0392	2848.0445	1424.5259	2847.0605	1424.0339	26
3	**383.0962**	192.0518	**366.0697**	183.5385	365.0857	183.0465	S	2764.0233	**1382.5153**	2746.9968	1374.0020	2746.0128	1373.5100	25
4	480.1490	240.5781	463.1224	232.0649	462.1384	231.5729	P	2597.0250	**1299.0161**	2579.9984	1290.5029	2579.0144	1290.0109	24

5	617.2079	309.1076	600.1814	300.5943	599.1973	300.1023	H	2499.9722	1250.4898	2482.9457	1241.9765	2481.9617	1241.4845	23
6	704.2399	352.6236	687.2134	344.1103	686.2294	343.6183	S	2362.9133	1181.9603	2345.8868	1173.4470	2344.9028	1172.9550	22
7	833.2825	417.1449	816.2560	408.6316	815.2720	408.1396	E	2275.8813	1138.4443	2258.8547	1129.9310	2257.8707	1129.4390	21
8	930.3353	465.6713	913.3087	457.1580	912.3247	456.6660	P	2146.8387	1073.9230	2129.8121	1065.4097	2128.8281	1064.9177	20
9	1090.3659	545.6866	1073.3394	537.1733	1072.3554	536.6813	C	2049.7859	1025.3966	2032.7594	1016.8833	2031.7754	1016.3913	19
10	1177.3980	589.2026	1160.3714	580.6893	1159.3874	580.1973	S	1889.7553	945.3813	1872.7287	936.8680	1871.7447	936.3760	18
11	1264.4300	632.7186	1247.4035	624.2054	1246.4194	623.7134	S	1802.7233	901.8653	1785.6967	893.3520	1784.7127	892.8600	17
12	1379.4569	690.2321	1362.4304	681.7188	1361.4464	681.2268	D	1715.6912	858.3493	1698.6647	849.8360	1697.6807	849.3440	16
13	1466.4890	733.7481	1449.4624	725.2348	1448.4784	724.7428	S	1600.6643	800.8358	1583.6377	792.3225	1582.6537	791.8305	15
14	1565.5574	783.2823	1548.5308	774.7691	1547.5468	774.2770	V	1513.6323	757.3198	1496.6057	748.8065	1495.6217	748.3145	14
15	1652.5894	826.7983	1635.5629	818.2851	1634.5788	817.7931	S	1414.5638	707.7856	1397.5373	699.2723	1396.5533	698.7803	13
16	1781.6320	891.3196	1764.6055	882.8064	1763.6214	882.3144	E	1327.5318	664.2695	1310.5053	655.7563	1309.5213	655.2643	12
17	1878.6848	939.8460	1861.6582	931.3327	1860.6742	930.8407	P	1198.4892	599.7482	1181.4627	591.2350	1180.4787	590.7430	11
18	2007.7274	1004.3673	1990.7008	995.8540	1989.7168	995.3620	E	1101.4365	551.2219	1084.4099	542.7086	1083.4259	542.2166	10
19	2167.7580	1084.3826	2150.7315	1075.8694	2149.7474	1075.3774	C	972.3939	486.7006	955.3673	478.1873	954.3833	477.6953	9
20	2268.8057	1134.9065	2251.7791	1126.3932	2250.7951	1125.9012	T	812.3632	406.6852	795.3367	398.1720	794.3527	397.6800	8
21	2369.8534	1185.4303	2352.8268	1176.9170	2351.8428	1176.4250	T	711.3155	356.1614	694.2890	347.6481	693.3050	347.1561	7
22	2484.8803	1242.9438	2467.8538	1234.4305	2466.8697	1233.9385	D	610.2679	305.6376	593.2413	297.1243	592.2573	296.6323	6
23	2571.9123	1286.4598	2554.8858	1277.9465	2553.9018	1277.4545	S	495.2409	248.1241	478.2144	239.6108	477.2304	239.1188	5
24	2658.9444	1329.9758	2641.9178	1321.4625	2640.9338	1320.9705	S	408.2089	204.6081	391.1823	196.0948	390.1983	195.6028	4
25	2745.9764	1373.4918	2728.9498	1364.9786	2727.9658	1364.4866	S	321.1769	161.0921	304.1503	152.5788	303.1663	152.0868	3
26	2833.0084	1417.0079	2815.9819	1408.4946	2814.9979	1408.0026	S	234.1448	117.5761	217.1183	109.0628	216.1343	108.5708	2
27							K	147.1128	74.0600	130.0863	65.5468			1

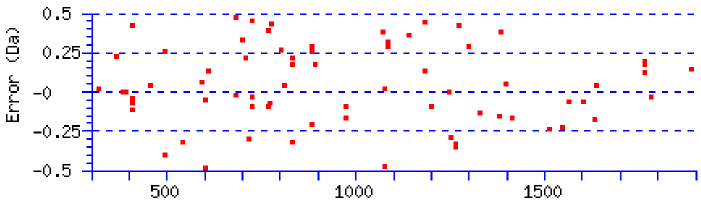

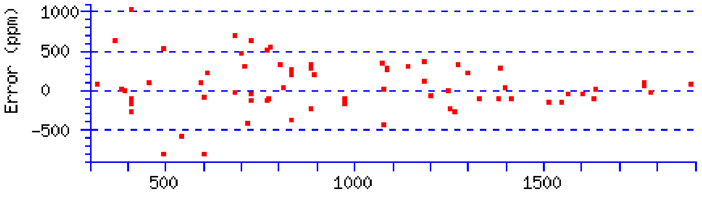

All matches to this query

Score	Mr(calc):	Delta	Sequence
51.8	2978.1067	0.0126	NTSPHSEPCSSDSVSEPECTTDSSSSK
45.9	2978.1067	0.0126	NTSPHSEPCSSDSVSEPECTTDSSSSK
38.7	2978.1067	0.0126	NTSPHSEPCSSDSVSEPECTTDSSSSK
29.3	2978.1067	0.0126	NTSPHSEPCSSDSVSEPECTTDSSSSK
29.3	2978.1067	0.0126	NTSPHSEPCSSDSVSEPECTTDSSSSK
29.3	2978.1067	0.0126	NTSPHSEPCSSDSVSEPECTTDSSSSK
3.8	2978.1067	0.0126	NTSPHSEPCSSDSVSEPECTTDSSSSK
3.4	2978.1318	-0.0125	CSSDANMLGEMMFGSVAMSYKGSTLK
3.4	2978.1318	-0.0125	CSSDANMLGEMMFGSVAMSYKGSTLK
3.4	2978.1318	-0.0125	CSSDANMLGEMMFGSVAMSYKGSTLK

Spectrum No: 274; Query: 2060; Rank: 1

Peptide View

MS/MS Fragmentation of **TQEISRPNSPSEGEGESSDSR**

Found in **IPI00210113**, Tax_Id=10116 Gene_Symbol=Rere Arginine-glutamic acid dipeptide repeats protein

Match to Query 2060: 2327.960292 from(776.994040,3+)
Title: 100101RatKid_NS_deglyco_19.593.593.3.dta
Data file K:\NewmanPaper\Piliang\3SubProteomes\Piliang3SP\mgf5ppm\ERLIC_3SubProteomes5ppm.mgf

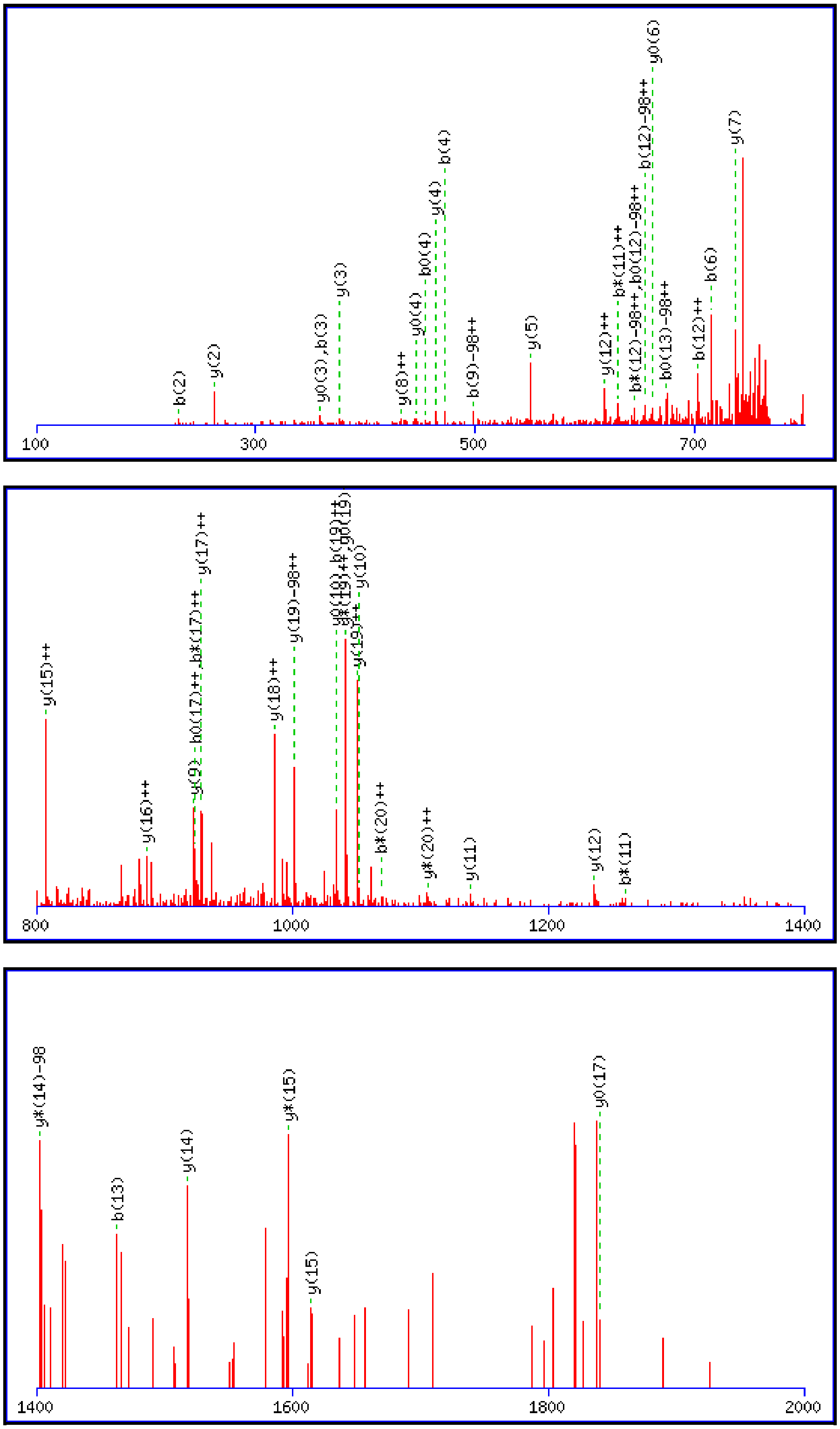

Monoisotopic mass of neutral peptide Mr(calc): 2327.9503
Fixed modifications: Carbamidomethyl (C)
Variable modifications:
S9 : Phospho (ST), with neutral losses 0.0000(shown in table), 97.9769
Ions Score: 52 **Expect:** 0.0016
Matches (Bold Red): 47/356 fragment ions using 72 most intense peaks

#	b	b++	b*	b*++	b0	b0++	Seq.	y	y++	y*	y*++	y0	y0++	#
1	102.0550	51.5311			84.0444	42.5258	T							21

2	230.1135	115.5604	213.0870	107.0471	212.1030	106.5551	Q	2227.9099	1114.4586	2210.8833	1105.9453	2209.8993	1105.4533	20
3	359.1561	180.0817	342.1296	171.5684	341.1456	171.0764	E	2099.8513	1050.4293	2082.8248	1041.9160	2081.8408	1041.4240	19
4	472.2402	236.6237	455.2136	228.1105	454.2296	227.6185	I	1970.8087	985.9080	1953.7822	977.3947	1952.7982	976.9027	18
5	559.2722	280.1397	542.2457	271.6265	541.2617	271.1345	S	1857.7247	929.3660	1840.6981	920.8527	1839.7141	920.3607	17
6	715.3733	358.1903	698.3468	349.6770	697.3628	349.1850	R	1770.6926	885.8500	1753.6661	877.3367	1752.6821	876.8447	16
7	812.4261	406.7167	795.3995	398.2034	794.4155	397.7114	P	1614.5915	807.7994	1597.5650	799.2861	1596.5810	798.7941	15
8	926.4690	463.7381	909.4425	455.2249	908.4585	454.7329	N	1517.5388	759.2730	1500.5122	750.7597	1499.5282	750.2677	14
9	1093.4674	547.2373	1076.4408	538.7240	1075.4568	538.2320	S	1403.4958	702.2516	1386.4693	693.7383	1385.4853	693.2463	13
10	1190.5201	595.7637	1173.4936	587.2504	1172.5096	586.7584	P	1236.4975	618.7524	1219.4709	610.2391	1218.4869	609.7471	12
11	1277.5522	639.2797	1260.5256	630.7664	1259.5416	630.2744	S	1139.4447	570.2260	1122.4182	561.7127	1121.4341	561.2207	11
12	1406.5948	703.8010	1389.5682	695.2877	1388.5842	694.7957	E	1052.4127	526.7100	1035.3861	518.1967	1034.4021	517.7047	10
13	1463.6162	732.3117	1446.5897	723.7985	1445.6057	723.3065	G	923.3701	462.1887	906.3435	453.6754	905.3595	453.1834	9
14	1592.6588	796.8330	1575.6323	788.3198	1574.6482	787.8278	E	866.3486	433.6780	849.3221	425.1647	848.3381	424.6727	8
15	1649.6803	825.3438	1632.6537	816.8305	1631.6697	816.3385	G	737.3060	369.1567	720.2795	360.6434	719.2955	360.1514	7
16	1778.7229	889.8651	1761.6963	881.3518	1760.7123	880.8598	E	680.2846	340.6459	663.2580	332.1326	662.2740	331.6406	6
17	1865.7549	933.3811	1848.7284	924.8678	1847.7443	924.3758	S	551.2420	276.1246	534.2154	267.6114	533.2314	267.1193	5
18	1952.7869	976.8971	1935.7604	968.3838	1934.7764	967.8918	S	464.2100	232.6086	447.1834	224.0953	446.1994	223.6033	4
19	2067.8139	1034.4106	2050.7873	1025.8973	2049.8033	1025.4053	D	377.1779	189.0926	360.1514	180.5793	359.1674	180.0873	3
20	2154.8459	1077.9266	2137.8193	1069.4133	2136.8353	1068.9213	S	262.1510	131.5791	245.1244	123.0659	244.1404	122.5738	2
21							R	175.1190	88.0631	158.0924	79.5498			1

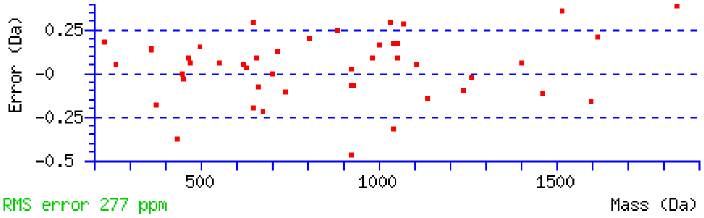

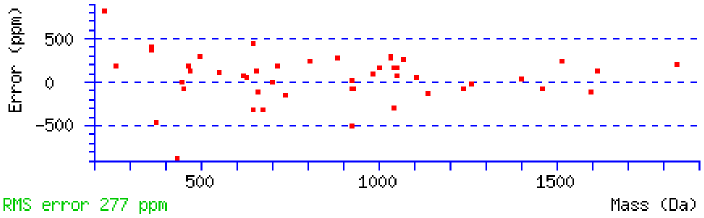

All matches to this query

Score	Mr(calc):	Delta	Sequence
51.8	2327.9503	0.0100	TQEISRPNSPSEGEGESSDSR
35.2	2327.9503	0.0100	TQEISRPNSPSEGEGESSDSR
32.5	2327.9503	0.0100	TQEISRPNSPSEGEGESSDSR
18.6	2327.9503	0.0100	TQEISRPNSPSEGEGESSDSR
15.3	2327.9503	0.0100	TQEISRPNSPSEGEGESSDSR
14.7	2327.9503	0.0100	TQEISRPNSPSEGEGESSDSR
9.3	2327.9503	0.0100	TQEISRPNSPSEGEGESSDSR
3.7	2326.9386	1.0217	NTSRTIYDPVHAAPTTSTR
3.6	2327.9779	-0.0176	EKTISGMKNIIAEMEQASR
0.4	2327.9552	0.0051	NIFGNGSRSAGEICYTEMNK

Spectrum No: 275; Query: 2703; Rank: 1

Peptide View

MS/MS Fragmentation of **CGSGPVHISGQHLVAVEEDAESEDEDEEDVK**
Found in **IPI00197553**, Tax_Id=10116 Gene_Symbol=Npm1 Isoform B23.1 of Nucleophosmin

Match to Query 2703: 3446.427912 from(1149.816580,3+)
Title: 100101RatKid_NS_deglyco_21.2872.2872.3.dta

Data file K:\NewmanPaper\Piliang\3SubProteomes\Piliang3SP\mgf5ppm\ERLIC_3SubProteomes5ppm.mgf

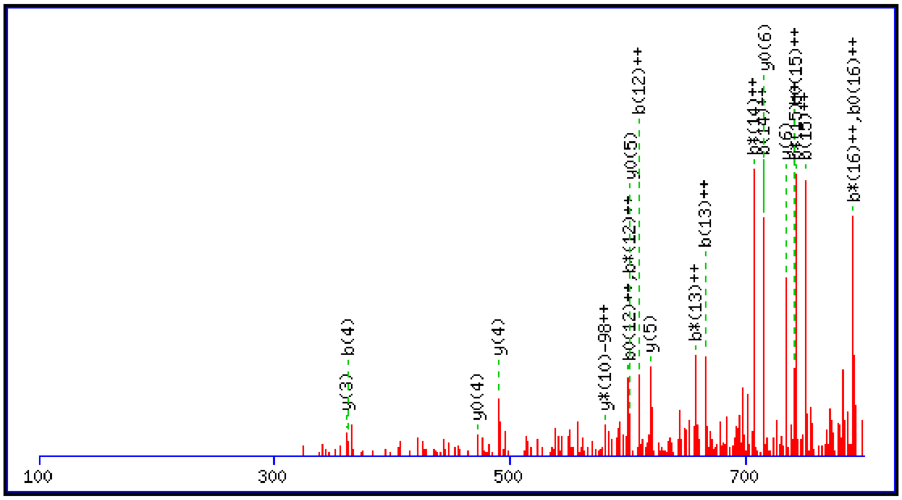

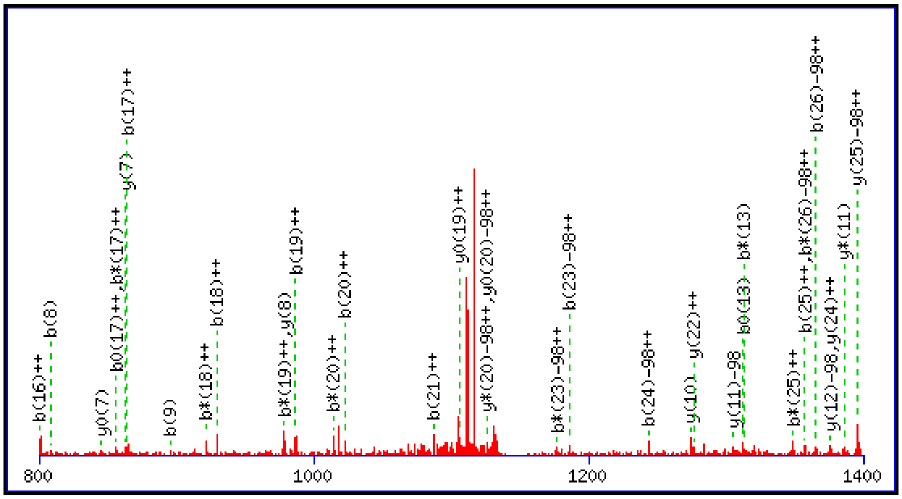

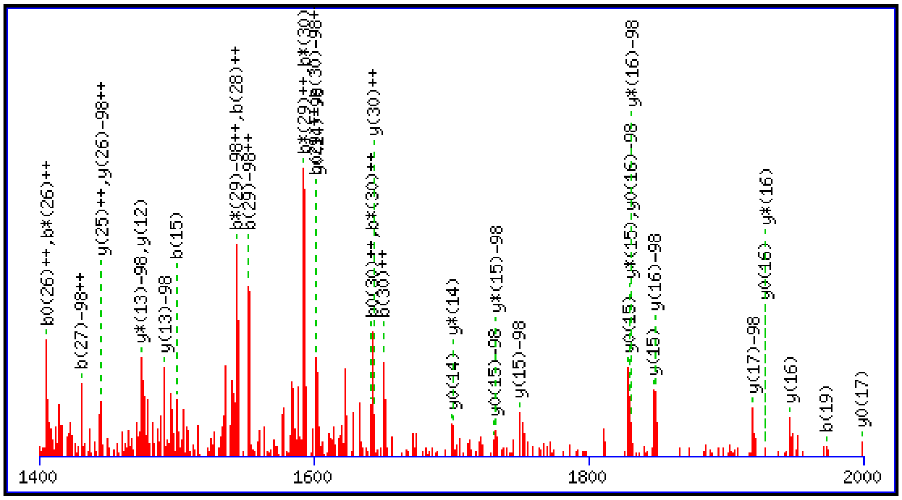

Monoisotopic mass of neutral peptide Mr(calc): 3445.4141
Fixed modifications: Carbamidomethyl (C)
Variable modifications:
S22 : Phospho (ST), with neutral losses 97.9769(shown in table), 0.0000
Ions Score: 52 **Expect:** 0.0028
Matches (Bold Red): 95/512 fragment ions using 164 most intense peaks

#	b	b++	b*	b*++	b0	b0++	Seq.	y	y++	y*	y*++	y0	y0++	#
1	161.0379	81.0226					C							31
2	218.0594	109.5333					G	3188.4138	1594.7105	3171.3873	1586.1973	3170.4032	1585.7053	30
3	305.0914	153.0493			287.0809	144.0441	S	3131.3923	1566.1998	3114.3658	1557.6865	3113.3818	1557.1945	29
4	**362.1129**	181.5601			344.1023	172.5548	G	3044.3603	1522.6838	3027.3338	1514.1705	3026.3498	1513.6785	28

5	459.1656	230.0865			441.1551	221.0812	P	2987.3389	1494.1731	2970.3123	1485.6598	2969.3283	1485.1678	27
6	558.2341	279.6207			540.2235	270.6154	V	2890.2861	**1445.6467**	2873.2595	1437.1334	2872.2755	1436.6414	26
7	695.2930	348.1501			677.2824	339.1448	H	2791.2177	**1396.1125**	2774.1911	1387.5992	2773.2071	1387.1072	25
8	**808.3770**	404.6922			790.3665	395.6869	I	2654.1588	1327.5830	2637.1322	1319.0697	2636.1482	1318.5777	24
9	**895.4091**	448.2082			877.3985	439.2029	S	2541.0747	1271.0410	2524.0481	1262.5277	2523.0641	1262.0357	23
10	952.4305	476.7189			934.4200	467.7136	G	2454.0427	1227.5250	2437.0161	1219.0117	2436.0321	1218.5197	22
11	1080.4891	540.7482	1063.4626	532.2349	1062.4785	531.7429	Q	2397.0212	1199.0142	2379.9947	1190.5010	2379.0106	1190.0090	21
12	1217.5480	**609.2776**	1200.5215	**600.7644**	1199.5375	**600.2724**	H	2268.9626	1134.9850	2251.9361	**1126.4717**	2250.9521	**1125.9797**	20
13	1330.6321	**665.8197**	**1313.6055**	**657.3064**	**1312.6215**	656.8144	L	2131.9037	1066.4555	2114.8772	1057.9422	2113.8932	1057.4502	19
14	1429.7005	**715.3539**	1412.6739	**706.8406**	1411.6899	706.3486	V	2018.8197	1009.9135	2001.7931	1001.4002	2000.8091	1000.9082	18
15	**1500.7376**	**750.8724**	1483.7111	**742.3592**	1482.7270	**741.8672**	A	**1919.7512**	960.3793	1902.7247	951.8660	1901.7407	951.3740	17
16	1599.8060	**800.4066**	1582.7795	**791.8934**	1581.7955	**791.4014**	V	**1848.7141**	924.8607	**1831.6876**	916.3474	**1830.7036**	915.8554	16
17	1728.8486	**864.9279**	1711.8221	**856.4147**	1710.8381	**855.9227**	E	**1749.6457**	875.3265	**1732.6192**	866.8132	**1731.6351**	866.3212	15
18	1857.8912	**929.4492**	1840.8647	**920.9360**	1839.8806	920.4440	E	1620.6031	810.8052	1603.5766	802.2919	**1602.5926**	801.7999	14
19	**1972.9182**	**986.9627**	1955.8916	**978.4494**	1954.9076	977.9574	D	**1491.5605**	746.2839	**1474.5340**	737.7706	1473.5500	737.2786	13
20	2043.9553	**1022.4813**	2026.9287	**1013.9680**	2025.9447	1013.4760	A	**1376.5336**	688.7704	1359.5070	680.2572	1358.5230	679.7651	12
21	2172.9979	**1087.0026**	2155.9713	1078.4893	2154.9873	1077.9973	E	**1305.4965**	653.2519	1288.4699	644.7386	1287.4859	644.2466	11
22	2242.0193	1121.5133	2224.9928	1113.0000	2224.0088	1112.5080	S	1176.4539	588.7306	1159.4273	**580.2173**	1158.4433	579.7253	10
23	2371.0619	**1186.0346**	2354.0354	**1177.5213**	2353.0513	1177.0293	E	1107.4324	554.2198	1090.4059	545.7066	1089.4219	545.2146	9
24	2486.0889	**1243.5481**	2469.0623	1235.0348	2468.0783	1234.5428	D	**978.3898**	489.6986	961.3633	481.1853	960.3793	480.6933	8
25	2615.1314	1308.0694	2598.1049	1299.5561	2597.1209	1299.0641	E	**863.3629**	432.1851	846.3363	423.6718	**845.3523**	423.1798	7
26	2730.1584	**1365.5828**	2713.1318	**1357.0696**	2712.1478	1356.5775	D	**734.3203**	367.6638	717.2937	359.1505	**716.3097**	358.6585	6
27	2859.2010	**1430.1041**	2842.1744	1421.5909	2841.1904	1421.0988	E	**619.2933**	310.1503	602.2668	301.6370	**601.2828**	301.1450	5
28	2988.2436	1494.6254	2971.2170	1486.1121	2970.2330	1485.6201	E	**490.2508**	245.6290	473.2242	237.1157	**472.2402**	236.6237	4
29	3103.2705	**1552.1389**	3086.2440	**1543.6256**	3085.2600	1543.1336	D	**361.2082**	181.1077	344.1816	172.5944	343.1976	172.1024	3
30	3202.3389	**1601.6731**	3185.3124	**1593.1598**	3184.3284	**1592.6678**	V	246.1812	123.5942	229.1547	115.0810			2
31							K	147.1128	74.0600	130.0863	65.5468			1

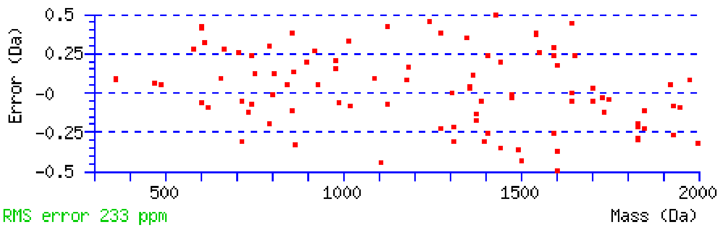

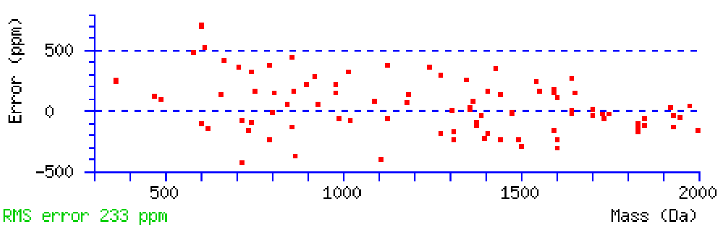

All matches to this query

Score	Mr(calc):	Delta	Sequence
51.7	3445.4141	1.0138	CGSGPVHISGQHLVAVEEDAESEDEDEEDVK
6.1	3445.4141	1.0138	CGSGPVHISGQHLVAVEEDAESEDEDEEDVK
5.4	3445.4141	1.0138	CGSGPVHISGQHLVAVEEDAESEDEDEEDVK
5.1	3445.4572	0.9707	KPSSGTQDNSKHYNHATANQNSNALSNIRK
3.2	3444.4326	1.9954	EGMAVAKPEATTMAMTAAPTPTSPSLSSGHGANP
3.1	3444.4326	1.9954	EGMAVAKPEATTMAMTAAPTPTSPSLSSGHGANP
3.0	3445.4161	1.0118	VNGELRQEGKTSSMIFSIPYIISYVSK
3.0	3445.4161	1.0118	VNGELRQEGKTSSMIFSIPYIISYVSK
2.6	3445.4572	0.9707	KPSSGTQDNSKHYNHATANQNSNALSNIRK
2.6	3445.4572	0.9707	KPSSGTQDNSKHYNHATANQNSNALSNIRK

Spectrum No: 276; Query: 1689; Rank: 1

Peptide View

MS/MS Fragmentation of **GTGDCSDEEVDGKADGADAK**
Found in **IPI00209113**, Tax_Id=10116 Gene_Symbol=Myh9 Myosin-9

Match to Query 1689: 2075.767328 from(1038.890940,2+)
Title: 100101RatKid_NS_deglyco_23.418.418.2.dta
Data file K:\NewmanPaper\Piliang\3SubProteomes\Piliang3SP\mgf5ppm\ERLIC_3SubProteomes5ppm.mgf

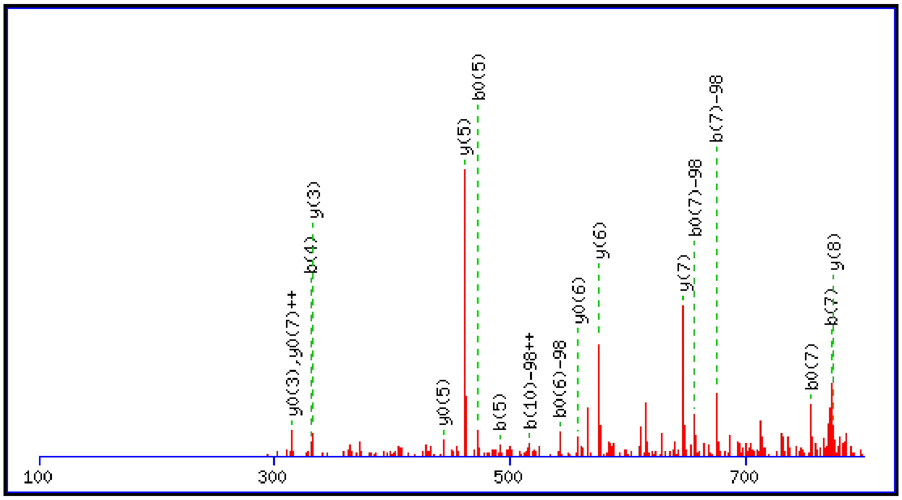

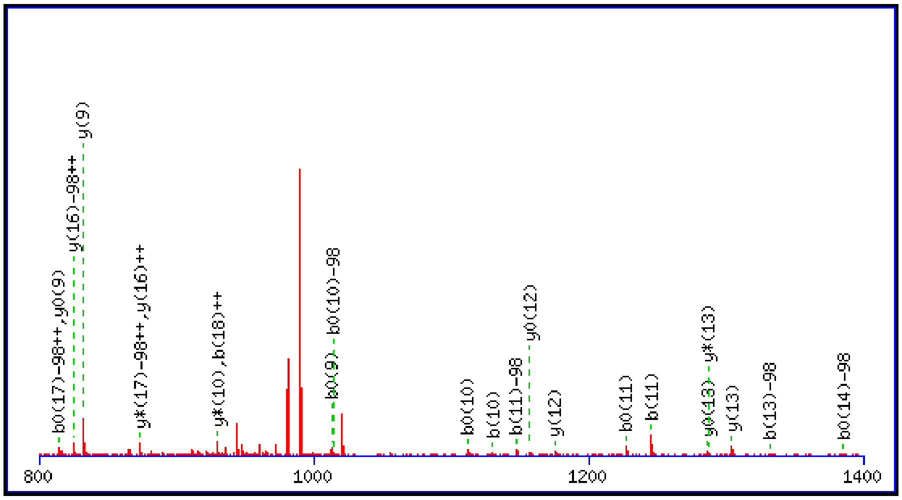

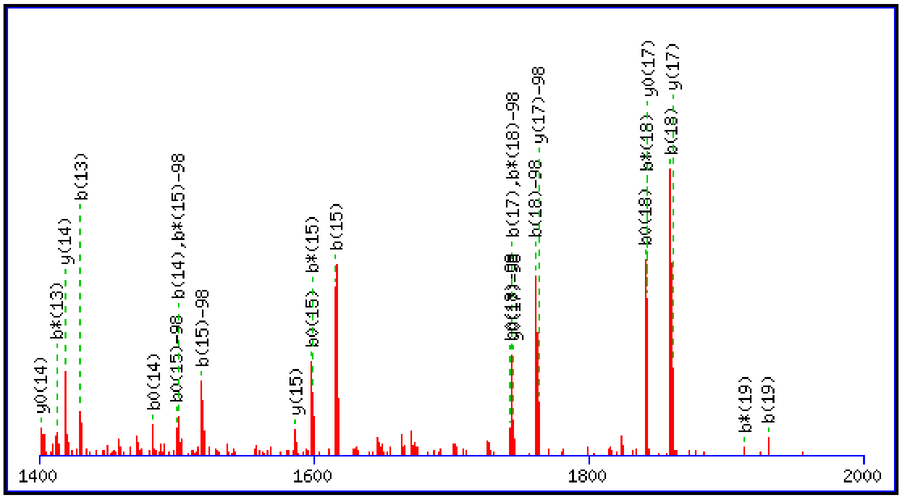

Monoisotopic mass of neutral peptide Mr(calc): 2075.7627
Fixed modifications: Carbamidomethyl (C)
Variable modifications:

S6 : Phospho (ST), with neutral losses 0.0000(shown in table), 97.9769
Ions Score: 52 **Expect:** 0.00083
Matches (Bold Red): 66/298 fragment ions using 103 most intense peaks

#	b	b^{++}	b*	b^{*++}	b^{0}	b^{0++}	Seq.	y	y^{++}	y*	y^{*++}	y^{0}	y^{0++}	#
1	58.0287	29.5180					G							20
2	159.0764	80.0418			141.0659	71.0366	T	2019.7485	1010.3779	2002.7219	1001.8646	2001.7379	1001.3726	19
3	216.0979	108.5526			198.0873	99.5473	G	1918.7008	959.8540	1901.6743	951.3408	1900.6903	950.8488	18
4	**331.1248**	166.0661			313.1143	157.0608	D	**1861.6794**	931.3433	1844.6528	922.8300	**1843.6688**	922.3380	17
5	**491.1555**	246.0814			**473.1449**	237.0761	C	1746.6524	**873.8298**	1729.6259	865.3166	1728.6418	864.8246	16
6	658.1538	329.5806			640.1433	320.5753	S	**1586.6218**	793.8145	1569.5952	785.3012	1568.6112	784.8092	15
7	**773.1808**	387.0940			**755.1702**	378.0887	D	**1419.6234**	710.3153	1402.5969	701.8021	**1401.6128**	701.3101	14
8	902.2234	451.6153			884.2128	442.6100	E	**1304.5965**	652.8019	**1287.5699**	644.2886	**1286.5859**	643.7966	13
9	1031.2660	516.1366			**1013.2554**	507.1313	E	**1175.5539**	588.2806	1158.5273	579.7673	**1157.5433**	579.2753	12
10	**1130.3344**	565.6708			**1112.3238**	556.6655	V	1046.5113	523.7593	1029.4847	515.2460	1028.5007	514.7540	11
11	**1245.3613**	623.1843			**1227.3507**	614.1790	D	947.4429	474.2251	**930.4163**	465.7118	929.4323	465.2198	10
12	1302.3828	651.6950			1284.3722	642.6897	G	**832.4159**	416.7116	815.3894	408.1983	**814.4054**	407.7063	9
13	**1430.4777**	715.7425	**1413.4512**	707.2292	1412.4672	706.7372	K	**775.3945**	388.2009	758.3679	379.6876	757.3839	379.1956	8
14	**1501.5149**	751.2611	1484.4883	742.7478	**1483.5043**	742.2558	A	**647.2995**	324.1534	630.2729	315.6401	629.2889	**315.1481**	7
15	**1616.5418**	808.7745	**1599.5152**	800.2613	**1598.5312**	799.7693	D	**576.2624**	288.6348	559.2358	280.1216	**558.2518**	279.6295	6
16	1673.5633	837.2853	1656.5367	828.7720	1655.5527	828.2800	G	**461.2354**	231.1214	444.2089	222.6081	**443.2249**	222.1161	5
17	**1744.6004**	872.8038	1727.5738	864.2906	1726.5898	863.7985	A	404.2140	202.6106	387.1874	194.0974	386.2034	193.6053	4
18	**1859.6273**	**930.3173**	**1842.6008**	921.8040	**1841.6168**	921.3120	D	**333.1769**	167.0921	316.1503	158.5788	**315.1663**	158.0868	3
19	**1930.6644**	965.8359	**1913.6379**	957.3226	1912.6539	956.8306	A	218.1499	109.5786	201.1234	101.0653			2
20							K	147.1128	74.0600	130.0863	65.5468			1

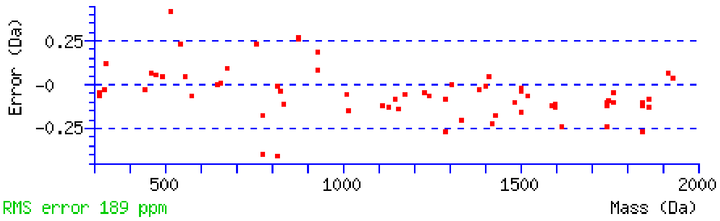

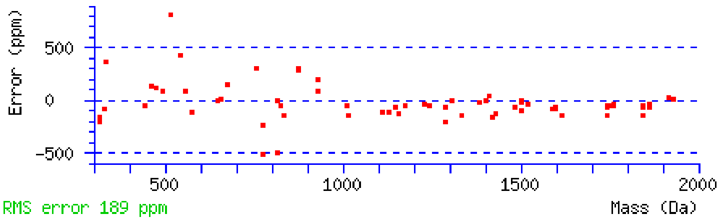

All matches to this query

Score	Mr(calc):	Delta	Sequence
51.7	2075.7627	0.0046	GTGDCSDEEVDGKADGADAK
37.9	2075.7627	0.0046	GTGDCSDEEVDGKADGADAK
3.8	2073.7582	2.0091	SSEPSTQQGTLGSLEGEK
3.8	2073.7582	2.0091	SSEPSTQQGTLGSLEGEK
3.4	2075.7695	-0.0022	SSGVRGSGSGSGGGGYSSGGGSR
2.4	2075.7618	0.0055	SNFLNATPTMMAGNESSK
2.4	2075.7618	0.0055	SNFLNATPTMMAGNESSK
2.0	2075.7472	0.0201	DQNNNCGIATYAIYPTV
2.0	2075.7472	0.0201	DQNNNCGIATYAIYPTV
2.0	2073.7751	1.9922	MSNPSPQAPEEEASTSPR

Spectrum No: 277; Query: 2607; Rank: 1

Peptide View

MS/MS Fragmentation of **MLAESDESGDEESVSQTDKTELQNTLR**
Found in **IPI00870224**, Tax_Id=10116 Gene_Symbol=Osbp_predicted oxysterol binding protein

Match to Query 2607: 3187.294212 from(1063.438680,3+)
Title: 100101RatKid_NS_deglyco_25.2768.2768.3.dta
Data file K:\NewmanPaper\Piliang\3SubProteomes\Piliang3SP\mgf5ppm\ERLIC_3SubProteomes5ppm.mgf

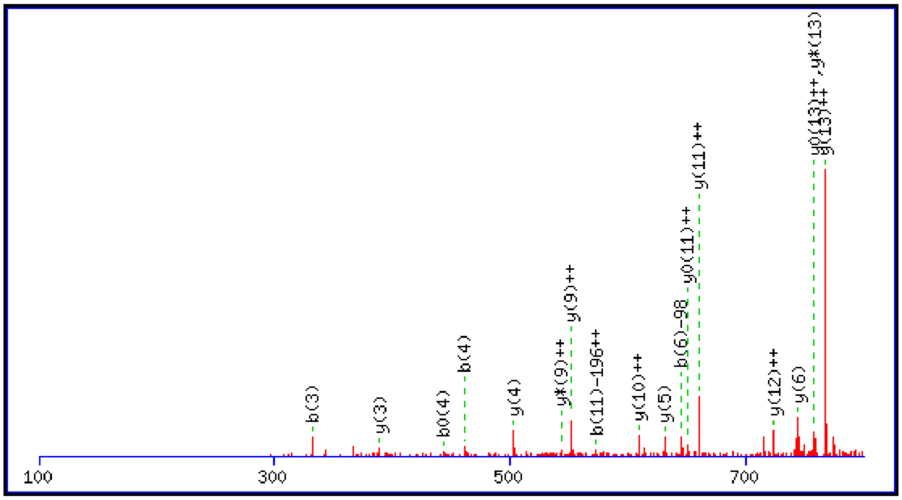

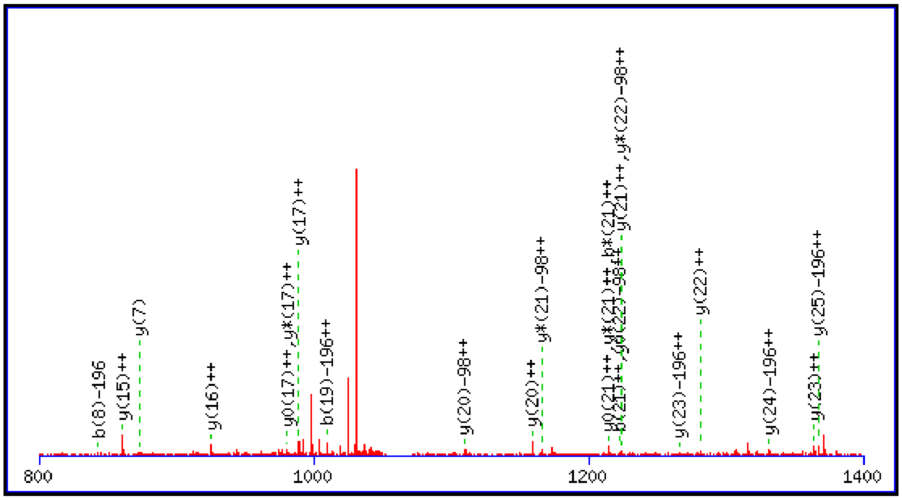

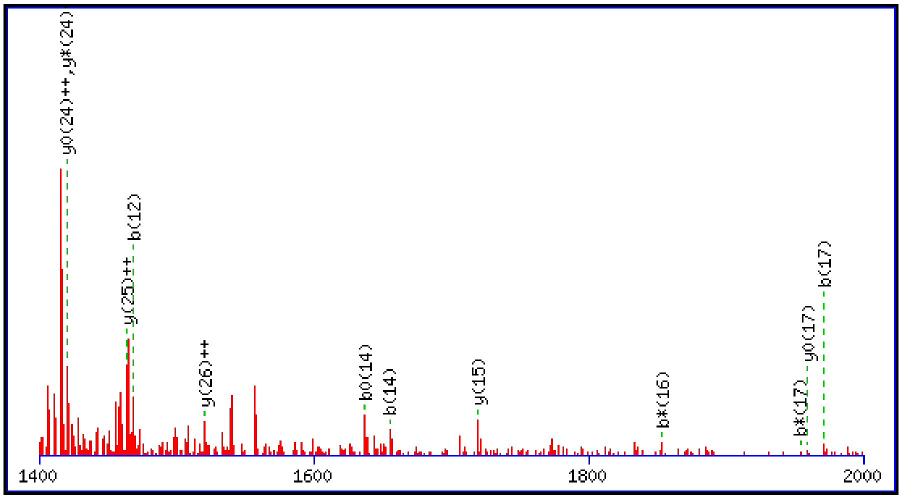

Monoisotopic mass of neutral peptide Mr(calc): 3187.2789
Fixed modifications: Carbamidomethyl (C)
Variable modifications:
M1 : Oxidation (M)
S5 : Phospho (ST), with neutral losses 0.0000(shown in table), 97.9769
S8 : Phospho (ST), with neutral losses 0.0000(shown in table), 97.9769
Ions Score: 52 **Expect:** 0.0025
Matches (Bold Red): 53/424 fragment ions using 97 most intense peaks

#	b	b^{++}	b*	b^{*++}	b^0	b^{0++}	Seq.	y	y^{++}	y*	y^{*++}	y^0	y^{0++}	#
1	148.0427	74.5250					M							27
2	261.1267	131.0670					L	3041.2508	**1521.1290**	3024.2242	1512.6157	3023.2402	1512.1237	26
3	**332.1639**	166.5856					A	2928.1667	**1464.5870**	2911.1402	1456.0737	2910.1561	1455.5817	25
4	**461.2065**	231.1069			**443.1959**	222.1016	E	2857.1296	1429.0684	2840.1030	**1420.5552**	2839.1190	**1420.0632**	24
5	628.2048	314.6060			610.1942	305.6008	S	2728.0870	**1364.5471**	2711.0605	1356.0339	2710.0764	1355.5419	23
6	743.2317	372.1195			725.2212	363.1142	D	2561.0886	**1281.0480**	2544.0621	1272.5347	2543.0781	1272.0427	22
7	872.2743	436.6408			854.2638	427.6355	E	2446.0617	**1223.5345**	2429.0352	**1215.0212**	2428.0511	**1214.5292**	21
8	1039.2727	520.1400			1021.2621	511.1347	S	2317.0191	**1159.0132**	2299.9926	1150.4999	2299.0085	1150.0079	20
9	1096.2942	548.6507			1078.2836	539.6454	G	2150.0208	1075.5140	2132.9942	1067.0007	2132.0102	1066.5087	19
10	1211.3211	606.1642			1193.3105	597.1589	D	2092.9993	1047.0033	2075.9727	1038.4900	2074.9887	1037.9980	18
11	1340.3637	670.6855			1322.3531	661.6802	E	1977.9724	**989.4898**	1960.9458	**980.9765**	**1959.9618**	**980.4845**	17
12	**1469.4063**	735.2068			1451.3957	726.2015	E	1848.9298	**924.9685**	1831.9032	916.4552	1830.9192	915.9632	16
13	1556.4383	778.7228			1538.4277	769.7175	S	**1719.8872**	**860.4472**	1702.8606	851.9339	1701.8766	851.4419	15
14	**1655.5067**	828.2570			**1637.4962**	819.2517	V	1632.8551	816.9312	1615.8286	808.4179	1614.8446	807.9259	14
15	1742.5388	871.7730			1724.5282	862.7677	S	1533.7867	**767.3970**	1516.7602	**758.8837**	1515.7762	**758.3917**	13
16	1870.5973	935.8023	**1853.5708**	927.2890	1852.5868	926.7970	Q	1446.7547	**723.8810**	1429.7282	715.3677	1428.7441	714.8757	12
17	**1971.6450**	986.3261	**1954.6185**	977.8129	1953.6344	977.3209	T	1318.6961	**659.8517**	1301.6696	651.3384	1300.6856	**650.8464**	11
18	2086.6720	1043.8396	2069.6454	1035.3263	2068.6614	1034.8343	D	1217.6484	**609.3279**	1200.6219	600.8146	1199.6379	600.3226	10
19	2214.7669	1107.8871	2197.7404	1099.3738	2196.7564	1098.8818	K	1102.6215	**551.8144**	1085.5950	**543.3011**	1084.6109	542.8091	9
20	2315.8146	1158.4109	2298.7880	1149.8977	2297.8040	1149.4057	T	974.5265	487.7669	957.5000	479.2536	956.5160	478.7616	8
21	2444.8572	**1222.9322**	2427.8306	**1214.4190**	2426.8466	1213.9269	E	**873.4789**	437.2431	856.4523	428.7298	855.4683	428.2378	7
22	2557.9413	1279.4743	2540.9147	1270.9610	2539.9307	1270.4690	L	**744.4363**	372.7218	727.4097	364.2085	726.4257	363.7165	6
23	2685.9998	1343.5036	2668.9733	1334.9903	2667.9893	1334.4983	Q	**631.3522**	316.1797	614.3257	307.6665	613.3416	307.1745	5
24	2800.0428	1400.5250	2783.0162	1392.0117	2782.0322	1391.5197	N	**503.2936**	252.1504	486.2671	243.6372	485.2831	243.1452	4
25	2901.0904	1451.0489	2884.0639	1442.5356	2883.0799	1442.0436	T	**389.2507**	195.1290	372.2241	186.6157	371.2401	186.1237	3
26	3014.1745	1507.5909	2997.1480	1499.0776	2996.1639	1498.5856	L	288.2030	144.6051	271.1765	136.0919			2
27							R	175.1190	88.0631	158.0924	79.5498			1

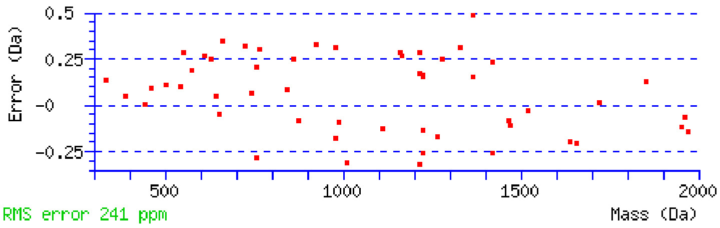

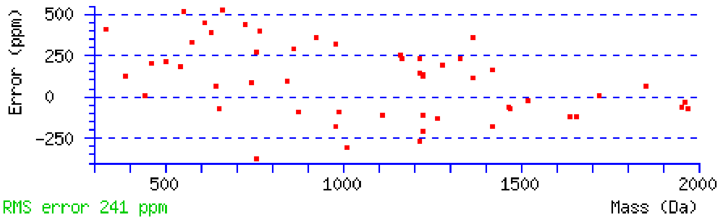

All matches to this query

Score	Mr(calc):	Delta	Sequence
51.6	3187.2789	0.0153	MLAESDESGDEESVSQTDKTELQNTLR
41.6	3187.2789	0.0153	MLAESDESGDEESVSQTDKTELQNTLR
12.5	3187.2789	0.0153	MLAESDESGDEESVSQTDKTELQNTLR
6.6	3185.2887	2.0055	NKTVSTLVIQAAYVSALYKCEAINK
3.9	3187.2789	0.0153	MLAESDESGDEESVSQTDKTELQNTLR
3.8	3185.3073	1.9869	AASVCGTPTSLESLPLGNMESNRQRNR
3.5	3187.2789	0.0153	MLAESDESGDEESVSQTDKTELQNTLR
2.8	3186.3144	0.9798	CGTLSSLPGASAAVSAQPGEVMTMDAMLAR
2.4	3185.3148	1.9794	RLSPTTSSNGNSPPIKCEDINTEEYSK
2.4	3185.3148	1.9794	RLSPTTSSNGNSPPIKCEDINTEEYSK

Spectrum No: 278; Query: 1648; Rank: 1

Peptide View

MS/MS Fragmentation of **KEESEESDDDMGFGLFD**
Found in **IPI00188804**, Tax_Id=10116 Gene_Symbol=Rplp2 60S acidic ribosomal protein P2

Match to Query 1648: 2044.713428 from(1023.363990,2+)
Title: 091008RatKidney_NoSalt_29.2946.2946.2.dta
Data file K:\NewmanPaper\Piliang\3SubProteomes\Piliang3SP\mgf5ppm\ERLIC_3SubProteomes5ppm.mgf

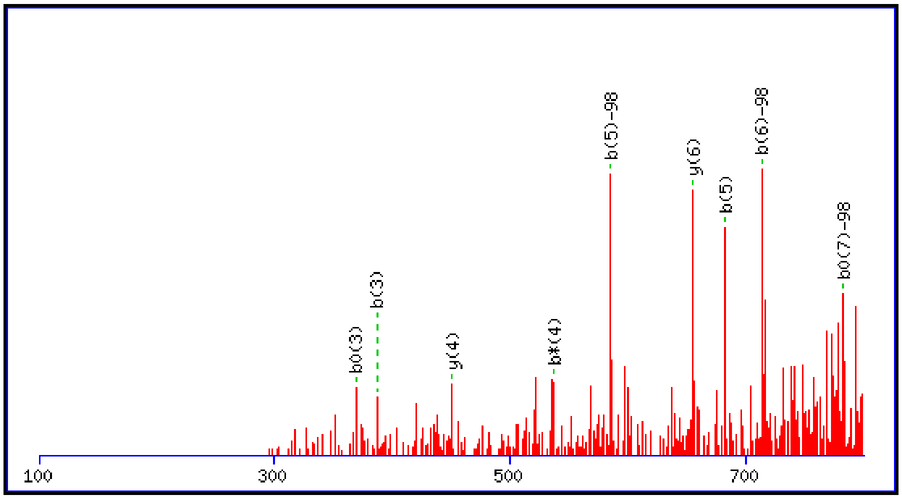

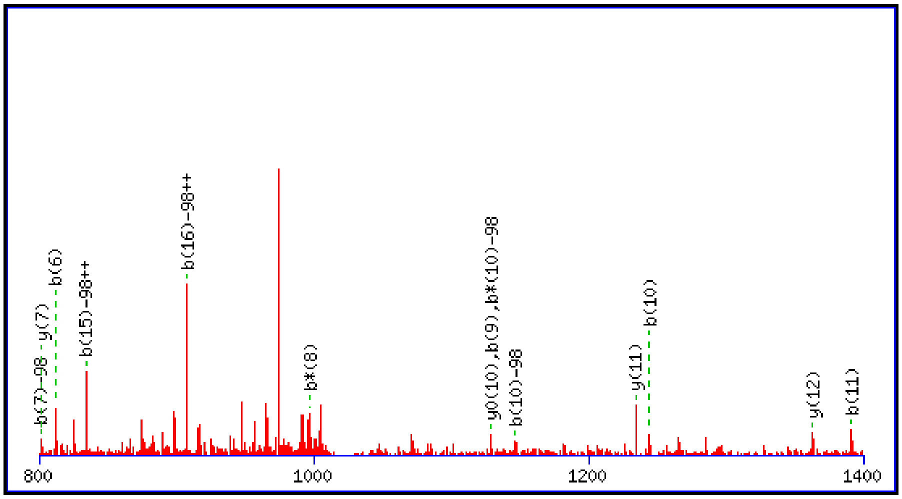

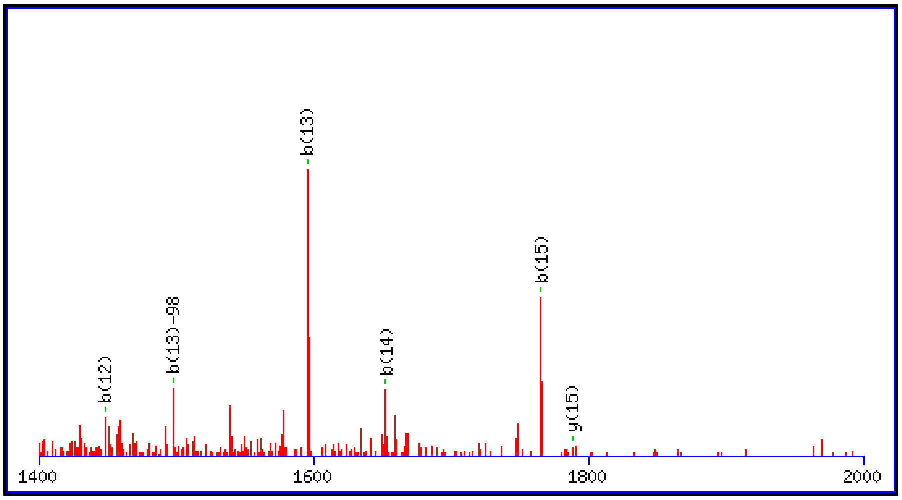

Monoisotopic mass of neutral peptide Mr(calc): 2044.7133

Fixed modifications: Carbamidomethyl (C)
Variable modifications:
S4 : Phospho (ST), with neutral losses 0.0000(shown in table), 97.9769
M11 : Oxidation (M)
Ions Score: 52 **Expect:** 0.00057
Matches (Bold Red): 29/248 fragment ions using 42 most intense peaks

#	b	b^{++}	b*	b^{*++}	b^0	b^{0++}	Seq.	y	y^{++}	y^0	y^{0++}	#
1	129.1022	65.0548	112.0757	56.5415			K					17
2	258.1448	129.5761	241.1183	121.0628	240.1343	120.5708	E	1917.6256	959.3164	1899.6150	950.3112	16
3	**387.1874**	194.0974	370.1609	185.5841	**369.1769**	185.0921	E	**1788.5830**	894.7951	1770.5724	885.7899	15
4	554.1858	277.5965	**537.1592**	269.0833	536.1752	268.5912	S	1659.5404	830.2738	1641.5298	821.2686	14
5	**683.2284**	342.1178	666.2018	333.6045	665.2178	333.1125	E	1492.5421	746.7747	1474.5315	737.7694	13
6	**812.2710**	406.6391	795.2444	398.1258	794.2604	397.6338	E	**1363.4995**	682.2534	1345.4889	673.2481	12
7	899.3030	450.1551	882.2764	441.6419	881.2924	441.1499	S	**1234.4569**	617.7321	1216.4463	608.7268	11
8	1014.3299	507.6686	**997.3034**	499.1553	996.3194	498.6633	D	1147.4248	574.2161	**1129.4143**	565.2108	10
9	**1129.3569**	565.1821	1112.3303	556.6688	1111.3463	556.1768	D	1032.3979	516.7026	1014.3873	507.6973	9
10	**1244.3838**	622.6955	1227.3573	614.1823	1226.3733	613.6903	D	917.3710	459.1891	899.3604	450.1838	8
11	**1391.4192**	696.2133	1374.3927	687.7000	1373.4087	687.2080	M	**802.3440**	401.6756	784.3334	392.6704	7
12	**1448.4407**	724.7240	1431.4141	716.2107	1430.4301	715.7187	G	**655.3086**	328.1579	637.2980	319.1527	6
13	**1595.5091**	798.2582	1578.4826	789.7449	1577.4985	789.2529	F	598.2871	299.6472	580.2766	290.6419	5
14	**1652.5306**	826.7689	1635.5040	818.2556	1634.5200	817.7636	G	**451.2187**	226.1130	433.2082	217.1077	4
15	**1765.6146**	883.3110	1748.5881	874.7977	1747.6041	874.3057	L	394.1973	197.6023	376.1867	188.5970	3
16	1912.6830	956.8452	1895.6565	948.3319	1894.6725	947.8399	F	281.1132	141.0602	263.1026	132.0550	2
17							D	134.0448	67.5260	116.0342	58.5207	1

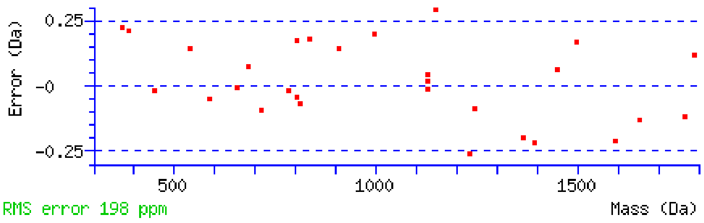

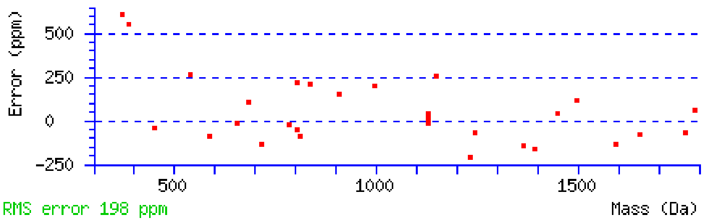

All matches to this query

Score	Mr(calc):	Delta	Sequence
51.6	2044.7133	0.0002	KEESEESDDDMGFGLFD
31.4	2044.7133	0.0002	KEESEESDDDMGFGLFD

Spectrum No: 279; Query: 1195; Rank: 1

Peptide View

MS/MS Fragmentation of **VIENTDGSEEEMDAR**
Found in **IPI00464464**, Tax_Id=10116 Gene_Symbol=Myh11 Isoform 1 of Myosin-11 (Fragment)

Match to Query 1195: 1773.674848 from(887.844700,2+)
Title: 091008RatKidney_NoSalt_28.924.924.2.dta
Data file K:\NewmanPaper\Piliang\3SubProteomes\Piliang3SP\mgf5ppm\ERLIC_3SubProteomes5ppm.mgf

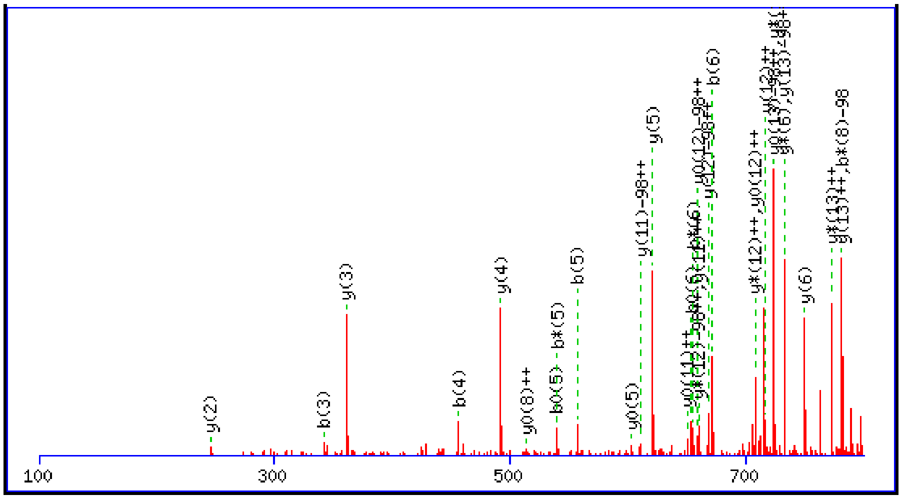

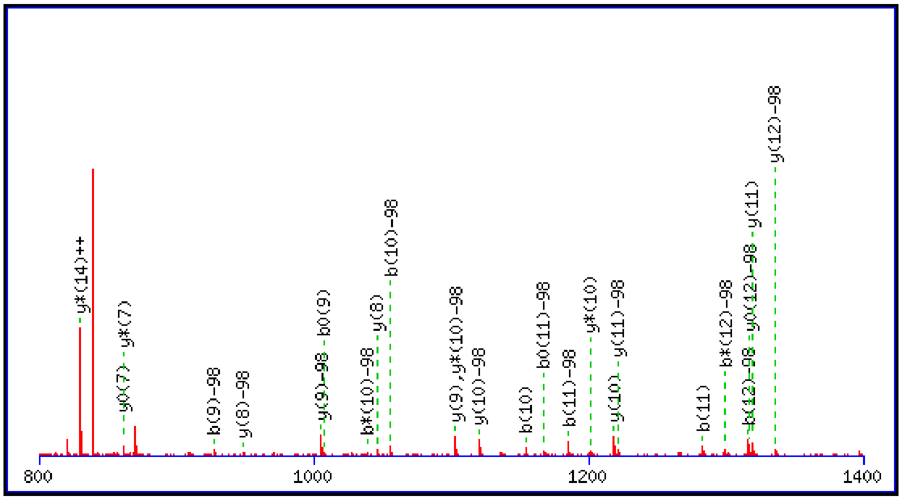

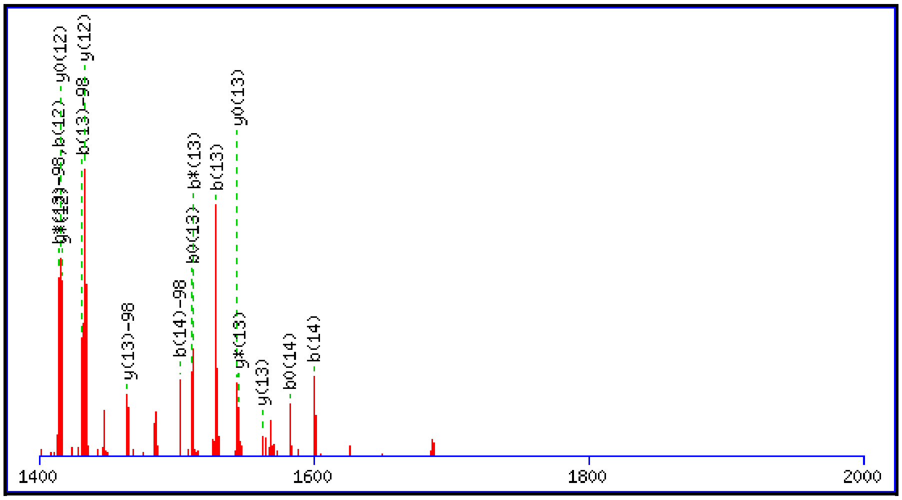

Monoisotopic mass of neutral peptide Mr(calc): 1773.6764
Fixed modifications: Carbamidomethyl (C)
Variable modifications:
S8 : Phospho (ST), with neutral losses 97.9769(shown in table), 0.0000
Ions Score: 52 **Expect:** 0.00087
Matches (Bold Red): 72/238 fragment ions using 112 most intense peaks

#	b	b^{++}	b*	b^{*++}	b^0	b^{0++}	Seq.	y	y^{++}	y*	y^{*++}	y^0	y^{0++}	#
1	100.0757	50.5415					V							15
2	213.1598	107.0835					I	1577.6384	789.3228	1560.6119	780.8096	1559.6278	780.3176	14
3	**342.2023**	171.6048			324.1918	162.5995	E	**1464.5543**	**732.7808**	1447.5278	**724.2675**	1446.5438	**723.7755**	13
4	**456.2453**	228.6263	439.2187	220.1130	438.2347	219.6210	N	**1335.5117**	**668.2595**	1318.4852	**659.7462**	**1317.5012**	**659.2542**	12
5	**557.2930**	279.1501	**540.2664**	270.6368	**539.2824**	270.1448	T	**1221.4688**	**611.2380**	1204.4423	602.7248	1203.4583	602.2328	11

**6**	**672.3199**	336.6636	**655.2933**	328.1503	**654.3093**	327.6583	**D**	**1120.4211**	560.7142	**1103.3946**	552.2009	1102.4106	551.7089	**10**
**7**	729.3414	365.1743	712.3148	356.6610	711.3308	356.1690	**G**	**1005.3942**	503.2007	988.3676	494.6875	987.3836	494.1955	**9**
**8**	798.3628	399.6850	**781.3363**	391.1718	780.3523	390.6798	**S**	**948.3727**	474.6900	931.3462	466.1767	930.3622	465.6847	**8**
**9**	**927.4054**	464.2063	910.3789	455.6931	909.3948	455.2011	**E**	879.3513	440.1793	**862.3247**	431.6660	**861.3407**	431.1740	**7**
**10**	**1056.4480**	528.7276	**1039.4215**	520.2144	1038.4374	519.7224	**E**	**750.3087**	375.6580	**733.2821**	367.1447	732.2981	366.6527	**6**
**11**	**1185.4906**	593.2489	1168.4640	584.7357	**1167.4800**	584.2437	**E**	**621.2661**	311.1367	604.2395	302.6234	**603.2555**	302.1314	**5**
**12**	**1316.5311**	658.7692	**1299.5045**	650.2559	1298.5205	649.7639	**M**	**492.2235**	246.6154	475.1969	238.1021	474.2129	237.6101	**4**
**13**	**1431.5580**	716.2826	**1414.5315**	707.7694	1413.5475	707.2774	**D**	**361.1830**	181.0951	344.1565	172.5819	343.1724	172.0899	**3**
**14**	**1502.5951**	751.8012	1485.5686	743.2879	1484.5846	742.7959	**A**	**246.1561**	123.5817	229.1295	115.0684			**2**
**15**							**R**	175.1190	88.0631	158.0924	79.5498			**1**

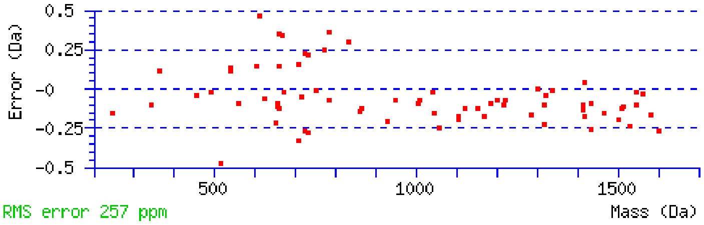

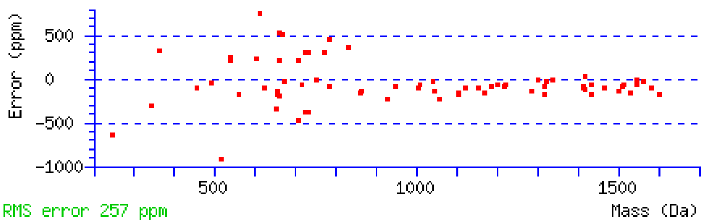

All matches to this query

Score	Mr(calc):	Delta	Sequence
51.5	1773.6764	-0.0016	VIENTDGSEEEMDAR
38.0	1773.6764	-0.0016	VIENTDGSEEEMDAR
2.3	1772.6617	1.0132	SGNSLPLSNSTMFIQ
2.3	1772.6617	1.0132	SGNSLPLSNSTMFIQ
2.3	1772.6617	1.0132	SGNSLPLSNSTMFIQ
2.3	1772.6617	1.0132	SGNSLPLSNSTMFIQ
1.9	1772.6620	1.0129	CPGLXPSRRGQSR
1.7	1771.6815	1.9934	SKDHEEKNLDGPSR
1.5	1773.6907	-0.0158	TCRPDGGAPQLDTAR
1.2	1773.6737	0.0012	SVRLEVSSVDNSSR

Spectrum No: 280; Query: 2846; Rank: 1

Peptide View

MS/MS Fragmentation of **TSVTVYPGDVFCLHTPGGGGYGDPEDPAPPPGSPPLFPAFPER**
Found in **IPI00326436**, Tax_Id=10116 Gene_Symbol=Oplah 5-oxoprolinase

Match to Query 2846: 4532.067882 from(1511.696570,3+)
Title: 100101RatKid_NS_deglyco_13.6253.6253.3.dta
Data file K:\NewmanPaper\Piliang\3SubProteomes\Piliang3SP\mgf5ppm\ERLIC_3SubProteomes5ppm.mgf

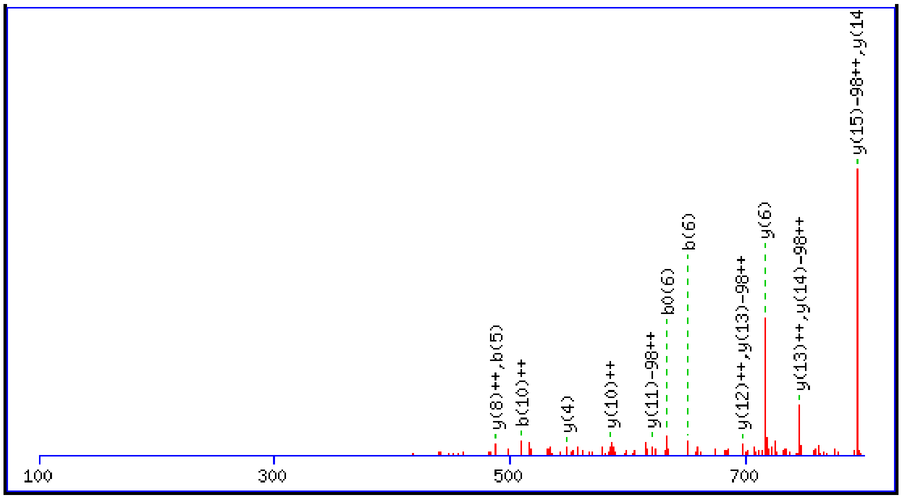

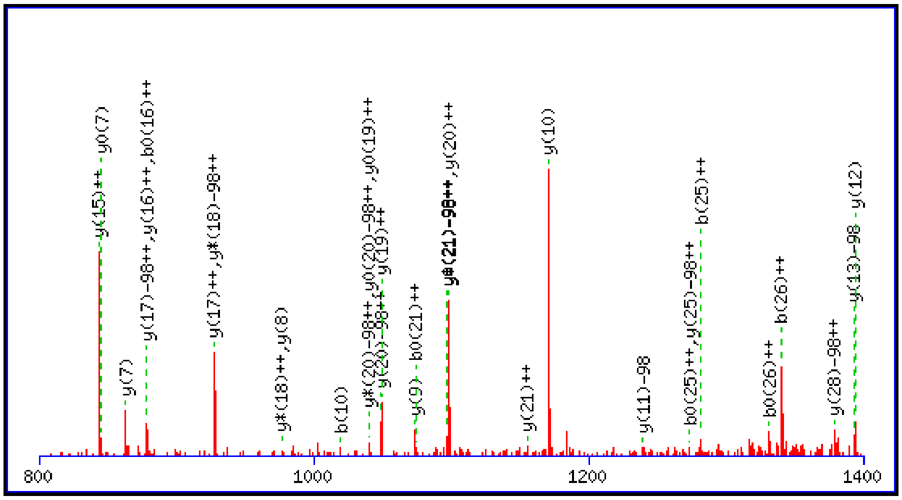

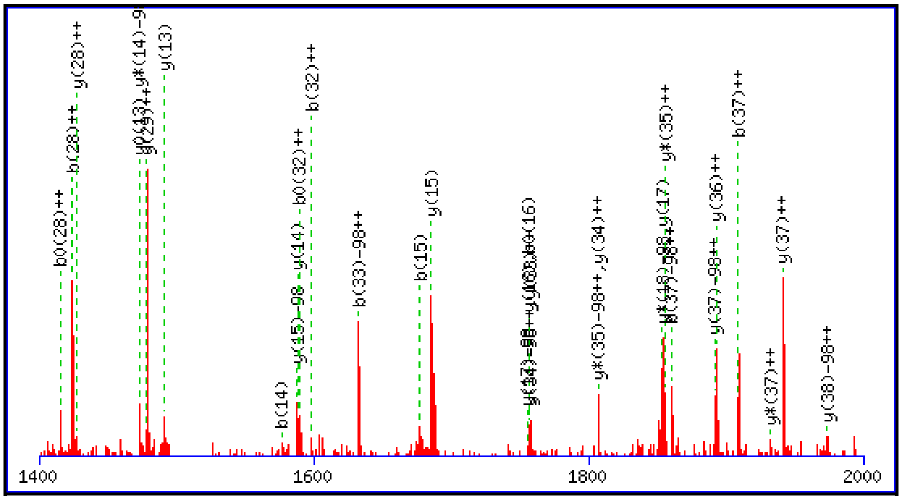

Monoisotopic mass of neutral peptide Mr(calc): 4531.0509
Fixed modifications: Carbamidomethyl (C)
Variable modifications:
S33 : Phospho (ST), with neutral losses 0.0000(shown in table), 97.9769
Ions Score: 51 **Expect:** 0.0037
Matches (Bold Red): 79/650 fragment ions using 131 most intense peaks

#	b	b^{++}	b^{0}	b^{0++}	Seq.	y	y^{++}	y*	y^{*++}	y^{0}	y^{0++}	#
1	102.0550	51.5311	84.0444	42.5258	T							43
2	189.0870	95.0471	171.0764	86.0418	S	4431.0104	2216.0088	4413.9839	2207.4956	4412.9999	2207.0036	42
3	288.1554	144.5813	270.1448	135.5761	V	4343.9784	2172.4928	4326.9518	2163.9796	4325.9678	2163.4875	41
4	389.2031	195.1052	371.1925	186.0999	T	4244.9100	2122.9586	4227.8834	2114.4454	4226.8994	2113.9533	40
5	**488.2715**	244.6394	470.2609	235.6341	V	4143.8623	2072.4348	4126.8357	2063.9215	4125.8517	2063.4295	39

6	**651.3348**	326.1710	**633.3243**	317.1658	Y	4044.7939	2022.9006	4027.7673	2014.3873	4026.7833	2013.8953	38
7	748.3876	374.6974	730.3770	365.6921	P	3881.7306	**1941.3689**	3864.7040	**1932.8556**	3863.7200	1932.3636	37
8	805.4090	403.2082	787.3985	394.2029	G	3784.6778	**1892.8425**	3767.6512	1884.3293	3766.6672	1883.8372	36
9	920.4360	460.7216	902.4254	451.7164	D	3727.6563	1864.3318	3710.6298	**1855.8185**	3709.6458	1855.3265	35
10	**1019.5044**	**510.2558**	1001.4938	501.2506	V	3612.6294	**1806.8183**	3595.6028	1798.3051	3594.6188	1797.8130	34
11	1166.5728	583.7900	1148.5623	574.7848	F	3513.5610	**1757.2841**	3496.5344	1748.7708	3495.5504	1748.2788	33
12	1326.6035	663.8054	1308.5929	654.8001	C	3366.4926	1683.7499	3349.4660	1675.2366	3348.4820	1674.7446	32
13	1439.6875	720.3474	1421.6770	711.3421	L	3206.4619	1603.7346	3189.4354	1595.2213	3188.4513	1594.7293	31
14	**1576.7464**	788.8769	1558.7359	779.8716	H	3093.3778	1547.1926	3076.3513	1538.6793	3075.3673	1538.1873	30
15	**1677.7941**	839.4007	1659.7836	830.3954	T	2956.3189	**1478.6631**	2939.2924	1470.1498	2938.3084	1469.6578	29
16	1774.8469	887.9271	**1756.8363**	**878.9218**	P	2855.2712	**1428.1393**	2838.2447	1419.6260	2837.2607	1419.1340	28
17	1831.8684	916.4378	1813.8578	907.4325	G	2758.2185	1379.6129	2741.1919	1371.0996	2740.2079	1370.6076	27
18	1888.8898	944.9485	1870.8792	935.9433	G	2701.1970	1351.1021	2684.1705	1342.5889	2683.1865	1342.0969	26
19	1945.9113	973.4593	1927.9007	964.4540	G	2644.1756	1322.5914	2627.1490	1314.0781	2626.1650	1313.5861	25
20	2002.9327	1001.9700	1984.9222	992.9647	G	2587.1541	1294.0807	2570.1275	1285.5674	2569.1435	1285.0754	24
21	2165.9961	1083.5017	2147.9855	**1074.4964**	Y	2530.1326	1265.5700	2513.1061	1257.0567	2512.1221	1256.5647	23
22	2223.0175	1112.0124	2205.0070	1103.0071	G	2367.0693	1184.0383	2350.0428	1175.5250	2349.0587	1175.0330	22
23	2338.0445	1169.5259	2320.0339	1160.5206	D	2310.0478	**1155.5276**	2293.0213	1147.0143	2292.0373	1146.5223	21
24	2435.0972	1218.0523	2417.0867	1209.0470	P	2195.0209	**1098.0141**	2177.9943	1089.5008	2177.0103	1089.0088	20
25	2564.1398	**1282.5736**	2546.1293	**1273.5683**	E	2097.9681	**1049.4877**	2080.9416	1040.9744	2079.9576	**1040.4824**	19
26	2679.1668	**1340.0870**	2661.1562	**1331.0817**	D	1968.9255	984.9664	1951.8990	**976.4531**	1950.9150	975.9611	18
27	2776.2195	1388.6134	2758.2090	1379.6081	P	**1853.8986**	**927.4529**	1836.8720	918.9397	1835.8880	918.4477	17
28	2847.2567	**1424.1320**	2829.2461	**1415.1267**	A	**1756.8458**	**878.9266**	1739.8193	870.4133	1738.8353	869.9213	16
29	2944.3094	1472.6583	2926.2989	1463.6531	P	**1685.8087**	**843.4080**	1668.7822	834.8947	1667.7982	834.4027	15
30	3041.3622	1521.1847	3023.3516	1512.1794	P	**1588.7560**	**794.8816**	1571.7294	786.3683	1570.7454	785.8763	14
31	3138.4149	1569.7111	3120.4044	1560.7058	P	**1491.7032**	**746.3552**	1474.6766	737.8420	**1473.6926**	737.3499	13
32	3195.4364	**1598.2218**	3177.4258	**1589.2166**	G	**1394.6504**	**697.8288**	1377.6239	689.3156	1376.6399	688.8236	12
33	3362.4348	1681.7210	3344.4242	1672.7157	S	1337.6290	669.3181	1320.6024	660.8048	1319.6184	660.3128	11
34	3459.4875	1730.2474	3441.4770	1721.2421	P	**1170.6306**	**585.8189**	1153.6041	577.3057	1152.6200	576.8137	10
35	3556.5403	1778.7738	3538.5297	1769.7685	P	**1073.5778**	537.2926	1056.5513	528.7793	1055.5673	528.2873	9
36	3669.6244	1835.3158	3651.6138	1826.3105	L	**976.5251**	**488.7662**	959.4985	480.2529	958.5145	479.7609	8
37	3816.6928	**1908.8500**	3798.6822	1899.8447	F	**863.4410**	432.2241	846.4145	423.7109	**845.4305**	423.2189	7
38	3913.7455	1957.3764	3895.7350	1948.3711	P	**716.3726**	358.6899	699.3461	350.1767	698.3620	349.6847	6
39	3984.7826	1992.8950	3966.7721	1983.8897	A	619.3198	310.1636	602.2933	301.6503	601.3093	301.1583	5
40	4131.8511	2066.4292	4113.8405	2057.4239	F	**548.2827**	274.6450	531.2562	266.1317	530.2722	265.6397	4
41	4228.9038	2114.9556	4210.8933	2105.9503	P	401.2143	201.1108	384.1878	192.5975	383.2037	192.1055	3
42	4357.9464	2179.4768	4339.9359	2170.4716	E	304.1615	152.5844	287.1350	144.0711	286.1510	143.5791	2
43					R	175.1190	88.0631	158.0924	79.5498			1

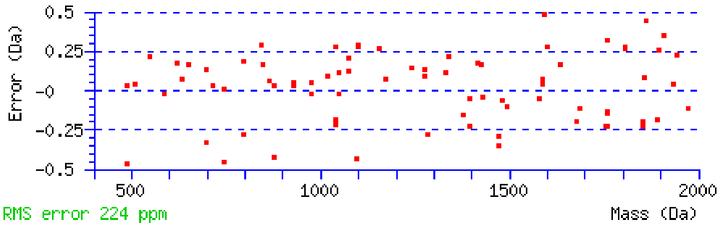

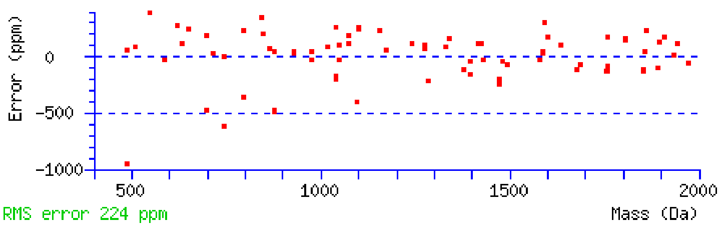

All matches to this query

Score	Mr(calc):	Delta	Sequence
51.4	4531.0509	1.0170	TSVTVYPGDVFCLHTPGGGGYGDPEDPAPPPGSPPLFPAFPER
23.0	4531.0509	1.0170	TSVTVYPGDVFCLHTPGGGGYGDPEDPAPPPGSPPLFPAFPER

8.4	4531.0509	1.0170	TSVTVYPGDVFCLHTPGGGGYGDPEDPAPPPGSPPLFPAFPER
8.4	4531.0509	1.0170	TSVTVYPGDVFCLHTPGGGGYGDPEDPAPPPGSPPLFPAFPER
8.4	4531.0509	1.0170	TSVTVYPGDVFCLHTPGGGGYGDPEDPAPPPGSPPLFPAFPER
8.4	4531.0509	1.0170	TSVTVYPGDVFCLHTPGGGGYGDPEDPAPPPGSPPLFPAFPER
7.0	4532.1119	-0.0441	GPLFSAPVVAVSPALLEAGLKPQVASSTMAQSENDDLFMMPR
6.4	4532.1119	-0.0441	GPLFSAPVVAVSPALLEAGLKPQVASSTMAQSENDDLFMMPR
6.4	4532.1119	-0.0441	GPLFSAPVVAVSPALLEAGLKPQVASSTMAQSENDDLFMMPR
5.7	4531.0350	1.0328	MASTPASYGNTTTKPMGLLSRVMNTGSQFVMEGVKNLVLK

Spectrum No: 281; Query: 2830; Rank: 1

Peptide View

MS/MS Fragmentation of **LIPLLEQSATEDDSEEEFFDAPCSPLEEYPQVSCR**
Found in **IPI00476312**, Tax_Id=10116 Gene_Symbol=RGD1311340_predicted 360 kDa protein

Match to Query 2830: 4180.808576 from(1046.209420,4+)
Title: 100101RatKid_NS_deglyco_23.5882.5882.4.dta
Data file K:\NewmanPaper\Piliang\3SubProteomes\Piliang3SP\mgf5ppm\ERLIC_3SubProteomes5ppm.mgf

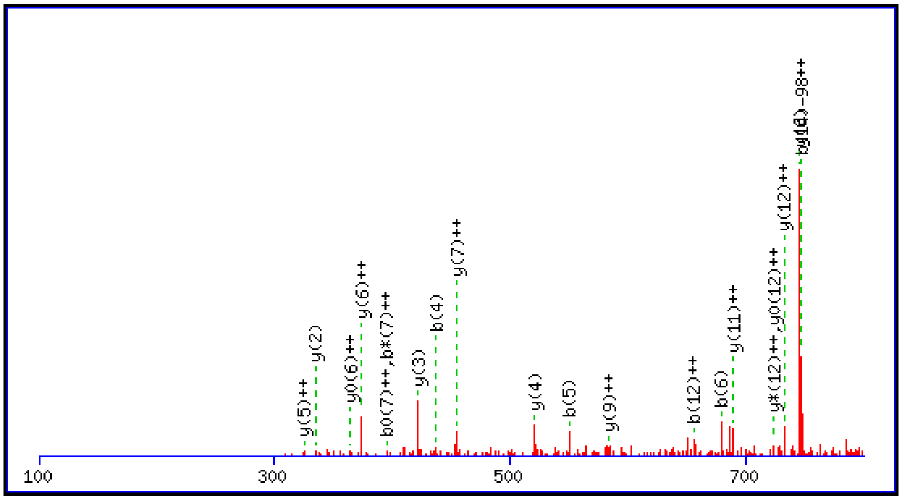

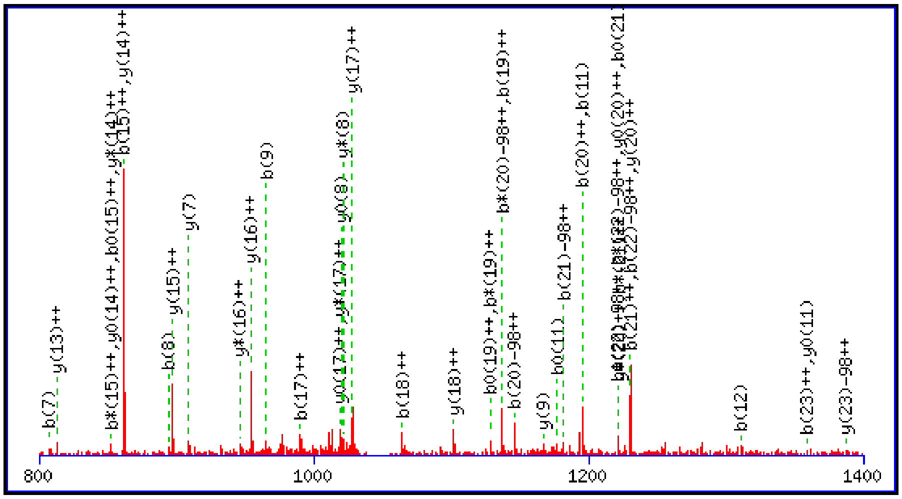

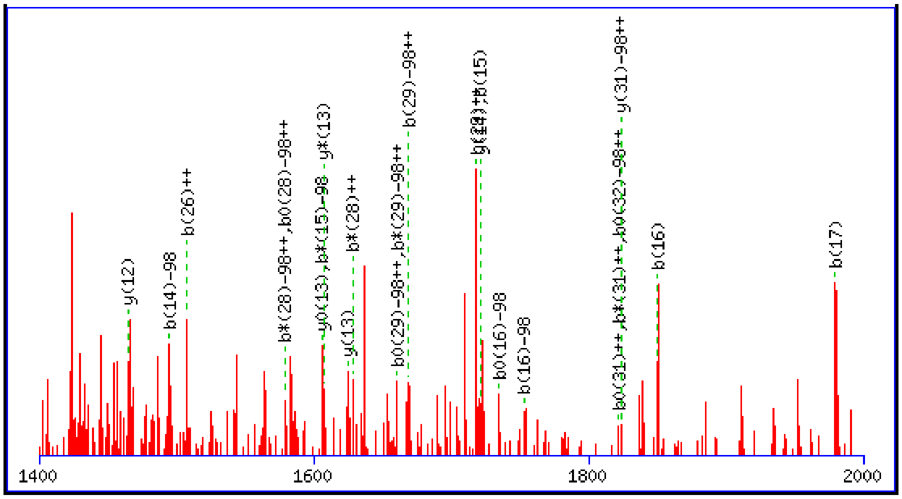

Monoisotopic mass of neutral peptide Mr(calc): 4179.7854
Fixed modifications: Carbamidomethyl (C)
Variable modifications:
S14 : Phospho (ST), with neutral losses 97.9769(shown in table), 0.0000
Ions Score: 51 **Expect:** 0.0041
Matches (Bold Red): 89/586 fragment ions using 155 most intense peaks

#	b	b++	b*	b*++	b0	b0++	Seq.	y	y++	y*	y*++	y0	y0++	#
1	114.0913	57.5493					L							35
2	227.1754	114.0913					I	3969.7317	1985.3695	3952.7052	1976.8562	3951.7212	1976.3642	34
3	324.2282	162.6177					P	3856.6477	1928.8275	3839.6211	1920.3142	3838.6371	1919.8222	33
4	**437.3122**	219.1598					L	3759.5949	1880.3011	3742.5683	1871.7878	3741.5843	1871.2958	32
5	**550.3963**	275.7018					L	3646.5108	**1823.7591**	3629.4843	1815.2458	3628.5003	1814.7538	31
6	**679.4389**	340.2231			661.4283	331.2178	E	3533.4268	1767.2170	3516.4002	1758.7037	3515.4162	1758.2117	30
7	**807.4975**	404.2524	790.4709	**395.7391**	789.4869	**395.2471**	Q	3404.3842	1702.6957	3387.3576	1694.1824	3386.3736	1693.6904	29
8	**894.5295**	447.7684	877.5029	439.2551	876.5189	438.7631	S	3276.3256	1638.6664	3259.2990	1630.1532	3258.3150	1629.6612	28
9	**965.5666**	483.2869	948.5401	474.7737	947.5560	474.2817	A	3189.2936	1595.1504	3172.2670	1586.6371	3171.2830	1586.1451	27
10	1066.6143	533.8108	1049.5877	525.2975	1048.6037	524.8055	T	3118.2564	1559.6319	3101.2299	1551.1186	3100.2459	1550.6266	26
11	**1195.6569**	598.3321	1178.6303	589.8188	**1177.6463**	589.3268	E	3017.2088	1509.1080	3000.1822	1500.5947	2999.1982	1500.1027	25
12	**1310.6838**	**655.8456**	1293.6573	647.3323	1292.6733	646.8403	D	2888.1662	1444.5867	2871.1396	1436.0735	2870.1556	1435.5814	24
13	1425.7108	713.3590	1408.6842	704.8457	1407.7002	704.3537	D	2773.1392	**1387.0733**	2756.1127	1378.5600	2755.1287	1378.0680	23
14	**1494.7322**	**747.8697**	1477.7057	739.3565	1476.7217	738.8645	S	2658.1123	1329.5598	2641.0857	1321.0465	2640.1017	1320.5545	22
15	1623.7748	812.3910	**1606.7483**	803.8778	1605.7643	803.3858	E	2589.0908	1295.0491	2572.0643	1286.5358	2571.0803	1286.0438	21
16	**1752.8174**	876.9123	1735.7909	868.3991	**1734.8068**	867.9071	E	2460.0482	**1230.5278**	2443.0217	**1222.0145**	2442.0377	**1221.5225**	20
17	1881.8600	941.4336	1864.8335	932.9204	1863.8494	932.4284	E	2331.0056	1166.0065	2313.9791	1157.4932	2312.9951	1157.0012	19
18	2028.9284	1014.9678	2011.9019	1006.4546	2010.9179	1005.9626	F	2201.9631	**1101.4852**	2184.9365	1092.9719	2183.9525	1092.4799	18
19	2175.9968	1088.5021	2158.9703	1079.9888	2157.9863	1079.4968	F	2054.8946	**1027.9510**	2037.8681	**1019.4377**	2036.8841	**1018.9457**	17
20	2291.0238	**1146.0155**	2273.9972	**1137.5022**	2273.0132	1137.0102	D	1907.8262	**954.4168**	1890.7997	**945.9035**	1889.8157	945.4115	16
21	2362.0609	**1181.5341**	2345.0343	1173.0208	2344.0503	1172.5288	A	1792.7993	**896.9033**	1775.7727	888.3900	1774.7887	887.8980	15
22	2459.1137	**1230.0605**	2442.0871	**1221.5472**	2441.1031	**1221.0552**	P	**1721.7622**	**861.3847**	1704.7356	**852.8714**	1703.7516	**852.3794**	14
23	2619.1443	1310.0758	2602.1178	1301.5625	2601.1337	1301.0705	C	**1624.7094**	**812.8583**	**1607.6829**	804.3451	**1606.6988**	803.8531	13
24	2706.1763	1353.5918	2689.1498	1345.0785	2688.1658	1344.5865	S	**1464.6788**	**732.8430**	1447.6522	**724.3297**	1446.6682	**723.8377**	12
25	2803.2291	1402.1182	2786.2025	1393.6049	2785.2185	1393.1129	P	1377.6467	**689.3270**	1360.6202	680.8137	**1359.6362**	680.3217	11
26	2916.3132	1458.6602	2899.2866	1450.1469	2898.3026	1449.6549	L	1280.5940	640.8006	1263.5674	632.2873	1262.5834	631.7953	10
27	3045.3557	1523.1815	3028.3292	1514.6682	3027.3452	1514.1762	E	**1167.5099**	**584.2586**	1150.4834	575.7453	1149.4993	575.2533	9
28	3174.3983	1587.7028	3157.3718	**1579.1895**	3156.3878	**1578.6975**	E	1038.4673	519.7373	**1021.4408**	511.2240	**1020.4567**	510.7320	8
29	3337.4617	**1669.2345**	3320.4351	**1660.7212**	3319.4511	**1660.2292**	Y	**909.4247**	**455.2160**	892.3982	446.7027	891.4141	446.2107	7
30	3434.5144	1717.7609	3417.4879	1709.2476	3416.5039	1708.7556	P	**746.3614**	**373.6843**	729.3348	365.1711	728.3508	**364.6790**	6
31	3562.5730	1781.7901	3545.5465	1773.2769	3544.5624	1772.7849	Q	649.3086	**325.1579**	632.2821	316.6447	631.2981	316.1527	5
32	3661.6414	1831.3244	3644.6149	1822.8111	3643.6309	**1822.3191**	V	**521.2500**	261.1287	504.2235	252.6154	503.2395	252.1234	4

33	3748.6735	1874.8404	3731.6469	1866.3271	3730.6629	1865.8351	S	422.1816	211.5945	405.1551	203.0812	404.1711	202.5892	3
34	3908.7041	1954.8557	3891.6776	1946.3424	3890.6935	1945.8504	C	335.1496	168.0784	318.1231	159.5652			2
35							R	175.1190	88.0631	158.0924	79.5498			1

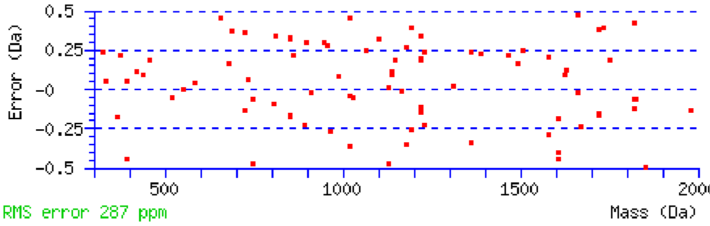

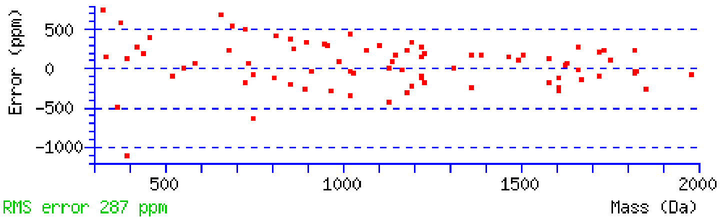

All matches to this query

Score	Mr(calc):	Delta	Sequence
51.3	4179.7854	1.0232	LIPLLEQSATEDDSEEEFFDAPCSPLEEYPQVSCR
49.6	4179.7854	1.0232	LIPLLEQSATEDDSEEEFFDAPCSPLEEYPQVSCR
42.2	4179.7854	1.0232	LIPLLEQSATEDDSEEEFFDAPCSPLEEYPQVSCR
18.7	4179.7854	1.0232	LIPLLEQSATEDDSEEEFFDAPCSPLEEYPQVSCR
7.8	4179.8196	0.9890	AYTTQSLASVAYLINTLANNVLQMLDIQASQLRR
7.5	4180.7848	0.0237	YISRFNADIAPYTTCLINGIYWEQNTPRLLTR
7.5	4180.7848	0.0237	YISRFNADIAPYTTCLINGIYWEQNTPRLLTR
7.1	4179.7670	1.0416	AYNTAGAGPFSATVNATTKKTPPSQPPGNVVWNATDTK
6.4	4178.8184	1.9902	GLMLSVMLASLMSSLTSIFNSASTLFTMDLYTKIR
6.4	4178.8184	1.9902	GLMLSVMLASLMSSLTSIFNSASTLFTMDLYTKIR

Spectrum No: 282; Query: 799; Rank: 1

Peptide View

MS/MS Fragmentation of **WLDESDAEMELR**
Found in **IPI00766722**, Tax_Id=10116 Gene_Symbol=LOC688570 similar to butyrate-induced transcript 1

Match to Query 799: 1572.619268 from(787.316910,2+)
Title: 100101RatKid_NS_deglyco_19.3852.3852.2.dta
Data file K:\NewmanPaper\Piliang\3SubProteomes\Piliang3SP\mgf5ppm\ERLIC_3SubProteomes5ppm.mgf

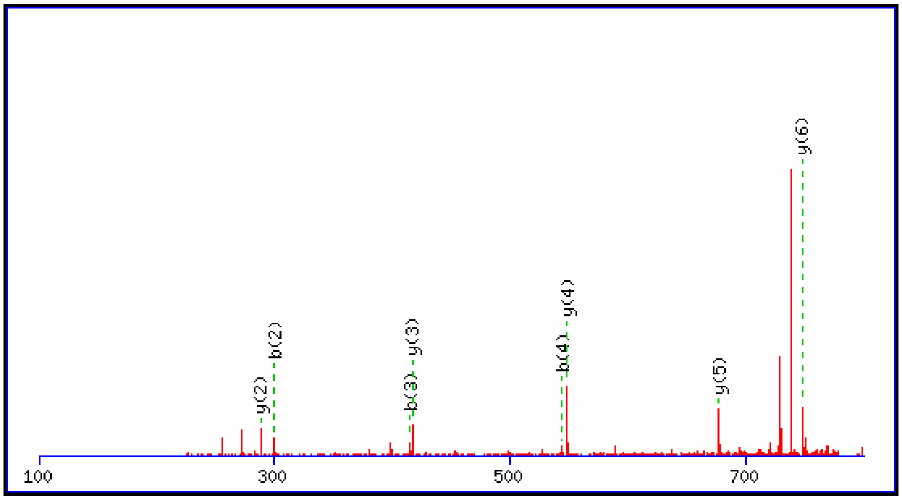

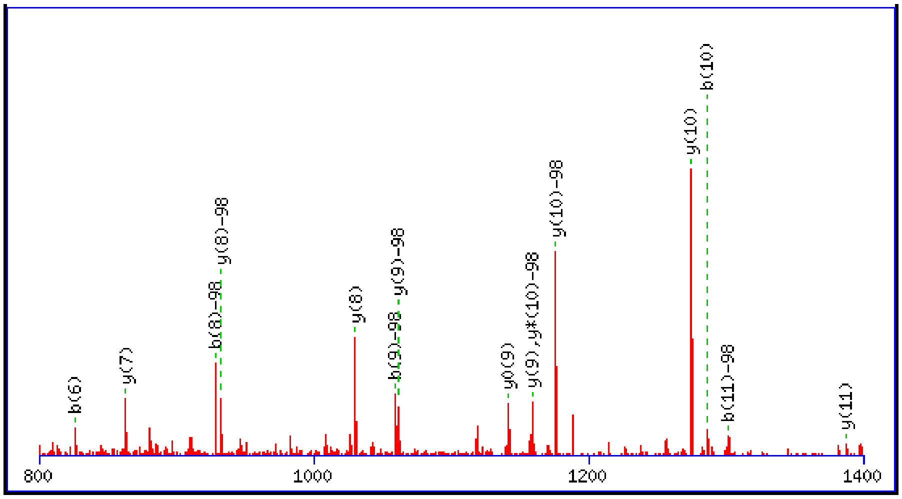

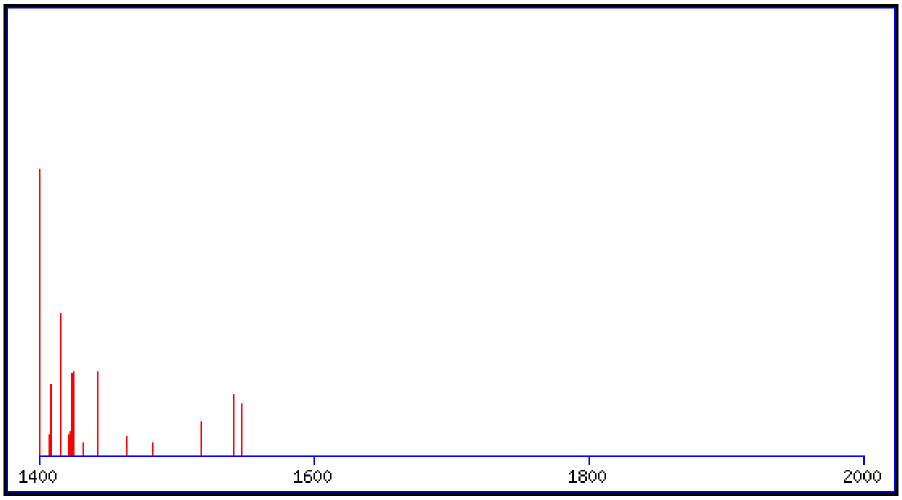

Monoisotopic mass of neutral peptide Mr(calc): 1572.6167
Fixed modifications: Carbamidomethyl (C)
Variable modifications:
S5 : Phospho (ST), with neutral losses 97.9769(shown in table), 0.0000
Ions Score: 51 **Expect:** 0.00079
Matches (Bold Red): 23/154 fragment ions using 50 most intense peaks

#	b	b^{++}	b^0	b^{0++}	Seq.	y	y^{++}	y*	y^{*++}	y^0	y^{0++}	#
1	187.0866	94.0469			W							12
2	**300.1707**	150.5890			L	1289.5678	645.2875	1272.5413	636.7743	1271.5572	636.2823	11
3	**415.1976**	208.1024	397.1870	199.0972	D	**1176.4837**	588.7455	**1159.4572**	580.2322	1158.4732	579.7402	10
4	**544.2402**	272.6237	526.2296	263.6185	E	**1061.4568**	531.2320	1044.4302	522.7188	1043.4462	522.2268	9
5	613.2616	307.1345	595.2511	298.1292	S	**932.4142**	466.7107	915.3877	458.1975	914.4036	457.7055	8
6	728.2886	364.6479	710.2780	355.6426	D	**863.3927**	432.2000	846.3662	423.6867	845.3822	423.1947	7
7	799.3257	400.1665	781.3151	391.1612	A	**748.3658**	374.6865	731.3393	366.1733	730.3552	365.6813	6
8	**928.3683**	464.6878	910.3577	455.6825	E	**677.3287**	339.1680	660.3021	330.6547	659.3181	330.1627	5
9	**1059.4088**	530.2080	1041.3982	521.2027	M	**548.2861**	274.6467	531.2595	266.1334	530.2755	265.6414	4
10	1188.4514	594.7293	1170.4408	585.7240	E	**417.2456**	209.1264	400.2191	200.6132	399.2350	200.1212	3
11	**1301.5354**	651.2714	1283.5249	642.2661	L	**288.2030**	144.6051	271.1765	136.0919			2
12					R	175.1190	88.0631	158.0924	79.5498			1

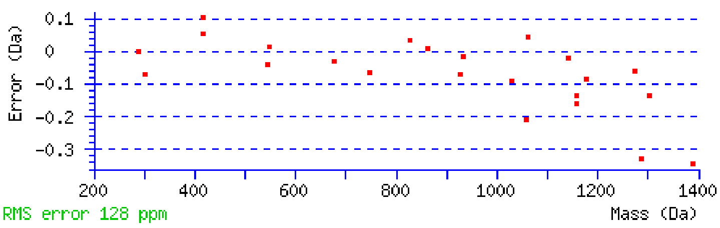

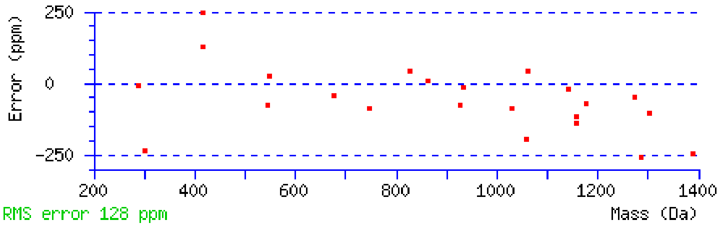

All matches to this query

Score	Mr(calc):	Delta	Sequence
51.3	1572.6167	0.0026	WLDESDAEMELR
0.1	1572.6123	0.0070	YRSIRGEDHPPS

Spectrum No: 283; Query: 512; Rank: 1

Peptide View

MS/MS Fragmentation of **KVMDSDEDDDY**
Found in **IPI00193547**, Tax_Id=10116 Gene_Symbol=Pdcd5_predicted programmed cell death 5

Match to Query 512: 1410.452048 from(706.233300,2+)
Title: 091008RatKidney_NoSalt_33.773.773.2.dta
Data file K:\NewmanPaper\Piliang\3SubProteomes\Piliang3SP\mgf5ppm\ERLIC_3SubProteomes5ppm.mgf

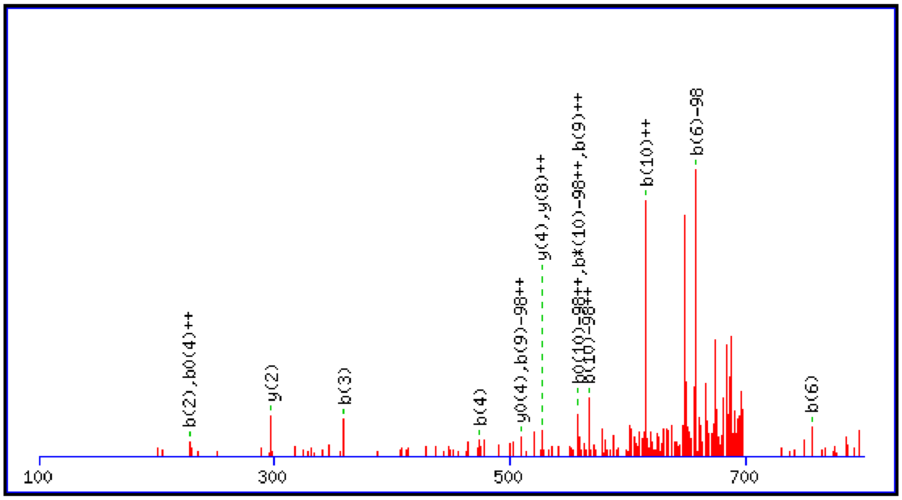

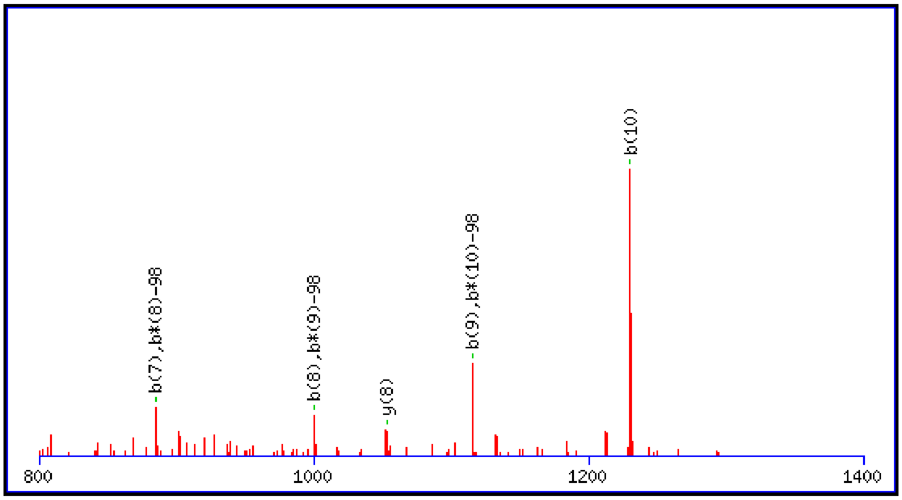

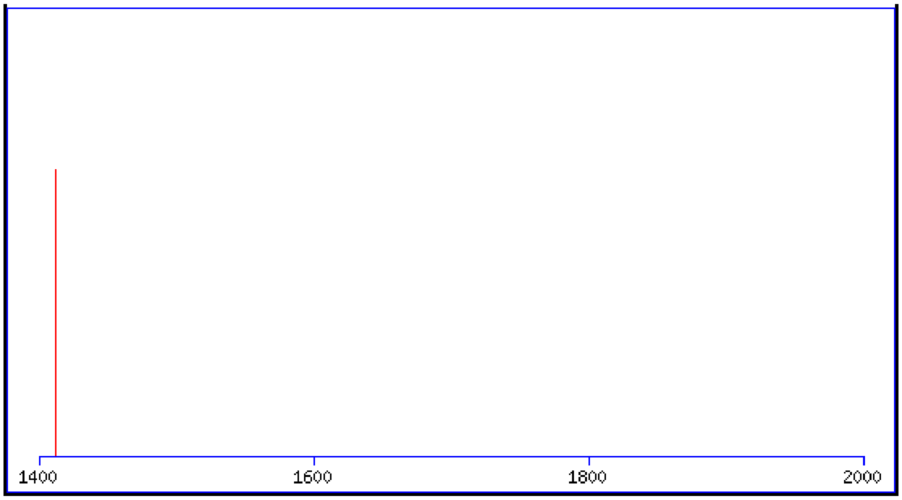

Monoisotopic mass of neutral peptide Mr(calc): 1410.4534
Fixed modifications: Carbamidomethyl (C)
Variable modifications:
S5 : Phospho (ST), with neutral losses 0.0000(shown in table), 97.9769
Ions Score: 51 **Expect:** 0.0002
Matches (Bold Red): 24/144 fragment ions using 44 most intense peaks

#	b	b^{++}	b*	b^{*++}	b^0	b^{0++}	Seq.	y	y^{++}	y^0	y^{0++}	#
1	129.1022	65.0548	112.0757	56.5415			K					11
2	**228.1707**	114.5890	211.1441	106.0757			V	1283.3657	642.1865	1265.3552	633.1812	10
3	**359.2111**	180.1092	342.1846	171.5959			M	1184.2973	592.6523	1166.2868	583.6470	9
4	**474.2381**	237.6227	457.2115	229.1094	456.2275	**228.6174**	D	**1053.2568**	**527.1321**	1035.2463	518.1268	8
5	641.2364	321.1219	624.2099	312.6086	623.2259	312.1166	S	938.2299	469.6186	920.2193	460.6133	7
6	**756.2634**	378.6353	739.2368	370.1221	738.2528	369.6300	D	771.2315	386.1194	753.2210	377.1141	6
7	**885.3060**	443.1566	868.2794	434.6433	867.2954	434.1513	E	656.2046	328.6059	638.1940	319.6007	5
8	**1000.3329**	500.6701	983.3064	492.1568	982.3223	491.6648	D	**527.1620**	264.0846	**509.1514**	255.0794	4
9	**1115.3599**	**558.1836**	1098.3333	549.6703	1097.3493	549.1783	D	412.1351	206.5712	394.1245	197.5659	3
10	**1230.3868**	**615.6970**	1213.3602	607.1838	1212.3762	606.6918	D	**297.1081**	149.0577	279.0975	140.0524	2
11							Y	182.0812	91.5442			1

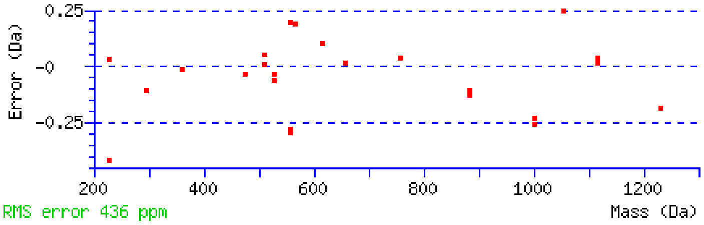

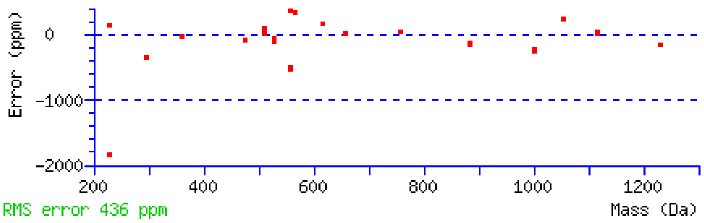

All matches to this query

Score	Mr(calc):	Delta	Sequence
51.3	1410.4534	-0.0014	KVMDSDEDDDY

Spectrum No: 284; Query: 1934; Rank: 1

Peptide View

MS/MS Fragmentation of **KLSGDQITLPTTVDYSSVPK**
Found in **IPI00366400**, Tax_Id=10116 Gene_Symbol=Ebag9 Receptor-binding cancer antigen expressed on SiSo cells (Fragment)

Match to Query 1934: 2228.096628 from(1115.055590,2+)

Title: 091008RatKidney_NH4Format02_23.3680.3680.2.dta
Data file K:\NewmanPaper\Piliang\3SubProteomes\Piliang3SP\mgf5ppm\ERLIC_3SubProteomes5ppm.mgf

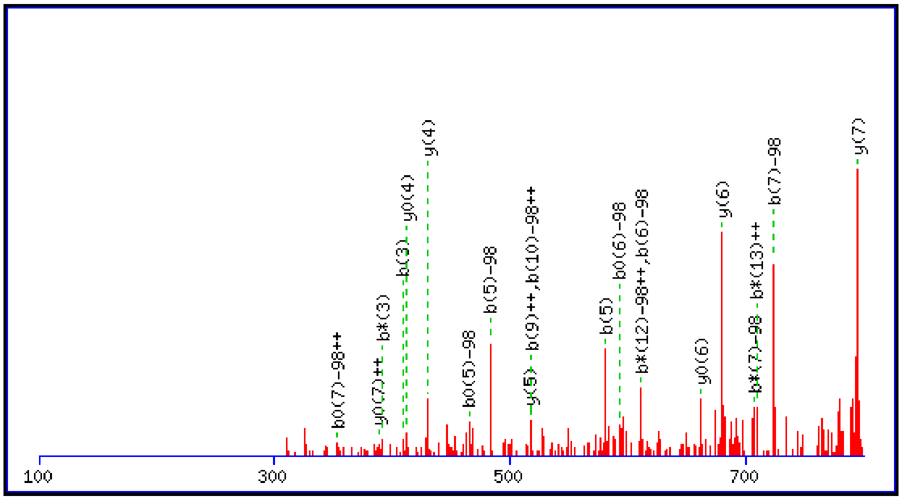

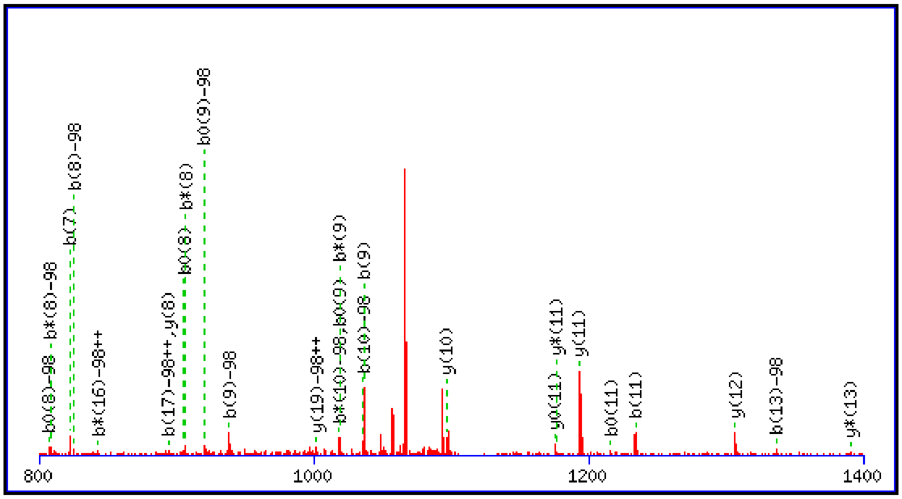

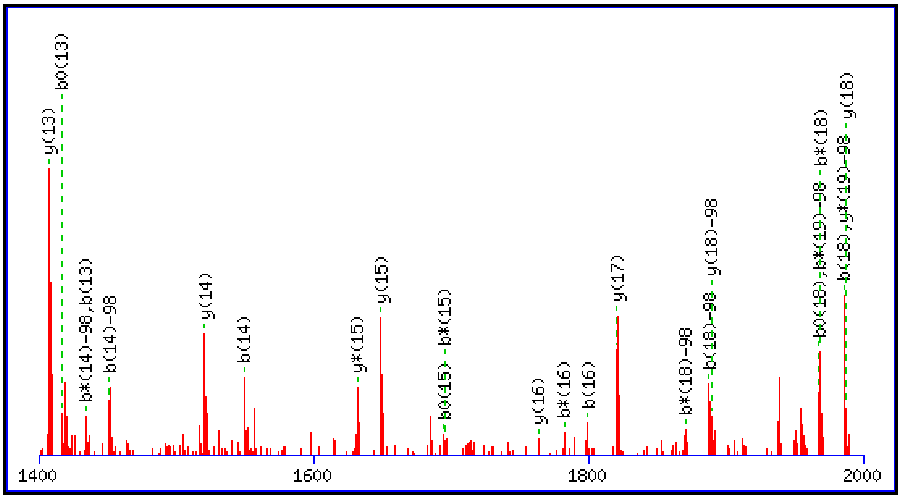

Monoisotopic mass of neutral peptide Mr(calc): 2228.0977
Fixed modifications: Carbamidomethyl (C)
Variable modifications:
S3 : Phospho (ST), with neutral losses 97.9769(shown in table), 0.0000
Ions Score: 51 **Expect:** 0.0016
Matches (Bold Red): 71/332 fragment ions using 133 most intense peaks

#	b	b++	b*	b*++	b0	b0++	Seq.	y	y++	y*	y*++	y0	y0++	#
1	129.1022	65.0548	112.0757	56.5415			K							20
2	242.1863	121.5968	225.1598	113.0835			L	2003.0332	**1002.0202**	**1986.0066**	993.5069	1985.0226	993.0149	19
3	311.2078	156.1075	294.1812	147.5942	293.1972	147.1022	S	**1889.9491**	945.4782	1872.9225	936.9649	1871.9385	936.4729	18

4	368.2292	184.6182	351.2027	176.1050	350.2187	175.6130	G	**1820.9276**	910.9675	1803.9011	902.4542	1802.9171	901.9622	17
5	**483.2562**	242.1317	466.2296	233.6184	**465.2456**	233.1264	D	**1763.9062**	882.4567	1746.8796	873.9435	1745.8956	873.4514	16
6	**611.3147**	306.1610	594.2882	297.6477	**593.3042**	297.1557	Q	**1648.8792**	824.9433	**1631.8527**	816.4300	1630.8687	815.9380	15
7	**724.3988**	362.7030	**707.3723**	354.1898	706.3882	**353.6978**	I	**1520.8207**	760.9140	1503.7941	752.4007	1502.8101	751.9087	14
8	**825.4465**	413.2269	**808.4199**	404.7136	**807.4359**	404.2216	T	**1407.7366**	704.3719	**1390.7100**	695.8587	1389.7260	695.3667	13
9	**938.5306**	469.7689	921.5040	461.2556	**920.5200**	460.7636	L	**1306.6889**	653.8481	1289.6624	645.3348	1288.6783	644.8428	12
10	**1035.5833**	**518.2953**	**1018.5568**	509.7820	1017.5728	509.2900	P	**1193.6048**	597.3061	**1176.5783**	588.7928	**1175.5943**	588.3008	11
11	1136.6310	568.8191	1119.6044	560.3059	1118.6204	559.8139	T	**1096.5521**	548.7797	1079.5255	540.2664	1078.5415	539.7744	10
12	1237.6787	619.3430	1220.6521	**610.8297**	1219.6681	610.3377	T	995.5044	498.2558	978.4779	489.7426	977.4938	489.2506	9
13	**1336.7471**	668.8772	1319.7205	660.3639	1318.7365	659.8719	V	**894.4567**	447.7320	877.4302	439.2187	876.4462	438.7267	8
14	**1451.7740**	726.3907	**1434.7475**	717.8774	1433.7635	717.3854	D	**795.3883**	398.1978	778.3618	389.6845	777.3777	**389.1925**	7
15	1614.8374	807.9223	1597.8108	799.4090	1596.8268	798.9170	Y	**680.3614**	340.6843	663.3348	332.1710	**662.3508**	331.6790	6
16	1701.8694	851.4383	1684.8428	**842.9251**	1683.8588	842.4330	S	**517.2980**	259.1527	500.2715	250.6394	499.2875	250.1474	5
17	1788.9014	**894.9543**	1771.8749	886.4411	1770.8909	885.9491	S	**430.2660**	215.6366	413.2395	207.1234	**412.2554**	206.6314	4
18	**1887.9698**	944.4886	**1870.9433**	935.9753	1869.9593	935.4833	V	343.2340	172.1206	326.2074	163.6074			3
19	1985.0226	993.0149	**1967.9960**	984.5017	1967.0120	984.0097	P	244.1656	122.5864	227.1390	114.0731			2
20							K	147.1128	74.0600	130.0863	65.5468			1

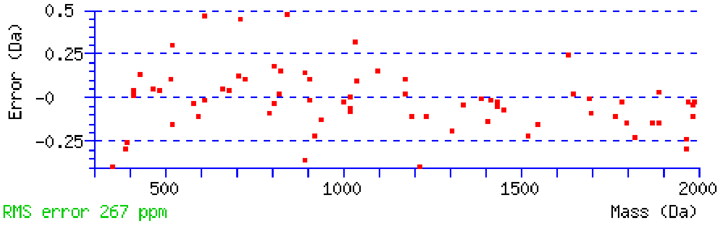

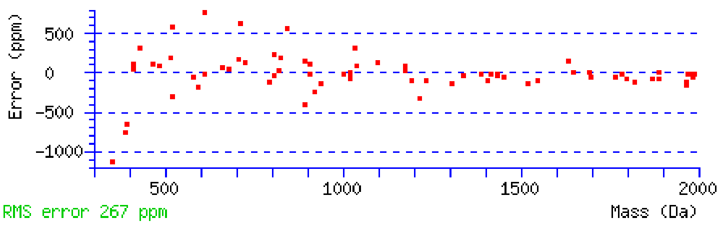

All matches to this query

Score	Mr(calc):	Delta	Sequence
51.3	2228.0977	-0.0011	KLSGDQITLPTTVDYSSVPK
18.6	2228.0977	-0.0011	KLSGDQITLPTTVDYSSVPK
14.7	2228.0895	0.0072	NKTVSTLVIQAAYVSALYK
14.7	2228.0895	0.0072	NKTVSTLVIQAAYVSALYK
14.7	2228.0895	0.0072	NKTVSTLVIQAAYVSALYK
12.9	2228.0977	-0.0011	KLSGDQITLPTTVDYSSVPK
12.5	2228.0977	-0.0011	KLSGDQITLPTTVDYSSVPK
7.7	2228.1177	-0.0211	MPKGPSNKLSHLTFITHPK
6.8	2228.0895	0.0072	NKTVSTLVIQAAYVSALYK
6.8	2228.0895	0.0072	NKTVSTLVIQAAYVSALYK

Spectrum No: 285; Query: 1287; Rank: 1

Peptide View

MS/MS Fragmentation of **SGSGSPPPTTEEVAATTR**
Found in **IPI00201120**, Tax_Id=10116 Gene_Symbol=Slc5a2 Low affinity Na-dependent glucose transporter SGLT2 delta e trans

Match to Query 1287: 1823.798188 from(912.906370,2+)
Title: 100101RatKid_NS_deglyco_18.1602.1602.2.dta
Data file K:\NewmanPaper\Piliang\3SubProteomes\Piliang3SP\mgf5ppm\ERLIC_3SubProteomes5ppm.mgf

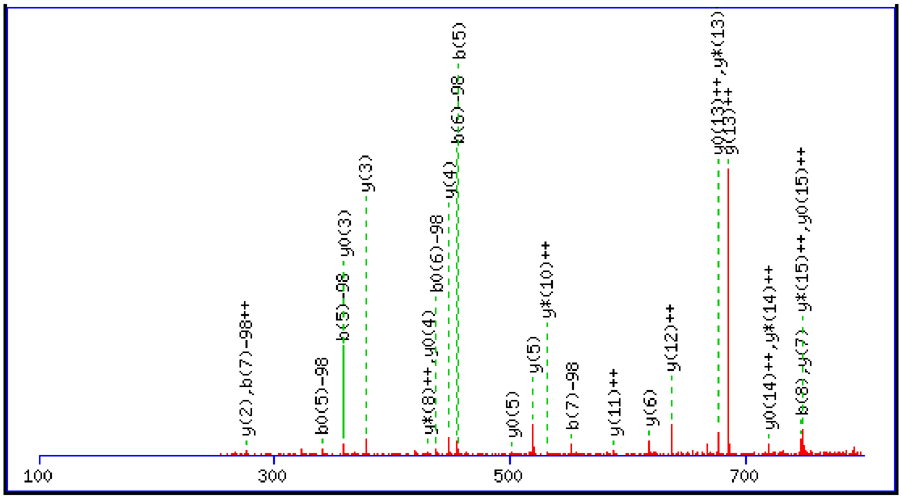

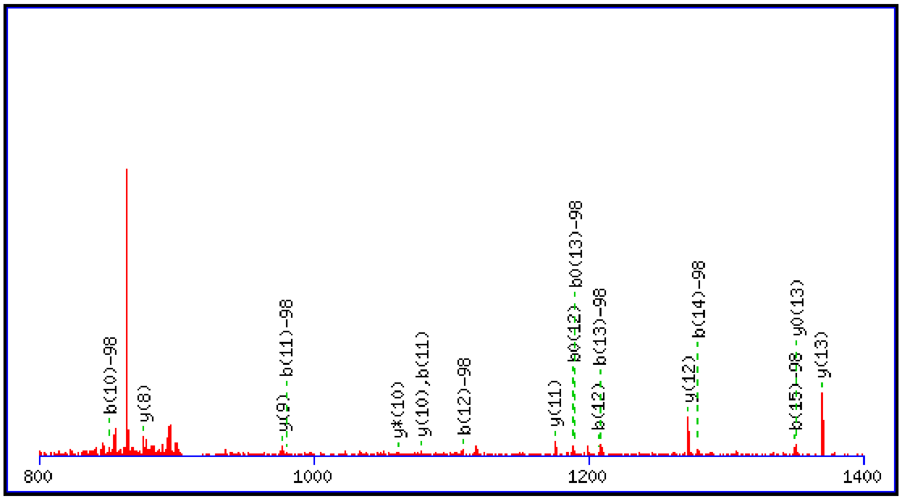

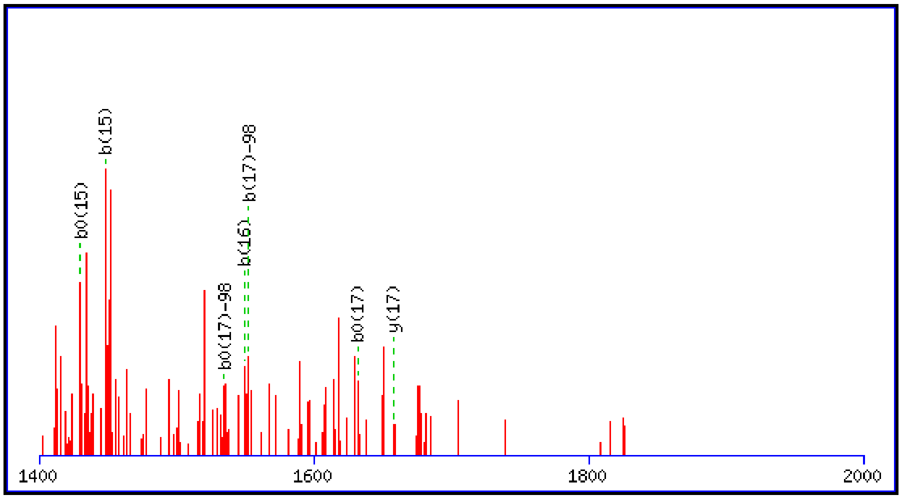

Monoisotopic mass of neutral peptide Mr(calc): 1823.7939
Fixed modifications: Carbamidomethyl (C)
Variable modifications:
S1 : Phospho (ST), with neutral losses 97.9769(shown in table), 0.0000
Ions Score: 51 **Expect:** 0.0016
Matches (Bold Red): 53/236 fragment ions using 134 most intense peaks

#	b	b^{++}	b^0	b^{0++}	Seq.	y	y^{++}	y*	y^{*++}	y^0	y^{0++}	#
**1**	70.0287	35.5180	52.0182	26.5127	**S**							**18**
**2**	127.0502	64.0287	109.0396	55.0235	**G**	**1657.8028**	829.4050	1640.7762	820.8917	1639.7922	820.3997	**17**
**3**	214.0822	107.5448	196.0717	98.5395	**S**	1600.7813	800.8943	1583.7548	792.3810	1582.7707	791.8890	**16**
**4**	271.1037	136.0555	253.0931	127.0502	**G**	1513.7493	757.3783	1496.7227	**748.8650**	1495.7387	**748.3730**	**15**
**5**	**358.1357**	179.5715	**340.1252**	170.5662	**S**	1456.7278	728.8675	1439.7013	**720.3543**	1438.7173	**719.8623**	**14**

**6**	**455.1885**	228.0979	**437.1779**	219.0926	**P**	**1369.6958**	**685.3515**	1352.6692	**676.8383**	**1351.6852**	**676.3462**	**13**
**7**	**552.2412**	**276.6243**	534.2307	267.6190	**P**	**1272.6430**	**636.8252**	1255.6165	628.3119	1254.6325	627.8199	**12**
**8**	649.2940	325.1506	631.2834	316.1454	**P**	**1175.5903**	**588.2988**	1158.5637	579.7855	1157.5797	579.2935	**11**
**9**	750.3417	375.6745	732.3311	366.6692	**T**	**1078.5375**	539.7724	**1061.5109**	**531.2591**	1060.5269	530.7671	**10**
**10**	**851.3894**	426.1983	833.3788	417.1930	**T**	**977.4898**	489.2485	960.4633	480.7353	959.4793	480.2433	**9**
**11**	**980.4320**	490.7196	962.4214	481.7143	**E**	**876.4421**	438.7247	859.4156	**430.2114**	858.4316	429.7194	**8**
**12**	**1109.4746**	555.2409	1091.4640	546.2356	**E**	**747.3995**	374.2034	730.3730	365.6901	729.3890	365.1981	**7**
**13**	**1208.5430**	604.7751	**1190.5324**	595.7698	**V**	**618.3570**	309.6821	601.3304	301.1688	600.3464	300.6768	**6**
**14**	**1279.5801**	640.2937	1261.5695	631.2884	**A**	**519.2885**	260.1479	502.2620	251.6346	**501.2780**	251.1426	**5**
**15**	**1350.6172**	675.8122	1332.6066	666.8070	**A**	**448.2514**	224.6293	431.2249	216.1161	**430.2409**	215.6241	**4**
**16**	1451.6649	726.3361	1433.6543	717.3308	**T**	**377.2143**	189.1108	360.1878	180.5975	**359.2037**	180.1055	**3**
**17**	**1552.7126**	776.8599	**1534.7020**	767.8546	**T**	**276.1666**	138.5870	259.1401	130.0737	258.1561	129.5817	**2**
**18**					**R**	175.1190	88.0631	158.0924	79.5498			**1**

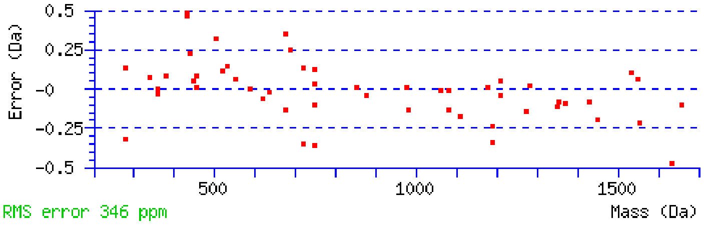

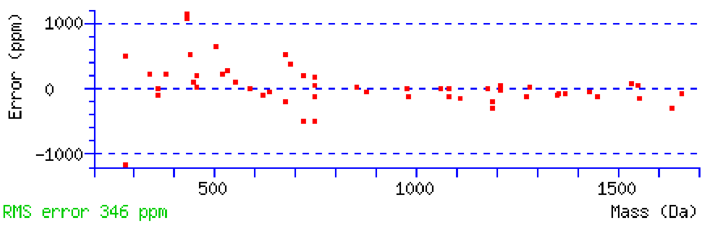

All matches to this query

Score	Mr(calc):	Delta	Sequence
51.3	1823.7939	0.0043	SGSGSPPPTTEEVAATTR
50.7	1823.7939	0.0043	SGSGSPPPTTEEVAATTR
50.7	1823.7939	0.0043	SGSGSPPPTTEEVAATTR
23.9	1823.7939	0.0043	SGSGSPPPTTEEVAATTR
18.9	1823.7939	0.0043	SGSGSPPPTTEEVAATTR
11.9	1823.7896	0.0086	NVLDTSWPTPPPALR
7.6	1822.7838	1.0144	IPMPPSSPQPRSTPR
6.3	1823.7882	0.0100	MIISAHPNLMSCNTR
6.3	1823.7896	0.0086	NVLDTSWPTPPPALR
6.3	1823.7896	0.0086	NVLDTSWPTPPPALR

Spectrum No: 286; Query: 1970; Rank: 1

Peptide View

MS/MS Fragmentation of **SDSSTEESGQDVLENTFSQK**
Found in **IPI00211776**, Tax_Id=10116 Gene_Symbol=Golgb1 Rat GCP360

Match to Query 1970: 2266.911608 from(1134.463080,2+)
Title: 091008RatKidney_NoSalt_27.2940.2940.2.dta
Data file K:\NewmanPaper\Piliang\3SubProteomes\Piliang3SP\mgf5ppm\ERLIC_3SubProteomes5ppm.mgf

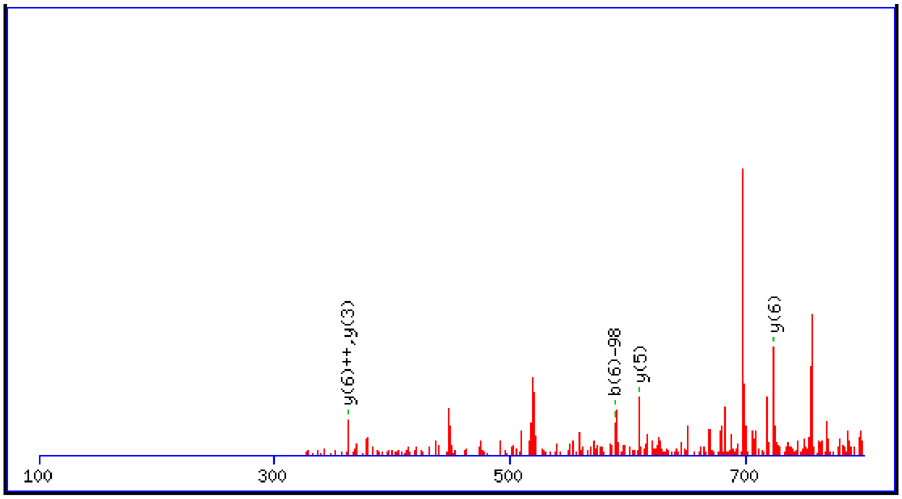

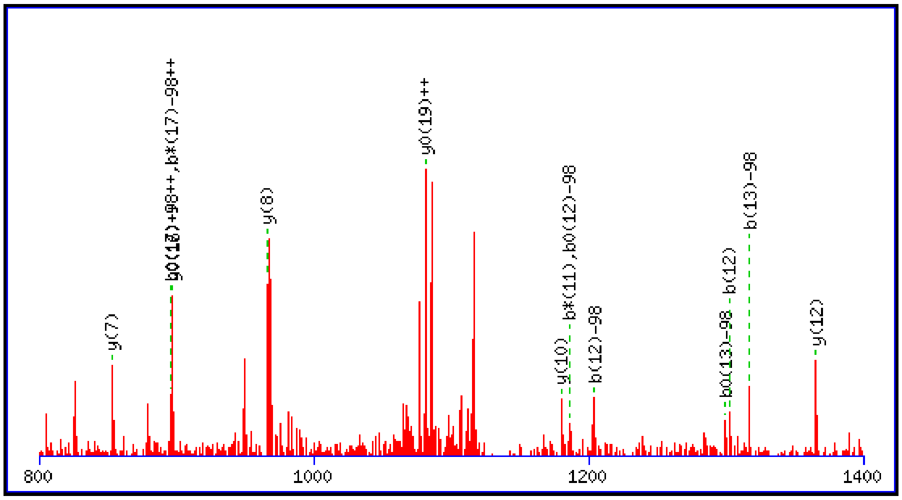

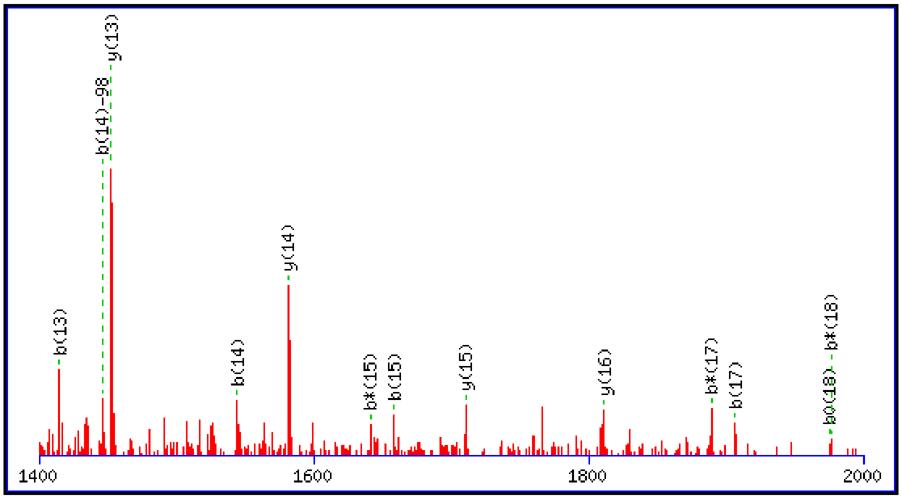

Monoisotopic mass of neutral peptide Mr(calc): 2266.9115
Fixed modifications: Carbamidomethyl (C)
Variable modifications:
S4 : Phospho (ST), with neutral losses 0.0000(shown in table), 97.9769
Ions Score: 51 **Expect:** 0.0015
Matches (Bold Red): 32/308 fragment ions using 54 most intense peaks

#	b	b^{++}	b*	b^{*++}	b^0	b^{0++}	Seq.	y	y^{++}	y*	y^{*++}	y^0	y^{0++}	#
1	88.0393	44.5233			70.0287	35.5180	S							20
2	203.0662	102.0368			185.0557	93.0315	D	2180.8867	1090.9470	2163.8602	1082.4337	2162.8761	**1081.9417**	19
3	290.0983	145.5528			272.0877	136.5475	S	2065.8598	1033.4335	2048.8332	1024.9202	2047.8492	1024.4282	18
4	457.0966	229.0520			439.0861	220.0467	S	1978.8277	989.9175	1961.8012	981.4042	1960.8172	980.9122	17
5	558.1443	279.5758			540.1337	270.5705	T	**1811.8294**	906.4183	1794.8028	897.9051	1793.8188	**897.4130**	16

**6**	687.1869	344.0971			669.1763	335.0918	**E**	**1710.7817**	855.8945	1693.7552	847.3812	1692.7711	846.8892	**15**
**7**	816.2295	408.6184			798.2189	399.6131	**E**	**1581.7391**	791.3732	1564.7126	782.8599	1563.7285	782.3679	**14**
**8**	903.2615	452.1344			885.2510	443.1291	**S**	**1452.6965**	726.8519	1435.6700	718.3386	1434.6859	717.8466	**13**
**9**	960.2830	480.6451			942.2724	471.6398	**G**	**1365.6645**	683.3359	1348.6379	674.8226	1347.6539	674.3306	**12**
**10**	1088.3416	544.6744	1071.3150	536.1611	1070.3310	535.6691	**Q**	1308.6430	654.8251	1291.6165	646.3119	1290.6325	645.8199	**11**
**11**	1203.3685	602.1879	**1186.3420**	593.6746	1185.3579	593.1826	**D**	**1180.5844**	590.7959	1163.5579	582.2826	1162.5739	581.7906	**10**
**12**	**1302.4369**	651.7221	1285.4104	643.2088	1284.4264	642.7168	**V**	1065.5575	533.2824	1048.5310	524.7691	1047.5469	524.2771	**9**
**13**	**1415.5210**	708.2641	1398.4944	699.7509	1397.5104	699.2588	**L**	**966.4891**	483.7482	949.4625	475.2349	948.4785	474.7429	**8**
**14**	**1544.5636**	772.7854	1527.5370	764.2722	1526.5530	763.7801	**E**	**853.4050**	427.2061	836.3785	418.6929	835.3945	418.2009	**7**
**15**	**1658.6065**	829.8069	**1641.5800**	821.2936	1640.5959	820.8016	**N**	**724.3624**	**362.6849**	707.3359	354.1716	706.3519	353.6796	**6**
**16**	1759.6542	880.3307	1742.6276	871.8175	1741.6436	871.3254	**T**	**610.3195**	305.6634	593.2930	297.1501	592.3089	296.6581	**5**
**17**	**1906.7226**	953.8649	**1889.6960**	945.3517	1888.7120	944.8597	**F**	509.2718	255.1396	492.2453	246.6263	491.2613	246.1343	**4**
**18**	1993.7546	997.3810	**1976.7281**	988.8677	**1975.7441**	988.3757	**S**	**362.2034**	181.6053	345.1769	173.0921	344.1928	172.6001	**3**
**19**	2121.8132	1061.4102	2104.7867	1052.8970	2103.8026	1052.4050	**Q**	275.1714	138.0893	258.1448	129.5761			**2**
**20**							**K**	147.1128	74.0600	130.0863	65.5468			**1**

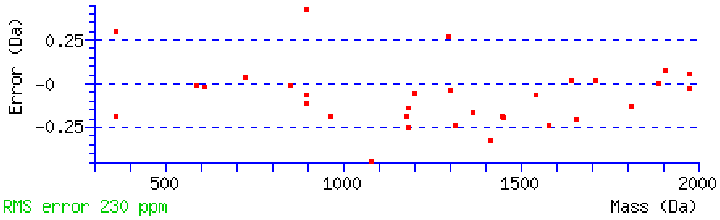

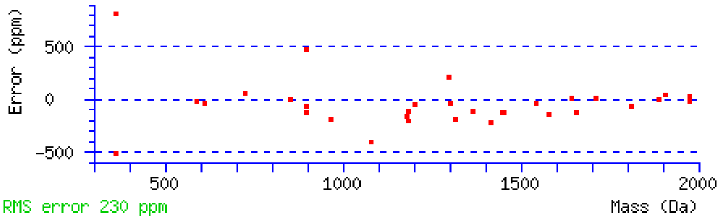

All matches to this query

Score	Mr(calc):	Delta	Sequence
51.3	2266.9115	0.0002	SDSSTEESGQDVLENTFSQK
51.3	2266.9115	0.0002	SDSSTEESGQDVLENTFSQK
50.4	2266.9115	0.0002	SDSSTEESGQDVLENTFSQK
39.7	2266.9115	0.0002	SDSSTEESGQDVLENTFSQK
29.5	2266.9115	0.0002	SDSSTEESGQDVLENTFSQK

Spectrum No: 287; Query: 2348; Rank: 1

Peptide View

MS/MS Fragmentation of **DSDRVAQSDGEESPAEEGQLLGER**
Found in **IPI00210147**, Tax_Id=10116 Gene_Symbol=Sec31l1 Isoform 1 of Protein transport protein Sec31A

Match to Query 2348: 2653.126782 from(885.382870,3+)
Title: 100101RatKid_NS_deglyco_23.2130.2130.3.dta
Data file K:\NewmanPaper\Piliang\3SubProteomes\Piliang3SP\mgf5ppm\ERLIC_3SubProteomes5ppm.mgf

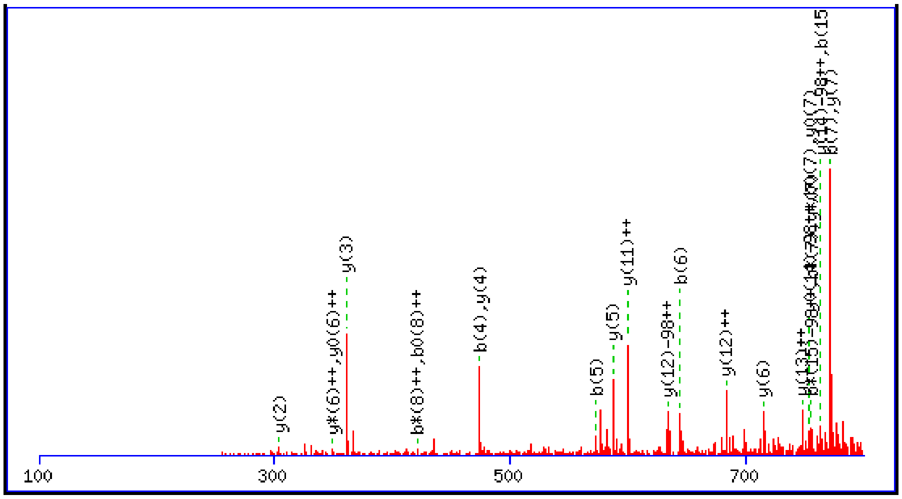

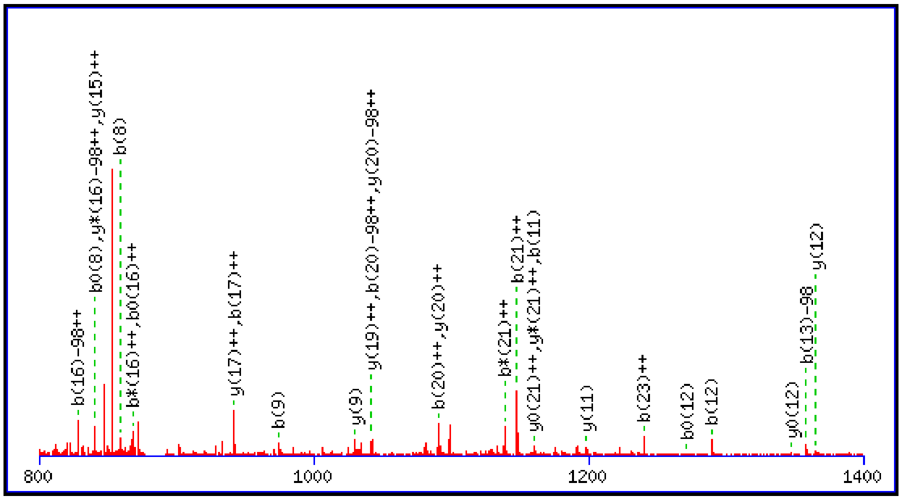

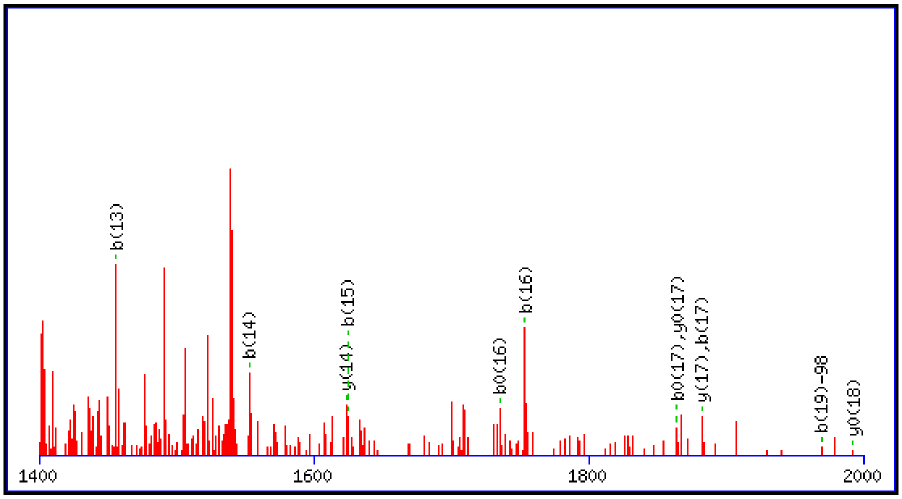

Monoisotopic mass of neutral peptide Mr(calc): 2653.1140
Fixed modifications: Carbamidomethyl (C)
Variable modifications:
S13 : Phospho (ST), with neutral losses 0.0000(shown in table), 97.9769
Ions Score: 51 **Expect:** 0.0028
Matches (Bold Red): 66/406 fragment ions using 99 most intense peaks

#	b	b^{++}	b*	b^{*++}	b^0	b^{0++}	Seq.	y	y^{++}	y*	y^{*++}	y^0	y^{0++}	#
1	116.0342	58.5207			98.0237	49.5155	D							24
2	203.0662	102.0368			185.0557	93.0315	S	2539.0944	1270.0508	2522.0679	1261.5376	2521.0838	1261.0456	23
3	318.0932	159.5502			300.0826	150.5450	D	2452.0624	1226.5348	2435.0358	1218.0216	2434.0518	1217.5295	22
4	**474.1943**	237.6008	457.1678	229.0875	456.1837	228.5955	R	2337.0354	1169.0214	2320.0089	**1160.5081**	2319.0249	**1160.0161**	21
5	**573.2627**	287.1350	556.2362	278.6217	555.2522	278.1297	V	2180.9343	**1090.9708**	2163.9078	1082.4575	2162.9238	1081.9655	20

**6**	**644.2998**	322.6536	627.2733	314.1403	626.2893	313.6483	**A**	2081.8659	**1041.4366**	2064.8394	1032.9233	2063.8553	1032.4313	**19**
**7**	**772.3584**	386.6828	**755.3319**	378.1696	**754.3478**	377.6776	**Q**	2010.8288	1005.9180	1993.8022	997.4048	**1992.8182**	996.9128	**18**
**8**	**859.3904**	430.1989	842.3639	**421.6856**	**841.3799**	**421.1936**	**S**	**1882.7702**	**941.8887**	1865.7437	933.3755	**1864.7597**	932.8835	**17**
**9**	**974.4174**	487.7123	957.3908	479.1991	956.4068	478.7070	**D**	1795.7382	898.3727	1778.7116	889.8595	1777.7276	889.3674	**16**
**10**	1031.4388	516.2231	1014.4123	507.7098	1013.4283	507.2178	**G**	1680.7112	**840.8593**	1663.6847	832.3460	1662.7007	831.8540	**15**
**11**	**1160.4814**	580.7444	1143.4549	572.2311	1142.4709	571.7391	**E**	**1623.6898**	812.3485	1606.6632	803.8353	1605.6792	803.3432	**14**
**12**	**1289.5240**	645.2657	1272.4975	636.7524	**1271.5135**	636.2604	**E**	1494.6472	**747.8272**	1477.6206	739.3140	1476.6366	738.8219	**13**
**13**	**1456.5224**	728.7648	1439.4958	720.2516	1438.5118	719.7595	**S**	**1365.6046**	**683.3059**	1348.5780	674.7927	**1347.5940**	674.3007	**12**
**14**	**1553.5751**	777.2912	1536.5486	768.7779	1535.5646	768.2859	**P**	**1198.6062**	**599.8068**	1181.5797	591.2935	1180.5957	590.8015	**11**
**15**	**1624.6123**	812.8098	1607.5857	804.2965	1606.6017	803.8045	**A**	1101.5535	551.2804	1084.5269	542.7671	1083.5429	542.2751	**10**
**16**	**1753.6549**	877.3311	1736.6283	**868.8178**	**1735.6443**	**868.3258**	**E**	**1030.5164**	515.7618	1013.4898	507.2485	1012.5058	506.7565	**9**
**17**	**1882.6974**	**941.8524**	1865.6709	933.3391	**1864.6869**	932.8471	**E**	901.4738	451.2405	884.4472	442.7272	883.4632	442.2352	**8**
**18**	1939.7189	970.3631	1922.6924	961.8498	1921.7083	961.3578	**G**	**772.4312**	386.7192	**755.4046**	378.2060	**754.4206**	377.7139	**7**
**19**	2067.7775	1034.3924	2050.7509	1025.8791	2049.7669	1025.3871	**Q**	**715.4097**	358.2085	698.3832	**349.6952**	697.3992	**349.2032**	**6**
**20**	2180.8616	**1090.9344**	2163.8350	1082.4211	2162.8510	1081.9291	**L**	**587.3511**	294.1792	570.3246	285.6659	569.3406	285.1739	**5**
**21**	2293.9456	**1147.4764**	2276.9191	**1138.9632**	2275.9350	1138.4712	**L**	**474.2671**	237.6372	457.2405	229.1239	456.2565	228.6319	**4**
**22**	2350.9671	1175.9872	2333.9405	1167.4739	2332.9565	1166.9819	**G**	**361.1830**	181.0951	344.1565	172.5819	343.1724	172.0899	**3**
**23**	2480.0097	**1240.5085**	2462.9831	1231.9952	2461.9991	1231.5032	**E**	**304.1615**	152.5844	287.1350	144.0711	286.1510	143.5791	**2**
**24**							**R**	175.1190	88.0631	158.0924	79.5498			**1**

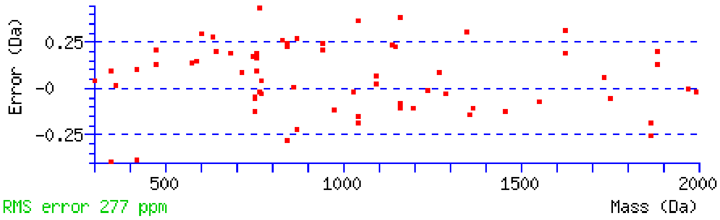

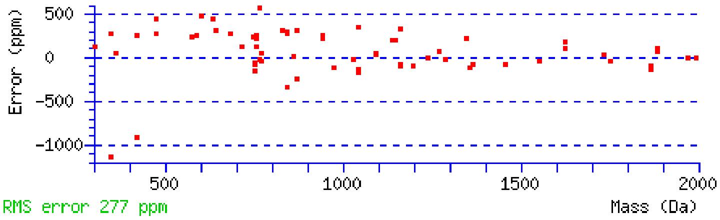

All matches to this query

Score	Mr(calc):	Delta	Sequence
50.9	2653.1140	0.0127	DSDRVAQSDGEESPAEEGQLLGER
24.2	2653.1140	0.0127	DSDRVAQSDGEESPAEEGQLLGER
14.9	2653.1140	0.0127	DSDRVAQSDGEESPAEEGQLLGER
6.5	2652.1496	0.9771	GDPPLTGETLVVVNVSDINDNPPK
2.4	2652.1427	0.9841	SEFEAILVYRVSSRTAWVDGK
2.3	2652.1427	0.9841	SEFEAILVYRVSSRTAWVDGK
2.1	2653.1342	-0.0074	GLQCIGMHENGIIFNNNPSLWR
1.4	2653.1302	-0.0034	VCTVGTHNGLEHSTFNMTIEPGR
1.0	2653.1446	-0.0178	QEINSTFQSDFVPDNGFRETSR
1.0	2653.1383	-0.0115	TNRTESYEMKSVKPPSINNPSI

Spectrum No: 288; Query: 741; Rank: 1

Peptide View

MS/MS Fragmentation of **SQSDSPPASPSPTAK**
Found in **IPI00777106**, Tax_Id=10116 Gene_Symbol=Ppp1r12b_predicted 21 kDa protein

Match to Query 741: 1535.654788 from(768.834670,2+)
Title: 100101RatKid_NS_deglyco_20.474.474.2.dta
Data file K:\NewmanPaper\Piliang\3SubProteomes\Piliang3SP\mgf5ppm\ERLIC_3SubProteomes5ppm.mgf

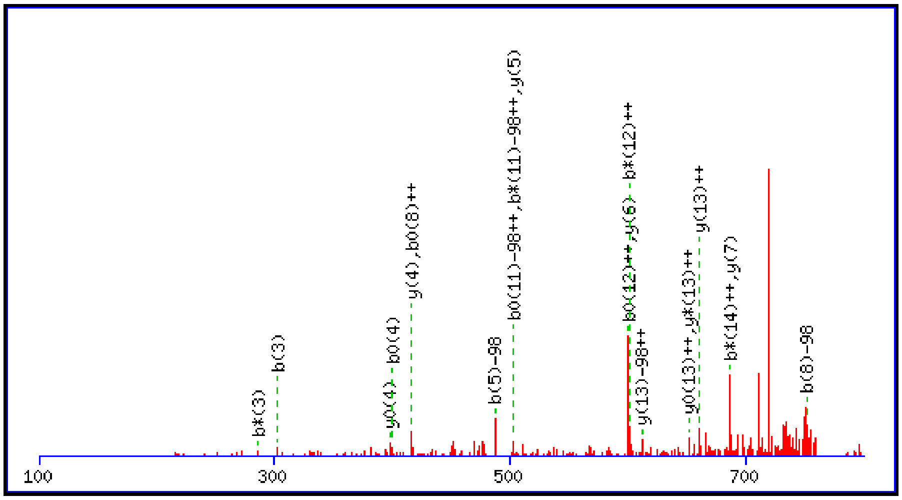

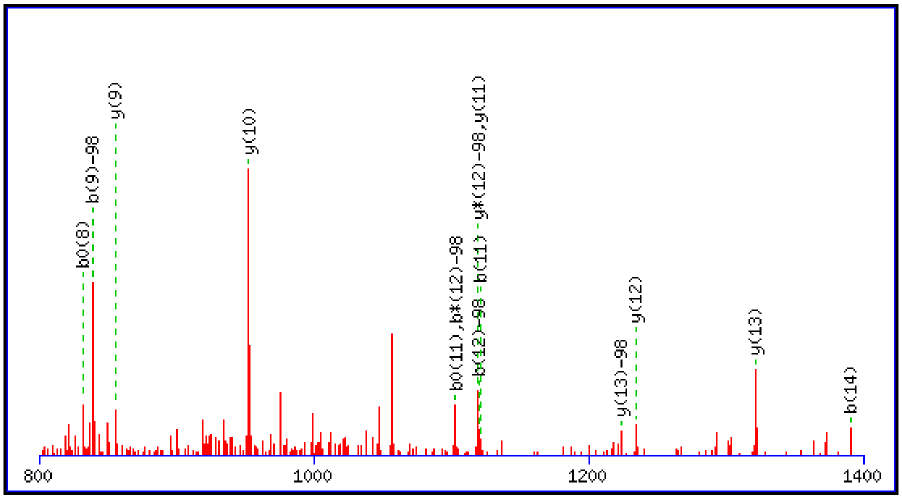

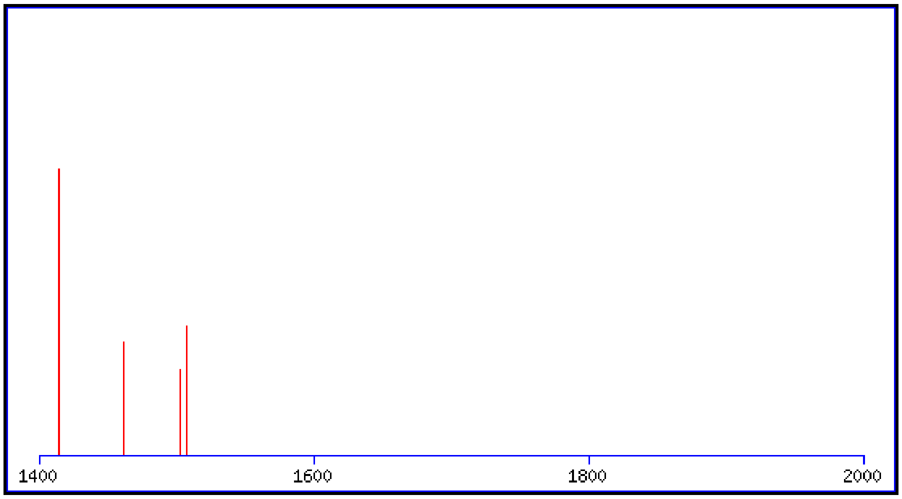

Monoisotopic mass of neutral peptide Mr(calc): 1535.6505
Fixed modifications: Carbamidomethyl (C)
Variable modifications:
S5 : Phospho (ST), with neutral losses 0.0000(shown in table), 97.9769
Ions Score: 51 **Expect:** 0.0012
Matches (Bold Red): 34/246 fragment ions using 44 most intense peaks

#	b	b^{++}	b*	b^{*++}	b^0	b^{0++}	Seq.	y	y^{++}	y*	y^{*++}	y^0	y^{0++}	#
1	88.0393	44.5233			70.0287	35.5180	S							15
2	216.0979	108.5526	199.0713	100.0393	198.0873	99.5473	Q	1449.6257	725.3165	1432.5992	716.8032	1431.6152	716.3112	14
3	**303.1299**	152.0686	**286.1034**	143.5553	285.1193	143.0633	S	**1321.5671**	**661.2872**	1304.5406	**652.7739**	1303.5566	**652.2819**	13
4	418.1569	209.5821	401.1303	201.0688	**400.1463**	200.5768	D	**1234.5351**	617.7712	1217.5086	609.2579	1216.5246	608.7659	12
5	585.1552	293.0812	568.1287	284.5680	567.1446	284.0760	S	**1119.5082**	560.2577	1102.4816	551.7445	1101.4976	551.2524	11

**6**	682.2080	341.6076	665.1814	333.0943	664.1974	332.6023	**P**	**952.5098**	476.7585	935.4833	468.2453	934.4993	467.7533	**10**
**7**	779.2607	390.1340	762.2342	381.6207	761.2502	381.1287	**P**	**855.4571**	428.2322	838.4305	419.7189	837.4465	419.2269	**9**
**8**	850.2978	425.6526	833.2713	417.1393	**832.2873**	**416.6473**	**A**	758.4043	379.7058	741.3777	371.1925	740.3937	370.7005	**8**
**9**	937.3299	469.1686	920.3033	460.6553	919.3193	460.1633	**S**	**687.3672**	344.1872	670.3406	335.6740	669.3566	335.1819	**7**
**10**	1034.3826	517.6950	1017.3561	509.1817	1016.3721	508.6897	**P**	**600.3352**	300.6712	583.3086	292.1579	582.3246	291.6659	**6**
**11**	**1121.4147**	561.2110	1104.3881	552.6977	**1103.4041**	552.2057	**S**	**503.2824**	252.1448	486.2558	243.6316	485.2718	243.1396	**5**
**12**	1218.4674	609.7374	1201.4409	**601.2241**	1200.4569	**600.7321**	**P**	**416.2504**	208.6288	399.2238	200.1155	**398.2398**	199.6235	**4**
**13**	1319.5151	660.2612	1302.4886	651.7479	1301.5045	651.2559	**T**	319.1976	160.1024	302.1710	151.5892	301.1870	151.0972	**3**
**14**	**1390.5522**	695.7798	1373.5257	**687.2665**	1372.5417	686.7745	**A**	218.1499	109.5786	201.1234	101.0653			**2**
**15**							**K**	147.1128	74.0600	130.0863	65.5468			**1**

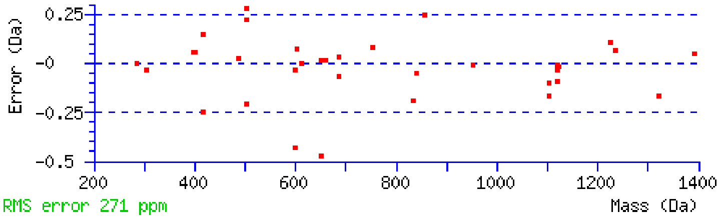

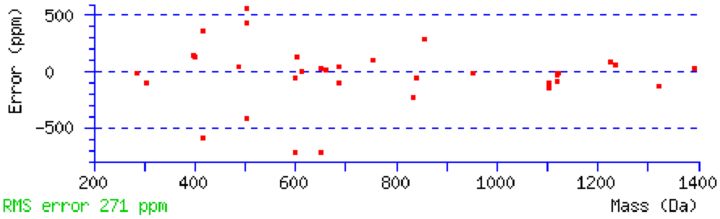

All matches to this query

Score	Mr(calc):	Delta	Sequence
50.8	1535.6505	0.0043	SQSDSPPASPSPTAK
31.9	1535.6505	0.0043	SQSDSPPASPSPTAK
24.8	1535.6505	0.0043	SQSDSPPASPSPTAK
23.9	1535.6505	0.0043	SQSDSPPASPSPTAK
12.1	1535.6552	-0.0004	CKDTLLHNGNQR
12.1	1535.6552	-0.0004	CKDTLLHNGNQR
11.5	1535.6505	0.0043	SQSDSPPASPSPTAK
9.3	1535.6673	-0.0126	AASVSPEKSLYPK
9.3	1535.6673	-0.0126	AASVSPEKSLYPK
3.1	1535.6673	-0.0126	AASVSPEKSLYPK

Spectrum No: 289; Query: 910; Rank: 1

Peptide View

MS/MS Fragmentation of **ATEEPSGTGSDELIK**
Found in **IPI00201300**, Tax_Id=10116 Gene_Symbol=Ptrf_predicted Polymerase I and transcript release factor

Match to Query 910: 1612.684948 from(807.349750,2+)
Title: 091008RatKidney_NH4Format01_22.1293.1293.2.dta
Data file K:\NewmanPaper\Piliang\3SubProteomes\Piliang3SP\mgf5ppm\ERLIC_3SubProteomes5ppm.mgf

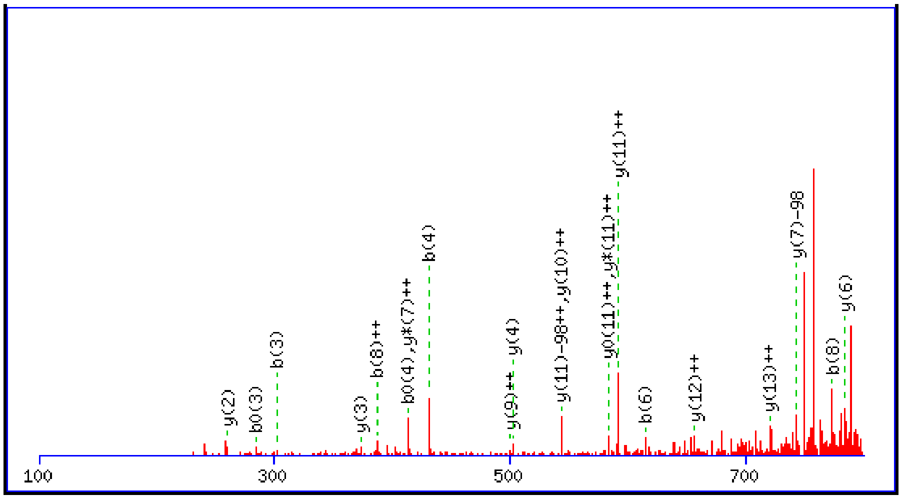

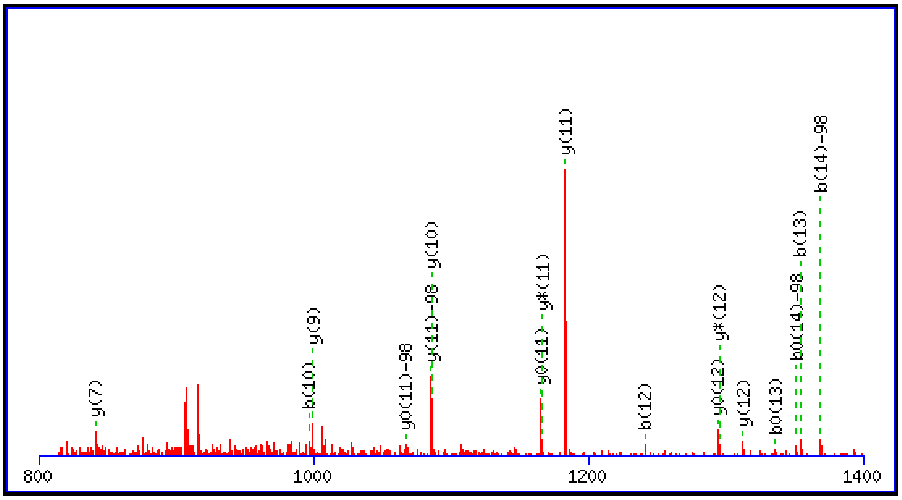

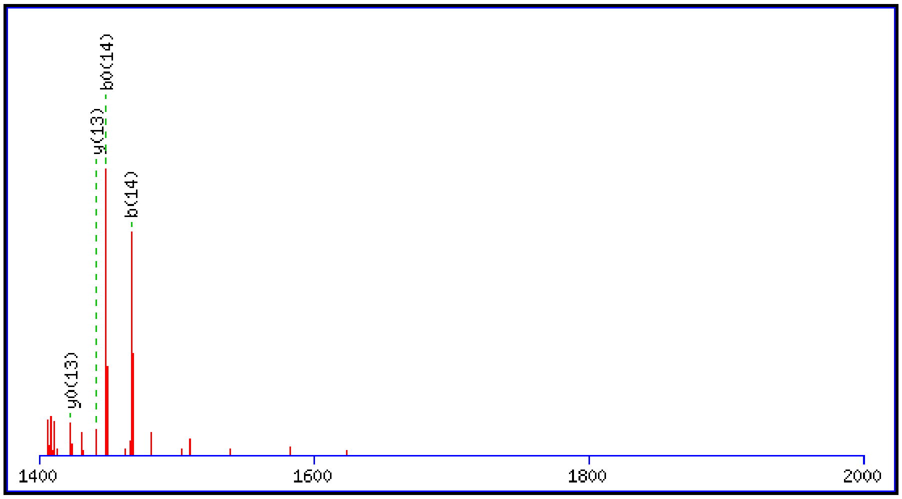

Monoisotopic mass of neutral peptide Mr(calc): 1612.6869
Fixed modifications: Carbamidomethyl (C)
Variable modifications:
S10 : Phospho (ST), with neutral losses 0.0000(shown in table), 97.9769
Ions Score: 51 **Expect:** 0.0013
Matches (Bold Red): 42/206 fragment ions using 79 most intense peaks

#	b	b^{++}	b^{0}	b^{0++}	Seq.	y	y^{++}	y*	y^{*++}	y^{0}	y^{0++}	#
1	72.0444	36.5258			A							15
2	173.0921	87.0497	155.0815	78.0444	T	1542.6571	771.8322	1525.6305	763.3189	1524.6465	762.8269	14
3	**302.1347**	151.5710	**284.1241**	142.5657	E	**1441.6094**	**721.3083**	1424.5829	712.7951	**1423.5988**	712.3031	13
4	**431.1773**	216.0923	**413.1667**	207.0870	E	**1312.5668**	**656.7870**	1295.5403	648.2738	**1294.5562**	647.7818	12
5	528.2300	264.6186	510.2195	255.6134	P	**1183.5242**	**592.2657**	**1166.4977**	**583.7525**	**1165.5137**	**583.2605**	11

**6**	**615.2620**	308.1347	597.2515	299.1294	**S**	**1086.4715**	**543.7394**	1069.4449	535.2261	1068.4609	534.7341	**10**
**7**	672.2835	336.6454	654.2729	327.6401	**G**	**999.4394**	**500.2234**	982.4129	491.7101	981.4289	491.2181	**9**
**8**	**773.3312**	**387.1692**	755.3206	378.1640	**T**	942.4180	471.7126	925.3914	463.1993	924.4074	462.7073	**8**
**9**	830.3527	415.6800	812.3421	406.6747	**G**	**841.3703**	421.1888	824.3437	**412.6755**	823.3597	412.1835	**7**
**10**	**997.3510**	499.1791	979.3404	490.1739	**S**	**784.3488**	392.6780	767.3223	384.1648	766.3383	383.6728	**6**
**11**	1112.3779	556.6926	1094.3674	547.6873	**D**	617.3505	309.1789	600.3239	300.6656	599.3399	300.1736	**5**
**12**	**1241.4205**	621.2139	1223.4100	612.2086	**E**	**502.3235**	251.6654	485.2970	243.1521	484.3130	242.6601	**4**
**13**	**1354.5046**	677.7559	**1336.4940**	668.7507	**L**	**373.2809**	187.1441	356.2544	178.6308			**3**
**14**	**1467.5887**	734.2980	**1449.5781**	725.2927	**I**	**260.1969**	130.6021	243.1703	122.0888			**2**
**15**					**K**	147.1128	74.0600	130.0863	65.5468			**1**

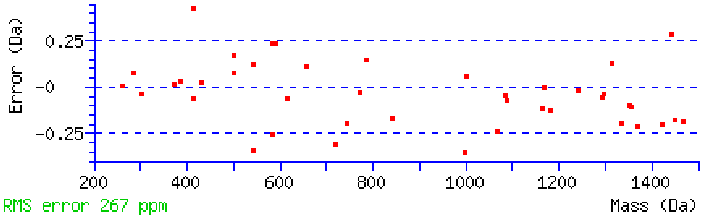

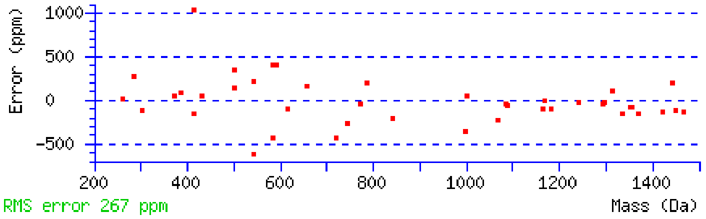

All matches to this query

Score	Mr(calc):	Delta	Sequence
50.7	1612.6869	-0.0020	ATEEPSGTGSDELIK
32.7	1612.6869	-0.0020	ATEEPSGTGSDELIK
23.3	1612.6869	-0.0020	ATEEPSGTGSDELIK
17.3	1612.6869	-0.0019	ASVENASLEDLAEGK
14.8	1612.6869	-0.0020	ATEEPSGTGSDELIK
11.8	1612.6939	-0.0090	DWKTKLFSSNVK
10.2	1612.6939	-0.0090	DWKTKLFSSNVK
3.5	1612.6939	-0.0090	DWKTKLFSSNVK
2.5	1612.6869	-0.0019	ASVENASLEDLAEGK

Spectrum No: 290; Query: 2468; Rank: 1

Peptide View

MS/MS Fragmentation of **SGDETPGSEAPGDKAVEEQGDDQDSEK**
Found in **IPI00360386**, Tax_Id=10116 Gene_Symbol=RGD1562784_predicted similar to heterogeneous nuclear ribonucleoprotein U isoform a

Match to Query 2468: 2856.114012 from(953.045280,3+)
Title: 091008RatKidney_NoSalt_28.891.891.3.dta
Data file K:\NewmanPaper\Piliang\3SubProteomes\Piliang3SP\mgf5ppm\ERLIC_3SubProteomes5ppm.mgf

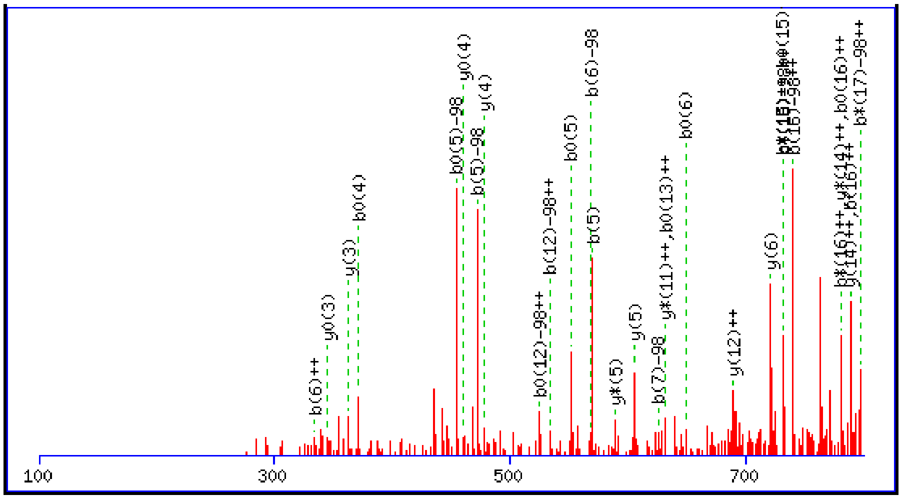

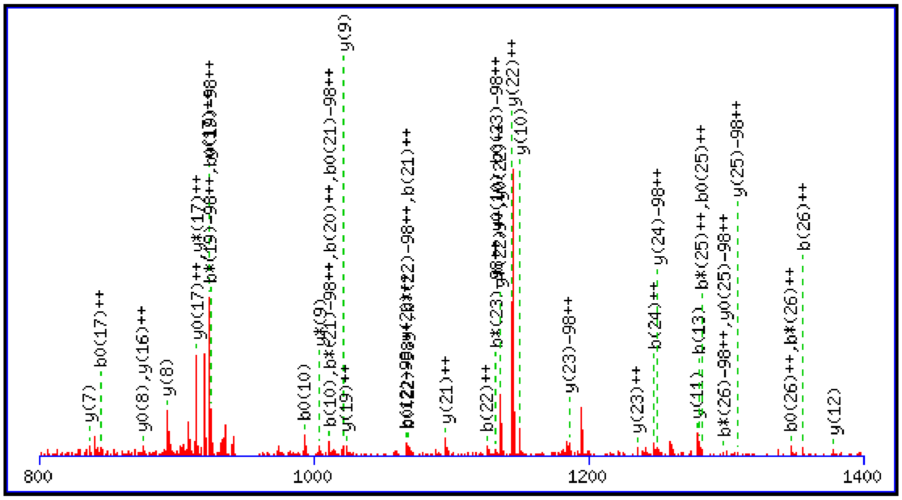

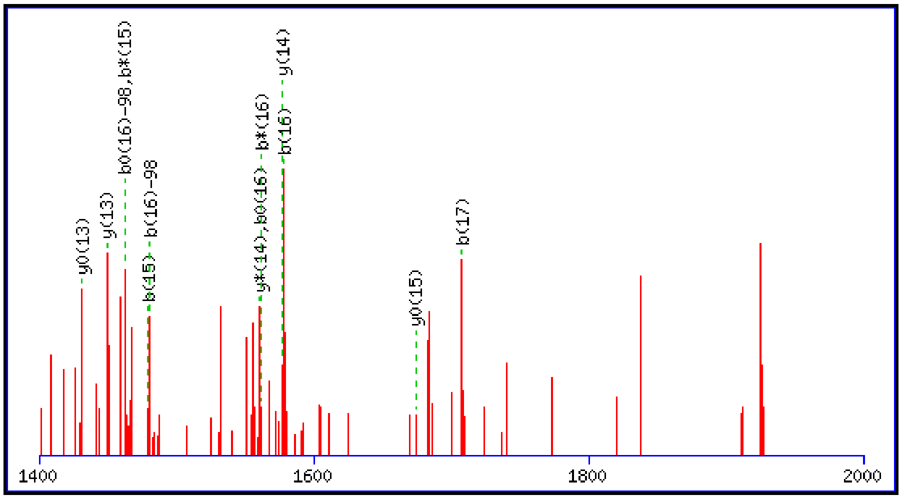

Monoisotopic mass of neutral peptide Mr(calc): 2856.1094
Fixed modifications: Carbamidomethyl (C)
Variable modifications:
T5 : Phospho (ST), with neutral losses 97.9769(shown in table), 0.0000
Ions Score: 51 **Expect:** 0.0023
Matches (Bold Red): 92/422 fragment ions using 162 most intense peaks

#	b	b^{++}	b*	b^{*++}	b^0	b^{0++}	Seq.	y	y^{++}	y*	y^{*++}	y^0	y^{0++}	#
1	88.0393	44.5233			70.0287	35.5180	S							27
2	145.0608	73.0340			127.0502	64.0287	G	2672.1078	1336.5575	2655.0812	1328.0443	2654.0972	1327.5523	26
3	260.0877	130.5475			242.0771	121.5422	D	2615.0863	**1308.0468**	2598.0598	1299.5335	2597.0758	**1299.0415**	25
4	389.1303	195.0688			**371.1197**	186.0635	E	2500.0594	**1250.5333**	2483.0328	1242.0201	2482.0488	1241.5280	24
5	**472.1674**	236.5873			**454.1568**	227.5821	T	2371.0168	**1186.0120**	2353.9902	1177.4988	2353.0062	1177.0068	23

6	**569.2202**	285.1137			551.2096	276.1084	P	2287.9797	**1144.4935**	2270.9531	**1135.9802**	2269.9691	**1135.4882**	22
7	**626.2416**	313.6245			608.2311	304.6192	G	2190.9269	**1095.9671**	2173.9004	1087.4538	2172.9164	1086.9618	21
8	713.2737	357.1405			695.2631	348.1352	S	2133.9055	**1067.4564**	2116.8789	1058.9431	2115.8949	1058.4511	20
9	842.3163	421.6618			824.3057	412.6565	E	2046.8734	**1023.9404**	2029.8469	1015.4271	2028.8629	1014.9351	19
10	913.3534	457.1803			895.3428	448.1750	A	1917.8308	959.4191	1900.8043	950.9058	1899.8203	950.4138	18
11	1010.4061	505.7067			992.3956	496.7014	P	1846.7937	**923.9005**	1829.7672	**915.3872**	1828.7832	**914.8952**	17
12	**1067.4276**	**534.2174**			1049.4170	**525.2122**	G	1749.7410	**875.3741**	1732.7144	866.8608	1731.7304	866.3688	16
13	1182.4545	591.7309			1164.4440	582.7256	D	1692.7195	846.8634	1675.6929	838.3501	**1674.7089**	837.8581	15
14	1310.5495	655.7784	1293.5230	647.2651	1292.5389	646.7731	K	**1577.6926**	**789.3499**	**1560.6660**	**780.8366**	1559.6820	780.3446	14
15	1381.5866	691.2969	1364.5601	682.7837	1363.5761	682.2917	A	**1449.5976**	725.3024	1432.5710	716.7892	**1431.5870**	716.2971	13
16	**1480.6550**	**740.8312**	1463.6285	**732.3179**	**1462.6445**	**731.8259**	V	**1378.5605**	**689.7839**	1361.5339	681.2706	1360.5499	680.7786	12
17	1609.6976	805.3525	1592.6711	**796.8392**	1591.6871	796.3472	E	**1279.4921**	640.2497	1262.4655	**631.7364**	1261.4815	631.2444	11
18	1738.7402	869.8737	1721.7137	861.3605	1720.7297	860.8685	E	**1150.4495**	575.7284	1133.4229	567.2151	**1132.4389**	566.7231	10
19	1866.7988	933.9030	1849.7723	**925.3898**	1848.7882	**924.8978**	Q	**1021.4069**	511.2071	**1004.3803**	502.6938	1003.3963	502.2018	9
20	1923.8203	962.4138	1906.7937	953.9005	1905.8097	953.4085	G	**893.3483**	447.1778	876.3217	438.6645	**875.3377**	438.1725	8
21	2038.8472	1019.9272	2021.8207	**1011.4140**	2020.8366	**1010.9220**	D	**836.3268**	418.6671	819.3003	410.1538	818.3163	409.6618	7
22	2153.8741	1077.4407	2136.8476	**1068.9274**	2135.8636	**1068.4354**	D	**721.2999**	361.1536	704.2733	352.6403	703.2893	352.1483	6
23	2281.9327	1141.4700	2264.9062	**1132.9567**	2263.9222	**1132.4647**	Q	**606.2729**	303.6401	**589.2464**	295.1268	588.2624	294.6348	5
24	2396.9597	1198.9835	2379.9331	1190.4702	2378.9491	1189.9782	D	**478.2144**	239.6108	461.1878	231.0975	**460.2038**	230.6055	4
25	2483.9917	1242.4995	2466.9651	1233.9862	2465.9811	1233.4942	S	**363.1874**	182.0974	346.1609	173.5841	**345.1769**	173.0921	3
26	2613.0343	1307.0208	2596.0077	**1298.5075**	2595.0237	1298.0155	E	276.1554	138.5813	259.1288	130.0681	258.1448	129.5761	2
27							K	147.1128	74.0600	130.0863	65.5468			1

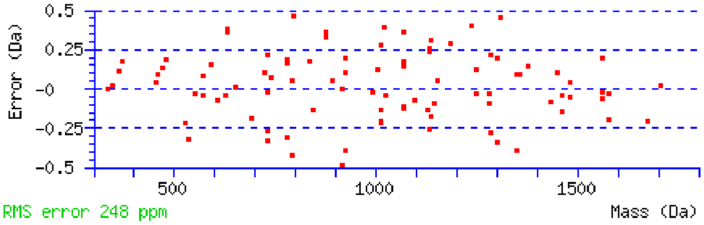

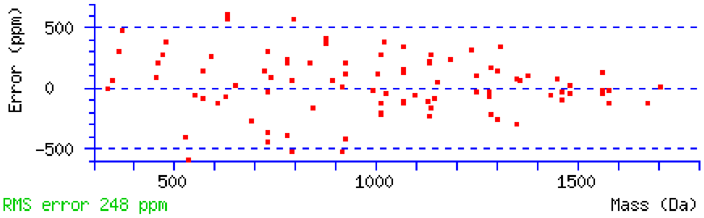

All matches to this query

Score	Mr(calc):	Delta	Sequence
50.6	2856.1094	0.0046	SGDETPGSEAPGDKAVEEQGDDQDSEK
49.4	2856.1094	0.0046	SGDETPGSEAPGDKAVEEQGDDQDSEK
33.9	2856.1094	0.0046	SGDETPGSEAPGDKAVEEQGDDQDSEK
3.0	2856.1157	-0.0017	NASSIPRSESASKGLNQMNNSNGSNK
2.3	2855.1317	0.9823	NASSIPRSESASKGLNQMNNSNGSNK
2.3	2855.1317	0.9823	NASSIPRSESASKGLNQMNNSNGSNK
1.4	2854.1024	2.0116	QKTNYGGLSSEDENEPEGLGVTYK
1.4	2855.0864	1.0276	QKTNYGGLSSEDENEPEGLGVTYK
1.2	2856.1317	-0.0176	LWSDGNEEENINDTLNGWYKNGK
1.1	2854.0921	2.0219	GDYDIPNVSPGLMPEIFSQSMSSK

Spectrum No: 291; Query: 2314; Rank: 1

Peptide View

MS/MS Fragmentation of **SKEPSSPGTDDVFTPGPSDSPGSQR**
Found in **IPI00193369**, Tax_Id=10116 Gene_Symbol=Slc9a1 Sodium/hydrogen exchanger 1

Match to Query 2314: 2611.118472 from(871.380100,3+)
Title: 091008RatKidney_NH4Format01_23.1474.1474.3.dta
Data file K:\NewmanPaper\Piliang\3SubProteomes\Piliang3SP\mgf5ppm\ERLIC_3SubProteomes5ppm.mgf

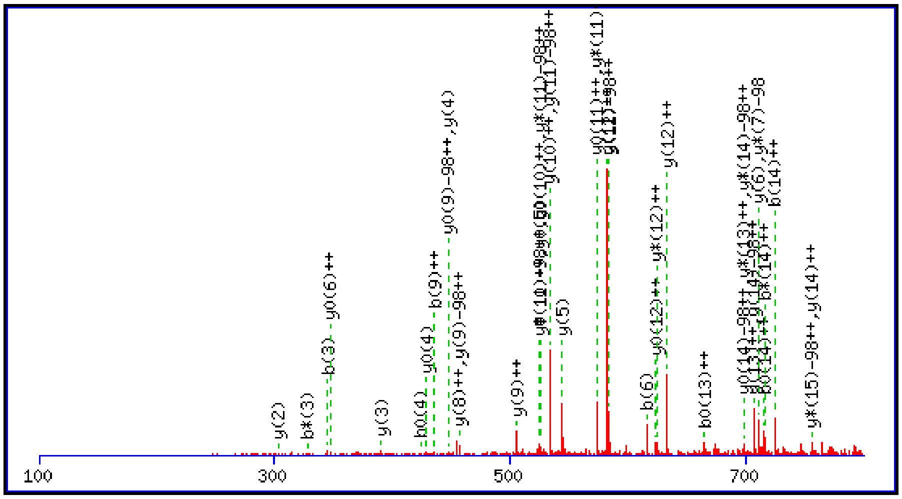

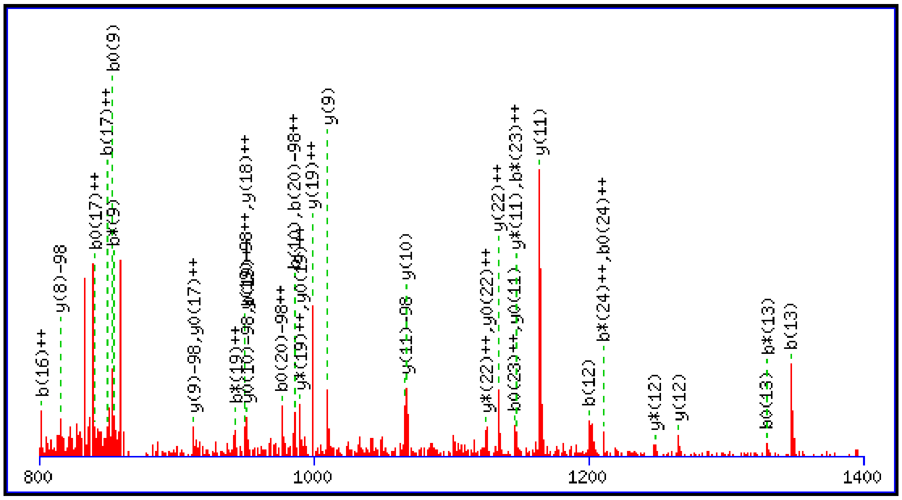

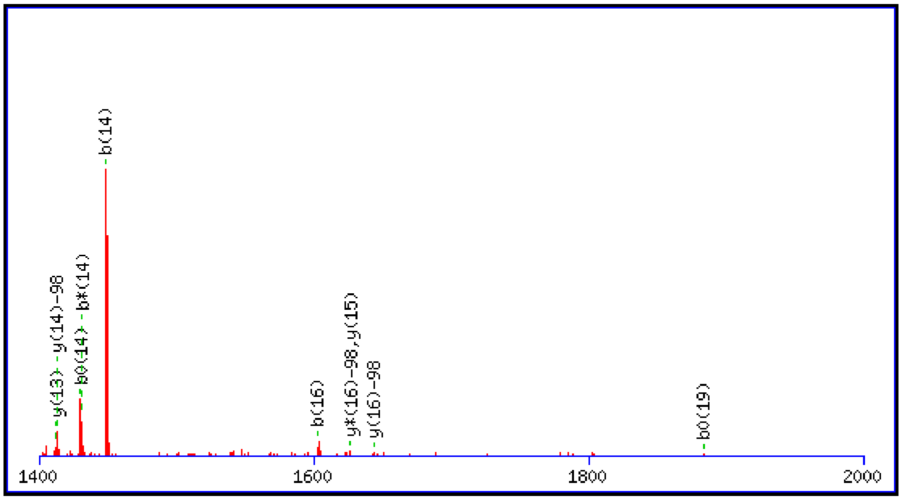

Monoisotopic mass of neutral peptide Mr(calc): 2611.1076
Fixed modifications: Carbamidomethyl (C)
Variable modifications:
S20 : Phospho (ST), with neutral losses 0.0000(shown in table), 97.9769
Ions Score: 50 **Expect:** 0.0033
Matches (Bold Red): 90/426 fragment ions using 124 most intense peaks

#	b	b^{++}	b*	b*$^{++}$	b^0	b^{0++}	Seq.	y	y^{++}	y*	y*$^{++}$	y^0	y^{0++}	#
1	88.0393	44.5233			70.0287	35.5180	S							25
2	216.1343	108.5708	199.1077	100.0575	198.1237	99.5655	K	2525.0828	1263.0450	2508.0562	1254.5318	2507.0722	1254.0397	24

3	**345.1769**	173.0921	**328.1503**	164.5788	327.1663	164.0868	E	2396.9878	1198.9975	2379.9613	1190.4843	2378.9772	1189.9923	23
4	442.2296	221.6185	425.2031	213.1052	**424.2191**	212.6132	P	2267.9452	**1134.4762**	2250.9187	**1125.9630**	2249.9347	**1125.4710**	22
5	529.2617	265.1345	512.2351	256.6212	511.2511	256.1292	S	2170.8925	1085.9499	2153.8659	1077.4366	2152.8819	1076.9446	21
6	**616.2937**	308.6505	599.2671	300.1372	598.2831	299.6452	S	2083.8604	1042.4339	2066.8339	1033.9206	2065.8499	1033.4286	20
7	713.3464	357.1769	696.3199	348.6636	695.3359	348.1716	P	1996.8284	**998.9178**	1979.8019	**990.4046**	1978.8178	**989.9126**	19
8	770.3679	385.6876	753.3414	377.1743	752.3573	376.6823	G	1899.7756	**950.3915**	1882.7491	941.8782	1881.7651	941.3862	18
9	871.4156	**436.2114**	**854.3890**	427.6982	**853.4050**	427.2061	T	1842.7542	921.8807	1825.7276	913.3675	1824.7436	**912.8754**	17
10	**986.4425**	493.7249	969.4160	485.2116	968.4320	484.7196	D	1741.7065	871.3569	1724.6799	862.8436	1723.6959	862.3516	16
11	1101.4695	551.2384	1084.4429	542.7251	1083.4589	542.2331	D	**1626.6796**	813.8434	1609.6530	805.3301	1608.6690	804.8381	15
12	**1200.5379**	600.7726	1183.5113	592.2593	1182.5273	591.7673	V	1511.6526	**756.3299**	1494.6261	747.8167	1493.6420	747.3247	14
13	**1347.6063**	674.3068	**1330.5798**	665.7935	**1329.5957**	**665.3015**	F	**1412.5842**	**706.7957**	1395.5576	**698.2825**	1394.5736	697.7905	13
14	**1448.6540**	**724.8306**	**1431.6274**	**716.3174**	**1430.6434**	**715.8253**	T	**1265.5158**	**633.2615**	**1248.4892**	**624.7483**	1247.5052	**624.2562**	12
15	1545.7067	773.3570	1528.6802	764.8437	1527.6962	764.3517	P	**1164.4681**	**582.7377**	**1147.4416**	**574.2244**	**1146.4575**	**573.7324**	11
16	**1602.7282**	**801.8677**	1585.7017	793.3545	1584.7176	792.8625	G	**1067.4153**	**534.2113**	1050.3888	**525.6980**	1049.4048	**525.2060**	10
17	1699.7810	**850.3941**	1682.7544	841.8808	1681.7704	**841.3888**	P	**1010.3939**	**505.7006**	993.3673	497.1873	992.3833	496.6953	9
18	1786.8130	893.9101	1769.7865	885.3969	1768.8024	884.9049	S	913.3411	**457.1742**	896.3146	448.6609	895.3305	448.1689	8
19	1901.8399	**951.4236**	1884.8134	**942.9103**	**1883.8294**	942.4183	D	826.3091	413.6582	809.2825	405.1449	808.2985	404.6529	7
20	2068.8383	1034.9228	2051.8117	1026.4095	2050.8277	1025.9175	S	**711.2821**	356.1447	694.2556	347.6314	693.2716	**347.1394**	6
21	2165.8911	1083.4492	2148.8645	1074.9359	2147.8805	1074.4439	P	**544.2838**	272.6455	527.2572	264.1323	**526.2732**	263.6402	5
22	2222.9125	1111.9599	2205.8860	1103.4466	2204.9020	1102.9546	G	**447.2310**	224.1191	430.2045	215.6059	**429.2205**	215.1139	4
23	2309.9446	1155.4759	2292.9180	**1146.9626**	2291.9340	**1146.4706**	S	**390.2096**	195.6084	373.1830	187.0951	372.1990	186.6031	3
24	2438.0031	1219.5052	2420.9766	**1210.9919**	2419.9926	**1210.4999**	Q	**303.1775**	152.0924	286.1510	143.5791			2
25							R	175.1190	88.0631	158.0924	79.5498			1

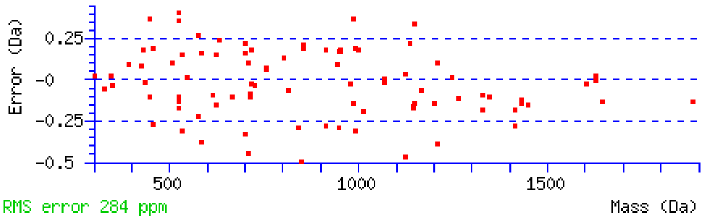

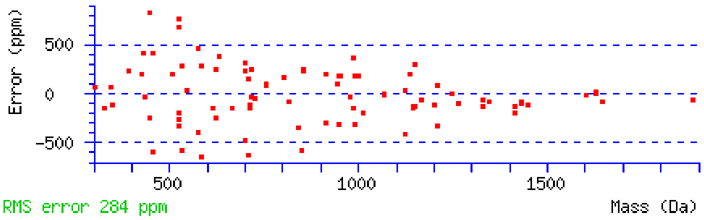

All matches to this query

Score	Mr(calc):	Delta	Sequence
50.5	2611.1076	0.0109	SKEPSSPGTDDVFTPGPSDSPGSQR
41.8	2611.1076	0.0109	SKEPSSPGTDDVFTPGPSDSPGSQR
37.5	2611.1076	0.0109	SKEPSSPGTDDVFTPGPSDSPGSQR
26.0	2611.1076	0.0109	SKEPSSPGTDDVFTPGPSDSPGSQR
25.5	2611.1076	0.0109	SKEPSSPGTDDVFTPGPSDSPGSQR
24.9	2611.1076	0.0109	SKEPSSPGTDDVFTPGPSDSPGSQR
18.6	2611.1076	0.0109	SKEPSSPGTDDVFTPGPSDSPGSQR
14.2	2611.1076	0.0109	SKEPSSPGTDDVFTPGPSDSPGSQR
9.1	2610.0943	1.0241	AYKMHQNISLTGGDMPSCLRR
8.1	2610.0943	1.0241	AYKMHQNISLTGGDMPSCLRR

Spectrum No: 292; Query: 2679; Rank: 1

Peptide View

MS/MS Fragmentation of **RTDPDHYETDYTTGGESCDELEEDWLR**

Found in **IPI00421524**, Tax_Id=10116 Gene_Symbol=Ocln;Tpmt Occludin

Match to Query 2679: 3368.324562 from(1123.782130,3+)
Title: 100101RatKid_NS_deglyco_20.3576.3576.3.dta
Data file K:\NewmanPaper\Piliang\3SubProteomes\Piliang3SP\mgf5ppm\ERLIC_3SubProteomes5ppm.mgf

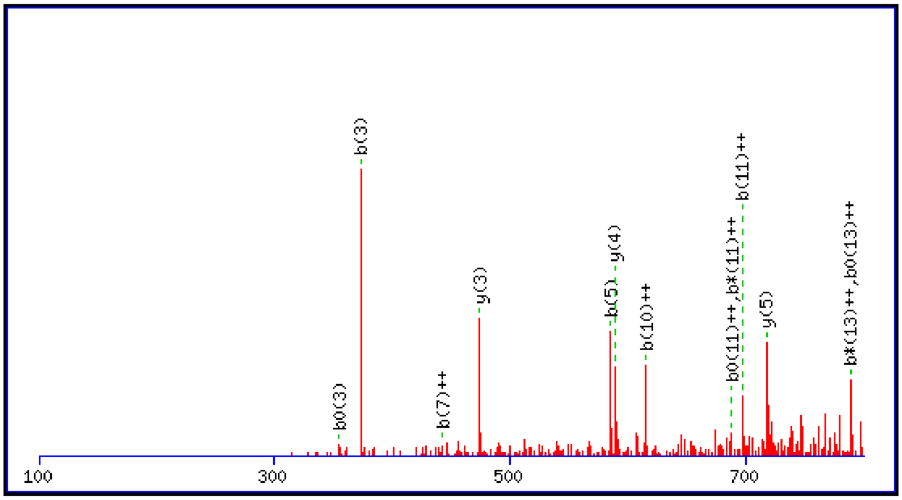

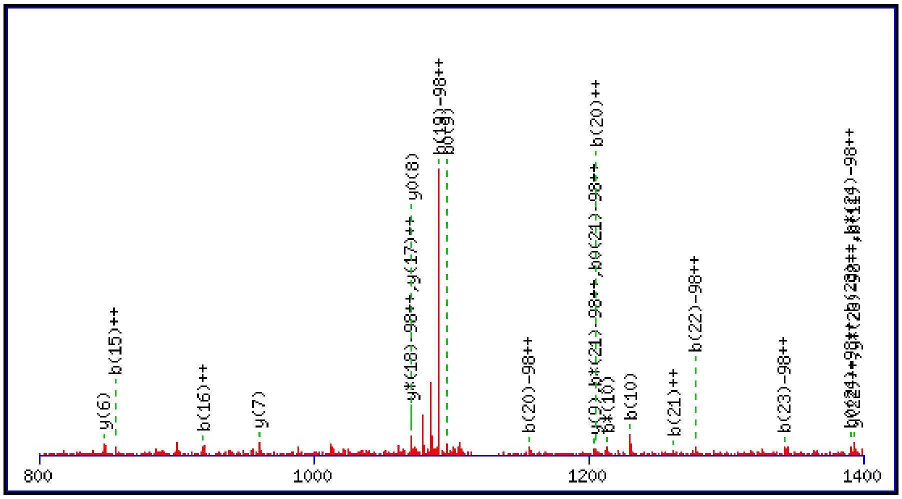

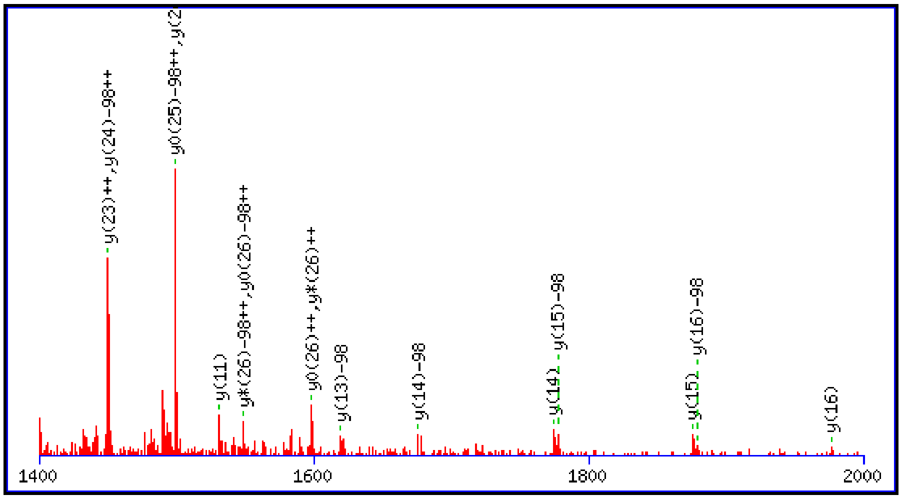

Monoisotopic mass of neutral peptide Mr(calc): 3368.3089
Fixed modifications: Carbamidomethyl (C)
Variable modifications:
S17 : Phospho (ST), with neutral losses 97.9769(shown in table), 0.0000
Ions Score: 51 **Expect:** 0.003
Matches (Bold Red): 57/460 fragment ions using 81 most intense peaks

#	b	b^{++}	b*	b^{*++}	b^{0}	b^{0++}	Seq.	y	y^{++}	y*	y^{*++}	y^{0}	y^{0++}	#
1	157.1084	79.0578	140.0818	70.5446			R							27

2	258.1561	129.5817	241.1295	121.0684	240.1455	120.5764	T	3115.2382	1558.1227	3098.2116	**1549.6094**	3097.2276	**1549.1174**	26
3	**373.1830**	187.0951	356.1565	178.5819	**355.1724**	178.0899	D	3014.1905	1507.5989	2997.1639	**1499.0856**	2996.1799	**1498.5936**	25
4	470.2358	235.6215	453.2092	227.1083	452.2252	226.6162	P	2899.1635	**1450.0854**	2882.1370	1441.5721	2881.1530	1441.0801	24
5	**585.2627**	293.1350	568.2362	284.6217	567.2522	284.1297	D	2802.1108	1401.5590	2785.0842	**1393.0457**	2784.1002	1392.5537	23
6	722.3216	361.6645	705.2951	353.1512	704.3111	352.6592	H	2687.0838	1344.0456	2670.0573	1335.5323	2669.0733	1335.0403	22
7	885.3850	**443.1961**	868.3584	434.6828	867.3744	434.1908	Y	2550.0249	1275.5161	2532.9984	1267.0028	2532.0143	1266.5108	21
8	1014.4276	507.7174	997.4010	499.2041	996.4170	498.7121	E	2386.9616	1193.9844	2369.9350	1185.4712	2368.9510	1184.9791	20
9	1115.4752	558.2413	1098.4487	549.7280	**1097.4647**	549.2360	T	2257.9190	1129.4631	2240.8924	1120.9499	2239.9084	1120.4579	19
10	**1230.5022**	**615.7547**	**1213.4756**	607.2414	1212.4916	606.7494	D	2156.8713	1078.9393	2139.8448	**1070.4260**	2138.8607	1069.9340	18
11	**1393.5655**	**697.2864**	1376.5390	**688.7731**	1375.5549	**688.2811**	Y	2041.8444	1021.4258	2024.8178	1012.9125	2023.8338	1012.4205	17
12	1494.6132	747.8102	1477.5866	739.2970	1476.6026	738.8049	T	**1878.7810**	939.8942	1861.7545	931.3809	1860.7705	930.8889	16
13	1595.6609	798.3341	1578.6343	**789.8208**	1577.6503	**789.3288**	T	**1777.7334**	889.3703	1760.7068	880.8570	1759.7228	880.3650	15
14	1652.6823	826.8448	1635.6558	818.3315	1634.6718	817.8395	G	**1676.6857**	838.8465	1659.6591	830.3332	1658.6751	829.8412	14
15	1709.7038	**855.3555**	1692.6772	846.8423	1691.6932	846.3502	G	**1619.6642**	810.3357	1602.6377	801.8225	1601.6537	801.3305	13
16	1838.7464	**919.8768**	1821.7198	911.3636	1820.7358	910.8715	E	1562.6428	781.8250	1545.6162	773.3117	1544.6322	772.8197	12
17	1907.7678	954.3876	1890.7413	945.8743	1889.7573	945.3823	S	1433.6002	717.3037	1416.5736	708.7904	1415.5896	708.2984	11
18	2067.7985	1034.4029	2050.7719	1025.8896	2049.7879	1025.3976	C	1364.5787	682.7930	1347.5522	674.2797	1346.5681	673.7877	10
19	2182.8254	**1091.9164**	2165.7989	1083.4031	2164.8149	1082.9111	D	**1204.5481**	602.7777	1187.5215	594.2644	1186.5375	593.7724	9
20	2311.8680	**1156.4376**	2294.8415	1147.9244	2293.8575	1147.4324	E	1089.5211	545.2642	1072.4946	536.7509	**1071.5106**	536.2589	8
21	2424.9521	1212.9797	2407.9255	**1204.4664**	2406.9415	**1203.9744**	L	**960.4785**	480.7429	943.4520	472.2296	942.4680	471.7376	7
22	2553.9947	**1277.5010**	2536.9681	1268.9877	2535.9841	1268.4957	E	**847.3945**	424.2009	830.3679	415.6876	829.3839	415.1956	6
23	2683.0373	**1342.0223**	2666.0107	1333.5090	2665.0267	1333.0170	E	**718.3519**	359.6796	701.3253	351.1663	700.3413	350.6743	5
24	2798.0642	1399.5357	2781.0377	**1391.0225**	2780.0536	**1390.5305**	D	**589.3093**	295.1583	572.2827	286.6450	571.2987	286.1530	4
25	2984.1435	1492.5754	2967.1170	1484.0621	2966.1330	1483.5701	W	**474.2823**	237.6448	457.2558	229.1315			3
26	3097.2276	**1549.1174**	3080.2010	1540.6042	3079.2170	1540.1122	L	288.2030	144.6051	271.1765	136.0919			2
27							R	175.1190	88.0631	158.0924	79.5498			1

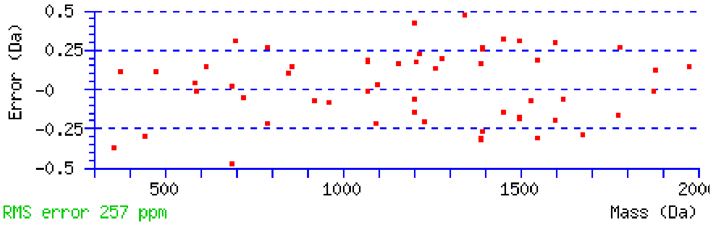

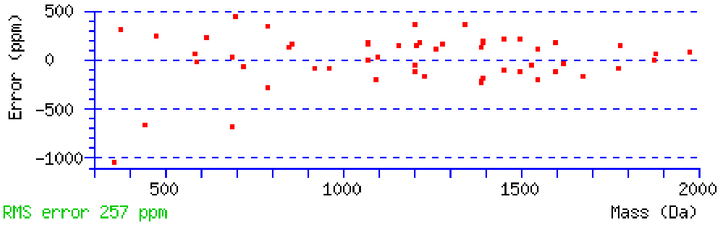

All matches to this query

Score	Mr(calc):	Delta	Sequence
50.5	3368.3089	0.0157	RTDPDHYETDYTTGGESCDELEEDWLR
42.7	3368.3089	0.0157	RTDPDHYETDYTTGGESCDELEEDWLR
35.8	3368.3089	0.0157	RTDPDHYETDYTTGGESCDELEEDWLR
33.4	3368.3089	0.0157	RTDPDHYETDYTTGGESCDELEEDWLR
18.7	3368.3089	0.0157	RTDPDHYETDYTTGGESCDELEEDWLR
13.9	3368.3089	0.0157	RTDPDHYETDYTTGGESCDELEEDWLR
12.5	3366.2940	2.0306	QIYSGKSYYFGGCPDNLTDSQCLNPIK
9.5	3368.3089	0.0157	RTDPDHYETDYTTGGESCDELEEDWLR
8.2	3366.2940	2.0306	QIYSGKSYYFGGCPDNLTDSQCLNPIK
7.8	3366.2940	2.0306	QIYSGKSYYFGGCPDNLTDSQCLNPIK

Spectrum No: 293; Query: 484; Rank: 1

Peptide View

MS/MS Fragmentation of **DIISLAPGSPNPK**
Found in **IPI00214373**, Tax_Id=10116 Gene_Symbol=Aadat Kynurenine/alpha-aminoadipate aminotransferase mitochondrial precursor

Match to Query 484: 1387.676668 from(694.845610,2+)
Title: 091008RatKidney_NH4Format02_25.2699.2699.2.dta
Data file K:\NewmanPaper\Piliang\3SubProteomes\Piliang3SP\mgf5ppm\ERLIC_3SubProteomes5ppm.mgf

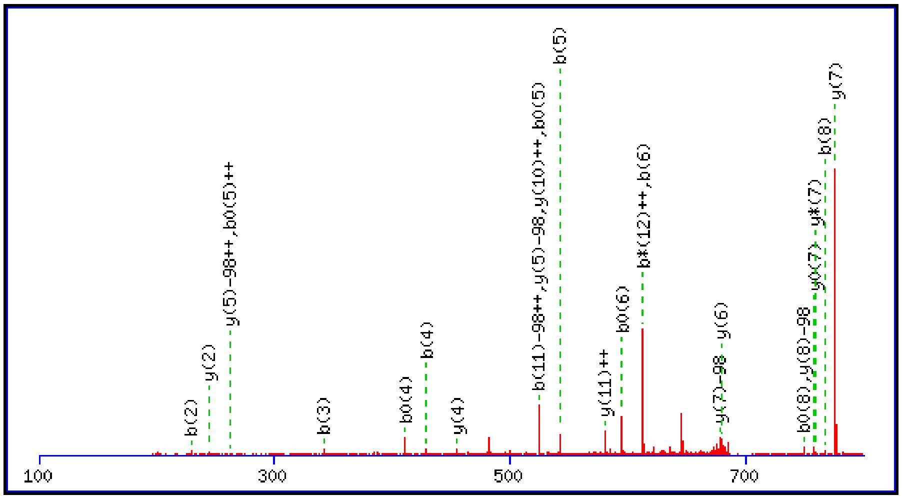

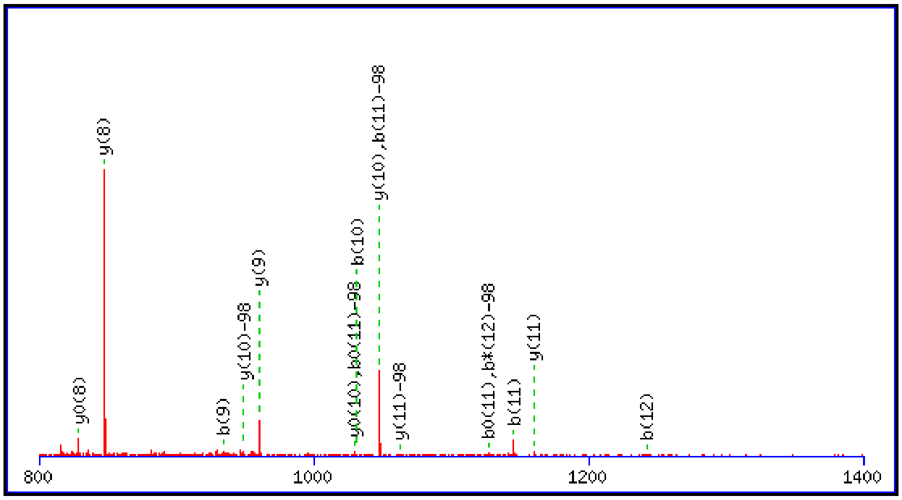

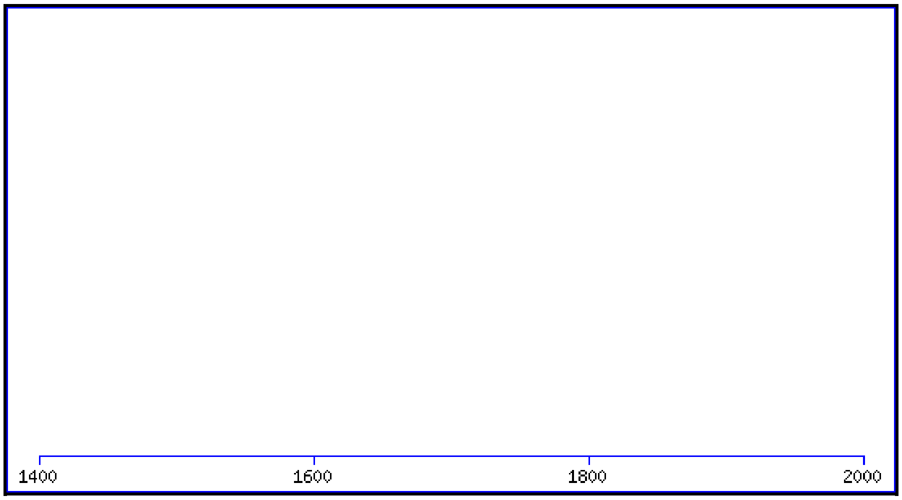

Monoisotopic mass of neutral peptide Mr(calc): 1387.6748
Fixed modifications: Carbamidomethyl (C)
Variable modifications:
S9 : Phospho (ST), with neutral losses 0.0000(shown in table), 97.9769
Ions Score: 50 **Expect:** 0.0013

Matches (Bold Red): 41/184 fragment ions using 84 most intense peaks

#	b	b^{++}	b*	b^{*++}	b^{0}	b^{0++}	Seq.	y	y^{++}	y*	y^{*++}	y^{0}	y^{0++}	#
1	116.0342	58.5207			98.0237	49.5155	D							13
2	**229.1183**	115.0628			211.1077	106.0575	I	1273.6552	637.3312	1256.6286	628.8180	1255.6446	628.3259	12
3	**342.2023**	171.6048			324.1918	162.5995	I	**1160.5711**	**580.7892**	1143.5446	572.2759	1142.5605	571.7839	11
4	**429.2344**	215.1208			**411.2238**	206.1155	S	**1047.4870**	**524.2472**	1030.4605	515.7339	**1029.4765**	515.2419	10
5	**542.3184**	271.6629			**524.3079**	**262.6576**	L	**960.4550**	480.7311	943.4285	472.2179	942.4445	471.7259	9
6	**613.3556**	307.1814			**595.3450**	298.1761	A	**847.3710**	424.1891	830.3444	415.6758	**829.3604**	415.1838	8
7	710.4083	355.7078			692.3978	346.7025	P	**776.3338**	388.6706	**759.3073**	380.1573	**758.3233**	379.6653	7
8	**767.4298**	384.2185			**749.4192**	375.2132	G	**679.2811**	340.1442	662.2545	331.6309	661.2705	331.1389	6
9	**934.4281**	467.7177			916.4176	458.7124	S	622.2596	311.6334	605.2331	303.1202	604.2490	302.6282	5
10	**1031.4809**	516.2441			1013.4703	507.2388	P	**455.2613**	228.1343	438.2347	219.6210			4
11	**1145.5238**	573.2655	1128.4973	564.7523	**1127.5133**	564.2603	N	358.2085	179.6079	341.1819	171.0946			3
12	**1242.5766**	621.7919	1225.5500	**613.2787**	1224.5660	612.7866	P	**244.1656**	122.5864	227.1390	114.0731			2
13							K	147.1128	74.0600	130.0863	65.5468			1

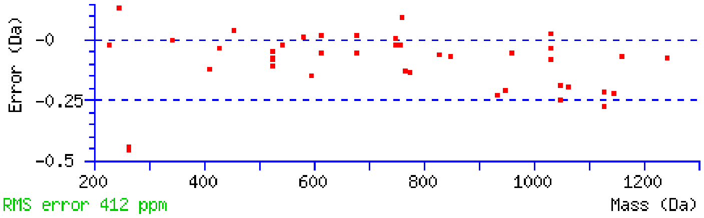

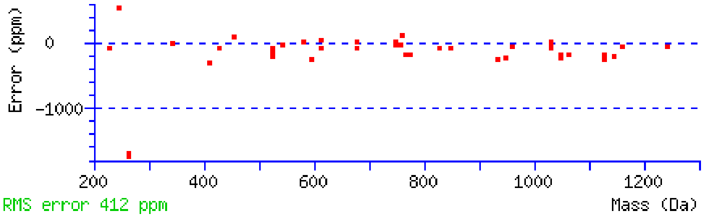

All matches to this query

Score	Mr(calc):	Delta	Sequence
50.4	1387.6748	0.0018	DIISLAPGSPNPK
29.8	1387.6748	0.0018	DIISLAPGSPNPK
11.9	1387.6765	0.0002	ILNSLTLPTKK
6.6	1387.6748	0.0018	ELTVGLTSVYAR
6.4	1385.6625	2.0141	KISLIKNCDSK
4.3	1387.6901	-0.0134	FHGIILPESPAK
4.1	1387.6901	-0.0134	WKVKFLSXSNK
2.2	1387.6683	0.0084	MPEPAKSRPAPK
2.2	1387.6748	0.0019	TAIQKAISEGYK
2.2	1387.6748	0.0019	TAIQKAISEGYK

Spectrum No: 294; Query: 385; Rank: 1

Peptide View

MS/MS Fragmentation of **SVDEVNYWDK**
Found in **IPI00365663**, Tax_Id=10116 Gene_Symbol=Bckdha branched chain ketoacid dehydrogenase E1, alpha polypeptide

Match to Query 385: 1333.520248 from(667.767400,2+)
Title: 091008RatKidney_NH4Format02_26.2213.2213.2.dta
Data file K:\NewmanPaper\Piliang\3SubProteomes\Piliang3SP\mgf5ppm\ERLIC_3SubProteomes5ppm.mgf

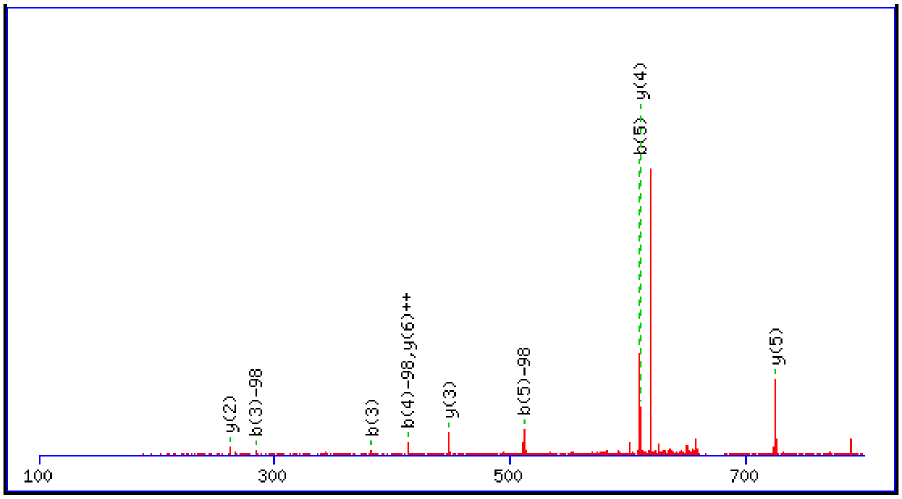

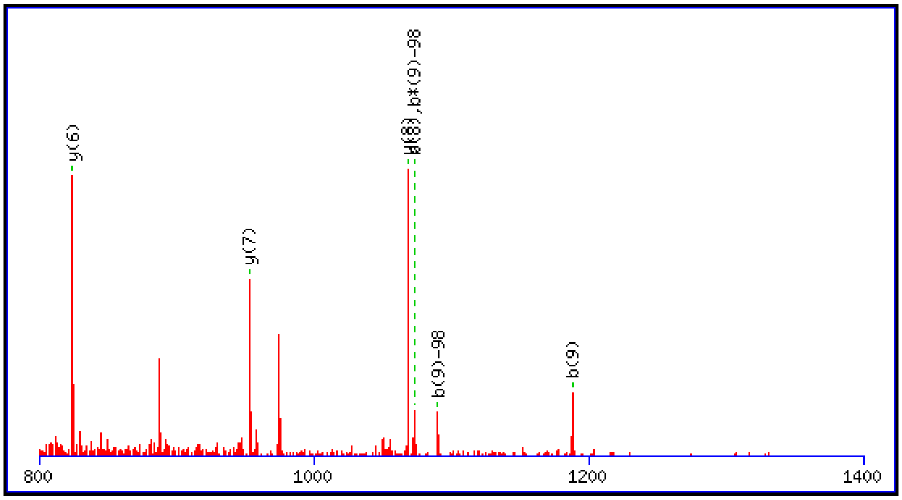

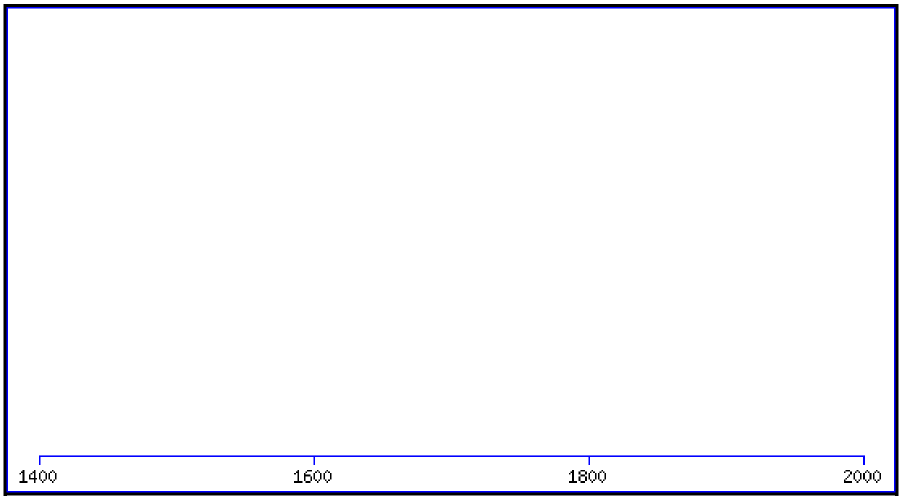

Monoisotopic mass of neutral peptide Mr(calc): 1333.5228
Fixed modifications: Carbamidomethyl (C)
Variable modifications:
S1 : Phospho (ST), with neutral losses 97.9769(shown in table), 0.0000
Ions Score: 50 **Expect:** 0.0007
Matches (Bold Red): 17/140 fragment ions using 22 most intense peaks

#	b	b++	b*	b*++	b0	b0++	Seq.	y	y++	y*	y*++	y0	y0++	#
1	70.0287	35.5180			52.0182	26.5127	S							10
2	169.0971	85.0522			151.0866	76.0469	V	1167.5317	584.2695	1150.5051	575.7562	1149.5211	575.2642	9
3	**284.1241**	142.5657			266.1135	133.5604	D	**1068.4633**	534.7353	1051.4367	526.2220	1050.4527	525.7300	8
4	**413.1667**	207.0870			395.1561	198.0817	E	**953.4363**	477.2218	936.4098	468.7085	935.4258	468.2165	7
5	**512.2351**	256.6212			494.2245	247.6159	V	**824.3937**	**412.7005**	807.3672	404.1872	806.3832	403.6952	6

**6**	626.2780	313.6426	609.2515	305.1294	608.2675	304.6374	**N**	**725.3253**	363.1663	708.2988	354.6530	707.3148	354.1610	**5**
**7**	789.3414	395.1743	772.3148	386.6610	771.3308	386.1690	**Y**	**611.2824**	306.1448	594.2558	297.6316	593.2718	297.1396	**4**
**8**	975.4207	488.2140	958.3941	479.7007	957.4101	479.2087	**W**	**448.2191**	224.6132	431.1925	216.0999	430.2085	215.6079	**3**
**9**	**1090.4476**	545.7274	**1073.4211**	537.2142	1072.4370	536.7222	**D**	**262.1397**	131.5735	245.1132	123.0602	244.1292	122.5682	**2**
**10**							**K**	147.1128	74.0600	130.0863	65.5468			**1**

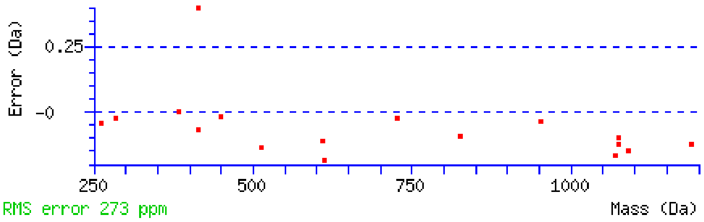

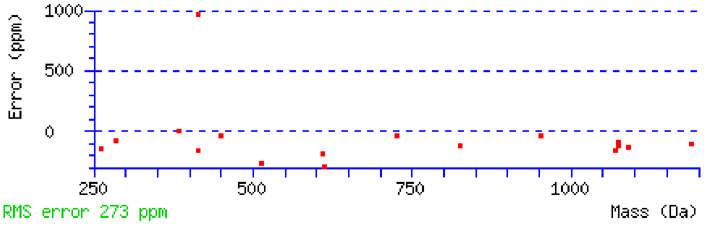

All matches to this query

Score	Mr(calc):	Delta	Sequence
50.3	1333.5228	-0.0025	SVDEVNYWDK
9.8	1333.5108	0.0094	ALENTMETSDK
8.3	1333.5088	0.0114	HGGYQPSDEHK
4.6	1333.5166	0.0036	LADMYGGMDSDK
3.3	1333.5187	0.0016	NLSYGRDADNK
2.7	1332.5251	0.9952	NETLALPAESK
2.0	1333.5187	0.0016	NLSYGRDADNK
1.5	1333.5105	0.0098	GVYELTHNNK
1.1	1333.5108	0.0094	ALENTMETSDK
1.1	1333.5108	0.0094	ALENTMETSDK

Spectrum No: 295; Query: 2770; Rank: 1

Peptide View

MS/MS Fragmentation of **YMAENPTAGVVQEEEEDNLEYDSDGNPIAPSKK**
Found in **IPI00361283**, Tax_Id=10116 Gene_Symbol=Ddx42_predicted DEAD (Asp-Glu-Ala-Asp) box polypeptide 42

Match to Query 2770: 3734.597562 from(1245.873130,3+)
Title: 100101RatKid_NS_deglyco_22.3223.3223.3.dta
Data file K:\NewmanPaper\Piliang\3SubProteomes\Piliang3SP\mgf5ppm\ERLIC_3SubProteomes5ppm.mgf

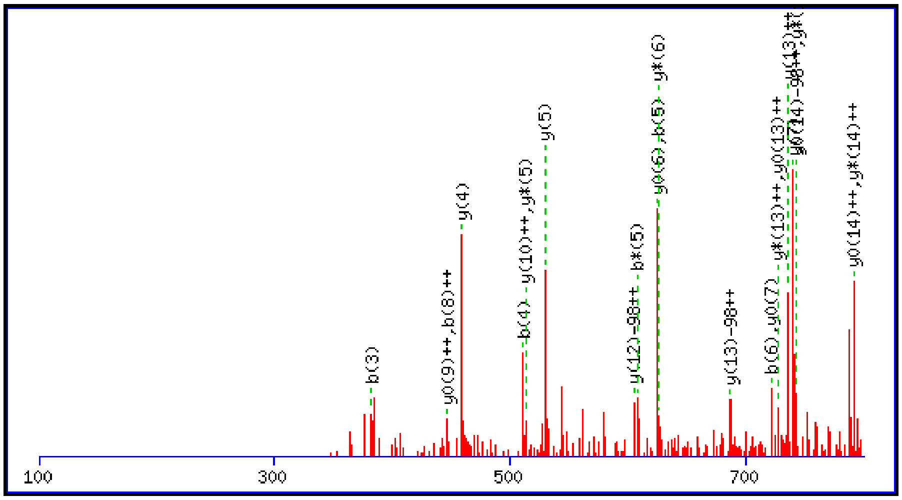

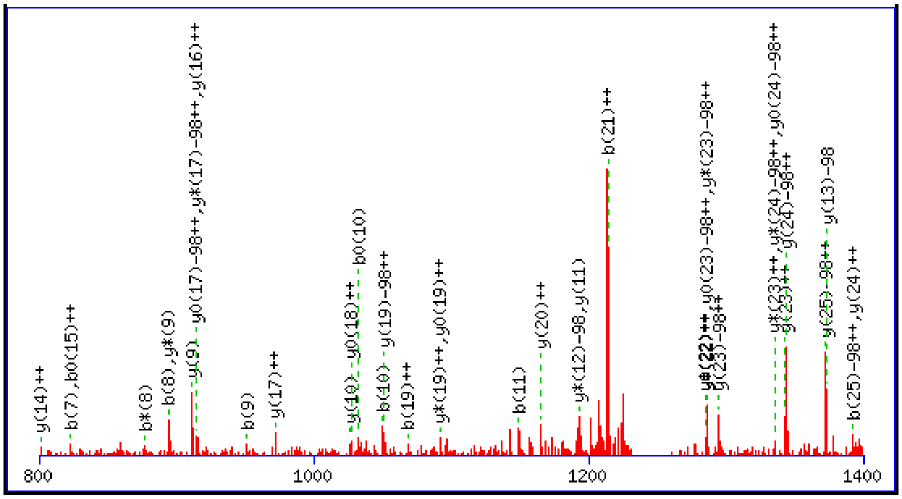

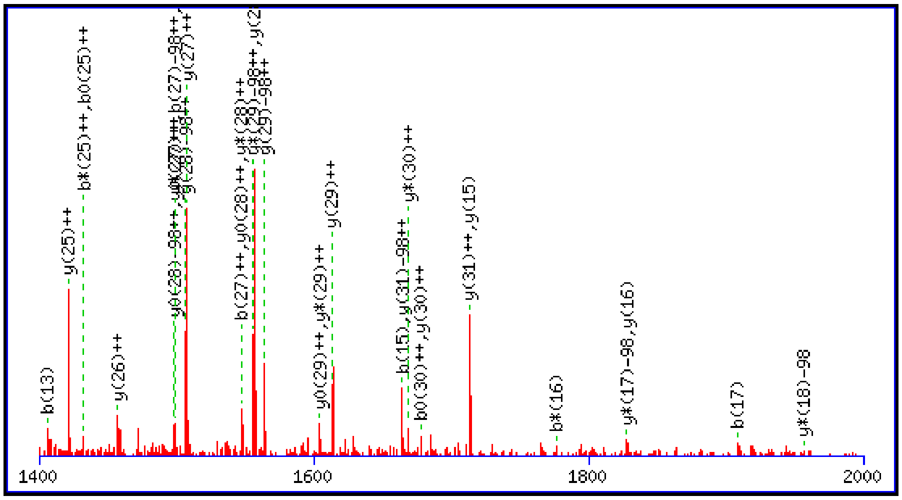

Monoisotopic mass of neutral peptide Mr(calc): 3734.5818
Fixed modifications: Carbamidomethyl (C)
Variable modifications:
M2 : Oxidation (M)
S23 : Phospho (ST), with neutral losses 0.0000(shown in table), 97.9769
Ions Score: 50 **Expect:** 0.0049
Matches (Bold Red): 97/558 fragment ions using 140 most intense peaks

#	b	b^{++}	b*	b^{*++}	b^{0}	b^{0++}	Seq.	y	y^{++}	y*	y^{*++}	y^{0}	y^{0++}	#
1	164.0706	82.5389					Y							33
2	311.1060	156.0566					M	3572.5258	1786.7665	3555.4993	1778.2533	3554.5153	1777.7613	32
3	**382.1431**	191.5752					A	3425.4904	**1713.2488**	3408.4639	1704.7356	3407.4799	1704.2436	31
4	**511.1857**	256.0965			493.1752	247.0912	E	3354.4533	**1677.7303**	3337.4268	**1669.2170**	3336.4427	1668.7250	30
5	**625.2286**	313.1180	**608.2021**	304.6047	607.2181	304.1127	N	3225.4107	**1613.2090**	3208.3842	**1604.6957**	3207.4001	**1604.2037**	29
6	**722.2814**	361.6443	705.2549	353.1311	704.2708	352.6391	P	3111.3678	**1556.1875**	3094.3412	**1547.6743**	3093.3572	**1547.1822**	28
7	**823.3291**	412.1682	806.3025	403.6549	805.3185	403.1629	T	3014.3150	**1507.6611**	2997.2885	**1499.1479**	2996.3045	**1498.6559**	27
8	**894.3662**	**447.6867**	**877.3397**	439.1735	876.3556	438.6815	A	2913.2673	**1457.1373**	2896.2408	1448.6240	2895.2568	1448.1320	26
9	**951.3877**	476.1975	934.3611	467.6842	933.3771	467.1922	G	2842.2302	**1421.6188**	2825.2037	1413.1055	2824.2197	1412.6135	25
10	**1050.4561**	525.7317	1033.4295	517.2184	**1032.4455**	516.7264	V	2785.2088	**1393.1080**	2768.1822	1384.5947	2767.1982	1384.1027	24
11	**1149.5245**	575.2659	1132.4979	566.7526	1131.5139	566.2606	V	2686.1404	**1343.5738**	2669.1138	**1335.0605**	2668.1298	1334.5685	23
12	1277.5831	639.2952	1260.5565	630.7819	1259.5725	630.2899	Q	2587.0719	1294.0396	2570.0454	**1285.5263**	2569.0614	**1285.0343**	22
13	**1406.6257**	703.8165	1389.5991	695.3032	1388.6151	694.8112	E	2459.0134	1230.0103	2441.9868	1221.4970	2441.0028	1221.0050	21
14	1535.6683	768.3378	1518.6417	759.8245	1517.6577	759.3325	E	2329.9708	**1165.4890**	2312.9442	1156.9757	2311.9602	1156.4837	20
15	**1664.7108**	832.8591	1647.6843	824.3458	1646.7003	**823.8538**	E	2200.9282	1100.9677	2183.9016	**1092.4544**	2182.9176	**1091.9624**	19
16	1793.7534	897.3804	**1776.7269**	888.8671	1775.7429	888.3751	E	2071.8856	1036.4464	2054.8590	1027.9332	2053.8750	**1027.4411**	18
17	**1908.7804**	954.8938	1891.7538	946.3806	1890.7698	945.8885	D	1942.8430	**971.9251**	1925.8164	963.4119	1924.8324	962.9198	17
18	2022.8233	1011.9153	2005.7968	1003.4020	2004.8127	1002.9100	N	**1827.8160**	**914.4117**	1810.7895	905.8984	1809.8055	905.4064	16

19	2135.9074	**1068.4573**	2118.8808	1059.9441	2117.8968	1059.4520	L	**1713.7731**	857.3902	1696.7466	848.8769	1695.7626	848.3849	15
20	2264.9500	1132.9786	2247.9234	1124.4653	2246.9394	1123.9733	E	1600.6891	**800.8482**	1583.6625	**792.3349**	1582.6785	**791.8429**	14
21	2428.0133	**1214.5103**	2410.9867	1205.9970	2410.0027	1205.5050	Y	1471.6465	**736.3269**	1454.6199	**727.8136**	1453.6359	**727.3216**	13
22	2543.0402	1272.0238	2526.0137	1263.5105	2525.0297	1263.0185	D	1308.5831	654.7952	1291.5566	646.2819	1290.5726	645.7899	12
23	2710.0386	1355.5229	2693.0120	1347.0097	2692.0280	1346.5177	S	**1193.5562**	597.2817	1176.5296	588.7685	1175.5456	588.2764	11
24	2825.0655	1413.0364	2808.0390	1404.5231	2807.0550	1404.0311	D	**1026.5578**	**513.7826**	1009.5313	505.2693	1008.5473	504.7773	10
25	2882.0870	1441.5471	2865.0605	**1433.0339**	2864.0764	**1432.5419**	G	**911.5309**	456.2691	**894.5043**	447.7558	893.5203	**447.2638**	9
26	2996.1299	**1498.5686**	2979.1034	1490.0553	2978.1194	1489.5633	N	854.5094	427.7584	837.4829	419.2451	836.4989	418.7531	8
27	3093.1827	**1547.0950**	3076.1561	1538.5817	3075.1721	1538.0897	P	**740.4665**	370.7369	723.4400	362.2236	**722.4559**	361.7316	7
28	3206.2668	1603.6370	3189.2402	1595.1237	3188.2562	1594.6317	I	643.4137	322.2105	**626.3872**	313.6972	**625.4032**	313.2052	6
29	3277.3039	1639.1556	3260.2773	1630.6423	3259.2933	1630.1503	A	**530.3297**	265.6685	**513.3031**	257.1552	512.3191	256.6632	5
30	3374.3566	1687.6820	3357.3301	1679.1687	3356.3461	**1678.6767**	P	**459.2926**	230.1499	442.2660	221.6366	441.2820	221.1446	4
31	3461.3887	1731.1980	3444.3621	1722.6847	3443.3781	1722.1927	S	362.2398	181.6235	345.2132	173.1103	344.2292	172.6183	3
32	3589.4836	1795.2455	3572.4571	1786.7322	3571.4731	1786.2402	K	275.2078	138.1075	258.1812	129.5942			2
33							K	147.1128	74.0600	130.0863	65.5468			1

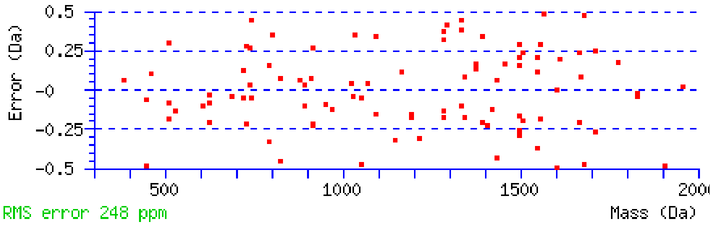

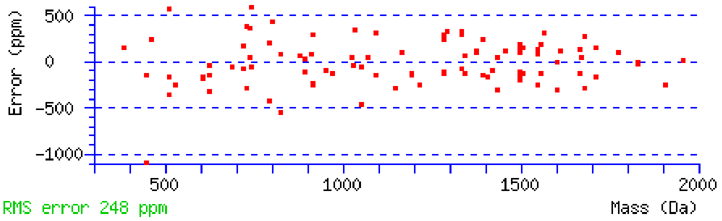

All matches to this query

Score	Mr(calc):	Delta	Sequence
50.3	3734.5818	0.0157	YMAENPTAGVVQEEEEDNLEYDSDGNPIAPSKK
45.6	3734.5818	0.0157	YMAENPTAGVVQEEEEDNLEYDSDGNPIAPSKK
36.9	3734.5818	0.0157	YMAENPTAGVVQEEEEDNLEYDSDGNPIAPSKK
9.4	3734.5818	0.0157	YMAENPTAGVVQEEEEDNLEYDSDGNPIAPSKK
4.7	3734.5989	-0.0014	ETLNSQYHLTEKALHHLAQIYAALVSSGLK
3.8	3734.6291	-0.0315	DHRFEYSHGPITVLTNSSDPSTGPESTHPLPAK
2.3	3733.6252	0.9724	GTETYGFIQVTLLDTMELATFCVRTFSLHK
1.7	3733.6027	0.9949	ENNMSNYNLMEKELILPGFTFMRNGTTGDWK
1.6	3733.5760	1.0216	LTIQNANNATVTTPNAPTSTPTVMPSTNGSYTVK
1.1	3733.6225	0.9750	GMRFVGFVPQHCSPSKHCNGASQDGVAESELHK

Spectrum No: 296; Query: 2692; Rank: 1

Peptide View

MS/MS Fragmentation of **KQTPPASPSPQPAEDRPPSSPIYEDAAPLK**
Found in **IPI00198498**, Tax_Id=10116 Gene_Symbol=Cttn Cortactin isoform C

Match to Query 2692: 3410.493912 from(1137.838580,3+)
Title: 100101RatKid_NS_deglyco_24.2589.2589.3.dta
Data file K:\NewmanPaper\Piliang\3SubProteomes\Piliang3SP\mgf5ppm\ERLIC_3SubProteomes5ppm.mgf

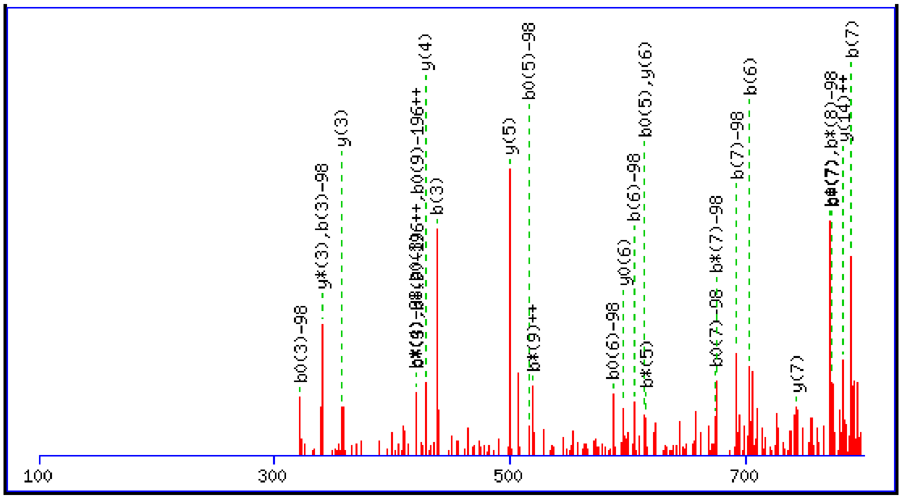

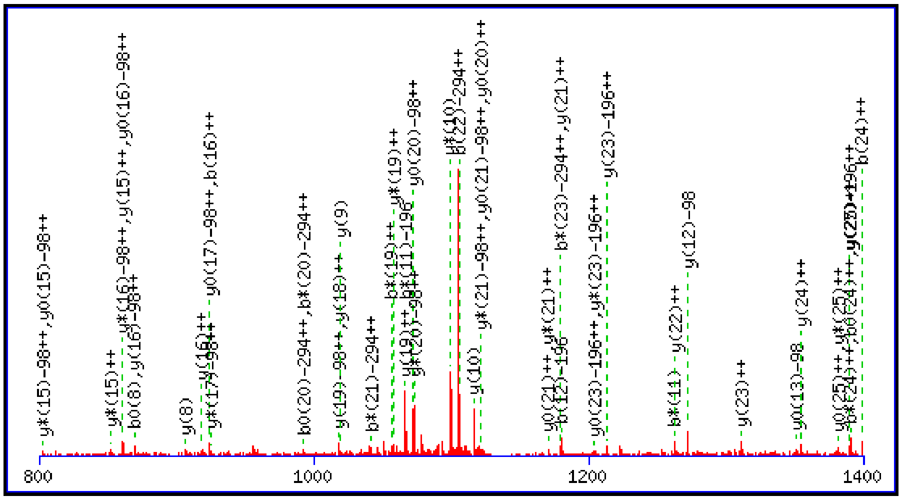

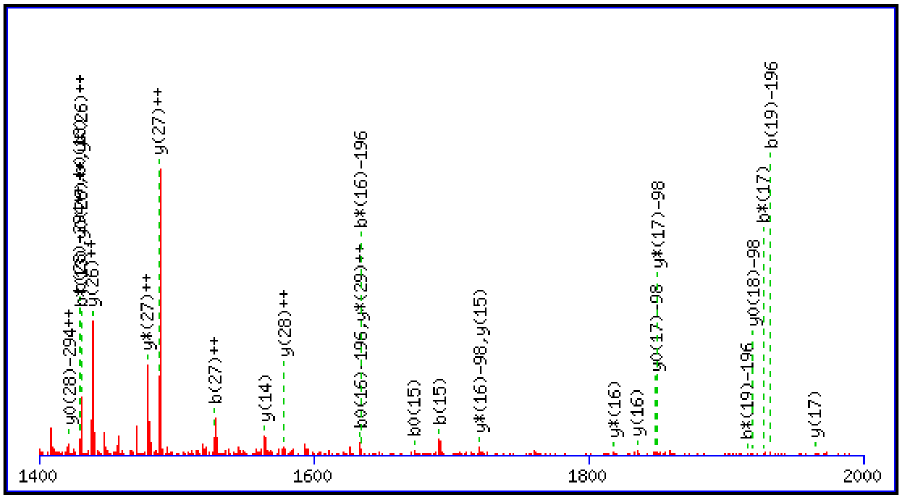

Monoisotopic mass of neutral peptide Mr(calc): 3410.4873
Fixed modifications: Carbamidomethyl (C)
Variable modifications:
T3 : Phospho (ST), with neutral losses 0.0000(shown in table), 97.9769
S9 : Phospho (ST), with neutral losses 0.0000(shown in table), 97.9769
S20 : Phospho (ST), with neutral losses 0.0000(shown in table), 97.9769
Ions Score: 50 **Expect:** 0.0045
Matches (Bold Red): 110/610 fragment ions using 162 most intense peaks

#	b	b^{++}	b^{*}	b^{*++}	b^{0}	b^{0++}	Seq.	y	y^{++}	y^{*}	y^{*++}	y^{0}	y^{0++}	#
1	129.1022	65.0548	112.0757	56.5415			K							30
2	257.1608	129.0840	240.1343	120.5708			Q	3283.3997	1642.2035	3266.3731	**1633.6902**	3265.3891	1633.1982	29
3	**438.1748**	219.5910	**421.1483**	211.0778	**420.1643**	210.5858	T	3155.3411	**1578.1742**	3138.3146	1569.6609	3137.3306	1569.1689	28
4	535.2276	268.1174	518.2010	259.6042	517.2170	259.1121	P	2974.3271	**1487.6672**	2957.3006	**1479.1539**	2956.3165	1478.6619	27

5	632.2803	316.6438	**615.2538**	308.1305	**614.2698**	307.6385	P	2877.2744	**1439.1408**	2860.2478	**1430.6275**	2859.2638	**1430.1355**	26
6	**703.3175**	352.1624	686.2909	343.6491	685.3069	343.1571	A	2780.2216	**1390.6144**	2763.1950	**1382.1012**	2762.2110	**1381.6091**	25
7	**790.3495**	395.6784	**773.3229**	387.1651	**772.3389**	386.6731	S	2709.1845	**1355.0959**	2692.1579	1346.5826	2691.1739	1346.0906	24
8	887.4023	444.2048	870.3757	435.6915	**869.3917**	435.1995	P	2622.1524	**1311.5799**	2605.1259	1303.0666	2604.1419	1302.5746	23
9	1054.4006	527.7039	1037.3741	**519.1907**	1036.3900	518.6987	S	2525.0997	**1263.0535**	2508.0731	1254.5402	2507.0891	1254.0482	22
10	1151.4534	576.2303	1134.4268	567.7170	1133.4428	567.2250	P	2358.1013	**1179.5543**	2341.0748	**1171.0410**	2340.0908	**1170.5490**	21
11	1279.5119	640.2596	**1262.4854**	631.7463	1261.5014	631.2543	Q	2261.0486	1131.0279	2244.0220	1122.5146	2243.0380	**1122.0226**	20
12	1376.5647	688.7860	1359.5382	680.2727	1358.5541	679.7807	P	2132.9900	**1066.9986**	2115.9634	**1058.4854**	2114.9794	1057.9933	19
13	1447.6018	724.3046	**1430.5753**	715.7913	**1429.5913**	715.2993	A	2035.9372	**1018.4722**	2018.9107	1009.9590	2017.9267	1009.4670	18
14	1576.6444	788.8258	1559.6179	780.3126	1558.6339	779.8206	E	**1964.9001**	982.9537	1947.8736	974.4404	1946.8895	973.9484	17
15	**1691.6714**	846.3393	1674.6448	837.8260	**1673.6608**	837.3340	D	**1835.8575**	**918.4324**	**1818.8310**	909.9191	1817.8470	909.4271	16
16	1847.7725	**924.3899**	1830.7459	915.8766	1829.7619	915.3846	R	**1720.8306**	**860.9189**	1703.8040	**852.4056**	1702.8200	851.9136	15
17	1944.8252	972.9163	**1927.7987**	964.4030	1926.8147	963.9110	P	**1564.7295**	**782.8684**	1547.7029	774.3551	1546.7189	773.8631	14
18	2041.8780	1021.4426	2024.8515	1012.9294	2023.8674	1012.4374	P	1467.6767	734.3420	1450.6501	725.8287	1449.6661	725.3367	13
19	2128.9100	1064.9587	2111.8835	**1056.4454**	2110.8995	1055.9534	S	1370.6239	685.8156	1353.5974	677.3023	1352.6134	676.8103	12
20	2295.9084	1148.4578	2278.8818	1139.9446	2277.8978	1139.4525	S	1283.5919	642.2996	1266.5654	633.7863	1265.5813	633.2943	11
21	2392.9611	1196.9842	2375.9346	1188.4709	2374.9506	1187.9789	P	**1116.5936**	558.8004	**1099.5670**	550.2871	1098.5830	549.7951	10
22	2506.0452	1253.5262	2489.0187	1245.0130	2488.0346	1244.5210	I	**1019.5408**	510.2740	1002.5142	501.7608	1001.5302	501.2688	9
23	2669.1085	1335.0579	2652.0820	1326.5446	2651.0980	1326.0526	Y	**906.4567**	453.7320	889.4302	445.2187	888.4462	444.7267	8
24	2798.1511	**1399.5792**	2781.1246	**1391.0659**	2780.1406	**1390.5739**	E	**743.3934**	372.2003	726.3668	363.6871	725.3828	363.1951	7
25	2913.1781	1457.0927	2896.1515	1448.5794	2895.1675	1448.0874	D	**614.3508**	307.6790	597.3243	299.1658	**596.3402**	298.6738	6
26	2984.2152	1492.6112	2967.1886	1484.0980	2966.2046	1483.6059	A	**499.3239**	250.1656	482.2973	241.6523			5
27	3055.2523	**1528.1298**	3038.2258	1519.6165	3037.2417	1519.1245	A	**428.2867**	214.6470	411.2602	206.1337			4
28	3152.3051	1576.6562	3135.2785	1568.1429	3134.2945	1567.6509	P	**357.2496**	179.1285	**340.2231**	170.6152			3
29	3265.3891	1633.1982	3248.3626	1624.6849	3247.3786	1624.1929	L	260.1969	130.6021	243.1703	122.0888			2
30							K	147.1128	74.0600	130.0863	65.5468			1

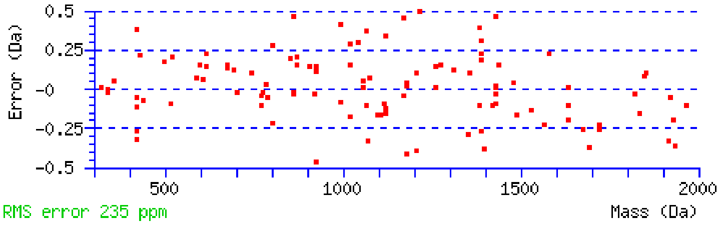

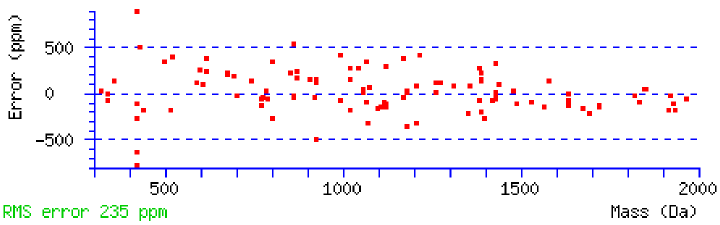

All matches to this query

Score	Mr(calc):	Delta	Sequence
50.3	3410.4873	0.0066	KQTPPASPSPQPAEDRPPSSPIYEDAAPLK
50.3	3410.4873	0.0066	KQTPPASPSPQPAEDRPPSSPIYEDAAPLK
45.8	3410.4873	0.0066	KQTPPASPSPQPAEDRPPSSPIYEDAAPLK
45.8	3410.4873	0.0066	KQTPPASPSPQPAEDRPPSSPIYEDAAPLK
44.5	3410.4873	0.0066	KQTPPASPSPQPAEDRPPSSPIYEDAAPLK
43.0	3410.4873	0.0066	KQTPPASPSPQPAEDRPPSSPIYEDAAPLK
39.0	3410.4873	0.0066	KQTPPASPSPQPAEDRPPSSPIYEDAAPLK
39.0	3410.4873	0.0066	KQTPPASPSPQPAEDRPPSSPIYEDAAPLK
31.1	3410.4873	0.0066	KQTPPASPSPQPAEDRPPSSPIYEDAAPLK
20.1	3410.4873	0.0066	KQTPPASPSPQPAEDRPPSSPIYEDAAPLK

Spectrum No: 297; Query: 2563; Rank: 1

Peptide View

MS/MS Fragmentation of **TGDLGIPPNPEDRSPSPEPIYNSEGKR**
Found in **IPI00558327**, Tax_Id=10116 Gene_Symbol=Sf1 splicing factor 1 isoform 2

Match to Query 2563: 3081.346632 from(1028.122820,3+)
Title: 091008RatKidney_NH4Format01_24.2234.2234.3.dta
Data file K:\NewmanPaper\Piliang\3SubProteomes\Piliang3SP\mgf5ppm\ERLIC_3SubProteomes5ppm.mgf

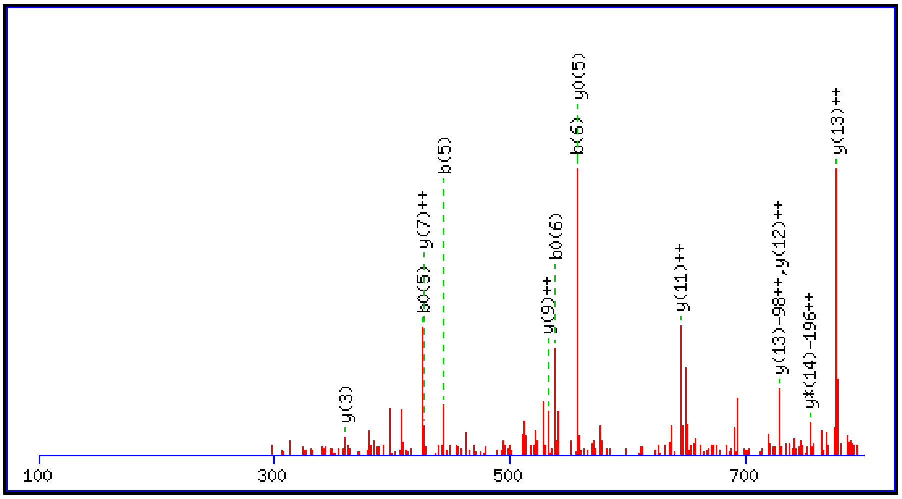

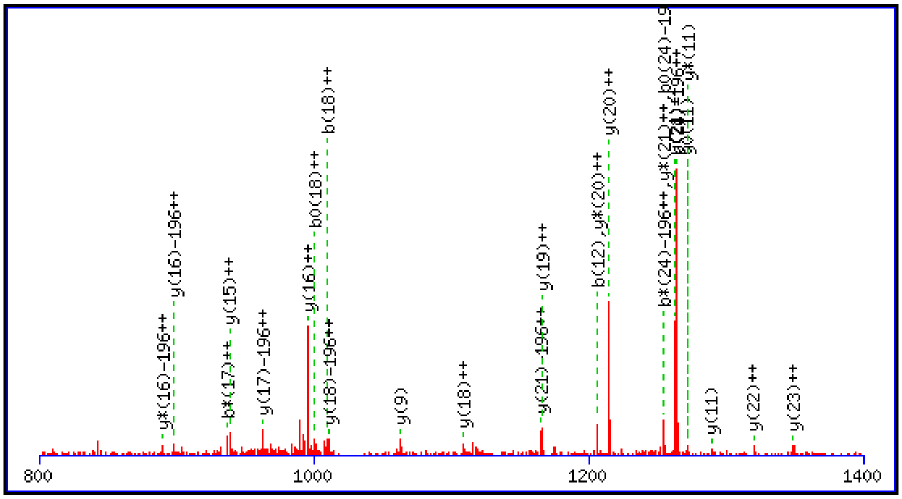

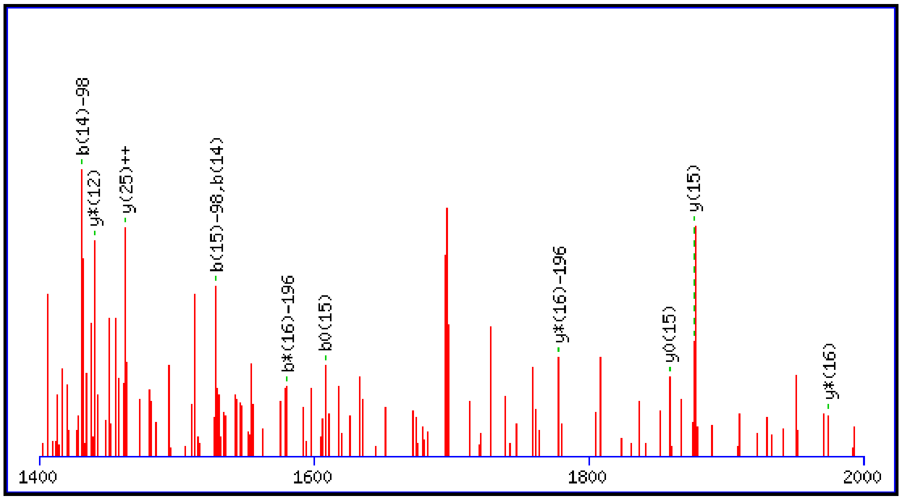

Monoisotopic mass of neutral peptide Mr(calc): 3081.3481
Fixed modifications: Carbamidomethyl (C)
Variable modifications:
S14 : Phospho (ST), with neutral losses 0.0000(shown in table), 97.9769
S16 : Phospho (ST), with neutral losses 0.0000(shown in table), 97.9769

Ions Score: 50 **Expect:** 0.0042
Matches (Bold Red): 50/458 fragment ions using 94 most intense peaks

#	b	b^{++}	b^{*}	b^{*++}	b^{0}	b^{0++}	Seq.	y	y^{++}	y^{*}	y^{*++}	y^{0}	y^{0++}	#
1	102.0550	51.5311			84.0444	42.5258	T							27
2	159.0764	80.0418			141.0659	71.0366	G	2981.3078	1491.1575	2964.2812	1482.6443	2963.2972	1482.1522	26
3	274.1034	137.5553			256.0928	128.5500	D	2924.2863	**1462.6468**	2907.2598	1454.1335	2906.2757	1453.6415	25
4	387.1874	194.0974			369.1769	185.0921	L	2809.2594	1405.1333	2792.2328	1396.6200	2791.2488	1396.1280	24
5	**444.2089**	222.6081			**426.1983**	213.6028	G	2696.1753	**1348.5913**	2679.1488	1340.0780	2678.1647	1339.5860	23
6	**557.2930**	279.1501			**539.2824**	270.1448	I	2639.1538	**1320.0806**	2622.1273	1311.5673	2621.1433	1311.0753	22
7	654.3457	327.6765			636.3352	318.6712	P	2526.0698	**1263.5385**	2509.0432	**1255.0253**	2508.0592	1254.5332	21
8	751.3985	376.2029			733.3879	367.1976	P	2429.0170	**1215.0121**	2411.9905	**1206.4989**	2411.0064	1206.0069	20
9	865.4414	433.2243	848.4149	424.7111	847.4308	424.2191	N	2331.9642	**1166.4858**	2314.9377	1157.9725	2313.9537	1157.4805	19
10	962.4942	481.7507	945.4676	473.2375	944.4836	472.7454	P	2217.9213	**1109.4643**	2200.8948	1100.9510	2199.9108	1100.4590	18
11	1091.5368	546.2720	1074.5102	537.7587	1073.5262	537.2667	E	2120.8686	1060.9379	2103.8420	1052.4246	2102.8580	1051.9326	17
12	**1206.5637**	603.7855	1189.5372	595.2722	1188.5531	594.7802	D	1991.8260	**996.4166**	**1974.7994**	987.9033	1973.8154	987.4113	16
13	1362.6648	681.8360	1345.6383	673.3228	1344.6543	672.8308	R	**1876.7990**	**938.9031**	1859.7725	930.3899	**1858.7885**	929.8979	15
14	**1529.6632**	765.3352	1512.6366	756.8219	1511.6526	756.3299	S	1720.6979	860.8526	1703.6714	852.3393	1702.6873	851.8473	14
15	1626.7159	813.8616	1609.6894	805.3483	**1608.7054**	804.8563	P	1553.6996	**777.3534**	1536.6730	768.8401	1535.6890	768.3481	13
16	1793.7143	897.3608	1776.6877	888.8475	1775.7037	888.3555	S	1456.6468	**728.8270**	**1439.6202**	720.3138	1438.6362	719.8218	12
17	1890.7671	945.8872	1873.7405	**937.3739**	1872.7565	936.8819	P	**1289.6484**	**645.3279**	**1272.6219**	636.8146	**1271.6379**	636.3226	11
18	2019.8096	**1010.4085**	2002.7831	1001.8952	2001.7991	**1001.4032**	E	1192.5957	596.8015	1175.5691	588.2882	1174.5851	587.7962	10
19	2116.8624	1058.9348	2099.8359	1050.4216	2098.8518	1049.9296	P	**1063.5531**	**532.2802**	1046.5265	523.7669	1045.5425	523.2749	9
20	2229.9465	1115.4769	2212.9199	1106.9636	2211.9359	1106.4716	I	966.5003	483.7538	949.4738	475.2405	948.4898	474.7485	8
21	2393.0098	1197.0085	2375.9833	1188.4953	2374.9992	1188.0033	Y	853.4163	**427.2118**	836.3897	418.6985	835.4057	418.2065	7
22	2507.0527	1254.0300	2490.0262	1245.5167	2489.0422	1245.0247	N	690.3529	345.6801	673.3264	337.1668	672.3424	336.6748	6
23	2594.0848	1297.5460	2577.0582	1289.0327	2576.0742	1288.5407	S	576.3100	288.6586	559.2835	280.1454	**558.2994**	279.6534	5
24	2723.1274	1362.0673	2706.1008	1353.5540	2705.1168	1353.0620	E	489.2780	245.1426	472.2514	236.6293	471.2674	236.1373	4
25	2780.1488	1390.5780	2763.1223	1382.0648	2762.1382	1381.5728	G	**360.2354**	180.6213	343.2088	172.1081			3
26	2908.2438	1454.6255	2891.2172	1446.1123	2890.2332	1445.6202	K	303.2139	152.1106	286.1874	143.5973			2
27							R	175.1190	88.0631	158.0924	79.5498			1

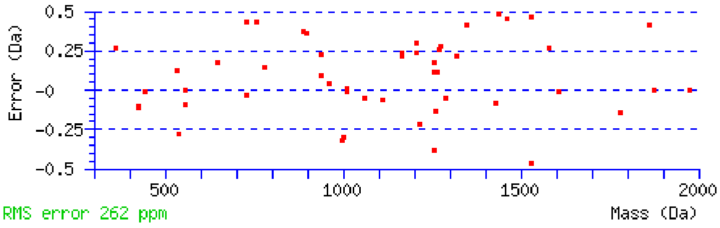

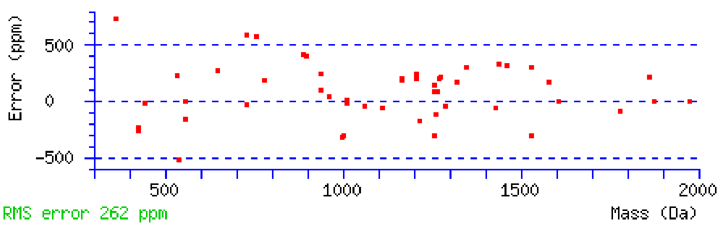

All matches to this query

Score	Mr(calc):	Delta	Sequence
50.2	3081.3481	-0.0015	TGDLGIPPNPEDRSPSPEPIYNSEGKR
28.4	3081.3481	-0.0015	TGDLGIPPNPEDRSPSPEPIYNSEGKR
23.1	3081.3481	-0.0015	TGDLGIPPNPEDRSPSPEPIYNSEGKR
22.3	3081.3481	-0.0015	TGDLGIPPNPEDRSPSPEPIYNSEGKR
21.0	3081.3481	-0.0015	TGDLGIPPNPEDRSPSPEPIYNSEGKR
17.6	3081.3481	-0.0015	TGDLGIPPNPEDRSPSPEPIYNSEGKR
9.8	3081.3724	-0.0258	NAVASLIYTAVSPMLNPFIYTLRNR
8.0	3081.3724	-0.0258	NAVASLIYTAVSPMLNPFIYTLRNR
5.9	3081.3415	0.0051	RHSFEHISLIPPETSTVLNSLKEK
5.9	3081.3415	0.0051	RHSFEHISLIPPETSTVLNSLKEK

Spectrum No: 298; Query: 1964; Rank: 1

Peptide View

MS/MS Fragmentation of **VQSQEEIHSDEEDQASEPK**
Found in **IPI00400666**, Tax_Id=10116 Gene_Symbol=Nhn1 Isoform A of Zinc finger CCCH domain-containing protein 18

Match to Query 1964: 2263.917612 from(755.646480,3+)
Title: 100101RatKid_NS_deglyco_21.864.864.3.dta
Data file K:\NewmanPaper\Piliang\3SubProteomes\Piliang3SP\mgf5ppm\ERLIC_3SubProteomes5ppm.mgf

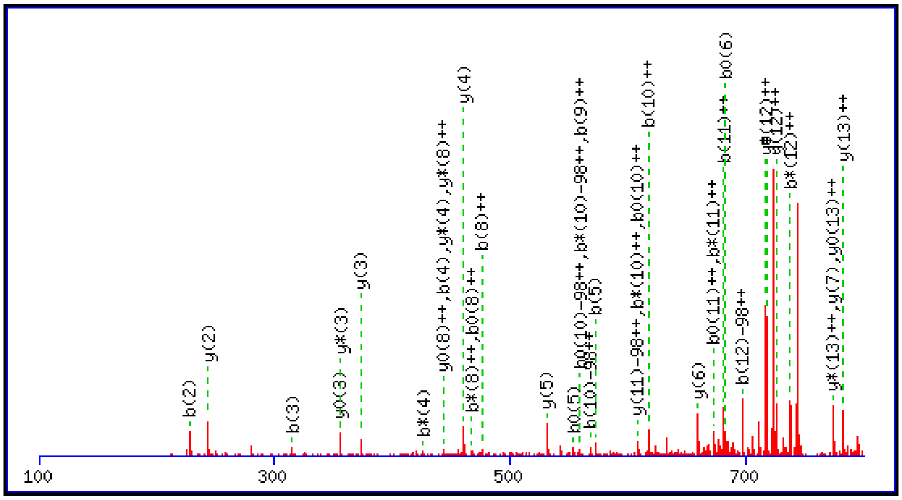

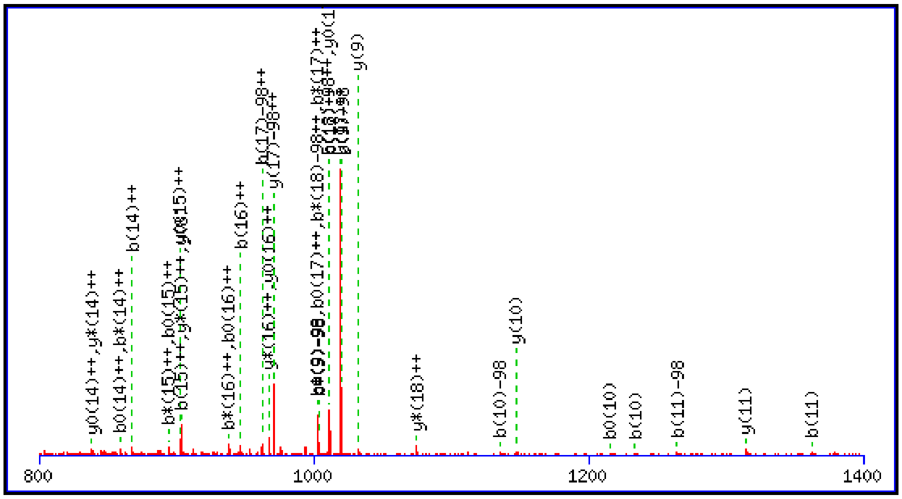

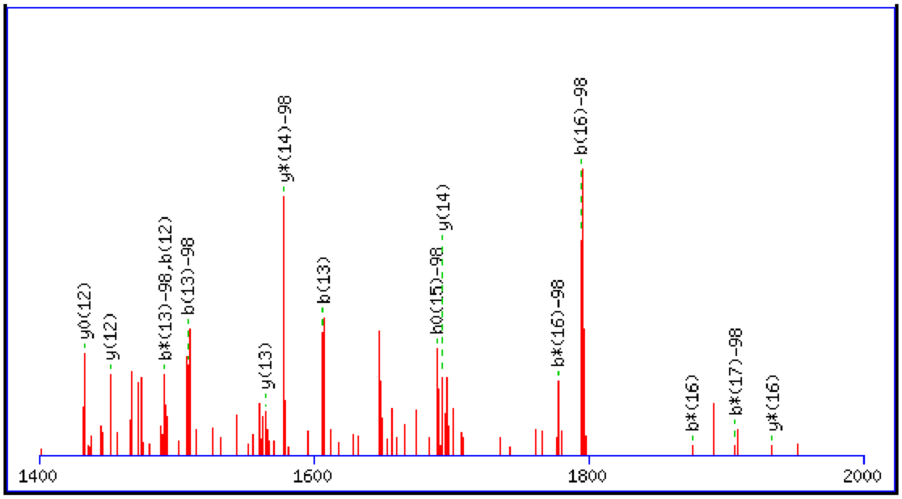

Monoisotopic mass of neutral peptide Mr(calc): 2263.9118
Fixed modifications: Carbamidomethyl (C)
Variable modifications:
S9 : Phospho (ST), with neutral losses 0.0000(shown in table), 97.9769
Ions Score: 50 **Expect:** 0.0023
Matches (Bold Red): 93/314 fragment ions using 128 most intense peaks

#	b	b^{++}	b*	b^{*++}	b^{0}	b^{0++}	Seq.	y	y^{++}	y*	y^{*++}	y^{0}	y^{0++}	#
1	100.0757	50.5415					V							19
2	**228.1343**	114.5708	211.1077	106.0575			Q	2165.8507	1083.4290	2148.8241	**1074.9157**	2147.8401	1074.4237	18
3	**315.1663**	158.0868	298.1397	149.5735	297.1557	149.0815	S	2037.7921	**1019.3997**	2020.7655	**1010.8864**	2019.7815	**1010.3944**	17
4	**443.2249**	222.1161	**426.1983**	213.6028	425.2143	213.1108	Q	1950.7600	975.8837	**1933.7335**	**967.3704**	1932.7495	**966.8784**	16
5	**572.2675**	286.6374	555.2409	278.1241	**554.2569**	277.6321	E	1822.7015	911.8544	1805.6749	**903.3411**	1804.6909	**902.8491**	15
6	701.3101	351.1587	684.2835	342.6454	**683.2995**	342.1534	E	**1693.6589**	847.3331	1676.6323	**838.8198**	1675.6483	**838.3278**	14
7	814.3941	407.7007	797.3676	399.1874	796.3836	398.6954	I	**1564.6163**	**782.8118**	1547.5897	**774.2985**	1546.6057	**773.8065**	13
8	951.4530	**476.2302**	934.4265	**467.7169**	933.4425	**467.2249**	H	**1451.5322**	**726.2697**	1434.5057	**717.7565**	**1433.5217**	**717.2645**	12
9	1118.4514	**559.7293**	1101.4248	551.2161	1100.4408	550.7240	S	**1314.4733**	657.7403	1297.4468	649.2270	1296.4627	648.7350	11
10	**1233.4783**	**617.2428**	1216.4518	**608.7295**	**1215.4678**	**608.2375**	D	**1147.4750**	574.2411	1130.4484	565.7278	1129.4644	565.2358	10
11	**1362.5209**	**681.7641**	1345.4944	**673.2508**	1344.5104	**672.7588**	E	**1032.4480**	516.7276	1015.4215	508.2144	1014.4374	507.7224	9
12	**1491.5635**	746.2854	1474.5370	**737.7721**	1473.5530	737.2801	E	**903.4054**	452.2063	886.3789	**443.6931**	885.3949	**443.2011**	8
13	**1606.5905**	803.7989	1589.5639	795.2856	1588.5799	794.7936	D	**774.3628**	387.6851	757.3363	379.1718	756.3523	378.6798	7
14	1734.6490	**867.8282**	1717.6225	**859.3149**	1716.6385	**858.8229**	Q	**659.3359**	330.1716	642.3093	321.6583	641.3253	321.1663	6
15	1805.6862	**903.3467**	1788.6596	**894.8334**	1787.6756	**894.3414**	A	**531.2773**	266.1423	514.2508	257.6290	513.2667	257.1370	5
16	1892.7182	**946.8627**	**1875.6916**	**938.3495**	1874.7076	**937.8574**	S	**460.2402**	230.6237	**443.2136**	222.1105	442.2296	221.6185	4
17	2021.7608	**1011.3840**	2004.7342	**1002.8708**	2003.7502	**1002.3787**	E	**373.2082**	187.1077	**356.1816**	178.5944	**355.1976**	178.1024	3
18	2118.8135	1059.9104	2101.7870	1051.3971	2100.8030	1050.9051	P	**244.1656**	122.5864	227.1390	114.0731			2
19							K	147.1128	74.0600	130.0863	65.5468			1

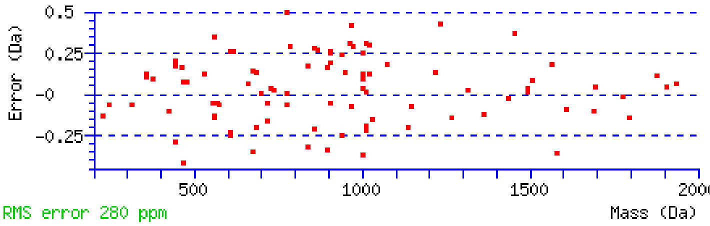

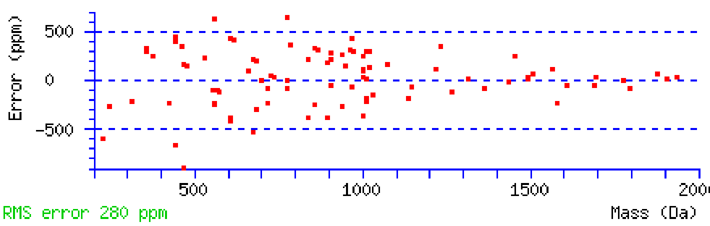

All matches to this query

Score	Mr(calc):	Delta	Sequence
50.2	2263.9118	0.0059	VQSQEEIHSDEEDQASEPK
25.2	2263.9118	0.0059	VQSQEEIHSDEEDQASEPK

14.5	2263.9118	0.0059	VQSQEEIHSDEEDQASEPK
8.2	2261.8993	2.0183	YAQEHDITNFYMLTLDK
5.0	2262.8960	1.0216	ANLHNGLSIPVPSSEGSSTR
5.0	2262.8960	1.0216	ANLHNGLSIPVPSSEGSSTR
4.5	2263.9181	-0.0004	INQSQTAGTVTSSIPSLLK
4.4	2263.9228	-0.0051	KGASCHHVTGACFCPPGWR
3.6	2261.9315	1.9861	NSSQNSANLGTPVDKEGAGTR
3.6	2261.9315	1.9861	NSSQNSANLGTPVDKEGAGTR

Spectrum No: 299; Query: 1117; Rank: 1

Peptide View

MS/MS Fragmentation of **SDVSDQEEDEESER**
Found in **IPI00359962**, Tax_Id=10116 Gene_Symbol=Pcm1 similar to pericentriolar material 1

Match to Query 1117: 1732.594028 from(867.304290,2+)
Title: 091008RatKidney_NoSalt_31.490.490.2.dta
Data file K:\NewmanPaper\Piliang\3SubProteomes\Piliang3SP\mgf5ppm\ERLIC_3SubProteomes5ppm.mgf

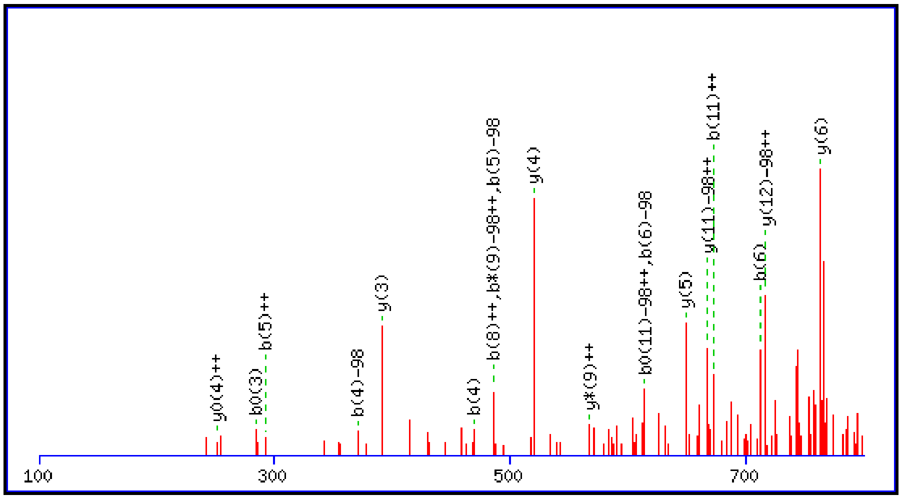

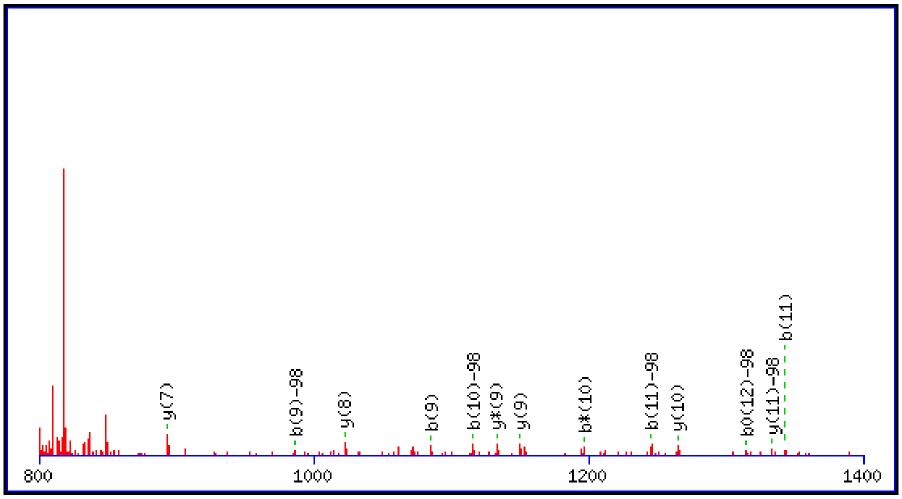

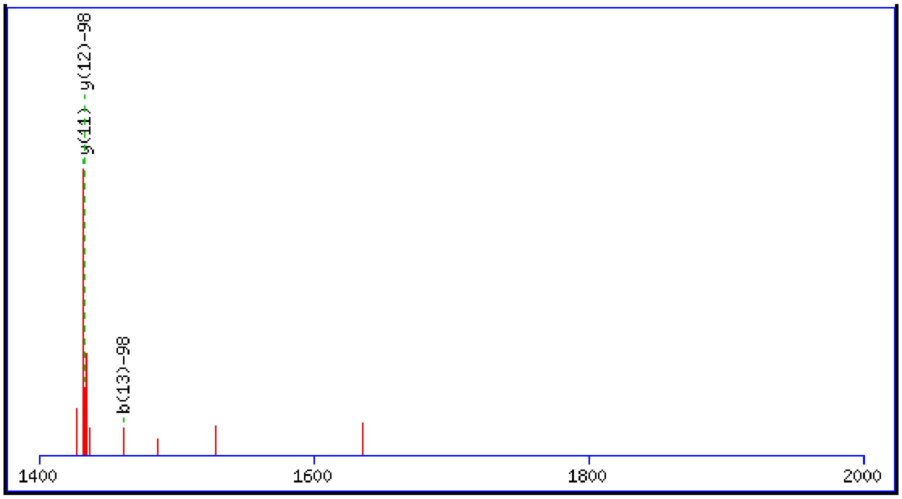

Monoisotopic mass of neutral peptide Mr(calc): 1732.5948
Fixed modifications: Carbamidomethyl (C)
Variable modifications:
S4 : Phospho (ST), with neutral losses 97.9769(shown in table), 0.0000
Ions Score: 50 **Expect:** 0.0006
Matches (Bold Red): 35/218 fragment ions using 67 most intense peaks

#	b	b++	b*	b*++	b0	b0++	Seq.	y	y++	y*	y*++	y0	y0++	#
1	88.0393	44.5233			70.0287	35.5180	S							14
2	203.0662	102.0368			185.0557	93.0315	D	1548.5932	774.8002	1531.5667	766.2870	1530.5827	765.7950	13
3	302.1347	151.5710			**284.1241**	142.5657	V	**1433.5663**	**717.2868**	1416.5397	708.7735	1415.5557	708.2815	12
4	**371.1561**	186.0817			353.1456	177.0764	S	**1334.4979**	**667.7526**	1317.4713	659.2393	1316.4873	658.7473	11
5	**486.1831**	243.5952			468.1725	234.5899	D	**1265.4764**	633.2418	1248.4499	624.7286	1247.4658	624.2366	10
6	**614.2416**	307.6245	597.2151	299.1112	596.2311	298.6192	Q	**1150.4495**	575.7284	**1133.4229**	**567.2151**	1132.4389	566.7231	9
7	743.2842	372.1458	726.2577	363.6325	725.2737	363.1405	E	**1022.3909**	511.6991	1005.3643	503.1858	1004.3803	502.6938	8
8	872.3268	436.6671	855.3003	428.1538	854.3163	427.6618	E	**893.3483**	447.1778	876.3217	438.6645	875.3377	438.1725	7
9	**987.3538**	494.1805	970.3272	**485.6672**	969.3432	485.1752	D	**764.3057**	382.6565	747.2792	374.1432	746.2951	373.6512	6
10	**1116.3964**	558.7018	1099.3698	550.1885	1098.3858	549.6965	E	**649.2788**	325.1430	632.2522	316.6297	631.2682	316.1377	5
11	**1245.4390**	623.2231	1228.4124	614.7098	1227.4284	**614.2178**	E	**520.2362**	260.6217	503.2096	252.1084	502.2256	**251.6164**	4
12	1332.4710	666.7391	1315.4444	658.2259	**1314.4604**	657.7338	S	**391.1936**	196.1004	374.1670	187.5872	373.1830	187.0951	3
13	**1461.5136**	731.2604	1444.4870	722.7472	1443.5030	722.2551	E	304.1615	152.5844	287.1350	144.0711	286.1510	143.5791	2
14							R	175.1190	88.0631	158.0924	79.5498			1

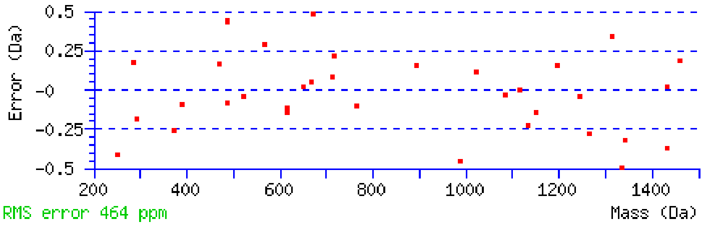

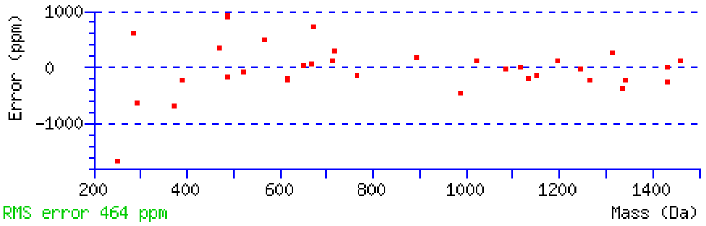

All matches to this query

Score	Mr(calc):	Delta	Sequence
50.0	1732.5948	-0.0008	SDVSDQEEDEESER
44.3	1732.5948	-0.0008	SDVSDQEEDEESER
6.5	1732.6101	-0.0161	GESNNFGESQEGNPGK
0.7	1732.6010	-0.0070	YFEKLSIYCDR
0.3	1732.5936	0.0004	YFDSNKDAYSKR
0.2	1731.5840	1.0100	FKDSMLASMFSGR

Spectrum No: 300; Query: 325; Rank: 1

Peptide View

MS/MS Fragmentation of **SKPVFSESLSD**
Found in **IPI00204831**, Tax_Id=10116 Gene_Symbol=Timm8a Mitochondrial import inner membrane translocase subunit Tim8 A

Match to Query 325: 1274.540748 from(638.277650,2+)
Title: 091008RatKidney_NH4Format02_28.1573.1573.2.dta
Data file K:\NewmanPaper\Piliang\3SubProteomes\Piliang3SP\mgf5ppm\ERLIC_3SubProteomes5ppm.mgf

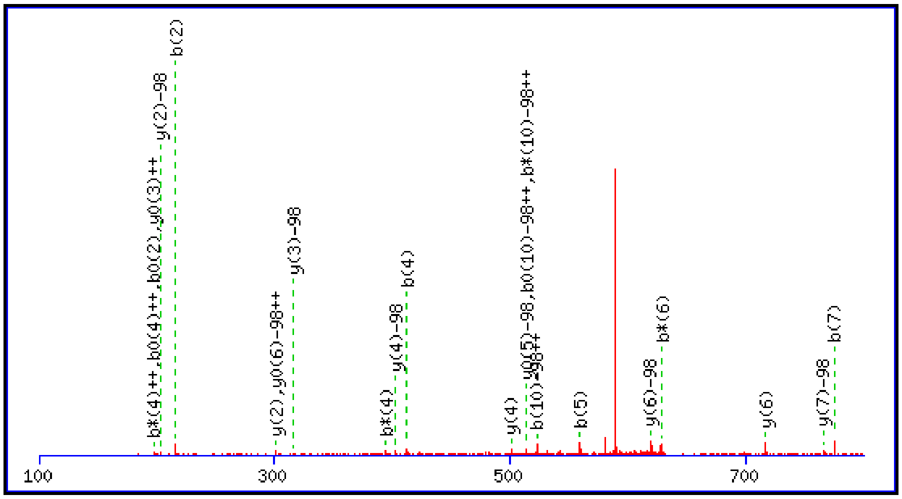

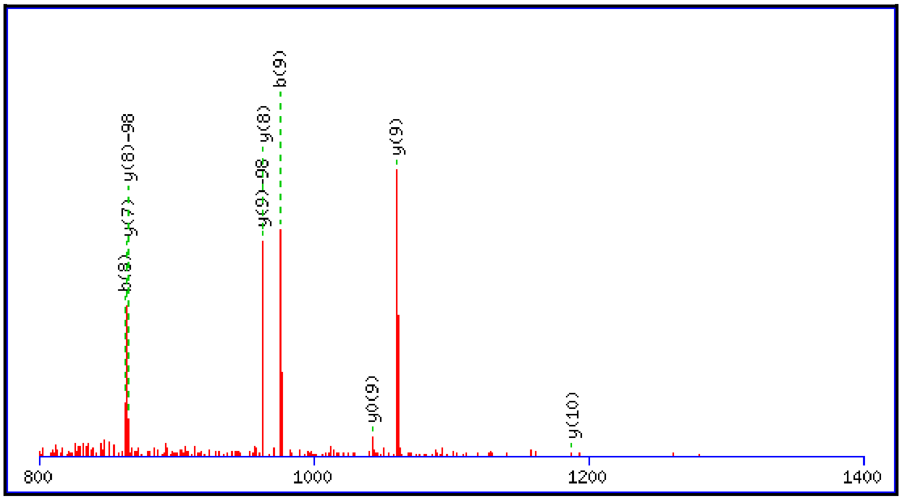

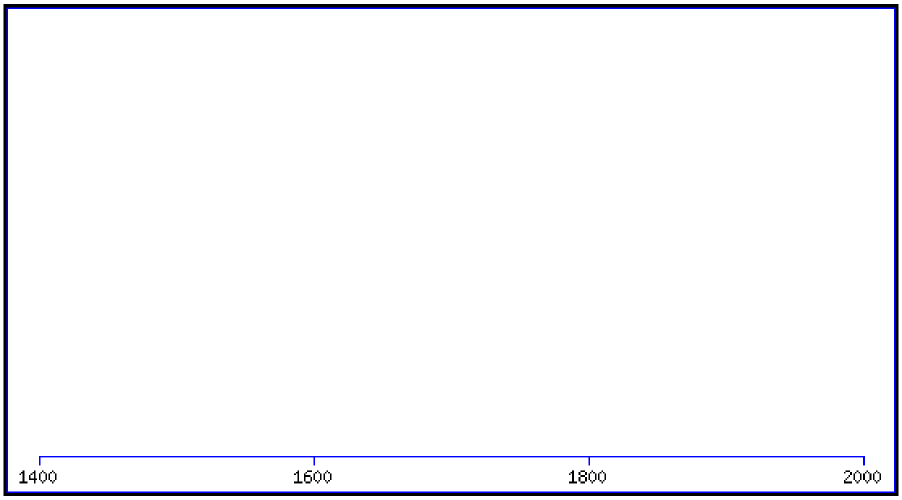

Monoisotopic mass of neutral peptide Mr(calc): 1274.5432

Fixed modifications: Carbamidomethyl (C)
Variable modifications:
S10 : Phospho (ST), with neutral losses 97.9769(shown in table), 0.0000
Ions Score: 50 **Expect:** 0.0013
Matches (Bold Red): 32/144 fragment ions using 38 most intense peaks

#	b	b^{++}	b*	b^{*++}	b^0	b^{0++}	Seq.	y	y^{++}	y*	y^{*++}	y^0	y^{0++}	#
1	88.0393	44.5233			70.0287	35.5180	S							11
2	**216.1343**	108.5708	199.1077	100.0575	**198.1237**	99.5655	K	1090.5415	545.7744	1073.5150	537.2611	1072.5309	536.7691	10
3	313.1870	157.0972	296.1605	148.5839	295.1765	148.0919	P	**962.4465**	481.7269			944.4360	472.7216	9
4	**412.2554**	206.6314	**395.2289**	**198.1181**	394.2449	**197.6261**	V	**865.3938**	433.2005			847.3832	424.1952	8
5	**559.3239**	280.1656	542.2973	271.6523	541.3133	271.1603	F	**766.3254**	383.6663			748.3148	374.6610	7
6	646.3559	323.6816	**629.3293**	315.1683	628.3453	314.6763	S	**619.2570**	310.1321			601.2464	**301.1268**	6
7	**775.3985**	388.2029	758.3719	379.6896	757.3879	379.1976	E	532.2249	266.6161			**514.2144**	257.6108	5
8	**862.4305**	431.7189	845.4040	423.2056	844.4199	422.7136	S	**403.1823**	202.0948			385.1718	193.0895	4
9	**975.5146**	488.2609	958.4880	479.7476	957.5040	479.2556	L	**316.1503**	158.5788			298.1397	149.5735	3
10	1044.5360	**522.7717**	1027.5095	**514.2584**	1026.5255	**513.7664**	S	**203.0662**	102.0368			185.0557	93.0315	2
11							D	134.0448	67.5260			116.0342	58.5207	1

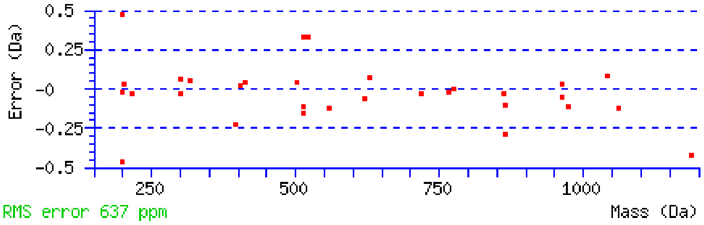

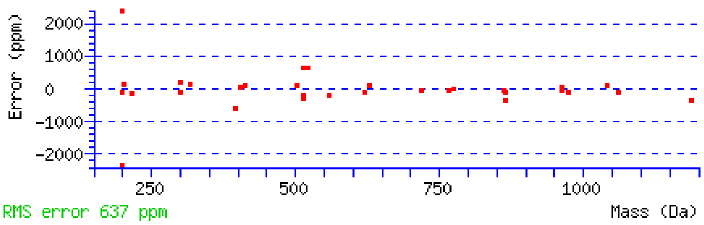

All matches to this query

Score	Mr(calc):	Delta	Sequence
50.0	1274.5432	-0.0024	SKPVFSESLSD
27.6	1274.5432	-0.0024	SKPVFSESLSD
26.0	1274.5432	-0.0024	SKPVFSESLSD
9.5	1274.5503	-0.0096	SNNSSKKQSSK
8.0	1274.5479	-0.0071	DSARMSLFPR
6.0	1273.5414	0.9994	SQILMWSANK
5.6	1274.5432	-0.0024	SKPVFSESLSD
5.2	1272.5405	2.0003	NHMVVSPDDSR
3.7	1274.5292	0.0115	KGQAVDYDGSR
3.1	1274.5332	0.0075	IAEYSYGHQK

Spectrum No: 301; Query: 1621; Rank: 1

Peptide View

MS/MS Fragmentation of **KEESEESDDDMGFGLFD**
Found in **IPI00188804**, Tax_Id=10116 Gene_Symbol=Rplp2 60S acidic ribosomal protein P2

Match to Query 1621: 2028.725208 from(1015.369880,2+)
Title: 100101RatKid_NS_deglyco_25.4362.4362.2.dta
Data file K:\NewmanPaper\Piliang\3SubProteomes\Piliang3SP\mgf5ppm\ERLIC_3SubProteomes5ppm.mgf

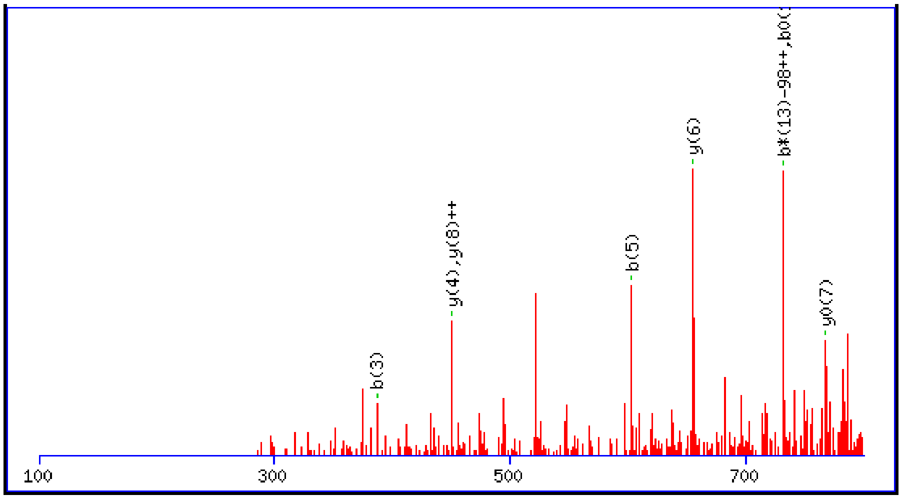

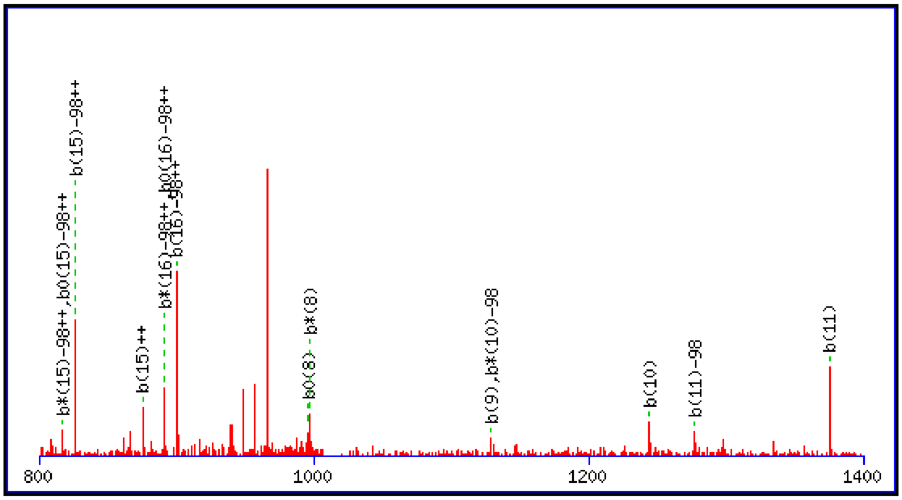

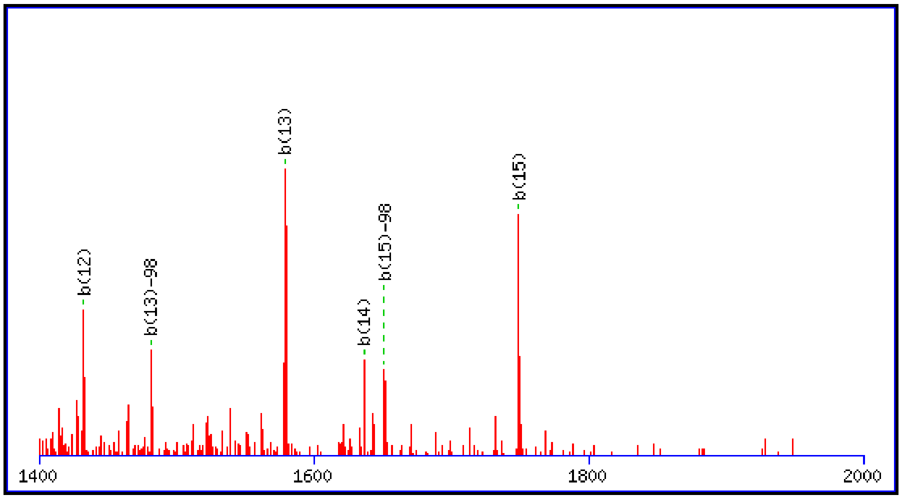

Monoisotopic mass of neutral peptide Mr(calc): 2028.7184
Fixed modifications: Carbamidomethyl (C)
Variable modifications:
S7 : Phospho (ST), with neutral losses 0.0000(shown in table), 97.9769
Ions Score: 50 **Expect:** 0.00094
Matches (Bold Red): 29/242 fragment ions using 41 most intense peaks

#	b	b^{++}	b*	b^{*++}	b^{0}	b^{0++}	Seq.	y	y^{++}	y^{0}	y^{0++}	#
1	129.1022	65.0548	112.0757	56.5415			K					17
2	258.1448	129.5761	241.1183	121.0628	240.1343	120.5708	E	1901.6307	951.3190	1883.6201	942.3137	16
3	**387.1874**	194.0974	370.1609	185.5841	369.1769	185.0921	E	1772.5881	886.7977	1754.5775	877.7924	15
4	474.2195	237.6134	457.1929	229.1001	456.2089	228.6081	S	1643.5455	822.2764	1625.5349	813.2711	14
5	**603.2620**	302.1347	586.2355	293.6214	585.2515	293.1294	E	1556.5135	778.7604	1538.5029	769.7551	13

**6**	**732.3046**	366.6560	715.2781	358.1427	714.2941	357.6507	**E**	1427.4709	714.2391	1409.4603	705.2338	**12**
**7**	899.3030	450.1551	882.2764	441.6419	881.2924	441.1499	**S**	1298.4283	649.7178	1280.4177	640.7125	**11**
**8**	1014.3299	507.6686	**997.3034**	499.1553	**996.3194**	498.6633	**D**	1131.4299	566.2186	1113.4194	557.2133	**10**
**9**	**1129.3569**	565.1821	1112.3303	556.6688	1111.3463	556.1768	**D**	1016.4030	508.7051	998.3924	499.6998	**9**
**10**	**1244.3838**	622.6955	1227.3573	614.1823	1226.3733	613.6903	**D**	901.3760	**451.1917**	883.3655	442.1864	**8**
**11**	**1375.4243**	688.2158	1358.3978	679.7025	1357.4137	679.2105	**M**	786.3491	393.6782	**768.3385**	384.6729	**7**
**12**	**1432.4458**	716.7265	1415.4192	708.2132	1414.4352	707.7212	**G**	**655.3086**	328.1579	637.2980	319.1527	**6**
**13**	**1579.5142**	790.2607	1562.4876	781.7475	1561.5036	781.2554	**F**	598.2871	299.6472	580.2766	290.6419	**5**
**14**	**1636.5356**	818.7715	1619.5091	810.2582	1618.5251	809.7662	**G**	**451.2187**	226.1130	433.2082	217.1077	**4**
**15**	**1749.6197**	**875.3135**	1732.5932	866.8002	1731.6091	866.3082	**L**	394.1973	197.6023	376.1867	188.5970	**3**
**16**	1896.6881	948.8477	1879.6616	940.3344	1878.6776	939.8424	**F**	281.1132	141.0602	263.1026	132.0550	**2**
**17**							**D**	134.0448	67.5260	116.0342	58.5207	**1**

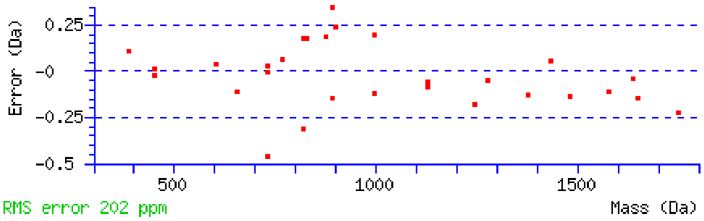

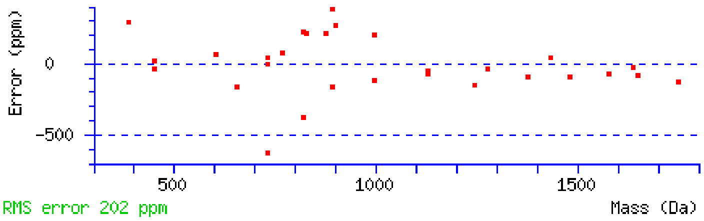

All matches to this query

Score	Mr(calc):	Delta	Sequence
50.0	2028.7184	0.0069	KEESEESDDDMGFGLFD
36.2	2028.7184	0.0069	KEESEESDDDMGFGLFD

Spectrum No: 302; Query: 2655; Rank: 1

Peptide View

MS/MS Fragmentation of **PGETEEPRSPEQQDQEGGPAAAADAASDELR**
Found in **IPI00197580**, Tax_Id=10116 Gene_Symbol=Zfp91 similar to Zinc finger protein 91

Match to Query 2655: 3287.389092 from(1096.803640,3+)
Title: 100101RatKid_NS_deglyco_21.2335.2335.3.dta
Data file K:\NewmanPaper\Piliang\3SubProteomes\Piliang3SP\mgf5ppm\ERLIC_3SubProteomes5ppm.mgf

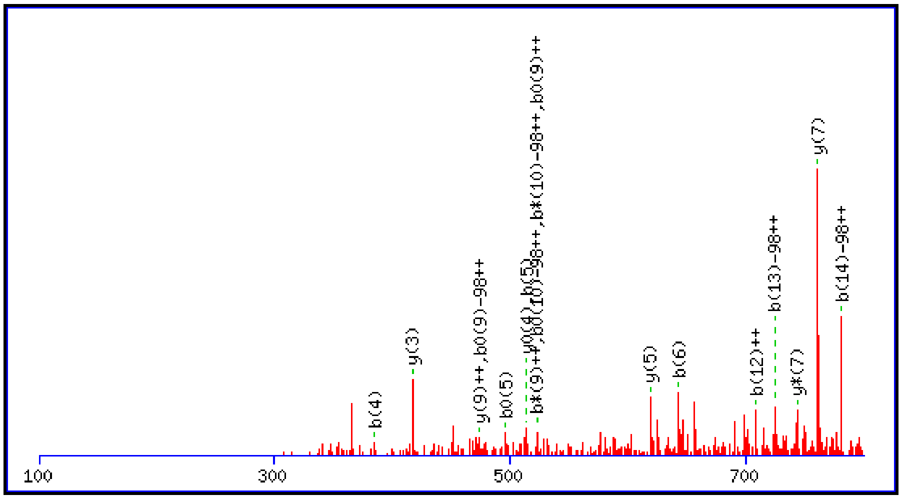

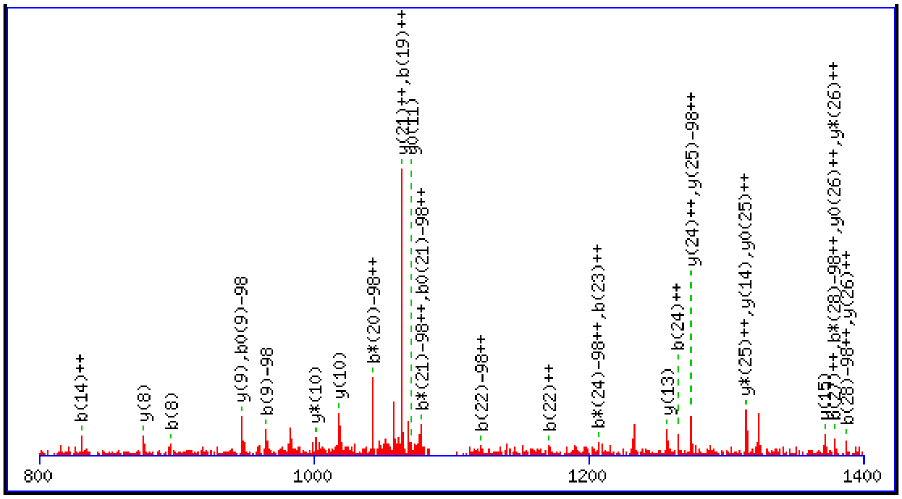

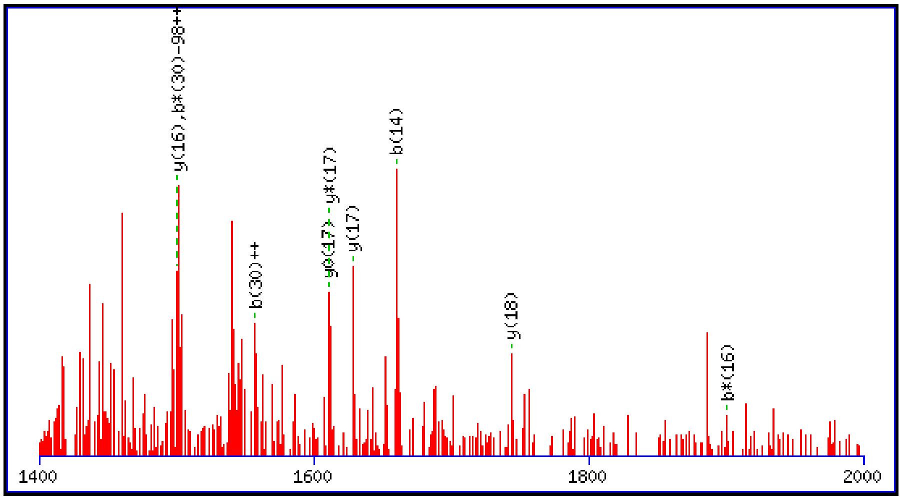

Monoisotopic mass of neutral peptide Mr(calc): 3287.3851
Fixed modifications: Carbamidomethyl (C)
Variable modifications:
S9 : Phospho (ST), with neutral losses 97.9769(shown in table), 0.0000
Ions Score: 50 **Expect:** 0.0044
Matches (Bold Red): 59/518 fragment ions using 89 most intense peaks

#	b	b^{++}	b*	b^{*++}	b^0	b^{0++}	Seq.	y	y^{++}	y*	y^{*++}	y^0	y^{0++}	#
1	98.0600	49.5337					P							31
2	155.0815	78.0444					G	3093.3628	1547.1850	3076.3362	1538.6718	3075.3522	1538.1797	30
3	284.1241	142.5657			266.1135	133.5604	E	3036.3413	1518.6743	3019.3148	1510.1610	3018.3308	1509.6690	29
4	**385.1718**	193.0895			367.1612	184.0842	T	2907.2987	1454.1530	2890.2722	1445.6397	2889.2882	1445.1477	28
5	**514.2144**	257.6108			**496.2038**	248.6055	E	2806.2510	1403.6292	2789.2245	1395.1159	2788.2405	1394.6239	27
6	**643.2570**	322.1321			625.2464	313.1268	E	2677.2085	1339.1079	2660.1819	1330.5946	2659.1979	1330.1026	26
7	740.3097	370.6585			722.2992	361.6532	P	2548.1659	**1274.5866**	2531.1393	1266.0733	2530.1553	1265.5813	25
8	**896.4108**	448.7091	879.3843	440.1958	878.4003	439.7038	R	2451.1131	1226.0602	2434.0865	1217.5469	2433.1025	1217.0549	24
9	**965.4323**	483.2198	948.4057	474.7065	**947.4217**	**474.2145**	S	2295.0120	1148.0096	2277.9854	1139.4964	2277.0014	1139.0043	23
10	1062.4851	531.7462	1045.4585	**523.2329**	1044.4745	**522.7409**	P	2225.9905	1113.4989	2208.9640	1104.9856	2207.9800	1104.4936	22
11	1191.5277	596.2675	1174.5011	587.7542	1173.5171	587.2622	E	2128.9378	**1064.9725**	2111.9112	1056.4592	2110.9272	1055.9672	21
12	1319.5862	660.2968	1302.5597	651.7835	1301.5757	651.2915	Q	1999.8952	1000.4512	1982.8686	991.9379	1981.8846	991.4459	20
13	1447.6448	**724.3260**	1430.6183	715.8128	1429.6342	715.3208	Q	1871.8366	936.4219	1854.8100	927.9087	1853.8260	927.4167	19
14	1562.6717	**781.8395**	1545.6452	773.3262	1544.6612	772.8342	D	**1743.7780**	872.3926	1726.7515	863.8794	1725.7674	863.3874	18
15	1690.7303	845.8688	1673.7038	837.3555	1672.7198	836.8635	Q	**1628.7511**	814.8792	**1611.7245**	806.3659	**1610.7405**	805.8739	17
16	1819.7729	910.3901	1802.7464	901.8768	1801.7624	901.3848	E	**1500.6925**	750.8499	1483.6659	742.3366	1482.6819	741.8446	16
17	1876.7944	938.9008	1859.7678	930.3876	1858.7838	929.8955	G	**1371.6499**	686.3286	1354.6234	677.8153	1353.6393	677.3233	15
18	1933.8158	967.4116	1916.7893	958.8983	1915.8053	958.4063	G	**1314.6284**	657.8179	1297.6019	649.3046	1296.6179	648.8126	14

**19**	2030.8686	1015.9379	2013.8421	1007.4247	2012.8580	1006.9327	**P**	**1257.6070**	629.3071	1240.5804	620.7938	1239.5964	620.3018	**13**
**20**	2101.9057	1051.4565	2084.8792	**1042.9432**	2083.8952	1042.4512	**A**	1160.5542	580.7807	1143.5277	572.2675	1142.5436	571.7755	**12**
**21**	2172.9428	1086.9751	2155.9163	**1078.4618**	2154.9323	**1077.9698**	**A**	1089.5171	545.2622	1072.4905	536.7489	**1071.5065**	536.2569	**11**
**22**	2243.9800	**1122.4936**	2226.9534	1113.9803	2225.9694	1113.4883	**A**	**1018.4800**	509.7436	**1001.4534**	501.2304	1000.4694	500.7383	**10**
**23**	2315.0171	1158.0122	2297.9905	1149.4989	2297.0065	1149.0069	**A**	**947.4429**	**474.2251**	930.4163	465.7118	929.4323	465.2198	**9**
**24**	2430.0440	1215.5256	2413.0175	**1207.0124**	2412.0334	1206.5204	**D**	**876.4058**	438.7065	859.3792	430.1932	858.3952	429.7012	**8**
**25**	2501.0811	1251.0442	2484.0546	1242.5309	2483.0706	1242.0389	**A**	**761.3788**	381.1930	**744.3523**	372.6798	743.3682	372.1878	**7**
**26**	2572.1182	1286.5628	2555.0917	1278.0495	2554.1077	1277.5575	**A**	690.3417	345.6745	673.3151	337.1612	672.3311	336.6692	**6**
**27**	2659.1503	1330.0788	2642.1237	1321.5655	2641.1397	1321.0735	**S**	**619.3046**	310.1559	602.2780	301.6427	601.2940	301.1506	**5**
**28**	2774.1772	**1387.5922**	2757.1507	**1379.0790**	2756.1666	1378.5870	**D**	532.2726	266.6399	515.2460	258.1266	**514.2620**	257.6346	**4**
**29**	2903.2198	1452.1135	2886.1933	1443.6003	2885.2092	1443.1083	**E**	**417.2456**	209.1264	400.2191	200.6132	399.2350	200.1212	**3**
**30**	3016.3039	1508.6556	2999.2773	**1500.1423**	2998.2933	1499.6503	**L**	288.2030	144.6051	271.1765	136.0919			**2**
**31**							**R**	175.1190	88.0631	158.0924	79.5498			**1**

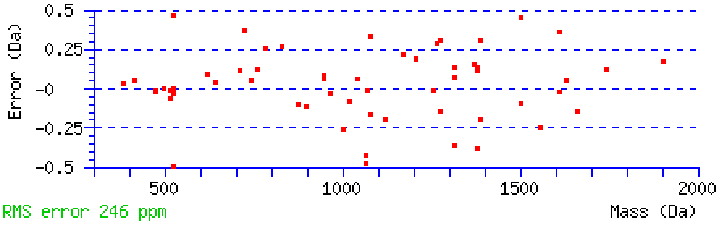

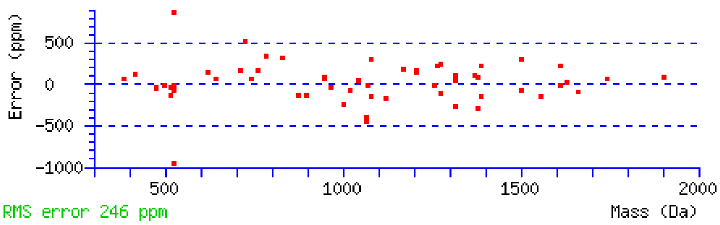

All matches to this query

Score	Mr(calc):	Delta	Sequence
49.9	3287.3851	0.0040	PGETEEPRSPEQQDQEGGPAAAADAASDELR
39.5	3287.3851	0.0040	PGETEEPRSPEQQDQEGGPAAAADAASDELR
8.3	3285.3592	2.0299	KIMGICLFFSQMPSSVCSADCSPGFRK
4.1	3287.3877	0.0014	NLGSPPNAVLGMSQGKDPDNMVQVRVDNR
4.0	3286.4172	0.9719	KLIAPASAPGSMGGPVPGSNSSSSFSAAVPFR
3.9	3285.3592	2.0299	KIMGICLFFSQMPSSVCSADCSPGFRK
3.9	3285.3592	2.0299	KIMGICLFFSQMPSSVCSADCSPGFRK
1.1	3287.3746	0.0145	QRTQESPNMPVPPLSMASSPRMGNRPSC
0.9	3286.4127	0.9764	KLTGTALHVSTPNMSALDLTCCLEKAAK
0.7	3285.3855	2.0036	STNILMIPNPHLHQYEVSSSLGYTSTR

Spectrum No: 303; Query: 91; Rank: 1

Peptide View

MS/MS Fragmentation of **DGGESPTVLK**
Found in **IPI00199585**, Tax_Id=10116 Gene_Symbol=Slc22a5 Organic cation/carnitine transporter 2

Match to Query 91: 1081.472368 from(541.743460,2+)
Title: 100101RatKid_NS_deglyco_22.1007.1007.2.dta
Data file K:\NewmanPaper\Piliang\3SubProteomes\Piliang3SP\mgf5ppm\ERLIC_3SubProteomes5ppm.mgf

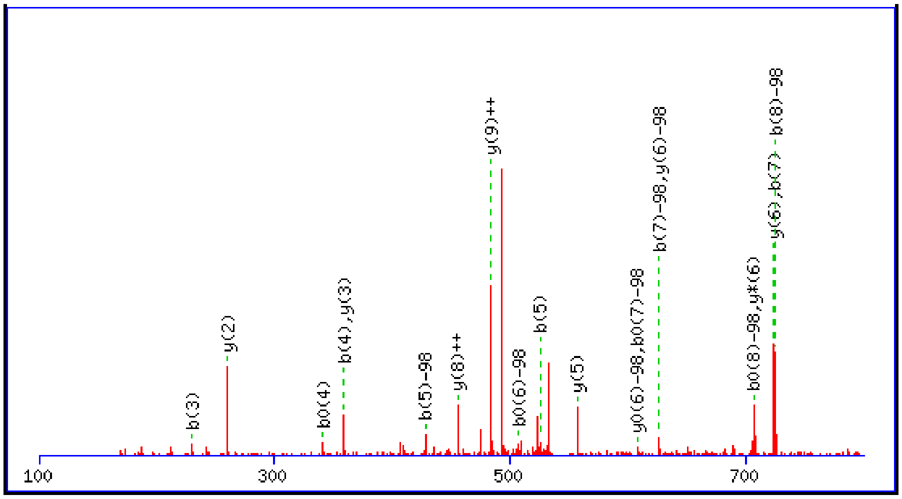

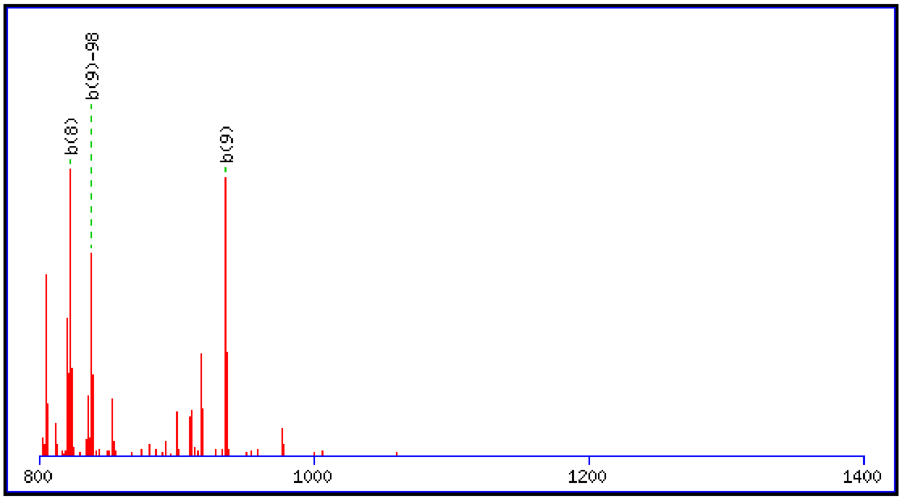

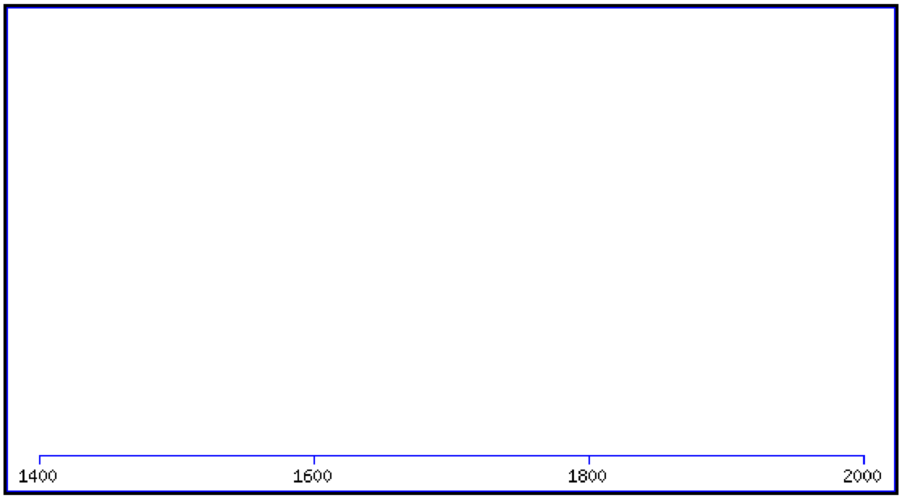

Monoisotopic mass of neutral peptide Mr(calc): 1081.4693
Fixed modifications: Carbamidomethyl (C)
Variable modifications:
S5 : Phospho (ST), with neutral losses 97.9769(shown in table), 0.0000
Ions Score: 50 **Expect:** 0.00093
Matches (Bold Red): 23/128 fragment ions using 31 most intense peaks

#	b	b^{++}	b^0	b^{0++}	Seq.	y	y^{++}	y*	y^{*++}	y^0	y^{0++}	#
1	116.0342	58.5207	98.0237	49.5155	D							10
2	173.0557	87.0315	155.0451	78.0262	G	869.4727	435.2400	852.4462	426.7267	851.4621	426.2347	9
3	**230.0771**	115.5422	212.0666	106.5369	G	812.4512	406.7293	795.4247	398.2160	794.4407	397.7240	8
4	**359.1197**	180.0635	**341.1092**	171.0582	E	755.4298	378.2185	738.4032	369.7053	737.4192	369.2132	7
5	**428.1412**	214.5742	410.1306	205.5690	S	**626.3872**	313.6972	609.3606	305.1840	**608.3766**	304.6919	6

6	525.1940	263.1006	507.1834	254.0953	P	557.3657	279.1865	540.3392	270.6732	539.3552	270.1812	5
7	626.2416	313.6245	608.2311	304.6192	T	460.3130	230.6601	443.2864	222.1468	442.3024	221.6548	4
8	725.3101	363.1587	707.2995	354.1534	V	359.2653	180.1363	342.2387	171.6230			3
9	838.3941	419.7007	820.3836	410.6954	L	260.1969	130.6021	243.1703	122.0888			2
10					K	147.1128	74.0600	130.0863	65.5468			1

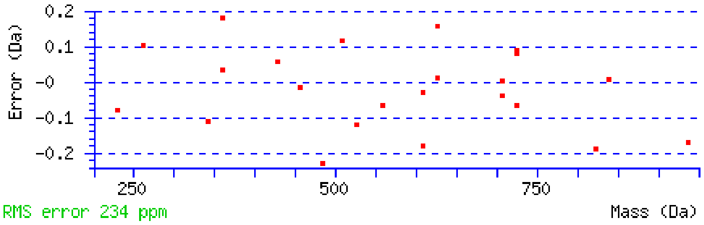

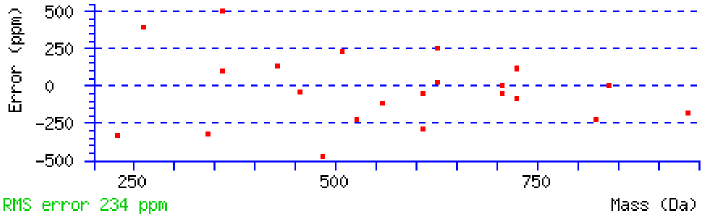

All matches to this query

Score	Mr(calc):	Delta	Sequence
49.8	1081.4693	0.0031	DGGESPTVLK
38.3	1081.4693	0.0031	DGGESPTVLK
8.3	1081.4636	0.0088	TASSQDGSSSR
8.2	1081.4783	-0.0060	NGSIVSMNMK
7.4	1081.4740	-0.0016	RTSTHCLK
7.4	1081.4740	-0.0016	RTSTHCLK
7.4	1081.4740	-0.0016	RTSTHCLK
7.3	1081.4693	0.0031	ALDSDPGSLK
6.0	1081.4693	0.0031	DGTSLLGNPK
5.9	1081.4693	0.0031	DGTSLLGNPK

Spectrum No: 304; Query: 2712; Rank: 1

Peptide View

MS/MS Fragmentation of **VKASPITNDGEDEFVPSDGIDKDEYAFSPGK**
Found in **IPI00364460**, Tax_Id=10116 Gene_Symbol=LOC361100 similar to topoisomerase (DNA) II beta

Match to Query 2712: 3486.478602 from(1163.166810,3+)
Title: 091008RatKidney_NH4Format01_24.3169.3169.3.dta
Data file K:\NewmanPaper\Piliang\3SubProteomes\Piliang3SP\mgf5ppm\ERLIC_3SubProteomes5ppm.mgf

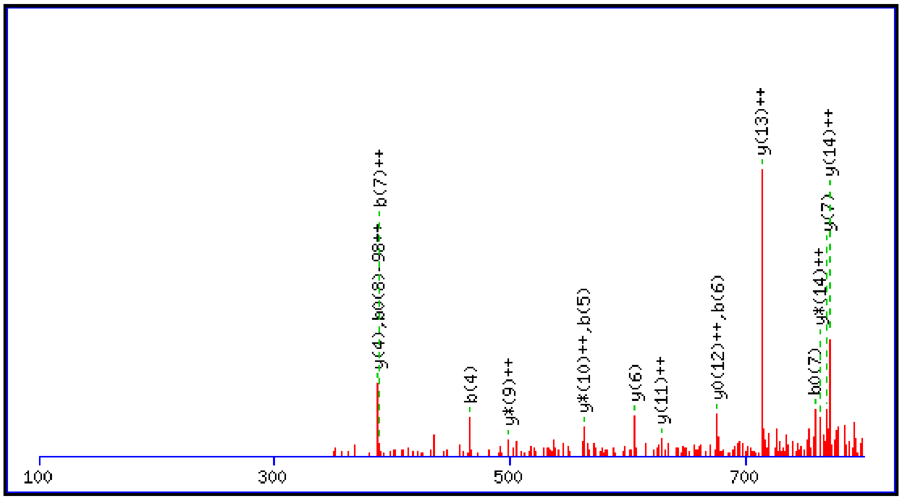

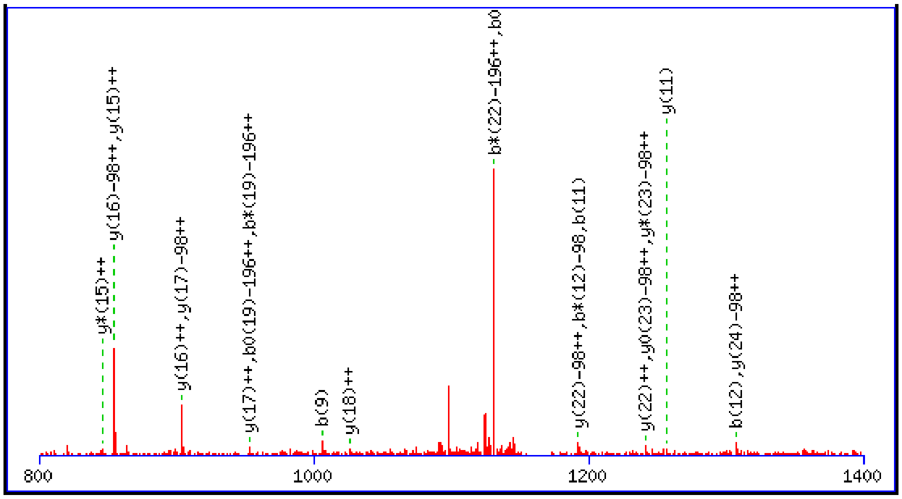

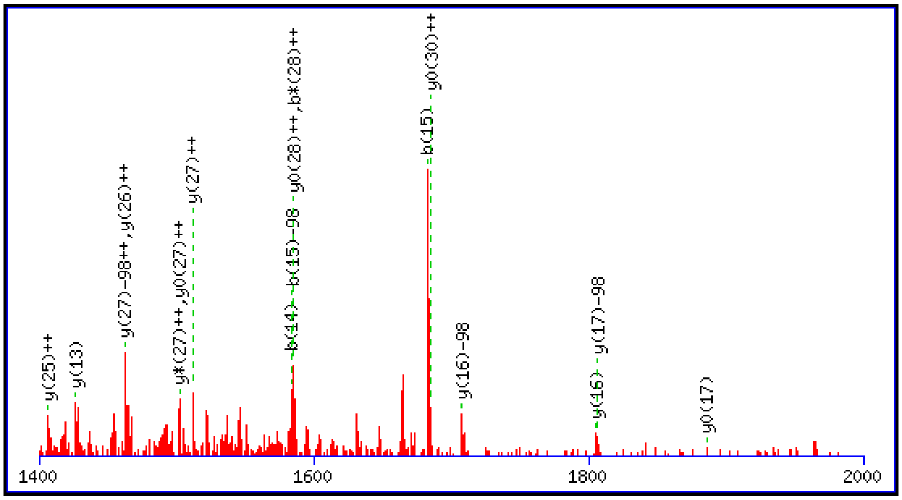

Monoisotopic mass of neutral peptide Mr(calc): 3486.4793
Fixed modifications: Carbamidomethyl (C)
Variable modifications:
S4 : Phospho (ST), with neutral losses 0.0000(shown in table), 97.9769
S17 : Phospho (ST), with neutral losses 0.0000(shown in table), 97.9769
Ions Score: 50 **Expect:** 0.0048
Matches (Bold Red): 54/604 fragment ions using 64 most intense peaks

#	b	b^{++}	b*	b^{*++}	b^0	b^{0++}	Seq.	y	y^{++}	y*	y^{*++}	y^0	y^{0++}	#
1	100.0757	50.5415					V							31
2	228.1707	114.5890	211.1441	106.0757			K	3388.4182	1694.7127	3371.3916	1686.1994	3370.4076	**1685.7074**	30
3	299.2078	150.1075	282.1812	141.5942			A	3260.3232	1630.6652	3243.2967	1622.1520	3242.3126	1621.6600	29
4	**466.2061**	233.6067	449.1796	225.0934	448.1956	224.6014	S	3189.2861	1595.1467	3172.2595	1586.6334	3171.2755	**1586.1414**	28
5	**563.2589**	282.1331	546.2323	273.6198	545.2483	273.1278	P	3022.2877	**1511.6475**	3005.2612	**1503.1342**	3004.2772	**1502.6422**	27
6	**676.3429**	338.6751	659.3164	330.1618	658.3324	329.6698	I	2925.2350	**1463.1211**	2908.2084	1454.6079	2907.2244	1454.1158	26
7	777.3906	**389.1990**	760.3641	380.6857	**759.3801**	380.1937	T	2812.1509	**1406.5791**	2795.1244	1398.0658	2794.1404	1397.5738	25
8	891.4336	446.2204	874.4070	437.7071	873.4230	437.2151	N	2711.1032	1356.0553	2694.0767	1347.5420	2693.0927	1347.0500	24
9	**1006.4605**	503.7339	989.4339	495.2206	988.4499	494.7286	D	2597.0603	1299.0338	2580.0338	1290.5205	2579.0497	1290.0285	23
10	1063.4820	532.2446	1046.4554	523.7313	1045.4714	523.2393	G	2482.0334	**1241.5203**	2465.0068	1233.0070	2464.0228	1232.5150	22
11	**1192.5246**	596.7659	1175.4980	588.2526	1174.5140	587.7606	E	2425.0119	1213.0096	2407.9854	1204.4963	2407.0013	1204.0043	21
12	**1307.5515**	654.2794	1290.5249	645.7661	1289.5409	645.2741	D	2295.9693	1148.4883	2278.9428	1139.9750	2277.9587	1139.4830	20
13	1436.5941	718.8007	1419.5675	710.2874	1418.5835	709.7954	E	2180.9424	1090.9748	2163.9158	1082.4615	2162.9318	1081.9695	19
14	**1583.6625**	792.3349	1566.6360	783.8216	1565.6519	783.3296	F	2051.8998	**1026.4535**	2034.8732	1017.9403	2033.8892	1017.4482	18
15	**1682.7309**	841.8691	1665.7044	833.3558	1664.7204	832.8638	V	1904.8314	**952.9193**	1887.8048	944.4060	**1886.8208**	943.9140	17
16	1779.7837	890.3955	1762.7571	881.8822	1761.7731	881.3902	P	**1805.7629**	**903.3851**	1788.7364	894.8718	1787.7524	894.3798	16
17	1946.7820	973.8947	1929.7555	965.3814	1928.7715	964.8894	S	1708.7102	**854.8587**	1691.6836	**846.3455**	1690.6996	845.8534	15
18	2061.8090	1031.4081	2044.7824	1022.8949	2043.7984	1022.4028	D	1541.7118	**771.3596**	1524.6853	**762.8463**	1523.7013	762.3543	14

19	2118.8304	1059.9189	2101.8039	1051.4056	2100.8199	1050.9136	G	**1426.6849**	**713.8461**	1409.6583	705.3328	1408.6743	704.8408	13
20	2231.9145	1116.4609	2214.8880	1107.9476	2213.9039	1107.4556	I	1369.6634	685.3353	1352.6369	676.8221	1351.6529	**676.3301**	12
21	2346.9414	1173.9744	2329.9149	1165.4611	2328.9309	1164.9691	D	**1256.5794**	**628.7933**	1239.5528	620.2800	1238.5688	619.7880	11
22	2475.0364	1238.0218	2458.0099	1229.5086	2457.0258	1229.0166	K	1141.5524	571.2798	1124.5259	**562.7666**	1123.5419	562.2746	10
23	2590.0634	1295.5353	2573.0368	1287.0220	2572.0528	1286.5300	D	1013.4575	507.2324	996.4309	**498.7191**	995.4469	498.2271	9
24	2719.1059	1360.0566	2702.0794	1351.5433	2701.0954	1351.0513	E	898.4305	449.7189	881.4040	441.2056	880.4199	440.7136	8
25	2882.1693	1441.5883	2865.1427	1433.0750	2864.1587	1432.5830	Y	**769.3879**	385.1976	752.3614	376.6843	751.3774	376.1923	7
26	2953.2064	1477.1068	2936.1798	1468.5936	2935.1958	1468.1015	A	**606.3246**	303.6659	589.2980	295.1527	588.3140	294.6606	6
27	3100.2748	1550.6410	3083.2483	1542.1278	3082.2642	1541.6358	F	535.2875	268.1474	518.2609	259.6341	517.2769	259.1421	5
28	3187.3068	1594.1571	3170.2803	**1585.6438**	3169.2963	1585.1518	S	**388.2191**	194.6132	371.1925	186.0999	370.2085	185.6079	4
29	3284.3596	1642.6834	3267.3330	1634.1702	3266.3490	1633.6782	P	301.1870	151.0972	284.1605	142.5839			3
30	3341.3811	1671.1942	3324.3545	1662.6809	3323.3705	1662.1889	G	204.1343	102.5708	187.1077	94.0575			2
31							K	147.1128	74.0600	130.0863	65.5468			1

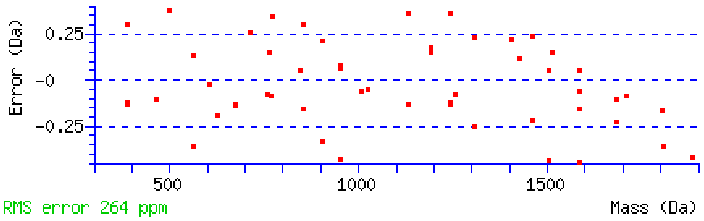

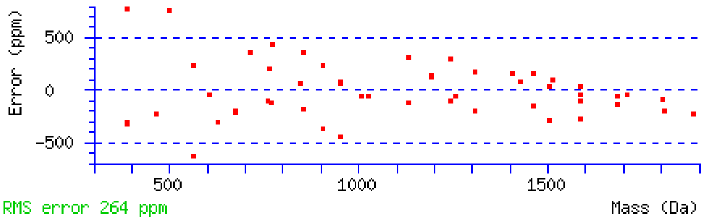

All matches to this query

Score	Mr(calc):	Delta	Sequence
49.8	3486.4793	-0.0007	VKASPITNDGEDEFVPSDGIDKDEYAFSPGK
30.4	3486.4793	-0.0007	VKASPITNDGEDEFVPSDGIDKDEYAFSPGK
30.0	3486.4793	-0.0007	VKASPITNDGEDEFVPSDGIDKDEYAFSPGK
26.2	3486.4793	-0.0007	VKASPITNDGEDEFVPSDGIDKDEYAFSPGK
14.5	3486.4793	-0.0007	VKASPITNDGEDEFVPSDGIDKDEYAFSPGK
13.2	3486.4793	-0.0007	VKASPITNDGEDEFVPSDGIDKDEYAFSPGK
12.1	3486.4793	-0.0007	VKASPITNDGEDEFVPSDGIDKDEYAFSPGK
4.3	3486.4766	0.0020	HIHSNLDFSPINSGSSEENVKFSSSQPEPR
3.6	3485.4809	0.9977	VGYWNDMDKLVLIQDMPTLGNDTAAIENR
3.4	3485.4914	0.9872	AEGESKASGLVVLVSVITSCLQTLKSCDSK

Spectrum No: 305; Query: 2768; Rank: 1

Peptide View

MS/MS Fragmentation of **QAEVADQQTTDLPAENGETENQSPASEEEKEAK**
Found in **IPI00373052**, Tax_Id=10116 Gene_Symbol=Hmgn1 High mobility group nucleosomal binding domain 1

Match to Query 2768: 3732.537222 from(1245.186350,3+)
Title: 100101RatKid_NS_deglyco_25.1714.1714.3.dta
Data file K:\NewmanPaper\Piliang\3SubProteomes\Piliang3SP\mgf5ppm\ERLIC_3SubProteomes5ppm.mgf

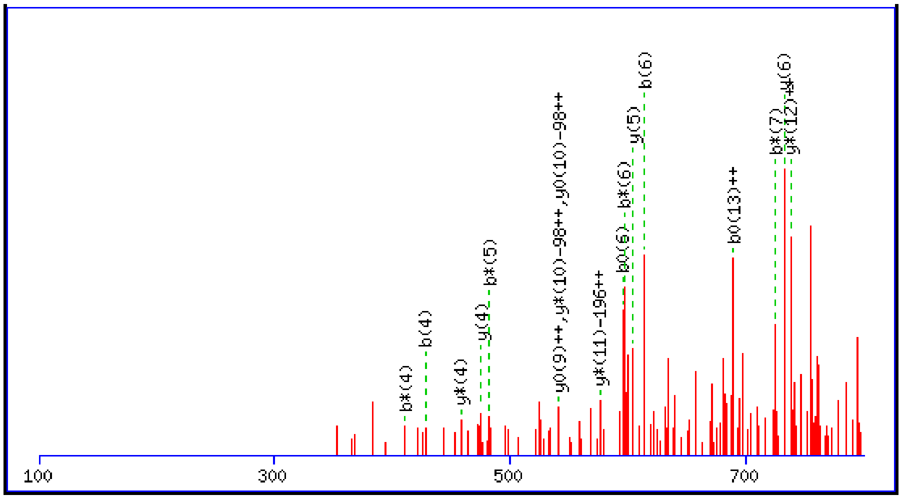

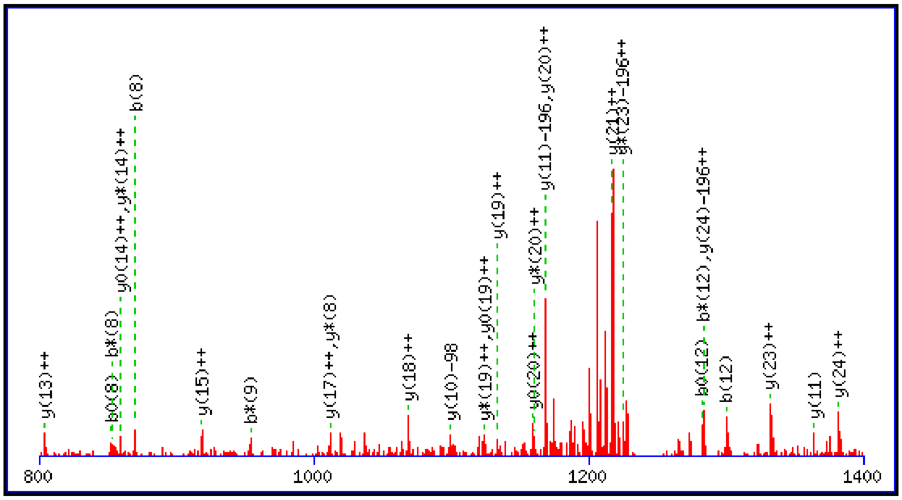

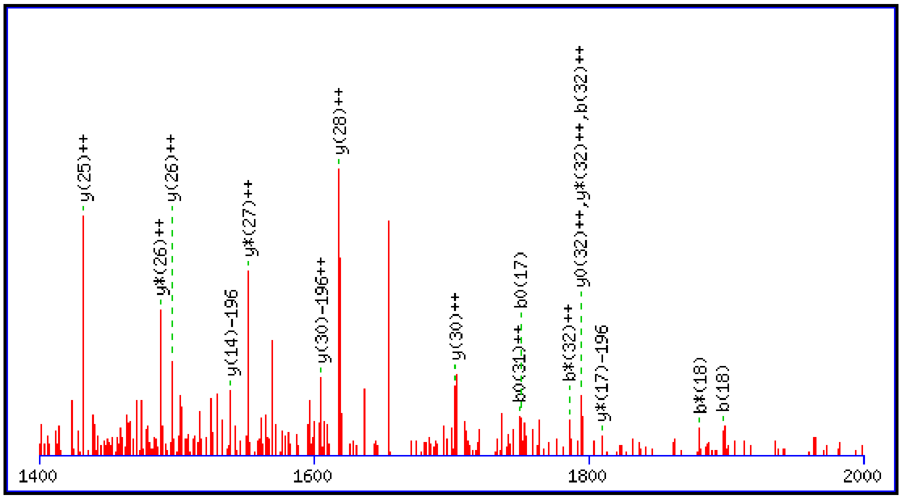

Monoisotopic mass of neutral peptide Mr(calc): 3732.5200
Fixed modifications: Carbamidomethyl (C)
Variable modifications:
S23 : Phospho (ST), with neutral losses 0.0000(shown in table), 97.9769
S26 : Phospho (ST), with neutral losses 0.0000(shown in table), 97.9769
Ions Score: 50 **Expect:** 0.005
Matches (Bold Red): 62/586 fragment ions using 97 most intense peaks

#	b	b^{++}	b*	b^{*++}	b^0	b^{0++}	Seq.	y	y^{++}	y*	y^{*++}	y^0	y^{0++}	#
**1**	129.0659	65.0366	112.0393	56.5233			**Q**							**33**
**2**	200.1030	100.5551	183.0764	92.0418			**A**	3605.4688	1803.2380	3588.4422	**1794.7247**	3587.4582	**1794.2327**	**32**
**3**	329.1456	165.0764	312.1190	156.5631	311.1350	156.0711	**E**	3534.4317	1767.7195	3517.4051	1759.2062	3516.4211	1758.7142	**31**
**4**	**428.2140**	214.6106	**411.1874**	206.0974	410.2034	205.6053	**V**	3405.3891	**1703.1982**	3388.3625	1694.6849	3387.3785	1694.1929	**30**

5	499.2511	250.1292	**482.2245**	241.6159	481.2405	241.1239	A	3306.3206	1653.6640	3289.2941	1645.1507	3288.3101	1644.6587	29
6	**614.2780**	307.6427	**597.2515**	299.1294	**596.2675**	298.6374	D	3235.2835	**1618.1454**	3218.2570	1609.6321	3217.2730	1609.1401	28
7	742.3366	371.6719	**725.3101**	363.1587	724.3260	362.6667	Q	3120.2566	1560.6319	3103.2300	**1552.1187**	3102.2460	1551.6266	27
8	**870.3952**	435.7012	**853.3686**	427.1880	**852.3846**	426.6959	Q	2992.1980	**1496.6026**	2975.1715	**1488.0894**	2974.1874	1487.5974	26
9	971.4429	486.2251	**954.4163**	477.7118	953.4323	477.2198	T	2864.1394	**1432.5734**	2847.1129	1424.0601	2846.1289	1423.5681	25
10	1072.4905	536.7489	1055.4640	528.2356	1054.4800	527.7436	T	2763.0918	**1382.0495**	2746.0652	1373.5362	2745.0812	1373.0442	24
11	1187.5175	594.2624	1170.4909	585.7491	1169.5069	585.2571	D	2662.0441	**1331.5257**	2645.0175	1323.0124	2644.0335	1322.5204	23
12	**1300.6016**	650.8044	**1283.5750**	642.2911	**1282.5910**	641.7991	L	2547.0171	1274.0122	2529.9906	1265.4989	2529.0066	1265.0069	22
13	1397.6543	699.3308	1380.6278	690.8175	1379.6438	**690.3255**	P	2433.9331	**1217.4702**	2416.9065	1208.9569	2415.9225	1208.4649	21
14	1468.6914	734.8494	1451.6649	726.3361	1450.6809	725.8441	A	2336.8803	**1168.9438**	2319.8538	**1160.4305**	2318.8697	**1159.9385**	20
15	1597.7340	799.3707	1580.7075	790.8574	1579.7235	790.3654	E	2265.8432	**1133.4252**	2248.8166	**1124.9120**	2247.8326	**1124.4200**	19
16	1711.7770	856.3921	1694.7504	847.8788	1693.7664	847.3868	N	2136.8006	**1068.9039**	2119.7740	1060.3907	2118.7900	1059.8987	18
17	1768.7984	884.9028	1751.7719	876.3896	**1750.7878**	875.8976	G	2022.7577	**1011.8825**	2005.7311	1003.3692	2004.7471	1002.8772	17
18	**1897.8410**	949.4241	**1880.8145**	940.9109	1879.8304	940.4189	E	1965.7362	983.3717	1948.7097	974.8585	1947.7256	974.3665	16
19	1998.8887	999.9480	1981.8621	991.4347	1980.8781	990.9427	T	1836.6936	**918.8504**	1819.6671	910.3372	1818.6830	909.8452	15
20	2127.9313	1064.4693	2110.9047	1055.9560	2109.9207	1055.4640	E	1735.6459	868.3266	1718.6194	**859.8133**	1717.6354	**859.3213**	14
21	2241.9742	1121.4907	2224.9477	1112.9775	2223.9636	1112.4855	N	1606.6033	**803.8053**	1589.5768	795.2920	1588.5928	794.8000	13
22	2370.0328	1185.5200	2353.0062	1177.0068	2352.0222	1176.5147	Q	1492.5604	746.7838	1475.5339	**738.2706**	1474.5498	737.7786	12
23	2537.0311	1269.0192	2520.0046	1260.5059	2519.0206	1260.0139	S	**1364.5018**	682.7546	1347.4753	674.2413	1346.4913	673.7493	11
24	2634.0839	1317.5456	2617.0574	1309.0323	2616.0733	1308.5403	P	1197.5035	599.2554	1180.4769	590.7421	1179.4929	590.2501	10
25	2705.1210	1353.0641	2688.0945	1344.5509	2687.1105	1344.0589	A	1100.4507	550.7290	1083.4242	542.2157	1082.4402	**541.7237**	9
26	2872.1194	1436.5633	2855.0928	1428.0500	2854.1088	1427.5580	S	1029.4136	515.2104	**1012.3871**	506.6972	1011.4030	506.2052	8
27	3001.1620	1501.0846	2984.1354	1492.5713	2983.1514	1492.0793	E	862.4153	431.7113	845.3887	423.1980	844.4047	422.7060	7
28	3130.2046	1565.6059	3113.1780	1557.0926	3112.1940	1556.6006	E	**733.3727**	367.1900	716.3461	358.6767	715.3621	358.1847	6
29	3259.2471	1630.1272	3242.2206	1621.6139	3241.2366	1621.1219	E	**604.3301**	302.6687	587.3035	294.1554	586.3195	293.6634	5
30	3387.3421	1694.1747	3370.3156	1685.6614	3369.3315	1685.1694	K	**475.2875**	238.1474	**458.2609**	229.6341	457.2769	229.1421	4
31	3516.3847	1758.6960	3499.3582	1750.1827	3498.3741	**1749.6907**	E	347.1925	174.0999	330.1660	165.5866	329.1819	165.0946	3
32	3587.4218	**1794.2145**	3570.3953	**1785.7013**	3569.4113	1785.2093	A	218.1499	109.5786	201.1234	101.0653			2
33							K	147.1128	74.0600	130.0863	65.5468			1

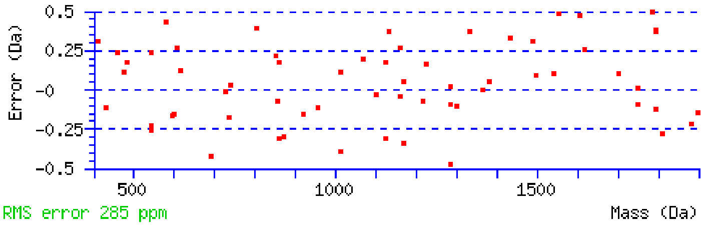

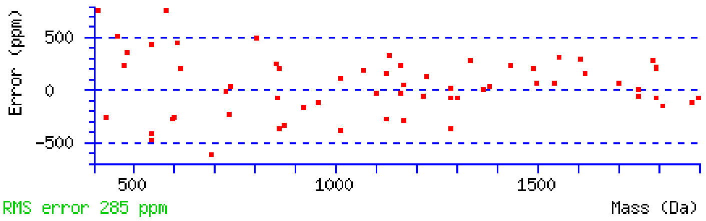

All matches to this query

Score	Mr(calc):	Delta	Sequence
49.6	3732.5200	0.0172	QAEVADQQTTDLPAENGETENQSPASEEEKEAK
48.5	3732.5200	0.0172	QAEVADQQTTDLPAENGETENQSPASEEEKEAK
40.5	3732.5200	0.0172	QAEVADQQTTDLPAENGETENQSPASEEEKEAK
8.4	3732.5200	0.0172	QAEVADQQTTDLPAENGETENQSPASEEEKEAK
8.0	3732.5200	0.0172	QAEVADQQTTDLPAENGETENQSPASEEEKEAK
6.5	3732.5200	0.0172	QAEVADQQTTDLPAENGETENQSPASEEEKEAK
6.0	3732.5189	0.0184	MGFVQMFWVITIVVSCFGRRASSPNVISK
6.0	3732.5189	0.0184	MGFVQMFWVITIVVSCFGRRASSPNVISK
5.7	3732.5200	0.0172	QAEVADQQTTDLPAENGETENQSPASEEEKEAK
5.0	3732.5200	0.0172	QAEVADQQTTDLPAENGETENQSPASEEEKEAK

Spectrum No: 306; Query: 2276; Rank: 1

Peptide View

MS/MS Fragmentation of **DTQTVHSNALNEDTQDELGDPR**
Found in **IPI00364124**, Tax_Id=10116 Gene_Symbol=Lrpap1 Alpha-2-macroglobulin receptor-associated protein precursor

Match to Query 2276: 2534.063712 from(845.695180,3+)
Title: 100101RatKid_NS_deglyco_22.2081.2081.3.dta
Data file K:\NewmanPaper\Piliang\3SubProteomes\Piliang3SP\mgf5ppm\ERLIC_3SubProteomes5ppm.mgf

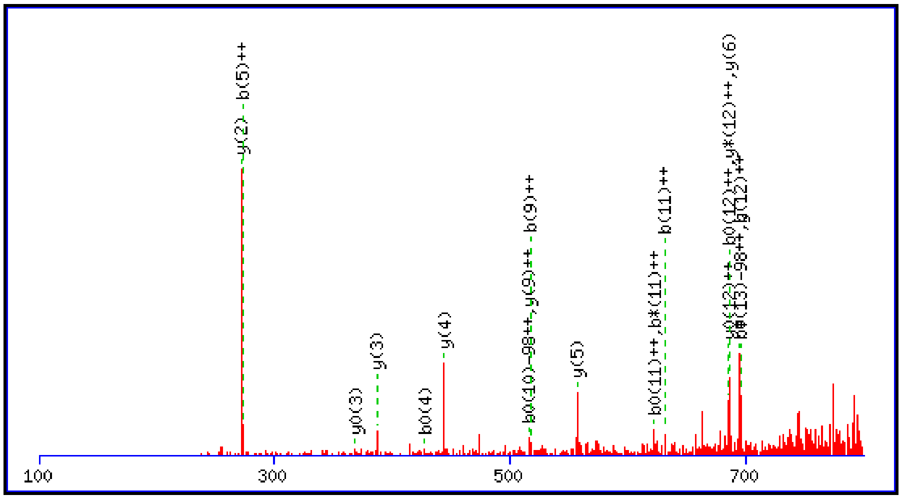

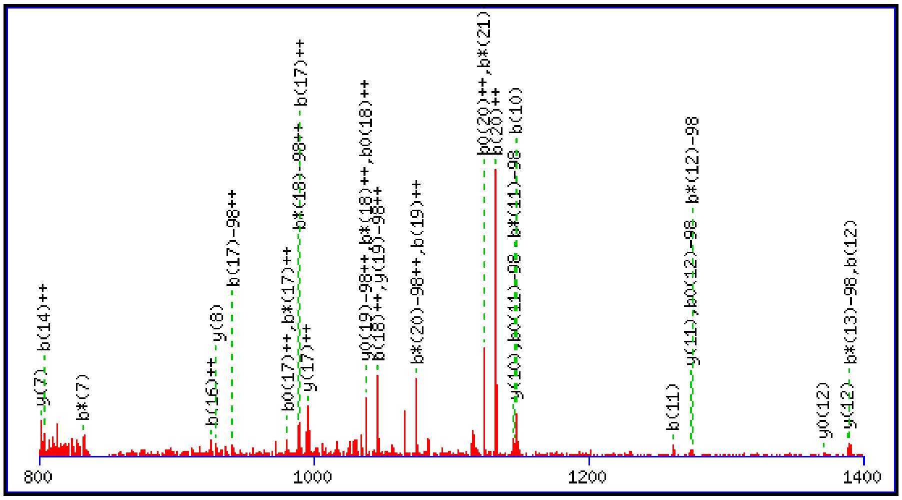

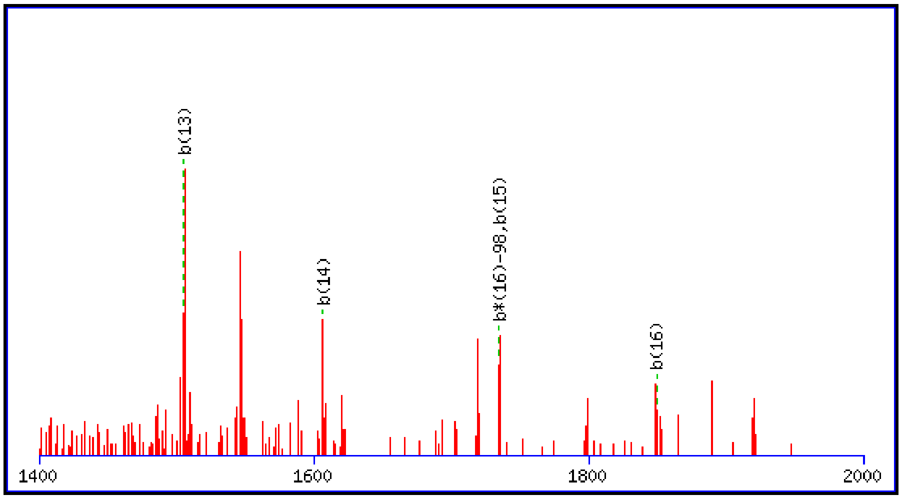

Monoisotopic mass of neutral peptide Mr(calc): 2534.0558
Fixed modifications: Carbamidomethyl (C)
Variable modifications:
S7 : Phospho (ST), with neutral losses 0.0000(shown in table), 97.9769
Ions Score: 50 **Expect:** 0.0033
Matches (Bold Red): 60/370 fragment ions using 81 most intense peaks

#	b	b^{++}	b^{*}	b^{*++}	b^{0}	b^{0++}	Seq.	y	y^{++}	y^{*}	y^{*++}	y^{0}	y^{0++}	#
1	116.0342	58.5207			98.0237	49.5155	D							22
2	217.0819	109.0446			199.0713	100.0393	T	2420.0362	1210.5217	2403.0096	1202.0084	2402.0256	1201.5164	21
3	345.1405	173.0739	328.1139	164.5606	327.1299	164.0686	Q	2318.9885	1159.9979	2301.9619	1151.4846	2300.9779	1150.9926	20
4	446.1882	223.5977	429.1616	215.0844	**428.1776**	214.5924	T	2190.9299	1095.9686	2173.9034	1087.4553	2172.9193	1086.9633	19
5	545.2566	**273.1319**	528.2300	264.6186	527.2460	264.1266	V	2089.8822	1045.4448	2072.8557	1036.9315	2071.8717	1036.4395	18
6	682.3155	341.6614	665.2889	333.1481	664.3049	332.6561	H	1990.8138	**995.9105**	1973.7873	987.3973	1972.8032	986.9053	17
7	849.3138	425.1606	**832.2873**	416.6473	831.3033	416.1553	S	1853.7549	927.3811	1836.7284	918.8678	1835.7443	918.3758	16
8	963.3568	482.1820	946.3302	473.6687	945.3462	473.1767	N	1686.7565	843.8819	1669.7300	835.3686	1668.7460	834.8766	15
9	1034.3939	**517.7006**	1017.3673	509.1873	1016.3833	508.6953	A	1572.7136	786.8604	1555.6871	778.3472	1554.7031	777.8552	14
10	**1147.4779**	574.2426	1130.4514	565.7293	1129.4674	565.2373	L	1501.6765	751.3419	1484.6500	742.8286	1483.6659	742.3366	13
11	**1261.5209**	**631.2641**	1244.4943	**622.7508**	1243.5103	**622.2588**	N	**1388.5924**	**694.7999**	1371.5659	**686.2866**	**1370.5819**	**685.7946**	12
12	**1390.5635**	**695.7854**	1373.5369	687.2721	1372.5529	**686.7801**	E	**1274.5495**	637.7784	1257.5230	629.2651	1256.5390	628.7731	11
13	**1505.5904**	753.2988	1488.5639	744.7856	1487.5798	744.2936	D	**1145.5069**	573.2571	1128.4804	564.7438	1127.4964	564.2518	10
14	**1606.6381**	**803.8227**	1589.6115	795.3094	1588.6275	794.8174	T	1030.4800	**515.7436**	1013.4534	507.2304	1012.4694	506.7383	9
15	**1734.6967**	867.8520	1717.6701	859.3387	1716.6861	858.8467	Q	**929.4323**	465.2198	912.4058	456.7065	911.4217	456.2145	8
16	**1849.7236**	**925.3654**	1832.6971	916.8522	1831.7130	916.3602	D	**801.3737**	401.1905	784.3472	392.6772	783.3632	392.1852	7
17	1978.7662	**989.8867**	1961.7396	**981.3735**	1960.7556	**980.8815**	E	**686.3468**	343.6770	669.3202	335.1638	668.3362	334.6717	6
18	2091.8503	**1046.4288**	2074.8237	**1037.9155**	2073.8397	**1037.4235**	L	**557.3042**	279.1557	540.2776	270.6425	539.2936	270.1504	5
19	2148.8717	**1074.9395**	2131.8452	1066.4262	2130.8612	1065.9342	G	**444.2201**	222.6137	427.1936	214.1004	426.2096	213.6084	4
20	2263.8987	**1132.4530**	2246.8721	**1123.9397**	2245.8881	**1123.4477**	D	**387.1987**	194.1030	370.1721	185.5897	**369.1881**	185.0977	3
21	2360.9514	1180.9794	2343.9249	1172.4661	2342.9409	1171.9741	P	**272.1717**	136.5895	255.1452	128.0762			2
22							R	175.1190	88.0631	158.0924	79.5498			1

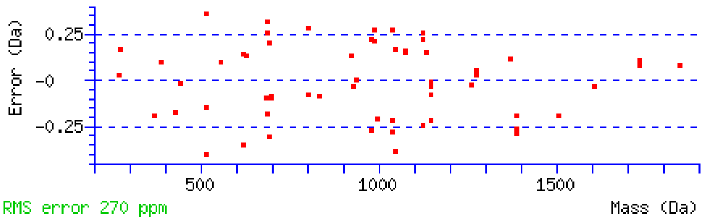

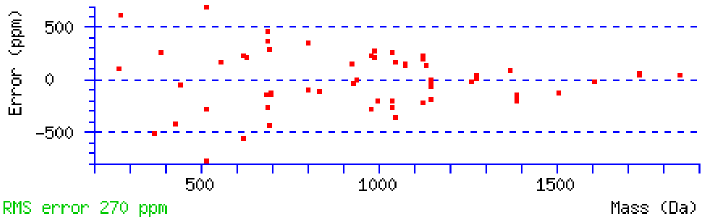

All matches to this query

Score	Mr(calc):	Delta	Sequence
49.6	2534.0558	0.0079	DTQTVHSNALNEDTQDELGDPR
40.1	2534.0558	0.0079	DTQTVHSNALNEDTQDELGDPR
40.1	2534.0558	0.0079	DTQTVHSNALNEDTQDELGDPR
15.4	2534.0558	0.0079	DTQTVHSNALNEDTQDELGDPR
12.8	2533.0705	0.9933	EVSAAEESQDTSSSSASVQATAPSK
9.4	2533.0705	0.9933	EVSAAEESQDTSSSSASVQATAPSK
9.0	2533.0705	0.9933	EVSAAEESQDTSSSSASVQATAPSK
7.7	2533.0705	0.9933	EVSAAEESQDTSSSSASVQATAPSK
7.5	2533.0705	0.9933	EVSAAEESQDTSSSSASVQATAPSK
7.5	2533.0705	0.9933	EVSAAEESQDTSSSSASVQATAPSK

Spectrum No: 307; Query: 2410; Rank: 1

Peptide View

MS/MS Fragmentation of **DSSSSSSSSSSDSDSDGEEHGSDIGPR**
Found in **IPI00197986**, Tax_Id=10116 Gene_Symbol=Ndufv3l;LOC685005 NADH dehydrogenase (ubiquinone) flavoprotein 3-like isoform 1

Match to Query 2410: 2736.985062 from(913.335630,3+)
Title: 091008RatKidney_NoSalt_29.444.444.3.dta
Data file K:\NewmanPaper\Piliang\3SubProteomes\Piliang3SP\mgf5ppm\ERLIC_3SubProteomes5ppm.mgf

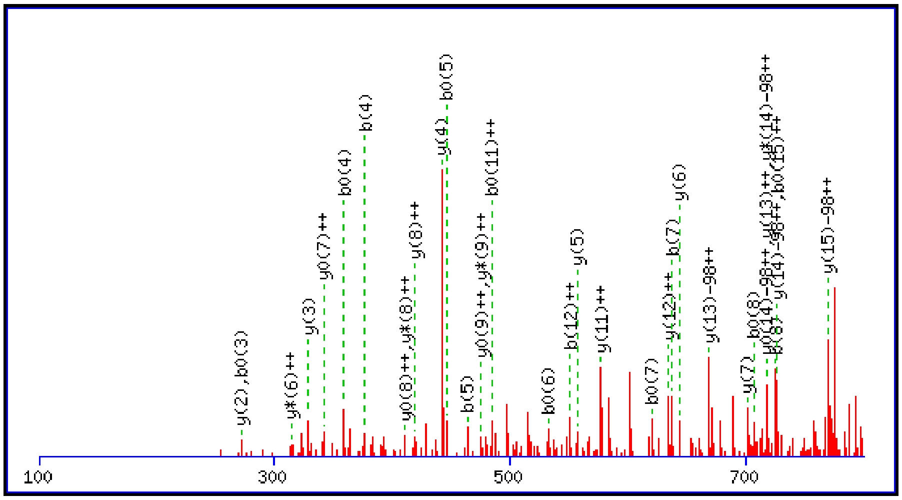

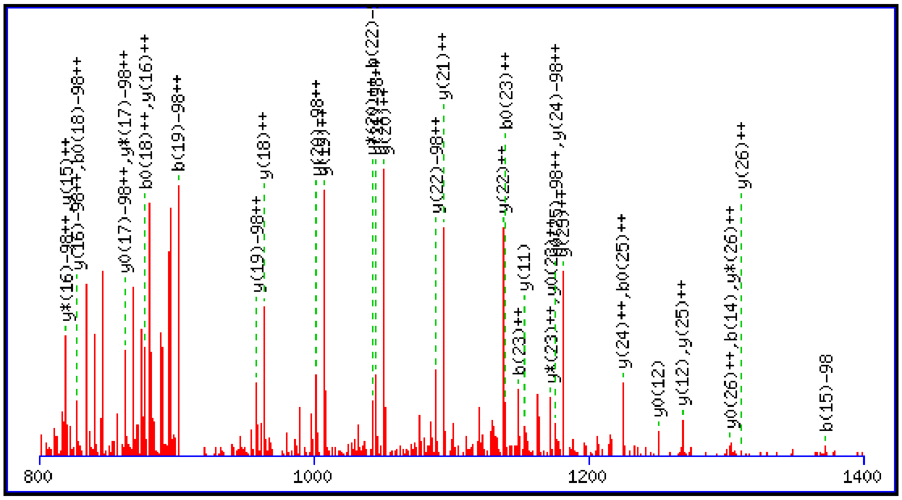

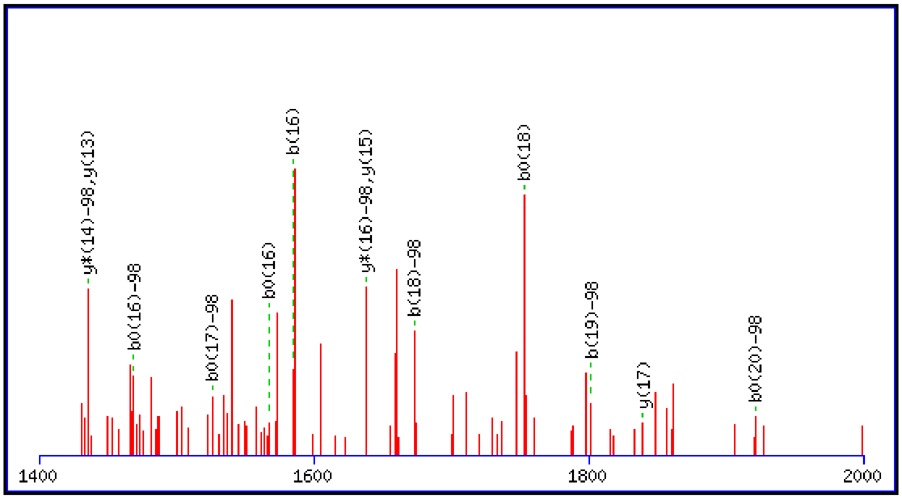

Monoisotopic mass of neutral peptide Mr(calc): 2736.9744
Fixed modifications: Carbamidomethyl (C)
Variable modifications:

```
S15    : Phospho (ST), with neutral losses 0.0000(shown in table), 97.9769
Ions Score: 50  Expect: 0.0016
Matches (Bold Red): 86/384 fragment ions using 170 most intense peaks
```

#	b	b^{++}	b^{0}	b^{0++}	Seq.	y	y^{++}	y^{*}	y^{*++}	y^{0}	y^{0++}	#
1	116.0342	58.5207	98.0237	49.5155	D							27
2	203.0662	102.0368	185.0557	93.0315	S	2622.9547	**1311.9810**	2605.9282	**1303.4677**	2604.9442	**1302.9757**	26
3	290.0983	145.5528	**272.0877**	136.5475	S	2535.9227	**1268.4650**	2518.8962	1259.9517	2517.9121	1259.4597	25
4	**377.1303**	189.0688	**359.1197**	180.0635	S	2448.8907	**1224.9490**	2431.8641	1216.4357	2430.8801	1215.9437	24
5	**464.1623**	232.5848	**446.1518**	223.5795	S	2361.8586	**1181.4330**	2344.8321	**1172.9197**	2343.8481	**1172.4277**	23
6	**551.1944**	276.1008	**533.1838**	267.0955	S	2274.8266	**1137.9169**	2257.8001	1129.4037	2256.8161	1128.9117	22
7	**638.2264**	319.6168	**620.2158**	310.6115	S	2187.7946	**1094.4009**	2170.7680	1085.8877	2169.7840	1085.3957	21
8	**725.2584**	363.1328	**707.2478**	354.1276	S	2100.7626	**1050.8849**	2083.7360	**1042.3716**	2082.7520	1041.8796	20
9	812.2904	406.6489	794.2799	397.6436	S	2013.7305	**1007.3689**	1996.7040	998.8556	1995.7200	998.3636	19
10	899.3225	450.1649	881.3119	441.1596	S	1926.6985	**963.8529**	1909.6720	955.3396	1908.6879	954.8476	18
11	986.3545	493.6809	968.3439	**484.6756**	S	**1839.6665**	920.3369	1822.6399	911.8236	1821.6559	911.3316	17
12	1101.3814	**551.1944**	1083.3709	542.1891	D	1752.6345	**876.8209**	1735.6079	868.3076	1734.6239	867.8156	16
13	1188.4135	594.7104	1170.4029	585.7051	S	**1637.6075**	**819.3074**	1620.5810	810.7941	1619.5969	810.3021	15
14	**1303.4404**	652.2238	1285.4298	643.2186	D	1550.5755	775.7914	1533.5489	767.2781	1532.5649	766.7861	14
15	1470.4388	735.7230	1452.4282	**726.7177**	S	**1435.5485**	**718.2779**	1418.5220	709.7646	1417.5380	709.2726	13
16	**1585.4657**	793.2365	**1567.4551**	784.2312	D	**1268.5502**	**634.7787**	1251.5236	626.2655	**1250.5396**	625.7734	12
17	1642.4872	821.7472	1624.4766	812.7419	G	**1153.5232**	**577.2653**	1136.4967	568.7520	1135.5127	568.2600	11
18	1771.5298	886.2685	**1753.5192**	**877.2632**	E	1096.5018	548.7545	1079.4752	540.2413	1078.4912	539.7492	10
19	1900.5724	950.7898	1882.5618	941.7845	E	967.4592	484.2332	950.4326	**475.7200**	949.4486	**475.2279**	9
20	2037.6313	1019.3193	2019.6207	1010.3140	H	838.4166	**419.7119**	821.3900	**411.1987**	820.4060	**410.7067**	8
21	2094.6527	1047.8300	2076.6422	1038.8247	G	**701.3577**	351.1825	684.3311	342.6692	683.3471	**342.1772**	7
22	2181.6848	1091.3460	2163.6742	1082.3407	S	**644.3362**	322.6717	627.3097	**314.1585**	626.3257	313.6665	6
23	2296.7117	**1148.8595**	2278.7011	**1139.8542**	D	**557.3042**	279.1557	540.2776	270.6425	539.2936	270.1504	5
24	2409.7958	1205.4015	2391.7852	1196.3962	I	**442.2772**	221.6423	425.2507	213.1290			4
25	2466.8172	1233.9123	2448.8067	**1224.9070**	G	**329.1932**	165.1002	312.1666	156.5870			3
26	2563.8700	1282.4386	2545.8594	1273.4334	P	**272.1717**	136.5895	255.1452	128.0762			2
27					R	175.1190	88.0631	158.0924	79.5498			1

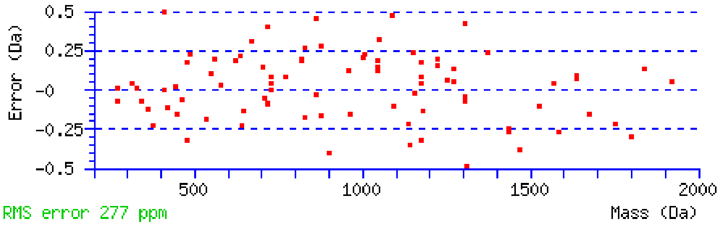

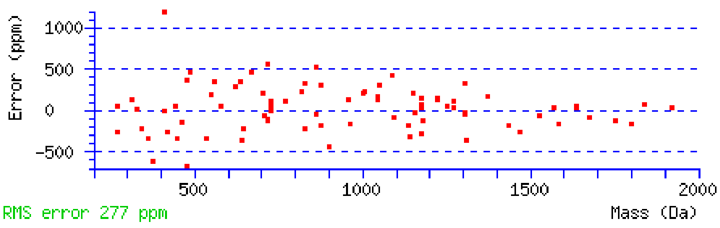

All matches to this query

Score	Mr(calc):	Delta	Sequence
49.5	2736.9744	0.0107	DSSSSSSSSSSDSDSDGEEHGSDIGPR
40.9	2736.9744	0.0107	DSSSSSSSSSSDSDSDGEEHGSDIGPR
34.0	2736.9744	0.0107	DSSSSSSSSSSDSDSDGEEHGSDIGPR
32.9	2736.9744	0.0107	DSSSSSSSSSSDSDSDGEEHGSDIGPR
31.6	2736.9744	0.0107	DSSSSSSSSSSDSDSDGEEHGSDIGPR
29.9	2736.9744	0.0107	DSSSSSSSSSSDSDSDGEEHGSDIGPR
27.7	2736.9744	0.0107	DSSSSSSSSSSDSDSDGEEHGSDIGPR
26.8	2736.9744	0.0107	DSSSSSSSSSSDSDSDGEEHGSDIGPR
21.8	2736.9744	0.0107	DSSSSSSSSSSDSDSDGEEHGSDIGPR

20.4	2736.9744	0.0107	DSSSSSSSSSDSDSDGEEHGSDIGPR

Spectrum No: 308; Query: 2357; Rank: 1

Peptide View

MS/MS Fragmentation of **SRSPVDSPVPASMFAPEPSSPGAAR**
Found in **IPI00366437**, Tax_Id=10116 Gene_Symbol=Gtpbp1_predicted 76 kDa protein

Match to Query 2357: 2656.143642 from(886.388490,3+)
Title: 100101RatKid_NS_deglyco_20.3292.3292.3.dta
Data file K:\NewmanPaper\Piliang\3SubProteomes\Piliang3SP\mgf5ppm\ERLIC_3SubProteomes5ppm.mgf

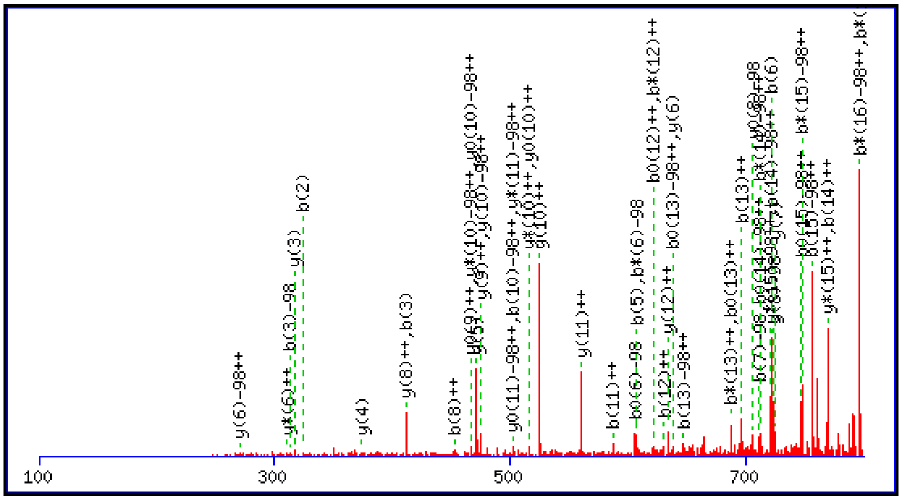

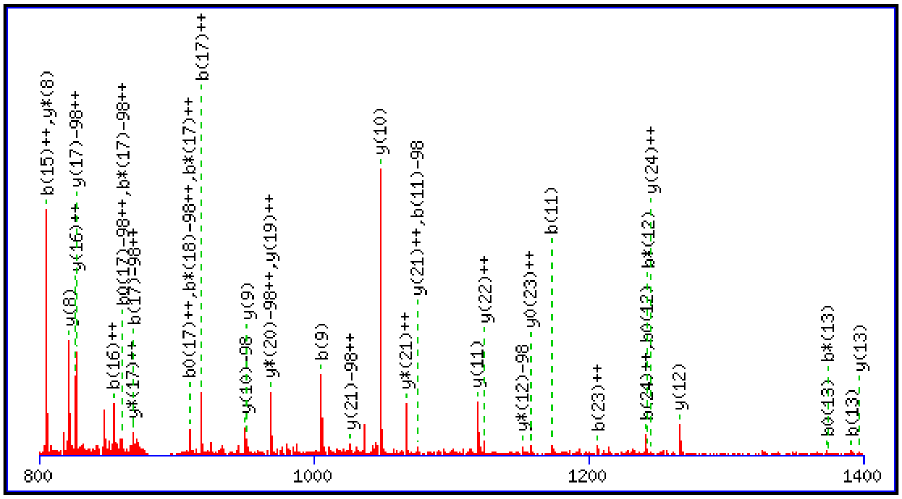

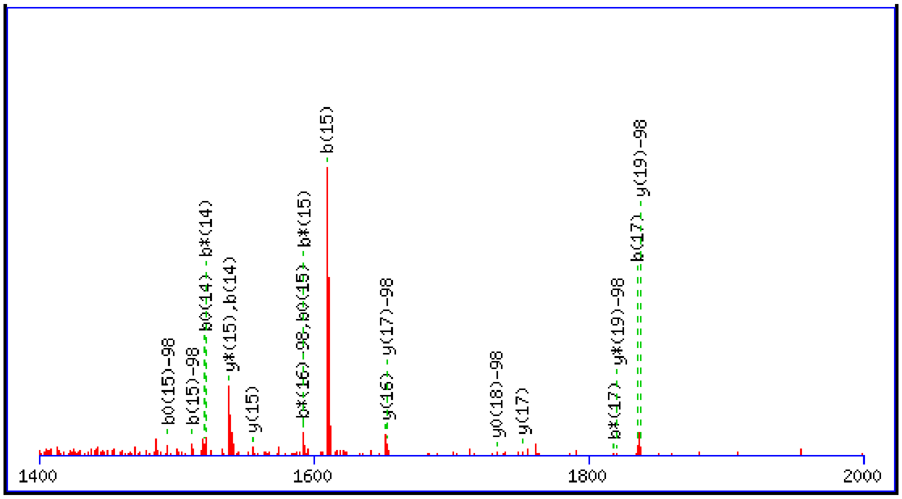

Monoisotopic mass of neutral peptide Mr(calc): 2656.1394
Fixed modifications: Carbamidomethyl (C)
Variable modifications:
S1 : Phospho (ST), with neutral losses 0.0000(shown in table), 97.9769
S20 : Phospho (ST), with neutral losses 0.0000(shown in table), 97.9769
Ions Score: 49 **Expect:** 0.0037
Matches (Bold Red): 111/532 fragment ions using 177 most intense peaks

#	b	b^{++}	b*	b^{*++}	b^0	b^{0++}	Seq.	y	y^{++}	y*	y^{*++}	y^0	y^{0++}	#
1	168.0056	84.5065			149.9951	75.5012	S							25
2	**324.1067**	162.5570	307.0802	154.0437	306.0962	153.5517	R	2490.1483	**1245.5778**	2473.1217	1237.0645	2472.1377	1236.5725	24
3	**411.1388**	206.0730	394.1122	197.5597	393.1282	197.0677	S	2334.0472	1167.5272	2317.0206	1159.0140	2316.0366	**1158.5219**	23
4	508.1915	254.5994	491.1650	246.0861	490.1810	245.5941	P	2247.0152	**1124.0112**	2229.9886	1115.4979	2229.0046	1115.0059	22
5	**607.2599**	304.1336	590.2334	295.6203	589.2494	295.1283	V	2149.9624	**1075.4848**	2132.9358	**1066.9716**	2131.9518	1066.4796	21
6	**722.2869**	361.6471	705.2603	353.1338	704.2763	352.6418	D	2050.8940	1025.9506	2033.8674	1017.4374	2032.8834	1016.9453	20
7	809.3189	405.1631	792.2924	396.6498	791.3084	396.1578	S	1935.8670	**968.4372**	1918.8405	959.9239	1917.8565	959.4319	19
8	906.3717	**453.6895**	889.3451	445.1762	888.3611	444.6842	P	1848.8350	924.9211	1831.8085	916.4079	1830.8244	915.9159	18
9	**1005.4401**	503.2237	988.4135	494.7104	987.4295	494.2184	V	**1751.7822**	876.3948	1734.7557	**867.8815**	1733.7717	867.3895	17
10	1102.4929	551.7501	1085.4663	543.2368	1084.4823	542.7448	P	**1652.7138**	**826.8606**	1635.6873	818.3473	1634.7033	817.8553	16
11	**1173.5300**	**587.2686**	1156.5034	578.7553	1155.5194	578.2633	A	**1555.6611**	778.3342	**1538.6345**	**769.8209**	1537.6505	769.3289	15
12	1260.5620	**630.7846**	**1243.5355**	**622.2714**	**1242.5514**	**621.7794**	S	1484.6240	742.8156	1467.5974	734.3023	1466.6134	733.8103	14
13	**1391.6025**	**696.3049**	**1374.5759**	**687.7916**	**1373.5919**	**687.2996**	M	**1397.5919**	699.2996	1380.5654	690.7863	1379.5814	690.2943	13
14	**1538.6709**	**769.8391**	**1521.6444**	761.3258	**1520.6603**	760.8338	F	**1266.5514**	**633.7794**	1249.5249	625.2661	1248.5409	624.7741	12
15	**1609.7080**	**805.3576**	**1592.6815**	**796.8444**	**1591.6974**	**796.3524**	A	**1119.4830**	**560.2451**	1102.4565	551.7319	1101.4725	551.2399	11
16	1706.7608	**853.8840**	1689.7342	845.3708	1688.7502	844.8787	P	**1048.4459**	**524.7266**	1031.4194	**516.2133**	1030.4353	**515.7213**	10
17	**1835.8034**	**918.4053**	**1818.7768**	**909.8920**	1817.7928	**909.4000**	E	**951.3931**	**476.2002**	934.3666	467.6869	933.3826	**467.1949**	9
18	1932.8561	966.9317	1915.8296	958.4184	1914.8456	957.9264	P	**822.3506**	**411.6789**	**805.3240**	403.1656	804.3400	402.6736	8
19	2019.8882	1010.4477	2002.8616	1001.9344	2001.8776	1001.4424	S	**725.2978**	363.1525	708.2712	354.6393	707.2872	354.1472	7
20	2186.8865	1093.9469	2169.8600	1085.4336	2168.8759	1084.9416	S	**638.2658**	319.6365	621.2392	**311.1232**	620.2552	310.6312	6
21	2283.9393	1142.4733	2266.9127	1133.9600	2265.9287	1133.4680	P	**471.2674**	236.1373	454.2409	227.6241			5
22	2340.9607	1170.9840	2323.9342	1162.4707	2322.9502	1161.9787	G	**374.2146**	187.6110	357.1881	179.0977			4
23	2411.9979	**1206.5026**	2394.9713	1197.9893	2393.9873	1197.4973	A	**317.1932**	159.1002	300.1666	150.5870			3
24	2483.0350	**1242.0211**	2466.0084	1233.5078	2465.0244	1233.0158	A	246.1561	123.5817	229.1295	115.0684			2
25							R	175.1190	88.0631	158.0924	79.5498			1

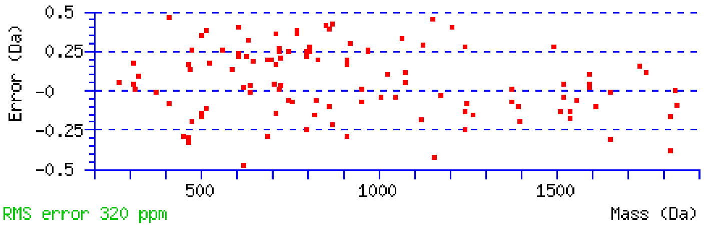

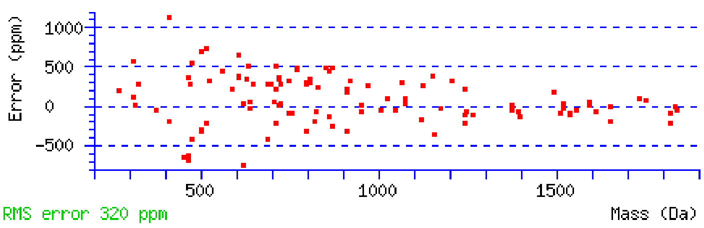

All matches to this query

Score	Mr(calc):	Delta	Sequence
49.5	2656.1394	0.0043	SRSPVDSPVPASMFAPEPSSPGAAR
46.1	2656.1394	0.0043	SRSPVDSPVPASMFAPEPSSPGAAR
45.8	2656.1394	0.0043	SRSPVDSPVPASMFAPEPSSPGAAR
45.6	2656.1394	0.0043	SRSPVDSPVPASMFAPEPSSPGAAR
38.6	2656.1394	0.0043	SRSPVDSPVPASMFAPEPSSPGAAR
38.3	2656.1394	0.0043	SRSPVDSPVPASMFAPEPSSPGAAR
25.8	2656.1394	0.0043	SRSPVDSPVPASMFAPEPSSPGAAR
24.3	2656.1394	0.0043	SRSPVDSPVPASMFAPEPSSPGAAR
10.1	2656.1298	0.0139	MENLVAYARKVEGDMYESANNR
10.1	2656.1298	0.0139	MENLVAYARKVEGDMYESANNR

Spectrum No: 309; Query: 404; Rank: 1

Peptide View

MS/MS Fragmentation of **AASPEACEVDSK**
Found in **IPI00366306**, Tax_Id=10116 Gene_Symbol=Recql ATP-dependent DNA helicase Q1

Match to Query 404: 1342.510428 from(672.262490,2+)
Title: 091008RatKidney_NH4Format02_27.409.409.2.dta
Data file K:\NewmanPaper\Piliang\3SubProteomes\Piliang3SP\mgf5ppm\ERLIC_3SubProteomes5ppm.mgf

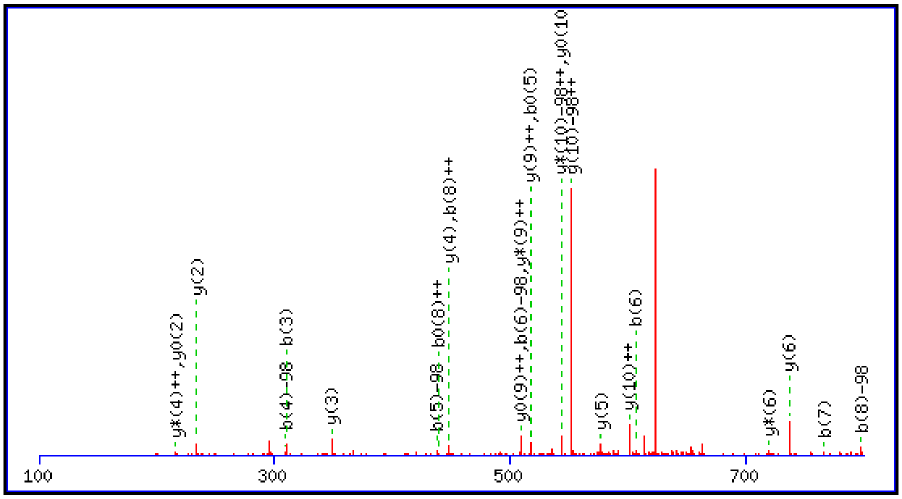

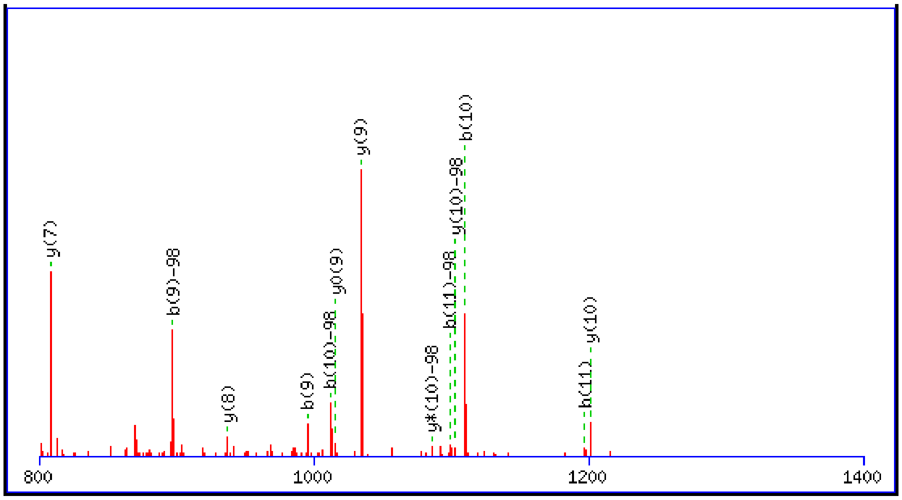

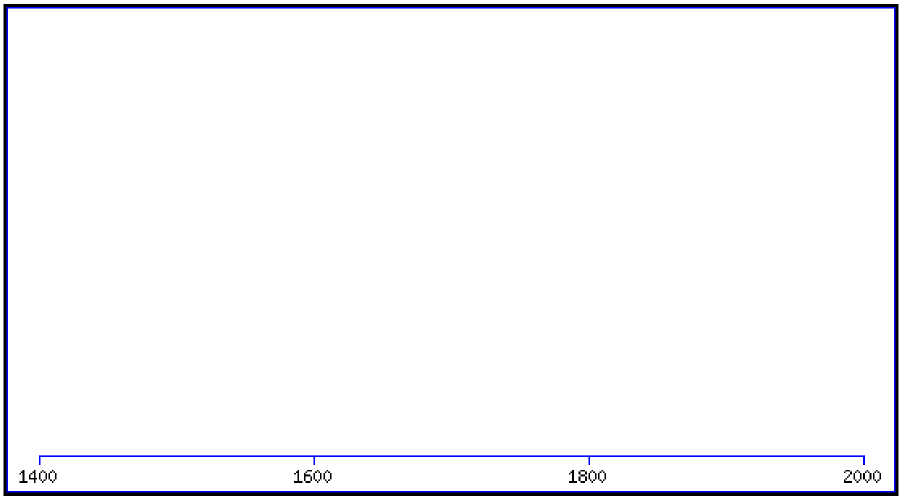

Monoisotopic mass of neutral peptide Mr(calc): 1342.5112
Fixed modifications: Carbamidomethyl (C)
Variable modifications:
S3 : Phospho (ST), with neutral losses 97.9769(shown in table), 0.0000
Ions Score: 49 **Expect:** 0.00085
Matches (Bold Red): 38/152 fragment ions using 65 most intense peaks

#	b	b^{++}	b^{0}	b^{0++}	Seq.	y	y^{++}	y*	y^{*++}	y^{0}	y^{0++}	#
1	72.0444	36.5258			A							12
2	143.0815	72.0444			A	1174.5045	587.7559	1157.4779	579.2426	1156.4939	578.7506	11
3	212.1030	106.5551	194.0924	97.5498	S	**1103.4674**	**552.2373**	**1086.4408**	**543.7240**	1085.4568	**543.2320**	10
4	**309.1557**	155.0815	291.1452	146.0762	P	**1034.4459**	**517.7266**	1017.4194	**509.2133**	**1016.4353**	**508.7213**	9
5	**438.1983**	219.6028	420.1878	210.5975	E	**937.3931**	469.2002	920.3666	460.6869	919.3826	460.1949	8
6	**509.2354**	255.1214	491.2249	246.1161	A	**808.3505**	404.6789	791.3240	396.1656	790.3400	395.6736	7
7	669.2661	335.1367	651.2555	326.1314	C	**737.3134**	369.1604	**720.2869**	360.6471	719.3029	360.1551	6
8	**798.3087**	399.6580	780.2981	390.6527	E	**577.2828**	289.1450	560.2562	280.6318	559.2722	280.1397	5
9	**897.3771**	449.1922	879.3665	440.1869	V	**448.2402**	224.6237	431.2136	**216.1105**	430.2296	215.6185	4
10	**1012.4040**	506.7057	994.3935	497.7004	D	**349.1718**	175.0895	332.1452	166.5763	331.1612	166.0842	3
11	**1099.4361**	550.2217	1081.4255	541.2164	S	**234.1448**	117.5761	217.1183	109.0628	**216.1343**	108.5708	2
12					K	147.1128	74.0600	130.0863	65.5468			1

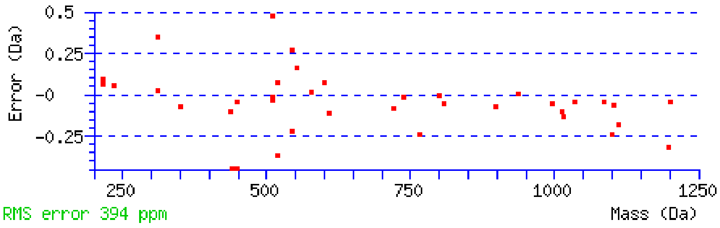

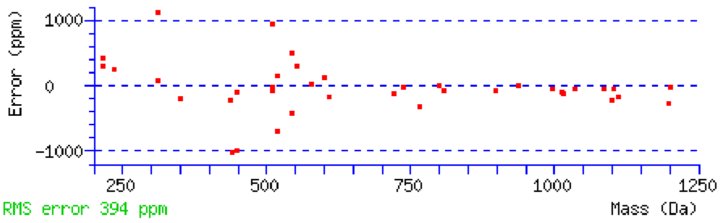

All matches to this query

Score	Mr(calc):	Delta	Sequence
49.4	1342.5112	-0.0008	AASPEACEVDSK
8.0	1342.5112	-0.0008	AASPEACEVDSK
7.7	1342.5112	-0.0008	SSEMEVHVDSK
7.7	1342.5112	-0.0008	SSEMEVHVDSK
5.3	1342.5207	-0.0103	TQEPGPAAPTSK
4.7	1342.5207	-0.0103	TQEPGPAAPTSK
3.6	1342.5191	-0.0086	TQQPQEDFDR
2.7	1341.5132	0.9973	SASSVRASLPK
2.7	1341.5132	0.9973	SASSVRASLPK
2.7	1341.5132	0.9973	SASSVRASLPK

Spectrum No: 310; Query: 1321; Rank: 1

Peptide View

MS/MS Fragmentation of **SASSDTSEELNAQDSPK**
Found in **IPI00200898**, Tax_Id=10116 Gene_Symbol=Slc9a3r1 Ezrin-radixin-moesin-binding phosphoprotein 50

Match to Query 1321: 1844.729528 from(923.372040,2+)
Title: 091008RatKidney_NH4Format01_26.672.672.2.dta
Data file K:\NewmanPaper\Piliang\3SubProteomes\Piliang3SP\mgf5ppm\ERLIC_3SubProteomes5ppm.mgf

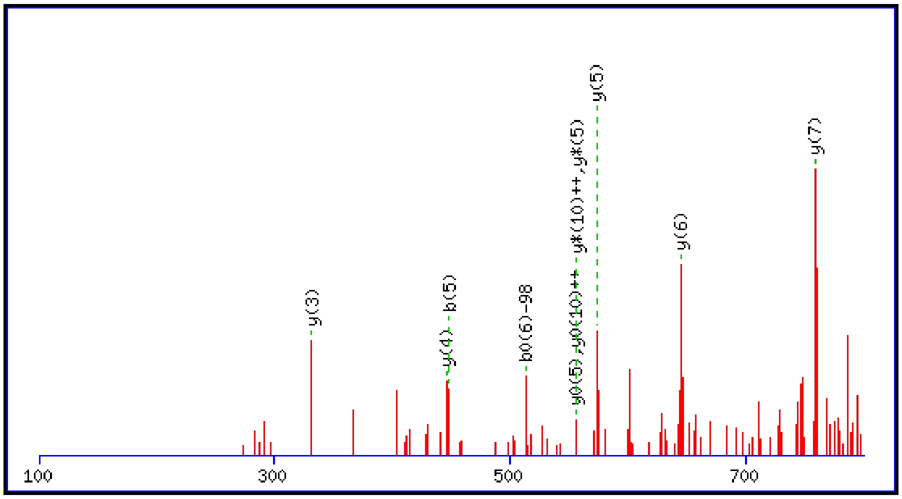

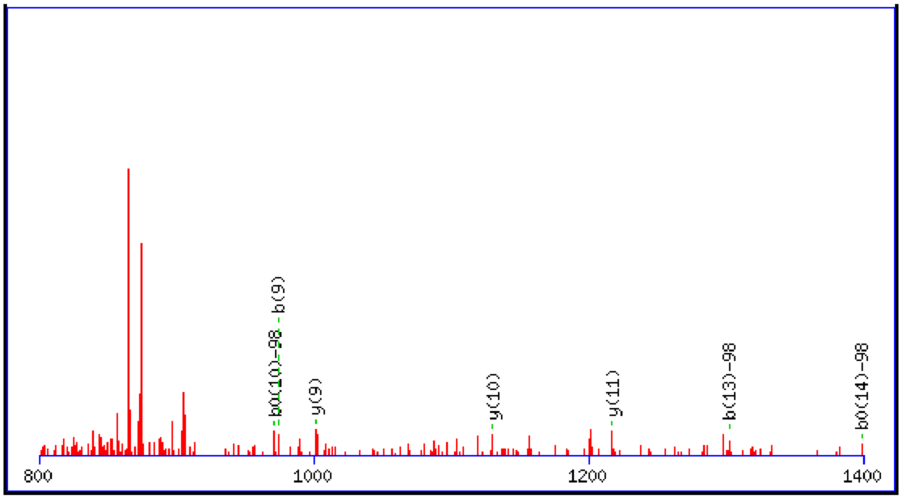

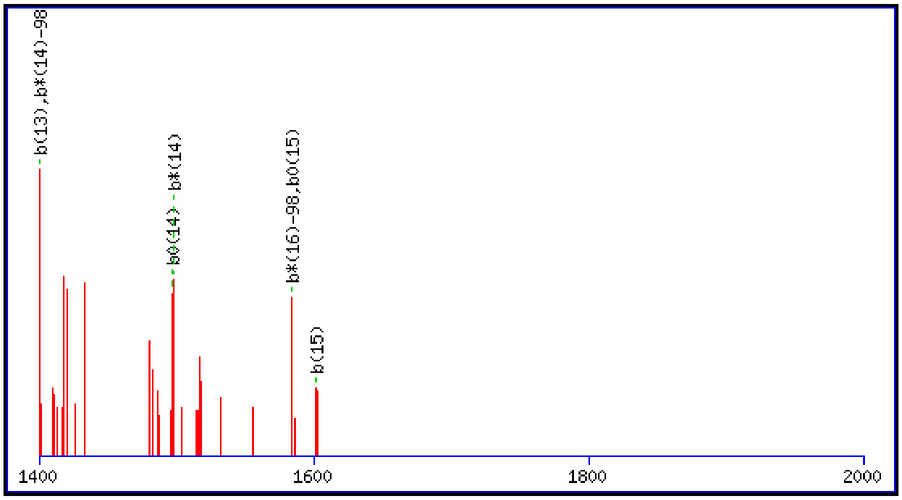

Monoisotopic mass of neutral peptide Mr(calc): 1844.7313
Fixed modifications: Carbamidomethyl (C)
Variable modifications:
T6 : Phospho (ST), with neutral losses 97.9769(shown in table), 0.0000
Ions Score: 49 **Expect:** 0.0017
Matches (Bold Red): 25/254 fragment ions using 37 most intense peaks

#	b	b^{++}	b*	b^{*++}	b^0	b^{0++}	Seq.	y	y^{++}	y*	y^{*++}	y^0	y^{0++}	#
1	88.0393	44.5233			70.0287	35.5180	S							17
2	159.0764	80.0418			141.0659	71.0366	A	1660.7297	830.8685	1643.7031	822.3552	1642.7191	821.8632	16
3	246.1084	123.5579			228.0979	114.5526	S	1589.6925	795.3499	1572.6660	786.8366	1571.6820	786.3446	15
4	333.1405	167.0739			315.1299	158.0686	S	1502.6605	751.8339	1485.6340	743.3206	1484.6499	742.8286	14
5	**448.1674**	224.5873			430.1569	215.5821	D	1415.6285	708.3179	1398.6019	699.8046	1397.6179	699.3126	13
6	531.2045	266.1059			**513.1940**	257.1006	T	1300.6015	650.8044	1283.5750	642.2911	1282.5910	641.7991	12
7	618.2366	309.6219			600.2260	300.6166	S	**1217.5644**	609.2859	1200.5379	600.7726	1199.5539	600.2806	11
8	747.2791	374.1432			729.2686	365.1379	E	**1130.5324**	565.7698	1113.5059	**557.2566**	1112.5218	**556.7646**	10
9	876.3217	438.6645			858.3112	429.6592	E	**1001.4898**	501.2485	984.4633	492.7353	983.4793	492.2433	9
10	989.4058	495.2065			**971.3952**	486.2013	L	872.4472	436.7272	855.4207	428.2140	854.4367	427.7220	8
11	1103.4487	552.2280	1086.4222	543.7147	1085.4382	543.2227	N	**759.3632**	380.1852	742.3366	371.6719	741.3526	371.1799	7
12	1174.4858	587.7466	1157.4593	579.2333	1156.4753	578.7413	A	**645.3202**	323.1638	628.2937	314.6505	627.3097	314.1585	6
13	**1302.5444**	651.7758	1285.5179	643.2626	1284.5339	642.7706	Q	**574.2831**	287.6452	**557.2566**	279.1319	**556.2726**	278.6399	5
14	1417.5714	709.2893	**1400.5448**	700.7760	**1399.5608**	700.2840	D	**446.2245**	223.6159	429.1980	215.1026	428.2140	214.6106	4
15	1504.6034	752.8053	1487.5768	744.2921	1486.5928	743.8001	S	**331.1976**	166.1024	314.1710	157.5892	313.1870	157.0972	3
16	1601.6562	801.3317	**1584.6296**	792.8184	1583.6456	792.3264	P	244.1656	122.5864	227.1390	114.0731			2
17							K	147.1128	74.0600	130.0863	65.5468			1

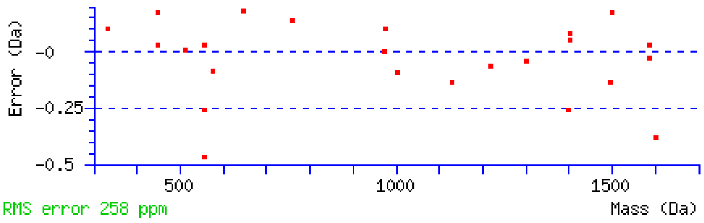

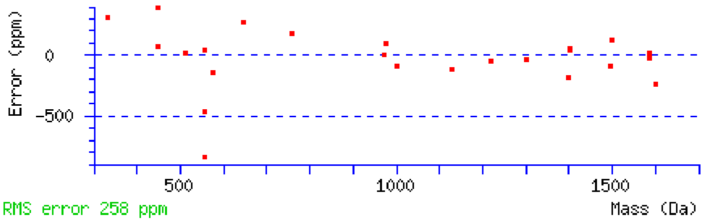

All matches to this query

Score	Mr(calc):	Delta	Sequence
49.4	1844.7313	-0.0017	SASSDTSEELNAQDSPK
44.7	1844.7313	-0.0017	SASSDTSEELNAQDSPK
44.7	1844.7313	-0.0017	SASSDTSEELNAQDSPK
44.5	1844.7313	-0.0017	SASSDTSEELNAQDSPK
36.3	1844.7313	-0.0017	SASSDTSEELNAQDSPK
5.2	1843.7261	1.0034	SYDEGPQNASSGEPGLR
4.5	1843.7139	1.0157	ESNPNVSQNSTNHKK
3.6	1843.7261	1.0034	SYDEGPQNASSGEPGLR
2.9	1844.7434	-0.0139	GDGDLSCINGDMEVRK
2.4	1844.7392	-0.0096	DIHGMHSLISGYXPK

Spectrum No: 311; Query: 1620; Rank: 1

Peptide View

MS/MS Fragmentation of **KEESEESDDDMGFGLFD**
Found in **IPI00188804**, Tax_Id=10116 Gene_Symbol=Rplp2 60S acidic ribosomal protein P2

Match to Query 1620: 2028.717928 from(1015.366240,2+)
Title: 091008RatKidney_NoSalt_29.3635.3635.2.dta
Data file K:\NewmanPaper\Piliang\3SubProteomes\Piliang3SP\mgf5ppm\ERLIC_3SubProteomes5ppm.mgf

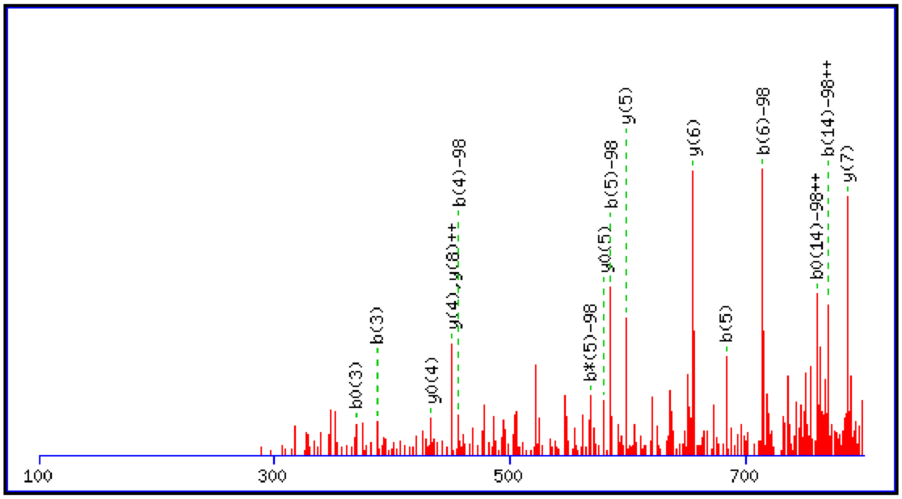

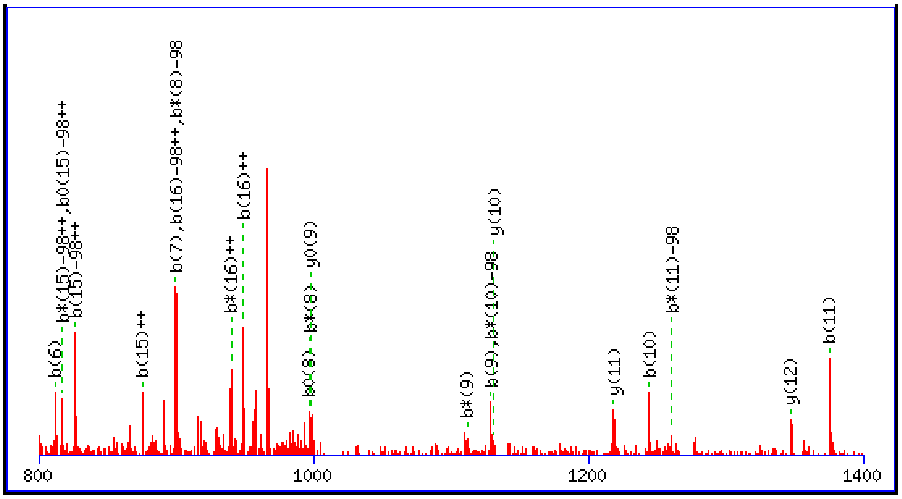

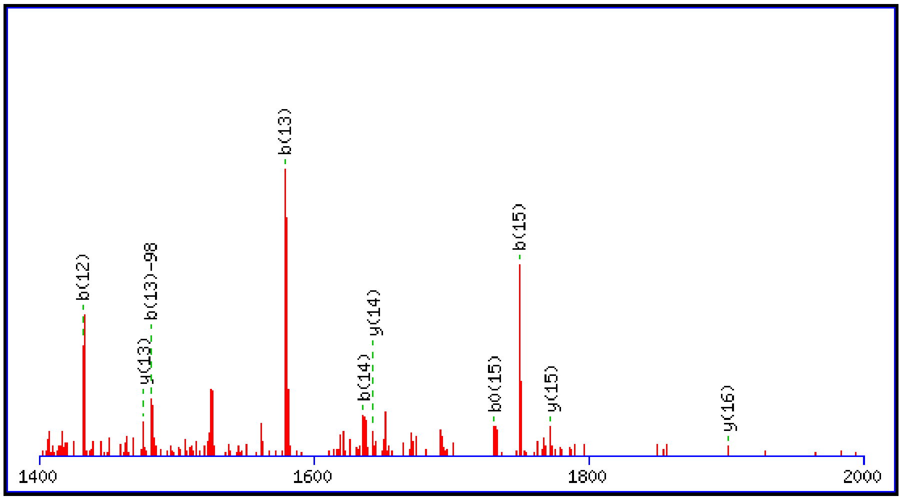

Monoisotopic mass of neutral peptide Mr(calc): 2028.7184
Fixed modifications: Carbamidomethyl (C)
Variable modifications:
S4 : Phospho (ST), with neutral losses 0.0000(shown in table), 97.9769
Ions Score: 49 **Expect:** 0.0011
Matches (Bold Red): 48/248 fragment ions using 88 most intense peaks

#	b	b^{++}	b*	b^{*++}	b^0	b^{0++}	Seq.	y	y^{++}	y^0	y^{0++}	#
1	129.1022	65.0548	112.0757	56.5415			K					17
2	258.1448	129.5761	241.1183	121.0628	240.1343	120.5708	E	**1901.6307**	951.3190	1883.6201	942.3137	16
3	**387.1874**	194.0974	370.1609	185.5841	**369.1769**	185.0921	E	**1772.5881**	886.7977	1754.5775	877.7924	15
4	554.1858	277.5965	537.1592	269.0833	536.1752	268.5912	S	**1643.5455**	822.2764	1625.5349	813.2711	14
5	**683.2284**	342.1178	666.2018	333.6045	665.2178	333.1125	E	**1476.5471**	738.7772	1458.5366	729.7719	13
6	**812.2710**	406.6391	795.2444	398.1258	794.2604	397.6338	E	**1347.5045**	674.2559	1329.4940	665.2506	12
7	**899.3030**	450.1551	882.2764	441.6419	881.2924	441.1499	S	**1218.4619**	609.7346	1200.4514	600.7293	11
8	1014.3299	507.6686	**997.3034**	499.1553	**996.3194**	498.6633	D	**1131.4299**	566.2186	1113.4194	557.2133	10
9	**1129.3569**	565.1821	**1112.3303**	556.6688	1111.3463	556.1768	D	1016.4030	508.7051	**998.3924**	499.6998	9
10	**1244.3838**	622.6955	1227.3573	614.1823	1226.3733	613.6903	D	901.3760	**451.1917**	883.3655	442.1864	8
11	**1375.4243**	688.2158	1358.3978	679.7025	1357.4137	679.2105	M	**786.3491**	393.6782	768.3385	384.6729	7
12	**1432.4458**	716.7265	1415.4192	708.2132	1414.4352	707.7212	G	**655.3086**	328.1579	637.2980	319.1527	6
13	**1579.5142**	790.2607	1562.4876	781.7475	1561.5036	781.2554	F	**598.2871**	299.6472	**580.2766**	290.6419	5
14	**1636.5356**	818.7715	1619.5091	810.2582	1618.5251	809.7662	G	**451.2187**	226.1130	**433.2082**	217.1077	4
15	**1749.6197**	**875.3135**	1732.5932	866.8002	**1731.6091**	866.3082	L	394.1973	197.6023	376.1867	188.5970	3
16	1896.6881	**948.8477**	1879.6616	**940.3344**	1878.6776	939.8424	F	281.1132	141.0602	263.1026	132.0550	2
17							D	134.0448	67.5260	116.0342	58.5207	1

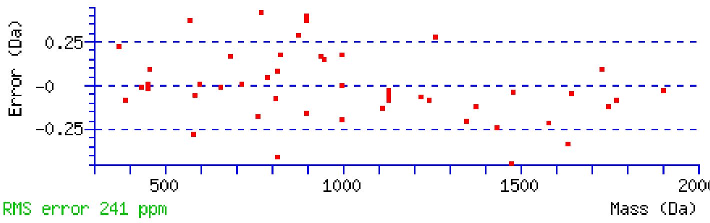

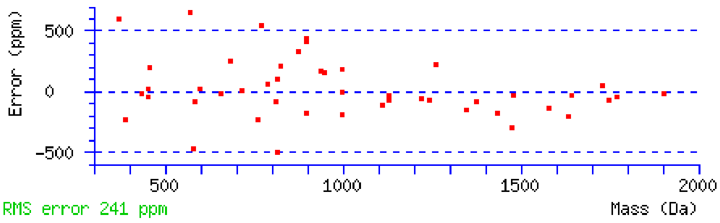

All matches to this query

Score	Mr(calc):	Delta	Sequence
49.3	2028.7184	-0.0004	KEESEESDDDMGFGLFD
33.5	2028.7184	-0.0004	KEESEESDDDMGFGLFD
1.6	2028.7367	-0.0188	KVGNPSANGTSTPDDLSK

Spectrum No: 312; Query: 2353; Rank: 1

Peptide View

MS/MS Fragmentation of **TENQPAVLEDAPDNTETGSVCTKV**
Found in **IPI00203446**, Tax_Id=10116 Gene_Symbol=Slc23a1 Solute carrier family 23 member 1

Match to Query 2353: 2654.142582 from(885.721470,3+)
Title: 091008RatKidney_NoSalt_26.2516.2516.3.dta
Data file K:\NewmanPaper\Piliang\3SubProteomes\Piliang3SP\mgf5ppm\ERLIC_3SubProteomes5ppm.mgf

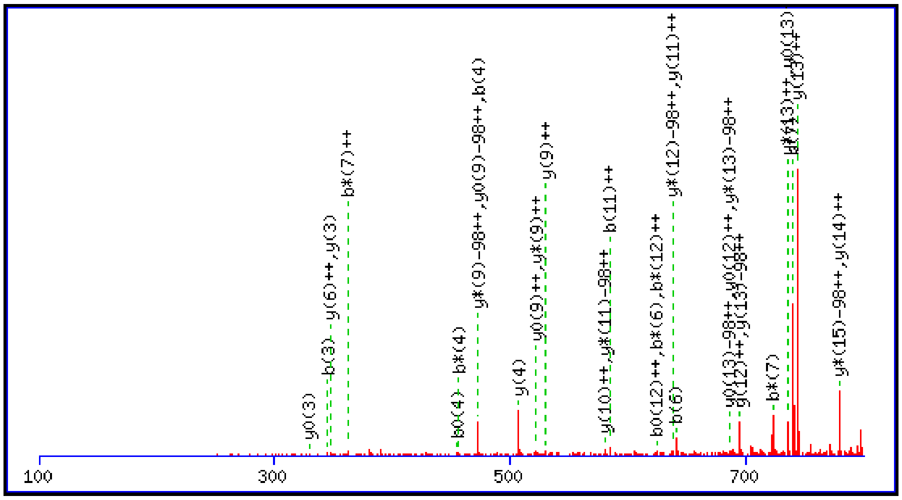

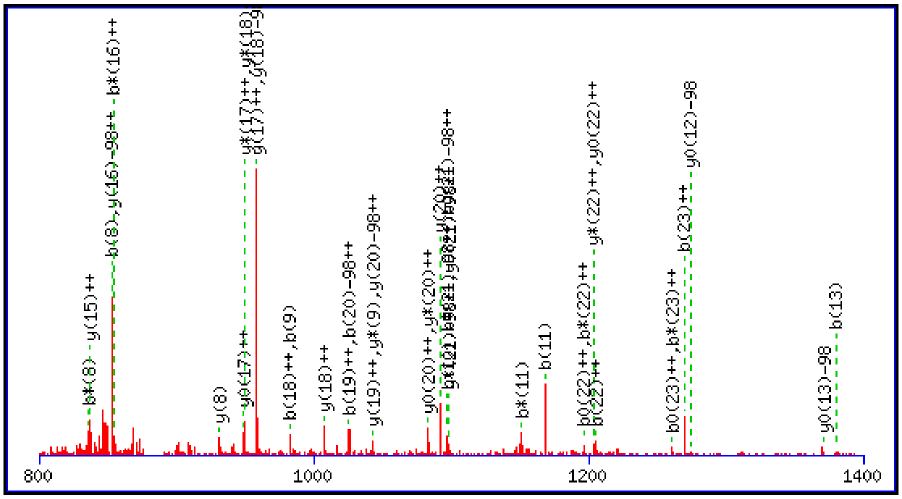

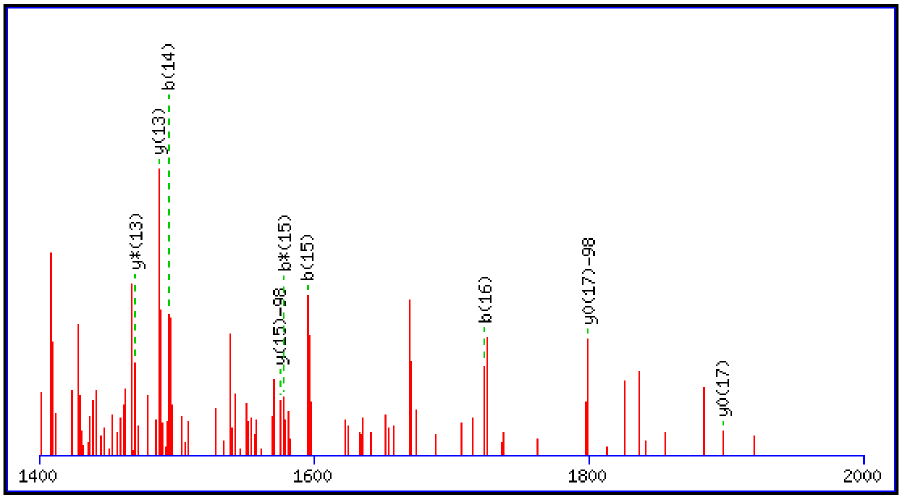

Monoisotopic mass of neutral peptide Mr(calc): 2654.1419
Fixed modifications: Carbamidomethyl (C)
Variable modifications:
T17 : Phospho (ST), with neutral losses 0.0000(shown in table), 97.9769
Ions Score: 49 **Expect:** 0.0041
Matches (Bold Red): 85/404 fragment ions using 124 most intense peaks

#	b	b^{++}	b*	b^{*++}	b^0	b^{0++}	Seq.	y	y^{++}	y*	y^{*++}	y^0	y^{0++}	#
1	102.0550	51.5311			84.0444	42.5258	T							24
2	231.0975	116.0524			213.0870	107.0471	E	2554.1015	1277.5544	2537.0749	1269.0411	2536.0909	1268.5491	23
3	**345.1405**	173.0739	328.1139	164.5606	327.1299	164.0686	N	2425.0589	1213.0331	2408.0323	**1204.5198**	2407.0483	**1204.0278**	22
4	**473.1991**	237.1032	**456.1725**	228.5899	**455.1885**	228.0979	Q	2311.0159	1156.0116	2293.9894	1147.4983	2293.0054	1147.0063	21
5	570.2518	285.6295	553.2253	277.1163	552.2413	276.6243	P	2182.9574	**1091.9823**	2165.9308	**1083.4690**	2164.9468	**1082.9770**	20
6	**641.2889**	321.1481	**624.2624**	312.6348	623.2784	312.1428	A	2085.9046	**1043.4559**	2068.8781	1034.9427	2067.8940	1034.4507	19
7	**740.3573**	370.6823	**723.3308**	**362.1690**	722.3468	361.6770	V	2014.8675	**1007.9374**	1997.8409	999.4241	1996.8569	998.9321	18
8	**853.4414**	427.2243	**836.4149**	418.7111	835.4308	418.2191	L	1915.7991	**958.4032**	1898.7725	**949.8899**	**1897.7885**	**949.3979**	17
9	**982.4840**	491.7456	965.4575	483.2324	964.4734	482.7404	E	1802.7150	901.8611	1785.6885	893.3479	1784.7044	892.8559	16
10	**1097.5109**	549.2591	1080.4844	540.7458	1079.5004	540.2538	D	1673.6724	**837.3398**	1656.6459	828.8266	1655.6619	828.3346	15
11	**1168.5481**	**584.7777**	**1151.5215**	576.2644	1150.5375	575.7724	A	1558.6455	**779.8264**	1541.6189	771.3131	1540.6349	770.8211	14
12	1265.6008	633.3040	1248.5743	**624.7908**	1247.5903	**624.2988**	P	**1487.6084**	**744.3078**	**1470.5818**	**735.7945**	1469.5978	**735.3025**	13
13	**1380.6278**	690.8175	1363.6012	682.3042	1362.6172	681.8122	D	1390.5556	**695.7814**	1373.5290	687.2682	1372.5450	**686.7762**	12
14	**1494.6707**	747.8390	1477.6441	739.3257	1476.6601	738.8337	N	1275.5287	**638.2680**	1258.5021	629.7547	1257.5181	629.2627	11
15	**1595.7184**	798.3628	**1578.6918**	789.8495	1577.7078	789.3575	T	1161.4857	**581.2465**	1144.4592	572.7332	1143.4752	572.2412	10
16	**1724.7610**	862.8841	1707.7344	**854.3708**	1706.7504	853.8788	E	1060.4380	**530.7227**	**1043.4115**	**522.2094**	1042.4275	**521.7174**	9
17	1905.7750	953.3911	1888.7484	944.8778	1887.7644	944.3858	T	**931.3955**	466.2014	914.3689	457.6881	913.3849	457.1961	8
18	1962.7964	**981.9019**	1945.7699	973.3886	1944.7859	972.8966	G	750.3815	375.6944	733.3549	367.1811	732.3709	366.6891	7
19	2049.8285	**1025.4179**	2032.8019	1016.9046	2031.8179	1016.4126	S	693.3600	**347.1836**	676.3334	338.6704	675.3494	338.1783	6
20	2148.8969	1074.9521	2131.8703	1066.4388	2130.8863	1065.9468	V	606.3280	303.6676	589.3014	295.1543	588.3174	294.6623	5
21	2308.9275	1154.9674	2291.9010	1146.4541	2290.9170	1145.9621	C	**507.2595**	254.1334	490.2330	245.6201	489.2490	245.1281	4
22	2409.9752	**1205.4912**	2392.9487	**1196.9780**	2391.9646	**1196.4860**	T	**347.2289**	174.1181	330.2023	165.6048	**329.2183**	165.1128	3
23	2538.0702	**1269.5387**	2521.0436	**1261.0254**	2520.0596	**1260.5334**	K	246.1812	123.5942	229.1547	115.0810			2
24							V	118.0863	59.5468					1

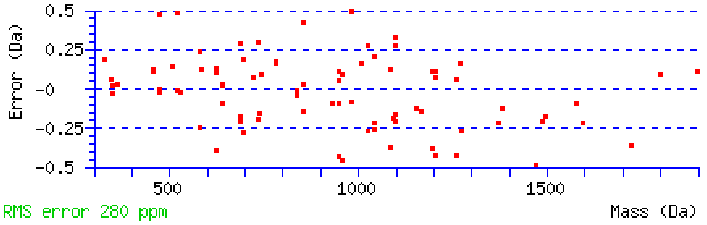

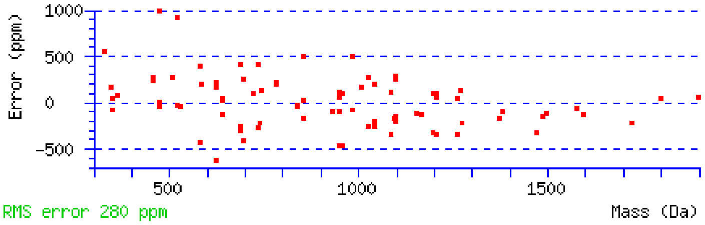

All matches to this query

Score	Mr(calc):	Delta	Sequence
49.3	2654.1419	0.0007	TENQPAVLEDAPDNTETGSVCTKV
46.8	2654.1419	0.0007	TENQPAVLEDAPDNTETGSVCTKV
38.0	2654.1419	0.0007	TENQPAVLEDAPDNTETGSVCTKV
35.3	2654.1419	0.0007	TENQPAVLEDAPDNTETGSVCTKV
3.6	2654.1587	-0.0161	EKANSKNPQVMYLEQELESLK
2.8	2654.1656	-0.0230	SNMLRGRNSATSADEQPHIGNYR
2.4	2654.1656	-0.0230	SNMLRGRNSATSADEQPHIGNYR
1.7	2654.1587	-0.0161	EKANSKNPQVMYLEQELESLK
1.7	2654.1367	0.0059	SQHAHVIEDLHCNLCDVDVSAR
1.4	2652.1412	2.0013	AQKHSSGSSNTSTANRSTHNELEK

Spectrum No: 313; Query: 2575; Rank: 1

Peptide View

MS/MS Fragmentation of **AALGLQDSDDEDAAVDIDEQIESMFNSK**
Found in **IPI00208266**, Tax_Id=10116 Gene_Symbol=Ddx46 Probable ATP-dependent RNA helicase DDX46

Match to Query 2575: 3106.313112 from(1036.444980,3+)
Title: 100101RatKid_NS_deglyco_24.6289.6289.3.dta
Data file K:\NewmanPaper\Piliang\3SubProteomes\Piliang3SP\mgf5ppm\ERLIC_3SubProteomes5ppm.mgf

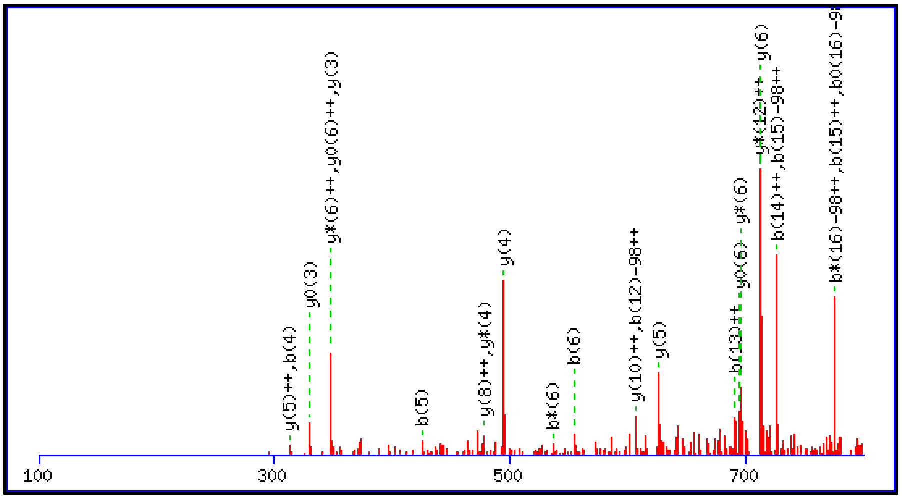

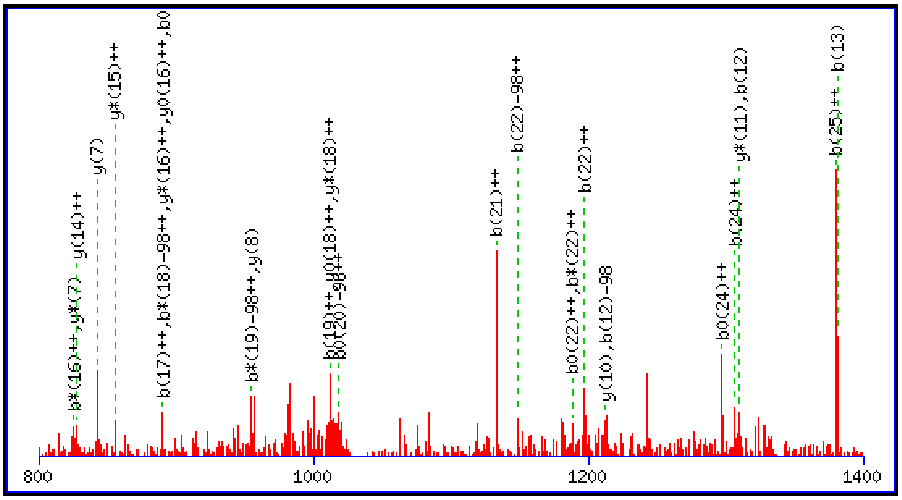

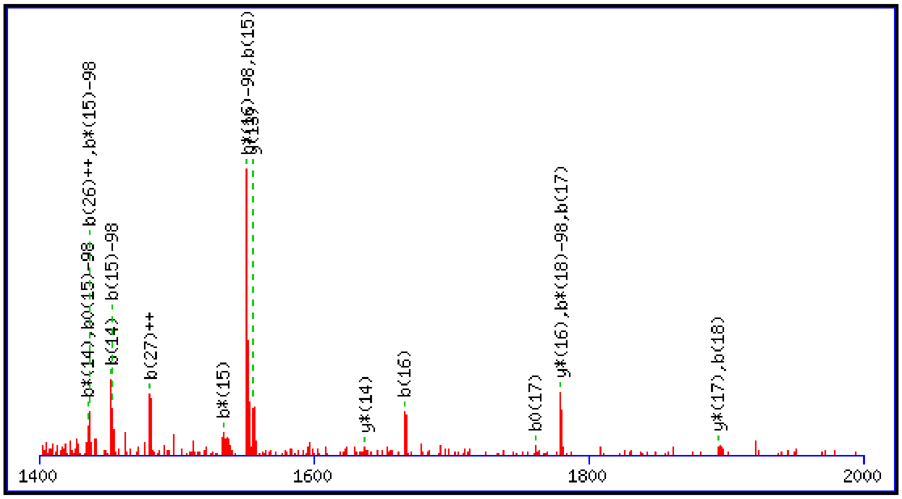

Monoisotopic mass of neutral peptide Mr(calc): 3105.3009
Fixed modifications: Carbamidomethyl (C)
Variable modifications:
S8 : Phospho (ST), with neutral losses 0.0000(shown in table), 97.9769
Ions Score: 49 **Expect:** 0.0048
Matches (Bold Red): 73/462 fragment ions using 104 most intense peaks

#	b	b^{++}	b*	b^{*++}	b^0	b^{0++}	Seq.	y	y^{++}	y*	y^{*++}	y^0	y^{0++}	#
1	72.0444	36.5258					A							28
2	143.0815	72.0444					A	3035.2711	1518.1392	3018.2446	1509.6259	3017.2605	1509.1339	27
3	256.1656	128.5864					L	2964.2340	1482.6206	2947.2074	1474.1074	2946.2234	1473.6154	26
4	**313.1870**	157.0972					G	2851.1499	1426.0786	2834.1234	1417.5653	2833.1394	1417.0733	25
5	**426.2711**	213.6392					L	2794.1285	1397.5679	2777.1019	1389.0546	2776.1179	1388.5626	24
6	**554.3297**	277.6685	**537.3031**	269.1552			Q	2681.0444	1341.0258	2664.0179	1332.5126	2663.0338	1332.0206	23
7	669.3566	335.1819	652.3301	326.6687	651.3461	326.1767	D	2552.9858	1276.9965	2535.9593	1268.4833	2534.9753	1267.9913	22
8	836.3550	418.6811	819.3284	410.1678	818.3444	409.6758	S	2437.9589	1219.4831	2420.9323	1210.9698	2419.9483	1210.4778	21
9	951.3819	476.1946	934.3554	467.6813	933.3713	467.1893	D	2270.9605	1135.9839	2253.9340	1127.4706	2252.9500	1126.9786	20
10	1066.4089	533.7081	1049.3823	525.1948	1048.3983	524.7028	D	2155.9336	1078.4704	2138.9070	1069.9572	2137.9230	1069.4651	19
11	1195.4514	598.2294	1178.4249	589.7161	1177.4409	589.2241	E	2040.9066	1020.9570	2023.8801	**1012.4437**	2022.8961	**1011.9517**	18
12	**1310.4784**	655.7428	1293.4518	647.2296	1292.4678	646.7376	D	1911.8640	956.4357	**1894.8375**	947.9224	1893.8535	947.4304	17
13	**1381.5155**	**691.2614**	1364.4890	682.7481	1363.5049	682.2561	A	1796.8371	898.9222	**1779.8106**	**890.4089**	1778.8265	**889.9169**	16
14	**1452.5526**	**726.7799**	**1435.5261**	718.2667	1434.5421	717.7747	A	1725.8000	863.4036	1708.7734	**854.8904**	1707.7894	854.3984	15
15	**1551.6210**	**776.3142**	**1534.5945**	767.8009	1533.6105	767.3089	V	1654.7629	**827.8851**	**1637.7363**	819.3718	1636.7523	818.8798	14
16	**1666.6480**	833.8276	1649.6214	**825.3144**	1648.6374	824.8223	D	**1555.6945**	778.3509	1538.6679	769.8376	1537.6839	769.3456	13
17	**1779.7320**	**890.3697**	1762.7055	881.8564	**1761.7215**	881.3644	I	1440.6675	720.8374	1423.6410	**712.3241**	1422.6570	711.8321	12
18	**1894.7590**	947.8831	1877.7324	939.3699	1876.7484	938.8778	D	1327.5835	664.2954	**1310.5569**	655.7821	1309.5729	655.2901	11
19	2023.8016	**1012.4044**	2006.7750	1003.8912	2005.7910	1003.3991	E	**1212.5565**	**606.7819**	1195.5300	598.2686	1194.5459	597.7766	10
20	2151.8602	1076.4337	2134.8336	1067.9204	2133.8496	1067.4284	Q	1083.5139	542.2606	1066.4874	533.7473	1065.5034	533.2553	9
21	2264.9442	**1132.9757**	2247.9177	1124.4625	2246.9337	1123.9705	I	**955.4553**	**478.2313**	938.4288	469.7180	937.4448	469.2260	8
22	2393.9868	**1197.4970**	2376.9603	**1188.9838**	2375.9762	**1188.4918**	E	**842.3713**	421.6893	**825.3447**	413.1760	824.3607	412.6840	7
23	2481.0188	1241.0131	2463.9923	1232.4998	2463.0083	1232.0078	S	**713.3287**	357.1680	**696.3021**	**348.6547**	**695.3181**	**348.1627**	6
24	2612.0593	**1306.5333**	2595.0328	1298.0200	2594.0488	**1297.5280**	M	**626.2967**	**313.6520**	609.2701	305.1387	608.2861	304.6467	5
25	2759.1277	**1380.0675**	2742.1012	1371.5542	2741.1172	1371.0622	F	**495.2562**	248.1317	**478.2296**	239.6184	477.2456	239.1264	4
26	2873.1707	**1437.0890**	2856.1441	1428.5757	2855.1601	1428.0837	N	**348.1878**	174.5975	331.1612	166.0842	**330.1772**	165.5922	3
27	2960.2027	**1480.6050**	2943.1761	1472.0917	2942.1921	1471.5997	S	234.1448	117.5761	217.1183	109.0628	216.1343	108.5708	2
28							K	147.1128	74.0600	130.0863	65.5468			1

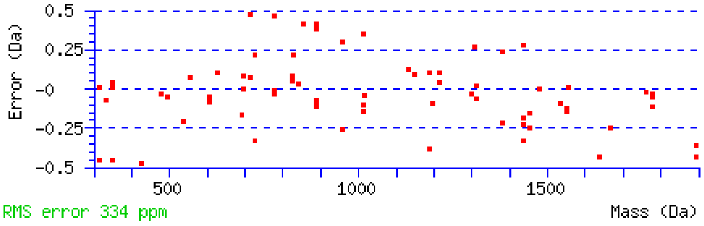

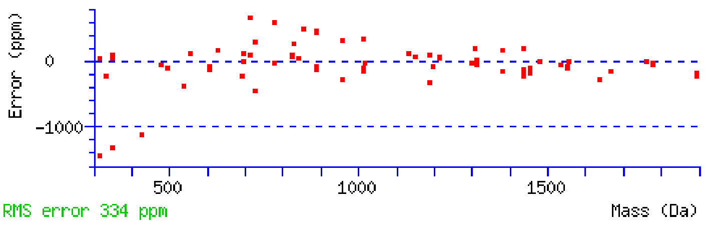

All matches to this query

Score	Mr(calc):	Delta	Sequence
49.2	3105.3009	1.0122	AALGLQDSDDEDAAVDIDEQIESMFNSK
42.0	3106.2849	0.0282	AALGLQDSDDEDAAVDIDEQIESMFNSK
6.5	3105.2867	1.0264	DLRSLASLNLSGCVHCLSADSLLRK
5.4	3104.2881	2.0250	TPCSSLLPLLNTHAVASGKQGNFSRK
4.6	3104.2881	2.0250	TPCSSLLPLLNTHAVASGKQGNFSRK
4.6	3104.2881	2.0250	TPCSSLLPLLNTHAVASGKQGNFSRK
4.5	3104.2777	2.0354	AATLSEAETRAEKSDYGAGAEGQSAAVXK
4.5	3104.2777	2.0354	AATLSEAETRAEKSDYGAGAEGQSAAVXK
2.0	3104.3027	2.0104	DLRSLASLNLSGCVHCLSADSLLRK
1.9	3106.3355	-0.0224	FCQENGFSSHFVSAKTGDSVFLCFQK

Spectrum No: 314; Query: 2693; Rank: 1

Peptide View

MS/MS Fragmentation of **KQTPPASPSPQPAEDRPPSSPIYEDAAPLK**
Found in **IPI00198498**, Tax_Id=10116 Gene_Symbol=Cttn Cortactin isoform C

Match to Query 2693: 3410.497722 from(1137.839850,3+)
Title: 100101RatKid_NS_deglyco_24.2577.2577.3.dta
Data file K:\NewmanPaper\Piliang\3SubProteomes\Piliang3SP\mgf5ppm\ERLIC_3SubProteomes5ppm.mgf

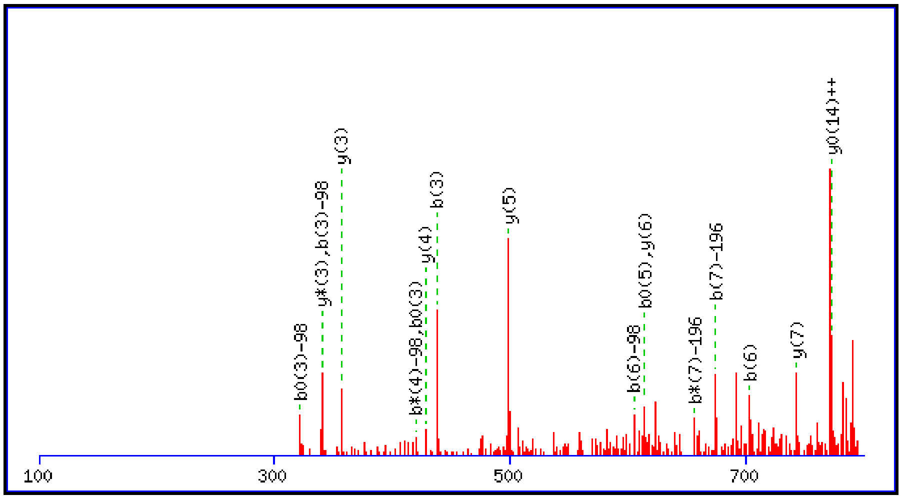

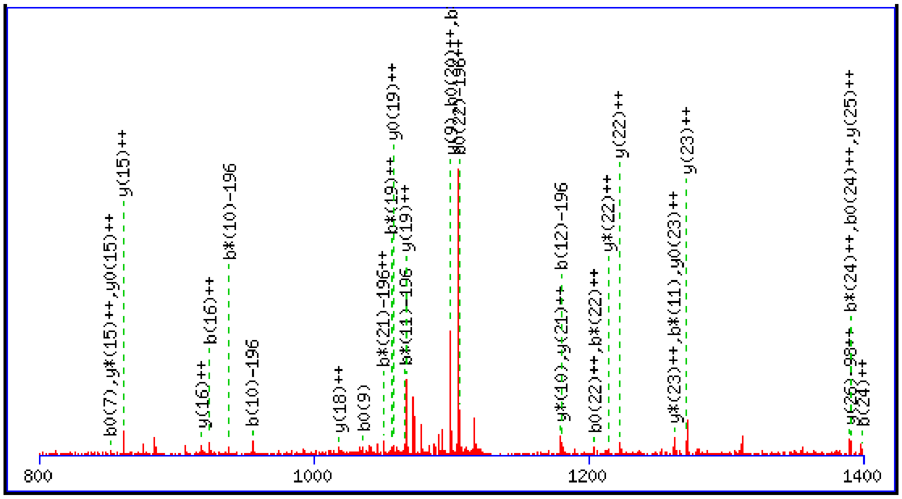

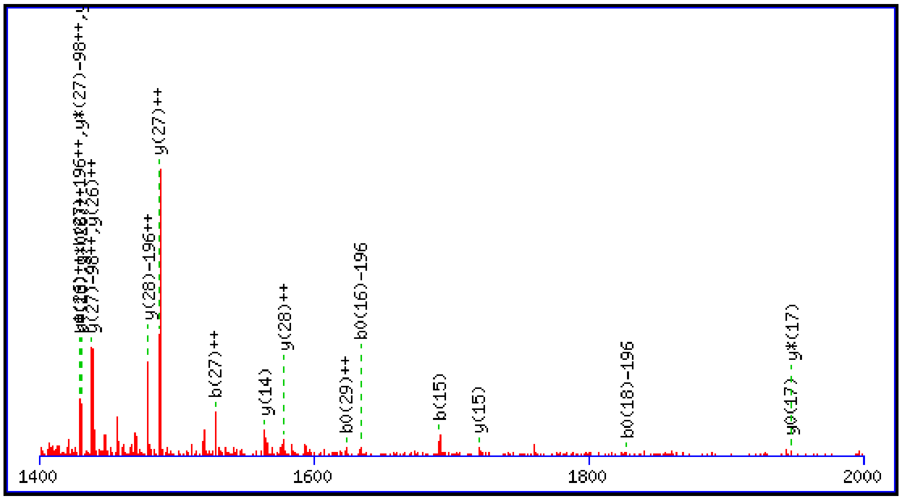

Monoisotopic mass of neutral peptide Mr(calc): 3410.4873
Fixed modifications: Carbamidomethyl (C)
Variable modifications:
T3 : Phospho (ST), with neutral losses 97.9769(shown in table), 0.0000
S7 : Phospho (ST), with neutral losses 97.9769(shown in table), 0.0000
Y23 : Phospho (Y)
Ions Score: 49 **Expect:** 0.0058
Matches (Bold Red): 73/532 fragment ions using 105 most intense peaks

#	b	b^{++}	b*	b^{*++}	b^0	b^{0++}	Seq.	y	y^{++}	y*	y^{*++}	y^0	y^{0++}	#
1	129.1022	65.0548	112.0757	56.5415			K							30
2	257.1608	129.0840	240.1343	120.5708			Q	3087.4459	1544.2266	3070.4194	1535.7133	3069.4353	1535.2213	29
3	**340.1979**	170.6026	323.1714	162.0893	**322.1874**	161.5973	T	2959.3873	**1480.1973**	2942.3608	1471.6840	2941.3768	1471.1920	28
4	437.2507	219.1290	**420.2241**	210.6157	419.2401	210.1237	P	2876.3502	**1438.6787**	2859.3237	**1430.1655**	2858.3397	**1429.6735**	27
5	534.3035	267.6554	517.2769	259.1421	516.2929	258.6501	P	2779.2975	**1390.1524**	2762.2709	1381.6391	2761.2869	1381.1471	26
6	**605.3406**	303.1739	588.3140	294.6606	587.3300	294.1686	A	2682.2447	1341.6260	2665.2181	1333.1127	2664.2341	1332.6207	25
7	**674.3620**	337.6846	**657.3355**	329.1714	656.3515	328.6794	S	2611.2076	1306.1074	2594.1810	1297.5942	2593.1970	1297.1021	24
8	771.4148	386.2110	754.3882	377.6978	753.4042	377.2057	P	2542.1861	**1271.5967**	2525.1596	**1263.0834**	2524.1756	**1262.5914**	23
9	858.4468	429.7270	841.4203	421.2138	840.4362	420.7218	S	2445.1334	**1223.0703**	2428.1068	**1214.5570**	2427.1228	1214.0650	22
10	**955.4996**	478.2534	**938.4730**	469.7402	937.4890	469.2481	P	2358.1013	**1179.5543**	2341.0748	1171.0410	2340.0908	1170.5490	21
11	1083.5582	542.2827	**1066.5316**	533.7694	1065.5476	533.2774	Q	2261.0486	1131.0279	2244.0220	1122.5146	2243.0380	1122.0226	20
12	**1180.6109**	590.8091	1163.5844	582.2958	1162.6004	581.8038	P	2132.9900	**1066.9986**	2115.9634	1058.4854	2114.9794	**1057.9933**	19
13	1251.6480	626.3277	1234.6215	617.8144	1233.6375	617.3224	A	2035.9372	**1018.4722**	2018.9107	1009.9590	2017.9267	1009.4670	18
14	1380.6906	690.8490	1363.6641	682.3357	1362.6801	681.8437	E	1964.9001	982.9537	**1947.8736**	974.4404	**1946.8895**	973.9484	17
15	1495.7176	748.3624	1478.6910	739.8491	1477.7070	739.3571	D	1835.8575	**918.4324**	1818.8310	909.9191	1817.8470	909.4271	16
16	1651.8187	826.4130	1634.7921	817.8997	**1633.8081**	817.4077	R	**1720.8306**	**860.9189**	1703.8040	**852.4056**	1702.8200	**851.9136**	15
17	1748.8714	874.9394	1731.8449	866.4261	1730.8609	865.9341	P	**1564.7295**	782.8684	1547.7029	774.3551	1546.7189	**773.8631**	14

**18**	1845.9242	923.4657	1828.8977	914.9525	**1827.9136**	914.4605	**P**	1467.6767	734.3420	1450.6501	725.8287	1449.6661	725.3367	**13**
**19**	1932.9562	966.9818	1915.9297	958.4685	1914.9457	957.9765	**S**	1370.6239	685.8156	1353.5974	677.3023	1352.6134	676.8103	**12**
**20**	2019.9883	1010.4978	2002.9617	1001.9845	2001.9777	1001.4925	**S**	1283.5919	642.2996	1266.5654	633.7863	1265.5813	633.2943	**11**
**21**	2117.0410	1059.0242	2100.0145	**1050.5109**	2099.0305	1050.0189	**P**	1196.5599	598.7836	**1179.5333**	590.2703	1178.5493	589.7783	**10**
**22**	2230.1251	1115.5662	2213.0985	1107.0529	2212.1145	**1106.5609**	**I**	**1099.5071**	550.2572	1082.4806	541.7439	1081.4965	541.2519	**9**
**23**	2473.1547	1237.0810	2456.1282	1228.5677	2455.1442	1228.0757	**Y**	986.4230	493.7152	969.3965	485.2019	968.4125	484.7099	**8**
**24**	2602.1973	1301.6023	2585.1708	1293.0890	2584.1868	1292.5970	**E**	**743.3934**	372.2003	726.3668	363.6871	725.3828	363.1951	**7**
**25**	2717.2243	1359.1158	2700.1977	1350.6025	2699.2137	1350.1105	**D**	**614.3508**	307.6790	597.3243	299.1658	596.3402	298.6738	**6**
**26**	2788.2614	1394.6343	2771.2348	1386.1211	2770.2508	1385.6291	**A**	**499.3239**	250.1656	482.2973	241.6523			**5**
**27**	2859.2985	**1430.1529**	2842.2720	1421.6396	2841.2879	1421.1476	**A**	**428.2867**	214.6470	411.2602	206.1337			**4**
**28**	2956.3513	1478.6793	2939.3247	1470.1660	2938.3407	1469.6740	**P**	**357.2496**	179.1285	**340.2231**	170.6152			**3**
**29**	3069.4353	1535.2213	3052.4088	1526.7080	3051.4248	1526.2160	**L**	260.1969	130.6021	243.1703	122.0888			**2**
**30**							**K**	147.1128	74.0600	130.0863	65.5468			**1**

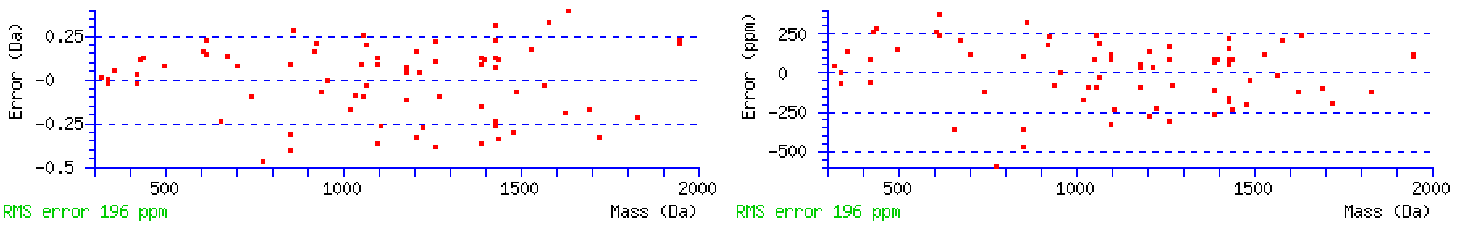

All matches to this query

Score	Mr(calc):	Delta	Sequence
49.2	3410.4873	0.0104	KQTPPASPSPQPAEDRPPSSPIYEDAAPLK
46.3	3410.4873	0.0104	KQTPPASPSPQPAEDRPPSSPIYEDAAPLK
46.3	3410.4873	0.0104	KQTPPASPSPQPAEDRPPSSPIYEDAAPLK
44.0	3410.4873	0.0104	KQTPPASPSPQPAEDRPPSSPIYEDAAPLK
44.0	3410.4873	0.0104	KQTPPASPSPQPAEDRPPSSPIYEDAAPLK
43.8	3410.4873	0.0104	KQTPPASPSPQPAEDRPPSSPIYEDAAPLK
32.9	3410.4873	0.0104	KQTPPASPSPQPAEDRPPSSPIYEDAAPLK
28.3	3410.4873	0.0104	KQTPPASPSPQPAEDRPPSSPIYEDAAPLK
28.3	3410.4873	0.0104	KQTPPASPSPQPAEDRPPSSPIYEDAAPLK
27.4	3410.4873	0.0104	KQTPPASPSPQPAEDRPPSSPIYEDAAPLK

Spectrum No: 315; Query: 2786; Rank: 1

Peptide View

MS/MS Fragmentation of **LAEGEEEKPEPDGSSEESISTVEEPENETPPAPSR**
Found in **IPI00400578**, Tax_Id=10116 Gene_Symbol=Hp1bp3 Heterochromatin protein 1, binding protein 3

Match to Query 2786: 3831.642162 from(1278.221330,3+)
Title: 100101RatKid_NS_deglyco_22.2297.2297.3.dta
Data file K:\NewmanPaper\Piliang\3SubProteomes\Piliang3SP\mgf5ppm\ERLIC_3SubProteomes5ppm.mgf

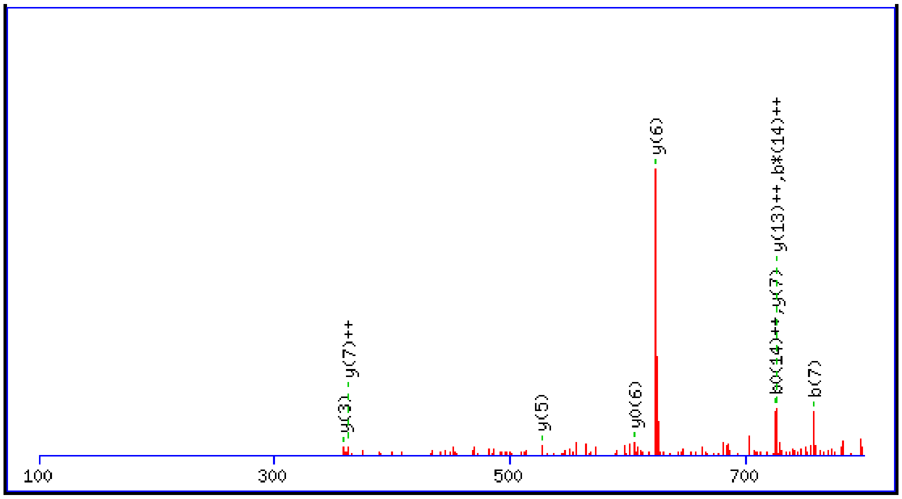

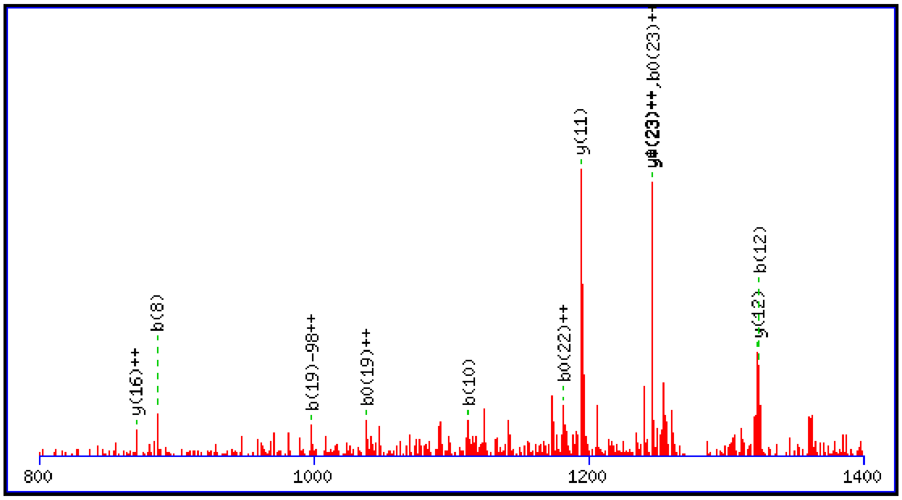

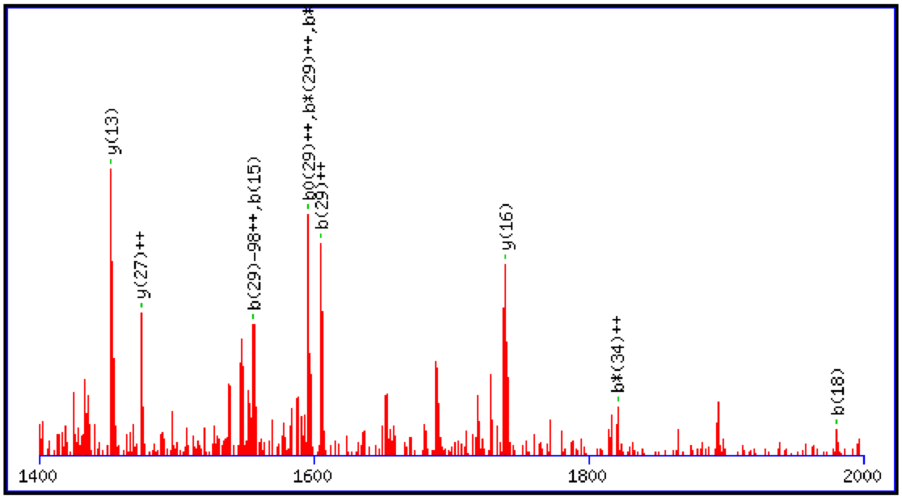

Monoisotopic mass of neutral peptide Mr(calc): 3831.6371
Fixed modifications: Carbamidomethyl (C)
Variable modifications:
S18 : Phospho (ST), with neutral losses 0.0000(shown in table), 97.9769
Ions Score: 49 **Expect:** 0.0063
Matches (Bold Red): 34/592 fragment ions using 42 most intense peaks

#	b	b++	b*	b*++	b0	b0++	Seq.	y	y++	y*	y*++	y0	y0++	#
1	114.0913	57.5493					L							35
2	185.1285	93.0679					A	3719.5603	1860.2838	3702.5338	1851.7705	3701.5498	1851.2785	34
3	314.1710	157.5892			296.1605	148.5839	E	3648.5232	1824.7653	3631.4967	1816.2520	3630.5127	1815.7600	33
4	371.1925	186.0999			353.1819	177.0946	G	3519.4806	1760.2440	3502.4541	1751.7307	3501.4701	1751.2387	32
5	500.2351	250.6212			482.2245	241.6159	E	3462.4592	1731.7332	3445.4326	1723.2200	3444.4486	1722.7279	31

6	629.2777	315.1425			611.2671	306.1372	E	3333.4166	1667.2119	3316.3900	1658.6987	3315.4060	1658.2066	30
7	**758.3203**	379.6638			740.3097	370.6585	E	3204.3740	1602.6906	3187.3474	1594.1774	3186.3634	1593.6854	29
8	**886.4153**	443.7113	869.3887	435.1980	868.4047	434.7060	K	3075.3314	1538.1693	3058.3048	1529.6561	3057.3208	1529.1641	28
9	983.4680	492.2376	966.4415	483.7244	965.4575	483.2324	P	2947.2364	**1474.1219**	2930.2099	1465.6086	2929.2259	1465.1166	27
10	**1112.5106**	556.7589	1095.4841	548.2457	1094.5000	547.7537	E	2850.1837	1425.5955	2833.1571	1417.0822	2832.1731	1416.5902	26
11	1209.5634	605.2853	1192.5368	596.7721	1191.5528	596.2800	P	2721.1411	1361.0742	2704.1145	1352.5609	2703.1305	1352.0689	25
12	**1324.5903**	662.7988	1307.5638	654.2855	1306.5798	653.7935	D	2624.0883	1312.5478	2607.0618	1304.0345	2606.0777	1303.5425	24
13	1381.6118	691.3095	1364.5852	682.7963	1363.6012	682.3042	G	2509.0614	1255.0343	2492.0348	**1246.5210**	2491.0508	**1246.0290**	23
14	1468.6438	734.8255	1451.6173	**726.3123**	1450.6332	**725.8203**	S	2452.0399	1226.5236	2435.0134	1218.0103	2434.0293	1217.5183	22
15	**1555.6758**	778.3416	1538.6493	769.8283	1537.6653	769.3363	S	2365.0079	1183.0076	2347.9813	1174.4943	2346.9973	1174.0023	21
16	1684.7184	842.8629	1667.6919	834.3496	1666.7079	833.8576	E	2277.9759	1139.4916	2260.9493	1130.9783	2259.9653	1130.4863	20
17	1813.7610	907.3841	1796.7345	898.8709	1795.7505	898.3789	E	2148.9333	1074.9703	2131.9067	1066.4570	2130.9227	1065.9650	19
18	**1980.7594**	990.8833	1963.7328	982.3701	1962.7488	981.8780	S	2019.8907	1010.4490	2002.8641	1001.9357	2001.8801	1001.4437	18
19	2093.8434	1047.4254	2076.8169	1038.9121	2075.8329	**1038.4201**	I	1852.8923	926.9498	1835.8658	918.4365	1834.8817	917.9445	17
20	2180.8755	1090.9414	2163.8489	1082.4281	2162.8649	1081.9361	S	**1739.8082**	**870.4078**	1722.7817	861.8945	1721.7977	861.4025	16
21	2281.9231	1141.4652	2264.8966	1132.9519	2263.9126	1132.4599	T	1652.7762	826.8917	1635.7497	818.3785	1634.7657	817.8865	15
22	2380.9916	1190.9994	2363.9650	1182.4861	2362.9810	**1181.9941**	V	1551.7285	776.3679	1534.7020	767.8546	1533.7180	767.3626	14
23	2510.0342	1255.5207	2493.0076	**1247.0074**	2492.0236	**1246.5154**	E	**1452.6601**	**726.8337**	1435.6336	718.3204	1434.6496	717.8284	13
24	2639.0767	1320.0420	2622.0502	1311.5287	2621.0662	1311.0367	E	**1323.6175**	662.3124	1306.5910	653.7991	1305.6070	653.3071	12
25	2736.1295	1368.5684	2719.1030	1360.0551	2718.1189	1359.5631	P	**1194.5749**	597.7911	1177.5484	589.2778	1176.5644	588.7858	11
26	2865.1721	1433.0897	2848.1456	1424.5764	2847.1615	1424.0844	E	1097.5222	549.2647	1080.4956	540.7515	1079.5116	540.2594	10
27	2979.2150	1490.1112	2962.1885	1481.5979	2961.2045	1481.1059	N	968.4796	484.7434	951.4530	476.2302	950.4690	475.7381	9
28	3108.2576	1554.6324	3091.2311	1546.1192	3090.2471	1545.6272	E	854.4367	427.7220	837.4101	419.2087	836.4261	418.7167	8
29	3209.3053	**1605.1563**	3192.2788	**1596.6430**	3191.2947	**1596.1510**	T	**725.3941**	**363.2007**	708.3675	354.6874	707.3835	354.1954	7
30	3306.3581	1653.6827	3289.3315	1645.1694	3288.3475	1644.6774	P	**624.3464**	312.6768	607.3198	304.1636	**606.3358**	303.6715	6
31	3403.4108	1702.2091	3386.3843	1693.6958	3385.4003	1693.2038	P	**527.2936**	264.1504	510.2671	255.6372	509.2831	255.1452	5
32	3474.4479	1737.7276	3457.4214	1729.2143	3456.4374	1728.7223	A	430.2409	215.6241	413.2143	207.1108	412.2303	206.6188	4
33	3571.5007	1786.2540	3554.4742	1777.7407	3553.4901	1777.2487	P	**359.2037**	180.1055	342.1772	171.5922	341.1932	171.1002	3
34	3658.5327	1829.7700	3641.5062	**1821.2567**	3640.5222	1820.7647	S	262.1510	131.5791	245.1244	123.0659	244.1404	122.5738	2
35							R	175.1190	88.0631	158.0924	79.5498			1

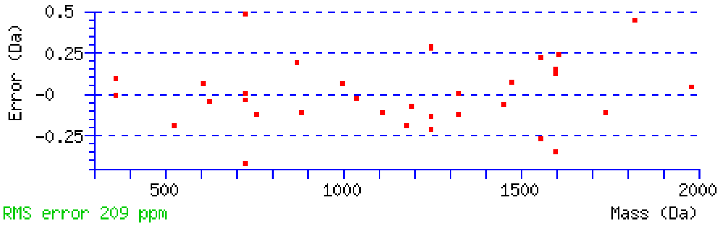

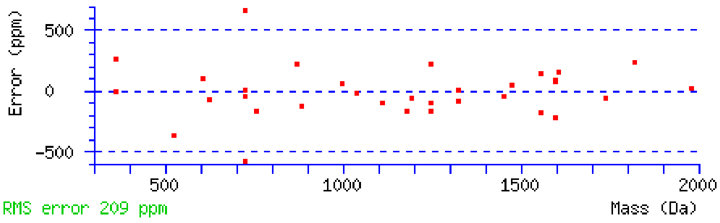

All matches to this query

Score	Mr(calc):	Delta	Sequence
49.2	3831.6371	0.0051	LAEGEEEKPEPDGSSEESISTVEEPENETPPAPSR
49.0	3831.6371	0.0051	LAEGEEEKPEPDGSSEESISTVEEPENETPPAPSR
49.0	3831.6371	0.0051	LAEGEEEKPEPDGSSEESISTVEEPENETPPAPSR
32.7	3831.6371	0.0051	LAEGEEEKPEPDGSSEESISTVEEPENETPPAPSR
32.7	3831.6371	0.0051	LAEGEEEKPEPDGSSEESISTVEEPENETPPAPSR
10.8	3831.6371	0.0051	LAEGEEEKPEPDGSSEESISTVEEPENETPPAPSR
5.4	3831.6061	0.0360	VYCNMETGQTCVFPTQPSVPQKNWYISPNPK
2.4	3829.6273	2.0148	VTEMLVNILNICSDEEMSLVEEEEEGATSVRK
1.8	3831.6229	0.0193	SGSLSSSRESALTNPVSVTKPVVLAAGVVLNSPK
1.6	3829.6511	1.9911	LTLQPVDNSTISLQMGTNKVASQKGMSVYGLGR

Spectrum No: 316; Query: 1557; Rank: 1

Peptide View

MS/MS Fragmentation of **TSLPDAPVGISSESPSDPR**
Found in **IPI00213334**, Tax_Id=10116 Gene_Symbol=- 36 kDa protein

Match to Query 1557: 1990.895148 from(996.454850,2+)
Title: 100101RatKid_NS_deglyco_20.2684.2684.2.dta
Data file K:\NewmanPaper\Piliang\3SubProteomes\Piliang3SP\mgf5ppm\ERLIC_3SubProteomes5ppm.mgf

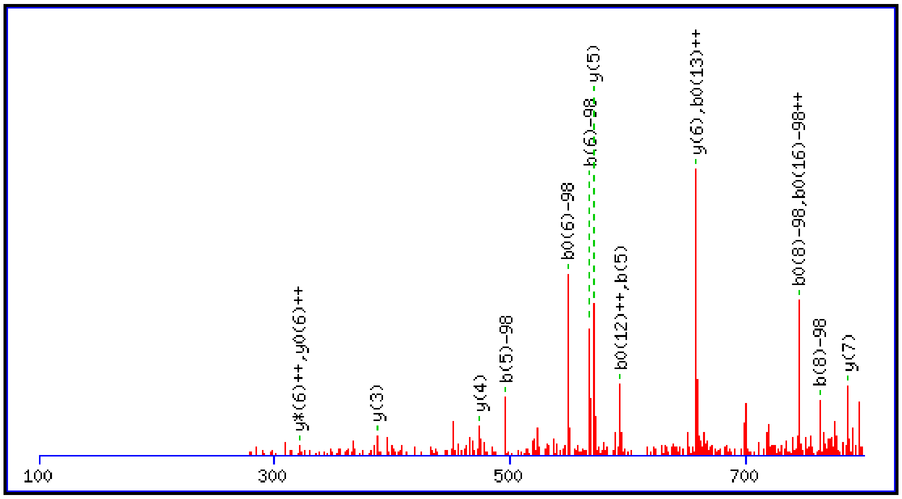

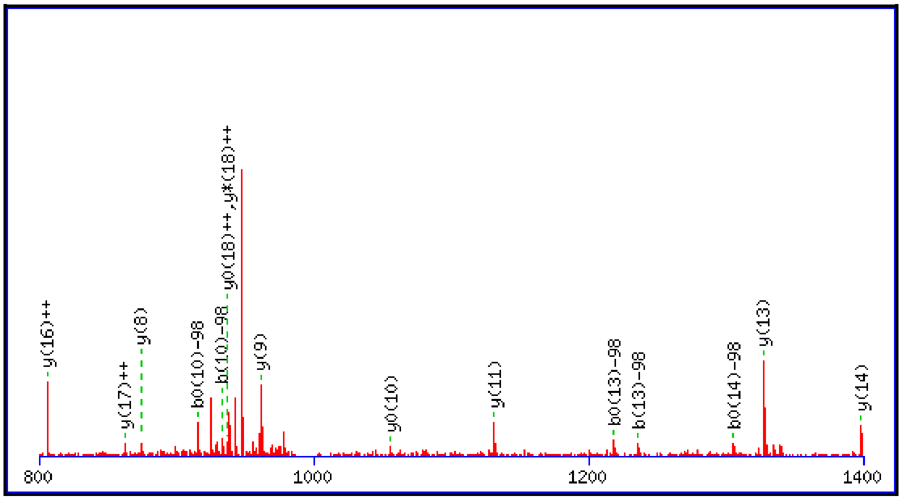

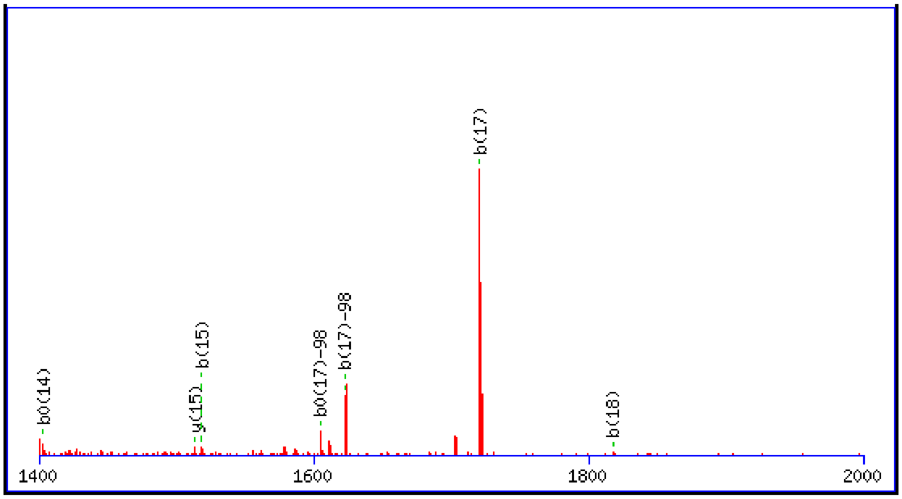

Monoisotopic mass of neutral peptide Mr(calc): 1990.8885
Fixed modifications: Carbamidomethyl (C)
Variable modifications:
S2 : Phospho (ST), with neutral losses 97.9769(shown in table), 0.0000
Ions Score: 49 **Expect:** 0.0032
Matches (Bold Red): 38/250 fragment ions using 75 most intense peaks

#	b	b^{++}	b^{0}	b^{0++}	Seq.	y	y^{++}	y*	y^{*++}	y^{0}	y^{0++}	#
1	102.0550	51.5311	84.0444	42.5258	T							19
2	171.0764	86.0418	153.0658	77.0366	S	1792.8712	896.9392	1775.8446	888.4260	1774.8606	887.9339	18
3	284.1605	142.5839	266.1499	133.5786	L	1723.8497	**862.4285**	1706.8232	853.9152	1705.8392	853.4232	17
4	381.2132	191.1103	363.2027	182.1050	P	1610.7657	**805.8865**	1593.7391	797.3732	1592.7551	796.8812	16
5	**496.2402**	248.6237	478.2296	239.6184	D	**1513.7129**	757.3601	1496.6863	748.8468	1495.7023	748.3548	15
6	**567.2773**	284.1423	**549.2667**	275.1370	A	**1398.6859**	699.8466	1381.6594	691.3333	1380.6754	690.8413	14
7	664.3301	332.6687	646.3195	323.6634	P	**1327.6488**	664.3281	1310.6223	655.8148	1309.6383	655.3228	13
8	**763.3985**	382.2029	**745.3879**	373.1976	V	1230.5961	615.8017	1213.5695	607.2884	1212.5855	606.7964	12
9	820.4199	410.7136	802.4094	401.7083	G	**1131.5277**	566.2675	1114.5011	557.7542	1113.5171	557.2622	11
10	**933.5040**	467.2556	**915.4934**	458.2504	I	1074.5062	537.7567	1057.4796	529.2435	**1056.4956**	528.7515	10
11	1020.5360	510.7717	1002.5255	501.7664	S	**961.4221**	481.2147	944.3956	472.7014	943.4116	472.2094	9
12	1107.5681	554.2877	1089.5575	545.2824	S	**874.3901**	437.6987	857.3636	429.1854	856.3795	428.6934	8
13	**1236.6107**	618.8090	**1218.6001**	609.8037	E	**787.3581**	394.1827	770.3315	385.6694	769.3475	385.1774	7
14	1323.6427	662.3250	**1305.6321**	653.3197	S	**658.3155**	329.6614	641.2889	**321.1481**	640.3049	**320.6561**	6
15	1420.6954	710.8514	1402.6849	701.8461	P	**571.2835**	286.1454	554.2569	277.6321	553.2729	277.1401	5
16	1507.7275	754.3674	1489.7169	**745.3621**	S	**474.2307**	237.6190	457.2041	229.1057	456.2201	228.6137	4
17	**1622.7544**	811.8808	**1604.7438**	802.8756	D	**387.1987**	194.1030	370.1721	185.5897	369.1881	185.0977	3
18	1719.8072	860.4072	1701.7966	851.4019	P	272.1717	136.5895	255.1452	128.0762			2
19					R	175.1190	88.0631	158.0924	79.5498			1

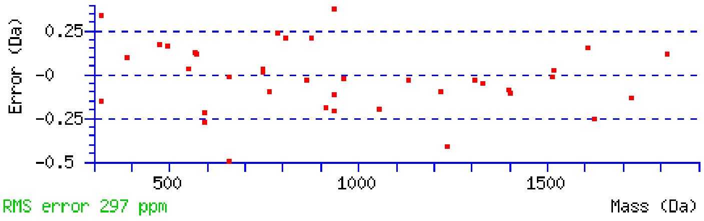

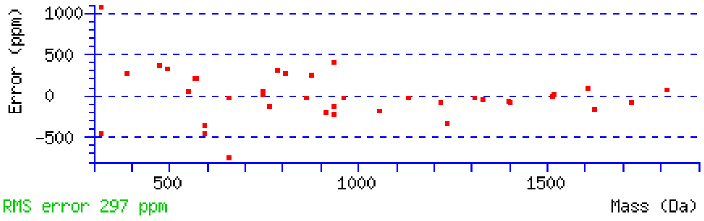

All matches to this query

Score	Mr(calc):	Delta	Sequence
49.1	1990.8885	0.0067	TSLPDAPVGISSESPSDPR
49.1	1990.8885	0.0067	TSLPDAPVGISSESPSDPR

11.5	1990.8885	0.0067	TSLPDAPVGISSESPSDPR
6.3	1990.8885	0.0067	TSLPDAPVGISSESPSDPR
2.1	1990.8972	-0.0020	YFDPKMLRSNDSSVPR
1.1	1989.8802	1.0150	ATSWSGSLSSAMRLMPGR
1.1	1989.8802	1.0150	ATSWSGSLSSAMRLMPGR
1.0	1990.8964	-0.0013	PPNWASMSMVTRCWPR
1.0	1990.8964	-0.0013	PPNWASMSMVTRCWPR
0.5	1989.8802	1.0150	ATSWSGSLSSAMRLMPGR

Spectrum No: 317; Query: 1016; Rank: 1

Peptide View

MS/MS Fragmentation of **DSSQSSDVSLPDCKA**
Found in **IPI00199635**, Tax_Id=10116 Gene_Symbol=RT1-A3 MHC class I protein precursor

Match to Query 1016: 1674.640448 from(838.327500,2+)
Title: 091008RatKidney_NoSalt_29.1164.1164.2.dta
Data file K:\NewmanPaper\Piliang\3SubProteomes\Piliang3SP\mgf5ppm\ERLIC_3SubProteomes5ppm.mgf

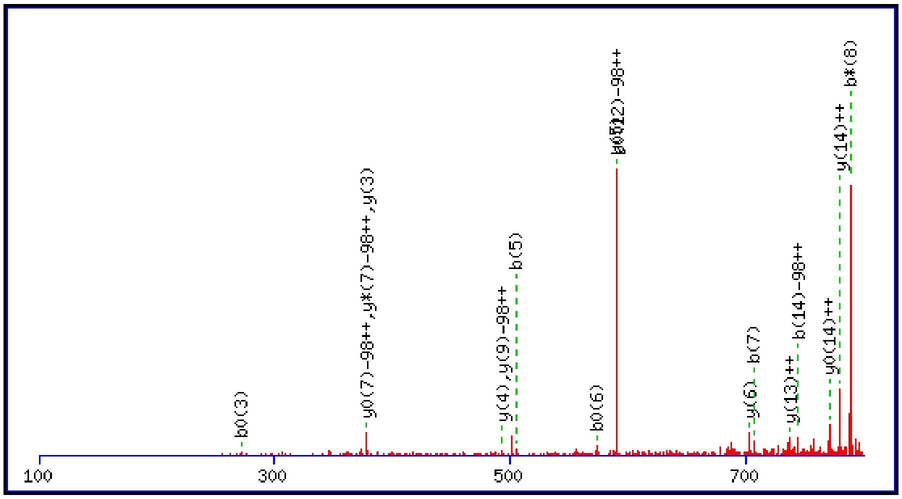

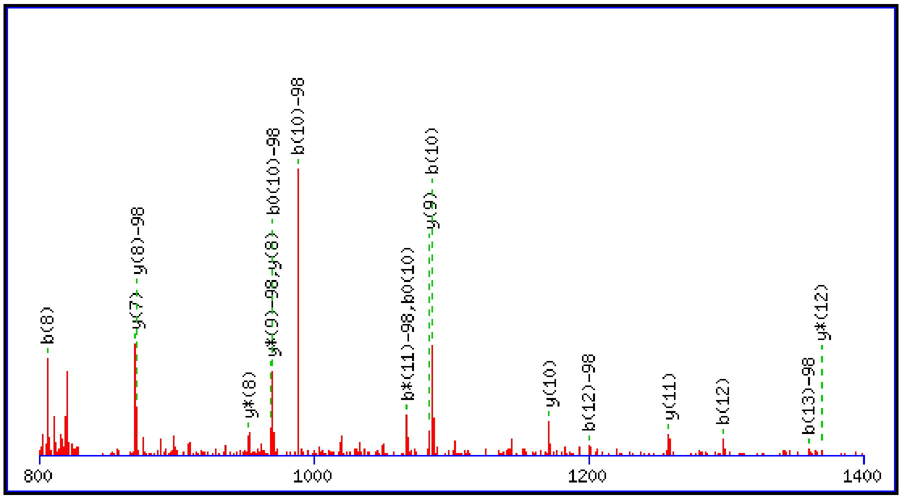

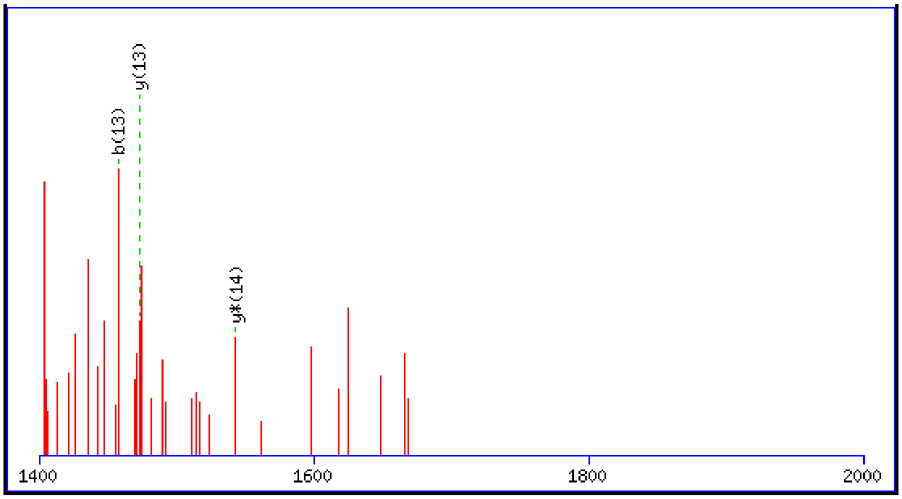

Monoisotopic mass of neutral peptide Mr(calc): 1674.6444
Fixed modifications: Carbamidomethyl (C)
Variable modifications:
S9 : Phospho (ST), with neutral losses 0.0000(shown in table), 97.9769
Ions Score: 49 **Expect:** 0.0012
Matches (Bold Red): 38/238 fragment ions using 59 most intense peaks

#	b	b^{++}	b*	b^{*++}	b^{0}	b^{0++}	Seq.	y	y^{++}	y*	y^{*++}	y^{0}	y^{0++}	#
1	116.0342	58.5207			98.0237	49.5155	D							15
2	203.0662	102.0368			185.0557	93.0315	S	1560.6247	**780.8160**	**1543.5982**	772.3027	1542.6142	**771.8107**	14
3	290.0983	145.5528			**272.0877**	136.5475	S	**1473.5927**	**737.3000**	1456.5662	728.7867	1455.5821	728.2947	13
4	418.1569	209.5821	401.1303	201.0688	400.1463	200.5768	Q	1386.5607	693.7840	**1369.5341**	685.2707	1368.5501	684.7787	12
5	**505.1889**	253.0981	488.1623	244.5848	487.1783	244.0928	S	**1258.5021**	629.7547	1241.4756	621.2414	1240.4915	620.7494	11
6	592.2209	296.6141	575.1944	288.1008	**574.2103**	287.6088	S	**1171.4701**	586.2387	1154.4435	577.7254	1153.4595	577.2334	10
7	**707.2479**	354.1276	690.2213	345.6143	689.2373	345.1223	D	**1084.4380**	542.7227	1067.4115	534.2094	1066.4275	533.7174	9
8	**806.3163**	403.6618	**789.2897**	395.1485	788.3057	394.6565	V	**969.4111**	485.2092	**952.3846**	476.6959	951.4005	476.2039	8
9	973.3146	487.1609	956.2881	478.6477	955.3041	478.1557	S	**870.3427**	435.6750	853.3161	427.1617	852.3321	426.6697	7
10	**1086.3987**	543.7030	1069.3721	535.1897	**1068.3881**	534.6977	L	**703.3443**	352.1758	686.3178	343.6625	685.3338	343.1705	6
11	1183.4514	592.2294	1166.4249	583.7161	1165.4409	583.2241	P	**590.2603**	295.6338	573.2337	287.1205	572.2497	286.6285	5
12	**1298.4784**	649.7428	1281.4518	641.2296	1280.4678	640.7375	D	**493.2075**	247.1074	476.1810	238.5941	475.1969	238.1021	4
13	**1458.5090**	729.7582	1441.4825	721.2449	1440.4985	720.7529	C	**378.1806**	189.5939	361.1540	181.0806			3
14	1586.6040	793.8056	1569.5775	785.2924	1568.5934	784.8004	K	218.1499	109.5786	201.1234	101.0653			2
15							A	90.0550	45.5311					1

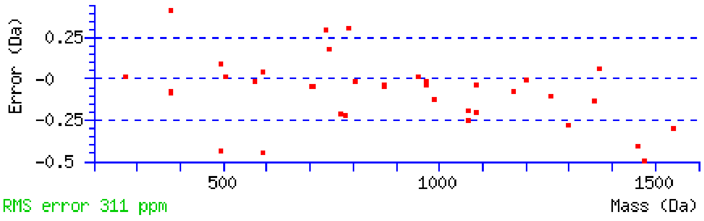

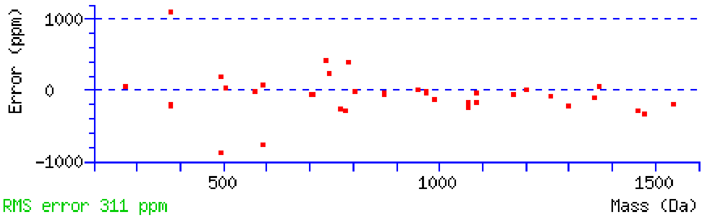

All matches to this query

Score	Mr(calc):	Delta	Sequence
49.0	1674.6444	-0.0040	DSSQSSDVSLPDCKA
28.9	1674.6444	-0.0040	DSSQSSDVSLPDCKA
28.2	1674.6444	-0.0040	DSSQSSDVSLPDCKA
21.6	1674.6444	-0.0040	DSSQSSDVSLPDCKA
20.0	1674.6444	-0.0040	DSSQSSDVSLPDCKA
4.3	1674.6385	0.0019	WQGETQEFCPNAK

3.1	1674.6362	0.0043	CSASTTDLRVDYK
3.1	1674.6362	0.0043	CSASTTDLRVDYK
0.9	1674.6362	0.0043	CSASTTDLRVDYK

Spectrum No: 318; Query: 2220; Rank: 1

Peptide View

MS/MS Fragmentation of **EGEEPTVYSDDEEPKDEAARK**
Found in **IPI00480820**, Tax_Id=10116 Gene_Symbol=Pgrmc1 Membrane-associated progesterone receptor component 1

Match to Query 2220: 2473.026222 from(825.349350,3+)
Title: 100101RatKid_NS_deglyco_21.1076.1076.3.dta
Data file K:\NewmanPaper\Piliang\3SubProteomes\Piliang3SP\mgf5ppm\ERLIC_3SubProteomes5ppm.mgf

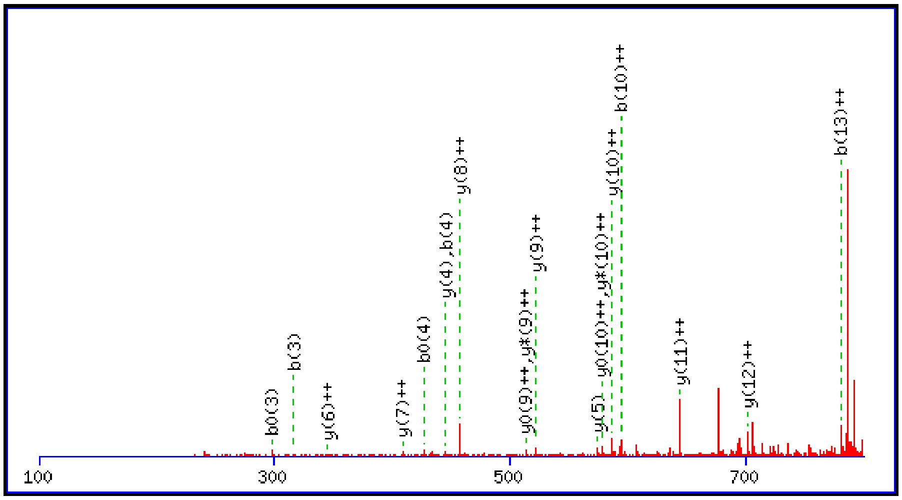

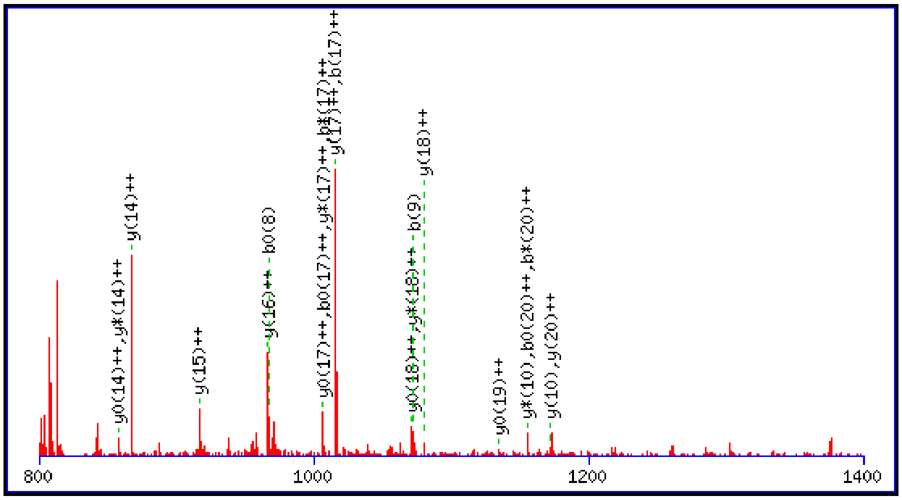

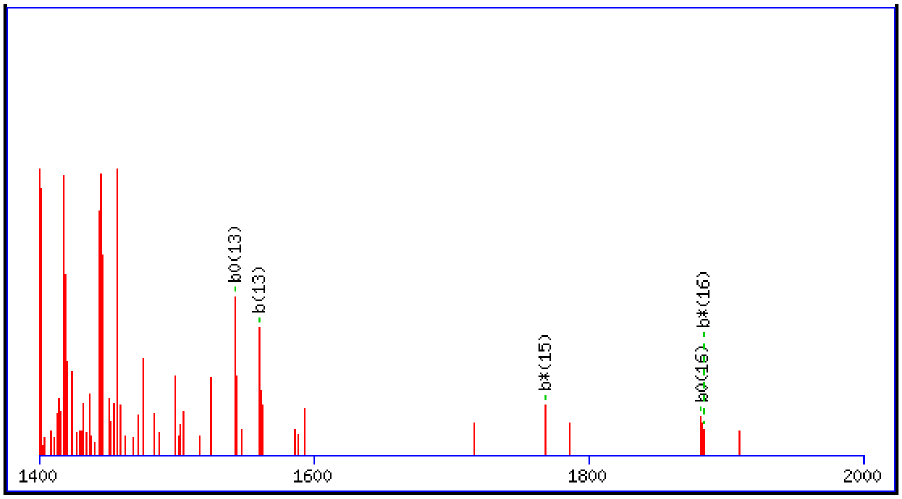

Monoisotopic mass of neutral peptide Mr(calc): 2473.0169
Fixed modifications: Carbamidomethyl (C)
Variable modifications:
Y8 : Phospho (Y)
Ions Score: 49 **Expect:** 0.0035
Matches (Bold Red): 46/204 fragment ions using 92 most intense peaks

#	b	b++	b*	b*++	b0	b0++	Seq.	y	y++	y*	y*++	y0	y0++	#
1	130.0499	65.5286			112.0393	56.5233	E							21
2	187.0713	94.0393			169.0608	85.0340	G	2344.9817	**1172.9945**	2327.9551	1164.4812	2326.9711	1163.9892	20
3	**316.1139**	158.5606			**298.1034**	149.5553	E	2287.9602	1144.4837	2270.9337	1135.9705	2269.9496	**1135.4785**	19
4	**445.1565**	223.0819			**427.1460**	214.0766	E	2158.9176	**1079.9624**	2141.8911	**1071.4492**	2140.9070	**1070.9572**	18
5	542.2093	271.6083			524.1987	262.6030	P	2029.8750	**1015.4411**	2012.8485	**1006.9279**	2011.8645	**1006.4359**	17
6	643.2570	322.1321			625.2464	313.1268	T	1932.8223	**966.9148**	1915.7957	958.4015	1914.8117	957.9095	16
7	742.3254	371.6663			724.3148	362.6610	V	1831.7746	**916.3909**	1814.7480	907.8777	1813.7640	907.3856	15
8	985.3550	493.1812			**967.3445**	484.1759	Y	1732.7062	**866.8567**	1715.6796	**858.3434**	1714.6956	**857.8514**	14
9	**1072.3871**	536.6972			1054.3765	527.6919	S	1489.6765	745.3419	1472.6500	736.8286	1471.6659	736.3366	13
10	1187.4140	**594.2106**			1169.4034	585.2054	D	1402.6445	**701.8259**	1385.6179	693.3126	1384.6339	692.8206	12
11	1302.4409	651.7241			1284.4304	642.7188	D	1287.6175	**644.3124**	1270.5910	635.7991	1269.6070	635.3071	11
12	1431.4835	716.2454			1413.4730	707.2401	E	**1172.5906**	**586.7989**	**1155.5640**	**578.2857**	1154.5800	**577.7937**	10
13	**1560.5261**	**780.7667**			**1542.5156**	771.7614	E	1043.5480	**522.2776**	1026.5215	**513.7644**	1025.5374	**513.2724**	9
14	1657.5789	829.2931			1639.5683	820.2878	P	914.5054	**457.7563**	897.4789	449.2431	896.4948	448.7511	8
15	1785.6739	893.3406	**1768.6473**	884.8273	1767.6633	884.3353	K	817.4526	**409.2300**	800.4261	400.7167	799.4421	400.2247	7
16	1900.7008	950.8540	**1883.6743**	942.3408	**1882.6902**	941.8488	D	689.3577	**345.1825**	672.3311	336.6692	671.3471	336.1772	6
17	2029.7434	**1015.3753**	2012.7168	**1006.8621**	2011.7328	**1006.3701**	E	**574.3307**	287.6690	557.3042	279.1557	556.3202	278.6637	5
18	2100.7805	1050.8939	2083.7540	1042.3806	2082.7699	1041.8886	A	**445.2881**	223.1477	428.2616	214.6344			4
19	2171.8176	1086.4124	2154.7911	1077.8992	2153.8071	1077.4072	A	374.2510	187.6292	357.2245	179.1159			3
20	2327.9187	1164.4630	2310.8922	**1155.9497**	2309.9082	**1155.4577**	R	303.2139	152.1106	286.1874	143.5973			2
21							K	147.1128	74.0600	130.0863	65.5468			1

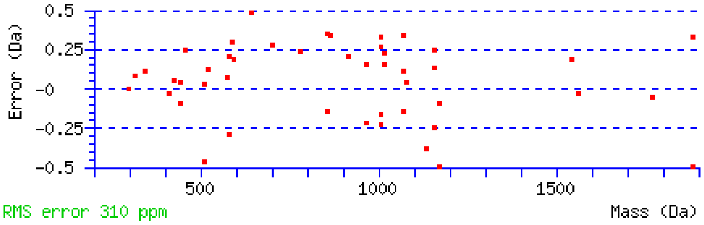

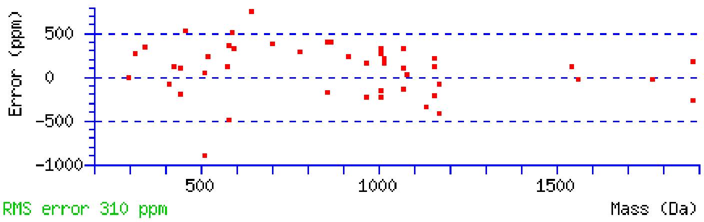

All matches to this query

Score	Mr(calc):	Delta	Sequence

49.0	2473.0169	0.0093	EGEEPTVYSDDEEPKDEAARK
47.8	2473.0169	0.0093	EGEEPTVYSDDEEPKDEAARK
33.7	2473.0169	0.0093	EGEEPTVYSDDEEPKDEAARK
1.4	2471.0125	2.0137	IGKPMFIMHLNSCPSASYNK
1.3	2471.0125	2.0137	IGKPMFIMHLNSCPSASYNK
1.2	2473.0064	0.0198	HCCISYLKEKSCVAGVMGAK
1.2	2471.0187	2.0075	FYRWISERYPCLSEVVK
0.7	2471.0342	1.9920	LNDTTYKVGHGCFQTTEGKR
0.7	2471.0359	1.9903	SSTSGMSTCWTQRXSKSWTAR
0.6	2471.0359	1.9903	SSTSGMSTCWTQRXSKSWTAR

Spectrum No: 319; Query: 2595; Rank: 1

Peptide View

MS/MS Fragmentation of **SPPDQSAVPNTPPSTPVKLEGGLPQEPTSR**
Found in **IPI00327244**, Tax_Id=10116 Gene_Symbol=Add1 Isoform 1 of Alpha-adducin

Match to Query 2595: 3162.528282 from(1055.183370,3+)
Title: 091008RatKidney_NH4Format01_19.2861.2861.3.dta
Data file K:\NewmanPaper\Piliang\3SubProteomes\Piliang3SP\mgf5ppm\ERLIC_3SubProteomes5ppm.mgf

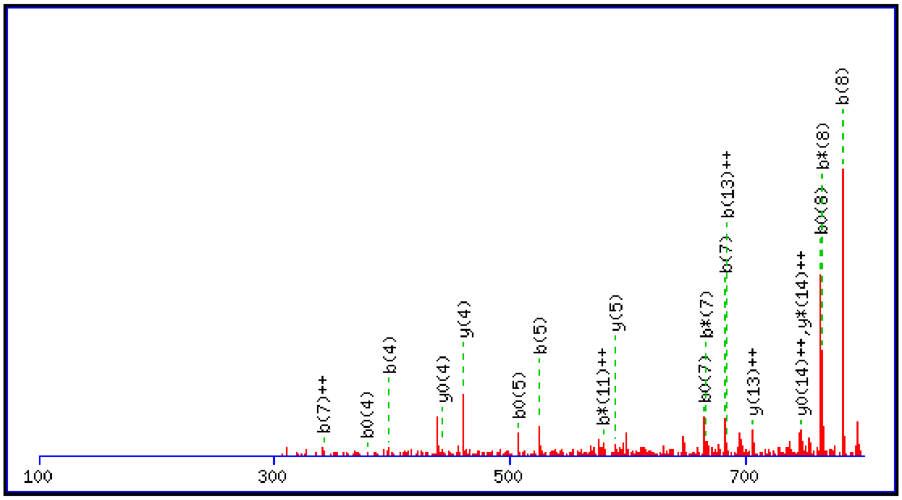

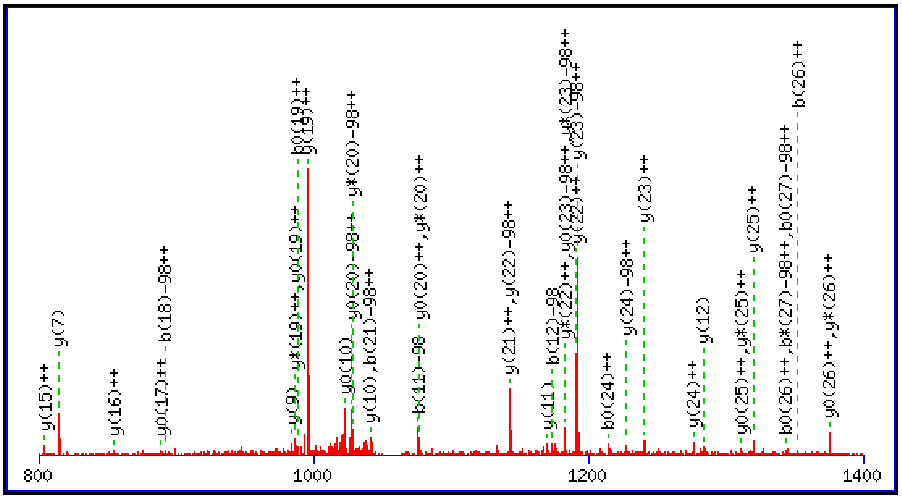

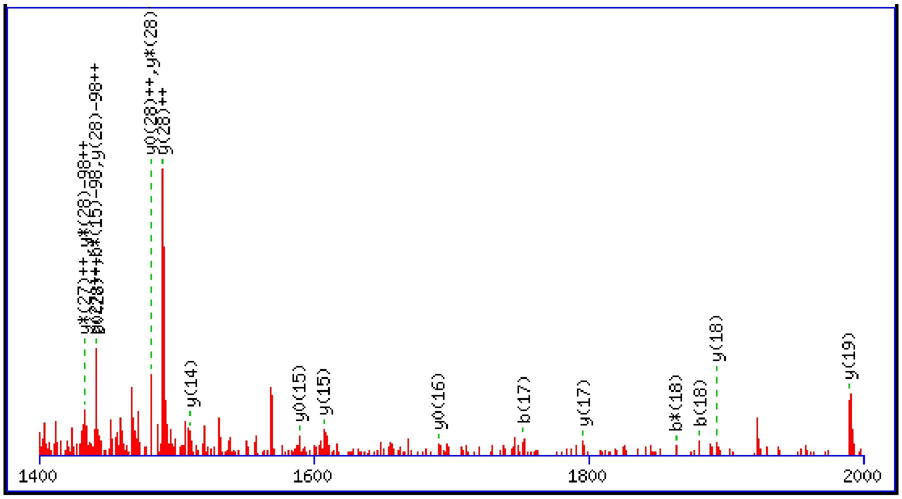

Monoisotopic mass of neutral peptide Mr(calc): 3162.5234
Fixed modifications: Carbamidomethyl (C)
Variable modifications:
T11 : Phospho (ST), with neutral losses 0.0000(shown in table), 97.9769
Ions Score: 49 **Expect:** 0.0039
Matches (Bold Red): 80/512 fragment ions using 161 most intense peaks

#	b	b^{++}	b*	b^{*++}	b^0	b^{0++}	Seq.	y	y^{++}	y*	y^{*++}	y^0	y^{0++}	#
1	88.0393	44.5233			70.0287	35.5180	S							30
2	185.0921	93.0497			167.0815	84.0444	P	3076.4987	1538.7530	3059.4721	1530.2397	3058.4881	1529.7477	29
3	282.1448	141.5761			264.1343	132.5708	P	2979.4459	**1490.2266**	2962.4194	**1481.7133**	2961.4354	**1481.2213**	28
4	**397.1718**	199.0895			**379.1612**	190.0842	D	2882.3932	**1441.7002**	2865.3666	**1433.1869**	2864.3826	1432.6949	27
5	**525.2304**	263.1188	508.2038	254.6055	**507.2198**	254.1135	Q	2767.3662	1384.1867	2750.3397	**1375.6735**	2749.3556	**1375.1815**	26
6	612.2624	306.6348	595.2358	298.1216	594.2518	297.6295	S	2639.3076	**1320.1575**	2622.2811	**1311.6442**	2621.2971	**1311.1522**	25
7	**683.2995**	**342.1534**	**666.2729**	333.6401	**665.2889**	333.1481	A	2552.2756	**1276.6414**	2535.2491	1268.1282	2534.2650	1267.6362	24
8	**782.3679**	391.6876	**765.3414**	383.1743	**764.3573**	382.6823	V	2481.2385	**1241.1229**	2464.2119	1232.6096	2463.2279	1232.1176	23
9	879.4207	440.2140	862.3941	431.7007	861.4101	431.2087	P	2382.1701	**1191.5887**	2365.1435	**1183.0754**	2364.1595	1182.5834	22
10	993.4636	497.2354	976.4371	488.7222	975.4530	488.2302	N	2285.1173	**1143.0623**	2268.0908	1134.5490	2267.1067	1134.0570	21
11	1174.4776	587.7424	1157.4511	**579.2292**	1156.4670	578.7372	T	2171.0744	1086.0408	2154.0478	**1077.5276**	2153.0638	**1077.0355**	20
12	1271.5304	636.2688	1254.5038	627.7555	1253.5198	627.2635	P	**1990.0604**	**995.5338**	1973.0338	**987.0206**	1972.0498	**986.5285**	19
13	1368.5831	**684.7952**	1351.5566	676.2819	1350.5726	675.7899	P	**1893.0076**	947.0074	1875.9811	938.4942	1874.9971	938.0022	18
14	1455.6152	728.3112	1438.5886	719.7979	1437.6046	719.3059	S	**1795.9549**	898.4811	1778.9283	889.9678	1777.9443	**889.4758**	17
15	1556.6628	778.8351	1539.6363	770.3218	1538.6523	769.8298	T	1708.9228	**854.9651**	1691.8963	846.4518	**1690.9123**	845.9598	16
16	1653.7156	827.3614	1636.6891	818.8482	1635.7050	818.3562	P	**1607.8751**	**804.4412**	1590.8486	795.9279	**1589.8646**	795.4359	15
17	**1752.7840**	876.8956	1735.7575	868.3824	1734.7735	867.8904	V	**1510.8224**	755.9148	1493.7958	**747.4016**	1492.8118	**746.9095**	14
18	**1880.8790**	940.9431	**1863.8524**	932.4299	1862.8684	931.9378	K	1411.7540	**706.3806**	1394.7274	697.8673	1393.7434	697.3753	13
19	1993.9630	997.4852	1976.9365	988.9719	1975.9525	**988.4799**	L	**1283.6590**	642.3331	1266.6325	633.8199	1265.6484	633.3279	12
20	2123.0056	1062.0065	2105.9791	1053.4932	2104.9951	1053.0012	E	**1170.5749**	585.7911	1153.5484	577.2778	1152.5644	576.7858	11
21	2180.0271	1090.5172	2163.0006	1082.0039	2162.0165	1081.5119	G	**1041.5324**	521.2698	1024.5058	512.7565	**1023.5218**	512.2645	10
22	2237.0486	1119.0279	2220.0220	1110.5146	2219.0380	1110.0226	G	**984.5109**	492.7591	967.4843	484.2458	966.5003	483.7538	9
23	2350.1326	1175.5700	2333.1061	1167.0567	2332.1221	1166.5647	L	927.4894	464.2483	910.4629	455.7351	909.4789	455.2431	8
24	2447.1854	1224.0963	2430.1588	1215.5831	2429.1748	**1215.0911**	P	**814.4054**	407.7063	797.3788	399.1930	796.3948	398.7010	7
25	2575.2440	1288.1256	2558.2174	1279.6123	2557.2334	1279.1203	Q	717.3526	359.1799	700.3260	350.6667	699.3420	350.1747	6
26	2704.2866	**1352.6469**	2687.2600	1344.1336	2686.2760	**1343.6416**	E	**589.2940**	295.1506	572.2675	286.6374	571.2835	286.1454	5
27	2801.3393	1401.1733	2784.3128	1392.6600	2783.3288	1392.1680	P	**460.2514**	230.6293	443.2249	222.1161	**442.2409**	221.6241	4
28	2902.3870	1451.6971	2885.3605	1443.1839	2884.3764	**1442.6919**	T	363.1987	182.1030	346.1721	173.5897	345.1881	173.0977	3
29	2989.4190	1495.2132	2972.3925	1486.6999	2971.4085	1486.2079	S	262.1510	131.5791	245.1244	123.0659	244.1404	122.5738	2
30							R	175.1190	88.0631	158.0924	79.5498			1

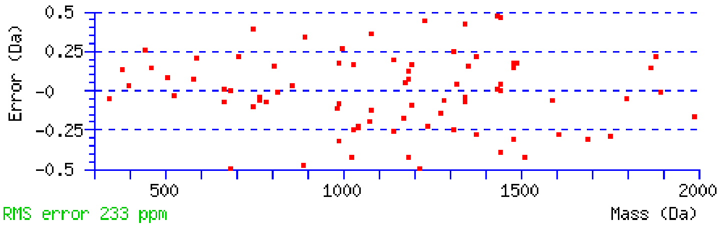

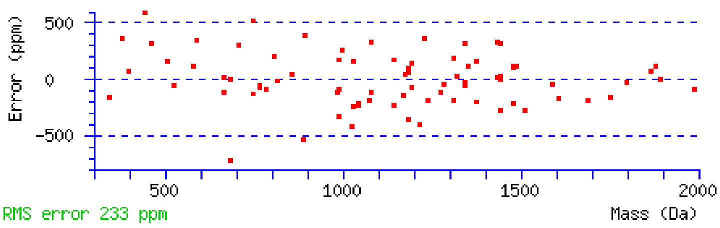

All matches to this query

Score	Mr(calc):	Delta	Sequence
49.0	3162.5234	0.0048	SPPDQSAVPNTPPSTPVKLEGGLPQEPTSR
32.0	3162.5234	0.0048	SPPDQSAVPNTPPSTPVKLEGGLPQEPTSR
29.0	3162.5234	0.0048	SPPDQSAVPNTPPSTPVKLEGGLPQEPTSR
27.1	3162.5234	0.0048	SPPDQSAVPNTPPSTPVKLEGGLPQEPTSR
18.5	3162.5234	0.0048	SPPDQSAVPNTPPSTPVKLEGGLPQEPTSR
7.8	3162.5234	0.0048	SPPDQSAVPNTPPSTPVKLEGGLPQEPTSR
7.5	3162.5234	0.0048	SPPDQSAVPNTPPSTPVKLEGGLPQEPTSR
2.7	3162.5147	0.0136	EVMSLLQNLEMRNIEHCEKFQELQK
2.7	3161.5307	0.9976	EVMSLLQNLEMRNIEHCEKFQELQK
2.7	3162.5147	0.0136	EVMSLLQNLEMRNIEHCEKFQELQK

Spectrum No: 320; Query: 2835; Rank: 1

Peptide View

MS/MS Fragmentation of **RPTLAWLEDSSSCSDIPKLEGDSDDLLEDSDSEEHSR**
Found in **IPI00360081**, Tax_Id=10116 Gene_Symbol=Birc6_predicted similar to baculoviral IAP repeat-containing 6

Match to Query 2835: 4269.831816 from(1068.465230,4+)
Title: 100101RatKid_NS_deglyco_18.4551.4551.4.dta
Data file K:\NewmanPaper\Piliang\3SubProteomes\Piliang3SP\mgf5ppm\ERLIC_3SubProteomes5ppm.mgf

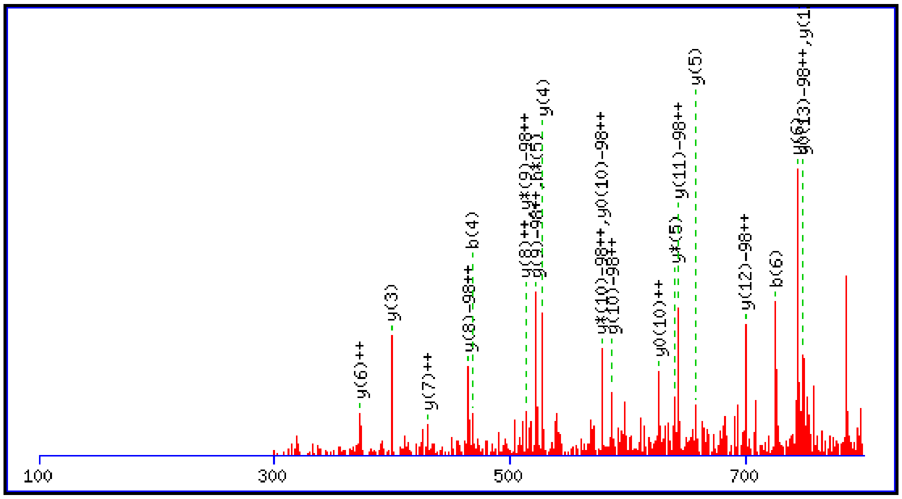

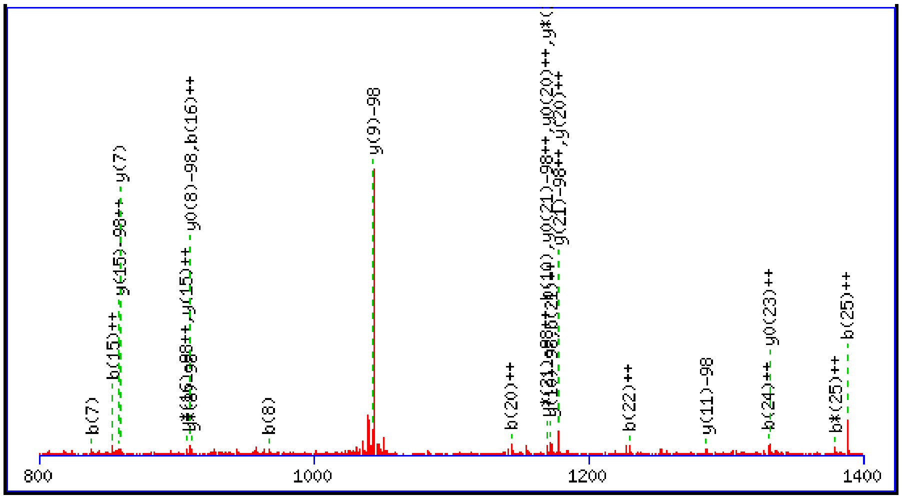

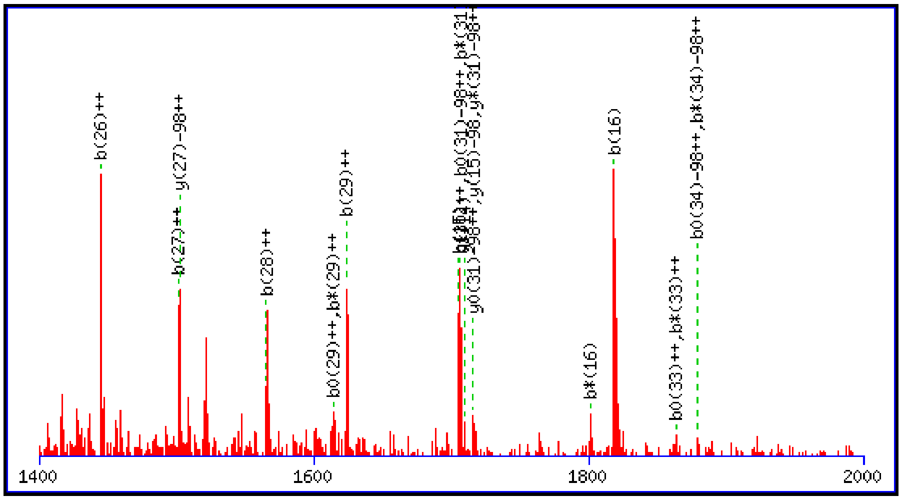

Monoisotopic mass of neutral peptide Mr(calc): 4269.8169
Fixed modifications: Carbamidomethyl (C)
Variable modifications:
S30 : Phospho (ST), with neutral losses 97.9769(shown in table), 0.0000
Ions Score: 49 **Expect:** 0.0068
Matches (Bold Red): 71/642 fragment ions using 97 most intense peaks

#	b	b^{++}	b*	b^{*++}	b^{0}	b^{0++}	Seq.	y	y^{++}	y*	y^{*++}	y^{0}	y^{0++}	#
1	157.1084	79.0578	140.0818	70.5446			R							37
2	254.1612	127.5842	237.1346	119.0709			P	4016.7462	2008.8767	3999.7196	2000.3635	3998.7356	1999.8714	36
3	355.2088	178.1081	338.1823	169.5948	337.1983	169.1028	T	3919.6934	1960.3503	3902.6669	1951.8371	3901.6828	1951.3451	35
4	**468.2929**	234.6501	451.2663	226.1368	450.2823	225.6448	L	3818.6457	1909.8265	3801.6192	1901.3132	3800.6352	1900.8212	34
5	539.3300	270.1686	**522.3035**	261.6554	521.3194	261.1634	A	3705.5617	1853.2845	3688.5351	1844.7712	3687.5511	1844.2792	33
6	**725.4093**	363.2083	708.3828	354.6950	707.3988	354.2030	W	3634.5246	1817.7659	3617.4980	1809.2526	3616.5140	1808.7606	32
7	**838.4934**	419.7503	821.4668	411.2371	820.4828	410.7450	L	3448.4452	1724.7263	3431.4187	**1716.2130**	3430.4347	**1715.7210**	31
8	**967.5360**	484.2716	950.5094	475.7584	949.5254	475.2663	E	3335.3612	1668.1842	3318.3346	1659.6710	3317.3506	1659.1789	30
9	1082.5629	541.7851	1065.5364	533.2718	1064.5524	532.7798	D	3206.3186	1603.6629	3189.2920	1595.1497	3188.3080	1594.6576	29
10	**1169.5949**	585.3011	1152.5684	576.7878	1151.5844	576.2958	S	3091.2916	1546.1495	3074.2651	1537.6362	3073.2811	1537.1442	28
11	1256.6270	628.8171	1239.6004	620.3039	1238.6164	619.8118	S	3004.2596	**1502.6334**	2987.2331	1494.1202	2986.2490	1493.6282	27
12	1343.6590	672.3331	1326.6325	663.8199	1325.6484	663.3279	S	2917.2276	1459.1174	2900.2010	1450.6042	2899.2170	1450.1121	26
13	1503.6897	752.3485	1486.6631	743.8352	1485.6791	743.3432	C	2830.1956	1415.6014	2813.1690	1407.0881	2812.1850	1406.5961	25
14	1590.7217	795.8645	1573.6951	787.3512	1572.7111	786.8592	S	2670.1649	1335.5861	2653.1384	1327.0728	2652.1543	1326.5808	24
15	**1705.7486**	**853.3780**	1688.7221	844.8647	1687.7381	844.3727	D	2583.1329	1292.0701	2566.1063	1283.5568	2565.1223	1283.0648	23
16	**1818.8327**	**909.9200**	**1801.8061**	901.4067	1800.8221	900.9147	I	2468.1059	1234.5566	2451.0794	1226.0433	2450.0954	1225.5513	22
17	1915.8855	958.4464	1898.8589	949.9331	1897.8749	949.4411	P	2355.0219	**1178.0146**	2337.9953	**1169.5013**	2337.0113	**1169.0093**	21
18	2043.9804	1022.4938	2026.9539	1013.9806	2025.9699	1013.4886	K	2257.9691	1129.4882	2240.9426	1120.9749	2239.9585	1120.4829	20

19	2157.0645	1079.0359	2140.0379	1070.5226	2139.0539	1070.0306	L	2129.8741	1065.4407	2112.8476	1056.9274	2111.8636	1056.4354	19
20	2286.1071	**1143.5572**	2269.0805	1135.0439	2268.0965	1134.5519	E	2016.7901	1008.8987	1999.7635	1000.3854	1998.7795	999.8934	18
21	2343.1285	**1172.0679**	2326.1020	1163.5546	2325.1180	1163.0626	G	1887.7475	944.3774	1870.7209	935.8641	1869.7369	935.3721	17
22	2458.1555	**1229.5814**	2441.1289	1221.0681	2440.1449	1220.5761	D	1830.7260	915.8667	1813.6995	**907.3534**	1812.7155	906.8614	16
23	2545.1875	1273.0974	2528.1610	1264.5841	2527.1769	1264.0921	S	**1715.6991**	**858.3532**	1698.6725	849.8399	1697.6885	849.3479	15
24	2660.2145	**1330.6109**	2643.1879	1322.0976	2642.2039	1321.6056	D	1628.6671	814.8372	1611.6405	806.3239	1610.6565	805.8319	14
25	2775.2414	**1388.1243**	2758.2148	**1379.6111**	2757.2308	1379.1191	D	1513.6401	757.3237	1496.6136	**748.8104**	1495.6295	**748.3184**	13
26	2888.3255	**1444.6664**	2871.2989	1436.1531	2870.3149	1435.6611	L	1398.6132	**699.8102**	1381.5866	691.2969	1380.6026	690.8049	12
27	3001.4095	**1501.2084**	2984.3830	1492.6951	2983.3990	1492.2031	L	**1285.5291**	**643.2682**	1268.5026	634.7549	1267.5185	634.2629	11
28	3130.4521	**1565.7297**	3113.4256	1557.2164	3112.4416	1556.7244	E	**1172.4450**	**586.7262**	1155.4185	**578.2129**	1154.4345	**577.7209**	10
29	3245.4791	**1623.2432**	3228.4525	**1614.7299**	3227.4685	**1614.2379**	D	**1043.4024**	**522.2049**	1026.3759	**513.6916**	1025.3919	513.1996	9
30	3314.5005	1657.7539	3297.4740	1649.2406	3296.4899	1648.7486	S	928.3755	**464.6914**	**911.3490**	456.1781	**910.3649**	455.6861	8
31	3429.5275	1715.2674	3412.5009	**1706.7541**	3411.5169	**1706.2621**	D	**859.3540**	**430.1807**	842.3275	421.6674	841.3435	421.1754	7
32	3516.5595	1758.7834	3499.5329	1750.2701	3498.5489	1749.7781	S	**744.3271**	**372.6672**	727.3006	364.1539	726.3165	363.6619	6
33	3645.6021	1823.3047	3628.5755	1814.7914	3627.5915	1814.2994	E	**657.2951**	329.1512	**640.2685**	320.6379	639.2845	320.1459	5
34	3774.6447	1887.8260	3757.6181	**1879.3127**	3756.6341	**1878.8207**	E	**528.2525**	264.6299	511.2259	256.1166	510.2419	255.6246	4
35	3911.7036	1956.3554	3894.6770	1947.8422	3893.6930	1947.3501	H	**399.2099**	200.1086	382.1833	191.5953	381.1993	191.1033	3
36	3998.7356	1999.8714	3981.7091	1991.3582	3980.7250	1990.8662	S	262.1510	131.5791	245.1244	123.0659	244.1404	122.5738	2
37							R	175.1190	88.0631	158.0924	79.5498			1

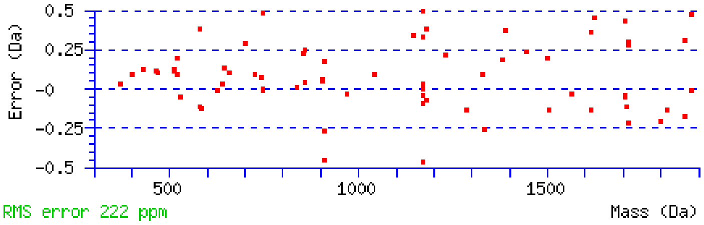

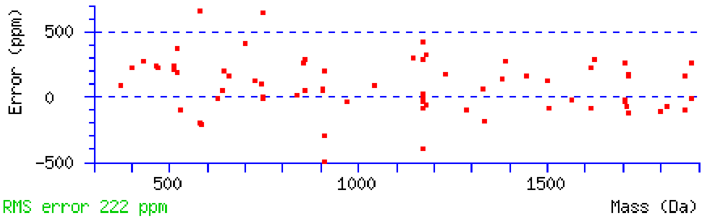

All matches to this query

Score	Mr(calc):	Delta	Sequence
48.9	4269.8169	0.0149	RPTLAWLEDSSSCSDIPKLEGDSDDLLEDSDSEEHSR
38.0	4269.8169	0.0149	RPTLAWLEDSSSCSDIPKLEGDSDDLLEDSDSEEHSR
35.4	4269.8169	0.0149	RPTLAWLEDSSSCSDIPKLEGDSDDLLEDSDSEEHSR
28.9	4269.8169	0.0149	RPTLAWLEDSSSCSDIPKLEGDSDDLLEDSDSEEHSR
11.8	4269.8169	0.0149	RPTLAWLEDSSSCSDIPKLEGDSDDLLEDSDSEEHSR
11.1	4269.8169	0.0149	RPTLAWLEDSSSCSDIPKLEGDSDDLLEDSDSEEHSR
11.1	4269.8169	0.0149	RPTLAWLEDSSSCSDIPKLEGDSDDLLEDSDSEEHSR
9.9	4269.8441	-0.0122	VGVSSSILQGLWISYSTEGLSMALASLRNLYTPNIK
9.7	4268.8707	0.9611	HQPPFTQEEAKETLIFDLKNMNVTLDEPIQITWS
9.7	4268.8707	0.9611	HQPPFTQEEAKETLIFDLKNMNVTLDEPIQITWS

Spectrum No: 321; Query: 1944; Rank: 1

Peptide View

MS/MS Fragmentation of **ESTQPPEDSSPPASSESSSTR**
Found in **IPI00201213**, Tax_Id=10116 Gene_Symbol=LOC501546 LOC501546 protein

Match to Query 1944: 2241.895008 from(1121.954780,2+)
Title: 091008RatKidney_NoSalt_28.627.627.2.dta
Data file K:\NewmanPaper\Piliang\3SubProteomes\Piliang3SP\mgf5ppm\ERLIC_3SubProteomes5ppm.mgf

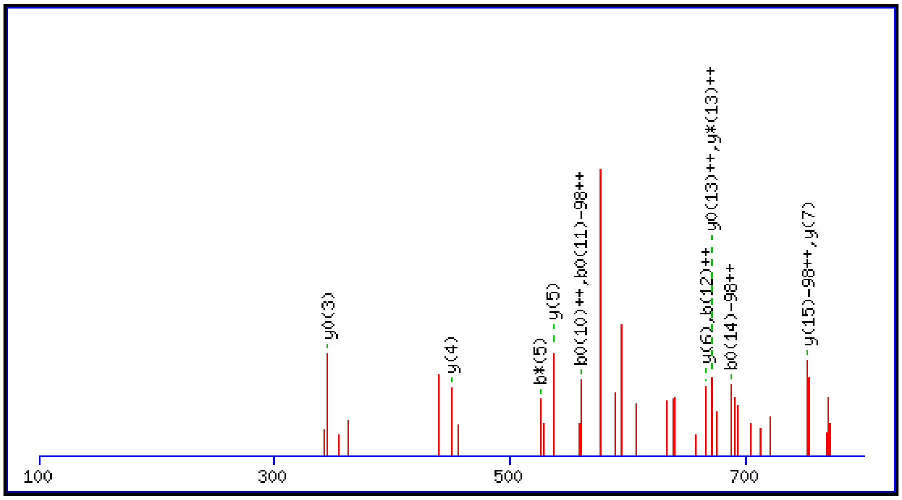

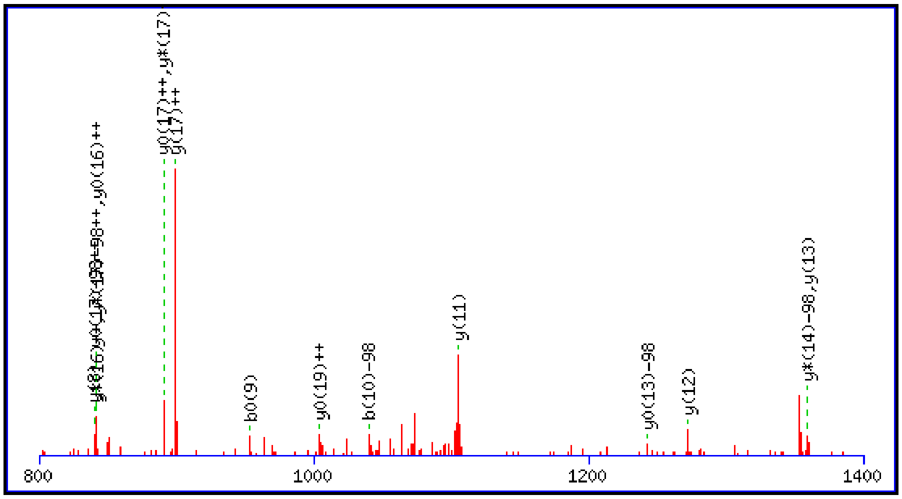

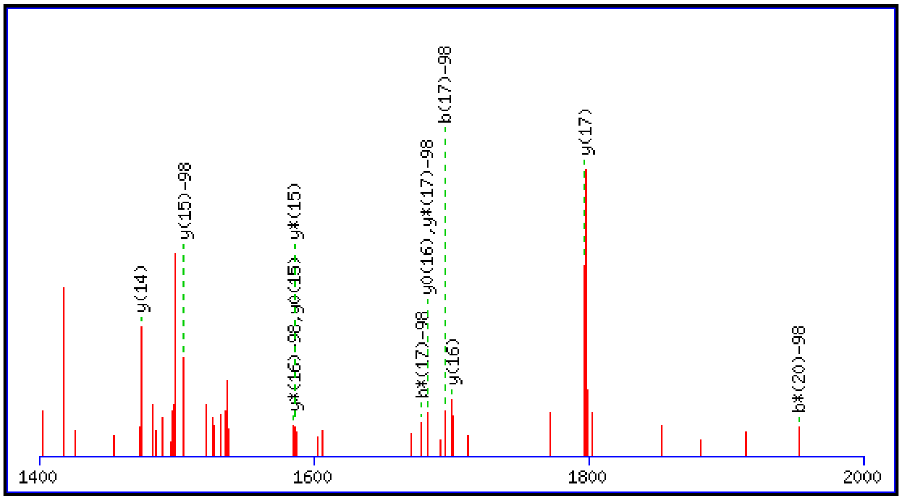

Monoisotopic mass of neutral peptide Mr(calc): 2241.8910
Fixed modifications: Carbamidomethyl (C)
Variable modifications:
S10 : Phospho (ST), with neutral losses 0.0000(shown in table), 97.9769
Ions Score: 49 **Expect:** 0.0028
Matches (Bold Red): 41/352 fragment ions using 63 most intense peaks

#	b	b++	b*	b*++	b0	b0++	Seq.	y	y++	y*	y*++	y0	y0++	#
1	130.0499	65.5286			112.0393	56.5233	E							21
2	217.0819	109.0446			199.0713	100.0393	S	2113.8557	1057.4315	2096.8292	1048.9182	2095.8452	1048.4262	20
3	318.1296	159.5684			300.1190	150.5631	T	2026.8237	1013.9155	2009.7972	1005.4022	2008.8131	**1004.9102**	19
4	446.1882	223.5977	429.1616	215.0844	428.1776	214.5924	Q	1925.7760	963.3917	1908.7495	954.8784	1907.7655	954.3864	18
5	543.2409	272.1241	**526.2144**	263.6108	525.2304	263.1188	P	**1797.7175**	**899.3624**	1780.6909	**890.8491**	1779.7069	**890.3571**	17

**6**	640.2937	320.6505	623.2671	312.1372	622.2831	311.6452	**P**	**1700.6647**	850.8360	1683.6381	**842.3227**	**1682.6541**	**841.8307**	**16**
**7**	769.3363	385.1718	752.3097	376.6585	751.3257	376.1665	**E**	1603.6119	802.3096	**1586.5854**	793.7963	**1585.6014**	793.3043	**15**
**8**	884.3632	442.6852	867.3367	434.1720	866.3527	433.6800	**D**	**1474.5693**	737.7883	1457.5428	729.2750	1456.5588	728.7830	**14**
**9**	971.3952	486.2013	954.3687	477.6880	**953.3847**	477.1960	**S**	**1359.5424**	680.2748	1342.5158	**671.7616**	1341.5318	**671.2695**	**13**
**10**	1138.3936	569.7004	1121.3670	561.1872	1120.3830	**560.6952**	**S**	**1272.5104**	636.7588	1255.4838	628.2455	1254.4998	627.7535	**12**
**11**	1235.4464	618.2268	1218.4198	609.7135	1217.4358	609.2215	**P**	**1105.5120**	553.2596	1088.4855	544.7464	1087.5014	544.2544	**11**
**12**	1332.4991	**666.7532**	1315.4726	658.2399	1314.4886	657.7479	**P**	1008.4592	504.7333	991.4327	496.2200	990.4487	495.7280	**10**
**13**	1403.5362	702.2718	1386.5097	693.7585	1385.5257	693.2665	**A**	911.4065	456.2069	894.3799	447.6936	893.3959	447.2016	**9**
**14**	1490.5683	745.7878	1473.5417	737.2745	1472.5577	736.7825	**S**	**840.3694**	420.6883	823.3428	412.1750	822.3588	411.6830	**8**
**15**	1577.6003	789.3038	1560.5737	780.7905	1559.5897	780.2985	**S**	**753.3373**	377.1723	736.3108	368.6590	735.3268	368.1670	**7**
**16**	1706.6429	853.8251	1689.6163	845.3118	1688.6323	844.8198	**E**	**666.3053**	333.6563	649.2788	325.1430	648.2947	324.6510	**6**
**17**	1793.6749	897.3411	1776.6484	888.8278	1775.6644	888.3358	**S**	**537.2627**	269.1350	520.2362	260.6217	519.2522	260.1297	**5**
**18**	1880.7069	940.8571	1863.6804	932.3438	1862.6964	931.8518	**S**	**450.2307**	225.6190	433.2041	217.1057	432.2201	216.6137	**4**
**19**	1967.7390	984.3731	1950.7124	975.8599	1949.7284	975.3678	**S**	363.1987	182.1030	346.1721	173.5897	**345.1881**	173.0977	**3**
**20**	2068.7867	1034.8970	2051.7601	1026.3837	2050.7761	1025.8917	**T**	276.1666	138.5870	259.1401	130.0737	258.1561	129.5817	**2**
**21**							**R**	175.1190	88.0631	158.0924	79.5498			**1**

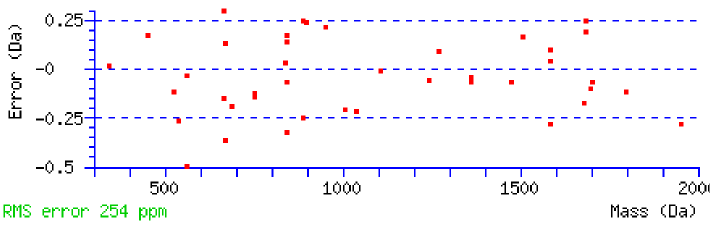

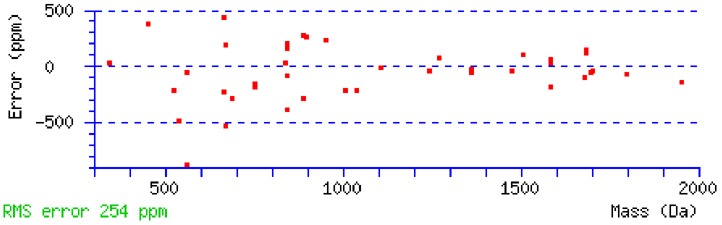

All matches to this query

Score	Mr(calc):	Delta	Sequence
48.9	2241.8910	0.0040	ESTQPPEDSSPPASSESSSTR
39.3	2241.8910	0.0040	ESTQPPEDSSPPASSESSSTR
29.8	2241.8910	0.0040	ESTQPPEDSSPPASSESSSTR
22.3	2241.8910	0.0040	ESTQPPEDSSPPASSESSSTR
10.7	2241.8910	0.0040	ESTQPPEDSSPPASSESSSTR
8.3	2241.8910	0.0040	ESTQPPEDSSPPASSESSSTR
8.3	2241.8910	0.0040	ESTQPPEDSSPPASSESSSTR
6.8	2241.8910	0.0040	ESTQPPEDSSPPASSESSSTR
5.3	2239.8943	2.0007	QVGRGHGGRPRPSSSSDPSC
4.2	2239.8943	2.0007	QVGRGHGGRPRPSSSSDPSC

Spectrum No: 322; Query: 2818; Rank: 1

Peptide View

MS/MS Fragmentation of **NEAAVPAAAAGSPVPVIAIPGILEDELEQTDAEAKDTPTE**
Found in **IPI00204776**, Tax_Id=10116 Gene_Symbol=Akap8 A-kinase anchor protein 8

Match to Query 2818: 4079.967072 from(1360.996300,3+)
Title: 091008RatKidney_NoSalt_25.5773.5773.3.dta
Data file K:\NewmanPaper\Piliang\3SubProteomes\Piliang3SP\mgf5ppm\ERLIC_3SubProteomes5ppm.mgf

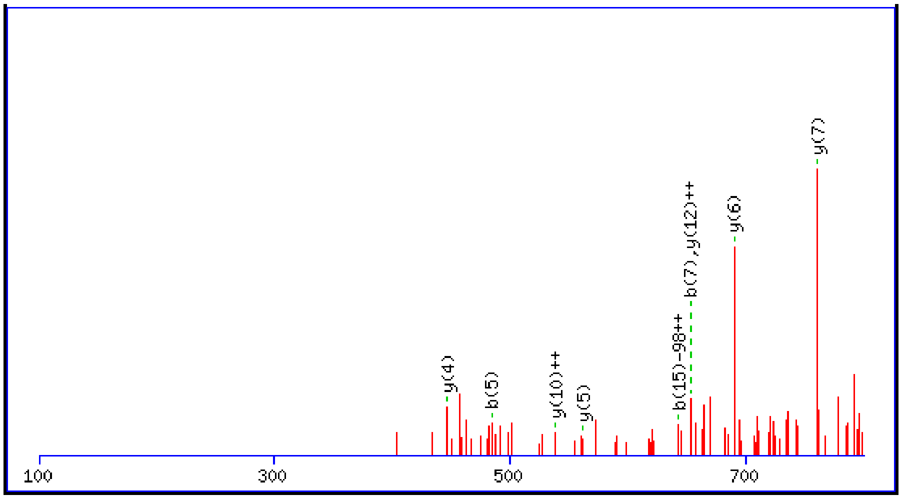

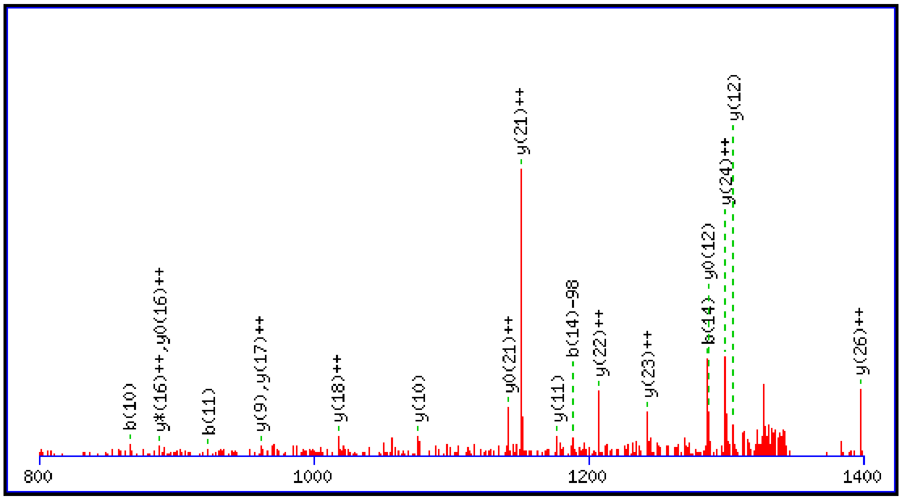

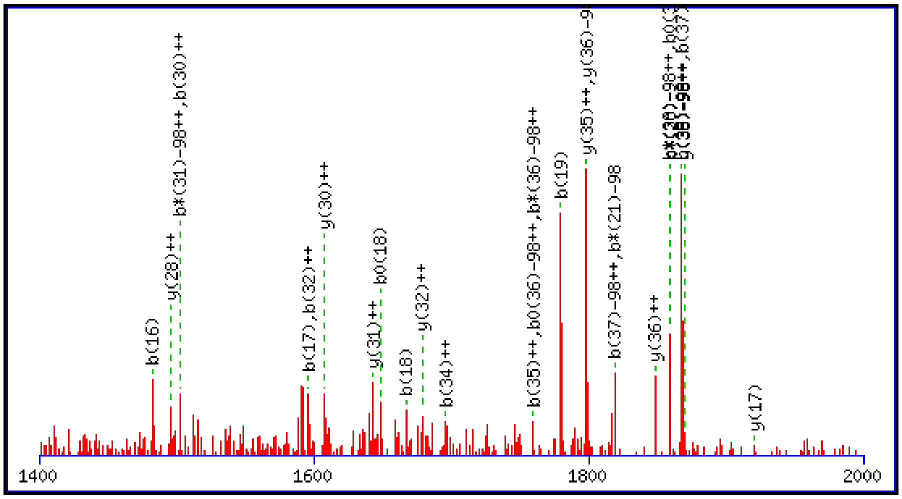

Monoisotopic mass of neutral peptide Mr(calc): 4078.9511
Fixed modifications: Carbamidomethyl (C)
Variable modifications:
S12 : Phospho (ST), with neutral losses 0.0000(shown in table), 97.9769
Ions Score: 49 **Expect:** 0.0043
Matches (Bold Red): 59/690 fragment ions using 109 most intense peaks

#	b	b++	b*	b*++	b0	b0++	Seq.	y	y++	y*	y*++	y0	y0++	#
1	115.0502	58.0287	98.0237	49.5155			N							40
2	244.0928	122.5500	227.0662	114.0368	226.0822	113.5448	E	3965.9155	1983.4614	3948.8889	1974.9481	3947.9049	1974.4561	39
3	315.1299	158.0686	298.1034	149.5553	297.1193	149.0633	A	3836.8729	1918.9401	3819.8464	1910.4268	3818.8623	1909.9348	38
4	386.1670	193.5872	369.1405	185.0739	368.1565	184.5819	A	3765.8358	1883.4215	3748.8092	1874.9083	3747.8252	1874.4162	37
5	**485.2354**	243.1214	468.2089	234.6081	467.2249	234.1161	V	3694.7987	**1847.9030**	3677.7721	1839.3897	3676.7881	1838.8977	36

6	582.2882	291.6477	565.2617	283.1345	564.2776	282.6425	P	3595.7303	1798.3688	3578.7037	1789.8555	3577.7197	1789.3635	35
7	653.3253	327.1663	636.2988	318.6530	635.3148	318.1610	A	3498.6775	1749.8424	3481.6509	1741.3291	3480.6669	1740.8371	34
8	724.3624	362.6849	707.3359	354.1716	706.3519	353.6796	A	3427.6404	1714.3238	3410.6138	1705.8106	3409.6298	1705.3185	33
9	795.3995	398.2034	778.3730	389.6901	777.3890	389.1981	A	3356.6033	1678.8053	3339.5767	1670.2920	3338.5927	1669.8000	32
10	866.4367	433.7220	849.4101	425.2087	848.4261	424.7167	A	3285.5662	1643.2867	3268.5396	1634.7734	3267.5556	1634.2814	31
11	923.4581	462.2327	906.4316	453.7194	905.4476	453.2274	G	3214.5290	1607.7682	3197.5025	1599.2549	3196.5185	1598.7629	30
12	1090.4565	545.7319	1073.4299	537.2186	1072.4459	536.7266	S	3157.5076	1579.2574	3140.4810	1570.7442	3139.4970	1570.2521	29
13	1187.5092	594.2583	1170.4827	585.7450	1169.4987	585.2530	P	2990.5092	1495.7582	2973.4827	1487.2450	2972.4987	1486.7530	28
14	1286.5777	643.7925	1269.5511	635.2792	1268.5671	634.7872	V	2893.4565	1447.2319	2876.4299	1438.7186	2875.4459	1438.2266	27
15	1383.6304	692.3188	1366.6039	683.8056	1365.6199	683.3136	P	2794.3880	1397.6977	2777.3615	1389.1844	2776.3775	1388.6924	26
16	1482.6988	741.8531	1465.6723	733.3398	1464.6883	732.8478	V	2697.3353	1349.1713	2680.3087	1340.6580	2679.3247	1340.1660	25
17	1595.7829	798.3951	1578.7563	789.8818	1577.7723	789.3898	I	2598.2669	1299.6371	2581.2403	1291.1238	2580.2563	1290.6318	24
18	1666.8200	833.9136	1649.7935	825.4004	1648.8094	824.9084	A	2485.1828	1243.0950	2468.1563	1234.5818	2467.1722	1234.0898	23
19	1779.9041	890.4557	1762.8775	881.9424	1761.8935	881.4504	I	2414.1457	1207.5765	2397.1191	1199.0632	2396.1351	1198.5712	22
20	1876.9568	938.9821	1859.9303	930.4688	1858.9463	929.9768	P	2301.0616	1151.0345	2284.0351	1142.5212	2283.0511	1142.0292	21
21	1933.9783	967.4928	1916.9518	958.9795	1915.9677	958.4875	G	2204.0089	1102.5081	2186.9823	1093.9948	2185.9983	1093.5028	20
22	2047.0624	1024.0348	2030.0358	1015.5215	2029.0518	1015.0295	I	2146.9874	1073.9973	2129.9608	1065.4841	2128.9768	1064.9921	19
23	2160.1464	1080.5769	2143.1199	1072.0636	2142.1359	1071.5716	L	2033.9033	1017.4553	2016.8768	1008.9420	2015.8928	1008.4500	18
24	2289.1890	1145.0981	2272.1625	1136.5849	2271.1785	1136.0929	E	1920.8193	960.9133	1903.7927	952.4000	1902.8087	951.9080	17
25	2404.2160	1202.6116	2387.1894	1194.0983	2386.2054	1193.6063	D	1791.7767	896.3920	1774.7501	887.8787	1773.7661	887.3867	16
26	2533.2586	1267.1329	2516.2320	1258.6196	2515.2480	1258.1276	E	1676.7497	838.8785	1659.7232	830.3652	1658.7392	829.8732	15
27	2646.3426	1323.6749	2629.3161	1315.1617	2628.3321	1314.6697	L	1547.7071	774.3572	1530.6806	765.8439	1529.6966	765.3519	14
28	2775.3852	1388.1962	2758.3587	1379.6830	2757.3746	1379.1910	E	1434.6231	717.8152	1417.5965	709.3019	1416.6125	708.8099	13
29	2903.4438	1452.2255	2886.4172	1443.7123	2885.4332	1443.2203	Q	1305.5805	653.2939	1288.5539	644.7806	1287.5699	644.2886	12
30	3004.4915	1502.7494	2987.4649	1494.2361	2986.4809	1493.7441	T	1177.5219	589.2646	1160.4954	580.7513	1159.5113	580.2593	11
31	3119.5184	1560.2628	3102.4919	1551.7496	3101.5078	1551.2576	D	1076.4742	538.7408	1059.4477	530.2275	1058.4637	529.7355	10
32	3190.5555	1595.7814	3173.5290	1587.2681	3172.5450	1586.7761	A	961.4473	481.2273	944.4207	472.7140	943.4367	472.2220	9
33	3319.5981	1660.3027	3302.5716	1651.7894	3301.5876	1651.2974	E	890.4102	445.7087	873.3836	437.1954	872.3996	436.7034	8
34	3390.6352	1695.8213	3373.6087	1687.3080	3372.6247	1686.8160	A	761.3676	381.1874	744.3410	372.6742	743.3570	372.1821	7
35	3518.7302	1759.8687	3501.7036	1751.3555	3500.7196	1750.8635	K	690.3305	345.6689	673.3039	337.1556	672.3199	336.6636	6
36	3633.7571	1817.3822	3616.7306	1808.8689	3615.7466	1808.3769	D	562.2355	281.6214			544.2249	272.6161	5
37	3734.8048	1867.9060	3717.7783	1859.3928	3716.7943	1858.9008	T	447.2086	224.1079			429.1980	215.1026	4
38	3831.8576	1916.4324	3814.8310	1907.9192	3813.8470	1907.4271	P	346.1609	173.5841			328.1503	164.5788	3
39	3932.9053	1966.9563	3915.8787	1958.4430	3914.8947	1957.9510	T	249.1081	125.0577			231.0975	116.0524	2
40							E	148.0604	74.5339			130.0499	65.5286	1

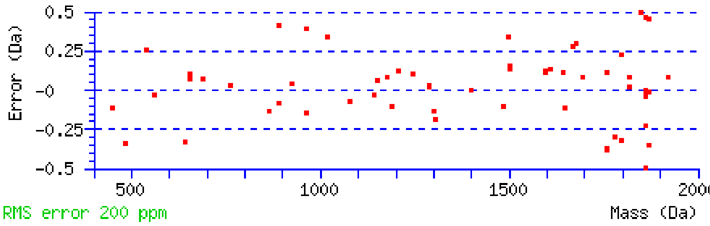

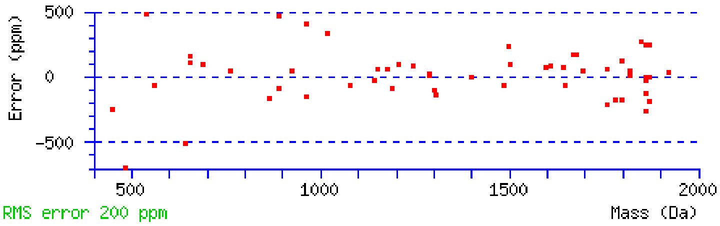

All matches to this query

Score	Mr(calc):	Delta	Sequence
48.9	4078.9511	1.0160	NEAAVPAAAAGSPVPVIAIPGILEDELEQTDAEAKDTPTE
47.5	4079.9351	0.0320	NEAAVPAAAAGSPVPVIAIPGILEDELEQTDAEAKDTPTE
14.0	4079.9351	0.0320	NEAAVPAAAAGSPVPVIAIPGILEDELEQTDAEAKDTPTE
12.7	4078.9511	1.0160	NEAAVPAAAAGSPVPVIAIPGILEDELEQTDAEAKDTPTE
5.7	4079.9295	0.0376	MVCSIQEDLPFVLKPSVENLYTLMTNTALDRETR

5.3	4078.9976	0.9695	WANSLHDEKPLSVPRDAHFQLETCPLTAVDALVLR
5.3	4079.9264	0.0406	NMMTILILMILDLLTTPITFSMTILTKLMYFR
4.7	4077.9467	2.0203	KVPEPEAIPPNILSYLVTGISVNCAYTSKILPPEK
4.0	4077.9260	2.0411	MLENYSNLVFLGLASSKPYLITLLEQIKEPSDMK
3.8	4077.9225	2.0446	VPQNAFIVSTVEILSNLPTFQTDGQSTKEDALQAVR

Spectrum No: 323; Query: 921; Rank: 1

Peptide View

MS/MS Fragmentation of **GGGSEDEPSPGGLTER**
Found in **IPI00211999**, Tax_Id=10116 Gene_Symbol=Rnmt 46 kDa protein

Match to Query 921: 1623.644908 from(812.829730,2+)
Title: 091008RatKidney_NoSalt_26.1027.1027.2.dta
Data file K:\NewmanPaper\Piliang\3SubProteomes\Piliang3SP\mgf5ppm\ERLIC_3SubProteomes5ppm.mgf

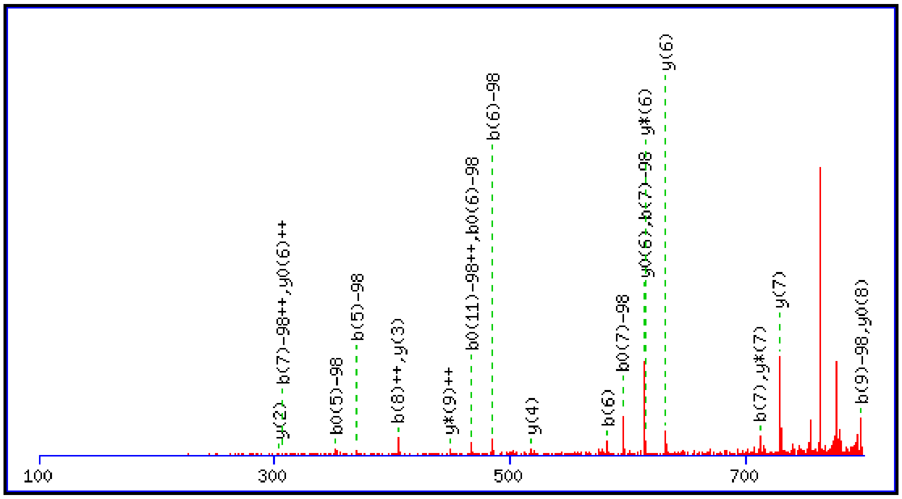

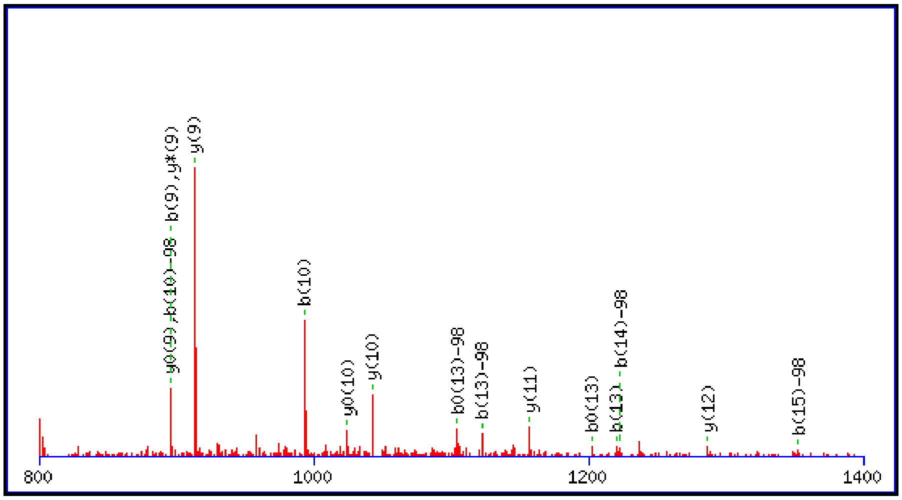

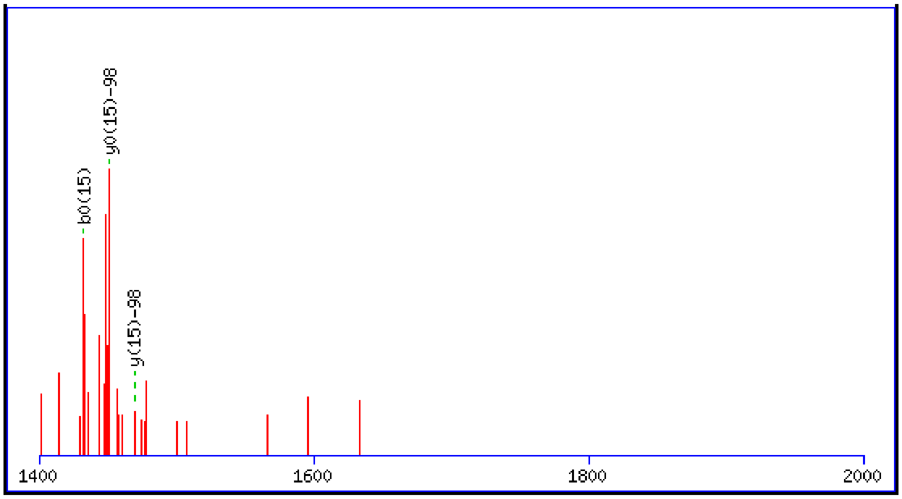

Monoisotopic mass of neutral peptide Mr(calc): 1623.6414
Fixed modifications: Carbamidomethyl (C)
Variable modifications:
S4 : Phospho (ST), with neutral losses 97.9769(shown in table), 0.0000
Ions Score: 49 **Expect:** 0.0015
Matches (Bold Red): 42/208 fragment ions using 60 most intense peaks

#	b	b^{++}	b^{0}	b^{0++}	Seq.	y	y^{++}	y*	y^{*++}	y^{0}	y^{0++}	#
1	58.0287	29.5180			G							16
2	115.0502	58.0287			G	**1469.6503**	735.3288	1452.6237	726.8155	**1451.6397**	726.3235	15
3	172.0717	86.5395			G	1412.6288	706.8180	1395.6023	698.3048	1394.6183	697.8128	14
4	241.0931	121.0502	223.0826	112.0449	S	1355.6074	678.3073	1338.5808	669.7940	1337.5968	669.3020	13
5	**370.1357**	185.5715	**352.1252**	176.5662	E	**1286.5859**	643.7966	1269.5594	635.2833	1268.5753	634.7913	12
6	**485.1627**	243.0850	**467.1521**	234.0797	D	**1157.5433**	579.2753	1140.5168	570.7620	1139.5327	570.2700	11
7	**614.2053**	**307.6063**	**596.1947**	298.6010	E	**1042.5164**	521.7618	1025.4898	513.2485	**1024.5058**	512.7565	10
8	711.2580	356.1326	693.2475	347.1274	P	**913.4738**	457.2405	**896.4472**	**448.7272**	**895.4632**	448.2352	9
9	**798.2900**	399.6487	780.2795	390.6434	S	816.4210	408.7141	799.3945	400.2009	**798.4104**	399.7089	8
10	**895.3428**	448.1750	877.3322	439.1698	P	**729.3890**	365.1981	**712.3624**	356.6849	711.3784	356.1928	7
11	952.3643	476.6858	934.3537	**467.6805**	G	**632.3362**	316.6717	**615.3097**	308.1585	**614.3257**	**307.6665**	6
12	1009.3857	505.1965	991.3752	496.1912	G	575.3148	288.1610	558.2882	279.6477	557.3042	279.1557	5
13	**1122.4698**	561.7385	**1104.4592**	552.7333	L	**518.2933**	259.6503	501.2667	251.1370	500.2827	250.6450	4
14	**1223.5175**	612.2624	1205.5069	603.2571	T	**405.2092**	203.1083	388.1827	194.5950	387.1987	194.1030	3
15	**1352.5601**	676.7837	1334.5495	667.7784	E	**304.1615**	152.5844	287.1350	144.0711	286.1510	143.5791	2
16					R	175.1190	88.0631	158.0924	79.5498			1

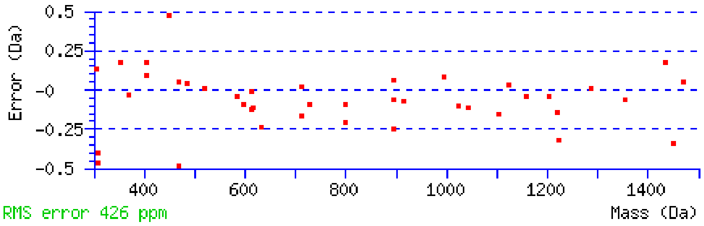

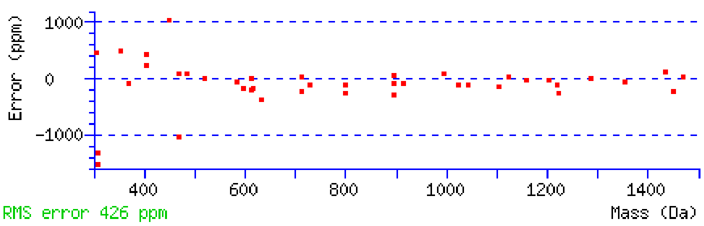

All matches to this query

Score	Mr(calc):	Delta	Sequence
48.9	1623.6414	0.0035	GGGSEDEPSPGGLTER
34.5	1623.6414	0.0035	GGGSEDEPSPGGLTER
22.7	1623.6414	0.0035	GGGSEDEPSPGGLTER
4.4	1623.6443	0.0006	YGREGLTGAGSGPSR
4.4	1623.6443	0.0006	YGREGLTGAGSGPSR

1.5	1623.6566	-0.0117	GGKSGAAFYATEDDR
1.4	1623.6566	-0.0117	GGKSGAAFYATEDDR
1.2	1623.6501	-0.0052	MADRFSRFNEDR

Spectrum No: 324; Query: 2061; Rank: 1

Peptide View

MS/MS Fragmentation of **RPGPEHSLGLEKESDEEPED**
Found in **IPI00360733**, Tax_Id=10116 Gene_Symbol=RGD1560168_predicted hypothetical protein LOC367307

Match to Query 2061: 2328.969342 from(777.330390,3+)
Title: 091008RatKidney_NoSalt_23.1032.1032.3.dta
Data file K:\NewmanPaper\Piliang\3SubProteomes\Piliang3SP\mgf5ppm\ERLIC_3SubProteomes5ppm.mgf

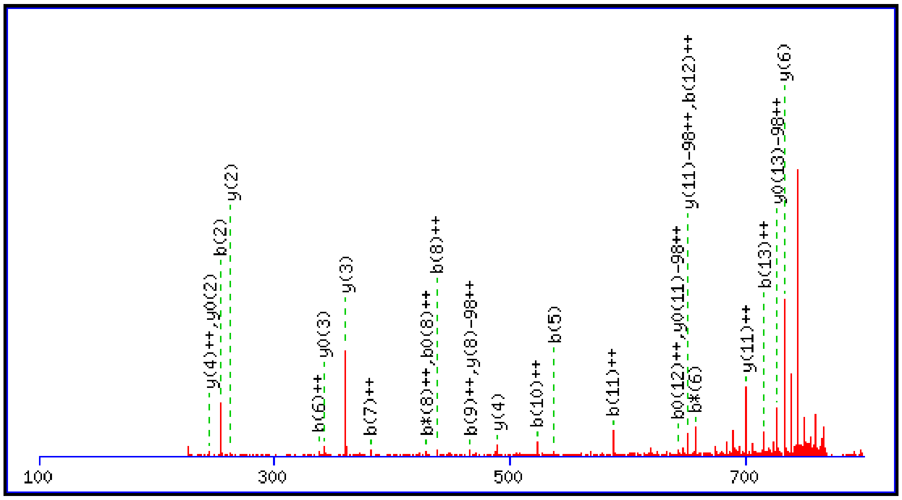

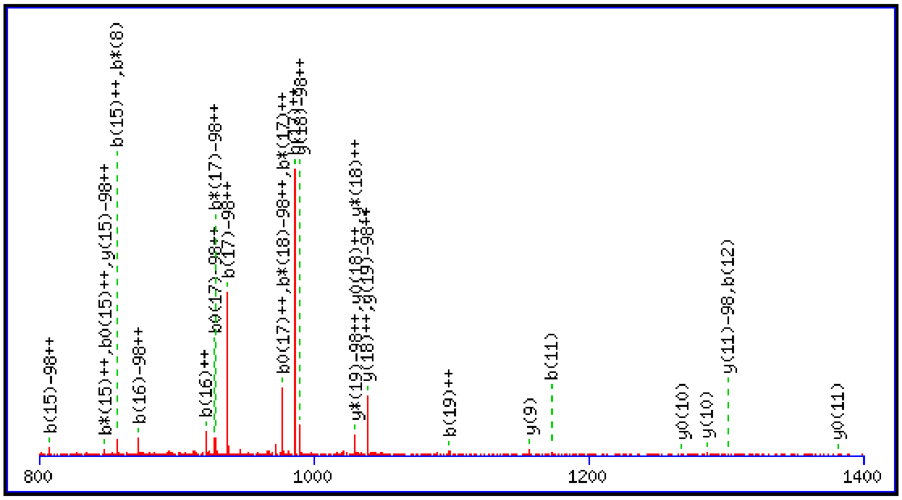

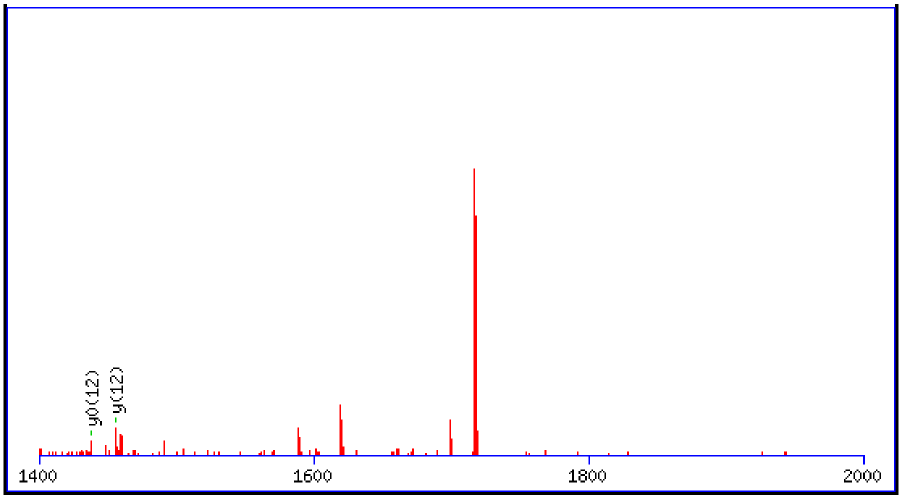

Monoisotopic mass of neutral peptide Mr(calc): 2328.9747
Fixed modifications: Carbamidomethyl (C)
Variable modifications:
S14 : Phospho (ST), with neutral losses 0.0000(shown in table), 97.9769
Ions Score: 49 **Expect:** 0.0038
Matches (Bold Red): 57/314 fragment ions using 96 most intense peaks

#	b	b^{++}	b*	b^{*++}	b^{0}	b^{0++}	Seq.	y	y^{++}	y*	y^{*++}	y^{0}	y^{0++}	#
1	157.1084	79.0578	140.0818	70.5446			R							20
2	**254.1612**	127.5842	237.1346	119.0709			P	2173.8809	1087.4441	2156.8543	1078.9308	2155.8703	1078.4388	19
3	311.1826	156.0949	294.1561	147.5817			G	2076.8281	**1038.9177**	2059.8016	**1030.4044**	2058.8176	**1029.9124**	18
4	408.2354	204.6213	391.2088	196.1081			P	2019.8067	1010.4070	2002.7801	1001.8937	2001.7961	1001.4017	17
5	**537.2780**	269.1426	520.2514	260.6293	519.2674	260.1373	E	1922.7539	961.8806	1905.7273	953.3673	1904.7433	952.8753	16
6	674.3369	**337.6721**	**657.3103**	329.1588	656.3263	328.6668	H	1793.7113	897.3593	1776.6848	888.8460	1775.7007	888.3540	15
7	761.3689	**381.1881**	744.3424	372.6748	743.3583	372.1828	S	1656.6524	828.8298	1639.6258	820.3166	1638.6418	819.8246	14
8	874.4530	**437.7301**	**857.4264**	**429.2169**	856.4424	**428.7248**	L	1569.6204	785.3138	1552.5938	776.8005	1551.6098	776.3085	13
9	931.4744	**466.2409**	914.4479	457.7276	913.4639	457.2356	G	**1456.5363**	728.7718	1439.5098	720.2585	**1438.5257**	719.7665	12
10	1044.5585	**522.7829**	1027.5320	514.2696	1026.5479	513.7776	L	1399.5148	**700.2611**	1382.4883	691.7478	**1381.5043**	691.2558	11
11	**1173.6011**	**587.3042**	1156.5745	578.7909	1155.5905	578.2989	E	**1286.4308**	643.7190	1269.4042	635.2057	**1268.4202**	634.7137	10
12	**1301.6961**	**651.3517**	1284.6695	642.8384	1283.6855	**642.3464**	K	**1157.3882**	579.1977	1140.3616	570.6845	1139.3776	570.1924	9
13	1430.7387	**715.8730**	1413.7121	707.3597	1412.7281	706.8677	E	1029.2932	515.1502			1011.2827	506.1450	8
14	1597.7370	799.3721	1580.7105	790.8589	1579.7264	790.3669	S	900.2506	450.6289			882.2401	441.6237	7
15	1712.7639	**856.8856**	1695.7374	**848.3723**	1694.7534	**847.8803**	D	**733.2523**	367.1298			715.2417	358.1245	6
16	1841.8065	**921.4069**	1824.7800	912.8936	1823.7960	912.4016	E	618.2253	309.6163			600.2148	300.6110	5
17	1970.8491	**985.9282**	1953.8226	**977.4149**	1952.8386	**976.9229**	E	**489.1827**	**245.0950**			471.1722	236.0897	4
18	2067.9019	1034.4546	2050.8753	1025.9413	2049.8913	1025.4493	P	**360.1401**	180.5737			**342.1296**	171.5684	3
19	2196.9445	**1098.9759**	2179.9179	1090.4626	2178.9339	1089.9706	E	**263.0874**	132.0473			**245.0768**	123.0420	2
20							D	134.0448	67.5260			116.0342	58.5207	1

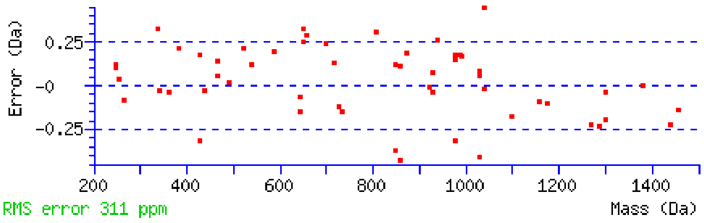

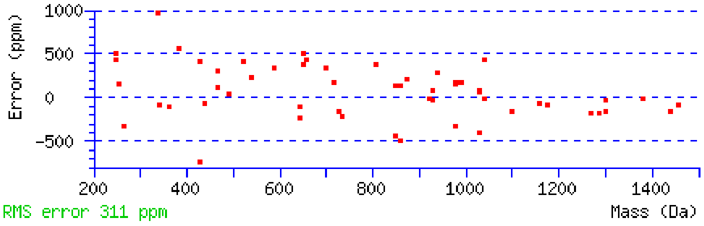

All matches to this query

Score	Mr(calc):	Delta	Sequence
48.8	2328.9747	-0.0053	RPGPEHSLGLEKESDEEPED

16.5	2328.9747	-0.0053	RPGPEHSLGLEKESDEEPED
1.6	2328.9626	0.0068	MIDPDEEKAKQDPSYYLK
1.6	2328.9626	0.0068	MIDPDEEKAKQDPSYYLK
1.6	2328.9626	0.0068	MIDPDEEKAKQDPSYYLK
1.3	2328.9821	-0.0127	TLAQAMLNNAQPEQYEAPDK
1.1	2328.9573	0.0121	MDIEDEENMSSSSIDVKENR
0.8	2328.9721	-0.0028	FNSKKLEIDTELSQFYK
0.8	2328.9811	-0.0118	TPCVEEEQSRGVGVPSSPVR
0.8	2328.9811	-0.0118	TPCVEEEQSRGVGVPSSPVR

Spectrum No: 325; Query: 2021; Rank: 1

Peptide View

MS/MS Fragmentation of **GGEFDEFVNDDTDDDLPISK**
Found in **IPI00371679**, Tax_Id=10116 Gene_Symbol=Sh2bp1 similar to SH2 domain binding protein 1

Match to Query 2021: 2306.917988 from(1154.466270,2+)
Title: 100101RatKid_NS_deglyco_25.3885.3885.2.dta
Data file K:\NewmanPaper\Piliang\3SubProteomes\Piliang3SP\mgf5ppm\ERLIC_3SubProteomes5ppm.mgf

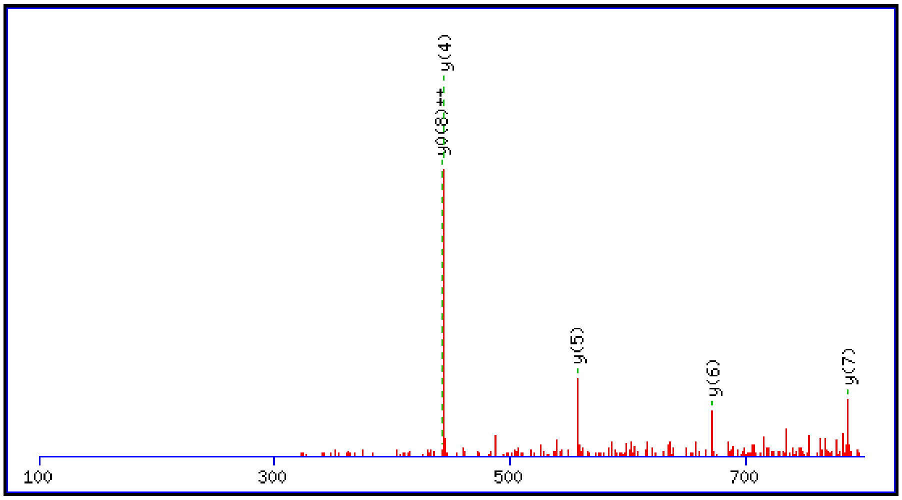

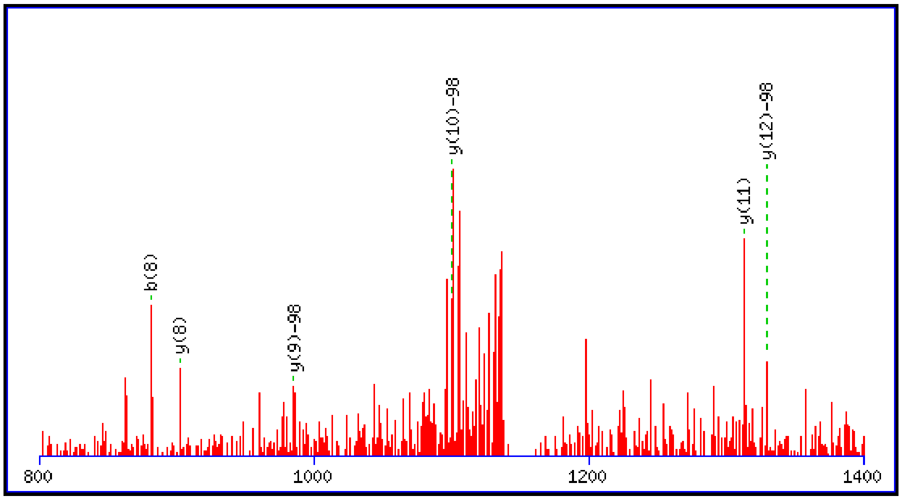

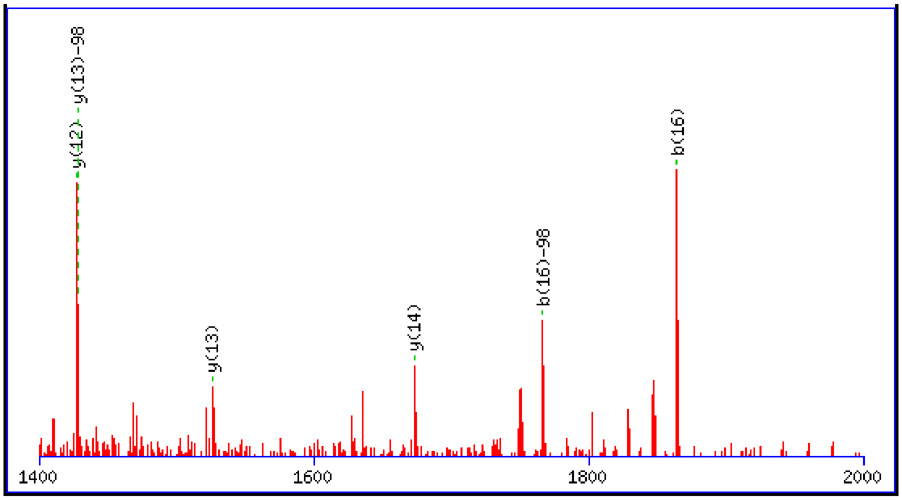

Monoisotopic mass of neutral peptide Mr(calc): 2306.9104
Fixed modifications: Carbamidomethyl (C)
Variable modifications:
T12 : Phospho (ST), with neutral losses 0.0000(shown in table), 97.9769
Ions Score: 49 **Expect:** 0.0029
Matches (Bold Red): 17/320 fragment ions using 25 most intense peaks

#	b	b++	b*	b*++	b0	b0++	Seq.	y	y++	y*	y*++	y0	y0++	#
1	58.0287	29.5180					G							20
2	115.0502	58.0287					G	2250.8962	1125.9517	2233.8697	1117.4385	2232.8856	1116.9465	19
3	244.0928	122.5500			226.0822	113.5448	E	2193.8747	1097.4410	2176.8482	1088.9277	2175.8642	1088.4357	18
4	391.1612	196.0842			373.1506	187.0790	F	2064.8321	1032.9197	2047.8056	1024.4064	2046.8216	1023.9144	17
5	506.1882	253.5977			488.1776	244.5924	D	1917.7637	959.3855	1900.7372	950.8722	1899.7532	950.3802	16
6	635.2307	318.1190			617.2202	309.1137	E	1802.7368	901.8720	1785.7102	893.3588	1784.7262	892.8668	15
7	782.2992	391.6532			764.2886	382.6479	F	**1673.6942**	837.3507	1656.6676	828.8375	1655.6836	828.3455	14
8	**881.3676**	441.1874			863.3570	432.1821	V	**1526.6258**	763.8165	1509.5992	755.3033	1508.6152	754.8112	13
9	995.4105	498.2089	978.3840	489.6956	977.3999	489.2036	N	**1427.5574**	714.2823	1410.5308	705.7690	1409.5468	705.2770	12
10	1110.4374	555.7224	1093.4109	547.2091	1092.4269	546.7171	D	**1313.5144**	657.2609	1296.4879	648.7476	1295.5039	648.2556	11
11	1225.4644	613.2358	1208.4378	604.7226	1207.4538	604.2305	D	1198.4875	599.7474	1181.4610	591.2341	1180.4769	590.7421	10
12	1406.4784	703.7428	1389.4518	695.2296	1388.4678	694.7376	T	1083.4606	542.2339	1066.4340	533.7206	1065.4500	533.2286	9
13	1521.5053	761.2563	1504.4788	752.7430	1503.4948	752.2510	D	**902.4466**	451.7269	885.4200	443.2136	884.4360	**442.7216**	8
14	1636.5323	818.7698	1619.5057	810.2565	1618.5217	809.7645	D	**787.4196**	394.2134	770.3931	385.7002	769.4090	385.2082	7
15	1751.5592	876.2832	1734.5327	867.7700	1733.5487	867.2780	D	**672.3927**	336.7000	655.3661	328.1867	654.3821	327.6947	6
16	**1864.6433**	932.8253	1847.6167	924.3120	1846.6327	923.8200	L	**557.3657**	279.1865	540.3392	270.6732	539.3552	270.1812	5
17	1961.6960	981.3517	1944.6695	972.8384	1943.6855	972.3464	P	**444.2817**	222.6445	427.2551	214.1312	426.2711	213.6392	4
18	2074.7801	1037.8937	2057.7536	1029.3804	2056.7695	1028.8884	I	347.2289	174.1181	330.2023	165.6048	329.2183	165.1128	3
19	2161.8121	1081.4097	2144.7856	1072.8964	2143.8016	1072.4044	S	234.1448	117.5761	217.1183	109.0628	216.1343	108.5708	2
20							K	147.1128	74.0600	130.0863	65.5468			1

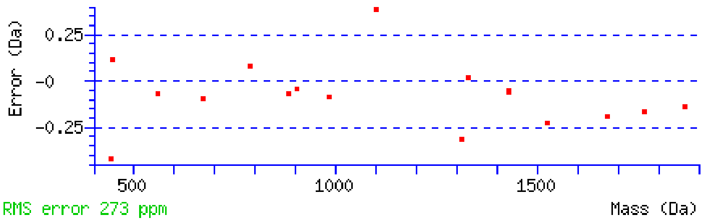

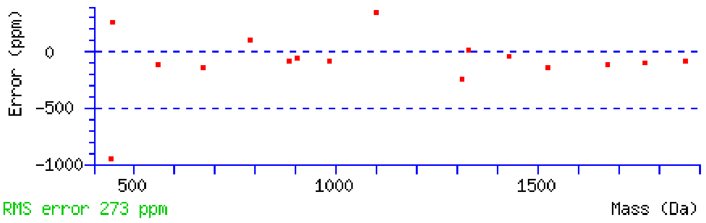

All matches to this query

Score	Mr(calc):	Delta	Sequence
48.8	2306.9104	0.0076	GGEFDEFVNDDTDDDLPISK

13.6	2306.9104	0.0076	GGEFDEFVNDDTDDDLPISK
6.4	2304.8928	2.0252	ARLEETVTLFSFYSSCR
3.1	2304.9156	2.0024	MPPRRDAMPSDANLNSINK
1.6	2306.9020	0.0160	AVCLRSATMWXTPSPSTR
0.8	2305.9269	0.9910	YDESDNVLIRASRALTDK

Spectrum No: 326; Query: 380; Rank: 1

Peptide View

MS/MS Fragmentation of **AIYQGPSSPDKS**
Found in **IPI00210280**, Tax_Id=10116 Gene_Symbol=Comt Isoform 1 of Catechol O-methyltransferase

Match to Query 380: 1328.564568 from(665.289560,2+)
Title: 091008RatKidney_NH4Format02_26.851.851.2.dta
Data file K:\NewmanPaper\Piliang\3SubProteomes\Piliang3SP\mgf5ppm\ERLIC_3SubProteomes5ppm.mgf

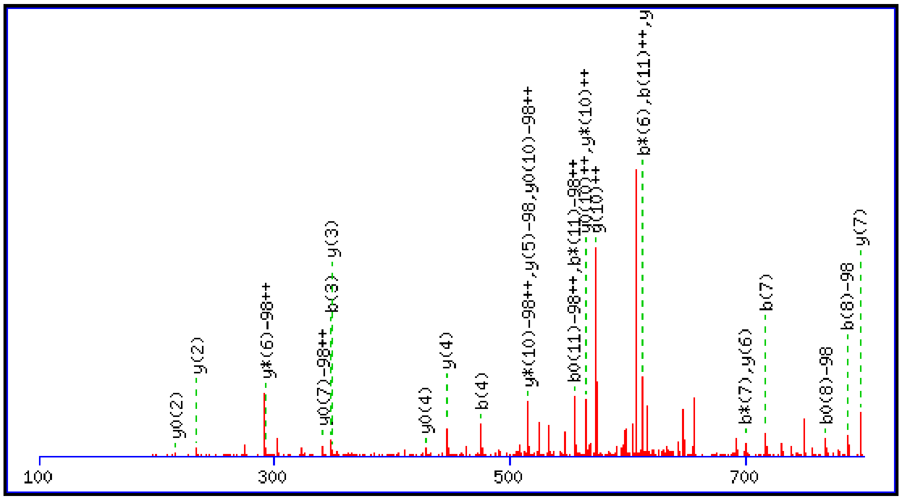

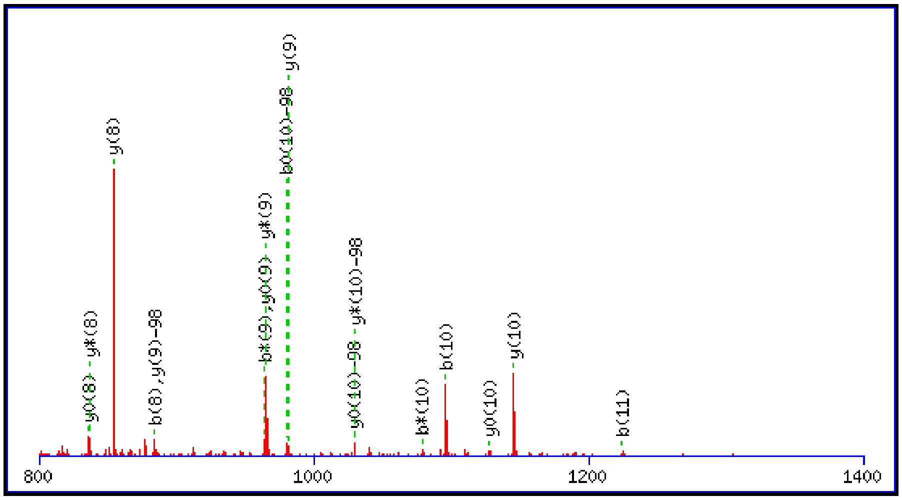

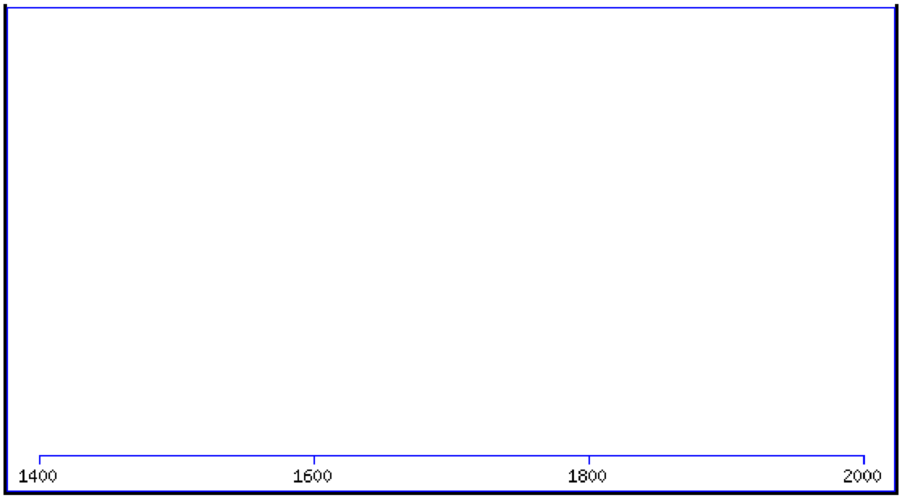

Monoisotopic mass of neutral peptide Mr(calc): 1328.5649
Fixed modifications: Carbamidomethyl (C)
Variable modifications:
S8 : Phospho (ST), with neutral losses 0.0000(shown in table), 97.9769
Ions Score: 49 **Expect:** 0.0015
Matches (Bold Red): 43/178 fragment ions using 70 most intense peaks

#	b	b++	b*	b*++	b0	b0++	Seq.	y	y++	y*	y*++	y0	y0++	#
1	72.0444	36.5258					A							12
2	185.1285	93.0679					I	1258.5351	629.7712	1241.5086	621.2579	1240.5246	620.7659	11
3	**348.1918**	174.5995					Y	**1145.4511**	**573.2292**	1128.4245	**564.7159**	**1127.4405**	**564.2239**	10
4	**476.2504**	238.6288	459.2238	230.1155			Q	**982.3877**	491.6975	**965.3612**	483.1842	**964.3772**	482.6922	9
5	533.2718	267.1396	516.2453	258.6263			G	**854.3291**	427.6682	**837.3026**	419.1549	**836.3186**	418.6629	8
6	630.3246	315.6659	**613.2980**	307.1527			P	**797.3077**	399.1575	780.2811	390.6442	779.2971	390.1522	7
7	**717.3566**	359.1819	**700.3301**	350.6687	699.3461	350.1767	S	**700.2549**	350.6311	683.2284	342.1178	682.2444	341.6258	6
8	**884.3550**	442.6811	867.3284	434.1678	866.3444	433.6758	S	**613.2229**	307.1151	596.1963	298.6018	595.2123	298.1098	5
9	981.4077	491.2075	**964.3812**	482.6942	963.3972	482.2022	P	**446.2245**	223.6159	429.1980	215.1026	**428.2140**	214.6106	4
10	**1096.4347**	548.7210	**1079.4081**	540.2077	1078.4241	539.7157	D	**349.1718**	175.0895	332.1452	166.5763	331.1612	166.0842	3
11	**1224.5296**	**612.7685**	1207.5031	604.2552	1206.5191	603.7632	K	**234.1448**	117.5761	217.1183	109.0628	**216.1343**	108.5708	2
12							S	106.0499	53.5286			88.0393	44.5233	1

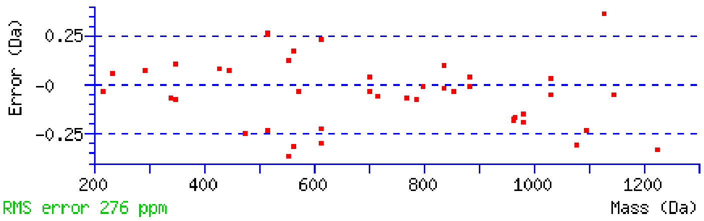

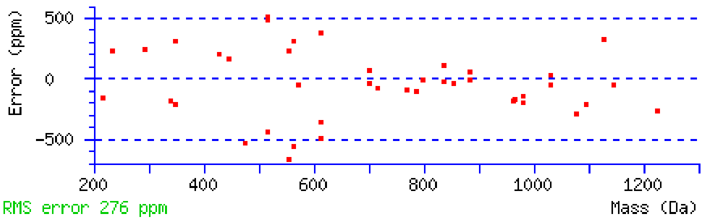

All matches to this query

Score	Mr(calc):	Delta	Sequence
48.7	1328.5649	-0.0004	AIYQGPSSPDKS
39.2	1328.5649	-0.0004	AIYQGPSSPDKS
18.2	1328.5649	-0.0004	AIYQGPSSPDKS
17.1	1328.5649	-0.0004	AIYQGPSSPDKS
4.7	1328.5683	-0.0037	DCEKAIAIDSK
2.8	1328.5567	0.0079	YSPKGVLYSR
1.9	1328.5567	0.0079	AINFSQSXKK
0.5	1326.5509	2.0136	TVYLAASSVEK

Spectrum No: 327; Query: 2528; Rank: 1

Peptide View

MS/MS Fragmentation of **AKDQPDGTSLSPVQSPSQSQPPAACTPR**
Found in **IPI00190868**, Tax_Id=10116 Gene_Symbol=Mrip Isoform 1 of Myosin phosphatase Rho-interacting protein

Match to Query 2528: 2986.355472 from(996.459100,3+)
Title: 091008RatKidney_NoSalt_21.1285.1285.3.dta
Data file K:\NewmanPaper\Piliang\3SubProteomes\Piliang3SP\mgf5ppm\ERLIC_3SubProteomes5ppm.mgf

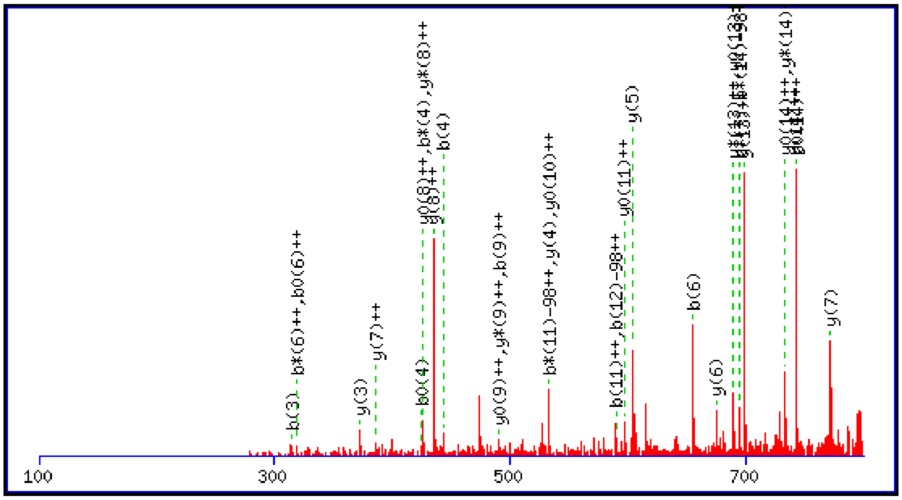

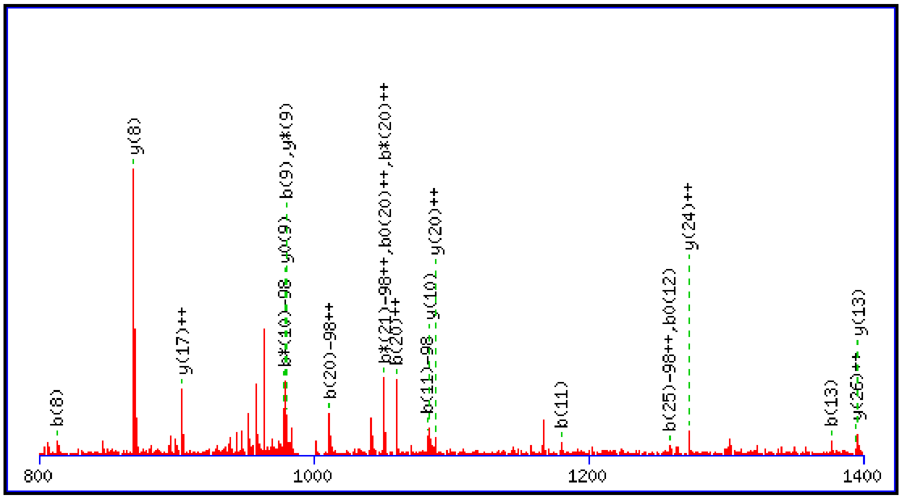

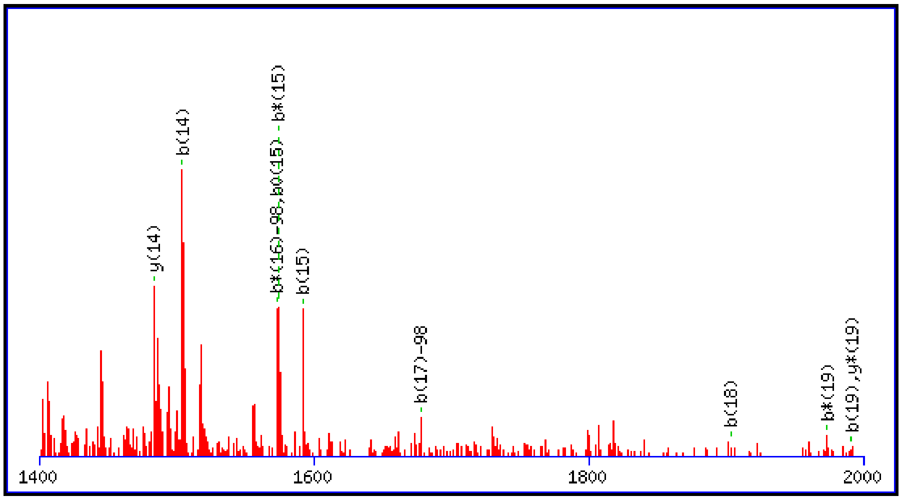

Monoisotopic mass of neutral peptide Mr(calc): 2986.3492

Fixed modifications: Carbamidomethyl (C)
Variable modifications:
S9 : Phospho (ST), with neutral losses 0.0000(shown in table), 97.9769
Ions Score: 49 **Expect:** 0.0056
Matches (Bold Red): 67/476 fragment ions using 102 most intense peaks

#	b	b^{++}	b^{*}	b^{*++}	b^{0}	b^{0++}	Seq.	y	y^{++}	y^{*}	y^{*++}	y^{0}	y^{0++}	#
1	72.0444	36.5258					A							28
2	200.1394	100.5733	183.1128	92.0600			K	2916.3193	1458.6633	2899.2928	1450.1500	2898.3088	1449.6580	27
3	**315.1663**	158.0868	298.1397	149.5735	297.1557	149.0815	D	2788.2244	**1394.6158**	2771.1978	1386.1026	2770.2138	1385.6105	26
4	**443.2249**	222.1161	**426.1983**	213.6028	**425.2143**	213.1108	Q	2673.1974	1337.1024	2656.1709	1328.5891	2655.1869	1328.0971	25
5	540.2776	270.6425	523.2511	262.1292	522.2671	261.6372	P	2545.1389	**1273.0731**	2528.1123	1264.5598	2527.1283	1264.0678	24
6	**655.3046**	328.1559	638.2780	**319.6427**	637.2940	**319.1506**	D	2448.0861	1224.5467	2431.0595	1216.0334	2430.0755	1215.5414	23
7	712.3260	356.6667	**695.2995**	348.1534	694.3155	347.6614	G	2333.0591	1167.0332	2316.0326	1158.5199	2315.0486	1158.0279	22
8	**813.3737**	407.1905	796.3472	398.6772	795.3632	398.1852	T	2276.0377	1138.5225	2259.0111	1130.0092	2258.0271	1129.5172	21
9	**980.3721**	**490.6897**	963.3455	482.1764	962.3615	481.6844	S	2174.9900	**1087.9986**	2157.9635	1079.4854	2156.9794	1078.9934	20
10	1093.4561	547.2317	1076.4296	538.7184	1075.4456	538.2264	L	2007.9917	1004.4995	**1990.9651**	995.9862	1989.9811	995.4942	19
11	**1180.4882**	**590.7477**	1163.4616	582.2344	1162.4776	581.7424	S	1894.9076	947.9574	1877.8810	939.4442	1876.8970	938.9521	18
12	1277.5409	639.2741	1260.5144	630.7608	**1259.5304**	630.2688	P	1807.8756	**904.4414**	1790.8490	895.9281	1789.8650	895.4361	17
13	**1376.6093**	688.8083	1359.5828	680.2950	1358.5988	679.8030	V	1710.8228	855.9150	1693.7962	847.4018	1692.8122	846.9098	16
14	**1504.6679**	752.8376	1487.6414	744.3243	1486.6574	**743.8323**	Q	1611.7544	806.3808	1594.7278	797.8676	1593.7438	797.3755	15
15	**1591.7000**	796.3536	**1574.6734**	787.8403	**1573.6894**	787.3483	S	**1483.6958**	**742.3515**	1466.6693	**733.8383**	1465.6852	**733.3463**	14
16	1688.7527	844.8800	1671.7262	836.3667	1670.7422	835.8747	P	**1396.6638**	**698.8355**	1379.6372	**690.3223**	1378.6532	**689.8302**	13
17	1775.7847	888.3960	1758.7582	879.8827	1757.7742	879.3907	S	1299.6110	650.3091	1282.5845	641.7959	1281.6004	641.3039	12
18	**1903.8433**	952.4253	1886.8168	943.9120	1885.8328	943.4200	Q	1212.5790	606.7931	1195.5524	598.2799	1194.5684	**597.7878**	11
19	**1990.8754**	995.9413	**1973.8488**	987.4280	1972.8648	986.9360	S	**1084.5204**	542.7638	1067.4939	534.2506	1066.5098	**533.7586**	10
20	2118.9339	**1059.9706**	2101.9074	**1051.4573**	2100.9234	**1050.9653**	Q	997.4884	499.2478	**980.4618**	**490.7346**	**979.4778**	**490.2425**	9
21	2215.9867	1108.4970	2198.9601	1099.9837	2197.9761	1099.4917	P	**869.4298**	**435.2185**	852.4033	**426.7053**	851.4192	**426.2133**	8
22	2313.0395	1157.0234	2296.0129	1148.5101	2295.0289	1148.0181	P	**772.3770**	**386.6922**	755.3505	378.1789	754.3665	377.6869	7
23	2384.0766	1192.5419	2367.0500	1184.0286	2366.0660	1183.5366	A	**675.3243**	338.1658	658.2977	329.6525	657.3137	329.1605	6
24	2455.1137	1228.0605	2438.0871	1219.5472	2437.1031	1219.0552	A	**604.2872**	302.6472	587.2606	294.1339	586.2766	293.6419	5
25	2615.1443	1308.0758	2598.1178	1299.5625	2597.1338	1299.0705	C	**533.2500**	267.1287	516.2235	258.6154	515.2395	258.1234	4
26	2716.1920	1358.5996	2699.1655	1350.0864	2698.1814	1349.5944	T	**373.2194**	187.1133	356.1928	178.6001	355.2088	178.1081	3
27	2813.2448	1407.1260	2796.2182	1398.6128	2795.2342	1398.1207	P	272.1717	136.5895	255.1452	128.0762			2
28							R	175.1190	88.0631	158.0924	79.5498			1

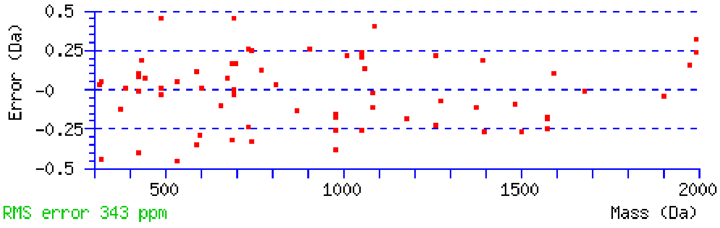

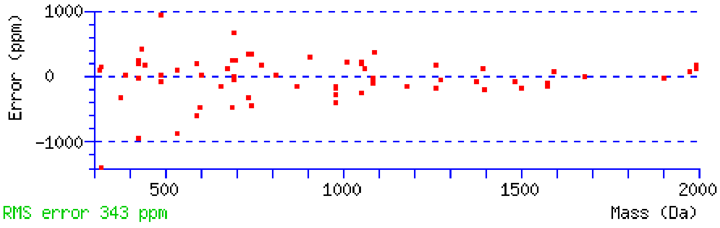

All matches to this query

Score	Mr(calc):	Delta	Sequence
48.7	2986.3492	0.0063	AKDQPDGTSLSPVQSPSQSQPPAACTPR
48.3	2986.3492	0.0063	AKDQPDGTSLSPVQSPSQSQPPAACTPR
41.5	2986.3492	0.0063	AKDQPDGTSLSPVQSPSQSQPPAACTPR
30.0	2986.3492	0.0063	AKDQPDGTSLSPVQSPSQSQPPAACTPR
28.3	2986.3492	0.0063	AKDQPDGTSLSPVQSPSQSQPPAACTPR
21.1	2986.3492	0.0063	AKDQPDGTSLSPVQSPSQSQPPAACTPR
8.4	2986.3605	-0.0050	LMPLLGYGTFQNPEIPASKILESTK

6.0	2986.3605	-0.0050	LMPLLGYGTFQNPEIPASKILESTK
5.0	2986.3605	-0.0050	LMPLLGYGTFQNPEIPASKILESTK
2.0	2986.3492	0.0063	ASPTTESDLQSQACQGVLAEGRVSPYR

Spectrum No: 328; Query: 595; Rank: 1

Peptide View

MS/MS Fragmentation of **IVSAQSLAEDDVE**
Found in **IPI00208207**, Tax_Id=10116 Gene_Symbol=Tomm20 Mitochondrial import receptor subunit TOM20 homolog

Match to Query 595: 1454.618988 from(728.316770,2+)
Title: 100101RatKid_NS_deglyco_26.2664.2664.2.dta
Data file K:\NewmanPaper\Piliang\3SubProteomes\Piliang3SP\mgf5ppm\ERLIC_3SubProteomes5ppm.mgf

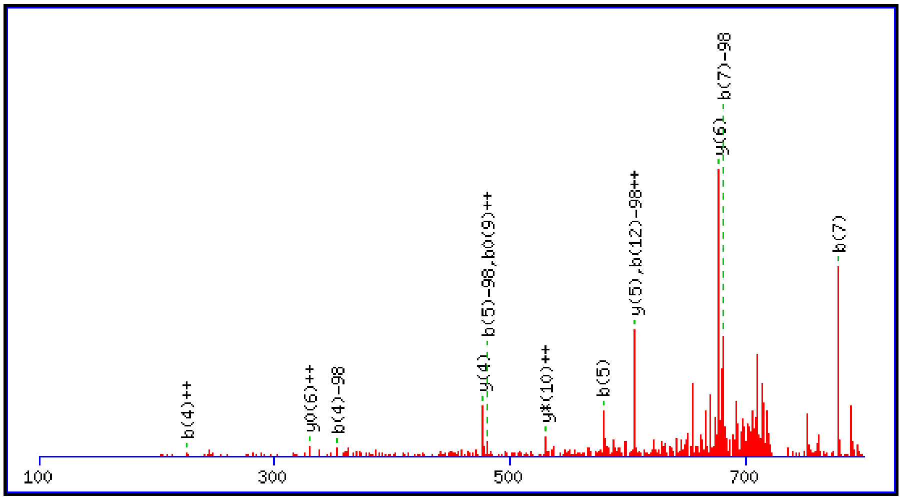

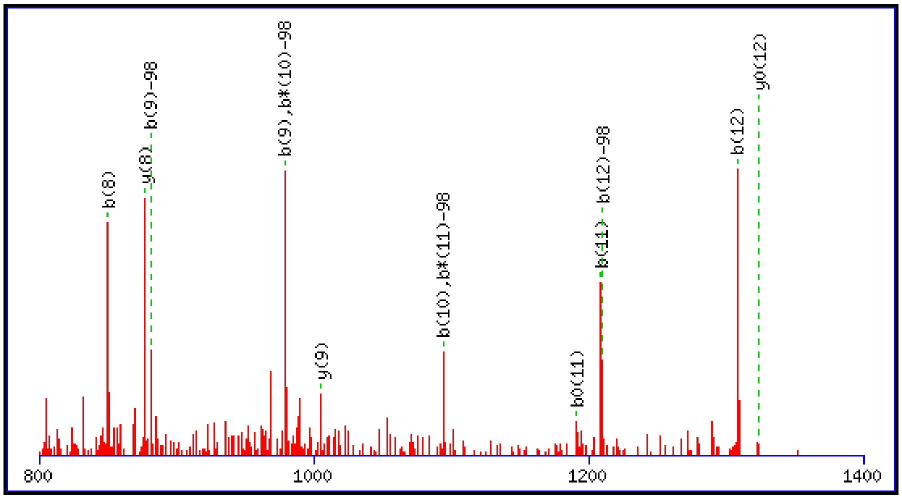

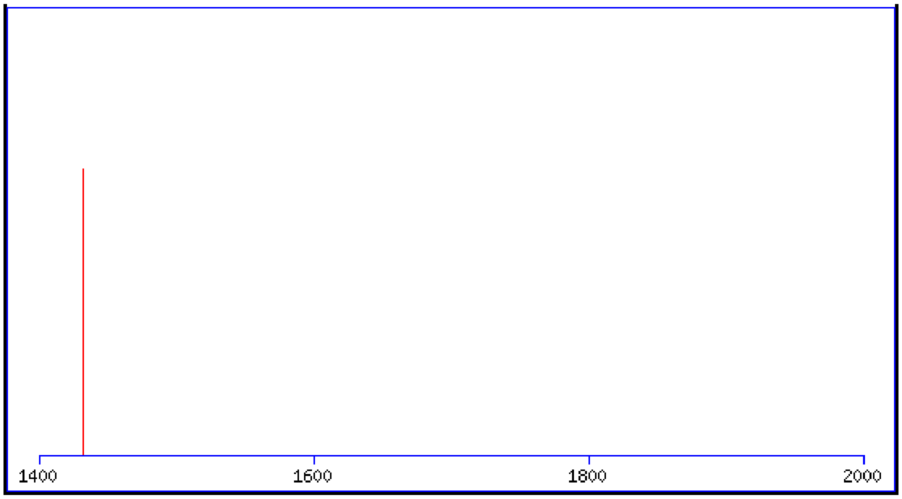

Monoisotopic mass of neutral peptide Mr(calc): 1454.6178
Fixed modifications: Carbamidomethyl (C)
Variable modifications:
S3 : Phospho (ST), with neutral losses 0.0000(shown in table), 97.9769
Ions Score: 49 **Expect:** 0.0017
Matches (Bold Red): 26/184 fragment ions using 30 most intense peaks

#	b	b++	b*	b*++	b0	b0++	Seq.	y	y++	y*	y*++	y0	y0++	#
1	114.0913	57.5493					I							13
2	213.1598	107.0835					V	1342.5410	671.7741	1325.5144	663.2609	**1324.5304**	662.7689	12
3	380.1581	190.5827			362.1475	181.5774	S	1243.4726	622.2399	1226.4460	613.7267	1225.4620	613.2346	11
4	451.1952	**226.1012**			433.1847	217.0960	A	1076.4742	538.7408	1059.4477	**530.2275**	1058.4637	529.7355	10
5	**579.2538**	290.1305	562.2272	281.6173	561.2432	281.1253	Q	**1005.4371**	503.2222	988.4106	494.7089	987.4265	494.2169	9
6	666.2858	333.6466	649.2593	325.1333	648.2753	324.6413	S	**877.3785**	439.1929			859.3680	430.1876	8
7	**779.3699**	390.1886	762.3433	381.6753	761.3593	381.1833	L	790.3465	395.6769			772.3359	386.6716	7
8	**850.4070**	425.7071	833.3805	417.1939	832.3964	416.7019	A	**677.2624**	339.1349			659.2519	**330.1296**	6
9	**979.4496**	490.2284	962.4230	481.7152	961.4390	**481.2232**	E	**606.2253**	303.6163			588.2148	294.6110	5
10	**1094.4765**	547.7419	1077.4500	539.2286	1076.4660	538.7366	D	**477.1827**	239.0950			459.1722	230.0897	4
11	**1209.5035**	605.2554	1192.4769	596.7421	**1191.4929**	596.2501	D	362.1558	181.5815			344.1452	172.5763	3
12	**1308.5719**	654.7896	1291.5453	646.2763	1290.5613	645.7843	V	247.1288	124.0681			229.1183	115.0628	2
13							E	148.0604	74.5339			130.0499	65.5286	1

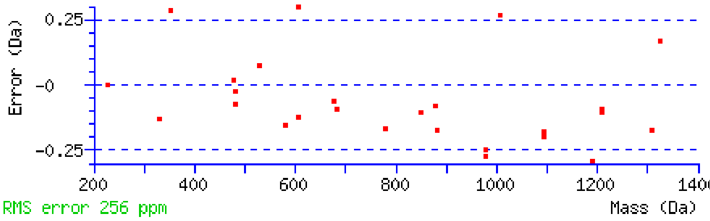

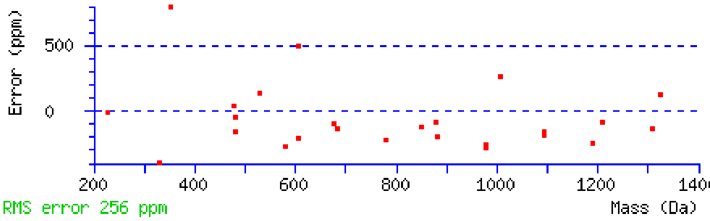

All matches to this query

Score	Mr(calc):	Delta	Sequence
48.6	1454.6178	0.0012	IVSAQSLAEDDVE
33.0	1454.6178	0.0012	IVSAQSLAEDDVE
6.5	1453.6143	1.0047	VVVDSDSLAYVK
2.9	1454.6095	0.0095	FVKTDALTSGEK
2.0	1453.6143	1.0047	VVVDSDSLAYVK
1.5	1454.6113	0.0077	EGLPSPGLSSSCGK
1.5	1454.6113	0.0077	EGLPSPGLSSSCGK
1.1	1454.6177	0.0013	MVNRGNCSKGCR

0.5	1453.6143	1.0047	VVVDSDSLAYVK

Spectrum No: 329; Query: 313; Rank: 1

Peptide View

MS/MS Fragmentation of **TVQAGDSPSAVR**
Found in **IPI00200640**, Tax_Id=10116 Gene_Symbol=Mucdhl Isoform 1 of Mucin and cadherin-like protein precursor

Match to Query 313: 1266.556208 from(634.285380,2+)
Title: 091008RatKidney_NH4Format02_26.439.439.2.dta
Data file K:\NewmanPaper\Piliang\3SubProteomes\Piliang3SP\mgf5ppm\ERLIC_3SubProteomes5ppm.mgf

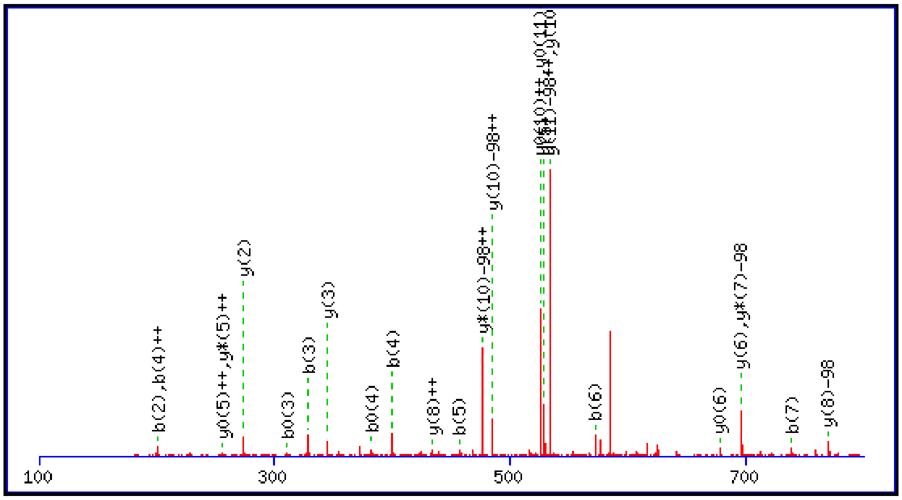

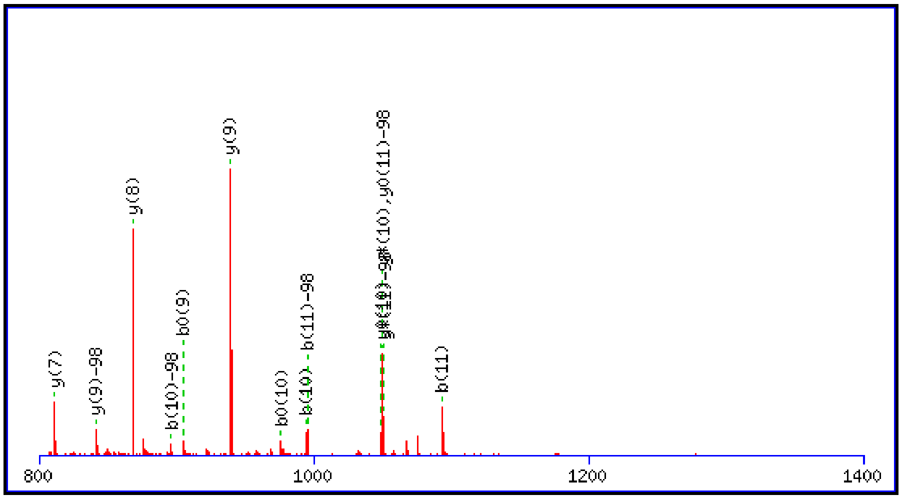

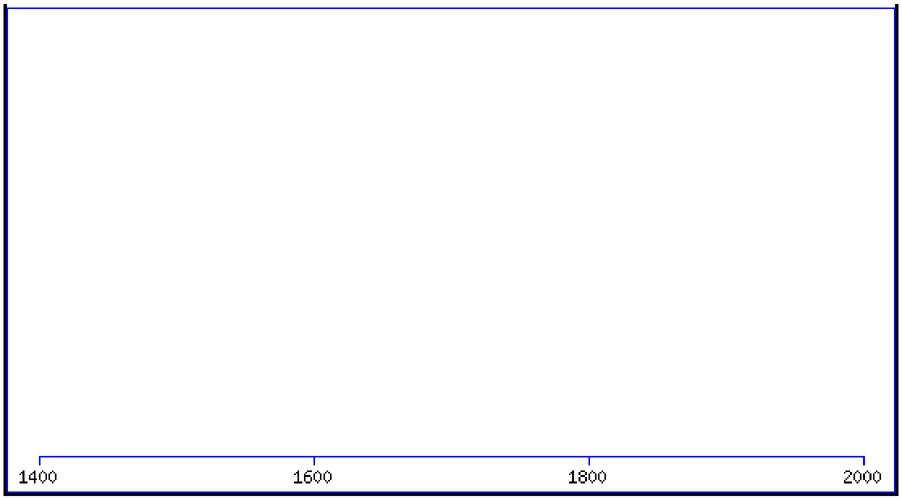

Monoisotopic mass of neutral peptide Mr(calc): 1266.5605
Fixed modifications: Carbamidomethyl (C)
Variable modifications:
S7 : Phospho (ST), with neutral losses 0.0000(shown in table), 97.9769
Ions Score: 49 **Expect:** 0.0015
Matches (Bold Red): 40/188 fragment ions using 51 most intense peaks

#	b	b^{++}	b*	b^{*++}	b^{0}	b^{0++}	Seq.	y	y^{++}	y*	y^{*++}	y^{0}	y^{0++}	#
1	102.0550	51.5311			84.0444	42.5258	T							12
2	**201.1234**	101.0653			183.1128	92.0600	V	1166.5201	583.7637	1149.4936	575.2504	1148.5096	574.7584	11
3	**329.1819**	165.0946	312.1554	156.5813	**311.1714**	156.0893	Q	1067.4517	**534.2295**	**1050.4252**	**525.7162**	**1049.4412**	**525.2242**	10
4	**400.2191**	**200.6132**	383.1925	192.0999	**382.2085**	191.6079	A	**939.3931**	470.2002	922.3666	461.6869	921.3826	461.1949	9
5	**457.2405**	229.1239	440.2140	220.6106	439.2300	220.1186	G	**868.3560**	**434.6817**	851.3295	426.1684	850.3455	425.6764	8
6	**572.2675**	286.6374	555.2409	278.1241	554.2569	277.6321	D	**811.3346**	406.1709	794.3080	397.6576	793.3240	397.1656	7
7	**739.2658**	370.1365	722.2393	361.6233	721.2553	361.1313	S	**696.3076**	348.6575	679.2811	340.1442	**678.2971**	339.6522	6
8	836.3186	418.6629	819.2920	410.1497	818.3080	409.6576	P	**529.3093**	265.1583	512.2827	**256.6450**	511.2987	**256.1530**	5
9	923.3506	462.1789	906.3241	453.6657	**905.3400**	453.1737	S	432.2565	216.6319	415.2300	208.1186	414.2459	207.6266	4
10	**994.3877**	497.6975	977.3612	489.1842	**976.3772**	488.6922	A	**345.2245**	173.1159	328.1979	164.6026			3
11	**1093.4561**	547.2317	1076.4296	538.7184	1075.4456	538.2264	V	**274.1874**	137.5973	257.1608	129.0840			2
12							R	175.1190	88.0631	158.0924	79.5498			1

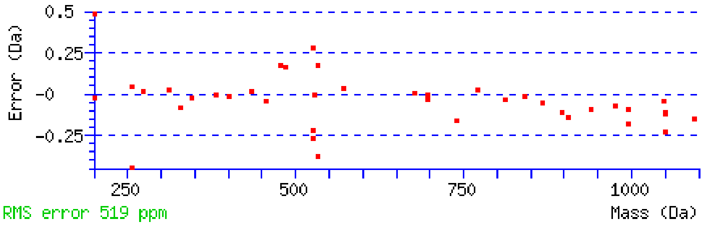

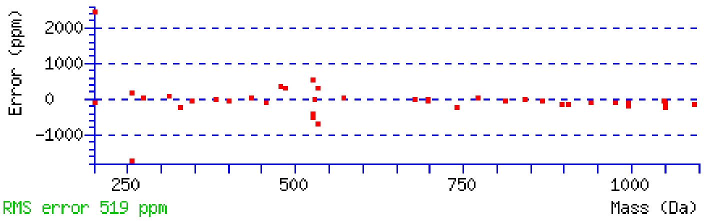

All matches to this query

Score	Mr(calc):	Delta	Sequence
48.6	1266.5605	-0.0043	TVQAGDSPSAVR
41.6	1266.5605	-0.0043	TVQAGDSPSAVR
21.1	1266.5605	-0.0043	TVQAGDSPSAVR
20.6	1266.5605	-0.0043	QAEKPVASDSR
13.7	1266.5605	-0.0043	QAEKPVASDSR
12.7	1265.5514	1.0048	TGAPAQADSRGR
12.3	1265.5514	1.0048	AGAEGTPGARGSR
8.8	1264.5449	2.0113	VQAVDSGGPAER
4.3	1265.5513	1.0049	RGSTAANEAGPR

2.1	1266.5493	0.0069	TEAGXTVLPDR

Spectrum No: 330; Query: 2242; Rank: 1

Peptide View

MS/MS Fragmentation of **QKSDAEEDGGTGSQDEEDSKPK**
Found in **IPI00199636**, Tax_Id=10116 Gene_Symbol=Canx Calnexin precursor

Match to Query 2242: 2495.915472 from(832.979100,3+)
Title: 091008RatKidney_NoSalt_28.156.156.3.dta
Data file K:\NewmanPaper\Piliang\3SubProteomes\Piliang3SP\mgf5ppm\ERLIC_3SubProteomes5ppm.mgf

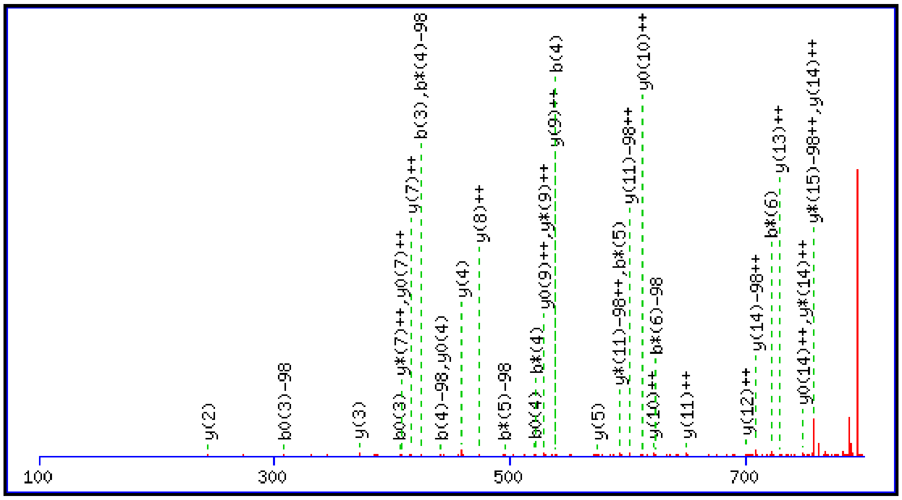

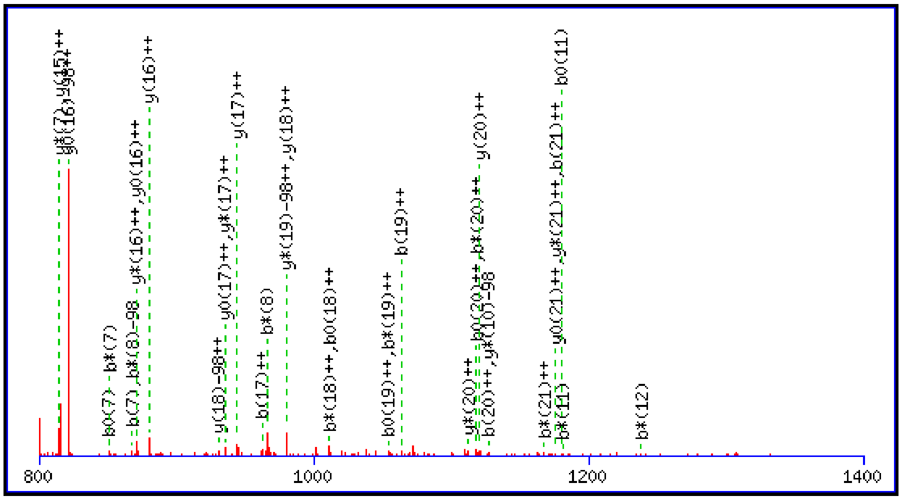

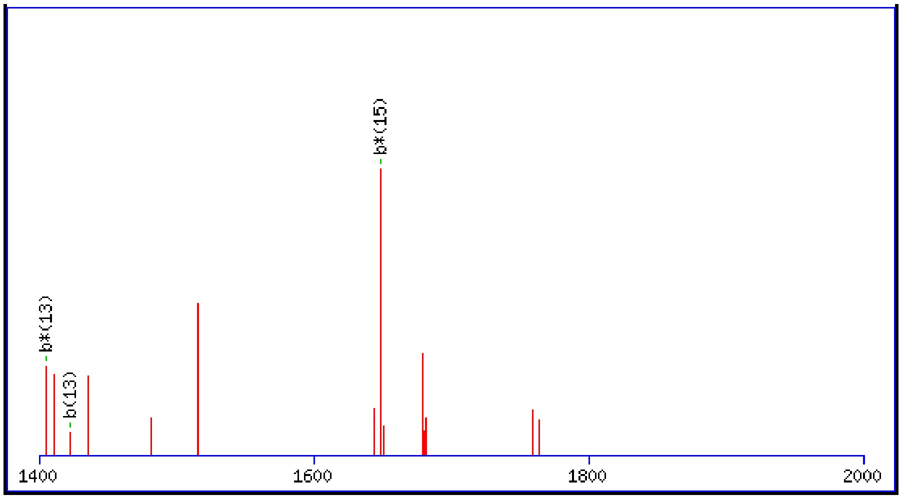

Monoisotopic mass of neutral peptide Mr(calc): 2495.9214
Fixed modifications: Carbamidomethyl (C)
Variable modifications:
S3 : Phospho (ST), with neutral losses 0.0000(shown in table), 97.9769
S13 : Phospho (ST), with neutral losses 0.0000(shown in table), 97.9769
Ions Score: 48 **Expect:** 0.0021
Matches (Bold Red): 75/428 fragment ions using 116 most intense peaks

#	b	b^{++}	b*	b*$^{++}$	b^{0}	b^{0++}	Seq.	y	y^{++}	y*	y*$^{++}$	y^{0}	y^{0++}	#
1	129.0659	65.0366	112.0393	56.5233			Q							22
2	257.1608	129.0840	240.1343	120.5708			K	2368.8701	1184.9387	2351.8436	**1176.4254**	2350.8596	**1175.9334**	21
3	**424.1592**	212.5832	407.1326	204.0699	**406.1486**	203.5779	S	2240.7752	**1120.8912**	2223.7486	**1112.3779**	2222.7646	1111.8859	20
4	**539.1861**	270.0967	**522.1596**	261.5834	**521.1755**	261.0914	D	2073.7768	1037.3920	2056.7503	1028.8788	2055.7663	1028.3868	19
5	610.2232	305.6153	**593.1967**	297.1020	592.2127	296.6100	A	1958.7499	**979.8786**	1941.7233	971.3653	1940.7393	970.8733	18
6	739.2658	370.1365	**722.2393**	361.6233	721.2553	361.1313	E	1887.7128	**944.3600**	1870.6862	**935.8467**	1869.7022	**935.3547**	17
7	**868.3084**	434.6578	**851.2819**	426.1446	**850.2978**	425.6526	E	1758.6702	**879.8387**	1741.6436	**871.3254**	1740.6596	**870.8334**	16
8	983.3354	492.1713	**966.3088**	483.6580	965.3248	483.1660	D	1629.6276	**815.3174**	1612.6010	806.8042	1611.6170	806.3121	15
9	1040.3568	520.6820	1023.3303	512.1688	1022.3463	511.6768	G	1514.6006	**757.8040**	1497.5741	**749.2907**	1496.5901	**748.7987**	14
10	1097.3783	549.1928	1080.3517	540.6795	1079.3677	540.1875	G	1457.5792	**729.2932**	1440.5526	720.7799	1439.5686	720.2879	13
11	1198.4260	599.7166	**1181.3994**	591.2033	**1180.4154**	590.7113	T	1400.5577	**700.7825**	1383.5312	692.2692	1382.5471	691.7772	12
12	1255.4474	628.2274	**1238.4209**	619.7141	1237.4369	619.2221	G	1299.5100	**650.2587**	1282.4835	641.7454	1281.4995	641.2534	11
13	**1422.4458**	711.7265	**1405.4192**	703.2133	1404.4352	702.7212	S	1242.4886	**621.7479**	1225.4620	613.2346	1224.4780	**612.7426**	10
14	1550.5044	775.7558	1533.4778	767.2425	1532.4938	766.7505	Q	1075.4902	**538.2487**	1058.4637	**529.7355**	1057.4796	**529.2435**	9
15	1665.5313	833.2693	**1648.5047**	824.7560	1647.5207	824.2640	D	947.4316	**474.2195**	930.4051	465.7062	929.4211	465.2142	8
16	1794.5739	897.7906	1777.5473	889.2773	1776.5633	888.7853	E	832.4047	**416.7060**	**815.3781**	**408.1927**	814.3941	**407.7007**	7
17	1923.6165	**962.3119**	1906.5899	953.7986	1905.6059	953.3066	E	703.3621	352.1847	686.3355	343.6714	685.3515	343.1794	6
18	2038.6434	1019.8254	2021.6169	**1011.3121**	2020.6329	**1010.8201**	D	**574.3195**	287.6634	557.2930	279.1501	556.3089	278.6581	5
19	2125.6755	**1063.3414**	2108.6489	**1054.8281**	2107.6649	**1054.3361**	S	**459.2926**	230.1499	442.2660	221.6366	**441.2820**	221.1446	4
20	2253.7704	**1127.3888**	2236.7439	**1118.8756**	2235.7599	**1118.3836**	K	**372.2605**	186.6339	355.2340	178.1206			3
21	2350.8232	**1175.9152**	2333.7966	**1167.4020**	2332.8126	1166.9099	P	**244.1656**	122.5864	227.1390	114.0731			2
22							K	147.1128	74.0600	130.0863	65.5468			1

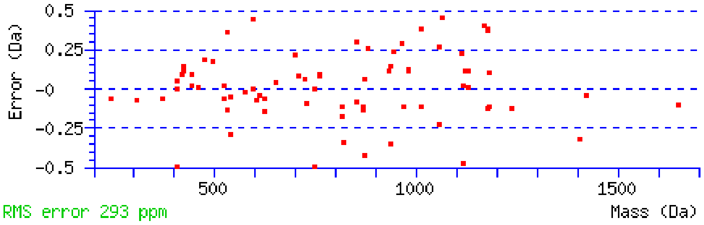

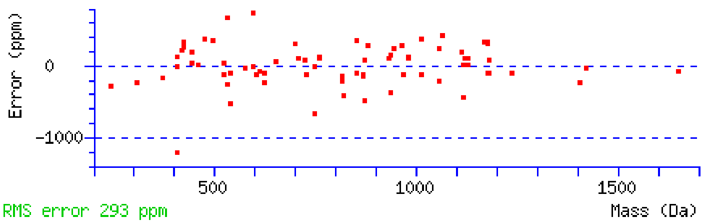

All matches to this query

Score	Mr(calc):	Delta	Sequence
48.5	2495.9214	-0.0059	QKSDAEEDGGTGSQDEEDSKPK
31.6	2495.9214	-0.0059	QKSDAEEDGGTGSQDEEDSKPK
15.3	2495.9214	-0.0059	QKSDAEEDGGTGSQDEEDSKPK
7.6	2495.9130	0.0025	FSQFFHISYSSSLPSLSDK
5.2	2495.9214	-0.0059	QKSDAEEDGGTGSQDEEDSKPK
4.5	2494.8957	1.0198	NSKKRPASPSHNSSGGGYGASK
4.0	2493.9264	1.9891	QTVMSGDGVSQRSQSPXQRR
4.0	2493.9264	1.9891	QTVMSGDGVSQRSQSPXQRR
4.0	2493.9264	1.9891	QTVMSGDGVSQRSQSPXQRR
4.0	2495.9039	0.0116	SKSQSSGSSATHPISVPGARR

Spectrum No: 331; Query: 2473; Rank: 1

Peptide View

MS/MS Fragmentation of **EGINPGYDDYADSDEDQHDAYLER**
Found in **IPI00231263**, Tax_Id=10116 Gene_Symbol=Ssrp1 FACT complex subunit SSRP1

Match to Query 2473: 2866.093452 from(956.371760,3+)
Title: 091008RatKidney_NoSalt_27.2485.2485.3.dta
Data file K:\NewmanPaper\Piliang\3SubProteomes\Piliang3SP\mgf5ppm\ERLIC_3SubProteomes5ppm.mgf

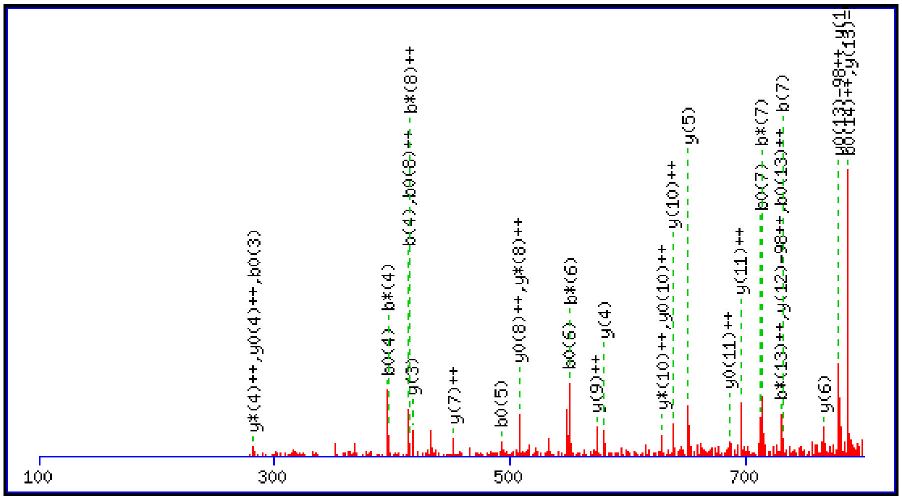

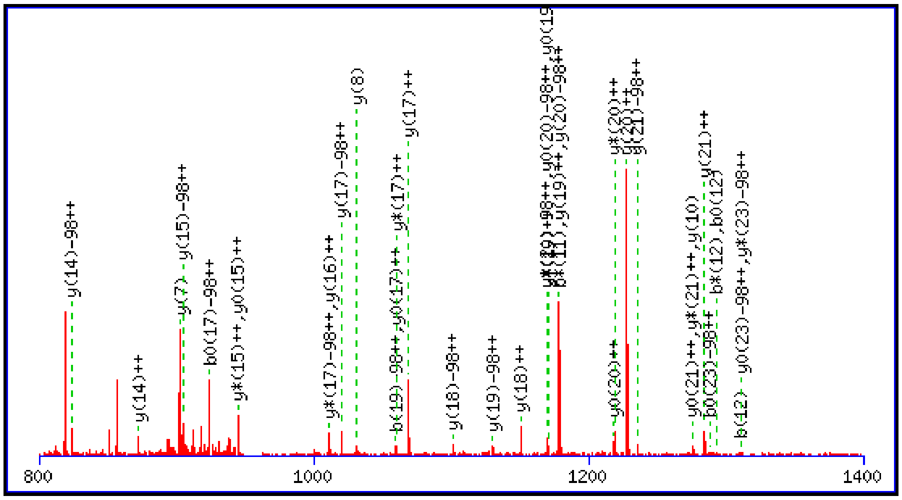

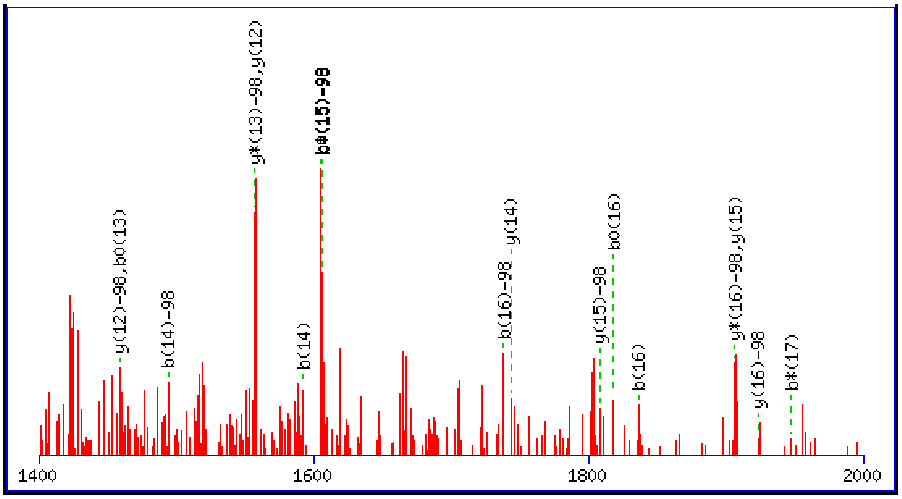

Monoisotopic mass of neutral peptide Mr(calc): 2866.0879
Fixed modifications: Carbamidomethyl (C)
Variable modifications:
S13 : Phospho (ST), with neutral losses 97.9769(shown in table), 0.0000
Ions Score: 49 **Expect:** 0.0034
Matches (Bold Red): 91/406 fragment ions using 160 most intense peaks

#	b	b^{++}	b*	b^{*++}	b^{0}	b^{0++}	Seq.	y	y^{++}	y*	y^{*++}	y^{0}	y^{0++}	#
1	130.0499	65.5286			112.0393	56.5233	E							24
2	187.0713	94.0393			169.0608	85.0340	G	2640.0757	1320.5415	2623.0492	**1312.0282**	2622.0651	**1311.5362**	23
3	300.1554	150.5813			**282.1448**	141.5761	I	2583.0542	1292.0308	2566.0277	1283.5175	2565.0437	1283.0255	22
4	**414.1983**	207.6028	**397.1718**	199.0895	**396.1878**	198.5975	N	2469.9702	**1235.4887**	2452.9436	1226.9755	2451.9596	1226.4834	21
5	511.2511	256.1292	494.2245	247.6159	**493.2405**	247.1239	P	2355.9272	**1178.4673**	2338.9007	**1169.9540**	2337.9167	**1169.4620**	20
6	568.2726	284.6399	**551.2460**	276.1266	**550.2620**	275.6346	G	2258.8745	**1129.9409**	2241.8479	1121.4276	2240.8639	1120.9356	19
7	**731.3359**	366.1716	**714.3093**	357.6583	**713.3253**	357.1663	Y	2201.8530	**1101.4301**	2184.8265	1092.9169	2183.8425	1092.4249	18
8	846.3628	423.6851	829.3363	**415.1718**	828.3523	**414.6798**	D	2038.7897	**1019.8985**	2021.7631	**1011.3852**	2020.7791	1010.8932	17
9	961.3898	481.1985	944.3632	472.6852	943.3792	472.1932	D	**1923.7627**	962.3850	**1906.7362**	953.8717	1905.7522	953.3797	16
10	1124.4531	562.7302	1107.4265	554.2169	1106.4425	553.7249	Y	**1808.7358**	**904.8715**	1791.7093	896.3583	1790.7252	895.8663	15
11	1195.4902	598.2487	**1178.4637**	589.7355	1177.4796	589.2435	A	1645.6725	**823.3399**	1628.6459	814.8266	1627.6619	814.3346	14
12	**1310.5172**	655.7622	**1293.4906**	647.2489	**1292.5066**	646.7569	D	1574.6354	**787.8213**	**1557.6088**	**779.3080**	1556.6248	**778.8160**	13
13	1379.5386	690.2729	1362.5121	681.7597	1361.5280	681.2677	S	**1459.6084**	**730.3078**	1442.5819	721.7946	1441.5979	721.3026	12
14	**1494.5656**	747.7864	1477.5390	739.2731	1476.5550	738.7811	D	1390.5870	**695.7971**	1373.5604	687.2838	1372.5764	**686.7918**	11
15	1623.6081	812.3077	**1606.5816**	803.7944	**1605.5976**	803.3024	E	**1275.5600**	**638.2836**	1258.5335	**629.7704**	1257.5495	**629.2784**	10
16	**1738.6351**	869.8212	1721.6085	861.3079	1720.6245	860.8159	D	1146.5174	**573.7624**	1129.4909	565.2491	1128.5069	564.7571	9
17	1866.6937	933.8505	1849.6671	925.3372	1848.6831	**924.8452**	Q	**1031.4905**	516.2489	1014.4639	**507.7356**	1013.4799	**507.2436**	8
18	2003.7526	1002.3799	1986.7260	993.8667	1985.7420	993.3746	H	**903.4319**	**452.2196**	886.4054	443.7063	885.4213	443.2143	7
19	2118.7795	**1059.8934**	2101.7530	1051.3801	2100.7690	1050.8881	D	**766.3730**	383.6901	749.3464	375.1769	748.3624	374.6849	6
20	2189.8166	1095.4120	2172.7901	1086.8987	2171.8061	1086.4067	A	**651.3461**	326.1767	634.3195	317.6634	633.3355	317.1714	5
21	2352.8800	1176.9436	2335.8534	1168.4303	2334.8694	1167.9383	Y	**580.3089**	290.6581	563.2824	**282.1448**	562.2984	**281.6528**	4
22	2465.9640	1233.4857	2448.9375	1224.9724	2447.9535	1224.4804	L	**417.2456**	209.1264	400.2191	200.6132	399.2350	200.1212	3
23	2595.0066	1298.0069	2577.9801	1289.4937	2576.9961	**1289.0017**	E	304.1615	152.5844	287.1350	144.0711	286.1510	143.5791	2
24							R	175.1190	88.0631	158.0924	79.5498			1

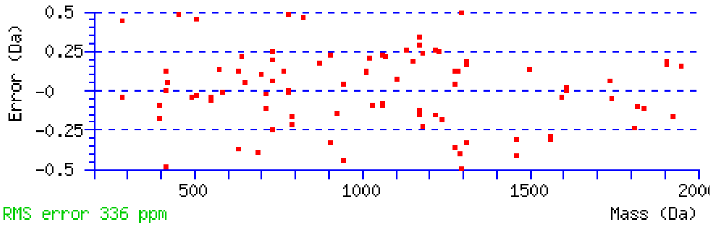

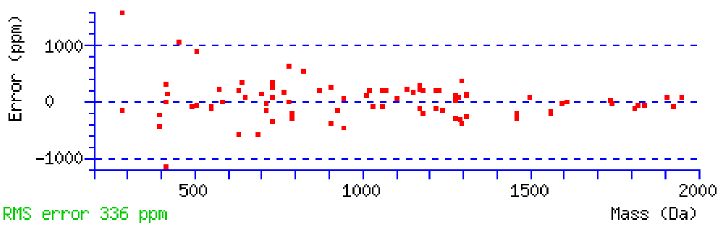

All matches to this query

Score	Mr(calc):	Delta	Sequence
48.5	2866.0879	0.0056	EGINPGYDDYADSDEDQHDAYLER
25.2	2866.0879	0.0056	EGINPGYDDYADSDEDQHDAYLER
13.4	2866.0879	0.0056	EGINPGYDDYADSDEDQHDAYLER
6.6	2866.0879	0.0056	EGINPGYDDYADSDEDQHDAYLER
0.3	2865.0939	0.9996	STSSGXQGLSGSLSGWLTSSGKLRR
0.3	2865.0657	1.0277	ADIISTQQSDGNQSGPVARSVNGSR
0.3	2864.1029	1.9905	EGWDPPMKTGVYGSQWVMSSAPSK
0.3	2864.1029	1.9905	EGWDPPMKTGVYGSQWVMSSAPSK
0.0	2864.0754	2.0181	EEYHVVIQAKDMGGHMGGLSGTTK

Spectrum No: 332; Query: 1790; Rank: 1

Peptide View

MS/MS Fragmentation of **AEETETASSPPEKDRENR**
Found in **IPI00364538**, Tax_Id=10116 Gene_Symbol=Ppp4r2_predicted protein phosphatase 4, regulatory subunit 2

Match to Query 1790: 2124.897702 from(709.306510,3+)
Title: 091008RatKidney_NH4Format01_22.265.265.3.dta
Data file K:\NewmanPaper\Piliang\3SubProteomes\Piliang3SP\mgf5ppm\ERLIC_3SubProteomes5ppm.mgf

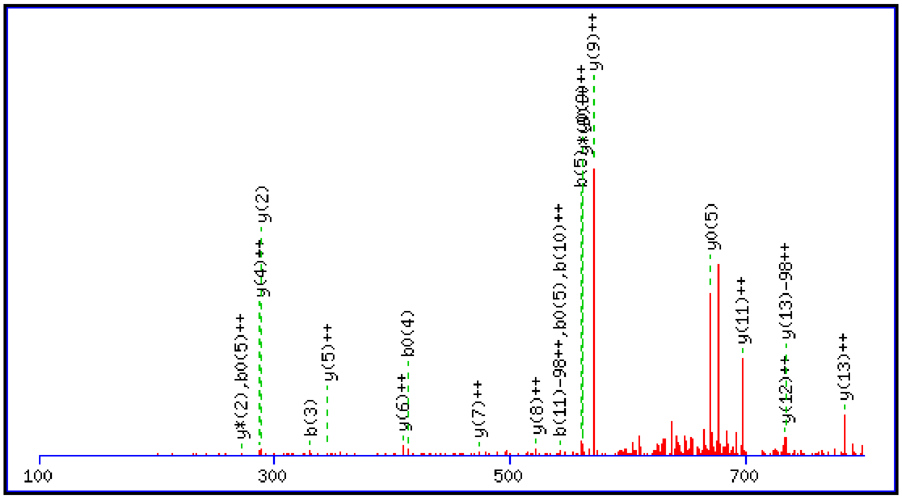

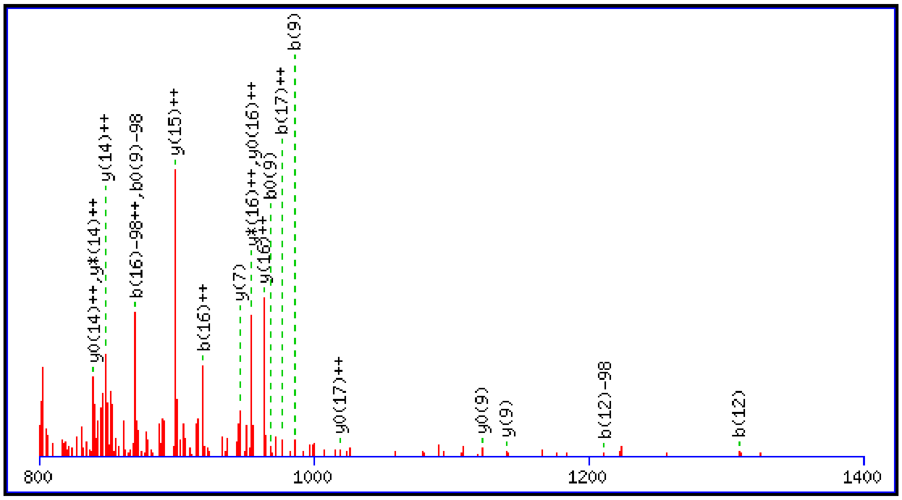

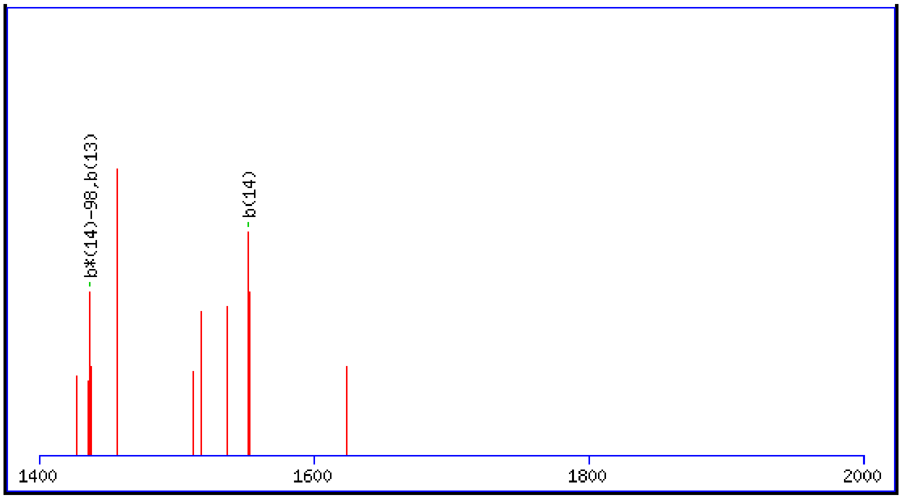

Monoisotopic mass of neutral peptide Mr(calc): 2124.8960
Fixed modifications: Carbamidomethyl (C)
Variable modifications:
S9 : Phospho (ST), with neutral losses 0.0000(shown in table), 97.9769
Ions Score: 48 **Expect:** 0.0033
Matches (Bold Red): 44/268 fragment ions using 85 most intense peaks

#	b	b^{++}	b*	b^{*++}	b^0	b^{0++}	Seq.	y	y^{++}	y*	y^{*++}	y^0	y^{0++}	#
1	72.0444	36.5258					A							18
2	201.0870	101.0471			183.0764	92.0418	E	2054.8662	1027.9368	2037.8397	1019.4235	2036.8557	**1018.9315**	17
3	**330.1296**	165.5684			312.1190	156.5631	E	1925.8236	**963.4155**	1908.7971	**954.9022**	1907.8131	**954.4102**	16
4	431.1773	216.0923			**413.1667**	207.0870	T	1796.7811	**898.8942**	1779.7545	890.3809	1778.7705	889.8889	15
5	**560.2198**	280.6136			**542.2093**	**271.6083**	E	1695.7334	**848.3703**	1678.7068	**839.8571**	1677.7228	**839.3650**	14
6	661.2675	331.1374			643.2570	322.1321	T	1566.6908	**783.8490**	1549.6642	775.3358	1548.6802	774.8437	13
7	732.3046	366.6560			714.2941	357.6507	A	1465.6431	**733.3252**	1448.6166	724.8119	1447.6325	724.3199	12
8	819.3367	410.1720			801.3261	401.1667	S	1394.6060	**697.8066**	1377.5794	689.2934	1376.5954	688.8014	11
9	**986.3350**	493.6711			**968.3245**	484.6659	S	1307.5740	654.2906	1290.5474	645.7773	1289.5634	645.2853	10
10	1083.3878	**542.1975**			1065.3772	533.1922	P	**1140.5756**	**570.7914**	1123.5491	**562.2782**	**1122.5650**	**561.7862**	9
11	1180.4405	590.7239			1162.4300	581.7186	P	1043.5228	**522.2651**	1026.4963	513.7518	1025.5123	513.2598	8
12	**1309.4831**	655.2452			1291.4726	646.2399	E	**946.4701**	**473.7387**	929.4435	465.2254	928.4595	464.7334	7
13	**1437.5781**	719.2927	1420.5516	710.7794	1419.5675	710.2874	K	817.4275	**409.2174**	800.4009	400.7041	799.4169	400.2121	6
14	**1552.6050**	776.8062	1535.5785	768.2929	1534.5945	767.8009	D	689.3325	**345.1699**	672.3060	336.6566	**671.3220**	336.1646	5
15	1708.7062	854.8567	1691.6796	846.3434	1690.6956	845.8514	R	574.3056	**287.6564**	557.2790	279.1432	556.2950	278.6511	4
16	1837.7488	**919.3780**	1820.7222	910.8647	1819.7382	910.3727	E	418.2045	209.6059	401.1779	201.0926	400.1939	200.6006	3
17	1951.7917	**976.3995**	1934.7651	967.8862	1933.7811	967.3942	N	**289.1619**	145.0846	**272.1353**	136.5713			2
18							R	175.1190	88.0631	158.0924	79.5498			1

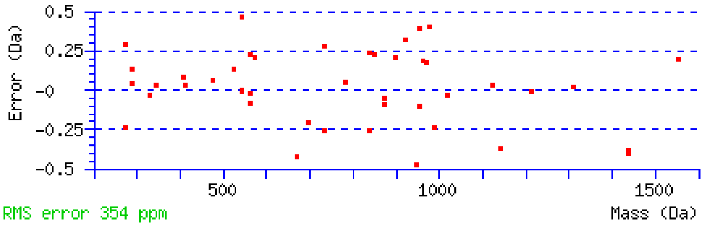

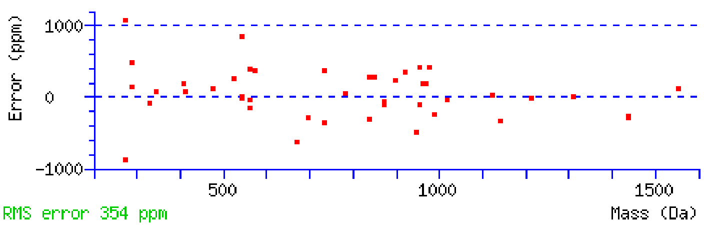

All matches to this query

Score	Mr(calc):	Delta	Sequence
48.4	2124.8960	0.0017	AEETETASSPPEKDRENR
48.4	2124.8960	0.0017	AEETETASSPPEKDRENR
30.8	2124.8960	0.0017	AEETETASSPPEKDRENR

16.3	2124.8960	0.0017	AEETETASSPPEKDRENR
7.9	2123.9099	0.9878	GSKTEMTVATGDVVDVVEK
7.9	2123.9099	0.9878	GSKTEMTVATGDVVDVVEK
7.9	2123.9099	0.9878	GSKTEMTVATGDVVDVVEK
2.9	2123.8981	0.9996	KLSGNGTDSXNGRNGGSTAGR
2.9	2123.8981	0.9996	KLSGNGTDSXNGRNGGSTAGR
2.8	2124.8914	0.0063	TTTLPVEFKCPDGEIMK

Spectrum No: 333; Query: 1489; Rank: 1

Peptide View

MS/MS Fragmentation of **SQSFPDVEPQLPQAPAR**
Found in **IPI00362755**, Tax_Id=10116 Gene_Symbol=Tns similar to tensin

Match to Query 1489: 1945.895928 from(973.955240,2+)
Title: 091008RatKidney_NH4Format01_19.3038.3038.2.dta
Data file K:\NewmanPaper\Piliang\3SubProteomes\Piliang3SP\mgf5ppm\ERLIC_3SubProteomes5ppm.mgf

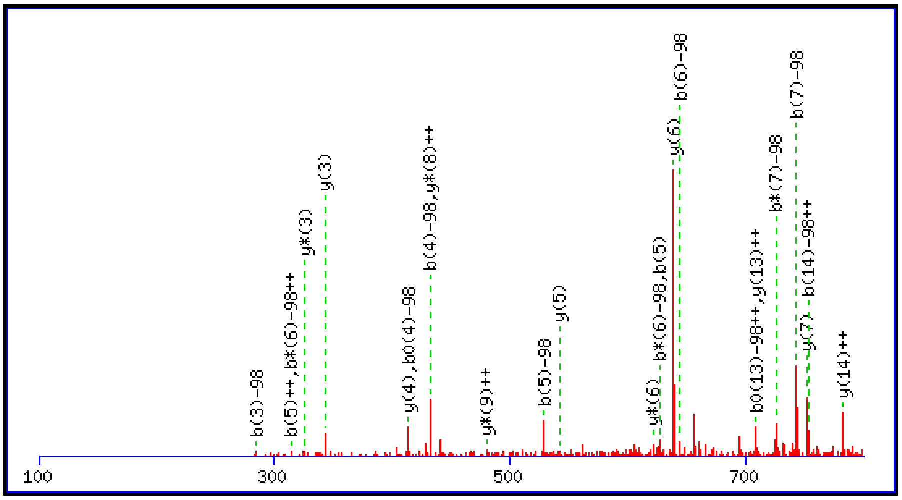

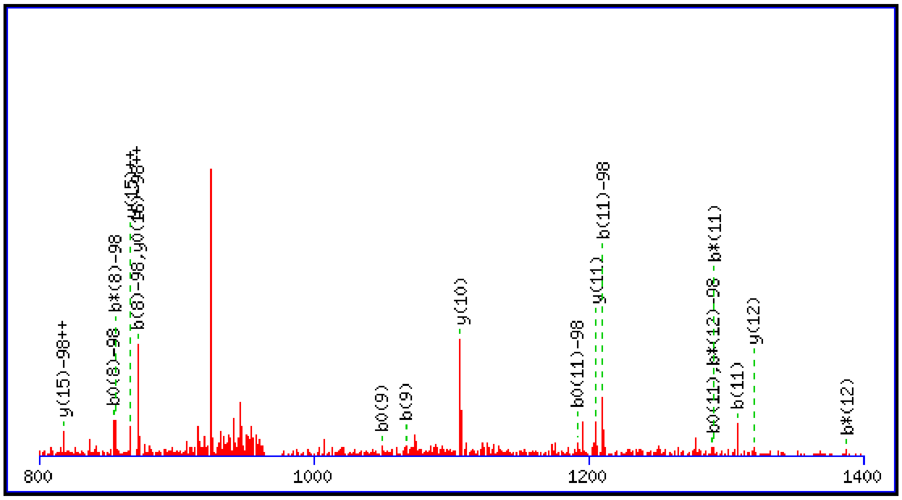

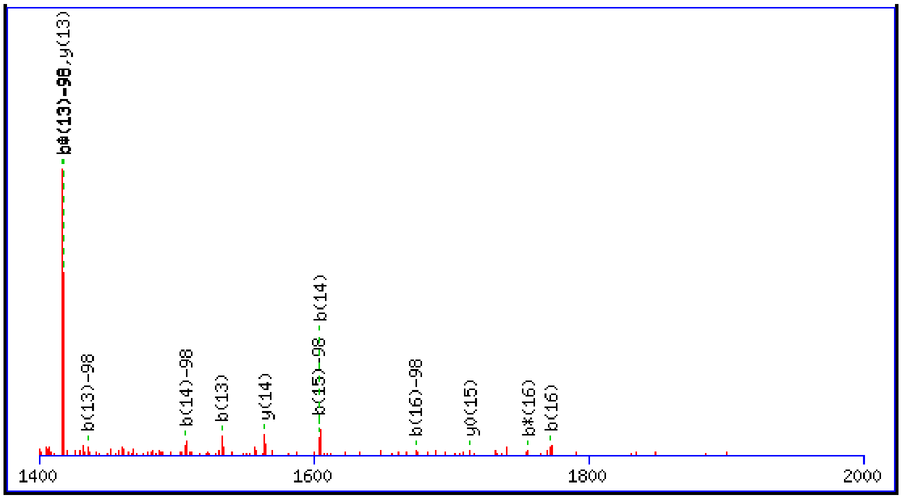

Monoisotopic mass of neutral peptide Mr(calc): 1945.8935
Fixed modifications: Carbamidomethyl (C)
Variable modifications:
S3 : Phospho (ST), with neutral losses 97.9769(shown in table), 0.0000
Ions Score: 48 **Expect:** 0.0035
Matches (Bold Red): 55/268 fragment ions using 100 most intense peaks

#	b	b^{++}	b*	b^{*++}	b^{0}	b^{0++}	Seq.	y	y^{++}	y*	y^{*++}	y^{0}	y^{0++}	#
1	88.0393	44.5233			70.0287	35.5180	S							17
2	216.0979	108.5526	199.0713	100.0393	198.0873	99.5473	Q	1761.8919	881.4496	1744.8653	872.9363	1743.8813	**872.4443**	16
3	**285.1193**	143.0633	268.0928	134.5500	267.1088	134.0580	S	1633.8333	**817.4203**	1616.8067	808.9070	1615.8227	808.4150	15
4	**432.1878**	216.5975	415.1612	208.0842	**414.1772**	207.5922	F	**1564.8118**	**782.9095**	1547.7853	774.3963	1546.8013	773.9043	14
5	**529.2405**	265.1239	512.2140	256.6106	511.2300	256.1186	P	**1417.7434**	**709.3753**	1400.7169	700.8621	1399.7328	700.3701	13
6	**644.2675**	322.6374	**627.2409**	**314.1241**	626.2569	313.6321	D	**1320.6906**	660.8490	1303.6641	652.3357	1302.6801	651.8437	12
7	**743.3359**	372.1716	**726.3093**	363.6583	725.3253	363.1663	V	**1205.6637**	603.3355	1188.6372	594.8222	1187.6531	594.3302	11
8	**872.3785**	436.6929	**855.3519**	428.1796	**854.3679**	427.6876	E	**1106.5953**	553.8013	1089.5687	545.2880	1088.5847	544.7960	10
9	969.4312	485.2193	952.4047	476.7060	951.4207	476.2140	P	977.5527	489.2800	960.5261	**480.7667**			9
10	1097.4898	549.2485	1080.4633	540.7353	1079.4792	540.2433	Q	880.4999	440.7536	863.4734	**432.2403**			8
11	**1210.5739**	605.7906	1193.5473	597.2773	**1192.5633**	596.7853	L	**752.4413**	376.7243	735.4148	368.2110			7
12	1307.6266	654.3170	**1290.6001**	645.8037	1289.6161	645.3117	P	**639.3573**	320.1823	**622.3307**	311.6690			6
13	**1435.6852**	718.3462	**1418.6587**	709.8330	**1417.6746**	**709.3410**	Q	**542.3045**	271.6559	525.2780	263.1426			5
14	**1506.7223**	**753.8648**	1489.6958	745.3515	1488.7118	744.8595	A	**414.2459**	207.6266	397.2194	199.1133			4
15	**1603.7751**	802.3912	1586.7485	793.8779	1585.7645	793.3859	P	**343.2088**	172.1081	**326.1823**	163.5948			3
16	**1674.8122**	837.9097	1657.7857	829.3965	1656.8016	828.9045	A	246.1561	123.5817	229.1295	115.0684			2
17							R	175.1190	88.0631	158.0924	79.5498			1

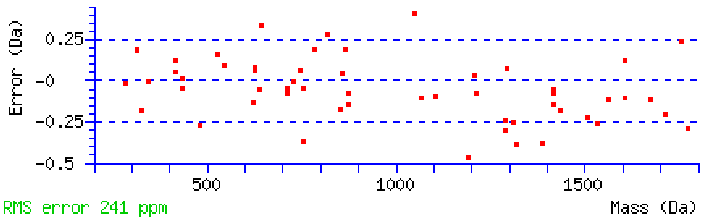

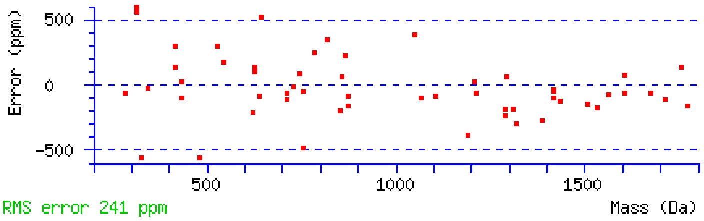

All matches to this query

Score	Mr(calc):	Delta	Sequence
48.4	1945.8935	0.0024	SQSFPDVEPQLPQAPAR
46.0	1945.8935	0.0024	SQSFPDVEPQLPQAPAR
3.0	1944.8863	1.0096	TLKAQNSELASTANMLR
2.5	1945.8822	0.0137	AQYYLPDGSTIEIGPSR

2.1	1944.8863	1.0096	TLKAQNSELASTANMLR
1.9	1945.9016	-0.0057	QSPRVASCLVHSLCPR
1.1	1945.8935	0.0024	HRTDEELQPPPTTFAK
1.1	1945.8935	0.0024	HRTDEELQPPPTTFAK
0.9	1945.9016	-0.0057	QSPRVASCLVHSLCPR
0.4	1943.9023	1.9936	TLKAQNSELASTANMLR

Spectrum No: 334; Query: 2498; Rank: 1

Peptide View

MS/MS Fragmentation of **LEREDSSEEEEEEEIDDEEIERR**
Found in **IPI00209527**, Tax_Id=10116 Gene_Symbol=RGD1562232_predicted similar to microfibrillar-associated protein 1

Match to Query 2498: 2924.116512 from(975.712780,3+)
Title: 100101RatKid_NS_deglyco_24.1910.1910.3.dta
Data file K:\NewmanPaper\Piliang\3SubProteomes\Piliang3SP\mgf5ppm\ERLIC_3SubProteomes5ppm.mgf

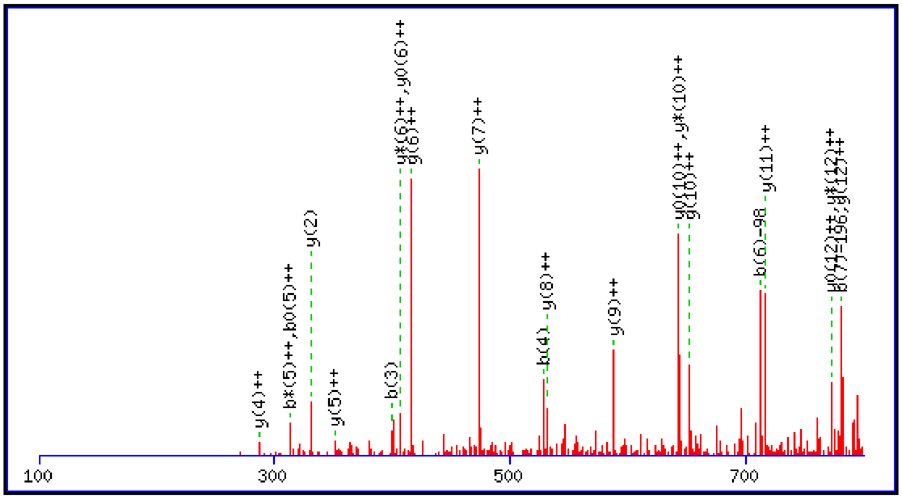

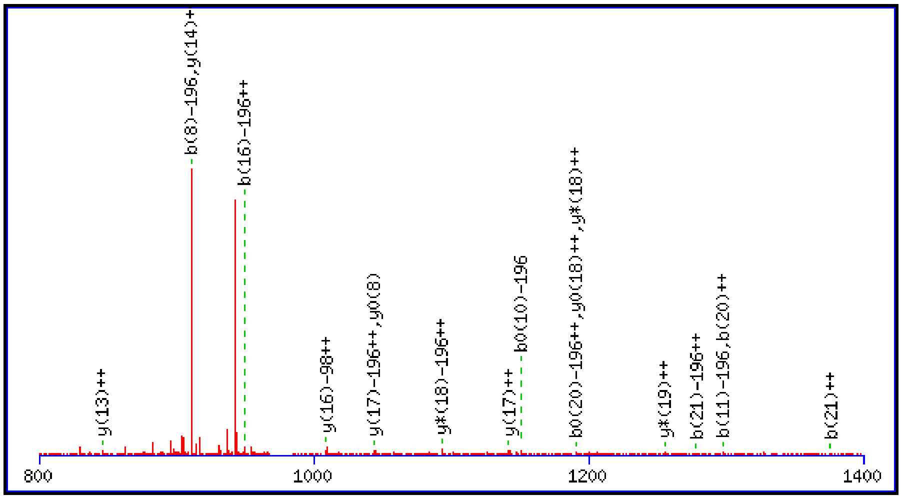

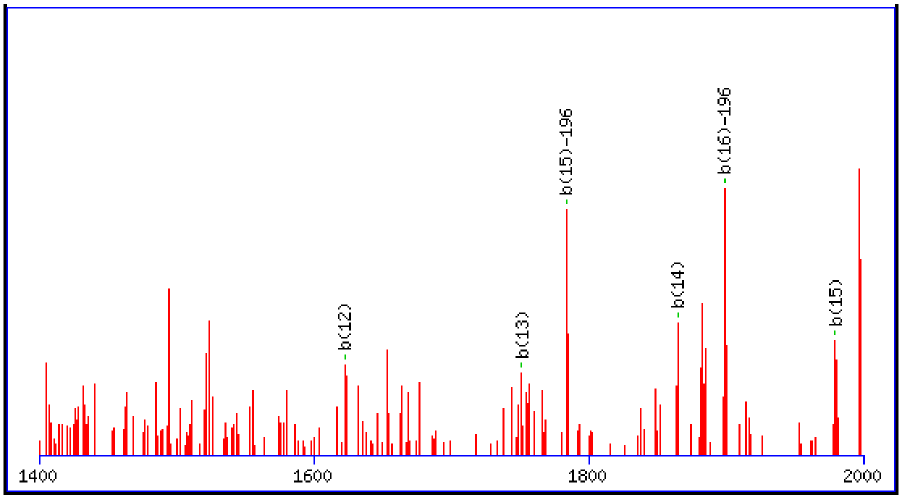

Monoisotopic mass of neutral peptide Mr(calc): 2924.1121
Fixed modifications: Carbamidomethyl (C)
Variable modifications:
S6 : Phospho (ST), with neutral losses 97.9769(shown in table), 0.0000
S7 : Phospho (ST), with neutral losses 97.9769(shown in table), 0.0000
Ions Score: 48 **Expect:** 0.0034
Matches (Bold Red): 47/374 fragment ions using 80 most intense peaks

#	b	b++	b*	b*++	b0	b0++	Seq.	y	y++	y*	y*++	y0	y0++	#
1	114.0913	57.5493					L							22
2	243.1339	122.0706			225.1234	113.0653	E	2616.0816	1308.5444	2599.0550	1300.0311	2598.0710	1299.5391	21
3	**399.2350**	200.1212	382.2085	191.6079	381.2245	191.1159	R	2487.0390	1244.0231	2470.0124	1235.5099	2469.0284	1235.0178	20
4	**528.2776**	264.6425	511.2511	256.1292	510.2671	255.6372	E	2330.9379	1165.9726	2313.9113	1157.4593	2312.9273	1156.9673	19
5	**643.3046**	322.1559	626.2780	**313.6427**	625.2940	**313.1506**	D	2201.8953	1101.4513	2184.8687	**1092.9380**	2183.8847	1092.4460	18
6	**712.3260**	356.6667	695.2995	348.1534	694.3155	347.6614	S	2086.8683	**1043.9378**	2069.8418	1035.4245	2068.8578	1034.9325	17
7	**781.3475**	391.1774	764.3209	382.6641	763.3369	382.1721	S	2017.8469	**1009.4271**	2000.8203	1000.9138	1999.8363	1000.4218	16
8	**910.3901**	455.6987	893.3635	447.1854	892.3795	446.6934	E	1948.8254	974.9163	1931.7989	966.4031	1930.8148	965.9111	15
9	1039.4327	520.2200	1022.4061	511.7067	1021.4221	511.2147	E	1819.7828	**910.3950**	1802.7563	901.8818	1801.7723	901.3898	14
10	1168.4753	584.7413	1151.4487	576.2280	**1150.4647**	575.7360	E	1690.7402	**845.8738**	1673.7137	837.3605	1672.7297	836.8685	13
11	**1297.5179**	649.2626	1280.4913	640.7493	1279.5073	640.2573	E	1561.6976	**781.3525**	1544.6711	**772.8392**	1543.6871	**772.3472**	12
12	1426.5605	713.7839	1409.5339	705.2706	1408.5499	704.7786	E	1432.6550	**716.8312**	1415.6285	708.3179	1414.6445	707.8259	11
13	1555.6031	778.3052	1538.5765	769.7919	1537.5925	769.2999	E	1303.6124	**652.3099**	1286.5859	**643.7966**	1285.6019	**643.3046**	10
14	1668.6871	834.8472	1651.6606	826.3339	1650.6765	825.8419	I	1174.5699	**587.7886**	1157.5433	579.2753	1156.5593	578.7833	9
15	**1783.7141**	892.3607	1766.6875	883.8474	1765.7035	883.3554	D	1061.4858	**531.2465**	1044.4592	522.7333	**1043.4752**	522.2413	8
16	**1898.7410**	**949.8741**	1881.7145	941.3609	1880.7304	940.8689	D	946.4588	**473.7331**	929.4323	465.2198	928.4483	464.7278	7
17	2027.7836	1014.3954	2010.7570	1005.8822	2009.7730	1005.3902	E	831.4319	**416.2196**	814.4054	**407.7063**	813.4213	**407.2143**	6
18	2156.8262	1078.9167	2139.7996	1070.4035	2138.8156	1069.9114	E	702.3893	**351.6983**	685.3628	343.1850	684.3787	342.6930	5
19	2269.9103	1135.4588	2252.8837	1126.9455	2251.8997	1126.4535	I	573.3467	**287.1770**	556.3202	278.6637	555.3362	278.1717	4
20	2398.9528	1199.9801	2381.9263	1191.4668	2380.9423	**1190.9748**	E	460.2627	230.6350	443.2361	222.1217	442.2521	221.6297	3
21	2555.0540	**1278.0306**	2538.0274	1269.5173	2537.0434	1269.0253	R	**331.2201**	166.1137	314.1935	157.6004			2
22							R	175.1190	88.0631	158.0924	79.5498			1

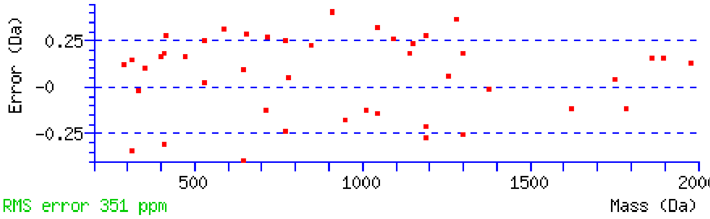

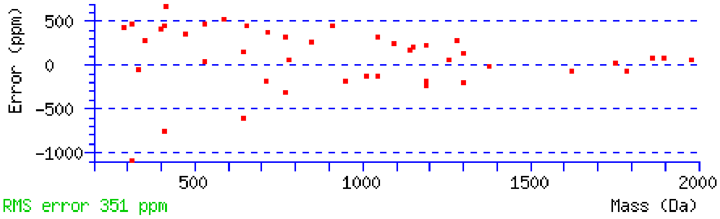

All matches to this query

Score	Mr(calc):	Delta	Sequence
48.4	2924.1121	0.0045	LEREDSSEEEEEEIDDEEIERR
9.9	2923.1355	0.9810	NSDSNLLSLDGLDNEVKDGLPDNCR
9.5	2924.1061	0.0104	KSNIFCSNTNHRTPAQTNIYCR
9.5	2924.1061	0.0104	KSNIFCSNTNHRTPAQTNIYCR
9.5	2924.1061	0.0104	KSNIFCSNTNHRTPAQTNIYCR
7.7	2924.1435	-0.0270	SQPYFAEGTCSSNLKSVWPMNTSR
5.3	2924.1061	0.0104	KSNIFCSNTNHRTPAQTNIYCR
5.3	2924.1061	0.0104	KSNIFCSNTNHRTPAQTNIYCR
5.3	2924.1061	0.0104	KSNIFCSNTNHRTPAQTNIYCR
5.3	2923.1221	0.9945	KSNIFCSNTNHRTPAQTNIYCR

Spectrum No: 335; Query: 2802; Rank: 1

Peptide View

MS/MS Fragmentation of **QAEVADQQTTDLPAENGETENQSPASEEEKEAKSD**
Found in **IPI00373052**, Tax_Id=10116 Gene_Symbol=Hmgn1 High mobility group nucleosomal binding domain 1

Match to Query 2802: 3934.596372 from(1312.539400,3+)
Title: 100101RatKid_NS_deglyco_26.1808.1808.3.dta
Data file K:\NewmanPaper\Piliang\3SubProteomes\Piliang3SP\mgf5ppm\ERLIC_3SubProteomes5ppm.mgf

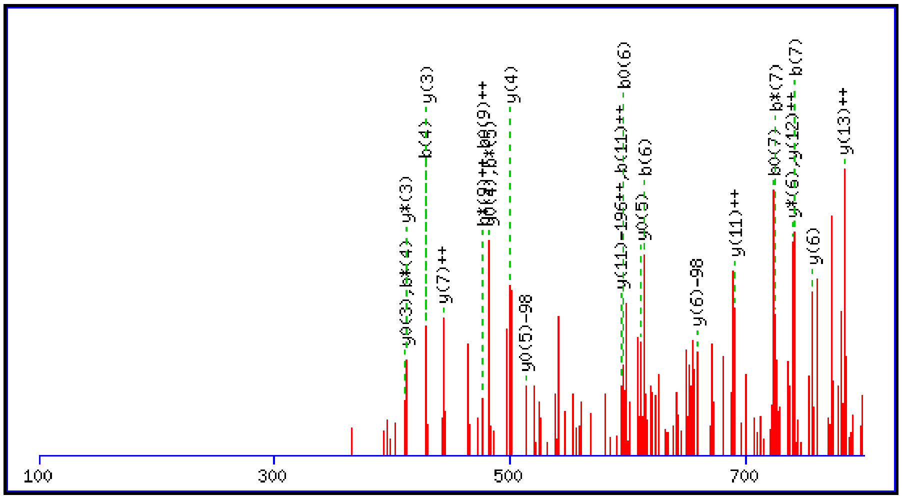

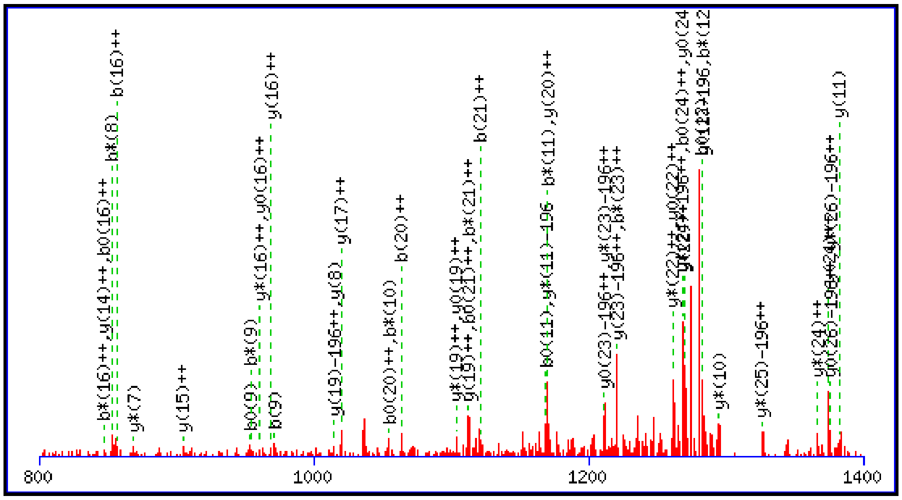

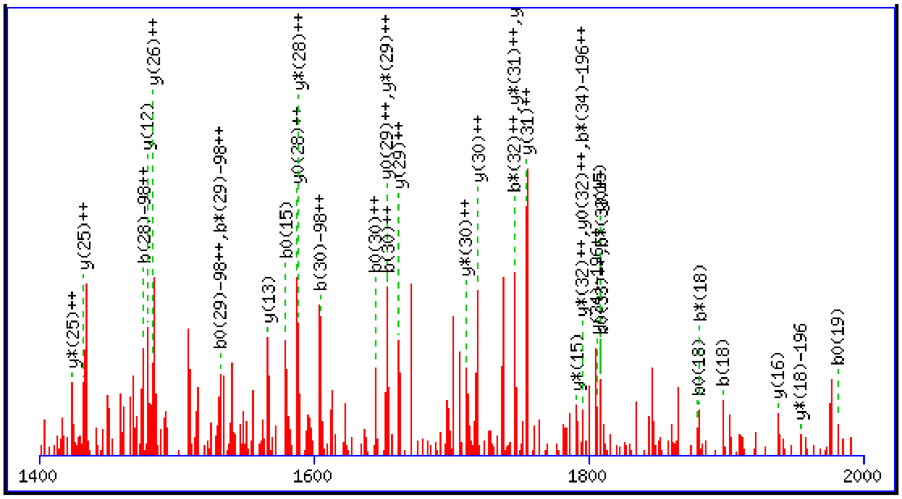

Monoisotopic mass of neutral peptide Mr(calc): 3934.5790
Fixed modifications: Carbamidomethyl (C)
Variable modifications:
S26 : Phospho (ST), with neutral losses 0.0000(shown in table), 97.9769
S34 : Phospho (ST), with neutral losses 0.0000(shown in table), 97.9769
Ions Score: 48 **Expect:** 0.0064
Matches (Bold Red): 112/650 fragment ions using 180 most intense peaks

#	b	b^{++}	b*	b^{*++}	b^0	b^{0++}	Seq.	y	y^{++}	y*	y^{*++}	y^0	y^{0++}	#
1	129.0659	65.0366	112.0393	56.5233			Q							35
2	200.1030	100.5551	183.0764	92.0418			A	3807.5277	1904.2675	3790.5012	1895.7542	3789.5172	1895.2622	34
3	329.1456	165.0764	312.1190	156.5631	311.1350	156.0711	E	3736.4906	1868.7489	3719.4641	1860.2357	3718.4801	1859.7437	33
4	**428.2140**	214.6106	**411.1874**	206.0974	410.2034	205.6053	V	3607.4480	1804.2277	3590.4215	**1795.7144**	3589.4375	**1795.2224**	32
5	499.2511	250.1292	**482.2245**	241.6159	481.2405	241.1239	A	3508.3796	**1754.6934**	3491.3531	**1746.1802**	3490.3691	**1745.6882**	31
6	**614.2780**	307.6427	597.2515	299.1294	**596.2675**	298.6374	D	3437.3425	**1719.1749**	3420.3160	**1710.6616**	3419.3319	1710.1696	30
7	**742.3366**	371.6719	**725.3101**	363.1587	**724.3260**	362.6667	Q	3322.3156	**1661.6614**	3305.2890	**1653.1481**	3304.3050	**1652.6561**	29
8	870.3952	435.7012	**853.3686**	427.1880	852.3846	426.6959	Q	3194.2570	1597.6321	3177.2304	**1589.1189**	3176.2464	**1588.6268**	28
9	**971.4429**	486.2251	**954.4163**	**477.7118**	**953.4323**	**477.2198**	T	3066.1984	1533.6028	3049.1719	1525.0896	3048.1878	1524.5976	27
10	1072.4905	536.7489	**1055.4640**	528.2356	1054.4800	527.7436	T	2965.1507	**1483.0790**	2948.1242	1474.5657	2947.1402	1474.0737	26
11	1187.5175	**594.2624**	**1170.4909**	585.7491	**1169.5069**	585.2571	D	2864.1030	**1432.5552**	2847.0765	**1424.0419**	2846.0925	1423.5499	25
12	1300.6016	650.8044	**1283.5750**	642.2911	**1282.5910**	641.7991	L	2749.0761	**1375.0417**	2732.0496	**1366.5284**	2731.0655	1366.0364	24
13	1397.6543	699.3308	1380.6278	690.8175	1379.6438	690.3255	P	2635.9920	1318.4997	2618.9655	1309.9864	2617.9815	1309.4944	23
14	1468.6914	734.8494	1451.6649	726.3361	1450.6809	725.8441	A	2538.9393	**1269.9733**	2521.9127	**1261.4600**	2520.9287	**1260.9680**	22
15	1597.7340	799.3707	1580.7075	790.8574	**1579.7235**	790.3654	E	2467.9022	1234.4547	2450.8756	1225.9414	2449.8916	1225.4494	21
16	1711.7770	**856.3921**	1694.7504	**847.8788**	1693.7664	**847.3868**	N	2338.8596	**1169.9334**	2321.8330	1161.4201	2320.8490	1160.9281	20
17	1768.7984	884.9028	1751.7719	876.3896	1750.7878	875.8976	G	2224.8166	**1112.9120**	2207.7901	**1104.3987**	2206.8061	**1103.9067**	19
18	**1897.8410**	949.4241	**1880.8145**	940.9109	**1879.8304**	940.4189	E	2167.7952	1084.4012	2150.7686	1075.8880	2149.7846	1075.3959	18
19	1998.8887	999.9480	1981.8621	991.4347	**1980.8781**	990.9427	T	2038.7526	**1019.8799**	2021.7260	1011.3667	2020.7420	1010.8746	17
20	2127.9313	**1064.4693**	2110.9047	1055.9560	2109.9207	**1055.4640**	E	**1937.7049**	**969.3561**	1920.6784	**960.8428**	1919.6943	**960.3508**	16
21	2241.9742	**1121.4907**	2224.9477	**1112.9775**	2223.9636	**1112.4855**	N	**1808.6623**	**904.8348**	**1791.6358**	896.3215	1790.6517	895.8295	15
22	2370.0328	1185.5200	2353.0062	1177.0068	2352.0222	1176.5147	Q	1694.6194	**847.8133**	1677.5928	839.3001	1676.6088	838.8080	14
23	2457.0648	1229.0360	2440.0383	**1220.5228**	2439.0542	1220.0308	S	**1566.5608**	**783.7840**	1549.5343	775.2708	1548.5502	774.7788	13
24	2554.1176	1277.5624	2537.0910	1269.0492	2536.1070	**1268.5571**	P	**1479.5288**	**740.2680**	1462.5022	731.7548	1461.5182	731.2627	12
25	2625.1547	1313.0810	2608.1281	1304.5677	2607.1441	1304.0757	A	**1382.4760**	**691.7416**	1365.4495	683.2284	1364.4654	682.7364	11
26	2792.1530	1396.5802	2775.1265	1388.0669	2774.1425	1387.5749	S	1311.4389	656.2231	**1294.4124**	647.7098	1293.4283	647.2178	10
27	2921.1956	1461.1015	2904.1691	1452.5882	2903.1851	1452.0962	E	1144.4405	572.7239	1127.4140	564.2106	1126.4300	563.7186	9
28	3050.2382	1525.6228	3033.2117	1517.1095	3032.2277	1516.6175	E	**1015.3980**	508.2026	998.3714	499.6893	997.3874	499.1973	8
29	3179.2808	1590.1440	3162.2543	1581.6308	3161.2703	1581.1388	E	886.3554	**443.6813**	**869.3288**	435.1680	868.3448	434.6760	7
30	3307.3758	**1654.1915**	3290.3492	1645.6783	3289.3652	**1645.1862**	K	**757.3128**	379.1600	**740.2862**	370.6467	739.3022	370.1547	6
31	3436.4184	1718.7128	3419.3918	1710.1996	3418.4078	1709.7075	E	629.2178	315.1125	612.1913	306.5993	**611.2072**	306.1073	5

32	3507.4555	1754.2314	3490.4289	1745.7181	3489.4449	1745.2261	A	500.1752	250.5912	483.1487	242.0780	482.1646	241.5860	4
33	3635.5505	1818.2789	3618.5239	1809.7656	3617.5399	1809.2736	K	429.1381	215.0727	412.1115	206.5594	411.1275	206.0674	3
34	3802.5488	1901.7780	3785.5223	1893.2648	3784.5382	1892.7728	S	301.0431	151.0252			283.0326	142.0199	2
35							D	134.0448	67.5260			116.0342	58.5207	1

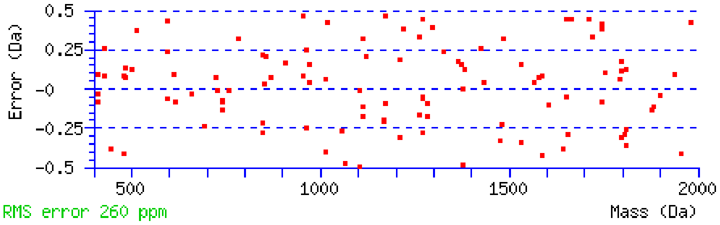

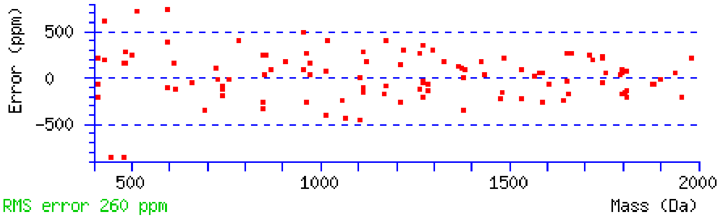

All matches to this query

Score	Mr(calc):	Delta	Sequence
48.3	3934.5790	0.0174	QAEVADQQTTDLPAENGETENQSPASEEEKEAKSD
36.6	3934.5790	0.0174	QAEVADQQTTDLPAENGETENQSPASEEEKEAKSD
26.2	3934.5790	0.0174	QAEVADQQTTDLPAENGETENQSPASEEEKEAKSD
15.9	3934.5790	0.0174	QAEVADQQTTDLPAENGETENQSPASEEEKEAKSD
7.8	3934.5790	0.0174	QAEVADQQTTDLPAENGETENQSPASEEEKEAKSD
5.0	3934.5790	0.0174	QAEVADQQTTDLPAENGETENQSPASEEEKEAKSD
4.2	3932.5899	2.0065	CDVMYGIEDTQNTMSLAVDGXGVVFHYRAATSR
4.2	3932.5899	2.0065	CDVMYGIEDTQNTMSLAVDGXGVVFHYRAATSR
3.0	3932.6086	1.9877	IGSSGFLNGSLFLEEMNVYNPEKCSYDGVEDKR
3.0	3932.6086	1.9877	IGSSGFLNGSLFLEEMNVYNPEKCSYDGVEDKR

Spectrum No: 336; Query: 440; Rank: 1

Peptide View

MS/MS Fragmentation of **NFSDNQLQEGK**
Found in **IPI00555171**, Tax_Id=10116 Gene_Symbol=Tagln2 Transgelin 2

Match to Query 440: 1358.553128 from(680.283840,2+)
Title: 091008RatKidney_NH4Format01_21.1184.1184.2.dta
Data file K:\NewmanPaper\Piliang\3SubProteomes\Piliang3SP\mgf5ppm\ERLIC_3SubProteomes5ppm.mgf

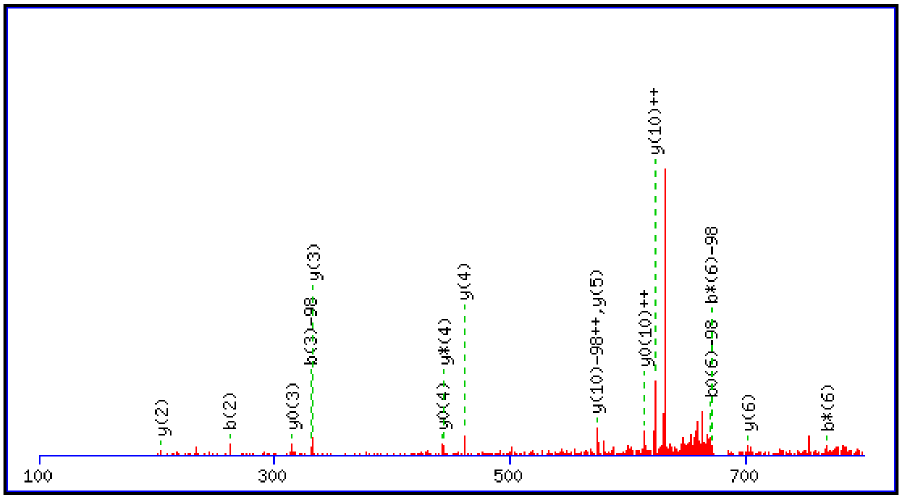

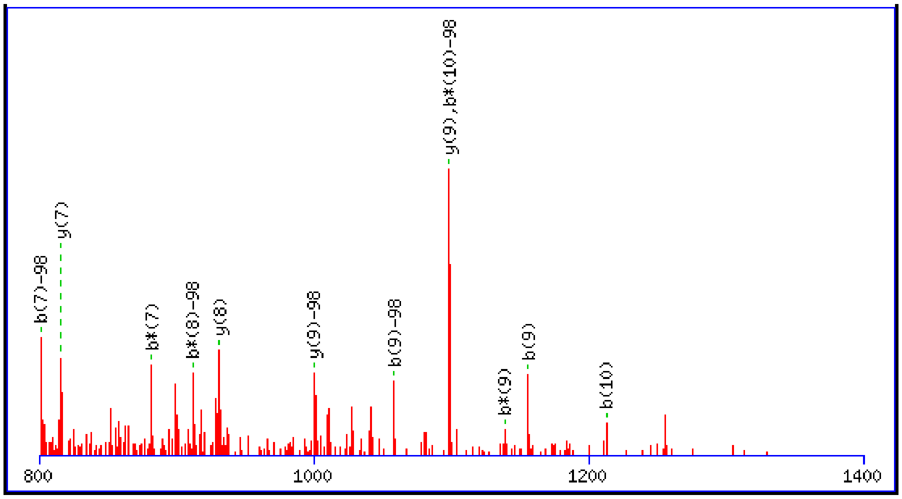

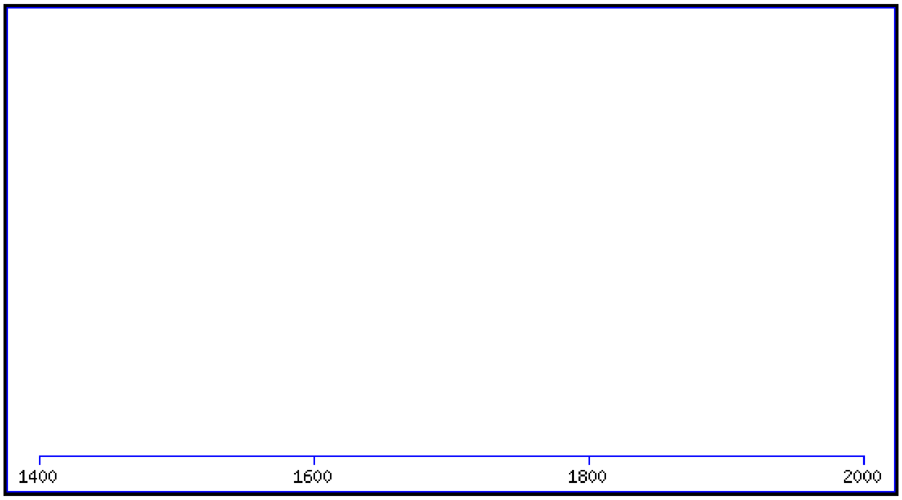

Monoisotopic mass of neutral peptide Mr(calc): 1358.5504
Fixed modifications: Carbamidomethyl (C)
Variable modifications:
S3 : Phospho (ST), with neutral losses 97.9769(shown in table), 0.0000
Ions Score: 48 **Expect:** 0.0015
Matches (Bold Red): 28/172 fragment ions using 44 most intense peaks

#	b	b++	b*	b*++	b0	b0++	Seq.	y	y++	y*	y*++	y0	y0++	#
1	115.0502	58.0287	98.0237	49.5155			N							11
2	**262.1186**	131.5629	245.0921	123.0497			F	1147.5378	**574.2725**	1130.5113	565.7593	1129.5273	565.2673	10
3	**331.1401**	166.0737	314.1135	157.5604	313.1295	157.0684	S	**1000.4694**	500.7383	983.4429	492.2251	982.4588	491.7331	9
4	446.1670	223.5871	429.1405	215.0739	428.1565	214.5819	D	**931.4480**	466.2276	914.4214	457.7143	913.4374	457.2223	8
5	560.2099	280.6086	543.1834	272.0953	542.1994	271.6033	N	**816.4210**	408.7141	799.3945	400.2009	798.4104	399.7089	7
6	688.2685	344.6379	**671.2420**	336.1246	**670.2580**	335.6326	Q	**702.3781**	351.6927	685.3515	343.1794	684.3675	342.6874	6
7	**801.3526**	401.1799	784.3260	392.6667	783.3420	392.1746	L	**574.3195**	287.6634	557.2930	279.1501	556.3089	278.6581	5
8	929.4112	465.2092	**912.3846**	456.6959	911.4006	456.2039	Q	**461.2354**	231.1214	**444.2089**	222.6081	**443.2249**	222.1161	4
9	**1058.4538**	529.7305	1041.4272	521.2172	1040.4432	520.7252	E	**333.1769**	167.0921	316.1503	158.5788	**315.1663**	158.0868	3
10	1115.4752	558.2412	**1098.4487**	549.7280	1097.4647	549.2360	G	**204.1343**	102.5708	187.1077	94.0575			2
11							K	147.1128	74.0600	130.0863	65.5468			1

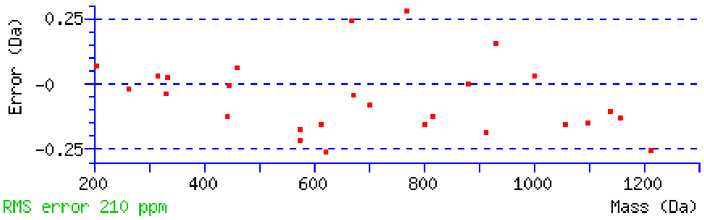

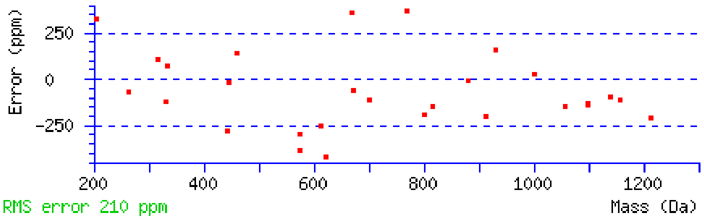

All matches to this query

Score	Mr(calc):	Delta	Sequence
48.3	1358.5504	0.0028	NFSDNQLQEGK
3.0	1358.5504	0.0028	DHGPNSLQPEGK
1.3	1358.5649	-0.0118	RRAVSMDNSNK
0.3	1357.5391	1.0140	TCGLCGNYNGNK
0.3	1357.5391	1.0140	TCGLCGNYNGNK
0.3	1358.5425	0.0106	EDGESADKLMGK

Spectrum No: 337; Query: 1012; Rank: 1

Peptide View

MS/MS Fragmentation of **TNPSPTNPFSSDAQK**
Found in **IPI00202443**, Tax_Id=10116 Gene_Symbol=Numb Numb isoform o/i

Match to Query 1012: 1669.694068 from(835.854310,2+)
Title: 091008RatKidney_NH4Format01_21.1612.1612.2.dta
Data file K:\NewmanPaper\Piliang\3SubProteomes\Piliang3SP\mgf5ppm\ERLIC_3SubProteomes5ppm.mgf

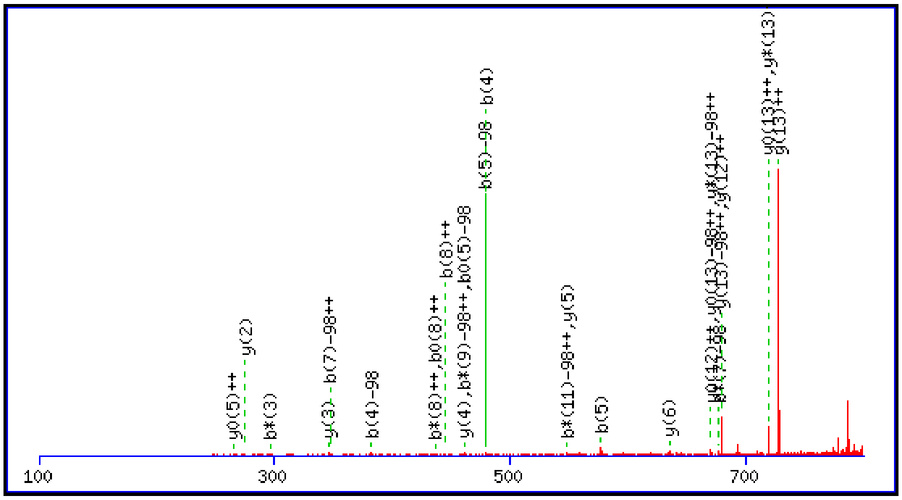

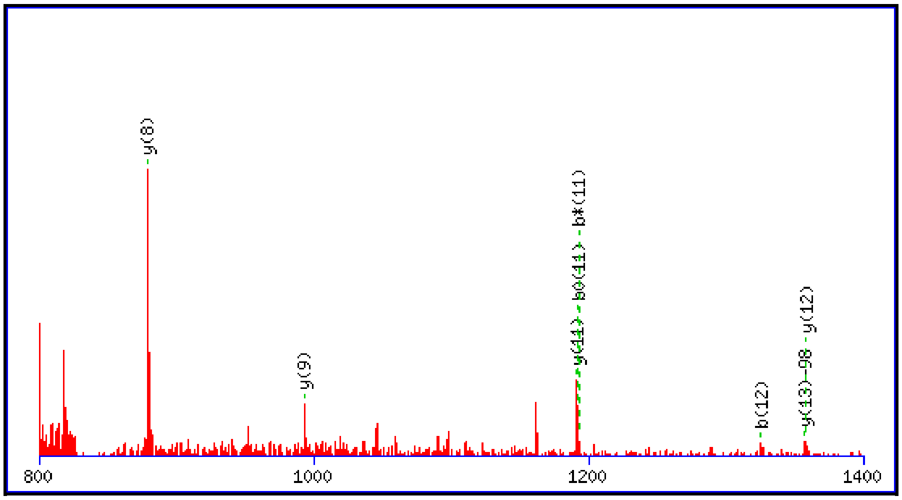

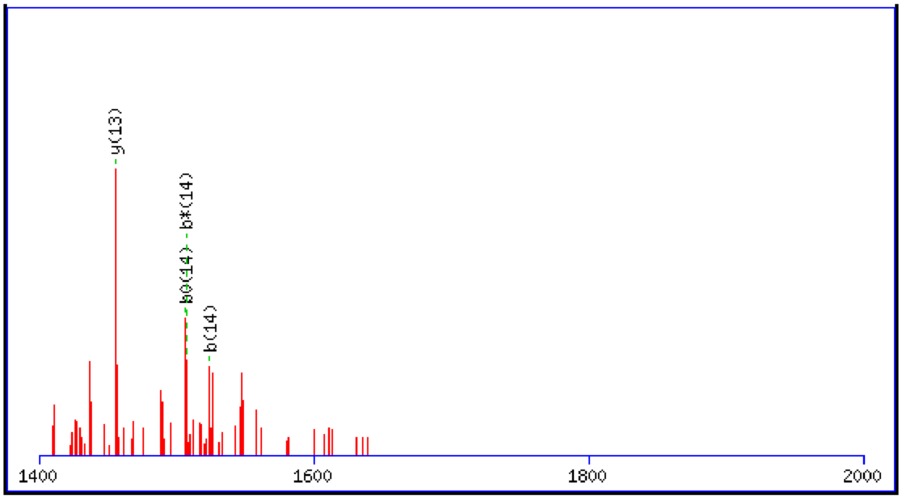

Monoisotopic mass of neutral peptide Mr(calc): 1669.6985
Fixed modifications: Carbamidomethyl (C)
Variable modifications:
S4 : Phospho (ST), with neutral losses 0.0000(shown in table), 97.9769
Ions Score: 48 **Expect:** 0.0026
Matches (Bold Red): 39/244 fragment ions using 62 most intense peaks

#	b	b^{++}	b*	b^{*++}	b^0	b^{0++}	Seq.	y	y^{++}	y*	y^{*++}	y^0	y^{0++}	#
1	102.0550	51.5311			84.0444	42.5258	T							15
2	216.0979	108.5526	199.0713	100.0393	198.0873	99.5473	N	1569.6581	785.3327	1552.6315	776.8194	1551.6475	776.3274	14
3	313.1506	157.0790	**296.1241**	148.5657	295.1401	148.0737	P	**1455.6152**	**728.3112**	1438.5886	**719.7979**	1437.6046	**719.3059**	13
4	**480.1490**	240.5781	463.1224	232.0649	462.1384	231.5729	S	**1358.5624**	**679.7848**	1341.5358	671.2716	1340.5518	**670.7796**	12
5	**577.2018**	289.1045	560.1752	280.5912	559.1912	280.0992	P	**1191.5640**	596.2857	1174.5375	587.7724	1173.5535	587.2804	11
6	678.2494	339.6284	661.2229	331.1151	660.2389	330.6231	T	1094.5113	547.7593	1077.4847	539.2460	1076.5007	538.7540	10
7	792.2924	396.6498	775.2658	388.1365	774.2818	387.6445	N	**993.4636**	497.2354	976.4371	488.7222	975.4530	488.2302	9
8	889.3451	**445.1762**	872.3186	**436.6629**	871.3346	**436.1709**	P	**879.4207**	440.2140	862.3941	431.7007	861.4101	431.2087	8
9	1036.4135	518.7104	1019.3870	510.1971	1018.4030	509.7051	F	782.3679	391.6876	765.3414	383.1743	764.3573	382.6823	7
10	1123.4456	562.2264	1106.4190	553.7132	1105.4350	553.2211	S	**635.2995**	318.1534	618.2729	309.6401	617.2889	309.1481	6
11	1210.4776	605.7424	**1193.4511**	597.2292	**1192.4670**	596.7372	S	**548.2675**	274.6374	531.2409	266.1241	530.2569	**265.6321**	5
12	**1325.5045**	663.2559	1308.4780	654.7426	1307.4940	654.2506	D	**461.2354**	231.1214	444.2089	222.6081	443.2249	222.1161	4
13	1396.5417	698.7745	1379.5151	690.2612	1378.5311	689.7692	A	**346.2085**	173.6079	329.1819	165.0946			3
14	**1524.6002**	762.8038	**1507.5737**	754.2905	**1506.5897**	753.7985	Q	**275.1714**	138.0893	258.1448	129.5761			2
15							K	147.1128	74.0600	130.0863	65.5468			1

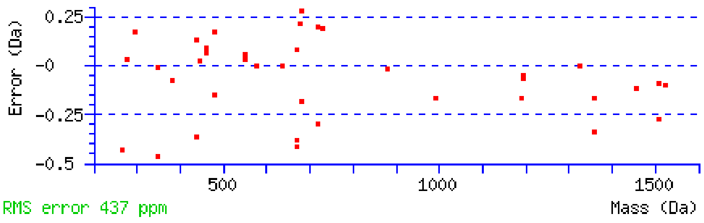

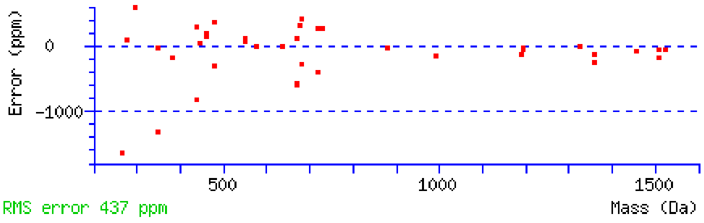

All matches to this query

Score	Mr(calc):	Delta	Sequence
48.3	1669.6985	-0.0044	TNPSPTNPFSSDAQK
31.6	1669.6985	-0.0044	TNPSPTNPFSSDAQK
25.3	1669.6985	-0.0044	TNPSPTNPFSSDAQK
11.8	1669.6985	-0.0044	TNPSPTNPFSSDAQK
8.4	1667.6892	2.0049	NVVSGRIINGYCR
7.8	1669.6985	-0.0044	TNPSPTNPFSSDAQK

7.5	1667.6804	2.0136	DSKSTSKNLNSAQK
6.7	1667.6804	2.0136	DSKSTSKNLNSAQK
6.3	1667.6804	2.0136	DSKSTSKNLNSAQK
6.1	1668.6943	0.9998	SRSAATIMAASTAAGK

Spectrum No: 338; Query: 2598; Rank: 1

Peptide View

MS/MS Fragmentation of **AKPAAQSEEETAASPAASPTPQSAQEPSAPGK**
Found in **IPI00363771**, Tax_Id=10116 Gene_Symbol=Eif3s9 108 kDa protein

Match to Query 2598: 3170.450772 from(1057.824200,3+)
Title: 100101RatKid_NS_deglyco_18.1374.1374.3.dta
Data file K:\NewmanPaper\Piliang\3SubProteomes\Piliang3SP\mgf5ppm\ERLIC_3SubProteomes5ppm.mgf

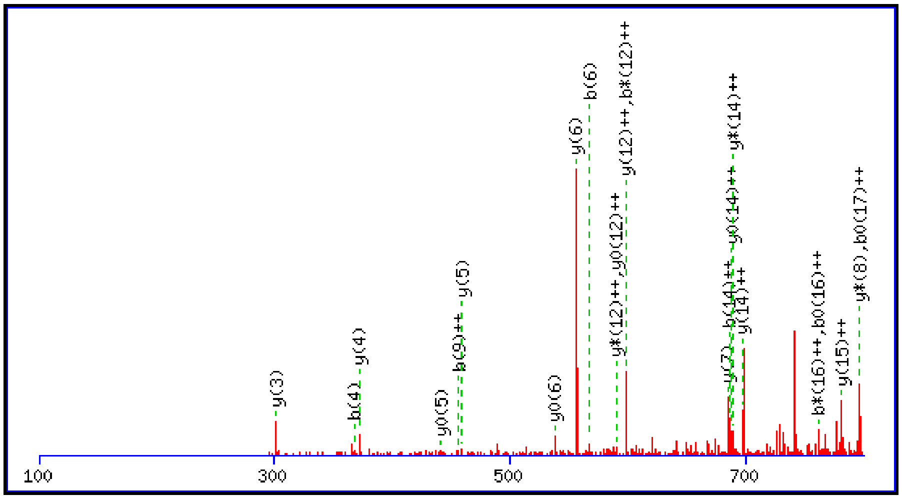

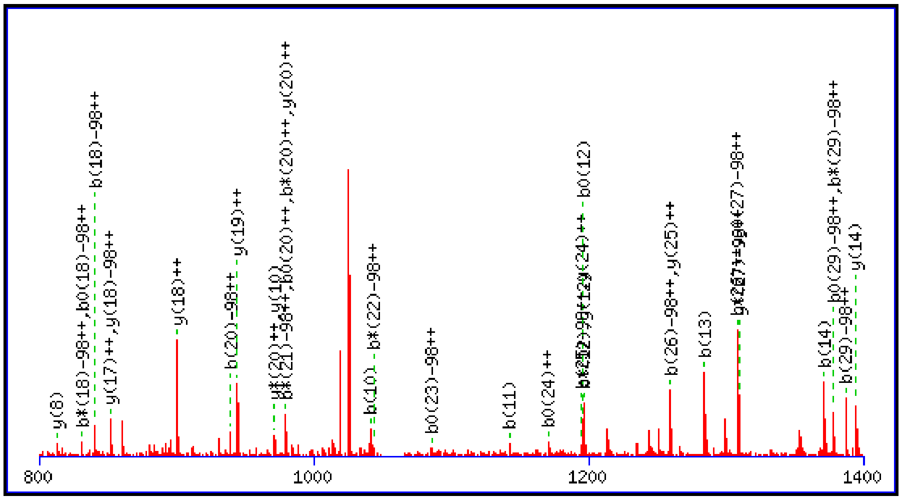

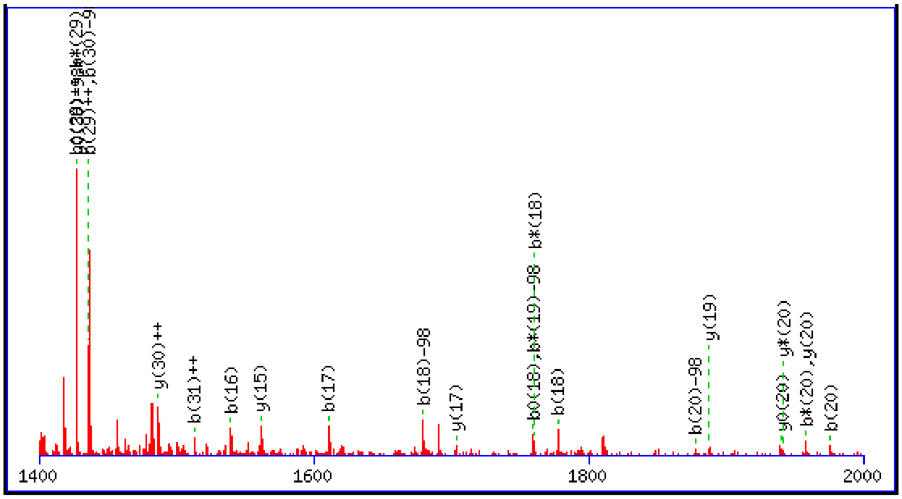

Monoisotopic mass of neutral peptide Mr(calc): 3170.4404
Fixed modifications: Carbamidomethyl (C)
Variable modifications:
S18 : Phospho (ST), with neutral losses 0.0000(shown in table), 97.9769
Ions Score: 48 **Expect:** 0.0063
Matches (Bold Red): 83/536 fragment ions using 130 most intense peaks

#	b	b^{++}	b*	b^{*++}	b^0	b^{0++}	Seq.	y	y^{++}	y*	y^{*++}	y^0	y^{0++}	#
1	72.0444	36.5258					A							32
2	200.1394	100.5733	183.1128	92.0600			K	3100.4107	1550.7090	3083.3841	1542.1957	3082.4001	1541.7037	31
3	297.1921	149.0997	280.1656	140.5864			P	2972.3157	**1486.6615**	2955.2891	1478.1482	2954.3051	1477.6562	30
4	**368.2292**	184.6183	351.2027	176.1050			A	2875.2629	1438.1351	2858.2364	1429.6218	2857.2524	1429.1298	29
5	439.2663	220.1368	422.2398	211.6235			A	2804.2258	1402.6165	2787.1993	1394.1033	2786.2152	1393.6113	28
6	**567.3249**	284.1661	550.2984	275.6528			Q	2733.1887	1367.0980	2716.1622	1358.5847	2715.1781	1358.0927	27
7	654.3570	327.6821	637.3304	319.1688	636.3464	318.6768	S	2605.1301	1303.0687	2588.1036	1294.5554	2587.1196	1294.0634	26
8	783.3995	392.2034	766.3730	383.6901	765.3890	383.1981	E	2518.0981	**1259.5527**	2501.0715	1251.0394	2500.0875	1250.5474	25
9	912.4421	**456.7247**	895.4156	448.2114	894.4316	447.7194	E	2389.0555	**1195.0314**	2372.0290	1186.5181	2371.0449	1186.0261	24
10	**1041.4847**	521.2460	1024.4582	512.7327	1023.4742	512.2407	E	2260.0129	1130.5101	2242.9864	1121.9968	2242.0023	1121.5048	23
11	**1142.5324**	571.7698	1125.5059	563.2566	1124.5218	562.7646	T	2130.9703	1065.9888	2113.9438	1057.4755	2112.9598	1056.9835	22
12	1213.5695	607.2884	**1196.5430**	**598.7751**	**1195.5590**	598.2831	A	2029.9226	1015.4650	2012.8961	1006.9517	2011.9121	1006.4597	21
13	**1284.6066**	642.8070	1267.5801	634.2937	1266.5961	633.8017	A	**1958.8855**	**979.9464**	**1941.8590**	**971.4331**	**1940.8750**	970.9411	20
14	**1371.6387**	**686.3230**	1354.6121	677.8097	1353.6281	677.3177	S	**1887.8484**	**944.4278**	1870.8219	935.9146	1869.8378	935.4226	19
15	1468.6914	734.8494	1451.6649	726.3361	1450.6809	725.8441	P	1800.8164	**900.9118**	1783.7898	892.3986	1782.8058	891.9065	18
16	**1539.7285**	770.3679	1522.7020	**761.8546**	1521.7180	**761.3626**	A	**1703.7636**	**852.3854**	1686.7371	843.8722	1685.7531	843.3802	17
17	**1610.7657**	805.8865	1593.7391	797.3732	1592.7551	**796.8812**	A	1632.7265	816.8669	1615.7000	808.3536	1614.7159	807.8616	16
18	**1777.7640**	889.3856	**1760.7375**	880.8724	**1759.7534**	880.3804	S	**1561.6894**	**781.3483**	1544.6628	772.8351	1543.6788	772.3430	15
19	1874.8168	937.9120	1857.7902	929.3988	1856.8062	928.9067	P	**1394.6910**	**697.8492**	1377.6645	**689.3359**	1376.6805	**688.8439**	14
20	**1975.8645**	988.4359	**1958.8379**	**979.9226**	1957.8539	**979.4306**	T	1297.6383	649.3228	1280.6117	640.8095	1279.6277	640.3175	13
21	2072.9172	1036.9622	2055.8907	1028.4490	2054.9067	1027.9570	P	**1196.5906**	**598.7989**	1179.5640	**590.2857**	1178.5800	**589.7937**	12
22	2200.9758	1100.9915	2183.9492	1092.4783	2182.9652	1091.9863	Q	1099.5378	550.2726	1082.5113	541.7593	1081.5273	541.2673	11
23	2288.0078	1144.5075	2270.9813	1135.9943	2269.9973	1135.5023	S	**971.4793**	486.2433	954.4527	477.7300	953.4687	477.2380	10
24	2359.0449	1180.0261	2342.0184	1171.5128	2341.0344	**1171.0208**	A	884.4472	442.7272	867.4207	434.2140	866.4367	433.7220	9
25	2487.1035	1244.0554	2470.0770	1235.5421	2469.0929	1235.0501	Q	**813.4101**	407.2087	**796.3836**	398.6954	795.3995	398.2034	8
26	2616.1461	**1308.5767**	2599.1196	1300.0634	2598.1355	1299.5714	E	**685.3515**	343.1794	668.3250	334.6661	667.3410	334.1741	7
27	2713.1989	1357.1031	2696.1723	1348.5898	2695.1883	1348.0978	P	**556.3089**	278.6581	539.2824	270.1448	**538.2984**	269.6528	6
28	2800.2309	1400.6191	2783.2044	1392.1058	2782.2203	1391.6138	S	**459.2562**	230.1317	442.2296	221.6185	**441.2456**	221.1264	5
29	2871.2680	**1436.1376**	2854.2415	**1427.6244**	2853.2574	**1427.1324**	A	**372.2241**	186.6157	355.1976	178.1024			4
30	2968.3208	1484.6640	2951.2942	1476.1508	2950.3102	1475.6587	P	**301.1870**	151.0972	284.1605	142.5839			3
31	3025.3422	**1513.1748**	3008.3157	1504.6615	3007.3317	1504.1695	G	204.1343	102.5708	187.1077	94.0575			2
32							K	147.1128	74.0600	130.0863	65.5468			1

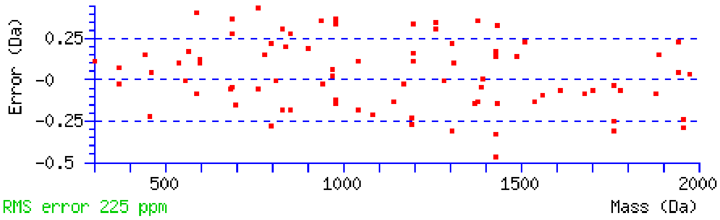

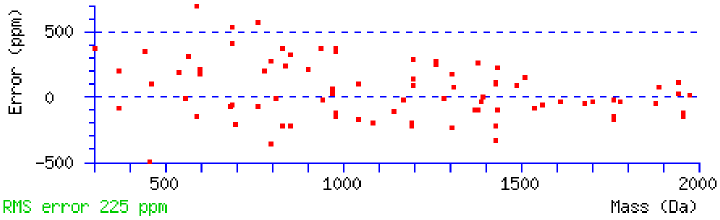

All matches to this query

Score	Mr(calc):	Delta	Sequence
48.1	3170.4404	0.0103	AKPAAQSEEETAASPAASPTPQSAQEPSAPGK
46.4	3170.4404	0.0103	AKPAAQSEEETAASPAASPTPQSAQEPSAPGK
40.0	3170.4404	0.0103	AKPAAQSEEETAASPAASPTPQSAQEPSAPGK
38.8	3170.4404	0.0103	AKPAAQSEEETAASPAASPTPQSAQEPSAPGK
28.7	3170.4404	0.0103	AKPAAQSEEETAASPAASPTPQSAQEPSAPGK
24.7	3170.4404	0.0103	AKPAAQSEEETAASPAASPTPQSAQEPSAPGK
23.5	3170.4404	0.0103	AKPAAQSEEETAASPAASPTPQSAQEPSAPGK
5.9	3170.4551	-0.0043	RNAITMQPQSVQGLSKISEEPSTSSDER
5.9	3170.4551	-0.0043	RNAITMQPQSVQGLSKISEEPSTSSDER
3.8	3169.4268	1.0240	VGANFDHSSNTSAGWLPSFGRVWNNGRR

Spectrum No: 339; Query: 1930; Rank: 1

Peptide View

MS/MS Fragmentation of **KSLDSDESEDEDDDYQQK**
Found in **IPI00208277**, Tax_Id=10116 Gene_Symbol=Pdap1 28 kDa heat- and acid-stable phosphoprotein

Match to Query 1930: 2224.822962 from(742.614930,3+)
Title: 091008RatKidney_NoSalt_28.829.829.3.dta
Data file K:\NewmanPaper\Piliang\3SubProteomes\Piliang3SP\mgf5ppm\ERLIC_3SubProteomes5ppm.mgf

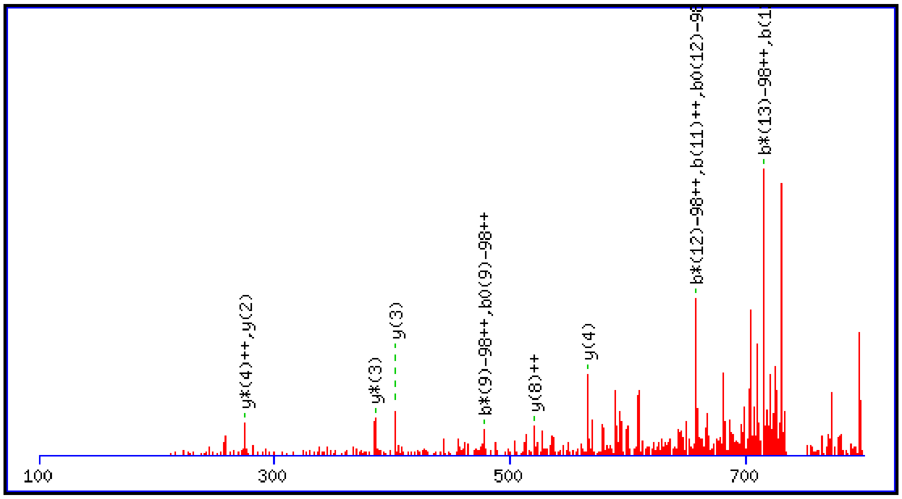

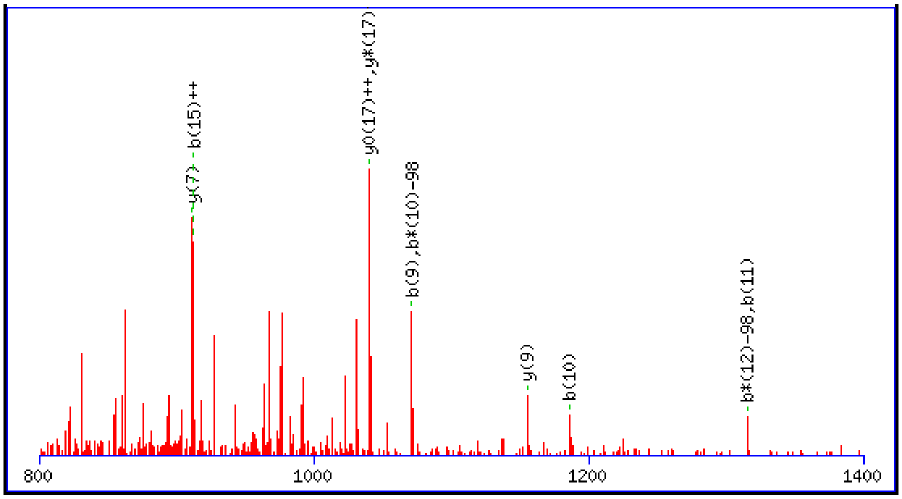

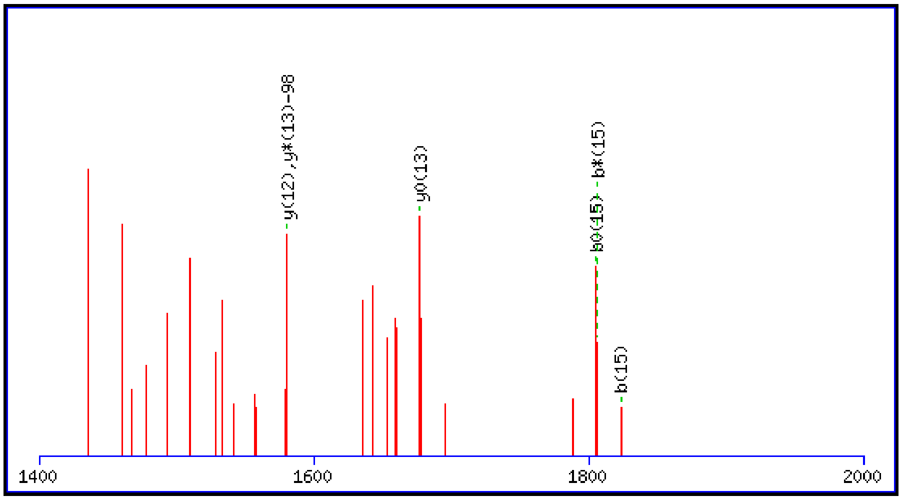

Monoisotopic mass of neutral peptide Mr(calc): 2224.8169
Fixed modifications: Carbamidomethyl (C)
Variable modifications:
S8 : Phospho (ST), with neutral losses 0.0000(shown in table), 97.9769
Ions Score: 48 **Expect:** 0.002
Matches (Bold Red): 31/296 fragment ions using 29 most intense peaks

#	b	b^{++}	b*	b^{*++}	b^0	b^{0++}	Seq.	y	y^{++}	y*	y^{*++}	y^0	y^{0++}	#
1	129.1022	65.0548	112.0757	56.5415			K							18
2	216.1343	108.5708	199.1077	100.0575	198.1237	99.5655	S	2097.7292	1049.3682	2080.7026	**1040.8550**	2079.7186	**1040.3630**	17
3	329.2183	165.1128	312.1918	156.5995	311.2078	156.1075	L	2010.6972	1005.8522	1993.6706	997.3389	1992.6866	996.8469	16
4	444.2453	222.6263	427.2187	214.1130	426.2347	213.6210	D	1897.6131	949.3102	1880.5866	940.7969	1879.6025	940.3049	15
5	531.2773	266.1423	514.2508	257.6290	513.2667	257.1370	S	1782.5862	891.7967	1765.5596	883.2834	1764.5756	882.7914	14
6	646.3042	323.6558	629.2777	315.1425	628.2937	314.6505	D	1695.5541	848.2807	1678.5276	839.7674	**1677.5436**	839.2754	13
7	775.3468	388.1771	758.3203	379.6638	757.3363	379.1718	E	**1580.5272**	790.7672	1563.5006	782.2540	1562.5166	781.7620	12
8	942.3452	471.6762	925.3186	463.1630	924.3346	462.6710	S	1451.4846	726.2459	1434.4581	717.7327	1433.4740	717.2407	11
9	**1071.3878**	536.1975	1054.3612	527.6843	1053.3772	527.1922	E	1284.4862	642.7468	1267.4597	634.2335	1266.4757	633.7415	10
10	**1186.4147**	593.7110	1169.3882	585.1977	1168.4042	584.7057	D	**1155.4437**	578.2255	1138.4171	569.7122	1137.4331	569.2202	9
11	**1315.4573**	**658.2323**	1298.4308	649.7190	1297.4468	649.2270	E	**1040.4167**	**520.7120**	1023.3902	512.1987	1022.4061	511.7067	8
12	1430.4843	**715.7458**	1413.4577	707.2325	1412.4737	706.7405	D	**911.3741**	456.1907	894.3476	447.6774	893.3636	447.1854	7
13	1545.5112	773.2592	1528.4847	764.7460	1527.5006	764.2540	D	796.3472	398.6772	779.3206	390.1640	778.3366	389.6719	6
14	1660.5381	830.7727	1643.5116	822.2594	1642.5276	821.7674	D	681.3202	341.1638	664.2937	332.6505	663.3097	332.1585	5
15	**1823.6015**	**912.3044**	**1806.5749**	903.7911	**1805.5909**	903.2991	Y	**566.2933**	283.6503	549.2667	**275.1370**			4
16	1951.6601	976.3337	1934.6335	967.8204	1933.6495	967.3284	Q	**403.2300**	202.1186	**386.2034**	193.6053			3
17	2079.7186	**1040.3630**	2062.6921	1031.8497	2061.7081	1031.3577	Q	**275.1714**	138.0893	258.1448	129.5761			2
18							K	147.1128	74.0600	130.0863	65.5468			1

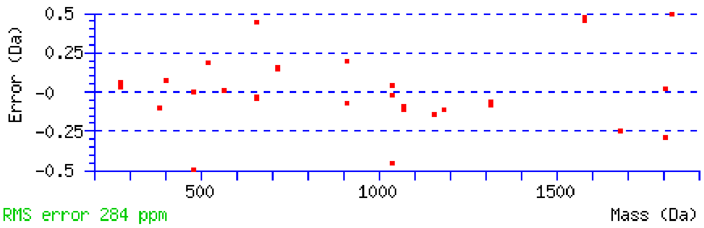

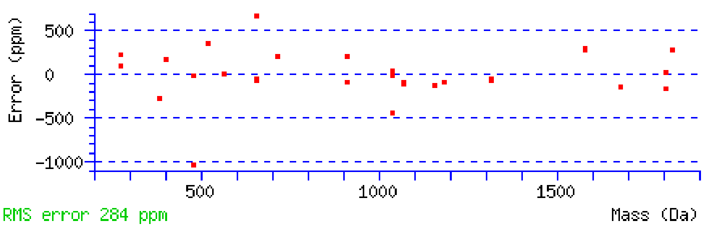

All matches to this query

Score	Mr(calc):	Delta	Sequence
48.0	2224.8169	0.0061	KSLDSDESEDEDDDYQQK
33.1	2224.8169	0.0061	KSLDSDESEDEDDDYQQK
33.1	2224.8169	0.0061	KSLDSDESEDEDDDYQQK
4.5	2224.8439	-0.0210	GTPGTGLSPENNNNNNKKK
4.5	2224.8439	-0.0210	GTPGTGLSPENNNNNNKKK
4.5	2224.8439	-0.0210	GTPGTGLSPENNNNNNKKK
4.2	2224.8401	-0.0172	DMDATSELKNKTFDTLR
3.6	2224.8401	-0.0172	DMDATSELKNKTFDTLR
2.1	2224.8169	0.0061	KSLDSDESEDEDDDYQQK
1.9	2223.8280	0.9950	VEQSSSENSELYTMTSTR

Spectrum No: 340; Query: 2664; Rank: 1

Peptide View

MS/MS Fragmentation of **DSLGDFIEHYAQLGPSSPEQLAAGAEEGGGPR**
Found in **IPI00189358**, Tax_Id=10116 Gene_Symbol=pur-beta Transcriptional activator protein Pur-beta

Match to Query 2664: 3334.485522 from(1112.502450,3+)
Title: 091008RatKidney_NoSalt_23.5307.5307.3.dta
Data file K:\NewmanPaper\Piliang\3SubProteomes\Piliang3SP\mgf5ppm\ERLIC_3SubProteomes5ppm.mgf

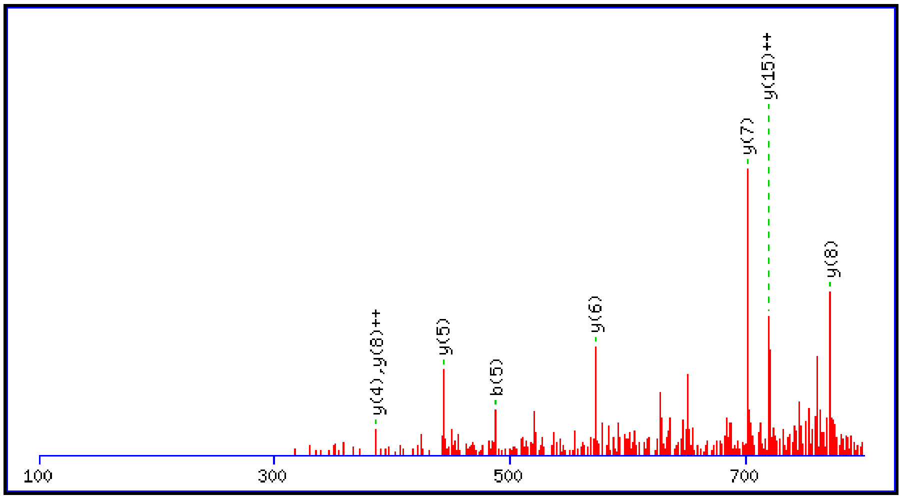

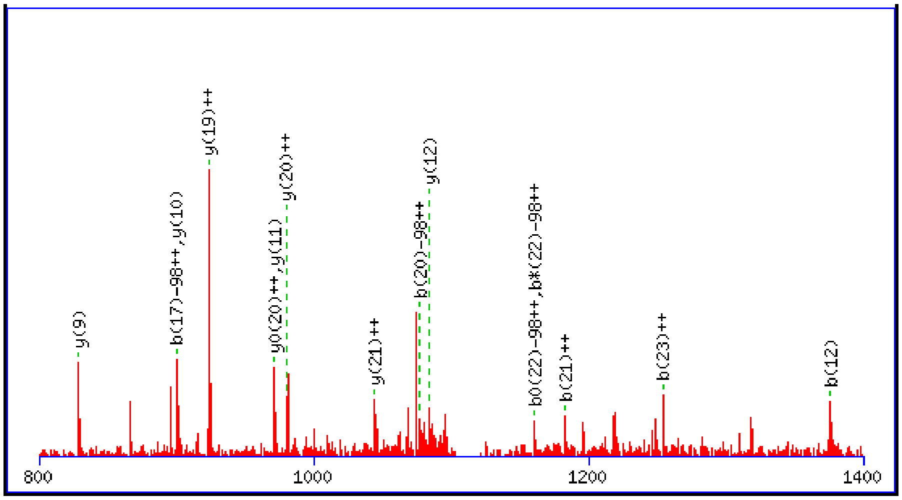

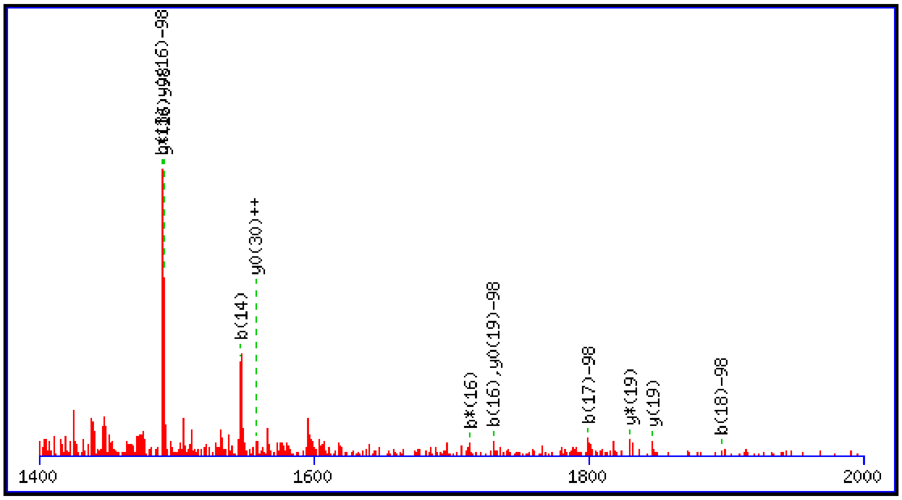

Monoisotopic mass of neutral peptide Mr(calc): 3334.4779
Fixed modifications: Carbamidomethyl (C)
Variable modifications:
S17 : Phospho (ST), with neutral losses 97.9769(shown in table), 0.0000
Ions Score: 48 **Expect:** 0.0075
Matches (Bold Red): 35/526 fragment ions using 61 most intense peaks

#	b	b++	b*	b*++	b0	b0++	Seq.	y	y++	y*	y*++	y0	y0++	#
1	116.0342	58.5207			98.0237	49.5155	D							32
2	203.0662	102.0368			185.0557	93.0315	S	3122.4814	1561.7443	3105.4548	1553.2311	3104.4708	1552.7390	31
3	316.1503	158.5788			298.1397	149.5735	L	3035.4493	1518.2283	3018.4228	1509.7150	3017.4388	1509.2230	30
4	373.1718	187.0895			355.1612	178.0842	G	2922.3653	1461.6863	2905.3387	1453.1730	2904.3547	1452.6810	29
5	**488.1987**	244.6030			470.1882	235.5977	D	2865.3438	1433.1755	2848.3173	1424.6623	2847.3333	1424.1703	28
6	635.2671	318.1372			617.2566	309.1319	F	2750.3169	1375.6621	2733.2903	1367.1488	2732.3063	1366.6568	27
7	748.3512	374.6792			730.3406	365.6740	I	2603.2485	1302.1279	2586.2219	1293.6146	2585.2379	1293.1226	26
8	877.3938	439.2005			859.3832	430.1953	E	2490.1644	1245.5858	2473.1379	1237.0726	2472.1538	1236.5806	25
9	1014.4527	507.7300			996.4421	498.7247	H	2361.1218	1181.0645	2344.0953	1172.5513	2343.1112	1172.0593	24
10	1177.5160	589.2617			1159.5055	580.2564	Y	2224.0629	1112.5351	2207.0363	1104.0218	2206.0523	1103.5298	23
11	1248.5531	624.7802			1230.5426	615.7749	A	2060.9996	1031.0034	2043.9730	1022.4901	2042.9890	1021.9981	22
12	**1376.6117**	688.8095	1359.5852	680.2962	1358.6012	679.8042	Q	1989.9625	995.4849	1972.9359	986.9716	1971.9519	986.4796	21
13	**1489.6958**	745.3515	1472.6692	736.8383	1471.6852	736.3462	L	1861.9039	931.4556	1844.8773	922.9423	1843.8933	922.4503	20
14	**1546.7172**	773.8623	1529.6907	765.3490	1528.7067	764.8570	G	1748.8198	874.9135	1731.7933	866.4003	**1730.8092**	865.9083	19
15	1643.7700	822.3886	1626.7435	813.8754	1625.7594	813.3834	P	1691.7983	846.4028	1674.7718	837.8895	1673.7878	837.3975	18
16	**1730.8020**	865.9047	**1713.7755**	857.3914	1712.7915	856.8994	S	1594.7456	797.8764	1577.7190	789.3632	1576.7350	788.8711	17
17	**1799.8235**	**900.4154**	1782.7969	891.9021	1781.8129	891.4101	S	1507.7136	754.3604	**1490.6870**	745.8471	**1489.7030**	745.3551	16
18	**1896.8763**	948.9418	1879.8497	940.4285	1878.8657	939.9365	P	1438.6921	**719.8497**	1421.6656	711.3364	1420.6815	710.8444	15

**19**	2025.9189	1013.4631	2008.8923	1004.9498	2007.9083	1004.4578	**E**	1341.6393	671.3233	1324.6128	662.8100	1323.6288	662.3180	**14**
**20**	2153.9774	**1077.4924**	2136.9509	1068.9791	2135.9669	1068.4871	**Q**	1212.5967	606.8020	1195.5702	598.2887	1194.5862	597.7967	**13**
**21**	2267.0615	1134.0344	2250.0349	1125.5211	2249.0509	1125.0291	**L**	**1084.5382**	542.7727	1067.5116	534.2594	1066.5276	533.7674	**12**
**22**	2338.0986	1169.5529	2321.0721	**1161.0397**	2320.0880	**1160.5477**	**A**	**971.4541**	486.2307	954.4276	477.7174	953.4435	477.2254	**11**
**23**	2409.1357	1205.0715	2392.1092	1196.5582	2391.1252	1196.0662	**A**	**900.4170**	450.7121	883.3904	442.1989	882.4064	441.7068	**10**
**24**	2466.1572	1233.5822	2449.1306	1225.0690	2448.1466	1224.5769	**G**	**829.3799**	415.1936	812.3533	406.6803	811.3693	406.1883	**9**
**25**	2537.1943	1269.1008	2520.1678	1260.5875	2519.1837	1260.0955	**A**	**772.3584**	**386.6828**	755.3319	378.1696	754.3478	377.6776	**8**
**26**	2666.2369	1333.6221	2649.2103	1325.1088	2648.2263	1324.6168	**E**	**701.3213**	351.1643	684.2947	342.6510	683.3107	342.1590	**7**
**27**	2795.2795	1398.1434	2778.2529	1389.6301	2777.2689	1389.1381	**E**	**572.2787**	286.6430	555.2522	278.1297	554.2681	277.6377	**6**
**28**	2852.3010	1426.6541	2835.2744	1418.1408	2834.2904	1417.6488	**G**	**443.2361**	222.1217	426.2096	213.6084			**5**
**29**	2909.3224	1455.1648	2892.2959	1446.6516	2891.3119	1446.1596	**G**	**386.2146**	193.6110	369.1881	185.0977			**4**
**30**	2966.3439	1483.6756	2949.3173	1475.1623	2948.3333	1474.6703	**G**	329.1932	165.1002	312.1666	156.5870			**3**
**31**	3063.3966	1532.2020	3046.3701	1523.6887	3045.3861	1523.1967	**P**	272.1717	136.5895	255.1452	128.0762			**2**
**32**							**R**	175.1190	88.0631	158.0924	79.5498			**1**

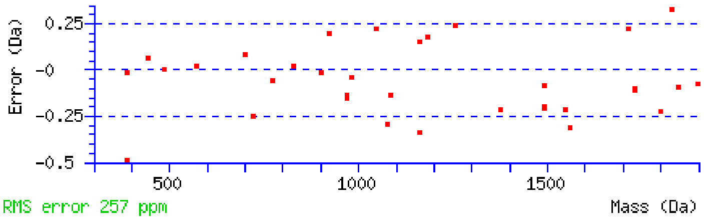

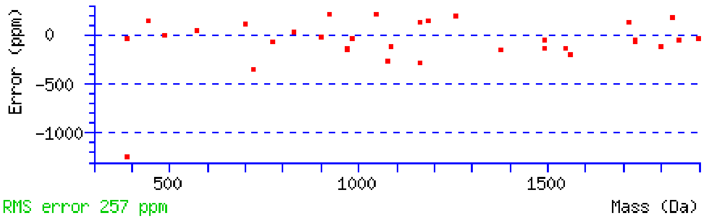

All matches to this query

Score	Mr(calc):	Delta	Sequence
48.0	3334.4779	0.0076	DSLGDFIEHYAQLGPSSPEQLAAGAEEGGGPR
38.6	3334.4779	0.0076	DSLGDFIEHYAQLGPSSPEQLAAGAEEGGGPR
20.5	3334.4779	0.0076	DSLGDFIEHYAQLGPSSPEQLAAGAEEGGGPR
18.5	3334.4779	0.0076	DSLGDFIEHYAQLGPSSPEQLAAGAEEGGGPR
11.6	3332.4495	2.0360	QSVMEAKQRYVNGLDQLAFAESQVGEMK
4.2	3332.4656	2.0199	NDQDSSPVKPCYLNILENEQHLNSATHK
4.1	3333.4951	0.9904	KFSIDWSKLVSVASTGTPAMMDANSGLVTK
2.7	3333.4511	1.0344	KVACLIKPSVSISFNGERMDIQAGSACR
2.4	3332.4663	2.0192	GSTPTRDMVTHGKAEPGSGLVNVSTPKPTR
2.3	3333.4513	1.0342	DNTTVTRAFNINPSDKYSGTCGAQLVTLK

Spectrum No: 341; Query: 707; Rank: 1

Peptide View

MS/MS Fragmentation of **DGATSEEETELEK**
Found in **IPI00368799**, Tax_Id=10116 Gene_Symbol=RGD1306067 ESF1 homolog

Match to Query 707: 1516.585208 from(759.299880,2+)
Title: 091008RatKidney_NoSalt_29.1017.1017.2.dta
Data file K:\NewmanPaper\Piliang\3SubProteomes\Piliang3SP\mgf5ppm\ERLIC_3SubProteomes5ppm.mgf

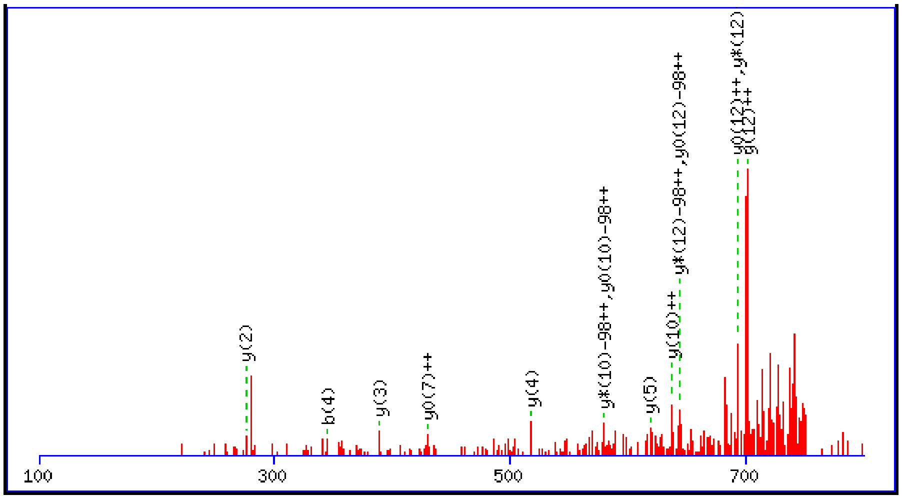

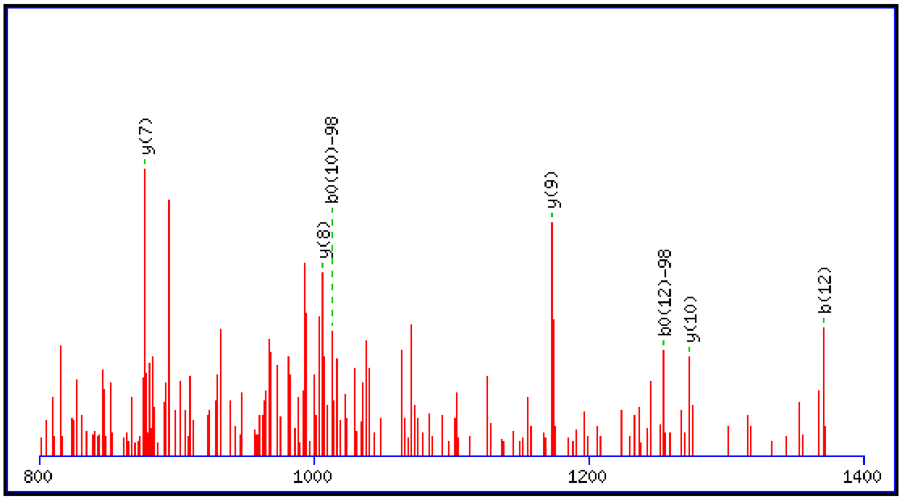

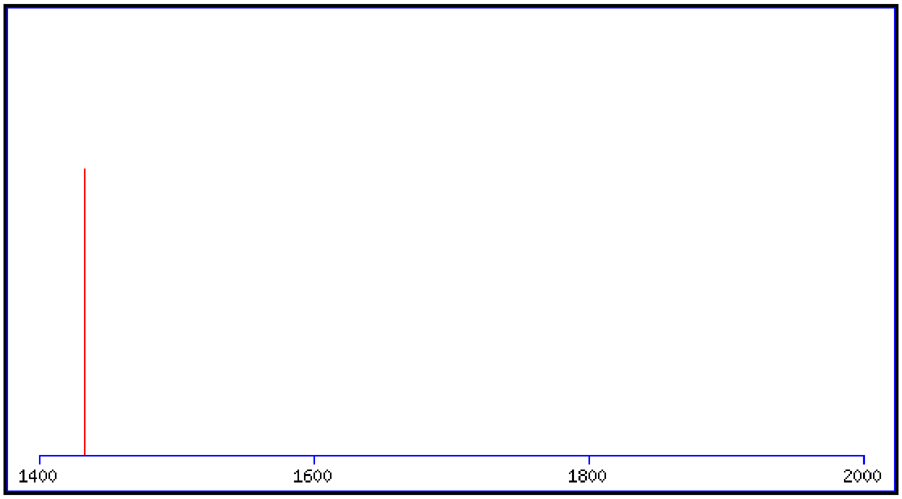

Monoisotopic mass of neutral peptide Mr(calc): 1516.5818
Fixed modifications: Carbamidomethyl (C)
Variable modifications:
S5 : Phospho (ST), with neutral losses 0.0000(shown in table), 97.9769
Ions Score: 48 **Expect:** 0.0016
Matches (Bold Red): 21/174 fragment ions using 36 most intense peaks

#	b	b^{++}	b^{0}	b^{0++}	Seq.	y	y^{++}	y^{*}	y^{*++}	y^{0}	y^{0++}	#
1	116.0342	58.5207	98.0237	49.5155	D							13
2	173.0557	87.0315	155.0451	78.0262	G	1402.5621	**701.7847**	1385.5356	**693.2714**	1384.5516	**692.7794**	12
3	244.0928	122.5500	226.0822	113.5448	A	1345.5407	673.2740	1328.5141	664.7607	1327.5301	664.2687	11
4	**345.1405**	173.0739	327.1299	164.0686	T	**1274.5035**	**637.7554**	1257.4770	629.2421	1256.4930	628.7501	10
5	512.1388	256.5731	494.1283	247.5678	S	**1173.4559**	587.2316	1156.4293	578.7183	1155.4453	578.2263	9

**6**	641.1814	321.0943	623.1709	312.0891	**E**	**1006.4575**	503.7324	989.4310	495.2191	988.4469	494.7271	**8**
**7**	770.2240	385.6156	752.2134	376.6104	**E**	**877.4149**	439.2111	860.3884	430.6978	859.4044	**430.2058**	**7**
**8**	899.2666	450.1369	881.2560	441.1317	**E**	748.3723	374.6898	731.3458	366.1765	730.3618	365.6845	**6**
**9**	1000.3143	500.6608	982.3037	491.6555	**T**	**619.3297**	310.1685	602.3032	301.6552	601.3192	301.1632	**5**
**10**	1129.3569	565.1821	1111.3463	556.1768	**E**	**518.2821**	259.6447	501.2555	251.1314	500.2715	250.6394	**4**
**11**	1242.4409	621.7241	1224.4304	612.7188	**L**	**389.2395**	195.1234	372.2129	186.6101	371.2289	186.1181	**3**
**12**	**1371.4835**	686.2454	1353.4730	677.2401	**E**	**276.1554**	138.5813	259.1288	130.0681	258.1448	129.5761	**2**
**13**					**K**	147.1128	74.0600	130.0863	65.5468			**1**

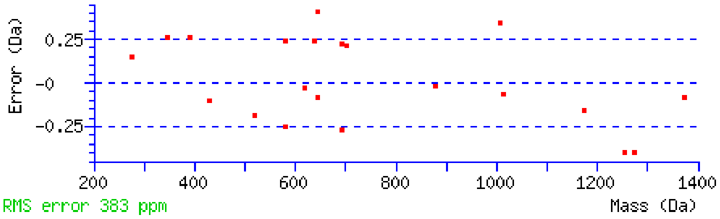

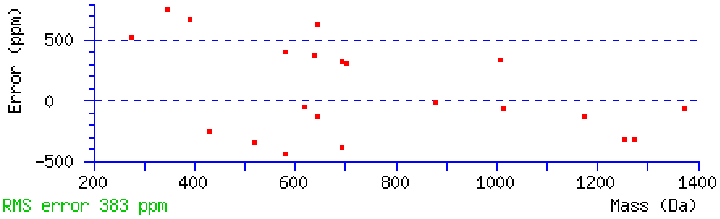

All matches to this query

Score	Mr(calc):	Delta	Sequence
48.0	1516.5818	0.0035	DGATSEEETELEK
26.5	1516.5818	0.0035	DGATSEEETELEK
16.5	1516.5818	0.0035	DGATSEEETELEK
7.5	1516.5871	-0.0019	WQDANSTEFDPK
5.9	1516.5810	0.0042	DMMNEAAPQDGPPQ
0.5	1516.5939	-0.0087	VTENMLCAGGEAGK
0.4	1516.5871	-0.0019	WQDANSTEFDPK

Spectrum No: 342; Query: 457; Rank: 1

Peptide View

MS/MS Fragmentation of **DLDQPADSVPLK**
Found in **IPI00471800**, Tax_Id=10116 Gene_Symbol=Cdh16 Cadherin 16

Match to Query 457: 1376.624668 from(689.319610,2+)
Title: 100101RatKid_NS_deglyco_23.2049.2049.2.dta
Data file K:\NewmanPaper\Piliang\3SubProteomes\Piliang3SP\mgf5ppm\ERLIC_3SubProteomes5ppm.mgf

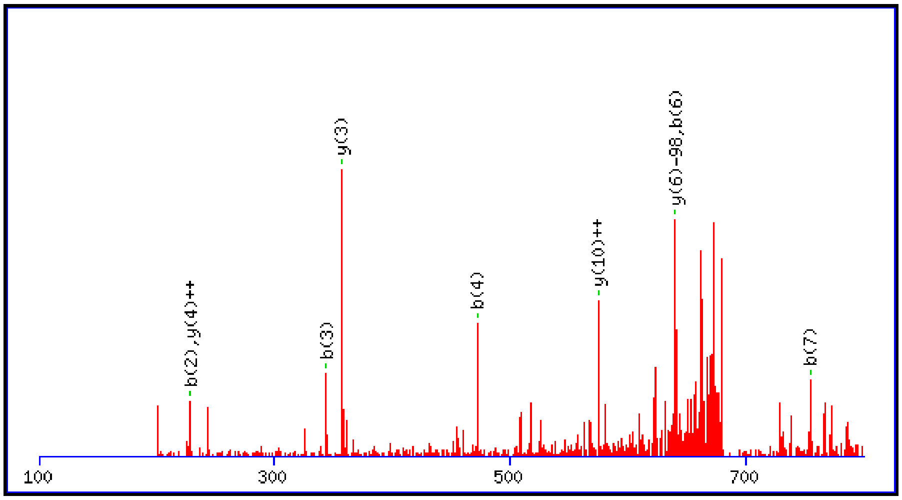

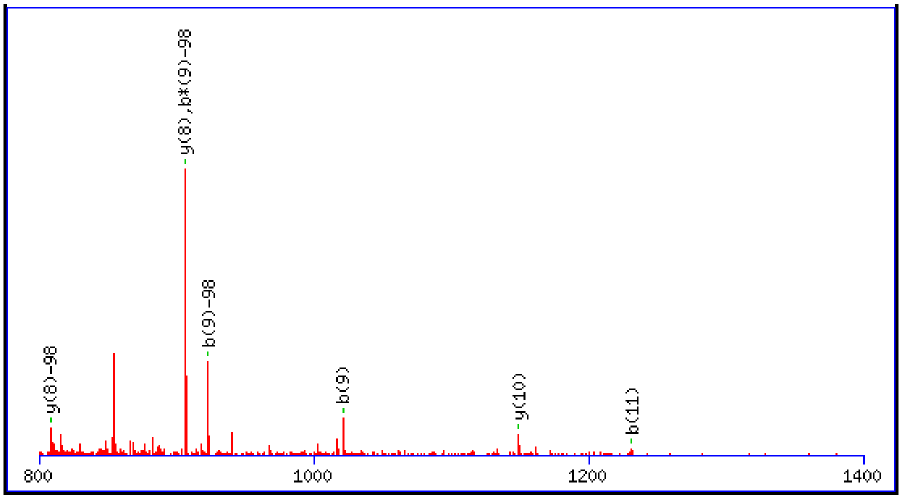

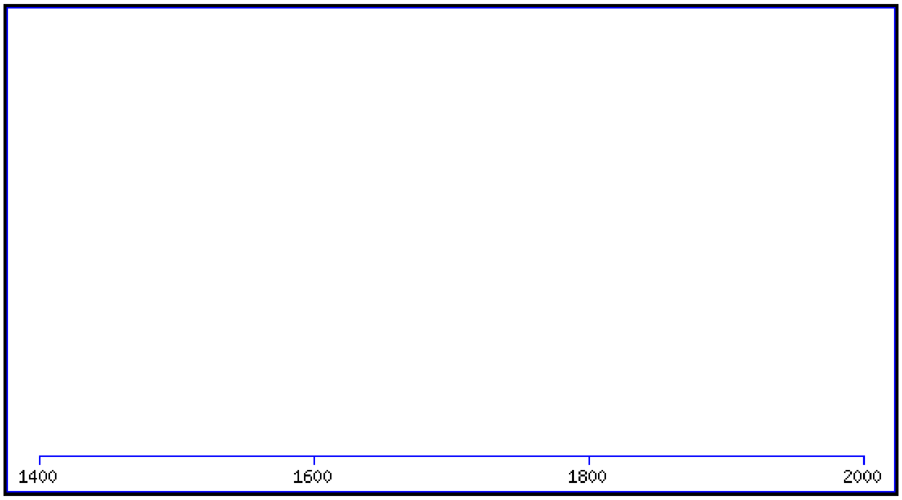

Monoisotopic mass of neutral peptide Mr(calc): 1376.6225
Fixed modifications: Carbamidomethyl (C)
Variable modifications:
S8 : Phospho (ST), with neutral losses 0.0000(shown in table), 97.9769
Ions Score: 48 **Expect:** 0.0025
Matches (Bold Red): 16/184 fragment ions using 30 most intense peaks

#	b	b^{++}	b*	b^{*++}	b^0	b^{0++}	Seq.	y	y^{++}	y*	y^{*++}	y^0	y^{0++}	#
1	116.0342	58.5207			98.0237	49.5155	D							12
2	**229.1183**	115.0628			211.1077	106.0575	L	1262.6028	631.8050	1245.5763	623.2918	1244.5922	622.7998	11
3	**344.1452**	172.5763			326.1347	163.5710	D	**1149.5187**	**575.2630**	1132.4922	566.7497	1131.5082	566.2577	10
4	**472.2038**	236.6055	455.1773	228.0923	454.1932	227.6003	Q	1034.4918	517.7495	1017.4652	509.2363	1016.4812	508.7443	9
5	569.2566	285.1319	552.2300	276.6186	551.2460	276.1266	P	**906.4332**	453.7202	889.4067	445.2070	888.4227	444.7150	8
6	**640.2937**	320.6505	623.2671	312.1372	622.2831	311.6452	A	809.3805	405.1939	792.3539	396.6806	791.3699	396.1886	7
7	**755.3206**	378.1640	738.2941	369.6507	737.3101	369.1587	D	738.3433	369.6753	721.3168	361.1620	720.3328	360.6700	6
8	922.3190	461.6631	905.2924	453.1499	904.3084	452.6578	S	623.3164	312.1618	606.2898	303.6486	605.3058	303.1566	5
9	**1021.3874**	511.1973	1004.3608	502.6841	1003.3768	502.1921	V	456.3180	**228.6627**	439.2915	220.1494			4
10	1118.4402	559.7237	1101.4136	551.2104	1100.4296	550.7184	P	**357.2496**	179.1285	340.2231	170.6152			3
11	**1231.5242**	616.2657	1214.4977	607.7525	1213.5137	607.2605	L	260.1969	130.6021	243.1703	122.0888			2
12							K	147.1128	74.0600	130.0863	65.5468			1

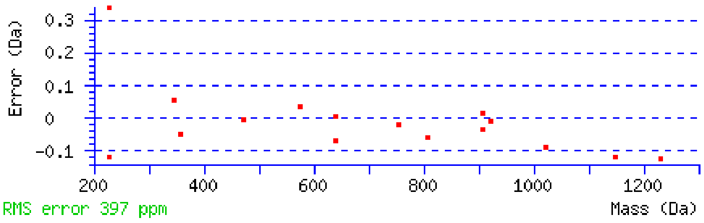

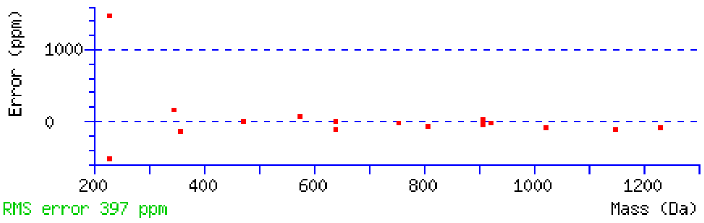

All matches to this query

Score	Mr(calc):	Delta	Sequence
48.0	1376.6225	0.0022	DLDQPADSVPLK
9.2	1376.6199	0.0047	WGLNGYMKIAK
7.6	1376.6216	0.0031	LYGVVLTPPMK
6.9	1376.6159	0.0087	MPEPSRSTPAPK
6.2	1376.6159	0.0087	MPEPSRSTPAPK
5.7	1374.6058	2.0189	ILQDSLGGRTR
5.5	1376.6200	0.0047	XPQGTSLFAFAK
5.5	1376.6200	0.0047	XPQGTSLFAFAK
5.1	1376.6159	0.0087	MPEPSRSTPAPK
4.7	1376.6159	0.0087	EVKEGFKAMSR

Spectrum No: 343; Query: 2483; Rank: 1

Peptide View

MS/MS Fragmentation of **IYQFPDCDSDEDEDFKLQDQALK**
Found in **IPI00421642**, Tax_Id=10116 Gene_Symbol=Sept4 Septin

Match to Query 2483: 2899.207948 from(1450.611250,2+)
Title: 100101RatKid_NS_deglyco_21.3709.3709.2.dta
Data file K:\NewmanPaper\Piliang\3SubProteomes\Piliang3SP\mgf5ppm\ERLIC_3SubProteomes5ppm.mgf

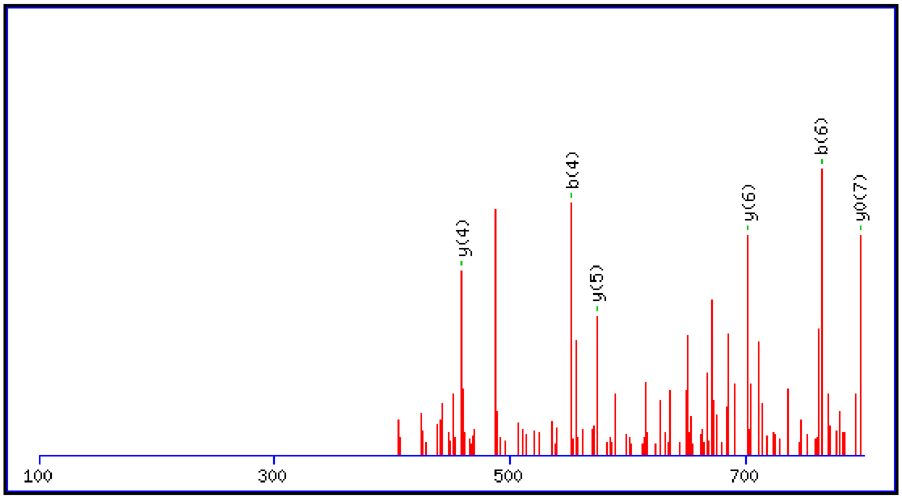

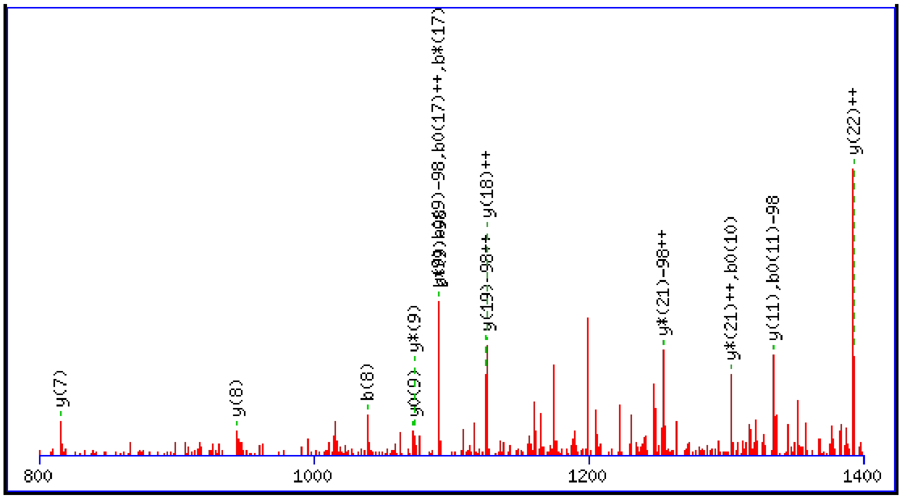

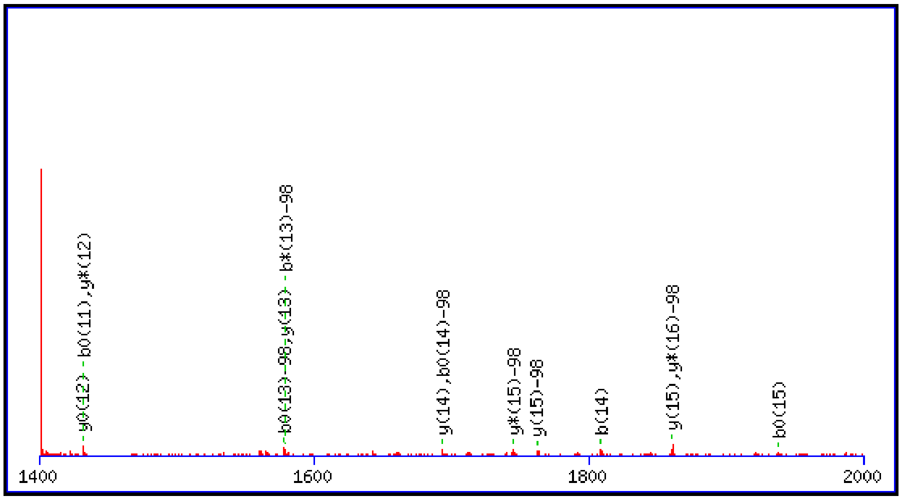

Monoisotopic mass of neutral peptide Mr(calc): 2898.1943
Fixed modifications: Carbamidomethyl (C)
Variable modifications:
S9 : Phospho (ST), with neutral losses 97.9769(shown in table), 0.0000
Ions Score: 48 **Expect:** 0.0053
Matches (Bold Red): 38/374 fragment ions using 45 most intense peaks

#	b	b^{++}	b*	b^{*++}	b^0	b^{0++}	Seq.	y	y^{++}	y*	y^{*++}	y^0	y^{0++}	#
1	114.0913	57.5493					I							23
2	277.1547	139.0810					Y	2688.1406	1344.5739	2671.1141	1336.0607	2670.1300	1335.5687	22
3	405.2132	203.1103	388.1867	194.5970			Q	2525.0773	1263.0423	2508.0507	**1254.5290**	2507.0667	1254.0370	21
4	**552.2817**	276.6445	535.2551	268.1312			F	2397.0187	1199.0130	2379.9922	1190.4997	2379.0081	1190.0077	20
5	649.3344	325.1709	632.3079	316.6576			P	2249.9503	**1125.4788**	2232.9237	1116.9655	2231.9397	1116.4735	19
6	**764.3614**	382.6843	747.3348	374.1710	746.3508	373.6790	D	2152.8975	1076.9524	2135.8710	1068.4391	2134.8870	1067.9471	18
7	924.3920	462.6996	907.3655	454.1864	906.3815	453.6944	C	2037.8706	1019.4389	2020.8440	1010.9257	2019.8600	1010.4336	17
8	**1039.4190**	520.2131	1022.3924	511.6998	1021.4084	511.2078	D	1877.8399	939.4236	**1860.8134**	930.9103	1859.8294	930.4183	16
9	1108.4404	554.7238	**1091.4139**	546.2106	**1090.4299**	545.7186	S	**1762.8130**	881.9101	**1745.7864**	873.3969	1744.8024	872.9049	15
10	1223.4674	612.2373	1206.4408	603.7240	1205.4568	603.2320	D	**1693.7915**	847.3994	1676.7650	838.8861	1675.7810	838.3941	14
11	1352.5100	676.7586	1335.4834	668.2453	**1334.4994**	667.7533	E	**1578.7646**	789.8859	1561.7380	781.3727	1560.7540	780.8807	13
12	1467.5369	734.2721	1450.5103	725.7588	1449.5263	725.2668	D	1449.7220	725.3646	**1432.6955**	716.8514	**1431.7114**	716.3594	12
13	1596.5795	798.7934	**1579.5529**	790.2801	**1578.5689**	789.7881	E	**1334.6951**	667.8512	1317.6685	659.3379	1316.6845	658.8459	11
14	1711.6064	856.3069	1694.5799	847.7936	**1693.5959**	847.3016	D	1205.6525	603.3299	1188.6259	594.8166	1187.6419	594.3246	10
15	1858.6748	929.8411	1841.6483	921.3278	1840.6643	920.8358	F	**1090.6255**	545.8164	**1073.5990**	537.3031	**1072.6150**	536.8111	9
16	1986.7698	993.8885	1969.7433	985.3753	1968.7592	984.8833	K	**943.5571**	472.2822	926.5306	463.7689	925.5465	463.2769	8
17	2099.8539	1050.4306	2082.8273	1041.9173	2081.8433	1041.4253	L	**815.4621**	408.2347	798.4356	399.7214	**797.4516**	399.2294	7
18	2227.9125	1114.4599	2210.8859	1105.9466	2209.9019	1105.4546	Q	**702.3781**	351.6927	685.3515	343.1794	684.3675	342.6874	6

19	2342.9394	1171.9733	2325.9128	1163.4601	2324.9288	1162.9681	D	574.3195	287.6634	557.2930	279.1501	556.3089	278.6581	5
20	2470.9980	1236.0026	2453.9714	1227.4893	2452.9874	1226.9973	Q	459.2926	230.1499	442.2660	221.6366			4
21	2542.0351	1271.5212	2525.0085	1263.0079	2524.0245	1262.5159	A	331.2340	166.1206	314.2074	157.6074			3
22	2655.1191	1328.0632	2638.0926	1319.5499	2637.1086	1319.0579	L	260.1969	130.6021	243.1703	122.0888			2
23							K	147.1128	74.0600	130.0863	65.5468			1

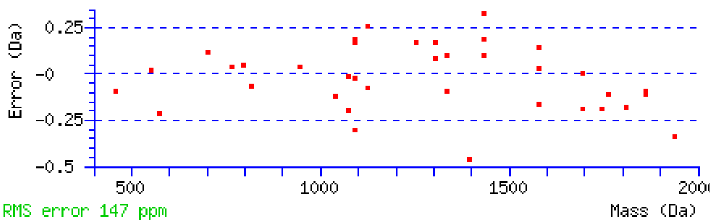

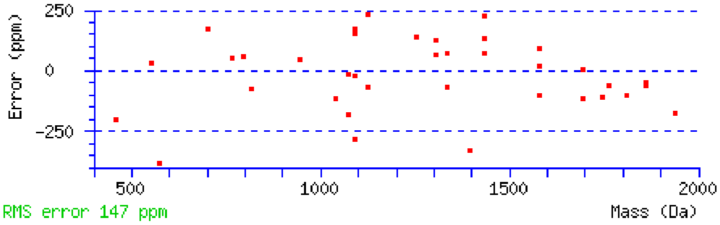

All matches to this query

Score	Mr(calc):	Delta	Sequence
47.9	2898.1943	1.0137	IYQFPDCDSDEDEDFKLQDQALK
24.6	2898.1943	1.0137	IYQFPDCDSDEDEDFKLQDQALK
7.5	2898.2171	0.9908	FATAACYCNMYASIMLMTVISIDR
7.2	2897.1843	2.0236	ENSKYLDEELMVLNSNSISLTSR
7.2	2897.1843	2.0236	ENSKYLDEELMVLNSNSISLTSR
7.2	2897.1843	2.0236	ENSKYLDEELMVLNSNSISLTSR
7.2	2897.1843	2.0236	ENSKYLDEELMVLNSNSISLTSR
7.2	2897.1843	2.0236	ENSKYLDEELMVLNSNSISLTSR
7.2	2897.1843	2.0236	ENSKYLDEELMVLNSNSISLTSR
7.1	2897.2204	1.9875	RINDNSEAPAHLLTDFQMLHQDR

Spectrum No: 344; Query: 2142; Rank: 1

Peptide View

MS/MS Fragmentation of **SLAPDLSDDEHDPVDSISRPR**
Found in **IPI00568245**, Tax_Id=10116 Gene_Symbol=LOC360570 similar to myosin XVIIIa

Match to Query 2142: 2400.059172 from(801.027000,3+)
Title: 091008RatKidney_NH4Format02_24.2973.2973.3.dta
Data file K:\NewmanPaper\Piliang\3SubProteomes\Piliang3SP\mgf5ppm\ERLIC_3SubProteomes5ppm.mgf

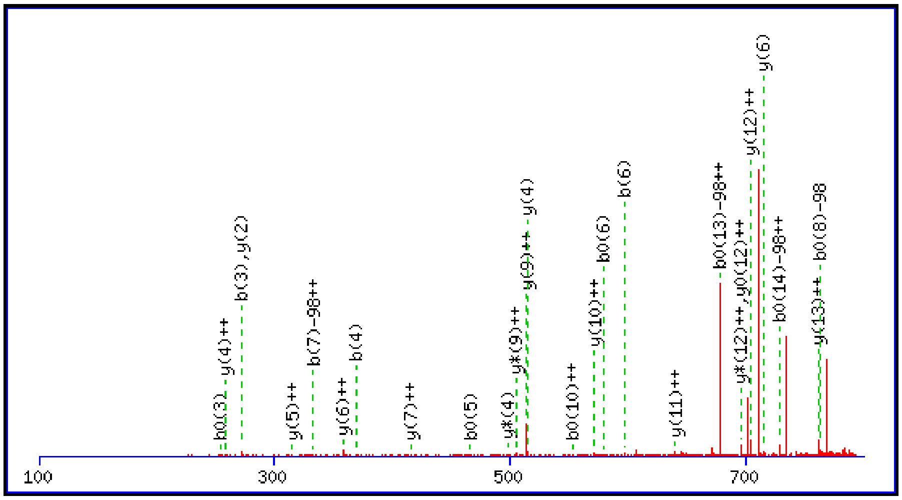

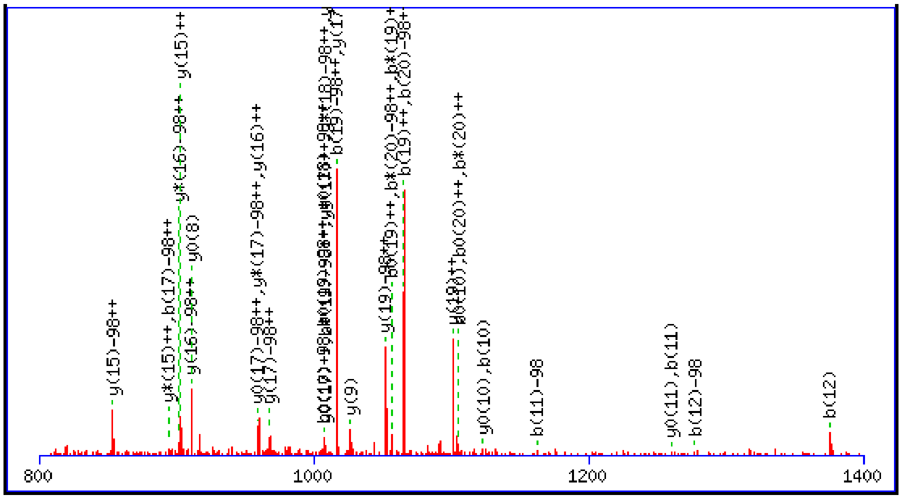

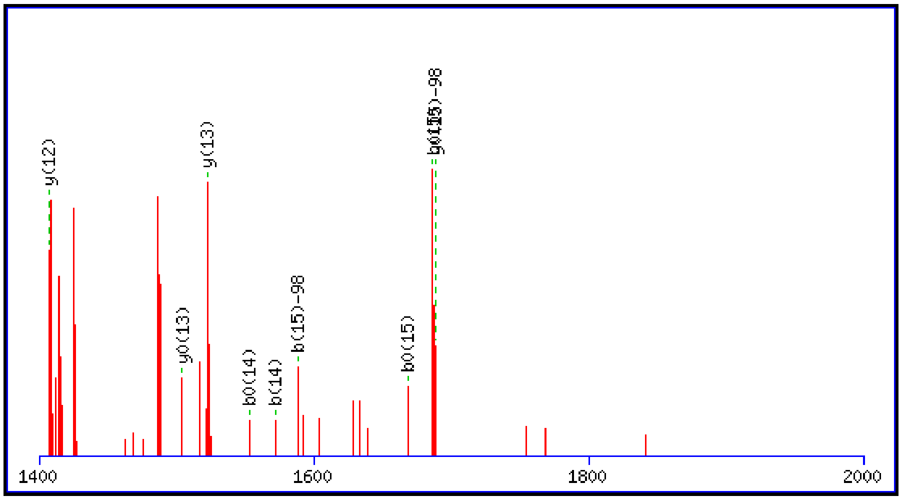

Monoisotopic mass of neutral peptide Mr(calc): 2400.0594
Fixed modifications: Carbamidomethyl (C)
Variable modifications:
S7 : Phospho (ST), with neutral losses 97.9769(shown in table), 0.0000
Ions Score: 48 **Expect:** 0.0053
Matches (Bold Red): 79/294 fragment ions using 153 most intense peaks

#	b	b^{++}	b*	b*$^{++}$	b^{0}	b^{0++}	Seq.	y	y^{++}	y*	y*$^{++}$	y^{0}	y^{0++}	#
1	88.0393	44.5233			70.0287	35.5180	S							21
2	201.1234	101.0653			183.1128	92.0600	L	2216.0578	1108.5325	2199.0313	1100.0193	2198.0472	1099.5273	20
3	**272.1605**	136.5839			**254.1499**	127.5786	A	2102.9737	**1051.9905**	2085.9472	1043.4772	2084.9632	1042.9852	19
4	**369.2132**	185.1103			351.2027	176.1050	P	2031.9366	**1016.4720**	2014.9101	**1007.9587**	2013.9261	**1007.4667**	18
5	484.2402	242.6237			**466.2296**	233.6185	D	1934.8839	**967.9456**	1917.8573	**959.4323**	1916.8733	**958.9403**	17
6	**597.3243**	299.1658			**579.3137**	290.1605	L	1819.8569	**910.4321**	1802.8304	**901.9188**	1801.8464	901.4268	16
7	666.3457	**333.6765**			648.3351	324.6712	S	1706.7729	**853.8901**	1689.7463	845.3768	**1688.7623**	844.8848	15
8	781.3727	391.1900			**763.3621**	382.1847	D	1637.7514	819.3793	1620.7249	810.8661	1619.7408	810.3741	14
9	896.3996	448.7034			878.3890	439.6982	D	**1522.7245**	**761.8659**	1505.6979	753.3526	**1504.7139**	752.8606	13
10	1025.4422	513.2247			**1007.4316**	504.2194	E	**1407.6975**	**704.3524**	1390.6710	**695.8391**	1389.6870	**695.3471**	12
11	**1162.5011**	581.7542			1144.4905	572.7489	H	1278.6549	**639.8311**	1261.6284	631.3178	**1260.6444**	630.8258	11
12	**1277.5280**	639.2677			1259.5175	630.2624	D	1141.5960	**571.3016**	1124.5695	562.7884	**1123.5854**	562.2964	10
13	1374.5808	687.7940			1356.5702	**678.7888**	P	**1026.5691**	**513.7882**	1009.5425	**505.2749**	**1008.5585**	504.7829	9
14	1473.6492	737.3282			1455.6387	**728.3230**	V	929.5163	465.2618	912.4898	456.7485	**911.5057**	456.2565	8
15	**1588.6762**	794.8417			1570.6656	785.8364	D	830.4479	**415.7276**	813.4213	407.2143	812.4373	406.7223	7
16	1675.7082	838.3577			1657.6976	829.3525	S	**715.4209**	**358.2141**	698.3944	349.7008	697.4104	349.2088	6
17	1788.7923	**894.8998**			1770.7817	885.8945	I	628.3889	**314.6981**	611.3624	306.1848	610.3784	305.6928	5
18	1875.8243	938.4158			1857.8137	929.4105	S	**515.3049**	**258.1561**	**498.2783**	249.6428	497.2943	249.1508	4

19	2031.9254	1016.4663	2014.8988	1007.9531	2013.9148	1007.4611	R	428.2728	214.6401	411.2463	206.1268			3
20	2128.9782	1064.9927	2111.9516	1056.4794	2110.9676	1055.9874	P	272.1717	136.5895	255.1452	128.0762			2
21							R	175.1190	88.0631	158.0924	79.5498			1

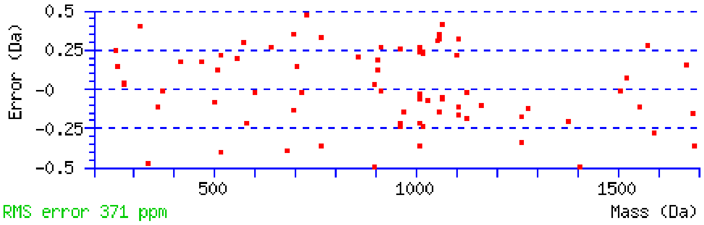

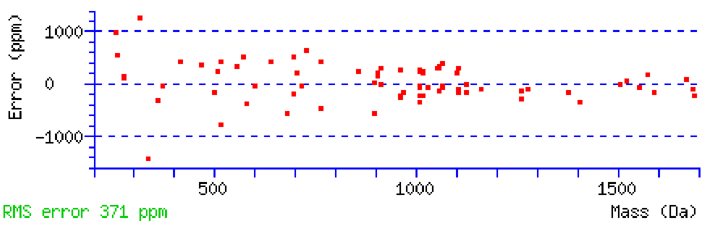

All matches to this query

Score	Mr(calc):	Delta	Sequence
47.8	2400.0594	-0.0003	SLAPDLSDDEHDPVDSISRPR
24.5	2400.0594	-0.0003	SLAPDLSDDEHDPVDSISRPR
17.4	2400.0594	-0.0003	SLAPDLSDDEHDPVDSISRPR
9.9	2400.0594	-0.0003	SLAPDLSDDEHDPVDSISRPR
4.7	2398.0484	2.0107	DTHSLPPAQILGIQSGQGIAR
2.3	2398.0738	1.9853	DYSDVIVAAYCLKHCYTLK
2.3	2398.0738	1.9853	DYSDVIVAAYCLKHCYTLK
1.1	2398.0307	2.0285	SCSSPLVASRAGKFLGATPPR
1.1	2398.0307	2.0285	SCSSPLVASRAGKFLGATPPR
1.1	2398.0307	2.0285	SCSSPLVASRAGKFLGATPPR

Spectrum No: 345; Query: 2698; Rank: 1

Peptide View

MS/MS Fragmentation of **VSEEAESQQWDTSKGDQVSQNGLPAEQGSPR**
Found in **IPI00373419**, Tax_Id=10116 Gene_Symbol=Spnb2 Non-erythrocyte beta-spectrin

Match to Query 2698: 3423.499452 from(1142.173760,3+)
Title: 100101RatKid_NS_deglyco_19.2129.2129.3.dta
Data file K:\NewmanPaper\Piliang\3SubProteomes\Piliang3SP\mgf5ppm\ERLIC_3SubProteomes5ppm.mgf

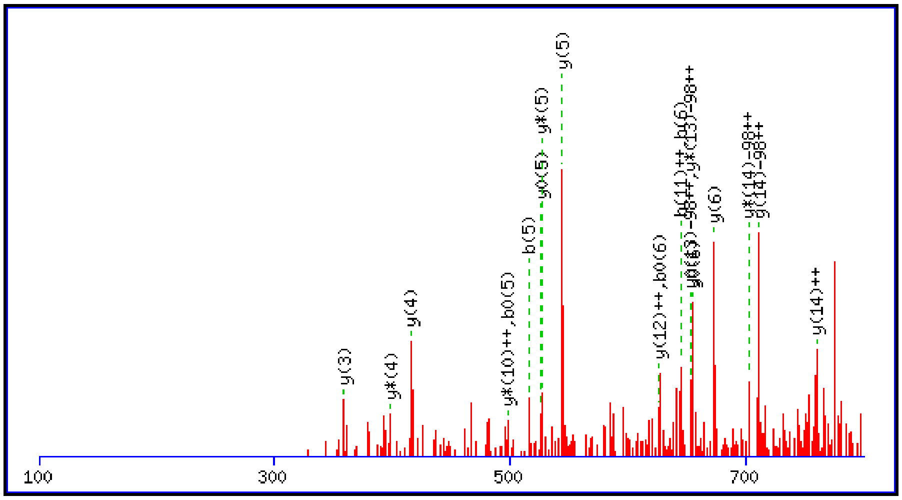

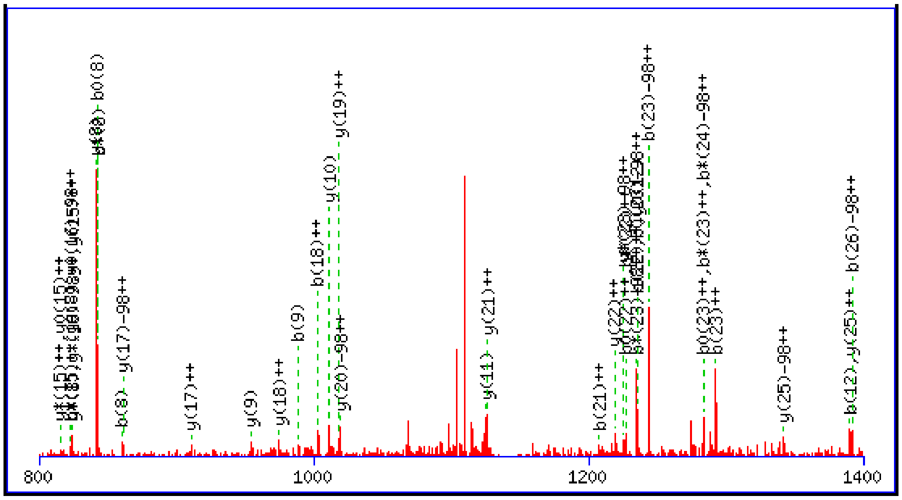

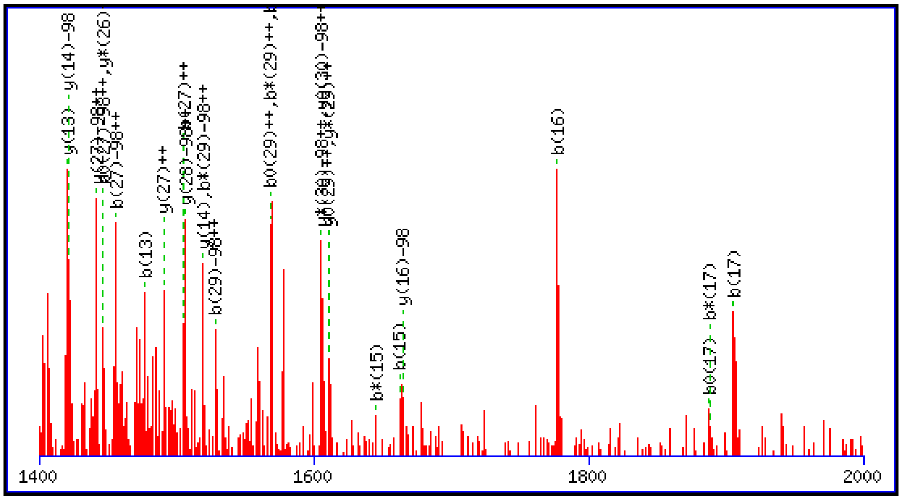

Monoisotopic mass of neutral peptide Mr(calc): 3423.4852
Fixed modifications: Carbamidomethyl (C)
Variable modifications:
S19 : Phospho (ST), with neutral losses 97.9769(shown in table), 0.0000
Ions Score: 48 **Expect:** 0.0078
Matches (Bold Red): 89/520 fragment ions using 139 most intense peaks

#	b	b++	b*	b*++	b0	b0++	Seq.	y	y++	y*	y*++	y0	y0++	#
1	100.0757	50.5415					V							31
2	187.1077	94.0575			169.0972	85.0522	S	3227.4472	1614.2272	3210.4206	**1605.7140**	3209.4366	**1605.2219**	30
3	316.1503	158.5788			298.1397	149.5735	E	3140.4151	1570.7112	3123.3886	1562.1979	3122.4046	1561.7059	29
4	445.1929	223.1001			427.1823	214.0948	E	3011.3726	**1506.1899**	2994.3460	1497.6766	2993.3620	1497.1846	28
5	**516.2300**	258.6186			**498.2195**	249.6134	A	2882.3300	**1441.6686**	2865.3034	1433.1553	2864.3194	1432.6633	27
6	**645.2726**	323.1399			**627.2620**	314.1347	E	2811.2928	1406.1501	2794.2663	1397.6368	2793.2823	1397.1448	26
7	732.3046	366.6560			714.2941	357.6507	S	2682.2503	**1341.6288**	2665.2237	1333.1155	2664.2397	1332.6235	25
8	**860.3632**	430.6852	**843.3367**	422.1720	**842.3527**	421.6800	Q	2595.2182	1298.1128	2578.1917	1289.5995	2577.2077	1289.1075	24
9	**988.4218**	494.7145	971.3952	486.2013	970.4112	485.7093	Q	2467.1596	1234.0835	2450.1331	**1225.5702**	2449.1491	1225.0782	23
10	1174.5011	587.7542	1157.4746	579.2409	1156.4905	578.7489	W	2339.1011	1170.0542	2322.0745	1161.5409	2321.0905	1161.0489	22
11	1289.5281	**645.2677**	1272.5015	636.7544	1271.5175	636.2624	D	2153.0218	1077.0145	2135.9952	1068.5012	2135.0112	1068.0092	21
12	**1390.5757**	695.7915	1373.5492	687.2782	1372.5652	686.7862	T	2037.9948	**1019.5010**	2020.9683	1010.9878	2019.9843	1010.4958	20
13	**1477.6078**	739.3075	1460.5812	730.7942	1459.5972	730.3022	S	1936.9471	968.9772	1919.9206	960.4639	1918.9366	959.9719	19
14	**1605.7027**	803.3550	1588.6762	794.8417	1587.6922	794.3497	K	1849.9151	925.4612	1832.8886	916.9479	1831.9045	916.4559	18
15	**1662.7242**	831.8657	**1645.6976**	**823.3525**	1644.7136	822.8604	G	1721.8201	**861.4137**	1704.7936	852.9004	1703.8096	852.4084	17
16	**1777.7511**	889.3792	1760.7246	880.8659	1759.7406	880.3739	D	**1664.7987**	832.9030	1647.7721	**824.3897**	1646.7881	**823.8977**	16
17	**1905.8097**	953.4085	**1888.7832**	944.8952	**1887.7991**	944.4032	Q	1549.7717	775.3895	1532.7452	766.8762	1531.7612	766.3842	15
18	2004.8781	**1002.9427**	1987.8516	994.4294	1986.8676	993.9374	V	**1421.7132**	**711.3602**	1404.6866	**702.8469**	1403.7026	702.3549	14

**19**	2073.8996	1037.4534	2056.8730	1028.9402	2055.8890	1028.4481	**S**	1322.6447	661.8260	1305.6182	**653.3127**	1304.6342	**652.8207**	**13**
**20**	2201.9582	1101.4827	2184.9316	1092.9694	2183.9476	1092.4774	**Q**	1253.6233	**627.3153**	1236.5967	618.8020	**1235.6127**	618.3100	**12**
**21**	2316.0011	1158.5042	2298.9745	1149.9909	2297.9905	1149.4989	**N**	**1125.5647**	563.2860	1108.5382	554.7727	1107.5541	554.2807	**11**
**22**	2373.0225	1187.0149	2355.9960	1178.5016	2355.0120	1178.0096	**G**	**1011.5218**	506.2645	994.4952	**497.7513**	993.5112	497.2592	**10**
**23**	2486.1066	**1243.5569**	2469.0801	**1235.0437**	2468.0960	**1234.5517**	**L**	**954.5003**	477.7538	937.4738	469.2405	936.4898	468.7485	**9**
**24**	2583.1594	1292.0833	2566.1328	**1283.5700**	2565.1488	1283.0780	**P**	**841.4163**	421.2118	**824.3897**	412.6985	**823.4057**	412.2065	**8**
**25**	2654.1965	1327.6019	2637.1699	1319.0886	2636.1859	1318.5966	**A**	744.3635	372.6854	727.3369	364.1721	726.3529	363.6801	**7**
**26**	2783.2391	**1392.1232**	2766.2125	1383.6099	2765.2285	1383.1179	**E**	**673.3264**	337.1668	656.2998	328.6536	**655.3158**	328.1615	**6**
**27**	2911.2977	**1456.1525**	2894.2711	1447.6392	2893.2871	**1447.1472**	**Q**	**544.2838**	272.6455	**527.2572**	264.1323	**526.2732**	263.6402	**5**
**28**	2968.3191	1484.6632	2951.2926	1476.1499	2950.3086	1475.6579	**G**	**416.2252**	208.6162	**399.1987**	200.1030	398.2146	199.6110	**4**
**29**	3055.3512	**1528.1792**	3038.3246	**1519.6659**	3037.3406	1519.1739	**S**	**359.2037**	180.1055	342.1772	171.5922	341.1932	171.1002	**3**
**30**	3152.4039	1576.7056	3135.3774	**1568.1923**	3134.3933	1567.7003	**P**	272.1717	136.5895	255.1452	128.0762			**2**
**31**							**R**	175.1190	88.0631	158.0924	79.5498			**1**

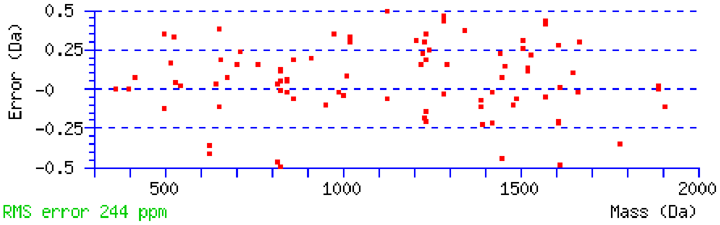

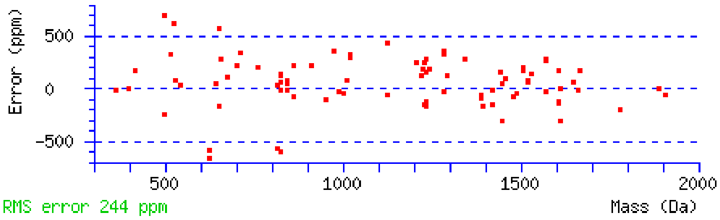

All matches to this query

Score	Mr(calc):	Delta	Sequence
47.8	3423.4852	0.0143	VSEEAESQQWDTSKGDQVSQNGLPAEQGSPR
29.1	3423.4852	0.0143	VSEEAESQQWDTSKGDQVSQNGLPAEQGSPR
28.6	3423.4852	0.0143	VSEEAESQQWDTSKGDQVSQNGLPAEQGSPR
24.4	3423.4852	0.0143	VSEEAESQQWDTSKGDQVSQNGLPAEQGSPR
18.9	3423.4852	0.0143	VSEEAESQQWDTSKGDQVSQNGLPAEQGSPR
11.1	3423.4852	0.0143	VSEEAESQQWDTSKGDQVSQNGLPAEQGSPR
6.2	3422.4716	1.0278	MATNVTNKTAPLSMNSYVLTENLNTPVIK
5.0	3422.5138	0.9856	APELKQGLYELSANNFELHVSQGNHFIK
4.9	3421.4686	2.0309	ETTGTSQQLHPSPFELNNTIIYITKIR
3.6	3423.4919	0.0076	VSANKVTTTSPQTMSTTTGKTNASKPGTGTTK

Spectrum No: 346; Query: 1167; Rank: 1

Peptide View

MS/MS Fragmentation of **SRTASGSSVTSLEGTR**
Found in **IPI00421389**, Tax_Id=10116 Gene_Symbol=Ndrg1 Protein NDRG1

Match to Query 1167: 1754.720532 from(585.914120,3+)
Title: 091008RatKidney_NH4Format01_26.931.931.3.dta
Data file K:\NewmanPaper\Piliang\3SubProteomes\Piliang3SP\mgf5ppm\ERLIC_3SubProteomes5ppm.mgf

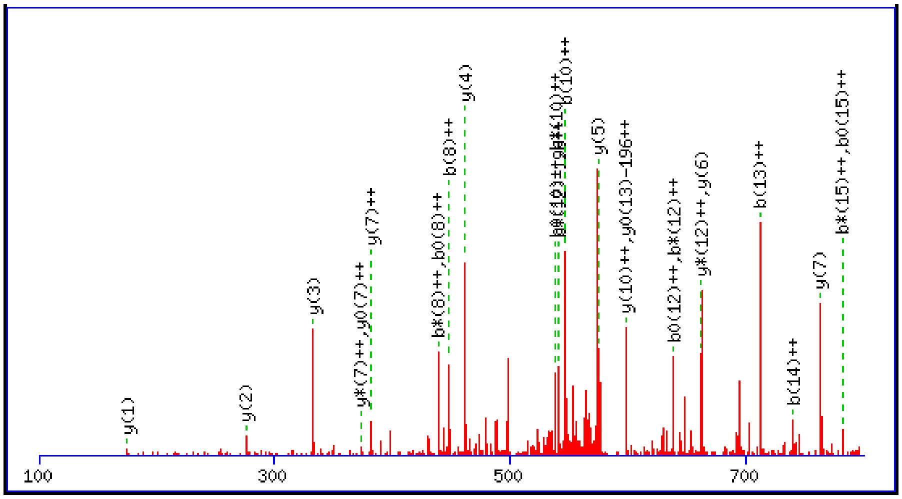

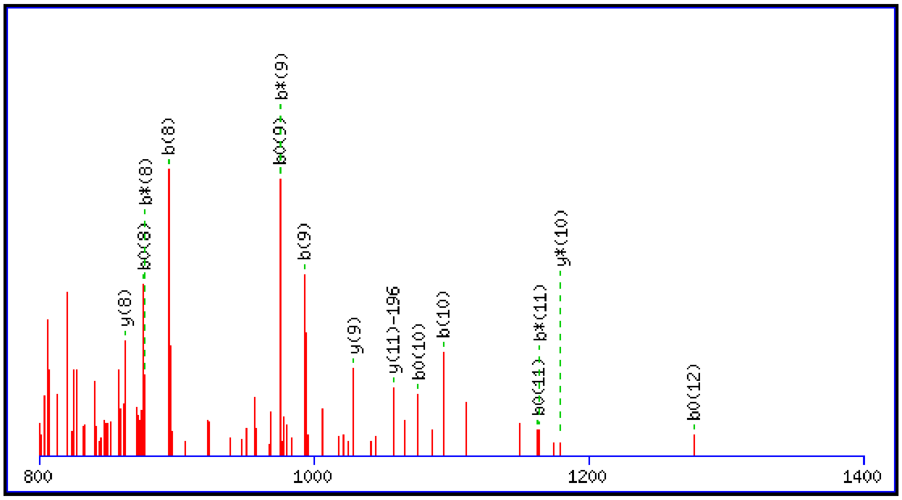

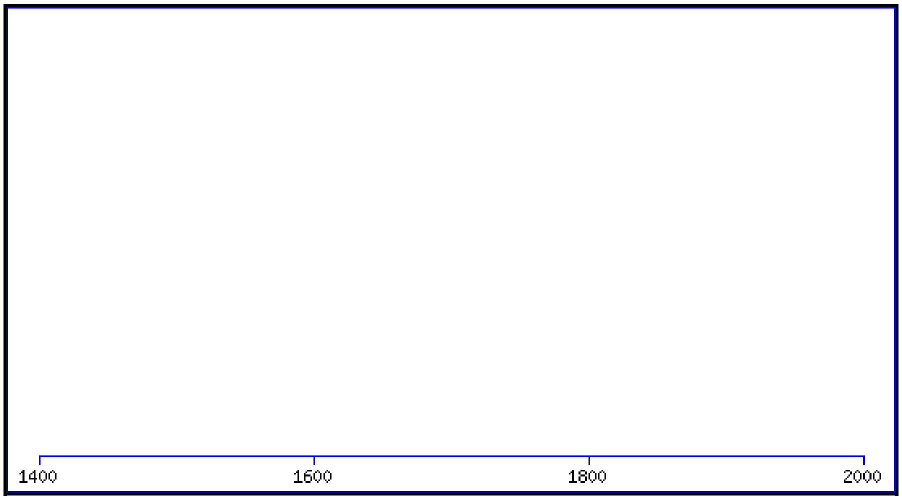

Monoisotopic mass of neutral peptide Mr(calc): 1754.7237
Fixed modifications: Carbamidomethyl (C)
Variable modifications:
S7 : Phospho (ST), with neutral losses 0.0000(shown in table), 97.9769
S8 : Phospho (ST), with neutral losses 0.0000(shown in table), 97.9769
Ions Score: 48 **Expect:** 0.0025
Matches (Bold Red): 41/272 fragment ions using 56 most intense peaks

#	b	b^{++}	b*	b^{*++}	b^0	b^{0++}	Seq.	y	y^{++}	y*	y^{*++}	y^0	y^{0++}	#
1	88.0393	44.5233			70.0287	35.5180	S							16
2	244.1404	122.5738	227.1139	114.0606	226.1298	113.5686	R	1668.6990	834.8531	1651.6724	826.3398	1650.6884	825.8478	15
3	345.1881	173.0977	328.1615	164.5844	327.1775	164.0924	T	1512.5979	756.8026	1495.5713	748.2893	1494.5873	747.7973	14
4	416.2252	208.6162	399.1987	200.1030	398.2146	199.6110	A	1411.5502	706.2787	1394.5236	697.7655	1393.5396	697.2734	13

**5**	503.2572	252.1323	486.2307	243.6190	485.2467	243.1270	**S**	1340.5131	670.7602	1323.4865	**662.2469**	1322.5025	661.7549	**12**
**6**	560.2787	280.6430	543.2522	272.1297	542.2681	271.6377	**G**	1253.4810	627.2442	1236.4545	618.7309	1235.4705	618.2389	**11**
**7**	727.2771	364.1422	710.2505	355.6289	709.2665	355.1369	**S**	1196.4596	**598.7334**	**1179.4330**	590.2202	1178.4490	589.7281	**10**
**8**	**894.2754**	**447.6413**	**877.2489**	**439.1281**	**876.2648**	**438.6361**	**S**	**1029.4612**	515.2343	1012.4347	506.7210	1011.4507	506.2290	**9**
**9**	**993.3438**	497.1755	**976.3173**	488.6623	**975.3333**	488.1703	**V**	**862.4629**	431.7351	845.4363	423.2218	844.4523	422.7298	**8**
**10**	**1094.3915**	**547.6994**	1077.3649	**539.1861**	**1076.3809**	**538.6941**	**T**	**763.3945**	**382.2009**	746.3679	**373.6876**	745.3839	**373.1956**	**7**
**11**	1181.4235	591.2154	**1164.3970**	582.7021	**1163.4130**	582.2101	**S**	**662.3468**	331.6770	645.3202	323.1638	644.3362	322.6717	**6**
**12**	1294.5076	647.7574	1277.4810	**639.2442**	**1276.4970**	**638.7521**	**L**	**575.3148**	288.1610	558.2882	279.6477	557.3042	279.1557	**5**
**13**	1423.5502	**712.2787**	1406.5236	703.7655	1405.5396	703.2734	**E**	**462.2307**	231.6190	445.2041	223.1057	444.2201	222.6137	**4**
**14**	1480.5716	**740.7895**	1463.5451	732.2762	1462.5611	731.7842	**G**	**333.1881**	167.0977	316.1615	158.5844	315.1775	158.0924	**3**
**15**	1581.6193	791.3133	1564.5928	**782.8000**	1563.6088	**782.3080**	**T**	**276.1666**	138.5870	259.1401	130.0737	258.1561	129.5817	**2**
**16**							**R**	**175.1190**	88.0631	158.0924	79.5498			**1**

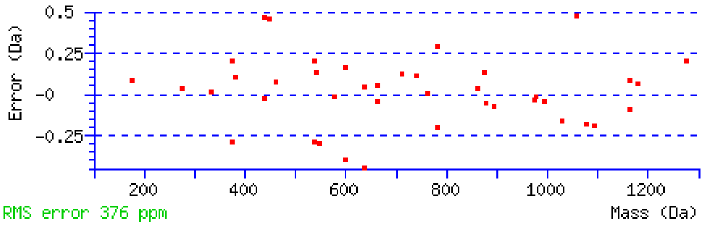

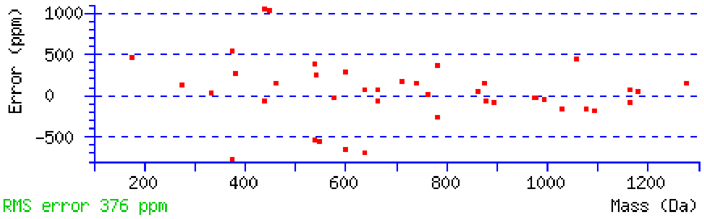

All matches to this query

Score	Mr(calc):	Delta	Sequence
47.9	1754.7237	-0.0032	SRTASGSSVTSLEGTR
38.9	1754.7237	-0.0032	SRTASGSSVTSLEGTR
38.0	1754.7237	-0.0032	SRTASGSSVTSLEGTR
36.6	1754.7237	-0.0032	SRTASGSSVTSLEGTR
12.6	1754.7352	-0.0147	DVSCSLTNTHFCASR
9.3	1754.7237	-0.0032	SRTASGSSVTSLEGTR
9.2	1752.7291	1.9915	FSLQFQRTCSENR
6.7	1753.7087	1.0119	RGHQPWNATGNSLR
6.7	1754.7237	-0.0032	SRTASGSSVTSLEGTR
6.0	1753.7091	1.0115	VTCHTEPSSSQQSAR

Spectrum No: 347; Query: 1355; Rank: 1

Peptide View

MS/MS Fragmentation of **VSEEDQSLENSEADVK**
Found in **IPI00192286**, Tax_Id=10116 Gene_Symbol=Abca2 ATP-binding cassette sub-family A member 2

Match to Query 1355: 1857.756308 from(929.885430,2+)
Title: 100101RatKid_NS_deglyco_24.1261.1261.2.dta
Data file K:\NewmanPaper\Piliang\3SubProteomes\Piliang3SP\mgf5ppm\ERLIC_3SubProteomes5ppm.mgf

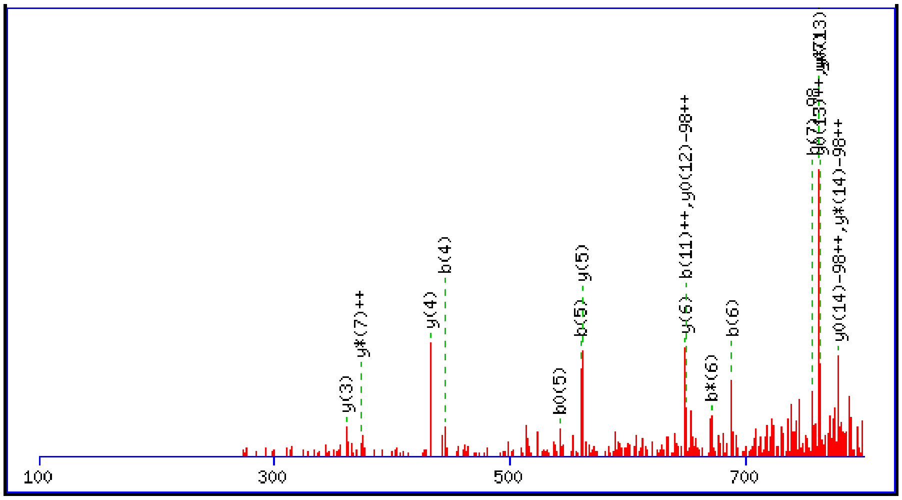

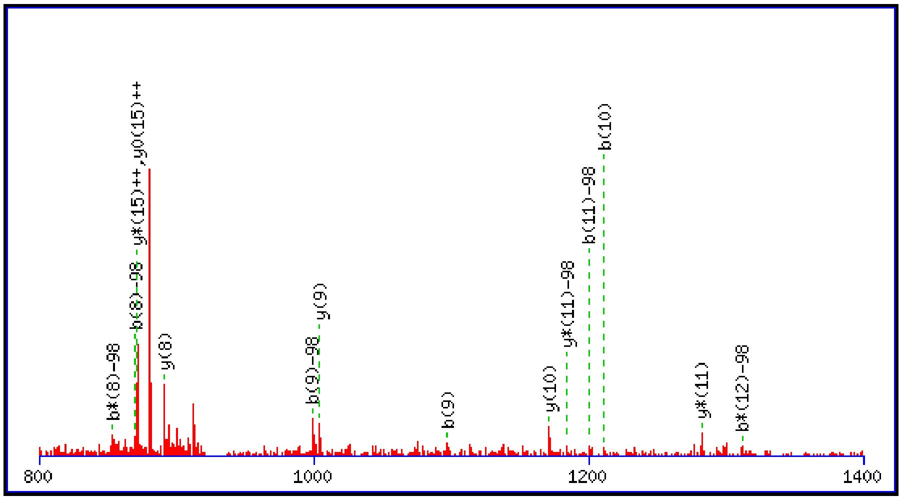

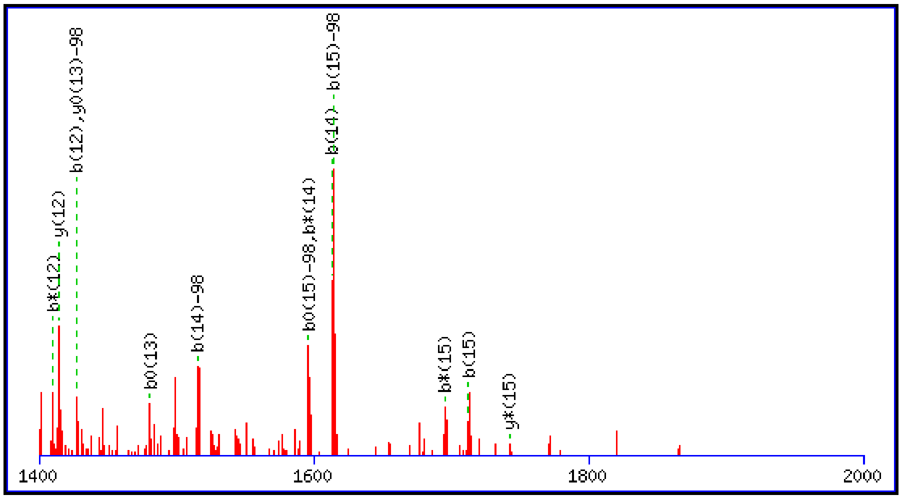

Monoisotopic mass of neutral peptide Mr(calc): 1857.7517
Fixed modifications: Carbamidomethyl (C)
Variable modifications:
S7 : Phospho (ST), with neutral losses 97.9769(shown in table), 0.0000
Ions Score: 48 **Expect:** 0.0028
Matches (Bold Red): 45/254 fragment ions using 71 most intense peaks

#	b	b++	b*	b*++	b0	b0++	Seq.	y	y++	y*	y*++	y0	y0++	#
1	100.0757	50.5415					V							16
2	187.1077	94.0575			169.0972	85.0522	S	1661.7137	831.3605	1644.6871	822.8472	1643.7031	822.3552	15
3	316.1503	158.5788			298.1397	149.5735	E	1574.6816	787.8445	1557.6551	**779.3312**	1556.6711	**778.8392**	14
4	**445.1929**	223.1001			427.1823	214.0948	E	1445.6391	723.3232	1428.6125	714.8099	**1427.6285**	714.3179	13
5	**560.2198**	280.6136			**542.2093**	271.6083	D	1316.5965	658.8019	1299.5699	650.2886	1298.5859	**649.7966**	12

**6**	**688.2784**	344.6429	**671.2519**	336.1296	670.2679	335.6376	**Q**	1201.5695	601.2884	**1184.5430**	592.7751	1183.5589	592.2831	**11**
**7**	**757.2999**	379.1536	740.2733	370.6403	739.2893	370.1483	**S**	1073.5109	537.2591	1056.4844	528.7458	1055.5004	528.2538	**10**
**8**	**870.3839**	435.6956	**853.3574**	427.1823	852.3734	426.6903	**L**	**1004.4895**	502.7484	987.4629	494.2351	986.4789	493.7431	**9**
**9**	**999.4265**	500.2169	982.4000	491.7036	981.4160	491.2116	**E**	**891.4054**	446.2063	874.3789	437.6931	873.3949	437.2011	**8**
**10**	1113.4695	557.2384	1096.4429	548.7251	1095.4589	548.2331	**N**	**762.3628**	381.6850	745.3363	**373.1718**	744.3523	372.6798	**7**
**11**	**1200.5015**	600.7544	1183.4749	592.2411	1182.4909	591.7491	**S**	**648.3199**	324.6636	631.2933	316.1503	630.3093	315.6583	**6**
**12**	1329.5441	665.2757	**1312.5175**	656.7624	1311.5335	656.2704	**E**	**561.2879**	281.1476	544.2613	272.6343	543.2773	272.1423	**5**
**13**	1400.5812	700.7942	1383.5547	692.2810	1382.5706	691.7890	**A**	**432.2453**	216.6263	415.2187	208.1130	414.2347	207.6210	**4**
**14**	**1515.6081**	758.3077	1498.5816	749.7944	1497.5976	749.3024	**D**	**361.2082**	181.1077	344.1816	172.5944	343.1976	172.1024	**3**
**15**	**1614.6766**	807.8419	1597.6500	799.3286	**1596.6660**	798.8366	**V**	246.1812	123.5942	229.1547	115.0810			**2**
**16**							**K**	147.1128	74.0600	130.0863	65.5468			**1**

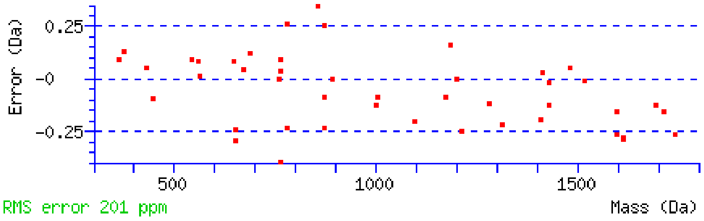

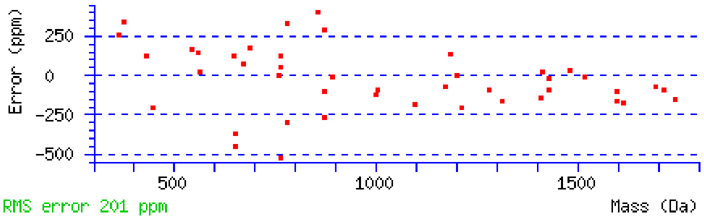

All matches to this query

Score	Mr(calc):	Delta	Sequence
47.8	1857.7517	0.0046	VSEEDQSLENSEADVK
34.9	1857.7517	0.0046	VSEEDQSLENSEADVK
21.0	1857.7452	0.0111	SASQDCIETTPGAQEGK
21.0	1857.7452	0.0111	SASQDCIETTPGAQEGK
16.8	1857.7517	0.0046	VSEEDQSLENSEADVK
6.6	1857.7532	0.0031	FPSESTFMQEFGDLK
6.6	1857.7532	0.0031	FPSESTFMQEFGDLK
5.3	1857.7434	0.0129	NSPATPPNTLNLXSEK
5.3	1857.7434	0.0129	NSPATPPNTLNLXSEK
4.6	1857.7434	0.0129	NSPATPPNTLNLXSEK

Spectrum No: 348; Query: 2698; Rank: 1

Peptide View

MS/MS Fragmentation of **VSEEAESQQWDTSKGDQVSQNGLPAEQGSPR**
Found in **IPI00373419**, Tax_Id=10116 Gene_Symbol=Spnb2 Non-erythrocyte beta-spectrin

Match to Query 2698: 3423.499452 from(1142.173760,3+)
Title: 100101RatKid_NS_deglyco_19.2129.2129.3.dta
Data file K:\NewmanPaper\Piliang\3SubProteomes\Piliang3SP\mgf5ppm\ERLIC_3SubProteomes5ppm.mgf

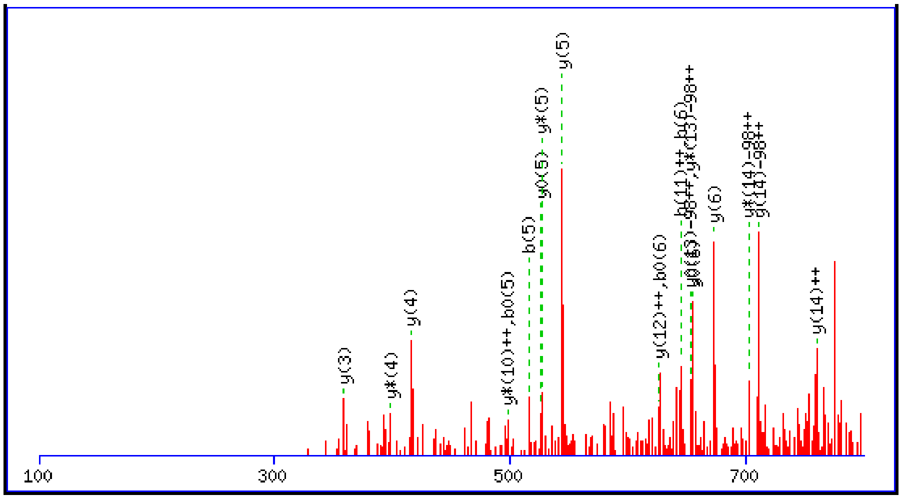

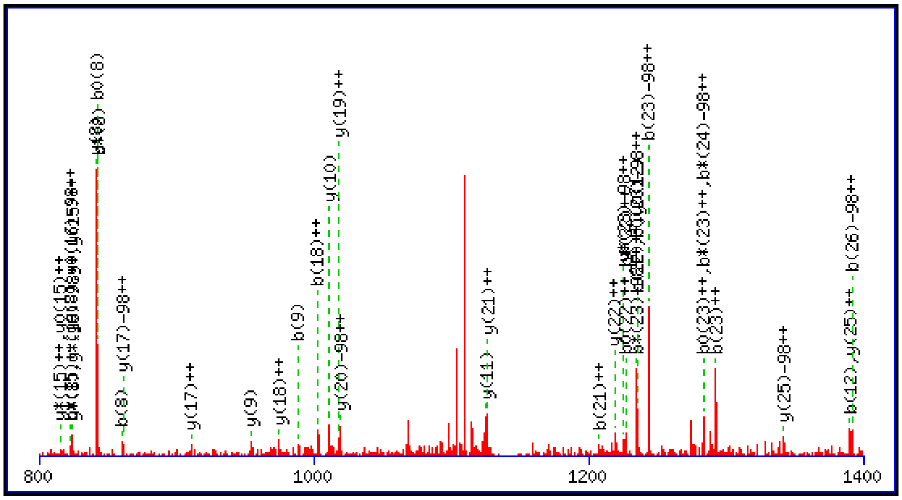

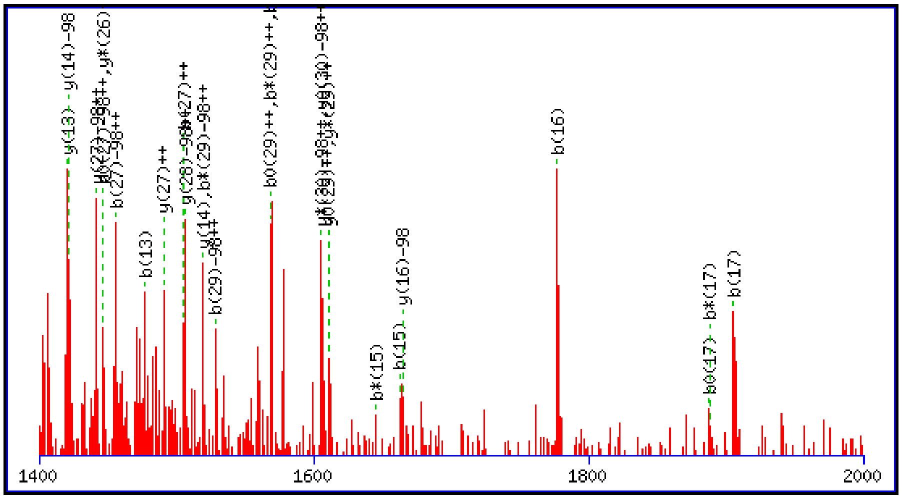

Monoisotopic mass of neutral peptide Mr(calc): 3423.4852
Fixed modifications: Carbamidomethyl (C)
Variable modifications:
S19 : Phospho (ST), with neutral losses 97.9769(shown in table), 0.0000
Ions Score: 48 **Expect:** 0.0078
Matches (Bold Red): 89/520 fragment ions using 139 most intense peaks

#	b	b^{++}	b*	b^{*++}	b^0	b^{0++}	Seq.	y	y^{++}	y*	y^{*++}	y^0	y^{0++}	#
1	100.0757	50.5415					V							31
2	187.1077	94.0575			169.0972	85.0522	S	3227.4472	1614.2272	3210.4206	**1605.7140**	3209.4366	**1605.2219**	30
3	316.1503	158.5788			298.1397	149.5735	E	3140.4151	1570.7112	3123.3886	1562.1979	3122.4046	1561.7059	29
4	445.1929	223.1001			427.1823	214.0948	E	3011.3726	**1506.1899**	2994.3460	1497.6766	2993.3620	1497.1846	28
5	**516.2300**	258.6186			**498.2195**	249.6134	A	2882.3300	**1441.6686**	2865.3034	1433.1553	2864.3194	1432.6633	27

6	**645.2726**	323.1399			**627.2620**	314.1347	E	2811.2928	1406.1501	2794.2663	1397.6368	2793.2823	1397.1448	26
7	732.3046	366.6560			714.2941	357.6507	S	2682.2503	**1341.6288**	2665.2237	1333.1155	2664.2397	1332.6235	25
8	**860.3632**	430.6852	**843.3367**	422.1720	**842.3527**	421.6800	Q	2595.2182	1298.1128	2578.1917	1289.5995	2577.2077	1289.1075	24
9	**988.4218**	494.7145	971.3952	486.2013	970.4112	485.7093	Q	2467.1596	1234.0835	2450.1331	**1225.5702**	2449.1491	1225.0782	23
10	1174.5011	587.7542	1157.4746	579.2409	1156.4905	578.7489	W	2339.1011	1170.0542	2322.0745	1161.5409	2321.0905	1161.0489	22
11	1289.5281	**645.2677**	1272.5015	636.7544	1271.5175	636.2624	D	2153.0218	1077.0145	2135.9952	1068.5012	2135.0112	1068.0092	21
12	**1390.5757**	695.7915	1373.5492	687.2782	1372.5652	686.7862	T	2037.9948	**1019.5010**	2020.9683	1010.9878	2019.9843	1010.4958	20
13	**1477.6078**	739.3075	1460.5812	730.7942	1459.5972	730.3022	S	1936.9471	968.9772	1919.9206	960.4639	1918.9366	959.9719	19
14	**1605.7027**	803.3550	1588.6762	794.8417	1587.6922	794.3497	K	1849.9151	925.4612	1832.8886	916.9479	1831.9045	916.4559	18
15	**1662.7242**	831.8657	**1645.6976**	**823.3525**	1644.7136	822.8604	G	1721.8201	**861.4137**	1704.7936	852.9004	1703.8096	852.4084	17
16	**1777.7511**	889.3792	1760.7246	880.8659	1759.7406	880.3739	D	**1664.7987**	832.9030	1647.7721	**824.3897**	1646.7881	**823.8977**	16
17	**1905.8097**	953.4085	**1888.7832**	944.8952	**1887.7991**	944.4032	Q	1549.7717	775.3895	1532.7452	766.8762	1531.7612	766.3842	15
18	2004.8781	**1002.9427**	1987.8516	994.4294	1986.8676	993.9374	V	**1421.7132**	**711.3602**	1404.6866	**702.8469**	1403.7026	702.3549	14
19	2073.8996	1037.4534	2056.8730	1028.9402	2055.8890	1028.4481	S	1322.6447	661.8260	1305.6182	**653.3127**	1304.6342	**652.8207**	13
20	2201.9582	1101.4827	2184.9316	1092.9694	2183.9476	1092.4774	Q	1253.6233	**627.3153**	1236.5967	618.8020	**1235.6127**	618.3100	12
21	2316.0011	1158.5042	2298.9745	1149.9909	2297.9905	1149.4989	N	**1125.5647**	563.2860	1108.5382	554.7727	1107.5541	554.2807	11
22	2373.0225	1187.0149	2355.9960	1178.5016	2355.0120	1178.0096	G	**1011.5218**	506.2645	994.4952	**497.7513**	993.5112	497.2592	10
23	2486.1066	**1243.5569**	2469.0801	**1235.0437**	2468.0960	**1234.5517**	L	**954.5003**	477.7538	937.4738	469.2405	936.4898	468.7485	9
24	2583.1594	1292.0833	2566.1328	**1283.5700**	2565.1488	1283.0780	P	**841.4163**	421.2118	**824.3897**	412.6985	**823.4057**	412.2065	8
25	2654.1965	1327.6019	2637.1699	1319.0886	2636.1859	1318.5966	A	744.3635	372.6854	727.3369	364.1721	726.3529	363.6801	7
26	2783.2391	**1392.1232**	2766.2125	1383.6099	2765.2285	1383.1179	E	**673.3264**	337.1668	656.2998	328.6536	**655.3158**	328.1615	6
27	2911.2977	**1456.1525**	2894.2711	1447.6392	2893.2871	**1447.1472**	Q	**544.2838**	272.6455	**527.2572**	264.1323	**526.2732**	263.6402	5
28	2968.3191	1484.6632	2951.2926	1476.1499	2950.3086	1475.6579	G	**416.2252**	208.6162	**399.1987**	200.1030	398.2146	199.6110	4
29	3055.3512	**1528.1792**	3038.3246	**1519.6659**	3037.3406	1519.1739	S	**359.2037**	180.1055	342.1772	171.5922	341.1932	171.1002	3
30	3152.4039	1576.7056	3135.3774	**1568.1923**	3134.3933	1567.7003	P	272.1717	136.5895	255.1452	128.0762			2
31							R	175.1190	88.0631	158.0924	79.5498			1

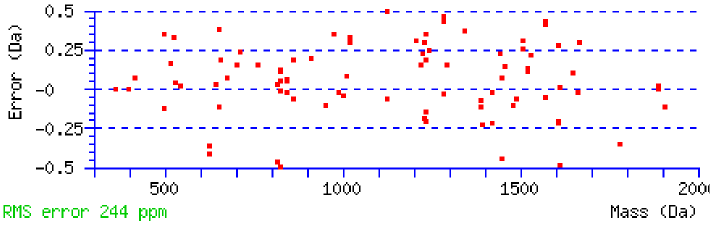

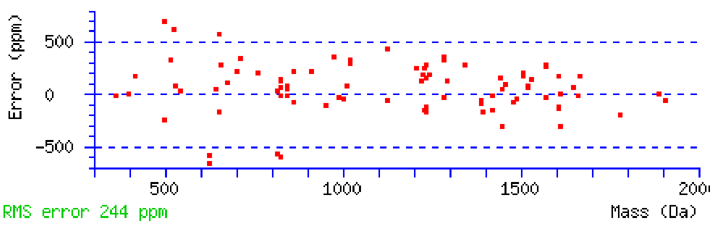

All matches to this query

Score	Mr(calc):	Delta	Sequence
47.8	3423.4852	0.0143	VSEEAESQQWDTSKGDQVSQNGLPAEQGSPR
29.1	3423.4852	0.0143	VSEEAESQQWDTSKGDQVSQNGLPAEQGSPR
28.6	3423.4852	0.0143	VSEEAESQQWDTSKGDQVSQNGLPAEQGSPR
24.4	3423.4852	0.0143	VSEEAESQQWDTSKGDQVSQNGLPAEQGSPR
18.9	3423.4852	0.0143	VSEEAESQQWDTSKGDQVSQNGLPAEQGSPR
11.1	3423.4852	0.0143	VSEEAESQQWDTSKGDQVSQNGLPAEQGSPR
6.2	3422.4716	1.0278	MATNVTNKTAPLSMNSYVLTENLNTPVIK
5.0	3422.5138	0.9856	APELKQGLYELSANNFELHVSQGNHFIK
4.9	3421.4686	2.0309	ETTGTSQQLHPSPFELNNTIIYITKIR
3.6	3423.4919	0.0076	VSANKVTTTSPQTMSTTTGKTNASKPGTGTTK

Spectrum No: 349; Query: 2065; Rank: 1

Peptide View

MS/MS Fragmentation of **AHNLQDGQISDTGDPGEDSTSN**
Found in **IPI00392092**, Tax_Id=10116 Gene_Symbol=Kif21a_predicted 185 kDa protein

Match to Query 2065: 2336.898192 from(779.973340,3+)
Title: 091008RatKidney_NH4Format01_31.684.684.3.dta
Data file K:\NewmanPaper\Piliang\3SubProteomes\Piliang3SP\mgf5ppm\ERLIC_3SubProteomes5ppm.mgf

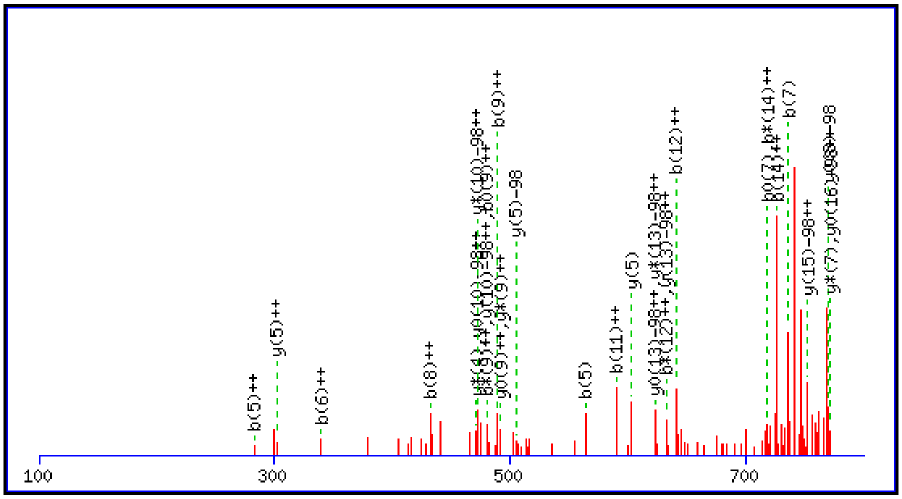

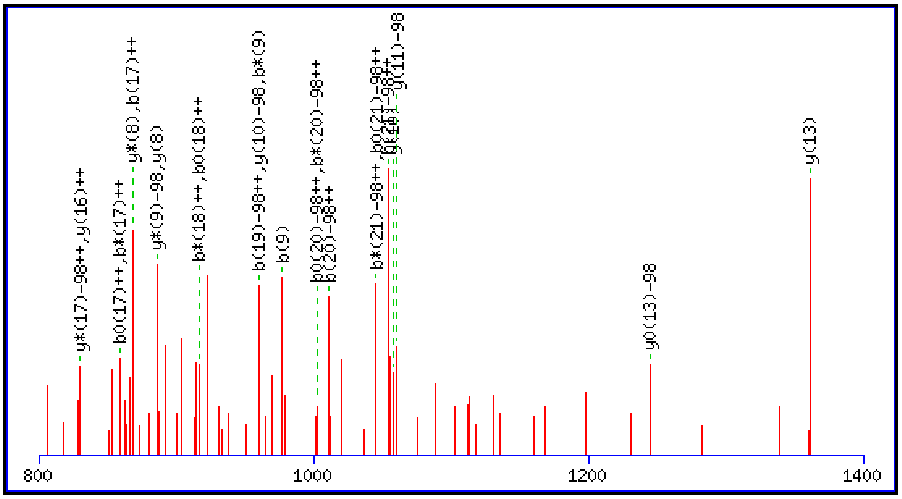

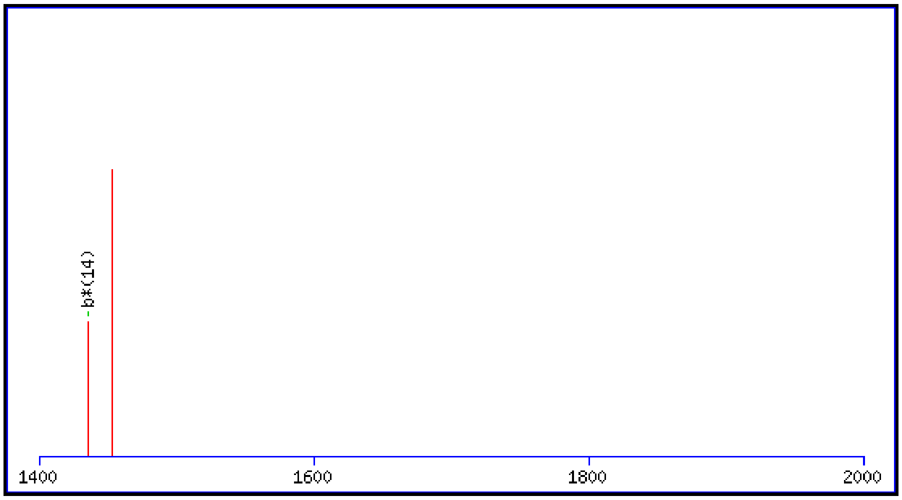

Monoisotopic mass of neutral peptide Mr(calc): 2336.9030
Fixed modifications: Carbamidomethyl (C)
Variable modifications:
S19 : Phospho (ST), with neutral losses 97.9769(shown in table), 0.0000
Ions Score: 48 **Expect:** 0.0032

Matches (Bold Red): 55/362 fragment ions using 85 most intense peaks

#	b	b^{++}	b*	b^{*++}	b^{0}	b^{0++}	Seq.	y	y^{++}	y*	y^{*++}	y^{0}	y^{0++}	#
1	72.0444	36.5258					A							22
2	209.1033	105.0553					H	2168.8963	1084.9518	2151.8697	1076.4385	2150.8857	1075.9465	21
3	323.1462	162.0768	306.1197	153.5635			N	2031.8374	1016.4223	2014.8108	1007.9090	2013.8268	1007.4170	20
4	436.2303	218.6188	419.2037	210.1055			L	1917.7944	959.4009	1900.7679	950.8876	1899.7839	950.3956	19
5	**564.2889**	**282.6481**	547.2623	274.1348			Q	1804.7104	902.8588	1787.6838	894.3456	1786.6998	893.8535	18
6	679.3158	**340.1615**	662.2893	331.6483	661.3052	331.1563	D	1676.6518	838.8295	1659.6253	**830.3163**	1658.6412	829.8243	17
7	**736.3373**	368.6723	719.3107	360.1590	**718.3267**	359.6670	G	1561.6249	781.3161	1544.5983	772.8028	1543.6143	**772.3108**	16
8	864.3959	**432.7016**	847.3693	424.1883	846.3853	423.6963	Q	1504.6034	**752.8053**	1487.5768	744.2921	1486.5928	743.8001	15
9	**977.4799**	**489.2436**	**960.4534**	**480.7303**	959.4694	**480.2383**	I	1376.5448	688.7760	1359.5183	680.2628	1358.5343	679.7708	14
10	1064.5119	532.7596	1047.4854	524.2463	1046.5014	523.7543	S	1263.4608	**632.2340**	1246.4342	**623.7207**	**1245.4502**	**623.2287**	13
11	1179.5389	**590.2731**	1162.5123	581.7598	1161.5283	581.2678	D	1176.4287	588.7180	1159.4022	580.2047	1158.4182	579.7127	12
12	1280.5866	**640.7969**	1263.5600	**632.2836**	1262.5760	631.7916	T	**1061.4018**	531.2045	1044.3752	522.6913	1043.3912	522.1992	11
13	1337.6080	669.3077	1320.5815	660.7944	1319.5975	660.3024	G	**960.3541**	**480.6807**	943.3276	**472.1674**	942.3435	**471.6754**	10
14	1452.6350	**726.8211**	**1435.6084**	**718.3079**	1434.6244	717.8158	D	903.3326	452.1700	**886.3061**	443.6567	885.3221	443.1647	9
15	1549.6877	775.3475	1532.6612	766.8342	1531.6772	766.3422	P	788.3057	394.6565	771.2791	386.1432	**770.2951**	385.6512	8
16	1606.7092	803.8582	1589.6827	795.3450	1588.6986	794.8530	G	691.2529	346.1301	674.2264	337.6168	673.2424	337.1248	7
17	1735.7518	**868.3795**	1718.7252	**859.8663**	1717.7412	**859.3743**	E	634.2315	317.6194	617.2049	309.1061	616.2209	308.6141	6
18	1850.7787	925.8930	1833.7522	**917.3797**	1832.7682	**916.8877**	D	**505.1889**	253.0981	488.1623	244.5848	487.1783	244.0928	5
19	1919.8002	**960.4037**	1902.7736	951.8905	1901.7896	951.3985	S	390.1619	195.5846	373.1354	187.0713	372.1514	186.5793	4
20	2020.8479	**1010.9276**	2003.8213	**1002.4143**	2002.8373	**1001.9223**	T	321.1405	161.0739	304.1139	152.5606	303.1299	152.0686	3
21	2107.8799	**1054.4436**	2090.8534	**1045.9303**	2089.8693	**1045.4383**	S	220.0928	110.5500	203.0662	102.0368	202.0822	101.5448	2
22							N	133.0608	67.0340	116.0342	58.5207			1

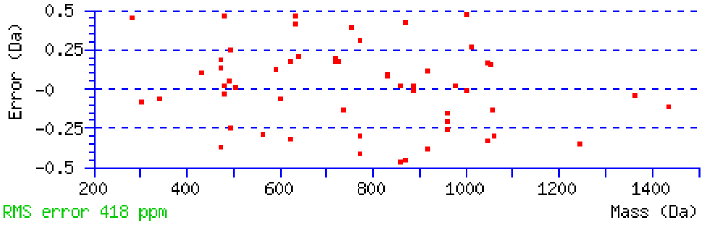

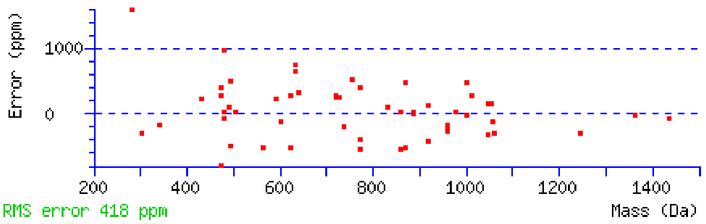

All matches to this query

Score	Mr(calc):	Delta	Sequence
47.7	2336.9030	-0.0048	AHNLQDGQISDTGDPGEDSTSN
41.0	2336.9030	-0.0048	AHNLQDGQISDTGDPGEDSTSN
40.0	2336.9030	-0.0048	AHNLQDGQISDTGDPGEDSTSN
24.4	2336.9030	-0.0048	AHNLQDGQISDTGDPGEDSTSN
17.2	2336.9030	-0.0048	AHNLQDGQISDTGDPGEDSTSN
4.0	2336.8938	0.0044	GHSGGSSGGGGGGSGGGPGIKKTER
3.6	2336.8938	0.0044	GHSGGSSGGGGGGSGGGPGIKKTER
0.5	2334.8851	2.0131	DAMGNTRQEMTHMVNAMDR
0.2	2335.8985	0.9997	TALAPGNDRNSDTHGTLGSGR

Spectrum No: 350; Query: 1660; Rank: 1

Peptide View

MS/MS Fragmentation of **TDSREDEISPPPPNPVVK**
Found in **IPI00231770**, Tax_Id=10116 Gene_Symbol=Prkar1a cAMP-dependent protein kinase type I-alpha regulatory subunit

Match to Query 1660: 2055.944772 from(686.322200,3+)
Title: 091008RatKidney_NoSalt_22.1333.1333.3.dta
Data file K:\NewmanPaper\Piliang\3SubProteomes\Piliang3SP\mgf5ppm\ERLIC_3SubProteomes5ppm.mgf

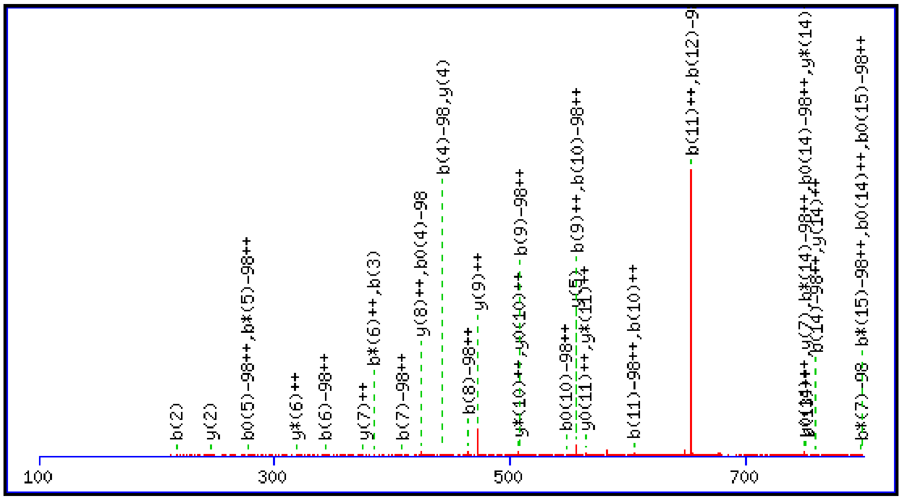

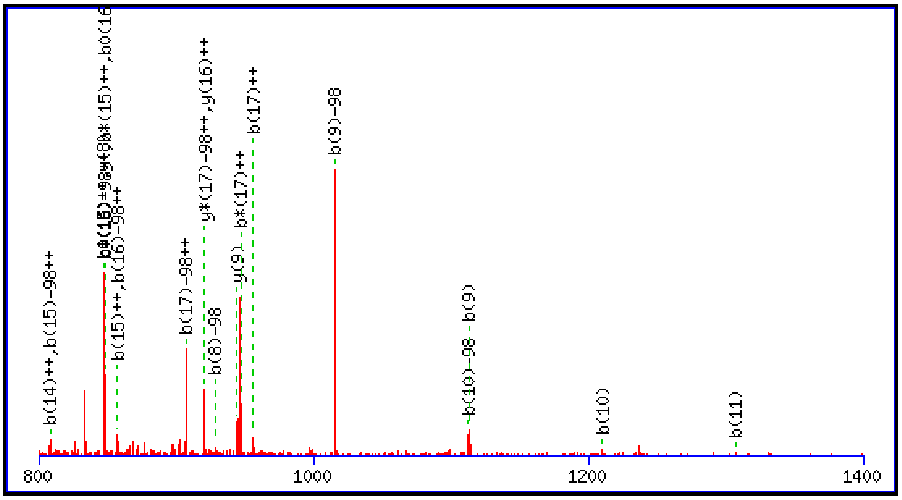

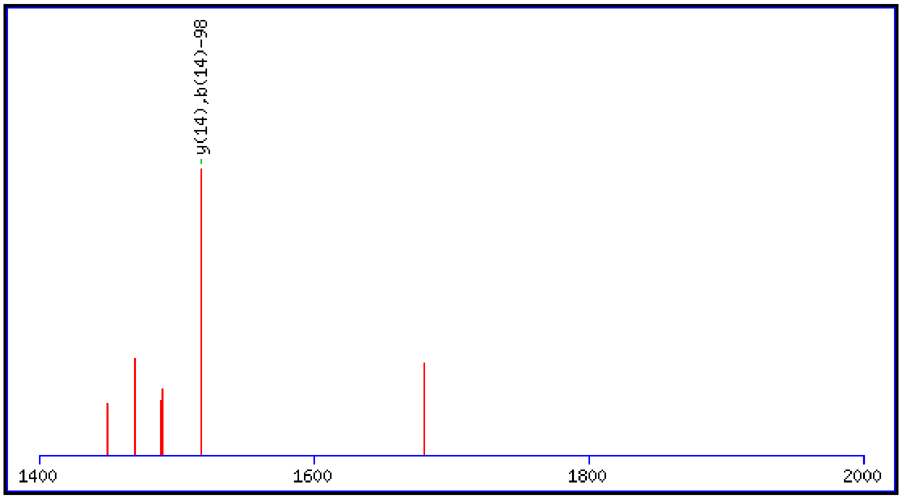

Monoisotopic mass of neutral peptide Mr(calc): 2055.9514
Fixed modifications: Carbamidomethyl (C)
Variable modifications:
S3 : Phospho (ST), with neutral losses 97.9769(shown in table), 0.0000
Ions Score: 48 **Expect:** 0.0042
Matches (Bold Red): 64/280 fragment ions using 90 most intense peaks

#	b	b^{++}	b*	b*$^{++}$	b^{0}	b^{0++}	Seq.	y	y^{++}	y*	y*$^{++}$	y^{0}	y^{0++}	#

1	102.0550	51.5311			84.0444	42.5258	T							18
2	**217.0819**	109.0446			199.0713	100.0393	D	1857.9341	929.4707	1840.9076	**920.9574**	1839.9235	920.4654	17
3	286.1034	143.5553			268.0928	134.5500	S	1742.9072	871.9572	1725.8806	863.4439	1724.8966	862.9519	16
4	**442.2045**	221.6059	425.1779	213.0926	**424.1939**	212.6006	R	1673.8857	837.4465	1656.8592	828.9332	1655.8751	828.4412	15
5	571.2471	286.1272	554.2205	**277.6139**	553.2365	**277.1219**	E	**1517.7846**	**759.3959**	1500.7581	**750.8827**	1499.7740	**750.3907**	14
6	686.2740	**343.6406**	669.2475	335.1274	668.2634	334.6354	D	1388.7420	694.8746	1371.7155	686.3614	1370.7314	685.8694	13
7	815.3166	**408.1619**	**798.2900**	399.6487	797.3060	399.1567	E	1273.7151	637.3612	1256.6885	628.8479	1255.7045	628.3559	12
8	**928.4007**	**464.7040**	911.3741	456.1907	910.3901	455.6987	I	1144.6725	572.8399	1127.6459	**564.3266**	1126.6619	**563.8346**	11
9	**1015.4327**	**508.2200**	998.4061	499.7067	997.4221	499.2147	S	1031.5884	516.2978	1014.5619	**507.7846**	1013.5778	**507.2926**	10
10	**1112.4855**	**556.7464**	1095.4589	548.2331	1094.4749	**547.7411**	P	**944.5564**	**472.7818**	927.5298	464.2686			9
11	1209.5382	**605.2727**	1192.5117	596.7595	1191.5276	596.2675	P	**847.5036**	**424.2554**	830.4771	415.7422			8
12	1306.5910	**653.7991**	1289.5644	645.2859	1288.5804	644.7938	P	**750.4509**	**375.7291**	733.4243	367.2158			7
13	1403.6437	702.3255	1386.6172	693.8122	1385.6332	693.3202	P	653.3981	327.2027	636.3715	**318.6894**			6
14	**1517.6867**	**759.3470**	1500.6601	**750.8337**	1499.6761	**750.3417**	N	**556.3453**	278.6763	539.3188	270.1630			5
15	1614.7394	**807.8734**	1597.7129	**799.3601**	1596.7289	**798.8681**	P	**442.3024**	221.6548	425.2758	213.1416			4
16	1713.8078	**857.4076**	1696.7813	**848.8943**	1695.7973	**848.4023**	V	345.2496	173.1285	328.2231	164.6152			3
17	1812.8763	**906.9418**	1795.8497	898.4285	1794.8657	897.9365	V	**246.1812**	123.5942	229.1547	115.0810			2
18							K	147.1128	74.0600	130.0863	65.5468			1

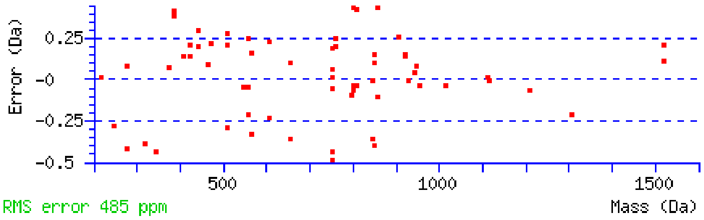

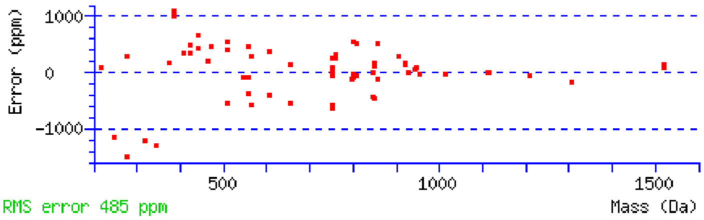

All matches to this query

Score	Mr(calc):	Delta	Sequence
47.7	2055.9514	-0.0066	TDSREDEISPPPPNPVVK
45.9	2055.9514	-0.0066	TDSREDEISPPPPNPVVK
38.0	2055.9514	-0.0066	TDSREDEISPPPPNPVVK
11.0	2053.9196	2.0251	YVGTNLVPLSTCKNTVK
6.5	2054.9254	1.0194	EETLTYLNIVSPFELK
6.2	2054.9228	1.0220	NLFTPQGKSVHDLSVPR
5.9	2054.9227	1.0220	SIDKTSNGFTYKPPGRK
5.9	2054.9227	1.0220	SIDKTSNGFTYKPPGRK
5.9	2054.9228	1.0220	NLFTPQGKSVHDLSVPR
5.8	2054.9227	1.0220	SIDKTSNGFTYKPPGRK

Spectrum No: 351; Query: 2330; Rank: 1

Peptide View

MS/MS Fragmentation of **KGSLDSDNDDSDCPYSEKVPSIK**
Found in **IPI00199985**, Tax_Id=10116 Gene_Symbol=Slc4a4 Isoform 1 of Electrogenic sodium bicarbonate cotransporter 1

Match to Query 2330: 2635.098642 from(879.373490,3+)
Title: 091008RatKidney_NoSalt_22.1558.1558.3.dta
Data file K:\NewmanPaper\Piliang\3SubProteomes\Piliang3SP\mgf5ppm\ERLIC_3SubProteomes5ppm.mgf

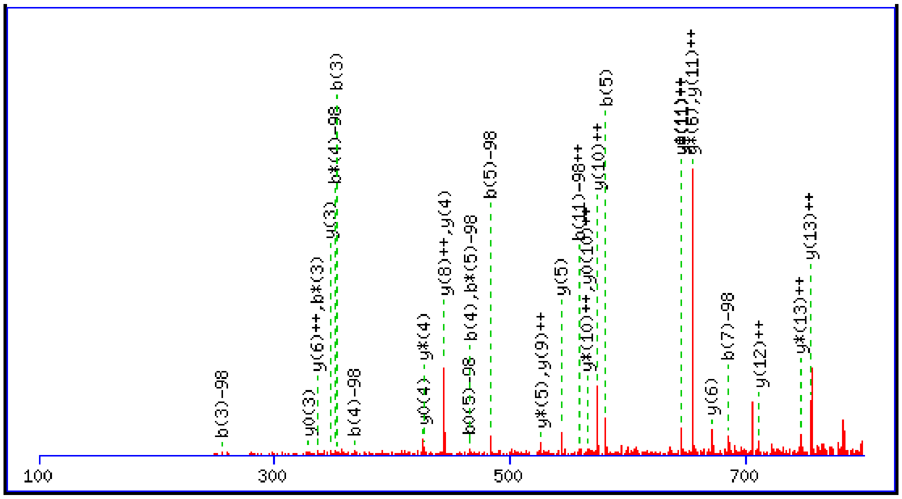

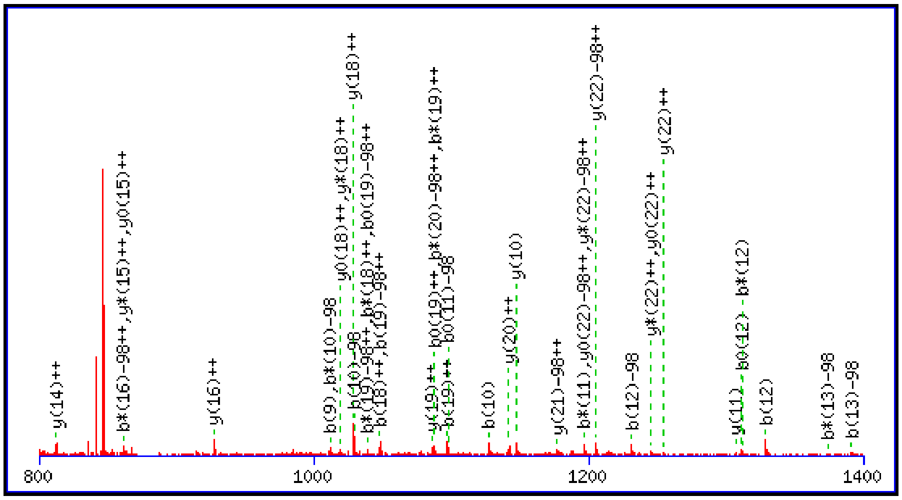

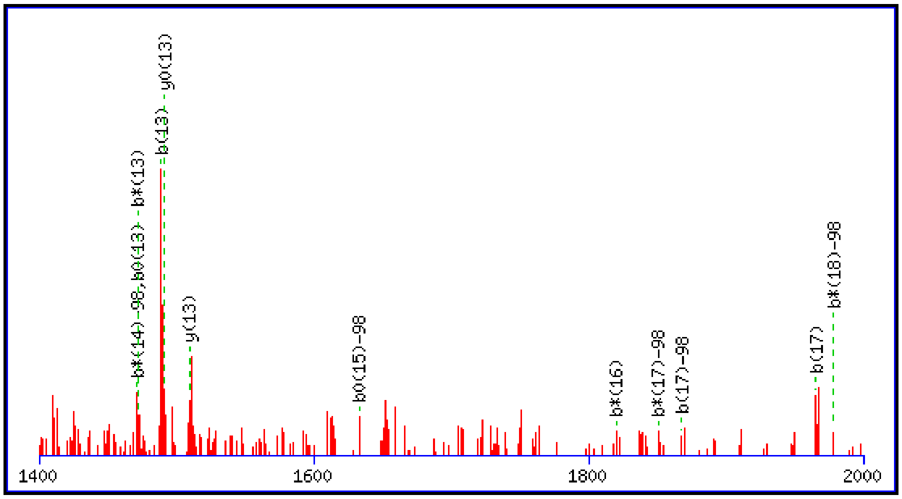

Monoisotopic mass of neutral peptide Mr(calc): 2635.0997
Fixed modifications: Carbamidomethyl (C)
Variable modifications:
S3 : Phospho (ST), with neutral losses 97.9769(shown in table), 0.0000
Ions Score: 48 **Expect:** 0.0054
Matches (Bold Red): 87/388 fragment ions using 154 most intense peaks

#	b	b^{++}	b*	b^{*++}	b^0	b^{0++}	Seq.	y	y^{++}	y*	y^{*++}	y^0	y^{0++}	#
1	129.1022	65.0548	112.0757	56.5415			K							23
2	186.1237	93.5655	169.0972	85.0522			G	2410.0351	**1205.5212**	2393.0085	**1197.0079**	2392.0245	**1196.5159**	22
3	**255.1452**	128.0762	238.1186	119.5629	237.1346	119.0709	S	2353.0136	**1177.0104**	2335.9871	1168.4972	2335.0031	1168.0052	21
4	**368.2292**	184.6182	**351.2027**	176.1050	350.2187	175.6130	L	2283.9922	**1142.4997**	2266.9656	1133.9864	2265.9816	1133.4944	20
5	**483.2562**	242.1317	**466.2296**	233.6184	**465.2456**	233.1264	D	2170.9081	**1085.9577**	2153.8815	1077.4444	2152.8975	1076.9524	19

**6**	570.2882	285.6477	553.2616	277.1345	552.2776	276.6425	**S**	2055.8812	**1028.4442**	2038.8546	**1019.9309**	2037.8706	**1019.4389**	**18**
**7**	**685.3151**	343.1612	668.2886	334.6479	667.3046	334.1559	**D**	1968.8491	984.9282	1951.8226	976.4149	1950.8386	975.9229	**17**
**8**	799.3581	400.1827	782.3315	391.6694	781.3475	391.1774	**N**	1853.8222	**927.4147**	1836.7956	918.9015	1835.8116	918.4094	**16**
**9**	914.3850	457.6961	897.3585	449.1829	896.3744	448.6909	**D**	1739.7793	870.3933	1722.7527	**861.8800**	1721.7687	**861.3880**	**15**
**10**	**1029.4119**	515.2096	**1012.3854**	506.6963	1011.4014	506.2043	**D**	1624.7523	**812.8798**	1607.7258	804.3665	1606.7417	803.8745	**14**
**11**	1116.4440	**558.7256**	1099.4174	550.2124	**1098.4334**	549.7203	**S**	**1509.7254**	**755.3663**	1492.6988	**746.8530**	**1491.7148**	746.3610	**13**
**12**	**1231.4709**	616.2391	1214.4444	607.7258	1213.4604	607.2338	**D**	1422.6933	**711.8503**	1405.6668	703.3370	1404.6828	702.8450	**12**
**13**	**1391.5016**	696.2544	**1374.4750**	687.7411	1373.4910	687.2491	**C**	**1307.6664**	**654.3368**	1290.6399	**645.8236**	1289.6558	**645.3316**	**11**
**14**	1488.5543	744.7808	**1471.5278**	736.2675	1470.5438	735.7755	**P**	**1147.6358**	**574.3215**	1130.6092	**565.8082**	1129.6252	**565.3162**	**10**
**15**	1651.6177	826.3125	1634.5911	817.7992	**1633.6071**	817.3072	**Y**	1050.5830	**525.7951**	1033.5564	517.2819	1032.5724	516.7898	**9**
**16**	1738.6497	869.8285	1721.6231	**861.3152**	1720.6391	860.8232	**S**	887.5197	**444.2635**	870.4931	435.7502	869.5091	435.2582	**8**
**17**	**1867.6923**	934.3498	**1850.6657**	925.8365	1849.6817	925.3445	**E**	800.4876	400.7475	783.4611	392.2342	782.4771	391.7422	**7**
**18**	1995.7872	998.3973	**1978.7607**	989.8840	1977.7767	989.3920	**K**	**671.4450**	**336.2262**	**654.4185**	327.7129	653.4345	327.2209	**6**
**19**	2094.8557	**1047.9315**	2077.8291	**1039.4182**	2076.8451	**1038.9262**	**V**	**543.3501**	272.1787	**526.3235**	263.6654	525.3395	263.1734	**5**
**20**	2191.9084	1096.4579	2174.8819	**1087.9446**	2173.8979	1087.4526	**P**	**444.2817**	222.6445	**427.2551**	214.1312	**426.2711**	213.6392	**4**
**21**	2278.9405	1139.9739	2261.9139	1131.4606	2260.9299	1130.9686	**S**	**347.2289**	174.1181	330.2023	165.6048	**329.2183**	165.1128	**3**
**22**	2392.0245	**1196.5159**	2374.9980	1188.0026	2374.0140	1187.5106	**I**	260.1969	130.6021	243.1703	122.0888			**2**
**23**							**K**	147.1128	74.0600	130.0863	65.5468			**1**

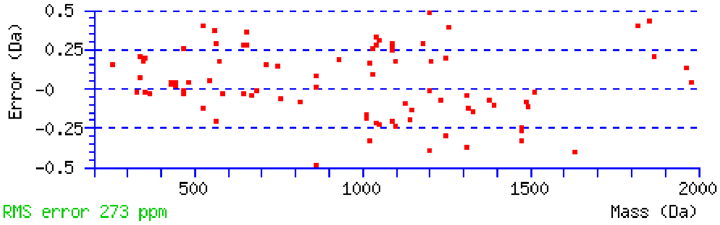

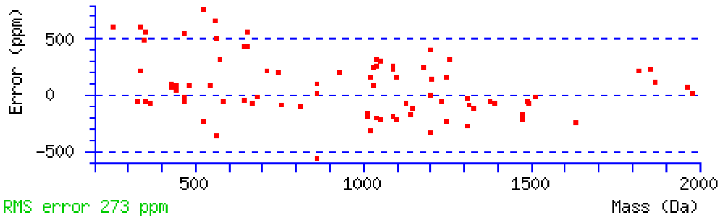

All matches to this query

Score	Mr(calc):	Delta	Sequence
47.5	2635.0997	-0.0010	KGSLDSDNDDSDCPYSEKVPSIK
31.2	2635.0997	-0.0010	KGSLDSDNDDSDCPYSEKVPSIK
24.8	2635.0997	-0.0010	KGSLDSDNDDSDCPYSEKVPSIK
12.6	2635.0997	-0.0010	KGSLDSDNDDSDCPYSEKVPSIK
2.5	2634.1181	0.9805	IYMTIDKTTIQELNRCMAEK
2.1	2633.0675	2.0311	MEEPPAVSLLQDWSKHPQGTK
1.8	2633.1022	1.9964	SNLDRCGFRGSSYLGIPFNPSK
1.6	2635.1077	-0.0091	DASIRGCSLSEDGGMLAQCQGLSK
1.2	2634.1090	0.9896	FEIVDNEGVGRITVSLMGSLNK
1.0	2633.0911	2.0075	LQLGSSLDSSNASVSSNLSLASQK

Spectrum No: 352; Query: 2504; Rank: 1

Peptide View

MS/MS Fragmentation of **ESDDKPEIEDVGSDEEEEEKKDGDK**
Found in **IPI00210566**, Tax_Id=10116 Gene_Symbol=Hspca Heat shock protein HSP 90-alpha

Match to Query 2504: 2930.170302 from(977.730710,3+)
Title: 091008RatKidney_NoSalt_25.1064.1064.3.dta
Data file K:\NewmanPaper\Piliang\3SubProteomes\Piliang3SP\mgf5ppm\ERLIC_3SubProteomes5ppm.mgf

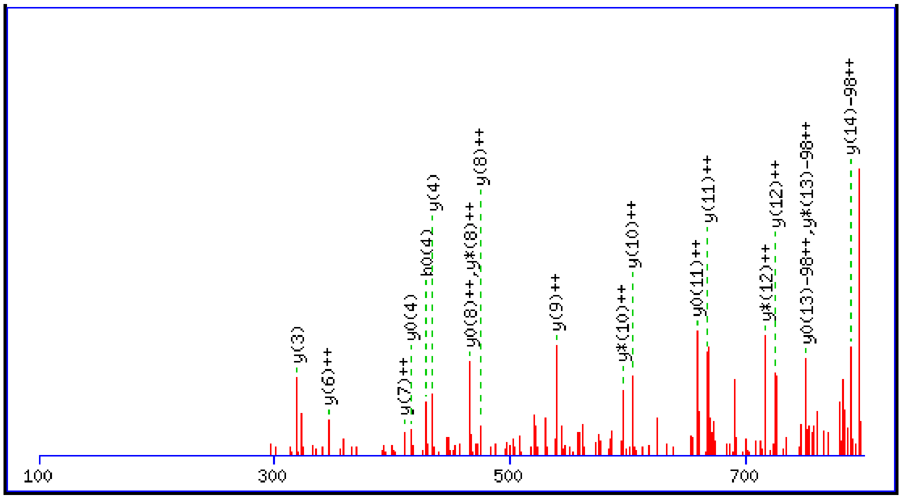

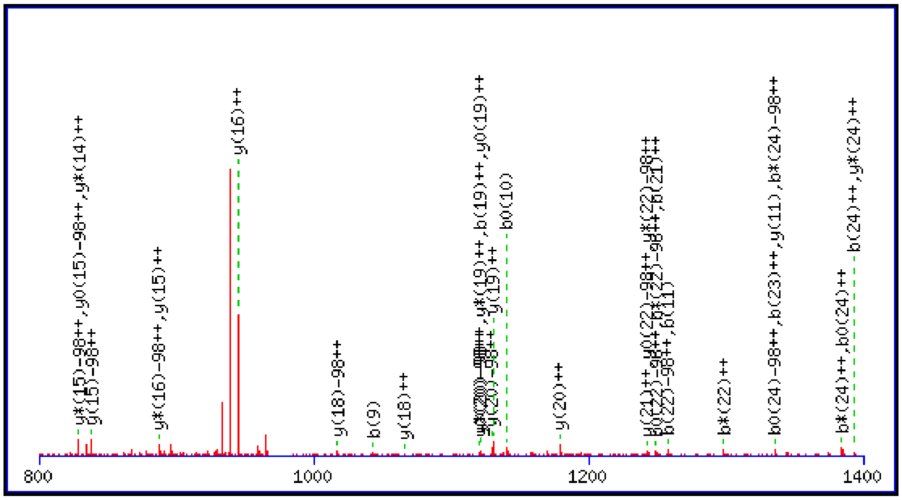

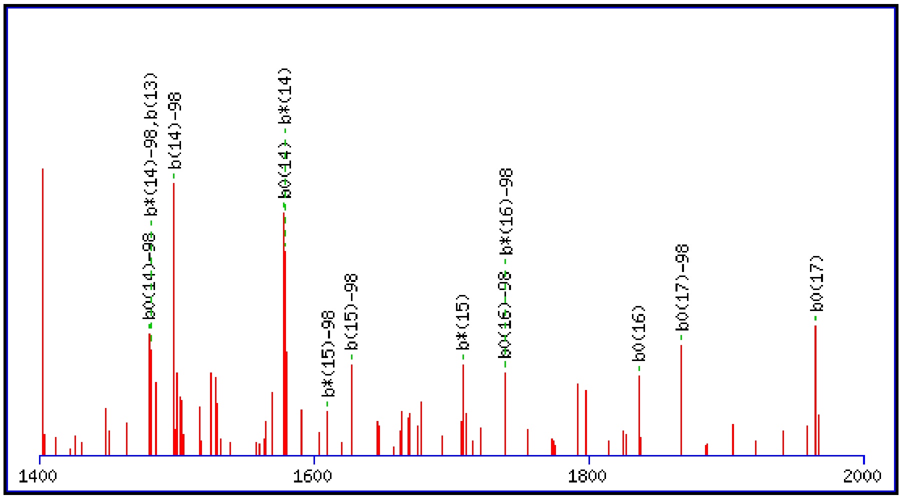

Monoisotopic mass of neutral peptide Mr(calc): 2930.1713
Fixed modifications: Carbamidomethyl (C)
Variable modifications:
S13 : Phospho (ST), with neutral losses 0.0000(shown in table), 97.9769
Ions Score: 48 **Expect:** 0.0055
Matches (Bold Red): 69/422 fragment ions using 78 most intense peaks

#	b	b^{++}	b*	b^{*++}	b^0	b^{0++}	Seq.	y	y^{++}	y*	y^{*++}	y^0	y^{0++}	#
1	130.0499	65.5286			112.0393	56.5233	E							25
2	217.0819	109.0446			199.0713	100.0393	S	2802.1361	1401.5717	2785.1095	**1393.0584**	2784.1255	1392.5664	24
3	332.1088	166.5581			314.0983	157.5528	D	2715.1040	1358.0556	2698.0775	1349.5424	2697.0935	1349.0504	23
4	447.1358	224.0715			**429.1252**	215.0662	D	2600.0771	1300.5422	2583.0505	1292.0289	2582.0665	1291.5369	22
5	575.2307	288.1190	558.2042	279.6057	557.2202	279.1137	K	2485.0501	**1243.0287**	2468.0236	1234.5154	2467.0396	1234.0234	21

6	672.2835	336.6454	655.2570	328.1321	654.2729	327.6401	P	2356.9552	**1178.9812**	2339.9286	1170.4680	2338.9446	1169.9759	20
7	801.3261	401.1667	784.2996	392.6534	783.3155	392.1614	E	2259.9024	**1130.4548**	2242.8759	**1121.9416**	2241.8918	**1121.4496**	19
8	914.4102	457.7087	897.3836	449.1954	896.3996	448.7034	I	2130.8598	**1065.9335**	2113.8333	1057.4203	2112.8493	1056.9283	18
9	**1043.4528**	522.2300	1026.4262	513.7167	1025.4422	513.2247	E	2017.7758	1009.3915	2000.7492	1000.8782	1999.7652	1000.3862	17
10	1158.4797	579.7435	1141.4532	571.2302	**1140.4691**	570.7382	D	1888.7332	**944.8702**	1871.7066	936.3569	1870.7226	935.8649	16
11	**1257.5481**	629.2777	1240.5216	620.7644	1239.5376	620.2724	V	1773.7062	**887.3567**	1756.6797	878.8435	1755.6957	878.3515	15
12	1314.5696	657.7884	1297.5430	649.2752	1296.5590	648.7831	G	1674.6378	837.8225	1657.6113	**829.3093**	1656.6272	828.8173	14
13	**1481.5679**	741.2876	1464.5414	732.7743	1463.5574	732.2823	S	1617.6163	809.3118	1600.5898	800.7985	1599.6058	800.3065	13
14	1596.5949	798.8011	**1579.5683**	790.2878	**1578.5843**	789.7958	D	1450.6180	**725.8126**	1433.5914	**717.2994**	1432.6074	716.8073	12
15	1725.6375	863.3224	**1708.6109**	854.8091	1707.6269	854.3171	E	**1335.5910**	**668.2992**	1318.5645	659.7859	1317.5805	**659.2939**	11
16	1854.6801	927.8437	1837.6535	919.3304	**1836.6695**	918.8384	E	1206.5485	**603.7779**	1189.5219	**595.2646**	1188.5379	594.7726	10
17	1983.7227	992.3650	1966.6961	983.8517	**1965.7121**	983.3597	E	1077.5059	**539.2566**	1060.4793	530.7433	1059.4953	530.2513	9
18	2112.7652	1056.8863	2095.7387	1048.3730	2094.7547	1047.8810	E	948.4633	**474.7353**	931.4367	**466.2220**	930.4527	**465.7300**	8
19	2241.8078	**1121.4076**	2224.7813	1112.8943	2223.7973	1112.4023	E	819.4207	**410.2140**	802.3941	401.7007	801.4101	401.2087	7
20	2369.9028	1185.4550	2352.8763	1176.9418	2351.8922	1176.4498	K	690.3781	**345.6927**	673.3515	337.1794	672.3675	336.6874	6
21	2497.9978	**1249.5025**	2480.9712	1240.9892	2479.9872	1240.4972	K	562.2831	281.6452	545.2566	273.1319	544.2726	272.6399	5
22	2613.0247	1307.0160	2595.9982	**1298.5027**	2595.0141	1298.0107	D	**434.1882**	217.5977	417.1616	209.0844	**416.1776**	208.5924	4
23	2670.0462	**1335.5267**	2653.0196	1327.0135	2652.0356	1326.5214	G	**319.1612**	160.0842	302.1347	151.5710	301.1506	151.0790	3
24	2785.0731	**1393.0402**	2768.0466	**1384.5269**	2767.0626	**1384.0349**	D	262.1397	131.5735	245.1132	123.0602	244.1292	122.5682	2
25							K	147.1128	74.0600	130.0863	65.5468			1

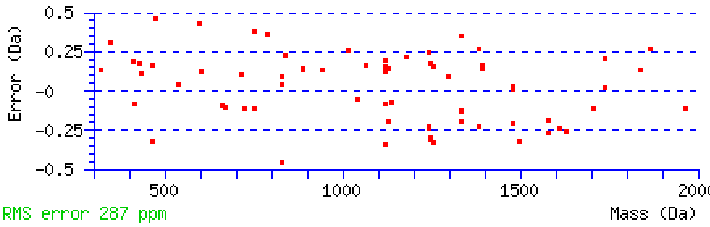

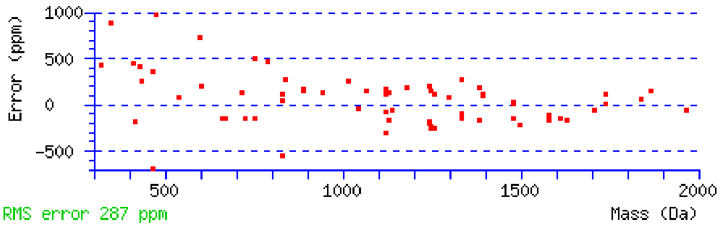

All matches to this query

Score	Mr(calc):	Delta	Sequence
47.5	2930.1713	-0.0010	ESDDKPEIEDVGSDEEEEEKKDGDK
16.4	2930.1713	-0.0010	ESDDKPEIEDVGSDEEEEEKKDGDK
2.9	2929.1642	1.0061	NIYYKSDLTKDITTSVLTVNNK
2.8	2930.1482	0.0221	NIYYKSDLTKDITTSVLTVNNK
2.6	2929.1531	1.0172	GMYALSHTRDSSTDITLTQFLSTE
2.2	2928.1513	2.0190	KDATNVGDMMSVSFTLTVASWEQR
2.2	2929.1779	0.9924	AWDELQDVTCRNANAGTKETFWR
2.1	2928.1691	2.0012	KVGPSEEETLDYSDCLTHRPVEK
2.0	2928.1763	1.9940	SHPTQSAQTLQSTGSSMENQTILSR
2.0	2928.1683	2.0020	IMHASPEDAGNYFCIAQNSVGSAMAK

Spectrum No: 353; Query: 1057; Rank: 1

Peptide View

MS/MS Fragmentation of **RESGEGEEEVADSAR**
Found in **IPI00359172**, Tax_Id=10116 Gene_Symbol=Lrrc47_predicted similar to leucine rich repeat containing 47

Match to Query 1057: 1699.664952 from(567.562260,3+)
Title: 091008RatKidney_NH4Format01_23.527.527.3.dta

Data file K:\NewmanPaper\Piliang\3SubProteomes\Piliang3SP\mgf5ppm\ERLIC_3SubProteomes5ppm.mgf

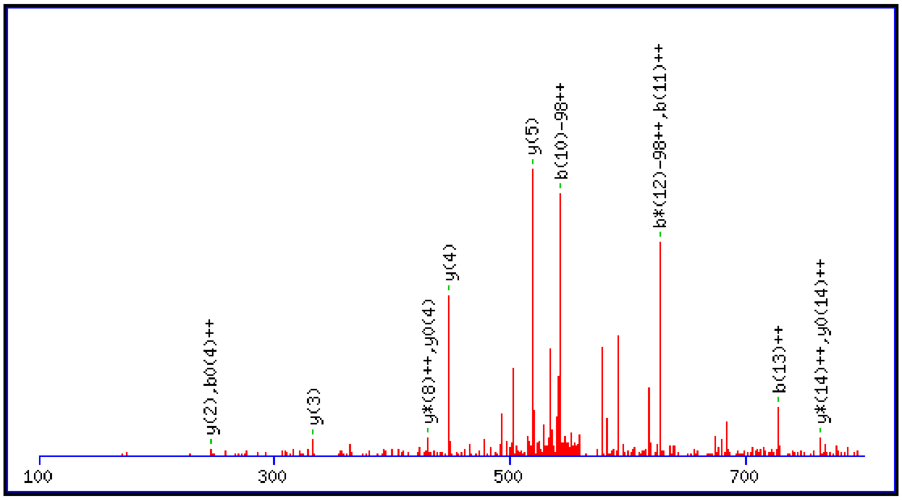

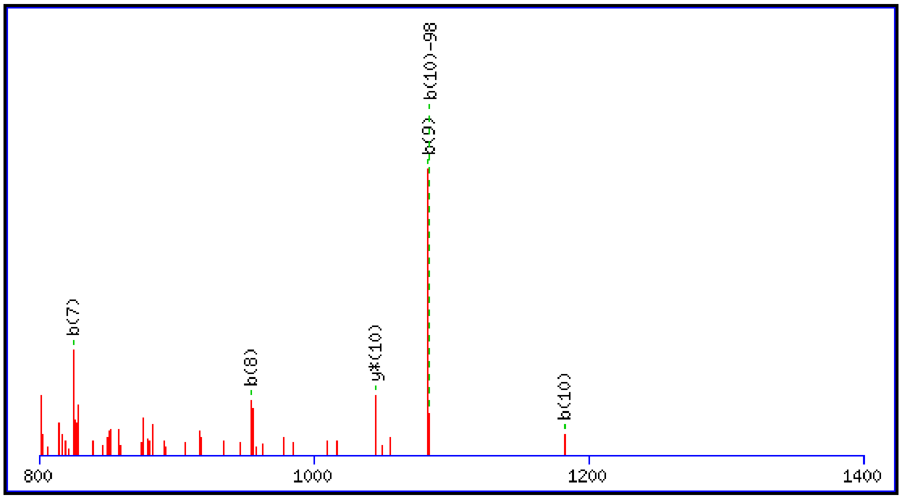

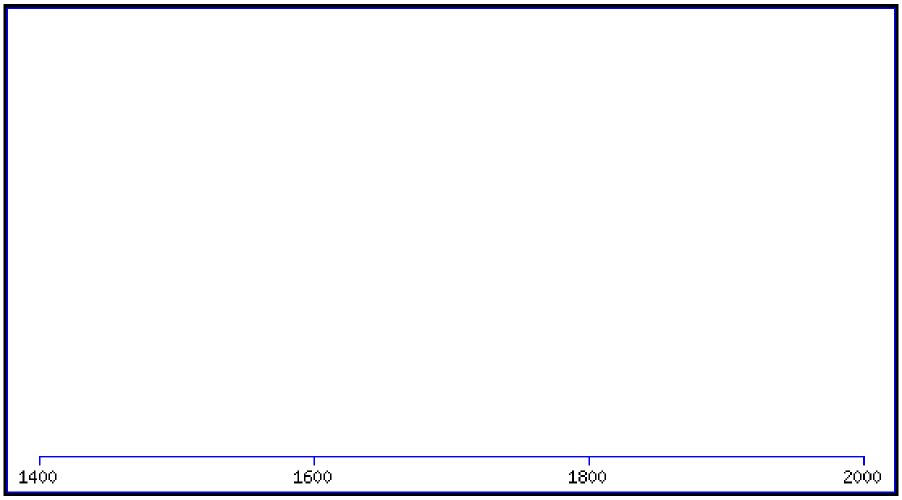

Monoisotopic mass of neutral peptide Mr(calc): 1699.6686
Fixed modifications: Carbamidomethyl (C)
Variable modifications:
S3 : Phospho (ST), with neutral losses 0.0000(shown in table), 97.9769
Ions Score: 47 **Expect:** 0.0024
Matches (Bold Red): 20/246 fragment ions using 15 most intense peaks

#	b	b++	b*	b*++	b0	b0++	Seq.	y	y++	y*	y*++	y0	y0++	#
1	157.1084	79.0578	140.0818	70.5446			R							15
2	286.1510	143.5791	269.1244	135.0659	268.1404	134.5738	E	1544.5748	772.7910	1527.5483	**764.2778**	1526.5642	**763.7858**	14
3	453.1493	227.0783	436.1228	218.5650	435.1388	218.0730	S	1415.5322	708.2697	1398.5057	699.7565	1397.5217	699.2645	13
4	510.1708	255.5890	493.1442	247.0758	492.1602	**246.5838**	G	1248.5339	624.7706	1231.5073	616.2573	1230.5233	615.7653	12

**5**	639.2134	320.1103	622.1868	311.5971	621.2028	311.1051	**E**	1191.5124	596.2598	1174.4859	587.7466	1173.5018	587.2546	**11**
**6**	696.2349	348.6211	679.2083	340.1078	678.2243	339.6158	**G**	1062.4698	531.7385	**1045.4433**	523.2253	1044.4592	522.7333	**10**
**7**	**825.2774**	413.1424	808.2509	404.6291	807.2669	404.1371	**E**	1005.4483	503.2278	988.4218	494.7145	987.4378	494.2225	**9**
**8**	**954.3200**	477.6637	937.2935	469.1504	936.3095	468.6584	**E**	876.4058	438.7065	859.3792	**430.1932**	858.3952	429.7012	**8**
**9**	**1083.3626**	542.1850	1066.3361	533.6717	1065.3521	533.1797	**E**	747.3632	374.1852	730.3366	365.6719	729.3526	365.1799	**7**
**10**	**1182.4310**	591.7192	1165.4045	583.2059	1164.4205	582.7139	**V**	618.3206	309.6639	601.2940	301.1506	600.3100	300.6586	**6**
**11**	1253.4682	**627.2377**	1236.4416	618.7244	1235.4576	618.2324	**A**	**519.2522**	260.1297	502.2256	251.6164	501.2416	251.1244	**5**
**12**	1368.4951	684.7512	1351.4686	676.2379	1350.4845	675.7459	**D**	**448.2150**	224.6112	431.1885	216.0979	**430.2045**	215.6059	**4**
**13**	1455.5271	**728.2672**	1438.5006	719.7539	1437.5166	719.2619	**S**	**333.1881**	167.0977	316.1615	158.5844	315.1775	158.0924	**3**
**14**	1526.5642	**763.7858**	1509.5377	755.2725	1508.5537	754.7805	**A**	**246.1561**	123.5817	229.1295	115.0684			**2**
**15**							**R**	175.1190	88.0631	158.0924	79.5498			**1**

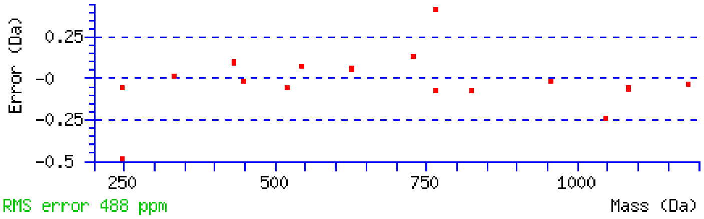

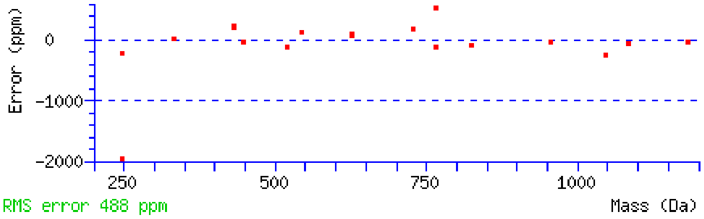

All matches to this query

Score	Mr(calc):	Delta	Sequence
47.3	1699.6686	-0.0037	RESGEGEEEVADSAR
15.7	1698.6611	1.0038	ASTSTSVSSGRNTGAR
13.9	1698.6457	1.0192	VHYMTHGTNNNSAR
13.9	1698.6457	1.0192	VHYMTHGTNNNSAR
13.5	1699.6678	-0.0028	AMTTLNIQYGDSAR
13.5	1699.6678	-0.0028	AMTTLNIQYGDSAR
11.8	1698.6611	1.0038	ASTSTSVSSGRNTGAR
11.7	1697.6617	2.0032	VHYMTHGTNNNSAR
11.6	1698.6457	1.0192	VHYMTHGTNNNSAR
11.6	1698.6457	1.0192	VHYMTHGTNNNSAR

Spectrum No: 354; Query: 295; Rank: 1

Peptide View

MS/MS Fragmentation of **TYSDEASQLR**
Found in **IPI00365119**, Tax_Id=10116 Gene_Symbol=LOC312502 70 kDa protein

Match to Query 295: 1248.500928 from(625.257740,2+)
Title: 091008RatKidney_NH4Format02_26.1300.1300.2.dta
Data file K:\NewmanPaper\Piliang\3SubProteomes\Piliang3SP\mgf5ppm\ERLIC_3SubProteomes5ppm.mgf

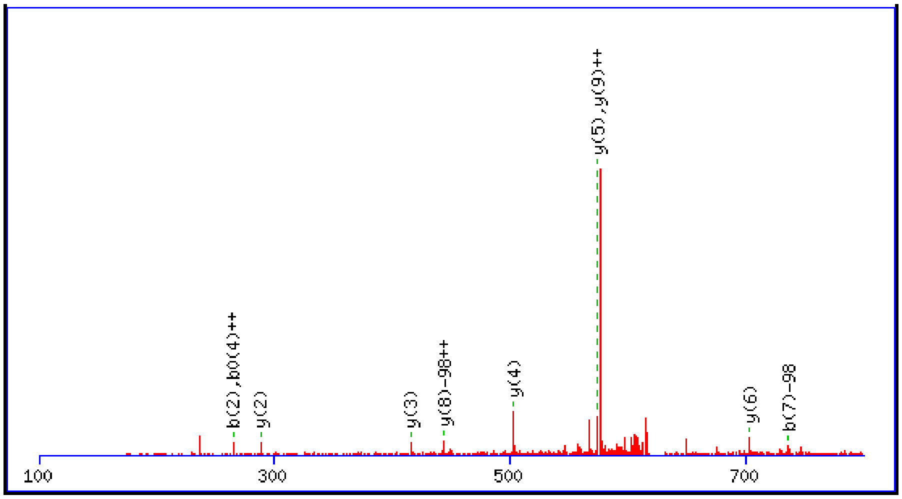

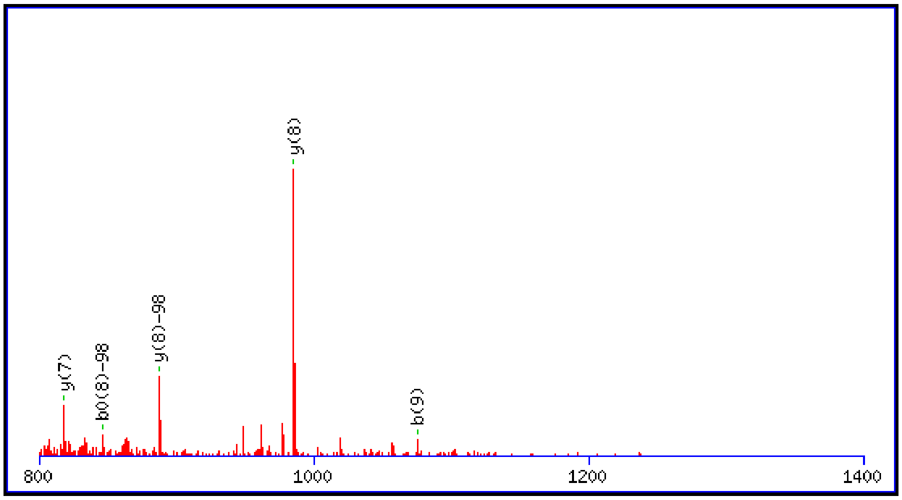

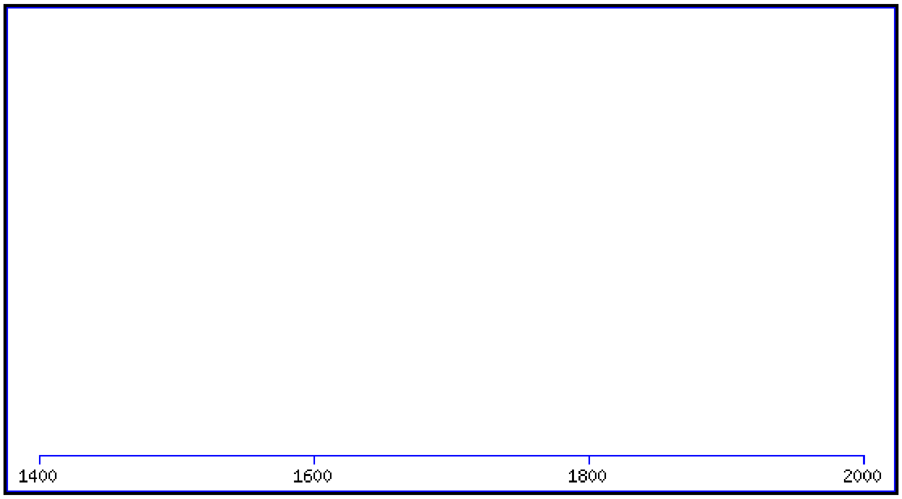

Monoisotopic mass of neutral peptide Mr(calc): 1248.5023
Fixed modifications: Carbamidomethyl (C)
Variable modifications:
S3 : Phospho (ST), with neutral losses 97.9769(shown in table), 0.0000
Ions Score: 47 **Expect:** 0.0023
Matches (Bold Red): 15/132 fragment ions using 28 most intense peaks

#	b	b^{++}	b*	b^{*++}	b^0	b^{0++}	Seq.	y	y^{++}	y*	y^{*++}	y^0	y^{0++}	#
1	102.0550	51.5311			84.0444	42.5258	T							10
2	**265.1183**	133.0628			247.1077	124.0575	Y	1050.4851	525.7462	1033.4585	517.2329	1032.4745	516.7409	9
3	334.1397	167.5735			316.1292	158.5682	S	**887.4217**	**444.2145**	870.3952	435.7012	869.4112	435.2092	8
4	449.1667	225.0870			431.1561	216.0817	D	**818.4003**	409.7038	801.3737	401.1905	800.3897	400.6985	7
5	578.2093	289.6083			560.1987	280.6030	E	**703.3733**	352.1903	686.3468	343.6770	685.3628	343.1850	6

**6**	649.2464	325.1268			631.2358	316.1216	**A**	**574.3307**	287.6690	557.3042	279.1557	556.3202	278.6637	**5**
**7**	**736.2784**	368.6428			718.2679	359.6376	**S**	**503.2936**	252.1504	486.2671	243.6372	485.2831	243.1452	**4**
**8**	864.3370	432.6721	847.3104	424.1589	**846.3264**	423.6669	**Q**	**416.2616**	208.6344	399.2350	200.1212			**3**
**9**	977.4211	489.2142	960.3945	480.7009	959.4105	480.2089	**L**	**288.2030**	144.6051	271.1765	136.0919			**2**
**10**							**R**	175.1190	88.0631	158.0924	79.5498			**1**

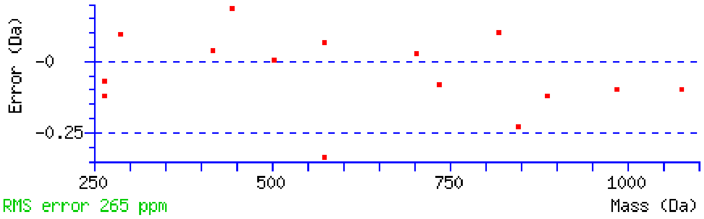

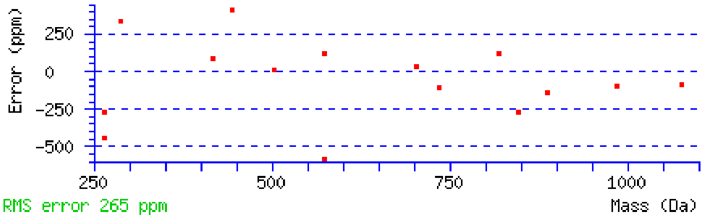

All matches to this query

Score	Mr(calc):	Delta	Sequence
47.3	1248.5023	-0.0014	TYSDEASQLR
31.6	1248.5023	-0.0014	TYSDEASQLR
27.4	1248.5023	-0.0014	TYSDEASQLR
10.0	1248.5023	-0.0014	TYSDEASQLR
1.0	1247.4883	1.0126	STARPCAGLR
0.4	1248.5098	-0.0088	DTPCSTFTIK
0.4	1248.5098	-0.0088	DTPCSTFTIK
0.4	1248.5098	-0.0088	DTPCSTFTIK
0.4	1248.5131	-0.0122	MTQIKMEEK
0.3	1248.4911	0.0098	EELFGESSSGK

Spectrum No: 355; Query: 1812; Rank: 1

Peptide View

MS/MS Fragmentation of **AQTLPTSVVTITSESSPGKR**
Found in **IPI00555287**, Tax_Id=10116 Gene_Symbol=Spnb2 Non-erythroid spectrin beta

Match to Query 1812: 2138.060708 from(1070.037630,2+)
Title: 091008RatKidney_NH4Format01_16.2364.2364.2.dta
Data file K:\NewmanPaper\Piliang\3SubProteomes\Piliang3SP\mgf5ppm\ERLIC_3SubProteomes5ppm.mgf

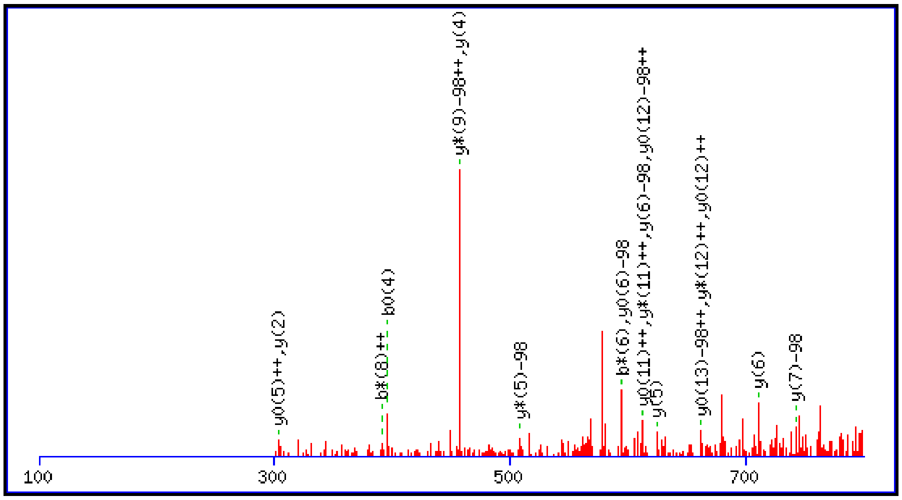

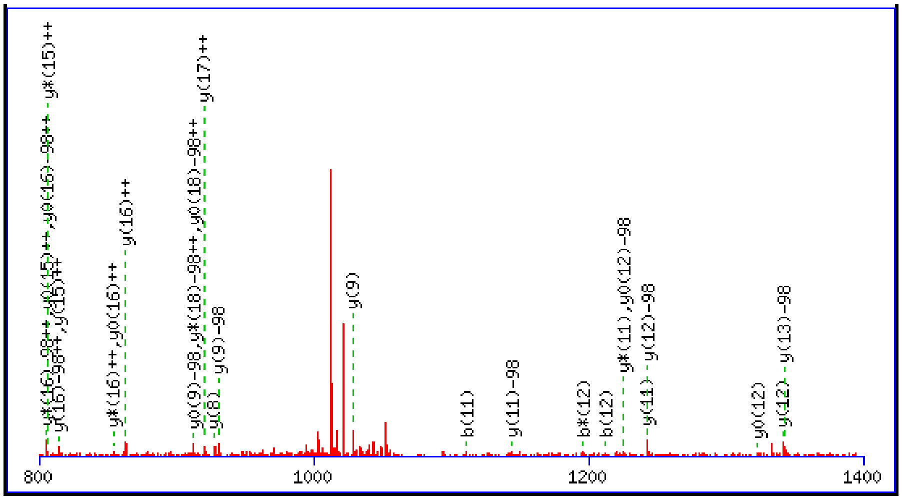

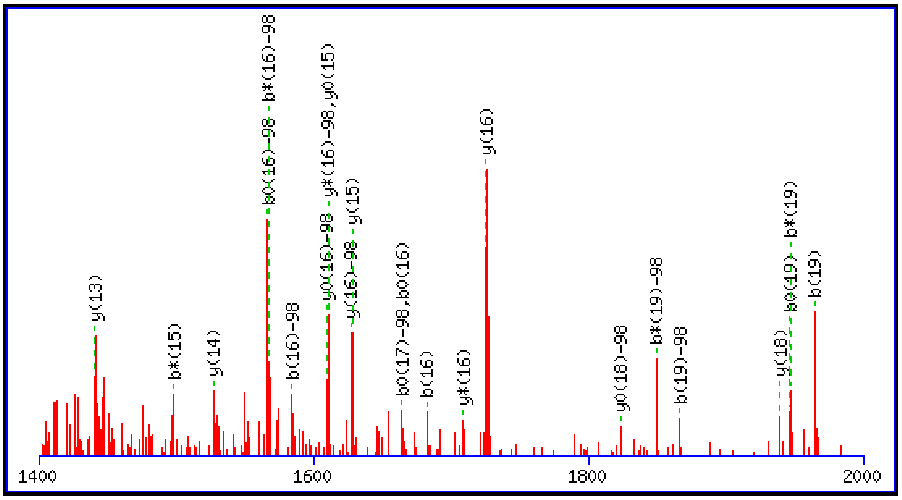

Monoisotopic mass of neutral peptide Mr(calc): 2138.0620
Fixed modifications: Carbamidomethyl (C)
Variable modifications:
S16 : Phospho (ST), with neutral losses 0.0000(shown in table), 97.9769
Ions Score: 47 **Expect:** 0.0041
Matches (Bold Red): 69/328 fragment ions using 111 most intense peaks

#	b	b^{++}	b*	b^{*++}	b^0	b^{0++}	Seq.	y	y^{++}	y*	y^{*++}	y^0	y^{0++}	#
1	72.0444	36.5258					A							20
2	200.1030	100.5551	183.0764	92.0418			Q	2068.0322	1034.5197	2051.0056	1026.0065	2050.0216	1025.5144	19
3	301.1506	151.0790	284.1241	142.5657	283.1401	142.0737	T	**1939.9736**	970.4904	1922.9471	961.9772	1921.9630	961.4852	18
4	414.2347	207.6210	397.2082	199.1077	**396.2241**	198.6157	L	1838.9259	**919.9666**	1821.8994	911.4533	1820.9154	910.9613	17
5	511.2875	256.1474	494.2609	247.6341	493.2769	247.1421	P	**1725.8419**	**863.4246**	**1708.8153**	**854.9113**	1707.8313	**854.4193**	16
6	612.3352	306.6712	**595.3086**	298.1579	594.3246	297.6659	T	**1628.7891**	**814.8982**	1611.7626	**806.3849**	**1610.7785**	**805.8929**	15
7	699.3672	350.1872	682.3406	341.6740	681.3566	341.1819	S	**1527.7414**	764.3743	1510.7149	755.8611	1509.7309	755.3691	14
8	798.4356	399.7214	781.4090	**391.2082**	780.4250	390.7162	V	**1440.7094**	720.8583	1423.6828	712.3451	1422.6988	711.8531	13
9	897.5040	449.2556	880.4775	440.7424	879.4934	440.2504	V	**1341.6410**	671.3241	1324.6144	**662.8109**	**1323.6304**	**662.3188**	12
10	998.5517	499.7795	981.5251	491.2662	980.5411	490.7742	T	**1242.5726**	621.7899	**1225.5460**	**613.2766**	1224.5620	**612.7846**	11
11	**1111.6358**	556.3215	1094.6092	547.8082	1093.6252	547.3162	I	1141.5249	571.2661	1124.4983	562.7528	1123.5143	562.2608	10
12	**1212.6834**	606.8454	**1195.6569**	598.3321	1194.6729	597.8401	T	**1028.4408**	514.7240	1011.4143	506.2108	1010.4303	505.7188	9
13	1299.7155	650.3614	1282.6889	641.8481	1281.7049	641.3561	S	**927.3931**	464.2002	910.3666	455.6869	909.3826	455.1949	8
14	1428.7581	714.8827	1411.7315	706.3694	1410.7475	705.8774	E	840.3611	420.6842	823.3346	412.1709	822.3506	411.6789	7
15	1515.7901	758.3987	**1498.7635**	749.8854	1497.7795	749.3934	S	**711.3185**	356.1629	694.2920	347.6496	693.3080	347.1576	6
16	**1682.7884**	841.8979	1665.7619	833.3846	**1664.7779**	832.8926	S	**624.2865**	312.6469	607.2599	304.1336	606.2759	**303.6416**	5
17	1779.8412	890.4242	1762.8146	881.9110	1761.8306	881.4190	P	**457.2881**	229.1477	440.2616	220.6344			4
18	1836.8627	918.9350	1819.8361	910.4217	1818.8521	909.9297	G	360.2354	180.6213	343.2088	172.1081			3

19	1964.9576	982.9825	1947.9311	974.4692	1946.9471	973.9772	K	303.2139	152.1106	286.1874	143.5973			2
20							R	175.1190	88.0631	158.0924	79.5498			1

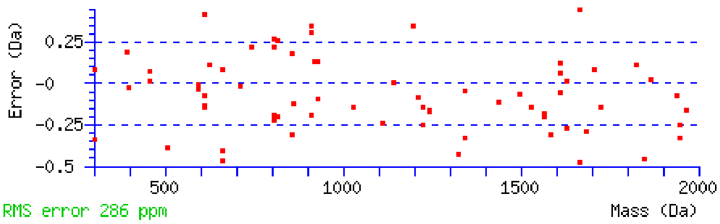

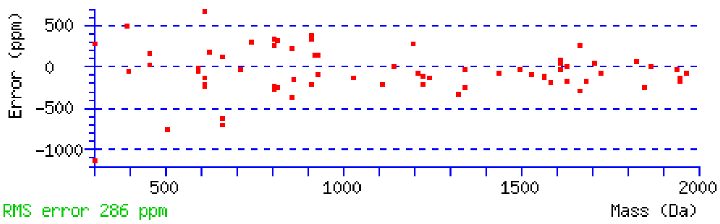

All matches to this query

Score	Mr(calc):	Delta	Sequence
47.2	2138.0620	-0.0013	AQTLPTSVVTITSESSPGKR
38.8	2138.0620	-0.0013	AQTLPTSVVTITSESSPGKR
37.7	2138.0620	-0.0013	AQTLPTSVVTITSESSPGKR
28.9	2138.0620	-0.0013	AQTLPTSVVTITSESSPGKR
18.9	2138.0620	-0.0013	AQTLPTSVVTITSESSPGKR
12.0	2138.0620	-0.0013	AQTLPTSVVTITSESSPGKR
11.6	2138.0620	-0.0013	AQTLPTSVVTITSESSPGKR
9.5	2138.0667	-0.0060	LESISSTVVGRPARMAATGR
9.5	2138.0667	-0.0060	LESISSTVVGRPARMAATGR
8.3	2138.0667	-0.0060	LESISSTVVGRPARMAATGR

Spectrum No: 356; Query: 1517; Rank: 1

Peptide View

MS/MS Fragmentation of **GLLYDSDEEDEERPAR**
Found in **IPI00870825**, Tax_Id=10116 Gene_Symbol=Mcm2_predicted minichromosome maintenance deficient 2 mitotin

Match to Query 1517: 1972.812028 from(987.413290,2+)
Title: 100101RatKid_NS_deglyco_20.1981.1981.2.dta
Data file K:\NewmanPaper\Piliang\3SubProteomes\Piliang3SP\mgf5ppm\ERLIC_3SubProteomes5ppm.mgf

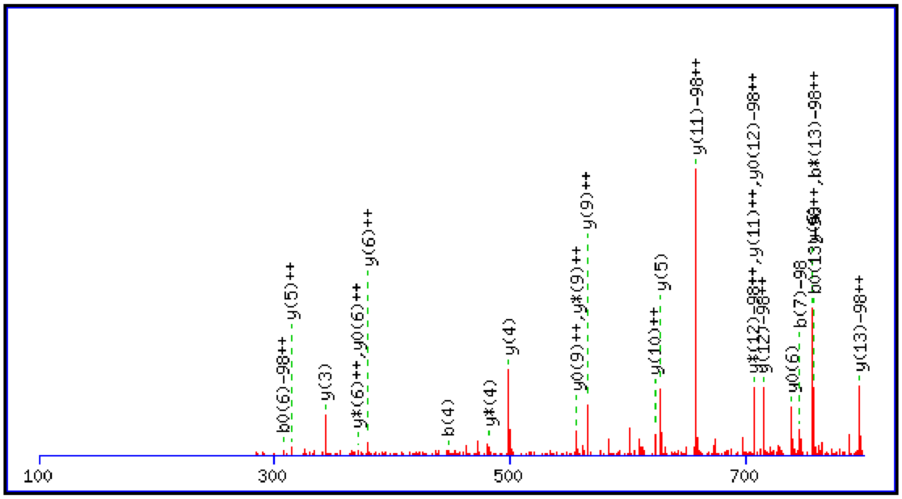

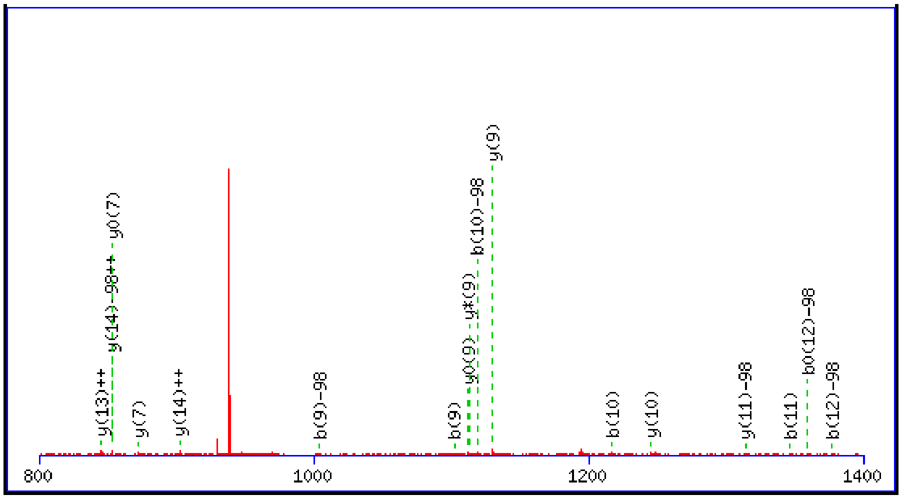

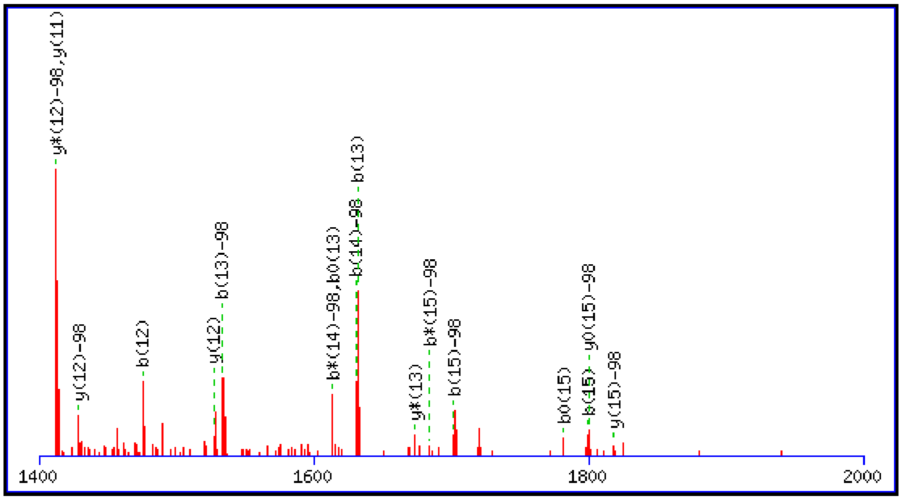

Monoisotopic mass of neutral peptide Mr(calc): 1972.8051
Fixed modifications: Carbamidomethyl (C)
Variable modifications:
S6 : Phospho (ST), with neutral losses 97.9769(shown in table), 0.0000
Ions Score: 47 **Expect:** 0.004
Matches (Bold Red): 59/216 fragment ions using 102 most intense peaks

#	b	b^{++}	b*	b^{*++}	b^0	b^{0++}	Seq.	y	y^{++}	y*	y^{*++}	y^0	y^{0++}	#
1	58.0287	29.5180					G							16
2	171.1128	86.0600					L	**1818.8141**	909.9107	1801.7875	901.3974	**1800.8035**	900.9054	15
3	284.1969	142.6021					L	1705.7300	**853.3686**	1688.7034	844.8554	1687.7194	844.3634	14
4	**447.2602**	224.1337					Y	1592.6459	**796.8266**	1575.6194	788.3133	1574.6354	787.8213	13
5	562.2871	281.6472			544.2766	272.6419	D	**1429.5826**	**715.2949**	**1412.5560**	**706.7817**	1411.5720	**706.2897**	12
6	631.3086	316.1579			613.2980	**307.1527**	S	**1314.5557**	**657.7815**	1297.5291	649.2682	1296.5451	648.7762	11
7	**746.3355**	373.6714			728.3250	364.6661	D	**1245.5342**	**623.2707**	1228.5077	614.7575	1227.5236	614.2655	10
8	875.3781	438.1927			857.3676	429.1874	E	**1130.5073**	**565.7573**	**1113.4807**	**557.2440**	**1112.4967**	**556.7520**	9
9	**1004.4207**	502.7140			986.4102	493.7087	E	1001.4647	501.2360	984.4381	492.7227	983.4541	492.2307	8
10	**1119.4477**	560.2275			1101.4371	551.2222	D	**872.4221**	436.7147	855.3955	428.2014	**854.4115**	427.7094	7
11	1248.4903	624.7488			1230.4797	615.7435	E	**757.3951**	**379.2012**	740.3686	**370.6879**	**739.3846**	**370.1959**	6
12	**1377.5329**	689.2701			**1359.5223**	680.2648	E	**628.3525**	**314.6799**	611.3260	306.1666	610.3420	305.6746	5
13	**1533.6340**	767.3206	1516.6074	**758.8073**	1515.6234	**758.3153**	R	**499.3099**	250.1586	**482.2834**	241.6453			4
14	**1630.6867**	815.8470	**1613.6602**	807.3337	1612.6762	806.8417	P	**343.2088**	172.1081	326.1823	163.5948			3
15	**1701.7238**	851.3656	**1684.6973**	842.8523	1683.7133	842.3603	A	246.1561	123.5817	229.1295	115.0684			2
16							R	175.1190	88.0631	158.0924	79.5498			1

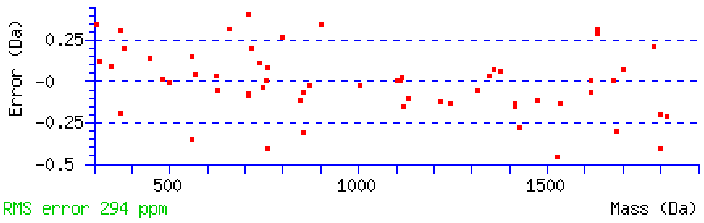

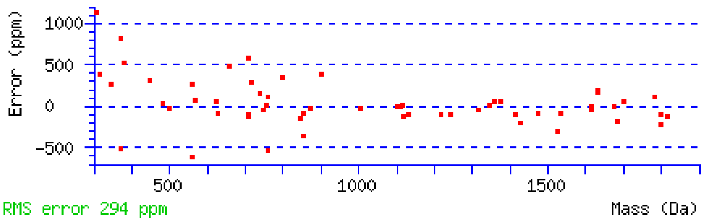

All matches to this query

Score	Mr(calc):	Delta	Sequence
47.2	1972.8051	0.0069	GLLYDSDEEDEERPAR
11.4	1972.8051	0.0069	GLLYDSDEEDEERPAR
6.8	1972.8155	-0.0035	TKSNFKYVADCPINR
6.8	1972.8155	-0.0035	TKSNFKYVADCPINR
6.2	1972.8197	-0.0077	DRQLENTEQMASGLER
4.3	1970.8152	1.9968	IATEGINGTVGGSKVATR
4.2	1972.8237	-0.0117	EDPVAADSVYAASQMPSR
2.9	1972.8194	-0.0073	FGSQGEAQAAIQGLHGSR
2.4	1972.8246	-0.0126	NLNERYKFCITMCK
2.4	1972.8246	-0.0126	NLNERYKFCITMCK

Spectrum No: 357; Query: 2580; Rank: 1

Peptide View

MS/MS Fragmentation of **KVEEEQEADEEDVSEEETENREGESK**
Found in **IPI00365626**, Tax_Id=10116 Gene_Symbol=Txndc1 Thioredoxin domain containing 1

Match to Query 2580: 3132.252282 from(1045.091370,3+)
Title: 100101RatKid_NS_deglyco_22.1107.1107.3.dta
Data file K:\NewmanPaper\Piliang\3SubProteomes\Piliang3SP\mgf5ppm\ERLIC_3SubProteomes5ppm.mgf

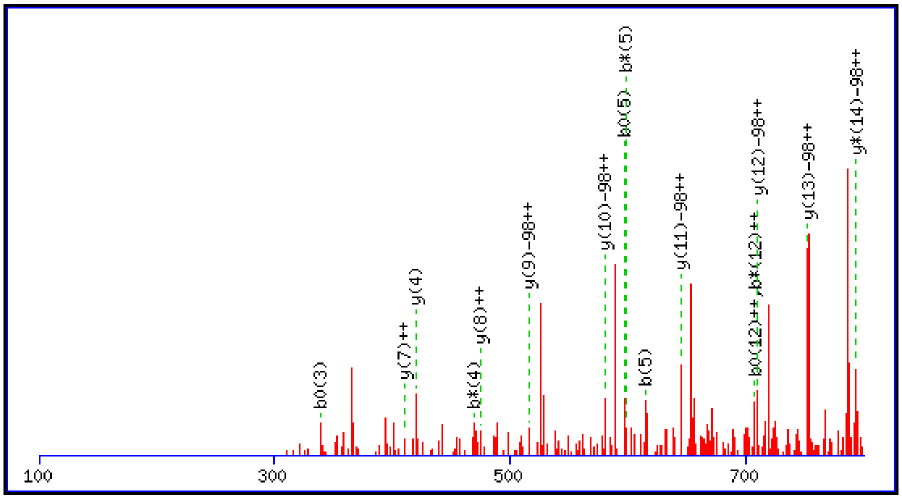

Mascot Search Results: Peptide View

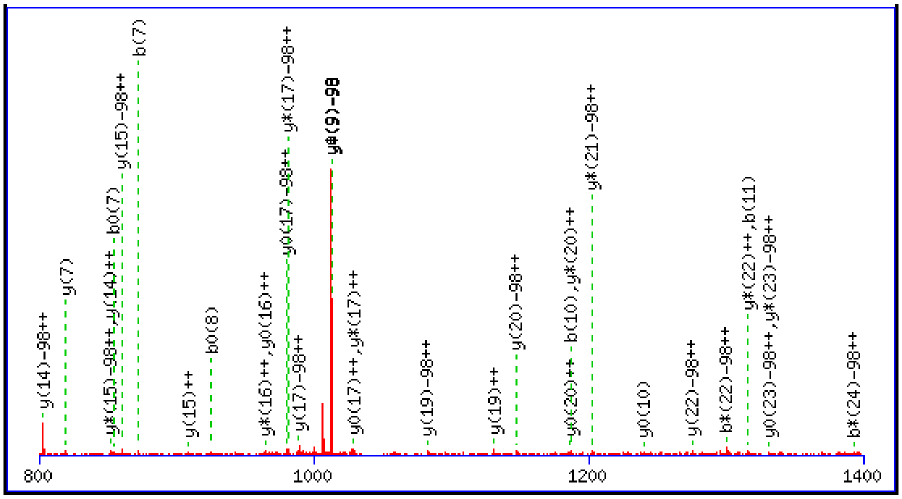

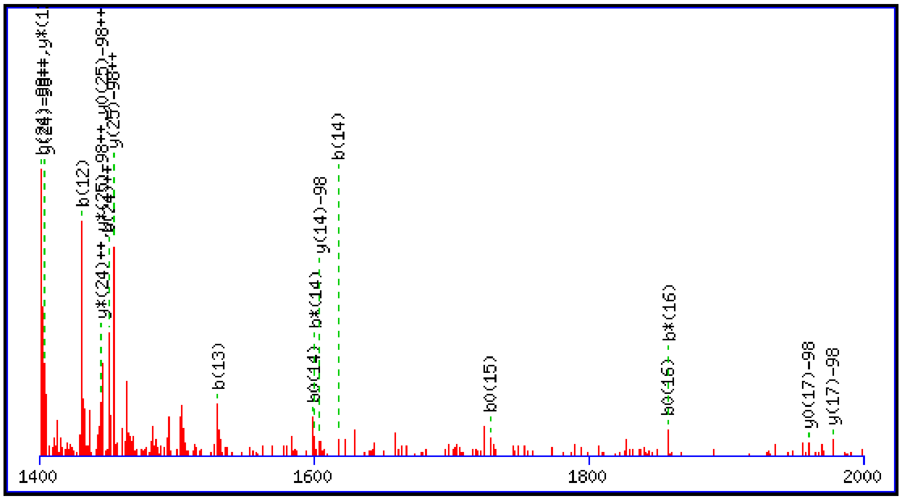

Monoisotopic mass of neutral peptide Mr(calc): 3132.2415
Fixed modifications: Carbamidomethyl (C)
Variable modifications:
T18 : Phospho (ST), with neutral losses 97.9769(shown in table), 0.0000
Ions Score: 47 **Expect:** 0.0062
Matches (Bold Red): 69/444 fragment ions using 143 most intense peaks

#	b	b++	b*	b*++	b0	b0++	Seq.	y	y++	y*	y*++	y0	y0++	#
1	129.1022	65.0548	112.0757	56.5415			K							26
2	228.1707	114.5890	211.1441	106.0757			V	2907.1770	**1454.0921**	2890.1504	**1445.5789**	2889.1664	**1445.0869**	25
3	357.2132	179.1103	340.1867	170.5970	**339.2027**	170.1050	E	2808.1086	**1404.5579**	2791.0820	1396.0447	2790.0980	1395.5526	24
4	486.2558	243.6316	**469.2293**	235.1183	468.2453	234.6263	E	2679.0660	1340.0366	2662.0394	**1331.5234**	2661.0554	**1331.0313**	23
5	**615.2984**	308.1529	**598.2719**	299.6396	**597.2879**	299.1476	E	2550.0234	**1275.5153**	2532.9968	1267.0021	2532.0128	1266.5101	22
6	743.3570	372.1821	726.3305	363.6689	725.3464	363.1769	Q	2420.9808	1210.9940	2403.9542	**1202.4808**	2402.9702	1201.9888	21
7	**872.3996**	436.7034	855.3731	428.1902	**854.3890**	427.6982	E	2292.9222	**1146.9647**	2275.8957	1138.4515	2274.9117	1137.9595	20
8	943.4367	472.2220	926.4102	463.7087	**925.4262**	463.2167	A	2163.8796	**1082.4435**	2146.8531	1073.9302	2145.8691	1073.4382	19
9	1058.4637	529.7355	1041.4371	521.2222	1040.4531	520.7302	D	2092.8425	1046.9249	2075.8160	1038.4116	2074.8319	1037.9196	18
10	**1187.5063**	594.2568	1170.4797	585.7435	1169.4957	585.2515	E	**1977.8156**	**989.4114**	1960.7890	**980.8981**	**1959.8050**	**980.4061**	17
11	**1316.5488**	658.7781	1299.5223	650.2648	1298.5383	649.7728	E	1848.7730	924.8901	1831.7464	916.3769	1830.7624	915.8848	16
12	**1431.5758**	716.2915	1414.5492	**707.7783**	1413.5652	**707.2863**	D	1719.7304	**860.3688**	1702.7038	**851.8556**	1701.7198	851.3635	15
13	**1530.6442**	765.8257	1513.6177	757.3125	1512.6336	756.8205	V	**1604.7034**	**802.8554**	1587.6769	**794.3421**	1586.6929	793.8501	14
14	**1617.6762**	809.3418	**1600.6497**	800.8285	**1599.6657**	800.3365	S	1505.6350	**753.3212**	1488.6085	744.8079	1487.6245	744.3159	13
15	1746.7188	873.8631	1729.6923	865.3498	**1728.7083**	864.8578	E	1418.6030	**709.8051**	**1401.5765**	701.2919	1400.5924	700.7999	12
16	1875.7614	938.3843	**1858.7349**	929.8711	**1857.7509**	929.3791	E	1289.5604	**645.2838**	1272.5339	636.7706	1271.5498	636.2786	11
17	2004.8040	1002.9056	1987.7775	994.3924	1986.7934	993.9004	E	1160.5178	**580.7625**	1143.4913	572.2493	1142.5072	571.7573	10
18	2087.8411	1044.4242	2070.8146	1035.9109	2069.8306	1035.4189	T	1031.4752	**516.2412**	**1014.4487**	507.7280	**1013.4647**	507.2360	9

**19**	2216.8837	1108.9455	2199.8572	1100.4322	2198.8731	1099.9402	**E**	948.4381	**474.7227**	931.4116	466.2094	930.4275	465.7174	**8**
**20**	2330.9266	1165.9670	2313.9001	1157.4537	2312.9161	1156.9617	**N**	**819.3955**	**410.2014**	802.3690	401.6881	801.3850	401.1961	**7**
**21**	2487.0277	1244.0175	2470.0012	1235.5042	2469.0172	1235.0122	**R**	705.3526	353.1799	688.3260	344.6667	687.3420	344.1747	**6**
**22**	2616.0703	1308.5388	2599.0438	**1300.0255**	2598.0598	1299.5335	**E**	549.2515	275.1294	532.2249	266.6161	531.2409	266.1241	**5**
**23**	2673.0918	1337.0495	2656.0653	1328.5363	2655.0812	1328.0443	**G**	**420.2089**	210.6081	403.1823	202.0948	402.1983	201.6028	**4**
**24**	2802.1344	**1401.5708**	2785.1078	**1393.0576**	2784.1238	1392.5656	**E**	363.1874	182.0974	346.1609	173.5841	345.1769	173.0921	**3**
**25**	2889.1664	**1445.0869**	2872.1399	1436.5736	2871.1559	1436.0816	**S**	234.1448	117.5761	217.1183	109.0628	216.1343	108.5708	**2**
**26**							**K**	147.1128	74.0600	130.0863	65.5468			**1**

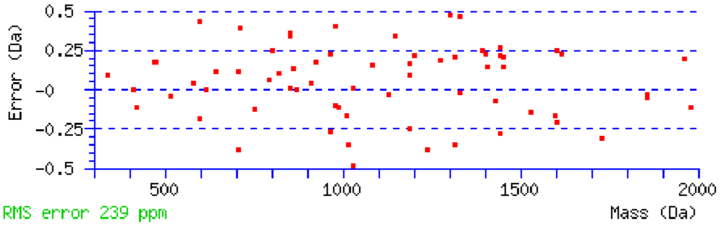

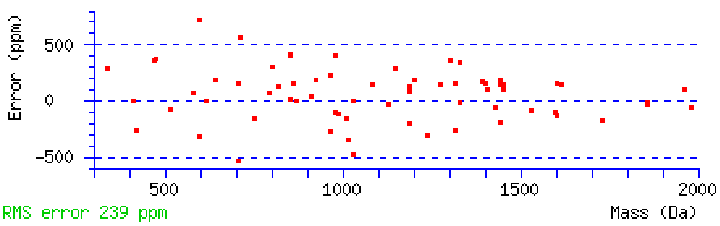

All matches to this query

Score	Mr(calc):	Delta	Sequence
47.1	3132.2415	0.0108	KVEEEQEADEEDVSEEETENREGESK
46.3	3132.2415	0.0108	KVEEEQEADEEDVSEEETENREGESK
17.1	3132.2415	0.0108	KVEEEQEADEEDVSEEETENREGESK
10.4	3130.2249	2.0274	NRNSSPLVASSLESMDMNPECLVSPR
7.1	3130.2249	2.0274	NRNSSPLVASSLESMDMNPECLVSPR
4.1	3130.2443	2.0080	KTESIDVMDAVGSNIVVSTRTGEVMR
3.4	3131.2292	1.0231	LLAAGVVSAMTCMVKTESPVLTNSCR
3.4	3130.2452	2.0071	LLAAGVVSAMTCMVKTESPVLTNSCR
3.3	3132.2834	-0.0311	LSTKTLTGKTIMLNVEPSDTIENVK
3.3	3131.2767	0.9756	SMDQDFGVVKEEISDDSARLPCFNGR

Spectrum No: 358; Query: 1959; Rank: 1

Peptide View

MS/MS Fragmentation of **VAQSDGEESPAEEGQLLGER**
Found in **IPI00210147**, Tax_Id=10116 Gene_Symbol=Sec31l1 Isoform 1 of Protein transport protein Sec31A

Match to Query 1959: 2259.889548 from(1130.952050,2+)
Title: 091008RatKidney_NH4Format01_36.1390.1390.2.dta
Data file K:\NewmanPaper\Piliang\3SubProteomes\Piliang3SP\mgf5ppm\ERLIC_3SubProteomes5ppm.mgf

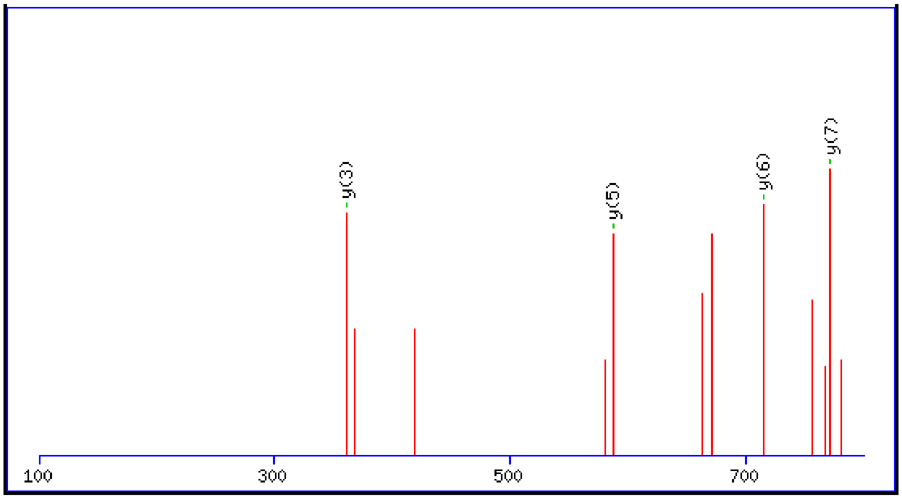

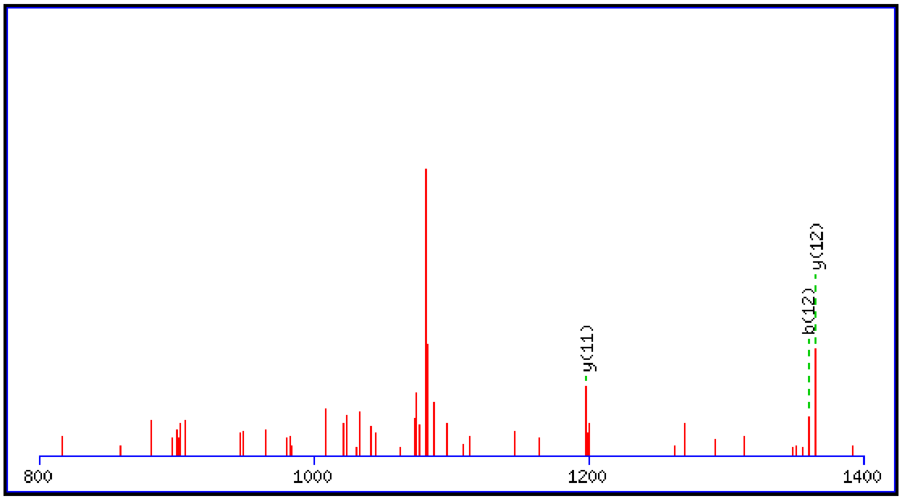

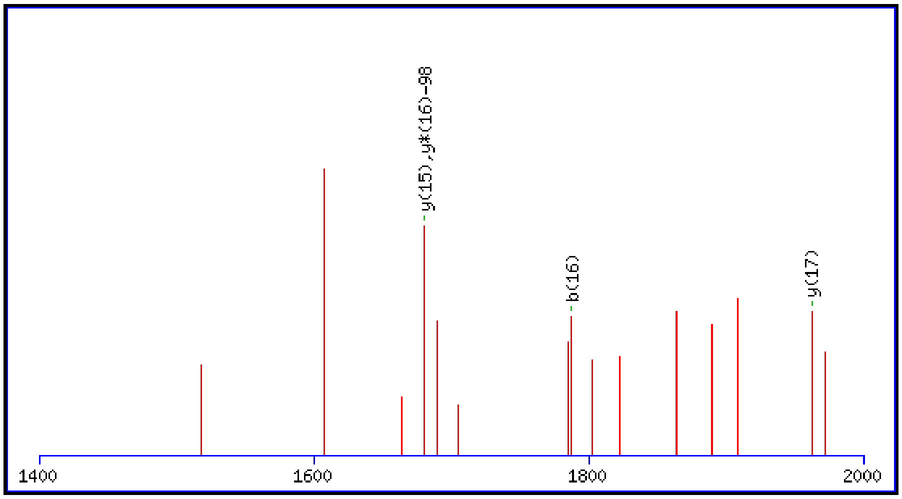

Monoisotopic mass of neutral peptide Mr(calc): 2259.8933
Fixed modifications: Carbamidomethyl (C)
Variable modifications:
S4 : Phospho (ST), with neutral losses 0.0000(shown in table), 97.9769
S9 : Phospho (ST), with neutral losses 0.0000(shown in table), 97.9769
Ions Score: 47 **Expect:** 0.0036
Matches (Bold Red): 11/360 fragment ions using 22 most intense peaks

#	b	b^{++}	b*	b^{*++}	b^{0}	b^{0++}	Seq.	y	y^{++}	y*	y^{*++}	y^{0}	y^{0++}	#
1	100.0757	50.5415					V							20
2	171.1128	86.0600					A	2161.8322	1081.4198	2144.8057	1072.9065	2143.8217	1072.4145	19
3	299.1714	150.0893	282.1448	141.5761			Q	2090.7951	1045.9012	2073.7686	1037.3879	2072.7846	1036.8959	18
4	466.1697	233.5885	449.1432	225.0752	448.1592	224.5832	S	**1962.7365**	981.8719	1945.7100	973.3586	1944.7260	972.8666	17

**5**	581.1967	291.1020	564.1701	282.5887	563.1861	282.0967	**D**	1795.7382	898.3727	1778.7116	889.8595	1777.7276	889.3674	**16**
**6**	638.2181	319.6127	621.1916	311.0994	620.2076	310.6074	**G**	**1680.7112**	840.8593	1663.6847	832.3460	1662.7007	831.8540	**15**
**7**	767.2607	384.1340	750.2342	375.6207	749.2502	375.1287	**E**	1623.6898	812.3485	1606.6632	803.8353	1605.6792	803.3432	**14**
**8**	896.3033	448.6553	879.2768	440.1420	878.2928	439.6500	**E**	1494.6472	747.8272	1477.6206	739.3140	1476.6366	738.8219	**13**
**9**	1063.3017	532.1545	1046.2751	523.6412	1045.2911	523.1492	**S**	**1365.6046**	683.3059	1348.5780	674.7927	1347.5940	674.3007	**12**
**10**	1160.3544	580.6809	1143.3279	572.1676	1142.3439	571.6756	**P**	**1198.6062**	599.8068	1181.5797	591.2935	1180.5957	590.8015	**11**
**11**	1231.3916	616.1994	1214.3650	607.6861	1213.3810	607.1941	**A**	1101.5535	551.2804	1084.5269	542.7671	1083.5429	542.2751	**10**
**12**	**1360.4342**	680.7207	1343.4076	672.2074	1342.4236	671.7154	**E**	1030.5164	515.7618	1013.4898	507.2485	1012.5058	506.7565	**9**
**13**	1489.4767	745.2420	1472.4502	736.7287	1471.4662	736.2367	**E**	901.4738	451.2405	884.4472	442.7272	883.4632	442.2352	**8**
**14**	1546.4982	773.7527	1529.4717	765.2395	1528.4876	764.7475	**G**	**772.4312**	386.7192	755.4046	378.2060	754.4206	377.7139	**7**
**15**	1674.5568	837.7820	1657.5302	829.2688	1656.5462	828.7767	**Q**	**715.4097**	358.2085	698.3832	349.6952	697.3991	349.2032	**6**
**16**	**1787.6408**	894.3241	1770.6143	885.8108	1769.6303	885.3188	**L**	**587.3511**	294.1792	570.3246	285.6659	569.3406	285.1739	**5**
**17**	1900.7249	950.8661	1883.6984	942.3528	1882.7143	941.8608	**L**	474.2671	237.6372	457.2405	229.1239	456.2565	228.6319	**4**
**18**	1957.7464	979.3768	1940.7198	970.8636	1939.7358	970.3715	**G**	**361.1830**	181.0951	344.1565	172.5819	343.1724	172.0899	**3**
**19**	2086.7890	1043.8981	2069.7624	1035.3848	2068.7784	1034.8928	**E**	304.1615	152.5844	287.1350	144.0711	286.1510	143.5791	**2**
**20**							**R**	175.1190	88.0631	158.0924	79.5498			**1**

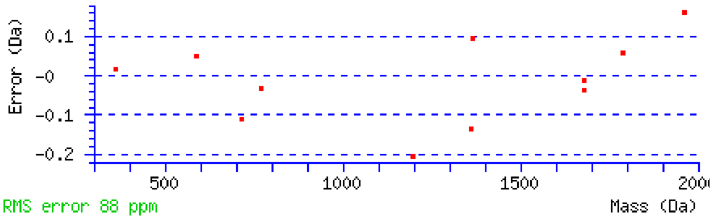

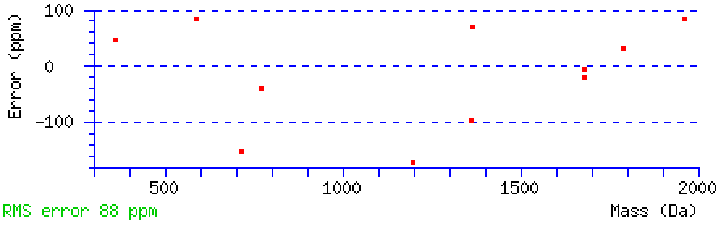

All matches to this query

Score	Mr(calc):	Delta	Sequence
47.1	2259.8933	-0.0038	VAQSDGEESPAEEGQLLGER
0.2	2258.9028	0.9868	MITENQVSNHQRLNAGER

Spectrum No: 359; Query: 978; Rank: 1

Peptide View

MS/MS Fragmentation of **VLHGAQTSDEEKDF**
Found in **IPI00365864**, Tax_Id=10116 Gene_Symbol=LOC688717 hypothetical protein LOC688717

Match to Query 978: 1654.695888 from(828.355220,2+)
Title: 100101RatKid_NS_deglyco_16.1376.1376.2.dta
Data file K:\NewmanPaper\Piliang\3SubProteomes\Piliang3SP\mgf5ppm\ERLIC_3SubProteomes5ppm.mgf

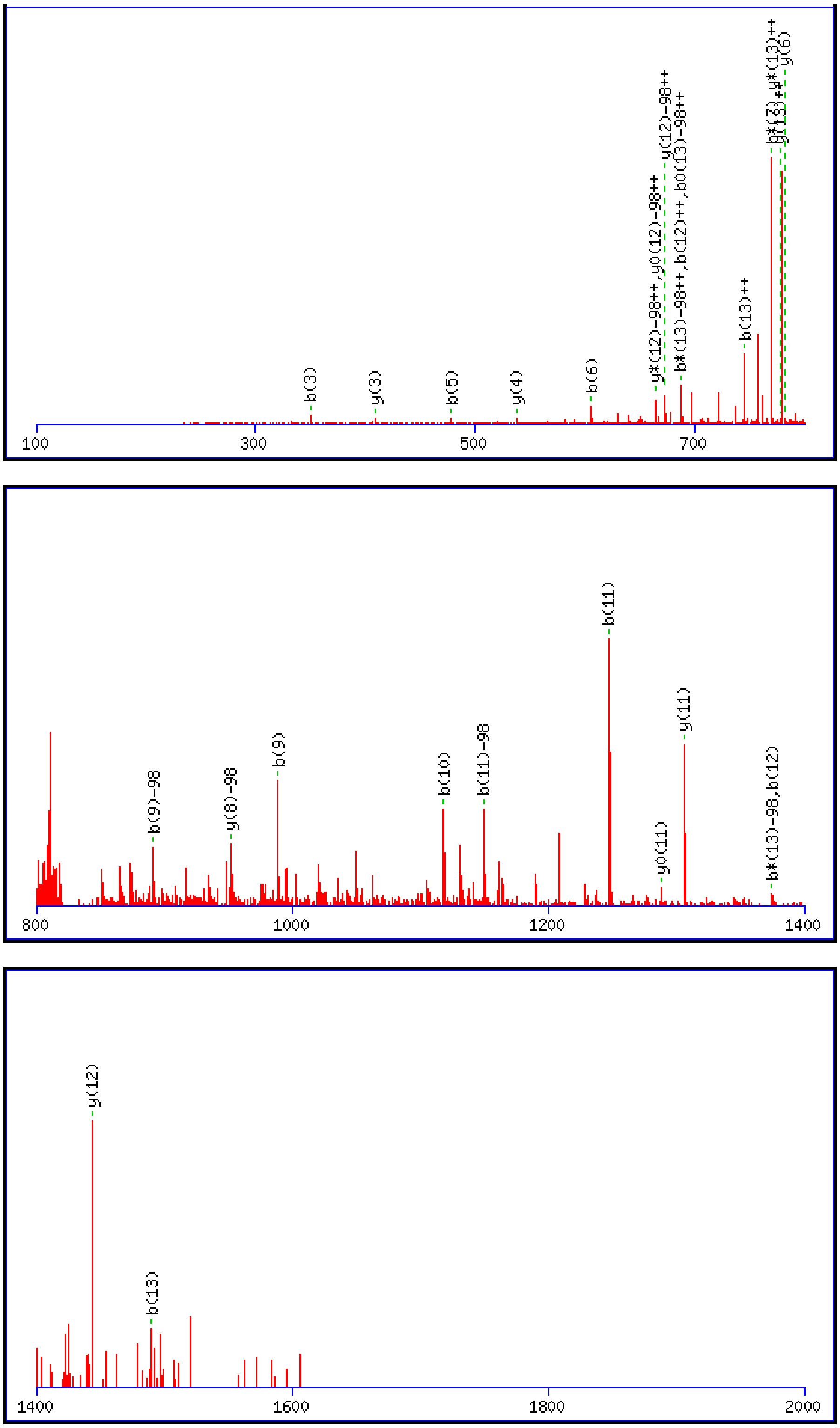

Monoisotopic mass of neutral peptide Mr(calc): 1654.6876
Fixed modifications: Carbamidomethyl (C)
Variable modifications:
T7 : Phospho (ST), with neutral losses 0.0000(shown in table), 97.9769
Ions Score: 47 **Expect:** 0.0031
Matches (Bold Red): 28/206 fragment ions using 42 most intense peaks

#	b	b++	b*	b*++	b0	b0++	Seq.	y	y++	y*	y*++	y0	y0++	#
1	100.0757	50.5415					V							14
2	213.1598	107.0835					L	1556.6265	**778.8169**	1539.5999	**770.3036**	1538.6159	769.8116	13
3	**350.2187**	175.6130					H	**1443.5424**	722.2748	1426.5158	713.7616	1425.5318	713.2696	12
4	407.2401	204.1237					G	**1306.4835**	653.7454	1289.4569	645.2321	**1288.4729**	644.7401	11
5	**478.2772**	239.6423					A	1249.4620	625.2346	1232.4355	616.7214	1231.4514	616.2294	10

6	**606.3358**	303.6715	589.3093	295.1583			Q	1178.4249	589.7161	1161.3984	581.2028	1160.4143	580.7108	9
7	787.3498	394.1786	**770.3233**	385.6653	769.3393	385.1733	T	1050.3663	525.6868	1033.3398	517.1735	1032.3558	516.6815	8
8	874.3819	437.6946	857.3553	429.1813	856.3713	428.6893	S	869.3523	435.1798	852.3258	426.6665	851.3418	426.1745	7
9	**989.4088**	495.2080	972.3822	486.6948	971.3982	486.2028	D	**782.3203**	391.6638	765.2937	383.1505	764.3097	382.6585	6
10	**1118.4514**	559.7293	1101.4248	551.2161	1100.4408	550.7241	E	667.2933	334.1503	650.2668	325.6370	649.2828	325.1450	5
11	**1247.4940**	624.2506	1230.4674	615.7374	1229.4834	615.2453	E	**538.2508**	269.6290	521.2242	261.1157	520.2402	260.6237	4
12	**1375.5889**	**688.2981**	1358.5624	679.7848	1357.5784	679.2928	K	**409.2082**	205.1077	392.1816	196.5944	391.1976	196.1024	3
13	**1490.6159**	**745.8116**	1473.5893	737.2983	1472.6053	736.8063	D	281.1132	141.0602			263.1026	132.0550	2
14							F	166.0863	83.5468					1

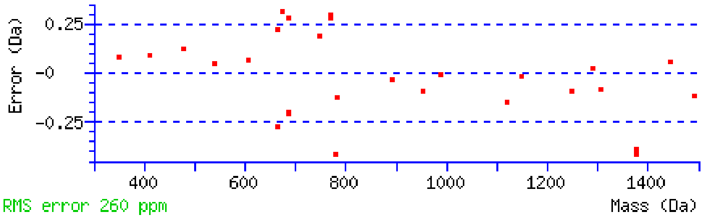

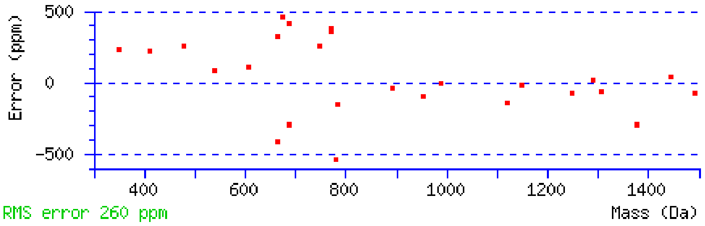

All matches to this query

Score	Mr(calc):	Delta	Sequence
47.1	1654.6876	0.0083	VLHGAQTSDEEKDF
44.0	1654.6876	0.0083	VLHGAQTSDEEKDF
2.1	1653.6997	0.9962	DPPEEEIPFCTLK
2.1	1654.7062	-0.0103	LSNCFSSFLESRK
2.0	1653.7052	0.9907	LLTIYKENGSNNK
2.0	1653.7052	0.9907	LLTIYKENGSNNK
2.0	1653.7052	0.9907	LLTIYKENGSNNK
1.6	1654.6865	0.0094	LSQDTYRRNSVR
1.6	1653.6968	0.9991	NNQSRRAQNNQSR
1.6	1654.6808	0.0151	NNQSRRAQNNQSR

Spectrum No: 360; Query: 1002; Rank: 1

Peptide View

MS/MS Fragmentation of **DGQDAIAQSPEKEAK**
Found in **IPI00196639**, Tax_Id=10116 Gene_Symbol=RGD1307526 39 kDa protein

Match to Query 1002: 1665.726102 from(556.249310,3+)
Title: 091008RatKidney_NH4Format01_22.657.657.3.dta
Data file K:\NewmanPaper\Piliang\3SubProteomes\Piliang3SP\mgf5ppm\ERLIC_3SubProteomes5ppm.mgf

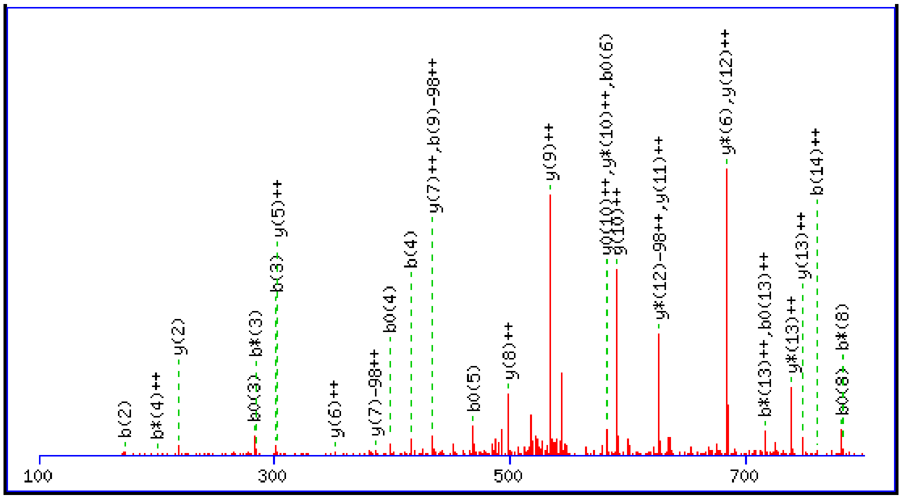

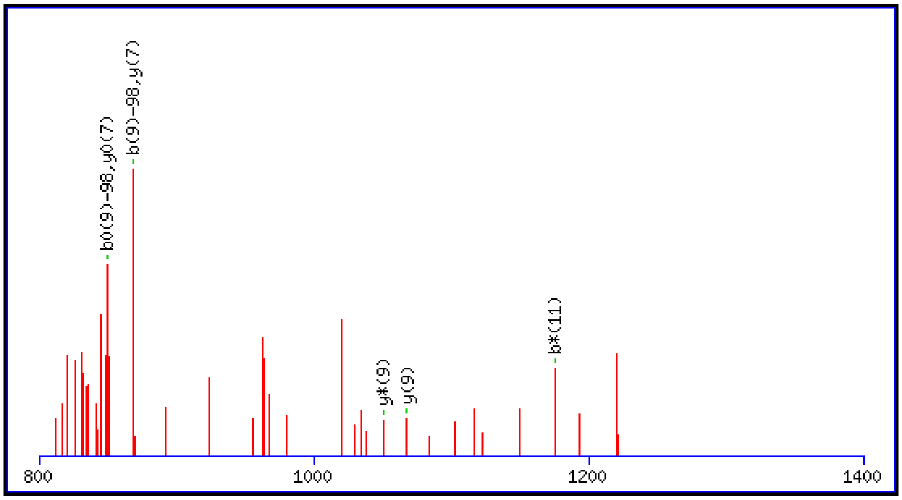

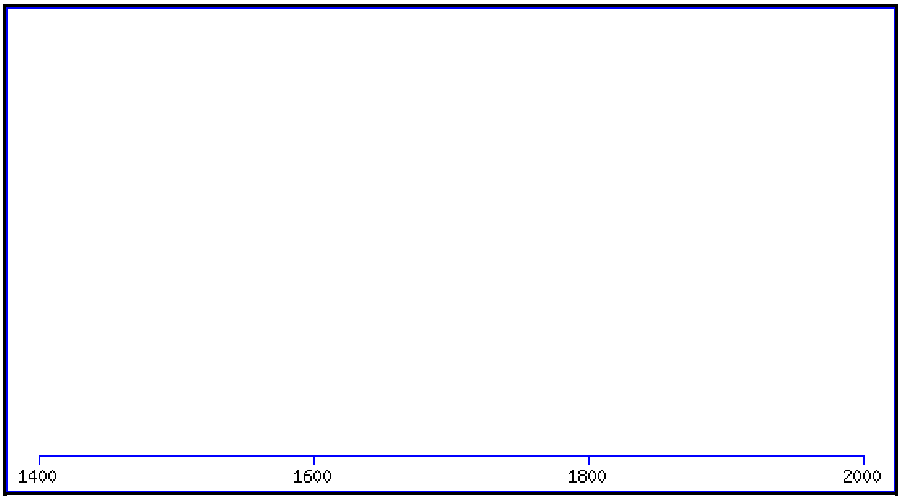

Monoisotopic mass of neutral peptide Mr(calc): 1665.7247
Fixed modifications: Carbamidomethyl (C)
Variable modifications:
S9 : Phospho (ST), with neutral losses 0.0000(shown in table), 97.9769
Ions Score: 47 **Expect:** 0.0032
Matches (Bold Red): 38/244 fragment ions using 57 most intense peaks

#	b	b^{++}	b*	b^{*++}	b^0	b^{0++}	Seq.	y	y^{++}	y*	y^{*++}	y^0	y^{0++}	#
1	116.0342	58.5207			98.0237	49.5155	D							15
2	**173.0557**	87.0315			155.0451	78.0262	G	1551.7050	776.3562	1534.6785	767.8429	1533.6945	767.3509	14
3	**301.1143**	151.0608	**284.0877**	142.5475	**283.1037**	142.0555	Q	1494.6836	**747.8454**	1477.6570	**739.3322**	1476.6730	738.8401	13
4	**416.1412**	208.5742	399.1147	**200.0610**	**398.1306**	199.5690	D	1366.6250	**683.8161**	1349.5984	675.3029	1348.6144	674.8109	12
5	487.1783	244.0928	470.1518	235.5795	**469.1678**	235.0875	A	1251.5981	**626.3027**	1234.5715	617.7894	1233.5875	617.2974	11

6	600.2624	300.6348	583.2358	292.1216	582.2518	291.6295	I	1180.5609	590.7841	1163.5344	582.2708	1162.5504	581.7788	10
7	671.2995	336.1534	654.2729	327.6401	653.2889	327.1481	A	1067.4769	534.2421	1050.4503	525.7288	1049.4663	525.2368	9
8	799.3581	400.1827	782.3315	391.6694	781.3475	391.1774	Q	996.4398	498.7235	979.4132	490.2102	978.4292	489.7182	8
9	966.3564	483.6819	949.3299	475.1686	948.3459	474.6766	S	868.3812	434.6942	851.3546	426.1810	850.3706	425.6889	7
10	1063.4092	532.2082	1046.3826	523.6950	1045.3986	523.2030	P	701.3828	351.1951	684.3563	342.6818	683.3723	342.1898	6
11	1192.4518	596.7295	1175.4252	588.2163	1174.4412	587.7242	E	604.3301	302.6687	587.3035	294.1554	586.3195	293.6634	5
12	1320.5467	660.7770	1303.5202	652.2637	1302.5362	651.7717	K	475.2875	238.1474	458.2609	229.6341	457.2769	229.1421	4
13	1449.5893	725.2983	1432.5628	716.7850	1431.5788	716.2930	E	347.1925	174.0999	330.1660	165.5866	329.1819	165.0946	3
14	1520.6265	760.8169	1503.5999	752.3036	1502.6159	751.8116	A	218.1499	109.5786	201.1234	101.0653			2
15							K	147.1128	74.0600	130.0863	65.5468			1

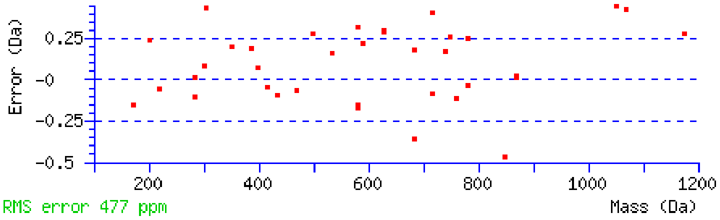

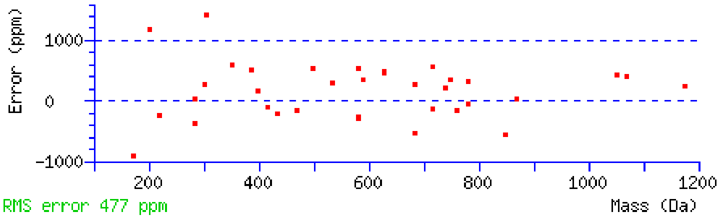

All matches to this query

Score	Mr(calc):	Delta	Sequence
47.1	1665.7247	0.0014	DGQDAIAQSPEKEAK
7.2	1664.7267	0.9994	SGGNGAGARAGAPSEGAAK
7.2	1665.7108	0.0153	SGGNGAGARAGAPSEGAAK
6.3	1663.7219	2.0042	SLNANTDITSLARK
5.5	1664.7348	0.9913	WIANNWINVDPSR
5.5	1665.7188	0.0073	WIANNWINVDPSR
5.3	1664.7324	0.9937	SGYLSSERLIPQR
5.3	1664.7324	0.9937	SGYLSSERLIPQR
4.2	1665.7400	-0.0139	GYNATVLAYGQTGSGK
4.2	1663.7259	2.0002	KIGNAYAVLSNPEK

Spectrum No: 361; Query: 1891; Rank: 1

Peptide View

MS/MS Fragmentation of **SGEDEQQEQTIAEDLVVTK**
Found in **IPI00371270**, Tax_Id=10116 Gene_Symbol=Pa2g4 Proliferation-associated 2G4

Match to Query 1891: 2197.958262 from(733.660030,3+)
Title: 100101RatKid_NS_deglyco_08.4054.4054.3.dta
Data file K:\NewmanPaper\Piliang\3SubProteomes\Piliang3SP\mgf5ppm\ERLIC_3SubProteomes5ppm.mgf

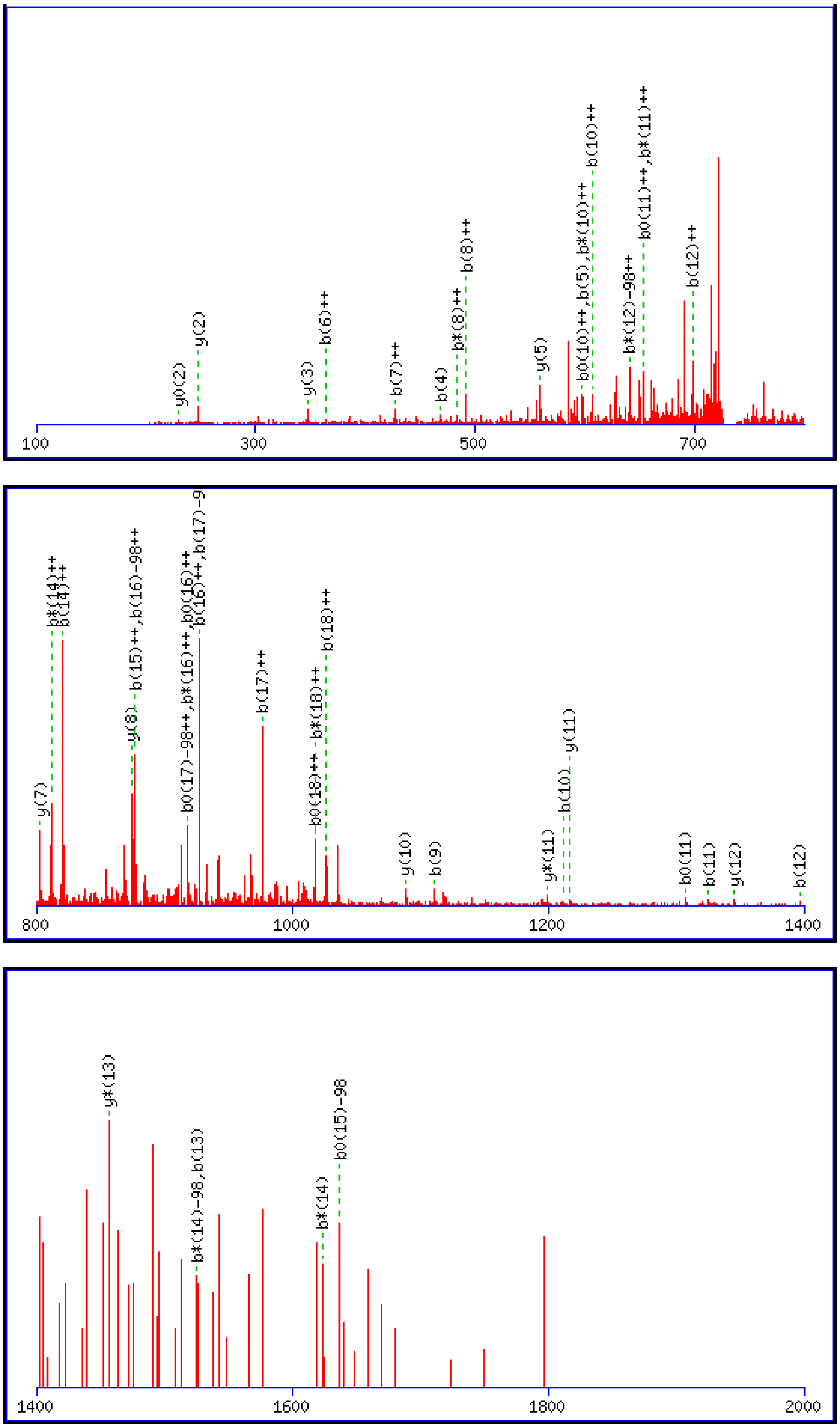

Monoisotopic mass of neutral peptide Mr(calc): 2197.9628
Fixed modifications: Carbamidomethyl (C)
Variable modifications:
S1 : Phospho (ST), with neutral losses 0.0000(shown in table), 97.9769
Ions Score: 47 **Expect:** 0.0062
Matches (Bold Red): 46/302 fragment ions using 86 most intense peaks

#	b	b^{++}	b*	b^{*++}	b^0	b^{0++}	Seq.	y	y^{++}	y*	y^{*++}	y^0	y^{0++}	#
1	168.0056	84.5065			149.9951	75.5012	S							19
2	225.0271	113.0172			207.0165	104.0119	G	2031.9717	1016.4895	2014.9451	1007.9762	2013.9611	1007.4842	18
3	354.0697	177.5385			336.0591	168.5332	E	1974.9502	987.9787	1957.9237	979.4655	1956.9397	978.9735	17
4	**469.0966**	235.0520			451.0861	226.0467	D	1845.9076	923.4575	1828.8811	914.9442	1827.8971	914.4522	16
5	**598.1392**	299.5732			580.1287	290.5680	E	1730.8807	865.9440	1713.8541	857.4307	1712.8701	856.9387	15

6	726.1978	**363.6025**	709.1713	355.0893	708.1872	354.5973	Q	1601.8381	801.4227	1584.8115	792.9094	1583.8275	792.4174	14
7	854.2564	**427.6318**	837.2298	419.1186	836.2458	418.6265	Q	1473.7795	737.3934	**1456.7530**	728.8801	1455.7690	728.3881	13
8	983.2990	**492.1531**	966.2724	**483.6398**	965.2884	483.1478	E	**1345.7209**	673.3641	1328.6944	664.8508	1327.7104	664.3588	12
9	**1111.3575**	556.1824	1094.3310	547.6691	1093.3470	547.1771	Q	**1216.6783**	608.8428	**1199.6518**	600.3295	1198.6678	599.8375	11
10	**1212.4052**	**606.7063**	1195.3787	**598.1930**	1194.3947	**597.7010**	T	**1088.6198**	544.8135	1071.5932	536.3002	1070.6092	535.8082	10
11	**1325.4893**	663.2483	1308.4627	**654.7350**	**1307.4787**	**654.2430**	I	987.5721	494.2897	970.5455	485.7764	969.5615	485.2844	9
12	**1396.5264**	**698.7668**	1379.4999	690.2536	1378.5158	689.7616	A	**874.4880**	437.7477	857.4615	429.2344	856.4775	428.7424	8
13	**1525.5690**	763.2881	1508.5424	754.7749	1507.5584	754.2829	E	**803.4509**	402.2291	786.4244	393.7158	785.4403	393.2238	7
14	1640.5959	**820.8016**	**1623.5694**	**812.2883**	1622.5854	811.7963	D	674.4083	337.7078	657.3818	329.1945	656.3978	328.7025	6
15	1753.6800	**877.3436**	1736.6535	868.8304	1735.6694	868.3384	L	**559.3814**	280.1943	542.3548	271.6811	541.3708	271.1890	5
16	1852.7484	**926.8778**	1835.7219	**918.3646**	1834.7379	**917.8726**	V	446.2973	223.6523	429.2708	215.1390	428.2867	214.6470	4
17	1951.8168	**976.4121**	1934.7903	967.8988	1933.8063	967.4068	V	**347.2289**	174.1181	330.2023	165.6048	329.2183	165.1128	3
18	2052.8645	**1026.9359**	2035.8380	**1018.4226**	2034.8539	**1017.9306**	T	**248.1605**	124.5839	231.1339	116.0706	**230.1499**	115.5786	2
19							K	147.1128	74.0600	130.0863	65.5468			1

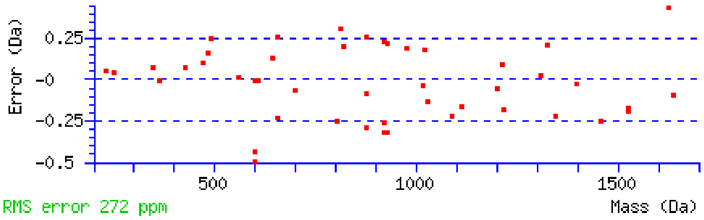

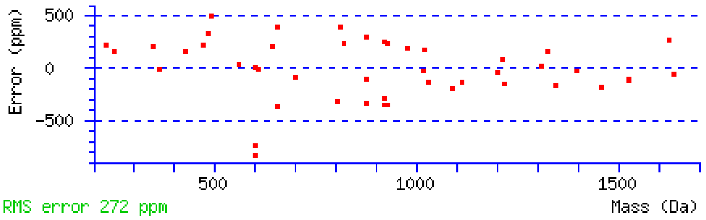

All matches to this query

Score	Mr(calc):	Delta	Sequence
47.0	2197.9628	-0.0045	SGEDEQQEQTIAEDLVVTK
13.9	2197.9628	-0.0045	SGEDEQQEQTIAEDLVVTK
1.8	2197.9406	0.0177	DESKVEHSAHSRSELISK
0.4	2196.9358	1.0225	EDQPSGVCNKTVENLQAAR

Spectrum No: 362; Query: 2527; Rank: 1

Peptide View

MS/MS Fragmentation of **SNSFSDEREFSAPSTPTGTLEFAGGEGK**
Found in **IPI00207069**, Tax_Id=10116 Gene_Symbol=Ahnak similar to AHNAK nucleoprotein isoform 1

Match to Query 2527: 2985.280062 from(996.100630,3+)
Title: 091008RatKidney_NoSalt_20.3541.3541.3.dta
Data file K:\NewmanPaper\Piliang\3SubProteomes\Piliang3SP\mgf5ppm\ERLIC_3SubProteomes5ppm.mgf

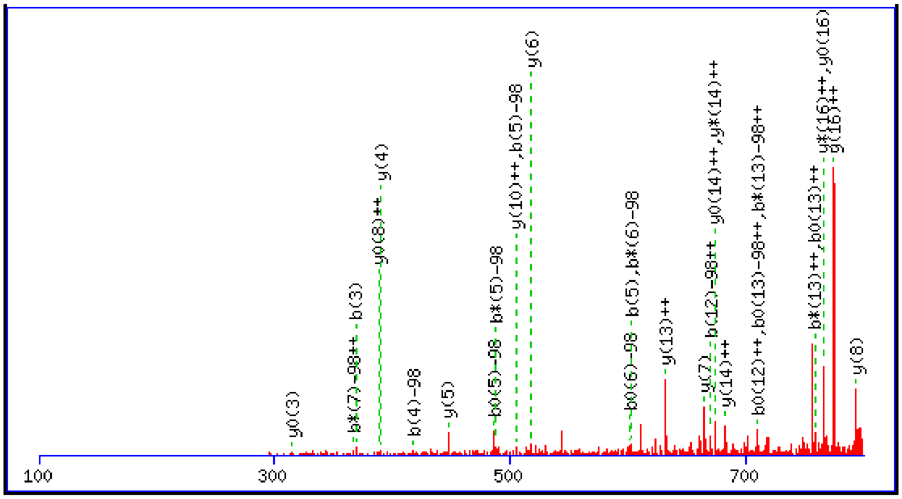

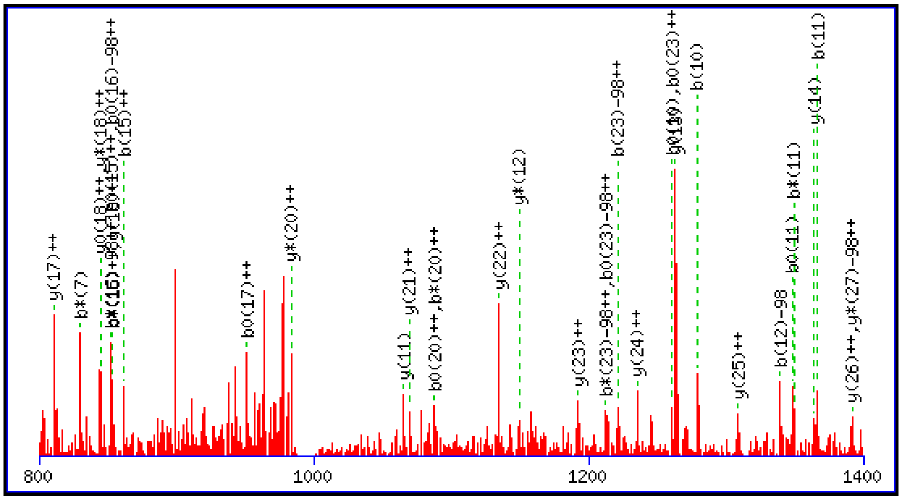

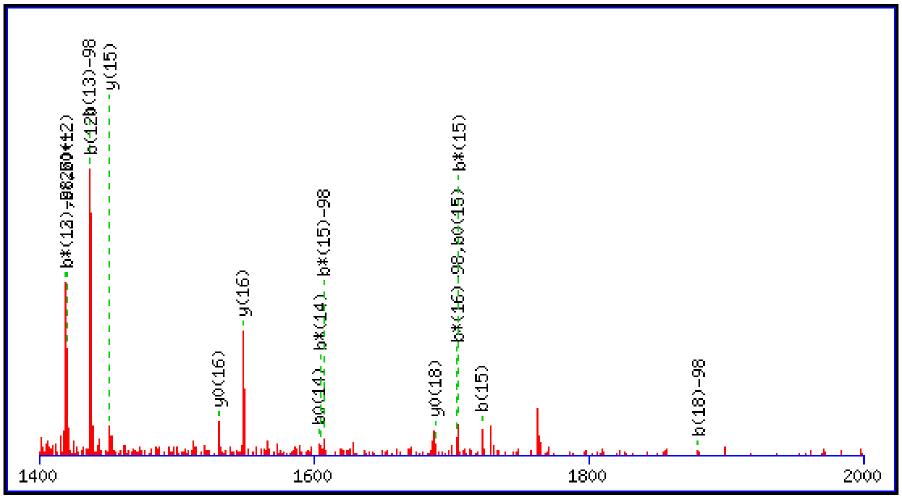

Monoisotopic mass of neutral peptide Mr(calc): 2984.2713
Fixed modifications: Carbamidomethyl (C)
Variable modifications:
S3 : Phospho (ST), with neutral losses 0.0000(shown in table), 97.9769
Ions Score: 47 **Expect:** 0.0081
Matches (Bold Red): 83/480 fragment ions using 134 most intense peaks

#	b	b^{++}	b*	b^{*++}	b^0	b^{0++}	Seq.	y	y^{++}	y*	y^{*++}	y^0	y^{0++}	#
1	88.0393	44.5233			70.0287	35.5180	S							28
2	202.0822	101.5448	185.0557	93.0315	184.0717	92.5395	N	2898.2465	1449.6269	2881.2200	1441.1136	2880.2360	1440.6216	27
3	**369.0806**	185.0439	352.0540	176.5307	351.0700	176.0386	S	2784.2036	**1392.6054**	2767.1771	1384.0922	2766.1931	1383.6002	26
4	516.1490	258.5781	499.1224	250.0649	498.1384	249.5729	F	2617.2053	**1309.1063**	2600.1787	1300.5930	2599.1947	1300.1010	25
5	**603.1810**	302.0942	586.1545	293.5809	585.1705	293.0889	S	2470.1369	**1235.5721**	2453.1103	1227.0588	2452.1263	1226.5668	24

6	718.2080	359.6076	701.1814	351.0943	700.1974	350.6023	D	2383.1048	**1192.0561**	2366.0783	1183.5428	2365.0943	1183.0508	23
7	847.2506	424.1289	**830.2240**	415.6156	829.2400	415.1236	E	2268.0779	**1134.5426**	2251.0513	1126.0293	2250.0673	1125.5373	22
8	1003.3517	502.1795	986.3251	493.6662	985.3411	493.1742	R	2139.0353	**1070.0213**	2122.0087	1061.5080	2121.0247	1061.0160	21
9	1132.3943	566.7008	1115.3677	558.1875	1114.3837	557.6955	E	1982.9342	991.9707	1965.9076	**983.4575**	1964.9236	982.9654	20
10	**1279.4627**	640.2350	1262.4361	631.7217	**1261.4521**	631.2297	F	1853.8916	927.4494	1836.8650	918.9362	1835.8810	918.4441	19
11	**1366.4947**	683.7510	**1349.4682**	675.2377	**1348.4841**	674.7457	S	1706.8232	**853.9152**	1689.7966	**845.4020**	**1688.8126**	**844.9099**	18
12	**1437.5318**	719.2695	**1420.5053**	710.7563	**1419.5213**	**710.2643**	A	1619.7911	**810.3992**	1602.7646	801.8859	1601.7806	801.3939	17
13	1534.5846	767.7959	1517.5580	**759.2827**	1516.5740	**758.7906**	P	**1548.7540**	**774.8807**	1531.7275	**766.3674**	**1530.7435**	**765.8754**	16
14	1621.6166	811.3119	**1604.5901**	802.7987	**1603.6060**	802.3067	S	**1451.7013**	726.3543	1434.6747	717.8410	1433.6907	717.3490	15
15	**1722.6643**	**861.8358**	**1705.6377**	**853.3225**	**1704.6537**	**852.8305**	T	**1364.6692**	**682.8383**	1347.6427	**674.3250**	1346.6587	**673.8330**	14
16	1819.7171	910.3622	1802.6905	901.8489	1801.7065	901.3569	P	**1263.6216**	**632.3144**	1246.5950	623.8011	1245.6110	623.3091	13
17	1920.7647	960.8860	1903.7382	952.3727	1902.7542	**951.8807**	T	1166.5688	583.7880	**1149.5422**	575.2748	1148.5582	574.7828	12
18	1977.7862	989.3967	1960.7597	980.8835	1959.7756	980.3915	G	**1065.5211**	533.2642	1048.4946	524.7509	1047.5106	524.2589	11
19	2078.8339	1039.9206	2061.8073	1031.4073	2060.8233	1030.9153	T	1008.4997	**504.7535**	991.4731	496.2402	990.4891	495.7482	10
20	2191.9179	1096.4626	2174.8914	**1087.9493**	2173.9074	**1087.4573**	L	907.4520	454.2296	890.4254	445.7164	889.4414	445.2243	9
21	2320.9605	1160.9839	2303.9340	1152.4706	2302.9500	1151.9786	E	**794.3679**	397.6876	777.3414	389.1743	776.3573	**388.6823**	8
22	2468.0289	1234.5181	2451.0024	1226.0048	2450.0184	1225.5128	F	**665.3253**	333.1663	648.2988	324.6530	647.3148	324.1610	7
23	2539.0661	1270.0367	2522.0395	1261.5234	2521.0555	**1261.0314**	A	**518.2569**	259.6321	501.2304	251.1188	500.2463	250.6268	6
24	2596.0875	1298.5474	2579.0610	1290.0341	2578.0770	1289.5421	G	**447.2198**	224.1135	430.1932	215.6003	429.2092	215.1082	5
25	2653.1090	1327.0581	2636.0824	1318.5449	2635.0984	1318.0529	G	**390.1983**	195.6028	373.1718	187.0895	372.1878	186.5975	4
26	2782.1516	1391.5794	2765.1250	1383.0662	2764.1410	1382.5741	E	333.1769	167.0921	316.1503	158.5788	**315.1663**	158.0868	3
27	2839.1730	**1420.0902**	2822.1465	1411.5769	2821.1625	1411.0849	G	204.1343	102.5708	187.1077	94.0575			2
28							K	147.1128	74.0600	130.0863	65.5468			1

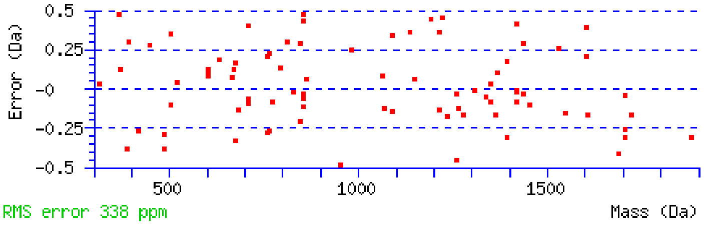

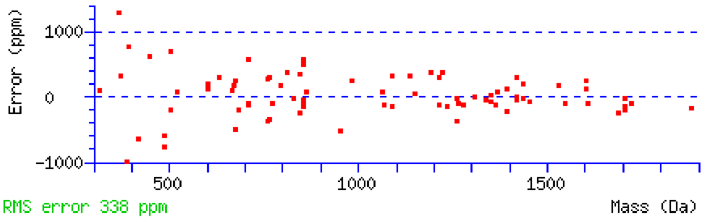

All matches to this query

Score	Mr(calc):	Delta	Sequence
46.9	2984.2713	1.0088	SNSFSDEREFSAPSTPTGTLEFAGGEGK
46.1	2984.2713	1.0088	SNSFSDEREFSAPSTPTGTLEFAGGEGK
41.2	2984.2713	1.0088	SNSFSDEREFSAPSTPTGTLEFAGGEGK
36.5	2985.2553	0.0248	SNSFSDEREFSAPSTPTGTLEFAGGEGK
36.5	2985.2553	0.0248	SNSFSDEREFSAPSTPTGTLEFAGGEGK
31.9	2985.2553	0.0248	SNSFSDEREFSAPSTPTGTLEFAGGEGK
21.8	2985.2553	0.0248	SNSFSDEREFSAPSTPTGTLEFAGGEGK
13.3	2984.2713	1.0088	SNSFSDEREFSAPSTPTGTLEFAGGEGK
12.9	2985.2553	0.0248	SNSFSDEREFSAPSTPTGTLEFAGGEGK
8.9	2985.2553	0.0248	SNSFSDEREFSAPSTPTGTLEFAGGEGK

Spectrum No: 363; Query: 1691; Rank: 1

Peptide View

MS/MS Fragmentation of **AERGGSSGEELEDEEPVK**

Found in **IPI00391633**, Tax_Id=10116 Gene_Symbol=Hdgfrp2 hepatoma-derived growth factor-related protein 2

Match to Query 1691: 2076.797628 from(1039.406090,2+)
Title: 100101RatKid_NS_deglyco_25.1454.1454.2.dta
Data file K:\NewmanPaper\Piliang\3SubProteomes\Piliang3SP\mgf5ppm\ERLIC_3SubProteomes5ppm.mgf

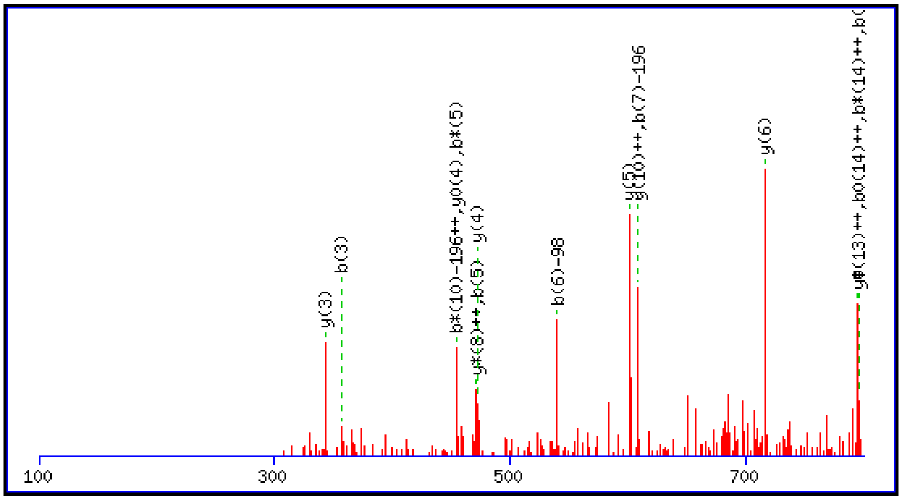

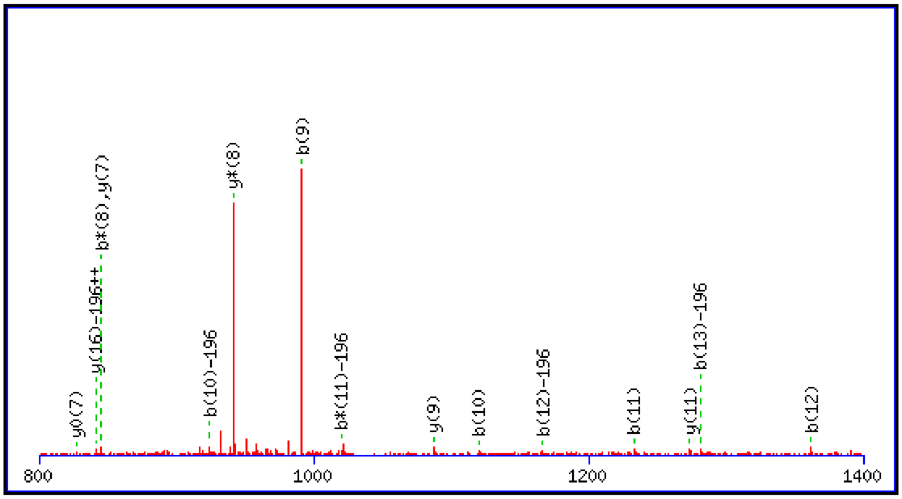

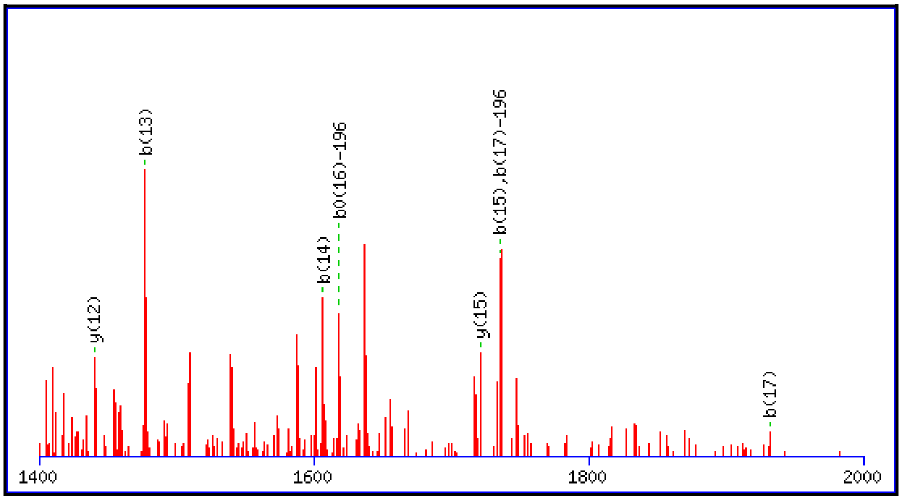

Monoisotopic mass of neutral peptide Mr(calc): 2076.7926
Fixed modifications: Carbamidomethyl (C)
Variable modifications:
S6 : Phospho (ST), with neutral losses 0.0000(shown in table), 97.9769
S7 : Phospho (ST), with neutral losses 0.0000(shown in table), 97.9769
Ions Score: 47 **Expect:** 0.0032
Matches (Bold Red): 41/300 fragment ions using 63 most intense peaks

#	b	b^{++}	b*	b^{*++}	b^0	b^{0++}	Seq.	y	y^{++}	y*	y^{*++}	y^0	y^{0++}	#

**1**	72.0444	36.5258					**A**							**18**
**2**	201.0870	101.0471			183.0764	92.0418	**E**	2006.7628	1003.8850	1989.7362	995.3717	1988.7522	994.8797	**17**
**3**	**357.1881**	179.0977	340.1615	170.5844	339.1775	170.0924	**R**	1877.7202	939.3637	1860.6936	930.8504	1859.7096	930.3584	**16**
**4**	414.2096	207.6084	397.1830	199.0951	396.1990	198.6031	**G**	**1721.6191**	861.3132	1704.5925	852.7999	1703.6085	852.3079	**15**
**5**	**471.2310**	236.1191	**454.2045**	227.6059	453.2205	227.1139	**G**	1664.5976	832.8024	1647.5710	824.2892	1646.5870	823.7971	**14**
**6**	638.2294	319.6183	621.2028	311.1051	620.2188	310.6130	**S**	1607.5761	804.2917	1590.5496	**795.7784**	1589.5656	**795.2864**	**13**
**7**	805.2277	403.1175	788.2012	394.6042	787.2172	394.1122	**S**	**1440.5778**	720.7925	1423.5512	712.2792	1422.5672	711.7872	**12**
**8**	862.2492	431.6282	**845.2226**	423.1150	844.2386	422.6230	**G**	**1273.5794**	637.2933	1256.5529	628.7801	1255.5689	628.2881	**11**
**9**	**991.2918**	496.1495	974.2652	487.6363	973.2812	487.1442	**E**	1216.5580	**608.7826**	1199.5314	600.2693	1198.5474	599.7773	**10**
**10**	**1120.3344**	560.6708	1103.3078	552.1576	1102.3238	551.6655	**E**	**1087.5154**	544.2613	1070.4888	535.7480	1069.5048	535.2560	**9**
**11**	**1233.4184**	617.2129	1216.3919	608.6996	1215.4079	608.2076	**L**	958.4728	479.7400	**941.4462**	**471.2267**	940.4622	470.7347	**8**
**12**	**1362.4610**	681.7342	1345.4345	673.2209	1344.4505	672.7289	**E**	**845.3887**	423.1980	828.3622	414.6847	**827.3781**	414.1927	**7**
**13**	**1477.4880**	739.2476	1460.4614	730.7344	1459.4774	730.2423	**D**	**716.3461**	358.6767	699.3196	350.1634	698.3355	349.6714	**6**
**14**	**1606.5306**	803.7689	1589.5040	**795.2556**	1588.5200	**794.7636**	**E**	**601.3192**	301.1632	584.2926	292.6499	583.3086	292.1579	**5**
**15**	**1735.5732**	868.2902	1718.5466	859.7769	1717.5626	859.2849	**E**	**472.2766**	236.6419	455.2500	228.1287	**454.2660**	227.6366	**4**
**16**	1832.6259	916.8166	1815.5994	908.3033	1814.6154	907.8113	**P**	**343.2340**	172.1206	326.2074	163.6074			**3**
**17**	**1931.6943**	966.3508	1914.6678	957.8375	1913.6838	957.3455	**V**	246.1812	123.5942	229.1547	115.0810			**2**
**18**							**K**	147.1128	74.0600	130.0863	65.5468			**1**

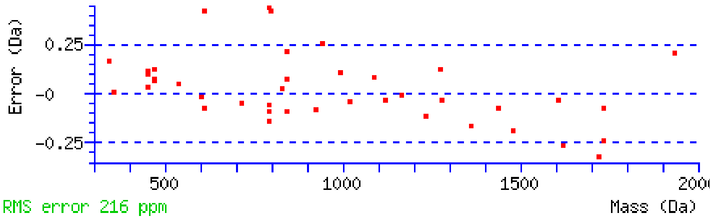

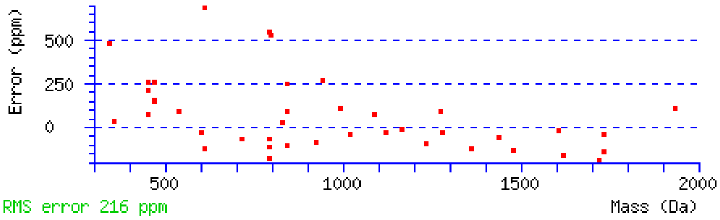

All matches to this query

Score	Mr(calc):	Delta	Sequence
46.9	2076.7926	0.0051	AERGGSSGEELEDEEPVK
7.0	2076.8143	-0.0167	EEEVQQHSHCVNCVSR
6.9	2076.8158	-0.0182	VLSSRIHSIEEKMTK
6.8	2076.7957	0.0019	ENSKSSMYYVLQFLK
6.8	2076.7957	0.0019	ENSKSSMYYVLQFLK
6.8	2076.7957	0.0019	ENSKSSMYYVLQFLK
4.8	2076.8176	-0.0200	CTELDMARGGNRGESCGK
3.9	2076.8008	-0.0032	VEESYAMPTKTMEVMR
3.8	2076.8071	-0.0095	CMVSGWGTTSSSHNNFPK
3.8	2076.8071	-0.0095	CMVSGWGTTSSSHNNFPK

Spectrum No: 364; Query: 147; Rank: 1

Peptide View

MS/MS Fragmentation of **AGSPQLDDIR**
Found in **IPI00211730**, Tax_Id=10116 Gene_Symbol=Eif2b5 Translation initiation factor eIF-2B subunit epsilon

Match to Query 147: 1150.498988 from(576.256770,2+)
Title: 091008RatKidney_NH4Format02_25.1542.1542.2.dta
Data file K:\NewmanPaper\Piliang\3SubProteomes\Piliang3SP\mgf5ppm\ERLIC_3SubProteomes5ppm.mgf

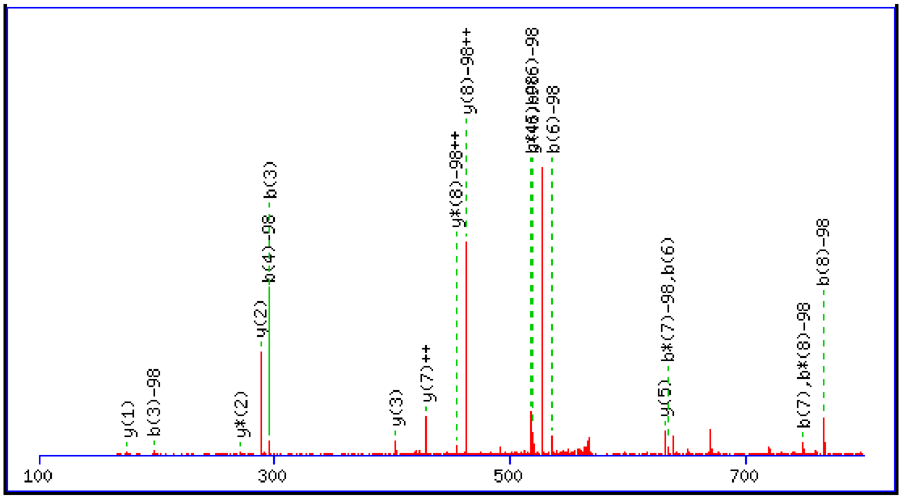

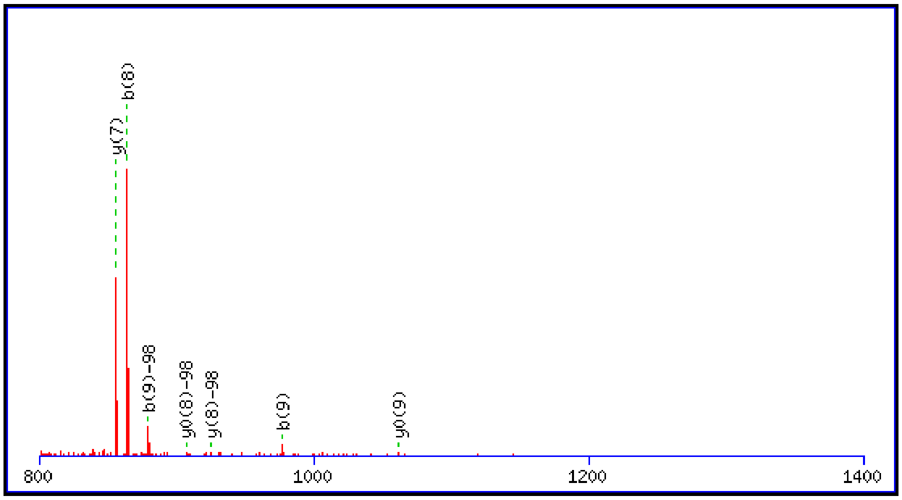

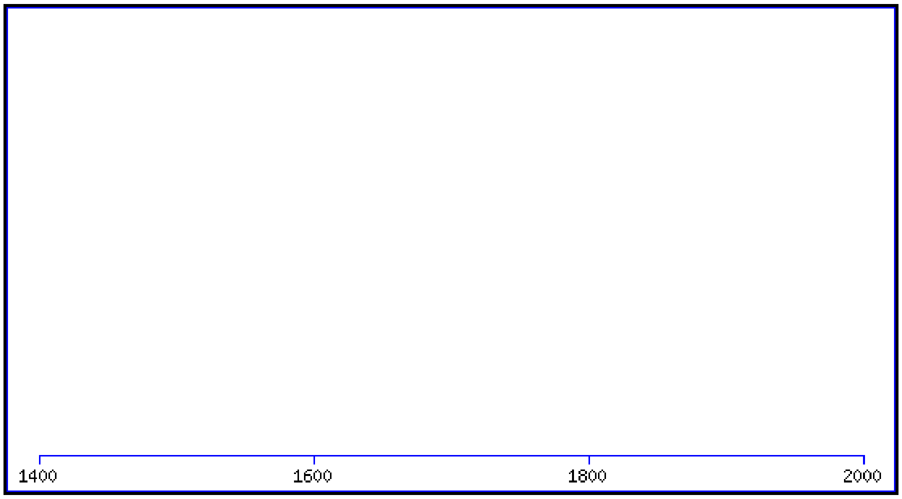

Monoisotopic mass of neutral peptide Mr(calc): 1150.5020
Fixed modifications: Carbamidomethyl (C)
Variable modifications:
S3 : Phospho (ST), with neutral losses 97.9769(shown in table), 0.0000
Ions Score: 47 **Expect:** 0.0019
Matches (Bold Red): 27/142 fragment ions using 41 most intense peaks

#	b	b^{++}	b*	b^{*++}	b^0	b^{0++}	Seq.	y	y^{++}	y*	y^{*++}	y^0	y^{0++}	#
1	72.0444	36.5258					A							10
2	129.0659	65.0366					G	982.4952	491.7513	965.4687	483.2380	964.4847	482.7460	9
3	**198.0873**	99.5473			180.0767	90.5420	S	**925.4738**	**463.2405**	908.4472	**454.7272**	**907.4632**	454.2352	8
4	**295.1401**	148.0737			277.1295	139.0684	P	**856.4523**	**428.7298**	839.4258	420.2165	838.4417	419.7245	7
5	423.1987	212.1030	406.1721	203.5897	405.1881	203.0977	Q	759.3995	380.2034	742.3730	371.6901	741.3890	371.1981	6

6	**536.2827**	268.6450	**519.2562**	260.1317	**518.2722**	259.6397	**L**	**631.3410**	316.1741	614.3144	307.6608	613.3304	307.1688	5
7	651.3097	326.1585	**634.2831**	317.6452	633.2991	317.1532	**D**	**518.2569**	259.6321	501.2304	251.1188	500.2463	250.6268	4
8	**766.3366**	383.6719	**749.3101**	375.1587	748.3260	374.6667	**D**	**403.2300**	202.1186	386.2034	193.6053	385.2194	193.1133	3
9	**879.4207**	440.2140	862.3941	431.7007	861.4101	431.2087	**I**	**288.2030**	144.6051	**271.1765**	136.0919			2
10							**R**	**175.1190**	88.0631	158.0924	79.5498			1

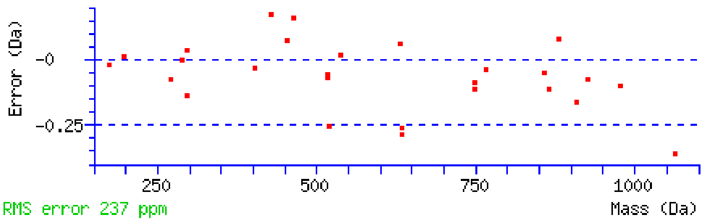

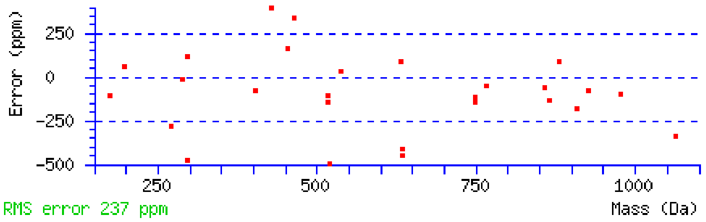

All matches to this query

Score	Mr(calc):	Delta	Sequence
46.9	1150.5020	-0.0030	AGSPQLDDIR
12.9	1150.5060	-0.0070	TFGLNSYLR
10.5	1150.5020	-0.0030	AGSLGPETPSR
8.7	1150.5019	-0.0029	SSEPPAQSLR
8.7	1148.4937	2.0053	QMYLTEIR
7.9	1150.5060	-0.0070	EAIYSGYIR
7.8	1150.5060	-0.0070	QYYTLLNR
7.2	1150.4907	0.0083	QSPASEPNLK
5.9	1150.5028	-0.0038	KMCVPSPPR
5.2	1150.4907	0.0083	TSSFVSQSTK

Spectrum No: 365; Query: 2831; Rank: 1

Peptide View

MS/MS Fragmentation of **SKSPPPPEEEAKDEEEDQTLVNLDTYTSDLHFQISK**
Found in **IPI00360386**, Tax_Id=10116 Gene_Symbol=RGD1562784_predicted similar to heterogeneous nuclear ribonucleoprotein U isoform a

Match to Query 2831: 4197.922216 from(1050.487830,4+)
Title: 100101RatKid_NS_deglyco_13.5146.5146.4.dta
Data file K:\NewmanPaper\Piliang\3SubProteomes\Piliang3SP\mgf5ppm\ERLIC_3SubProteomes5ppm.mgf

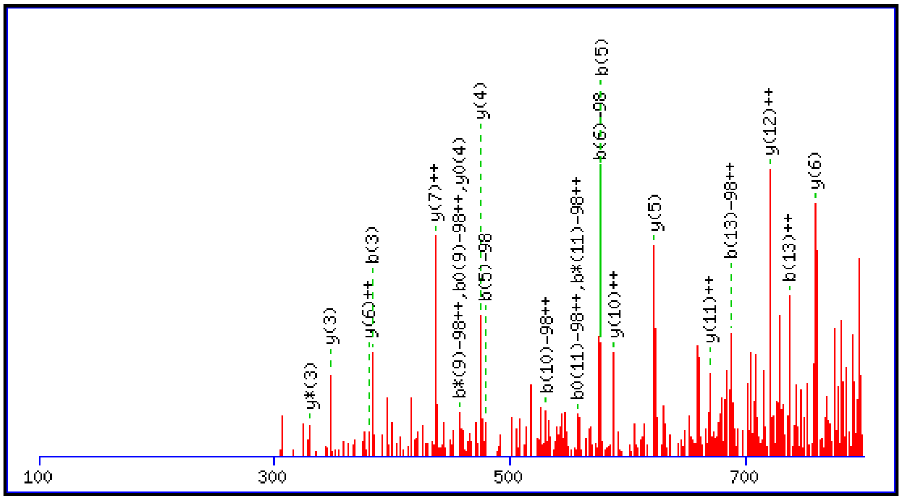

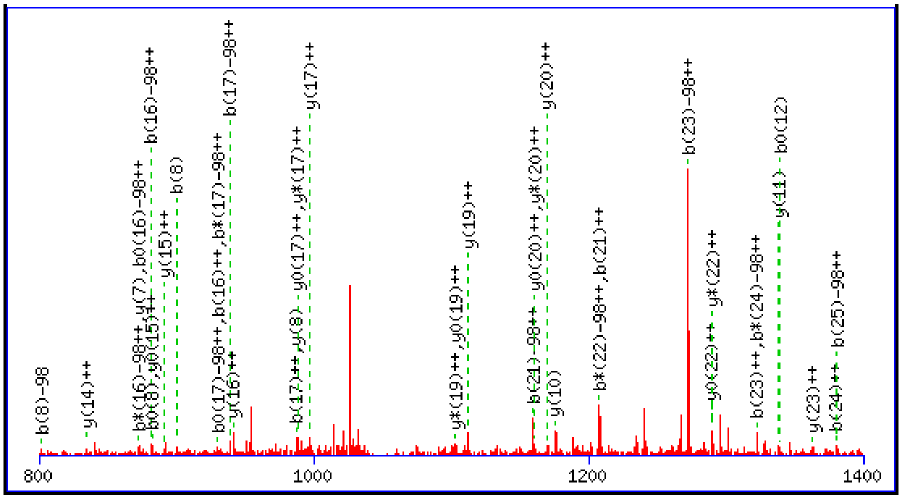

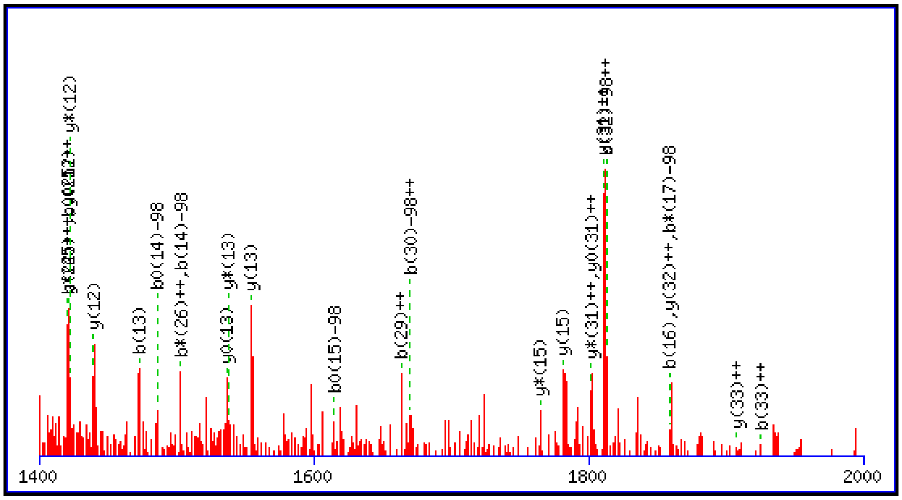

Monoisotopic mass of neutral peptide Mr(calc): 4195.8998
Fixed modifications: Carbamidomethyl (C)
Variable modifications:
S3 : Phospho (ST), with neutral losses 97.9769(shown in table), 0.0000
Ions Score: 47 **Expect:** 0.01
Matches (Bold Red): 89/626 fragment ions using 178 most intense peaks

#	b	b++	b*	b*++	b0	b0++	Seq.	y	y++	y*	y*++	y0	y0++	#
1	88.0393	44.5233			70.0287	35.5180	S							36
2	216.1343	108.5708	199.1077	100.0575	198.1237	99.5655	K	4011.8982	2006.4527	3994.8716	1997.9395	3993.8876	1997.4474	35
3	285.1557	143.0815	268.1292	134.5682	267.1452	134.0762	S	3883.8032	1942.4052	3866.7767	1933.8920	3865.7927	1933.4000	34
4	382.2085	191.6079	365.1819	183.0946	364.1979	182.6026	P	3814.7818	**1907.8945**	3797.7552	1899.3812	3796.7712	1898.8892	33
5	**479.2613**	240.1343	462.2347	231.6210	461.2507	231.1290	P	3717.7290	**1859.3681**	3700.7025	1850.8549	3699.7184	1850.3629	32
6	**576.3140**	288.6606	559.2875	280.1474	558.3035	279.6554	P	3620.6762	**1810.8418**	3603.6497	**1802.3285**	3602.6657	**1801.8365**	31
7	673.3668	337.1870	656.3402	328.6738	655.3562	328.1817	P	3523.6235	1762.3154	3506.5969	1753.8021	3505.6129	1753.3101	30
8	**802.4094**	401.7083	785.3828	393.1950	784.3988	392.7030	E	3426.5707	1713.7890	3409.5442	1705.2757	3408.5601	1704.7837	29
9	931.4520	466.2296	914.4254	**457.7163**	913.4414	**457.2243**	E	3297.5281	1649.2677	3280.5016	1640.7544	3279.5176	1640.2624	28
10	1060.4946	**530.7509**	1043.4680	522.2376	1042.4840	521.7456	E	3168.4855	1584.7464	3151.4590	1576.2331	3150.4750	1575.7411	27
11	1131.5317	566.2695	1114.5051	**557.7562**	1113.5211	**557.2642**	A	3039.4429	1520.2251	3022.4164	1511.7118	3021.4324	1511.2198	26
12	1259.6266	630.3170	1242.6001	621.8037	1241.6161	621.3117	K	2968.4058	1484.7065	2951.3793	1476.1933	2950.3953	1475.7013	25
13	1374.6536	**687.8304**	1357.6270	679.3172	1356.6430	678.8251	D	2840.3109	**1420.6591**	2823.2843	1412.1458	2822.3003	1411.6538	24
14	**1503.6962**	752.3517	1486.6696	743.8384	**1485.6856**	743.3464	E	2725.2839	**1363.1456**	2708.2574	1354.6323	2707.2733	1354.1403	23
15	1632.7388	816.8730	1615.7122	808.3597	**1614.7282**	807.8677	E	2596.2413	1298.6243	2579.2148	**1290.1110**	2578.2308	**1289.6190**	22
16	1761.7814	**881.3943**	1744.7548	**872.8810**	1743.7708	**872.3890**	E	2467.1987	1234.1030	2450.1722	1225.5897	2449.1882	1225.0977	21
17	1876.8083	**938.9078**	**1859.7818**	**930.3945**	1858.7977	**929.9025**	D	2338.1561	**1169.5817**	2321.1296	**1161.0684**	2320.1456	**1160.5764**	20
18	2004.8669	1002.9371	1987.8403	994.4238	1986.8563	993.9318	Q	2223.1292	**1112.0682**	2206.1026	**1103.5550**	2205.1186	**1103.0630**	19

**19**	2105.9146	1053.4609	2088.8880	1044.9476	2087.9040	1044.4556	**T**	2095.0706	1048.0389	2078.0441	1039.5257	2077.0600	1039.0337	**18**
**20**	2218.9986	1110.0029	2201.9721	1101.4897	2200.9881	1100.9977	**L**	1994.0229	**997.5151**	1976.9964	**989.0018**	1976.0124	**988.5098**	**17**
**21**	2318.0670	**1159.5372**	2301.0405	1151.0239	2300.0565	1150.5319	**V**	1880.9389	**940.9731**	1863.9123	932.4598	1862.9283	931.9678	**16**
**22**	2432.1100	1216.5586	2415.0834	**1208.0453**	2414.0994	1207.5533	**N**	**1781.8705**	**891.4389**	**1764.8439**	882.9256	1763.8599	**882.4336**	**15**
**23**	2545.1940	**1273.1007**	2528.1675	1264.5874	2527.1835	1264.0954	**L**	1667.8275	**834.4174**	1650.8010	825.9041	1649.8170	825.4121	**14**
**24**	2660.2210	1330.6141	2643.1944	**1322.1008**	2642.2104	1321.6088	**D**	**1554.7435**	777.8754	**1537.7169**	769.3621	**1536.7329**	768.8701	**13**
**25**	2761.2686	**1381.1380**	2744.2421	1372.6247	2743.2581	1372.1327	**T**	**1439.7165**	**720.3619**	**1422.6900**	711.8486	**1421.7060**	711.3566	**12**
**26**	2924.3320	1462.6696	2907.3054	1454.1564	2906.3214	1453.6643	**Y**	**1338.6688**	**669.8381**	1321.6423	661.3248	1320.6583	660.8328	**11**
**27**	3025.3797	1513.1935	3008.3531	1504.6802	3007.3691	1504.1882	**T**	**1175.6055**	**588.3064**	1158.5790	579.7931	1157.5949	579.3011	**10**
**28**	3112.4117	1556.7095	3095.3851	1548.1962	3094.4011	1547.7042	**S**	1074.5578	537.7826	1057.5313	529.2693	1056.5473	528.7773	**9**
**29**	3227.4386	1614.2230	3210.4121	1605.7097	3209.4281	1605.2177	**D**	**987.5258**	494.2665	970.4993	485.7533	969.5152	485.2613	**8**
**30**	3340.5227	**1670.7650**	3323.4961	1662.2517	3322.5121	1661.7597	**L**	**872.4989**	**436.7531**	855.4723	428.2398	854.4883	427.7478	**7**
**31**	3477.5816	1739.2944	3460.5551	1730.7812	3459.5710	1730.2892	**H**	**759.4148**	**380.2110**	742.3883	371.6978	741.4042	371.2058	**6**
**32**	3624.6500	**1812.8286**	3607.6235	1804.3154	3606.6395	1803.8234	**F**	**622.3559**	311.6816	605.3293	303.1683	604.3453	302.6763	**5**
**33**	3752.7086	1876.8579	3735.6820	1868.3447	3734.6980	1867.8527	**Q**	**475.2875**	238.1474	458.2609	229.6341	**457.2769**	229.1421	**4**
**34**	3865.7927	1933.4000	3848.7661	1924.8867	3847.7821	1924.3947	**I**	**347.2289**	174.1181	**330.2023**	165.6048	329.2183	165.1128	**3**
**35**	3952.8247	1976.9160	3935.7981	1968.4027	3934.8141	1967.9107	**S**	234.1448	117.5761	217.1183	109.0628	216.1343	108.5708	**2**
**36**							**K**	147.1128	74.0600	130.0863	65.5468			**1**

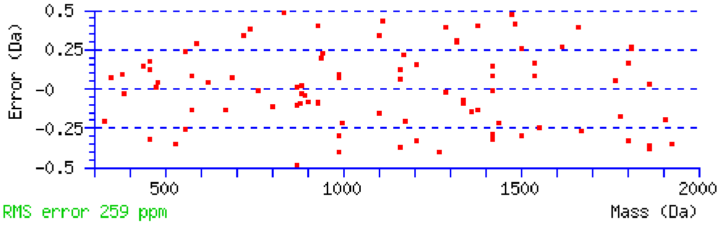

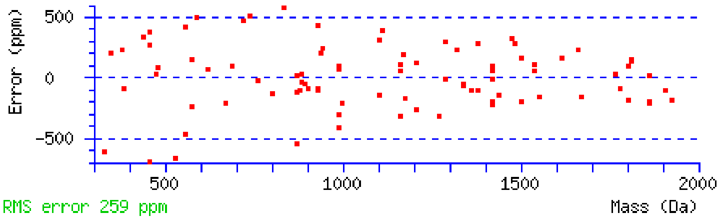

All matches to this query

Score	Mr(calc):	Delta	Sequence
46.9	4195.8998	2.0224	SKSPPPPEEEAKDEEEDQTLVNLDTYTSDLHFQISK
46.9	4195.8998	2.0224	SKSPPPPEEEAKDEEEDQTLVNLDTYTSDLHFQISK
41.4	4196.8838	1.0384	SKSPPPPEEEAKDEEEDQTLVNLDTYTSDLHFQISK
41.4	4196.8838	1.0384	SKSPPPPEEEAKDEEEDQTLVNLDTYTSDLHFQISK
22.9	4196.8838	1.0384	SKSPPPPEEEAKDEEEDQTLVNLDTYTSDLHFQISK
21.9	4195.8998	2.0224	SKSPPPPEEEAKDEEEDQTLVNLDTYTSDLHFQISK
6.2	4196.8838	1.0384	SKSPPPPEEEAKDEEEDQTLVNLDTYTSDLHFQISK
5.9	4195.8998	2.0224	SKSPPPPEEEAKDEEEDQTLVNLDTYTSDLHFQISK
5.2	4195.9105	2.0117	VMAENEYGIGEPTETTEPVRASEAPLPPDSLNIMDITK
4.5	4196.9139	1.0083	MATWNRPHPRLPVAPEPVAEGESQQPLGRELSEANR

Spectrum No: 366; Query: 2365; Rank: 1

Peptide View

MS/MS Fragmentation of **DMPGGFLFDGLSDDEDDFHPSTR**
Found in **IPI00191651**, Tax_Id=10116 Gene_Symbol=Bmsc-UbP Bone marrow stromal cell-derived ubiquitin-like protein

Match to Query 2365: 2665.034652 from(889.352160,3+)
Title: 100101RatKid_NS_deglyco_21.4568.4568.3.dta
Data file K:\NewmanPaper\Piliang\3SubProteomes\Piliang3SP\mgf5ppm\ERLIC_3SubProteomes5ppm.mgf

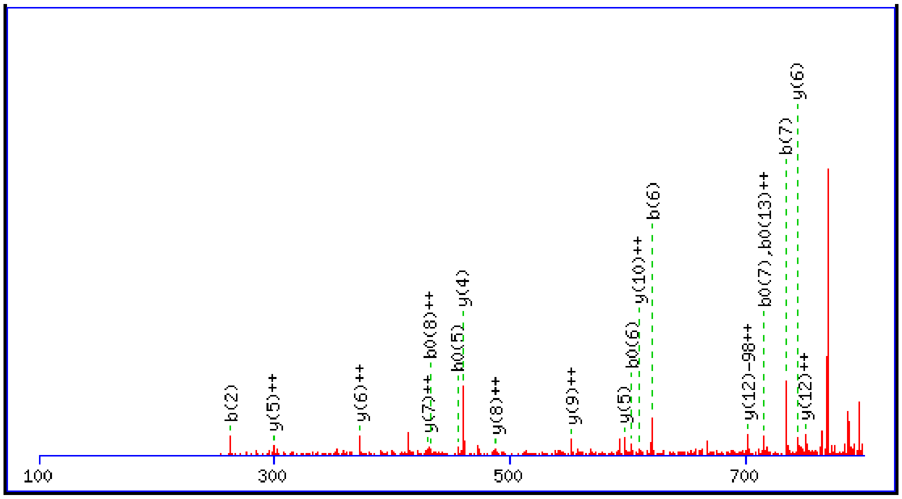

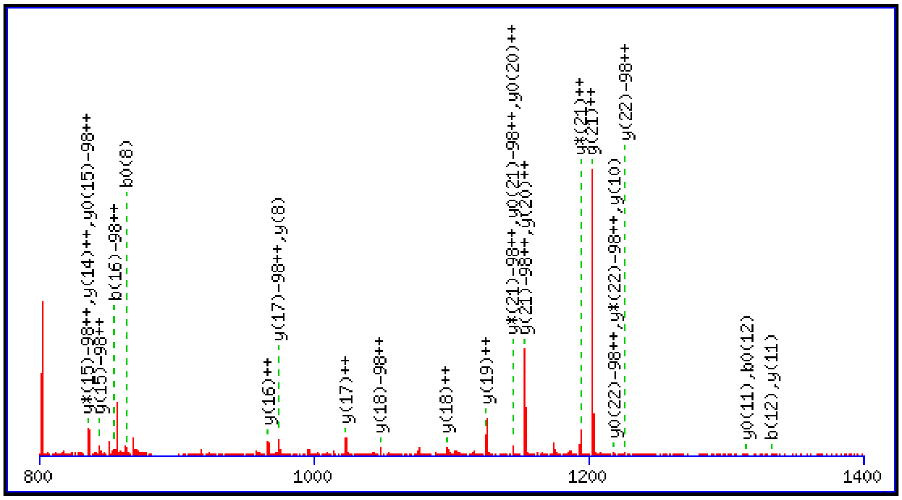

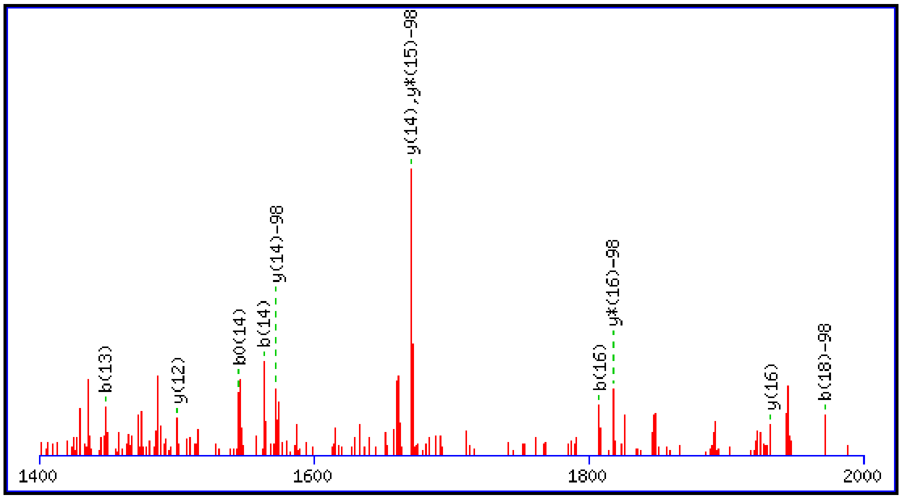

Monoisotopic mass of neutral peptide Mr(calc): 2665.0316
Fixed modifications: Carbamidomethyl (C)
Variable modifications:
M2 : Oxidation (M)
S12 : Phospho (ST), with neutral losses 0.0000(shown in table), 97.9769
Ions Score: 47 **Expect:** 0.0045
Matches (Bold Red): 58/328 fragment ions using 115 most intense peaks

#	b	b^{++}	b^{0}	b^{0++}	Seq.	y	y^{++}	y*	y^{*++}	y^{0}	y^{0++}	#
1	116.0342	58.5207	98.0237	49.5155	D							23
2	**263.0696**	132.0384	245.0591	123.0332	M	2551.0119	1276.0096	2533.9854	1267.4963	2533.0014	1267.0043	22
3	360.1224	180.5648	342.1118	171.5595	P	2403.9765	**1202.4919**	2386.9500	**1193.9786**	2385.9660	1193.4866	21
4	417.1439	209.0756	399.1333	200.0703	G	2306.9238	**1153.9655**	2289.8972	1145.4522	2288.9132	**1144.9602**	20

5	474.1653	237.5863	**456.1547**	228.5810	G	2249.9023	**1125.4548**	2232.8757	1116.9415	2231.8917	1116.4495	19
6	**621.2337**	311.1205	**603.2232**	302.1152	F	2192.8808	**1096.9441**	2175.8543	1088.4308	2174.8703	1087.9388	18
7	**734.3178**	367.6625	**716.3072**	358.6573	L	2045.8124	**1023.4098**	2028.7859	1014.8966	2027.8019	1014.4046	17
8	881.3862	441.1967	**863.3756**	**432.1915**	F	**1932.7284**	**966.8678**	1915.7018	958.3545	1914.7178	957.8625	16
9	996.4131	498.7102	978.4026	489.7049	D	1785.6599	893.3336	1768.6334	884.8203	1767.6494	884.3283	15
10	1053.4346	527.2209	1035.4240	518.2157	G	**1670.6330**	**835.8201**	1653.6064	827.3069	1652.6224	826.8149	14
11	1166.5187	583.7630	1148.5081	574.7577	L	1613.6115	807.3094	1596.5850	798.7961	1595.6010	798.3041	13
12	**1333.5170**	667.2622	**1315.5065**	658.2569	S	**1500.5275**	**750.7674**	1483.5009	742.2541	1482.5169	741.7621	12
13	**1448.5440**	724.7756	1430.5334	**715.7703**	D	**1333.5291**	667.2682	1316.5026	658.7549	**1315.5185**	658.2629	11
14	**1563.5709**	782.2891	**1545.5604**	773.2838	D	**1218.5022**	**609.7547**	1201.4756	601.2414	1200.4916	600.7494	10
15	1692.6135	846.8104	1674.6029	837.8051	E	1103.4752	**552.2413**	1086.4487	543.7280	1085.4647	543.2360	9
16	**1807.6405**	904.3239	1789.6299	895.3186	D	**974.4326**	**487.7200**	957.4061	479.2067	956.4221	478.7147	8
17	1922.6674	961.8373	1904.6568	952.8321	D	859.4057	**430.2065**	842.3791	421.6932	841.3951	421.2012	7
18	2069.7358	1035.3715	2051.7252	1026.3663	F	**744.3787**	**372.6930**	727.3522	364.1797	726.3682	363.6877	6
19	2206.7947	1103.9010	2188.7842	1094.8957	H	**597.3103**	**299.1588**	580.2838	290.6455	579.2998	290.1535	5
20	2303.8475	1152.4274	2285.8369	1143.4221	P	**460.2514**	230.6293	443.2249	222.1161	442.2409	221.6241	4
21	2390.8795	1195.9434	2372.8689	1186.9381	S	363.1987	182.1030	346.1721	173.5897	345.1881	173.0977	3
22	2491.9272	1246.4672	2473.9166	1237.4620	T	276.1666	138.5870	259.1401	130.0737	258.1561	129.5817	2
23					R	175.1190	88.0631	158.0924	79.5498			1

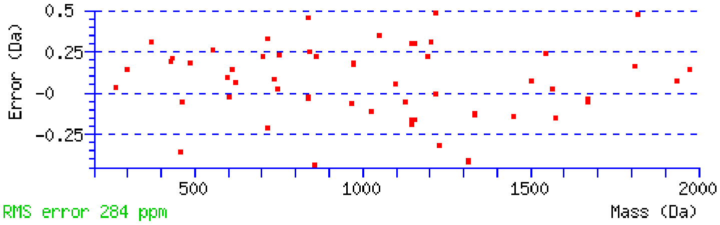

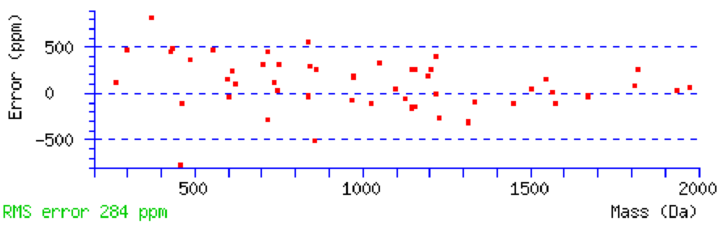

All matches to this query

Score	Mr(calc):	Delta	Sequence
46.9	2665.0316	0.0030	DMPGGFLFDGLSDDEDDFHPSTR
8.1	2665.0316	0.0030	DMPGGFLFDGLSDDEDDFHPSTR
8.1	2665.0316	0.0030	DMPGGFLFDGLSDDEDDFHPSTR
3.9	2663.0118	2.0229	DFKSNSSLYLKSDGNNDYLDK
3.9	2663.0118	2.0229	DFKSNSSLYLKSDGNNDYLDK
3.4	2663.0118	2.0229	DFKSNSSLYLKSDGNNDYLDK
3.3	2663.0118	2.0229	DFKSNSSLYLKSDGNNDYLDK
3.0	2664.0118	1.0229	DFETLYNDCTNASSRPGRPPK
3.0	2664.0118	1.0229	DFETLYNDCTNASSRPGRPPK
2.8	2663.0118	2.0229	DFKSNSSLYLKSDGNNDYLDK

Spectrum No: 367; Query: 2121; Rank: 1

Peptide View

MS/MS Fragmentation of **ASALFSSDEEDQWSVADSQTK**
Found in **IPI00331898**, Tax_Id=10116 Gene_Symbol=LOC297530 hypothetical protein LOC297530

Match to Query 2121: 2380.974608 from(1191.494580,2+)
Title: 091008RatKidney_NoSalt_27.3256.3256.2.dta
Data file K:\NewmanPaper\Piliang\3SubProteomes\Piliang3SP\mgf5ppm\ERLIC_3SubProteomes5ppm.mgf

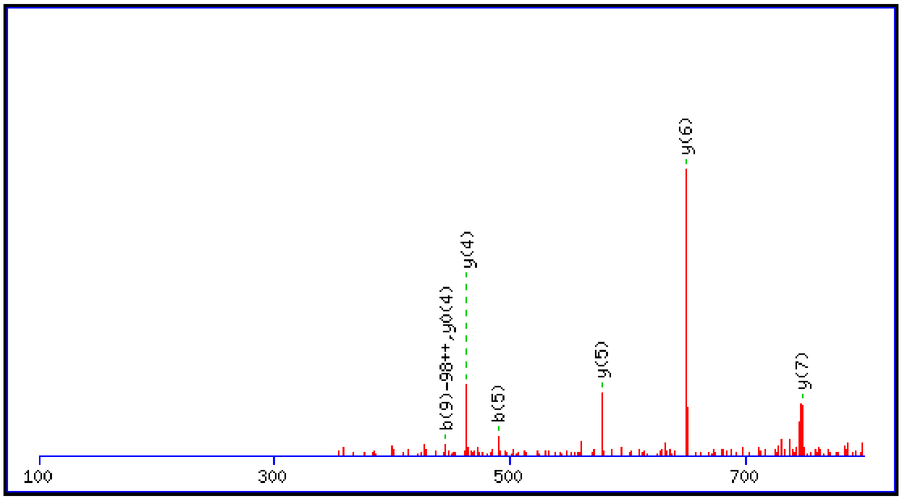

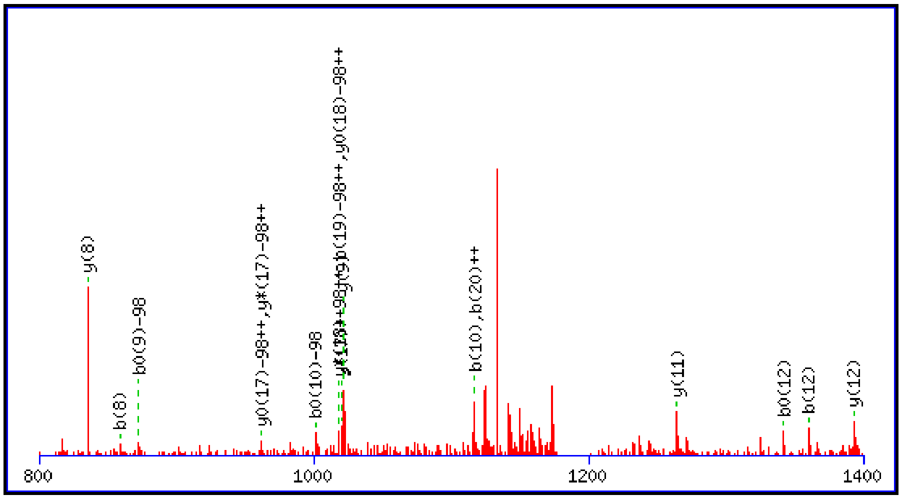

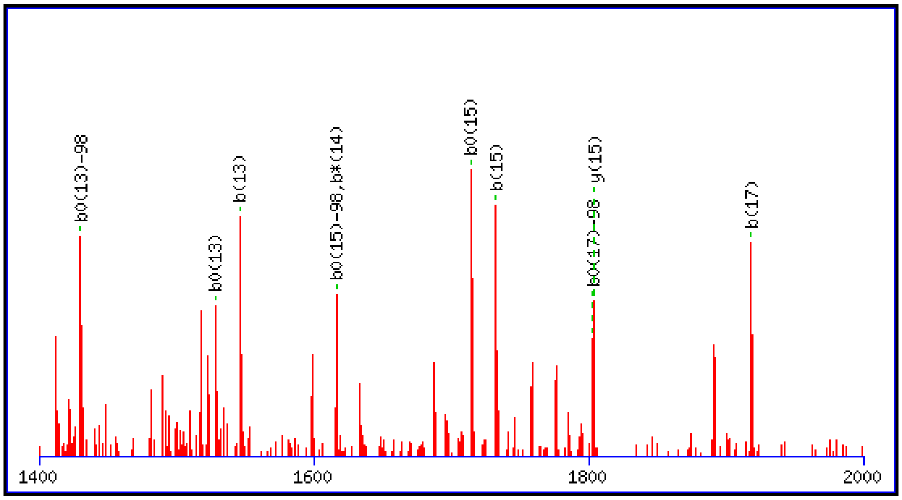

Monoisotopic mass of neutral peptide Mr(calc): 2379.9744
Fixed modifications: Carbamidomethyl (C)
Variable modifications:
S7 : Phospho (ST), with neutral losses 0.0000(shown in table), 97.9769
Ions Score: 47 **Expect:** 0.0051
Matches (Bold Red): 34/324 fragment ions using 42 most intense peaks

#	b	b^{++}	b*	b^{*++}	b^{0}	b^{0++}	Seq.	y	y^{++}	y*	y^{*++}	y^{0}	y^{0++}	#
1	72.0444	36.5258					A							21
2	159.0764	80.0418			141.0659	71.0366	S	2309.9446	1155.4759	2292.9180	1146.9626	2291.9340	1146.4706	20
3	230.1135	115.5604			212.1030	106.5551	A	2222.9125	1111.9599	2205.8860	1103.4466	2204.9020	1102.9546	19
4	343.1976	172.1024			325.1870	163.0972	L	2151.8754	1076.4413	2134.8489	1067.9281	2133.8648	1067.4361	18
5	**490.2660**	245.6366			472.2554	236.6314	F	2038.7913	**1019.8993**	2021.7648	1011.3860	2020.7808	1010.8940	17

file:///R|/wwwroot/SupportingData/3SubProteomes/ERLIC_3SubProteo_Phospho/ERLIC_3SubProteo_Phospho_002.html[4/27/2010 12:34:58 PM]

**6**	577.2980	289.1527			559.2875	280.1474	**S**	1891.7229	946.3651	1874.6964	937.8518	1873.7124	937.3598	**16**
**7**	744.2964	372.6518			726.2858	363.6466	**S**	**1804.6909**	902.8491	1787.6644	894.3358	1786.6803	893.8438	**15**
**8**	**859.3233**	430.1653			841.3128	421.1600	**D**	1637.6926	819.3499	1620.6660	810.8366	1619.6820	810.3446	**14**
**9**	988.3659	494.6866			970.3554	485.6813	**E**	1522.6656	761.8364	1505.6391	753.3232	1504.6550	752.8312	**13**
**10**	**1117.4085**	559.2079			1099.3980	550.2026	**E**	**1393.6230**	697.3151	1376.5965	688.8019	1375.6125	688.3099	**12**
**11**	1232.4355	616.7214			1214.4249	607.7161	**D**	**1264.5804**	632.7938	1247.5539	624.2806	1246.5699	623.7886	**11**
**12**	**1360.4940**	680.7507	1343.4675	672.2374	**1342.4835**	671.7454	**Q**	1149.5535	575.2804	1132.5269	566.7671	1131.5429	566.2751	**10**
**13**	**1546.5734**	773.7903	1529.5468	765.2770	**1528.5628**	764.7850	**W**	**1021.4949**	511.2511	1004.4684	502.7378	1003.4843	502.2458	**9**
**14**	1633.6054	817.3063	**1616.5788**	808.7931	1615.5948	808.3010	**S**	**835.4156**	418.2114	818.3890	409.6982	817.4050	409.2061	**8**
**15**	**1732.6738**	866.8405	1715.6472	858.3273	**1714.6632**	857.8353	**V**	**748.3836**	374.6954	731.3570	366.1821	730.3730	365.6901	**7**
**16**	1803.7109	902.3591	1786.6844	893.8458	1785.7003	893.3538	**A**	**649.3151**	325.1612	632.2886	316.6479	631.3046	316.1559	**6**
**17**	**1918.7379**	959.8726	1901.7113	951.3593	1900.7273	950.8673	**D**	**578.2780**	289.6427	561.2515	281.1294	560.2675	280.6374	**5**
**18**	2005.7699	1003.3886	1988.7433	994.8753	1987.7593	994.3833	**S**	**463.2511**	232.1292	446.2245	223.6159	**445.2405**	223.1239	**4**
**19**	2133.8285	1067.4179	2116.8019	1058.9046	2115.8179	1058.4126	**Q**	376.2191	188.6132	359.1925	180.0999	358.2085	179.6079	**3**
**20**	2234.8761	**1117.9417**	2217.8496	1109.4284	2216.8656	1108.9364	**T**	248.1605	124.5839	231.1339	116.0706	230.1499	115.5786	**2**
**21**							**K**	147.1128	74.0600	130.0863	65.5468			**1**

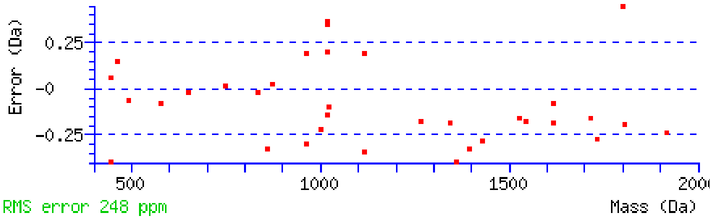

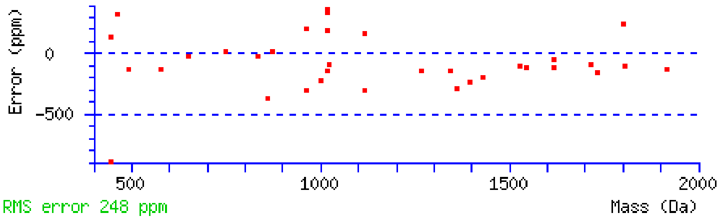

All matches to this query

Score	Mr(calc):	Delta	Sequence
46.9	2379.9744	1.0002	ASALFSSDEEDQWSVADSQTK
37.0	2379.9744	1.0002	ASALFSSDEEDQWSVADSQTK
35.0	2379.9744	1.0002	ASALFSSDEEDQWSVADSQTK
9.4	2379.9744	1.0002	ASALFSSDEEDQWSVADSQTK
6.4	2379.9509	1.0237	EIQESSVEFAPSDHSDKSTK
4.7	2379.9509	1.0237	EIQESSVEFAPSDHSDKSTK
2.7	2379.9509	1.0237	EIQESSVEFAPSDHSDKSTK
2.6	2380.9679	0.0067	KGMFSMGWPAFLSITPNIK
2.1	2379.9744	1.0002	ASALFSSDEEDQWSVADSQTK
1.5	2379.9572	1.0174	RGTIYGLLPSMTPTSSSSXR

Spectrum No: 368; Query: 2173; Rank: 1

Peptide View

MS/MS Fragmentation of **EVEDKESEGEEEDEDEDLSK**
Found in **IPI00214258**, Tax_Id=10116 Gene_Symbol=Zfp265 Zinc finger Ran-binding domain-containing protein 2

Match to Query 2173: 2418.898568 from(1210.456560,2+)
Title: 091008RatKidney_NoSalt_29.838.838.2.dta
Data file K:\NewmanPaper\Piliang\3SubProteomes\Piliang3SP\mgf5ppm\ERLIC_3SubProteomes5ppm.mgf

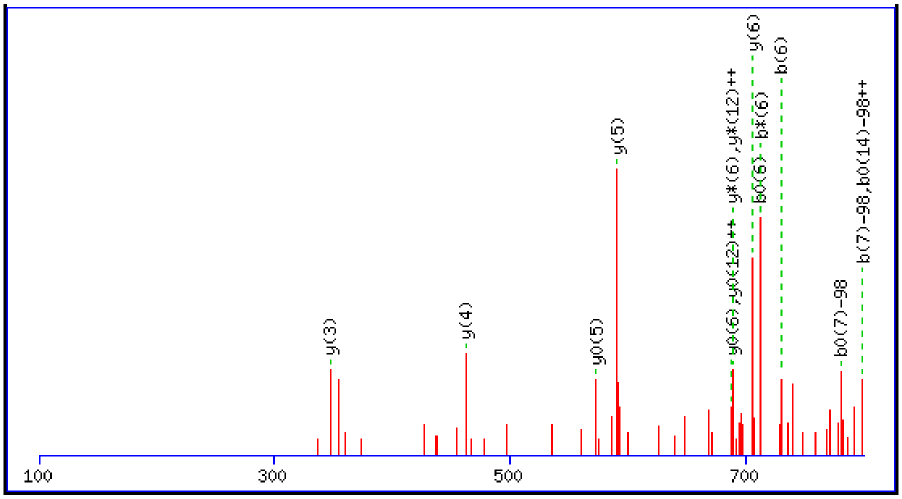

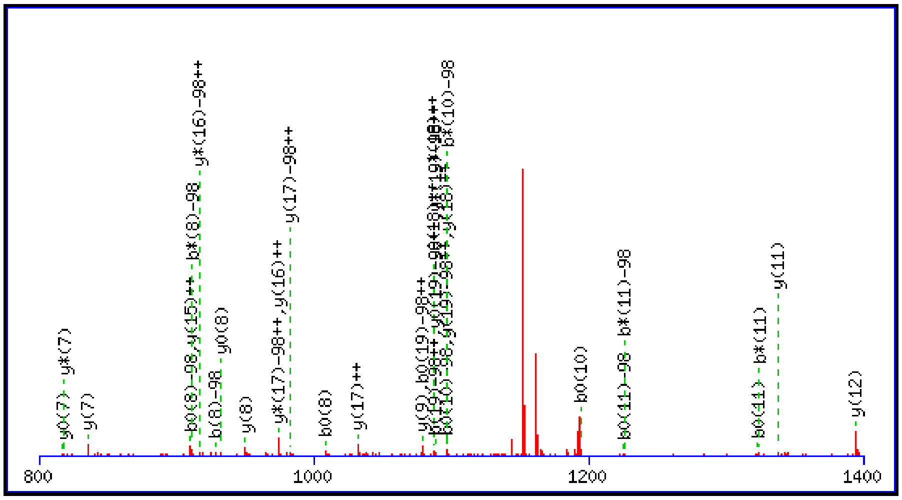

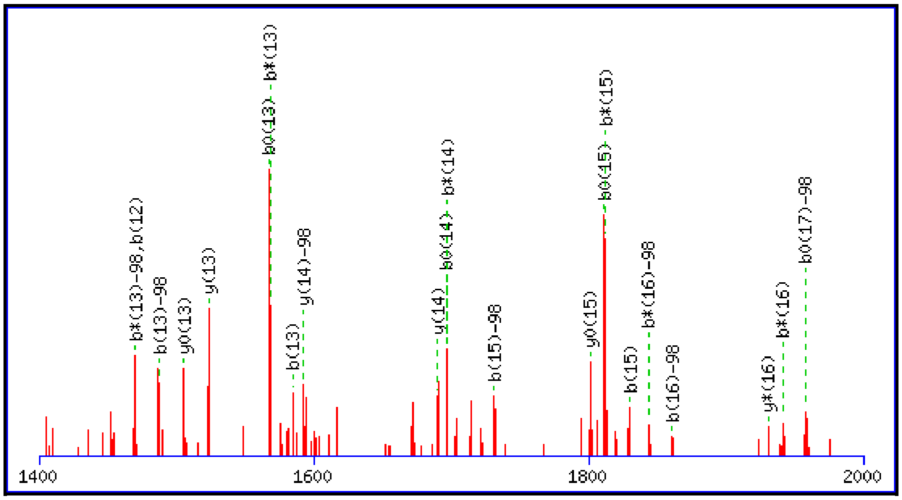

Monoisotopic mass of neutral peptide Mr(calc): 2418.8959
Fixed modifications: Carbamidomethyl (C)
Variable modifications:
S7 : Phospho (ST), with neutral losses 97.9769(shown in table), 0.0000
Ions Score: 47 **Expect:** 0.0032
Matches (Bold Red): 70/332 fragment ions using 91 most intense peaks

#	b	b++	b*	b*++	b0	b0++	Seq.	y	y++	y*	y*++	y0	y0++	#
1	130.0499	65.5286			112.0393	56.5233	E							20
2	229.1183	115.0628			211.1077	106.0575	V	2192.8837	**1096.9455**	2175.8572	**1088.4322**	2174.8731	**1087.9402**	19
3	358.1609	179.5841			340.1503	170.5788	E	2093.8153	1047.4113	2076.7887	1038.8980	2075.8047	1038.4060	18
4	473.1878	237.0975			455.1773	228.0923	D	1964.7727	**982.8900**	1947.7462	**974.3767**	1946.7621	973.8847	17
5	601.2828	301.1450	584.2562	292.6318	583.2722	292.1397	K	1849.7458	925.3765	1832.7192	**916.8632**	1831.7352	916.3712	16

6	**730.3254**	365.6663	**713.2988**	357.1531	**712.3148**	356.6610	E	1721.6508	861.3290	1704.6242	852.8158	1703.6402	852.3238	15
7	**799.3468**	400.1771	782.3203	391.6638	**781.3363**	391.1718	S	**1592.6082**	796.8077	1575.5817	788.2945	1574.5976	787.8025	14
8	**928.3894**	464.6984	**911.3629**	456.1851	**910.3789**	455.6931	E	**1523.5867**	762.2970	1506.5602	753.7837	**1505.5762**	753.2917	13
9	985.4109	493.2091	968.3843	484.6958	967.4003	484.2038	G	**1394.5442**	697.7757	1377.5176	**689.2624**	1376.5336	**688.7704**	12
10	1114.4535	557.7304	**1097.4269**	549.2171	**1096.4429**	548.7251	E	**1337.5227**	669.2650	1320.4961	660.7517	1319.5121	660.2597	11
11	1243.4961	622.2517	**1226.4695**	613.7384	**1225.4855**	613.2464	E	1208.4801	604.7437	1191.4535	596.2304	1190.4695	595.7384	10
12	1372.5387	686.7730	1355.5121	678.2597	1354.5281	677.7677	E	**1079.4375**	540.2224	1062.4110	531.7091	1061.4269	531.2171	9
13	**1487.5656**	744.2864	**1470.5391**	735.7732	1469.5550	735.2812	D	**950.3949**	475.7011	933.3684	467.1878	**932.3843**	466.6958	8
14	1616.6082	808.8077	1599.5817	800.2945	1598.5976	**799.8025**	E	**835.3680**	418.1876	**818.3414**	409.6743	**817.3574**	409.1823	7
15	**1731.6351**	866.3212	1714.6086	857.8079	1713.6246	857.3159	D	**706.3254**	353.6663	**689.2988**	345.1531	**688.3148**	344.6610	6
16	**1860.6777**	930.8425	**1843.6512**	922.3292	1842.6672	921.8372	E	**591.2984**	296.1529	574.2719	287.6396	**573.2879**	287.1476	5
17	1975.7047	988.3560	1958.6781	979.8427	**1957.6941**	979.3507	D	**462.2558**	231.6316	445.2293	223.1183	444.2453	222.6263	4
18	2088.7887	1044.8980	2071.7622	1036.3847	2070.7782	1035.8927	L	**347.2289**	174.1181	330.2023	165.6048	329.2183	165.1128	3
19	2175.8208	**1088.4140**	2158.7942	1079.9008	2157.8102	**1079.4087**	S	234.1448	117.5761	217.1183	109.0628	216.1343	108.5708	2
20							K	147.1128	74.0600	130.0863	65.5468			1

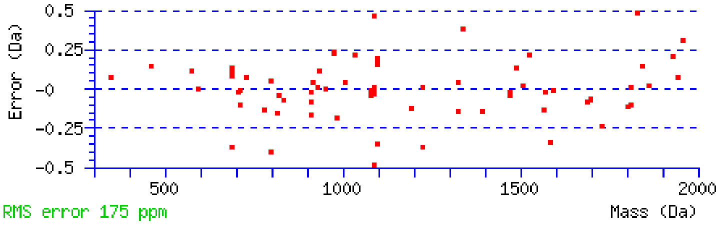

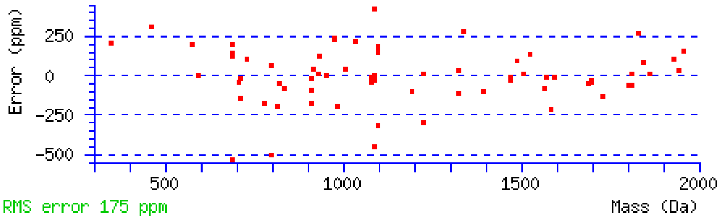

All matches to this query

Score	Mr(calc):	Delta	Sequence
46.8	2418.8959	0.0027	EVEDKESEGEEEDEDEDLSK
7.7	2418.8959	0.0027	EVEDKESEGEEEDEDEDLSK
6.3	2418.8770	0.0216	FSFDQFSSACHDVNECSSSK
3.5	2418.8770	0.0216	FSFDQFSSACHDVNECSSSK
3.4	2417.8930	1.0056	FSFDQFSSACHDVNECSSSK
3.3	2418.8770	0.0216	FSFDQFSSACHDVNECSSSK
2.9	2416.9055	1.9931	ITGYYGYYPGYSSQKSTSR
2.3	2418.8826	0.0160	DYNEAYNYYTKAIDMCPK
2.3	2418.8826	0.0160	DYNEAYNYYTKAIDMCPK
2.3	2418.8826	0.0160	DYNEAYNYYTKAIDMCPK

Spectrum No: 369; Query: 1125; Rank: 1

Peptide View

MS/MS Fragmentation of **AELGMNDSPSQSPPVK**
Found in **IPI00869599**, Tax_Id=10116 Gene_Symbol=Acss2_predicted acyl-CoA synthetase short-chain family member 2

Match to Query 1125: 1735.747548 from(868.881050,2+)
Title: 091008RatKidney_NoSalt_22.1506.1506.2.dta
Data file K:\NewmanPaper\Piliang\3SubProteomes\Piliang3SP\mgf5ppm\ERLIC_3SubProteomes5ppm.mgf

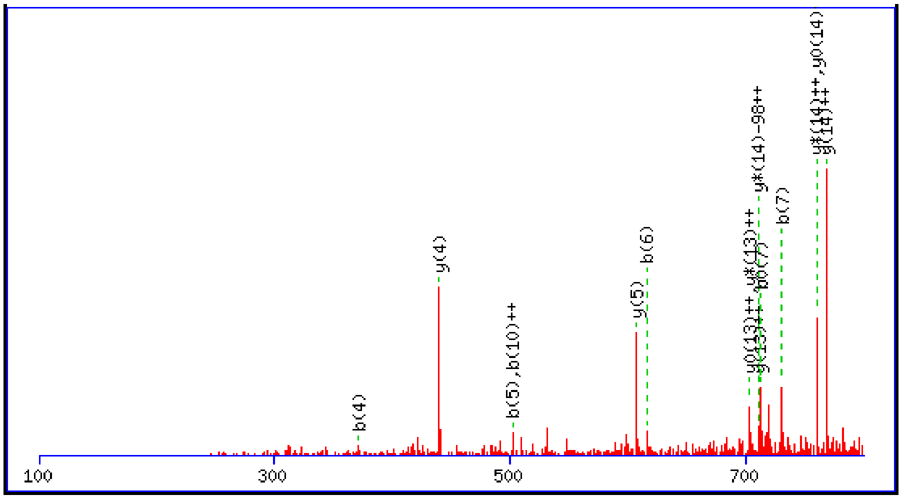

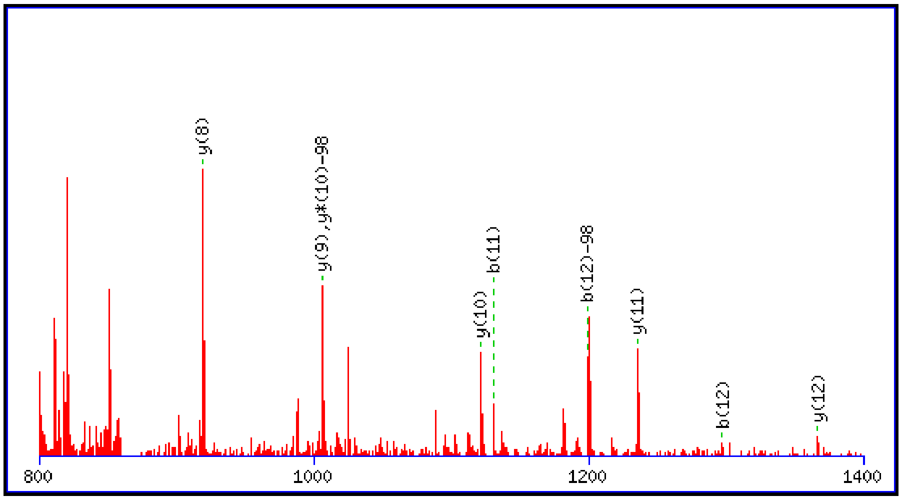

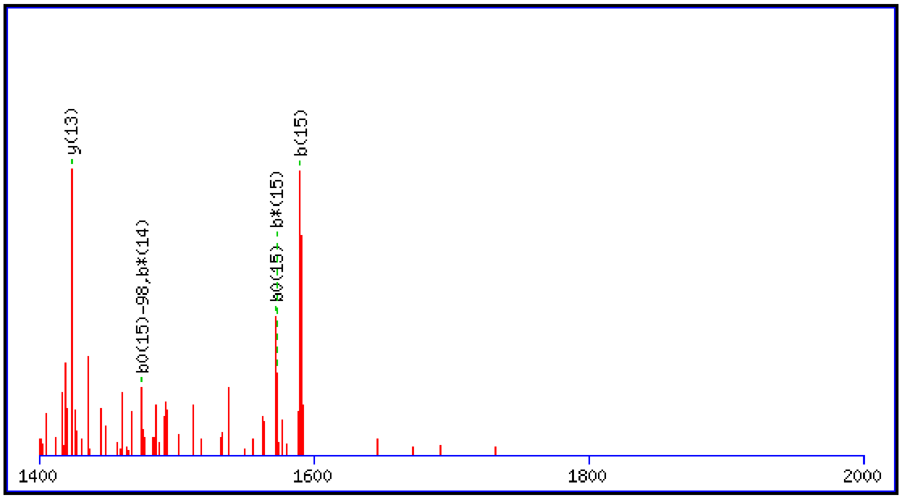

Monoisotopic mass of neutral peptide Mr(calc): 1735.7488
Fixed modifications: Carbamidomethyl (C)
Variable modifications:
S12 : Phospho (ST), with neutral losses 0.0000(shown in table), 97.9769
Ions Score: 47 **Expect:** 0.0039
Matches (Bold Red): 30/250 fragment ions using 45 most intense peaks

#	b	b^{++}	b*	b^{*++}	b^0	b^{0++}	Seq.	y	y^{++}	y*	y^{*++}	y^0	y^{0++}	#
1	72.0444	36.5258					A							16
2	201.0870	101.0471			183.0764	92.0418	E	1665.7190	833.3631	1648.6924	824.8498	1647.7084	824.3578	15
3	314.1710	157.5892			296.1605	148.5839	L	1536.6764	**768.8418**	1519.6498	**760.3286**	1518.6658	**759.8365**	14
4	**371.1925**	186.0999			353.1819	177.0946	G	**1423.5923**	**712.2998**	1406.5658	**703.7865**	1405.5818	**703.2945**	13
5	**502.2330**	251.6201			484.2224	242.6149	M	**1366.5709**	683.7891	1349.5443	675.2758	1348.5603	674.7838	12

**6**	**616.2759**	308.6416	599.2494	300.1283	598.2654	299.6363	**N**	**1235.5304**	618.2688	1218.5038	609.7555	1217.5198	609.2635	**11**
**7**	**731.3029**	366.1551	714.2763	357.6418	**713.2923**	357.1498	**D**	**1121.4874**	561.2474	1104.4609	552.7341	1103.4769	552.2421	**10**
**8**	818.3349	409.6711	801.3083	401.1578	800.3243	400.6658	**S**	**1006.4605**	503.7339	989.4339	495.2206	988.4499	494.7286	**9**
**9**	915.3877	458.1975	898.3611	449.6842	897.3771	449.1922	**P**	**919.4285**	460.2179	902.4019	451.7046	901.4179	451.2126	**8**
**10**	1002.4197	**501.7135**	985.3931	493.2002	984.4091	492.7082	**S**	822.3757	411.6915	805.3492	403.1782	804.3651	402.6862	**7**
**11**	**1130.4783**	565.7428	1113.4517	557.2295	1112.4677	556.7375	**Q**	735.3437	368.1755	718.3171	359.6622	717.3331	359.1702	**6**
**12**	**1297.4766**	649.2419	1280.4501	640.7287	1279.4661	640.2367	**S**	**607.2851**	304.1462	590.2585	295.6329	589.2745	295.1409	**5**
**13**	1394.5294	697.7683	1377.5028	689.2551	1376.5188	688.7630	**P**	**440.2867**	220.6470	423.2602	212.1337			**4**
**14**	1491.5821	746.2947	**1474.5556**	737.7814	1473.5716	737.2894	**P**	343.2340	172.1206	326.2074	163.6074			**3**
**15**	**1590.6506**	795.8289	**1573.6240**	787.3156	**1572.6400**	786.8236	**V**	246.1812	123.5942	229.1547	115.0810			**2**
**16**							**K**	147.1128	74.0600	130.0863	65.5468			**1**

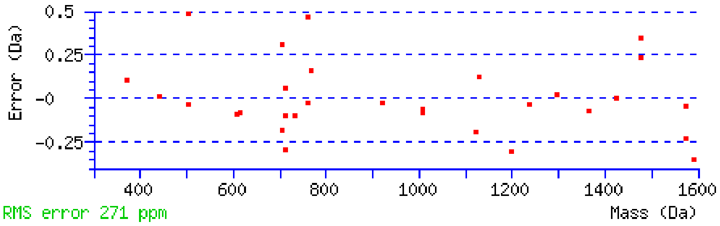

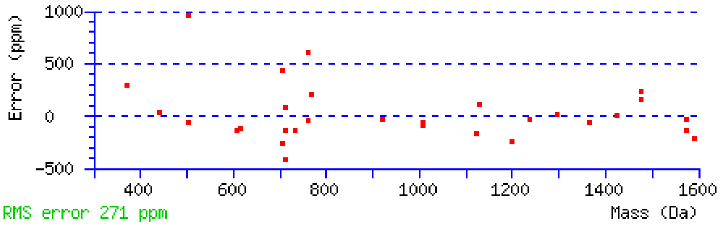

All matches to this query

Score	Mr(calc):	Delta	Sequence
46.7	1735.7488	-0.0012	AELGMNDSPSQSPPVK
35.7	1735.7488	-0.0012	AELGMNDSPSQSPPVK
26.9	1735.7488	-0.0012	AELGMNDSPSQSPPVK
10.4	1735.7349	0.0127	NIHCVTNSNPDRTK
0.3	1735.7430	0.0045	VTSAVEALLSADSASR
0.1	1735.7349	0.0127	NIHCVTNSNPDRTK
0.1	1733.7410	2.0066	SGYKRLNWNSLGNGS

Spectrum No: 370; Query: 1398; Rank: 1

Peptide View

MS/MS Fragmentation of **SLSTSGESLYHVLGLDK**
Found in **IPI00210881**, Tax_Id=10116 Gene_Symbol=Dnajc5 DnaJ homolog subfamily C member 5

Match to Query 1398: 1884.882128 from(943.448340,2+)
Title: 091008RatKidney_NH4Format01_12.4844.4844.2.dta
Data file K:\NewmanPaper\Piliang\3SubProteomes\Piliang3SP\mgf5ppm\ERLIC_3SubProteomes5ppm.mgf

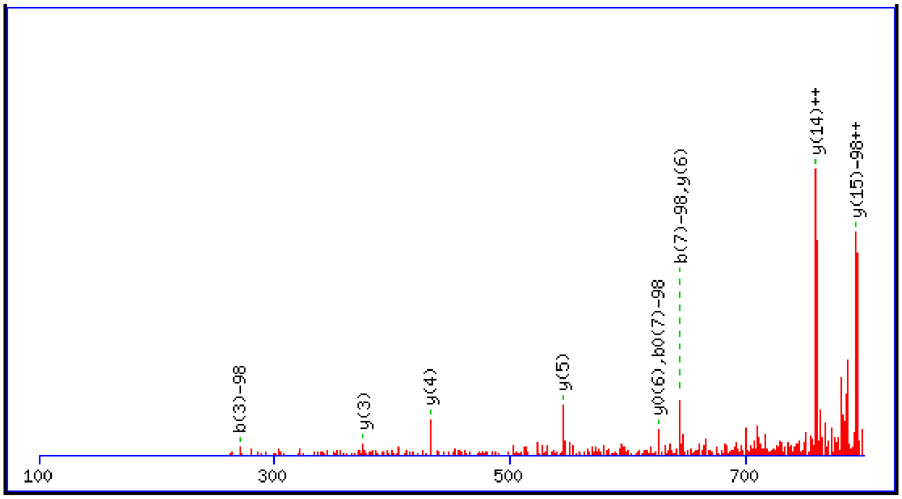

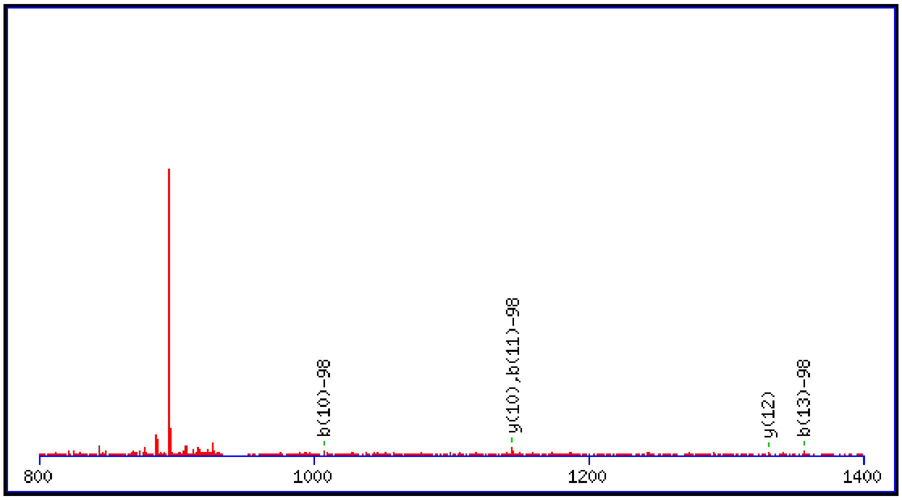

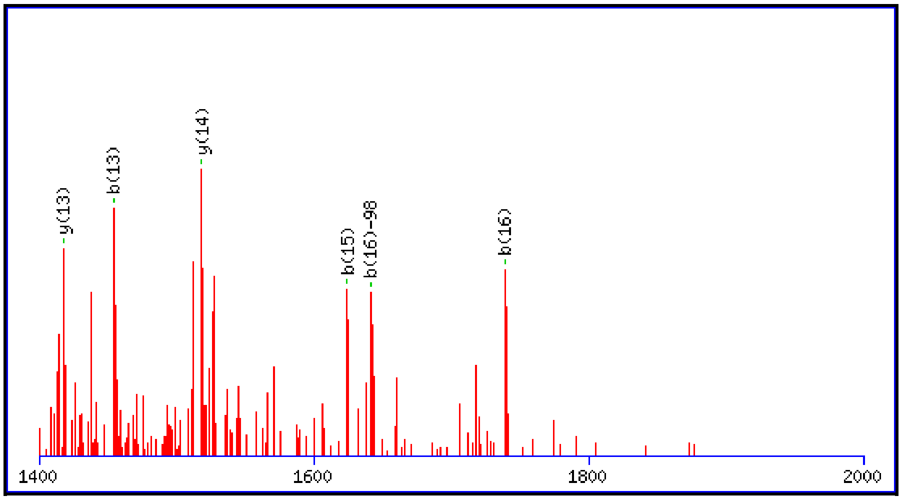

Monoisotopic mass of neutral peptide Mr(calc): 1884.8870
Fixed modifications: Carbamidomethyl (C)
Variable modifications:
S3 : Phospho (ST), with neutral losses 97.9769(shown in table), 0.0000
Ions Score: 47 **Expect:** 0.0051
Matches (Bold Red): 21/226 fragment ions using 47 most intense peaks

#	b	b^{++}	b^0	b^{0++}	Seq.	y	y^{++}	y^*	y^{*++}	y^0	y^{0++}	#
1	88.0393	44.5233	70.0287	35.5180	S							17
2	201.1234	101.0653	183.1128	92.0600	L	1700.8854	850.9463	1683.8588	842.4330	1682.8748	841.9410	16
3	**270.1448**	135.5760	252.1343	126.5708	S	1587.8013	**794.4043**	1570.7748	785.8910	1569.7907	785.3990	15
4	371.1925	186.0999	353.1819	177.0946	T	**1518.7799**	**759.8936**	1501.7533	751.3803	1500.7693	750.8883	14
5	458.2245	229.6159	440.2140	220.6106	S	**1417.7322**	709.3697	1400.7056	700.8564	1399.7216	700.3644	13

6	515.2460	258.1266	497.2354	249.1214	G	**1330.7001**	665.8537	1313.6736	657.3404	1312.6896	656.8484	12
7	**644.2886**	322.6479	**626.2780**	313.6426	E	1273.6787	637.3430	1256.6521	628.8297	1255.6681	628.3377	11
8	731.3206	366.1639	713.3101	357.1587	S	**1144.6361**	572.8217	1127.6095	564.3084	1126.6255	563.8164	10
9	844.4047	422.7060	826.3941	413.7007	L	1057.6041	529.3057	1040.5775	520.7924	1039.5935	520.3004	9
10	**1007.4680**	504.2376	989.4574	495.2324	Y	944.5200	472.7636	927.4934	464.2504	926.5094	463.7584	8
11	**1144.5269**	572.7671	1126.5164	563.7618	H	781.4567	391.2320	764.4301	382.7187	763.4461	382.2267	7
12	1243.5953	622.3013	1225.5848	613.2960	V	**644.3978**	322.7025	627.3712	314.1892	**626.3872**	313.6972	6
13	**1356.6794**	678.8433	1338.6688	669.8381	L	**545.3293**	273.1683	528.3028	264.6550	527.3188	264.1630	5
14	1413.7009	707.3541	1395.6903	698.3488	G	**432.2453**	216.6263	415.2187	208.1130	414.2347	207.6210	4
15	1526.7849	763.8961	1508.7744	754.8908	L	**375.2238**	188.1155	358.1973	179.6023	357.2132	179.1103	3
16	**1641.8119**	821.4096	1623.8013	812.4043	D	262.1397	131.5735	245.1132	123.0602	244.1292	122.5682	2
17					K	147.1128	74.0600	130.0863	65.5468			1

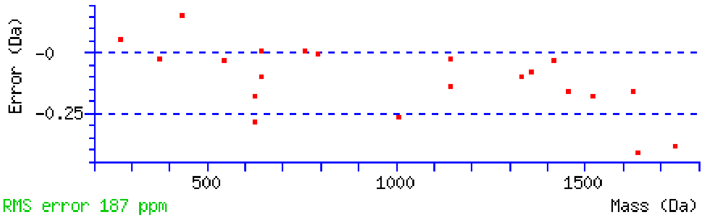

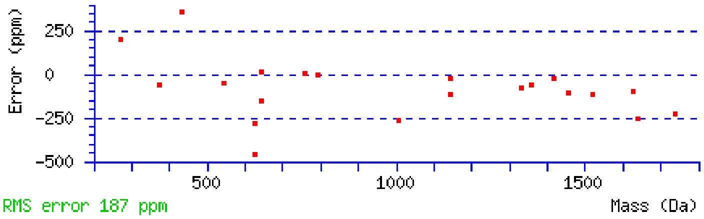

All matches to this query

Score	Mr(calc):	Delta	Sequence
46.6	1884.8870	-0.0049	SLSTSGESLYHVLGLDK
45.1	1884.8870	-0.0049	SLSTSGESLYHVLGLDK
33.5	1884.8870	-0.0049	SLSTSGESLYHVLGLDK
27.1	1884.8870	-0.0049	SLSTSGESLYHVLGLDK
14.6	1884.8870	-0.0049	SLSTSGESLYHVLGLDK
9.0	1884.8692	0.0129	TICGTPNYLSPEVLNK
8.2	1884.8699	0.0122	RLERACSLNNCTIAK
7.7	1884.8952	-0.0131	IDGAEPLTPQESEEKDK
7.7	1884.8991	-0.0170	FNVATTARMSCLPPLK
5.8	1884.8692	0.0129	TICGTPNYLSPEVLNK

Spectrum No: 371; Query: 2306; Rank: 1

Peptide View

MS/MS Fragmentation of **EDDGVEDNEKNFYDSDDEEKK**
Found in **IPI00360117**, Tax_Id=10116 Gene_Symbol=Ap3b1_predicted adaptor-related protein complex 3, beta 1 subunit

Match to Query 2306: 2598.986232 from(867.336020,3+)
Title: 100101RatKid_NS_deglyco_23.1275.1275.3.dta
Data file K:\NewmanPaper\Piliang\3SubProteomes\Piliang3SP\mgf5ppm\ERLIC_3SubProteomes5ppm.mgf

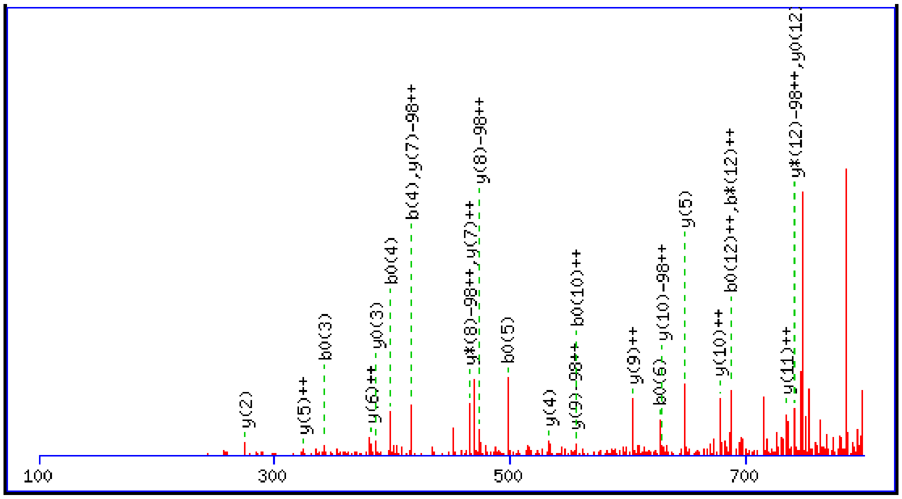

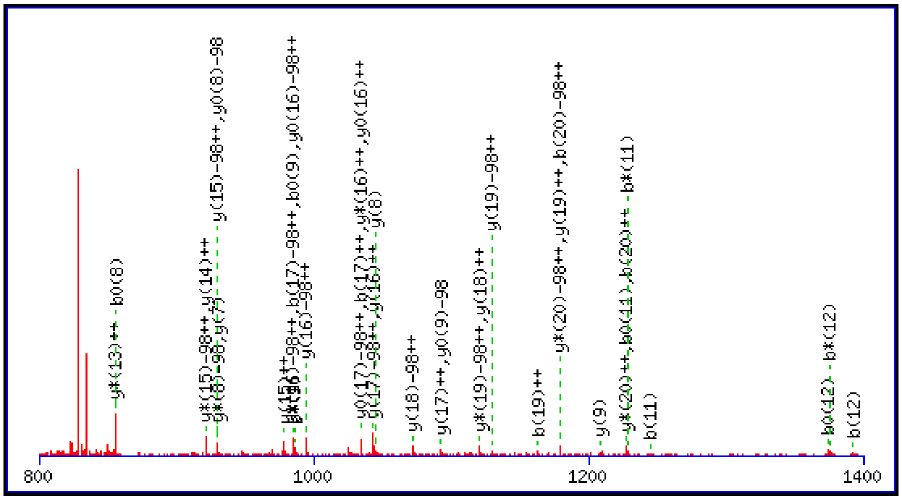

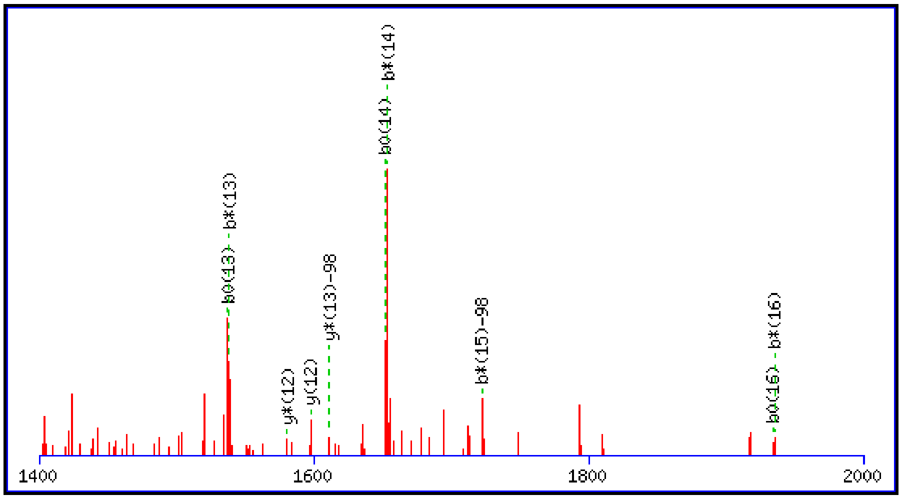

Monoisotopic mass of neutral peptide Mr(calc): 2598.9759
Fixed modifications: Carbamidomethyl (C)
Variable modifications:
S15 : Phospho (ST), with neutral losses 0.0000(shown in table), 97.9769
Ions Score: 47 **Expect:** 0.0048
Matches (Bold Red): 76/342 fragment ions using 105 most intense peaks

#	b	b^{++}	b*	b^{*++}	b^0	b^{0++}	Seq.	y	y^{++}	y*	y^{*++}	y^0	y^{0++}	#
1	130.0499	65.5286			112.0393	56.5233	E							21
2	245.0768	123.0420			227.0662	114.0368	D	2470.9406	1235.9739	2453.9140	**1227.4607**	2452.9300	1226.9686	20
3	360.1038	180.5555			**342.0932**	171.5502	D	2355.9136	**1178.4605**	2338.8871	1169.9472	2337.9031	1169.4552	19
4	**417.1252**	209.0662			**399.1147**	200.0610	G	2240.8867	**1120.9470**	2223.8602	1112.4337	2222.8761	1111.9417	18
5	516.1936	258.6005			**498.1831**	249.5952	V	2183.8652	**1092.4363**	2166.8387	1083.9230	2165.8547	1083.4310	17

6	645.2362	323.1218			**627.2257**	314.1165	E	2084.7968	**1042.9020**	2067.7703	**1034.3888**	2066.7863	**1033.8968**	16
7	760.2632	380.6352			742.2526	371.6299	D	1955.7542	**978.3808**	1938.7277	969.8675	1937.7437	969.3755	15
8	874.3061	437.6567	857.2795	429.1434	**856.2955**	428.6514	N	1840.7273	**920.8673**	1823.7007	912.3540	1822.7167	911.8620	14
9	1003.3487	502.1780	**986.3221**	493.6647	**985.3381**	493.1727	E	1726.6844	863.8458	1709.6578	**855.3325**	1708.6738	854.8405	13
10	1131.4437	566.2255	1114.4171	557.7122	1113.4331	**557.2202**	K	**1597.6418**	799.3245	**1580.6152**	790.8112	1579.6312	790.3192	12
11	**1245.4866**	623.2469	**1228.4600**	614.7337	**1227.4760**	614.2416	N	1469.5468	**735.2770**	1452.5203	726.7638	1451.5362	726.2718	11
12	**1392.5550**	696.7811	**1375.5284**	**688.2679**	**1374.5444**	**687.7759**	F	1355.5039	**678.2556**	1338.4773	669.7423	1337.4933	669.2503	10
13	1555.6183	778.3128	**1538.5918**	769.7995	**1537.6078**	769.3075	Y	**1208.4355**	**604.7214**	1191.4089	596.2081	1190.4249	595.7161	9
14	1670.6453	835.8263	**1653.6187**	827.3130	**1652.6347**	826.8210	D	**1045.3721**	523.1897	1028.3456	514.6764	1027.3616	514.1844	8
15	1837.6436	919.3254	1820.6171	910.8122	1819.6331	910.3202	S	**930.3452**	**465.6762**	913.3186	457.1630	912.3346	456.6710	7
16	1952.6706	976.8389	**1935.6440**	968.3256	**1934.6600**	967.8336	D	763.3468	**382.1771**	746.3203	373.6638	745.3363	373.1718	6
17	2067.6975	**1034.3524**	2050.6710	1025.8391	2049.6869	1025.3471	D	**648.3199**	**324.6636**	631.2933	316.1503	630.3093	315.6583	5
18	2196.7401	1098.8737	2179.7135	1090.3604	2178.7295	1089.8684	E	**533.2930**	267.1501	516.2664	258.6368	515.2824	258.1448	4
19	2325.7827	**1163.3950**	2308.7561	1154.8817	2307.7721	1154.3897	E	404.2504	202.6288	387.2238	194.1155	**386.2398**	193.6235	3
20	2453.8777	**1227.4425**	2436.8511	1218.9292	2435.8671	1218.4372	K	**275.2078**	138.1075	258.1812	129.5942			2
21							K	147.1128	74.0600	130.0863	65.5468			1

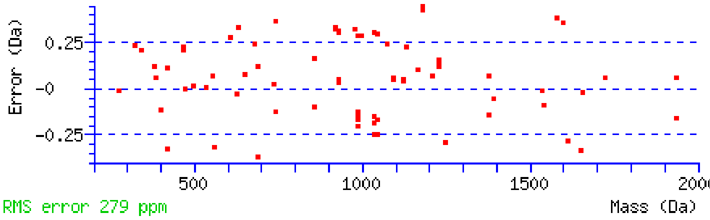

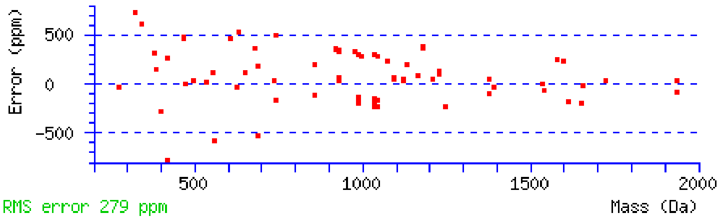

All matches to this query

Score	Mr(calc):	Delta	Sequence
46.6	2598.9759	0.0104	EDDGVEDNEKNFYDSDDEEKK
36.0	2598.9759	0.0104	EDDGVEDNEKNFYDSDDEEKK
2.4	2597.9917	0.9945	ENDQATHFCNICLSTTEDRK
2.4	2598.9757	0.0105	ENDQATHFCNICLSTTEDRK
2.4	2598.9757	0.0105	ENDQATHFCNICLSTTEDRK
2.1	2597.9722	1.0140	NSTYSRSSVDMLYSFAKCSR
2.1	2597.9722	1.0140	NSTYSRSSVDMLYSFAKCSR
2.1	2597.9722	1.0140	NSTYSRSSVDMLYSFAKCSR
2.1	2596.9795	2.0068	SPVFLSAAAGSSTSSSLCDAATNR
2.1	2597.9870	0.9993	NSEYNSSTCSLSSSENPYATIK

Spectrum No: 372; Query: 2650; Rank: 1

Peptide View

MS/MS Fragmentation of **WVEENIPSSFTDVALPVLVDSDEEIMTR**
Found in **IPI00357921**, Tax_Id=10116 Gene_Symbol=RGD1307907_predicted similar to CG17660-PA

Match to Query 2650: 3270.515682 from(1091.179170,3+)
Title: 100101RatKid_NS_deglyco_20.6062.6062.3.dta
Data file K:\NewmanPaper\Piliang\3SubProteomes\Piliang3SP\mgf5ppm\ERLIC_3SubProteomes5ppm.mgf

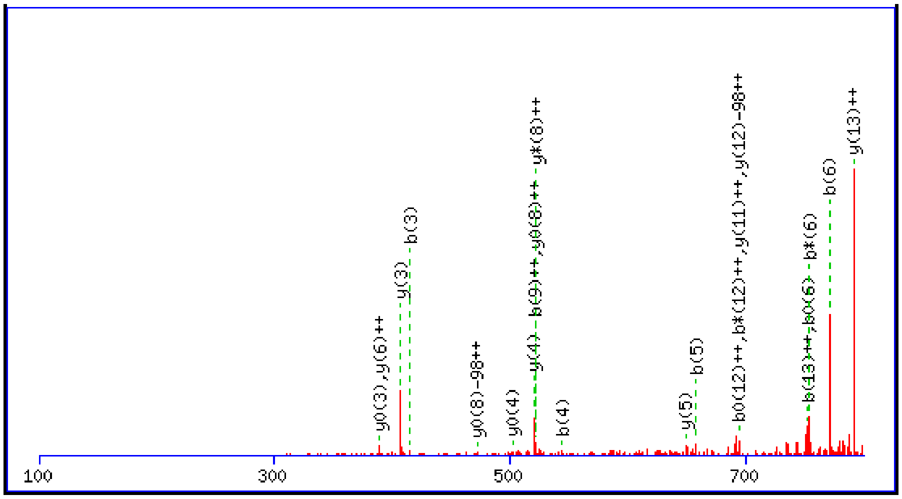

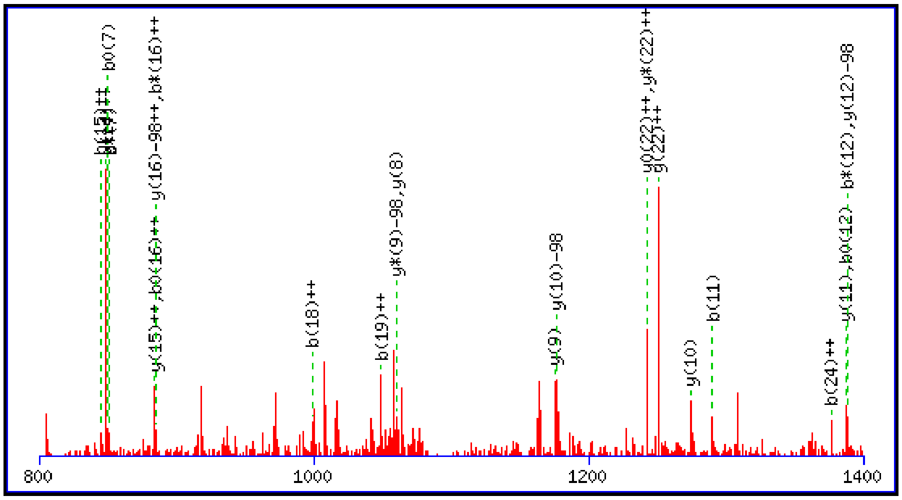

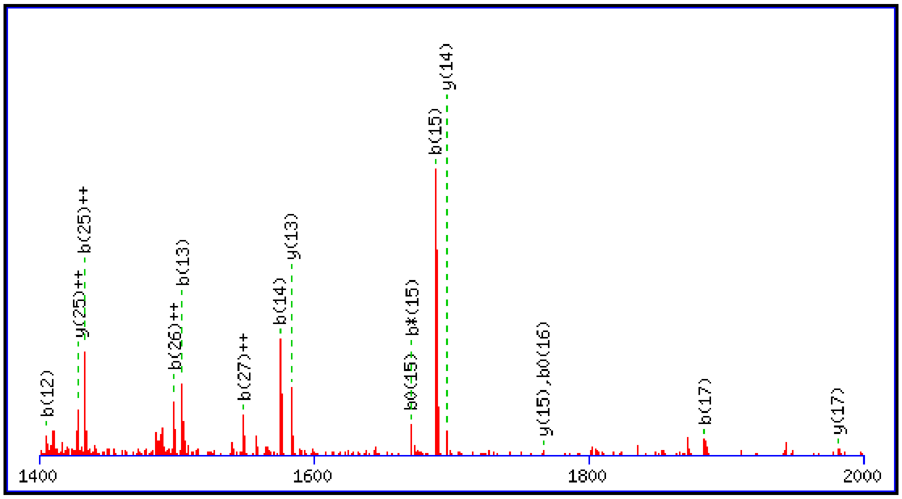

Monoisotopic mass of neutral peptide Mr(calc): 3270.5043
Fixed modifications: Carbamidomethyl (C)
Variable modifications:
S21 : Phospho (ST), with neutral losses 0.0000(shown in table), 97.9769
Ions Score: 47 **Expect:** 0.009
Matches (Bold Red): 62/472 fragment ions using 113 most intense peaks

#	b	b++	b*	b*++	b0	b0++	Seq.	y	y++	y*	y*++	y0	y0++	#
1	187.0866	94.0469					W							28
2	286.1550	143.5811					V	3085.4323	1543.2198	3068.4057	1534.7065	3067.4217	1534.2145	27
3	**415.1976**	208.1024			397.1870	199.0972	E	2986.3639	1493.6856	2969.3373	1485.1723	2968.3533	1484.6803	26
4	**544.2402**	272.6237			526.2296	263.6185	E	2857.3213	**1429.1643**	2840.2947	1420.6510	2839.3107	1420.1590	25
5	**658.2831**	329.6452	641.2566	321.1319	640.2726	320.6399	N	2728.2787	1364.6430	2711.2521	1356.1297	2710.2681	1355.6377	24

6	771.3672	386.1872	754.3406	377.6740	753.3566	377.1819	I	2614.2358	1307.6215	2597.2092	1299.1082	2596.2252	1298.6162	23
7	868.4199	434.7136	851.3934	426.2003	850.4094	425.7083	P	2501.1517	1251.0795	2484.1252	1242.5662	2483.1411	1242.0742	22
8	955.4520	478.2296	938.4254	469.7163	937.4414	469.2243	S	2404.0989	1202.5531	2387.0724	1194.0398	2386.0884	1193.5478	21
9	1042.4840	521.7456	1025.4575	513.2324	1024.4734	512.7404	S	2317.0669	1159.0371	2300.0404	1150.5238	2299.0564	1150.0318	20
10	1189.5524	595.2798	1172.5259	586.7666	1171.5418	586.2746	F	2230.0349	1115.5211	2213.0083	1107.0078	2212.0243	1106.5158	19
11	1290.6001	645.8037	1273.5735	637.2904	1272.5895	636.7984	T	2082.9665	1041.9869	2065.9399	1033.4736	2064.9559	1032.9816	18
12	1405.6270	703.3172	1388.6005	694.8039	1387.6165	694.3119	D	1981.9188	991.4630	1964.8922	982.9498	1963.9082	982.4578	17
13	1504.6955	752.8514	1487.6689	744.3381	1486.6849	743.8461	V	1866.8919	933.9496	1849.8653	925.4363	1848.8813	924.9443	16
14	1575.7326	788.3699	1558.7060	779.8566	1557.7220	779.3646	A	1767.8234	884.4154	1750.7969	875.9021	1749.8129	875.4101	15
15	1688.8166	844.9120	1671.7901	836.3987	1670.8061	835.9067	L	1696.7863	848.8968	1679.7598	840.3835	1678.7758	839.8915	14
16	1785.8694	893.4383	1768.8428	884.9251	1767.8588	884.4331	P	1583.7023	792.3548	1566.6757	783.8415	1565.6917	783.3495	13
17	1884.9378	942.9725	1867.9113	934.4593	1866.9272	933.9673	V	1486.6495	743.8284	1469.6229	735.3151	1468.6389	734.8231	12
18	1998.0219	999.5146	1980.9953	991.0013	1980.0113	990.5093	L	1387.5811	694.2942	1370.5545	685.7809	1369.5705	685.2889	11
19	2097.0903	1049.0488	2080.0637	1040.5355	2079.0797	1040.0435	V	1274.4970	637.7521	1257.4705	629.2389	1256.4865	628.7469	10
20	2212.1172	1106.5623	2195.0907	1098.0490	2194.1067	1097.5570	D	1175.4286	588.2179	1158.4021	579.7047	1157.4180	579.2127	9
21	2379.1156	1190.0614	2362.0890	1181.5482	2361.1050	1181.0561	S	1060.4017	530.7045	1043.3751	522.1912	1042.3911	521.6992	8
22	2494.1425	1247.5749	2477.1160	1239.0616	2476.1320	1238.5696	D	893.4033	447.2053	876.3768	438.6920	875.3927	438.2000	7
23	2623.1851	1312.0962	2606.1586	1303.5829	2605.1745	1303.0909	E	778.3764	389.6918	761.3498	381.1785	760.3658	380.6865	6
24	2752.2277	1376.6175	2735.2012	1368.1042	2734.2171	1367.6122	E	649.3338	325.1705	632.3072	316.6573	631.3232	316.1652	5
25	2865.3118	1433.1595	2848.2852	1424.6462	2847.3012	1424.1542	I	520.2912	260.6492	503.2646	252.1360	502.2806	251.6439	4
26	2996.3523	1498.6798	2979.3257	1490.1665	2978.3417	1489.6745	M	407.2071	204.1072	390.1806	195.5939	389.1966	195.1019	3
27	3097.3999	1549.2036	3080.3734	1540.6903	3079.3894	1540.1983	T	276.1666	138.5870	259.1401	130.0737	258.1561	129.5817	2
28							R	175.1190	88.0631	158.0924	79.5498			1

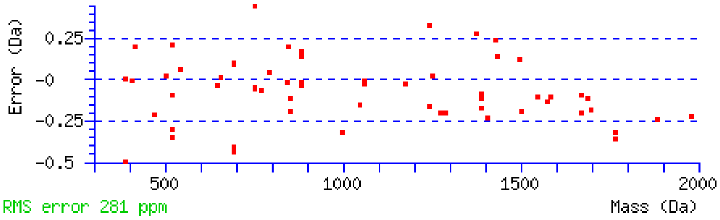

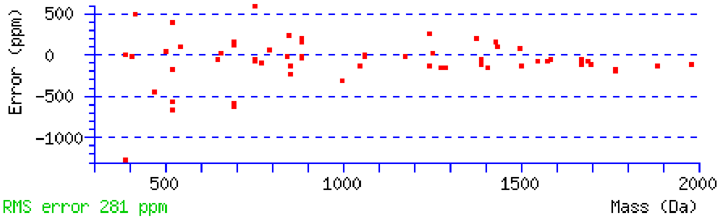

All matches to this query

Score	Mr(calc):	Delta	Sequence
46.6	3270.5043	0.0113	WVEENIPSSFTDVALPVLVDSDEEIMTR
25.7	3270.5043	0.0113	WVEENIPSSFTDVALPVLVDSDEEIMTR
14.9	3270.5043	0.0113	WVEENIPSSFTDVALPVLVDSDEEIMTR
10.3	3270.5043	0.0113	WVEENIPSSFTDVALPVLVDSDEEIMTR
9.9	3270.5043	0.0113	WVEENIPSSFTDVALPVLVDSDEEIMTR
6.0	3270.5145	0.0012	LPAPVLTAEPSSHNLEPGSTVQLRCTAHK
4.8	3270.5172	-0.0015	NRLSFMAIMILVAMVLSLMSYALLWK
4.5	3270.5172	-0.0015	NRLSFMAIMILVAMVLSLMSYALLWK
3.4	3270.4830	0.0327	QELASDGEGESTVLSSPPVPDAHGHLKYDR
3.4	3270.4830	0.0327	QELASDGEGESTVLSSPPVPDAHGHLKYDR

Spectrum No: 373; Query: 1250; Rank: 1

Peptide View

MS/MS Fragmentation of **EGSPVADDFVPSALGTR**

Found in **IPI00212797**, Tax_Id=10116 Gene_Symbol=RGD1306300_predicted hypothetical protein LOC361664

Match to Query 1250: 1796.797468 from(899.406010,2+)
Title: 091008RatKidney_NoSalt_23.3505.3505.2.dta
Data file K:\NewmanPaper\Piliang\3SubProteomes\Piliang3SP\mgf5ppm\ERLIC_3SubProteomes5ppm.mgf

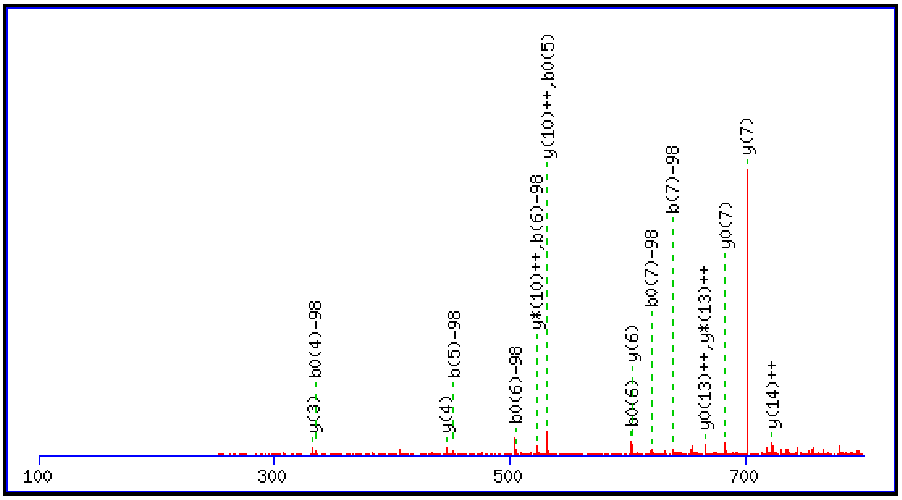

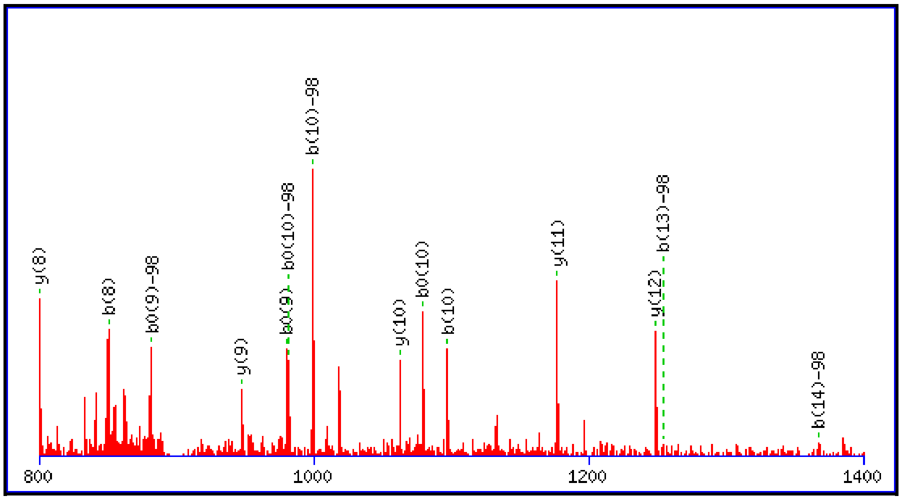

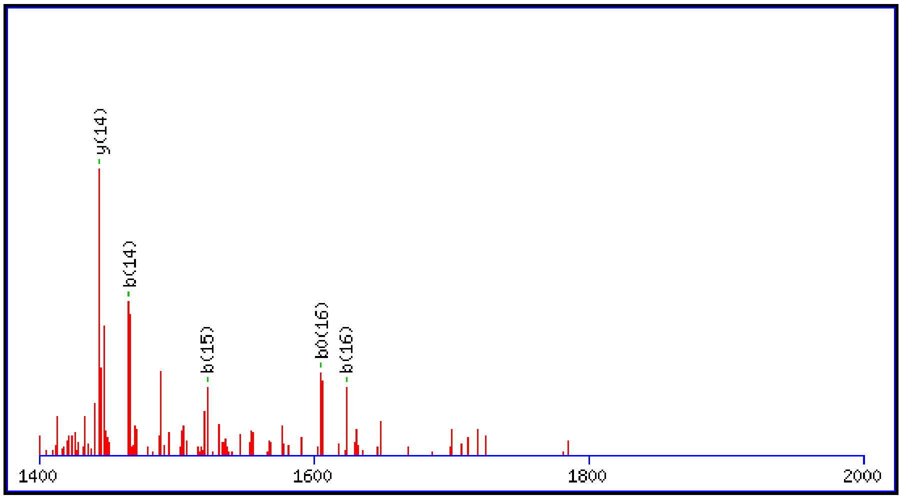

Monoisotopic mass of neutral peptide Mr(calc): 1796.7982
Fixed modifications: Carbamidomethyl (C)
Variable modifications:
S3 : Phospho (ST), with neutral losses 97.9769(shown in table), 0.0000
Ions Score: 47 **Expect:** 0.0052
Matches (Bold Red): 37/226 fragment ions using 67 most intense peaks

#	b	b^{++}	b^{0}	b^{0++}	Seq.	y	y^{++}	y*	y^{*++}	y^{0}	y^{0++}	#
1	130.0499	65.5286	112.0393	56.5233	E							17

2	187.0713	94.0393	169.0608	85.0340	G	1570.7860	785.8966	1553.7594	777.3834	1552.7754	776.8914	16
3	256.0928	128.5500	238.0822	119.5448	S	1513.7645	757.3859	1496.7380	748.8726	1495.7540	748.3806	15
4	353.1456	177.0764	**335.1350**	168.0711	P	**1444.7431**	**722.8752**	1427.7165	714.3619	1426.7325	713.8699	14
5	**452.2140**	226.6106	434.2034	217.6053	V	1347.6903	674.3488	1330.6638	**665.8355**	1329.6797	**665.3435**	13
6	**523.2511**	262.1292	**505.2405**	253.1239	A	**1248.6219**	624.8146	1231.5953	616.3013	1230.6113	615.8093	12
7	**638.2780**	319.6427	**620.2675**	310.6374	D	**1177.5848**	589.2960	1160.5582	580.7828	1159.5742	580.2907	11
8	753.3050	377.1561	735.2944	368.1508	D	**1062.5578**	**531.7826**	1045.5313	**523.2693**	1044.5473	522.7773	10
9	900.3734	450.6903	**882.3628**	441.6850	F	**947.5309**	474.2691	930.5043	465.7558	929.5203	465.2638	9
10	**999.4418**	500.2245	**981.4312**	491.2193	V	**800.4625**	400.7349	783.4359	392.2216	782.4519	391.7296	8
11	1096.4946	548.7509	1078.4840	539.7456	P	**701.3941**	351.2007	684.3675	342.6874	**683.3835**	342.1954	7
12	1183.5266	592.2669	1165.5160	583.2616	S	**604.3413**	302.6743	587.3148	294.1610	586.3307	293.6690	6
13	**1254.5637**	627.7855	1236.5531	618.7802	A	517.3093	259.1583	500.2827	250.6450	499.2987	250.1530	5
14	**1367.6478**	684.3275	1349.6372	675.3222	L	**446.2722**	223.6397	429.2456	215.1264	428.2616	214.6344	4
15	1424.6692	712.8383	1406.6587	703.8330	G	**333.1881**	167.0977	316.1615	158.5844	315.1775	158.0924	3
16	1525.7169	763.3621	1507.7063	754.3568	T	276.1666	138.5870	259.1401	130.0737	258.1561	129.5817	2
17					R	175.1190	88.0631	158.0924	79.5498			1

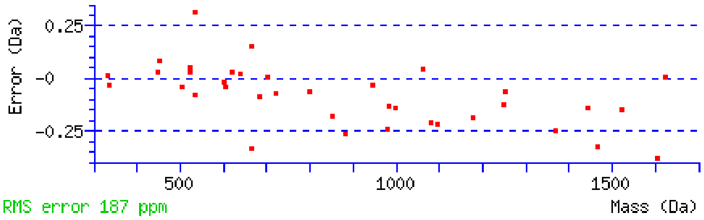

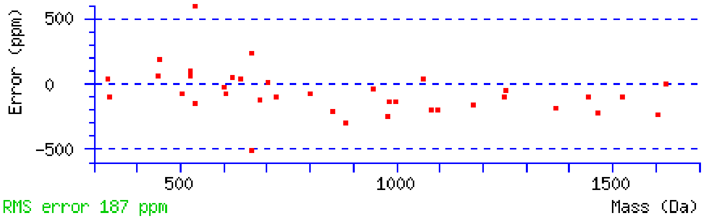

All matches to this query

Score	Mr(calc):	Delta	Sequence
46.6	1796.7982	-0.0008	EGSPVADDFVPSALGTR
6.5	1795.7971	1.0004	GFQTWEYSPVYAIR
1.4	1795.8085	0.9890	SSSAATAAAWSEGSSRDR
0.3	1795.7841	1.0133	VNSNFRPTARKAMK
0.3	1795.7841	1.0133	VNSNFRPTARKAMK

Spectrum No: 374; Query: 2608; Rank: 1

Peptide View

MS/MS Fragmentation of **AVAVATPAPAAPAAVSAAATPSAQEEPAAAPEPR**
Found in **IPI00359359**, Tax_Id=10116 Gene_Symbol=Srrm1_predicted serine/arginine repetitive matrix 1

Match to Query 2608: 3187.558542 from(1063.526790,3+)
Title: 100101RatKid_NS_deglyco_18.2794.2794.3.dta
Data file K:\NewmanPaper\Piliang\3SubProteomes\Piliang3SP\mgf5ppm\ERLIC_3SubProteomes5ppm.mgf

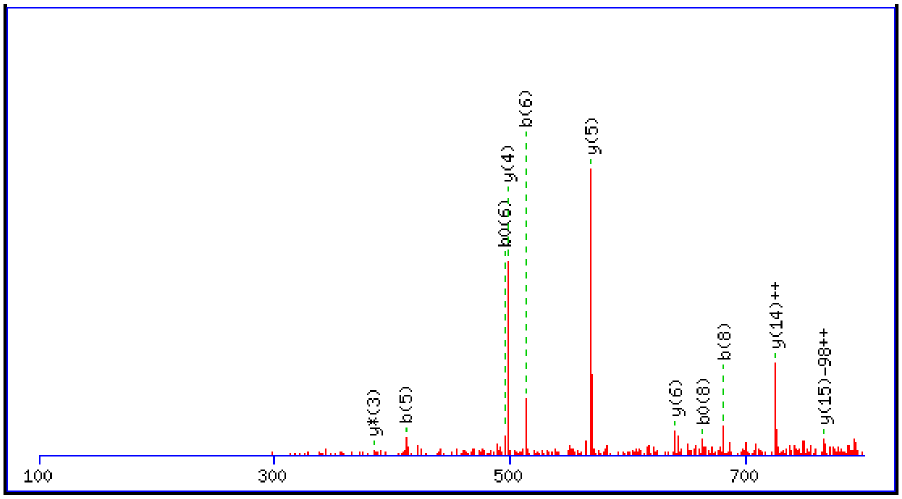

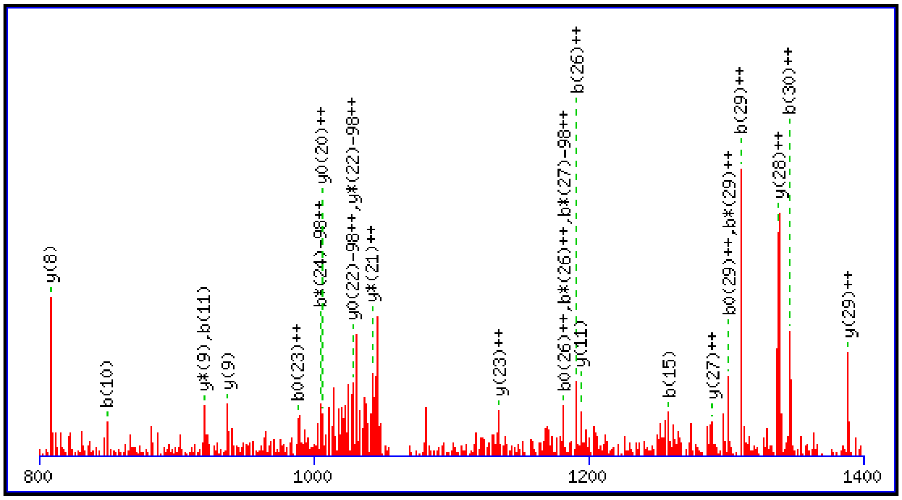

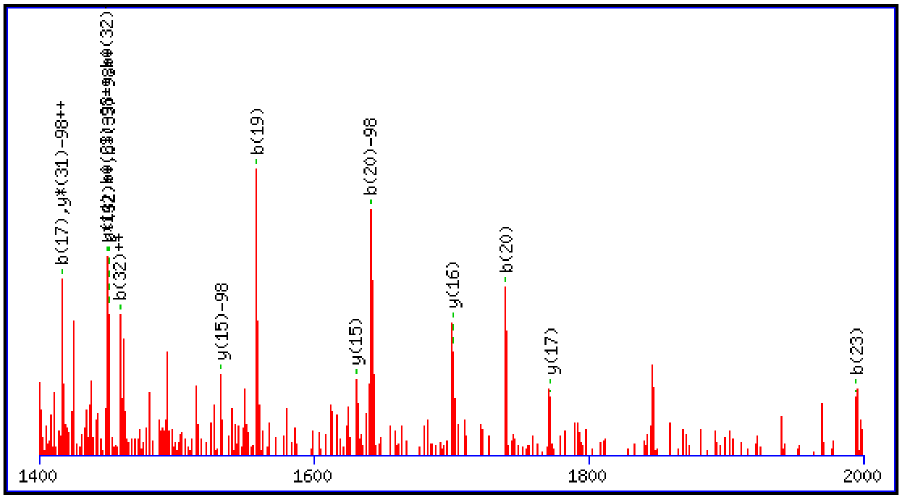

Monoisotopic mass of neutral peptide Mr(calc): 3187.5550
Fixed modifications: Carbamidomethyl (C)
Variable modifications:
T20 : Phospho (ST), with neutral losses 0.0000(shown in table), 97.9769
Ions Score: 47 **Expect:** 0.0063
Matches (Bold Red): 52/526 fragment ions using 91 most intense peaks

#	b	b^{++}	b*	b^{*++}	b^0	b^{0++}	Seq.	y	y^{++}	y*	y^{*++}	y^0	y^{0++}	#
1	72.0444	36.5258					A							34
2	171.1128	86.0600					V	3117.5252	1559.2663	3100.4987	1550.7530	3099.5147	1550.2610	33
3	242.1499	121.5786					A	3018.4568	1509.7320	3001.4303	1501.2188	3000.4463	1500.7268	32
4	341.2183	171.1128					V	2947.4197	1474.2135	2930.3932	1465.7002	2929.4091	1465.2082	31
5	**412.2554**	206.6314					A	2848.3513	1424.6793	2831.3247	1416.1660	2830.3407	1415.6740	30

6	**513.3031**	257.1552			**495.2926**	248.1499	T	2777.3142	**1389.1607**	2760.2876	1380.6475	2759.3036	1380.1554	29
7	610.3559	305.6816			592.3453	296.6763	P	2676.2665	**1338.6369**	2659.2399	1330.1236	2658.2559	1329.6316	28
8	**681.3930**	341.2001			**663.3824**	332.1949	A	2579.2137	**1290.1105**	2562.1872	1281.5972	2561.2032	1281.1052	27
9	778.4458	389.7265			760.4352	380.7212	P	2508.1766	1254.5919	2491.1501	1246.0787	2490.1661	1245.5867	26
10	**849.4829**	425.2451			831.4723	416.2398	A	2411.1239	1206.0656	2394.0973	1197.5523	2393.1133	1197.0603	25
11	**920.5200**	460.7636			902.5094	451.7584	A	2340.0867	1170.5470	2323.0602	1162.0337	2322.0762	1161.5417	24
12	1017.5728	509.2900			999.5622	500.2847	P	2269.0496	**1135.0285**	2252.0231	1126.5152	2251.0391	1126.0232	23
13	1088.6099	544.8086			1070.5993	535.8033	A	2171.9969	1086.5021	2154.9703	1077.9888	2153.9863	1077.4968	22
14	1159.6470	580.3271			1141.6364	571.3218	A	2100.9598	1050.9835	2083.9332	**1042.4702**	2082.9492	1041.9782	21
15	**1258.7154**	629.8613			1240.7048	620.8561	V	2029.9226	1015.4650	2012.8961	1006.9517	2011.9121	**1006.4597**	20
16	1345.7474	673.3774			1327.7369	664.3721	S	1930.8542	965.9307	1913.8277	957.4175	1912.8437	956.9255	19
17	**1416.7845**	708.8959			1398.7740	699.8906	A	1843.8222	922.4147	1826.7956	913.9015	1825.8116	913.4095	18
18	1487.8217	744.4145			1469.8111	735.4092	A	**1772.7851**	886.8962	1755.7585	878.3829	1754.7745	877.8909	17
19	**1558.8588**	779.9330			1540.8482	770.9277	A	**1701.7480**	851.3776	1684.7214	842.8643	1683.7374	842.3723	16
20	**1739.8728**	870.4400			1721.8622	861.4347	T	**1630.7109**	815.8591	1613.6843	807.3458	1612.7003	806.8538	15
21	1836.9255	918.9664			1818.9150	909.9611	P	**1449.6968**	**725.3521**	1432.6703	716.8388	1431.6863	716.3468	14
22	1923.9576	962.4824			1905.9470	953.4771	S	1352.6441	676.8257	1335.6175	668.3124	1334.6335	667.8204	13
23	**1994.9947**	998.0010			1976.9841	**988.9957**	A	1265.6121	633.3097	1248.5855	624.7964	1247.6015	624.3044	12
24	2123.0533	1062.0303	2106.0267	1053.5170	2105.0427	1053.0250	Q	**1194.5749**	597.7911	1177.5484	589.2778	1176.5644	588.7858	11
25	2252.0959	1126.5516	2235.0693	1118.0383	2234.0853	1117.5463	E	1066.5164	533.7618	1049.4898	525.2485	1048.5058	524.7565	10
26	2381.1384	**1191.0729**	2364.1119	**1182.5596**	2363.1279	**1182.0676**	E	**937.4738**	469.2405	**920.4472**	460.7272	919.4632	460.2352	9
27	2478.1912	1239.5992	2461.1647	1231.0860	2460.1806	1230.5940	P	**808.4312**	404.7192	791.4046	396.2060	790.4206	395.7139	8
28	2549.2283	1275.1178	2532.2018	1266.6045	2531.2178	1266.1125	A	711.3784	356.1928	694.3519	347.6796	693.3679	347.1876	7
29	2620.2654	**1310.6364**	2603.2389	**1302.1231**	2602.2549	**1301.6311**	A	**640.3413**	320.6743	623.3148	312.1610	622.3307	311.6690	6
30	2691.3026	**1346.1549**	2674.2760	1337.6416	2673.2920	1337.1496	A	**569.3042**	285.1557	552.2776	276.6425	551.2936	276.1504	5
31	2788.3553	1394.6813	2771.3288	1386.1680	2770.3447	1385.6760	P	**498.2671**	249.6372	481.2405	241.1239	480.2565	240.6319	4
32	2917.3979	**1459.2026**	2900.3714	**1450.6893**	2899.3873	**1450.1973**	E	401.2143	201.1108	**384.1878**	192.5975	383.2037	192.1055	3
33	3014.4507	1507.7290	2997.4241	1499.2157	2996.4401	1498.7237	P	272.1717	136.5895	255.1452	128.0762			2
34							R	175.1190	88.0631	158.0924	79.5498			1

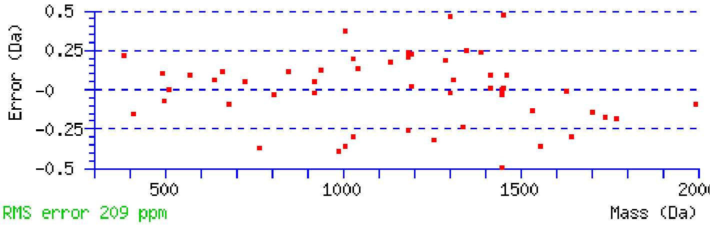

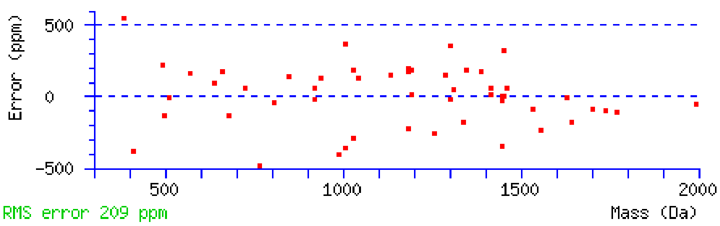

All matches to this query

Score	Mr(calc):	Delta	Sequence
46.5	3187.5550	0.0035	AVAVATPAPAAPAAVSAAATPSAQEEPAAAPEPR
38.4	3187.5550	0.0035	AVAVATPAPAAPAAVSAAATPSAQEEPAAAPEPR
28.4	3187.5550	0.0035	AVAVATPAPAAPAAVSAAATPSAQEEPAAAPEPR
17.1	3187.5550	0.0035	AVAVATPAPAAPAAVSAAATPSAQEEPAAAPEPR
5.8	3186.5249	1.0337	NPATLGSRPISRGLMVTMAPAVPTKPLR
3.2	3186.5249	1.0337	NPATLGSRPISRGLMVTMAPAVPTKPLR
0.4	3186.5251	1.0335	AIGNIVTGTDEQTQKVIDAGFPSLLTNPK
0.1	3187.5778	-0.0192	AAAAAAAAAASTSASVASASSSPTTVSPIVLIKK
0.1	3187.5778	-0.0192	AAAAAAAAAASTSASVASASSSPTTVSPIVLIKK

Spectrum No: 375; Query: 2854; Rank: 1

Peptide View

MS/MS Fragmentation of **DSVVLHNSDPNLHLLAEGAPIDWGEQYGDGGDGSDSGGSPCPSEAATLTEK**
Found in **IPI00210691**, Tax_Id=10116 Gene_Symbol=RGD1307018 hypothetical protein LOC362115

Match to Query 2854: 5301.325296 from(1326.338600,4+)
Title: 100101RatKid_NS_deglyco_19.4678.4678.4.dta
Data file K:\NewmanPaper\Piliang\3SubProteomes\Piliang3SP\mgf5ppm\ERLIC_3SubProteomes5ppm.mgf

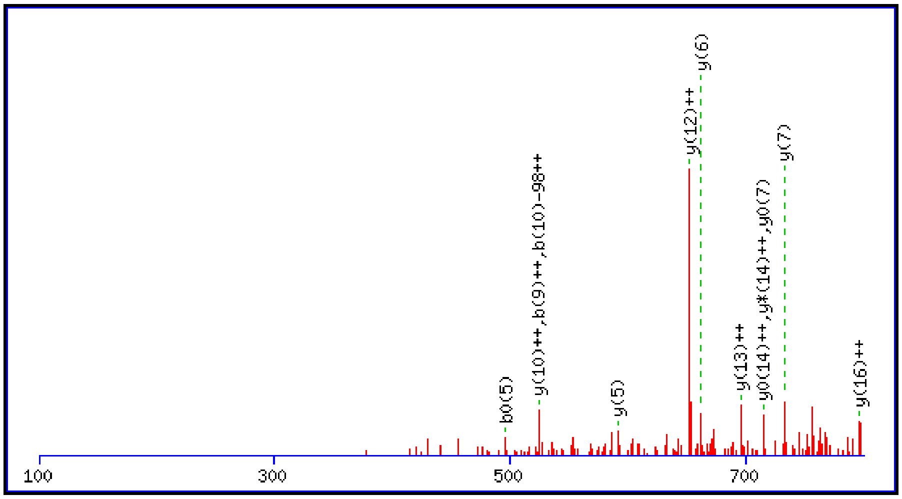

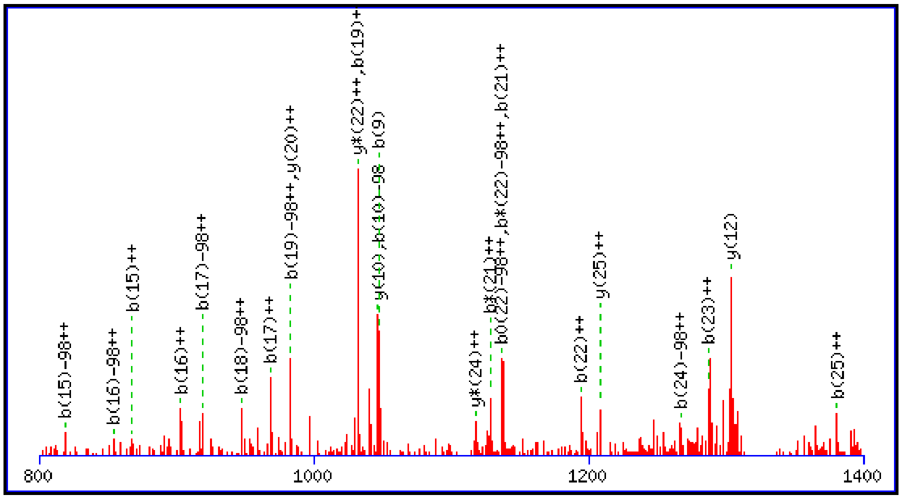

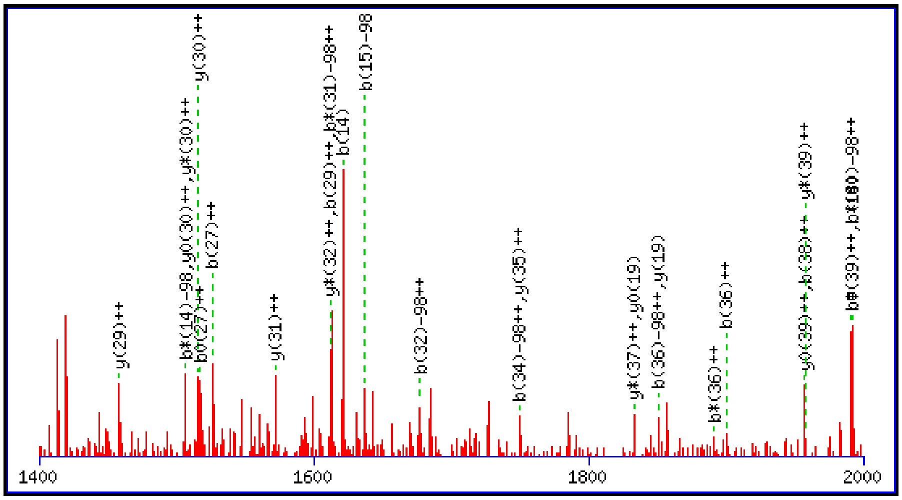

Monoisotopic mass of neutral peptide Mr(calc): 5301.2994
Fixed modifications: Carbamidomethyl (C)

```
Variable modifications:
S8      : Phospho (ST), with neutral losses 0.0000(shown in table), 97.9769
Ions Score: 46  Expect: 0.013
Matches (Bold Red): 68/886 fragment ions using 91 most intense peaks
```

#	b	b^{++}	b*	b^{*++}	b^{0}	b^{0++}	Seq.	y	y^{++}	y*	y^{*++}	y^{0}	y^{0++}	#
1	116.0342	58.5207			98.0237	49.5155	D							51
2	203.0662	102.0368			185.0557	93.0315	S	5187.2797	2594.1435	5170.2532	2585.6302	5169.2692	2585.1382	50
3	302.1347	151.5710			284.1241	142.5657	V	5100.2477	2550.6275	5083.2211	2542.1142	5082.2371	2541.6222	49
4	401.2031	201.1052			383.1925	192.0999	V	5001.1793	2501.0933	4984.1527	2492.5800	4983.1687	2492.0880	48
5	514.2871	257.6472			**496.2766**	248.6419	L	4902.1109	2451.5591	4885.0843	2443.0458	4884.1003	2442.5538	47
6	651.3461	326.1767			633.3355	317.1714	H	4789.0268	2395.0170	4772.0003	2386.5038	4771.0162	2386.0118	46
7	765.3890	383.1981	748.3624	374.6849	747.3784	374.1928	N	4651.9679	2326.4876	4634.9413	2317.9743	4633.9573	2317.4823	45
8	932.3873	466.6973	915.3608	458.1840	914.3768	457.6920	S	4537.9250	2269.4661	4520.8984	2260.9528	4519.9144	2260.4608	44
9	**1047.4143**	**524.2108**	1030.3877	515.6975	1029.4037	515.2055	D	4370.9266	2185.9669	4353.9001	2177.4537	4352.9160	2176.9617	43
10	1144.4670	572.7372	1127.4405	564.2239	1126.4565	563.7319	P	4255.8997	2128.4535	4238.8731	2119.9402	4237.8891	2119.4482	42
11	1258.5100	629.7586	1241.4834	621.2453	1240.4994	620.7533	N	4158.8469	2079.9271	4141.8204	2071.4138	4140.8363	2070.9218	41
12	1371.5940	686.3007	1354.5675	677.7874	1353.5835	677.2954	L	4044.8040	2022.9056	4027.7774	2014.3924	4026.7934	2013.9003	40
13	1508.6529	754.8301	1491.6264	746.3168	1490.6424	745.8248	H	3931.7199	1966.3636	3914.6934	**1957.8503**	3913.7093	**1957.3583**	39
14	**1621.7370**	811.3721	1604.7105	802.8589	1603.7264	802.3669	L	3794.6610	1897.8341	3777.6345	1889.3209	3776.6504	1888.8289	38
15	1734.8211	**867.9142**	1717.7945	859.4009	1716.8105	858.9089	L	3681.5769	1841.2921	3664.5504	**1832.7788**	3663.5664	1832.2868	37
16	1805.8582	**903.4327**	1788.8316	894.9195	1787.8476	894.4274	A	3568.4929	1784.7501	3551.4663	1776.2368	3550.4823	1775.7448	36
17	1934.9008	**967.9540**	1917.8742	959.4408	1916.8902	958.9487	E	3497.4558	**1749.2315**	3480.4292	1740.7182	3479.4452	1740.2262	35
18	**1991.9222**	996.4648	1974.8957	987.9515	1973.9117	987.4595	G	3368.4132	1684.7102	3351.3866	1676.1969	3350.4026	1675.7049	34
19	2062.9594	**1031.9833**	2045.9328	1023.4700	2044.9488	1022.9780	A	3311.3917	1656.1995	3294.3652	1647.6862	3293.3811	1647.1942	33
20	2160.0121	1080.5097	2142.9856	1071.9964	2142.0016	1071.5044	P	3240.3546	1620.6809	3223.3280	**1612.1677**	3222.3440	1611.6757	32
21	2273.0962	**1137.0517**	2256.0696	**1128.5385**	2255.0856	1128.0464	I	3143.3018	**1572.1546**	3126.2753	1563.6413	3125.2913	1563.1493	31
22	2388.1231	**1194.5652**	2371.0966	1186.0519	2370.1126	1185.5599	D	3030.2178	**1515.6125**	3013.1912	**1507.0992**	3012.2072	**1506.6072**	30
23	2574.2024	**1287.6049**	2557.1759	1279.0916	2556.1919	1278.5996	W	2915.1908	**1458.0990**	2898.1643	1449.5858	2897.1803	1449.0938	29
24	2631.2239	1316.1156	2614.1974	1307.6023	2613.2133	1307.1103	G	2729.1115	1365.0594	2712.0850	1356.5461	2711.1009	1356.0541	28
25	2760.2665	**1380.6369**	2743.2399	1372.1236	2742.2559	1371.6316	E	2672.0900	1336.5487	2655.0635	1328.0354	2654.0795	1327.5434	27
26	2888.3251	1444.6662	2871.2985	1436.1529	2870.3145	1435.6609	Q	2543.0474	1272.0274	2526.0209	1263.5141	2525.0369	1263.0221	26
27	3051.3884	**1526.1978**	3034.3619	1517.6846	3033.3778	**1517.1926**	Y	2414.9889	**1207.9981**	2397.9623	1199.4848	2396.9783	1198.9928	25
28	3108.4099	1554.7086	3091.3833	1546.1953	3090.3993	1545.7033	G	2251.9255	1126.4664	2234.8990	**1117.9531**	2233.9150	1117.4611	24
29	3223.4368	**1612.2220**	3206.4103	1603.7088	3205.4262	1603.2168	D	2194.9041	1097.9557	2177.8775	1089.4424	2176.8935	1088.9504	23
30	3280.4583	1640.7328	3263.4317	1632.2195	3262.4477	1631.7275	G	2079.8771	1040.4422	2062.8506	**1031.9289**	2061.8666	1031.4369	22
31	3337.4797	1669.2435	3320.4532	1660.7302	3319.4692	1660.2382	G	2022.8557	1011.9315	2005.8291	1003.4182	2004.8451	1002.9262	21
32	3452.5067	1726.7570	3435.4801	1718.2437	3434.4961	1717.7517	D	1965.8342	**983.4207**	1948.8077	974.9075	1947.8236	974.4155	20
33	3509.5281	1755.2677	3492.5016	1746.7544	3491.5176	1746.2624	G	**1850.8073**	925.9073	1833.7807	917.3940	**1832.7967**	916.9020	19
34	3596.5602	1798.7837	3579.5336	1790.2704	3578.5496	1789.7784	S	1793.7858	897.3965	1776.7593	888.8833	1775.7752	888.3913	18
35	3711.5871	1856.2972	3694.5606	1847.7839	3693.5765	1847.2919	D	1706.7538	853.8805	1689.7272	845.3672	1688.7432	844.8752	17
36	3798.6191	**1899.8132**	3781.5926	**1891.2999**	3780.6086	1890.8079	S	1591.7268	**796.3671**	1574.7003	787.8538	1573.7163	787.3618	16
37	3855.6406	1928.3239	3838.6141	1919.8107	3837.6300	1919.3187	G	1504.6948	752.8510	1487.6683	744.3378	1486.6842	743.8458	15
38	3912.6621	**1956.8347**	3895.6355	1948.3214	3894.6515	1947.8294	G	1447.6733	724.3403	1430.6468	**715.8270**	1429.6628	**715.3350**	14
39	3999.6941	2000.3507	3982.6675	**1991.8374**	3981.6835	**1991.3454**	S	1390.6519	**695.8296**	1373.6253	687.3163	1372.6413	686.8243	13
40	4096.7469	2048.8771	4079.7203	2040.3638	4078.7363	2039.8718	P	**1303.6198**	**652.3136**	1286.5933	643.8003	1285.6093	643.3083	12
41	4256.7775	2128.8924	4239.7510	2120.3791	4238.7669	2119.8871	C	1206.5671	603.7872	1189.5405	595.2739	1188.5565	594.7819	11
42	4353.8303	2177.4188	4336.8037	2168.9055	4335.8197	2168.4135	P	**1046.5364**	**523.7719**	1029.5099	515.2586	1028.5259	514.7666	10
43	4440.8623	2220.9348	4423.8358	2212.4215	4422.8517	2211.9295	S	949.4837	475.2455	932.4571	466.7322	931.4731	466.2402	9
44	4569.9049	2285.4561	4552.8783	2276.9428	4551.8943	2276.4508	E	862.4516	431.7295	845.4251	423.2162	844.4411	422.7242	8
45	4640.9420	2320.9746	4623.9155	2312.4614	4622.9314	2311.9694	A	**733.4090**	367.2082	716.3825	358.6949	**715.3985**	358.2029	7
46	4711.9791	2356.4932	4694.9526	2347.9799	4693.9686	2347.4879	A	**662.3719**	331.6896	645.3454	323.1763	644.3614	322.6843	6

**47**	4813.0268	2407.0170	4796.0003	2398.5038	4795.0162	2398.0118	**T**	**591.3348**	296.1710	574.3083	287.6578	573.3243	287.1658	**5**
**48**	4926.1109	2463.5591	4909.0843	2455.0458	4908.1003	2454.5538	**L**	490.2871	245.6472	473.2606	237.1339	472.2766	236.6419	**4**
**49**	5027.1585	2514.0829	5010.1320	2505.5696	5009.1480	2505.0776	**T**	377.2031	189.1052	360.1765	180.5919	359.1925	180.0999	**3**
**50**	5156.2011	2578.6042	5139.1746	2570.0909	5138.1906	2569.5989	**E**	276.1554	138.5813	259.1288	130.0681	258.1448	129.5761	**2**
**51**							**K**	147.1128	74.0600	130.0863	65.5468			**1**

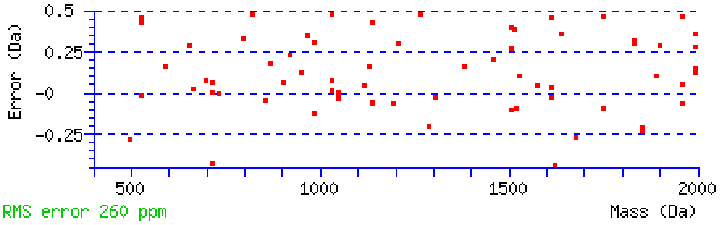

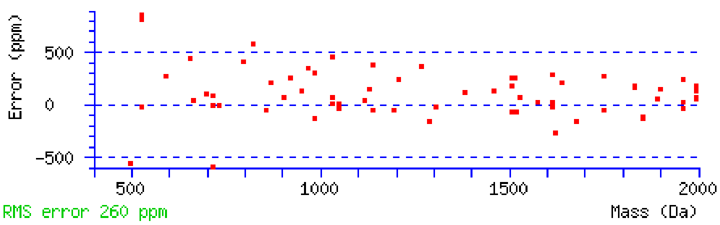

All matches to this query

Score	Mr(calc):	Delta	Sequence
46.5	5301.2994	0.0259	DSVVLHNSDPNLHLLAEGAPIDWGEQYGDGGDGSDSGGSPCPSEAATLTEK
45.4	5301.2994	0.0259	DSVVLHNSDPNLHLLAEGAPIDWGEQYGDGGDGSDSGGSPCPSEAATLTEK
5.1	5301.2994	0.0259	DSVVLHNSDPNLHLLAEGAPIDWGEQYGDGGDGSDSGGSPCPSEAATLTEK
4.9	5299.3041	2.0212	AQASGSTAQSSSSESEDEDMIPATQPPTLAIRTNVTTPTALSQTAAQPSK
4.7	5299.3041	2.0212	AQASGSTAQSSSSESEDEDMIPATQPPTLAIRTNVTTPTALSQTAAQPSK
3.9	5300.3026	1.0227	QSTQVMAASMSAFDPLKNQDEINKNVMSAFGLTDDQVSGPPSAPTEDR
3.9	5301.2994	0.0259	DSVVLHNSDPNLHLLAEGAPIDWGEQYGDGGDGSDSGGSPCPSEAATLTEK
3.9	5301.2994	0.0259	DSVVLHNSDPNLHLLAEGAPIDWGEQYGDGGDGSDSGGSPCPSEAATLTEK
3.4	5299.3041	2.0212	AQASGSTAQSSSSESEDEDMIPATQPPTLAIRTNVTTPTALSQTAAQPSK
3.4	5299.3041	2.0212	AQASGSTAQSSSSESEDEDMIPATQPPTLAIRTNVTTPTALSQTAAQPSK

Spectrum No: 376; Query: 2662; Rank: 1

Peptide View

MS/MS Fragmentation of **GENEETLGRPAQPPSAGETPHSPGVEDAPIAK**
Found in **IPI00373011**, Tax_Id=10116 Gene_Symbol=eplin similar to Epithelial protein lost in neoplasm

Match to Query 2662: 3317.534652 from(1106.852160,3+)
Title: 091008RatKidney_NH4Format01_18.1783.1783.3.dta
Data file K:\NewmanPaper\Piliang\3SubProteomes\Piliang3SP\mgf5ppm\ERLIC_3SubProteomes5ppm.mgf

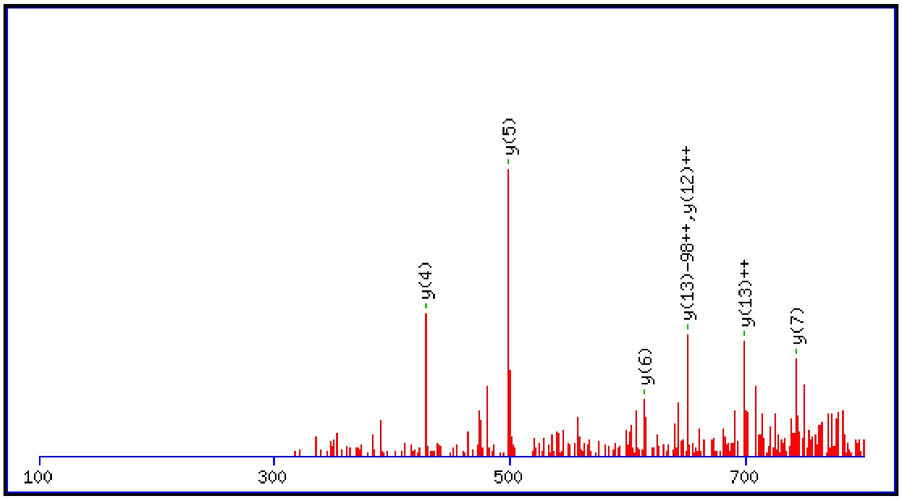

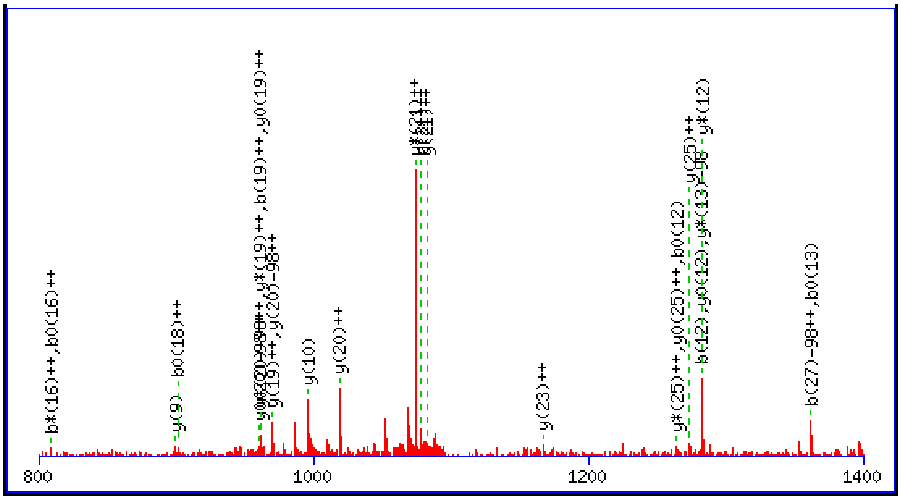

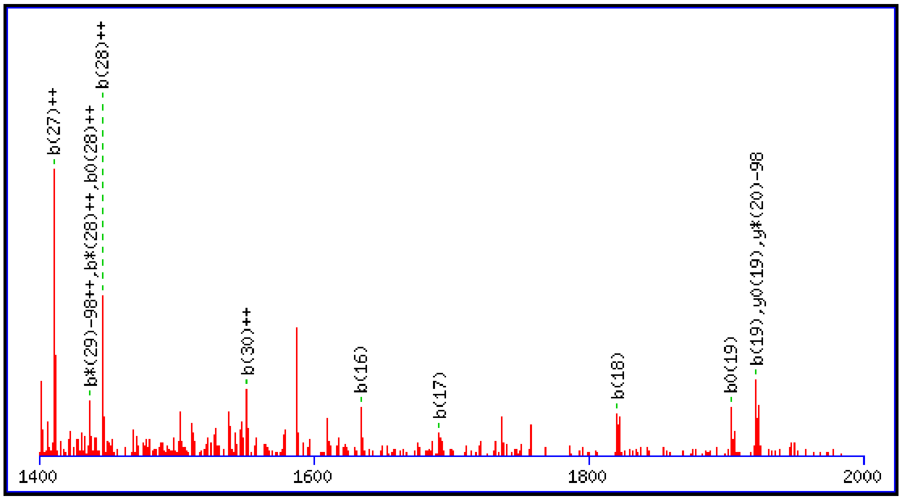

Monoisotopic mass of neutral peptide Mr(calc): 3317.5201
Fixed modifications: Carbamidomethyl (C)
Variable modifications:
S22 : Phospho (ST), with neutral losses 0.0000(shown in table), 97.9769
Ions Score: 46 **Expect:** 0.0098
Matches (Bold Red): 47/542 fragment ions using 58 most intense peaks

#	b	b^{++}	b*	b^{*++}	b^0	b^{0++}	Seq.	y	y^{++}	y*	y^{*++}	y^0	y^{0++}	#
1	58.0287	29.5180					G							32
2	187.0713	94.0393			169.0608	85.0340	E	3261.5060	1631.2566	3244.4794	1622.7433	3243.4954	1622.2513	31
3	301.1143	151.0608	284.0877	142.5475	283.1037	142.0555	N	3132.4634	1566.7353	3115.4368	1558.2220	3114.4528	1557.7300	30
4	430.1569	215.5821	413.1303	207.0688	412.1463	206.5768	E	3018.4204	1509.7139	3001.3939	1501.2006	3000.4099	1500.7086	29
5	559.1994	280.1034	542.1729	271.5901	541.1889	271.0981	E	2889.3778	1445.1926	2872.3513	1436.6793	2871.3673	1436.1873	28
6	660.2471	330.6272	643.2206	322.1139	642.2366	321.6219	T	2760.3352	1380.6713	2743.3087	1372.1580	2742.3247	1371.6660	27
7	773.3312	387.1692	756.3046	378.6560	755.3206	378.1640	L	2659.2876	1330.1474	2642.2610	1321.6341	2641.2770	1321.1421	26
8	830.3527	415.6800	813.3261	407.1667	812.3421	406.6747	G	2546.2035	**1273.6054**	2529.1770	**1265.0921**	2528.1929	**1264.6001**	25
9	986.4538	493.7305	969.4272	485.2172	968.4432	484.7252	R	2489.1820	1245.0947	2472.1555	1236.5814	2471.1715	1236.0894	24
10	1083.5065	542.2569	1066.4800	533.7436	1065.4960	533.2516	P	2333.0809	**1167.0441**	2316.0544	1158.5308	2315.0704	1158.0388	23
11	1154.5436	577.7755	1137.5171	569.2622	1136.5331	568.7702	A	2236.0282	1118.5177	2219.0016	1110.0044	2218.0176	1109.5124	22
12	**1282.6022**	641.8047	1265.5757	633.2915	**1264.5917**	632.7995	Q	2164.9911	**1082.9992**	2147.9645	**1074.4859**	2146.9805	1073.9939	21
13	1379.6550	690.3311	1362.6284	681.8179	**1361.6444**	681.3258	P	2036.9325	**1018.9699**	2019.9059	1010.4566	2018.9219	1009.9646	20
14	1476.7077	738.8575	1459.6812	730.3442	1458.6972	729.8522	P	1939.8797	**970.4435**	1922.8532	**961.9302**	**1921.8691**	**961.4382**	19
15	1563.7398	782.3735	1546.7132	773.8603	1545.7292	773.3682	S	1842.8269	921.9171	1825.8004	913.4038	1824.8164	912.9118	18
16	**1634.7769**	817.8921	1617.7503	**809.3788**	1616.7663	**808.8868**	A	1755.7949	878.4011	1738.7684	869.8878	1737.7844	869.3958	17
17	**1691.7984**	846.4028	1674.7718	837.8895	1673.7878	837.3975	G	1684.7578	842.8825	1667.7313	834.3693	1666.7472	833.8773	16
18	**1820.8409**	910.9241	1803.8144	902.4108	1802.8304	**901.9188**	E	1627.7363	814.3718	1610.7098	805.8585	1609.7258	805.3665	15

19	1921.8886	961.4480	1904.8621	952.9347	1903.8781	952.4427	T	1498.6937	749.8505	1481.6672	741.3372	1480.6832	740.8452	14
20	2018.9414	1009.9743	2001.9148	1001.4611	2000.9308	1000.9691	P	1397.6461	699.3267	1380.6195	690.8134	1379.6355	690.3214	13
21	2156.0003	1078.5038	2138.9738	1069.9905	2137.9897	1069.4985	H	1300.5933	650.8003	1283.5668	642.2870	1282.5827	641.7950	12
22	2322.9987	1162.0030	2305.9721	1153.4897	2304.9881	1152.9977	S	1163.5344	582.2708	1146.5078	573.7576	1145.5238	573.2656	11
23	2420.0514	1210.5293	2403.0249	1202.0161	2402.0409	1201.5241	P	996.5360	498.7717	979.5095	490.2584	978.5255	489.7664	10
24	2477.0729	1239.0401	2460.0463	1230.5268	2459.0623	1230.0348	G	899.4833	450.2453	882.4567	441.7320	881.4727	441.2400	9
25	2576.1413	1288.5743	2559.1147	1280.0610	2558.1307	1279.5690	V	842.4618	421.7345	825.4353	413.2213	824.4512	412.7293	8
26	2705.1839	1353.0956	2688.1573	1344.5823	2687.1733	1344.0903	E	743.3934	372.2003	726.3668	363.6871	725.3828	363.1951	7
27	2820.2108	1410.6091	2803.1843	1402.0958	2802.2003	1401.6038	D	614.3508	307.6790	597.3243	299.1658	596.3402	298.6738	6
28	2891.2479	1446.1276	2874.2214	1437.6143	2873.2374	1437.1223	A	499.3239	250.1656	482.2973	241.6523			5
29	2988.3007	1494.6540	2971.2742	1486.1407	2970.2901	1485.6487	P	428.2867	214.6470	411.2602	206.1337			4
30	3101.3848	1551.1960	3084.3582	1542.6828	3083.3742	1542.1907	I	331.2340	166.1206	314.2074	157.6074			3
31	3172.4219	1586.7146	3155.3953	1578.2013	3154.4113	1577.7093	A	218.1499	109.5786	201.1234	101.0653			2
32							K	147.1128	74.0600	130.0863	65.5468			1

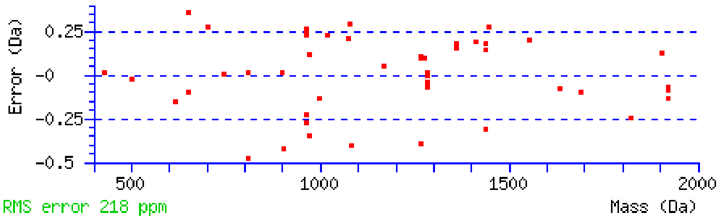

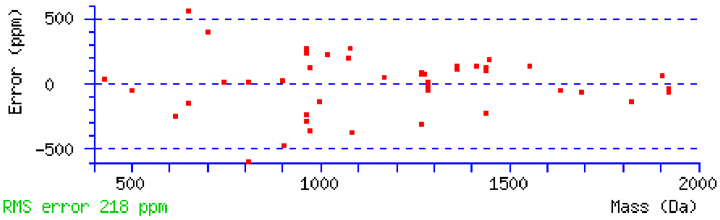

All matches to this query

Score	Mr(calc):	Delta	Sequence
46.4	3317.5201	0.0145	GENEETLGRPAQPPSAGETPHSPGVEDAPIAK
34.1	3317.5201	0.0145	GENEETLGRPAQPPSAGETPHSPGVEDAPIAK
22.5	3317.5201	0.0145	GENEETLGRPAQPPSAGETPHSPGVEDAPIAK
14.2	3317.5201	0.0145	GENEETLGRPAQPPSAGETPHSPGVEDAPIAK
5.3	3316.5378	0.9969	SSNPTGENFQEKTTPVQLIPSLVRSPSSR
4.8	3316.5378	0.9969	SSNPTGENFQEKTTPVQLIPSLVRSPSSR
4.4	3317.5209	0.0137	MTDAAVSFAKDFLAGGVAAAISKTAVAPIER
3.3	3316.5475	0.9872	SKPSEGLSAIISRGHMYLLWVGSEEFEAQ
3.2	3315.5328	2.0018	SALNWKPFVYGGLASITAECGTFPIDLTK
3.0	3316.5475	0.9872	SKPSEGLSAIISRGHMYLLWVGSEEFEAQ

Spectrum No: 377; Query: 2740; Rank: 1

Peptide View

MS/MS Fragmentation of **AEPEKSEGAAEEQPEPAPAPEQEAAAPGPAAGGEAPK**
Found in **IPI00231651**, Tax_Id=10116 Gene_Symbol=Basp1 Brain acid soluble protein 1

Match to Query 2740: 3645.628752 from(1216.216860,3+)
Title: 100101RatKid_NS_deglyco_19.1567.1567.3.dta
Data file K:\NewmanPaper\Piliang\3SubProteomes\Piliang3SP\mgf5ppm\ERLIC_3SubProteomes5ppm.mgf

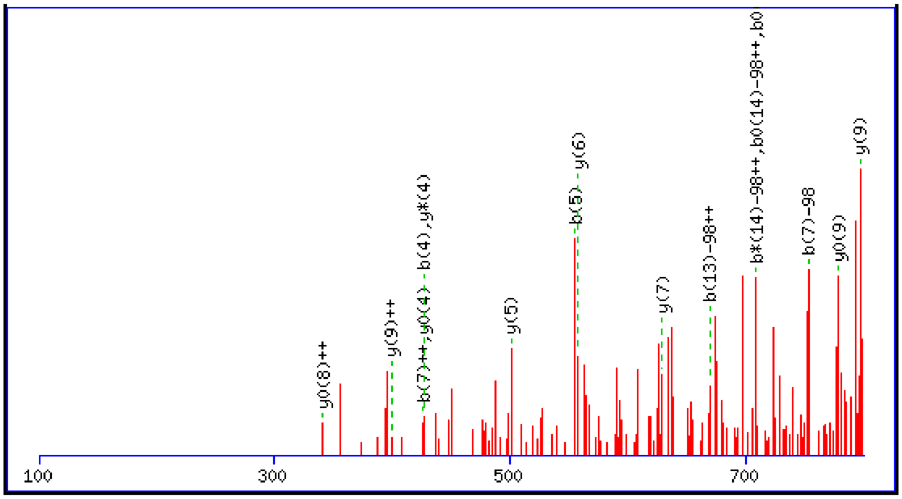

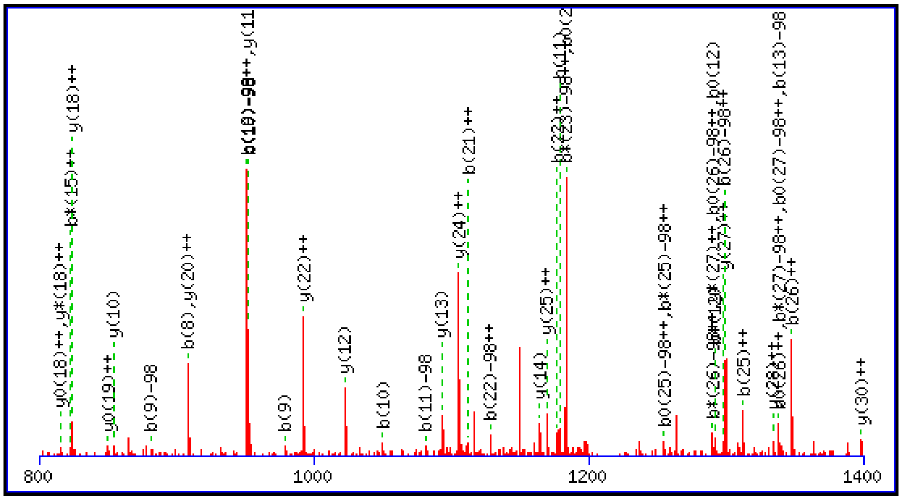

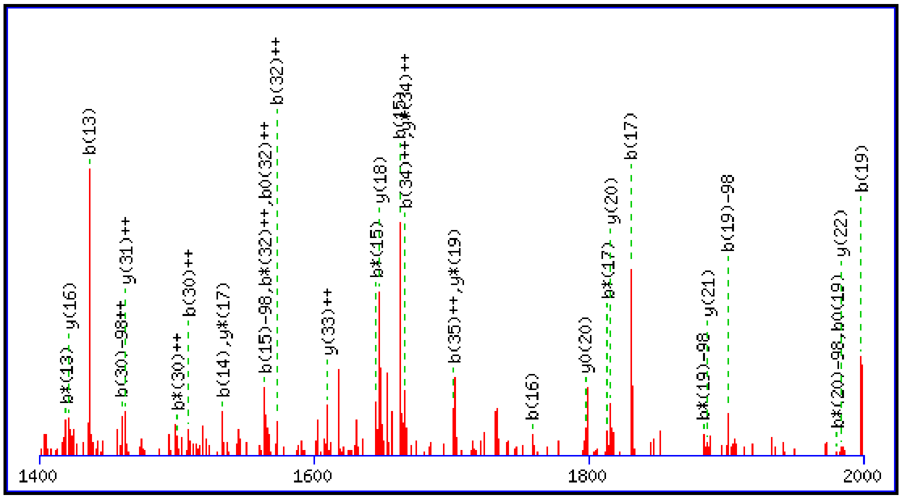

Monoisotopic mass of neutral peptide Mr(calc): 3644.6155
Fixed modifications: Carbamidomethyl (C)
Variable modifications:
S6 : Phospho (ST), with neutral losses 0.0000(shown in table), 97.9769
Ions Score: 46 **Expect:** 0.011
Matches (Bold Red): 94/632 fragment ions using 171 most intense peaks

#	b	b++	b*	b*++	b0	b0++	Seq.	y	y++	y*	y*++	y0	y0++	#
1	72.0444	36.5258					A							37
2	201.0870	101.0471			183.0764	92.0418	E	3574.5857	1787.7965	3557.5592	1779.2832	3556.5752	1778.7912	36
3	298.1397	149.5735			280.1292	140.5682	P	3445.5431	1723.2752	3428.5166	1714.7619	3427.5326	1714.2699	35
4	**427.1823**	214.0948			409.1718	205.0895	E	3348.4904	1674.7488	3331.4638	**1666.2355**	3330.4798	1665.7435	34
5	**555.2773**	278.1423	538.2508	269.6290	537.2667	269.1370	K	3219.4478	**1610.2275**	3202.4212	1601.7142	3201.4372	1601.2222	33

6	722.2757	361.6415	705.2491	353.1282	704.2651	352.6362	S	3091.3528	1546.1800	3074.3263	1537.6668	3073.3422	1537.1748	32
7	851.3182	426.1628	834.2917	417.6495	833.3077	417.1575	E	2924.3545	1462.6809	2907.3279	1454.1676	2906.3439	1453.6756	31
8	908.3397	454.6735	891.3132	446.1602	890.3291	445.6682	G	2795.3119	1398.1596	2778.2853	1389.6463	2777.3013	1389.1543	30
9	979.3768	490.1921	962.3503	481.6788	961.3663	481.1868	A	2738.2904	1369.6488	2721.2638	1361.1356	2720.2798	1360.6436	29
10	1050.4139	525.7106	1033.3874	517.1973	1032.4034	516.7053	A	2667.2533	1334.1303	2650.2267	1325.6170	2649.2427	1325.1250	28
11	1179.4565	590.2319	1162.4300	581.7186	1161.4460	581.2266	E	2596.2162	1298.6117	2579.1896	1290.0984	2578.2056	1289.6064	27
12	1308.4991	654.7532	1291.4726	646.2399	1290.4886	645.7479	E	2467.1736	1234.0904	2450.1470	1225.5772	2449.1630	1225.0851	26
13	1436.5577	718.7825	1419.5312	710.2692	1418.5471	709.7772	Q	2338.1310	1169.5691	2321.1044	1161.0559	2320.1204	1160.5638	25
14	1533.6105	767.3089	1516.5839	758.7956	1515.5999	758.3036	P	2210.0724	1105.5398	2193.0459	1097.0266	2192.0618	1096.5346	24
15	1662.6531	831.8302	1645.6265	823.3169	1644.6425	822.8249	E	2113.0196	1057.0135	2095.9931	1048.5002	2095.0091	1048.0082	23
16	1759.7058	880.3566	1742.6793	871.8433	1741.6953	871.3513	P	1983.9770	992.4922	1966.9505	983.9789	1965.9665	983.4869	22
17	1830.7429	915.8751	1813.7164	907.3618	1812.7324	906.8698	A	1886.9243	943.9658	1869.8977	935.4525	1868.9137	934.9605	21
18	1927.7957	964.4015	1910.7692	955.8882	1909.7851	955.3962	P	1815.8872	908.4472	1798.8606	899.9339	1797.8766	899.4419	20
19	1998.8328	999.9200	1981.8063	991.4068	1980.8223	990.9148	A	1718.8344	859.9208	1701.8079	851.4076	1700.8238	850.9156	19
20	2095.8856	1048.4464	2078.8590	1039.9332	2077.8750	1039.4411	P	1647.7973	824.4023	1630.7707	815.8890	1629.7867	815.3970	18
21	2224.9282	1112.9677	2207.9016	1104.4545	2206.9176	1103.9624	E	1550.7445	775.8759	1533.7180	767.3626	1532.7340	766.8706	17
22	2352.9868	1176.9970	2335.9602	1168.4837	2334.9762	1167.9917	Q	1421.7019	711.3546	1404.6754	702.8413	1403.6914	702.3493	16
23	2482.0293	1241.5183	2465.0028	1233.0050	2464.0188	1232.5130	E	1293.6434	647.3253	1276.6168	638.8120	1275.6328	638.3200	15
24	2553.0665	1277.0369	2536.0399	1268.5236	2535.0559	1268.0316	A	1164.6008	582.8040	1147.5742	574.2907	1146.5902	573.7987	14
25	2624.1036	1312.5554	2607.0770	1304.0422	2606.0930	1303.5501	A	1093.5637	547.2855	1076.5371	538.7722	1075.5531	538.2802	13
26	2695.1407	1348.0740	2678.1141	1339.5607	2677.1301	1339.0687	A	1022.5265	511.7669	1005.5000	503.2536	1004.5160	502.7616	12
27	2792.1935	1396.6004	2775.1669	1388.0871	2774.1829	1387.5951	P	951.4894	476.2483	934.4629	467.7351	933.4789	467.2431	11
28	2849.2149	1425.1111	2832.1884	1416.5978	2831.2044	1416.1058	G	854.4367	427.7220	837.4101	419.2087	836.4261	418.7167	10
29	2946.2677	1473.6375	2929.2411	1465.1242	2928.2571	1464.6322	P	797.4152	399.2112	780.3886	390.6980	779.4046	390.2060	9
30	3017.3048	1509.1560	3000.2782	1500.6428	2999.2942	1500.1508	A	700.3624	350.6849	683.3359	342.1716	682.3519	341.6796	8
31	3088.3419	1544.6746	3071.3154	1536.1613	3070.3313	1535.6693	A	629.3253	315.1663	612.2988	306.6530	611.3148	306.1610	7
32	3145.3634	1573.1853	3128.3368	1564.6720	3127.3528	1564.1800	G	558.2882	279.6477	541.2617	271.1345	540.2776	270.6425	6
33	3202.3848	1601.6961	3185.3583	1593.1828	3184.3743	1592.6908	G	501.2667	251.1370	484.2402	242.6237	483.2562	242.1317	5
34	3331.4274	1666.2174	3314.4009	1657.7041	3313.4169	1657.2121	E	444.2453	222.6263	427.2187	214.1130	426.2347	213.6210	4
35	3402.4645	1701.7359	3385.4380	1693.2226	3384.4540	1692.7306	A	315.2027	158.1050	298.1761	149.5917			3
36	3499.5173	1750.2623	3482.4908	1741.7490	3481.5067	1741.2570	P	244.1656	122.5864	227.1390	114.0731			2
37							K	147.1128	74.0600	130.0863	65.5468			1

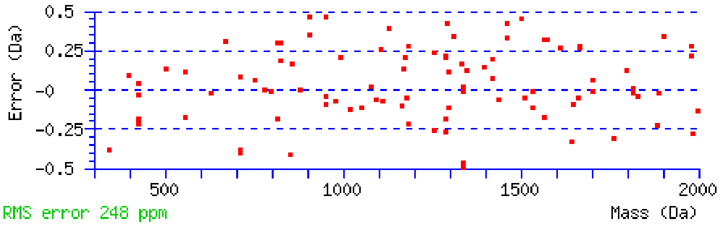

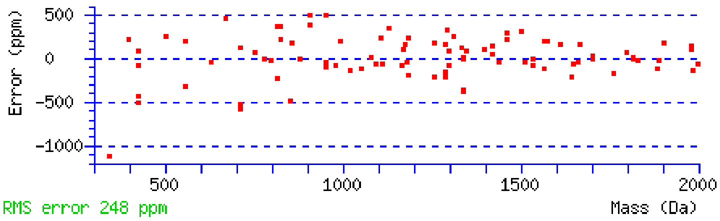

All matches to this query

Score	Mr(calc):	Delta	Sequence
46.4	3644.6155	1.0133	AEPEKSEGAAEEQPEPAPAPEQEAAAPGPAAGGEAPK
4.7	3644.6113	1.0174	ARCLEGVESHTRGLVASLDMQLEQAQGTQER
4.5	3644.6518	0.9770	AHKGLGIAAQMSHLSGSSGNMSVLSDSAGIVFPSR
4.2	3644.6113	1.0174	ARCLEGVESHTRGLVASLDMQLEQAQGTQER
3.3	3645.6131	0.0157	SGATSAPRHQSTLNLNNPASVCSGSSANTTQKLK
3.0	3644.6545	0.9743	VIHDNFGIMEELMTTVHVITATQKTVDGPSGK
3.0	3645.6355	-0.0068	YVINPWVLTDRMGMYRILLNETATYFAR
3.0	3645.6355	-0.0068	YVINPWVLTDRMGMYRILLNETATYFAR

3.0	3645.6355	-0.0068	YVINPWVLTDRMGMYRILLNETATYFAR
2.9	3643.6322	1.9965	SCAEAFFLGLRVKCLPSVASASINCCSRPAR

Spectrum No: 378; Query: 1160; Rank: 1

Peptide View

MS/MS Fragmentation of **GQSQTWPDTSSPEVR**
Found in **IPI00368692**, Tax_Id=10116 Gene_Symbol=Srrm2_predicted similar to serine/arginine repetitive matrix 2

Match to Query 1160: 1753.731908 from(877.873230,2+)
Title: 091008RatKidney_NoSalt_24.1395.1395.2.dta
Data file K:\NewmanPaper\Piliang\3SubProteomes\Piliang3SP\mgf5ppm\ERLIC_3SubProteomes5ppm.mgf

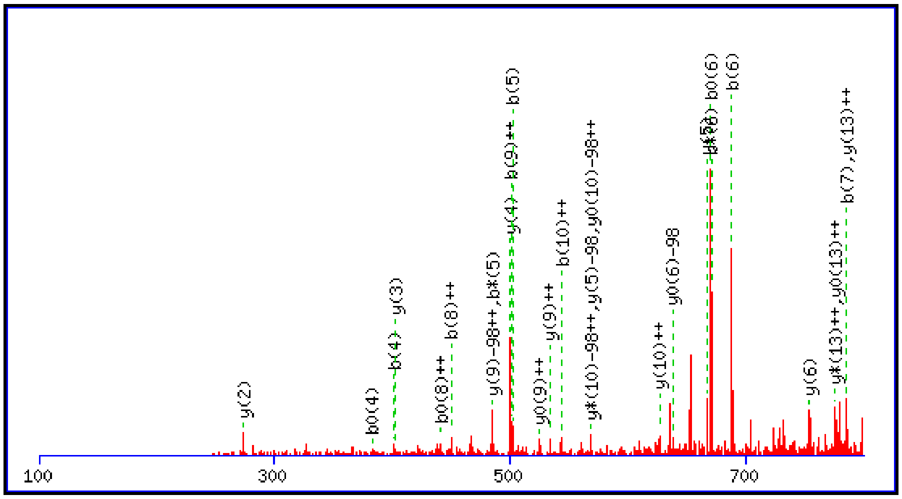

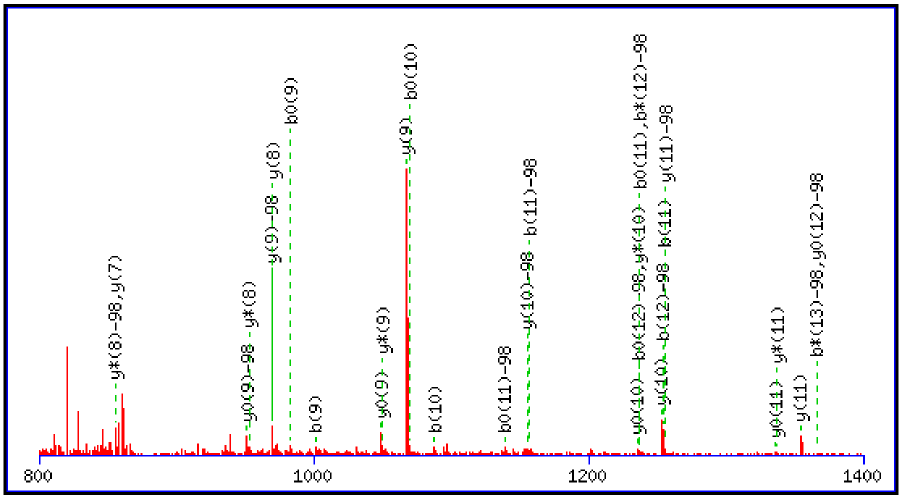

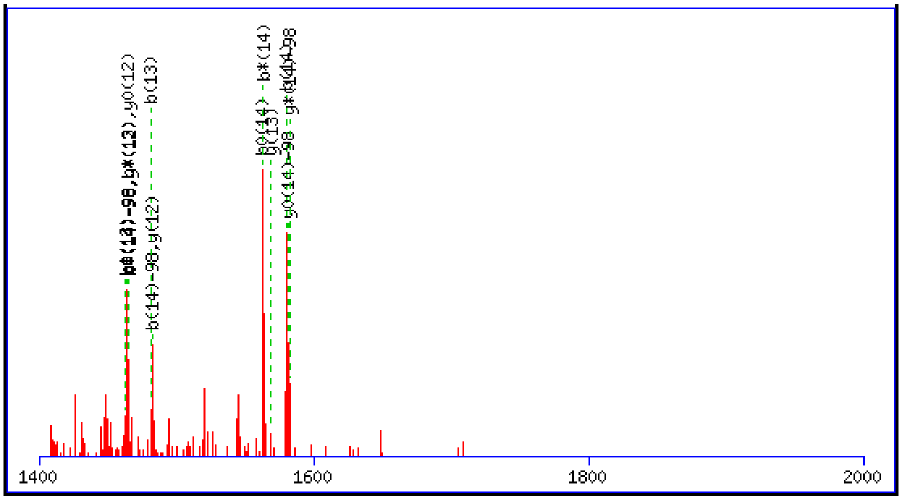

Monoisotopic mass of neutral peptide Mr(calc): 1753.7309
Fixed modifications: Carbamidomethyl (C)
Variable modifications:
S11 : Phospho (ST), with neutral losses 0.0000(shown in table), 97.9769
Ions Score: 46 **Expect:** 0.0038
Matches (Bold Red): 73/242 fragment ions using 130 most intense peaks

#	b	b^{++}	b^{*}	b^{*++}	b^{0}	b^{0++}	Seq.	y	y^{++}	y^{*}	y^{*++}	y^{0}	y^{0++}	#
1	58.0287	29.5180					G							15
2	186.0873	93.5473	169.0608	85.0340			Q	1697.7167	849.3620	1680.6901	840.8487	1679.7061	840.3567	14
3	273.1193	137.0633	256.0928	128.5500	255.1088	128.0580	S	**1569.6581**	**785.3327**	1552.6315	**776.8194**	1551.6475	**776.3274**	13
4	**401.1779**	201.0926	384.1514	192.5793	**383.1674**	192.0873	Q	**1482.6261**	741.8167	**1465.5995**	733.3034	**1464.6155**	732.8114	12
5	**502.2256**	251.6164	**485.1991**	243.1032	484.2150	242.6112	T	**1354.5675**	677.7874	**1337.5409**	669.2741	**1336.5569**	668.7821	11
6	**688.3049**	344.6561	**671.2784**	336.1428	**670.2944**	335.6508	W	**1253.5198**	**627.2635**	**1236.4933**	618.7503	**1235.5092**	618.2583	10
7	**785.3577**	393.1825	768.3311	384.6692	767.3471	384.1772	P	**1067.4405**	**534.2239**	**1050.4139**	525.7106	**1049.4299**	**525.2186**	9
8	900.3846	**450.6959**	883.3581	442.1827	882.3741	**441.6907**	D	**970.3877**	485.6975	**953.3612**	477.1842	952.3772	476.6922	8
9	**1001.4323**	**501.2198**	984.4058	492.7065	**983.4217**	492.2145	T	**855.3608**	428.1840	838.3342	419.6708	837.3502	419.1787	7
10	**1088.4643**	**544.7358**	1071.4378	536.2225	**1070.4538**	535.7305	S	**754.3131**	377.6602	737.2866	369.1469	736.3025	368.6549	6
11	**1255.4627**	628.2350	1238.4361	619.7217	**1237.4521**	619.2297	S	**667.2811**	334.1442	650.2545	325.6309	649.2705	325.1389	5
12	1352.5154	676.7614	1335.4889	668.2481	1334.5049	667.7561	P	**500.2827**	250.6450	483.2562	242.1317	482.2722	241.6397	4
13	**1481.5580**	741.2827	**1464.5315**	732.7694	**1463.5475**	732.2774	E	**403.2300**	202.1186	386.2034	193.6053	385.2194	193.1133	3
14	**1580.6265**	790.8169	**1563.5999**	782.3036	**1562.6159**	781.8116	V	**274.1874**	137.5973	257.1608	129.0840			2
15							R	175.1190	88.0631	158.0924	79.5498			1

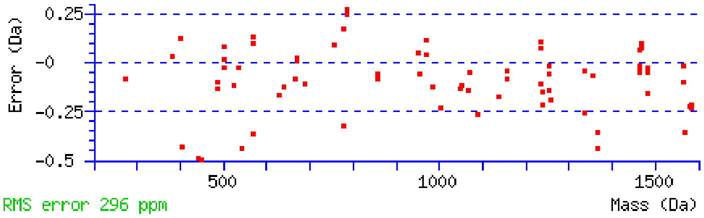

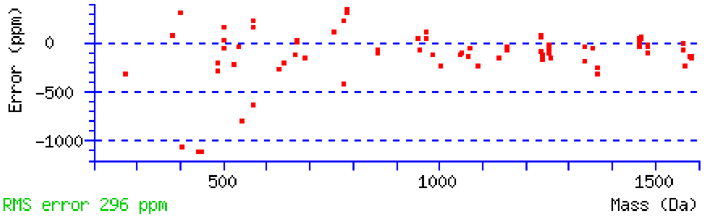

All matches to this query

Score	Mr(calc):	Delta	Sequence
46.4	1753.7309	0.0010	GQSQTWPDTSSPEVR
37.2	1753.7309	0.0010	GQSQTWPDTSSPEVR
34.1	1753.7309	0.0010	GQSQTWPDTSSPEVR
9.9	1753.7309	0.0010	GQSQTWPDTSSPEVR
5.8	1753.7342	-0.0023	SNAAGVPCDLVTGEER
4.1	1751.7103	2.0216	TTTWQRPTMESVR

4.0	1753.7309	0.0010	GQSQTWPDTSSPEVR
2.9	1753.7277	0.0042	LPADCACVTSGPRDR
2.7	1753.7381	-0.0061	SHSQSETRSSSPTQR
2.7	1753.7381	-0.0061	SHSQSETRSSSPTQR

Spectrum No: 379; Query: 2426; Rank: 1

Peptide View

MS/MS Fragmentation of **KLPSESDLLEGEVTDEDEEAEMSR**
Found in **IPI00207181**, Tax_Id=10116 Gene_Symbol=Plcl1 130kDa-Ins(1,4,5)P3 binding protein

Match to Query 2426: 2787.175272 from(930.065700,3+)
Title: 100101RatKid_NS_deglyco_22.3408.3408.3.dta
Data file K:\NewmanPaper\Piliang\3SubProteomes\Piliang3SP\mgf5ppm\ERLIC_3SubProteomes5ppm.mgf

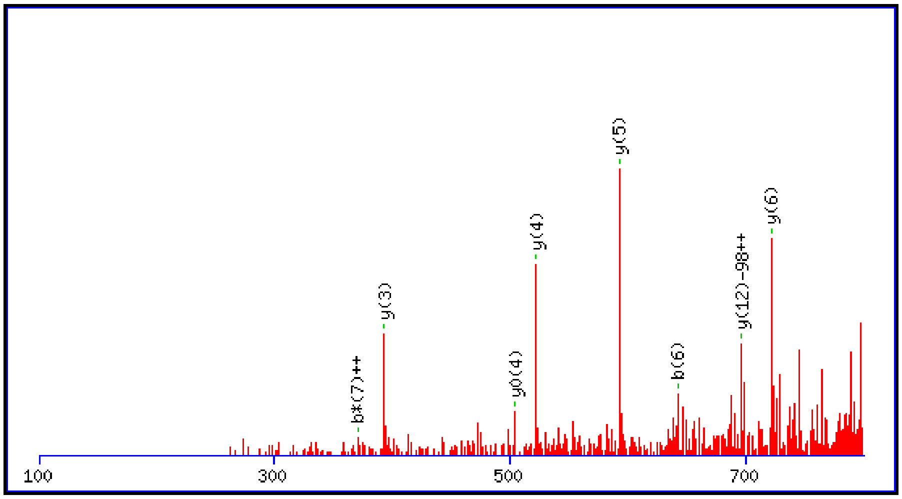

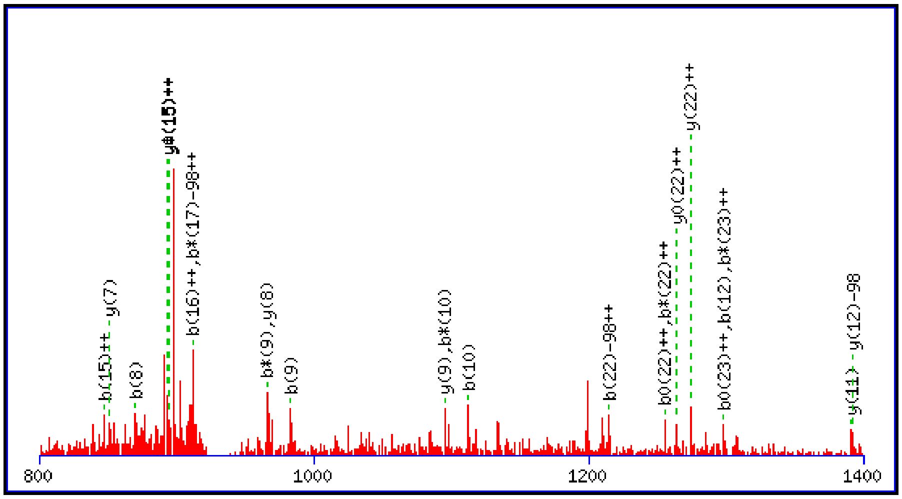

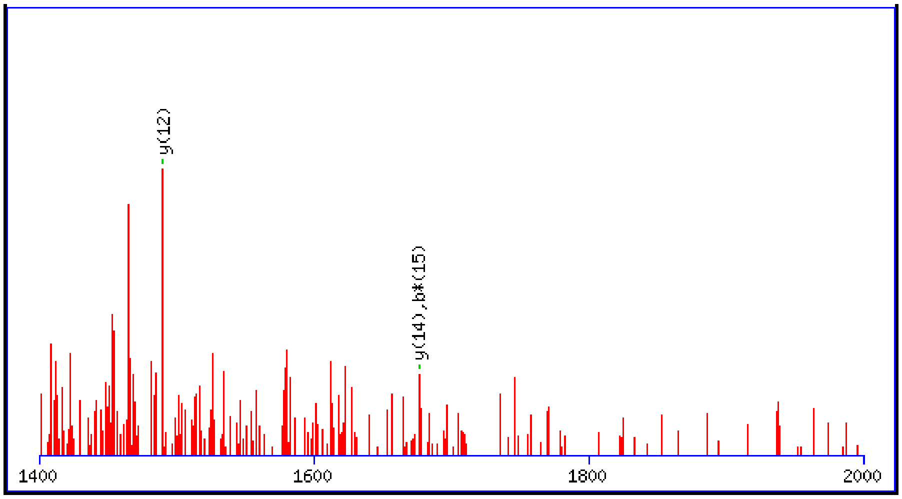

Monoisotopic mass of neutral peptide Mr(calc): 2787.1681
Fixed modifications: Carbamidomethyl (C)
Variable modifications:
T14 : Phospho (ST), with neutral losses 0.0000(shown in table), 97.9769
Ions Score: 46 **Expect:** 0.0079
Matches (Bold Red): 34/406 fragment ions using 47 most intense peaks

#	b	b++	b*	b*++	b0	b0++	Seq.	y	y++	y*	y*++	y0	y0++	#
1	129.1022	65.0548	112.0757	56.5415			K							24
2	242.1863	121.5968	225.1598	113.0835			L	2660.0805	1330.5439	2643.0539	1322.0306	2642.0699	1321.5386	23
3	339.2391	170.1232	322.2125	161.6099			P	2546.9964	**1274.0018**	2529.9698	1265.4886	2528.9858	**1264.9965**	22
4	426.2711	213.6392	409.2445	205.1259	408.2605	204.6339	S	2449.9436	1225.4754	2432.9171	1216.9622	2431.9331	1216.4702	21
5	555.3137	278.1605	538.2871	269.6472	537.3031	269.1552	E	2362.9116	1181.9594	2345.8850	1173.4462	2344.9010	1172.9542	20
6	**642.3457**	321.6765	625.3192	313.1632	624.3352	312.6712	S	2233.8690	1117.4381	2216.8425	1108.9249	2215.8584	1108.4329	19
7	757.3727	379.1900	740.3461	**370.6767**	739.3621	370.1847	D	2146.8370	1073.9221	2129.8104	1065.4089	2128.8264	1064.9168	18
8	**870.4567**	435.7320	853.4302	427.2187	852.4462	426.7267	L	2031.8100	1016.4087	2014.7835	1007.8954	2013.7995	1007.4034	17
9	**983.5408**	492.2740	**966.5142**	483.7608	965.5302	483.2687	L	1918.7260	959.8666	1901.6994	951.3533	1900.7154	950.8613	16
10	**1112.5834**	556.7953	**1095.5568**	548.2821	1094.5728	547.7900	E	1805.6419	903.3246	1788.6154	**894.8113**	1787.6313	**894.3193**	15
11	1169.6048	585.3061	1152.5783	576.7928	1151.5943	576.3008	G	**1676.5993**	838.8033	1659.5728	830.2900	1658.5887	829.7980	14
12	**1298.6474**	649.8274	1281.6209	641.3141	1280.6369	640.8221	E	1619.5778	810.2926	1602.5513	801.7793	1601.5673	801.2873	13
13	1397.7159	699.3616	1380.6893	690.8483	1379.7053	690.3563	V	**1490.5353**	745.7713	1473.5087	737.2580	1472.5247	736.7660	12
14	1578.7299	789.8686	1561.7033	781.3553	1560.7193	780.8633	T	**1391.4668**	696.2371	1374.4403	687.7238	1373.4563	687.2318	11
15	1693.7568	**847.3820**	**1676.7302**	838.8688	1675.7462	838.3768	D	1210.4528	605.7301	1193.4263	597.2168	1192.4423	596.7248	10
16	1822.7994	**911.9033**	1805.7728	903.3901	1804.7888	902.8981	E	**1095.4259**	548.2166	1078.3993	539.7033	1077.4153	539.2113	9
17	1937.8263	969.4168	1920.7998	960.9035	1919.8158	960.4115	D	**966.3833**	483.6953	949.3568	475.1820	948.3727	474.6900	8
18	2066.8689	1033.9381	2049.8424	1025.4248	2048.8584	1024.9328	E	**851.3564**	426.1818	834.3298	417.6685	833.3458	417.1765	7
19	2195.9115	1098.4594	2178.8850	1089.9461	2177.9010	1089.4541	E	**722.3138**	361.6605	705.2872	353.1472	704.3032	352.6552	6
20	2266.9486	1133.9780	2249.9221	1125.4647	2248.9381	1124.9727	A	**593.2712**	297.1392	576.2446	288.6259	575.2606	288.1339	5
21	2395.9912	1198.4993	2378.9647	1189.9860	2377.9807	1189.4940	E	**522.2341**	261.6207	505.2075	253.1074	**504.2235**	252.6154	4
22	2527.0317	1264.0195	2510.0052	**1255.5062**	2509.0211	**1255.0142**	M	**393.1915**	197.0994	376.1649	188.5861	375.1809	188.0941	3
23	2614.0637	1307.5355	2597.0372	**1299.0222**	2596.0532	**1298.5302**	S	262.1510	131.5791	245.1244	123.0659	244.1404	122.5738	2
24							R	175.1190	88.0631	158.0924	79.5498			1

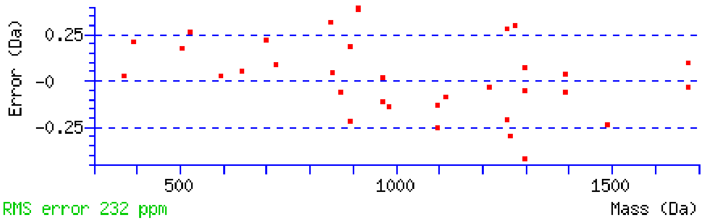

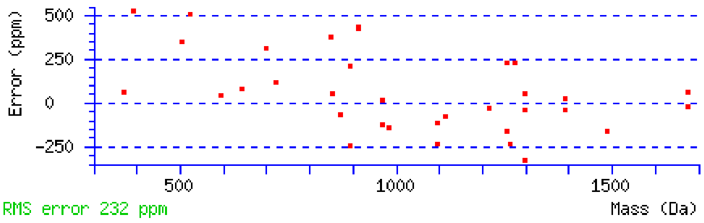

All matches to this query

Score	Mr(calc):	Delta	Sequence
46.3	2787.1681	0.0072	KLPSESDLLEGEVTDEDEEAEMSR
18.5	2787.1681	0.0072	KLPSESDLLEGEVTDEDEEAEMSR
18.5	2787.1681	0.0072	KLPSESDLLEGEVTDEDEEAEMSR
4.1	2787.1668	0.0084	YQVMNNVNKFGINRLVSSGIYK
1.2	2787.1711	0.0042	DPMEDGPPEEAARALSGSATLVSSPK
1.2	2787.1711	0.0042	DPMEDGPPEEAARALSGSATLVSSPK
0.7	2786.1828	0.9924	YQVMNNVNKFGINRLVSSGIYK
0.7	2787.1681	0.0072	KLPSESDLLEGEVTDEDEEAEMSR
0.3	2787.1711	0.0042	DPMEDGPPEEAARALSGSATLVSSPK
0.3	2786.1828	0.9924	YQVMNNVNKFGINRLVSSGIYK

Spectrum No: 380; Query: 1848; Rank: 1

Peptide View

MS/MS Fragmentation of **QLHIEGASLELSDDDTESK**
Found in **IPI00211813**, Tax_Id=10116 Gene_Symbol=Myh10 Myosin-10

Match to Query 1848: 2165.943582 from(722.988470,3+)
Title: 100101RatKid_NS_deglyco_20.3022.3022.3.dta
Data file K:\NewmanPaper\Piliang\3SubProteomes\Piliang3SP\mgf5ppm\ERLIC_3SubProteomes5ppm.mgf

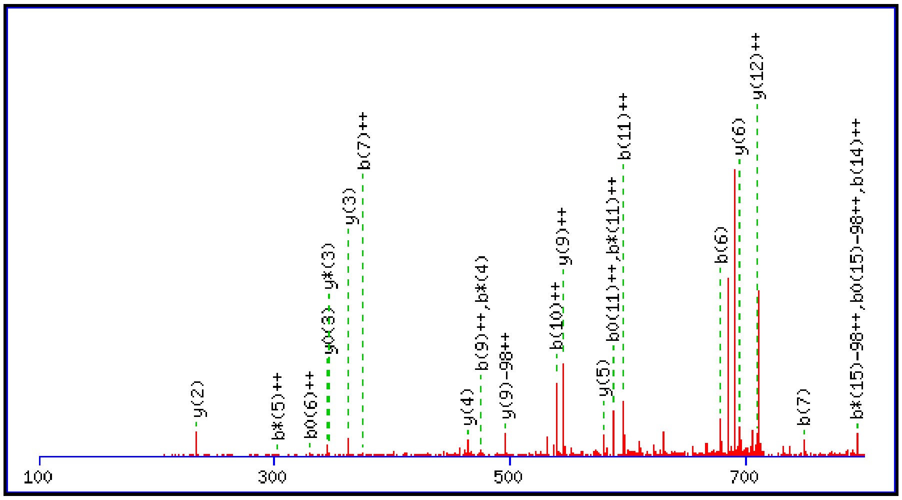

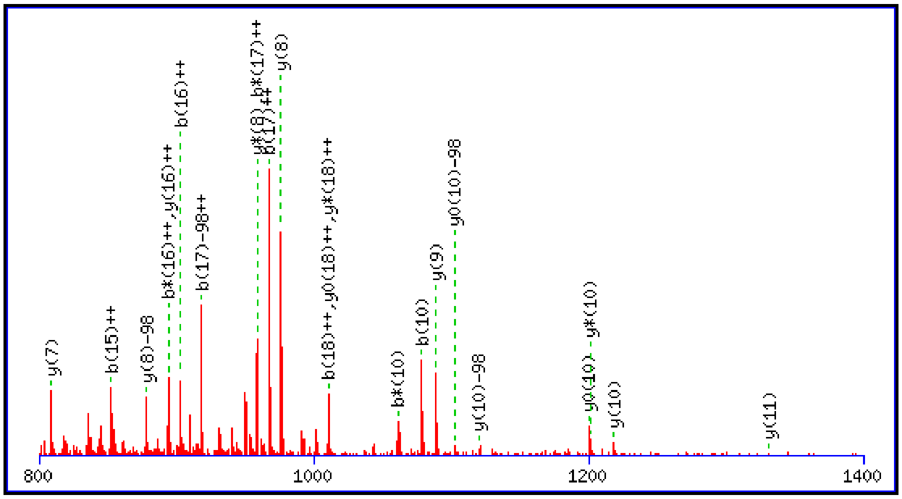

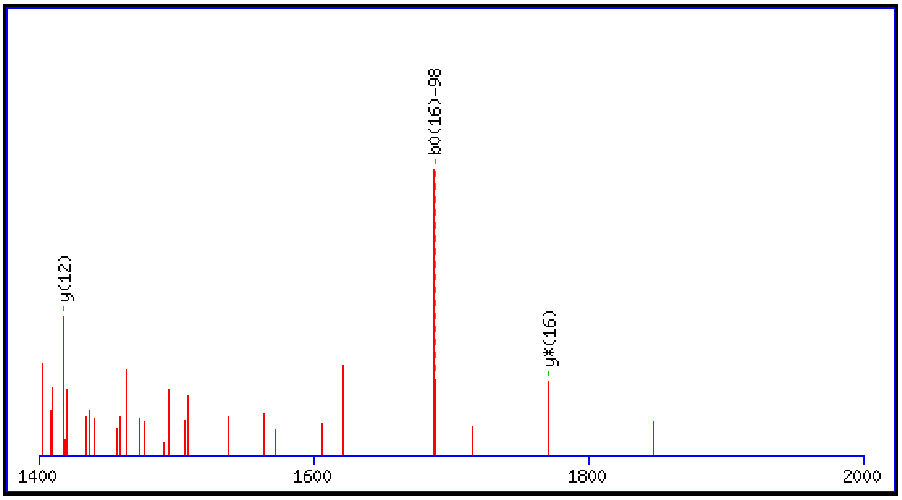

Monoisotopic mass of neutral peptide Mr(calc): 2165.9365
Fixed modifications: Carbamidomethyl (C)
Variable modifications:
S12 : Phospho (ST), with neutral losses 0.0000(shown in table), 97.9769
Ions Score: 46 **Expect:** 0.006
Matches (Bold Red): 50/314 fragment ions using 87 most intense peaks

#	b	b^{++}	b*	b^{*++}	b^0	b^{0++}	Seq.	y	y^{++}	y*	y^{*++}	y^0	y^{0++}	#
1	129.0659	65.0366	112.0393	56.5233			Q							19
2	242.1499	121.5786	225.1234	113.0653			L	2038.8852	1019.9463	2021.8587	**1011.4330**	2020.8747	**1010.9410**	18
3	379.2088	190.1081	362.1823	181.5948			H	1925.8012	963.4042	1908.7746	954.8910	1907.7906	954.3989	17
4	492.2929	246.6501	**475.2663**	238.1368			I	1788.7423	**894.8748**	**1771.7157**	886.3615	1770.7317	885.8695	16
5	621.3355	311.1714	604.3089	**302.6581**	603.3249	302.1661	E	1675.6582	838.3327	1658.6317	829.8195	1657.6476	829.3275	15
6	**678.3570**	339.6821	661.3304	331.1688	660.3464	**330.6768**	G	1546.6156	773.8114	1529.5891	765.2982	1528.6050	764.8062	14
7	**749.3941**	**375.2007**	732.3675	366.6874	731.3835	366.1954	A	1489.5941	745.3007	1472.5676	736.7874	1471.5836	736.2954	13
8	836.4261	418.7167	819.3995	410.2034	818.4155	409.7114	S	**1418.5570**	**709.7822**	1401.5305	701.2689	1400.5465	700.7769	12
9	949.5102	**475.2587**	932.4836	466.7454	931.4996	466.2534	L	**1331.5250**	666.2661	1314.4985	657.7529	1313.5144	657.2609	11
10	**1078.5528**	**539.7800**	**1061.5262**	531.2667	1060.5422	530.7747	E	**1218.4409**	609.7241	**1201.4144**	601.2108	**1200.4304**	600.7188	10
11	1191.6368	**596.3220**	1174.6103	**587.8088**	1173.6262	**587.3168**	L	**1089.3983**	**545.2028**	1072.3718	536.6895	1071.3878	536.1975	9
12	1358.6352	679.8212	1341.6086	671.3079	1340.6246	670.8159	S	**976.3143**	488.6608	**959.2877**	480.1475	958.3037	479.6555	8
13	1473.6621	737.3347	1456.6356	728.8214	1455.6515	728.3294	D	**809.3159**	405.1616	792.2894	396.6483	791.3054	396.1563	7
14	1588.6891	**794.8482**	1571.6625	786.3349	1570.6785	785.8429	D	**694.2890**	347.6481	677.2624	339.1349	676.2784	338.6429	6
15	1703.7160	**852.3616**	1686.6894	843.8484	1685.7054	843.3564	D	**579.2620**	290.1347	562.2355	281.6214	561.2515	281.1294	5
16	1804.7637	**902.8855**	1787.7371	**894.3722**	1786.7531	893.8802	T	**464.2351**	232.6212	447.2086	224.1079	446.2245	223.6159	4
17	1933.8063	**967.4068**	1916.7797	**958.8935**	1915.7957	958.4015	E	**363.1874**	182.0974	**346.1609**	173.5841	**345.1769**	173.0921	3
18	2020.8383	**1010.9228**	2003.8117	1002.4095	2002.8277	1001.9175	S	**234.1448**	117.5761	217.1183	109.0628	216.1343	108.5708	2
19							K	147.1128	74.0600	130.0863	65.5468			1

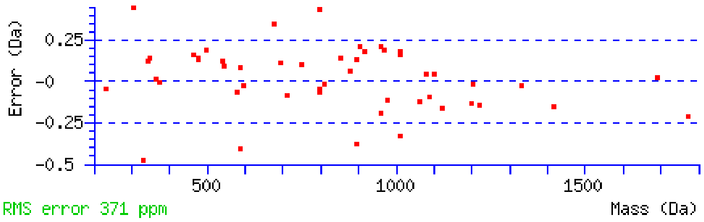

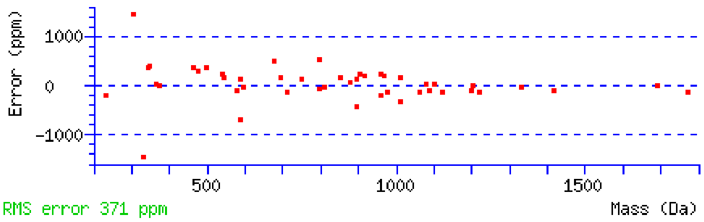

All matches to this query

Score	Mr(calc):	Delta	Sequence
46.3	2165.9365	0.0071	QLHIEGASLELSDDDTESK
24.7	2165.9365	0.0071	QLHIEGASLELSDDDTESK

15.4	2165.9365	0.0071	QLHIEGASLELSDDDTESK
15.0	2165.9365	0.0071	QLHIEGASLELSDDDTESK
3.3	2165.9452	-0.0017	QSMNESAHLGWELEQLSK
3.2	2163.9428	2.0008	QSFQKAMGNPCEFFVDIM
0.5	2164.9268	1.0168	QSFQKAMGNPCEFFVDIM
0.5	2164.9572	0.9864	SRATHCVADQTNQAALNVK

Spectrum No: 381; Query: 2512; Rank: 1

Peptide View

MS/MS Fragmentation of **LNEAAAGLNQAATELVQASRGTPQDLAR**
Found in **IPI00362014**, Tax_Id=10116 Gene_Symbol=Tln1 Tln1 protein

Match to Query 2512: 2944.442172 from(982.488000,3+)
Title: 091008RatKidney_NH4Format01_15.4154.4154.3.dta
Data file K:\NewmanPaper\Piliang\3SubProteomes\Piliang3SP\mgf5ppm\ERLIC_3SubProteomes5ppm.mgf

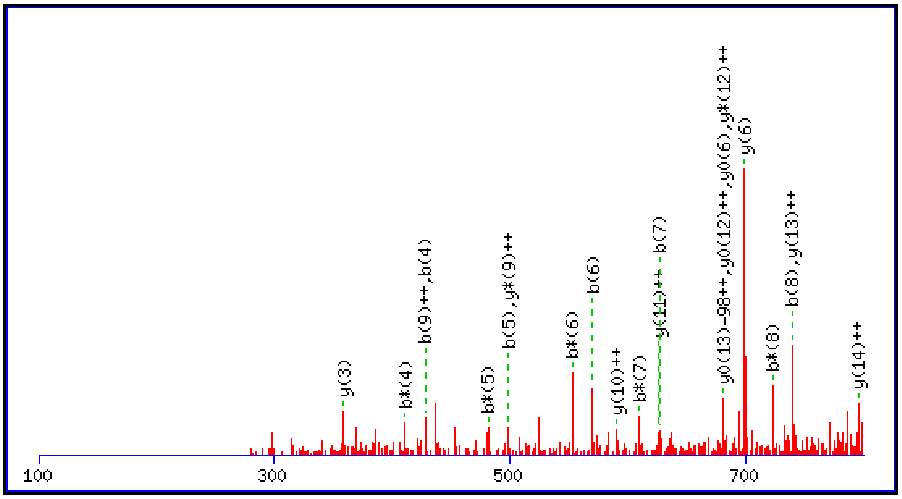

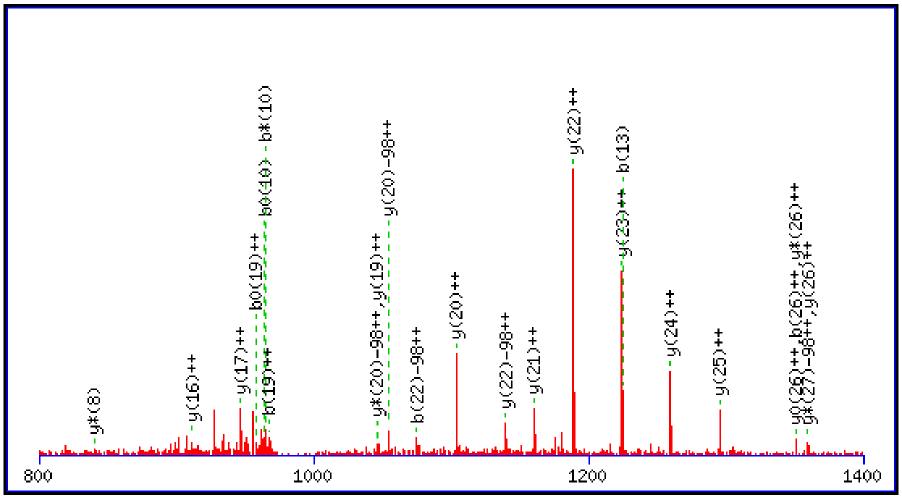

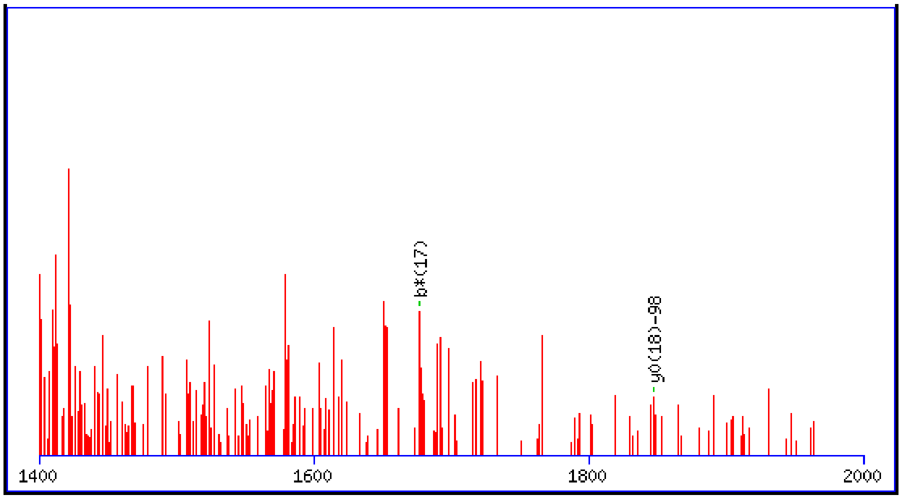

Monoisotopic mass of neutral peptide Mr(calc): 2944.4403
Fixed modifications: Carbamidomethyl (C)
Variable modifications:
S19 : Phospho (ST), with neutral losses 0.0000(shown in table), 97.9769
Ions Score: 46 **Expect:** 0.006
Matches (Bold Red): 48/474 fragment ions using 90 most intense peaks

#	b	b^{++}	b*	b^{*++}	b^{0}	b^{0++}	Seq.	y	y^{++}	y*	y^{*++}	y^{0}	y^{0++}	#
1	114.0913	57.5493					L							28
2	228.1343	114.5708	211.1077	106.0575			N	2832.3636	1416.6854	2815.3370	1408.1722	2814.3530	1407.6801	27
3	357.1769	179.0921	340.1503	170.5788	339.1663	170.0868	E	2718.3207	**1359.6640**	2701.2941	**1351.1507**	2700.3101	**1350.6587**	26
4	**428.2140**	214.6106	**411.1874**	206.0974	410.2034	205.6053	A	2589.2781	**1295.1427**	2572.2515	1286.6294	2571.2675	1286.1374	25
5	**499.2511**	250.1292	**482.2245**	241.6159	481.2405	241.1239	A	2518.2410	**1259.6241**	2501.2144	1251.1108	2500.2304	1250.6188	24
6	**570.2882**	285.6477	**553.2617**	277.1345	552.2776	276.6425	A	2447.2038	**1224.1056**	2430.1773	1215.5923	2429.1933	1215.1003	23
7	**627.3097**	314.1585	**610.2831**	305.6452	609.2991	305.1532	G	2376.1667	**1188.5870**	2359.1402	1180.0737	2358.1562	1179.5817	22
8	**740.3937**	370.7005	**723.3672**	362.1872	722.3832	361.6952	L	2319.1453	**1160.0763**	2302.1187	1151.5630	2301.1347	1151.0710	21
9	854.4367	**427.7220**	837.4101	419.2087	836.4261	418.7167	N	2206.0612	**1103.5342**	2189.0346	1095.0210	2188.0506	1094.5290	20
10	982.4952	491.7513	**965.4687**	483.2380	**964.4847**	482.7460	Q	2092.0183	**1046.5128**	2074.9917	1037.9995	2074.0077	1037.5075	19
11	1053.5323	527.2698	1036.5058	518.7565	1035.5218	518.2645	A	1963.9597	982.4835	1946.9331	973.9702	1945.9491	973.4782	18
12	1124.5695	562.7884	1107.5429	554.2751	1106.5589	553.7831	A	1892.9226	**946.9649**	1875.8960	938.4517	1874.9120	937.9596	17
13	**1225.6171**	613.3122	1208.5906	604.7989	1207.6066	604.3069	T	1821.8855	**911.4464**	1804.8589	902.9331	1803.8749	902.4411	16
14	1354.6597	677.8335	1337.6332	669.3202	1336.6492	668.8282	E	1720.8378	860.9225	1703.8112	852.4093	1702.8272	851.9172	15
15	1467.7438	734.3755	1450.7172	725.8623	1449.7332	725.3703	L	1591.7952	**796.4012**	1574.7686	787.8880	1573.7846	787.3960	14
16	1566.8122	783.9097	1549.7857	775.3965	1548.8016	774.9045	V	1478.7111	**739.8592**	1461.6846	731.3459	1460.7006	730.8539	13
17	1694.8708	847.9390	**1677.8442**	839.4258	1676.8602	838.9338	Q	1379.6427	690.3250	1362.6162	**681.8117**	1361.6321	**681.3197**	12
18	1765.9079	883.4576	1748.8814	874.9443	1747.8973	874.4523	A	1251.5841	**626.2957**	1234.5576	617.7824	1233.5736	617.2904	11
19	1932.9063	**966.9568**	1915.8797	958.4435	1914.8957	**957.9515**	S	1180.5470	**590.7771**	1163.5205	582.2639	1162.5365	581.7719	10
20	2089.0074	1045.0073	2071.9808	1036.4940	2070.9968	1036.0020	R	1013.5487	507.2780	996.5221	**498.7647**	995.5381	498.2727	9
21	2146.0288	1073.5181	2129.0023	1065.0048	2128.0183	1064.5128	G	857.4476	429.2274	**840.4210**	420.7141	839.4370	420.2221	8
22	2247.0765	1124.0419	2230.0500	1115.5286	2229.0659	1115.0366	T	800.4261	400.7167	783.3995	392.2034	782.4155	391.7114	7
23	2344.1293	1172.5683	2327.1027	1164.0550	2326.1187	1163.5630	P	**699.3784**	350.1928	682.3519	341.6796	**681.3679**	341.1876	6
24	2472.1879	1236.5976	2455.1613	1228.0843	2454.1773	1227.5923	Q	602.3257	301.6665	585.2991	293.1532	584.3151	292.6612	5
25	2587.2148	1294.1110	2570.1882	1285.5978	2569.2042	1285.1058	D	474.2671	237.6372	457.2405	229.1239	456.2565	228.6319	4
26	2700.2989	**1350.6531**	2683.2723	1342.1398	2682.2883	1341.6478	L	**359.2401**	180.1237	342.2136	171.6104			3
27	2771.3360	1386.1716	2754.3094	1377.6584	2753.3254	1377.1663	A	246.1561	123.5817	229.1295	115.0684			2
28							R	175.1190	88.0631	158.0924	79.5498			1

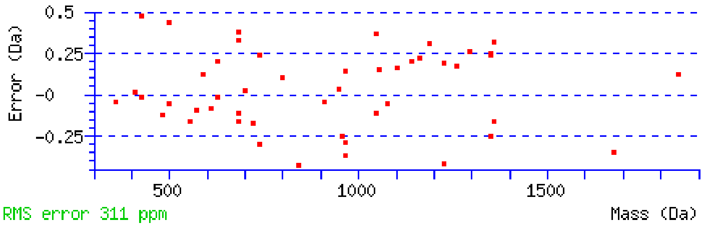

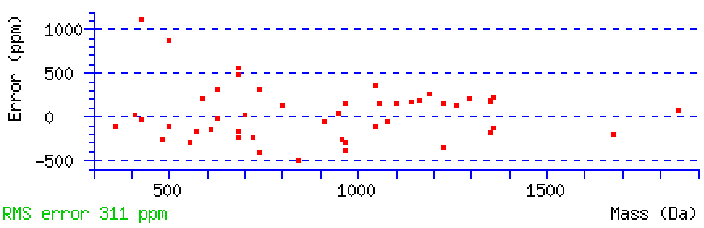

All matches to this query

Score	Mr(calc):	Delta	Sequence
46.2	2944.4403	0.0018	LNEAAAGLNQAATELVQASRGTPQDLAR
46.2	2944.4403	0.0018	LNEAAAGLNQAATELVQASRGTPQDLAR
25.5	2944.4403	0.0018	LNEAAAGLNQAATELVQASRGTPQDLAR
8.2	2944.4565	-0.0143	RPPVPARGSGMPASMFSIDNILAARPR
1.6	2944.4565	-0.0143	RPPVPARGSGMPASMFSIDNILAARPR
1.5	2944.4565	-0.0143	RPPVPARGSGMPASMFSIDNILAARPR
1.1	2944.4167	0.0254	IERVIGAGLGSEFXHQCSHRLTWPK
1.1	2944.4167	0.0254	IERVIGAGLGSEFXHQCSHRLTWPK
0.9	2942.4144	2.0278	VVRVLGCNPGPMTLQGTNTYLVGTGSR
0.9	2942.4144	2.0278	VVRVLGCNPGPMTLQGTNTYLVGTGSR

Spectrum No: 382; Query: 2691; Rank: 1

Peptide View

MS/MS Fragmentation of **KQTPPASPSPQPAEDRPPSSPIYEDAAPLK**
Found in **IPI00198498**, Tax_Id=10116 Gene_Symbol=Cttn Cortactin isoform C

Match to Query 2691: 3410.477382 from(1137.833070,3+)
Title: 091008RatKidney_NoSalt_27.2452.2452.3.dta
Data file K:\NewmanPaper\Piliang\3SubProteomes\Piliang3SP\mgf5ppm\ERLIC_3SubProteomes5ppm.mgf

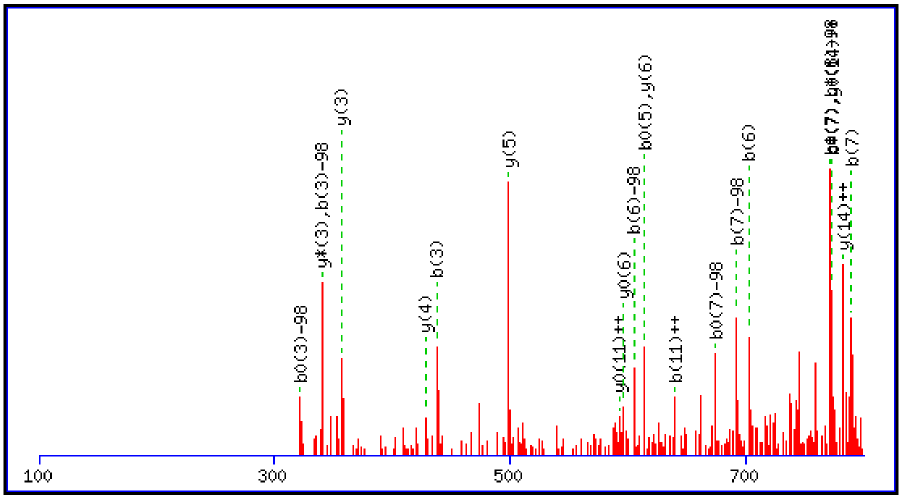

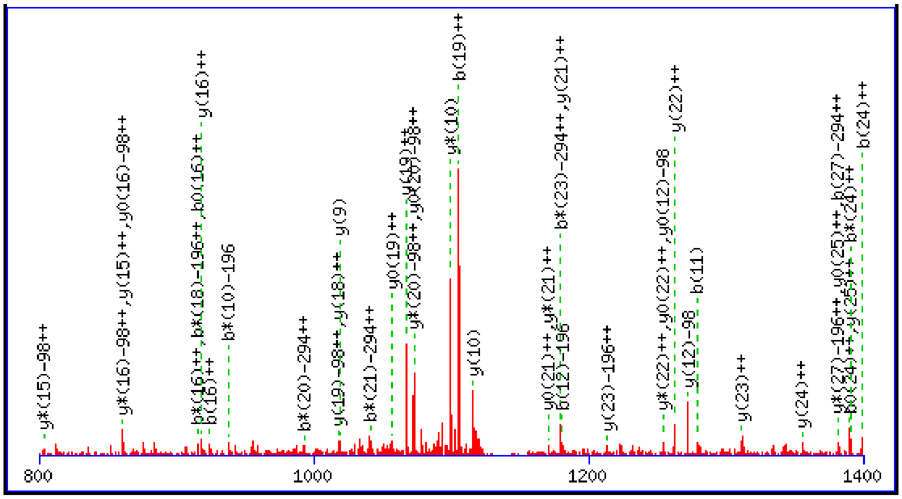

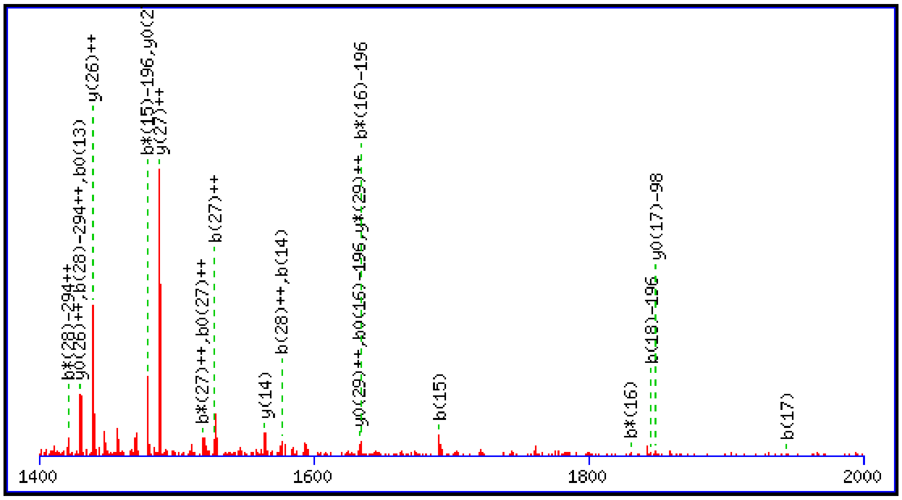

Monoisotopic mass of neutral peptide Mr(calc): 3410.4873
Fixed modifications: Carbamidomethyl (C)
Variable modifications:
T3 : Phospho (ST), with neutral losses 0.0000(shown in table), 97.9769
S9 : Phospho (ST), with neutral losses 0.0000(shown in table), 97.9769
S19 : Phospho (ST), with neutral losses 0.0000(shown in table), 97.9769
Ions Score: 46 **Expect:** 0.011
Matches (Bold Red): 90/604 fragment ions using 120 most intense peaks

#	b	b^{++}	b*	b^{*++}	b^{0}	b^{0++}	Seq.	y	y^{++}	y*	y^{*++}	y^{0}	y^{0++}	#
1	129.1022	65.0548	112.0757	56.5415			K							30
2	257.1608	129.0840	240.1343	120.5708			Q	3283.3997	1642.2035	3266.3731	**1633.6902**	3265.3891	**1633.1982**	29
3	**438.1748**	219.5910	421.1483	211.0778	420.1643	210.5858	T	3155.3411	1578.1742	3138.3146	1569.6609	3137.3306	1569.1689	28
4	535.2276	268.1174	518.2010	259.6042	517.2170	259.1121	P	2974.3271	**1487.6672**	2957.3006	**1479.1539**	2956.3165	**1478.6619**	27
5	632.2803	316.6438	615.2538	308.1305	**614.2698**	307.6385	P	2877.2744	**1439.1408**	2860.2478	1430.6275	2859.2638	**1430.1355**	26
6	**703.3175**	352.1624	686.2909	343.6491	685.3069	343.1571	A	2780.2216	**1390.6144**	2763.1950	1382.1012	2762.2110	**1381.6091**	25
7	**790.3495**	395.6784	**773.3229**	387.1651	**772.3389**	386.6731	S	2709.1845	**1355.0959**	2692.1579	1346.5826	2691.1739	1346.0906	24
8	887.4023	444.2048	870.3757	435.6915	869.3917	435.1995	P	2622.1524	**1311.5799**	2605.1259	1303.0666	2604.1419	1302.5746	23
9	1054.4006	527.7039	1037.3741	519.1907	1036.3900	518.6987	S	2525.0997	**1263.0535**	2508.0731	**1254.5402**	2507.0891	**1254.0482**	22
10	1151.4534	576.2303	1134.4268	567.7170	1133.4428	567.2250	P	2358.1013	**1179.5543**	2341.0748	**1171.0410**	2340.0908	**1170.5490**	21
11	**1279.5119**	**640.2596**	1262.4854	631.7463	1261.5014	631.2543	Q	2261.0486	1131.0279	2244.0220	1122.5146	2243.0380	1122.0226	20
12	1376.5647	688.7860	1359.5382	680.2727	1358.5541	679.7807	P	2132.9900	**1066.9986**	2115.9634	1058.4854	2114.9794	**1057.9933**	19
13	1447.6018	724.3046	1430.5753	715.7913	**1429.5913**	715.2993	A	2035.9372	**1018.4722**	2018.9107	1009.9590	2017.9267	1009.4670	18
14	**1576.6444**	788.8258	1559.6179	780.3126	1558.6339	779.8206	E	1964.9001	982.9537	1947.8736	974.4404	1946.8895	973.9484	17
15	**1691.6714**	846.3393	1674.6448	837.8260	1673.6608	837.3340	D	1835.8575	**918.4324**	1818.8310	909.9191	1817.8470	909.4271	16
16	1847.7725	**924.3899**	**1830.7459**	**915.8766**	1829.7619	**915.3846**	R	1720.8306	**860.9189**	1703.8040	852.4056	1702.8200	851.9136	15
17	**1944.8252**	972.9163	1927.7987	964.4030	1926.8147	963.9110	P	**1564.7295**	**782.8684**	1547.7029	774.3551	1546.7189	**773.8631**	14

**18**	2041.8780	1021.4426	2024.8515	1012.9294	2023.8674	1012.4374	**P**	1467.6767	734.3420	1450.6501	725.8287	1449.6661	725.3367	**13**
**19**	2208.8764	**1104.9418**	2191.8498	1096.4285	2190.8658	1095.9365	**S**	1370.6239	685.8156	1353.5974	677.3023	1352.6134	676.8103	**12**
**20**	2295.9084	1148.4578	2278.8818	1139.9446	2277.8978	1139.4525	**S**	1203.6256	602.3164	1186.5990	593.8032	1185.6150	**593.3111**	**11**
**21**	2392.9611	1196.9842	2375.9346	1188.4709	2374.9506	1187.9789	**P**	**1116.5936**	558.8004	**1099.5670**	550.2871	1098.5830	549.7951	**10**
**22**	2506.0452	1253.5262	2489.0187	1245.0130	2488.0346	1244.5210	**I**	**1019.5408**	510.2740	1002.5142	501.7608	1001.5302	501.2688	**9**
**23**	2669.1085	1335.0579	2652.0820	1326.5446	2651.0980	1326.0526	**Y**	906.4567	453.7320	889.4302	445.2187	888.4462	444.7267	**8**
**24**	2798.1511	**1399.5792**	2781.1246	**1391.0659**	2780.1406	**1390.5739**	**E**	743.3934	372.2003	726.3668	363.6871	725.3828	363.1951	**7**
**25**	2913.1781	1457.0927	2896.1515	1448.5794	2895.1675	1448.0874	**D**	**614.3508**	307.6790	597.3243	299.1658	**596.3402**	298.6738	**6**
**26**	2984.2152	1492.6112	2967.1886	1484.0980	2966.2046	1483.6059	**A**	**499.3239**	250.1656	482.2973	241.6523			**5**
**27**	3055.2523	**1528.1298**	3038.2258	**1519.6165**	3037.2417	**1519.1245**	**A**	**428.2867**	214.6470	411.2602	206.1337			**4**
**28**	3152.3051	**1576.6562**	3135.2785	1568.1429	3134.2945	1567.6509	**P**	**357.2496**	179.1285	**340.2231**	170.6152			**3**
**29**	3265.3891	**1633.1982**	3248.3626	1624.6849	3247.3786	1624.1929	**L**	260.1969	130.6021	243.1703	122.0888			**2**
**30**							**K**	147.1128	74.0600	130.0863	65.5468			**1**

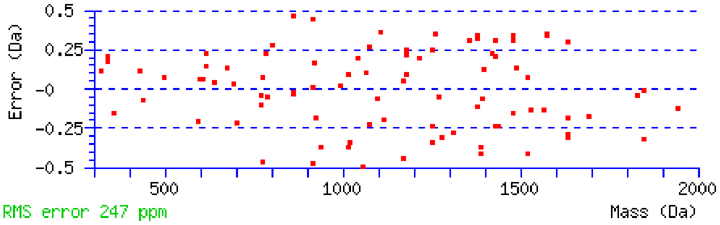

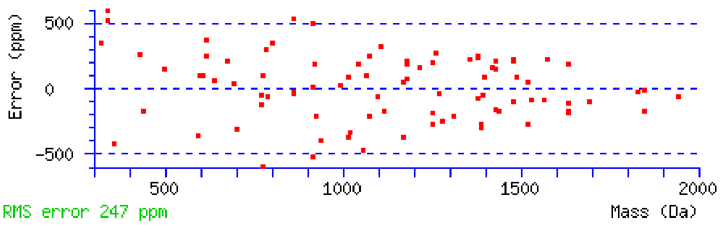

All matches to this query

Score	Mr(calc):	Delta	Sequence
46.1	3410.4873	-0.0100	KQTPPASPSPQPAEDRPPSSPIYEDAAPLK
43.5	3410.4873	-0.0100	KQTPPASPSPQPAEDRPPSSPIYEDAAPLK
42.0	3410.4873	-0.0100	KQTPPASPSPQPAEDRPPSSPIYEDAAPLK
40.5	3410.4873	-0.0100	KQTPPASPSPQPAEDRPPSSPIYEDAAPLK
39.1	3410.4873	-0.0100	KQTPPASPSPQPAEDRPPSSPIYEDAAPLK
36.6	3410.4873	-0.0100	KQTPPASPSPQPAEDRPPSSPIYEDAAPLK
36.1	3410.4873	-0.0100	KQTPPASPSPQPAEDRPPSSPIYEDAAPLK
32.9	3410.4873	-0.0100	KQTPPASPSPQPAEDRPPSSPIYEDAAPLK
31.7	3410.4873	-0.0100	KQTPPASPSPQPAEDRPPSSPIYEDAAPLK
21.5	3410.4873	-0.0100	KQTPPASPSPQPAEDRPPSSPIYEDAAPLK

Spectrum No: 383; Query: 2202; Rank: 1

Peptide View

MS/MS Fragmentation of **DVEDSLPIKESDETVTVALSPK**
Found in **IPI00421709**, Tax_Id=10116 Gene_Symbol=Son similar to SON protein

Match to Query 2202: 2451.169388 from(1226.591970,2+)
Title: 100101RatKid_NS_deglyco_19.3821.3821.2.dta
Data file K:\NewmanPaper\Piliang\3SubProteomes\Piliang3SP\mgf5ppm\ERLIC_3SubProteomes5ppm.mgf

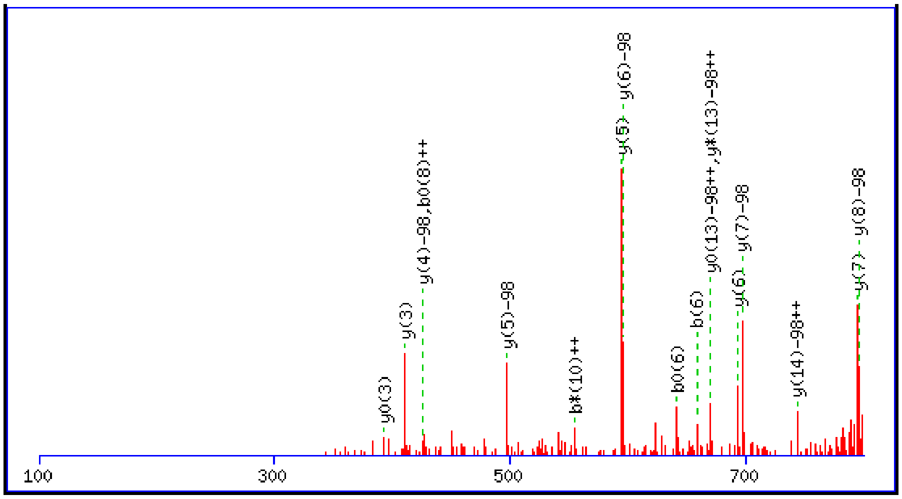

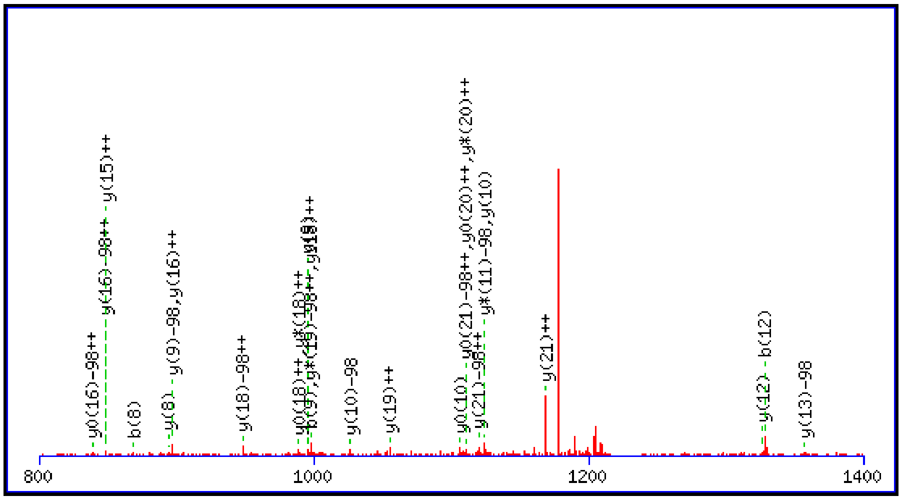

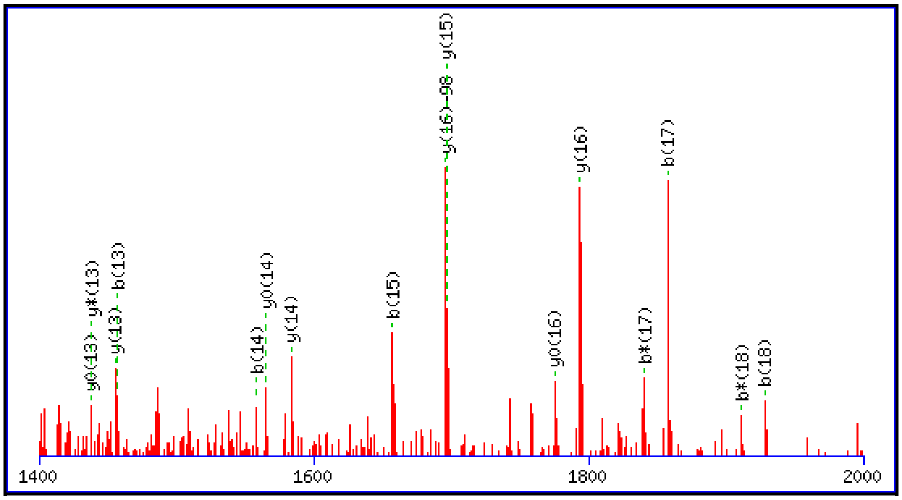

Monoisotopic mass of neutral peptide Mr(calc): 2451.1669
Fixed modifications: Carbamidomethyl (C)
Variable modifications:
S20 : Phospho (ST), with neutral losses 0.0000(shown in table), 97.9769
Ions Score: 46 **Expect:** 0.0073
Matches (Bold Red): 60/358 fragment ions using 90 most intense peaks

#	b	b^{++}	b*	b^{*++}	b^0	b^{0++}	Seq.	y	y^{++}	y*	y^{*++}	y^0	y^{0++}	#
1	116.0342	58.5207			98.0237	49.5155	D							22
2	215.1026	108.0550			197.0921	99.0497	V	2337.1473	**1169.0773**	2320.1207	1160.5640	2319.1367	1160.0720	21
3	344.1452	172.5763			326.1347	163.5710	E	2238.0789	1119.5431	2221.0523	**1111.0298**	2220.0683	**1110.5378**	20
4	459.1722	230.0897			441.1616	221.0844	D	2109.0363	**1055.0218**	2092.0097	1046.5085	2091.0257	1046.0165	19
5	546.2042	273.6057			528.1936	264.6005	S	1994.0093	**997.5083**	1976.9828	**988.9950**	1975.9988	**988.5030**	18

**6**	**659.2883**	330.1478			**641.2777**	321.1425	**L**	1906.9773	953.9923	1889.9507	945.4790	1888.9667	944.9870	**17**
**7**	756.3410	378.6742			738.3305	369.6689	**P**	**1793.8932**	**897.4503**	1776.8667	888.9370	**1775.8827**	888.4450	**16**
**8**	**869.4251**	435.2162			851.4145	**426.2109**	**I**	**1696.8405**	**848.9239**	1679.8139	840.4106	1678.8299	839.9186	**15**
**9**	**997.5201**	499.2637	980.4935	490.7504	979.5095	490.2584	**K**	**1583.7564**	792.3818	1566.7299	783.8686	**1565.7458**	783.3766	**14**
**10**	1126.5626	563.7850	1109.5361	**555.2717**	1108.5521	554.7797	**E**	**1455.6614**	728.3344	**1438.6349**	719.8211	**1437.6509**	719.3291	**13**
**11**	1213.5947	607.3010	1196.5681	598.7877	1195.5841	598.2957	**S**	**1326.6188**	663.8131	1309.5923	655.2998	1308.6083	654.8078	**12**
**12**	**1328.6216**	664.8144	1311.5951	656.3012	1310.6111	655.8092	**D**	1239.5868	620.2970	1222.5603	611.7838	1221.5763	611.2918	**11**
**13**	**1457.6642**	729.3357	1440.6377	720.8225	1439.6536	720.3305	**E**	**1124.5599**	562.7836	1107.5333	554.2703	**1106.5493**	553.7783	**10**
**14**	**1558.7119**	779.8596	1541.6853	771.3463	1540.7013	770.8543	**T**	**995.5173**	498.2623	978.4907	489.7490	977.5067	489.2570	**9**
**15**	**1657.7803**	829.3938	1640.7538	820.8805	1639.7697	820.3885	**V**	**894.4696**	447.7384	877.4431	439.2252	876.4590	438.7332	**8**
**16**	1758.8280	879.9176	1741.8014	871.4044	1740.8174	870.9123	**T**	**795.4012**	398.2042	778.3746	389.6910	777.3906	389.1990	**7**
**17**	**1857.8964**	929.4518	**1840.8698**	920.9386	1839.8858	920.4466	**V**	**694.3535**	347.6804	677.3270	339.1671	676.3429	338.6751	**6**
**18**	**1928.9335**	964.9704	**1911.9070**	956.4571	1910.9229	955.9651	**A**	**595.2851**	298.1462	578.2585	289.6329	577.2745	289.1409	**5**
**19**	2042.0176	1021.5124	2024.9910	1012.9992	2024.0070	1012.5071	**L**	524.2480	262.6276	507.2214	254.1144	506.2374	253.6223	**4**
**20**	2209.0159	1105.0116	2191.9894	1096.4983	2191.0054	1096.0063	**S**	**411.1639**	206.0856	394.1374	197.5723	**393.1534**	197.0803	**3**
**21**	2306.0687	1153.5380	2289.0421	1145.0247	2288.0581	1144.5327	**P**	244.1656	122.5864	227.1390	114.0731			**2**
**22**							**K**	147.1128	74.0600	130.0863	65.5468			**1**

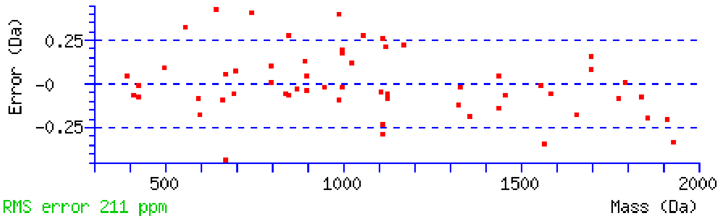

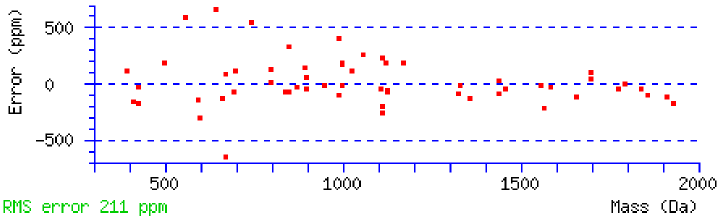

All matches to this query

Score	Mr(calc):	Delta	Sequence
45.9	2451.1669	0.0025	DVEDSLPIKESDETVTVALSPK
24.6	2451.1669	0.0025	DVEDSLPIKESDETVTVALSPK
18.4	2451.1669	0.0025	DVEDSLPIKESDETVTVALSPK
11.2	2451.1669	0.0025	DVEDSLPIKESDETVTVALSPK
7.0	2450.1747	0.9947	SLILVSFVRSNEDGTLGELLK
5.8	2450.1747	0.9947	SLILVSFVRSNEDGTLGELLK
2.8	2451.1790	-0.0096	TMKLSDSYTPSMVTDLLRVSK
2.3	2451.1505	0.0189	GPDRAWSLLQECGLALELSEK
2.2	2450.1470	1.0224	LKAKYAAATGGPPPTPLPHMEK
2.2	2450.1470	1.0224	LKAKYAAATGGPPPTPLPHMEK

Spectrum No: 384; Query: 2614; Rank: 1

Peptide View

MS/MS Fragmentation of **TNTNVNCPIECFMPLDVQADRDDSRE**
Found in **IPI00200757**, Tax_Id=10116 Gene_Symbol=Fn1 Isoform 1 of Fibronectin precursor

Match to Query 2614: 3191.313672 from(1064.778500,3+)
Title: 100101RatKid_NS_deglyco_19.3677.3677.3.dta
Data file K:\NewmanPaper\Piliang\3SubProteomes\Piliang3SP\mgf5ppm\ERLIC_3SubProteomes5ppm.mgf

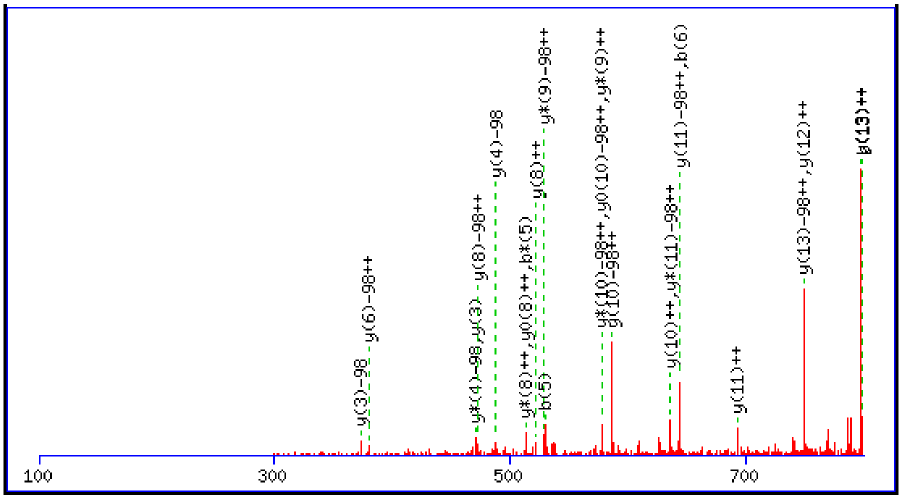

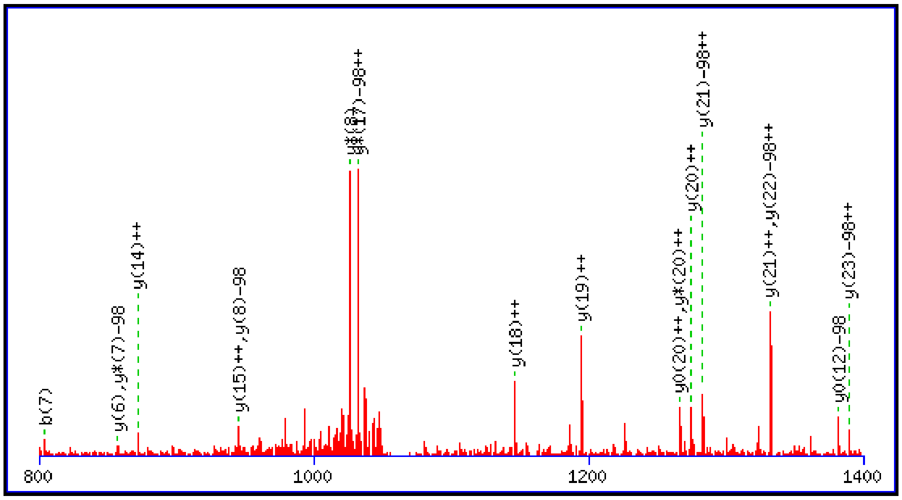

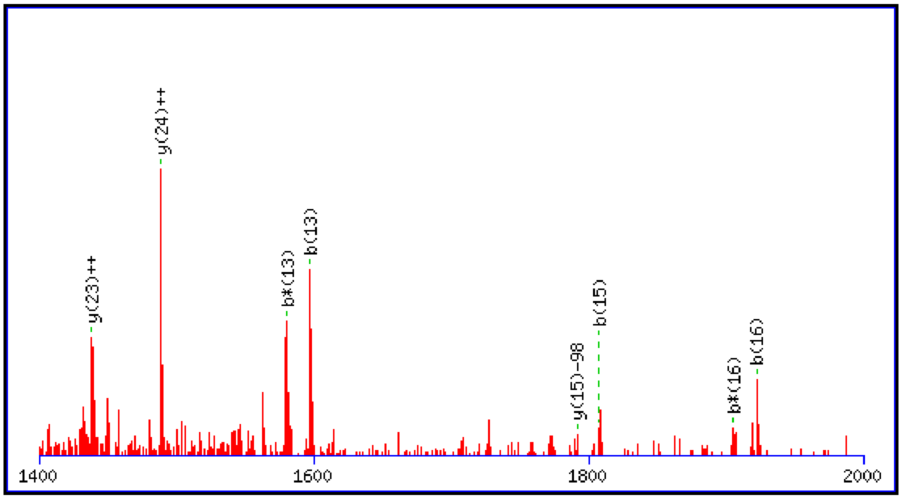

Monoisotopic mass of neutral peptide Mr(calc): 3191.2995
Fixed modifications: Carbamidomethyl (C)
Variable modifications:
M13 : Oxidation (M)
S24 : Phospho (ST), with neutral losses 0.0000(shown in table), 97.9769
Ions Score: 46 **Expect:** 0.01
Matches (Bold Red): 51/446 fragment ions using 60 most intense peaks

#	b	b++	b*	b*++	b0	b0++	Seq.	y	y++	y*	y*++	y0	y0++	#
1	102.0550	51.5311			84.0444	42.5258	T							26
2	216.0979	108.5526	199.0713	100.0393	198.0873	99.5473	N	3091.2591	1546.1332	3074.2326	1537.6199	3073.2486	1537.1279	25
3	317.1456	159.0764	300.1190	150.5631	299.1350	150.0711	T	2977.2162	**1489.1117**	2960.1896	1480.5985	2959.2056	1480.1065	24
4	431.1885	216.0979	414.1619	207.5846	413.1779	207.0926	N	2876.1685	**1438.5879**	2859.1420	1430.0746	2858.1580	1429.5826	23

5	530.2569	265.6321	513.2304	257.1188	512.2463	256.6268	V	2762.1256	1381.5664	2745.0990	1373.0532	2744.1150	1372.5612	22
6	644.2998	322.6536	627.2733	314.1403	626.2893	313.6483	N	2663.0572	1332.0322	2646.0306	1323.5190	2645.0466	1323.0269	21
7	804.3305	402.6689	787.3039	394.1556	786.3199	393.6636	C	2549.0143	1275.0108	2531.9877	1266.4975	2531.0037	1266.0055	20
8	901.3832	451.1953	884.3567	442.6820	883.3727	442.1900	P	2388.9836	1194.9954	2371.9571	1186.4822	2370.9730	1185.9902	19
9	1014.4673	507.7373	997.4408	499.2240	996.4567	498.7320	I	2291.9308	1146.4691	2274.9043	1137.9558	2273.9203	1137.4638	18
10	1143.5099	572.2586	1126.4834	563.7453	1125.4993	563.2533	E	2178.8468	1089.9270	2161.8202	1081.4138	2160.8362	1080.9217	17
11	1303.5405	652.2739	1286.5140	643.7606	1285.5300	643.2686	C	2049.8042	1025.4057	2032.7776	1016.8925	2031.7936	1016.4004	16
12	1450.6090	725.8081	1433.5824	717.2948	1432.5984	716.8028	F	1889.7735	945.3904	1872.7470	936.8771	1871.7630	936.3851	15
13	1597.6444	799.3258	1580.6178	790.8125	1579.6338	790.3205	M	1742.7051	871.8562	1725.6786	863.3429	1724.6946	862.8509	14
14	1694.6971	847.8522	1677.6706	839.3389	1676.6866	838.8469	P	1595.6697	798.3385	1578.6432	789.8252	1577.6591	789.3332	13
15	1807.7812	904.3942	1790.7546	895.8810	1789.7706	895.3890	L	1498.6169	749.8121	1481.5904	741.2988	1480.6064	740.8068	12
16	1922.8081	961.9077	1905.7816	953.3944	1904.7976	952.9024	D	1385.5329	693.2701	1368.5063	684.7568	1367.5223	684.2648	11
17	2021.8766	1011.4419	2004.8500	1002.9286	2003.8660	1002.4366	V	1270.5059	635.7566	1253.4794	627.2433	1252.4954	626.7513	10
18	2149.9351	1075.4712	2132.9086	1066.9579	2131.9246	1066.4659	Q	1171.4375	586.2224	1154.4110	577.7091	1153.4270	577.2171	9
19	2220.9722	1110.9898	2203.9457	1102.4765	2202.9617	1101.9845	A	1043.3790	522.1931	1026.3524	513.6798	1025.3684	513.1878	8
20	2335.9992	1168.5032	2318.9726	1159.9900	2317.9886	1159.4979	D	972.3418	486.6746	955.3153	478.1613	954.3313	477.6693	7
21	2492.1003	1246.5538	2475.0737	1238.0405	2474.0897	1237.5485	R	857.3149	429.1611	840.2883	420.6478	839.3043	420.1558	6
22	2607.1272	1304.0673	2590.1007	1295.5540	2589.1167	1295.0620	D	701.2138	351.1105	684.1872	342.5973	683.2032	342.1052	5
23	2722.1542	1361.5807	2705.1276	1353.0675	2704.1436	1352.5754	D	586.1868	293.5971	569.1603	285.0838	568.1763	284.5918	4
24	2889.1525	1445.0799	2872.1260	1436.5666	2871.1420	1436.0746	S	471.1599	236.0836	454.1333	227.5703	453.1493	227.0783	3
25	3045.2536	1523.1305	3028.2271	1514.6172	3027.2431	1514.1252	R	304.1615	152.5844	287.1350	144.0711	286.1510	143.5791	2
26							E	148.0604	74.5339			130.0499	65.5286	1

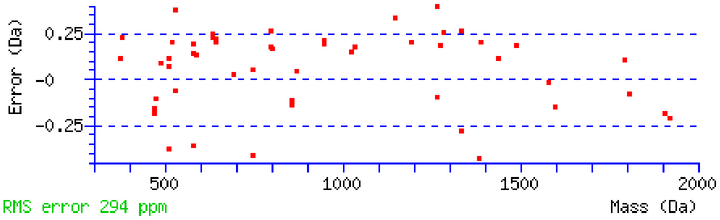

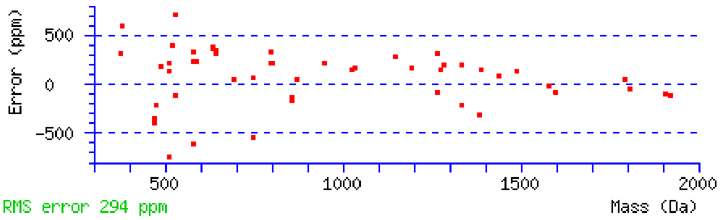

All matches to this query

Score	Mr(calc):	Delta	Sequence
45.9	3191.2995	0.0141	TNTNVNCPIECFMPLDVQADRDDSRE
8.1	3189.3386	1.9751	VPSDSLWIPDIVLFDNADGRFEGASTK
8.1	3189.3386	1.9751	VPSDSLWIPDIVLFDNADGRFEGASTK
2.4	3191.3263	-0.0126	MTQEEVEMLVVGHEDSNGGINCVNILK
2.1	3191.2904	0.0233	MAHARISMYMPPDIDPTKAAIAYGCR
1.8	3191.3263	-0.0126	MTQEEVEMLVVGHEDSNGGINCVNILK
1.4	3190.3161	0.9976	CSLSDIPHHIPEEVTAWFQETVLDK
1.4	3190.3161	0.9976	CSLSDIPHHIPEEVTAWFQETVLDK
1.1	3190.2802	1.0334	SMMKMLLDPMMTNDGNAILQEIQNSR
1.0	3189.2787	2.0350	FKDYDLETLLSSLQEIDTVXSFNR

Spectrum No: 385; Query: 1686; Rank: 1

Peptide View

MS/MS Fragmentation of **DMDEPSPVPNVEEVTLPK**
Found in **IPI00393081**, Tax_Id=10116 Gene_Symbol=LOC685179 similar to SWI/SNF-related matrix-associated actin-dependent regulator of

chromatin c2 isoform b isoform 2

Match to Query 1686: 2074.914808 from(1038.464680,2+)
Title: 091008RatKidney_NH4Format02_26.3318.3318.2.dta
Data file K:\NewmanPaper\Piliang\3SubProteomes\Piliang3SP\mgf5ppm\ERLIC_3SubProteomes5ppm.mgf

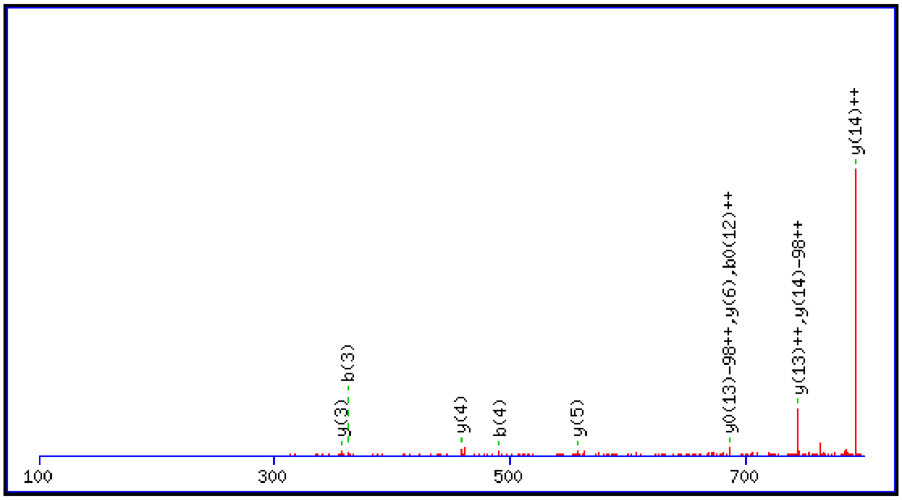

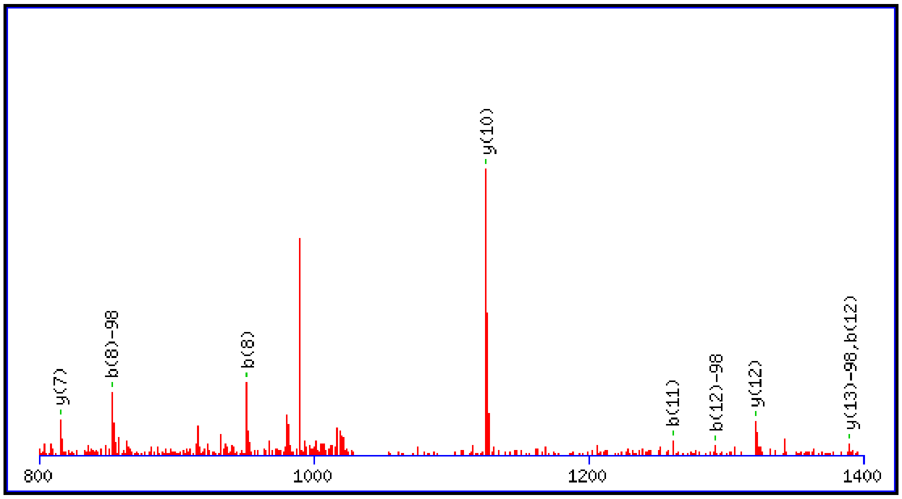

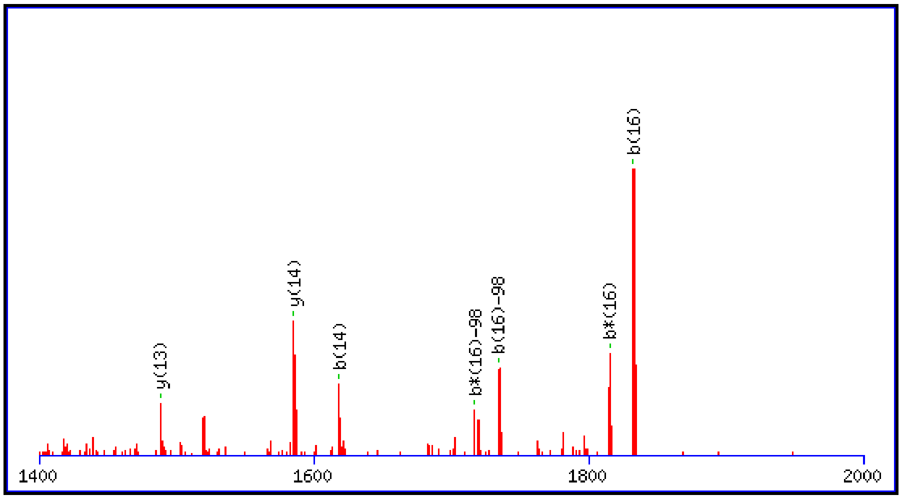

Monoisotopic mass of neutral peptide Mr(calc): 2074.9170
Fixed modifications: Carbamidomethyl (C)
Variable modifications:
S6 : Phospho (ST), with neutral losses 0.0000(shown in table), 97.9769
Ions Score: 46 **Expect:** 0.0078
Matches (Bold Red): 27/274 fragment ions using 46 most intense peaks

#	b	b^{++}	b*	b^{*++}	b^0	b^{0++}	Seq.	y	y^{++}	y*	y^{*++}	y^0	y^{0++}	#
1	116.0342	58.5207			98.0237	49.5155	D							18

2	247.0747	124.0410			229.0641	115.0357	M	1960.8973	980.9523	1943.8708	972.4390	1942.8868	971.9470	17
3	362.1016	181.5545			344.0911	172.5492	D	1829.8568	915.4321	1812.8303	906.9188	1811.8463	906.4268	16
4	491.1442	246.0758			473.1337	237.0705	E	1714.8299	857.9186	1697.8034	849.4053	1696.8193	848.9133	15
5	588.1970	294.6021			570.1864	285.5969	P	1585.7873	793.3973	1568.7608	784.8840	1567.7767	784.3920	14
6	755.1954	378.1013			737.1848	369.0960	S	1488.7345	744.8709	1471.7080	736.3576	1470.7240	735.8656	13
7	852.2481	426.6277			834.2376	417.6224	P	1321.7362	661.3717	1304.7096	652.8585	1303.7256	652.3665	12
8	951.3165	476.1619			933.3060	467.1566	V	1224.6834	612.8454	1207.6569	604.3321	1206.6729	603.8401	11
9	1048.3693	524.6883			1030.3587	515.6830	P	1125.6150	563.3111	1108.5885	554.7979	1107.6045	554.3059	10
10	1162.4122	581.7098	1145.3857	573.1965	1144.4017	572.7045	N	1028.5623	514.7848	1011.5357	506.2715	1010.5517	505.7795	9
11	1261.4806	631.2440	1244.4541	622.7307	1243.4701	622.2387	V	914.5193	457.7633	897.4928	449.2500	896.5088	448.7580	8
12	1390.5232	695.7653	1373.4967	687.2520	1372.5127	686.7600	E	815.4509	408.2291	798.4244	399.7158	797.4403	399.2238	7
13	1519.5658	760.2866	1502.5393	751.7733	1501.5553	751.2813	E	686.4083	343.7078	669.3818	335.1945	668.3978	334.7025	6
14	1618.6342	809.8208	1601.6077	801.3075	1600.6237	800.8155	V	557.3657	279.1865	540.3392	270.6732	539.3552	270.1812	5
15	1719.6819	860.3446	1702.6554	851.8313	1701.6714	851.3393	T	458.2973	229.6523	441.2708	221.1390	440.2867	220.6470	4
16	1832.7660	916.8866	1815.7394	908.3734	1814.7554	907.8813	L	357.2496	179.1285	340.2231	170.6152			3
17	1929.8187	965.4130	1912.7922	956.8997	1911.8082	956.4077	P	244.1656	122.5864	227.1390	114.0731			2
18							K	147.1128	74.0600	130.0863	65.5468			1

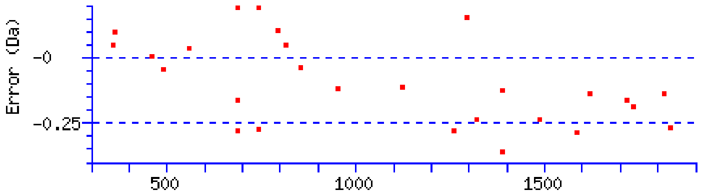

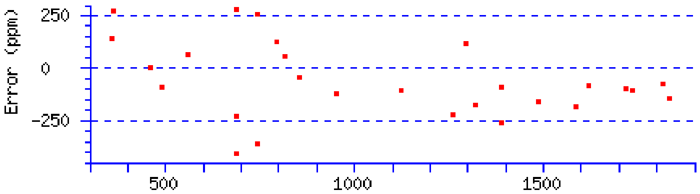

All matches to this query

Score	Mr(calc):	Delta	Sequence
45.8	2074.9170	-0.0022	DMDEPSPVPNVEEVTLPK
6.0	2074.9337	-0.0189	AVAEVSESTKAEAVAAVQR
5.8	2074.9170	-0.0022	DMDEPSPVPNVEEVTLPK
5.0	2073.9060	1.0088	MAGACGKHHMSPGSLPGKR
3.4	2073.9190	0.9958	QKLYTVNAEECAVGQER
3.4	2073.9190	0.9958	QKLYTVNAEECAVGQER
2.1	2074.9272	-0.0124	CGTLRLDLTGTSGAAVPAR
1.7	2074.9238	-0.0090	SNQFRSPSGHLITDKTK
1.7	2074.9238	-0.0090	SNQFRSPSGHLITDKTK
0.9	2074.9213	-0.0065	AFGQSPSLYKHMRIHK

Spectrum No: 386; Query: 975; Rank: 1

Peptide View

MS/MS Fragmentation of **VLHGAQTSDEEKDF**
Found in **IPI00365864**, Tax_Id=10116 Gene_Symbol=LOC688717 hypothetical protein LOC688717

Match to Query 975: 1654.684548 from(828.349550,2+)
Title: 091008RatKidney_NoSalt_21.883.883.2.dta
Data file K:\NewmanPaper\Piliang\3SubProteomes\Piliang3SP\mgf5ppm\ERLIC_3SubProteomes5ppm.mgf

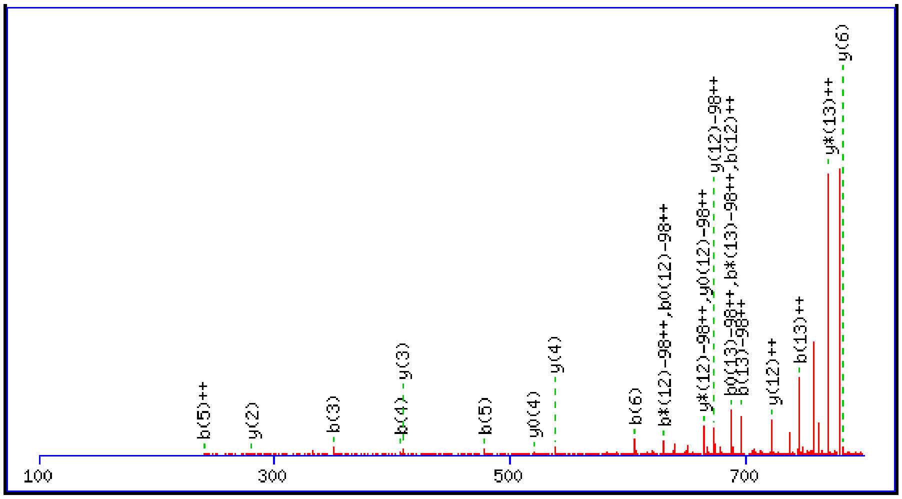

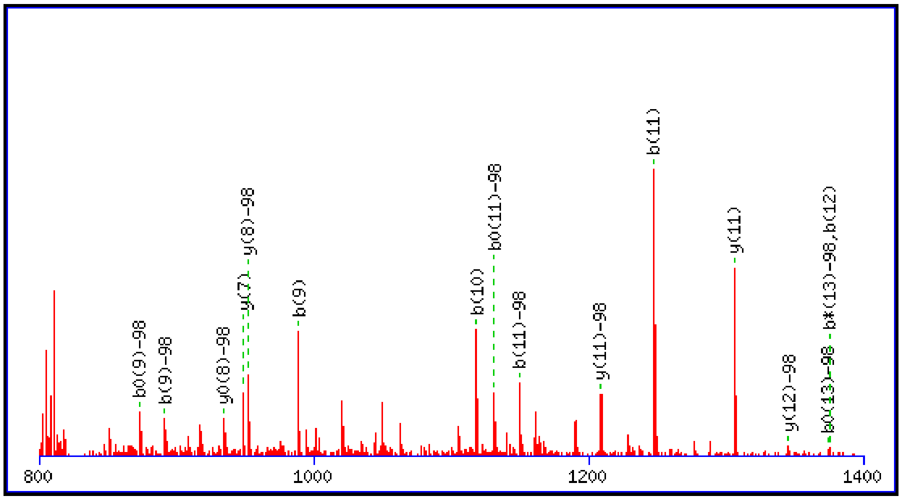

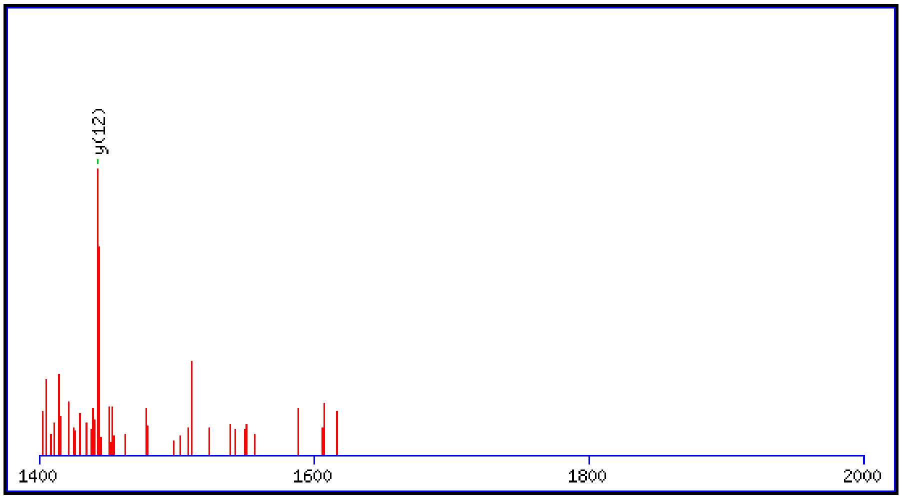

Monoisotopic mass of neutral peptide Mr(calc): 1654.6876
Fixed modifications: Carbamidomethyl (C)
Variable modifications:
S8 : Phospho (ST), with neutral losses 97.9769(shown in table), 0.0000
Ions Score: 46 **Expect:** 0.004
Matches (Bold Red): 39/206 fragment ions using 50 most intense peaks

#	b	b^{++}	b*	b^{*++}	b^0	b^{0++}	Seq.	y	y^{++}	y*	y^{*++}	y^0	y^{0++}	#
1	100.0757	50.5415					V							14
2	213.1598	107.0835					L	1458.6496	729.8284	1441.6230	721.3151	1440.6390	720.8231	13
3	**350.2187**	175.6130					H	**1345.5655**	**673.2864**	1328.5389	**664.7731**	1327.5549	**664.2811**	12
4	**407.2401**	204.1237					G	**1208.5066**	604.7569	1191.4800	596.2437	1190.4960	595.7516	11
5	**478.2772**	**239.6423**					A	1151.4851	576.2462	1134.4586	567.7329	1133.4746	567.2409	10

**6**	**606.3358**	303.6715	589.3093	295.1583			**Q**	1080.4480	540.7276	1063.4215	532.2144	1062.4374	531.7224	**9**
**7**	707.3835	354.1954	690.3570	345.6821	689.3729	345.1901	**T**	**952.3894**	476.6984	935.3629	468.1851	**934.3789**	467.6931	**8**
**8**	776.4050	388.7061	759.3784	380.1928	758.3944	379.7008	**S**	851.3417	426.1745	834.3152	417.6612	833.3312	417.1692	**7**
**9**	**891.4319**	446.2196	874.4054	437.7063	**873.4213**	437.2143	**D**	**782.3203**	391.6638	765.2937	383.1505	764.3097	382.6585	**6**
**10**	1020.4745	510.7409	1003.4479	502.2276	1002.4639	501.7356	**E**	667.2933	334.1503	650.2668	325.6370	649.2828	325.1450	**5**
**11**	**1149.5171**	575.2622	1132.4905	566.7489	**1131.5065**	566.2569	**E**	**538.2508**	269.6290	521.2242	261.1157	**520.2402**	260.6237	**4**
**12**	1277.6120	639.3097	1260.5855	**630.7964**	1259.6015	**630.3044**	**K**	**409.2082**	205.1077	392.1816	196.5944	391.1976	196.1024	**3**
**13**	1392.6390	**696.8231**	**1375.6124**	**688.3099**	**1374.6284**	**687.8179**	**D**	**281.1132**	141.0602			263.1026	132.0550	**2**
**14**							**F**	166.0863	83.5468					**1**

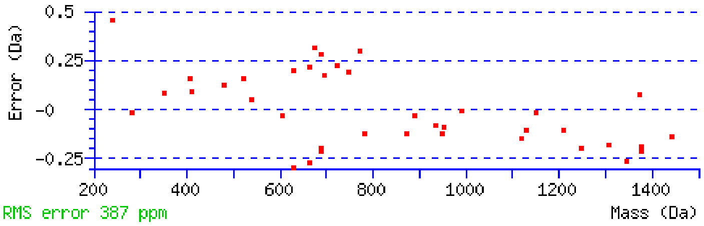

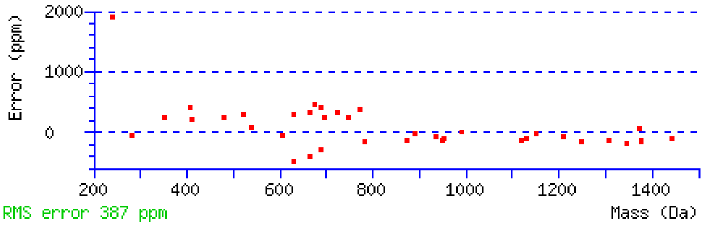

All matches to this query

Score	Mr(calc):	Delta	Sequence
45.8	1654.6876	-0.0030	VLHGAQTSDEEKDF
44.4	1654.6876	-0.0030	VLHGAQTSDEEKDF
2.1	1654.6947	-0.0102	NNSRNNSSGRAPSVI
1.5	1654.6865	-0.0020	LSQDTYRRNSVR
1.4	1654.6745	0.0100	CDLCGKAFQHPSR
0.9	1654.6925	-0.0079	QFVPMSDMKWYK
0.7	1654.6947	-0.0102	NNSRNNSSGRAPSVI
0.6	1654.6762	0.0084	ISPVNRMYAICR
0.6	1654.6947	-0.0102	NNSRNNSSGRAPSVI
0.5	1654.6865	-0.0020	LSKSNSQNVEPHR

Spectrum No: 387; Query: 2787; Rank: 1

Peptide View

MS/MS Fragmentation of **QAEVADQQTTDLPAENGETENQSPASEEEKEAKSD**
Found in **IPI00373052**, Tax_Id=10116 Gene_Symbol=Hmgn1 High mobility group nucleosomal binding domain 1

Match to Query 2787: 3854.630052 from(1285.883960,3+)
Title: 100101RatKid_NS_deglyco_24.1648.1648.3.dta
Data file K:\NewmanPaper\Piliang\3SubProteomes\Piliang3SP\mgf5ppm\ERLIC_3SubProteomes5ppm.mgf

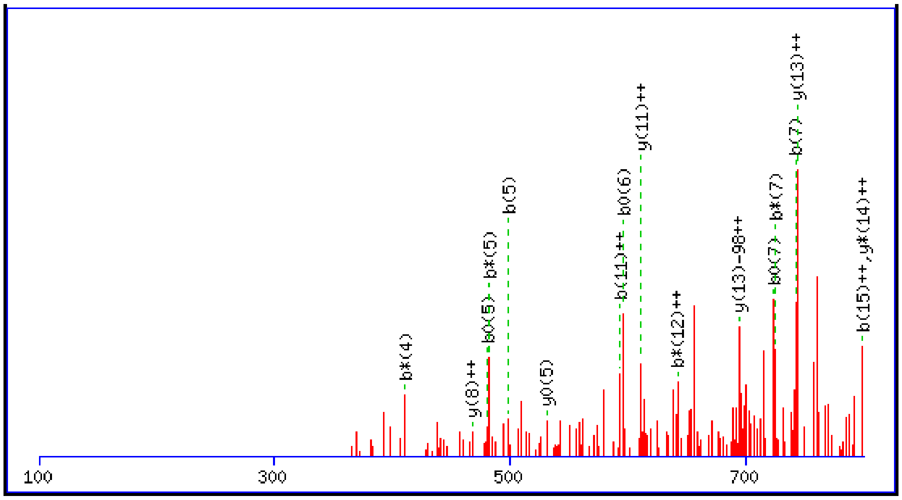

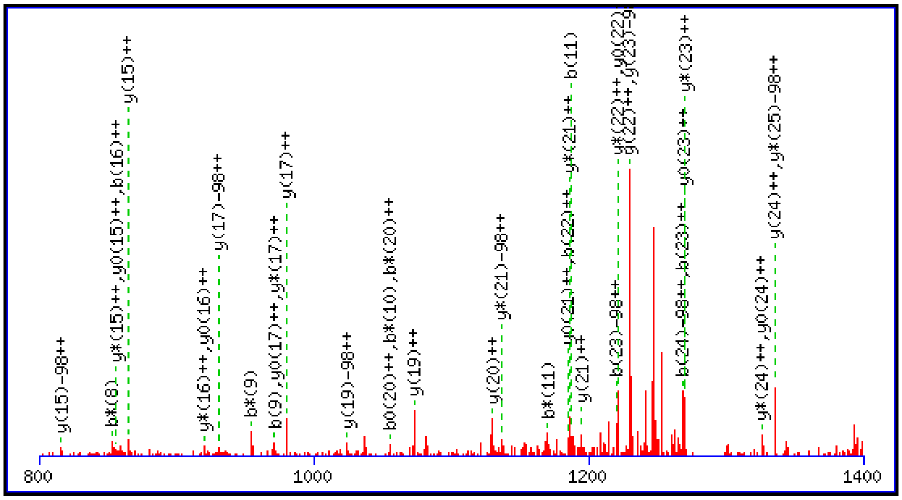

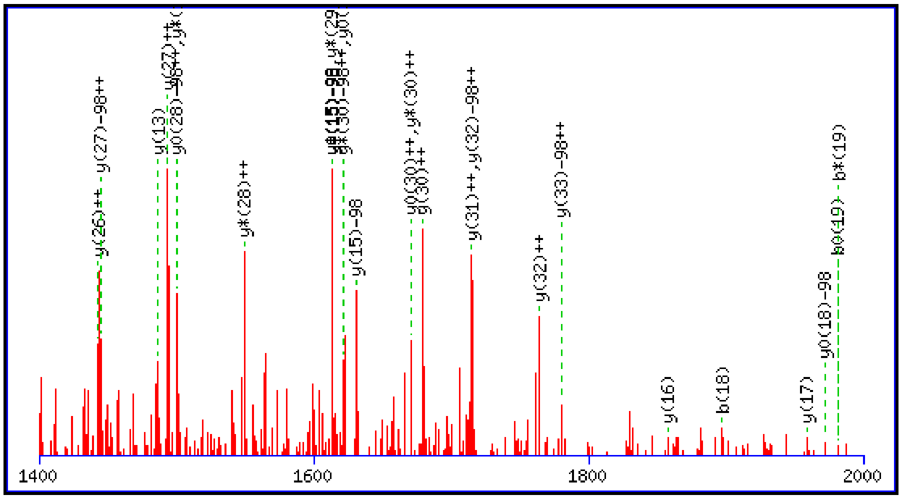

Monoisotopic mass of neutral peptide Mr(calc): 3854.6127
Fixed modifications: Carbamidomethyl (C)
Variable modifications:
S23 : Phospho (ST), with neutral losses 0.0000(shown in table), 97.9769
Ions Score: 46 **Expect:** 0.013
Matches (Bold Red): 86/604 fragment ions using 112 most intense peaks

#	b	b++	b*	b*++	b0	b0++	Seq.	y	y++	y*	y*++	y0	y0++	#
1	129.0659	65.0366	112.0393	56.5233			Q							35
2	200.1030	100.5551	183.0764	92.0418			A	3727.5614	1864.2843	3710.5349	1855.7711	3709.5508	1855.2791	34
3	329.1456	165.0764	312.1190	156.5631	311.1350	156.0711	E	3656.5243	1828.7658	3639.4978	1820.2525	3638.5137	1819.7605	33
4	428.2140	214.6106	**411.1874**	206.0974	410.2034	205.6053	V	3527.4817	**1764.2445**	3510.4552	1755.7312	3509.4711	1755.2392	32
5	**499.2511**	250.1292	**482.2245**	241.6159	**481.2405**	241.1239	A	3428.4133	**1714.7103**	3411.3867	1706.1970	3410.4027	1705.7050	31

6	614.2780	307.6427	597.2515	299.1294	**596.2675**	298.6374	D	3357.3762	**1679.1917**	3340.3496	**1670.6785**	3339.3656	**1670.1864**	30
7	**742.3366**	371.6719	**725.3101**	363.1587	**724.3260**	362.6667	Q	3242.3492	**1621.6783**	3225.3227	**1613.1650**	3224.3387	1612.6730	29
8	870.3952	435.7012	**853.3686**	427.1880	852.3846	426.6959	Q	3114.2907	1557.6490	3097.2641	**1549.1357**	3096.2801	1548.6437	28
9	**971.4429**	486.2251	**954.4163**	477.7118	953.4323	477.2198	T	2986.2321	**1493.6197**	2969.2055	1485.1064	2968.2215	1484.6144	27
10	1072.4905	536.7489	**1055.4640**	528.2356	1054.4800	527.7436	T	2885.1844	**1443.0958**	2868.1579	1434.5826	2867.1738	1434.0906	26
11	**1187.5175**	**594.2624**	**1170.4909**	585.7491	1169.5069	585.2571	D	2784.1367	1392.5720	2767.1102	1384.0587	2766.1262	1383.5667	25
12	1300.6016	650.8044	1283.5750	**642.2911**	1282.5910	641.7991	L	2669.1098	**1335.0585**	2652.0832	**1326.5453**	2651.0992	**1326.0532**	24
13	1397.6543	699.3308	1380.6278	690.8175	1379.6438	690.3255	P	2556.0257	1278.5165	2538.9992	**1270.0032**	2538.0151	**1269.5112**	23
14	1468.6914	734.8494	1451.6649	726.3361	1450.6809	725.8441	A	2458.9730	**1229.9901**	2441.9464	**1221.4768**	2440.9624	**1220.9848**	22
15	1597.7340	**799.3707**	1580.7075	790.8574	1579.7235	790.3654	E	2387.9358	**1194.4716**	2370.9093	**1185.9583**	2369.9253	**1185.4663**	21
16	1711.7770	**856.3921**	1694.7504	847.8788	1693.7664	847.3868	N	2258.8932	**1129.9503**	2241.8667	1121.4370	2240.8827	1120.9450	20
17	1768.7984	884.9028	1751.7719	876.3896	1750.7878	875.8976	G	2144.8503	**1072.9288**	2127.8238	1064.4155	2126.8398	1063.9235	19
18	**1897.8410**	949.4241	1880.8145	940.9109	1879.8304	940.4189	E	2087.8289	1044.4181	2070.8023	1035.9048	2069.8183	1035.4128	18
19	1998.8887	999.9480	**1981.8621**	991.4347	**1980.8781**	990.9427	T	**1958.7863**	**979.8968**	1941.7597	**971.3835**	1940.7757	**970.8915**	17
20	2127.9313	1064.4693	2110.9047	**1055.9560**	2109.9207	**1055.4640**	E	**1857.7386**	929.3729	1840.7120	**920.8597**	1839.7280	**920.3676**	16
21	2241.9742	1121.4907	2224.9477	1112.9775	2223.9636	1112.4855	N	1728.6960	**864.8516**	1711.6694	**856.3384**	1710.6854	**855.8463**	15
22	2370.0328	**1185.5200**	2353.0062	1177.0068	2352.0222	1176.5147	Q	1614.6531	807.8302	1597.6265	**799.3169**	1596.6425	798.8249	14
23	2537.0311	**1269.0192**	2520.0046	1260.5059	2519.0206	1260.0139	S	**1486.5945**	**743.8009**	1469.5679	735.2876	1468.5839	734.7956	13
24	2634.0839	1317.5456	2617.0574	1309.0323	2616.0733	1308.5403	P	1319.5961	660.3017	1302.5696	651.7884	1301.5856	651.2964	12
25	2705.1210	1353.0641	2688.0945	1344.5509	2687.1105	1344.0589	A	1222.5434	**611.7753**	1205.5168	603.2620	1204.5328	602.7700	11
26	2792.1530	1396.5802	2775.1265	1388.0669	2774.1425	1387.5749	S	1151.5063	576.2568	1134.4797	567.7435	1133.4957	567.2515	10
27	2921.1956	1461.1015	2904.1691	1452.5882	2903.1851	1452.0962	E	1064.4742	532.7408	1047.4477	524.2275	1046.4637	523.7355	9
28	3050.2382	1525.6228	3033.2117	1517.1095	3032.2277	1516.6175	E	935.4316	**468.2195**	918.4051	459.7062	917.4211	459.2142	8
29	3179.2808	1590.1440	3162.2543	1581.6308	3161.2703	1581.1388	E	806.3890	403.6982	789.3625	395.1849	788.3785	394.6929	7
30	3307.3758	1654.1915	3290.3492	1645.6783	3289.3652	1645.1862	K	677.3464	339.1769	660.3199	330.6636	659.3359	330.1716	6
31	3436.4184	1718.7128	3419.3918	1710.1996	3418.4078	1709.7075	E	549.2515	275.1294	532.2249	266.6161	**531.2409**	266.1241	5
32	3507.4555	1754.2314	3490.4289	1745.7181	3489.4449	1745.2261	A	420.2089	210.6081	403.1823	202.0948	402.1983	201.6028	4
33	3635.5505	1818.2789	3618.5239	1809.7656	3617.5399	1809.2736	K	349.1718	175.0895	332.1452	166.5763	331.1612	166.0842	3
34	3722.5825	1861.7949	3705.5559	1853.2816	3704.5719	1852.7896	S	221.0768	111.0420			203.0662	102.0368	2
35							D	134.0448	67.5260			116.0342	58.5207	1

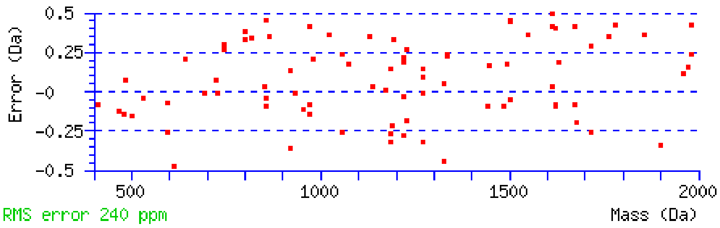

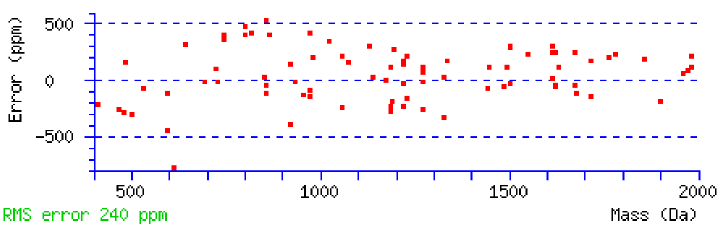

All matches to this query

Score	Mr(calc):	Delta	Sequence
45.6	3854.6127	0.0174	QAEVADQQTTDLPAENGETENQSPASEEEKEAKSD
44.5	3854.6127	0.0174	QAEVADQQTTDLPAENGETENQSPASEEEKEAKSD
41.4	3854.6127	0.0174	QAEVADQQTTDLPAENGETENQSPASEEEKEAKSD
27.1	3854.6127	0.0174	QAEVADQQTTDLPAENGETENQSPASEEEKEAKSD
6.6	3853.6365	0.9936	EARKYIFNSLDDIVHVNTVMDLEGSDLLAEK
5.3	3854.6301	-0.0000	DTESHFPVAELNHTRDYCVNQDLLALALTSGK
3.3	3854.6650	-0.0349	RVIISSSANAPMFVMGVNHVSLKIVSNASCTTK
2.5	3852.6073	2.0228	YDAVDMQDMNNPLSHYWISSSHNTYLTGDQLR
2.5	3852.6073	2.0228	YDAVDMQDMNNPLSHYWISSSHNTYLTGDQLR
2.5	3852.6073	2.0228	YDAVDMQDMNNPLSHYWISSSHNTYLTGDQLR

Spectrum No: 388; Query: 2570; Rank: 1

Peptide View

MS/MS Fragmentation of **WAHDKFSGEEGEIEDDESGTENREEK**
Found in **IPI00359323**, Tax_Id=10116 Gene_Symbol=Thrap3 Thyroid hormone receptor-associated protein 3

Match to Query 2570: 3102.242856 from(776.567990,4+)
Title: 100101RatKid_NS_deglyco_18.1993.1993.4.dta
Data file K:\NewmanPaper\Piliang\3SubProteomes\Piliang3SP\mgf5ppm\ERLIC_3SubProteomes5ppm.mgf

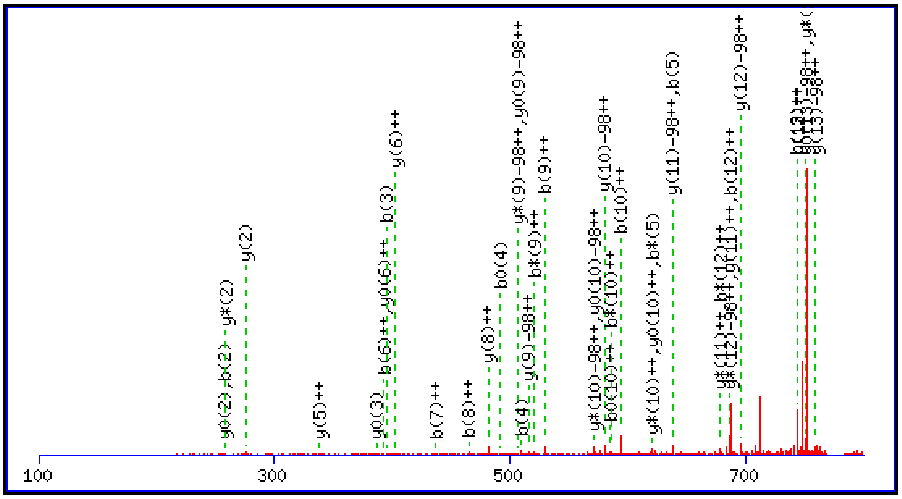

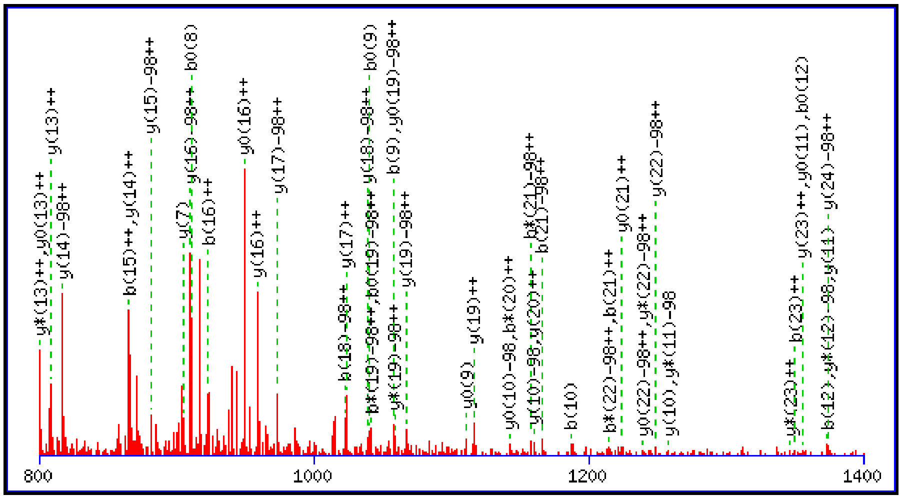

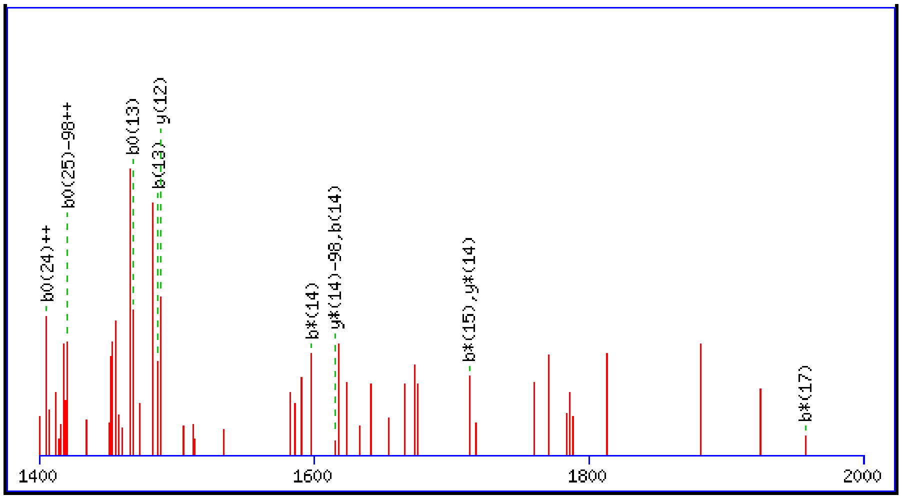

Monoisotopic mass of neutral peptide Mr(calc): 3102.2363
Fixed modifications: Carbamidomethyl (C)
Variable modifications:
S18 : Phospho (ST), with neutral losses 97.9769(shown in table), 0.0000
Ions Score: 46 **Expect:** 0.0085
Matches (Bold Red): 103/434 fragment ions using 160 most intense peaks

#	b	b^{++}	b*	b^{*++}	b^0	b^{0++}	Seq.	y	y^{++}	y*	y^{*++}	y^0	y^{0++}	#
1	187.0866	94.0469					W							26
2	**258.1237**	129.5655					A	2819.1874	1410.0974	2802.1609	1401.5841	2801.1769	1401.0921	25
3	**395.1826**	198.0949					H	2748.1503	**1374.5788**	2731.1238	1366.0655	2730.1398	1365.5735	24
4	**510.2096**	255.6084			**492.1990**	246.6031	D	2611.0914	1306.0493	2594.0649	1297.5361	2593.0808	1297.0441	23
5	**638.3045**	319.6559	**621.2780**	311.1426	620.2940	310.6506	K	2496.0645	**1248.5359**	2479.0379	**1240.0226**	2478.0539	**1239.5306**	22
6	785.3729	**393.1901**	768.3464	384.6768	767.3624	384.1848	F	2367.9695	1184.4884	2350.9430	1175.9751	2349.9589	1175.4831	21
7	872.4050	**436.7061**	855.3784	428.1928	854.3944	427.7008	S	2220.9011	1110.9542	2203.8745	1102.4409	2202.8905	1101.9489	20
8	929.4264	**465.2169**	912.3999	456.7036	**911.4159**	456.2116	G	2133.8691	**1067.4382**	2116.8425	**1058.9249**	2115.8585	**1058.4329**	19
9	**1058.4690**	**529.7381**	1041.4425	**521.2249**	**1040.4585**	520.7329	E	2076.8476	**1038.9274**	2059.8210	1030.4142	2058.8370	1029.9222	18
10	**1187.5116**	**594.2594**	1170.4851	**585.7462**	1169.5010	**585.2542**	E	1947.8050	**974.4061**	1930.7785	965.8929	1929.7944	965.4009	17
11	1244.5331	622.7702	1227.5065	614.2569	1226.5225	613.7649	G	1818.7624	**909.8848**	1801.7359	901.3716	1800.7518	900.8796	16
12	**1373.5757**	**687.2915**	1356.5491	**678.7782**	**1355.5651**	678.2862	E	1761.7409	**881.3741**	1744.7144	872.8608	1743.7304	872.3688	15
13	**1486.6597**	**743.8335**	1469.6332	735.3202	**1468.6492**	734.8282	I	1632.6984	**816.8528**	**1615.6718**	808.3395	1614.6878	807.8475	14
14	**1615.7023**	808.3548	**1598.6758**	799.8415	1597.6918	799.3495	E	1519.6143	**760.3108**	1502.5877	**751.7975**	1501.6037	**751.3055**	13
15	1730.7293	**865.8683**	**1713.7027**	857.3550	1712.7187	856.8630	D	1390.5717	**695.7895**	**1373.5452**	**687.2762**	1372.5611	686.7842	12
16	1845.7562	**923.3817**	1828.7297	914.8685	1827.7456	914.3765	D	1275.5448	**638.2760**	**1258.5182**	629.7627	1257.5342	629.2707	11
17	1974.7988	987.9030	**1957.7723**	979.3898	1956.7882	978.8978	E	**1160.5178**	**580.7625**	1143.4913	**572.2493**	**1142.5072**	**571.7573**	10
18	2043.8203	**1022.4138**	2026.7937	1013.9005	2025.8097	1013.4085	S	1031.4752	**516.2412**	1014.4487	**507.7280**	1013.4647	**507.2360**	9
19	2100.8417	1050.9245	2083.8152	**1042.4112**	2082.8312	**1041.9192**	G	962.4538	**481.7305**	945.4272	473.2172	944.4432	472.7252	8
20	2201.8894	1101.4483	2184.8629	1092.9351	2183.8788	1092.4431	T	**905.4323**	453.2198	888.4058	444.7065	887.4217	444.2145	7
21	2330.9320	**1165.9696**	2313.9054	**1157.4564**	2312.9214	1156.9644	E	804.3846	**402.6959**	787.3581	394.1827	786.3741	**393.6907**	6
22	2444.9749	1222.9911	2427.9484	**1214.4778**	2426.9644	1213.9858	N	675.3420	**338.1747**	658.3155	329.6614	657.3315	329.1694	5
23	2601.0760	1301.0417	2584.0495	1292.5284	2583.0655	1292.0364	R	561.2991	281.1532	544.2726	272.6399	543.2885	272.1479	4
24	2730.1186	1365.5630	2713.0921	1357.0497	2712.1081	1356.5577	E	405.1980	203.1026	388.1714	194.5894	**387.1874**	194.0974	3
25	2859.1612	1430.0842	2842.1347	1421.5710	2841.1507	**1421.0790**	E	**276.1554**	138.5813	**259.1288**	130.0681	**258.1448**	129.5761	2
26							K	147.1128	74.0600	130.0863	65.5468			1

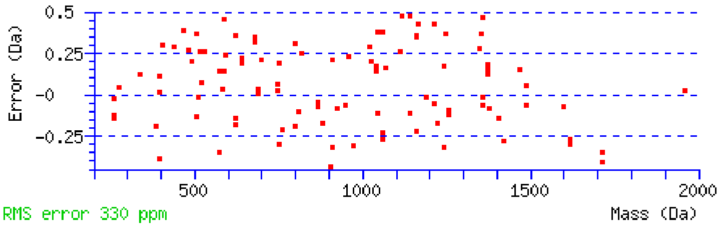

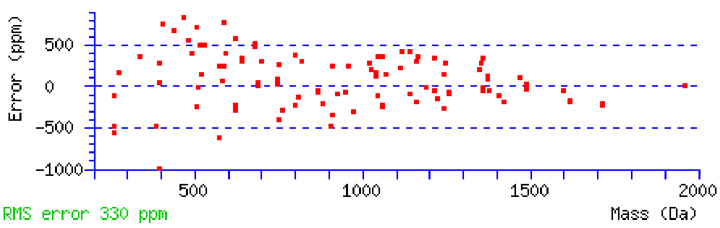

All matches to this query

Score	Mr(calc):	Delta	Sequence
45.7	3102.2363	0.0065	WAHDKFSGEEGEIEDDESGTENREEK
37.7	3102.2363	0.0065	WAHDKFSGEEGEIEDDESGTENREEK
9.6	3100.2395	2.0034	TEDMKMSVPRQAVMEEPSTEPLSPR
4.3	3100.2075	2.0354	TLETHIKIFHAPNSSAPSSSLSTFK
3.2	3100.2221	2.0207	TNVTLTKTTASPKTPMNPAPTAWTR
2.9	3102.2273	0.0156	VMGGYDFPGSNSNIDLHALTGWIPER
2.9	3102.2273	0.0156	VMGGYDFPGSNSNIDLHALTGWIPER
2.3	3101.2102	1.0326	AAGEYICNQCGAKYTSLDSFQTHLK
2.3	3101.2102	1.0326	AAGEYICNQCGAKYTSLDSFQTHLK
1.8	3101.2102	1.0326	AAGEYICNQCGAKYTSLDSFQTHLK

Spectrum No: 389; Query: 2343; Rank: 1

Peptide View

MS/MS Fragmentation of **YGVYEDENYEVESDDEEIPFK**
Found in **IPI00367989**, Tax_Id=10116 Gene_Symbol=Znf183 zinc finger protein 183

Match to Query 2343: 2649.046728 from(1325.530640,2+)
Title: 100101RatKid_NS_deglyco_24.3881.3881.2.dta
Data file K:\NewmanPaper\Piliang\3SubProteomes\Piliang3SP\mgf5ppm\ERLIC_3SubProteomes5ppm.mgf

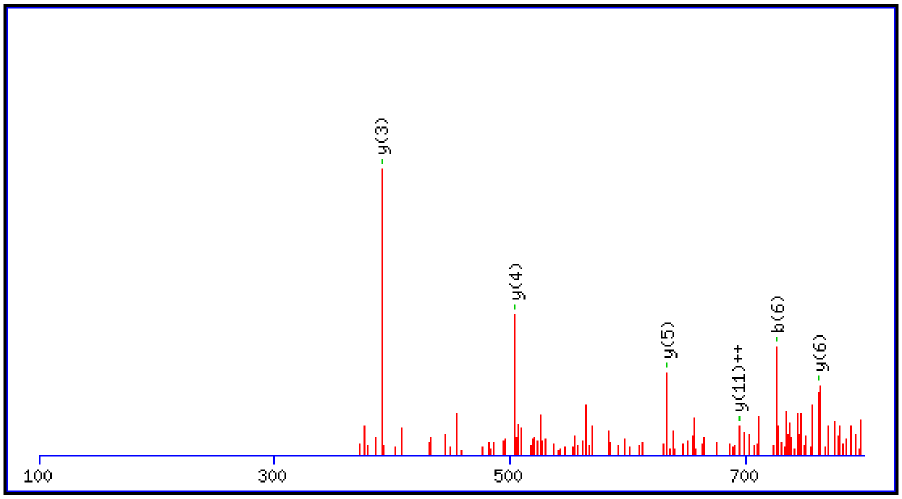

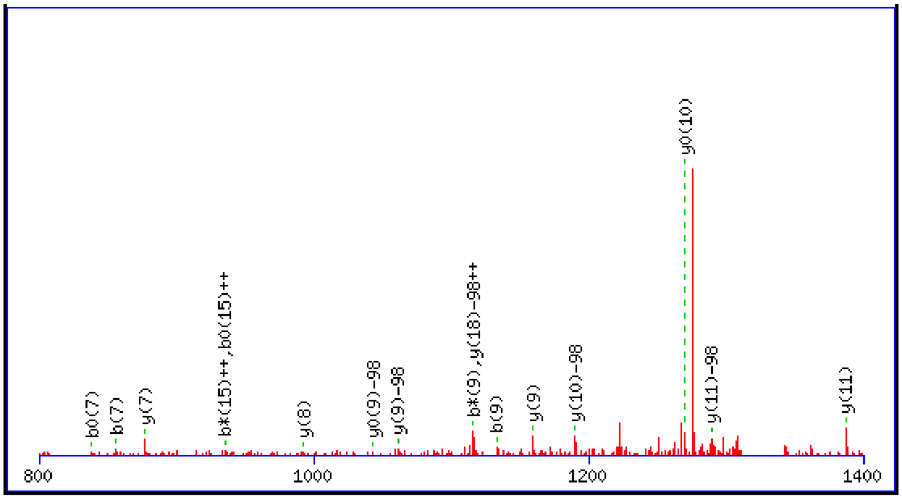

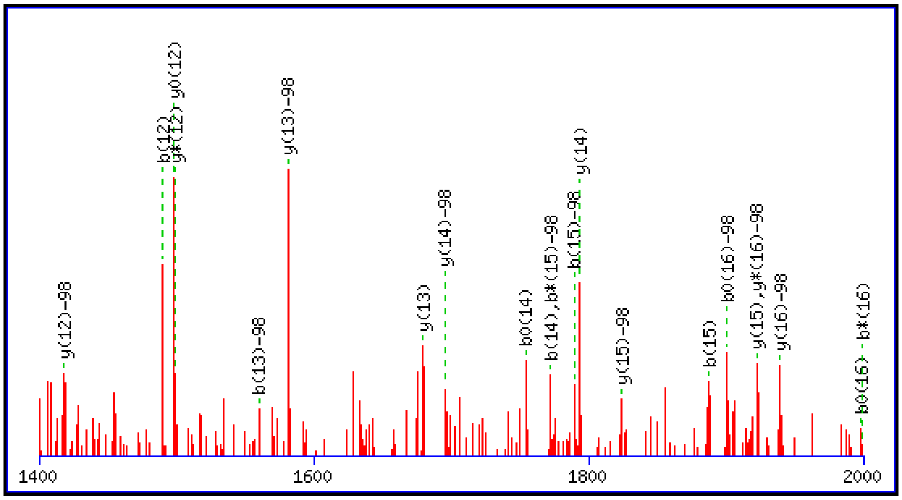

Monoisotopic mass of neutral peptide Mr(calc): 2648.0367
Fixed modifications: Carbamidomethyl (C)
Variable modifications:
S13 : Phospho (ST), with neutral losses 97.9769(shown in table), 0.0000
Ions Score: 46 **Expect:** 0.0067
Matches (Bold Red): 43/330 fragment ions using 113 most intense peaks

#	b	b^{++}	b*	b^{*++}	b^{0}	b^{0++}	Seq.	y	y^{++}	y*	y^{*++}	y^{0}	y^{0++}	#
1	164.0706	82.5389					Y							21
2	221.0921	111.0497					G	2388.0038	1194.5055	2370.9772	1185.9922	2369.9932	1185.5002	20
3	320.1605	160.5839					V	2330.9823	1165.9948	2313.9558	1157.4815	2312.9717	1156.9895	19
4	483.2238	242.1155					Y	2231.9139	**1116.4606**	2214.8873	1107.9473	2213.9033	1107.4553	18
5	612.2664	306.6368			594.2558	297.6316	E	2068.8506	1034.9289	2051.8240	1026.4156	2050.8400	1025.9236	17
6	**727.2933**	364.1503			709.2828	355.1450	D	**1939.8080**	970.4076	**1922.7814**	961.8943	1921.7974	961.4023	16
7	**856.3359**	428.6716			**838.3254**	419.6663	E	**1824.7810**	912.8941	1807.7545	904.3809	1806.7705	903.8889	15
8	970.3789	485.6931	953.3523	477.1798	952.3683	476.6878	N	**1695.7384**	848.3729	1678.7119	839.8596	1677.7279	839.3676	14
9	**1133.4422**	567.2247	**1116.4156**	558.7115	1115.4316	558.2195	Y	**1581.6955**	791.3514	1564.6690	782.8381	1563.6849	782.3461	13
10	1262.4848	631.7460	1245.4582	623.2328	1244.4742	622.7408	E	**1418.6322**	709.8197	1401.6056	701.3065	1400.6216	700.8144	12
11	1361.5532	681.2802	1344.5267	672.7670	1343.5426	672.2750	V	**1289.5896**	645.2984	1272.5630	636.7852	1271.5790	636.2931	11
12	**1490.5958**	745.8015	1473.5692	737.2883	1472.5852	736.7963	E	**1190.5212**	595.7642	1173.4946	587.2509	1172.5106	586.7589	10
13	**1559.6173**	780.3123	1542.5907	771.7990	1541.6067	771.3070	S	**1061.4786**	531.2429	1044.4520	522.7297	**1043.4680**	522.2376	9
14	1674.6442	837.8257	1657.6176	829.3125	1656.6336	828.8205	D	**992.4571**	496.7322	975.4306	488.2189	974.4466	487.7269	8
15	**1789.6711**	895.3392	**1772.6446**	886.8259	1771.6606	886.3339	D	**877.4302**	439.2187	860.4036	430.7055	859.4196	430.2134	7
16	1918.7137	959.8605	1901.6872	951.3472	**1900.7032**	950.8552	E	**762.4032**	381.7053	745.3767	373.1920	744.3927	372.7000	6
17	2047.7563	1024.3818	2030.7298	1015.8685	2029.7458	1015.3765	E	**633.3606**	317.1840	616.3341	308.6707	615.3501	308.1787	5
18	2160.8404	1080.9238	2143.8138	1072.4106	2142.8298	1071.9186	I	**504.3180**	252.6627	487.2915	244.1494			4

**19**	2257.8932	1129.4502	2240.8666	1120.9369	2239.8826	1120.4449	**P**	**391.2340**	196.1206	374.2074	187.6074			**3**
**20**	2404.9616	1202.9844	2387.9350	1194.4711	2386.9510	1193.9791	**F**	294.1812	147.5942	277.1547	139.0810			**2**
**21**							**K**	147.1128	74.0600	130.0863	65.5468			**1**

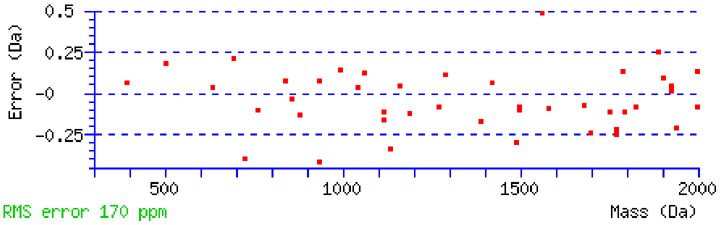

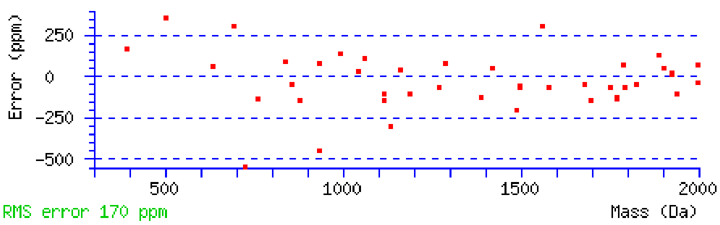

All matches to this query

Score	Mr(calc):	Delta	Sequence
45.6	2648.0367	1.0100	YGVYEDENYEVESDDEEIPFK
30.6	2649.0207	0.0260	YGVYEDENYEVESDDEEIPFK
19.9	2648.0367	1.0100	YGVYEDENYEVESDDEEIPFK
10.6	2649.0207	0.0260	YGVYEDENYEVESDDEEIPFK
4.9	2648.0367	1.0100	YGVYEDENYEVESDDEEIPFK
1.7	2647.0151	2.0316	IHTGEKPYKCSECGKSFSHR
1.6	2647.0151	2.0316	IHTGEKPYKCSECGKSFSHR
1.6	2647.0151	2.0316	IHTGEKPYKCSECGKSFSHR
1.3	2649.0239	0.0228	TNPEDIYPSNPTDDDVSSGSSSER
1.3	2649.0239	0.0228	TNPEDIYPSNPTDDDVSSGSSSER

Spectrum No: 390; Query: 430; Rank: 1

Peptide View

MS/MS Fragmentation of **LCLSTVDLEVK**
Found in **IPI00369678**, Tax_Id=10116 Gene_Symbol=Slc43a2_predicted solute carrier family 43, member 2

Match to Query 430: 1355.643348 from(678.828950,2+)
Title: 091008RatKidney_NoSalt_18.3105.3105.2.dta
Data file K:\NewmanPaper\Piliang\3SubProteomes\Piliang3SP\mgf5ppm\ERLIC_3SubProteomes5ppm.mgf

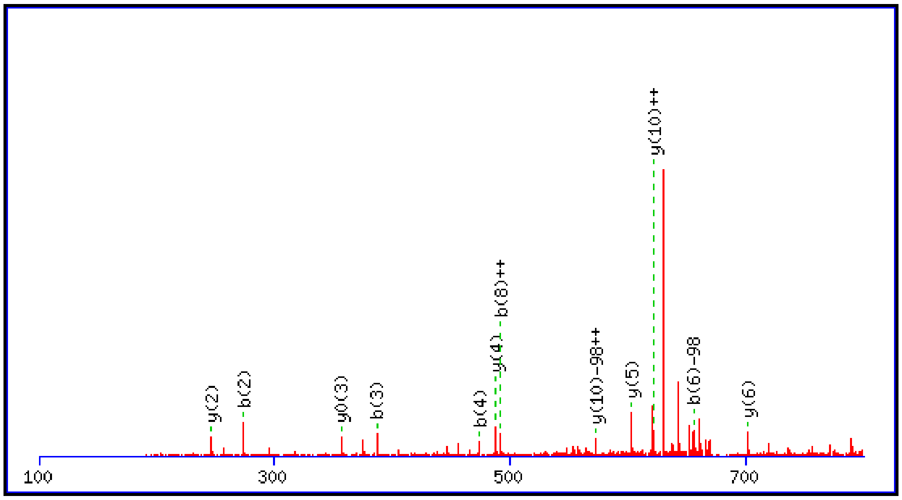

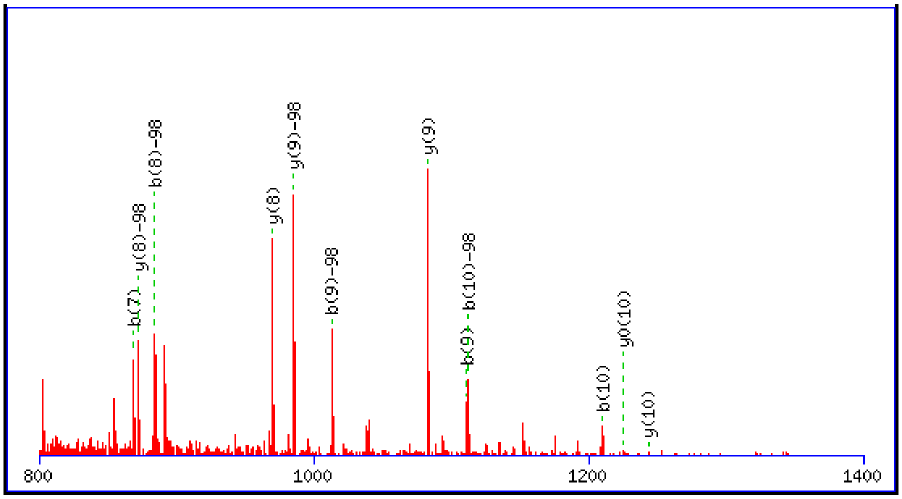

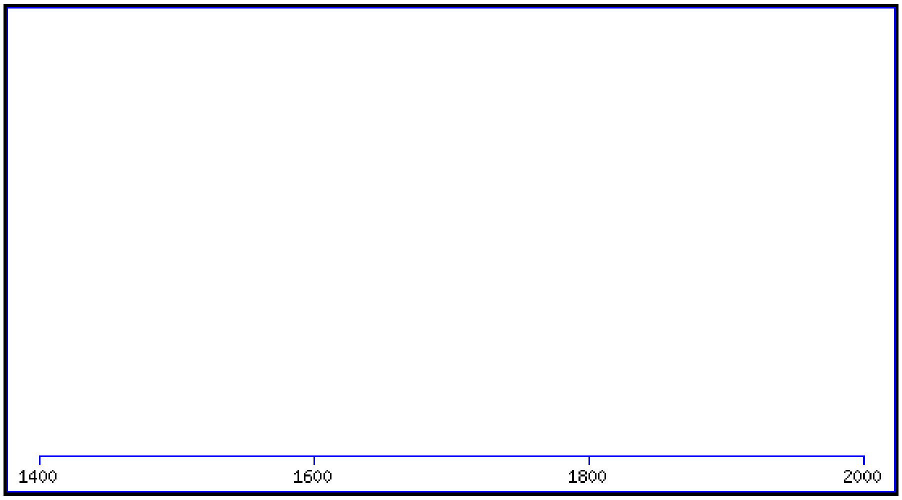

Monoisotopic mass of neutral peptide Mr(calc): 1355.6408
Fixed modifications: Carbamidomethyl (C)
Variable modifications:
T5 : Phospho (ST), with neutral losses 97.9769(shown in table), 0.0000
Ions Score: 45 **Expect:** 0.0043
Matches (Bold Red): 24/138 fragment ions using 38 most intense peaks

#	b	b^{++}	b^{0}	b^{0++}	Seq.	y	y^{++}	y*	y^{*++}	y^{0}	y^{0++}	#
1	114.0913	57.5493			L							11
2	**274.1220**	137.5646			C	1145.5871	**573.2972**	1128.5605	564.7839	1127.5765	564.2919	10
3	**387.2061**	194.1067			L	**985.5564**	493.2819	968.5299	484.7686	967.5459	484.2766	9
4	**474.2381**	237.6227	456.2275	228.6174	S	**872.4724**	436.7398	855.4458	428.2265	854.4618	427.7345	8
5	557.2752	279.1412	539.2646	270.1359	T	785.4403	393.2238	768.4138	384.7105	767.4298	384.2185	7
6	**656.3436**	328.6754	638.3330	319.6702	V	**702.4032**	351.7053	685.3767	343.1920	684.3927	342.7000	6
7	771.3705	386.1889	753.3600	377.1836	D	**603.3348**	302.1710	586.3083	293.6578	585.3243	293.1658	5
8	**884.4546**	442.7309	866.4440	433.7257	L	**488.3079**	244.6576	471.2813	236.1443	470.2973	235.6523	4
9	**1013.4972**	507.2522	995.4866	498.2470	E	375.2238	188.1155	358.1973	179.6023	**357.2132**	179.1103	3
10	**1112.5656**	556.7864	1094.5551	547.7812	V	**246.1812**	123.5942	229.1547	115.0810			2
11					K	147.1128	74.0600	130.0863	65.5468			1

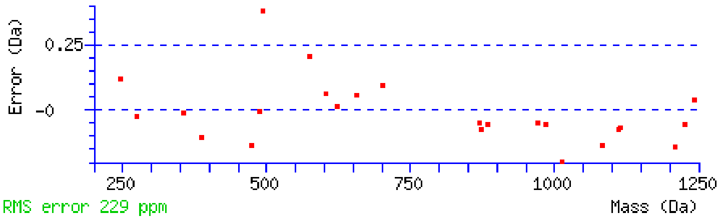

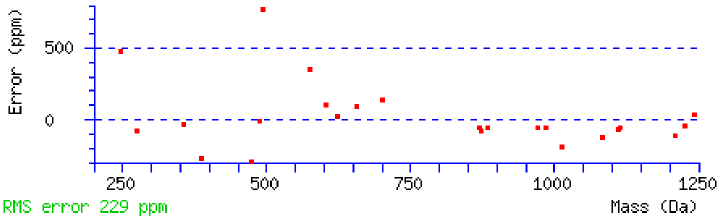

All matches to this query

Score	Mr(calc):	Delta	Sequence
45.5	1355.6408	0.0026	LCLSTVDLEVK
42.2	1355.6408	0.0026	LCLSTVDLEVK
13.3	1353.6235	2.0199	NDSFTACIELGK
9.8	1353.6476	1.9958	AMPSLERSLVR
9.3	1355.6446	-0.0012	ASSARANITLSGK
7.7	1355.6407	0.0026	KMPNILTSNLK
7.7	1355.6407	0.0026	KMPNILTSNLK
7.5	1355.6407	0.0026	MNATSGEILLVK
7.5	1355.6407	0.0026	MNATSGEILLVK
6.5	1355.6380	0.0053	AVREMRLSNGK

Spectrum No: 391; Query: 460; Rank: 1

Peptide View

MS/MS Fragmentation of **NVDNAEGSDTDY**
Found in **IPI00765979**, Tax_Id=10116 Gene_Symbol=Herc1_predicted similar to guanine nucleotide exchange factor p532

Match to Query 460: 1378.451968 from(690.233260,2+)
Title: 091008RatKidney_NoSalt_43.430.430.2.dta
Data file K:\NewmanPaper\Piliang\3SubProteomes\Piliang3SP\mgf5ppm\ERLIC_3SubProteomes5ppm.mgf

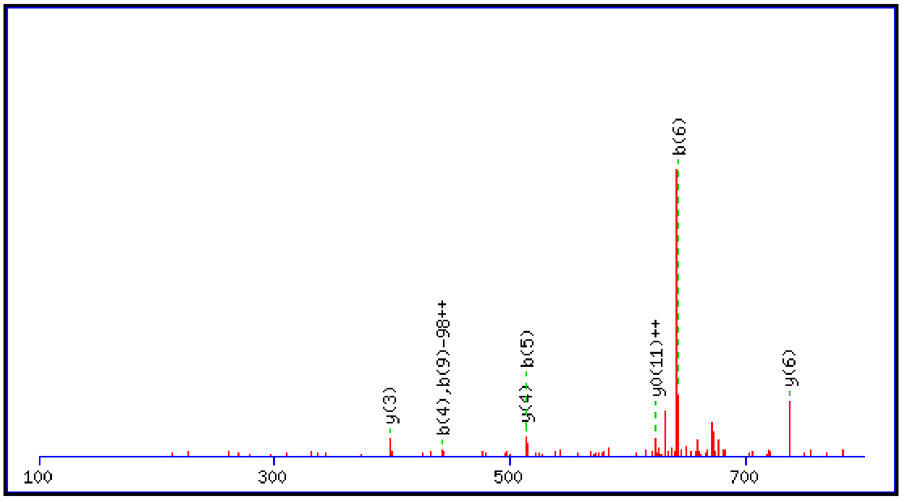

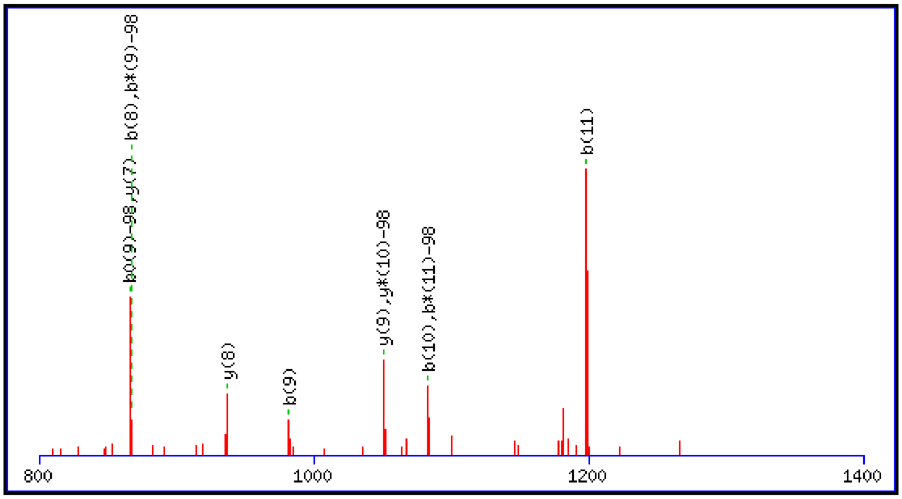

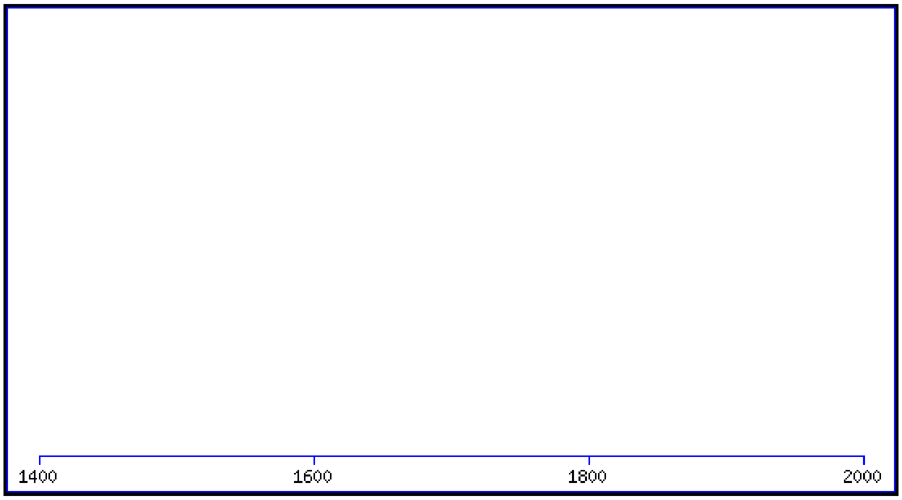

Monoisotopic mass of neutral peptide Mr(calc): 1378.4562
Fixed modifications: Carbamidomethyl (C)
Variable modifications:
S8 : Phospho (ST), with neutral losses 0.0000(shown in table), 97.9769
Ions Score: 45 **Expect:** 0.0011
Matches (Bold Red): 19/168 fragment ions using 25 most intense peaks

#	b	b^{++}	b^{*}	b^{*++}	b^{0}	b^{0++}	Seq.	y	y^{++}	y^{*}	y^{*++}	y^{0}	y^{0++}	#
1	115.0502	58.0287	98.0237	49.5155			N							12
2	214.1186	107.5629	197.0921	99.0497			V	1265.4205	633.2139	1248.3940	624.7006	1247.4100	**624.2086**	11
3	329.1456	165.0764	312.1190	156.5631	311.1350	156.0711	D	1166.3521	583.6797	1149.3256	575.1664	1148.3416	574.6744	10
4	**443.1885**	222.0979	426.1619	213.5846	425.1779	213.0926	N	**1051.3252**	526.1662	1034.2986	517.6530	1033.3146	517.1609	9
5	**514.2256**	257.6164	497.1991	249.1032	496.2150	248.6112	A	**937.2823**	469.1448			919.2717	460.1395	8
6	**643.2682**	322.1377	626.2416	313.6245	625.2576	313.1325	E	**866.2451**	433.6262			848.2346	424.6209	7
7	700.2897	350.6485	683.2631	342.1352	682.2791	341.6432	G	**737.2026**	369.1049			719.1920	360.0996	6
8	**867.2880**	434.1476	850.2615	425.6344	849.2774	425.1424	S	680.1811	340.5942			662.1705	331.5889	5
9	**982.3150**	491.6611	965.2884	483.1478	964.3044	482.6558	D	**513.1827**	257.0950			495.1722	248.0897	4
10	**1083.3626**	542.1850	1066.3361	533.6717	1065.3521	533.1797	T	**398.1558**	199.5815			380.1452	190.5763	3
11	**1198.3896**	599.6984	1181.3630	591.1852	1180.3790	590.6931	D	297.1081	149.0577			279.0975	140.0524	2
12							Y	182.0812	91.5442					1

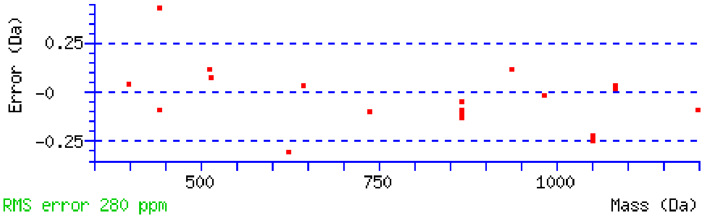

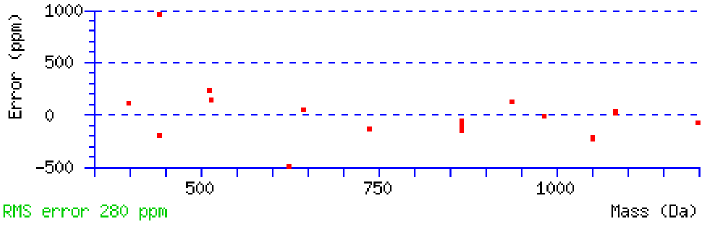

All matches to this query

Score	Mr(calc):	Delta	Sequence
45.4	1378.4562	-0.0042	NVDNAEGSDTDY
19.6	1378.4562	-0.0042	NVDNAEGSDTDY
11.3	1378.4562	-0.0042	NVDNAEGSDTDY
4.7	1378.4496	0.0024	TFSNSLNLNK
1.6	1376.4564	1.9956	SDLSRGSPYR
0.6	1376.4485	2.0034	CTNSLQKTGK
0.2	1377.4438	1.0082	MKSQSEGSIR
0.1	1376.4386	2.0133	HYCKSISSR
0.1	1376.4386	2.0133	HYCKSISSR

0.1	1376.4386	2.0133	HYCKSISSR

Spectrum No: 392; Query: 1715; Rank: 1

Peptide View

MS/MS Fragmentation of **YVESDDEKPTDENVNEK**
Found in **IPI00569556**, Tax_Id=10116 Gene_Symbol=Atrx 38 kDa protein

Match to Query 1715: 2089.846188 from(1045.930370,2+)
Title: 100101RatKid_NS_deglyco_22.874.874.2.dta
Data file K:\NewmanPaper\Piliang\3SubProteomes\Piliang3SP\mgf5ppm\ERLIC_3SubProteomes5ppm.mgf

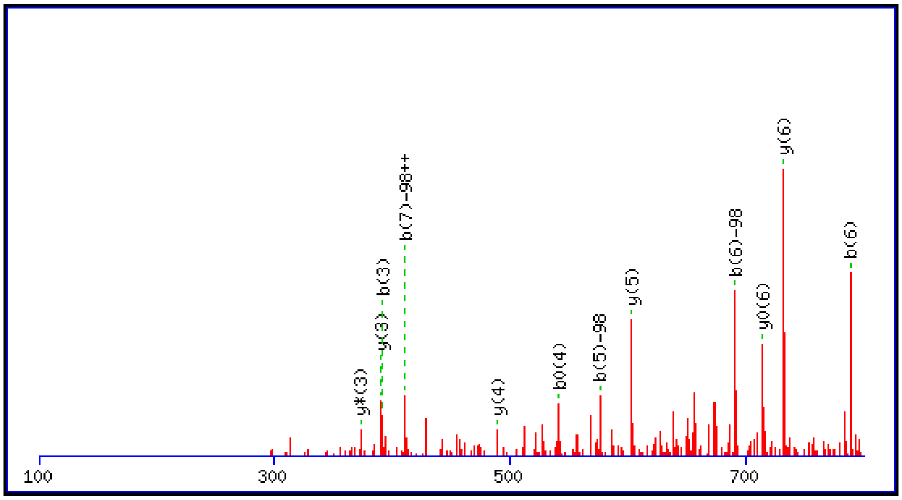

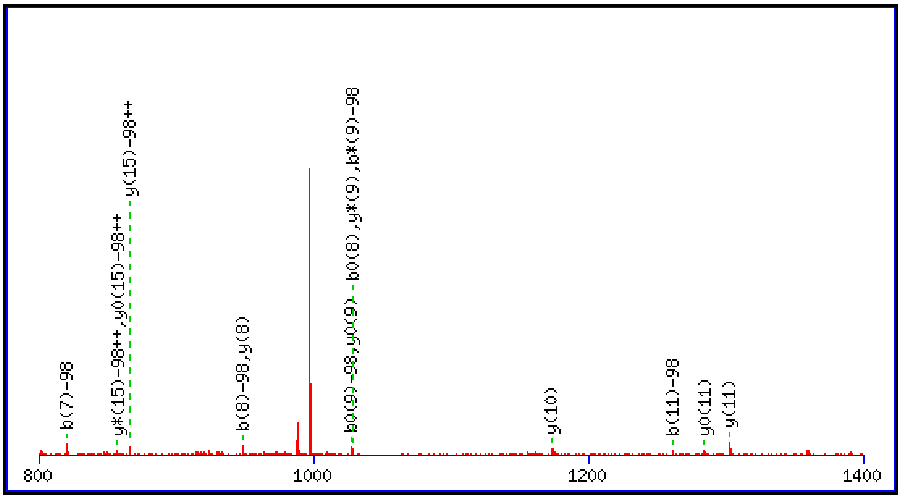

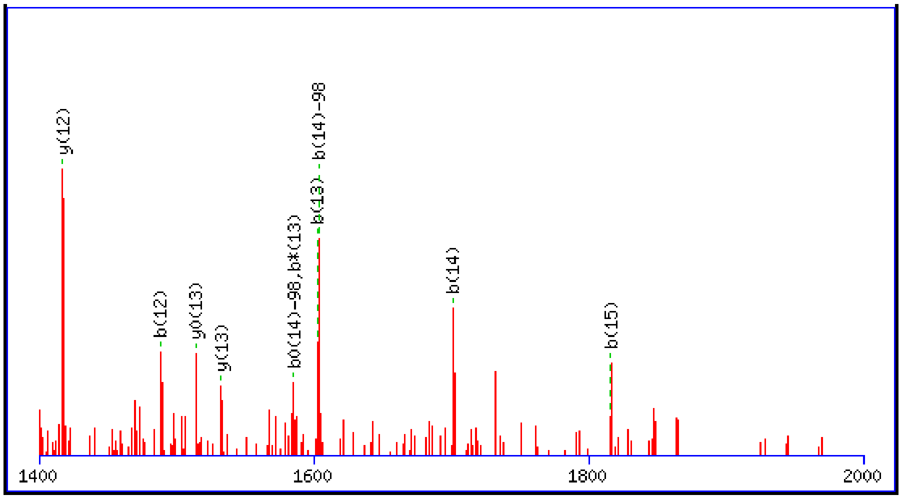

Monoisotopic mass of neutral peptide Mr(calc): 2089.8365
Fixed modifications: Carbamidomethyl (C)
Variable modifications:
S4 : Phospho (ST), with neutral losses 97.9769(shown in table), 0.0000
Ions Score: 45 **Expect:** 0.0061
Matches (Bold Red): 37/260 fragment ions using 59 most intense peaks

#	b	b++	b*	b*++	b0	b0++	Seq.	y	y++	y*	y*++	y0	y0++	#
1	164.0706	82.5389					Y							17
2	263.1390	132.0731					V	1829.8035	915.4054	1812.7770	906.8921	1811.7930	906.4001	16
3	**392.1816**	196.5944			374.1710	187.5892	E	1730.7351	**865.8712**	1713.7086	**857.3579**	1712.7246	**856.8659**	15
4	461.2031	231.1052			443.1925	222.0999	S	1601.6925	801.3499	1584.6660	792.8366	1583.6820	792.3446	14
5	**576.2300**	288.6186			558.2194	279.6134	D	**1532.6711**	766.8392	1515.6445	758.3259	**1514.6605**	757.8339	13
6	**691.2570**	346.1321			673.2464	337.1268	D	**1417.6441**	709.3257	1400.6176	700.8124	1399.6336	700.3204	12
7	**820.2995**	**410.6534**			802.2890	401.6481	E	**1302.6172**	651.8122	1285.5907	643.2990	**1284.6066**	642.8070	11
8	**948.3945**	474.7009	931.3680	466.1876	930.3839	465.6956	K	**1173.5746**	587.2909	1156.5481	578.7777	1155.5640	578.2857	10
9	1045.4473	523.2273	**1028.4207**	514.7140	**1027.4367**	514.2220	P	1045.4796	523.2435	**1028.4531**	514.7302	**1027.4691**	514.2382	9
10	1146.4950	573.7511	1129.4684	565.2378	1128.4844	564.7458	T	**948.4269**	474.7171	931.4003	466.2038	930.4163	465.7118	8
11	**1261.5219**	631.2646	1244.4953	622.7513	1243.5113	622.2593	D	847.3792	424.1932	830.3527	415.6800	829.3686	415.1880	7
12	1390.5645	695.7859	1373.5379	687.2726	1372.5539	686.7806	E	**732.3523**	366.6798	715.3257	358.1665	**714.3417**	357.6745	6
13	1504.6074	752.8073	1487.5809	744.2941	1486.5969	743.8021	N	**603.3097**	302.1585	586.2831	293.6452	585.2991	293.1532	5
14	**1603.6758**	802.3416	1586.6493	793.8283	**1585.6653**	793.3363	V	**489.2667**	245.1370	472.2402	236.6237	471.2562	236.1317	4
15	1717.7188	859.3630	1700.6922	850.8497	1699.7082	850.3577	N	**390.1983**	195.6028	**373.1718**	187.0895	372.1878	186.5975	3
16	1846.7614	923.8843	1829.7348	915.3710	1828.7508	914.8790	E	276.1554	138.5813	259.1288	130.0681	258.1448	129.5761	2
17							K	147.1128	74.0600	130.0863	65.5468			1

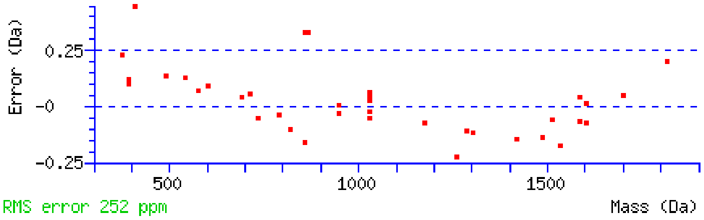

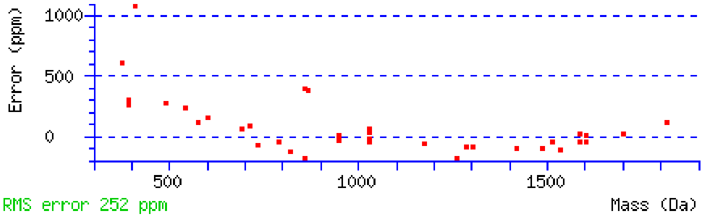

All matches to this query

Score	Mr(calc):	Delta	Sequence
45.4	2089.8365	0.0097	YVESDDEKPTDENVNEK
40.3	2089.8365	0.0097	YVESDDEKPTDENVNEK
34.5	2089.8365	0.0097	YVESDDEKPTDENVNEK
9.4	2089.8603	-0.0141	GSSWSAQNTPRGNYNESR

3.9	2089.8510	-0.0048	MEEAETDATENKRASEAK
3.5	2089.8510	-0.0048	MEEAETDATENKRASEAK
3.4	2087.8342	2.0120	TMDFPTPAAAFRSPLAR
3.1	2088.8472	0.9990	APSLPDAPSPPPAFSRSR
3.1	2088.8472	0.9990	APSLPDAPSPPPAFSRSR
3.1	2088.8595	0.9867	TAESFHALGTGEKGFSYK

Spectrum No: 393; Query: 1231; Rank: 1

Peptide View

MS/MS Fragmentation of **STSPPPSPEVWAESR**
Found in **IPI00778976**, Tax_Id=10116 Gene_Symbol=Epb4.9_predicted 45 kDa protein

Match to Query 1231: 1785.710288 from(893.862420,2+)
Title: 091008RatKidney_NoSalt_33.1720.1720.2.dta
Data file K:\NewmanPaper\Piliang\3SubProteomes\Piliang3SP\mgf5ppm\ERLIC_3SubProteomes5ppm.mgf

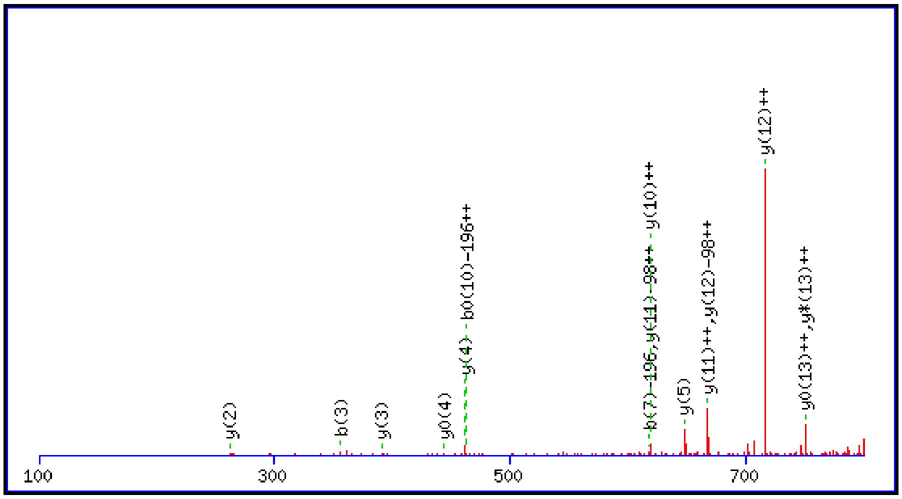

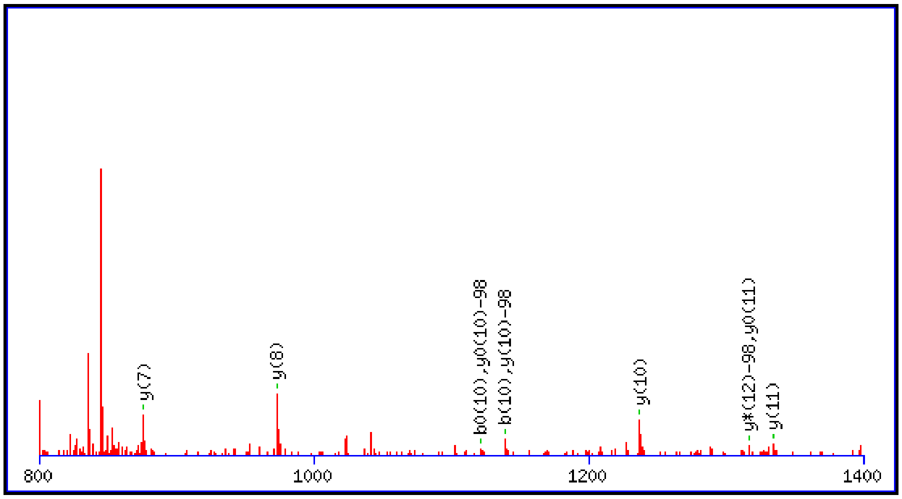

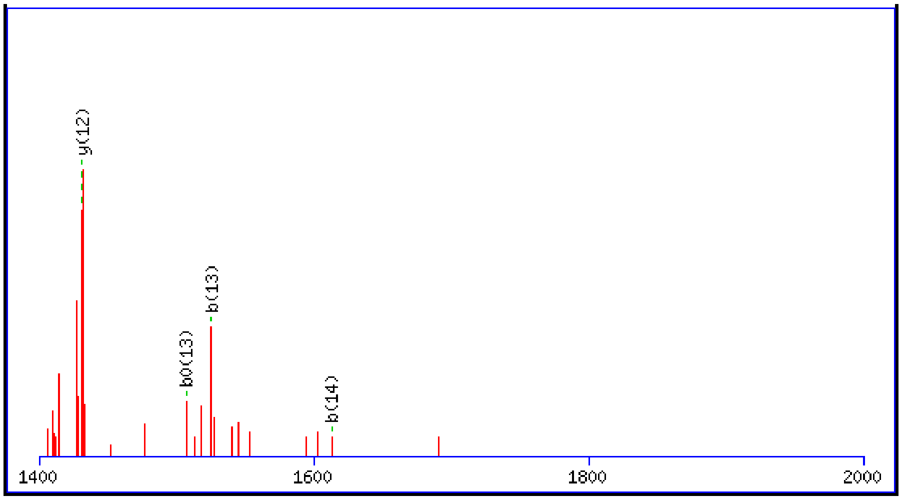

Monoisotopic mass of neutral peptide Mr(calc): 1785.7012
Fixed modifications: Carbamidomethyl (C)
Variable modifications:
T2 : Phospho (ST), with neutral losses 0.0000(shown in table), 97.9769
S7 : Phospho (ST), with neutral losses 0.0000(shown in table), 97.9769
Ions Score: 45 **Expect:** 0.0046
Matches (Bold Red): 29/226 fragment ions using 50 most intense peaks

#	b	b++	b0	b0++	Seq.	y	y++	y*	y*++	y0	y0++	#
1	88.0393	44.5233	70.0287	35.5180	S							15
2	269.0533	135.0303	251.0427	126.0250	T	1699.6764	850.3419	1682.6499	841.8286	1681.6659	841.3366	14
3	**356.0853**	178.5463	338.0748	169.5410	S	1518.6624	759.8349	1501.6359	**751.3216**	1500.6519	**750.8296**	13
4	453.1381	227.0727	435.1275	218.0674	P	**1431.6304**	**716.3188**	1414.6039	707.8056	1413.6199	707.3136	12
5	550.1909	275.5991	532.1803	266.5938	P	**1334.5777**	**667.7925**	1317.5511	659.2792	**1316.5671**	658.7872	11
6	647.2436	324.1255	629.2331	315.1202	P	**1237.5249**	**619.2661**	1220.4983	610.7528	1219.5143	610.2608	10
7	814.2420	407.6246	796.2314	398.6193	S	1140.4721	570.7397	1123.4456	562.2264	1122.4616	561.7344	9
8	911.2947	456.1510	893.2842	447.1457	P	**973.4738**	487.2405	956.4472	478.7272	955.4632	478.2352	8
9	1040.3373	520.6723	1022.3268	511.6670	E	**876.4210**	438.7141	859.3945	430.2009	858.4104	429.7089	7
10	**1139.4058**	570.2065	**1121.3952**	561.2012	V	747.3784	374.1928	730.3519	365.6796	729.3678	365.1876	6
11	1325.4851	663.2462	1307.4745	654.2409	W	**648.3100**	324.6586	631.2835	316.1454	630.2994	315.6534	5
12	1396.5222	698.7647	1378.5116	689.7594	A	**462.2307**	231.6190	445.2041	223.1057	**444.2201**	222.6137	4
13	**1525.5648**	763.2860	**1507.5542**	754.2807	E	**391.1936**	196.1004	374.1670	187.5872	373.1830	187.0951	3
14	**1612.5968**	806.8020	1594.5862	797.7968	S	**262.1510**	131.5791	245.1244	123.0659	244.1404	122.5738	2
15					R	175.1190	88.0631	158.0924	79.5498			1

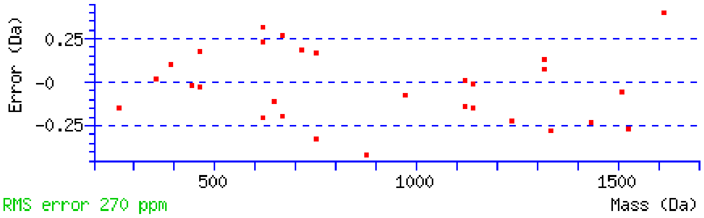

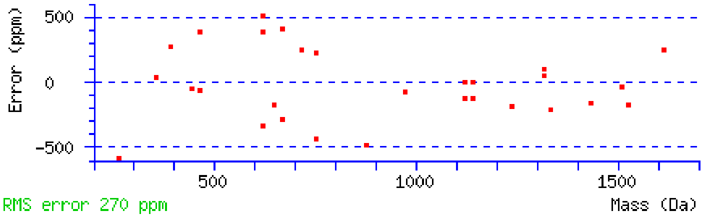

All matches to this query

Score	Mr(calc):	Delta	Sequence
45.4	1785.7012	0.0091	STSPPPSPEVWAESR
45.4	1785.7012	0.0091	STSPPPSPEVWAESR
45.3	1785.7012	0.0091	STSPPPSPEVWAESR
14.3	1785.7012	0.0091	STSPPPSPEVWAESR
14.3	1785.7012	0.0091	STSPPPSPEVWAESR
12.6	1785.7012	0.0091	STSPPPSPEVWAESR

9.2	1785.7012	0.0091	STSPPPSPEVWAESR
6.4	1785.7012	0.0091	STSPPPSPEVWAESR
6.4	1785.7012	0.0091	STSPPPSPEVWAESR
4.5	1783.7137	1.9965	NHTTYNQSCKWHK

Spectrum No: 394; Query: 2376; Rank: 1

Peptide View

MS/MS Fragmentation of **ENVEYIEREESDGEYDEFGRK**
Found in **IPI00214258**, Tax_Id=10116 Gene_Symbol=Zfp265 Zinc finger Ran-binding domain-containing protein 2

Match to Query 2376: 2672.095992 from(891.705940,3+)
Title: 100101RatKid_NS_deglyco_16.2598.2598.3.dta
Data file K:\NewmanPaper\Piliang\3SubProteomes\Piliang3SP\mgf5ppm\ERLIC_3SubProteomes5ppm.mgf

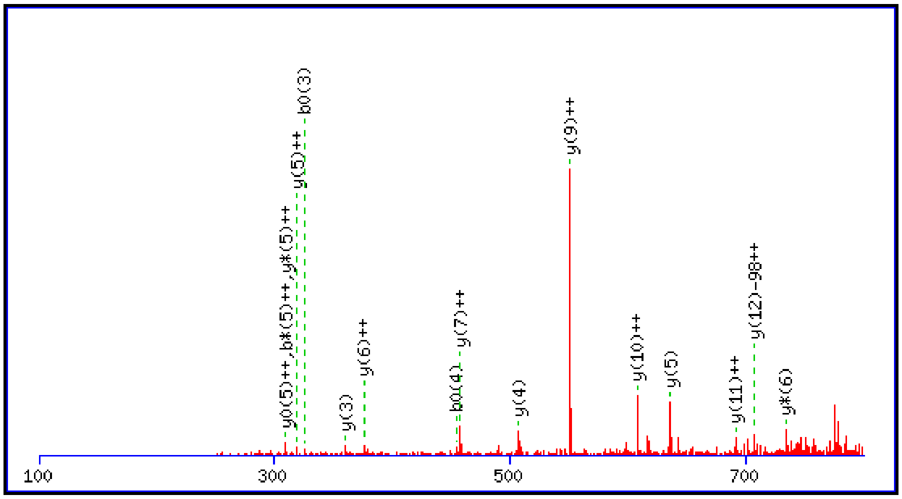

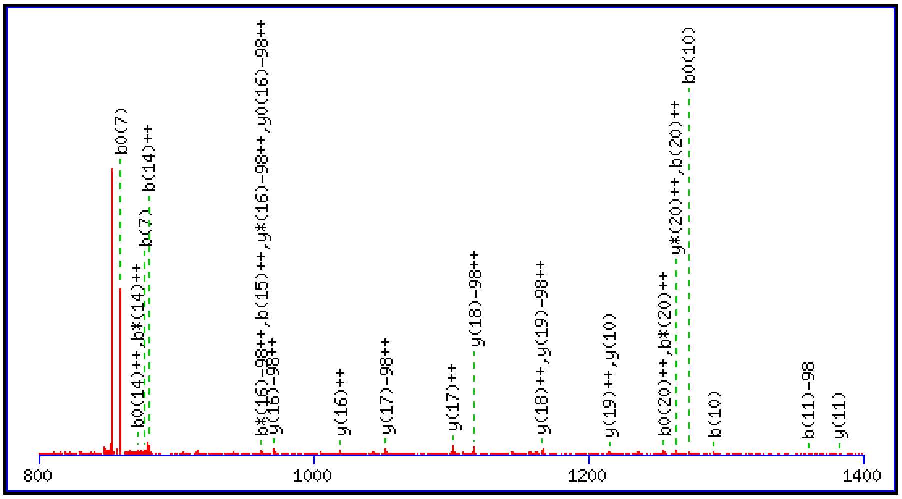

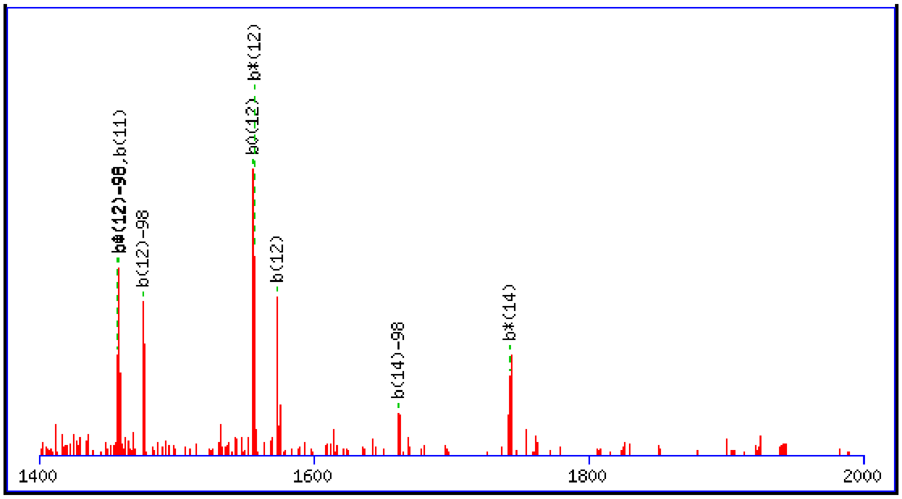

Monoisotopic mass of neutral peptide Mr(calc): 2672.0915
Fixed modifications: Carbamidomethyl (C)
Variable modifications:
S11 : Phospho (ST), with neutral losses 97.9769(shown in table), 0.0000
Ions Score: 45 **Expect:** 0.0087
Matches (Bold Red): 51/350 fragment ions using 68 most intense peaks

#	b	b^{++}	b*	b^{*++}	b^0	b^{0++}	Seq.	y	y^{++}	y*	y^{*++}	y^0	y^{0++}	#
1	130.0499	65.5286			112.0393	56.5233	E							21
2	244.0928	122.5500	227.0662	114.0368	226.0822	113.5448	N	2446.0793	1223.5433	2429.0528	1215.0300	2428.0688	1214.5380	20
3	343.1612	172.0842	326.1347	163.5710	**325.1506**	163.0790	V	2332.0364	**1166.5218**	2315.0099	1158.0086	2314.0258	1157.5166	19
4	472.2038	236.6055	455.1773	228.0923	**454.1932**	227.6003	E	2232.9680	**1116.9876**	2215.9414	1108.4744	2214.9574	1107.9824	18
5	635.2671	318.1372	618.2406	**309.6239**	617.2566	309.1319	Y	2103.9254	**1052.4663**	2086.8988	1043.9531	2085.9148	1043.4611	17
6	748.3512	374.6792	731.3246	366.1660	730.3406	365.6740	I	1940.8621	**970.9347**	1923.8355	**962.4214**	1922.8515	**961.9294**	16
7	**877.3938**	439.2005	860.3672	430.6873	**859.3832**	430.1953	E	1827.7780	914.3926	1810.7515	905.8794	1809.7674	905.3874	15
8	1033.4949	517.2511	1016.4684	508.7378	1015.4843	508.2458	R	1698.7354	849.8713	1681.7089	841.3581	1680.7248	840.8661	14
9	1162.5375	581.7724	1145.5109	573.2591	1144.5269	572.7671	E	1542.6343	771.8208	1525.6078	763.3075	1524.6237	762.8155	13
10	**1291.5801**	646.2937	1274.5535	637.7804	**1273.5695**	637.2884	E	1413.5917	**707.2995**	1396.5652	698.7862	1395.5811	698.2942	12
11	**1360.6015**	680.8044	1343.5750	672.2911	1342.5910	671.7991	S	1284.5491	642.7782	1267.5226	634.2649	1266.5385	633.7729	11
12	**1475.6285**	738.3179	**1458.6019**	729.8046	**1457.6179**	729.3126	D	**1215.5277**	**608.2675**	1198.5011	599.7542	1197.5171	599.2622	10
13	1532.6499	766.8286	1515.6234	758.3153	1514.6394	757.8233	G	1100.5007	**550.7540**	1083.4742	542.2407	1082.4901	541.7487	9
14	**1661.6925**	831.3499	1644.6660	822.8366	1643.6820	822.3446	E	1043.4793	522.2433	1026.4527	513.7300	1025.4687	513.2380	8
15	1824.7559	912.8816	1807.7293	904.3683	1806.7453	903.8763	Y	914.4367	**457.7220**	897.4101	449.2087	896.4261	448.7167	7
16	1939.7828	970.3950	1922.7563	**961.8818**	1921.7722	961.3898	D	751.3733	**376.1903**	**734.3468**	367.6770	733.3628	367.1850	6
17	2068.8254	1034.9163	2051.7989	1026.4031	2050.8148	1025.9111	E	**636.3464**	**318.6768**	619.3198	**310.1636**	618.3358	**309.6715**	5
18	2215.8938	1108.4505	2198.8673	1099.9373	2197.8833	1099.4453	F	**507.3038**	254.1555	490.2772	245.6423			4
19	2272.9153	1136.9613	2255.8887	1128.4480	2254.9047	1127.9560	G	**360.2354**	180.6213	343.2088	172.1081			3
20	2429.0164	1215.0118	2411.9898	1206.4986	2411.0058	1206.0066	R	303.2139	152.1106	286.1874	143.5973			2
21							K	147.1128	74.0600	130.0863	65.5468			1

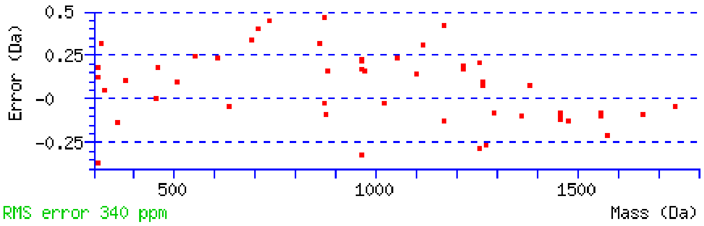

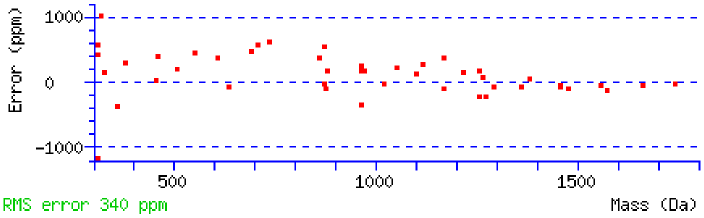

All matches to this query

Score	Mr(calc):	Delta	Sequence

45.3	2672.0915	0.0045	ENVEYIEREESDGEYDEFGRK
20.0	2672.0915	0.0045	ENVEYIEREESDGEYDEFGRK
12.3	2672.0915	0.0045	ENVEYIEREESDGEYDEFGRK
2.7	2672.1190	-0.0231	SDVDSDILAGQCVQFTRSDGSKK
2.7	2672.1190	-0.0231	SDVDSDILAGQCVQFTRSDGSKK
2.5	2671.0753	1.0207	SKPVMIVTEYMENGSLDSFLR
2.4	2670.0989	1.9971	ASAPPDAPARSPAATAGPRSPSDGAR
2.4	2670.0989	1.9971	ASAPPDAPARSPAATAGPRSPSDGAR
2.4	2671.0753	1.0207	SKPVMIVTEYMENGSLDSFLR
2.4	2670.0989	1.9971	ASAPPDAPARSPAATAGPRSPSDGAR

Spectrum No: 395; Query: 896; Rank: 1

Peptide View

MS/MS Fragmentation of **VDSTTCLFPVEEK**
Found in **IPI00190908**, Tax_Id=10116 Gene_Symbol=Gfpt1 79 kDa protein

Match to Query 896: 1603.682928 from(802.848740,2+)
Title: 091008RatKidney_NoSalt_22.2716.2716.2.dta
Data file K:\NewmanPaper\Piliang\3SubProteomes\Piliang3SP\mgf5ppm\ERLIC_3SubProteomes5ppm.mgf

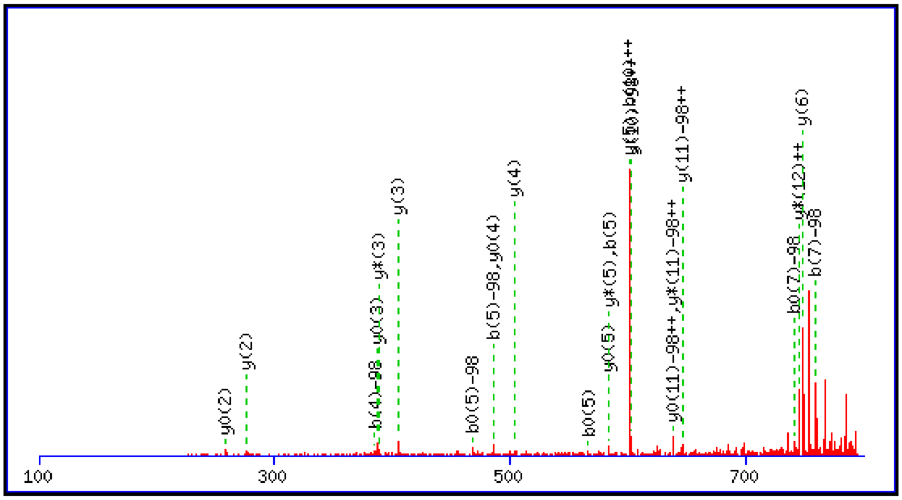

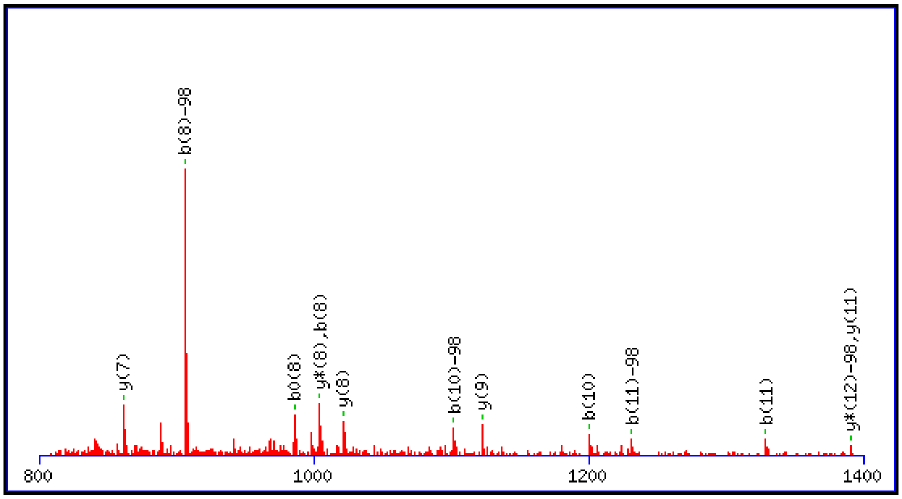

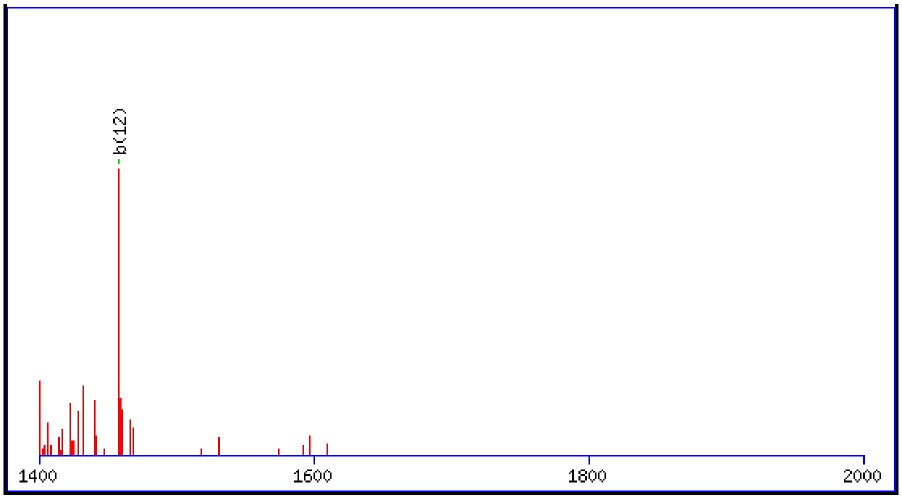

Monoisotopic mass of neutral peptide Mr(calc): 1603.6841
Fixed modifications: Carbamidomethyl (C)
Variable modifications:
T4 : Phospho (ST), with neutral losses 97.9769(shown in table), 0.0000
Ions Score: 45 **Expect:** 0.0044
Matches (Bold Red): 38/170 fragment ions using 66 most intense peaks

#	b	b^{++}	b^0	b^{0++}	Seq.	y	y^{++}	y*	y^{*++}	y^0	y^{0++}	#
1	100.0757	50.5415			V							13
2	215.1026	108.0550	197.0921	99.0497	D	1407.6461	704.3267	**1390.6195**	695.8134	1389.6355	695.3214	12
3	302.1347	151.5710	284.1241	142.5657	S	1292.6191	**646.8132**	1275.5926	**638.2999**	1274.6085	**637.8079**	11
4	**385.1718**	193.0895	367.1612	184.0842	T	1205.5871	**603.2972**	1188.5605	594.7839	1187.5765	594.2919	10
5	**486.2194**	243.6134	**468.2089**	234.6081	T	**1122.5500**	561.7786	1105.5234	553.2654	1104.5394	552.7733	9
6	646.2501	323.6287	628.2395	314.6234	C	**1021.5023**	511.2548	**1004.4757**	502.7415	1003.4917	502.2495	8
7	**759.3342**	380.1707	**741.3236**	371.1654	L	**861.4716**	431.2395	844.4451	422.7262	843.4611	422.2342	7
8	**906.4026**	453.7049	888.3920	444.6996	F	**748.3876**	374.6974	731.3610	366.1842	730.3770	365.6921	6
9	1003.4553	502.2313	985.4448	493.2260	P	**601.3192**	301.1632	**584.2926**	292.6499	**583.3086**	292.1579	5
10	**1102.5238**	551.7655	1084.5132	542.7602	V	**504.2664**	252.6368	487.2399	244.1236	**486.2558**	243.6316	4
11	**1231.5663**	616.2868	1213.5558	607.2815	E	**405.1980**	203.1026	**388.1714**	194.5894	**387.1874**	194.0974	3
12	1360.6089	680.8081	1342.5984	671.8028	E	**276.1554**	138.5813	259.1288	130.0681	**258.1448**	129.5761	2
13					K	147.1128	74.0600	130.0863	65.5468			1

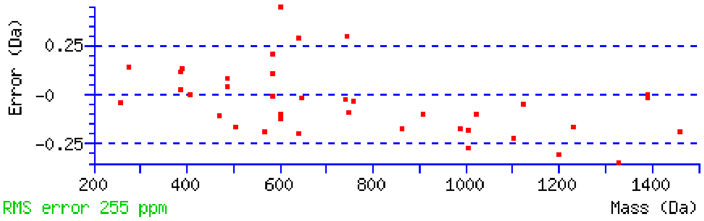

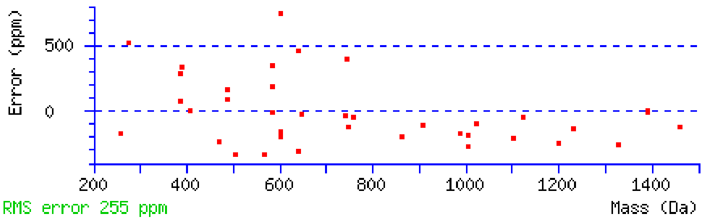

All matches to this query

Score	Mr(calc):	Delta	Sequence
45.3	1603.6841	-0.0012	VDSTTCLFPVEEK
44.1	1603.6841	-0.0012	VDSTTCLFPVEEK
34.9	1603.6841	-0.0012	VDSTTCLFPVEEK
11.3	1603.6888	-0.0058	NCFSHLMKSEKK
7.5	1603.6987	-0.0157	DAKMAETCVVKEK
5.6	1603.6953	-0.0124	CQKSLNEFLEEK
5.4	1601.6852	1.9978	DQTKKSPTGPLDR
4.9	1603.6966	-0.0137	CNKAFRQQGTWK

4.4	1601.6852	1.9978	DQTKKSPTGPLDR
2.1	1601.6852	1.9978	DQTKKSPTGPLDR

Spectrum No: 396; Query: 2755; Rank: 1

Peptide View

MS/MS Fragmentation of **ALENGEADEPSFSDPEDFVDDVSEEELLGDVLK**
Found in **IPI00363771**, Tax_Id=10116 Gene_Symbol=Eif3s9 108 kDa protein

Match to Query 2755: 3688.587792 from(1230.536540,3+)
Title: 100101RatKid_NS_deglyco_20.6357.6357.3.dta
Data file K:\NewmanPaper\Piliang\3SubProteomes\Piliang3SP\mgf5ppm\ERLIC_3SubProteomes5ppm.mgf

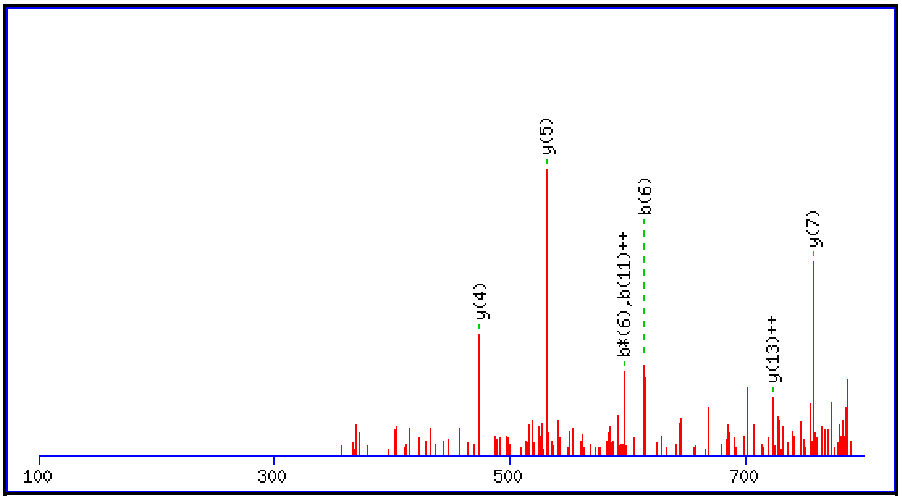

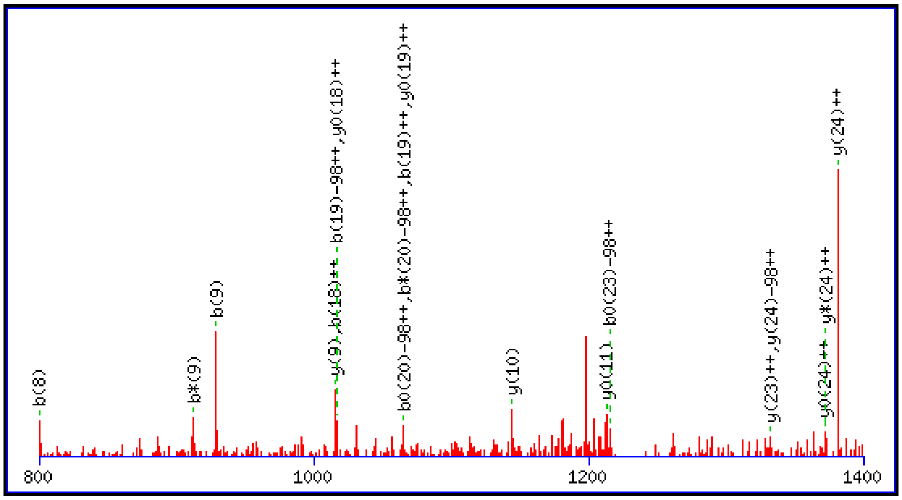

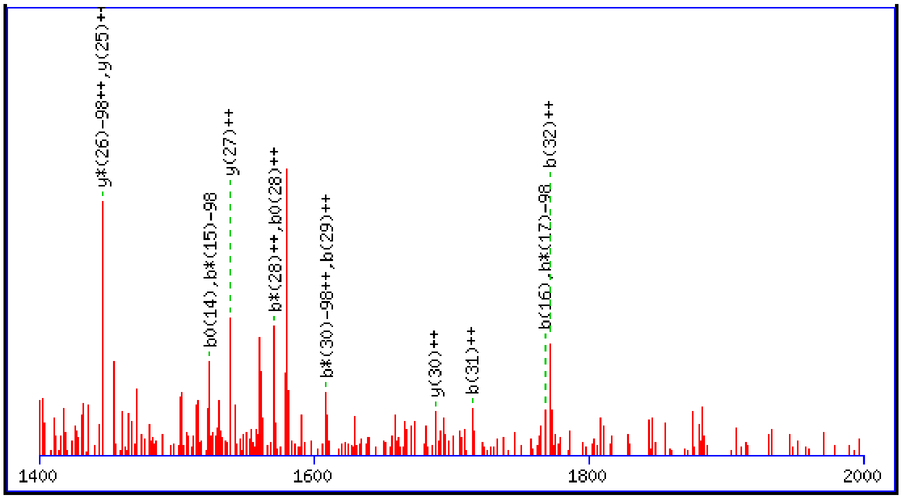

Monoisotopic mass of neutral peptide Mr(calc): 3688.5717
Fixed modifications: Carbamidomethyl (C)
Variable modifications:
S11 : Phospho (ST), with neutral losses 0.0000(shown in table), 97.9769
Ions Score: 45 **Expect:** 0.014
Matches (Bold Red): 41/560 fragment ions using 45 most intense peaks

#	b	b^{++}	b*	b*$^{++}$	b^{0}	b^{0++}	Seq.	y	y^{++}	y*	y*$^{++}$	y^{0}	y^{0++}	#
1	72.0444	36.5258					A							33
2	185.1285	93.0679					L	3618.5418	1809.7746	3601.5153	1801.2613	3600.5313	1800.7693	32
3	314.1710	157.5892			296.1605	148.5839	E	3505.4578	1753.2325	3488.4312	1744.7193	3487.4472	1744.2272	31
4	428.2140	214.6106	411.1874	206.0974	410.2034	205.6053	N	3376.4152	**1688.7112**	3359.3886	1680.1980	3358.4046	1679.7059	30
5	485.2354	243.1214	468.2089	234.6081	467.2249	234.1161	G	3262.3723	1631.6898	3245.3457	1623.1765	3244.3617	1622.6845	29
6	**614.2780**	307.6427	**597.2515**	299.1294	596.2675	298.6374	E	3205.3508	1603.1790	3188.3242	1594.6658	3187.3402	1594.1738	28
7	685.3151	343.1612	668.2886	334.6479	667.3046	334.1559	A	3076.3082	**1538.6577**	3059.2817	1530.1445	3058.2976	1529.6525	27
8	**800.3421**	400.6747	783.3155	392.1614	782.3315	391.6694	D	3005.2711	1503.1392	2988.2445	1494.6259	2987.2605	1494.1339	26
9	**929.3847**	465.1960	**912.3581**	456.6827	911.3741	456.1907	E	2890.2441	**1445.6257**	2873.2176	1437.1124	2872.2336	1436.6204	25
10	1026.4374	513.7224	1009.4109	505.2091	1008.4269	504.7171	P	2761.2016	**1381.1044**	2744.1750	**1372.5911**	2743.1910	**1372.0991**	24
11	1193.4358	**597.2215**	1176.4092	588.7083	1175.4252	588.2163	S	2664.1488	**1332.5780**	2647.1222	1324.0648	2646.1382	1323.5727	23
12	1340.5042	670.7557	1323.4777	662.2425	1322.4936	661.7505	F	2497.1504	1249.0789	2480.1239	1240.5656	2479.1399	1240.0736	22
13	1427.5362	714.2718	1410.5097	705.7585	1409.5257	705.2665	S	2350.0820	1175.5446	2333.0555	1167.0314	2332.0715	1166.5394	21
14	1542.5632	771.7852	1525.5366	763.2720	**1524.5526**	762.7799	D	2263.0500	1132.0286	2246.0234	1123.5154	2245.0394	1123.0234	20
15	1639.6159	820.3116	1622.5894	811.7983	1621.6054	811.3063	P	2148.0231	1074.5152	2130.9965	1066.0019	2130.0125	**1065.5099**	19
16	**1768.6585**	884.8329	1751.6320	876.3196	1750.6480	875.8276	E	2050.9703	1025.9888	2033.9437	1017.4755	2032.9597	**1016.9835**	18
17	1883.6855	942.3464	1866.6589	933.8331	1865.6749	933.3411	D	1921.9277	961.4675	1904.9011	952.9542	1903.9171	952.4622	17
18	2030.7539	**1015.8806**	2013.7273	1007.3673	2012.7433	1006.8753	F	1806.9008	903.9540	1789.8742	895.4407	1788.8902	894.9487	16
19	2129.8223	**1065.4148**	2112.7958	1056.9015	2111.8117	1056.4095	V	1659.8323	830.4198	1642.8058	821.9065	1641.8218	821.4145	15
20	2244.8493	1122.9283	2227.8227	1114.4150	2226.8387	1113.9230	D	1560.7639	780.8856	1543.7374	772.3723	1542.7534	771.8803	14
21	2359.8762	1180.4417	2342.8496	1171.9285	2341.8656	1171.4365	D	**1445.7370**	**723.3721**	1428.7104	714.8589	1427.7264	714.3668	13
22	2458.9446	1229.9759	2441.9181	1221.4627	2440.9340	1220.9707	V	1330.7100	665.8587	1313.6835	657.3454	1312.6995	656.8534	12
23	2545.9766	1273.4920	2528.9501	1264.9787	2527.9661	1264.4867	S	1231.6416	616.3245	1214.6151	607.8112	**1213.6311**	607.3192	11
24	2675.0192	1338.0133	2657.9927	1329.5000	2657.0087	1329.0080	E	**1144.6096**	572.8084	1127.5830	564.2952	1126.5990	563.8032	10
25	2804.0618	1402.5345	2787.0353	1394.0213	2786.0513	1393.5293	E	**1015.5670**	508.2871	998.5405	499.7739	997.5564	499.2819	9
26	2933.1044	1467.0558	2916.0779	1458.5426	2915.0939	1458.0506	E	886.5244	443.7658	869.4979	435.2526	868.5138	434.7606	8
27	3046.1885	1523.5979	3029.1619	1515.0846	3028.1779	1514.5926	L	**757.4818**	379.2445	740.4553	370.7313	739.4713	370.2393	7
28	3159.2725	1580.1399	3142.2460	**1571.6266**	3141.2620	**1571.1346**	L	644.3978	322.7025	627.3712	314.1892	626.3872	313.6972	6
29	3216.2940	**1608.6506**	3199.2675	1600.1374	3198.2834	1599.6454	G	**531.3137**	266.1605	514.2871	257.6472	513.3031	257.1552	5
30	3331.3210	1666.1641	3314.2944	1657.6508	3313.3104	1657.1588	D	**474.2922**	237.6498	457.2657	229.1365	456.2817	228.6445	4
31	3430.3894	**1715.6983**	3413.3628	1707.1850	3412.3788	1706.6930	V	359.2653	180.1363	342.2387	171.6230			3
32	3543.4734	**1772.2404**	3526.4469	1763.7271	3525.4629	1763.2351	L	260.1969	130.6021	243.1703	122.0888			2

33							K	147.1128	74.0600	130.0863	65.5468			1

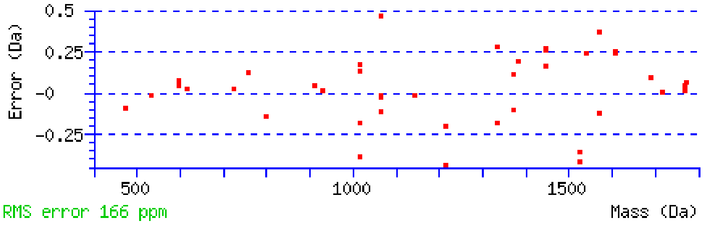

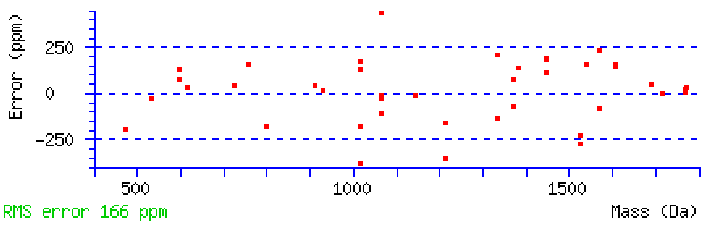

All matches to this query

Score	Mr(calc):	Delta	Sequence
45.3	3688.5717	0.0161	ALENGEADEPSFSDPEDFVDDVSEEELLGDVLK
40.8	3688.5717	0.0161	ALENGEADEPSFSDPEDFVDDVSEEELLGDVLK
26.4	3688.5717	0.0161	ALENGEADEPSFSDPEDFVDDVSEEELLGDVLK
7.3	3686.5808	2.0070	QTSITSFVTSQPGMANGGNQKNASSLKENQINR
7.2	3687.5648	1.0230	QTSITSFVTSQPGMANGGNQKNASSLKENQINR
6.6	3686.5808	2.0070	QTSITSFVTSQPGMANGGNQKNASSLKENQINR
6.3	3686.5808	2.0070	QTSITSFVTSQPGMANGGNQKNASSLKENQINR
5.6	3686.5808	2.0070	QTSITSFVTSQPGMANGGNQKNASSLKENQINR
5.4	3688.6210	-0.0332	MCIEIVSLAFCPEADVMADENIRQVYVEYK
5.3	3686.5808	2.0070	QTSITSFVTSQPGMANGGNQKNASSLKENQINR

Spectrum No: 397; Query: 2469; Rank: 1

Peptide View

MS/MS Fragmentation of **VLEIPDRDSEEELEHVIEQIAYR**
Found in **IPI00231379**, Tax_Id=10116 Gene_Symbol=Slc4a1 solute carrier family 4, member 1

Match to Query 2469: 2861.338002 from(954.786610,3+)
Title: 091008RatKidney_NH4Format01_11.5840.5840.3.dta
Data file K:\NewmanPaper\Piliang\3SubProteomes\Piliang3SP\mgf5ppm\ERLIC_3SubProteomes5ppm.mgf

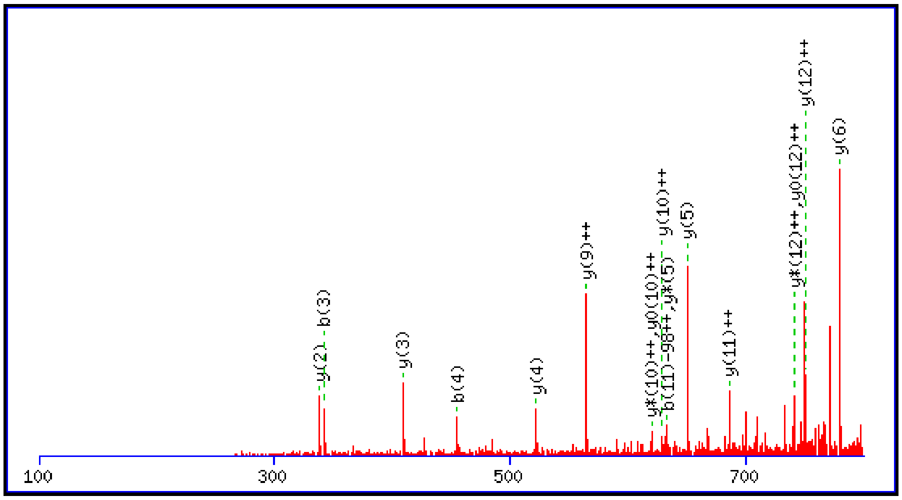

Mascot Search Results: Peptide View

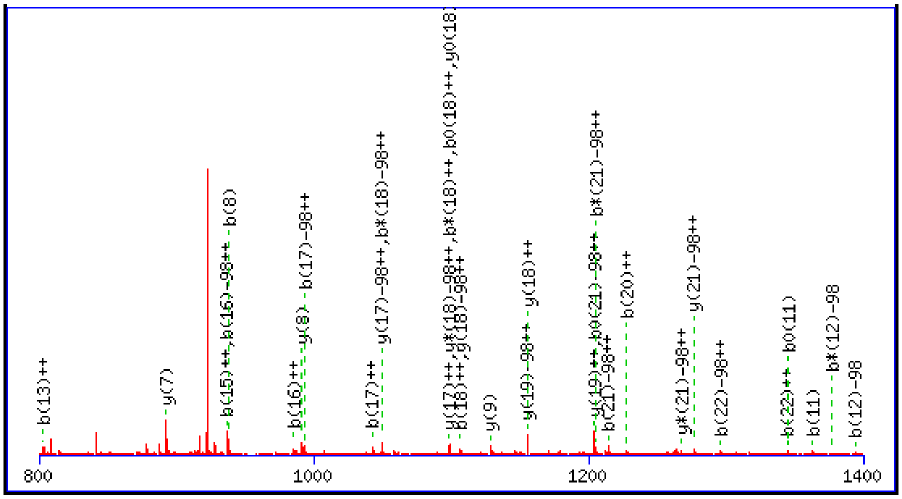

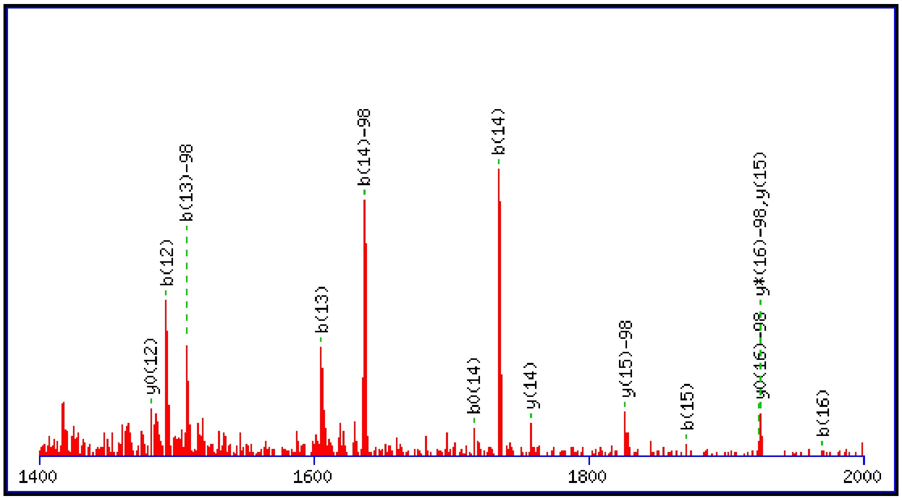

Monoisotopic mass of neutral peptide Mr(calc): 2861.3484
Fixed modifications: Carbamidomethyl (C)
Variable modifications:
S9 : Phospho (ST), with neutral losses 97.9769(shown in table), 0.0000
Ions Score: 45 **Expect:** 0.01
Matches (Bold Red): 65/370 fragment ions using 120 most intense peaks

#	b	b^{++}	b*	b^{*++}	b^0	b^{0++}	Seq.	y	y^{++}	y*	y^{*++}	y^0	y^{0++}	#
1	100.0757	50.5415					V							23
2	213.1598	107.0835					L	2665.3104	1333.1588	2648.2838	1324.6456	2647.2998	1324.1536	22
3	**342.2023**	171.6048			324.1918	162.5995	E	2552.2263	**1276.6168**	2535.1998	**1268.1035**	2534.2158	1267.6115	21
4	**455.2864**	228.1468			437.2758	219.1416	I	2423.1837	1212.0955	2406.1572	1203.5822	2405.1732	1203.0902	20
5	552.3392	276.6732			534.3286	267.6679	P	2310.0997	**1155.5535**	2293.0731	1147.0402	2292.0891	1146.5482	19
6	667.3661	334.1867			649.3556	325.1814	D	2213.0469	**1107.0271**	2196.0204	**1098.5138**	2195.0363	**1098.0218**	18
7	823.4672	412.2373	806.4407	403.7240	805.4567	403.2320	R	2098.0200	**1049.5136**	2080.9934	1041.0003	2080.0094	1040.5083	17
8	**938.4942**	469.7507	921.4676	461.2374	920.4836	460.7454	D	1941.9189	971.4631	**1924.8923**	962.9498	**1923.9083**	962.4578	16
9	1007.5156	504.2615	990.4891	495.7482	989.5051	495.2562	S	**1826.8919**	913.9496	1809.8654	905.4363	1808.8813	904.9443	15
10	1136.5582	568.7827	1119.5317	560.2695	1118.5477	559.7775	E	**1757.8705**	879.4389	1740.8439	870.9256	1739.8599	870.4336	14
11	1265.6008	**633.3040**	1248.5743	624.7908	1247.5902	624.2988	E	1628.8279	814.9176	1611.8013	806.4043	1610.8173	805.9123	13
12	**1394.6434**	697.8253	**1377.6169**	689.3121	1376.6328	688.8201	E	1499.7853	**750.3963**	1482.7587	**741.8830**	**1481.7747**	**741.3910**	12
13	**1507.7275**	754.3674	1490.7009	745.8541	1489.7169	745.3621	L	1370.7427	**685.8750**	1353.7161	677.3617	1352.7321	676.8697	11
14	**1636.7701**	818.8887	1619.7435	810.3754	1618.7595	809.8834	E	1257.6586	**629.3329**	1240.6321	**620.8197**	1239.6480	**620.3277**	10
15	1773.8290	887.4181	1756.8024	878.9049	1755.8184	878.4128	H	**1128.6160**	**564.8116**	1111.5895	556.2984	1110.6055	555.8064	9
16	1872.8974	**936.9523**	1855.8708	928.4391	1854.8868	927.9471	V	**991.5571**	496.2822	974.5306	487.7689	973.5465	487.2769	8
17	1985.9815	**993.4944**	1968.9549	984.9811	1967.9709	984.4891	I	**892.4887**	446.7480	875.4621	438.2347	874.4781	437.7427	7
18	2115.0240	1058.0157	2097.9975	**1049.5024**	2097.0135	1049.0104	E	**779.4046**	390.2060	762.3781	381.6927	761.3941	381.2007	6

**19**	2243.0826	1122.0450	2226.0561	1113.5317	2225.0721	1113.0397	**Q**	**650.3620**	325.6847	**633.3355**	317.1714			**5**
**20**	2356.1667	1178.5870	2339.1401	1170.0737	2338.1561	1169.5817	**I**	**522.3035**	261.6554	505.2769	253.1421			**4**
**21**	2427.2038	**1214.1055**	2410.1773	**1205.5923**	2409.1932	**1205.1003**	**A**	**409.2194**	205.1133	392.1928	196.6001			**3**
**22**	2590.2671	**1295.6372**	2573.2406	1287.1239	2572.2566	1286.6319	**Y**	**338.1823**	169.5948	321.1557	161.0815			**2**
**23**							**R**	175.1190	88.0631	158.0924	79.5498			**1**

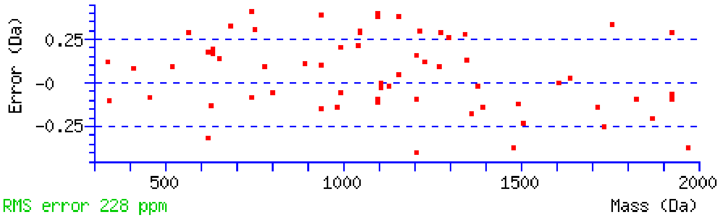

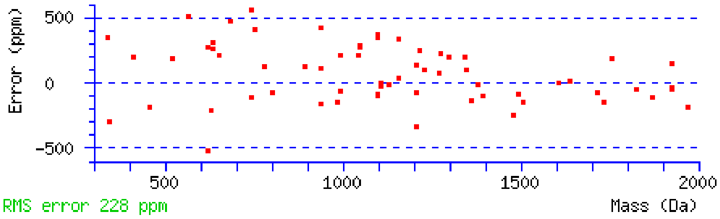

All matches to this query

Score	Mr(calc):	Delta	Sequence
45.2	2861.3484	-0.0104	VLEIPDRDSEEELEHVIEQIAYR
5.9	2859.3522	1.9858	LPSGPPEAEGQADLGPSTSEQNLASPGPR
5.8	2861.3258	0.0122	EVKPPGMTVMKDVAIAKQGYAPSER
5.8	2861.3258	0.0122	EVKPPGMTVMKDVAIAKQGYAPSER
3.7	2860.3597	0.9783	DKIMSVFYTVVTPMLNPIIYSLR
2.5	2861.3513	-0.0133	QEPAPKPNNKTPAILYTYSGLRSR
2.5	2861.3513	-0.0133	QEPAPKPNNKTPAILYTYSGLRSR
2.5	2860.3198	1.0182	SRAISPETQILAALGFYTSGSFQTR
2.2	2861.3101	0.0279	ILKLKNLRPQDYANYSCIASVR
2.2	2861.3101	0.0279	ILKLKNLRPQDYANYSCIASVR

Spectrum No: 398; Query: 1935; Rank: 1

Peptide View

MS/MS Fragmentation of **ESVIGASEVAPPAAPPSSPGSSR**
Found in **IPI00566819**, Tax_Id=10116 Gene_Symbol=Mlh1 DNA mismatch repair protein Mlh1

Match to Query 1935: 2229.038892 from(744.020240,3+)
Title: 100101RatKid_NS_deglyco_19.2405.2405.3.dta
Data file K:\NewmanPaper\Piliang\3SubProteomes\Piliang3SP\mgf5ppm\ERLIC_3SubProteomes5ppm.mgf

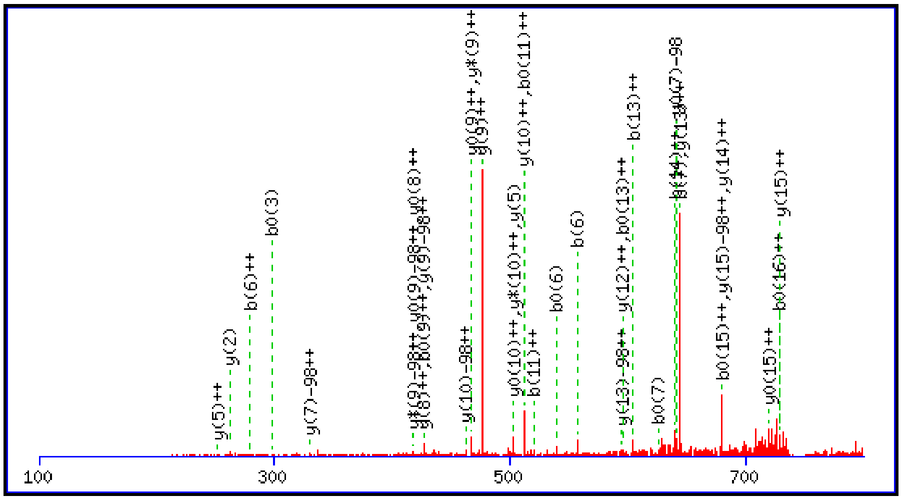

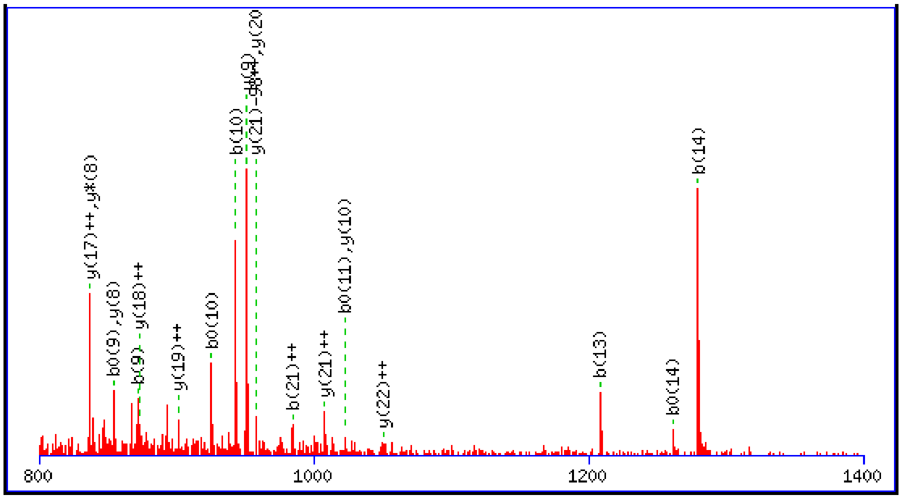

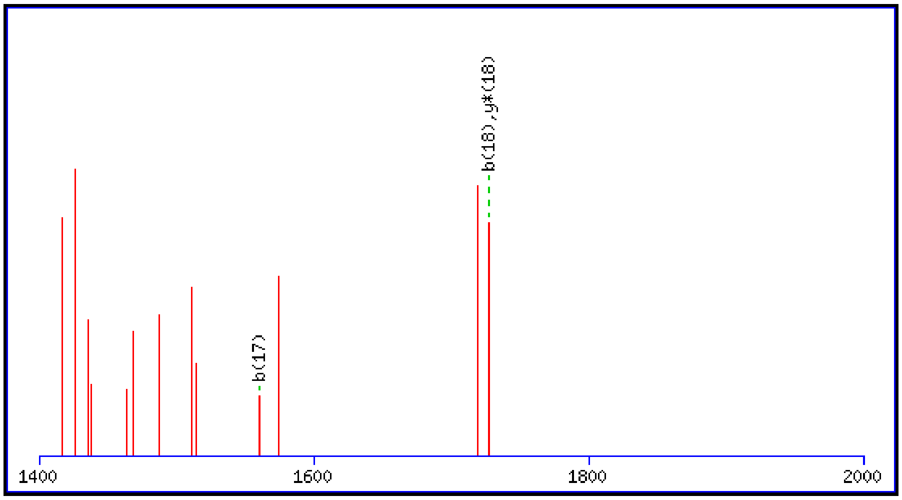

Monoisotopic mass of neutral peptide Mr(calc): 2229.0314
Fixed modifications: Carbamidomethyl (C)
Variable modifications:
S18 : Phospho (ST), with neutral losses 0.0000(shown in table), 97.9769
Ions Score: 45 **Expect:** 0.0083
Matches (Bold Red): 61/340 fragment ions using 128 most intense peaks

#	b	b^{++}	b^0	b^{0++}	Seq.	y	y^{++}	y*	y^{*++}	y^0	y^{0++}	#
1	130.0499	65.5286	112.0393	56.5233	E							23
2	217.0819	109.0446	199.0713	100.0393	S	2100.9961	**1051.0017**	2083.9696	1042.4884	2082.9856	1041.9964	22
3	316.1503	158.5788	**298.1397**	149.5735	V	2013.9641	**1007.4857**	1996.9376	998.9724	1995.9535	998.4804	21
4	429.2344	215.1208	411.2238	206.1155	I	1914.8957	**957.9515**	1897.8691	949.4382	1896.8851	948.9462	20
5	486.2558	243.6316	468.2453	234.6263	G	1801.8116	**901.4095**	1784.7851	892.8962	1783.8011	892.4042	19
6	**557.2930**	**279.1501**	**539.2824**	270.1448	A	1744.7902	**872.8987**	**1727.7636**	864.3854	1726.7796	863.8934	18
7	**644.3250**	322.6661	**626.3144**	313.6608	S	1673.7530	**837.3802**	1656.7265	828.8669	1655.7425	828.3749	17
8	773.3676	387.1874	755.3570	378.1821	E	1586.7210	793.8641	1569.6945	785.3509	1568.7105	784.8589	16
9	**872.4360**	436.7216	**854.4254**	**427.7163**	V	1457.6784	**729.3429**	1440.6519	720.8296	1439.6679	**720.3376**	15
10	**943.4731**	472.2402	**925.4625**	463.2349	A	1358.6100	**679.8086**	1341.5835	671.2954	1340.5994	670.8034	14
11	1040.5259	**520.7666**	**1022.5153**	**511.7613**	P	1287.5729	**644.2901**	1270.5464	635.7768	1269.5623	635.2848	13
12	1137.5786	569.2930	1119.5681	560.2877	P	1190.5201	**595.7637**	1173.4936	587.2504	1172.5096	586.7584	12
13	**1208.6157**	**604.8115**	1190.6052	**595.8062**	A	1093.4674	547.2373	1076.4408	538.7240	1075.4568	538.2320	11
14	**1279.6529**	**640.3301**	**1261.6423**	631.3248	A	**1022.4303**	**511.7188**	1005.4037	**503.2055**	1004.4197	**502.7135**	10
15	1376.7056	688.8564	1358.6951	**679.8512**	P	**951.3931**	**476.2002**	934.3666	**467.6869**	933.3826	**467.1949**	9
16	1473.7584	737.3828	1455.7478	**728.3775**	P	**854.3404**	**427.6738**	**837.3138**	419.1606	836.3298	**418.6685**	8
17	**1560.7904**	780.8988	1542.7798	771.8936	S	757.2876	379.1474	740.2611	370.6342	739.2771	370.1422	7
18	**1727.7888**	864.3980	1709.7782	855.3927	S	670.2556	335.6314	653.2290	327.1182	652.2450	326.6261	6

19	1824.8415	912.9244	1806.8310	903.9191	P	503.2572	252.1323	486.2307	243.6190	485.2467	243.1270	5
20	1881.8630	941.4351	1863.8524	932.4299	G	406.2045	203.6059	389.1779	195.0926	388.1939	194.6006	4
21	1968.8950	984.9511	1950.8845	975.9459	S	349.1830	175.0951	332.1565	166.5819	331.1724	166.0899	3
22	2055.9270	1028.4672	2037.9165	1019.4619	S	262.1510	131.5791	245.1244	123.0659	244.1404	122.5738	2
23					R	175.1190	88.0631	158.0924	79.5498			1

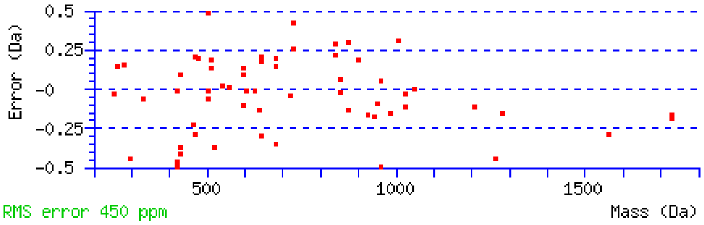

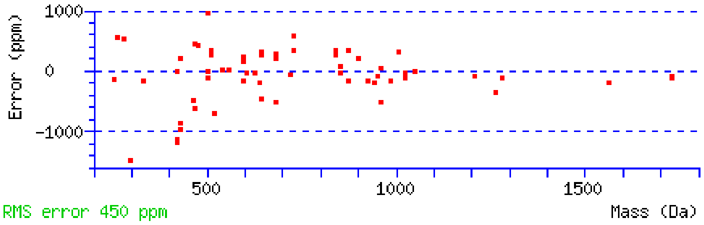

All matches to this query

Score	Mr(calc):	Delta	Sequence
45.2	2229.0314	0.0075	ESVIGASEVAPPAAPPSSPGSSR
44.3	2229.0314	0.0075	ESVIGASEVAPPAAPPSSPGSSR
38.6	2229.0314	0.0075	ESVIGASEVAPPAAPPSSPGSSR
31.6	2229.0314	0.0075	ESVIGASEVAPPAAPPSSPGSSR
27.8	2229.0314	0.0075	ESVIGASEVAPPAAPPSSPGSSR
12.0	2229.0314	0.0075	ESVIGASEVAPPAAPPSSPGSSR
5.6	2229.0427	-0.0038	GEGTPAAGDGLGKPLGPTPSQSR
2.5	2227.0474	1.9915	QQKDSGCFRSSGSLLNNAMK
2.5	2228.0314	1.0075	QQKDSGCFRSSGSLLNNAMK
2.5	2228.0314	1.0075	QQKDSGCFRSSGSLLNNAMK

Spectrum No: 399; Query: 2464; Rank: 1

Peptide View

MS/MS Fragmentation of **SQSSEGVSSLSSSPSNSLETQSQSLSR**
Found in **IPI00359635**, Tax_Id=10116 Gene_Symbol=Ube4b_predicted similar to Ubiquitin conjugation factor E4 B

Match to Query 2464: 2836.253652 from(946.425160,3+)
Title: 100101RatKid_NS_deglyco_20.2612.2612.3.dta
Data file K:\NewmanPaper\Piliang\3SubProteomes\Piliang3SP\mgf5ppm\ERLIC_3SubProteomes5ppm.mgf

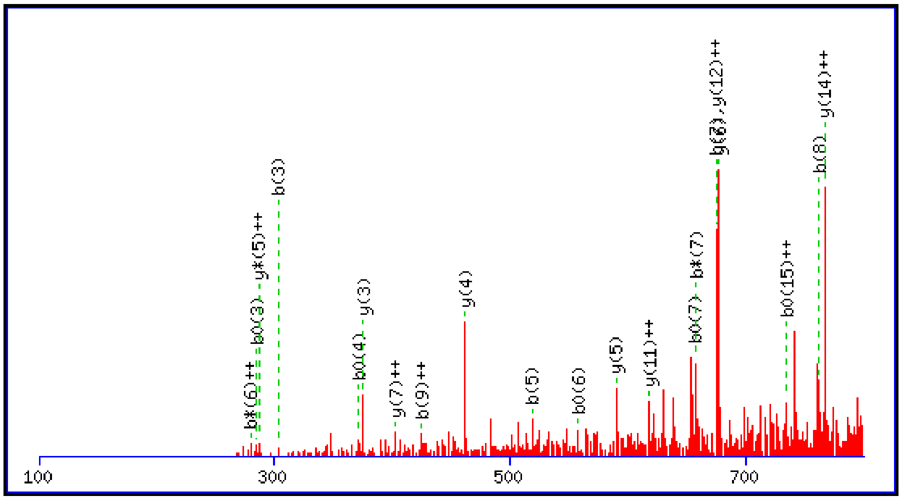

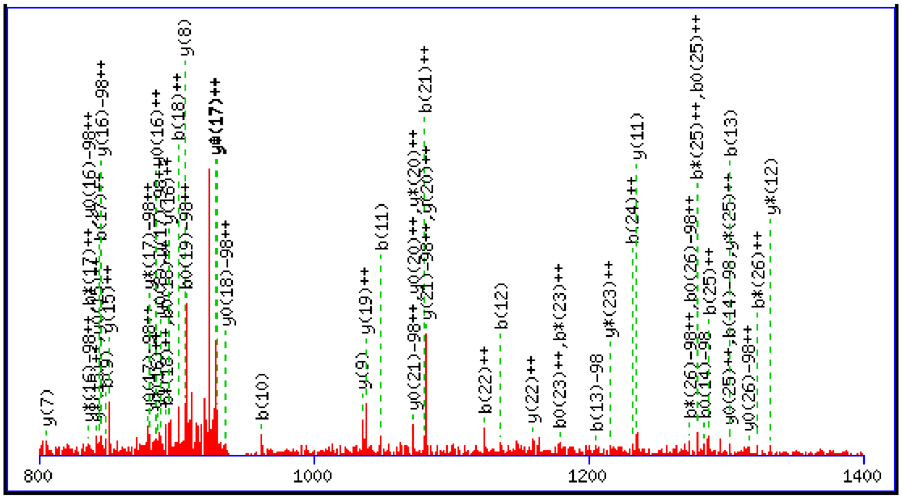

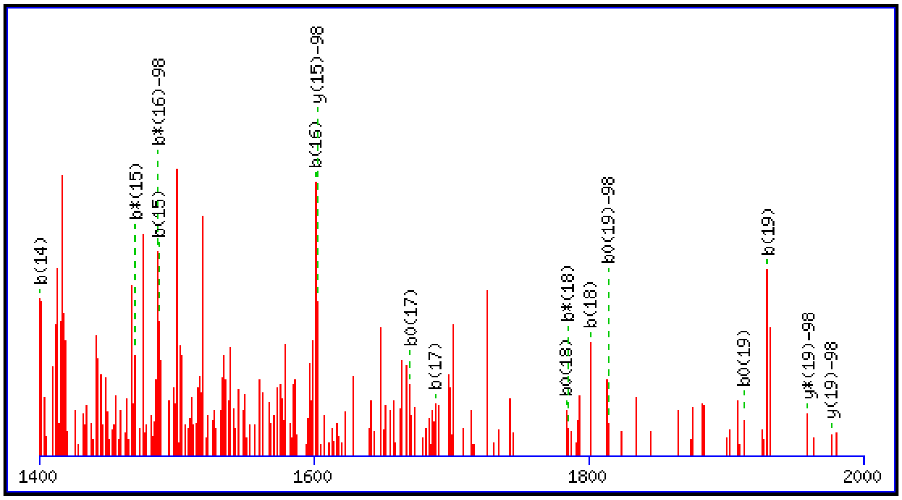

Monoisotopic mass of neutral peptide Mr(calc): 2835.2407
Fixed modifications: Carbamidomethyl (C)
Variable modifications:
S13 : Phospho (ST), with neutral losses 0.0000(shown in table), 97.9769
Ions Score: 45 **Expect:** 0.011
Matches (Bold Red): 94/464 fragment ions using 179 most intense peaks

#	b	b++	b*	b*++	b0	b0++	Seq.	y	y++	y*	y*++	y0	y0++	#
1	88.0393	44.5233			70.0287	35.5180	S							27
2	216.0979	108.5526	199.0713	100.0393	198.0873	99.5473	Q	2749.2160	1375.1116	2732.1894	1366.5983	2731.2054	1366.1063	26
3	**303.1299**	152.0686	286.1034	143.5553	**285.1193**	143.0633	S	2621.1574	1311.0823	2604.1308	**1302.5691**	2603.1468	**1302.0771**	25
4	390.1619	195.5846	373.1354	187.0713	**372.1514**	186.5793	S	2534.1254	1267.5663	2517.0988	1259.0530	2516.1148	1258.5610	24
5	**519.2045**	260.1059	502.1780	251.5926	501.1940	251.1006	E	2447.0933	1224.0503	2430.0668	**1215.5370**	2429.0828	1215.0450	23
6	576.2260	288.6166	559.1994	**280.1034**	**558.2154**	279.6114	G	2318.0507	**1159.5290**	2301.0242	1151.0157	2300.0402	1150.5237	22
7	**675.2944**	338.1508	**658.2679**	329.6376	**657.2838**	329.1456	V	2261.0293	1131.0183	2244.0027	1122.5050	2243.0187	1122.0130	21
8	**762.3264**	381.6669	745.2999	373.1536	744.3159	372.6616	S	2161.9609	**1081.4841**	2144.9343	**1072.9708**	2143.9503	**1072.4788**	20
9	**849.3585**	**425.1829**	832.3319	416.6696	831.3479	416.1776	S	2074.9288	**1037.9681**	2057.9023	1029.4548	2056.9183	1028.9628	19
10	**962.4425**	481.7249	945.4160	473.2116	944.4320	472.7196	L	1987.8968	994.4520	1970.8703	985.9388	1969.8862	985.4468	18
11	**1049.4746**	525.2409	1032.4480	516.7276	1031.4640	516.2356	S	1874.8127	937.9100	1857.7862	**929.3967**	1856.8022	**928.9047**	17
12	**1136.5066**	568.7569	1119.4800	560.2437	1118.4960	559.7516	S	1787.7807	**894.3940**	1770.7542	**885.8807**	1769.7702	**885.3887**	16
13	**1303.5049**	652.2561	1286.4784	643.7428	1285.4944	643.2508	S	1700.7487	**850.8780**	1683.7221	**842.3647**	1682.7381	**841.8727**	15
14	**1400.5577**	700.7825	1383.5312	692.2692	1382.5471	691.7772	P	1533.7503	**767.3788**	1516.7238	758.8655	1515.7398	758.3735	14
15	**1487.5897**	744.2985	**1470.5632**	735.7852	1469.5792	**735.2932**	S	1436.6976	718.8524	1419.6710	710.3392	1418.6870	709.8471	13
16	**1601.6327**	801.3200	1584.6061	792.8067	1583.6221	792.3147	N	1349.6655	**675.3364**	**1332.6390**	666.8231	1331.6550	666.3311	12
17	**1688.6647**	**844.8360**	1671.6381	**836.3227**	**1670.6541**	835.8307	S	**1235.6226**	**618.3149**	1218.5961	609.8017	1217.6121	609.3097	11
18	**1801.7487**	**901.3780**	**1784.7222**	**892.8647**	**1783.7382**	**892.3727**	L	1148.5906	574.7989	1131.5640	566.2857	1130.5800	565.7937	10

**19**	**1930.7913**	965.8993	1913.7648	957.3860	**1912.7808**	956.8940	**E**	**1035.5065**	518.2569	1018.4800	509.7436	1017.4960	509.2516	**9**
**20**	2031.8390	1016.4231	2014.8125	1007.9099	2013.8285	1007.4179	**T**	**906.4639**	453.7356	889.4374	445.2223	**888.4534**	444.7303	**8**
**21**	2159.8976	**1080.4524**	2142.8710	1071.9392	2141.8870	1071.4472	**Q**	**805.4163**	**403.2118**	788.3897	394.6985	787.4057	394.2065	**7**
**22**	2246.9296	**1123.9685**	2229.9031	1115.4552	2228.9191	1114.9632	**S**	**677.3577**	339.1825	660.3311	330.6692	659.3471	330.1772	**6**
**23**	2374.9882	1187.9977	2357.9617	**1179.4845**	2356.9776	**1178.9925**	**Q**	**590.3257**	295.6665	573.2991	**287.1532**	572.3151	286.6612	**5**
**24**	2462.0202	**1231.5138**	2444.9937	1223.0005	2444.0097	1222.5085	**S**	**462.2671**	231.6372	445.2405	223.1239	444.2565	222.6319	**4**
**25**	2575.1043	**1288.0558**	2558.0777	**1279.5425**	2557.0937	**1279.0505**	**L**	**375.2350**	188.1212	358.2085	179.6079	357.2245	179.1159	**3**
**26**	2662.1363	1331.5718	2645.1098	**1323.0585**	2644.1258	1322.5665	**S**	262.1510	131.5791	245.1244	123.0659	244.1404	122.5738	**2**
**27**							**R**	175.1190	88.0631	158.0924	79.5498			**1**

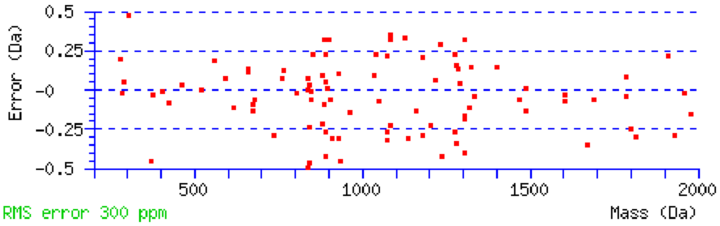

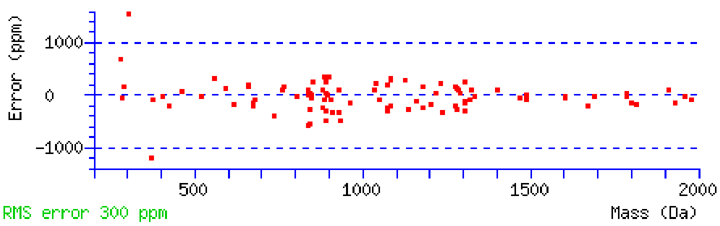

All matches to this query

Score	Mr(calc):	Delta	Sequence
45.1	2835.2407	1.0129	SQSSEGVSSLSSSPSNSLETQSQSLSR
39.2	2835.2407	1.0129	SQSSEGVSSLSSSPSNSLETQSQSLSR
35.8	2835.2407	1.0129	SQSSEGVSSLSSSPSNSLETQSQSLSR
35.1	2835.2407	1.0129	SQSSEGVSSLSSSPSNSLETQSQSLSR
33.4	2835.2407	1.0129	SQSSEGVSSLSSSPSNSLETQSQSLSR
33.2	2835.2407	1.0129	SQSSEGVSSLSSSPSNSLETQSQSLSR
26.7	2835.2407	1.0129	SQSSEGVSSLSSSPSNSLETQSQSLSR
24.1	2835.2407	1.0129	SQSSEGVSSLSSSPSNSLETQSQSLSR
22.4	2835.2407	1.0129	SQSSEGVSSLSSSPSNSLETQSQSLSR
21.9	2835.2407	1.0129	SQSSEGVSSLSSSPSNSLETQSQSLSR

Spectrum No: 400; Query: 1248; Rank: 1

Peptide View

MS/MS Fragmentation of **DHSPTPSVFNSDEER**
Found in **IPI00193470**, Tax_Id=10116 Gene_Symbol=Fip1l1 65 kDa protein

Match to Query 1248: 1795.706188 from(898.860370,2+)
Title: 091008RatKidney_NH4Format02_26.1375.1375.2.dta
Data file K:\NewmanPaper\Piliang\3SubProteomes\Piliang3SP\mgf5ppm\ERLIC_3SubProteomes5ppm.mgf

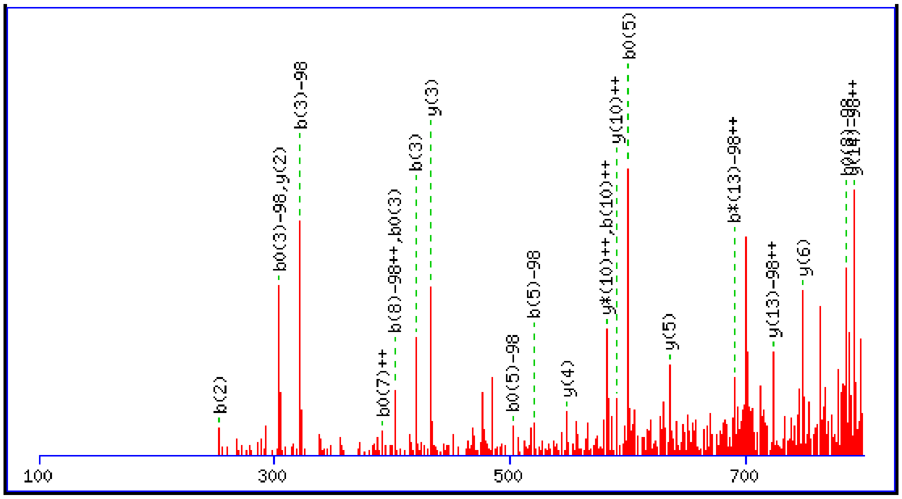

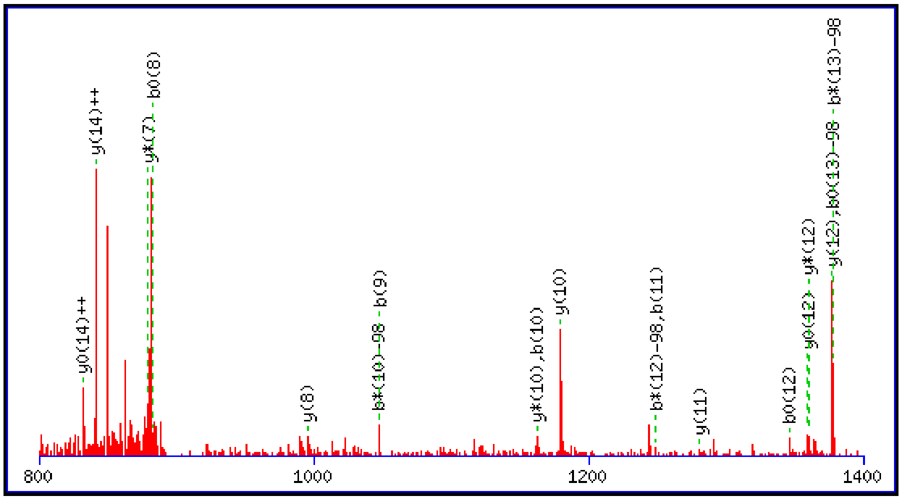

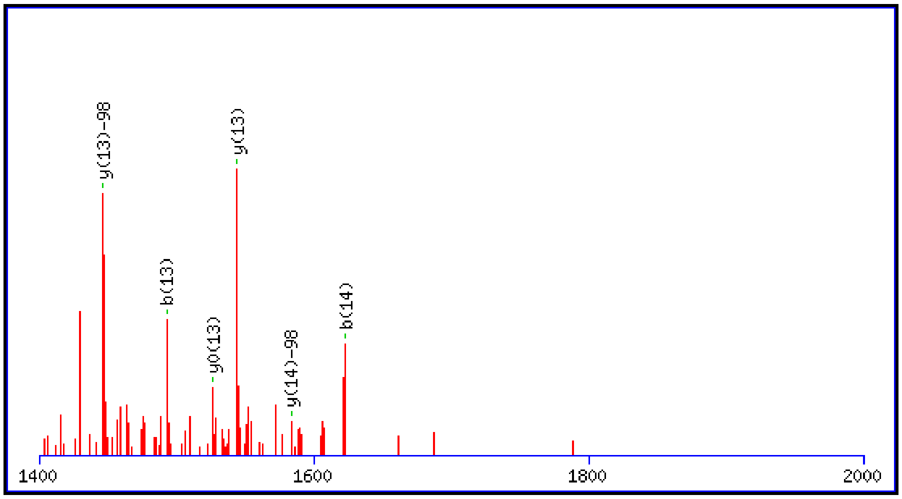

Monoisotopic mass of neutral peptide Mr(calc): 1795.7050
Fixed modifications: Carbamidomethyl (C)
Variable modifications:
S3 : Phospho (ST), with neutral losses 97.9769(shown in table), 0.0000
Ions Score: 45 **Expect:** 0.0043
Matches (Bold Red): 47/218 fragment ions using 97 most intense peaks

#	b	b^{++}	b*	b^{*++}	b^0	b^{0++}	Seq.	y	y^{++}	y*	y^{*++}	y^0	y^{0++}	#
1	116.0342	58.5207			98.0237	49.5155	D							15
2	**253.0931**	127.0502			235.0826	118.0449	H	**1583.7085**	**792.3579**	1566.6819	783.8446	1565.6979	783.3526	14
3	**322.1146**	161.5609			**304.1040**	152.5556	S	**1446.6496**	**723.8284**	1429.6230	715.3151	1428.6390	714.8231	13
4	419.1674	210.0873			401.1568	201.0820	P	**1377.6281**	689.3177	**1360.6016**	680.8044	**1359.6175**	680.3124	12
5	**520.2150**	260.6112			**502.2045**	251.6059	T	**1280.5753**	640.7913	1263.5488	632.2780	1262.5648	631.7860	11

6	617.2678	309.1375			599.2572	300.1323	P	**1179.5277**	**590.2675**	**1162.5011**	**581.7542**	1161.5171	581.2622	10
7	704.2998	352.6535			686.2893	343.6483	S	1082.4749	541.7411	1065.4483	533.2278	1064.4643	532.7358	9
8	803.3682	**402.1878**			**785.3577**	393.1825	V	**995.4429**	498.2251	978.4163	489.7118	977.4323	489.2198	8
9	950.4366	475.7220			932.4261	466.7167	F	896.3745	448.6909	**879.3479**	440.1776	878.3639	439.6856	7
10	1064.4796	532.7434	**1047.4530**	524.2302	1046.4690	523.7381	N	**749.3060**	375.1567	732.2795	366.6434	731.2955	366.1514	6
11	1151.5116	576.2594	1134.4851	567.7462	1133.5010	567.2542	S	**635.2631**	318.1352	618.2366	309.6219	617.2525	309.1299	5
12	1266.5385	633.7729	**1249.5120**	625.2596	1248.5280	624.7676	D	**548.2311**	274.6192	531.2045	266.1059	530.2205	265.6139	4
13	1395.5811	698.2942	**1378.5546**	**689.7809**	**1377.5706**	689.2889	E	**433.2041**	217.1057	416.1776	208.5924	415.1936	208.1004	3
14	1524.6237	762.8155	1507.5972	754.3022	1506.6132	753.8102	E	**304.1615**	152.5844	287.1350	144.0711	286.1510	143.5791	2
15							R	175.1190	88.0631	158.0924	79.5498			1

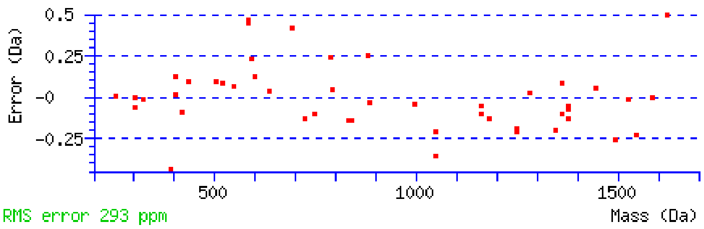

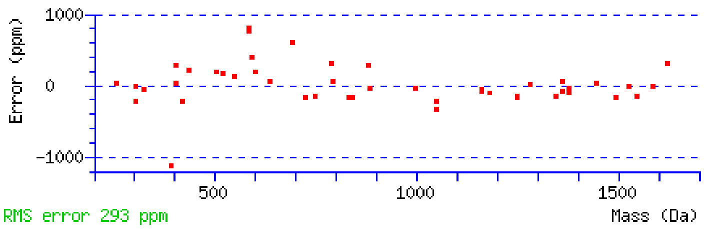

All matches to this query

Score	Mr(calc):	Delta	Sequence
45.1	1795.7050	0.0012	DHSPTPSVFNSDEER
30.8	1795.7050	0.0012	DHSPTPSVFNSDEER
26.1	1795.7050	0.0012	DHSPTPSVFNSDEER
7.5	1795.7050	0.0012	DHSPTPSVFNSDEER
5.2	1795.6906	0.0156	RMEFTSSCPNVSER
5.1	1795.6906	0.0156	RMEFTSSCPNVSER
5.1	1795.6906	0.0156	RMEFTSSCPNVSER
4.9	1795.7158	-0.0096	DSMYQSVCLISEER
4.4	1793.6845	2.0217	SYSSRNGSIPTYMR
4.2	1795.7188	-0.0126	LTLLCCTWREER

Mascot: http://www.matrixscience.com/

Mascot Search Results

Results Generated by a Script Modified from Mascot Pepitde View
by Newman Sze, School of Biological Sciences, Nanyang Technological University

---

Spectrum No: 401; Query: 2066; Rank: 1

Peptide View

MS/MS Fragmentation of **NKSEVDLYNSDPLICHAGVK**
Found in **IPI00197344**, Tax_Id=10116 Gene_Symbol=Mgll Monoglyceride lipase

Match to Query 2066: 2338.069992 from(780.363940,3+)
Title: 091008RatKidney_NH4Format01_14.2812.2812.3.dta
Data file K:\NewmanPaper\Piliang\3SubProteomes\Piliang3SP\mgf5ppm\ERLIC_3SubProteomes5ppm.mgf

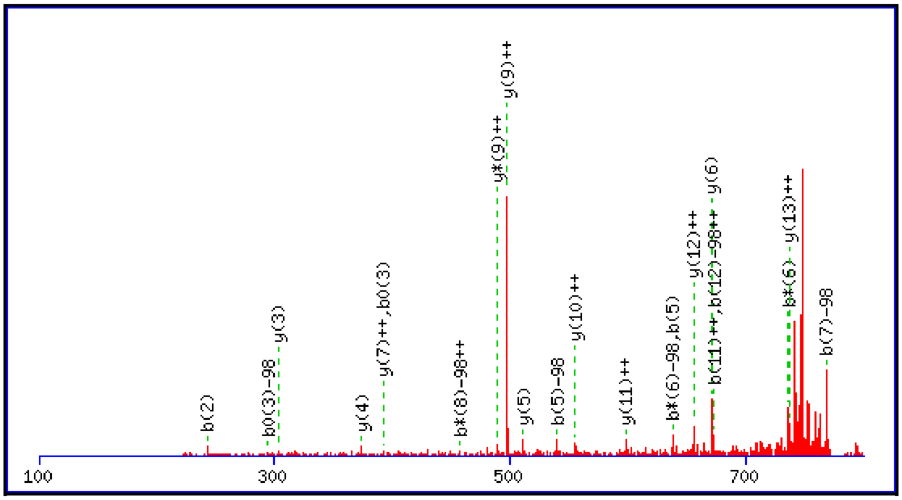

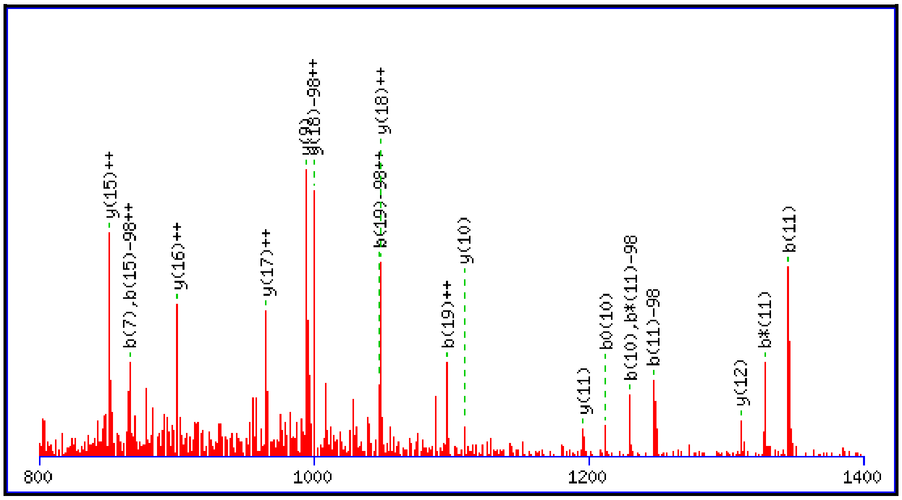

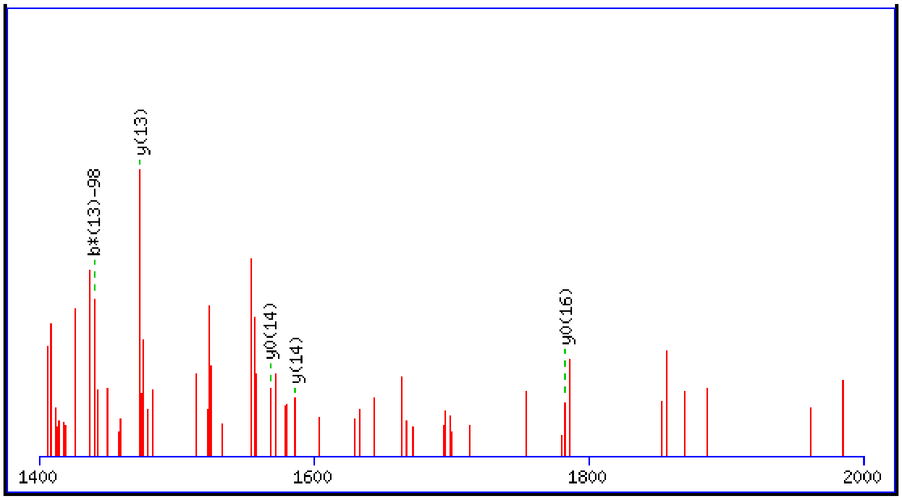

Monoisotopic mass of neutral peptide Mr(calc): 2338.0664
Fixed modifications: Carbamidomethyl (C)
Variable modifications:
S3 : Phospho (ST), with neutral losses 97.9769(shown in table), 0.0000
Ions Score: 45 **Expect:** 0.0093
Matches (Bold Red): 46/320 fragment ions using 91 most intense peaks

#	b	b^{++}	b*	b^{*++}	b^{0}	b^{0++}	Seq.	y	y^{++}	y*	y^{*++}	y^{0}	y^{0++}	#
1	115.0502	58.0287	98.0237	49.5155			N							20
2	**243.1452**	122.0762	226.1186	113.5629			K	2127.0539	1064.0306	2110.0274	1055.5173	2109.0433	1055.0253	19
3	312.1666	156.5869	295.1401	148.0737	**294.1561**	147.5817	S	1998.9589	**999.9831**	1981.9324	991.4698	1980.9484	990.9778	18
4	441.2092	221.1082	424.1827	212.5950	423.1987	212.1030	E	1929.9375	**965.4724**	1912.9109	956.9591	1911.9269	956.4671	17
5	**540.2776**	270.6425	523.2511	262.1292	522.2671	261.6372	V	1800.8949	**900.9511**	1783.8683	892.4378	**1782.8843**	891.9458	16
6	655.3046	328.1559	**638.2780**	319.6426	637.2940	319.1506	D	1701.8265	**851.4169**	1684.7999	842.9036	1683.8159	842.4116	15
7	**768.3886**	384.6980	751.3621	376.1847	750.3781	375.6927	L	**1586.7995**	793.9034	1569.7730	785.3901	**1568.7890**	784.8981	14
8	931.4520	466.2296	914.4254	**457.7163**	913.4414	457.2243	Y	**1473.7155**	**737.3614**	1456.6889	728.8481	1455.7049	728.3561	13
9	1045.4949	523.2511	1028.4683	514.7378	1027.4843	514.2458	N	**1310.6521**	**655.8297**	1293.6256	647.3164	1292.6416	646.8244	12
10	1132.5269	566.7671	1115.5004	558.2538	1114.5164	557.7618	S	**1196.6092**	**598.8082**	1179.5827	590.2950	1178.5987	589.8030	11
11	**1247.5539**	624.2806	**1230.5273**	615.7673	1229.5433	615.2753	D	**1109.5772**	**555.2922**	1092.5506	546.7790	1091.5666	546.2870	10
12	1344.6066	**672.8070**	1327.5801	664.2937	1326.5961	663.8017	P	**994.5502**	**497.7788**	977.5237	**489.2655**			9
13	1457.6907	729.3490	**1440.6641**	720.8357	1439.6801	720.3437	L	897.4975	449.2524	880.4709	440.7391			8
14	1570.7748	785.8910	1553.7482	777.3777	1552.7642	776.8857	I	784.4134	**392.7103**	767.3869	384.1971			7
15	1730.8054	**865.9063**	1713.7789	857.3931	1712.7948	856.9011	C	**671.3294**	336.1683	654.3028	327.6550			6
16	1867.8643	934.4358	1850.8378	925.9225	1849.8538	925.4305	H	**511.2987**	256.1530	494.2722	247.6397			5
17	1938.9014	969.9544	1921.8749	961.4411	1920.8909	960.9491	A	**374.2398**	187.6235	357.2132	179.1103			4
18	1995.9229	998.4651	1978.8963	989.9518	1977.9123	989.4598	G	**303.2027**	152.1050	286.1761	143.5917			3
19	2094.9913	**1047.9993**	2077.9648	1039.4860	2076.9807	1038.9940	V	246.1812	123.5942	229.1547	115.0810			2
20							K	147.1128	74.0600	130.0863	65.5468			1

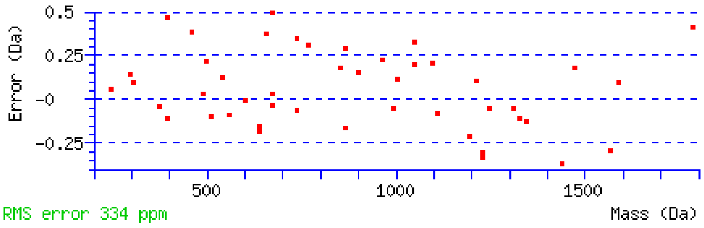

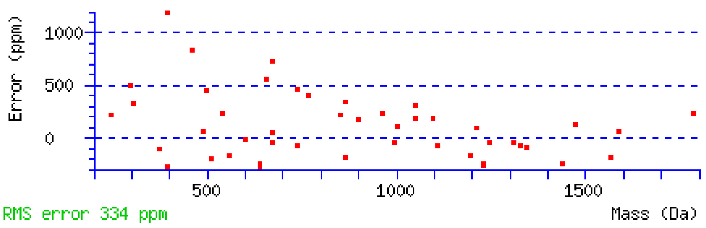

All matches to this query

Score	Mr(calc):	Delta	Sequence
45.1	2338.0664	0.0036	NKSEVDLYNSDPLICHAGVK

18.7	2338.0664	0.0036	NKSEVDLYNSDPLICHAGVK
16.8	2338.0664	0.0036	NKSEVDLYNSDPLICHAGVK

Spectrum No: 402; Query: 2434; Rank: 1

Peptide View

MS/MS Fragmentation of **GGPEPPASGEMDDNSLSPEACYECK**
Found in **IPI00204006**, Tax_Id=10116 Gene_Symbol=Fbn1 Fibrillin-1

Match to Query 2434: 2792.022612 from(931.681480,3+)
Title: 091008RatKidney_NoSalt_27.1874.1874.3.dta
Data file K:\NewmanPaper\Piliang\3SubProteomes\Piliang3SP\mgf5ppm\ERLIC_3SubProteomes5ppm.mgf

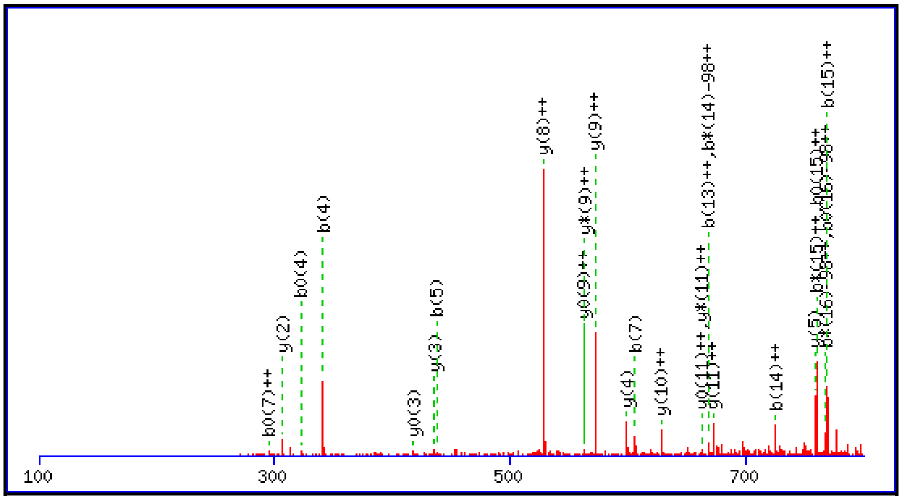

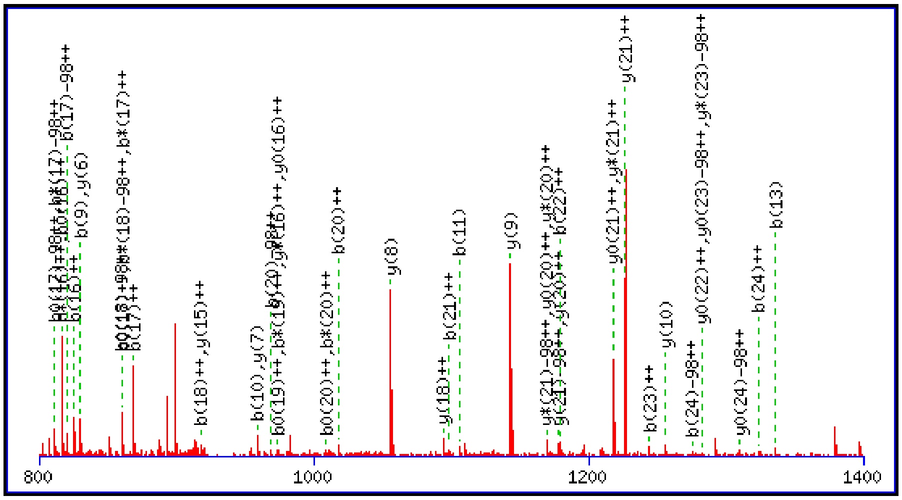

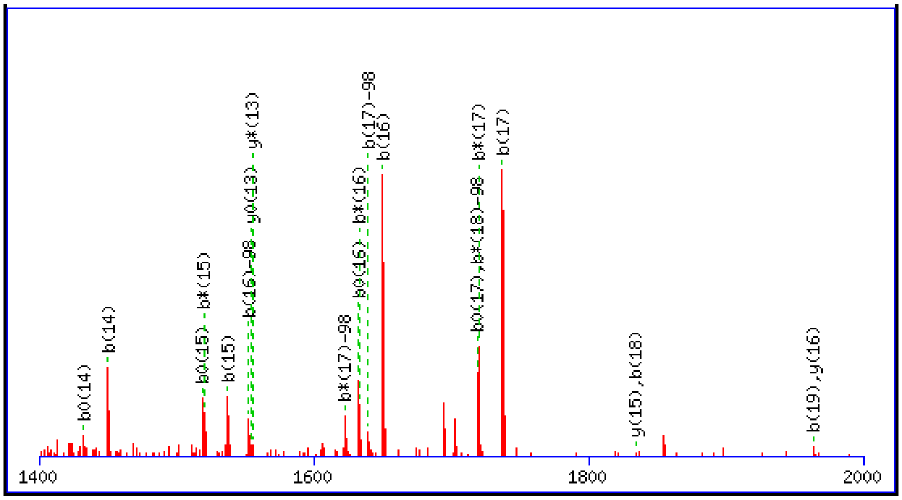

Monoisotopic mass of neutral peptide Mr(calc): 2792.0289
Fixed modifications: Carbamidomethyl (C)
Variable modifications:
S8 : Phospho (ST), with neutral losses 0.0000(shown in table), 97.9769
M11 : Oxidation (M)
Ions Score: 45 **Expect:** 0.0048
Matches (Bold Red): 95/384 fragment ions using 174 most intense peaks

#	b	b^{++}	b*	b^{*++}	b^0	b^{0++}	Seq.	y	y^{++}	y*	y^{*++}	y^0	y^{0++}	#
1	58.0287	29.5180					G							25
2	115.0502	58.0287					G	2736.0147	1368.5110	2718.9882	1359.9977	2718.0041	1359.5057	24
3	212.1030	106.5551					P	2678.9932	1340.0003	2661.9667	1331.4870	2660.9827	1330.9950	23
4	**341.1456**	171.0764			**323.1350**	162.0711	E	2581.9405	1291.4739	2564.9139	1282.9606	2563.9299	**1282.4686**	22
5	**438.1983**	219.6028			420.1878	210.5975	P	2452.8979	**1226.9526**	2435.8713	**1218.4393**	2434.8873	**1217.9473**	21
6	535.2511	268.1292			517.2405	259.1239	P	2355.8451	**1178.4262**	2338.8186	**1169.9129**	2337.8346	**1169.4209**	20
7	**606.2882**	303.6477			588.2776	**294.6425**	A	2258.7924	1129.8998	2241.7658	1121.3865	2240.7818	1120.8945	19
8	773.2866	387.1469			755.2760	378.1416	S	2187.7552	**1094.3813**	2170.7287	1085.8680	2169.7447	1085.3760	18
9	**830.3080**	415.6576			812.2975	406.6524	G	2020.7569	1010.8821	2003.7303	1002.3688	2002.7463	1001.8768	17
10	**959.3506**	480.1789			941.3400	471.1737	E	**1963.7354**	982.3714	1946.7089	**973.8581**	1945.7249	**973.3661**	16
11	**1106.3860**	553.6966			1088.3755	544.6914	M	**1834.6928**	**917.8501**	1817.6663	909.3368	1816.6823	908.8448	15
12	1221.4130	611.2101			1203.4024	602.2048	D	1687.6574	844.3324	1670.6309	835.8191	1669.6469	835.3271	14
13	**1336.4399**	**668.7236**			1318.4293	659.7183	D	1572.6305	786.8189	**1555.6039**	778.3056	**1554.6199**	777.8136	13
14	**1450.4828**	**725.7451**	1433.4563	717.2318	**1432.4723**	716.7398	N	1457.6035	729.3054	1440.5770	720.7921	1439.5930	720.3001	12
15	**1537.5149**	**769.2611**	**1520.4883**	**760.7478**	**1519.5043**	**760.2558**	S	1343.5606	**672.2839**	1326.5341	**663.7707**	1325.5501	**663.2787**	11
16	**1650.5989**	**825.8031**	**1633.5724**	**817.2898**	**1632.5884**	**816.7978**	L	**1256.5286**	**628.7679**	1239.5020	620.2547	1238.5180	619.7626	10
17	**1737.6309**	**869.3191**	**1720.6044**	**860.8058**	**1719.6204**	**860.3138**	S	**1143.4445**	**572.2259**	1126.4180	**563.7126**	1125.4340	**563.2206**	9
18	**1834.6837**	**917.8455**	1817.6572	909.3322	1816.6731	908.8402	P	**1056.4125**	**528.7099**	1039.3859	520.1966	1038.4019	519.7046	8
19	**1963.7263**	982.3668	1946.6998	**973.8535**	1945.7157	**973.3615**	E	**959.3597**	480.1835	942.3332	471.6702	941.3492	471.1782	7
20	2034.7634	**1017.8853**	2017.7369	**1009.3721**	2016.7529	**1008.8801**	A	**830.3171**	415.6622	813.2906	407.1489	812.3066	406.6569	6
21	2194.7941	**1097.9007**	2177.7675	1089.3874	2176.7835	1088.8954	C	**759.2800**	380.1437	742.2535	371.6304	741.2695	371.1384	5
22	2357.8574	**1179.4323**	2340.8308	1170.9191	2339.8468	1170.4271	Y	**599.2494**	300.1283	582.2228	291.6151	581.2388	291.1230	4
23	2486.9000	**1243.9536**	2469.8734	1235.4404	2468.8894	1234.9484	E	**436.1860**	218.5967	419.1595	210.0834	**418.1755**	209.5914	3
24	2646.9306	**1323.9690**	2629.9041	1315.4557	2628.9201	1314.9637	C	**307.1435**	154.0754	290.1169	145.5621			2
25							K	147.1128	74.0600	130.0863	65.5468			1

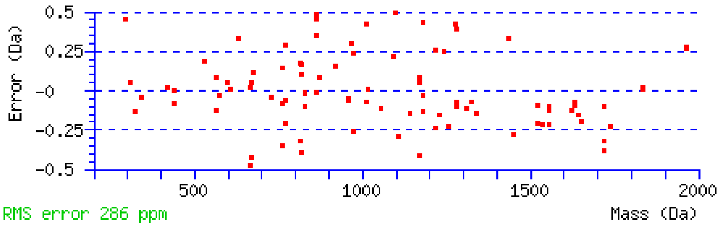

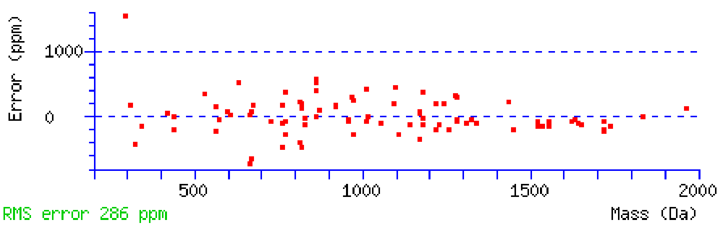

All matches to this query

Score	Mr(calc):	Delta	Sequence
45.1	2792.0289	-0.0063	GGPEPPASGEMDDNSLSPEACYECK
29.9	2792.0289	-0.0063	GGPEPPASGEMDDNSLSPEACYECK
23.9	2792.0289	-0.0063	GGPEPPASGEMDDNSLSPEACYECK
8.2	2790.0367	1.9859	LGSSGSQVSNPGSRPYARXTKAR
8.2	2790.0367	1.9859	LGSSGSQVSNPGSRPYARXTKAR
5.6	2790.0367	1.9859	LGSSGSQVSNPGSRPYARXTKAR
4.8	2792.0317	-0.0091	TKEYTACELMNIYKTDNHLK
3.7	2791.0270	0.9956	NPDLLPNMSLTMDIPSCNWTSK
3.6	2790.0430	1.9796	NPDLLPNMSLTMDIPSCNWTSK
2.0	2792.0034	0.0192	RLSEGSSHSDSSESSDSLAPMGASR

Spectrum No: 403; Query: 1788; Rank: 1

Peptide View

MS/MS Fragmentation of **LGSTGSQPSSEAEPGPEQASK**
Found in **IPI00764230**, Tax_Id=10116 Gene_Symbol=LOC682920 similar to phosphatase and actin regulator 4

Match to Query 1788: 2122.911808 from(1062.463180,2+)
Title: 100101RatKid_NS_deglyco_20.977.977.2.dta
Data file K:\NewmanPaper\Piliang\3SubProteomes\Piliang3SP\mgf5ppm\ERLIC_3SubProteomes5ppm.mgf

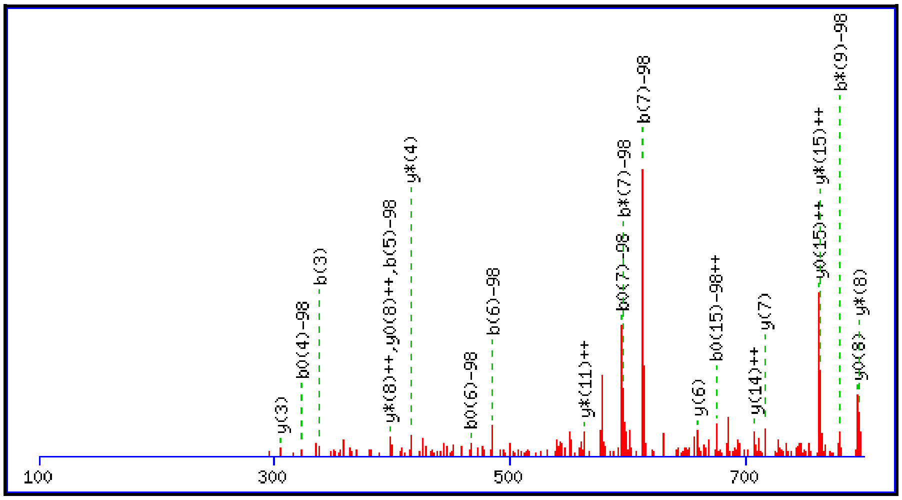

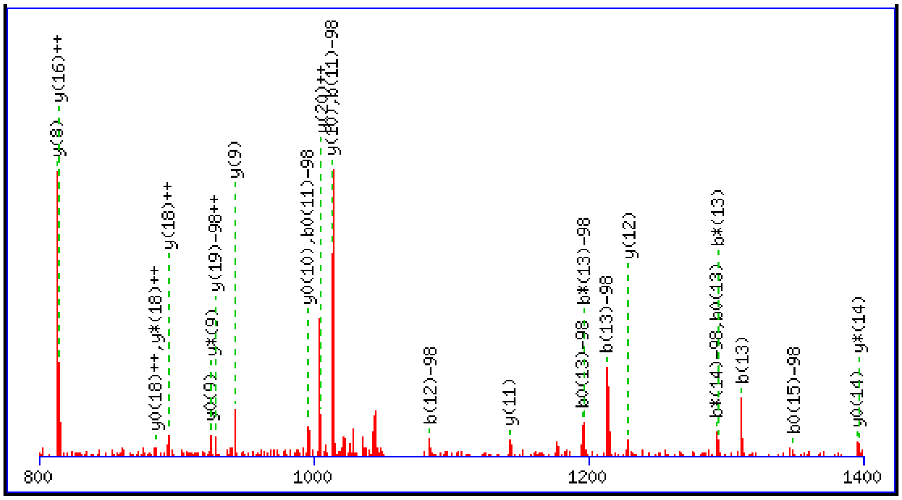

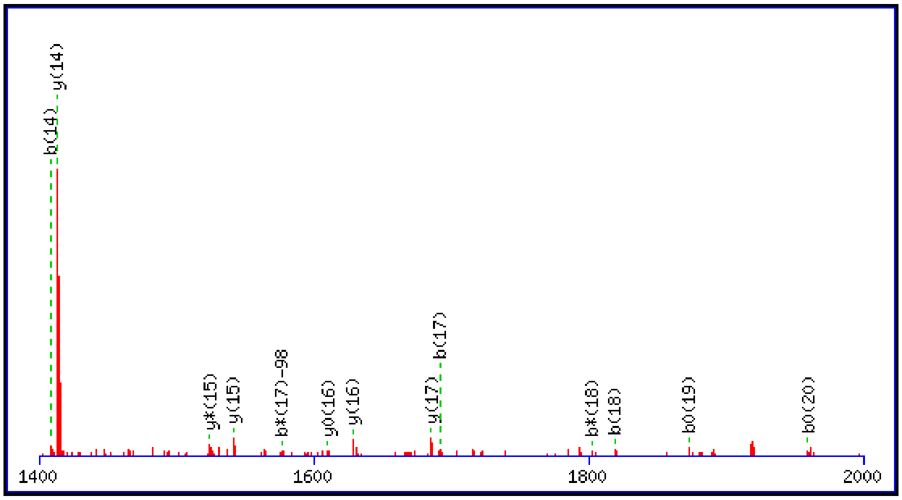

Monoisotopic mass of neutral peptide Mr(calc): 2122.9056
Fixed modifications: Carbamidomethyl (C)
Variable modifications:
S3 : Phospho (ST), with neutral losses 97.9769(shown in table), 0.0000
Ions Score: 45 **Expect:** 0.0078
Matches (Bold Red): 62/334 fragment ions using 125 most intense peaks

#	b	b^{++}	b*	b^{*++}	b^{0}	b^{0++}	Seq.	y	y^{++}	y*	y^{*++}	y^{0}	y^{0++}	#
1	114.0913	57.5493					L							21
2	171.1128	86.0600					G	1912.8519	956.9296	1895.8253	948.4163	1894.8413	947.9243	20
3	240.1343	120.5708			222.1237	111.5655	S	1855.8304	**928.4189**	1838.8039	919.9056	1837.8199	919.4136	19
4	341.1819	171.0946			**323.1714**	162.0893	T	1786.8090	**893.9081**	1769.7824	**885.3949**	1768.7984	**884.9028**	18
5	**398.2034**	199.6053			380.1928	190.6001	G	**1685.7613**	843.3843	1668.7348	834.8710	1667.7507	834.3790	17
6	**485.2354**	243.1214			**467.2249**	234.1161	S	**1628.7398**	**814.8736**	1611.7133	806.3603	**1610.7293**	805.8683	16
7	**613.2940**	307.1506	**596.2675**	298.6374	**595.2834**	298.1454	Q	**1541.7078**	771.3575	**1524.6813**	**762.8443**	1523.6972	**762.3523**	15
8	710.3468	355.6770	693.3202	347.1638	692.3362	346.6717	P	**1413.6492**	**707.3283**	**1396.6227**	698.8150	**1395.6387**	698.3230	14
9	797.3788	399.1930	**780.3523**	390.6798	779.3682	390.1878	S	1316.5965	658.8019	1299.5699	650.2886	1298.5859	649.7966	13
10	884.4108	442.7091	867.3843	434.1958	866.4003	433.7038	S	**1229.5644**	615.2859	1212.5379	606.7726	1211.5539	606.2806	12
11	**1013.4534**	507.2303	996.4269	498.7171	**995.4429**	498.2251	E	**1142.5324**	571.7698	1125.5059	**563.2566**	1124.5218	562.7646	11
12	**1084.4905**	542.7489	1067.4640	534.2356	1066.4800	533.7436	A	**1013.4898**	507.2485	996.4633	498.7353	**995.4793**	498.2433	10
13	**1213.5331**	607.2702	**1196.5066**	598.7569	**1195.5226**	598.2649	E	**942.4527**	471.7300	**925.4262**	463.2167	**924.4421**	462.7247	9
14	1310.5859	655.7966	**1293.5593**	647.2833	1292.5753	646.7913	P	**813.4101**	407.2087	**796.3836**	**398.6954**	**795.3995**	**398.2034**	8
15	1367.6074	684.3073	1350.5808	675.7940	**1349.5968**	**675.3020**	G	**716.3573**	358.6823	699.3308	350.1690	698.3468	349.6770	7
16	1464.6601	732.8337	1447.6336	724.3204	1446.6496	723.8284	P	**659.3359**	330.1716	642.3093	321.6583	641.3253	321.1663	6
17	1593.7027	797.3550	**1576.6762**	788.8417	1575.6921	788.3497	E	562.2831	281.6452	545.2566	273.1319	544.2726	272.6399	5
18	1721.7613	861.3843	1704.7347	852.8710	1703.7507	852.3790	Q	433.2405	217.1239	**416.2140**	208.6106	415.2300	208.1186	4

19	1792.7984	896.9028	1775.7719	888.3896	1774.7878	887.8976	A	305.1819	153.0946	288.1554	144.5813	287.1714	144.0893	3
20	1879.8304	940.4189	1862.8039	931.9056	1861.8199	931.4136	S	234.1448	117.5761	217.1183	109.0628	216.1343	108.5708	2
21							K	147.1128	74.0600	130.0863	65.5468			1

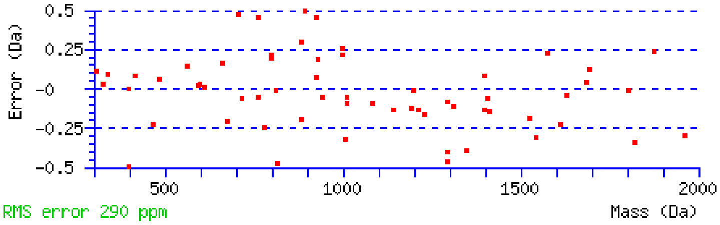

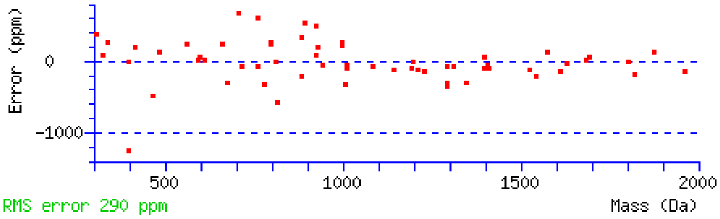

All matches to this query

Score	Mr(calc):	Delta	Sequence
45.0	2122.9056	0.0062	LGSTGSQPSSEAEPGPEQASK
42.9	2122.9056	0.0062	LGSTGSQPSSEAEPGPEQASK
31.9	2122.9056	0.0062	LGSTGSQPSSEAEPGPEQASK
21.3	2122.9056	0.0062	LGSTGSQPSSEAEPGPEQASK
21.0	2122.9056	0.0062	LGSTGSQPSSEAEPGPEQASK
7.7	2122.9056	0.0062	LGSTGSQPSSEAEPGPEQASK
4.4	2120.9140	1.9978	ALLSEDSDSNVESVRGKR
3.1	2122.9087	0.0031	GIYLSPISSISFGYSGMGK
2.3	2122.8952	0.0167	AMNVLTEAEERYNDLMK
1.6	2120.9140	1.9978	ALLSEDSDSNVESVRGKR

Spectrum No: 404; Query: 1130; Rank: 1

Peptide View

MS/MS Fragmentation of **AEEEGGSDGSAAEAEPR**
Found in **IPI00363771**, Tax_Id=10116 Gene_Symbol=Eif3s9 108 kDa protein

Match to Query 1130: 1740.649228 from(871.331890,2+)
Title: 091008RatKidney_NoSalt_28.391.391.2.dta
Data file K:\NewmanPaper\Piliang\3SubProteomes\Piliang3SP\mgf5ppm\ERLIC_3SubProteomes5ppm.mgf

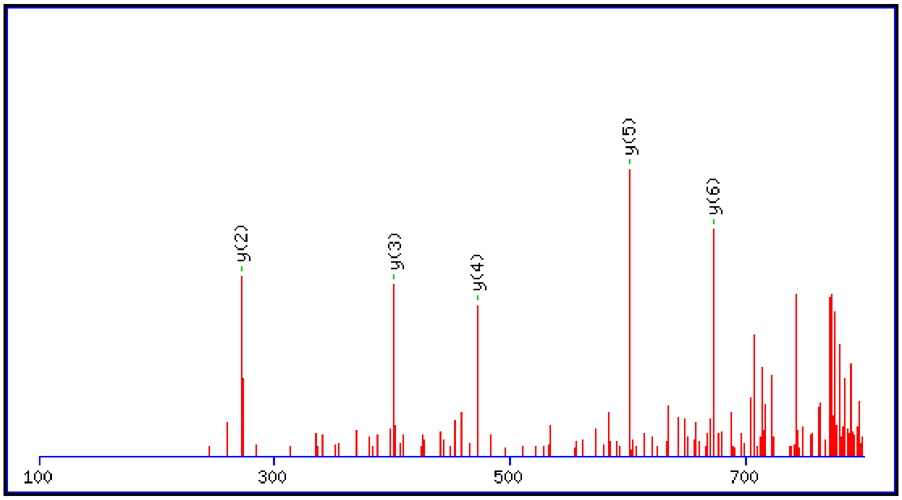

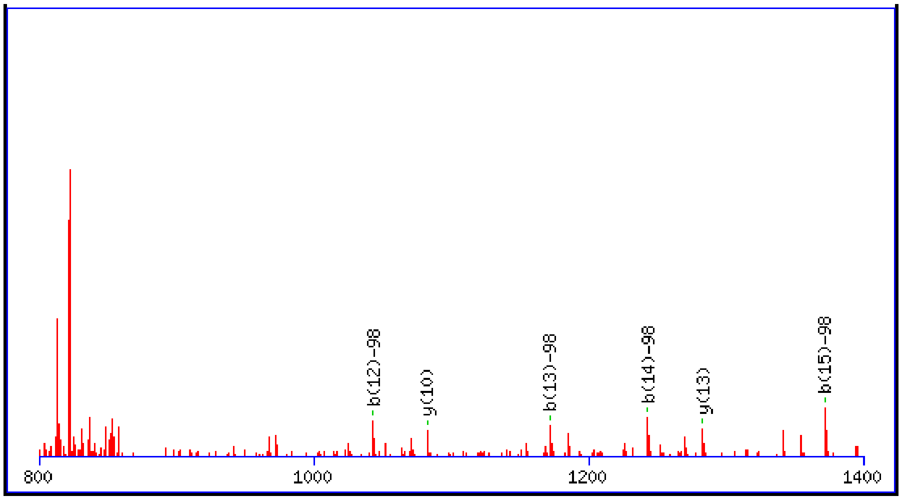

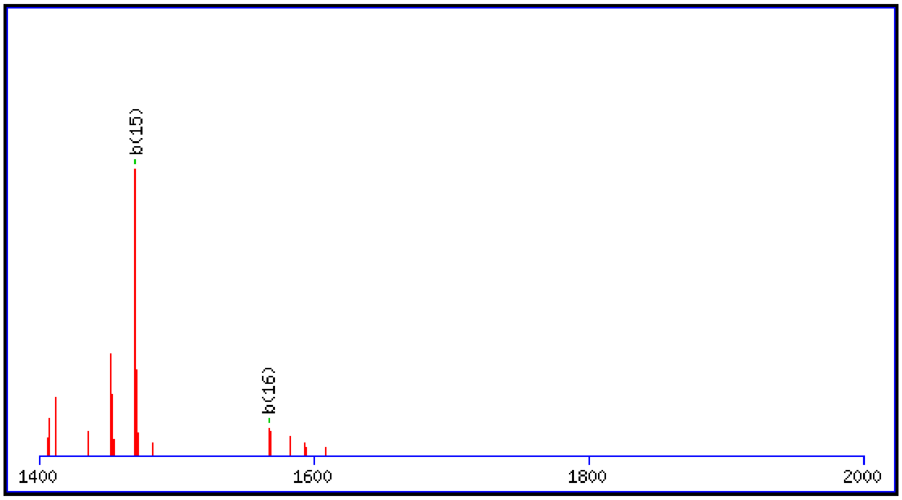

Monoisotopic mass of neutral peptide Mr(calc): 1740.6475
Fixed modifications: Carbamidomethyl (C)
Variable modifications:
S10 : Phospho (ST), with neutral losses 0.0000(shown in table), 97.9769
Ions Score: 45 **Expect:** 0.0034
Matches (Bold Red): 13/236 fragment ions using 19 most intense peaks

#	b	b^{++}	b^{0}	b^{0++}	Seq.	y	y^{++}	y*	y^{*++}	y^{0}	y^{0++}	#
1	72.0444	36.5258			A							17
2	201.0870	101.0471	183.0764	92.0418	E	1670.6177	835.8125	1653.5912	827.2992	1652.6072	826.8072	16
3	330.1296	165.5684	312.1190	156.5631	E	1541.5751	771.2912	1524.5486	762.7779	1523.5646	762.2859	15
4	459.1722	230.0897	441.1616	221.0844	E	1412.5326	706.7699	1395.5060	698.2566	1394.5220	697.7646	14
5	516.1936	258.6005	498.1831	249.5952	G	**1283.4900**	642.2486	1266.4634	633.7353	1265.4794	633.2433	13
6	573.2151	287.1112	555.2045	278.1059	G	1226.4685	613.7379	1209.4419	605.2246	1208.4579	604.7326	12
7	660.2471	330.6272	642.2366	321.6219	S	1169.4470	585.2272	1152.4205	576.7139	1151.4365	576.2219	11
8	775.2741	388.1407	757.2635	379.1354	D	**1082.4150**	541.7111	1065.3885	533.1979	1064.4044	532.7059	10
9	832.2955	416.6514	814.2850	407.6461	G	967.3881	484.1977	950.3615	475.6844	949.3775	475.1924	9
10	999.2939	500.1506	981.2833	491.1453	S	910.3666	455.6869	893.3400	447.1737	892.3560	446.6817	8
11	1070.3310	535.6691	1052.3204	526.6639	A	743.3682	372.1878	726.3417	363.6745	725.3577	363.1825	7
12	1141.3681	571.1877	1123.3575	562.1824	A	**672.3311**	336.6692	655.3046	328.1559	654.3206	327.6639	6
13	1270.4107	635.7090	1252.4001	626.7037	E	**601.2940**	301.1506	584.2675	292.6374	583.2835	292.1454	5
14	1341.4478	671.2275	1323.4373	662.2223	A	**472.2514**	236.6293	455.2249	228.1161	454.2409	227.6241	4
15	**1470.4904**	735.7488	1452.4798	726.7436	E	**401.2143**	201.1108	384.1878	192.5975	383.2037	192.1055	3
16	**1567.5432**	784.2752	1549.5326	775.2699	P	**272.1717**	136.5895	255.1452	128.0762			2
17					R	175.1190	88.0631	158.0924	79.5498			1

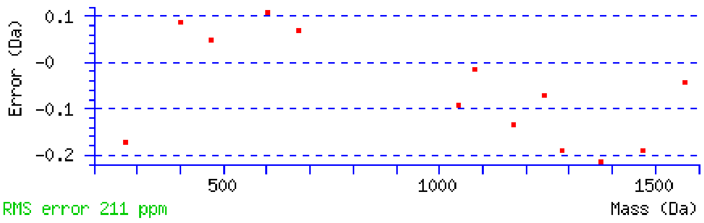

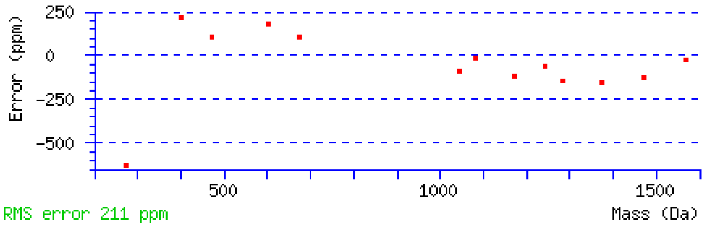

All matches to this query

Score	Mr(calc):	Delta	Sequence
45.0	1740.6475	0.0017	AEEEGGSDGSAAEAEPR
38.3	1740.6475	0.0017	AEEEGGSDGSAAEAEPR
3.3	1740.6410	0.0083	LGTEATSNGLVHSSK
3.3	1740.6410	0.0083	LGTEATSNGLVHSSK
2.9	1739.6610	0.9883	SSYEVLPVQEPPR
2.7	1740.6410	0.0083	LGTEATSNGLVHSSK
2.7	1740.6410	0.0083	LGTEATSNGLVHSSK
2.2	1739.6570	0.9923	LGTEATSNGLVHSSK
0.6	1740.6562	-0.0070	SDSWSTVAPLSVPR
0.6	1740.6562	-0.0070	SDSWSTVAPLSVPR

Spectrum No: 405; Query: 1107; Rank: 1

Peptide View

MS/MS Fragmentation of **ETAVQEGSSSPVADIR**
Found in **IPI00454563**, Tax_Id=10116 Gene_Symbol=Mdc1 similar to Mediator of DNA damage checkpoint protein 1

Match to Query 1107: 1724.769328 from(863.391940,2+)
Title: 100101RatKid_NS_deglyco_21.1681.1681.2.dta
Data file K:\NewmanPaper\Piliang\3SubProteomes\Piliang3SP\mgf5ppm\ERLIC_3SubProteomes5ppm.mgf

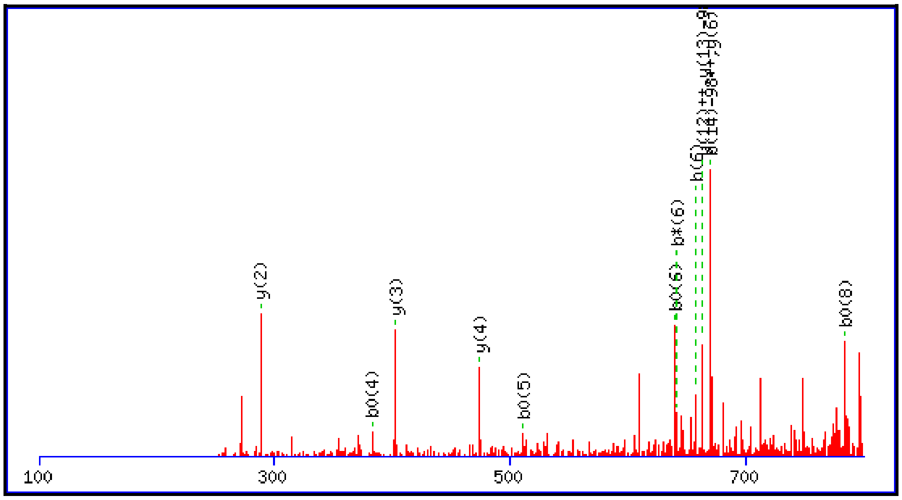

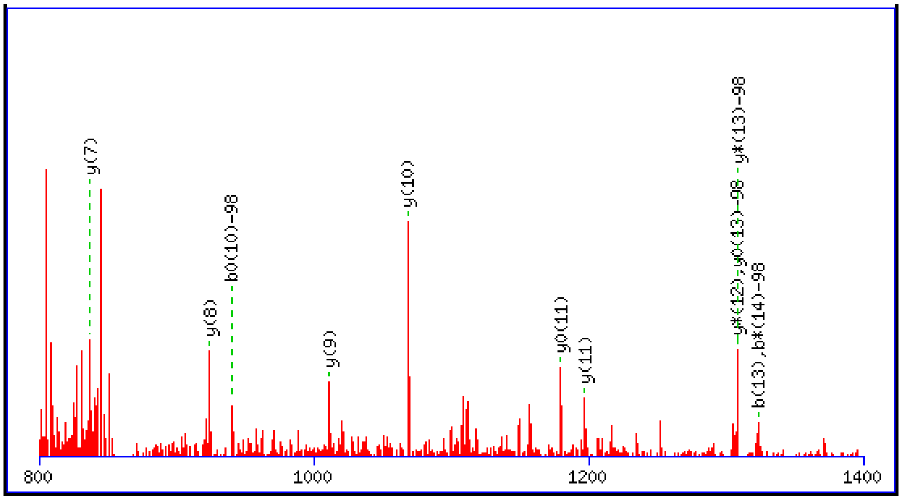

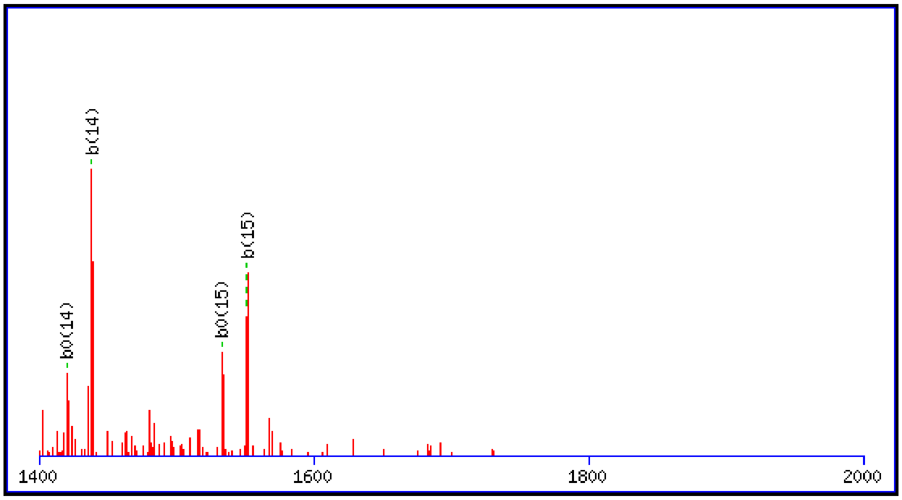

Monoisotopic mass of neutral peptide Mr(calc): 1724.7618
Fixed modifications: Carbamidomethyl (C)
Variable modifications:
S10 : Phospho (ST), with neutral losses 0.0000(shown in table), 97.9769
Ions Score: 45 **Expect:** 0.0066
Matches (Bold Red): 29/258 fragment ions using 52 most intense peaks

#	b	b^{++}	b^{*}	b^{*++}	b^{0}	b^{0++}	Seq.	y	y^{++}	y^{*}	y^{*++}	y^{0}	y^{0++}	#
1	130.0499	65.5286			112.0393	56.5233	E							16
2	231.0975	116.0524			213.0870	107.0471	T	1596.7265	798.8669	1579.7000	790.3536	1578.7159	789.8616	15
3	302.1347	151.5710			284.1241	142.5657	A	1495.6788	748.3430	1478.6523	739.8298	1477.6683	739.3378	14
4	401.2031	201.1052			**383.1925**	192.0999	V	1424.6417	712.8245	1407.6152	704.3112	1406.6311	703.8192	13
5	529.2617	265.1345	512.2351	256.6212	**511.2511**	256.1292	Q	1325.5733	**663.2903**	**1308.5467**	654.7770	1307.5627	654.2850	12
6	**658.3042**	329.6558	**641.2777**	321.1425	**640.2937**	320.6505	E	**1197.5147**	599.2610	1180.4882	590.7477	**1179.5042**	590.2557	11
7	715.3257	358.1665	698.2992	349.6532	697.3151	349.1612	G	**1068.4721**	534.7397	1051.4456	526.2264	1050.4616	525.7344	10
8	802.3577	401.6825	785.3312	393.1692	**784.3472**	392.6772	S	**1011.4507**	506.2290	994.4241	497.7157	993.4401	497.2237	9
9	889.3898	445.1985	872.3632	436.6852	871.3792	436.1932	S	**924.4186**	462.7130	907.3921	454.1997	906.4081	453.7077	8
10	1056.3881	528.6977	1039.3616	520.1844	1038.3776	519.6924	S	**837.3866**	419.1969	820.3601	410.6837	819.3760	410.1917	7
11	1153.4409	577.2241	1136.4143	568.7108	1135.4303	568.2188	P	**670.3883**	335.6978	653.3617	327.1845	652.3777	326.6925	6
12	1252.5093	626.7583	1235.4827	618.2450	1234.4987	617.7530	V	573.3355	287.1714	556.3089	278.6581	555.3249	278.1661	5
13	**1323.5464**	662.2768	1306.5199	653.7636	1305.5358	653.2716	A	**474.2671**	237.6372	457.2405	229.1239	456.2565	228.6319	4
14	**1438.5734**	719.7903	1421.5468	711.2770	**1420.5628**	710.7850	D	**403.2300**	202.1186	386.2034	193.6053	385.2194	193.1133	3
15	**1551.6574**	776.3323	1534.6309	767.8191	**1533.6469**	767.3271	I	**288.2030**	144.6051	271.1765	136.0919			2
16							R	175.1190	88.0631	158.0924	79.5498			1

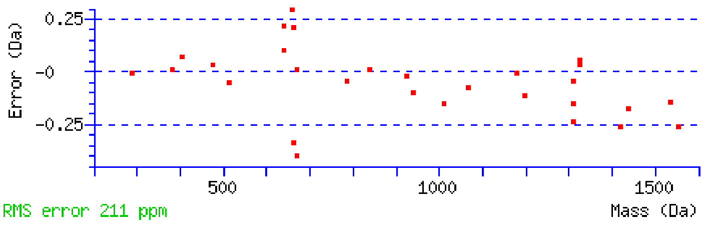

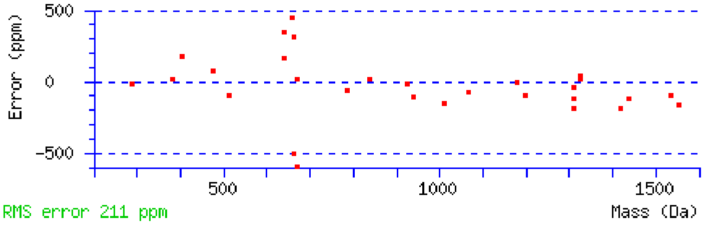

All matches to this query

Score	Mr(calc):	Delta	Sequence
44.9	1724.7618	0.0075	ETAVQEGSSSPVADIR
44.8	1724.7618	0.0075	ETAVQEGSSSPVADIR
33.8	1724.7618	0.0075	ETAVQEGSSSPVADIR
13.3	1724.7618	0.0075	ETAVQEGSSSPVADIR
1.5	1722.7701	1.9992	RMDPGYPAQGPLWR

Spectrum No: 406; Query: 1463; Rank: 1

Peptide View

MS/MS Fragmentation of **LRSEDGVEGDLGETQSR**
Found in **IPI00769072**, Tax_Id=10116 Gene_Symbol=Ahnak similar to AHNAK nucleoprotein isoform 1 isoform 2

Match to Query 1463: 1926.834192 from(643.285340,3+)
Title: 091008RatKidney_NH4Format01_21.1337.1337.3.dta
Data file K:\NewmanPaper\Piliang\3SubProteomes\Piliang3SP\mgf5ppm\ERLIC_3SubProteomes5ppm.mgf

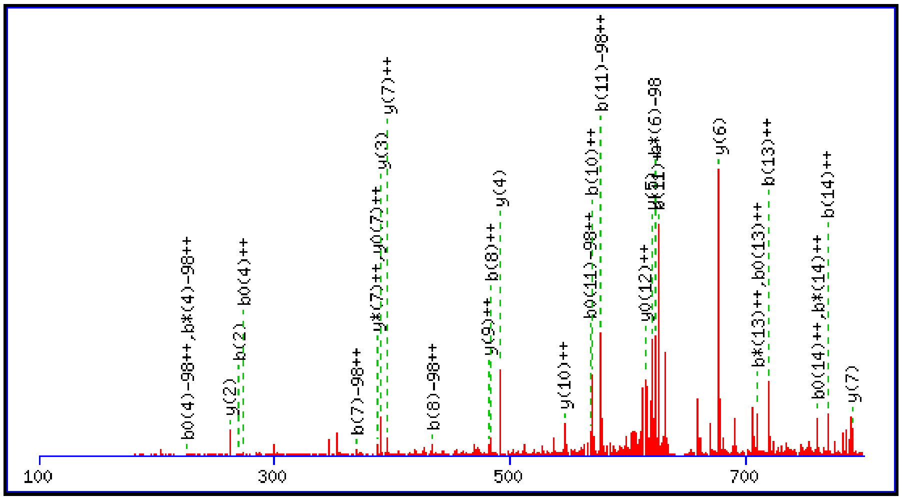

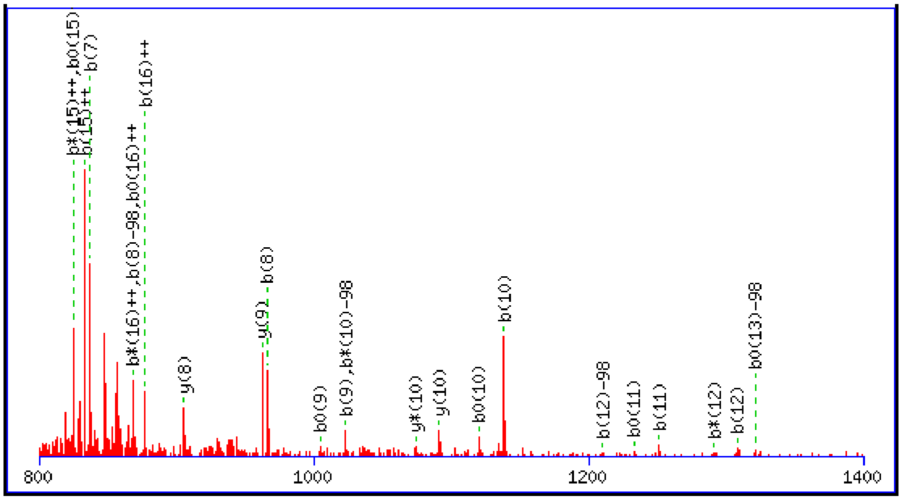

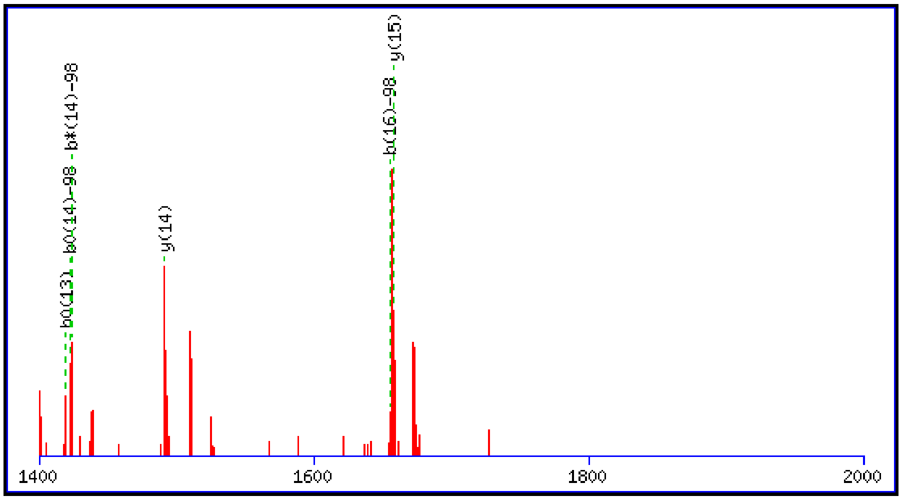

Monoisotopic mass of neutral peptide Mr(calc): 1926.8320
Fixed modifications: Carbamidomethyl (C)
Variable modifications:
S3 : Phospho (ST), with neutral losses 0.0000(shown in table), 97.9769
Ions Score: 45 **Expect:** 0.0071
Matches (Bold Red): 60/280 fragment ions using 105 most intense peaks

#	b	b++	b*	b*++	b0	b0++	Seq.	y	y++	y*	y*++	y0	y0++	#
1	114.0913	57.5493					L							17
2	**270.1925**	135.5999	253.1659	127.0866			R	1814.7552	907.8813	1797.7287	899.3680	1796.7447	898.8760	16
3	437.1908	219.0990	420.1643	210.5858	419.1802	210.0938	S	**1658.6541**	829.8307	1641.6276	821.3174	1640.6436	820.8254	15
4	566.2334	283.6203	549.2068	275.1071	548.2228	**274.6151**	E	**1491.6558**	746.3315	1474.6292	737.8182	1473.6452	737.3262	14
5	681.2603	341.1338	664.2338	332.6205	663.2498	332.1285	D	1362.6132	681.8102	1345.5866	673.2970	1344.6026	672.8049	13
6	738.2818	369.6445	721.2553	361.1313	720.2712	360.6393	G	1247.5862	624.2968	1230.5597	615.7835	1229.5757	**615.2915**	12
7	**837.3502**	419.1787	820.3237	410.6655	819.3397	410.1735	V	1190.5648	595.7860	1173.5382	587.2727	1172.5542	586.7807	11
8	**966.3928**	**483.7000**	949.3663	475.1868	948.3822	474.6948	E	**1091.4964**	**546.2518**	**1074.4698**	537.7385	1073.4858	537.2465	10
9	**1023.4143**	512.2108	1006.3877	503.6975	**1005.4037**	503.2055	G	**962.4538**	**481.7305**	945.4272	473.2172	944.4432	472.7252	9
10	**1138.4412**	**569.7242**	1121.4147	561.2110	**1120.4307**	560.7190	D	**905.4323**	453.2198	888.4058	444.7065	887.4217	444.2145	8
11	**1251.5253**	**626.2663**	1234.4987	617.7530	**1233.5147**	617.2610	L	**790.4054**	**395.7063**	773.3788	**387.1930**	772.3948	**386.7010**	7
12	**1308.5467**	654.7770	**1291.5202**	646.2637	1290.5362	645.7717	G	**677.3213**	339.1643	660.2947	330.6510	659.3107	330.1590	6
13	1437.5893	**719.2983**	1420.5628	**710.7850**	**1419.5788**	**710.2930**	E	**620.2998**	310.6536	603.2733	302.1403	602.2893	301.6483	5
14	1538.6370	**769.8221**	1521.6105	**761.3089**	1520.6265	**760.8169**	T	**491.2572**	246.1323	474.2307	237.6190	473.2467	237.1270	4
15	1666.6956	**833.8514**	1649.6690	**825.3382**	1648.6850	**824.8462**	Q	**390.2096**	195.6084	373.1830	187.0951	372.1990	186.6031	3
16	1753.7276	**877.3674**	1736.7011	**868.8542**	1735.7171	**868.3622**	S	**262.1510**	131.5791	245.1244	123.0659	244.1404	122.5738	2
17							R	175.1190	88.0631	158.0924	79.5498			1

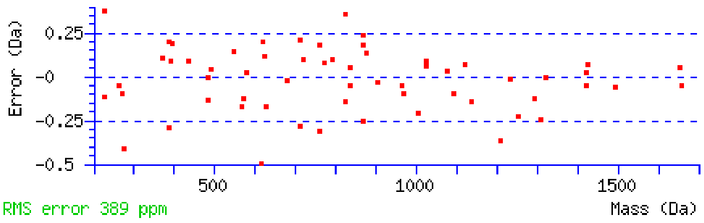

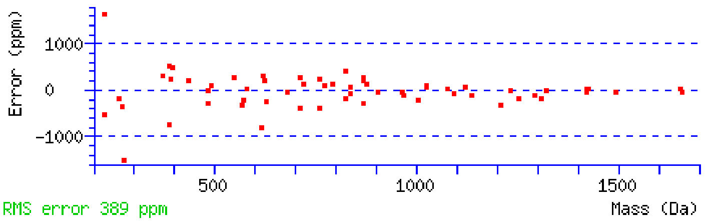

All matches to this query

Score	Mr(calc):	Delta	Sequence
44.9	1926.8320	0.0022	LRSEDGVEGDLGETQSR
6.3	1926.8387	-0.0045	CVCAEGFRQEDGICVK
4.2	1925.8304	1.0038	YENLGLVGEGSYGMVMK
2.8	1924.8349	1.9992	SEENHLNMGKADLKSR
0.9	1925.8226	1.0116	VEMLFGETMGIDTMLK
0.7	1926.8320	0.0022	GPXQGQTETGAKNTSGAQK
0.4	1925.8336	1.0006	ATFEEVSVLGFEEFDK
0.3	1926.8175	0.0167	EYRPCGPMYPASCSPR

Spectrum No: 407; Query: 287; Rank: 1

Peptide View

MS/MS Fragmentation of **ATISDEEIER**
Found in **IPI00368144**, Tax_Id=10116 Gene_Symbol=RGD1306781_predicted similar to chromatin modifying protein 2B

Match to Query 287: 1241.520208 from(621.767380,2+)
Title: 091008RatKidney_NoSalt_25.1013.1013.2.dta
Data file K:\NewmanPaper\Piliang\3SubProteomes\Piliang3SP\mgf5ppm\ERLIC_3SubProteomes5ppm.mgf

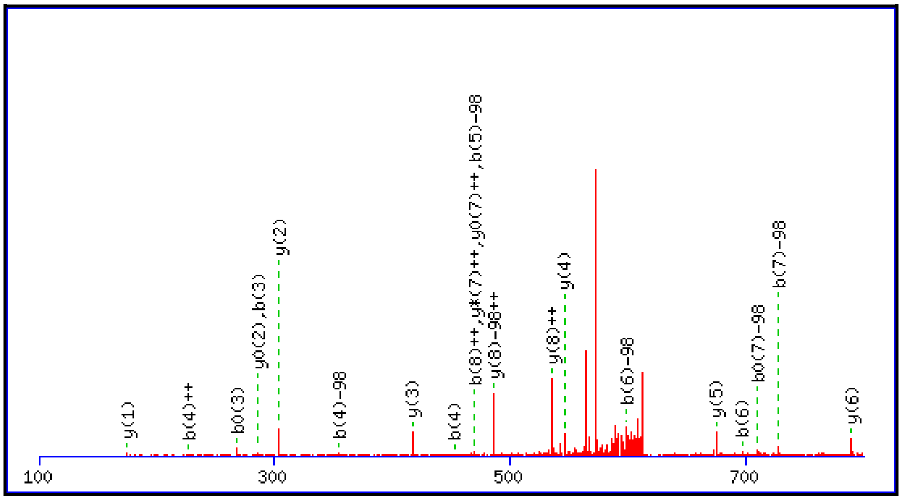

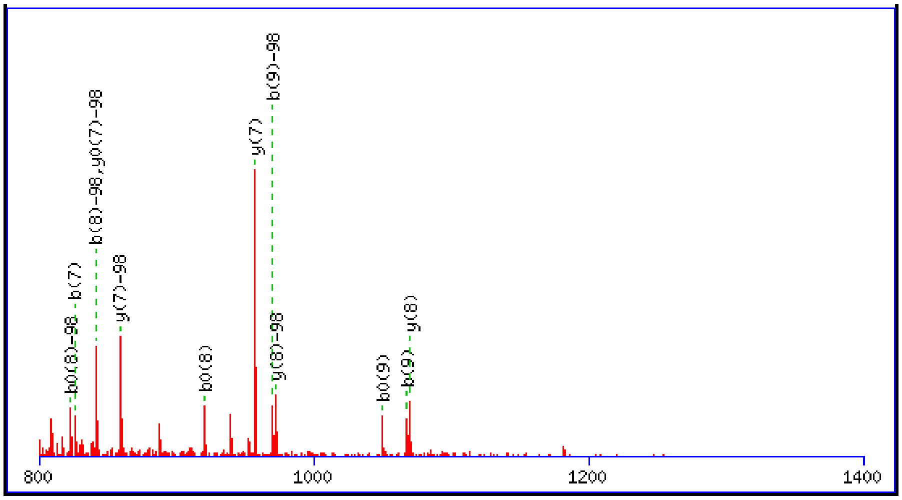

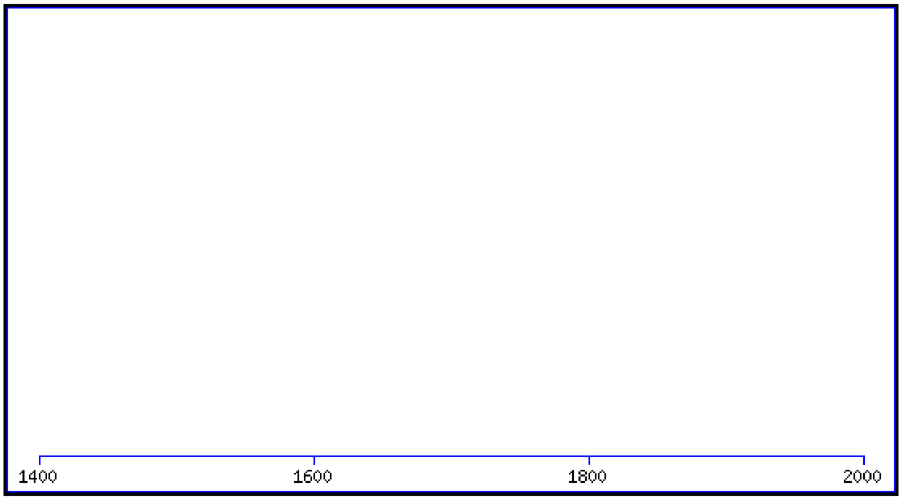

Monoisotopic mass of neutral peptide Mr(calc): 1241.5176
Fixed modifications: Carbamidomethyl (C)
Variable modifications:
S4 : Phospho (ST), with neutral losses 97.9769(shown in table), 0.0000
Ions Score: 45 **Expect:** 0.003
Matches (Bold Red): 34/128 fragment ions using 55 most intense peaks

#	b	b^{++}	b^0	b^{0++}	Seq.	y	y^{++}	y*	y^{*++}	y^0	y^{0++}	#
1	72.0444	36.5258			A							10
2	173.0921	87.0497	155.0815	78.0444	T	1073.5109	537.2591	1056.4844	528.7458	1055.5004	528.2538	9
3	**286.1761**	143.5917	**268.1656**	134.5864	I	**972.4633**	**486.7353**	955.4367	478.2220	954.4527	477.7300	8
4	**355.1976**	178.1024	337.1870	169.0971	S	**859.3792**	430.1932	842.3526	421.6800	**841.3686**	421.1880	7
5	**470.2245**	235.6159	452.2140	226.6106	D	**790.3577**	395.6825	773.3312	387.1692	772.3472	386.6772	6
6	**599.2671**	300.1372	581.2566	291.1319	E	**675.3308**	338.1690	658.3042	329.6558	657.3202	329.1638	5
7	**728.3097**	364.6585	**710.2992**	355.6532	E	**546.2882**	273.6477	529.2617	265.1345	528.2776	264.6425	4
8	**841.3938**	421.2005	**823.3832**	412.1952	I	**417.2456**	209.1264	400.2191	200.6132	399.2350	200.1212	3
9	**970.4364**	485.7218	952.4258	476.7165	E	**304.1615**	152.5844	287.1350	144.0711	**286.1510**	143.5791	2
10					R	**175.1190**	88.0631	158.0924	79.5498			1

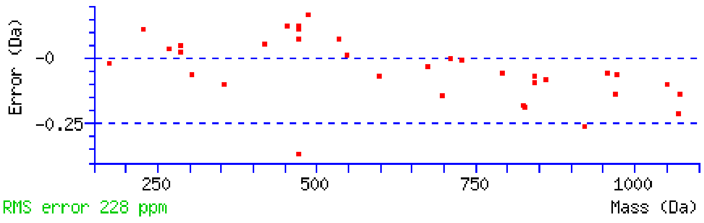

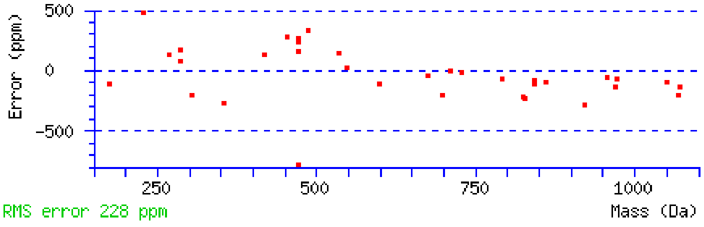

All matches to this query

Score	Mr(calc):	Delta	Sequence
44.9	1241.5176	0.0026	ATISDEEIER
33.3	1241.5176	0.0026	ATISDEEIER
13.7	1241.5264	-0.0062	HTCVTFIER
13.7	1241.5264	-0.0062	HTCVTFIER
13.7	1241.5264	-0.0062	HTCVTFLER
13.7	1241.5264	-0.0062	HTCVTFLER
8.7	1241.5302	-0.0100	GQHQAHSLER
8.2	1240.5125	1.0077	WDETPKTER
8.1	1241.5289	-0.0087	XNVQTSESGLR
8.1	1241.5289	-0.0087	XNVQTSESGLR

Spectrum No: 408; Query: 2633; Rank: 1

Peptide View

MS/MS Fragmentation of **IAESHLQTISNLSENQASEEEDELGELR**
Found in **IPI00421955**, Tax_Id=10116 Gene_Symbol=Ppp1r1b Protein phosphatase 1 regulatory subunit 1B

Match to Query 2633: 3220.447002 from(1074.489610,3+)
Title: 100101RatKid_NS_deglyco_08.4029.4029.3.dta
Data file K:\NewmanPaper\Piliang\3SubProteomes\Piliang3SP\mgf5ppm\ERLIC_3SubProteomes5ppm.mgf

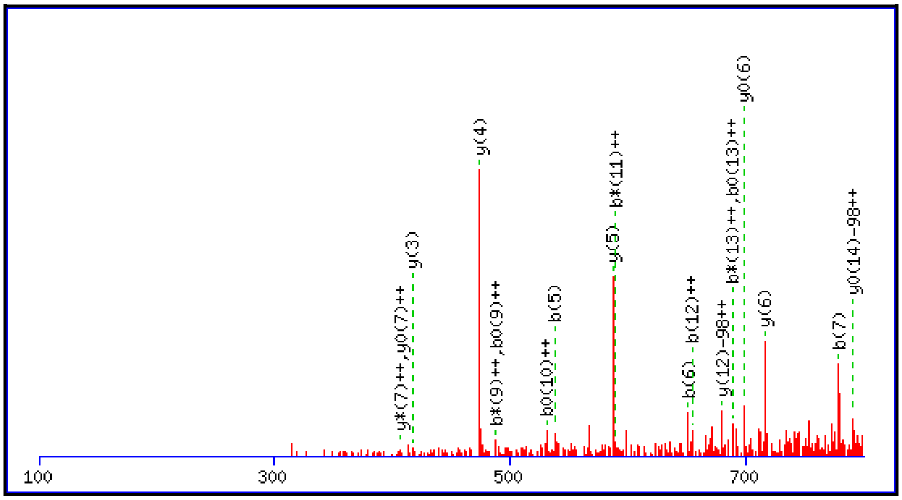

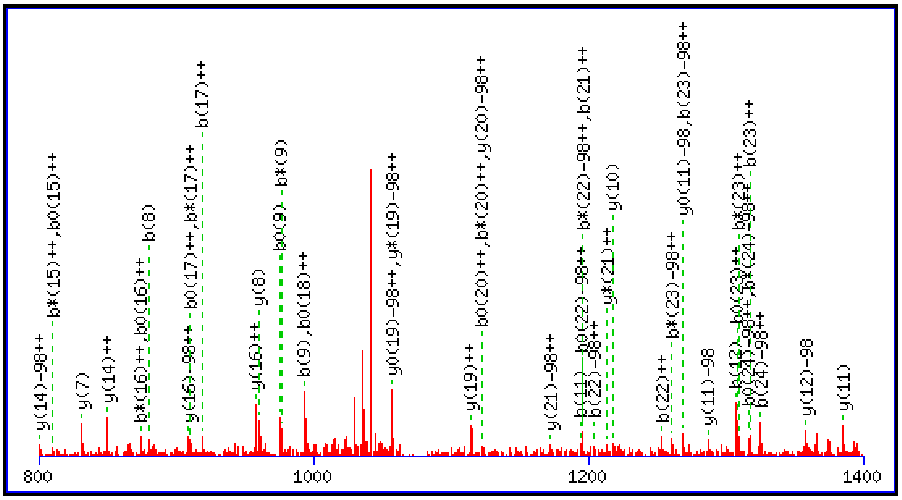

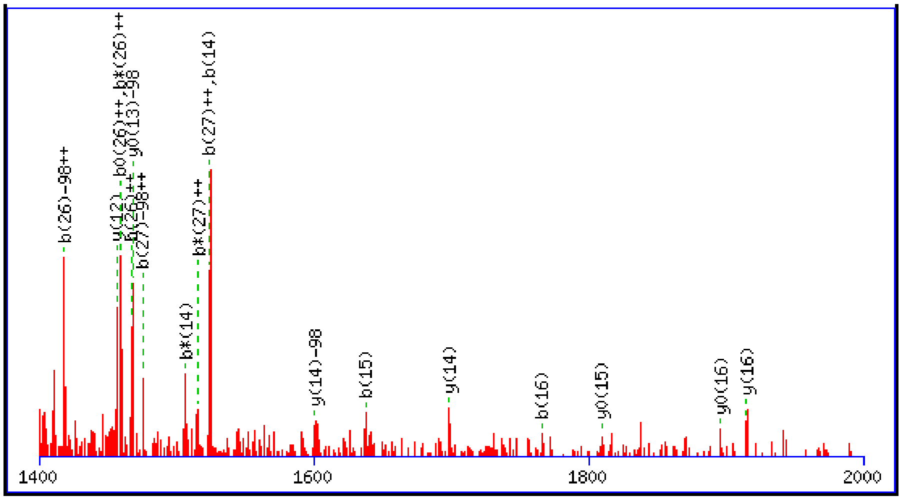

Monoisotopic mass of neutral peptide Mr(calc): 3220.4408
Fixed modifications: Carbamidomethyl (C)
Variable modifications:
S18 : Phospho (ST), with neutral losses 97.9769(shown in table), 0.0000
Ions Score: 45 **Expect:** 0.014
Matches (Bold Red): 83/466 fragment ions using 138 most intense peaks

#	b	b^{++}	b*	b^{*++}	b^{0}	b^{0++}	Seq.	y	y^{++}	y*	y^{*++}	y^{0}	y^{0++}	#
1	114.0913	57.5493					I							28
2	185.1285	93.0679					A	3010.3872	1505.6972	2993.3606	1497.1840	2992.3766	1496.6920	27
3	314.1710	157.5892			296.1605	148.5839	E	2939.3501	1470.1787	2922.3235	1461.6654	2921.3395	1461.1734	26
4	401.2031	201.1052			383.1925	192.0999	S	2810.3075	1405.6574	2793.2809	1397.1441	2792.2969	1396.6521	25
5	**538.2620**	269.6346			520.2514	260.6293	H	2723.2755	1362.1414	2706.2489	1353.6281	2705.2649	1353.1361	24
6	**651.3461**	326.1767			633.3355	317.1714	L	2586.2166	1293.6119	2569.1900	1285.0986	2568.2060	1284.6066	23
7	**779.4046**	390.2060	762.3781	381.6927	761.3941	381.2007	Q	2473.1325	1237.0699	2456.1059	1228.5566	2455.1219	1228.0646	22
8	**880.4523**	440.7298	863.4258	432.2165	862.4417	431.7245	T	2345.0739	**1173.0406**	2328.0474	1164.5273	2327.0633	1164.0353	21
9	**993.5364**	497.2718	**976.5098**	**488.7585**	**975.5258**	**488.2665**	I	2244.0262	**1122.5168**	2226.9997	1114.0035	2226.0157	1113.5115	20
10	1080.5684	540.7878	1063.5419	532.2746	1062.5578	**531.7826**	S	2130.9422	1065.9747	2113.9156	**1057.4614**	2112.9316	**1056.9694**	19
11	**1194.6113**	597.8093	1177.5848	**589.2960**	1176.6008	588.8040	N	2043.9101	1022.4587	2026.8836	1013.9454	2025.8996	1013.4534	18
12	**1307.6954**	**654.3513**	1290.6688	645.8381	1289.6848	645.3461	L	1929.8672	965.4372	1912.8407	956.9240	1911.8566	956.4320	17
13	1394.7274	697.8673	1377.7009	**689.3541**	1376.7169	**688.8621**	S	1816.7831	**908.8952**	1799.7566	900.3819	1798.7726	899.8899	16
14	**1523.7700**	762.3886	**1506.7435**	753.8754	1505.7594	753.3834	E	1729.7511	865.3792	1712.7246	856.8659	1711.7406	856.3739	15
15	**1637.8129**	819.4101	1620.7864	**810.8968**	1619.8024	**810.4048**	N	**1600.7085**	**800.8579**	1583.6820	792.3446	1582.6980	**791.8526**	14
16	**1765.8715**	883.4394	1748.8450	**874.9261**	1747.8610	**874.4341**	Q	1486.6656	743.8364	1469.6391	735.3232	**1468.6550**	734.8312	13
17	1836.9086	**918.9580**	1819.8821	**910.4447**	1818.8981	**909.9527**	A	**1358.6070**	**679.8071**	1341.5805	671.2939	1340.5965	670.8019	12
18	1905.9301	953.4687	1888.9035	944.9554	1887.9195	944.4634	S	**1287.5699**	644.2886	1270.5434	635.7753	**1269.5593**	635.2833	11
19	2034.9727	1017.9900	2017.9461	1009.4767	2016.9621	1008.9847	E	**1218.5485**	609.7779	1201.5219	601.2646	1200.5379	600.7726	10
20	2164.0153	1082.5113	2146.9887	1073.9980	2146.0047	1073.5060	E	1089.5059	545.2566	1072.4793	536.7433	1071.4953	536.2513	9
21	2293.0579	1147.0326	2276.0313	1138.5193	2275.0473	1138.0273	E	**960.4633**	480.7353	943.4367	472.2220	942.4527	471.7300	8
22	2408.0848	**1204.5460**	2391.0583	**1196.0328**	2390.0742	**1195.5408**	D	**831.4207**	416.2140	814.3941	**407.7007**	813.4101	**407.2087**	7
23	2537.1274	**1269.0673**	2520.1009	**1260.5541**	2519.1168	1260.0621	E	**716.3937**	358.7005	699.3672	350.1872	**698.3832**	349.6952	6
24	2650.2115	**1325.6094**	2633.1849	**1317.0961**	2632.2009	**1316.6041**	L	**587.3511**	294.1792	570.3246	285.6659	569.3406	285.1739	5
25	2707.2329	1354.1201	2690.2064	1345.6068	2689.2224	1345.1148	G	**474.2671**	237.6372	457.2405	229.1239	456.2565	228.6319	4
26	2836.2755	**1418.6414**	2819.2490	1410.1281	2818.2650	1409.6361	E	**417.2456**	209.1264	400.2191	200.6132	399.2350	200.1212	3
27	2949.3596	**1475.1834**	2932.3330	1466.6702	2931.3490	1466.1781	L	288.2030	144.6051	271.1765	136.0919			2
28							R	175.1190	88.0631	158.0924	79.5498			1

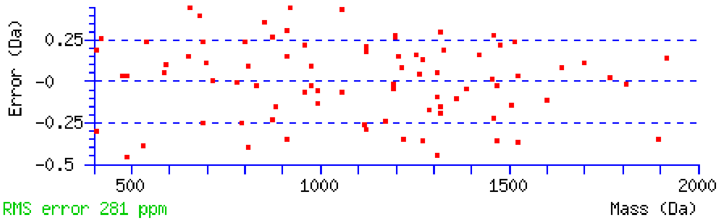

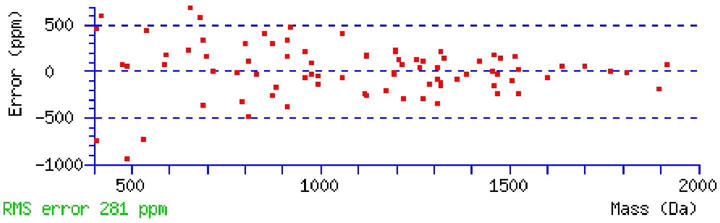

All matches to this query

Score	Mr(calc):	Delta	Sequence
44.8	3220.4408	0.0062	IAESHLQTISNLSENQASEEEDELGELR
34.7	3220.4408	0.0062	IAESHLQTISNLSENQASEEEDELGELR
21.3	3220.4408	0.0062	IAESHLQTISNLSENQASEEEDELGELR
21.2	3220.4408	0.0062	IAESHLQTISNLSENQASEEEDELGELR
11.8	3220.4408	0.0062	IAESHLQTISNLSENQASEEEDELGELR
5.7	3220.4707	-0.0237	AMKELTEEQQNLQKELESLQNEHAQR
4.6	3218.4451	2.0019	RKVEVVSPATPVPSETAPASVFPQNGSAR
4.3	3220.4367	0.0103	MANFWNPISTLHFRSXMTPMRSVPMK
1.7	3220.4367	0.0103	MANFWNPISTLHFRSXMTPMRSVPMK
0.5	3220.4681	-0.0211	ISTATPYMNGETATKSLWVINSALRIK

Spectrum No: 409; Query: 2592; Rank: 1

Peptide View

MS/MS Fragmentation of **ASAPEKLSDGEYISDGEEGEDAITGDTDK**
Found in **IPI00199445**, Tax_Id=10116 Gene_Symbol=Ank3 ankyrin 3, epithelial isoform 2

Match to Query 2592: 3158.245032 from(1053.755620,3+)
Title: 100101RatKid_NS_deglyco_26.2753.2753.3.dta
Data file K:\NewmanPaper\Piliang\3SubProteomes\Piliang3SP\mgf5ppm\ERLIC_3SubProteomes5ppm.mgf

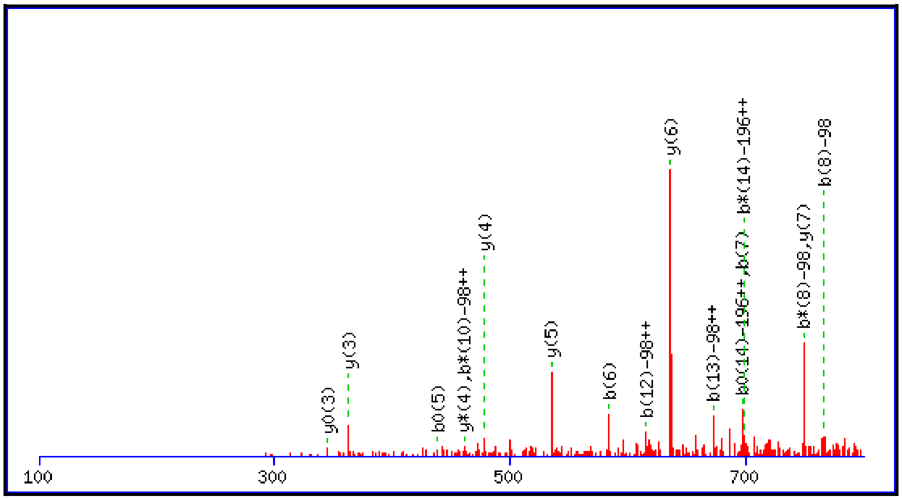

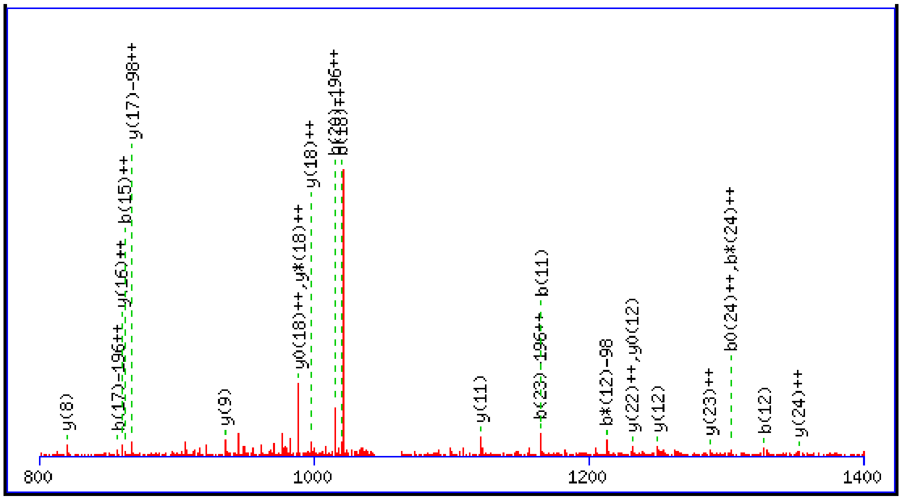

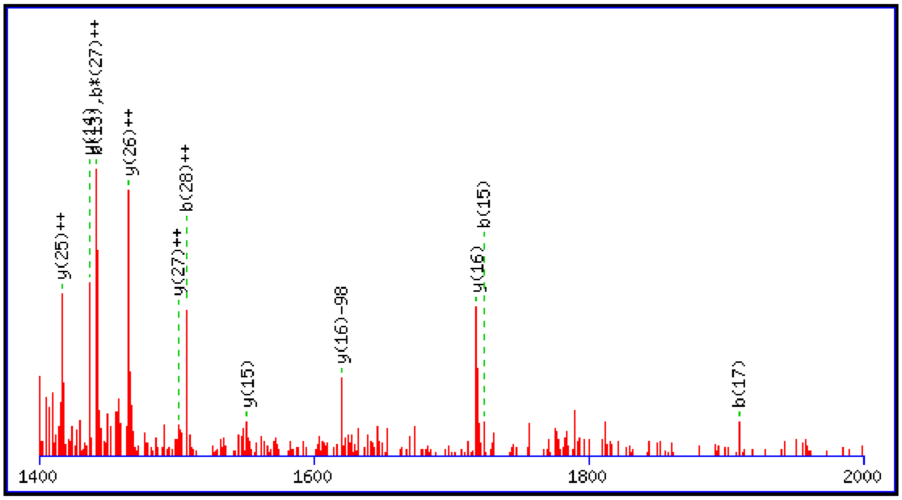

Monoisotopic mass of neutral peptide Mr(calc): 3158.2377
Fixed modifications: Carbamidomethyl (C)
Variable modifications:
S8 : Phospho (ST), with neutral losses 0.0000(shown in table), 97.9769
S14 : Phospho (ST), with neutral losses 0.0000(shown in table), 97.9769
Ions Score: 45 **Expect:** 0.011
Matches (Bold Red): 52/526 fragment ions using 100 most intense peaks

#	b	b++	b*	b*++	b0	b0++	Seq.	y	y++	y*	y*++	y0	y0++	#
1	72.0444	36.5258					A							29
2	159.0764	80.0418			141.0659	71.0366	S	3088.2079	1544.6076	3071.1814	1536.0943	3070.1973	1535.6023	28
3	230.1135	115.5604			212.1030	106.5551	A	3001.1759	**1501.0916**	2984.1493	1492.5783	2983.1653	1492.0863	27
4	327.1663	164.0868			309.1557	155.0815	P	2930.1388	**1465.5730**	2913.1122	1457.0597	2912.1282	1456.5677	26
5	456.2089	228.6081			**438.1983**	219.6028	E	2833.0860	**1417.0466**	2816.0595	1408.5334	2815.0754	1408.0414	25
6	**584.3039**	292.6556	567.2773	284.1423	566.2933	283.6503	K	2704.0434	**1352.5253**	2687.0169	1344.0121	2686.0328	1343.5201	24
7	**697.3879**	349.1976	680.3614	340.6843	679.3774	340.1923	L	2575.9484	**1288.4779**	2558.9219	1279.9646	2557.9379	1279.4726	23
8	864.3863	432.6968	847.3597	424.1835	846.3757	423.6915	S	2462.8644	**1231.9358**	2445.8378	1223.4226	2444.8538	1222.9305	22
9	979.4132	490.2102	962.3867	481.6970	961.4026	481.2050	D	2295.8660	1148.4367	2278.8395	1139.9234	2277.8555	1139.4314	21
10	1036.4347	518.7210	1019.4081	510.2077	1018.4241	509.7157	G	2180.8391	1090.9232	2163.8125	1082.4099	2162.8285	1081.9179	20
11	**1165.4773**	583.2423	1148.4507	574.7290	1147.4667	574.2370	E	2123.8176	1062.4124	2106.7911	1053.8992	2105.8071	1053.4072	19
12	**1328.5406**	664.7739	1311.5140	656.2607	1310.5300	655.7687	Y	1994.7750	**997.8912**	1977.7485	**989.3779**	1976.7645	**988.8859**	18
13	**1441.6247**	721.3160	1424.5981	712.8027	1423.6141	712.3107	I	1831.7117	916.3595	1814.6851	907.8462	1813.7011	907.3542	17
14	1608.6230	804.8151	1591.5965	796.3019	1590.6124	795.8099	S	**1718.6276**	**859.8175**	1701.6011	851.3042	1700.6171	850.8122	16
15	**1723.6500**	**862.3286**	1706.6234	853.8153	1705.6394	853.3233	D	**1551.6293**	776.3183	1534.6027	767.8050	1533.6187	767.3130	15
16	1780.6714	890.8393	1763.6449	882.3261	1762.6609	881.8341	G	**1436.6023**	718.8048	1419.5758	710.2915	1418.5918	709.7995	14
17	**1909.7140**	955.3606	1892.6875	946.8474	1891.7034	946.3554	E	1379.5809	690.2941	1362.5543	681.7808	1361.5703	681.2888	13
18	2038.7566	**1019.8819**	2021.7301	1011.3687	2020.7460	1010.8767	E	**1250.5383**	625.7728	1233.5117	617.2595	**1232.5277**	616.7675	12

**19**	2095.7781	1048.3927	2078.7515	1039.8794	2077.7675	1039.3874	**G**	**1121.4957**	561.2515	1104.4691	552.7382	1103.4851	552.2462	**11**
**20**	2224.8207	1112.9140	2207.7941	1104.4007	2206.8101	1103.9087	**E**	1064.4742	532.7408	1047.4477	524.2275	1046.4637	523.7355	**10**
**21**	2339.8476	1170.4274	2322.8211	1161.9142	2321.8370	1161.4222	**D**	**935.4316**	468.2195	918.4051	459.7062	917.4211	459.2142	**9**
**22**	2410.8847	1205.9460	2393.8582	1197.4327	2392.8742	1196.9407	**A**	**820.4047**	410.7060	803.3781	402.1927	802.3941	401.7007	**8**
**23**	2523.9688	1262.4880	2506.9422	1253.9748	2505.9582	1253.4827	**I**	**749.3676**	375.1874	732.3410	366.6742	731.3570	366.1821	**7**
**24**	2625.0165	1313.0119	2607.9899	**1304.4986**	2607.0059	**1304.0066**	**T**	**636.2835**	318.6454	619.2570	310.1321	618.2729	309.6401	**6**
**25**	2682.0379	1341.5226	2665.0114	1333.0093	2664.0274	1332.5173	**G**	**535.2358**	268.1216	518.2093	259.6083	517.2253	259.1163	**5**
**26**	2797.0649	1399.0361	2780.0383	1390.5228	2779.0543	1390.0308	**D**	**478.2144**	239.6108	**461.1878**	231.0975	460.2038	230.6055	**4**
**27**	2898.1125	1449.5599	2881.0860	**1441.0466**	2880.1020	1440.5546	**T**	**363.1874**	182.0974	346.1609	173.5841	**345.1769**	173.0921	**3**
**28**	3013.1395	**1507.0734**	2996.1129	1498.5601	2995.1289	1498.0681	**D**	262.1397	131.5735	245.1132	123.0602	244.1292	122.5682	**2**
**29**							**K**	147.1128	74.0600	130.0863	65.5468			**1**

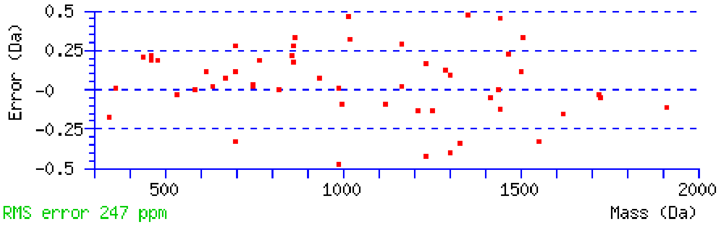

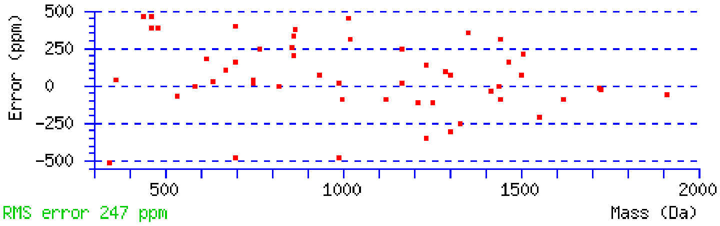

All matches to this query

Score	Mr(calc):	Delta	Sequence
44.8	3158.2377	0.0073	ASAPEKLSDGEYISDGEEGEDAITGDTDK
40.4	3158.2377	0.0073	ASAPEKLSDGEYISDGEEGEDAITGDTDK
37.3	3158.2377	0.0073	ASAPEKLSDGEYISDGEEGEDAITGDTDK
30.9	3158.2377	0.0073	ASAPEKLSDGEYISDGEEGEDAITGDTDK
23.1	3158.2377	0.0073	ASAPEKLSDGEYISDGEEGEDAITGDTDK
21.9	3158.2377	0.0073	ASAPEKLSDGEYISDGEEGEDAITGDTDK
20.4	3158.2377	0.0073	ASAPEKLSDGEYISDGEEGEDAITGDTDK
16.4	3158.2377	0.0073	ASAPEKLSDGEYISDGEEGEDAITGDTDK
15.8	3158.2377	0.0073	ASAPEKLSDGEYISDGEEGEDAITGDTDK
9.3	3158.2377	0.0073	ASAPEKLSDGEYISDGEEGEDAITGDTDK

Spectrum No: 410; Query: 1876; Rank: 1

Peptide View

MS/MS Fragmentation of **KGSLDSDNDDSDCPYSEK**
Found in **IPI00199985**, Tax_Id=10116 Gene_Symbol=Slc4a4 Isoform 1 of Electrogenic sodium bicarbonate cotransporter 1

Match to Query 1876: 2190.741588 from(1096.378070,2+)
Title: 100101RatKid_NS_deglyco_26.1095.1095.2.dta
Data file K:\NewmanPaper\Piliang\3SubProteomes\Piliang3SP\mgf5ppm\ERLIC_3SubProteomes5ppm.mgf

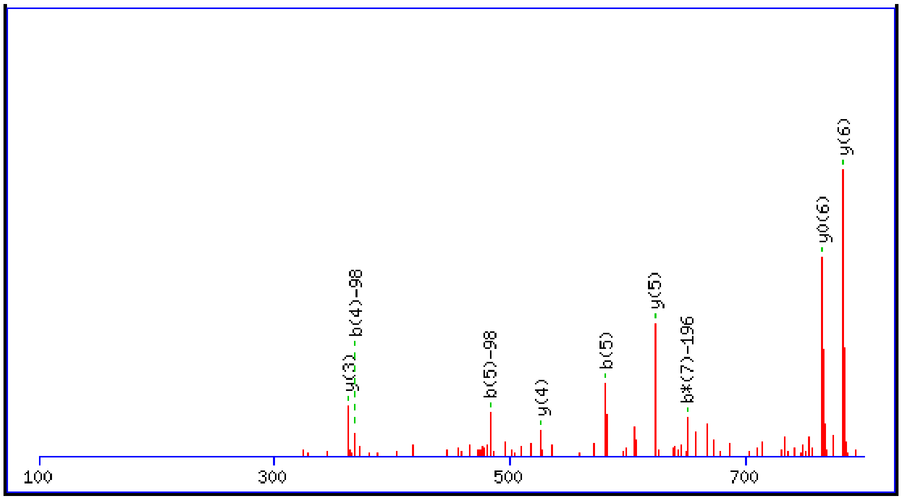

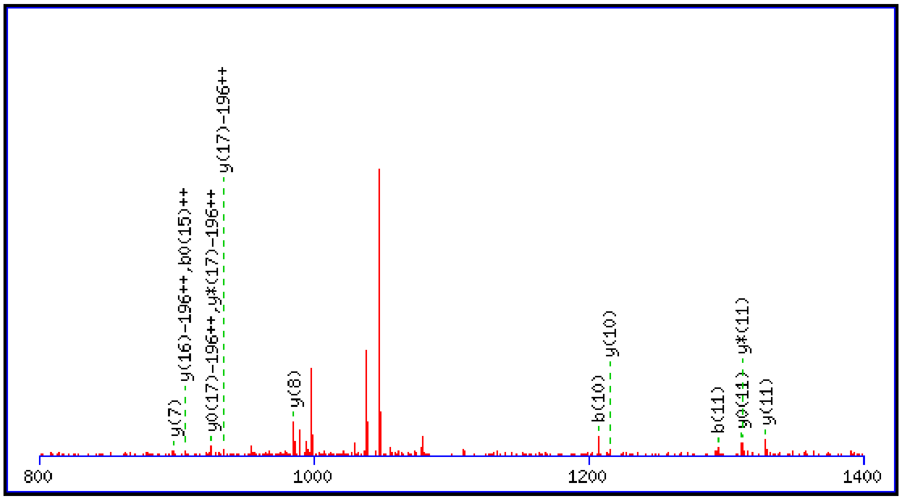

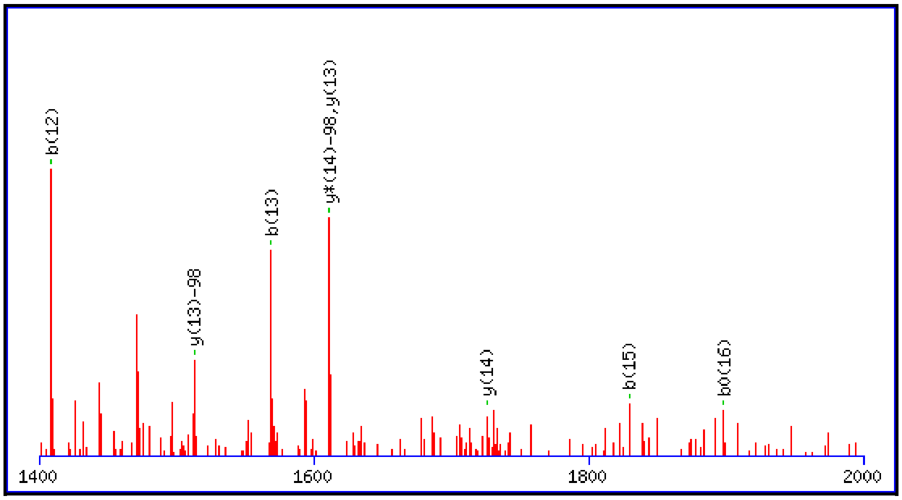

Monoisotopic mass of neutral peptide Mr(calc): 2190.7337
Fixed modifications: Carbamidomethyl (C)
Variable modifications:
S3 : Phospho (ST), with neutral losses 0.0000(shown in table), 97.9769
S6 : Phospho (ST), with neutral losses 0.0000(shown in table), 97.9769
Ions Score: 45 **Expect:** 0.0022
Matches (Bold Red): 31/318 fragment ions using 44 most intense peaks

#	b	b^{++}	b*	b^{*++}	b^{0}	b^{0++}	Seq.	y	y^{++}	y*	y^{*++}	y^{0}	y^{0++}	#
1	129.1022	65.0548	112.0757	56.5415			K							18
2	186.1237	93.5655	169.0972	85.0522			G	2063.6461	1032.3267	2046.6195	1023.8134	2045.6355	1023.3214	17
3	353.1221	177.0647	336.0955	168.5514	335.1115	168.0594	S	2006.6246	1003.8159	1989.5981	995.3027	1988.6140	994.8107	16
4	466.2061	233.6067	449.1796	225.0934	448.1956	224.6014	L	1839.6263	920.3168	1822.5997	911.8035	1821.6157	911.3115	15

5	581.2331	291.1202	564.2065	282.6069	563.2225	282.1149	D	1726.5422	863.7747	1709.5156	855.2615	1708.5316	854.7695	14
6	748.2314	374.6193	731.2049	366.1061	730.2208	365.6141	S	1611.5152	806.2613	1594.4887	797.7480	1593.5047	797.2560	13
7	863.2584	432.1328	846.2318	423.6195	845.2478	423.1275	D	1444.5169	722.7621	1427.4903	714.2488	1426.5063	713.7568	12
8	977.3013	489.1543	960.2747	480.6410	959.2907	480.1490	N	1329.4900	665.2486	1312.4634	656.7353	1311.4794	656.2433	11
9	1092.3282	546.6678	1075.3017	538.1545	1074.3177	537.6625	D	1215.4470	608.2272	1198.4205	599.7139	1197.4365	599.2219	10
10	1207.3552	604.1812	1190.3286	595.6679	1189.3446	595.1759	D	1100.4201	550.7137	1083.3935	542.2004	1082.4095	541.7084	9
11	1294.3872	647.6972	1277.3606	639.1840	1276.3766	638.6920	S	985.3931	493.2002	968.3666	484.6869	967.3826	484.1949	8
12	1409.4141	705.2107	1392.3876	696.6974	1391.4036	696.2054	D	898.3611	449.6842	881.3346	441.1709	880.3505	440.6789	7
13	1569.4448	785.2260	1552.4182	776.7128	1551.4342	776.2208	C	783.3342	392.1707	766.3076	383.6574	765.3236	383.1654	6
14	1666.4976	833.7524	1649.4710	825.2391	1648.4870	824.7471	P	623.3035	312.1554	606.2770	303.6421	605.2930	303.1501	5
15	1829.5609	915.2841	1812.5343	906.7708	1811.5503	906.2788	Y	526.2508	263.6290	509.2242	255.1157	508.2402	254.6237	4
16	1916.5929	958.8001	1899.5664	950.2868	1898.5823	949.7948	S	363.1874	182.0974	346.1609	173.5841	345.1769	173.0921	3
17	2045.6355	1023.3214	2028.6090	1014.8081	2027.6249	1014.3161	E	276.1554	138.5813	259.1288	130.0681	258.1448	129.5761	2
18							K	147.1128	74.0600	130.0863	65.5468			1

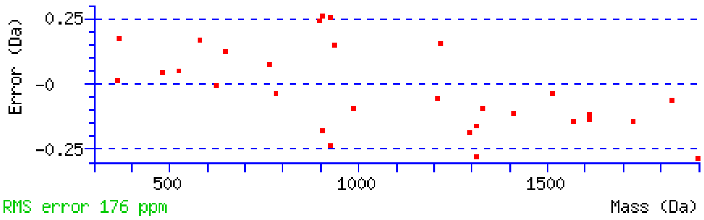

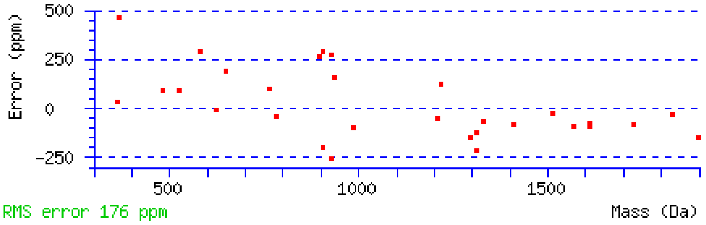

All matches to this query

Score	Mr(calc):	Delta	Sequence
44.8	2190.7337	0.0078	KGSLDSDNDDSDCPYSEK
25.5	2190.7337	0.0078	KGSLDSDNDDSDCPYSEK
17.1	2190.7337	0.0078	KGSLDSDNDDSDCPYSEK
13.4	2190.7337	0.0078	KGSLDSDNDDSDCPYSEK
6.9	2190.7337	0.0078	KGSLDSDNDDSDCPYSEK
5.8	2190.7337	0.0078	KGSLDSDNDDSDCPYSEK
5.5	2190.7337	0.0078	KGSLDSDNDDSDCPYSEK
1.9	2190.7337	0.0078	KGSLDSDNDDSDCPYSEK
1.5	2190.7285	0.0131	MXNFTPVNGSSANQSVR
1.5	2190.7285	0.0131	MXNFTPVNGSSANQSVR

Spectrum No: 411; Query: 2326; Rank: 1

Peptide View

MS/MS Fragmentation of **FIDKDQQPSGSEGEDDDAEAALK**
Found in **IPI00359917**, Tax_Id=10116 Gene_Symbol=Thumpd1 THUMP domain containing 1

Match to Query 2326: 2624.026632 from(875.682820,3+)
Title: 100101RatKid_NS_deglyco_26.1930.1930.3.dta
Data file K:\NewmanPaper\Piliang\3SubProteomes\Piliang3SP\mgf5ppm\ERLIC_3SubProteomes5ppm.mgf

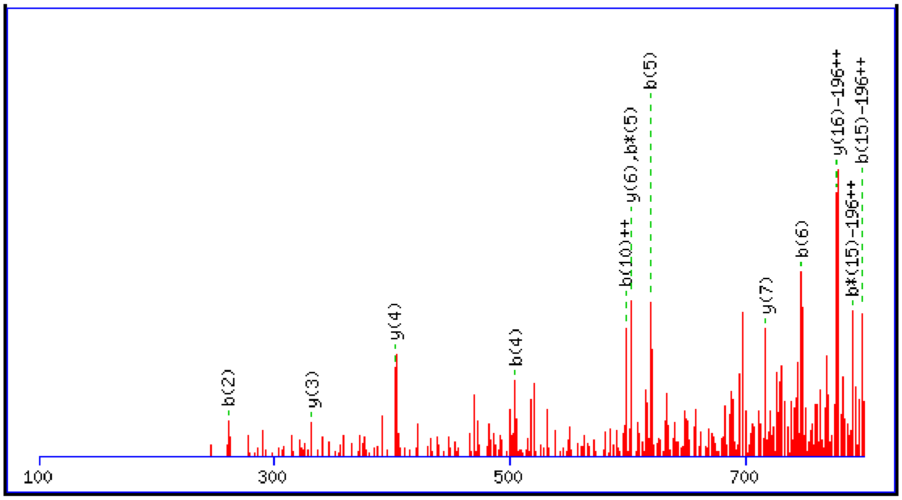

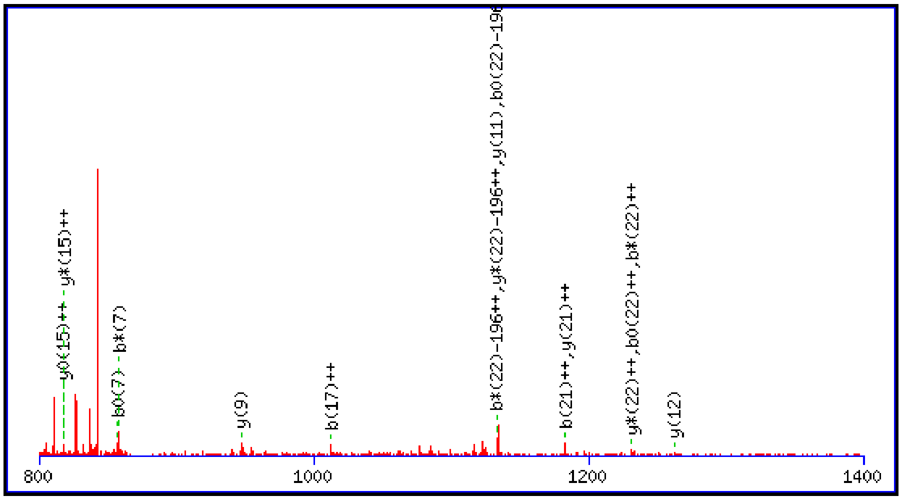

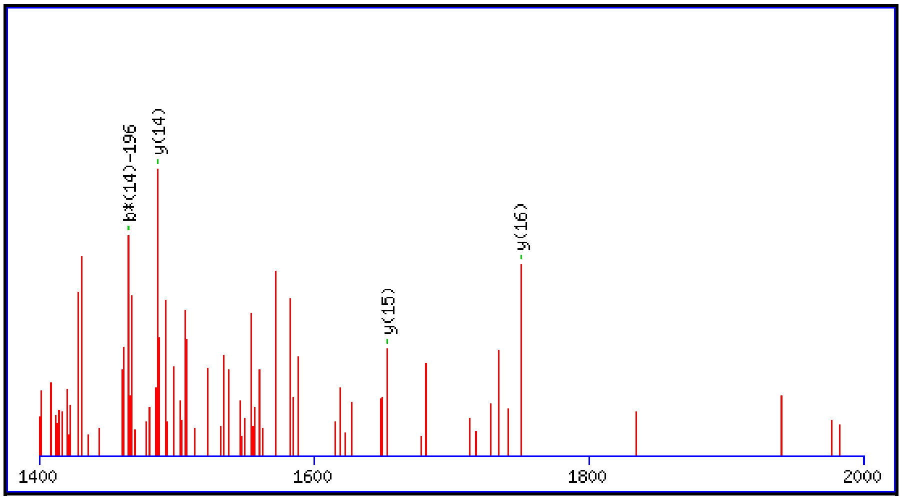

Monoisotopic mass of neutral peptide Mr(calc): 2624.0204
Fixed modifications: Carbamidomethyl (C)
Variable modifications:
S9 : Phospho (ST), with neutral losses 0.0000(shown in table), 97.9769
S11 : Phospho (ST), with neutral losses 0.0000(shown in table), 97.9769
Ions Score: 45 **Expect:** 0.0074
Matches (Bold Red): 33/390 fragment ions using 54 most intense peaks

#	b	b^{++}	b*	b^{*++}	b^0	b^{0++}	Seq.	y	y^{++}	y*	y^{*++}	y^0	y^{0++}	#
1	148.0757	74.5415					F							23
2	**261.1598**	131.0835					I	2477.9593	1239.4833	2460.9327	**1230.9700**	2459.9487	1230.4780	22
3	376.1867	188.5970			358.1761	179.5917	D	2364.8752	**1182.9412**	2347.8487	1174.4280	2346.8647	1173.9360	21
4	**504.2817**	252.6445	487.2551	244.1312	486.2711	243.6392	K	2249.8483	1125.4278	2232.8217	1116.9145	2231.8377	1116.4225	20

**5**	**619.3086**	310.1579	**602.2821**	301.6447	601.2980	301.1527	**D**	2121.7533	1061.3803	2104.7268	1052.8670	2103.7427	1052.3750	**19**
**6**	**747.3672**	374.1872	730.3406	365.6740	729.3566	365.1819	**Q**	2006.7264	1003.8668	1989.6998	995.3535	1988.7158	994.8615	**18**
**7**	875.4258	438.2165	**858.3992**	429.7032	**857.4152**	429.2112	**Q**	1878.6678	939.8375	1861.6412	931.3243	1860.6572	930.8323	**17**
**8**	972.4785	486.7429	955.4520	478.2296	954.4680	477.7376	**P**	**1750.6092**	875.8082	1733.5827	867.2950	1732.5986	866.8030	**16**
**9**	1139.4769	570.2421	1122.4503	561.7288	1121.4663	561.2368	**S**	**1653.5565**	827.2819	1636.5299	**818.7686**	1635.5459	**818.2766**	**15**
**10**	1196.4983	**598.7528**	1179.4718	590.2395	1178.4878	589.7475	**G**	**1486.5581**	743.7827	1469.5315	735.2694	1468.5475	734.7774	**14**
**11**	1363.4967	682.2520	1346.4701	673.7387	1345.4861	673.2467	**S**	1429.5366	715.2720	1412.5101	706.7587	1411.5261	706.2667	**13**
**12**	1492.5393	746.7733	1475.5127	738.2600	1474.5287	737.7680	**E**	**1262.5383**	631.7728	1245.5117	623.2595	1244.5277	622.7675	**12**
**13**	1549.5607	775.2840	1532.5342	766.7707	1531.5502	766.2787	**G**	**1133.4957**	567.2515	1116.4691	558.7382	1115.4851	558.2462	**11**
**14**	1678.6033	839.8053	1661.5768	831.2920	1660.5928	830.8000	**E**	1076.4742	538.7408	1059.4477	530.2275	1058.4637	529.7355	**10**
**15**	1793.6303	897.3188	1776.6037	888.8055	1775.6197	888.3135	**D**	**947.4316**	474.2195	930.4051	465.7062	929.4211	465.2142	**9**
**16**	1908.6572	954.8323	1891.6307	946.3190	1890.6467	945.8270	**D**	832.4047	416.7060	815.3781	408.1927	814.3941	407.7007	**8**
**17**	2023.6842	**1012.3457**	2006.6576	1003.8324	2005.6736	1003.3404	**D**	**717.3777**	359.1925	700.3512	350.6792	699.3672	350.1872	**7**
**18**	2094.7213	1047.8643	2077.6947	1039.3510	2076.7107	1038.8590	**A**	**602.3508**	301.6790	585.3243	293.1658	584.3402	292.6738	**6**
**19**	2223.7639	1112.3856	2206.7373	1103.8723	2205.7533	1103.3803	**E**	531.3137	266.1605	514.2871	257.6472	513.3031	257.1552	**5**
**20**	2294.8010	1147.9041	2277.7744	1139.3909	2276.7904	1138.8989	**A**	**402.2711**	201.6392	385.2445	193.1259			**4**
**21**	2365.8381	**1183.4227**	2348.8116	1174.9094	2347.8275	1174.4174	**A**	**331.2340**	166.1206	314.2074	157.6074			**3**
**22**	2478.9222	1239.9647	2461.8956	**1231.4514**	2460.9116	**1230.9594**	**L**	260.1969	130.6021	243.1703	122.0888			**2**
**23**							**K**	147.1128	74.0600	130.0863	65.5468			**1**

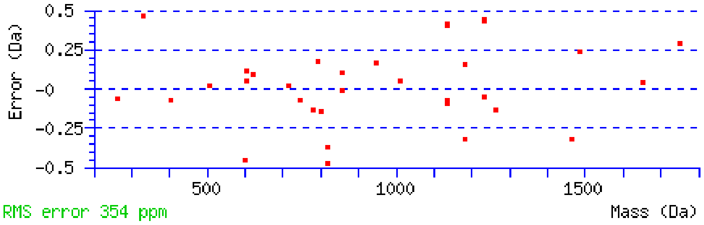

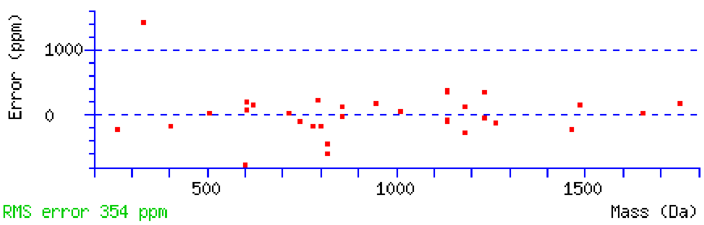

All matches to this query

Score	Mr(calc):	Delta	Sequence
44.9	2624.0204	0.0062	FIDKDQQPSGSEGEDDDAEAALK
6.3	2624.0032	0.0234	SGSLCASVPSNSVSQLQKANIR
6.0	2624.0032	0.0234	SGSLCASVPSNSVSQLQKANIR
4.7	2622.0086	2.0180	KSEQGMPYPMHNSTQIIAYR
4.6	2621.9995	2.0272	IFQLSLRNIQQYYNSSGDR
2.3	2622.0224	2.0043	MLRSDGSGAASSGLSPGLGFQESR
2.1	2624.0032	0.0234	SGSLCASVPSNSVSQLQKANIR
2.0	2624.0155	0.0111	SGDASINVTNLQLSDIGTYQCK
1.6	2622.0054	2.0213	KSNPPASTSSGSSRLPQPSTTSK
1.6	2624.0437	-0.0171	HCAQQPSQVCAYSSNTSLLDAK

Spectrum No: 412; Query: 1776; Rank: 1

Peptide View

MS/MS Fragmentation of **NVPQEESLEDSDVDADFK**
Found in **IPI00364414**, Tax_Id=10116 Gene_Symbol=Kpna3 Karyopherin alpha 3

Match to Query 1776: 2116.864448 from(1059.439500,2+)
Title: 100101RatKid_NS_deglyco_24.2810.2810.2.dta
Data file K:\NewmanPaper\Piliang\3SubProteomes\Piliang3SP\mgf5ppm\ERLIC_3SubProteomes5ppm.mgf

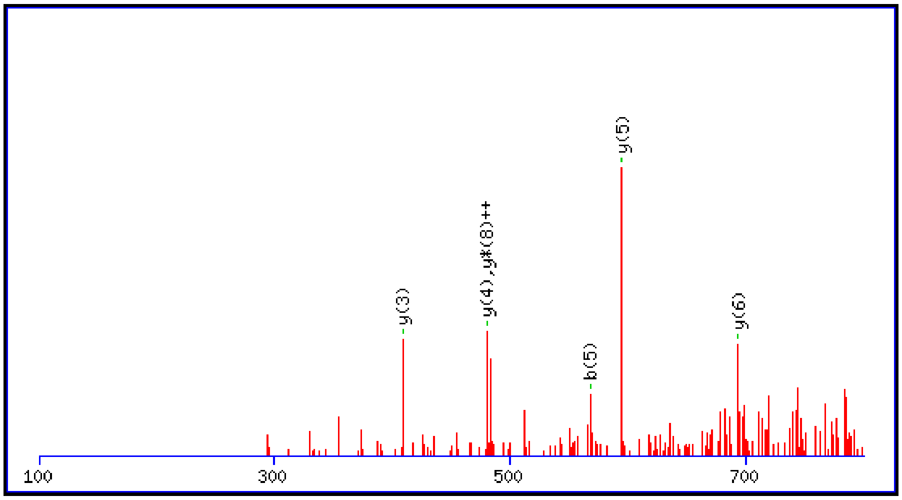

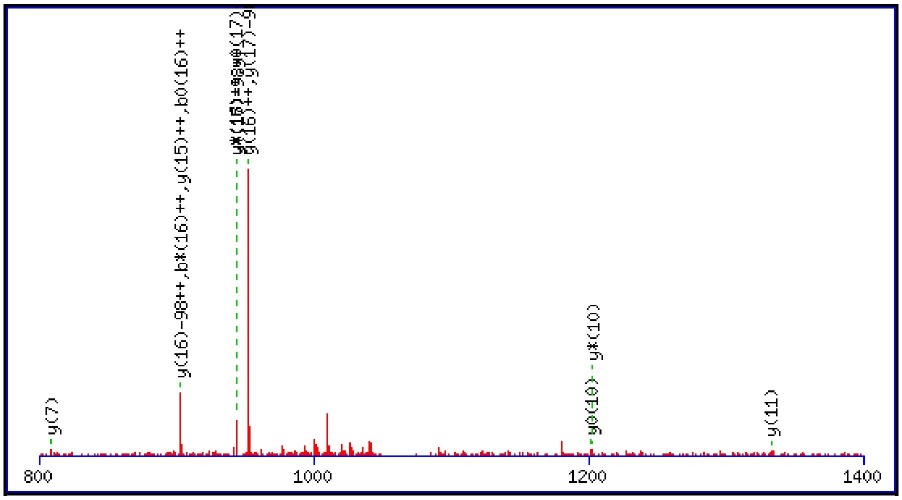

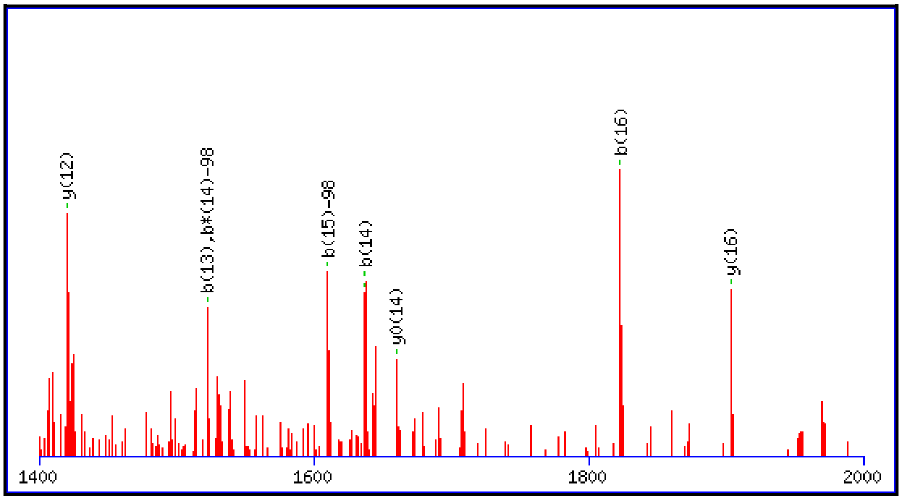

Monoisotopic mass of neutral peptide Mr(calc): 2115.8521
Fixed modifications: Carbamidomethyl (C)
Variable modifications:
S11 : Phospho (ST), with neutral losses 0.0000(shown in table), 97.9769
Ions Score: 45 **Expect:** 0.0072
Matches (Bold Red): 28/294 fragment ions using 32 most intense peaks

#	b	b^{++}	b*	b^{*++}	b^0	b^{0++}	Seq.	y	y^{++}	y*	y^{*++}	y^0	y^{0++}	#
1	115.0502	58.0287	98.0237	49.5155			N							18
2	214.1186	107.5629	197.0921	99.0497			V	2002.8165	1001.9119	1985.7899	993.3986	1984.8059	992.9066	17
3	311.1714	156.0893	294.1448	147.5761			P	**1903.7481**	**952.3777**	1886.7215	**943.8644**	1885.7375	**943.3724**	16
4	439.2300	220.1186	422.2034	211.6053			Q	1806.6953	**903.8513**	1789.6688	895.3380	1788.6848	894.8460	15
5	**568.2726**	284.6399	551.2460	276.1266	550.2620	275.6346	E	1678.6367	839.8220	1661.6102	831.3087	**1660.6262**	830.8167	14

6	697.3151	349.1612	680.2886	340.6479	679.3046	340.1559	E	1549.5941	775.3007	1532.5676	766.7874	1531.5836	766.2954	13
7	784.3472	392.6772	767.3206	384.1640	766.3366	383.6719	S	1420.5516	710.7794	1403.5250	702.2661	1402.5410	701.7741	12
8	897.4312	449.2193	880.4047	440.7060	879.4207	440.2140	L	1333.5195	667.2634	1316.4930	658.7501	1315.5090	658.2581	11
9	1026.4738	513.7406	1009.4473	505.2273	1008.4633	504.7353	E	1220.4355	610.7214	1203.4089	602.2081	1202.4249	601.7161	10
10	1141.5008	571.2540	1124.4742	562.7408	1123.4902	562.2487	D	1091.3929	546.2001	1074.3663	537.6868	1073.3823	537.1948	9
11	1308.4991	654.7532	1291.4726	646.2399	1290.4886	645.7479	S	976.3659	488.6866	959.3394	480.1733	958.3554	479.6813	8
12	1423.5261	712.2667	1406.4995	703.7534	1405.5155	703.2614	D	809.3676	405.1874	792.3410	396.6742	791.3570	396.1821	7
13	1522.5945	761.8009	1505.5679	753.2876	1504.5839	752.7956	V	694.3406	347.6740	677.3141	339.1607	676.3301	338.6687	6
14	1637.6214	819.3144	1620.5949	810.8011	1619.6109	810.3091	D	595.2722	298.1397	578.2457	289.6265	577.2617	289.1345	5
15	1708.6585	854.8329	1691.6320	846.3196	1690.6480	845.8276	A	480.2453	240.6263	463.2187	232.1130	462.2347	231.6210	4
16	1823.6855	912.3464	1806.6589	903.8331	1805.6749	903.3411	D	409.2082	205.1077	392.1816	196.5944	391.1976	196.1024	3
17	1970.7539	985.8806	1953.7273	977.3673	1952.7433	976.8753	F	294.1812	147.5942	277.1547	139.0810			2
18							K	147.1128	74.0600	130.0863	65.5468			1

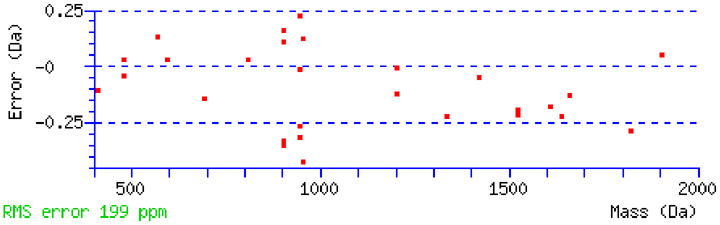

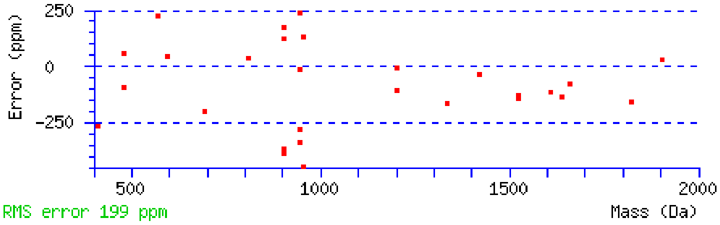

All matches to this query

Score	Mr(calc):	Delta	Sequence
44.6	2115.8521	1.0123	NVPQEESLEDSDVDADFK
30.7	2115.8521	1.0123	NVPQEESLEDSDVDADFK
4.7	2115.8694	0.9951	VLRADHAHLHSSTTGFK
2.8	2115.8608	1.0036	NDYFKGLMEGSAQYQER
2.6	2115.8486	1.0159	HTDAHSKIQEKSDMWK
2.5	2116.8544	0.0100	DTFGTSQTAVAHPTFYSK
2.3	2114.8768	1.9876	NDYFKGLMEGSAQYQER
1.4	2115.8746	0.9898	NTIYMRPVCLYLCPR
1.0	2116.8585	0.0060	GRGAQMKHSATSPHTTMK
1.0	2116.8585	0.0060	GRGAQMKHSATSPHTTMK

Spectrum No: 413; Query: 1545; Rank: 1

Peptide View

MS/MS Fragmentation of **GSLDSDNDDSDCPYSEK**
Found in **IPI00199985**, Tax_Id=10116 Gene_Symbol=Slc4a4 Isoform 1 of Electrogenic sodium bicarbonate cotransporter 1

Match to Query 1545: 1982.667268 from(992.340910,2+)
Title: 091008RatKidney_NH4Format01_27.942.942.2.dta
Data file K:\NewmanPaper\Piliang\3SubProteomes\Piliang3SP\mgf5ppm\ERLIC_3SubProteomes5ppm.mgf

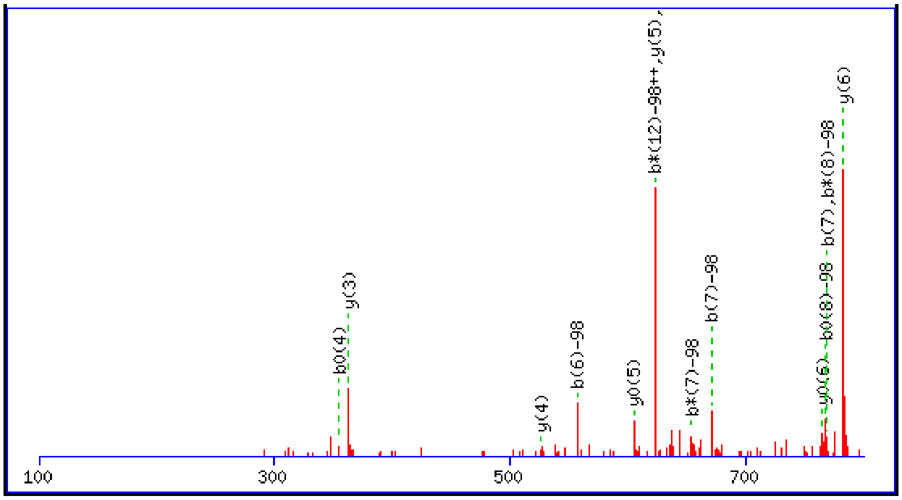

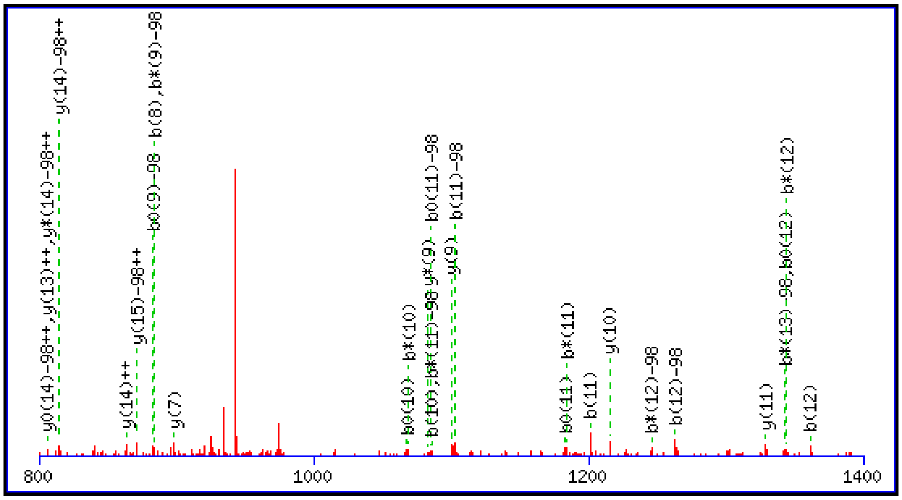

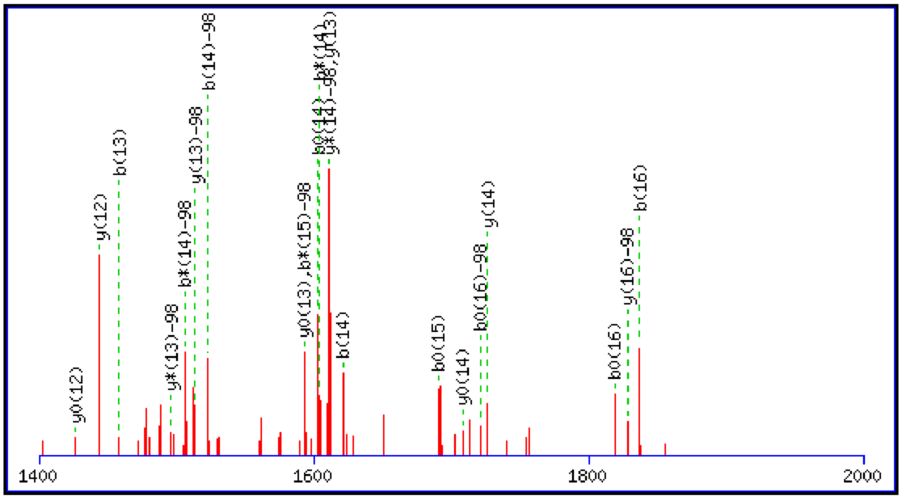

Monoisotopic mass of neutral peptide Mr(calc): 1982.6725
Fixed modifications: Carbamidomethyl (C)
Variable modifications:
S5 : Phospho (ST), with neutral losses 97.9769(shown in table), 0.0000
Ions Score: 45 **Expect:** 0.0021
Matches (Bold Red): 67/268 fragment ions using 115 most intense peaks

#	b	b++	b*	b*++	b0	b0++	Seq.	y	y++	y*	y*++	y0	y0++	#
1	58.0287	29.5180					G							17
2	145.0608	73.0340			127.0502	64.0287	S	**1828.6814**	914.8443	1811.6548	906.3311	1810.6708	905.8390	16
3	258.1448	129.5761			240.1343	120.5708	L	1741.6494	**871.3283**	1724.6228	862.8150	1723.6388	862.3230	15
4	373.1718	187.0895			**355.1612**	178.0842	D	1628.5653	**814.7863**	**1611.5387**	**806.2730**	1610.5547	**805.7810**	14
5	442.1932	221.6003			424.1827	212.5950	S	**1513.5384**	757.2728	**1496.5118**	748.7595	1495.5278	748.2675	13

**6**	**557.2202**	279.1137			539.2096	270.1084	**D**	**1444.5169**	722.7621	1427.4903	714.2488	**1426.5063**	713.7568	**12**
**7**	**671.2631**	336.1352	**654.2366**	327.6219	653.2525	327.1299	**N**	**1329.4900**	665.2486	1312.4634	656.7353	1311.4794	656.2433	**11**
**8**	786.2900	393.6487	**769.2635**	385.1354	**768.2795**	384.6434	**D**	**1215.4470**	608.2272	1198.4205	599.7139	1197.4365	599.2219	**10**
**9**	901.3170	451.1621	**884.2904**	442.6489	**883.3064**	442.1568	**D**	**1100.4201**	550.7137	**1083.3935**	542.2004	1082.4095	541.7084	**9**
**10**	988.3490	494.6781	971.3225	486.1649	970.3384	485.6729	**S**	985.3931	493.2002	968.3666	484.6869	967.3826	484.1949	**8**
**11**	**1103.3760**	552.1916	**1086.3494**	543.6783	**1085.3654**	543.1863	**D**	**898.3611**	449.6842	881.3346	441.1709	880.3505	440.6789	**7**
**12**	**1263.4066**	632.2069	**1246.3801**	**623.6937**	1245.3960	**623.2017**	**C**	**783.3342**	392.1707	766.3076	383.6574	**765.3236**	383.1654	**6**
**13**	1360.4594	680.7333	**1343.4328**	672.2200	1342.4488	671.7280	**P**	**623.3035**	312.1554	606.2770	303.6421	**605.2930**	303.1501	**5**
**14**	**1523.5227**	762.2650	**1506.4962**	753.7517	1505.5121	753.2597	**Y**	**526.2508**	263.6290	509.2242	255.1157	508.2402	254.6237	**4**
**15**	1610.5547	**805.7810**	**1593.5282**	797.2677	1592.5442	796.7757	**S**	**363.1874**	182.0974	346.1609	173.5841	345.1769	173.0921	**3**
**16**	1739.5973	870.3023	1722.5708	861.7890	**1721.5868**	861.2970	**E**	276.1554	138.5813	259.1288	130.0681	258.1448	129.5761	**2**
**17**							**K**	147.1128	74.0600	130.0863	65.5468			**1**

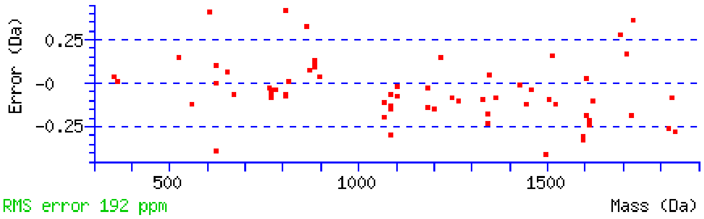

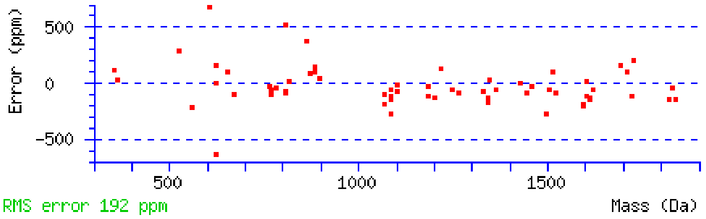

All matches to this query

Score	Mr(calc):	Delta	Sequence
44.7	1982.6725	-0.0052	GSLDSDNDDSDCPYSEK
35.1	1982.6725	-0.0052	GSLDSDNDDSDCPYSEK
13.8	1982.6725	-0.0052	GSLDSDNDDSDCPYSEK
2.8	1982.6794	-0.0121	TTGFGLIYDSLDYAK
2.8	1982.6794	-0.0121	TTGFGLIYDSLDYAK
2.7	1982.6794	-0.0121	TTGFGLIYDSLDYAK
1.9	1981.6488	1.0184	ETSSPGTDLSKNXAMK
1.7	1982.6714	-0.0041	TENTSGERGGVDIVTK
1.4	1982.6818	-0.0145	GNGSCRAKEXFSITR
1.3	1982.6686	-0.0014	TSRRSDEERPSTSR

Spectrum No: 414; Query: 1586; Rank: 1

Peptide View

MS/MS Fragmentation of **STPGVSDDSDQCDSTQDI**
Found in **IPI00454529**, Tax_Id=10116 Gene_Symbol=Dtnbp1 Dysbindin

Match to Query 1586: 2005.712068 from(1003.863310,2+)
Title: 100101RatKid_NS_deglyco_28.1515.1515.2.dta
Data file K:\NewmanPaper\Piliang\3SubProteomes\Piliang3SP\mgf5ppm\ERLIC_3SubProteomes5ppm.mgf

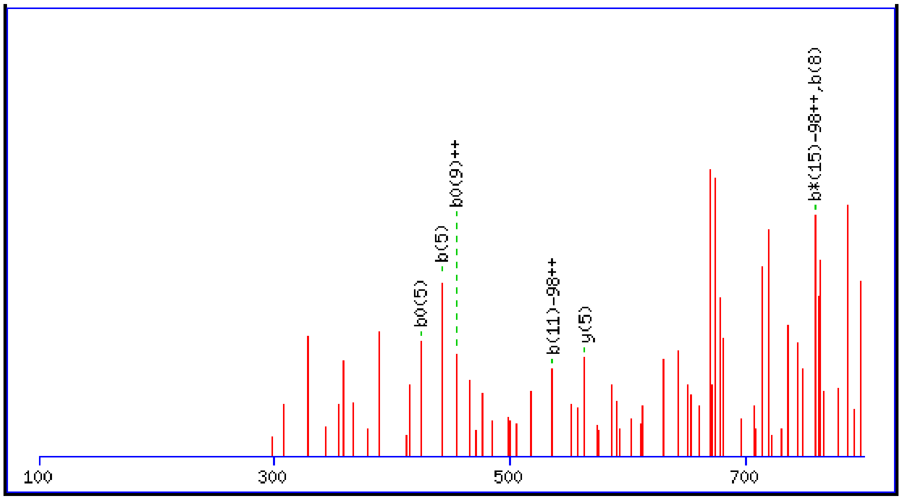

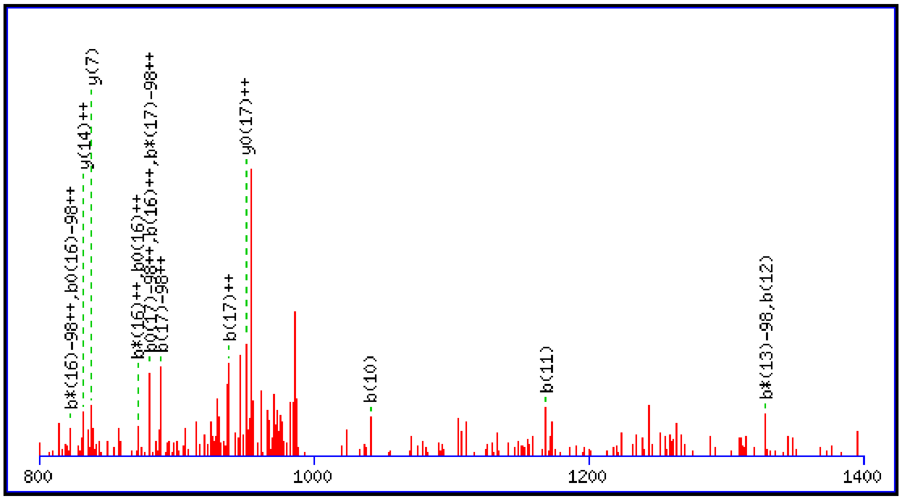

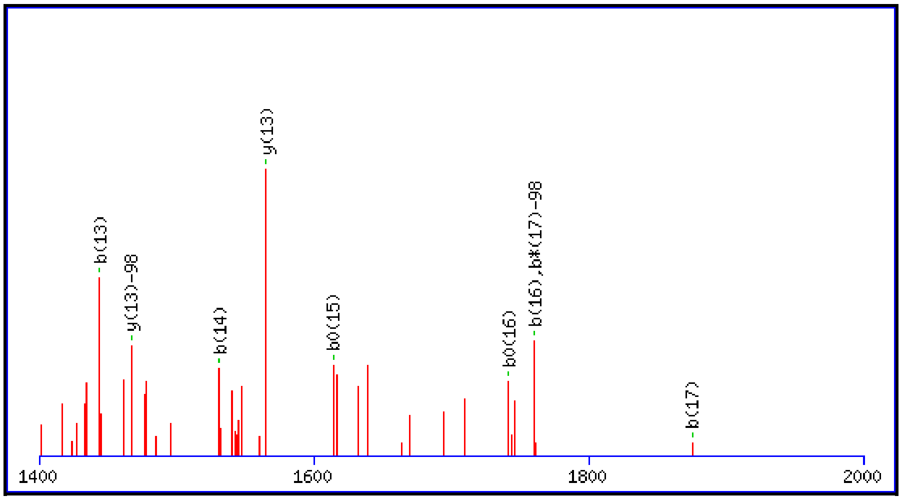

Monoisotopic mass of neutral peptide Mr(calc): 2005.7096
Fixed modifications: Carbamidomethyl (C)
Variable modifications:
S9 : Phospho (ST), with neutral losses 0.0000(shown in table), 97.9769
Ions Score: 45 **Expect:** 0.0028
Matches (Bold Red): 32/276 fragment ions using 46 most intense peaks

#	b	b^{++}	b*	b^{*++}	b^0	b^{0++}	Seq.	y	y^{++}	y*	y^{*++}	y^0	y^{0++}	#
1	88.0393	44.5233			70.0287	35.5180	S							18
2	189.0870	95.0471			171.0764	86.0418	T	1919.6848	960.3461	1902.6583	951.8328	1901.6743	**951.3408**	17
3	286.1397	143.5735			268.1292	134.5682	P	1818.6372	909.8222	1801.6106	901.3089	1800.6266	900.8169	16
4	343.1612	172.0842			325.1506	163.0790	G	1721.5844	861.2958	1704.5578	852.7826	1703.5738	852.2906	15
5	**442.2296**	221.6185			**424.2191**	212.6132	V	1664.5629	**832.7851**	1647.5364	824.2718	1646.5524	823.7798	14

**6**	529.2617	265.1345			511.2511	256.1292	**S**	**1565.4945**	783.2509	1548.4680	774.7376	1547.4839	774.2456	**13**
**7**	644.2886	322.6479			626.2780	313.6427	**D**	1478.4625	739.7349	1461.4359	731.2216	1460.4519	730.7296	**12**
**8**	**759.3155**	380.1614			741.3050	371.1561	**D**	1363.4355	682.2214	1346.4090	673.7081	1345.4250	673.2161	**11**
**9**	926.3139	463.6606			908.3033	**454.6553**	**S**	1248.4086	624.7079	1231.3820	616.1947	1230.3980	615.7027	**10**
**10**	**1041.3408**	521.1741			1023.3303	512.1688	**D**	1081.4102	541.2088	1064.3837	532.6955	1063.3997	532.2035	**9**
**11**	**1169.3994**	585.2033	1152.3729	576.6901	1151.3888	576.1981	**Q**	966.3833	483.6953	949.3568	475.1820	948.3727	474.6900	**8**
**12**	**1329.4301**	665.2187	1312.4035	656.7054	1311.4195	656.2134	**C**	**838.3247**	419.6660	821.2982	411.1527	820.3142	410.6607	**7**
**13**	**1444.4570**	722.7321	1427.4305	714.2189	1426.4464	713.7269	**D**	678.2941	339.6507	661.2675	331.1374	660.2835	330.6454	**6**
**14**	**1531.4890**	766.2482	1514.4625	757.7349	1513.4785	757.2429	**S**	**563.2671**	282.1372	546.2406	273.6239	545.2566	273.1319	**5**
**15**	1632.5367	816.7720	1615.5102	808.2587	**1614.5261**	807.7667	**T**	476.2351	238.6212	459.2086	230.1079	458.2245	229.6159	**4**
**16**	**1760.5953**	**880.8013**	1743.5687	**872.2880**	**1742.5847**	**871.7960**	**Q**	375.1874	188.0974	358.1609	179.5841	357.1769	179.0921	**3**
**17**	**1875.6222**	**938.3148**	1858.5957	929.8015	1857.6117	929.3095	**D**	247.1288	124.0681			229.1183	115.0628	**2**
**18**							**I**	132.1019	66.5546					**1**

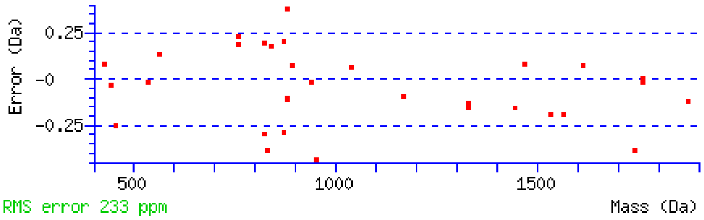

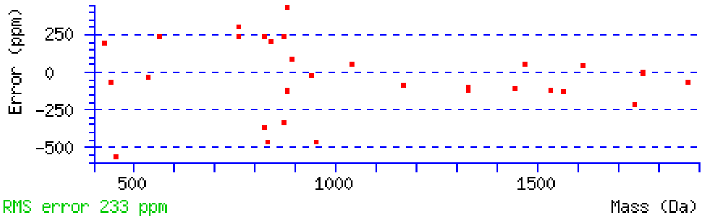

All matches to this query

Score	Mr(calc):	Delta	Sequence
44.7	2005.7096	0.0024	STPGVSDDSDQCDSTQDI
36.2	2005.7096	0.0024	STPGVSDDSDQCDSTQDI
19.8	2005.7096	0.0024	STPGVSDDSDQCDSTQDI
19.8	2005.7096	0.0024	STPGVSDDSDQCDSTQDI
12.1	2005.7096	0.0024	STPGVSDDSDQCDSTQDI
7.0	2005.7096	0.0024	STPGVSDDSDQCDSTQDI

Spectrum No: 415; Query: 2486; Rank: 1

Peptide View

MS/MS Fragmentation of **EELEQQTDGDCDEEDDDKDGEMPK**
Found in **IPI00382218**, Tax_Id=10116 Gene_Symbol=Tloc1 Ab2-292

Match to Query 2486: 2906.036022 from(969.685950,3+)
Title: 100101RatKid_NS_deglyco_25.1240.1240.3.dta
Data file K:\NewmanPaper\Piliang\3SubProteomes\Piliang3SP\mgf5ppm\ERLIC_3SubProteomes5ppm.mgf

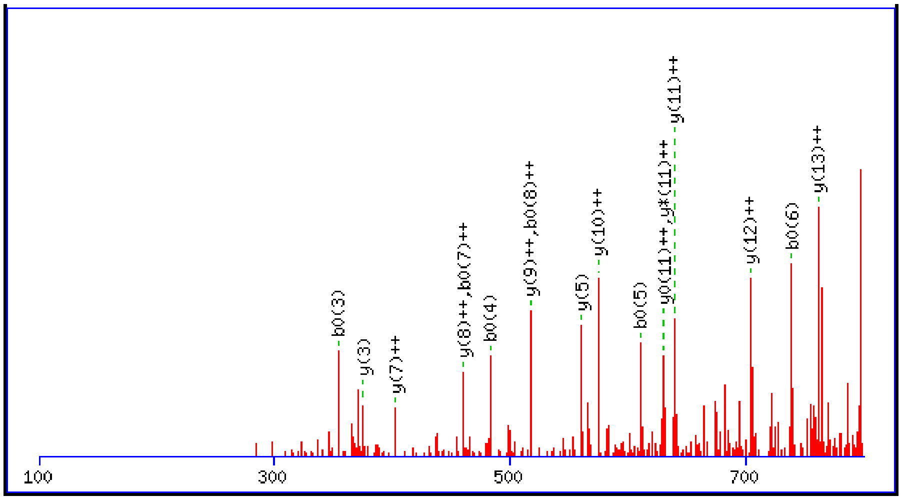

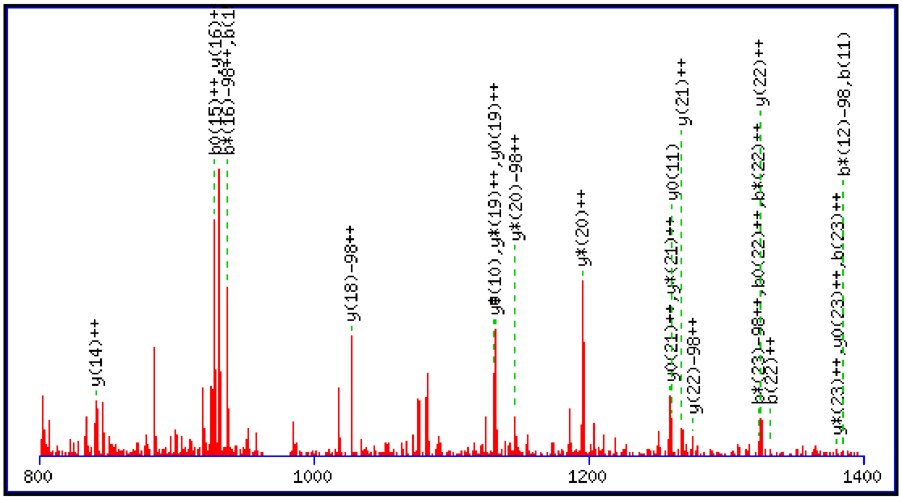

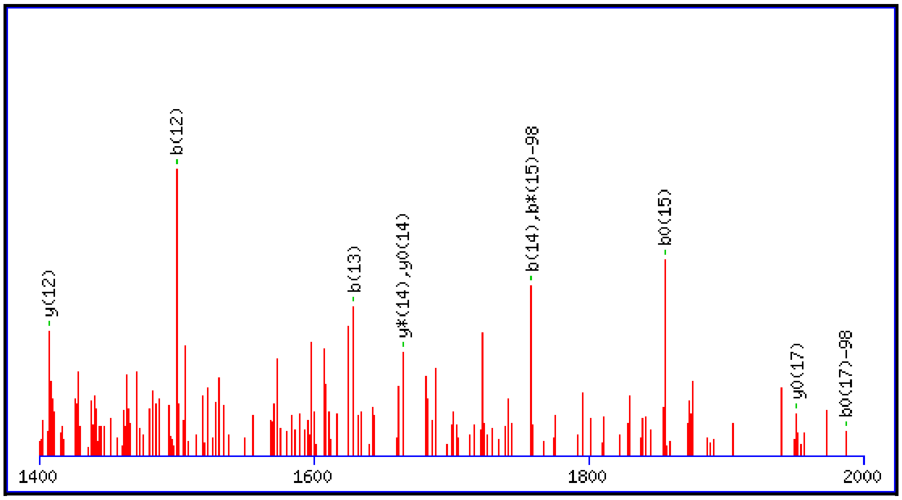

Monoisotopic mass of neutral peptide Mr(calc): 2906.0267
Fixed modifications: Carbamidomethyl (C)
Variable modifications:
T7 : Phospho (ST), with neutral losses 0.0000(shown in table), 97.9769
Ions Score: 45 **Expect:** 0.0052
Matches (Bold Red): 55/400 fragment ions using 67 most intense peaks

#	b	b^{++}	b*	b^{*++}	b^0	b^{0++}	Seq.	y	y^{++}	y*	y^{*++}	y^0	y^{0++}	#
1	130.0499	65.5286			112.0393	56.5233	E							24
2	259.0925	130.0499			241.0819	121.0446	E	2777.9914	1389.4993	2760.9648	**1380.9861**	2759.9808	**1380.4940**	23
3	372.1765	186.5919			**354.1660**	177.5866	L	2648.9488	**1324.9780**	2631.9222	1316.4648	2630.9382	1315.9727	22
4	501.2191	251.1132			**483.2086**	242.1079	E	2535.8647	**1268.4360**	2518.8382	**1259.9227**	2517.8542	**1259.4307**	21
5	629.2777	315.1425	612.2511	306.6292	**611.2671**	306.1372	Q	2406.8221	1203.9147	2389.7956	**1195.4014**	2388.8116	1194.9094	20

6	757.3363	379.1718	740.3097	370.6585	**739.3257**	370.1665	Q	2278.7636	1139.8854	2261.7370	**1131.3721**	2260.7530	**1130.8801**	19
7	938.3503	469.6788	921.3237	461.1655	920.3397	**460.6735**	T	2150.7050	1075.8561	2133.6784	1067.3429	2132.6944	1066.8508	18
8	1053.3772	527.1922	1036.3507	518.6790	1035.3667	**518.1870**	D	1969.6910	985.3491	1952.6644	976.8359	**1951.6804**	976.3438	17
9	1110.3987	555.7030	1093.3721	547.1897	1092.3881	546.6977	G	1854.6640	**927.8357**	1837.6375	919.3224	1836.6535	918.8304	16
10	1225.4256	613.2165	1208.3991	604.7032	1207.4151	604.2112	D	1797.6426	899.3249	1780.6160	890.8116	1779.6320	890.3196	15
11	**1385.4563**	693.2318	1368.4297	684.7185	1367.4457	684.2265	C	1682.6156	**841.8114**	**1665.5891**	833.2982	**1664.6051**	832.8062	14
12	**1500.4832**	750.7452	1483.4567	742.2320	1482.4727	741.7400	D	1522.5850	**761.7961**	1505.5584	753.2829	1504.5744	752.7908	13
13	**1629.5258**	815.2665	1612.4993	806.7533	1611.5152	806.2613	E	**1407.5580**	**704.2827**	1390.5315	695.7694	1389.5475	695.2774	12
14	**1758.5684**	879.7878	1741.5419	871.2746	1740.5578	870.7826	E	1278.5154	**639.7614**	1261.4889	**631.2481**	**1260.5049**	**630.7561**	11
15	1873.5953	**937.3013**	1856.5688	928.7880	**1855.5848**	**928.2960**	D	1149.4728	**575.2401**	**1132.4463**	566.7268	**1131.4623**	566.2348	10
16	1988.6223	994.8148	1971.5957	986.3015	1970.6117	985.8095	D	1034.4459	**517.7266**	1017.4194	509.2133	1016.4353	508.7213	9
17	2103.6492	1052.3283	2086.6227	1043.8150	2085.6387	1043.3230	D	919.4190	**460.2131**	902.3924	451.6998	901.4084	451.2078	8
18	2231.7442	1116.3757	2214.7176	1107.8625	2213.7336	1107.3705	K	804.3920	**402.6996**	787.3655	394.1864	786.3815	393.6944	7
19	2346.7711	1173.8892	2329.7446	1165.3759	2328.7606	1164.8839	D	676.2971	338.6522	659.2705	330.1389	658.2865	329.6469	6
20	2403.7926	1202.3999	2386.7661	1193.8867	2385.7820	1193.3947	G	**561.2701**	281.1387	544.2436	272.6254	543.2595	272.1334	5
21	2532.8352	1266.9212	2515.8086	1258.4080	2514.8246	1257.9160	E	504.2486	252.6280	487.2221	244.1147	486.2381	243.6227	4
22	2663.8757	**1332.4415**	2646.8491	**1323.9282**	2645.8651	**1323.4362**	M	**375.2061**	188.1067	358.1795	179.5934			3
23	2760.9284	**1380.9679**	2743.9019	1372.4546	2742.9179	1371.9626	P	244.1656	122.5864	227.1390	114.0731			2
24							K	147.1128	74.0600	130.0863	65.5468			1

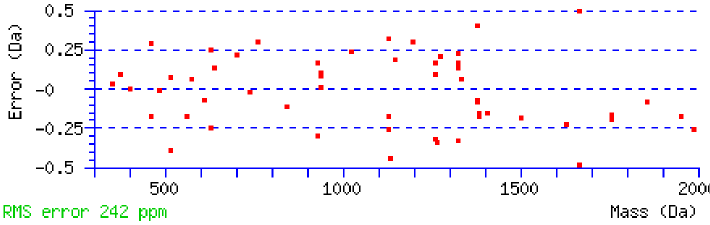

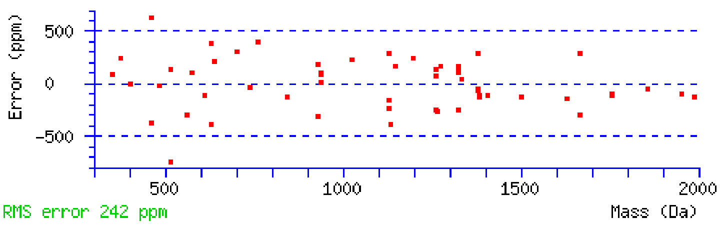

All matches to this query

Score	Mr(calc):	Delta	Sequence
44.7	2906.0267	0.0093	EELEQQTDGDCDEEDDDKDGEMPK

Spectrum No: 416; Query: 2511; Rank: 1

Peptide View

MS/MS Fragmentation of **IQQQAGAVPPSQEEDSQEEEEEASSR**
Found in **IPI00197321**, Tax_Id=10116 Gene_Symbol=RGD1565452_predicted hypothetical protein LOC500578

Match to Query 2511: 2937.208242 from(980.076690,3+)
Title: 091008RatKidney_NoSalt_28.1126.1126.3.dta
Data file K:\NewmanPaper\Piliang\3SubProteomes\Piliang3SP\mgf5ppm\ERLIC_3SubProteomes5ppm.mgf

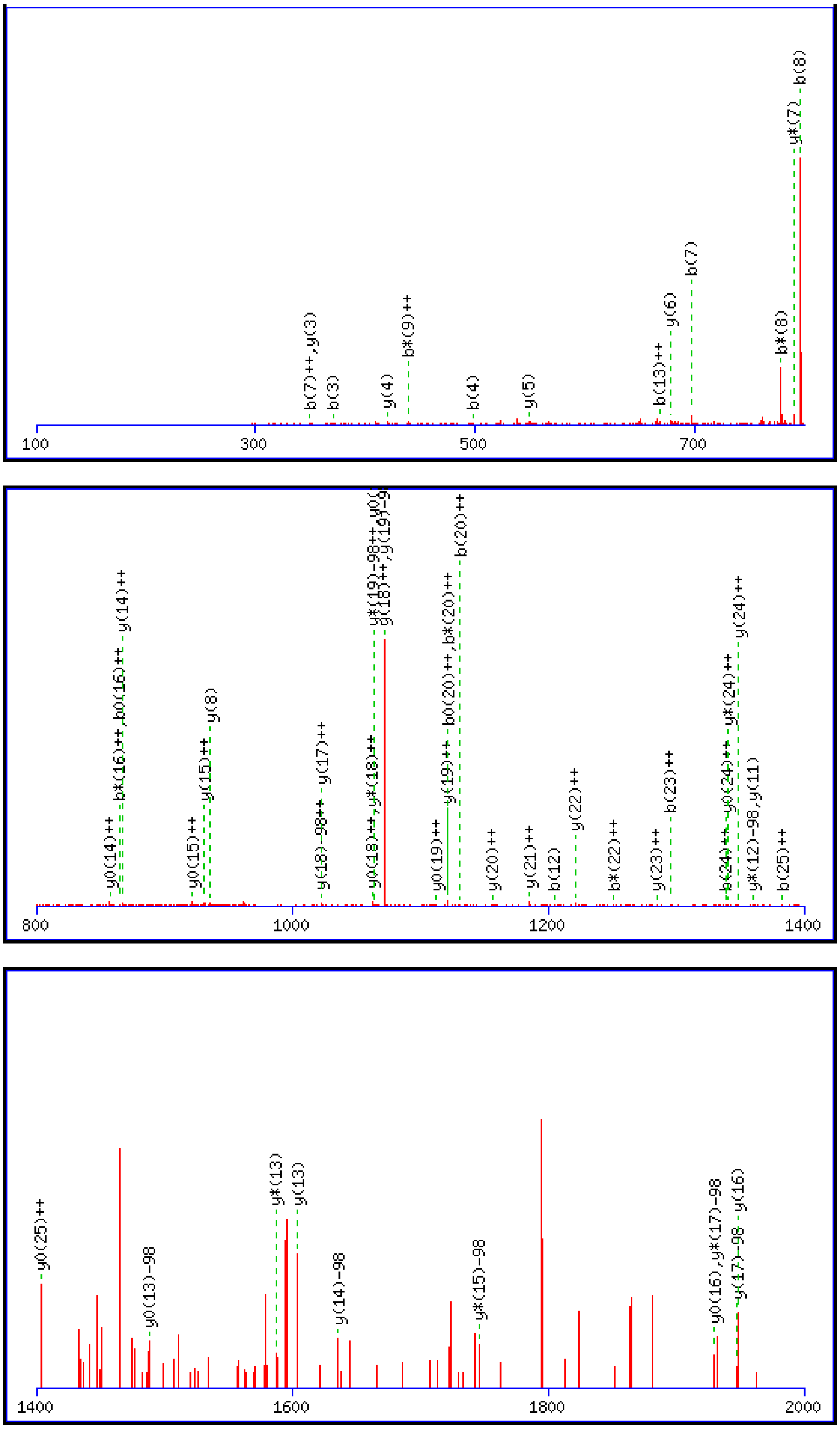

Monoisotopic mass of neutral peptide Mr(calc): 2937.2149
Fixed modifications: Carbamidomethyl (C)
Variable modifications:
S16 : Phospho (ST), with neutral losses 0.0000(shown in table), 97.9769
Ions Score: 45 **Expect:** 0.012
Matches (Bold Red): 57/426 fragment ions using 119 most intense peaks

#	b	b++	b*	b*++	b0	b0++	Seq.	y	y++	y*	y*++	y0	y0++	#
1	114.0913	57.5493					I							26
2	242.1499	121.5786	225.1234	113.0653			Q	2825.1381	1413.0727	2808.1116	1404.5594	2807.1276	**1404.0674**	25
3	**370.2085**	185.6079	353.1819	177.0946			Q	2697.0795	**1349.0434**	2680.0530	**1340.5301**	2679.0690	**1340.0381**	24
4	**498.2671**	249.6372	481.2405	241.1239			Q	2569.0210	**1285.0141**	2551.9944	1276.5008	2551.0104	1276.0088	23
5	569.3042	285.1557	552.2776	276.6425			A	2440.9624	**1220.9848**	2423.9358	1212.4716	2422.9518	1211.9795	22

6	626.3257	313.6665	609.2991	305.1532			G	2369.9253	**1185.4663**	2352.8987	1176.9530	2351.9147	1176.4610	21
7	**697.3628**	**349.1850**	680.3362	340.6717			A	2312.9038	**1156.9555**	2295.8773	1148.4423	2294.8932	1147.9503	20
8	**796.4312**	398.7192	**779.4046**	390.2060			V	2241.8667	**1121.4370**	2224.8401	1112.9237	2223.8561	**1112.4317**	19
9	893.4839	447.2456	876.4574	**438.7323**			P	2142.7983	**1071.9028**	2125.7717	**1063.3895**	2124.7877	**1062.8975**	18
10	990.5367	495.7720	973.5102	487.2587			P	2045.7455	**1023.3764**	2028.7190	1014.8631	2027.7350	1014.3711	17
11	1077.5687	539.2880	1060.5422	530.7747	1059.5582	530.2827	S	**1948.6928**	974.8500	1931.6662	966.3367	**1930.6822**	965.8447	16
12	**1205.6273**	603.3173	1188.6008	594.8040	1187.6167	594.3120	Q	1861.6607	**931.3340**	1844.6342	922.8207	1843.6502	**922.3287**	15
13	1334.6699	**667.8386**	1317.6434	659.3253	1316.6593	658.8333	E	1733.6021	**867.3047**	1716.5756	858.7914	1715.5916	**858.2994**	14
14	1463.7125	732.3599	1446.6860	723.8466	1445.7019	723.3546	E	**1604.5596**	802.7834	**1587.5330**	794.2701	1586.5490	793.7781	13
15	1578.7394	789.8734	1561.7129	781.3601	1560.7289	780.8681	D	1475.5170	738.2621	1458.4904	729.7488	1457.5064	729.2568	12
16	1745.7378	873.3725	1728.7112	**864.8593**	1727.7272	**864.3673**	S	**1360.4900**	680.7486	1343.4635	672.2354	1342.4795	671.7434	11
17	1873.7964	937.4018	1856.7698	928.8886	1855.7858	928.3965	Q	1193.4917	597.2495	1176.4651	588.7362	1175.4811	588.2442	10
18	2002.8390	1001.9231	1985.8124	993.4098	1984.8284	992.9178	E	1065.4331	533.2202	1048.4065	524.7069	1047.4225	524.2149	9
19	2131.8816	1066.4444	2114.8550	1057.9311	2113.8710	1057.4391	E	**936.3905**	468.6989	919.3639	460.1856	918.3799	459.6936	8
20	2260.9242	**1130.9657**	2243.8976	**1122.4524**	2242.9136	**1121.9604**	E	807.3479	404.1776	**790.3214**	395.6643	789.3373	395.1723	7
21	2389.9667	1195.4870	2372.9402	1186.9737	2371.9562	1186.4817	E	**678.3053**	339.6563	661.2788	331.1430	660.2947	330.6510	6
22	2519.0093	1260.0083	2501.9828	**1251.4950**	2500.9988	1251.0030	E	**549.2627**	275.1350	532.2362	266.6217	531.2522	266.1297	5
23	2590.0465	**1295.5269**	2573.0199	1287.0136	2572.0359	1286.5216	A	**420.2201**	210.6137	403.1936	202.1004	402.2096	201.6084	4
24	2677.0785	**1339.0429**	2660.0519	1330.5296	2659.0679	1330.0376	S	**349.1830**	175.0951	332.1565	166.5819	331.1724	166.0899	3
25	2764.1105	**1382.5589**	2747.0840	1374.0456	2746.0999	1373.5536	S	262.1510	131.5791	245.1244	123.0659	244.1404	122.5738	2
26							R	175.1190	88.0631	158.0924	79.5498			1

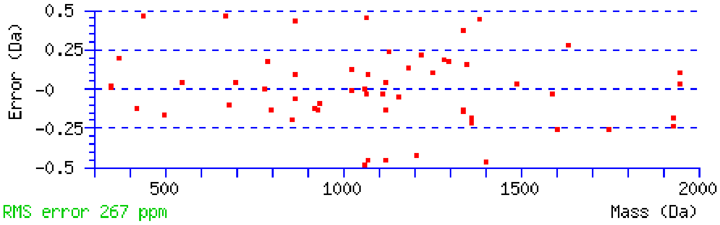

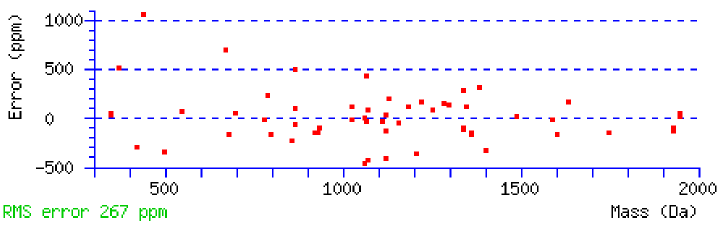

All matches to this query

Score	Mr(calc):	Delta	Sequence
44.6	2937.2149	-0.0066	IQQQAGAVPPSQEEDSQEEEEEASSR
29.7	2937.2149	-0.0066	IQQQAGAVPPSQEEDSQEEEEEASSR
20.3	2937.2149	-0.0066	IQQQAGAVPPSQEEDSQEEEEEASSR
18.1	2937.2149	-0.0066	IQQQAGAVPPSQEEDSQEEEEEASSR
5.5	2935.1976	2.0106	GGYMDDGGYSMNFNMSSSRGPLPVKR
5.5	2935.2276	1.9807	WIISDGPVDALWIENMNTVLDDNK
5.5	2935.2276	1.9807	WIISDGPVDALWIENMNTVLDDNK
5.5	2935.2276	1.9807	WIISDGPVDALWIENMNTVLDDNK
5.0	2935.1976	2.0106	GGYMDDGGYSMNFNMSSSRGPLPVKR
5.0	2936.1816	1.0266	GGYMDDGGYSMNFNMSSSRGPLPVKR

Spectrum No: 417; Query: 797; Rank: 1

Peptide View

MS/MS Fragmentation of **QGDNISDDEDEVR**
Found in **IPI00204739**, Tax_Id=10116 Gene_Symbol=Stx4a Syntaxin-4

Match to Query 797: 1570.579908 from(786.297230,2+)

Title: 100101RatKid_NS_deglyco_25.1166.1166.2.dta
Data file K:\NewmanPaper\Piliang\3SubProteomes\Piliang3SP\mgf5ppm\ERLIC_3SubProteomes5ppm.mgf

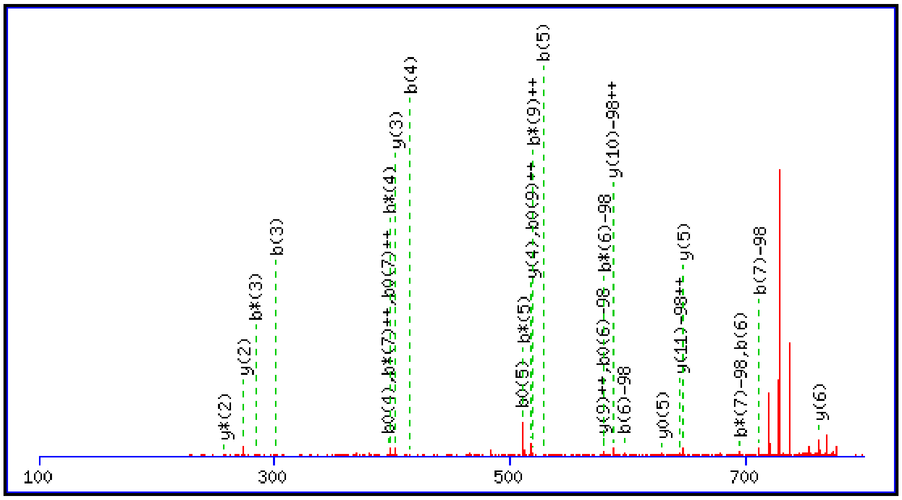

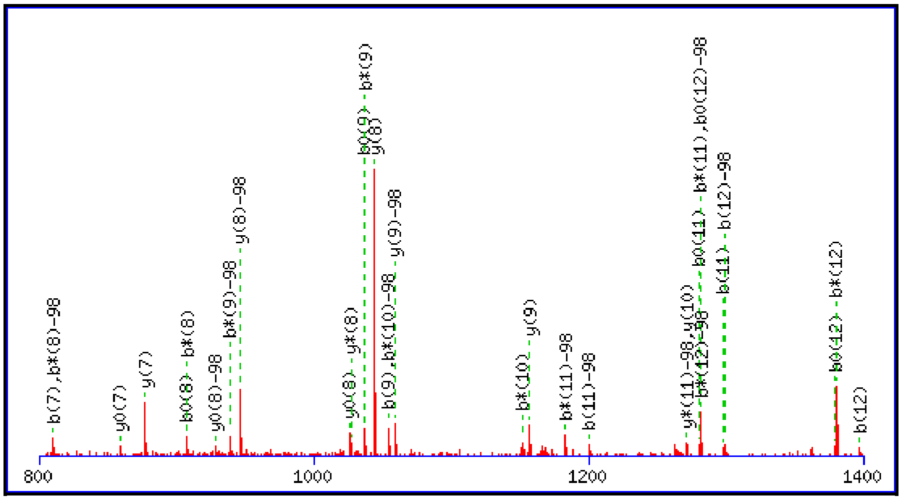

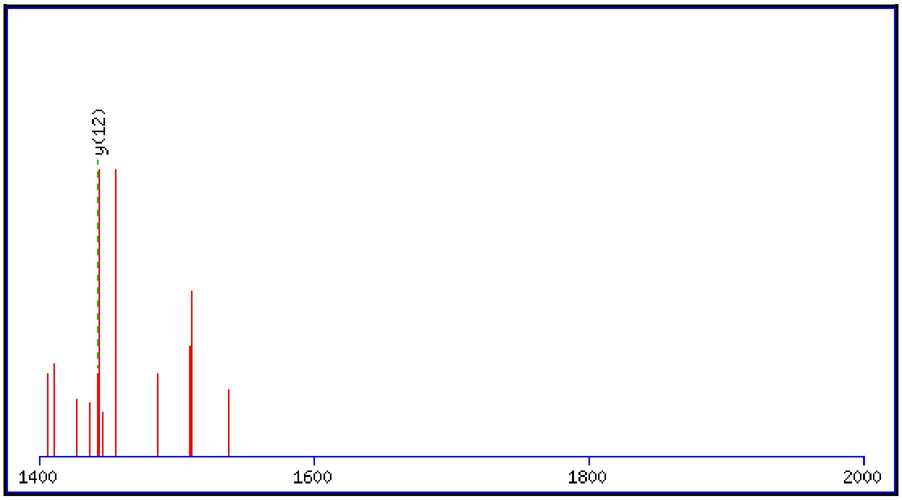

Monoisotopic mass of neutral peptide Mr(calc): 1570.5784
Fixed modifications: Carbamidomethyl (C)
Variable modifications:
S6 : Phospho (ST), with neutral losses 0.0000(shown in table), 97.9769
Ions Score: 45 **Expect:** 0.0029
Matches (Bold Red): 61/208 fragment ions using 94 most intense peaks

#	b	b^{++}	b*	b^{*++}	b^0	b^{0++}	Seq.	y	y^{++}	y*	y^{*++}	y^0	y^{0++}	#
1	129.0659	65.0366	112.0393	56.5233			Q							13
2	186.0873	93.5473	169.0608	85.0340			G	**1443.5271**	722.2672	1426.5006	713.7539	1425.5166	713.2619	12
3	**301.1143**	151.0608	**284.0877**	142.5475	283.1037	142.0555	D	1386.5057	693.7565	1369.4791	685.2432	1368.4951	684.7512	11

4	415.1572	208.0822	398.1306	199.5690	397.1466	199.0769	N	1271.4787	636.2430	1254.4522	627.7297	1253.4682	627.2377	10
5	528.2413	264.6243	511.2147	256.1110	510.2307	255.6190	I	1157.4358	579.2215	1140.4092	570.7083	1139.4252	570.2163	9
6	695.2396	348.1234	678.2131	339.6102	677.2290	339.1182	S	1044.3517	522.6795	1027.3252	514.1662	1026.3412	513.6742	8
7	810.2665	405.6369	793.2400	397.1236	792.2560	396.6316	D	877.3534	439.1803	860.3268	430.6671	859.3428	430.1750	7
8	925.2935	463.1504	908.2669	454.6371	907.2829	454.1451	D	762.3264	381.6669	745.2999	373.1536	744.3159	372.6616	6
9	1054.3361	527.6717	1037.3095	519.1584	1036.3255	518.6664	E	647.2995	324.1534	630.2729	315.6401	629.2889	315.1481	5
10	1169.3630	585.1852	1152.3365	576.6719	1151.3525	576.1799	D	518.2569	259.6321	501.2304	251.1188	500.2463	250.6268	4
11	1298.4056	649.7064	1281.3791	641.1932	1280.3951	640.7012	E	403.2300	202.1186	386.2034	193.6053	385.2194	193.1133	3
12	1397.4740	699.2407	1380.4475	690.7274	1379.4635	690.2354	V	274.1874	137.5973	257.1608	129.0840			2
13							R	175.1190	88.0631	158.0924	79.5498			1

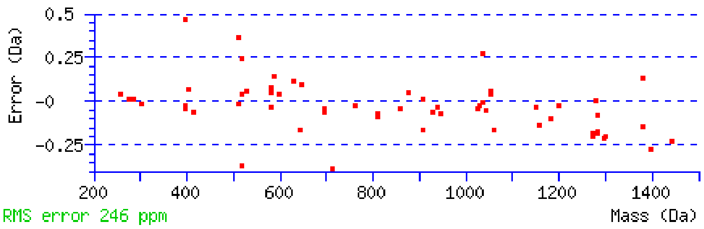

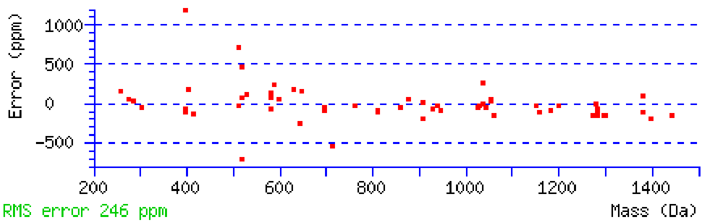

All matches to this query

Score	Mr(calc):	Delta	Sequence
44.6	1570.5784	0.0015	QGDNISDDEDEVR
3.5	1570.5776	0.0023	GVDSGLYLGMNER
3.3	1569.5862	0.9937	GFSTVGDTEGERR
3.3	1569.5862	0.9937	GFSTVGDTEGERR
3.2	1570.5697	0.0102	SILTLSPMDSSTC
3.2	1570.5697	0.0102	SILTLSPMDSSTC
3.2	1570.5697	0.0102	SILTLSPMDSSTC
1.4	1568.5644	2.0155	ITNSESGEKNGGSK
1.2	1568.5748	2.0051	MSVNINHSISVK
0.6	1570.5912	-0.0113	SWEEPNWMGSPR

Spectrum No: 418; Query: 2441; Rank: 1

Peptide View

MS/MS Fragmentation of **KLPSESDLLEGEVTDEDEEAEMSR**
Found in **IPI00207181**, Tax_Id=10116 Gene_Symbol=Plcl1 130kDa-Ins(1,4,5)P3 binding protein

Match to Query 2441: 2804.168592 from(935.730140,3+)
Title: 100101RatKid_NS_deglyco_22.3157.3157.3.dta
Data file K:\NewmanPaper\Piliang\3SubProteomes\Piliang3SP\mgf5ppm\ERLIC_3SubProteomes5ppm.mgf

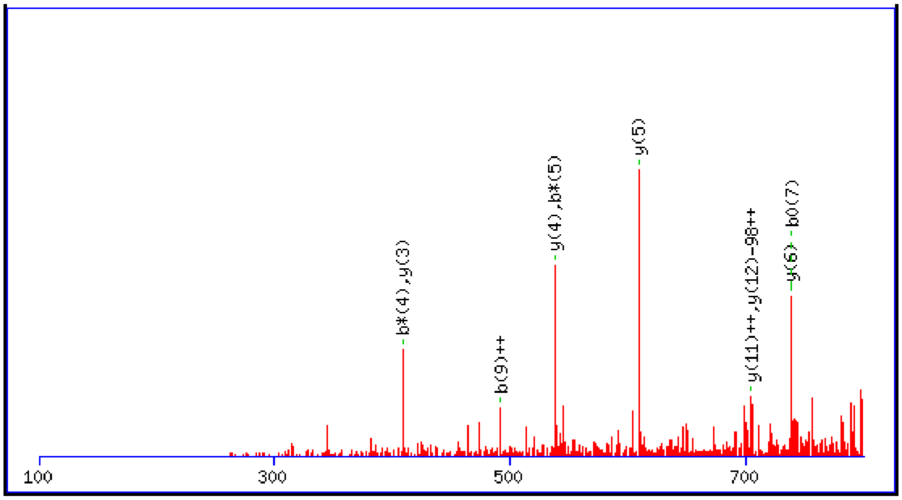

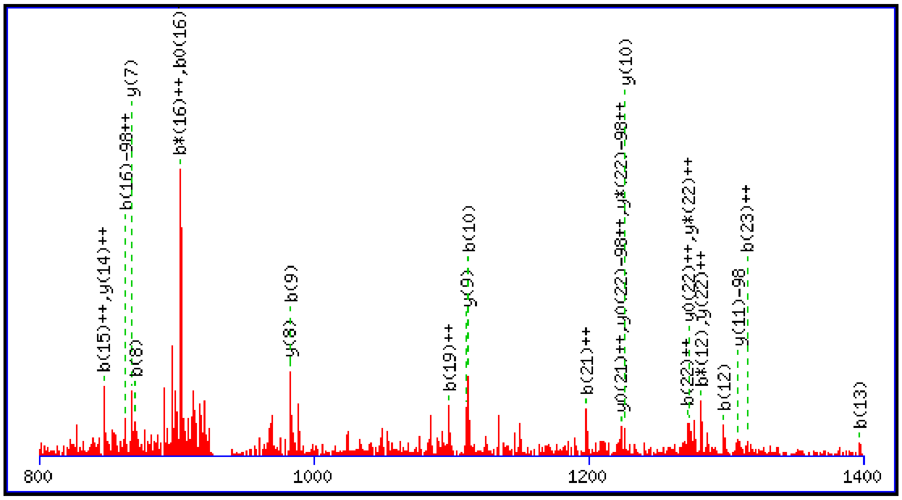

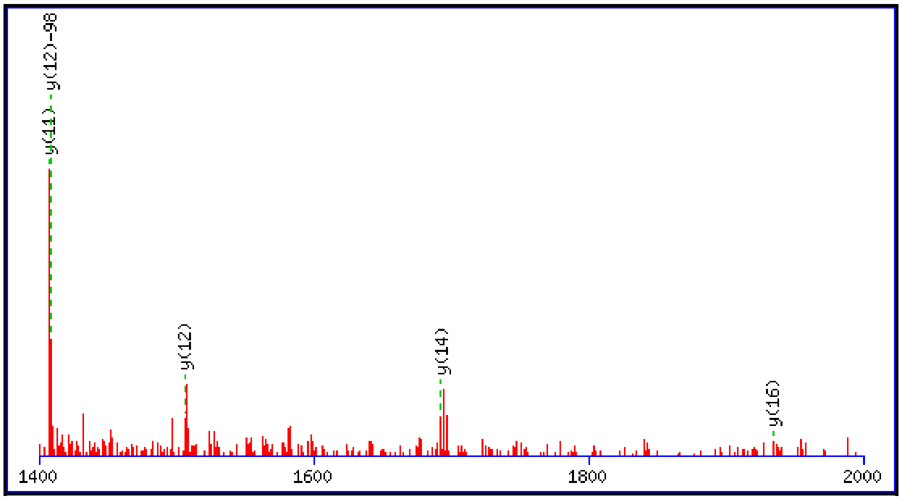

Monoisotopic mass of neutral peptide Mr(calc): 2803.1630
Fixed modifications: Carbamidomethyl (C)
Variable modifications:
T14 : Phospho (ST), with neutral losses 0.0000(shown in table), 97.9769
M22 : Oxidation (M)
Ions Score: 45 **Expect:** 0.012
Matches (Bold Red): 42/406 fragment ions using 72 most intense peaks

#	b	b^{++}	b*	b^{*++}	b^{0}	b^{0++}	Seq.	y	y^{++}	y*	y^{*++}	y^{0}	y^{0++}	#
1	129.1022	65.0548	112.0757	56.5415			K							24
2	242.1863	121.5968	225.1598	113.0835			L	2676.0754	1338.5413	2659.0488	1330.0280	2658.0648	1329.5360	23
3	339.2391	170.1232	322.2125	161.6099			P	2562.9913	**1281.9993**	2545.9648	**1273.4860**	2544.9807	**1272.9940**	22
4	426.2711	213.6392	**409.2445**	205.1259	408.2605	204.6339	S	2465.9385	1233.4729	2448.9120	1224.9596	2447.9280	**1224.4676**	21

5	555.3137	278.1605	**538.2871**	269.6472	537.3031	269.1552	E	2378.9065	1189.9569	2361.8800	1181.4436	2360.8960	1180.9516	20
6	642.3457	321.6765	625.3192	313.1632	624.3352	312.6712	S	2249.8639	1125.4356	2232.8374	1116.9223	2231.8534	1116.4303	19
7	757.3727	379.1900	740.3461	370.6767	**739.3621**	370.1847	D	2162.8319	1081.9196	2145.8053	1073.4063	2144.8213	1072.9143	18
8	**870.4567**	435.7320	853.4302	427.2187	852.4462	426.7267	L	2047.8050	1024.4061	2030.7784	1015.8928	2029.7944	1015.4008	17
9	**983.5408**	**492.2740**	966.5142	483.7608	965.5302	483.2687	L	**1934.7209**	967.8641	1917.6943	959.3508	1916.7103	958.8588	16
10	**1112.5834**	556.7953	1095.5568	548.2821	1094.5728	547.7900	E	1821.6368	911.3220	1804.6103	**902.8088**	1803.6263	902.3168	15
11	1169.6048	585.3061	1152.5783	576.7928	1151.5943	576.3008	G	**1692.5942**	**846.8008**	1675.5677	838.2875	1674.5837	837.7955	14
12	**1298.6474**	649.8274	**1281.6209**	641.3141	1280.6369	640.8221	E	1635.5728	818.2900	1618.5462	809.7767	1617.5622	809.2847	13
13	**1397.7159**	699.3616	1380.6893	690.8483	1379.7053	690.3563	V	**1506.5302**	753.7687	1489.5036	745.2555	1488.5196	744.7634	12
14	1578.7299	789.8686	1561.7033	781.3553	1560.7193	780.8633	T	**1407.4618**	**704.2345**	1390.4352	695.7212	1389.4512	695.2292	11
15	1693.7568	**847.3820**	1676.7302	838.8688	1675.7462	838.3768	D	**1226.4478**	613.7275	1209.4212	605.2142	1208.4372	604.7222	10
16	1822.7994	911.9033	1805.7728	**903.3901**	1804.7888	**902.8981**	E	**1111.4208**	556.2140	1094.3943	547.7008	1093.4102	547.2088	9
17	1937.8263	969.4168	1920.7998	960.9035	1919.8158	960.4115	D	**982.3782**	491.6927	965.3517	483.1795	964.3677	482.6875	8
18	2066.8689	1033.9381	2049.8424	1025.4248	2048.8584	1024.9328	E	**867.3513**	434.1793	850.3247	425.6660	849.3407	425.1740	7
19	2195.9115	**1098.4594**	2178.8850	1089.9461	2177.9010	1089.4541	E	**738.3087**	369.6580	721.2821	361.1447	720.2981	360.6527	6
20	2266.9486	1133.9780	2249.9221	1125.4647	2248.9381	1124.9727	A	**609.2661**	305.1367	592.2395	296.6234	591.2555	296.1314	5
21	2395.9912	**1198.4993**	2378.9647	1189.9860	2377.9807	1189.4940	E	**538.2290**	269.6181	521.2024	261.1049	520.2184	260.6128	4
22	2543.0266	**1272.0170**	2526.0001	1263.5037	2525.0161	1263.0117	M	**409.1864**	205.0968	392.1598	196.5836	391.1758	196.0915	3
23	2630.0587	**1315.5330**	2613.0321	1307.0197	2612.0481	1306.5277	S	262.1510	131.5791	245.1244	123.0659	244.1404	122.5738	2
24							R	175.1190	88.0631	158.0924	79.5498			1

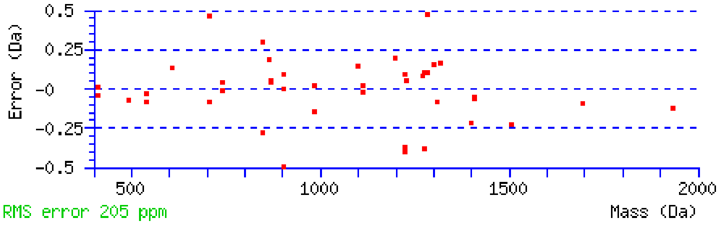

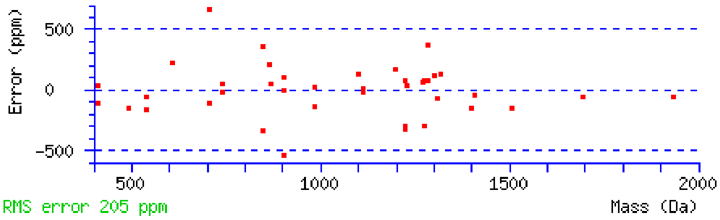

All matches to this query

Score	Mr(calc):	Delta	Sequence
44.5	2803.1630	1.0056	KLPSESDLLEGEVTDEDEEAEMSR
12.5	2803.1630	1.0056	KLPSESDLLEGEVTDEDEEAEMSR
12.5	2803.1630	1.0056	KLPSESDLLEGEVTDEDEEAEMSR
8.8	2803.1630	1.0056	KLPSESDLLEGEVTDEDEEAEMSR
4.3	2802.1746	1.9940	IEASPGHSVRSSAKDGLQENEESSK
1.1	2803.1792	0.9894	ALNQLSLTHGLSRTSMRPRSSR
0.3	2803.1557	1.0128	IGSGGWSPAVSNAHQWLQIDLGNR
0.0	2802.1382	2.0303	RSMISAPSADAPMFVMGVNHTSLK

Spectrum No: 419; Query: 1283; Rank: 1

Peptide View

MS/MS Fragmentation of **TSSKESSPVPSPTSDR**
Found in **IPI00555287**, Tax_Id=10116 Gene_Symbol=Spnb2 Non-erythroid spectrin beta

Match to Query 1283: 1820.720188 from(911.367370,2+)
Title: 091008RatKidney_NoSalt_27.516.516.2.dta
Data file K:\NewmanPaper\Piliang\3SubProteomes\Piliang3SP\mgf5ppm\ERLIC_3SubProteomes5ppm.mgf

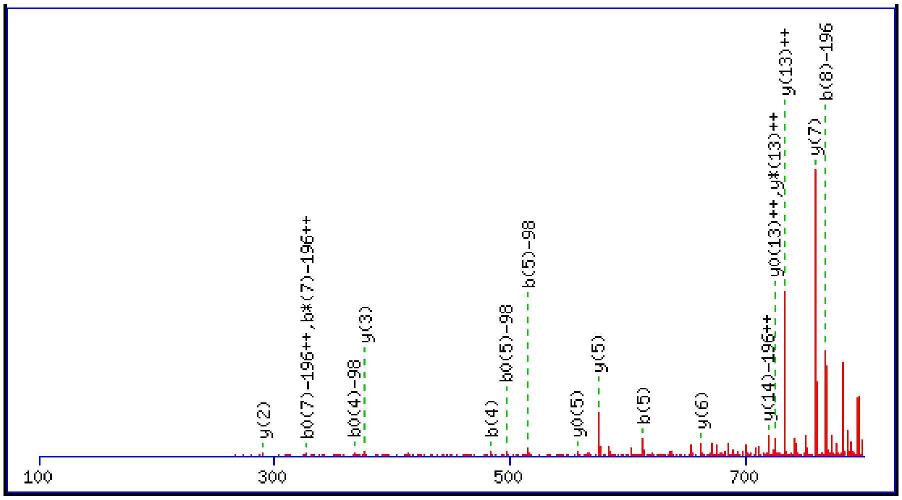

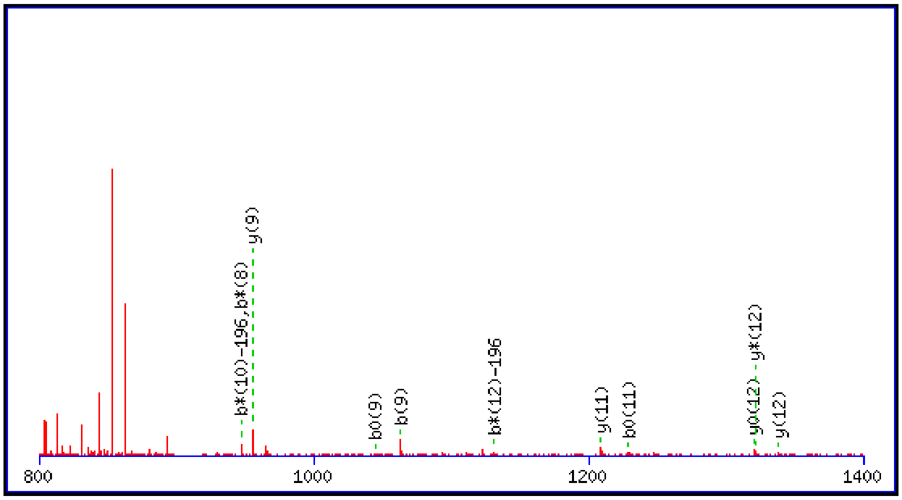

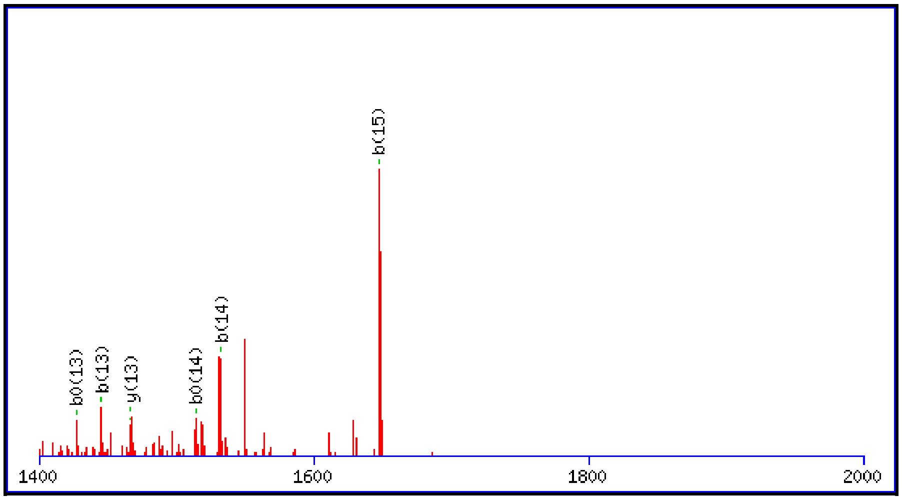

Monoisotopic mass of neutral peptide Mr(calc): 1820.7231
Fixed modifications: Carbamidomethyl (C)
Variable modifications:
S3 : Phospho (ST), with neutral losses 0.0000(shown in table), 97.9769
S6 : Phospho (ST), with neutral losses 0.0000(shown in table), 97.9769
Ions Score: 44 **Expect:** 0.0052
Matches (Bold Red): 35/278 fragment ions using 60 most intense peaks

#	b	b^{++}	b*	b^{*++}	b^0	b^{0++}	Seq.	y	y^{++}	y*	y^{*++}	y^0	y^{0++}	#
1	102.0550	51.5311			84.0444	42.5258	T							16
2	189.0870	95.0471			171.0764	86.0418	S	1720.6827	860.8450	1703.6561	852.3317	1702.6721	851.8397	15
3	356.0853	178.5463			338.0748	169.5410	S	1633.6506	817.3290	1616.6241	808.8157	1615.6401	808.3237	14
4	**484.1803**	242.5938	467.1537	234.0805	466.1697	233.5885	K	**1466.6523**	**733.8298**	1449.6257	**725.3165**	1448.6417	**724.8245**	13

**5**	**613.2229**	307.1151	596.1963	298.6018	595.2123	298.1098	**E**	**1338.5573**	669.7823	**1321.5308**	661.2690	**1320.5467**	660.7770	**12**
**6**	780.2212	390.6143	763.1947	382.1010	762.2107	381.6090	**S**	**1209.5147**	605.2610	1192.4882	596.7477	1191.5042	596.2557	**11**
**7**	867.2533	434.1303	850.2267	425.6170	849.2427	425.1250	**S**	1042.5164	521.7618	1025.4898	513.2485	1024.5058	512.7565	**10**
**8**	964.3060	482.6567	**947.2795**	474.1434	946.2955	473.6514	**P**	**955.4843**	478.2458	938.4578	469.7325	937.4738	469.2405	**9**
**9**	**1063.3744**	532.1909	1046.3479	523.6776	**1045.3639**	523.1856	**V**	858.4316	429.7194	841.4050	421.2061	840.4210	420.7141	**8**
**10**	1160.4272	580.7172	1143.4007	572.2040	1142.4166	571.7120	**P**	**759.3632**	380.1852	742.3366	371.6719	741.3526	371.1799	**7**
**11**	1247.4592	624.2333	1230.4327	615.7200	**1229.4487**	615.2280	**S**	**662.3104**	331.6588	645.2838	323.1456	644.2998	322.6536	**6**
**12**	1344.5120	672.7596	1327.4855	664.2464	1326.5014	663.7544	**P**	**575.2784**	288.1428	558.2518	279.6295	**557.2678**	279.1375	**5**
**13**	**1445.5597**	723.2835	1428.5331	714.7702	**1427.5491**	714.2782	**T**	478.2256	239.6164	461.1991	231.1032	460.2150	230.6112	**4**
**14**	**1532.5917**	766.7995	1515.5652	758.2862	**1514.5811**	757.7942	**S**	**377.1779**	189.0926	360.1514	180.5793	359.1674	180.0873	**3**
**15**	**1647.6187**	824.3130	1630.5921	815.7997	1629.6081	815.3077	**D**	**290.1459**	145.5766	273.1193	137.0633	272.1353	136.5713	**2**
**16**							**R**	175.1190	88.0631	158.0924	79.5498			**1**

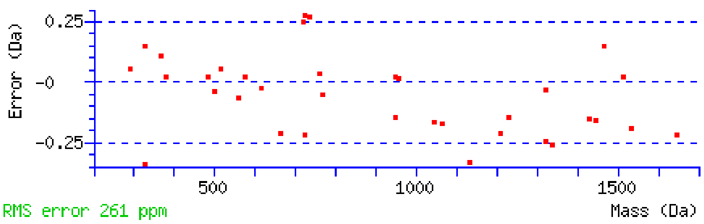

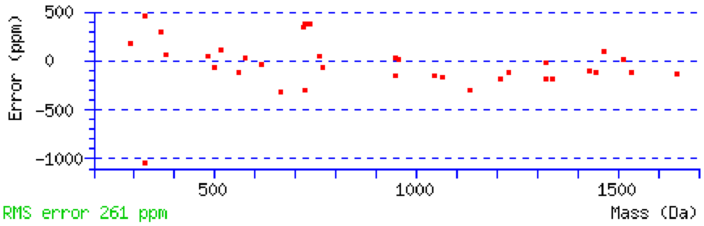

All matches to this query

Score	Mr(calc):	Delta	Sequence
44.5	1820.7231	-0.0029	TSSKESSPVPSPTSDR
20.9	1820.7231	-0.0029	TSSKESSPVPSPTSDR
18.8	1820.7231	-0.0029	TSSKESSPVPSPTSDR
18.3	1820.7231	-0.0029	TSSKESSPVPSPTSDR
17.1	1820.7231	-0.0029	TSSKESSPVPSPTSDR
10.0	1820.7321	-0.0120	TNTGSAMLEQIAMSDR
10.0	1820.7321	-0.0120	TNTGSAMLEQIAMSDR
10.0	1820.7321	-0.0120	TNTGSAMLEQIAMSDR
9.0	1820.7036	0.0166	CSRGYEPNEDGTSLR
9.0	1820.7036	0.0166	CSRGYEPNEDGTSLR

Spectrum No: 420; Query: 2611; Rank: 1

Peptide View

MS/MS Fragmentation of **KKPSEDESDLEDEEEKSQEEDTEQTR**
Found in **IPI00361669**, Tax_Id=10116 Gene_Symbol=LOC367976 similar to DNA replication licensing factor MCM3

Match to Query 2611: 3189.305976 from(798.333770,4+)
Title: 100101RatKid_NS_deglyco_20.1283.1283.4.dta
Data file K:\NewmanPaper\Piliang\3SubProteomes\Piliang3SP\mgf5ppm\ERLIC_3SubProteomes5ppm.mgf

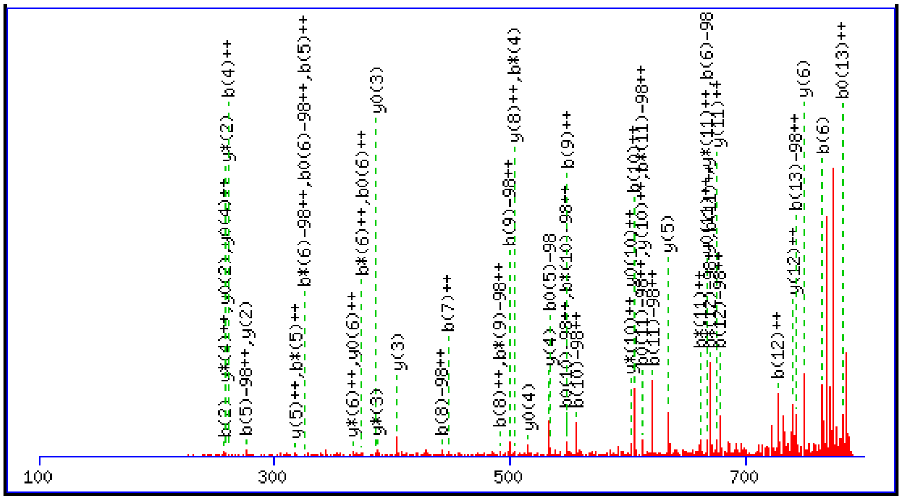

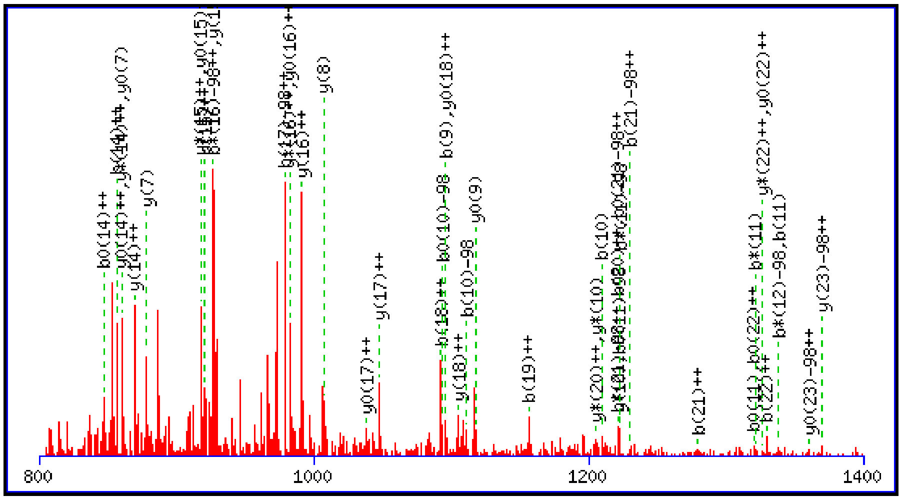

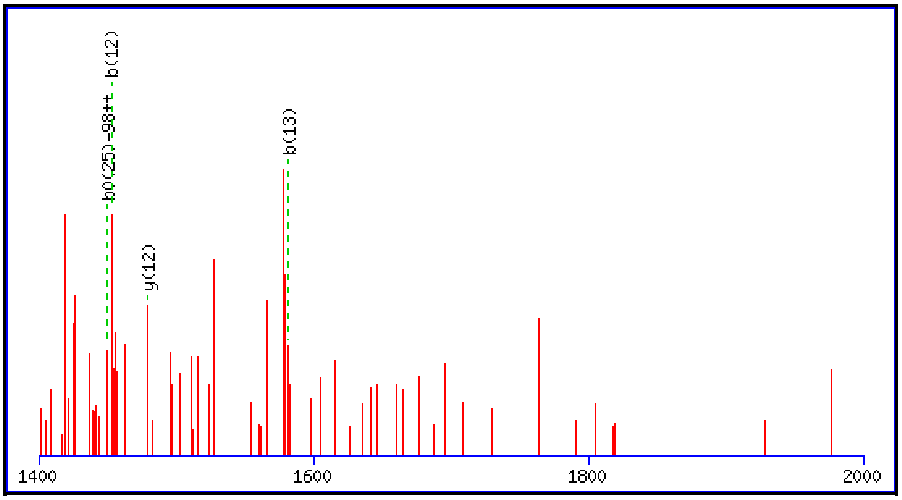

Monoisotopic mass of neutral peptide Mr(calc): 3189.2994
Fixed modifications: Carbamidomethyl (C)
Variable modifications:
S4 : Phospho (ST), with neutral losses 0.0000(shown in table), 97.9769
Ions Score: 44 **Expect:** 0.014
Matches (Bold Red): 109/442 fragment ions using 169 most intense peaks

#	b	b++	b*	b*++	b0	b0++	Seq.	y	y++	y*	y*++	y0	y0++	#
1	129.1022	65.0548	112.0757	56.5415			K							26
2	**257.1972**	129.1022	240.1707	120.5890			K	3062.2117	1531.6095	3045.1852	1523.0962	3044.2012	1522.6042	25
3	354.2500	177.6286	337.2234	169.1153			P	2934.1168	1467.5620	2917.0902	1459.0488	2916.1062	1458.5567	24
4	521.2483	**261.1278**	**504.2218**	252.6145	503.2378	252.1225	S	2837.0640	1419.0356	2820.0375	1410.5224	2819.0534	1410.0304	23
5	650.2909	**325.6491**	633.2644	**317.1358**	632.2803	316.6438	E	2670.0657	1335.5365	2653.0391	**1327.0232**	2652.0551	**1326.5312**	22

6	765.3179	383.1626	748.2913	374.6493	747.3073	374.1573	D	2541.0231	1271.0152	2523.9965	1262.5019	2523.0125	1262.0099	21
7	894.3604	447.6839	877.3339	439.1706	876.3499	438.6786	E	2425.9961	1213.5017	2408.9696	1204.9884	2407.9856	1204.4964	20
8	981.3925	491.1999	964.3659	482.6866	963.3819	482.1946	S	2296.9535	1148.9804	2279.9270	1140.4671	2278.9430	1139.9751	19
9	1096.4194	548.7133	1079.3929	540.2001	1078.4089	539.7081	D	2209.9215	1105.4644	2192.8950	1096.9511	2191.9109	1096.4591	18
10	1209.5035	605.2554	1192.4769	596.7421	1191.4929	596.2501	L	2094.8946	1047.9509	2077.8680	1039.4376	2076.8840	1038.9456	17
11	1338.5461	669.7767	1321.5195	661.2634	1320.5355	660.7714	E	1981.8105	991.4089	1964.7839	982.8956	1963.7999	982.4036	16
12	1453.5730	727.2901	1436.5465	718.7769	1435.5625	718.2849	D	1852.7679	926.8876	1835.7414	918.3743	1834.7573	917.8823	15
13	1582.6156	791.8114	1565.5891	783.2982	1564.6050	782.8062	E	1737.7410	869.3741	1720.7144	860.8608	1719.7304	860.3688	14
14	1711.6582	856.3327	1694.6317	847.8195	1693.6476	847.3275	E	1608.6984	804.8528	1591.6718	796.3395	1590.6878	795.8475	13
15	1840.7008	920.8540	1823.6742	912.3408	1822.6902	911.8488	E	1479.6558	740.3315	1462.6292	731.8182	1461.6452	731.3262	12
16	1968.7958	984.9015	1951.7692	976.3882	1950.7852	975.8962	K	1350.6132	675.8102	1333.5866	667.2970	1332.6026	666.8049	11
17	2055.8278	1028.4175	2038.8012	1019.9043	2037.8172	1019.4122	S	1222.5182	611.7627	1205.4917	603.2495	1204.5077	602.7575	10
18	2183.8864	1092.4468	2166.8598	1083.9335	2165.8758	1083.4415	Q	1135.4862	568.2467	1118.4596	559.7335	1117.4756	559.2414	9
19	2312.9290	1156.9681	2295.9024	1148.4548	2294.9184	1147.9628	E	1007.4276	504.2174	990.4011	495.7042	989.4170	495.2122	8
20	2441.9716	1221.4894	2424.9450	1212.9761	2423.9610	1212.4841	E	878.3850	439.6961	861.3585	431.1829	860.3745	430.6909	7
21	2556.9985	1279.0029	2539.9719	1270.4896	2538.9879	1269.9976	D	749.3424	375.1748	732.3159	366.6616	731.3319	366.1696	6
22	2658.0462	1329.5267	2641.0196	1321.0135	2640.0356	1320.5214	T	634.3155	317.6614	617.2889	309.1481	616.3049	308.6561	5
23	2787.0888	1394.0480	2770.0622	1385.5347	2769.0782	1385.0427	E	533.2678	267.1375	516.2413	258.6243	515.2572	258.1323	4
24	2915.1473	1458.0773	2898.1208	1449.5640	2897.1368	1449.0720	Q	404.2252	202.6162	387.1987	194.1030	386.2146	193.6110	3
25	3016.1950	1508.6012	2999.1685	1500.0879	2998.1845	1499.5959	T	276.1666	138.5870	259.1401	130.0737	258.1561	129.5817	2
26							R	175.1190	88.0631	158.0924	79.5498			1

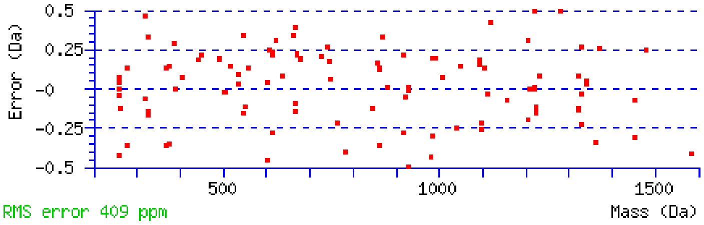

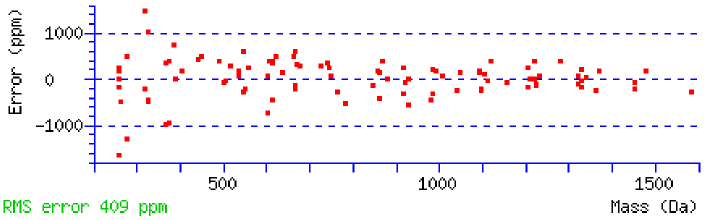

All matches to this query

Score	Mr(calc):	Delta	Sequence
44.5	3189.2994	0.0066	KKPSEDESDLEDEEEKSQEEDTEQTR
43.6	3189.2994	0.0066	KKPSEDESDLEDEEEKSQEEDTEQTR
10.3	3189.2994	0.0066	KKPSEDESDLEDEEEKSQEEDTEQTR
5.7	3189.3138	-0.0079	GVVSDFELEEQSKASGSVMWGLDSDTEK
5.7	3189.3138	-0.0079	GVVSDFELEEQSKASGSVMWGLDSDTEK
3.5	3189.2994	0.0066	KKPSEDESDLEDEEEKSQEEDTEQTR
3.5	3189.2994	0.0066	KKPSEDESDLEDEEEKSQEEDTEQTR
2.9	3188.3221	0.9838	YATSKHAKISTLAQLNCTLVQNLSK
2.9	3188.3221	0.9838	YATSKHAKISTLAQLNCTLVQNLSK
2.0	3187.2829	2.0231	SLKYSYLSLPVSHLGKGFAGFXPAR

Spectrum No: 421; Query: 773; Rank: 1

Peptide View

MS/MS Fragmentation of **RDSDGVDGFEAEGK**
Found in **IPI00367952**, Tax_Id=10116 Gene_Symbol=Zfr Zinc finger RNA-binding protein

Match to Query 773: 1560.613568 from(781.314060,2+)

Title: 100101RatKid_NS_deglyco_19.1286.1286.2.dta
Data file K:\NewmanPaper\Piliang\3SubProteomes\Piliang3SP\mgf5ppm\ERLIC_3SubProteomes5ppm.mgf

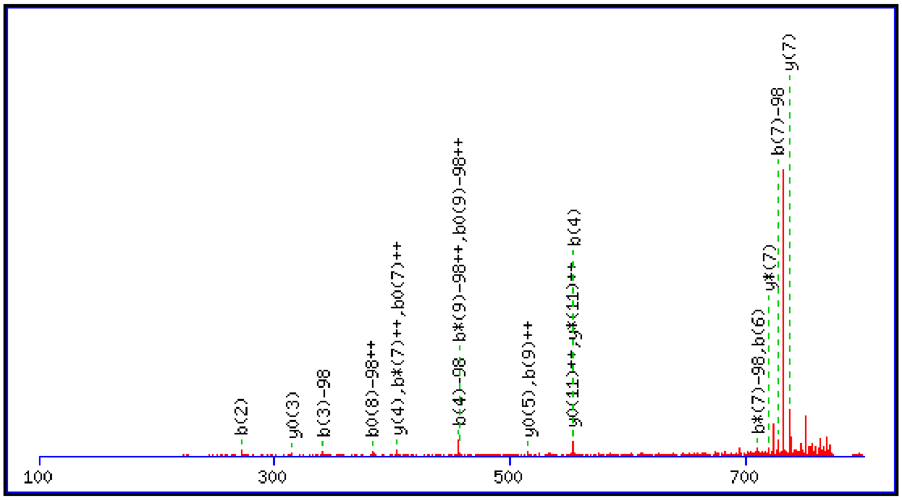

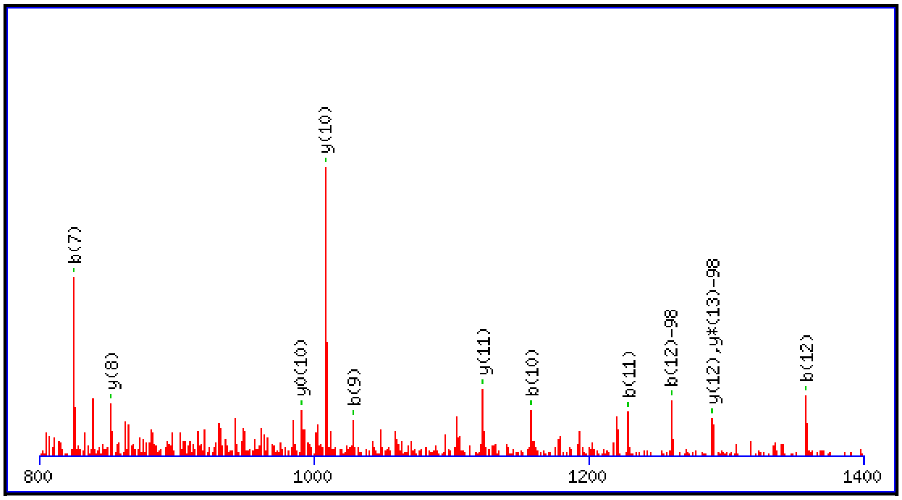

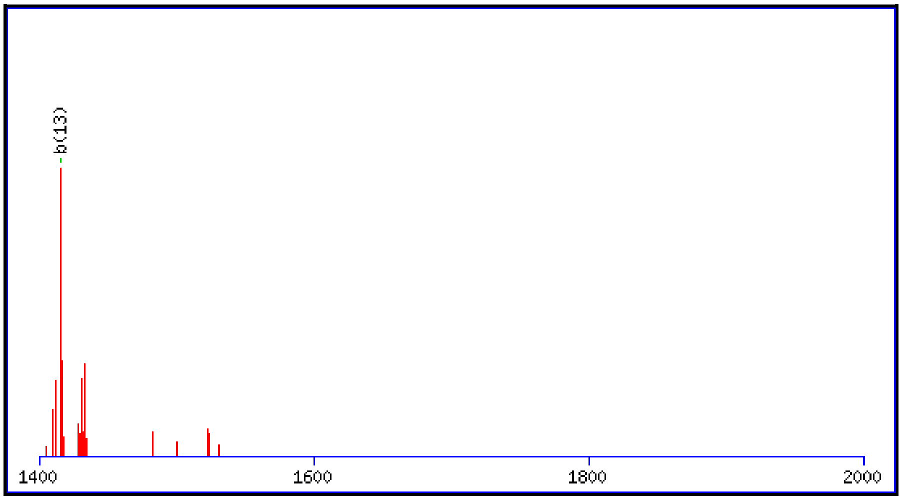

Monoisotopic mass of neutral peptide Mr(calc): 1560.6093
Fixed modifications: Carbamidomethyl (C)
Variable modifications:
S3 : Phospho (ST), with neutral losses 0.0000(shown in table), 97.9769
Ions Score: 45 **Expect:** 0.0042
Matches (Bold Red): 33/228 fragment ions using 45 most intense peaks

#	b	b^{++}	b*	b^{*++}	b^0	b^{0++}	Seq.	y	y^{++}	y*	y^{*++}	y^0	y^{0++}	#
1	157.1084	79.0578	140.0818	70.5446			R							14
2	**272.1353**	136.5713	255.1088	128.0580	254.1248	127.5660	D	1405.5155	703.2614	1388.4890	694.7481	1387.5049	694.2561	13
3	439.1337	220.0705	422.1071	211.5572	421.1231	211.0652	S	**1290.4886**	645.7479	1273.4620	637.2346	1272.4780	636.7426	12

4	554.1606	277.5840	537.1341	269.0707	536.1501	268.5787	D	1123.4902	562.2487	1106.4637	553.7355	1105.4796	553.2435	11
5	611.1821	306.0947	594.1555	297.5814	593.1715	297.0894	G	1008.4633	504.7353	991.4367	496.2220	990.4527	495.7300	10
6	710.2505	355.6289	693.2240	347.1156	692.2399	346.6236	V	951.4418	476.2245	934.4153	467.7113	933.4312	467.2193	9
7	825.2774	413.1424	808.2509	404.6291	807.2669	404.1371	D	852.3734	426.6903	835.3468	418.1771	834.3628	417.6851	8
8	882.2989	441.6531	865.2724	433.1398	864.2883	432.6478	G	737.3464	369.1769	720.3199	360.6636	719.3359	360.1716	7
9	1029.3673	515.1873	1012.3408	506.6740	1011.3568	506.1820	F	680.3250	340.6661	663.2984	332.1529	662.3144	331.6608	6
10	1158.4099	579.7086	1141.3834	571.1953	1140.3994	570.7033	E	533.2566	267.1319	516.2300	258.6186	515.2460	258.1266	5
11	1229.4470	615.2272	1212.4205	606.7139	1211.4365	606.2219	A	404.2140	202.6106	387.1874	194.0974	386.2034	193.6053	4
12	1358.4896	679.7485	1341.4631	671.2352	1340.4791	670.7432	E	333.1769	167.0921	316.1503	158.5788	315.1663	158.0868	3
13	1415.5111	708.2592	1398.4845	699.7459	1397.5005	699.2539	G	204.1343	102.5708	187.1077	94.0575			2
14							K	147.1128	74.0600	130.0863	65.5468			1

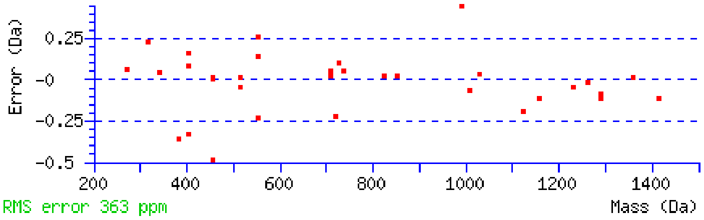

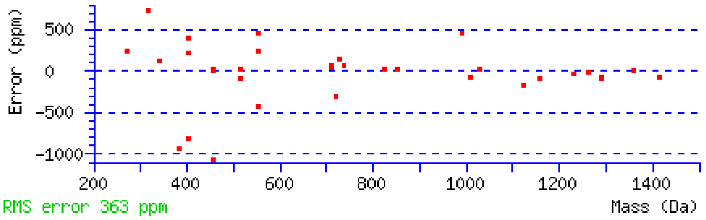

All matches to this query

Score	Mr(calc):	Delta	Sequence
44.5	1560.6093	0.0042	RDSDGVDGFEAEGK
2.9	1559.6187	0.9949	ILTRSLYSNPR
0.7	1560.6103	0.0032	NEIDNYEEDYQK

Spectrum No: 422; Query: 2838; Rank: 1

Peptide View

MS/MS Fragmentation of **SAAGSEKEEEPEEEEEEEEEEYDEEEEEEDDDRPPK**
Found in **IPI00198210**, Tax_Id=10116 Gene_Symbol=Supt5h suppressor of Ty 5 homolog

Match to Query 2838: 4379.622056 from(1095.912790,4+)
Title: 100101RatKid_NS_deglyco_24.2135.2135.4.dta
Data file K:\NewmanPaper\Piliang\3SubProteomes\Piliang3SP\mgf5ppm\ERLIC_3SubProteomes5ppm.mgf

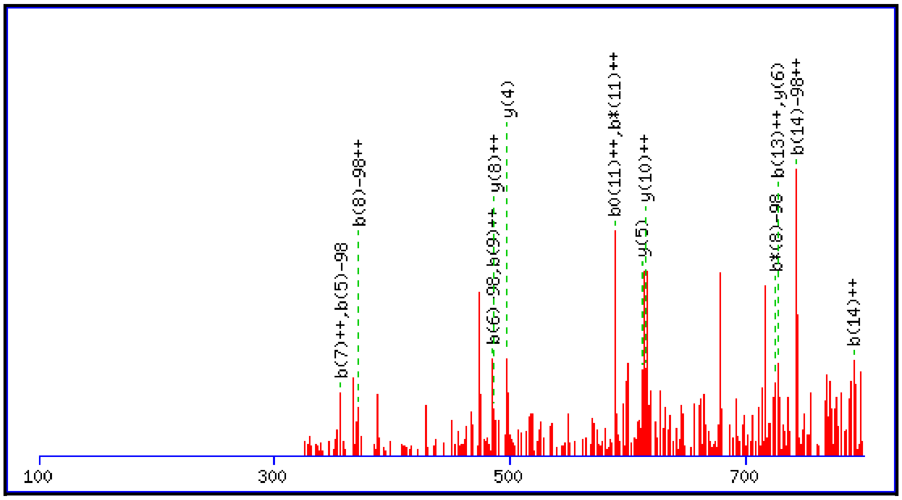

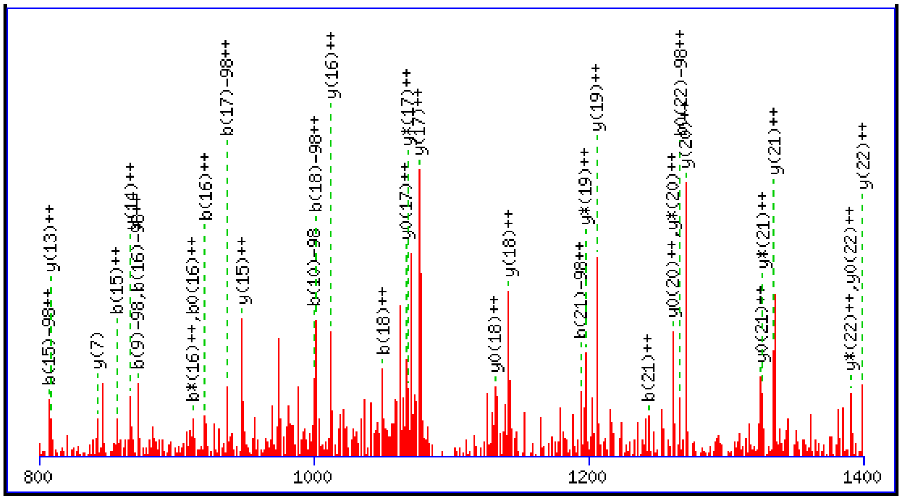

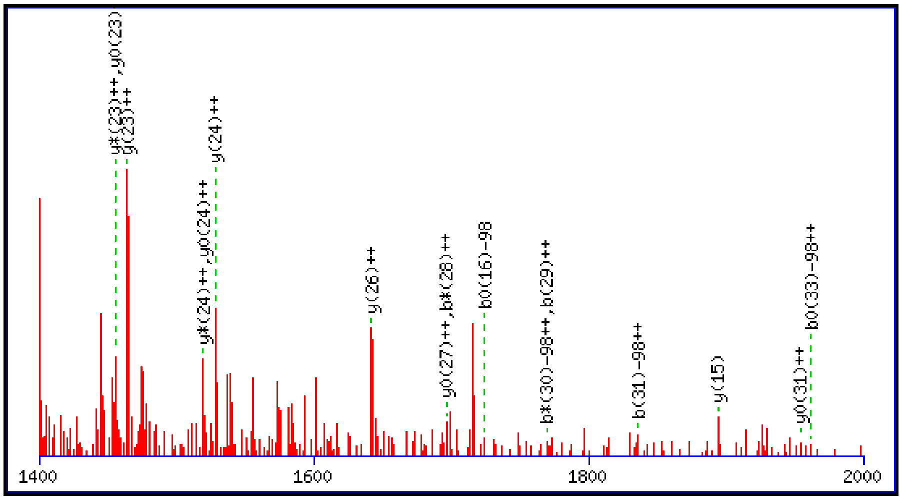

Monoisotopic mass of neutral peptide Mr(calc): 4378.6088
Fixed modifications: Carbamidomethyl (C)
Variable modifications:
S5 : Phospho (ST), with neutral losses 97.9769(shown in table), 0.0000
Ions Score: 44 **Expect:** 0.0096
Matches (Bold Red): 67/606 fragment ions using 123 most intense peaks

#	b	b++	b*	b*++	b0	b0++	Seq.	y	y++	y*	y*++	y0	y0++	#
1	88.0393	44.5233			70.0287	35.5180	S							36
2	159.0764	80.0418			141.0659	71.0366	A	4194.6073	2097.8073	4177.5807	2089.2940	4176.5967	2088.8020	35
3	230.1135	115.5604			212.1030	106.5551	A	4123.5702	2062.2887	4106.5436	2053.7754	4105.5596	2053.2834	34
4	287.1350	144.0711			269.1244	135.0659	G	4052.5331	2026.7702	4035.5065	2018.2569	4034.5225	2017.7649	33
5	**356.1565**	178.5819			338.1459	169.5766	S	3995.5116	1998.2594	3978.4850	1989.7462	3977.5010	1989.2541	32
6	**485.1990**	243.1032			467.1885	234.0979	E	3926.4901	1963.7487	3909.4636	1955.2354	3908.4796	**1954.7434**	31
7	613.2940	307.1506	596.2675	298.6374	595.2834	298.1454	K	3797.4475	1899.2274	3780.4210	1890.7141	3779.4370	1890.2221	30
8	742.3366	**371.6719**	**725.3101**	363.1587	724.3260	362.6667	E	3669.3526	1835.1799	3652.3260	1826.6667	3651.3420	1826.1746	29
9	**871.3792**	436.1932	854.3526	427.6800	853.3686	427.1880	E	3540.3100	1770.6586	3523.2834	1762.1454	3522.2994	1761.6533	28
10	**1000.4218**	500.7145	983.3952	492.2013	982.4112	491.7092	E	3411.2674	1706.1373	3394.2408	1697.6241	3393.2568	**1697.1320**	27
11	1097.4746	549.2409	1080.4480	540.7276	1079.4640	540.2356	P	3282.2248	**1641.6160**	3265.1982	1633.1028	3264.2142	1632.6108	26
12	1226.5171	613.7622	1209.4906	605.2489	1208.5066	604.7569	E	3185.1720	1593.0897	3168.1455	1584.5764	3167.1615	1584.0844	25
13	1355.5597	678.2835	1338.5332	669.7702	1337.5492	669.2782	E	3056.1294	**1528.5684**	3039.1029	**1520.0551**	3038.1189	**1519.5631**	24
14	1484.6023	**742.8048**	1467.5758	734.2915	1466.5918	733.7995	E	2927.0868	**1464.0471**	2910.0603	**1455.5338**	2909.0763	**1455.0418**	23
15	1613.6449	**807.3261**	1596.6184	798.8128	1595.6344	798.3208	E	2798.0443	**1399.5258**	2781.0177	**1391.0125**	2780.0337	**1390.5205**	22
16	1742.6875	**871.8474**	1725.6610	863.3341	**1724.6770**	862.8421	E	2669.0017	**1335.0045**	2651.9751	**1326.4912**	2650.9911	**1325.9992**	21
17	1871.7301	**936.3687**	1854.7036	927.8554	1853.7195	927.3634	E	2539.9591	**1270.4832**	2522.9325	**1261.9699**	2521.9485	**1261.4779**	20
18	2000.7727	**1000.8900**	1983.7462	992.3767	1982.7621	991.8847	E	2410.9165	**1205.9619**	2393.8899	**1197.4486**	2392.9059	1196.9566	19

**19**	2129.8153	1065.4113	2112.7887	1056.8980	2111.8047	1056.4060	**E**	2281.8739	**1141.4406**	2264.8473	1132.9273	2263.8633	**1132.4353**	**18**
**20**	2258.8579	1129.9326	2241.8313	1121.4193	2240.8473	1120.9273	**E**	2152.8313	**1076.9193**	2135.8047	**1068.4060**	2134.8207	**1067.9140**	**17**
**21**	2387.9005	**1194.4539**	2370.8739	1185.9406	2369.8899	1185.4486	**E**	2023.7887	**1012.3980**	2006.7621	1003.8847	2005.7781	1003.3927	**16**
**22**	2550.9638	1275.9855	2533.9373	1267.4723	2532.9532	**1266.9803**	**Y**	**1894.7461**	**947.8767**	1877.7196	939.3634	1876.7355	938.8714	**15**
**23**	2665.9908	1333.4990	2648.9642	1324.9857	2647.9802	1324.4937	**D**	1731.6828	**866.3450**	1714.6562	857.8317	1713.6722	857.3397	**14**
**24**	2795.0333	1398.0203	2778.0068	1389.5070	2777.0228	1389.0150	**E**	1616.6558	**808.8316**	1599.6293	800.3183	1598.6453	799.8263	**13**
**25**	2924.0759	1462.5416	2907.0494	1454.0283	2906.0654	1453.5363	**E**	1487.6132	744.3103	1470.5867	735.7970	1469.6027	735.3050	**12**
**26**	3053.1185	1527.0629	3036.0920	1518.5496	3035.1080	1518.0576	**E**	1358.5706	679.7890	1341.5441	671.2757	1340.5601	670.7837	**11**
**27**	3182.1611	1591.5842	3165.1346	1583.0709	3164.1506	1582.5789	**E**	1229.5281	**615.2677**	1212.5015	606.7544	1211.5175	606.2624	**10**
**28**	3311.2037	1656.1055	3294.1772	1647.5922	3293.1932	1647.1002	**E**	1100.4855	550.7464	1083.4589	542.2331	1082.4749	541.7411	**9**
**29**	3440.2463	1720.6268	3423.2198	1712.1135	3422.2357	1711.6215	**E**	971.4429	**486.2251**	954.4163	477.7118	953.4323	477.2198	**8**
**30**	3555.2733	1778.1403	3538.2467	**1769.6270**	3537.2627	1769.1350	**D**	**842.4003**	421.7038	825.3737	413.1905	824.3897	412.6985	**7**
**31**	3670.3002	**1835.6537**	3653.2736	1827.1405	3652.2896	1826.6485	**D**	**727.3733**	364.1903	710.3468	355.6770	709.3628	355.1850	**6**
**32**	3785.3271	1893.1672	3768.3006	1884.6539	3767.3166	1884.1619	**D**	**612.3464**	306.6768	595.3198	298.1636	594.3358	297.6715	**5**
**33**	3941.4283	1971.2178	3924.4017	1962.7045	3923.4177	**1962.2125**	**R**	**497.3194**	249.1634	480.2929	240.6501			**4**
**34**	4038.4810	2019.7441	4021.4545	2011.2309	4020.4705	2010.7389	**P**	341.2183	171.1128	324.1918	162.5995			**3**
**35**	4135.5338	2068.2705	4118.5072	2059.7573	4117.5232	2059.2652	**P**	244.1656	122.5864	227.1390	114.0731			**2**
**36**							**K**	147.1128	74.0600	130.0863	65.5468			**1**

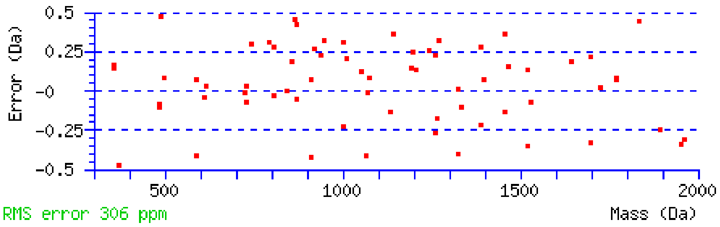

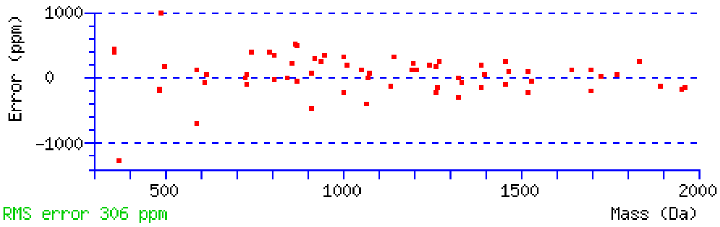

All matches to this query

Score	Mr(calc):	Delta	Sequence
44.4	4378.6088	1.0133	SAAGSEKEEEPEEEEEEEEEEYDEEEEEEDDDRPPK
44.4	4378.6088	1.0133	SAAGSEKEEEPEEEEEEEEEEYDEEEEEEDDDRPPK
4.5	4377.5972	2.0248	AAGQSFSQVRVGEXGETAGTAPAPAPTTQSQPYCLIR
4.5	4377.5972	2.0248	AAGQSFSQVRVGEXGETAGTAPAPAPTTQSQPYCLIR
4.0	4378.5815	1.0405	SASANKTMVIVSASGTVEIVNEKEEGSSAPDGSVFIK
4.0	4378.5815	1.0405	SASANKTMVIVSASGTVEIVNEKEEGSSAPDGSVFIK
3.5	4379.6522	-0.0301	NGGSMDFPPYYLSSMLKNIKFMNPGGDPISLNPSEK
2.3	4377.5853	2.0368	LPPPLPQNLLYTVSENTYEPDGYNPEAPSITSSGR
2.3	4377.5853	2.0368	LPPPLPQNLLYTVSENTYEPDGYNPEAPSITSSGR
1.4	4377.5744	2.0476	CVAGYHGSNCSEEINECLSQPCQNGGTCIDLTNTYK

Spectrum No: 423; Query: 1289; Rank: 1

Peptide View

MS/MS Fragmentation of **TLNEWSSQISPDLVR**
Found in **IPI00191738**, Tax_Id=10116 Gene_Symbol=Gclm Glutamate--cysteine ligase regulatory subunit

Match to Query 1289: 1823.852808 from(912.933680,2+)
Title: 100101RatKid_NS_deglyco_15.4215.4215.2.dta
Data file K:\NewmanPaper\Piliang\3SubProteomes\Piliang3SP\mgf5ppm\ERLIC_3SubProteomes5ppm.mgf

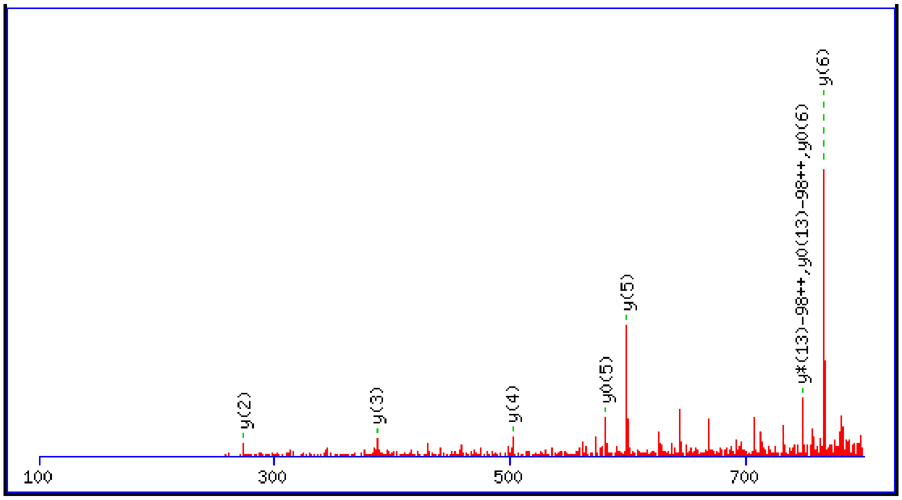

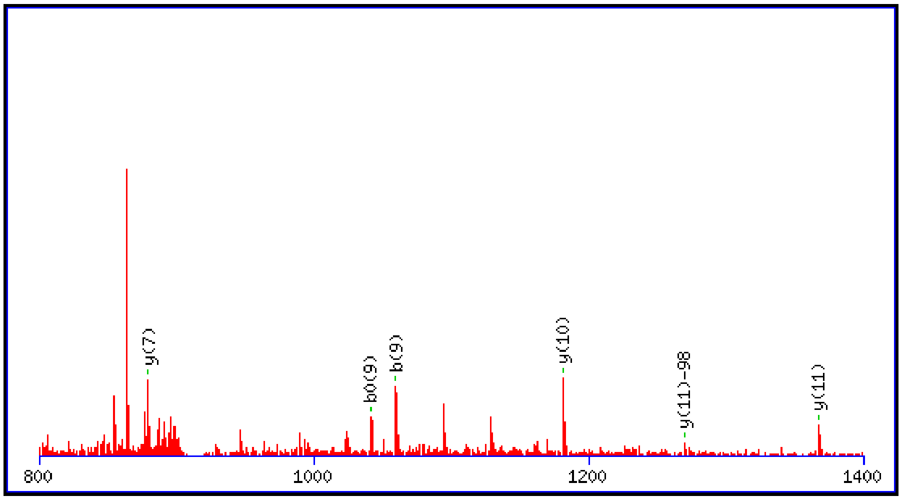

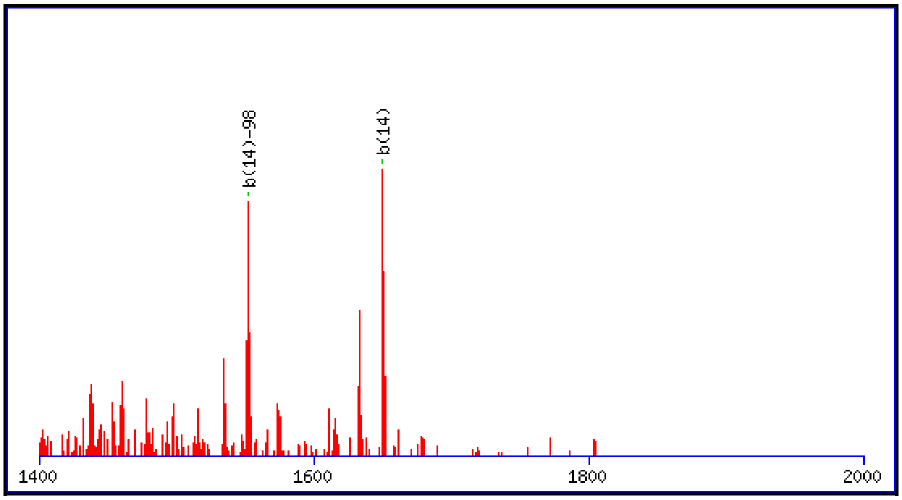

Monoisotopic mass of neutral peptide Mr(calc): 1823.8455
Fixed modifications: Carbamidomethyl (C)
Variable modifications:
S10 : Phospho (ST), with neutral losses 0.0000(shown in table), 97.9769
Ions Score: 44 **Expect:** 0.0078
Matches (Bold Red): 17/242 fragment ions using 25 most intense peaks

#	b	b^{++}	b*	b^{*++}	b^0	b^{0++}	Seq.	y	y^{++}	y*	y^{*++}	y^0	y^{0++}	#
1	102.0550	51.5311			84.0444	42.5258	T							15
2	215.1390	108.0731			197.1285	99.0679	L	1723.8051	862.4062	1706.7785	853.8929	1705.7945	853.4009	14
3	329.1819	165.0946	312.1554	156.5813	311.1714	156.0893	N	1610.7210	805.8641	1593.6945	797.3509	1592.7105	796.8589	13
4	458.2245	229.6159	441.1980	221.1026	440.2140	220.6106	E	1496.6781	748.8427	1479.6515	740.3294	1478.6675	739.8374	12
5	644.3039	322.6556	627.2773	314.1423	626.2933	313.6503	W	**1367.6355**	684.3214	1350.6090	675.8081	1349.6249	675.3161	11

**6**	731.3359	366.1716	714.3093	357.6583	713.3253	357.1663	**S**	**1181.5562**	591.2817	1164.5296	582.7685	1163.5456	582.2764	**10**
**7**	818.3679	409.6876	801.3414	401.1743	800.3573	400.6823	**S**	1094.5242	547.7657	1077.4976	539.2524	1076.5136	538.7604	**9**
**8**	946.4265	473.7169	929.3999	465.2036	928.4159	464.7116	**Q**	1007.4921	504.2497	990.4656	495.7364	989.4816	495.2444	**8**
**9**	**1059.5106**	530.2589	1042.4840	521.7456	**1041.5000**	521.2536	**I**	**879.4336**	440.2204	862.4070	431.7071	861.4230	431.2151	**7**
**10**	1226.5089	613.7581	1209.4824	605.2448	1208.4983	604.7528	**S**	**766.3495**	383.6784	749.3229	375.1651	**748.3389**	374.6731	**6**
**11**	1323.5617	662.2845	1306.5351	653.7712	1305.5511	653.2792	**P**	**599.3511**	300.1792	582.3246	291.6659	**581.3406**	291.1739	**5**
**12**	1438.5886	719.7979	1421.5621	711.2847	1420.5780	710.7927	**D**	**502.2984**	251.6528	485.2718	243.1395	484.2878	242.6475	**4**
**13**	1551.6727	776.3400	1534.6461	767.8267	1533.6621	767.3347	**L**	**387.2714**	194.1394	370.2449	185.6261			**3**
**14**	**1650.7411**	825.8742	1633.7145	817.3609	1632.7305	816.8689	**V**	**274.1874**	137.5973	257.1608	129.0840			**2**
**15**							**R**	175.1190	88.0631	158.0924	79.5498			**1**

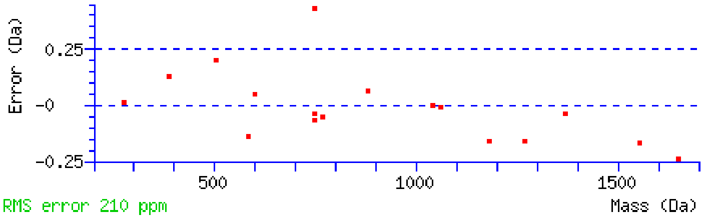

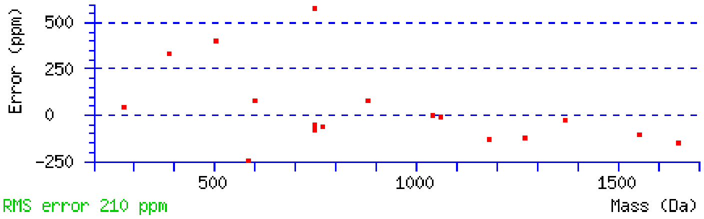

All matches to this query

Score	Mr(calc):	Delta	Sequence
44.4	1823.8455	0.0073	TLNEWSSQISPDLVR
26.0	1823.8455	0.0073	TLNEWSSQISPDLVR
26.0	1823.8455	0.0073	TLNEWSSQISPDLVR
14.5	1823.8455	0.0073	TLNEWSSQISPDLVR
4.1	1823.8389	0.0139	LCSLSQGNLSLNWPR
1.9	1823.8639	-0.0111	GAREVLTGSRGDXSLGGR
1.3	1823.8414	0.0114	ASKENSSLTQSKPDPR
1.3	1823.8414	0.0114	ASKENSSLTQSKPDPR
0.6	1823.8390	0.0138	KACDVVHNFTDAVIR

Spectrum No: 424; Query: 2567; Rank: 1

Peptide View

MS/MS Fragmentation of **EYVSNDATQSDDEEKLQSQQTDTDGGR**
Found in **IPI00205633**, Tax_Id=10116 Gene_Symbol=Wdr44 99 kDa protein

Match to Query 2567: 3095.255202 from(1032.759010,3+)
Title: 100101RatKid_NS_deglyco_24.1368.1368.3.dta
Data file K:\NewmanPaper\Piliang\3SubProteomes\Piliang3SP\mgf5ppm\ERLIC_3SubProteomes5ppm.mgf

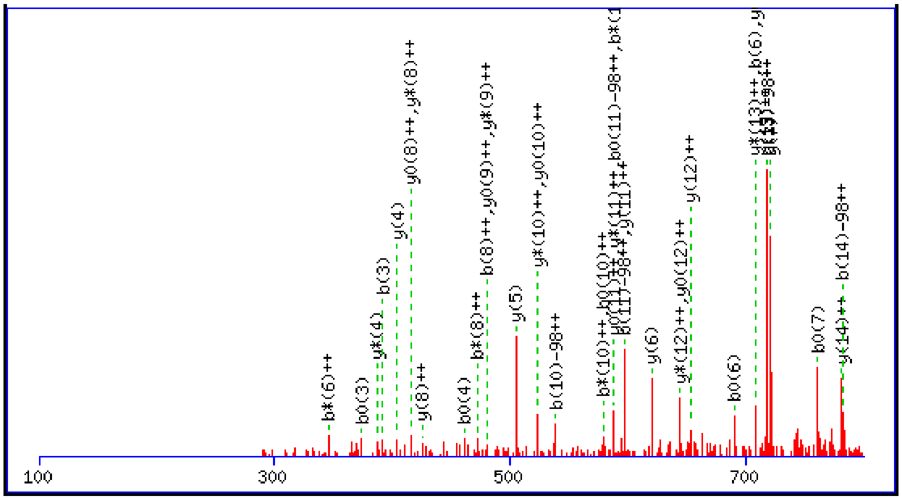

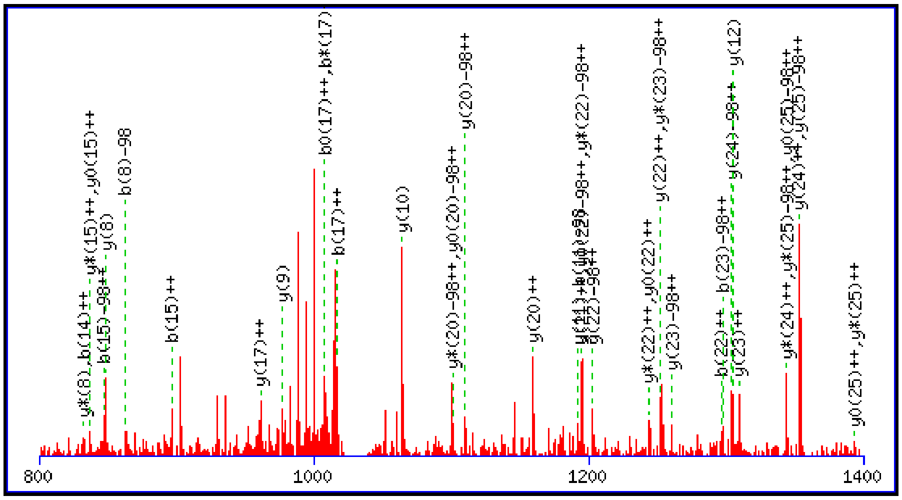

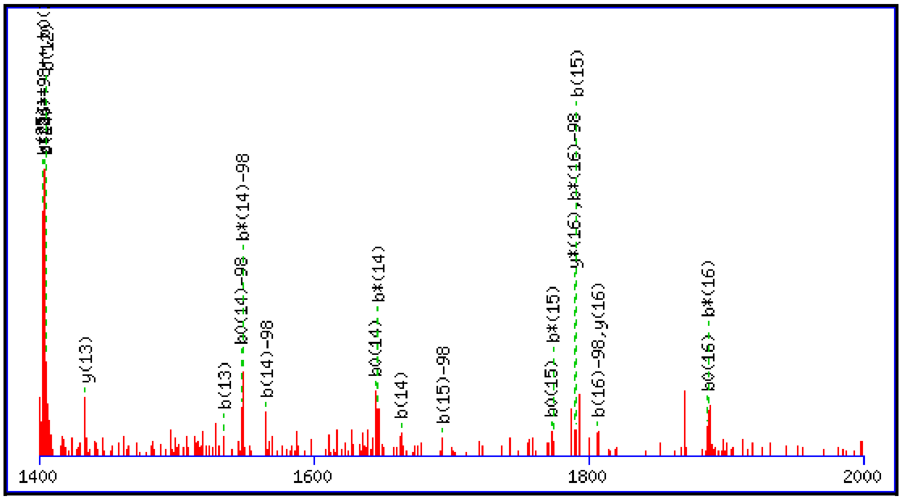

Monoisotopic mass of neutral peptide Mr(calc): 3095.2477
Fixed modifications: Carbamidomethyl (C)
Variable modifications:
T8 : Phospho (ST), with neutral losses 0.0000(shown in table), 97.9769
Ions Score: 44 **Expect:** 0.013
Matches (Bold Red): 104/454 fragment ions using 163 most intense peaks

#	b	b^{++}	b*	b^{*++}	b^0	b^{0++}	Seq.	y	y^{++}	y*	y^{*++}	y^0	y^{0++}	#
1	130.0499	65.5286			112.0393	56.5233	E							27
2	293.1132	147.0602			275.1026	138.0550	Y	2967.2123	1484.1098	2950.1858	1475.5965	2949.2018	1475.1045	26
3	**392.1816**	196.5944			**374.1710**	187.5892	V	2804.1490	**1402.5781**	2787.1225	**1394.0649**	2786.1385	**1393.5729**	25
4	479.2136	240.1105			**461.2031**	231.1052	S	2705.0806	**1353.0439**	2688.0541	**1344.5307**	2687.0700	1344.0387	24
5	593.2566	297.1319	576.2300	288.6186	575.2460	288.1266	N	2618.0486	**1309.5279**	2601.0220	1301.0147	2600.0380	1300.5226	23

6	708.2835	354.6454	691.2570	346.1321	690.2729	345.6401	D	2504.0057	1252.5065	2486.9791	1243.9932	2485.9951	1243.5012	22
7	779.3206	390.1640	762.2941	381.6507	761.3101	381.1587	A	2388.9787	1194.9930	2371.9522	1186.4797	2370.9681	1185.9877	21
8	960.3346	480.6710	943.3081	472.1577	942.3241	471.6657	T	2317.9416	1159.4744	2300.9150	1150.9612	2299.9310	1150.4692	20
9	1088.3932	544.7002	1071.3667	536.1870	1070.3826	535.6950	Q	2136.9276	1068.9674	2119.9010	1060.4542	2118.9170	1059.9622	19
10	1175.4252	588.2163	1158.3987	579.7030	1157.4147	579.2110	S	2008.8690	1004.9381	1991.8425	996.4249	1990.8584	995.9329	18
11	1290.4522	645.7297	1273.4256	637.2165	1272.4416	636.7244	D	1921.8370	961.4221	1904.8104	952.9089	1903.8264	952.4168	17
12	1405.4791	703.2432	1388.4526	694.7299	1387.4686	694.2379	D	1806.8100	903.9087	1789.7835	895.3954	1788.7995	894.9034	16
13	1534.5217	767.7645	1517.4952	759.2512	1516.5111	758.7592	E	1691.7831	846.3952	1674.7565	837.8819	1673.7725	837.3899	15
14	1663.5643	832.2858	1646.5378	823.7725	1645.5537	823.2805	E	1562.7405	781.8739	1545.7140	773.3606	1544.7299	772.8686	14
15	1791.6593	896.3333	1774.6327	887.8200	1773.6487	887.3280	K	1433.6979	717.3526	1416.6714	708.8393	1415.6873	708.3473	13
16	1904.7433	952.8753	1887.7168	944.3620	1886.7328	943.8700	L	1305.6029	653.3051	1288.5764	644.7918	1287.5924	644.2998	12
17	2032.8019	1016.9046	2015.7754	1008.3913	2014.7913	1007.8993	Q	1192.5189	596.7631	1175.4923	588.2498	1174.5083	587.7578	11
18	2119.8339	1060.4206	2102.8074	1051.9073	2101.8234	1051.4153	S	1064.4603	532.7338	1047.4338	524.2205	1046.4497	523.7285	10
19	2247.8925	1124.4499	2230.8660	1115.9366	2229.8820	1115.4446	Q	977.4283	489.2178	960.4017	480.7045	959.4177	480.2125	9
20	2375.9511	1188.4792	2358.9245	1179.9659	2357.9405	1179.4739	Q	849.3697	425.1885	832.3432	416.6752	831.3591	416.1832	8
21	2476.9988	1239.0030	2459.9722	1230.4898	2458.9882	1229.9977	T	721.3111	361.1592	704.2846	352.6459	703.3006	352.1539	7
22	2592.0257	1296.5165	2574.9992	1288.0032	2574.0152	1287.5112	D	620.2634	310.6354	603.2369	302.1221	602.2529	301.6301	6
23	2693.0734	1347.0403	2676.0468	1338.5271	2675.0628	1338.0351	T	505.2365	253.1219	488.2100	244.6086	487.2259	244.1166	5
24	2808.1003	1404.5538	2791.0738	1396.0405	2790.0898	1395.5485	D	404.1888	202.5980	387.1623	194.0848	386.1783	193.5928	4
25	2865.1218	1433.0645	2848.0953	1424.5513	2847.1112	1424.0593	G	289.1619	145.0846	272.1353	136.5713			3
26	2922.1433	1461.5753	2905.1167	1453.0620	2904.1327	1452.5700	G	232.1404	116.5738	215.1139	108.0606			2
27							R	175.1190	88.0631	158.0924	79.5498			1

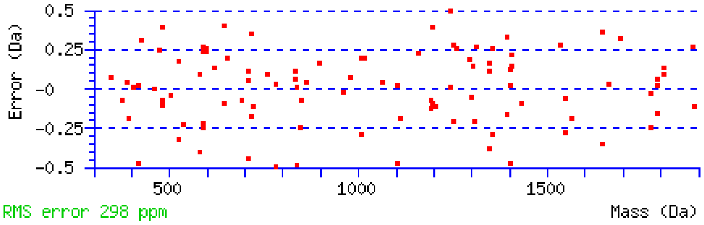

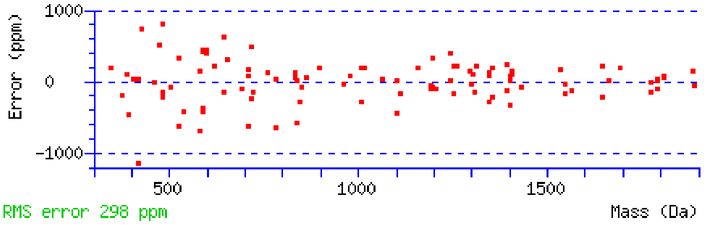

All matches to this query

Score	Mr(calc):	Delta	Sequence
44.3	3095.2477	0.0075	EYVSNDATQSDDEEKLQSQQTDTDGGR
41.4	3095.2477	0.0075	EYVSNDATQSDDEEKLQSQQTDTDGGR
33.0	3095.2477	0.0075	EYVSNDATQSDDEEKLQSQQTDTDGGR
19.9	3095.2477	0.0075	EYVSNDATQSDDEEKLQSQQTDTDGGR
19.8	3095.2477	0.0075	EYVSNDATQSDDEEKLQSQQTDTDGGR
12.7	3095.2477	0.0075	EYVSNDATQSDDEEKLQSQQTDTDGGR
10.2	3095.2477	0.0075	EYVSNDATQSDDEEKLQSQQTDTDGGR
3.0	3093.2593	1.9959	AQVLANINGMSFITAGDTSSEERWQK
0.1	3094.2433	1.0119	AQVLANINGMSFITAGDTSSEERWQK

Spectrum No: 425; Query: 525; Rank: 1

Peptide View

MS/MS Fragmentation of **LDQPVSAPPSPR**
Found in **IPI00360771**, Tax_Id=10116 Gene_Symbol=LOC691155 similar to coiled-coil domain containing 6

Match to Query 525: 1422.592888 from(712.303720,2+)

Title: 091008RatKidney_NH4Format01_31.609.609.2.dta
Data file K:\NewmanPaper\Piliang\3SubProteomes\Piliang3SP\mgf5ppm\ERLIC_3SubProteomes5ppm.mgf

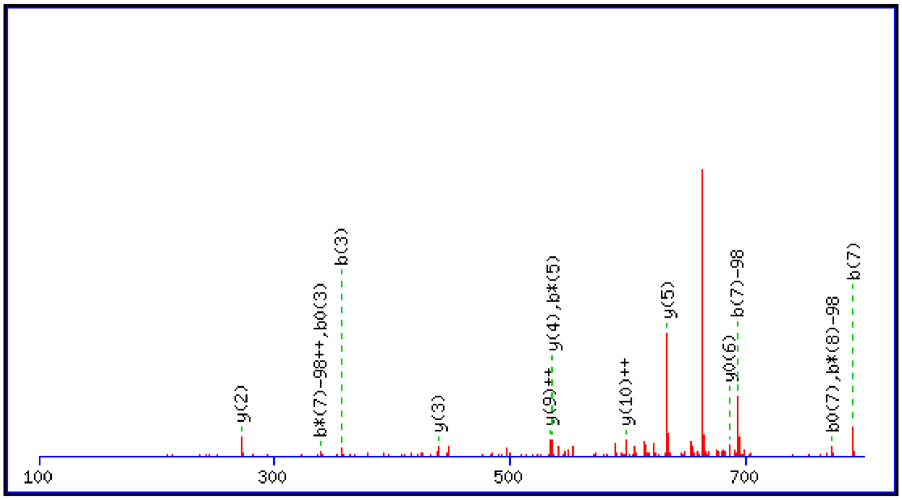

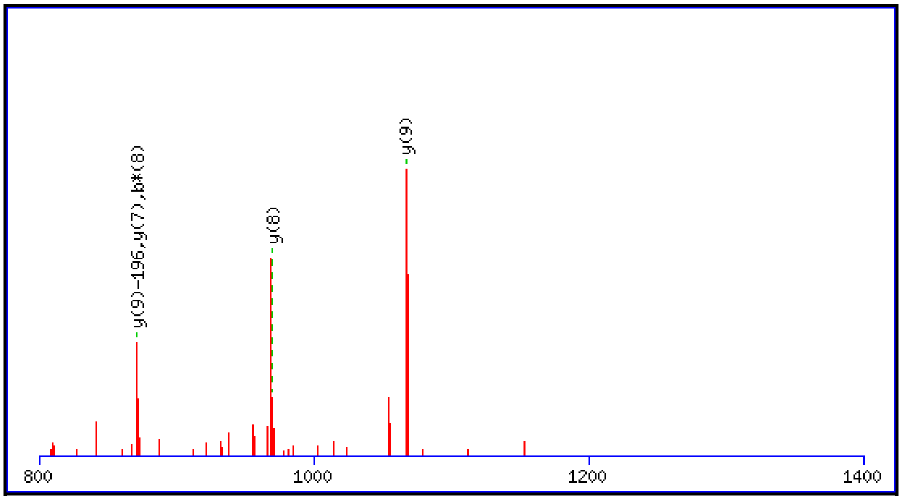

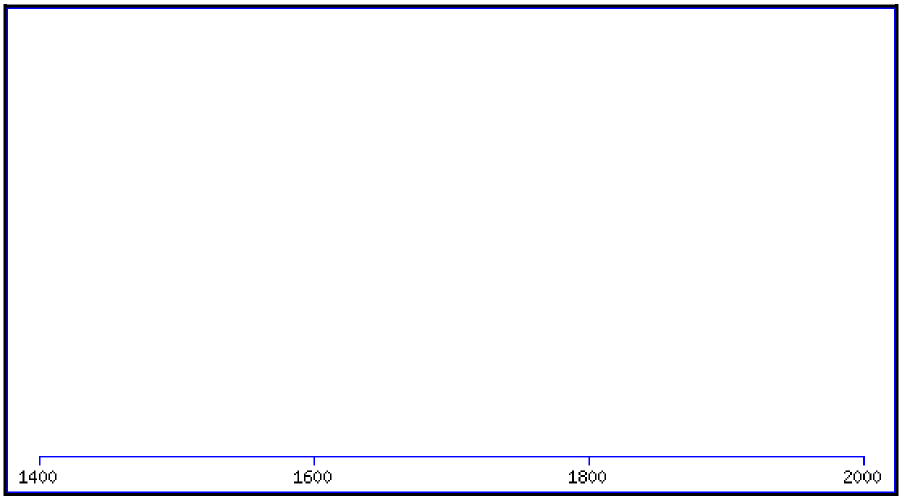

Monoisotopic mass of neutral peptide Mr(calc): 1422.5945
Fixed modifications: Carbamidomethyl (C)
Variable modifications:
S6 : Phospho (ST), with neutral losses 0.0000(shown in table), 97.9769
S10 : Phospho (ST), with neutral losses 0.0000(shown in table), 97.9769
Ions Score: 44 **Expect:** 0.0048
Matches (Bold Red): 20/212 fragment ions using 28 most intense peaks

#	b	b^{++}	b*	b^{*++}	b^{0}	b^{0++}	Seq.	y	y^{++}	y*	y^{*++}	y^{0}	y^{0++}	#
1	114.0913	57.5493					L							12
2	229.1183	115.0628			211.1077	106.0575	D	1310.5178	655.7625	1293.4912	647.2492	1292.5072	646.7572	11

3	**357.1769**	179.0921	340.1503	170.5788	**339.1663**	170.0868	Q	1195.4908	**598.2490**	1178.4643	589.7358	1177.4803	589.2438	10
4	454.2296	227.6185	437.2031	219.1052	436.2191	218.6132	P	**1067.4322**	**534.2198**	1050.4057	525.7065	1049.4217	525.2145	9
5	553.2980	277.1527	**536.2715**	268.6394	535.2875	268.1474	V	**970.3795**	485.6934	953.3529	477.1801	952.3689	476.6881	8
6	720.2964	360.6518	703.2698	352.1386	702.2858	351.6466	S	**871.3111**	436.1592	854.2845	427.6459	853.3005	427.1539	7
7	**791.3335**	396.1704	774.3070	387.6571	**773.3229**	387.1651	A	704.3127	352.6600	687.2862	344.1467	**686.3021**	343.6547	6
8	888.3863	444.6968	**871.3597**	436.1835	870.3757	435.6915	P	**633.2756**	317.1414	616.2490	308.6282	615.2650	308.1362	5
9	985.4390	493.2232	968.4125	484.7099	967.4285	484.2179	P	**536.2228**	268.6151	519.1963	260.1018	518.2123	259.6098	4
10	1152.4374	576.7223	1135.4108	568.2091	1134.4268	567.7170	S	**439.1701**	220.0887	422.1435	211.5754	421.1595	211.0834	3
11	1249.4901	625.2487	1232.4636	616.7354	1231.4796	616.2434	P	**272.1717**	136.5895	255.1452	128.0762			2
12							R	175.1190	88.0631	158.0924	79.5498			1

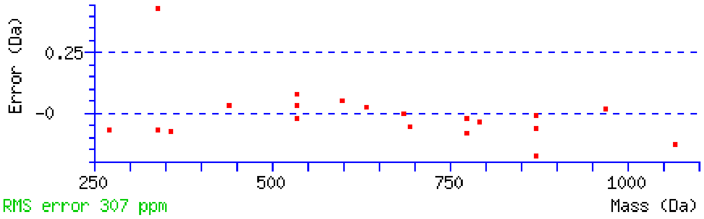

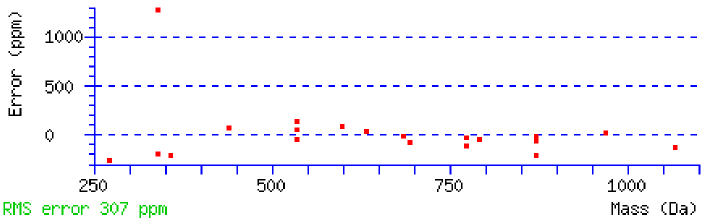

All matches to this query

Score	Mr(calc):	Delta	Sequence
44.3	1422.5945	-0.0017	LDQPVSAPPSPR
9.1	1422.6058	-0.0129	REAPGSPPLSPR
0.5	1421.5840	1.0089	KASESKEAVGEK
0.5	1422.5933	-0.0004	NNCSNAKAVETDV
0.5	1422.5933	-0.0004	NNCSNAKAVETDV
0.5	1422.5933	-0.0004	NNCSNAKAVETDV
0.3	1422.6045	-0.0116	EKEMEADERDR
0.3	1420.5742	2.0187	SPAGENSDEHYSK

Spectrum No: 426; Query: 938; Rank: 1

Peptide View

MS/MS Fragmentation of **EKEISDDEAEEEK**
Found in **IPI00471584**, Tax_Id=10116 Gene_Symbol=Hspcb Heat shock protein HSP 90-beta

Match to Query 938: 1629.632188 from(815.823370,2+)
Title: 100101RatKid_NS_deglyco_23.444.444.2.dta
Data file K:\NewmanPaper\Piliang\3SubProteomes\Piliang3SP\mgf5ppm\ERLIC_3SubProteomes5ppm.mgf

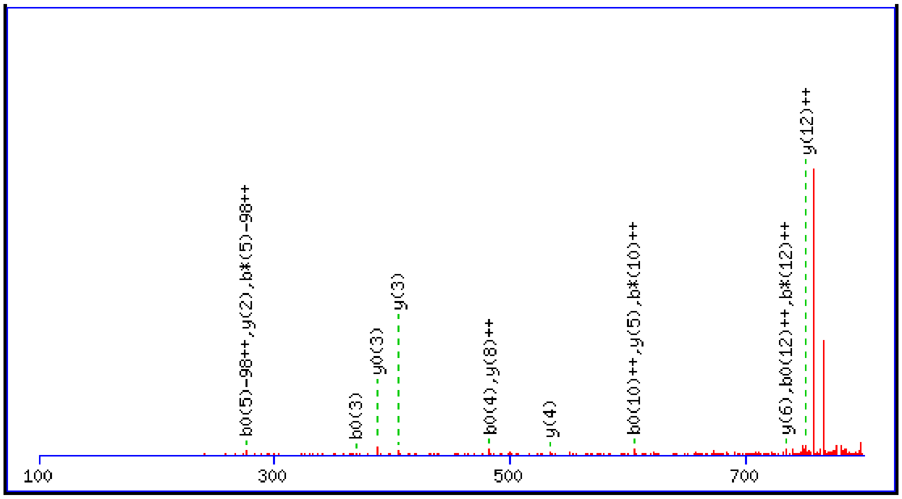

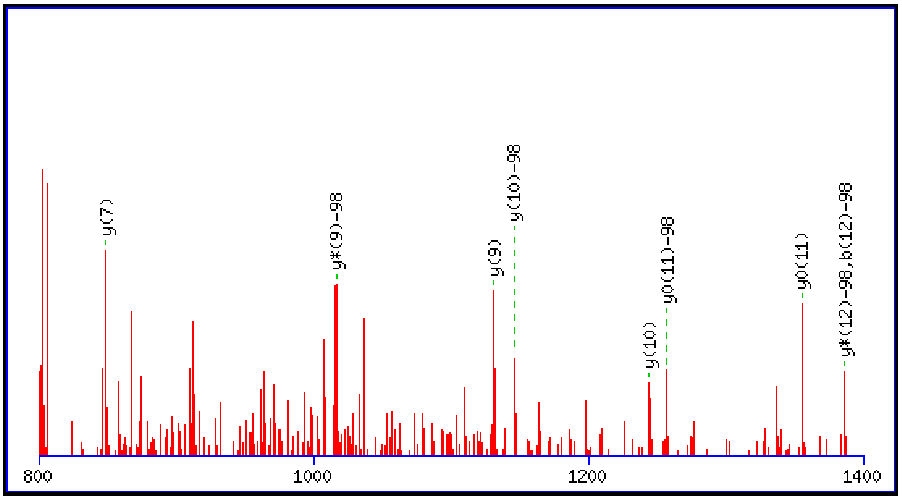

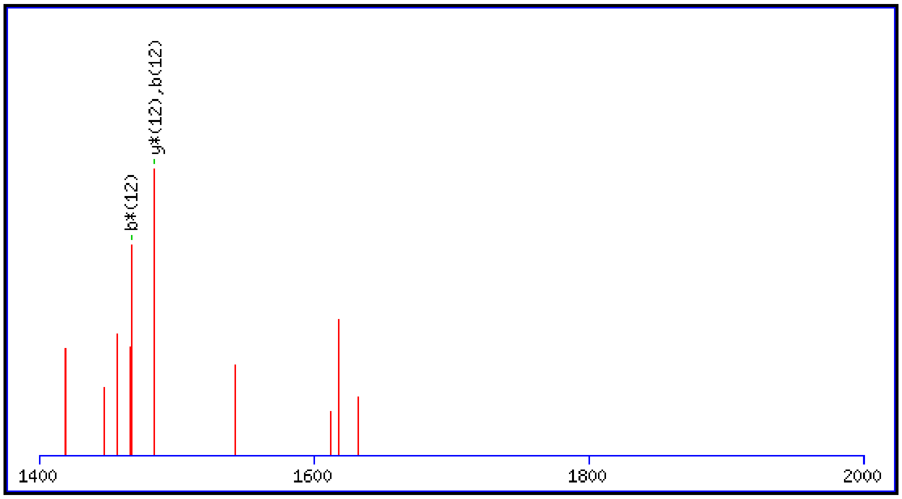

Monoisotopic mass of neutral peptide Mr(calc): 1629.6294
Fixed modifications: Carbamidomethyl (C)
Variable modifications:
S5 : Phospho (ST), with neutral losses 97.9769(shown in table), 0.0000
Ions Score: 44 **Expect:** 0.0043
Matches (Bold Red): 31/212 fragment ions using 44 most intense peaks

#	b	b^{++}	b*	b^{*++}	b^{0}	b^{0++}	Seq.	y	y^{++}	y*	y^{*++}	y^{0}	y^{0++}	#
1	130.0499	65.5286			112.0393	56.5233	E							13
2	258.1448	129.5761	241.1183	121.0628	240.1343	120.5708	K	1403.6173	702.3123	**1386.5907**	693.7990	1385.6067	693.3070	12
3	**387.1874**	194.0974	370.1609	185.5841	**369.1769**	185.0921	E	1275.5223	638.2648	1258.4957	629.7515	**1257.5117**	629.2595	11
4	500.2715	250.6394	483.2449	242.1261	**482.2609**	241.6341	I	**1146.4797**	573.7435	1129.4531	565.2302	1128.4691	564.7382	10
5	569.2929	285.1501	552.2664	**276.6368**	551.2824	**276.1448**	S	1033.3956	517.2015	**1016.3691**	508.6882	1015.3851	508.1962	9

**6**	684.3199	342.6636	667.2933	334.1503	666.3093	333.6583	**D**	964.3742	**482.6907**	947.3476	474.1775	946.3636	473.6854	**8**
**7**	799.3468	400.1771	782.3203	391.6638	781.3363	391.1718	**D**	**849.3472**	425.1773	832.3207	416.6640	831.3367	416.1720	**7**
**8**	928.3894	464.6983	911.3629	456.1851	910.3789	455.6931	**E**	**734.3203**	367.6638	717.2937	359.1505	716.3097	358.6585	**6**
**9**	999.4265	500.2169	982.4000	491.7036	981.4160	491.2116	**A**	**605.2777**	303.1425	588.2511	294.6292	587.2671	294.1372	**5**
**10**	1128.4691	564.7382	1111.4426	556.2249	1110.4586	555.7329	**E**	**534.2406**	267.6239	517.2140	259.1107	516.2300	258.6186	**4**
**11**	**1257.5117**	629.2595	1240.4852	620.7462	1239.5012	620.2542	**E**	**405.1980**	203.1026	388.1714	194.5894	**387.1874**	194.0974	**3**
**12**	**1386.5543**	693.7808	1369.5278	685.2675	1368.5438	684.7755	**E**	**276.1554**	138.5813	259.1288	130.0681	258.1448	129.5761	**2**
**13**							**K**	147.1128	74.0600	130.0863	65.5468			**1**

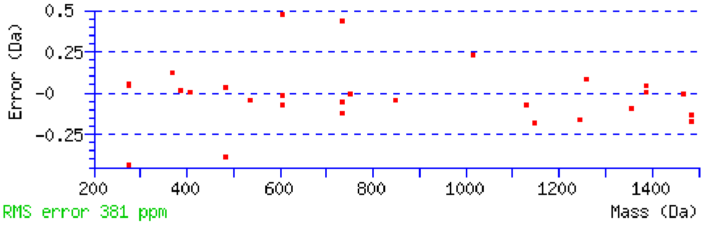

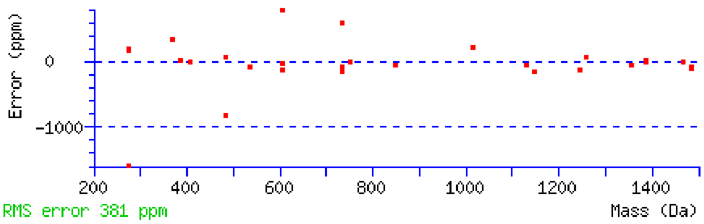

All matches to this query

Score	Mr(calc):	Delta	Sequence
44.2	1629.6294	0.0028	EKEISDDEAEEEK
5.5	1629.6287	0.0035	NCSGDGPSMYPTAEK
4.5	1629.6308	0.0014	DATNVGDESGFAPNR
2.7	1629.6315	0.0007	MAGGGGSSDNGGRAANR
2.4	1629.6398	-0.0076	MYTLETLNINNK
2.4	1629.6398	-0.0076	MYTLETLNINNK
2.4	1629.6398	-0.0076	MYTLETLNINNK
2.3	1629.6308	0.0014	ESHQIGDAYSNSDK
2.3	1629.6308	0.0014	ESHQIGDAYSNSDK
1.8	1629.6229	0.0093	AMENGEIDQASEEK

Spectrum No: 427; Query: 1757; Rank: 1

Peptide View

MS/MS Fragmentation of **ESESEAKLDGETASDSESR**
Found in **IPI00208200**, Tax_Id=10116 Gene_Symbol=Cds2 Phosphatidate cytidylyltransferase 2

Match to Query 1757: 2105.833932 from(702.951920,3+)
Title: 100101RatKid_NS_deglyco_23.610.610.3.dta
Data file K:\NewmanPaper\Piliang\3SubProteomes\Piliang3SP\mgf5ppm\ERLIC_3SubProteomes5ppm.mgf

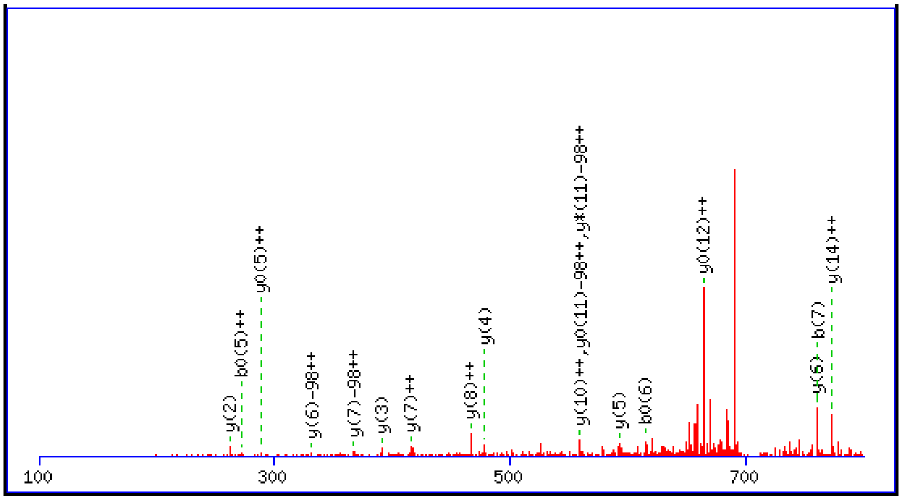

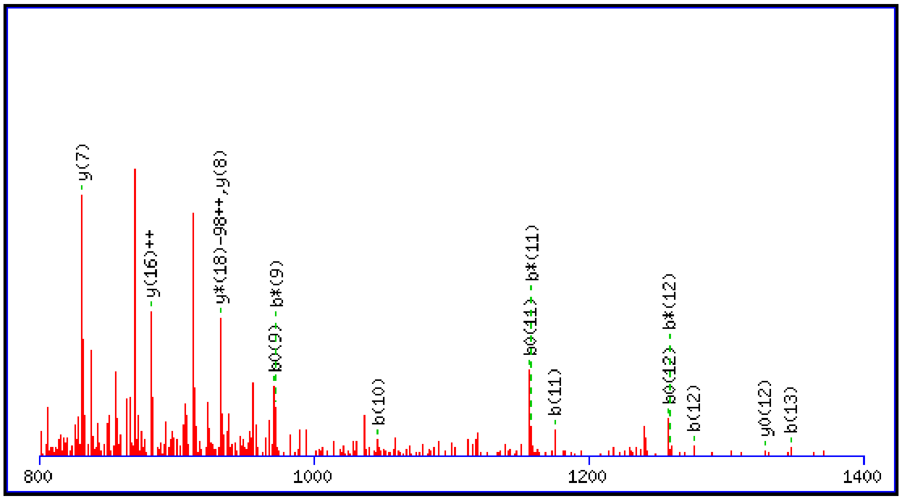

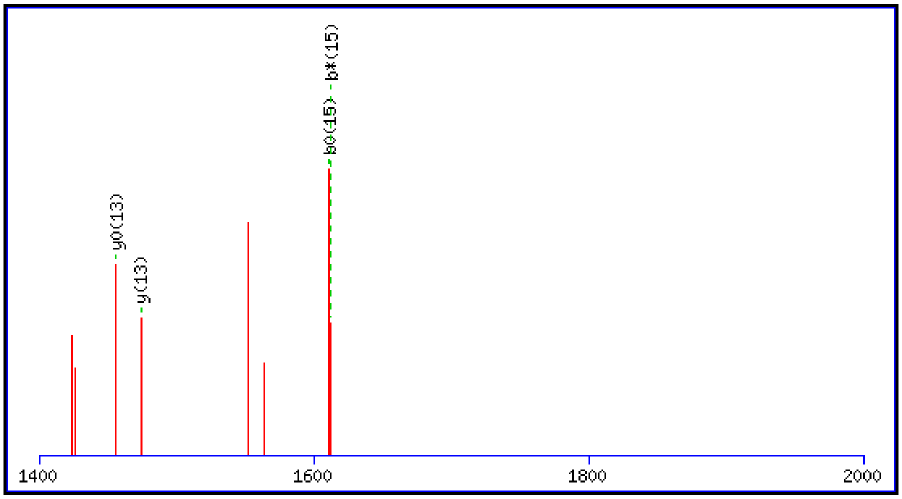

Monoisotopic mass of neutral peptide Mr(calc): 2105.8273
Fixed modifications: Carbamidomethyl (C)
Variable modifications:
S14 : Phospho (ST), with neutral losses 0.0000(shown in table), 97.9769
Ions Score: 44 **Expect:** 0.0069
Matches (Bold Red): 37/310 fragment ions using 59 most intense peaks

#	b	b^{++}	b*	b^{*++}	b^{0}	b^{0++}	Seq.	y	y^{++}	y*	y^{*++}	y^{0}	y^{0++}	#
1	130.0499	65.5286			112.0393	56.5233	E							19
2	217.0819	109.0446			199.0713	100.0393	S	1977.7921	989.3997	1960.7655	980.8864	1959.7815	980.3944	18
3	346.1245	173.5659			328.1139	164.5606	E	1890.7600	945.8837	1873.7335	937.3704	1872.7495	936.8784	17
4	433.1565	217.0819			415.1460	208.0766	S	1761.7175	**881.3624**	1744.6909	872.8491	1743.7069	872.3571	16
5	562.1991	281.6032			544.1885	**272.5979**	E	1674.6854	837.8463	1657.6589	829.3331	1656.6749	828.8411	15

6	633.2362	317.1218			**615.2257**	308.1165	A	1545.6428	**773.3251**	1528.6163	764.8118	1527.6323	764.3198	14
7	**761.3312**	381.1692	744.3046	372.6560	743.3206	372.1639	K	**1474.6057**	737.8065	1457.5792	729.2932	**1456.5952**	728.8012	13
8	874.4153	437.7113	857.3887	429.1980	856.4047	428.7060	L	1346.5108	673.7590	1329.4842	665.2457	**1328.5002**	**664.7537**	12
9	989.4422	495.2247	**972.4156**	486.7115	**971.4316**	486.2195	D	1233.4267	617.2170	1216.4001	608.7037	1215.4161	608.2117	11
10	**1046.4637**	523.7355	1029.4371	515.2222	1028.4531	514.7302	G	1118.3997	**559.7035**	1101.3732	551.1902	1100.3892	550.6982	10
11	**1175.5063**	588.2568	**1158.4797**	579.7435	**1157.4957**	579.2515	E	1061.3783	531.1928	1044.3517	522.6795	1043.3677	522.1875	9
12	**1276.5539**	638.7806	**1259.5274**	630.2673	**1258.5434**	629.7753	T	**932.3357**	**466.6715**	915.3091	458.1582	914.3251	457.6662	8
13	**1347.5910**	674.2992	1330.5645	665.7859	1329.5805	665.2939	A	**831.2880**	**416.1476**	814.2615	407.6344	813.2774	407.1424	7
14	1514.5894	757.7983	1497.5628	749.2851	1496.5788	748.7931	S	**760.2509**	380.6291	743.2243	372.1158	742.2403	371.6238	6
15	1629.6163	815.3118	**1612.5898**	806.7985	**1611.6058**	806.3065	D	**593.2525**	297.1299	576.2260	288.6166	575.2420	**288.1246**	5
16	1716.6484	858.8278	1699.6218	850.3145	1698.6378	849.8225	S	**478.2256**	239.6164	461.1991	231.1032	460.2150	230.6112	4
17	1845.6910	923.3491	1828.6644	914.8358	1827.6804	914.3438	E	**391.1936**	196.1004	374.1670	187.5871	373.1830	187.0951	3
18	1932.7230	966.8651	1915.6964	958.3519	1914.7124	957.8599	S	**262.1510**	131.5791	245.1244	123.0659	244.1404	122.5738	2
19							R	175.1190	88.0631	158.0924	79.5498			1

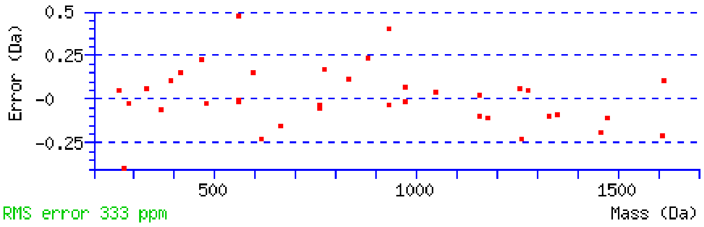

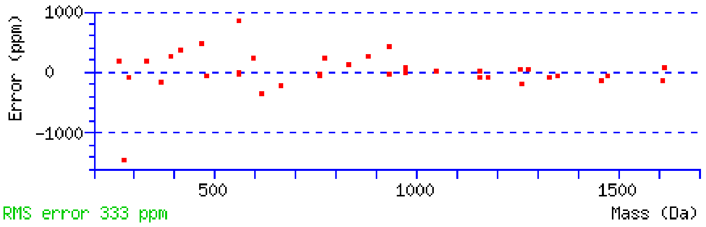

All matches to this query

Score	Mr(calc):	Delta	Sequence
44.2	2105.8273	0.0066	ESESEAKLDGETASDSESR
28.3	2105.8273	0.0066	ESESEAKLDGETASDSESR
27.2	2105.8273	0.0066	ESESEAKLDGETASDSESR
24.8	2105.8273	0.0066	ESESEAKLDGETASDSESR
18.9	2104.8408	0.9932	SLTGIEYKPVSATGAEDK
16.5	2104.8408	0.9932	SLTGIEYKPVSATGAEDK
9.3	2104.8408	0.9932	SLTGIEYKPVSATGAEDK
7.7	2105.8547	-0.0207	RISVVTIFGPVNNSSMK
6.4	2105.8273	0.0066	ESESEAKLDGETASDSESR
5.3	2104.8408	0.9932	SLTGIEYKPVSATGAEDK

Spectrum No: 428; Query: 405; Rank: 1

Peptide View

MS/MS Fragmentation of **SLDGAAAAESTDR**
Found in **IPI00366799**, Tax_Id=10116 Gene_Symbol=RGD1564762_predicted similar to Protein 4.1

Match to Query 405: 1342.537628 from(672.276090,2+)
Title: 091008RatKidney_NoSalt_26.769.769.2.dta
Data file K:\NewmanPaper\Piliang\3SubProteomes\Piliang3SP\mgf5ppm\ERLIC_3SubProteomes5ppm.mgf

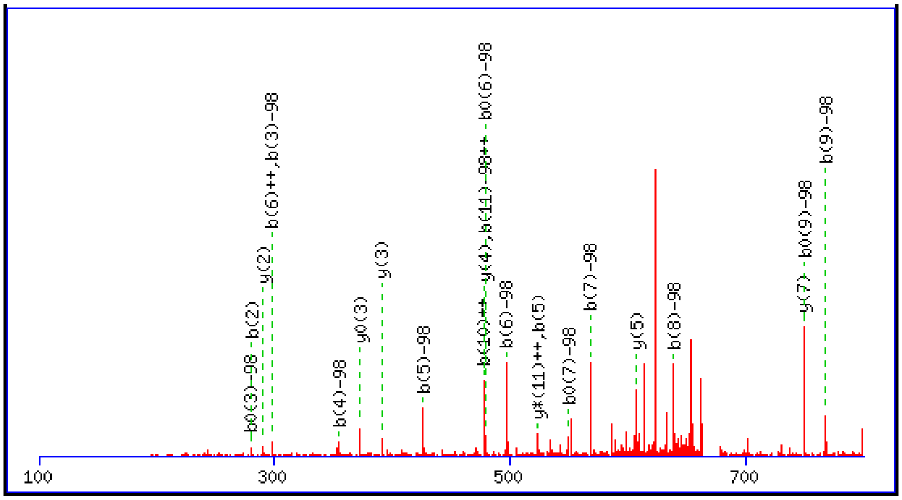

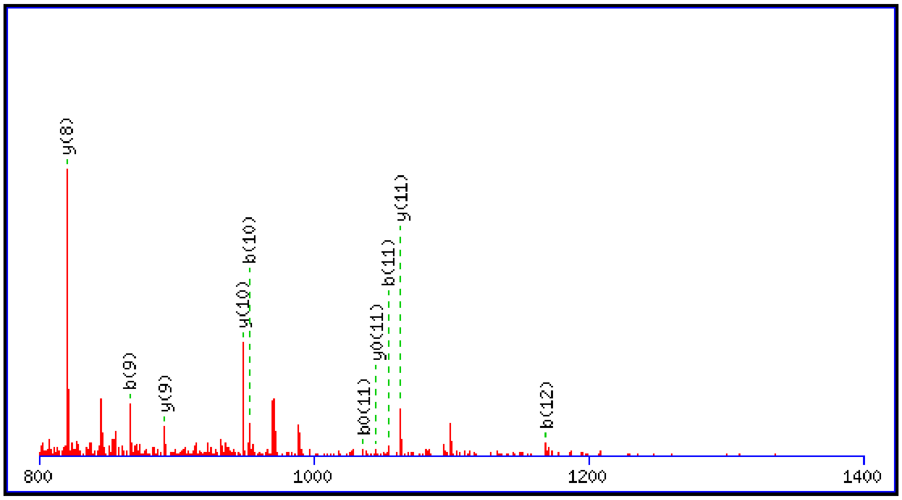

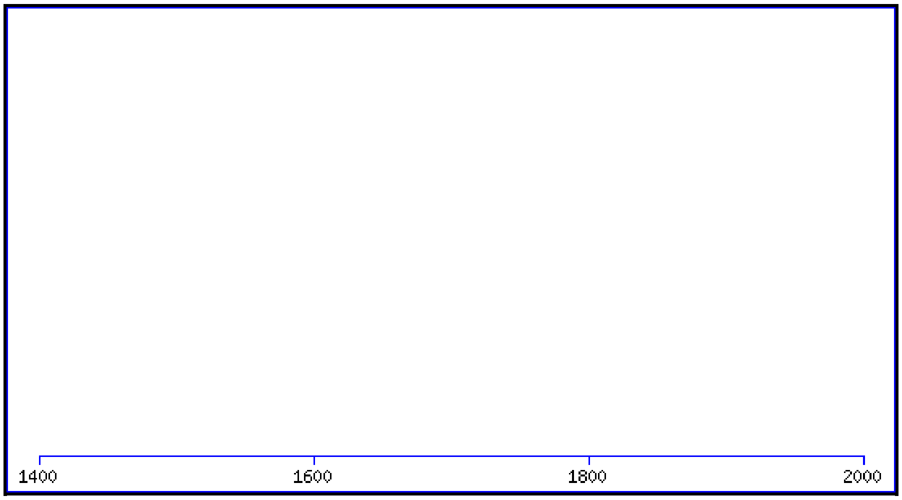

Monoisotopic mass of neutral peptide Mr(calc): 1342.5402
Fixed modifications: Carbamidomethyl (C)
Variable modifications:
S1 : Phospho (ST), with neutral losses 97.9769(shown in table), 0.0000
Ions Score: 44 **Expect:** 0.0044
Matches (Bold Red): 33/166 fragment ions using 72 most intense peaks

#	b	b^{++}	b^{0}	b^{0++}	Seq.	y	y^{++}	y^{*}	y^{*++}	y^{0}	y^{0++}	#
1	70.0287	35.5180	52.0182	26.5127	S							13
2	183.1128	92.0600	165.1022	83.0548	L	1176.5491	588.7782	1159.5226	580.2649	1158.5386	579.7729	12
3	**298.1397**	149.5735	**280.1292**	140.5682	D	**1063.4651**	532.2362	1046.4385	**523.7229**	**1045.4545**	523.2309	11
4	**355.1612**	178.0842	337.1506	169.0790	G	**948.4381**	474.7227	931.4116	466.2094	930.4275	465.7174	10
5	**426.1983**	213.6028	408.1878	204.5975	A	**891.4167**	446.2120	874.3901	437.6987	873.4061	437.2067	9

6	497.2354	249.1214	479.2249	240.1161	A	820.3795	410.6934	803.3530	402.1801	802.3690	401.6881	8
7	568.2725	284.6399	550.2620	275.6346	A	749.3424	375.1748	732.3159	366.6616	731.3319	366.1696	7
8	639.3097	320.1585	621.2991	311.1532	A	678.3053	339.6563	661.2788	331.1430	660.2947	330.6510	6
9	768.3523	384.6798	750.3417	375.6745	E	607.2682	304.1377	590.2416	295.6245	589.2576	295.1325	5
10	855.3843	428.1958	837.3737	419.1905	S	478.2256	239.6164	461.1991	231.1032	460.2150	230.6112	4
11	956.4320	478.7196	938.4214	469.7143	T	391.1936	196.1004	374.1670	187.5872	373.1830	187.0951	3
12	1071.4589	536.2331	1053.4483	527.2278	D	290.1459	145.5766	273.1193	137.0633	272.1353	136.5713	2
13					R	175.1190	88.0631	158.0924	79.5498			1

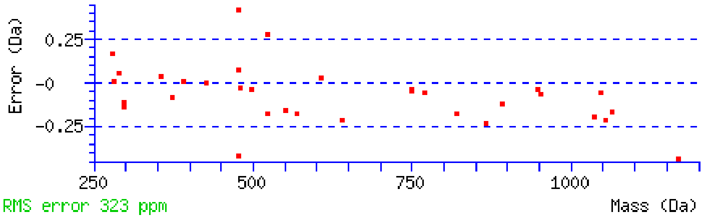

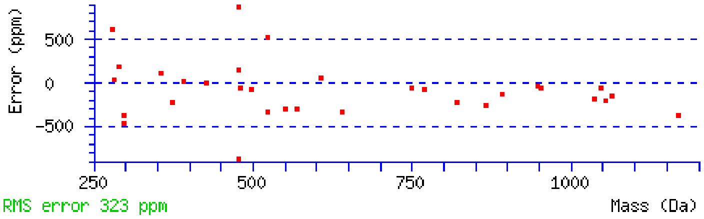

All matches to this query

Score	Mr(calc):	Delta	Sequence
44.1	1342.5402	-0.0025	SLDGAAAAESTDR
8.6	1342.5402	-0.0025	SLNDGLGQSSER
5.5	1342.5402	-0.0025	SLDGAAAAESTDR
4.8	1342.5281	0.0095	YMSVNSLQLL
2.4	1342.5402	-0.0025	SLDGAAAAESTDR
2.1	1342.5442	-0.0066	TVNGHLDSYEK

Spectrum No: 429; Query: 2295; Rank: 1

Peptide View

MS/MS Fragmentation of **WKAEGHSDEESDSEGSDSESSSR**
Found in **IPI00559178**, Tax_Id=10116 Gene_Symbol=RGD1563568_predicted similar to serine/threonine protein kinase MASK

Match to Query 2295: 2576.951502 from(859.991110,3+)
Title: 100101RatKid_NS_deglyco_21.549.551.3.dta
Data file K:\NewmanPaper\Piliang\3SubProteomes\Piliang3SP\mgf5ppm\ERLIC_3SubProteomes5ppm.mgf

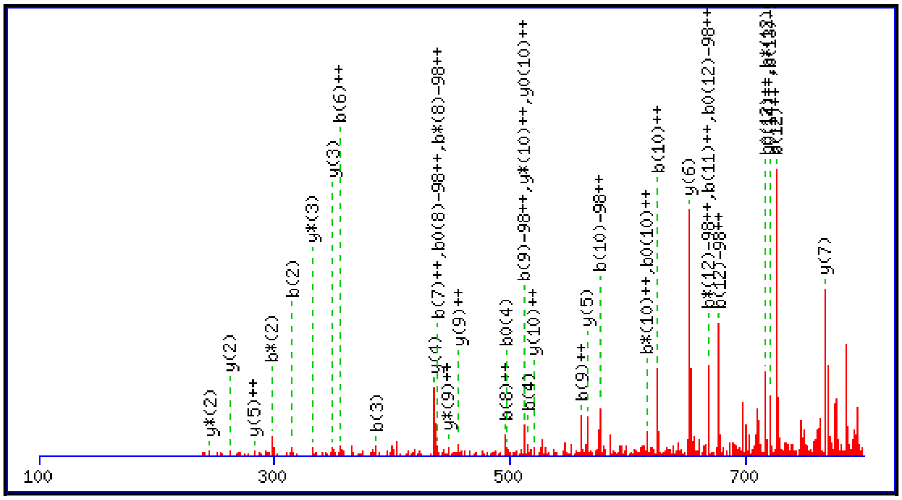

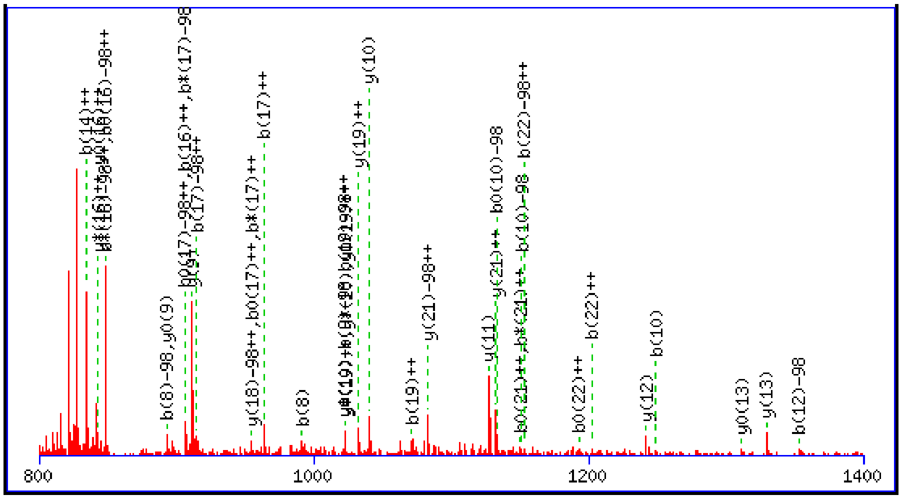

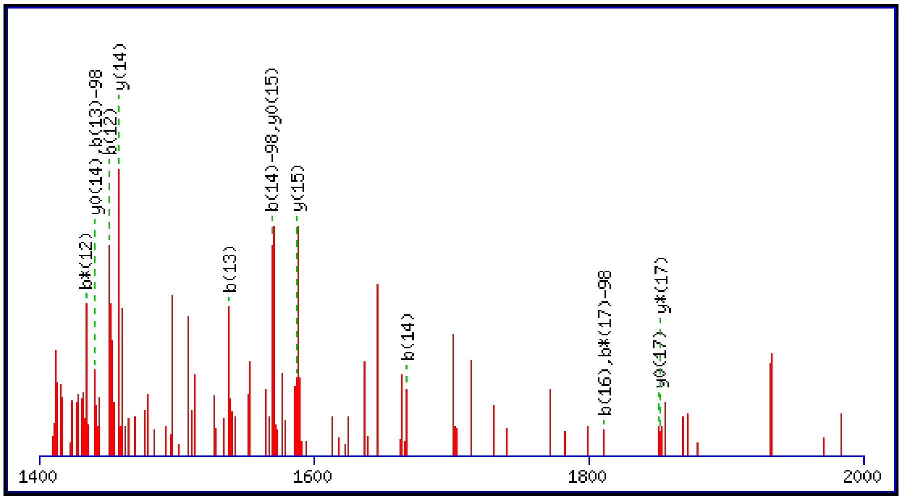

Monoisotopic mass of neutral peptide Mr(calc): 2576.9412
Fixed modifications: Carbamidomethyl (C)
Variable modifications:
S7 : Phospho (ST), with neutral losses 0.0000(shown in table), 97.9769
Ions Score: 44 **Expect:** 0.0061
Matches (Bold Red): 95/386 fragment ions using 154 most intense peaks

#	b	b^{++}	b*	b^{*++}	b^{0}	b^{0++}	Seq.	y	y^{++}	y*	y^{*++}	y^{0}	y^{0++}	#
1	187.0866	94.0469					W							23
2	**315.1816**	158.0944	**298.1550**	149.5811			K	2391.8692	1196.4382	2374.8427	1187.9250	2373.8586	1187.4330	22
3	**386.2187**	193.6130	369.1921	185.0997			A	2263.7742	**1132.3908**	2246.7477	1123.8775	2245.7637	1123.3855	21
4	**515.2613**	258.1343	498.2347	249.6210	**497.2507**	249.1290	E	2192.7371	1096.8722	2175.7106	1088.3589	2174.7266	1087.8669	20
5	572.2827	286.6450	555.2562	278.1317	554.2722	277.6397	G	2063.6945	**1032.3509**	2046.6680	**1023.8376**	2045.6840	**1023.3456**	19
6	709.3416	**355.1745**	692.3151	346.6612	691.3311	346.1692	H	2006.6731	1003.8402	1989.6465	995.3269	1988.6625	994.8349	18
7	876.3400	**438.6736**	859.3134	430.1604	858.3294	429.6683	S	1869.6142	935.3107	**1852.5876**	926.7974	**1851.6036**	926.3054	17
8	**991.3669**	**496.1871**	974.3404	487.6738	973.3564	487.1818	D	1702.6158	851.8115	1685.5893	**843.2983**	1684.6052	**842.8063**	16
9	1120.4095	**560.7084**	1103.3830	552.1951	1102.3990	551.7031	E	**1587.5889**	794.2981	1570.5623	785.7848	**1569.5783**	785.2928	15
10	**1249.4521**	**625.2297**	1232.4256	**616.7164**	1231.4416	**616.2244**	E	**1458.5463**	729.7768	1441.5197	**721.2635**	**1440.5357**	**720.7715**	14
11	1336.4841	**668.7457**	1319.4576	660.2324	1318.4736	659.7404	S	**1329.5037**	665.2555	1312.4771	656.7422	**1311.4931**	656.2502	13
12	**1451.5111**	**726.2592**	**1434.4845**	**717.7459**	1433.5005	**717.2539**	D	**1242.4717**	621.7395	1225.4451	613.2262	1224.4611	612.7342	12
13	**1538.5431**	769.7752	1521.5166	761.2619	1520.5326	760.7699	S	**1127.4447**	564.2260	1110.4182	555.7127	1109.4341	555.2207	11
14	**1667.5857**	**834.2965**	1650.5592	825.7832	1649.5751	825.2912	E	**1040.4127**	**520.7100**	**1023.3861**	**512.1967**	**1022.4021**	**511.7047**	10
15	1724.6072	862.8072	1707.5806	854.2939	1706.5966	853.8019	G	**911.3701**	**456.1887**	894.3435	**447.6754**	**893.3595**	447.1834	9
16	**1811.6392**	**906.3232**	1794.6127	897.8100	1793.6286	897.3180	S	854.3486	427.6780	837.3221	419.1647	836.3381	418.6727	8
17	1926.6661	**963.8367**	1909.6396	**955.3234**	1908.6556	**954.8314**	D	**767.3166**	384.1619	750.2901	375.6487	749.3060	375.1567	7
18	2013.6982	1007.3527	1996.6716	998.8394	1995.6876	998.3474	S	**652.2897**	326.6485	635.2631	318.1352	634.2791	317.6432	6

**19**	2142.7408	**1071.8740**	2125.7142	1063.3607	2124.7302	1062.8687	**E**	**565.2576**	**283.1325**	548.2311	274.6192	547.2471	274.1272	**5**
**20**	2229.7728	1115.3900	2212.7462	1106.8768	2211.7622	1106.3848	**S**	**436.2150**	218.6112	419.1885	210.0979	418.2045	209.6059	**4**
**21**	2316.8048	1158.9060	2299.7783	**1150.3928**	2298.7943	**1149.9008**	**S**	**349.1830**	175.0951	**332.1565**	166.5819	331.1724	166.0899	**3**
**22**	2403.8368	**1202.4221**	2386.8103	1193.9088	2385.8263	**1193.4168**	**S**	**262.1510**	131.5791	**245.1244**	123.0659	244.1404	122.5738	**2**
**23**							**R**	175.1190	88.0631	158.0924	79.5498			**1**

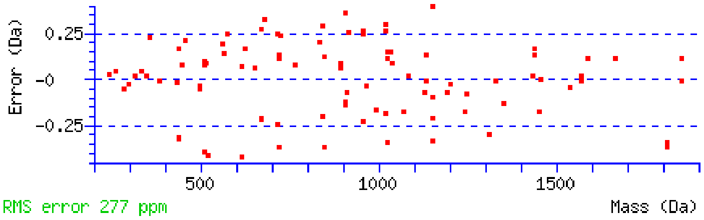

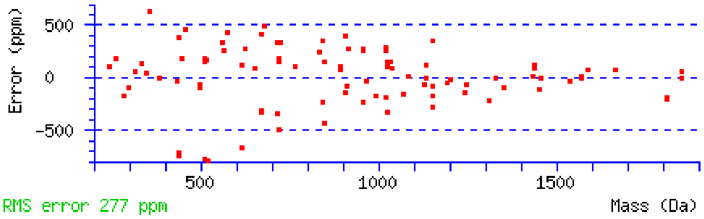

All matches to this query

Score	Mr(calc):	Delta	Sequence
44.1	2576.9412	0.0103	WKAEGHSDEESDSEGSDSESSSR
38.9	2576.9412	0.0103	WKAEGHSDEESDSEGSDSESSSR
34.4	2576.9412	0.0103	WKAEGHSDEESDSEGSDSESSSR
31.5	2576.9412	0.0103	WKAEGHSDEESDSEGSDSESSSR
23.6	2576.9412	0.0103	WKAEGHSDEESDSEGSDSESSSR
19.6	2576.9412	0.0103	WKAEGHSDEESDSEGSDSESSSR
15.8	2576.9412	0.0103	WKAEGHSDEESDSEGSDSESSSR
14.8	2576.9412	0.0103	WKAEGHSDEESDSEGSDSESSSR
4.7	2575.9518	0.9997	VTETVMNGGMKETLSLTVDAK
3.0	2574.9215	2.0300	VGVGTCGIADKPMTQYQDTSK

Spectrum No: 430; Query: 2848; Rank: 1

Peptide View

MS/MS Fragmentation of **LPPPFPGMDPESEGAAGGSEPEAGDSDTEGEDIFTGAAAASKPQSPK**
Found in **IPI00198369**, Tax_Id=10116 Gene_Symbol=Snx1 Sorting nexin-1

Match to Query 2848: 4679.023736 from(1170.763210,4+)
Title: 100101RatKid_NS_deglyco_21.4353.4353.4.dta
Data file K:\NewmanPaper\Piliang\3SubProteomes\Piliang3SP\mgf5ppm\ERLIC_3SubProteomes5ppm.mgf

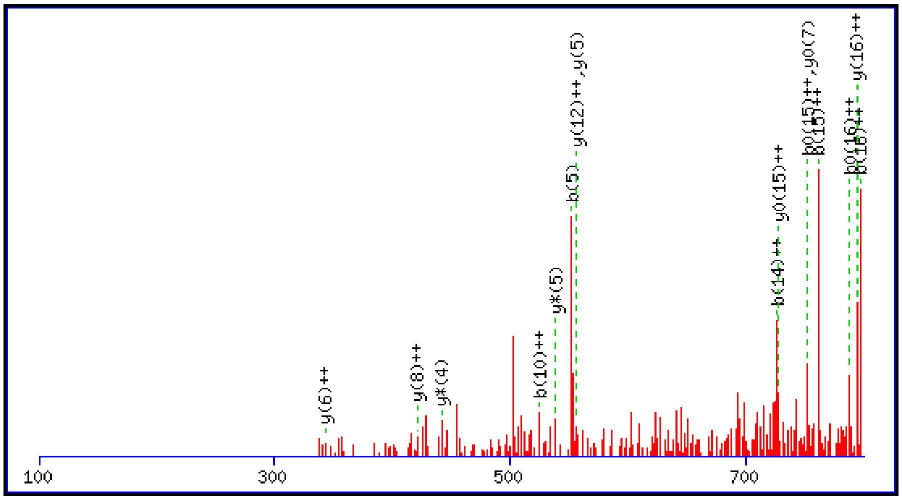

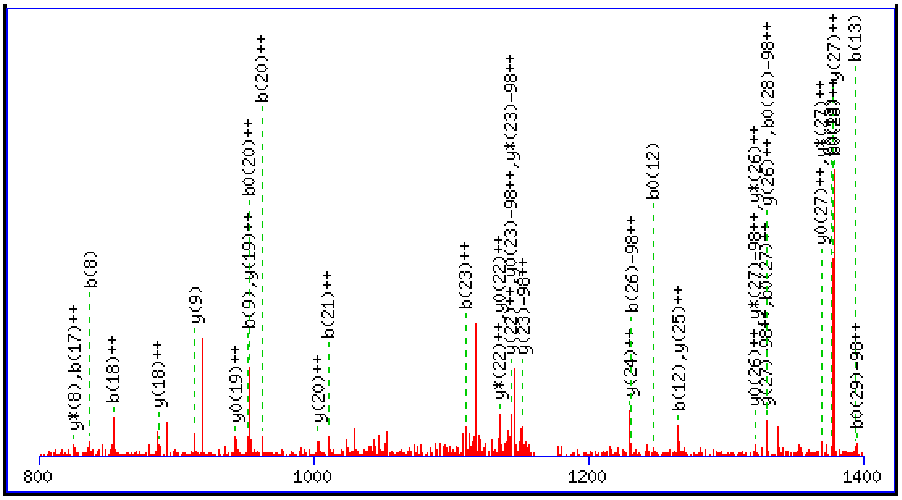

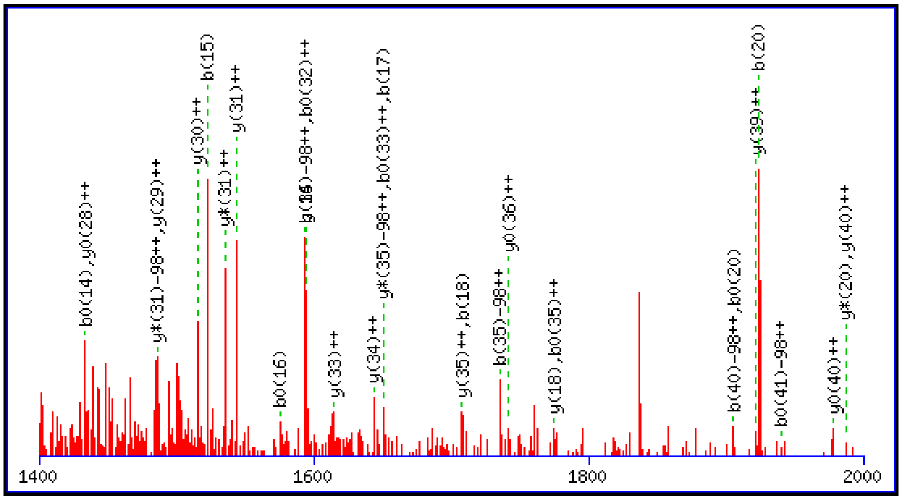

Monoisotopic mass of neutral peptide Mr(calc): 4678.0218
Fixed modifications: Carbamidomethyl (C)
Variable modifications:
S26 : Phospho (ST), with neutral losses 0.0000(shown in table), 97.9769
Ions Score: 44 **Expect:** 0.023
Matches (Bold Red): 86/694 fragment ions using 169 most intense peaks

#	b	b^{++}	b*	b^{*++}	b^0	b^{0++}	Seq.	y	y^{++}	y*	y^{*++}	y^0	y^{0++}	#
1	114.0913	57.5493					L							47
2	211.1441	106.0757					P	4565.9450	2283.4762	4548.9185	2274.9629	4547.9345	2274.4709	46
3	308.1969	154.6021					P	4468.8923	2234.9498	4451.8657	2226.4365	4450.8817	2225.9445	45
4	405.2496	203.1285					P	4371.8395	2186.4234	4354.8130	2177.9101	4353.8289	2177.4181	44
5	**552.3180**	276.6627					F	4274.7867	2137.8970	4257.7602	2129.3837	4256.7762	2128.8917	43
6	649.3708	325.1890					P	4127.7183	2064.3628	4110.6918	2055.8495	4109.7078	2055.3575	42
7	706.3923	353.6998					G	4030.6656	2015.8364	4013.6390	2007.3231	4012.6550	2006.8311	41
8	**837.4328**	419.2200					M	3973.6441	**1987.3257**	3956.6175	1978.8124	3955.6335	**1978.3204**	40
9	**952.4597**	476.7335			934.4491	467.7282	D	3842.6036	**1921.8054**	3825.5771	1913.2922	3824.5930	1912.8002	39
10	1049.5125	**525.2599**			1031.5019	516.2546	P	3727.5767	1864.2920	3710.5501	1855.7787	3709.5661	1855.2867	38
11	1178.5551	589.7812			1160.5445	580.7759	E	3630.5239	1815.7656	3613.4974	1807.2523	3612.5133	1806.7603	37
12	**1265.5871**	633.2972			**1247.5765**	624.2919	S	3501.4813	1751.2443	3484.4548	1742.7310	3483.4707	**1742.2390**	36
13	**1394.6297**	697.8185			**1376.6191**	688.8132	E	3414.4493	**1707.7283**	3397.4227	1699.2150	3396.4387	1698.7230	35
14	1451.6511	**726.3292**			**1433.6406**	717.3239	G	3285.4067	**1643.2070**	3268.3801	1634.6937	3267.3961	1634.2017	34
15	**1522.6883**	**761.8478**			1504.6777	**752.8425**	A	3228.3852	**1614.6963**	3211.3587	1606.1830	3210.3747	1605.6910	33
16	**1593.7254**	**797.3663**			**1575.7148**	**788.3610**	A	3157.3481	1579.1777	3140.3216	1570.6644	3139.3375	1570.1724	32
17	**1650.7468**	**825.8771**			1632.7363	816.8718	G	3086.3110	**1543.6591**	3069.2845	**1535.1459**	3068.3004	1534.6539	31
18	**1707.7683**	**854.3878**			1689.7577	845.3825	G	3029.2895	**1515.1484**	3012.2630	1506.6351	3011.2790	1506.1431	30

19	1794.8003	897.9038			1776.7898	888.8985	S	2972.2681	**1486.6377**	2955.2415	1478.1244	2954.2575	1477.6324	29
20	**1923.8429**	**962.4251**			**1905.8324**	**953.4198**	E	2885.2360	1443.1217	2868.2095	1434.6084	2867.2255	**1434.1164**	28
21	2020.8957	**1010.9515**			2002.8851	1001.9462	P	2756.1935	**1378.6004**	2739.1669	**1370.0871**	2738.1829	**1369.5951**	27
22	2149.9383	1075.4728			2131.9277	1066.4675	E	2659.1407	**1330.0740**	2642.1141	**1321.5607**	2641.1301	**1321.0687**	26
23	2220.9754	**1110.9913**			2202.9648	1101.9861	A	2530.0981	**1265.5527**	2513.0715	1257.0394	2512.0875	1256.5474	25
24	2277.9969	1139.5021			2259.9863	1130.4968	G	2459.0610	**1230.0341**	2442.0344	1221.5209	2441.0504	1221.0288	24
25	2393.0238	1197.0155			2375.0132	1188.0103	D	2402.0395	1201.5234	2385.0130	1193.0101	2384.0290	1192.5181	23
26	2560.0222	1280.5147			2542.0116	1271.5094	S	2287.0126	**1144.0099**	2269.9860	**1135.4966**	2269.0020	**1135.0046**	22
27	2675.0491	1338.0282			2657.0385	**1329.0229**	D	2120.0142	1060.5107	2102.9877	1051.9975	2102.0037	1051.5055	21
28	2776.0968	1388.5520			2758.0862	**1379.5467**	T	2004.9873	**1002.9973**	**1987.9607**	994.4840	1986.9767	993.9920	20
29	2905.1394	1453.0733			2887.1288	1444.0680	E	1903.9396	**952.4734**	1886.9130	943.9602	1885.9290	**943.4682**	19
30	2962.1608	1481.5841			2944.1503	1472.5788	G	**1774.8970**	**887.9521**	1757.8705	879.4389	1756.8864	878.9469	18
31	3091.2034	1546.1053			3073.1929	1537.1001	E	1717.8755	859.4414	1700.8490	850.9281	1699.8650	850.4361	17
32	3206.2304	1603.6188			3188.2198	**1594.6135**	D	1588.8329	**794.9201**	1571.8064	786.4068	1570.8224	785.9148	16
33	3319.3144	1660.1609			3301.3039	**1651.1556**	I	1473.8060	737.4066	1456.7795	728.8934	1455.7954	**728.4014**	15
34	3466.3828	1733.6951			3448.3723	1724.6898	F	1360.7219	680.8646	1343.6954	672.3513	1342.7114	671.8593	14
35	3567.4305	1784.2189			3549.4200	**1775.2136**	T	1213.6535	607.3304	1196.6270	598.8171	1195.6430	598.3251	13
36	3624.4520	1812.7296			3606.4414	1803.7243	G	1112.6058	**556.8066**	1095.5793	548.2933	1094.5953	547.8013	12
37	3695.4891	1848.2482			3677.4785	1839.2429	A	1055.5844	528.2958	1038.5578	519.7826	1037.5738	519.2905	11
38	3766.5262	1883.7667			3748.5156	1874.7615	A	984.5473	492.7773	967.5207	484.2640	966.5367	483.7720	10
39	3837.5633	1919.2853			3819.5528	1910.2800	A	**913.5102**	457.2587	896.4836	448.7454	895.4996	448.2534	9
40	3908.6004	1954.8039			3890.5899	1945.7986	A	842.4730	**421.7402**	**825.4465**	413.2269	824.4625	412.7349	8
41	3995.6325	1998.3199			3977.6219	1989.3146	S	771.4359	386.2216	754.4094	377.7083	**753.4254**	377.2163	7
42	4123.7274	2062.3674	4106.7009	2053.8541	4105.7169	2053.3621	K	684.4039	**342.7056**	667.3774	334.1923	666.3933	333.7003	6
43	4220.7802	2110.8937	4203.7536	2102.3805	4202.7696	2101.8885	P	**556.3089**	278.6581	**539.2824**	270.1448	538.2984	269.6528	5
44	4348.8388	2174.9230	4331.8122	2166.4098	4330.8282	2165.9177	Q	459.2562	230.1317	**442.2296**	221.6185	441.2456	221.1264	4
45	4435.8708	2218.4390	4418.8443	2209.9258	4417.8602	2209.4338	S	331.1976	166.1024	314.1710	157.5892	313.1870	157.0972	3
46	4532.9236	2266.9654	4515.8970	2258.4521	4514.9130	2257.9601	P	244.1656	122.5864	227.1390	114.0731			2
47							K	147.1128	74.0600	130.0863	65.5468			1

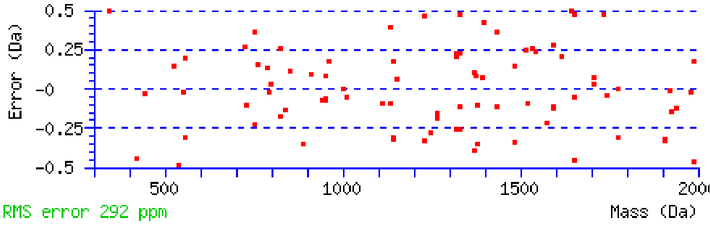

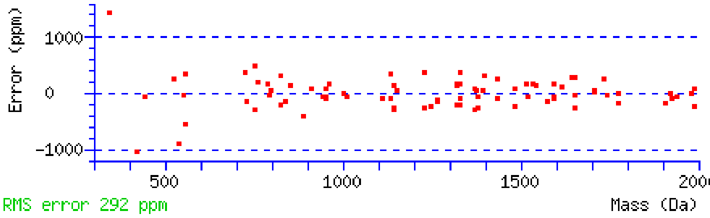

All matches to this query

Score	Mr(calc):	Delta	Sequence
44.0	4678.0218	1.0019	LPPPFPGMDPESEGAAGGSEPEAGDSDTEGEDIFTGAAAASKPQSPK
42.1	4678.0218	1.0019	LPPPFPGMDPESEGAAGGSEPEAGDSDTEGEDIFTGAAAASKPQSPK
27.5	4678.0218	1.0019	LPPPFPGMDPESEGAAGGSEPEAGDSDTEGEDIFTGAAAASKPQSPK
24.3	4678.0218	1.0019	LPPPFPGMDPESEGAAGGSEPEAGDSDTEGEDIFTGAAAASKPQSPK
24.3	4678.0218	1.0019	LPPPFPGMDPESEGAAGGSEPEAGDSDTEGEDIFTGAAAASKPQSPK
18.5	4678.0218	1.0019	LPPPFPGMDPESEGAAGGSEPEAGDSDTEGEDIFTGAAAASKPQSPK
8.5	4677.9926	1.0311	SAFSPLQPNASHFGADAGAYPLGAPLGLSPLRLAYSAAAAHSR
7.9	4677.0146	2.0091	VPVKHRLGHATTNQSDTSHLLNQTGGSSGDDCPVFSTPGHPK
6.7	4678.9797	0.0441	SHRLERGLPYGGSCGGGIDGGGSSVTTSGEFSNDITELEDSFSK
6.3	4678.9797	0.0441	SHRLERGLPYGGSCGGGIDGGGSSVTTSGEFSNDITELEDSFSK

Spectrum No: 431; Query: 1836; Rank: 1

Peptide View

MS/MS Fragmentation of **LTSIGSDEDEETETYQEK**
Found in **IPI00209590**, Tax_Id=10116 Gene_Symbol=Slc12a6 solute carrier family 12, member 6

Match to Query 1836: 2152.865248 from(1077.439900,2+)
Title: 100101RatKid_NS_deglyco_24.1855.1855.2.dta
Data file K:\NewmanPaper\Piliang\3SubProteomes\Piliang3SP\mgf5ppm\ERLIC_3SubProteomes5ppm.mgf

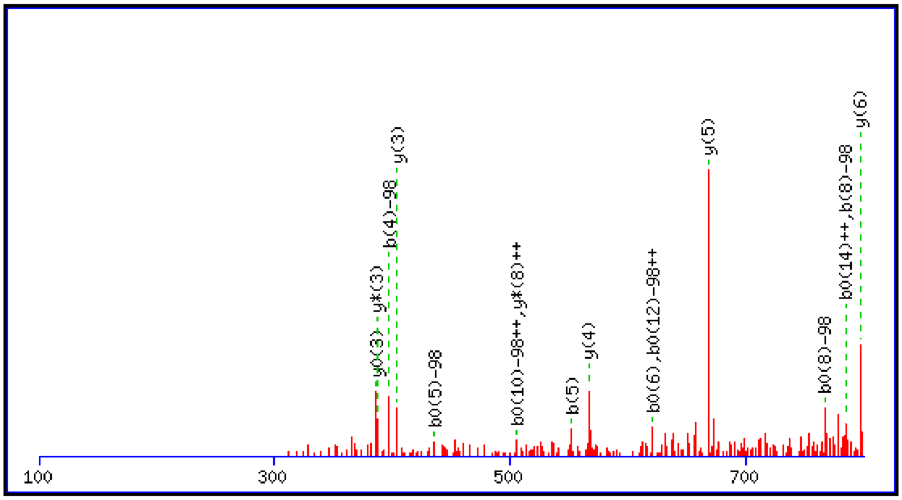

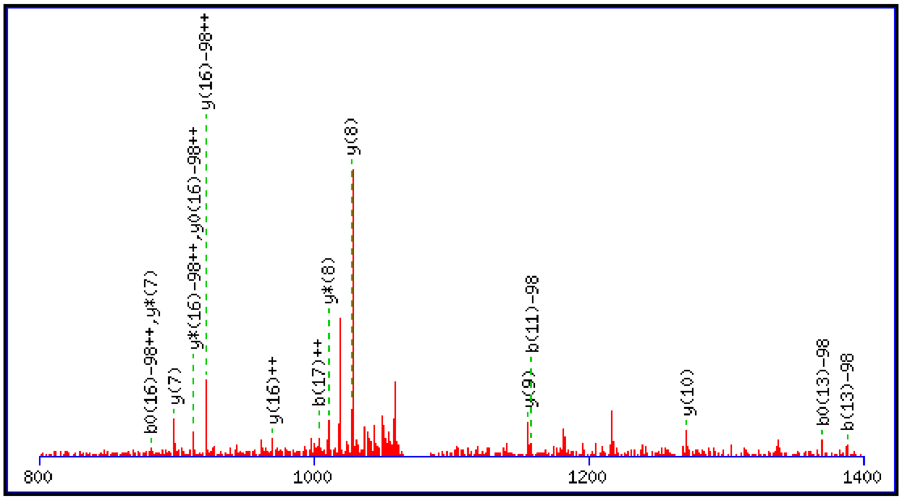

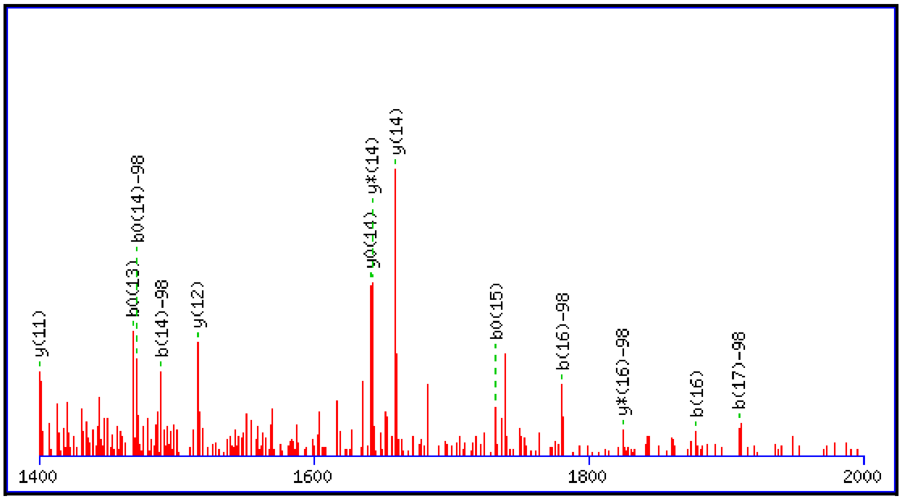

Monoisotopic mass of neutral peptide Mr(calc): 2152.8573
Fixed modifications: Carbamidomethyl (C)
Variable modifications:
S3 : Phospho (ST), with neutral losses 97.9769(shown in table), 0.0000
Ions Score: 44 **Expect:** 0.0087
Matches (Bold Red): 44/246 fragment ions using 89 most intense peaks

#	b	b^{++}	b*	b*$^{++}$	b^{0}	b^{0++}	Seq.	y	y^{++}	y*	y*$^{++}$	y^{0}	y^{0++}	#
1	114.0913	57.5493					L							18
2	215.1390	108.0731			197.1285	99.0679	T	1942.8036	971.9054	1925.7771	963.3922	1924.7930	962.9002	17
3	284.1605	142.5839			266.1499	133.5786	S	1841.7559	**921.3816**	**1824.7294**	**912.8683**	1823.7454	**912.3763**	16
4	**397.2445**	199.1259			379.2340	190.1206	I	1772.7345	886.8709	1755.7079	878.3576	1754.7239	877.8656	15
5	454.2660	227.6366			**436.2554**	218.6314	G	**1659.6504**	830.3288	**1642.6239**	821.8156	**1641.6398**	821.3236	14
6	541.2980	271.1527			523.2875	262.1474	S	1602.6289	801.8181	1585.6024	793.3048	1584.6184	792.8128	13
7	656.3250	328.6661			638.3144	319.6608	D	**1515.5969**	758.3021	1498.5704	749.7888	1497.5864	749.2968	12
8	**785.3676**	393.1874			**767.3570**	384.1821	E	**1400.5700**	700.7886	1383.5434	692.2754	1382.5594	691.7833	11
9	900.3945	450.7009			882.3839	441.6956	D	**1271.5274**	636.2673	1254.5008	627.7541	1253.5168	627.2620	10
10	1029.4371	515.2222			1011.4265	**506.2169**	E	**1156.5004**	578.7539	1139.4739	570.2406	1138.4899	569.7486	9
11	**1158.4797**	579.7435			1140.4691	570.7382	E	**1027.4578**	514.2326	**1010.4313**	**505.7193**	1009.4473	505.2273	8
12	1259.5274	630.2673			1241.5168	**621.2620**	T	**898.4153**	449.7113	**881.3887**	441.1980	880.4047	440.7060	7
13	**1388.5700**	694.7886			**1370.5594**	685.7833	E	**797.3676**	399.1874	780.3410	390.6742	779.3570	390.1821	6
14	**1489.6176**	745.3125			**1471.6071**	736.3072	T	**668.3250**	334.6661	651.2984	326.1529	650.3144	325.6608	5
15	1652.6810	826.8441			1634.6704	817.8388	Y	**567.2773**	284.1423	550.2508	275.6290	549.2667	275.1370	4
16	**1780.7396**	890.8734	1763.7130	882.3601	1762.7290	**881.8681**	Q	**404.2140**	202.6106	**387.1874**	194.0974	**386.2034**	193.6053	3
17	**1909.7821**	955.3947	1892.7556	946.8814	1891.7716	946.3894	E	276.1554	138.5813	259.1288	130.0681	258.1448	129.5761	2
18							K	147.1128	74.0600	130.0863	65.5468			1

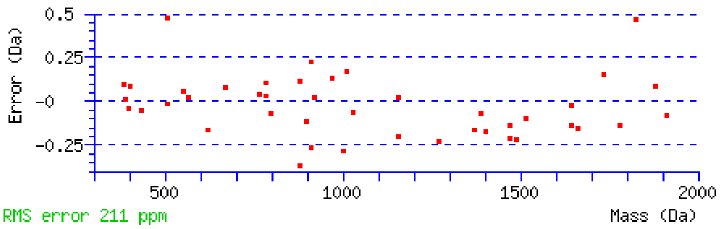

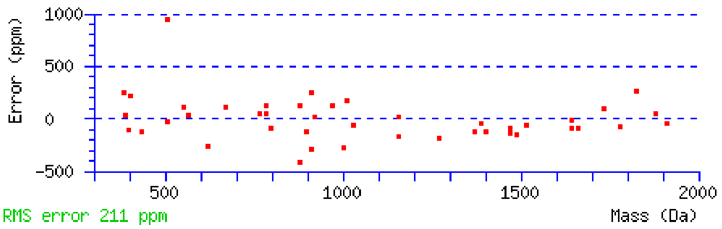

All matches to this query

Score	Mr(calc):	Delta	Sequence
44.0	2152.8573	0.0080	LTSIGSDEDEETETYQEK
42.0	2152.8573	0.0080	LTSIGSDEDEETETYQEK
42.0	2152.8573	0.0080	LTSIGSDEDEETETYQEK
10.1	2152.8573	0.0080	LTSIGSDEDEETETYQEK
7.0	2151.8571	1.0081	TCQRWSEQTPHRHNR
3.3	2152.8573	0.0080	LTSIGSDEDEETETYQEK
2.0	2151.8571	1.0081	TCQRWSEQTPHRHNR
1.4	2152.8474	0.0179	VNYETEDSESSFVTHNSK
0.9	2152.8474	0.0179	VNYETEDSESSFVTHNSK
0.9	2152.8474	0.0179	VNYETEDSESSFVTHNSK

Spectrum No: 432; Query: 2816; Rank: 1

Peptide View

MS/MS Fragmentation of **RTFQQIQEEEDDDYPGSYSPQDPSAGPLLTEELIK**
Found in **IPI00364827**, Tax_Id=10116 Gene_Symbol=RGD1304782_predicted hypothetical protein LOC311591

Match to Query 2816: 4076.838612 from(1359.953480,3+)
Title: 100101RatKid_NS_deglyco_19.4539.4539.3.dta
Data file K:\NewmanPaper\Piliang\3SubProteomes\Piliang3SP\mgf5ppm\ERLIC_3SubProteomes5ppm.mgf

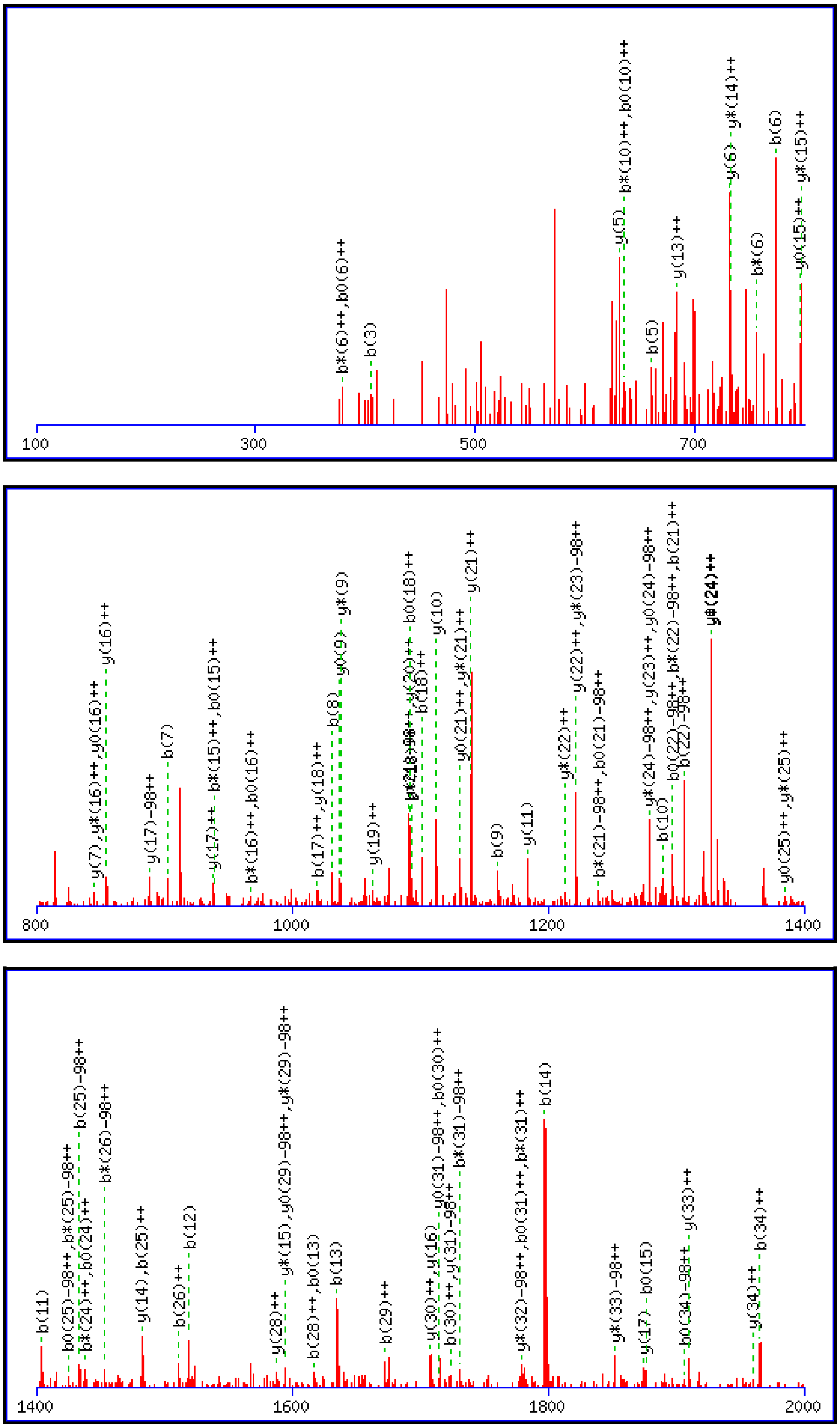

Monoisotopic mass of neutral peptide Mr(calc): 4074.8259
Fixed modifications: Carbamidomethyl (C)
Variable modifications:
S19 : Phospho (ST), with neutral losses 0.0000(shown in table), 97.9769
Ions Score: 44 **Expect:** 0.02
Matches (Bold Red): 96/604 fragment ions using 168 most intense peaks

#	b	b^{++}	b*	b^{*++}	b^0	b^{0++}	Seq.	y	y^{++}	y*	y^{*++}	y^0	y^{0++}	#

1	157.1084	79.0578	140.0818	70.5446			R							35
2	258.1561	129.5817	241.1295	121.0684	240.1455	120.5764	T	3919.7321	1960.3697	3902.7056	1951.8564	3901.7215	1951.3644	34
3	405.2245	203.1159	388.1979	194.6026	387.2139	194.1106	F	3818.6844	1909.8459	3801.6579	1901.3326	3800.6739	1900.8406	33
4	533.2831	267.1452	516.2565	258.6319	515.2725	258.1399	Q	3671.6160	1836.3116	3654.5895	1827.7984	3653.6055	1827.3064	32
5	661.3416	331.1745	644.3151	322.6612	643.3311	322.1692	Q	3543.5574	1772.2824	3526.5309	1763.7691	3525.5469	1763.2771	31
6	774.4257	387.7165	757.3992	379.2032	756.4151	378.7112	I	3415.4989	1708.2531	3398.4723	1699.7398	3397.4883	1699.2478	30
7	902.4843	451.7458	885.4577	443.2325	884.4737	442.7405	Q	3302.4148	1651.7110	3285.3882	1643.1978	3284.4042	1642.7058	29
8	1031.5269	516.2671	1014.5003	507.7538	1013.5163	507.2618	E	3174.3562	1587.6817	3157.3297	1579.1685	3156.3457	1578.6765	28
9	1160.5695	580.7884	1143.5429	572.2751	1142.5589	571.7831	E	3045.3136	1523.1605	3028.2871	1514.6472	3027.3031	1514.1552	27
10	1289.6121	645.3097	1272.5855	636.7964	1271.6015	636.3044	E	2916.2710	1458.6392	2899.2445	1450.1259	2898.2605	1449.6339	26
11	1404.6390	702.8231	1387.6125	694.3099	1386.6284	693.8179	D	2787.2284	1394.1179	2770.2019	1385.6046	2769.2179	1385.1126	25
12	1519.6659	760.3366	1502.6394	751.8233	1501.6554	751.3313	D	2672.2015	1336.6044	2655.1749	1328.0911	2654.1909	1327.5991	24
13	1634.6929	817.8501	1617.6663	809.3368	1616.6823	808.8448	D	2557.1746	1279.0909	2540.1480	1270.5776	2539.1640	1270.0856	23
14	1797.7562	899.3817	1780.7297	890.8685	1779.7457	890.3765	Y	2442.1476	1221.5774	2425.1211	1213.0642	2424.1370	1212.5722	22
15	1894.8090	947.9081	1877.7824	939.3949	1876.7984	938.9028	P	2279.0843	1140.0458	2262.0577	1131.5325	2261.0737	1131.0405	21
16	1951.8304	976.4189	1934.8039	967.9056	1933.8199	967.4136	G	2182.0315	1091.5194	2165.0050	1083.0061	2164.0210	1082.5141	20
17	2038.8625	1019.9349	2021.8359	1011.4216	2020.8519	1010.9296	S	2125.0101	1063.0087	2107.9835	1054.4954	2106.9995	1054.0034	19
18	2201.9258	1101.4665	2184.8993	1092.9533	2183.9152	1092.4613	Y	2037.9780	1019.4927	2020.9515	1010.9794	2019.9675	1010.4874	18
19	2368.9242	1184.9657	2351.8976	1176.4524	2350.9136	1175.9604	S	1874.9147	937.9610	1857.8881	929.4477	1856.9041	928.9557	17
20	2465.9769	1233.4921	2448.9504	1224.9788	2447.9664	1224.4868	P	1707.9163	854.4618	1690.8898	845.9485	1689.9058	845.4565	16
21	2594.0355	1297.5214	2577.0089	1289.0081	2576.0249	1288.5161	Q	1610.8636	805.9354	1593.8370	797.4222	1592.8530	796.9301	15
22	2709.0624	1355.0349	2692.0359	1346.5216	2691.0519	1346.0296	D	1482.8050	741.9061	1465.7785	733.3929	1464.7944	732.9009	14
23	2806.1152	1403.5612	2789.0887	1395.0480	2788.1046	1394.5560	P	1367.7781	684.3927	1350.7515	675.8794	1349.7675	675.3874	13
24	2893.1472	1447.0773	2876.1207	1438.5640	2875.1367	1438.0720	S	1270.7253	635.8663	1253.6987	627.3530	1252.7147	626.8610	12
25	2964.1843	1482.5958	2947.1578	1474.0825	2946.1738	1473.5905	A	1183.6933	592.3503	1166.6667	583.8370	1165.6827	583.3450	11
26	3021.2058	1511.1065	3004.1793	1502.5933	3003.1952	1502.1013	G	1112.6562	556.8317	1095.6296	548.3184	1094.6456	547.8264	10
27	3118.2586	1559.6329	3101.2320	1551.1196	3100.2480	1550.6276	P	1055.6347	528.3210	1038.6081	519.8077	1037.6241	519.3157	9
28	3231.3426	1616.1750	3214.3161	1607.6617	3213.3321	1607.1697	L	958.5819	479.7946	941.5554	471.2813	940.5714	470.7893	8
29	3344.4267	1672.7170	3327.4002	1664.2037	3326.4161	1663.7117	L	845.4979	423.2526	828.4713	414.7393	827.4873	414.2473	7
30	3445.4744	1723.2408	3428.4478	1714.7276	3427.4638	1714.2355	T	732.4138	366.7105	715.3872	358.1973	714.4032	357.7053	6
31	3574.5170	1787.7621	3557.4904	1779.2488	3556.5064	1778.7568	E	631.3661	316.1867	614.3396	307.6734	613.3556	307.1814	5
32	3703.5596	1852.2834	3686.5330	1843.7701	3685.5490	1843.2781	E	502.3235	251.6654	485.2970	243.1521	484.3130	242.6601	4
33	3816.6436	1908.8255	3799.6171	1900.3122	3798.6331	1899.8202	L	373.2809	187.1441	356.2544	178.6308			3
34	3929.7277	1965.3675	3912.7011	1956.8542	3911.7171	1956.3622	I	260.1969	130.6021	243.1703	122.0888			2
35							K	147.1128	74.0600	130.0863	65.5468			1

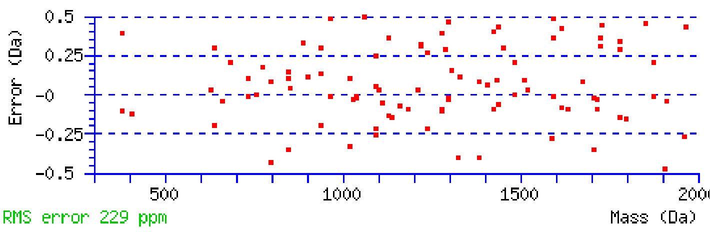

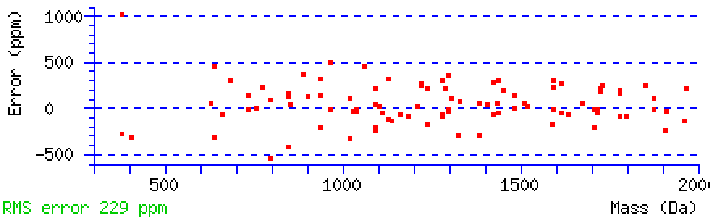

All matches to this query

Score	Mr(calc):	Delta	Sequence
43.9	4074.8259	2.0127	RTFQQIQEEEDDDYPGSYSPQDPSAGPLLTEELIK
37.4	4074.8259	2.0127	RTFQQIQEEEDDDYPGSYSPQDPSAGPLLTEELIK
35.9	4074.8259	2.0127	RTFQQIQEEEDDDYPGSYSPQDPSAGPLLTEELIK
33.3	4074.8259	2.0127	RTFQQIQEEEDDDYPGSYSPQDPSAGPLLTEELIK
23.5	4074.8259	2.0127	RTFQQIQEEEDDDYPGSYSPQDPSAGPLLTEELIK

19.5	4074.8259	2.0127	RTFQQIQEEEDDDYPGSYSPQDPSAGPLLTEELIK
3.4	4076.8122	0.0264	SSQASLASSQPVTPRSSTMDRHSLSLPDPMSFQTQR
3.4	4076.8577	-0.0191	GINGSVNGNSAASVHGVNTSGLSTPASSSMGQNQSTSSGGGNLK
3.4	4076.8577	-0.0191	GINGSVNGNSAASVHGVNTSGLSTPASSSMGQNQSTSSGGGNLK
3.2	4076.8577	-0.0191	GINGSVNGNSAASVHGVNTSGLSTPASSSMGQNQSTSSGGGNLK

Spectrum No: 433; Query: 1622; Rank: 1

Peptide View

MS/MS Fragmentation of **EQESSGEEDNDLSPEER**
Found in **IPI00400646**, Tax_Id=10116 Gene_Symbol=Ppp1r2 Protein phosphatase inhibitor 2

Match to Query 1622: 2028.741008 from(1015.377780,2+)
Title: 091008RatKidney_NoSalt_30.694.694.2.dta
Data file K:\NewmanPaper\Piliang\3SubProteomes\Piliang3SP\mgf5ppm\ERLIC_3SubProteomes5ppm.mgf

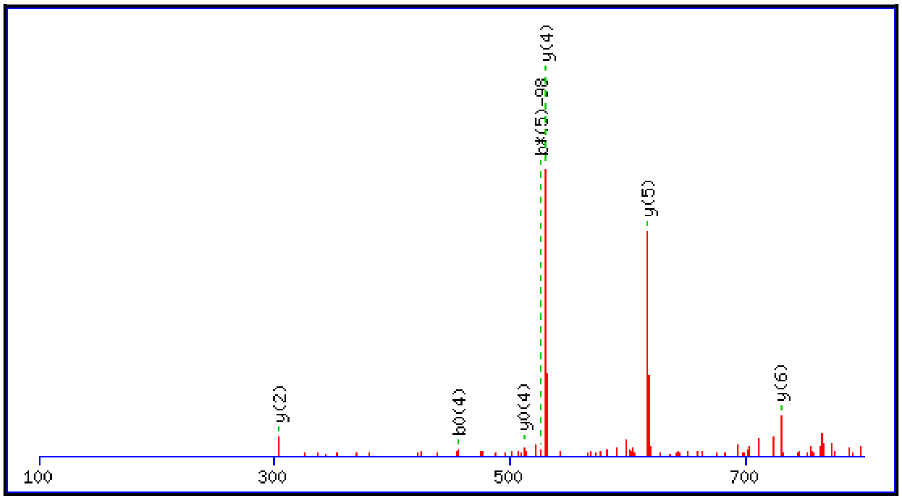

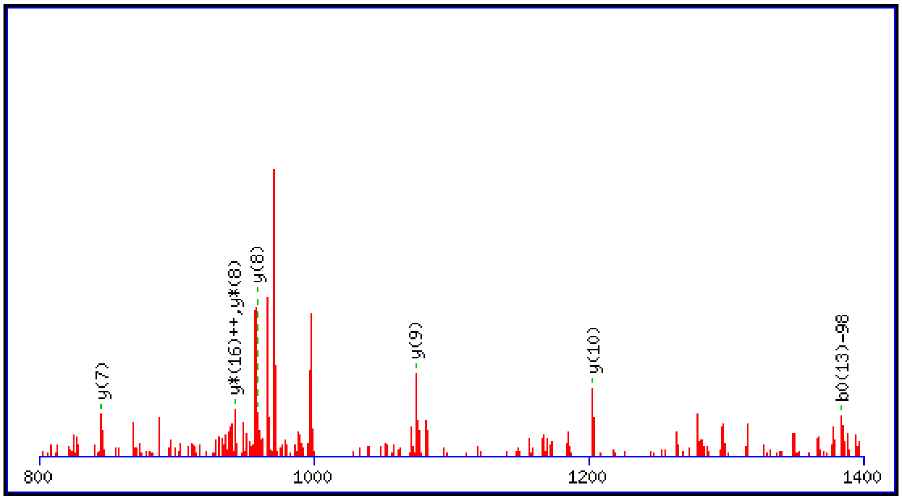

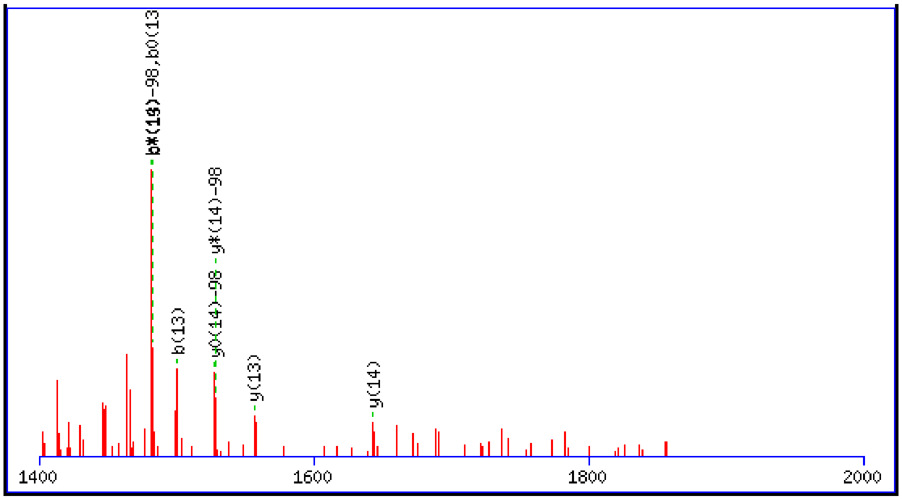

Monoisotopic mass of neutral peptide Mr(calc): 2028.7433
Fixed modifications: Carbamidomethyl (C)
Variable modifications:
S5 : Phospho (ST), with neutral losses 0.0000(shown in table), 97.9769
Ions Score: 44 **Expect:** 0.0046
Matches (Bold Red): 22/284 fragment ions using 38 most intense peaks

#	b	b^{++}	b*	b^{*++}	b^0	b^{0++}	Seq.	y	y^{++}	y*	y^{*++}	y^0	y^{0++}	#
1	130.0499	65.5286			112.0393	56.5233	E							17
2	258.1084	129.5579	241.0819	121.0446	240.0979	120.5526	Q	1900.7080	950.8576	1883.6815	**942.3444**	1882.6974	941.8524	16
3	387.1510	194.0792	370.1245	185.5659	369.1405	185.0739	E	1772.6494	886.8284	1755.6229	878.3151	1754.6389	877.8231	15
4	474.1831	237.5952	457.1565	229.0819	**456.1725**	228.5899	S	**1643.6068**	822.3071	1626.5803	813.7938	1625.5963	813.3018	14
5	641.1814	321.0943	624.1549	312.5811	623.1709	312.0891	S	**1556.5748**	778.7910	1539.5483	770.2778	1538.5642	769.7858	13
[truncated: 834,374 more chars]
