# Supplementary material for: Simultaneous Analysis of Proteome, Phospho- and Glycoproteome of Rat Kidney Tissue with Electrostatic Repulsion Hydrophilic Interaction Chromatography
Source: PLoS One. 2011 Feb 23;6(2):e16884. doi: 10.1371/journal.pone.0016884 (PMC3044146; doi:10.1371/journal.pone.0016884)
Supplement: Data S3 — Tandem MS spectra_of glycopeptides from SCX enrichment. (PDF) [file pone.0016884.s003.pdf]

Mascot Search Results

Results Generated by a Script Modified from Mascot Pepitde View
by Newman Sze, School of Biological Sciences, Nanyang Technological University

---

Spectrum No: 1; Query: 645; Rank: 1

Peptide View

MS/MS Fragmentation of **SNSTAGELDALQAEAGSLDK**
Found in **IPI00365542**, Tax_Id=10116 Gene_Symbol=Lamb1_predicted laminin, beta 1

Match to Query 645: 1976.925148 from(989.469850,2+)
Title: 091127RatKid_SCX01_16.3803.3803.2.dta
Data file K:\NewmanPaper\Piliang\3SubProteomes\Piliang3SP\mgf5ppm\SCX_3SubProteomes5ppm.mgf

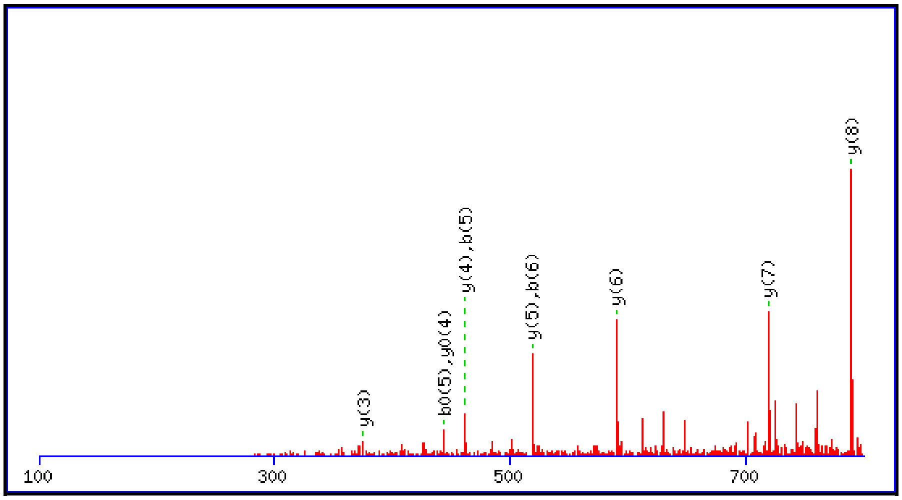

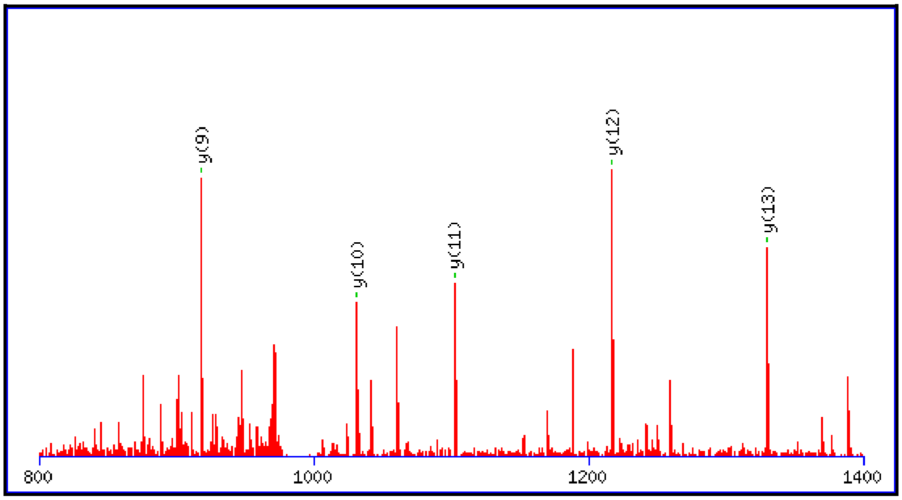

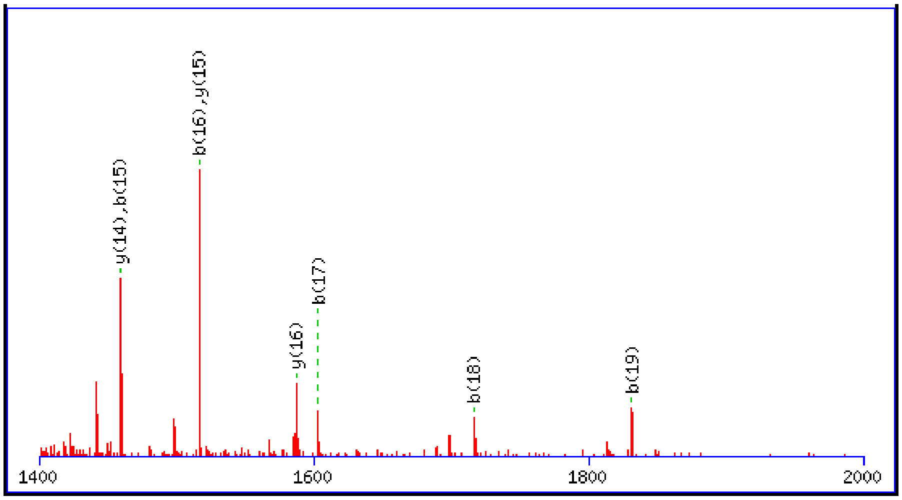

Monoisotopic mass of neutral peptide Mr(calc): 1976.9174
Fixed modifications: Carbamidomethyl (C)
Variable modifications:
N2 : Deamidated_N (N)
Ions Score: 155 **Expect:** 8.2e-014
Matches (Bold Red): 23/224 fragment ions using 23 most intense peaks

#	b	b^{++}	b*	b^{*++}	b^0	b^{0++}	Seq.	y	y^{++}	y*	y^{*++}	y^0	y^{0++}	#
1	88.0393	44.5233			70.0287	35.5180	S							20
2	203.0662	102.0368	186.0397	93.5235	185.0557	93.0315	N	1890.8927	945.9500	1873.8662	937.4367	1872.8821	936.9447	19
3	290.0983	145.5528	273.0717	137.0395	272.0877	136.5475	S	1775.8658	888.4365	1758.8392	879.9232	1757.8552	879.4312	18
4	391.1459	196.0766	374.1194	187.5633	373.1354	187.0713	T	1688.8337	844.9205	1671.8072	836.4072	1670.8232	835.9152	17
5	**462.1831**	231.5952	445.1565	223.0819	**444.1725**	222.5899	A	**1587.7861**	794.3967	1570.7595	785.8834	1569.7755	785.3914	16
6	**519.2045**	260.1059	502.1780	251.5926	501.1940	251.1006	G	**1516.7489**	758.8781	1499.7224	750.3648	1498.7384	749.8728	15
7	648.2471	324.6272	631.2206	316.1139	630.2366	315.6219	E	**1459.7275**	730.3674	1442.7009	721.8541	1441.7169	721.3621	14
8	761.3312	381.1692	744.3046	372.6560	743.3206	372.1639	L	**1330.6849**	665.8461	1313.6583	657.3328	1312.6743	656.8408	13
9	876.3581	438.6827	859.3316	430.1694	858.3476	429.6774	D	**1217.6008**	609.3040	1200.5743	600.7908	1199.5903	600.2988	12
10	947.3952	474.2013	930.3687	465.6880	929.3847	465.1960	A	**1102.5739**	551.7906	1085.5473	543.2773	1084.5633	542.7853	11
11	1060.4793	530.7433	1043.4528	522.2300	1042.4687	521.7380	L	**1031.5368**	516.2720	1014.5102	507.7587	1013.5262	507.2667	10
12	1188.5379	594.7726	1171.5113	586.2593	1170.5273	585.7673	Q	**918.4527**	459.7300	901.4262	451.2167	900.4421	450.7247	9
13	1259.5750	630.2911	1242.5484	621.7779	1241.5644	621.2859	A	**790.3941**	395.7007	773.3676	387.1874	772.3836	386.6954	8
14	1388.6176	694.8124	1371.5910	686.2992	1370.6070	685.8071	E	**719.3570**	360.1821	702.3305	351.6689	701.3464	351.1769	7
15	**1459.6547**	730.3310	1442.6282	721.8177	1441.6441	721.3257	A	**590.3144**	295.6608	573.2879	287.1476	572.3039	286.6556	6
16	**1516.6762**	758.8417	1499.6496	750.3284	1498.6656	749.8364	G	**519.2773**	260.1423	502.2508	251.6290	501.2667	251.1370	5
17	**1603.7082**	802.3577	1586.6816	793.8445	1585.6976	793.3525	S	**462.2558**	231.6316	445.2293	223.1183	**444.2453**	222.6263	4
18	**1716.7923**	858.8998	1699.7657	850.3865	1698.7817	849.8945	L	**375.2238**	188.1155	358.1973	179.6023	357.2132	179.1103	3
19	**1831.8192**	916.4132	1814.7927	907.9000	1813.8086	907.4080	D	262.1397	131.5735	245.1132	123.0602	244.1292	122.5682	2
20							K	147.1128	74.0600	130.0863	65.5468			1

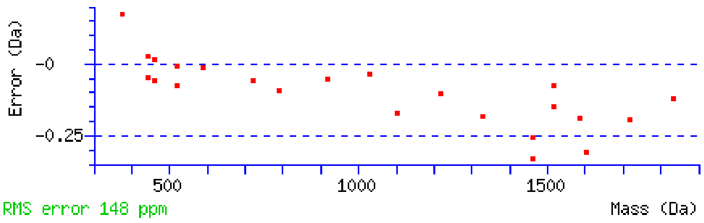

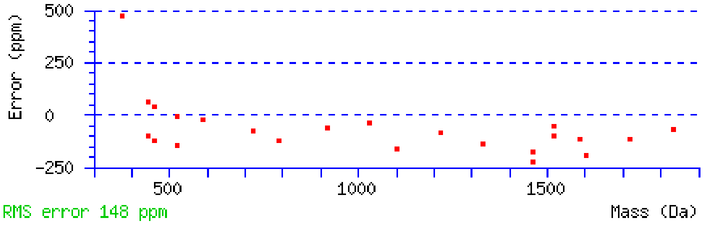

All matches to this query

Score	Mr(calc):	Delta	Sequence
154.6	1976.9174	0.0077	SNSTAGELDALQAEAGSLDK

140.6	1975.9334	0.9917	SNSTAGELDALQAEAGSLDK
9.6	1976.9327	-0.0075	QSLDGFLDETNKENLPR
7.0	1974.9201	2.0051	SDWIHDTPVSLGKADVR
6.8	1976.9206	0.0045	YMVLVPSQLYTETPEK
6.8	1976.9206	0.0045	YMVLVPSQLYTETPEK
6.8	1976.9206	0.0045	YMVLVPSQLYTETPEK
6.5	1975.9308	0.9943	LKFEEITSVINPALDK
5.6	1975.9244	1.0007	GSDLSKWNVSPCKSHMK
5.3	1976.9196	0.0056	ILMHHIPEQTTQLLK

Spectrum No: 2; Query: 792; Rank: 1

Peptide View

MS/MS Fragmentation of **DGGSTTAGNSSQVSDGAAAVLLAR**
Found in **IPI00370596**, Tax_Id=10116 Gene_Symbol=RGD1562373_predicted 3-ketoacyl-CoA thiolase B, peroxisomal precursor

Match to Query 792: 2205.057788 from(1103.536170,2+)
Title: 091127RatKid_SCX01_16.3232.3232.2.dta
Data file K:\NewmanPaper\Piliang\3SubProteomes\Piliang3SP\mgf5ppm\SCX_3SubProteomes5ppm.mgf

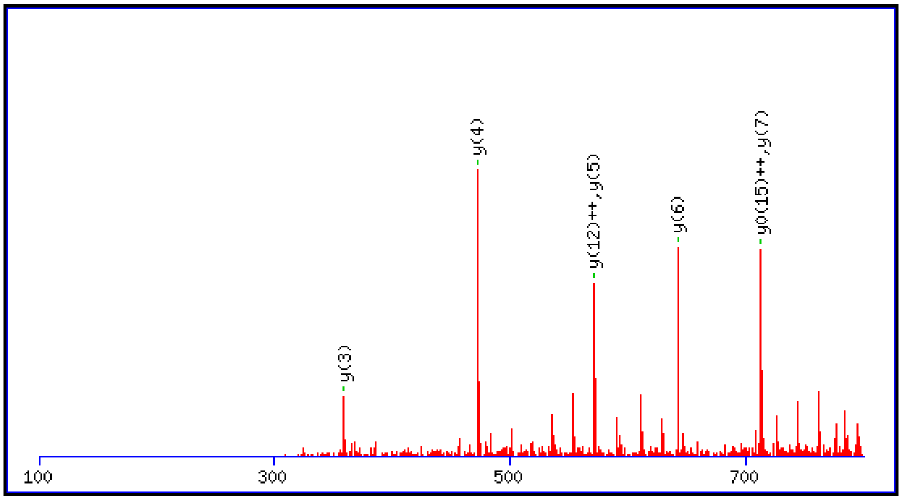

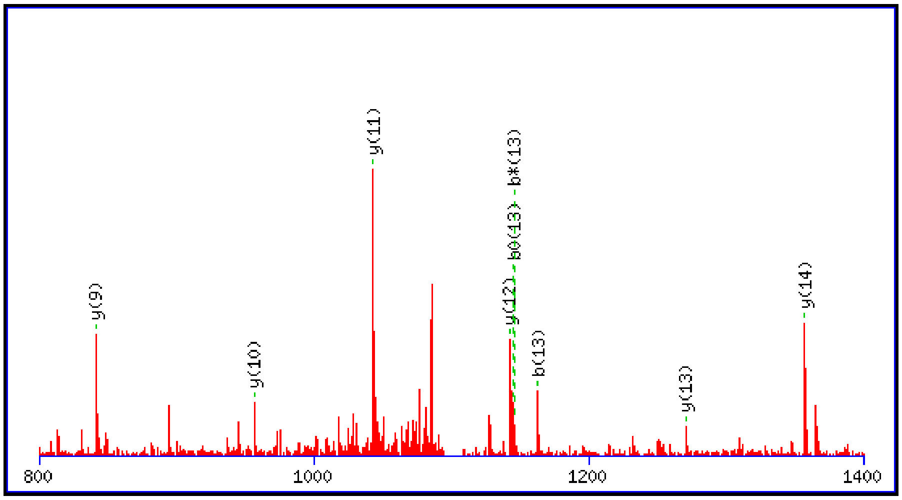

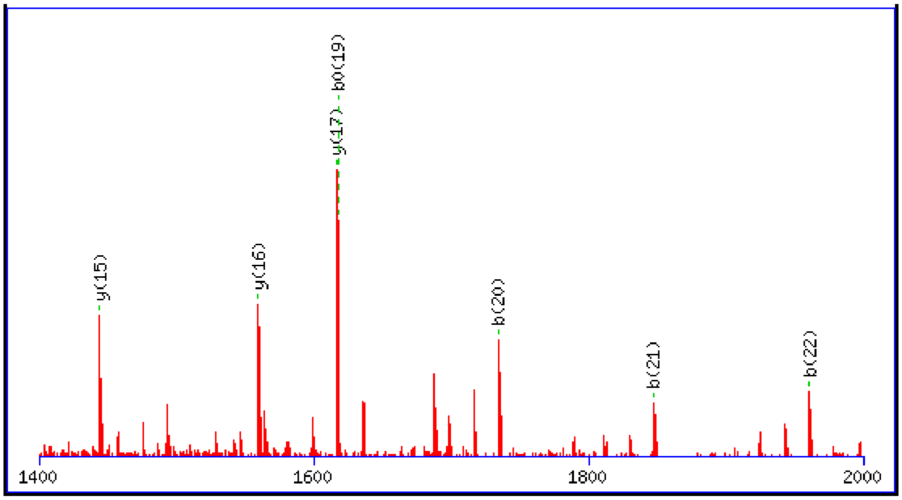

Monoisotopic mass of neutral peptide Mr(calc): 2205.0509
Fixed modifications: Carbamidomethyl (C)
Variable modifications:
N9 : Deamidated_N (N)
Ions Score: 146 **Expect:** 6.7e-013
Matches (Bold Red): 23/242 fragment ions using 26 most intense peaks

#	b	b^{++}	b*	b^{*++}	b^0	b^{0++}	Seq.	y	y^{++}	y*	y^{*++}	y^0	y^{0++}	#
1	116.0342	58.5207			98.0237	49.5155	D							24
2	173.0557	87.0315			155.0451	78.0262	G	2091.0313	1046.0193	2074.0047	1037.5060	2073.0207	1037.0140	23
3	230.0771	115.5422			212.0666	106.5369	G	2034.0098	1017.5085	2016.9832	1008.9953	2015.9992	1008.5033	22
4	317.1092	159.0582			299.0986	150.0529	S	1976.9883	988.9978	1959.9618	980.4845	1958.9778	979.9925	21
5	418.1569	209.5821			400.1463	200.5768	T	1889.9563	945.4818	1872.9298	936.9685	1871.9457	936.4765	20
6	519.2045	260.1059			501.1940	251.1006	T	1788.9086	894.9580	1771.8821	886.4447	1770.8981	885.9527	19
7	590.2416	295.6245			572.2311	286.6192	A	1687.8609	844.4341	1670.8344	835.9208	1669.8504	835.4288	18
8	647.2631	324.1352			629.2525	315.1299	G	**1616.8238**	808.9156	1599.7973	800.4023	1598.8133	799.9103	17
9	762.2900	381.6487	745.2635	373.1354	744.2795	372.6434	N	**1559.8024**	780.4048	1542.7758	771.8915	1541.7918	771.3995	16
10	849.3221	425.1647	832.2955	416.6514	831.3115	416.1594	S	**1444.7754**	722.8914	1427.7489	714.3781	1426.7649	**713.8861**	15
11	936.3541	468.6807	919.3276	460.1674	918.3435	459.6754	S	**1357.7434**	679.3753	1340.7169	670.8621	1339.7328	670.3701	14
12	1064.4127	532.7100	1047.3861	524.1967	1046.4021	523.7047	Q	**1270.7114**	635.8593	1253.6848	627.3461	1252.7008	626.8540	13
13	**1163.4811**	582.2442	**1146.4545**	573.7309	**1145.4705**	573.2389	V	**1142.6528**	**571.8300**	1125.6263	563.3168	1124.6422	562.8248	12
14	1250.5131	625.7602	1233.4866	617.2469	1232.5026	616.7549	S	**1043.5844**	522.2958	1026.5578	513.7826	1025.5738	513.2905	11
15	1365.5401	683.2737	1348.5135	674.7604	1347.5295	674.2684	D	**956.5524**	478.7798	939.5258	470.2665	938.5418	469.7745	10
16	1422.5615	711.7844	1405.5350	703.2711	1404.5510	702.7791	G	**841.5254**	421.2663	824.4989	412.7531			9
17	1493.5986	747.3030	1476.5721	738.7897	1475.5881	738.2977	A	784.5040	392.7556	767.4774	384.2423			8
18	1564.6358	782.8215	1547.6092	774.3082	1546.6252	773.8162	A	**713.4668**	357.2371	696.4403	348.7238			7
19	1635.6729	818.3401	1618.6463	809.8268	**1617.6623**	809.3348	A	**642.4297**	321.7185	625.4032	313.2052			6
20	**1734.7413**	867.8743	1717.7147	859.3610	1716.7307	858.8690	V	**571.3926**	286.1999	554.3661	277.6867			5
21	**1847.8254**	924.4163	1830.7988	915.9030	1829.8148	915.4110	L	**472.3242**	236.6657	455.2976	228.1525			4
22	**1960.9094**	980.9583	1943.8829	972.4451	1942.8988	971.9531	L	**359.2401**	180.1237	342.2136	171.6104			3
23	2031.9465	1016.4769	2014.9200	1007.9636	2013.9360	1007.4716	A	246.1561	123.5817	229.1295	115.0684			2
24							R	175.1190	88.0631	158.0924	79.5498			1

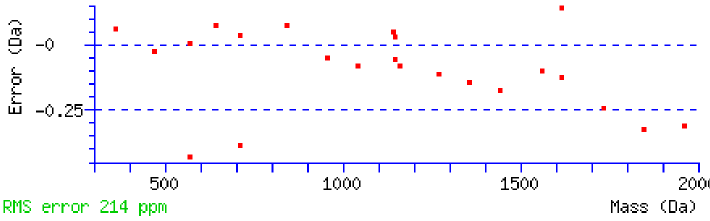

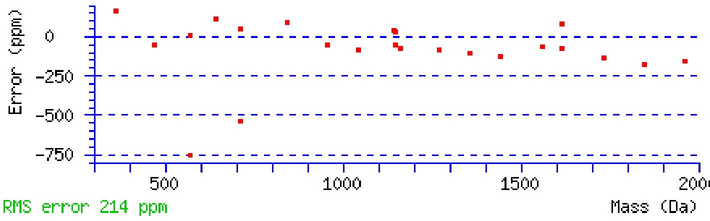

All matches to this query

Score	Mr(calc):	Delta	Sequence
145.8	2205.0509	0.0069	DGGSTTAGNSSQVSDGAAAVLLAR
91.2	2204.0669	0.9909	DGGSTTAGNSSQVSDGAAAVLLAR
12.2	2203.0438	2.0140	GFSWWPAMVVSWKATSKR
11.7	2203.0438	2.0140	GFSWWPAMVVSWKATSKR
6.4	2204.0588	0.9990	IMASFGNFANDNEIKVLLK
6.4	2204.0588	0.9990	IMASFGNFANDNEIKVLLK
6.2	2204.0667	0.9911	TIYIANRFPQNGLYTPQK
6.2	2204.0667	0.9911	TIYIANRFPQNGLYTPQK
3.5	2205.0694	-0.0116	GYFTLRQPVVLWKEMDK
3.5	2204.0643	0.9935	LILTLTHGNVVSTRTYEK

Spectrum No: 3; Query: 854; Rank: 1

Peptide View

MS/MS Fragmentation of **NNLSFIETSALDSTNVEEAFK**
Found in **IPI00210381**, Tax_Id=10116 Gene_Symbol=Rab11b Ras-related protein Rab-11B

Match to Query 854: 2329.100688 from(1165.557620,2+)
Title: 091127RatKid_SCX01_12.5090.5090.2.dta
Data file K:\NewmanPaper\Piliang\3SubProteomes\Piliang3SP\mgf5ppm\SCX_3SubProteomes5ppm.mgf

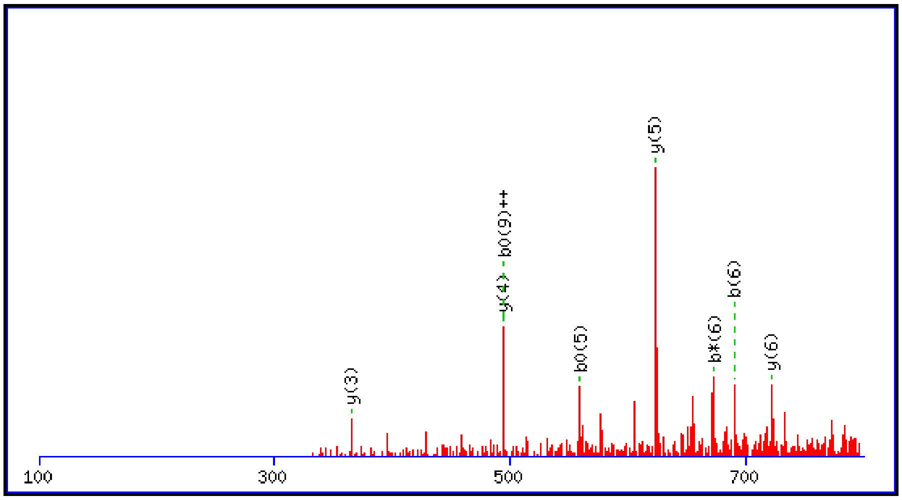

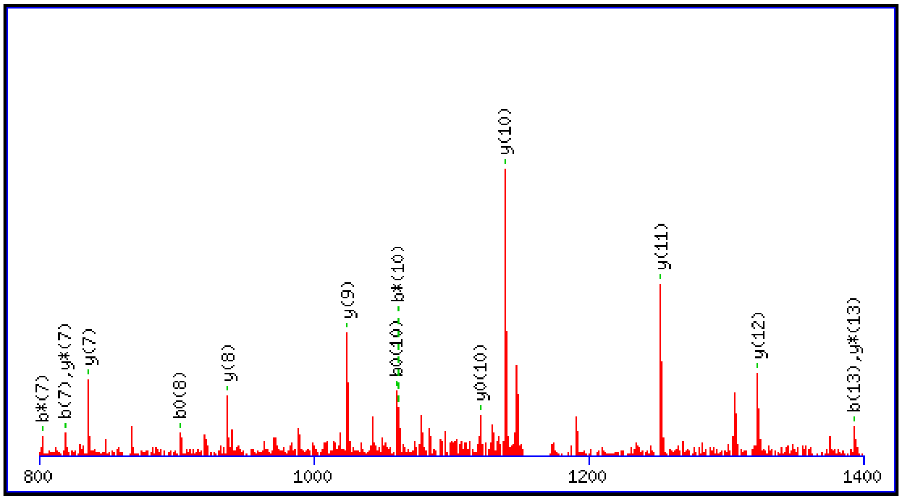

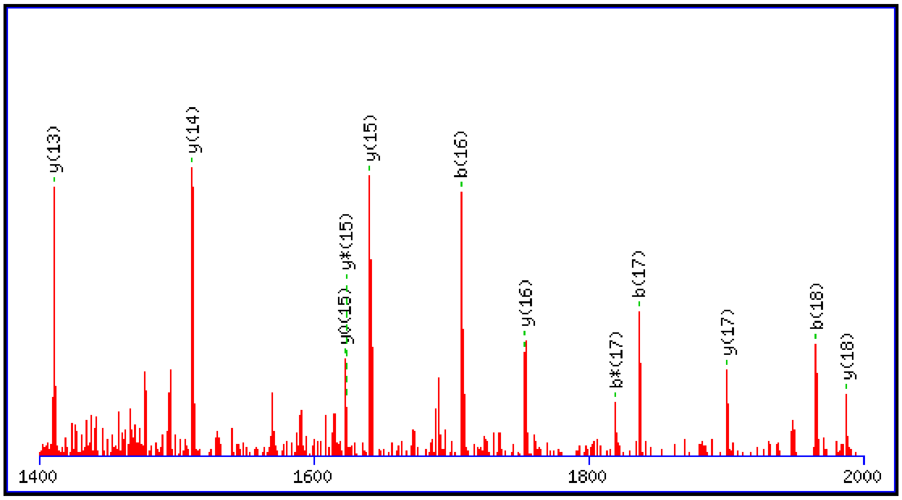

Monoisotopic mass of neutral peptide Mr(calc): 2329.0961
Fixed modifications: Carbamidomethyl (C)
Variable modifications:
N2 : Deamidated_N (N)
Ions Score: 125 **Expect:** 9e-011
Matches (Bold Red): 35/228 fragment ions using 53 most intense peaks

#	b	b^{++}	b*	b^{*++}	b^0	b^{0++}	Seq.	y	y^{++}	y*	y^{*++}	y^0	y^{0++}	#
1	115.0502	58.0287	98.0237	49.5155			N							21
2	230.0771	115.5422	213.0506	107.0289			N	2216.0605	1108.5339	2199.0339	1100.0206	2198.0499	1099.5286	20
3	343.1612	172.0842	326.1347	163.5710			L	2101.0336	1051.0204	2084.0070	1042.5071	2083.0230	1042.0151	19
4	430.1932	215.6003	413.1667	207.0870	412.1827	206.5950	S	**1987.9495**	994.4784	1970.9229	985.9651	1969.9389	985.4731	18
5	577.2616	289.1345	560.2351	280.6212	**559.2511**	280.1292	F	**1900.9175**	950.9624	1883.8909	942.4491	1882.9069	941.9571	17
6	**690.3457**	345.6765	**673.3192**	337.1632	672.3351	336.6712	I	**1753.8491**	877.4282	1736.8225	868.9149	1735.8385	868.4229	16
7	**819.3883**	410.1978	**802.3618**	401.6845	801.3777	401.1925	E	**1640.7650**	820.8861	**1623.7384**	812.3729	**1622.7544**	811.8808	15
8	920.4360	460.7216	903.4094	452.2084	**902.4254**	451.7163	T	**1511.7224**	756.3648	1494.6958	747.8516	1493.7118	747.3596	14
9	1007.4680	504.2376	990.4415	495.7244	989.4574	**495.2324**	S	**1410.6747**	705.8410	**1393.6482**	697.3277	1392.6642	696.8357	13
10	1078.5051	539.7562	**1061.4786**	531.2429	**1060.4946**	530.7509	A	**1323.6427**	662.3250	1306.6161	653.8117	1305.6321	653.3197	12
11	1191.5892	596.2982	1174.5626	587.7850	1173.5786	587.2929	L	**1252.6056**	626.8064	1235.5790	618.2932	1234.5950	617.8011	11
12	1306.6161	653.8117	1289.5896	645.2984	1288.6056	644.8064	D	**1139.5215**	570.2644	1122.4950	561.7511	**1121.5109**	561.2591	10
13	**1393.6482**	697.3277	1376.6216	688.8144	1375.6376	688.3224	S	**1024.4946**	512.7509	1007.4680	504.2376	1006.4840	503.7456	9
14	1494.6958	747.8516	1477.6693	739.3383	1476.6853	738.8463	T	**937.4625**	469.2349	920.4360	460.7216	919.4520	460.2296	8
15	1608.7388	804.8730	1591.7122	796.3597	1590.7282	795.8677	N	**836.4149**	418.7111	**819.3883**	410.1978	818.4043	409.7058	7
16	**1707.8072**	854.4072	1690.7806	845.8940	1689.7966	845.4019	V	**722.3719**	361.6896	705.3454	353.1763	704.3614	352.6843	6
17	**1836.8498**	918.9285	**1819.8232**	910.4152	1818.8392	909.9232	E	**623.3035**	312.1554	606.2770	303.6421	605.2930	303.1501	5
18	**1965.8924**	983.4498	1948.8658	974.9365	1947.8818	974.4445	E	**494.2609**	247.6341	477.2344	239.1208	476.2504	238.6288	4
19	2036.9295	1018.9684	2019.9029	1010.4551	2018.9189	1009.9631	A	**365.2183**	183.1128	348.1918	174.5995			3
20	2183.9979	1092.5026	2166.9713	1083.9893	2165.9873	1083.4973	F	294.1812	147.5942	277.1547	139.0810			2
21							K	147.1128	74.0600	130.0863	65.5468			1

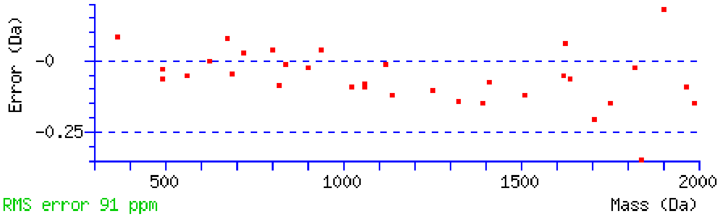

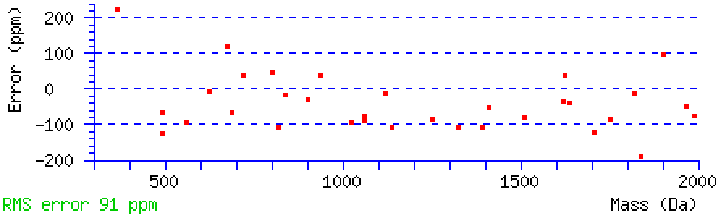

All matches to this query

Score	Mr(calc):	Delta	Sequence

125.3	2329.0961	0.0046	NNLSFIETSALDSTNVEEAFK
125.3	2329.0961	0.0046	NNLSFIETSALDSTNVEEAFK
94.7	2328.1121	0.9886	NNLSFIETSALDSTNVEEAFK
69.8	2329.0961	0.0046	NNLSFIETSALDSTNVEEAFK

Spectrum No: 4; Query: 902; Rank: 1

Peptide View

MS/MS Fragmentation of **VILILDPAISGNETEPYPAFTR**
Found in **IPI00191437**, Tax_Id=10116 Gene_Symbol=- 64 kDa protein

Match to Query 902: 2416.263048 from(1209.138800,2+)
Title: 091129RatKid_SCX02_14.4369.4369.2.dta
Data file K:\NewmanPaper\Piliang\3SubProteomes\Piliang3SP\mgf5ppm\SCX_3SubProteomes5ppm.mgf

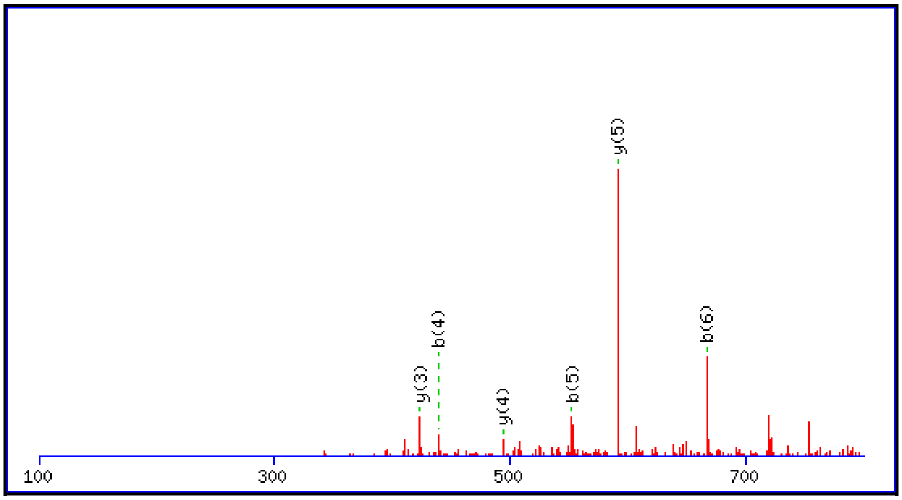

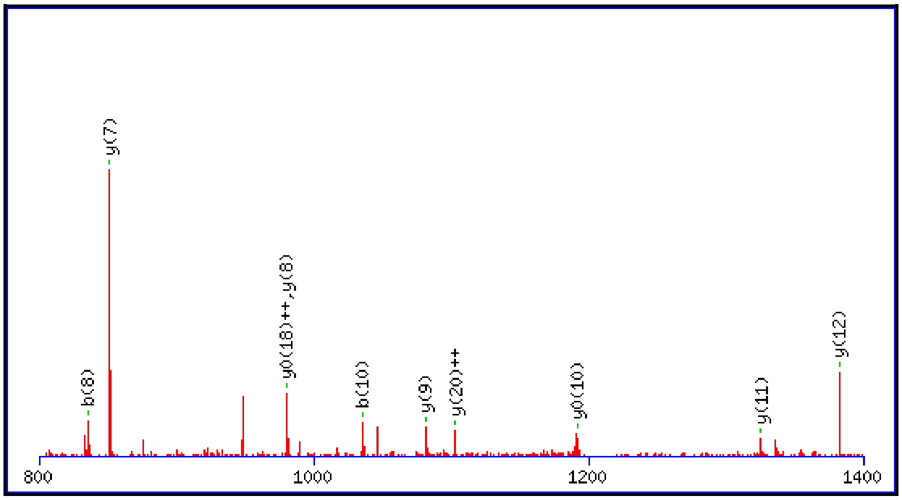

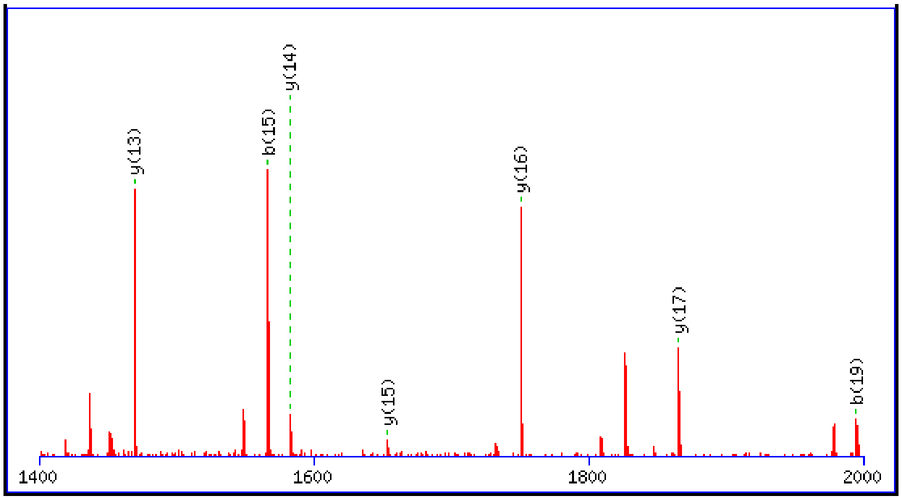

Monoisotopic mass of neutral peptide Mr(calc): 2416.2526
Fixed modifications: Carbamidomethyl (C)
Variable modifications:
N12 : Deamidated_N (N)
Ions Score: 124 **Expect:** 4.6e-011
Matches (Bold Red): 23/218 fragment ions using 29 most intense peaks

#	b	b^{++}	b*	b^{*++}	b^0	b^{0++}	Seq.	y	y^{++}	y*	y^{*++}	y^0	y^{0++}	#
1	100.0757	50.5415					V							22
2	213.1598	107.0835					I	2318.1914	1159.5994	2301.1649	1151.0861	2300.1809	1150.5941	21
3	326.2438	163.6255					L	2205.1074	**1103.0573**	2188.0808	1094.5441	2187.0968	1094.0520	20
4	**439.3279**	220.1676					I	2092.0233	1046.5153	2074.9968	1038.0020	2074.0128	1037.5100	19
5	**552.4119**	276.7096					L	1978.9393	989.9733	1961.9127	981.4600	1960.9287	**980.9680**	18
6	**667.4389**	334.2231			649.4283	325.2178	D	**1865.8552**	933.4312	1848.8286	924.9180	1847.8446	924.4260	17
7	764.4917	382.7495			746.4811	373.7442	P	**1750.8283**	875.9178	1733.8017	867.4045	1732.8177	866.9125	16
8	**835.5288**	418.2680			817.5182	409.2627	A	**1653.7755**	827.3914	1636.7489	818.8781	1635.7649	818.3861	15
9	948.6128	474.8101			930.6023	465.8048	I	**1582.7384**	791.8728	1565.7118	783.3596	1564.7278	782.8675	14
10	**1035.6449**	518.3261			1017.6343	509.3208	S	**1469.6543**	735.3308	1452.6278	726.8175	1451.6437	726.3255	13
11	1092.6663	546.8368			1074.6558	537.8315	G	**1382.6223**	691.8148	1365.5957	683.3015	1364.6117	682.8095	12
12	1207.6933	604.3503	1190.6667	595.8370	1189.6827	595.3450	N	**1325.6008**	663.3040	1308.5743	654.7908	1307.5903	654.2988	11
13	1336.7359	668.8716	1319.7093	660.3583	1318.7253	659.8663	E	1210.5739	605.7906	1193.5473	597.2773	**1192.5633**	596.7853	10
14	1437.7835	719.3954	1420.7570	710.8821	1419.7730	710.3901	T	**1081.5313**	541.2693	1064.5047	532.7560	1063.5207	532.2640	9
15	**1566.8261**	783.9167	1549.7996	775.4034	1548.8156	774.9114	E	**980.4836**	490.7454	963.4571	482.2322	962.4730	481.7402	8
16	1663.8789	832.4431	1646.8523	823.9298	1645.8683	823.4378	P	**851.4410**	426.2241	834.4145	417.7109	833.4305	417.2189	7
17	1826.9422	913.9747	1809.9157	905.4615	1808.9317	904.9695	Y	754.3883	377.6978	737.3617	369.1845	736.3777	368.6925	6
18	1923.9950	962.5011	1906.9684	953.9879	1905.9844	953.4958	P	**591.3249**	296.1661	574.2984	287.6528	573.3144	287.1608	5
19	**1995.0321**	998.0197	1978.0055	989.5064	1977.0215	989.0144	A	**494.2722**	247.6397	477.2456	239.1264	476.2616	238.6344	4
20	2142.1005	1071.5539	2125.0740	1063.0406	2124.0899	1062.5486	F	**423.2350**	212.1212	406.2085	203.6079	405.2245	203.1159	3
21	2243.1482	1122.0777	2226.1216	1113.5645	2225.1376	1113.0724	T	276.1666	138.5870	259.1401	130.0737	258.1561	129.5817	2
22							R	175.1190	88.0631	158.0924	79.5498			1

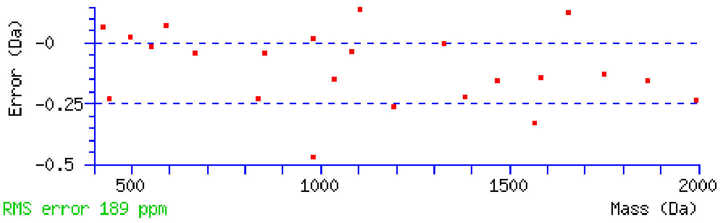

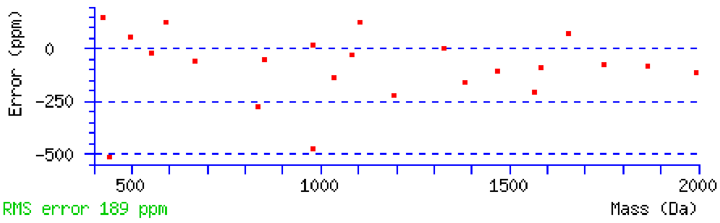

All matches to this query

Score	Mr(calc):	Delta	Sequence
123.8	2416.2526	0.0105	VILILDPAISGNETEPYPAFTR
25.2	2415.2686	0.9945	VILILDPAISGNETEPYPAFTR
8.7	2416.2646	-0.0016	FAFQAEVNRMMKLIINSLYK
4.9	2416.2515	0.0115	EIQRLLPSPLKDAVEVSTWR
0.1	2415.2831	0.9799	QLSIALPQEFAALTKELNLCR
0.1	2415.2546	1.0084	RPSENLGQVLFGERIEPSPYK

Spectrum No: 5; Query: 897; Rank: 1

Peptide View

MS/MS Fragmentation of **IYYSDFSNQTINSMAEDGSNR**
Found in **IPI00205325**, Tax_Id=10116 Gene_Symbol=Lrp2 Low-density lipoprotein receptor-related protein 2 precursor

Match to Query 897: 2412.025688 from(1207.020120,2+)
Title: 091127RatKid_SCX01_12.3028.3028.2.dta
Data file K:\NewmanPaper\Piliang\3SubProteomes\Piliang3SP\mgf5ppm\SCX_3SubProteomes5ppm.mgf

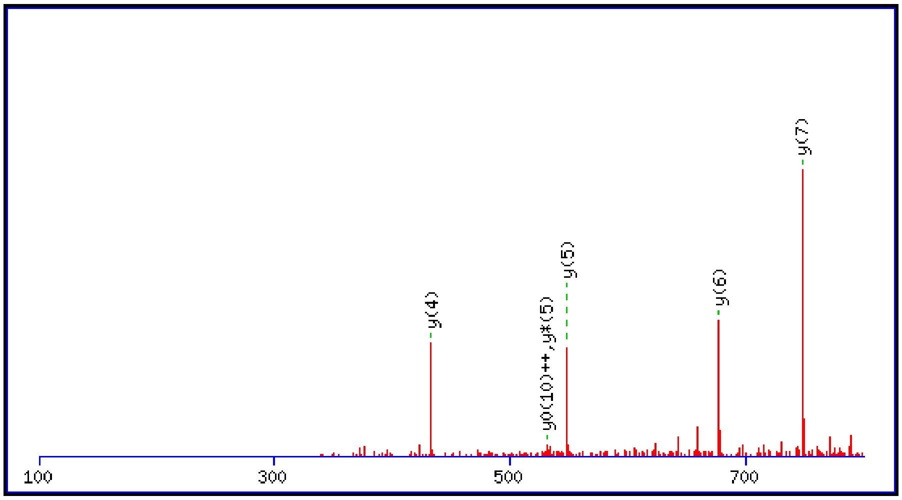

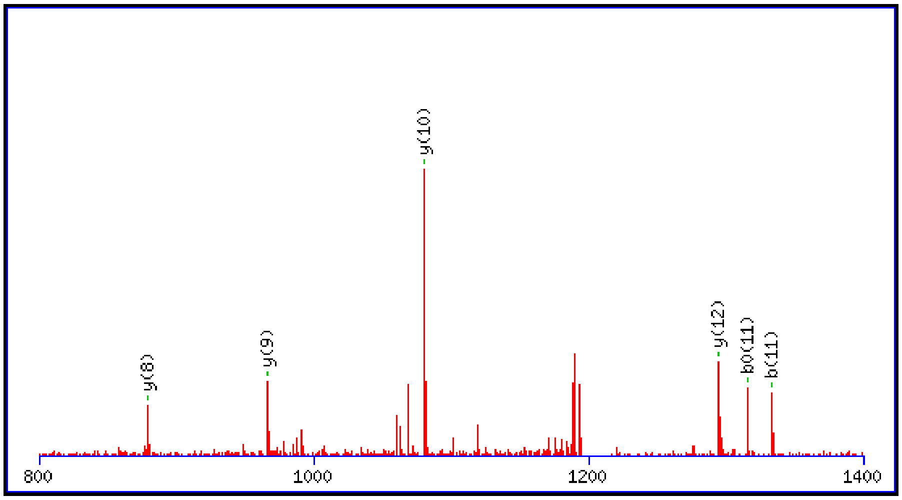

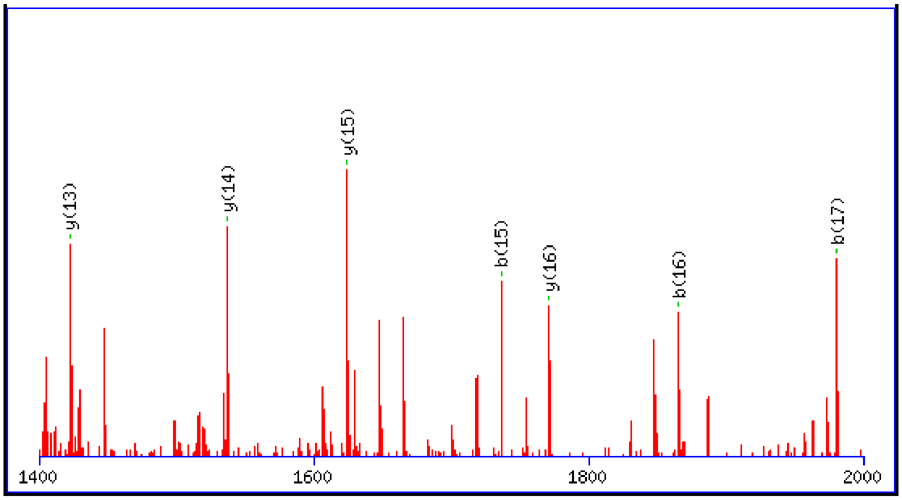

Monoisotopic mass of neutral peptide Mr(calc): 2412.0175
Fixed modifications: Carbamidomethyl (C)
Variable modifications:
N8 : Deamidated_N (N)
Ions Score: 123 **Expect:** 1.4e-010
Matches (Bold Red): 19/216 fragment ions using 19 most intense peaks

#	b	b^{++}	b*	b^{*++}	b^{0}	b^{0++}	Seq.	y	y^{++}	y*	y^{*++}	y^{0}	y^{0++}	#
1	114.0913	57.5493					I							21
2	277.1547	139.0810					Y	2299.9408	1150.4740	2282.9142	1141.9608	2281.9302	1141.4687	20
3	440.2180	220.6126					Y	2136.8775	1068.9424	2119.8509	1060.4291	2118.8669	1059.9371	19
4	527.2500	264.1287			509.2395	255.1234	S	1973.8141	987.4107	1956.7876	978.8974	1955.8036	978.4054	18
5	642.2770	321.6421			624.2664	312.6368	D	1886.7821	943.8947	1869.7556	935.3814	1868.7715	934.8894	17
6	789.3454	395.1763			771.3348	386.1710	F	**1771.7552**	886.3812	1754.7286	877.8679	1753.7446	877.3759	16
7	876.3774	438.6923			858.3668	429.6871	S	**1624.6867**	812.8470	1607.6602	804.3337	1606.6762	803.8417	15
8	991.4043	496.2058	974.3778	487.6925	973.3938	487.2005	N	**1537.6547**	769.3310	1520.6282	760.8177	1519.6442	760.3257	14
9	1119.4629	560.2351	1102.4364	551.7218	1101.4524	551.2298	Q	**1422.6278**	711.8175	1405.6012	703.3043	1404.6172	702.8122	13
10	1220.5106	610.7589	1203.4841	602.2457	1202.5000	601.7537	T	**1294.5692**	647.7882	1277.5427	639.2750	1276.5586	638.7830	12
11	**1333.5947**	667.3010	1316.5681	658.7877	**1315.5841**	658.2957	I	1193.5215	597.2644	1176.4950	588.7511	1175.5110	588.2591	11
12	1447.6376	724.3224	1430.6110	715.8092	1429.6270	715.3172	N	**1080.4375**	540.7224	1063.4109	532.2091	1062.4269	**531.7171**	10
13	1534.6696	767.8384	1517.6431	759.3252	1516.6591	758.8332	S	**966.3945**	483.7009	949.3680	475.1876	948.3840	474.6956	9
14	1665.7101	833.3587	1648.6836	824.8454	1647.6995	824.3534	M	**879.3625**	440.1849	862.3360	431.6716	861.3519	431.1796	8
15	**1736.7472**	868.8772	1719.7207	860.3640	1718.7367	859.8720	A	**748.3220**	374.6646	731.2955	366.1514	730.3115	365.6594	7
16	**1865.7898**	933.3985	1848.7633	924.8853	1847.7792	924.3933	E	**677.2849**	339.1461	660.2584	330.6328	659.2743	330.1408	6
17	**1980.8168**	990.9120	1963.7902	982.3987	1962.8062	981.9067	D	**548.2423**	274.6248	**531.2158**	266.1115	530.2317	265.6195	5
18	2037.8382	1019.4227	2020.8117	1010.9095	2019.8277	1010.4175	G	**433.2154**	217.1113	416.1888	208.5980	415.2048	208.1060	4
19	2124.8702	1062.9388	2107.8437	1054.4255	2106.8597	1053.9335	S	376.1939	188.6006	359.1674	180.0873	358.1833	179.5953	3
20	2238.9132	1119.9602	2221.8866	1111.4470	2220.9026	1110.9549	N	289.1619	145.0846	272.1353	136.5713			2
21							R	175.1190	88.0631	158.0924	79.5498			1

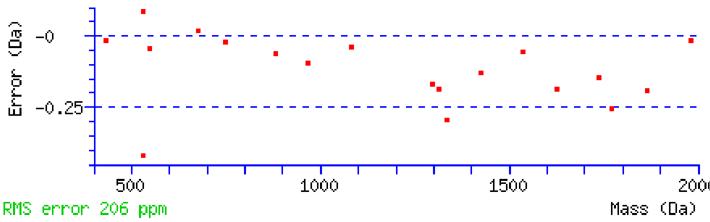

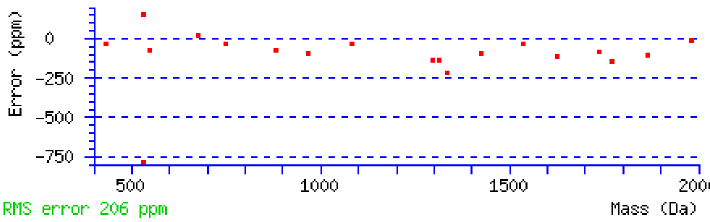

All matches to this query

Score	Mr(calc):	Delta	Sequence

123.3	2412.0175	0.0081	IYYSDFSNQTINSMAEDGSNR
92.7	2412.0175	0.0081	IYYSDFSNQTINSMAEDGSNR
65.3	2412.0175	0.0081	IYYSDFSNQTINSMAEDGSNR
55.1	2411.0335	0.9921	IYYSDFSNQTINSMAEDGSNR
5.9	2412.0403	-0.0146	EGICGSCAMNINGGNTLACTRR
4.1	2412.0378	-0.0121	IMNGNLSAEIERFSAMFQEK
4.0	2412.0161	0.0096	HRRLQSSFTAFANSHYSSR
2.3	2411.0287	0.9970	QSEWHKMTVSKNCPDQDLK
1.4	2412.0287	-0.0030	DATEISVLSFWKAQEQSADK
1.2	2412.0279	-0.0023	TNYLLSSTFPEXMALCWNR

Spectrum No: 6; Query: 882; Rank: 1

Peptide View

MS/MS Fragmentation of **FKLDWLGNCSGLNDESYGYK**
Found in **IPI00339124**, Tax_Id=10116 Gene_Symbol=Atp1b1 Sodium/potassium-transporting ATPase subunit beta-1

Match to Query 882: 2366.060428 from(1184.037490,2+)
Title: 091127RatKid_SCX01_21.5044.5044.2.dta
Data file K:\NewmanPaper\Piliang\3SubProteomes\Piliang3SP\mgf5ppm\SCX_3SubProteomes5ppm.mgf

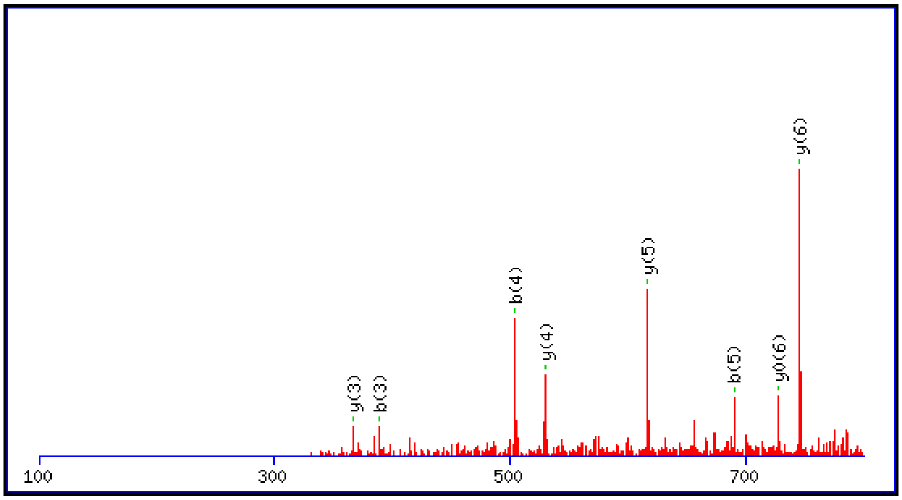

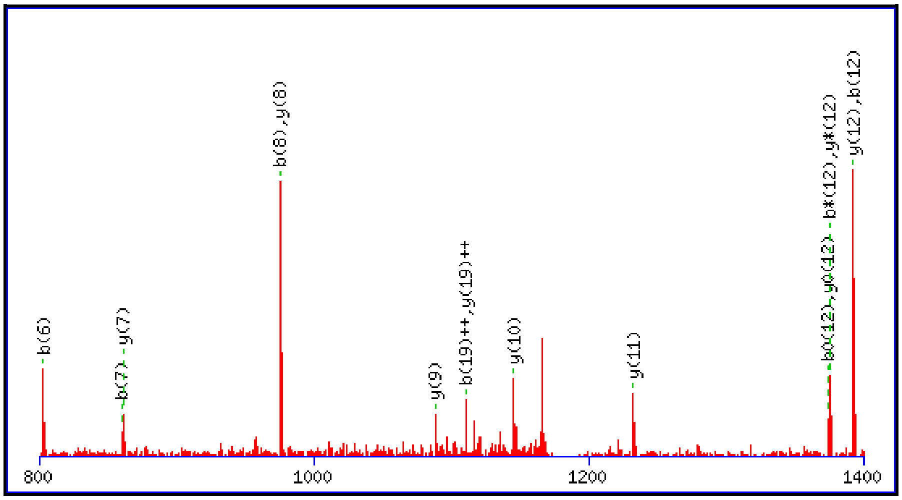

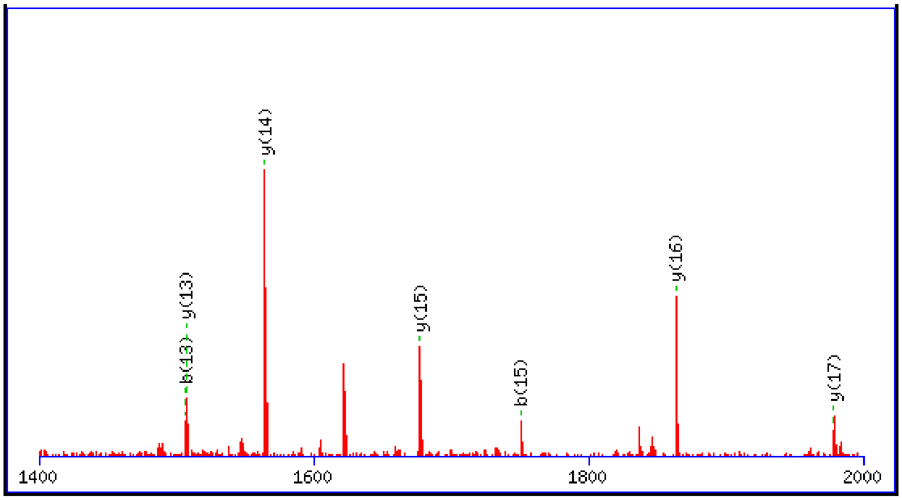

Monoisotopic mass of neutral peptide Mr(calc): 2366.0525
Fixed modifications: Carbamidomethyl (C)
Variable modifications:
N8 : Deamidated_N (N)
Ions Score: 123 **Expect:** 1.6e-010
Matches (Bold Red): 31/212 fragment ions using 41 most intense peaks

#	b	b^{++}	b*	b^{*++}	b^0	b^{0++}	Seq.	y	y^{++}	y*	y^{*++}	y^0	y^{0++}	#
1	148.0757	74.5415					F							20
2	276.1707	138.5890	259.1441	130.0757			K	2219.9914	**1110.4993**	2202.9648	1101.9860	2201.9808	1101.4940	19
3	**389.2547**	195.1310	372.2282	186.6177			L	2091.8964	1046.4518	2074.8699	1037.9386	2073.8858	1037.4466	18
4	**504.2817**	252.6445	487.2551	244.1312	486.2711	243.6392	D	**1978.8123**	989.9098	1961.7858	981.3965	1960.8018	980.9045	17
5	**690.3610**	345.6841	673.3344	337.1709	672.3504	336.6788	W	**1863.7854**	932.3963	1846.7589	923.8831	1845.7748	923.3911	16
6	**803.4450**	402.2262	786.4185	393.7129	785.4345	393.2209	L	**1677.7061**	839.3567	1660.6795	830.8434	1659.6955	830.3514	15
7	**860.4665**	430.7369	843.4400	422.2236	842.4559	421.7316	G	**1564.6220**	782.8146	1547.5955	774.3014	1546.6115	773.8094	14
8	**975.4934**	488.2504	958.4669	479.7371	957.4829	479.2451	N	**1507.6006**	754.3039	1490.5740	745.7906	1489.5900	745.2986	13
9	1135.5241	568.2657	1118.4975	559.7524	1117.5135	559.2604	C	**1392.5736**	696.7904	**1375.5471**	688.2772	**1374.5631**	687.7852	12
10	1222.5561	611.7817	1205.5296	603.2684	1204.5455	602.7764	S	**1232.5430**	616.7751	1215.5164	608.2619	1214.5324	607.7698	11
11	1279.5776	640.2924	1262.5510	631.7792	1261.5670	631.2871	G	**1145.5109**	573.2591	1128.4844	564.7458	1127.5004	564.2538	10
12	**1392.6616**	696.8345	**1375.6351**	688.3212	**1374.6511**	687.8292	L	**1088.4895**	544.7484	1071.4629	536.2351	1070.4789	535.7431	9
13	**1506.7046**	753.8559	1489.6780	745.3426	1488.6940	744.8506	N	**975.4054**	488.2063	958.3789	479.6931	957.3949	479.2011	8
14	1621.7315	811.3694	1604.7050	802.8561	1603.7209	802.3641	D	**861.3625**	431.1849	844.3359	422.6716	843.3519	422.1796	7
15	**1750.7741**	875.8907	1733.7476	867.3774	1732.7635	866.8854	E	**746.3355**	373.6714	729.3090	365.1581	**728.3250**	364.6661	6
16	1837.8061	919.4067	1820.7796	910.8934	1819.7956	910.4014	S	**617.2930**	309.1501	600.2664	300.6368	599.2824	300.1448	5
17	2000.8695	1000.9384	1983.8429	992.4251	1982.8589	991.9331	Y	**530.2609**	265.6341	513.2344	257.1208			4
18	2057.8909	1029.4491	2040.8644	1020.9358	2039.8804	1020.4438	G	**367.1976**	184.1024	350.1710	175.5892			3
19	2220.9543	**1110.9808**	2203.9277	1102.4675	2202.9437	1101.9755	Y	310.1761	155.5917	293.1496	147.0784			2
20							K	147.1128	74.0600	130.0863	65.5468			1

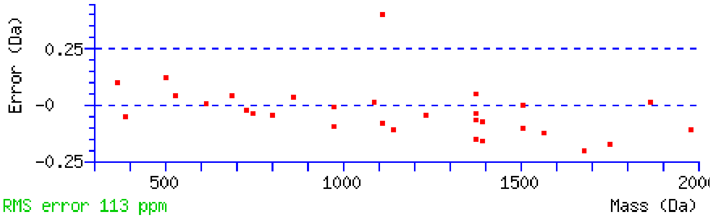

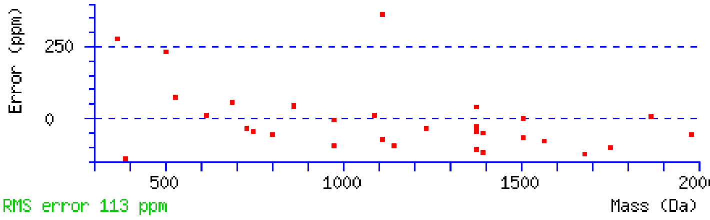

All matches to this query

Score	Mr(calc):	Delta	Sequence
122.9	2366.0525	0.0079	FKLDWLGNCSGLNDESYGYK

95.1	2366.0525	0.0079	FKLDWLGNCSGLNDESYGYK
60.1	2365.0685	0.9919	FKLDWLGNCSGLNDESYGYK
7.2	2366.0630	-0.0026	LQGGKDFNMPLTISSLKDGGK
7.2	2366.0630	-0.0026	LQGGKDFNMPLTISSLKDGGK
7.2	2366.0630	-0.0026	LQGGKDFNMPLTISSLKDGGK
5.8	2366.0432	0.0172	GLYASHADLHRLKYQCFK
5.3	2366.0759	-0.0155	SFSFSVNWNEIINFPLEIK
5.3	2366.0759	-0.0155	SFSFSVNWNEIINFPLEIK
4.8	2366.0840	-0.0236	EKMYNVLLFVDGGWMVDVR

Spectrum No: 7; Query: 914; Rank: 1

Peptide View

MS/MS Fragmentation of **SLGEVNFTATAEALQSPELCGNK**
Found in **IPI00326140**, Tax_Id=10116 Gene_Symbol=Pzp Alpha-1-macroglobulin precursor

Match to Query 914: 2437.163228 from(1219.588890,2+)
Title: 091127RatKid_SCX01_12.3808.3808.2.dta
Data file K:\NewmanPaper\Piliang\3SubProteomes\Piliang3SP\mgf5ppm\SCX_3SubProteomes5ppm.mgf

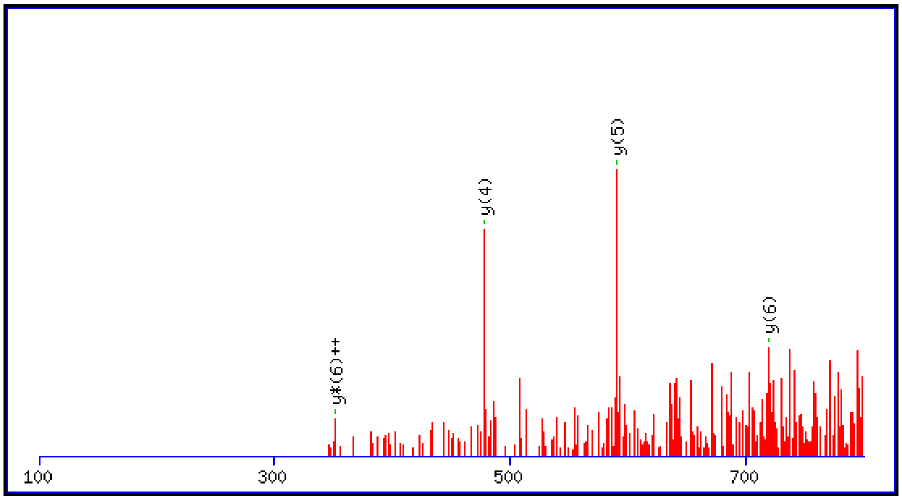

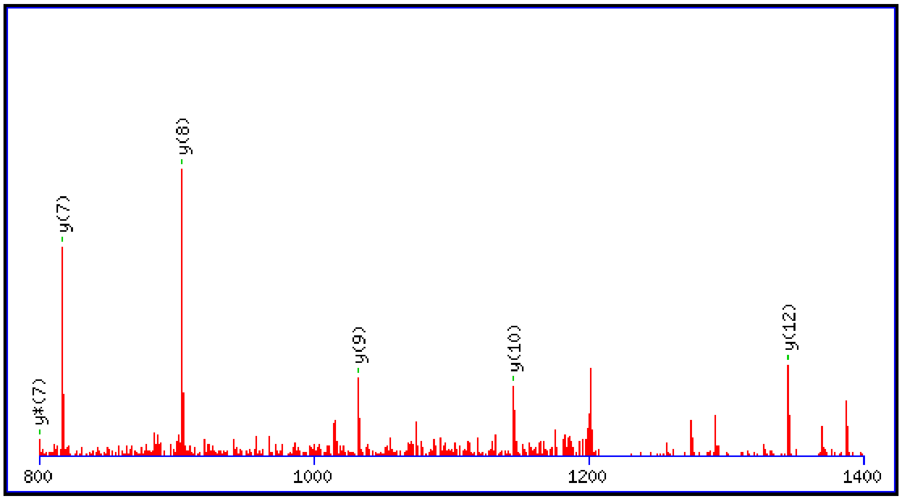

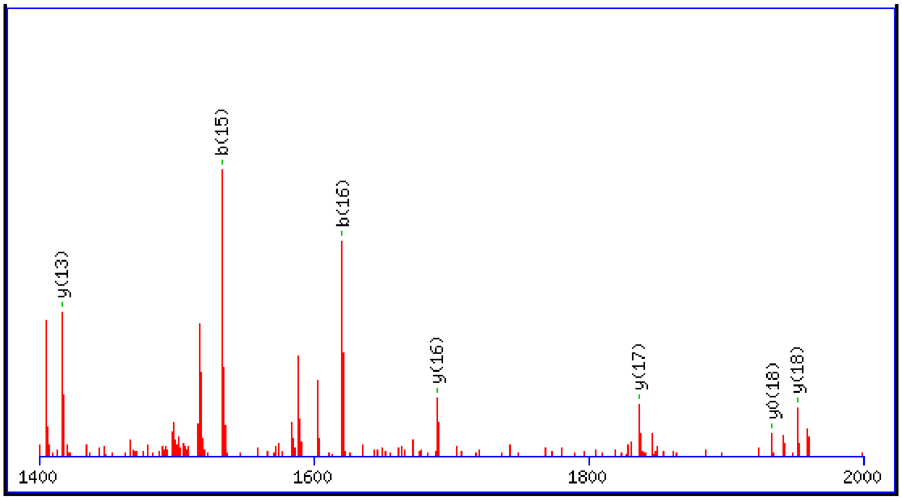

Monoisotopic mass of neutral peptide Mr(calc): 2436.1478
Fixed modifications: Carbamidomethyl (C)
Variable modifications:
N6 : Deamidated_N (N)
Ions Score: 122 **Expect:** 1.9e-010
Matches (Bold Red): 17/244 fragment ions using 20 most intense peaks

#	b	b++	b*	b*++	b0	b0++	Seq.	y	y++	y*	y*++	y0	y0++	#
1	88.0393	44.5233			70.0287	35.5180	S							23
2	201.1234	101.0653			183.1128	92.0600	L	2350.1231	1175.5652	2333.0966	1167.0519	2332.1125	1166.5599	22
3	258.1448	129.5761			240.1343	120.5708	G	2237.0390	1119.0232	2220.0125	1110.5099	2219.0285	1110.0179	21
4	387.1874	194.0974			369.1769	185.0921	E	2180.0176	1090.5124	2162.9910	1081.9992	2162.0070	1081.5071	20
5	486.2558	243.6316			468.2453	234.6263	V	2050.9750	1025.9911	2033.9484	1017.4779	2032.9644	1016.9859	19
6	601.2828	301.1450	584.2562	292.6318	583.2722	292.1397	N	**1951.9066**	976.4569	1934.8800	967.9437	**1933.8960**	967.4516	18
7	748.3512	374.6792	731.3246	366.1660	730.3406	365.6739	F	**1836.8796**	918.9435	1819.8531	910.4302	1818.8691	909.9382	17
8	849.3989	425.2031	832.3723	416.6898	831.3883	416.1978	T	**1689.8112**	845.4093	1672.7847	836.8960	1671.8007	836.4040	16
9	920.4360	460.7216	903.4094	452.2084	902.4254	451.7163	A	1588.7635	794.8854	1571.7370	786.3721	1570.7530	785.8801	15
10	1021.4837	511.2455	1004.4571	502.7322	1003.4731	502.2402	T	1517.7264	759.3669	1500.6999	750.8536	1499.7159	750.3616	14
11	1092.5208	546.7640	1075.4942	538.2508	1074.5102	537.7587	A	**1416.6788**	708.8430	1399.6522	700.3297	1398.6682	699.8377	13
12	1221.5634	611.2853	1204.5368	602.7720	1203.5528	602.2800	E	**1345.6416**	673.3245	1328.6151	664.8112	1327.6311	664.3192	12
13	1292.6005	646.8039	1275.5739	638.2906	1274.5899	637.7986	A	1216.5990	608.8032	1199.5725	600.2899	1198.5885	599.7979	11
14	1405.6845	703.3459	1388.6580	694.8326	1387.6740	694.3406	L	**1145.5619**	573.2846	1128.5354	564.7713	1127.5514	564.2793	10
15	**1533.7431**	767.3752	1516.7166	758.8619	1515.7326	758.3699	Q	**1032.4779**	516.7426	1015.4513	508.2293	1014.4673	507.7373	9
16	**1620.7752**	810.8912	1603.7486	802.3779	1602.7646	801.8859	S	**904.4193**	452.7133	887.3927	444.2000	886.4087	443.7080	8
17	1717.8279	859.4176	1700.8014	850.9043	1699.8174	850.4123	P	**817.3873**	409.1973	**800.3607**	400.6840	799.3767	400.1920	7
18	1846.8705	923.9389	1829.8440	915.4256	1828.8599	914.9336	E	**720.3345**	360.6709	703.3080	**352.1576**	702.3239	351.6656	6
19	1959.9546	980.4809	1942.9280	971.9677	1941.9440	971.4756	L	**591.2919**	296.1496	574.2654	287.6363			5
20	2119.9852	1060.4962	2102.9587	1051.9830	2101.9747	1051.4910	C	**478.2078**	239.6076	461.1813	231.0943			4
21	2177.0067	1089.0070	2159.9801	1080.4937	2158.9961	1080.0017	G	318.1772	159.5922	301.1506	151.0790			3
22	2291.0496	1146.0284	2274.0231	1137.5152	2273.0390	1137.0232	N	261.1557	131.0815	244.1292	122.5682			2
23							K	147.1128	74.0600	130.0863	65.5468			1

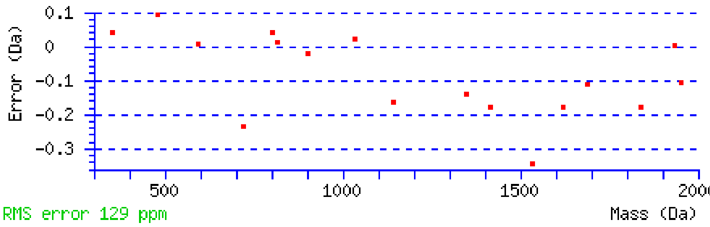

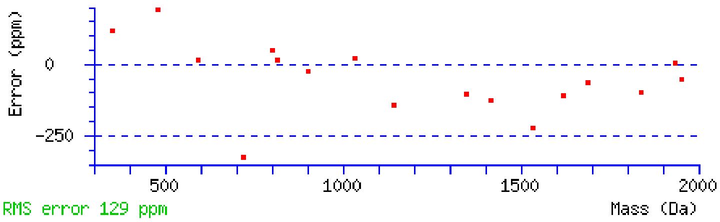

All matches to this query

Score	Mr(calc):	Delta	Sequence
121.6	2436.1478	1.0154	SLGEVNFTATAEALQSPELCGNK
86.2	2435.1638	1.9994	SLGEVNFTATAEALQSPELCGNK
16.6	2436.1478	1.0154	SLGEVNFTATAEALQSPELCGNK
6.6	2436.1461	1.0171	YDGGSEIINYVLESRLIGTEK
5.5	2437.1638	-0.0006	SATFPSEKARHLLDDSVLESR
5.5	2437.1638	-0.0006	SATFPSEKARHLLDDSVLESR
4.6	2436.1378	1.0254	LSSGTKWIIEQNLSQLPYAK
4.6	2436.1378	1.0254	LSSGTKWIIEQNLSQLPYAK
4.6	2436.1378	1.0254	LSSGTKWIIEQNLSQLPYAK
4.1	2437.1783	-0.0151	SLIRSTSQLLSRPLSAVELK

Spectrum No: 8; Query: 674; Rank: 1

Peptide View

MS/MS Fragmentation of **TQLTCFLNSSGIDIVGHR**
Found in **IPI00193425**, Tax_Id=10116 Gene_Symbol=Bsg Isoform 2 of Basigin precursor

Match to Query 674: 2017.989368 from(1010.001960,2+)
Title: 091129RatKid_SCX02_14.3755.3755.2.dta
Data file K:\NewmanPaper\Piliang\3SubProteomes\Piliang3SP\mgf5ppm\SCX_3SubProteomes5ppm.mgf

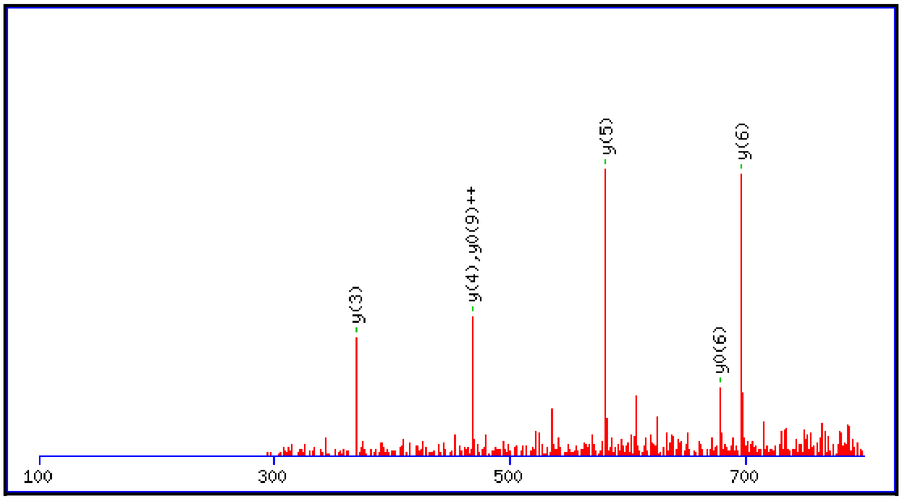

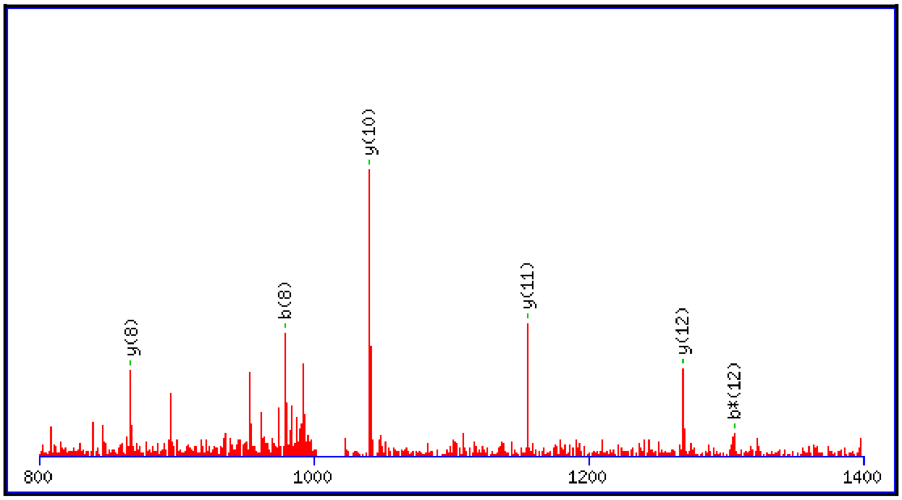

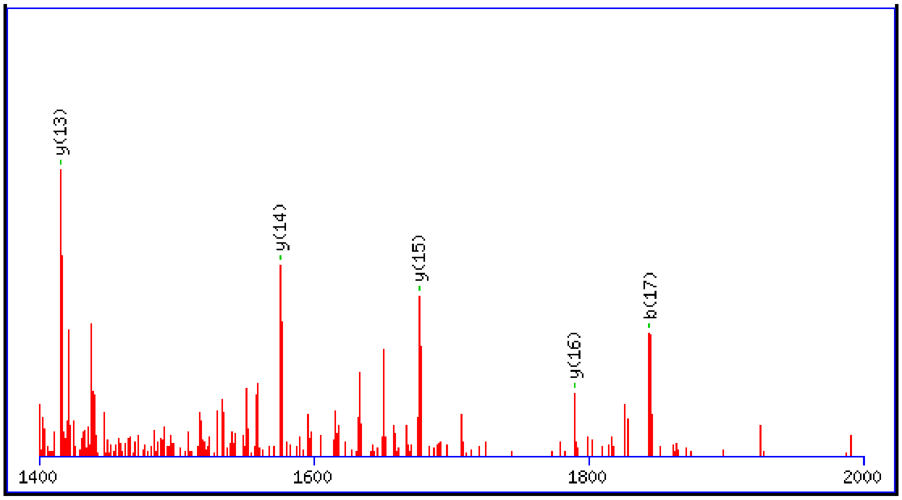

Monoisotopic mass of neutral peptide Mr(calc): 2017.9891
Fixed modifications: Carbamidomethyl (C)
Variable modifications:
N8 : Deamidated_N (N)
Ions Score: 121 **Expect:** 1.6e-010
Matches (Bold Red): 17/192 fragment ions using 22 most intense peaks

#	b	b^{++}	b*	b^{*++}	b^0	b^{0++}	Seq.	y	y^{++}	y*	y^{*++}	y^0	y^{0++}	#
1	102.0550	51.5311			84.0444	42.5258	T							18
2	230.1135	115.5604	213.0870	107.0471	212.1030	106.5551	Q	1917.9487	959.4780	1900.9222	950.9647	1899.9382	950.4727	17
3	343.1976	172.1024	326.1710	163.5892	325.1870	163.0972	L	**1789.8901**	895.4487	1772.8636	886.9354	1771.8796	886.4434	16
4	444.2453	222.6263	427.2187	214.1130	426.2347	213.6210	T	**1676.8061**	838.9067	1659.7795	830.3934	1658.7955	829.9014	15
5	604.2759	302.6416	587.2494	294.1283	586.2654	293.6363	C	**1575.7584**	788.3828	1558.7318	779.8696	1557.7478	779.3776	14
6	751.3443	376.1758	734.3178	367.6625	733.3338	367.1705	F	**1415.7277**	708.3675	1398.7012	699.8542	1397.7172	699.3622	13
7	864.4284	432.7178	847.4019	424.2046	846.4178	423.7126	L	**1268.6593**	634.8333	1251.6328	626.3200	1250.6488	625.8280	12
8	**979.4553**	490.2313	962.4288	481.7180	961.4448	481.2260	N	**1155.5753**	578.2913	1138.5487	569.7780	1137.5647	569.2860	11
9	1066.4874	533.7473	1049.4608	525.2340	1048.4768	524.7420	S	**1040.5483**	520.7778	1023.5218	512.2645	1022.5378	511.7725	10
10	1153.5194	577.2633	1136.4928	568.7501	1135.5088	568.2581	S	953.5163	477.2618	936.4898	468.7485	935.5057	**468.2565**	9
11	1210.5409	605.7741	1193.5143	597.2608	1192.5303	596.7688	G	**866.4843**	433.7458	849.4577	425.2325	848.4737	424.7405	8
12	1323.6249	662.3161	**1306.5984**	653.8028	1305.6144	653.3108	I	809.4628	405.2350	792.4363	396.7218	791.4522	396.2298	7
13	1438.6519	719.8296	1421.6253	711.3163	1420.6413	710.8243	D	**696.3787**	348.6930	679.3522	340.1797	**678.3682**	339.6877	6
14	1551.7359	776.3716	1534.7094	767.8583	1533.7254	767.3663	I	**581.3518**	291.1795	564.3253	282.6663			5
15	1650.8043	825.9058	1633.7778	817.3925	1632.7938	816.9005	V	**468.2677**	234.6375	451.2412	226.1242			4
16	1707.8258	854.4165	1690.7993	845.9033	1689.8152	845.4113	G	**369.1993**	185.1033	352.1728	176.5900			3
17	**1844.8847**	922.9460	1827.8582	914.4327	1826.8742	913.9407	H	312.1779	156.5926	295.1513	148.0793			2
18							R	175.1190	88.0631	158.0924	79.5498			1

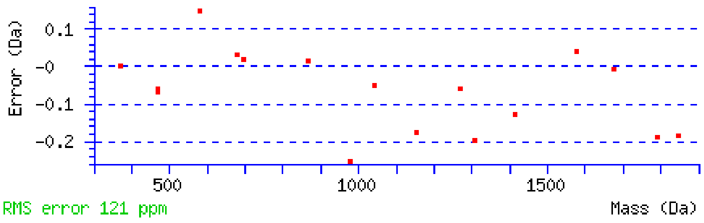

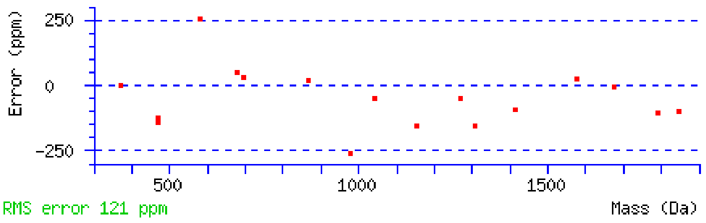

All matches to this query

Score	Mr(calc):	Delta	Sequence
121.1	2017.9891	0.0002	TQLTCFLNSSGIDIVGHR
22.4	2017.0051	0.9842	TQLTCFLNSSGIDIVGHR
2.7	2017.0010	0.9884	NANVSITIASSALRTLVK

0.8	2015.9985	1.9908	ASINVLDLIIPPSFTKK
0.8	2015.9985	1.9908	ASINVLDLIIPPSFTKK

Spectrum No: 9; Query: 869; Rank: 1

Peptide View

MS/MS Fragmentation of **FVLLMTNCYATPSSNSTDPVK**
Found in **IPI00198021**, Tax_Id=10116 Gene_Symbol=Umod Uromodulin precursor

Match to Query 869: 2347.108568 from(1174.561560,2+)
Title: 091127RatKid_SCX01_02.3715.3715.2.dta
Data file K:\NewmanPaper\Piliang\3SubProteomes\Piliang3SP\mgf5ppm\SCX_3SubProteomes5ppm.mgf

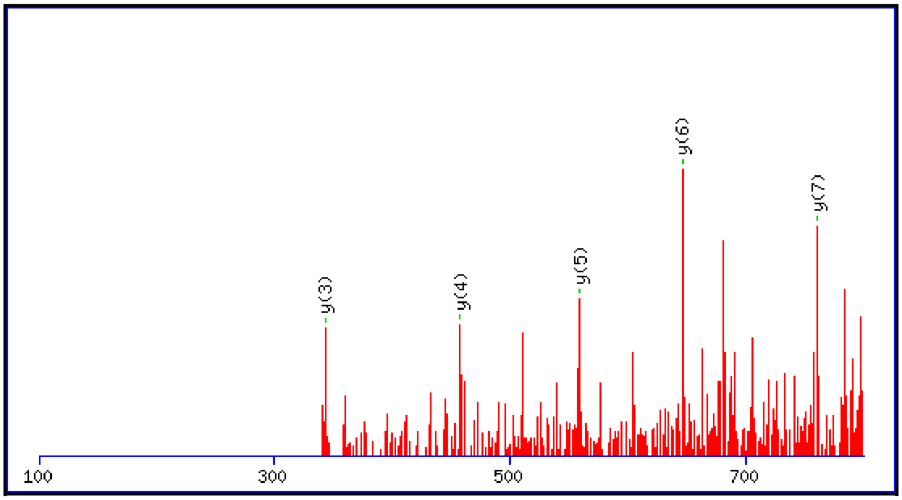

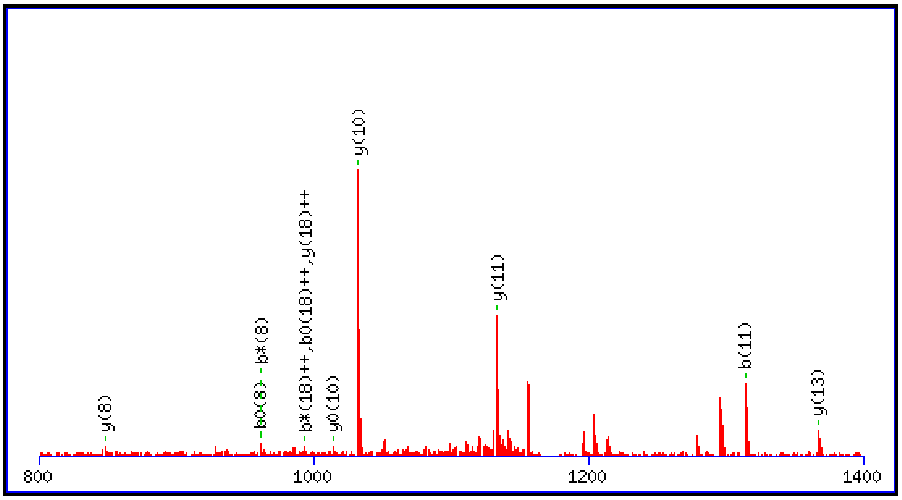

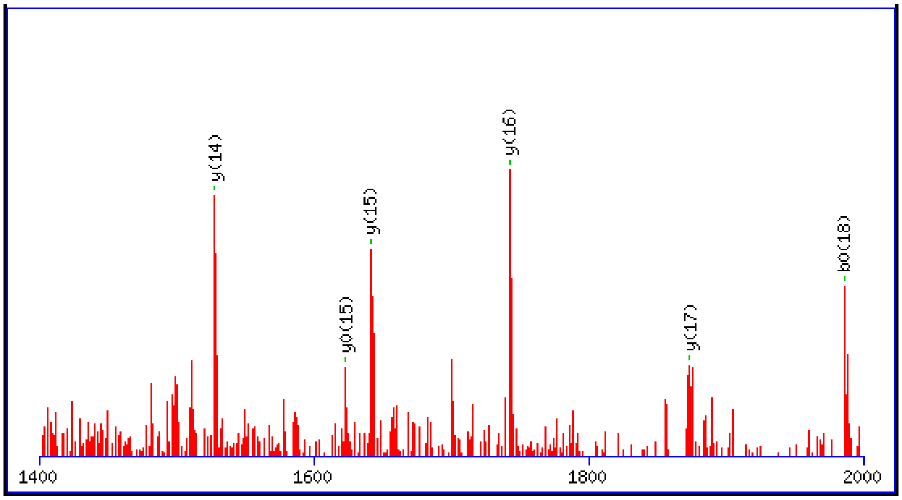

Monoisotopic mass of neutral peptide Mr(calc): 2345.0919
Fixed modifications: Carbamidomethyl (C)
Variable modifications:
N15 : Deamidated_N (N)
Ions Score: 118 **Expect:** 4.9e-010
Matches (Bold Red): 22/212 fragment ions using 26 most intense peaks

#	b	b^{++}	b*	b^{*++}	b^0	b^{0++}	Seq.	y	y^{++}	y*	y^{*++}	y^0	y^{0++}	#
1	148.0757	74.5415					F							21
2	247.1441	124.0757					V	2199.0308	1100.0190	2182.0042	1091.5058	2181.0202	1091.0138	20
3	360.2282	180.6177					L	2099.9624	1050.4848	2082.9358	1041.9716	2081.9518	1041.4795	19
4	473.3122	237.1598					L	1986.8783	**993.9428**	1969.8518	985.4295	1968.8677	984.9375	18
5	604.3527	302.6800					M	**1873.7943**	937.4008	1856.7677	928.8875	1855.7837	928.3955	17
6	705.4004	353.2038			687.3898	344.1986	T	**1742.7538**	871.8805	1725.7272	863.3672	1724.7432	862.8752	16
7	819.4433	410.2253	802.4168	401.7120	801.4328	401.2200	N	**1641.7061**	821.3567	1624.6795	812.8434	**1623.6955**	812.3514	15
8	979.4740	490.2406	**962.4474**	481.7273	**961.4634**	481.2353	C	**1527.6632**	764.3352	1510.6366	755.8219	1509.6526	755.3299	14
9	1142.5373	571.7723	1125.5108	563.2590	1124.5267	562.7670	Y	**1367.6325**	684.3199	1350.6060	675.8066	1349.6219	675.3146	13
10	1213.5744	607.2908	1196.5479	598.7776	1195.5639	598.2856	A	1204.5692	602.7882	1187.5426	594.2750	1186.5586	593.7829	12
11	**1314.6221**	657.8147	1297.5955	649.3014	1296.6115	648.8094	T	**1133.5321**	567.2697	1116.5055	558.7564	1115.5215	558.2644	11
12	1411.6749	706.3411	1394.6483	697.8278	1393.6643	697.3358	P	**1032.4844**	516.7458	1015.4578	508.2326	**1014.4738**	507.7405	10
13	1498.7069	749.8571	1481.6803	741.3438	1480.6963	740.8518	S	935.4316	468.2195	918.4051	459.7062	917.4211	459.2142	9
14	1585.7389	793.3731	1568.7124	784.8598	1567.7283	784.3678	S	**848.3996**	424.7034	831.3730	416.1902	830.3890	415.6982	8
15	1700.7659	850.8866	1683.7393	842.3733	1682.7553	841.8813	N	**761.3676**	381.1874	744.3410	372.6741	743.3570	372.1821	7
16	1787.7979	894.4026	1770.7713	885.8893	1769.7873	885.3973	S	**646.3406**	323.6740	629.3141	315.1607	628.3301	314.6687	6
17	1888.8456	944.9264	1871.8190	936.4131	1870.8350	935.9211	T	**559.3086**	280.1579	542.2821	271.6447	541.2980	271.1527	5
18	2003.8725	1002.4399	1986.8460	**993.9266**	**1985.8619**	**993.4346**	D	**458.2609**	229.6341	441.2344	221.1208	440.2504	220.6288	4
19	2100.9253	1050.9663	2083.8987	1042.4530	2082.9147	1041.9610	P	**343.2340**	172.1206	326.2074	163.6074			3
20	2199.9937	1100.5005	2182.9671	1091.9872	2181.9831	1091.4952	V	246.1812	123.5942	229.1547	115.0810			2
21							K	147.1128	74.0600	130.0863	65.5468			1

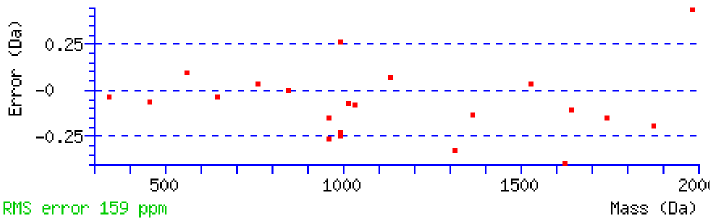

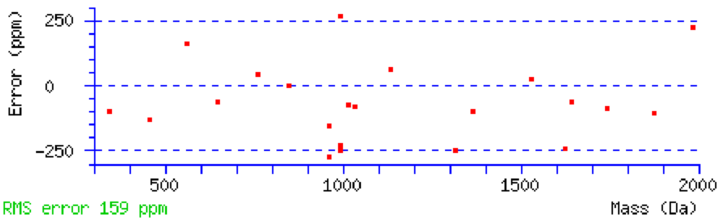

All matches to this query

Score	Mr(calc):	Delta	Sequence

117.5	2345.0919	2.0166	FVLLMTNCYATPSSNSTDPVK
25.9	2345.0919	2.0166	FVLLMTNCYATPSSNSTDPVK
3.6	2345.1008	2.0078	TENCVAKTKLANGTSSMIVPK
3.6	2345.1008	2.0078	TENCVAKTKLANGTSSMIVPK
3.6	2345.1008	2.0078	TENCVAKTKLANGTSSMIVPK
0.5	2345.0796	2.0290	NMLNIPVANILMVSNYASER
0.1	2345.1008	2.0078	TENCVAKTKLANGTSSMIVPK
0.1	2346.1022	1.0064	LSRDTLVSTPESADRLWLK
0.1	2346.1022	1.0064	LSRDTLVSTPESADRLWLK
0.0	2346.0884	1.0201	APPPLQIPMAVTWPLTDPAR

Spectrum No: 10; Query: 811; Rank: 1

Peptide View

MS/MS Fragmentation of **YGYENDTEIANLYDEMVAK**
Found in **IPI00191437**, Tax_Id=10116 Gene_Symbol=- 64 kDa protein

Match to Query 811: 2237.975948 from(1119.995250,2+)
Title: 091127RatKid_SCX01_12.4610.4610.2.dta
Data file K:\NewmanPaper\Piliang\3SubProteomes\Piliang3SP\mgf5ppm\SCX_3SubProteomes5ppm.mgf

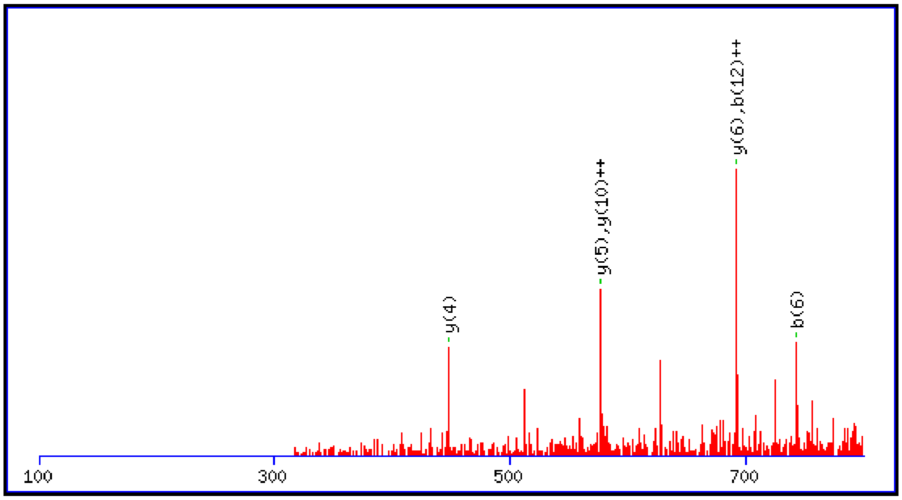

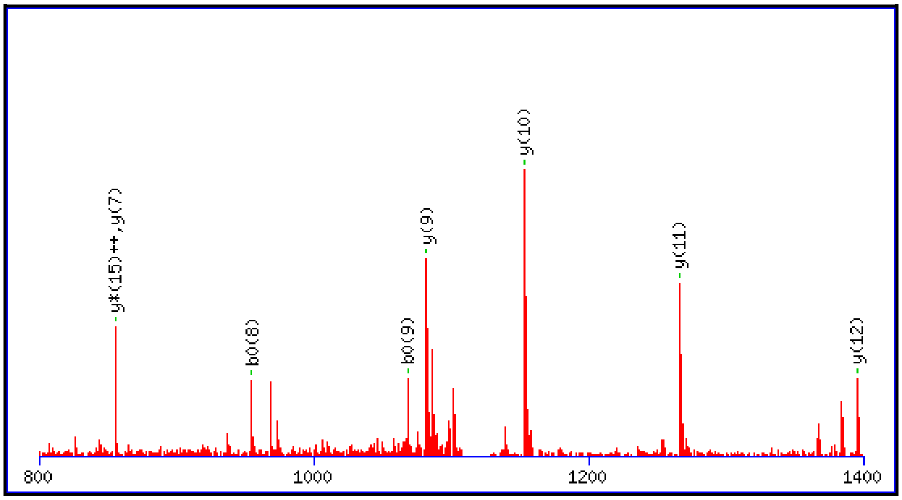

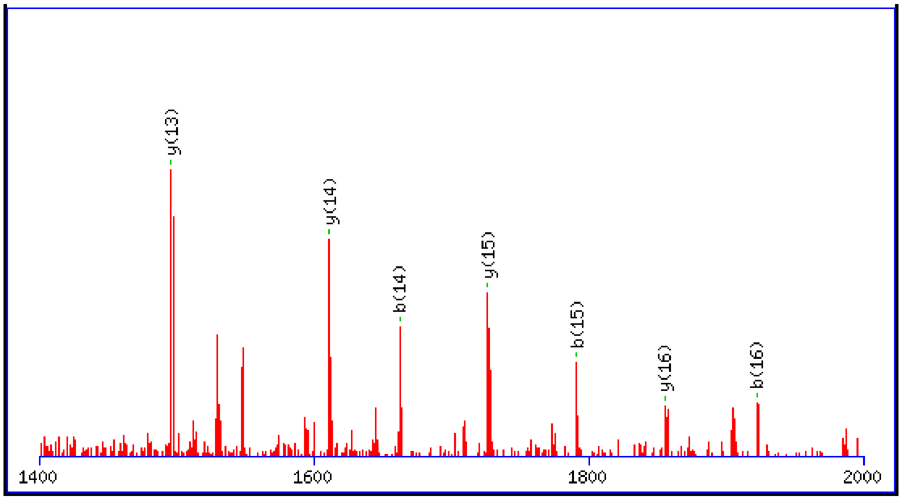

Monoisotopic mass of neutral peptide Mr(calc): 2237.9674
Fixed modifications: Carbamidomethyl (C)
Variable modifications:
N5 : Deamidated_N (N)
Ions Score: 117 **Expect:** 5.2e-010
Matches (Bold Red): 21/194 fragment ions using 27 most intense peaks

#	b	b^{++}	b*	b^{*++}	b^{0}	b^{0++}	Seq.	y	y^{++}	y*	y^{*++}	y^{0}	y^{0++}	#
1	164.0706	82.5389					Y							19
2	221.0921	111.0497					G	2075.9114	1038.4593	2058.8848	1029.9461	2057.9008	1029.4540	18
3	384.1554	192.5813					Y	2018.8899	1009.9486	2001.8634	1001.4353	2000.8794	1000.9433	17
4	513.1980	257.1026			495.1874	248.0974	E	**1855.8266**	928.4169	1838.8000	919.9037	1837.8160	919.4117	16
5	628.2249	314.6161	611.1984	306.1028	610.2144	305.6108	N	**1726.7840**	863.8956	1709.7575	**855.3824**	1708.7734	854.8904	15
6	**743.2519**	372.1296	726.2253	363.6163	725.2413	363.1243	D	**1611.7571**	806.3822	1594.7305	797.8689	1593.7465	797.3769	14
7	844.2995	422.6534	827.2730	414.1401	826.2890	413.6481	T	**1496.7301**	748.8687	1479.7036	740.3554	1478.7196	739.8634	13
8	973.3421	487.1747	956.3156	478.6614	**955.3316**	478.1694	E	**1395.6824**	698.3449	1378.6559	689.8316	1377.6719	689.3396	12
9	1086.4262	543.7167	1069.3997	535.2035	**1068.4156**	534.7115	I	**1266.6399**	633.8236	1249.6133	625.3103	1248.6293	624.8183	11
10	1157.4633	579.2353	1140.4368	570.7220	1139.4528	570.2300	A	**1153.5558**	**577.2815**	1136.5292	568.7683	1135.5452	568.2762	10
11	1271.5062	636.2568	1254.4797	627.7435	1253.4957	627.2515	N	**1082.5187**	541.7630	1065.4921	533.2497	1064.5081	532.7577	9
12	1384.5903	**692.7988**	1367.5638	684.2855	1366.5797	683.7935	L	968.4757	484.7415	951.4492	476.2282	950.4652	475.7362	8
13	1547.6536	774.3305	1530.6271	765.8172	1529.6431	765.3252	Y	**855.3917**	428.1995	838.3651	419.6862	837.3811	419.1942	7
14	**1662.6806**	831.8439	1645.6540	823.3307	1644.6700	822.8386	D	**692.3284**	346.6678	675.3018	338.1545	674.3178	337.6625	6
15	**1791.7232**	896.3652	1774.6966	887.8520	1773.7126	887.3599	E	**577.3014**	289.1543	560.2749	280.6411	559.2908	280.1491	5
16	**1922.7637**	961.8855	1905.7371	953.3722	1904.7531	952.8802	M	**448.2588**	224.6330	431.2323	216.1198			4
17	2021.8321	1011.4197	2004.8055	1002.9064	2003.8215	1002.4144	V	317.2183	159.1128	300.1918	150.5995			3
18	2092.8692	1046.9382	2075.8426	1038.4250	2074.8586	1037.9329	A	218.1499	109.5786	201.1234	101.0653			2
19							K	147.1128	74.0600	130.0863	65.5468			1

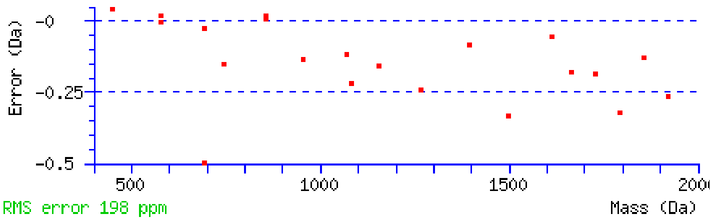

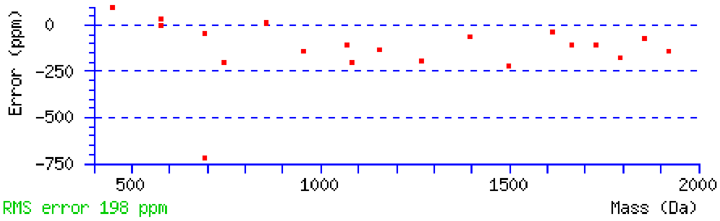

All matches to this query

Score	Mr(calc):	Delta	Sequence
117.3	2237.9674	0.0086	YGYENDTEIANLYDEMVAK
66.6	2236.9834	0.9926	YGYENDTEIANLYDEMVAK

64.5	2237.9674	0.0086	YGYENDTEIANLYDEMVAK
5.5	2236.9729	1.0031	TCTNPAPLNGGAFCEGQSVQK
4.0	2235.9889	1.9871	TCTNPAPLNGGAFCEGQSVQK
3.6	2236.9879	0.9881	TPALQPTTDAHNGLQVASTR
2.2	2235.9792	1.9967	SMLGNGNYDVNVIMAALQTK
1.3	2236.9717	1.0042	RQSQLLSSMLSRTLSYK
0.9	2237.9719	0.0041	ASSVNSPLGNKPQTLSPSHR
0.8	2235.9844	1.9916	KATGAVSSNVLQWGSHXKK

Spectrum No: 11; Query: 878; Rank: 1

Peptide View

MS/MS Fragmentation of **GPNVPISTEVNATQFLALVQEK**
Found in **IPI00471666**, Tax_Id=10116 Gene_Symbol=MGC94145 Uncharacterized protein C1orf179 homolog

Match to Query 878: 2355.236568 from(1178.625560,2+)
Title: 091127RatKid_SCX01_14.5110.5110.2.dta
Data file K:\NewmanPaper\Piliang\3SubProteomes\Piliang3SP\mgf5ppm\SCX_3SubProteomes5ppm.mgf

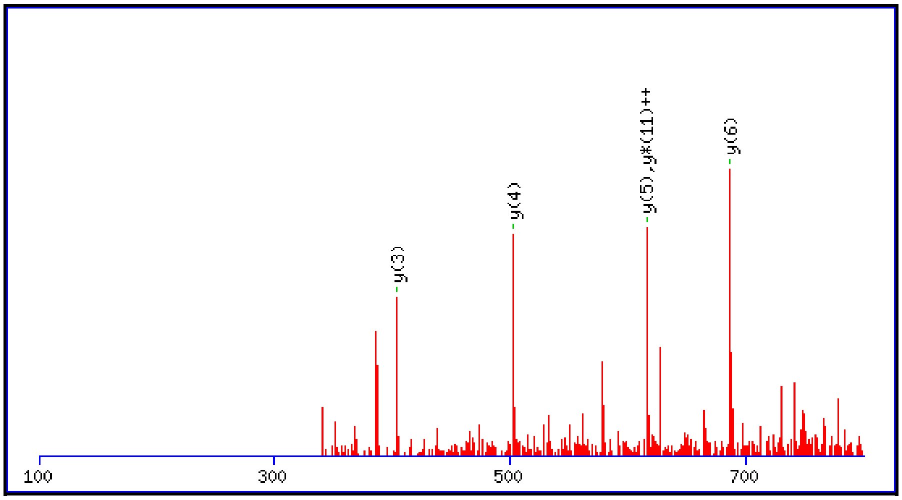

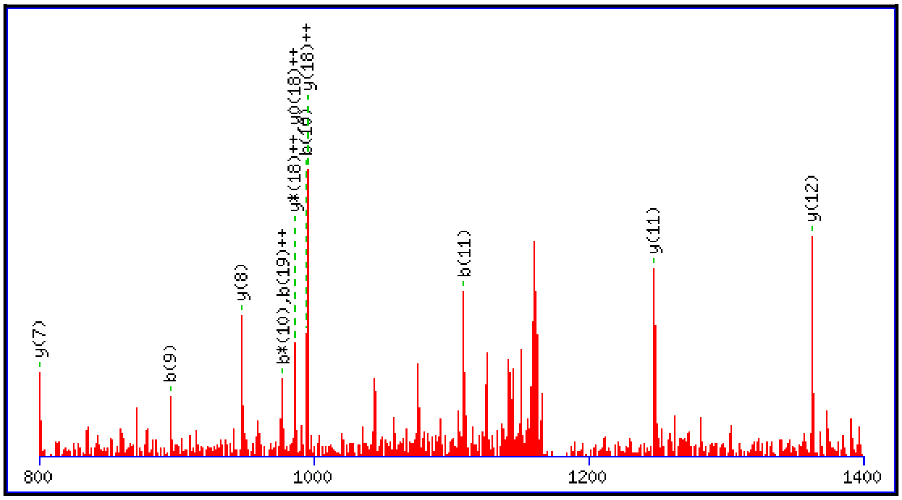

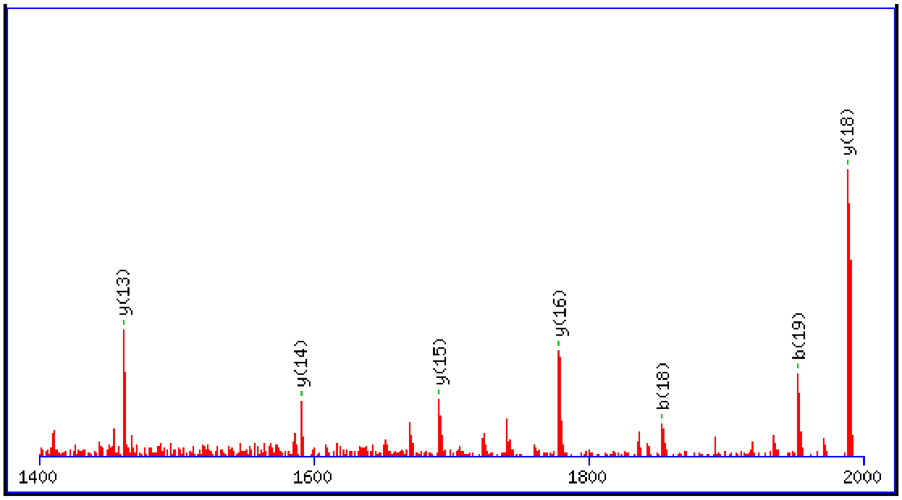

Monoisotopic mass of neutral peptide Mr(calc): 2355.2322
Fixed modifications: Carbamidomethyl (C)
Variable modifications:
N11 : Deamidated_N (N)
Ions Score: 115 **Expect:** 3.4e-010
Matches (Bold Red): 24/234 fragment ions using 26 most intense peaks

#	b	b++	b*	b*++	b0	b0++	Seq.	y	y++	y*	y*++	y0	y0++	#
1	58.0287	29.5180					G							22
2	155.0815	78.0444					P	2299.2180	1150.1126	2282.1914	1141.5994	2281.2074	1141.1074	21
3	269.1244	135.0659	252.0979	126.5526			N	2202.1652	1101.5863	2185.1387	1093.0730	2184.1547	1092.5810	20
4	368.1928	184.6001	351.1663	176.0868			V	2088.1223	1044.5648	2071.0958	1036.0515	2070.1117	1035.5595	19
5	465.2456	233.1264	448.2191	224.6132			P	**1989.0539**	**995.0306**	1972.0273	**986.5173**	1971.0433	**986.0253**	18
6	578.3297	289.6685	561.3031	281.1552			I	1892.0011	946.5042	1874.9746	937.9909	1873.9906	937.4989	17
7	665.3617	333.1845	648.3352	324.6712	647.3511	324.1792	S	**1778.9171**	889.9622	1761.8905	881.4489	1760.9065	880.9569	16
8	766.4094	383.7083	749.3828	375.1951	748.3988	374.7030	T	**1691.8850**	846.4462	1674.8585	837.9329	1673.8745	837.4409	15
9	**895.4520**	448.2296	878.4254	439.7164	877.4414	439.2243	E	**1590.8374**	795.9223	1573.8108	787.4090	1572.8268	786.9170	14
10	**994.5204**	497.7638	**977.4938**	489.2506	976.5098	488.7585	V	**1461.7948**	731.4010	1444.7682	722.8877	1443.7842	722.3957	13
11	**1109.5473**	555.2773	1092.5208	546.7640	1091.5368	546.2720	N	**1362.7264**	681.8668	1345.6998	673.3535	1344.7158	672.8615	12
12	1180.5844	590.7959	1163.5579	582.2826	1162.5739	581.7906	A	**1247.6994**	624.3533	1230.6729	**615.8401**	1229.6889	615.3481	11
13	1281.6321	641.3197	1264.6056	632.8064	1263.6216	632.3144	T	1176.6623	588.8348	1159.6358	580.3215	1158.6517	579.8295	10
14	1409.6907	705.3490	1392.6641	696.8357	1391.6801	696.3437	Q	1075.6146	538.3109	1058.5881	529.7977	1057.6041	529.3057	9
15	1556.7591	778.8832	1539.7326	770.3699	1538.7485	769.8779	F	**947.5560**	474.2817	930.5295	465.7684	929.5455	465.2764	8
16	1669.8432	835.4252	1652.8166	826.9119	1651.8326	826.4199	L	**800.4876**	400.7475	783.4611	392.2342	782.4771	391.7422	7
17	1740.8803	870.9438	1723.8537	862.4305	1722.8697	861.9385	A	**687.4036**	344.2054	670.3770	335.6921	669.3930	335.2001	6
18	**1853.9644**	927.4858	1836.9378	918.9725	1835.9538	918.4805	L	**616.3665**	308.6869	599.3399	300.1736	598.3559	299.6816	5
19	**1953.0328**	**977.0200**	1936.0062	968.5067	1935.0222	968.0147	V	**503.2824**	252.1448	486.2558	243.6316	485.2718	243.1396	4
20	2081.0913	1041.0493	2064.0648	1032.5360	2063.0808	1032.0440	Q	**404.2140**	202.6106	387.1874	194.0974	386.2034	193.6053	3
21	2210.1339	1105.5706	2193.1074	1097.0573	2192.1234	1096.5653	E	276.1554	138.5813	259.1288	130.0681	258.1448	129.5761	2
22							K	147.1128	74.0600	130.0863	65.5468			1

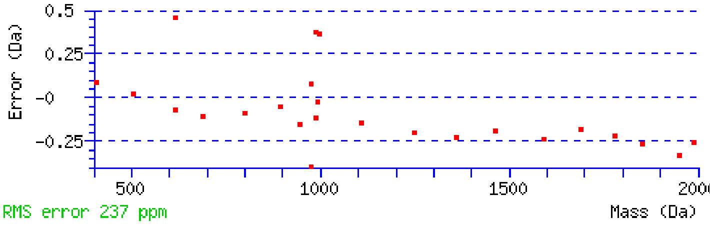

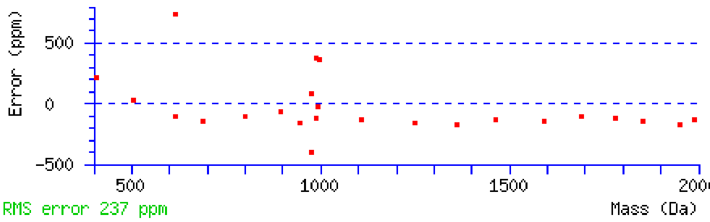

All matches to this query

Score	Mr(calc):	Delta	Sequence
114.9	2355.2322	0.0044	GPNVPISTEVNATQFLALVQEK
34.0	2355.2322	0.0044	GPNVPISTEVNATQFLALVQEK
22.1	2354.2482	0.9884	GPNVPISTEVNATQFLALVQEK
1.0	2354.2270	1.0095	ITFDVYKQNPRDLFGSLNVK
0.2	2353.2430	1.9935	ITFDVYKQNPRDLFGSLNVK

Spectrum No: 12; Query: 467; Rank: 1

Peptide View

MS/MS Fragmentation of **AITCGGIYNESSGILR**
Found in **IPI00196620**, Tax_Id=10116 Gene_Symbol=Cubn Cubilin precursor

Match to Query 467: 1710.827788 from(856.421170,2+)
Title: 091127RatKid_SCX01_17.2699.2699.2.dta
Data file K:\NewmanPaper\Piliang\3SubProteomes\Piliang3SP\mgf5ppm\SCX_3SubProteomes5ppm.mgf

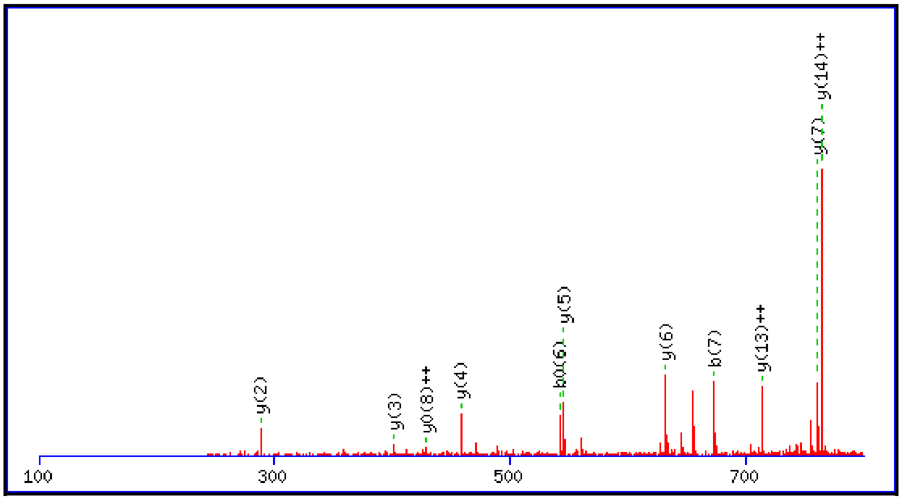

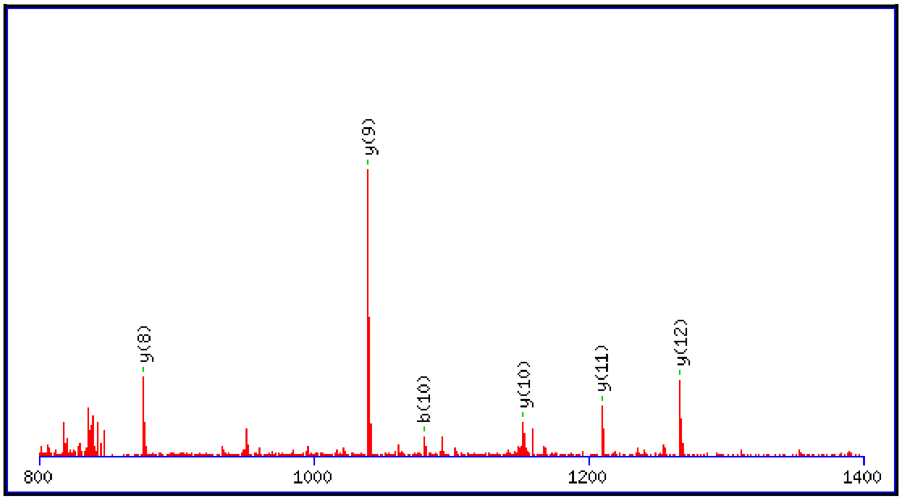

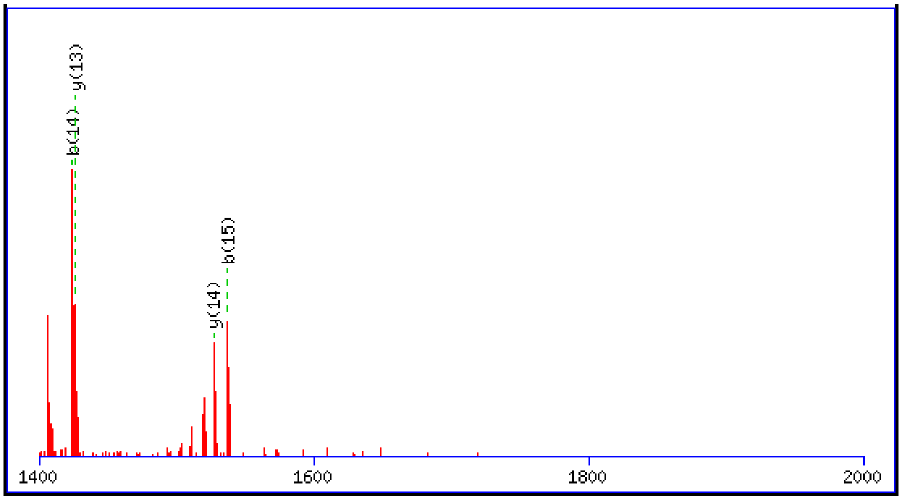

Monoisotopic mass of neutral peptide Mr(calc): 1710.8247
Fixed modifications: Carbamidomethyl (C)
Variable modifications:
N9 : Deamidated_N (N)
Ions Score: 114 **Expect:** 7.3e-010
Matches (Bold Red): 21/152 fragment ions using 34 most intense peaks

#	b	b^{++}	b*	b^{*++}	b^{0}	b^{0++}	Seq.	y	y^{++}	y*	y^{*++}	y^{0}	y^{0++}	#
1	72.0444	36.5258					A							16
2	185.1285	93.0679					I	1640.7948	820.9011	1623.7683	812.3878	1622.7843	811.8958	15
3	286.1761	143.5917			268.1656	134.5864	T	**1527.7108**	**764.3590**	1510.6842	755.8458	1509.7002	755.3537	14
4	446.2068	223.6070			428.1962	214.6017	C	**1426.6631**	**713.8352**	1409.6365	705.3219	1408.6525	704.8299	13
5	503.2282	252.1178			485.2177	243.1125	G	**1266.6324**	633.8199	1249.6059	625.3066	1248.6219	624.8146	12
6	560.2497	280.6285			**542.2391**	271.6232	G	**1209.6110**	605.3091	1192.5844	596.7959	1191.6004	596.3038	11
7	**673.3338**	337.1705			655.3232	328.1652	I	**1152.5895**	576.7984	1135.5630	568.2851	1134.5790	567.7931	10
8	836.3971	418.7022			818.3865	409.6969	Y	**1039.5055**	520.2564	1022.4789	511.7431	1021.4949	511.2511	9
9	951.4240	476.2157	934.3975	467.7024	933.4135	467.2104	N	**876.4421**	438.7247	859.4156	430.2114	858.4316	**429.7194**	8
10	**1080.4666**	540.7370	1063.4401	532.2237	1062.4561	531.7317	E	**761.4152**	381.2112	744.3886	372.6980	743.4046	372.2060	7
11	1167.4987	584.2530	1150.4721	575.7397	1149.4881	575.2477	S	**632.3726**	316.6899	615.3461	308.1767	614.3620	307.6847	6
12	1254.5307	627.7690	1237.5041	619.2557	1236.5201	618.7637	S	**545.3406**	273.1739	528.3140	264.6606	527.3300	264.1686	5
13	1311.5522	656.2797	1294.5256	647.7664	1293.5416	647.2744	G	**458.3085**	229.6579	441.2820	221.1446			4
14	**1424.6362**	712.8217	1407.6097	704.3085	1406.6257	703.8165	I	**401.2871**	201.1472	384.2605	192.6339			3
15	**1537.7203**	769.3638	1520.6937	760.8505	1519.7097	760.3585	L	**288.2030**	144.6051	271.1765	136.0919			2
16							R	175.1190	88.0631	158.0924	79.5498			1

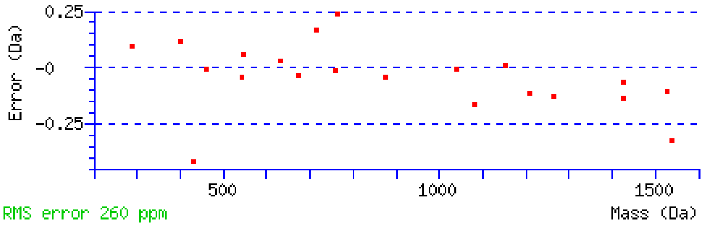

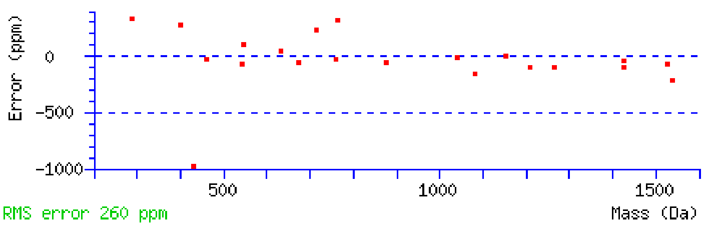

All matches to this query

Score	Mr(calc):	Delta	Sequence
114.3	1710.8247	0.0031	AITCGGIYNESSGILR
17.3	1709.8407	0.9871	AITCGGIYNESSGILR
7.7	1710.8375	-0.0098	KSSEQLLGSMPVSLR
6.8	1710.8375	-0.0098	KSSEQLLGSMPVSLR
2.9	1710.8189	0.0089	QELILSNSEDKSIR

2.9	1709.8359	0.9919	TKEYELLSDELNQK
0.9	1710.8342	-0.0064	TFEVNPVTGEIRLR
0.4	1710.8237	0.0041	VVMLLTAGSGGGAGGGRR

Spectrum No: 13; Query: 590; Rank: 1

Peptide View

MS/MS Fragmentation of **FTSDTSVAGTGFNASFHK**
Found in **IPI00196620**, Tax_Id=10116 Gene_Symbol=Cubn Cubilin precursor

Match to Query 590: 1873.851948 from(937.933250,2+)
Title: 091129RatKid_SCX02_24.1967.1967.2.dta
Data file K:\NewmanPaper\Piliang\3SubProteomes\Piliang3SP\mgf5ppm\SCX_3SubProteomes5ppm.mgf

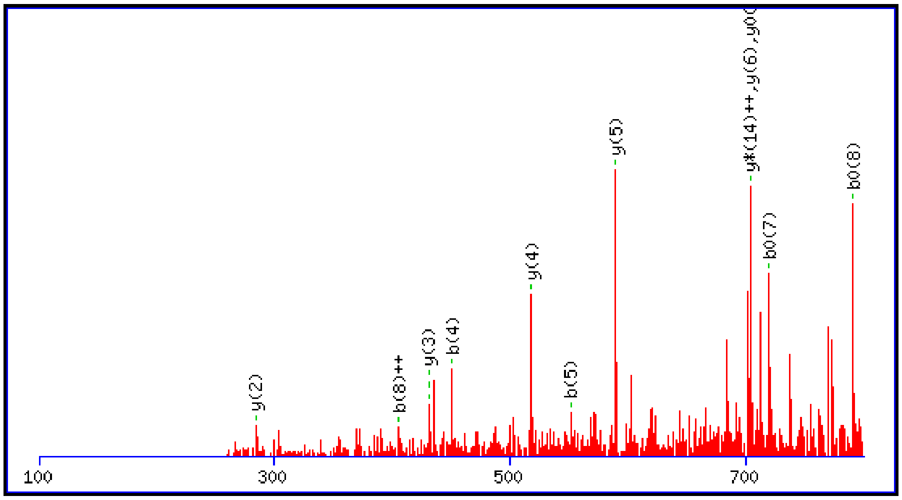

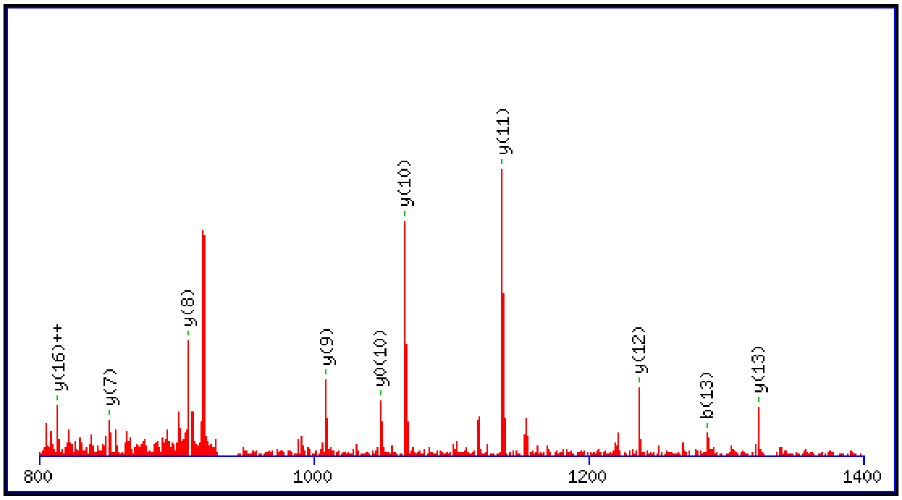

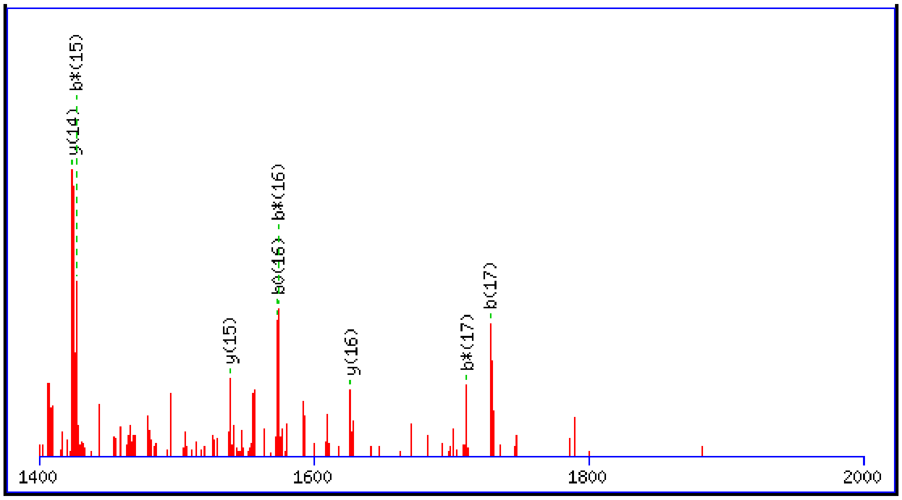

Monoisotopic mass of neutral peptide Mr(calc): 1873.8483
Fixed modifications: Carbamidomethyl (C)
Variable modifications:
N13 : Deamidated_N (N)
Ions Score: 114 **Expect:** 8.4e-010
Matches (Bold Red): 30/172 fragment ions using 59 most intense peaks

#	b	b^{++}	b^*	b^{*++}	b^0	b^{0++}	Seq.	y	y^{++}	y^*	y^{*++}	y^0	y^{0++}	#
1	148.0757	74.5415					F							18
2	249.1234	125.0653			231.1128	116.0600	T	1727.7871	864.3972	1710.7606	855.8839	1709.7765	855.3919	17
3	336.1554	168.5813			318.1448	159.5761	S	**1626.7394**	**813.8734**	1609.7129	805.3601	1608.7289	804.8681	16
4	**451.1823**	226.0948			433.1718	217.0895	D	**1539.7074**	770.3573	1522.6809	761.8441	1521.6968	761.3521	15
5	**552.2300**	276.6186			534.2195	267.6134	T	**1424.6805**	712.8439	1407.6539	**704.3306**	1406.6699	**703.8386**	14
6	639.2620	320.1347			621.2515	311.1294	S	**1323.6328**	662.3200	1306.6062	653.8068	1305.6222	653.3147	13
7	738.3305	369.6689			**720.3199**	360.6636	V	**1236.6008**	618.8040	1219.5742	610.2907	1218.5902	609.7987	12
8	809.3676	**405.1874**			**791.3570**	396.1821	A	**1137.5323**	569.2698	1120.5058	560.7565	1119.5218	560.2645	11
9	866.3890	433.6982			848.3785	424.6929	G	**1066.4952**	533.7513	1049.4687	525.2380	**1048.4847**	524.7460	10
10	967.4367	484.2220			949.4262	475.2167	T	**1009.4738**	505.2405	992.4472	496.7272	991.4632	496.2352	9
11	1024.4582	512.7327			1006.4476	503.7274	G	**908.4261**	454.7167	891.3995	446.2034	890.4155	445.7114	8
12	1171.5266	586.2669			1153.5160	577.2617	F	**851.4046**	426.2059	834.3781	417.6927	833.3941	417.2007	7
13	**1286.5535**	643.7804	1269.5270	635.2671	1268.5430	634.7751	N	**704.3362**	352.6717	687.3097	344.1585	686.3256	343.6665	6
14	1357.5906	679.2990	1340.5641	670.7857	1339.5801	670.2937	A	**589.3093**	295.1583	572.2827	286.6450	571.2987	286.1530	5
15	1444.6227	722.8150	**1427.5961**	714.3017	1426.6121	713.8097	S	**518.2722**	259.6397	501.2456	251.1264	500.2616	250.6344	4
16	1591.6911	796.3492	**1574.6645**	787.8359	**1573.6805**	787.3439	F	**431.2401**	216.1237	414.2136	207.6104			3
17	**1728.7500**	864.8786	**1711.7235**	856.3654	1710.7394	855.8734	H	**284.1717**	142.5895	267.1452	134.0762			2
18							K	147.1128	74.0600	130.0863	65.5468			1

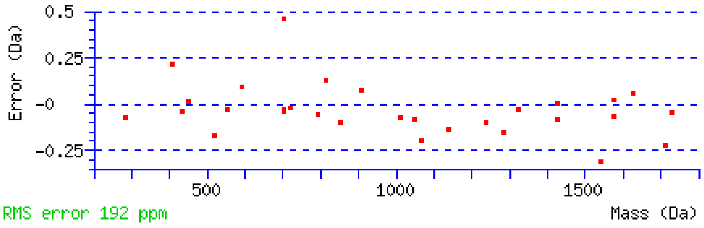

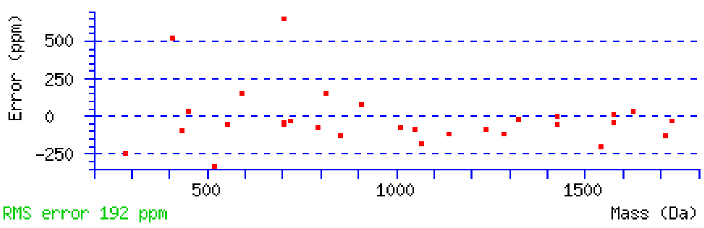

All matches to this query

Score	Mr(calc):	Delta	Sequence
114.2	1873.8483	0.0037	FTSDTSVAGTGFNASFHK
8.2	1872.8492	1.0028	MERTSVMERTSVMER

Spectrum No: 14; Query: 732; Rank: 1

Peptide View

MS/MS Fragmentation of **MYVTNDTEVAENNYQALK**
Found in **IPI00464785**, Tax_Id=10116 Gene_Symbol=Ppgb Protective protein for beta-galactosidase

Match to Query 732: 2118.945288 from(1060.479920,2+)
Title: 091127RatKid_SCX01_16.2414.2414.2.dta
Data file K:\NewmanPaper\Piliang\3SubProteomes\Piliang3SP\mgf5ppm\SCX_3SubProteomes5ppm.mgf

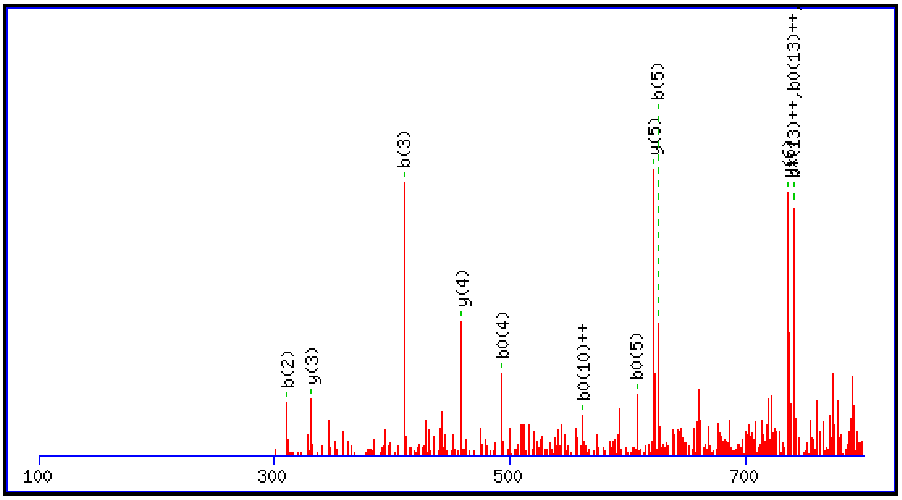

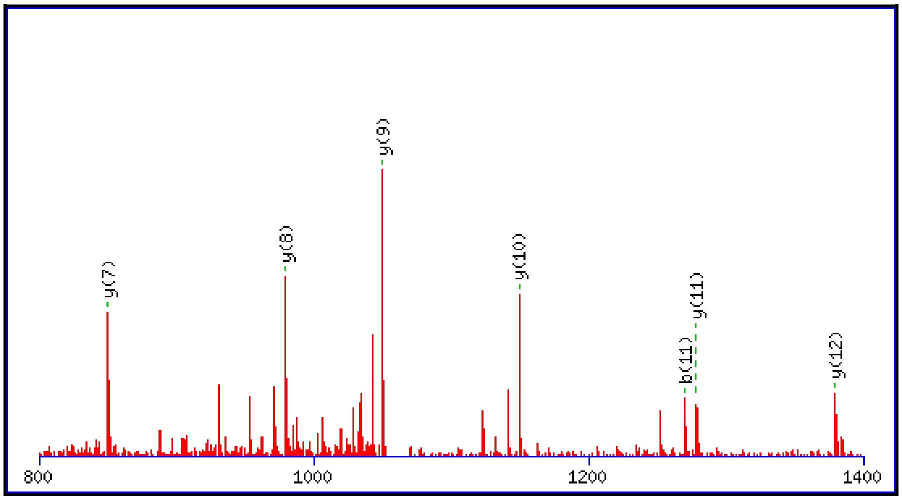

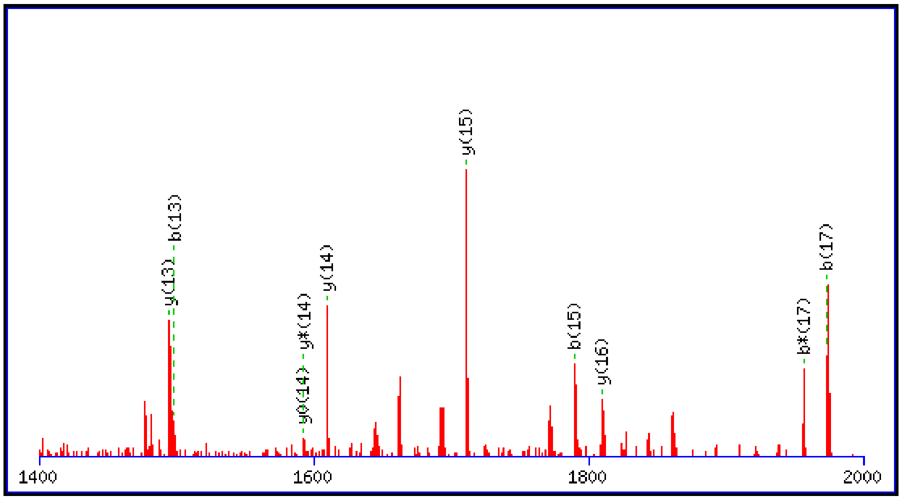

Monoisotopic mass of neutral peptide Mr(calc): 2118.9415

Fixed modifications: Carbamidomethyl (C)
Variable modifications:
M1 : Oxidation (M)
N5 : Deamidated_N (N)
Ions Score: 114 **Expect:** 1.1e-009
Matches (Bold Red): 30/176 fragment ions using 44 most intense peaks

#	b	b^{++}	b*	b^{*++}	b^0	b^{0++}	Seq.	y	y^{++}	y*	y^{*++}	y^0	y^{0++}	#
1	148.0427	74.5250					M							18
2	**311.1060**	156.0566					Y	1972.9134	986.9604	1955.8869	978.4471	1954.9029	977.9551	17
3	**410.1744**	205.5908					V	**1809.8501**	905.4287	1792.8236	896.9154	1791.8395	896.4234	16
4	511.2221	256.1147			**493.2115**	247.1094	T	**1710.7817**	855.8945	1693.7551	847.3812	1692.7711	846.8892	15
5	**626.2490**	313.6282	609.2225	305.1149	**608.2385**	304.6229	N	**1609.7340**	805.3706	**1592.7075**	796.8574	**1591.7235**	796.3654	14
6	**741.2760**	371.1416	724.2494	362.6284	723.2654	362.1363	D	**1494.7071**	747.8572	1477.6805	739.3439	1476.6965	738.8519	13
7	842.3237	421.6655	825.2971	413.1522	824.3131	412.6602	T	**1379.6801**	690.3437	1362.6536	681.8304	1361.6696	681.3384	12
8	971.3663	486.1868	954.3397	477.6735	953.3557	477.1815	E	**1278.6325**	639.8199	1261.6059	631.3066	1260.6219	630.8146	11
9	1070.4347	535.7210	1053.4081	527.2077	1052.4241	526.7157	V	**1149.5899**	575.2986	1132.5633	566.7853	1131.5793	566.2933	10
10	1141.4718	571.2395	1124.4452	562.7263	1123.4612	**562.2342**	A	**1050.5215**	525.7644	1033.4949	517.2511	1032.5109	516.7591	9
11	**1270.5144**	635.7608	1253.4878	627.2476	1252.5038	626.7555	E	**979.4843**	490.2458	962.4578	481.7325	961.4738	481.2405	8
12	1384.5573	692.7823	1367.5308	684.2690	1366.5467	683.7770	N	**850.4417**	425.7245	833.4152	417.2112			7
13	**1498.6002**	749.8038	1481.5737	**741.2905**	1480.5897	**740.7985**	N	**736.3988**	368.7030	719.3723	360.1898			6
14	1661.6636	831.3354	1644.6370	822.8221	1643.6530	822.3301	Y	**622.3559**	311.6816	605.3293	303.1683			5
15	**1789.7221**	895.3647	1772.6956	886.8514	1771.7116	886.3594	Q	**459.2926**	230.1499	442.2660	221.6366			4
16	1860.7592	930.8833	1843.7327	922.3700	1842.7487	921.8780	A	**331.2340**	166.1206	314.2074	157.6074			3
17	**1973.8433**	987.4253	**1956.8168**	978.9120	1955.8327	978.4200	L	260.1969	130.6021	243.1703	122.0888			2
18							K	147.1128	74.0600	130.0863	65.5468			1

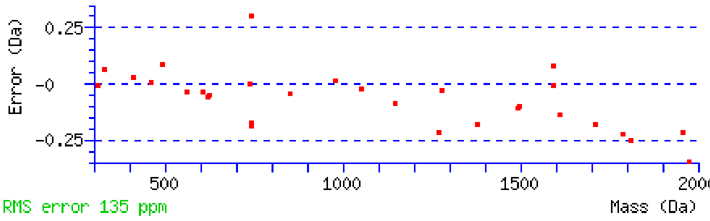

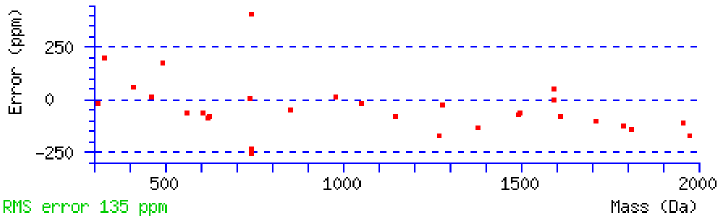

All matches to this query

Score	Mr(calc):	Delta	Sequence
114.0	2118.9415	0.0038	MYVTNDTEVAENNYQALK
89.5	2118.9415	0.0038	MYVTNDTEVAENNYQALK
87.4	2118.9415	0.0038	MYVTNDTEVAENNYQALK
61.5	2117.9575	0.9878	MYVTNDTEVAENNYQALK
6.7	2116.9384	2.0069	FYTVISHYSVAKQSTAR
5.8	2118.9400	0.0053	KTVXNLLGMAGMTPNVNSK
5.6	2118.9591	-0.0138	VMDLSNRELKHGHLNMK
3.0	2117.9453	1.0000	SDMPSITQGESSFXPQRK
3.0	2118.9391	0.0062	YMFSPDTPYISHSKDMGK
2.5	2118.9400	0.0053	KTVXNLLGMAGMTPNVNSK

Spectrum No: 15; Query: 200; Rank: 1

Peptide View

MS/MS Fragmentation of **ALGYENATQALSR**

Found in **IPI00199448**, Tax_Id=10116 Gene_Symbol=Lgals3bp Mama

Match to Query 200: 1393.687168 from(697.850860,2+)
Title: 091127RatKid_SCX01_02.2437.2437.2.dta
Data file K:\NewmanPaper\Piliang\3SubProteomes\Piliang3SP\mgf5ppm\SCX_3SubProteomes5ppm.mgf

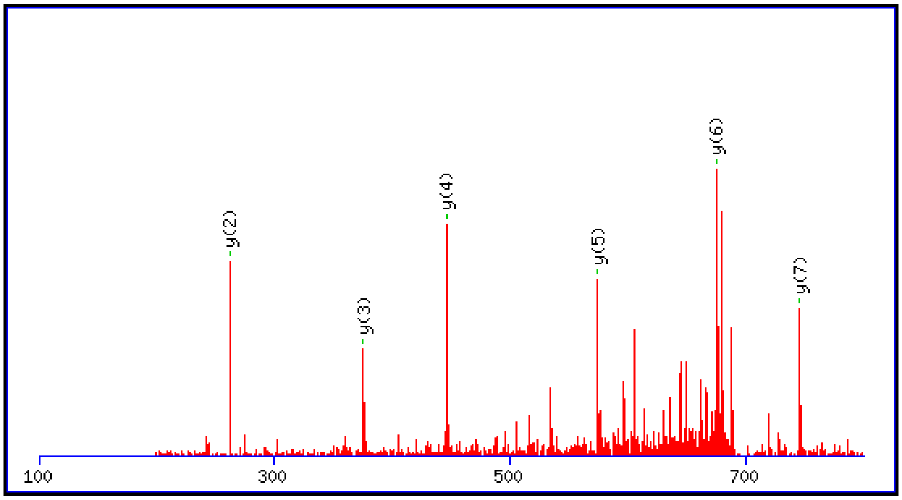

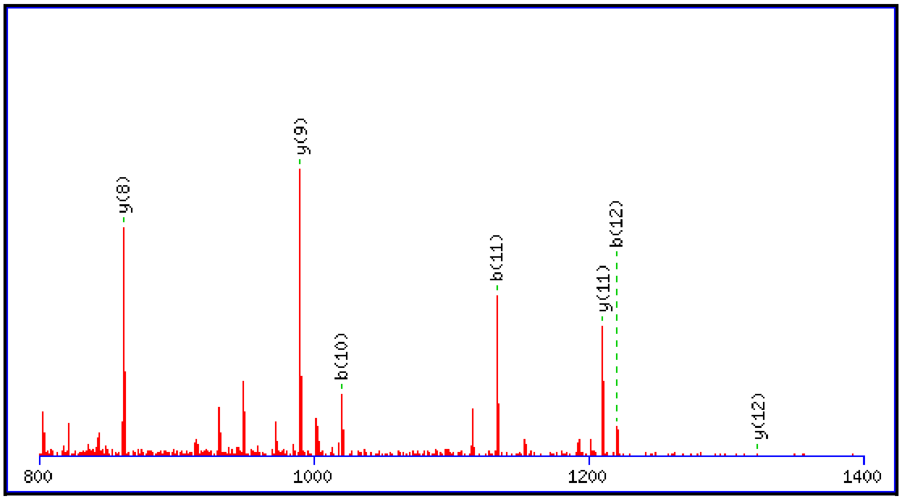

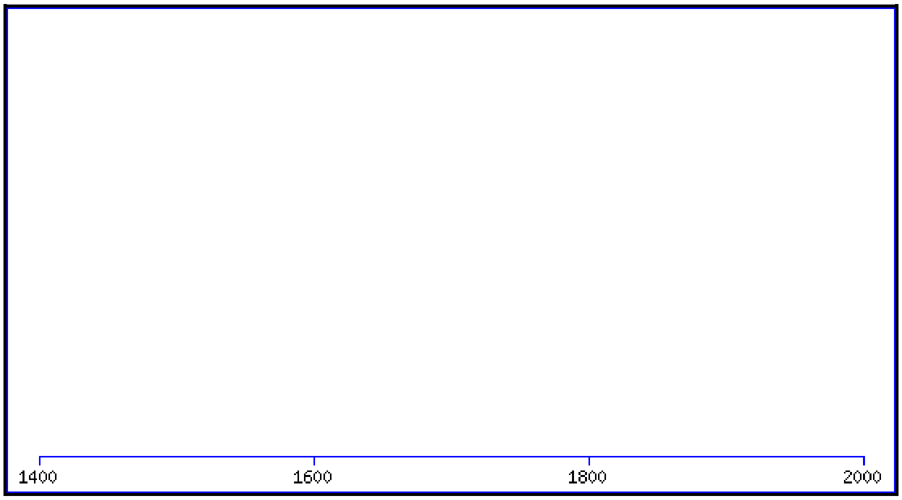

Monoisotopic mass of neutral peptide Mr(calc): 1393.6837
Fixed modifications: Carbamidomethyl (C)
Variable modifications:
N6 : Deamidated_N (N)
Ions Score: 113 **Expect:** 6.5e-010
Matches (Bold Red): 13/124 fragment ions using 29 most intense peaks

#	b	b^{++}	b*	b^{*++}	b^0	b^{0++}	Seq.	y	y^{++}	y*	y^{*++}	y^0	y^{0++}	#
1	72.0444	36.5258					A							13

2	185.1285	93.0679					L	**1323.6539**	662.3306	1306.6274	653.8173	1305.6434	653.3253	12
3	242.1499	121.5786					G	**1210.5699**	605.7886	1193.5433	597.2753	1192.5593	596.7833	11
4	405.2132	203.1103					Y	1153.5484	577.2778	1136.5218	568.7646	1135.5378	568.2725	10
5	534.2558	267.6316			516.2453	258.6263	E	**990.4851**	495.7462	973.4585	487.2329	972.4745	486.7409	9
6	649.2828	325.1450	632.2562	316.6318	631.2722	316.1397	N	**861.4425**	431.2249	844.4159	422.7116	843.4319	422.2196	8
7	720.3199	360.6636	703.2933	352.1503	702.3093	351.6583	A	**746.4155**	373.7114	729.3890	365.1981	728.4050	364.7061	7
8	821.3676	411.1874	804.3410	402.6741	803.3570	402.1821	T	**675.3784**	338.1928	658.3519	329.6796	657.3679	329.1876	6
9	949.4261	475.2167	932.3996	466.7034	931.4156	466.2114	Q	**574.3307**	287.6690	557.3042	279.1557	556.3202	278.6637	5
10	**1020.4633**	510.7353	1003.4367	502.2220	1002.4527	501.7300	A	**446.2722**	223.6397	429.2456	215.1264	428.2616	214.6344	4
11	**1133.5473**	567.2773	1116.5208	558.7640	1115.5368	558.2720	L	**375.2350**	188.1212	358.2085	179.6079	357.2245	179.1159	3
12	**1220.5794**	610.7933	1203.5528	602.2800	1202.5688	601.7880	S	**262.1510**	131.5791	245.1244	123.0659	244.1404	122.5738	2
13							R	175.1190	88.0631	158.0924	79.5498			1

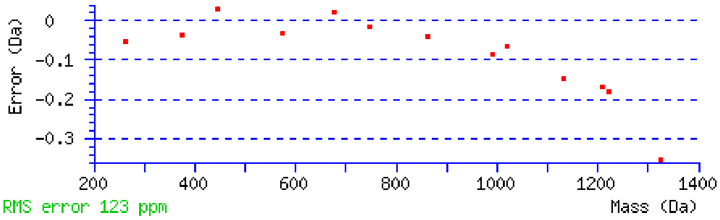

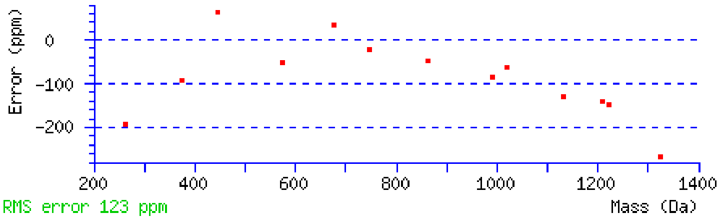

All matches to this query

Score	Mr(calc):	Delta	Sequence
113.3	1393.6837	0.0035	ALGYENATQALSR
10.8	1393.6945	-0.0073	MMLVTLSENLSR
8.9	1393.6827	0.0045	ASPSNARKQISR
8.9	1393.6827	0.0045	ASPSNARKQISR
8.7	1393.6759	0.0113	AGTSTLTECVINK
8.0	1393.6936	-0.0065	SDATASISLSSNLK
7.5	1393.6949	-0.0078	IQENYNTRLSR
7.1	1393.6949	-0.0078	IQENYNTRLSR
5.5	1393.6827	0.0045	NSQQRRVALDK
5.3	1393.6741	0.0131	LTPEKEEILNK

Spectrum No: 16; Query: 426; Rank: 1

Peptide View

MS/MS Fragmentation of **NLQVYNATSNSLTVK**
Found in **IPI00189714**, Tax_Id=10116 Gene_Symbol=Col12a1 similar to Collagen alpha-1(XII) chain precursor

Match to Query 426: 1651.842528 from(826.928540,2+)
Title: 091127RatKid_SCX01_17.2118.2118.2.dta
Data file K:\NewmanPaper\Piliang\3SubProteomes\Piliang3SP\mgf5ppm\SCX_3SubProteomes5ppm.mgf

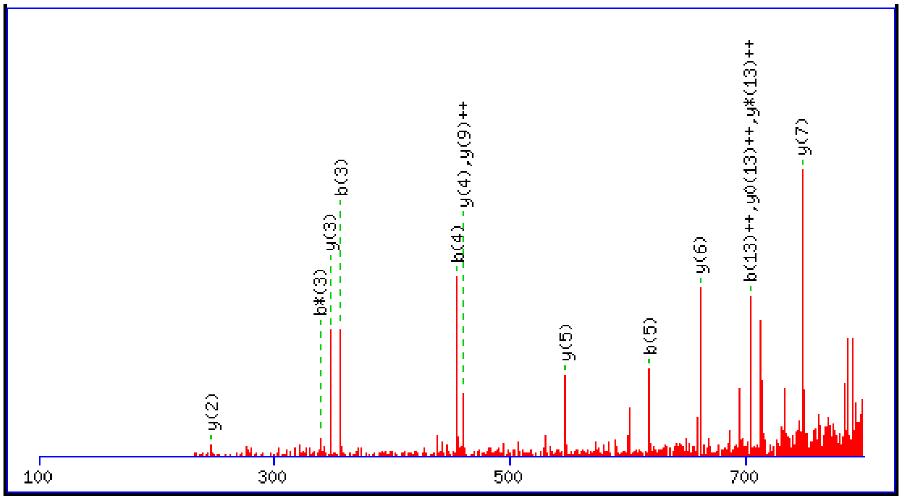

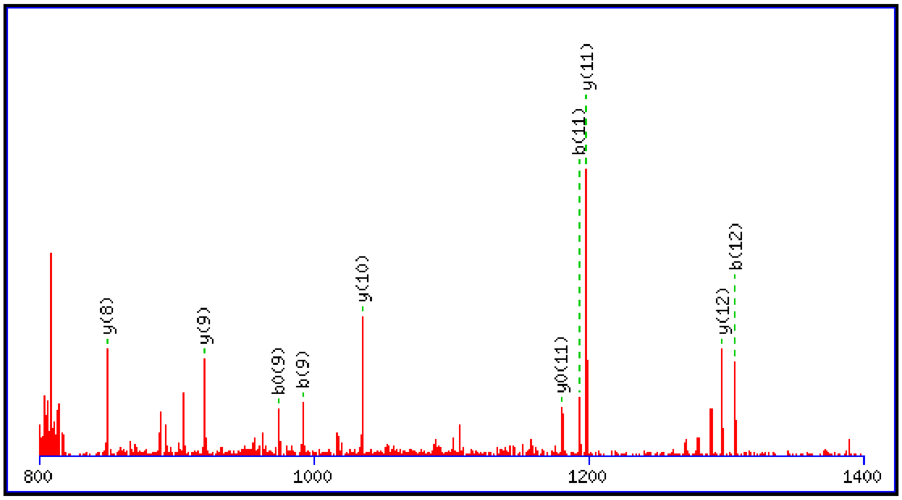

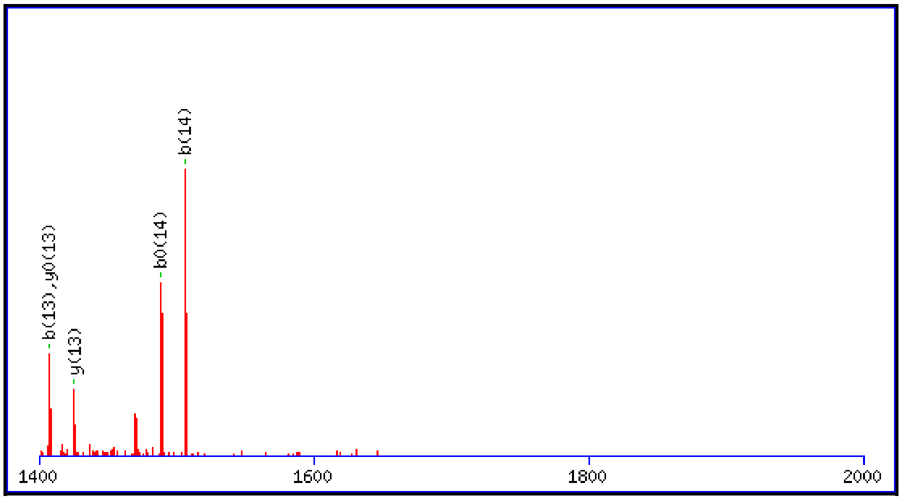

Monoisotopic mass of neutral peptide Mr(calc): 1651.8417
Fixed modifications: Carbamidomethyl (C)
Variable modifications:
N6 : Deamidated_N (N)
Ions Score: 113 **Expect:** 8.8e-010
Matches (Bold Red): 29/150 fragment ions using 33 most intense peaks

#	b	b^{++}	b*	b^{*++}	b^0	b^{0++}	Seq.	y	y^{++}	y*	y^{*++}	y^0	y^{0++}	#
1	115.0502	58.0287	98.0237	49.5155			N							15
2	228.1343	114.5708	211.1077	106.0575			L	1538.8061	769.9067	1521.7795	761.3934	1520.7955	760.9014	14
3	**356.1928**	178.6001	**339.1663**	170.0868			Q	**1425.7220**	713.3646	1408.6954	**704.8514**	**1407.7114**	**704.3594**	13
4	**455.2613**	228.1343	438.2347	219.6210			V	**1297.6634**	649.3353	1280.6369	640.8221	1279.6529	640.3301	12
5	**618.3246**	309.6659	601.2980	301.1527			Y	**1198.5950**	599.8011	1181.5685	591.2879	**1180.5844**	590.7959	11

**6**	733.3515	367.1794	716.3250	358.6661			**N**	**1035.5317**	518.2695	1018.5051	509.7562	1017.5211	509.2642	**10**
**7**	804.3886	402.6980	787.3621	394.1847			**A**	**920.5047**	**460.7560**	903.4782	452.2427	902.4942	451.7507	**9**
**8**	905.4363	453.2218	888.4098	444.7085	887.4258	444.2165	**T**	**849.4676**	425.2374	832.4411	416.7242	831.4571	416.2322	**8**
**9**	**992.4683**	496.7378	975.4418	488.2245	**974.4578**	487.7325	**S**	**748.4199**	374.7136	731.3934	366.2003	730.4094	365.7083	**7**
**10**	1106.5113	553.7593	1089.4847	545.2460	1088.5007	544.7540	**N**	**661.3879**	331.1976	644.3614	322.6843	643.3774	322.1923	**6**
**11**	**1193.5433**	597.2753	1176.5168	588.7620	1175.5327	588.2700	**S**	**547.3450**	274.1761	530.3184	265.6629	529.3344	265.1709	**5**
**12**	**1306.6274**	653.8173	1289.6008	645.3040	1288.6168	644.8120	**L**	**460.3130**	230.6601	443.2864	222.1468	442.3024	221.6548	**4**
**13**	**1407.6750**	**704.3412**	1390.6485	695.8279	1389.6645	695.3359	**T**	**347.2289**	174.1181	330.2023	165.6048	329.2183	165.1128	**3**
**14**	**1506.7435**	753.8754	1489.7169	745.3621	**1488.7329**	744.8701	**V**	**246.1812**	123.5942	229.1547	115.0810			**2**
**15**							**K**	147.1128	74.0600	130.0863	65.5468			**1**

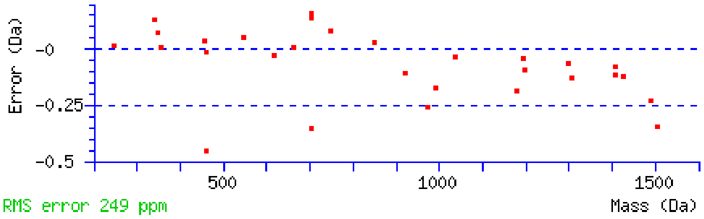

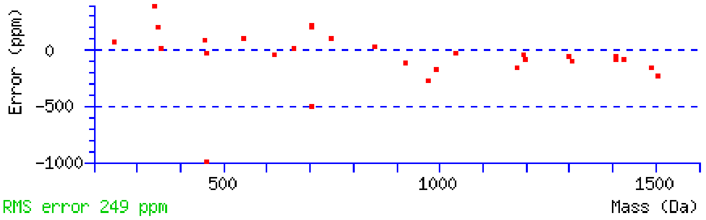

All matches to this query

Score	Mr(calc):	Delta	Sequence
112.6	1651.8417	0.0008	NLQVYNATSNSLTVK
51.5	1651.8417	0.0008	NLQVYNATSNSLTVK
51.2	1651.8417	0.0008	NLQVYNATSNSLTVK
16.3	1651.8529	-0.0103	EPEKPNIINANREK
5.2	1651.8529	-0.0103	EPEKPNIINANREK
4.3	1651.8433	-0.0008	GITQALNLVDSLSLK
3.1	1651.8464	-0.0038	MNTASLAVRKYNER
2.6	1651.8525	-0.0099	LNTLDMDTVKKMVK
1.8	1651.8529	-0.0103	EPEKPNIINANREK
1.7	1651.8442	-0.0017	VKMPSLTMPKVDVK

Spectrum No: 17; Query: 1168; Rank: 1

Peptide View

MS/MS Fragmentation of **VSGILFNDEMDDFSSPNFTNQFGVAPSPANFIKPGK**
Found in **IPI00206254**, Tax_Id=10116 Gene_Symbol=Ggt1 Gamma-glutamyltranspeptidase 1 precursor

Match to Query 1168: 3887.854572 from(1296.958800,3+)
Title: 091127RatKid_SCX01_12.5110.5110.3.dta
Data file K:\NewmanPaper\Piliang\3SubProteomes\Piliang3SP\mgf5ppm\SCX_3SubProteomes5ppm.mgf

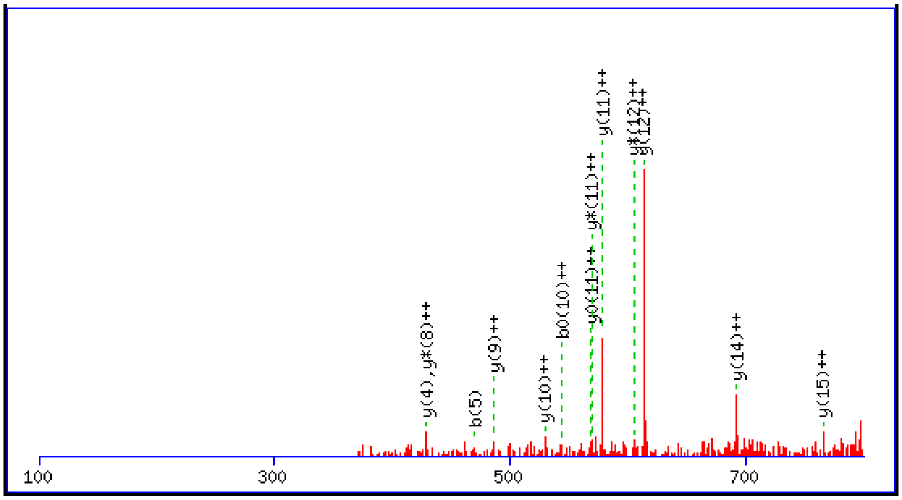

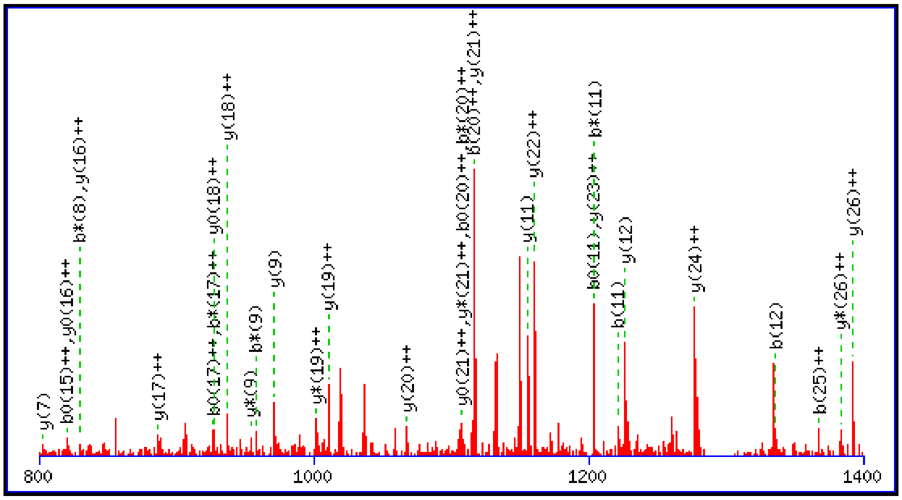

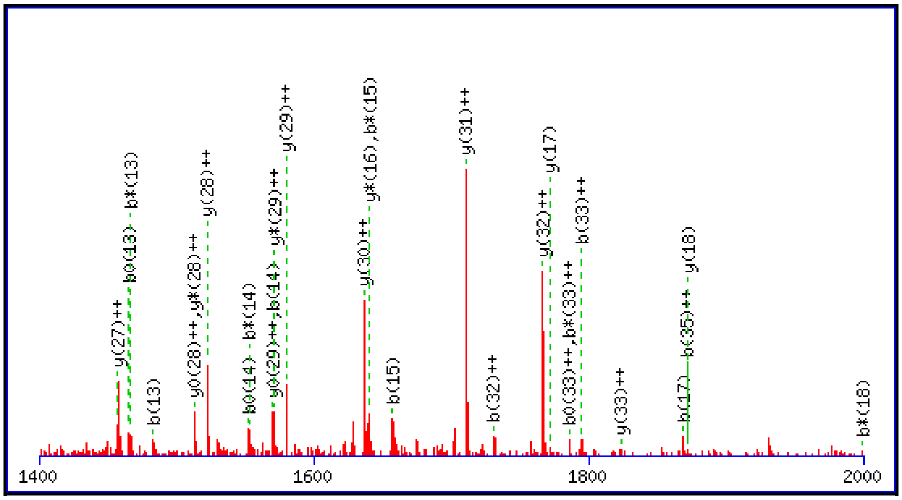

Monoisotopic mass of neutral peptide Mr(calc): 3887.8353
Fixed modifications: Carbamidomethyl (C)
Variable modifications:
N17 : Deamidated_N (N)
Ions Score: 110 **Expect:** 3.5e-009
Matches (Bold Red): 76/388 fragment ions using 142 most intense peaks

#	b	b^{++}	b*	b^{*++}	b^0	b^{0++}	Seq.	y	y^{++}	y*	y^{*++}	y^0	y^{0++}	#
1	100.0757	50.5415					V							36
2	187.1077	94.0575			169.0972	85.0522	S	3789.7741	1895.3907	3772.7476	1886.8774	3771.7635	1886.3854	35
3	244.1292	122.5682			226.1186	113.5629	G	3702.7421	1851.8747	3685.7155	1843.3614	3684.7315	1842.8694	34
4	357.2132	179.1103			339.2027	170.1050	I	3645.7206	**1823.3639**	3628.6941	1814.8507	3627.7101	1814.3587	33
5	**470.2973**	235.6523			452.2867	226.6470	L	3532.6366	**1766.8219**	3515.6100	1758.3086	3514.6260	1757.8166	32

6	617.3657	309.1865			599.3552	300.1812	F	3419.5525	**1710.2799**	3402.5259	1701.7666	3401.5419	1701.2746	31
7	731.4087	366.2080	714.3821	357.6947	713.3981	357.2027	N	3272.4841	**1636.7457**	3255.4575	1628.2324	3254.4735	1627.7404	30
8	846.4356	423.7214	**829.4090**	415.2082	828.4250	414.7162	D	3158.4412	**1579.7242**	3141.4146	**1571.2109**	3140.4306	**1570.7189**	29
9	975.4782	488.2427	**958.4516**	479.7295	957.4676	479.2374	E	3043.4142	**1522.2107**	3026.3877	**1513.6975**	3025.4036	**1513.2055**	28
10	1106.5187	553.7630	1089.4921	545.2497	1088.5081	**544.7577**	M	2914.3716	**1457.6894**	2897.3451	1449.1762	2896.3611	1448.6842	27
11	**1221.5456**	611.2764	**1204.5191**	602.7632	**1203.5351**	602.2712	D	2783.3311	**1392.1692**	2766.3046	**1383.6559**	2765.3206	1383.1639	26
12	**1336.5726**	668.7899	1319.5460	660.2766	1318.5620	659.7846	D	2668.3042	1334.6557	2651.2776	1326.1425	2650.2936	1325.6504	25
13	**1483.6410**	742.3241	**1466.6144**	733.8108	**1465.6304**	733.3188	F	2553.2772	**1277.1423**	2536.2507	1268.6290	2535.2667	1268.1370	24
14	**1570.6730**	785.8401	**1553.6465**	777.3269	**1552.6624**	776.8349	S	2406.2088	**1203.6081**	2389.1823	1195.0948	2388.1983	1194.6028	23
15	**1657.7050**	829.3562	**1640.6785**	820.8429	1639.6945	**820.3509**	S	2319.1768	**1160.0920**	2302.1503	1151.5788	2301.1662	1151.0868	22
16	1754.7578	877.8825	1737.7312	869.3693	1736.7472	868.8773	P	2232.1448	**1116.5760**	2215.1182	**1108.0628**	2214.1342	**1107.5707**	21
17	**1869.7847**	935.3960	1852.7582	**926.8827**	1851.7742	**926.3907**	N	2135.0920	**1068.0496**	2118.0655	1059.5364	2117.0814	1059.0444	20
18	2016.8531	1008.9302	**1999.8266**	1000.4169	1998.8426	999.9249	F	2020.0651	**1010.5362**	2003.0385	**1002.0229**	2002.0545	1001.5309	19
19	2117.9008	1059.4540	2100.8743	1050.9408	2099.8903	1050.4488	T	**1872.9967**	**937.0020**	1855.9701	928.4887	1854.9861	**927.9967**	18
20	2231.9437	**1116.4755**	2214.9172	**1107.9622**	2213.9332	**1107.4702**	N	**1771.9490**	**886.4781**	1754.9224	877.9649	1753.9384	877.4728	17
21	2360.0023	1180.5048	2342.9758	1171.9915	2341.9918	1171.4995	Q	1657.9061	**829.4567**	**1640.8795**	820.9434	1639.8955	**820.4514**	16
22	2507.0707	1254.0390	2490.0442	1245.5257	2489.0602	1245.0337	F	1529.8475	**765.4274**	1512.8209	756.9141	1511.8369	756.4221	15
23	2564.0922	1282.5497	2547.0657	1274.0365	2546.0816	1273.5445	G	1382.7791	**691.8932**	1365.7525	683.3799	1364.7685	682.8879	14
24	2663.1606	1332.0839	2646.1341	1323.5707	2645.1501	1323.0787	V	1325.7576	663.3824	1308.7310	654.8692	1307.7470	654.3772	13
25	2734.1977	**1367.6025**	2717.1712	1359.0892	2716.1872	1358.5972	A	**1226.6892**	**613.8482**	1209.6626	**605.3350**	1208.6786	604.8429	12
26	2831.2505	1416.1289	2814.2239	1407.6156	2813.2399	1407.1236	P	**1155.6521**	**578.3297**	1138.6255	**569.8164**	1137.6415	**569.3244**	11
27	2918.2825	1459.6449	2901.2560	1451.1316	2900.2720	1450.6396	S	1058.5993	**529.8033**	1041.5728	521.2900	1040.5887	520.7980	10
28	3015.3353	1508.1713	2998.3087	1499.6580	2997.3247	1499.1660	P	**971.5673**	**486.2873**	**954.5407**	477.7740			9
29	3086.3724	1543.6898	3069.3459	1535.1766	3068.3618	1534.6846	A	874.5145	437.7609	857.4880	**429.2476**			8
30	3200.4153	1600.7113	3183.3888	1592.1980	3182.4048	1591.7060	N	**803.4774**	402.2423	786.4509	393.7291			7
31	3347.4837	1674.2455	3330.4572	1665.7322	3329.4732	1665.2402	F	689.4345	345.2209	672.4079	336.7076			6
32	3460.5678	**1730.7875**	3443.5413	1722.2743	3442.5572	1721.7823	I	542.3661	271.6867	525.3395	263.1734			5
33	3588.6628	**1794.8350**	3571.6362	**1786.3217**	3570.6522	**1785.8297**	K	**429.2820**	215.1446	412.2554	206.6314			4
34	3685.7155	1843.3614	3668.6890	1834.8481	3667.7050	1834.3561	P	301.1870	151.0972	284.1605	142.5839			3
35	3742.7370	**1871.8721**	3725.7104	1863.3589	3724.7264	1862.8669	G	204.1343	102.5708	187.1077	94.0575			2
36							K	147.1128	74.0600	130.0863	65.5468			1

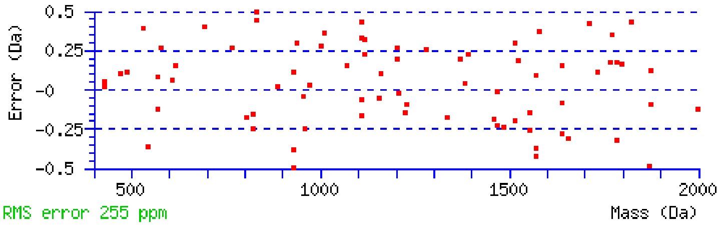

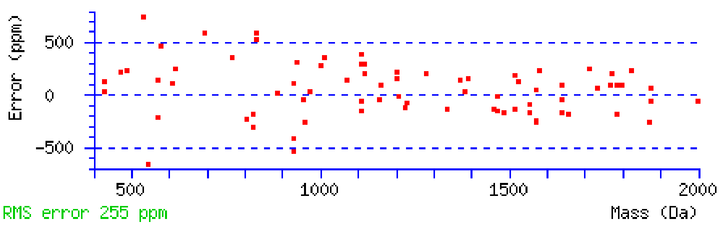

All matches to this query

Score	Mr(calc):	Delta	Sequence
110.3	3887.8353	0.0193	VSGILFNDEMDDFSSPNFTNQFGVAPSPANFIKPGK
106.2	3887.8353	0.0193	VSGILFNDEMDDFSSPNFTNQFGVAPSPANFIKPGK
105.3	3887.8353	0.0193	VSGILFNDEMDDFSSPNFTNQFGVAPSPANFIKPGK
44.4	3887.8353	0.0193	VSGILFNDEMDDFSSPNFTNQFGVAPSPANFIKPGK
32.6	3886.8513	1.0033	VSGILFNDEMDDFSSPNFTNQFGVAPSPANFIKPGK
3.1	3887.8439	0.0106	STYDGLHVITGTTENSPADRSQKIHAGDEVIQVNR
3.0	3886.8716	0.9830	IMFDVNGDLVTKFDILQGQKTPTVPFNGVYFMR
1.8	3886.8245	1.0300	FVGNAQNTLAQPTVWLTIVLTTAVCIMPVVAFR
1.8	3886.8245	1.0300	FVGNAQNTLAQPTVWLTIVLTTAVCIMPVVAFR

1.8	3886.8245	1.0300	FVGNAQNTLAQPTVWLTIVLTTAVCIMPVVAFR

Spectrum No: 18; Query: 1136; Rank: 1

Peptide View

MS/MS Fragmentation of **QQADFVDLSFPTISSTGPNGAIIHYAPIPETNR**
Found in **IPI00200054**, Tax_Id=10116 Gene_Symbol=Xpnpep1 Xaa-Pro aminopeptidase 1

Match to Query 1136: 3556.755732 from(1186.592520,3+)
Title: 091129RatKid_SCX02_21.5675.5675.3.dta
Data file K:\NewmanPaper\Piliang\3SubProteomes\Piliang3SP\mgf5ppm\SCX_3SubProteomes5ppm.mgf

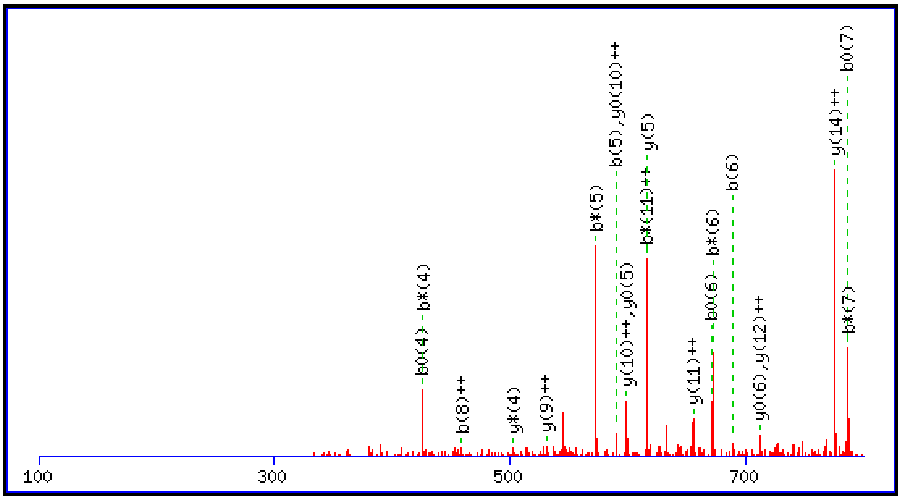

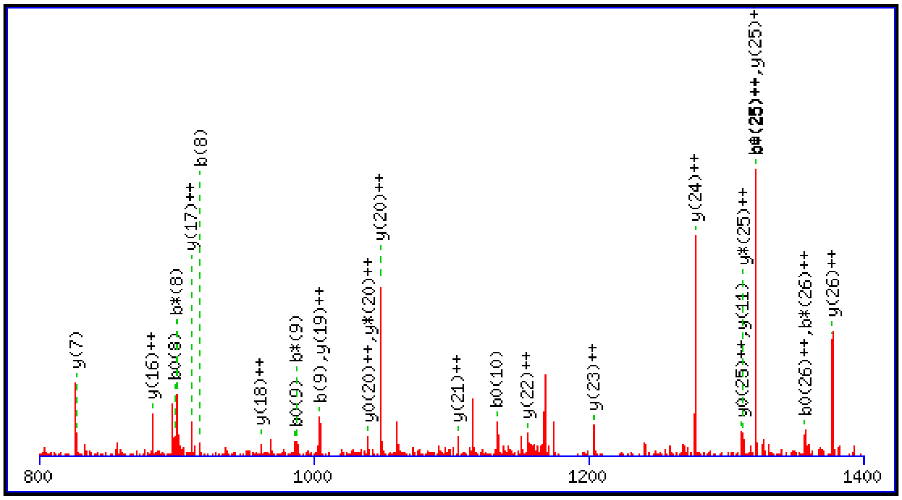

Mascot Search Results: Peptide View

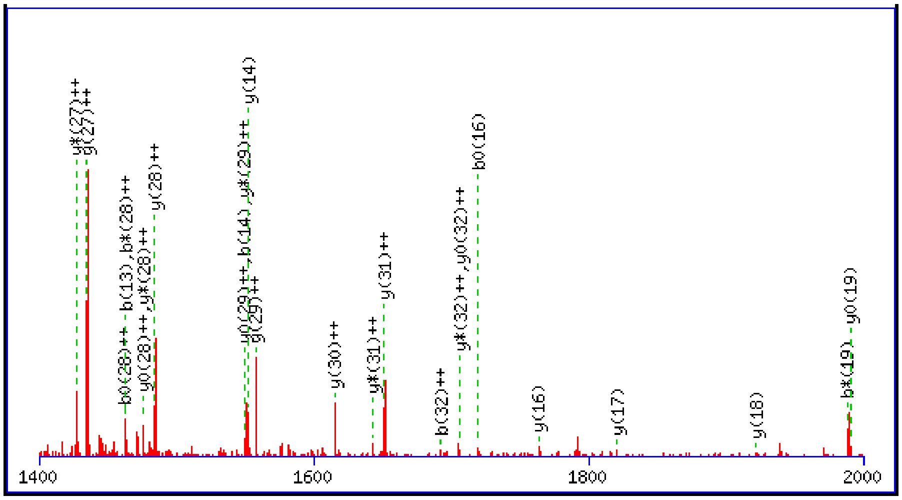

Monoisotopic mass of neutral peptide Mr(calc): 3556.7474
Fixed modifications: Carbamidomethyl (C)
Variable modifications:
N32 : Deamidated_N (N)
Ions Score: 108 **Expect:** 4e-009
Matches (Bold Red): 74/374 fragment ions using 120 most intense peaks

#	b	b^{++}	b*	b^{*++}	b^{0}	b^{0++}	Seq.	y	y^{++}	y*	y^{*++}	y^{0}	y^{0++}	#
1	129.0659	65.0366	112.0393	56.5233			Q							33
2	257.1244	129.0659	240.0979	120.5526			Q	3429.6961	1715.3517	3412.6696	**1706.8384**	3411.6856	**1706.3464**	32
3	328.1615	164.5844	311.1350	156.0711			A	3301.6375	**1651.3224**	3284.6110	**1642.8091**	3283.6270	1642.3171	31
4	443.1885	222.0979	**426.1619**	213.5846	**425.1779**	213.0926	D	3230.6004	**1615.8039**	3213.5739	1607.2906	3212.5899	1606.7986	30
5	**590.2569**	295.6321	**573.2304**	287.1188	572.2463	286.6268	F	3115.5735	**1558.2904**	3098.5469	**1549.7771**	3097.5629	**1549.2851**	29
6	**689.3253**	345.1663	**672.2988**	336.6530	**671.3148**	336.1610	V	2968.5051	**1484.7562**	2951.4785	**1476.2429**	2950.4945	**1475.7509**	28
7	804.3523	402.6798	**787.3257**	394.1665	**786.3417**	393.6745	D	2869.4367	**1435.2220**	2852.4101	**1426.7087**	2851.4261	1426.2167	27
8	**917.4363**	**459.2218**	**900.4098**	450.7085	**899.4258**	450.2165	L	2754.4097	**1377.7085**	2737.3832	1369.1952	2736.3992	1368.7032	26
9	**1004.4684**	502.7378	**987.4418**	494.2245	**986.4578**	493.7325	S	2641.3257	**1321.1665**	2624.2991	**1312.6532**	2623.3151	**1312.1612**	25
10	1151.5368	576.2720	1134.5102	567.7587	**1133.5262**	567.2667	F	2554.2936	**1277.6504**	2537.2671	1269.1372	2536.2831	1268.6452	24
11	1248.5895	624.7984	1231.5630	**616.2851**	1230.5790	615.7931	P	2407.2252	**1204.1162**	2390.1987	1195.6030	2389.2146	1195.1110	23
12	1349.6372	675.3222	1332.6107	666.8090	1331.6266	666.3170	T	2310.1724	**1155.5899**	2293.1459	1147.0766	2292.1619	1146.5846	22
13	**1462.7213**	731.8643	1445.6947	723.3510	1444.7107	722.8590	I	2209.1248	**1105.0660**	2192.0982	1096.5527	2191.1142	1096.0607	21
14	**1549.7533**	775.3803	1532.7268	766.8670	1531.7427	766.3750	S	2096.0407	**1048.5240**	2079.0142	**1040.0107**	2078.0301	**1039.5187**	20
15	1636.7853	818.8963	1619.7588	810.3830	1618.7748	809.8910	S	2009.0087	**1005.0080**	1991.9821	996.4947	**1990.9981**	996.0027	19
16	1737.8330	869.4201	1720.8065	860.9069	**1719.8224**	860.4149	T	**1921.9766**	**961.4920**	1904.9501	952.9787	1903.9661	952.4867	18
17	1794.8545	897.9309	1777.8279	889.4176	1776.8439	888.9256	G	**1820.9290**	**910.9681**	1803.9024	902.4548	1802.9184	901.9628	17
18	1891.9072	946.4573	1874.8807	937.9440	1873.8967	937.4520	P	**1763.9075**	**882.4574**	1746.8810	873.9441	1745.8969	873.4521	16
19	2005.9502	1003.4787	**1988.9236**	994.9654	1987.9396	994.4734	N	1666.8547	833.9310	1649.8282	825.4177	1648.8442	824.9257	15
20	2062.9716	1031.9895	2045.9451	1023.4762	2044.9611	1022.9842	G	**1552.8118**	**776.9095**	1535.7853	768.3963	1534.8012	767.9043	14
21	2134.0087	1067.5080	2116.9822	1058.9947	2115.9982	1058.5027	A	1495.7903	748.3988	1478.7638	739.8855	1477.7798	739.3935	13
22	2247.0928	1124.0500	2230.0663	1115.5368	2229.0822	1115.0448	I	1424.7532	**712.8803**	1407.7267	704.3670	1406.7427	703.8750	12
23	2360.1769	1180.5921	2343.1503	1172.0788	2342.1663	1171.5868	I	**1311.6692**	**656.3382**	1294.6426	647.8249	1293.6586	647.3329	11
24	2497.2358	1249.1215	2480.2092	1240.6083	2479.2252	1240.1162	H	1198.5851	**599.7962**	1181.5586	591.2829	1180.5745	**590.7909**	10
25	2660.2991	1330.6532	2643.2726	**1322.1399**	2642.2885	**1321.6479**	Y	1061.5262	**531.2667**	1044.4996	522.7535	1043.5156	522.2615	9
26	2731.3362	1366.1718	2714.3097	**1357.6585**	2713.3257	**1357.1665**	A	898.4629	449.7351	881.4363	441.2218	880.4523	440.7298	8
27	2828.3890	1414.6981	2811.3624	1406.1849	2810.3784	1405.6928	P	**827.4258**	414.2165	810.3992	405.7032	809.4152	405.2112	7
28	2941.4731	1471.2402	2924.4465	**1462.7269**	2923.4625	**1462.2349**	I	730.3730	365.6901	713.3464	357.1769	**712.3624**	356.6848	6
29	3038.5258	1519.7665	3021.4993	1511.2533	3020.5153	1510.7613	P	**617.2889**	309.1481	600.2624	300.6348	**599.2784**	300.1428	5
30	3167.5684	1584.2878	3150.5419	1575.7746	3149.5578	1575.2826	E	520.2362	260.6217	**503.2096**	252.1084	502.2256	251.6164	4
31	3268.6161	1634.8117	3251.5895	1626.2984	3250.6055	1625.8064	T	391.1936	196.1004	374.1670	187.5871	373.1830	187.0951	3
32	3383.6430	**1692.3252**	3366.6165	1683.8119	3365.6325	1683.3199	N	290.1459	145.5766	273.1193	137.0633			2

33							R	175.1190	88.0631	158.0924	79.5498				1

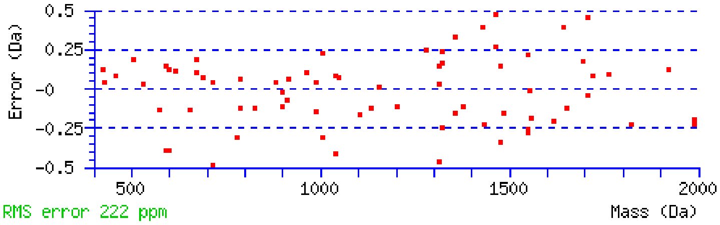

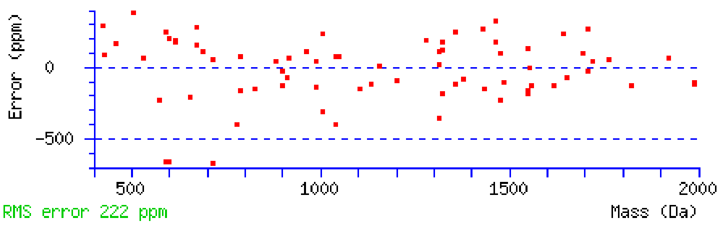

All matches to this query

Score	Mr(calc):	Delta	Sequence
108.3	3556.7474	0.0083	QQADFVDLSFPTISSTGPNGAIIHYAPIPETNR
103.0	3556.7474	0.0083	QQADFVDLSFPTISSTGPNGAIIHYAPIPETNR
49.4	3555.7634	0.9923	QQADFVDLSFPTISSTGPNGAIIHYAPIPETNR
4.6	3556.7442	0.0116	VVAALYTLVTPTLNPIVYSFRNKEMQSGIR
2.0	3555.7524	1.0034	GDVAFGLMFALLGSVSVGLSLLLNTVSVATLCR
2.0	3555.7524	1.0034	GDVAFGLMFALLGSVSVGLSLLLNTVSVATLCR
1.9	3554.7439	2.0118	IKLGICKAATEEENSHGQVNGILNAPSLGSPIR
1.6	3555.7524	1.0034	GDVAFGLMFALLGSVSVGLSLLLNTVSVATLCR
1.5	3555.7602	0.9956	VVAALYTLVTPTLNPIVYSFRNKEMQSGIR
1.2	3555.7619	0.9938	GFSFAPEVCESTTHVLVGKSVRTLNVLMGIAR

Spectrum No: 19; Query: 350; Rank: 1

Peptide View

MS/MS Fragmentation of **KNSTLCDLCIGPAK**
Found in **IPI00196656**, Tax_Id=10116 Gene_Symbol=Srprb Ba1-667

Match to Query 350: 1576.762488 from(789.388520,2+)
Title: 091127RatKid_SCX01_13.1817.1817.2.dta
Data file K:\NewmanPaper\Piliang\3SubProteomes\Piliang3SP\mgf5ppm\SCX_3SubProteomes5ppm.mgf

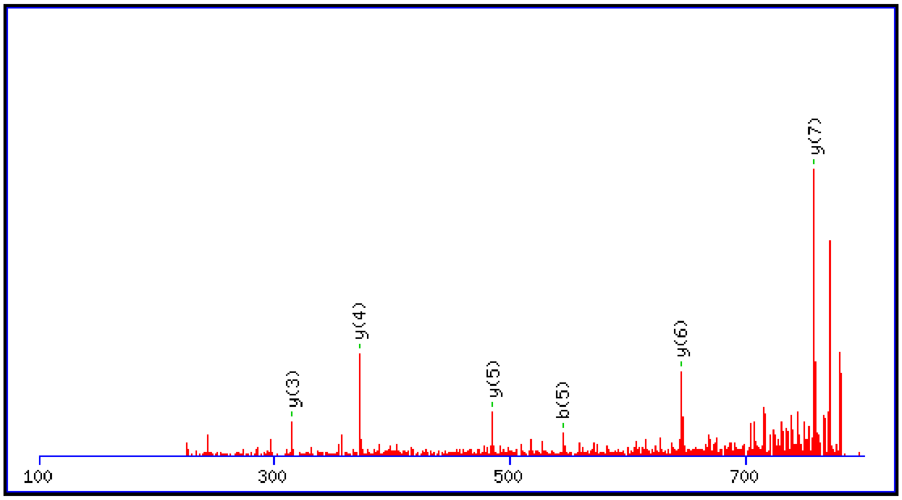

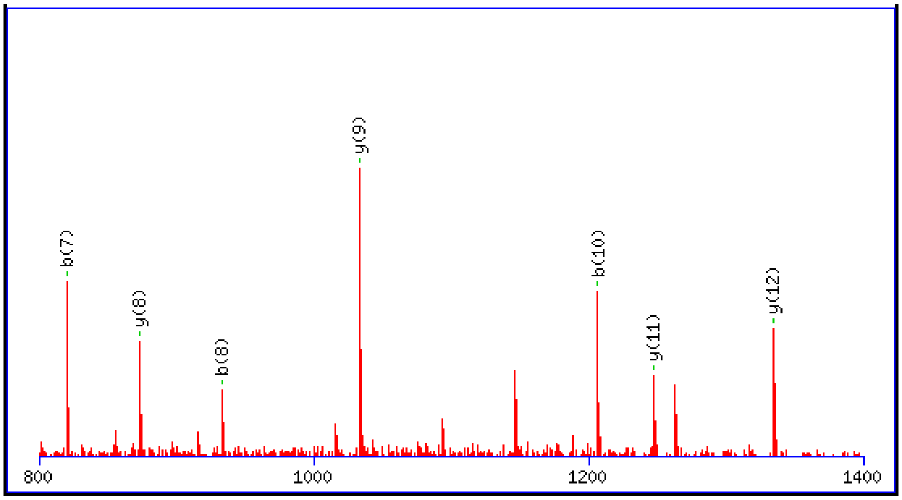

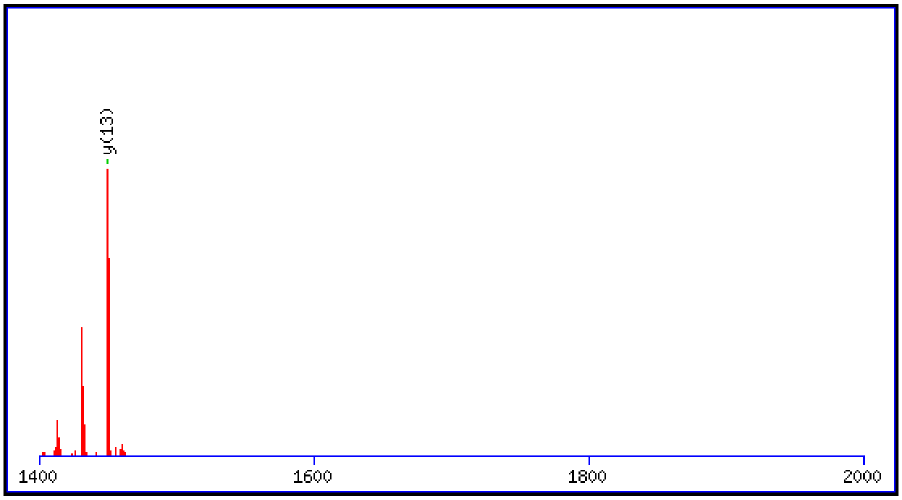

Monoisotopic mass of neutral peptide Mr(calc): 1576.7589
Fixed modifications: Carbamidomethyl (C)
Variable modifications:
N2 : Deamidated_N (N)
Ions Score: 108 **Expect:** 2.8e-009
Matches (Bold Red): 14/138 fragment ions using 17 most intense peaks

#	b	b^{++}	b*	b^{*++}	b^0	b^{0++}	Seq.	y	y^{++}	y*	y^{*++}	y^0	y^{0++}	#
1	129.1022	65.0548	112.0757	56.5415			K							14
2	244.1292	122.5682	227.1026	114.0550			N	**1449.6712**	725.3392	1432.6447	716.8260	1431.6607	716.3340	13
3	331.1612	166.0842	314.1347	157.5710	313.1506	157.0790	S	**1334.6443**	667.8258	1317.6177	659.3125	1316.6337	658.8205	12
4	432.2089	216.6081	415.1823	208.0948	414.1983	207.6028	T	**1247.6123**	624.3098	1230.5857	615.7965	1229.6017	615.3045	11
5	**545.2929**	273.1501	528.2664	264.6368	527.2824	264.1448	L	1146.5646	573.7859	1129.5380	565.2727	1128.5540	564.7806	10
6	705.3236	353.1654	688.2970	344.6522	687.3130	344.1602	C	**1033.4805**	517.2439	1016.4540	508.7306	1015.4699	508.2386	9
7	**820.3505**	410.6789	803.3240	402.1656	802.3400	401.6736	D	**873.4499**	437.2286	856.4233	428.7153	855.4393	428.2233	8
8	**933.4346**	467.2209	916.4081	458.7077	915.4240	458.2157	L	**758.4229**	379.7151	741.3964	371.2018			7
9	1093.4653	547.2363	1076.4387	538.7230	1075.4547	538.2310	C	**645.3389**	323.1731	628.3123	314.6598			6
10	**1206.5493**	603.7783	1189.5228	595.2650	1188.5387	594.7730	I	**485.3082**	243.1577	468.2817	234.6445			5
11	1263.5708	632.2890	1246.5442	623.7758	1245.5602	623.2837	G	**372.2241**	186.6157	355.1976	178.1024			4
12	1360.6235	680.8154	1343.5970	672.3021	1342.6130	671.8101	P	**315.2027**	158.1050	298.1761	149.5917			3
13	1431.6607	716.3340	1414.6341	707.8207	1413.6501	707.3287	A	218.1499	109.5786	201.1234	101.0653			2
14							K	147.1128	74.0600	130.0863	65.5468			1

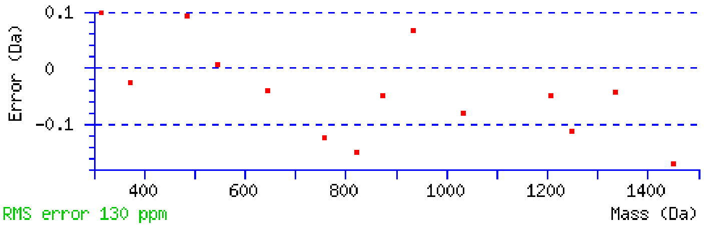

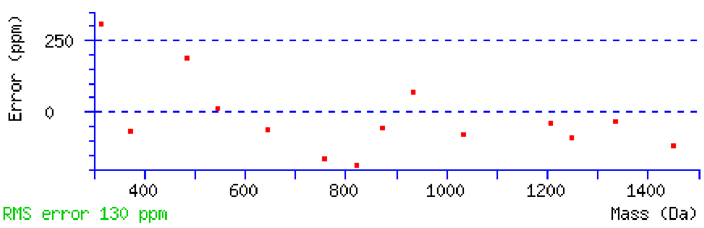

All matches to this query

Score	Mr(calc):	Delta	Sequence
107.9	1576.7589	0.0036	KNSTLCDLCIGPAK
72.1	1575.7749	0.9876	KNSTLCDLCIGPAK
9.9	1576.7571	0.0053	NSVAILMKNLYSK
7.7	1576.7722	-0.0097	KDQSNLRQLNGPK
7.4	1576.7538	0.0087	QYEKSIVNYKPK
7.0	1576.7668	-0.0043	GGQELSWVSRPMSK
6.6	1576.7610	0.0015	ENLEKTRHTLEK
5.6	1576.7498	0.0127	FSGSSSGTTAILTIR
5.5	1576.7538	0.0087	QYEKSIVNYKPK
4.7	1576.7766	-0.0141	KNEDLKQMLESNK

Spectrum No: 20; Query: 925; Rank: 1

Peptide View

MS/MS Fragmentation of **LVSDDLDSSLANLVGNLGIGNGTTK**
Found in **IPI00194958**, Tax_Id=10116 Gene_Symbol=Picalm Isoform 2 of Phosphatidylinositol-binding clathrin assembly protein

Match to Query 925: 2473.257028 from(1237.635790,2+)
Title: 091127RatKid_SCX01_16.6401.6401.2.dta
Data file K:\NewmanPaper\Piliang\3SubProteomes\Piliang3SP\mgf5ppm\SCX_3SubProteomes5ppm.mgf

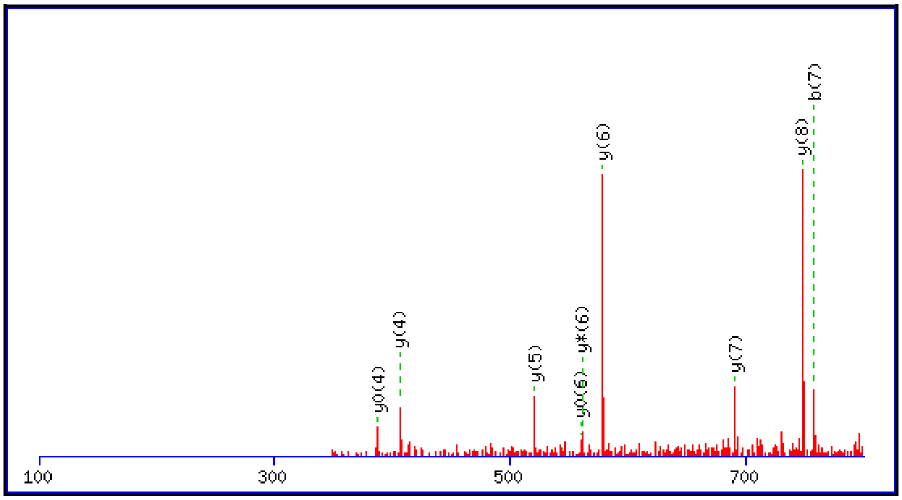

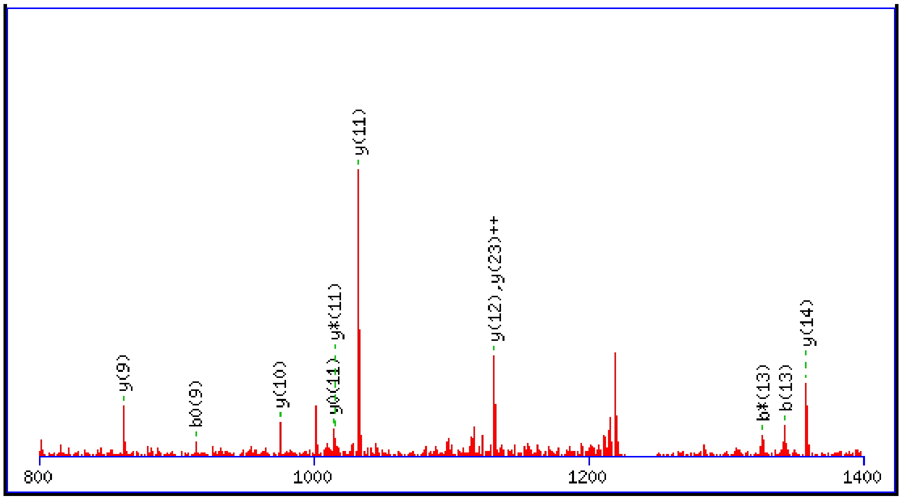

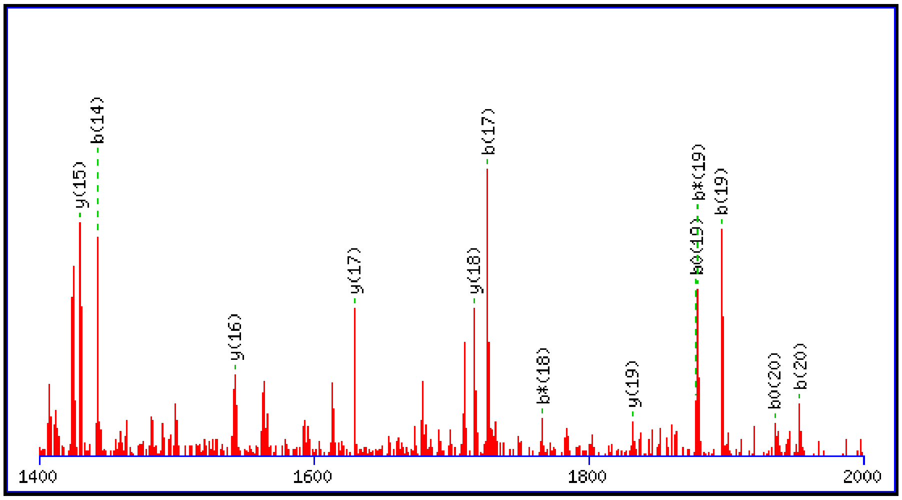

Monoisotopic mass of neutral peptide Mr(calc): 2473.2548
Fixed modifications: Carbamidomethyl (C)
Variable modifications:
N21 : Deamidated_N (N)
Ions Score: 108 **Expect:** 2.8e-009
Matches (Bold Red): 33/260 fragment ions using 51 most intense peaks

#	b	b^{++}	b*	b^{*++}	b^0	b^{0++}	Seq.	y	y^{++}	y*	y^{*++}	y^0	y^{0++}	#
1	114.0913	57.5493					L							25
2	213.1598	107.0835					V	2361.1780	1181.0926	2344.1514	1172.5794	2343.1674	1172.0873	24
3	300.1918	150.5995			282.1812	141.5942	S	2262.1096	**1131.5584**	2245.0830	1123.0451	2244.0990	1122.5531	23
4	415.2187	208.1130			397.2082	199.1077	D	2175.0775	1088.0424	2158.0510	1079.5291	2157.0670	1079.0371	22
5	530.2457	265.6265			512.2351	256.6212	D	2060.0506	1030.5289	2043.0240	1022.0157	2042.0400	1021.5237	21
6	643.3297	322.1685			625.3192	313.1632	L	1945.0237	973.0155	1927.9971	964.5022	1927.0131	964.0102	20
7	**758.3567**	379.6820			740.3461	370.6767	D	**1831.9396**	916.4734	1814.9130	907.9602	1813.9290	907.4682	19
8	845.3887	423.1980			827.3781	414.1927	S	**1716.9126**	858.9600	1699.8861	850.4467	1698.9021	849.9547	18
9	932.4207	466.7140			**914.4102**	457.7087	S	**1629.8806**	815.4439	1612.8541	806.9307	1611.8701	806.4387	17
10	1045.5048	523.2560			1027.4942	514.2508	L	**1542.8486**	771.9279	1525.8220	763.4147	1524.8380	762.9227	16
11	1116.5419	558.7746			1098.5313	549.7693	A	**1429.7645**	715.3859	1412.7380	706.8726	1411.7540	706.3806	15
12	1230.5848	615.7961	1213.5583	607.2828	1212.5743	606.7908	N	**1358.7274**	679.8673	1341.7009	671.3541	1340.7168	670.8621	14
13	**1343.6689**	672.3381	**1326.6424**	663.8248	1325.6583	663.3328	L	1244.6845	622.8459	1227.6579	614.3326	1226.6739	613.8406	13
14	**1442.7373**	721.8723	1425.7108	713.3590	1424.7267	712.8670	V	**1131.6004**	566.3038	1114.5739	557.7906	1113.5899	557.2986	12
15	1499.7588	750.3830	1482.7322	741.8698	1481.7482	741.3777	G	**1032.5320**	516.7696	**1015.5055**	508.2564	**1014.5214**	507.7644	11
16	1613.8017	807.4045	1596.7752	798.8912	1595.7911	798.3992	N	**975.5105**	488.2589	958.4840	479.7456	957.5000	479.2536	10
17	**1726.8858**	863.9465	1709.8592	855.4332	1708.8752	854.9412	L	**861.4676**	431.2374	844.4411	422.7242	843.4571	422.2322	9
18	1783.9072	892.4573	**1766.8807**	883.9440	1765.8967	883.4520	G	**748.3836**	374.6954	731.3570	366.1821	730.3730	365.6901	8

**19**	**1896.9913**	948.9993	**1879.9647**	940.4860	**1878.9807**	939.9940	**I**	**691.3621**	346.1847	674.3355	337.6714	673.3515	337.1794	**7**
**20**	**1954.0128**	977.5100	1936.9862	968.9967	**1936.0022**	968.5047	**G**	**578.2780**	289.6427	**561.2515**	281.1294	**560.2675**	280.6374	**6**
**21**	2069.0397	1035.0235	2052.0131	1026.5102	2051.0291	1026.0182	**N**	**521.2566**	261.1319	504.2300	252.6186	503.2460	252.1266	**5**
**22**	2126.0612	1063.5342	2109.0346	1055.0209	2108.0506	1054.5289	**G**	**406.2296**	203.6185	389.2031	195.1052	**388.2191**	194.6132	**4**
**23**	2227.1088	1114.0581	2210.0823	1105.5448	2209.0983	1105.0528	**T**	349.2082	175.1077	332.1816	166.5944	331.1976	166.1024	**3**
**24**	2328.1565	1164.5819	2311.1300	1156.0686	2310.1460	1155.5766	**T**	248.1605	124.5839	231.1339	116.0706	230.1499	115.5786	**2**
**25**							**K**	147.1128	74.0600	130.0863	65.5468			**1**

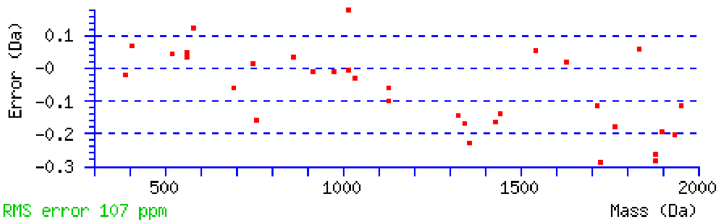

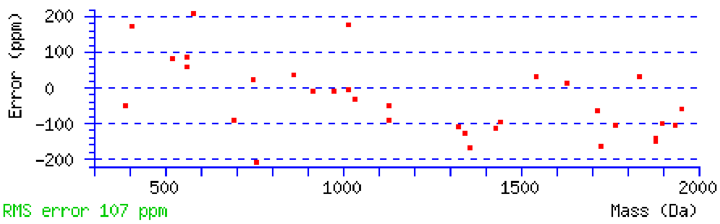

All matches to this query

Score	Mr(calc):	Delta	Sequence
107.8	2473.2548	0.0023	LVSDDLDSSLANLVGNLGIGNGTTK
45.7	2473.2548	0.0023	LVSDDLDSSLANLVGNLGIGNGTTK
28.4	2473.2548	0.0023	LVSDDLDSSLANLVGNLGIGNGTTK
27.1	2472.2708	0.9863	LVSDDLDSSLANLVGNLGIGNGTTK
2.9	2473.2770	-0.0200	GRLPPGPTPLPFIGNYLQLNTK
2.9	2473.2770	-0.0200	GRLPPGPTPLPFIGNYLQLNTK
2.9	2473.2770	-0.0200	GRLPPGPTPLPFIGNYLQLNTK
2.9	2473.2770	-0.0200	GRLPPGPTPLPFIGNYLQLNTK

Spectrum No: 21; Query: 483; Rank: 1

Peptide View

MS/MS Fragmentation of **TALAAFNAQNNGTYFK**
Found in **IPI00327469**, Tax_Id=10116 Gene_Symbol=Ahsg Alpha-2-HS-glycoprotein precursor

Match to Query 483: 1730.826328 from(866.420440,2+)
Title: 091129RatKid_SCX02_11.1990.1990.2.dta
Data file K:\NewmanPaper\Piliang\3SubProteomes\Piliang3SP\mgf5ppm\SCX_3SubProteomes5ppm.mgf

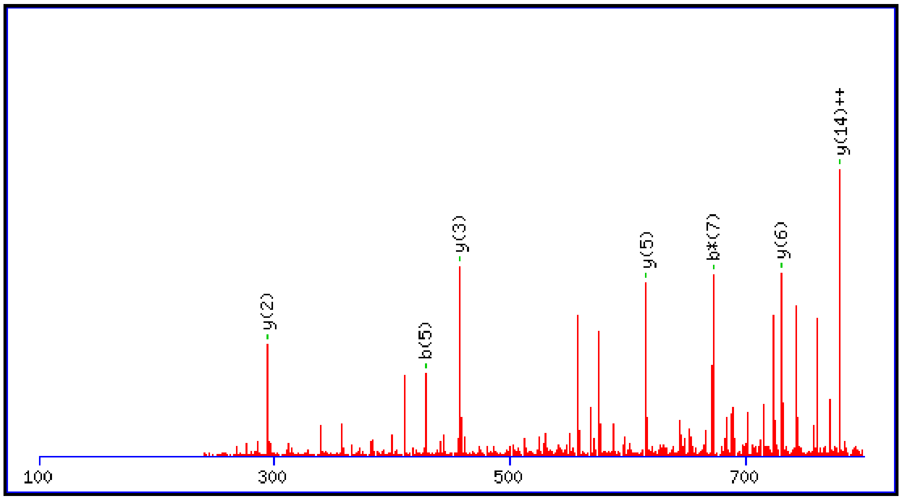

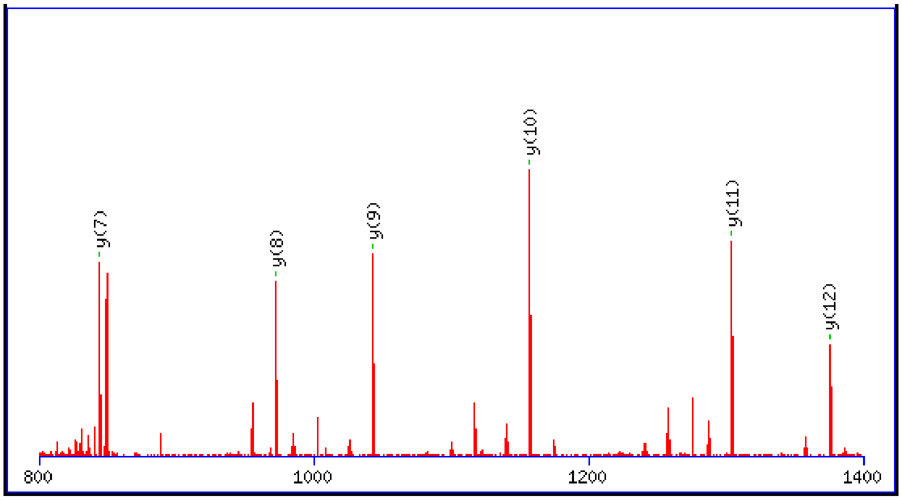

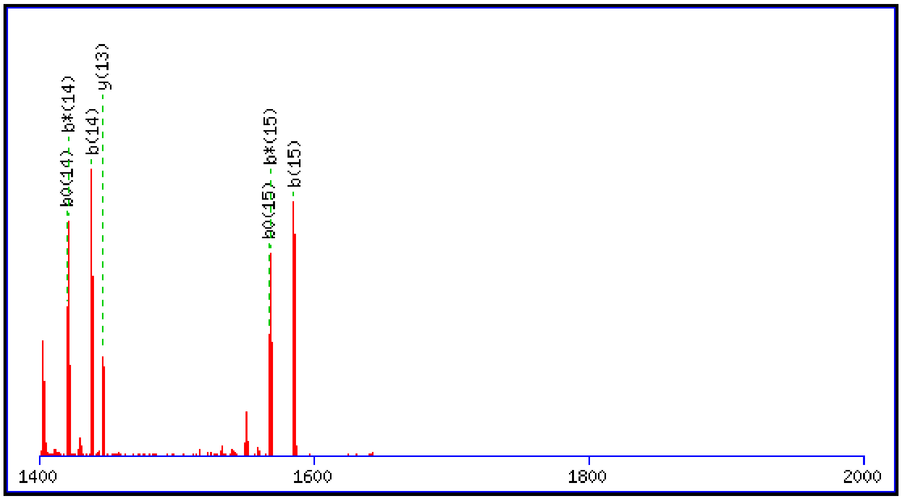

Monoisotopic mass of neutral peptide Mr(calc): 1730.8264
Fixed modifications: Carbamidomethyl (C)
Variable modifications:
N11 : Deamidated_N (N)
Ions Score: 108 **Expect:** 4.1e-009
Matches (Bold Red): 21/162 fragment ions using 29 most intense peaks

#	b	b^{++}	b*	b^{*++}	b^0	b^{0++}	Seq.	y	y^{++}	y*	y^{*++}	y^0	y^{0++}	#
1	102.0550	51.5311			84.0444	42.5258	T							16
2	173.0921	87.0497			155.0815	78.0444	A	1630.7860	815.8966	1613.7594	807.3834	1612.7754	806.8914	15
3	286.1761	143.5917			268.1656	134.5864	L	1559.7489	**780.3781**	1542.7223	771.8648	1541.7383	771.3728	14
4	357.2132	179.1103			339.2027	170.1050	A	**1446.6648**	723.8360	1429.6383	715.3228	1428.6542	714.8308	13
5	**428.2504**	214.6288			410.2398	205.6235	A	**1375.6277**	688.3175	1358.6012	679.8042	1357.6171	679.3122	12
6	575.3188	288.1630			557.3082	279.1577	F	**1304.5906**	652.7989	1287.5640	644.2857	1286.5800	643.7936	11
7	689.3617	345.1845	**672.3352**	336.6712	671.3511	336.1792	N	**1157.5222**	579.2647	1140.4956	570.7514	1139.5116	570.2594	10
8	760.3988	380.7030	743.3723	372.1898	742.3883	371.6978	A	**1043.4792**	522.2433	1026.4527	513.7300	1025.4687	513.2380	9
9	888.4574	444.7323	871.4308	436.2191	870.4468	435.7271	Q	**972.4421**	486.7247	955.4156	478.2114	954.4316	477.7194	8
10	1002.5003	501.7538	985.4738	493.2405	984.4898	492.7485	N	**844.3836**	422.6954	827.3570	414.1821	826.3730	413.6901	7
11	1117.5273	559.2673	1100.5007	550.7540	1099.5167	550.2620	N	**730.3406**	365.6740	713.3141	357.1607	712.3301	356.6687	6
12	1174.5487	587.7780	**1157.5222**	579.2647	1156.5382	578.7727	G	**615.3137**	308.1605	598.2871	299.6472	597.3031	299.1552	5
13	1275.5964	638.3018	1258.5699	629.7886	1257.5858	629.2966	T	558.2922	279.6498	541.2657	271.1365	540.2817	270.6445	4
14	**1438.6597**	719.8335	**1421.6332**	711.3202	**1420.6492**	710.8282	Y	**457.2445**	229.1259	440.2180	220.6126			3
15	**1585.7281**	793.3677	**1568.7016**	784.8544	**1567.7176**	784.3624	F	**294.1812**	147.5942	277.1547	139.0810			2
16							K	147.1128	74.0600	130.0863	65.5468			1

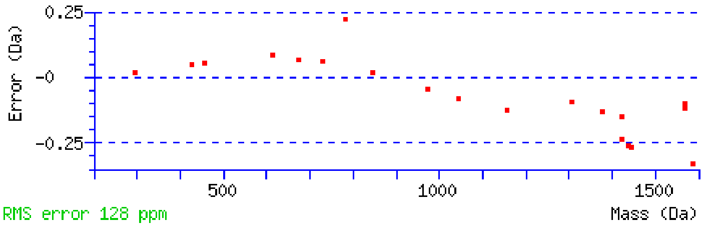

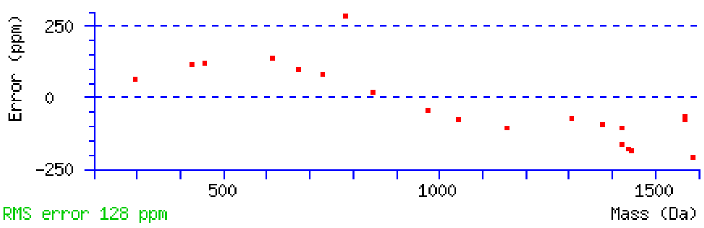

All matches to this query

Score	Mr(calc):	Delta	Sequence
107.6	1730.8264	-0.0000	TALAAFNAQNNGTYFK
89.2	1730.8264	-0.0000	TALAAFNAQNNGTYFK
49.0	1730.8264	-0.0000	TALAAFNAQNNGTYFK
5.7	1730.8253	0.0010	TQTRAGSHSLRYFK
5.7	1730.8253	0.0010	TQTRAGSHSLRYFK
5.1	1730.8185	0.0078	TPTGPDLGTSYKGYMK
4.5	1728.8117	2.0146	TTAAMLSQQAQVSSVK
4.5	1728.8117	2.0146	TTAAMLSQQAQVSSVK
2.5	1730.8103	0.0161	LFSSKSSAQFKMYK
2.3	1729.8288	0.9976	QESLGVIFSDSLSLR

Spectrum No: 22; Query: 1174; Rank: 1

Peptide View

MS/MS Fragmentation of **QAIDDDCNQTGQMTAGLLDWPQGTFASQVTLEGDKVK**
Found in **IPI00364321**, Tax_Id=10116 Gene_Symbol=Etfb Electron transfer flavoprotein subunit beta

Match to Query 1174: 4037.882202 from(1346.968010,3+)
Title: 091127RatKid_SCX01_21.6341.6341.3.dta
Data file K:\NewmanPaper\Piliang\3SubProteomes\Piliang3SP\mgf5ppm\SCX_3SubProteomes5ppm.mgf

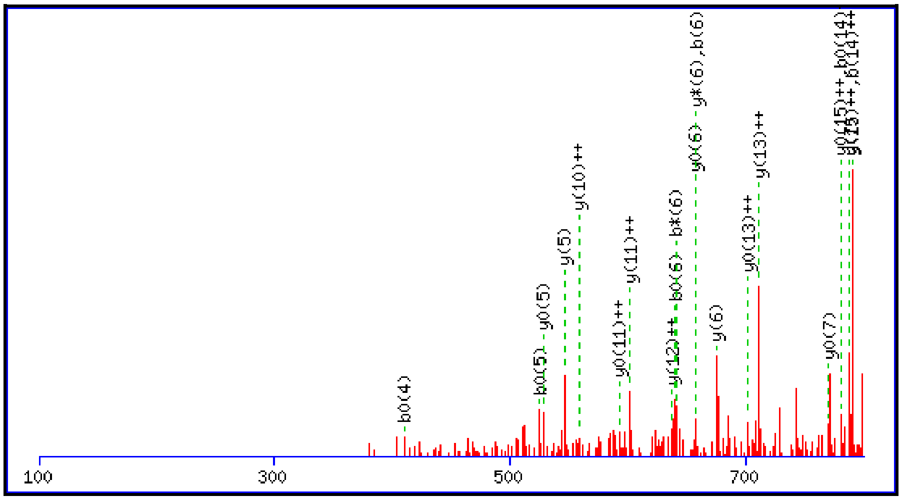

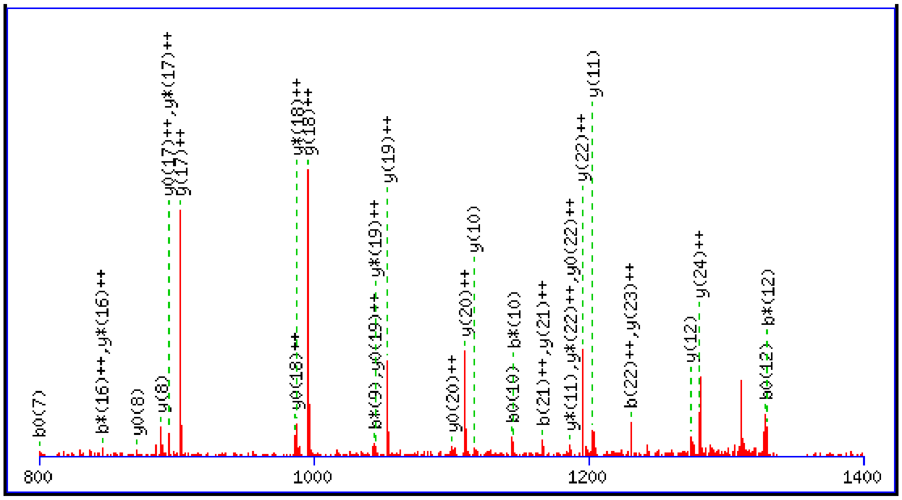

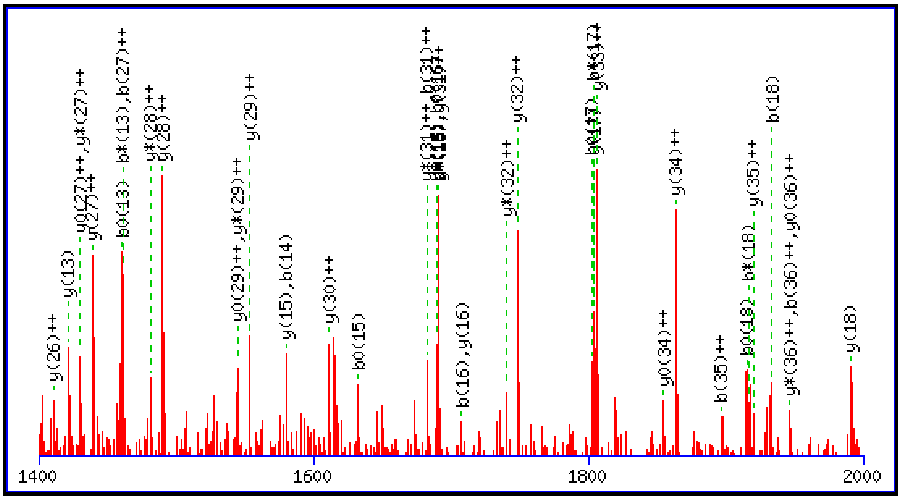

Monoisotopic mass of neutral peptide Mr(calc): 4037.8623
Fixed modifications: Carbamidomethyl (C)
Variable modifications:
N8 : Deamidated_N (N)
Ions Score: 104 **Expect:** 1.6e-008
Matches (Bold Red): 99/420 fragment ions using 176 most intense peaks

#	b	b^{++}	b*	b^{*++}	b^0	b^{0++}	Seq.	y	y^{++}	y*	y^{*++}	y^0	y^{0++}	#
1	129.0659	65.0366	112.0393	56.5233			Q							37
2	200.1030	100.5551	183.0764	92.0418			A	3910.8110	1955.9091	3893.7844	**1947.3959**	3892.8004	**1946.9038**	36
3	313.1870	157.0972	296.1605	148.5839			I	3839.7739	**1920.3906**	3822.7473	1911.8773	3821.7633	1911.3853	35
4	428.2140	214.6106	411.1874	206.0974	**410.2034**	205.6053	D	3726.6898	**1863.8485**	3709.6632	1855.3353	3708.6792	**1854.8433**	34
5	543.2409	272.1241	526.2144	263.6108	**525.2304**	263.1188	D	3611.6629	**1806.3351**	3594.6363	1797.8218	3593.6523	1797.3298	33
6	**658.2679**	329.6376	**641.2413**	321.1243	**640.2573**	320.6323	D	3496.6359	**1748.8216**	3479.6094	**1740.3083**	3478.6253	1739.8163	32
7	818.2985	409.6529	801.2720	401.1396	**800.2879**	400.6476	C	3381.6090	**1691.3081**	3364.5824	**1682.7948**	3363.5984	1682.3028	31
8	933.3254	467.1664	916.2989	458.6531	915.3149	458.1611	N	3221.5783	**1611.2928**	3204.5518	1602.7795	3203.5678	1602.2875	30
9	1061.3840	531.1957	**1044.3575**	522.6824	1043.3735	522.1904	Q	3106.5514	**1553.7793**	3089.5248	**1545.2661**	3088.5408	**1544.7740**	29
10	1162.4317	581.7195	**1145.4052**	573.2062	**1144.4211**	572.7142	T	2978.4928	**1489.7500**	2961.4663	**1481.2368**	2960.4822	1480.7448	28
11	1219.4532	610.2302	1202.4266	601.7169	1201.4426	601.2249	G	2877.4451	**1439.2262**	2860.4186	**1430.7129**	2859.4346	**1430.2209**	27
12	1347.5117	674.2595	**1330.4852**	665.7462	**1329.5012**	665.2542	Q	2820.4237	**1410.7155**	2803.3971	1402.2022	2802.4131	1401.7102	26
13	1478.5522	739.7798	**1461.5257**	731.2665	**1460.5417**	730.7745	M	2692.3651	1346.6862	2675.3385	1338.1729	2674.3545	1337.6809	25
14	**1579.5999**	**790.3036**	1562.5734	**781.7903**	1561.5893	**781.2983**	T	2561.3246	**1281.1659**	2544.2980	1272.6527	2543.3140	1272.1607	24
15	1650.6370	825.8221	1633.6105	817.3089	**1632.6265**	816.8169	A	2460.2769	**1230.6421**	2443.2504	1222.1288	2442.2664	1221.6368	23
16	**1707.6585**	854.3329	**1690.6319**	**845.8196**	**1689.6479**	845.3276	G	2389.2398	**1195.1235**	2372.2133	**1186.6103**	2371.2292	**1186.1183**	22
17	1820.7426	910.8749	**1803.7160**	902.3616	**1802.7320**	901.8696	L	2332.2183	**1166.6128**	2315.1918	1158.0995	2314.2078	1157.6075	21
18	**1933.8266**	967.4169	**1916.8001**	958.9037	**1915.8160**	958.4117	L	2219.1343	**1110.0708**	2202.1077	1101.5575	2201.1237	**1101.0655**	20

19	2048.8536	1024.9304	2031.8270	1016.4171	2030.8430	1015.9251	D	2106.0502	**1053.5287**	2089.0237	**1045.0155**	2088.0396	**1044.5235**	19
20	2234.9329	1117.9701	2217.9063	1109.4568	2216.9223	1108.9648	W	**1991.0233**	**996.0153**	1973.9967	**987.5020**	1973.0127	**987.0100**	18
21	2331.9856	**1166.4965**	2314.9591	1157.9832	2313.9751	1157.4912	P	**1804.9440**	**902.9756**	1787.9174	**894.4623**	1786.9334	**893.9703**	17
22	2460.0442	**1230.5257**	2443.0177	1222.0125	2442.0336	1221.5205	Q	**1707.8912**	854.4492	**1690.8646**	**845.9360**	**1689.8806**	845.4440	16
23	2517.0657	1259.0365	2500.0391	1250.5232	2499.0551	1250.0312	G	**1579.8326**	**790.4199**	1562.8061	781.9067	1561.8220	**781.4147**	15
24	2618.1134	1309.5603	2601.0868	1301.0470	2600.1028	1300.5550	T	1522.8112	761.9092	1505.7846	753.3959	1504.8006	752.9039	14
25	2765.1818	1383.0945	2748.1552	1374.5812	2747.1712	1374.0892	F	**1421.7635**	**711.3854**	1404.7369	702.8721	1403.7529	**702.3801**	13
26	2836.2189	1418.6131	2819.1923	1410.0998	2818.2083	1409.6078	A	**1274.6951**	**637.8512**	1257.6685	629.3379	1256.6845	628.8459	12
27	2923.2509	**1462.1291**	2906.2244	1453.6158	2905.2403	1453.1238	S	**1203.6579**	**602.3326**	**1186.6314**	593.8193	1185.6474	**593.3273**	11
28	3051.3095	1526.1584	3034.2829	1517.6451	3033.2989	1517.1531	Q	**1116.6259**	**558.8166**	1099.5994	550.3033	1098.6154	549.8113	10
29	3150.3779	1575.6926	3133.3514	1567.1793	3132.3673	1566.6873	V	988.5673	494.7873	971.5408	486.2740	970.5568	485.7820	9
30	3251.4256	1626.2164	3234.3990	1617.7032	3233.4150	1617.2111	T	**889.4989**	445.2531	872.4724	436.7398	**871.4884**	436.2478	8
31	3364.5096	**1682.7585**	3347.4831	1674.2452	3346.4991	1673.7532	L	**788.4512**	394.7293	771.4247	386.2160	**770.4407**	385.7240	7
32	3493.5522	1747.2798	3476.5257	1738.7665	3475.5417	1738.2745	E	**675.3672**	338.1872	**658.3406**	329.6740	**657.3566**	329.1819	6
33	3550.5737	1775.7905	3533.5472	1767.2772	3532.5631	1766.7852	G	**546.3246**	273.6659	529.2980	265.1527	**528.3140**	264.6606	5
34	3665.6006	1833.3040	3648.5741	1824.7907	3647.5901	1824.2987	D	489.3031	245.1552	472.2766	236.6419	471.2926	236.1499	4
35	3793.6956	**1897.3514**	3776.6691	1888.8382	3775.6850	1888.3462	K	374.2762	187.6417	357.2496	179.1285			3
36	3892.7640	**1946.8856**	3875.7375	1938.3724	3874.7535	1937.8804	V	246.1812	123.5942	229.1547	115.0810			2
37							K	147.1128	74.0600	130.0863	65.5468			1

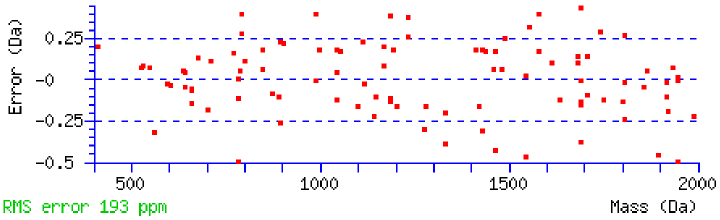

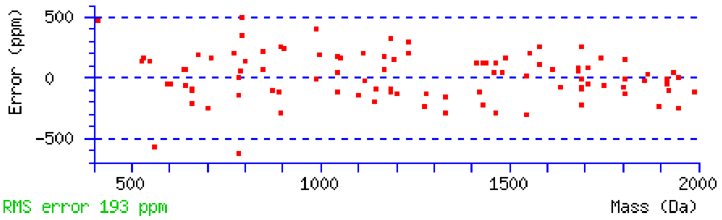

All matches to this query

Score	Mr(calc):	Delta	Sequence
104.2	4037.8623	0.0199	QAIDDDCNQTGQMTAGLLDWPQGTFASQVTLEGDKVK
85.6	4036.8783	1.0039	QAIDDDCNQTGQMTAGLLDWPQGTFASQVTLEGDKVK
9.7	4037.9178	-0.0356	ETFFPIQLLSGAFKPFKGDLVLVEYSMNAGTSNIK
7.3	4036.9088	0.9734	DNSNIILLGDSQGDLRMADGVANVEHILKIGYLNDR
6.8	4035.9142	1.9680	FYTEDGNWDLVGNNTPIFFIRDAMLFPSFIHSQK
6.8	4035.9142	1.9680	FYTEDGNWDLVGNNTPIFFIRDAMLFPSFIHSQK
6.8	4035.9142	1.9680	FYTEDGNWDLVGNNTPIFFIRDAMLFPSFIHSQK
6.4	4035.8631	2.0191	GSYLRNGWNVMDFIVVLSGILATAGTHFNTHVDLR
6.4	4035.8631	2.0191	GSYLRNGWNVMDFIVVLSGILATAGTHFNTHVDLR
6.3	4037.9178	-0.0356	ETFFPIQLLSGAFKPFKGDLVLVEYSMNAGTSNIK

Spectrum No: 23; Query: 607; Rank: 1

Peptide View

MS/MS Fragmentation of **TLAGENQTALEIEELNR**
Found in **IPI00363849**, Tax_Id=10116 Gene_Symbol=Lamc1 similar to Laminin gamma-1 chain precursor

Match to Query 607: 1900.944868 from(951.479710,2+)
Title: 091129RatKid_SCX02_16.2616.2616.2.dta
Data file K:\NewmanPaper\Piliang\3SubProteomes\Piliang3SP\mgf5ppm\SCX_3SubProteomes5ppm.mgf

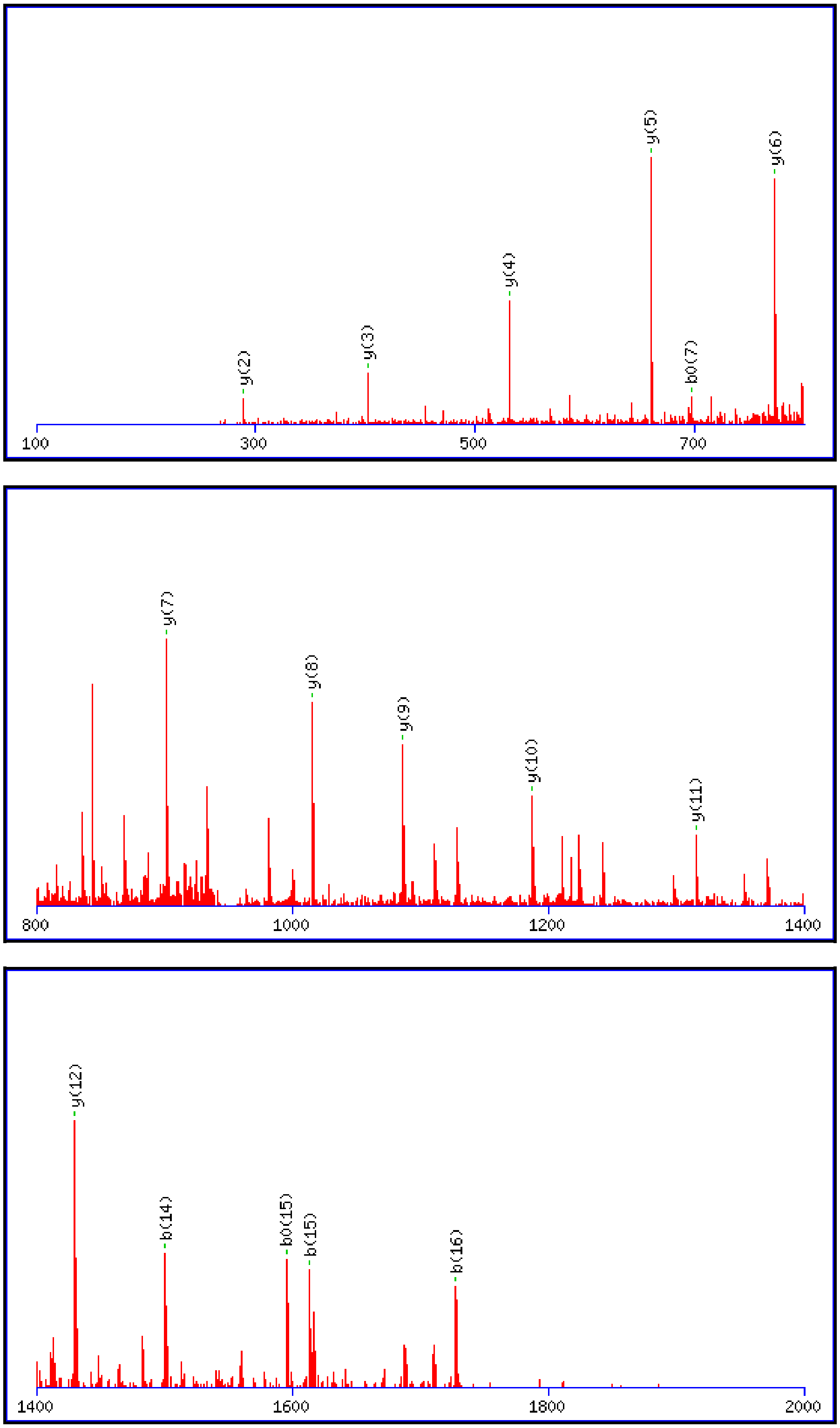

Monoisotopic mass of neutral peptide Mr(calc): 1900.9377
Fixed modifications: Carbamidomethyl (C)
Variable modifications:
N6 : Deamidated_N (N)
Ions Score: 104 **Expect:** 8.6e-009
Matches (Bold Red): 16/176 fragment ions using 25 most intense peaks

#	b	b^{++}	b*	b^{*++}	b^0	b^{0++}	Seq.	y	y^{++}	y*	y^{*++}	y^0	y^{0++}	#
1	102.0550	51.5311			84.0444	42.5258	T							17
2	215.1390	108.0731			197.1285	99.0679	L	1800.8974	900.9523	1783.8708	892.4391	1782.8868	891.9471	16
3	286.1761	143.5917			268.1656	134.5864	A	1687.8133	844.4103	1670.7868	835.8970	1669.8028	835.4050	15
4	343.1976	172.1024			325.1870	163.0972	G	1616.7762	808.8917	1599.7497	800.3785	1598.7656	799.8865	14
5	472.2402	236.6237			454.2296	227.6185	E	1559.7548	780.3810	1542.7282	771.8677	1541.7442	771.3757	13

6	587.2671	294.1372	570.2406	285.6239	569.2566	285.1319	N	1430.7122	715.8597	1413.6856	707.3464	1412.7016	706.8544	12
7	715.3257	358.1665	698.2992	349.6532	697.3151	349.1612	Q	1315.6852	658.3462	1298.6587	649.8330	1297.6747	649.3410	11
8	816.3734	408.6903	799.3468	400.1771	798.3628	399.6850	T	1187.6266	594.3170	1170.6001	585.8037	1169.6161	585.3117	10
9	887.4105	444.2089	870.3839	435.6956	869.3999	435.2036	A	1086.5790	543.7931	1069.5524	535.2798	1068.5684	534.7878	9
10	1000.4946	500.7509	983.4680	492.2376	982.4840	491.7456	L	1015.5419	508.2746	998.5153	499.7613	997.5313	499.2693	8
11	1129.5372	565.2722	1112.5106	556.7589	1111.5266	556.2669	E	902.4578	451.7325	885.4312	443.2193	884.4472	442.7272	7
12	1242.6212	621.8142	1225.5947	613.3010	1224.6107	612.8090	I	773.4152	387.2112	756.3886	378.6980	755.4046	378.2060	6
13	1371.6638	686.3355	1354.6373	677.8223	1353.6532	677.3303	E	660.3311	330.6692	643.3046	322.1559	642.3206	321.6639	5
14	1500.7064	750.8568	1483.6799	742.3436	1482.6958	741.8516	E	531.2885	266.1479	514.2620	257.6346	513.2780	257.1426	4
15	1613.7905	807.3989	1596.7639	798.8856	1595.7799	798.3936	L	402.2459	201.6266	385.2194	193.1133			3
16	1727.8334	864.4203	1710.8068	855.9071	1709.8228	855.4151	N	289.1619	145.0846	272.1353	136.5713			2
17							R	175.1190	88.0631	158.0924	79.5498			1

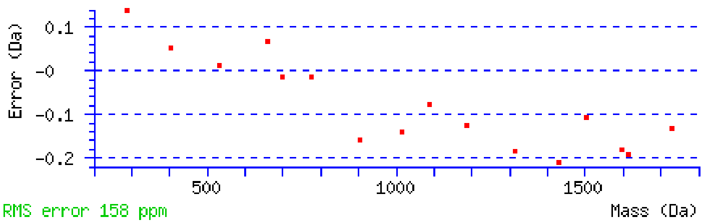

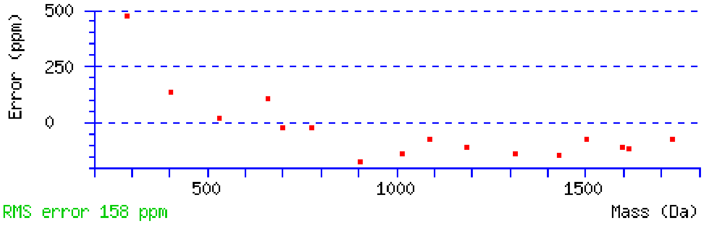

All matches to this query

Score	Mr(calc):	Delta	Sequence
103.6	1900.9377	0.0071	TLAGENQTALEIEELNR
70.1	1899.9537	0.9911	TLAGENQTALEIEELNR
45.9	1900.9377	0.0071	TLAGENQTALEIEELNR
8.8	1899.9482	0.9967	TELDSFLIEITANILK
8.8	1899.9482	0.9967	TELDSFLIEITANILK
6.2	1900.9274	0.0175	SVSTMLYLMALQELNR
1.3	1900.9560	-0.0111	VYTLTPFLANNREKR
1.3	1900.9560	-0.0111	VYTLTPFLANNREKR
1.3	1900.9560	-0.0111	VYTLTPFLANNREKR
0.3	1900.9369	0.0080	KCIDILIEKEYLER

Spectrum No: 24; Query: 859; Rank: 1

Peptide View

MS/MS Fragmentation of **EASQYFINNITLSYNTNDTK**
Found in **IPI00212730**, Tax_Id=10116 Gene_Symbol=Lamp2 Lysosome-associated membrane glycoprotein 2 precursor

Match to Query 859: 2337.067688 from(1169.541120,2+)
Title: 091127RatKid_SCX01_14.3819.3819.2.dta
Data file K:\NewmanPaper\Piliang\3SubProteomes\Piliang3SP\mgf5ppm\SCX_3SubProteomes5ppm.mgf

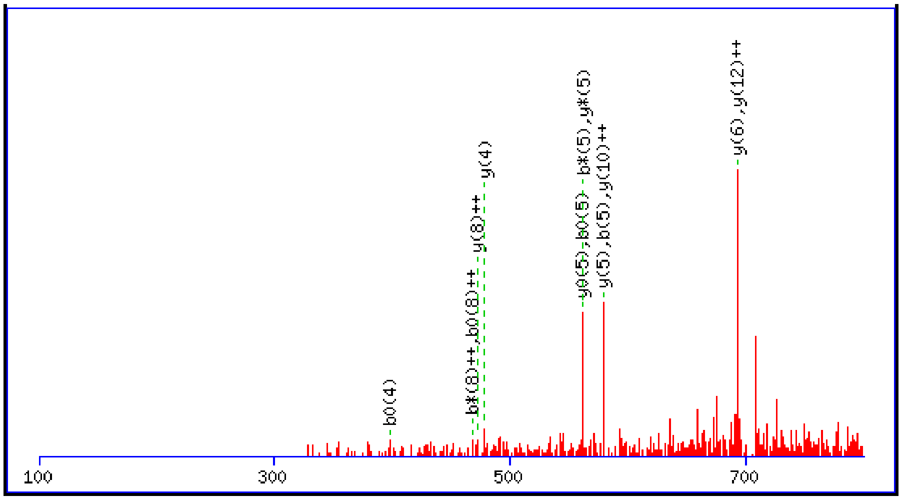

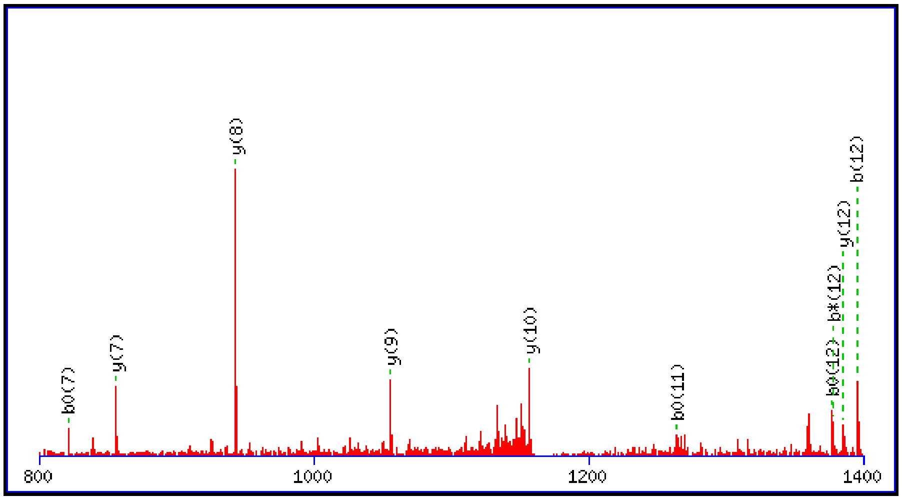

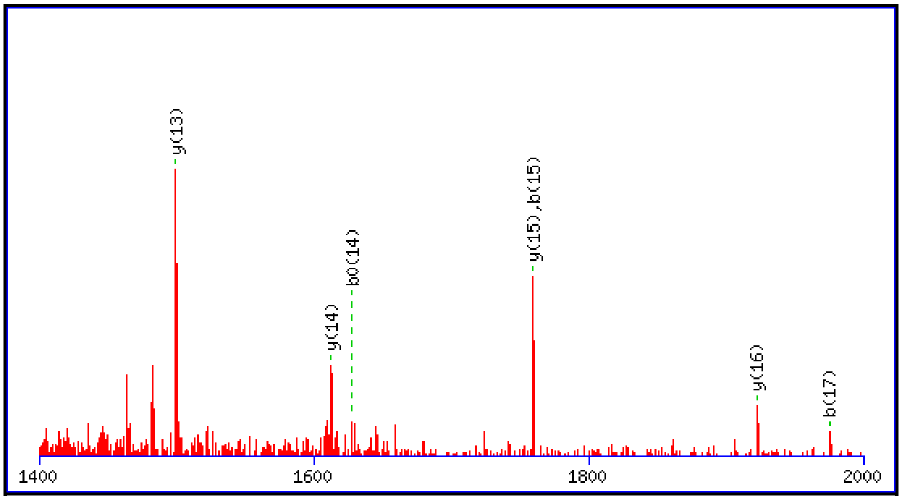

Monoisotopic mass of neutral peptide Mr(calc): 2337.0648
Fixed modifications: Carbamidomethyl (C)
Variable modifications:
N9 : Deamidated_N (N)
N17 : Deamidated_N (N)
Ions Score: 103 **Expect:** 1.5e-008
Matches (Bold Red): 31/220 fragment ions using 31 most intense peaks

#	b	b++	b*	b*++	b0	b0++	Seq.	y	y++	y*	y*++	y0	y0++	#
1	130.0499	65.5286			112.0393	56.5233	E							20
2	201.0870	101.0471			183.0764	92.0418	A	2209.0295	1105.0184	2192.0030	1096.5051	2191.0190	1096.0131	19
3	288.1190	144.5631			270.1084	135.5579	S	2137.9924	1069.4998	2120.9659	1060.9866	2119.9818	1060.4946	18
4	416.1776	208.5924	399.1510	200.0792	**398.1670**	199.5872	Q	2050.9604	1025.9838	2033.9338	1017.4706	2032.9498	1016.9785	17

**5**	**579.2409**	290.1241	**562.2144**	281.6108	**561.2304**	281.1188	**Y**	**1922.9018**	961.9545	1905.8753	953.4413	1904.8912	952.9493	**16**
**6**	726.3093	363.6583	709.2828	355.1450	708.2988	354.6530	**F**	**1759.8385**	880.4229	1742.8119	871.9096	1741.8279	871.4176	**15**
**7**	839.3934	420.2003	822.3668	411.6871	**821.3828**	411.1951	**I**	**1612.7701**	806.8887	1595.7435	798.3754	1594.7595	797.8834	**14**
**8**	953.4363	477.2218	936.4098	**468.7085**	935.4258	**468.2165**	**N**	**1499.6860**	750.3466	1482.6594	741.8334	1481.6754	741.3414	**13**
**9**	1068.4633	534.7353	1051.4367	526.2220	1050.4527	525.7300	**N**	**1385.6431**	**693.3252**	1368.6165	684.8119	1367.6325	684.3199	**12**
**10**	1181.5473	591.2773	1164.5208	582.7640	1163.5368	582.2720	**I**	1270.6161	635.8117	1253.5896	627.2984	1252.6056	626.8064	**11**
**11**	1282.5950	641.8011	1265.5685	633.2879	**1264.5844**	632.7959	**T**	**1157.5321**	**579.2697**	1140.5055	570.7564	1139.5215	570.2644	**10**
**12**	**1395.6791**	698.3432	**1378.6525**	689.8299	**1377.6685**	689.3379	**L**	**1056.4844**	528.7458	1039.4578	520.2326	1038.4738	519.7405	**9**
**13**	1482.7111	741.8592	1465.6845	733.3459	1464.7005	732.8539	**S**	**943.4003**	**472.2038**	926.3738	463.6905	925.3898	463.1985	**8**
**14**	1645.7744	823.3908	1628.7479	814.8776	**1627.7639**	814.3856	**Y**	**856.3683**	428.6878	839.3417	420.1745	838.3577	419.6825	**7**
**15**	**1759.8174**	880.4123	1742.7908	871.8990	1741.8068	871.4070	**N**	**693.3050**	347.1561	676.2784	338.6428	675.2944	338.1508	**6**
**16**	1860.8650	930.9362	1843.8385	922.4229	1842.8545	921.9309	**T**	**579.2620**	290.1347	**562.2355**	281.6214	**561.2515**	281.1294	**5**
**17**	**1975.8920**	988.4496	1958.8654	979.9363	1957.8814	979.4443	**N**	**478.2144**	239.6108	461.1878	231.0975	460.2038	230.6055	**4**
**18**	2090.9189	1045.9631	2073.8924	1037.4498	2072.9083	1036.9578	**D**	363.1874	182.0974	346.1609	173.5841	345.1769	173.0921	**3**
**19**	2191.9666	1096.4869	2174.9400	1087.9737	2173.9560	1087.4816	**T**	248.1605	124.5839	231.1339	116.0706	230.1499	115.5786	**2**
**20**							**K**	147.1128	74.0600	130.0863	65.5468			**1**

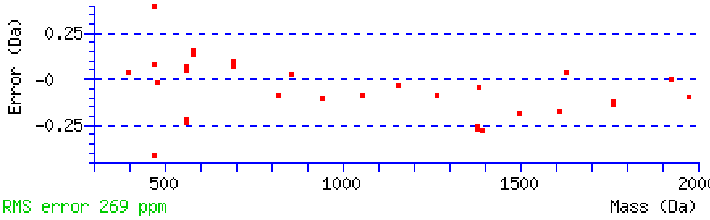

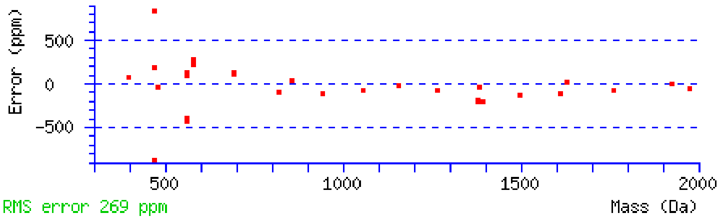

All matches to this query

Score	Mr(calc):	Delta	Sequence
103.1	2337.0648	0.0029	EASQYFINNITLSYNTNDTK
103.1	2337.0648	0.0029	EASQYFINNITLSYNTNDTK
74.1	2337.0648	0.0029	EASQYFINNITLSYNTNDTK
74.1	2337.0648	0.0029	EASQYFINNITLSYNTNDTK
33.6	2337.0648	0.0029	EASQYFINNITLSYNTNDTK
29.0	2336.0808	0.9869	EASQYFINNITLSYNTNDTK
24.9	2337.0648	0.0029	EASQYFINNITLSYNTNDTK
20.8	2336.0808	0.9869	EASQYFINNITLSYNTNDTK
12.5	2336.0808	0.9869	EASQYFINNITLSYNTNDTK
10.9	2336.0808	0.9869	EASQYFINNITLSYNTNDTK

Spectrum No: 25; Query: 657; Rank: 1

Peptide View

MS/MS Fragmentation of **VNASTTDPNSTVEQSALTR**
Found in **IPI00365542**, Tax_Id=10116 Gene_Symbol=Lamb1_predicted laminin, beta 1

Match to Query 657: 1991.921068 from(996.967810,2+)
Title: 091129RatKid_SCX02_14.1398.1398.2.dta
Data file K:\NewmanPaper\Piliang\3SubProteomes\Piliang3SP\mgf5ppm\SCX_3SubProteomes5ppm.mgf

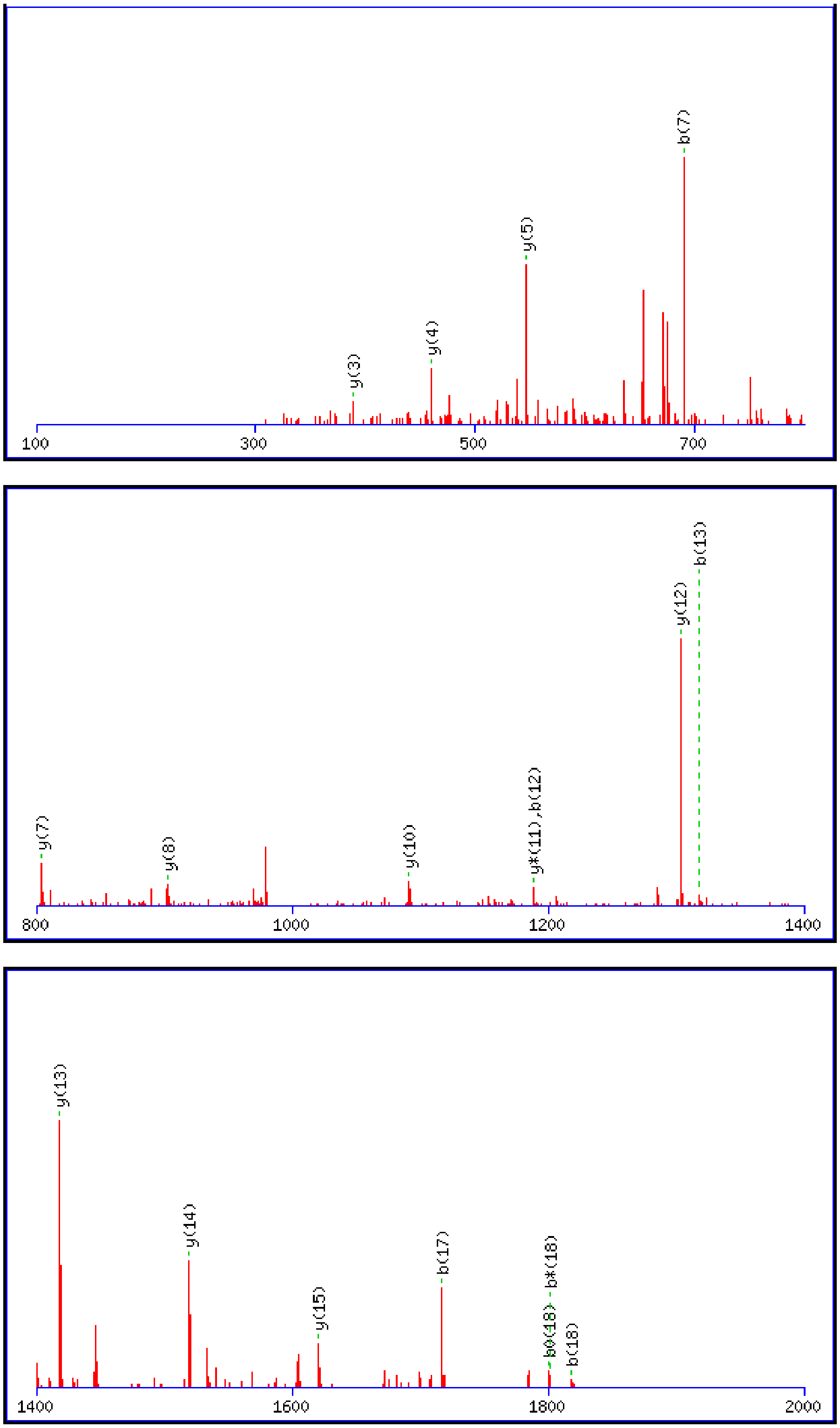

Monoisotopic mass of neutral peptide Mr(calc): 1991.9283
Fixed modifications: Carbamidomethyl (C)
Variable modifications:
N2 : Deamidated_N (N)
N9 : Deamidated_N (N)
Ions Score: 103 **Expect:** 1.3e-008
Matches (Bold Red): 18/206 fragment ions using 25 most intense peaks

#	b	b^{++}	b*	b^{*++}	b^{0}	b^{0++}	Seq.	y	y^{++}	y*	y^{*++}	y^{0}	y^{0++}	#
1	100.0757	50.5415					V							19
2	215.1026	108.0550	198.0761	99.5417			N	1893.8672	947.4372	1876.8407	938.9240	1875.8566	938.4320	18
3	286.1397	143.5735	269.1132	135.0602			A	1778.8403	889.9238	1761.8137	881.4105	1760.8297	880.9185	17
4	373.1718	187.0895	356.1452	178.5762	355.1612	178.0842	S	1707.8032	854.4052	1690.7766	845.8919	1689.7926	845.3999	16

5	474.2194	237.6134	457.1929	229.1001	456.2089	228.6081	T	**1620.7711**	810.8892	1603.7446	802.3759	1602.7606	801.8839	15
6	575.2671	288.1372	558.2406	279.6239	557.2566	279.1319	T	**1519.7235**	760.3654	1502.6969	751.8521	1501.7129	751.3601	14
7	**690.2941**	345.6507	673.2675	337.1374	672.2835	336.6454	D	**1418.6758**	709.8415	1401.6492	701.3282	1400.6652	700.8362	13
8	787.3468	394.1771	770.3203	385.6638	769.3363	385.1718	P	**1303.6488**	652.3281	1286.6223	643.8148	1285.6383	643.3228	12
9	902.3738	451.6905	885.3472	443.1772	884.3632	442.6852	N	1206.5961	603.8017	**1189.5695**	595.2884	1188.5855	594.7964	11
10	989.4058	495.2065	972.3792	486.6933	971.3952	486.2013	S	**1091.5691**	546.2882	1074.5426	537.7749	1073.5586	537.2829	10
11	1090.4535	545.7304	1073.4269	537.2171	1072.4429	536.7251	T	1004.5371	502.7722	987.5106	494.2589	986.5265	493.7669	9
12	**1189.5219**	595.2646	1172.4953	586.7513	1171.5113	586.2593	V	**903.4894**	452.2483	886.4629	443.7351	885.4789	443.2431	8
13	**1318.5645**	659.7859	1301.5379	651.2726	1300.5539	650.7806	E	**804.4210**	402.7141	787.3945	394.2009	786.4104	393.7089	7
14	1446.6231	723.8152	1429.5965	715.3019	1428.6125	714.8099	Q	675.3784	338.1928	658.3519	329.6796	657.3679	329.1876	6
15	1533.6551	767.3312	1516.6285	758.8179	1515.6445	758.3259	S	**547.3198**	274.1636	530.2933	265.6503	529.3093	265.1583	5
16	1604.6922	802.8497	1587.6657	794.3365	1586.6816	793.8445	A	**460.2878**	230.6475	443.2613	222.1343	442.2772	221.6423	4
17	**1717.7763**	859.3918	1700.7497	850.8785	1699.7657	850.3865	L	**389.2507**	195.1290	372.2241	186.6157	371.2401	186.1237	3
18	**1818.8239**	909.9156	**1801.7974**	901.4023	**1800.8134**	900.9103	T	276.1666	138.5870	259.1401	130.0737	258.1561	129.5817	2
19							R	175.1190	88.0631	158.0924	79.5498			1

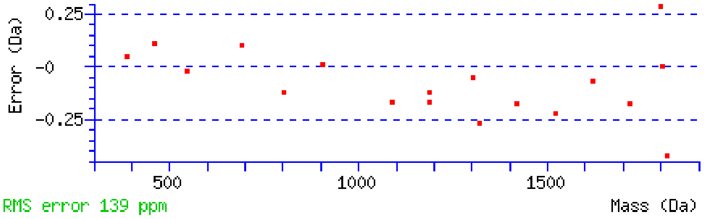

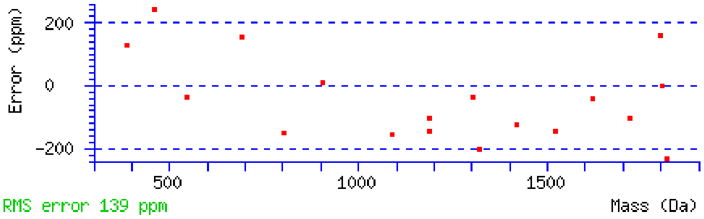

All matches to this query

Score	Mr(calc):	Delta	Sequence
103.1	1991.9283	-0.0073	VNASTTDPNSTVEQSALTR
17.8	1991.9201	0.0010	QNSESAVSSTVNPITIHK
15.9	1989.9296	1.9915	SPSTYNALESGLTSLTIR
12.9	1989.9296	1.9915	SPSTYNALESGLTSLTIR
8.6	1989.9296	1.9915	SPSTYNALESGLTSLTIR
8.2	1990.9361	0.9850	QNSESAVSSTVNPITIHK
5.4	1991.9061	0.0149	NAQKSNQNGKGLNPSTPR
5.3	1989.9296	1.9915	SPSTYNALESGLTSLTIR
5.0	1991.9061	0.0149	NAQKSNQNGKGLNPSTPR
5.0	1989.9296	1.9915	SPSTYNALESGLTSLTIR

Spectrum No: 26; Query: 936; Rank: 1

Peptide View

MS/MS Fragmentation of **YGMQNSGNEAAWNYTLEQYQK**
Found in **IPI00327398**, Tax_Id=10116 Gene_Symbol=Enpep Isoform 1 of Glutamyl aminopeptidase

Match to Query 936: 2495.081388 from(1248.547970,2+)
Title: 091127RatKid_SCX01_12.3214.3214.2.dta
Data file K:\NewmanPaper\Piliang\3SubProteomes\Piliang3SP\mgf5ppm\SCX_3SubProteomes5ppm.mgf

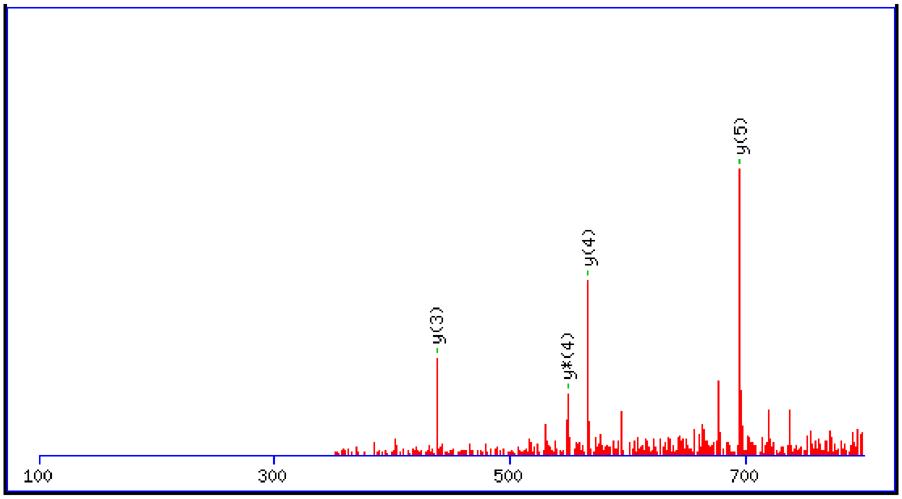

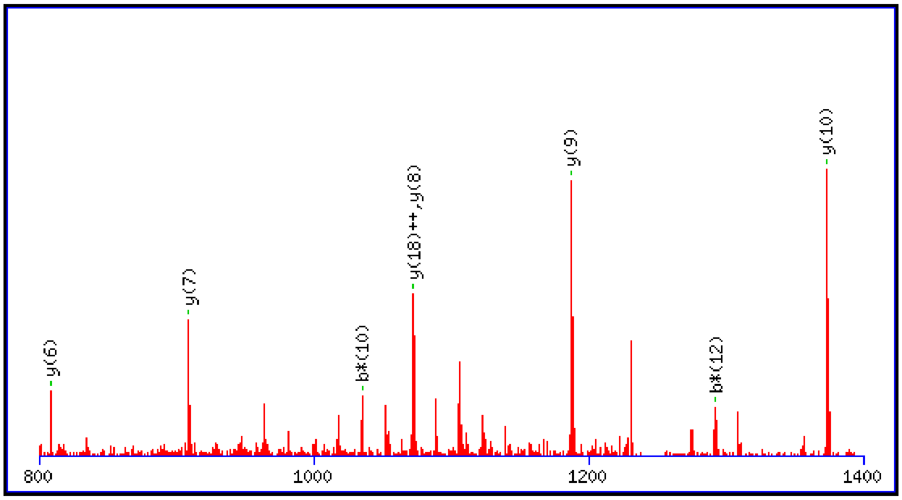

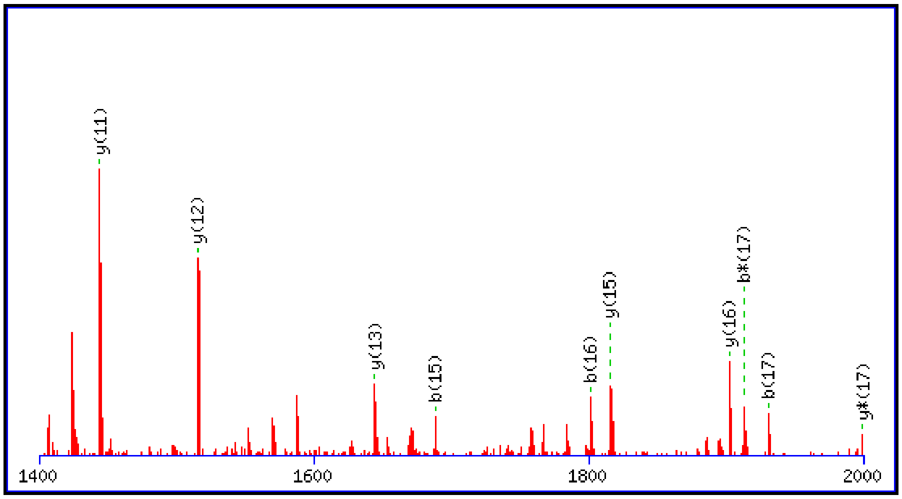

Monoisotopic mass of neutral peptide Mr(calc): 2495.0699
Fixed modifications: Carbamidomethyl (C)
Variable modifications:
N13 : Deamidated_N (N)
Ions Score: 103 **Expect:** 1.5e-008
Matches (Bold Red): 22/216 fragment ions using 31 most intense peaks

#	b	b^{++}	b*	b^{*++}	b^0	b^{0++}	Seq.	y	y^{++}	y*	y^{*++}	y^0	y^{0++}	#
1	164.0706	82.5389					Y							21
2	221.0921	111.0497					G	2333.0139	1167.0106	2315.9873	1158.4973	2315.0033	1158.0053	20
3	352.1326	176.5699					M	2275.9924	1138.4999	2258.9659	1129.9866	2257.9819	1129.4946	19
4	480.1911	240.5992	463.1646	232.0859			Q	2144.9519	**1072.9796**	2127.9254	1064.4663	2126.9414	1063.9743	18
5	594.2341	297.6207	577.2075	289.1074			N	2016.8934	1008.9503	**1999.8668**	1000.4370	1998.8828	999.9450	17

6	681.2661	341.1367	664.2395	332.6234	663.2555	332.1314	S	**1902.8504**	951.9289	1885.8239	943.4156	1884.8399	942.9236	16
7	738.2876	369.6474	721.2610	361.1341	720.2770	360.6421	G	**1815.8184**	908.4128	1798.7919	899.8996	1797.8079	899.4076	15
8	852.3305	426.6689	835.3039	418.1556	834.3199	417.6636	N	1758.7970	879.9021	1741.7704	871.3888	1740.7864	870.8968	14
9	981.3731	491.1902	964.3465	482.6769	963.3625	482.1849	E	**1644.7540**	822.8807	1627.7275	814.3674	1626.7435	813.8754	13
10	1052.4102	526.7087	**1035.3836**	518.1955	1034.3996	517.7034	A	**1515.7114**	758.3594	1498.6849	749.8461	1497.7009	749.3541	12
11	1123.4473	562.2273	1106.4208	553.7140	1105.4367	553.2220	A	**1444.6743**	722.8408	1427.6478	714.3275	1426.6638	713.8355	11
12	1309.5266	655.2669	**1292.5001**	646.7537	1291.5160	646.2617	W	**1373.6372**	687.3222	1356.6107	678.8090	1355.6266	678.3170	10
13	1424.5535	712.7804	1407.5270	704.2671	1406.5430	703.7751	N	**1187.5579**	594.2826	1170.5313	585.7693	1169.5473	585.2773	9
14	1587.6169	794.3121	1570.5903	785.7988	1569.6063	785.3068	Y	**1072.5310**	536.7691	1055.5044	528.2558	1054.5204	527.7638	8
15	**1688.6646**	844.8359	1671.6380	836.3226	1670.6540	835.8306	T	**909.4676**	455.2375	892.4411	446.7242	891.4571	446.2322	7
16	**1801.7486**	901.3779	1784.7221	892.8647	1783.7381	892.3727	L	**808.4199**	404.7136	791.3934	396.2003	790.4094	395.7083	6
17	**1930.7912**	965.8992	**1913.7647**	957.3860	1912.7806	956.8940	E	**695.3359**	348.1716	678.3093	339.6583	677.3253	339.1663	5
18	2058.8498	1029.9285	2041.8232	1021.4153	2040.8392	1020.9233	Q	**566.2933**	283.6503	**549.2667**	275.1370			4
19	2221.9131	1111.4602	2204.8866	1102.9469	2203.9026	1102.4549	Y	**438.2347**	219.6210	421.2082	211.1077			3
20	2349.9717	1175.4895	2332.9451	1166.9762	2331.9611	1166.4842	Q	275.1714	138.0893	258.1448	129.5761			2
21							K	147.1128	74.0600	130.0863	65.5468			1

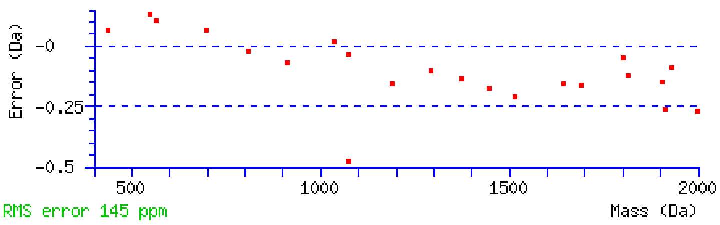

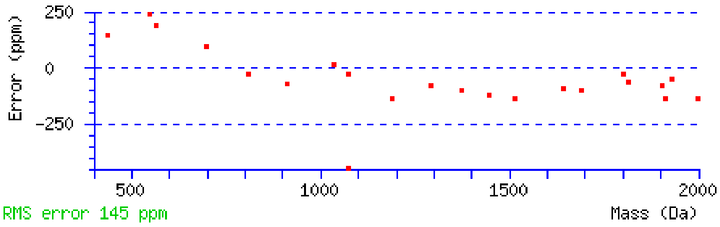

All matches to this query

Score	Mr(calc):	Delta	Sequence
103.0	2495.0699	0.0115	YGMQNSGNEAAWNYTLEQYQK
42.5	2495.0699	0.0115	YGMQNSGNEAAWNYTLEQYQK
20.9	2495.0699	0.0115	YGMQNSGNEAAWNYTLEQYQK
18.6	2494.0859	0.9955	YGMQNSGNEAAWNYTLEQYQK
9.1	2495.1013	-0.0199	AFDMISQRSSGGHPGSGPQLGTGR
3.1	2494.1022	0.9792	VTMSCKASQNVGINVGWYQQK
3.0	2494.1022	0.9792	VTMSCKASQNVGINVGWYQQK
2.1	2494.1022	0.9792	VTMSCKASQNVGINVGWYQQK
1.0	2494.1022	0.9792	VTMSCKASQNVGINVGWYQQK
0.1	2495.0723	0.0091	MPTPSSQPTSMSHRSYQPPQR

Spectrum No: 27; Query: 872; Rank: 1

Peptide View

MS/MS Fragmentation of **YLQPLLAVQFTNLTLDTEIR**
Found in **IPI00339124**, Tax_Id=10116 Gene_Symbol=Atp1b1 Sodium/potassium-transporting ATPase subunit beta-1

Match to Query 872: 2348.273088 from(1175.143820,2+)
Title: 091129RatKid_SCX02_02.5925.5925.2.dta
Data file K:\NewmanPaper\Piliang\3SubProteomes\Piliang3SP\mgf5ppm\SCX_3SubProteomes5ppm.mgf

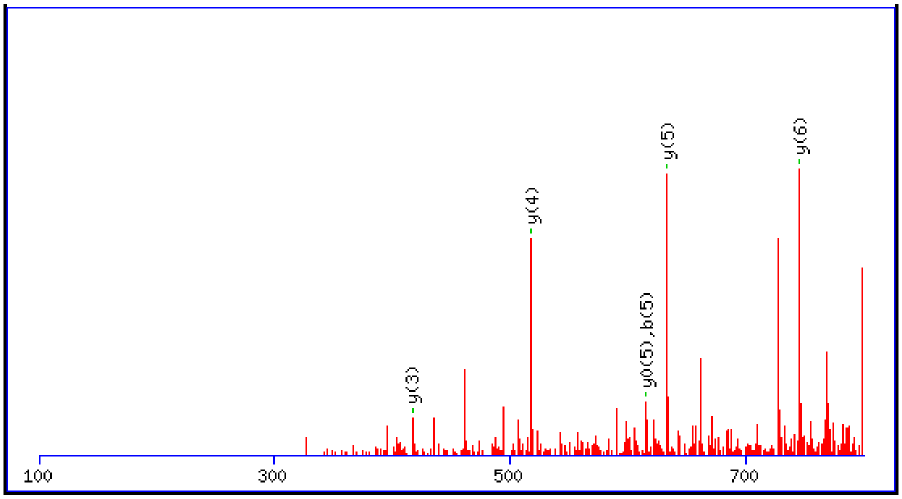

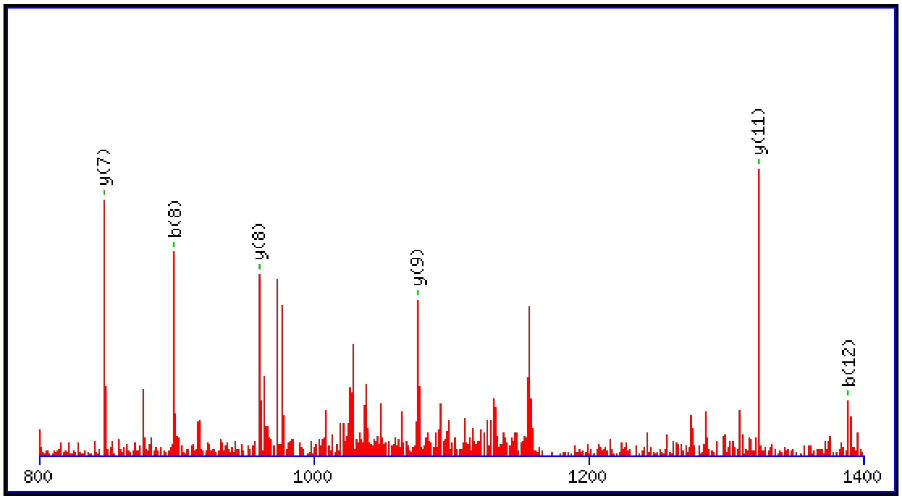

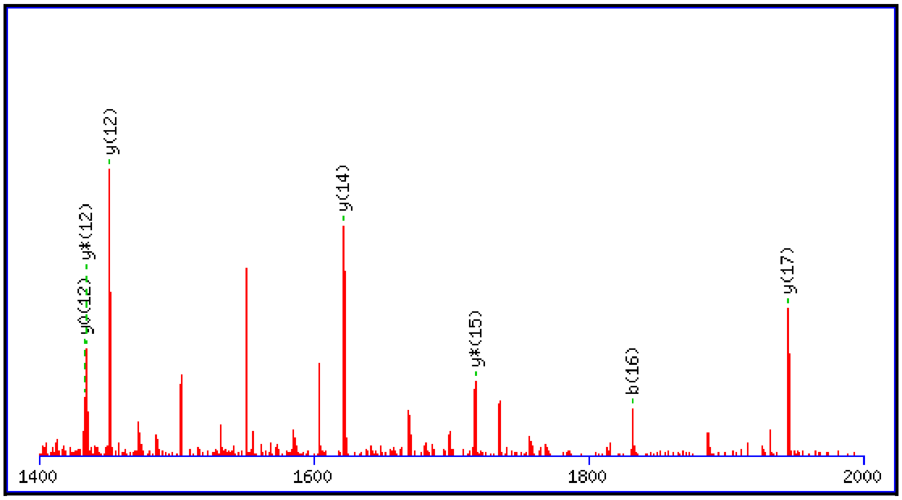

Monoisotopic mass of neutral peptide Mr(calc): 2348.2627
Fixed modifications: Carbamidomethyl (C)
Variable modifications:
N12 : Deamidated_N (N)
Ions Score: 102 **Expect:** 3.5e-009
Matches (Bold Red): 19/200 fragment ions using 26 most intense peaks

#	b	b^{++}	b*	b^{*++}	b^0	b^{0++}	Seq.	y	y^{++}	y*	y^{*++}	y^0	y^{0++}	#
1	164.0706	82.5389					Y							20
2	277.1547	139.0810					L	2186.2067	1093.6070	2169.1802	1085.0937	2168.1961	1084.6017	19
3	405.2132	203.1103	388.1867	194.5970			Q	2073.1226	1037.0650	2056.0961	1028.5517	2055.1121	1028.0597	18
4	502.2660	251.6366	485.2395	243.1234			P	**1945.0641**	973.0357	1928.0375	964.5224	1927.0535	964.0304	17
5	**615.3501**	308.1787	598.3235	299.6654			L	1848.0113	924.5093	1830.9848	915.9960	1830.0007	915.5040	16

6	728.4341	364.7207	711.4076	356.2074			L	1734.9272	867.9673	**1717.9007**	859.4540	1716.9167	858.9620	15
7	799.4713	400.2393	782.4447	391.7260			A	**1621.8432**	811.4252	1604.8166	802.9120	1603.8326	802.4199	14
8	**898.5397**	449.7735	881.5131	441.2602			V	1550.8061	775.9067	1533.7795	767.3934	1532.7955	766.9014	13
9	1026.5982	513.8028	1009.5717	505.2895			Q	**1451.7376**	726.3725	**1434.7111**	717.8592	**1433.7271**	717.3672	12
10	1173.6667	587.3370	1156.6401	578.8237			F	**1323.6791**	662.3432	1306.6525	653.8299	1305.6685	653.3379	11
11	1274.7143	637.8608	1257.6878	629.3475	1256.7038	628.8555	T	1176.6107	588.8090	1159.5841	580.2957	1158.6001	579.8037	10
12	**1389.7413**	695.3743	1372.7147	686.8610	1371.7307	686.3690	N	**1075.5630**	538.2851	1058.5364	529.7719	1057.5524	529.2798	9
13	1502.8253	751.9163	1485.7988	743.4030	1484.8148	742.9110	L	**960.5360**	480.7717	943.5095	472.2584	942.5255	471.7664	8
14	1603.8730	802.4401	1586.8465	793.9269	1585.8625	793.4349	T	**847.4520**	424.2296	830.4254	415.7164	829.4414	415.2243	7
15	1716.9571	858.9822	1699.9305	850.4689	1698.9465	849.9769	L	**746.4043**	373.7058	729.3777	365.1925	728.3937	364.7005	6
16	**1831.9840**	916.4956	1814.9575	907.9824	1813.9735	907.4904	D	**633.3202**	317.1638	616.2937	308.6505	**615.3097**	308.1585	5
17	1933.0317	967.0195	1916.0052	958.5062	1915.0211	958.0142	T	**518.2933**	259.6503	501.2667	251.1370	500.2827	250.6450	4
18	2062.0743	1031.5408	2045.0477	1023.0275	2044.0637	1022.5355	E	**417.2456**	209.1264	400.2191	200.6132	399.2350	200.1212	3
19	2175.1584	1088.0828	2158.1318	1079.5695	2157.1478	1079.0775	I	288.2030	144.6051	271.1765	136.0919			2
20							R	175.1190	88.0631	158.0924	79.5498			1

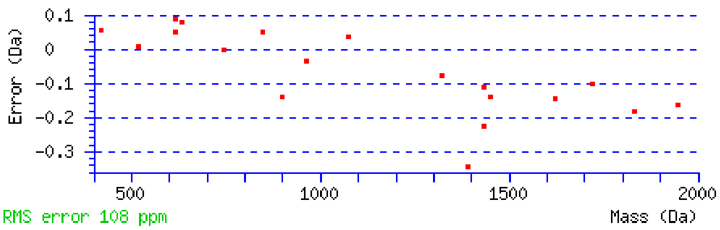

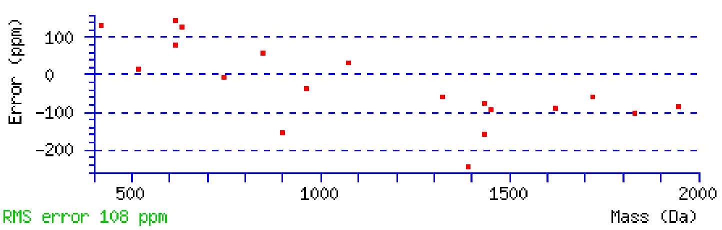

All matches to this query

Score	Mr(calc):	Delta	Sequence
102.3	2348.2627	0.0103	YLQPLLAVQFTNLTLDTEIR
20.7	2347.2787	0.9944	YLQPLLAVQFTNLTLDTEIR

Spectrum No: 28; Query: 662; Rank: 1

Peptide View

MS/MS Fragmentation of **TNSTCSPNLDACLVAVSGK**
Found in **IPI00195173**, Tax_Id=10116 Gene_Symbol=Cd59 CD59 glycoprotein precursor

Match to Query 662: 1993.914548 from(997.964550,2+)
Title: 091129RatKid_SCX02_02.2334.2334.2.dta
Data file K:\NewmanPaper\Piliang\3SubProteomes\Piliang3SP\mgf5ppm\SCX_3SubProteomes5ppm.mgf

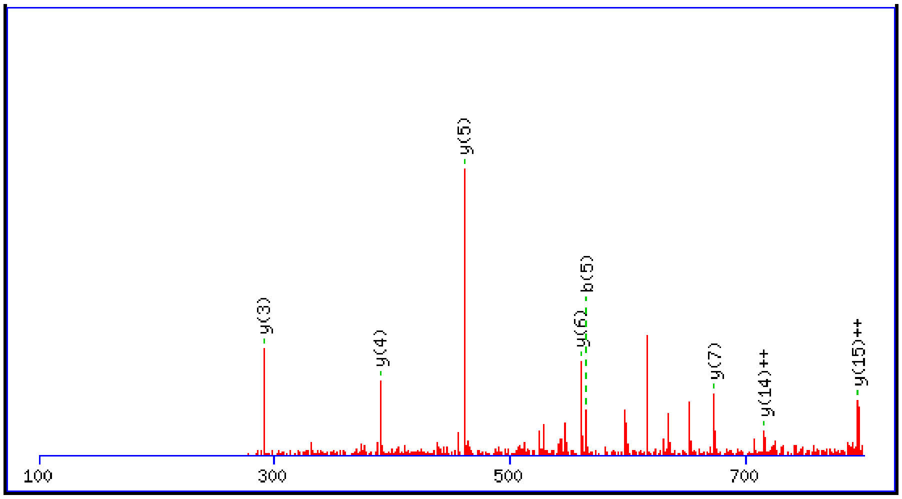

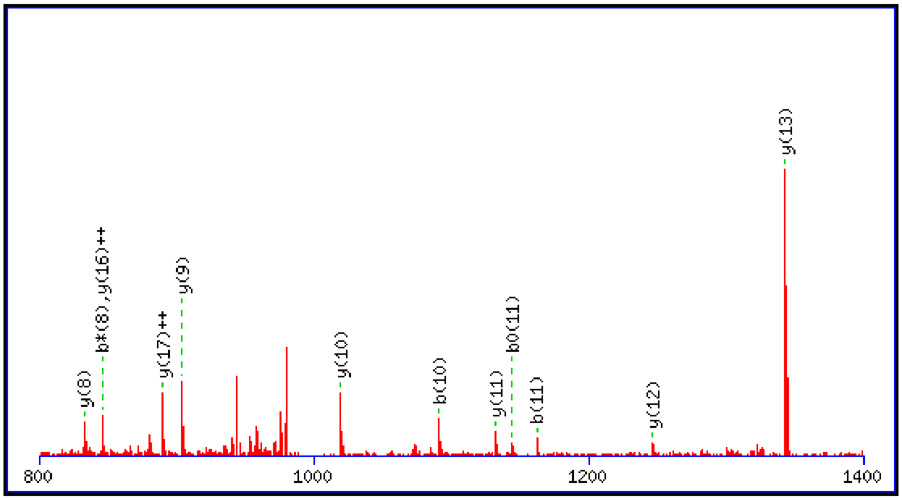

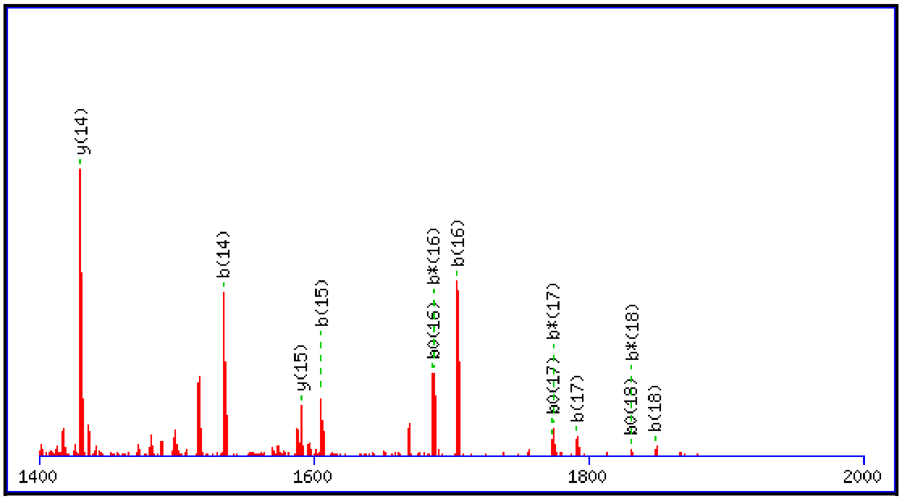

Monoisotopic mass of neutral peptide Mr(calc): 1993.9085
Fixed modifications: Carbamidomethyl (C)
Variable modifications:
N2 : Deamidated_N (N)
Ions Score: 101 **Expect:** 2.2e-008
Matches (Bold Red): 33/210 fragment ions using 57 most intense peaks

#	b	b^{++}	b*	b*$^{++}$	b^{0}	b^{0++}	Seq.	y	y^{++}	y*	y*$^{++}$	y^{0}	y^{0++}	#
1	102.0550	51.5311			84.0444	42.5258	T							19
2	217.0819	109.0446	200.0553	100.5313	199.0713	100.0393	N	1893.8681	947.4377	1876.8415	938.9244	1875.8575	938.4324	18
3	304.1139	152.5606	287.0874	144.0473	286.1034	143.5553	S	1778.8411	**889.9242**	1761.8146	881.4109	1760.8306	880.9189	17
4	405.1616	203.0844	388.1350	194.5712	387.1510	194.0792	T	1691.8091	**846.4082**	1674.7826	837.8949	1673.7986	837.4029	16
5	**565.1922**	283.0998	548.1657	274.5865	547.1817	274.0945	C	**1590.7614**	**795.8844**	1573.7349	787.3711	1572.7509	786.8791	15

**6**	652.2243	326.6158	635.1977	318.1025	634.2137	317.6105	**S**	**1430.7308**	**715.8690**	1413.7042	707.3558	1412.7202	706.8638	**14**
**7**	749.2770	375.1422	732.2505	366.6289	731.2665	366.1369	**P**	**1343.6988**	672.3530	1326.6722	663.8397	1325.6882	663.3477	**13**
**8**	863.3200	432.1636	**846.2934**	423.6503	845.3094	423.1583	**N**	**1246.6460**	623.8266	1229.6195	615.3134	1228.6354	614.8214	**12**
**9**	976.4040	488.7057	959.3775	480.1924	958.3935	479.7004	**L**	**1132.6031**	566.8052	1115.5765	558.2919	1114.5925	557.7999	**11**
**10**	**1091.4310**	546.2191	1074.4044	537.7059	1073.4204	537.2138	**D**	**1019.5190**	510.2631	1002.4925	501.7499	1001.5084	501.2579	**10**
**11**	**1162.4681**	581.7377	1145.4415	573.2244	**1144.4575**	572.7324	**A**	**904.4921**	452.7497	887.4655	444.2364	886.4815	443.7444	**9**
**12**	1322.4987	661.7530	1305.4722	653.2397	1304.4882	652.7477	**C**	**833.4550**	417.2311	816.4284	408.7178	815.4444	408.2258	**8**
**13**	1435.5828	718.2950	1418.5563	709.7818	1417.5722	709.2898	**L**	**673.4243**	337.2158	656.3978	328.7025	655.4137	328.2105	**7**
**14**	**1534.6512**	767.8292	1517.6247	759.3160	1516.6406	758.8240	**V**	**560.3402**	280.6738	543.3137	272.1605	542.3297	271.6685	**6**
**15**	**1605.6883**	803.3478	1588.6618	794.8345	1587.6778	794.3425	**A**	**461.2718**	231.1396	444.2453	222.6263	443.2613	222.1343	**5**
**16**	**1704.7567**	852.8820	**1687.7302**	844.3687	**1686.7462**	843.8767	**V**	**390.2347**	195.6210	373.2082	187.1077	372.2241	186.6157	**4**
**17**	**1791.7888**	896.3980	**1774.7622**	887.8847	**1773.7782**	887.3927	**S**	**291.1663**	146.0868	274.1397	137.5735	273.1557	137.0815	**3**
**18**	**1848.8102**	924.9088	**1831.7837**	916.3955	**1830.7997**	915.9035	**G**	204.1343	102.5708	187.1077	94.0575			**2**
**19**							**K**	147.1128	74.0600	130.0863	65.5468			**1**

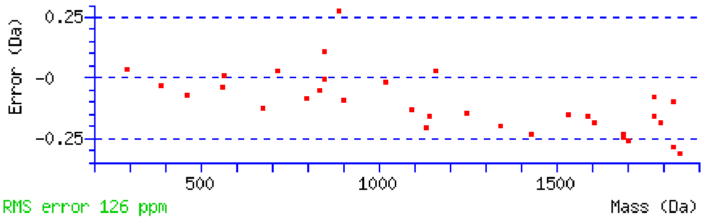

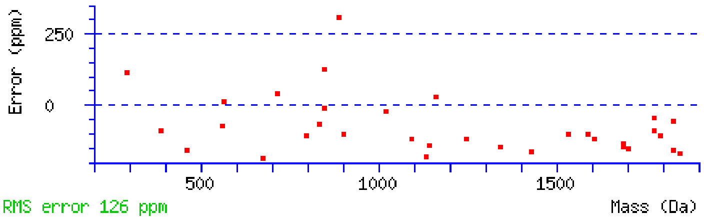

All matches to this query

Score	Mr(calc):	Delta	Sequence
100.8	1993.9085	0.0061	TNSTCSPNLDACLVAVSGK
88.3	1993.9085	0.0061	TNSTCSPNLDACLVAVSGK
80.2	1992.9245	0.9901	TNSTCSPNLDACLVAVSGK
5.2	1993.9197	-0.0051	MSEIAEQSNTRDNLMVR
0.2	1993.9076	0.0069	FNSTMKVLMLNSNMVGK

Spectrum No: 29; Query: 189; Rank: 1

Peptide View

MS/MS Fragmentation of **VSLTNVSISDEGR**
Found in **IPI00464765**, Tax_Id=10116 Gene_Symbol=Igsf4a Immunoglobulin superfamily, member 4A

Match to Query 189: 1376.680828 from(689.347690,2+)
Title: 091127RatKid_SCX01_02.2308.2308.2.dta
Data file K:\NewmanPaper\Piliang\3SubProteomes\Piliang3SP\mgf5ppm\SCX_3SubProteomes5ppm.mgf

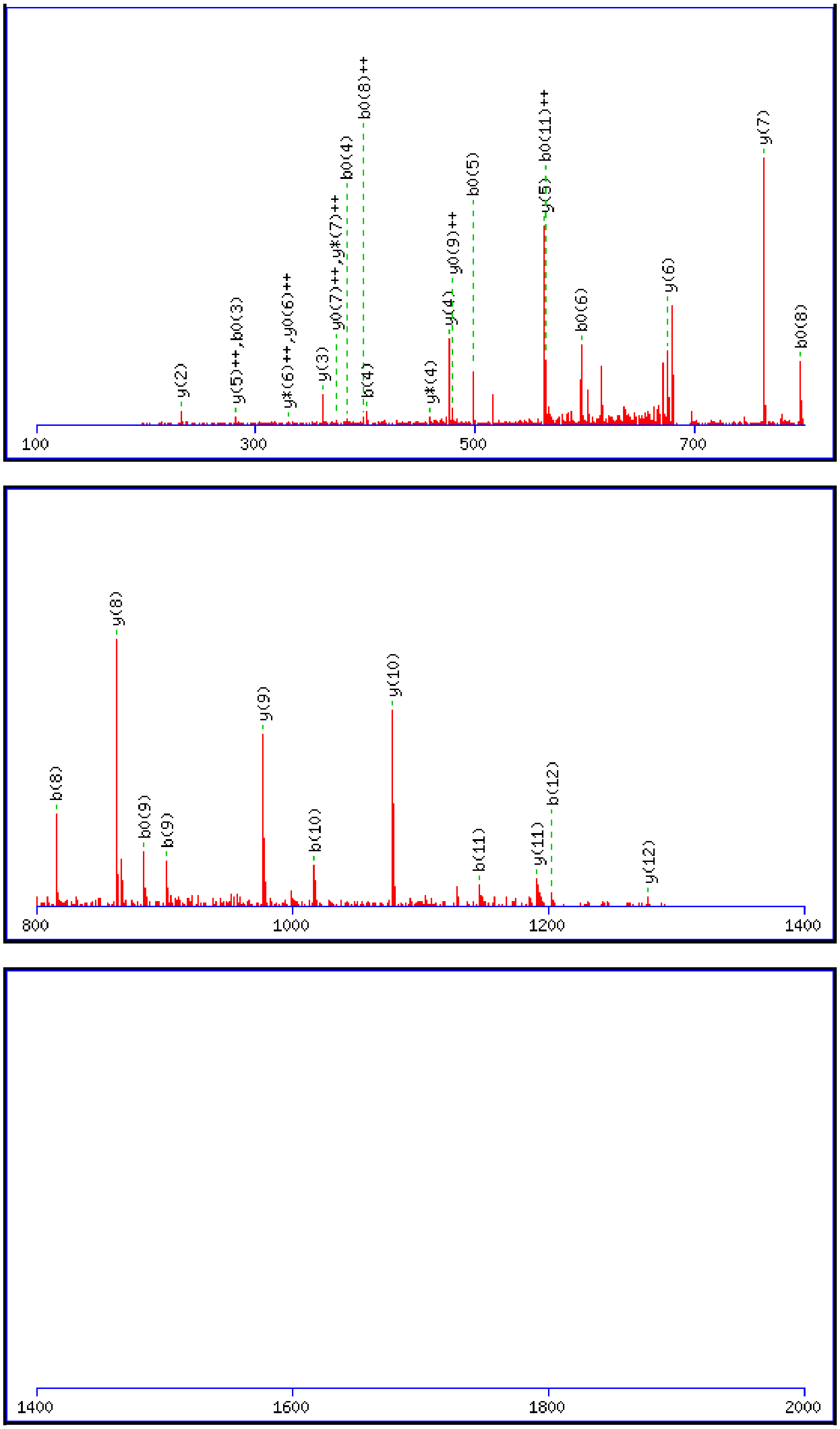

Monoisotopic mass of neutral peptide Mr(calc): 1376.6783
Fixed modifications: Carbamidomethyl (C)
Variable modifications:
N5 : Deamidated_N (N)
Ions Score: 101 **Expect:** 1.2e-008
Matches (Bold Red): 32/130 fragment ions using 42 most intense peaks

#	b	b++	b*	b*++	b0	b0++	Seq.	y	y++	y*	y*++	y0	y0++	#
1	100.0757	50.5415					V							13
2	187.1077	94.0575			169.0972	85.0522	S	**1278.6172**	639.8122	1261.5906	631.2990	1260.6066	630.8070	12
3	300.1918	150.5995			**282.1812**	141.5942	L	**1191.5852**	596.2962	1174.5586	587.7829	1173.5746	587.2909	11
4	**401.2395**	201.1234			**383.2289**	192.1181	T	**1078.5011**	539.7542	1061.4746	531.2409	1060.4905	530.7489	10
5	516.2664	258.6368	499.2398	250.1236	**498.2558**	249.6316	N	**977.4534**	489.2303	960.4269	480.7171	959.4429	**480.2251**	9

**6**	615.3348	308.1710	598.3083	299.6578	**597.3242**	299.1658	**V**	**862.4265**	431.7169	845.3999	423.2036	844.4159	422.7116	**8**
**7**	702.3668	351.6871	685.3403	343.1738	684.3563	342.6818	**S**	**763.3581**	382.1827	746.3315	**373.6694**	745.3475	**373.1774**	**7**
**8**	**815.4509**	408.2291	798.4244	399.7158	**797.4403**	**399.2238**	**I**	**676.3260**	338.6667	659.2995	**330.1534**	658.3155	**329.6614**	**6**
**9**	**902.4829**	451.7451	885.4564	443.2318	**884.4724**	442.7398	**S**	**563.2420**	**282.1246**	546.2154	273.6114	545.2314	273.1193	**5**
**10**	**1017.5099**	509.2586	1000.4833	500.7453	999.4993	500.2533	**D**	**476.2100**	238.6086	**459.1834**	230.0953	458.1994	229.6033	**4**
**11**	**1146.5525**	573.7799	1129.5259	565.2666	1128.5419	**564.7746**	**E**	**361.1830**	181.0951	344.1565	172.5819	343.1724	172.0899	**3**
**12**	**1203.5739**	602.2906	1186.5474	593.7773	1185.5634	593.2853	**G**	**232.1404**	116.5738	215.1139	108.0606			**2**
**13**							**R**	175.1190	88.0631	158.0924	79.5498			**1**

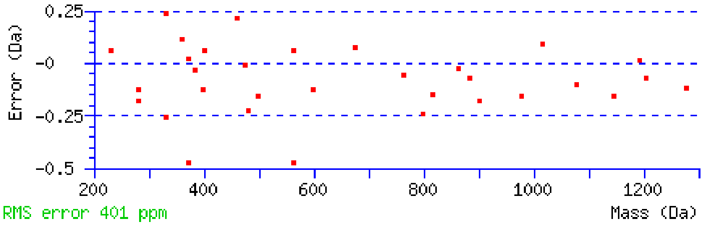

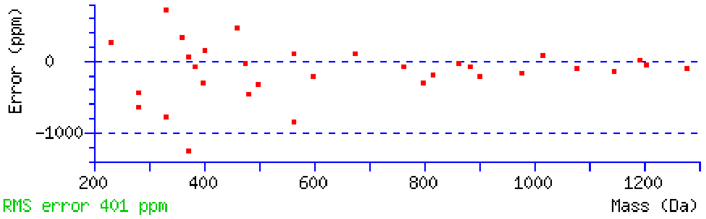

All matches to this query

Score	Mr(calc):	Delta	Sequence
100.8	1376.6783	0.0025	VSLTNVSISDEGR
6.9	1375.6732	1.0076	SHFNKVSTNVDK
6.3	1375.6766	1.0043	TSEPKAMVVGEGR
5.7	1376.6718	0.0091	SSPEERERMIK
3.6	1376.6718	0.0091	KNIEEKSGMEGR
3.1	1376.6693	0.0115	MHIASLQYMAGR
3.0	1376.6758	0.0050	WTLLPTNMSTGR
2.6	1375.6878	0.9931	ERLPCASSTISR
1.7	1374.6853	1.9955	YLELMIVNDHK
1.6	1376.6871	-0.0062	IFKSSDHIQMR

Spectrum No: 30; Query: 718; Rank: 1

Peptide View

MS/MS Fragmentation of **MYVTNDTEVAENNYQALK**
Found in **IPI00464785**, Tax_Id=10116 Gene_Symbol=Ppgb Protective protein for beta-galactosidase

Match to Query 718: 2102.951448 from(1052.483000,2+)
Title: 091127RatKid_SCX01_17.2644.2644.2.dta
Data file K:\NewmanPaper\Piliang\3SubProteomes\Piliang3SP\mgf5ppm\SCX_3SubProteomes5ppm.mgf

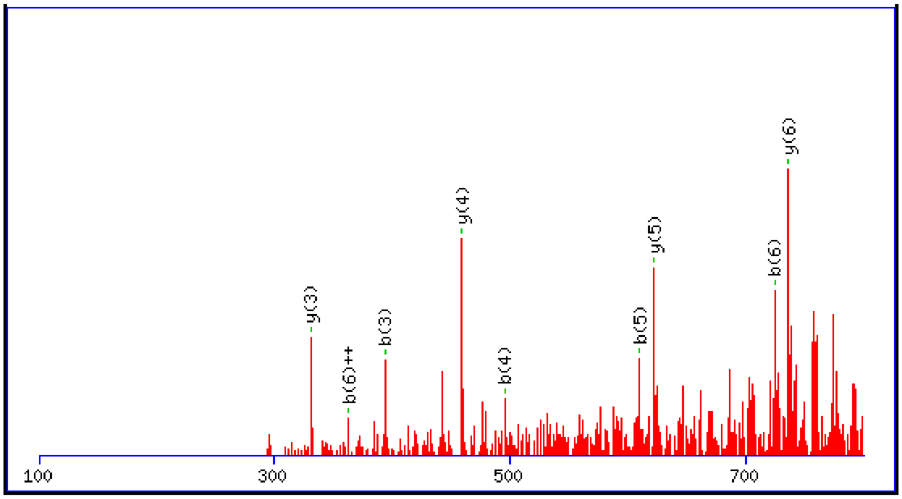

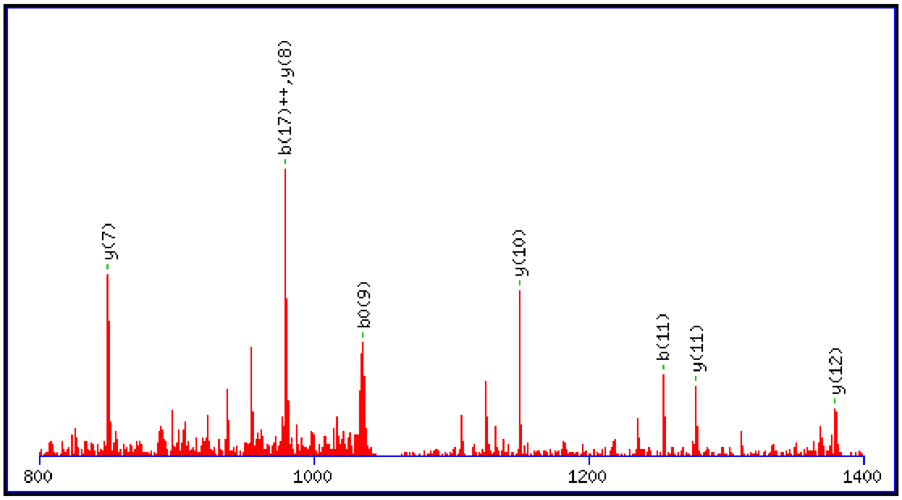

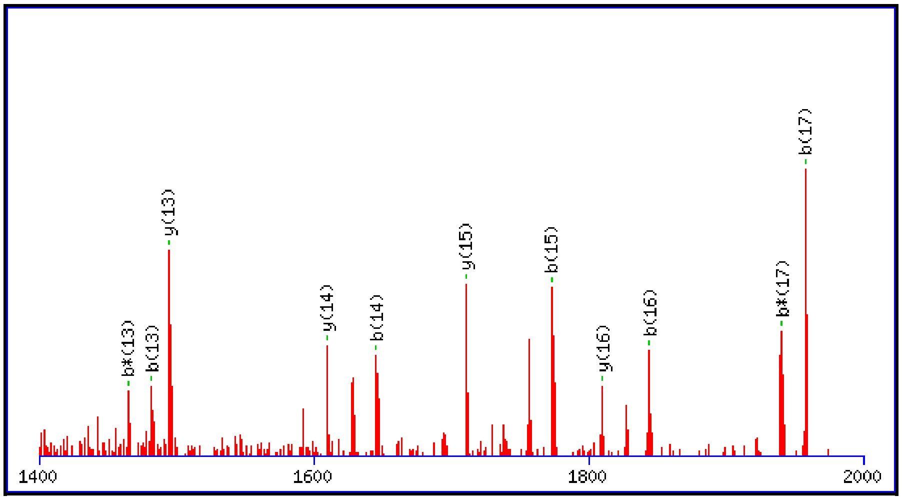

Monoisotopic mass of neutral peptide Mr(calc): 2102.9466
Fixed modifications: Carbamidomethyl (C)
Variable modifications:
N5 : Deamidated_N (N)
Ions Score: 101 **Expect:** 2.2e-008
Matches (Bold Red): 28/176 fragment ions using 39 most intense peaks

#	b	b^{++}	b*	b*$^{++}$	b^{0}	b^{0++}	Seq.	y	y^{++}	y*	y*$^{++}$	y^{0}	y^{0++}	#
1	132.0478	66.5275					M							18
2	295.1111	148.0592					Y	1972.9134	986.9604	1955.8869	978.4471	1954.9029	977.9551	17
3	**394.1795**	197.5934					V	**1809.8501**	905.4287	1792.8236	896.9154	1791.8395	896.4234	16
4	**495.2272**	248.1172			477.2166	239.1119	T	**1710.7817**	855.8945	1693.7551	847.3812	1692.7711	846.8892	15
5	**610.2541**	305.6307	593.2276	297.1174	592.2436	296.6254	N	**1609.7340**	805.3706	1592.7075	796.8574	1591.7234	796.3654	14

6	725.2811	363.1442	708.2545	354.6309	707.2705	354.1389	D	1494.7071	747.8572	1477.6805	739.3439	1476.6965	738.8519	13
7	826.3287	413.6680	809.3022	405.1547	808.3182	404.6627	T	1379.6801	690.3437	1362.6536	681.8304	1361.6696	681.3384	12
8	955.3713	478.1893	938.3448	469.6760	937.3608	469.1840	E	1278.6325	639.8199	1261.6059	631.3066	1260.6219	630.8146	11
9	1054.4397	527.7235	1037.4132	519.2102	1036.4292	518.7182	V	1149.5899	575.2986	1132.5633	566.7853	1131.5793	566.2933	10
10	1125.4769	563.2421	1108.4503	554.7288	1107.4663	554.2368	A	1050.5215	525.7644	1033.4949	517.2511	1032.5109	516.7591	9
11	1254.5195	627.7634	1237.4929	619.2501	1236.5089	618.7581	E	979.4843	490.2458	962.4578	481.7325	961.4738	481.2405	8
12	1368.5624	684.7848	1351.5358	676.2716	1350.5518	675.7795	N	850.4417	425.7245	833.4152	417.2112			7
13	1482.6053	741.8063	1465.5788	733.2930	1464.5947	732.8010	N	736.3988	368.7030	719.3723	360.1898			6
14	1645.6686	823.3380	1628.6421	814.8247	1627.6581	814.3327	Y	622.3559	311.6816	605.3293	303.1683			5
15	1773.7272	887.3672	1756.7007	878.8540	1755.7167	878.3620	Q	459.2926	230.1499	442.2660	221.6366			4
16	1844.7643	922.8858	1827.7378	914.3725	1826.7538	913.8805	A	331.2340	166.1206	314.2074	157.6074			3
17	1957.8484	979.4278	1940.8218	970.9146	1939.8378	970.4226	L	260.1969	130.6021	243.1703	122.0888			2
18							K	147.1128	74.0600	130.0863	65.5468			1

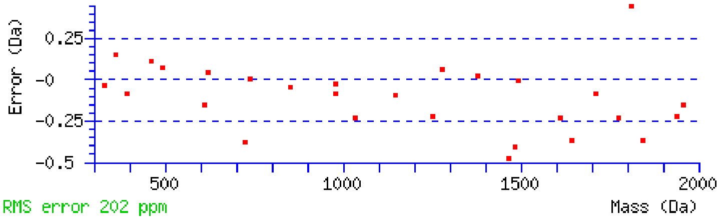

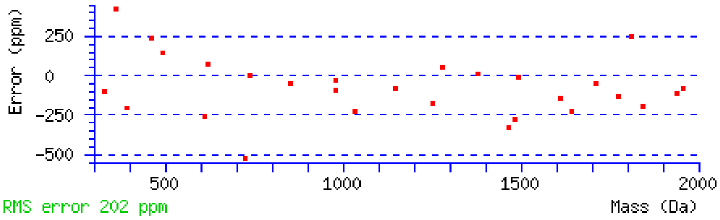

All matches to this query

Score	Mr(calc):	Delta	Sequence
100.7	2102.9466	0.0048	MYVTNDTEVAENNYQALK
74.4	2102.9466	0.0048	MYVTNDTEVAENNYQALK
64.2	2102.9466	0.0048	MYVTNDTEVAENNYQALK
53.5	2101.9626	0.9888	MYVTNDTEVAENNYQALK
2.8	2102.9496	0.0019	SAQYPFVASLTVVAVALK
1.1	2100.9256	2.0259	HTKPSSHLSVLQGGHNSR
1.1	2100.9256	2.0259	HTKPSSHLSVLQGGHNSR
1.1	2102.9570	-0.0056	MSKFAMPSSTPLYHDAIK
0.9	2102.9620	-0.0106	SDAAVDSSSEITTKDLKEK
0.9	2102.9620	-0.0106	SDAAVDSSSEITTKDLKEK

Spectrum No: 31; Query: 703; Rank: 1

Peptide View

MS/MS Fragmentation of **LDWLGNCSGLNDESYGYK**
Found in **IPI00339124**, Tax_Id=10116 Gene_Symbol=Atp1b1 Sodium/potassium-transporting ATPase subunit beta-1

Match to Query 703: 2090.887528 from(1046.451040,2+)
Title: 091129RatKid_SCX02_15.2838.2838.2.dta
Data file K:\NewmanPaper\Piliang\3SubProteomes\Piliang3SP\mgf5ppm\SCX_3SubProteomes5ppm.mgf

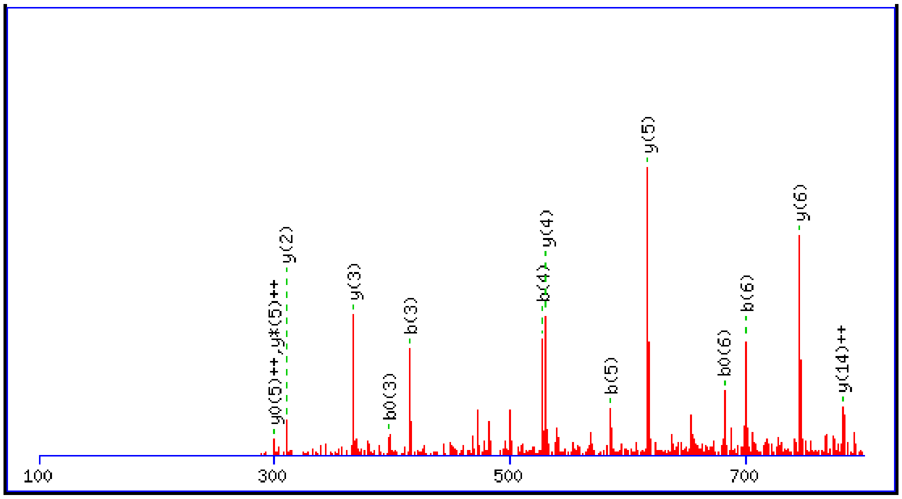

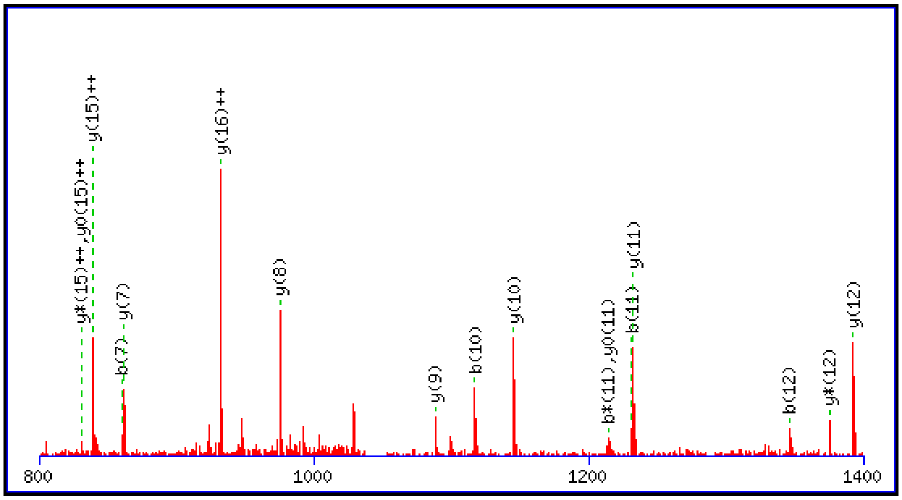

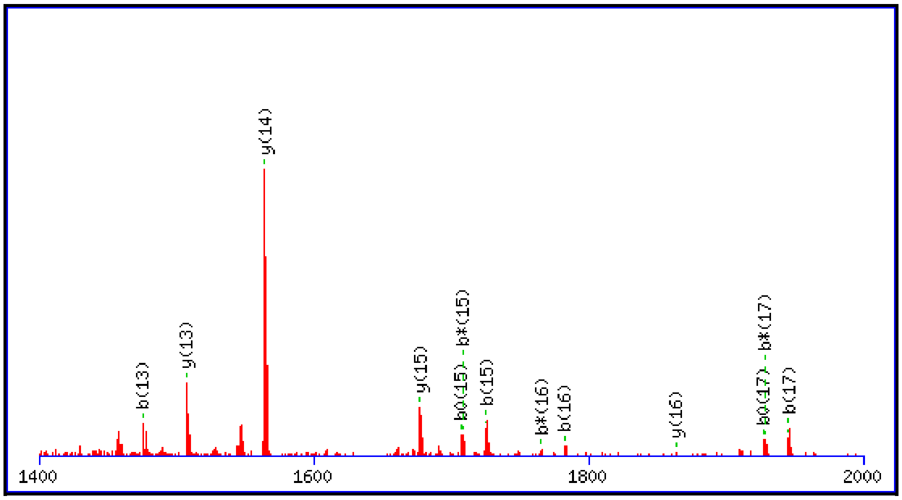

Monoisotopic mass of neutral peptide Mr(calc): 2090.8891
Fixed modifications: Carbamidomethyl (C)
Variable modifications:
N6 : Deamidated_N (N)
Ions Score: 100 **Expect:** 2.9e-008
Matches (Bold Red): 44/184 fragment ions using 90 most intense peaks

#	b	b^{++}	b*	b^{*++}	b^0	b^{0++}	Seq.	y	y^{++}	y*	y^{*++}	y^0	y^{0++}	#
1	114.0913	57.5493					L							18
2	229.1183	115.0628			211.1077	106.0575	D	1978.8123	989.9098	1961.7858	981.3965	1960.8018	980.9045	17
3	**415.1976**	208.1024			**397.1870**	199.0972	W	**1863.7854**	**932.3963**	1846.7589	923.8831	1845.7748	923.3911	16
4	**528.2817**	264.6445			510.2711	255.6392	L	**1677.7061**	**839.3567**	1660.6795	**830.8434**	1659.6955	**830.3514**	15
5	**585.3031**	293.1552			567.2926	284.1499	G	**1564.6220**	**782.8146**	1547.5955	774.3014	1546.6115	773.8094	14

**6**	**700.3301**	350.6687	683.3035	342.1554	**682.3195**	341.6634	**N**	**1507.6006**	754.3039	1490.5740	745.7906	1489.5900	745.2986	**13**
**7**	**860.3607**	430.6840	843.3342	422.1707	842.3501	421.6787	**C**	**1392.5736**	696.7904	**1375.5471**	688.2772	1374.5631	687.7852	**12**
**8**	947.3927	474.2000	930.3662	465.6867	929.3822	465.1947	**S**	**1232.5430**	616.7751	1215.5164	608.2619	**1214.5324**	607.7698	**11**
**9**	1004.4142	502.7107	987.3877	494.1975	986.4036	493.7055	**G**	**1145.5109**	573.2591	1128.4844	564.7458	1127.5004	564.2538	**10**
**10**	**1117.4983**	559.2528	1100.4717	550.7395	1099.4877	550.2475	**L**	**1088.4895**	544.7484	1071.4629	536.2351	1070.4789	535.7431	**9**
**11**	**1231.5412**	616.2742	**1214.5146**	607.7610	1213.5306	607.2690	**N**	**975.4054**	488.2063	958.3789	479.6931	957.3949	479.2011	**8**
**12**	**1346.5681**	673.7877	1329.5416	665.2744	1328.5576	664.7824	**D**	**861.3625**	431.1849	844.3359	422.6716	843.3519	422.1796	**7**
**13**	**1475.6107**	738.3090	1458.5842	729.7957	1457.6002	729.3037	**E**	**746.3355**	373.6714	729.3090	365.1581	728.3250	364.6661	**6**
**14**	1562.6428	781.8250	1545.6162	773.3117	1544.6322	772.8197	**S**	**617.2930**	309.1501	600.2664	**300.6368**	599.2824	**300.1448**	**5**
**15**	**1725.7061**	863.3567	**1708.6795**	854.8434	**1707.6955**	854.3514	**Y**	**530.2609**	265.6341	513.2344	257.1208			**4**
**16**	**1782.7275**	891.8674	**1765.7010**	883.3541	1764.7170	882.8621	**G**	**367.1976**	184.1024	350.1710	175.5892			**3**
**17**	**1945.7909**	973.3991	**1928.7643**	964.8858	**1927.7803**	964.3938	**Y**	**310.1761**	155.5917	293.1496	147.0784			**2**
**18**							**K**	147.1128	74.0600	130.0863	65.5468			**1**

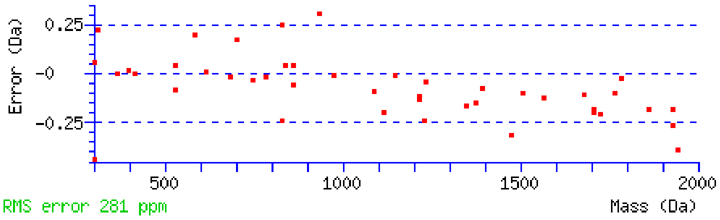

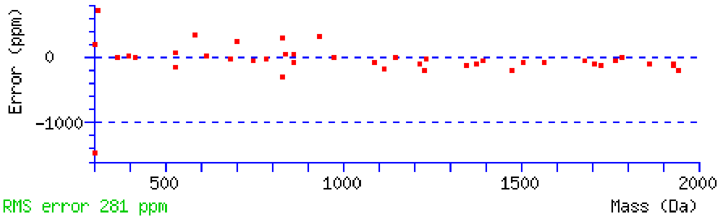

All matches to this query

Score	Mr(calc):	Delta	Sequence
99.6	2090.8891	-0.0016	LDWLGNCSGLNDESYGYK
83.3	2090.8891	-0.0016	LDWLGNCSGLNDESYGYK
2.2	2090.9043	-0.0168	ASISRSQHGILQMICSK
1.7	2089.9017	0.9858	RASDPVRTVSENMSLPR
1.6	2089.9017	0.9858	RASDPVRTVSENMSLPR
1.6	2090.9043	-0.0168	ASISRSQHGILQMICSK
1.6	2090.9043	-0.0168	ASISRSQHGILQMICSK
0.7	2090.8931	-0.0056	ELHQKSSGGMLQMLASSK
0.7	2090.8931	-0.0056	ELHQKSSGGMLQMLASSK
0.7	2090.8931	-0.0056	ELHQKSSGGMLQMLASSK

Spectrum No: 32; Query: 799; Rank: 1

Peptide View

MS/MS Fragmentation of **IGCFALSEPGNGSDAGAASTTAR**
Found in **IPI00231359**, Tax_Id=10116 Gene_Symbol=Acads Acetyl-Coenzyme A dehydrogenase, short chain

Match to Query 799: 2209.987668 from(1106.001110,2+)
Title: 091129RatKid_SCX02_18.1677.1677.2.dta
Data file K:\NewmanPaper\Piliang\3SubProteomes\Piliang3SP\mgf5ppm\SCX_3SubProteomes5ppm.mgf

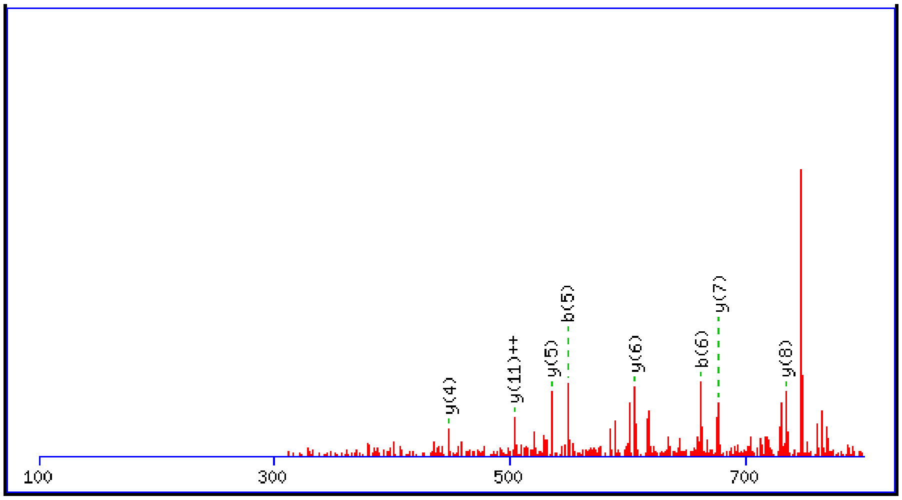

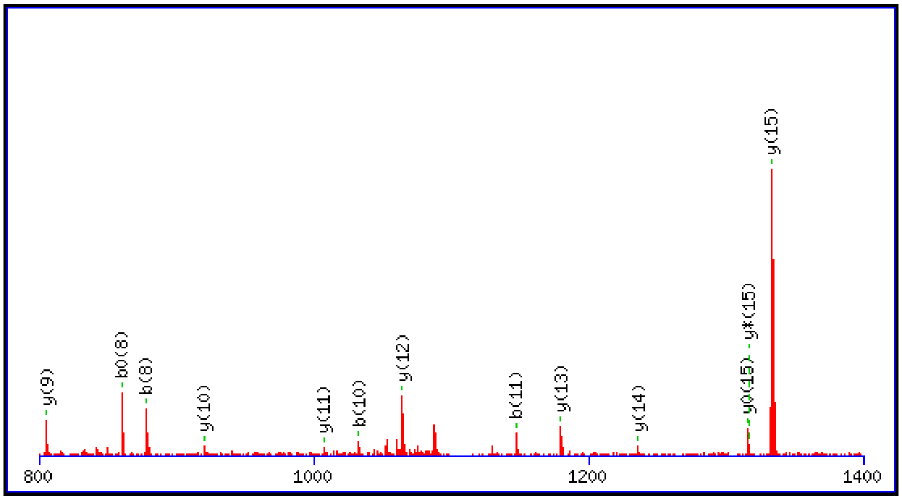

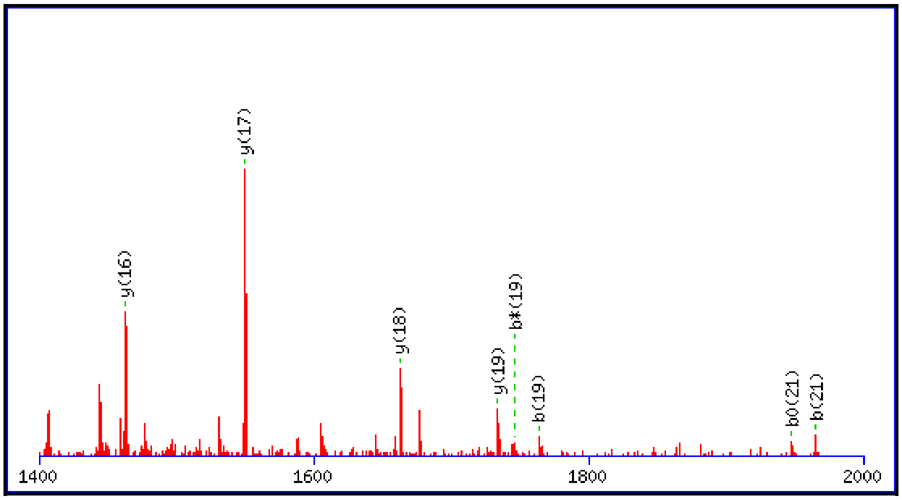

Monoisotopic mass of neutral peptide Mr(calc): 2209.9910
Fixed modifications: Carbamidomethyl (C)
Variable modifications:
N11 : Deamidated_N (N)
Ions Score: 99 **Expect:** 3.1e-008
Matches (Bold Red): 29/228 fragment ions using 53 most intense peaks

#	b	b^{++}	b*	b^{*++}	b^0	b^{0++}	Seq.	y	y^{++}	y*	y^{*++}	y^0	y^{0++}	#
1	114.0913	57.5493					I							23
2	171.1128	86.0600					G	2097.9142	1049.4607	2080.8876	1040.9475	2079.9036	1040.4554	22
3	331.1435	166.0754					C	2040.8927	1020.9500	2023.8662	1012.4367	2022.8822	1011.9447	21
4	478.2119	239.6096					F	1880.8621	940.9347	1863.8355	932.4214	1862.8515	931.9294	20
5	**549.2490**	275.1281					A	**1733.7937**	867.4005	1716.7671	858.8872	1715.7831	858.3952	19

6	**662.3330**	331.6702					L	**1662.7565**	831.8819	1645.7300	823.3686	1644.7460	822.8766	18
7	749.3651	375.1862			731.3545	366.1809	S	**1549.6725**	775.3399	1532.6459	766.8266	1531.6619	766.3346	17
8	**878.4077**	439.7075			**860.3971**	430.7022	E	**1462.6405**	731.8239	1445.6139	723.3106	1444.6299	722.8186	16
9	975.4604	488.2339			957.4499	479.2286	P	**1333.5979**	667.3026	**1316.5713**	658.7893	**1315.5873**	658.2973	15
10	**1032.4819**	516.7446			1014.4713	507.7393	G	**1236.5451**	618.7762	1219.5185	610.2629	1218.5345	609.7709	14
11	**1147.5088**	574.2581	1130.4823	565.7448	1129.4983	565.2528	N	**1179.5236**	590.2655	1162.4971	581.7522	1161.5131	581.2602	13
12	1204.5303	602.7688	1187.5037	594.2555	1186.5197	593.7635	G	**1064.4967**	532.7520	1047.4701	524.2387	1046.4861	523.7467	12
13	1291.5623	646.2848	1274.5358	637.7715	1273.5518	637.2795	S	**1007.4752**	**504.2413**	990.4487	495.7280	989.4647	495.2360	11
14	1406.5893	703.7983	1389.5627	695.2850	1388.5787	694.7930	D	**920.4432**	460.7252	903.4167	452.2120	902.4326	451.7200	10
15	1477.6264	739.3168	1460.5998	730.8036	1459.6158	730.3115	A	**805.4163**	403.2118	788.3897	394.6985	787.4057	394.2065	9
16	1534.6478	767.8276	1517.6213	759.3143	1516.6373	758.8223	G	**734.3791**	367.6932	717.3526	359.1799	716.3686	358.6879	8
17	1605.6850	803.3461	1588.6584	794.8328	1587.6744	794.3408	A	**677.3577**	339.1825	660.3311	330.6692	659.3471	330.1772	7
18	1676.7221	838.8647	1659.6955	830.3514	1658.7115	829.8594	A	**606.3206**	303.6639	589.2940	295.1506	588.3100	294.6586	6
19	**1763.7541**	882.3807	**1746.7276**	873.8674	1745.7435	873.3754	S	**535.2835**	268.1454	518.2569	259.6321	517.2729	259.1401	5
20	1864.8018	932.9045	1847.7752	924.3913	1846.7912	923.8992	T	**448.2514**	224.6294	431.2249	216.1161	430.2409	215.6241	4
21	**1965.8495**	983.4284	1948.8229	974.9151	**1947.8389**	974.4231	T	347.2037	174.1055	330.1772	165.5922	329.1932	165.1002	3
22	2036.8866	1018.9469	2019.8600	1010.4336	2018.8760	1009.9416	A	246.1561	123.5817	229.1295	115.0684			2
23							R	175.1190	88.0631	158.0924	79.5498			1

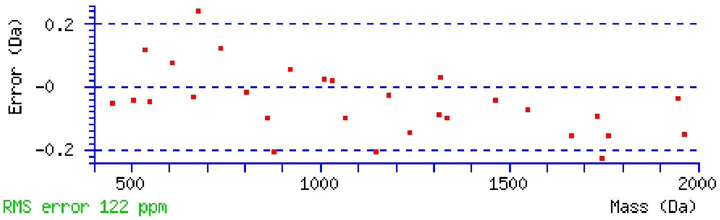

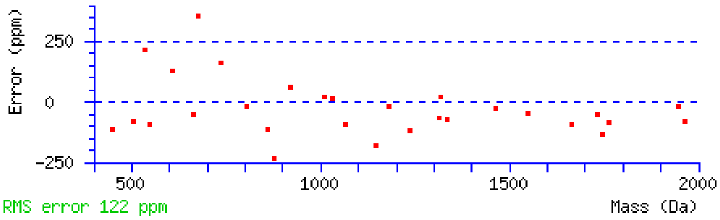

All matches to this query

Score	Mr(calc):	Delta	Sequence
99.5	2209.9910	-0.0033	IGCFALSEPGNGSDAGAASTTAR
9.0	2208.9673	1.0203	LNATTDVMRGLLSNVLCR
3.7	2208.9762	1.0114	RQFTVFNCTLPSNTSIDK
2.4	2208.9631	1.0245	HPSARAEEEGGSDGSAAEAEPR
1.5	2207.9786	2.0091	DRLLAEETAATISAIEAMK
1.5	2209.0052	0.9824	ALSPPPVDPGYSKGEQASSSR
0.6	2209.9674	0.0203	EPRKSAEEALAAAGDMDNAGK

Spectrum No: 33; Query: 213; Rank: 1

Peptide View

MS/MS Fragmentation of **AFYNGSLTAQIVK**
Found in **IPI00206254**, Tax_Id=10116 Gene_Symbol=Ggt1 Gamma-glutamyltranspeptidase 1 precursor

Match to Query 213: 1411.737048 from(706.875800,2+)
Title: 091127RatKid_SCX01_16.3135.3135.2.dta
Data file K:\NewmanPaper\Piliang\3SubProteomes\Piliang3SP\mgf5ppm\SCX_3SubProteomes5ppm.mgf

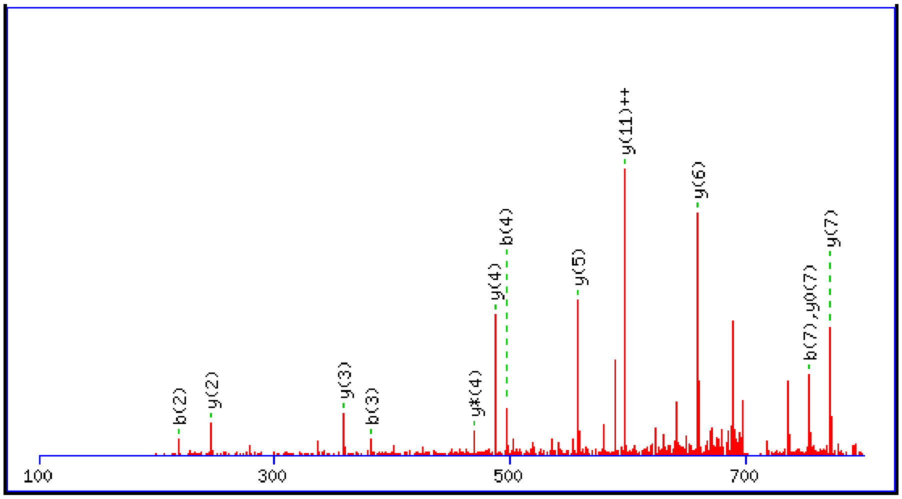

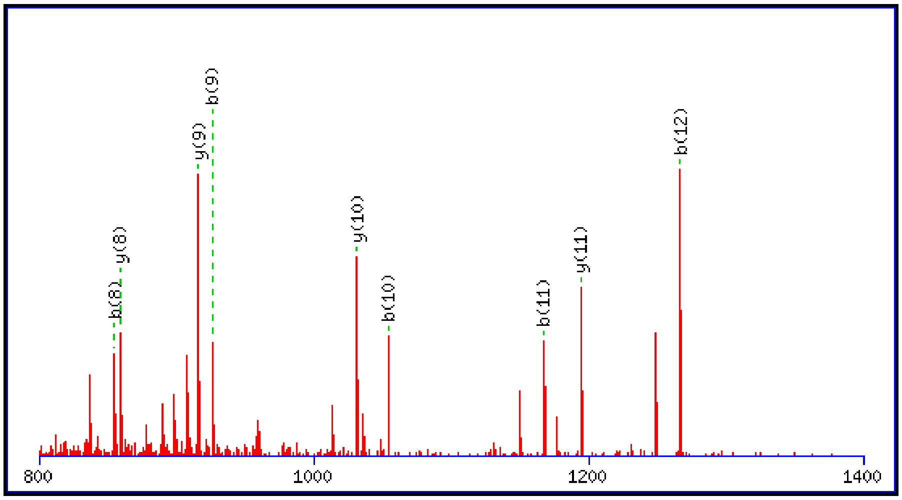

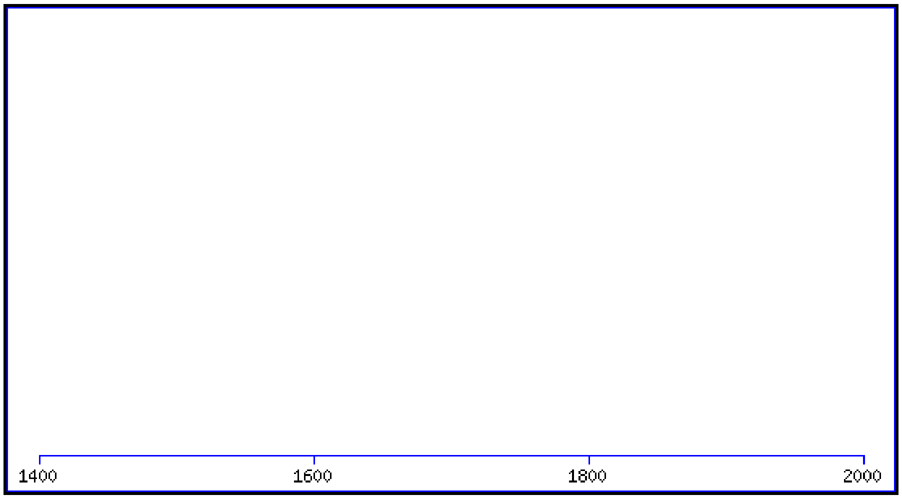

Monoisotopic mass of neutral peptide Mr(calc): 1411.7347
Fixed modifications: Carbamidomethyl (C)
Variable modifications:
N4 : Deamidated_N (N)
Ions Score: 99 **Expect:** 1.4e-008
Matches (Bold Red): 22/118 fragment ions using 27 most intense peaks

#	b	b^{++}	b*	b^{*++}	b^{0}	b^{0++}	Seq.	y	y^{++}	y*	y^{*++}	y^{0}	y^{0++}	#
1	72.0444	36.5258					A							13
2	**219.1128**	110.0600					F	1341.7049	671.3561	1324.6783	662.8428	1323.6943	662.3508	12
3	**382.1761**	191.5917					Y	**1194.6365**	**597.8219**	1177.6099	589.3086	1176.6259	588.8166	11
4	**497.2031**	249.1052	480.1765	240.5919			N	**1031.5731**	516.2902	1014.5466	507.7769	1013.5626	507.2849	10
5	554.2245	277.6159	537.1980	269.1026			G	**916.5462**	458.7767	899.5197	450.2635	898.5356	449.7715	9

6	641.2566	321.1319	624.2300	312.6186	623.2460	312.1266	S	**859.5247**	430.2660	842.4982	421.7527	841.5142	421.2607	8
7	**754.3406**	377.6740	737.3141	369.1607	736.3301	368.6687	L	**772.4927**	386.7500	755.4662	378.2367	**754.4822**	377.7447	7
8	**855.3883**	428.1978	838.3618	419.6845	837.3777	419.1925	T	**659.4087**	330.2080	642.3821	321.6947	641.3981	321.2027	6
9	**926.4254**	463.7163	909.3989	455.2031	908.4149	454.7111	A	**558.3610**	279.6841	541.3344	271.1709			5
10	**1054.4840**	527.7456	1037.4574	519.2324	1036.4734	518.7404	Q	**487.3239**	244.1656	**470.2973**	235.6523			4
11	**1167.5681**	584.2877	1150.5415	575.7744	1149.5575	575.2824	I	**359.2653**	180.1363	342.2387	171.6230			3
12	**1266.6365**	633.8219	1249.6099	625.3086	1248.6259	624.8166	V	**246.1812**	123.5942	229.1547	115.0810			2
13							K	147.1128	74.0600	130.0863	65.5468			1

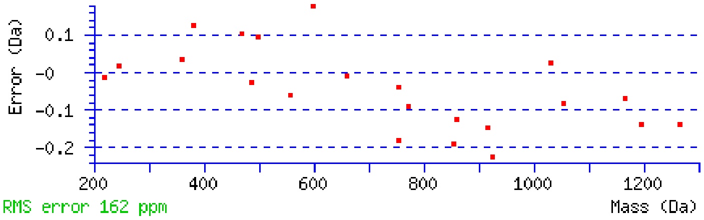

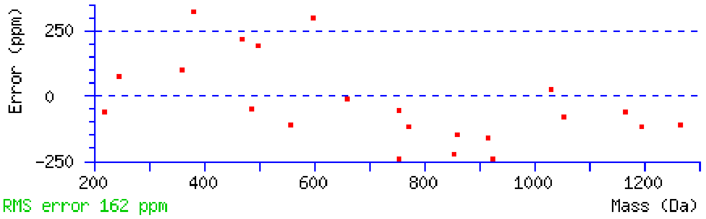

All matches to this query

Score	Mr(calc):	Delta	Sequence
98.7	1411.7347	0.0023	AFYNGSLTAQIVK
6.5	1411.7476	-0.0106	LTGQIFLGGSIVK
5.6	1411.7364	0.0007	FLISDLTLSPVK
4.6	1411.7364	0.0007	FLISDLTLSPVK
4.2	1410.7467	0.9904	QSIPNVLQGEVAR
3.9	1411.7436	-0.0065	SSISKQGSKVLAK
3.4	1411.7321	0.0050	SGQITAFPRHGGGK
3.3	1411.7323	0.0047	EISADKKTTVIK
2.9	1411.7258	0.0112	SVVTTARMQVLK
2.9	1411.7258	0.0112	SVVTTARMQVLK

Spectrum No: 34; Query: 290; Rank: 1

Peptide View

MS/MS Fragmentation of **TAFQNGTESTIITK**
Found in **IPI00204808**, Tax_Id=10116 Gene_Symbol=Mep1b Meprin A subunit beta precursor

Match to Query 290: 1510.753228 from(756.383890,2+)
Title: 091127RatKid_SCX01_02.1954.1954.2.dta
Data file K:\NewmanPaper\Piliang\3SubProteomes\Piliang3SP\mgf5ppm\SCX_3SubProteomes5ppm.mgf

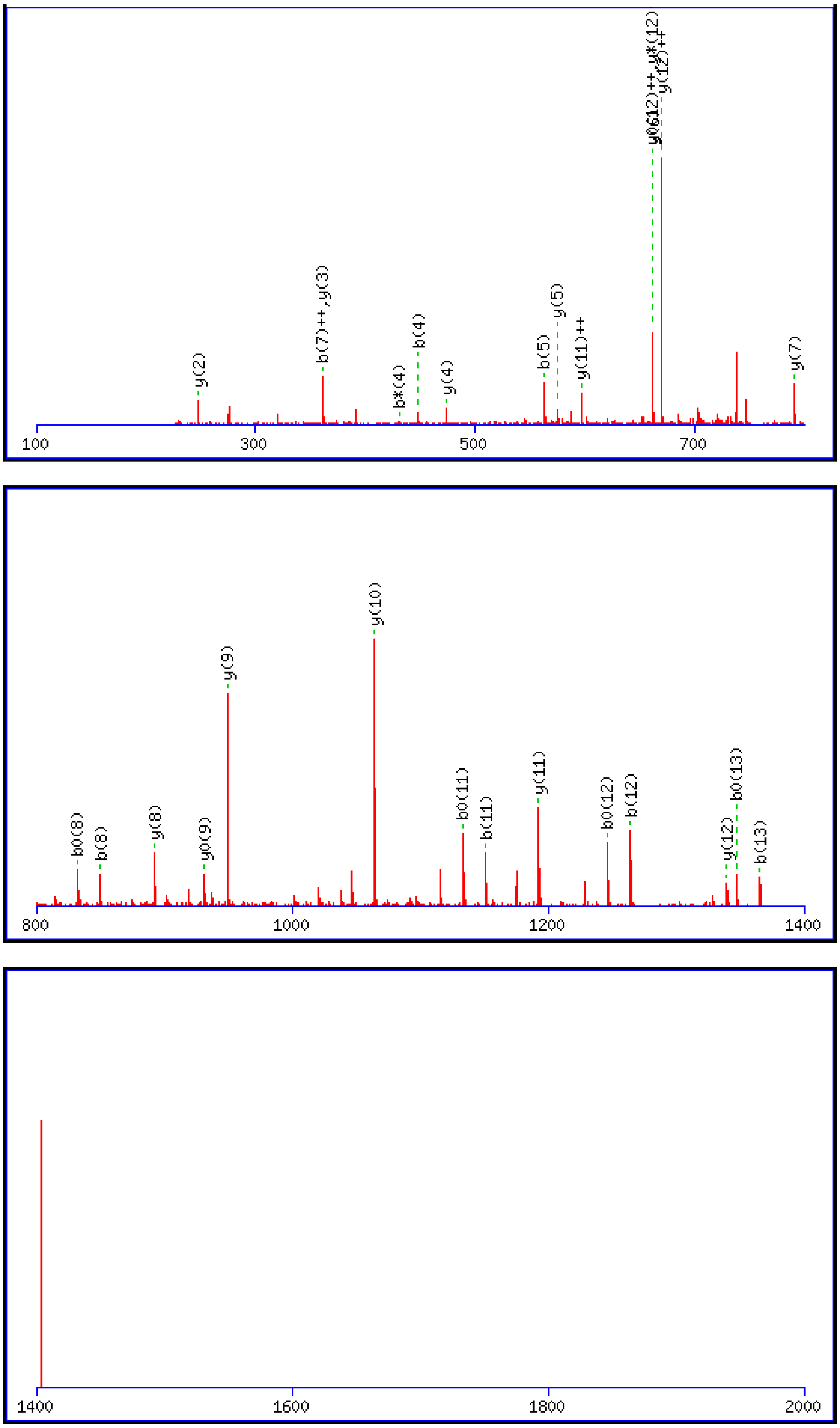

Monoisotopic mass of neutral peptide Mr(calc): 1510.7515
Fixed modifications: Carbamidomethyl (C)
Variable modifications:
N5 : Deamidated_N (N)
Ions Score: 99 **Expect:** 2e-008
Matches (Bold Red): 28/148 fragment ions using 37 most intense peaks

#	b	b^{++}	b*	b^{*++}	b^0	b^{0++}	Seq.	y	y^{++}	y*	y^{*++}	y^0	y^{0++}	#
1	102.0550	51.5311			84.0444	42.5258	T							14
2	173.0921	87.0497			155.0815	78.0444	A	1410.7111	705.8592	1393.6845	697.3459	1392.7005	696.8539	13
3	320.1605	160.5839			302.1499	151.5786	F	**1339.6740**	**670.3406**	1322.6474	**661.8274**	1321.6634	**661.3353**	12
4	**448.2191**	224.6132	**431.1925**	216.0999	430.2085	215.6079	Q	**1192.6056**	**596.8064**	1175.5790	588.2931	1174.5950	587.8011	11
5	**563.2460**	282.1266	546.2194	273.6134	545.2354	273.1214	N	**1064.5470**	532.7771	1047.5204	524.2639	1046.5364	523.7719	10

**6**	620.2675	310.6374	603.2409	302.1241	602.2569	301.6321	**G**	**949.5201**	475.2637	932.4935	466.7504	**931.5095**	466.2584	**9**
**7**	721.3151	**361.1612**	704.2886	352.6479	703.3046	352.1559	**T**	**892.4986**	446.7529	875.4720	438.2397	874.4880	437.7477	**8**
**8**	**850.3577**	425.6825	833.3312	417.1692	**832.3472**	416.6772	**E**	**791.4509**	396.2291	774.4244	387.7158	773.4403	387.2238	**7**
**9**	937.3898	469.1985	920.3632	460.6852	919.3792	460.1932	**S**	**662.4083**	331.7078	645.3818	323.1945	644.3978	322.7025	**6**
**10**	1038.4374	519.7224	1021.4109	511.2091	1020.4269	510.7171	**T**	**575.3763**	288.1918	558.3497	279.6785	557.3657	279.1865	**5**
**11**	**1151.5215**	576.2644	1134.4950	567.7511	**1133.5109**	567.2591	**I**	**474.3286**	237.6679	457.3021	229.1547	456.3180	228.6627	**4**
**12**	**1264.6056**	632.8064	1247.5790	624.2931	**1246.5950**	623.8011	**I**	**361.2445**	181.1259	344.2180	172.6126	343.2340	172.1206	**3**
**13**	**1365.6532**	683.3303	1348.6267	674.8170	**1347.6427**	674.3250	**T**	**248.1605**	124.5839	231.1339	116.0706	230.1499	115.5786	**2**
**14**							**K**	147.1128	74.0600	130.0863	65.5468			**1**

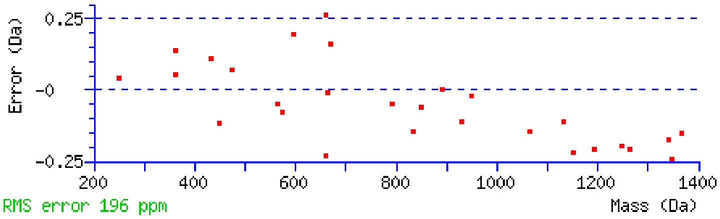

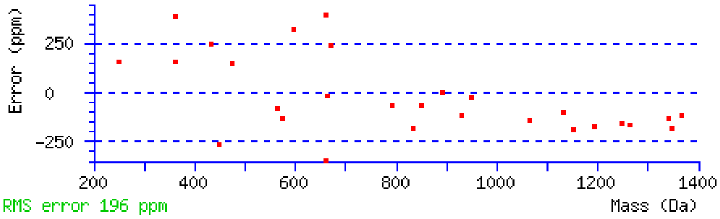

All matches to this query

Score	Mr(calc):	Delta	Sequence
98.6	1510.7515	0.0017	TAFQNGTESTIITK
4.5	1510.7456	0.0076	TAFQEEFVHYLK
4.5	1510.7502	0.0031	TVPHQGTEGHHRR
1.8	1510.7504	0.0028	TELKQRLNSQSK
1.8	1508.7505	2.0028	SGNVTTVMETIGRK
1.3	1508.7500	2.0032	AFNIRSNLTKHK
1.2	1510.7504	0.0028	ENRKTLAAQTATK
0.8	1508.7500	2.0032	AFSQKSNLLRHK

Spectrum No: 35; Query: 816; Rank: 1

Peptide View

MS/MS Fragmentation of **YGYENDTEIANLYDEMVAK**
Found in **IPI00191437**, Tax_Id=10116 Gene_Symbol=- 64 kDa protein

Match to Query 816: 2253.971068 from(1127.992810,2+)
Title: 091127RatKid_SCX01_15.3102.3102.2.dta
Data file K:\NewmanPaper\Piliang\3SubProteomes\Piliang3SP\mgf5ppm\SCX_3SubProteomes5ppm.mgf

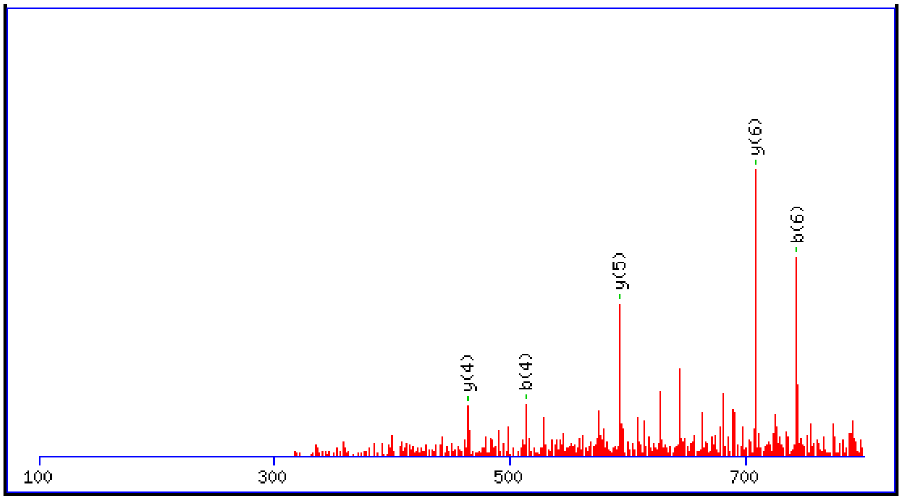

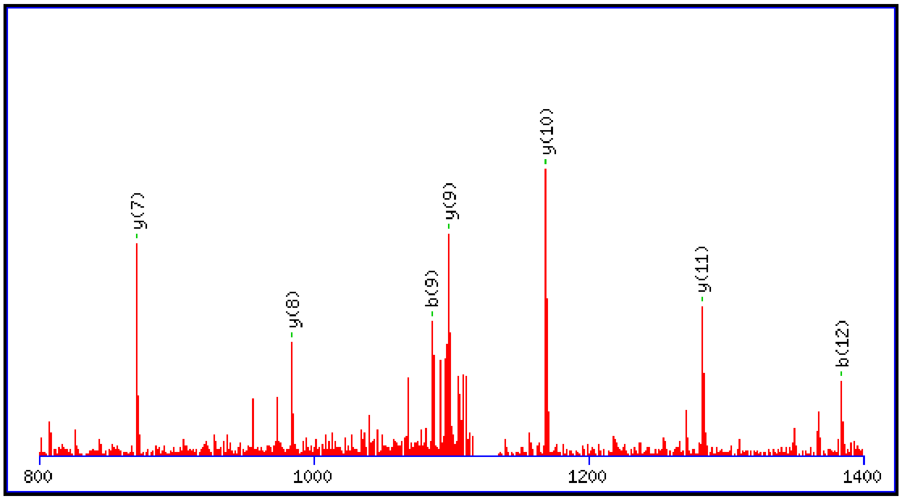

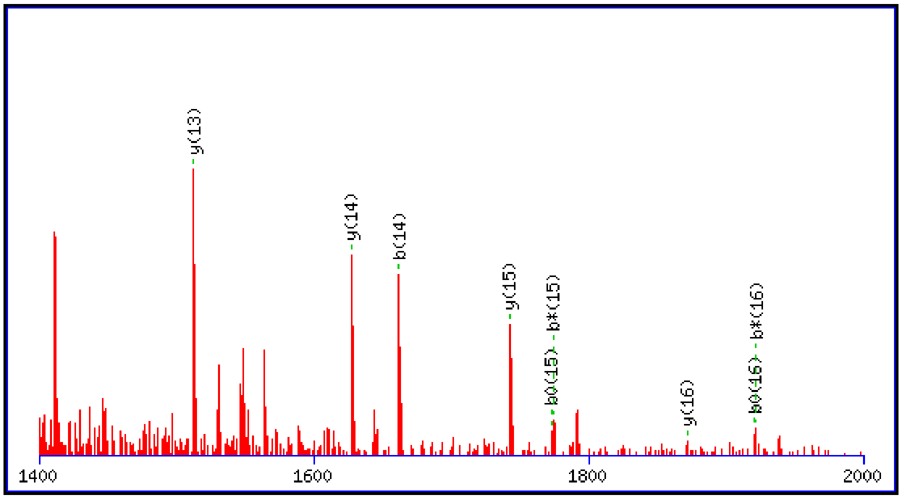

Monoisotopic mass of neutral peptide Mr(calc): 2253.9623
Fixed modifications: Carbamidomethyl (C)
Variable modifications:
N5 : Deamidated_N (N)
M16 : Oxidation (M)
Ions Score: 99 **Expect:** 3.9e-008
Matches (Bold Red): 21/194 fragment ions using 34 most intense peaks

#	b	b^{++}	b*	b^{*++}	b^{0}	b^{0++}	Seq.	y	y^{++}	y*	y^{*++}	y^{0}	y^{0++}	#
1	164.0706	82.5389					Y							19
2	221.0921	111.0497					G	2091.9063	1046.4568	2074.8798	1037.9435	2073.8957	1037.4515	18
3	384.1554	192.5813					Y	2034.8848	1017.9461	2017.8583	1009.4328	2016.8743	1008.9408	17
4	**513.1980**	257.1026			495.1874	248.0974	E	**1871.8215**	936.4144	1854.7950	927.9011	1853.8109	927.4091	16

**5**	628.2249	314.6161	611.1984	306.1028	610.2144	305.6108	**N**	**1742.7789**	871.8931	1725.7524	863.3798	1724.7684	862.8878	**15**
**6**	**743.2519**	372.1296	726.2253	363.6163	725.2413	363.1243	**D**	**1627.7520**	814.3796	1610.7254	805.8664	1609.7414	805.3743	**14**
**7**	844.2995	422.6534	827.2730	414.1401	826.2890	413.6481	**T**	**1512.7250**	756.8662	1495.6985	748.3529	1494.7145	747.8609	**13**
**8**	973.3421	487.1747	956.3156	478.6614	955.3316	478.1694	**E**	1411.6774	706.3423	1394.6508	697.8290	1393.6668	697.3370	**12**
**9**	**1086.4262**	543.7167	1069.3997	535.2035	1068.4156	534.7115	**I**	**1282.6348**	641.8210	1265.6082	633.3077	1264.6242	632.8157	**11**
**10**	1157.4633	579.2353	1140.4368	570.7220	1139.4528	570.2300	**A**	**1169.5507**	585.2790	1152.5242	576.7657	1151.5401	576.2737	**10**
**11**	1271.5062	636.2568	1254.4797	627.7435	1253.4957	627.2515	**N**	**1098.5136**	549.7604	1081.4870	541.2472	1080.5030	540.7552	**9**
**12**	**1384.5903**	692.7988	1367.5638	684.2855	1366.5797	683.7935	**L**	**984.4707**	492.7390	967.4441	484.2257	966.4601	483.7337	**8**
**13**	1547.6536	774.3305	1530.6271	765.8172	1529.6431	765.3252	**Y**	**871.3866**	436.1969	854.3601	427.6837	853.3760	427.1917	**7**
**14**	**1662.6806**	831.8439	1645.6540	823.3307	1644.6700	822.8386	**D**	**708.3233**	354.6653	691.2967	346.1520	690.3127	345.6600	**6**
**15**	1791.7232	896.3652	**1774.6966**	887.8520	**1773.7126**	887.3599	**E**	**593.2963**	297.1518	576.2698	288.6385	575.2858	288.1465	**5**
**16**	1938.7586	969.8829	**1921.7320**	961.3697	**1920.7480**	960.8776	**M**	**464.2537**	232.6305	447.2272	224.1172			**4**
**17**	2037.8270	1019.4171	2020.8004	1010.9039	2019.8164	1010.4119	**V**	317.2183	159.1128	300.1918	150.5995			**3**
**18**	2108.8641	1054.9357	2091.8376	1046.4224	2090.8535	1045.9304	**A**	218.1499	109.5786	201.1234	101.0653			**2**
**19**							**K**	147.1128	74.0600	130.0863	65.5468			**1**

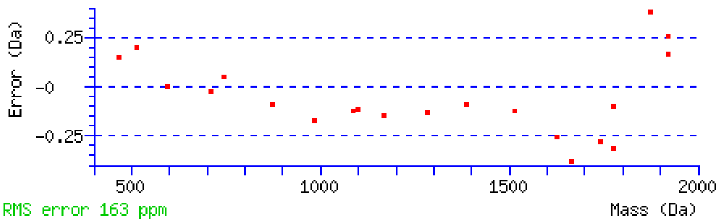

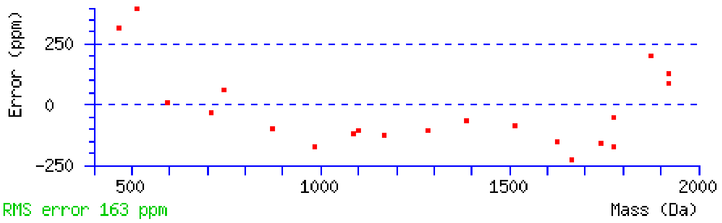

All matches to this query

Score	Mr(calc):	Delta	Sequence
98.5	2253.9623	0.0088	YGYENDTEIANLYDEMVAK
69.5	2253.9623	0.0088	YGYENDTEIANLYDEMVAK
52.6	2252.9783	0.9928	YGYENDTEIANLYDEMVAK
11.1	2251.9482	2.0229	GNQSDEGSDIDSEPDLPLKR
10.9	2251.9568	2.0143	SIPLVTNNSIEVPTFPQR
7.9	2251.9482	2.0229	GNQSDEGSDIDSEPDLPLKR
4.7	2251.9568	2.0143	SIPLVTNNSIEVPTFPQR
3.1	2252.9500	1.0211	GKFYSCNDLSKMTEEECR
3.0	2252.9787	0.9924	GCQFRGAGSGGLNLGNFFASR
1.6	2251.9511	2.0199	DQRILDYPSEVSGKNSQR

Spectrum No: 36; Query: 551; Rank: 1

Peptide View

MS/MS Fragmentation of **GVGIISEGNETVEDIAAR**
Found in **IPI00205693**, Tax_Id=10116 Gene_Symbol=Atp1a2 Sodium/potassium-transporting ATPase subunit alpha-2 precursor

Match to Query 551: 1829.903588 from(915.959070,2+)
Title: 091127RatKid_SCX01_18.2819.2819.2.dta
Data file K:\NewmanPaper\Piliang\3SubProteomes\Piliang3SP\mgf5ppm\SCX_3SubProteomes5ppm.mgf

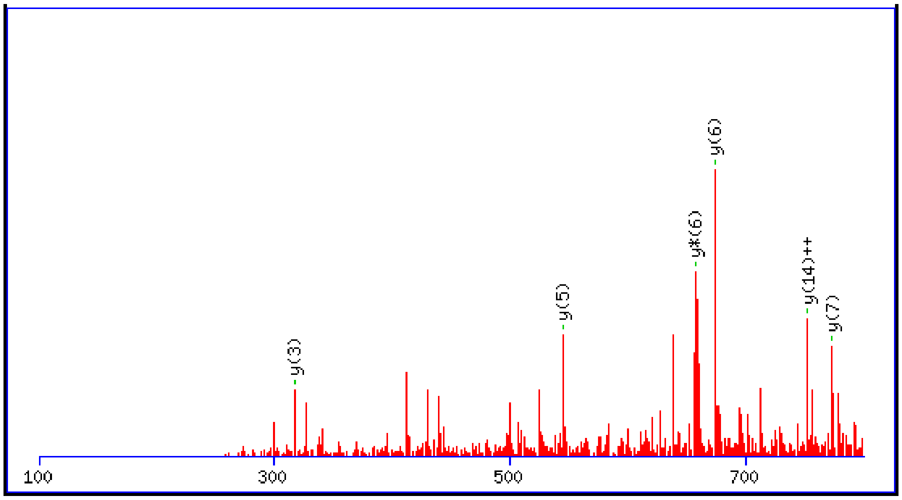

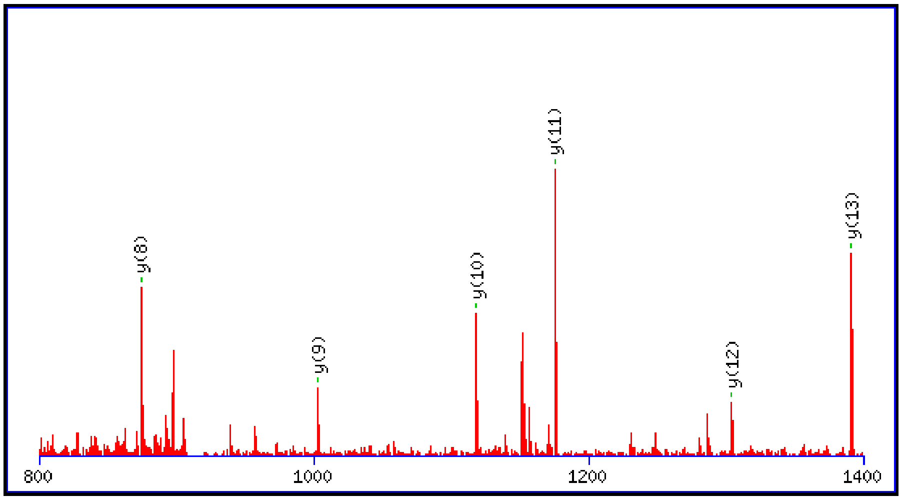

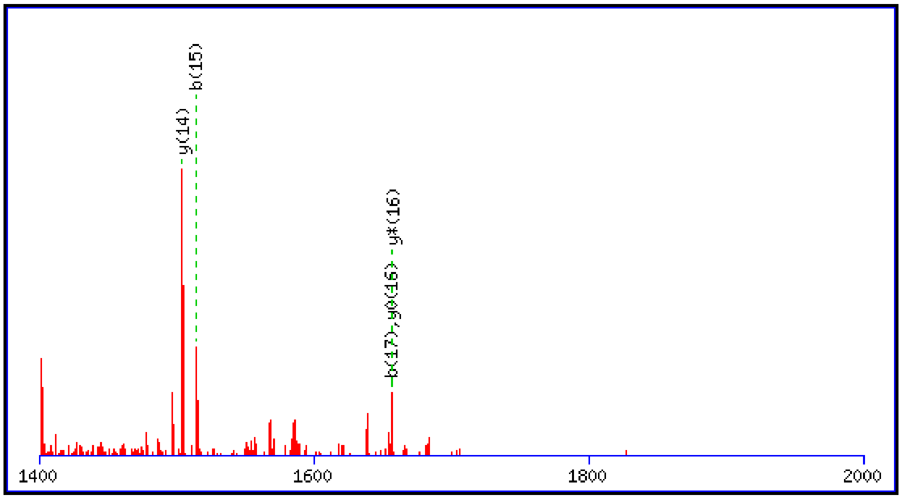

Monoisotopic mass of neutral peptide Mr(calc): 1829.9007
Fixed modifications: Carbamidomethyl (C)
Variable modifications:
N9 : Deamidated_N (N)
Ions Score: 98 **Expect:** 2.7e-008
Matches (Bold Red): 17/170 fragment ions using 26 most intense peaks

#	b	b^{++}	b*	b^{*++}	b^0	b^{0++}	Seq.	y	y^{++}	y*	y^{*++}	y^0	y^{0++}	#
1	58.0287	29.5180					G							18
2	157.0972	79.0522					V	1773.8865	887.4469	1756.8599	878.9336	1755.8759	878.4416	17
3	214.1186	107.5629					G	1674.8181	837.9127	**1657.7915**	829.3994	**1656.8075**	828.9074	16
4	327.2027	164.1050					I	1617.7966	809.4019	1600.7701	800.8887	1599.7861	800.3967	15
5	440.2867	220.6470					I	**1504.7126**	**752.8599**	1487.6860	744.3466	1486.7020	743.8546	14

**6**	527.3188	264.1630			509.3082	255.1577	**S**	**1391.6285**	696.3179	1374.6019	687.8046	1373.6179	687.3126	**13**
**7**	656.3614	328.6843			638.3508	319.6790	**E**	**1304.5965**	652.8019	1287.5699	644.2886	1286.5859	643.7966	**12**
**8**	713.3828	357.1951			695.3723	348.1898	**G**	**1175.5539**	588.2806	1158.5273	579.7673	1157.5433	579.2753	**11**
**9**	828.4098	414.7085	811.3832	406.1952	810.3992	405.7032	**N**	**1118.5324**	559.7698	1101.5059	551.2566	1100.5218	550.7646	**10**
**10**	957.4524	479.2298	940.4258	470.7165	939.4418	470.2245	**E**	**1003.5055**	502.2564	986.4789	493.7431	985.4949	493.2511	**9**
**11**	1058.5000	529.7537	1041.4735	521.2404	1040.4895	520.7484	**T**	**874.4629**	437.7351	857.4363	429.2218	856.4523	428.7298	**8**
**12**	1157.5685	579.2879	1140.5419	570.7746	1139.5579	570.2826	**V**	**773.4152**	387.2112	756.3886	378.6980	755.4046	378.2060	**7**
**13**	1286.6110	643.8092	1269.5845	635.2959	1268.6005	634.8039	**E**	**674.3468**	337.6770	**657.3202**	329.1638	656.3362	328.6717	**6**
**14**	1401.6380	701.3226	1384.6114	692.8094	1383.6274	692.3174	**D**	**545.3042**	273.1557	528.2776	264.6425	527.2936	264.1504	**5**
**15**	**1514.7221**	757.8647	1497.6955	749.3514	1496.7115	748.8594	**I**	430.2772	215.6423	413.2507	207.1290			**4**
**16**	1585.7592	793.3832	1568.7326	784.8699	1567.7486	784.3779	**A**	**317.1932**	159.1002	300.1666	150.5870			**3**
**17**	**1656.7963**	828.9018	1639.7697	820.3885	1638.7857	819.8965	**A**	246.1561	123.5817	229.1295	115.0684			**2**
**18**							**R**	175.1190	88.0631	158.0924	79.5498			**1**

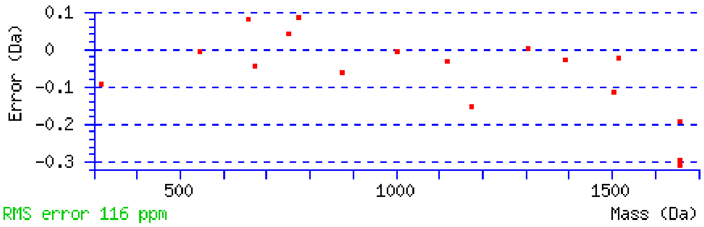

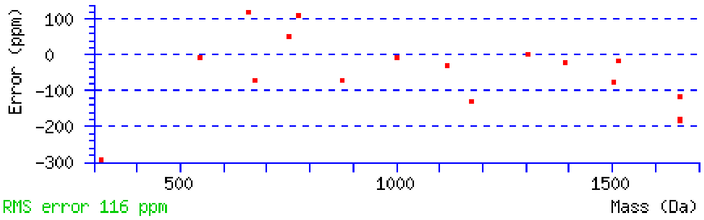

All matches to this query

Score	Mr(calc):	Delta	Sequence
98.4	1829.9007	0.0029	GVGIISEGNETVEDIAAR
24.6	1828.9167	0.9869	GVGIISEGNETVEDIAAR
5.1	1828.8877	1.0159	TKTITIESEGCGSTFAK
0.7	1828.9108	0.9928	DWRIFREDYSITTK

Spectrum No: 37; Query: 149; Rank: 1

Peptide View

MS/MS Fragmentation of **LNLSGGEVSATEK**
Found in **IPI00326462**, Tax_Id=10116 Gene_Symbol=Enpp3 Ectonucleotide pyrophosphatase/phosphodiesterase family member 3

Match to Query 149: 1304.645608 from(653.330080,2+)
Title: 091129RatKid_SCX02_12.1008.1008.2.dta
Data file K:\NewmanPaper\Piliang\3SubProteomes\Piliang3SP\mgf5ppm\SCX_3SubProteomes5ppm.mgf

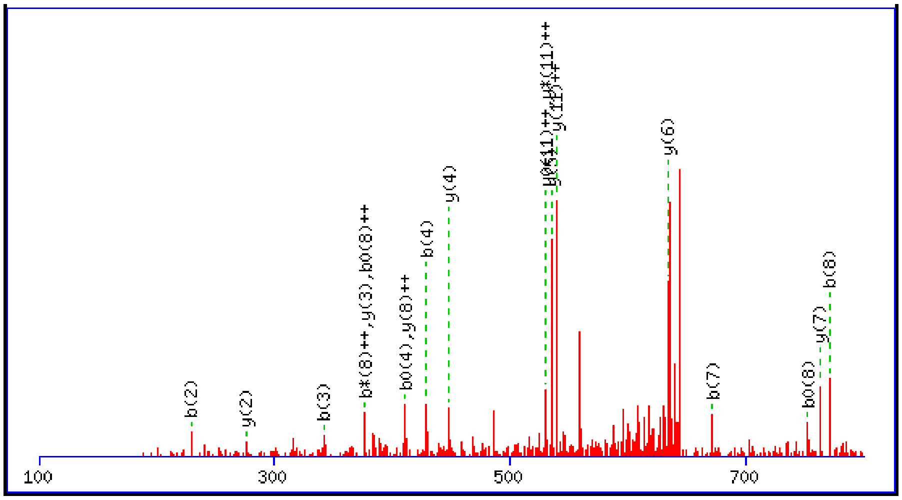

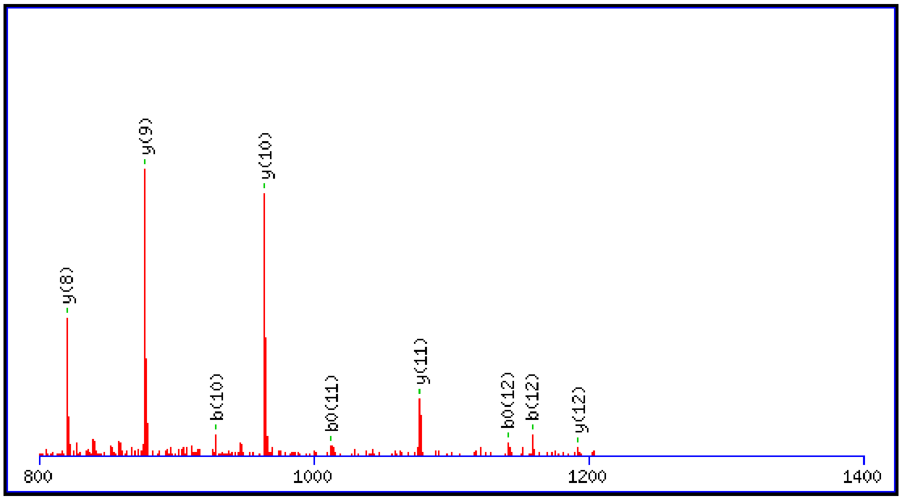

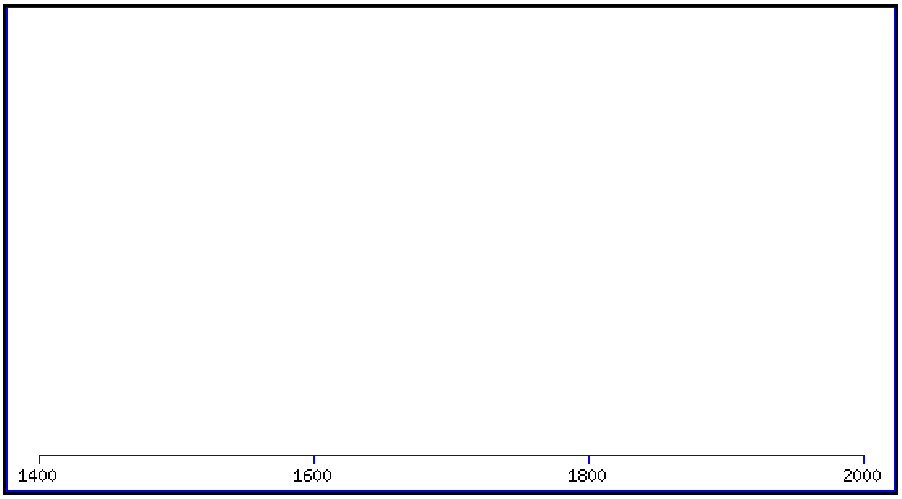

Monoisotopic mass of neutral peptide Mr(calc): 1304.6460
Fixed modifications: Carbamidomethyl (C)
Variable modifications:
N2 : Deamidated_N (N)
Ions Score: 98 **Expect:** 1.9e-008
Matches (Bold Red): 28/134 fragment ions using 39 most intense peaks

#	b	b^{++}	b*	b^{*++}	b^0	b^{0++}	Seq.	y	y^{++}	y*	y^{*++}	y^0	y^{0++}	#
1	114.0913	57.5493					L							13
2	**229.1183**	115.0628	212.0917	106.5495			N	**1192.5692**	596.7882	1175.5426	588.2750	1174.5586	587.7829	12
3	**342.2023**	171.6048	325.1758	163.0915			L	**1077.5422**	**539.2748**	1060.5157	**530.7615**	1059.5317	**530.2695**	11
4	**429.2344**	215.1208	412.2078	206.6075	**411.2238**	206.1155	S	**964.4582**	482.7327	947.4316	474.2195	946.4476	473.7274	10
5	486.2558	243.6316	469.2293	235.1183	468.2453	234.6263	G	**877.4262**	439.2167	860.3996	430.7034	859.4156	430.2114	9

**6**	543.2773	272.1423	526.2507	263.6290	525.2667	263.1370	**G**	**820.4047**	**410.7060**	803.3781	402.1927	802.3941	401.7007	**8**
**7**	**672.3199**	336.6636	655.2933	328.1503	654.3093	327.6583	**E**	**763.3832**	382.1953	746.3567	373.6820	745.3727	373.1900	**7**
**8**	**771.3883**	386.1978	754.3618	**377.6845**	**753.3777**	**377.1925**	**V**	**634.3406**	317.6740	617.3141	309.1607	616.3301	308.6687	**6**
**9**	858.4203	429.7138	841.3938	421.2005	840.4098	420.7085	**S**	**535.2722**	268.1397	518.2457	259.6265	517.2617	259.1345	**5**
**10**	**929.4574**	465.2324	912.4309	456.7191	911.4469	456.2271	**A**	**448.2402**	224.6237	431.2136	216.1105	430.2296	215.6185	**4**
**11**	1030.5051	515.7562	1013.4786	507.2429	**1012.4946**	506.7509	**T**	**377.2031**	189.1052	360.1765	180.5919	359.1925	180.0999	**3**
**12**	**1159.5477**	580.2775	1142.5212	571.7642	**1141.5372**	571.2722	**E**	**276.1554**	138.5813	259.1288	130.0681	258.1448	129.5761	**2**
**13**							**K**	147.1128	74.0600	130.0863	65.5468			**1**

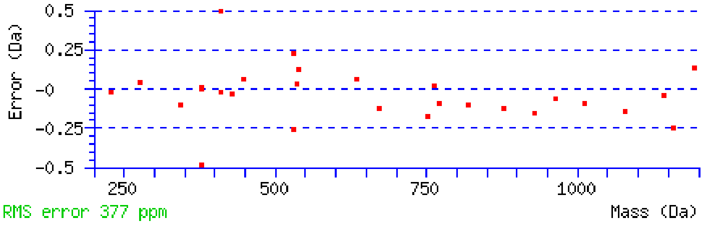

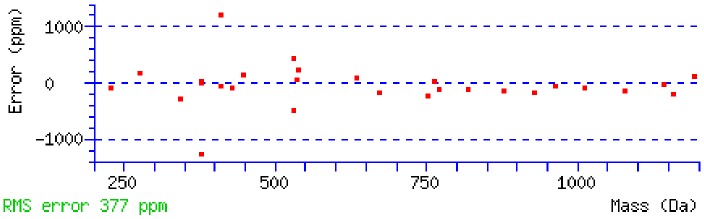

All matches to this query

Score	Mr(calc):	Delta	Sequence
98.1	1304.6460	-0.0003	LNLSGGEVSATEK
11.3	1304.6489	-0.0033	TVINNKAHLSK
10.6	1304.6394	0.0062	DLEALSKQCNK
10.5	1304.6572	-0.0116	VEEAKSSLATNR
6.8	1304.6460	-0.0003	LSVELEASDSQK
3.7	1304.6401	0.0055	LPYHLFGNNTK
3.4	1304.6507	-0.0050	LDDRARMEATK
2.3	1304.6433	0.0023	RASDRGAGETSAK
2.0	1304.6489	-0.0033	TVINNKAHLSK
1.4	1304.6394	0.0062	NIRAEGLQMEK

Spectrum No: 38; Query: 707; Rank: 1

Peptide View

MS/MS Fragmentation of **AEYAVANDTGFVDIPQQEK**
Found in **IPI00326070**, Tax_Id=10116 Gene_Symbol=Ctsl Cathepsin L1 precursor

Match to Query 707: 2094.979148 from(1048.496850,2+)
Title: 091127RatKid_SCX01_15.2555.2555.2.dta
Data file K:\NewmanPaper\Piliang\3SubProteomes\Piliang3SP\mgf5ppm\SCX_3SubProteomes5ppm.mgf

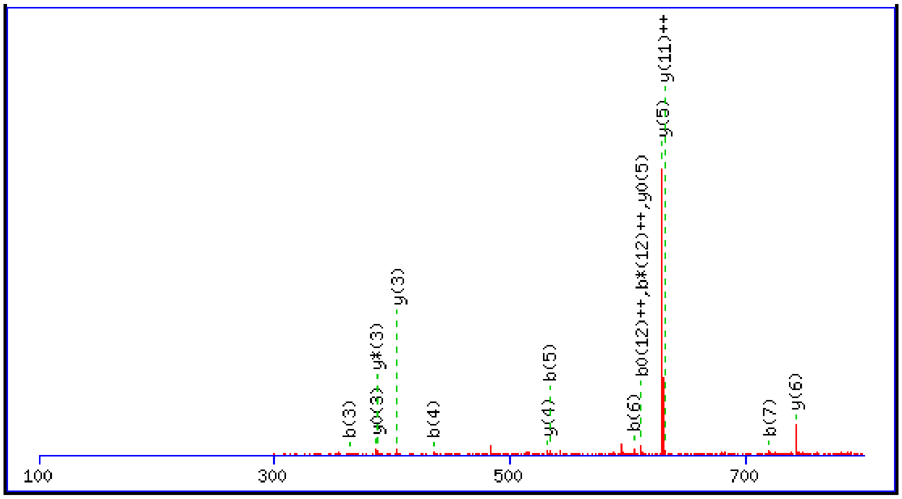

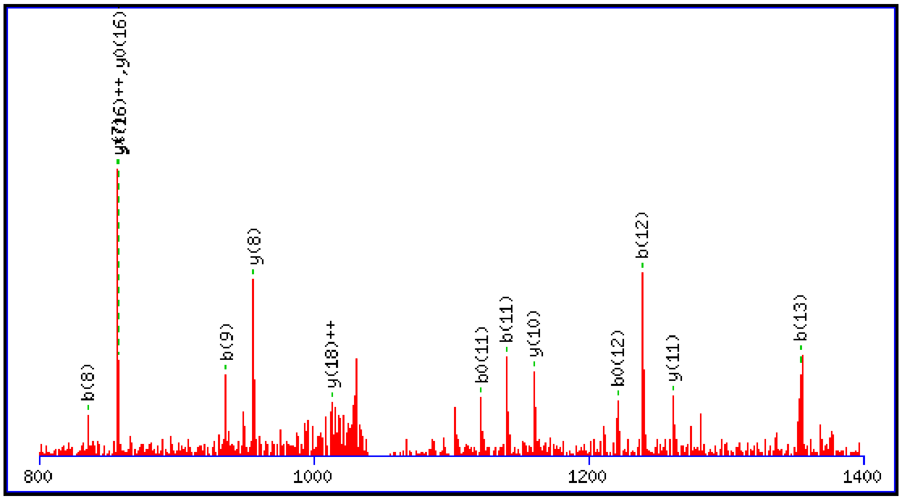

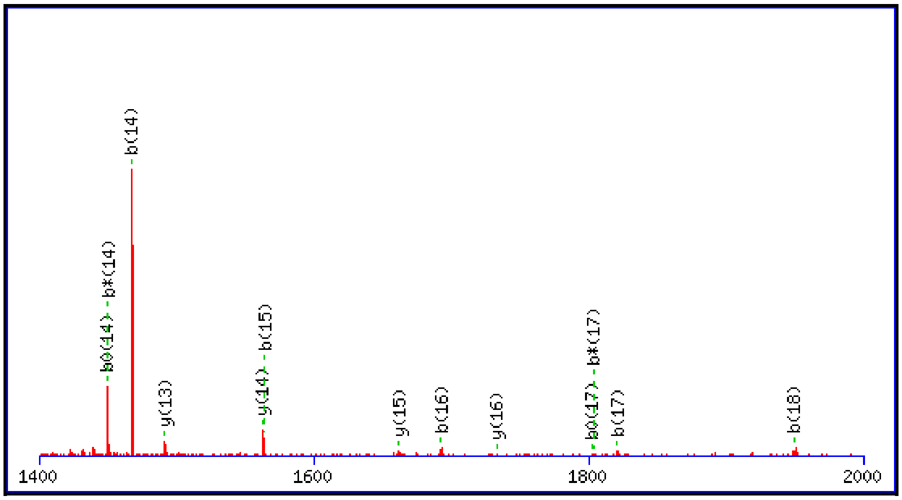

Monoisotopic mass of neutral peptide Mr(calc): 2094.9746
Fixed modifications: Carbamidomethyl (C)
Variable modifications:
N7 : Deamidated_N (N)
Ions Score: 98 **Expect:** 3.8e-008
Matches (Bold Red): 42/200 fragment ions using 77 most intense peaks

#	b	b++	b*	b*++	b0	b0++	Seq.	y	y++	y*	y*++	y0	y0++	#
1	72.0444	36.5258					A							19
2	201.0870	101.0471			183.0764	92.0418	E	2024.9447	**1012.9760**	2007.9182	1004.4627	2006.9342	1003.9707	18
3	**364.1503**	182.5788			346.1397	173.5735	Y	1895.9021	948.4547	1878.8756	939.9414	1877.8916	939.4494	17
4	**435.1874**	218.0974			417.1769	209.0921	A	**1732.8388**	866.9230	1715.8123	**858.4098**	1714.8283	**857.9178**	16
5	**534.2558**	267.6316			516.2453	258.6263	V	**1661.8017**	831.4045	1644.7752	822.8912	1643.7911	822.3992	15

6	605.2930	303.1501			587.2824	294.1448	A	1562.7333	781.8703	1545.7067	773.3570	1544.7227	772.8650	14
7	720.3199	360.6636	703.2933	352.1503	702.3093	351.6583	N	1491.6962	746.3517	1474.6696	737.8385	1473.6856	737.3464	13
8	835.3468	418.1771	818.3203	409.6638	817.3363	409.1718	D	1376.6692	688.8383	1359.6427	680.3250	1358.6587	679.8330	12
9	936.3945	468.7009	919.3680	460.1876	918.3839	459.6956	T	1261.6423	631.3248	1244.6157	622.8115	1243.6317	622.3195	11
10	993.4160	497.2116	976.3894	488.6984	975.4054	488.2063	G	1160.5946	580.8009	1143.5681	572.2877	1142.5841	571.7957	10
11	1140.4844	570.7458	1123.4578	562.2326	1122.4738	561.7406	F	1103.5732	552.2902	1086.5466	543.7769	1085.5626	543.2849	9
12	1239.5528	620.2800	1222.5263	611.7668	1221.5422	611.2748	V	956.5047	478.7560	939.4782	470.2427	938.4942	469.7507	8
13	1354.5797	677.7935	1337.5532	669.2802	1336.5692	668.7882	D	857.4363	429.2218	840.4098	420.7085	839.4258	420.2165	7
14	1467.6638	734.3355	1450.6373	725.8223	1449.6532	725.3303	I	742.4094	371.7083	725.3828	363.1951	724.3988	362.7030	6
15	1564.7166	782.8619	1547.6900	774.3487	1546.7060	773.8566	P	629.3253	315.1663	612.2988	306.6530	611.3148	306.1610	5
16	1692.7752	846.8912	1675.7486	838.3779	1674.7646	837.8859	Q	532.2726	266.6399	515.2460	258.1266	514.2620	257.6346	4
17	1820.8337	910.9205	1803.8072	902.4072	1802.8232	901.9152	Q	404.2140	202.6106	387.1874	194.0974	386.2034	193.6053	3
18	1949.8763	975.4418	1932.8498	966.9285	1931.8658	966.4365	E	276.1554	138.5813	259.1288	130.0681	258.1448	129.5761	2
19							K	147.1128	74.0600	130.0863	65.5468			1

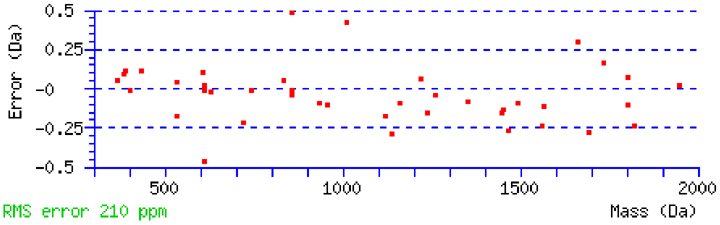

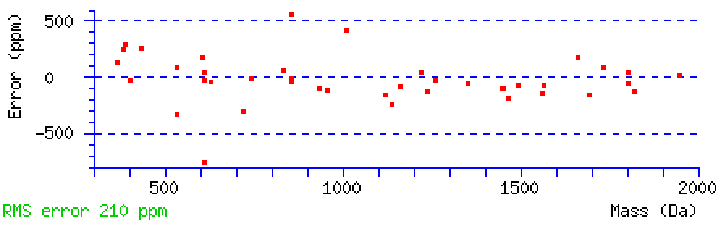

All matches to this query

Score	Mr(calc):	Delta	Sequence
98.1	2094.9746	0.0046	AEYAVANDTGFVDIPQQEK
37.1	2093.9906	0.9886	AEYAVANDTGFVDIPQQEK
13.9	2094.9834	-0.0042	KEIEELQSQAQALSQEGK
13.4	2094.9760	0.0031	FKGPMTVLRLNNLMASK
9.8	2094.9832	-0.0040	IITGEFYRIYYLKEK
7.4	2094.9926	-0.0134	MSAVMSISSVTENGGSPQGIK
6.9	2094.9583	0.0209	EEAGVSRELPGTEERTQK
5.4	2094.9921	-0.0130	RKILQAFPDMHNSNISK
4.6	2094.9663	0.0128	SFPSDKQNTPWPLLQEK
3.6	2094.9850	-0.0058	SYITPGMFFILTPVSGNR

Spectrum No: 39; Query: 1180; Rank: 1

Peptide View

MS/MS Fragmentation of **ALAGLVYNSSGMEPCFDIYQMYQSCADPTGCGTGSNAR**
Found in **IPI00230946**, Tax_Id=10116 Gene_Symbol=Dpp7 Dipeptidyl-peptidase 2 precursor

Match to Query 1180: 4148.752242 from(1383.924690,3+)
Title: 091129RatKid_SCX02_13.4449.4449.3.dta
Data file K:\NewmanPaper\Piliang\3SubProteomes\Piliang3SP\mgf5ppm\SCX_3SubProteomes5ppm.mgf

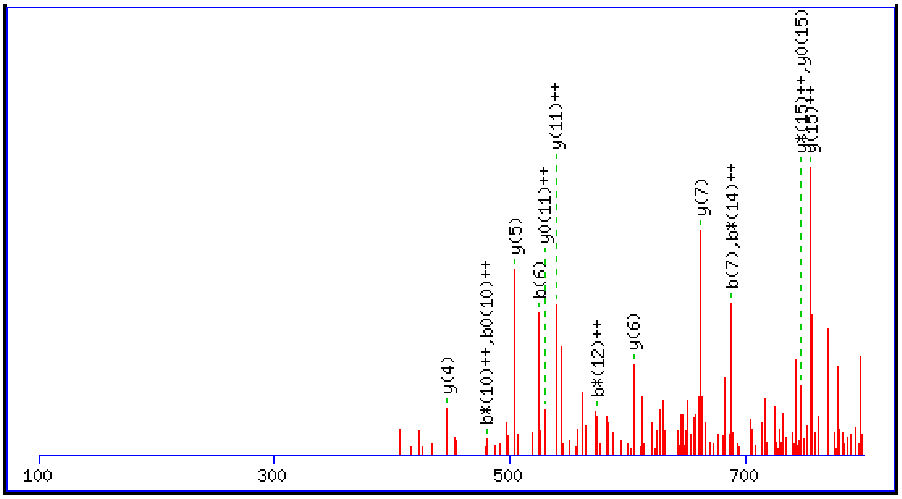

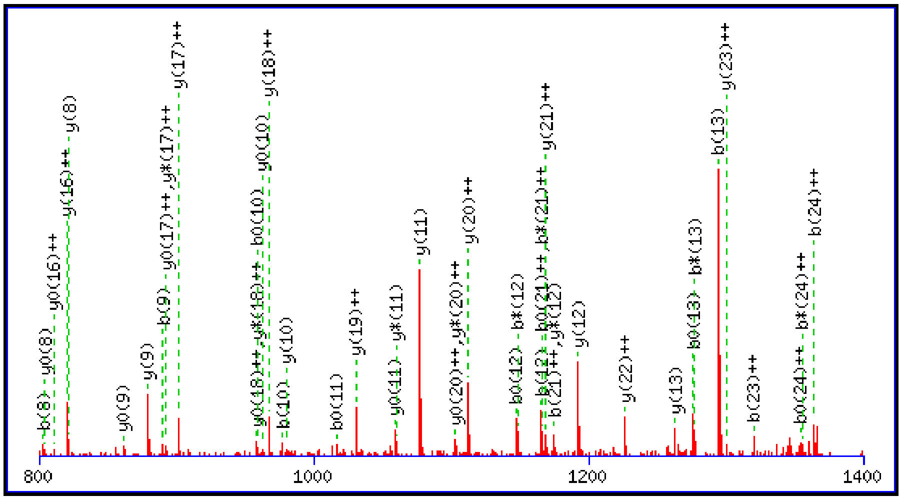

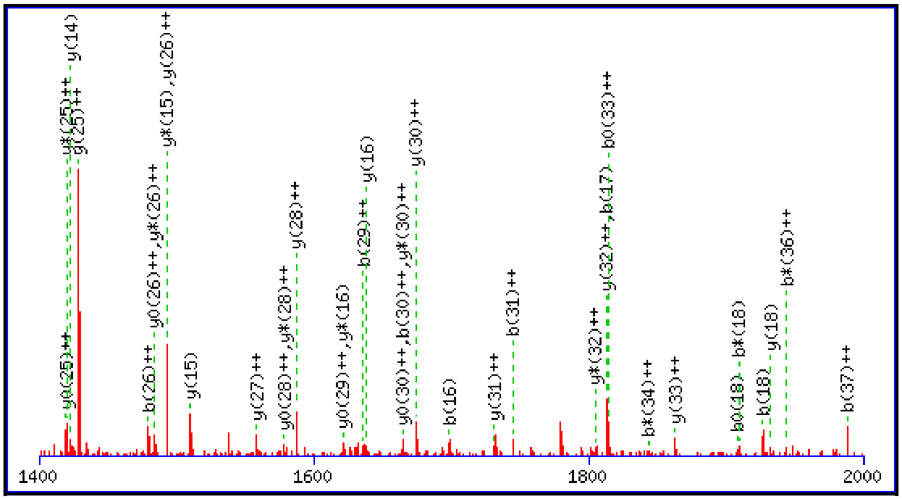

Monoisotopic mass of neutral peptide Mr(calc): 4148.7319
Fixed modifications: Carbamidomethyl (C)
Variable modifications:
N8 : Deamidated_N (N)
Ions Score: 98 **Expect:** 8e-008
Matches (Bold Red): 97/408 fragment ions using 160 most intense peaks

#	b	b^{++}	b*	b*$^{++}$	b^{0}	b^{0++}	Seq.	y	y^{++}	y*	y*$^{++}$	y^{0}	y^{0++}	#
1	72.0444	36.5258					A							38
2	185.1285	93.0679					L	4078.7020	2039.8547	4061.6755	2031.3414	4060.6915	2030.8494	37
3	256.1656	128.5864					A	3965.6180	1983.3126	3948.5914	1974.7993	3947.6074	1974.3073	36
4	313.1870	157.0972					G	3894.5809	1947.7941	3877.5543	1939.2808	3876.5703	1938.7888	35
5	426.2711	213.6392					L	3837.5594	1919.2833	3820.5328	1910.7701	3819.5488	1910.2781	34

6	525.3395	263.1734					V	3724.4753	1862.7413	3707.4488	1854.2280	3706.4648	1853.7360	33
7	688.4028	344.7051					Y	3625.4069	1813.2071	3608.3804	1804.6938	3607.3963	1804.2018	32
8	803.4298	402.2185	786.4032	393.7053			N	3462.3436	1731.6754	3445.3170	1723.1622	3444.3330	1722.6701	31
9	890.4618	445.7345	873.4353	437.2213	872.4512	436.7293	S	3347.3166	1674.1620	3330.2901	1665.6487	3329.3061	1665.1567	30
10	977.4938	489.2506	960.4673	480.7373	959.4833	480.2453	S	3260.2846	1630.6459	3243.2581	1622.1327	3242.2741	1621.6407	29
11	1034.5153	517.7613	1017.4887	509.2480	1016.5047	508.7560	G	3173.2526	1587.1299	3156.2260	1578.6167	3155.2420	1578.1247	28
12	1165.5558	583.2815	1148.5292	574.7683	1147.5452	574.2762	M	3116.2311	1558.6192	3099.2046	1550.1059	3098.2206	1549.6139	27
13	1294.5984	647.8028	1277.5718	639.2896	1276.5878	638.7975	E	2985.1906	1493.0990	2968.1641	1484.5857	2967.1801	1484.0937	26
14	1391.6511	696.3292	1374.6246	687.8159	1373.6406	687.3239	P	2856.1481	1428.5777	2839.1215	1420.0644	2838.1375	1419.5724	25
15	1551.6818	776.3445	1534.6552	767.8313	1533.6712	767.3392	C	2759.0953	1380.0513	2742.0687	1371.5380	2741.0847	1371.0460	24
16	1698.7502	849.8787	1681.7237	841.3655	1680.7396	840.8735	F	2599.0646	1300.0360	2582.0381	1291.5227	2581.0541	1291.0307	23
17	1813.7771	907.3922	1796.7506	898.8789	1795.7666	898.3869	D	2451.9962	1226.5017	2434.9697	1217.9885	2433.9857	1217.4965	22
18	1926.8612	963.9342	1909.8347	955.4210	1908.8506	954.9290	I	2336.9693	1168.9883	2319.9427	1160.4750	2318.9587	1159.9830	21
19	2089.9245	1045.4659	2072.8980	1036.9526	2071.9140	1036.4606	Y	2223.8852	1112.4462	2206.8587	1103.9330	2205.8747	1103.4410	20
20	2217.9831	1109.4952	2200.9566	1100.9819	2199.9725	1100.4899	Q	2060.8219	1030.9146	2043.7953	1022.4013	2042.8113	1021.9093	19
21	2349.0236	1175.0154	2331.9970	1166.5022	2331.0130	1166.0102	M	1932.7633	966.8853	1915.7368	958.3720	1914.7527	957.8800	18
22	2512.0869	1256.5471	2495.0604	1248.0338	2494.0764	1247.5418	Y	1801.7228	901.3651	1784.6963	892.8518	1783.7123	892.3598	17
23	2640.1455	1320.5764	2623.1190	1312.0631	2622.1349	1311.5711	Q	1638.6595	819.8334	1621.6329	811.3201	1620.6489	810.8281	16
24	2727.1775	1364.0924	2710.1510	1355.5791	2709.1670	1355.0871	S	1510.6009	755.8041	1493.5744	747.2908	1492.5904	746.7988	15
25	2887.2082	1444.1077	2870.1816	1435.5945	2869.1976	1435.1024	C	1423.5689	712.2881	1406.5423	703.7748	1405.5583	703.2828	14
26	2958.2453	1479.6263	2941.2187	1471.1130	2940.2347	1470.6210	A	1263.5382	632.2728	1246.5117	623.7595	1245.5277	623.2675	13
27	3073.2722	1537.1398	3056.2457	1528.6265	3055.2617	1528.1345	D	1192.5011	596.7542	1175.4746	588.2409	1174.4906	587.7489	12
28	3170.3250	1585.6661	3153.2985	1577.1529	3152.3144	1576.6609	P	1077.4742	539.2407	1060.4476	530.7275	1059.4636	530.2354	11
29	3271.3727	1636.1900	3254.3461	1627.6767	3253.3621	1627.1847	T	980.4214	490.7143	963.3949	482.2011	962.4109	481.7091	10
30	3328.3941	1664.7007	3311.3676	1656.1874	3310.3836	1655.6954	G	879.3737	440.1905	862.3472	431.6772	861.3632	431.1852	9
31	3488.4248	1744.7160	3471.3982	1736.2028	3470.4142	1735.7108	C	822.3523	411.6798	805.3257	403.1665	804.3417	402.6745	8
32	3545.4463	1773.2268	3528.4197	1764.7135	3527.4357	1764.2215	G	662.3216	331.6645	645.2951	323.1512	644.3111	322.6592	7
33	3646.4939	1823.7506	3629.4674	1815.2373	3628.4834	1814.7453	T	605.3002	303.1537	588.2736	294.6404	587.2896	294.1484	6
34	3703.5154	1852.2613	3686.4889	1843.7481	3685.5048	1843.2561	G	504.2525	252.6299	487.2259	244.1166	486.2419	243.6246	5
35	3790.5474	1895.7774	3773.5209	1887.2641	3772.5369	1886.7721	S	447.2310	224.1191	430.2045	215.6059	429.2205	215.1139	4
36	3904.5904	1952.7988	3887.5638	1944.2855	3886.5798	1943.7935	N	360.1990	180.6031	343.1724	172.0899			3
37	3975.6275	1988.3174	3958.6009	1979.8041	3957.6169	1979.3121	A	246.1561	123.5817	229.1295	115.0684			2
38							R	175.1190	88.0631	158.0924	79.5498			1

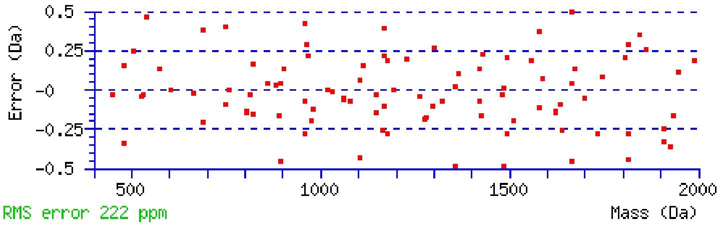

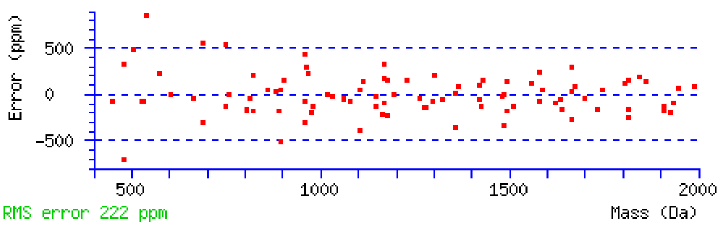

All matches to this query

Score	Mr(calc):	Delta	Sequence
97.9	4148.7319	0.0204	ALAGLVYNSSGMEPCFDIYQMYQSCADPTGCGTGSNAR
60.6	4147.7479	1.0044	ALAGLVYNSSGMEPCFDIYQMYQSCADPTGCGTGSNAR
44.5	4148.7319	0.0204	ALAGLVYNSSGMEPCFDIYQMYQSCADPTGCGTGSNAR
18.4	4147.7078	1.0444	TNQSHESMAEEQHMATASENVPQQPPDSPPHKKFR
10.4	4146.7502	2.0020	EPPPYQEPRPRGTPTHSAPCVPNGSALLLSNPAYR
10.4	4146.7502	2.0020	EPPPYQEPRPRGTPTHSAPCVPNGSALLLSNPAYR
8.1	4146.7238	2.0285	TNQSHESMAEEQHMATASENVPQQPPDSPPHKKFR

7.1	4146.7238	2.0285	TNQSHESMAEEQHMATASENVPQQPPDSPPHKKFR
6.5	4148.7930	-0.0408	ETKTVIPSFQHTAMSNLGLNPPNLATQMISSLNLK
5.7	4148.7654	-0.0131	MAMRTESALGSKNNTIHFVTEFILLGFSIQGEMR

Spectrum No: 40; Query: 301; Rank: 1

Peptide View

MS/MS Fragmentation of **VFGANQSLSAAQLGR**
Found in **IPI00392474**, Tax_Id=10116 Gene_Symbol=Slc39a8 Zinc transporter ZIP8

Match to Query 301: 1518.779368 from(760.396960,2+)
Title: 091127RatKid_SCX01_12.2229.2229.2.dta
Data file K:\NewmanPaper\Piliang\3SubProteomes\Piliang3SP\mgf5ppm\SCX_3SubProteomes5ppm.mgf

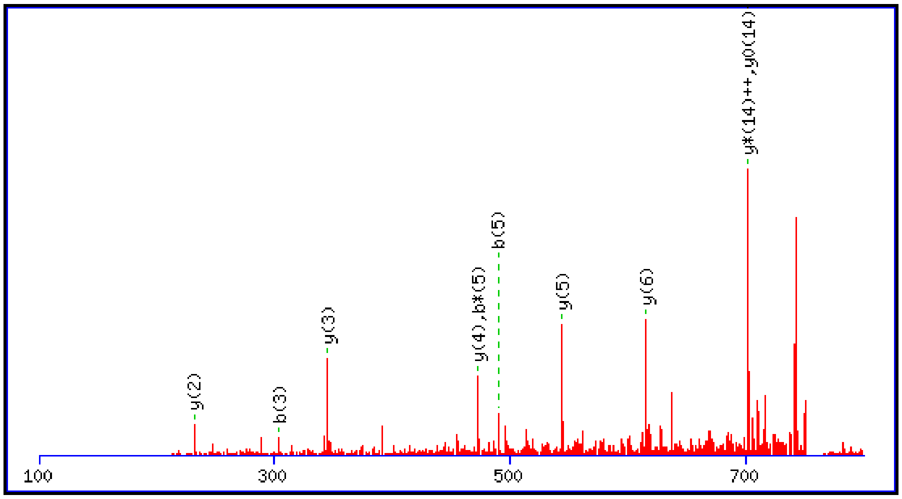

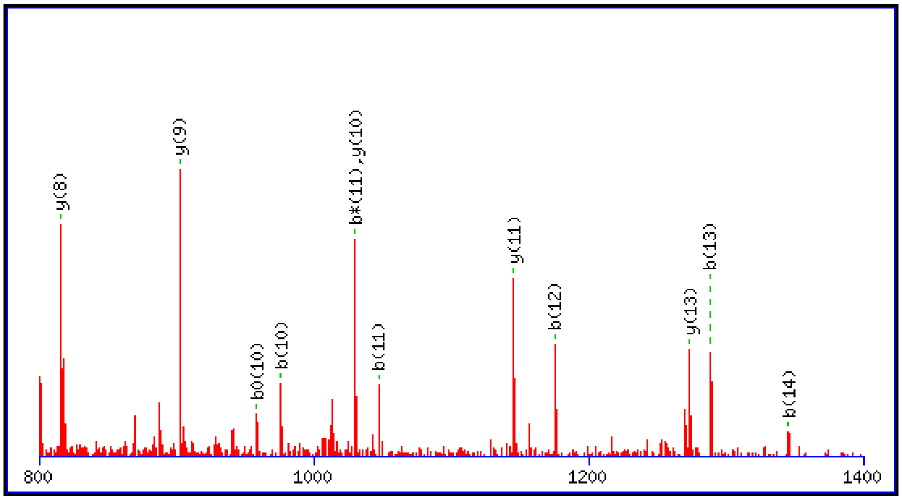

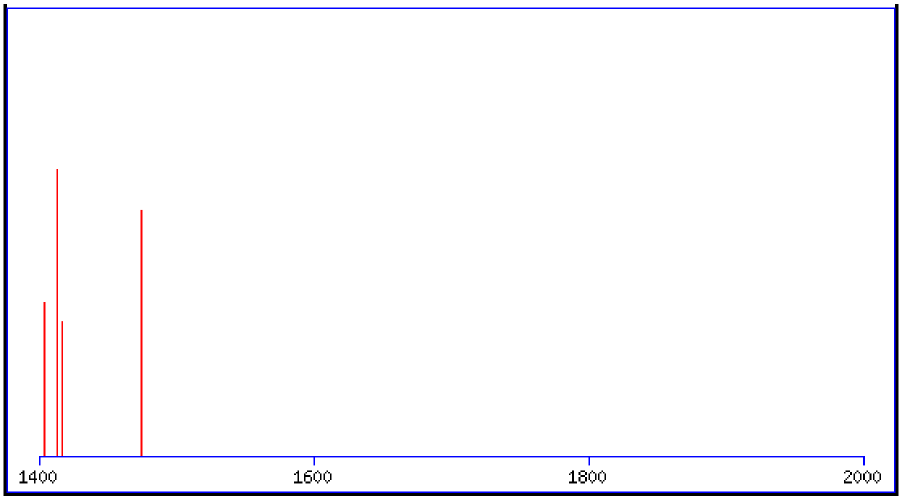

Monoisotopic mass of neutral peptide Mr(calc): 1518.7790
Fixed modifications: Carbamidomethyl (C)
Variable modifications:
N5 : Deamidated_N (N)
Ions Score: 98 **Expect:** 2.3e-008
Matches (Bold Red): 23/136 fragment ions using 36 most intense peaks

#	b	b++	b*	b*++	b0	b0++	Seq.	y	y++	y*	y*++	y0	y0++	#
1	100.0757	50.5415					V							15
2	247.1441	124.0757					F	1420.7179	710.8626	1403.6914	**702.3493**	1402.7073	**701.8573**	14
3	**304.1656**	152.5864					G	**1273.6495**	637.3284	1256.6229	628.8151	1255.6389	628.3231	13
4	375.2027	188.1050					A	1216.6280	608.8177	1199.6015	600.3044	1198.6175	599.8124	12
5	**490.2296**	245.6184	**473.2031**	237.1052			N	**1145.5909**	573.2991	1128.5644	564.7858	1127.5804	564.2938	11
6	618.2882	309.6477	601.2616	301.1345			Q	**1030.5640**	515.7856	1013.5374	507.2724	1012.5534	506.7803	10
7	705.3202	353.1637	688.2937	344.6505	687.3097	344.1585	S	**902.5054**	451.7563	885.4789	443.2431	884.4948	442.7511	9
8	818.4043	409.7058	801.3777	401.1925	800.3937	400.7005	L	**815.4734**	408.2403	798.4468	399.7271	797.4628	399.2350	8
9	905.4363	453.2218	888.4098	444.7085	887.4258	444.2165	S	**702.3893**	351.6983	685.3628	343.1850	684.3787	342.6930	7
10	**976.4734**	488.7404	959.4469	480.2271	**958.4629**	479.7351	A	**615.3573**	308.1823	598.3307	299.6690			6
11	**1047.5105**	524.2589	**1030.4840**	515.7456	1029.5000	515.2536	A	**544.3202**	272.6637	527.2936	264.1504			5
12	**1175.5691**	588.2882	1158.5426	579.7749	1157.5586	579.2829	Q	**473.2831**	237.1452	456.2565	228.6319			4
13	**1288.6532**	644.8302	1271.6266	636.3170	1270.6426	635.8249	L	**345.2245**	173.1159	328.1979	164.6026			3
14	**1345.6746**	673.3410	1328.6481	664.8277	1327.6641	664.3357	G	**232.1404**	116.5738	215.1139	108.0606			2
15							R	175.1190	88.0631	158.0924	79.5498			1

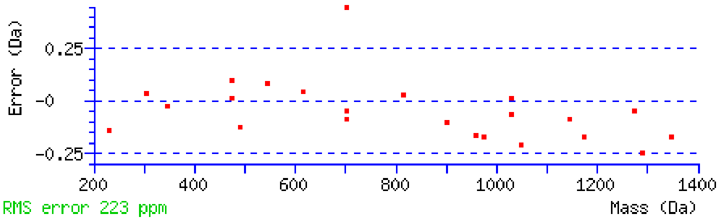

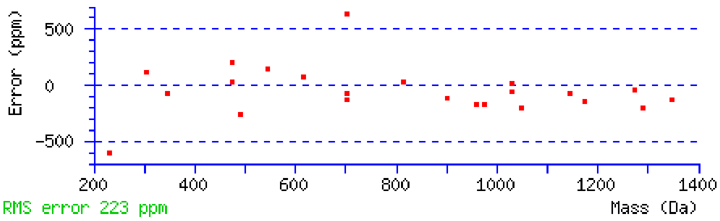

All matches to this query

Score	Mr(calc):	Delta	Sequence
97.9	1518.7790	0.0003	VFGANQSLSAAQLGR
6.7	1518.7748	0.0046	FASIWYAKKLGR
6.7	1518.7748	0.0046	FASIWYAKKLGR
5.8	1517.7912	0.9882	VMLTNVLWTDLGR
5.5	1518.7711	0.0082	NKTAGEEEMIKIR
4.2	1517.7732	1.0062	SKAGAGAASRLMNER

4.2	1518.7807	-0.0013	VSVPGKPTVEASLR
4.1	1518.7712	0.0082	ESLLLDMASLQQR
3.9	1517.7620	1.0174	LMKDGRSVANSPSR
3.9	1517.7759	1.0035	SQNVMAAASIANIVK

Spectrum No: 41; Query: 561; Rank: 1

Peptide View

MS/MS Fragmentation of **LENLSSTESGYTATLTR**
Found in **IPI00400579**, Tax_Id=10116 Gene_Symbol=Gaa Lysosomal alpha-glucosidase precursor

Match to Query 561: 1842.890108 from(922.452330,2+)
Title: 091127RatKid_SCX01_17.2318.2318.2.dta
Data file K:\NewmanPaper\Piliang\3SubProteomes\Piliang3SP\mgf5ppm\SCX_3SubProteomes5ppm.mgf

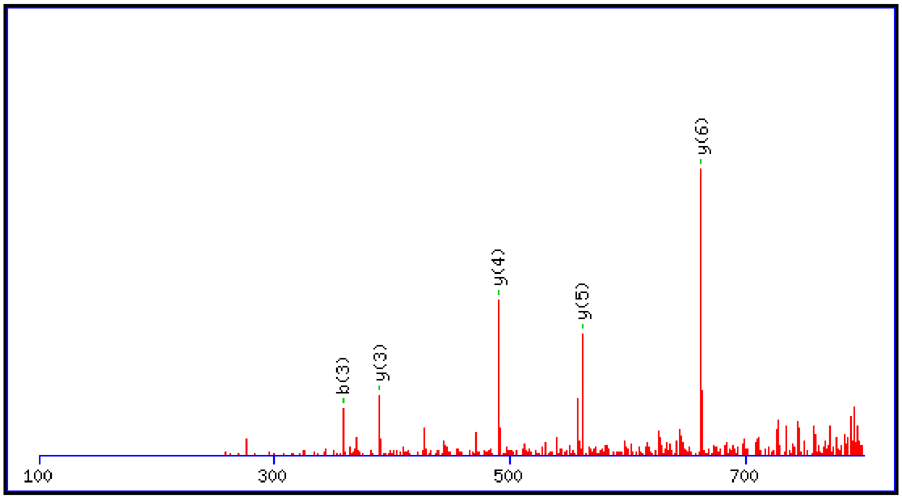

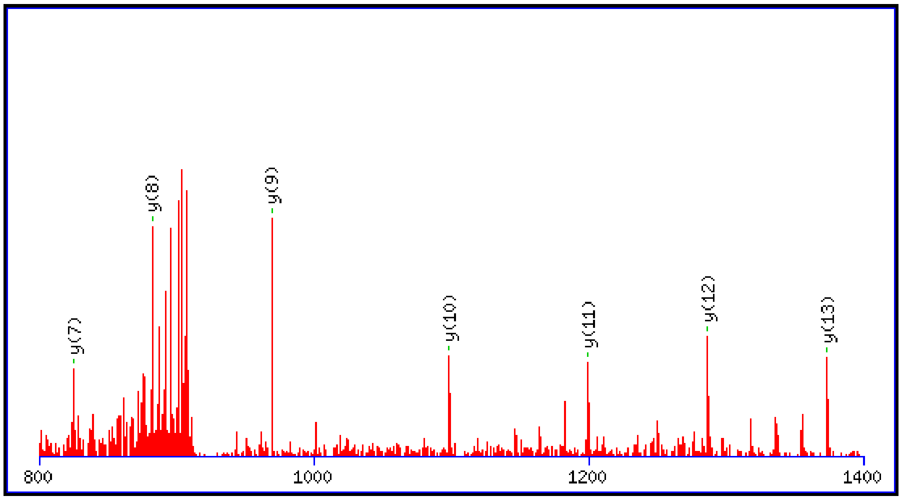

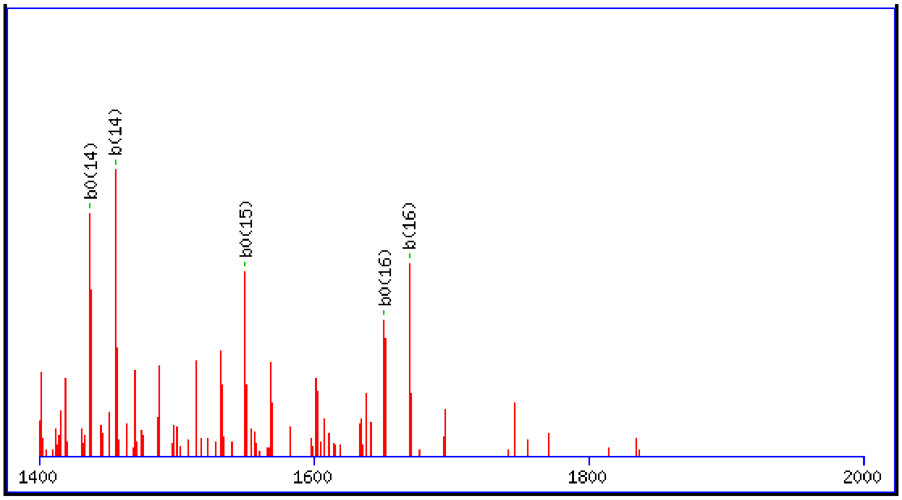

Monoisotopic mass of neutral peptide Mr(calc): 1842.8847
Fixed modifications: Carbamidomethyl (C)
Variable modifications:
N3 : Deamidated_N (N)
Ions Score: 97 **Expect:** 4e-008
Matches (Bold Red): 17/184 fragment ions using 30 most intense peaks

#	b	b^{++}	b*	b^{*++}	b^0	b^{0++}	Seq.	y	y^{++}	y*	y^{*++}	y^0	y^{0++}	#
1	114.0913	57.5493					L							17
2	243.1339	122.0706			225.1234	113.0653	E	1730.8079	865.9076	1713.7814	857.3943	1712.7973	856.9023	16
3	**358.1609**	179.5841	341.1343	171.0708	340.1503	170.5788	N	1601.7653	801.3863	1584.7388	792.8730	1583.7548	792.3810	15
4	471.2449	236.1261	454.2184	227.6128	453.2344	227.1208	L	1486.7384	743.8728	1469.7118	735.3596	1468.7278	734.8675	14
5	558.2770	279.6421	541.2504	271.1288	540.2664	270.6368	S	**1373.6543**	687.3308	1356.6278	678.8175	1355.6438	678.3255	13
6	645.3090	323.1581	628.2824	314.6449	627.2984	314.1529	S	**1286.6223**	643.8148	1269.5957	635.3015	1268.6117	634.8095	12
7	746.3567	373.6820	729.3301	365.1687	728.3461	364.6767	T	**1199.5903**	600.2988	1182.5637	591.7855	1181.5797	591.2935	11
8	875.3993	438.2033	858.3727	429.6900	857.3887	429.1980	E	**1098.5426**	549.7749	1081.5160	541.2617	1080.5320	540.7696	10
9	962.4313	481.7193	945.4047	473.2060	944.4207	472.7140	S	**969.5000**	485.2536	952.4734	476.7404	951.4894	476.2483	9
10	1019.4528	510.2300	1002.4262	501.7167	1001.4422	501.2247	G	**882.4680**	441.7376	865.4414	433.2243	864.4574	432.7323	8
11	1182.5161	591.7617	1165.4895	583.2484	1164.5055	582.7564	Y	**825.4465**	413.2269	808.4199	404.7136	807.4359	404.2216	7
12	1283.5638	642.2855	1266.5372	633.7722	1265.5532	633.2802	T	**662.3832**	331.6952	645.3566	323.1819	644.3726	322.6899	6
13	1354.6009	677.8041	1337.5743	669.2908	1336.5903	668.7988	A	**561.3355**	281.1714	544.3089	272.6581	543.3249	272.1661	5
14	**1455.6486**	728.3279	1438.6220	719.8146	**1437.6380**	719.3226	T	**490.2984**	245.6528	473.2718	237.1396	472.2878	236.6475	4
15	1568.7326	784.8699	1551.7061	776.3567	**1550.7221**	775.8647	L	**389.2507**	195.1290	372.2241	186.6157	371.2401	186.1237	3
16	**1669.7803**	835.3938	1652.7537	826.8805	**1651.7697**	826.3885	T	276.1666	138.5870	259.1401	130.0737	258.1561	129.5817	2
17							R	175.1190	88.0631	158.0924	79.5498			1

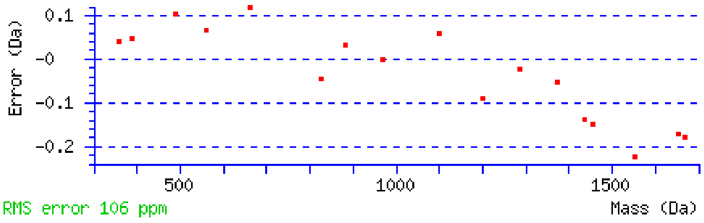

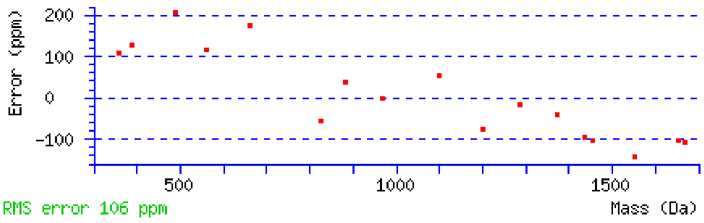

All matches to this query

Score	Mr(calc):	Delta	Sequence
97.0	1842.8847	0.0054	LENLSSTESGYTATLTR
73.7	1841.9007	0.9894	LENLSSTESGYTATLTR
4.7	1842.8877	0.0024	FKSDQNLQTALELTR
3.8	1842.8950	-0.0049	MKDYQLNALLPALTR

3.8	1841.8893	1.0009	LMPQAMKTDSSLLRR
1.3	1842.8911	-0.0010	QTKVTEISLVVSGCSR
0.6	1842.8765	0.0137	KFINQIVDTLDVSDR

Spectrum No: 42; Query: 481; Rank: 1

Peptide View

MS/MS Fragmentation of **NYTDDAIETDDLTIK**
Found in **IPI00215243**, Tax_Id=10116 Gene_Symbol=Psma7 Isoform RC6-IL of Proteasome subunit alpha type-7

Match to Query 481: 1726.779928 from(864.397240,2+)
Title: 091127RatKid_SCX01_13.3067.3067.2.dta
Data file K:\NewmanPaper\Piliang\3SubProteomes\Piliang3SP\mgf5ppm\SCX_3SubProteomes5ppm.mgf

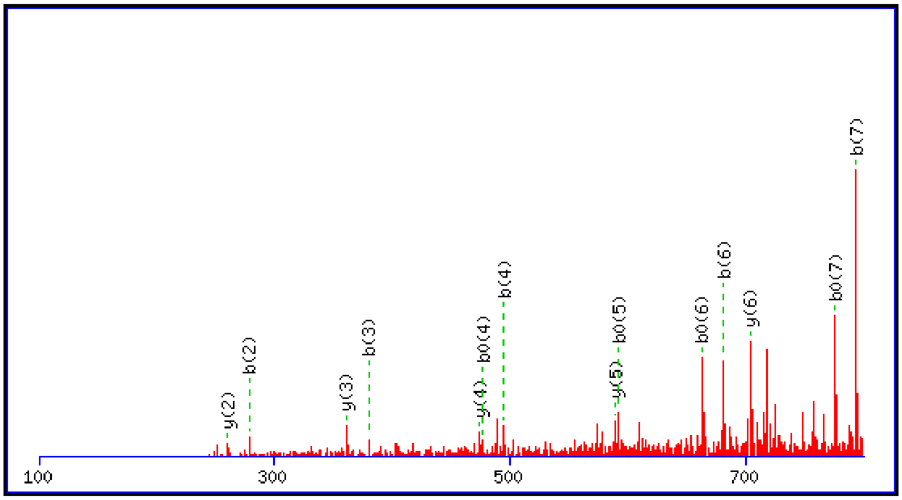

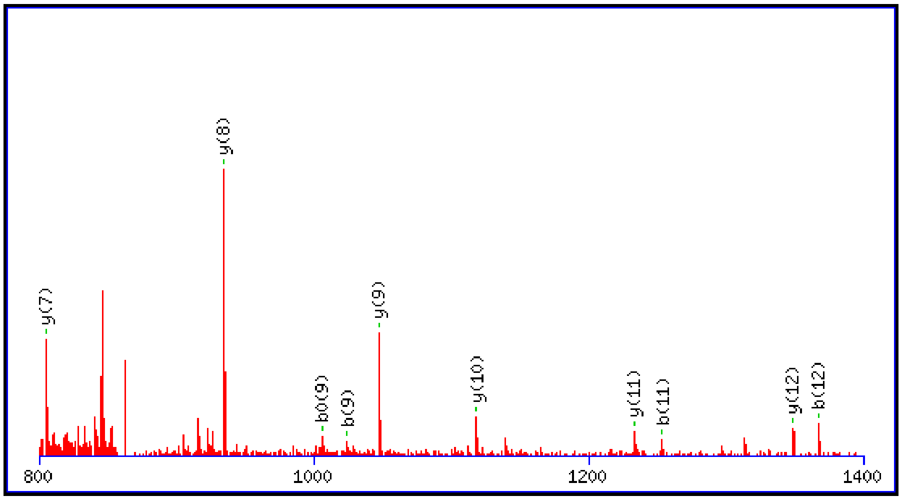

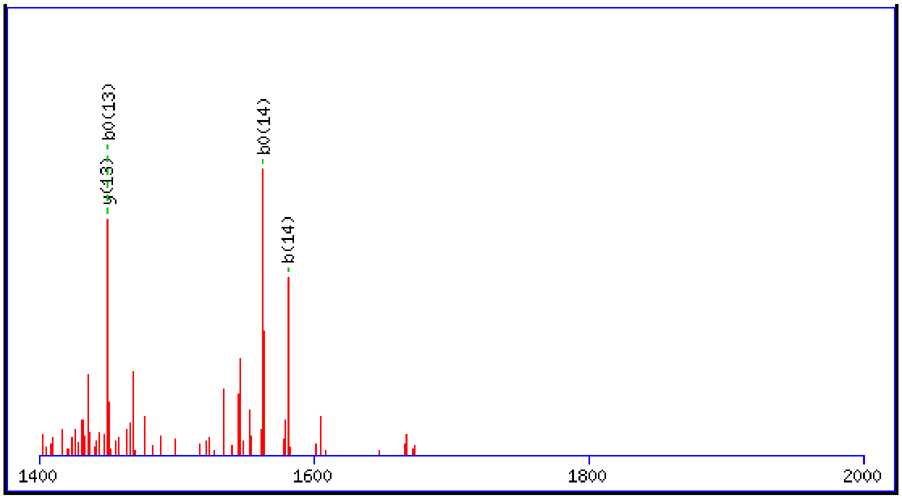

Monoisotopic mass of neutral peptide Mr(calc): 1726.7785
Fixed modifications: Carbamidomethyl (C)
Variable modifications:
N1 : Deamidated_N (N)
Ions Score: 97 **Expect:** 4.3e-008
Matches (Bold Red): 28/160 fragment ions using 41 most intense peaks

#	b	b^{++}	b*	b^{*++}	b^{0}	b^{0++}	Seq.	y	y^{++}	y*	y^{*++}	y^{0}	y^{0++}	#
1	116.0342	58.5207	99.0077	50.0075			N							15
2	**279.0975**	140.0524	262.0710	131.5391			Y	1612.7588	806.8831	1595.7323	798.3698	1594.7483	797.8778	14
3	**380.1452**	190.5762	363.1187	182.0630	362.1347	181.5710	T	**1449.6955**	725.3514	1432.6690	716.8381	1431.6849	716.3461	13
4	**495.1722**	248.0897	478.1456	239.5764	**477.1616**	239.0844	D	**1348.6478**	674.8276	1331.6213	666.3143	1330.6373	665.8223	12
5	610.1991	305.6032	593.1726	297.0899	**592.1885**	296.5979	D	**1233.6209**	617.3141	1216.5943	608.8008	1215.6103	608.3088	11
6	**681.2362**	341.1217	664.2097	332.6085	**663.2257**	332.1165	A	**1118.5939**	559.8006	1101.5674	551.2873	1100.5834	550.7953	10
7	**794.3203**	397.6638	777.2937	389.1505	**776.3097**	388.6585	I	**1047.5568**	524.2821	1030.5303	515.7688	1029.5463	515.2768	9
8	923.3629	462.1851	906.3363	453.6718	905.3523	453.1798	E	**934.4728**	467.7400	917.4462	459.2267	916.4622	458.7347	8
9	**1024.4106**	512.7089	1007.3840	504.1956	**1006.4000**	503.7036	T	**805.4302**	403.2187	788.4036	394.7055	787.4196	394.2134	7
10	1139.4375	570.2224	1122.4110	561.7091	1121.4269	561.2171	D	**704.3825**	352.6949	687.3559	344.1816	686.3719	343.6896	6
11	**1254.4644**	627.7359	1237.4379	619.2226	1236.4539	618.7306	D	**589.3556**	295.1814	572.3290	286.6681	571.3450	286.1761	5
12	**1367.5485**	684.2779	1350.5220	675.7646	1349.5379	675.2726	L	**474.3286**	237.6679	457.3021	229.1547	456.3180	228.6627	4
13	1468.5962	734.8017	1451.5696	726.2885	**1450.5856**	725.7964	T	**361.2445**	181.1259	344.2180	172.6126	343.2340	172.1206	3
14	**1581.6802**	791.3438	1564.6537	782.8305	**1563.6697**	782.3385	I	**260.1969**	130.6021	243.1703	122.0888			2
15							K	147.1128	74.0600	130.0863	65.5468			1

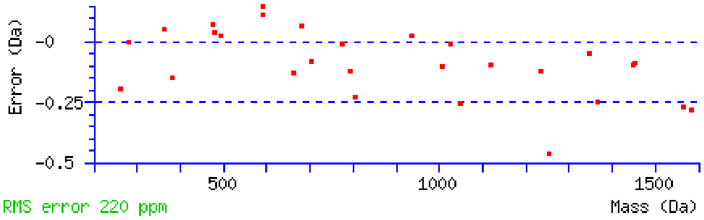

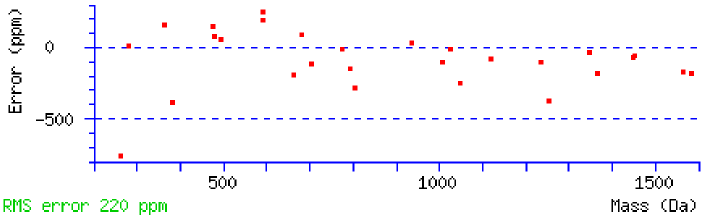

All matches to this query

Score	Mr(calc):	Delta	Sequence
96.9	1726.7785	0.0014	NYTDDAIETDDLTIK
9.2	1725.7767	1.0033	MEKAEESEANIFAEK
0.2	1725.7723	1.0076	AASTNHLDTFDLSVR

Spectrum No: 43; Query: 252; Rank: 1

Peptide View

MS/MS Fragmentation of **VEAIGSLANTTMEK**
Found in **IPI00193397**, Tax_Id=10116 Gene_Symbol=Slc34a1 Sodium-dependent phosphate transport protein 2A

Match to Query 252: 1463.720028 from(732.867290,2+)
Title: 091127RatKid_SCX01_02.2407.2407.2.dta
Data file K:\NewmanPaper\Piliang\3SubProteomes\Piliang3SP\mgf5ppm\SCX_3SubProteomes5ppm.mgf

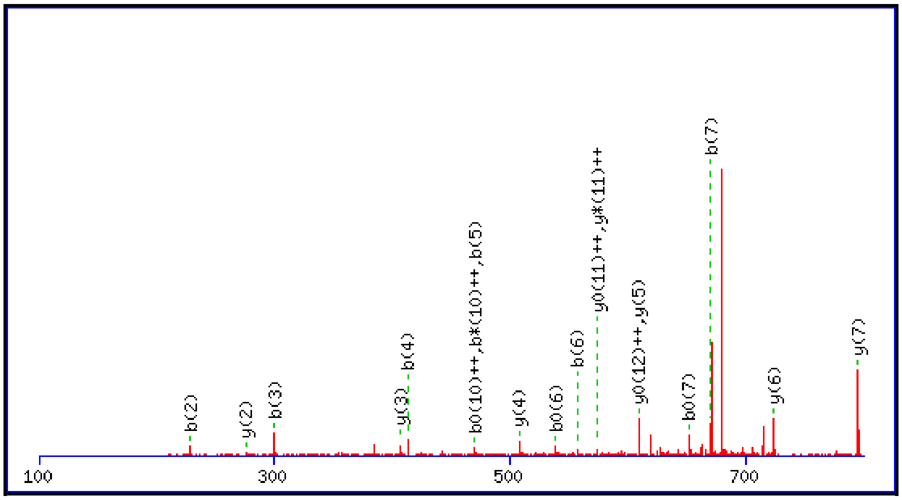

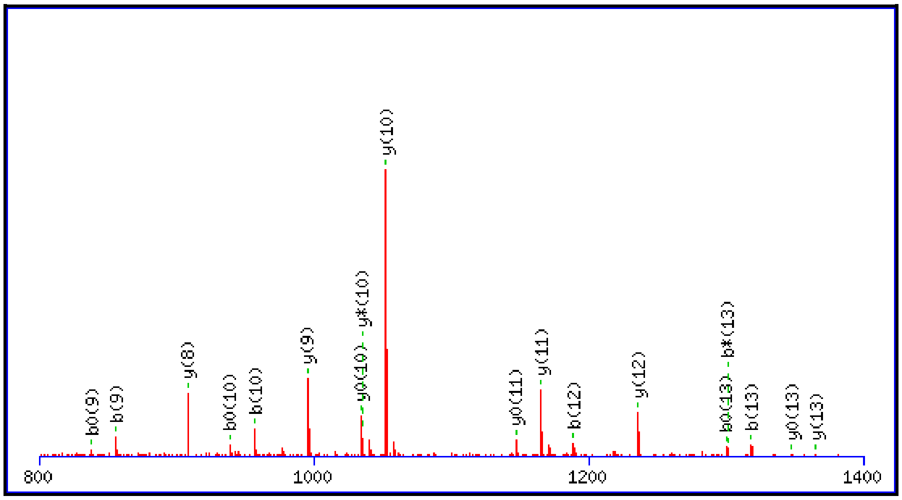

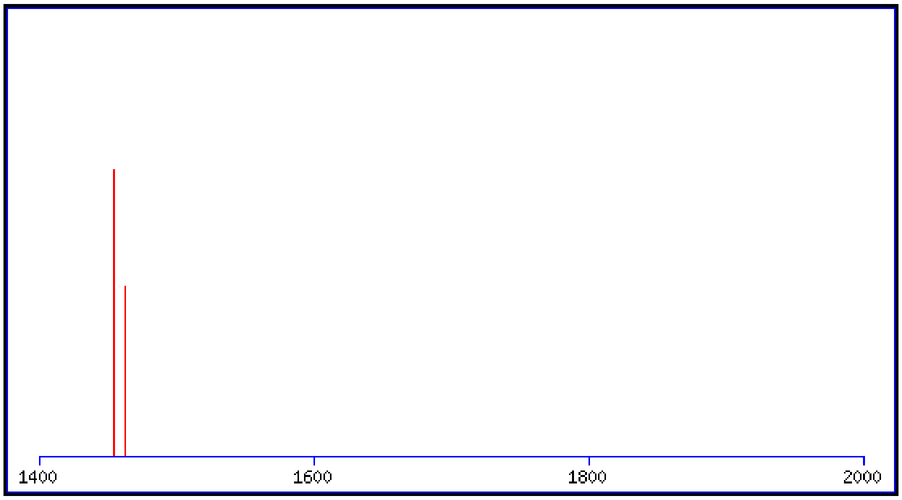

Monoisotopic mass of neutral peptide Mr(calc): 1463.7177
Fixed modifications: Carbamidomethyl (C)
Variable modifications:
N9 : Deamidated_N (N)
Ions Score: 97 **Expect:** 3.1e-008

Matches (Bold Red): 37/136 fragment ions using 50 most intense peaks

#	b	b^{++}	b^{*}	b^{*++}	b^{0}	b^{0++}	Seq.	y	y^{++}	y^{*}	y^{*++}	y^{0}	y^{0++}	#
1	100.0757	50.5415					V							14
2	**229.1183**	115.0628			211.1077	106.0575	E	**1365.6566**	683.3319	1348.6301	674.8187	**1347.6461**	674.3267	13
3	**300.1554**	150.5813			282.1448	141.5761	A	**1236.6140**	618.8107	1219.5875	610.2974	1218.6035	**609.8054**	12
4	**413.2395**	207.1234			395.2289	198.1181	I	**1165.5769**	583.2921	1148.5504	**574.7788**	**1147.5663**	**574.2868**	11
5	**470.2609**	235.6341			452.2504	226.6288	G	**1052.4928**	526.7501	**1035.4663**	518.2368	**1034.4823**	517.7448	10
6	**557.2930**	279.1501			**539.2824**	270.1448	S	**995.4714**	498.2393	978.4448	489.7261	977.4608	489.2340	9
7	**670.3770**	335.6921			**652.3665**	326.6869	L	**908.4394**	454.7233	891.4128	446.2100	890.4288	445.7180	8
8	741.4141	371.2107			723.4036	362.2054	A	**795.3553**	398.1813	778.3287	389.6680	777.3447	389.1760	7
9	**856.4411**	428.7242	839.4145	420.2109	**838.4305**	419.7189	N	**724.3182**	362.6627	707.2916	354.1495	706.3076	353.6574	6
10	**957.4887**	479.2480	940.4622	**470.7347**	**939.4782**	**470.2427**	T	**609.2912**	305.1493	592.2647	296.6360	591.2807	296.1440	5
11	1058.5364	529.7719	1041.5099	521.2586	1040.5259	520.7666	T	**508.2436**	254.6254	491.2170	246.1121	490.2330	245.6201	4
12	**1189.5769**	595.2921	1172.5504	586.7788	1171.5663	586.2868	M	**407.1959**	204.1016	390.1693	195.5883	389.1853	195.0963	3
13	**1318.6195**	659.8134	**1301.5930**	651.3001	**1300.6089**	650.8081	E	**276.1554**	138.5813	259.1288	130.0681	258.1448	129.5761	2
14							K	147.1128	74.0600	130.0863	65.5468			1

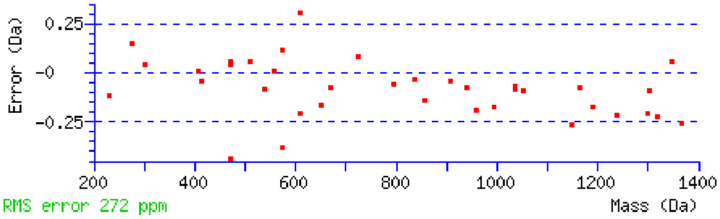

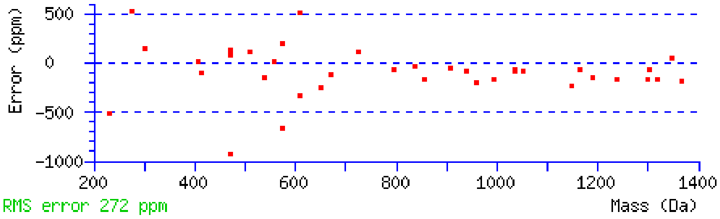

All matches to this query

Score	Mr(calc):	Delta	Sequence
96.8	1463.7177	0.0023	VEAIGSLANTTMEK
14.4	1463.7256	-0.0056	NNKLSLQFEEDK
10.1	1463.7133	0.0067	RVSTISALSHEGK
8.0	1463.7272	-0.0072	IDPVEKLQESVK
7.5	1463.7133	0.0067	RVSTISALSHEGK
7.5	1463.7133	0.0067	RVSTISALSHEGK
4.3	1463.7061	0.0139	DAILKYNVAYSK
4.3	1463.7061	0.0139	DAILKYNVAYSK
4.3	1463.7061	0.0139	DAILKYNVAYSK
4.2	1463.7273	-0.0072	EEGPVLVADTVKK

Spectrum No: 44; Query: 413; Rank: 1

Peptide View

MS/MS Fragmentation of **DDALLETTDGFNTTK**
Found in **IPI00212546**, Tax_Id=10116 Gene_Symbol=Emb Embigin precursor

Match to Query 413: 1640.745028 from(821.379790,2+)
Title: 091127RatKid_SCX01_02.2968.2968.2.dta
Data file K:\NewmanPaper\Piliang\3SubProteomes\Piliang3SP\mgf5ppm\SCX_3SubProteomes5ppm.mgf

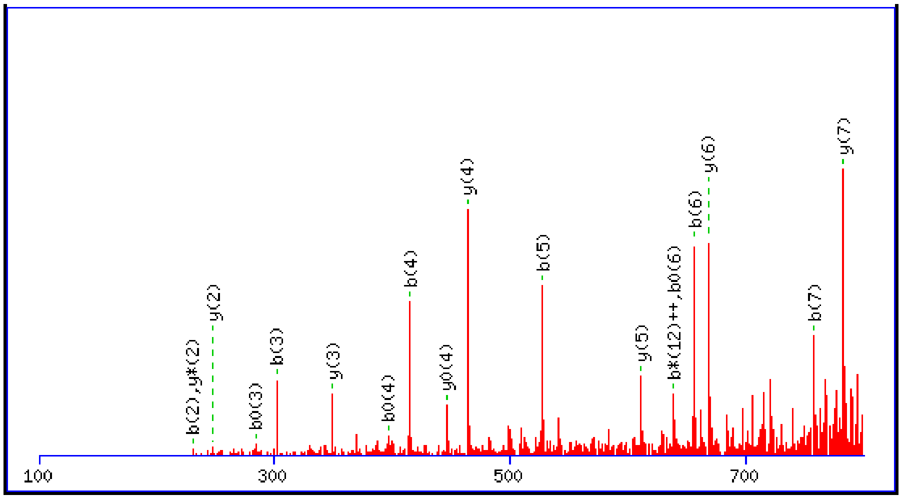

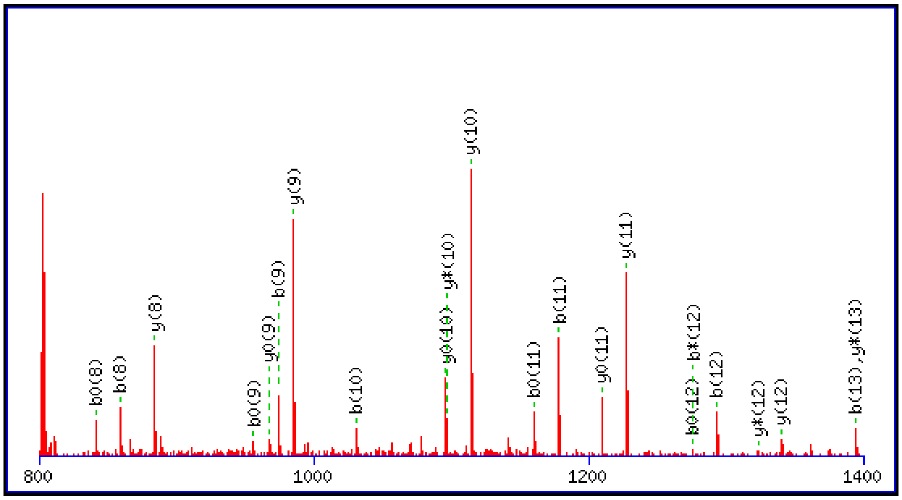

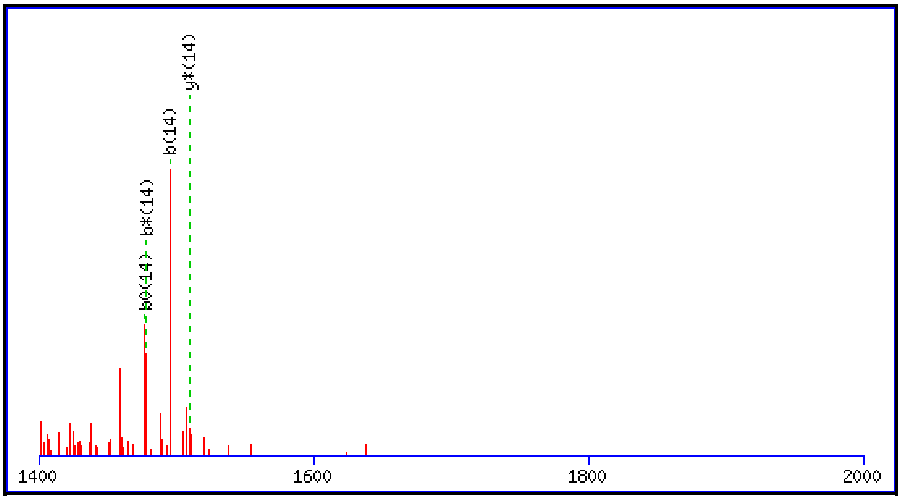

Monoisotopic mass of neutral peptide Mr(calc): 1640.7417
Fixed modifications: Carbamidomethyl (C)
Variable modifications:
N12 : Deamidated_N (N)
Ions Score: 97 **Expect:** 3.9e-008
Matches (Bold Red): 44/144 fragment ions using 74 most intense peaks

#	b	b^{++}	b*	b^{*++}	b^{0}	b^{0++}	Seq.	y	y^{++}	y*	y^{*++}	y^{0}	y^{0++}	#
1	116.0342	58.5207			98.0237	49.5155	D							15
2	**231.0612**	116.0342			213.0506	107.0289	D	1526.7221	763.8647	**1509.6955**	755.3514	1508.7115	754.8594	14
3	**302.0983**	151.5528			**284.0877**	142.5475	A	1411.6951	706.3512	**1394.6686**	697.8379	1393.6845	697.3459	13
4	**415.1823**	208.0948			**397.1718**	199.0895	L	**1340.6580**	670.8326	**1323.6314**	662.3194	1322.6474	661.8274	12
5	**528.2664**	264.6368			510.2558	255.6316	L	**1227.5739**	614.2906	1210.5474	605.7773	**1209.5634**	605.2853	11

**6**	**657.3090**	329.1581			**639.2984**	320.1529	**E**	**1114.4899**	557.7486	**1097.4633**	549.2353	**1096.4793**	548.7433	**10**
**7**	**758.3567**	379.6820			740.3461	370.6767	**T**	**985.4473**	493.2273	968.4207	484.7140	**967.4367**	484.2220	**9**
**8**	**859.4044**	430.2058			**841.3938**	421.2005	**T**	**884.3996**	442.7034	867.3730	434.1902	866.3890	433.6982	**8**
**9**	**974.4313**	487.7193			**956.4207**	478.7140	**D**	**783.3519**	392.1796	766.3254	383.6663	765.3414	383.1743	**7**
**10**	**1031.4528**	516.2300			1013.4422	507.2247	**G**	**668.3250**	334.6661	651.2984	326.1529	650.3144	325.6608	**6**
**11**	**1178.5212**	589.7642			**1160.5106**	580.7589	**F**	**611.3035**	306.1554	594.2770	297.6421	593.2929	297.1501	**5**
**12**	**1293.5481**	647.2777	**1276.5216**	**638.7644**	**1275.5375**	638.2724	**N**	**464.2351**	232.6212	447.2085	224.1079	**446.2245**	223.6159	**4**
**13**	**1394.5958**	697.8015	1377.5692	689.2883	1376.5852	688.7963	**T**	**349.2082**	175.1077	332.1816	166.5944	331.1976	166.1024	**3**
**14**	**1495.6435**	748.3254	**1478.6169**	739.8121	**1477.6329**	739.3201	**T**	**248.1605**	124.5839	**231.1339**	116.0706	230.1499	115.5786	**2**
**15**							**K**	147.1128	74.0600	130.0863	65.5468			**1**

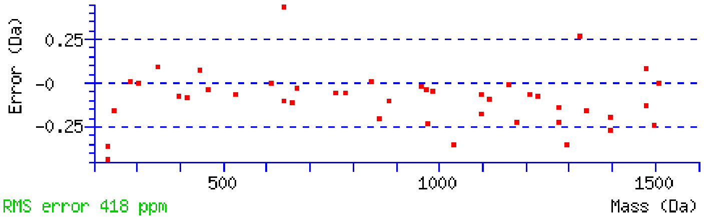

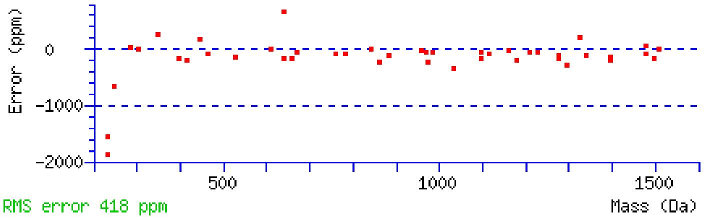

All matches to this query

Score	Mr(calc):	Delta	Sequence
96.8	1640.7417	0.0033	DDALLETTDGFNTTK
31.6	1639.7577	0.9873	DDALLETTDGFNTTK
0.9	1638.7324	2.0127	KANLHSSLENMGLK
0.9	1638.7324	2.0127	KANLHSSLENMGLK

Spectrum No: 45; Query: 763; Rank: 1

Peptide View

MS/MS Fragmentation of **SLTQGSMIVGNLAPVNGTSQGK**
Found in **IPI00388323**, Tax_Id=10116 Gene_Symbol=Hspg2 396 kDa protein

Match to Query 763: 2159.094048 from(1080.554300,2+)
Title: 091127RatKid_SCX01_15.2658.2658.2.dta
Data file K:\NewmanPaper\Piliang\3SubProteomes\Piliang3SP\mgf5ppm\SCX_3SubProteomes5ppm.mgf

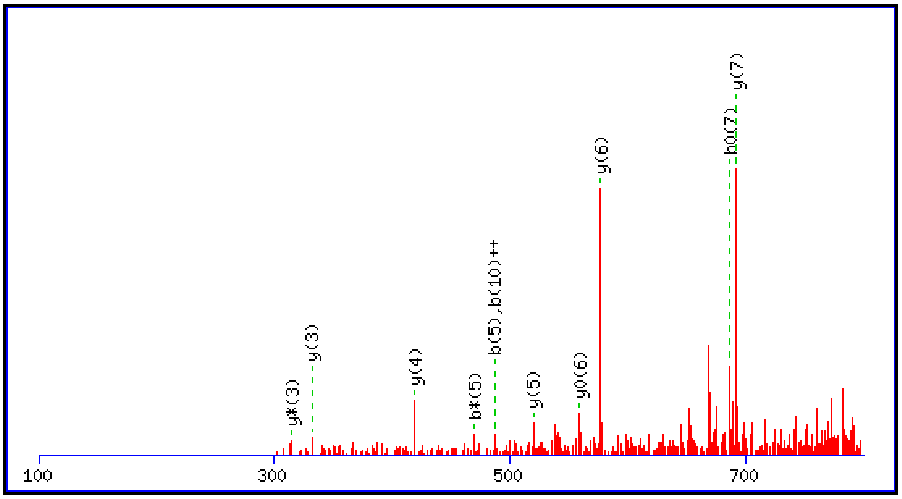

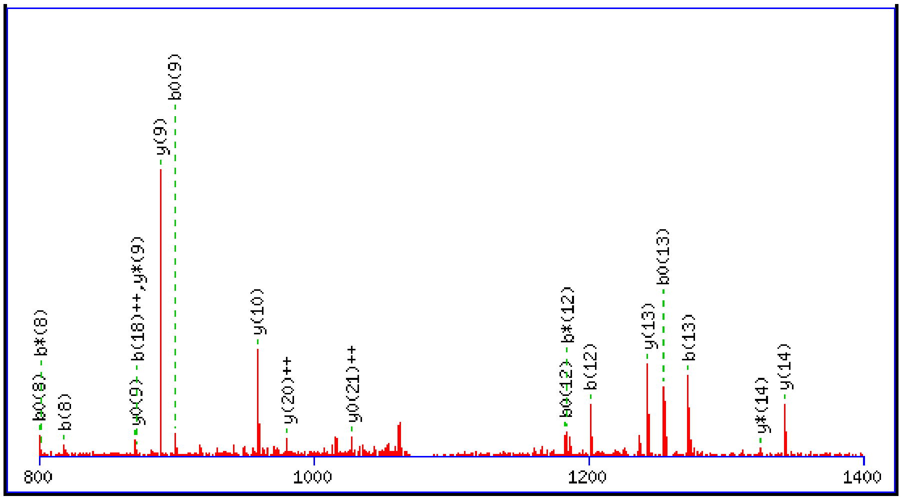

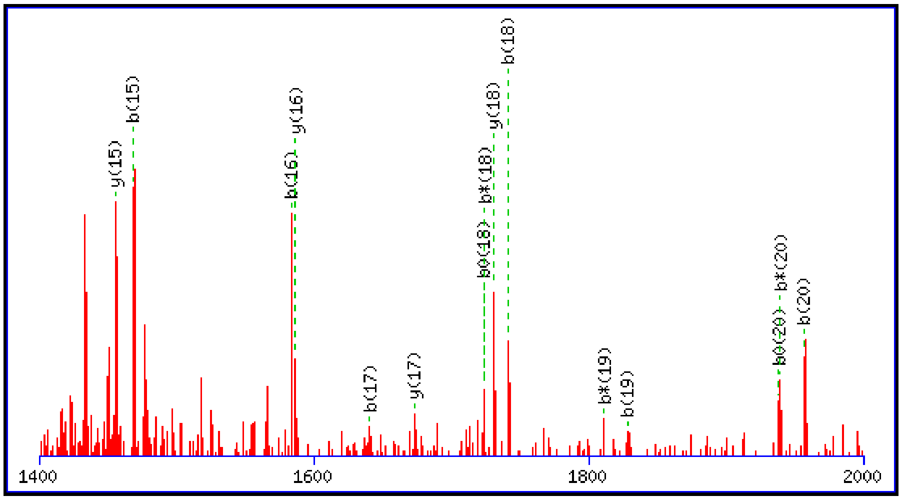

Monoisotopic mass of neutral peptide Mr(calc): 2159.0892
Fixed modifications: Carbamidomethyl (C)
Variable modifications:
N16 : Deamidated_N (N)
Ions Score: 97 **Expect:** 3.7e-008
Matches (Bold Red): 45/240 fragment ions using 65 most intense peaks

#	b	b^{++}	b*	b*$^{++}$	b^{0}	b^{0++}	Seq.	y	y^{++}	y*	y*$^{++}$	y^{0}	y^{0++}	#
1	88.0393	44.5233			70.0287	35.5180	S							22
2	201.1234	101.0653			183.1128	92.0600	L	2073.0645	1037.0359	2056.0379	1028.5226	2055.0539	**1028.0306**	21
3	302.1710	151.5892			284.1605	142.5839	T	1959.9804	**980.4938**	1942.9539	971.9806	1941.9698	971.4886	20
4	430.2296	215.6185	413.2031	207.1052	412.2191	206.6132	Q	1858.9327	929.9700	1841.9062	921.4567	1840.9222	920.9647	19
5	**487.2511**	244.1292	**470.2245**	235.6159	469.2405	235.1239	G	**1730.8742**	865.9407	1713.8476	857.4274	1712.8636	856.9354	18
6	574.2831	287.6452	557.2566	279.1319	556.2726	278.6399	S	**1673.8527**	837.4300	1656.8261	828.9167	1655.8421	828.4247	17
7	705.3236	353.1654	688.2971	344.6522	**687.3130**	344.1602	M	**1586.8207**	793.9140	1569.7941	785.4007	1568.8101	784.9087	16
8	**818.4077**	409.7075	**801.3811**	401.1942	**800.3971**	400.7022	I	**1455.7802**	728.3937	1438.7536	719.8805	1437.7696	719.3884	15
9	917.4761	459.2417	900.4495	450.7284	**899.4655**	450.2364	V	**1342.6961**	671.8517	**1325.6696**	663.3384	1324.6855	662.8464	14
10	974.4975	**487.7524**	957.4710	479.2391	956.4870	478.7471	G	**1243.6277**	622.3175	1226.6012	613.8042	1225.6171	613.3122	13
11	1088.5405	544.7739	1071.5139	536.2606	1070.5299	535.7686	N	1186.6062	593.8068	1169.5797	585.2935	1168.5957	584.8015	12
12	**1201.6245**	601.3159	**1184.5980**	592.8026	**1183.6140**	592.3106	L	1072.5633	536.7853	1055.5368	528.2720	1054.5527	527.7800	11
13	**1272.6616**	636.8345	1255.6351	628.3212	**1254.6511**	627.8292	A	**959.4792**	480.2433	942.4527	471.7300	941.4687	471.2380	10
14	1369.7144	685.3608	1352.6879	676.8476	1351.7038	676.3556	P	**888.4421**	444.7247	**871.4156**	436.2114	**870.4316**	435.7194	9
15	**1468.7828**	734.8951	1451.7563	726.3818	1450.7723	725.8898	V	791.3894	396.1983	774.3628	387.6850	773.3788	387.1930	8
16	**1583.8098**	792.4085	1566.7832	783.8952	1565.7992	783.4032	N	**692.3210**	346.6641	675.2944	338.1508	674.3104	337.6588	7
17	**1640.8312**	820.9193	1623.8047	812.4060	1622.8207	811.9140	G	**577.2940**	289.1506	560.2675	280.6374	**559.2835**	280.1454	6
18	**1741.8789**	**871.4431**	**1724.8524**	862.9298	**1723.8683**	862.4378	T	**520.2726**	260.6399	503.2460	252.1266	502.2620	251.6346	5

19	1828.9109	914.9591	1811.8844	906.4458	1810.9004	905.9538	S	419.2249	210.1161	402.1983	201.6028	401.2143	201.1108	4
20	1956.9695	978.9884	1939.9430	970.4751	1938.9589	969.9831	Q	332.1928	166.6001	315.1663	158.0868			3
21	2013.9910	1007.4991	1996.9644	998.9859	1995.9804	998.4938	G	204.1343	102.5708	187.1077	94.0575			2
22							K	147.1128	74.0600	130.0863	65.5468			1

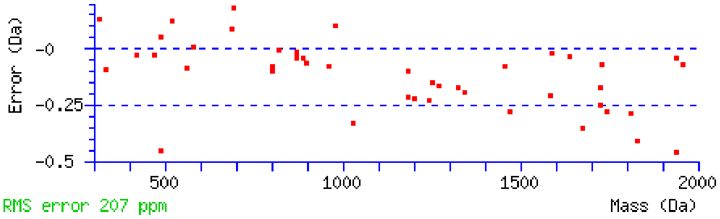

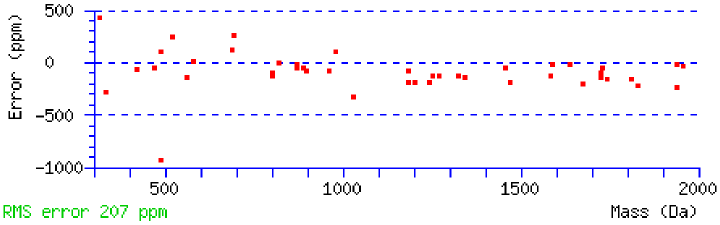

All matches to this query

Score	Mr(calc):	Delta	Sequence
96.6	2159.0892	0.0048	SLTQGSMIVGNLAPVNGTSQGK
58.7	2159.0892	0.0048	SLTQGSMIVGNLAPVNGTSQGK
13.4	2158.1052	0.9888	SLTQGSMIVGNLAPVNGTSQGK
6.5	2159.0888	0.0052	LHNLNAGPSRTVGLFLQGGK
3.1	2159.1070	-0.0129	NDEQSSKTDVQKLLELGQK
2.4	2159.0888	0.0052	LHNLNAGPSRTVGLFLQGGK
1.3	2159.0888	0.0052	LHNLNAGPSRTVGLFLQGGK

Spectrum No: 46; Query: 239; Rank: 1

Peptide View

MS/MS Fragmentation of **SLGNVNFSVSAEAR**
Found in **IPI00201262**, Tax_Id=10116 Gene_Symbol=LOC297568;Mug2;Mug1 Alpha-1-inhibitor 3 precursor

Match to Query 239: 1451.708408 from(726.861480,2+)
Title: 091127RatKid_SCX01_12.2955.2955.2.dta
Data file K:\NewmanPaper\Piliang\3SubProteomes\Piliang3SP\mgf5ppm\SCX_3SubProteomes5ppm.mgf

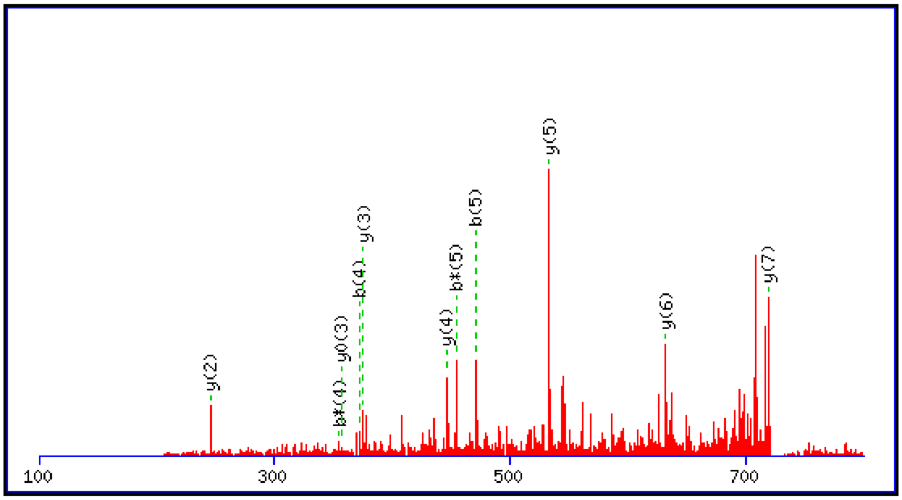

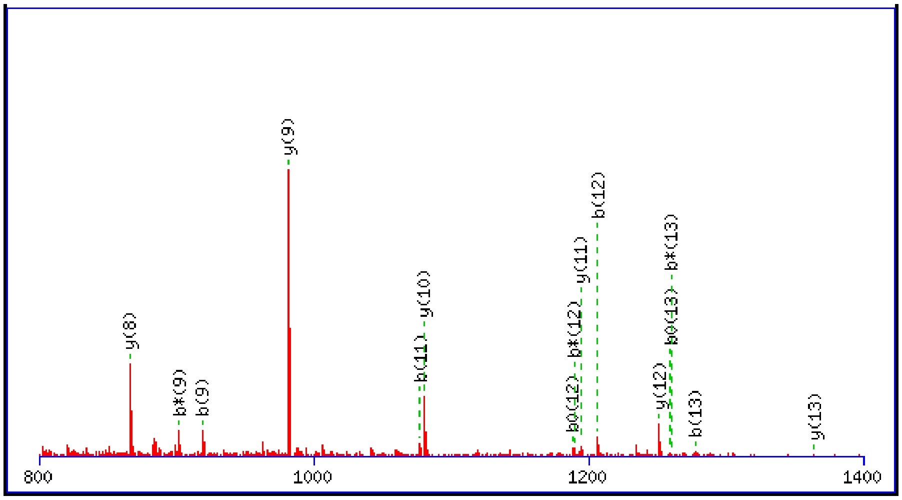

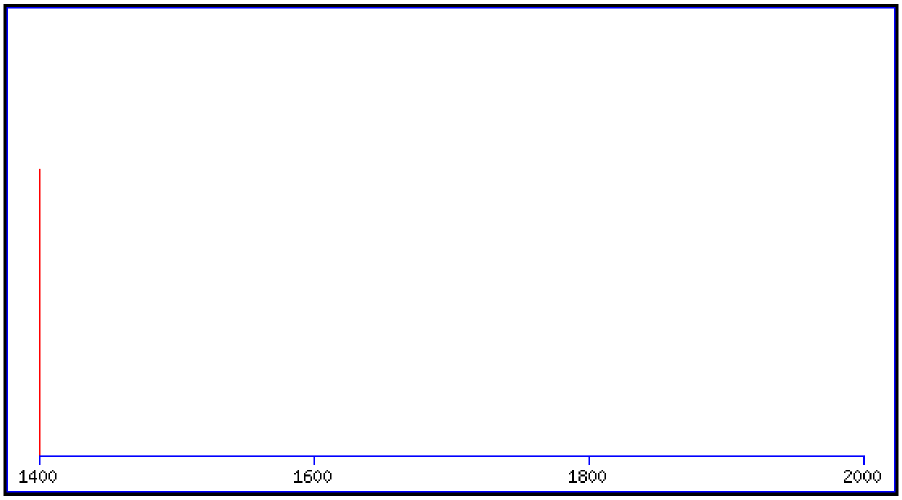

Monoisotopic mass of neutral peptide Mr(calc): 1450.7052
Fixed modifications: Carbamidomethyl (C)
Variable modifications:
N6 : Deamidated_N (N)
Ions Score: 96 **Expect:** 3.5e-008
Matches (Bold Red): 26/146 fragment ions using 72 most intense peaks

#	b	b^{++}	b*	b^{*++}	b^0	b^{0++}	Seq.	y	y^{++}	y*	y^{*++}	y^0	y^{0++}	#
1	88.0393	44.5233			70.0287	35.5180	S							14
2	201.1234	101.0653			183.1128	92.0600	L	**1364.6805**	682.8439	1347.6539	674.3306	1346.6699	673.8386	13
3	258.1448	129.5761			240.1343	120.5708	G	**1251.5964**	626.3018	1234.5698	617.7886	1233.5858	617.2966	12
4	**372.1878**	186.5975	**355.1612**	178.0842	354.1772	177.5922	N	**1194.5749**	597.7911	1177.5484	589.2778	1176.5644	588.7858	11
5	**471.2562**	236.1317	**454.2296**	227.6185	453.2456	227.1264	V	**1080.5320**	540.7696	1063.5055	532.2564	1062.5214	531.7644	10
6	586.2831	293.6452	569.2566	285.1319	568.2725	284.6399	N	**981.4636**	491.2354	964.4370	482.7222	963.4530	482.2302	9
7	733.3515	367.1794	716.3250	358.6661	715.3410	358.1741	F	**866.4367**	433.7220	849.4101	425.2087	848.4261	424.7167	8
8	820.3836	410.6954	803.3570	402.1821	802.3730	401.6901	S	**719.3682**	360.1878	702.3417	351.6745	701.3577	351.1825	7
9	**919.4520**	460.2296	**902.4254**	451.7163	901.4414	451.2243	V	**632.3362**	316.6717	615.3097	308.1585	614.3256	307.6665	6
10	1006.4840	503.7456	989.4574	495.2324	988.4734	494.7404	S	**533.2678**	267.1375	516.2413	258.6243	515.2572	258.1323	5
11	**1077.5211**	539.2642	1060.4946	530.7509	1059.5105	530.2589	A	**446.2358**	223.6215	429.2092	215.1082	428.2252	214.6162	4
12	**1206.5637**	603.7855	**1189.5372**	595.2722	**1188.5531**	594.7802	E	**375.1987**	188.1030	358.1721	179.5897	**357.1881**	179.0977	3
13	**1277.6008**	639.3040	**1260.5743**	630.7908	**1259.5902**	630.2988	A	**246.1561**	123.5817	229.1295	115.0684			2
14							R	175.1190	88.0631	158.0924	79.5498			1

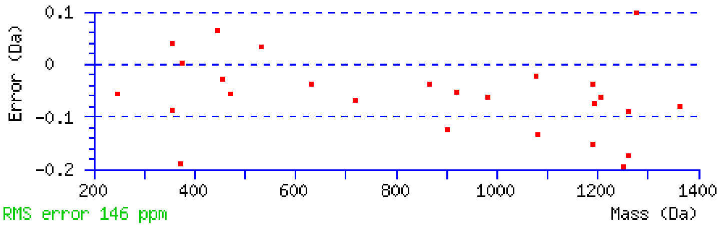

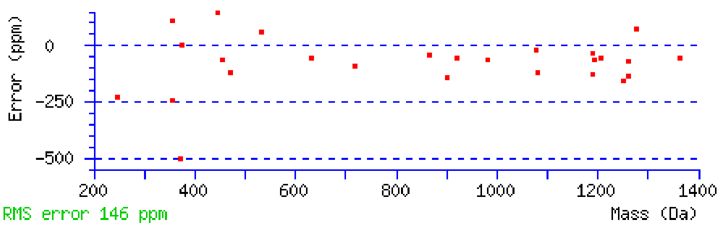

All matches to this query

Score	Mr(calc):	Delta	Sequence
96.3	1450.7052	1.0032	SLGNVNFSVSAEAR
61.7	1450.7052	1.0032	SLGNVNFSVSAEAR
5.2	1450.6969	1.0115	TYHALSNLPKAR
4.3	1450.6969	1.0115	TYHALSNLPKAR
4.3	1450.6969	1.0115	TYHALSNLPKAR
2.5	1449.7017	2.0067	ITKFEGQPPTPR
1.8	1451.6991	0.0093	SDVAVDTSSEITTK
1.6	1451.7173	-0.0089	DYLNLIKHLSR
1.2	1451.6966	0.0118	LPFDGGMVSTGNLK
0.4	1450.6931	1.0153	LGFGTGVSVYLMK

Spectrum No: 47; Query: 778; Rank: 1

Peptide View

MS/MS Fragmentation of **VPFNPNDTFESEFYLDEK**
Found in **IPI00200591**, Tax_Id=10116 Gene_Symbol=LOC299282 Serine protease inhibitor A3L precursor

Match to Query 778: 2190.970828 from(1096.492690,2+)
Title: 091127RatKid_SCX01_02.4307.4307.2.dta
Data file K:\NewmanPaper\Piliang\3SubProteomes\Piliang3SP\mgf5ppm\SCX_3SubProteomes5ppm.mgf

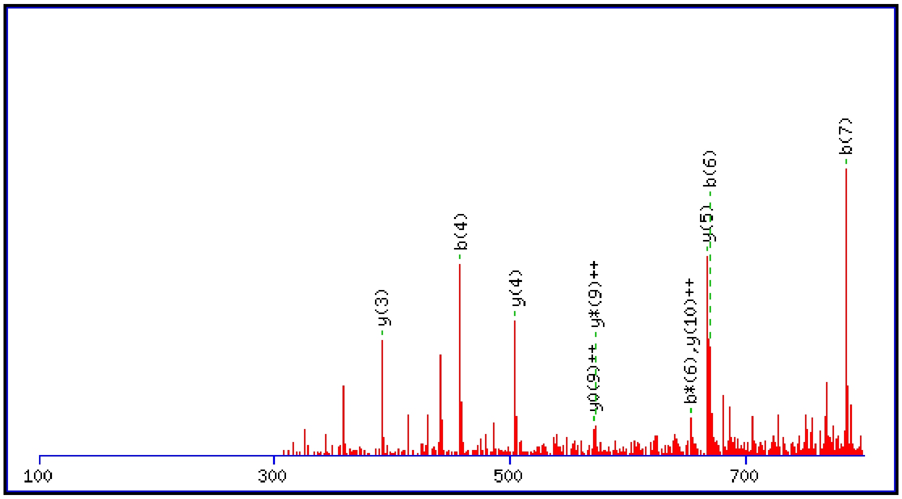

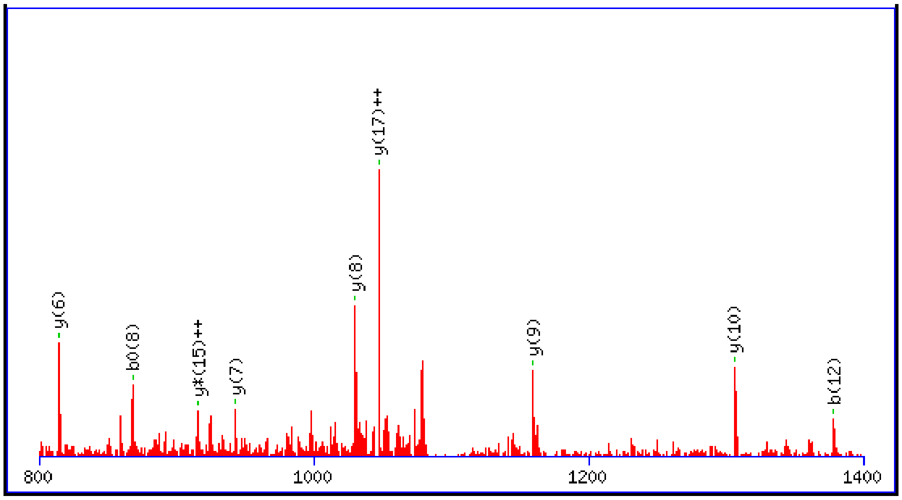

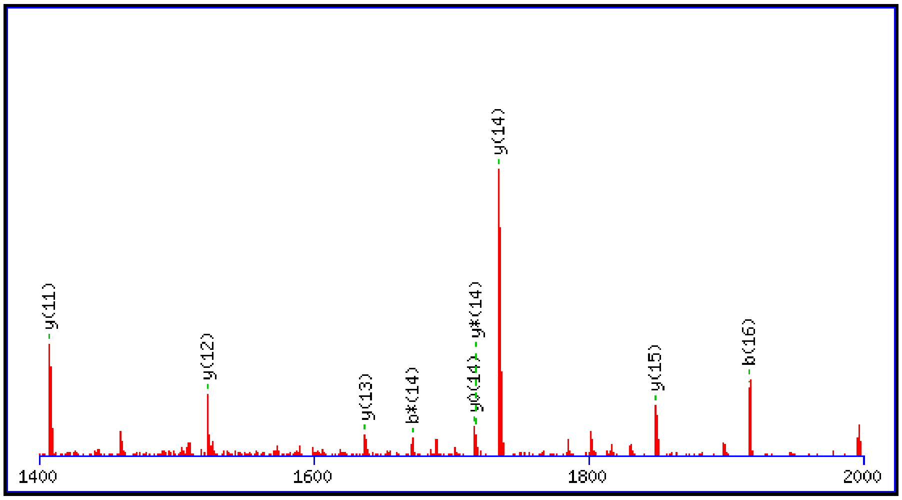

Monoisotopic mass of neutral peptide Mr(calc): 2190.9633
Fixed modifications: Carbamidomethyl (C)
Variable modifications:
N6 : Deamidated_N (N)
Ions Score: 96 **Expect:** 6.9e-008
Matches (Bold Red): 28/184 fragment ions using 42 most intense peaks

#	b	b^{++}	b*	b^{*++}	b^0	b^{0++}	Seq.	y	y^{++}	y*	y^{*++}	y^0	y^{0++}	#
1	100.0757	50.5415					V							18
2	197.1285	99.0679					P	2092.9022	**1046.9547**	2075.8757	1038.4415	2074.8916	1037.9495	17
3	344.1969	172.6021					F	1995.8494	998.4284	1978.8229	989.9151	1977.8389	989.4231	16
4	**458.2398**	229.6235	441.2132	221.1103			N	**1848.7810**	924.8942	1831.7545	**916.3809**	1830.7705	915.8889	15
5	555.2926	278.1499	538.2660	269.6366			P	**1734.7381**	867.8727	**1717.7115**	859.3594	**1716.7275**	858.8674	14
6	**670.3195**	335.6634	**653.2929**	327.1501			N	**1637.6853**	819.3463	1620.6588	810.8330	1619.6748	810.3410	13
7	**785.3464**	393.1769	768.3199	384.6636	767.3359	384.1716	D	**1522.6584**	761.8328	1505.6318	753.3196	1504.6478	752.8276	12
8	886.3941	443.7007	869.3676	435.1874	**868.3836**	434.6954	T	**1407.6315**	704.3194	1390.6049	695.8061	1389.6209	695.3141	11
9	1033.4625	517.2349	1016.4360	508.7216	1015.4520	508.2296	F	**1306.5838**	**653.7955**	1289.5572	645.2823	1288.5732	644.7902	10
10	1162.5051	581.7562	1145.4786	573.2429	1144.4946	572.7509	E	**1159.5154**	580.2613	1142.4888	**571.7480**	1141.5048	**571.2560**	9
11	1249.5372	625.2722	1232.5106	616.7589	1231.5266	616.2669	S	**1030.4728**	515.7400	1013.4462	507.2267	1012.4622	506.7347	8
12	**1378.5797**	689.7935	1361.5532	681.2802	1360.5692	680.7882	E	**943.4407**	472.2240	926.4142	463.7107	925.4302	463.2187	7
13	1525.6482	763.3277	1508.6216	754.8144	1507.6376	754.3224	F	**814.3981**	407.7027	797.3716	399.1894	796.3876	398.6974	6
14	1688.7115	844.8594	**1671.6849**	836.3461	1670.7009	835.8541	Y	**667.3297**	334.1685	650.3032	325.6552	649.3192	325.1632	5
15	1801.7956	901.4014	1784.7690	892.8881	1783.7850	892.3961	L	**504.2664**	252.6368	487.2399	244.1236	486.2558	243.6316	4
16	**1916.8225**	958.9149	1899.7959	950.4016	1898.8119	949.9096	D	**391.1823**	196.0948	374.1558	187.5815	373.1718	187.0895	3
17	2045.8651	1023.4362	2028.8385	1014.9229	2027.8545	1014.4309	E	276.1554	138.5813	259.1288	130.0681	258.1448	129.5761	2
18							K	147.1128	74.0600	130.0863	65.5468			1

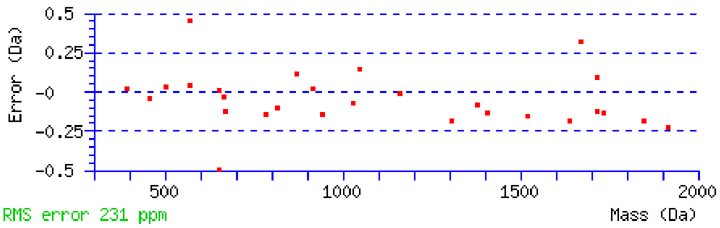

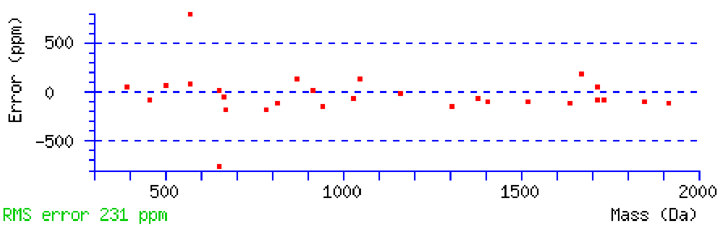

All matches to this query

Score	Mr(calc):	Delta	Sequence
96.2	2190.9633	0.0075	VPFNPNDTFESEFYLDEK
65.9	2190.9633	0.0075	VPFNPNDTFESEFYLDEK
41.2	2189.9793	0.9915	VPFNPNDTFESEFYLDEK
10.1	2190.9731	-0.0022	EFNLMYDGTKEVPMNPVK
9.8	2190.9792	-0.0083	FLKLSPQINASNFYFNK
6.8	2189.9581	1.0127	MQNTLYLSLTRFIGDSR
5.2	2190.9745	-0.0037	IEVMNTDGTGRRVLVEDK
5.2	2190.9792	-0.0083	FLKLSPQINASNFYFNK
5.2	2190.9792	-0.0083	FLKLSPQINASNFYFNK
4.6	2188.9476	2.0232	SRNTDEMVELHILLQSK

Spectrum No: 48; Query: 541; Rank: 1

Peptide View

MS/MS Fragmentation of **NEIQVNTSQIPDTVTR**
Found in **IPI00211648**, Tax_Id=10116 Gene_Symbol=Slc3a1 Neutral and basic amino acid transport protein rBAT

Match to Query 541: 1814.897848 from(908.456200,2+)
Title: 091129RatKid_SCX02_16.2007.2007.2.dta
Data file K:\NewmanPaper\Piliang\3SubProteomes\Piliang3SP\mgf5ppm\SCX_3SubProteomes5ppm.mgf

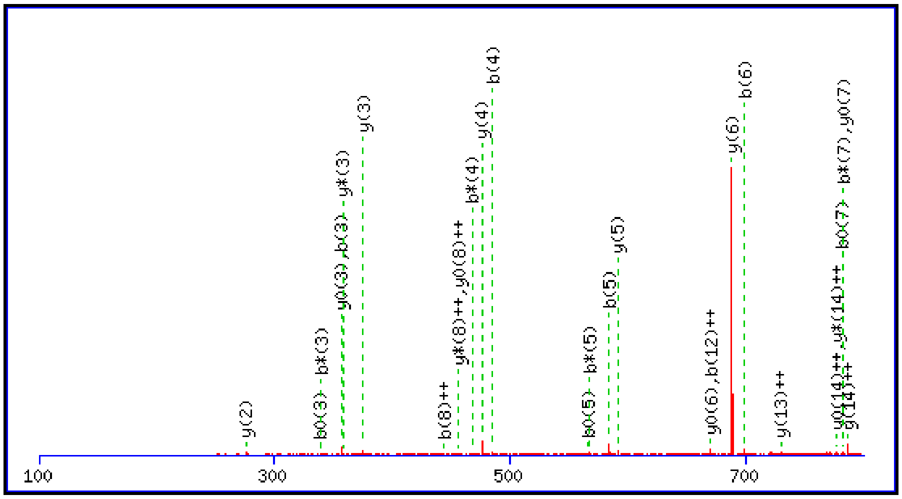

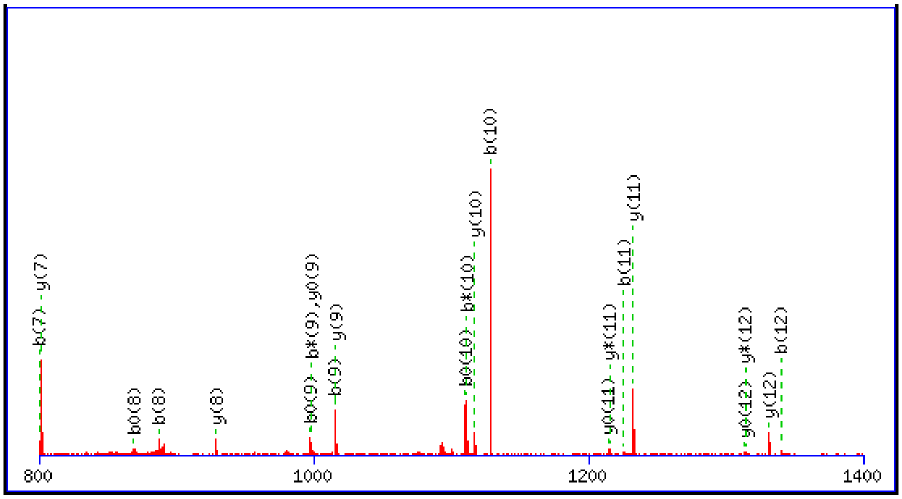

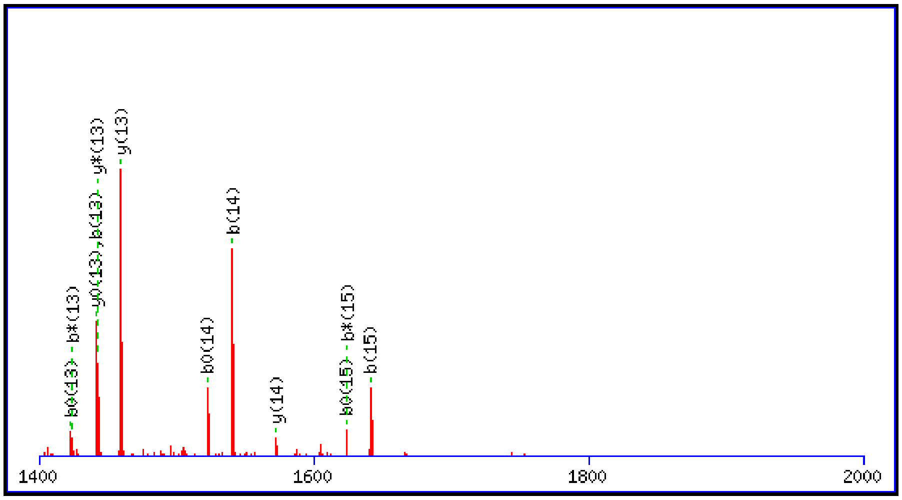

Monoisotopic mass of neutral peptide Mr(calc): 1814.9010
Fixed modifications: Carbamidomethyl (C)
Variable modifications:
N6 : Deamidated_N (N)
Ions Score: 96 **Expect:** 4.5e-008
Matches (Bold Red): 62/176 fragment ions using 79 most intense peaks

#	b	b^{++}	b*	b^{*++}	b^{0}	b^{0++}	Seq.	y	y^{++}	y*	y^{*++}	y^{0}	y^{0++}	#
1	115.0502	58.0287	98.0237	49.5155			N							16
2	244.0928	122.5500	227.0662	114.0368	226.0822	113.5448	E	1701.8654	851.4363	1684.8388	842.9230	1683.8548	842.4310	15
3	**357.1769**	179.0921	**340.1503**	170.5788	**339.1663**	170.0868	I	**1572.8228**	**786.9150**	1555.7962	**778.4017**	1554.8122	**777.9097**	14
4	**485.2354**	243.1214	**468.2089**	234.6081	467.2249	234.1161	Q	**1459.7387**	**730.3730**	**1442.7122**	721.8597	**1441.7281**	721.3677	13
5	**584.3039**	292.6556	**567.2773**	284.1423	**566.2933**	283.6503	V	**1331.6801**	666.3437	**1314.6536**	657.8304	**1313.6696**	657.3384	12
6	**699.3308**	350.1690	682.3042	341.6558	681.3202	341.1637	N	**1232.6117**	616.8095	**1215.5852**	608.2962	**1214.6012**	607.8042	11
7	**800.3785**	400.6929	**783.3519**	392.1796	**782.3679**	391.6876	T	**1117.5848**	559.2960	1100.5582	550.7828	1099.5742	550.2907	10
8	**887.4105**	**444.2089**	870.3839	435.6956	**869.3999**	435.2036	S	**1016.5371**	508.7722	999.5106	500.2589	**998.5265**	499.7669	9
9	**1015.4691**	508.2382	**998.4425**	499.7249	**997.4585**	499.2329	Q	**929.5051**	465.2562	912.4785	**456.7429**	911.4945	**456.2509**	8
10	**1128.5531**	564.7802	**1111.5266**	556.2669	**1110.5426**	555.7749	I	**801.4465**	401.2269	784.4199	392.7136	**783.4359**	392.2216	7
11	**1225.6059**	613.3066	1208.5794	604.7933	1207.5953	604.3013	P	**688.3624**	344.6849	671.3359	336.1716	**670.3519**	335.6796	6
12	**1340.6328**	**670.8201**	1323.6063	662.3068	1322.6223	661.8148	D	**591.3097**	296.1585	574.2831	287.6452	573.2991	287.1532	5
13	**1441.6805**	721.3439	**1424.6540**	712.8306	**1423.6700**	712.3386	T	**476.2827**	238.6450	459.2562	230.1317	458.2722	229.6397	4
14	**1540.7489**	770.8781	1523.7224	762.3648	**1522.7384**	761.8728	V	**375.2350**	188.1212	**358.2085**	179.6079	**357.2245**	179.1159	3
15	**1641.7966**	821.4019	**1624.7701**	812.8887	**1623.7861**	812.3967	T	**276.1666**	138.5870	259.1401	130.0737	258.1561	129.5817	2
16							R	175.1190	88.0631	158.0924	79.5498			1

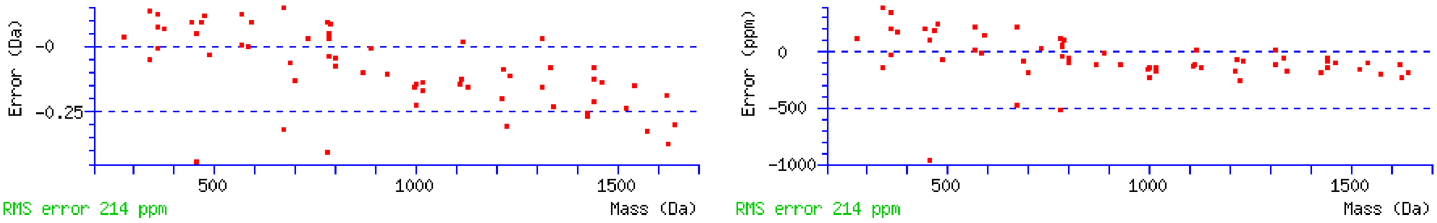

All matches to this query

Score	Mr(calc):	Delta	Sequence
96.1	1814.9010	-0.0032	NEIQVNTSQIPDTVTR
71.7	1814.9010	-0.0032	NEIQVNTSQIPDTVTR
16.0	1812.8870	2.0109	EEKLSLTKSLDDVTR
11.3	1814.9040	-0.0061	AQSALHEQKTLPGVTR
9.6	1814.8981	-0.0003	AFRVWESATPLRFR
7.6	1814.8985	-0.0007	LGPAQSGPCLFLSNPTR
7.0	1814.8981	-0.0003	AFRVWESATPLRFR
6.2	1814.8927	0.0051	DLGAHLVIINSVAEQR
3.6	1814.9131	-0.0152	MAGGPTRMEGNLPAKLR
3.4	1812.8870	2.0109	EEKLSLTKSLDDVTR

Spectrum No: 49; Query: 831; Rank: 1

Peptide View

MS/MS Fragmentation of **KYPVPFQNENVTAAFVELGR**
Found in **IPI00213569**, Tax_Id=10116 Gene_Symbol=Ugt1a1 UDP-glucuronosyltransferase 1-1 precursor

Match to Query 831: 2279.168128 from(1140.591340,2+)
Title: 091129RatKid_SCX02_26.4831.4831.2.dta
Data file K:\NewmanPaper\Piliang\3SubProteomes\Piliang3SP\mgf5ppm\SCX_3SubProteomes5ppm.mgf

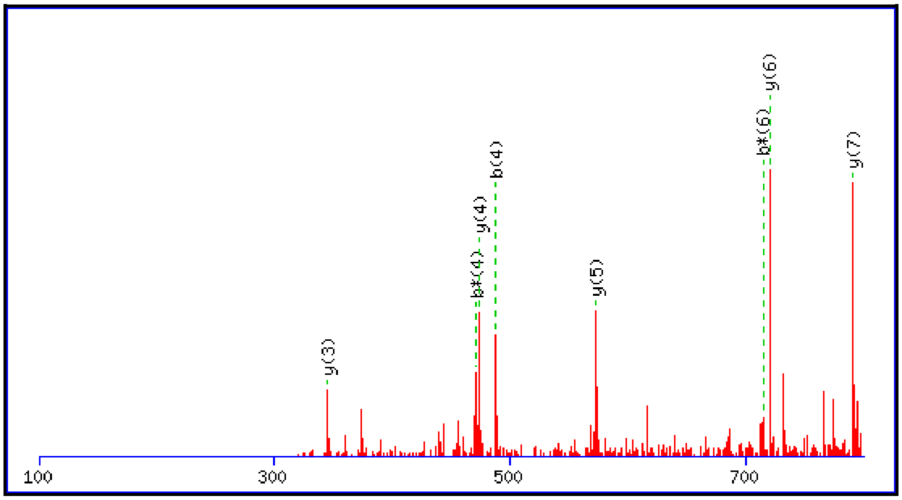

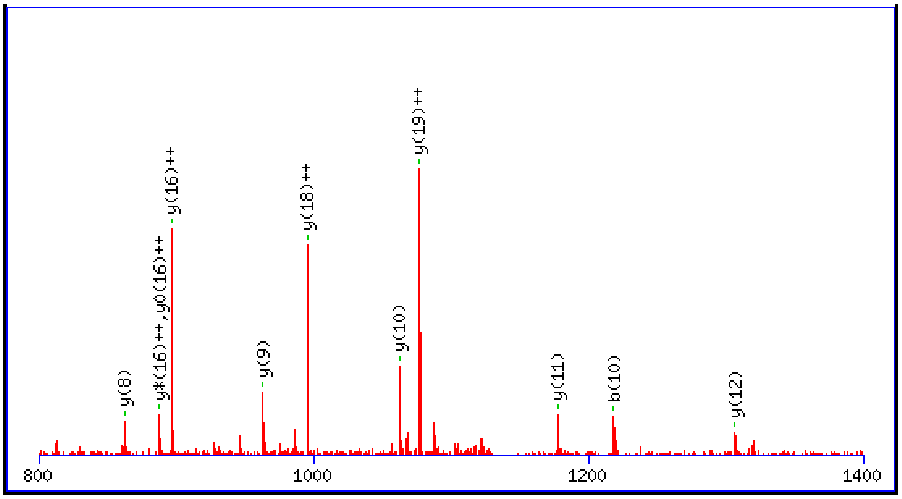

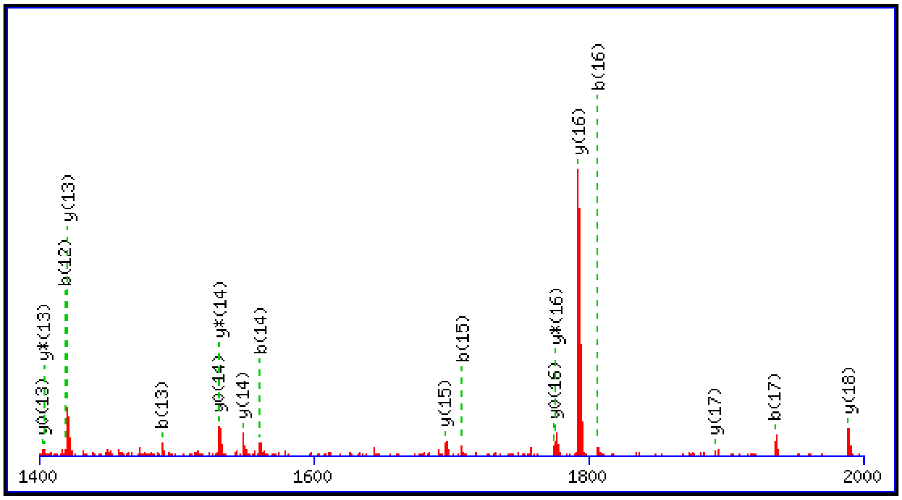

Monoisotopic mass of neutral peptide Mr(calc): 2279.1586
Fixed modifications: Carbamidomethyl (C)
Variable modifications:
N10 : Deamidated_N (N)
Ions Score: 96 **Expect:** 3.6e-008
Matches (Bold Red): 37/206 fragment ions using 80 most intense peaks

#	b	b^{++}	b*	b^{*++}	b^{0}	b^{0++}	Seq.	y	y^{++}	y*	y^{*++}	y^{0}	y^{0++}	#
1	129.1022	65.0548	112.0757	56.5415			K							20
2	292.1656	146.5864	275.1390	138.0731			Y	2152.0709	**1076.5391**	2135.0444	1068.0258	2134.0604	1067.5338	19
3	389.2183	195.1128	372.1918	186.5995			P	**1989.0076**	**995.0074**	1971.9811	986.4942	1970.9970	986.0022	18
4	**488.2867**	244.6470	**471.2602**	236.1337			V	**1891.9548**	946.4811	1874.9283	937.9678	1873.9443	937.4758	17
5	585.3395	293.1734	568.3130	284.6601			P	**1792.8864**	**896.9469**	**1775.8599**	**888.4336**	**1774.8759**	**887.9416**	16
6	732.4079	366.7076	**715.3814**	358.1943			F	**1695.8337**	848.4205	1678.8071	839.9072	1677.8231	839.4152	15
7	860.4665	430.7369	843.4400	422.2236			Q	**1548.7653**	774.8863	**1531.7387**	766.3730	**1530.7547**	765.8810	14
8	974.5094	487.7584	957.4829	479.2451			N	**1420.7067**	710.8570	**1403.6801**	702.3437	**1402.6961**	701.8517	13
9	1103.5520	552.2796	1086.5255	543.7664	1085.5415	543.2744	E	**1306.6638**	653.8355	1289.6372	645.3222	1288.6532	644.8302	12
10	**1218.5790**	609.7931	1201.5524	601.2798	1200.5684	600.7878	N	**1177.6212**	589.3142	1160.5946	580.8009	1159.6106	580.3089	11
11	1317.6474	659.3273	1300.6208	650.8140	1299.6368	650.3220	V	**1062.5942**	531.8007	1045.5677	523.2875	1044.5837	522.7955	10
12	**1418.6951**	709.8512	1401.6685	701.3379	1400.6845	700.8459	T	**963.5258**	482.2665	946.4993	473.7533	945.5152	473.2613	9
13	**1489.7322**	745.3697	1472.7056	736.8564	1471.7216	736.3644	A	**862.4781**	431.7427	845.4516	423.2294	844.4676	422.7374	8
14	**1560.7693**	780.8883	1543.7427	772.3750	1542.7587	771.8830	A	**791.4410**	396.2241	774.4145	387.7109	773.4305	387.2189	7
15	**1707.8377**	854.4225	1690.8111	845.9092	1689.8271	845.4172	F	**720.4039**	360.7056	703.3774	352.1923	702.3933	351.7003	6
16	**1806.9061**	903.9567	1789.8796	895.4434	1788.8955	894.9514	V	**573.3355**	287.1714	556.3089	278.6581	555.3249	278.1661	5
17	**1935.9487**	968.4780	1918.9222	959.9647	1917.9381	959.4727	E	**474.2671**	237.6372	457.2405	229.1239	456.2565	228.6319	4
18	2049.0328	1025.0200	2032.0062	1016.5067	2031.0222	1016.0147	L	**345.2245**	173.1159	328.1979	164.6026			3

**19**	2106.0542	1053.5308	2089.0277	1045.0175	2088.0437	1044.5255	**G**	232.1404	116.5738	215.1139	108.0606			**2**
**20**							**R**	175.1190	88.0631	158.0924	79.5498			**1**

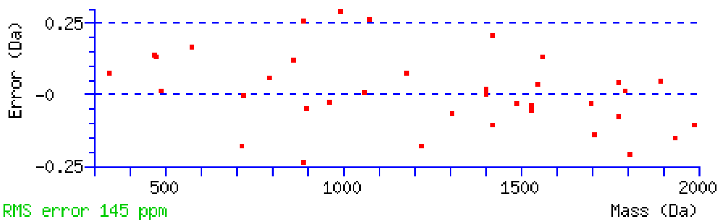

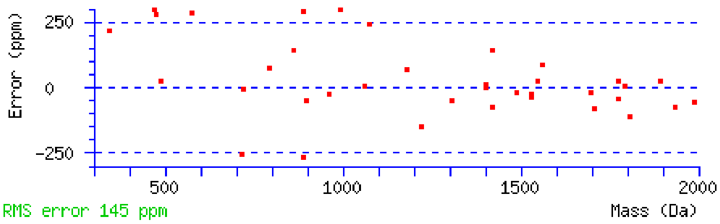

All matches to this query

Score	Mr(calc):	Delta	Sequence
96.1	2279.1586	0.0095	KYPVPFQNENVTAAFVELGR
72.3	2279.1586	0.0095	KYPVPFQNENVTAAFVELGR
6.8	2277.1626	2.0055	TLIRFKTTLMNTLMDVLR
6.8	2277.1626	2.0055	TLIRFKTTLMNTLMDVLR
6.0	2278.1746	0.9935	KYPVPFQNENVTAAFVELGR
1.4	2277.1518	2.0163	TIINQVLSQTQIEFASHLR
0.1	2277.1448	2.0233	SSDLTSDLGNVLTSSNAKAVNGK

Spectrum No: 50; Query: 548; Rank: 1

Peptide View

MS/MS Fragmentation of **TSDTGDQTISNGTEANSK**
Found in **IPI00193425**, Tax_Id=10116 Gene_Symbol=Bsg Isoform 2 of Basigin precursor

Match to Query 548: 1825.785428 from(913.899990,2+)
Title: 091127RatKid_SCX01_02.385.385.2.dta
Data file K:\NewmanPaper\Piliang\3SubProteomes\Piliang3SP\mgf5ppm\SCX_3SubProteomes5ppm.mgf

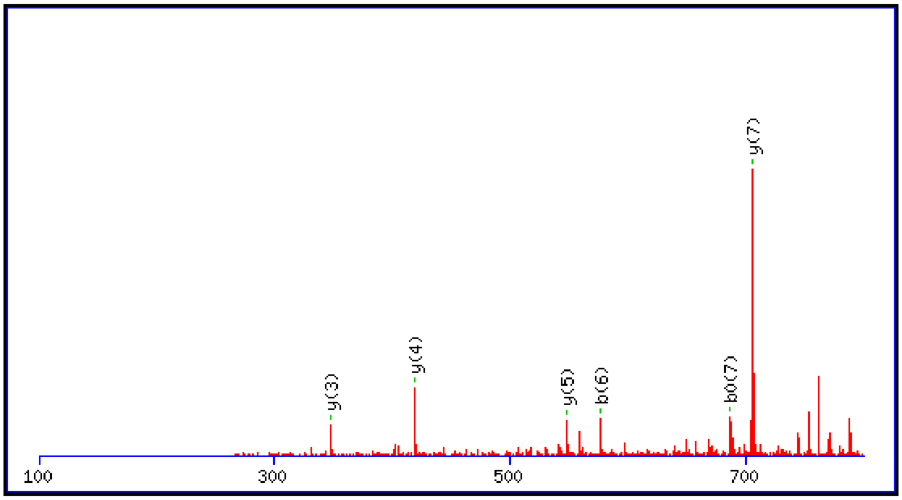

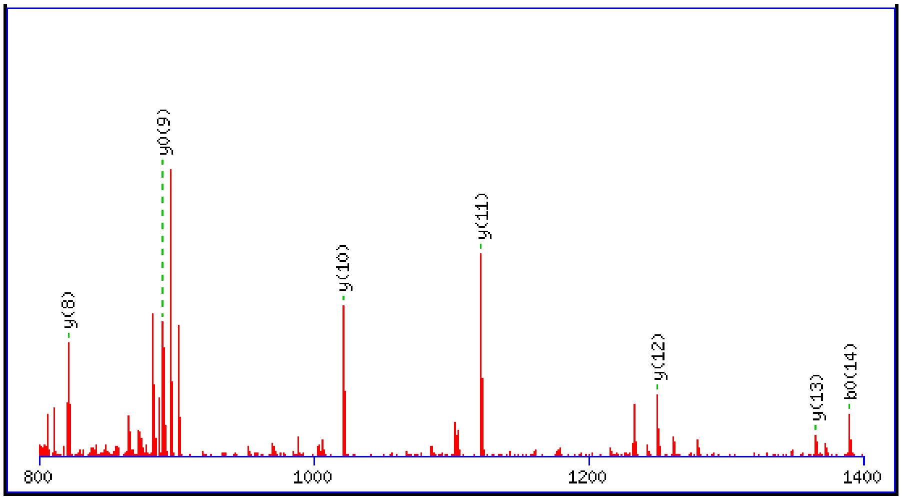

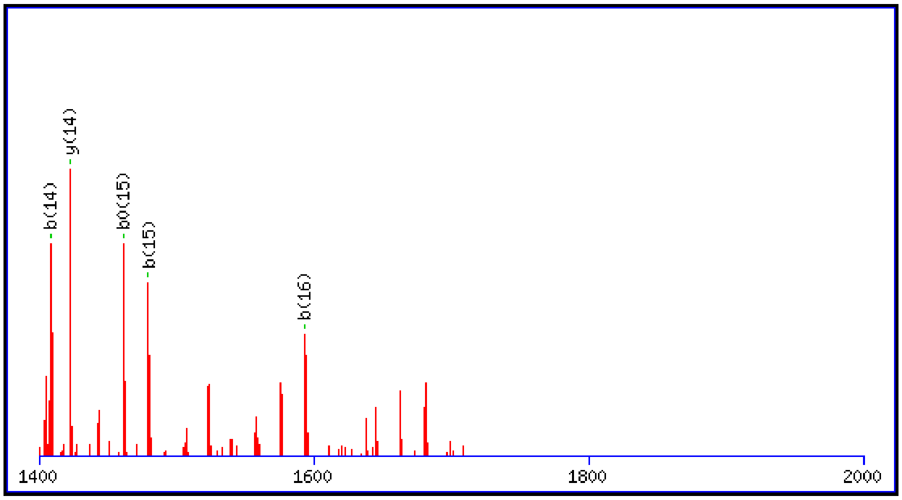

Monoisotopic mass of neutral peptide Mr(calc): 1825.7814
Fixed modifications: Carbamidomethyl (C)
Variable modifications:
N11 : Deamidated_N (N)
Ions Score: 96 **Expect:** 6.4e-008
Matches (Bold Red): 18/190 fragment ions using 24 most intense peaks

#	b	b^{++}	b*	b^{*++}	b^0	b^{0++}	Seq.	y	y^{++}	y*	y^{*++}	y^0	y^{0++}	#
1	102.0550	51.5311			84.0444	42.5258	T							18
2	189.0870	95.0471			171.0764	86.0418	S	1725.7410	863.3741	1708.7144	854.8608	1707.7304	854.3688	17
3	304.1139	152.5606			286.1034	143.5553	D	1638.7089	819.8581	1621.6824	811.3448	1620.6984	810.8528	16
4	405.1616	203.0844			387.1510	194.0792	T	1523.6820	762.3446	1506.6554	753.8314	1505.6714	753.3393	15
5	462.1831	231.5952			444.1725	222.5899	G	**1422.6343**	711.8208	1405.6078	703.3075	1404.6237	702.8155	14
6	**577.2100**	289.1086			559.1994	280.1034	D	**1365.6128**	683.3101	1348.5863	674.7968	1347.6023	674.3048	13
7	705.2686	353.1379	688.2420	344.6247	**687.2580**	344.1327	Q	**1250.5859**	625.7966	1233.5593	617.2833	1232.5753	616.7913	12
8	806.3163	403.6618	789.2897	395.1485	788.3057	394.6565	T	**1122.5273**	561.7673	1105.5008	553.2540	1104.5168	552.7620	11
9	919.4003	460.2038	902.3738	451.6905	901.3898	451.1985	I	**1021.4796**	511.2435	1004.4531	502.7302	1003.4691	502.2382	10
10	1006.4324	503.7198	989.4058	495.2065	988.4218	494.7145	S	908.3956	454.7014	891.3690	446.1882	**890.3850**	445.6961	9
11	1121.4593	561.2333	1104.4327	552.7200	1103.4487	552.2280	N	**821.3635**	411.1854	804.3370	402.6721	803.3530	402.1801	8
12	1178.4808	589.7440	1161.4542	581.2307	1160.4702	580.7387	G	**706.3366**	353.6719	689.3101	345.1587	688.3260	344.6667	7
13	1279.5284	640.2679	1262.5019	631.7546	1261.5179	631.2626	T	649.3151	325.1612	632.2886	316.6479	631.3046	316.1559	6
14	**1408.5710**	704.7892	1391.5445	696.2759	**1390.5605**	695.7839	E	**548.2675**	274.6374	531.2409	266.1241	530.2569	265.6321	5
15	**1479.6081**	740.3077	1462.5816	731.7944	**1461.5976**	731.3024	A	**419.2249**	210.1161	402.1983	201.6028	401.2143	201.1108	4
16	**1593.6511**	797.3292	1576.6245	788.8159	1575.6405	788.3239	N	**348.1878**	174.5975	331.1612	166.0842	330.1772	165.5922	3
17	1680.6831	840.8452	1663.6566	832.3319	1662.6725	831.8399	S	234.1448	117.5761	217.1183	109.0628	216.1343	108.5708	2
18							K	147.1128	74.0600	130.0863	65.5468			1

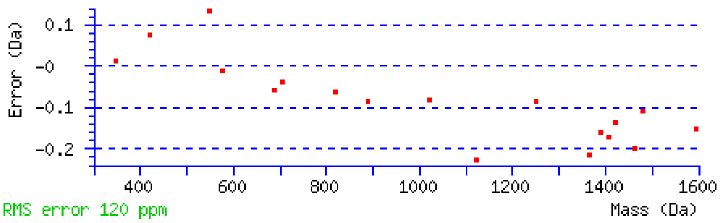

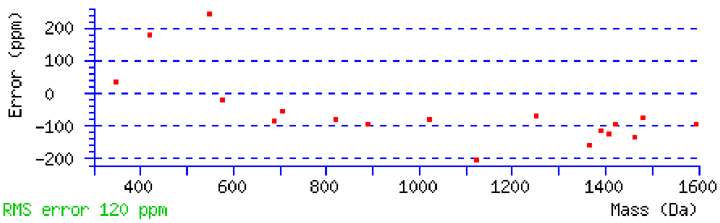

All matches to this query

Score	Mr(calc):	Delta	Sequence
95.9	1825.7814	0.0041	TSDTGDQTISNGTEANSK
59.8	1825.7814	0.0041	TSDTGDQTISNGTEANSK
28.6	1824.7973	0.9881	TSDTGDQTISNGTEANSK
7.4	1824.7832	1.0022	AFEADPFYSRTASQR
3.7	1825.8012	-0.0158	GKYSPTVQTRSISNK
3.0	1825.8012	-0.0158	GKYSPTVQTRSISNK
2.7	1825.8012	-0.0158	GKYSPTVQTRSISNK
0.5	1825.7709	0.0145	MMKDISNLEENDETR

Spectrum No: 51; Query: 905; Rank: 1

Peptide View

MS/MS Fragmentation of **IYYSDFSNQTINSMAEDGSNR**
Found in **IPI00205325**, Tax_Id=10116 Gene_Symbol=Lrp2 Low-density lipoprotein receptor-related protein 2 precursor

Match to Query 905: 2428.016948 from(1215.015750,2+)
Title: 091127RatKid_SCX01_16.2632.2632.2.dta
Data file K:\NewmanPaper\Piliang\3SubProteomes\Piliang3SP\mgf5ppm\SCX_3SubProteomes5ppm.mgf

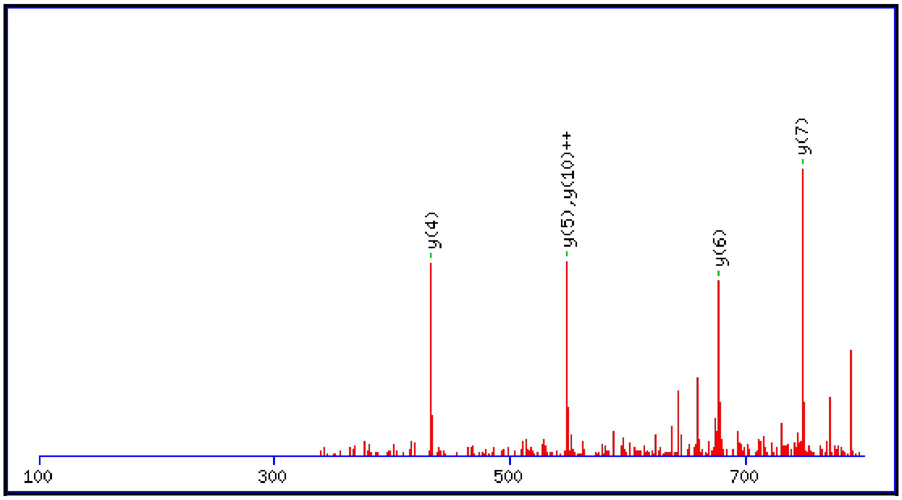

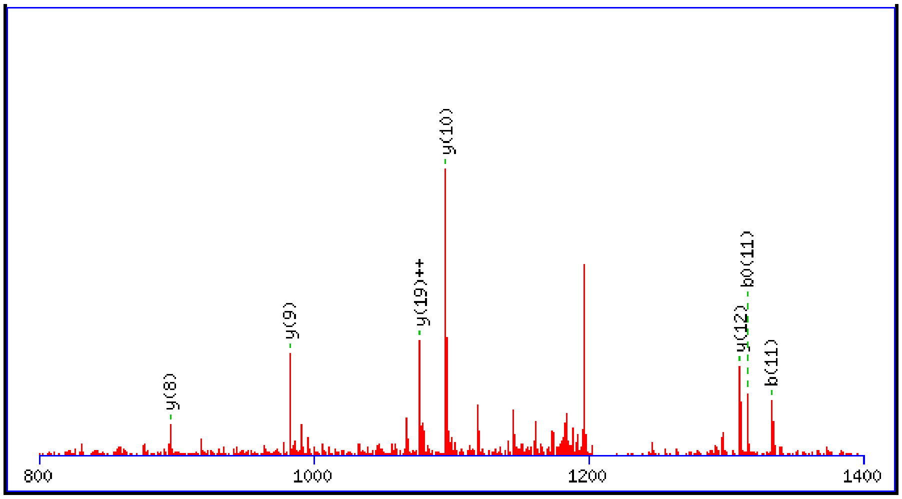

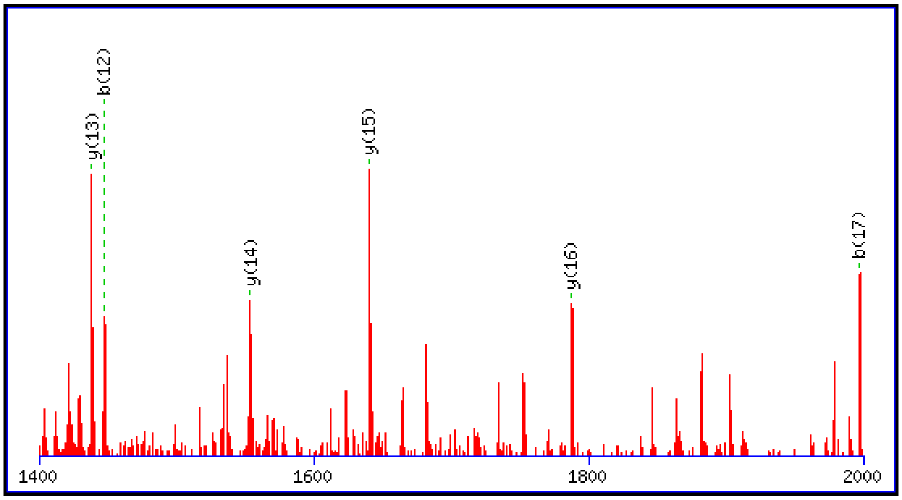

Monoisotopic mass of neutral peptide Mr(calc): 2428.0125
Fixed modifications: Carbamidomethyl (C)
Variable modifications:
N8 : Deamidated_N (N)
M14 : Oxidation (M)
Ions Score: 96 **Expect:** 9.2e-008
Matches (Bold Red): 18/216 fragment ions using 24 most intense peaks

#	b	b^{++}	b*	b^{*++}	b^0	b^{0++}	Seq.	y	y^{++}	y*	y^{*++}	y^0	y^{0++}	#
1	114.0913	57.5493					I							21
2	277.1547	139.0810					Y	2315.9357	1158.4715	2298.9092	1149.9582	2297.9251	1149.4662	20
3	440.2180	220.6126					Y	2152.8724	**1076.9398**	2135.8458	1068.4266	2134.8618	1067.9345	19
4	527.2500	264.1287			509.2395	255.1234	S	1989.8091	995.4082	1972.7825	986.8949	1971.7985	986.4029	18
5	642.2770	321.6421			624.2664	312.6368	D	1902.7770	951.8921	1885.7505	943.3789	1884.7665	942.8869	17
6	789.3454	395.1763			771.3348	386.1710	F	**1787.7501**	894.3787	1770.7235	885.8654	1769.7395	885.3734	16
7	876.3774	438.6923			858.3668	429.6871	S	**1640.6817**	820.8445	1623.6551	812.3312	1622.6711	811.8392	15
8	991.4043	496.2058	974.3778	487.6925	973.3938	487.2005	N	**1553.6496**	777.3285	1536.6231	768.8152	1535.6391	768.3232	14
9	1119.4629	560.2351	1102.4364	551.7218	1101.4524	551.2298	Q	**1438.6227**	719.8150	1421.5962	711.3017	1420.6121	710.8097	13
10	1220.5106	610.7589	1203.4841	602.2457	1202.5000	601.7537	T	**1310.5641**	655.7857	1293.5376	647.2724	1292.5536	646.7804	12
11	**1333.5947**	667.3010	1316.5681	658.7877	**1315.5841**	658.2957	I	1209.5164	605.2619	1192.4899	596.7486	1191.5059	596.2566	11
12	**1447.6376**	724.3224	1430.6110	715.8092	1429.6270	715.3172	N	**1096.4324**	**548.7198**	1079.4058	540.2066	1078.4218	539.7145	10
13	1534.6696	767.8384	1517.6431	759.3252	1516.6591	758.8332	S	**982.3895**	491.6984	965.3629	483.1851	964.3789	482.6931	9
14	1681.7050	841.3562	1664.6785	832.8429	1663.6945	832.3509	M	**895.3574**	448.1824	878.3309	439.6691	877.3469	439.1771	8
15	1752.7421	876.8747	1735.7156	868.3614	1734.7316	867.8694	A	**748.3220**	374.6646	731.2955	366.1514	730.3115	365.6594	7
16	1881.7847	941.3960	1864.7582	932.8827	1863.7742	932.3907	E	**677.2849**	339.1461	660.2584	330.6328	659.2743	330.1408	6
17	**1996.8117**	998.9095	1979.7851	990.3962	1978.8011	989.9042	D	**548.2423**	274.6248	531.2158	266.1115	530.2317	265.6195	5
18	2053.8331	1027.4202	2036.8066	1018.9069	2035.8226	1018.4149	G	**433.2154**	217.1113	416.1888	208.5980	415.2048	208.1060	4

**19**	2140.8652	1070.9362	2123.8386	1062.4229	2122.8546	1061.9309	**S**	376.1939	188.6006	359.1674	180.0873	358.1833	179.5953	**3**
**20**	2254.9081	1127.9577	2237.8815	1119.4444	2236.8975	1118.9524	**N**	289.1619	145.0846	272.1353	136.5713			**2**
**21**							**R**	175.1190	88.0631	158.0924	79.5498			**1**

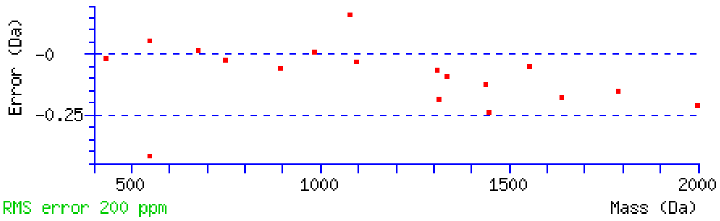

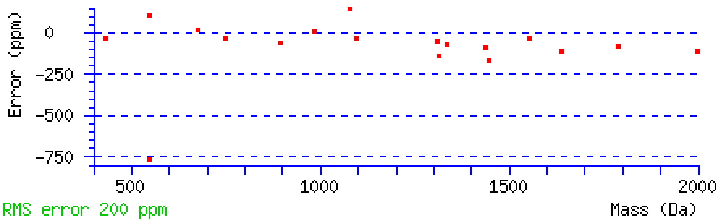

All matches to this query

Score	Mr(calc):	Delta	Sequence
95.6	2428.0125	0.0045	IYYSDFSNQTINSMAEDGSNR
87.0	2428.0125	0.0045	IYYSDFSNQTINSMAEDGSNR
62.6	2428.0125	0.0045	IYYSDFSNQTINSMAEDGSNR
52.0	2427.0285	0.9885	IYYSDFSNQTINSMAEDGSNR
4.5	2428.0352	-0.0182	EGICGSCAMNINGGNTLACTRR
3.1	2425.9876	2.0294	GPGGVEGSINAGGCNFNSFLRR
3.1	2425.9876	2.0294	GPGGVEGSINAGGCNFNSFLRR
3.0	2428.0171	-0.0002	VQVSLDEHFSKMAAETDPHK
2.2	2428.0325	-0.0155	KLSKATQDSLAEATQLAEER
2.1	2427.0236	0.9934	QSEWHKMTVSKNCPDQDLK

Spectrum No: 52; Query: 486; Rank: 1

Peptide View

MS/MS Fragmentation of **LDAPTNLQFVNETDR**
Found in **IPI00200757**, Tax_Id=10116 Gene_Symbol=Fn1 Isoform 1 of Fibronectin precursor

Match to Query 486: 1732.831488 from(867.423020,2+)
Title: 091127RatKid_SCX01_11.2303.2303.2.dta
Data file K:\NewmanPaper\Piliang\3SubProteomes\Piliang3SP\mgf5ppm\SCX_3SubProteomes5ppm.mgf

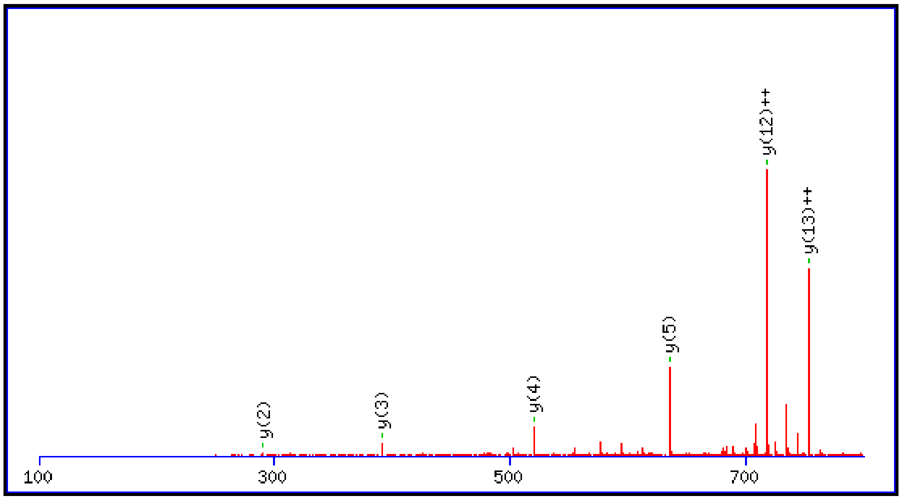

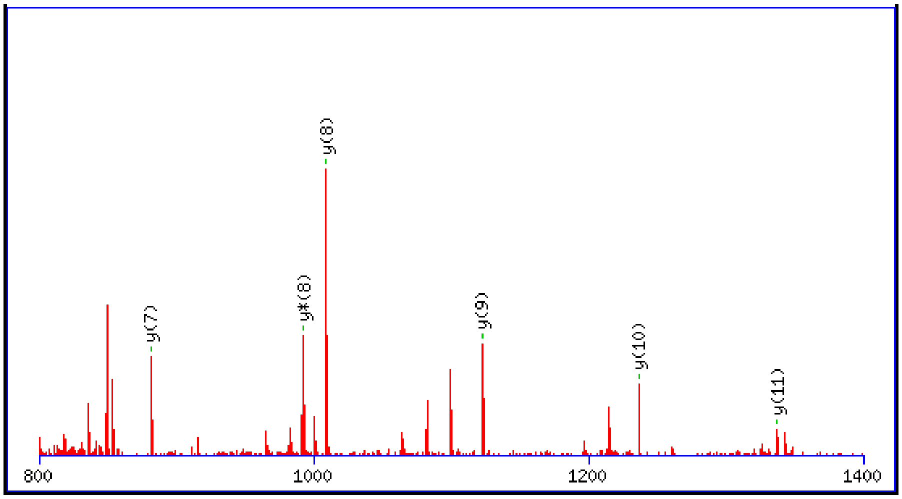

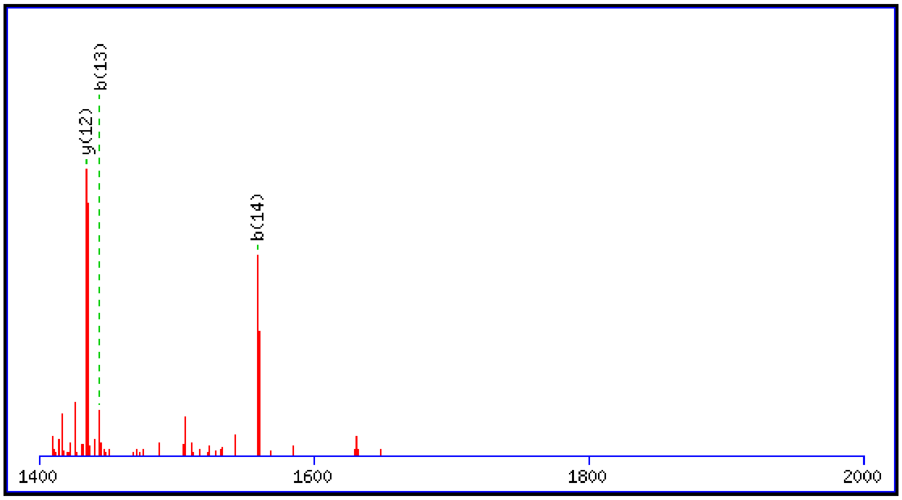

Monoisotopic mass of neutral peptide Mr(calc): 1732.8268
Fixed modifications: Carbamidomethyl (C)
Variable modifications:
N11 : Deamidated_N (N)
Ions Score: 95 **Expect:** 6.6e-008
Matches (Bold Red): 15/154 fragment ions using 20 most intense peaks

#	b	b^{++}	b*	b^{*++}	b^0	b^{0++}	Seq.	y	y^{++}	y*	y^{*++}	y^0	y^{0++}	#
1	114.0913	57.5493					L							15
2	229.1183	115.0628			211.1077	106.0575	D	1620.7500	810.8786	1603.7235	802.3654	1602.7394	801.8734	14
3	300.1554	150.5813			282.1448	141.5761	A	1505.7231	**753.3652**	1488.6965	744.8519	1487.7125	744.3599	13
4	397.2082	199.1077			379.1976	190.1024	P	**1434.6859**	**717.8466**	1417.6594	709.3333	1416.6754	708.8413	12
5	498.2558	249.6316			480.2453	240.6263	T	**1337.6332**	669.3202	1320.6066	660.8070	1319.6226	660.3149	11
6	612.2988	306.6530	595.2722	298.1397	594.2882	297.6477	N	**1236.5855**	618.7964	1219.5590	610.2831	1218.5749	609.7911	10
7	725.3828	363.1951	708.3563	354.6818	707.3723	354.1898	L	**1122.5426**	561.7749	1105.5160	553.2617	1104.5320	552.7696	9
8	853.4414	427.2243	836.4149	418.7111	835.4308	418.2191	Q	**1009.4585**	505.2329	**992.4320**	496.7196	991.4479	496.2276	8
9	1000.5098	500.7585	983.4833	492.2453	982.4993	491.7533	F	**881.3999**	441.2036	864.3734	432.6903	863.3894	432.1983	7
10	1099.5782	550.2928	1082.5517	541.7795	1081.5677	541.2875	V	734.3315	367.6694	717.3050	359.1561	716.3210	358.6641	6
11	1214.6052	607.8062	1197.5786	599.2930	1196.5946	598.8009	N	**635.2631**	318.1352	618.2366	309.6219	617.2525	309.1299	5
12	1343.6478	672.3275	1326.6212	663.8142	1325.6372	663.3222	E	**520.2362**	260.6217	503.2096	252.1084	502.2256	251.6164	4
13	**1444.6954**	722.8514	1427.6689	714.3381	1426.6849	713.8461	T	**391.1936**	196.1004	374.1670	187.5872	373.1830	187.0951	3
14	**1559.7224**	780.3648	1542.6958	771.8516	1541.7118	771.3595	D	**290.1459**	145.5766	273.1193	137.0633	272.1353	136.5713	2
15							R	175.1190	88.0631	158.0924	79.5498			1

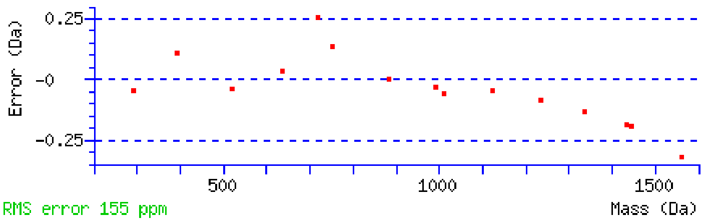

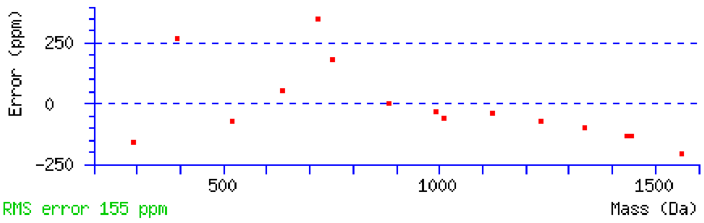

All matches to this query

Score	Mr(calc):	Delta	Sequence
95.5	1732.8268	0.0047	LDAPTNLQFVNETDR
34.2	1732.8268	0.0047	LDAPTNLQFVNETDR
20.4	1731.8428	0.9887	LDAPTNLQFVNETDR
3.6	1731.8120	1.0195	EPNGLIVLYEVSYR
2.3	1732.8331	-0.0016	EASLLVSCRTSKFR
2.3	1732.8331	-0.0016	EASLLVSCRTSKFR
1.8	1730.8410	1.9905	ECGKTFKYQSNLTR
1.7	1732.8331	-0.0016	EASLLVSCRTSKFR
1.1	1731.8122	1.0192	MSPKSMKMALSGVASK
1.0	1732.8156	0.0159	DSPDELPVYVGTNEAK

Spectrum No: 53; Query: 832; Rank: 1

Peptide View

MS/MS Fragmentation of **NVIIWGNHSSTQYPDVNHAK**
Found in **IPI00198717**, Tax_Id=10116 Gene_Symbol=Mdh1 Malate dehydrogenase, cytoplasmic

Match to Query 832: 2280.095488 from(1141.055020,2+)
Title: 091129RatKid_SCX02_35.1941.1941.2.dta
Data file K:\NewmanPaper\Piliang\3SubProteomes\Piliang3SP\mgf5ppm\SCX_3SubProteomes5ppm.mgf

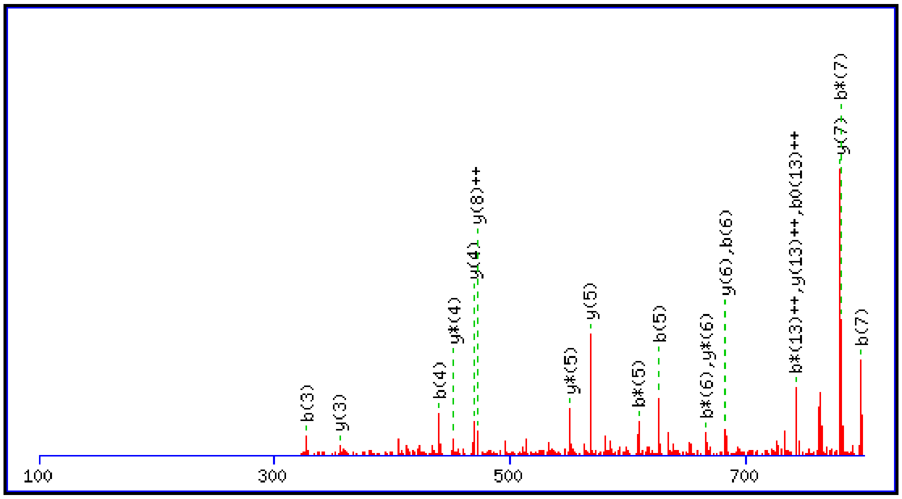

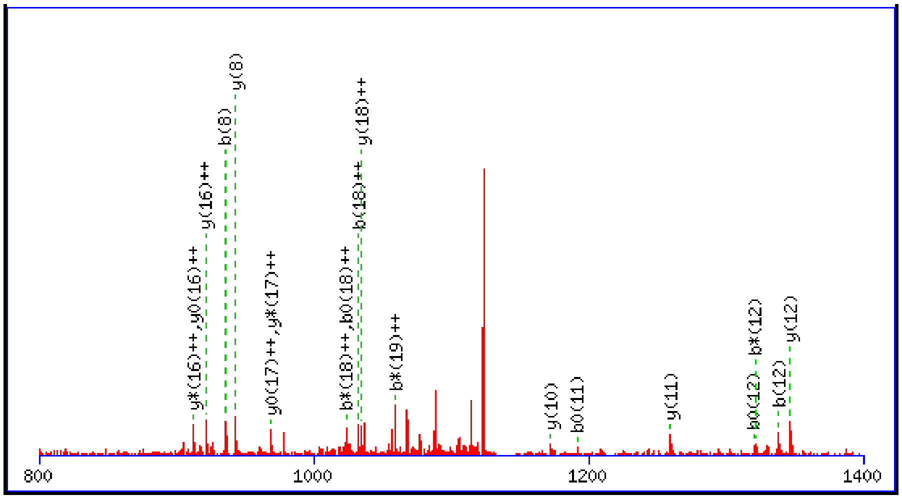

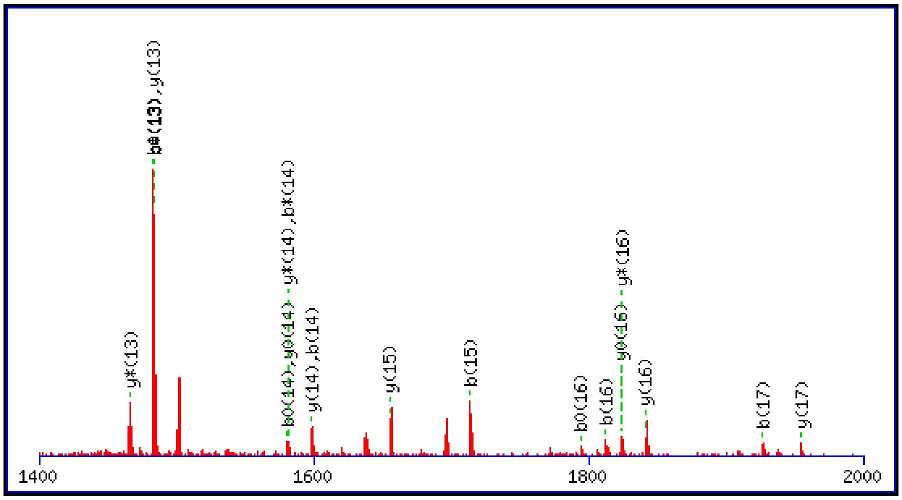

Monoisotopic mass of neutral peptide Mr(calc): 2280.0923
Fixed modifications: Carbamidomethyl (C)
Variable modifications:
N7 : Deamidated_N (N)
Ions Score: 95 **Expect:** 8.3e-008
Matches (Bold Red): 58/202 fragment ions using 77 most intense peaks

#	b	b^{++}	b*	b^{*++}	b^0	b^{0++}	Seq.	y	y^{++}	y*	y^{*++}	y^0	y^{0++}	#
1	115.0502	58.0287	98.0237	49.5155			N							20
2	214.1186	107.5629	197.0921	99.0497			V	2167.0567	1084.0320	2150.0301	1075.5187	2149.0461	1075.0267	19
3	**327.2027**	164.1050	310.1761	155.5917			I	2067.9883	**1034.4978**	2050.9617	1025.9845	2049.9777	1025.4925	18
4	**440.2867**	220.6470	423.2602	212.1337			I	**1954.9042**	977.9557	1937.8777	**969.4425**	1936.8936	**968.9505**	17
5	**626.3661**	313.6867	**609.3395**	305.1734			W	**1841.8201**	**921.4137**	**1824.7936**	**912.9004**	**1823.8096**	**912.4084**	16
6	**683.3875**	342.1974	**666.3610**	333.6841			G	**1655.7408**	828.3741	1638.7143	819.8608	1637.7303	819.3688	15
7	**798.4145**	399.7109	**781.3879**	391.1976			N	**1598.7194**	799.8633	**1581.6928**	791.3500	**1580.7088**	790.8580	14
8	**935.4734**	468.2403	918.4468	459.7270			H	**1483.6924**	**742.3499**	**1466.6659**	733.8366	1465.6819	733.3446	13
9	1022.5054	511.7563	1005.4788	503.2431	1004.4948	502.7511	S	**1346.6335**	673.8204	1329.6070	665.3071	1328.6230	664.8151	12
10	1109.5374	555.2724	1092.5109	546.7591	1091.5269	546.2671	S	**1259.6015**	630.3044	1242.5749	621.7911	1241.5909	621.2991	11
11	1210.5851	605.7962	1193.5586	597.2829	**1192.5745**	596.7909	T	**1172.5695**	586.7884	1155.5429	578.2751	1154.5589	577.7831	10
12	**1338.6437**	669.8255	**1321.6171**	661.3122	**1320.6331**	660.8202	Q	1071.5218	536.2645	1054.4952	527.7513	1053.5112	527.2592	9
13	1501.7070	751.3571	**1484.6805**	**742.8439**	**1483.6964**	**742.3519**	Y	**943.4632**	**472.2352**	926.4367	463.7220	925.4526	463.2300	8
14	**1598.7598**	799.8835	**1581.7332**	791.3703	**1580.7492**	790.8782	P	**780.3999**	390.7036	763.3733	382.1903	762.3893	381.6983	7
15	**1713.7867**	857.3970	1696.7602	848.8837	1695.7762	848.3917	D	**683.3471**	342.1772	**666.3206**	333.6639	665.3365	333.1719	6
16	**1812.8551**	906.9312	1795.8286	898.4179	**1794.8446**	897.9259	V	**568.3202**	284.6637	**551.2936**	276.1504			5
17	**1926.8981**	963.9527	1909.8715	955.4394	1908.8875	954.9474	N	**469.2518**	235.1295	**452.2252**	226.6162			4
18	2063.9570	**1032.4821**	2046.9304	**1023.9688**	2045.9464	**1023.4768**	H	**355.2088**	178.1081	338.1823	169.5948			3

19	2134.9941	1068.0007	2117.9675	**1059.4874**	2116.9835	1058.9954	**A**	218.1499	109.5786	201.1234	101.0653			2
20							**K**	147.1128	74.0600	130.0863	65.5468			1

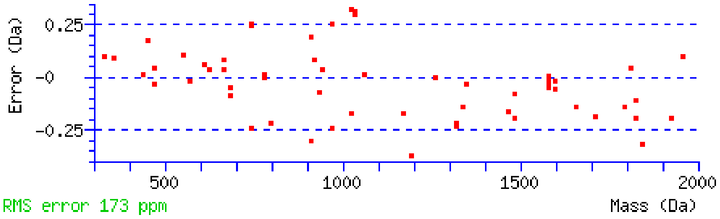

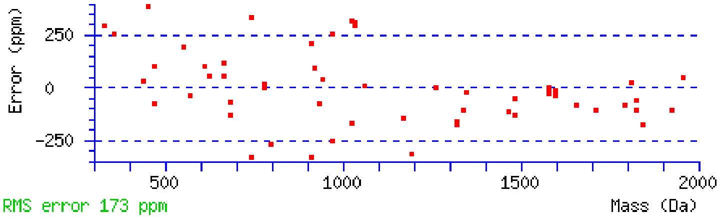

All matches to this query

Score	Mr(calc):	Delta	Sequence
95.3	2280.0923	0.0032	NVIIWGNHSSTQYPDVNHAK
58.0	2280.0923	0.0032	NVIIWGNHSSTQYPDVNHAK
51.3	2280.0923	0.0032	NVIIWGNHSSTQYPDVNHAK
33.3	2279.1083	0.9872	NVIIWGNHSSTQYPDVNHAK
0.3	2280.0791	0.0164	VNLSVALVDMVDSNSTLTDNR

Spectrum No: 54; Query: 981; Rank: 1

Peptide View

MS/MS Fragmentation of **YEVDWNQSTLDCVDPLSSLAADR**
Found in **IPI00188881**, Tax_Id=10116 Gene_Symbol=Slc22a2 Organic cation transporter OCT2

Match to Query 981: 2654.189188 from(1328.101870,2+)
Title: 091127RatKid_SCX01_02.4736.4736.2.dta
Data file K:\NewmanPaper\Piliang\3SubProteomes\Piliang3SP\mgf5ppm\SCX_3SubProteomes5ppm.mgf

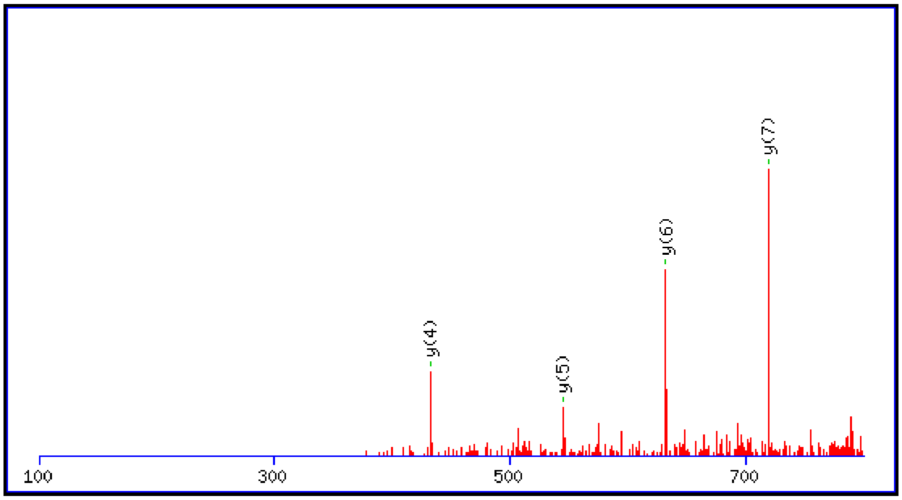

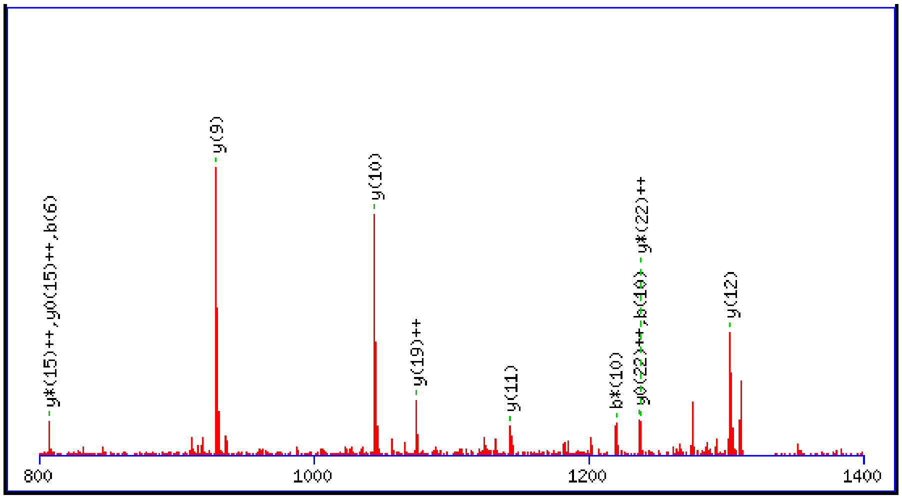

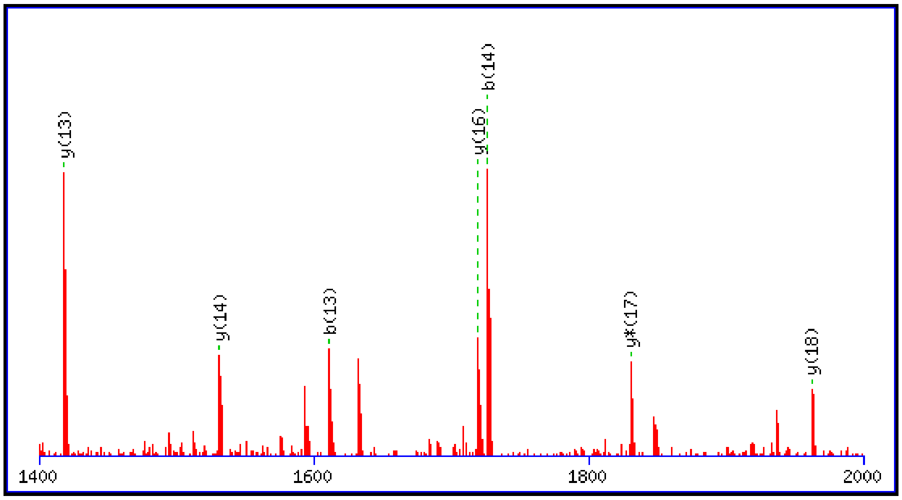

Monoisotopic mass of neutral peptide Mr(calc): 2654.1806
Fixed modifications: Carbamidomethyl (C)
Variable modifications:
N6 : Deamidated_N (N)
Ions Score: 95 **Expect:** 1.1e-007
Matches (Bold Red): 23/250 fragment ions using 29 most intense peaks

#	b	b++	b*	b*++	b0	b0++	Seq.	y	y++	y*	y*++	y0	y0++	#
1	164.0706	82.5389					Y							23
2	293.1132	147.0602			275.1026	138.0550	E	2492.1246	1246.5659	2475.0980	**1238.0526**	2474.1140	**1237.5606**	22
3	392.1816	196.5944			374.1710	187.5892	V	2363.0820	1182.0446	2346.0554	1173.5314	2345.0714	1173.0393	21
4	507.2086	254.1079			489.1980	245.1026	D	2264.0136	1132.5104	2246.9870	1123.9971	2246.0030	1123.5051	20
5	693.2879	347.1476			675.2773	338.1423	W	2148.9866	**1074.9969**	2131.9601	1066.4837	2130.9761	1065.9917	19
6	**808.3148**	404.6610	791.2883	396.1478	790.3042	395.6558	N	**1962.9073**	981.9573	1945.8808	973.4440	1944.8967	972.9520	18
7	936.3734	468.6903	919.3468	460.1771	918.3628	459.6850	Q	1847.8804	924.4438	**1830.8538**	915.9305	1829.8698	915.4385	17
8	1023.4054	512.2063	1006.3789	503.6931	1005.3948	503.2011	S	**1719.8218**	860.4145	1702.7952	851.9013	1701.8112	851.4093	16
9	1124.4531	562.7302	1107.4265	554.2169	1106.4425	553.7249	T	1632.7898	816.8985	1615.7632	**808.3852**	1614.7792	**807.8932**	15
10	**1237.5372**	619.2722	**1220.5106**	610.7589	1219.5266	610.2669	L	**1531.7421**	766.3747	1514.7155	757.8614	1513.7315	757.3694	14
11	1352.5641	676.7857	1335.5375	668.2724	1334.5535	667.7804	D	**1418.6580**	709.8326	1401.6315	701.3194	1400.6475	700.8274	13
12	1512.5947	756.8010	1495.5682	748.2877	1494.5842	747.7957	C	**1303.6311**	652.3192	1286.6045	643.8059	1285.6205	643.3139	12
13	**1611.6632**	806.3352	1594.6366	797.8219	1593.6526	797.3299	V	**1143.6004**	572.3039	1126.5739	563.7906	1125.5899	563.2986	11
14	**1726.6901**	863.8487	1709.6636	855.3354	1708.6795	854.8434	D	**1044.5320**	522.7696	1027.5055	514.2564	1026.5214	513.7644	10
15	1823.7429	912.3751	1806.7163	903.8618	1805.7323	903.3698	P	**929.5051**	465.2562	912.4785	456.7429	911.4945	456.2509	9
16	1936.8269	968.9171	1919.8004	960.4038	1918.8164	959.9118	L	832.4523	416.7298	815.4258	408.2165	814.4417	407.7245	8
17	2023.8590	1012.4331	2006.8324	1003.9198	2005.8484	1003.4278	S	**719.3682**	360.1878	702.3417	351.6745	701.3577	351.1825	7
18	2110.8910	1055.9491	2093.8644	1047.4359	2092.8804	1046.9438	S	**632.3362**	316.6717	615.3097	308.1585	614.3257	307.6665	6

**19**	2223.9751	1112.4912	2206.9485	1103.9779	2205.9645	1103.4859	**L**	545.3042	273.1557	528.2776	264.6425	527.2936	264.1504	**5**
**20**	2295.0122	1148.0097	2277.9856	1139.4964	2277.0016	1139.0044	**A**	432.2201	216.6137	415.1936	208.1004	414.2096	207.6084	**4**
**21**	2366.0493	1183.5283	2349.0227	1175.0150	2348.0387	1174.5230	**A**	361.1830	181.0951	344.1565	172.5819	343.1724	172.0899	**3**
**22**	2481.0762	1241.0417	2464.0497	1232.5285	2463.0657	1232.0365	**D**	290.1459	145.5766	273.1193	137.0633	272.1353	136.5713	**2**
**23**							**R**	175.1190	88.0631	158.0924	79.5498			**1**

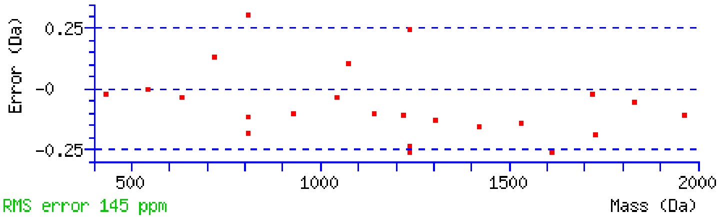

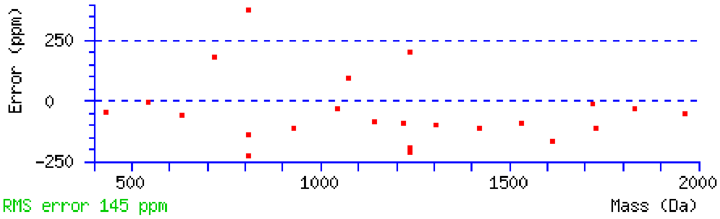

All matches to this query

Score	Mr(calc):	Delta	Sequence
95.2	2654.1806	0.0086	YEVDWNQSTLDCVDPLSSLAADR
70.4	2653.1966	0.9926	YEVDWNQSTLDCVDPLSSLAADR
5.2	2654.1763	0.0129	FSPCCVVCFXVSLRPIGLAWR
2.5	2652.1737	2.0155	SISIAALLDVNKEEPNKELNSK
2.5	2652.1737	2.0155	SISIAALLDVNKEEPNKELNSK
2.5	2652.1737	2.0155	SISIAALLDVNKEEPNKELNSK
1.8	2652.1672	2.0220	TKELIAEIGSLSLHCTIDEKR
0.4	2653.2056	0.9836	TMSKSDMLNALEEVINNPFYKK
0.4	2653.2056	0.9836	TMSKSDMLNALEEVINNPFYKK
0.4	2653.2056	0.9836	TMSKSDMLNALEEVINNPFYKK

Spectrum No: 55; Query: 438; Rank: 1

Peptide View

MS/MS Fragmentation of **TCVSNCTASQFVCK**
Found in **IPI00369995**, Tax_Id=10116 Gene_Symbol=Lrp1 similar to low density lipoprotein receptor-related protein 1

Match to Query 438: 1661.688108 from(831.851330,2+)
Title: 091127RatKid_SCX01_12.1573.1573.2.dta
Data file K:\NewmanPaper\Piliang\3SubProteomes\Piliang3SP\mgf5ppm\SCX_3SubProteomes5ppm.mgf

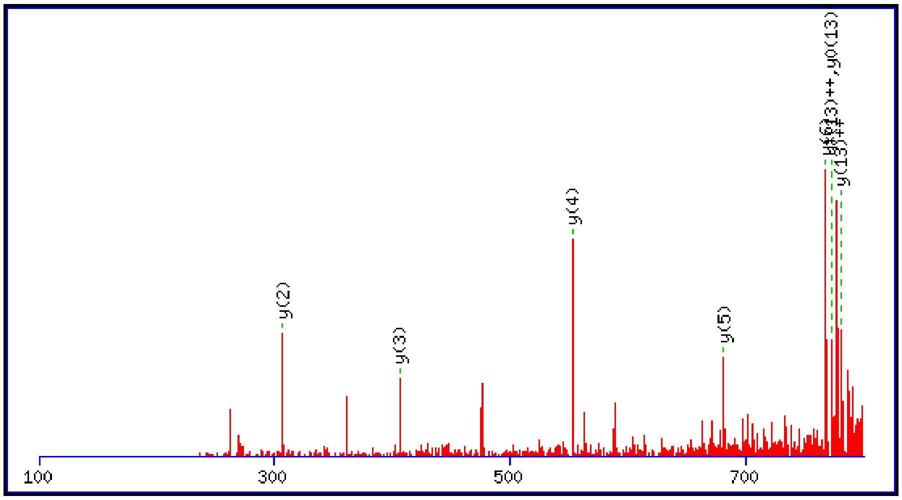

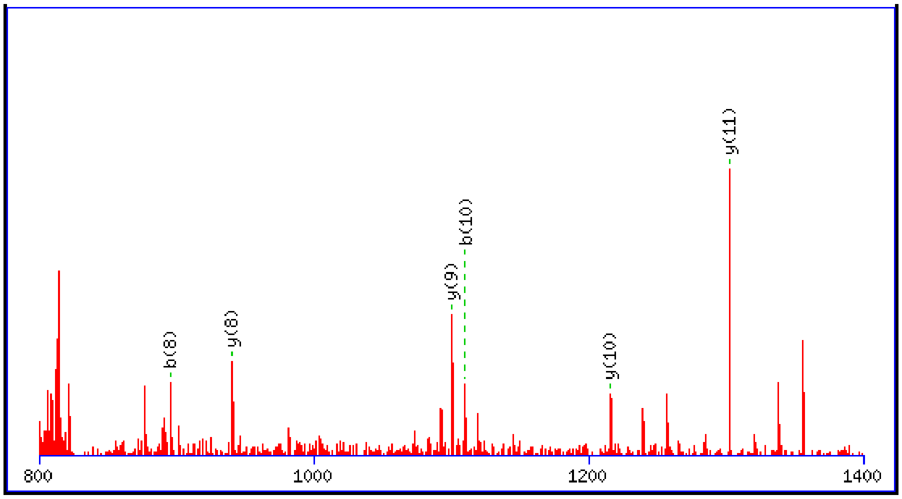

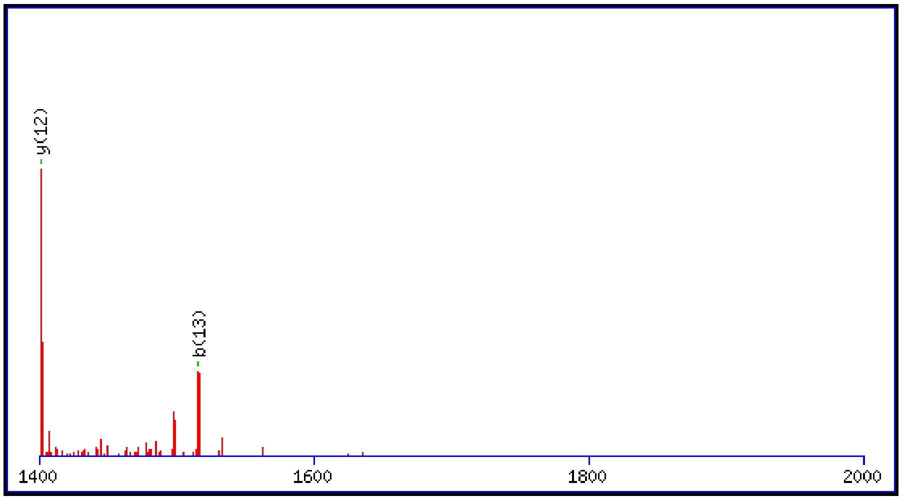

Monoisotopic mass of neutral peptide Mr(calc): 1661.6848
Fixed modifications: Carbamidomethyl (C)
Variable modifications:
N5 : Deamidated_N (N)
Ions Score: 95 **Expect:** 5.3e-008
Matches (Bold Red): 16/138 fragment ions using 29 most intense peaks

#	b	b^{++}	b*	b^{*++}	b^0	b^{0++}	Seq.	y	y^{++}	y*	y^{*++}	y^0	y^{0++}	#
1	102.0550	51.5311			84.0444	42.5258	T							14
2	262.0856	131.5464			244.0750	122.5412	C	1561.6444	**781.3258**	1544.6178	**772.8125**	1543.6338	**772.3205**	13
3	361.1540	181.0806			343.1435	172.0754	V	**1401.6137**	701.3105	1384.5872	692.7972	1383.6031	692.3052	12
4	448.1860	224.5967			430.1755	215.5914	S	**1302.5453**	651.7763	1285.5187	643.2630	1284.5347	642.7710	11
5	563.2130	282.1101	546.1864	273.5969	545.2024	273.1048	N	**1215.5133**	608.2603	1198.4867	599.7470	1197.5027	599.2550	10
6	723.2436	362.1255	706.2171	353.6122	705.2331	353.1202	C	**1100.4863**	550.7468	1083.4598	542.2335	1082.4758	541.7415	9
7	824.2913	412.6493	807.2648	404.1360	806.2807	403.6440	T	**940.4557**	470.7315	923.4291	462.2182	922.4451	461.7262	8
8	**895.3284**	448.1679	878.3019	439.6546	877.3179	439.1626	A	839.4080	420.2076	822.3815	411.6944	821.3974	411.2024	7
9	982.3605	491.6839	965.3339	483.1706	964.3499	482.6786	S	**768.3709**	384.6891	751.3443	376.1758	750.3603	375.6838	6
10	**1110.4190**	555.7132	1093.3925	547.1999	1092.4085	546.7079	Q	**681.3389**	341.1731	664.3123	332.6598			5
11	1257.4874	629.2474	1240.4609	620.7341	1239.4769	620.2421	F	**553.2803**	277.1438	536.2537	268.6305			4
12	1356.5559	678.7816	1339.5293	670.2683	1338.5453	669.7763	V	**406.2119**	203.6096	389.1853	195.0963			3
13	**1516.5865**	758.7969	1499.5600	750.2836	1498.5759	749.7916	C	**307.1435**	154.0754	290.1169	145.5621			2
14							K	147.1128	74.0600	130.0863	65.5468			1

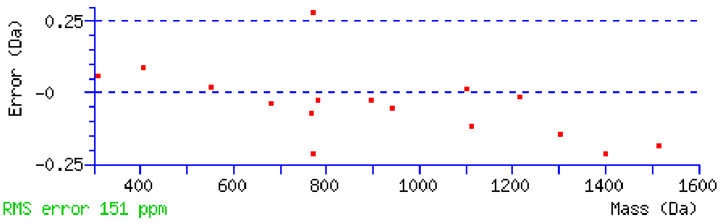

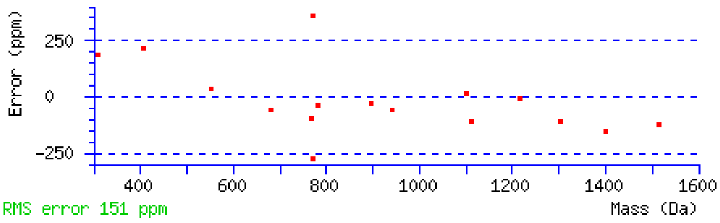

All matches to this query

Score	Mr(calc):	Delta	Sequence
95.0	1661.6848	0.0033	TCVSNCTASQFVCK
38.4	1660.7008	0.9873	TCVSNCTASQFVCK
2.0	1661.6967	-0.0086	STGNETTEMPKKSR
0.3	1661.6913	-0.0032	TCSVCITEYTEGNK

Spectrum No: 56; Query: 421; Rank: 1

Peptide View

MS/MS Fragmentation of **NCNSTTEFTCSNGR**
Found in **IPI00205325**, Tax_Id=10116 Gene_Symbol=Lrp2 Low-density lipoprotein receptor-related protein 2 precursor

Match to Query 421: 1647.629528 from(824.822040,2+)
Title: 091127RatKid_SCX01_13.550.550.2.dta
Data file K:\NewmanPaper\Piliang\3SubProteomes\Piliang3SP\mgf5ppm\SCX_3SubProteomes5ppm.mgf

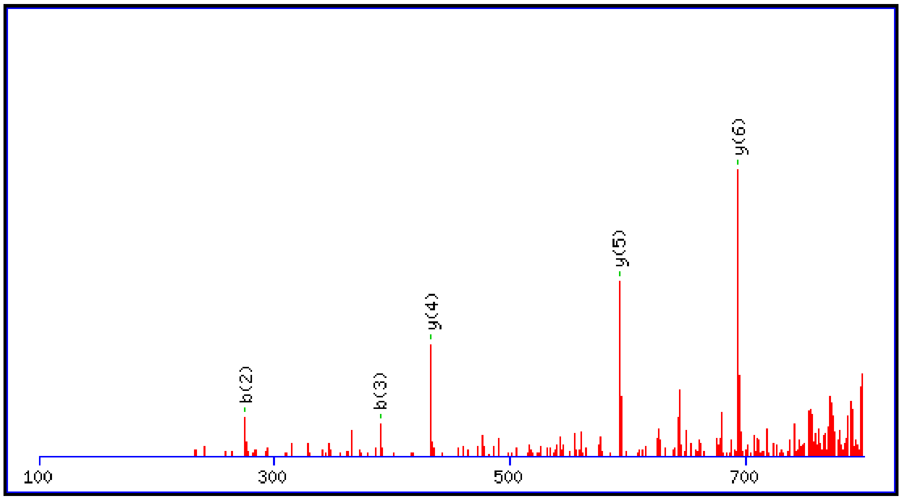

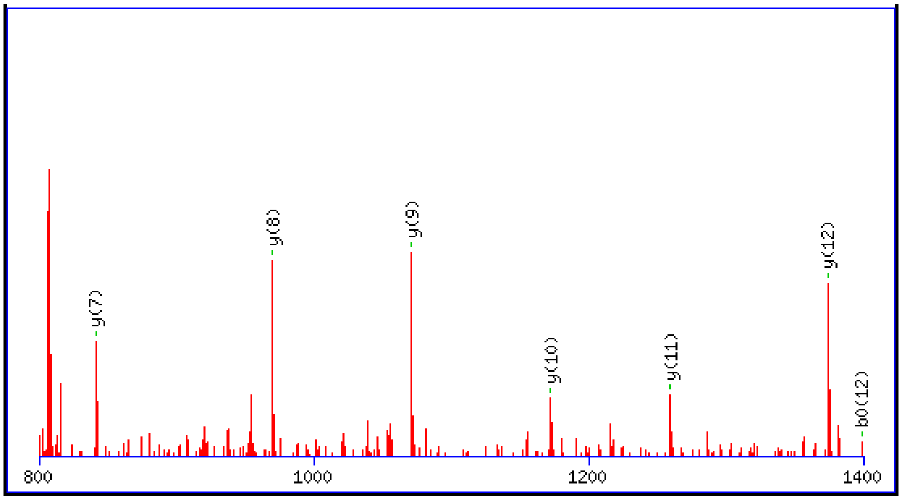

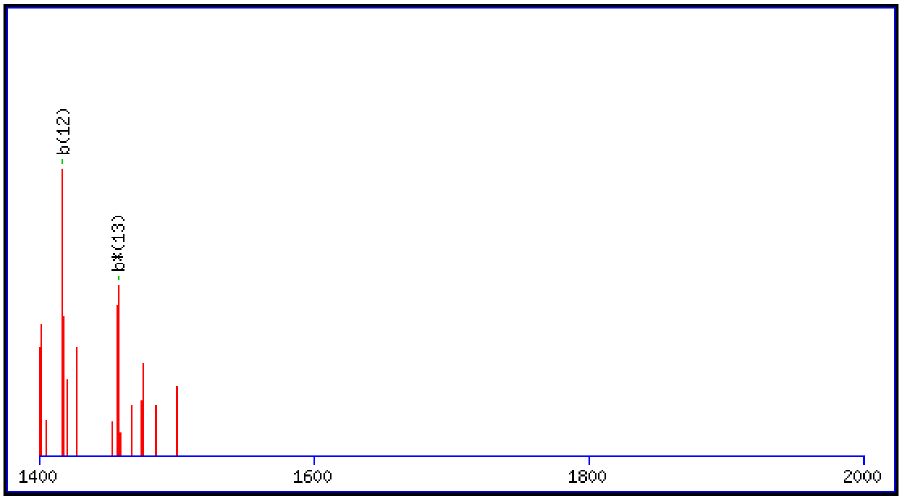

Monoisotopic mass of neutral peptide Mr(calc): 1647.6253
Fixed modifications: Carbamidomethyl (C)
Variable modifications:
N3 : Deamidated_N (N)
Ions Score: 94 **Expect:** 3.6e-008
Matches (Bold Red): 14/144 fragment ions using 18 most intense peaks

#	b	b^{++}	b*	b^{*++}	b^0	b^{0++}	Seq.	y	y^{++}	y*	y^{*++}	y^0	y^{0++}	#
1	115.0502	58.0287	98.0237	49.5155			N							14
2	**275.0809**	138.0441	258.0543	129.5308			C	1534.5897	767.7985	1517.5631	759.2852	1516.5791	758.7932	13
3	**390.1078**	195.5575	373.0812	187.0443			N	**1374.5590**	687.7832	1357.5325	679.2699	1356.5485	678.7779	12
4	477.1398	239.0735	460.1133	230.5603	459.1293	230.0683	S	**1259.5321**	630.2697	1242.5055	621.7564	1241.5215	621.2644	11
5	578.1875	289.5974	561.1609	281.0841	560.1769	280.5921	T	**1172.5001**	586.7537	1155.4735	578.2404	1154.4895	577.7484	10
6	679.2352	340.1212	662.2086	331.6080	661.2246	331.1159	T	**1071.4524**	536.2298	1054.4258	527.7166	1053.4418	527.2245	9
7	808.2778	404.6425	791.2512	396.1292	790.2672	395.6372	E	**970.4047**	485.7060	953.3782	477.1927	952.3941	476.7007	8
8	955.3462	478.1767	938.3196	469.6635	937.3356	469.1714	F	**841.3621**	421.1847	824.3356	412.6714	823.3515	412.1794	7
9	1056.3939	528.7006	1039.3673	520.1873	1038.3833	519.6953	T	**694.2937**	347.6505	677.2672	339.1372	676.2831	338.6452	6
10	1216.4245	608.7159	1199.3980	600.2026	1198.4139	599.7106	C	**593.2460**	297.1266	576.2195	288.6134	575.2355	288.1214	5
11	1303.4565	652.2319	1286.4300	643.7186	1285.4460	643.2266	S	**433.2154**	217.1113	416.1888	208.5980	415.2048	208.1060	4
12	**1417.4995**	709.2534	1400.4729	700.7401	**1399.4889**	700.2481	N	346.1833	173.5953	329.1568	165.0820			3
13	1474.5209	737.7641	**1457.4944**	729.2508	1456.5104	728.7588	G	232.1404	116.5738	215.1139	108.0606			2
14							R	175.1190	88.0631	158.0924	79.5498			1

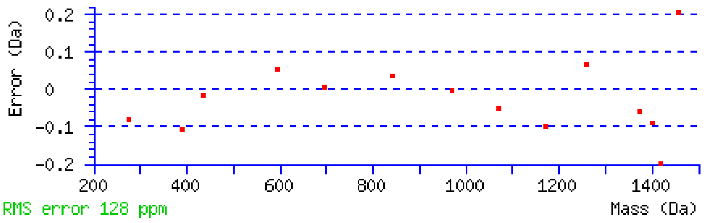

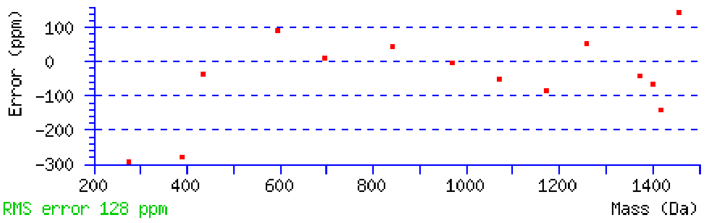

All matches to this query

Score	Mr(calc):	Delta	Sequence
94.5	1647.6253	0.0042	NCNSTTEFTCSNGR
65.5	1646.6413	0.9882	NCNSTTEFTCSNGR
57.6	1647.6253	0.0042	NCNSTTEFTCSNGR
42.3	1647.6253	0.0042	NCNSTTEFTCSNGR
4.8	1647.6365	-0.0069	MSKQTARASDSYK
4.4	1646.6177	1.0118	SSSSRPLSMSQLK
4.4	1646.6177	1.0118	SSSSRPLSMSQLK
2.3	1647.6365	-0.0070	AVTCSTPPQISNGR
1.9	1647.6331	-0.0036	IFSGNSHQNLNQK
0.8	1647.6331	-0.0036	IFSGNSHQNLNQK

Spectrum No: 57; Query: 1161; Rank: 1

Peptide View

MS/MS Fragmentation of **CISPLYVCDGDKDCADGSDEAGCVLNCTSAQFK**
Found in **IPI00205325**, Tax_Id=10116 Gene_Symbol=Lrp2 Low-density lipoprotein receptor-related protein 2 precursor

Match to Query 1161: 3712.514952 from(1238.512260,3+)
Title: 091129RatKid_SCX02_13.2776.2776.3.dta
Data file K:\NewmanPaper\Piliang\3SubProteomes\Piliang3SP\mgf5ppm\SCX_3SubProteomes5ppm.mgf

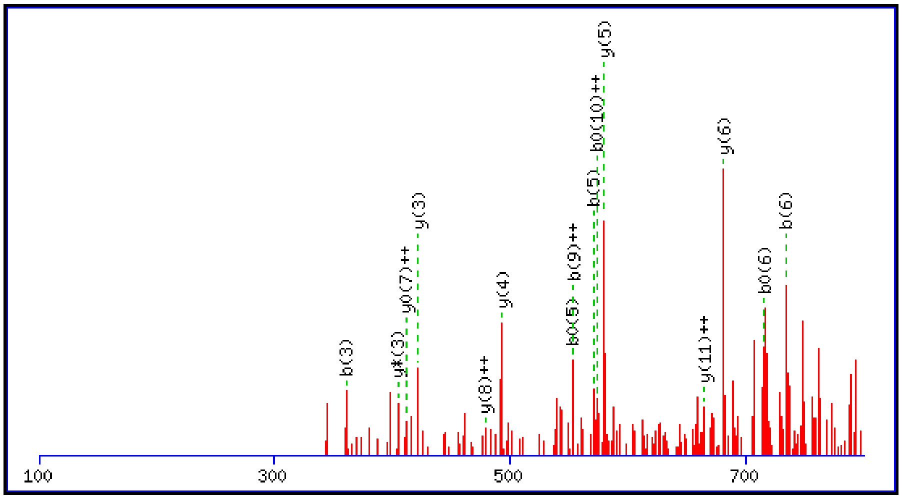

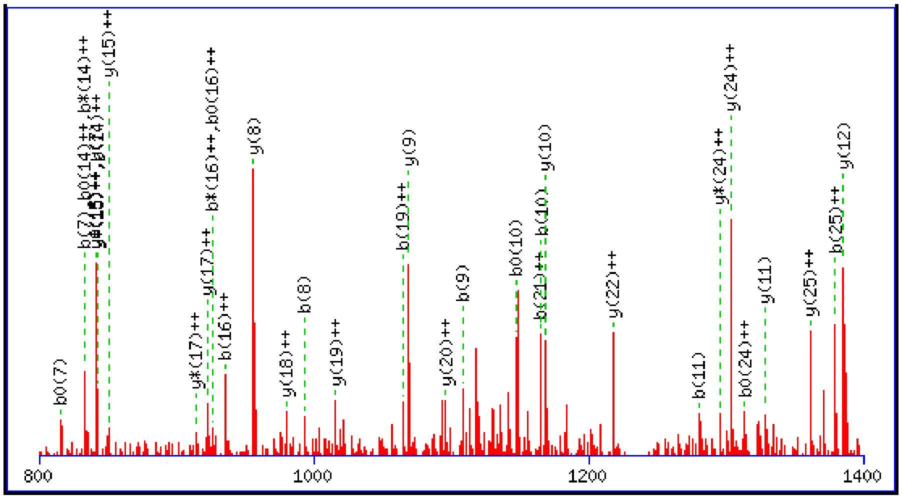

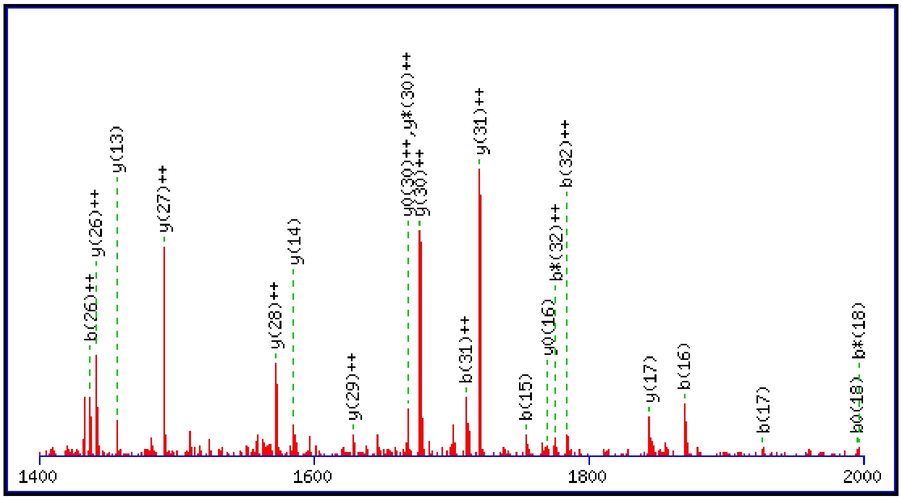

Monoisotopic mass of neutral peptide Mr(calc): 3712.5096
Fixed modifications: Carbamidomethyl (C)
Variable modifications:
N26 : Deamidated_N (N)
Ions Score: 94 **Expect:** 1.5e-007
Matches (Bold Red): 71/350 fragment ions using 128 most intense peaks

#	b	b^{++}	b*	b^{*++}	b^{0}	b^{0++}	Seq.	y	y^{++}	y*	y^{*++}	y^{0}	y^{0++}	#
1	161.0379	81.0226					C							33
2	274.1220	137.5646					I	3553.4862	1777.2467	3536.4597	1768.7335	3535.4756	1768.2415	32
3	**361.1540**	181.0806			343.1435	172.0754	S	3440.4021	**1720.7047**	3423.3756	1712.1914	3422.3916	1711.6994	31
4	458.2068	229.6070			440.1962	220.6017	P	3353.3701	**1677.1887**	3336.3436	**1668.6754**	3335.3596	**1668.1834**	30
5	**571.2908**	286.1491			**553.2803**	277.1438	L	3256.3174	**1628.6623**	3239.2908	1620.1490	3238.3068	1619.6570	29
6	**734.3542**	367.6807			**716.3436**	358.6754	Y	3143.2333	**1572.1203**	3126.2067	1563.6070	3125.2227	1563.1150	28
7	**833.4226**	417.2149			**815.4120**	408.2096	V	2980.1700	**1490.5886**	2963.1434	1482.0753	2962.1594	1481.5833	27
8	**993.4532**	497.2303			975.4427	488.2250	C	2881.1015	**1441.0544**	2864.0750	1432.5411	2863.0910	1432.0491	26
9	**1108.4802**	**554.7437**			1090.4696	545.7384	D	2721.0709	**1361.0391**	2704.0443	1352.5258	2703.0603	1352.0338	25
10	**1165.5016**	583.2545			**1147.4911**	**574.2492**	G	2606.0440	**1303.5256**	2589.0174	**1295.0123**	2588.0334	1294.5203	24
11	**1280.5286**	640.7679			1262.5180	631.7626	D	2549.0225	1275.0149	2531.9959	1266.5016	2531.0119	1266.0096	23
12	1408.6236	704.8154	1391.5970	696.3021	1390.6130	695.8101	K	2433.9955	**1217.5014**	2416.9690	1208.9881	2415.9850	1208.4961	22
13	1523.6505	762.3289	1506.6239	753.8156	1505.6399	753.3236	D	2305.9006	1153.4539	2288.8740	1144.9407	2287.8900	1144.4486	21
14	1683.6811	**842.3442**	1666.6546	**833.8309**	1665.6706	**833.3389**	C	2190.8736	**1095.9405**	2173.8471	1087.4272	2172.8631	1086.9352	20
15	**1754.7183**	877.8628	1737.6917	869.3495	1736.7077	868.8575	A	2030.8430	**1015.9251**	2013.8164	1007.4119	2012.8324	1006.9199	19
16	**1869.7452**	**935.3762**	1852.7187	**926.8630**	1851.7346	**926.3710**	D	1959.8059	**980.4066**	1942.7793	971.8933	1941.7953	971.4013	18
17	**1926.7667**	963.8870	1909.7401	955.3737	1908.7561	954.8817	G	**1844.7789**	**922.8931**	1827.7524	**914.3798**	1826.7684	913.8878	17
18	2013.7987	1007.4030	**1996.7721**	998.8897	**1995.7881**	998.3977	S	1787.7575	894.3824	1770.7309	885.8691	**1769.7469**	885.3771	16

19	2128.8256	1064.9165	2111.7991	1056.4032	2110.8151	1055.9112	D	1700.7254	850.8664	1683.6989	842.3531	1682.7149	841.8611	15
20	2257.8682	1129.4378	2240.8417	1120.9245	2239.8577	1120.4325	E	1585.6985	793.3529	1568.6720	784.8396	1567.6879	784.3476	14
21	2328.9053	1164.9563	2311.8788	1156.4430	2310.8948	1155.9510	A	1456.6559	728.8316	1439.6294	720.3183	1438.6453	719.8263	13
22	2385.9268	1193.4670	2368.9003	1184.9538	2367.9162	1184.4618	G	1385.6188	693.3130	1368.5922	684.7998	1367.6082	684.3078	12
23	2545.9575	1273.4824	2528.9309	1264.9691	2527.9469	1264.4771	C	1328.5973	664.8023	1311.5708	656.2890	1310.5868	655.7970	11
24	2645.0259	1323.0166	2627.9993	1314.5033	2627.0153	1314.0113	V	1168.5667	584.7870	1151.5401	576.2737	1150.5561	575.7817	10
25	2758.1099	1379.5586	2741.0834	1371.0453	2740.0994	1370.5533	L	1069.4983	535.2528	1052.4717	526.7395	1051.4877	526.2475	9
26	2873.1369	1437.0721	2856.1103	1428.5588	2855.1263	1428.0668	N	956.4142	478.7107	939.3877	470.1975	938.4036	469.7055	8
27	3033.1675	1517.0874	3016.1410	1508.5741	3015.1570	1508.0821	C	841.3873	421.1973	824.3607	412.6840	823.3767	412.1920	7
28	3134.2152	1567.6112	3117.1886	1559.0980	3116.2046	1558.6060	T	681.3566	341.1819	664.3301	332.6687	663.3461	332.1767	6
29	3221.2472	1611.1272	3204.2207	1602.6140	3203.2367	1602.1220	S	580.3089	290.6581	563.2824	282.1448	562.2984	281.6528	5
30	3292.2843	1646.6458	3275.2578	1638.1325	3274.2738	1637.6405	A	493.2769	247.1421	476.2504	238.6288			4
31	3420.3429	1710.6751	3403.3164	1702.1618	3402.3324	1701.6698	Q	422.2398	211.6235	405.2132	203.1103			3
32	3567.4113	1784.2093	3550.3848	1775.6960	3549.4008	1775.2040	F	294.1812	147.5942	277.1547	139.0810			2
33							K	147.1128	74.0600	130.0863	65.5468			1

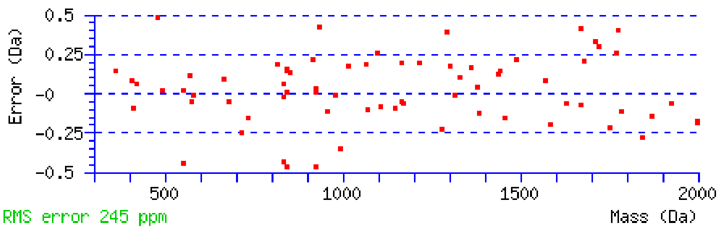

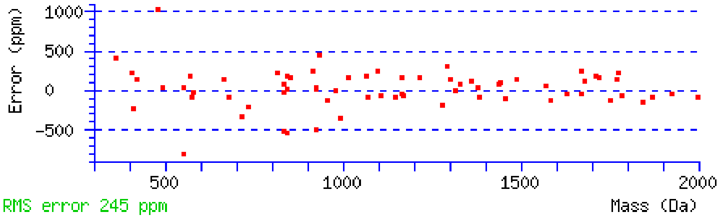

All matches to this query

Score	Mr(calc):	Delta	Sequence
94.3	3712.5096	0.0053	CISPLYVCDGDKDCADGSDEAGCVLNCTSAQFK
23.2	3711.5256	0.9893	CISPLYVCDGDKDCADGSDEAGCVLNCTSAQFK
1.4	3711.5088	1.0062	EAEKYSGAELVWCQEEHKNMGYYDYISPR
1.4	3711.5088	1.0062	EAEKYSGAELVWCQEEHKNMGYYDYISPR
1.4	3712.4928	0.0222	EAEKYSGAELVWCQEEHKNMGYYDYISPR
1.4	3712.4928	0.0222	EAEKYSGAELVWCQEEHKNMGYYDYISPR
0.9	3711.5427	0.9723	ARTHTNVMNATSPPAGSNGNSVTTPGDSGPPPLPR
0.9	3711.5427	0.9723	ARTHTNVMNATSPPAGSNGNSVTTPGDSGPPPLPR
0.5	3711.4892	1.0258	FGAYIMETENAAAGDGPHPDSGGVSWRXGQPALR
0.3	3711.5427	0.9723	ARTHTNVMNATSPPAGSNGNSVTTPGDSGPPPLPR

Spectrum No: 58; Query: 633; Rank: 1

Peptide View

MS/MS Fragmentation of **DQDPASFGEDSLLLNSSR**
Found in **IPI00193894**, Tax_Id=10116 Gene_Symbol=Mgam_predicted 198 kDa protein

Match to Query 633: 1950.880448 from(976.447500,2+)
Title: 091129RatKid_SCX02_12.2947.2947.2.dta
Data file K:\NewmanPaper\Piliang\3SubProteomes\Piliang3SP\mgf5ppm\SCX_3SubProteomes5ppm.mgf

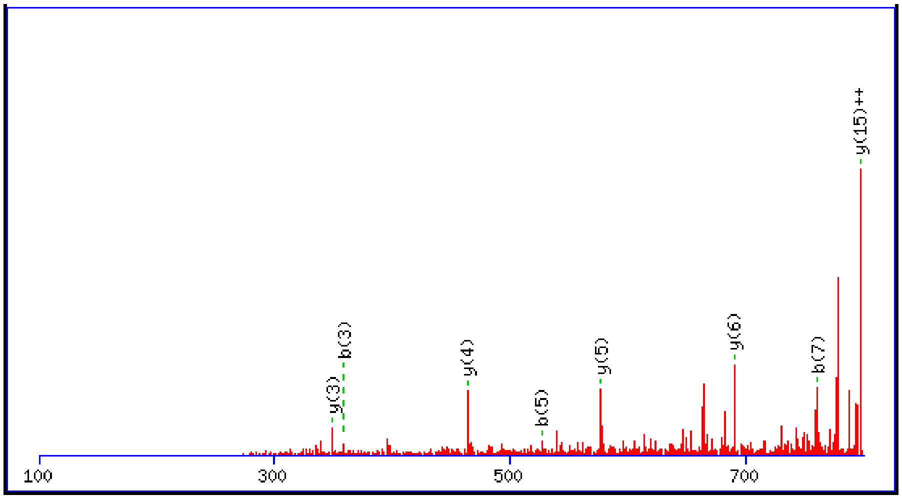

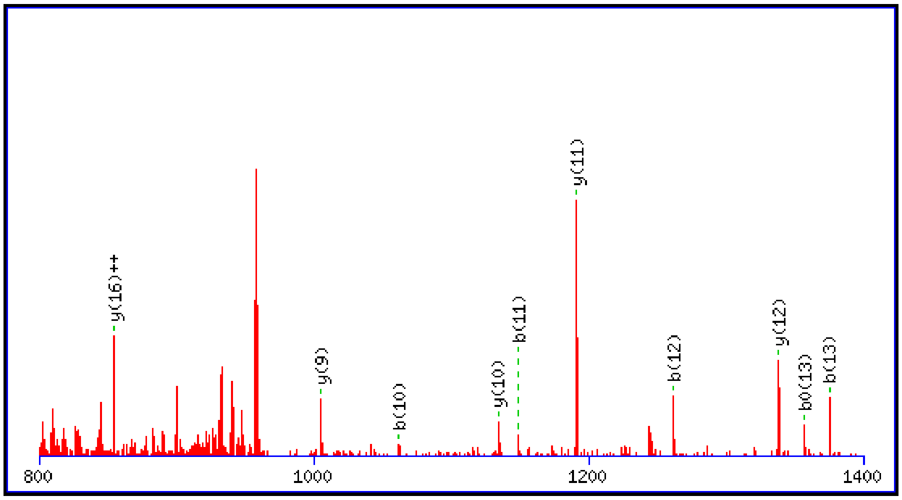

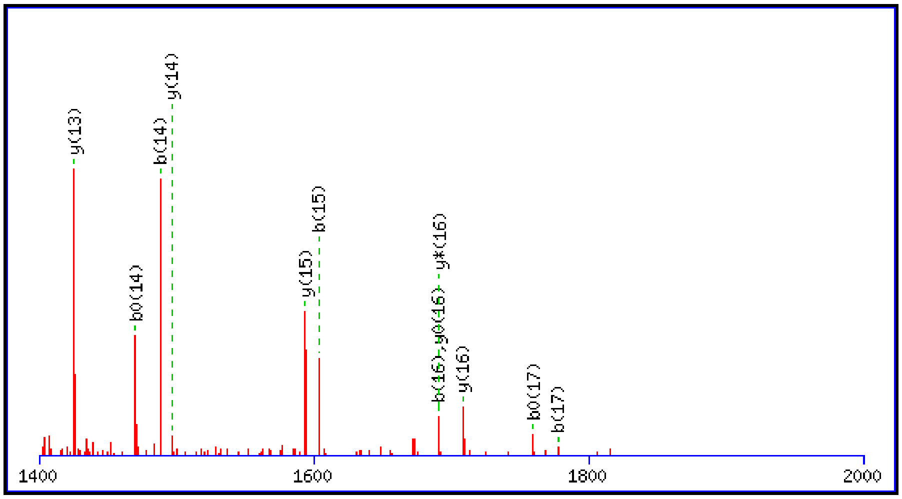

Monoisotopic mass of neutral peptide Mr(calc): 1950.8807
Fixed modifications: Carbamidomethyl (C)
Variable modifications:
N15 : Deamidated_N (N)
Ions Score: 94 **Expect:** 1e-007
Matches (Bold Red): 30/200 fragment ions using 51 most intense peaks

#	b	b^{++}	b*	b^{*++}	b^{0}	b^{0++}	Seq.	y	y^{++}	y*	y^{*++}	y^{0}	y^{0++}	#
1	116.0342	58.5207			98.0237	49.5155	D							18
2	244.0928	122.5500	227.0662	114.0368	226.0822	113.5448	Q	1836.8610	918.9341	1819.8345	910.4209	1818.8504	909.9289	17
3	**359.1197**	180.0635	342.0932	171.5502	341.1092	171.0582	D	**1708.8024**	**854.9049**	**1691.7759**	846.3916	**1690.7919**	845.8996	16
4	456.1725	228.5899	439.1460	220.0766	438.1619	219.5846	P	**1593.7755**	**797.3914**	1576.7489	788.8781	1575.7649	788.3861	15
5	**527.2096**	264.1084	510.1831	255.5952	509.1991	255.1032	A	**1496.7227**	748.8650	1479.6962	740.3517	1478.7122	739.8597	14

**6**	614.2416	307.6245	597.2151	299.1112	596.2311	298.6192	**S**	**1425.6856**	713.3464	1408.6591	704.8332	1407.6750	704.3412	**13**
**7**	**761.3101**	381.1587	744.2835	372.6454	743.2995	372.1534	**F**	**1338.6536**	669.8304	1321.6270	661.3172	1320.6430	660.8251	**12**
**8**	818.3315	409.6694	801.3050	401.1561	800.3210	400.6641	**G**	**1191.5852**	596.2962	1174.5586	587.7829	1173.5746	587.2909	**11**
**9**	947.3741	474.1907	930.3476	465.6774	929.3636	465.1854	**E**	**1134.5637**	567.7855	1117.5372	559.2722	1116.5531	558.7802	**10**
**10**	**1062.4011**	531.7042	1045.3745	523.1909	1044.3905	522.6989	**D**	**1005.5211**	503.2642	988.4946	494.7509	987.5105	494.2589	**9**
**11**	**1149.4331**	575.2202	1132.4065	566.7069	1131.4225	566.2149	**S**	890.4942	445.7507	873.4676	437.2374	872.4836	436.7454	**8**
**12**	**1262.5172**	631.7622	1245.4906	623.2489	1244.5066	622.7569	**L**	803.4621	402.2347	786.4356	393.7214	785.4516	393.2294	**7**
**13**	**1375.6012**	688.3042	1358.5747	679.7910	**1357.5907**	679.2990	**L**	**690.3781**	345.6927	673.3515	337.1794	672.3675	336.6874	**6**
**14**	**1488.6853**	744.8463	1471.6587	736.3330	**1470.6747**	735.8410	**L**	**577.2940**	289.1506	560.2675	280.6374	559.2834	280.1454	**5**
**15**	**1603.7122**	802.3597	1586.6857	793.8465	1585.7017	793.3545	**N**	**464.2099**	232.6086	447.1834	224.0953	446.1994	223.6033	**4**
**16**	**1690.7442**	845.8758	1673.7177	837.3625	1672.7337	836.8705	**S**	**349.1830**	175.0951	332.1565	166.5819	331.1724	166.0899	**3**
**17**	**1777.7763**	889.3918	1760.7497	880.8785	**1759.7657**	880.3865	**S**	262.1510	131.5791	245.1244	123.0659	244.1404	122.5738	**2**
**18**							**R**	175.1190	88.0631	158.0924	79.5498			**1**

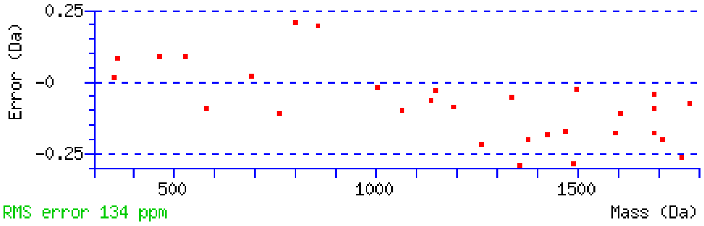

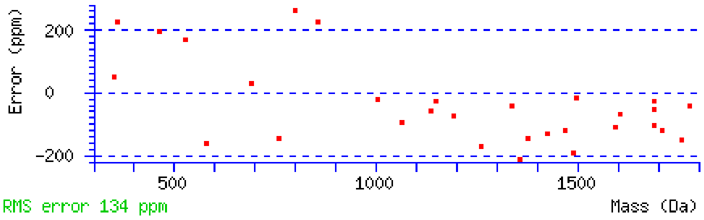

All matches to this query

Score	Mr(calc):	Delta	Sequence
93.9	1950.8807	-0.0002	DQDPASFGEDSLLLNSSR
10.4	1950.8870	-0.0066	VSKSPSHGSPLIDMASSR
10.4	1950.8870	-0.0066	VSKSPSHGSPLIDMASSR
3.0	1950.8870	-0.0066	VSKSPSHGSPLIDMASSR
2.4	1950.8989	-0.0184	GTKWIANNWINVDPSR
2.4	1950.8989	-0.0184	GTKWIANNWINVDPSR
2.1	1950.8710	0.0094	VKHCRCMSVNLSDSDK
1.3	1950.8737	0.0067	RSLSPWGYSINHDPSR
0.3	1950.8870	-0.0066	NIDINDVTPNCRVALR
0.2	1950.8740	0.0064	SEYIIIPKSTLQEDR

Spectrum No: 59; Query: 457; Rank: 1

Peptide View

MS/MS Fragmentation of **TINISNLYIGGLPEGK**
Found in **IPI00363534**, Tax_Id=10116 Gene_Symbol=Lama1 laminin, alpha 1

Match to Query 457: 1688.901908 from(845.458230,2+)
Title: 091127RatKid_SCX01_15.3635.3635.2.dta
Data file K:\NewmanPaper\Piliang\3SubProteomes\Piliang3SP\mgf5ppm\SCX_3SubProteomes5ppm.mgf

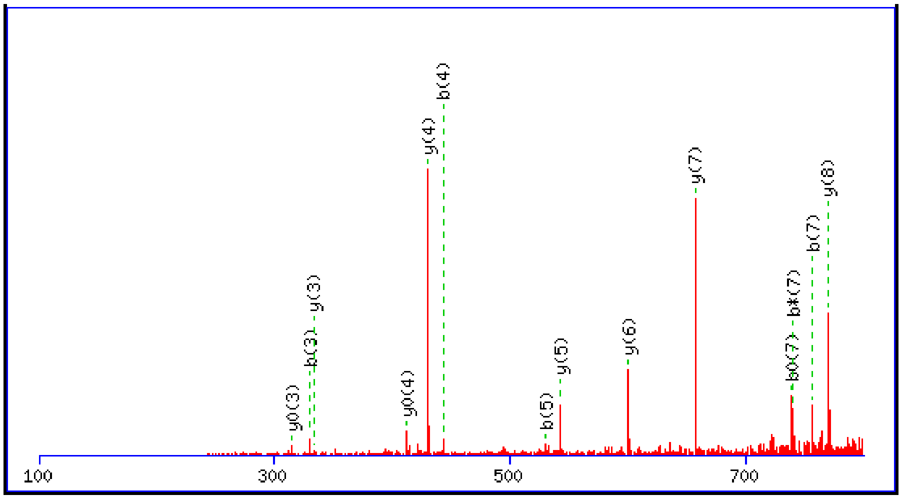

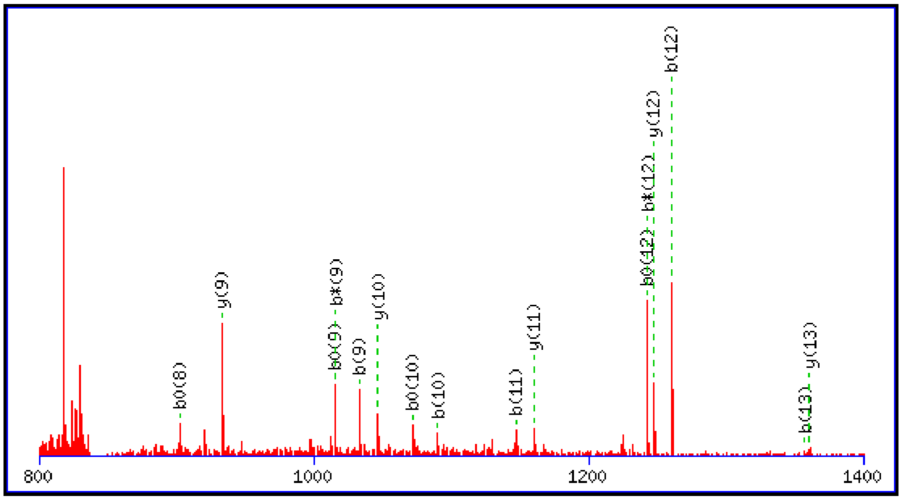

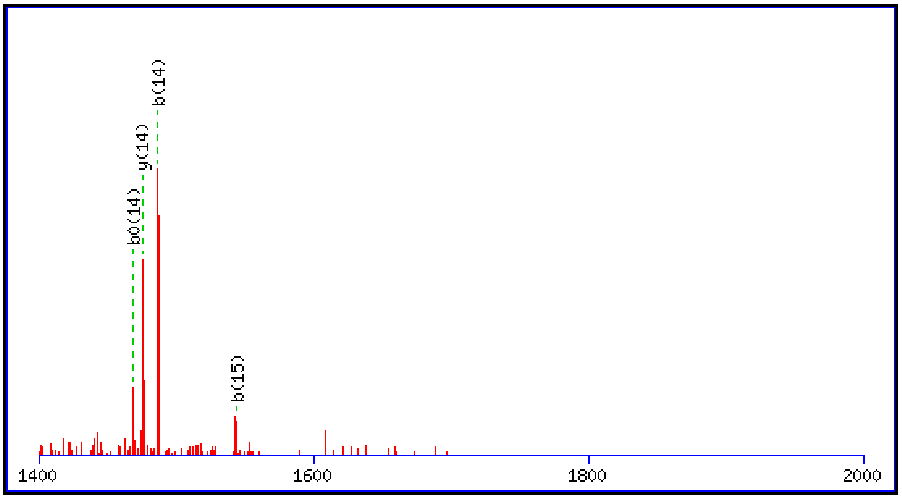

Monoisotopic mass of neutral peptide Mr(calc): 1688.8985
Fixed modifications: Carbamidomethyl (C)
Variable modifications:
N3 : Deamidated_N (N)
Ions Score: 94 **Expect:** 4.3e-008
Matches (Bold Red): 34/172 fragment ions using 58 most intense peaks

#	b	b^{++}	b*	b^{*++}	b^0	b^{0++}	Seq.	y	y^{++}	y*	y^{*++}	y^0	y^{0++}	#
1	102.0550	51.5311			84.0444	42.5258	T							16
2	215.1390	108.0731			197.1285	99.0679	I	1588.8581	794.9327	1571.8315	786.4194	1570.8475	785.9274	15
3	**330.1660**	165.5866	313.1394	157.0733	312.1554	156.5813	N	**1475.7740**	738.3907	1458.7475	729.8774	1457.7635	729.3854	14
4	**443.2500**	222.1286	426.2235	213.6154	425.2395	213.1234	I	**1360.7471**	680.8772	1343.7205	672.3639	1342.7365	671.8719	13
5	**530.2820**	265.6447	513.2555	257.1314	512.2715	256.6394	S	**1247.6630**	624.3352	1230.6365	615.8219	1229.6525	615.3299	12

**6**	644.3250	322.6661	627.2984	314.1529	626.3144	313.6608	**N**	**1160.6310**	580.8191	1143.6045	572.3059	1142.6204	571.8139	**11**
**7**	**757.4090**	379.2082	**740.3825**	370.6949	**739.3985**	370.2029	**L**	**1046.5881**	523.7977	1029.5615	515.2844	1028.5775	514.7924	**10**
**8**	920.4724	460.7398	903.4458	452.2265	**902.4618**	451.7345	**Y**	**933.5040**	467.2556	916.4775	458.7424	915.4934	458.2504	**9**
**9**	**1033.5564**	517.2819	**1016.5299**	508.7686	**1015.5459**	508.2766	**I**	**770.4407**	385.7240	753.4141	377.2107	752.4301	376.7187	**8**
**10**	**1090.5779**	545.7926	1073.5513	537.2793	**1072.5673**	536.7873	**G**	**657.3566**	329.1819	640.3301	320.6687	639.3461	320.1767	**7**
**11**	**1147.5994**	574.3033	1130.5728	565.7900	1129.5888	565.2980	**G**	**600.3352**	300.6712	583.3086	292.1579	582.3246	291.6659	**6**
**12**	**1260.6834**	630.8453	**1243.6569**	622.3321	**1242.6729**	621.8401	**L**	**543.3137**	272.1605	526.2871	263.6472	525.3031	263.1552	**5**
**13**	**1357.7362**	679.3717	1340.7096	670.8585	1339.7256	670.3664	**P**	**430.2296**	215.6185	413.2031	207.1052	**412.2191**	206.6132	**4**
**14**	**1486.7788**	743.8930	1469.7522	735.3798	**1468.7682**	734.8877	**E**	**333.1769**	167.0921	316.1503	158.5788	**315.1663**	158.0868	**3**
**15**	**1543.8002**	772.4038	1526.7737	763.8905	1525.7897	763.3985	**G**	204.1343	102.5708	187.1077	94.0575			**2**
**16**							**K**	147.1128	74.0600	130.0863	65.5468			**1**

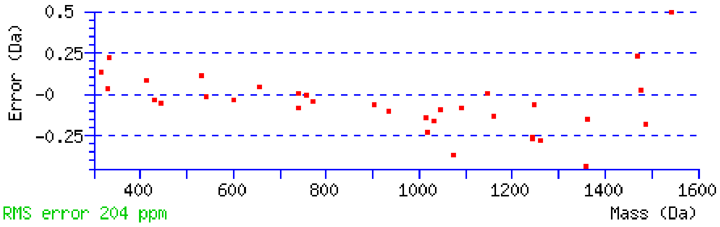

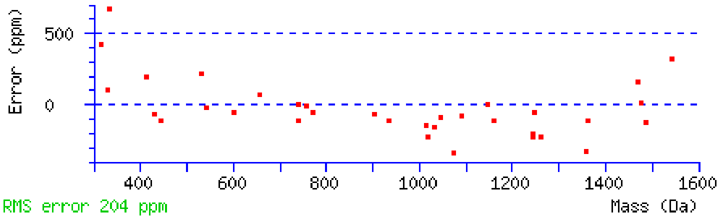

All matches to this query

Score	Mr(calc):	Delta	Sequence
93.8	1688.8985	0.0034	TINISNLYIGGLPEGK
64.4	1688.8985	0.0034	TINISNLYIGGLPEGK
48.0	1687.9145	0.9874	TINISNLYIGGLPEGK
3.6	1688.8944	0.0075	ATRSQLLEEELSSVK
3.1	1686.8957	2.0062	SKITILKTFNASGVK
1.9	1687.9006	1.0014	TTEPNGLLLFSQGRR
0.1	1686.9043	1.9976	LRASGGPLGAAGLGVRR

Spectrum No: 60; Query: 610; Rank: 1

Peptide View

MS/MS Fragmentation of **NPCTSEQNCTSPFSYK**
Found in **IPI00191681**, Tax_Id=10116 Gene_Symbol=Itgb1 Integrin beta-1 precursor

Match to Query 610: 1919.771168 from(960.892860,2+)
Title: 091127RatKid_SCX01_12.1630.1630.2.dta
Data file K:\NewmanPaper\Piliang\3SubProteomes\Piliang3SP\mgf5ppm\SCX_3SubProteomes5ppm.mgf

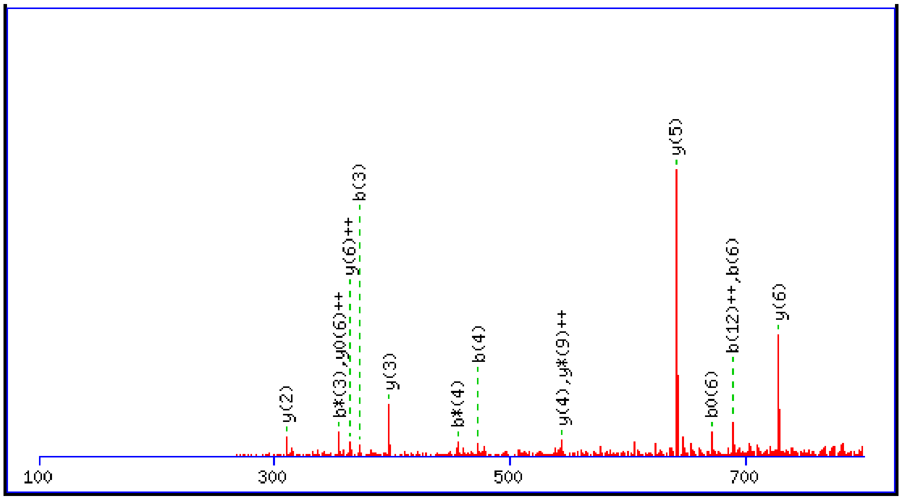

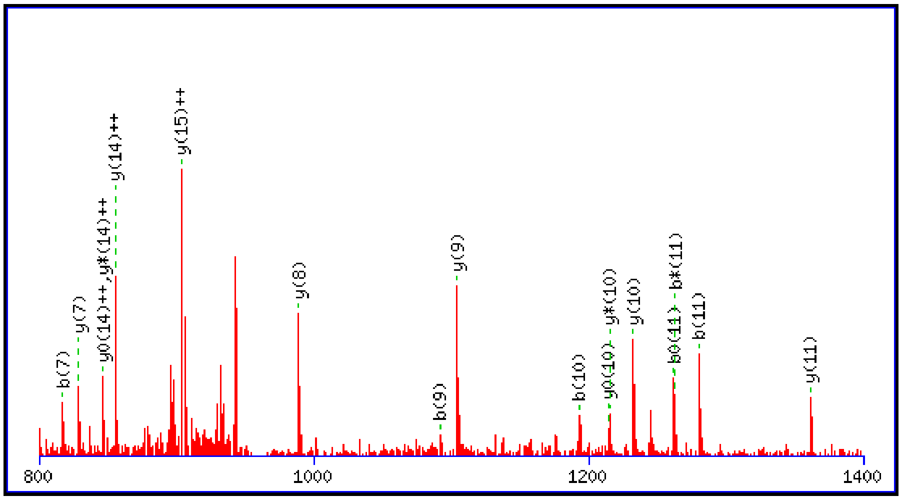

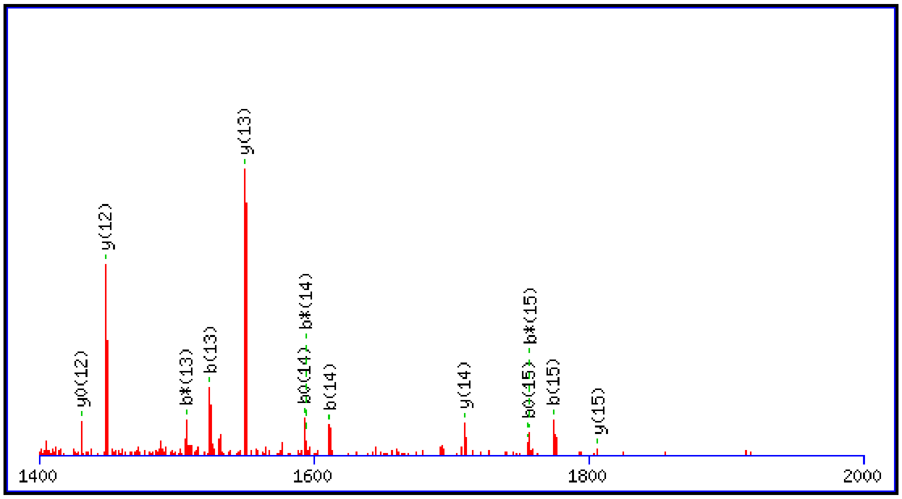

Monoisotopic mass of neutral peptide Mr(calc): 1919.7666
Fixed modifications: Carbamidomethyl (C)
Variable modifications:
N8 : Deamidated_N (N)
Ions Score: 93 **Expect:** 8.7e-008
Matches (Bold Red): 45/170 fragment ions using 75 most intense peaks

#	b	b^{++}	b*	b^{*++}	b^0	b^{0++}	Seq.	y	y^{++}	y*	y^{*++}	y^0	y^{0++}	#
1	115.0502	58.0287	98.0237	49.5155			N							16
2	212.1030	106.5551	195.0764	98.0418			P	**1806.7309**	**903.8691**	1789.7044	895.3558	1788.7204	894.8638	15
3	**372.1336**	186.5704	**355.1071**	178.0572			C	**1709.6782**	**855.3427**	1692.6516	**846.8294**	1691.6676	**846.3374**	14
4	**473.1813**	237.0943	**456.1547**	228.5810	455.1707	228.0890	T	**1549.6475**	775.3274	1532.6210	766.8141	1531.6369	766.3221	13
5	560.2133	280.6103	543.1868	272.0970	542.2028	271.6050	S	**1448.5998**	724.8036	1431.5733	716.2903	**1430.5893**	715.7983	12

**6**	**689.2559**	345.1316	672.2294	336.6183	**671.2454**	336.1263	**E**	**1361.5678**	681.2875	1344.5413	672.7743	1343.5572	672.2823	**11**
**7**	**817.3145**	409.1609	800.2879	400.6476	799.3039	400.1556	**Q**	**1232.5252**	616.7662	**1215.4987**	608.2530	**1214.5146**	607.7610	**10**
**8**	932.3414	466.6744	915.3149	458.1611	914.3309	457.6691	**N**	**1104.4666**	552.7370	1087.4401	**544.2237**	1086.4561	543.7317	**9**
**9**	**1092.3721**	546.6897	1075.3455	538.1764	1074.3615	537.6844	**C**	**989.4397**	495.2235	972.4131	486.7102	971.4291	486.2182	**8**
**10**	**1193.4198**	597.2135	1176.3932	588.7002	1175.4092	588.2082	**T**	**829.4090**	415.2082	812.3825	406.6949	811.3985	406.2029	**7**
**11**	**1280.4518**	640.7295	**1263.4252**	632.2163	**1262.4412**	631.7242	**S**	**728.3614**	**364.6843**	711.3348	356.1710	710.3508	**355.6790**	**6**
**12**	1377.5046	**689.2559**	1360.4780	680.7426	1359.4940	680.2506	**P**	**641.3293**	321.1683	624.3028	312.6550	623.3188	312.1630	**5**
**13**	**1524.5730**	762.7901	**1507.5464**	754.2768	1506.5624	753.7848	**F**	**544.2766**	272.6419	527.2500	264.1287	526.2660	263.6366	**4**
**14**	**1611.6050**	806.3061	**1594.5784**	797.7929	**1593.5944**	797.3009	**S**	**397.2082**	199.1077	380.1816	190.5944	379.1976	190.1024	**3**
**15**	**1774.6683**	887.8378	**1757.6418**	879.3245	**1756.6578**	878.8325	**Y**	**310.1761**	155.5917	293.1496	147.0784			**2**
**16**							**K**	147.1128	74.0600	130.0863	65.5468			**1**

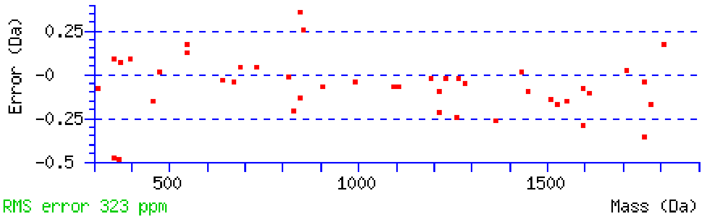

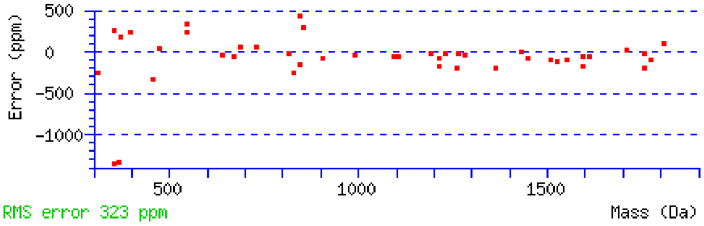

All matches to this query

Score	Mr(calc):	Delta	Sequence
93.1	1919.7666	0.0046	NPCTSEQNCTSPFSYK
22.3	1919.7666	0.0046	NPCTSEQNCTSPFSYK
18.6	1918.7826	0.9886	NPCTSEQNCTSPFSYK
5.7	1919.7614	0.0097	KTSNVQSTAGMSVRAK
5.7	1919.7614	0.0097	KTSNVQSTAGMSVRAK
3.7	1919.7614	0.0097	KTSNVQSTAGMSVRAK
3.6	1919.7645	0.0067	VQSGTWVGYQYPGYR
1.3	1919.7720	-0.0008	ASGPGTPPAAEPSVLTTK
0.3	1919.7889	-0.0178	LCYFKKSSAQTADNK
0.1	1918.7793	0.9919	DPTSSNDDSKSNLSTSGK

Spectrum No: 61; Query: 471; Rank: 1

Peptide View

MS/MS Fragmentation of **DSSDEANCTTLCSQK**
Found in **IPI00205325**, Tax_Id=10116 Gene_Symbol=Lrp2 Low-density lipoprotein receptor-related protein 2 precursor

Match to Query 471: 1715.667248 from(858.840900,2+)
Title: 091127RatKid_SCX01_16.689.689.2.dta
Data file K:\NewmanPaper\Piliang\3SubProteomes\Piliang3SP\mgf5ppm\SCX_3SubProteomes5ppm.mgf

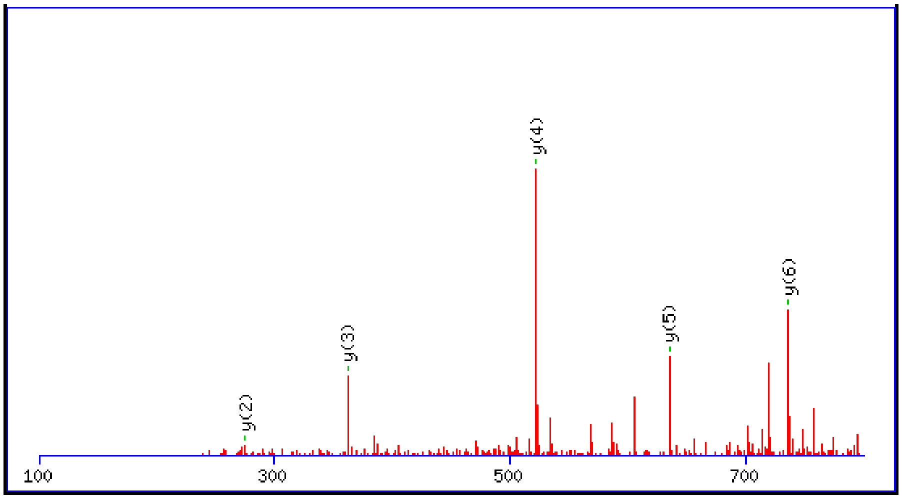

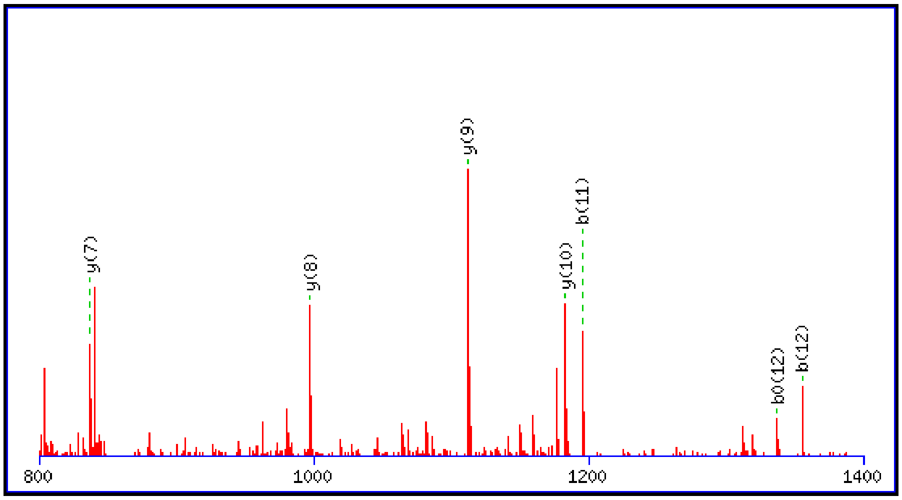

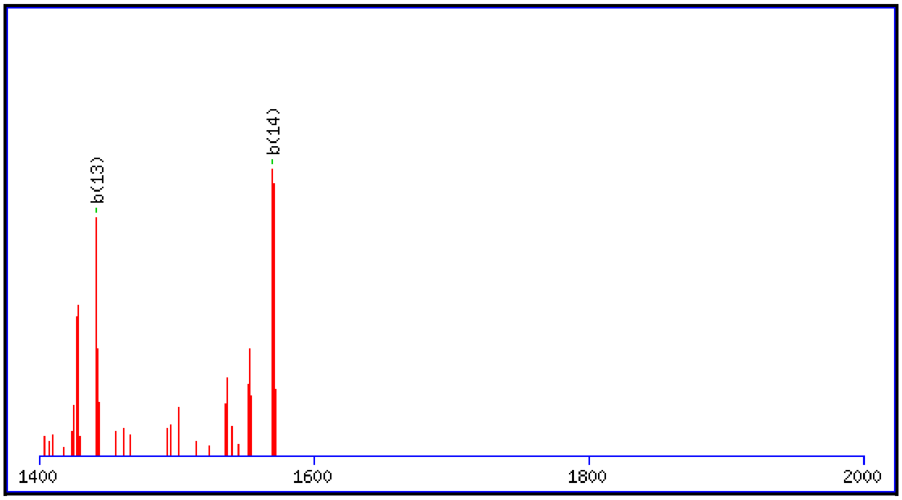

Monoisotopic mass of neutral peptide Mr(calc): 1715.6614
Fixed modifications: Carbamidomethyl (C)
Variable modifications:
N7 : Deamidated_N (N)
Ions Score: 93 **Expect:** 6.8e-008
Matches (Bold Red): 14/152 fragment ions using 18 most intense peaks

#	b	b++	b*	b*++	b0	b0++	Seq.	y	y++	y*	y*++	y0	y0++	#
1	116.0342	58.5207			98.0237	49.5155	D							15
2	203.0662	102.0368			185.0557	93.0315	S	1601.6418	801.3245	1584.6152	792.8112	1583.6312	792.3192	14
3	290.0983	145.5528			272.0877	136.5475	S	1514.6097	757.8085	1497.5832	749.2952	1496.5992	748.8032	13
4	405.1252	203.0662			387.1147	194.0610	D	1427.5777	714.2925	1410.5512	705.7792	1409.5672	705.2872	12
5	534.1678	267.5875			516.1572	258.5823	E	1312.5508	656.7790	1295.5242	648.2658	1294.5402	647.7737	11

**6**	605.2049	303.1061			587.1944	294.1008	**A**	**1183.5082**	592.2577	1166.4816	583.7445	1165.4976	583.2524	**10**
**7**	720.2319	360.6196	703.2053	352.1063	702.2213	351.6143	**N**	**1112.4711**	556.7392	1095.4445	548.2259	1094.4605	547.7339	**9**
**8**	880.2625	440.6349	863.2360	432.1216	862.2519	431.6296	**C**	**997.4441**	499.2257	980.4176	490.7124	979.4336	490.2204	**8**
**9**	981.3102	491.1587	964.2836	482.6455	963.2996	482.1535	**T**	**837.4135**	419.2104	820.3869	410.6971	819.4029	410.2051	**7**
**10**	1082.3579	541.6826	1065.3313	533.1693	1064.3473	532.6773	**T**	**736.3658**	368.6865	719.3393	360.1733	718.3552	359.6813	**6**
**11**	**1195.4419**	598.2246	1178.4154	589.7113	1177.4314	589.2193	**L**	**635.3181**	318.1627	618.2916	309.6494	617.3076	309.1574	**5**
**12**	**1355.4726**	678.2399	1338.4460	669.7267	**1337.4620**	669.2346	**C**	**522.2341**	261.6207	505.2075	253.1074	504.2235	252.6154	**4**
**13**	**1442.5046**	721.7559	1425.4781	713.2427	1424.4940	712.7507	**S**	**362.2034**	181.6053	345.1769	173.0921	344.1928	172.6001	**3**
**14**	**1570.5632**	785.7852	1553.5366	777.2720	1552.5526	776.7799	**Q**	**275.1714**	138.0893	258.1448	129.5761			**2**
**15**							**K**	147.1128	74.0600	130.0863	65.5468			**1**

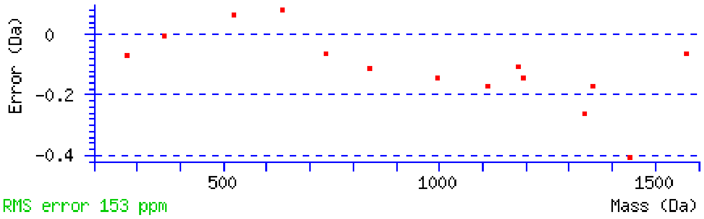

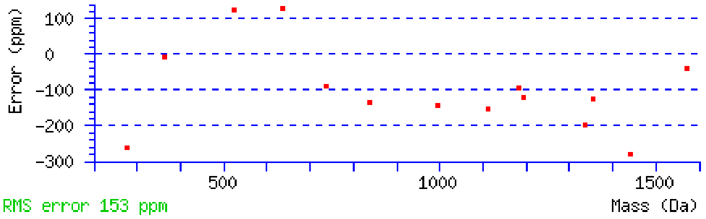

All matches to this query

Score	Mr(calc):	Delta	Sequence
93.0	1715.6614	0.0058	DSSDEANCTTLCSQK
39.2	1714.6774	0.9898	DSSDEANCTTLCSQK
7.4	1715.6593	0.0079	NQNFNESFTISQK
6.5	1715.6706	-0.0033	RFYRGDSPTDSQK
6.5	1715.6706	-0.0033	RFYRGDSPTDSQK
4.6	1713.6746	1.9927	FVDSGEMAESFPYR
4.0	1715.6706	-0.0033	RFYRGDSPTDSQK
2.1	1714.6641	1.0032	YTHAANTVVYSSNK
2.1	1714.6641	1.0032	YTHAANTVVYSSNK
1.7	1715.6831	-0.0158	NSKSCPCCGTPIQK

Spectrum No: 62; Query: 660; Rank: 1

Peptide View

MS/MS Fragmentation of **MGAGEALGNASQLFEAWLK**
Found in **IPI00327398**, Tax_Id=10116 Gene_Symbol=Enpep Isoform 1 of Glutamyl aminopeptidase

Match to Query 660: 1992.968548 from(997.491550,2+)
Title: 091129RatKid_SCX02_12.5494.5494.2.dta
Data file K:\NewmanPaper\Piliang\3SubProteomes\Piliang3SP\mgf5ppm\SCX_3SubProteomes5ppm.mgf

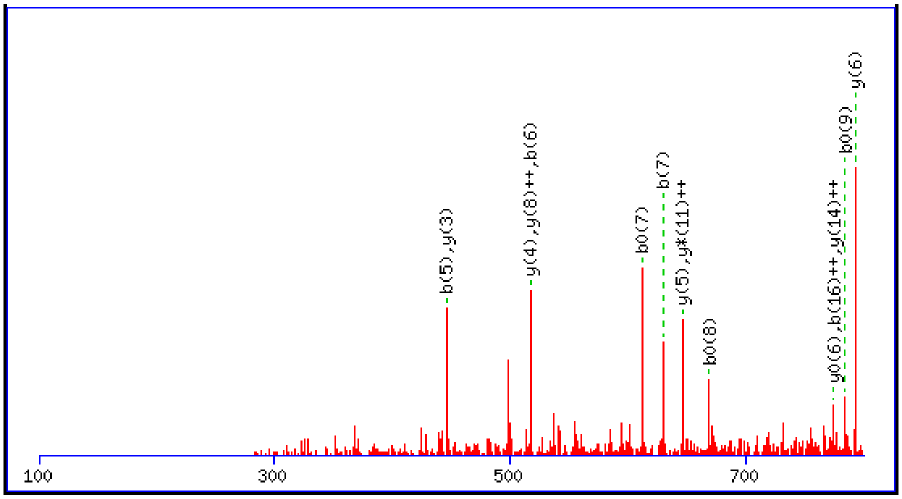

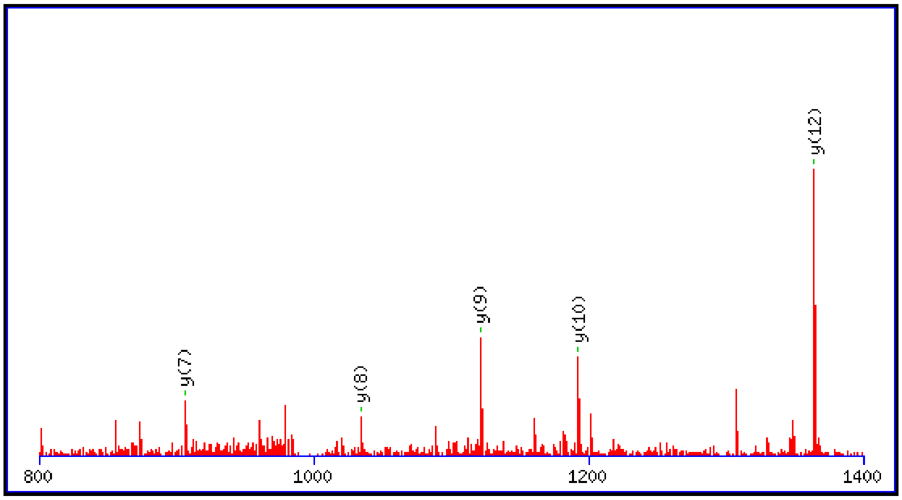

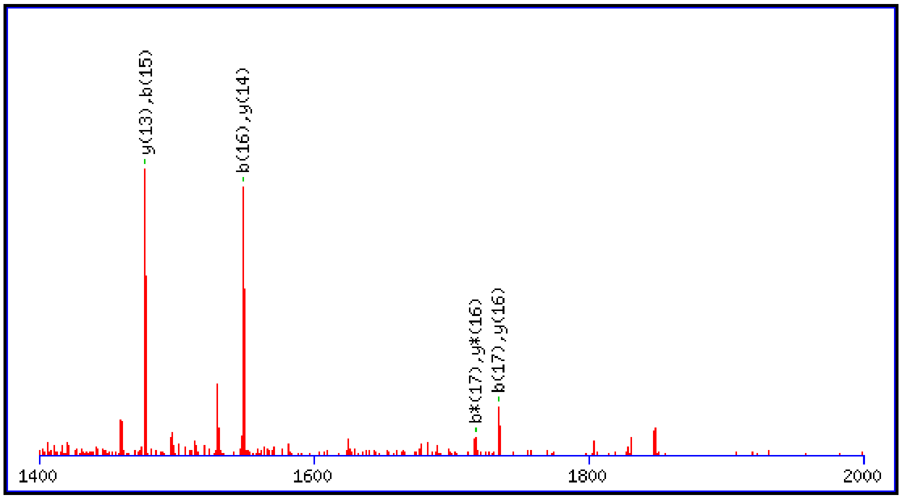

Monoisotopic mass of neutral peptide Mr(calc): 1992.9615
Fixed modifications: Carbamidomethyl (C)
Variable modifications:
N9 : Deamidated_N (N)
Ions Score: 93 **Expect:** 1.1e-007
Matches (Bold Red): 28/184 fragment ions using 27 most intense peaks

#	b	b^{++}	b*	b^{*++}	b^0	b^{0++}	Seq.	y	y^{++}	y*	y^{*++}	y^0	y^{0++}	#
1	132.0478	66.5275					M							19
2	189.0692	95.0383					G	1862.9283	931.9678	1845.9018	923.4545	1844.9177	922.9625	18
3	260.1063	130.5568					A	1805.9068	903.4571	1788.8803	894.9438	1787.8963	894.4518	17
4	317.1278	159.0675					G	**1734.8697**	867.9385	**1717.8432**	859.4252	1716.8592	858.9332	16
5	**446.1704**	223.5888			428.1598	214.5836	E	1677.8483	839.4278	1660.8217	830.9145	1659.8377	830.4225	15

6	**517.2075**	259.1074			499.1969	250.1021	A	**1548.8057**	**774.9065**	1531.7791	766.3932	1530.7951	765.9012	14
7	**630.2916**	315.6494			**612.2810**	306.6441	L	**1477.7686**	739.3879	1460.7420	730.8746	1459.7580	730.3826	13
8	687.3130	344.1602			**669.3025**	335.1549	G	**1364.6845**	682.8459	1347.6579	674.3326	1346.6739	673.8406	12
9	802.3400	401.6736	785.3134	393.1604	**784.3294**	392.6683	N	1307.6630	654.3351	1290.6365	**645.8219**	1289.6525	645.3299	11
10	873.3771	437.1922	856.3505	428.6789	855.3665	428.1869	A	**1192.6361**	596.8217	1175.6095	588.3084	1174.6255	587.8164	10
11	960.4091	480.7082	943.3826	472.1949	942.3986	471.7029	S	**1121.5990**	561.3031	1104.5724	552.7898	1103.5884	552.2978	9
12	1088.4677	544.7375	1071.4411	536.2242	1070.4571	535.7322	Q	**1034.5669**	**517.7871**	1017.5404	509.2738	1016.5564	508.7818	8
13	1201.5518	601.2795	1184.5252	592.7662	1183.5412	592.2742	L	**906.5084**	453.7578	889.4818	445.2445	888.4978	444.7525	7
14	1348.6202	674.8137	1331.5936	666.3004	1330.6096	665.8084	F	**793.4243**	397.2158	776.3978	388.7025	**775.4137**	388.2105	6
15	**1477.6628**	739.3350	1460.6362	730.8217	1459.6522	730.3297	E	**646.3559**	323.6816	629.3293	315.1683	628.3453	314.6763	5
16	**1548.6999**	**774.8536**	1531.6733	766.3403	1530.6893	765.8483	A	**517.3133**	259.1603	500.2867	250.6470			4
17	**1734.7792**	867.8932	**1717.7526**	859.3800	1716.7686	858.8880	W	**446.2762**	223.6417	429.2496	215.1285			3
18	1847.8633	924.4353	1830.8367	915.9220	1829.8527	915.4300	L	260.1969	130.6021	243.1703	122.0888			2
19							K	147.1128	74.0600	130.0863	65.5468			1

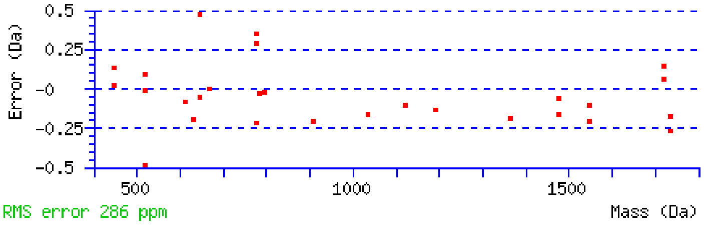

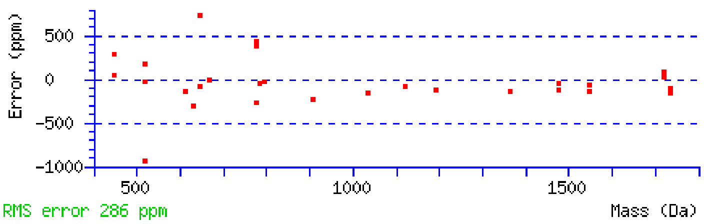

All matches to this query

Score	Mr(calc):	Delta	Sequence
92.9	1992.9615	0.0071	MGAGEALGNASQLFEAWLK
34.3	1991.9775	0.9911	MGAGEALGNASQLFEAWLK
10.0	1991.9679	1.0006	GSILQFMPFTTVSELVK
7.7	1991.9679	1.0006	GSILQFMPFTTVSELVK
6.9	1992.9686	-0.0001	KVTVTTYNTSITIHKK
6.2	1992.9686	-0.0001	KVTVTTYNTSITIHKK
5.0	1991.9679	1.0006	GSILQFMPFTTVSELVK
4.3	1992.9686	-0.0001	KVTVTTYNTSITIHKK
4.2	1992.9496	0.0190	MGALEEKNSLSEEIANMK
4.2	1992.9629	0.0056	DLNTLREAQENINKQK

Spectrum No: 63; Query: 511; Rank: 1

Peptide View

MS/MS Fragmentation of **KDDALLETTDGFNTTK**
Found in **IPI00212546**, Tax_Id=10116 Gene_Symbol=Emb Embigin precursor

Match to Query 511: 1769.844408 from(885.929480,2+)
Title: 091127RatKid_SCX01_13.2014.2014.2.dta
Data file K:\NewmanPaper\Piliang\3SubProteomes\Piliang3SP\mgf5ppm\SCX_3SubProteomes5ppm.mgf

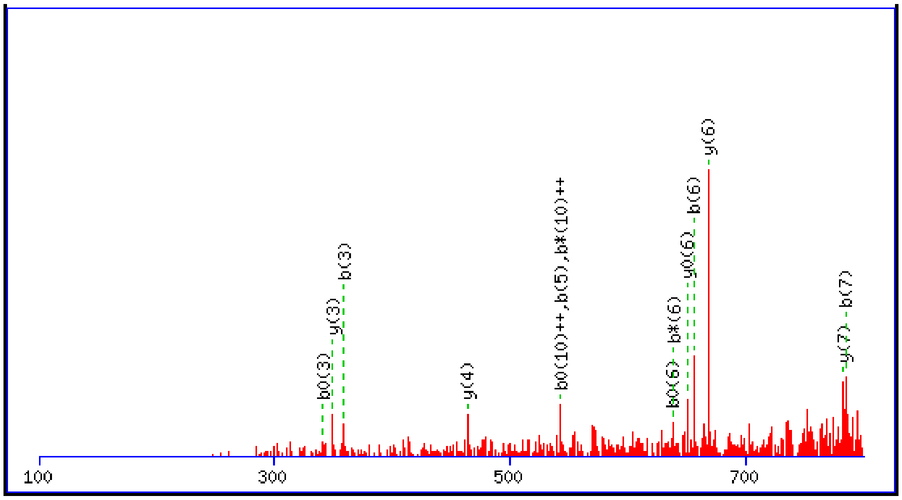

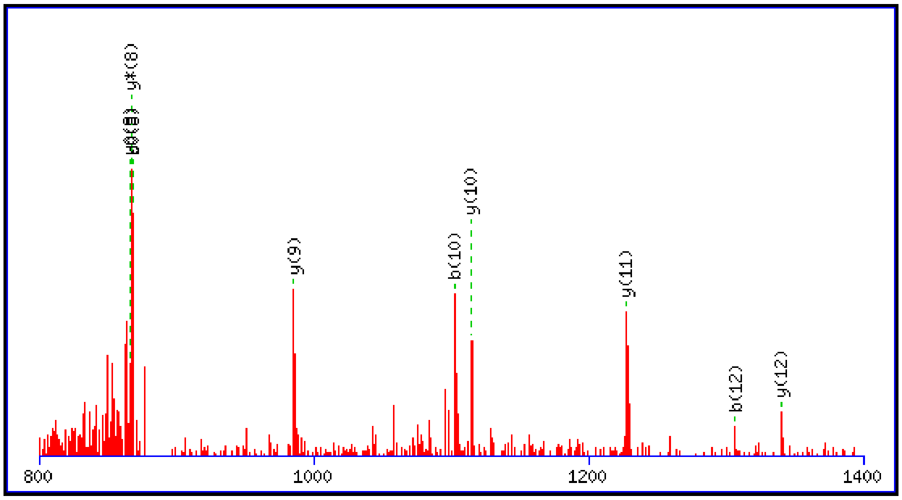

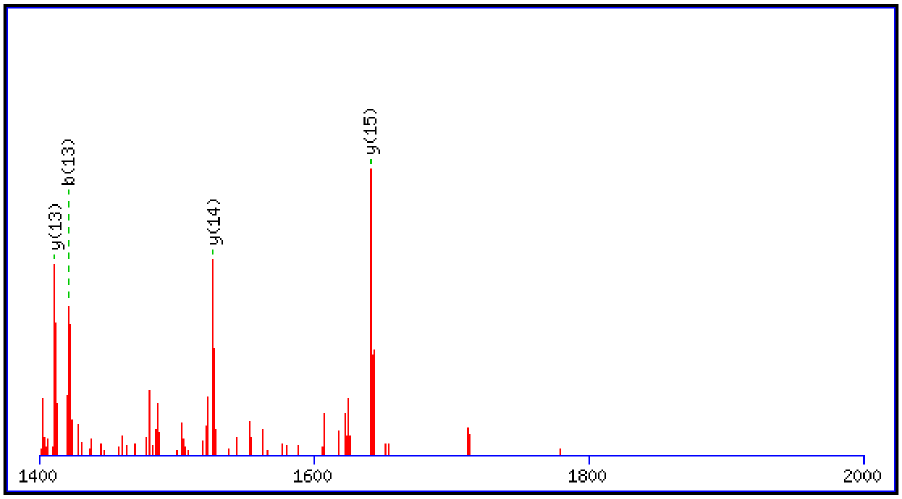

Monoisotopic mass of neutral peptide Mr(calc): 1768.8367
Fixed modifications: Carbamidomethyl (C)
Variable modifications:
N13 : Deamidated_N (N)
Ions Score: 93 **Expect:** 1.1e-007
Matches (Bold Red): 27/176 fragment ions using 37 most intense peaks

#	b	b++	b*	b*++	b0	b0++	Seq.	y	y++	y*	y*++	y0	y0++	#
1	129.1022	65.0548	112.0757	56.5415			K							16
2	244.1292	122.5682	227.1026	114.0550	226.1186	113.5629	D	**1641.7490**	821.3781	1624.7224	812.8649	1623.7384	812.3729	15
3	**359.1561**	180.0817	342.1296	171.5684	**341.1456**	171.0764	D	**1526.7221**	763.8647	1509.6955	755.3514	1508.7115	754.8594	14
4	430.1932	215.6003	413.1667	207.0870	412.1827	206.5950	A	**1411.6951**	706.3512	1394.6686	697.8379	1393.6845	697.3459	13
5	**543.2773**	272.1423	526.2508	263.6290	525.2667	263.1370	L	**1340.6580**	670.8326	1323.6314	662.3194	1322.6474	661.8274	12

6	656.3614	328.6843	639.3348	320.1710	638.3508	319.6790	L	1227.5739	614.2906	1210.5474	605.7773	1209.5634	605.2853	11
7	785.4040	393.2056	768.3774	384.6923	767.3934	384.2003	E	1114.4899	557.7486	1097.4633	549.2353	1096.4793	548.7433	10
8	886.4516	443.7295	869.4251	435.2162	868.4411	434.7242	T	985.4473	493.2273	968.4207	484.7140	967.4367	484.2220	9
9	987.4993	494.2533	970.4728	485.7400	969.4888	485.2480	T	884.3996	442.7034	867.3730	434.1902	866.3890	433.6982	8
10	1102.5263	551.7668	1085.4997	543.2535	1084.5157	542.7615	D	783.3519	392.1796	766.3254	383.6663	765.3414	383.1743	7
11	1159.5477	580.2775	1142.5212	571.7642	1141.5372	571.2722	G	668.3250	334.6661	651.2984	326.1529	650.3144	325.6608	6
12	1306.6161	653.8117	1289.5896	645.2984	1288.6056	644.8064	F	611.3035	306.1554	594.2770	297.6421	593.2929	297.1501	5
13	1421.6431	711.3252	1404.6165	702.8119	1403.6325	702.3199	N	464.2351	232.6212	447.2085	224.1079	446.2245	223.6159	4
14	1522.6908	761.8490	1505.6642	753.3357	1504.6802	752.8437	T	349.2082	175.1077	332.1816	166.5944	331.1976	166.1024	3
15	1623.7384	812.3729	1606.7119	803.8596	1605.7279	803.3676	T	248.1605	124.5839	231.1339	116.0706	230.1499	115.5786	2
16							K	147.1128	74.0600	130.0863	65.5468			1

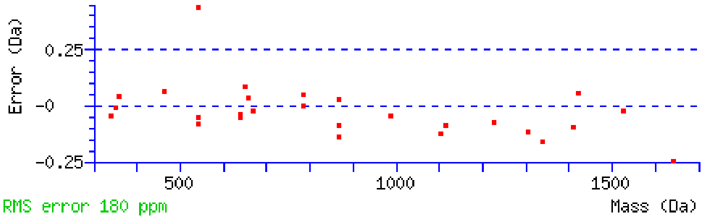

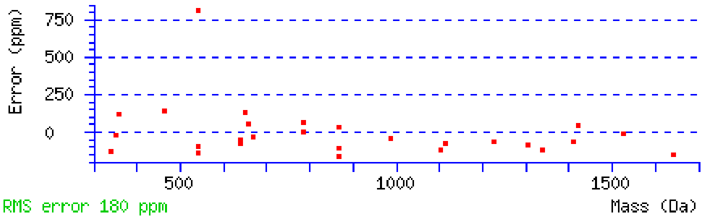

All matches to this query

Score	Mr(calc):	Delta	Sequence
92.7	1768.8367	1.0077	KDDALLETTDGFNTTK
17.1	1767.8527	1.9917	KDDALLETTDGFNTTK
3.2	1768.8498	0.9946	KPSAKRAPTRPSGTR
3.2	1768.8498	0.9946	KPSAKRAPTRPSGTR
2.2	1768.8498	0.9946	KPSAKRAPTRPSGTR
0.8	1768.8365	1.0079	ICLREAELTGRMPK
0.1	1769.8270	0.0174	TLKGNAIGLNMLSNNK
0.1	1769.8284	0.0160	RRMNVLSFQPAXQK

Spectrum No: 64; Query: 170; Rank: 1

Peptide View

MS/MS Fragmentation of **KLDFIVLNETR**
Found in **IPI00208422**, Tax_Id=10116 Gene_Symbol=Dpp4 Dipeptidyl peptidase 4

Match to Query 170: 1347.740508 from(674.877530,2+)
Title: 091129RatKid_SCX02_30.3075.3075.2.dta
Data file K:\NewmanPaper\Piliang\3SubProteomes\Piliang3SP\mgf5ppm\SCX_3SubProteomes5ppm.mgf

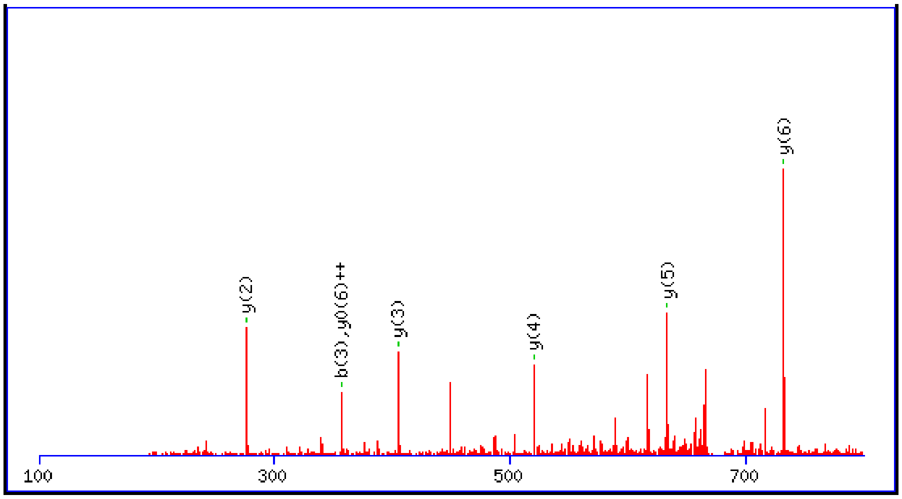

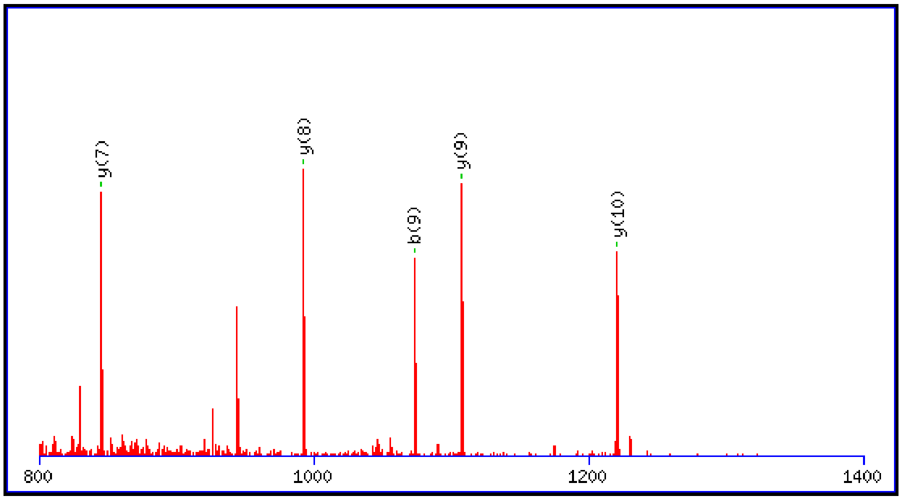

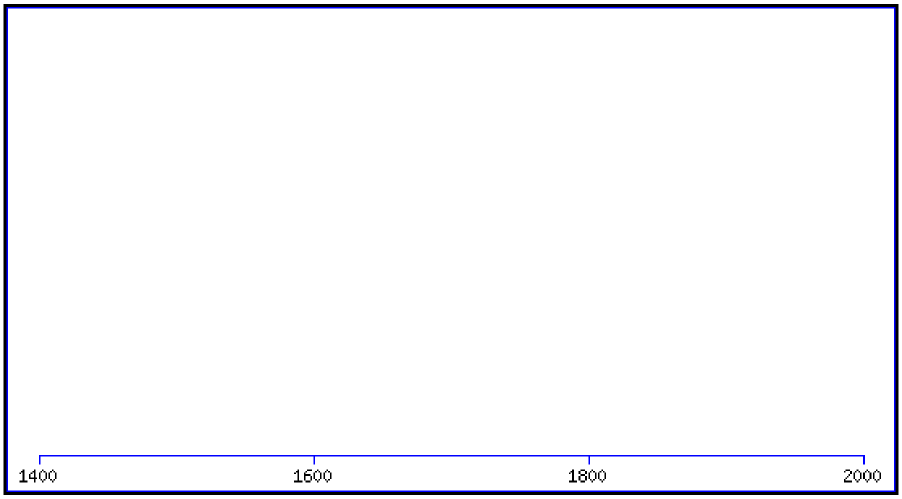

Monoisotopic mass of neutral peptide Mr(calc): 1347.7398
Fixed modifications: Carbamidomethyl (C)
Variable modifications:
N8 : Deamidated_N (N)
Ions Score: 93 **Expect:** 4.9e-008
Matches (Bold Red): 12/114 fragment ions using 17 most intense peaks

#	b	b^{++}	b*	b^{*++}	b^0	b^{0++}	Seq.	y	y^{++}	y*	y^{*++}	y^0	y^{0++}	#
1	129.1022	65.0548	112.0757	56.5415			K							11
2	242.1863	121.5968	225.1598	113.0835			L	**1220.6521**	610.8297	1203.6256	602.3164	1202.6416	601.8244	10
3	**357.2132**	179.1103	340.1867	170.5970	339.2027	170.1050	D	**1107.5681**	554.2877	1090.5415	545.7744	1089.5575	545.2824	9
4	504.2817	252.6445	487.2551	244.1312	486.2711	243.6392	F	**992.5411**	496.7742	975.5146	488.2609	974.5306	487.7689	8
5	617.3657	309.1865	600.3392	300.6732	599.3552	300.1812	I	**845.4727**	423.2400	828.4462	414.7267	827.4621	414.2347	7

**6**	716.4341	358.7207	699.4076	350.2074	698.4236	349.7154	**V**	**732.3886**	366.6980	715.3621	358.1847	714.3781	**357.6927**	**6**
**7**	829.5182	415.2627	812.4917	406.7495	811.5076	406.2575	**L**	**633.3202**	317.1638	616.2937	308.6505	615.3097	308.1585	**5**
**8**	944.5451	472.7762	927.5186	464.2629	926.5346	463.7709	**N**	**520.2362**	260.6217	503.2096	252.1084	502.2256	251.6164	**4**
**9**	**1073.5877**	537.2975	1056.5612	528.7842	1055.5772	528.2922	**E**	**405.2092**	203.1082	388.1827	194.5950	387.1987	194.1030	**3**
**10**	1174.6354	587.8213	1157.6089	579.3081	1156.6248	578.8161	**T**	**276.1666**	138.5870	259.1401	130.0737	258.1561	129.5817	**2**
**11**							**R**	175.1190	88.0631	158.0924	79.5498			**1**

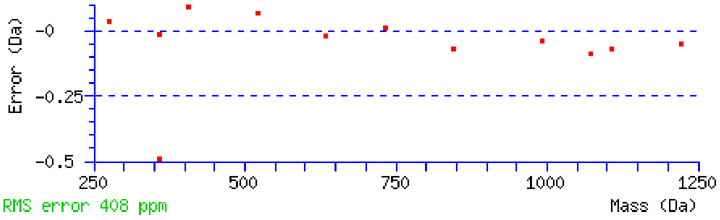

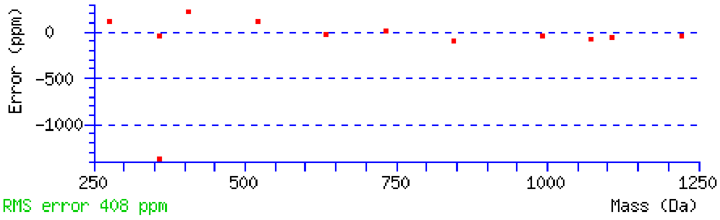

All matches to this query

Score	Mr(calc):	Delta	Sequence
92.7	1347.7398	0.0007	KLDFIVLNETR
17.8	1347.7510	-0.0105	LKNVDFEVKTR
9.6	1347.7428	-0.0023	ITQPLVKKWR
7.6	1347.7431	-0.0026	KITNLKITCEK
5.5	1347.7431	-0.0026	QIVSLKEKMEK
4.5	1347.7431	-0.0026	KMKLVESQLEK
3.9	1347.7398	0.0007	QIYEKGLELQK
3.5	1347.7387	0.0018	QLLSIPRASKR
2.4	1346.7446	0.9959	ATDDVTLLPFKK
1.6	1346.7306	1.0099	IGAVGRENYLQK

Spectrum No: 65; Query: 249; Rank: 1

Peptide View

MS/MS Fragmentation of **NVSLNNLSAAEVTK**
Found in **IPI00191103**, Tax_Id=10116 Gene_Symbol=Ndufa2_predicted NADH dehydrogenase (ubiquinone) 1 alpha subcomplex, 2

Match to Query 249: 1459.755428 from(730.884990,2+)
Title: 091127RatKid_SCX01_15.2303.2303.2.dta
Data file K:\NewmanPaper\Piliang\3SubProteomes\Piliang3SP\mgf5ppm\SCX_3SubProteomes5ppm.mgf

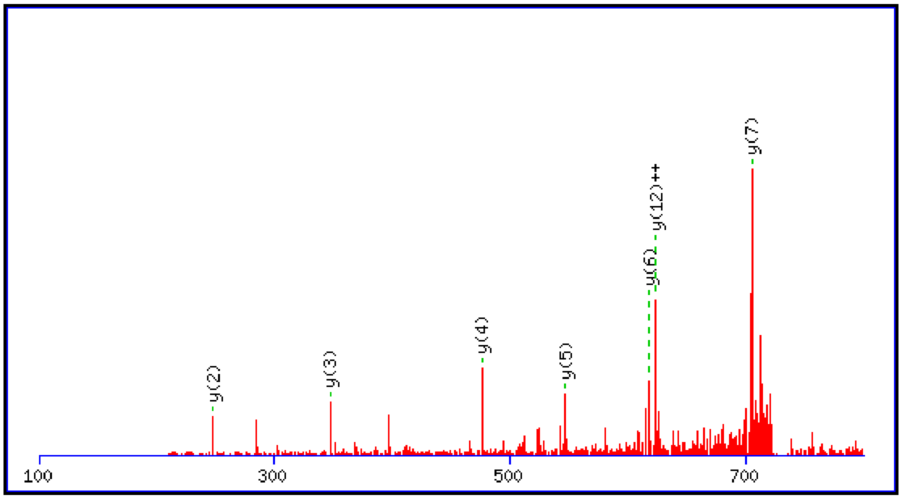

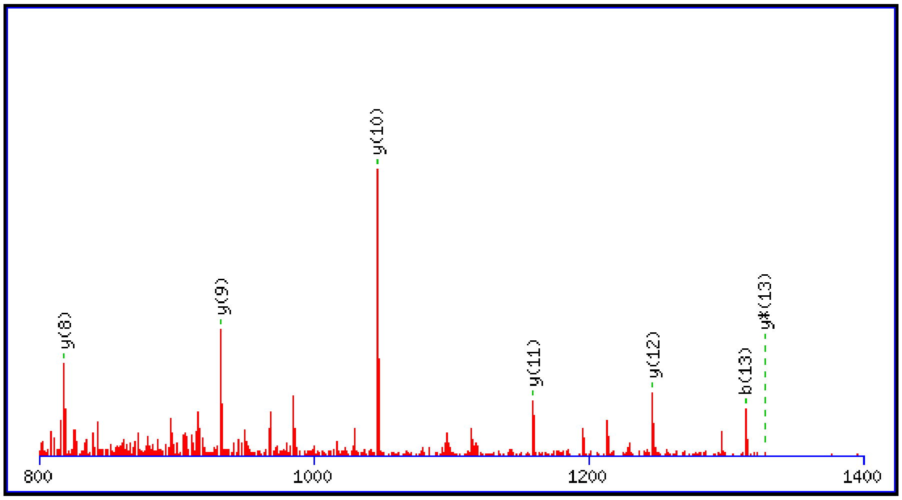

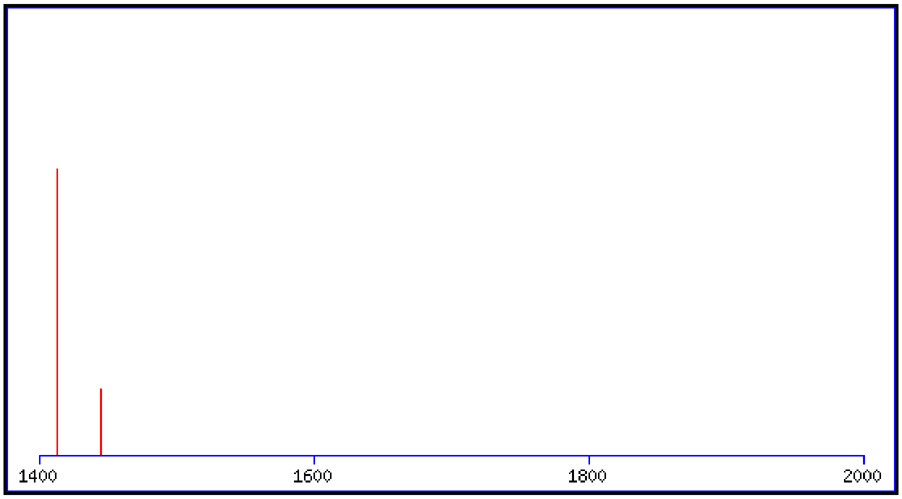

Monoisotopic mass of neutral peptide Mr(calc): 1459.7518
Fixed modifications: Carbamidomethyl (C)
Variable modifications:
N1 : Deamidated_N (N)
Ions Score: 92 **Expect:** 7.1e-008
Matches (Bold Red): 14/150 fragment ions using 19 most intense peaks

#	b	b^{++}	b*	b*$^{++}$	b^{0}	b^{0++}	Seq.	y	y^{++}	y*	y*$^{++}$	y^{0}	y^{0++}	#
1	116.0342	58.5207	99.0077	50.0075			N							14
2	215.1026	108.0550	198.0761	99.5417			V	1345.7322	673.3697	**1328.7056**	664.8564	1327.7216	664.3644	13
3	302.1347	151.5710	285.1081	143.0577	284.1241	142.5657	S	**1246.6638**	**623.8355**	1229.6372	615.3222	1228.6532	614.8302	12
4	415.2187	208.1130	398.1922	199.5997	397.2082	199.1077	L	**1159.6317**	580.3195	1142.6052	571.8062	1141.6212	571.3142	11
5	529.2616	265.1345	512.2351	256.6212	511.2511	256.1292	N	**1046.5477**	523.7775	1029.5211	515.2642	1028.5371	514.7722	10
6	643.3046	322.1559	626.2780	313.6426	625.2940	313.1506	N	**932.5047**	466.7560	915.4782	458.2427	914.4942	457.7507	9
7	756.3886	378.6980	739.3621	370.1847	738.3781	369.6927	L	**818.4618**	409.7345	801.4353	401.2213	800.4512	400.7293	8
8	843.4207	422.2140	826.3941	413.7007	825.4101	413.2087	S	**705.3777**	353.1925	688.3512	344.6792	687.3672	344.1872	7
9	914.4578	457.7325	897.4312	449.2193	896.4472	448.7272	A	**618.3457**	309.6765	601.3192	301.1632	600.3352	300.6712	6
10	985.4949	493.2511	968.4683	484.7378	967.4843	484.2458	A	**547.3086**	274.1579	530.2821	265.6447	529.2980	265.1527	5
11	1114.5375	557.7724	1097.5109	549.2591	1096.5269	548.7671	E	**476.2715**	238.6394	459.2449	230.1261	458.2609	229.6341	4
12	1213.6059	607.3066	1196.5794	598.7933	1195.5953	598.3013	V	**347.2289**	174.1181	330.2023	165.6048	329.2183	165.1128	3
13	**1314.6536**	657.8304	1297.6270	649.3172	1296.6430	648.8251	T	**248.1605**	124.5839	231.1339	116.0706	230.1499	115.5786	2
14							K	147.1128	74.0600	130.0863	65.5468			1

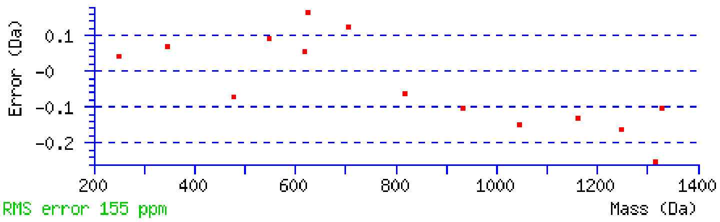

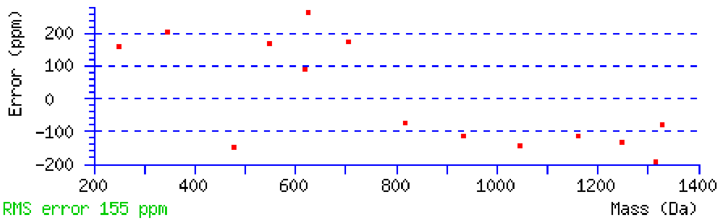

All matches to this query

Score	Mr(calc):	Delta	Sequence
92.3	1459.7518	0.0036	NVSLNNLSAAEVTK
68.3	1459.7518	0.0036	NVSLNNLSAAEVTK
67.0	1459.7518	0.0036	NVSLNNLSAAEVTK
11.5	1458.7500	1.0054	AGATLDILVENMGR
5.5	1458.7606	0.9949	WIIDNIIDSIEK
1.3	1457.7626	1.9928	NLAANRPPNTYVK
0.2	1459.7493	0.0061	AFVPIDLQCIER

Spectrum No: 66; Query: 769; Rank: 1

Peptide View

MS/MS Fragmentation of **SLTQGSMIVGNLAPVNGTSQGK**
Found in **IPI00388323**, Tax_Id=10116 Gene_Symbol=Hspg2 396 kDa protein

Match to Query 769: 2175.087848 from(1088.551200,2+)
Title: 091127RatKid_SCX01_15.2268.2268.2.dta
Data file K:\NewmanPaper\Piliang\3SubProteomes\Piliang3SP\mgf5ppm\SCX_3SubProteomes5ppm.mgf

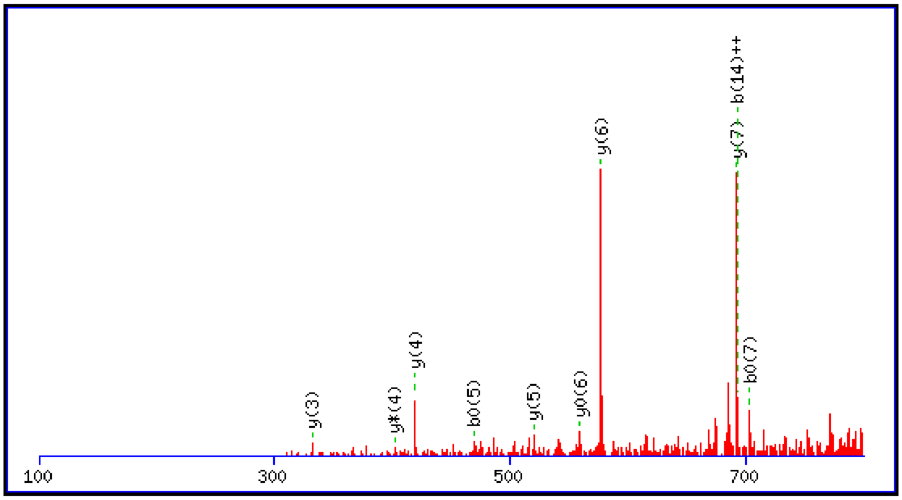

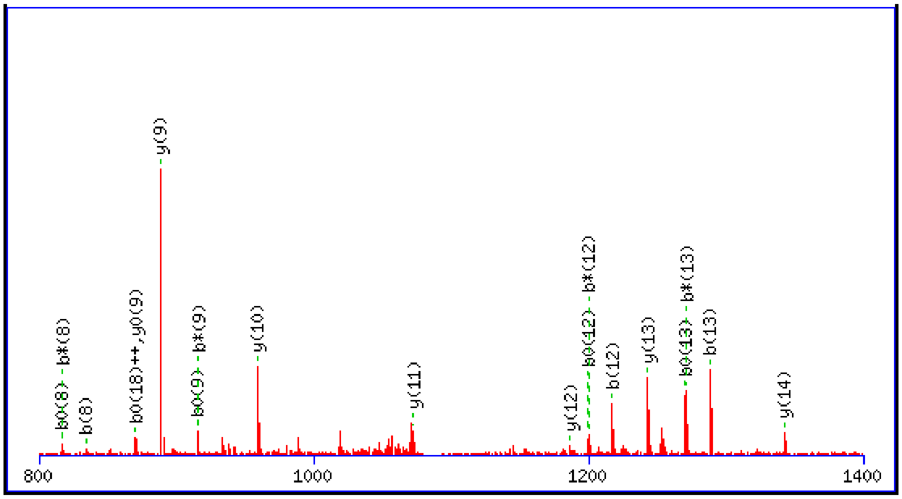

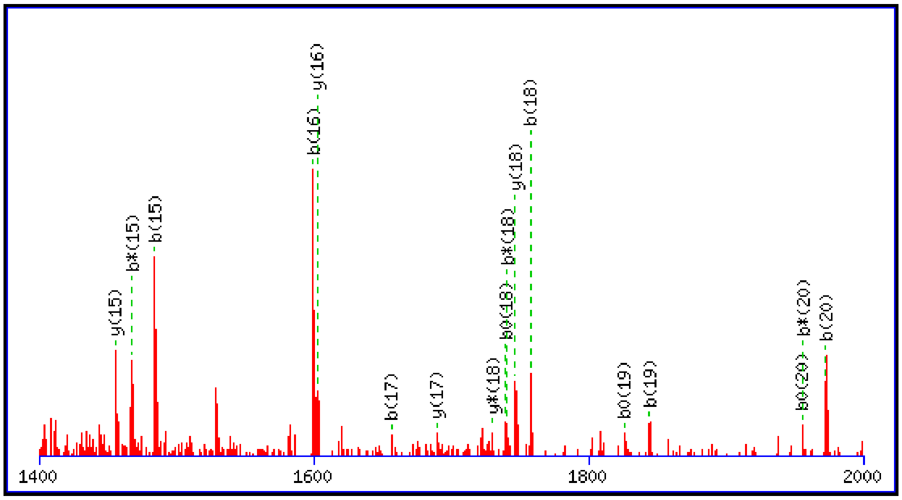

Monoisotopic mass of neutral peptide Mr(calc): 2175.0842
Fixed modifications: Carbamidomethyl (C)
Variable modifications:
M7 : Oxidation (M)
N16 : Deamidated_N (N)
Ions Score: 92 **Expect:** 1.3e-007
Matches (Bold Red): 46/240 fragment ions using 90 most intense peaks

#	b	b^{++}	b*	b^{*++}	b^0	b^{0++}	Seq.	y	y^{++}	y*	y^{*++}	y^0	y^{0++}	#
1	88.0393	44.5233			70.0287	35.5180	S							22
2	201.1234	101.0653			183.1128	92.0600	L	2089.0594	1045.0333	2072.0328	1036.5201	2071.0488	1036.0281	21
3	302.1710	151.5892			284.1605	142.5839	T	1975.9753	988.4913	1958.9488	979.9780	1957.9648	979.4860	20
4	430.2296	215.6185	413.2031	207.1052	412.2191	206.6132	Q	1874.9277	937.9675	1857.9011	929.4542	1856.9171	928.9622	19
5	487.2511	244.1292	470.2245	235.6159	**469.2405**	235.1239	G	**1746.8691**	873.9382	**1729.8425**	865.4249	1728.8585	864.9329	18
6	574.2831	287.6452	557.2566	279.1319	556.2726	278.6399	S	**1689.8476**	845.4274	1672.8211	836.9142	1671.8370	836.4222	17
7	721.3185	361.1629	704.2920	352.6496	**703.3080**	352.1576	M	**1602.8156**	801.9114	1585.7890	793.3982	1584.8050	792.9061	16
8	**834.4026**	417.7049	**817.3760**	409.1917	**816.3920**	408.6996	I	**1455.7802**	728.3937	1438.7536	719.8805	1437.7696	719.3884	15
9	933.4710	467.2391	**916.4444**	458.7259	**915.4604**	458.2339	V	**1342.6961**	671.8517	1325.6696	663.3384	1324.6855	662.8464	14
10	990.4925	495.7499	973.4659	487.2366	972.4819	486.7446	G	**1243.6277**	622.3175	1226.6012	613.8042	1225.6171	613.3122	13
11	1104.5354	552.7713	1087.5088	544.2581	1086.5248	543.7661	N	**1186.6062**	593.8068	1169.5797	585.2935	1168.5957	584.8015	12
12	**1217.6195**	609.3134	**1200.5929**	600.8001	**1199.6089**	600.3081	L	**1072.5633**	536.7853	1055.5368	528.2720	1054.5527	527.7800	11
13	**1288.6566**	644.8319	**1271.6300**	636.3186	**1270.6460**	635.8266	A	**959.4792**	480.2433	942.4527	471.7300	941.4687	471.2380	10
14	1385.7093	**693.3583**	1368.6828	684.8450	1367.6988	684.3530	P	**888.4421**	444.7247	871.4156	436.2114	**870.4316**	435.7194	9
15	**1484.7777**	742.8925	**1467.7512**	734.3792	1466.7672	733.8872	V	791.3894	396.1983	774.3628	387.6850	773.3788	387.1930	8
16	**1599.8047**	800.4060	1582.7781	791.8927	1581.7941	791.4007	N	**692.3210**	346.6641	675.2944	338.1508	674.3104	337.6588	7
17	**1656.8261**	828.9167	1639.7996	820.4034	1638.8156	819.9114	G	**577.2940**	289.1506	560.2675	280.6374	**559.2835**	280.1454	6
18	**1757.8738**	879.4406	**1740.8473**	870.9273	**1739.8633**	**870.4353**	T	**520.2726**	260.6399	503.2460	252.1266	502.2620	251.6346	5

**19**	**1844.9059**	922.9566	1827.8793	914.4433	**1826.8953**	913.9513	**S**	**419.2249**	210.1161	**402.1983**	201.6028	401.2143	201.1108	**4**
**20**	**1972.9644**	986.9859	**1955.9379**	978.4726	**1954.9539**	977.9806	**Q**	**332.1928**	166.6001	315.1663	158.0868			**3**
**21**	2029.9859	1015.4966	2012.9593	1006.9833	2011.9753	1006.4913	**G**	204.1343	102.5708	187.1077	94.0575			**2**
**22**							**K**	147.1128	74.0600	130.0863	65.5468			**1**

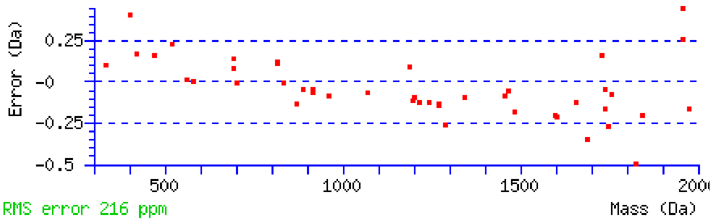

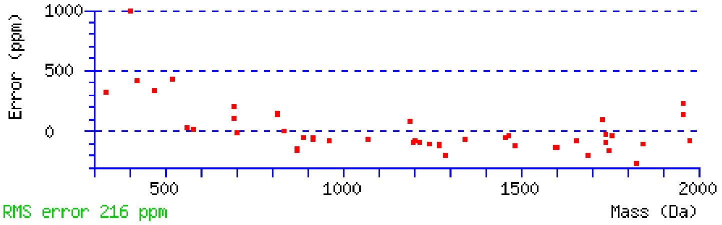

All matches to this query

Score	Mr(calc):	Delta	Sequence
91.8	2175.0842	0.0037	SLTQGSMIVGNLAPVNGTSQGK
48.0	2175.0842	0.0037	SLTQGSMIVGNLAPVNGTSQGK
9.6	2174.1001	0.9877	SLTQGSMIVGNLAPVNGTSQGK
9.2	2173.0854	2.0025	YKKAVTLAIGDGANDVSMIK

Spectrum No: 67; Query: 369; Rank: 1

Peptide View

MS/MS Fragmentation of **GVAEVTENVTEGGVTK**
Found in **IPI00214499**, Tax_Id=10116 Gene_Symbol=P2rx7 P2X purinoceptor 7

Match to Query 369: 1589.782928 from(795.898740,2+)
Title: 091127RatKid_SCX01_12.1884.1884.2.dta
Data file K:\NewmanPaper\Piliang\3SubProteomes\Piliang3SP\mgf5ppm\SCX_3SubProteomes5ppm.mgf

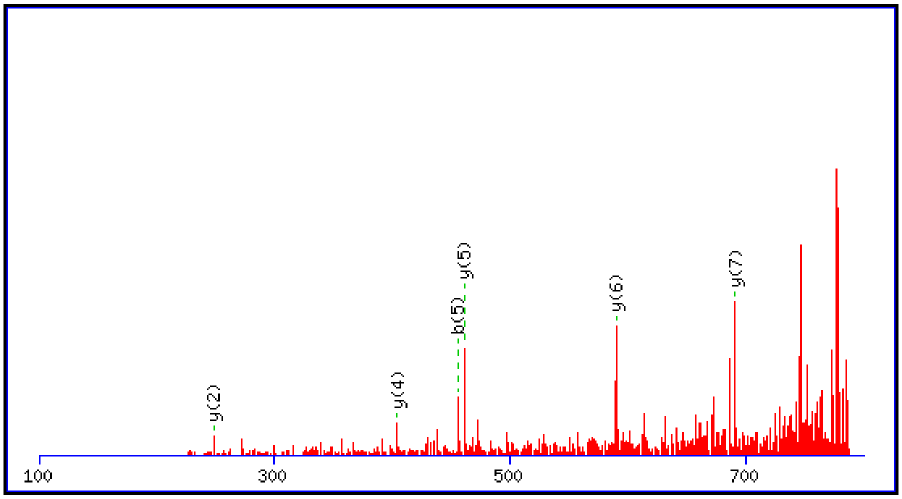

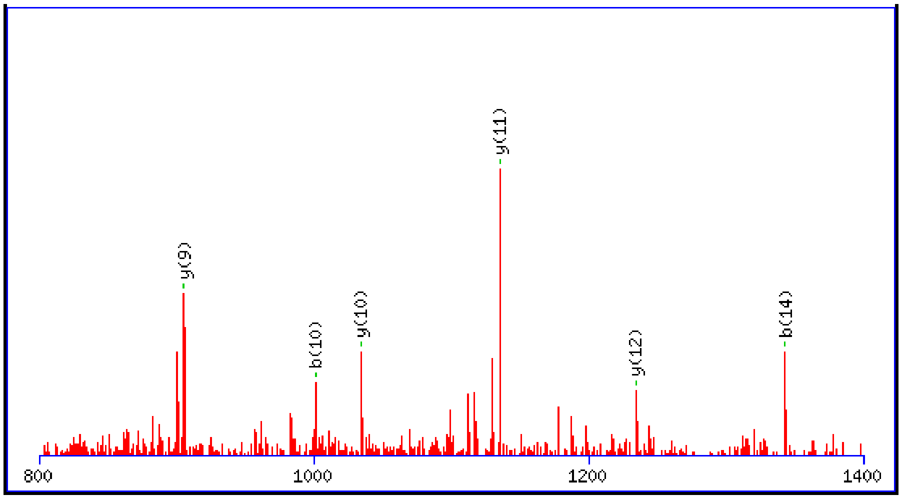

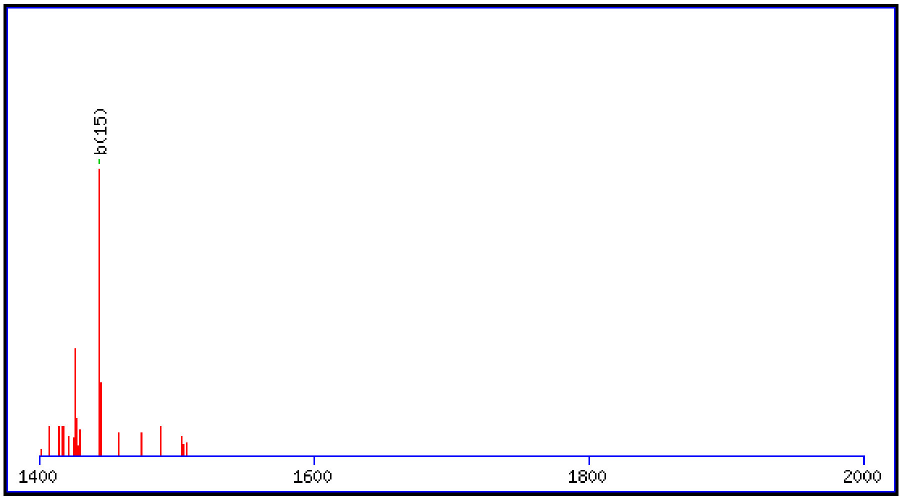

Monoisotopic mass of neutral peptide Mr(calc): 1589.7785
Fixed modifications: Carbamidomethyl (C)
Variable modifications:
N8 : Deamidated_N (N)
Ions Score: 91 **Expect:** 1.2e-007
Matches (Bold Red): 13/158 fragment ions using 18 most intense peaks

#	b	b^{++}	b*	b^{*++}	b^0	b^{0++}	Seq.	y	y^{++}	y*	y^{*++}	y^0	y^{0++}	#
1	58.0287	29.5180					G							16
2	157.0972	79.0522					V	1533.7643	767.3858	1516.7377	758.8725	1515.7537	758.3805	15
3	228.1343	114.5708					A	1434.6958	717.8516	1417.6693	709.3383	1416.6853	708.8463	14
4	357.1769	179.0921			339.1663	170.0868	E	1363.6587	682.3330	1346.6322	673.8197	1345.6482	673.3277	13
5	**456.2453**	228.6263			438.2347	219.6210	V	**1234.6161**	617.8117	1217.5896	609.2984	1216.6056	608.8064	12
6	557.2930	279.1501			539.2824	270.1448	T	**1135.5477**	568.2775	1118.5212	559.7642	1117.5372	559.2722	11
7	686.3355	343.6714			668.3250	334.6661	E	**1034.5000**	517.7537	1017.4735	509.2404	1016.4895	508.7484	10
8	801.3625	401.1849	784.3359	392.6716	783.3519	392.1796	N	**905.4574**	453.2324	888.4309	444.7191	887.4469	444.2271	9
9	900.4309	450.7191	883.4043	442.2058	882.4203	441.7138	V	790.4305	395.7189	773.4040	387.2056	772.4199	386.7136	8
10	**1001.4786**	501.2429	984.4520	492.7297	983.4680	492.2376	T	**691.3621**	346.1847	674.3355	337.6714	673.3515	337.1794	7
11	1130.5212	565.7642	1113.4946	557.2509	1112.5106	556.7589	E	**590.3144**	295.6608	573.2879	287.1476	572.3039	286.6556	6
12	1187.5426	594.2750	1170.5161	585.7617	1169.5321	585.2697	G	**461.2718**	231.1396	444.2453	222.6263	443.2613	222.1343	5
13	1244.5641	622.7857	1227.5375	614.2724	1226.5535	613.7804	G	**404.2504**	202.6288	387.2238	194.1155	386.2398	193.6235	4
14	**1343.6325**	672.3199	1326.6060	663.8066	1325.6219	663.3146	V	347.2289	174.1181	330.2023	165.6048	329.2183	165.1128	3
15	**1444.6802**	722.8437	1427.6536	714.3305	1426.6696	713.8385	T	**248.1605**	124.5839	231.1339	116.0706	230.1499	115.5786	2
16							K	147.1128	74.0600	130.0863	65.5468			1

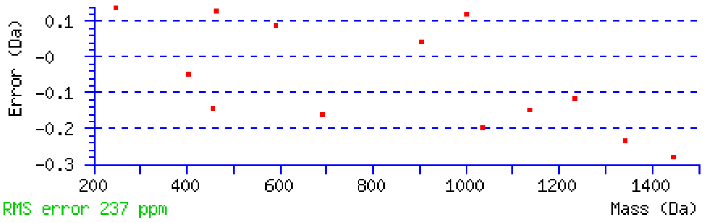

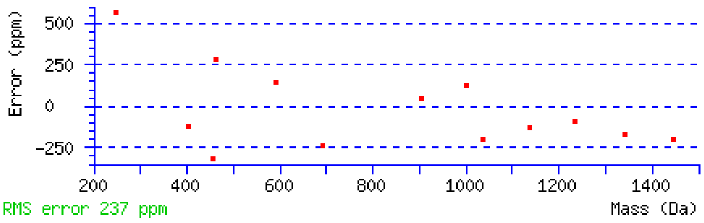

All matches to this query

Score	Mr(calc):	Delta	Sequence
91.4	1589.7785	0.0045	GVAEVTENVTEGGVTK
16.7	1588.7944	0.9885	GVAEVTENVTEGGVTK
9.3	1589.7742	0.0087	GVFTKEYELSPIK
9.3	1589.7742	0.0087	GVFTKEYELSPIK
8.4	1589.7793	0.0037	KELENMCNPIITK
7.3	1589.7971	-0.0141	QVEEALTMVIQADK
6.5	1589.7854	-0.0025	YIVNDLVLRNYK
5.3	1589.7912	-0.0082	ALYLAGPNCLSPWK
4.4	1589.7793	0.0037	KELENMCNPIITK
3.7	1589.7854	-0.0025	YIVNDLVLRNYK

Spectrum No: 68; Query: 829; Rank: 1

Peptide View

MS/MS Fragmentation of **IVNDNPYGNGTAIFTTNGAIAR**
Found in **IPI00205018**, Tax_Id=10116 Gene_Symbol=Aldh6a1 Methylmalonate-semialdehyde dehydrogenase [acylating], mitochondrial precursor

Match to Query 829: 2279.126388 from(1140.570470,2+)
Title: 091129RatKid_SCX02_18.2614.2614.2.dta
Data file K:\NewmanPaper\Piliang\3SubProteomes\Piliang3SP\mgf5ppm\SCX_3SubProteomes5ppm.mgf

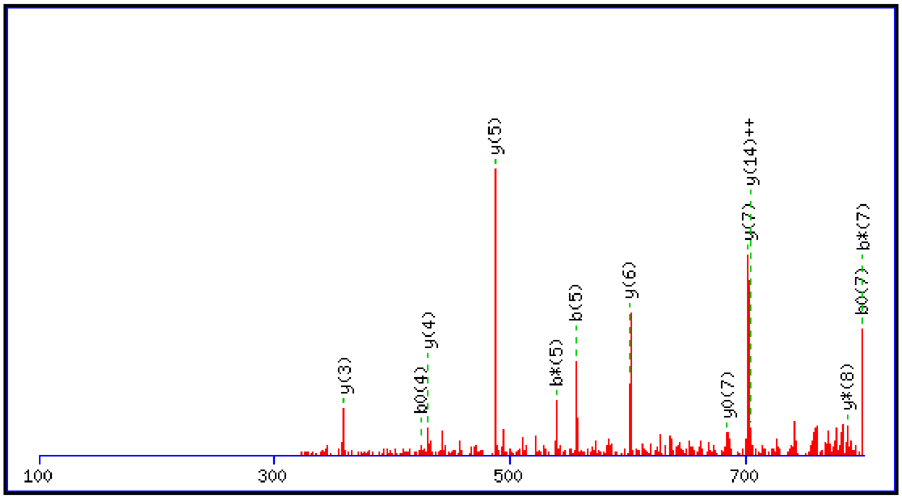

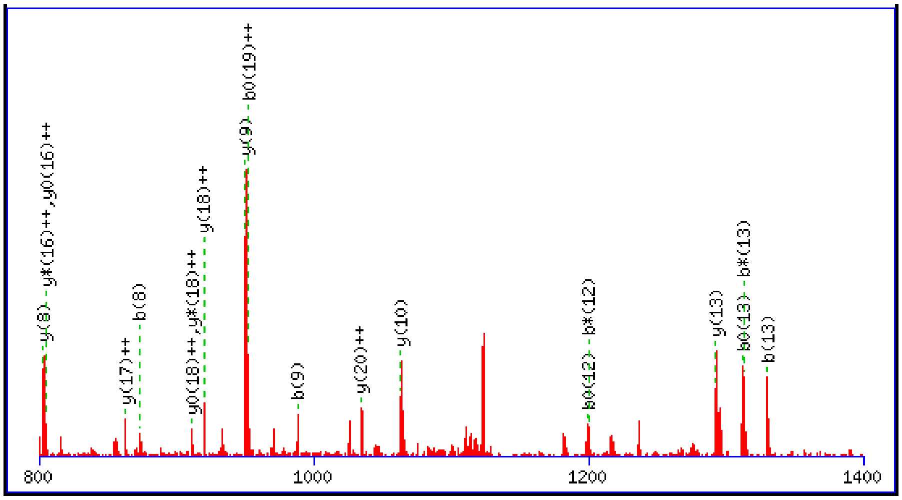

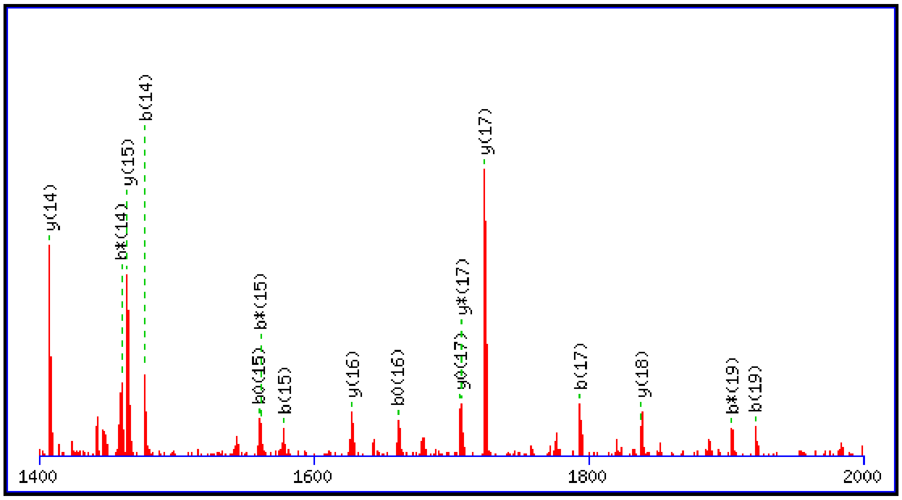

Monoisotopic mass of neutral peptide Mr(calc): 2279.1182
Fixed modifications: Carbamidomethyl (C)
Variable modifications:
N9 : Deamidated_N (N)
Ions Score: 91 **Expect:** 1.7e-007
Matches (Bold Red): 48/230 fragment ions using 79 most intense peaks

#	b	b^{++}	b*	b^{*++}	b^0	b^{0++}	Seq.	y	y^{++}	y*	y^{*++}	y^0	y^{0++}	#
1	114.0913	57.5493					I							22
2	213.1598	107.0835					V	2167.0414	1084.0244	2150.0149	1075.5111	2149.0309	1075.0191	21
3	327.2027	164.1050	310.1761	155.5917			N	2067.9730	**1034.4901**	2050.9465	1025.9769	2049.9625	1025.4849	20
4	442.2296	221.6185	425.2031	213.1052	**424.2191**	212.6132	D	1953.9301	977.4687	1936.9035	968.9554	1935.9195	968.4634	19
5	**556.2726**	278.6399	**539.2460**	270.1266	538.2620	269.6346	N	**1838.9031**	**919.9552**	1821.8766	**911.4419**	1820.8926	**910.9499**	18
6	653.3253	327.1663	636.2988	318.6530	635.3148	318.1610	P	**1724.8602**	**862.9337**	**1707.8337**	854.4205	**1706.8497**	853.9285	17
7	816.3886	408.6980	**799.3621**	400.1847	**798.3781**	399.6927	Y	**1627.8075**	814.4074	1610.7809	**805.8941**	1609.7969	**805.4021**	16
8	**873.4101**	437.2087	856.3836	428.6954	855.3995	428.2034	G	**1464.7441**	732.8757	1447.7176	724.3624	1446.7336	723.8704	15
9	**988.4370**	494.7222	971.4105	486.2089	970.4265	485.7169	N	**1407.7227**	**704.3650**	1390.6961	695.8517	1389.7121	695.3597	14
10	1045.4585	523.2329	1028.4320	514.7196	1027.4479	514.2276	G	**1292.6957**	646.8515	1275.6692	638.3382	1274.6852	637.8462	13
11	1146.5062	573.7567	1129.4796	565.2435	1128.4956	564.7514	T	1235.6743	618.3408	1218.6477	609.8275	1217.6637	609.3355	12
12	1217.5433	609.2753	**1200.5168**	600.7620	**1199.5327**	600.2700	A	1134.6266	567.8169	1117.6000	559.3037	1116.6160	558.8116	11
13	**1330.6274**	665.8173	**1313.6008**	657.3040	**1312.6168**	656.8120	I	**1063.5895**	532.2984	1046.5629	523.7851	1045.5789	523.2931	10
14	**1477.6958**	739.3515	**1460.6692**	730.8383	1459.6852	730.3462	F	**950.5054**	475.7563	933.4789	467.2431	932.4948	466.7511	9
15	**1578.7435**	789.8754	**1561.7169**	781.3621	**1560.7329**	780.8701	T	**803.4370**	402.2221	**786.4104**	393.7089	785.4264	393.2169	8
16	1679.7911	840.3992	1662.7646	831.8859	**1661.7806**	831.3939	T	**702.3893**	351.6983	685.3628	343.1850	**684.3787**	342.6930	7
17	**1793.8341**	897.4207	1776.8075	888.9074	1775.8235	888.4154	N	**601.3416**	301.1745	584.3151	292.6612			6
18	1850.8555	925.9314	1833.8290	917.4181	1832.8450	916.9261	G	**487.2987**	244.1530	470.2722	235.6397			5

**19**	**1921.8926**	961.4500	**1904.8661**	952.9367	1903.8821	**952.4447**	**A**	**430.2772**	215.6423	413.2507	207.1290			**4**
**20**	2034.9767	1017.9920	2017.9502	1009.4787	2016.9661	1008.9867	**I**	**359.2401**	180.1237	342.2136	171.6104			**3**
**21**	2106.0138	1053.5105	2088.9873	1044.9973	2088.0033	1044.5053	**A**	246.1561	123.5817	229.1295	115.0684			**2**
**22**							**R**	175.1190	88.0631	158.0924	79.5498			**1**

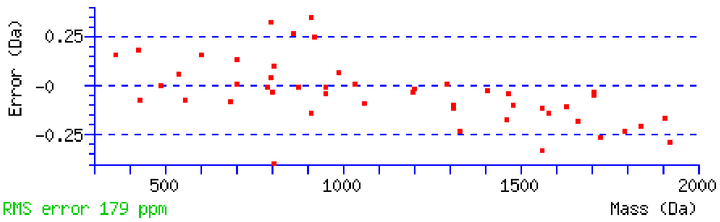

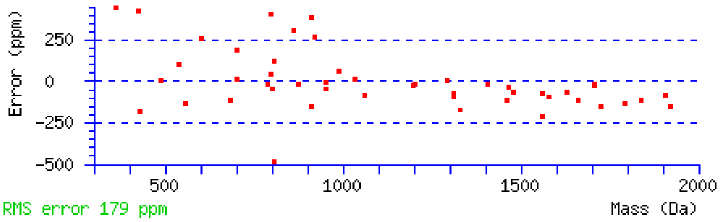

All matches to this query

Score	Mr(calc):	Delta	Sequence
91.2	2279.1182	0.0082	IVNDNPYGNGTAIFTTNGAIAR
79.1	2279.1182	0.0082	IVNDNPYGNGTAIFTTNGAIAR
43.9	2279.1182	0.0082	IVNDNPYGNGTAIFTTNGAIAR
32.4	2279.1182	0.0082	IVNDNPYGNGTAIFTTNGAIAR
30.9	2278.1342	0.9922	IVNDNPYGNGTAIFTTNGAIAR
0.1	2279.1046	0.0218	GKASIEIANESGETPLDIAKR

Spectrum No: 69; Query: 693; Rank: 1

Peptide View

MS/MS Fragmentation of **YCGNSTPSSVDTSSNVASVK**
Found in **IPI00196620**, Tax_Id=10116 Gene_Symbol=Cubn Cubilin precursor

Match to Query 693: 2059.904248 from(1030.959400,2+)
Title: 091127RatKid_SCX01_16.1278.1278.2.dta
Data file K:\NewmanPaper\Piliang\3SubProteomes\Piliang3SP\mgf5ppm\SCX_3SubProteomes5ppm.mgf

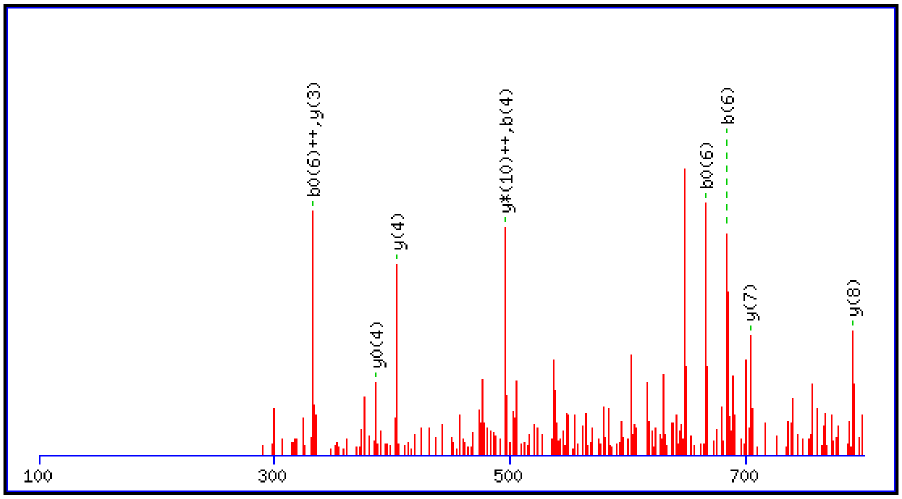

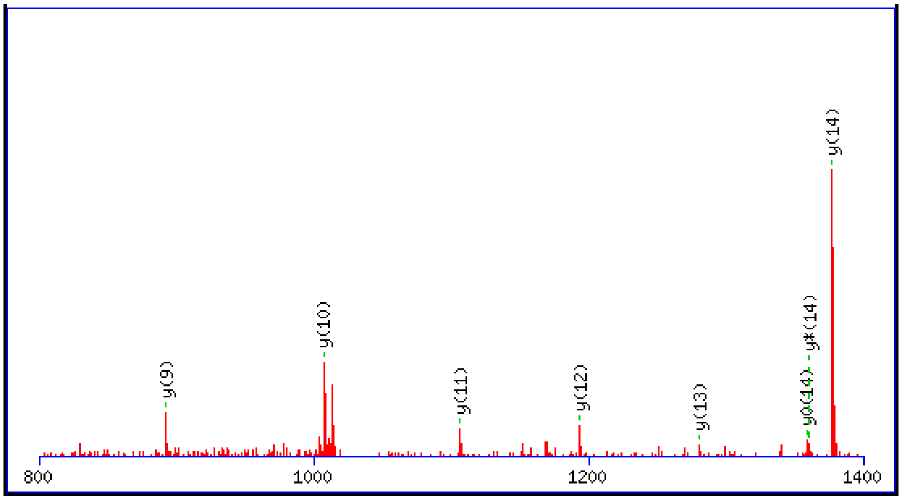

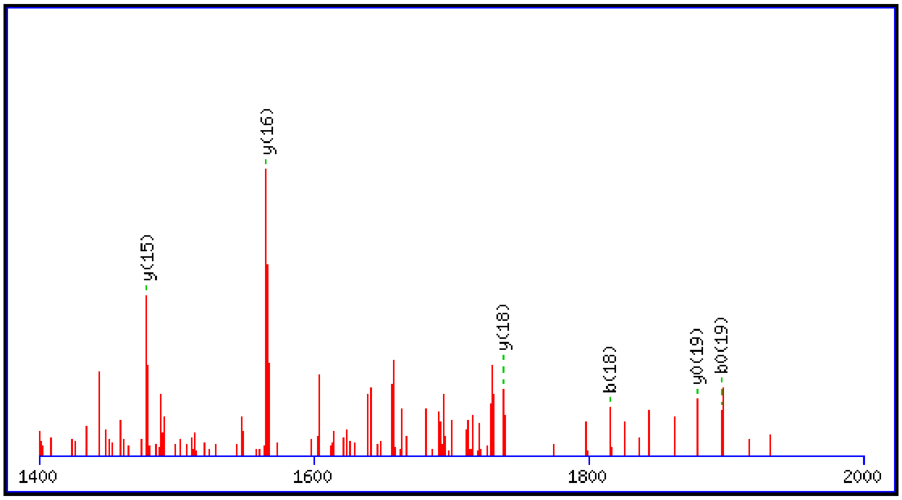

Monoisotopic mass of neutral peptide Mr(calc): 2059.9004
Fixed modifications: Carbamidomethyl (C)
Variable modifications:
N4 : Deamidated_N (N)
Ions Score: 91 **Expect:** 2.1e-007
Matches (Bold Red): 24/210 fragment ions using 45 most intense peaks

#	b	b^{++}	b^{*}	b^{*++}	b^{0}	b^{0++}	Seq.	y	y^{++}	y^{*}	y^{*++}	y^{0}	y^{0++}	#
1	164.0706	82.5389					Y							20
2	324.1013	162.5543					C	1897.8444	949.4258	1880.8178	940.9125	**1879.8338**	940.4205	19
3	381.1227	191.0650					G	**1737.8137**	869.4105	1720.7872	860.8972	1719.8032	860.4052	18
4	**496.1497**	248.5785	479.1231	240.0652			N	1680.7923	840.8998	1663.7657	832.3865	1662.7817	831.8945	17
5	583.1817	292.0945	566.1551	283.5812	565.1711	283.0892	S	**1565.7653**	783.3863	1548.7388	774.8730	1547.7548	774.3810	16
6	**684.2294**	342.6183	667.2028	334.1050	**666.2188**	**333.6130**	T	**1478.7333**	739.8703	1461.7067	731.3570	1460.7227	730.8650	15
7	781.2821	391.1447	764.2556	382.6314	763.2716	382.1394	P	**1377.6856**	689.3464	**1360.6591**	680.8332	**1359.6750**	680.3412	14
8	868.3142	434.6607	851.2876	426.1474	850.3036	425.6554	S	**1280.6328**	640.8201	1263.6063	632.3068	1262.6223	631.8148	13
9	955.3462	478.1767	938.3196	469.6635	937.3356	469.1714	S	**1193.6008**	597.3040	1176.5743	588.7908	1175.5903	588.2988	12
10	1054.4146	527.7109	1037.3880	519.1977	1036.4040	518.7057	V	**1106.5688**	553.7880	1089.5422	545.2748	1088.5582	544.7828	11
11	1169.4415	585.2244	1152.4150	576.7111	1151.4310	576.2191	D	**1007.5004**	504.2538	990.4738	**495.7406**	989.4898	495.2485	10
12	1270.4892	635.7482	1253.4627	627.2350	1252.4787	626.7430	T	**892.4734**	446.7404	875.4469	438.2271	874.4629	437.7351	9
13	1357.5212	679.2643	1340.4947	670.7510	1339.5107	670.2590	S	**791.4258**	396.2165	774.3992	387.7032	773.4152	387.2112	8
14	1444.5533	722.7803	1427.5267	714.2670	1426.5427	713.7750	S	**704.3937**	352.7005	687.3672	344.1872	686.3832	343.6952	7
15	1558.5962	779.8017	1541.5697	771.2885	1540.5856	770.7965	N	617.3617	309.1845	600.3352	300.6712	599.3511	300.1792	6
16	1657.6646	829.3359	1640.6381	820.8227	1639.6540	820.3307	V	503.3188	252.1630	486.2922	243.6498	485.3082	243.1577	5
17	1728.7017	864.8545	1711.6752	856.3412	1710.6912	855.8492	A	**404.2504**	202.6288	387.2238	194.1155	**386.2398**	193.6235	4
18	**1815.7338**	908.3705	1798.7072	899.8572	1797.7232	899.3652	S	**333.2132**	167.1103	316.1867	158.5970	315.2027	158.1050	3

**19**	1914.8022	957.9047	1897.7756	949.3914	**1896.7916**	948.8994	**V**	246.1812	123.5942	229.1547	115.0810			**2**
**20**							**K**	147.1128	74.0600	130.0863	65.5468			**1**

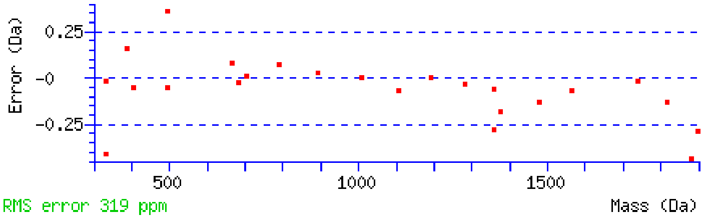

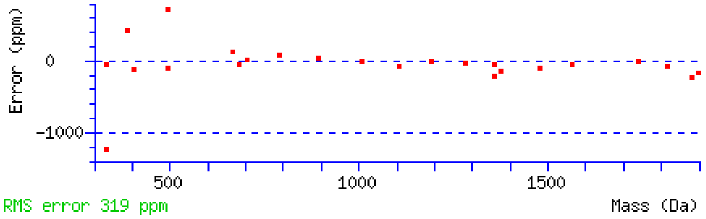

All matches to this query

Score	Mr(calc):	Delta	Sequence
90.9	2059.9004	0.0038	YCGNSTPSSVDTSSNVASVK
79.8	2058.9164	0.9878	YCGNSTPSSVDTSSNVASVK
48.8	2059.9004	0.0038	YCGNSTPSSVDTSSNVASVK
2.7	2059.9074	-0.0031	LHYIMGDYAEALSNQQK
0.1	2058.9160	0.9883	VNLRTALHYYNQSEDR

Spectrum No: 70; Query: 373; Rank: 1

Peptide View

MS/MS Fragmentation of **CNTTQGNEVTSILR**
Found in **IPI00327143**, Tax_Id=10116 Gene_Symbol=Alpl Alkaline phosphatase, tissue-nonspecific isozyme precursor

Match to Query 373: 1592.745228 from(797.379890,2+)
Title: 091127RatKid_SCX01_02.2363.2363.2.dta
Data file K:\NewmanPaper\Piliang\3SubProteomes\Piliang3SP\mgf5ppm\SCX_3SubProteomes5ppm.mgf

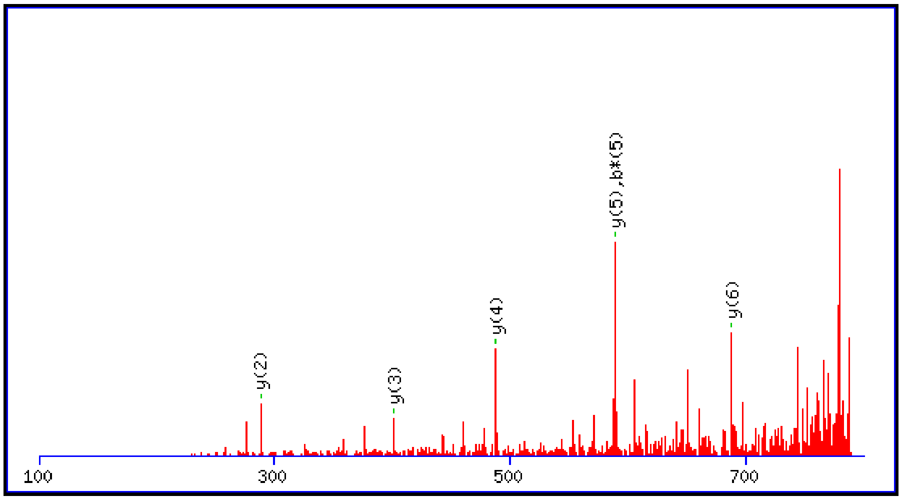

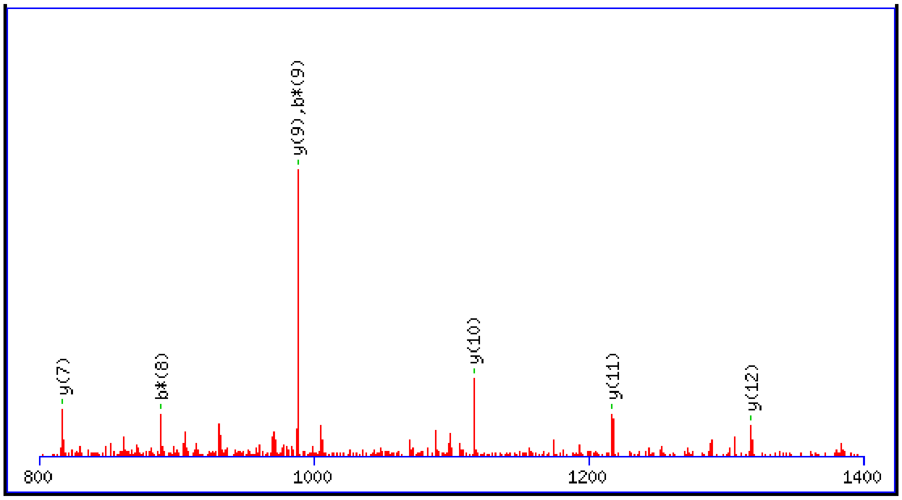

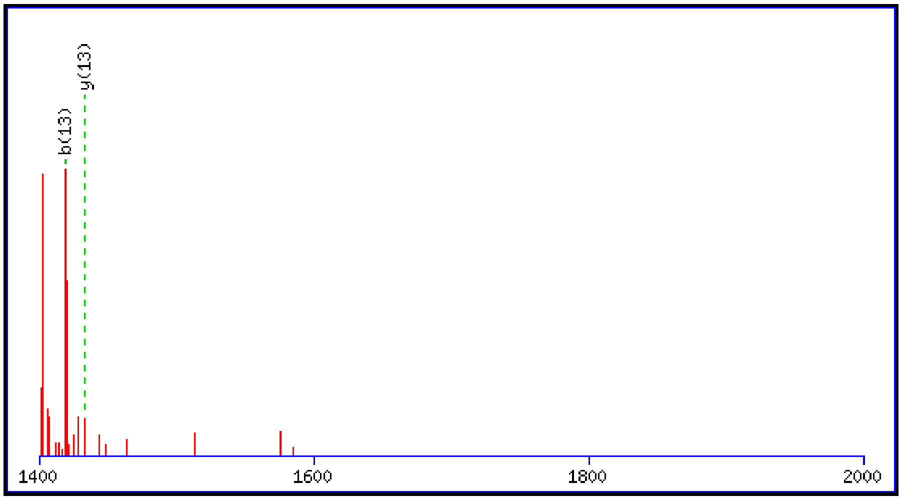

Monoisotopic mass of neutral peptide Mr(calc): 1592.7464
Fixed modifications: Carbamidomethyl (C)
Variable modifications:
N2 : Deamidated_N (N)
Ions Score: 91 **Expect:** 1.5e-007
Matches (Bold Red): 15/144 fragment ions using 21 most intense peaks

#	b	b++	b*	b*++	b0	b0++	Seq.	y	y++	y*	y*++	y0	y0++	#
1	161.0379	81.0226					C							14
2	276.0649	138.5361	259.0383	130.0228			N	**1433.7231**	717.3652	1416.6965	708.8519	1415.7125	708.3599	13
3	377.1125	189.0599	360.0860	180.5466	359.1020	180.0546	T	**1318.6961**	659.8517	1301.6696	651.3384	1300.6856	650.8464	12
4	478.1602	239.5837	461.1337	231.0705	460.1497	230.5785	T	**1217.6484**	609.3279	1200.6219	600.8146	1199.6379	600.3226	11
5	606.2188	303.6130	**589.1922**	295.0998	588.2082	294.6078	Q	**1116.6008**	558.8040	1099.5742	550.2907	1098.5902	549.7987	10
6	663.2403	332.1238	646.2137	323.6105	645.2297	323.1185	G	**988.5422**	494.7747	971.5156	486.2615	970.5316	485.7694	9
7	777.2832	389.1452	760.2566	380.6320	759.2726	380.1399	N	931.5207	466.2640	914.4942	457.7507	913.5102	457.2587	8
8	906.3258	453.6665	**889.2992**	445.1533	888.3152	444.6612	E	**817.4778**	409.2425	800.4512	400.7293	799.4672	400.2373	7
9	1005.3942	503.2007	**988.3676**	494.6875	987.3836	494.1955	V	**688.4352**	344.7212	671.4087	336.2080	670.4246	335.7160	6
10	1106.4419	553.7246	1089.4153	545.2113	1088.4313	544.7193	T	**589.3668**	295.1870	572.3402	286.6738	571.3562	286.1817	5
11	1193.4739	597.2406	1176.4474	588.7273	1175.4633	588.2353	S	**488.3191**	244.6632	471.2926	236.1499	470.3085	235.6579	4
12	1306.5580	653.7826	1289.5314	645.2693	1288.5474	644.7773	I	**401.2871**	201.1472	384.2605	192.6339			3
13	**1419.6420**	710.3247	1402.6155	701.8114	1401.6315	701.3194	L	**288.2030**	144.6051	271.1765	136.0919			2
14							R	175.1190	88.0631	158.0924	79.5498			1

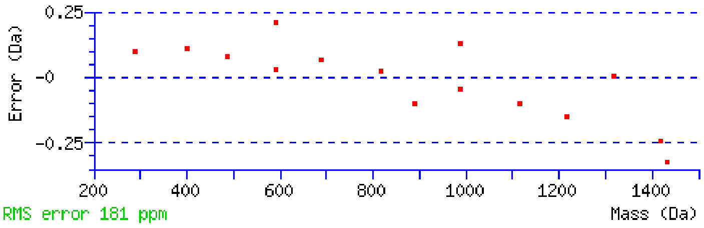

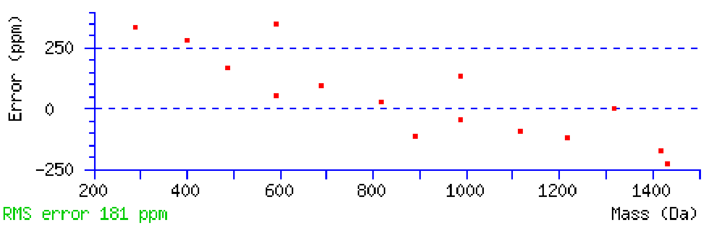

All matches to this query

Score	Mr(calc):	Delta	Sequence
90.8	1592.7464	-0.0012	CNTTQGNEVTSILR
64.8	1592.7464	-0.0012	CNTTQGNEVTSILR
14.8	1592.7577	-0.0124	TLGESQRTDCSLAR
14.1	1592.7318	0.0134	NFIETDNEGNGILR
14.1	1592.7318	0.0134	NFIETDNEGNGILR
10.1	1592.7390	0.0062	NRERVESSEETTR
8.8	1592.7487	-0.0035	IENFTTDYKIIR
8.8	1592.7487	-0.0035	IENFTTDYKIIR
8.0	1592.7365	0.0087	NRYGDVPCLDQTR
8.0	1592.7352	0.0101	MDTQEETAAELSLR

Spectrum No: 71; Query: 1012; Rank: 1

Peptide View

MS/MS Fragmentation of **DLVVGLYSQWMNNSDNNPIHPNLR**
Found in **IPI00230862**, Tax_Id=10116 Gene_Symbol=Anpep Aminopeptidase N

Match to Query 1012: 2796.335502 from(933.119110,3+)
Title: 091127RatKid_SCX01_13.4801.4801.3.dta
Data file K:\NewmanPaper\Piliang\3SubProteomes\Piliang3SP\mgf5ppm\SCX_3SubProteomes5ppm.mgf

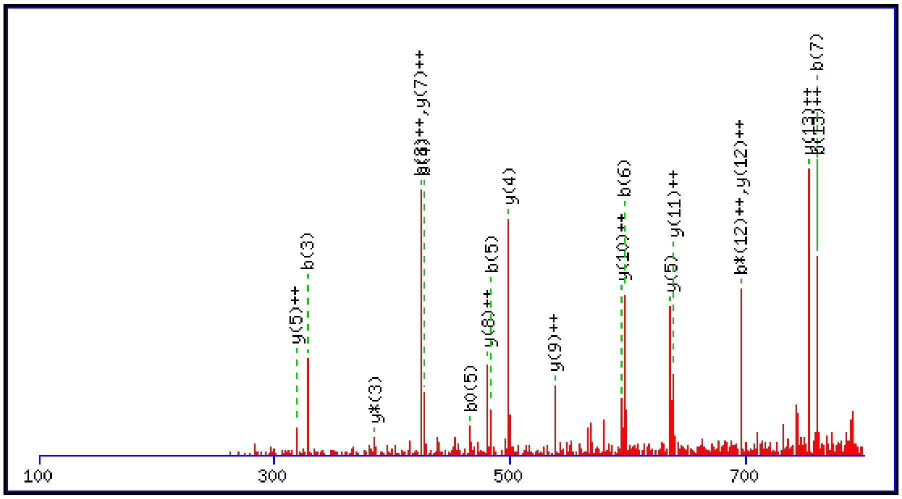

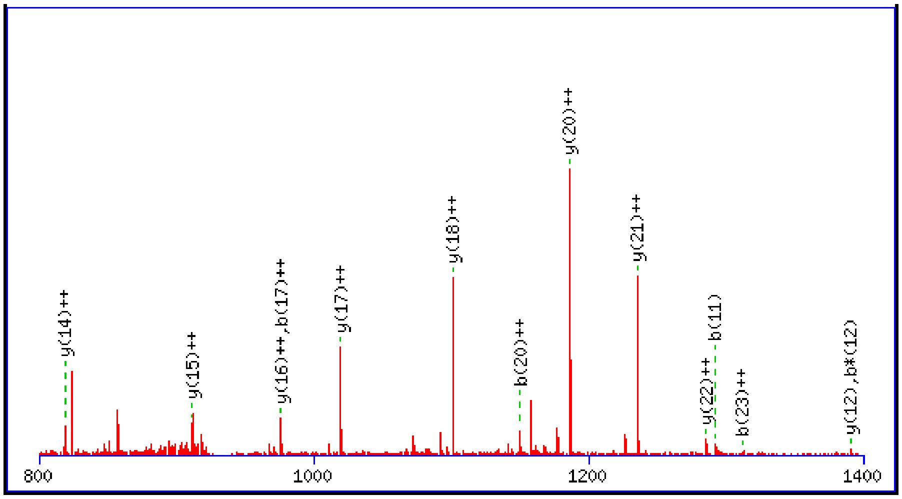

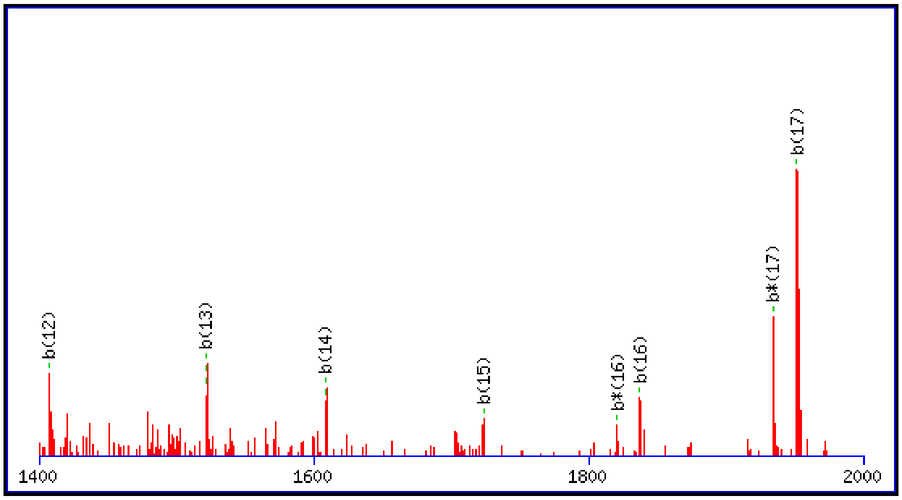

Monoisotopic mass of neutral peptide Mr(calc): 2796.3289
Fixed modifications: Carbamidomethyl (C)
Variable modifications:
N12 : Deamidated_N (N)
Ions Score: 90 **Expect:** 2.9e-007
Matches (Bold Red): 42/242 fragment ions using 78 most intense peaks

#	b	b^{++}	b*	b^{*++}	b^0	b^{0++}	Seq.	y	y^{++}	y*	y^{*++}	y^0	y^{0++}	#
1	116.0342	58.5207			98.0237	49.5155	D							24
2	229.1183	115.0628			211.1077	106.0575	L	2682.3093	1341.6583	2665.2827	1333.1450	2664.2987	1332.6530	23
3	**328.1867**	164.5970			310.1761	155.5917	V	2569.2252	**1285.1162**	2552.1987	1276.6030	2551.2147	1276.1110	22
4	**427.2551**	214.1312			409.2445	205.1259	V	2470.1568	**1235.5820**	2453.1303	1227.0688	2452.1462	1226.5768	21
5	**484.2766**	242.6419			**466.2660**	233.6366	G	2371.0884	**1186.0478**	2354.0618	1177.5346	2353.0778	1177.0426	20
6	**597.3606**	299.1840			579.3501	290.1787	L	2314.0669	1157.5371	2297.0404	1149.0238	2296.0564	1148.5318	19
7	**760.4240**	380.7156			742.4134	371.7103	Y	2200.9829	**1100.9951**	2183.9563	1092.4818	2182.9723	1091.9898	18
8	847.4560	**424.2316**			829.4454	415.2264	S	2037.9195	**1019.4634**	2020.8930	1010.9501	2019.9090	1010.4581	17
9	975.5146	488.2609	958.4880	479.7477	957.5040	479.2556	Q	1950.8875	**975.9474**	1933.8610	967.4341	1932.8769	966.9421	16
10	1161.5939	581.3006	1144.5673	572.7873	1143.5833	572.2953	W	1822.8289	**911.9181**	1805.8024	903.4048	1804.8184	902.9128	15
11	**1292.6344**	646.8208	1275.6078	638.3075	1274.6238	637.8155	M	1636.7496	**818.8784**	1619.7231	810.3652	1618.7391	809.8732	14
12	**1407.6613**	704.3343	**1390.6348**	**695.8210**	1389.6507	695.3290	N	1505.7091	**753.3582**	1488.6826	744.8449	1487.6986	744.3529	13
13	**1521.7042**	**761.3558**	1504.6777	752.8425	1503.6937	752.3505	N	**1390.6822**	**695.8447**	1373.6557	687.3315	1372.6716	686.8395	12
14	**1608.7363**	804.8718	1591.7097	796.3585	1590.7257	795.8665	S	1276.6393	**638.8233**	1259.6127	630.3100	1258.6287	629.8180	11
15	**1723.7632**	862.3852	1706.7367	853.8720	1705.7526	853.3800	D	1189.6072	**595.3073**	1172.5807	586.7940	1171.5967	586.3020	10
16	**1837.8061**	919.4067	**1820.7796**	910.8934	1819.7956	910.4014	N	1074.5803	**537.7938**	1057.5538	529.2805			9
17	**1951.8491**	**976.4282**	**1934.8225**	967.9149	1933.8385	967.4229	N	960.5374	**480.7723**	943.5108	472.2591			8
18	2048.9018	1024.9545	2031.8753	1016.4413	2030.8913	1015.9493	P	846.4944	**423.7509**	829.4679	415.2376			7

**19**	2161.9859	1081.4966	2144.9593	1072.9833	2143.9753	1072.4913	**I**	749.4417	375.2245	732.4151	366.7112			**6**
**20**	2299.0448	**1150.0260**	2282.0183	1141.5128	2281.0342	1141.0208	**H**	**636.3576**	**318.6824**	619.3311	310.1692			**5**
**21**	2396.0976	1198.5524	2379.0710	1190.0391	2378.0870	1189.5471	**P**	**499.2987**	250.1530	482.2722	241.6397			**4**
**22**	2510.1405	1255.5739	2493.1139	1247.0606	2492.1299	1246.5686	**N**	402.2459	201.6266	**385.2194**	193.1133			**3**
**23**	2623.2246	**1312.1159**	2606.1980	1303.6026	2605.2140	1303.1106	**L**	288.2030	144.6051	271.1765	136.0919			**2**
**24**							**R**	175.1190	88.0631	158.0924	79.5498			**1**

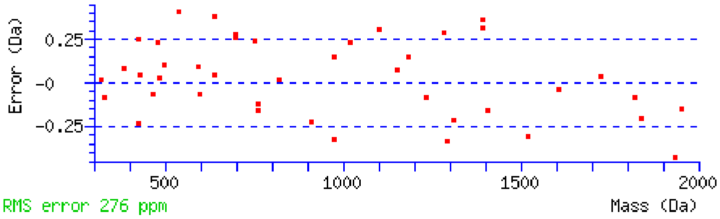

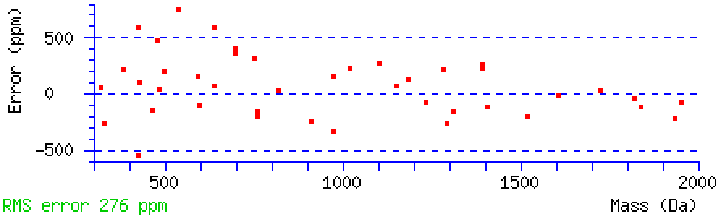

All matches to this query

Score	Mr(calc):	Delta	Sequence
90.2	2796.3289	0.0066	DLVVGLYSQWMNNSDNNPIHPNLR
82.1	2796.3289	0.0066	DLVVGLYSQWMNNSDNNPIHPNLR
78.8	2796.3289	0.0066	DLVVGLYSQWMNNSDNNPIHPNLR
75.5	2796.3289	0.0066	DLVVGLYSQWMNNSDNNPIHPNLR
69.9	2796.3289	0.0066	DLVVGLYSQWMNNSDNNPIHPNLR
26.8	2795.3449	0.9906	DLVVGLYSQWMNNSDNNPIHPNLR
1.7	2796.3192	0.0163	QVTAPQRVPVASPSAHNISSSSGAPDR
1.7	2796.3192	0.0163	QVTAPQRVPVASPSAHNISSSSGAPDR
1.6	2796.3500	-0.0145	GAVTYAENIPAGEKRSLIYSSGILK
1.5	2794.3278	2.0077	IGRGQFSEVYRASCLLDGVPVALK

Spectrum No: 72; Query: 842; Rank: 1

Peptide View

MS/MS Fragmentation of **QILEGLEFNLTQIPEADIHK**
Found in **IPI00324019**, Tax_Id=10116 Gene_Symbol=Serpina1 Alpha-1-antiproteinase precursor

Match to Query 842: 2308.204048 from(1155.109300,2+)
Title: 091127RatKid_SCX01_13.4773.4773.2.dta
Data file K:\NewmanPaper\Piliang\3SubProteomes\Piliang3SP\mgf5ppm\SCX_3SubProteomes5ppm.mgf

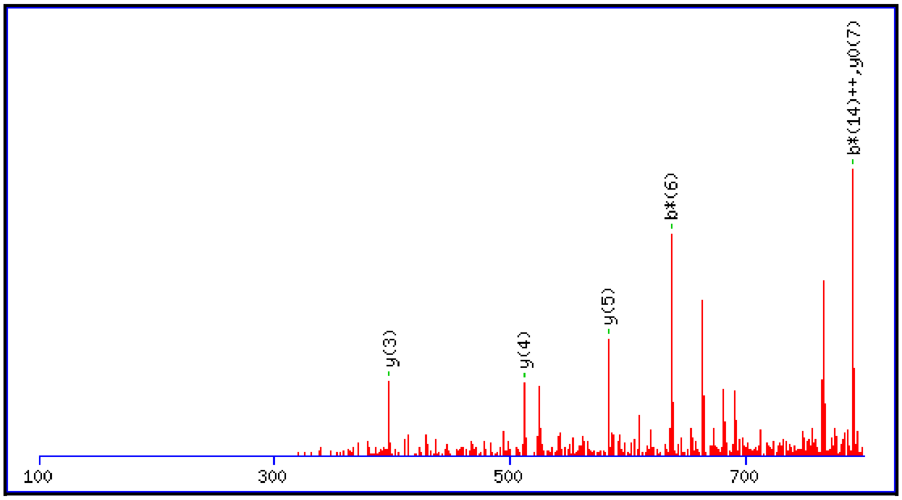

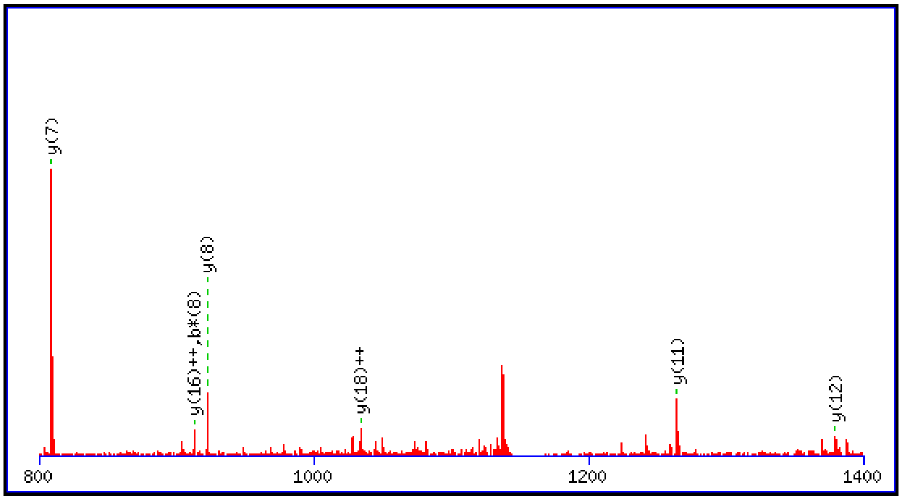

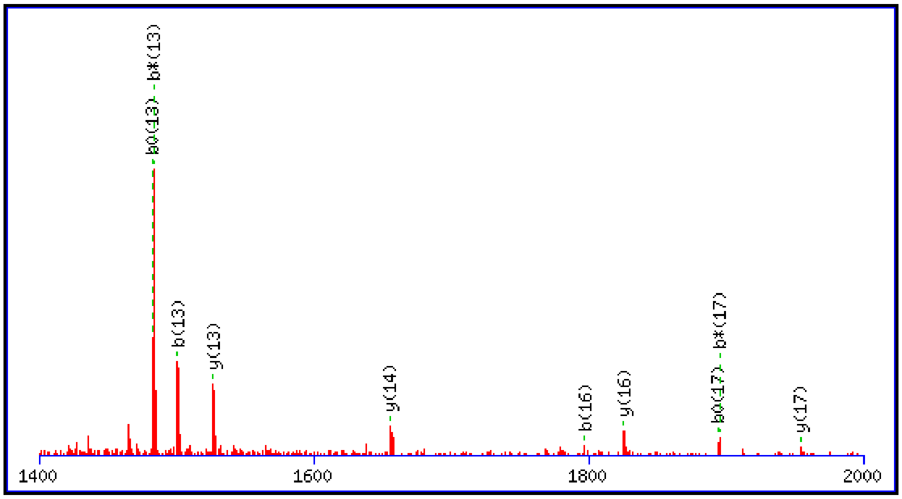

Monoisotopic mass of neutral peptide Mr(calc): 2308.1950
Fixed modifications: Carbamidomethyl (C)
Variable modifications:
N9 : Deamidated_N (N)
Ions Score: 90 **Expect:** 1.1e-007
Matches (Bold Red): 23/216 fragment ions using 32 most intense peaks

#	b	b^{++}	b*	b^{*++}	b^0	b^{0++}	Seq.	y	y^{++}	y*	y^{*++}	y^0	y^{0++}	#
1	129.0659	65.0366	112.0393	56.5233			Q							20
2	242.1499	121.5786	225.1234	113.0653			I	2181.1438	1091.0755	2164.1172	1082.5622	2163.1332	1082.0702	19
3	355.2340	178.1206	338.2074	169.6074			L	2068.0597	**1034.5335**	2051.0332	1026.0202	2050.0491	1025.5282	18
4	484.2766	242.6419	467.2500	234.1287	466.2660	233.6366	E	**1954.9756**	977.9915	1937.9491	969.4782	1936.9651	968.9862	17
5	541.2980	271.1527	524.2715	262.6394	523.2875	262.1474	G	**1825.9330**	**913.4702**	1808.9065	904.9569	1807.9225	904.4649	16
6	654.3821	327.6947	**637.3556**	319.1814	636.3715	318.6894	L	1768.9116	884.9594	1751.8850	876.4462	1750.9010	875.9541	15
7	783.4247	392.2160	766.3981	383.7027	765.4141	383.2107	E	**1655.8275**	828.4174	1638.8010	819.9041	1637.8170	819.4121	14
8	930.4931	465.7502	**913.4666**	457.2369	912.4825	456.7449	F	**1526.7849**	763.8961	1509.7584	755.3828	1508.7744	754.8908	13
9	1045.5200	523.2637	1028.4935	514.7504	1027.5095	514.2584	N	**1379.7165**	690.3619	1362.6900	681.8486	1361.7059	681.3566	12
10	1158.6041	579.8057	1141.5776	571.2924	1140.5935	570.8004	L	**1264.6896**	632.8484	1247.6630	624.3352	1246.6790	623.8431	11
11	1259.6518	630.3295	1242.6252	621.8163	1241.6412	621.3243	T	1151.6055	576.3064	1134.5790	567.7931	1133.5949	567.3011	10
12	1387.7104	694.3588	1370.6838	685.8455	1369.6998	685.3535	Q	1050.5578	525.7826	1033.5313	517.2693	1032.5473	516.7773	9
13	**1500.7944**	750.9009	**1483.7679**	742.3876	**1482.7839**	741.8956	I	**922.4993**	461.7533	905.4727	453.2400	904.4887	452.7480	8
14	1597.8472	799.4272	1580.8206	**790.9140**	1579.8366	790.4220	P	**809.4152**	405.2112	792.3886	396.6980	**791.4046**	396.2060	7
15	1726.8898	863.9485	1709.8632	855.4353	1708.8792	854.9432	E	712.3624	356.6849	695.3359	348.1716	694.3519	347.6796	6
16	**1797.9269**	899.4671	1780.9004	890.9538	1779.9163	890.4618	A	**583.3198**	292.1636	566.2933	283.6503	565.3093	283.1583	5
17	1912.9538	956.9806	**1895.9273**	948.4673	**1894.9433**	947.9753	D	**512.2827**	256.6450	495.2562	248.1317	494.2722	247.6397	4
18	2026.0379	1013.5226	2009.0114	1005.0093	2008.0273	1004.5173	I	**397.2558**	199.1315	380.2292	190.6183			3

**19**	2163.0968	1082.0520	2146.0703	1073.5388	2145.0863	1073.0468	**H**	284.1717	142.5895	267.1452	134.0762			**2**
**20**							**K**	147.1128	74.0600	130.0863	65.5468			**1**

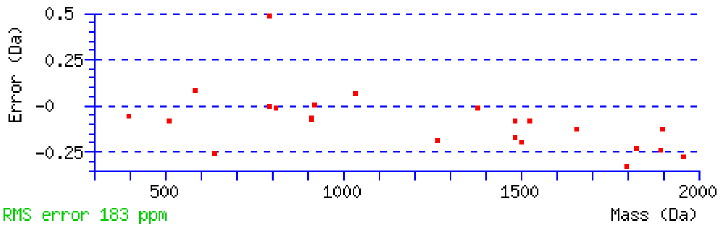

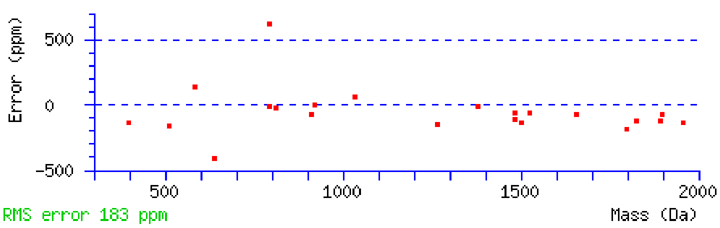

All matches to this query

Score	Mr(calc):	Delta	Sequence
90.1	2308.1950	0.0090	QILEGLEFNLTQIPEADIHK
19.5	2307.2110	0.9930	QILEGLEFNLTQIPEADIHK
1.3	2308.1918	0.0122	TLEITSRLMILVLFSATLK

Spectrum No: 73; Query: 1064; Rank: 1

Peptide View

MS/MS Fragmentation of **LQAALDNEAGGRPAMEPGNGSLDLGGDAAGR**
Found in **IPI00210090**, Tax_Id=10116 Gene_Symbol=Hnrpu SP120

Match to Query 1064: 2980.404192 from(994.475340,3+)
Title: 091129RatKid_SCX02_23.1975.1975.3.dta
Data file K:\NewmanPaper\Piliang\3SubProteomes\Piliang3SP\mgf5ppm\SCX_3SubProteomes5ppm.mgf

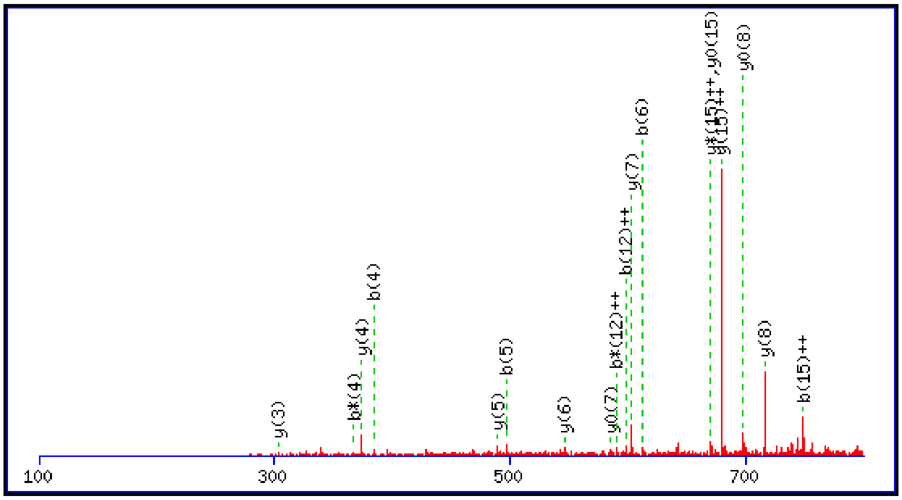

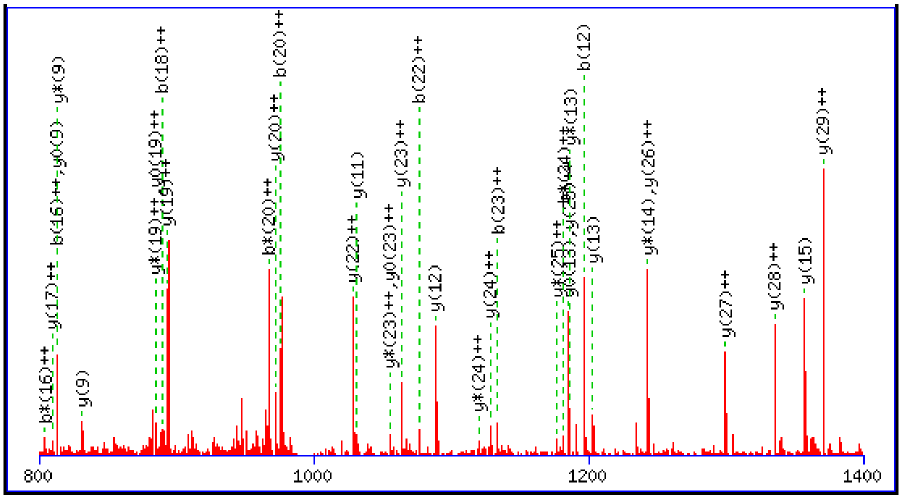

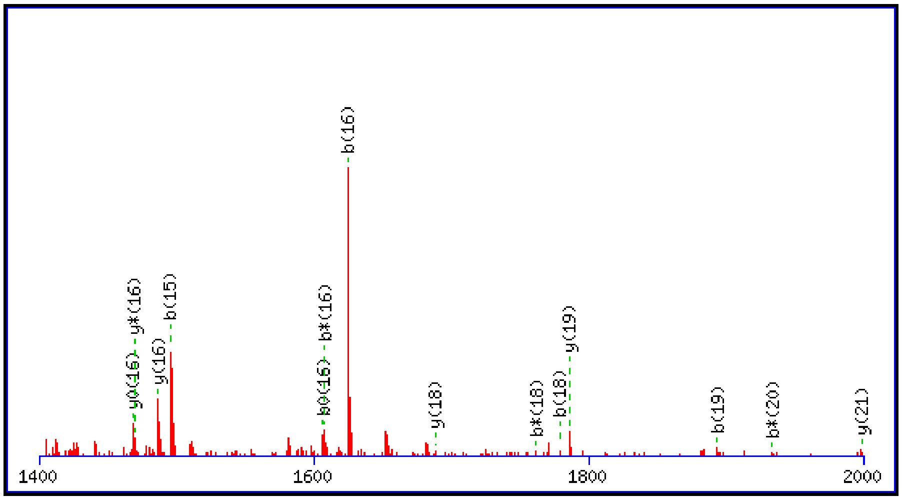

Monoisotopic mass of neutral peptide Mr(calc): 2980.3944
Fixed modifications: Carbamidomethyl (C)
Variable modifications:
N19 : Deamidated_N (N)
Ions Score: 90 **Expect:** 3.5e-007
Matches (Bold Red): 68/340 fragment ions using 132 most intense peaks

#	b	b^{++}	b*	b^{*++}	b^0	b^{0++}	Seq.	y	y^{++}	y*	y^{*++}	y^0	y^{0++}	#
1	114.0913	57.5493					L							31
2	242.1499	121.5786	225.1234	113.0653			Q	2868.3177	1434.6625	2851.2911	1426.1492	2850.3071	1425.6572	30
3	313.1870	157.0972	296.1605	148.5839			A	2740.2591	**1370.6332**	2723.2326	1362.1199	2722.2485	1361.6279	29
4	**384.2241**	192.6157	**367.1976**	184.1024			A	2669.2220	**1335.1146**	2652.1954	1326.6014	2651.2114	1326.1094	28
5	**497.3082**	249.1577	480.2817	240.6445			L	2598.1849	**1299.5961**	2581.1583	1291.0828	2580.1743	1290.5908	27
6	**612.3352**	306.6712	595.3086	298.1579	594.3246	297.6659	D	2485.1008	**1243.0540**	2468.0743	1234.5408	2467.0902	1234.0488	26
7	726.3781	363.6927	709.3515	355.1794	708.3675	354.6874	N	2370.0739	**1185.5406**	2353.0473	**1177.0273**	2352.0633	1176.5353	25
8	855.4207	428.2140	838.3941	419.7007	837.4101	419.2087	E	2256.0309	**1128.5191**	2239.0044	**1120.0058**	2238.0204	1119.5138	24
9	926.4578	463.7325	909.4312	455.2193	908.4472	454.7272	A	2126.9884	**1063.9978**	2109.9618	**1055.4845**	2108.9778	**1054.9925**	23
10	983.4793	492.2433	966.4527	483.7300	965.4687	483.2380	G	2055.9512	**1028.4793**	2038.9247	1019.9660	2037.9407	1019.4740	22
11	1040.5007	520.7540	1023.4742	512.2407	1022.4901	511.7487	G	**1998.9298**	999.9685	1981.9032	991.4552	1980.9192	990.9632	21
12	**1196.6018**	**598.8046**	1179.5753	**590.2913**	1178.5913	589.7993	R	1941.9083	**971.4578**	1924.8818	962.9445	1923.8977	962.4525	20
13	1293.6546	647.3309	1276.6280	638.8177	1275.6440	638.3257	P	**1785.8072**	**893.4072**	1768.7806	**884.8940**	1767.7966	**884.4020**	19
14	1364.6917	682.8495	1347.6652	674.3362	1346.6811	673.8442	A	**1688.7544**	844.8809	1671.7279	836.3676	1670.7439	835.8756	18
15	**1495.7322**	**748.3697**	1478.7056	739.8565	1477.7216	739.3644	M	1617.7173	**809.3623**	1600.6908	800.8490	1599.7068	800.3570	17
16	**1624.7748**	**812.8910**	**1607.7482**	**804.3778**	**1606.7642**	803.8857	E	**1486.6768**	743.8421	**1469.6503**	735.3288	**1468.6663**	734.8368	16
17	1721.8275	861.4174	1704.8010	852.9041	1703.8170	852.4121	P	**1357.6342**	**679.3208**	1340.6077	**670.8075**	1339.6237	**670.3155**	15
18	**1778.8490**	**889.9281**	**1761.8225**	881.4149	1760.8384	880.9229	G	1260.5815	630.7944	**1243.5549**	622.2811	1242.5709	621.7891	14

19	1893.8759	947.4416	1876.8494	938.9283	1875.8654	938.4363	N	1203.5600	602.2836	1186.5335	593.7704	1185.5494	593.2784	13
20	1950.8974	975.9523	1933.8709	967.4391	1932.8868	966.9471	G	1088.5331	544.7702	1071.5065	536.2569	1070.5225	535.7649	12
21	2037.9294	1019.4684	2020.9029	1010.9551	2019.9189	1010.4631	S	1031.5116	516.2594	1014.4851	507.7462	1013.5010	507.2542	11
22	2151.0135	1076.0104	2133.9870	1067.4971	2133.0029	1067.0051	L	944.4796	472.7434	927.4530	464.2302	926.4690	463.7381	10
23	2266.0404	1133.5239	2249.0139	1125.0106	2248.0299	1124.5186	D	831.3955	416.2014	814.3690	407.6881	813.3850	407.1961	9
24	2379.1245	1190.0659	2362.0980	1181.5526	2361.1139	1181.0606	L	716.3686	358.6879	699.3420	350.1747	698.3580	349.6826	8
25	2436.1460	1218.5766	2419.1194	1210.0633	2418.1354	1209.5713	G	603.2845	302.1459	586.2580	293.6326	585.2740	293.1406	7
26	2493.1674	1247.0874	2476.1409	1238.5741	2475.1569	1238.0821	G	546.2631	273.6352	529.2365	265.1219	528.2525	264.6299	6
27	2608.1944	1304.6008	2591.1678	1296.0876	2590.1838	1295.5955	D	489.2416	245.1244	472.2150	236.6112	471.2310	236.1191	5
28	2679.2315	1340.1194	2662.2049	1331.6061	2661.2209	1331.1141	A	374.2146	187.6110	357.1881	179.0977			4
29	2750.2686	1375.6379	2733.2421	1367.1247	2732.2580	1366.6327	A	303.1775	152.0924	286.1510	143.5791			3
30	2807.2901	1404.1487	2790.2635	1395.6354	2789.2795	1395.1434	G	232.1404	116.5738	215.1139	108.0606			2
31							R	175.1190	88.0631	158.0924	79.5498			1

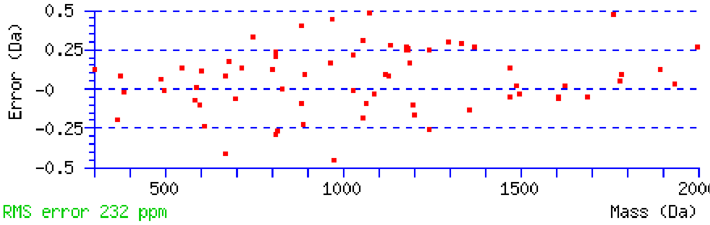

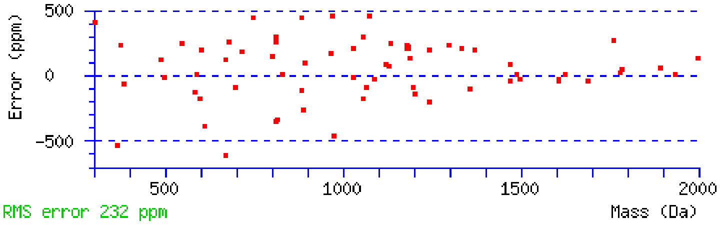

All matches to this query

Score	Mr(calc):	Delta	Sequence
90.0	2980.3944	0.0098	LQAALDNEAGGRPAMEPGNGSLDLGGDAAGR
31.0	2980.3944	0.0098	LQAALDNEAGGRPAMEPGNGSLDLGGDAAGR
22.1	2979.4104	0.9938	LQAALDNEAGGRPAMEPGNGSLDLGGDAAGR
7.7	2980.3808	0.0234	GSASLNLCIKETLRLRPPIMTMMR
5.5	2980.3808	0.0234	GSASLNLCIKETLRLRPPIMTMMR
5.5	2980.3808	0.0234	GSASLNLCIKETLRLRPPIMTMMR
3.7	2979.3968	1.0074	GSASLNLCIKETLRLRPPIMTMMR
3.7	2980.4147	-0.0105	VQCAARAVNTNGNEGLELMSPIVTIAR
3.5	2980.3808	0.0234	GSASLNLCIKETLRLRPPIMTMMR
3.4	2980.4147	-0.0105	VQCAARAVNTNGNEGLELMSPIVTIAR

Spectrum No: 74; Query: 759; Rank: 1

Peptide View

MS/MS Fragmentation of **ISASGAELEALEAQVLNLSLK**
Found in **IPI00188541**, Tax_Id=10116 Gene_Symbol=Itih4 inter-alpha-inhibitor H4 heavy chain

Match to Query 759: 2156.161168 from(1079.087860,2+)
Title: 091127RatKid_SCX01_02.5733.5733.2.dta
Data file K:\NewmanPaper\Piliang\3SubProteomes\Piliang3SP\mgf5ppm\SCX_3SubProteomes5ppm.mgf

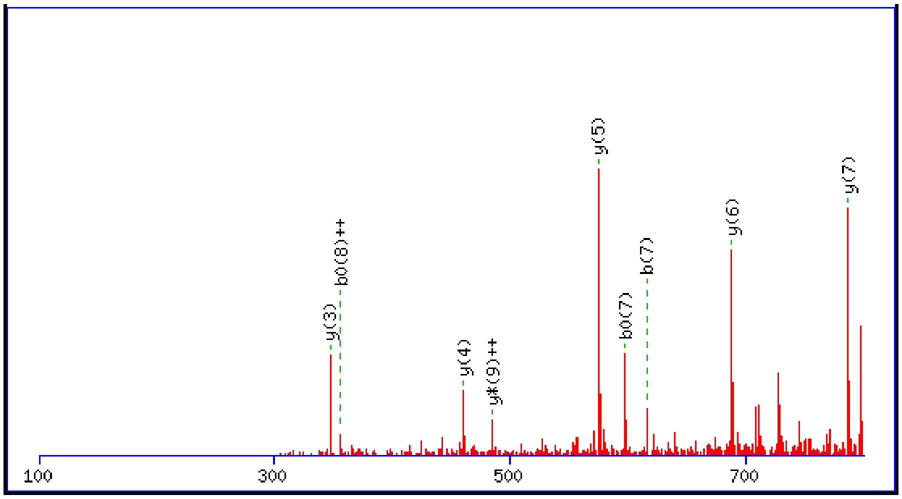

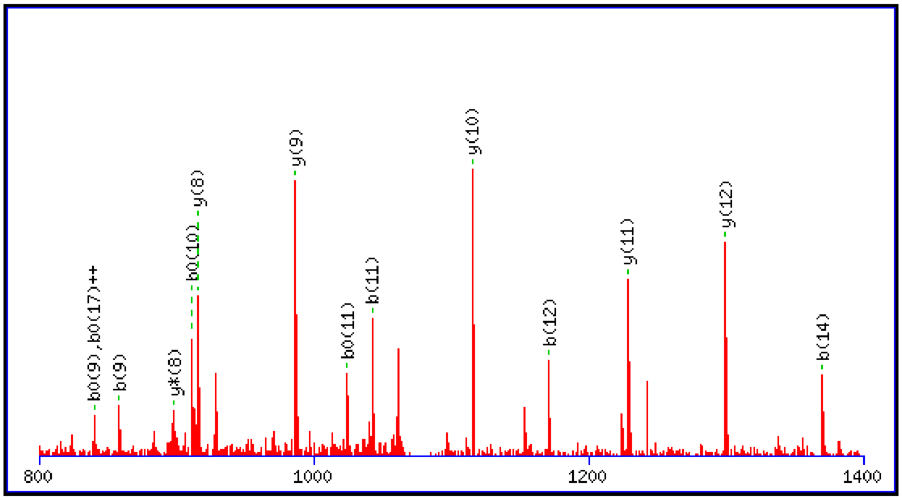

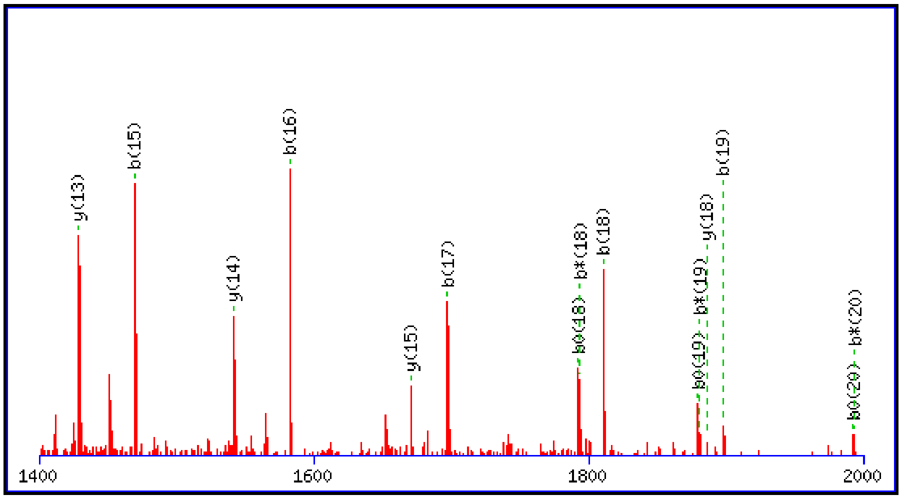

Monoisotopic mass of neutral peptide Mr(calc): 2156.1576
Fixed modifications: Carbamidomethyl (C)
Variable modifications:
N17 : Deamidated_N (N)
Ions Score: 90 **Expect:** 7.3e-008
Matches (Bold Red): 38/208 fragment ions using 61 most intense peaks

#	b	b^{++}	b*	b*$^{++}$	b^{0}	b^{0++}	Seq.	y	y^{++}	y*	y*$^{++}$	y^{0}	y^{0++}	#
1	114.0913	57.5493					I							21
2	201.1234	101.0653			183.1128	92.0600	S	2044.0808	1022.5441	2027.0543	1014.0308	2026.0703	1013.5388	20
3	272.1605	136.5839			254.1499	127.5786	A	1957.0488	979.0280	1940.0223	970.5148	1939.0382	970.0228	19
4	359.1925	180.0999			341.1819	171.0946	S	**1886.0117**	943.5095	1868.9851	934.9962	1868.0011	934.5042	18
5	416.2140	208.6106			398.2034	199.6053	G	1798.9797	899.9935	1781.9531	891.4802	1780.9691	890.9882	17

**6**	487.2511	244.1292			469.2405	235.1239	**A**	1741.9582	871.4827	1724.9317	862.9695	1723.9476	862.4775	**16**
**7**	**616.2937**	308.6505			**598.2831**	299.6452	**E**	**1670.9211**	835.9642	1653.8945	827.4509	1652.9105	826.9589	**15**
**8**	729.3777	365.1925			711.3672	**356.1872**	**L**	**1541.8785**	771.4429	1524.8519	762.9296	1523.8679	762.4376	**14**
**9**	**858.4203**	429.7138			**840.4098**	420.7085	**E**	**1428.7944**	714.9009	1411.7679	706.3876	1410.7839	705.8956	**13**
**10**	929.4575	465.2324			**911.4469**	456.2271	**A**	**1299.7518**	650.3796	1282.7253	641.8663	1281.7413	641.3743	**12**
**11**	**1042.5415**	521.7744			**1024.5310**	512.7691	**L**	**1228.7147**	614.8610	1211.6882	606.3477	1210.7042	605.8557	**11**
**12**	**1171.5841**	586.2957			1153.5735	577.2904	**E**	**1115.6307**	558.3190	1098.6041	549.8057	1097.6201	549.3137	**10**
**13**	1242.6212	621.8142			1224.6107	612.8090	**A**	**986.5881**	493.7977	969.5615	**485.2844**	968.5775	484.7924	**9**
**14**	**1370.6798**	685.8435	1353.6533	677.3303	1352.6692	676.8383	**Q**	**915.5510**	458.2791	**898.5244**	449.7658	897.5404	449.2738	**8**
**15**	**1469.7482**	735.3777	1452.7217	726.8645	1451.7377	726.3725	**V**	**787.4924**	394.2498	770.4658	385.7366	769.4818	385.2445	**7**
**16**	**1582.8323**	791.9198	1565.8057	783.4065	1564.8217	782.9145	**L**	**688.4240**	344.7156	671.3974	336.2023	670.4134	335.7103	**6**
**17**	**1697.8592**	849.4332	1680.8327	840.9200	1679.8486	**840.4280**	**N**	**575.3399**	288.1736	558.3133	279.6603	557.3293	279.1683	**5**
**18**	**1810.9433**	905.9753	**1793.9167**	897.4620	**1792.9327**	896.9700	**L**	**460.3130**	230.6601	443.2864	222.1468	442.3024	221.6548	**4**
**19**	**1897.9753**	949.4913	**1880.9488**	940.9780	**1879.9647**	940.4860	**S**	**347.2289**	174.1181	330.2023	165.6048	329.2183	165.1128	**3**
**20**	2011.0594	1006.0333	**1994.0328**	997.5200	**1993.0488**	997.0280	**L**	260.1969	130.6021	243.1703	122.0888			**2**
**21**							**K**	147.1128	74.0600	130.0863	65.5468			**1**

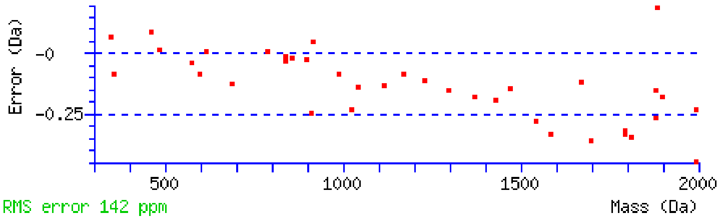

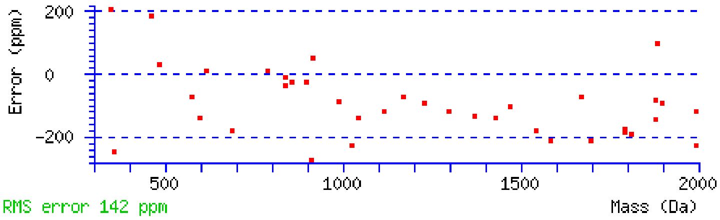

All matches to this query

Score	Mr(calc):	Delta	Sequence
90.0	2156.1576	0.0036	ISASGAELEALEAQVLNLSLK
18.2	2155.1736	0.9876	ISASGAELEALEAQVLNLSLK
5.1	2156.1412	0.0200	WCGDPALLNSLKQKSALVR
4.5	2156.1801	-0.0189	TVQIEASTVEIEERGVKLR
3.3	2156.1814	-0.0202	NAEIFLALQNQRGGRISLR
2.8	2154.1644	1.9967	AGSQAPSRSSPATSSVLITLPK

Spectrum No: 75; Query: 518; Rank: 1

Peptide View

MS/MS Fragmentation of **TSVGNSSFYQDTVVFK**
Found in **IPI00195241**, Tax_Id=10116 Gene_Symbol=Apoh Beta-2-glycoprotein 1 precursor

Match to Query 518: 1778.842408 from(890.428480,2+)
Title: 091127RatKid_SCX01_02.3381.3381.2.dta
Data file K:\NewmanPaper\Piliang\3SubProteomes\Piliang3SP\mgf5ppm\SCX_3SubProteomes5ppm.mgf

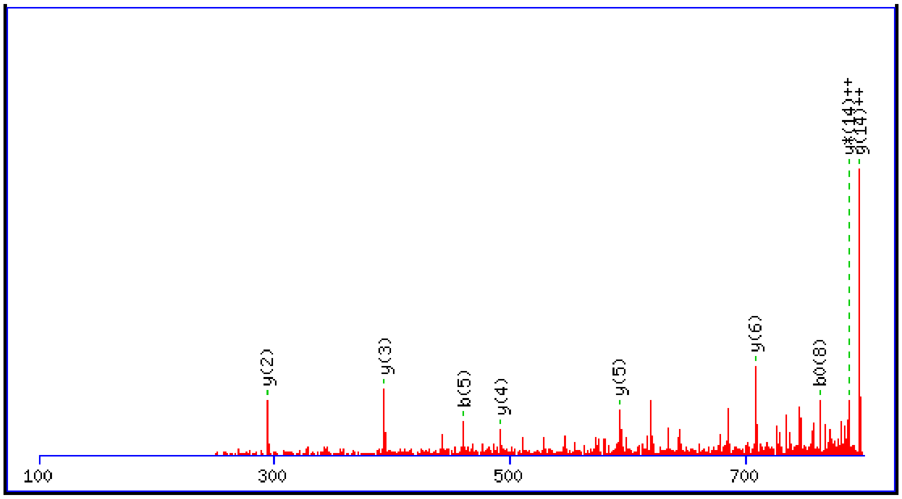

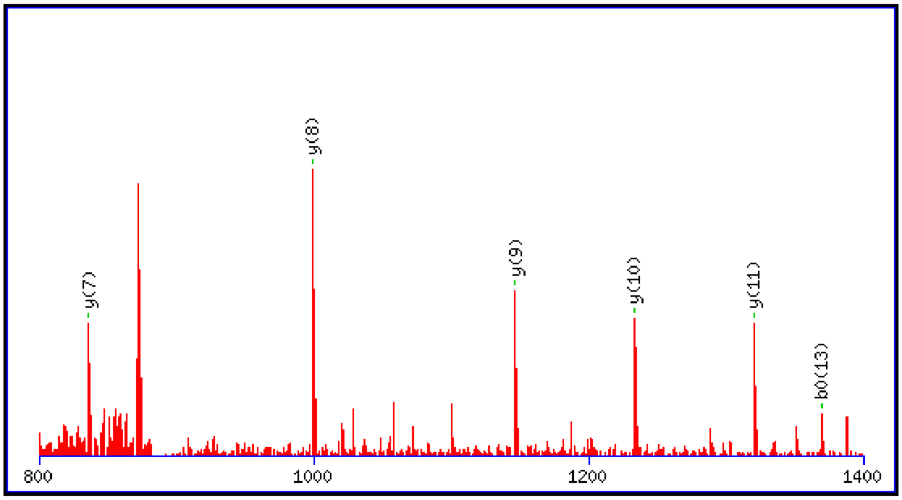

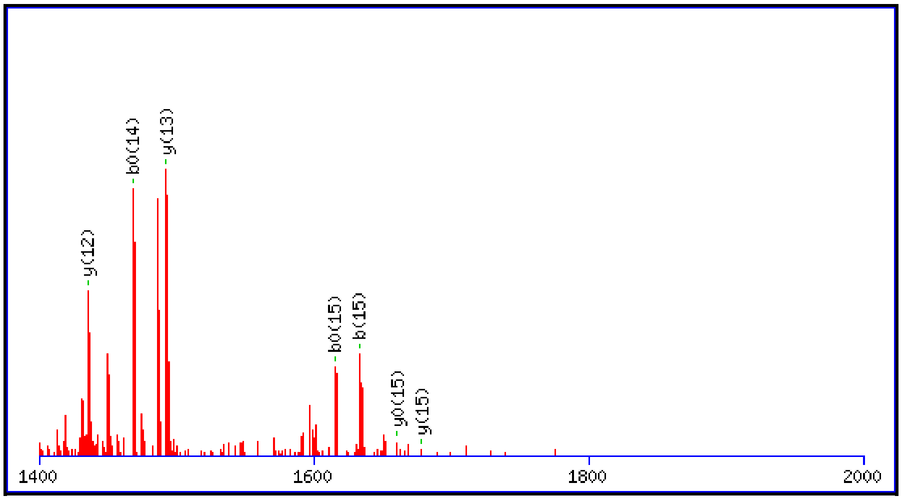

Monoisotopic mass of neutral peptide Mr(calc): 1778.8363
Fixed modifications: Carbamidomethyl (C)
Variable modifications:
N5 : Deamidated_N (N)
Ions Score: 90 **Expect:** 2.2e-007
Matches (Bold Red): 22/164 fragment ions using 41 most intense peaks

#	b	b^{++}	b*	b^{*++}	b^{0}	b^{0++}	Seq.	y	y^{++}	y*	y^{*++}	y^{0}	y^{0++}	#
1	102.0550	51.5311			84.0444	42.5258	T							16
2	189.0870	95.0471			171.0764	86.0418	S	**1678.7959**	839.9016	1661.7693	831.3883	**1660.7853**	830.8963	15
3	288.1554	144.5813			270.1448	135.5761	V	1591.7639	**796.3856**	1574.7373	**787.8723**	1573.7533	787.3803	14
4	345.1769	173.0921			327.1663	164.0868	G	**1492.6954**	746.8514	1475.6689	738.3381	1474.6849	737.8461	13
5	**460.2038**	230.6055	443.1772	222.0923	442.1932	221.6003	N	**1435.6740**	718.3406	1418.6474	709.8274	1417.6634	709.3353	12

6	547.2358	274.1216	530.2093	265.6083	529.2253	265.1163	S	**1320.6470**	660.8272	1303.6205	652.3139	1302.6365	651.8219	11
7	634.2679	317.6376	617.2413	309.1243	616.2573	308.6323	S	**1233.6150**	617.3111	1216.5885	608.7979	1215.6045	608.3059	10
8	781.3363	391.1718	764.3097	382.6585	**763.3257**	382.1665	F	**1146.5830**	573.7951	1129.5564	565.2819	1128.5724	564.7899	9
9	944.3996	472.7034	927.3730	464.1902	926.3890	463.6982	Y	**999.5146**	500.2609	982.4880	491.7477	981.5040	491.2556	8
10	1072.4582	536.7327	1055.4316	528.2195	1054.4476	527.7274	Q	**836.4512**	418.7293	819.4247	410.2160	818.4407	409.7240	7
11	1187.4851	594.2462	1170.4586	585.7329	1169.4746	585.2409	D	**708.3927**	354.7000	691.3661	346.1867	690.3821	345.6947	6
12	1288.5328	644.7700	1271.5062	636.2568	1270.5222	635.7648	T	**593.3657**	297.1865	576.3392	288.6732	575.3552	288.1812	5
13	1387.6012	694.3042	1370.5747	685.7910	**1369.5906**	685.2990	V	**492.3180**	246.6627	475.2915	238.1494			4
14	1486.6696	743.8385	1469.6431	735.3252	**1468.6591**	734.8332	V	**393.2496**	197.1285	376.2231	188.6152			3
15	**1633.7380**	817.3727	1616.7115	808.8594	**1615.7275**	808.3674	F	**294.1812**	147.5942	277.1547	139.0810			2
16							K	147.1128	74.0600	130.0863	65.5468			1

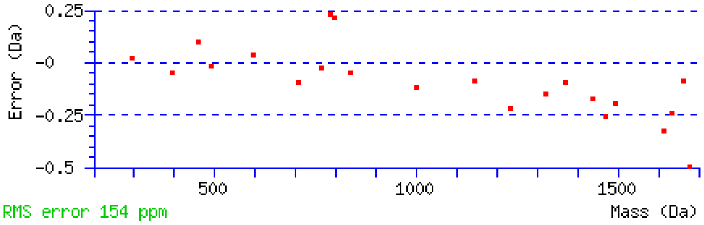

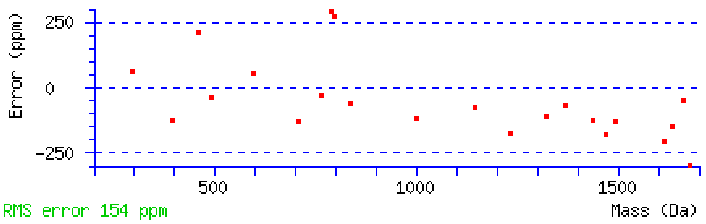

All matches to this query

Score	Mr(calc):	Delta	Sequence
89.6	1778.8363	0.0061	TSVGNSSFYQDTVVFK
43.1	1777.8523	0.9901	TSVGNSSFYQDTVVFK
5.9	1778.8465	-0.0041	SVLHGVNDNRSQVFK
5.6	1777.8264	1.0160	AGTKTSSASLSSLALPK
3.6	1778.8543	-0.0119	TASSXGPALATGNVVVMK

Spectrum No: 76; Query: 1031; Rank: 1

Peptide View

MS/MS Fragmentation of **TVVTEAGNLLKDNATEEEILHYLEK**
Found in **IPI00195160**, Tax_Id=10116 Gene_Symbol=Psap Sulfated glycoprotein 1 precursor

Match to Query 1031: 2829.437922 from(944.153250,3+)
Title: 091127RatKid_SCX01_31.5725.5725.3.dta
Data file K:\NewmanPaper\Piliang\3SubProteomes\Piliang3SP\mgf5ppm\SCX_3SubProteomes5ppm.mgf

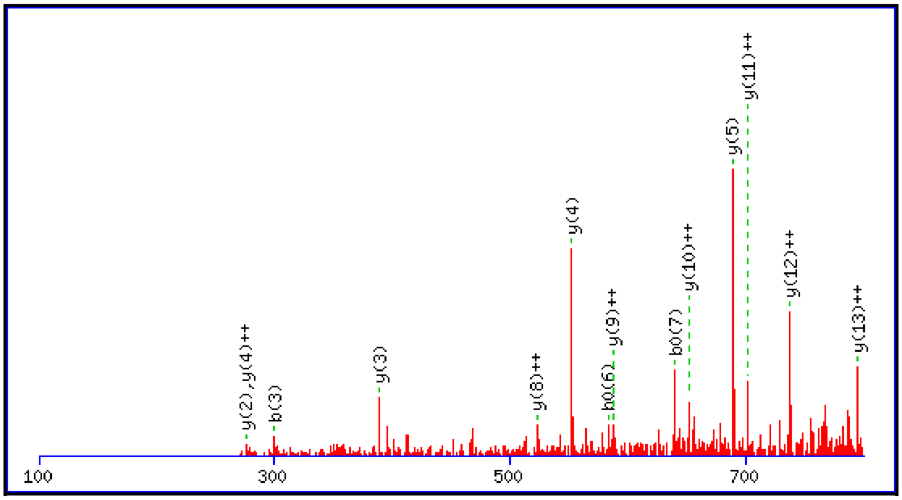

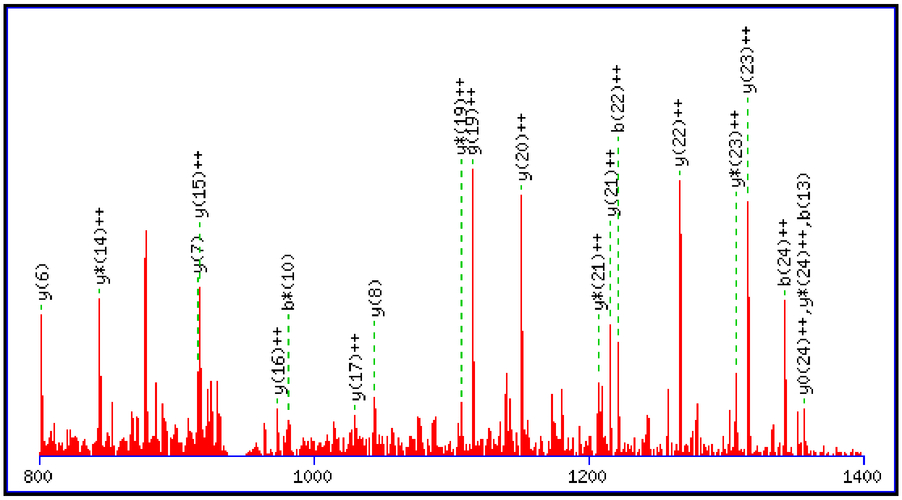

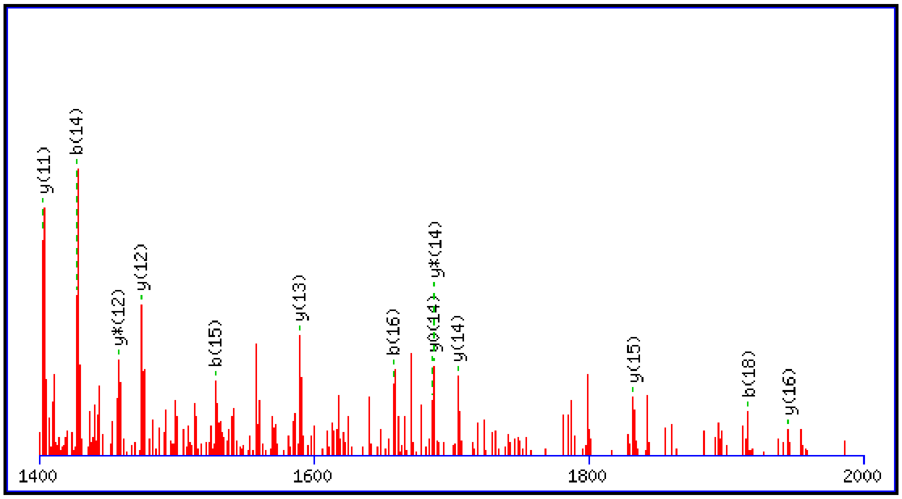

Monoisotopic mass of neutral peptide Mr(calc): 2829.4283
Fixed modifications: Carbamidomethyl (C)
Variable modifications:
N13 : Deamidated_N (N)
Ions Score: 90 **Expect:** 1.8e-007
Matches (Bold Red): 48/272 fragment ions using 93 most intense peaks

#	b	b++	b*	b*++	b0	b0++	Seq.	y	y++	y*	y*++	y0	y0++	#
1	102.0550	51.5311			84.0444	42.5258	T							25
2	201.1234	101.0653			183.1128	92.0600	V	2729.3880	1365.1976	2712.3614	**1356.6844**	2711.3774	**1356.1923**	24
3	**300.1918**	150.5995			282.1812	141.5942	V	2630.3196	**1315.6634**	2613.2930	**1307.1501**	2612.3090	1306.6581	23
4	401.2395	201.1234			383.2289	192.1181	T	2531.2511	**1266.1292**	2514.2246	1257.6159	2513.2406	1257.1239	22
5	530.2821	265.6447			512.2715	256.6394	E	2430.2035	**1215.6054**	2413.1769	**1207.0921**	2412.1929	1206.6001	21
6	601.3192	301.1632			**583.3086**	292.1579	A	2301.1609	**1151.0841**	2284.1343	1142.5708	2283.1503	1142.0788	20
7	658.3406	329.6740			**640.3301**	320.6687	G	2230.1238	**1115.5655**	2213.0972	**1107.0522**	2212.1132	1106.5602	19
8	772.3836	386.6954	755.3570	378.1821	754.3730	377.6901	N	2173.1023	1087.0548	2156.0758	1078.5415	2155.0917	1078.0495	18
9	885.4676	443.2375	868.4411	434.7242	867.4571	434.2322	L	2059.0594	**1030.0333**	2042.0328	1021.5200	2041.0488	1021.0280	17
10	998.5517	499.7795	**981.5251**	491.2662	980.5411	490.7742	L	**1945.9753**	**973.4913**	1928.9488	964.9780	1927.9647	964.4860	16
11	1126.6467	563.8270	1109.6201	555.3137	1108.6361	554.8217	K	**1832.8912**	**916.9493**	1815.8647	908.4360	1814.8807	907.9440	15
12	1241.6736	621.3404	1224.6470	612.8272	1223.6630	612.3352	D	**1704.7963**	852.9018	**1687.7697**	**844.3885**	**1686.7857**	843.8965	14
13	**1356.7005**	678.8539	1339.6740	670.3406	1338.6900	669.8486	N	**1589.7693**	**795.3883**	1572.7428	786.8750	1571.7588	786.3830	13
14	**1427.7376**	714.3725	1410.7111	705.8592	1409.7271	705.3672	A	**1474.7424**	**737.8748**	**1457.7159**	729.3616	1456.7318	728.8696	12
15	**1528.7853**	764.8963	1511.7588	756.3830	1510.7748	755.8910	T	**1403.7053**	**702.3563**	1386.6787	693.8430	1385.6947	693.3510	11
16	**1657.8279**	829.4176	1640.8014	820.9043	1639.8174	820.4123	E	1302.6576	**651.8324**	1285.6311	643.3192	1284.6470	642.8272	10
17	1786.8705	893.9389	1769.8440	885.4256	1768.8599	884.9336	E	1173.6150	**587.3111**	1156.5885	578.7979	1155.6045	578.3059	9
18	**1915.9131**	958.4602	1898.8866	949.9469	1897.9025	949.4549	E	**1044.5724**	**522.7898**	1027.5459	514.2766	1026.5619	513.7846	8

19	2028.9972	1015.0022	2011.9706	1006.4889	2010.9866	1005.9969	I	915.5298	458.2686	898.5033	449.7553	897.5193	449.2633	7
20	2142.0812	1071.5443	2125.0547	1063.0310	2124.0707	1062.5390	L	802.4458	401.7265	785.4192	393.2132	784.4352	392.7212	6
21	2279.1401	1140.0737	2262.1136	1131.5604	2261.1296	1131.0684	H	689.3617	345.1845	672.3352	336.6712	671.3511	336.1792	5
22	2442.2035	1221.6054	2425.1769	1213.0921	2424.1929	1212.6001	Y	552.3028	276.6550	535.2762	268.1418	534.2922	267.6498	4
23	2555.2875	1278.1474	2538.2610	1269.6341	2537.2770	1269.1421	L	389.2395	195.1234	372.2129	186.6101	371.2289	186.1181	3
24	2684.3301	1342.6687	2667.3036	1334.1554	2666.3196	1333.6634	E	276.1554	138.5813	259.1288	130.0681	258.1448	129.5761	2
25							K	147.1128	74.0600	130.0863	65.5468			1

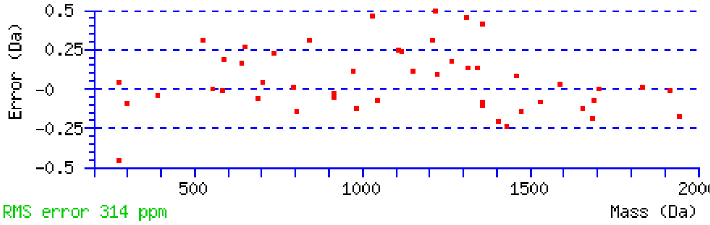

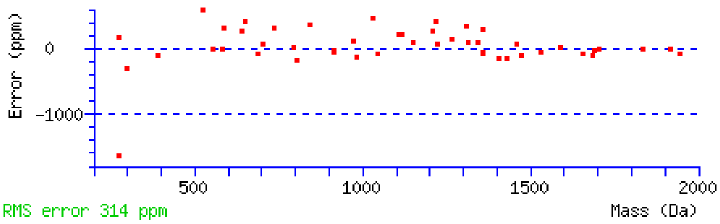

All matches to this query

Score	Mr(calc):	Delta	Sequence
89.6	2829.4283	0.0096	TVVTEAGNLLKDNATEEEILHYLEK
50.4	2829.4283	0.0096	TVVTEAGNLLKDNATEEEILHYLEK
9.3	2828.4443	0.9936	TVVTEAGNLLKDNATEEEILHYLEK
0.8	2827.4036	2.0343	VATVFYTVVIPMLNPLIYSLRNK

Spectrum No: 77; Query: 574; Rank: 1

Peptide View

MS/MS Fragmentation of **ANVQFGENGTTISAVTNK**
Found in **IPI00231478**, Tax_Id=10116 Gene_Symbol=Scarb2 Lysosome membrane protein 2

Match to Query 574: 1850.906288 from(926.460420,2+)
Title: 091129RatKid_SCX02_02.1744.1744.2.dta
Data file K:\NewmanPaper\Piliang\3SubProteomes\Piliang3SP\mgf5ppm\SCX_3SubProteomes5ppm.mgf

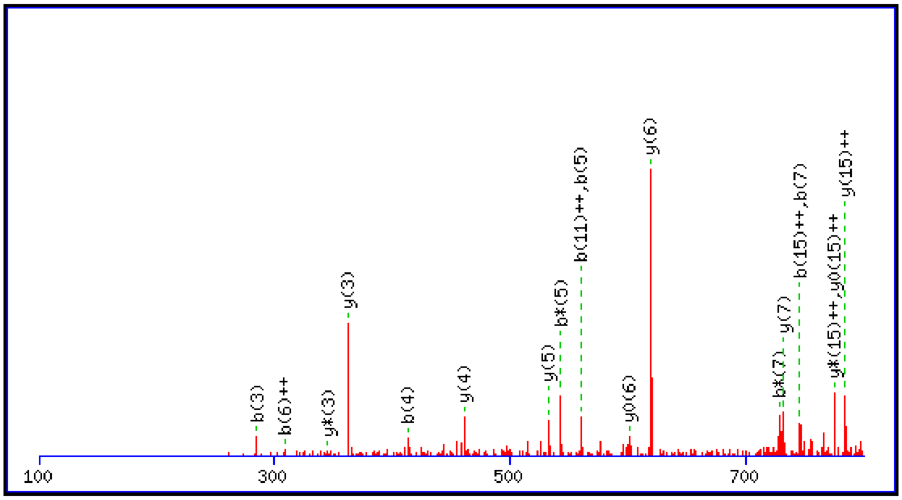

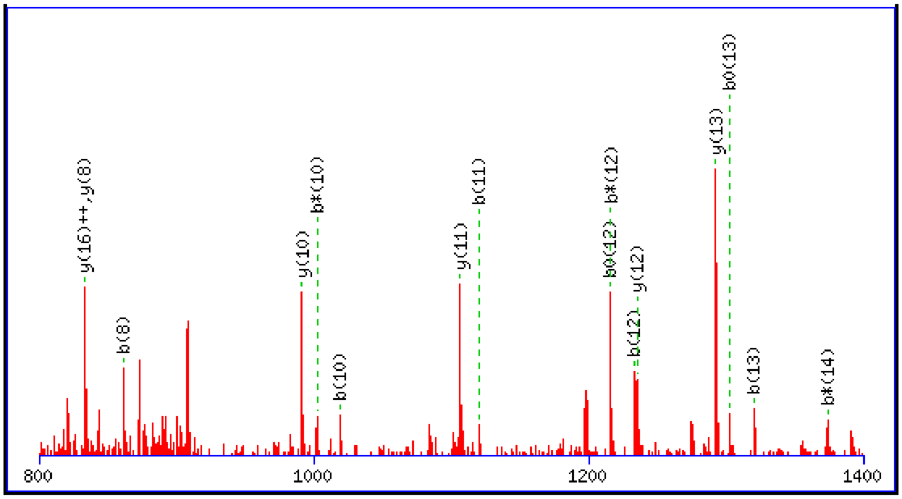

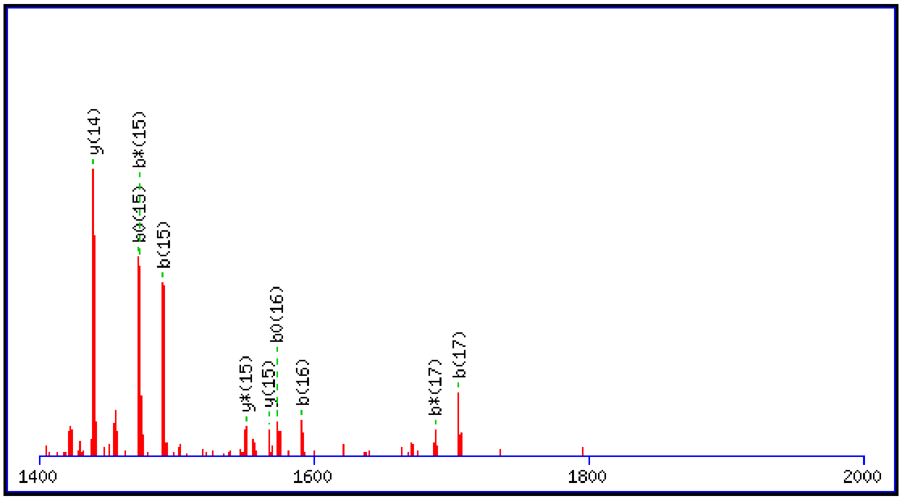

Monoisotopic mass of neutral peptide Mr(calc): 1850.9010
Fixed modifications: Carbamidomethyl (C)
Variable modifications:
N8 : Deamidated_N (N)
Ions Score: 90 **Expect:** 2.4e-007
Matches (Bold Red): 45/186 fragment ions using 65 most intense peaks

#	b	b^{++}	b*	b^{*++}	b^0	b^{0++}	Seq.	y	y^{++}	y*	y^{*++}	y^0	y^{0++}	#
1	72.0444	36.5258					A							18
2	186.0873	93.5473	169.0608	85.0340			N	1780.8712	890.9392	1763.8446	882.4260	1762.8606	881.9339	17
3	**285.1557**	143.0815	268.1292	134.5682			V	1666.8283	**833.9178**	1649.8017	825.4045	1648.8177	824.9125	16
4	**413.2143**	207.1108	396.1878	198.5975			Q	**1567.7598**	**784.3836**	**1550.7333**	**775.8703**	1549.7493	**775.3783**	15
5	**560.2827**	280.6450	**543.2562**	272.1317			F	**1439.7013**	720.3543	1422.6747	711.8410	1421.6907	711.3490	14
6	617.3042	**309.1557**	600.2776	300.6425			G	**1292.6328**	646.8201	1275.6063	638.3068	1274.6223	637.8148	13
7	**746.3468**	373.6770	**729.3202**	365.1638	728.3362	364.6717	E	**1235.6114**	618.3093	1218.5848	609.7961	1217.6008	609.3040	12
8	**861.3737**	431.1905	844.3472	422.6772	843.3632	422.1852	N	**1106.5688**	553.7880	1089.5422	545.2748	1088.5582	544.7827	11
9	918.3952	459.7012	901.3686	451.1880	900.3846	450.6959	G	**991.5419**	496.2746	974.5153	487.7613	973.5313	487.2693	10
10	**1019.4429**	510.2251	**1002.4163**	501.7118	1001.4323	501.2198	T	934.5204	467.7638	917.4938	459.2506	916.5098	458.7585	9
11	**1120.4905**	**560.7489**	1103.4640	552.2356	1102.4800	551.7436	T	**833.4727**	417.2400	816.4462	408.7267	815.4621	408.2347	8
12	**1233.5746**	617.2909	**1216.5481**	608.7777	**1215.5640**	608.2857	I	**732.4250**	366.7162	715.3985	358.2029	714.4145	357.7109	7
13	**1320.6066**	660.8070	1303.5801	652.2937	**1302.5961**	651.8017	S	**619.3410**	310.1741	602.3144	301.6608	**601.3304**	301.1688	6
14	1391.6437	696.3255	**1374.6172**	687.8122	1373.6332	687.3202	A	**532.3089**	266.6581	515.2824	258.1448	514.2984	257.6528	5
15	**1490.7122**	**745.8597**	**1473.6856**	737.3464	**1472.7016**	736.8544	V	**461.2718**	231.1396	444.2453	222.6263	443.2613	222.1343	4
16	**1591.7598**	796.3836	1574.7333	787.8703	**1573.7493**	787.3783	T	**362.2034**	181.6053	**345.1769**	173.0921	344.1928	172.6001	3
17	**1705.8028**	853.4050	**1688.7762**	844.8917	1687.7922	844.3997	N	261.1557	131.0815	244.1292	122.5682			2
18							K	147.1128	74.0600	130.0863	65.5468			1

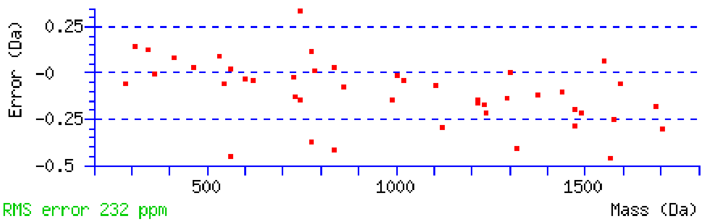

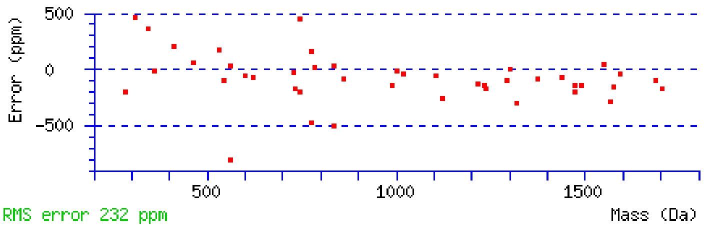

All matches to this query

Score	Mr(calc):	Delta	Sequence
89.5	1850.9010	0.0053	ANVQFGENGTTISAVTNK
43.5	1850.9010	0.0053	ANVQFGENGTTISAVTNK
32.9	1850.9010	0.0053	ANVQFGENGTTISAVTNK
21.2	1849.9170	0.9893	ANVQFGENGTTISAVTNK
2.8	1850.8975	0.0088	SNPLILCNQIHHGLR
1.5	1850.9196	-0.0133	QYAEQELEQVRMALK
1.4	1850.8889	0.0174	NDMAVPTPPPPPVLPTK
0.0	1850.9230	-0.0167	IEGITQRMMVIEGTNK

Spectrum No: 78; Query: 571; Rank: 1

Peptide View

MS/MS Fragmentation of **VIDLWDLAQSANFTEK**
Found in **IPI00364124**, Tax_Id=10116 Gene_Symbol=Lrpap1 Alpha-2-macroglobulin receptor-associated protein precursor

Match to Query 571: 1849.915388 from(925.964970,2+)
Title: 091127RatKid_SCX01_12.5081.5081.2.dta
Data file K:\NewmanPaper\Piliang\3SubProteomes\Piliang3SP\mgf5ppm\SCX_3SubProteomes5ppm.mgf

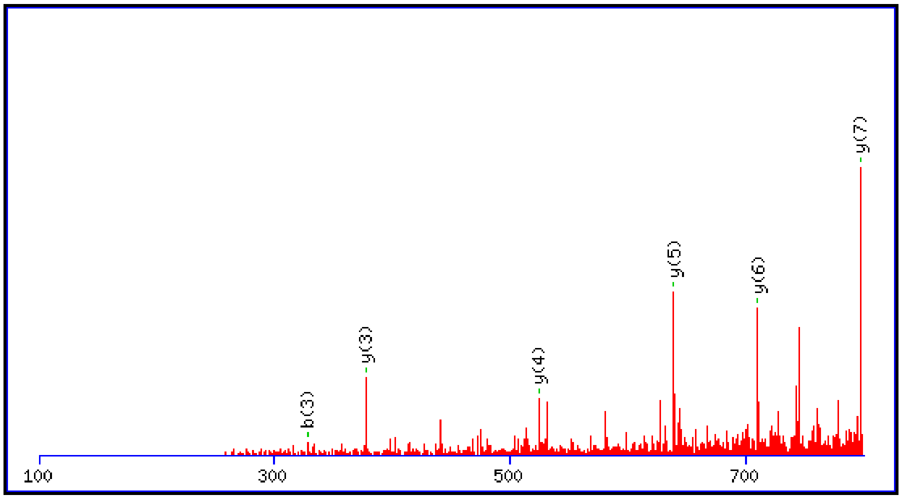

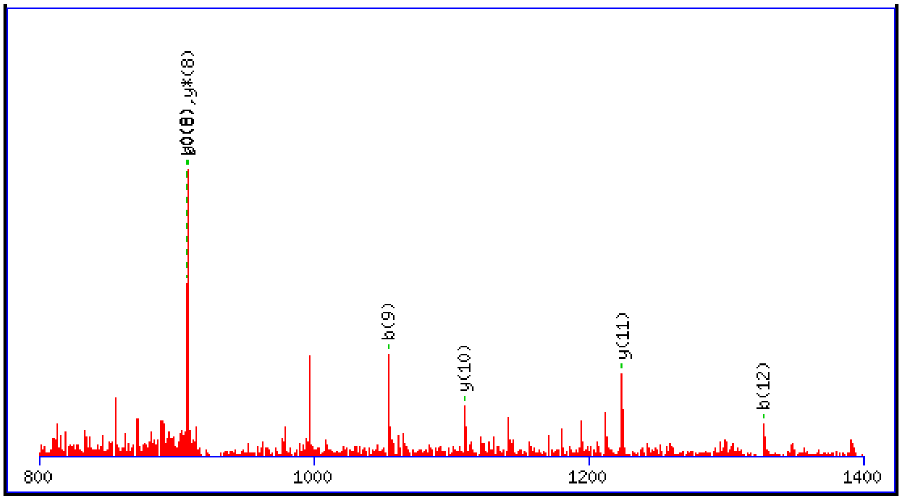

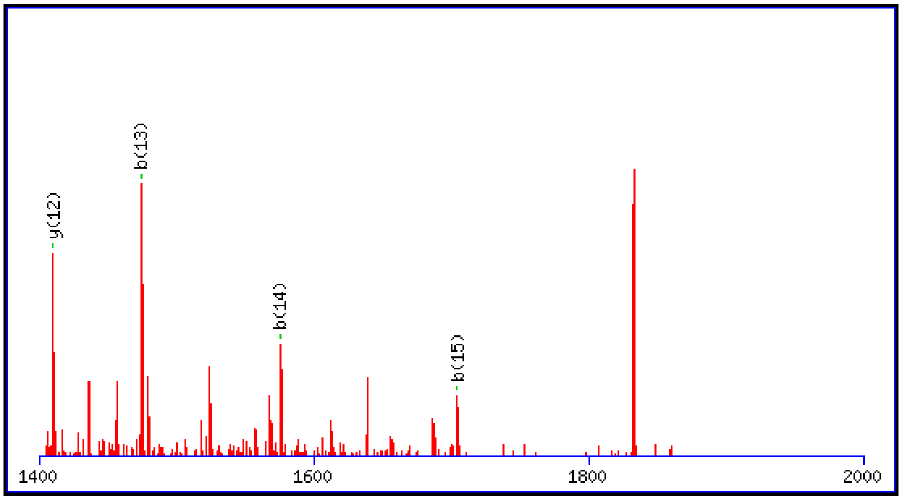

Monoisotopic mass of neutral peptide Mr(calc): 1849.9098
Fixed modifications: Carbamidomethyl (C)
Variable modifications:
N12 : Deamidated_N (N)
Ions Score: 89 **Expect:** 2.7e-007
Matches (Bold Red): 17/158 fragment ions using 23 most intense peaks

#	b	b^{++}	b*	b^{*++}	b^0	b^{0++}	Seq.	y	y^{++}	y*	y^{*++}	y^0	y^{0++}	#
1	100.0757	50.5415					V							16
2	213.1598	107.0835					I	1751.8487	876.4280	1734.8221	867.9147	1733.8381	867.4227	15
3	**328.1867**	164.5970			310.1761	155.5917	D	1638.7646	819.8859	1621.7380	811.3727	1620.7540	810.8806	14
4	441.2708	221.1390			423.2602	212.1337	L	1523.7376	762.3725	1506.7111	753.8592	1505.7271	753.3672	13
5	627.3501	314.1787			609.3395	305.1734	W	**1410.6536**	705.8304	1393.6270	697.3172	1392.6430	696.8251	12
6	742.3770	371.6921			724.3665	362.6869	D	**1224.5743**	612.7908	1207.5477	604.2775	1206.5637	603.7855	11
7	855.4611	428.2342			837.4505	419.2289	L	**1109.5473**	555.2773	1092.5208	546.7640	1091.5368	546.2720	10
8	926.4982	463.7527			**908.4876**	454.7475	A	996.4633	498.7353	979.4367	490.2220	978.4527	489.7300	9
9	**1054.5568**	527.7820	1037.5302	519.2688	1036.5462	518.7767	Q	925.4261	463.2167	**908.3996**	454.7034	**907.4156**	454.2114	8
10	1141.5888	571.2980	1124.5623	562.7848	1123.5782	562.2928	S	**797.3676**	399.1874	780.3410	390.6741	779.3570	390.1821	7
11	1212.6259	606.8166	1195.5994	598.3033	1194.6154	597.8113	A	**710.3355**	355.6714	693.3090	347.1581	692.3250	346.6661	6
12	**1327.6529**	664.3301	1310.6263	655.8168	1309.6423	655.3248	N	**639.2984**	320.1529	622.2719	311.6396	621.2879	311.1476	5
13	**1474.7213**	737.8643	1457.6947	729.3510	1456.7107	728.8590	F	**524.2715**	262.6394	507.2449	254.1261	506.2609	253.6341	4
14	**1575.7689**	788.3881	1558.7424	779.8748	1557.7584	779.3828	T	**377.2031**	189.1052	360.1765	180.5919	359.1925	180.0999	3
15	**1704.8115**	852.9094	1687.7850	844.3961	1686.8010	843.9041	E	276.1554	138.5813	259.1288	130.0681	258.1448	129.5761	2
16							K	147.1128	74.0600	130.0863	65.5468			1

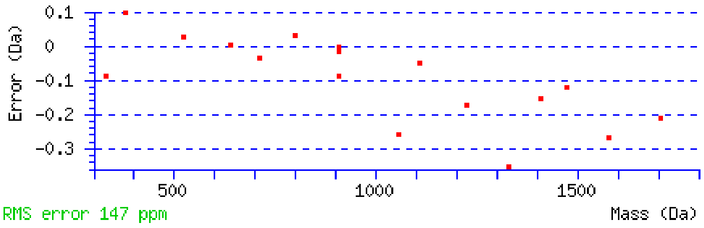

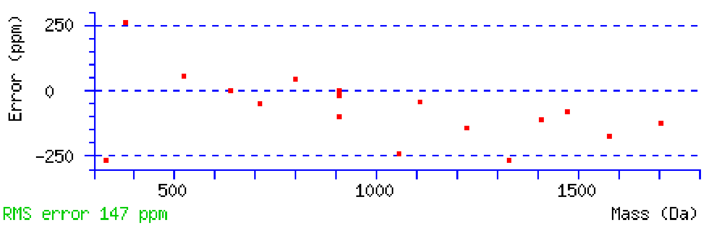

All matches to this query

Score	Mr(calc):	Delta	Sequence
89.4	1849.9098	0.0056	VIDLWDLAQSANFTEK
19.0	1848.9258	0.9896	VIDLWDLAQSANFTEK
12.2	1849.9244	-0.0090	NLVHYLTTVERMETK
8.8	1849.9298	-0.0145	KDLRNDLNTIQELAK
2.0	1849.9226	-0.0072	RYEEIIKEVSTYIK
1.7	1849.9267	-0.0113	VLYESLKYPSFPLSK
1.4	1849.8975	0.0179	TRSPIPAQPYPSSLEK
0.5	1849.9033	0.0121	YAAATGGPPPTPLPHMEK

Spectrum No: 79; Query: 643; Rank: 1

Peptide View

MS/MS Fragmentation of **AQEAGYTNATTTDQHGLAK**
Found in **IPI00212666**, Tax_Id=10116 Gene_Symbol=Mug1 Isoform 1 of Murinoglobulin-1 precursor

Match to Query 643: 1976.911428 from(989.462990,2+)
Title: 091127RatKid_SCX01_14.883.883.2.dta
Data file K:\NewmanPaper\Piliang\3SubProteomes\Piliang3SP\mgf5ppm\SCX_3SubProteomes5ppm.mgf

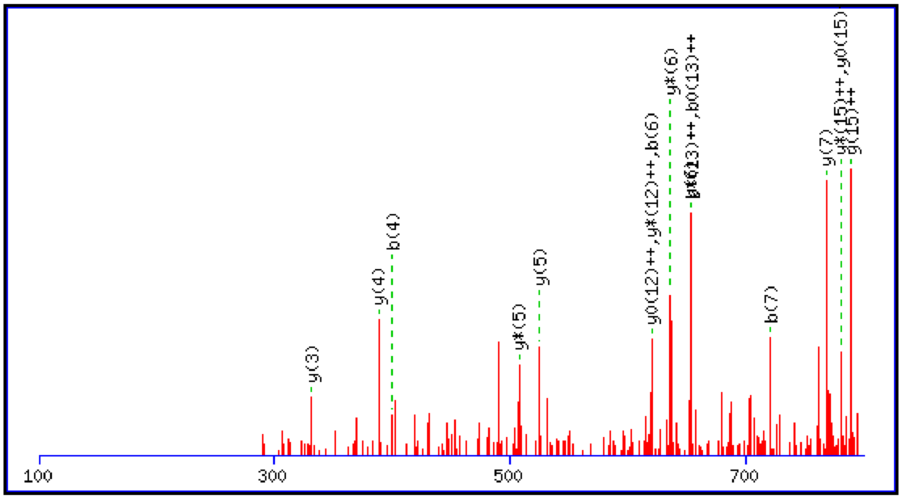

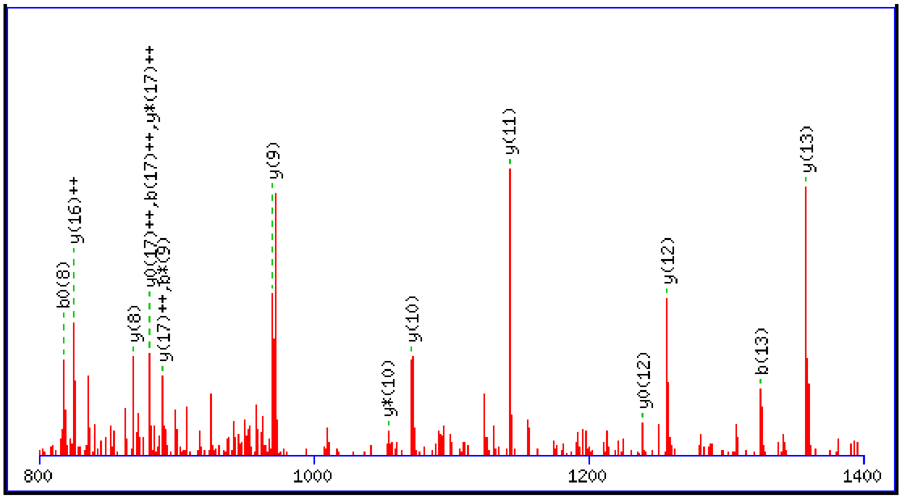

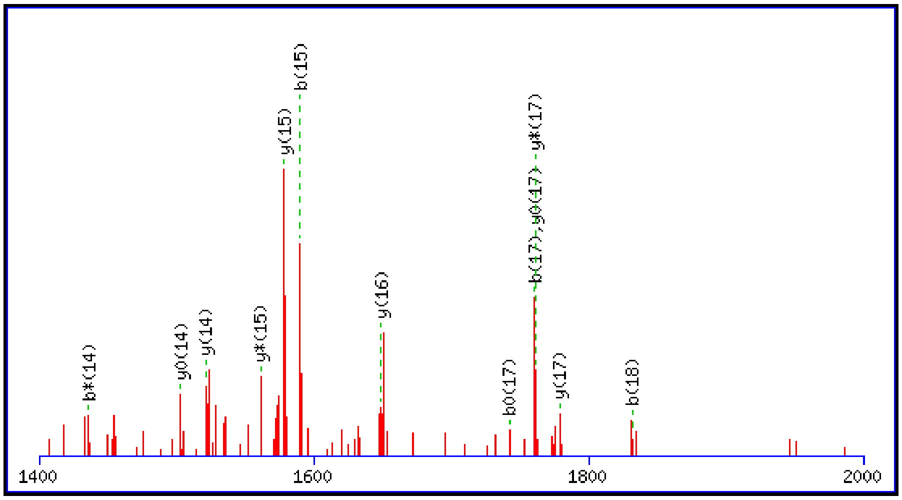

Monoisotopic mass of neutral peptide Mr(calc): 1976.9075
Fixed modifications: Carbamidomethyl (C)
Variable modifications:
N8 : Deamidated_N (N)
Ions Score: 89 **Expect:** 2.7e-007
Matches (Bold Red): 46/198 fragment ions using 86 most intense peaks

#	b	b++	b*	b*++	b0	b0++	Seq.	y	y++	y*	y*++	y0	y0++	#
1	72.0444	36.5258					A							19
2	200.1030	100.5551	183.0764	92.0418			Q	1906.8777	953.9425	1889.8512	945.4292	1888.8672	944.9372	18
3	329.1456	165.0764	312.1190	156.5631	311.1350	156.0711	E	**1778.8191**	**889.9132**	**1761.7926**	**881.3999**	**1760.8086**	**880.9079**	17
4	**400.1827**	200.5950	383.1561	192.0817	382.1721	191.5897	A	**1649.7766**	**825.3919**	1632.7500	816.8786	1631.7660	816.3866	16
5	457.2041	229.1057	440.1776	220.5924	439.1936	220.1004	G	**1578.7394**	**789.8734**	**1561.7129**	**781.3601**	1560.7289	**780.8681**	15
6	**620.2675**	310.6374	603.2409	302.1241	602.2569	301.6321	Y	**1521.7180**	761.3626	1504.6914	752.8494	**1503.7074**	752.3573	14
7	**721.3151**	361.1612	704.2886	352.6479	703.3046	352.1559	T	**1358.6546**	679.8310	1341.6281	671.3177	1340.6441	670.8257	13
8	836.3421	418.6747	819.3155	410.1614	**818.3315**	409.6694	N	**1257.6070**	629.3071	1240.5804	**620.7938**	**1239.5964**	**620.3018**	12
9	907.3792	454.1932	**890.3526**	445.6800	889.3686	445.1880	A	**1142.5800**	571.7937	1125.5535	563.2804	1124.5695	562.7884	11
10	1008.4269	504.7171	991.4003	496.2038	990.4163	495.7118	T	**1071.5429**	536.2751	**1054.5164**	527.7618	1053.5324	527.2698	10
11	1109.4746	555.2409	1092.4480	546.7276	1091.4640	546.2356	T	**970.4952**	485.7513	953.4687	477.2380	952.4847	476.7460	9
12	1210.5222	605.7648	1193.4957	597.2515	1192.5117	596.7595	T	**869.4476**	435.2274	852.4210	426.7141	851.4370	426.2221	8
13	**1325.5492**	663.2782	1308.5226	**654.7650**	1307.5386	**654.2729**	D	**768.3999**	384.7036	751.3733	376.1903	750.3893	375.6983	7
14	1453.6078	727.3075	**1436.5812**	718.7942	1435.5972	718.3022	Q	**653.3729**	327.1901	**636.3464**	318.6768			6
15	**1590.6667**	795.8370	1573.6401	787.3237	1572.6561	786.8317	H	**525.3144**	263.1608	**508.2878**	254.6475			5
16	1647.6881	824.3477	1630.6616	815.8344	1629.6776	815.3424	G	**388.2554**	194.6314	371.2289	186.1181			4
17	**1760.7722**	**880.8897**	1743.7456	872.3765	**1742.7616**	871.8845	L	**331.2340**	166.1206	314.2074	157.6074			3
18	**1831.8093**	916.4083	1814.7828	907.8950	1813.7987	907.4030	A	218.1499	109.5786	201.1234	101.0653			2

19							K	147.1128	74.0600	130.0863	65.5468			1

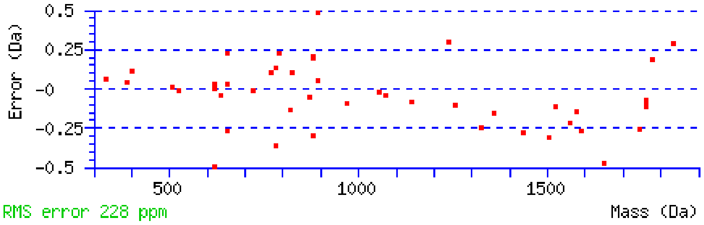

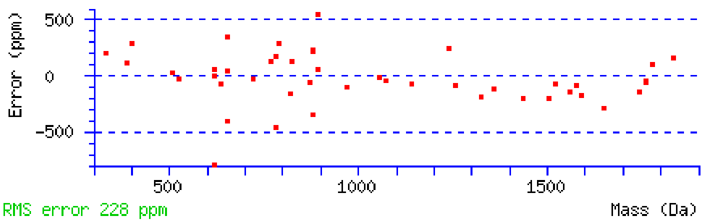

All matches to this query

Score	Mr(calc):	Delta	Sequence
89.4	1976.9075	0.0039	AQEAGYTNATTTDQHGLAK
25.8	1975.9235	0.9879	AQEAGYTNATTTDQHGLAK
1.4	1976.9261	-0.0146	ASSISKSPLLSLEWATK
1.4	1976.9261	-0.0146	ASSISKSPLLSLEWATK
1.4	1976.9261	-0.0146	ASSISKSPLLSLEWATK

Spectrum No: 80; Query: 1156; Rank: 1

Peptide View

MS/MS Fragmentation of **VTEPCIDGWVYDNSTFPSTIVTEWNLVCSHR**
Found in **IPI00214158**, Tax_Id=10116 Gene_Symbol=Slc22a6 Renal organic anion transporter 1

Match to Query 1156: 3682.670112 from(1228.563980,3+)
Title: 091127RatKid_SCX01_20.7644.7644.3.dta
Data file K:\NewmanPaper\Piliang\3SubProteomes\Piliang3SP\mgf5ppm\SCX_3SubProteomes5ppm.mgf

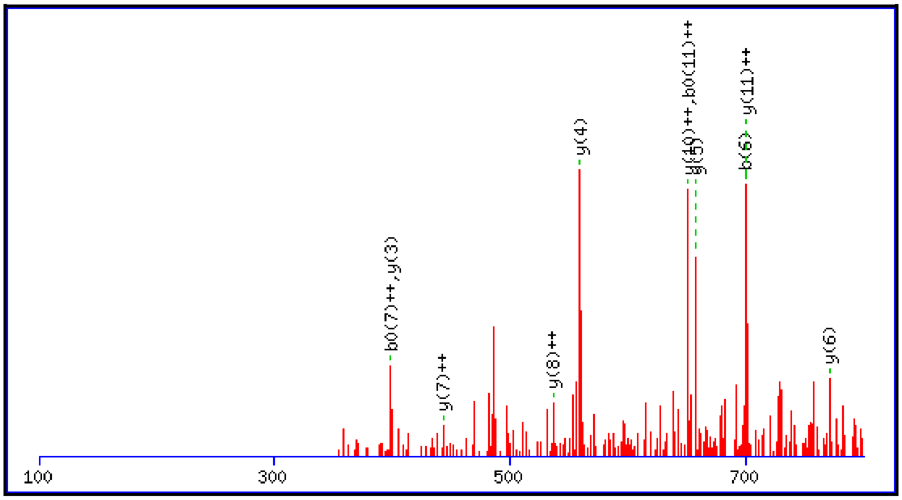

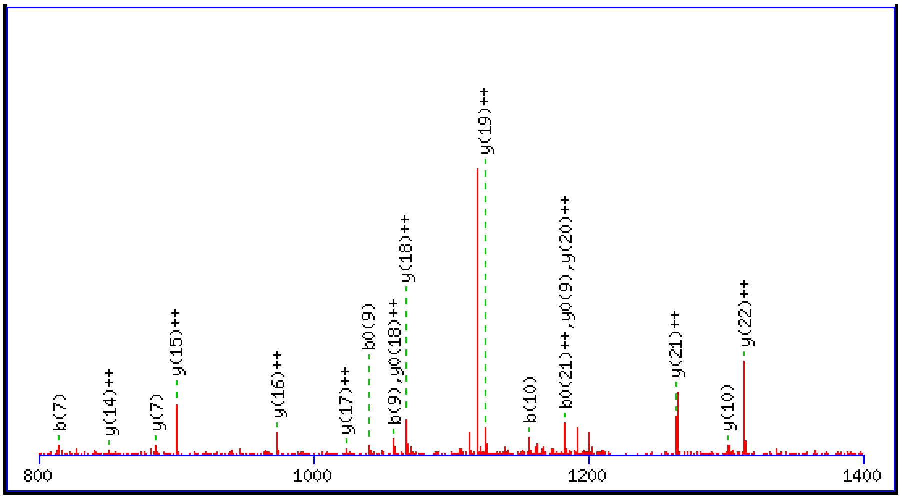

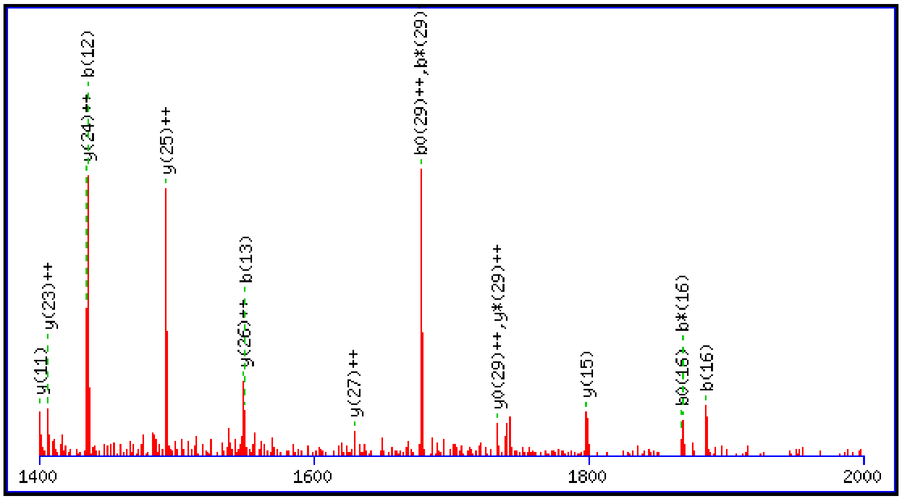

Monoisotopic mass of neutral peptide Mr(calc): 3682.6709
Fixed modifications: Carbamidomethyl (C)
Variable modifications:
N13 : Deamidated_N (N)
Ions Score: 89 **Expect:** 5.8e-007
Matches (Bold Red): 46/330 fragment ions using 91 most intense peaks

#	b	b^{++}	b*	b^{*++}	b^0	b^{0++}	Seq.	y	y^{++}	y*	y^{*++}	y^0	y^{0++}	#
1	100.0757	50.5415					V							31
2	201.1234	101.0653			183.1128	92.0600	T	3584.6097	1792.8085	3567.5831	1784.2952	3566.5991	1783.8032	30
3	330.1660	165.5866			312.1554	156.5813	E	3483.5620	1742.2846	3466.5355	**1733.7714**	3465.5514	**1733.2794**	29
4	427.2187	214.1130			409.2082	205.1077	P	3354.5194	**1677.7633**	3337.4929	1669.2501	3336.5089	1668.7581	28
5	587.2494	294.1283			569.2388	285.1230	C	3257.4667	**1629.2370**	3240.4401	1620.7237	3239.4561	1620.2317	27
6	**700.3334**	350.6704			682.3229	341.6651	I	3097.4360	**1549.2216**	3080.4095	1540.7084	3079.4254	1540.2164	26
7	**815.3604**	408.1838			797.3498	**399.1785**	D	2984.3519	**1492.6796**	2967.3254	1484.1663	2966.3414	1483.6743	25
8	872.3818	436.6946			854.3713	427.6893	G	2869.3250	**1435.1661**	2852.2985	1426.6529	2851.3144	1426.1609	24
9	**1058.4612**	529.7342			**1040.4506**	520.7289	W	2812.3035	**1406.6554**	2795.2770	1398.1421	2794.2930	1397.6501	23
10	**1157.5296**	579.2684			1139.5190	570.2631	V	2626.2242	**1313.6157**	2609.1977	1305.1025	2608.2137	1304.6105	22
11	1320.5929	660.8001			1302.5823	**651.7948**	Y	2527.1558	**1264.0815**	2510.1293	1255.5683	2509.1452	1255.0763	21
12	**1435.6198**	718.3136			1417.6093	709.3083	D	2364.0925	**1182.5499**	2347.0659	1174.0366	2346.0819	1173.5446	20
13	**1550.6468**	775.8270	1533.6202	767.3138	1532.6362	766.8217	N	2249.0655	**1125.0364**	2232.0390	1116.5231	2231.0550	1116.0311	19
14	1637.6788	819.3430	1620.6523	810.8298	1619.6682	810.3378	S	2134.0386	**1067.5229**	2117.0121	1059.0097	2116.0280	**1058.5177**	18
15	1738.7265	869.8669	1721.6999	861.3536	1720.7159	860.8616	T	2047.0066	**1024.0069**	2029.9800	1015.4936	2028.9960	1015.0016	17
16	**1885.7949**	943.4011	**1868.7684**	934.8878	**1867.7843**	934.3958	F	1945.9589	**973.4831**	1928.9323	964.9698	1927.9483	964.4778	16
17	1982.8477	991.9275	1965.8211	983.4142	1964.8371	982.9222	P	**1798.8905**	**899.9489**	1781.8639	891.4356	1780.8799	890.9436	15
18	2069.8797	1035.4435	2052.8531	1026.9302	2051.8691	1026.4382	S	1701.8377	**851.4225**	1684.8112	842.9092	1683.8272	842.4172	14

19	2170.9274	1085.9673	2153.9008	1077.4540	2152.9168	1076.9620	T	1614.8057	807.9065	1597.7791	799.3932	1596.7951	798.9012	13
20	2284.0114	1142.5094	2266.9849	1133.9961	2266.0009	1133.5041	I	1513.7580	757.3826	1496.7315	748.8694	1495.7474	748.3774	12
21	2383.0799	1192.0436	2366.0533	1183.5303	2365.0693	**1183.0383**	V	**1400.6739**	**700.8406**	1383.6474	692.3273	1382.6634	691.8353	11
22	2484.1275	1242.5674	2467.1010	1234.0541	2466.1170	1233.5621	T	**1301.6055**	**651.3064**	1284.5790	642.7931	1283.5950	642.3011	10
23	2613.1701	1307.0887	2596.1436	1298.5754	2595.1596	1298.0834	E	1200.5579	600.7826	1183.5313	592.2693	**1182.5473**	591.7773	9
24	2799.2494	1400.1284	2782.2229	1391.6151	2781.2389	1391.1231	W	1071.5153	**536.2613**	1054.4887	527.7480	1053.5047	527.2560	8
25	2913.2924	1457.1498	2896.2658	1448.6365	2895.2818	1448.1445	N	**885.4359**	**443.2216**	868.4094	434.7083	867.4254	434.2163	7
26	3026.3764	1513.6919	3009.3499	1505.1786	3008.3659	1504.6866	L	**771.3930**	386.2001	754.3665	377.6869	753.3825	377.1949	6
27	3125.4448	1563.2261	3108.4183	1554.7128	3107.4343	1554.2208	V	**658.3090**	329.6581	641.2824	321.1448	640.2984	320.6528	5
28	3285.4755	1643.2414	3268.4489	1634.7281	3267.4649	1634.2361	C	**559.2405**	280.1239	542.2140	271.6106	541.2300	271.1186	4
29	3372.5075	1686.7574	3355.4810	**1678.2441**	3354.4970	**1677.7521**	S	**399.2099**	200.1086	382.1833	191.5953	381.1993	191.1033	3
30	3509.5664	1755.2869	3492.5399	1746.7736	3491.5559	1746.2816	H	312.1779	156.5926	295.1513	148.0793			2
31							R	175.1190	88.0631	158.0924	79.5498			1

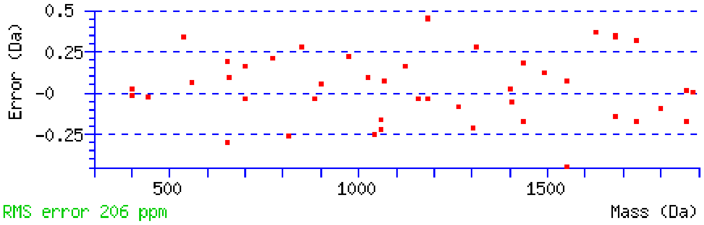

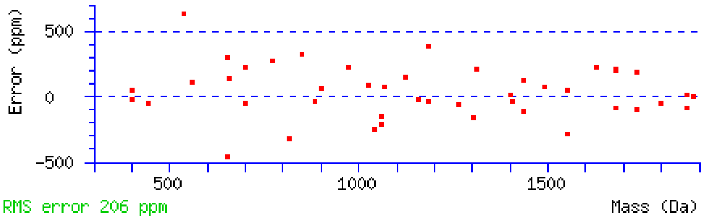

All matches to this query

Score	Mr(calc):	Delta	Sequence
89.1	3682.6709	-0.0008	VTEPCIDGWVYDNSTFPSTIVTEWNLVCSHR
74.5	3682.6709	-0.0008	VTEPCIDGWVYDNSTFPSTIVTEWNLVCSHR
31.0	3681.6869	0.9833	VTEPCIDGWVYDNSTFPSTIVTEWNLVCSHR
7.6	3682.6878	-0.0177	QASNLADLDTGNMEKIIKDENVTLYVWANLK
7.4	3682.6878	-0.0177	QASNLADLDTGNMEKIIKDENVTLYVWANLK
7.3	3682.6878	-0.0177	QASNLADLDTGNMEKIIKDENVTLYVWANLK
6.5	3682.6423	0.0279	HFHASQCPRPTSPVSTDSNMSAAVIQKARPTK
5.6	3681.6604	1.0097	QLSQQLNGLVSESTSYINGEGLTSSNMKELESR
5.6	3682.6878	-0.0177	QASNLADLDTGNMEKIIKDENVTLYVWANLK
3.6	3682.6423	0.0279	HFHASQCPRPTSPVSTDSNMSAAVIQKARPTK

Spectrum No: 81; Query: 930; Rank: 1

Peptide View

MS/MS Fragmentation of **ENYFEQYMDHFNFESFSNK**
Found in **IPI00230946**, Tax_Id=10116 Gene_Symbol=Dpp7 Dipeptidyl-peptidase 2 precursor

Match to Query 930: 2475.997628 from(1239.006090,2+)
Title: 091127RatKid_SCX01_26.6064.6064.2.dta
Data file K:\NewmanPaper\Piliang\3SubProteomes\Piliang3SP\mgf5ppm\SCX_3SubProteomes5ppm.mgf

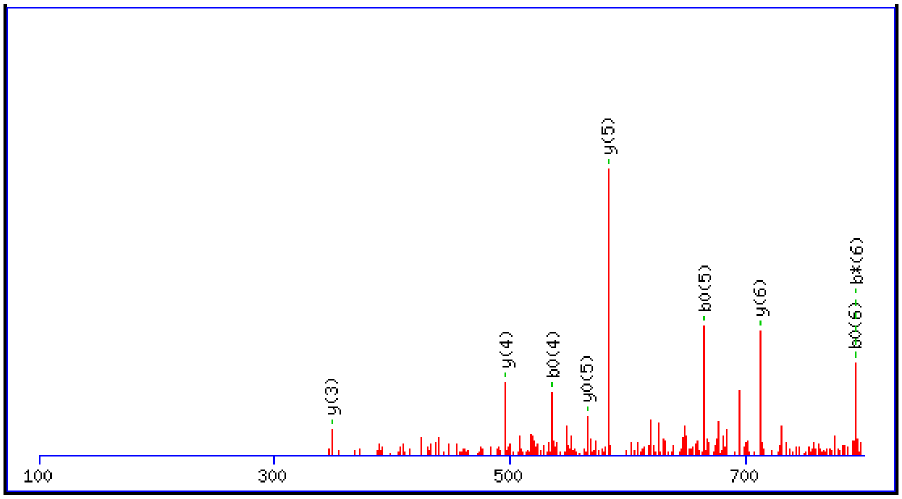

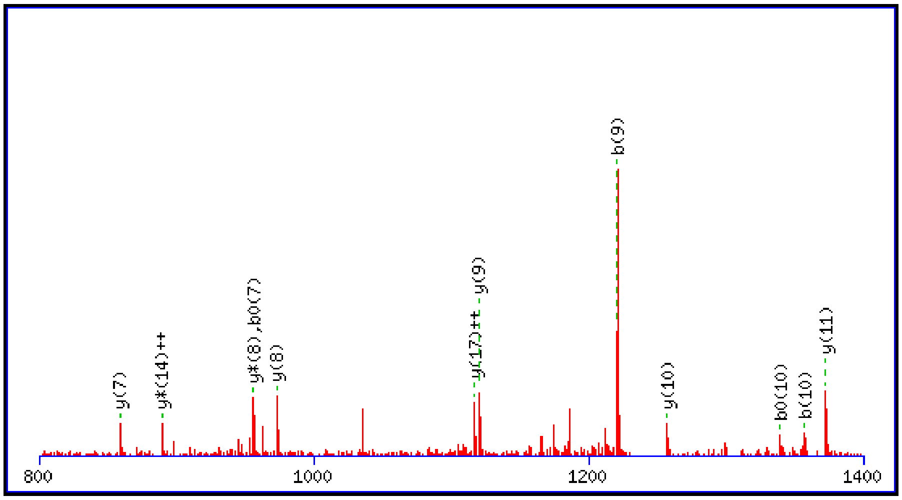

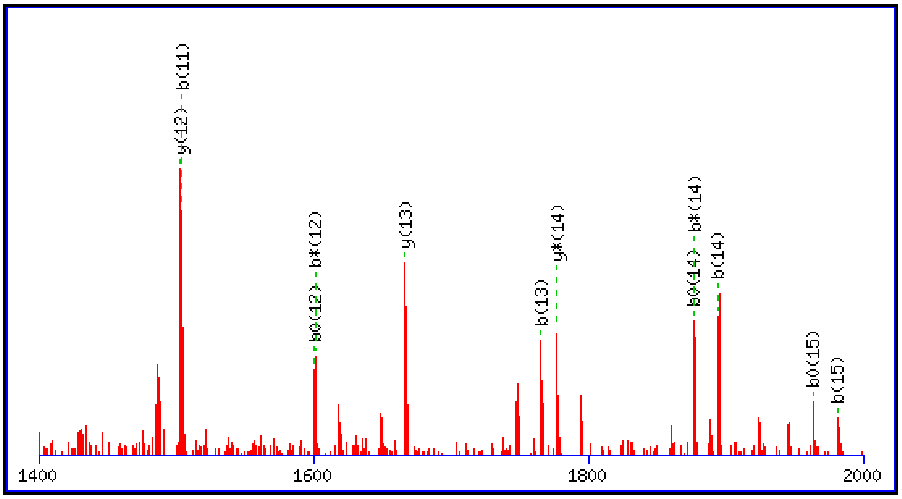

Monoisotopic mass of neutral peptide Mr(calc): 2475.9954
Fixed modifications: Carbamidomethyl (C)
Variable modifications:
N18 : Deamidated_N (N)
Ions Score: 89 **Expect:** 3.6e-007
Matches (Bold Red): 33/210 fragment ions using 39 most intense peaks

#	b	b^{++}	b*	b^{*++}	b^{0}	b^{0++}	Seq.	y	y^{++}	y*	y^{*++}	y^{0}	y^{0++}	#
1	130.0499	65.5286			112.0393	56.5233	E							19
2	244.0928	122.5500	227.0662	114.0368	226.0822	113.5448	N	2347.9601	1174.4837	2330.9335	1165.9704	2329.9495	1165.4784	18
3	407.1561	204.0817	390.1296	195.5684	389.1456	195.0764	Y	2233.9171	**1117.4622**	2216.8906	1108.9489	2215.9066	1108.4569	17
4	554.2245	277.6159	537.1980	269.1026	**536.2140**	268.6106	F	2070.8538	1035.9305	2053.8273	1027.4173	2052.8432	1026.9253	16
5	683.2671	342.1372	666.2406	333.6239	**665.2566**	333.1319	E	1923.7854	962.3963	1906.7588	953.8831	1905.7748	953.3911	15

**6**	811.3257	406.1665	**794.2992**	397.6532	**793.3151**	397.1612	**Q**	1794.7428	897.8750	**1777.7163**	**889.3618**	1776.7322	888.8698	**14**
**7**	974.3890	487.6982	957.3625	479.1849	**956.3785**	478.6929	**Y**	**1666.6842**	833.8458	1649.6577	825.3325	1648.6737	824.8405	**13**
**8**	1105.4295	553.2184	1088.4030	544.7051	1087.4190	544.2131	**M**	**1503.6209**	752.3141	1486.5943	743.8008	1485.6103	743.3088	**12**
**9**	**1220.4565**	610.7319	1203.4299	602.2186	1202.4459	601.7266	**D**	**1372.5804**	686.7938	1355.5539	678.2806	1354.5698	677.7886	**11**
**10**	**1357.5154**	679.2613	1340.4888	670.7481	**1339.5048**	670.2560	**H**	**1257.5535**	629.2804	1240.5269	620.7671	1239.5429	620.2751	**10**
**11**	**1504.5838**	752.7955	1487.5572	744.2823	1486.5732	743.7903	**F**	**1120.4946**	560.7509	1103.4680	552.2376	1102.4840	551.7456	**9**
**12**	1618.6267	809.8170	**1601.6002**	801.3037	**1600.6162**	800.8117	**N**	**973.4261**	487.2167	**956.3996**	478.7034	955.4156	478.2114	**8**
**13**	**1765.6951**	883.3512	1748.6686	874.8379	1747.6846	874.3459	**F**	**859.3832**	430.1952	842.3567	421.6820	841.3727	421.1900	**7**
**14**	**1894.7377**	947.8725	**1877.7112**	939.3592	**1876.7272**	938.8672	**E**	**712.3148**	356.6610	695.2883	348.1478	694.3042	347.6558	**6**
**15**	**1981.7698**	991.3885	1964.7432	982.8752	**1963.7592**	982.3832	**S**	**583.2722**	292.1397	566.2457	283.6265	**565.2616**	283.1345	**5**
**16**	2128.8382	1064.9227	2111.8116	1056.4094	2110.8276	1055.9174	**F**	**496.2402**	248.6237	479.2136	240.1105	478.2296	239.6184	**4**
**17**	2215.8702	1108.4387	2198.8436	1099.9255	2197.8596	1099.4335	**S**	**349.1718**	175.0895	332.1452	166.5762	331.1612	166.0842	**3**
**18**	2330.8971	1165.9522	2313.8706	1157.4389	2312.8866	1156.9469	**N**	262.1397	131.5735	245.1132	123.0602			**2**
**19**							**K**	147.1128	74.0600	130.0863	65.5468			**1**

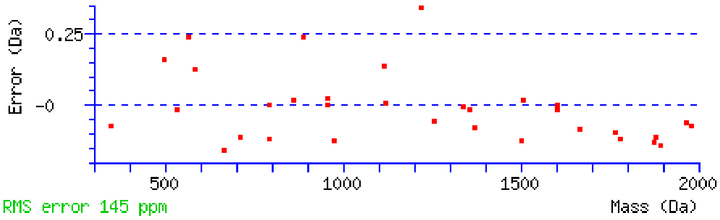

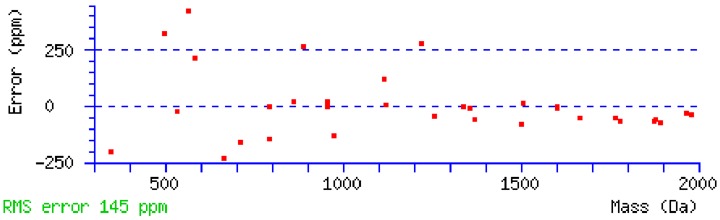

All matches to this query

Score	Mr(calc):	Delta	Sequence
88.6	2475.9954	0.0023	ENYFEQYMDHFNFESFSNK
36.6	2475.9954	0.0023	ENYFEQYMDHFNFESFSNK
13.7	2475.0114	0.9863	ENYFEQYMDHFNFESFSNK
13.3	2475.9742	0.0234	CPSASIYNHNINTYARFCK
13.3	2475.9742	0.0234	CPSASIYNHNINTYARFCK
13.3	2475.9742	0.0234	CPSASIYNHNINTYARFCK
13.3	2475.9742	0.0234	CPSASIYNHNINTYARFCK
8.1	2475.9742	0.0234	CPSASIYNHNINTYARFCK
8.1	2475.9742	0.0234	CPSASIYNHNINTYARFCK
6.5	2474.9902	1.0075	CPSASIYNHNINTYARFCK

Spectrum No: 82; Query: 801; Rank: 1

Peptide View

MS/MS Fragmentation of **LDNNGCSTQEVNITEFQLK**
Found in **IPI00201262**, Tax_Id=10116 Gene_Symbol=LOC297568;Mug2;Mug1 Alpha-1-inhibitor 3 precursor

Match to Query 801: 2210.022468 from(1106.018510,2+)
Title: 091127RatKid_SCX01_02.3374.3374.2.dta
Data file K:\NewmanPaper\Piliang\3SubProteomes\Piliang3SP\mgf5ppm\SCX_3SubProteomes5ppm.mgf

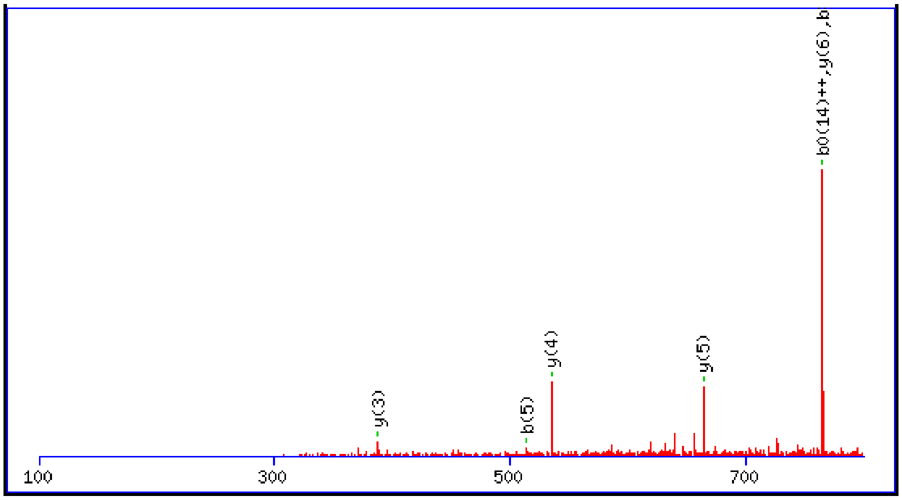

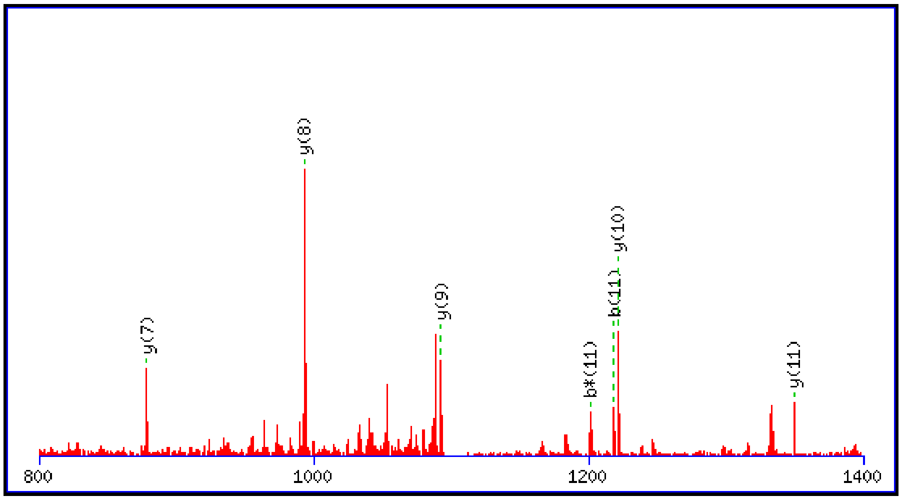

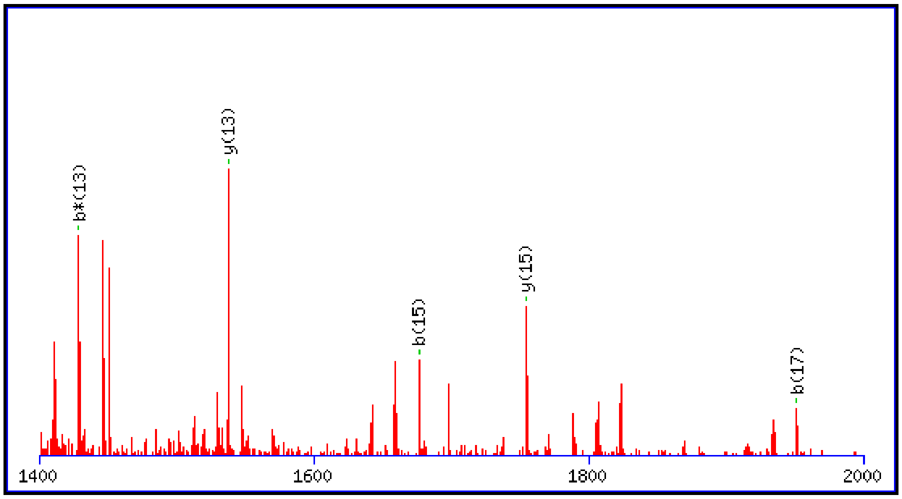

Monoisotopic mass of neutral peptide Mr(calc): 2210.0161
Fixed modifications: Carbamidomethyl (C)
Variable modifications:
N12 : Deamidated_N (N)
Ions Score: 88 **Expect:** 4e-007
Matches (Bold Red): 19/202 fragment ions using 24 most intense peaks

#	b	b++	b*	b*++	b0	b0++	Seq.	y	y++	y*	y*++	y0	y0++	#
1	114.0913	57.5493					L							19
2	229.1183	115.0628			211.1077	106.0575	D	2097.9393	1049.4733	2080.9128	1040.9600	2079.9288	1040.4680	18
3	343.1612	172.0842	326.1347	163.5710	325.1506	163.0790	N	1982.9124	991.9598	1965.8858	983.4466	1964.9018	982.9546	17
4	457.2041	229.1057	440.1776	220.5924	439.1936	220.1004	N	1868.8695	934.9384	1851.8429	926.4251	1850.8589	925.9331	16
5	**514.2256**	257.6164	497.1991	249.1032	496.2150	248.6112	G	**1754.8265**	877.9169	1737.8000	869.4036	1736.8160	868.9116	15

**6**	674.2563	337.6318	657.2297	329.1185	656.2457	328.6265	**C**	1697.8051	849.4062	1680.7785	840.8929	1679.7945	840.4009	**14**
**7**	761.2883	381.1478	744.2617	372.6345	743.2777	372.1425	**S**	**1537.7744**	769.3909	1520.7479	760.8776	1519.7639	760.3856	**13**
**8**	862.3360	431.6716	845.3094	423.1583	844.3254	422.6663	**T**	1450.7424	725.8748	1433.7158	717.3616	1432.7318	716.8696	**12**
**9**	990.3945	495.7009	973.3680	487.1876	972.3840	486.6956	**Q**	**1349.6947**	675.3510	1332.6682	666.8377	1331.6842	666.3457	**11**
**10**	1119.4371	560.2222	1102.4106	551.7089	1101.4266	551.2169	**E**	**1221.6361**	611.3217	1204.6096	602.8084	1203.6256	602.3164	**10**
**11**	**1218.5055**	609.7564	**1201.4790**	601.2431	1200.4950	600.7511	**V**	**1092.5935**	546.8004	1075.5670	538.2871	1074.5830	537.7951	**9**
**12**	1333.5325	667.2699	1316.5059	658.7566	1315.5219	658.2646	**N**	**993.5251**	497.2662	976.4986	488.7529	975.5146	488.2609	**8**
**13**	1446.6165	723.8119	**1429.5900**	715.2986	1428.6060	714.8066	**I**	**878.4982**	439.7527	861.4716	431.2395	860.4876	430.7475	**7**
**14**	1547.6642	774.3357	1530.6377	**765.8225**	1529.6537	**765.3305**	**T**	**765.4141**	383.2107	748.3876	374.6974	747.4036	374.2054	**6**
**15**	**1676.7068**	838.8570	1659.6803	830.3438	1658.6962	829.8518	**E**	**664.3665**	332.6869	647.3399	324.1736	646.3559	323.6816	**5**
**16**	1823.7752	912.3913	1806.7487	903.8780	1805.7647	903.3860	**F**	**535.3239**	268.1656	518.2973	259.6523			**4**
**17**	**1951.8338**	976.4205	1934.8073	967.9073	1933.8232	967.4153	**Q**	**388.2554**	194.6314	371.2289	186.1181			**3**
**18**	2064.9179	1032.9626	2047.8913	1024.4493	2046.9073	1023.9573	**L**	260.1969	130.6021	243.1703	122.0888			**2**
**19**							**K**	147.1128	74.0600	130.0863	65.5468			**1**

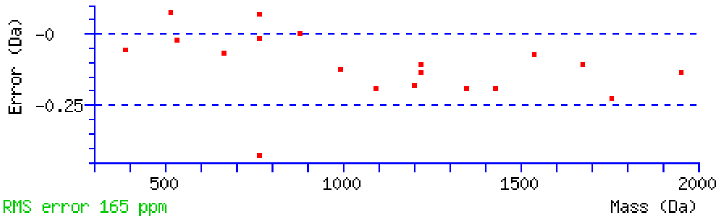

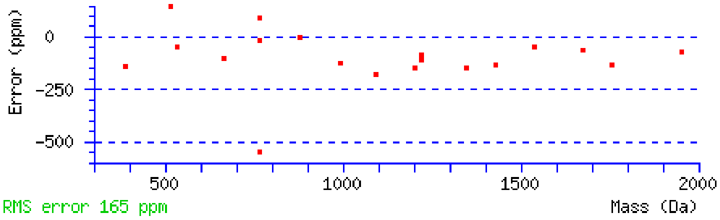

All matches to this query

Score	Mr(calc):	Delta	Sequence
88.2	2210.0161	0.0064	LDNNGCSTQEVNITEFQLK
20.0	2210.0161	0.0064	LDNNGCSTQEVNITEFQLK
20.0	2210.0161	0.0064	LDNNGCSTQEVNITEFQLK
11.2	2209.0321	0.9904	LDNNGCSTQEVNITEFQLK
4.0	2208.0230	1.9995	GVGGVSVTSFPARCDGEEQTR
3.4	2209.0205	1.0020	LSHNPLKSIPDNAFQSFGR
3.0	2208.0059	2.0165	NRVTLTLDNDAASPAPETSR
2.6	2210.0248	-0.0023	LKSHPXTVMNLKNYFLK
1.7	2208.0365	1.9860	LSHNPLKSIPDNAFQSFGR
1.7	2210.0207	0.0018	FGALNSSRLLMEQAKELK

Spectrum No: 83; Query: 801; Rank: 1

Peptide View

MS/MS Fragmentation of **LDNNGCSTQEVNITEFQLK**
Found in **IPI00201262**, Tax_Id=10116 Gene_Symbol=LOC297568;Mug2;Mug1 Alpha-1-inhibitor 3 precursor

Match to Query 801: 2210.022468 from(1106.018510,2+)
Title: 091127RatKid_SCX01_02.3374.3374.2.dta
Data file K:\NewmanPaper\Piliang\3SubProteomes\Piliang3SP\mgf5ppm\SCX_3SubProteomes5ppm.mgf

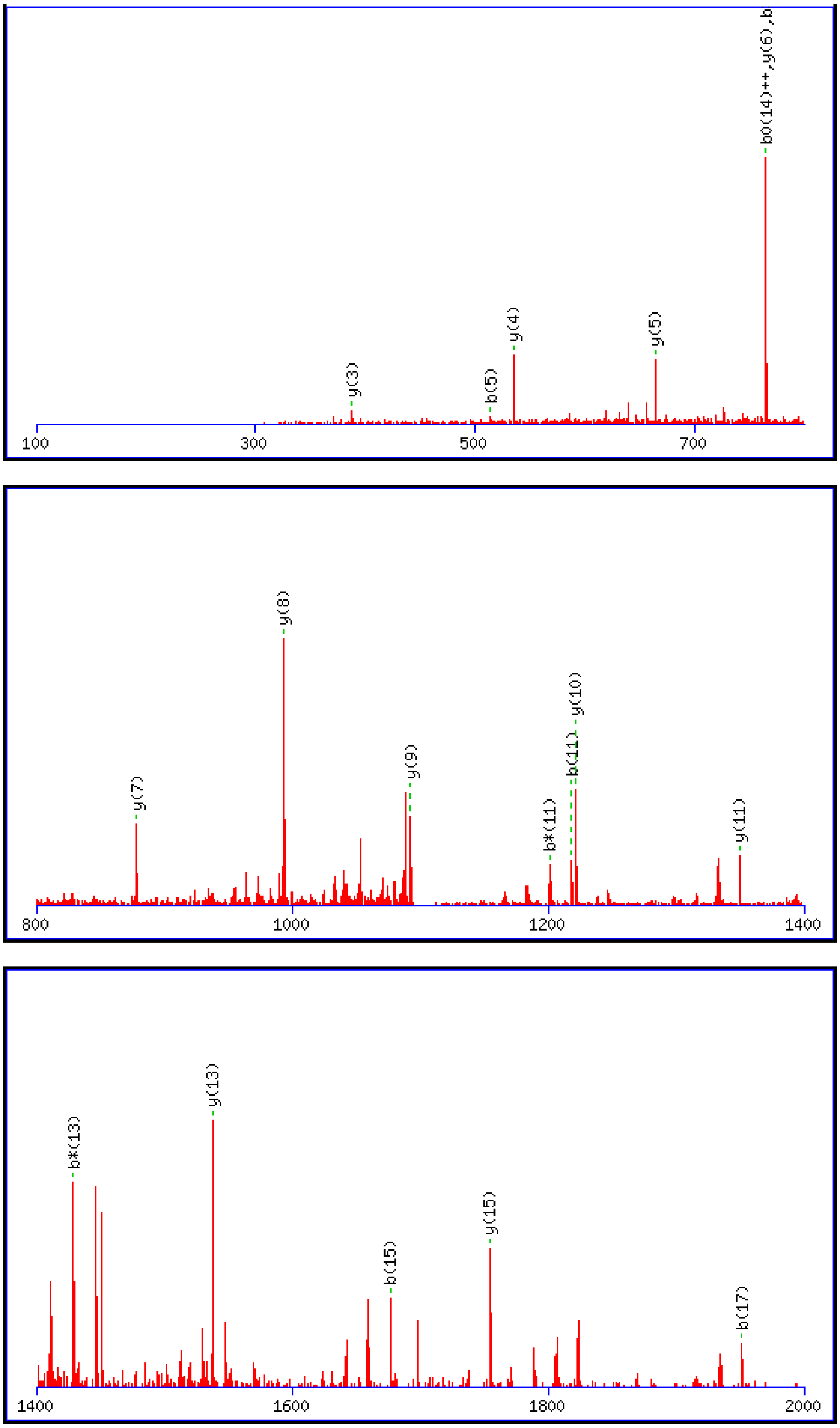

Monoisotopic mass of neutral peptide Mr(calc): 2210.0161
Fixed modifications: Carbamidomethyl (C)
Variable modifications:
N12 : Deamidated_N (N)
Ions Score: 88 **Expect:** 4e-007
Matches (Bold Red): 19/202 fragment ions using 24 most intense peaks

#	b	b^{++}	b*	b^{*++}	b^0	b^{0++}	Seq.	y	y^{++}	y*	y^{*++}	y^0	y^{0++}	#
1	114.0913	57.5493					L							19
2	229.1183	115.0628			211.1077	106.0575	D	2097.9393	1049.4733	2080.9128	1040.9600	2079.9288	1040.4680	18
3	343.1612	172.0842	326.1347	163.5710	325.1506	163.0790	N	1982.9124	991.9598	1965.8858	983.4466	1964.9018	982.9546	17
4	457.2041	229.1057	440.1776	220.5924	439.1936	220.1004	N	1868.8695	934.9384	1851.8429	926.4251	1850.8589	925.9331	16
5	**514.2256**	257.6164	497.1991	249.1032	496.2150	248.6112	G	**1754.8265**	877.9169	1737.8000	869.4036	1736.8160	868.9116	15

**6**	674.2563	337.6318	657.2297	329.1185	656.2457	328.6265	**C**	1697.8051	849.4062	1680.7785	840.8929	1679.7945	840.4009	**14**
**7**	761.2883	381.1478	744.2617	372.6345	743.2777	372.1425	**S**	**1537.7744**	769.3909	1520.7479	760.8776	1519.7639	760.3856	**13**
**8**	862.3360	431.6716	845.3094	423.1583	844.3254	422.6663	**T**	1450.7424	725.8748	1433.7158	717.3616	1432.7318	716.8696	**12**
**9**	990.3945	495.7009	973.3680	487.1876	972.3840	486.6956	**Q**	**1349.6947**	675.3510	1332.6682	666.8377	1331.6842	666.3457	**11**
**10**	1119.4371	560.2222	1102.4106	551.7089	1101.4266	551.2169	**E**	**1221.6361**	611.3217	1204.6096	602.8084	1203.6256	602.3164	**10**
**11**	**1218.5055**	609.7564	**1201.4790**	601.2431	1200.4950	600.7511	**V**	**1092.5935**	546.8004	1075.5670	538.2871	1074.5830	537.7951	**9**
**12**	1333.5325	667.2699	1316.5059	658.7566	1315.5219	658.2646	**N**	**993.5251**	497.2662	976.4986	488.7529	975.5146	488.2609	**8**
**13**	1446.6165	723.8119	**1429.5900**	715.2986	1428.6060	714.8066	**I**	**878.4982**	439.7527	861.4716	431.2395	860.4876	430.7475	**7**
**14**	1547.6642	774.3357	1530.6377	**765.8225**	1529.6537	**765.3305**	**T**	**765.4141**	383.2107	748.3876	374.6974	747.4036	374.2054	**6**
**15**	**1676.7068**	838.8570	1659.6803	830.3438	1658.6962	829.8518	**E**	**664.3665**	332.6869	647.3399	324.1736	646.3559	323.6816	**5**
**16**	1823.7752	912.3913	1806.7487	903.8780	1805.7647	903.3860	**F**	**535.3239**	268.1656	518.2973	259.6523			**4**
**17**	**1951.8338**	976.4205	1934.8073	967.9073	1933.8232	967.4153	**Q**	**388.2554**	194.6314	371.2289	186.1181			**3**
**18**	2064.9179	1032.9626	2047.8913	1024.4493	2046.9073	1023.9573	**L**	260.1969	130.6021	243.1703	122.0888			**2**
**19**							**K**	147.1128	74.0600	130.0863	65.5468			**1**

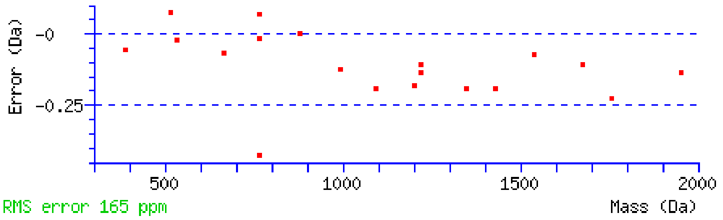

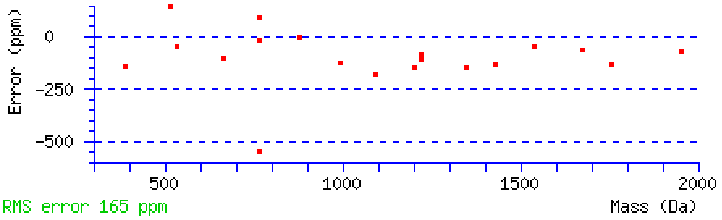

All matches to this query

Score	Mr(calc):	Delta	Sequence
88.2	2210.0161	0.0064	LDNNGCSTQEVNITEFQLK
20.0	2210.0161	0.0064	LDNNGCSTQEVNITEFQLK
20.0	2210.0161	0.0064	LDNNGCSTQEVNITEFQLK
11.2	2209.0321	0.9904	LDNNGCSTQEVNITEFQLK
4.0	2208.0230	1.9995	GVGGVSVTSFPARCDGEEQTR
3.4	2209.0205	1.0020	LSHNPLKSIPDNAFQSFGR
3.0	2208.0059	2.0165	NRVTLTLDNDAASPAPETSR
2.6	2210.0248	-0.0023	LKSHPXTVMNLKNYFLK
1.7	2208.0365	1.9860	LSHNPLKSIPDNAFQSFGR
1.7	2210.0207	0.0018	FGALNSSRLLMEQAKELK

Spectrum No: 84; Query: 275; Rank: 1

Peptide View

MS/MS Fragmentation of **DASTQLSTNLTSVR**
Found in **IPI00325312**, Tax_Id=10116 Gene_Symbol=Prom1 prominin 1 isoform 1

Match to Query 275: 1492.740088 from(747.377320,2+)
Title: 091127RatKid_SCX01_16.2649.2649.2.dta
Data file K:\NewmanPaper\Piliang\3SubProteomes\Piliang3SP\mgf5ppm\SCX_3SubProteomes5ppm.mgf

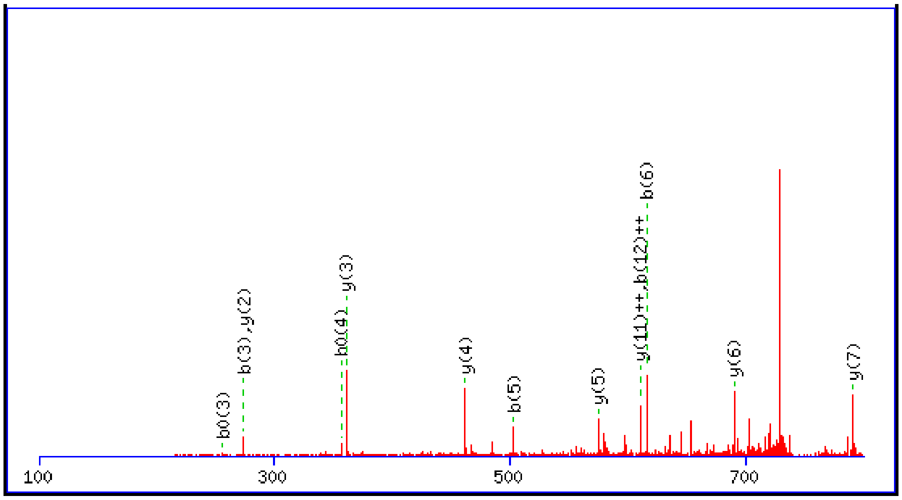

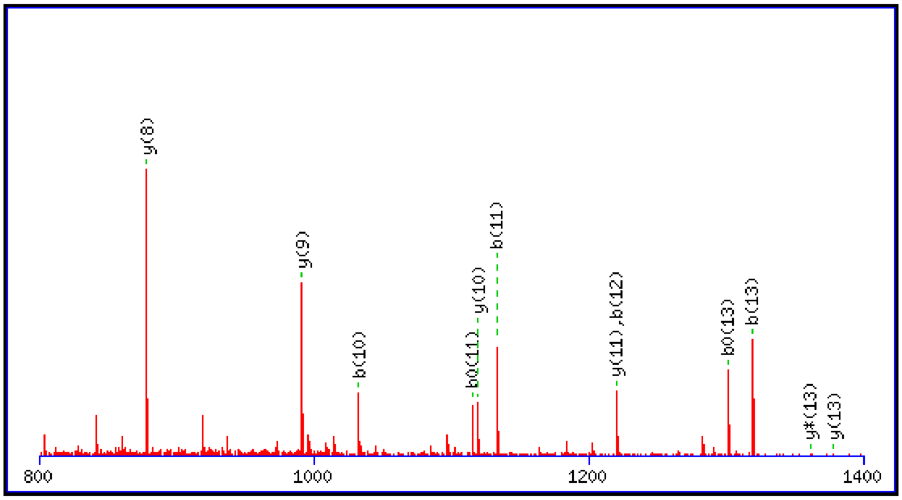

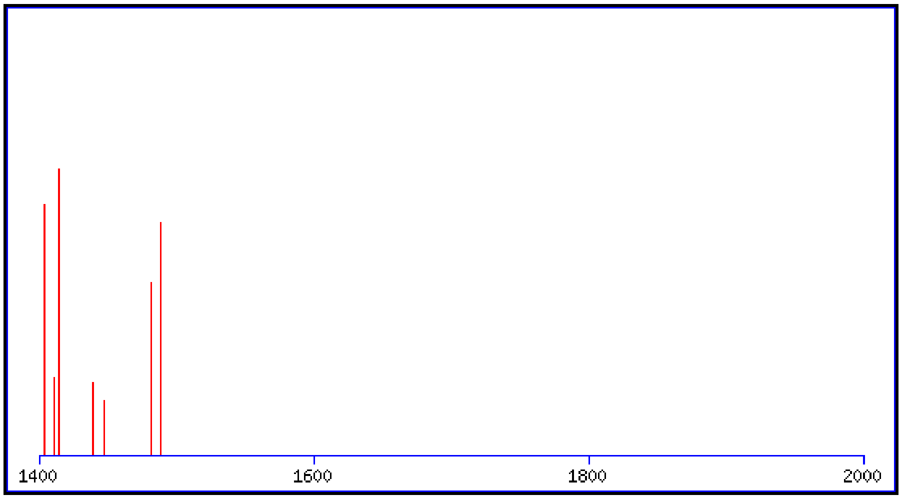

Monoisotopic mass of neutral peptide Mr(calc): 1492.7369
Fixed modifications: Carbamidomethyl (C)
Variable modifications:
N9 : Deamidated_N (N)
Ions Score: 88 **Expect:** 2.8e-007
Matches (Bold Red): 25/144 fragment ions using 36 most intense peaks

#	b	b^{++}	b*	b^{*++}	b^0	b^{0++}	Seq.	y	y^{++}	y*	y^{*++}	y^0	y^{0++}	#
1	116.0342	58.5207			98.0237	49.5155	D							14
2	187.0713	94.0393			169.0608	85.0340	A	**1378.7172**	689.8623	**1361.6907**	681.3490	1360.7067	680.8570	13
3	**274.1034**	137.5553			**256.0928**	128.5500	S	1307.6801	654.3437	1290.6536	645.8304	1289.6696	645.3384	12
4	375.1510	188.0792			**357.1405**	179.0739	T	**1220.6481**	**610.8277**	1203.6216	602.3144	1202.6375	601.8224	11
5	**503.2096**	252.1084	486.1831	243.5952	485.1991	243.1032	Q	**1119.6004**	560.3038	1102.5739	551.7906	1101.5899	551.2986	10

**6**	**616.2937**	308.6505	599.2671	300.1372	598.2831	299.6452	**L**	**991.5418**	496.2746	974.5153	487.7613	973.5313	487.2693	**9**
**7**	703.3257	352.1665	686.2992	343.6532	685.3151	343.1612	**S**	**878.4578**	439.7325	861.4312	431.2193	860.4472	430.7272	**8**
**8**	804.3734	402.6903	787.3468	394.1771	786.3628	393.6851	**T**	**791.4258**	396.2165	774.3992	387.7032	773.4152	387.2112	**7**
**9**	919.4003	460.2038	902.3738	451.6905	901.3898	451.1985	**N**	**690.3781**	345.6927	673.3515	337.1794	672.3675	336.6874	**6**
**10**	**1032.4844**	516.7458	1015.4578	508.2326	1014.4738	507.7405	**L**	**575.3511**	288.1792	558.3246	279.6659	557.3406	279.1739	**5**
**11**	**1133.5321**	567.2697	1116.5055	558.7564	**1115.5215**	558.2644	**T**	**462.2671**	231.6372	445.2405	223.1239	444.2565	222.6319	**4**
**12**	**1220.5641**	**610.7857**	1203.5375	602.2724	1202.5535	601.7804	**S**	**361.2194**	181.1133	344.1928	172.6001	343.2088	172.1081	**3**
**13**	**1319.6325**	660.3199	1302.6060	651.8066	**1301.6219**	651.3146	**V**	**274.1874**	137.5973	257.1608	129.0840			**2**
**14**							**R**	175.1190	88.0631	158.0924	79.5498			**1**

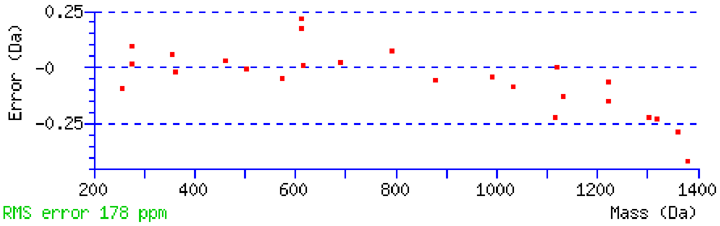

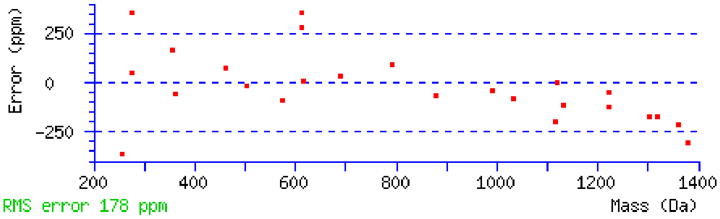

All matches to this query

Score	Mr(calc):	Delta	Sequence
88.2	1492.7369	0.0032	DASTQLSTNLTSVR
9.7	1492.7286	0.0115	AENGLGSPAIKSIR
8.8	1492.7286	0.0115	EREVPISLEVSR
7.6	1492.7522	-0.0121	VSENPYTSGIIASR
7.2	1492.7490	-0.0089	CLSGSLQVMNKTR
6.9	1492.7344	0.0057	NFAKKEMGTPDVR
5.4	1491.7417	0.9984	SEGIDSITNTTVVR
4.9	1491.7219	1.0182	TATSWSSGXRTWR
4.7	1490.7283	2.0118	YTVGLRNXFSVK
2.3	1492.7443	-0.0042	LEQNSCSVITTLK

Spectrum No: 85; Query: 1028; Rank: 1

Peptide View

MS/MS Fragmentation of **LANTSMFNNSKDSEEGGLSVAVPGEIR**
Found in **IPI00206254**, Tax_Id=10116 Gene_Symbol=Ggt1 Gamma-glutamyltranspeptidase 1 precursor

Match to Query 1028: 2823.338828 from(1412.676690,2+)
Title: 091127RatKid_SCX01_12.3115.3115.2.dta
Data file K:\NewmanPaper\Piliang\3SubProteomes\Piliang3SP\mgf5ppm\SCX_3SubProteomes5ppm.mgf

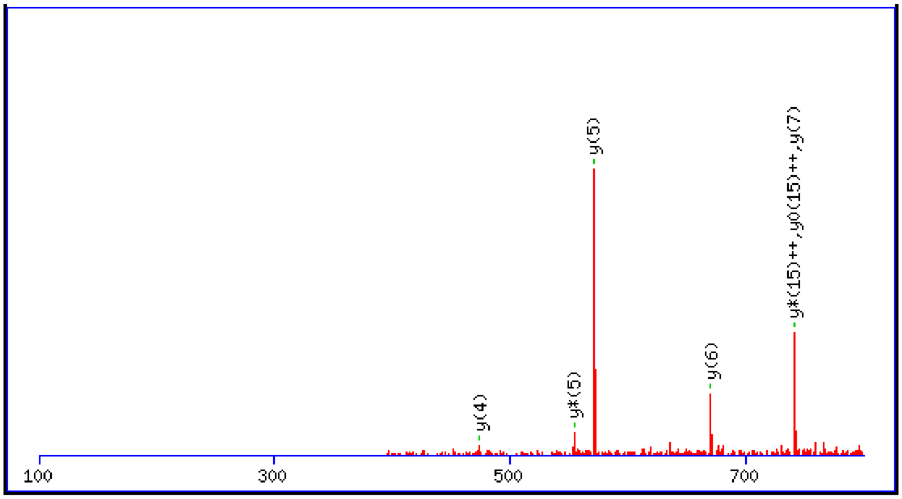

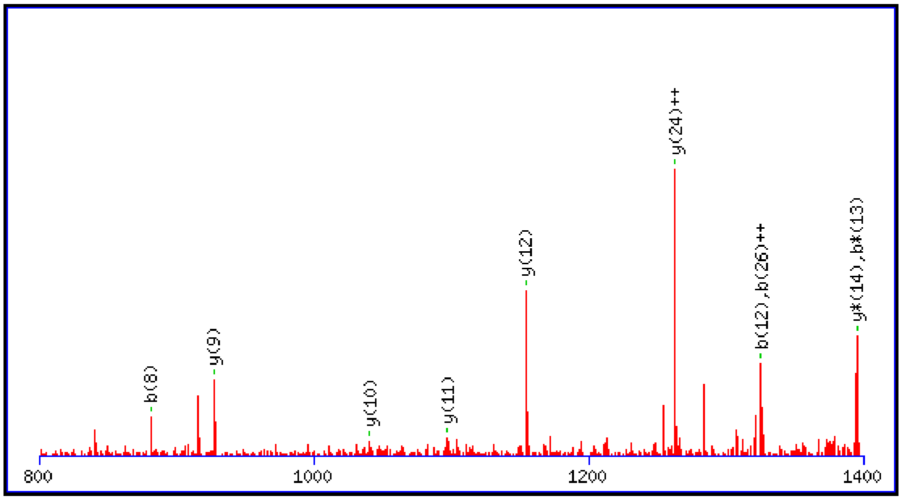

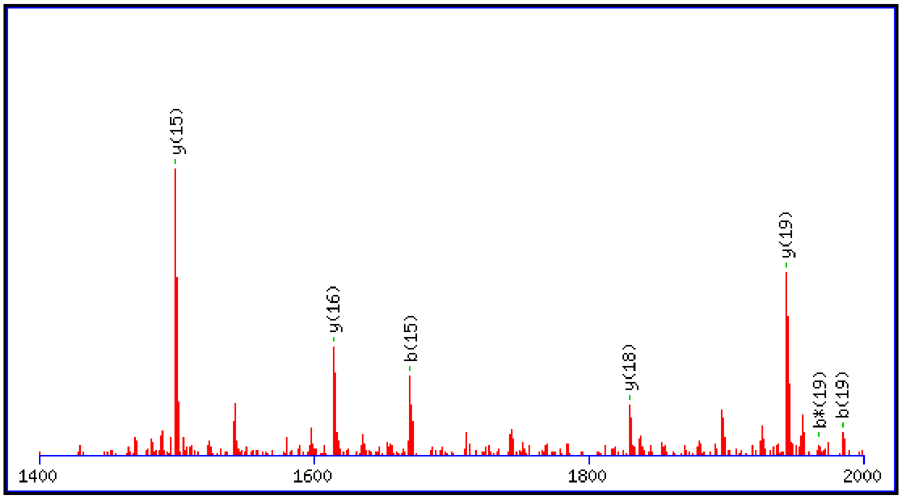

Monoisotopic mass of neutral peptide Mr(calc): 2823.3232
Fixed modifications: Carbamidomethyl (C)
Variable modifications:
N3 : Deamidated_N (N)
N8 : Deamidated_N (N)
Ions Score: 88 **Expect:** 5e-007
Matches (Bold Red): 24/298 fragment ions using 43 most intense peaks

#	b	b^{++}	b*	b^{*++}	b^{0}	b^{0++}	Seq.	y	y^{++}	y*	y^{*++}	y^{0}	y^{0++}	#
1	114.0913	57.5493					L							27
2	185.1285	93.0679					A	2711.2465	1356.1269	2694.2199	1347.6136	2693.2359	1347.1216	26
3	300.1554	150.5813	283.1288	142.0681			N	2640.2094	1320.6083	2623.1828	1312.0950	2622.1988	1311.6030	25
4	401.2031	201.1052	384.1765	192.5919	383.1925	192.0999	T	2525.1824	**1263.0948**	2508.1559	1254.5816	2507.1719	1254.0896	24

5	488.2351	244.6212	471.2085	236.1079	470.2245	235.6159	S	2424.1347	1212.5710	2407.1082	1204.0577	2406.1242	1203.5657	23
6	619.2756	310.1414	602.2490	301.6282	601.2650	301.1361	M	2337.1027	1169.0550	2320.0762	1160.5417	2319.0921	1160.0497	22
7	766.3440	383.6756	749.3174	375.1624	748.3334	374.6704	F	2206.0622	1103.5347	2189.0357	1095.0215	2188.0517	1094.5295	21
8	881.3709	441.1891	864.3444	432.6758	863.3604	432.1838	N	2058.9938	1030.0005	2041.9673	1021.4873	2040.9832	1020.9953	20
9	995.4139	498.2106	978.3873	489.6973	977.4033	489.2053	N	1943.9669	972.4871	1926.9403	963.9738	1925.9563	963.4818	19
10	1082.4459	541.7266	1065.4193	533.2133	1064.4353	532.7213	S	1829.9239	915.4656	1812.8974	906.9523	1811.9134	906.4603	18
11	1210.5409	605.7741	1193.5143	597.2608	1192.5303	596.7688	K	1742.8919	871.9496	1725.8654	863.4363	1724.8814	862.9443	17
12	1325.5678	663.2875	1308.5412	654.7743	1307.5572	654.2823	D	1614.7970	807.9021	1597.7704	799.3888	1596.7864	798.8968	16
13	1412.5998	706.8035	1395.5733	698.2903	1394.5893	697.7983	S	1499.7700	750.3886	1482.7435	741.8754	1481.7594	741.3834	15
14	1541.6424	771.3248	1524.6159	762.8116	1523.6318	762.3196	E	1412.7380	706.8726	1395.7114	698.3594	1394.7274	697.8673	14
15	1670.6850	835.8461	1653.6585	827.3329	1652.6744	826.8409	E	1283.6954	642.3513	1266.6688	633.8381	1265.6848	633.3461	13
16	1727.7065	864.3569	1710.6799	855.8436	1709.6959	855.3516	G	1154.6528	577.8300	1137.6263	569.3168	1136.6422	568.8248	12
17	1784.7279	892.8676	1767.7014	884.3543	1766.7174	883.8623	G	1097.6313	549.3193	1080.6048	540.8060	1079.6208	540.3140	11
18	1897.8120	949.4096	1880.7855	940.8964	1879.8014	940.4044	L	1040.6099	520.8086	1023.5833	512.2953	1022.5993	511.8033	10
19	1984.8440	992.9257	1967.8175	984.4124	1966.8335	983.9204	S	927.5258	464.2665	910.4993	455.7533	909.5152	455.2613	9
20	2083.9124	1042.4599	2066.8859	1033.9466	2065.9019	1033.4546	V	840.4938	420.7505	823.4672	412.2373	822.4832	411.7452	8
21	2154.9496	1077.9784	2137.9230	1069.4651	2136.9390	1068.9731	A	741.4254	371.2163	724.3988	362.7030	723.4148	362.2110	7
22	2254.0180	1127.5126	2236.9914	1118.9993	2236.0074	1118.5073	V	670.3883	335.6978	653.3617	327.1845	652.3777	326.6925	6
23	2351.0707	1176.0390	2334.0442	1167.5257	2333.0602	1167.0337	P	571.3198	286.1636	554.2933	277.6503	553.3093	277.1583	5
24	2408.0922	1204.5497	2391.0656	1196.0365	2390.0816	1195.5445	G	474.2671	237.6372	457.2405	229.1239	456.2565	228.6319	4
25	2537.1348	1269.0710	2520.1082	1260.5578	2519.1242	1260.0658	E	417.2456	209.1264	400.2191	200.6132	399.2350	200.1212	3
26	2650.2189	1325.6131	2633.1923	1317.0998	2632.2083	1316.6078	I	288.2030	144.6051	271.1765	136.0919			2
27							R	175.1190	88.0631	158.0924	79.5498			1

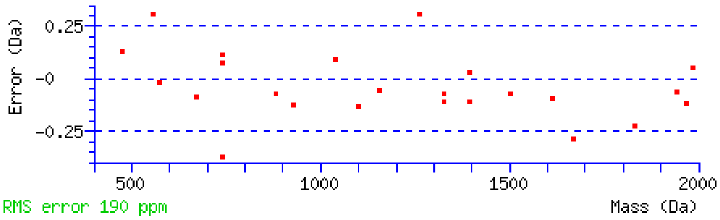

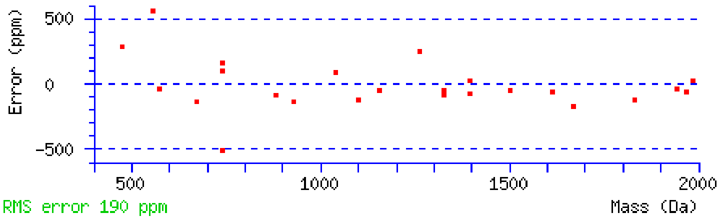

All matches to this query

Score	Mr(calc):	Delta	Sequence
88.1	2823.3232	0.0156	LANTSMFNNSKDSEEGGLSVAVPGEIR
79.5	2822.3392	0.9996	LANTSMFNNSKDSEEGGLSVAVPGEIR
78.6	2822.3392	0.9996	LANTSMFNNSKDSEEGGLSVAVPGEIR
78.3	2821.3552	1.9836	LANTSMFNNSKDSEEGGLSVAVPGEIR
68.3	2823.3232	0.0156	LANTSMFNNSKDSEEGGLSVAVPGEIR
68.3	2823.3232	0.0156	LANTSMFNNSKDSEEGGLSVAVPGEIR
68.3	2822.3392	0.9996	LANTSMFNNSKDSEEGGLSVAVPGEIR
7.9	2822.3487	0.9901	NITGSNNHYSLISLNINGLNSPIKR
6.5	2822.3487	0.9901	NITGSNNHYSLISLNINGLNSPIKR
4.6	2822.3252	1.0136	LVINGKXKGILGYTEDQVVSSDSNR

Spectrum No: 86; Query: 359; Rank: 1

Peptide View

MS/MS Fragmentation of **CISDLTLNVSTTEK**

Found in **IPI00324585**, Tax_Id=10116 Gene_Symbol=Itga1 Integrin alpha-1 precursor

Match to Query 359: 1580.763328 from(791.388940,2+)
Title: 091127RatKid_SCX01_17.2411.2411.2.dta
Data file K:\NewmanPaper\Piliang\3SubProteomes\Piliang3SP\mgf5ppm\SCX_3SubProteomes5ppm.mgf

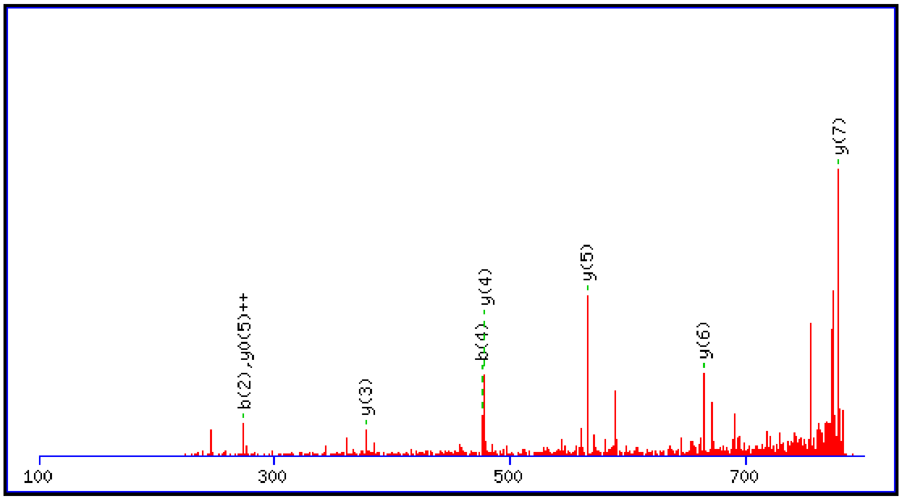

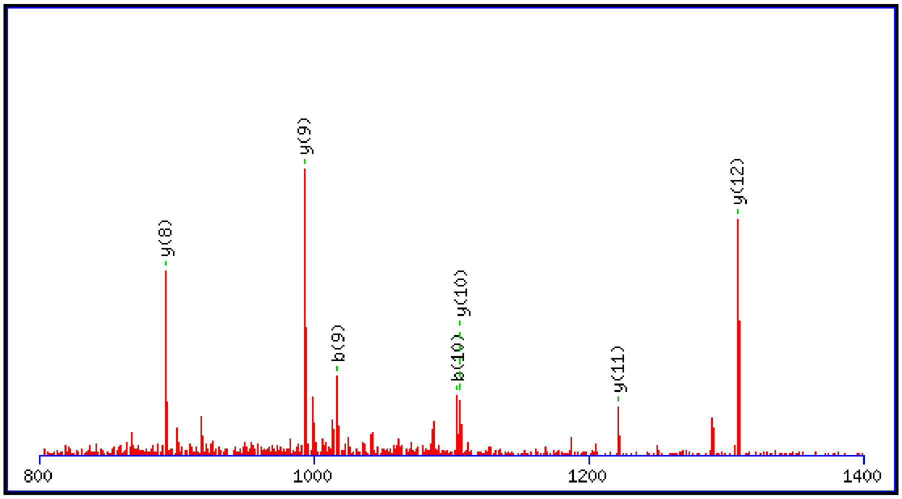

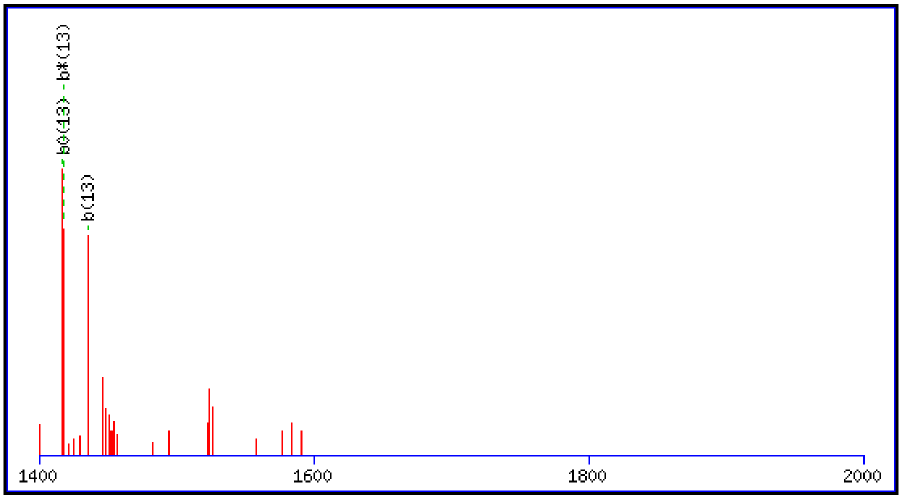

Monoisotopic mass of neutral peptide Mr(calc): 1580.7603
Fixed modifications: Carbamidomethyl (C)
Variable modifications:
N8 : Deamidated_N (N)
Ions Score: 88 **Expect:** 2.5e-007
Matches (Bold Red): 18/136 fragment ions using 26 most intense peaks

#	b	b++	b*	b*++	b0	b0++	Seq.	y	y++	y*	y*++	y0	y0++	#
1	161.0379	81.0226					C							14

2	274.1220	137.5646					I	1421.7370	711.3721	1404.7104	702.8589	1403.7264	702.3668	13
3	361.1540	181.0806			343.1435	172.0754	S	1308.6529	654.8301	1291.6264	646.3168	1290.6423	645.8248	12
4	476.1810	238.5941			458.1704	229.5888	D	1221.6209	611.3141	1204.5943	602.8008	1203.6103	602.3088	11
5	589.2650	295.1362			571.2545	286.1309	L	1106.5939	553.8006	1089.5674	545.2873	1088.5834	544.7953	10
6	690.3127	345.6600			672.3021	336.6547	T	993.5099	497.2586	976.4833	488.7453	975.4993	488.2533	9
7	803.3968	402.2020			785.3862	393.1967	L	892.4622	446.7347	875.4356	438.2215	874.4516	437.7295	8
8	918.4237	459.7155	901.3972	451.2022	900.4131	450.7102	N	779.3781	390.1927	762.3516	381.6794	761.3676	381.1874	7
9	1017.4921	509.2497	1000.4656	500.7364	999.4816	500.2444	V	664.3512	332.6792	647.3246	324.1660	646.3406	323.6740	6
10	1104.5241	552.7657	1087.4976	544.2524	1086.5136	543.7604	S	565.2828	283.1450	548.2562	274.6318	547.2722	274.1397	5
11	1205.5718	603.2896	1188.5453	594.7763	1187.5613	594.2843	T	478.2508	239.6290	461.2242	231.1157	460.2402	230.6237	4
12	1306.6195	653.8134	1289.5930	645.3001	1288.6089	644.8081	T	377.2031	189.1052	360.1765	180.5919	359.1925	180.0999	3
13	1435.6621	718.3347	1418.6355	709.8214	1417.6515	709.3294	E	276.1554	138.5813	259.1288	130.0681	258.1448	129.5761	2
14							K	147.1128	74.0600	130.0863	65.5468			1

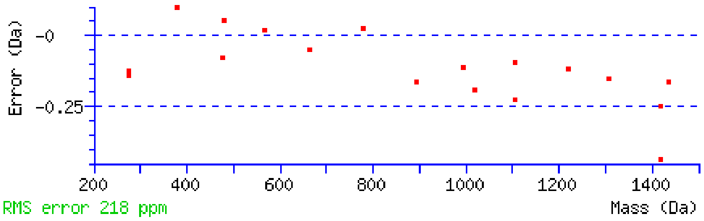

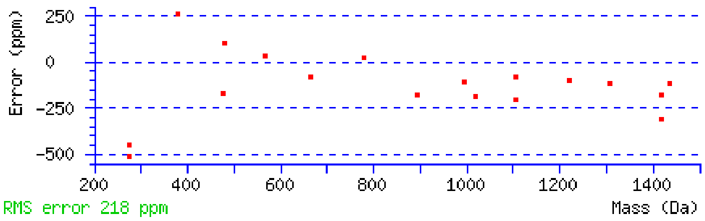

All matches to this query

Score	Mr(calc):	Delta	Sequence
88.0	1580.7603	0.0030	CISDLTLNVSTTEK
18.3	1580.7603	0.0030	GLKDMELDTSSIEK
11.2	1580.7477	0.0156	RKLSVGAYVSSVR
8.9	1580.7477	0.0156	RKLSVGAYVSSVR
5.8	1580.7521	0.0113	MPQLLNSILNVNK
4.5	1580.7559	0.0074	TPQAVSLSSKSGPSR
2.9	1578.7443	2.0191	NRTLQLWLDNPK
2.7	1580.7633	0.0000	GALTPSIRNLAMEK
2.1	1579.7746	0.9887	AEIVPVTTTVLDDK
1.5	1579.7746	0.9887	AEIVPVTTTVLDDK

Spectrum No: 87; Query: 688; Rank: 1

Peptide View

MS/MS Fragmentation of **ADNLYSQVVGLSAAQANLSK**
Found in **IPI00204504**, Tax_Id=10116 Gene_Symbol=Plvap Plasmalemma vesicle-associated protein

Match to Query 688: 2049.037068 from(1025.525810,2+)
Title: 091129RatKid_SCX02_15.3200.3200.2.dta
Data file K:\NewmanPaper\Piliang\3SubProteomes\Piliang3SP\mgf5ppm\SCX_3SubProteomes5ppm.mgf

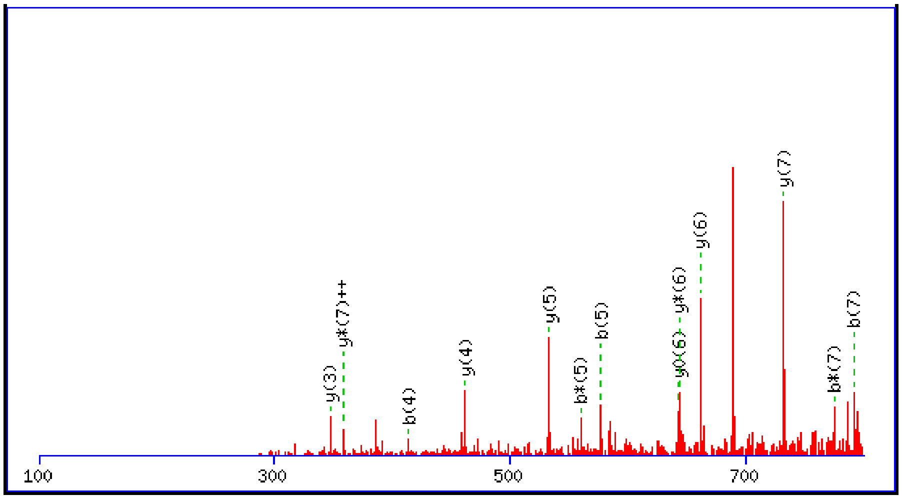

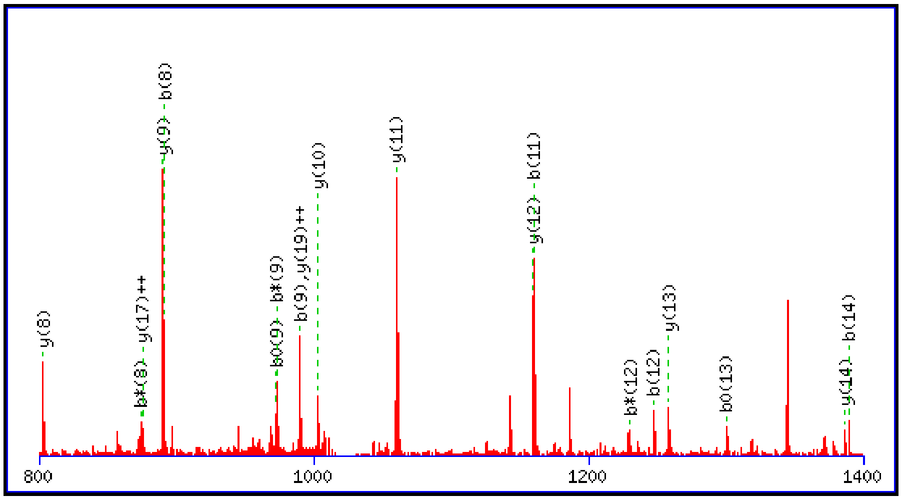

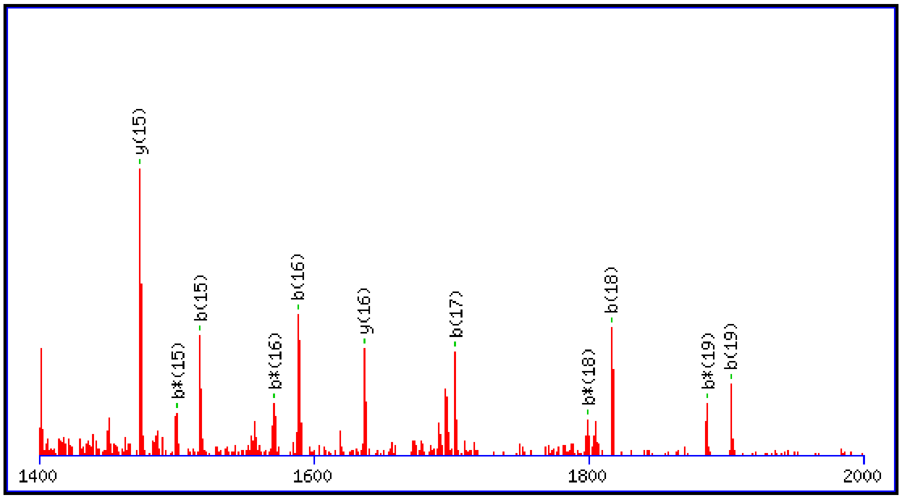

Monoisotopic mass of neutral peptide Mr(calc): 2049.0378
Fixed modifications: Carbamidomethyl (C)
Variable modifications:
N17 : Deamidated_N (N)
Ions Score: 88 **Expect:** 2.9e-007
Matches (Bold Red): 43/220 fragment ions using 76 most intense peaks

#	b	b^{++}	b*	b^{*++}	b^0	b^{0++}	Seq.	y	y^{++}	y*	y^{*++}	y^0	y^{0++}	#
1	72.0444	36.5258					A							20
2	187.0713	94.0393			169.0608	85.0340	D	1979.0080	**990.0076**	1961.9815	981.4944	1960.9974	981.0024	19
3	301.1143	151.0608	284.0877	142.5475	283.1037	142.0555	N	1863.9811	932.4942	1846.9545	923.9809	1845.9705	923.4889	18
4	**414.1983**	207.6028	397.1718	199.0895	396.1878	198.5975	L	1749.9381	**875.4727**	1732.9116	866.9594	1731.9276	866.4674	17
5	**577.2617**	289.1345	**560.2351**	280.6212	559.2511	280.1292	Y	**1636.8541**	818.9307	1619.8275	810.4174	1618.8435	809.9254	16

6	664.2937	332.6505	647.2671	324.1372	646.2831	323.6452	S	**1473.7907**	737.3990	1456.7642	728.8857	1455.7802	728.3937	15
7	**792.3523**	396.6798	**775.3257**	388.1665	774.3417	387.6745	Q	**1386.7587**	693.8830	1369.7322	685.3697	1368.7481	684.8777	14
8	**891.4207**	446.2140	**874.3941**	437.7007	873.4101	437.2087	V	**1258.7001**	629.8537	1241.6736	621.3404	1240.6896	620.8484	13
9	**990.4891**	495.7482	**973.4625**	487.2349	**972.4785**	486.7429	V	**1159.6317**	580.3195	1142.6052	571.8062	1141.6212	571.3142	12
10	1047.5106	524.2589	1030.4840	515.7456	1029.5000	515.2536	G	**1060.5633**	530.7853	1043.5368	522.2720	1042.5527	521.7800	11
11	**1160.5946**	580.8009	1143.5681	572.2877	1142.5840	571.7957	L	**1003.5418**	502.2746	986.5153	493.7613	985.5313	493.2693	10
12	**1247.6266**	624.3170	**1230.6001**	615.8037	1229.6161	615.3117	S	**890.4578**	445.7325	873.4312	437.2193	872.4472	436.7272	9
13	1318.6638	659.8355	1301.6372	651.3222	**1300.6532**	650.8302	A	**803.4258**	402.2165	786.3992	393.7032	785.4152	393.2112	8
14	**1389.7009**	695.3541	1372.6743	686.8408	1371.6903	686.3488	A	**732.3886**	366.6980	715.3621	**358.1847**	714.3781	357.6927	7
15	**1517.7594**	759.3834	**1500.7329**	750.8701	1499.7489	750.3781	Q	**661.3515**	331.1794	**644.3250**	322.6661	**643.3410**	322.1741	6
16	**1588.7966**	794.9019	**1571.7700**	786.3886	1570.7860	785.8966	A	**533.2929**	267.1501	516.2664	258.6368	515.2824	258.1448	5
17	**1703.8235**	852.4154	1686.7969	843.9021	1685.8129	843.4101	N	**462.2558**	231.6316	445.2293	223.1183	444.2453	222.6263	4
18	**1816.9076**	908.9574	**1799.8810**	900.4441	1798.8970	899.9521	L	**347.2289**	174.1181	330.2023	165.6048	329.2183	165.1128	3
19	**1903.9396**	952.4734	**1886.9130**	943.9602	1885.9290	943.4682	S	234.1448	117.5761	217.1183	109.0628	216.1343	108.5708	2
20							K	147.1128	74.0600	130.0863	65.5468			1

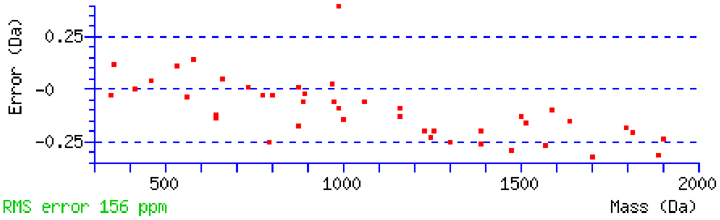

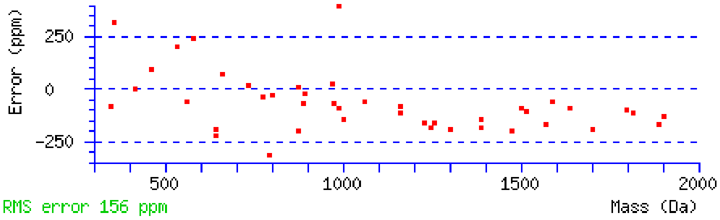

All matches to this query

Score	Mr(calc):	Delta	Sequence
87.9	2049.0378	-0.0007	ADNLYSQVVGLSAAQANLSK
27.2	2048.0538	0.9833	ADNLYSQVVGLSAAQANLSK
14.8	2049.0378	-0.0007	ADNLYSQVVGLSAAQANLSK
9.4	2048.0249	1.0122	FTATAGIQVVGDDLTMANPK
6.4	2047.0474	1.9897	FTATAGIQVVGDDLTVTNPK
0.9	2049.0565	-0.0194	ELRLYNTGLTEVPSGICK

Spectrum No: 88; Query: 713; Rank: 1

Peptide View

MS/MS Fragmentation of **TTLVDNNTWNNSHIALVGK**
Found in **IPI00382320**, Tax_Id=10116 Gene_Symbol=RGD1311563 93 kDa protein

Match to Query 713: 2097.047468 from(1049.531010,2+)
Title: 091129RatKid_SCX02_24.2826.2826.2.dta
Data file K:\NewmanPaper\Piliang\3SubProteomes\Piliang3SP\mgf5ppm\SCX_3SubProteomes5ppm.mgf

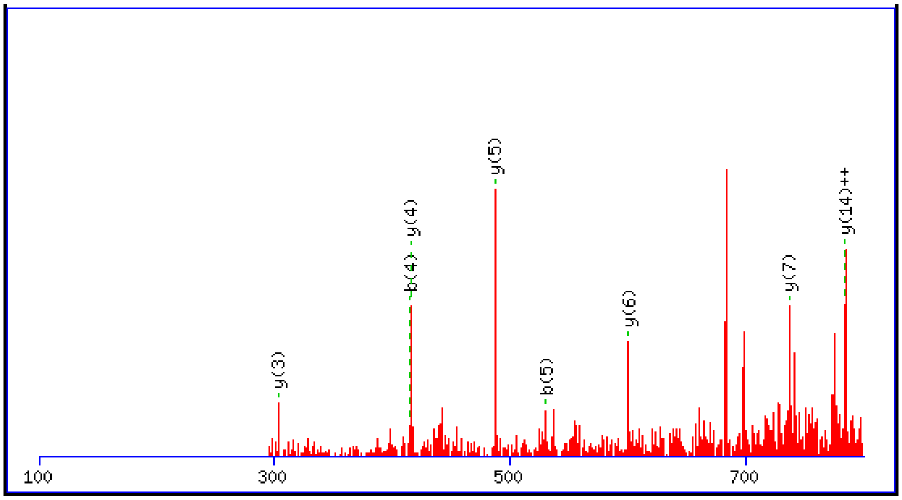

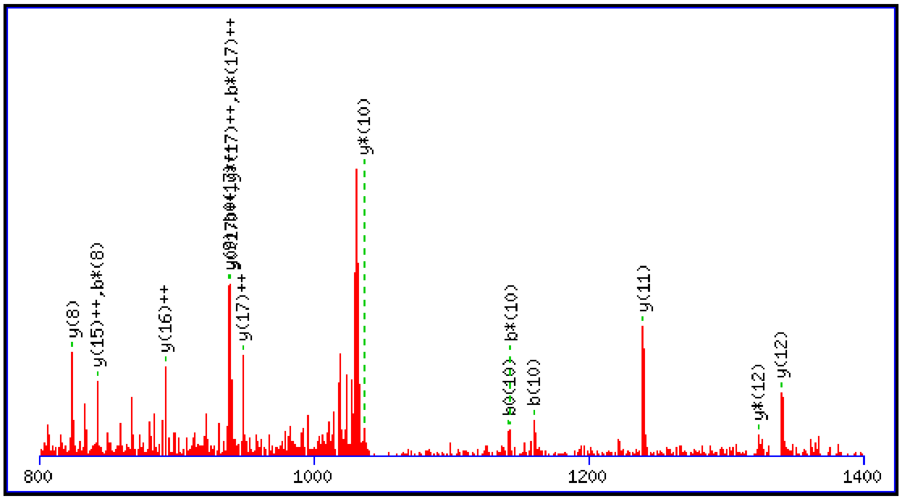

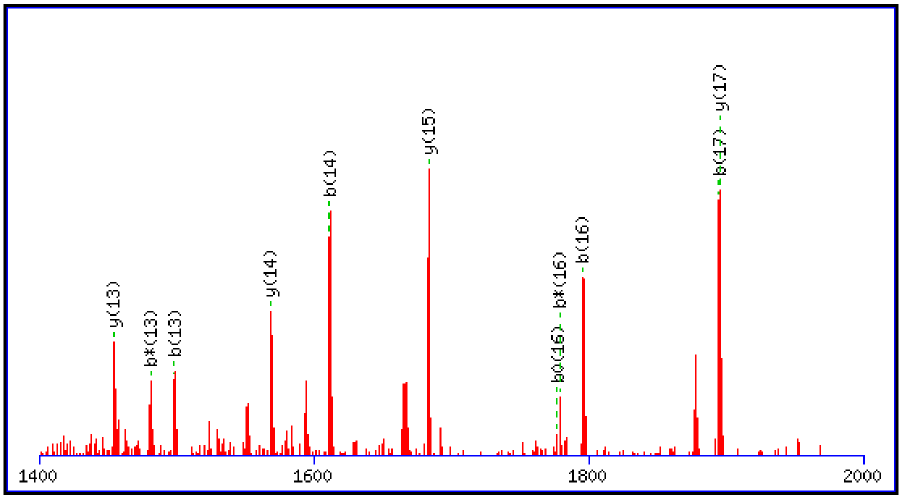

Monoisotopic mass of neutral peptide Mr(calc): 2097.0491
Fixed modifications: Carbamidomethyl (C)
Variable modifications:
N10 : Deamidated_N (N)
Ions Score: 88 **Expect:** 3e-007
Matches (Bold Red): 36/192 fragment ions using 61 most intense peaks

#	b	b^{++}	b*	b^{*++}	b^0	b^{0++}	Seq.	y	y^{++}	y*	y^{*++}	y^0	y^{0++}	#
1	102.0550	51.5311			84.0444	42.5258	T							19
2	203.1026	102.0550			185.0921	93.0497	T	1997.0087	999.0080	1979.9821	990.4947	1978.9981	990.0027	18
3	316.1867	158.5970			298.1761	149.5917	L	**1895.9610**	**948.4841**	1878.9344	**939.9709**	1877.9504	**939.4789**	17
4	**415.2551**	208.1312			397.2445	199.1259	V	1782.8769	**891.9421**	1765.8504	883.4288	1764.8664	882.9368	16
5	**530.2821**	265.6447			512.2715	256.6394	D	**1683.8085**	**842.4079**	1666.7820	833.8946	1665.7980	833.4026	15

**6**	644.3250	322.6661	627.2984	314.1529	626.3144	313.6608	**N**	**1568.7816**	**784.8944**	1551.7550	776.3812	1550.7710	775.8891	**14**
**7**	758.3679	379.6876	741.3414	371.1743	740.3573	370.6823	**N**	**1454.7386**	727.8730	1437.7121	719.3597	1436.7281	718.8677	**13**
**8**	859.4156	430.2114	**842.3890**	421.6982	841.4050	421.2061	**T**	**1340.6957**	670.8515	**1323.6692**	662.3382	1322.6852	661.8462	**12**
**9**	1045.4949	523.2511	1028.4684	514.7378	1027.4843	514.2458	**W**	**1239.6480**	620.3277	1222.6215	611.8144	1221.6375	611.3224	**11**
**10**	**1160.5218**	580.7646	**1143.4953**	572.2513	**1142.5113**	571.7593	**N**	1053.5687	527.2880	**1036.5422**	518.7747	1035.5582	518.2827	**10**
**11**	1274.5648	637.7860	1257.5382	629.2727	1256.5542	628.7807	**N**	**938.5418**	469.7745	921.5152	461.2613	920.5312	460.7693	**9**
**12**	1361.5968	681.3020	1344.5702	672.7888	1343.5862	672.2968	**S**	**824.4989**	412.7531	807.4723	404.2398	806.4883	403.7478	**8**
**13**	**1498.6557**	749.8315	**1481.6292**	741.3182	1480.6451	740.8262	**H**	**737.4668**	369.2371	720.4403	360.7238			**7**
**14**	**1611.7398**	806.3735	1594.7132	797.8602	1593.7292	797.3682	**I**	**600.4079**	300.7076	583.3814	292.1943			**6**
**15**	1682.7769	841.8921	1665.7503	833.3788	1664.7663	832.8868	**A**	**487.3239**	244.1656	470.2973	235.6523			**5**
**16**	**1795.8609**	898.4341	**1778.8344**	889.9208	**1777.8504**	889.4288	**L**	**416.2867**	208.6470	399.2602	200.1337			**4**
**17**	**1894.9294**	947.9683	1877.9028	**939.4550**	1876.9188	**938.9630**	**V**	**303.2027**	152.1050	286.1761	143.5917			**3**
**18**	1951.9508	976.4790	1934.9243	967.9658	1933.9403	967.4738	**G**	204.1343	102.5708	187.1077	94.0575			**2**
**19**							**K**	147.1128	74.0600	130.0863	65.5468			**1**

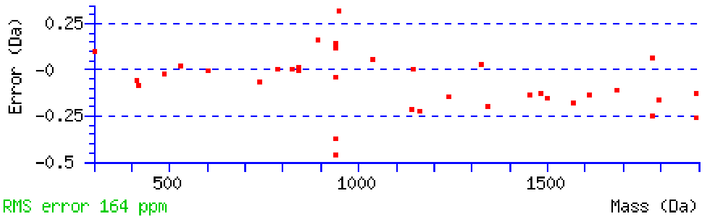

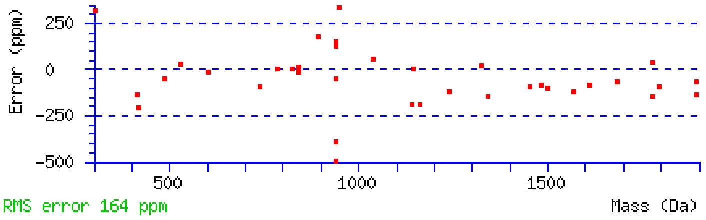

All matches to this query

Score	Mr(calc):	Delta	Sequence
87.8	2097.0491	-0.0016	TTLVDNNTWNNSHIALVGK
84.1	2097.0491	-0.0016	TTLVDNNTWNNSHIALVGK
61.5	2097.0491	-0.0016	TTLVDNNTWNNSHIALVGK
50.6	2097.0491	-0.0016	TTLVDNNTWNNSHIALVGK
12.7	2096.0651	0.9824	TTLVDNNTWNNSHIALVGK
9.8	2097.0531	-0.0056	WDNASGDWDLILLLVPDR
5.4	2097.0541	-0.0066	SDLIKTTLQCALERELK
3.5	2097.0507	-0.0032	TREAALDAQFLVLASDLGK
3.3	2097.0660	-0.0185	FNQPLDLSLFSLVGSPRK
3.3	2097.0660	-0.0185	FNQPLDLSLFSLVGSPRK

Spectrum No: 89; Query: 747; Rank: 1

Peptide View

MS/MS Fragmentation of **GQIIGISCQSVNGTAPITYR**
Found in **IPI00206467**, Tax_Id=10116 Gene_Symbol=Pecam Platelet endothelial cell adhesion molecule precursor

Match to Query 747: 2135.074768 from(1068.544660,2+)
Title: 091127RatKid_SCX01_12.2837.2837.2.dta
Data file K:\NewmanPaper\Piliang\3SubProteomes\Piliang3SP\mgf5ppm\SCX_3SubProteomes5ppm.mgf

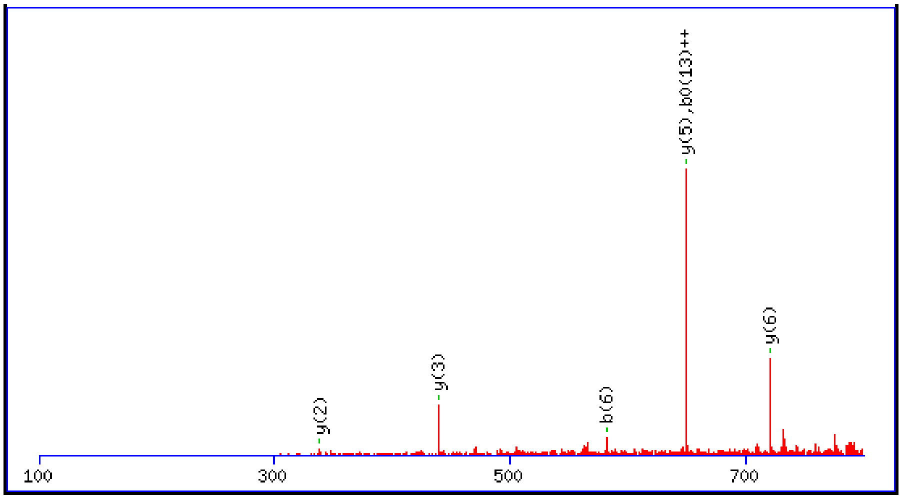

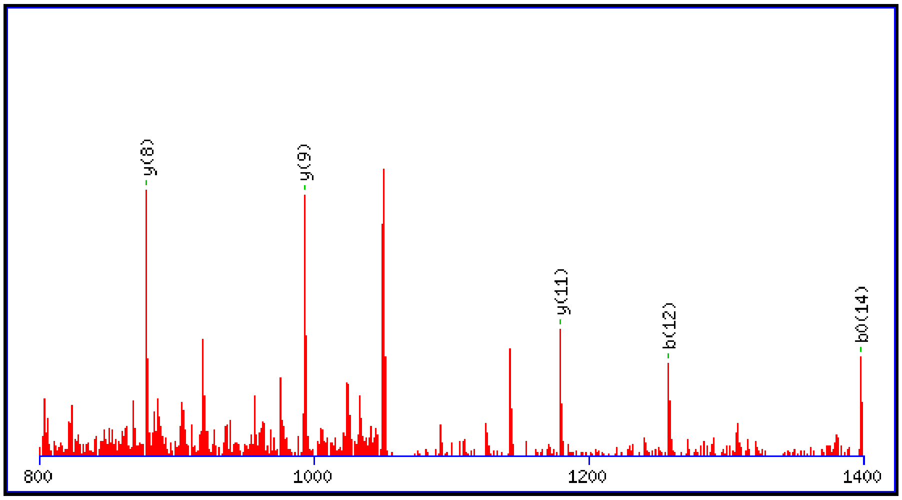

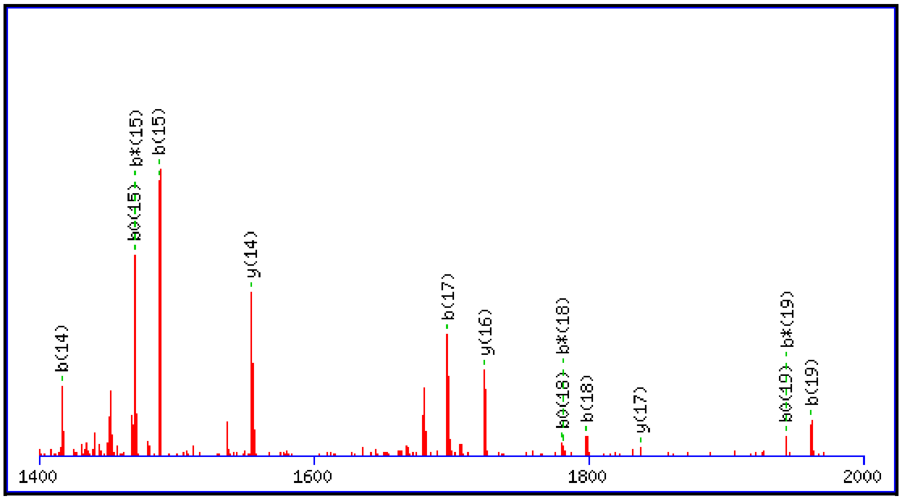

Monoisotopic mass of neutral peptide Mr(calc): 2135.0681
Fixed modifications: Carbamidomethyl (C)
Variable modifications:
N12 : Deamidated_N (N)
Ions Score: 88 **Expect:** 3.3e-007
Matches (Bold Red): 25/210 fragment ions using 29 most intense peaks

#	b	b^{++}	b*	b^{*++}	b^0	b^{0++}	Seq.	y	y^{++}	y*	y^{*++}	y^0	y^{0++}	#
1	58.0287	29.5180					G							20
2	186.0873	93.5473	169.0608	85.0340			Q	2079.0539	1040.0306	2062.0274	1031.5173	2061.0433	1031.0253	19
3	299.1714	150.0893	282.1448	141.5761			I	1950.9953	976.0013	1933.9688	967.4880	1932.9848	966.9960	18
4	412.2554	206.6314	395.2289	198.1181			I	**1837.9113**	919.4593	1820.8847	910.9460	1819.9007	910.4540	17
5	469.2769	235.1421	452.2504	226.6288			G	**1724.8272**	862.9172	1707.8007	854.4040	1706.8166	853.9120	16

**6**	**582.3610**	291.6841	565.3344	283.1709			**I**	1667.8057	834.4065	1650.7792	825.8932	1649.7952	825.4012	**15**
**7**	669.3930	335.2001	652.3665	326.6869	651.3824	326.1949	**S**	**1554.7217**	777.8645	1537.6951	769.3512	1536.7111	768.8592	**14**
**8**	829.4237	415.2155	812.3971	406.7022	811.4131	406.2102	**C**	1467.6897	734.3485	1450.6631	725.8352	1449.6791	725.3432	**13**
**9**	957.4822	479.2448	940.4557	470.7315	939.4717	470.2395	**Q**	1307.6590	654.3331	1290.6325	645.8199	1289.6484	645.3279	**12**
**10**	1044.5143	522.7608	1027.4877	514.2475	1026.5037	513.7555	**S**	**1179.6004**	590.3038	1162.5739	581.7906	1161.5899	581.2986	**11**
**11**	1143.5827	572.2950	1126.5561	563.7817	1125.5721	563.2897	**V**	1092.5684	546.7878	1075.5418	538.2746	1074.5578	537.7826	**10**
**12**	**1258.6096**	629.8084	1241.5831	621.2952	1240.5990	620.8032	**N**	**993.5000**	497.2536	976.4734	488.7404	975.4894	488.2483	**9**
**13**	1315.6311	658.3192	1298.6045	649.8059	1297.6205	**649.3139**	**G**	**878.4730**	439.7402	861.4465	431.2269	860.4625	430.7349	**8**
**14**	**1416.6788**	708.8430	1399.6522	700.3297	**1398.6682**	699.8377	**T**	821.4516	411.2294	804.4250	402.7162	803.4410	402.2241	**7**
**15**	**1487.7159**	744.3616	**1470.6893**	735.8483	**1469.7053**	735.3563	**A**	**720.4039**	360.7056	703.3774	352.1923	702.3933	351.7003	**6**
**16**	1584.7686	792.8880	1567.7421	784.3747	1566.7581	783.8827	**P**	**649.3668**	325.1870	632.3402	316.6738	631.3562	316.1817	**5**
**17**	**1697.8527**	849.4300	1680.8261	840.9167	1679.8421	840.4247	**I**	552.3140	276.6607	535.2875	268.1474	534.3035	267.6554	**4**
**18**	**1798.9004**	899.9538	**1781.8738**	891.4405	**1780.8898**	890.9485	**T**	**439.2300**	220.1186	422.2034	211.6053	421.2194	211.1133	**3**
**19**	**1961.9637**	981.4855	**1944.9372**	972.9722	**1943.9531**	972.4802	**Y**	**338.1823**	169.5948	321.1557	161.0815			**2**
**20**							**R**	175.1190	88.0631	158.0924	79.5498			**1**

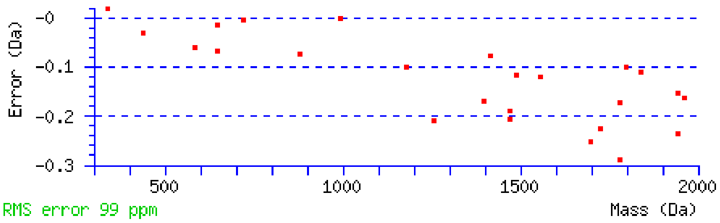

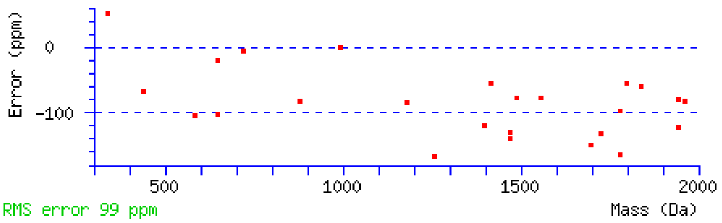

All matches to this query

Score	Mr(calc):	Delta	Sequence
87.8	2135.0681	0.0067	GQIIGISCQSVNGTAPITYR
11.7	2134.0841	0.9907	GQIIGISCQSVNGTAPITYR
6.0	2135.0535	0.0213	LNKSYIFIYDGNKNSTTR
6.0	2135.0535	0.0213	LNKSYIFIYDGNKNSTTR
4.4	2135.0535	0.0213	FPLEGASDADIGENAVLVYR
0.9	2135.0697	0.0051	LEPVLPSVAALSSAGTMERK
0.9	2135.0697	0.0051	LEPVLPSVAALSSAGTMERK

Spectrum No: 90; Query: 1040; Rank: 1

Peptide View

MS/MS Fragmentation of **HLNSSLPRPQPAILLLTAAQDAAEVLK**
Found in **IPI00203610**, Tax_Id=10116 Gene_Symbol=Tor1aip1 59 kDa protein

Match to Query 1040: 2866.597902 from(956.539910,3+)
Title: 091129RatKid_SCX02_30.7553.7553.3.dta
Data file K:\NewmanPaper\Piliang\3SubProteomes\Piliang3SP\mgf5ppm\SCX_3SubProteomes5ppm.mgf

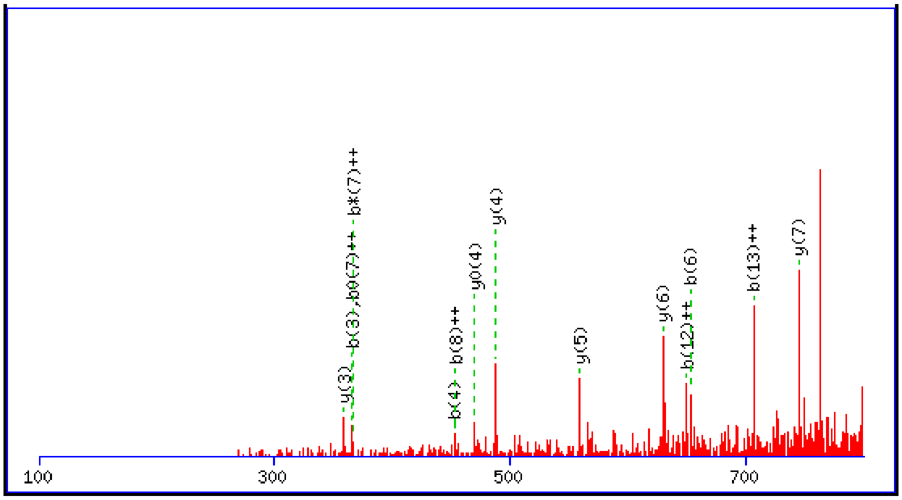

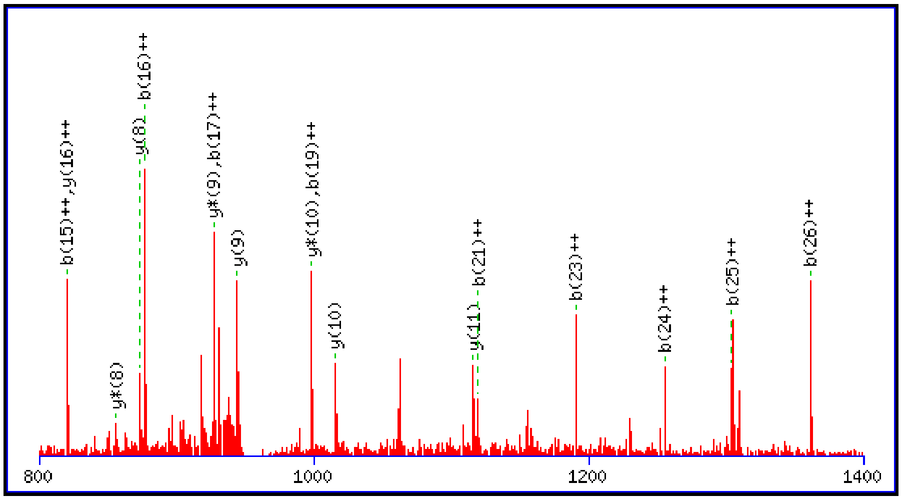

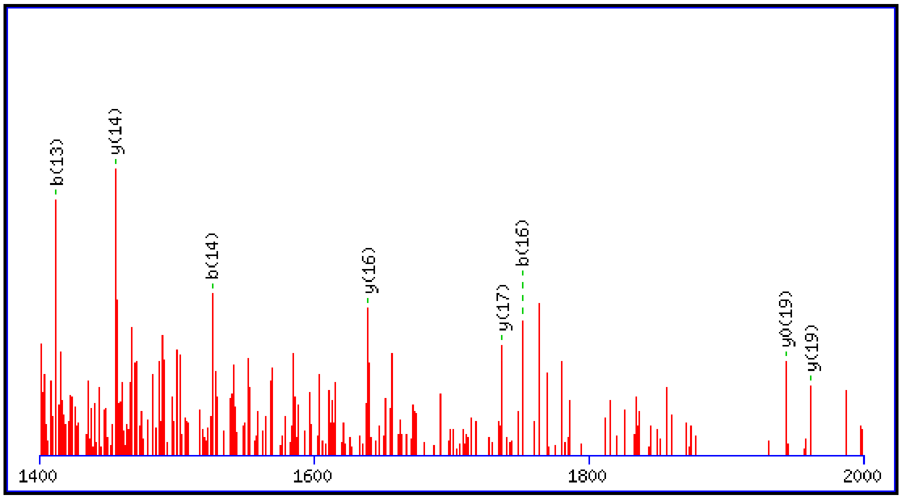

Monoisotopic mass of neutral peptide Mr(calc): 2866.5916
Fixed modifications: Carbamidomethyl (C)
Variable modifications:
N3 : Deamidated_N (N)
Ions Score: 88 **Expect:** 4.1e-008
Matches (Bold Red): 39/296 fragment ions using 60 most intense peaks

#	b	b++	b*	b*++	b0	b0++	Seq.	y	y++	y*	y*++	y0	y0++	#
1	138.0662	69.5367					H							27
2	251.1503	126.0788					L	2730.5400	1365.7736	2713.5135	1357.2604	2712.5294	1356.7684	26
3	**366.1772**	183.5922	349.1506	175.0790			N	2617.4559	1309.2316	2600.4294	1300.7183	2599.4454	1300.2263	25
4	**453.2092**	227.1082	436.1827	218.5950	435.1987	218.1030	S	2502.4290	1251.7181	2485.4025	1243.2049	2484.4184	1242.7129	24
5	540.2412	270.6243	523.2147	262.1110	522.2307	261.6190	S	2415.3970	1208.2021	2398.3704	1199.6889	2397.3864	1199.1968	23

6	**653.3253**	327.1663	636.2988	318.6530	635.3147	318.1610	L	2328.3649	1164.6861	2311.3384	1156.1728	2310.3544	1155.6808	22
7	750.3781	375.6927	733.3515	**367.1794**	732.3675	**366.6874**	P	2215.2809	1108.1441	2198.2543	1099.6308	2197.2703	1099.1388	21
8	906.4792	**453.7432**	889.4526	445.2300	888.4686	444.7379	R	2118.2281	1059.6177	2101.2016	1051.1044	2100.2176	1050.6124	20
9	1003.5319	502.2696	986.5054	493.7563	985.5214	493.2643	P	**1962.1270**	981.5671	1945.1005	973.0539	**1944.1164**	972.5619	19
10	1131.5905	566.2989	1114.5640	557.7856	1113.5800	557.2936	Q	1865.0742	933.0408	1848.0477	924.5275	1847.0637	924.0355	18
11	1228.6433	614.8253	1211.6167	606.3120	1210.6327	605.8200	P	**1737.0157**	869.0115	1719.9891	860.4982	1719.0051	860.0062	17
12	1299.6804	**650.3438**	1282.6539	641.8306	1281.6698	641.3386	A	**1639.9629**	**820.4851**	1622.9364	811.9718	1621.9523	811.4798	16
13	**1412.7645**	**706.8859**	1395.7379	698.3726	1394.7539	697.8806	I	1568.9258	784.9665	1551.8992	776.4533	1550.9152	775.9612	15
14	**1525.8485**	763.4279	1508.8220	754.9146	1507.8380	754.4226	L	**1455.8417**	728.4245	1438.8152	719.9112	1437.8312	719.4192	14
15	1638.9326	**819.9699**	1621.9060	811.4567	1620.9220	810.9647	L	1342.7577	671.8825	1325.7311	663.3692	1324.7471	662.8772	13
16	**1752.0167**	**876.5120**	1734.9901	867.9987	1734.0061	867.5067	L	1229.6736	615.3404	1212.6470	606.8272	1211.6630	606.3352	12
17	1853.0643	**927.0358**	1836.0378	918.5225	1835.0538	918.0305	T	**1116.5895**	558.7984	1099.5630	550.2851	1098.5790	549.7931	11
18	1924.1015	962.5544	1907.0749	954.0411	1906.0909	953.5491	A	**1015.5419**	508.2746	**998.5153**	499.7613	997.5313	499.2693	10
19	1995.1386	**998.0729**	1978.1120	989.5596	1977.1280	989.0676	A	**944.5047**	472.7560	**927.4782**	464.2427	926.4942	463.7507	9
20	2123.1971	1062.1022	2106.1706	1053.5889	2105.1866	1053.0969	Q	**873.4676**	437.2375	**856.4411**	428.7242	855.4571	428.2322	8
21	2238.2241	**1119.6157**	2221.1975	1111.1024	2220.2135	1110.6104	D	**745.4090**	373.2082	728.3825	364.6949	727.3985	364.2029	7
22	2309.2612	1155.1342	2292.2347	1146.6210	2291.2506	1146.1290	A	**630.3821**	315.6947	613.3556	307.1814	612.3715	306.6894	6
23	2380.2983	**1190.6528**	2363.2718	1182.1395	2362.2878	1181.6475	A	**559.3450**	280.1761	542.3184	271.6629	541.3344	271.1709	5
24	2509.3409	**1255.1741**	2492.3144	1246.6608	2491.3303	1246.1688	E	**488.3079**	244.6576	471.2813	236.1443	**470.2973**	235.6523	4
25	2608.4093	**1304.7083**	2591.3828	1296.1950	2590.3988	1295.7030	V	**359.2653**	180.1363	342.2387	171.6230			3
26	2721.4934	**1361.2503**	2704.4668	1352.7371	2703.4828	1352.2450	L	260.1969	130.6021	243.1703	122.0888			2
27							K	147.1128	74.0600	130.0863	65.5468			1

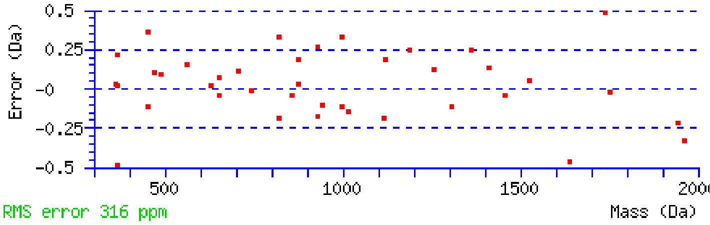

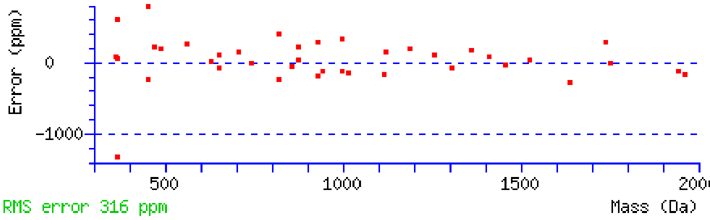

All matches to this query

Score	Mr(calc):	Delta	Sequence
87.5	2866.5916	0.0063	HLNSSLPRPQPAILLLTAAQDAAEVLK
43.3	2865.6076	0.9903	HLNSSLPRPQPAILLLTAAQDAAEVLK
1.9	2866.5853	0.0126	MAPVCLFAPLLLLLLGGFPVAPGESIR

Spectrum No: 91; Query: 1153; Rank: 1

Peptide View

MS/MS Fragmentation of **LADDVDLEQVANETHGHVGADLAALCSEAALQAIR**
Found in **IPI00212014**, Tax_Id=10116 Gene_Symbol=Vcp Transitional endoplasmic reticulum ATPase

Match to Query 1153: 3672.775632 from(1225.265820,3+)
Title: 091127RatKid_SCX01_29.5695.5695.3.dta
Data file K:\NewmanPaper\Piliang\3SubProteomes\Piliang3SP\mgf5ppm\SCX_3SubProteomes5ppm.mgf

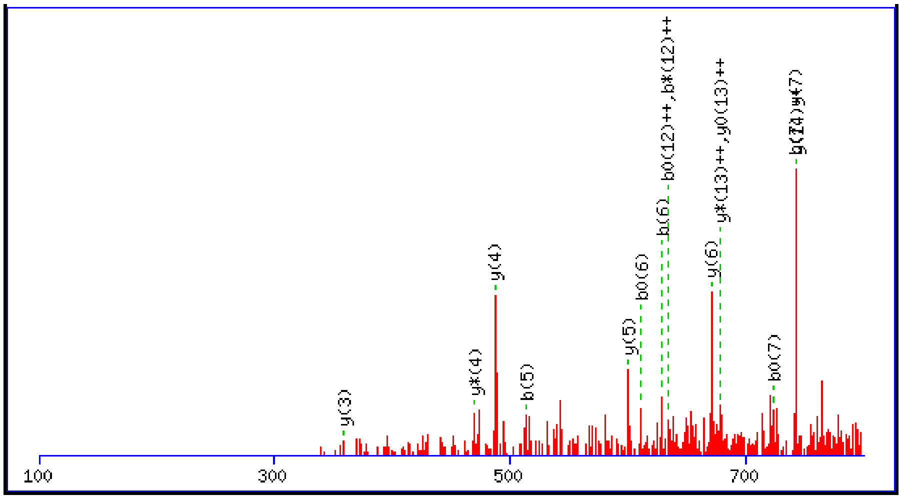

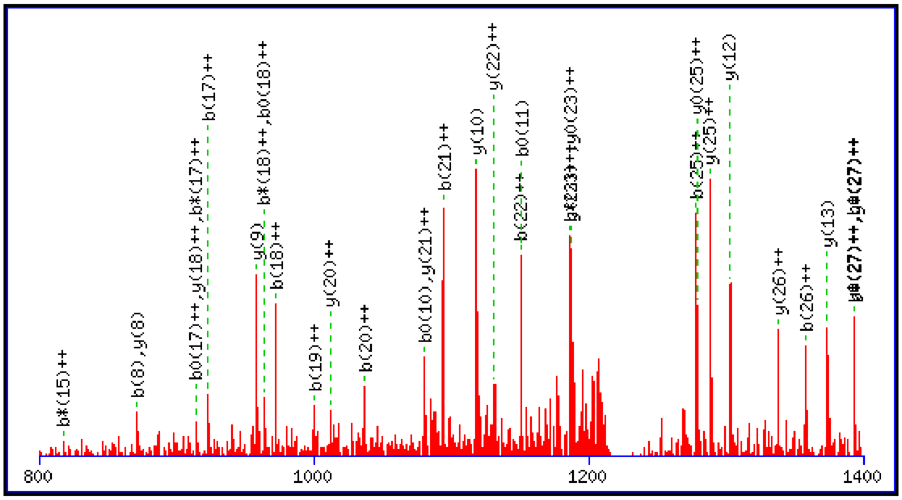

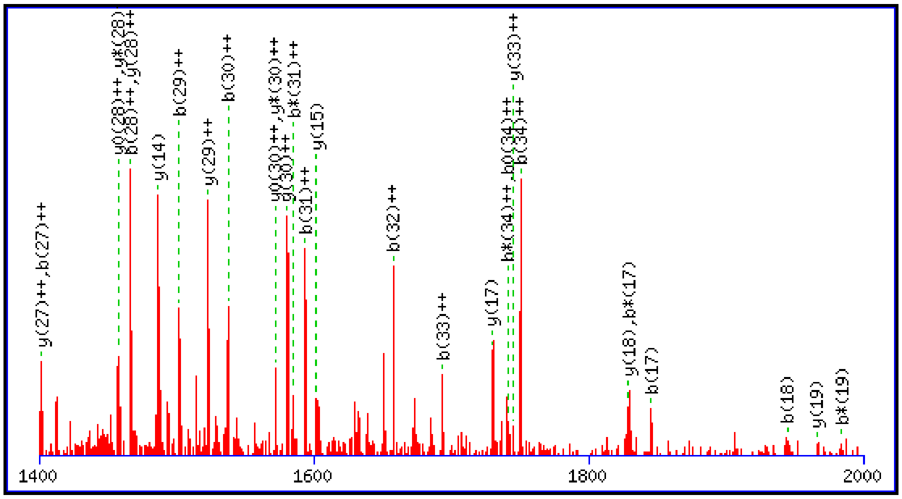

Monoisotopic mass of neutral peptide Mr(calc): 3672.7689
Fixed modifications: Carbamidomethyl (C)
Variable modifications:
N12 : Deamidated_N (N)
Ions Score: 87 **Expect:** 5.8e-007
Matches (Bold Red): 81/374 fragment ions using 138 most intense peaks

#	b	b^{++}	b*	b^{*++}	b^0	b^{0++}	Seq.	y	y^{++}	y*	y^{*++}	y^0	y^{0++}	#
1	114.0913	57.5493					L							35
2	185.1285	93.0679					A	3560.6922	1780.8497	3543.6656	1772.3365	3542.6816	1771.8444	34
3	300.1554	150.5813			282.1448	141.5761	D	3489.6551	**1745.3312**	3472.6285	1736.8179	3471.6445	1736.3259	33
4	415.1823	208.0948			397.1718	199.0895	D	3374.6281	1687.8177	3357.6016	1679.3044	3356.6176	1678.8124	32
5	**514.2508**	257.6290			496.2402	248.6237	V	3259.6012	1630.3042	3242.5746	1621.7910	3241.5906	1621.2989	31

6	**629.2777**	315.1425			**611.2671**	306.1372	D	3160.5328	**1580.7700**	3143.5062	**1572.2567**	3142.5222	**1571.7647**	30
7	**742.3618**	371.6845			**724.3512**	362.6792	L	3045.5058	**1523.2565**	3028.4793	1514.7433	3027.4953	1514.2513	29
8	**871.4044**	436.2058			853.3938	427.2005	E	2932.4218	**1466.7145**	2915.3952	**1458.2012**	2914.4112	**1457.7092**	28
9	999.4629	500.2351	982.4364	491.7218	981.4524	491.2298	Q	2803.3792	**1402.1932**	2786.3526	**1393.6799**	2785.3686	**1393.1879**	27
10	1098.5313	549.7693	1081.5048	541.2560	**1080.5208**	540.7640	V	2675.3206	**1338.1639**	2658.2940	1329.6507	2657.3100	1329.1586	26
11	1169.5685	585.2879	1152.5419	576.7746	**1151.5579**	576.2826	A	2576.2522	**1288.6297**	2559.2256	1280.1164	2558.2416	**1279.6244**	25
12	1284.5954	642.8013	1267.5688	**634.2881**	1266.5848	**633.7961**	N	2505.2151	1253.1112	2488.1885	1244.5979	2487.2045	1244.1059	24
13	1413.6380	707.3226	1396.6114	698.8094	1395.6274	698.3174	E	2390.1881	1195.5977	2373.1616	**1187.0844**	2372.1776	**1186.5924**	23
14	1514.6857	757.8465	1497.6591	749.3332	1496.6751	748.8412	T	2261.1455	**1131.0764**	2244.1190	1122.5631	2243.1350	1122.0711	22
15	1651.7446	826.3759	1634.7180	**817.8627**	1633.7340	817.3706	H	2160.0978	**1080.5526**	2143.0713	1072.0393	2142.0873	1071.5473	21
16	1708.7660	854.8867	1691.7395	846.3734	1690.7555	845.8814	G	2023.0389	**1012.0231**	2006.0124	1003.5098	2005.0284	1003.0178	20
17	**1845.8250**	**923.4161**	**1828.7984**	**914.9028**	1827.8144	**914.4108**	H	**1966.0175**	983.5124	1948.9909	974.9991	1948.0069	974.5071	19
18	**1944.8934**	**972.9503**	1927.8668	**964.4370**	1926.8828	**963.9450**	V	**1828.9586**	**914.9829**	1811.9320	906.4696	1810.9480	905.9776	18
19	2001.9148	**1001.4611**	**1984.8883**	992.9478	1983.9043	992.4558	G	**1729.8901**	865.4487	1712.8636	856.9354	1711.8796	856.4434	17
20	2072.9519	**1036.9796**	2055.9254	1028.4663	2054.9414	1027.9743	A	1672.8687	836.9380	1655.8421	828.4247	1654.8581	827.9327	16
21	2187.9789	**1094.4931**	2170.9523	1085.9798	2169.9683	1085.4878	D	**1601.8316**	801.4194	1584.8050	792.9061	1583.8210	792.4141	15
22	2301.0630	**1151.0351**	2284.0364	1142.5218	2283.0524	1142.0298	L	**1486.8046**	**743.9060**	1469.7781	735.3927	1468.7941	734.9007	14
23	2372.1001	**1186.5537**	2355.0735	1178.0404	2354.0895	1177.5484	A	**1373.7206**	687.3639	1356.6940	**678.8506**	1355.7100	**678.3586**	13
24	2443.1372	1222.0722	2426.1106	1213.5590	2425.1266	1213.0669	A	**1302.6834**	651.8454	1285.6569	643.3321	1284.6729	642.8401	12
25	2556.2212	**1278.6143**	2539.1947	1270.1010	2538.2107	1269.6090	L	1231.6463	616.3268	1214.6198	607.8135	1213.6358	607.3215	11
26	2716.2519	**1358.6296**	2699.2253	1350.1163	2698.2413	1349.6243	C	**1118.5623**	559.7848	1101.5357	551.2715	1100.5517	550.7795	10
27	2803.2839	**1402.1456**	2786.2574	**1393.6323**	2785.2734	**1393.1403**	S	**958.5316**	479.7694	941.5051	471.2562	940.5211	470.7642	9
28	2932.3265	**1466.6669**	2915.3000	**1458.1536**	2914.3160	1457.6616	E	**871.4996**	436.2534	854.4730	427.7402	853.4890	427.2482	8
29	3003.3636	**1502.1855**	2986.3371	1493.6722	2985.3531	1493.1802	A	**742.4570**	371.7321	725.4305	363.2189			7
30	3074.4007	**1537.7040**	3057.3742	1529.1907	3056.3902	1528.6987	A	**671.4199**	336.2136	654.3933	327.7003			6
31	3187.4848	**1594.2460**	3170.4583	**1585.7328**	3169.4742	1585.2408	L	**600.3828**	300.6950	583.3562	292.1817			5
32	3315.5434	**1658.2753**	3298.5168	1649.7621	3297.5328	1649.2700	Q	**487.2987**	244.1530	**470.2722**	235.6397			4
33	3386.5805	**1693.7939**	3369.5540	1685.2806	3368.5699	1684.7886	A	**359.2401**	180.1237	342.2136	171.6104			3
34	3499.6646	**1750.3359**	3482.6380	**1741.8226**	3481.6540	**1741.3306**	I	288.2030	144.6051	271.1765	136.0919			2
35							R	175.1190	88.0631	158.0924	79.5498			1

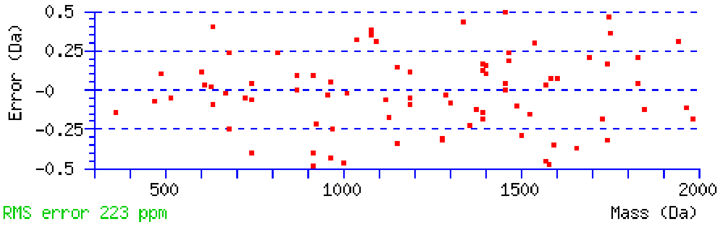

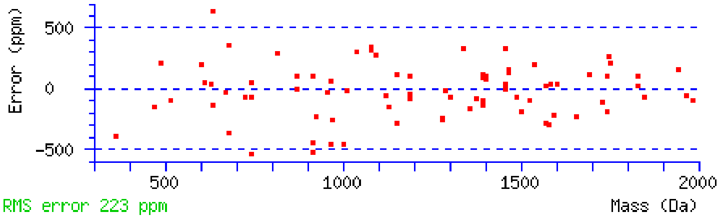

All matches to this query

Score	Mr(calc):	Delta	Sequence
87.3	3672.7689	0.0067	LADDVDLEQVANETHGHVGADLAALCSEAALQAIR
63.2	3671.7849	0.9907	LADDVDLEQVANETHGHVGADLAALCSEAALQAIR
9.3	3671.7410	1.0346	GATWVALHNGGGVGWGEVINGGFGLVLDGTPEAEQK
4.3	3671.7758	0.9998	MLLASYSLIVRPATPAPAPQAPATPGAQLAHDMR
2.9	3671.7678	1.0079	SLQAFSNPLAGISVDVKVFIVPVGNHSNIPFSR
2.7	3671.7735	1.0021	FLQALEFLVGSLEQASLPCHFNPSVNLLGSFR
2.5	3672.7468	0.0288	ASSRPCATSVTIVWTRLGSSGSQVSNPGSRPYAR
2.4	3672.7467	0.0289	ALGLPATPSLVGVGPSGKGPAAARDPAPDAAASLPAGR
1.7	3671.7943	0.9814	VGFKIFFFAALNHGVTATFPSTTPISISGATRR
1.2	3671.7753	1.0003	TIIMIVIGSGILAAMFLLTGVVLCLYSKIAR

Spectrum No: 92; Query: 434; Rank: 1

Peptide View

MS/MS Fragmentation of **DLNISLDTDAPLSVGK**
Found in **IPI00326990**, Tax_Id=10116 Gene_Symbol=Slc15a2 Oligopeptide transporter, kidney isoform

Match to Query 434: 1657.845288 from(829.929920,2+)
Title: 091127RatKid_SCX01_02.3717.3717.2.dta
Data file K:\NewmanPaper\Piliang\3SubProteomes\Piliang3SP\mgf5ppm\SCX_3SubProteomes5ppm.mgf

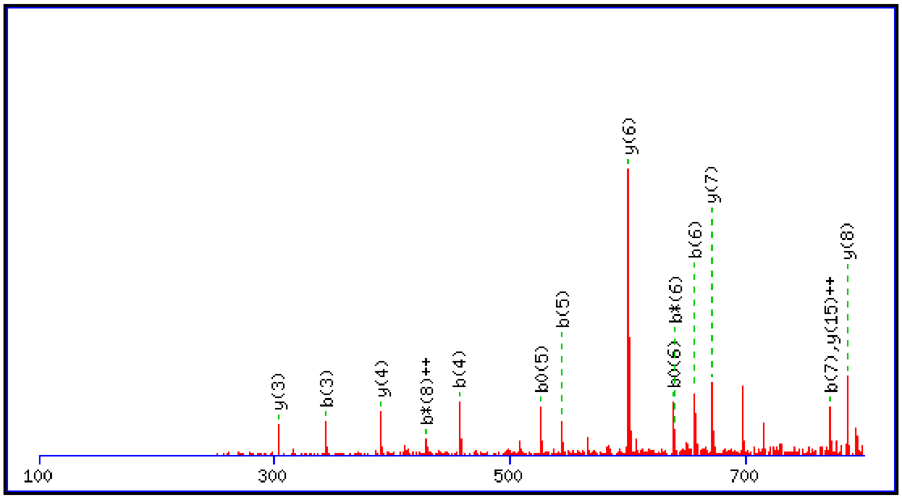

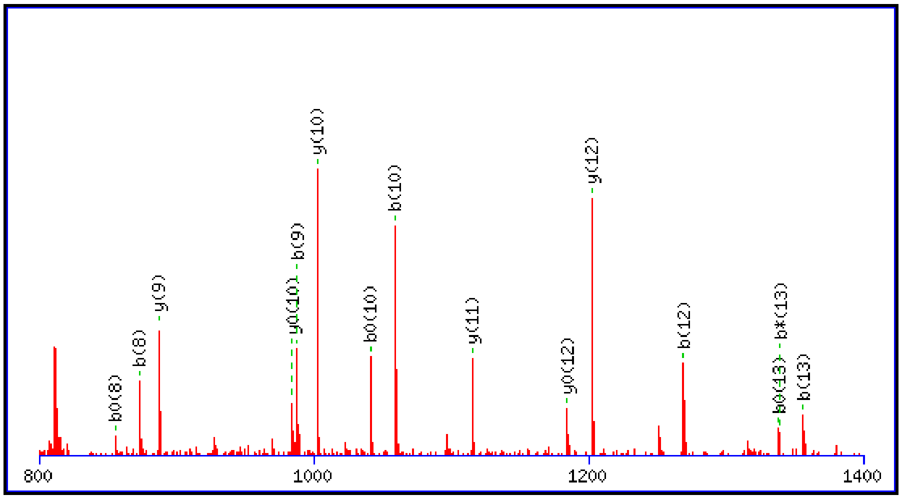

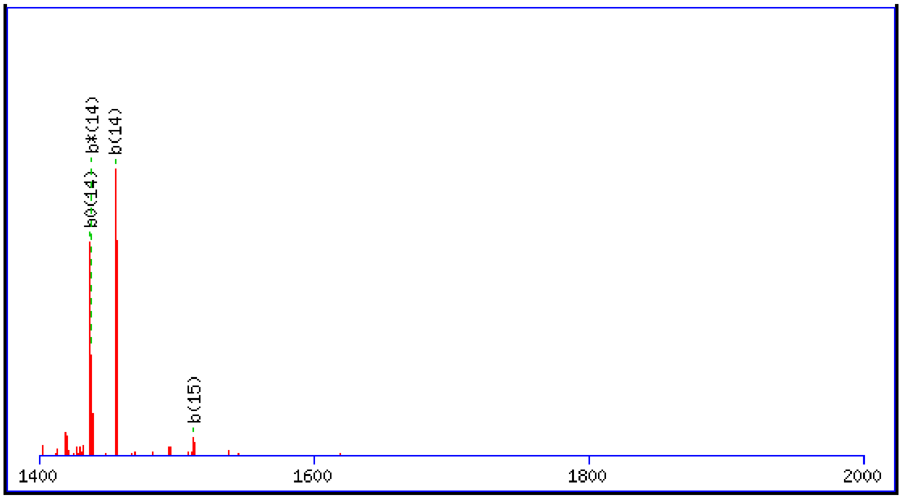

Monoisotopic mass of neutral peptide Mr(calc): 1657.8410
Fixed modifications: Carbamidomethyl (C)
Variable modifications:
N3 : Deamidated_N (N)
Ions Score: 87 **Expect:** 2.7e-007
Matches (Bold Red): 34/170 fragment ions using 58 most intense peaks

#	b	b^{++}	b*	b^{*++}	b^0	b^{0++}	Seq.	y	y^{++}	y*	y^{*++}	y^0	y^{0++}	#
1	116.0342	58.5207			98.0237	49.5155	D							16
2	229.1183	115.0628			211.1077	106.0575	L	1543.8214	**772.4143**	1526.7948	763.9011	1525.8108	763.4090	15
3	**344.1452**	172.5762	327.1187	164.0630	326.1347	163.5710	N	1430.7373	715.8723	1413.7108	707.3590	1412.7267	706.8670	14
4	**457.2293**	229.1183	440.2027	220.6050	439.2187	220.1130	I	1315.7104	658.3588	1298.6838	649.8456	1297.6998	649.3535	13
5	**544.2613**	272.6343	527.2348	264.1210	**526.2507**	263.6290	S	**1202.6263**	601.8168	1185.5998	593.3035	**1184.6157**	592.8115	12
6	**657.3454**	329.1763	**640.3188**	320.6631	**639.3348**	320.1710	L	**1115.5943**	558.3008	1098.5677	549.7875	1097.5837	549.2955	11
7	**772.3723**	386.6898	755.3458	378.1765	754.3618	377.6845	D	**1002.5102**	501.7587	985.4837	493.2455	**984.4997**	492.7535	10
8	**873.4200**	437.2136	856.3934	**428.7004**	**855.4094**	428.2084	T	**887.4833**	444.2453	870.4567	435.7320	869.4727	435.2400	9
9	**988.4469**	494.7271	971.4204	486.2138	970.4364	485.7218	D	**786.4356**	393.7214	769.4090	385.2082	768.4250	384.7162	8
10	**1059.4841**	530.2457	1042.4575	521.7324	**1041.4735**	521.2404	A	**671.4087**	336.2080	654.3821	327.6947	653.3981	327.2027	7
11	1156.5368	578.7720	1139.5103	570.2588	1138.5263	569.7668	P	**600.3715**	300.6894	583.3450	292.1761	582.3610	291.6841	6
12	**1269.6209**	635.3141	1252.5943	626.8008	1251.6103	626.3088	L	503.3188	252.1630	486.2922	243.6498	485.3082	243.1577	5
13	**1356.6529**	678.8301	**1339.6264**	670.3168	**1338.6423**	669.8248	S	**390.2347**	195.6210	373.2082	187.1077	372.2241	186.6157	4
14	**1455.7213**	728.3643	**1438.6948**	719.8510	**1437.7108**	719.3590	V	**303.2027**	152.1050	286.1761	143.5917			3
15	**1512.7428**	756.8750	1495.7162	748.3618	1494.7322	747.8697	G	204.1343	102.5708	187.1077	94.0575			2
16							K	147.1128	74.0600	130.0863	65.5468			1

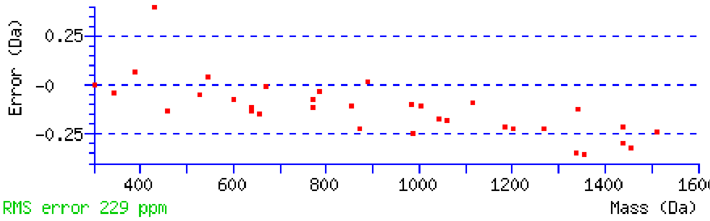

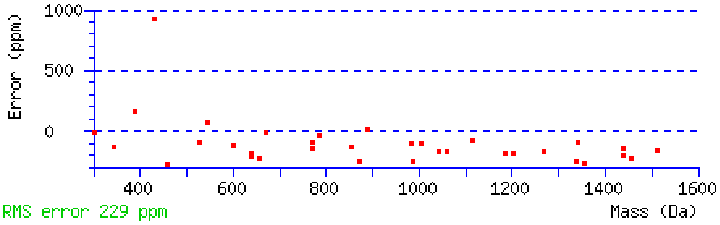

All matches to this query

Score	Mr(calc):	Delta	Sequence
87.2	1657.8410	0.0043	DLNISLDTDAPLSVGK
39.9	1656.8570	0.9883	DLNISLDTDAPLSVGK
11.9	1657.8383	0.0070	KASESPGGAASPAGTSKR
8.2	1656.8488	0.9965	GGGLFSVGETTAKILK
4.4	1657.8385	0.0068	NAPFLLNDPVLGGMAK

4.2	1657.8522	-0.0069	NILLTNEQLENARK
0.8	1657.8497	-0.0045	MELSLRNIYGYVGK
0.4	1657.8522	-0.0069	ASLLKSQEPISNDQK
0.3	1655.8478	1.9975	SSHETLNVVEEKKR

Spectrum No: 93; Query: 1103; Rank: 1

Peptide View

MS/MS Fragmentation of **TSLDWWTTDTCNMINGTDGDSFHPLISK**
Found in **IPI00231478**, Tax_Id=10116 Gene_Symbol=Scarb2 Lysosome membrane protein 2

Match to Query 1103: 3212.417892 from(1071.813240,3+)
Title: 091127RatKid_SCX01_21.6597.6597.3.dta
Data file K:\NewmanPaper\Piliang\3SubProteomes\Piliang3SP\mgf5ppm\SCX_3SubProteomes5ppm.mgf

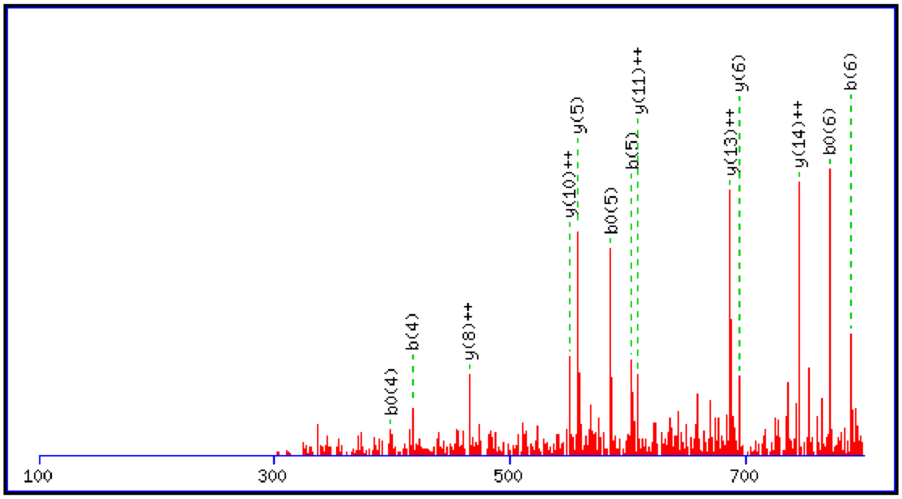

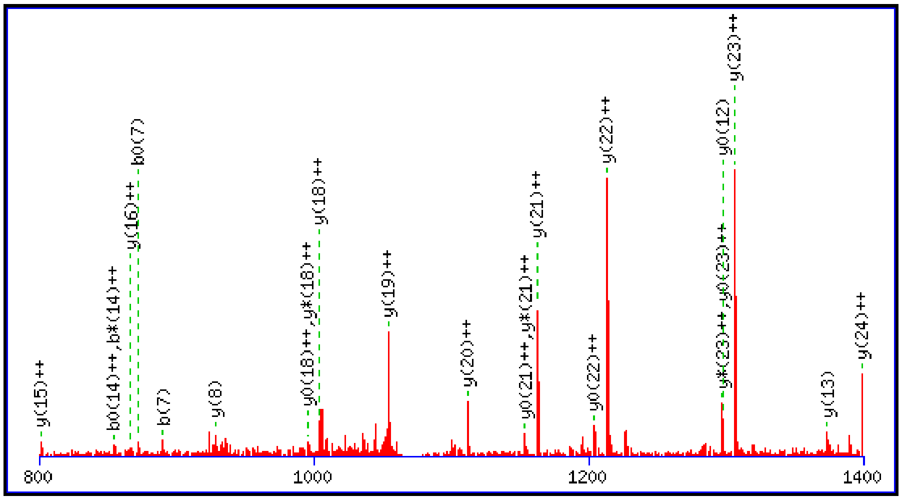

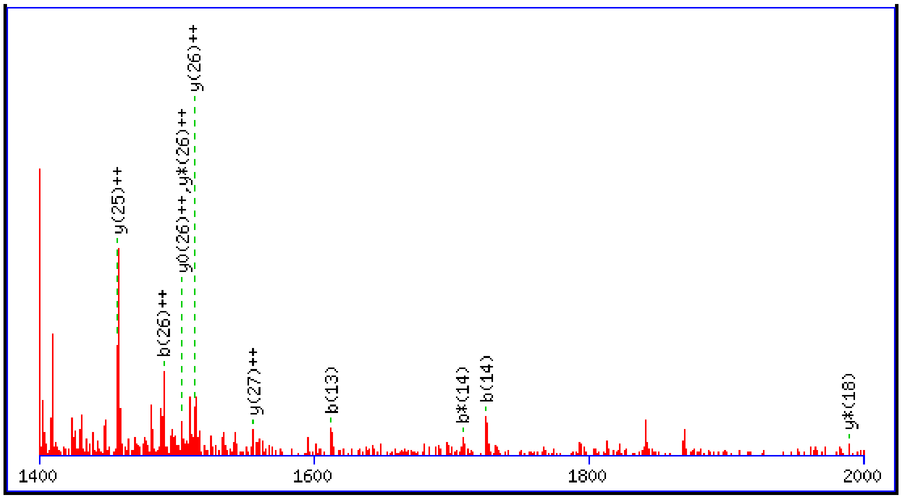

Monoisotopic mass of neutral peptide Mr(calc): 3212.4067
Fixed modifications: Carbamidomethyl (C)
Variable modifications:
N15 : Deamidated_N (N)
Ions Score: 87 **Expect:** 8.2e-007
Matches (Bold Red): 46/300 fragment ions using 92 most intense peaks

#	b	b^{++}	b*	b^{*++}	b^{0}	b^{0++}	Seq.	y	y^{++}	y*	y^{*++}	y^{0}	y^{0++}	#
1	102.0550	51.5311			84.0444	42.5258	T							28
2	189.0870	95.0471			171.0764	86.0418	S	3112.3663	**1556.6868**	3095.3397	1548.1735	3094.3557	1547.6815	27
3	302.1710	151.5892			284.1605	142.5839	L	3025.3342	**1513.1708**	3008.3077	**1504.6575**	3007.3237	**1504.1655**	26
4	**417.1980**	209.1026			**399.1874**	200.0974	D	2912.2502	**1456.6287**	2895.2236	1448.1155	2894.2396	1447.6234	25
5	**603.2773**	302.1423			**585.2667**	293.1370	W	2797.2232	**1399.1153**	2780.1967	1390.6020	2779.2127	1390.1100	24
6	**789.3566**	395.1819			**771.3461**	386.1767	W	2611.1439	**1306.0756**	2594.1174	**1297.5623**	2593.1334	**1297.0703**	23
7	**890.4043**	445.7058			**872.3937**	436.7005	T	2425.0646	**1213.0359**	2408.0381	1204.5227	2407.0540	**1204.0307**	22
8	991.4520	496.2296			973.4414	487.2243	T	2324.0169	**1162.5121**	2306.9904	**1153.9988**	2306.0064	**1153.5068**	21
9	1106.4789	553.7431			1088.4684	544.7378	D	2222.9693	**1111.9883**	2205.9427	1103.4750	2204.9587	1102.9830	20
10	1207.5266	604.2669			1189.5160	595.2617	T	2107.9423	**1054.4748**	2090.9158	1045.9615	2089.9317	1045.4695	19
11	1367.5572	684.2823			1349.5467	675.2770	C	2006.8946	**1003.9510**	**1989.8681**	**995.4377**	1988.8841	**994.9457**	18
12	1481.6002	741.3037	1464.5736	732.7904	1463.5896	732.2984	N	1846.8640	923.9356	1829.8374	915.4224	1828.8534	914.9303	17
13	**1612.6407**	806.8240	1595.6141	798.3107	1594.6301	797.8187	M	1732.8211	**866.9142**	1715.7945	858.4009	1714.8105	857.9089	16
14	**1725.7247**	863.3660	**1708.6982**	**854.8527**	1707.7142	**854.3607**	I	1601.7806	**801.3939**	1584.7540	792.8806	1583.7700	792.3886	15
15	1840.7517	920.8795	1823.7251	912.3662	1822.7411	911.8742	N	1488.6965	**744.8519**	1471.6700	736.3386	1470.6859	735.8466	14
16	1897.7731	949.3902	1880.7466	940.8769	1879.7626	940.3849	G	**1373.6696**	**687.3384**	1356.6430	678.8251	1355.6590	678.3331	13
17	1998.8208	999.9140	1981.7943	991.4008	1980.8102	990.9088	T	1316.6481	658.8277	1299.6216	650.3144	**1298.6375**	649.8224	12
18	2113.8477	1057.4275	2096.8212	1048.9142	2095.8372	1048.4222	D	1215.6004	**608.3039**	1198.5739	599.7906	1197.5899	599.2986	11
19	2170.8692	1085.9382	2153.8427	1077.4250	2152.8586	1076.9330	G	1100.5735	**550.7904**	1083.5469	542.2771	1082.5629	541.7851	10
20	2285.8961	1143.4517	2268.8696	1134.9384	2267.8856	1134.4464	D	1043.5520	522.2796	1026.5255	513.7664	1025.5415	513.2744	9
21	2372.9282	1186.9677	2355.9016	1178.4545	2354.9176	1177.9624	S	**928.5251**	**464.7662**	911.4985	456.2529	910.5145	455.7609	8
22	2519.9966	1260.5019	2502.9700	1251.9887	2501.9860	1251.4967	F	841.4931	421.2502	824.4665	412.7369	823.4825	412.2449	7
23	2657.0555	1329.0314	2640.0290	1320.5181	2639.0449	1320.0261	H	**694.4246**	347.7160	677.3981	339.2027	676.4141	338.7107	6
24	2754.1083	1377.5578	2737.0817	1369.0445	2736.0977	1368.5525	P	**557.3657**	279.1865	540.3392	270.6732	539.3552	270.1812	5
25	2867.1923	1434.0998	2850.1658	1425.5865	2849.1818	1425.0945	L	460.3130	230.6601	443.2864	222.1468	442.3024	221.6548	4
26	2980.2764	**1490.6418**	2963.2498	1482.1286	2962.2658	1481.6366	I	347.2289	174.1181	330.2023	165.6048	329.2183	165.1128	3
27	3067.3084	1534.1578	3050.2819	1525.6446	3049.2979	1525.1526	S	234.1448	117.5761	217.1183	109.0628	216.1343	108.5708	2
28							K	147.1128	74.0600	130.0863	65.5468			1

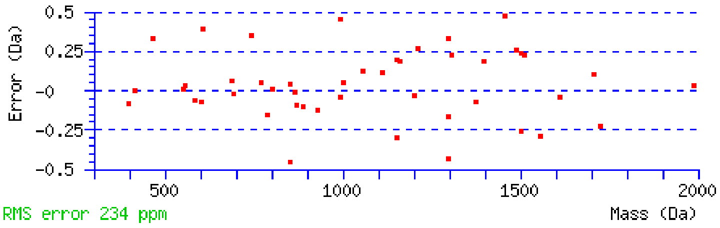

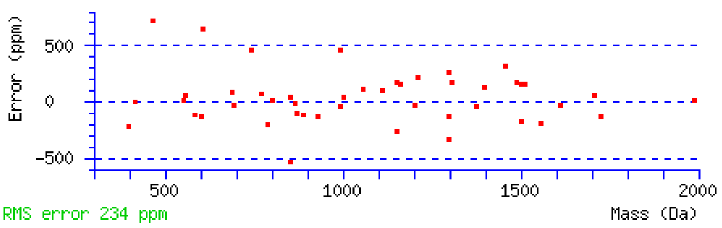

All matches to this query

Score	Mr(calc):	Delta	Sequence
87.2	3212.4067	0.0112	TSLDWWTTDTCNMINGTDGDSFHPLISK
76.8	3212.4067	0.0112	TSLDWWTTDTCNMINGTDGDSFHPLISK
21.9	3211.4227	0.9952	TSLDWWTTDTCNMINGTDGDSFHPLISK
4.2	3210.4127	2.0052	VDIWSAGVTLYNITTGLYPFEGDNIYK
4.2	3210.4127	2.0052	VDIWSAGVTLYNITTGLYPFEGDNIYK
4.1	3210.4358	1.9821	TMSAESVHYNALLDLINREQNRWYK
4.1	3210.4358	1.9821	TMSAESVHYNALLDLINREQNRWYK
3.7	3211.4411	0.9768	DGHCSPPAAGDPGPGAPAGPEAAAACNVIVNGTR
3.7	3211.4411	0.9768	DGHCSPPAAGDPGPGAPAGPEAAAACNVIVNGTR
3.7	3212.4251	-0.0072	DGHCSPPAAGDPGPGAPAGPEAAAACNVIVNGTR

Spectrum No: 94; Query: 269; Rank: 1

Peptide View

MS/MS Fragmentation of **VGPNGTLFVVDAYK**
Found in **IPI00382223**, Tax_Id=10116 Gene_Symbol=RGD1308874 Ab2-305

Match to Query 269: 1479.765268 from(740.889910,2+)
Title: 091129RatKid_SCX02_17.2916.2916.2.dta
Data file K:\NewmanPaper\Piliang\3SubProteomes\Piliang3SP\mgf5ppm\SCX_3SubProteomes5ppm.mgf

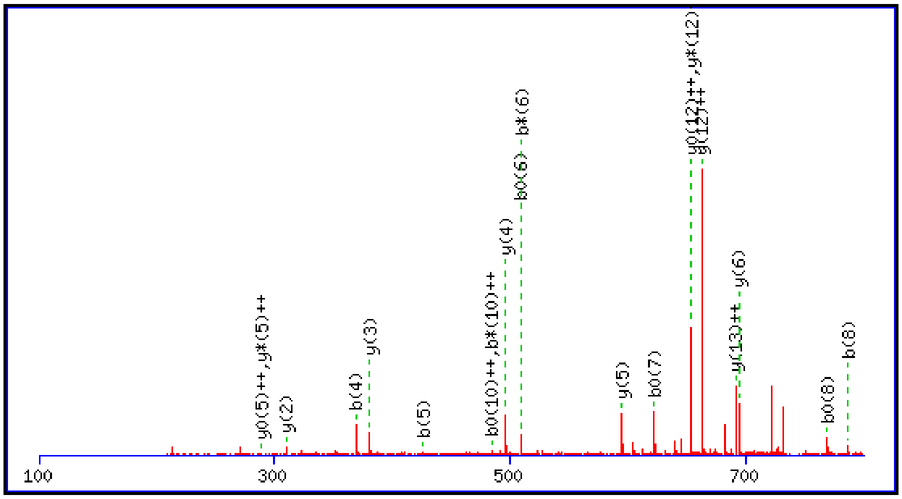

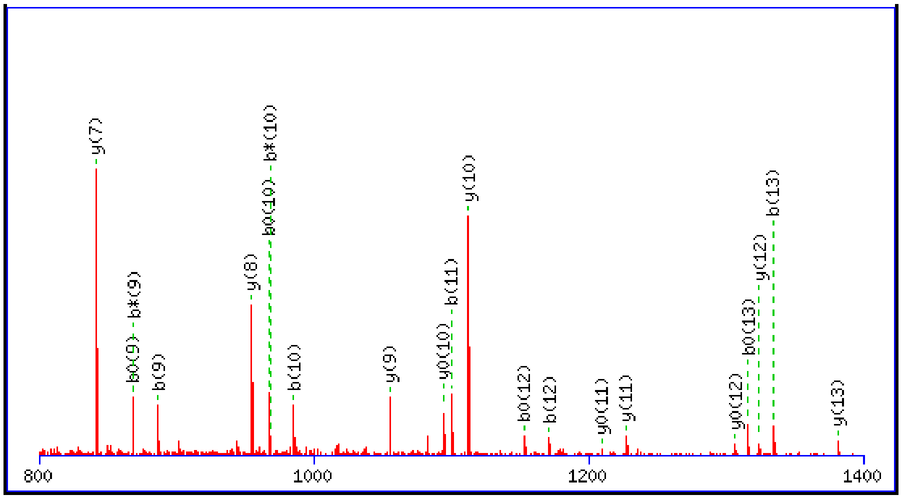

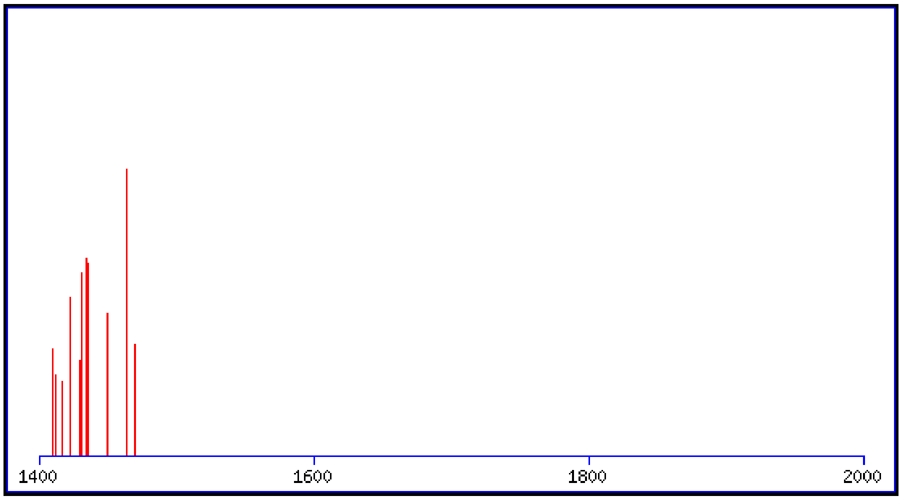

Monoisotopic mass of neutral peptide Mr(calc): 1479.7610
Fixed modifications: Carbamidomethyl (C)
Variable modifications:
N4 : Deamidated_N (N)
Ions Score: 87 **Expect:** 2.7e-007
Matches (Bold Red): 41/134 fragment ions using 68 most intense peaks

#	b	b^{++}	b*	b^{*++}	b^{0}	b^{0++}	Seq.	y	y^{++}	y*	y^{*++}	y^{0}	y^{0++}	#
1	100.0757	50.5415					V							14
2	157.0972	79.0522					G	**1381.6998**	**691.3535**	1364.6733	682.8403	1363.6892	682.3483	13
3	254.1499	127.5786					P	**1324.6783**	**662.8428**	1307.6518	**654.3295**	**1306.6678**	**653.8375**	12
4	**369.1769**	185.0921	352.1503	176.5788			N	**1227.6256**	614.3164	1210.5990	605.8032	**1209.6150**	605.3111	11
5	**426.1983**	213.6028	409.1718	205.0895			G	**1112.5986**	556.8030	1095.5721	548.2897	**1094.5881**	547.7977	10
6	527.2460	264.1266	**510.2194**	255.6134	**509.2354**	255.1214	T	**1055.5772**	528.2922	1038.5506	519.7790	1037.5666	519.2869	9
7	640.3301	320.6687	623.3035	312.1554	**622.3195**	311.6634	L	**954.5295**	477.7684	937.5029	469.2551	936.5189	468.7631	8
8	**787.3985**	394.2029	770.3719	385.6896	**769.3879**	385.1976	F	**841.4454**	421.2264	824.4189	412.7131	823.4349	412.2211	7
9	**886.4669**	443.7371	**869.4403**	435.2238	**868.4563**	434.7318	V	**694.3770**	347.6921	677.3505	339.1789	676.3665	338.6869	6
10	**985.5353**	493.2713	**968.5088**	**484.7580**	**967.5247**	**484.2660**	V	**595.3086**	298.1579	578.2821	**289.6447**	577.2980	**289.1527**	5
11	**1100.5622**	550.7848	1083.5357	542.2715	1082.5517	541.7795	D	**496.2402**	248.6237	479.2136	240.1105	478.2296	239.6185	4
12	**1171.5994**	586.3033	1154.5728	577.7900	**1153.5888**	577.2980	A	**381.2132**	191.1103	364.1867	182.5970			3
13	**1334.6627**	667.8350	1317.6361	659.3217	**1316.6521**	658.8297	Y	**310.1761**	155.5917	293.1496	147.0784			2
14							K	147.1128	74.0600	130.0863	65.5468			1

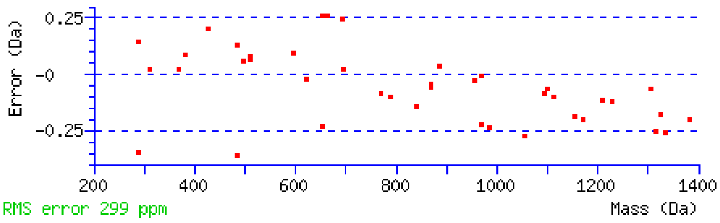

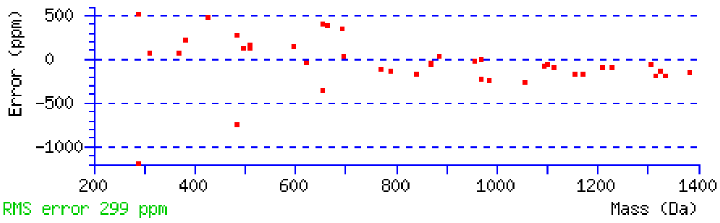

All matches to this query

Score	Mr(calc):	Delta	Sequence
87.1	1479.7610	0.0043	VGPNGTLFVVDAYK
5.0	1477.7443	2.0210	SVTVKFTPLHNR
5.0	1477.7443	2.0210	SVTVKFTPLHNR
4.0	1479.7735	-0.0082	LRVSAPFWHNPR
2.0	1479.7569	0.0084	TEGYSGADISIIVR
1.3	1479.7538	0.0115	RLQTICLSGTGMK
0.3	1479.7711	-0.0059	RLGTLAFTTRHK

Spectrum No: 95; Query: 695; Rank: 1

Peptide View

MS/MS Fragmentation of **FSPLLYCQSQELPNGSVK**
Found in **IPI00200998**, Tax_Id=10116 Gene_Symbol=Pdzk1 PDZ domain-containing protein 1

Match to Query 695: 2067.005028 from(1034.509790,2+)
Title: 091129RatKid_SCX02_18.2837.2837.2.dta
Data file K:\NewmanPaper\Piliang\3SubProteomes\Piliang3SP\mgf5ppm\SCX_3SubProteomes5ppm.mgf

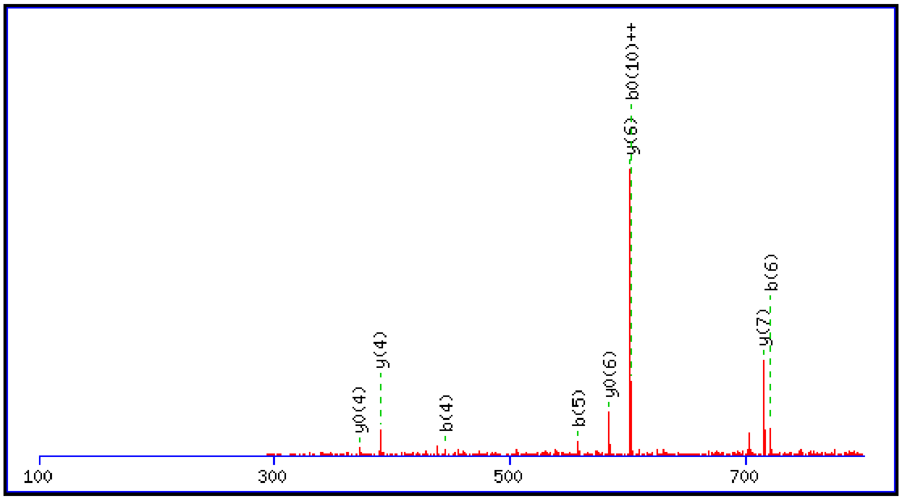

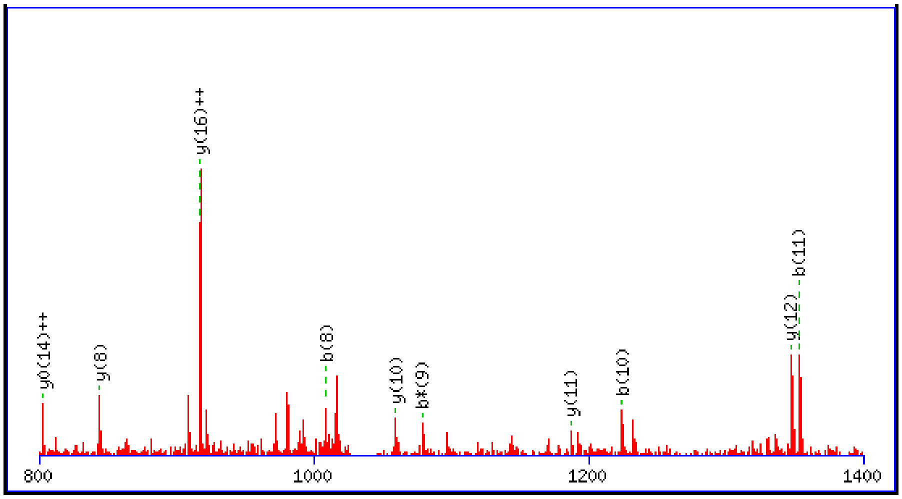

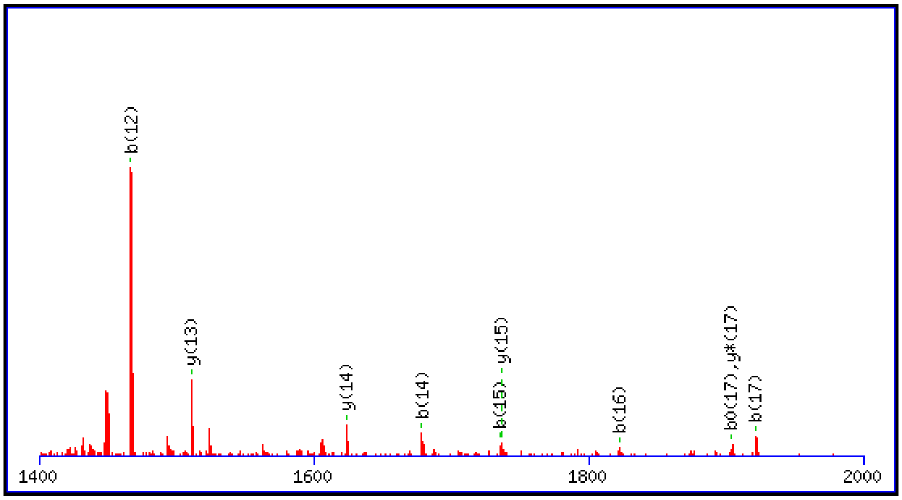

Monoisotopic mass of neutral peptide Mr(calc): 2066.9983
Fixed modifications: Carbamidomethyl (C)
Variable modifications:
N14 : Deamidated_N (N)
Ions Score: 87 **Expect:** 5e-007
Matches (Bold Red): 29/184 fragment ions using 43 most intense peaks

#	b	b^{++}	b*	b^{*++}	b^{0}	b^{0++}	Seq.	y	y^{++}	y*	y^{*++}	y^{0}	y^{0++}	#
1	148.0757	74.5415					F							18
2	235.1077	118.0575			217.0972	109.0522	S	1920.9371	960.9722	**1903.9106**	952.4589	1902.9266	951.9669	17
3	332.1605	166.5839			314.1499	157.5786	P	1833.9051	**917.4562**	1816.8786	908.9429	1815.8946	908.4509	16
4	**445.2445**	223.1259			427.2340	214.1206	L	**1736.8524**	868.9298	1719.8258	860.4165	1718.8418	859.9245	15
5	**558.3286**	279.6679			540.3180	270.6627	L	**1623.7683**	812.3878	1606.7417	803.8745	1605.7577	**803.3825**	14
6	**721.3919**	361.1996			703.3814	352.1943	Y	**1510.6842**	755.8458	1493.6577	747.3325	1492.6737	746.8405	13
7	881.4226	441.2149			863.4120	432.2096	C	**1347.6209**	674.3141	1330.5944	665.8008	1329.6103	665.3088	12
8	**1009.4812**	505.2442	992.4546	496.7309	991.4706	496.2389	Q	**1187.5903**	594.2988	1170.5637	585.7855	1169.5797	585.2935	11
9	1096.5132	548.7602	**1079.4866**	540.2470	1078.5026	539.7550	S	**1059.5317**	530.2695	1042.5051	521.7562	1041.5211	521.2642	10
10	**1224.5718**	612.7895	1207.5452	604.2762	1206.5612	**603.7842**	Q	972.4996	486.7535	955.4731	478.2402	954.4891	477.7482	9
11	**1353.6144**	677.3108	1336.5878	668.7975	1335.6038	668.3055	E	**844.4411**	422.7242	827.4145	414.2109	826.4305	413.7189	8
12	**1466.6984**	733.8529	1449.6719	725.3396	1448.6879	724.8476	L	**715.3985**	358.2029	698.3719	349.6896	697.3879	349.1976	7
13	1563.7512	782.3792	1546.7246	773.8660	1545.7406	773.3740	P	**602.3144**	301.6608	585.2879	293.1476	**584.3038**	292.6556	6
14	**1678.7781**	839.8927	1661.7516	831.3794	1660.7676	830.8874	N	505.2616	253.1345	488.2351	244.6212	487.2511	244.1292	5
15	**1735.7996**	868.4034	1718.7730	859.8902	1717.7890	859.3982	G	**390.2347**	195.6210	373.2082	187.1077	**372.2241**	186.6157	4
16	**1822.8316**	911.9194	1805.8051	903.4062	1804.8211	902.9142	S	333.2132	167.1103	316.1867	158.5970	315.2027	158.1050	3
17	**1921.9000**	961.4537	1904.8735	952.9404	**1903.8895**	952.4484	V	246.1812	123.5942	229.1547	115.0810			2
18							K	147.1128	74.0600	130.0863	65.5468			1

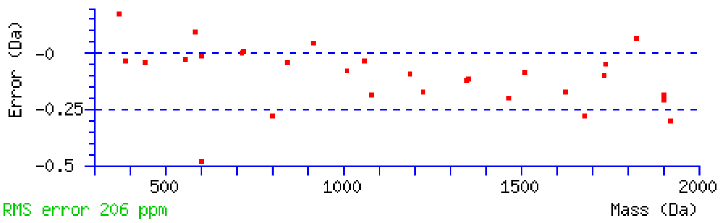

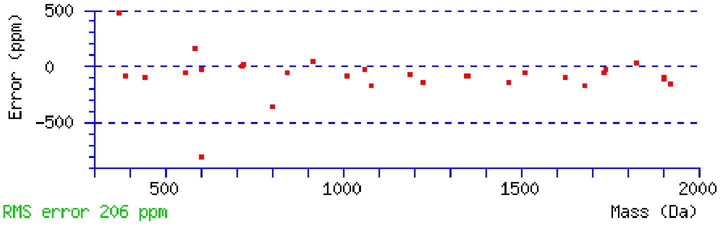

All matches to this query

Score	Mr(calc):	Delta	Sequence
87.0	2066.9983	0.0068	FSPLLYCQSQELPNGSVK
20.9	2066.0143	0.9908	FSPLLYCQSQELPNGSVK
5.1	2067.0016	0.0034	EAMCIGAEVYDNLKNVIK
4.1	2065.9905	1.0145	ISAAGNSGNESGVRELLRR
3.2	2066.9925	0.0125	QVLNFTLEDVLLPQSDR
3.0	2067.0207	-0.0157	IVDQKYHIINGHNCEVK
2.3	2066.9844	0.0207	EKAAHLASMFGHGDLPQDK
1.4	2067.0136	-0.0086	DSLEKSPSVKNDQLSLVK
0.9	2066.9860	0.0190	LEFGGSKGAVLNAQIHSMK
0.7	2067.0169	-0.0119	GDWNKLGILDMFLGCIAK

Spectrum No: 96; Query: 639; Rank: 1

Peptide View

MS/MS Fragmentation of **ALYLIATNGTPELQNPEK**
Found in **IPI00198685**, Tax_Id=10116 Gene_Symbol=Pak1 Serine/threonine-protein kinase PAK 1

Match to Query 639: 1972.022168 from(987.018360,2+)
Title: 091127RatKid_SCX01_17.3190.3190.2.dta
Data file K:\NewmanPaper\Piliang\3SubProteomes\Piliang3SP\mgf5ppm\SCX_3SubProteomes5ppm.mgf

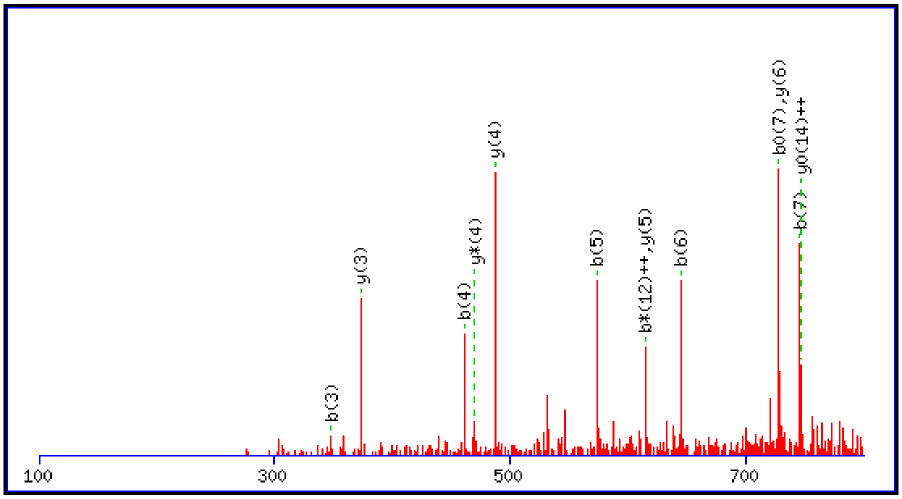

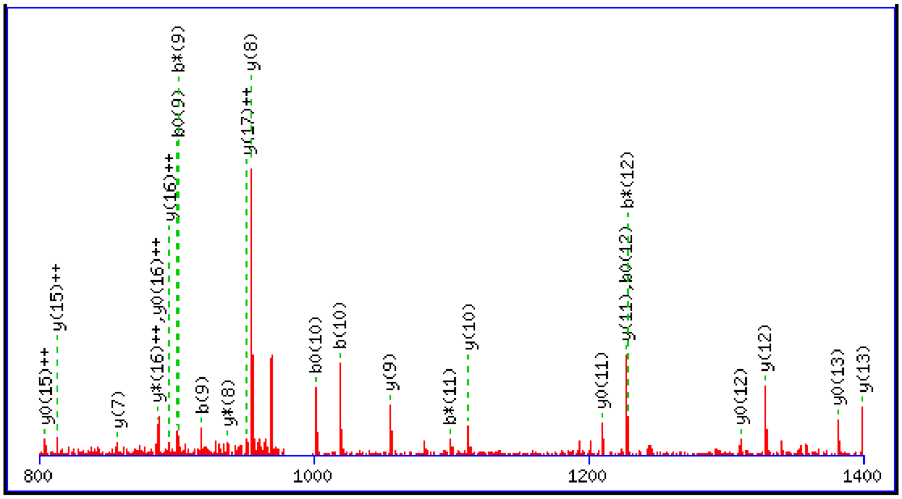

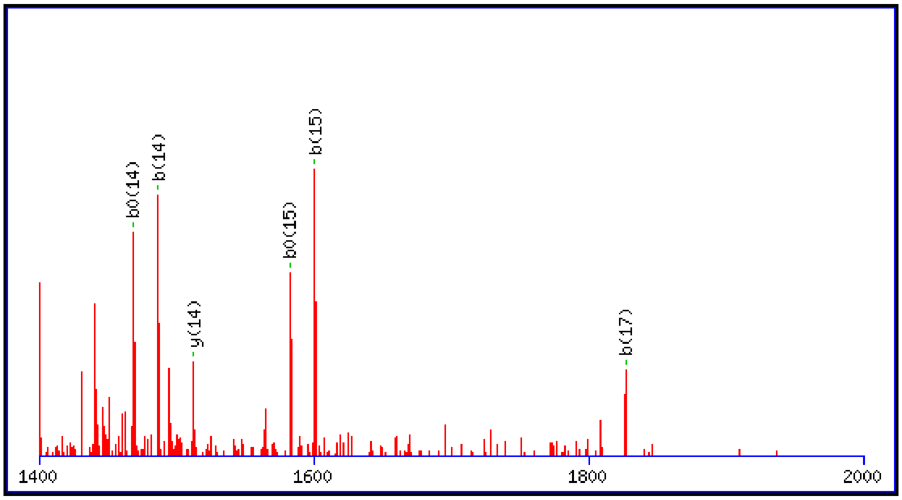

Monoisotopic mass of neutral peptide Mr(calc): 1972.0153
Fixed modifications: Carbamidomethyl (C)
Variable modifications:
N8 : Deamidated_N (N)
Ions Score: 87 **Expect:** 2.8e-007
Matches (Bold Red): 44/176 fragment ions using 63 most intense peaks

#	b	b^{++}	b*	b^{*++}	b^0	b^{0++}	Seq.	y	y^{++}	y*	y^{*++}	y^0	y^{0++}	#
1	72.0444	36.5258					A							18
2	185.1285	93.0679					L	1901.9855	**951.4964**	1884.9589	942.9831	1883.9749	942.4911	17
3	**348.1918**	174.5995					Y	1788.9014	**894.9543**	1771.8749	**886.4411**	1770.8909	**885.9491**	16
4	**461.2758**	231.1416					L	1625.8381	**813.4227**	1608.8115	804.9094	1607.8275	**804.4174**	15
5	**574.3599**	287.6836					I	**1512.7540**	756.8806	1495.7275	748.3674	1494.7435	**747.8754**	14
6	**645.3970**	323.2022					A	**1399.6700**	700.3386	1382.6434	691.8253	**1381.6594**	691.3333	13
7	**746.4447**	373.7260			**728.4341**	364.7207	T	**1328.6328**	664.8201	1311.6063	656.3068	**1310.6223**	655.8148	12
8	861.4716	431.2395	844.4451	422.7262	843.4611	422.2342	N	**1227.5852**	614.2962	1210.5586	605.7829	**1209.5746**	605.2909	11
9	**918.4931**	459.7502	**901.4666**	451.2369	**900.4825**	450.7449	G	**1112.5582**	556.7828	1095.5317	548.2695	1094.5477	547.7775	10
10	**1019.5408**	510.2740	1002.5142	501.7608	**1001.5302**	501.2687	T	**1055.5368**	528.2720	1038.5102	519.7587	1037.5262	519.2667	9
11	1116.5935	558.8004	**1099.5670**	550.2871	1098.5830	549.7951	P	**954.4891**	477.7482	**937.4625**	469.2349	936.4785	468.7429	8
12	1245.6361	623.3217	**1228.6096**	**614.8084**	**1227.6256**	614.3164	E	**857.4363**	429.2218	840.4098	420.7085	839.4258	420.2165	7
13	1358.7202	679.8637	1341.6937	671.3505	1340.7096	670.8585	L	**728.3937**	364.7005	711.3672	356.1872	710.3832	355.6952	6
14	**1486.7788**	743.8930	1469.7522	735.3798	**1468.7682**	734.8877	Q	**615.3097**	308.1585	598.2831	299.6452	597.2991	299.1532	5
15	**1600.8217**	800.9145	1583.7952	792.4012	**1582.8111**	791.9092	N	**487.2511**	244.1292	**470.2245**	235.6159	469.2405	235.1239	4
16	1697.8745	849.4409	1680.8479	840.9276	1679.8639	840.4356	P	**373.2082**	187.1077	356.1816	178.5944	355.1976	178.1024	3
17	**1826.9171**	913.9622	1809.8905	905.4489	1808.9065	904.9569	E	276.1554	138.5813	259.1288	130.0681	258.1448	129.5761	2
18							K	147.1128	74.0600	130.0863	65.5468			1

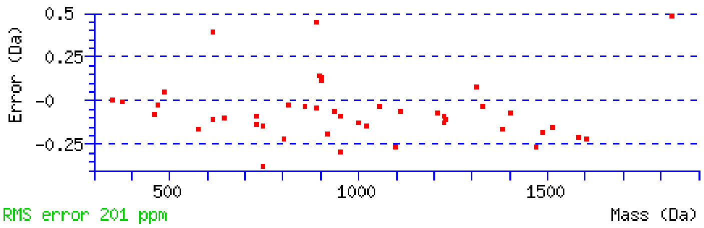

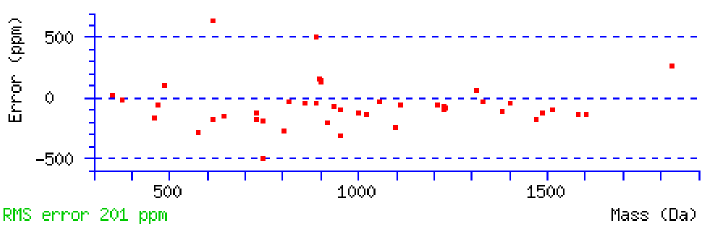

All matches to this query

Score	Mr(calc):	Delta	Sequence
86.8	1972.0153	0.0069	ALYLIATNGTPELQNPEK
64.0	1971.0313	0.9909	ALYLIATNGTPELQNPEK
50.3	1972.0153	0.0069	ALYLIATNGTPELQNPEK
9.9	1972.0088	0.0134	DNIGSQLLLNWCVQIAK
8.0	1970.0060	2.0162	QIERDITCFLKNIIK
3.9	1972.0166	0.0055	VALAPNPTAPHFLRDQEP
3.5	1972.0299	-0.0077	LLLDCQDVENQLAVKSK
2.8	1972.0143	0.0079	VQHILILGKSGVNDRDK
1.1	1971.0248	0.9974	DNIGSQLLLNWCVQIAK
0.9	1971.0095	1.0127	GPVTPTKVATTSVITIVK

Spectrum No: 97; Query: 932; Rank: 1

Peptide View

MS/MS Fragmentation of **HYSCACPSGWNLSDDSVNCVR**
Found in **IPI00205325**, Tax_Id=10116 Gene_Symbol=Lrp2 Low-density lipoprotein receptor-related protein 2 precursor

Match to Query 932: 2483.992568 from(1243.003560,2+)
Title: 091127RatKid_SCX01_24.2481.2481.2.dta
Data file K:\NewmanPaper\Piliang\3SubProteomes\Piliang3SP\mgf5ppm\SCX_3SubProteomes5ppm.mgf

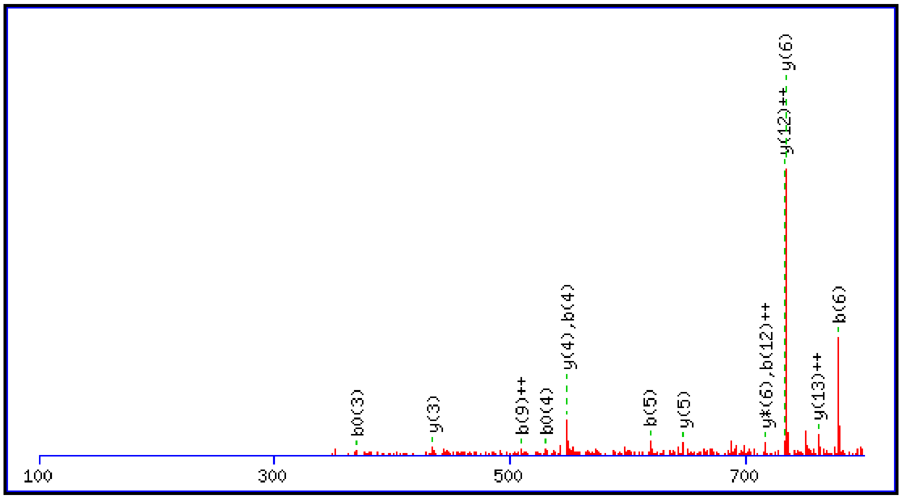

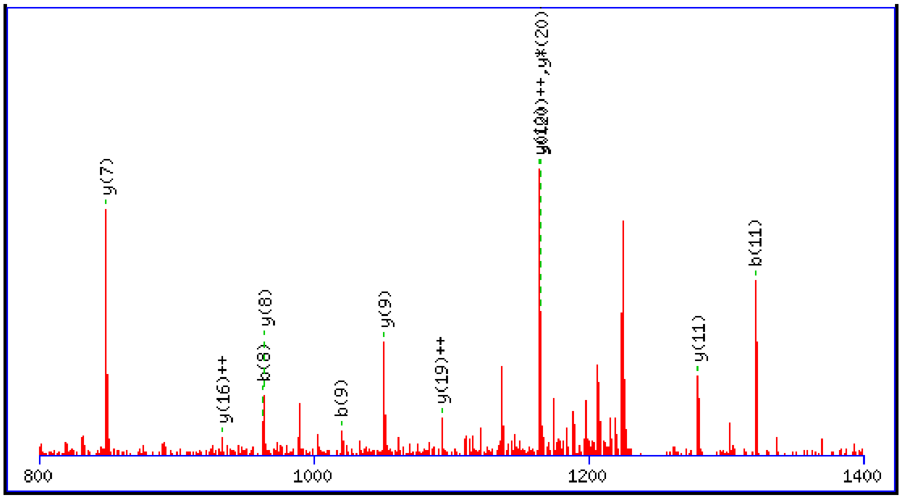

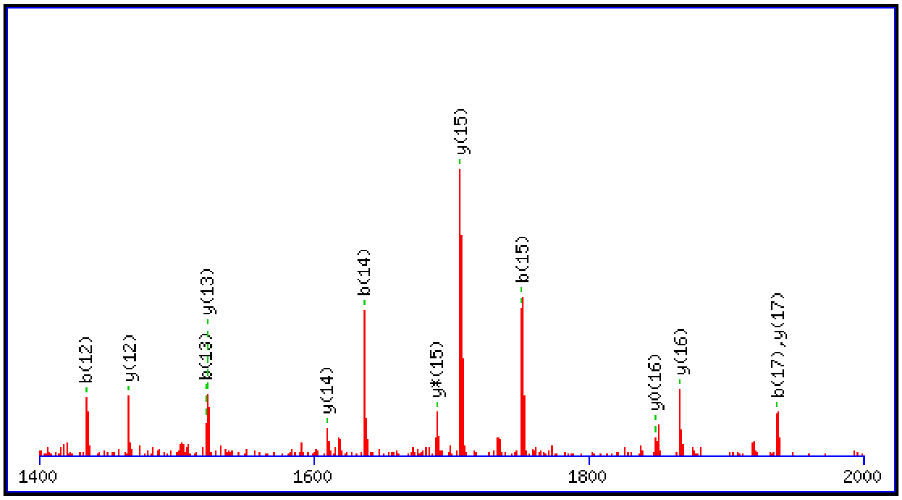

Monoisotopic mass of neutral peptide Mr(calc): 2483.9893
Fixed modifications: Carbamidomethyl (C)
Variable modifications:
N11 : Deamidated_N (N)
Ions Score: 86 **Expect:** 5.5e-007
Matches (Bold Red): 39/206 fragment ions using 74 most intense peaks

#	b	b^{++}	b*	b^{*++}	b^0	b^{0++}	Seq.	y	y^{++}	y*	y^{*++}	y^0	y^{0++}	#
1	138.0662	69.5367					H							21
2	301.1295	151.0684					Y	2347.9376	1174.4725	2330.9111	**1165.9592**	2329.9271	**1165.4672**	20
3	388.1615	194.5844			**370.1510**	185.5791	S	2184.8743	**1092.9408**	2167.8478	1084.4275	2166.8637	1083.9355	19
4	**548.1922**	274.5997			**530.1816**	265.5945	C	2097.8423	1049.4248	2080.8157	1040.9115	2079.8317	1040.4195	18
5	**619.2293**	310.1183			601.2187	301.1130	A	**1937.8116**	969.4095	1920.7851	960.8962	1919.8011	960.4042	17
6	**779.2600**	390.1336			761.2494	381.1283	C	**1866.7745**	**933.8909**	1849.7480	925.3776	**1848.7640**	924.8856	16
7	876.3127	438.6600			858.3022	429.6547	P	**1706.7439**	853.8756	**1689.7173**	845.3623	1688.7333	844.8703	15
8	**963.3447**	482.1760			945.3342	473.1707	S	**1609.6911**	805.3492	1592.6646	796.8359	1591.6805	796.3439	14
9	**1020.3662**	**510.6867**			1002.3556	501.6815	G	**1522.6591**	**761.8332**	1505.6325	753.3199	1504.6485	752.8279	13
10	1206.4455	603.7264			1188.4350	594.7211	W	**1465.6376**	**733.3224**	1448.6111	724.8092	1447.6270	724.3172	12
11	**1321.4725**	661.2399	1304.4459	652.7266	1303.4619	652.2346	N	**1279.5583**	640.2828	1262.5317	631.7695	1261.5477	631.2775	11
12	**1434.5565**	**717.7819**	1417.5300	709.2686	1416.5460	708.7766	L	**1164.5314**	582.7693	1147.5048	574.2560	1146.5208	573.7640	10
13	**1521.5886**	761.2979	1504.5620	752.7846	1503.5780	752.2926	S	**1051.4473**	526.2273	1034.4207	517.7140	1033.4367	517.2220	9
14	**1636.6155**	818.8114	1619.5889	810.2981	1618.6049	809.8061	D	**964.4153**	482.7113	947.3887	474.1980	946.4047	473.7060	8
15	**1751.6424**	876.3249	1734.6159	867.8116	1733.6319	867.3196	D	**849.3883**	425.1978	832.3618	416.6845	831.3778	416.1925	7
16	1838.6745	919.8409	1821.6479	911.3276	1820.6639	910.8356	S	**734.3614**	367.6843	**717.3348**	359.1711	716.3508	358.6790	6
17	**1937.7429**	969.3751	1920.7163	960.8618	1919.7323	960.3698	V	**647.3294**	324.1683	630.3028	315.6550			5
18	2051.7858	1026.3965	2034.7593	1017.8833	2033.7752	1017.3913	N	**548.2609**	274.6341	531.2344	266.1208			4

**19**	2211.8165	1106.4119	2194.7899	1097.8986	2193.8059	1097.4066	**C**	**434.2180**	217.6126	417.1915	209.0994			**3**
**20**	2310.8849	1155.9461	2293.8583	1147.4328	2292.8743	1146.9408	**V**	274.1874	137.5973	257.1608	129.0840			**2**
**21**							**R**	175.1190	88.0631	158.0924	79.5498			**1**

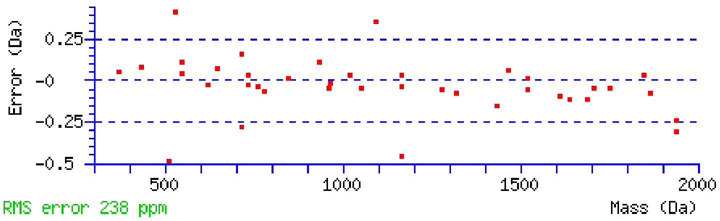

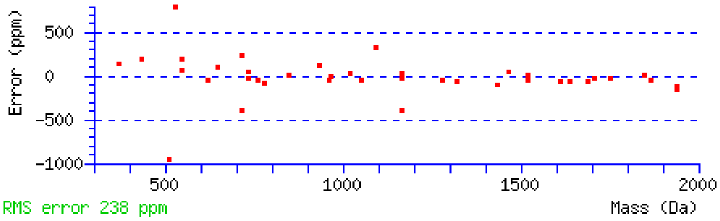

All matches to this query

Score	Mr(calc):	Delta	Sequence
86.5	2483.9893	0.0033	HYSCACPSGWNLSDDSVNCVR
55.3	2483.9893	0.0033	HYSCACPSGWNLSDDSVNCVR
29.8	2483.0053	0.9873	HYSCACPSGWNLSDDSVNCVR
10.9	2481.9938	1.9987	TTSSGVGGSTESNTDLFTYRNK
2.3	2481.9938	1.9987	TTSSGVGGSTESNTDLFTYRNK
2.1	2481.9938	1.9987	TTSSGVGGSTESNTDLFTYRNK
2.1	2481.9938	1.9987	TTSSGVGGSTESNTDLFTYRNK
1.3	2481.9938	1.9987	TTSSGVGGSTESNTDLFTYRNK
1.3	2482.9778	1.0147	TTSSGVGGSTESNTDLFTYRNK
1.1	2481.9938	1.9987	TTSSGVGGSTESNTDLFTYRNK

Spectrum No: 98; Query: 152; Rank: 1

Peptide View

MS/MS Fragmentation of **ILNETLYENAK**
Found in **IPI00195931**, Tax_Id=10116 Gene_Symbol=Tspan8 D6.1A protein

Match to Query 152: 1307.662728 from(654.838640,2+)
Title: 091129RatKid_SCX02_12.1389.1389.2.dta
Data file K:\NewmanPaper\Piliang\3SubProteomes\Piliang3SP\mgf5ppm\SCX_3SubProteomes5ppm.mgf

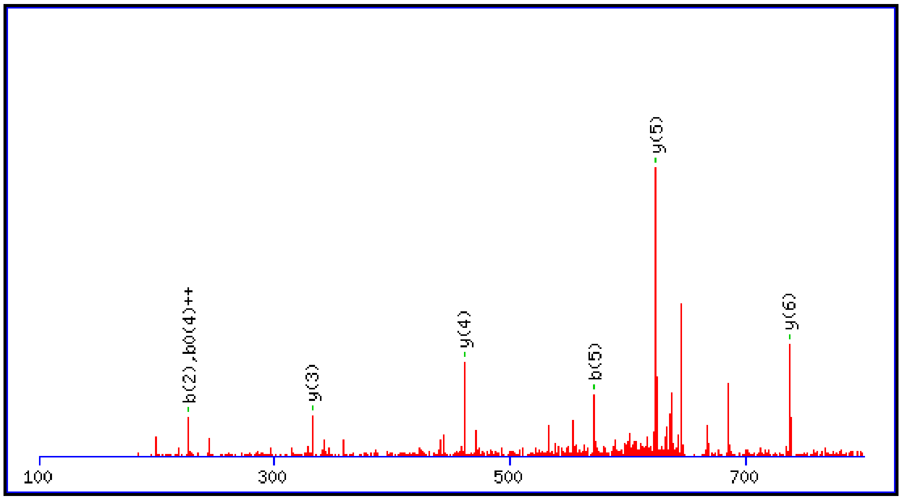

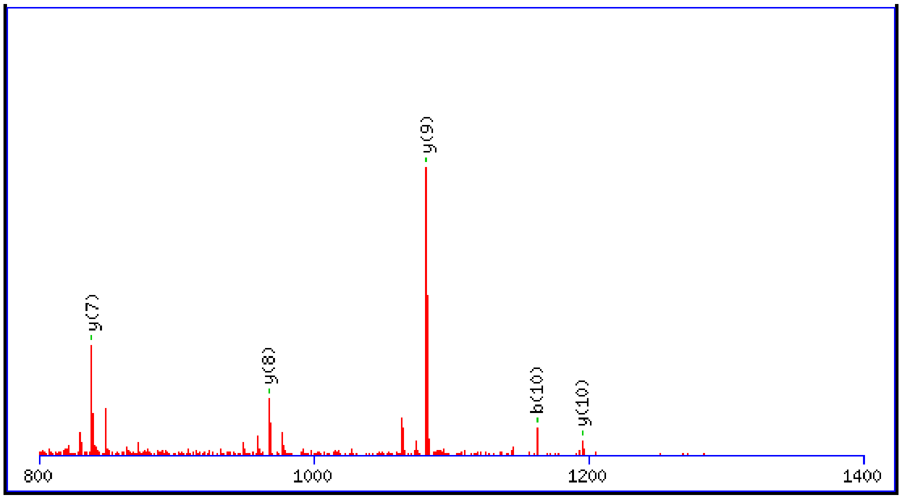

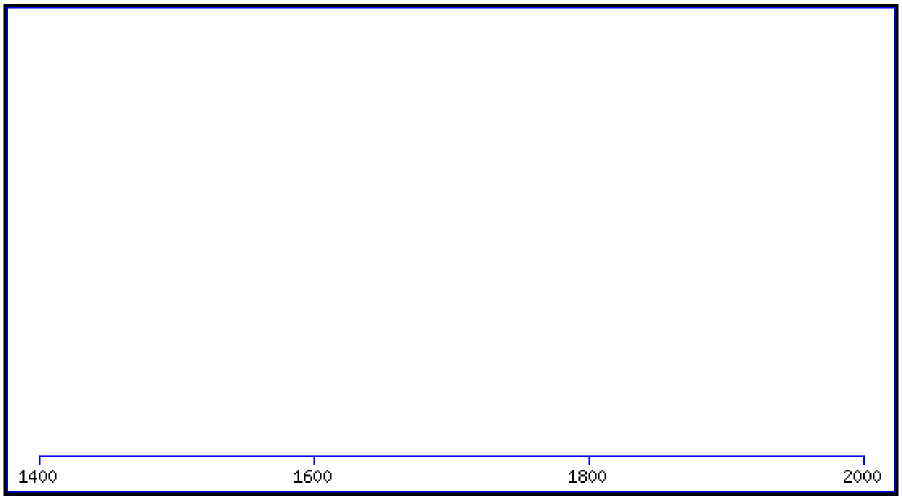

Monoisotopic mass of neutral peptide Mr(calc): 1307.6608
Fixed modifications: Carbamidomethyl (C)
Variable modifications:
N3 : Deamidated_N (N)
Ions Score: 87 **Expect:** 2.5e-007
Matches (Bold Red): 12/104 fragment ions using 14 most intense peaks

#	b	b^{++}	b*	b^{*++}	b^0	b^{0++}	Seq.	y	y^{++}	y*	y^{*++}	y^0	y^{0++}	#
1	114.0913	57.5493					I							11
2	**227.1754**	114.0913					L	**1195.5841**	598.2957	1178.5576	589.7824	1177.5735	589.2904	10
3	342.2023	171.6048	325.1758	163.0915			N	**1082.5000**	541.7537	1065.4735	533.2404	1064.4895	532.7484	9
4	471.2449	236.1261	454.2184	227.6128	453.2344	**227.1208**	E	**967.4731**	484.2402	950.4466	475.7269	949.4625	475.2349	8
5	**572.2926**	286.6499	555.2661	278.1367	554.2820	277.6447	T	**838.4305**	419.7189	821.4040	411.2056	820.4199	410.7136	7
6	685.3767	343.1920	668.3501	334.6787	667.3661	334.1867	L	**737.3828**	369.1951	720.3563	360.6818	719.3723	360.1898	6
7	848.4400	424.7236	831.4135	416.2104	830.4294	415.7184	Y	**624.2988**	312.6530	607.2722	304.1397	606.2882	303.6477	5
8	977.4826	489.2449	960.4560	480.7317	959.4720	480.2397	E	**461.2354**	231.1214	444.2089	222.6081	443.2249	222.1161	4
9	1091.5255	546.2664	1074.4990	537.7531	1073.5150	537.2611	N	**332.1928**	166.6001	315.1663	158.0868			3
10	**1162.5626**	581.7850	1145.5361	573.2717	1144.5521	572.7797	A	218.1499	109.5786	201.1234	101.0653			2
11							K	147.1128	74.0600	130.0863	65.5468			1

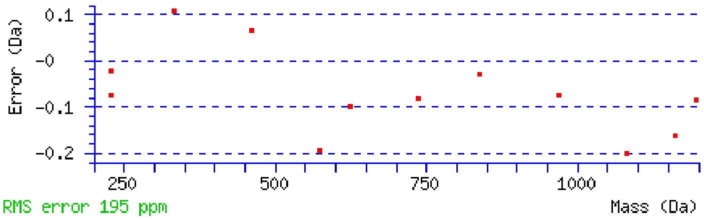

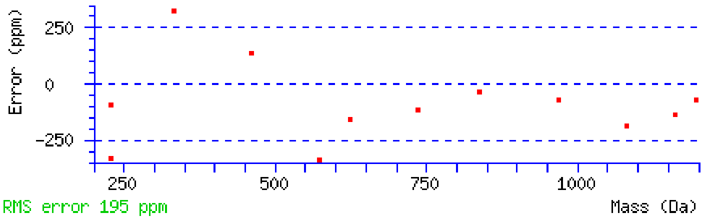

All matches to this query

Score	Mr(calc):	Delta	Sequence
86.5	1307.6608	0.0019	ILNETLYENAK
32.4	1307.6608	0.0019	ILNETLYENAK
9.7	1307.6526	0.0101	IINVIHYINK
8.7	1307.6609	0.0019	ILEDEGLYTQK
4.4	1307.6721	-0.0094	ITKQGNIYNEK
3.9	1307.6544	0.0084	LLAAGFDNSCIK
3.6	1307.6577	0.0050	NETMNMILKAK
3.1	1307.6577	0.0050	NETMNMILKAK
2.7	1307.6730	-0.0102	LLMQNTWMKK
2.0	1307.6609	0.0019	DELKLFNETAK

Spectrum No: 99; Query: 537; Rank: 1

Peptide View

MS/MS Fragmentation of **QGAIVAVTGDGVNDSPALK**
Found in **IPI00205693**, Tax_Id=10116 Gene_Symbol=Atp1a2 Sodium/potassium-transporting ATPase subunit alpha-2 precursor

Match to Query 537: 1811.921428 from(906.967990,2+)
Title: 091129RatKid_SCX02_18.1674.1674.2.dta
Data file K:\NewmanPaper\Piliang\3SubProteomes\Piliang3SP\mgf5ppm\SCX_3SubProteomes5ppm.mgf

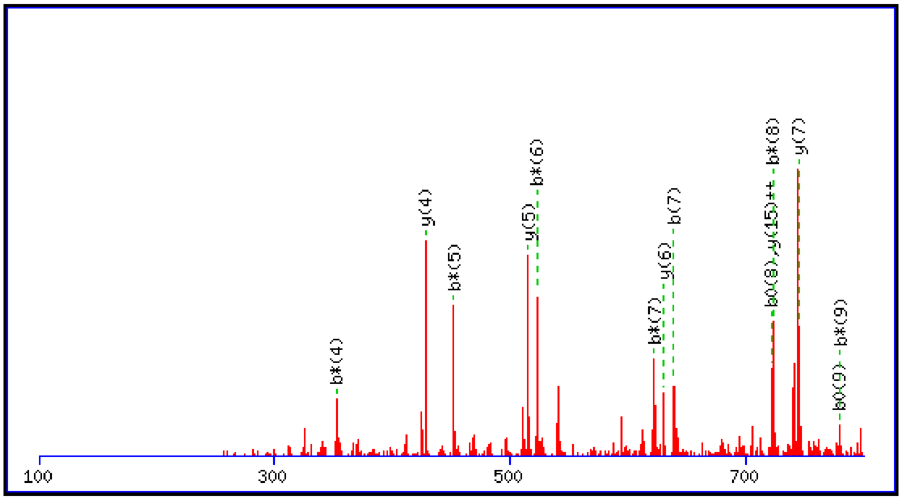

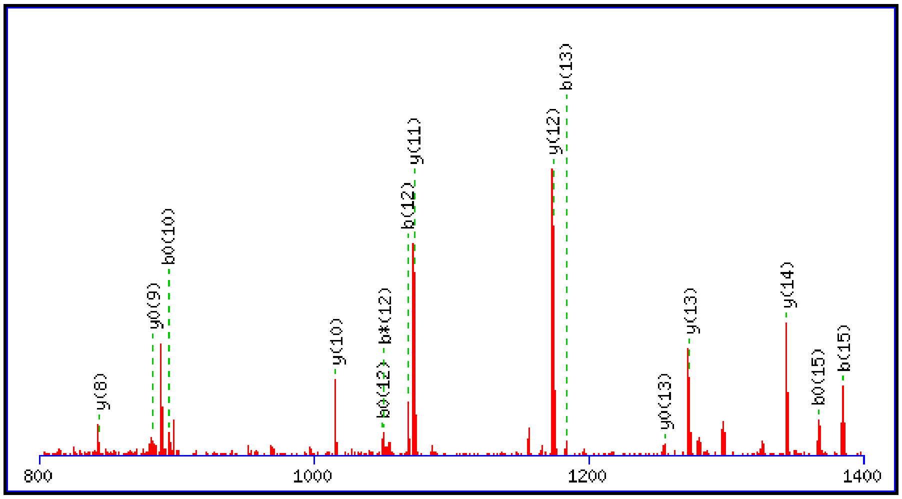

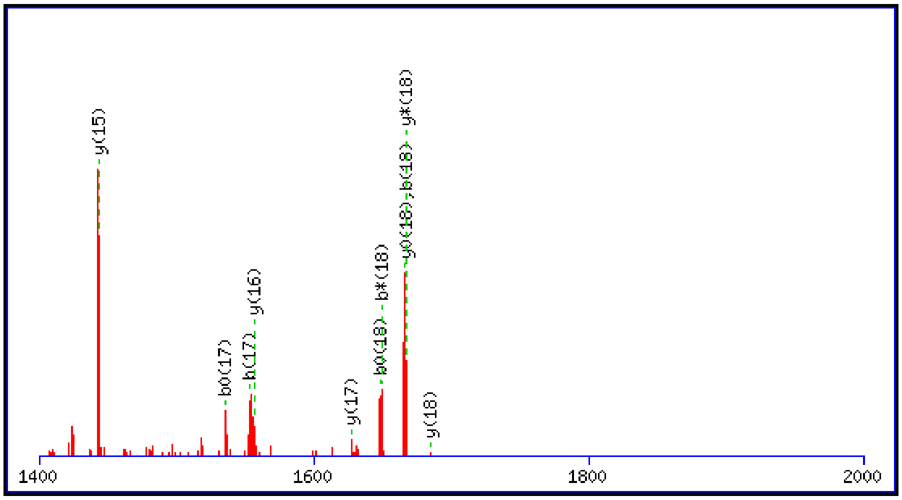

Monoisotopic mass of neutral peptide Mr(calc): 1811.9265
Fixed modifications: Carbamidomethyl (C)
Variable modifications:
N13 : Deamidated_N (N)
Ions Score: 86 **Expect:** 3.6e-007
Matches (Bold Red): 40/194 fragment ions using 79 most intense peaks

#	b	b^{++}	b*	b^{*++}	b^0	b^{0++}	Seq.	y	y^{++}	y*	y^{*++}	y^0	y^{0++}	#
1	129.0659	65.0366	112.0393	56.5233			Q							19
2	186.0873	93.5473	169.0608	85.0340			G	**1684.8752**	842.9412	**1667.8487**	834.4280	**1666.8646**	833.9360	18
3	257.1244	129.0659	240.0979	120.5526			A	**1627.8537**	814.4305	1610.8272	805.9172	1609.8432	805.4252	17
4	370.2085	185.6079	**353.1819**	177.0946			I	**1556.8166**	778.9119	1539.7901	770.3987	1538.8061	769.9067	16
5	469.2769	235.1421	**452.2504**	226.6288			V	**1443.7326**	**722.3699**	1426.7060	713.8566	1425.7220	713.3646	15
6	540.3140	270.6607	**523.2875**	262.1474			A	**1344.6641**	672.8357	1327.6376	664.3224	1326.6536	663.8304	14
7	**639.3824**	320.1949	**622.3559**	311.6816			V	**1273.6270**	637.3172	1256.6005	628.8039	**1255.6165**	628.3119	13
8	740.4301	370.7187	**723.4036**	362.2054	**722.4196**	361.7134	T	**1174.5586**	587.7829	1157.5321	579.2697	1156.5481	578.7777	12
9	797.4516	399.2294	**780.4250**	390.7162	**779.4410**	390.2241	G	**1073.5109**	537.2591	1056.4844	528.7458	1055.5004	528.2538	11
10	912.4785	456.7429	895.4520	448.2296	**894.4680**	447.7376	D	**1016.4895**	508.7484	999.4629	500.2351	998.4789	499.7431	10
11	969.5000	485.2536	952.4734	476.7404	951.4894	476.2483	G	901.4625	451.2349	884.4360	442.7216	**883.4520**	442.2296	9
12	**1068.5684**	534.7878	**1051.5419**	526.2746	**1050.5578**	525.7826	V	**844.4411**	422.7242	827.4145	414.2109	826.4305	413.7189	8
13	**1183.5953**	592.3013	1166.5688	583.7880	1165.5848	583.2960	N	**745.3727**	373.1900	728.3461	364.6767	727.3621	364.1847	7
14	1298.6223	649.8148	1281.5957	641.3015	1280.6117	640.8095	D	**630.3457**	315.6765	613.3192	307.1632	612.3352	306.6712	6
15	**1385.6543**	693.3308	1368.6278	684.8175	**1367.6437**	684.3255	S	**515.3188**	258.1630	498.2922	249.6498	497.3082	249.1577	5
16	1482.7071	741.8572	1465.6805	733.3439	1464.6965	732.8519	P	**428.2867**	214.6470	411.2602	206.1337			4
17	**1553.7442**	777.3757	1536.7176	768.8625	**1535.7336**	768.3704	A	331.2340	166.1206	314.2074	157.6074			3
18	**1666.8283**	833.9178	**1649.8017**	825.4045	**1648.8177**	824.9125	L	260.1969	130.6021	243.1703	122.0888			2
19							K	147.1128	74.0600	130.0863	65.5468			1

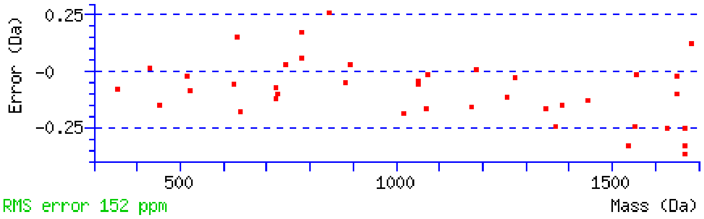

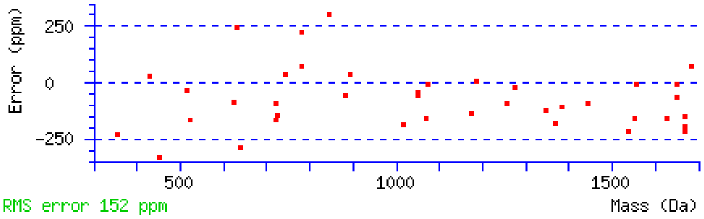

All matches to this query

Score	Mr(calc):	Delta	Sequence
86.5	1811.9265	-0.0051	QGAIVAVTGDGVNDSPALK
4.9	1811.9166	0.0048	EKGLSGRGPDTGPDWLK

3.2	1811.9182	0.0032	QLEGGPSDHIPLLKTK

Spectrum No: 100; Query: 181; Rank: 1

Peptide View

MS/MS Fragmentation of **AFINSSYTIDPK**
Found in **IPI00200067**, Tax_Id=10116 Gene_Symbol=F11r Junctional adhesion molecule A precursor

Match to Query 181: 1355.664528 from(678.839540,2+)
Title: 091127RatKid_SCX01_11.1850.1850.2.dta
Data file K:\NewmanPaper\Piliang\3SubProteomes\Piliang3SP\mgf5ppm\SCX_3SubProteomes5ppm.mgf

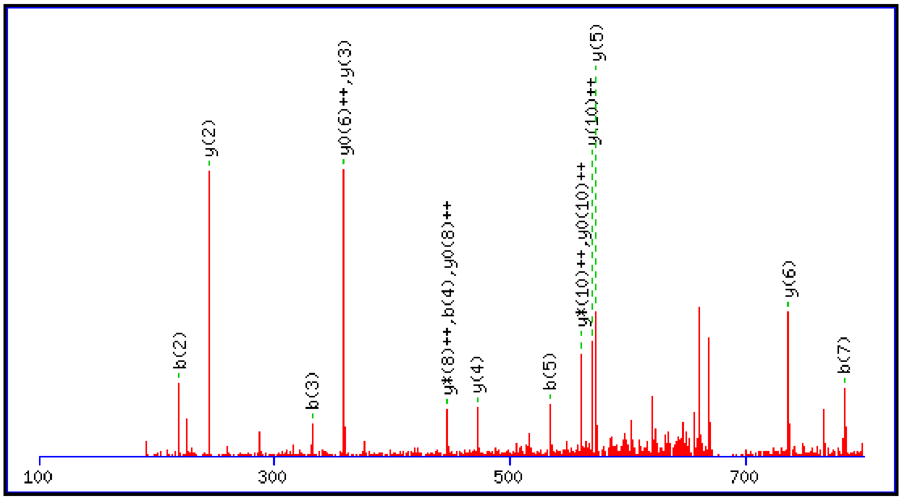

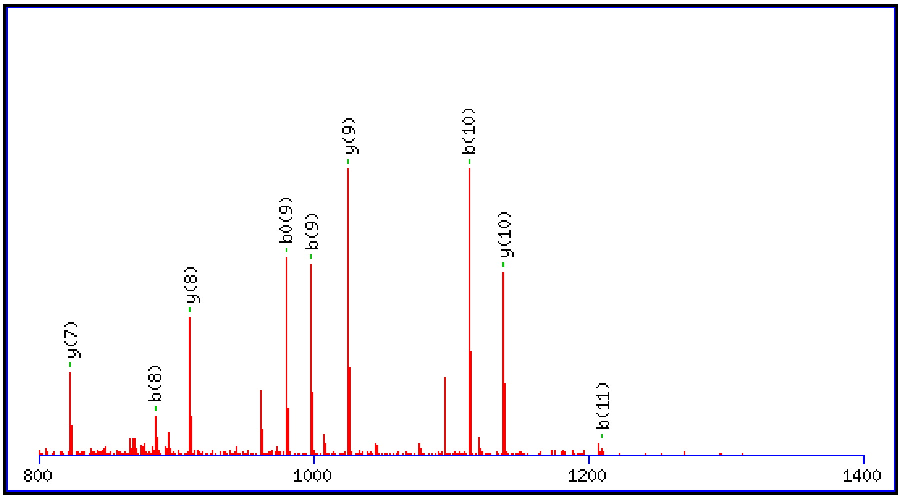

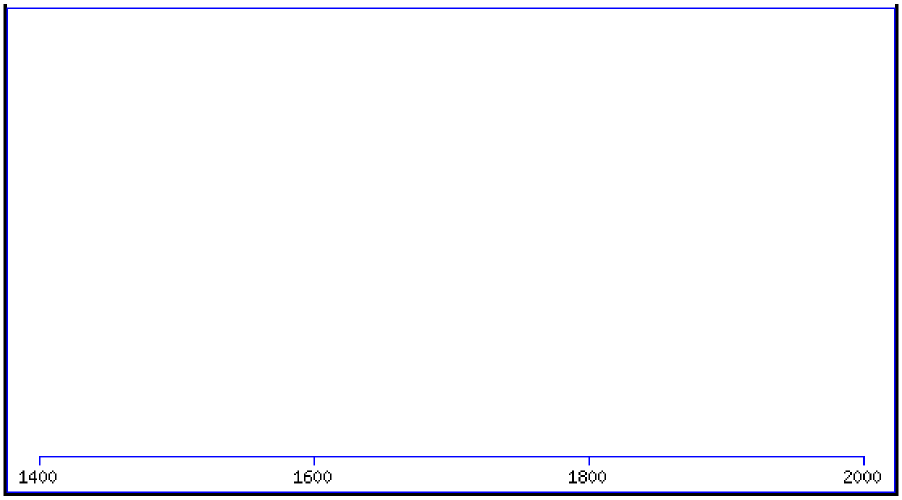

Monoisotopic mass of neutral peptide Mr(calc): 1355.6609
Fixed modifications: Carbamidomethyl (C)
Variable modifications:
N4 : Deamidated_N (N)
Ions Score: 86 **Expect:** 3.7e-007
Matches (Bold Red): 25/114 fragment ions using 31 most intense peaks

#	b	b^{++}	b^*	b^{*++}	b^0	b^{0++}	Seq.	y	y^{++}	y^*	y^{*++}	y^0	y^{0++}	#
1	72.0444	36.5258					A							12
2	**219.1128**	110.0600					F	1285.6311	643.3192	1268.6045	634.8059	1267.6205	634.3139	11
3	**332.1969**	166.6021					I	**1138.5626**	**569.7850**	1121.5361	**561.2717**	1120.5521	**560.7797**	10
4	**447.2238**	224.1155	430.1973	215.6023			N	**1025.4786**	513.2429	1008.4520	504.7297	1007.4680	504.2376	9
5	**534.2558**	267.6316	517.2293	259.1183	516.2453	258.6263	S	**910.4516**	455.7295	893.4251	**447.2162**	892.4411	**446.7242**	8
6	621.2879	311.1476	604.2613	302.6343	603.2773	302.1423	S	**823.4196**	412.2134	806.3931	403.7002	805.4090	403.2082	7
7	**784.3512**	392.6792	767.3246	384.1660	766.3406	383.6739	Y	**736.3876**	368.6974	719.3610	360.1842	718.3770	**359.6921**	6
8	**885.3989**	443.2031	868.3723	434.6898	867.3883	434.1978	T	**573.3243**	287.1658	556.2977	278.6525	555.3137	278.1605	5
9	**998.4829**	499.7451	981.4564	491.2318	**980.4724**	490.7398	I	**472.2766**	236.6419	455.2500	228.1287	454.2660	227.6366	4
10	**1113.5099**	557.2586	1096.4833	548.7453	1095.4993	548.2533	D	**359.1925**	180.0999	342.1660	171.5866	341.1819	171.0946	3
11	**1210.5626**	605.7850	1193.5361	597.2717	1192.5521	596.7797	P	**244.1656**	122.5864	227.1390	114.0731			2
12							K	147.1128	74.0600	130.0863	65.5468			1

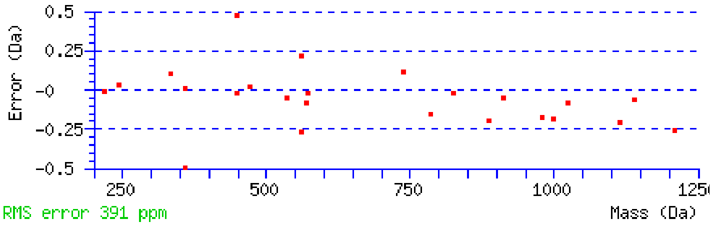

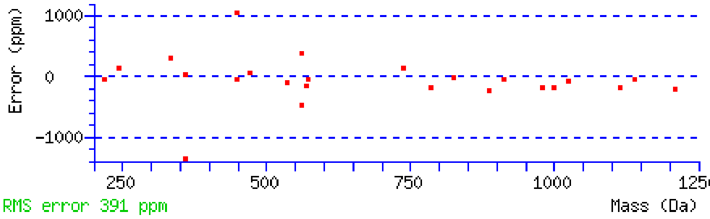

All matches to this query

Score	Mr(calc):	Delta	Sequence
85.8	1355.6609	0.0037	AFINSSYTIDPK
10.5	1355.6520	0.0126	KDMPTTKNTLK
9.1	1355.6569	0.0077	QNDVEIPSPTQK
9.0	1355.6520	0.0126	KDMPTTKNTLK
8.2	1355.6755	-0.0109	VKSPESCPLPNK
7.7	1355.6761	-0.0116	YGYIYPLQDPK
6.0	1353.6694	1.9952	ETVAVXFPLDK
5.8	1353.6637	2.0009	QSAGXRHENIDK
5.6	1353.6524	2.0121	NINENPVSQNPK

5.6	1353.6524	2.0121	NINENPVSQNPK

Spectrum No: 101; Query: 785; Rank: 1

Peptide View

MS/MS Fragmentation of **DQCIVDDITYNVNDTFHK**
Found in **IPI00200757**, Tax_Id=10116 Gene_Symbol=Fn1 Isoform 1 of Fibronectin precursor

Match to Query 785: 2196.969348 from(1099.491950,2+)
Title: 091127RatKid_SCX01_14.3778.3778.2.dta
Data file K:\NewmanPaper\Piliang\3SubProteomes\Piliang3SP\mgf5ppm\SCX_3SubProteomes5ppm.mgf

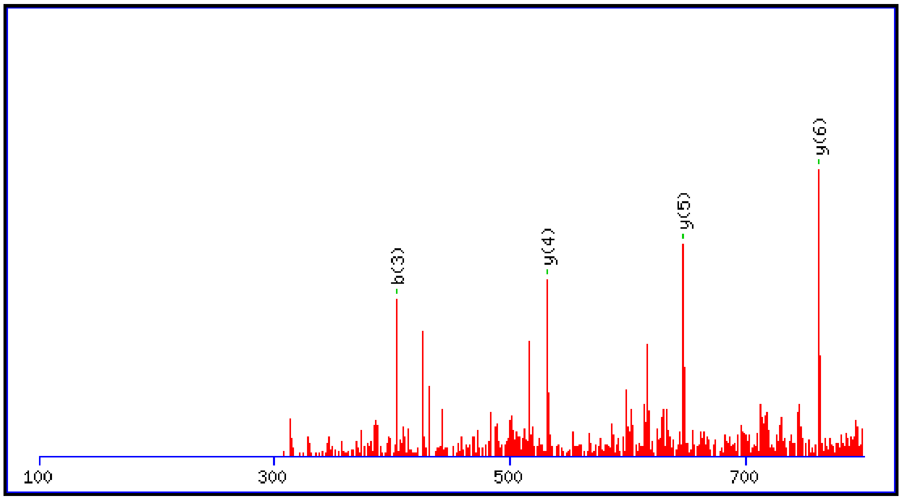

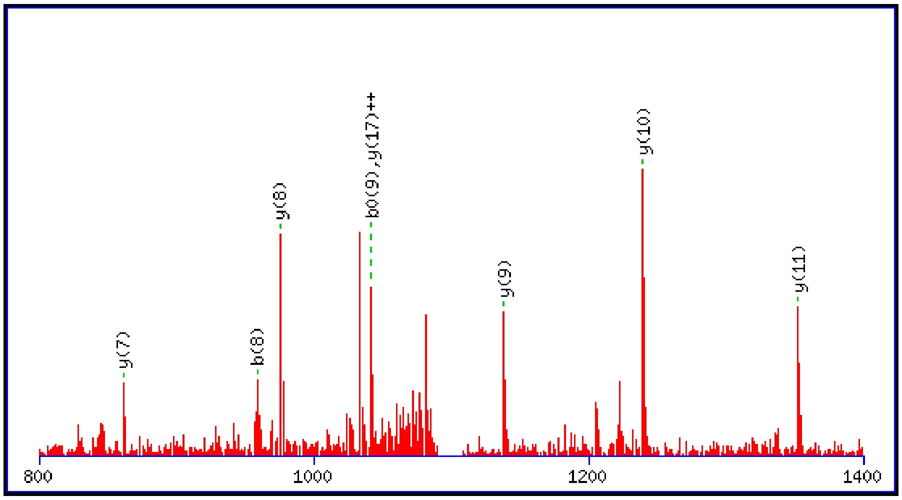

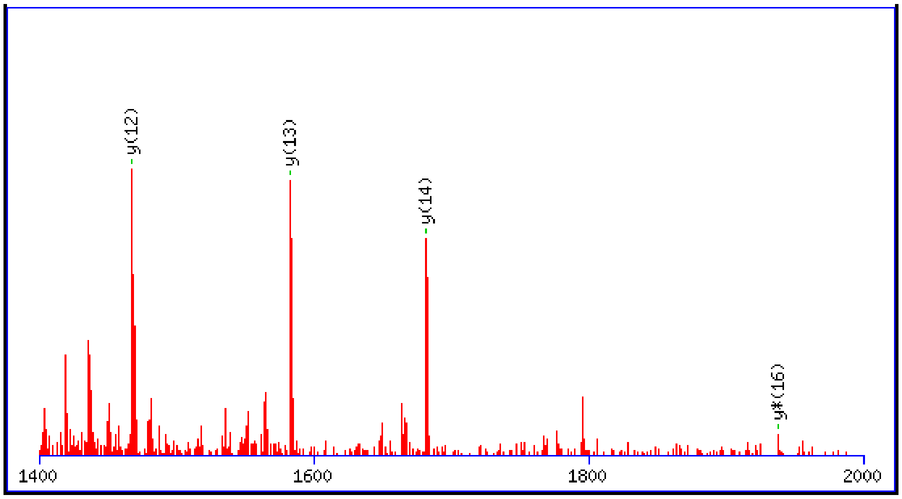

Monoisotopic mass of neutral peptide Mr(calc): 2196.9634
Fixed modifications: Carbamidomethyl (C)
Variable modifications:
N13 : Deamidated_N (N)
Ions Score: 86 **Expect:** 7.8e-007
Matches (Bold Red): 16/196 fragment ions using 25 most intense peaks

#	b	b^{++}	b*	b^{*++}	b^{0}	b^{0++}	Seq.	y	y^{++}	y*	y^{*++}	y^{0}	y^{0++}	#
1	116.0342	58.5207			98.0237	49.5155	D							18
2	244.0928	122.5500	227.0662	114.0368	226.0822	113.5448	Q	2082.9437	**1041.9755**	2065.9171	1033.4622	2064.9331	1032.9702	17
3	**404.1234**	202.5654	387.0969	194.0521	386.1129	193.5601	C	1954.8851	977.9462	**1937.8586**	969.4329	1936.8745	968.9409	16
4	517.2075	259.1074	500.1810	250.5941	499.1969	250.1021	I	1794.8545	897.9309	1777.8279	889.4176	1776.8439	888.9256	15
5	616.2759	308.6416	599.2494	300.1283	598.2654	299.6363	V	**1681.7704**	841.3888	1664.7439	832.8756	1663.7598	832.3836	14
6	731.3029	366.1551	714.2763	357.6418	713.2923	357.1498	D	**1582.7020**	791.8546	1565.6754	783.3414	1564.6914	782.8493	13
7	846.3298	423.6685	829.3033	415.1553	828.3192	414.6633	D	**1467.6750**	734.3412	1450.6485	725.8279	1449.6645	725.3359	12
8	**959.4139**	480.2106	942.3873	471.6973	941.4033	471.2053	I	**1352.6481**	676.8277	1335.6216	668.3144	1334.6375	667.8224	11
9	1060.4616	530.7344	1043.4350	522.2211	**1042.4510**	521.7291	T	**1239.5640**	620.2857	1222.5375	611.7724	1221.5535	611.2804	10
10	1223.5249	612.2661	1206.4983	603.7528	1205.5143	603.2608	Y	**1138.5164**	569.7618	1121.4898	561.2485	1120.5058	560.7565	9
11	1337.5678	669.2875	1320.5413	660.7743	1319.5572	660.2823	N	**975.4530**	488.2302	958.4265	479.7169	957.4425	479.2249	8
12	1436.6362	718.8217	1419.6097	710.3085	1418.6257	709.8165	V	**861.4101**	431.2087	844.3836	422.6954	843.3995	422.2034	7
13	1551.6632	776.3352	1534.6366	767.8219	1533.6526	767.3299	N	**762.3417**	381.6745	745.3151	373.1612	744.3311	372.6692	6
14	1666.6901	833.8487	1649.6636	825.3354	1648.6795	824.8434	D	**647.3148**	324.1610	630.2882	315.6477	629.3042	315.1557	5
15	1767.7378	884.3725	1750.7112	875.8593	1749.7272	875.3672	T	**532.2878**	266.6475	515.2613	258.1343	514.2772	257.6423	4
16	1914.8062	957.9067	1897.7796	949.3935	1896.7956	948.9015	F	431.2401	216.1237	414.2136	207.6104			3
17	2051.8651	1026.4362	2034.8386	1017.9229	2033.8545	1017.4309	H	284.1717	142.5895	267.1452	134.0762			2
18							K	147.1128	74.0600	130.0863	65.5468			1

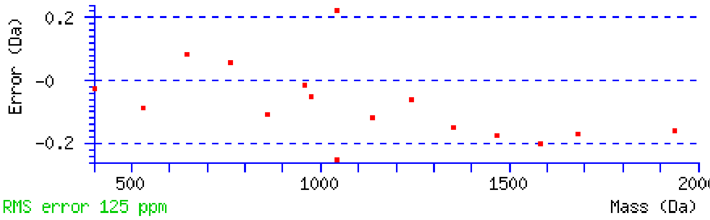

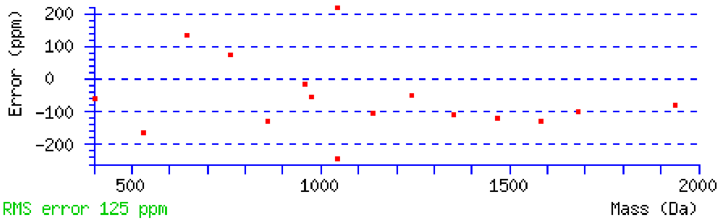

All matches to this query

Score	Mr(calc):	Delta	Sequence
85.7	2196.9634	0.0060	DQCIVDDITYNVNDTFHK
56.1	2196.9634	0.0060	DQCIVDDITYNVNDTFHK
20.4	2195.9794	0.9900	DQCIVDDITYNVNDTFHK

7.1	2196.9752	-0.0059	HGSLXLSVTPTLRGSMHNK
6.9	2196.9583	0.0111	DDQGKNQSNRNVSSAICPK
6.0	2196.9752	-0.0059	HGSLXLSVTPTLRGSMHNK
6.0	2196.9752	-0.0059	HGSLXLSVTPTLRGSMHNK
5.1	2196.9857	-0.0164	VIHLSNLPHSGYSDSAVLK
3.0	2196.9874	-0.0181	DSYDMATLLINHGANVNLR
2.3	2196.9705	-0.0011	DGTELSIQVSNQYKAEKK

Spectrum No: 102; Query: 331; Rank: 1

Peptide View

MS/MS Fragmentation of **NGSEADIDESLYSR**
Found in **IPI00368347**, Tax_Id=10116 Gene_Symbol=Ube1x Ubiquitin-activating enzyme E1, Chr X

Match to Query 331: 1555.664368 from(778.839460,2+)
Title: 091127RatKid_SCX01_14.2221.2221.2.dta
Data file K:\NewmanPaper\Piliang\3SubProteomes\Piliang3SP\mgf5ppm\SCX_3SubProteomes5ppm.mgf

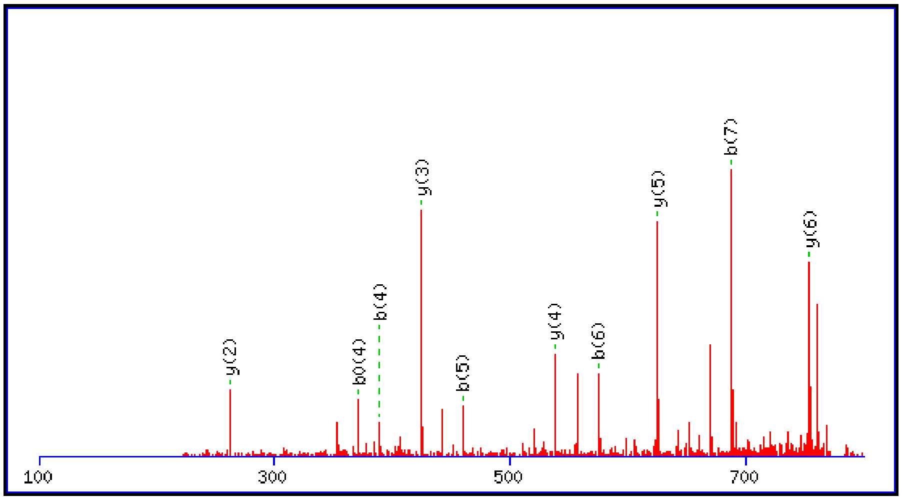

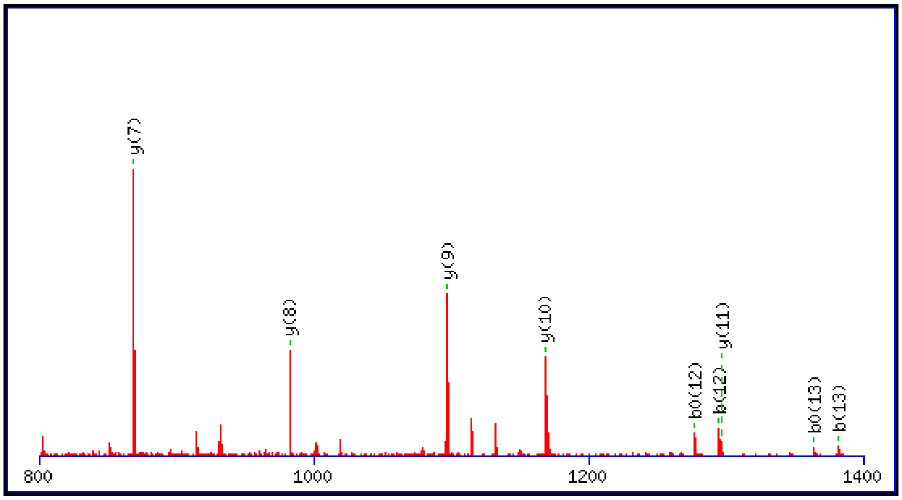

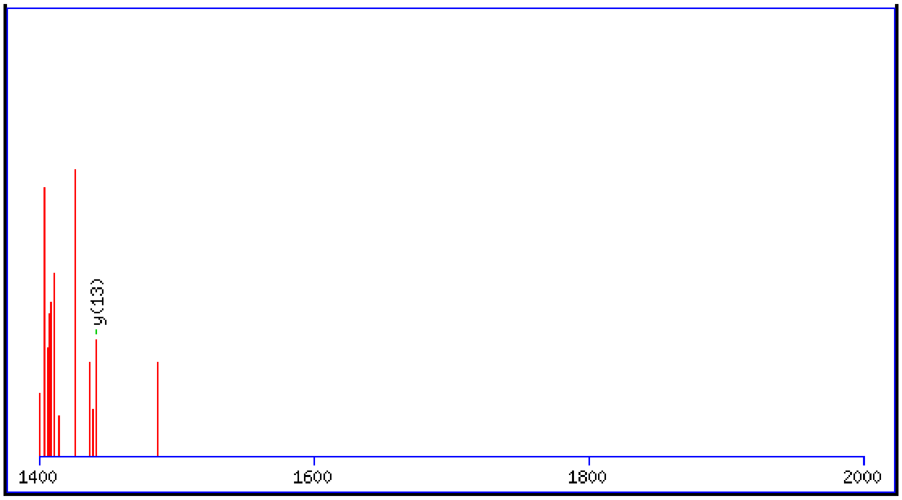

Monoisotopic mass of neutral peptide Mr(calc): 1555.6638
Fixed modifications: Carbamidomethyl (C)
Variable modifications:
N1 : Deamidated_N (N)
Ions Score: 85 **Expect:** 4.3e-007
Matches (Bold Red): 20/150 fragment ions using 35 most intense peaks

#	b	b^{++}	b*	b^{*++}	b^{0}	b^{0++}	Seq.	y	y^{++}	y*	y^{*++}	y^{0}	y^{0++}	#
1	116.0342	58.5207	99.0077	50.0075			N							14
2	173.0557	87.0315	156.0291	78.5182			G	**1441.6441**	721.3257	1424.6176	712.8124	1423.6336	712.3204	13
3	260.0877	130.5475	243.0612	122.0342	242.0771	121.5422	S	1384.6227	692.8150	1367.5961	684.3017	1366.6121	683.8097	12
4	**389.1303**	195.0688	372.1037	186.5555	**371.1197**	186.0635	E	**1297.5907**	649.2990	1280.5641	640.7857	1279.5801	640.2937	11
5	**460.1674**	230.5873	443.1409	222.0741	442.1568	221.5821	A	**1168.5481**	584.7777	1151.5215	576.2644	1150.5375	575.7724	10
6	**575.1944**	288.1008	558.1678	279.5875	557.1838	279.0955	D	**1097.5109**	549.2591	1080.4844	540.7458	1079.5004	540.2538	9
7	**688.2784**	344.6428	671.2519	336.1296	670.2679	335.6376	I	**982.4840**	491.7456	965.4575	483.2324	964.4734	482.7404	8
8	803.3054	402.1563	786.2788	393.6430	785.2948	393.1510	D	**869.3999**	435.2036	852.3734	426.6903	851.3894	426.1983	7
9	932.3480	466.6776	915.3214	458.1643	914.3374	457.6723	E	**754.3730**	377.6901	737.3464	369.1769	736.3624	368.6849	6
10	1019.3800	510.1936	1002.3534	501.6804	1001.3694	501.1883	S	**625.3304**	313.1688	608.3039	304.6556	607.3198	304.1636	5
11	1132.4640	566.7357	1115.4375	558.2224	1114.4535	557.7304	L	**538.2984**	269.6528	521.2718	261.1396	520.2878	260.6475	4
12	**1295.5274**	648.2673	1278.5008	639.7541	**1277.5168**	639.2620	Y	**425.2143**	213.1108	408.1878	204.5975	407.2037	204.1055	3
13	**1382.5594**	691.7833	1365.5329	683.2701	**1364.5488**	682.7781	S	**262.1510**	131.5791	245.1244	123.0659	244.1404	122.5738	2
14							R	175.1190	88.0631	158.0924	79.5498			1

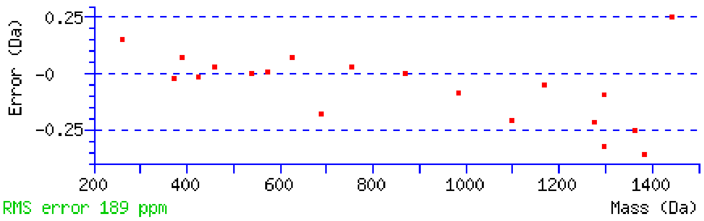

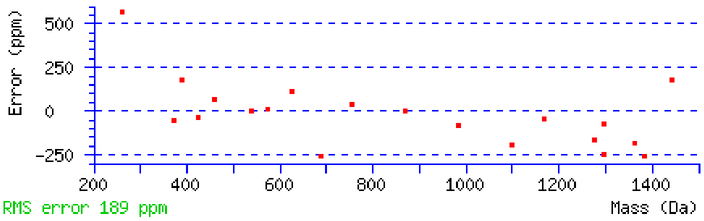

All matches to this query

Score	Mr(calc):	Delta	Sequence
85.3	1555.6638	0.0006	NGSEADIDESLYSR
15.7	1555.6784	-0.0140	QLETSETSSMGQSR
10.6	1555.6780	-0.0136	YQPRDEEVRER
9.2	1553.6507	2.0137	MKMFESLDSSATK
9.2	1553.6507	2.0137	MKMFESLDSSATK
9.2	1553.6507	2.0137	MKMFESLDSSATK
8.6	1555.6799	-0.0156	TLNMDMFTWEPR

6.8	1555.6603	0.0041	SHRLGGTMPFDSR
6.1	1554.6675	0.9969	EQLSSSDTAPRER
5.7	1555.6702	-0.0058	GTGATGKDTMGDLPR

Spectrum No: 103; Query: 655; Rank: 1

Peptide View

MS/MS Fragmentation of **VNASTTDPNSTVEQSALTR**
Found in **IPI00365542**, Tax_Id=10116 Gene_Symbol=Lamb1_predicted laminin, beta 1

Match to Query 655: 1990.944388 from(996.479470,2+)
Title: 091127RatKid_SCX01_16.1540.1540.2.dta
Data file K:\NewmanPaper\Piliang\3SubProteomes\Piliang3SP\mgf5ppm\SCX_3SubProteomes5ppm.mgf

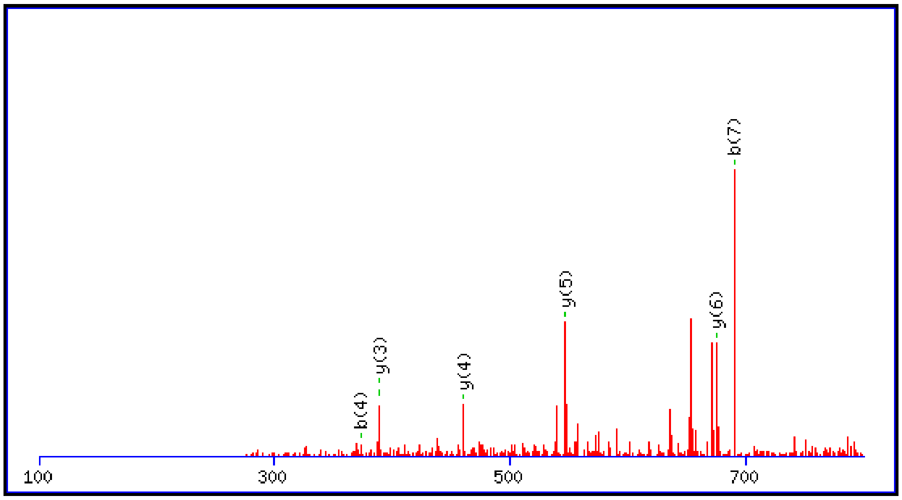

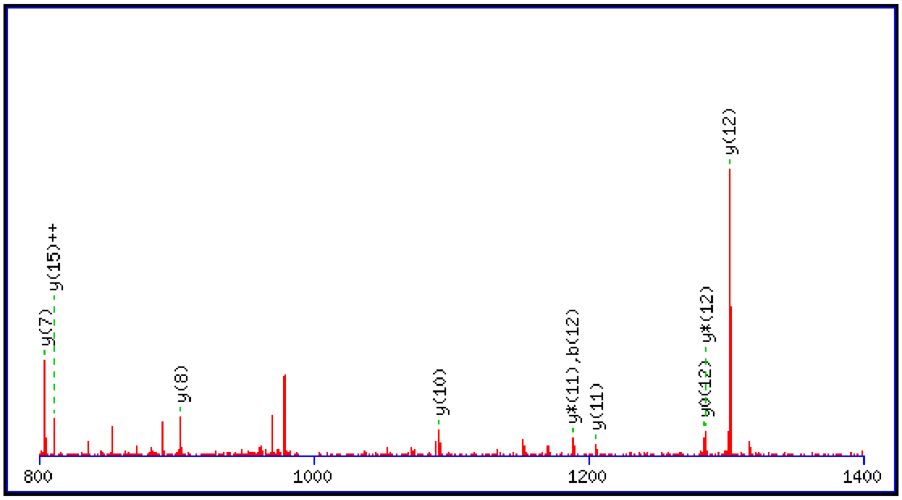

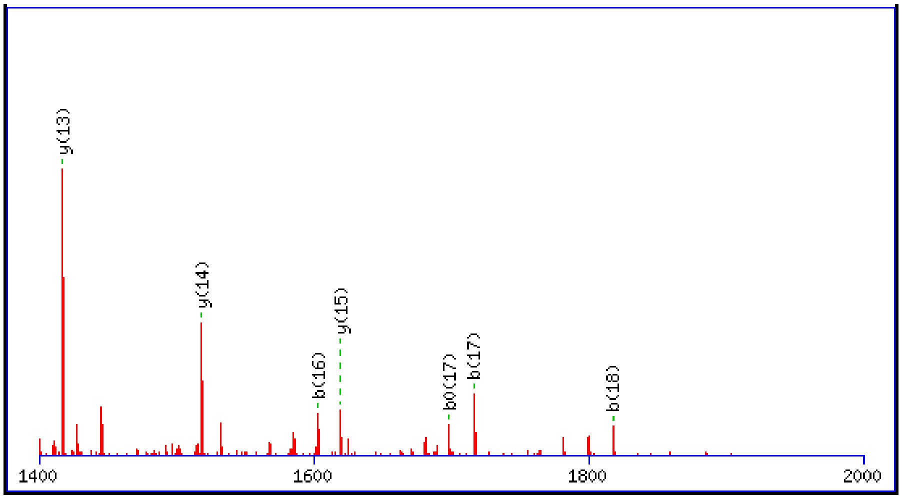

Monoisotopic mass of neutral peptide Mr(calc): 1990.9443
Fixed modifications: Carbamidomethyl (C)
Variable modifications:
N2 : Deamidated_N (N)
Ions Score: 85 **Expect:** 7.3e-007
Matches (Bold Red): 23/206 fragment ions using 40 most intense peaks

#	b	b^{++}	b*	b^{*++}	b^0	b^{0++}	Seq.	y	y^{++}	y*	y^{*++}	y^0	y^{0++}	#
1	100.0757	50.5415					V							19
2	215.1026	108.0550	198.0761	99.5417			N	1892.8832	946.9452	1875.8567	938.4320	1874.8726	937.9400	18
3	286.1397	143.5735	269.1132	135.0602			A	1777.8563	889.4318	1760.8297	880.9185	1759.8457	880.4265	17
4	**373.1718**	187.0895	356.1452	178.5762	355.1612	178.0842	S	1706.8191	853.9132	1689.7926	845.3999	1688.8086	844.9079	16
5	474.2194	237.6134	457.1929	229.1001	456.2089	228.6081	T	**1619.7871**	**810.3972**	1602.7606	801.8839	1601.7766	801.3919	15
6	575.2671	288.1372	558.2406	279.6239	557.2566	279.1319	T	**1518.7394**	759.8734	1501.7129	751.3601	1500.7289	750.8681	14
7	**690.2941**	345.6507	673.2675	337.1374	672.2835	336.6454	D	**1417.6918**	709.3495	1400.6652	700.8362	1399.6812	700.3442	13
8	787.3468	394.1771	770.3203	385.6638	769.3363	385.1718	P	**1302.6648**	651.8360	**1285.6383**	643.3228	**1284.6543**	642.8308	12
9	901.3898	451.1985	884.3632	442.6852	883.3792	442.1932	N	**1205.6121**	603.3097	**1188.5855**	594.7964	1187.6015	594.3044	11
10	988.4218	494.7145	971.3952	486.2013	970.4112	485.7092	S	**1091.5691**	546.2882	1074.5426	537.7749	1073.5586	537.2829	10
11	1089.4695	545.2384	1072.4429	536.7251	1071.4589	536.2331	T	1004.5371	502.7722	987.5106	494.2589	986.5265	493.7669	9
12	**1188.5379**	594.7726	1171.5113	586.2593	1170.5273	585.7673	V	**903.4894**	452.2483	886.4629	443.7351	885.4789	443.2431	8
13	1317.5805	659.2939	1300.5539	650.7806	1299.5699	650.2886	E	**804.4210**	402.7141	787.3945	394.2009	786.4104	393.7089	7
14	1445.6391	723.3232	1428.6125	714.8099	1427.6285	714.3179	Q	**675.3784**	338.1928	658.3519	329.6796	657.3679	329.1876	6
15	1532.6711	766.8392	1515.6445	758.3259	1514.6605	757.8339	S	**547.3198**	274.1636	530.2933	265.6503	529.3093	265.1583	5
16	**1603.7082**	802.3577	1586.6816	793.8445	1585.6976	793.3525	A	**460.2878**	230.6475	443.2613	222.1343	442.2772	221.6423	4
17	**1716.7923**	858.8998	1699.7657	850.3865	**1698.7817**	849.8945	L	**389.2507**	195.1290	372.2241	186.6157	371.2401	186.1237	3
18	**1817.8399**	909.4236	1800.8134	900.9103	1799.8294	900.4183	T	276.1666	138.5870	259.1401	130.0737	258.1561	129.5817	2
19							R	175.1190	88.0631	158.0924	79.5498			1

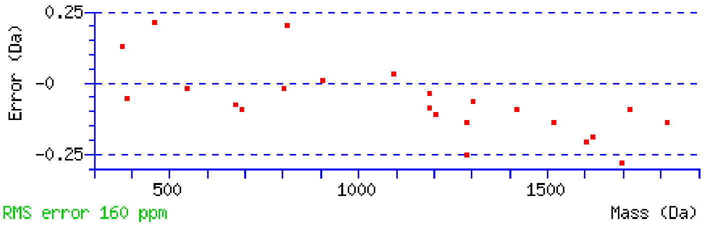

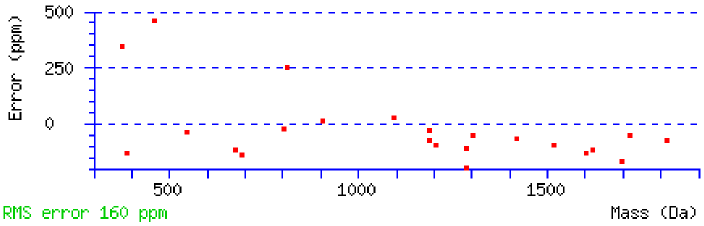

All matches to this query

Score	Mr(calc):	Delta	Sequence
85.3	1990.9443	0.0001	VNASTTDPNSTVEQSALTR
64.0	1989.9603	0.9841	VNASTTDPNSTVEQSALTR

55.7	1990.9443	0.0001	VNASTTDPNSTVEQSALTR
10.0	1990.9249	0.0195	SDTGLYSITAVNNLGTASK
10.0	1990.9249	0.0195	SDTGLYSITAVNNLGTASK
9.6	1988.9456	1.9988	SPSTYNALESGLTSLTIR
4.2	1988.9456	1.9988	SPSTYNALESGLTSLTIR
4.2	1990.9249	0.0195	SDTGLYSITAVNNLGTASK
4.2	1990.9249	0.0195	SDTGLYSITAVNNLGTASK
2.9	1990.9249	0.0195	SDTGLYSITAVNNLGTASK

Spectrum No: 104; Query: 880; Rank: 1

Peptide View

MS/MS Fragmentation of **ALNELASQGFPGNATTPSGEQLR**
Found in **IPI00190577**, Tax_Id=10116 Gene_Symbol=Lama5 similar to Laminin alpha-5 chain precursor

Match to Query 880: 2358.155568 from(1180.085060,2+)
Title: 091127RatKid_SCX01_02.3124.3124.2.dta
Data file K:\NewmanPaper\Piliang\3SubProteomes\Piliang3SP\mgf5ppm\SCX_3SubProteomes5ppm.mgf

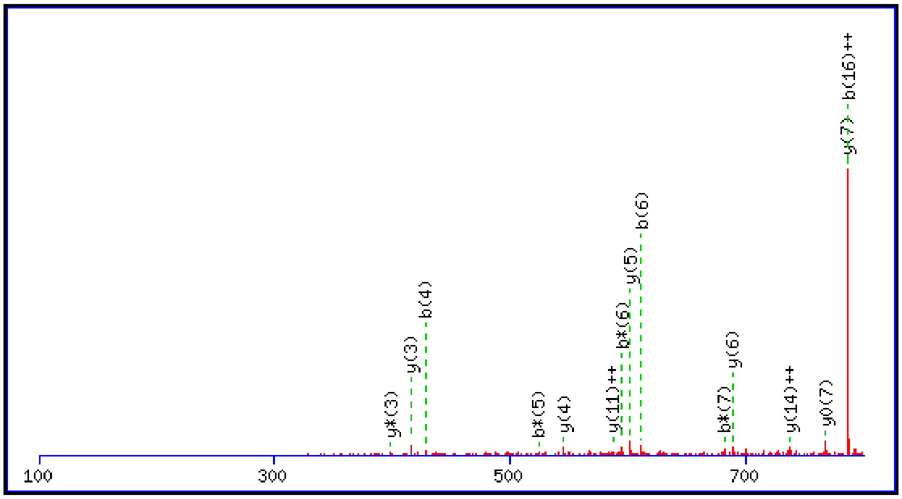

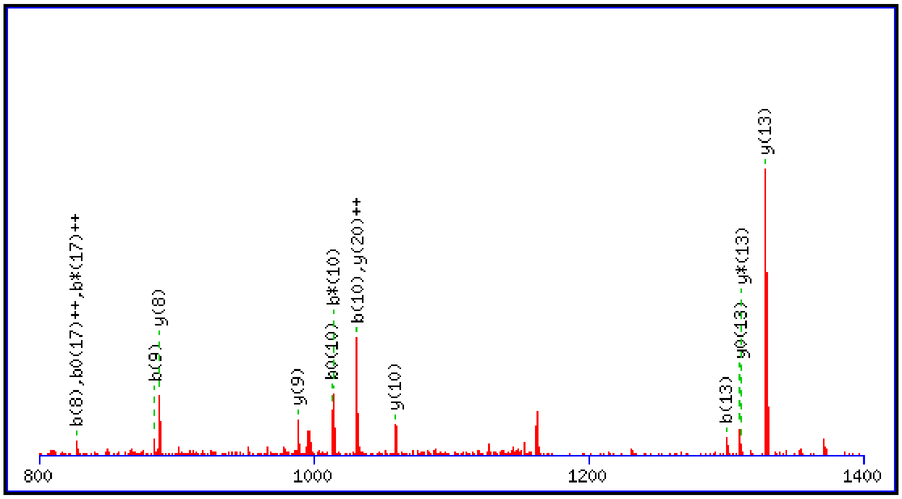

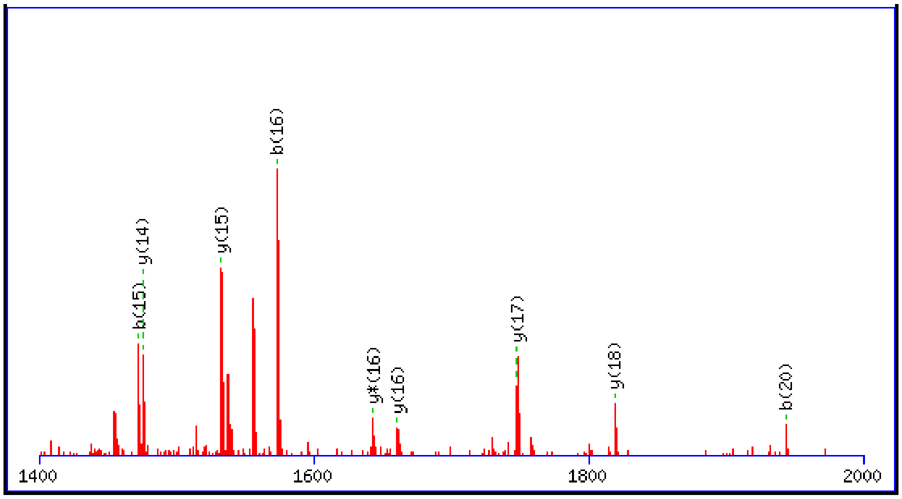

Monoisotopic mass of neutral peptide Mr(calc): 2358.1451
Fixed modifications: Carbamidomethyl (C)
Variable modifications:
N13 : Deamidated_N (N)
Ions Score: 85 **Expect:** 7e-007
Matches (Bold Red): 39/248 fragment ions using 62 most intense peaks

#	b	b^{++}	b*	b^{*++}	b^{0}	b^{0++}	Seq.	y	y^{++}	y*	y^{*++}	y^{0}	y^{0++}	#
1	72.0444	36.5258					A							23
2	185.1285	93.0679					L	2288.1153	1144.5613	2271.0888	1136.0480	2270.1048	1135.5560	22
3	299.1714	150.0893	282.1448	141.5761			N	2175.0313	1088.0193	2158.0047	1079.5060	2157.0207	1079.0140	21
4	**428.2140**	214.6106	411.1874	206.0974	410.2034	205.6053	E	2060.9883	**1030.9978**	2043.9618	1022.4845	2042.9778	1021.9925	20
5	541.2980	271.1527	**524.2715**	262.6394	523.2875	262.1474	L	1931.9457	966.4765	1914.9192	957.9632	1913.9352	957.4712	19
6	**612.3352**	306.6712	**595.3086**	298.1579	594.3246	297.6659	A	**1818.8617**	909.9345	1801.8351	901.4212	1800.8511	900.9292	18
7	699.3672	350.1872	**682.3406**	341.6740	681.3566	341.1819	S	**1747.8246**	874.4159	1730.7980	865.9026	1729.8140	865.4106	17
8	**827.4258**	414.2165	810.3992	405.7032	809.4152	405.2112	Q	**1660.7925**	830.8999	**1643.7660**	822.3866	1642.7820	821.8946	16
9	**884.4472**	442.7272	867.4207	434.2140	866.4367	433.7220	G	**1532.7340**	766.8706	1515.7074	758.3573	1514.7234	757.8653	15
10	**1031.5156**	516.2615	**1014.4891**	507.7482	**1013.5051**	507.2562	F	**1475.7125**	**738.3599**	1458.6859	729.8466	1457.7019	729.3546	14
11	1128.5684	564.7878	1111.5419	556.2746	1110.5578	555.7826	P	**1328.6441**	664.8257	**1311.6175**	656.3124	**1310.6335**	655.8204	13
12	1185.5899	593.2986	1168.5633	584.7853	1167.5793	584.2933	G	1231.5913	616.2993	1214.5648	607.7860	1213.5808	607.2940	12
13	**1300.6168**	650.8120	1283.5903	642.2988	1282.6062	641.8068	N	1174.5699	**587.7886**	1157.5433	579.2753	1156.5593	578.7833	11
14	1371.6539	686.3306	1354.6274	677.8173	1353.6433	677.3253	A	**1059.5429**	530.2751	1042.5164	521.7618	1041.5324	521.2698	10
15	**1472.7016**	736.8544	1455.6750	728.3412	1454.6910	727.8492	T	**988.5058**	494.7565	971.4793	486.2433	970.4952	485.7513	9
16	**1573.7493**	**787.3783**	1556.7227	778.8650	1555.7387	778.3730	T	**887.4581**	444.2327	870.4316	435.7194	869.4476	435.2274	8
17	1670.8020	835.9047	1653.7755	**827.3914**	1652.7915	**826.8994**	P	**786.4104**	393.7089	769.3839	385.1956	**768.3999**	384.7036	7
18	1757.8341	879.4207	1740.8075	870.9074	1739.8235	870.4154	S	**689.3577**	345.1825	672.3311	336.6692	671.3471	336.1772	6
19	1814.8555	907.9314	1797.8290	899.4181	1796.8450	898.9261	G	**602.3257**	301.6665	585.2991	293.1532	584.3151	292.6612	5
20	**1943.8981**	972.4527	1926.8716	963.9394	1925.8876	963.4474	E	**545.3042**	273.1557	528.2776	264.6425	527.2936	264.1504	4
21	2071.9567	1036.4820	2054.9301	1027.9687	2053.9461	1027.4767	Q	**416.2616**	208.6344	**399.2350**	200.1212			3
22	2185.0408	1093.0240	2168.0142	1084.5107	2167.0302	1084.0187	L	288.2030	144.6051	271.1765	136.0919			2
23							R	175.1190	88.0631	158.0924	79.5498			1

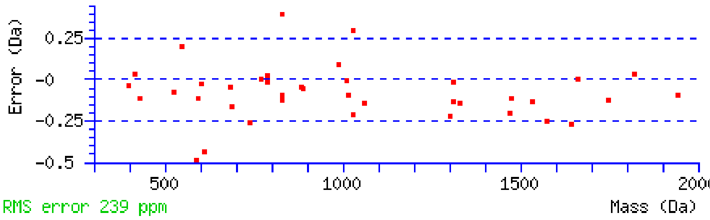

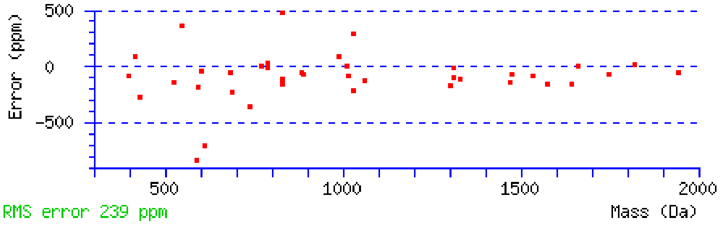

All matches to this query

Score	Mr(calc):	Delta	Sequence
85.1	2358.1451	0.0104	ALNELASQGFPGNATTPSGEQLR
27.1	2357.1611	0.9944	ALNELASQGFPGNATTPSGEQLR
22.4	2358.1451	0.0104	ALNELASQGFPGNATTPSGEQLR
12.5	2358.1597	-0.0041	ELRSTLDSIPSLSLPSSIKR
10.7	2358.1597	-0.0041	ELRSTLDSIPSLSLPSSIKR
5.9	2358.1597	-0.0041	ELRSTLDSIPSLSLPSSIKR
4.5	2358.1354	0.0202	KLVQLLASSPCESVMKRPR
2.6	2358.1402	0.0153	SQLSLSMANHDIPPDAVRAKK
2.3	2358.1597	-0.0041	ELRSTLDSIPSLSLPSSIKR
1.6	2358.1416	0.0139	WPRMTVVPTAHSLPSRSXQR

Spectrum No: 105; Query: 475; Rank: 1

Peptide View

MS/MS Fragmentation of **VFDKEGNGTVMGAEIR**
Found in **IPI00365944**, Tax_Id=10116 Gene_Symbol=Myl6_predicted Myosin light polypeptide 6

Match to Query 475: 1722.829108 from(862.421830,2+)
Title: 091129RatKid_SCX02_29.1812.1812.2.dta
Data file K:\NewmanPaper\Piliang\3SubProteomes\Piliang3SP\mgf5ppm\SCX_3SubProteomes5ppm.mgf

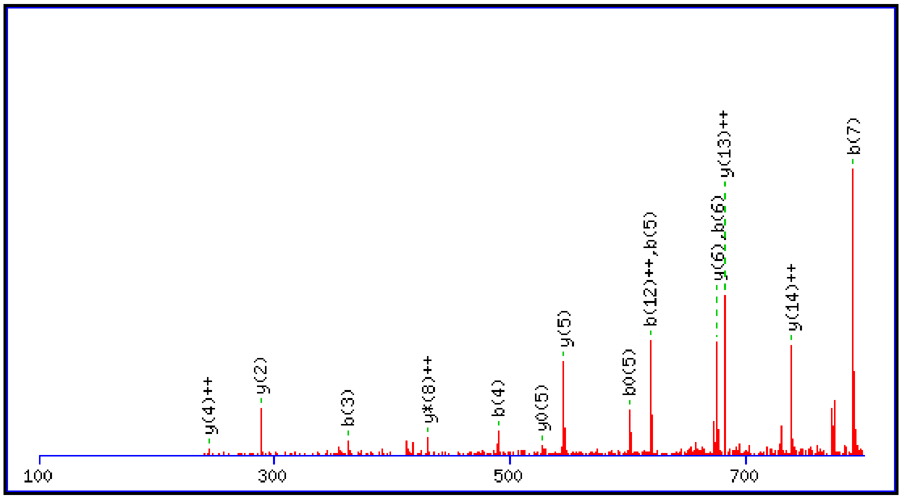

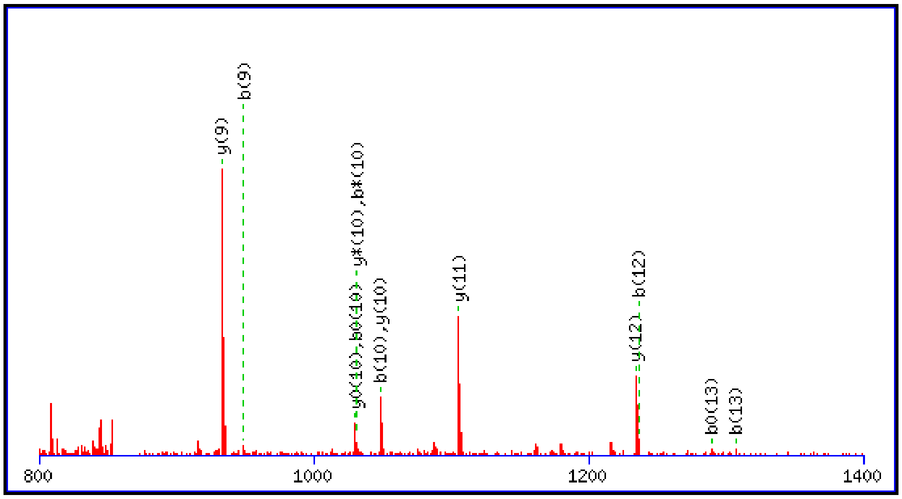

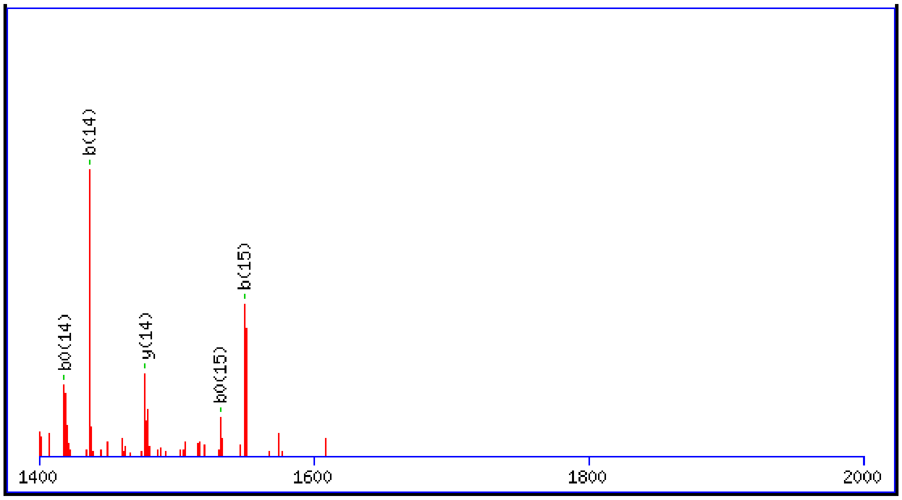

Monoisotopic mass of neutral peptide Mr(calc): 1722.8247
Fixed modifications: Carbamidomethyl (C)
Variable modifications:
N7 : Deamidated_N (N)
Ions Score: 85 **Expect:** 6.9e-007
Matches (Bold Red): 33/166 fragment ions using 45 most intense peaks

#	b	b++	b*	b*++	b0	b0++	Seq.	y	y++	y*	y*++	y0	y0++	#
1	100.0757	50.5415					V							16
2	247.1441	124.0757					F	1624.7635	812.8854	1607.7370	804.3721	1606.7530	803.8801	15
3	**362.1710**	181.5892			344.1605	172.5839	D	**1477.6951**	**739.3512**	1460.6686	730.8379	1459.6846	730.3459	14
4	**490.2660**	245.6366	473.2395	237.1234	472.2554	236.6314	K	1362.6682	**681.8377**	1345.6416	673.3245	1344.6576	672.8324	13
5	**619.3086**	310.1579	602.2821	301.6447	**601.2980**	301.1527	E	**1234.5732**	617.7902	1217.5467	609.2770	1216.5627	608.7850	12
6	**676.3301**	338.6687	659.3035	330.1554	658.3195	329.6634	G	**1105.5306**	553.2690	1088.5041	544.7557	1087.5201	544.2637	11
7	**791.3570**	396.1821	774.3305	387.6689	773.3464	387.1769	N	**1048.5092**	524.7582	**1031.4826**	516.2449	**1030.4986**	515.7529	10
8	848.3785	424.6929	831.3519	416.1796	830.3679	415.6876	G	**933.4822**	467.2448	916.4557	458.7315	915.4717	458.2395	9
9	**949.4261**	475.2167	932.3996	466.7034	931.4156	466.2114	T	876.4608	438.7340	859.4342	**430.2207**	858.4502	429.7287	8
10	**1048.4946**	524.7509	**1031.4680**	516.2376	**1030.4840**	515.7456	V	775.4131	388.2102	758.3865	379.6969	757.4025	379.2049	7
11	1179.5350	590.2712	1162.5085	581.7579	1161.5245	581.2659	M	**676.3447**	338.6760	659.3181	330.1627	658.3341	329.6707	6
12	**1236.5565**	**618.7819**	1219.5300	610.2686	1218.5459	609.7766	G	**545.3042**	273.1557	528.2776	264.6425	**527.2936**	264.1504	5
13	**1307.5936**	654.3004	1290.5671	645.7872	**1289.5831**	645.2952	A	488.2827	**244.6450**	471.2562	236.1317	470.2722	235.6397	4
14	**1436.6362**	718.8217	1419.6097	710.3085	**1418.6257**	709.8165	E	417.2456	209.1264	400.2191	200.6132	399.2350	200.1212	3
15	**1549.7203**	775.3638	1532.6937	766.8505	**1531.7097**	766.3585	I	**288.2030**	144.6051	271.1765	136.0919			2
16							R	175.1190	88.0631	158.0924	79.5498			1

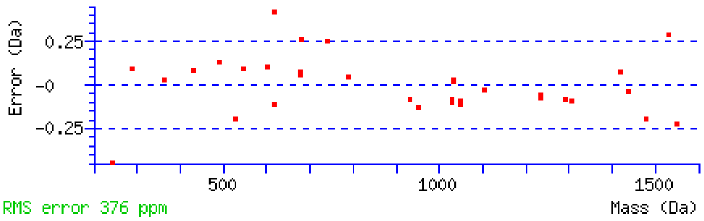

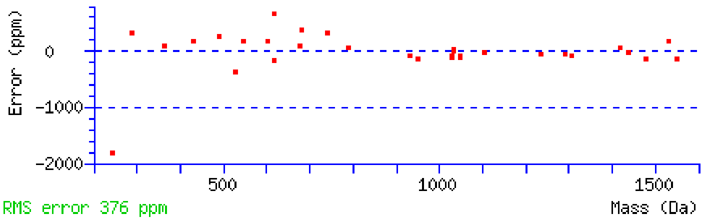

All matches to this query

Score	Mr(calc):	Delta	Sequence
85.0	1722.8247	0.0044	VFDKEGNGTVMGAEIR
85.0	1722.8247	0.0044	VFDKEGNGTVMGAELR
26.0	1721.8407	0.9884	VFDKEGNGTVMGAEIR
26.0	1721.8407	0.9884	VFDKEGNGTVMGAELR
15.0	1722.8213	0.0078	NEDLFSQPVSFNGIR

10.7	1720.8219	2.0072	STLKPNAPXPNSAAVK
9.8	1722.8276	0.0015	NKYHPDLRMVTIR
7.3	1722.8414	-0.0123	GTAQGTPRNSXGTVLR
7.1	1722.8280	0.0011	DKEVAALTSQMDMLR
6.8	1722.8124	0.0167	RMLVSGQGPLVENAR

Spectrum No: 106; Query: 475; Rank: 1

Peptide View

MS/MS Fragmentation of **VFDKEGNGTVMGAEIR**
Found in **IPI00365944**, Tax_Id=10116 Gene_Symbol=Myl6_predicted Myosin light polypeptide 6

Match to Query 475: 1722.829108 from(862.421830,2+)
Title: 091129RatKid_SCX02_29.1812.1812.2.dta
Data file K:\NewmanPaper\Piliang\3SubProteomes\Piliang3SP\mgf5ppm\SCX_3SubProteomes5ppm.mgf

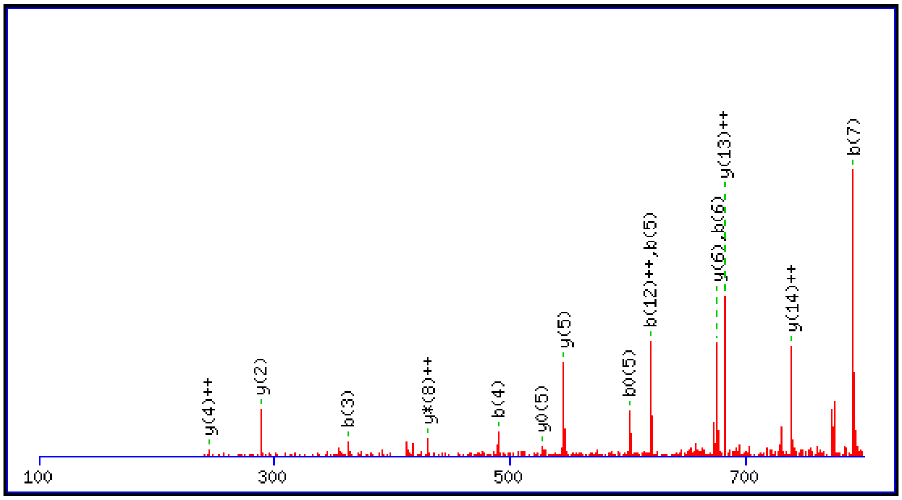

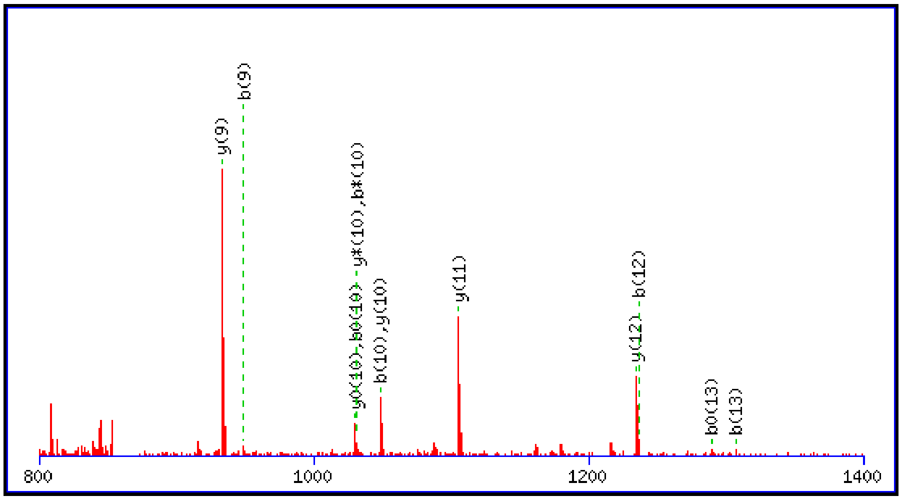

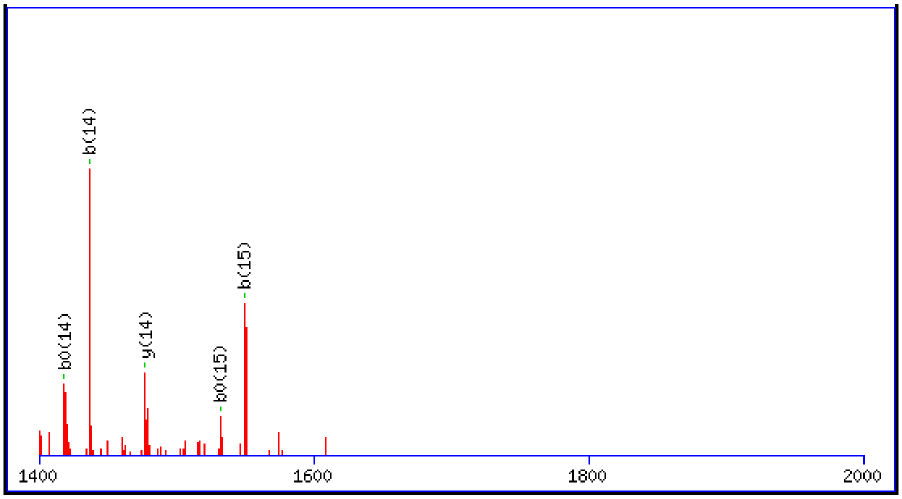

Monoisotopic mass of neutral peptide Mr(calc): 1722.8247
Fixed modifications: Carbamidomethyl (C)
Variable modifications:
N7 : Deamidated_N (N)
Ions Score: 85 **Expect:** 6.9e-007
Matches (Bold Red): 33/166 fragment ions using 45 most intense peaks

#	b	b^{++}	b*	b^{*++}	b^0	b^{0++}	Seq.	y	y^{++}	y*	y^{*++}	y^0	y^{0++}	#
1	100.0757	50.5415					V							16
2	247.1441	124.0757					F	1624.7635	812.8854	1607.7370	804.3721	1606.7530	803.8801	15
3	**362.1710**	181.5892			344.1605	172.5839	D	**1477.6951**	**739.3512**	1460.6686	730.8379	1459.6846	730.3459	14
4	**490.2660**	245.6366	473.2395	237.1234	472.2554	236.6314	K	1362.6682	**681.8377**	1345.6416	673.3245	1344.6576	672.8324	13
5	**619.3086**	310.1579	602.2821	301.6447	**601.2980**	301.1527	E	**1234.5732**	617.7902	1217.5467	609.2770	1216.5627	608.7850	12
6	**676.3301**	338.6687	659.3035	330.1554	658.3195	329.6634	G	**1105.5306**	553.2690	1088.5041	544.7557	1087.5201	544.2637	11
7	**791.3570**	396.1821	774.3305	387.6689	773.3464	387.1769	N	**1048.5092**	524.7582	**1031.4826**	516.2449	**1030.4986**	515.7529	10
8	848.3785	424.6929	831.3519	416.1796	830.3679	415.6876	G	**933.4822**	467.2448	916.4557	458.7315	915.4717	458.2395	9
9	**949.4261**	475.2167	932.3996	466.7034	931.4156	466.2114	T	876.4608	438.7340	859.4342	**430.2207**	858.4502	429.7287	8
10	**1048.4946**	524.7509	**1031.4680**	516.2376	**1030.4840**	515.7456	V	775.4131	388.2102	758.3865	379.6969	757.4025	379.2049	7
11	1179.5350	590.2712	1162.5085	581.7579	1161.5245	581.2659	M	**676.3447**	338.6760	659.3181	330.1627	658.3341	329.6707	6
12	**1236.5565**	**618.7819**	1219.5300	610.2686	1218.5459	609.7766	G	**545.3042**	273.1557	528.2776	264.6425	**527.2936**	264.1504	5
13	**1307.5936**	654.3004	1290.5671	645.7872	**1289.5831**	645.2952	A	488.2827	**244.6450**	471.2562	236.1317	470.2722	235.6397	4
14	**1436.6362**	718.8217	1419.6097	710.3085	**1418.6257**	709.8165	E	417.2456	209.1264	400.2191	200.6132	399.2350	200.1212	3
15	**1549.7203**	775.3638	1532.6937	766.8505	**1531.7097**	766.3585	I	**288.2030**	144.6051	271.1765	136.0919			2
16							R	175.1190	88.0631	158.0924	79.5498			1

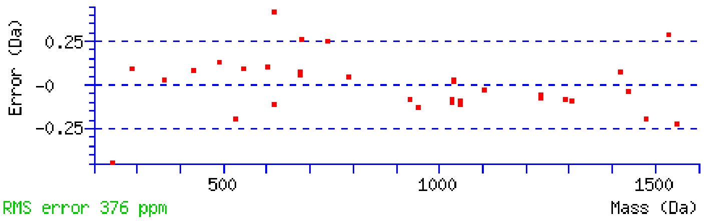

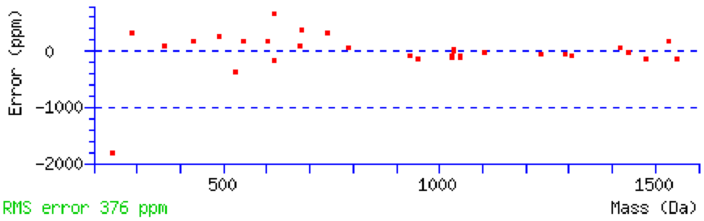

All matches to this query

Score	Mr(calc):	Delta	Sequence
85.0	1722.8247	0.0044	VFDKEGNGTVMGAEIR
85.0	1722.8247	0.0044	VFDKEGNGTVMGAELR
26.0	1721.8407	0.9884	VFDKEGNGTVMGAEIR
26.0	1721.8407	0.9884	VFDKEGNGTVMGAELR
15.0	1722.8213	0.0078	NEDLFSQPVSFNGIR

10.7	1720.8219	2.0072	STLKPNAPXPNSAAVK
9.8	1722.8276	0.0015	NKYHPDLRMVTIR
7.3	1722.8414	-0.0123	GTAQGTPRNSXGTVLR
7.1	1722.8280	0.0011	DKEVAALTSQMDMLR
6.8	1722.8124	0.0167	RMLVSGQGPLVENAR

Spectrum No: 107; Query: 727; Rank: 1

Peptide View

MS/MS Fragmentation of **AWGTPCELCPPVNTSEYK**
Found in **IPI00204006**, Tax_Id=10116 Gene_Symbol=Fbn1 Fibrillin-1

Match to Query 727: 2108.922228 from(1055.468390,2+)
Title: 091127RatKid_SCX01_12.2881.2881.2.dta
Data file K:\NewmanPaper\Piliang\3SubProteomes\Piliang3SP\mgf5ppm\SCX_3SubProteomes5ppm.mgf

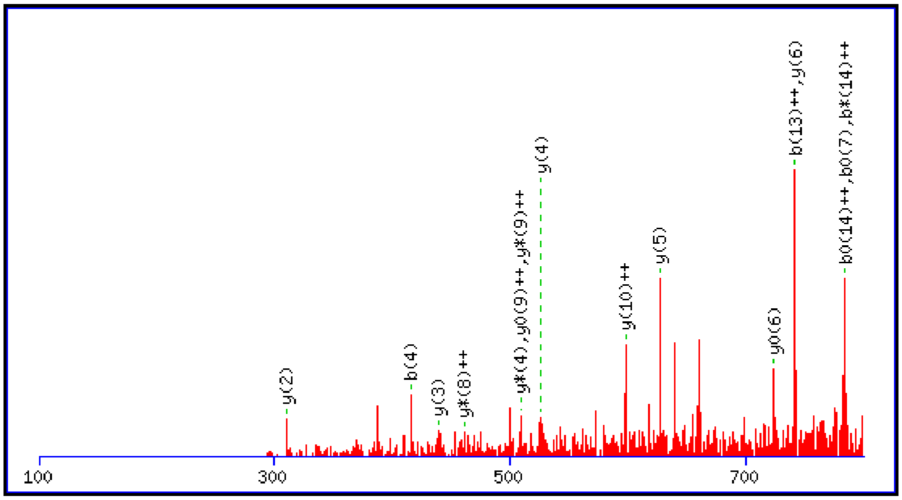

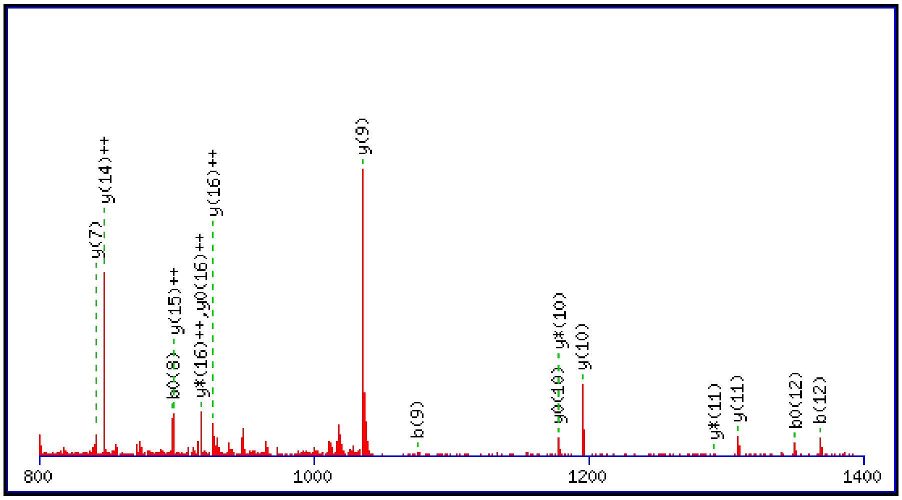

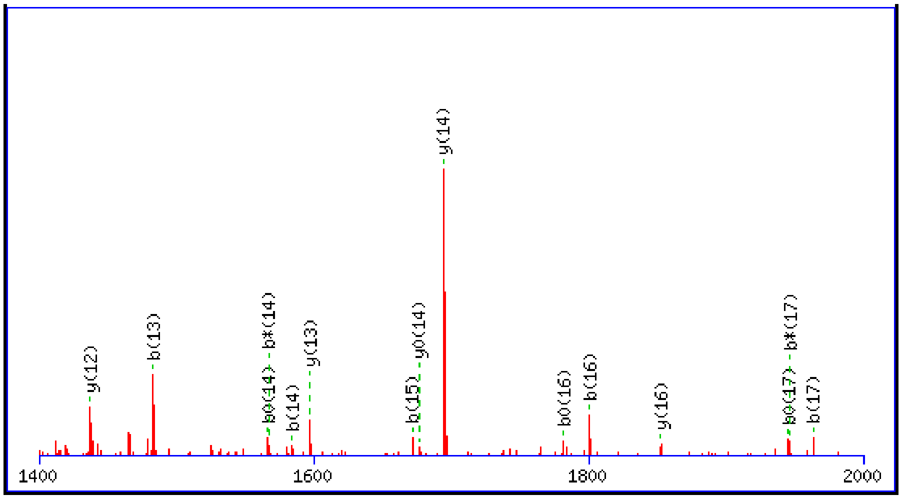

Monoisotopic mass of neutral peptide Mr(calc): 2108.9183
Fixed modifications: Carbamidomethyl (C)
Variable modifications:
N13 : Deamidated_N (N)
Ions Score: 85 **Expect:** 8.9e-007
Matches (Bold Red): 47/170 fragment ions using 75 most intense peaks

#	b	b^{++}	b*	b^{*++}	b^{0}	b^{0++}	Seq.	y	y^{++}	y*	y^{*++}	y^{0}	y^{0++}	#
1	72.0444	36.5258					A							18
2	258.1237	129.5655					W	2038.8885	1019.9479	2021.8619	1011.4346	2020.8779	1010.9426	17
3	315.1452	158.0762					G	**1852.8092**	**926.9082**	1835.7826	**918.3950**	1834.7986	**917.9029**	16
4	**416.1928**	208.6001			398.1823	199.5948	T	1795.7877	**898.3975**	1778.7612	889.8842	1777.7771	889.3922	15
5	513.2456	257.1264			495.2350	248.1212	P	**1694.7400**	**847.8737**	1677.7135	839.3604	**1676.7295**	838.8684	14
6	673.2763	337.1418			655.2657	328.1365	C	**1597.6873**	799.3473	1580.6607	790.8340	1579.6767	790.3420	13
7	802.3189	401.6631			**784.3083**	392.6578	E	**1437.6566**	719.3319	1420.6301	710.8187	1419.6461	710.3267	12
8	915.4029	458.2051			**897.3924**	449.1998	L	**1308.6140**	654.8107	**1291.5875**	646.2974	1290.6035	645.8054	11
9	**1075.4336**	538.2204			1057.4230	529.2151	C	**1195.5300**	**598.2686**	**1178.5034**	589.7553	**1177.5194**	589.2633	10
10	1172.4863	586.7468			1154.4758	577.7415	P	**1035.4993**	518.2533	1018.4728	**509.7400**	1017.4887	**509.2480**	9
11	1269.5391	635.2732			1251.5285	626.2679	P	938.4465	469.7269	921.4200	**461.2136**	920.4360	460.7216	8
12	**1368.6075**	684.8074			**1350.5969**	675.8021	V	**841.3938**	421.2005	824.3672	412.6873	823.3832	412.1952	7
13	**1483.6344**	**742.3209**	1466.6079	733.8076	1465.6239	733.3156	N	**742.3254**	371.6663	725.2988	363.1530	**724.3148**	362.6610	6
14	**1584.6821**	792.8447	**1567.6556**	**784.3314**	**1566.6716**	**783.8394**	T	**627.2984**	314.1529	610.2719	305.6396	609.2879	305.1476	5
15	**1671.7142**	836.3607	1654.6876	827.8474	1653.7036	827.3554	S	**526.2508**	263.6290	**509.2242**	255.1157	508.2402	254.6237	4
16	**1800.7567**	900.8820	1783.7302	892.3687	**1782.7462**	891.8767	E	**439.2187**	220.1130	422.1922	211.5997	421.2082	211.1077	3
17	**1963.8201**	982.4137	**1946.7935**	973.9004	**1945.8095**	973.4084	Y	**310.1761**	155.5917	293.1496	147.0784			2
18							K	147.1128	74.0600	130.0863	65.5468			1

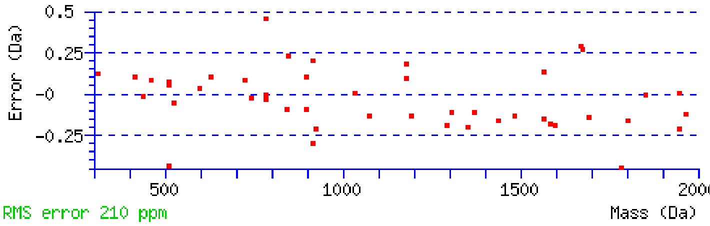

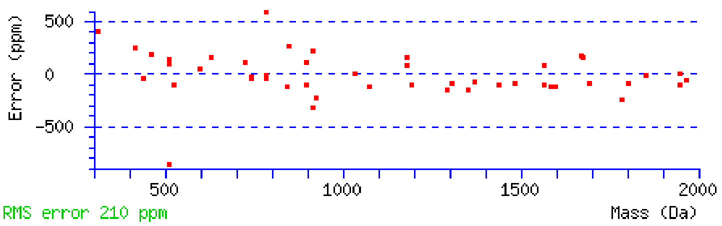

All matches to this query

Score	Mr(calc):	Delta	Sequence
84.6	2108.9183	0.0039	AWGTPCELCPPVNTSEYK
3.4	2107.9197	1.0026	MSVCTLSVPVSSISPGRR
3.4	2107.9197	1.0026	MSVCTLSVPVSSISPGRR

3.4	2107.9197	1.0026	MSVCTLSVPVSSISPGRR
2.0	2107.9268	0.9954	FIQNHLLADTTFVTEAK
2.0	2107.9268	0.9954	FIQNHLLADTTFVTEAK
0.1	2108.9126	0.0097	QFSQLPANVAVCSSETYK
0.1	2108.9405	-0.0183	LALDNVRPATVGGDHSTAR
0.1	2108.9405	-0.0183	LALDNVRPATVGGDHSTAR

Spectrum No: 108; Query: 491; Rank: 1

Peptide View

MS/MS Fragmentation of **VISNNCTSYGVLDIGK**
Found in **IPI00231197**, Tax_Id=10116 Gene_Symbol=Cd36 Platelet glycoprotein 4

Match to Query 491: 1739.845388 from(870.929970,2+)
Title: 091127RatKid_SCX01_16.2837.2837.2.dta
Data file K:\NewmanPaper\Piliang\3SubProteomes\Piliang3SP\mgf5ppm\SCX_3SubProteomes5ppm.mgf

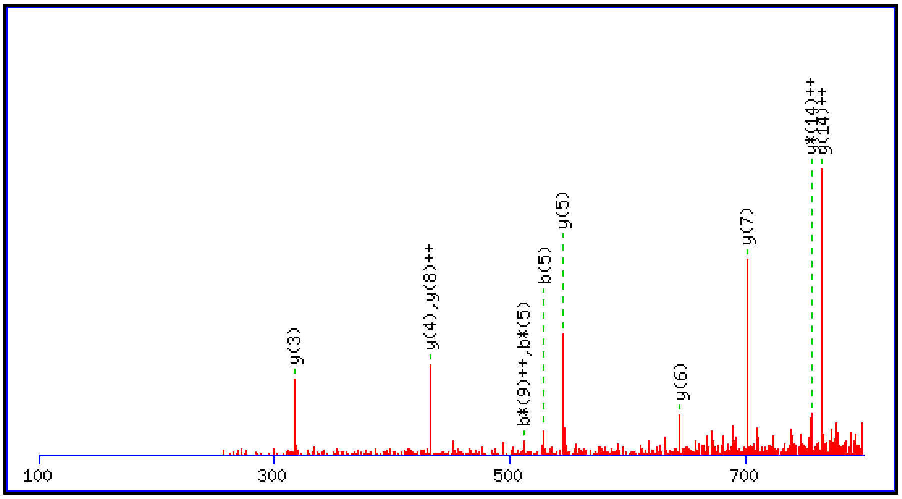

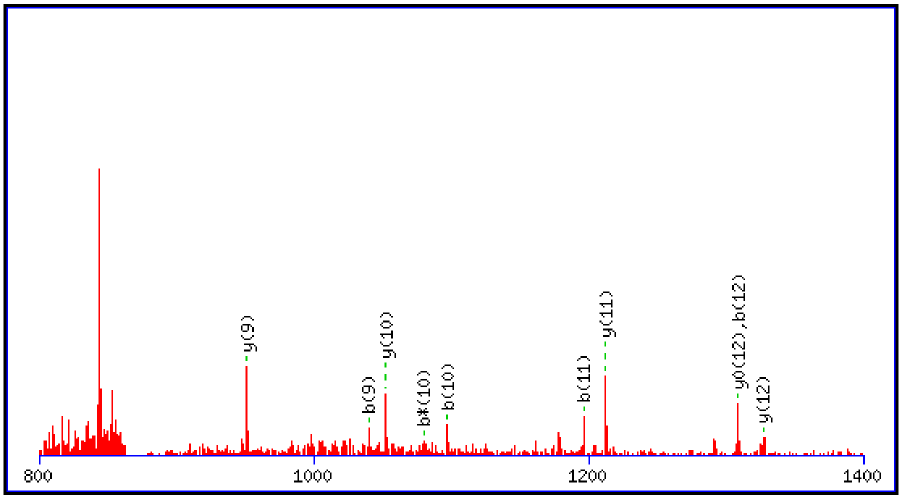

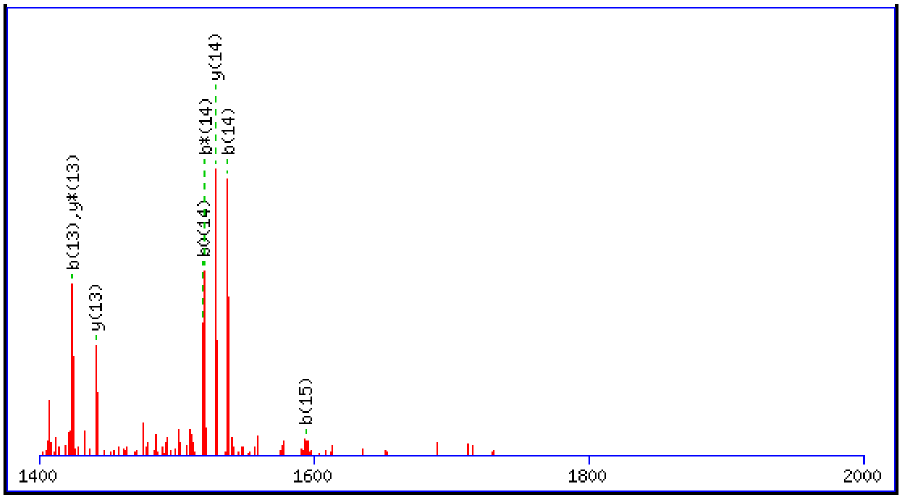

Monoisotopic mass of neutral peptide Mr(calc): 1739.8400
Fixed modifications: Carbamidomethyl (C)
Variable modifications:
N5 : Deamidated_N (N)
Ions Score: 85 **Expect:** 6.6e-007
Matches (Bold Red): 29/164 fragment ions using 38 most intense peaks

#	b	b^{++}	b*	b*$^{++}$	b^{0}	b^{0++}	Seq.	y	y^{++}	y*	y*$^{++}$	y^{0}	y^{0++}	#
1	100.0757	50.5415					V							16
2	213.1598	107.0835					I	1641.7789	821.3931	1624.7523	812.8798	1623.7683	812.3878	15
3	300.1918	150.5995			282.1812	141.5942	S	**1528.6948**	**764.8510**	1511.6682	**756.3378**	1510.6842	755.8458	14
4	414.2347	207.6210	397.2082	199.1077	396.2241	198.6157	N	**1441.6628**	721.3350	**1424.6362**	712.8217	1423.6522	712.3297	13
5	**529.2616**	265.1345	**512.2351**	256.6212	511.2511	256.1292	N	**1327.6198**	664.3136	1310.5933	655.8003	**1309.6093**	655.3083	12
6	689.2923	345.1498	672.2657	336.6365	671.2817	336.1445	C	**1212.5929**	606.8001	1195.5664	598.2868	1194.5823	597.7948	11
7	790.3400	395.6736	773.3134	387.1604	772.3294	386.6683	T	**1052.5623**	526.7848	1035.5357	518.2715	1034.5517	517.7795	10
8	877.3720	439.1896	860.3455	430.6764	859.3614	430.1844	S	**951.5146**	476.2609	934.4880	467.7477	933.5040	467.2556	9
9	**1040.4353**	520.7213	1023.4088	**512.2080**	1022.4248	511.7160	Y	864.4825	**432.7449**	847.4560	424.2316	846.4720	423.7396	8
10	**1097.4568**	549.2320	**1080.4302**	540.7188	1079.4462	540.2268	G	**701.4192**	351.2132	684.3927	342.7000	683.4087	342.2080	7
11	**1196.5252**	598.7662	1179.4987	590.2530	1178.5146	589.7610	V	**644.3978**	322.7025	627.3712	314.1892	626.3872	313.6972	6
12	**1309.6093**	655.3083	1292.5827	646.7950	1291.5987	646.3030	L	**545.3293**	273.1683	528.3028	264.6550	527.3188	264.1630	5
13	**1424.6362**	712.8217	1407.6097	704.3085	1406.6257	703.8165	D	**432.2453**	216.6263	415.2187	208.1130	414.2347	207.6210	4
14	**1537.7203**	769.3638	**1520.6937**	760.8505	**1519.7097**	760.3585	I	**317.2183**	159.1128	300.1918	150.5995			3
15	**1594.7417**	797.8745	1577.7152	789.3612	1576.7312	788.8692	G	204.1343	102.5708	187.1077	94.0575			2
16							K	147.1128	74.0600	130.0863	65.5468			1

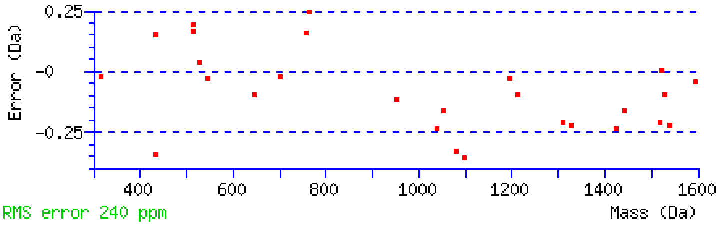

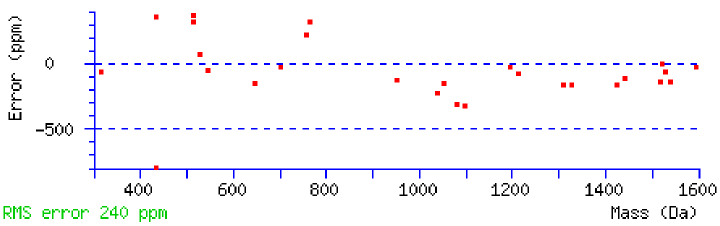

All matches to this query

Score	Mr(calc):	Delta	Sequence
84.5	1739.8400	0.0054	VISNNCTSYGVLDIGK
75.5	1739.8400	0.0054	VISNNCTSYGVLDIGK
52.2	1738.8560	0.9894	VISNNCTSYGVLDIGK
10.8	1739.8502	-0.0048	MRKPDGSTVTQRIR
10.8	1739.8502	-0.0048	MRKPDGSTVTQRIR

9.0	1738.8542	0.9912	GELAPDAKSFVLNLGK
7.7	1739.8502	-0.0048	MRKPDGSTVTQRIR
6.9	1739.8607	-0.0153	KIGNAYAVLSNPEKR
4.7	1739.8607	-0.0153	KIGNAYAVLSNPEKR
4.1	1739.8607	-0.0153	KIGNAYAVLSNPEKR

Spectrum No: 109; Query: 796; Rank: 1

Peptide View

MS/MS Fragmentation of **TNSSFVQGLVDHVKEDCDR**
Found in **IPI00195160**, Tax_Id=10116 Gene_Symbol=Psap Sulfated glycoprotein 1 precursor

Match to Query 796: 2205.999788 from(1104.007170,2+)
Title: 091127RatKid_SCX01_35.3485.3485.2.dta
Data file K:\NewmanPaper\Piliang\3SubProteomes\Piliang3SP\mgf5ppm\SCX_3SubProteomes5ppm.mgf

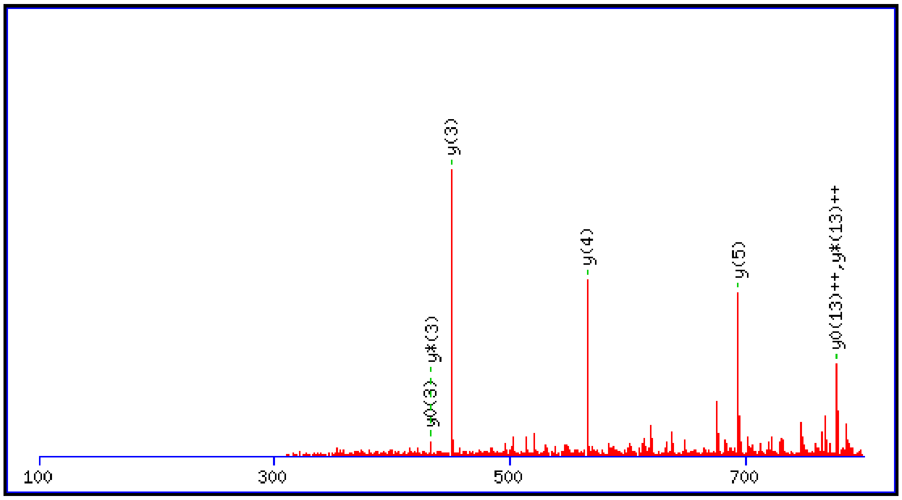

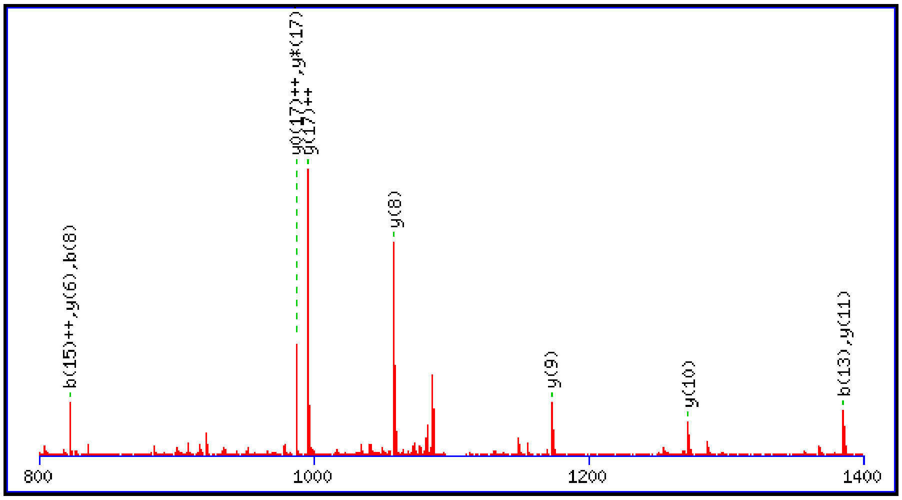

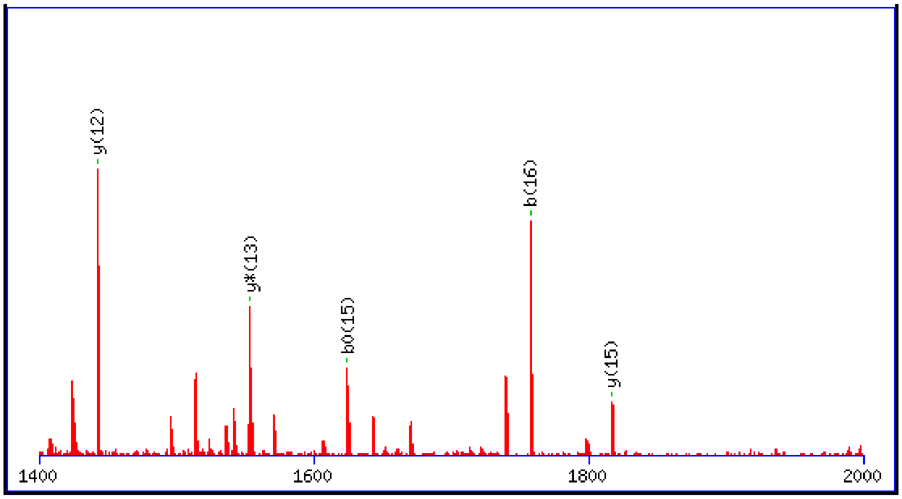

Monoisotopic mass of neutral peptide Mr(calc): 2205.9961
Fixed modifications: Carbamidomethyl (C)
Variable modifications:
N2 : Deamidated_N (N)
Ions Score: 84 **Expect:** 1e-006
Matches (Bold Red): 23/212 fragment ions using 30 most intense peaks

#	b	b^{++}	b*	b^{*++}	b^{0}	b^{0++}	Seq.	y	y^{++}	y*	y^{*++}	y^{0}	y^{0++}	#
1	102.0550	51.5311			84.0444	42.5258	T							19
2	217.0819	109.0446	200.0553	100.5313	199.0713	100.0393	N	2105.9557	1053.4815	2088.9291	1044.9682	2087.9451	1044.4762	18
3	304.1139	152.5606	287.0874	144.0473	286.1034	143.5553	S	1990.9287	**995.9680**	1973.9022	**987.4547**	1972.9182	**986.9627**	17
4	391.1459	196.0766	374.1194	187.5633	373.1354	187.0713	S	1903.8967	952.4520	1886.8701	943.9387	1885.8861	943.4467	16
5	538.2144	269.6108	521.1878	261.0975	520.2038	260.6055	F	**1816.8647**	908.9360	1799.8381	900.4227	1798.8541	899.9307	15
6	637.2828	319.1450	620.2562	310.6318	619.2722	310.1397	V	1669.7962	835.4018	1652.7697	826.8885	1651.7857	826.3965	14
7	765.3414	383.1743	748.3148	374.6610	747.3308	374.1690	Q	1570.7278	785.8676	**1553.7013**	**777.3543**	1552.7173	**776.8623**	13
8	**822.3628**	411.6850	805.3363	403.1718	804.3523	402.6798	G	**1442.6693**	721.8383	1425.6427	713.3250	1424.6587	712.8330	12
9	935.4469	468.2271	918.4203	459.7138	917.4363	459.2218	L	**1385.6478**	693.3275	1368.6212	684.8143	1367.6372	684.3223	11
10	1034.5153	517.7613	1017.4887	509.2480	1016.5047	508.7560	V	**1272.5637**	636.7855	1255.5372	628.2722	1254.5532	627.7802	10
11	1149.5422	575.2748	1132.5157	566.7615	1131.5317	566.2695	D	**1173.4953**	587.2513	1156.4688	578.7380	1155.4847	578.2460	9
12	1286.6012	643.8042	1269.5746	635.2909	1268.5906	634.7989	H	**1058.4684**	529.7378	1041.4418	521.2245	1040.4578	520.7325	8
13	**1385.6696**	693.3384	1368.6430	684.8251	1367.6590	684.3331	V	921.4095	461.2084	904.3829	452.6951	903.3989	452.2031	7
14	1513.7645	757.3859	1496.7380	748.8726	1495.7540	748.3806	K	**822.3410**	411.6742	805.3145	403.1609	804.3305	402.6689	6
15	1642.8071	**821.9072**	1625.7806	813.3939	**1624.7966**	812.9019	E	**694.2461**	347.6267	677.2195	339.1134	676.2355	338.6214	5
16	**1757.8341**	879.4207	1740.8075	870.9074	1739.8235	870.4154	D	**565.2035**	283.1054	548.1769	274.5921	547.1929	274.1001	4
17	1917.8647	959.4360	1900.8382	950.9227	1899.8541	950.4307	C	**450.1765**	225.5919	**433.1500**	217.0786	**432.1660**	216.5866	3
18	2032.8917	1016.9495	2015.8651	1008.4362	2014.8811	1007.9442	D	290.1459	145.5766	273.1193	137.0633	272.1353	136.5713	2
19							R	175.1190	88.0631	158.0924	79.5498			1

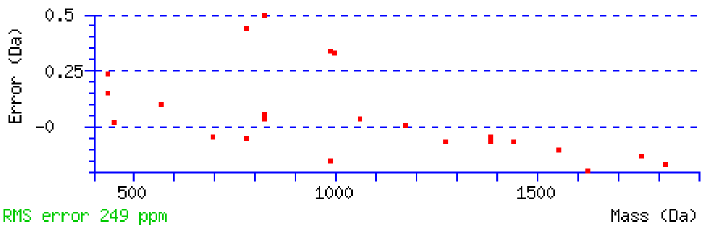

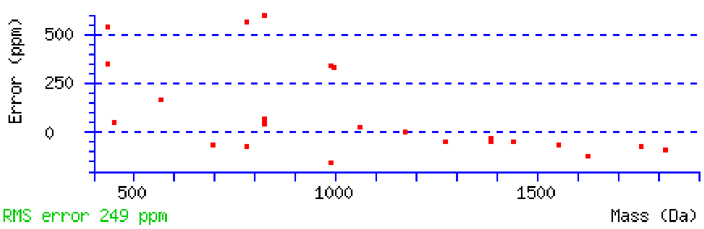

All matches to this query

Score	Mr(calc):	Delta	Sequence
84.4	2205.9961	0.0037	TNSSFVQGLVDHVKEDCDR
76.2	2205.0121	0.9877	TNSSFVQGLVDHVKEDCDR

5.7	2203.9816	2.0181	TFKAWEGLHENSVRLTR
4.8	2204.0127	1.9871	GLAPRAEASTSSAAVSSLIEK
4.5	2204.0127	1.9871	GLAPRAEASTSSAAVSSLIEK
4.4	2203.9763	2.0235	LENLTDTTVAQKAGGPSSVR
4.1	2204.0127	1.9871	GLAPRAEASTSSAAVSSLIEK
3.2	2205.0054	0.9944	VSIAPRTNAALGFSQMLPR
1.8	2203.9935	2.0063	AYTFSVAPPGLIMAEVPGMSS
1.8	2203.9935	2.0063	AYTFSVAPPGLIMAEVPGMSS

Spectrum No: 110; Query: 505; Rank: 1

Peptide View

MS/MS Fragmentation of **DAGYTNATTTDQHGLAK**
Found in **IPI00201262**, Tax_Id=10116 Gene_Symbol=LOC297568;Mug2;Mug1 Alpha-1-inhibitor 3 precursor

Match to Query 505: 1763.798148 from(882.906350,2+)
Title: 091127RatKid_SCX01_15.820.820.2.dta
Data file K:\NewmanPaper\Piliang\3SubProteomes\Piliang3SP\mgf5ppm\SCX_3SubProteomes5ppm.mgf

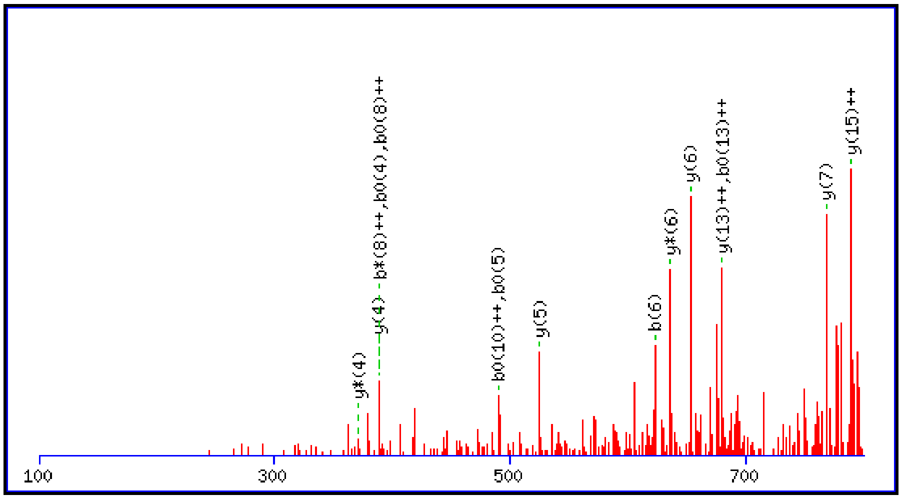

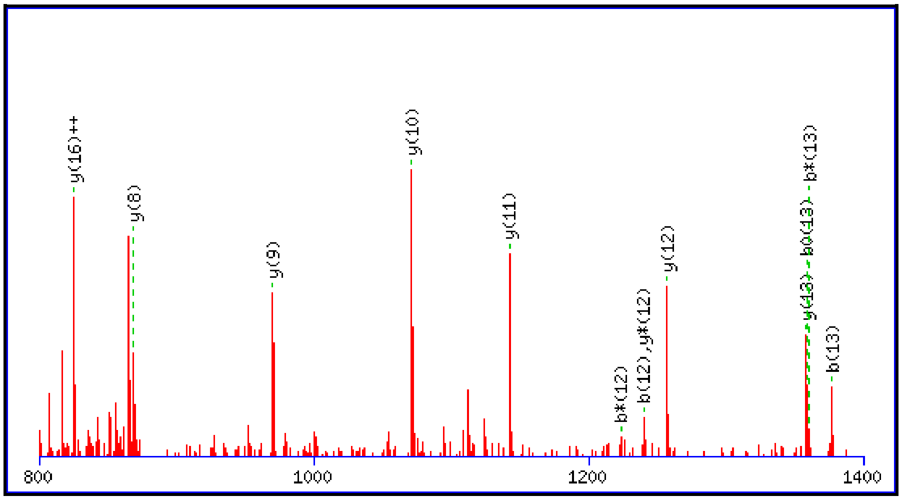

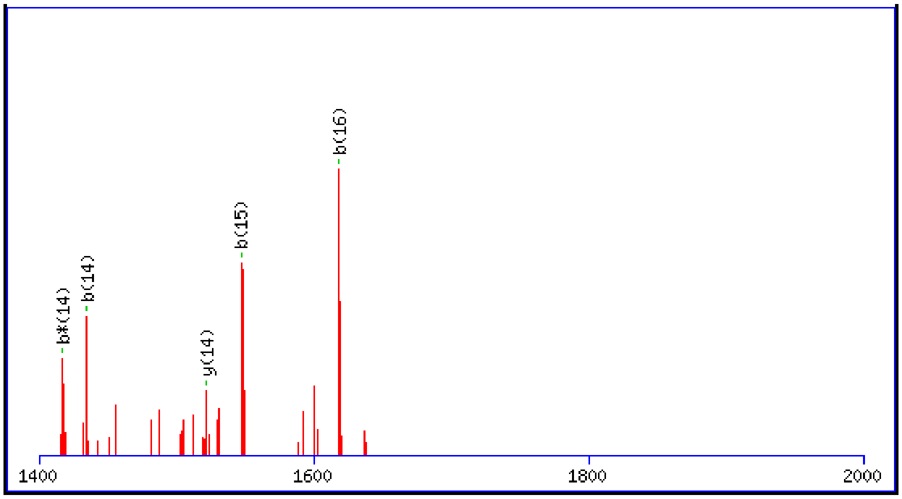

Monoisotopic mass of neutral peptide Mr(calc): 1763.7962
Fixed modifications: Carbamidomethyl (C)
Variable modifications:
N6 : Deamidated_N (N)
Ions Score: 84 **Expect:** 7.7e-007
Matches (Bold Red): 33/170 fragment ions using 43 most intense peaks

#	b	b^{++}	b*	b^{*++}	b^{0}	b^{0++}	Seq.	y	y^{++}	y*	y^{*++}	y^{0}	y^{0++}	#
1	116.0342	58.5207			98.0237	49.5155	D							17
2	187.0713	94.0393			169.0608	85.0340	A	1649.7766	**825.3919**	1632.7500	816.8786	1631.7660	816.3866	16
3	244.0928	122.5500			226.0822	113.5448	G	1578.7394	**789.8734**	1561.7129	781.3601	1560.7289	780.8681	15
4	407.1561	204.0817			**389.1456**	195.0764	Y	**1521.7180**	761.3626	1504.6914	752.8494	1503.7074	752.3573	14
5	508.2038	254.6055			**490.1932**	245.6003	T	**1358.6546**	**679.8310**	1341.6281	671.3177	1340.6441	670.8257	13
6	**623.2307**	312.1190	606.2042	303.6057	605.2202	303.1137	N	**1257.6070**	629.3071	**1240.5804**	620.7938	1239.5964	620.3018	12
7	694.2679	347.6376	677.2413	339.1243	676.2573	338.6323	A	**1142.5800**	571.7937	1125.5535	563.2804	1124.5695	562.7884	11
8	795.3155	398.1614	778.2890	**389.6481**	777.3050	**389.1561**	T	**1071.5429**	536.2751	1054.5164	527.7618	1053.5324	527.2698	10
9	896.3632	448.6852	879.3367	440.1720	878.3526	439.6800	T	**970.4952**	485.7513	953.4687	477.2380	952.4847	476.7460	9
10	997.4109	499.2091	980.3843	490.6958	979.4003	**490.2038**	T	**869.4476**	435.2274	852.4210	426.7141	851.4370	426.2221	8
11	1112.4378	556.7226	1095.4113	548.2093	1094.4273	547.7173	D	**768.3999**	384.7036	751.3733	376.1903	750.3893	375.6983	7
12	**1240.4964**	620.7518	**1223.4699**	612.2386	1222.4858	611.7466	Q	**653.3729**	327.1901	**636.3464**	318.6768			6
13	**1377.5553**	689.2813	**1360.5288**	680.7680	**1359.5448**	**680.2760**	H	**525.3144**	263.1608	508.2878	254.6475			5
14	**1434.5768**	717.7920	**1417.5502**	709.2788	1416.5662	708.7867	G	**388.2554**	194.6314	**371.2289**	186.1181			4
15	**1547.6609**	774.3341	1530.6343	765.8208	1529.6503	765.3288	L	331.2340	166.1206	314.2074	157.6074			3
16	**1618.6980**	809.8526	1601.6714	801.3393	1600.6874	800.8473	A	218.1499	109.5786	201.1234	101.0653			2
17							K	147.1128	74.0600	130.0863	65.5468			1

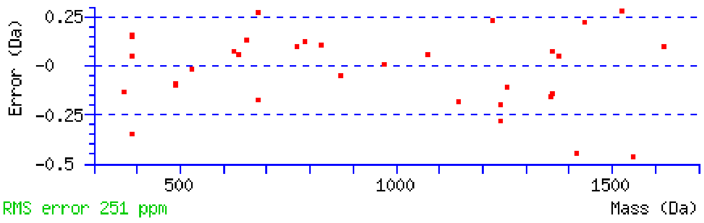

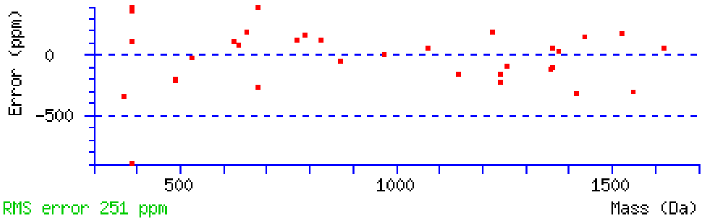

All matches to this query

Score	Mr(calc):	Delta	Sequence
84.4	1763.7962	0.0019	DAGYTNATTTDQHGLAK
21.8	1762.8122	0.9859	DAGYTNATTTDQHGLAK
4.7	1763.7896	0.0085	LSYVLGQPGLSLSDR
4.6	1763.8008	-0.0027	EGPDRSVSRLTFLK

2.8	1763.7987	-0.0006	GATSKEVMCFNAKLK
2.5	1761.8046	1.9935	LKNVLSHSEPNSNSR
2.5	1761.8046	1.9935	LKNVLSHSEPNSNSR
1.3	1763.7987	-0.0006	GATSKEVMCFNAKLK
0.6	1763.7880	0.0102	DRVNDDLISEFPHK
0.2	1763.7973	0.0008	MMTTTRMTTRMTMR

Spectrum No: 111; Query: 416; Rank: 1

Peptide View

MS/MS Fragmentation of **KFINDYVSNQTQGK**
Found in **IPI00200593**, Tax_Id=10116 Gene_Symbol=Serpina3k Serine protease inhibitor A3K precursor

Match to Query 416: 1641.800628 from(821.907590,2+)
Title: 091127RatKid_SCX01_13.1321.1321.2.dta
Data file K:\NewmanPaper\Piliang\3SubProteomes\Piliang3SP\mgf5ppm\SCX_3SubProteomes5ppm.mgf

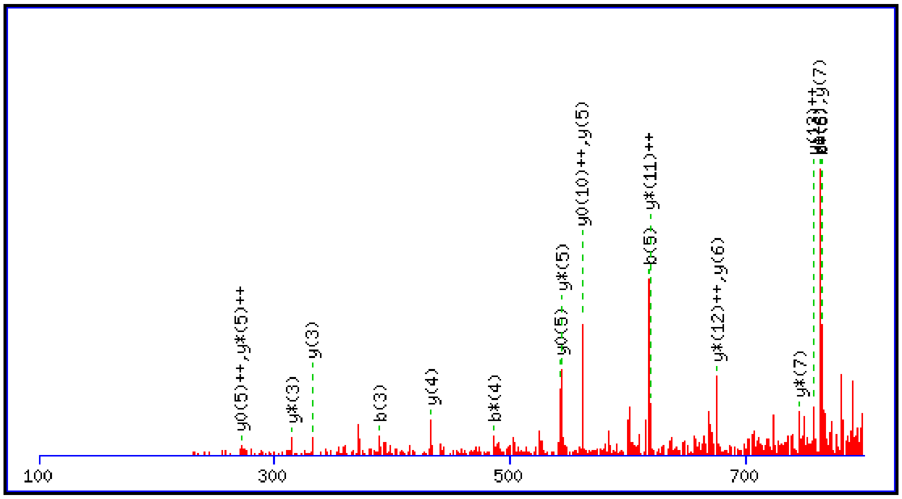

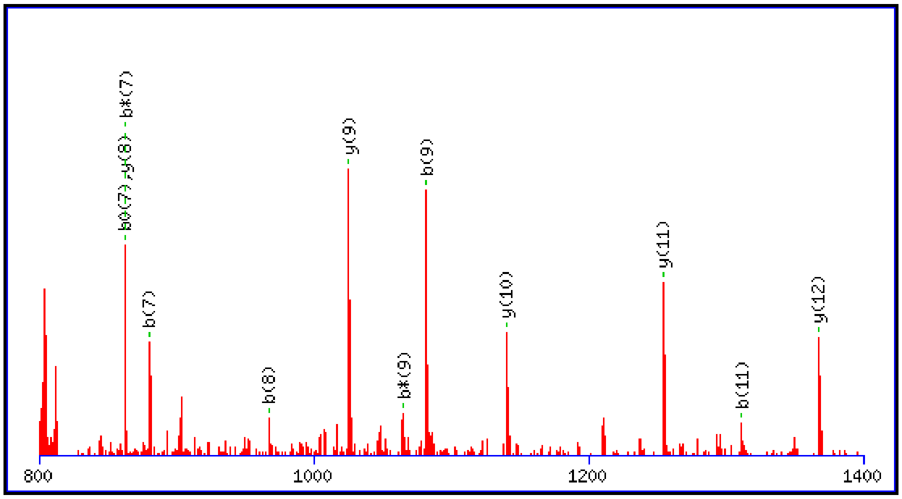

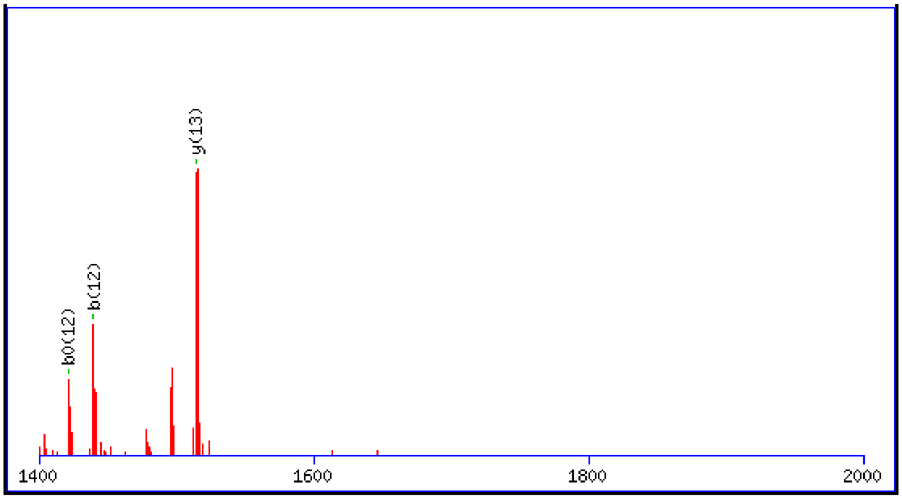

Monoisotopic mass of neutral peptide Mr(calc): 1641.7998
Fixed modifications: Carbamidomethyl (C)
Variable modifications:
N9 : Deamidated_N (N)
Ions Score: 84 **Expect:** 6.6e-007
Matches (Bold Red): 35/142 fragment ions using 50 most intense peaks

#	b	b^{++}	b^{*}	b^{*++}	b^{0}	b^{0++}	Seq.	y	y^{++}	y^{*}	y^{*++}	y^{0}	y^{0++}	#
1	129.1022	65.0548	112.0757	56.5415			K							14
2	276.1707	138.5890	259.1441	130.0757			F	**1514.7122**	**757.8597**	1497.6856	749.3464	1496.7016	748.8544	13
3	**389.2547**	195.1310	372.2282	186.6177			I	**1367.6437**	684.3255	1350.6172	**675.8122**	1349.6332	675.3202	12
4	503.2976	252.1525	**486.2711**	243.6392			N	**1254.5597**	627.7835	1237.5331	**619.2702**	1236.5491	618.7782	11
5	**618.3246**	309.6659	601.2980	301.1527	600.3140	300.6606	D	**1140.5168**	570.7620	1123.4902	562.2487	1122.5062	**561.7567**	10
6	781.3879	391.1976	**764.3614**	382.6843	**763.3774**	382.1923	Y	**1025.4898**	513.2485	1008.4633	504.7353	1007.4792	504.2433	9
7	**880.4563**	440.7318	**863.4298**	432.2185	**862.4458**	431.7265	V	**862.4265**	431.7169	845.3999	423.2036	844.4159	422.7116	8
8	**967.4884**	484.2478	950.4618	475.7345	949.4778	475.2425	S	**763.3581**	382.1827	**746.3315**	373.6694	745.3475	373.1774	7
9	**1082.5153**	541.7613	**1065.4887**	533.2480	1064.5047	532.7560	N	**676.3260**	338.6667	659.2995	330.1534	658.3155	329.6614	6
10	1210.5739	605.7906	1193.5473	597.2773	1192.5633	596.7853	Q	**561.2991**	281.1532	**544.2726**	**272.6399**	**543.2885**	**272.1479**	5
11	**1311.6216**	656.3144	1294.5950	647.8011	1293.6110	647.3091	T	**433.2405**	217.1239	416.2140	208.6106	415.2300	208.1186	4
12	**1439.6801**	720.3437	1422.6536	711.8304	**1421.6696**	711.3384	Q	**332.1928**	166.6001	**315.1663**	158.0868			3
13	1496.7016	748.8544	1479.6750	740.3412	1478.6910	739.8492	G	204.1343	102.5708	187.1077	94.0575			2
14							K	147.1128	74.0600	130.0863	65.5468			1

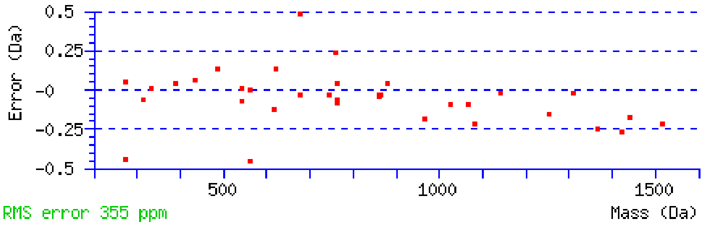

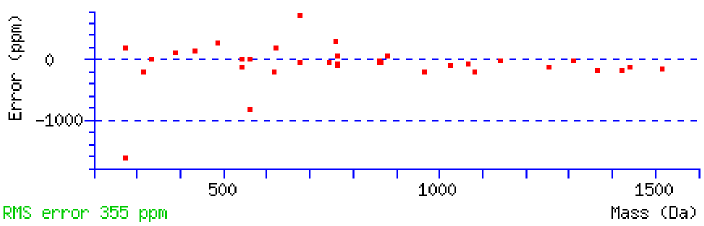

All matches to this query

Score	Mr(calc):	Delta	Sequence
84.3	1641.7998	0.0008	KFINDYVSNQTQGK
35.5	1641.7998	0.0008	KFINDYVSNQTQGK
8.8	1640.7957	1.0050	QELDLNSSMRLKK
6.5	1639.8093	1.9913	VFLEEEDSFSALVR
5.8	1641.8015	-0.0009	LTVGDPVTVEYITR
5.3	1641.7875	0.0131	WTTNXRSLNSNKK
5.3	1641.7875	0.0131	WTTNXRSLNSNKK

4.9	1639.7954	2.0052	ERVDYASGFQVDVR
4.9	1641.7886	0.0120	DLDDYKEFNDLKK
4.0	1639.7818	2.0188	VEISKASASLNGDLR

Spectrum No: 112; Query: 124; Rank: 1

Peptide View

MS/MS Fragmentation of **SCINESAIDSR**
Found in **IPI00231789**, Tax_Id=10116 Gene_Symbol=Mme Neprilysin

Match to Query 124: 1251.539068 from(626.776810,2+)
Title: 091129RatKid_SCX02_14.677.677.2.dta
Data file K:\NewmanPaper\Piliang\3SubProteomes\Piliang3SP\mgf5ppm\SCX_3SubProteomes5ppm.mgf

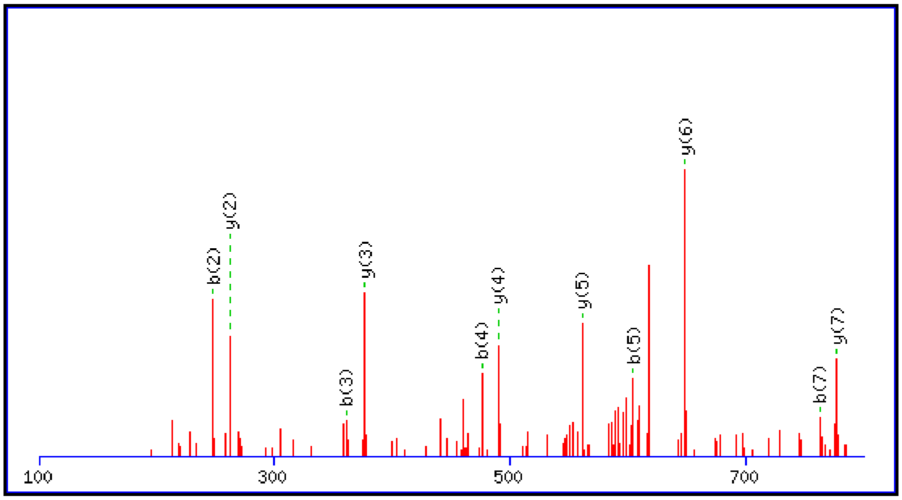

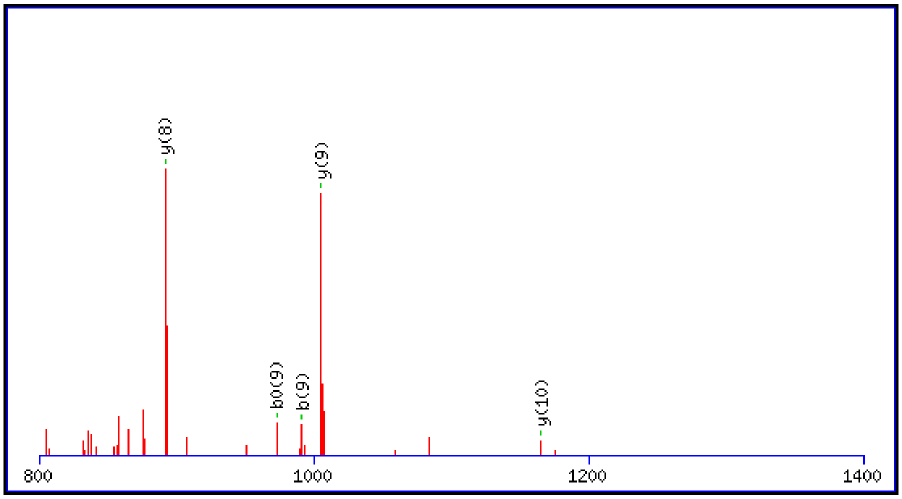

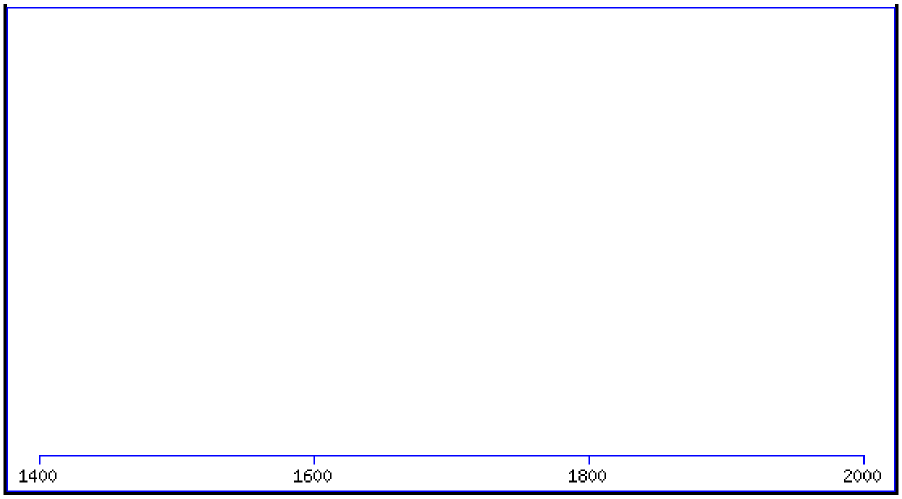

Monoisotopic mass of neutral peptide Mr(calc): 1251.5401
Fixed modifications: Carbamidomethyl (C)
Variable modifications:
N4 : Deamidated_N (N)
Ions Score: 84 **Expect:** 4.1e-007
Matches (Bold Red): 16/112 fragment ions using 27 most intense peaks

#	b	b^{++}	b*	b^{*++}	b^{0}	b^{0++}	Seq.	y	y^{++}	y*	y^{*++}	y^{0}	y^{0++}	#
1	88.0393	44.5233			70.0287	35.5180	S							11
2	**248.0700**	124.5386			230.0594	115.5333	C	**1165.5154**	583.2613	1148.4888	574.7480	1147.5048	574.2560	10
3	**361.1540**	181.0806			343.1435	172.0754	I	**1005.4847**	503.2460	988.4582	494.7327	987.4742	494.2407	9
4	**476.1810**	238.5941	459.1544	230.0808	458.1704	229.5888	N	**892.4007**	446.7040	875.3741	438.1907	874.3901	437.6987	8
5	**605.2235**	303.1154	588.1970	294.6021	587.2130	294.1101	E	**777.3737**	389.1905	760.3472	380.6772	759.3632	380.1852	7
6	692.2556	346.6314	675.2290	338.1182	674.2450	337.6261	S	**648.3311**	324.6692	631.3046	316.1559	630.3206	315.6639	6
7	**763.2927**	382.1500	746.2661	373.6367	745.2821	373.1447	A	**561.2991**	281.1532	544.2726	272.6399	543.2885	272.1479	5
8	876.3768	438.6920	859.3502	430.1787	858.3662	429.6867	I	**490.2620**	245.6346	473.2354	237.1214	472.2514	236.6293	4
9	**991.4037**	496.2055	974.3771	487.6922	**973.3931**	487.2002	D	**377.1779**	189.0926	360.1514	180.5793	359.1674	180.0873	3
10	1078.4357	539.7215	1061.4092	531.2082	1060.4252	530.7162	S	**262.1510**	131.5791	245.1244	123.0659	244.1404	122.5738	2
11							R	175.1190	88.0631	158.0924	79.5498			1

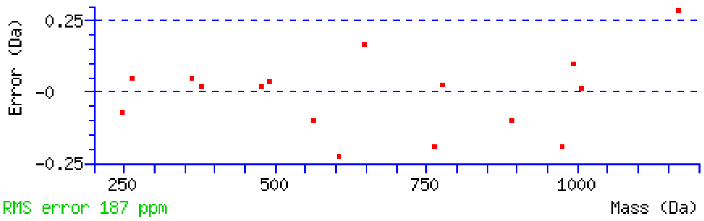

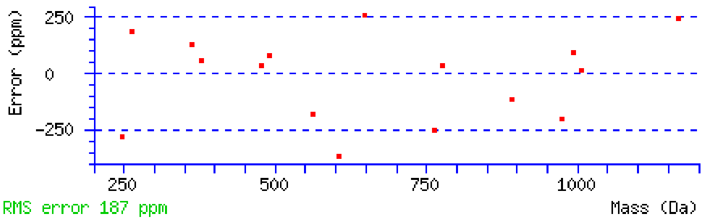

All matches to this query

Score	Mr(calc):	Delta	Sequence
84.1	1251.5401	-0.0010	SCINESAIDSR
8.5	1250.5449	0.9942	LDLMDEGTDAR
6.6	1251.5335	0.0055	MVSTILSVSR
6.6	1251.5335	0.0055	MVSTILSVSR
6.6	1251.5335	0.0055	MVSTILSVSR
6.5	1251.5401	-0.0010	KMADENSNVSR
5.0	1249.5268	2.0123	FNFTVEDGLK
4.7	1251.5280	0.0111	EYQMMLLTK
3.9	1251.5448	-0.0057	SCIRTKTVK
3.3	1250.5445	0.9946	FGGSFRTASNK

Spectrum No: 113; Query: 630; Rank: 1

Peptide View

MS/MS Fragmentation of **LNLTAAQLSQLEGLLQAR**
Found in **IPI00199867**, Tax_Id=10116 Gene_Symbol=Emilin1_predicted elastin microfibril interfacer 1

Match to Query 630: 1939.079888 from(970.547220,2+)
Title: 091127RatKid_SCX01_02.4983.4983.2.dta
Data file K:\NewmanPaper\Piliang\3SubProteomes\Piliang3SP\mgf5ppm\SCX_3SubProteomes5ppm.mgf

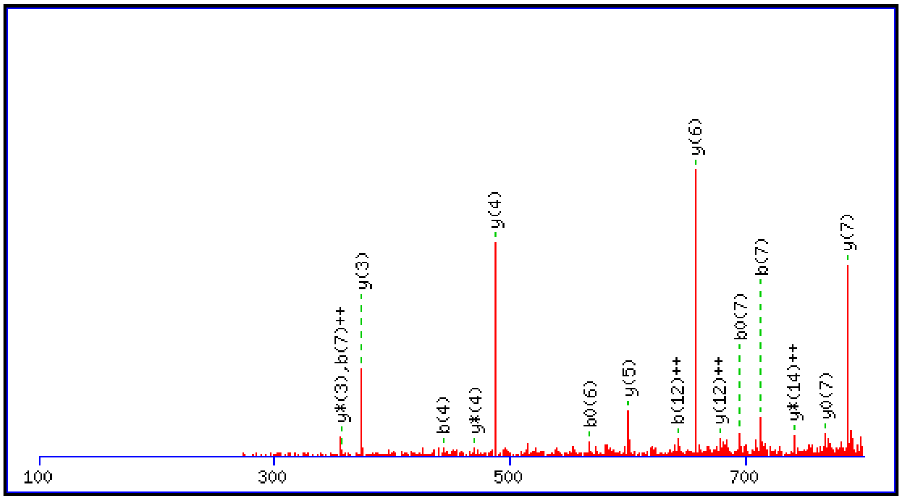

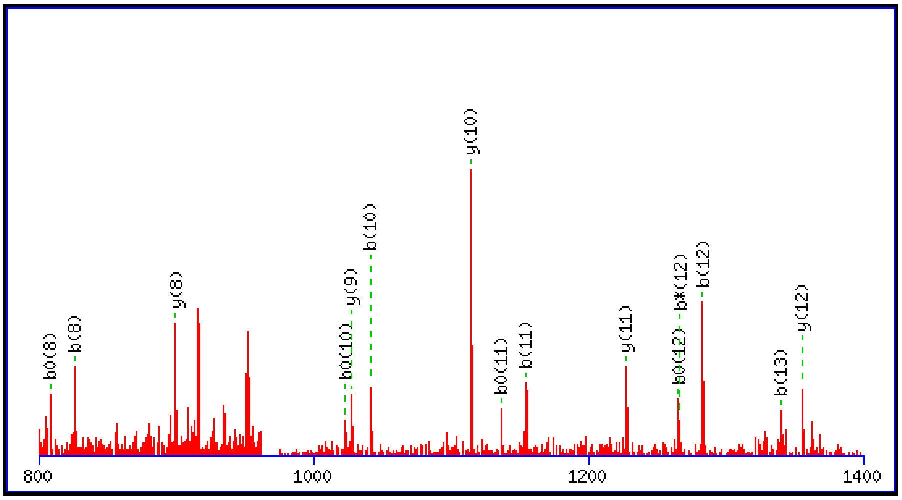

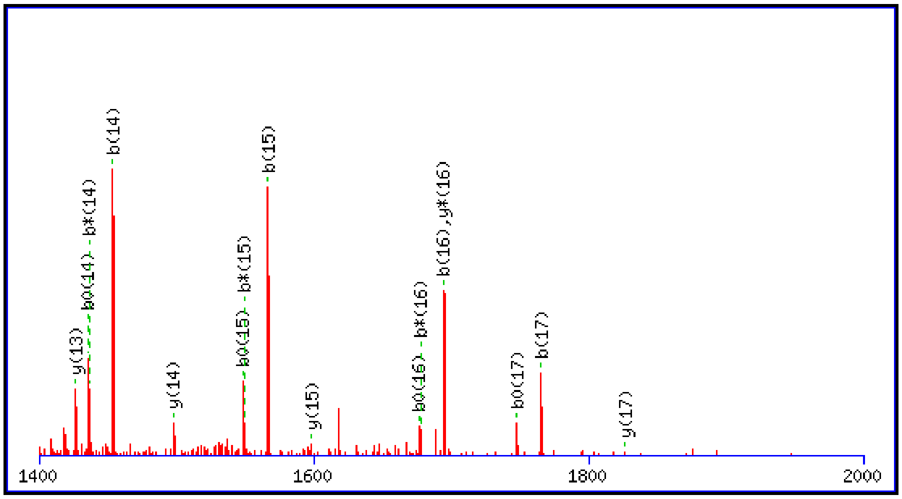

Monoisotopic mass of neutral peptide Mr(calc): 1939.0738
Fixed modifications: Carbamidomethyl (C)
Variable modifications:
N2 : Deamidated_N (N)
Ions Score: 84 **Expect:** 1.7e-007
Matches (Bold Red): 47/184 fragment ions using 89 most intense peaks

#	b	b^{++}	b*	b^{*++}	b^0	b^{0++}	Seq.	y	y^{++}	y*	y^{*++}	y^0	y^{0++}	#
1	114.0913	57.5493					L							18
2	229.1183	115.0628	212.0917	106.5495			N	**1826.9970**	914.0022	1809.9705	905.4889	1808.9865	904.9969	17
3	342.2023	171.6048	325.1758	163.0915			L	1711.9701	856.4887	**1694.9436**	847.9754	1693.9595	847.4834	16
4	**443.2500**	222.1286	426.2235	213.6154	425.2395	213.1234	T	**1598.8860**	799.9467	1581.8595	791.4334	1580.8755	790.9414	15
5	514.2871	257.6472	497.2606	249.1339	496.2766	248.6419	A	**1497.8384**	749.4228	1480.8118	**740.9095**	1479.8278	740.4175	14
6	585.3242	293.1658	568.2977	284.6525	**567.3137**	284.1605	A	**1426.8013**	713.9043	1409.7747	705.3910	1408.7907	704.8990	13
7	**713.3828**	**357.1951**	696.3563	348.6818	**695.3723**	348.1898	Q	**1355.7641**	**678.3857**	1338.7376	669.8724	1337.7536	669.3804	12
8	**826.4669**	413.7371	809.4403	405.2238	**808.4563**	404.7318	L	**1227.7056**	614.3564	1210.6790	605.8431	1209.6950	605.3511	11
9	913.4989	457.2531	896.4724	448.7398	895.4884	448.2478	S	**1114.6215**	557.8144	1097.5949	549.3011	1096.6109	548.8091	10
10	**1041.5575**	521.2824	1024.5309	512.7691	**1023.5469**	512.2771	Q	**1027.5895**	514.2984	1010.5629	505.7851	1009.5789	505.2931	9
11	**1154.6416**	577.8244	1137.6150	569.3111	**1136.6310**	568.8191	L	**899.5309**	450.2691	882.5043	441.7558	881.5203	441.2638	8
12	**1283.6842**	**642.3457**	**1266.6576**	633.8324	**1265.6736**	633.3404	E	**786.4468**	393.7271	769.4203	385.2138	**768.4363**	384.7218	7
13	**1340.7056**	670.8564	1323.6791	662.3432	1322.6951	661.8512	G	**657.4042**	329.2058	640.3777	320.6925			6
14	**1453.7897**	727.3985	**1436.7631**	718.8852	**1435.7791**	718.3932	L	**600.3828**	300.6950	583.3562	292.1817			5
15	**1566.8737**	783.9405	**1549.8472**	775.4272	**1548.8632**	774.9352	L	**487.2987**	244.1530	**470.2722**	235.6397			4
16	**1694.9323**	847.9698	**1677.9058**	839.4565	**1676.9218**	838.9645	Q	**374.2146**	187.6110	**357.1881**	179.0977			3
17	**1765.9694**	883.4884	1748.9429	874.9751	**1747.9589**	874.4831	A	246.1561	123.5817	229.1295	115.0684			2
18							R	175.1190	88.0631	158.0924	79.5498			1

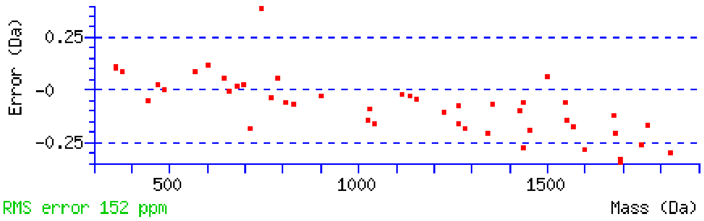

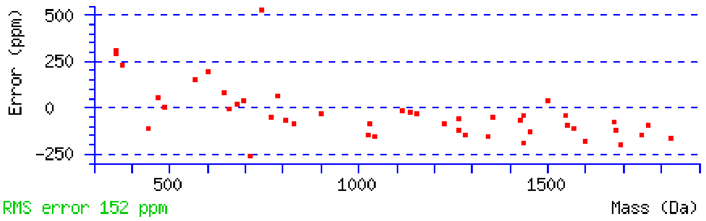

All matches to this query

Score	Mr(calc):	Delta	Sequence
83.7	1939.0738	0.0061	LNLTAAQLSQLEGLLQAR
40.4	1938.0898	0.9901	LNLTAAQLSQLEGLLQAR
3.5	1937.0694	2.0105	ELLSEVDARTLARHVTK
3.3	1939.0648	0.0151	ILFIRMLHNKHVMVR

Spectrum No: 114; Query: 190; Rank: 1

Peptide View

MS/MS Fragmentation of **CNVSEGVAQCTR**
Found in **IPI00231767**, Tax_Id=10116 Gene_Symbol=Tpi1 Triosephosphate isomerase

Match to Query 190: 1380.574188 from(691.294370,2+)
Title: 091129RatKid_SCX02_10.281.281.2.dta
Data file K:\NewmanPaper\Piliang\3SubProteomes\Piliang3SP\mgf5ppm\SCX_3SubProteomes5ppm.mgf

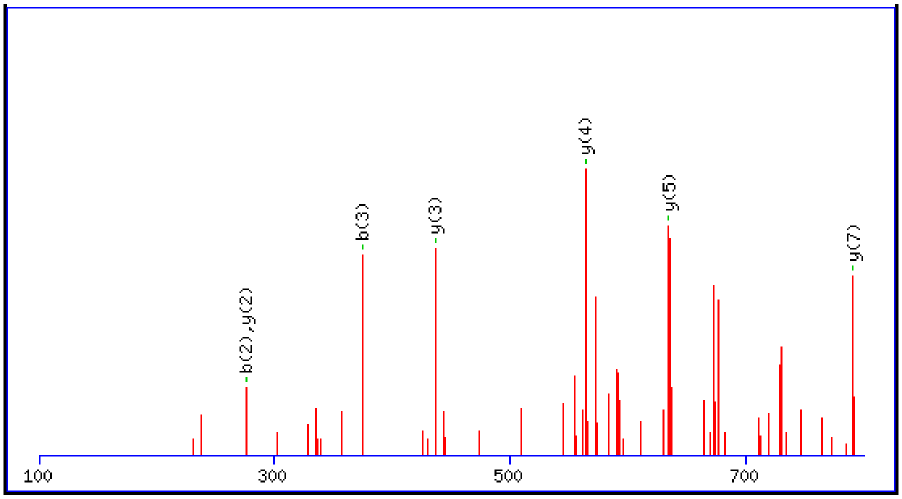

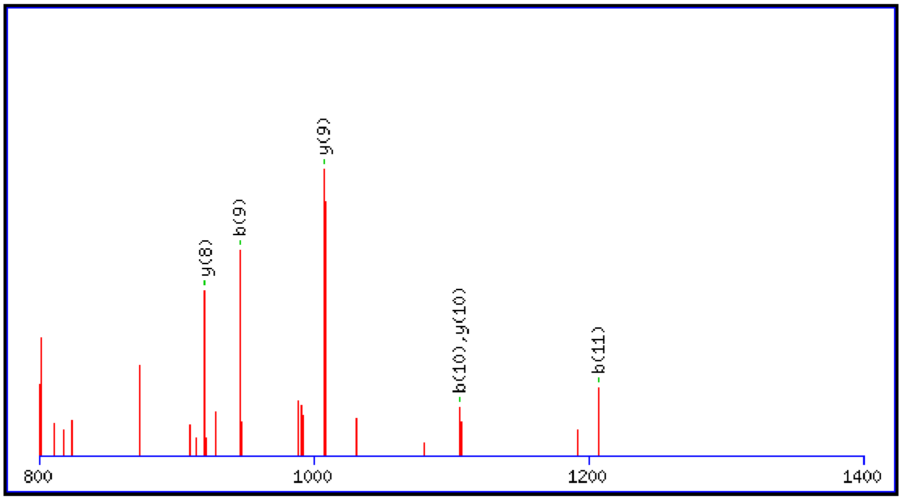

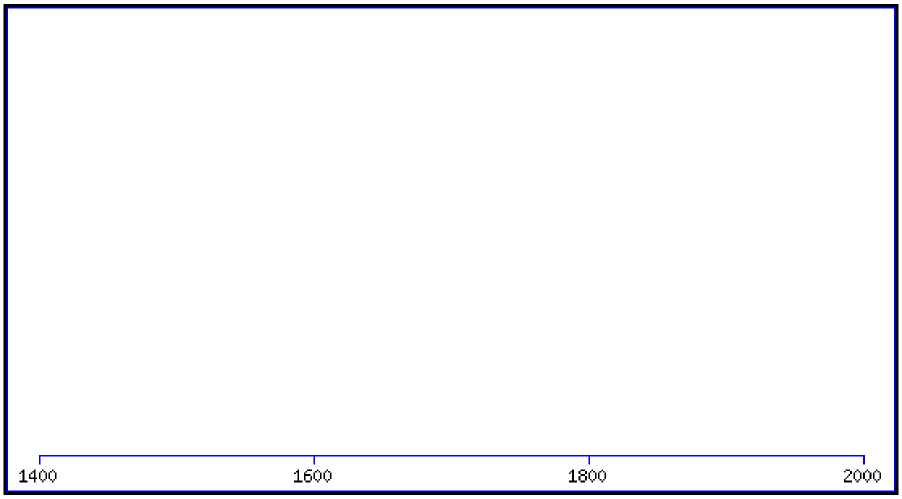

Monoisotopic mass of neutral peptide Mr(calc): 1380.5762
Fixed modifications: Carbamidomethyl (C)
Variable modifications:
N2 : Deamidated_N (N)
Ions Score: 84 **Expect:** 4.6e-007
Matches (Bold Red): 13/122 fragment ions using 13 most intense peaks

#	b	b^{++}	b*	b^{*++}	b^0	b^{0++}	Seq.	y	y^{++}	y*	y^{*++}	y^0	y^{0++}	#
1	161.0379	81.0226					C							12
2	**276.0649**	138.5361	259.0383	130.0228			N	1221.5528	611.2800	1204.5263	602.7668	1203.5423	602.2748	11
3	**375.1333**	188.0703	358.1067	179.5570			V	**1106.5259**	553.7666	1089.4993	545.2533	1088.5153	544.7613	10
4	462.1653	231.5863	445.1388	223.0730	444.1547	222.5810	S	**1007.4575**	504.2324	990.4309	495.7191	989.4469	495.2271	9
5	591.2079	296.1076	574.1813	287.5943	573.1973	287.1023	E	**920.4254**	460.7164	903.3989	452.2031	902.4149	451.7111	8

**6**	648.2294	324.6183	631.2028	316.1050	630.2188	315.6130	**G**	**791.3828**	396.1951	774.3563	387.6818	773.3723	387.1898	**7**
**7**	747.2978	374.1525	730.2712	365.6393	729.2872	365.1472	**V**	734.3614	367.6843	717.3348	359.1711	716.3508	358.6790	**6**
**8**	818.3349	409.6711	801.3083	401.1578	800.3243	400.6658	**A**	**635.2930**	318.1501	618.2664	309.6368	617.2824	309.1448	**5**
**9**	**946.3935**	473.7004	929.3669	465.1871	928.3829	464.6951	**Q**	**564.2559**	282.6316	547.2293	274.1183	546.2453	273.6263	**4**
**10**	**1106.4241**	553.7157	1089.3976	545.2024	1088.4136	544.7104	**C**	**436.1973**	218.6023	419.1707	210.0890	418.1867	209.5970	**3**
**11**	**1207.4718**	604.2395	1190.4452	595.7263	1189.4612	595.2343	**T**	**276.1666**	138.5870	259.1401	130.0737	258.1561	129.5817	**2**
**12**							**R**	175.1190	88.0631	158.0924	79.5498			**1**

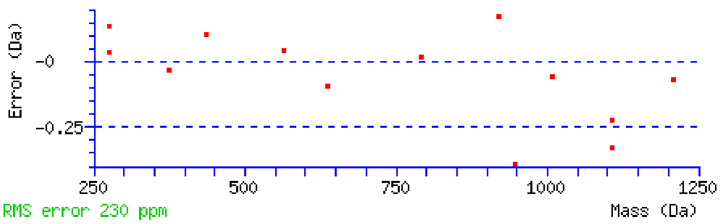

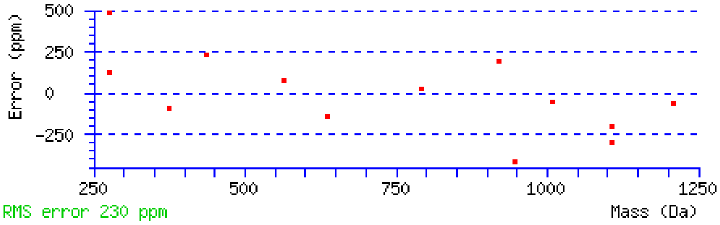

All matches to this query

Score	Mr(calc):	Delta	Sequence
83.7	1380.5762	-0.0020	CNVSEGVAQCTR
14.8	1380.5785	-0.0043	MQVLYEFEAR
7.4	1380.5840	-0.0098	KFVNQTVTER
5.5	1380.5836	-0.0094	MQNSTPMVCQGK
4.4	1380.5671	0.0071	NESHSNGTVTVR
3.4	1380.5605	0.0136	QNLTRDCHTR
2.7	1380.5662	0.0080	TKSPKFPCTR
2.0	1380.5671	0.0071	NESHSNGTVTVR
1.7	1380.5671	0.0071	NESHSNGTVTVR
1.5	1380.5605	0.0136	RPSSGGPCQAER

Spectrum No: 115; Query: 254; Rank: 1

Peptide View

MS/MS Fragmentation of **QSNGSIAIISSMAGK**
Found in **IPI00211096**, Tax_Id=10116 Gene_Symbol=Hsd11b1 Isoform 11-HSD1A of Corticosteroid 11-beta-dehydrogenase isozyme 1

Match to Query 254: 1463.730808 from(732.872680,2+)
Title: 091127RatKid_SCX01_17.2679.2679.2.dta
Data file K:\NewmanPaper\Piliang\3SubProteomes\Piliang3SP\mgf5ppm\SCX_3SubProteomes5ppm.mgf

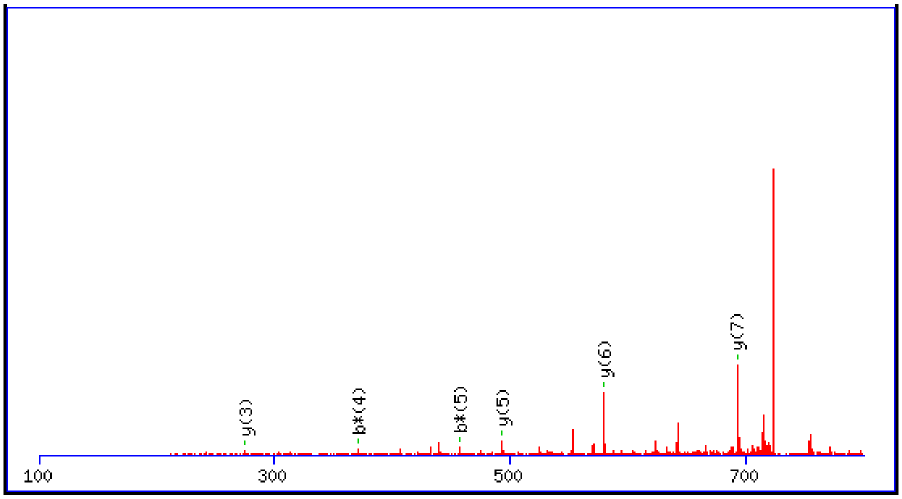

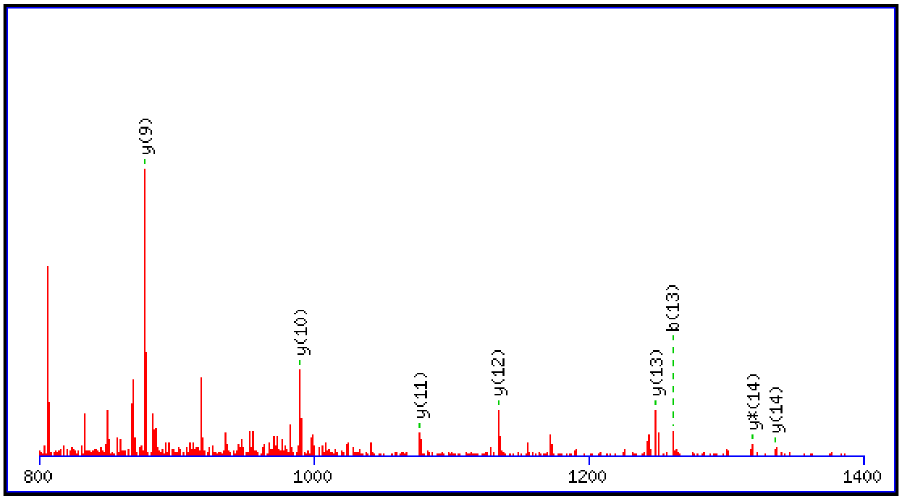

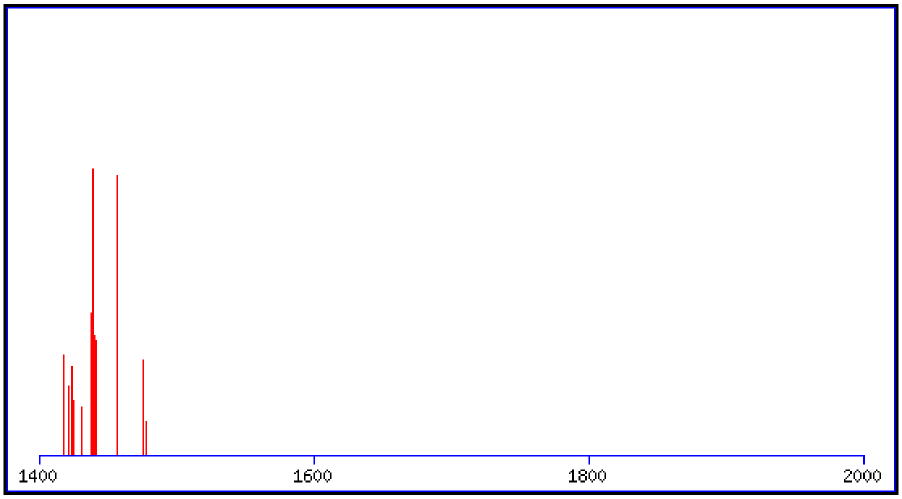

Monoisotopic mass of neutral peptide Mr(calc): 1463.7290
Fixed modifications: Carbamidomethyl (C)
Variable modifications:
N3 : Deamidated_N (N)
Ions Score: 84 **Expect:** 6e-007
Matches (Bold Red): 14/158 fragment ions using 23 most intense peaks

#	b	b^{++}	b*	b^{*++}	b^0	b^{0++}	Seq.	y	y^{++}	y*	y^{*++}	y^0	y^{0++}	#
1	129.0659	65.0366	112.0393	56.5233			Q							15
2	216.0979	108.5526	199.0713	100.0393	198.0873	99.5473	S	**1336.6777**	668.8425	**1319.6511**	660.3292	1318.6671	659.8372	14
3	331.1248	166.0660	314.0983	157.5528	313.1143	157.0608	N	**1249.6457**	625.3265	1232.6191	616.8132	1231.6351	616.3212	13
4	388.1463	194.5768	**371.1197**	186.0635	370.1357	185.5715	G	**1134.6187**	567.8130	1117.5922	559.2997	1116.6082	558.8077	12
5	475.1783	238.0928	**458.1518**	229.5795	457.1677	229.0875	S	**1077.5973**	539.3023	1060.5707	530.7890	1059.5867	530.2970	11

6	588.2624	294.6348	571.2358	286.1216	570.2518	285.6295	I	990.5652	495.7863	973.5387	487.2730	972.5547	486.7810	10
7	659.2995	330.1534	642.2729	321.6401	641.2889	321.1481	A	877.4812	439.2442	860.4546	430.7309	859.4706	430.2389	9
8	772.3836	386.6954	755.3570	378.1821	754.3730	377.6901	I	806.4441	403.7257	789.4175	395.2124	788.4335	394.7204	8
9	885.4676	443.2374	868.4411	434.7242	867.4571	434.2322	I	693.3600	347.1836	676.3334	338.6704	675.3494	338.1783	7
10	972.4996	486.7535	955.4731	478.2402	954.4891	477.7482	S	580.2759	290.6416	563.2494	282.1283	562.2654	281.6363	6
11	1059.5317	530.2695	1042.5051	521.7562	1041.5211	521.2642	S	493.2439	247.1256	476.2173	238.6123	475.2333	238.1203	5
12	1190.5722	595.7897	1173.5456	587.2764	1172.5616	586.7844	M	406.2119	203.6096	389.1853	195.0963			4
13	1261.6093	631.3083	1244.5827	622.7950	1243.5987	622.3030	A	275.1714	138.0893	258.1448	129.5761			3
14	1318.6307	659.8190	1301.6042	651.3057	1300.6202	650.8137	G	204.1343	102.5708	187.1077	94.0575			2
15							K	147.1128	74.0600	130.0863	65.5468			1

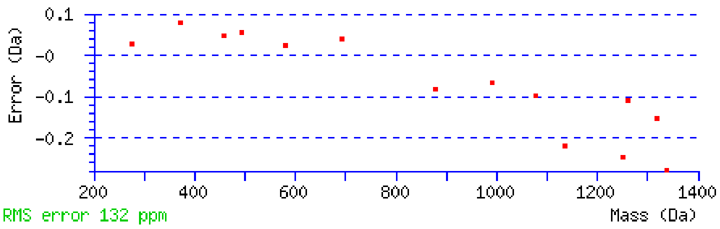

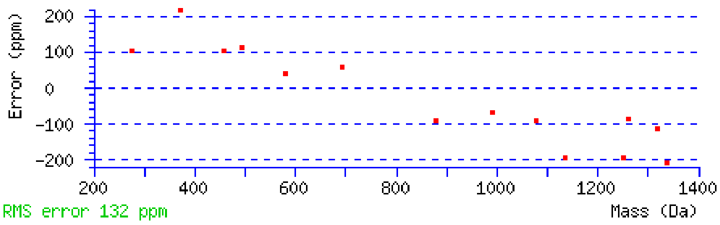

All matches to this query

Score	Mr(calc):	Delta	Sequence
83.6	1463.7290	0.0019	QSNGSIAIISSMAGK
18.5	1462.7272	1.0036	RMEEKPLSSMQK
7.3	1463.7425	-0.0117	YEFLTKNTLKK
5.9	1463.7247	0.0061	KYFMATVTKAPK
5.1	1463.7425	-0.0117	YEFLTKNTLKK
4.8	1462.7337	0.9971	KAEEQLALATNMK
2.4	1463.7442	-0.0134	LKEVGYSTHMVGK
0.5	1462.7238	1.0070	KQNAPMTLEEFR
0.4	1463.7319	-0.0011	HLLMSIDQRKK
0.4	1463.7330	-0.0022	LQEEAMVKPYEK

Spectrum No: 116; Query: 1146; Rank: 1

Peptide View

MS/MS Fragmentation of **GFDTYFGYLLGSEDYYTHEACAPIECLNGTR**
Found in **IPI00198405**, Tax_Id=10116 Gene_Symbol=Arsb Arylsulfatase B

Match to Query 1146: 3619.564092 from(1207.528640,3+)
Title: 091127RatKid_SCX01_20.7325.7325.3.dta
Data file K:\NewmanPaper\Piliang\3SubProteomes\Piliang3SP\mgf5ppm\SCX_3SubProteomes5ppm.mgf

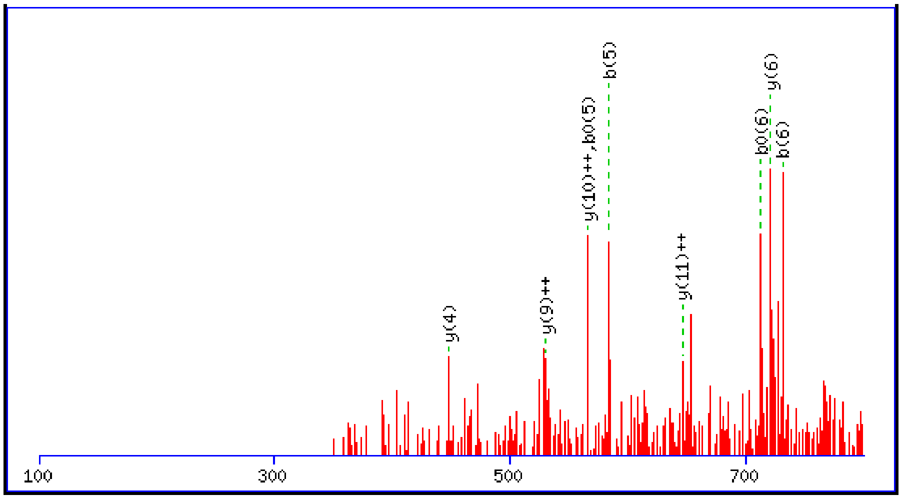

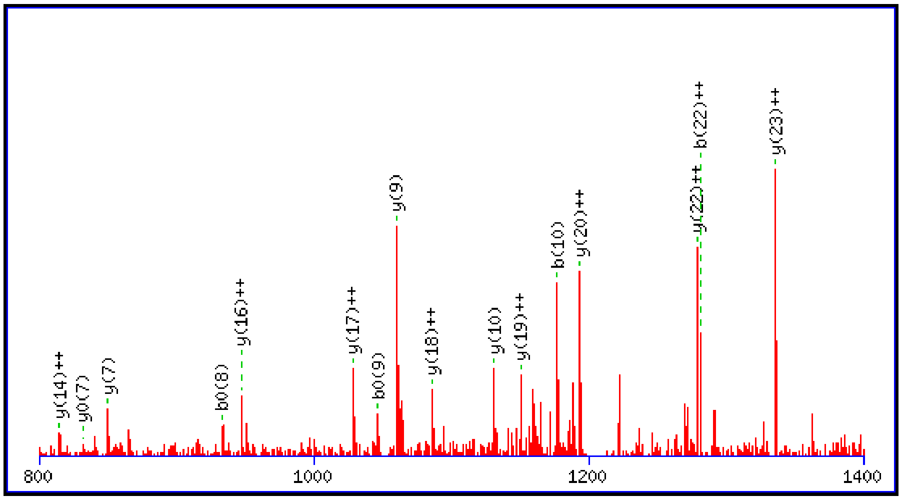

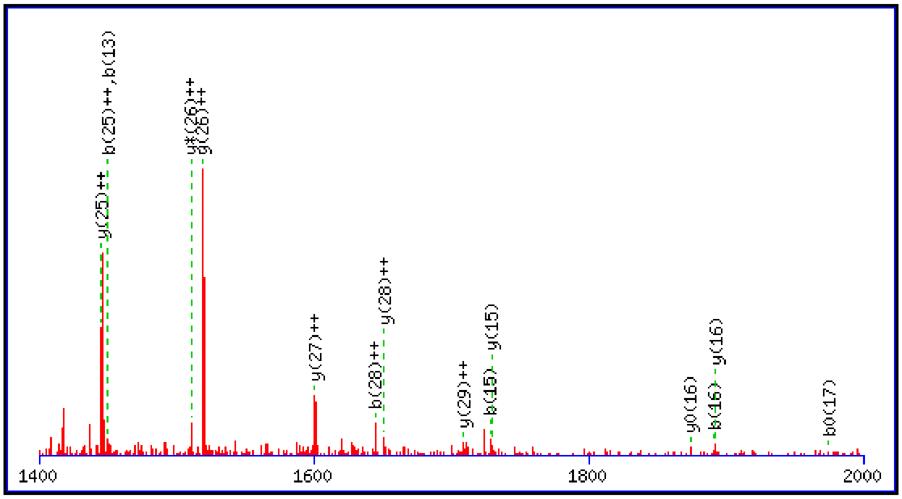

Monoisotopic mass of neutral peptide Mr(calc): 3619.5548
Fixed modifications: Carbamidomethyl (C)
Variable modifications:
N28 : Deamidated_N (N)
Ions Score: 83 **Expect:** 2.2e-006
Matches (Bold Red): 40/300 fragment ions using 71 most intense peaks

#	b	b^{++}	b*	b^{*++}	b^0	b^{0++}	Seq.	y	y^{++}	y*	y^{*++}	y^0	y^{0++}	#
1	58.0287	29.5180					G							31
2	205.0972	103.0522					F	3563.5406	1782.2739	3546.5141	1773.7607	3545.5300	1773.2687	30
3	320.1241	160.5657			302.1135	151.5604	D	3416.4722	**1708.7397**	3399.4456	1700.2265	3398.4616	1699.7345	29
4	421.1718	211.0895			403.1612	202.0842	T	3301.4453	**1651.2263**	3284.4187	1642.7130	3283.4347	1642.2210	28
5	**584.2351**	292.6212			**566.2245**	283.6159	Y	3200.3976	**1600.7024**	3183.3710	1592.1892	3182.3870	1591.6971	27

6	731.3035	366.1554			713.2930	357.1501	F	3037.3342	1519.1708	3020.3077	1510.6575	3019.3237	1510.1655	26
7	788.3250	394.6661			770.3144	385.6608	G	2890.2658	1445.6366	2873.2393	1437.1233	2872.2553	1436.6313	25
8	951.3883	476.1978			933.3777	467.1925	Y	2833.2444	1417.1258	2816.2178	1408.6125	2815.2338	1408.1205	24
9	1064.4724	532.7398			1046.4618	523.7345	L	2670.1810	1335.5942	2653.1545	1327.0809	2652.1705	1326.5889	23
10	1177.5564	589.2819			1159.5459	580.2766	L	2557.0970	1279.0521	2540.0704	1270.5389	2539.0864	1270.0468	22
11	1234.5779	617.7926			1216.5673	608.7873	G	2444.0129	1222.5101	2426.9864	1213.9968	2426.0023	1213.5048	21
12	1321.6099	661.3086			1303.5994	652.3033	S	2386.9914	1193.9994	2369.9649	1185.4861	2368.9809	1184.9941	20
13	1450.6525	725.8299			1432.6420	716.8246	E	2299.9594	1150.4833	2282.9329	1141.9701	2281.9489	1141.4781	19
14	1565.6795	783.3434			1547.6689	774.3381	D	2170.9168	1085.9621	2153.8903	1077.4488	2152.9063	1076.9568	18
15	1728.7428	864.8750			1710.7322	855.8698	Y	2055.8899	1028.4486	2038.8633	1019.9353	2037.8793	1019.4433	17
16	1891.8061	946.4067			1873.7956	937.4014	Y	1892.8266	946.9169	1875.8000	938.4036	1874.8160	937.9116	16
17	1992.8538	996.9305			1974.8432	987.9253	T	1729.7632	865.3853	1712.7367	856.8720	1711.7527	856.3800	15
18	2129.9127	1065.4600			2111.9022	1056.4547	H	1628.7155	814.8614	1611.6890	806.3481	1610.7050	805.8561	14
19	2258.9553	1129.9813			2240.9447	1120.9760	E	1491.6566	746.3320	1474.6301	737.8187	1473.6461	737.3267	13
20	2329.9924	1165.4998			2311.9819	1156.4946	A	1362.6140	681.8107	1345.5875	673.2974	1344.6035	672.8054	12
21	2490.0231	1245.5152			2472.0125	1236.5099	C	1291.5769	646.2921	1274.5504	637.7788	1273.5664	637.2868	11
22	2561.0602	1281.0337			2543.0496	1272.0284	A	1131.5463	566.2768	1114.5197	557.7635	1113.5357	557.2715	10
23	2658.1130	1329.5601			2640.1024	1320.5548	P	1060.5092	530.7582	1043.4826	522.2449	1042.4986	521.7529	9
24	2771.1970	1386.1021			2753.1865	1377.0969	I	963.4564	482.2318	946.4299	473.7186	945.4458	473.2266	8
25	2900.2396	1450.6234			2882.2290	1441.6182	E	850.3723	425.6898	833.3458	417.1765	832.3618	416.6845	7
26	3060.2703	1530.6388			3042.2597	1521.6335	C	721.3297	361.1685	704.3032	352.6552	703.3192	352.1632	6
27	3173.3543	1587.1808			3155.3438	1578.1755	L	561.2991	281.1532	544.2725	272.6399	543.2885	272.1479	5
28	3288.3813	1644.6943	3271.3547	1636.1810	3270.3707	1635.6890	N	448.2150	224.6112	431.1885	216.0979	430.2045	215.6059	4
29	3345.4027	1673.2050	3328.3762	1664.6917	3327.3922	1664.1997	G	333.1881	167.0977	316.1615	158.5844	315.1775	158.0924	3
30	3446.4504	1723.7288	3429.4239	1715.2156	3428.4398	1714.7236	T	276.1666	138.5870	259.1401	130.0737	258.1561	129.5817	2
31							R	175.1190	88.0631	158.0924	79.5498			1

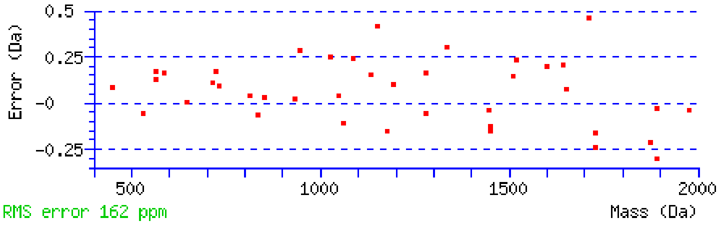

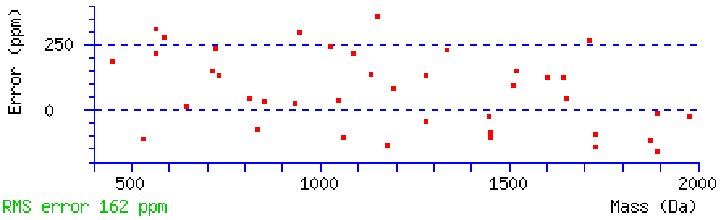

All matches to this query

Score	Mr(calc):	Delta	Sequence
83.3	3619.5548	0.0093	GFDTYFGYLLGSEDYYTHEACAPIECLNGTR
10.4	3618.5708	0.9933	GFDTYFGYLLGSEDYYTHEACAPIECLNGTR
9.3	3618.5895	0.9746	NTSVMMADPLAHTNYTFEIEAVNGVSDLSPGTR
4.5	3618.5895	0.9746	NTSVMMADPLAHTNYTFEIEAVNGVSDLSPGTR
4.4	3617.5242	2.0399	WNVNSSSKPLSALRLENDVSEGAQPSVGKAR
4.4	3617.5242	2.0399	WNVNSSSKPLSALRLENDVSEGAQPSVGKAR
3.0	3618.5895	0.9746	NTSVMMADPLAHTNYTFEIEAVNGVSDLSPGTR
2.8	3618.5895	0.9746	NTSVMMADPLAHTNYTFEIEAVNGVSDLSPGTR
2.3	3618.5925	0.9716	CENRGGVSPAFEVQLTNDLICFFDSSVKLR
1.7	3617.5673	1.9968	LCPIPMTPNGHLDPSLPLGAHLGPAGTMGTAPR

Spectrum No: 117; Query: 982; Rank: 1

Peptide View

MS/MS Fragmentation of **DGQQNISLLYTEPGASQTQTGASFR**
Found in **IPI00214859**, Tax_Id=10116 Gene_Symbol=Col18a1 similar to Collagen alpha-1(XVIII) chain precursor

Match to Query 982: 2669.263028 from(1335.638790,2+)
Title: 091129RatKid_SCX02_14.3160.3160.2.dta
Data file K:\NewmanPaper\Piliang\3SubProteomes\Piliang3SP\mgf5ppm\SCX_3SubProteomes5ppm.mgf

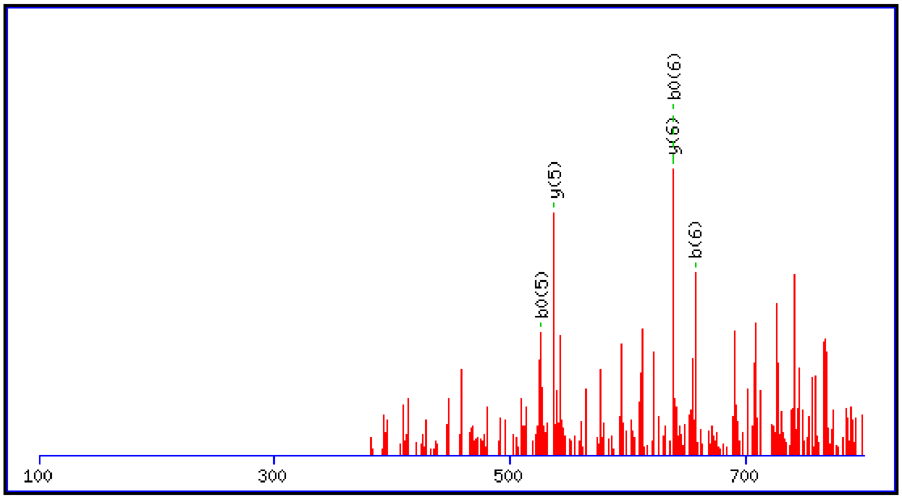

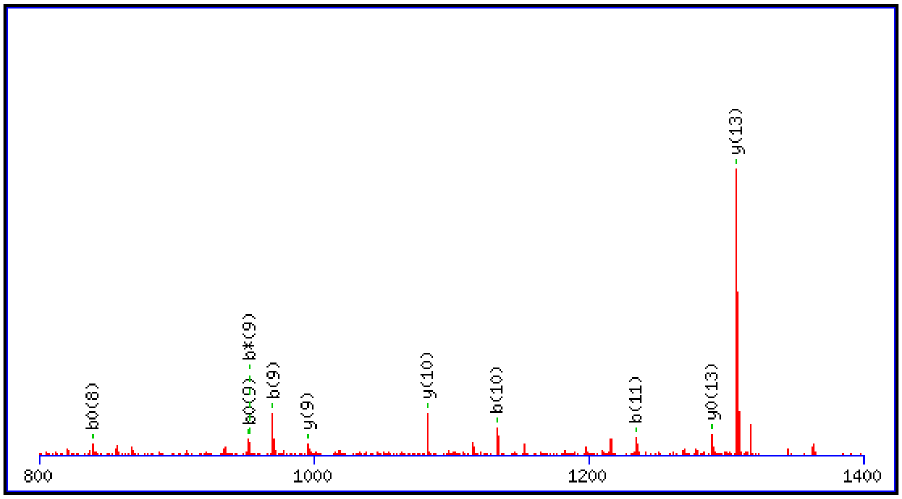

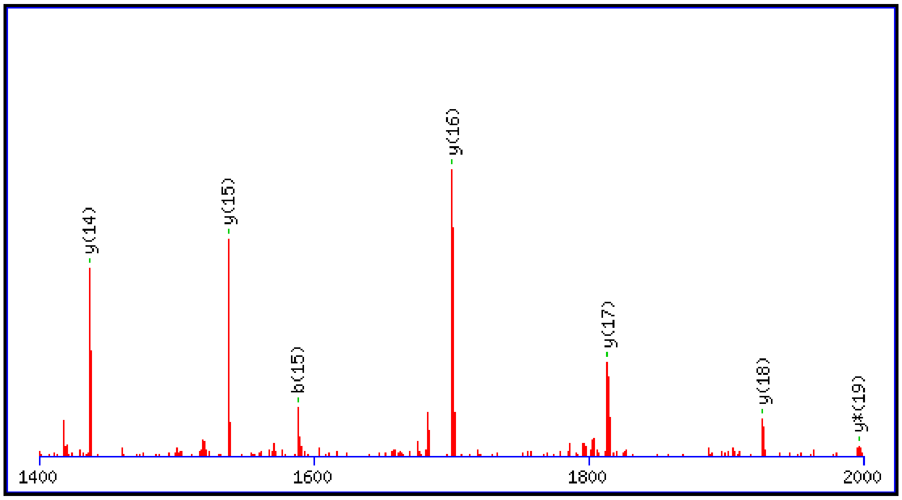

Monoisotopic mass of neutral peptide Mr(calc): 2669.2569
Fixed modifications: Carbamidomethyl (C)
Variable modifications:
N5 : Deamidated_N (N)
Ions Score: 83 **Expect:** 1.5e-006

Matches (Bold Red): 22/280 fragment ions using 34 most intense peaks

#	b	b^{++}	b*	b^{*++}	b^0	b^{0++}	Seq.	y	y^{++}	y*	y^{*++}	y^0	y^{0++}	#
1	116.0342	58.5207			98.0237	49.5155	D							25
2	173.0557	87.0315			155.0451	78.0262	G	2555.2372	1278.1223	2538.2107	1269.6090	2537.2267	1269.1170	24
3	301.1143	151.0608	284.0877	142.5475	283.1037	142.0555	Q	2498.2158	1249.6115	2481.1892	1241.0982	2480.2052	1240.6062	23
4	429.1728	215.0901	412.1463	206.5768	411.1623	206.0848	Q	2370.1572	1185.5822	2353.1306	1177.0690	2352.1466	1176.5770	22
5	544.1998	272.6035	527.1732	264.0903	**526.1892**	263.5982	N	2242.0986	1121.5529	2225.0721	1113.0397	2224.0880	1112.5477	21
6	**657.2838**	329.1456	640.2573	320.6323	**639.2733**	320.1403	I	2127.0717	1064.0395	2110.0451	1055.5262	2109.0611	1055.0342	20
7	744.3159	372.6616	727.2893	364.1483	726.3053	363.6563	S	2013.9876	1007.4974	**1996.9611**	998.9842	1995.9770	998.4922	19
8	857.3999	429.2036	840.3734	420.6903	**839.3894**	420.1983	L	**1926.9556**	963.9814	1909.9290	955.4682	1908.9450	954.9761	18
9	**970.4840**	485.7456	**953.4574**	477.2324	**952.4734**	476.7404	L	**1813.8715**	907.4394	1796.8450	898.9261	1795.8610	898.4341	17
10	**1133.5473**	567.2773	1116.5208	558.7640	1115.5368	558.2720	Y	**1700.7875**	850.8974	1683.7609	842.3841	1682.7769	841.8921	16
11	**1234.5950**	617.8011	1217.5685	609.2879	1216.5844	608.7959	T	**1537.7241**	769.3657	1520.6976	760.8524	1519.7136	760.3604	15
12	1363.6376	682.3224	1346.6110	673.8092	1345.6270	673.3172	E	**1436.6764**	718.8419	1419.6499	710.3286	1418.6659	709.8366	14
13	1460.6904	730.8488	1443.6638	722.3355	1442.6798	721.8435	P	**1307.6339**	654.3206	1290.6073	645.8073	**1289.6233**	645.3153	13
14	1517.7118	759.3596	1500.6853	750.8463	1499.7013	750.3543	G	1210.5811	605.7942	1193.5545	597.2809	1192.5705	596.7889	12
15	**1588.7489**	794.8781	1571.7224	786.3648	1570.7384	785.8728	A	1153.5596	577.2835	1136.5331	568.7702	1135.5491	568.2782	11
16	1675.7810	838.3941	1658.7544	829.8808	1657.7704	829.3888	S	**1082.5225**	541.7649	1065.4960	533.2516	1064.5119	532.7596	10
17	1803.8395	902.4234	1786.8130	893.9101	1785.8290	893.4181	Q	**995.4905**	498.2489	978.4639	489.7356	977.4799	489.2436	9
18	1904.8872	952.9472	1887.8607	944.4340	1886.8767	943.9420	T	867.4319	434.2196	850.4054	425.7063	849.4213	425.2143	8
19	2032.9458	1016.9765	2015.9193	1008.4633	2014.9352	1007.9713	Q	766.3842	383.6958	749.3577	375.1825	748.3737	374.6905	7
20	2133.9935	1067.5004	2116.9669	1058.9871	2115.9829	1058.4951	T	**638.3257**	319.6665	621.2991	311.1532	620.3151	310.6612	6
21	2191.0149	1096.0111	2173.9884	1087.4978	2173.0044	1087.0058	G	**537.2780**	269.1426	520.2514	260.6293	519.2674	260.1373	5
22	2262.0521	1131.5297	2245.0255	1123.0164	2244.0415	1122.5244	A	480.2565	240.6319	463.2300	232.1186	462.2459	231.6266	4
23	2349.0841	1175.0457	2332.0575	1166.5324	2331.0735	1166.0404	S	409.2194	205.1133	392.1928	196.6001	391.2088	196.1081	3
24	2496.1525	1248.5799	2479.1260	1240.0666	2478.1419	1239.5746	F	322.1874	161.5973	305.1608	153.0840			2
25							R	175.1190	88.0631	158.0924	79.5498			1

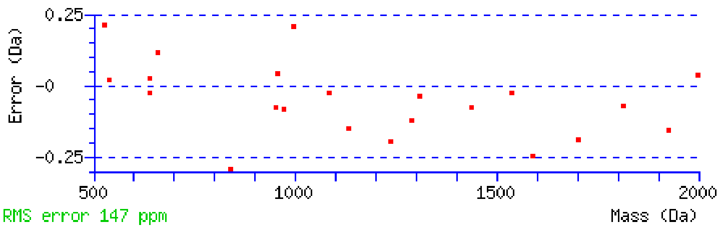

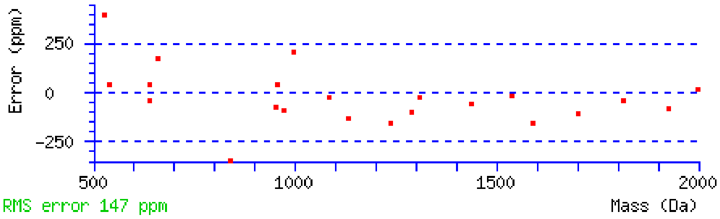

All matches to this query

Score	Mr(calc):	Delta	Sequence
83.2	2669.2569	0.0061	DGQQNISLLYTEPGASQTQTGASFR
66.6	2668.2729	0.9901	DGQQNISLLYTEPGASQTQTGASFR
6.5	2669.2808	-0.0177	YIAWYQQKPGKAPGLLIGYTSR
6.5	2669.2808	-0.0177	YIAWYQQKPGKAPGLLIGYTSR
6.5	2669.2808	-0.0177	YIAWYQQKPGKAPGLLIGYTSR
3.5	2667.2370	2.0260	TVSVWSLINSNKDEFTNPFYAR
1.5	2669.2382	0.0248	LLSLSIFFSPLQQVMESHILK
1.1	2667.2370	2.0260	TVSVWSLINSNKDEFTNPFYAR
0.8	2667.2370	2.0260	TVSVWSLINSNKDEFTNPFYAR
0.8	2668.2580	1.0050	LAPLSSSRSRFHAFILGLLNTK

Spectrum No: 118; Query: 298; Rank: 1

Peptide View

MS/MS Fragmentation of **CCGASNYTDWER**
Found in **IPI00231686**, Tax_Id=10116 Gene_Symbol=Cd63 CD63 antigen

Match to Query 298: 1518.545648 from(760.280100,2+)
Title: 091129RatKid_SCX02_02.1206.1206.2.dta
Data file K:\NewmanPaper\Piliang\3SubProteomes\Piliang3SP\mgf5ppm\SCX_3SubProteomes5ppm.mgf

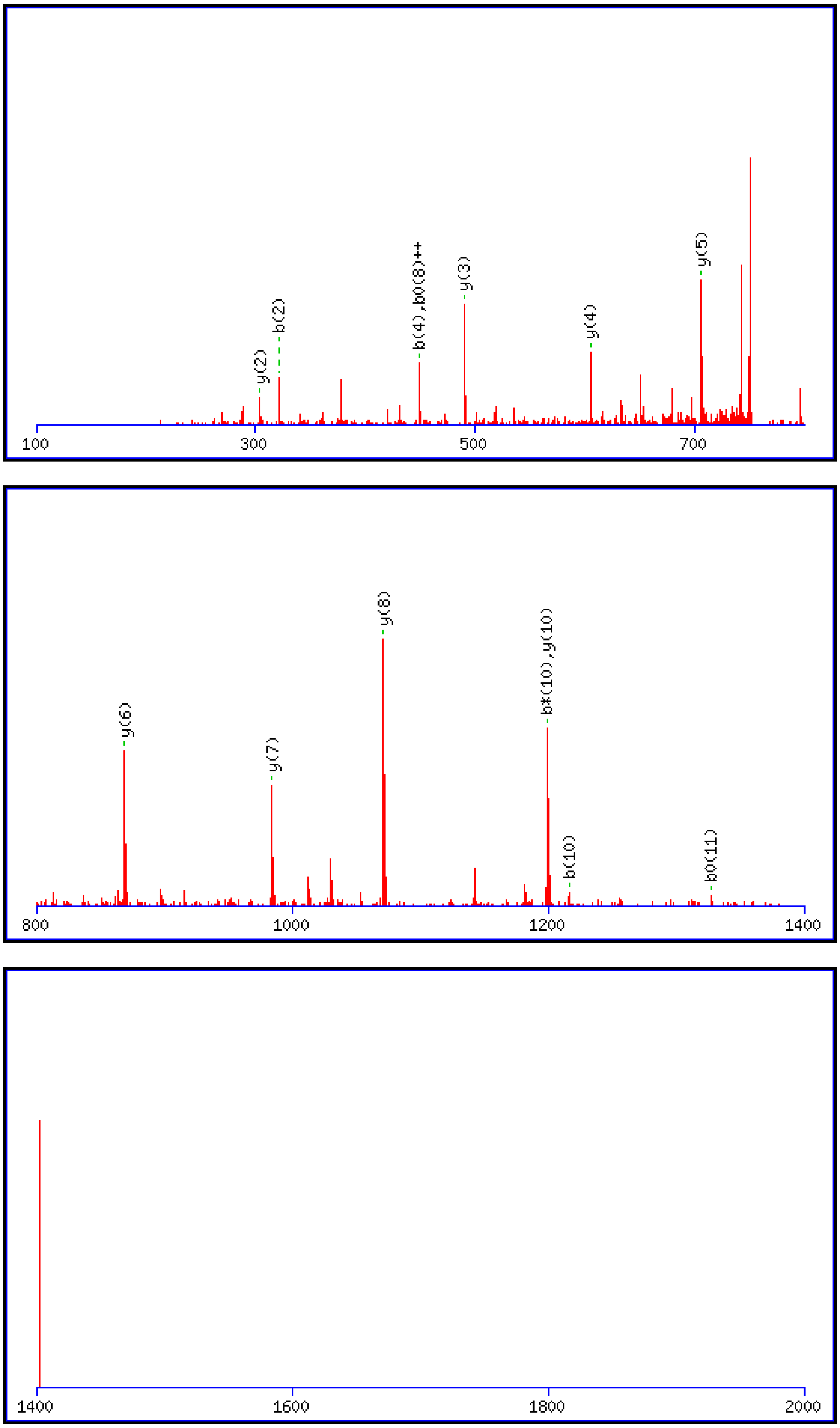

Monoisotopic mass of neutral peptide Mr(calc): 1518.5504
Fixed modifications: Carbamidomethyl (C)
Variable modifications:

N6 : Deamidated_N (N)
Ions Score: 83 **Expect:** 3.1e-007
Matches (Bold Red): 14/112 fragment ions using 16 most intense peaks

#	b	b^{++}	b*	b^{*++}	b^{0}	b^{0++}	Seq.	y	y^{++}	y*	y^{*++}	y^{0}	y^{0++}	#
1	161.0379	81.0226					C							12
2	**321.0686**	161.0379					C	1359.5270	680.2671	1342.5005	671.7539	1341.5164	671.2619	11
3	378.0900	189.5487					G	**1199.4964**	600.2518	1182.4698	591.7385	1181.4858	591.2465	10
4	**449.1272**	225.0672					A	1142.4749	571.7411	1125.4483	563.2278	1124.4643	562.7358	9
5	536.1592	268.5832			518.1486	259.5779	S	**1071.4378**	536.2225	1054.4112	527.7093	1053.4272	527.2172	8
6	651.1861	326.0967	634.1596	317.5834	633.1756	317.0914	N	**984.4057**	492.7065	967.3792	484.1932	966.3952	483.7012	7
7	814.2494	407.6284	797.2229	399.1151	796.2389	398.6231	Y	**869.3788**	435.1930	852.3523	426.6798	851.3682	426.1878	6
8	915.2971	458.1522	898.2706	449.6389	897.2866	**449.1469**	T	**706.3155**	353.6614	689.2889	345.1481	688.3049	344.6561	5
9	1030.3241	515.6657	1013.2975	507.1524	1012.3135	506.6604	D	**605.2678**	303.1375	588.2413	294.6243	587.2572	294.1323	4
10	**1216.4034**	608.7053	**1199.3768**	600.1921	1198.3928	599.7000	W	**490.2409**	245.6241	473.2143	237.1108	472.2303	236.6188	3
11	1345.4460	673.2266	1328.4194	664.7134	**1327.4354**	664.2213	E	**304.1615**	152.5844	287.1350	144.0711	286.1510	143.5791	2
12							R	175.1190	88.0631	158.0924	79.5498			1

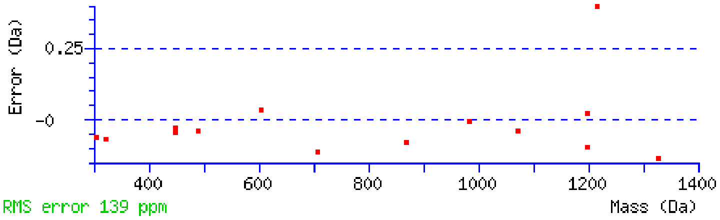

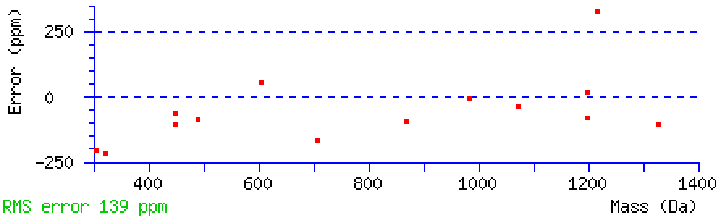

All matches to this query

Score	Mr(calc):	Delta	Sequence
83.0	1518.5504	-0.0047	CCGASNYTDWER
4.8	1518.5541	-0.0085	DVPHSNRYPTSS
3.2	1518.5479	-0.0023	TASSVIELTCAK

Spectrum No: 119; Query: 619; Rank: 1

Peptide View

MS/MS Fragmentation of **KSEANLSALAETHTNIPK**
Found in **IPI00327697**, Tax_Id=10116 Gene_Symbol=Dpep1 Dipeptidase 1 precursor

Match to Query 619: 1923.990068 from(963.002310,2+)
Title: 091127RatKid_SCX01_32.1936.1936.2.dta
Data file K:\NewmanPaper\Piliang\3SubProteomes\Piliang3SP\mgf5ppm\SCX_3SubProteomes5ppm.mgf

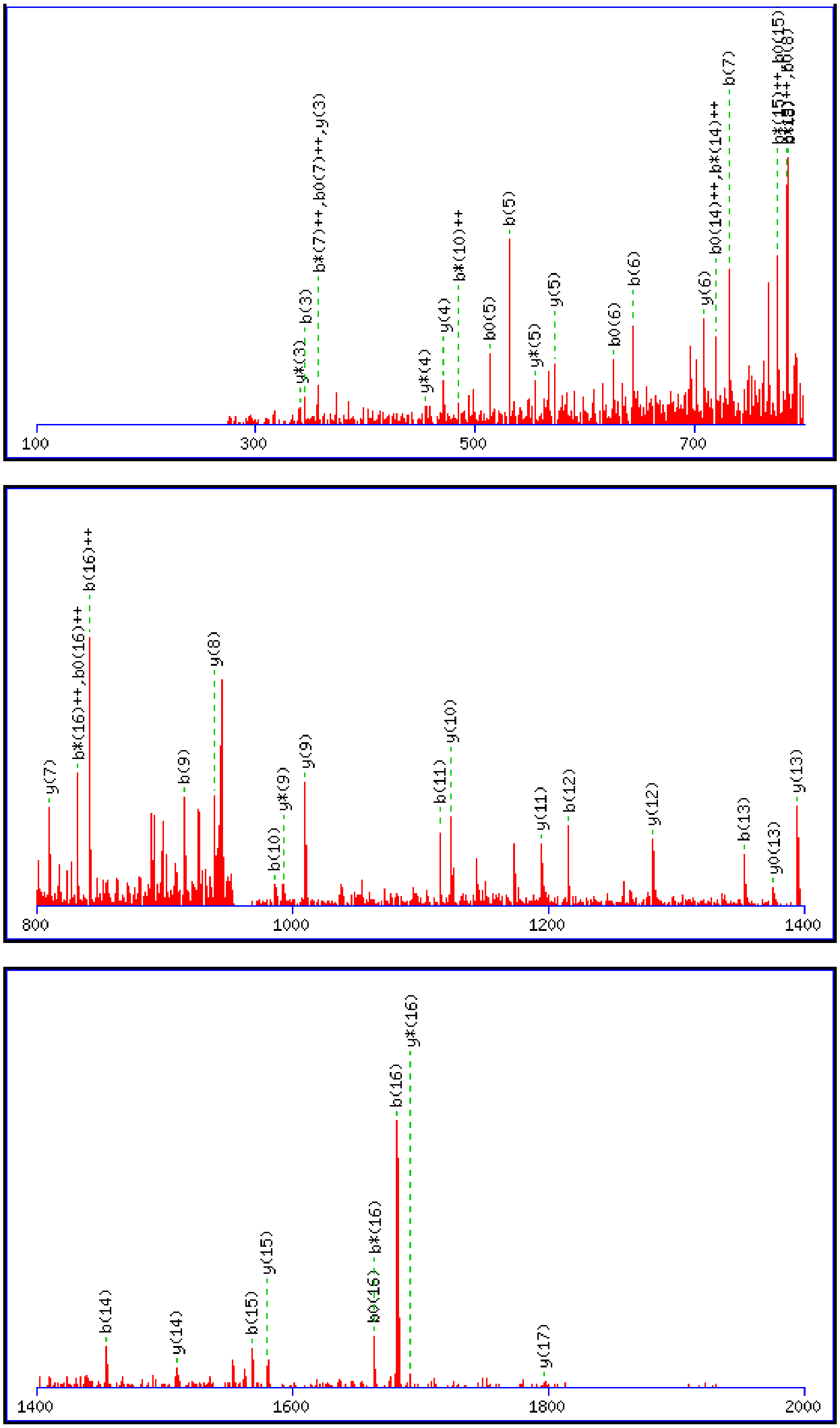

Monoisotopic mass of neutral peptide Mr(calc): 1923.9901
Fixed modifications: Carbamidomethyl (C)
Variable modifications:
N5 : Deamidated_N (N)
Ions Score: 83 **Expect:** 7.9e-007
Matches (Bold Red): 49/194 fragment ions using 97 most intense peaks

#	b	b^{++}	b*	b^{*++}	b^0	b^{0++}	Seq.	y	y^{++}	y*	y^{*++}	y^0	y^{0++}	#
1	129.1022	65.0548	112.0757	56.5415			K							18
2	216.1343	108.5708	199.1077	100.0575	198.1237	99.5655	S	**1796.9025**	898.9549	1779.8759	890.4416	1778.8919	889.9496	17
3	**345.1769**	173.0921	328.1503	164.5788	327.1663	164.0868	E	1709.8704	855.4389	**1692.8439**	846.9256	1691.8599	846.4336	16
4	416.2140	208.6106	399.1874	200.0974	398.2034	199.6053	A	**1580.8279**	790.9176	1563.8013	782.4043	1562.8173	781.9123	15
5	**531.2409**	266.1241	514.2144	257.6108	**513.2303**	257.1188	N	**1509.7907**	755.3990	1492.7642	746.8857	1491.7802	746.3937	14

**6**	**644.3250**	322.6661	627.2984	314.1529	**626.3144**	313.6608	**L**	**1394.7638**	697.8855	1377.7373	689.3723	**1376.7532**	688.8803	**13**
**7**	**731.3570**	366.1821	714.3305	**357.6689**	713.3464	**357.1769**	**S**	**1281.6797**	641.3435	1264.6532	632.8302	1263.6692	632.3382	**12**
**8**	802.3941	401.7007	**785.3676**	393.1874	**784.3836**	392.6954	**A**	**1194.6477**	597.8275	1177.6212	589.3142	1176.6371	588.8222	**11**
**9**	**915.4782**	458.2427	898.4516	449.7295	897.4676	449.2374	**L**	**1123.6106**	562.3089	1106.5841	553.7957	1105.6000	553.3037	**10**
**10**	**986.5153**	493.7613	969.4887	**485.2480**	968.5047	484.7560	**A**	**1010.5265**	505.7669	**993.5000**	497.2536	992.5160	496.7616	**9**
**11**	**1115.5579**	558.2826	1098.5313	549.7693	1097.5473	549.2773	**E**	**939.4894**	470.2483	922.4629	461.7351	921.4789	461.2431	**8**
**12**	**1216.6056**	608.8064	1199.5790	600.2931	1198.5950	599.8011	**T**	**810.4468**	405.7271	793.4203	397.2138	792.4363	396.7218	**7**
**13**	**1353.6645**	677.3359	1336.6379	668.8226	1335.6539	668.3306	**H**	**709.3991**	355.2032	692.3726	346.6899	691.3886	346.1979	**6**
**14**	**1454.7122**	727.8597	1437.6856	**719.3464**	1436.7016	**718.8544**	**T**	**572.3402**	286.6738	**555.3137**	278.1605	554.3297	277.6685	**5**
**15**	**1568.7551**	**784.8812**	1551.7285	**776.3679**	1550.7445	**775.8759**	**N**	**471.2926**	236.1499	**454.2660**	227.6366			**4**
**16**	**1681.8391**	**841.4232**	**1664.8126**	**832.9099**	**1663.8286**	**832.4179**	**I**	**357.2496**	179.1285	**340.2231**	170.6152			**3**
**17**	1778.8919	889.9496	1761.8654	881.4363	1760.8813	880.9443	**P**	244.1656	122.5864	227.1390	114.0731			**2**
**18**							**K**	147.1128	74.0600	130.0863	65.5468			**1**

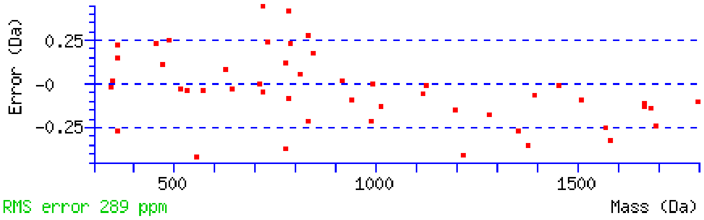

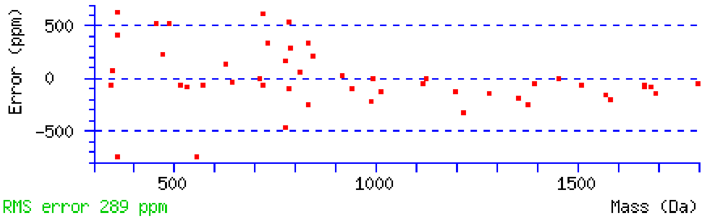

All matches to this query

Score	Mr(calc):	Delta	Sequence
82.5	1923.9901	-0.0000	KSEANLSALAETHTNIPK
28.6	1923.9901	-0.0000	KSEANLSALAETHTNIPK
7.8	1923.9779	0.0122	TRKLLENSTASIQQQK
6.2	1923.9779	0.0122	TRKLLENSTASIQQQK
6.1	1923.9779	0.0122	TRKLLENSTASIQQQK
4.2	1924.0054	-0.0153	STNAFTTGKLTHPPNIPK

Spectrum No: 120; Query: 349; Rank: 1

Peptide View

MS/MS Fragmentation of **TNGSVDLGEEEEAAR**
Found in **IPI00464886**, Tax_Id=10116 Gene_Symbol=LOC652956 p55 protein

Match to Query 349: 1576.686848 from(789.350700,2+)
Title: 091127RatKid_SCX01_20.1534.1534.2.dta
Data file K:\NewmanPaper\Piliang\3SubProteomes\Piliang3SP\mgf5ppm\SCX_3SubProteomes5ppm.mgf

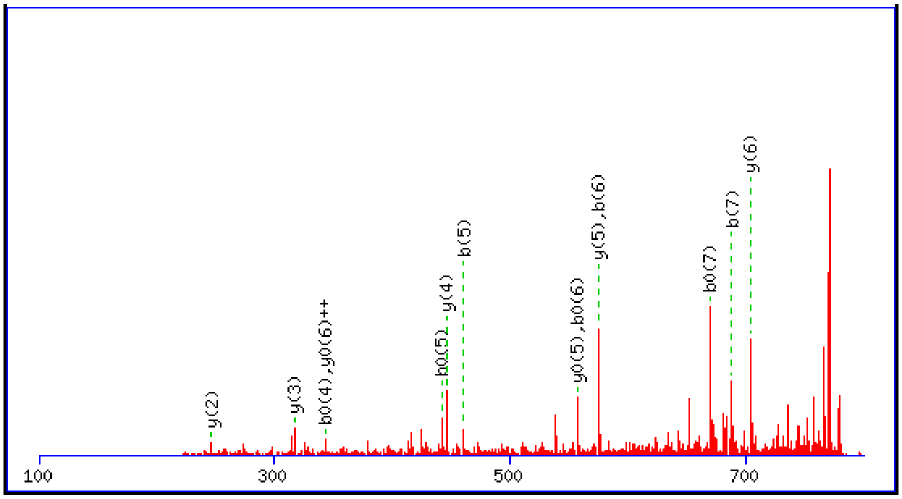

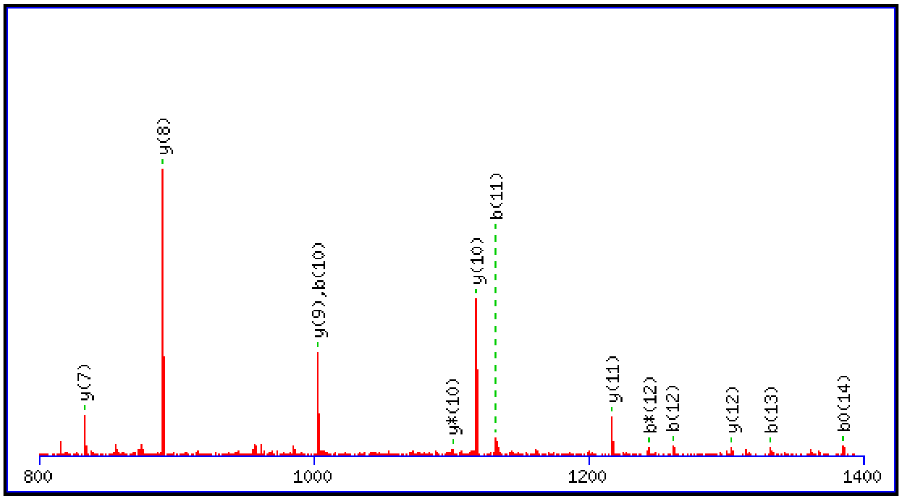

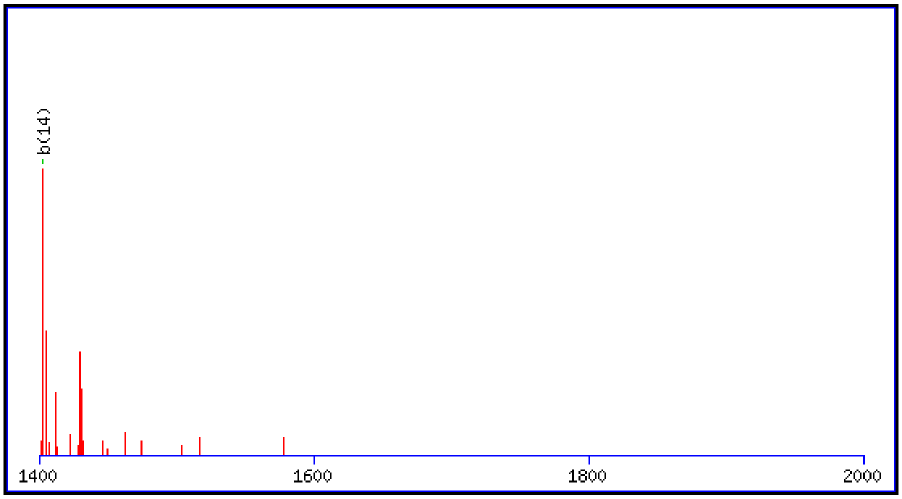

Monoisotopic mass of neutral peptide Mr(calc): 1576.6852
Fixed modifications: Carbamidomethyl (C)
Variable modifications:
N2 : Deamidated_N (N)
Ions Score: 82 **Expect:** 9.8e-007
Matches (Bold Red): 28/160 fragment ions using 41 most intense peaks

#	b	b^{++}	b*	b^{*++}	b^0	b^{0++}	Seq.	y	y^{++}	y*	y^{*++}	y^0	y^{0++}	#
1	102.0550	51.5311			84.0444	42.5258	T							15
2	217.0819	109.0446	200.0553	100.5313	199.0713	100.0393	N	1476.6449	738.8261	1459.6183	730.3128	1458.6343	729.8208	14
3	274.1034	137.5553	257.0768	129.0420	256.0928	128.5500	G	1361.6179	681.3126	1344.5914	672.7993	1343.6074	672.3073	13
4	361.1354	181.0713	344.1088	172.5581	**343.1248**	172.0660	S	**1304.5965**	652.8019	1287.5699	644.2886	1286.5859	643.7966	12
5	**460.2038**	230.6055	443.1772	222.0923	**442.1932**	221.6003	V	**1217.5644**	609.2859	1200.5379	600.7726	1199.5539	600.2806	11

**6**	**575.2307**	288.1190	558.2042	279.6057	**557.2202**	279.1137	**D**	**1118.4960**	559.7516	**1101.4695**	551.2384	1100.4855	550.7464	**10**
**7**	**688.3148**	344.6610	671.2883	336.1478	**670.3042**	335.6558	**L**	**1003.4691**	502.2382	986.4425	493.7249	985.4585	493.2329	**9**
**8**	745.3363	373.1718	728.3097	364.6585	727.3257	364.1665	**G**	**890.3850**	445.6961	873.3585	437.1829	872.3745	436.6909	**8**
**9**	874.3789	437.6931	857.3523	429.1798	856.3683	428.6878	**E**	**833.3636**	417.1854	816.3370	408.6721	815.3530	408.1801	**7**
**10**	**1003.4215**	502.2144	986.3949	493.7011	985.4109	493.2091	**E**	**704.3210**	352.6641	687.2944	344.1508	686.3104	**343.6588**	**6**
**11**	**1132.4640**	566.7357	1115.4375	558.2224	1114.4535	557.7304	**E**	**575.2784**	288.1428	558.2518	279.6295	**557.2678**	279.1375	**5**
**12**	**1261.5066**	631.2570	**1244.4801**	622.7437	1243.4961	622.2517	**E**	**446.2358**	223.6215	429.2092	215.1083	428.2252	214.6162	**4**
**13**	**1332.5438**	666.7755	1315.5172	658.2622	1314.5332	657.7702	**A**	**317.1932**	159.1002	300.1666	150.5870			**3**
**14**	**1403.5809**	702.2941	1386.5543	693.7808	**1385.5703**	693.2888	**A**	**246.1561**	123.5817	229.1295	115.0684			**2**
**15**							**R**	175.1190	88.0631	158.0924	79.5498			**1**

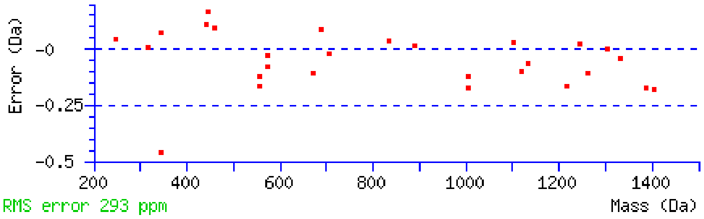

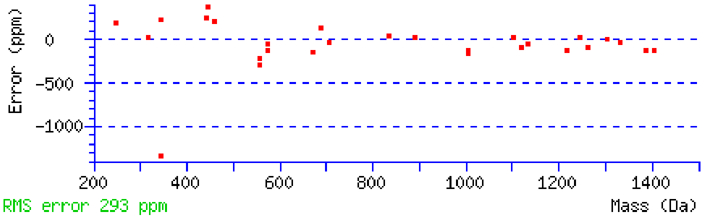

All matches to this query

Score	Mr(calc):	Delta	Sequence
82.3	1576.6852	0.0016	TNGSVDLGEEEEAAR
8.6	1576.6899	-0.0030	AGQNASIVSLKSDK
8.6	1575.6680	1.0188	MSEFWLISAPGDK
7.9	1576.6899	-0.0030	AGQNASIVSLKSDK
7.4	1576.6995	-0.0126	YSEDIQSRSTRR
7.4	1576.6995	-0.0126	YSEDIQSRSTRR
5.7	1576.6770	0.0098	SPEQIESHEVTNK
5.6	1576.6787	0.0082	NASSESAVVPKTVK
5.3	1576.6779	0.0090	NTPFMGIMDKTAR
5.1	1576.6844	0.0025	MKAEDGENYAIKK

Spectrum No: 121; Query: 748; Rank: 1

Peptide View

MS/MS Fragmentation of **YSNESQDLSVNGYNCTMR**
Found in **IPI00191789**, Tax_Id=10116 Gene_Symbol=Hrg Histidine-rich glycoprotein

Match to Query 748: 2138.850448 from(1070.432500,2+)
Title: 091127RatKid_SCX01_02.2289.2289.2.dta
Data file K:\NewmanPaper\Piliang\3SubProteomes\Piliang3SP\mgf5ppm\SCX_3SubProteomes5ppm.mgf

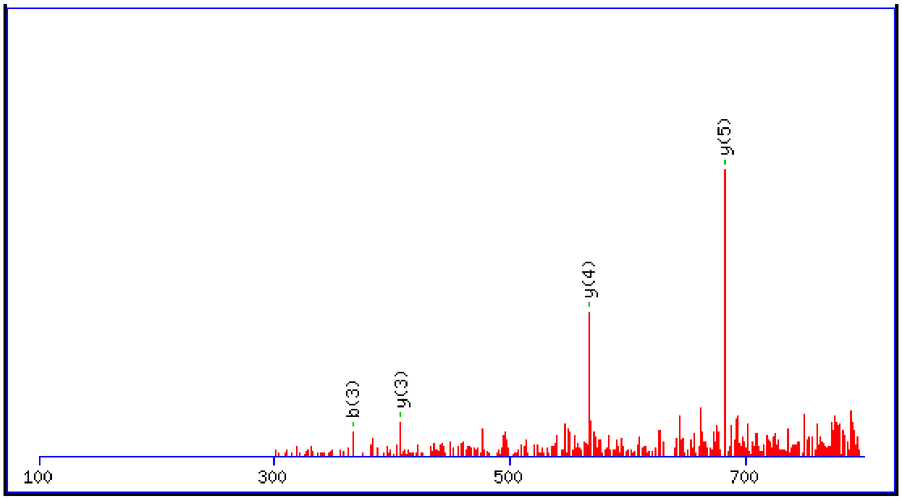

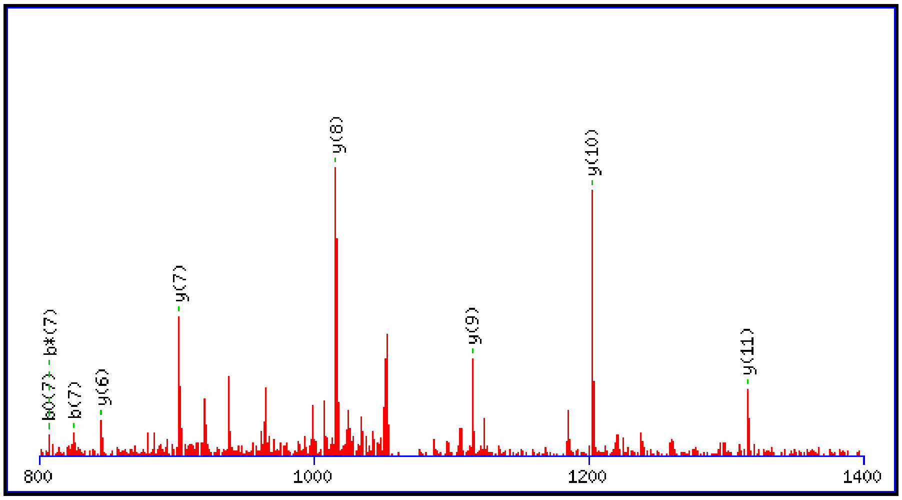

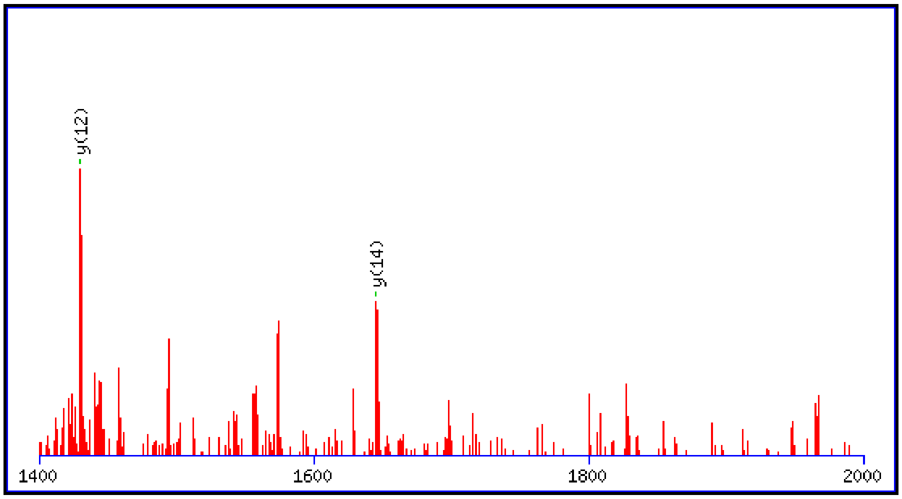

Monoisotopic mass of neutral peptide Mr(calc): 2138.8521
Fixed modifications: Carbamidomethyl (C)
Variable modifications:
N3 : Deamidated_N (N)
N14 : Deamidated_N (N)
Ions Score: 82 **Expect:** 1.1e-006
Matches (Bold Red): 15/194 fragment ions using 28 most intense peaks

#	b	b^{++}	b*	b^{*++}	b^{0}	b^{0++}	Seq.	y	y^{++}	y*	y^{*++}	y^{0}	y^{0++}	#
**1**	164.0706	82.5389					**Y**							**18**
**2**	251.1026	126.0550			233.0921	117.0497	**S**	1976.7960	988.9017	1959.7695	980.3884	1958.7855	979.8964	**17**
**3**	**366.1296**	183.5684	349.1030	175.0551	348.1190	174.5631	**N**	1889.7640	945.3856	1872.7375	936.8724	1871.7534	936.3804	**16**
**4**	495.1722	248.0897	478.1456	239.5764	477.1616	239.0844	**E**	1774.7371	887.8722	1757.7105	879.3589	1756.7265	878.8669	**15**

**5**	582.2042	291.6057	565.1776	283.0925	564.1936	282.6005	**S**	**1645.6945**	823.3509	1628.6679	814.8376	1627.6839	814.3456	**14**
**6**	710.2628	355.6350	693.2362	347.1217	692.2522	346.6297	**Q**	1558.6624	779.8349	1541.6359	771.3216	1540.6519	770.8296	**13**
**7**	**825.2897**	413.1485	**808.2632**	404.6352	**807.2791**	404.1432	**D**	**1430.6039**	715.8056	1413.5773	707.2923	1412.5933	706.8003	**12**
**8**	938.3738	469.6905	921.3472	461.1773	920.3632	460.6852	**L**	**1315.5769**	658.2921	1298.5504	649.7788	1297.5664	649.2868	**11**
**9**	1025.4058	513.2065	1008.3793	504.6933	1007.3952	504.2013	**S**	**1202.4929**	601.7501	1185.4663	593.2368	1184.4823	592.7448	**10**
**10**	1124.4742	562.7407	1107.4477	554.2275	1106.4637	553.7355	**V**	**1115.4608**	558.2341	1098.4343	549.7208	1097.4503	549.2288	**9**
**11**	1238.5171	619.7622	1221.4906	611.2489	1220.5066	610.7569	**N**	**1016.3924**	508.6998	999.3659	500.1866	998.3819	499.6946	**8**
**12**	1295.5386	648.2729	1278.5121	639.7597	1277.5280	639.2677	**G**	**902.3495**	451.6784	885.3229	443.1651	884.3389	442.6731	**7**
**13**	1458.6019	729.8046	1441.5754	721.2913	1440.5914	720.7993	**Y**	**845.3280**	423.1677	828.3015	414.6544	827.3175	414.1624	**6**
**14**	1573.6289	787.3181	1556.6023	778.8048	1555.6183	778.3128	**N**	**682.2647**	341.6360	665.2382	333.1227	664.2541	332.6307	**5**
**15**	1733.6595	867.3334	1716.6330	858.8201	1715.6490	858.3281	**C**	**567.2378**	284.1225	550.2112	275.6092	549.2272	275.1172	**4**
**16**	1834.7072	917.8572	1817.6807	909.3440	1816.6966	908.8520	**T**	**407.2071**	204.1072	390.1806	195.5939	389.1966	195.1019	**3**
**17**	1965.7477	983.3775	1948.7211	974.8642	1947.7371	974.3722	**M**	306.1594	153.5834	289.1329	145.0701			**2**
**18**							**R**	175.1190	88.0631	158.0924	79.5498			**1**

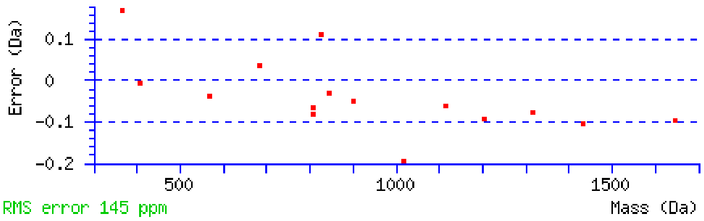

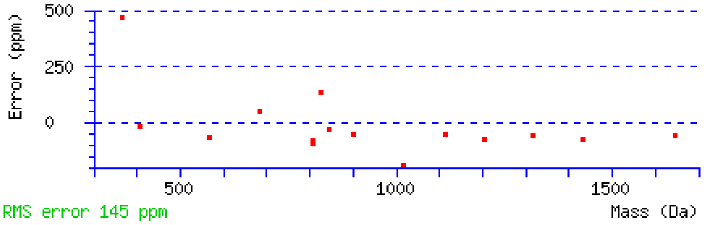

All matches to this query

Score	Mr(calc):	Delta	Sequence
82.1	2138.8521	-0.0016	YSNESQDLSVNGYNCTMR
82.1	2137.8681	0.9824	YSNESQDLSVNGYNCTMR
58.5	2138.8521	-0.0016	YSNESQDLSVNGYNCTMR
40.3	2138.8521	-0.0016	YSNESQDLSVNGYNCTMR
40.3	2137.8681	0.9824	YSNESQDLSVNGYNCTMR
7.4	2138.8540	-0.0036	CSSGASLDSHLHRMLHR
4.1	2137.8681	0.9824	YSNESQDLSVNGYNCTMR
3.3	2138.8531	-0.0027	SGSAEPTNNHANGTQNKPR
3.3	2138.8531	-0.0027	SGSAEPTNNHANGTQNKPR
2.8	2136.8246	2.0258	GNYSGNNNNNSNNDFQKR

Spectrum No: 122; Query: 539; Rank: 1

Peptide View

MS/MS Fragmentation of **LSPNCCNSSTVADAYR**
Found in **IPI00203176**, Tax_Id=10116 Gene_Symbol=Tpcn1 Two pore calcium channel protein 1

Match to Query 539: 1814.760548 from(908.387550,2+)
Title: 091127RatKid_SCX01_02.1432.1432.2.dta
Data file K:\NewmanPaper\Piliang\3SubProteomes\Piliang3SP\mgf5ppm\SCX_3SubProteomes5ppm.mgf

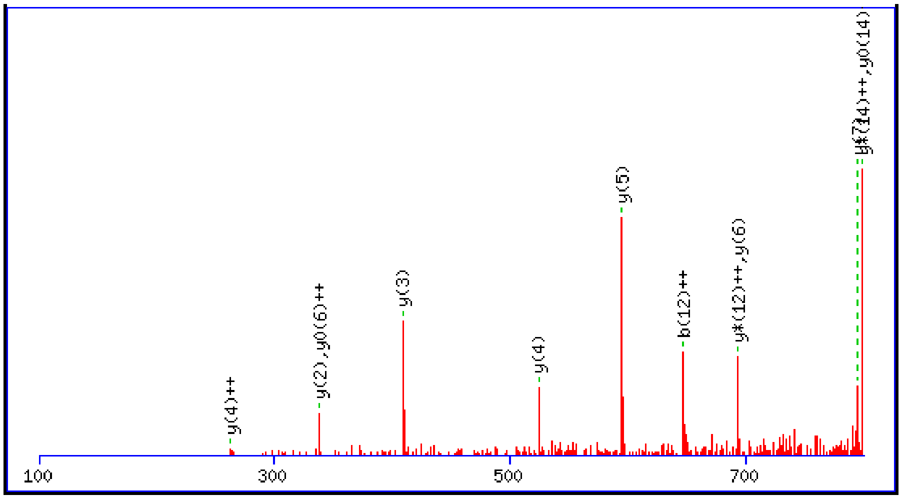

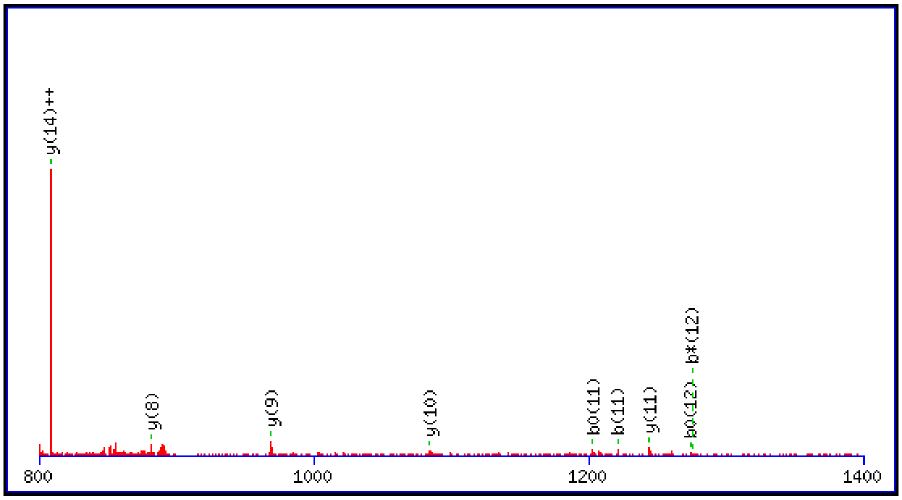

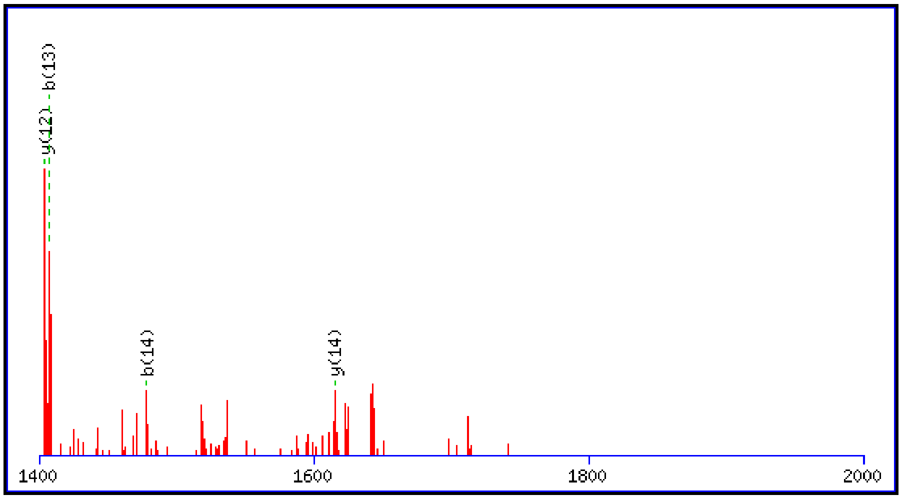

Monoisotopic mass of neutral peptide Mr(calc): 1814.7563
Fixed modifications: Carbamidomethyl (C)
Variable modifications:
N7 : Deamidated_N (N)
Ions Score: 82 **Expect:** 1.3e-006
Matches (Bold Red): 25/166 fragment ions using 44 most intense peaks

#	b	b^{++}	b*	b^{*++}	b^0	b^{0++}	Seq.	y	y^{++}	y*	y^{*++}	y^0	y^{0++}	#
1	114.0913	57.5493					L							16
2	201.1234	101.0653			183.1128	92.0600	S	1702.6796	851.8434	1685.6530	843.3301	1684.6690	842.8381	15
3	298.1761	149.5917			280.1656	140.5864	P	**1615.6475**	**808.3274**	1598.6210	**799.8141**	1597.6370	**799.3221**	14
4	412.2191	206.6132	395.1925	198.0999	394.2085	197.6079	N	1518.5948	759.8010	1501.5682	751.2877	1500.5842	750.7957	13
5	572.2497	286.6285	555.2232	278.1152	554.2391	277.6232	C	**1404.5518**	702.7796	1387.5253	**694.2663**	1386.5413	693.7743	12

**6**	732.2804	366.6438	715.2538	358.1305	714.2698	357.6385	**C**	**1244.5212**	622.7642	1227.4946	614.2510	1226.5106	613.7589	**11**
**7**	847.3073	424.1573	830.2807	415.6440	829.2967	415.1520	**N**	**1084.4905**	542.7489	1067.4640	534.2356	1066.4800	533.7436	**10**
**8**	934.3393	467.6733	917.3128	459.1600	916.3288	458.6680	**S**	**969.4636**	485.2354	952.4371	476.7222	951.4530	476.2302	**9**
**9**	1021.3713	511.1893	1004.3448	502.6760	1003.3608	502.1840	**S**	**882.4316**	441.7194	865.4050	433.2062	864.4210	432.7141	**8**
**10**	1122.4190	561.7132	1105.3925	553.1999	1104.4085	552.7079	**T**	**795.3995**	398.2034	778.3730	389.6901	777.3890	389.1981	**7**
**11**	**1221.4874**	611.2474	1204.4609	602.7341	**1203.4769**	602.2421	**V**	**694.3519**	347.6796	677.3253	339.1663	676.3413	**338.6743**	**6**
**12**	1292.5246	**646.7659**	**1275.4980**	638.2526	**1274.5140**	637.7606	**A**	**595.2835**	298.1454	578.2569	289.6321	577.2729	289.1401	**5**
**13**	**1407.5515**	704.2794	1390.5250	695.7661	1389.5409	695.2741	**D**	**524.2463**	**262.6268**	507.2198	254.1135	506.2358	253.6215	**4**
**14**	**1478.5886**	739.7979	1461.5621	731.2847	1460.5780	730.7927	**A**	**409.2194**	205.1133	392.1928	196.6001			**3**
**15**	1641.6519	821.3296	1624.6254	812.8163	1623.6414	812.3243	**Y**	**338.1823**	169.5948	321.1557	161.0815			**2**
**16**							**R**	175.1190	88.0631	158.0924	79.5498			**1**

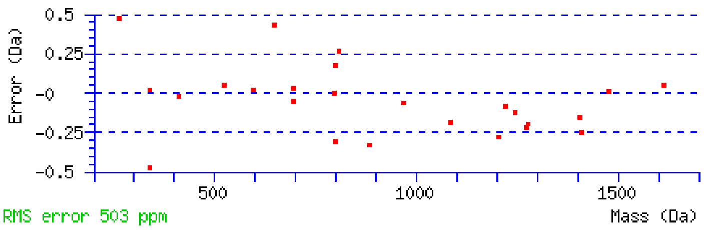

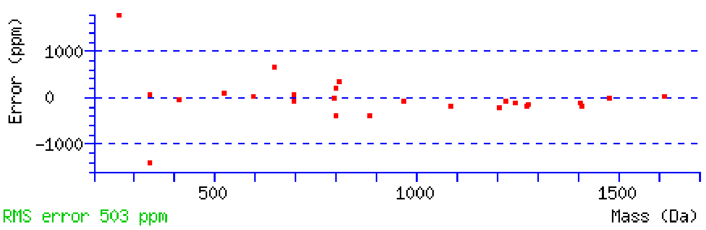

All matches to this query

Score	Mr(calc):	Delta	Sequence
81.7	1814.7563	0.0042	LSPNCCNSSTVADAYR
44.1	1814.7563	0.0042	LSPNCCNSSTVADAYR
21.1	1813.7723	0.9882	LSPNCCNSSTVADAYR
12.6	1813.7416	1.0190	TELFCSINCLSAYR
5.0	1813.7413	1.0192	QVTDAETKPKSSRK
4.9	1812.7567	2.0038	TTYVNTNDEVVSHPR
4.3	1812.7576	2.0029	VETCCVIGNYYSLR
3.7	1812.7567	2.0038	TTYVNTNDEVVSHPR
3.7	1812.7567	2.0038	TTYVNTNDEVVSHPR
3.7	1812.7567	2.0038	TTYVNTNDEVVSHPR

Spectrum No: 123; Query: 460; Rank: 1

Peptide View

MS/MS Fragmentation of **YTGNASALFILPDQGK**
Found in **IPI00200591**, Tax_Id=10116 Gene_Symbol=LOC299282 Serine protease inhibitor A3L precursor

Match to Query 460: 1694.855468 from(848.435010,2+)
Title: 091127RatKid_SCX01_03.3558.3558.2.dta
Data file K:\NewmanPaper\Piliang\3SubProteomes\Piliang3SP\mgf5ppm\SCX_3SubProteomes5ppm.mgf

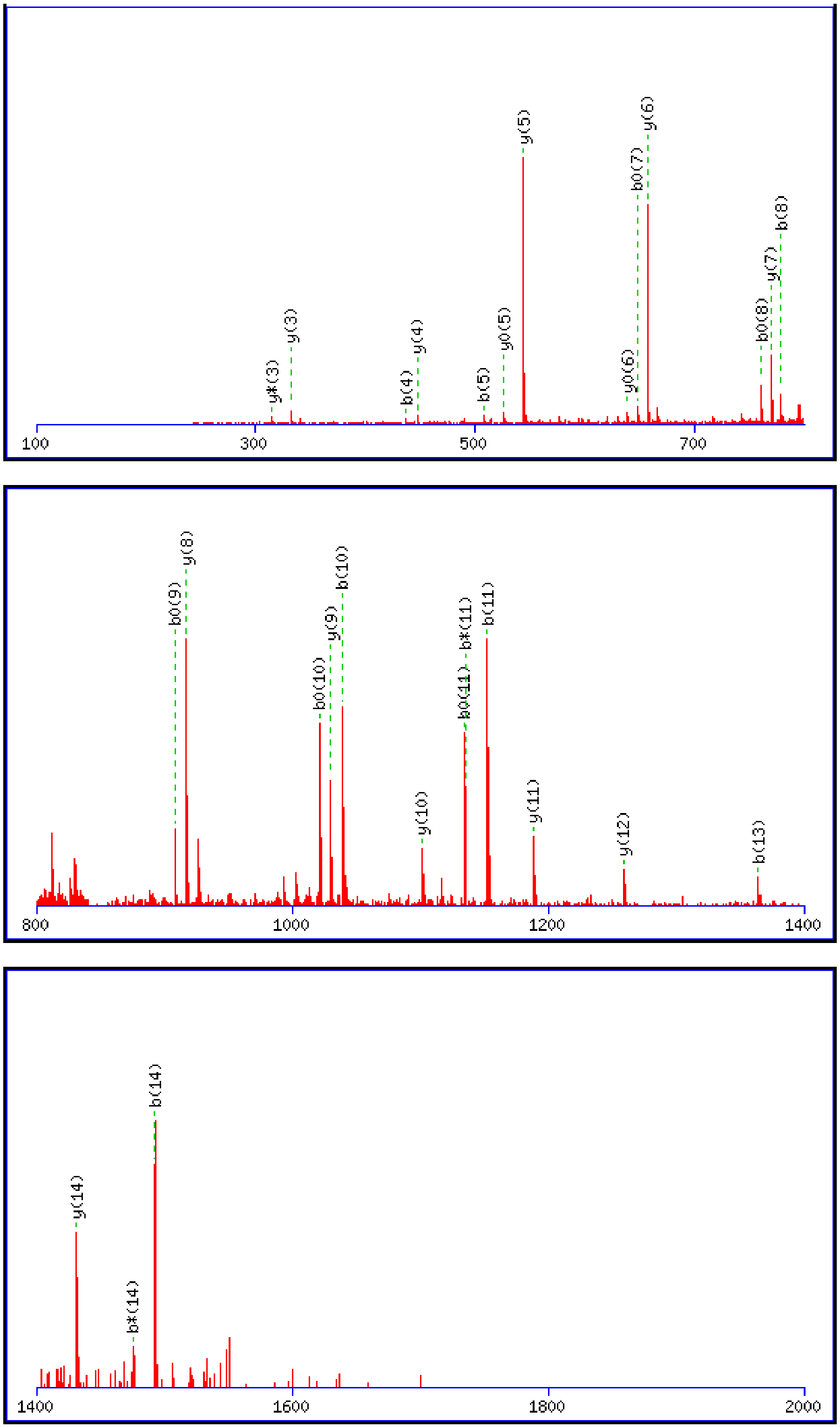

Monoisotopic mass of neutral peptide Mr(calc): 1694.8515
Fixed modifications: Carbamidomethyl (C)
Variable modifications:
N4 : Deamidated_N (N)
Ions Score: 82 **Expect:** 1.1e-006
Matches (Bold Red): 28/166 fragment ions using 56 most intense peaks

#	b	b++	b*	b*++	b0	b0++	Seq.	y	y++	y*	y*++	y0	y0++	#
1	164.0706	82.5389					Y							16
2	265.1183	133.0628			247.1077	124.0575	T	1532.7955	766.9014	1515.7689	758.3881	1514.7849	757.8961	15
3	322.1397	161.5735			304.1292	152.5682	G	**1431.7478**	716.3775	1414.7213	707.8643	1413.7372	707.3723	14
4	**437.1667**	219.0870	420.1401	210.5737	419.1561	210.0817	N	1374.7264	687.8668	1357.6998	679.3535	1356.7158	678.8615	13
5	**508.2038**	254.6055	491.1772	246.0923	490.1932	245.6003	A	**1259.6994**	630.3533	1242.6729	621.8401	1241.6888	621.3481	12

**6**	595.2358	298.1216	578.2093	289.6083	577.2253	289.1163	**S**	**1188.6623**	594.8348	1171.6358	586.3215	1170.6517	585.8295	**11**
**7**	666.2729	333.6401	649.2464	325.1268	**648.2624**	324.6348	**A**	**1101.6303**	551.3188	1084.6037	542.8055	1083.6197	542.3135	**10**
**8**	**779.3570**	390.1821	762.3305	381.6689	**761.3464**	381.1769	**L**	**1030.5932**	515.8002	1013.5666	507.2869	1012.5826	506.7949	**9**
**9**	926.4254	463.7163	909.3989	455.2031	**908.4149**	454.7111	**F**	**917.5091**	459.2582	900.4825	450.7449	899.4985	450.2529	**8**
**10**	**1039.5095**	520.2584	1022.4829	511.7451	**1021.4989**	511.2531	**I**	**770.4407**	385.7240	753.4141	377.2107	752.4301	376.7187	**7**
**11**	**1152.5935**	576.8004	**1135.5670**	568.2871	**1134.5830**	567.7951	**L**	**657.3566**	329.1819	640.3301	320.6687	**639.3461**	320.1767	**6**
**12**	1249.6463	625.3268	1232.6198	616.8135	1231.6357	616.3215	**P**	**544.2726**	272.6399	527.2460	264.1266	**526.2620**	263.6346	**5**
**13**	**1364.6733**	682.8403	1347.6467	674.3270	1346.6627	673.8350	**D**	**447.2198**	224.1135	430.1932	215.6003	429.2092	215.1082	**4**
**14**	**1492.7318**	746.8696	**1475.7053**	738.3563	1474.7213	737.8643	**Q**	**332.1928**	166.6001	**315.1663**	158.0868			**3**
**15**	1549.7533	775.3803	1532.7267	766.8670	1531.7427	766.3750	**G**	204.1343	102.5708	187.1077	94.0575			**2**
**16**							**K**	147.1128	74.0600	130.0863	65.5468			**1**

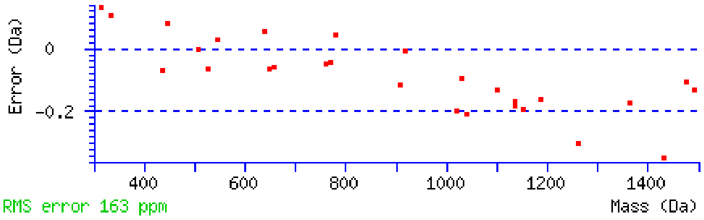

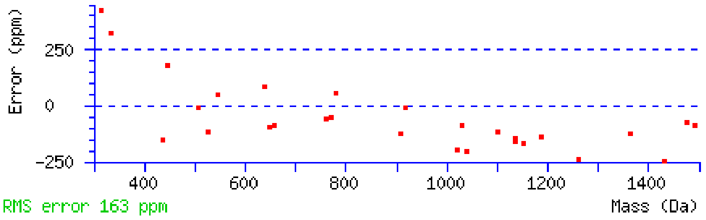

All matches to this query

Score	Mr(calc):	Delta	Sequence
81.7	1694.8515	0.0039	YTGNASALFILPDQGK
40.7	1693.8675	0.9879	YTGNASALFILPDQGK
10.3	1694.8662	-0.0107	SLITFEGGSMIQIQR
8.9	1693.8440	1.0115	FAENQGTPLLVIANK
2.7	1694.8562	-0.0007	NHYELTRKNYMVK
2.7	1694.8662	-0.0107	SQDNVIKVCASVTFK
2.3	1694.8406	0.0149	IHHRIHTGEKPYK
2.2	1694.8406	0.0149	IHHRIHTGEKPYK
1.9	1693.8675	0.9880	AYSPYVEDILQQLR
1.9	1693.8651	0.9904	LKVIENELIQASTR

Spectrum No: 124; Query: 370; Rank: 1

Peptide View

MS/MS Fragmentation of **ESNSTSLTQAALLEK**
Found in **IPI00199867**, Tax_Id=10116 Gene_Symbol=Emilin1_predicted elastin microfibril interfacer 1

Match to Query 370: 1591.793028 from(796.903790,2+)
Title: 091127RatKid_SCX01_16.2371.2371.2.dta
Data file K:\NewmanPaper\Piliang\3SubProteomes\Piliang3SP\mgf5ppm\SCX_3SubProteomes5ppm.mgf

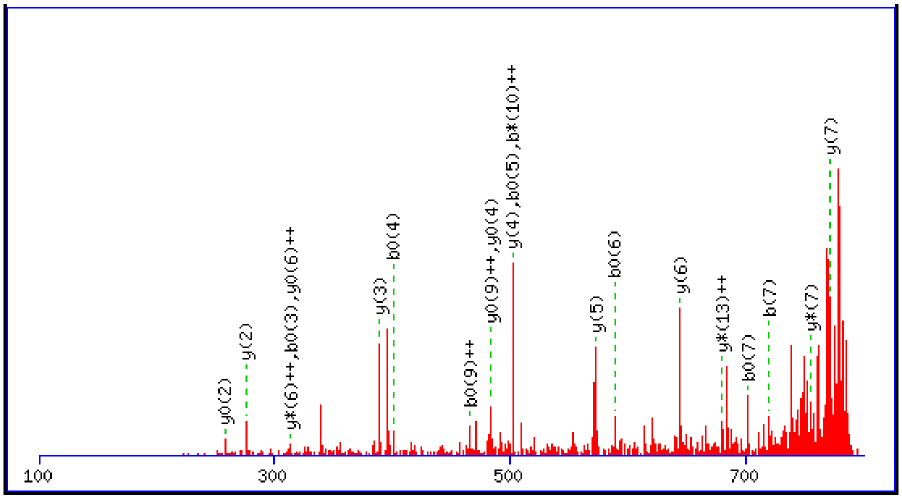

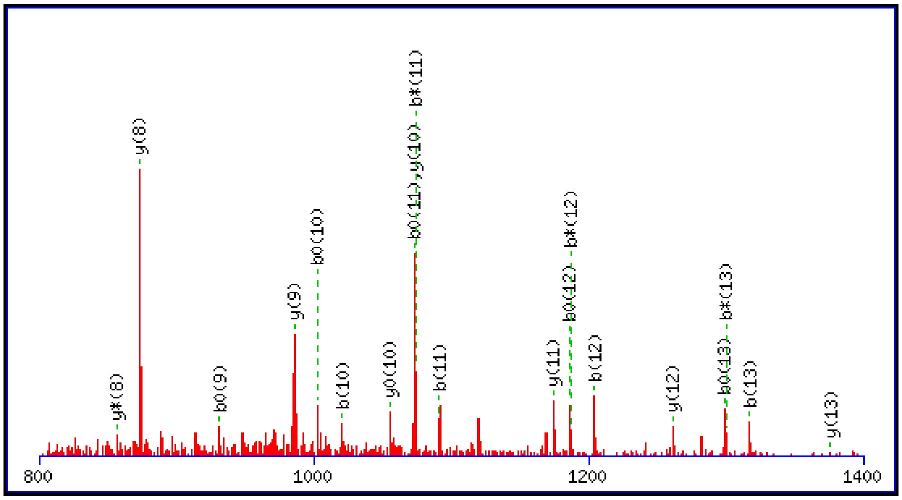

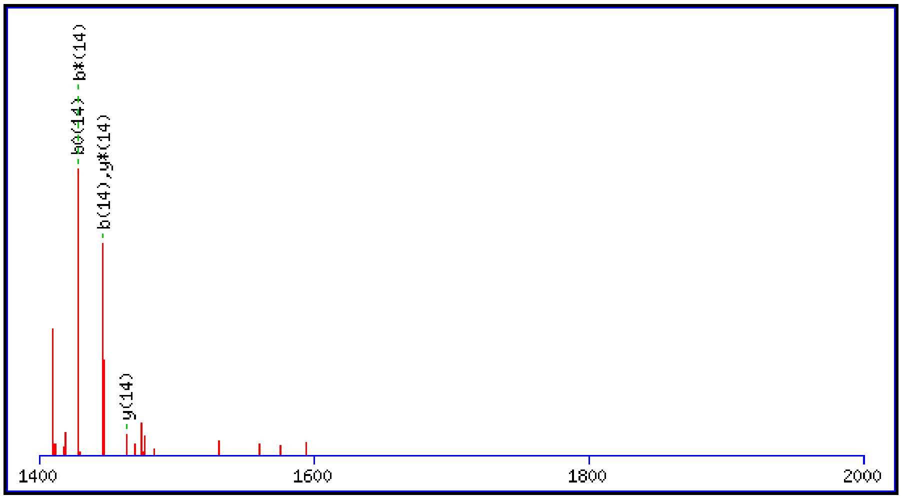

Monoisotopic mass of neutral peptide Mr(calc): 1591.7941
Fixed modifications: Carbamidomethyl (C)
Variable modifications:
N3 : Deamidated_N (N)
Ions Score: 81 **Expect:** 1.1e-006
Matches (Bold Red): 46/162 fragment ions using 105 most intense peaks

#	b	b^{++}	b*	b^{*++}	b^0	b^{0++}	Seq.	y	y^{++}	y*	y^{*++}	y^0	y^{0++}	#
1	130.0499	65.5286			112.0393	56.5233	E							15
2	217.0819	109.0446			199.0713	100.0393	S	**1463.7588**	732.3830	**1446.7322**	723.8697	1445.7482	723.3777	14
3	332.1088	166.5581	315.0823	158.0448	**314.0983**	157.5528	N	**1376.7267**	688.8670	1359.7002	**680.3537**	1358.7162	679.8617	13
4	419.1409	210.0741	402.1143	201.5608	**401.1303**	201.0688	S	**1261.6998**	631.3535	1244.6733	622.8403	1243.6892	622.3483	12
5	520.1885	260.5979	503.1620	252.0846	**502.1780**	251.5926	T	**1174.6678**	587.8375	1157.6412	579.3243	1156.6572	578.8322	11

**6**	607.2206	304.1139	590.1940	295.6006	**589.2100**	295.1086	**S**	**1073.6201**	537.3137	1056.5936	528.8004	**1055.6095**	528.3084	**10**
**7**	**720.3046**	360.6560	703.2781	352.1427	**702.2941**	351.6507	**L**	**986.5881**	493.7977	969.5615	485.2844	968.5775	**484.7924**	**9**
**8**	821.3523	411.1798	804.3258	402.6665	803.3417	402.1745	**T**	**873.5040**	437.2556	**856.4775**	428.7424	855.4934	428.2504	**8**
**9**	949.4109	475.2091	932.3843	466.6958	**931.4003**	**466.2038**	**Q**	**772.4563**	386.7318	**755.4298**	378.2185	754.4458	377.7265	**7**
**10**	**1020.4480**	510.7276	1003.4215	**502.2144**	**1002.4374**	501.7224	**A**	**644.3978**	322.7025	627.3712	**314.1892**	626.3872	**313.6972**	**6**
**11**	**1091.4851**	546.2462	**1074.4586**	537.7329	**1073.4746**	537.2409	**A**	**573.3606**	287.1840	556.3341	278.6707	555.3501	278.1787	**5**
**12**	**1204.5692**	602.7882	**1187.5426**	594.2750	**1186.5586**	593.7829	**L**	**502.3235**	251.6654	485.2970	243.1521	**484.3130**	242.6601	**4**
**13**	**1317.6532**	659.3303	**1300.6267**	650.8170	**1299.6427**	650.3250	**L**	**389.2395**	195.1234	372.2129	186.6101	371.2289	186.1181	**3**
**14**	**1446.6958**	723.8516	**1429.6693**	715.3383	**1428.6853**	714.8463	**E**	**276.1554**	138.5813	259.1288	130.0681	**258.1448**	129.5761	**2**
**15**							**K**	147.1128	74.0600	130.0863	65.5468			**1**

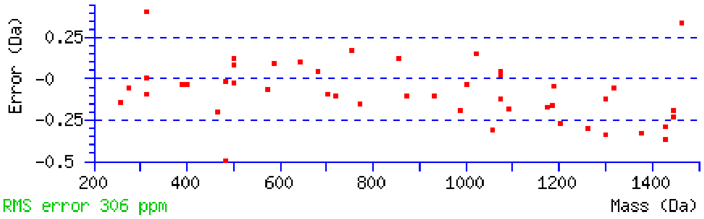

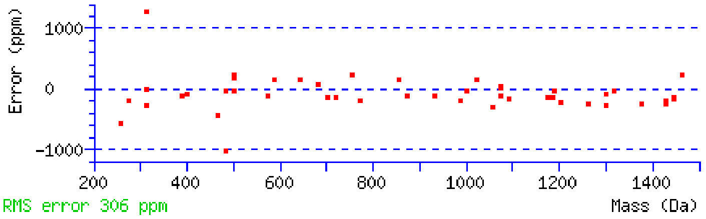

All matches to this query

Score	Mr(calc):	Delta	Sequence
81.4	1591.7941	-0.0010	ESNSTSLTQAALLEK
24.8	1591.7828	0.0102	TSSLLILTENENEK
10.9	1591.8062	-0.0132	MKLMRSEGPDVSVK
7.6	1591.7810	0.0120	CNKVKPMEGRTEK
7.4	1589.7970	1.9960	GESMALNEKQILEK
7.4	1591.7945	-0.0015	GNYRFMKLLLTR
6.2	1591.8062	-0.0131	EKMAATNKEVACLK
6.1	1589.7970	1.9960	GEAMALNEKQILEK
5.0	1590.7988	0.9942	TSSLLILTENENEK
4.3	1591.8053	-0.0123	EKVAGEDVGTSSSAKK

Spectrum No: 125; Query: 558; Rank: 1

Peptide View

MS/MS Fragmentation of **LANASLYLAEWQNITK**
Found in **IPI00211616**, Tax_Id=10116 Gene_Symbol=Slc3a2 4F2 cell-surface antigen heavy chain

Match to Query 558: 1835.936368 from(918.975460,2+)
Title: 091127RatKid_SCX01_02.4729.4729.2.dta
Data file K:\NewmanPaper\Piliang\3SubProteomes\Piliang3SP\mgf5ppm\SCX_3SubProteomes5ppm.mgf

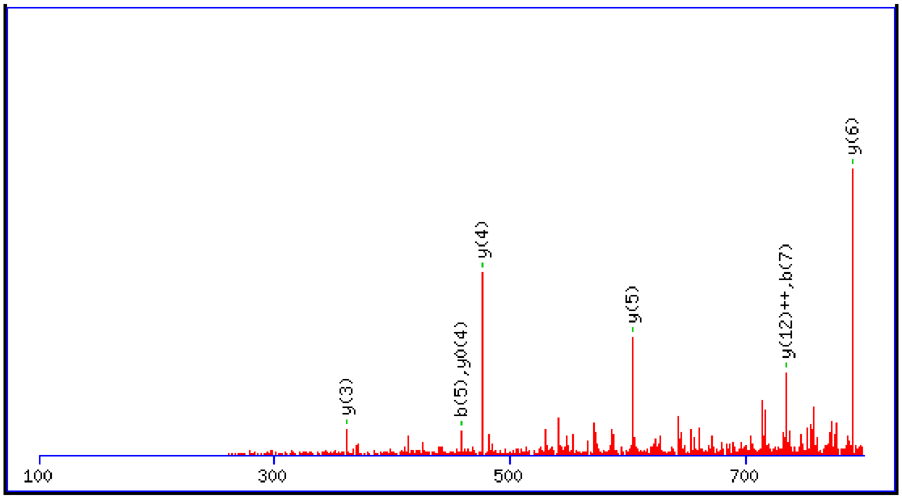

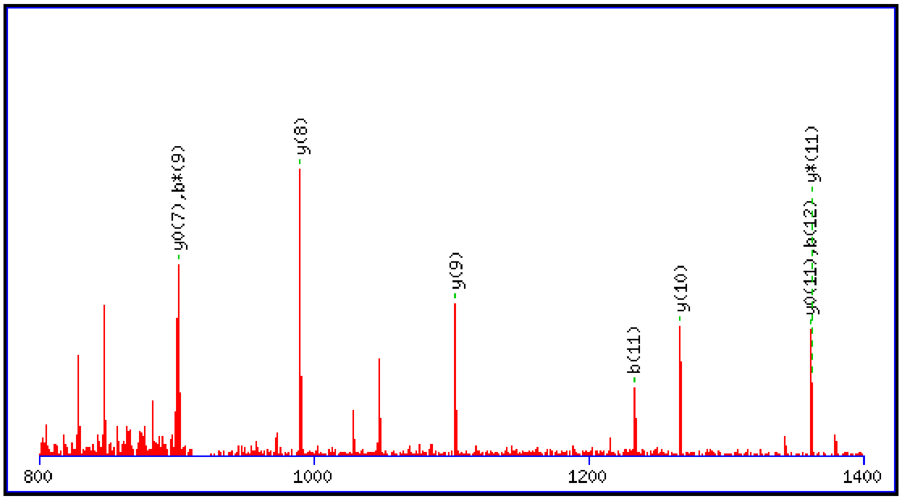

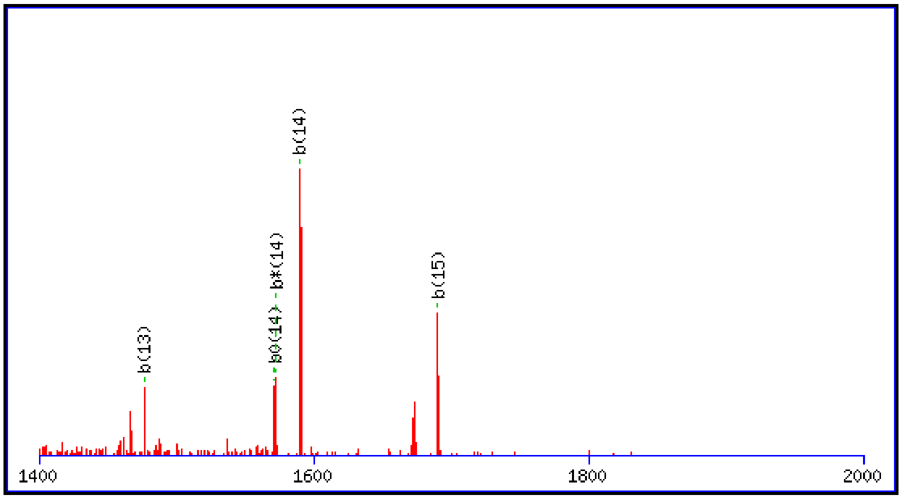

Monoisotopic mass of neutral peptide Mr(calc): 1835.9305
Fixed modifications: Carbamidomethyl (C)
Variable modifications:
N3 : Deamidated_N (N)
N13 : Deamidated_N (N)
Ions Score: 81 **Expect:** 1.2e-006
Matches (Bold Red): 22/166 fragment ions using 23 most intense peaks

#	b	b^{++}	b*	b^{*++}	b^0	b^{0++}	Seq.	y	y^{++}	y*	y^{*++}	y^0	y^{0++}	#
1	114.0913	57.5493					L							16
2	185.1285	93.0679					A	1723.8537	862.4305	1706.8272	853.9172	1705.8432	853.4252	15
3	300.1554	150.5813	283.1288	142.0681			N	1652.8166	826.9119	1635.7901	818.3987	1634.8061	817.9067	14
4	371.1925	186.0999	354.1660	177.5866			A	1537.7897	769.3985	1520.7631	760.8852	1519.7791	760.3932	13

**5**	**458.2245**	229.6159	441.1980	221.1026	440.2140	220.6106	**S**	1466.7526	**733.8799**	1449.7260	725.3666	1448.7420	724.8746	**12**
**6**	571.3086	286.1579	554.2820	277.6447	553.2980	277.1527	**L**	1379.7205	690.3639	**1362.6940**	681.8506	**1361.7100**	681.3586	**11**
**7**	**734.3719**	367.6896	717.3454	359.1763	716.3614	358.6843	**Y**	**1266.6365**	633.8219	1249.6099	625.3086	1248.6259	624.8166	**10**
**8**	847.4560	424.2316	830.4294	415.7184	829.4454	415.2264	**L**	**1103.5731**	552.2902	1086.5466	543.7769	1085.5626	543.2849	**9**
**9**	918.4931	459.7502	**901.4666**	451.2369	900.4825	450.7449	**A**	**990.4891**	495.7482	973.4625	487.2349	972.4785	486.7429	**8**
**10**	1047.5357	524.2715	1030.5091	515.7582	1029.5251	515.2662	**E**	919.4520	460.2296	902.4254	451.7163	**901.4414**	451.2243	**7**
**11**	**1233.6150**	617.3111	1216.5885	608.7979	1215.6044	608.3059	**W**	**790.4094**	395.7083	773.3828	387.1951	772.3988	386.7030	**6**
**12**	**1361.6736**	681.3404	1344.6470	672.8272	1343.6630	672.3351	**Q**	**604.3301**	302.6687	587.3035	294.1554	586.3195	293.6634	**5**
**13**	**1476.7005**	738.8539	1459.6740	730.3406	1458.6900	729.8486	**N**	**476.2715**	238.6394	459.2449	230.1261	**458.2609**	229.6341	**4**
**14**	**1589.7846**	795.3959	**1572.7580**	786.8827	**1571.7740**	786.3906	**I**	**361.2445**	181.1259	344.2180	172.6126	343.2340	172.1206	**3**
**15**	**1690.8323**	845.9198	1673.8057	837.4065	1672.8217	836.9145	**T**	248.1605	124.5839	231.1339	116.0706	230.1499	115.5786	**2**
**16**							**K**	147.1128	74.0600	130.0863	65.5468			**1**

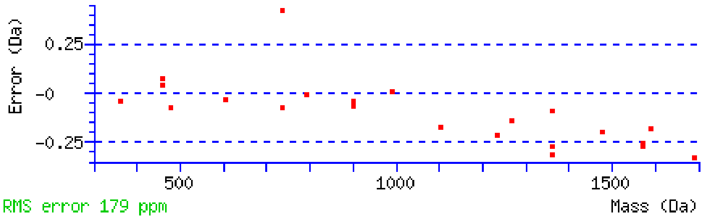

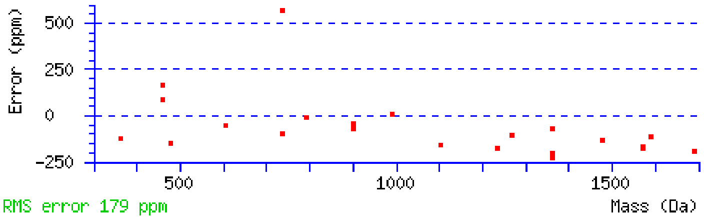

All matches to this query

Score	Mr(calc):	Delta	Sequence
81.1	1835.9305	0.0059	LANASLYLAEWQNITK
43.2	1834.9465	0.9899	LANASLYLAEWQNITK
16.1	1834.9465	0.9899	LANASLYLAEWQNITK
7.7	1835.9546	-0.0183	GKILHYQVTLQEVTK
7.7	1835.9546	-0.0183	GKILHYQVTLQEVTK
6.7	1835.9328	0.0035	GMKAGKPVVAASGAGNLTK
5.6	1835.9394	-0.0030	GSTIRQELVLPSXTLK
5.6	1835.9394	-0.0030	GSTIRQELVLPSXTLK
4.8	1835.9240	0.0124	MVQFHFTNKDLESLK
4.8	1834.9481	0.9882	FLLEYAVSTKKNTIK

Spectrum No: 126; Query: 794; Rank: 1

Peptide View

MS/MS Fragmentation of **VNCSTSCAAPEIGGLETPTNK**
Found in **IPI00372952**, Tax_Id=10116 Gene_Symbol=Icam2 Intercellular adhesion molecule 2

Match to Query 794: 2205.994168 from(1104.004360,2+)
Title: 091127RatKid_SCX01_12.1922.1922.2.dta
Data file K:\NewmanPaper\Piliang\3SubProteomes\Piliang3SP\mgf5ppm\SCX_3SubProteomes5ppm.mgf

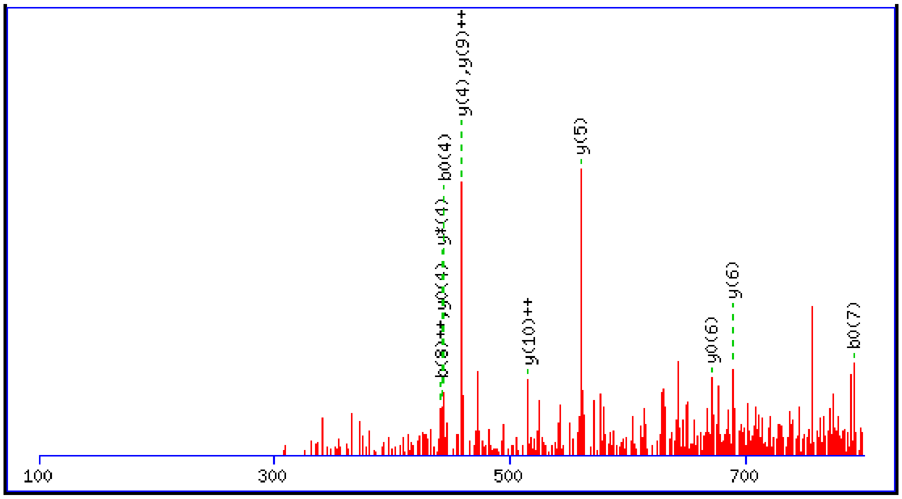

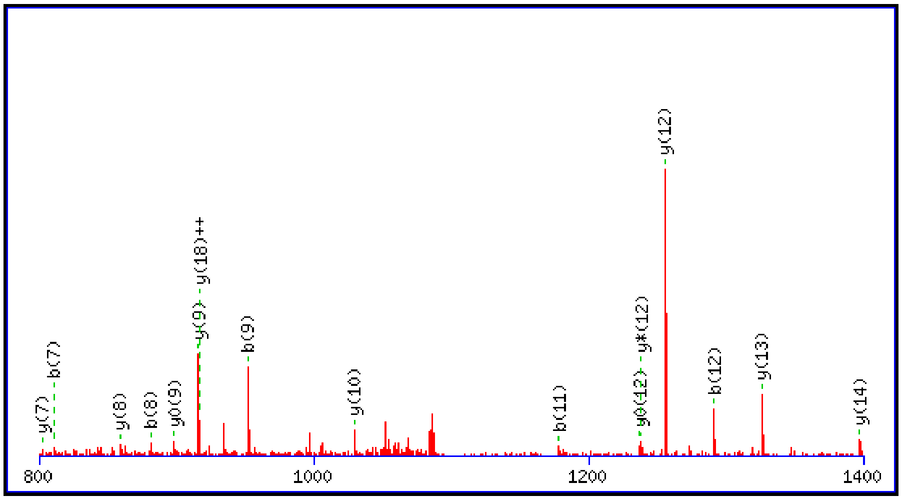

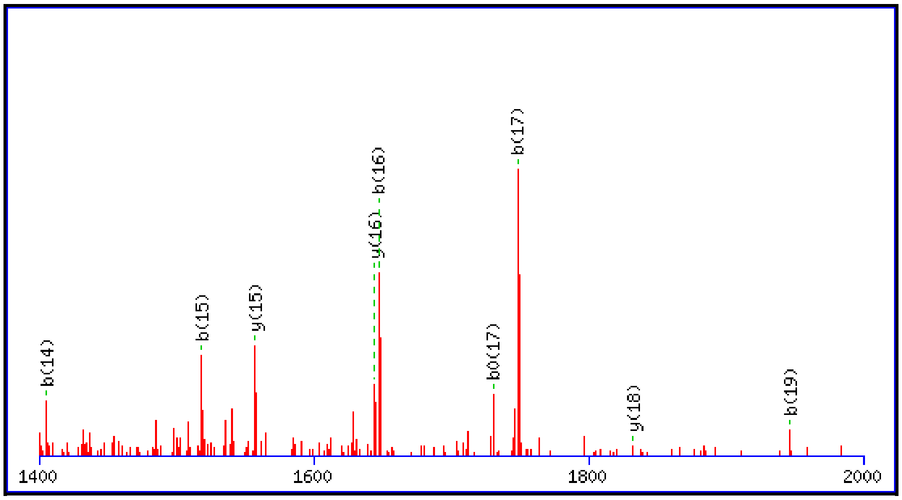

Monoisotopic mass of neutral peptide Mr(calc): 2205.9882
Fixed modifications: Carbamidomethyl (C)
Variable modifications:
N2 : Deamidated_N (N)
Ions Score: 81 **Expect:** 2.1e-006
Matches (Bold Red): 36/228 fragment ions using 71 most intense peaks

#	b	b^{++}	b*	b^{*++}	b^{0}	b^{0++}	Seq.	y	y^{++}	y*	y^{*++}	y^{0}	y^{0++}	#
1	100.0757	50.5415					V							21
2	215.1026	108.0550	198.0761	99.5417			N	2107.9271	1054.4672	2090.9005	1045.9539	2089.9165	1045.4619	20
3	375.1333	188.0703	358.1067	179.5570			C	1992.9001	996.9537	1975.8736	988.4404	1974.8896	987.9484	19
4	462.1653	231.5863	445.1388	223.0730	**444.1547**	222.5810	S	**1832.8695**	**916.9384**	1815.8429	908.4251	1814.8589	907.9331	18
5	563.2130	282.1101	546.1864	273.5969	545.2024	273.1048	T	1745.8374	873.4224	1728.8109	864.9091	1727.8269	864.4171	17

**6**	650.2450	325.6261	633.2185	317.1129	632.2344	316.6209	**S**	**1644.7898**	822.8985	1627.7632	814.3852	1626.7792	813.8932	**16**
**7**	**810.2757**	405.6415	793.2491	397.1282	**792.2651**	396.6362	**C**	**1557.7577**	779.3825	1540.7312	770.8692	1539.7472	770.3772	**15**
**8**	**881.3128**	**441.1600**	864.2862	432.6468	863.3022	432.1547	**A**	**1397.7271**	699.3672	1380.7005	690.8539	1379.7165	690.3619	**14**
**9**	**952.3499**	476.6786	935.3233	468.1653	934.3393	467.6733	**A**	**1326.6900**	663.8486	1309.6634	655.3353	1308.6794	654.8433	**13**
**10**	1049.4027	525.2050	1032.3761	516.6917	1031.3921	516.1997	**P**	**1255.6529**	628.3301	**1238.6263**	619.8168	**1237.6423**	619.3248	**12**
**11**	**1178.4452**	589.7263	1161.4187	581.2130	1160.4347	580.7210	**E**	1158.6001	579.8037	1141.5735	571.2904	1140.5895	570.7984	**11**
**12**	**1291.5293**	646.2683	1274.5028	637.7550	1273.5187	637.2630	**I**	**1029.5575**	**515.2824**	1012.5310	506.7691	1011.5469	506.2771	**10**
**13**	1348.5508	674.7790	1331.5242	666.2657	1330.5402	665.7737	**G**	**916.4734**	**458.7404**	899.4469	450.2271	**898.4629**	449.7351	**9**
**14**	**1405.5722**	703.2898	1388.5457	694.7765	1387.5617	694.2845	**G**	**859.4520**	430.2296	842.4254	421.7164	841.4414	421.2243	**8**
**15**	**1518.6563**	759.8318	1501.6298	751.3185	1500.6457	750.8265	**L**	**802.4305**	401.7189	785.4040	393.2056	784.4199	392.7136	**7**
**16**	**1647.6989**	824.3531	1630.6723	815.8398	1629.6883	815.3478	**E**	**689.3464**	345.1769	672.3199	336.6636	**671.3359**	336.1716	**6**
**17**	**1748.7466**	874.8769	1731.7200	866.3636	**1730.7360**	865.8716	**T**	**560.3039**	280.6556	543.2773	272.1423	542.2933	271.6503	**5**
**18**	1845.7993	923.4033	1828.7728	914.8900	1827.7888	914.3980	**P**	**459.2562**	230.1317	**442.2296**	221.6185	**441.2456**	221.1264	**4**
**19**	**1946.8470**	973.9271	1929.8205	965.4139	1928.8364	964.9219	**T**	362.2034	181.6053	345.1769	173.0921	344.1928	172.6001	**3**
**20**	2060.8899	1030.9486	2043.8634	1022.4353	2042.8794	1021.9433	**N**	261.1557	131.0815	244.1292	122.5682			**2**
**21**							**K**	147.1128	74.0600	130.0863	65.5468			**1**

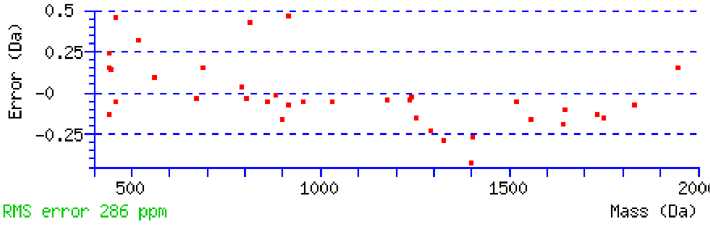

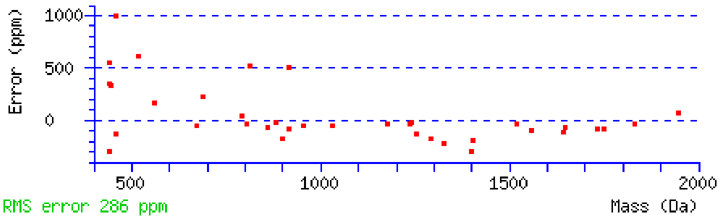

All matches to this query

Score	Mr(calc):	Delta	Sequence
81.0	2205.9882	0.0060	VNCSTSCAAPEIGGLETPTNK
57.1	2205.0042	0.9900	VNCSTSCAAPEIGGLETPTNK
9.0	2205.9882	0.0060	VNCSTSCAAPEIGGLETPTNK
1.7	2205.9749	0.0193	SFVGKQFSANDKVYTVEK
1.5	2205.9782	0.0159	VFTTLVLCDKPAVSENPR
1.5	2205.9782	0.0159	VFTTLVLCDKPAVSENPR
1.2	2205.9820	0.0122	GGGASAAAAAAAASGALLGAYLER
1.0	2204.0015	1.9927	STIATDSLTAVEPAQTKSPK
0.9	2206.0112	-0.0171	FTADVPIGAEPKPSPTXALV
0.9	2206.0112	-0.0171	FTADVPIGAEPKPSPTXALV

Spectrum No: 127; Query: 109; Rank: 1

Peptide View

MS/MS Fragmentation of **LANTSMFNNSK**
Found in **IPI00206254**, Tax_Id=10116 Gene_Symbol=Ggt1 Gamma-glutamyltranspeptidase 1 precursor

Match to Query 109: 1227.545348 from(614.779950,2+)
Title: 091127RatKid_SCX01_02.1609.1609.2.dta
Data file K:\NewmanPaper\Piliang\3SubProteomes\Piliang3SP\mgf5ppm\SCX_3SubProteomes5ppm.mgf

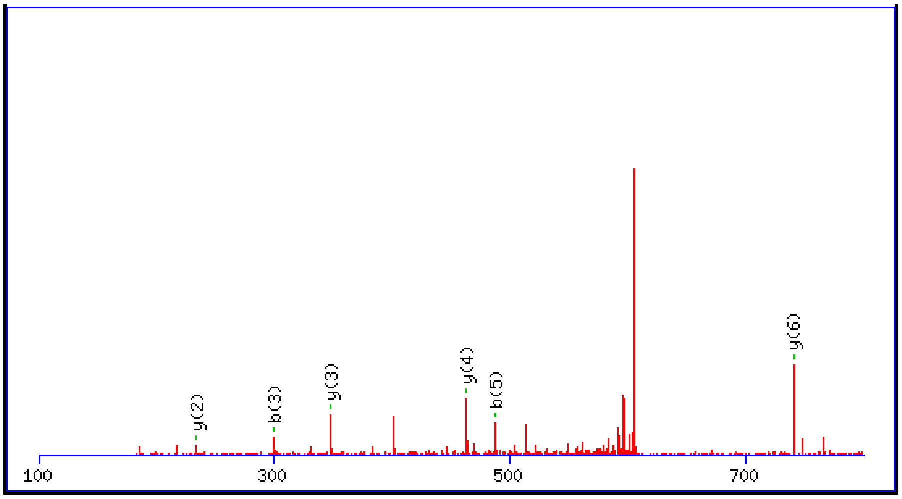

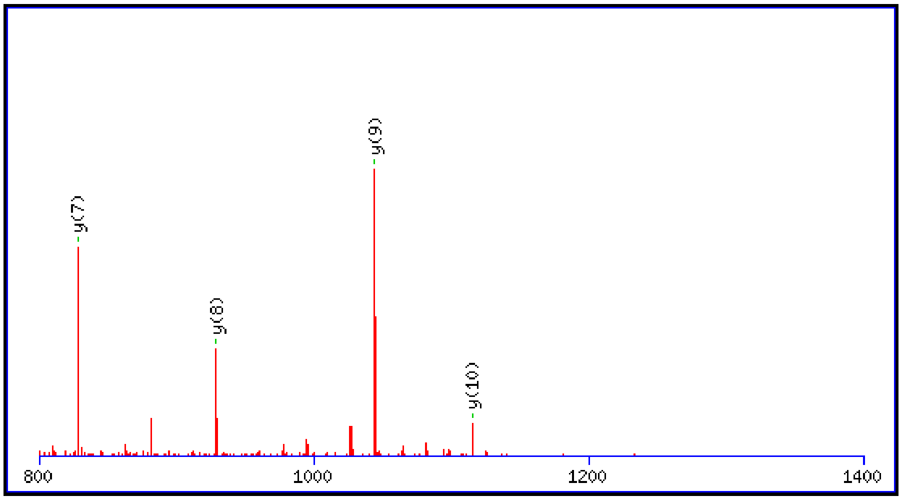

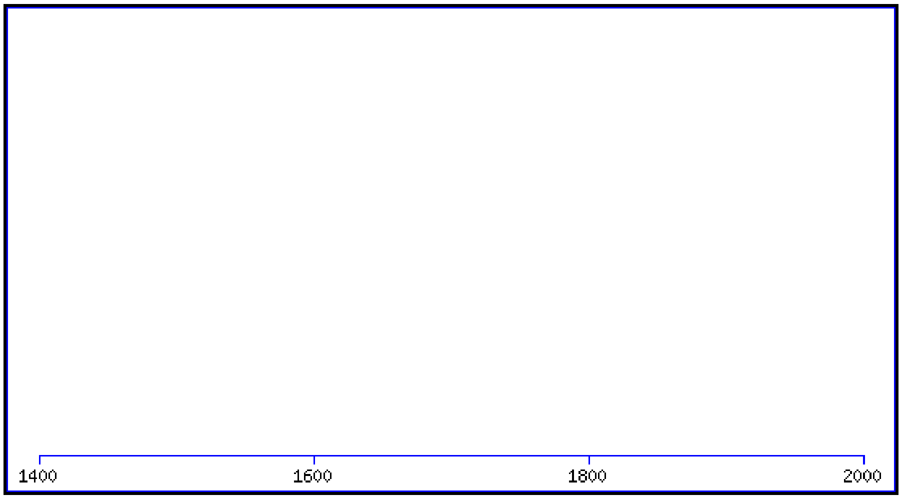

Monoisotopic mass of neutral peptide Mr(calc): 1227.5441
Fixed modifications: Carbamidomethyl (C)
Variable modifications:
N3 : Deamidated_N (N)
N8 : Deamidated_N (N)
Ions Score: 81 **Expect:** 1.1e-006
Matches (Bold Red): 10/108 fragment ions using 14 most intense peaks

#	b	b^{++}	b*	b^{*++}	b^0	b^{0++}	Seq.	y	y^{++}	y*	y^{*++}	y^0	y^{0++}	#
1	114.0913	57.5493					L							11
2	185.1285	93.0679					A	**1115.4674**	558.2373	1098.4408	549.7240	1097.4568	549.2320	10
3	**300.1554**	150.5813	283.1288	142.0681			N	**1044.4302**	522.7188	1027.4037	514.2055	1026.4197	513.7135	9
4	401.2031	201.1052	384.1765	192.5919	383.1925	192.0999	T	**929.4033**	465.2053	912.3768	456.6920	911.3927	456.2000	8

**5**	**488.2351**	244.6212	471.2085	236.1079	470.2245	235.6159	**S**	**828.3556**	414.6814	811.3291	406.1682	810.3451	405.6762	**7**
**6**	619.2756	310.1414	602.2490	301.6282	601.2650	301.1361	**M**	**741.3236**	371.1654	724.2970	362.6522	723.3130	362.1602	**6**
**7**	766.3440	383.6756	749.3174	375.1624	748.3334	374.6704	**F**	610.2831	305.6452	593.2566	297.1319	592.2725	296.6399	**5**
**8**	881.3709	441.1891	864.3444	432.6758	863.3604	432.1838	**N**	**463.2147**	232.1110	446.1881	223.5977	445.2041	223.1057	**4**
**9**	995.4139	498.2106	978.3873	489.6973	977.4033	489.2053	**N**	**348.1878**	174.5975	331.1612	166.0842	330.1772	165.5922	**3**
**10**	1082.4459	541.7266	1065.4193	533.2133	1064.4353	532.7213	**S**	**234.1448**	117.5761	217.1183	109.0628	216.1343	108.5708	**2**
**11**							**K**	147.1128	74.0600	130.0863	65.5468			**1**

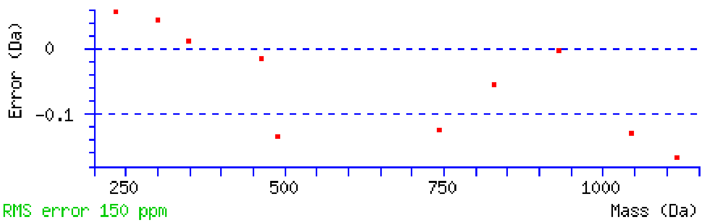

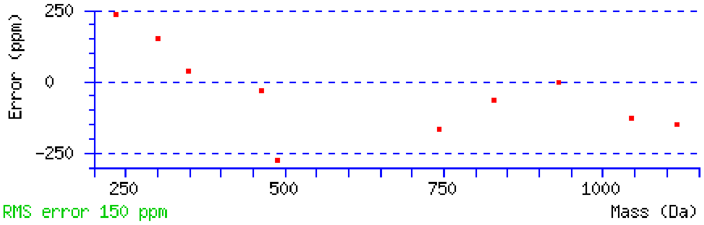

All matches to this query

Score	Mr(calc):	Delta	Sequence
81.0	1227.5441	0.0012	LANTSMFNNSK
62.5	1227.5441	0.0012	LANTSMFNNSK
42.0	1227.5441	0.0012	LANTSMFNNSK
12.2	1227.5384	0.0070	DEKPLTSGSSK
11.8	1227.5496	-0.0042	ENKNLASSKR
9.9	1226.5366	1.0087	MSTSIQPLNR
9.0	1226.5489	0.9965	ENEIMVFGGSK
5.4	1227.5384	0.0070	DEKPLTSGSSK
4.4	1225.5340	2.0114	AETRKPDNSK
2.6	1227.5384	0.0070	DEKPLTSGSSK

Spectrum No: 128; Query: 807; Rank: 1

Peptide View

MS/MS Fragmentation of **AGFAQCICPTLTCPEANSTK**
Found in **IPI00188885**, Tax_Id=10116 Gene_Symbol=Agrn Isoform 1 of Agrin precursor

Match to Query 807: 2225.983928 from(1113.999240,2+)
Title: 091127RatKid_SCX01_02.2989.2989.2.dta
Data file K:\NewmanPaper\Piliang\3SubProteomes\Piliang3SP\mgf5ppm\SCX_3SubProteomes5ppm.mgf

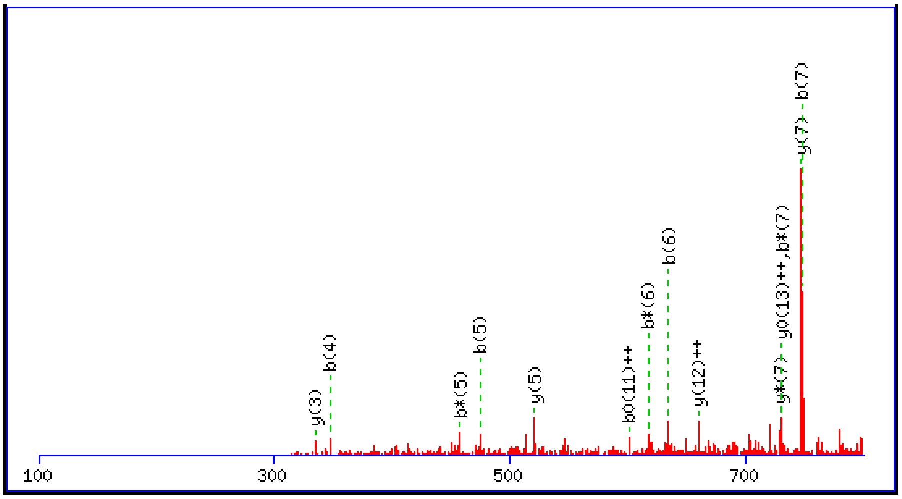

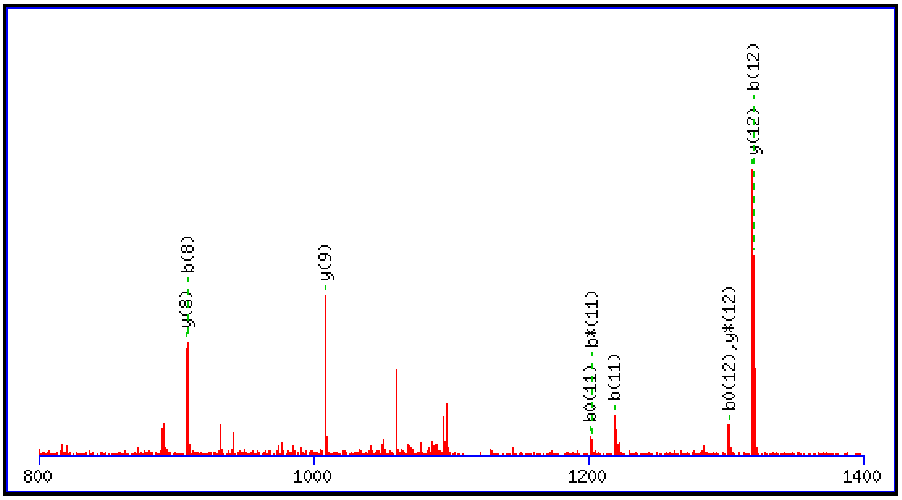

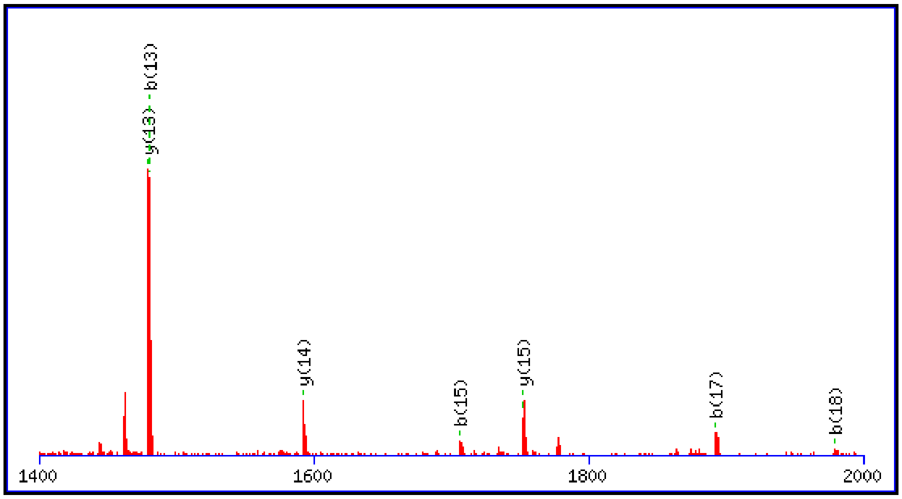

Monoisotopic mass of neutral peptide Mr(calc): 2225.9755
Fixed modifications: Carbamidomethyl (C)
Variable modifications:
N17 : Deamidated_N (N)
Ions Score: 81 **Expect:** 2.3e-006
Matches (Bold Red): 31/200 fragment ions using 42 most intense peaks

#	b	b^{++}	b*	b^{*++}	b^0	b^{0++}	Seq.	y	y^{++}	y*	y^{*++}	y^0	y^{0++}	#
1	72.0444	36.5258					A							20
2	129.0659	65.0366					G	2155.9457	1078.4765	2138.9191	1069.9632	2137.9351	1069.4712	19
3	276.1343	138.5708					F	2098.9242	1049.9657	2081.8977	1041.4525	2080.9137	1040.9605	18
4	**347.1714**	174.0893					A	1951.8558	976.4315	1934.8293	967.9183	1933.8452	967.4263	17
5	**475.2300**	238.1186	**458.2034**	229.6053			Q	1880.8187	940.9130	1863.7921	932.3997	1862.8081	931.9077	16

6	**635.2606**	318.1339	**618.2341**	309.6207			C	**1752.7601**	876.8837	1735.7336	868.3704	1734.7496	867.8784	15
7	**748.3447**	374.6760	**731.3181**	366.1627			I	**1592.7295**	796.8684	1575.7029	788.3551	1574.7189	787.8631	14
8	**908.3753**	454.6913	891.3488	446.1780			C	**1479.6454**	740.3263	1462.6189	731.8131	1461.6348	**731.3211**	13
9	1005.4281	503.2177	988.4015	494.7044			P	**1319.6148**	**660.3110**	**1302.5882**	651.7977	1301.6042	651.3057	12
10	1106.4758	553.7415	1089.4492	545.2282	1088.4652	544.7362	T	1222.5620	611.7846	1205.5354	603.2714	1204.5514	602.7794	11
11	**1219.5598**	610.2836	**1202.5333**	601.7703	**1201.5493**	**601.2783**	L	1121.5143	561.2608	1104.4878	552.7475	1103.5037	552.2555	10
12	**1320.6075**	660.8074	1303.5810	652.2941	**1302.5969**	651.8021	T	**1008.4302**	504.7188	991.4037	496.2055	990.4197	495.7135	9
13	**1480.6382**	740.8227	1463.6116	732.3094	1462.6276	731.8174	C	**907.3826**	454.1949	890.3560	445.6816	889.3720	445.1896	8
14	1577.6909	789.3491	1560.6644	780.8358	1559.6804	780.3438	P	**747.3519**	374.1796	**730.3254**	365.6663	729.3414	365.1743	7
15	**1706.7335**	853.8704	1689.7070	845.3571	1688.7229	844.8651	E	650.2992	325.6532	633.2726	317.1399	632.2886	316.6479	6
16	1777.7706	889.3890	1760.7441	880.8757	1759.7601	880.3837	A	**521.2566**	261.1319	504.2300	252.6186	503.2460	252.1266	5
17	**1892.7976**	946.9024	1875.7710	938.3891	1874.7870	937.8971	N	450.2194	225.6134	433.1929	217.1001	432.2089	216.6081	4
18	**1979.8296**	990.4184	1962.8030	981.9052	1961.8190	981.4132	S	**335.1925**	168.0999	318.1660	159.5866	317.1819	159.0946	3
19	2080.8773	1040.9423	2063.8507	1032.4290	2062.8667	1031.9370	T	248.1605	124.5839	231.1339	116.0706	230.1499	115.5786	2
20							K	147.1128	74.0600	130.0863	65.5468			1

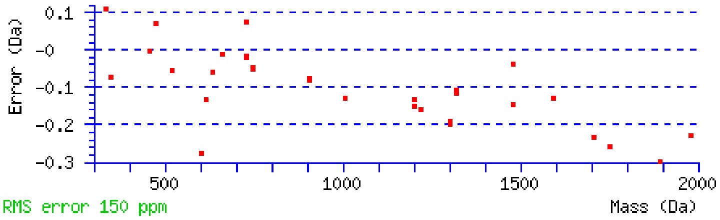

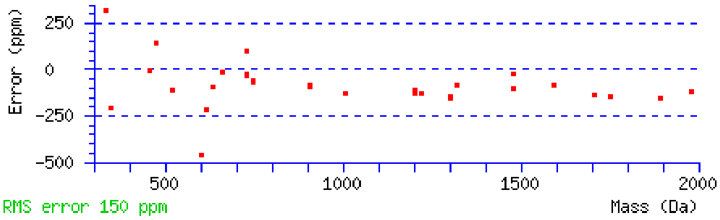

All matches to this query

Score	Mr(calc):	Delta	Sequence
80.9	2225.9755	0.0084	AGFAQCICPTLTCPEANSTK
36.0	2224.9915	0.9924	AGFAQCICPTLTCPEANSTK
7.8	2225.9720	0.0119	NAWEESQITLFLDILMK
5.3	2223.9829	2.0010	EYLKNFPLLTKYCGYR
3.1	2225.9892	-0.0053	LMENRSCDQELSEKGTEGK
1.6	2225.9867	-0.0028	VNQDMCMKVPDDPEHLAAR
0.6	2223.9660	2.0179	FSNWTNGAFLAQGSLLNMR
0.4	2223.9793	2.0046	IDVCLESQDFLMKTNTSK
0.4	2223.9660	2.0179	FSNWTNGAFLAQGSLLNMR

Spectrum No: 129; Query: 1090; Rank: 1

Peptide View

MS/MS Fragmentation of **RNENFSSGPSFETISASGLNAALAHYSPTK**
Found in **IPI00197684**, Tax_Id=10116 Gene_Symbol=Xpnpep2 Membrane-bound aminopeptidase P

Match to Query 1090: 3153.500056 from(789.382290,4+)
Title: 091129RatKid_SCX02_30.4804.4804.4.dta
Data file K:\NewmanPaper\Piliang\3SubProteomes\Piliang3SP\mgf5ppm\SCX_3SubProteomes5ppm.mgf

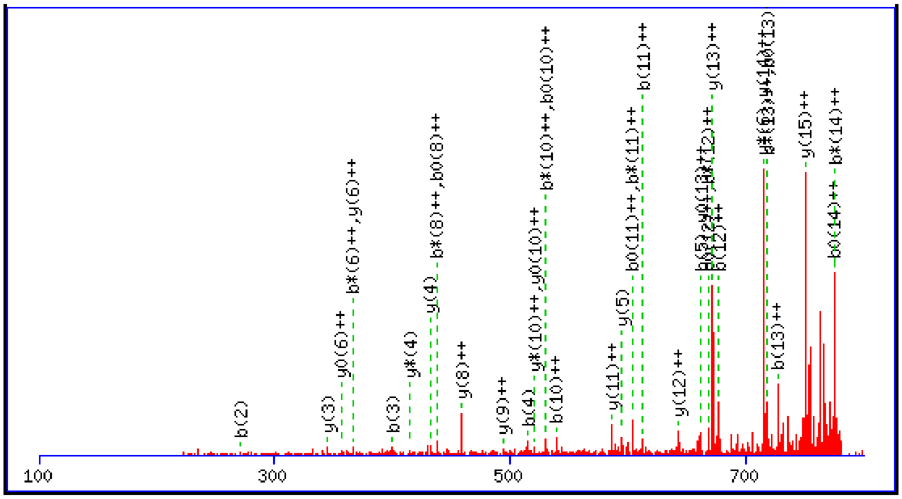

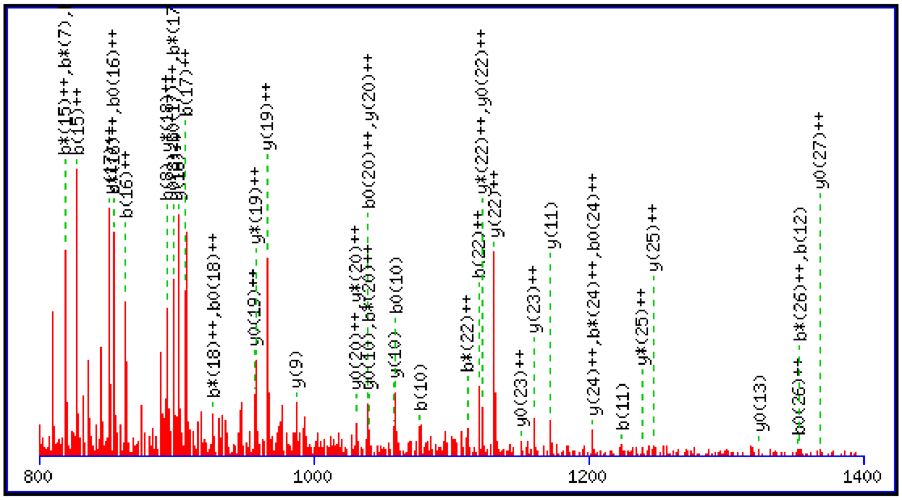

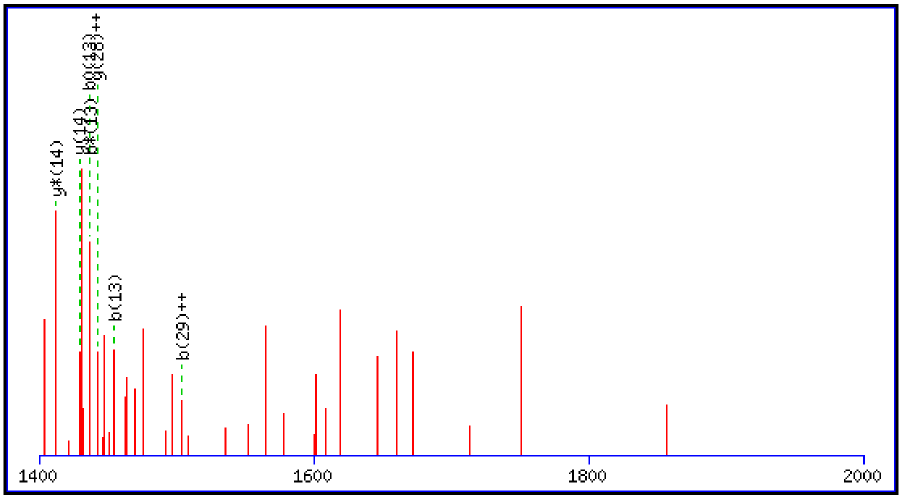

Monoisotopic mass of neutral peptide Mr(calc): 3153.5003
Fixed modifications: Carbamidomethyl (C)
Variable modifications:
N4 : Deamidated_N (N)
Ions Score: 81 **Expect:** 3e-006
Matches (Bold Red): 94/342 fragment ions using 134 most intense peaks

#	b	b++	b*	b*++	b0	b0++	Seq.	y	y++	y*	y*++	y0	y0++	#
1	157.1084	79.0578	140.0818	70.5446			R							30
2	**271.1513**	136.0793	254.1248	127.5660			N	2998.4065	1499.7069	2981.3799	1491.1936	2980.3959	1490.7016	29
3	**400.1939**	200.6006	383.1674	192.0873	382.1833	191.5953	E	2884.3636	**1442.6854**	2867.3370	1434.1721	2866.3530	1433.6801	28
4	**515.2208**	258.1141	498.1943	249.6008	497.2103	249.1088	N	2755.3210	1378.1641	2738.2944	1369.6508	2737.3104	**1369.1588**	27
5	**662.2893**	331.6483	645.2627	323.1350	644.2787	322.6430	F	2640.2940	1320.6506	2623.2675	1312.1374	2622.2835	1311.6454	26

6	749.3213	375.1643	732.2947	366.6510	731.3107	366.1590	S	2493.2256	1247.1164	2476.1991	1238.6032	2475.2150	1238.1112	25
7	836.3533	418.6803	819.3268	410.1670	818.3427	409.6750	S	2406.1936	1203.6004	2389.1670	1195.0872	2388.1830	1194.5951	24
8	893.3748	447.1910	876.3482	438.6778	875.3642	438.1857	G	2319.1616	1160.0844	2302.1350	1151.5711	2301.1510	1151.0791	23
9	990.4275	495.7174	973.4010	487.2041	972.4170	486.7121	P	2262.1401	1131.5737	2245.1135	1123.0604	2244.1295	1122.5684	22
10	1077.4596	539.2334	1060.4330	530.7201	1059.4490	530.2281	S	2165.0873	1083.0473	2148.0608	1074.5340	2147.0768	1074.0420	21
11	1224.5280	612.7676	1207.5014	604.2544	1206.5174	603.7623	F	2078.0553	1039.5313	2061.0287	1031.0180	2060.0447	1030.5260	20
12	1353.5706	677.2889	1336.5440	668.7757	1335.5600	668.2836	E	1930.9869	965.9971	1913.9603	957.4838	1912.9763	956.9918	19
13	1454.6183	727.8128	1437.5917	719.2995	1436.6077	718.8075	T	1801.9443	901.4758	1784.9177	892.9625	1783.9337	892.4705	18
14	1567.7023	784.3548	1550.6758	775.8415	1549.6918	775.3495	I	1700.8966	850.9519	1683.8701	842.4387	1682.8860	841.9467	17
15	1654.7343	827.8708	1637.7078	819.3575	1636.7238	818.8655	S	1587.8125	794.4099	1570.7860	785.8966	1569.8020	785.4046	16
16	1725.7715	863.3894	1708.7449	854.8761	1707.7609	854.3841	A	1500.7805	750.8939	1483.7540	742.3806	1482.7700	741.8886	15
17	1812.8035	906.9054	1795.7769	898.3921	1794.7929	897.9001	S	1429.7434	715.3753	1412.7169	706.8621	1411.7328	706.3701	14
18	1869.8250	935.4161	1852.7984	926.9028	1851.8144	926.4108	G	1342.7114	671.8593	1325.6848	663.3461	1324.7008	662.8540	13
19	1982.9090	991.9581	1965.8825	983.4449	1964.8985	982.9529	L	1285.6899	643.3486	1268.6634	634.8353	1267.6793	634.3433	12
20	2096.9519	1048.9796	2079.9254	1040.4663	2078.9414	1039.9743	N	1172.6058	586.8066	1155.5793	578.2933	1154.5953	577.8013	11
21	2167.9891	1084.4982	2150.9625	1075.9849	2149.9785	1075.4929	A	1058.5629	529.7851	1041.5364	521.2718	1040.5524	520.7798	10
22	2239.0262	1120.0167	2221.9996	1111.5034	2221.0156	1111.0114	A	987.5258	494.2665	970.4993	485.7533	969.5152	485.2613	9
23	2352.1102	1176.5588	2335.0837	1168.0455	2334.0997	1167.5535	L	916.4887	458.7480	899.4621	450.2347	898.4781	449.7427	8
24	2423.1474	1212.0773	2406.1208	1203.5640	2405.1368	1203.0720	A	803.4046	402.2060	786.3781	393.6927	785.3941	393.2007	7
25	2560.2063	1280.6068	2543.1797	1272.0935	2542.1957	1271.6015	H	732.3675	366.6874	715.3410	358.1741	714.3570	357.6821	6
26	2723.2696	1362.1384	2706.2430	1353.6252	2705.2590	1353.1332	Y	595.3086	298.1579	578.2821	289.6447	577.2980	289.1527	5
27	2810.3016	1405.6544	2793.2751	1397.1412	2792.2911	1396.6492	S	432.2453	216.6263	415.2187	208.1130	414.2347	207.6210	4
28	2907.3544	1454.1808	2890.3278	1445.6676	2889.3438	1445.1755	P	345.2132	173.1103	328.1867	164.5970	327.2027	164.1050	3
29	3008.4021	1504.7047	2991.3755	1496.1914	2990.3915	1495.6994	T	248.1605	124.5839	231.1339	116.0706	230.1499	115.5786	2
30							K	147.1128	74.0600	130.0863	65.5468			1

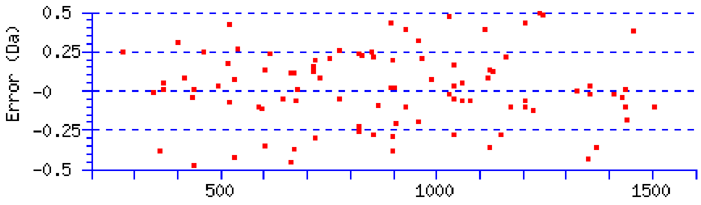

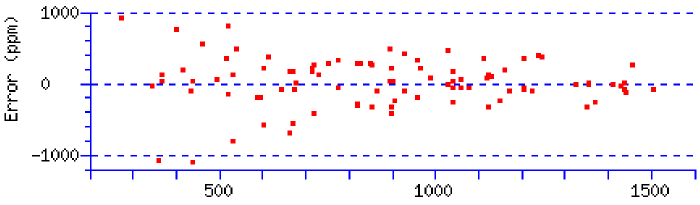

All matches to this query

Score	Mr(calc):	Delta	Sequence
80.7	3153.5003	-0.0002	RNENFSSGPSFETISASGLNAALAHYSPTK
78.9	3153.5003	-0.0002	RNENFSSGPSFETISASGLNAALAHYSPTK
57.7	3152.5163	0.9838	RNENFSSGPSFETISASGLNAALAHYSPTK
44.4	3153.5003	-0.0002	RNENFSSGPSFETISASGLNAALAHYSPTK
7.9	3152.4837	1.0164	AGVSTRVGFMAIYLAVTRWTFAVIQR
7.5	3152.4837	1.0164	AGVSTRVGFMAIYLAVTRWTFAVIQR
5.3	3152.4722	1.0279	AKVMICQLEISPAASLEALTMARSSGVK
5.3	3152.4722	1.0279	AKVMICQLEISPAASLEALTMARSSGVK
5.3	3152.4722	1.0279	AKVMICQLEISPAASLEALTMARSSGVK
3.1	3152.5101	0.9900	LTMFTSRGSGLGGTVGNTLGPPPETSPAELK

Spectrum No: 130; Query: 640; Rank: 1

Peptide View

MS/MS Fragmentation of **NGTSGSDSPGQAVEAEEIVK**
Found in **IPI00364187**, Tax_Id=10116 Gene_Symbol=RGD1309219 Putative uncharacterized protein RGD1309219

Match to Query 640: 1974.905308 from(988.459930,2+)
Title: 091127RatKid_SCX01_13.2177.2177.2.dta
Data file K:\NewmanPaper\Piliang\3SubProteomes\Piliang3SP\mgf5ppm\SCX_3SubProteomes5ppm.mgf

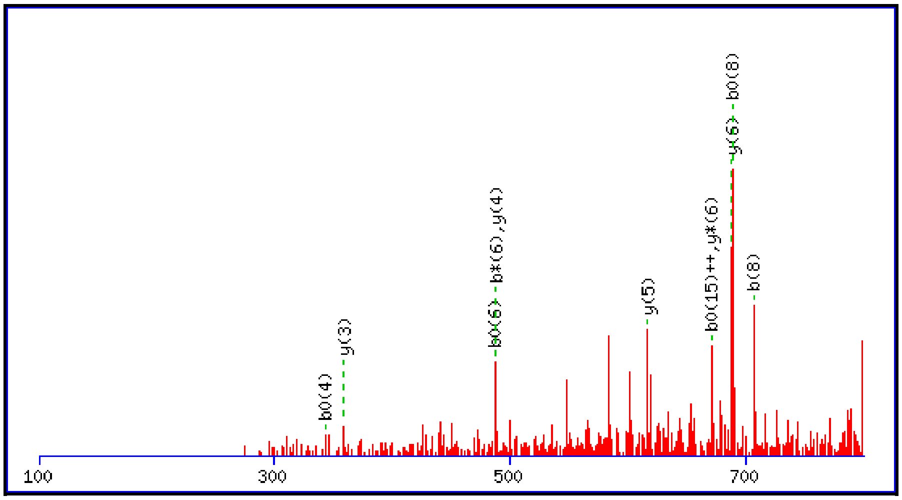

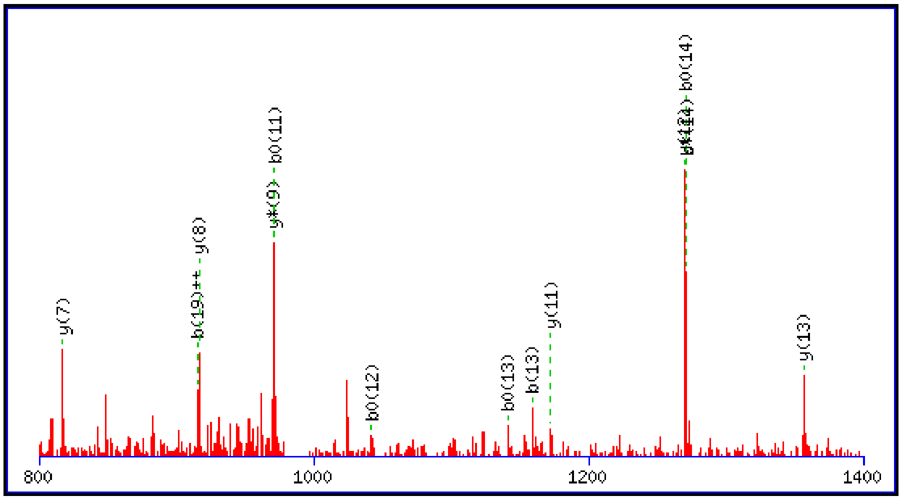

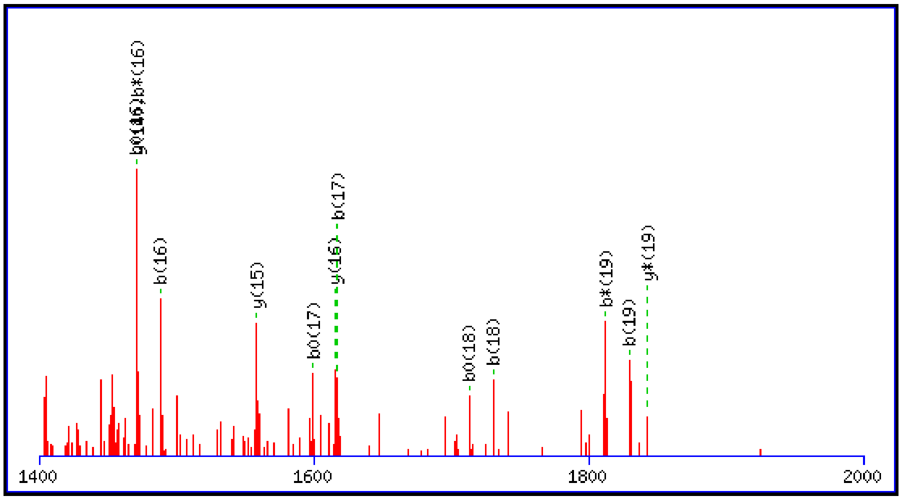

Monoisotopic mass of neutral peptide Mr(calc): 1974.9018
Fixed modifications: Carbamidomethyl (C)
Variable modifications:
N1 : Deamidated_N (N)
Ions Score: 80 **Expect:** 2.2e-006
Matches (Bold Red): 37/218 fragment ions using 57 most intense peaks

#	b	b^{++}	b*	b^{*++}	b^{0}	b^{0++}	Seq.	y	y^{++}	y*	y^{*++}	y^{0}	y^{0++}	#
1	116.0342	58.5207	99.0077	50.0075			N							20
2	173.0557	87.0315	156.0291	78.5182			G	1860.8821	930.9447	**1843.8556**	922.4314	1842.8716	921.9394	19
3	274.1034	137.5553	257.0768	129.0420	256.0928	128.5500	T	1803.8607	902.4340	1786.8341	893.9207	1785.8501	893.4287	18
4	361.1354	181.0713	344.1088	172.5581	**343.1248**	172.0660	S	1702.8130	851.9101	1685.7865	843.3969	1684.8024	842.9049	17
5	418.1568	209.5821	401.1303	201.0688	400.1463	200.5768	G	**1615.7810**	808.3941	1598.7544	799.8808	1597.7704	799.3888	16
6	505.1889	253.0981	**488.1623**	244.5848	**487.1783**	244.0928	S	**1558.7595**	779.8834	1541.7330	771.3701	1540.7489	770.8781	15
7	620.2158	310.6115	603.1893	302.0983	602.2053	301.6063	D	**1471.7275**	736.3674	1454.7009	727.8541	1453.7169	727.3621	14
8	**707.2478**	354.1276	690.2213	345.6143	**689.2373**	345.1223	S	**1356.7005**	678.8539	1339.6740	670.3406	1338.6900	669.8486	13
9	804.3006	402.6539	787.2741	394.1407	786.2900	393.6487	P	**1269.6685**	635.3379	1252.6420	626.8246	1251.6579	626.3326	12
10	861.3221	431.1647	844.2955	422.6514	843.3115	422.1594	G	**1172.6157**	586.8115	1155.5892	578.2982	1154.6052	577.8062	11
11	989.3807	495.1940	972.3541	486.6807	**971.3701**	486.1887	Q	1115.5943	558.3008	1098.5677	549.7875	1097.5837	549.2955	10
12	1060.4178	530.7125	1043.3912	522.1992	**1042.4072**	521.7072	A	987.5357	494.2715	**970.5092**	485.7582	969.5251	485.2662	9
13	**1159.4862**	580.2467	1142.4596	571.7335	**1141.4756**	571.2414	V	**916.4986**	458.7529	899.4720	450.2397	898.4880	449.7477	8
14	1288.5288	644.7680	**1271.5022**	636.2548	**1270.5182**	635.7627	E	**817.4302**	409.2187	800.4036	400.7055	799.4196	400.2134	7
15	1359.5659	680.2866	1342.5393	671.7733	1341.5553	**671.2813**	A	**688.3876**	344.6974	**671.3610**	336.1842	670.3770	335.6921	6
16	**1488.6085**	744.8079	**1471.5819**	736.2946	**1470.5979**	735.8026	E	**617.3505**	309.1789	600.3239	300.6656	599.3399	300.1736	5
17	**1617.6511**	809.3292	1600.6245	800.8159	**1599.6405**	800.3239	E	**488.3079**	244.6576	471.2813	236.1443	470.2973	235.6523	4
18	**1730.7351**	865.8712	1713.7086	857.3579	**1712.7246**	856.8659	I	**359.2653**	180.1363	342.2387	171.6230			3
19	**1829.8036**	**915.4054**	**1812.7770**	906.8921	1811.7930	906.4001	V	246.1812	123.5942	229.1547	115.0810			2
20							K	147.1128	74.0600	130.0863	65.5468			1

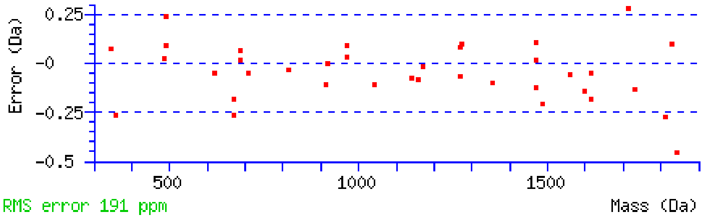

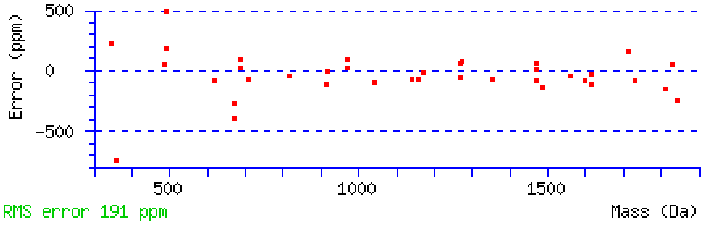

All matches to this query

Score	Mr(calc):	Delta	Sequence
80.2	1974.9018	0.0035	NGTSGSDSPGQAVEAEEIVK
45.4	1973.9178	0.9875	NGTSGSDSPGQAVEAEEIVK
13.9	1974.8978	0.0075	NQMLMTPTLASRSSVMK
13.0	1974.8978	0.0075	NQMLMTPTLASRSSVMK
8.6	1974.9064	-0.0011	TLISELSGKPTSQAEVR
6.3	1974.8978	0.0075	NQMLMTPTLASRSSVMK
6.3	1974.8978	0.0075	NQMLMTPTLASRSSVMK
6.1	1974.8978	0.0075	NQMLMTPTLASRSSVMK
4.9	1974.9138	-0.0085	MLVEAGPVPLGKSLSSSK
4.9	1974.9138	-0.0085	MLVEAGPVPLGKSLSSSK

Spectrum No: 131; Query: 599; Rank: 1

Peptide View

MS/MS Fragmentation of **IYAGMVSLLDEAVGNVTK**
Found in **IPI00198405**, Tax_Id=10116 Gene_Symbol=Arsb Arylsulfatase B

Match to Query 599: 1879.965308 from(940.989930,2+)
Title: 091129RatKid_SCX02_16.5743.5743.2.dta
Data file K:\NewmanPaper\Piliang\3SubProteomes\Piliang3SP\mgf5ppm\SCX_3SubProteomes5ppm.mgf

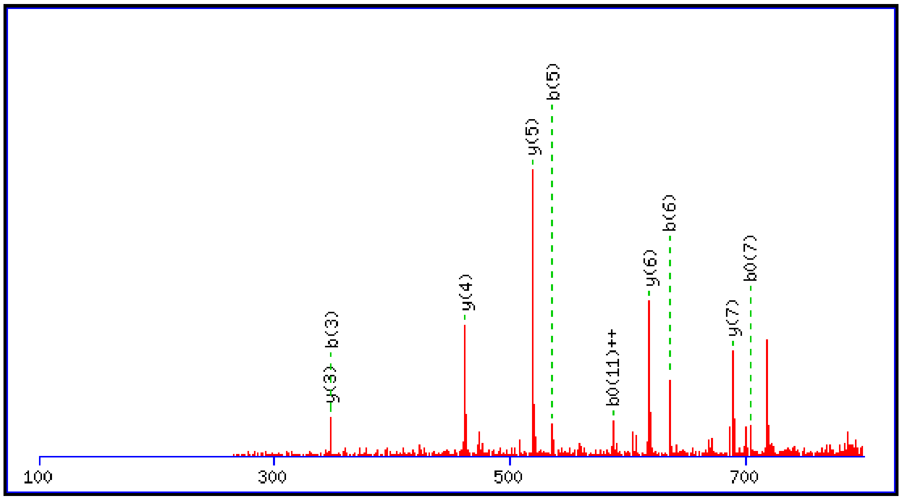

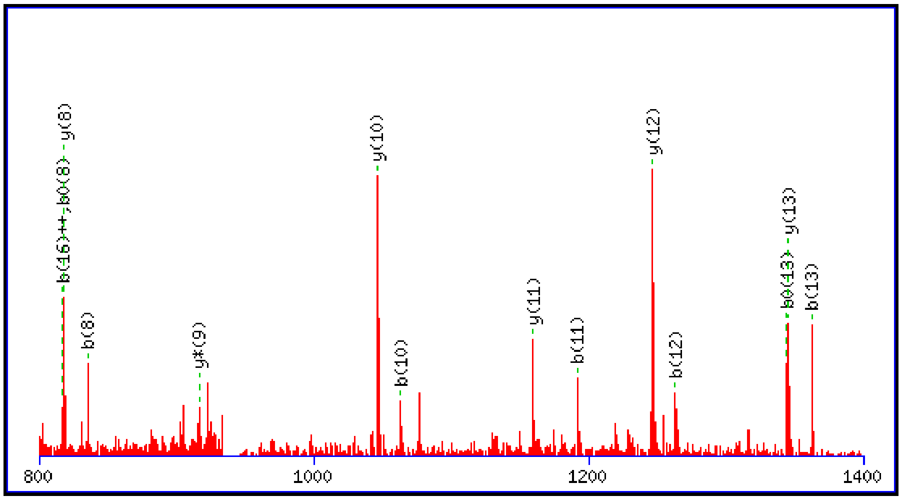

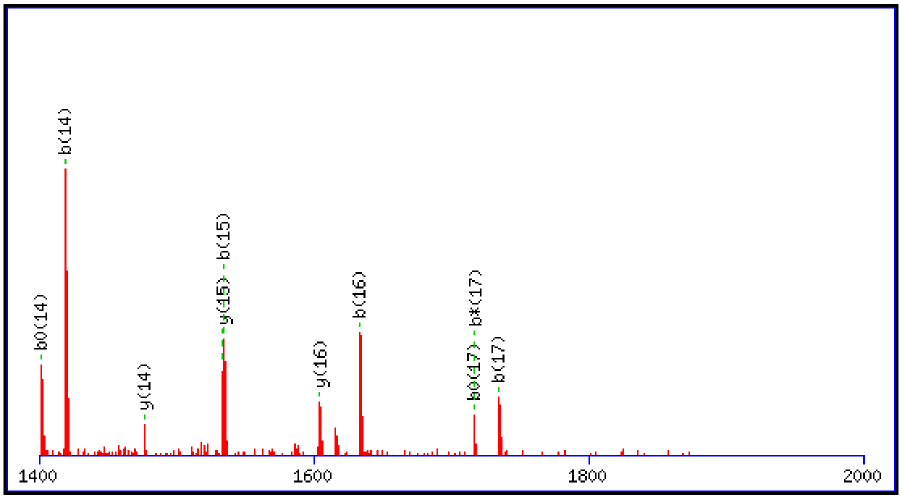

Monoisotopic mass of neutral peptide Mr(calc): 1879.9601
Fixed modifications: Carbamidomethyl (C)
Variable modifications:
N15 : Deamidated_N (N)
Ions Score: 80 **Expect:** 1.4e-006
Matches (Bold Red): 34/162 fragment ions using 68 most intense peaks

#	b	b^{++}	b*	b^{*++}	b^0	b^{0++}	Seq.	y	y^{++}	y*	y^{*++}	y^0	y^{0++}	#
1	114.0913	57.5493					I							18
2	277.1547	139.0810					Y	1767.8833	884.4453	1750.8568	875.9320	1749.8728	875.4400	17

3	**348.1918**	174.5995					A	**1604.8200**	802.9136	1587.7934	794.4004	1586.8094	793.9084	16
4	405.2132	203.1103					G	**1533.7829**	767.3951	1516.7563	758.8818	1515.7723	758.3898	15
5	**536.2537**	268.6305					M	**1476.7614**	738.8843	1459.7349	730.3711	1458.7509	729.8791	14
6	**635.3221**	318.1647					V	**1345.7209**	673.3641	1328.6944	664.8508	1327.7104	664.3588	13
7	722.3542	361.6807			**704.3436**	352.6754	S	**1246.6525**	623.8299	1229.6260	615.3166	1228.6420	614.8246	12
8	**835.4382**	418.2228			**817.4277**	409.2175	L	**1159.6205**	580.3139	1142.5939	571.8006	1141.6099	571.3086	11
9	948.5223	474.7648			930.5117	465.7595	L	**1046.5364**	523.7719	1029.5099	515.2586	1028.5259	514.7666	10
10	**1063.5492**	532.2783			1045.5387	523.2730	D	933.4524	467.2298	**916.4258**	458.7165	915.4418	458.2245	9
11	**1192.5918**	596.7996			1174.5813	**587.7943**	E	**818.4254**	409.7163	801.3989	401.2031	800.4149	400.7111	8
12	**1263.6290**	632.3181			1245.6184	623.3128	A	**689.3828**	345.1951	672.3563	336.6818	671.3723	336.1898	7
13	**1362.6974**	681.8523			**1344.6868**	672.8470	V	**618.3457**	309.6765	601.3192	301.1632	600.3351	300.6712	6
14	**1419.7188**	710.3631			**1401.7083**	701.3578	G	**519.2773**	260.1423	502.2507	251.6290	501.2667	251.1370	5
15	**1534.7458**	767.8765	1517.7192	759.3632	1516.7352	758.8712	N	**462.2558**	231.6316	445.2293	223.1183	444.2453	222.6263	4
16	**1633.8142**	**817.4107**	1616.7876	808.8975	1615.8036	808.4054	V	**347.2289**	174.1181	330.2023	165.6048	329.2183	165.1128	3
17	**1734.8619**	867.9346	**1717.8353**	859.4213	**1716.8513**	858.9293	T	248.1605	124.5839	231.1339	116.0706	230.1499	115.5786	2
18							K	147.1128	74.0600	130.0863	65.5468			1

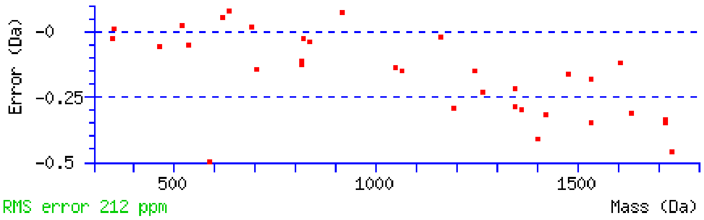

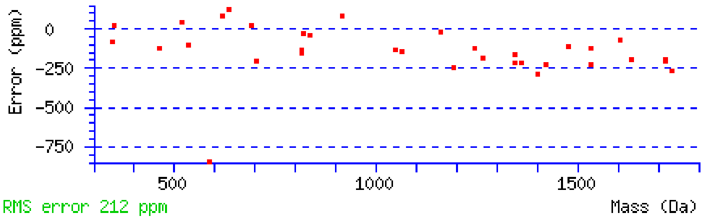

All matches to this query

Score	Mr(calc):	Delta	Sequence
80.2	1879.9601	0.0052	IYAGMVSLLDEAVGNVTK
26.0	1878.9761	0.9892	IYAGMVSLLDEAVGNVTK
7.3	1879.9502	0.0151	AWEDMLIQFLRNSLK
6.9	1879.9536	0.0117	SWVQCGISMKILNSLK
0.9	1879.9639	0.0014	FQSRTSEAAKLEAEVSK
0.4	1879.9536	0.0117	VMGPLIFNASIMTDLAR

Spectrum No: 132; Query: 402; Rank: 1

Peptide View

MS/MS Fragmentation of **VDLEDFENNTAYAK**
Found in **IPI00369539**, Tax_Id=10116 Gene_Symbol=Mfap4 Microfibrillar-associated protein 4

Match to Query 402: 1628.725908 from(815.370230,2+)
Title: 091127RatKid_SCX01_16.3037.3037.2.dta
Data file K:\NewmanPaper\Piliang\3SubProteomes\Piliang3SP\mgf5ppm\SCX_3SubProteomes5ppm.mgf

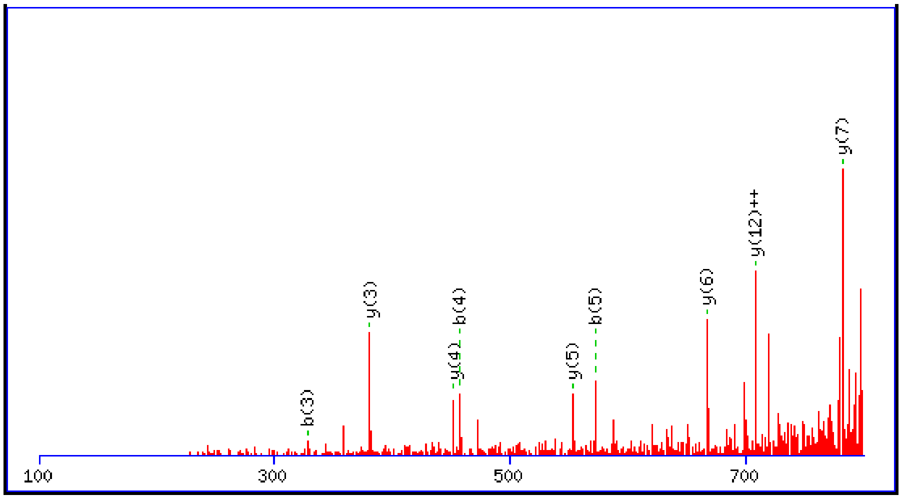

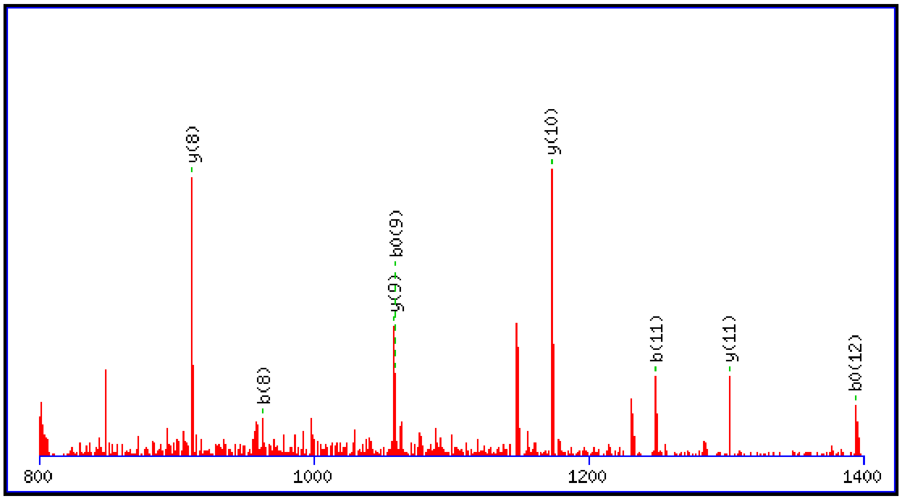

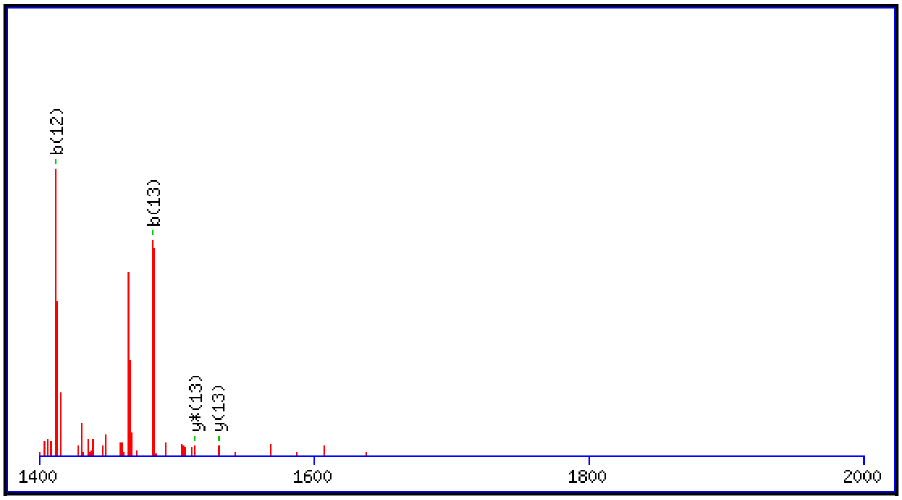

Monoisotopic mass of neutral peptide Mr(calc): 1628.7206
Fixed modifications: Carbamidomethyl (C)
Variable modifications:
N8 : Deamidated_N (N)
Ions Score: 80 **Expect:** 2.2e-006
Matches (Bold Red): 21/132 fragment ions using 33 most intense peaks

#	b	b^{++}	b*	b^{*++}	b^0	b^{0++}	Seq.	y	y^{++}	y*	y^{*++}	y^0	y^{0++}	#
1	100.0757	50.5415					V							14
2	215.1026	108.0550			197.0921	99.0497	D	**1530.6595**	765.8334	**1513.6329**	757.3201	1512.6489	756.8281	13
3	**328.1867**	164.5970			310.1761	155.5917	L	1415.6325	**708.3199**	1398.6060	699.8066	1397.6219	699.3146	12
4	**457.2293**	229.1183			439.2187	220.1130	E	**1302.5484**	651.7779	1285.5219	643.2646	1284.5379	642.7726	11
5	**572.2562**	286.6318			554.2457	277.6265	D	**1173.5059**	587.2566	1156.4793	578.7433	1155.4953	578.2513	10

**6**	719.3246	360.1660			701.3141	351.1607	**F**	**1058.4789**	529.7431	1041.4524	521.2298	1040.4683	520.7378	**9**
**7**	848.3672	424.6873			830.3567	415.6820	**E**	**911.4105**	456.2089	894.3839	447.6956	893.3999	447.2036	**8**
**8**	**963.3942**	482.2007	946.3676	473.6875	945.3836	473.1954	**N**	**782.3679**	391.6876	765.3414	383.1743	764.3573	382.6823	**7**
**9**	1077.4371	539.2222	1060.4106	530.7089	**1059.4265**	530.2169	**N**	**667.3410**	334.1741	650.3144	325.6608	649.3304	325.1688	**6**
**10**	1178.4848	589.7460	1161.4582	581.2328	1160.4742	580.7407	**T**	**553.2980**	277.1527	536.2715	268.6394	535.2875	268.1474	**5**
**11**	**1249.5219**	625.2646	1232.4953	616.7513	1231.5113	616.2593	**A**	**452.2504**	226.6288	435.2238	218.1155			**4**
**12**	**1412.5852**	706.7963	1395.5587	698.2830	**1394.5747**	697.7910	**Y**	**381.2132**	191.1103	364.1867	182.5970			**3**
**13**	**1483.6223**	742.3148	1466.5958	733.8015	1465.6118	733.3095	**A**	218.1499	109.5786	201.1234	101.0653			**2**
**14**							**K**	147.1128	74.0600	130.0863	65.5468			**1**

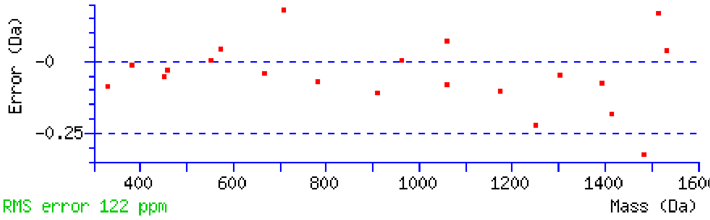

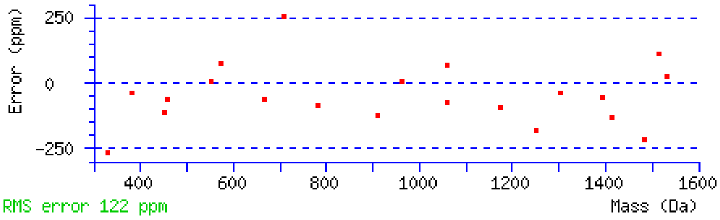

All matches to this query

Score	Mr(calc):	Delta	Sequence
79.8	1628.7206	0.0053	VDLEDFENNTAYAK
62.9	1628.7206	0.0053	VDLEDFENNTAYAK
21.3	1627.7366	0.9894	VDLEDFENNTAYAK
6.4	1628.7294	-0.0035	ADVNGDLNTVSTKSK
5.1	1628.7294	-0.0035	ADVNGDLNTVSTKSK
5.1	1628.7294	-0.0035	ADVNGDLNTVSTKSK
5.1	1628.7294	-0.0035	ADVNGDLNTVSTKSK
0.9	1627.7131	1.0129	KVVEDEVYSESHK
0.9	1627.7131	1.0129	KVVEDEVYSESHK
0.7	1628.7157	0.0102	THNTLYTPEMLTK

Spectrum No: 133; Query: 723; Rank: 1

Peptide View

MS/MS Fragmentation of **LAGGTNFNNSIQADPQTVTR**
Found in **IPI00192495**, Tax_Id=10116 Gene_Symbol=Ncstn Isoform 1 of Nicastrin precursor

Match to Query 723: 2104.024048 from(1053.019300,2+)
Title: 091127RatKid_SCX01_12.1959.1959.2.dta
Data file K:\NewmanPaper\Piliang\3SubProteomes\Piliang3SP\mgf5ppm\SCX_3SubProteomes5ppm.mgf

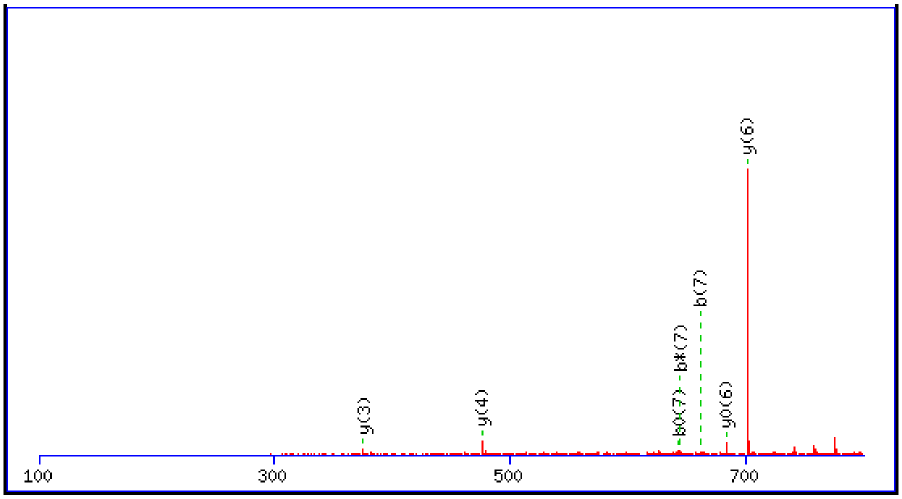

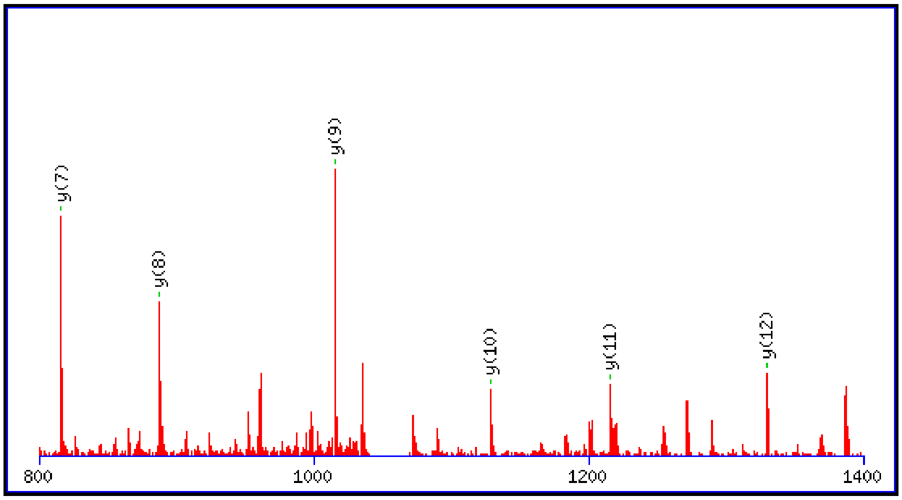

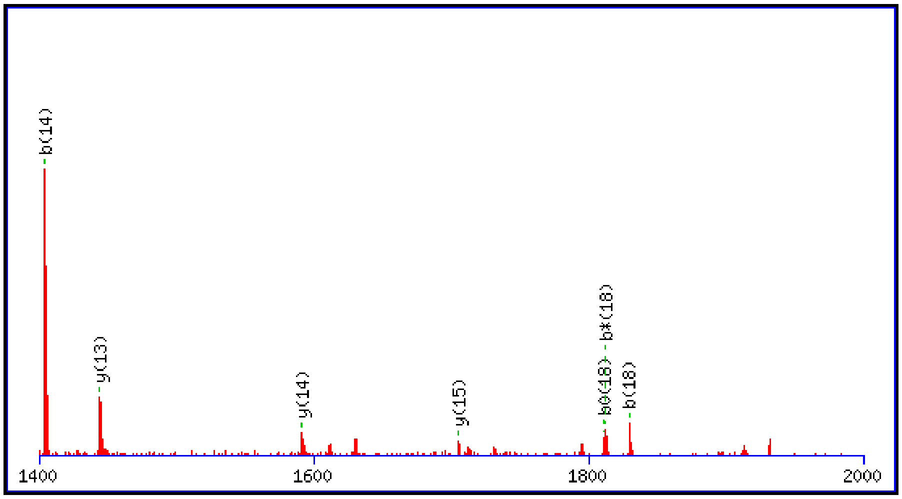

Monoisotopic mass of neutral peptide Mr(calc): 2104.0185
Fixed modifications: Carbamidomethyl (C)
Variable modifications:
N8 : Deamidated_N (N)
Ions Score: 80 **Expect:** 2.5e-006
Matches (Bold Red): 20/208 fragment ions using 37 most intense peaks

#	b	b^{++}	b*	b^{*++}	b^0	b^{0++}	Seq.	y	y^{++}	y*	y^{*++}	y^0	y^{0++}	#
1	114.0913	57.5493					L							20
2	185.1285	93.0679					A	1991.9417	996.4745	1974.9152	987.9612	1973.9312	987.4692	19
3	242.1499	121.5786					G	1920.9046	960.9559	1903.8781	952.4427	1902.8940	951.9507	18
4	299.1714	150.0893					G	1863.8831	932.4452	1846.8566	923.9319	1845.8726	923.4399	17
5	400.2191	200.6132			382.2085	191.6079	T	1806.8617	903.9345	1789.8351	895.4212	1788.8511	894.9292	16

6	514.2620	257.6346	497.2354	249.1214	496.2514	248.6293	N	**1705.8140**	853.4106	1688.7874	844.8974	1687.8034	844.4054	15
7	**661.3304**	331.1688	**644.3039**	322.6556	**643.3198**	322.1636	F	**1591.7711**	796.3892	1574.7445	787.8759	1573.7605	787.3839	14
8	776.3573	388.6823	759.3308	380.1690	758.3468	379.6770	N	**1444.7027**	722.8550	1427.6761	714.3417	1426.6921	713.8497	13
9	890.4003	445.7038	873.3737	437.1905	872.3897	436.6985	N	**1329.6757**	665.3415	1312.6492	656.8282	1311.6652	656.3362	12
10	977.4323	489.2198	960.4057	480.7065	959.4217	480.2145	S	**1215.6328**	608.3200	1198.6062	599.8068	1197.6222	599.3148	11
11	1090.5164	545.7618	1073.4898	537.2485	1072.5058	536.7565	I	**1128.6008**	564.8040	1111.5742	556.2907	1110.5902	555.7987	10
12	1218.5749	609.7911	1201.5484	601.2778	1200.5644	600.7858	Q	**1015.5167**	508.2620	998.4902	499.7487	997.5061	499.2567	9
13	1289.6120	645.3097	1272.5855	636.7964	1271.6015	636.3044	A	**887.4581**	444.2327	870.4316	435.7194	869.4476	435.2274	8
14	**1404.6390**	702.8231	1387.6124	694.3099	1386.6284	693.8179	D	**816.4210**	408.7141	799.3945	400.2009	798.4104	399.7089	7
15	1501.6918	751.3495	1484.6652	742.8362	1483.6812	742.3442	P	**701.3941**	351.2007	684.3675	342.6874	**683.3835**	342.1954	6
16	1629.7503	815.3788	1612.7238	806.8655	1611.7398	806.3735	Q	604.3413	302.6743	587.3148	294.1610	586.3307	293.6690	5
17	1730.7980	865.9026	1713.7715	857.3894	1712.7874	856.8974	T	**476.2827**	238.6450	459.2562	230.1317	458.2722	229.6397	4
18	**1829.8664**	915.4369	**1812.8399**	906.9236	**1811.8559**	906.4316	V	**375.2350**	188.1212	358.2085	179.6079	357.2245	179.1159	3
19	1930.9141	965.9607	1913.8876	957.4474	1912.9035	956.9554	T	276.1666	138.5870	259.1401	130.0737	258.1561	129.5817	2
20							R	175.1190	88.0631	158.0924	79.5498			1

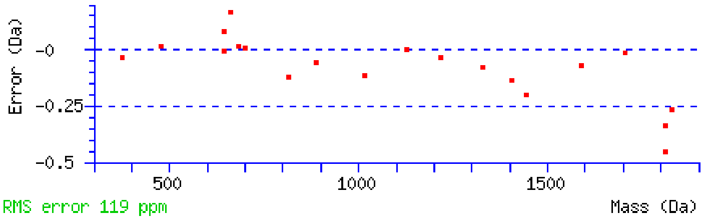

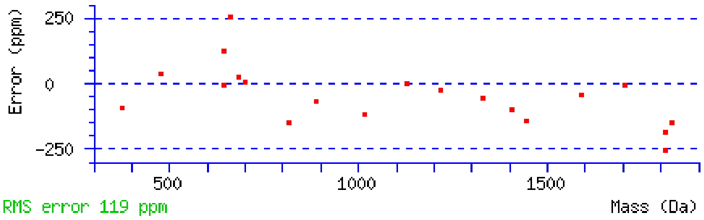

All matches to this query

Score	Mr(calc):	Delta	Sequence
79.8	2104.0185	0.0055	LAGGTNFNNSIQADPQTVTR
64.9	2104.0185	0.0055	LAGGTNFNNSIQADPQTVTR
64.8	2104.0185	0.0055	LAGGTNFNNSIQADPQTVTR
44.7	2103.0345	0.9895	LAGGTNFNNSIQADPQTVTR
5.9	2104.0354	-0.0113	SLFQDKNRTLEAFSLVR
4.7	2104.0201	0.0039	DASSSSKLFSAKLEQLASR
3.0	2102.0020	2.0220	AAMTTGAWIFTGGVNTGVIR
3.0	2102.0020	2.0220	AAMTTGAWIFTGGVNTGVIR
0.8	2103.0080	1.0161	KKSSDTAGSGAPNSDSQLPEK
0.5	2103.0037	1.0203	AKAYFPESQNDIGKQSIK

Spectrum No: 134; Query: 546; Rank: 1

Peptide View

MS/MS Fragmentation of **IVSNASCTTNCLAPLAK**
Found in **IPI00361708**, Tax_Id=10116 Gene_Symbol=- 36 kDa protein

Match to Query 546: 1819.884268 from(910.949410,2+)
Title: 091127RatKid_SCX01_18.2031.2031.2.dta
Data file K:\NewmanPaper\Piliang\3SubProteomes\Piliang3SP\mgf5ppm\SCX_3SubProteomes5ppm.mgf

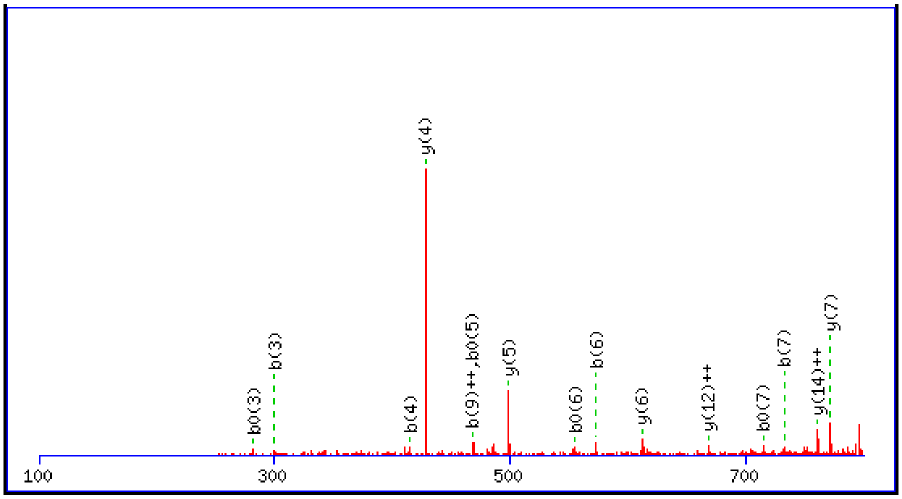

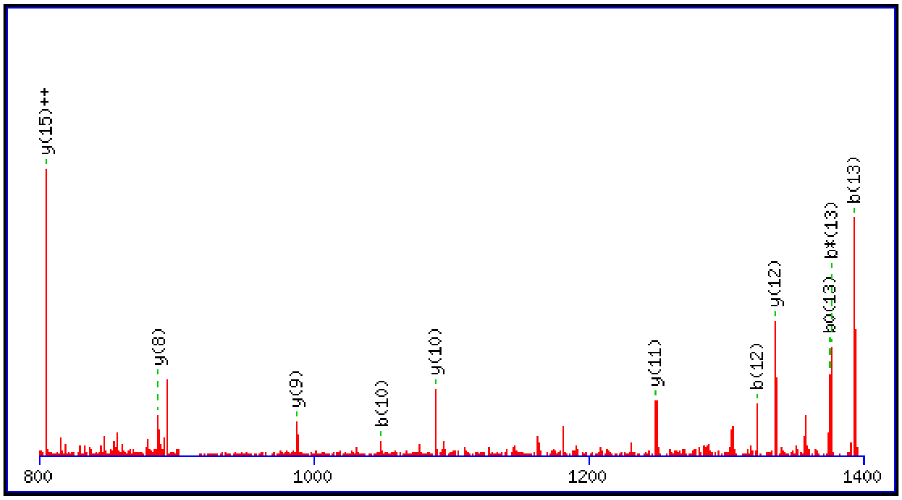

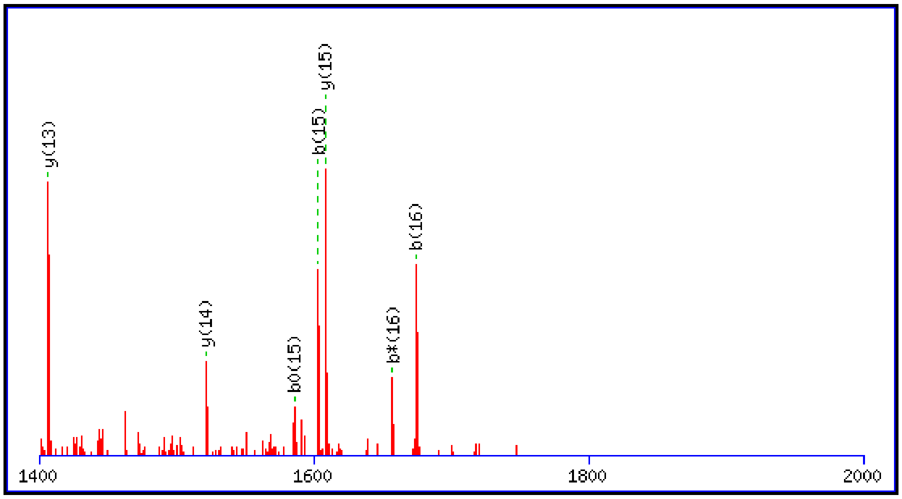

Monoisotopic mass of neutral peptide Mr(calc): 1819.8808
Fixed modifications: Carbamidomethyl (C)
Variable modifications:
N4 : Deamidated_N (N)
Ions Score: 80 **Expect:** 2.2e-006
Matches (Bold Red): 33/166 fragment ions using 56 most intense peaks

#	b	b^{++}	b*	b^{*++}	b^{0}	b^{0++}	Seq.	y	y^{++}	y*	y^{*++}	y^{0}	y^{0++}	#
1	114.0913	57.5493					I							17
2	213.1598	107.0835					V	1707.8040	854.4057	1690.7775	845.8924	1689.7935	845.4004	16
3	**300.1918**	150.5995			**282.1812**	141.5942	S	**1608.7356**	**804.8714**	1591.7091	796.3582	1590.7250	795.8662	15
4	**415.2187**	208.1130	398.1922	199.5997	397.2082	199.1077	N	**1521.7036**	**761.3554**	1504.6770	752.8422	1503.6930	752.3501	14
5	486.2558	243.6316	469.2293	235.1183	**468.2453**	234.6263	A	**1406.6766**	703.8420	1389.6501	695.3287	1388.6661	694.8367	13

6	573.2879	287.1476	556.2613	278.6343	555.2773	278.1423	S	1335.6395	668.3234	1318.6130	659.8101	1317.6290	659.3181	12
7	733.3185	367.1629	716.2920	358.6496	715.3079	358.1576	C	1248.6075	624.8074	1231.5810	616.2941	1230.5969	615.8021	11
8	834.3662	417.6867	817.3396	409.1735	816.3556	408.6814	T	1088.5769	544.7921	1071.5503	536.2788	1070.5663	535.7868	10
9	935.4139	468.2106	918.3873	459.6973	917.4033	459.2053	T	987.5292	494.2682	970.5026	485.7550	969.5186	485.2629	9
10	1049.4568	525.2320	1032.4302	516.7188	1031.4462	516.2268	N	886.4815	443.7444	869.4550	435.2311			8
11	1209.4874	605.2474	1192.4609	596.7341	1191.4769	596.2421	C	772.4386	386.7229	755.4120	378.2096			7
12	1322.5715	661.7894	1305.5450	653.2761	1304.5609	652.7841	L	612.4079	306.7076	595.3814	298.1943			6
13	1393.6086	697.3079	1376.5821	688.7947	1375.5981	688.3027	A	499.3239	250.1656	482.2973	241.6523			5
14	1490.6614	745.8343	1473.6348	737.3211	1472.6508	736.8290	P	428.2867	214.6470	411.2602	206.1337			4
15	1603.7454	802.3764	1586.7189	793.8631	1585.7349	793.3711	L	331.2340	166.1206	314.2074	157.6074			3
16	1674.7826	837.8949	1657.7560	829.3816	1656.7720	828.8896	A	218.1499	109.5786	201.1234	101.0653			2
17							K	147.1128	74.0600	130.0863	65.5468			1

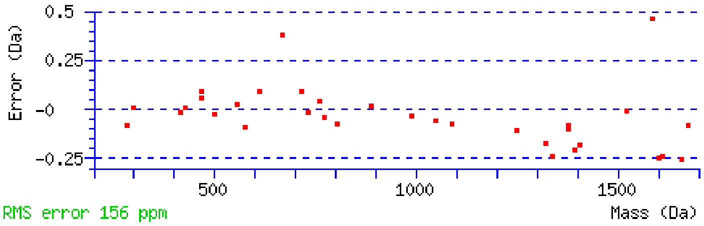

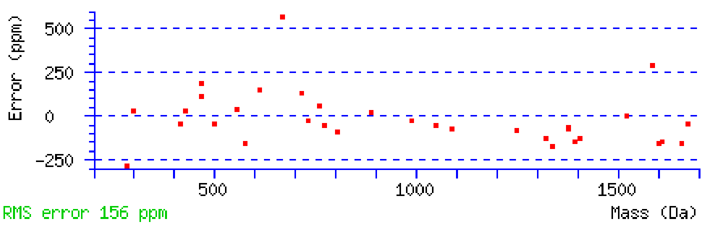

All matches to this query

Score	Mr(calc):	Delta	Sequence
79.6	1819.8808	0.0035	IVSNASCTTNCLAPLAK
68.1	1819.8808	0.0035	IVSNASCTTNCLAPLAK
44.4	1818.8968	0.9875	IVSNASCTTNCLAPLAK
6.0	1819.9015	-0.0173	RNVXISLHLELANSK
5.0	1818.8890	0.9953	GALDKSSPPPGAPHPRR
3.7	1819.8774	0.0069	SDPQAQLEAMEVYLAR
2.2	1818.8872	0.9971	LSGKIKMTLEMLSEK
2.2	1818.8733	1.0110	LMSNALSTVTRGSMKK
2.1	1819.8673	0.0169	REAAANTNRPSPGGHER
1.7	1819.8999	-0.0156	QRENYLGNSLMAPVGR

Spectrum No: 135; Query: 1165; Rank: 1

Peptide View

MS/MS Fragmentation of **EGNCSVQSGLTWQDCDFKDAEEAATGECTTTLGK**
Found in **IPI00327182**, Tax_Id=10116 Gene_Symbol=Kng1 T-kininogen 1 precursor

Match to Query 1165: 3766.583352 from(1256.535060,3+)
Title: 091129RatKid_SCX02_12.3145.3145.3.dta
Data file K:\NewmanPaper\Piliang\3SubProteomes\Piliang3SP\mgf5ppm\SCX_3SubProteomes5ppm.mgf

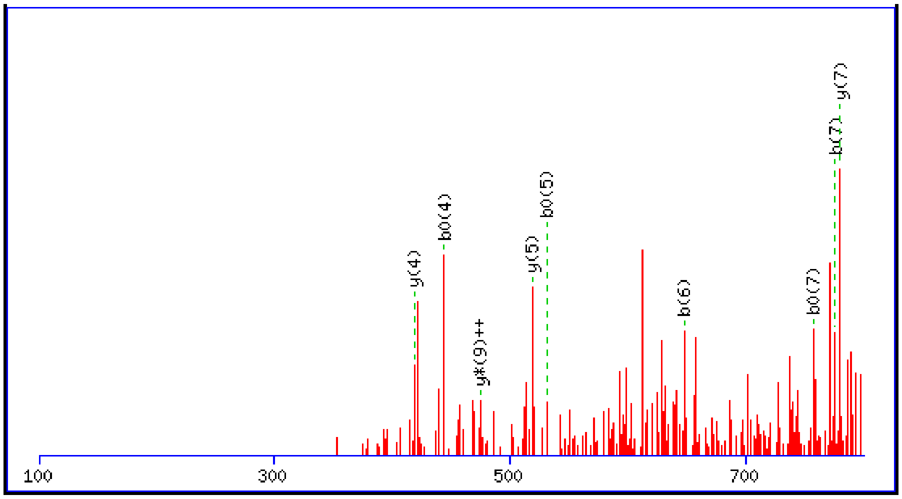

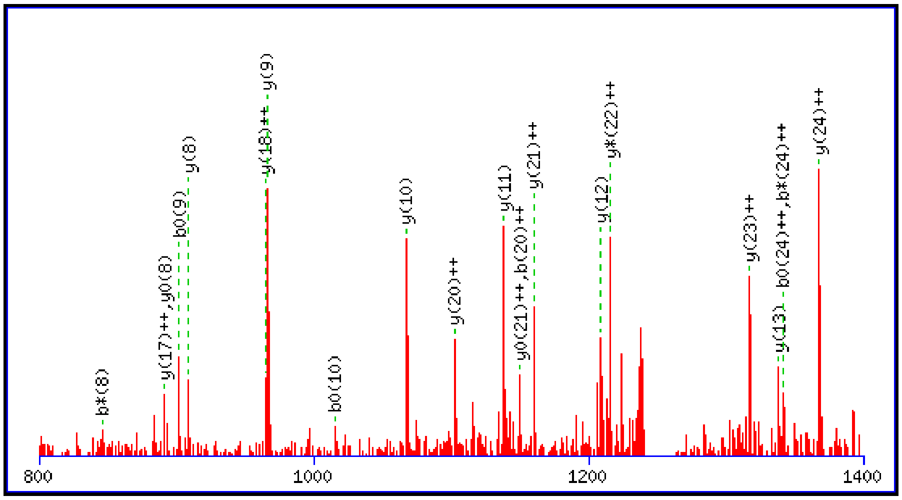

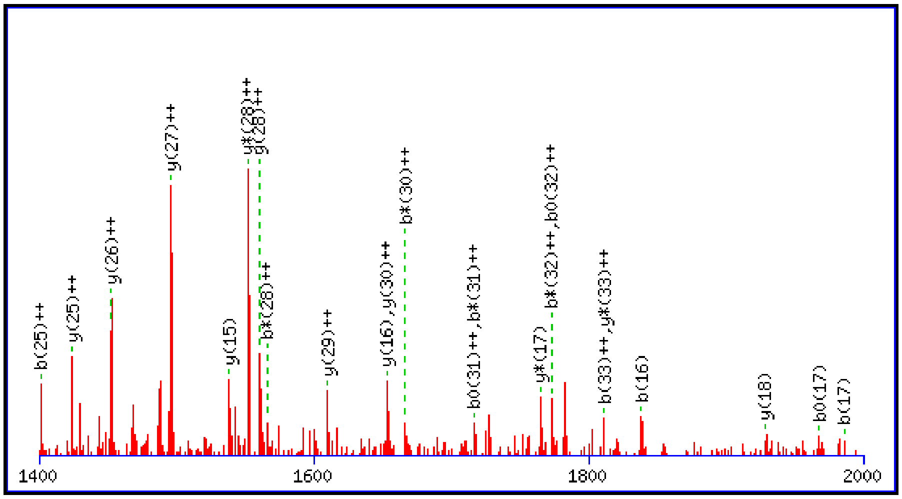

Monoisotopic mass of neutral peptide Mr(calc): 3765.5717
Fixed modifications: Carbamidomethyl (C)
Variable modifications:
N3 : Deamidated_N (N)
Ions Score: 80 **Expect:** 5.8e-006
Matches (Bold Red): 53/386 fragment ions using 86 most intense peaks

#	b	b^{++}	b*	b^{*++}	b^0	b^{0++}	Seq.	y	y^{++}	y*	y^{*++}	y^0	y^{0++}	#
1	130.0499	65.5286			112.0393	56.5233	E							34
2	187.0713	94.0393			169.0608	85.0340	G	3637.5363	1819.2718	3620.5098	**1810.7585**	3619.5258	1810.2665	33
3	302.0983	151.5528	285.0717	143.0395	284.0877	142.5475	N	3580.5149	1790.7611	3563.4883	1782.2478	3562.5043	1781.7558	32
4	462.1289	231.5681	445.1024	223.0548	**444.1184**	222.5628	C	3465.4879	1733.2476	3448.4614	1724.7343	3447.4774	1724.2423	31
5	549.1609	275.0841	532.1344	266.5708	**531.1504**	266.0788	S	3305.4573	**1653.2323**	3288.4307	1644.7190	3287.4467	1644.2270	30

6	**648.2294**	324.6183	631.2028	316.1050	630.2188	315.6130	V	3218.4253	**1609.7163**	3201.3987	1601.2030	3200.4147	1600.7110	29
7	**776.2879**	388.6476	759.2614	380.1343	**758.2774**	379.6423	Q	3119.3568	**1560.1821**	3102.3303	**1551.6688**	3101.3463	1551.1768	28
8	863.3200	432.1636	**846.2934**	423.6503	845.3094	423.1583	S	2991.2983	**1496.1528**	2974.2717	1487.6395	2973.2877	1487.1475	27
9	920.3414	460.6744	903.3149	452.1611	**902.3309**	451.6691	G	2904.2662	**1452.6368**	2887.2397	1444.1235	2886.2557	1443.6315	26
10	1033.4255	517.2164	1016.3989	508.7031	**1015.4149**	508.2111	L	2847.2448	**1424.1260**	2830.2182	1415.6127	2829.2342	1415.1207	25
11	1134.4732	567.7402	1117.4466	559.2270	1116.4626	558.7349	T	2734.1607	**1367.5840**	2717.1342	1359.0707	2716.1501	1358.5787	24
12	1320.5525	660.7799	1303.5259	652.2666	1302.5419	651.7746	W	2633.1130	**1317.0602**	2616.0865	1308.5469	2615.1025	1308.0549	23
13	1448.6111	724.8092	1431.5845	716.2959	1430.6005	715.8039	Q	2447.0337	1224.0205	2430.0072	**1215.5072**	2429.0231	1215.0152	22
14	1563.6380	782.3226	1546.6115	773.8094	1545.6274	773.3174	D	2318.9751	**1159.9912**	2301.9486	1151.4779	2300.9646	**1150.9859**	21
15	1723.6687	862.3380	1706.6421	853.8247	1705.6581	853.3327	C	2203.9482	**1102.4777**	2186.9216	1093.9645	2185.9376	1093.4725	20
16	**1838.6956**	919.8514	1821.6691	911.3382	1820.6850	910.8462	D	2043.9175	1022.4624	2026.8910	1013.9491	2025.9070	1013.4571	19
17	**1985.7640**	993.3856	1968.7375	984.8724	**1967.7534**	984.3804	F	**1928.8906**	**964.9489**	1911.8641	956.4357	1910.8800	955.9437	18
18	2113.8590	1057.4331	2096.8324	1048.9199	2095.8484	1048.4278	K	1781.8222	**891.4147**	**1764.7956**	882.9015	1763.8116	882.4094	17
19	2228.8859	1114.9466	2211.8594	1106.4333	2210.8754	1105.9413	D	**1653.7272**	827.3673	1636.7007	818.8540	1635.7167	818.3620	16
20	2299.9230	**1150.4652**	2282.8965	1141.9519	2281.9125	1141.4599	A	**1538.7003**	769.8538	1521.6737	761.3405	1520.6897	760.8485	15
21	2428.9656	1214.9865	2411.9391	1206.4732	2410.9551	1205.9812	E	1467.6632	734.3352	1450.6366	725.8219	1449.6526	725.3299	14
22	2558.0082	1279.5077	2540.9817	1270.9945	2539.9977	1270.5025	E	**1338.6206**	669.8139	1321.5940	661.3007	1320.6100	660.8086	13
23	2629.0453	1315.0263	2612.0188	1306.5130	2611.0348	1306.0210	A	**1209.5780**	605.2926	1192.5514	596.7794	1191.5674	596.2873	12
24	2700.0824	1350.5449	2683.0559	**1342.0316**	2682.0719	**1341.5396**	A	**1138.5409**	569.7741	1121.5143	561.2608	1120.5303	560.7688	11
25	2801.1301	**1401.0687**	2784.1036	1392.5554	2783.1196	1392.0634	T	**1067.5038**	534.2555	1050.4772	525.7422	1049.4932	525.2502	10
26	2858.1516	1429.5794	2841.1250	1421.0662	2840.1410	1420.5742	G	**966.4561**	483.7317	949.4295	**475.2184**	948.4455	474.7264	9
27	2987.1942	1494.1007	2970.1676	1485.5875	2969.1836	1485.0954	E	**909.4346**	455.2209	892.4081	446.7077	**891.4240**	446.2157	8
28	3147.2248	1574.1161	3130.1983	**1565.6028**	3129.2143	1565.1108	C	**780.3920**	390.6996	763.3655	382.1864	762.3815	381.6944	7
29	3248.2725	1624.6399	3231.2460	1616.1266	3230.2619	1615.6346	T	620.3614	310.6843	603.3348	302.1710	602.3508	301.6790	6
30	3349.3202	1675.1637	3332.2936	**1666.6505**	3331.3096	1666.1585	T	**519.3137**	260.1605	502.2871	251.6472	501.3031	251.1552	5
31	3450.3679	1725.6876	3433.3413	**1717.1743**	3432.3573	**1716.6823**	T	**418.2660**	209.6366	401.2395	201.1234	400.2554	200.6314	4
32	3563.4519	1782.2296	3546.4254	**1773.7163**	3545.4414	**1773.2243**	L	317.2183	159.1128	300.1918	150.5995			3
33	3620.4734	**1810.7403**	3603.4468	1802.2271	3602.4628	1801.7351	G	204.1343	102.5708	187.1077	94.0575			2
34							K	147.1128	74.0600	130.0863	65.5468			1

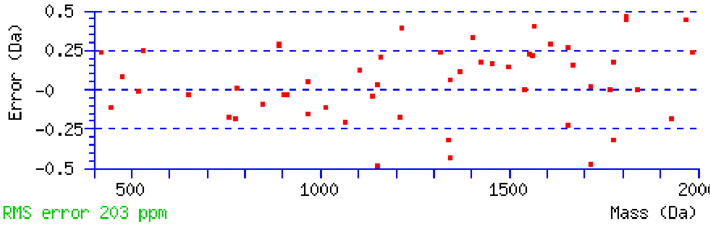

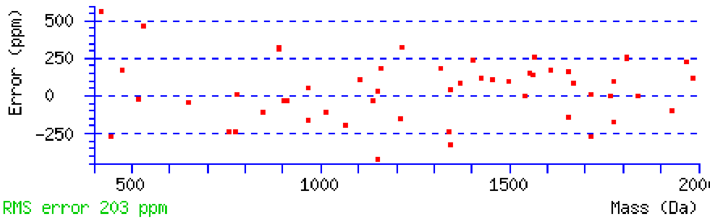

All matches to this query

Score	Mr(calc):	Delta	Sequence
79.5	3765.5717	1.0117	EGNCSVQSGLTWQDCDFKDAEEAATGECTTTLGK
60.9	3764.5877	1.9957	EGNCSVQSGLTWQDCDFKDAEEAATGECTTTLGK
3.4	3764.6091	1.9742	SSSEGESSWSLLNSSRSSFGSLTGQTSQEILEAR
1.6	3764.6091	1.9742	SSSEGESSWSLLNSSRSSFGSLTGQTSQEILEAR
0.8	3764.6091	1.9742	SSSEGESSWSLLNSSRSSFGSLTGQTSQEILEAR
0.7	3765.5445	1.0389	HNTVNLGMLPYGGISAMHAGRSMTLNLQTKSK
0.3	3766.6198	-0.0365	DAMERGDYTLDHVSACAGGGFQIGGNVYKVYLK
0.2	3766.6083	-0.0249	GTNVTISSLKPDTTYVFQIRARTAAGYGTNSR
0.1	3764.6091	1.9742	SSSEGESSWSLLNSSRSSFGSLTGQTSQEILEAR

Spectrum No: 136; Query: 775; Rank: 1

Peptide View

MS/MS Fragmentation of **LRNPCTSEQNCTSPFSYK**
Found in **IPI00191681**, Tax_Id=10116 Gene_Symbol=Itgb1 Integrin beta-1 precursor

Match to Query 775: 2188.957272 from(730.659700,3+)
Title: 091127RatKid_SCX01_13.1554.1554.3.dta
Data file K:\NewmanPaper\Piliang\3SubProteomes\Piliang3SP\mgf5ppm\SCX_3SubProteomes5ppm.mgf

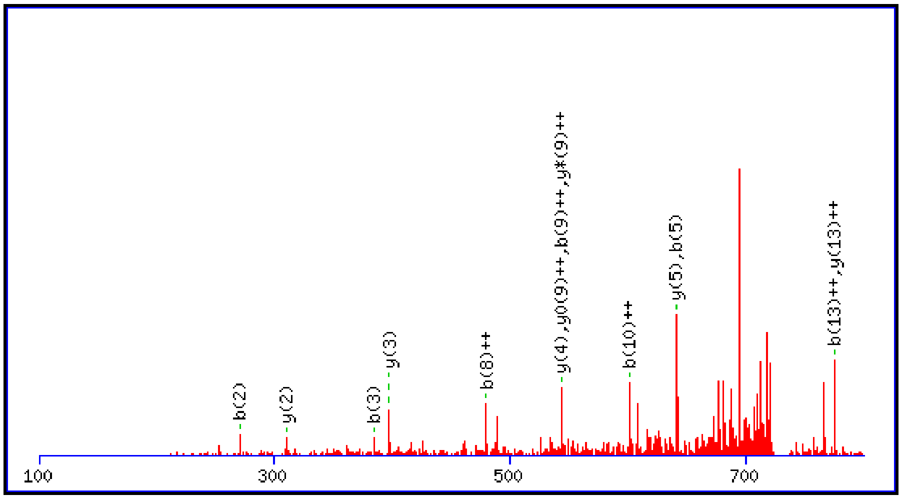

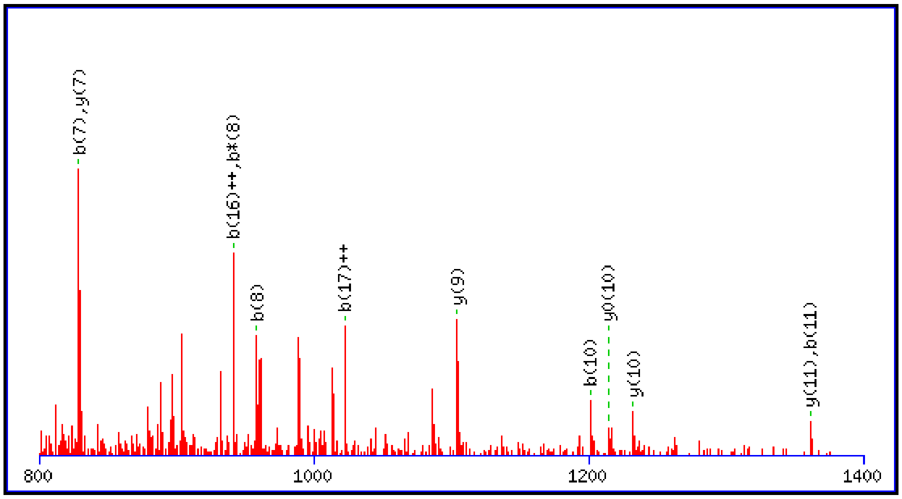

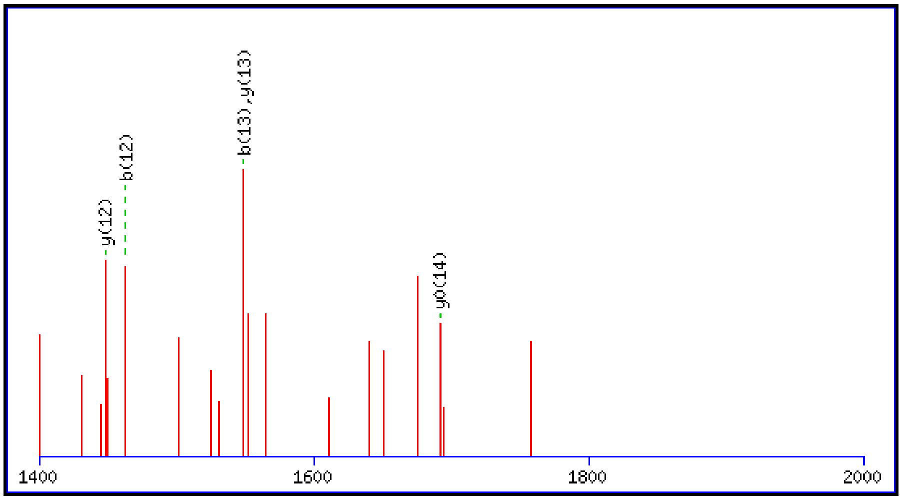

Monoisotopic mass of neutral peptide Mr(calc): 2188.9517
Fixed modifications: Carbamidomethyl (C)

Variable modifications:
N10 : Deamidated_N (N)
Ions Score: 80 **Expect:** 2.9e-006
Matches (Bold Red): 31/188 fragment ions using 33 most intense peaks

#	b	b^{++}	b*	b^{*++}	b^0	b^{0++}	Seq.	y	y^{++}	y*	y^{*++}	y^0	y^{0++}	#
1	114.0913	57.5493					L							18
2	**270.1925**	135.5999	253.1659	127.0866			R	2076.8750	1038.9411	2059.8484	1030.4278	2058.8644	1029.9358	17
3	**384.2354**	192.6213	367.2088	184.1081			N	1920.7738	960.8906	1903.7473	952.3773	1902.7633	951.8853	16
4	481.2881	241.1477	464.2616	232.6344			P	1806.7309	903.8691	1789.7044	895.3558	1788.7204	894.8638	15
5	**641.3188**	321.1630	624.2922	312.6498			C	1709.6782	855.3427	1692.6516	846.8294	**1691.6676**	846.3374	14
6	742.3665	371.6869	725.3399	363.1736	724.3559	362.6816	T	**1549.6475**	**775.3274**	1532.6210	766.8141	1531.6369	766.3221	13
7	**829.3985**	415.2029	812.3719	406.6896	811.3879	406.1976	S	**1448.5998**	724.8036	1431.5733	716.2903	1430.5893	715.7983	12
8	**958.4411**	**479.7242**	**941.4145**	471.2109	940.4305	470.7189	E	**1361.5678**	681.2875	1344.5413	672.7743	1343.5572	672.2823	11
9	1086.4997	**543.7535**	1069.4731	535.2402	1068.4891	534.7482	Q	**1232.5252**	616.7662	1215.4987	608.2530	**1214.5146**	607.7610	10
10	**1201.5266**	**601.2669**	1184.5001	592.7537	1183.5160	592.2617	N	**1104.4666**	552.7370	1087.4401	**544.2237**	1086.4561	**543.7317**	9
11	**1361.5573**	681.2823	1344.5307	672.7690	1343.5467	672.2770	C	989.4397	495.2235	972.4131	486.7102	971.4291	486.2182	8
12	**1462.6049**	731.8061	1445.5784	723.2928	1444.5944	722.8008	T	**829.4090**	415.2082	812.3825	406.6949	811.3985	406.2029	7
13	**1549.6370**	**775.3221**	1532.6104	766.8088	1531.6264	766.3168	S	728.3614	364.6843	711.3348	356.1710	710.3508	355.6790	6
14	1646.6897	823.8485	1629.6632	815.3352	1628.6792	814.8432	P	**641.3293**	321.1683	624.3028	312.6550	623.3188	312.1630	5
15	1793.7581	897.3827	1776.7316	888.8694	1775.7476	888.3774	F	**544.2766**	272.6419	527.2500	264.1287	526.2660	263.6366	4
16	1880.7902	**940.8987**	1863.7636	932.3854	1862.7796	931.8934	S	**397.2082**	199.1077	380.1816	190.5944	379.1976	190.1024	3
17	2043.8535	**1022.4304**	2026.8269	1013.9171	2025.8429	1013.4251	Y	**310.1761**	155.5917	293.1496	147.0784			2
18							K	147.1128	74.0600	130.0863	65.5468			1

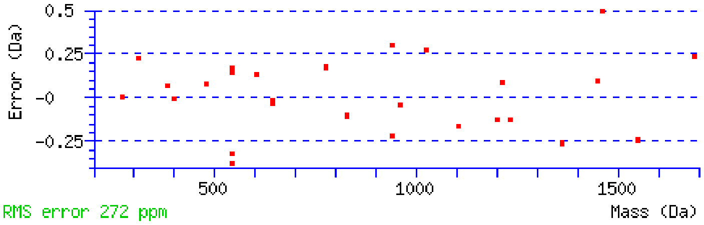

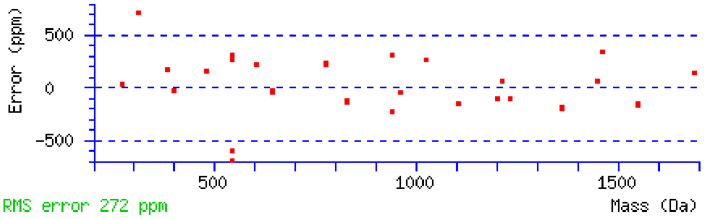

All matches to this query

Score	Mr(calc):	Delta	Sequence
79.5	2188.9517	0.0055	LRNPCTSEQNCTSPFSYK
37.2	2188.9517	0.0055	LRNPCTSEQNCTSPFSYK
14.5	2187.9677	0.9895	LRNPCTSEQNCTSPFSYK
7.4	2188.9357	0.0215	HVHAPTQHQTFSFSHIR
5.1	2188.9357	0.0215	HVHAPTQHQTFSFSHIR
3.8	2186.9665	1.9907	ESIMVAFKGVWTQAFWK
2.4	2188.9357	0.0215	HVHAPTQHQTFSFSHIR
1.4	2188.9731	-0.0159	LRDGDRFWWENPGVFSK

Spectrum No: 137; Query: 861; Rank: 1

Peptide View

MS/MS Fragmentation of **FFNDSIVDPVDSEWFGFYR**
Found in **IPI00208382**, Tax_Id=10116 Gene_Symbol=Ppt1 Palmitoyl-protein thioesterase 1 precursor

Match to Query 861: 2340.046728 from(1171.030640,2+)
Title: 091127RatKid_SCX01_15.6180.6180.2.dta

Data file K:\NewmanPaper\Piliang\3SubProteomes\Piliang3SP\mgf5ppm\SCX_3SubProteomes5ppm.mgf

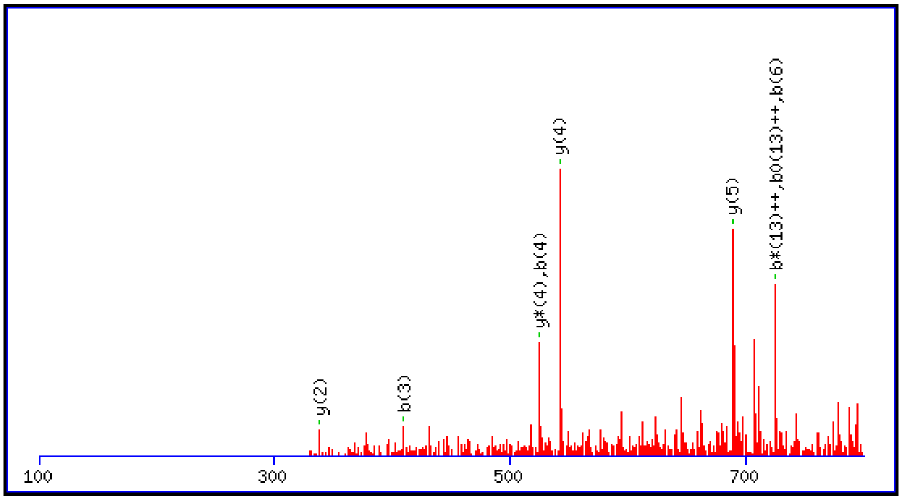

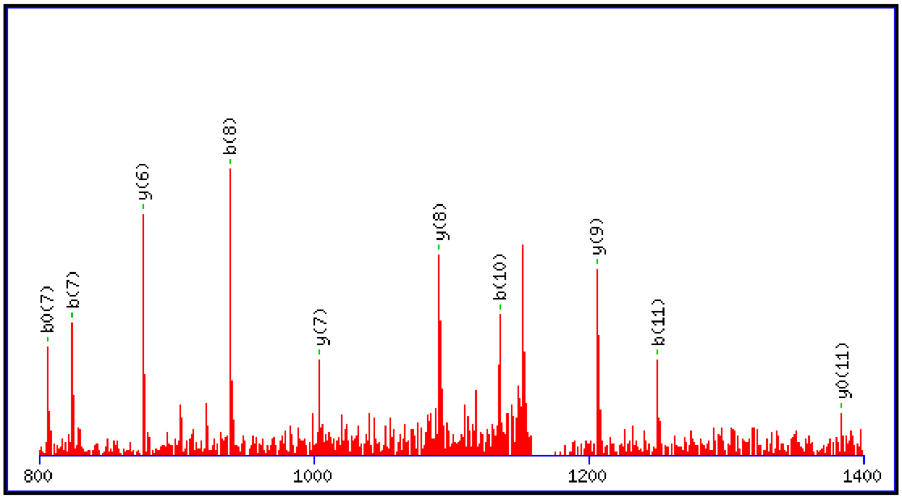

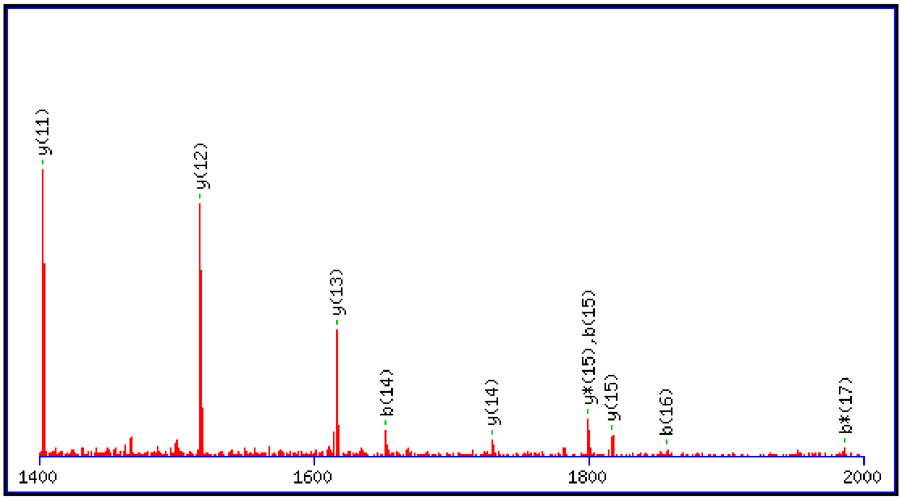

Monoisotopic mass of neutral peptide Mr(calc): 2340.0375
Fixed modifications: Carbamidomethyl (C)
Variable modifications:
N3 : Deamidated_N (N)
Ions Score: 80 **Expect:** 3.5e-006
Matches (Bold Red): 29/194 fragment ions using 43 most intense peaks

#	b	b^{++}	b*	b^{*++}	b^0	b^{0++}	Seq.	y	y^{++}	y*	y^{*++}	y^0	y^{0++}	#
1	148.0757	74.5415					F							19
2	295.1441	148.0757					F	2193.9764	1097.4918	2176.9498	1088.9785	2175.9658	1088.4865	18
3	**410.1710**	205.5892	393.1445	197.0759			N	2046.9080	1023.9576	2029.8814	1015.4443	2028.8974	1014.9523	17
4	**525.1980**	263.1026	508.1714	254.5894	507.1874	254.0973	D	1931.8810	966.4441	1914.8545	957.9309	1913.8705	957.4389	16

5	612.2300	306.6186	595.2035	298.1054	594.2194	297.6134	S	**1816.8541**	908.9307	**1799.8275**	900.4174	1798.8435	899.9254	15
6	**725.3141**	363.1607	708.2875	354.6474	707.3035	354.1554	I	**1729.8220**	865.4147	1712.7955	856.9014	1711.8115	856.4094	14
7	**824.3825**	412.6949	807.3559	404.1816	**806.3719**	403.6896	V	**1616.7380**	808.8726	1599.7114	800.3594	1598.7274	799.8673	13
8	**939.4094**	470.2084	922.3829	461.6951	921.3989	461.2031	D	**1517.6696**	759.3384	1500.6430	750.8251	1499.6590	750.3331	12
9	1036.4622	518.7347	1019.4356	510.2215	1018.4516	509.7295	P	**1402.6426**	701.8250	1385.6161	693.3117	**1384.6321**	692.8197	11
10	**1135.5306**	568.2689	1118.5041	559.7557	1117.5200	559.2637	V	1305.5899	653.2986	1288.5633	644.7853	1287.5793	644.2933	10
11	**1250.5576**	625.7824	1233.5310	617.2691	1232.5470	616.7771	D	**1206.5215**	603.7644	1189.4949	595.2511	1188.5109	594.7591	9
12	1337.5896	669.2984	1320.5630	660.7852	1319.5790	660.2931	S	**1091.4945**	546.2509	1074.4680	537.7376	1073.4839	537.2456	8
13	1466.6322	733.8197	1449.6056	**725.3065**	1448.6216	**724.8144**	E	**1004.4625**	502.7349	987.4359	494.2216	986.4519	493.7296	7
14	**1652.7115**	826.8594	1635.6849	818.3461	1634.7009	817.8541	W	**875.4199**	438.2136	858.3933	429.7003			6
15	**1799.7799**	900.3936	1782.7534	891.8803	1781.7693	891.3883	F	**689.3406**	345.1739	672.3140	336.6606			5
16	**1856.8014**	928.9043	1839.7748	920.3910	1838.7908	919.8990	G	**542.2722**	271.6397	**525.2456**	263.1264			4
17	2003.8698	1002.4385	**1986.8432**	993.9253	1985.8592	993.4332	F	485.2507	243.1290	468.2241	234.6157			3
18	2166.9331	1083.9702	2149.9066	1075.4569	2148.9225	1074.9649	Y	**338.1823**	169.5948	321.1557	161.0815			2
19							R	175.1190	88.0631	158.0924	79.5498			1

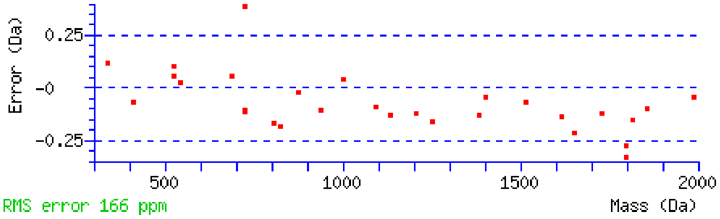

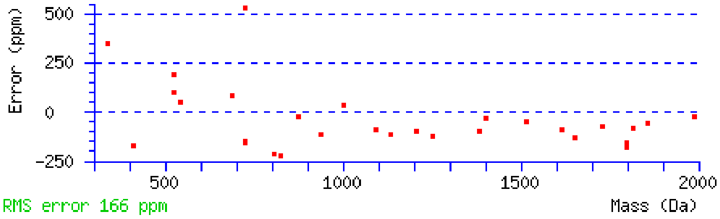

All matches to this query

Score	Mr(calc):	Delta	Sequence
79.5	2340.0375	0.0092	FFNDSIVDPVDSEWFGFYR
50.4	2339.0535	0.9932	FFNDSIVDPVDSEWFGFYR
8.6	2340.0512	-0.0045	LLSASSSPTLPPSPGGGGGSKGNR
4.1	2339.0439	1.0028	RMGLNDFIQKLANNSYACK
3.6	2339.0439	1.0028	RMGLNDFIQKLANNSYACK
3.3	2339.0471	0.9996	WPTTQENVDIRDLTWEQK
2.3	2338.0404	2.0063	MTSLLRHNCLILWYSXK
2.0	2339.0569	0.9898	RAPQTGIVDECCFRSCDLR
1.2	2338.0306	2.0161	ARRPNTKSTMPTSLPNLAK
1.2	2338.0306	2.0161	ARRPNTKSTMPTSLPNLAK

Spectrum No: 138; Query: 102; Rank: 1

Peptide View

MS/MS Fragmentation of **IEVEANNTVTK**
Found in **IPI00200640**, Tax_Id=10116 Gene_Symbol=Mucdhl Isoform 1 of Mucin and cadherin-like protein precursor

Match to Query 102: 1217.614068 from(609.814310,2+)
Title: 091127RatKid_SCX01_02.745.745.2.dta
Data file K:\NewmanPaper\Piliang\3SubProteomes\Piliang3SP\mgf5ppm\SCX_3SubProteomes5ppm.mgf

Mascot Search Results: Peptide View

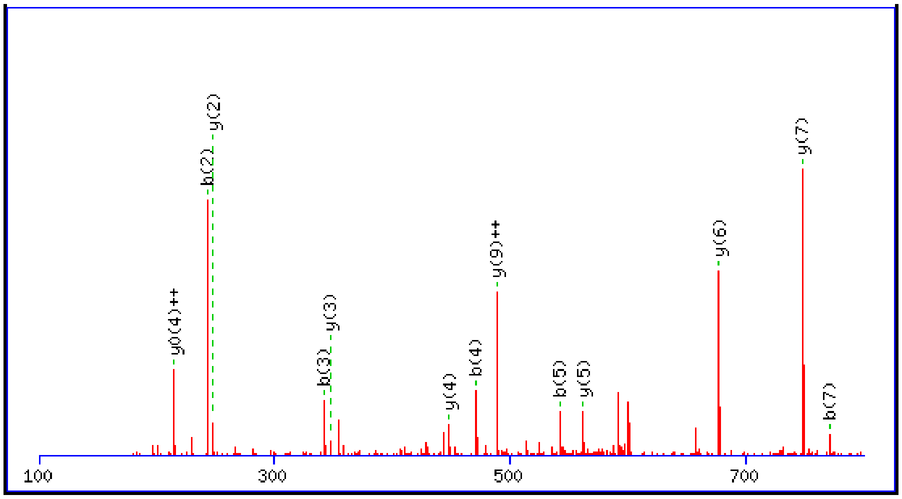

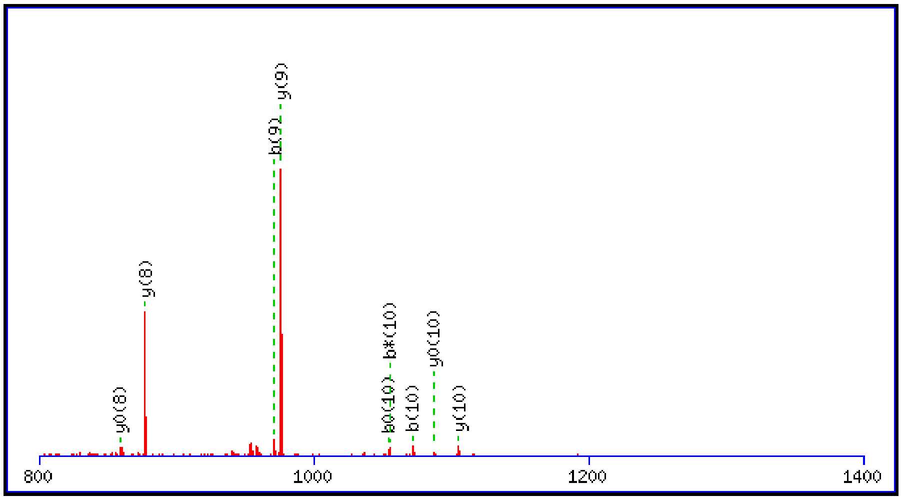

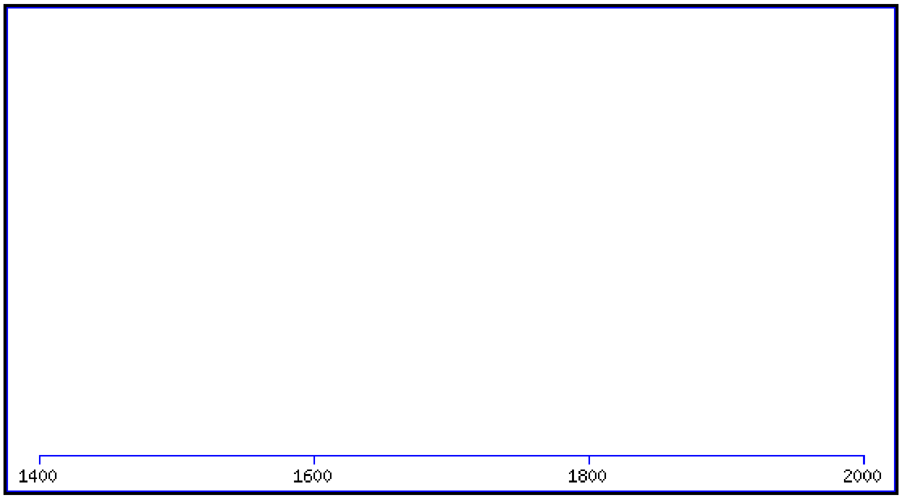

Monoisotopic mass of neutral peptide Mr(calc): 1217.6139
Fixed modifications: Carbamidomethyl (C)
Variable modifications:
N6 : Deamidated_N (N)
Ions Score: 80 **Expect:** 1.5e-006
Matches (Bold Red): 22/106 fragment ions using 33 most intense peaks

#	b	b^{++}	b*	b^{*++}	b^0	b^{0++}	Seq.	y	y^{++}	y*	y^{*++}	y^0	y^{0++}	#
1	114.0913	57.5493					I							11
2	**243.1339**	122.0706			225.1234	113.0653	E	**1105.5372**	553.2722	1088.5106	544.7589	**1087.5266**	544.2669	10
3	**342.2023**	171.6048			324.1918	162.5995	V	**976.4946**	**488.7509**	959.4680	480.2376	958.4840	479.7456	9
4	**471.2449**	236.1261			453.2344	227.1208	E	**877.4261**	439.2167	860.3996	430.7034	**859.4156**	430.2114	8
5	**542.2821**	271.6447			524.2715	262.6394	A	**748.3836**	374.6954	731.3570	366.1821	730.3730	365.6901	7

**6**	657.3090	329.1581	640.2824	320.6449	639.2984	320.1529	**N**	**677.3464**	339.1769	660.3199	330.6636	659.3359	330.1716	**6**
**7**	**771.3519**	386.1796	754.3254	377.6663	753.3414	377.1743	**N**	**562.3195**	281.6634	545.2930	273.1501	544.3089	272.6581	**5**
**8**	872.3996	436.7034	855.3730	428.1902	854.3890	427.6982	**T**	**448.2766**	224.6419	431.2500	216.1287	430.2660	**215.6366**	**4**
**9**	**971.4680**	486.2376	954.4415	477.7244	953.4574	477.2324	**V**	**347.2289**	174.1181	330.2023	165.6048	329.2183	165.1128	**3**
**10**	**1072.5157**	536.7615	**1055.4891**	528.2482	**1054.5051**	527.7562	**T**	**248.1605**	124.5839	231.1339	116.0706	230.1499	115.5786	**2**
**11**							**K**	147.1128	74.0600	130.0863	65.5468			**1**

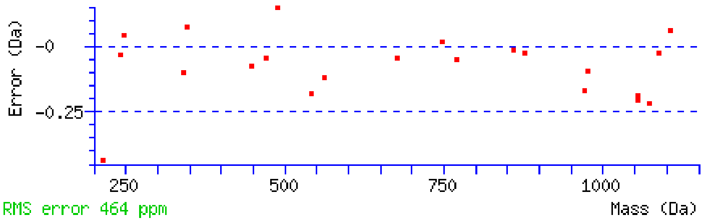

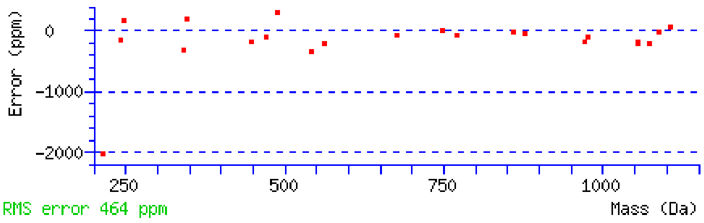

All matches to this query

Score	Mr(calc):	Delta	Sequence
79.5	1217.6139	0.0001	IEVEANNTVTK
64.9	1217.6139	0.0001	IEVEANNTVTK
17.3	1217.6179	-0.0039	LEDLNFPEIK
16.7	1217.6252	-0.0111	GKGEGDLSQLSK
16.2	1217.6169	-0.0028	NKLDHSAIIK
12.6	1217.6169	-0.0028	TKKLAESHPK
12.2	1217.6040	0.0100	VEISLNGSWGR
11.7	1217.6193	-0.0052	IEDFWGPAKR
9.3	1217.6139	0.0002	IDEIASNLQSK
8.3	1217.6057	0.0084	DLEALHSLLK

Spectrum No: 139; Query: 1128; Rank: 1

Peptide View

MS/MS Fragmentation of **LLYISPENFQIDNSPNSTAGPSCSLLLEAFR**
Found in **IPI00464518**, Tax_Id=10116 Gene_Symbol=Hexb Beta-hexosaminidase beta chain precursor

Match to Query 1128: 3453.686142 from(1152.235990,3+)
Title: 091127RatKid_SCX01_13.5963.5963.3.dta
Data file K:\NewmanPaper\Piliang\3SubProteomes\Piliang3SP\mgf5ppm\SCX_3SubProteomes5ppm.mgf

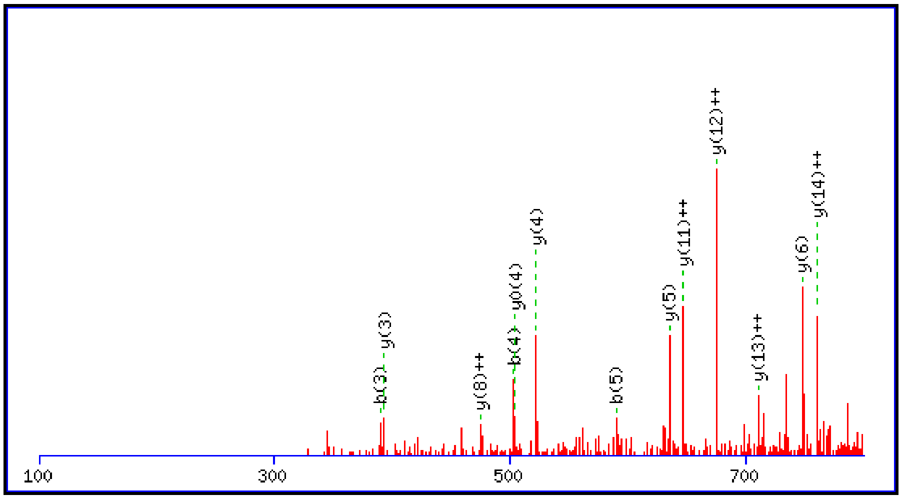

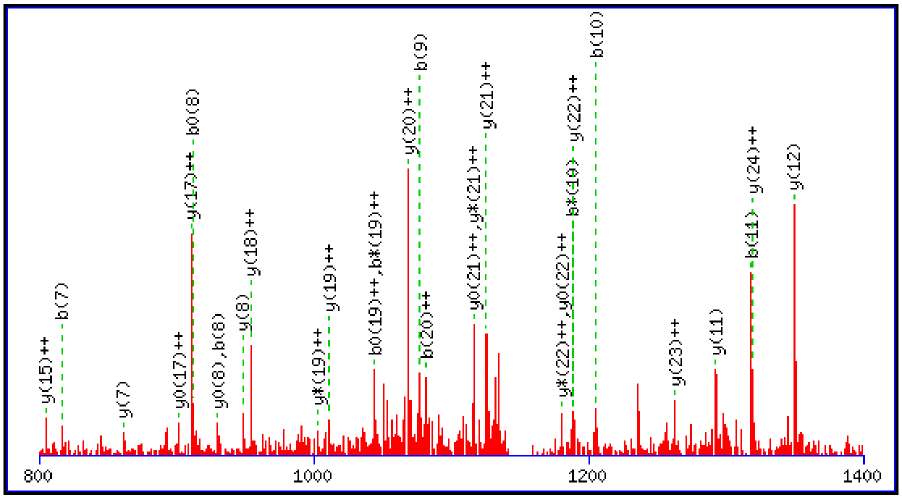

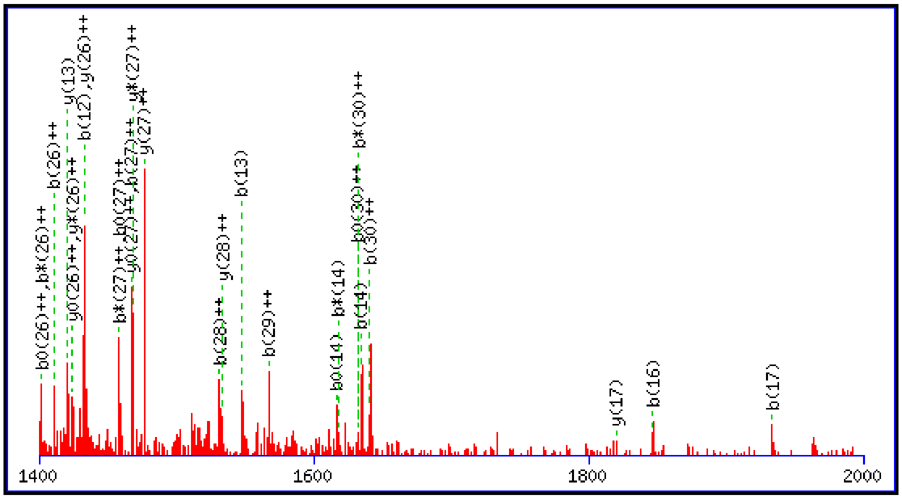

Monoisotopic mass of neutral peptide Mr(calc): 3453.6762
Fixed modifications: Carbamidomethyl (C)
Variable modifications:
N16 : Deamidated_N (N)
Ions Score: 79 **Expect:** 3.6e-006
Matches (Bold Red): 70/332 fragment ions using 123 most intense peaks

#	b	b++	b*	b*++	b0	b0++	Seq.	y	y++	y*	y*++	y0	y0++	#
1	114.0913	57.5493					L							31
2	227.1754	114.0913					L	3341.5994	1671.3034	3324.5729	1662.7901	3323.5889	1662.2981	30
3	**390.2387**	195.6230					Y	3228.5154	1614.7613	3211.4888	1606.2481	3210.5048	1605.7560	29
4	**503.3228**	252.1650					I	3065.4520	**1533.2297**	3048.4255	1524.7164	3047.4415	1524.2244	28
5	**590.3548**	295.6811			572.3443	286.6758	S	2952.3680	**1476.6876**	2935.3414	**1468.1744**	2934.3574	**1467.6823**	27
6	687.4076	344.2074			669.3970	335.2022	P	2865.3360	**1433.1716**	2848.3094	**1424.6583**	2847.3254	**1424.1663**	26
7	**816.4502**	408.7287			798.4396	399.7234	E	2768.2832	1384.6452	2751.2566	1376.1320	2750.2726	1375.6400	25
8	**930.4931**	465.7502	913.4666	457.2369	**912.4825**	456.7449	N	2639.2406	**1320.1239**	2622.2141	1311.6107	2621.2300	1311.1187	24
9	**1077.5615**	539.2844	1060.5350	530.7711	1059.5510	530.2791	F	2525.1977	**1263.1025**	2508.1711	1254.5892	2507.1871	1254.0972	23
10	**1205.6201**	603.3137	**1188.5936**	594.8004	1187.6095	594.3084	Q	2378.1293	**1189.5683**	2361.1027	**1181.0550**	2360.1187	**1180.5630**	22
11	**1318.7042**	659.8557	1301.6776	651.3424	1300.6936	650.8504	I	2250.0707	**1125.5390**	2233.0441	**1117.0257**	2232.0601	**1116.5337**	21
12	**1433.7311**	717.3692	1416.7046	708.8559	1415.7205	708.3639	D	2136.9866	**1068.9969**	2119.9601	1060.4837	2118.9761	1059.9917	20
13	**1547.7740**	774.3907	1530.7475	765.8774	1529.7635	765.3854	N	2021.9597	**1011.4835**	2004.9331	**1002.9702**	2003.9491	1002.4782	19
14	**1634.8061**	817.9067	**1617.7795**	809.3934	**1616.7955**	808.9014	S	1907.9167	**954.4620**	1890.8902	945.9487	1889.9062	945.4567	18
15	1731.8588	866.4331	1714.8323	857.9198	1713.8483	857.4278	P	**1820.8847**	**910.9460**	1803.8582	902.4327	1802.8742	**901.9407**	17
16	**1846.8858**	923.9465	1829.8592	915.4332	1828.8752	914.9412	N	1723.8320	862.4196	1706.8054	853.9063	1705.8214	853.4143	16
17	**1933.9178**	967.4625	1916.8912	958.9493	1915.9072	958.4573	S	1608.8050	**804.9061**	1591.7785	796.3929	1590.7945	795.9009	15
18	2034.9655	1017.9864	2017.9389	1009.4731	2016.9549	1008.9811	T	1521.7730	**761.3901**	1504.7464	752.8769	1503.7624	752.3849	14

**19**	2106.0026	1053.5049	2088.9760	**1044.9917**	2087.9920	**1044.4996**	**A**	**1420.7253**	**710.8663**	1403.6988	702.3530	1402.7147	701.8610	**13**
**20**	2163.0240	**1082.0157**	2145.9975	1073.5024	2145.0135	1073.0104	**G**	**1349.6882**	**675.3477**	1332.6616	666.8345	1331.6776	666.3425	**12**
**21**	2260.0768	1130.5420	2243.0503	1122.0288	2242.0662	1121.5368	**P**	**1292.6667**	**646.8370**	1275.6402	638.3237	1274.6562	637.8317	**11**
**22**	2347.1088	1174.0581	2330.0823	1165.5448	2329.0983	1165.0528	**S**	1195.6140	598.3106	1178.5874	589.7973	1177.6034	589.3053	**10**
**23**	2507.1395	1254.0734	2490.1129	1245.5601	2489.1289	1245.0681	**C**	1108.5819	554.7946	1091.5554	546.2813	1090.5714	545.7893	**9**
**24**	2594.1715	1297.5894	2577.1450	1289.0761	2576.1610	1288.5841	**S**	**948.5513**	**474.7793**	931.5247	466.2660	**930.5407**	465.7740	**8**
**25**	2707.2556	1354.1314	2690.2290	1345.6182	2689.2450	1345.1261	**L**	**861.5193**	431.2633	844.4927	422.7500	843.5087	422.2580	**7**
**26**	2820.3396	**1410.6735**	2803.3131	**1402.1602**	2802.3291	**1401.6682**	**L**	**748.4352**	374.7212	731.4087	366.2080	730.4246	365.7160	**6**
**27**	2933.4237	**1467.2155**	2916.3972	**1458.7022**	2915.4131	**1458.2102**	**L**	**635.3511**	318.1792	618.3246	309.6659	617.3406	309.1739	**5**
**28**	3062.4663	**1531.7368**	3045.4398	1523.2235	3044.4557	1522.7315	**E**	**522.2671**	261.6372	505.2405	253.1239	**504.2565**	252.6319	**4**
**29**	3133.5034	**1567.2553**	3116.4769	1558.7421	3115.4929	1558.2501	**A**	**393.2245**	197.1159	376.1979	188.6026			**3**
**30**	3280.5718	**1640.7896**	3263.5453	**1632.2763**	3262.5613	**1631.7843**	**F**	322.1874	161.5973	305.1608	153.0840			**2**
**31**							**R**	175.1190	88.0631	158.0924	79.5498			**1**

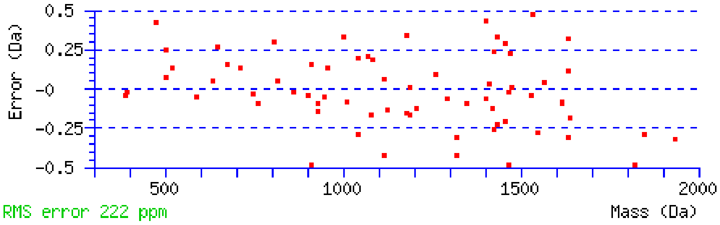

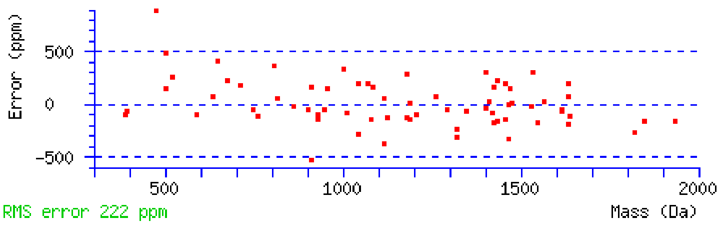

All matches to this query

Score	Mr(calc):	Delta	Sequence
79.0	3453.6762	0.0099	LLYISPENFQIDNSPNSTAGPSCSLLLEAFR
72.0	3453.6762	0.0099	LLYISPENFQIDNSPNSTAGPSCSLLLEAFR
54.2	3452.6922	0.9940	LLYISPENFQIDNSPNSTAGPSCSLLLEAFR
54.1	3453.6762	0.0099	LLYISPENFQIDNSPNSTAGPSCSLLLEAFR
1.2	3451.6829	2.0032	SQHFKNQTYTNLYHLIITLISSITVSLR
1.2	3453.6920	-0.0059	MFNIEFLARLDSQLEILPSSLSARIQFR
0.7	3451.6829	2.0032	SQHFKNQTYTNLYHLIITLISSITVSLR
0.7	3451.6829	2.0032	SQHFKNQTYTNLYHLIITLISSITVSLR
0.7	3451.6829	2.0032	SQHFKNQTYTNLYHLIITLISSITVSLR
0.7	3451.6829	2.0032	SQHFKNQTYTNLYHLIITLISSITVSLR

Spectrum No: 140; Query: 398; Rank: 1

Peptide View

MS/MS Fragmentation of **NLTNPNTVIILIGNK**
Found in **IPI00196794**, Tax_Id=10116 Gene_Symbol=Rab14 Ras-related protein Rab-14

Match to Query 398: 1623.925208 from(812.969880,2+)
Title: 091127RatKid_SCX01_13.4486.4486.2.dta
Data file K:\NewmanPaper\Piliang\3SubProteomes\Piliang3SP\mgf5ppm\SCX_3SubProteomes5ppm.mgf

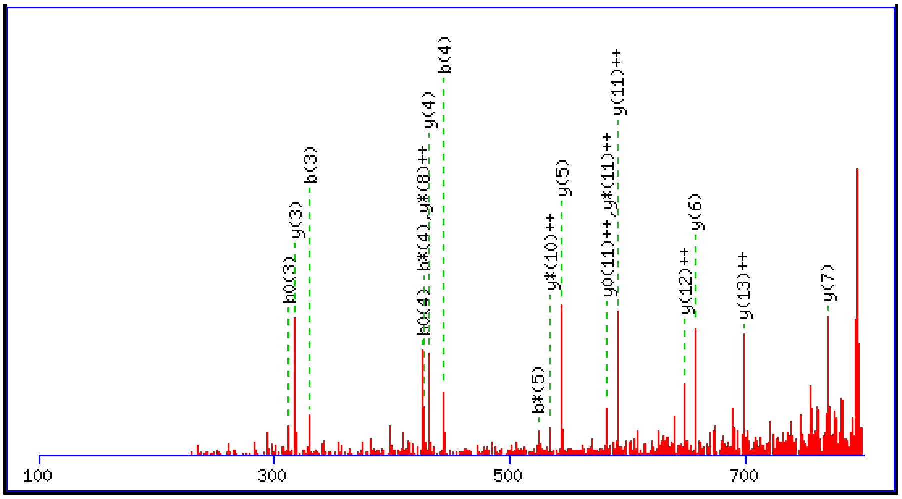

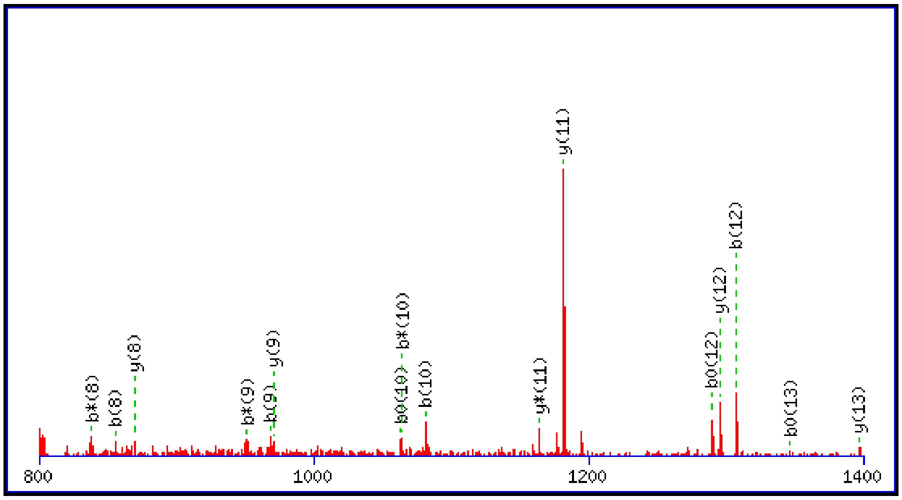

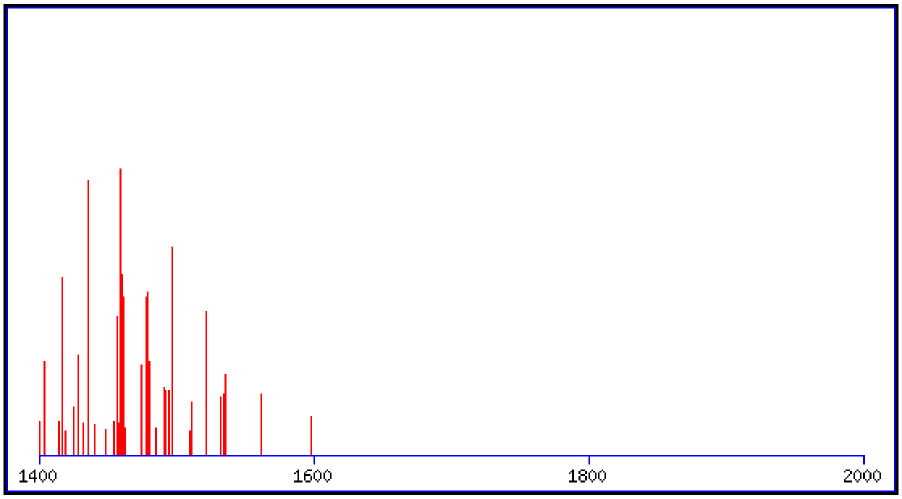

Monoisotopic mass of neutral peptide Mr(calc): 1623.9195
Fixed modifications: Carbamidomethyl (C)
Variable modifications:
N1 : Deamidated_N (N)
Ions Score: 79 **Expect:** 4.1e-007
Matches (Bold Red): 34/148 fragment ions using 49 most intense peaks

#	b	b++	b*	b*++	b0	b0++	Seq.	y	y++	y*	y*++	y0	y0++	#
1	116.0342	58.5207	99.0077	50.0075			N							15
2	229.1183	115.0628	212.0917	106.5495			L	1509.8999	755.4536	1492.8734	746.9403	1491.8893	746.4483	14
3	**330.1660**	165.5866	313.1394	157.0733	**312.1554**	156.5813	T	**1396.8158**	**698.9116**	1379.7893	690.3983	1378.8053	689.9063	13
4	**444.2089**	222.6081	**427.1823**	214.0948	**426.1983**	213.6028	N	**1295.7682**	**648.3877**	1278.7416	639.8744	1277.7576	639.3824	12
5	541.2616	271.1345	**524.2351**	262.6212	523.2511	262.1292	P	**1181.7252**	**591.3663**	**1164.6987**	**582.8530**	1163.7147	**582.3610**	11

6	655.3046	328.1559	638.2780	319.6427	637.2940	319.1506	N	1084.6725	542.8399	1067.6459	**534.3266**	1066.6619	533.8346	10
7	756.3523	378.6798	739.3257	370.1665	738.3417	369.6745	T	**970.6295**	485.8184	953.6030	477.3051	952.6190	476.8131	9
8	**855.4207**	428.2140	**838.3941**	419.7007	837.4101	419.2087	V	**869.5819**	435.2946	852.5553	**426.7813**			8
9	**968.5047**	484.7560	**951.4782**	476.2427	950.4942	475.7507	I	**770.5135**	385.7604	753.4869	377.2471			7
10	**1081.5888**	541.2980	**1064.5622**	532.7848	**1063.5782**	532.2928	I	**657.4294**	329.2183	640.4028	320.7051			6
11	1194.6729	597.8401	1177.6463	589.3268	1176.6623	588.8348	L	**544.3453**	272.6763	527.3188	264.1630			5
12	**1307.7569**	654.3821	1290.7304	645.8688	**1289.7464**	645.3768	I	**431.2613**	216.1343	414.2347	207.6210			4
13	1364.7784	682.8928	1347.7518	674.3796	**1346.7678**	673.8875	G	**318.1772**	159.5922	301.1506	151.0790			3
14	1478.8213	739.9143	1461.7948	731.4010	1460.8107	730.9090	N	261.1557	131.0815	244.1292	122.5682			2
15							K	147.1128	74.0600	130.0863	65.5468			1

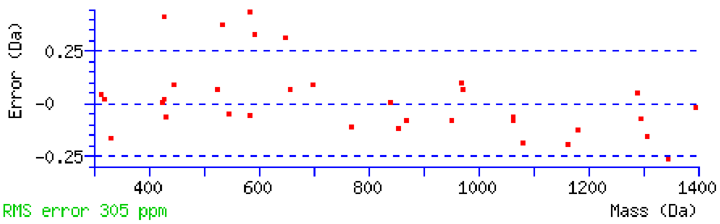

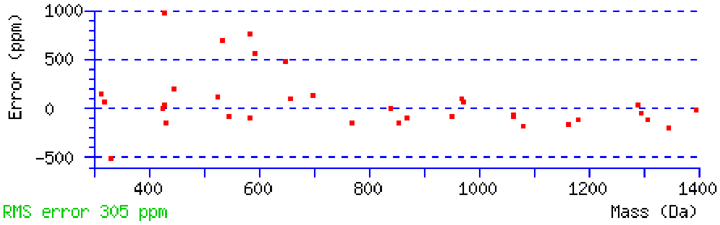

All matches to this query

Score	Mr(calc):	Delta	Sequence
79.0	1623.9195	0.0057	NLTNPNTVIILIGNK
69.0	1623.9195	0.0057	NLTNPNTVIILIGNK
59.5	1623.9195	0.0057	NLTNPNTVIILIGNK
49.0	1622.9355	0.9897	NLTNPNTVIILIGNK
9.4	1623.9195	0.0057	NLTNPNTVIILIGNK
3.6	1622.9144	1.0108	VWVLQDNILTAVPR

Spectrum No: 141; Query: 441; Rank: 1

Peptide View

MS/MS Fragmentation of **TANETSAEAYNLLLR**
Found in **IPI00363849**, Tax_Id=10116 Gene_Symbol=Lamc1 similar to Laminin gamma-1 chain precursor

Match to Query 441: 1665.824708 from(833.919630,2+)
Title: 091127RatKid_SCX01_17.3233.3233.2.dta
Data file K:\NewmanPaper\Piliang\3SubProteomes\Piliang3SP\mgf5ppm\SCX_3SubProteomes5ppm.mgf

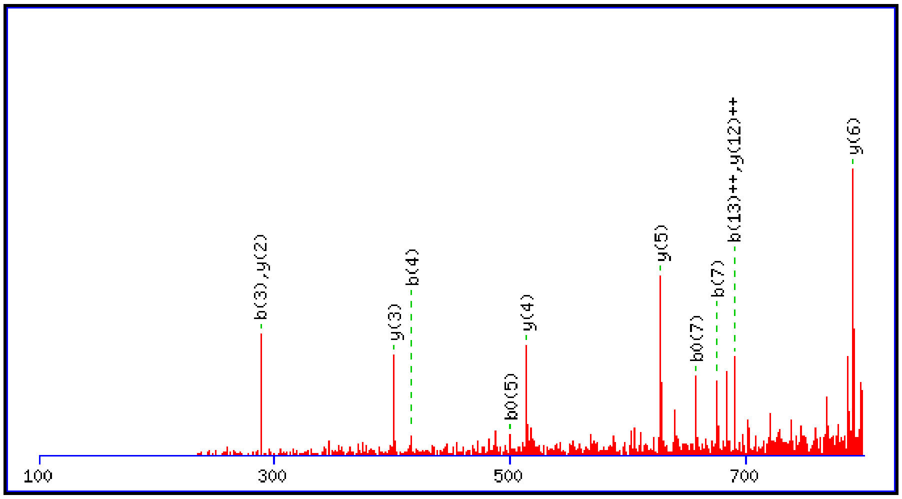

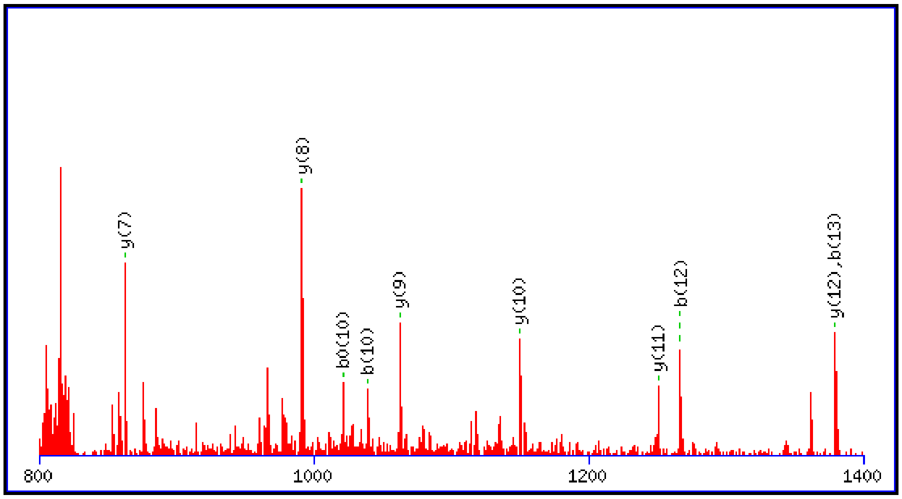

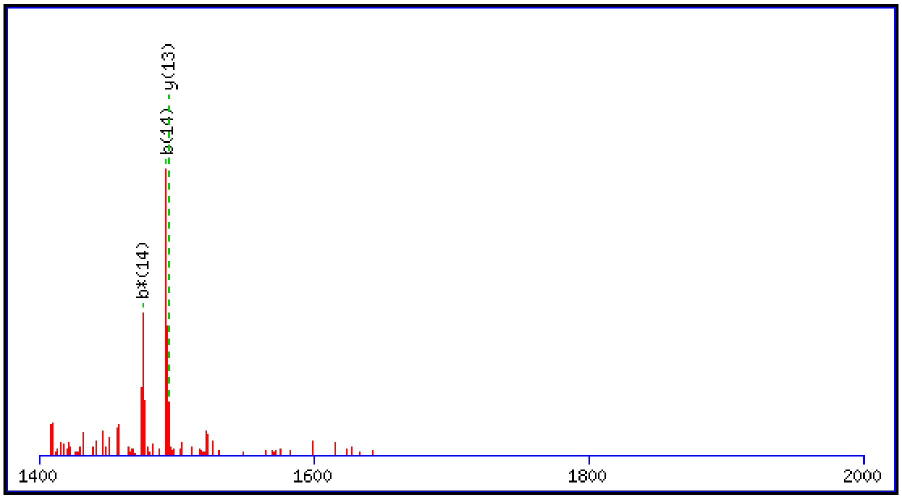

Monoisotopic mass of neutral peptide Mr(calc): 1665.8209
Fixed modifications: Carbamidomethyl (C)
Variable modifications:
N3 : Deamidated_N (N)
Ions Score: 79 **Expect:** 2.2e-006
Matches (Bold Red): 25/150 fragment ions using 43 most intense peaks

#	b	b^{++}	b*	b^{*++}	b^0	b^{0++}	Seq.	y	y^{++}	y*	y^{*++}	y^0	y^{0++}	#
1	102.0550	51.5311			84.0444	42.5258	T							15
2	173.0921	87.0497			155.0815	78.0444	A	1565.7806	783.3939	1548.7540	774.8806	1547.7700	774.3886	14
3	**288.1190**	144.5631	271.0925	136.0499	270.1084	135.5579	N	**1494.7435**	747.8754	1477.7169	739.3621	1476.7329	738.8701	13
4	**417.1616**	209.0844	400.1350	200.5712	399.1510	200.0792	E	**1379.7165**	**690.3619**	1362.6900	681.8486	1361.7060	681.3566	12
5	518.2093	259.6083	501.1827	251.0950	**500.1987**	250.6030	T	**1250.6739**	625.8406	1233.6474	617.3273	1232.6634	616.8353	11
6	605.2413	303.1243	588.2148	294.6110	587.2307	294.1190	S	**1149.6262**	575.3168	1132.5997	566.8035	1131.6157	566.3115	10
7	**676.2784**	338.6428	659.2519	330.1296	**658.2679**	329.6376	A	**1062.5942**	531.8007	1045.5677	523.2875	1044.5837	522.7955	9
8	805.3210	403.1641	788.2945	394.6509	787.3104	394.1589	E	**991.5571**	496.2822	974.5306	487.7689	973.5465	487.2769	8
9	876.3581	438.6827	859.3316	430.1694	858.3476	429.6774	A	**862.5145**	431.7609	845.4880	423.2476			7
10	**1039.4215**	520.2144	1022.3949	511.7011	**1021.4109**	511.2091	Y	**791.4774**	396.2423	774.4509	387.7291			6
11	1153.4644	577.2358	1136.4378	568.7226	1135.4538	568.2305	N	**628.4141**	314.7107	611.3875	306.1974			5
12	**1266.5484**	633.7779	1249.5219	625.2646	1248.5379	624.7726	L	**514.3711**	257.6892	497.3446	249.1759			4
13	**1379.6325**	**690.3199**	1362.6060	681.8066	1361.6219	681.3146	L	**401.2871**	201.1472	384.2605	192.6339			3
14	**1492.7166**	746.8619	**1475.6900**	738.3487	1474.7060	737.8566	L	**288.2030**	144.6051	271.1765	136.0919			2
15							R	175.1190	88.0631	158.0924	79.5498			1

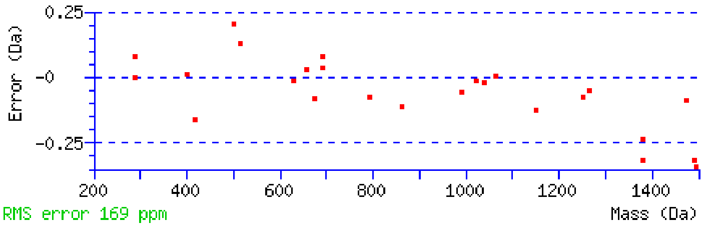

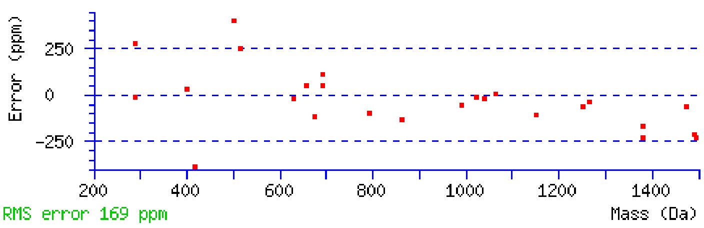

All matches to this query

Score	Mr(calc):	Delta	Sequence
78.9	1665.8209	0.0038	TANETSAEAYNLLLR
65.6	1665.8209	0.0038	TANETSAEAYNLLLR
64.4	1664.8369	0.9878	TANETSAEAYNLLLR
25.3	1665.8087	0.0160	NNSTPLNISETKLR
11.7	1665.8239	0.0008	NPDYRSNKPIILR
9.7	1665.8239	0.0008	NPDYRSNKPIILR
9.2	1665.8352	-0.0105	SVSRSLWRTPELR
9.2	1665.8087	0.0160	NNSTPLNISETKLR
9.1	1665.8087	0.0161	EIEQKDSEIARLR
8.4	1665.8239	0.0008	NPDYRSNKPIILR

Spectrum No: 142; Query: 222; Rank: 1

Peptide View

MS/MS Fragmentation of **IIYSIVQTNCSK**
Found in **IPI00327182**, Tax_Id=10116 Gene_Symbol=Kng1 T-kininogen 1 precursor

Match to Query 222: 1425.721888 from(713.868220,2+)
Title: 091127RatKid_SCX01_02.2125.2125.2.dta
Data file K:\NewmanPaper\Piliang\3SubProteomes\Piliang3SP\mgf5ppm\SCX_3SubProteomes5ppm.mgf

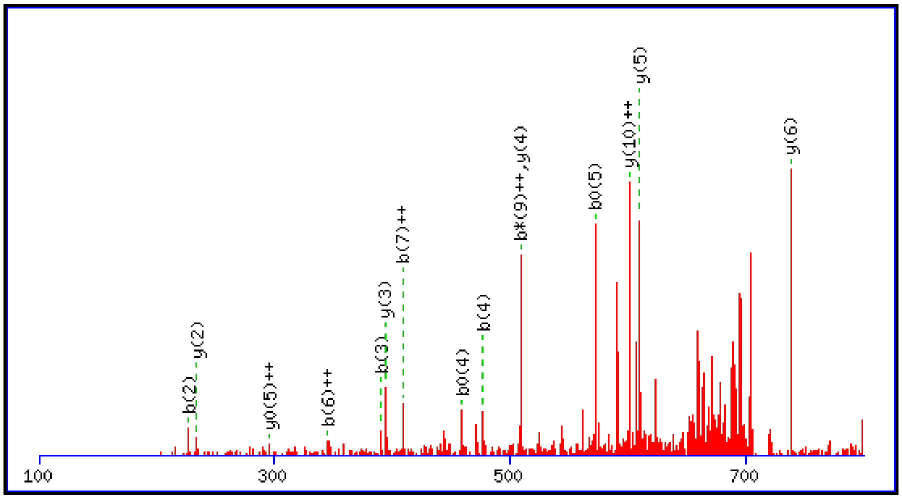

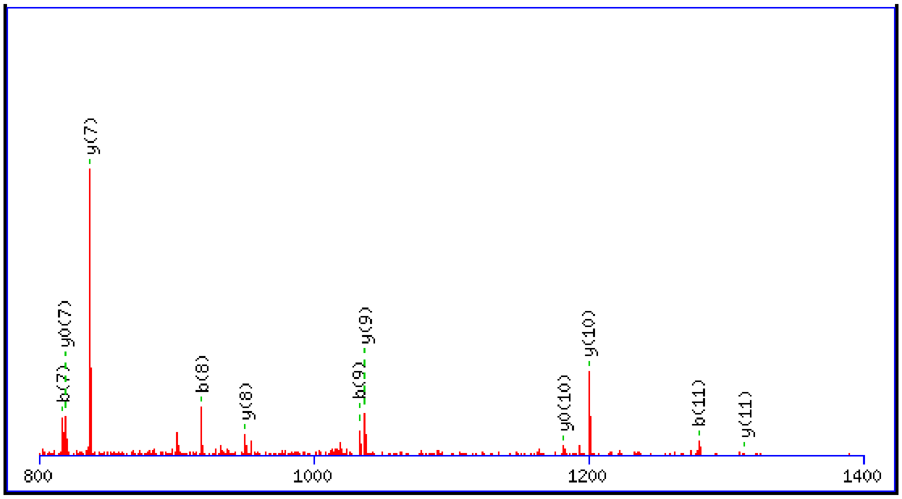

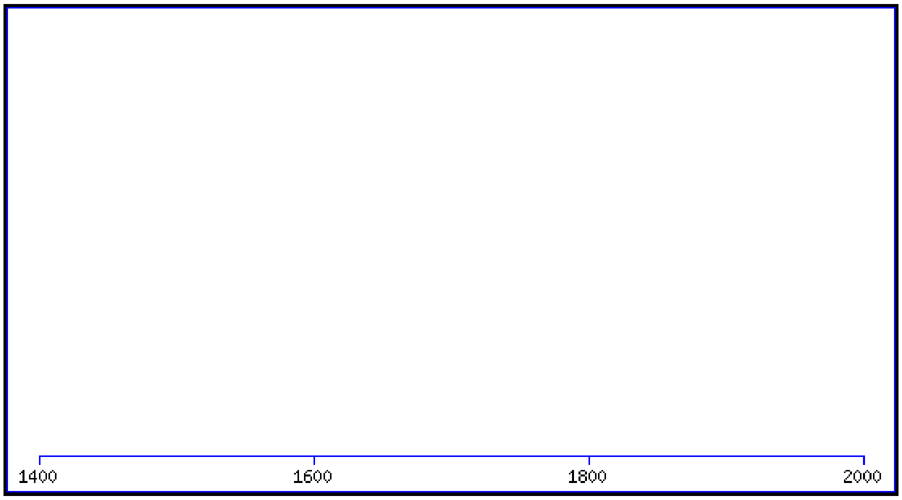

Monoisotopic mass of neutral peptide Mr(calc): 1425.7173
Fixed modifications: Carbamidomethyl (C)
Variable modifications:
N9 : Deamidated_N (N)
Ions Score: 79 **Expect:** 1.6e-006
Matches (Bold Red): 26/112 fragment ions using 41 most intense peaks

#	b	b^{++}	b*	b^{*++}	b^0	b^{0++}	Seq.	y	y^{++}	y*	y^{*++}	y^0	y^{0++}	#
1	114.0913	57.5493					I							12
2	**227.1754**	114.0913					I	**1313.6406**	657.3239	1296.6140	648.8107	1295.6300	648.3186	11
3	**390.2387**	195.6230					Y	**1200.5565**	**600.7819**	1183.5300	592.2686	**1182.5459**	591.7766	10
4	**477.2708**	239.1390			**459.2602**	230.1337	S	**1037.4932**	519.2502	1020.4666	510.7370	1019.4826	510.2449	9
5	590.3548	295.6811			**572.3443**	286.6758	I	**950.4612**	475.7342	933.4346	467.2209	932.4506	466.7289	8
6	689.4232	**345.2153**			671.4127	336.2100	V	**837.3771**	419.1922	820.3505	410.6789	**819.3665**	410.1869	7
7	**817.4818**	**409.2445**	800.4553	400.7313	799.4713	400.2393	Q	**738.3087**	369.6580	721.2821	361.1447	720.2981	360.6527	6
8	**918.5295**	459.7684	901.5029	451.2551	900.5189	450.7631	T	**610.2501**	305.6287	593.2235	297.1154	592.2395	**296.6234**	5
9	**1033.5564**	517.2819	1016.5299	**508.7686**	1015.5459	508.2766	N	**509.2024**	255.1048	492.1759	246.5916	491.1919	246.0996	4
10	1193.5871	597.2972	1176.5605	588.7839	1175.5765	588.2919	C	**394.1755**	197.5914	377.1489	189.0781	376.1649	188.5861	3
11	**1280.6191**	640.8132	1263.5926	632.2999	1262.6085	631.8079	S	**234.1448**	117.5761	217.1183	109.0628	216.1343	108.5708	2
12							K	147.1128	74.0600	130.0863	65.5468			1

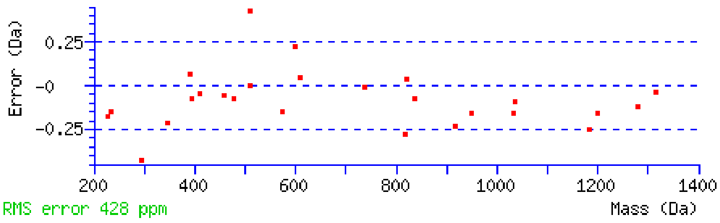

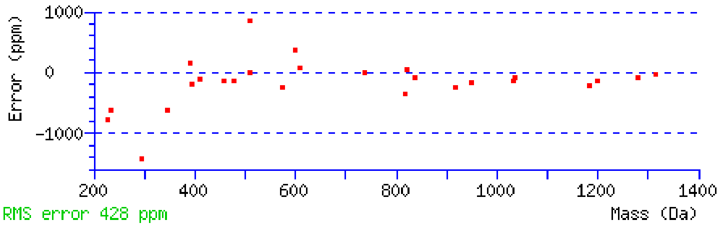

All matches to this query

Score	Mr(calc):	Delta	Sequence
78.6	1425.7173	0.0045	IIYSIVQTNCSK
9.2	1425.7351	-0.0132	IIAINVNDPEAEK
8.0	1425.7228	-0.0009	ITITNDKGRLSK
7.7	1425.7156	0.0063	IITPEAVNIFTK
4.7	1425.7351	-0.0132	IIAINVNDPEAEK
3.9	1424.7177	1.0042	RSIWDVISKNK
3.5	1423.7194	2.0025	TYEDQLSEAKIK
2.9	1425.7129	0.0090	SHVRELLDLHK
2.8	1425.7140	0.0079	LLYTDRYPEEK
2.6	1425.7097	0.0121	LLYYVFPYLR

Spectrum No: 143; Query: 725; Rank: 1

Peptide View

MS/MS Fragmentation of **HIDCASVYGNETEIGEALK**
Found in **IPI00230859**, Tax_Id=10116 Gene_Symbol=Akr1a1 Alcohol dehydrogenase

Match to Query 725: 2105.963888 from(1053.989220,2+)
Title: 091129RatKid_SCX02_30.2916.2916.2.dta
Data file K:\NewmanPaper\Piliang\3SubProteomes\Piliang3SP\mgf5ppm\SCX_3SubProteomes5ppm.mgf

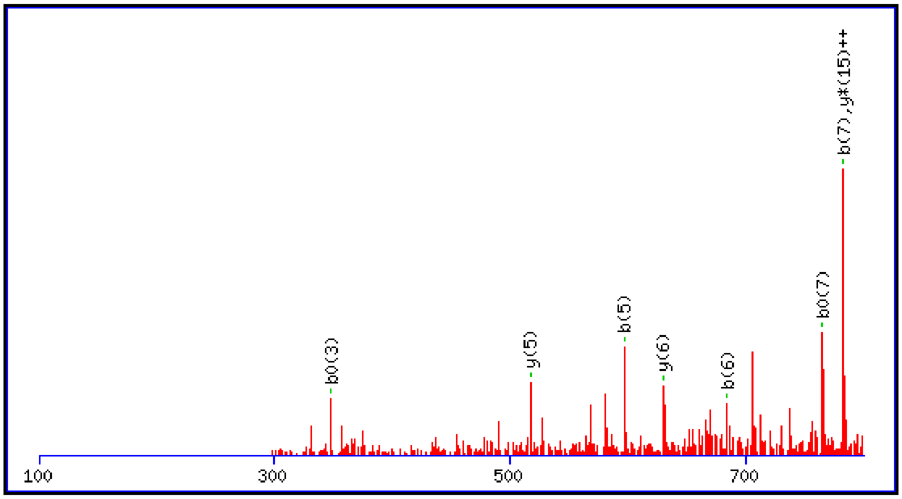

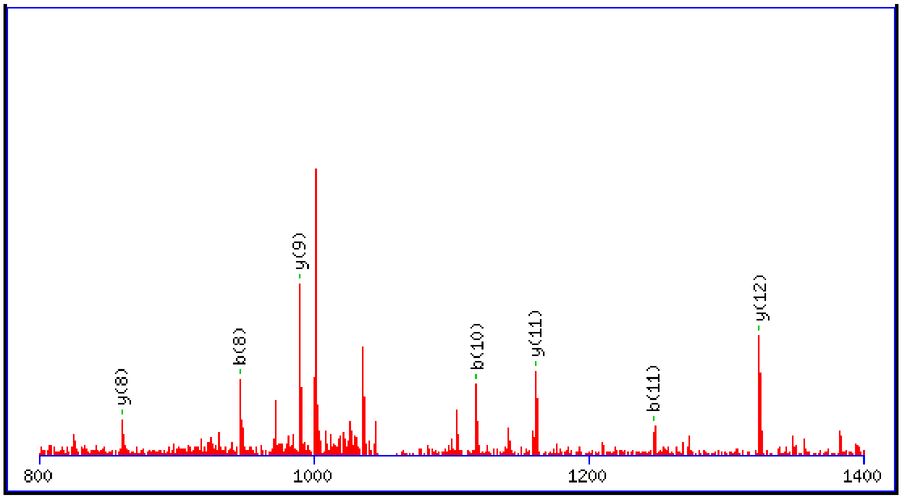

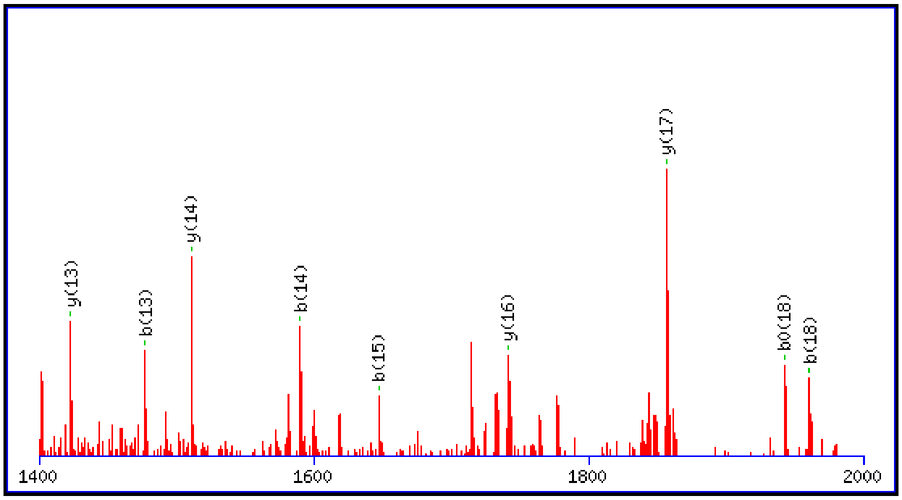

Monoisotopic mass of neutral peptide Mr(calc): 2105.9575
Fixed modifications: Carbamidomethyl (C)
Variable modifications:
N10 : Deamidated_N (N)
Ions Score: 78 **Expect:** 3.7e-006
Matches (Bold Red): 24/188 fragment ions using 39 most intense peaks

#	b	b^{++}	b*	b^{*++}	b^{0}	b^{0++}	Seq.	y	y^{++}	y*	y^{*++}	y^{0}	y^{0++}	#
1	138.0662	69.5367					H							19
2	251.1503	126.0788					I	1969.9059	985.4566	1952.8794	976.9433	1951.8953	976.4513	18
3	366.1772	183.5922			**348.1666**	174.5870	D	**1856.8218**	928.9146	1839.7953	920.4013	1838.8113	919.9093	17
4	526.2078	263.6076			508.1973	254.6023	C	**1741.7949**	871.4011	1724.7684	862.8878	1723.7843	862.3958	16
5	**597.2450**	299.1261			579.2344	290.1208	A	1581.7643	791.3858	1564.7377	**782.8725**	1563.7537	782.3805	15
6	**684.2770**	342.6421			666.2664	333.6368	S	**1510.7271**	755.8672	1493.7006	747.3539	1492.7166	746.8619	14
7	**783.3454**	392.1763			**765.3348**	383.1711	V	**1423.6951**	712.3512	1406.6686	703.8379	1405.6845	703.3459	13
8	**946.4087**	473.7080			928.3982	464.7027	Y	**1324.6267**	662.8170	1307.6001	654.3037	1306.6161	653.8117	12
9	1003.4302	502.2187			985.4196	493.2135	G	**1161.5634**	581.2853	1144.5368	572.7720	1143.5528	572.2800	11
10	**1118.4571**	559.7322	1101.4306	551.2189	1100.4466	550.7269	N	1104.5419	552.7746	1087.5154	544.2613	1086.5313	543.7693	10
11	**1247.4997**	624.2535	1230.4732	615.7402	1229.4892	615.2482	E	**989.5150**	495.2611	972.4884	486.7478	971.5044	486.2558	9
12	1348.5474	674.7773	1331.5209	666.2641	1330.5368	665.7721	T	**860.4724**	430.7398	843.4458	422.2266	842.4618	421.7345	8
13	**1477.5900**	739.2986	1460.5634	730.7854	1459.5794	730.2934	E	759.4247	380.2160	742.3981	371.7027	741.4141	371.2107	7
14	**1590.6741**	795.8407	1573.6475	787.3274	1572.6635	786.8354	I	**630.3821**	315.6947	613.3556	307.1814	612.3715	306.6894	6
15	**1647.6955**	824.3514	1630.6690	815.8381	1629.6850	815.3461	G	**517.2980**	259.1527	500.2715	250.6394	499.2875	250.1474	5
16	1776.7381	888.8727	1759.7116	880.3594	1758.7275	879.8674	E	460.2766	230.6419	443.2500	222.1287	442.2660	221.6366	4
17	1847.7752	924.3913	1830.7487	915.8780	1829.7647	915.3860	A	331.2340	166.1206	314.2074	157.6074			3
18	**1960.8593**	980.9333	1943.8327	972.4200	**1942.8487**	971.9280	L	260.1969	130.6021	243.1703	122.0888			2

19							K	147.1128	74.0600	130.0863	65.5468			1

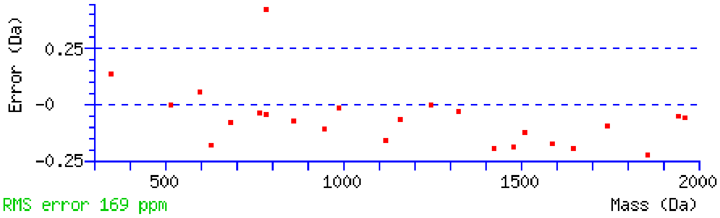

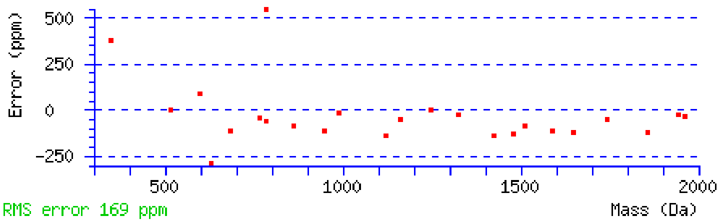

All matches to this query

Score	Mr(calc):	Delta	Sequence
78.5	2105.9575	0.0064	HIDCASVYGNETEIGEALK
20.2	2105.9753	-0.0114	EGRPSGEAFVELESEDEVK
10.9	2104.9735	0.9904	HIDCASVYGNETEIGEALK

Spectrum No: 144; Query: 184; Rank: 1

Peptide View

MS/MS Fragmentation of **YLNETQQLTEK**
Found in **IPI00201262**, Tax_Id=10116 Gene_Symbol=LOC297568;Mug2;Mug1 Alpha-1-inhibitor 3 precursor

Match to Query 184: 1366.662408 from(684.338480,2+)
Title: 091129RatKid_SCX02_11.648.648.2.dta
Data file K:\NewmanPaper\Piliang\3SubProteomes\Piliang3SP\mgf5ppm\SCX_3SubProteomes5ppm.mgf

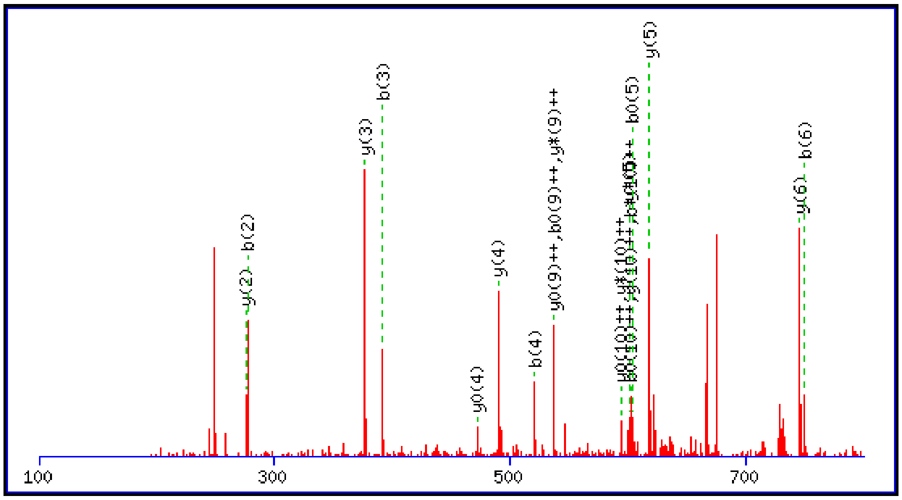

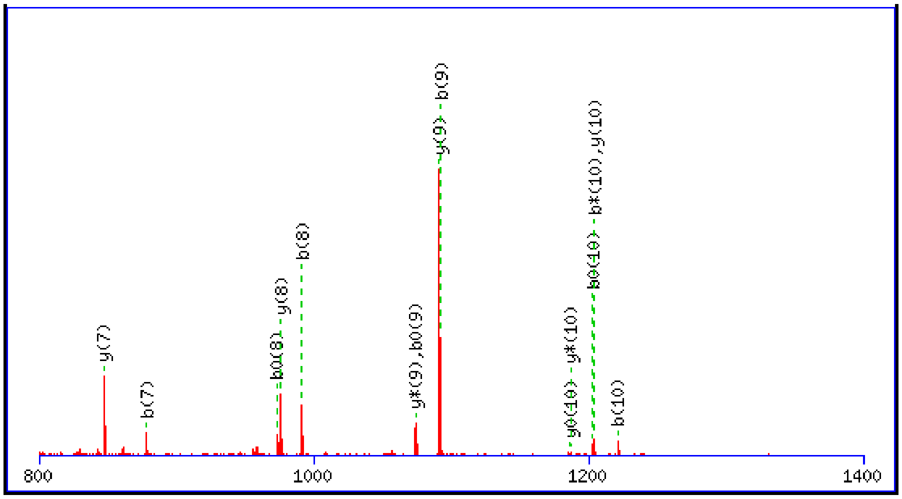

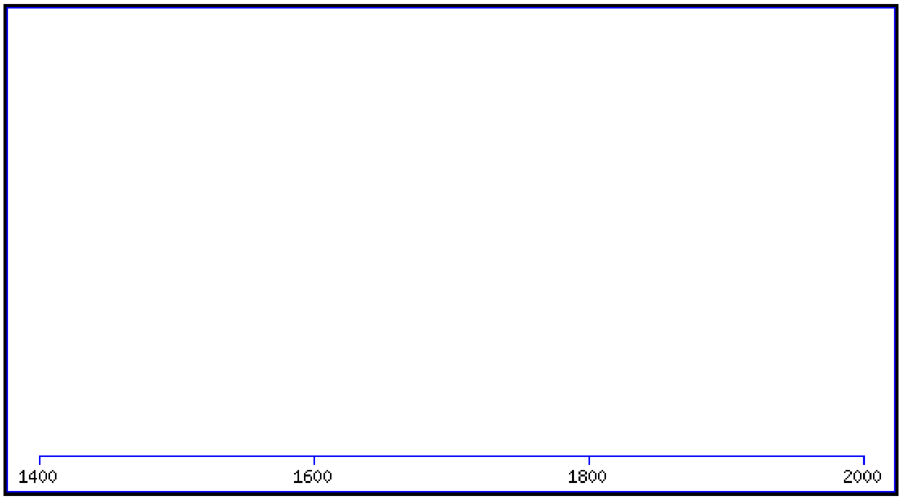

Monoisotopic mass of neutral peptide Mr(calc): 1366.6616
Fixed modifications: Carbamidomethyl (C)
Variable modifications:
N3 : Deamidated_N (N)
Ions Score: 78 **Expect:** 2.2e-006
Matches (Bold Red): 35/108 fragment ions using 44 most intense peaks

#	b	b^{++}	b*	b*$^{++}$	b^{0}	b^{0++}	Seq.	y	y^{++}	y*	y*$^{++}$	y^{0}	y^{0++}	#
1	164.0706	82.5389					Y							11
2	**277.1547**	139.0810					L	**1204.6056**	**602.8064**	**1187.5790**	**594.2931**	**1186.5950**	**593.8011**	10
3	**392.1816**	196.5944	375.1551	188.0812			N	**1091.5215**	546.2644	**1074.4950**	**537.7511**	1073.5109	**537.2591**	9
4	**521.2242**	261.1157	504.1976	252.6025	503.2136	252.1105	E	**976.4946**	488.7509	959.4680	480.2376	958.4840	479.7456	8
5	622.2719	311.6396	605.2453	303.1263	**604.2613**	302.6343	T	**847.4520**	424.2296	830.4254	415.7164	829.4414	415.2243	7
6	**750.3305**	375.6689	733.3039	367.1556	732.3199	366.6636	Q	**746.4043**	373.7058	729.3777	365.1925	728.3937	364.7005	6
7	**878.3890**	439.6982	861.3625	431.1849	860.3785	430.6929	Q	**618.3457**	309.6765	**601.3192**	301.1632	600.3352	300.6712	5
8	**991.4731**	496.2402	974.4465	487.7269	**973.4625**	487.2349	L	**490.2871**	245.6472	473.2606	237.1339	**472.2766**	236.6419	4
9	**1092.5208**	546.7640	1075.4942	538.2508	**1074.5102**	**537.7587**	T	**377.2031**	189.1052	360.1765	180.5919	359.1925	180.0999	3
10	**1221.5634**	611.2853	**1204.5368**	**602.7720**	**1203.5528**	**602.2800**	E	**276.1554**	138.5813	259.1288	130.0681	258.1448	129.5761	2
11							K	147.1128	74.0600	130.0863	65.5468			1

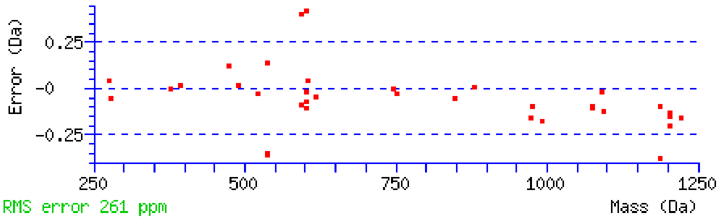

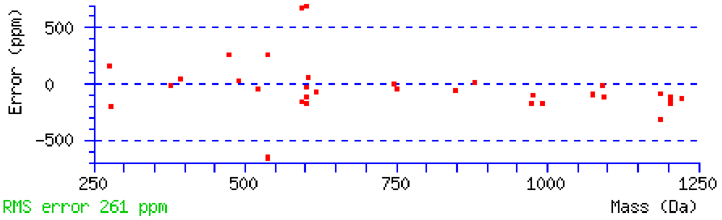

All matches to this query

Score	Mr(calc):	Delta	Sequence
78.4	1366.6616	0.0008	YLNETQQLTEK
19.2	1366.6728	-0.0104	YITENNRSEIK
18.9	1366.6728	-0.0104	YITENNRSEIK
11.4	1366.6680	-0.0055	ITDNLLQLMAR
10.0	1366.6517	0.0107	ALFTSGWNNTEK
8.9	1365.6524	1.0100	YNPNNSSAKITR
8.6	1365.6524	1.0100	YNPNNSSAKITR
8.5	1366.6517	0.0107	NNSTWFKLNDK
5.8	1366.6663	-0.0039	LYRGNNTCGIAK
5.7	1366.6728	-0.0104	EYTQNKKEAEK

Spectrum No: 145; Query: 737; Rank: 1

Peptide View

MS/MS Fragmentation of **TLQEDPSWNVTEFHPTPK**
Found in **IPI00230862**, Tax_Id=10116 Gene_Symbol=Anpep Aminopeptidase N

Match to Query 737: 2126.000508 from(1064.007530,2+)
Title: 091129RatKid_SCX02_23.2101.2101.2.dta
Data file K:\NewmanPaper\Piliang\3SubProteomes\Piliang3SP\mgf5ppm\SCX_3SubProteomes5ppm.mgf

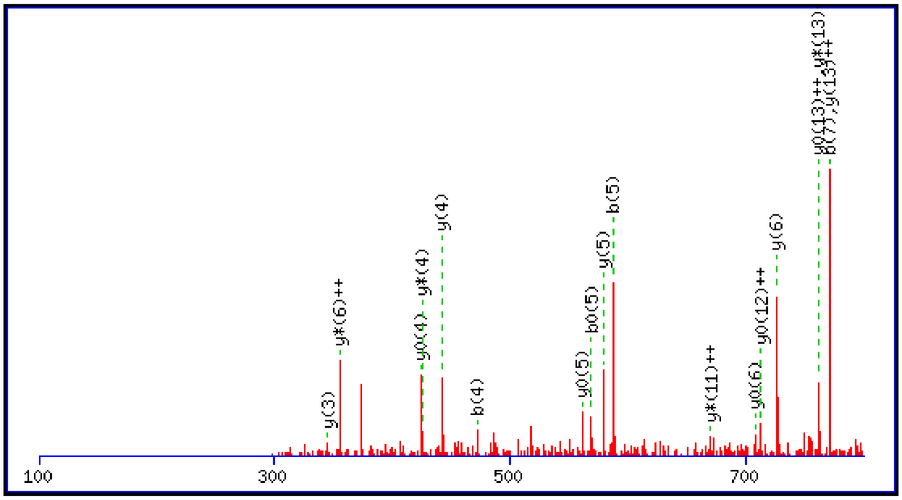

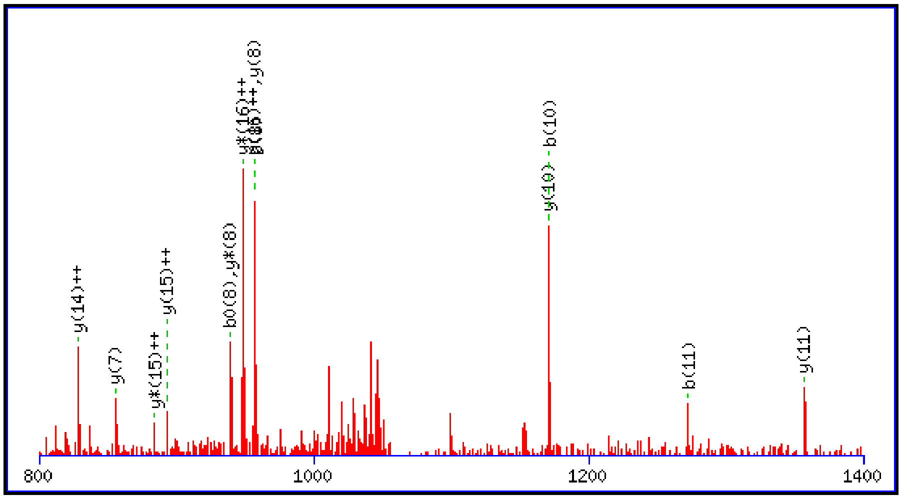

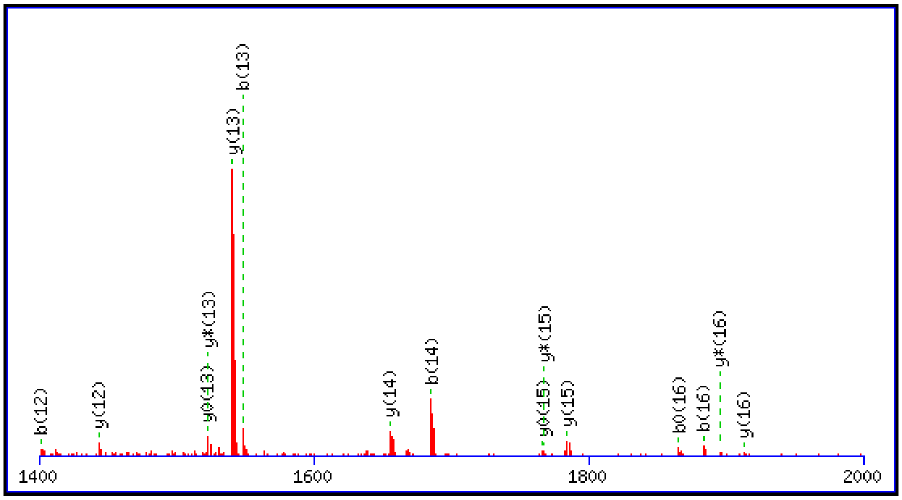

Monoisotopic mass of neutral peptide Mr(calc): 2125.9957
Fixed modifications: Carbamidomethyl (C)
Variable modifications:
N9 : Deamidated_N (N)
Ions Score: 78 **Expect:** 3.9e-006
Matches (Bold Red): 47/196 fragment ions using 85 most intense peaks

#	b	b^{++}	b^{*}	b^{*++}	b^{0}	b^{0++}	Seq.	y	y^{++}	y^{*}	y^{*++}	y^{0}	y^{0++}	#
1	102.0550	51.5311			84.0444	42.5258	T							18
2	215.1390	108.0731			197.1285	99.0679	L	2025.9552	1013.4813	2008.9287	1004.9680	2007.9447	1004.4760	17
3	343.1976	172.1024	326.1710	163.5892	325.1870	163.0972	Q	**1912.8712**	**956.9392**	**1895.8446**	**948.4260**	1894.8606	947.9339	16
4	**472.2402**	236.6237	455.2136	228.1105	454.2296	227.6185	E	**1784.8126**	**892.9099**	**1767.7861**	**884.3967**	**1766.8020**	883.9047	15
5	**587.2671**	294.1372	570.2406	285.6239	**569.2566**	285.1319	D	**1655.7700**	**828.3886**	1638.7435	819.8754	1637.7594	819.3834	14
6	684.3199	342.6636	667.2933	334.1503	666.3093	333.6583	P	**1540.7431**	**770.8752**	**1523.7165**	**762.3619**	**1522.7325**	**761.8699**	13
7	**771.3519**	386.1796	754.3254	377.6663	753.3414	377.1743	S	**1443.6903**	722.3488	1426.6638	713.8355	1425.6797	**713.3435**	12
8	**957.4312**	479.2193	940.4047	470.7060	**939.4207**	470.2140	W	**1356.6583**	678.8328	1339.6317	**670.3195**	1338.6477	669.8275	11
9	1072.4582	536.7327	1055.4316	528.2195	1054.4476	527.7274	N	**1170.5790**	585.7931	1153.5524	577.2798	1152.5684	576.7878	10
10	**1171.5266**	586.2669	1154.5000	577.7537	1153.5160	577.2616	V	1055.5520	528.2796	1038.5255	519.7664	1037.5415	519.2744	9
11	**1272.5743**	636.7908	1255.5477	628.2775	1254.5637	627.7855	T	**956.4836**	478.7454	**939.4571**	470.2322	938.4730	469.7402	8
12	**1401.6169**	701.3121	1384.5903	692.7988	1383.6063	692.3068	E	**855.4359**	428.2216	838.4094	419.7083	837.4254	419.2163	7
13	**1548.6853**	774.8463	1531.6587	766.3330	1530.6747	765.8410	F	**726.3933**	363.7003	709.3668	**355.1870**	**708.3828**	354.6950	6
14	**1685.7442**	843.3757	1668.7176	834.8625	1667.7336	834.3704	H	**579.3249**	290.1661	562.2984	281.6528	**561.3144**	281.1608	5
15	1782.7970	891.9021	1765.7704	883.3888	1764.7864	882.8968	P	**442.2660**	221.6366	**425.2395**	213.1234	**424.2554**	212.6314	4
16	**1883.8446**	942.4260	1866.8181	933.9127	**1865.8341**	933.4207	T	**345.2132**	173.1103	328.1867	164.5970	327.2027	164.1050	3
17	1980.8974	990.9523	1963.8708	982.4391	1962.8868	981.9471	P	244.1656	122.5864	227.1390	114.0731			2
18							K	147.1128	74.0600	130.0863	65.5468			1

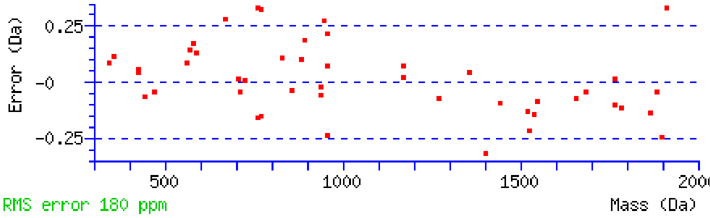

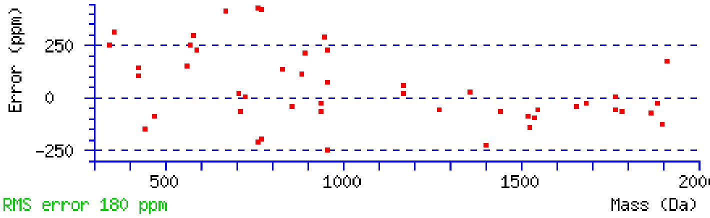

All matches to this query

Score	Mr(calc):	Delta	Sequence
78.0	2125.9957	0.0049	TLQEDPSWNVTEFHPTPK
11.4	2125.0116	0.9889	TLQEDPSWNVTEFHPTPK
6.3	2126.0144	-0.0139	KDENLVTGEKQEVSVSVGK

5.3	2125.9850	0.0155	LLRTLPADDVGKTYYNK
5.2	2126.0061	-0.0056	ETLSSTSTLGFRIEGIKK
4.1	2124.9827	1.0178	DEKLALSSKEETVQEDPK
3.8	2124.9827	1.0178	DEKLALSSKEETVQEDPK
3.7	2124.9827	1.0178	DEKLALSSKEETVQEDPK
3.5	2124.9827	1.0178	NKIQSELDGLQSDNEILK
3.5	2123.9922	2.0083	IKTMVTEQAAQGSSPPDLR

Spectrum No: 146; Query: 997; Rank: 1

Peptide View

MS/MS Fragmentation of **HIDCASVYGNETEIGEALKESVGAGK**
Found in **IPI00230859**, Tax_Id=10116 Gene_Symbol=Akr1a1 Alcohol dehydrogenase

Match to Query 997: 2734.285182 from(912.435670,3+)
Title: 091129RatKid_SCX02_34.3973.3973.3.dta
Data file K:\NewmanPaper\Piliang\3SubProteomes\Piliang3SP\mgf5ppm\SCX_3SubProteomes5ppm.mgf

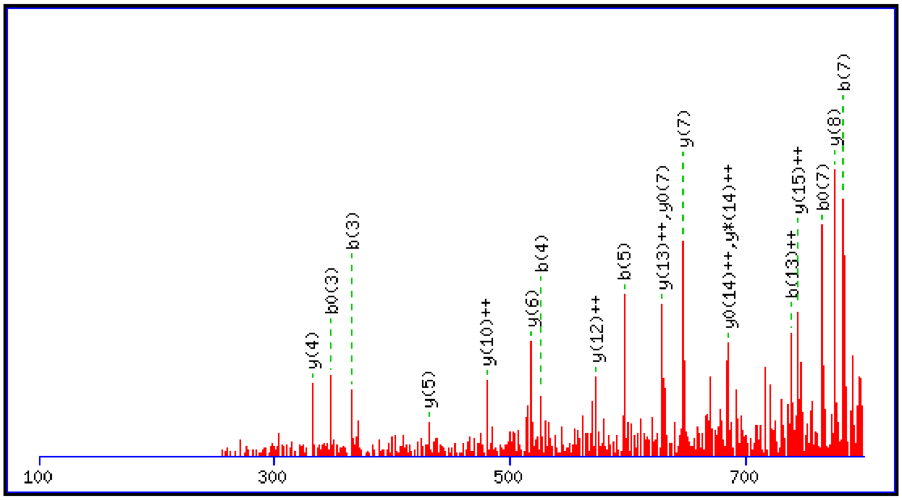

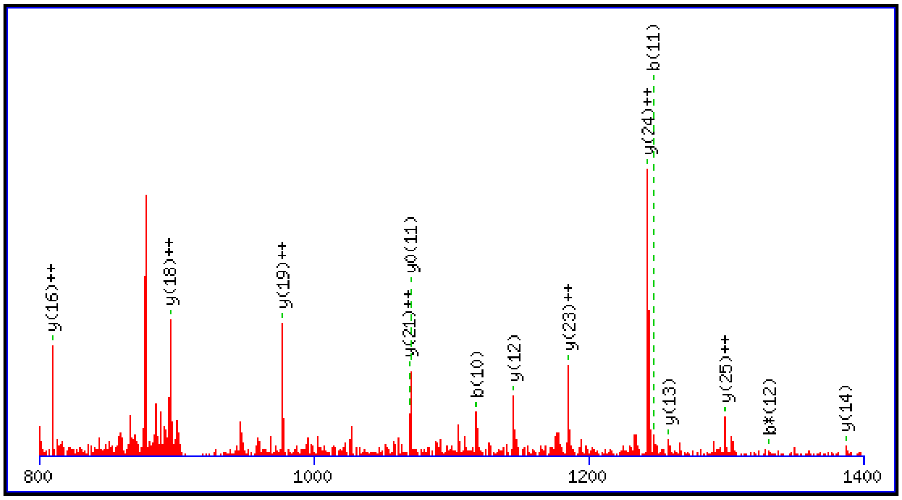

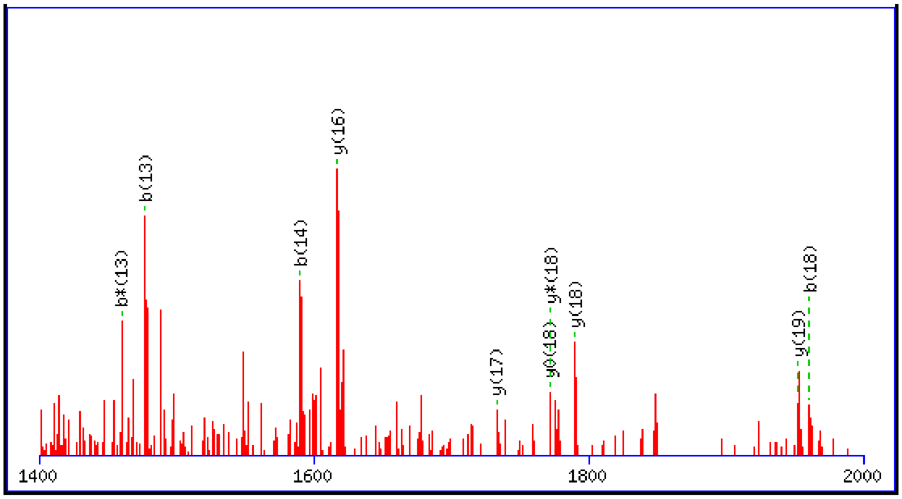

Monoisotopic mass of neutral peptide Mr(calc): 2734.2756
Fixed modifications: Carbamidomethyl (C)
Variable modifications:
N10 : Deamidated_N (N)
Ions Score: 78 **Expect:** 5e-006
Matches (Bold Red): 43/268 fragment ions using 70 most intense peaks

#	b	b^{++}	b*	b*$^{++}$	b^{0}	b^{0++}	Seq.	y	y^{++}	y*	y*$^{++}$	y^{0}	y^{0++}	#
1	138.0662	69.5367					H							26
2	251.1503	126.0788					I	2598.2239	**1299.6156**	2581.1974	1291.1023	2580.2134	1290.6103	25
3	**366.1772**	183.5922			**348.1666**	174.5870	D	2485.1399	**1243.0736**	2468.1133	1234.5603	2467.1293	1234.0683	24
4	**526.2078**	263.6076			508.1973	254.6023	C	2370.1129	**1185.5601**	2353.0864	1177.0468	2352.1024	1176.5548	23
5	**597.2450**	299.1261			579.2344	290.1208	A	2210.0823	1105.5448	2193.0557	1097.0315	2192.0717	1096.5395	22
6	684.2770	342.6421			666.2664	333.6368	S	2139.0452	**1070.0262**	2122.0186	1061.5130	2121.0346	1061.0209	21
7	**783.3454**	392.1763			**765.3348**	383.1711	V	2052.0132	1026.5102	2034.9866	1017.9969	2034.0026	1017.5049	20
8	946.4087	473.7080			928.3982	464.7027	Y	**1952.9447**	**976.9760**	1935.9182	968.4627	1934.9342	967.9707	19
9	1003.4302	502.2187			985.4196	493.2135	G	**1789.8814**	**895.4443**	**1772.8549**	886.9311	**1771.8708**	886.4391	18
10	**1118.4571**	559.7322	1101.4306	551.2189	1100.4466	550.7269	N	**1732.8599**	866.9336	1715.8334	858.4203	1714.8494	857.9283	17
11	**1247.4997**	624.2535	1230.4732	615.7402	1229.4892	615.2482	E	**1617.8330**	**809.4201**	1600.8065	800.9069	1599.8224	800.4149	16
12	1348.5474	674.7773	**1331.5209**	666.2641	1330.5368	665.7721	T	1488.7904	**744.8988**	1471.7639	736.3856	1470.7798	735.8936	15
13	**1477.5900**	**739.2986**	**1460.5634**	730.7854	1459.5794	730.2934	E	**1387.7427**	694.3750	1370.7162	**685.8617**	1369.7322	**685.3697**	14
14	**1590.6741**	795.8407	1573.6475	787.3274	1572.6635	786.8354	I	**1258.7001**	**629.8537**	1241.6736	621.3404	1240.6896	620.8484	13
15	1647.6955	824.3514	1630.6690	815.8381	1629.6850	815.3461	G	**1145.6161**	**573.3117**	1128.5895	564.7984	1127.6055	564.3064	12
16	1776.7381	888.8727	1759.7116	880.3594	1758.7275	879.8674	E	1088.5946	544.8009	1071.5681	536.2877	**1070.5840**	535.7957	11
17	1847.7752	924.3913	1830.7487	915.8780	1829.7647	915.3860	A	959.5520	**480.2796**	942.5255	471.7664	941.5415	471.2744	10
18	**1960.8593**	980.9333	1943.8327	972.4200	1942.8487	971.9280	L	888.5149	444.7611	871.4884	436.2478	870.5043	435.7558	9
19	2088.9543	1044.9808	2071.9277	1036.4675	2070.9437	1035.9755	K	**775.4308**	388.2191	758.4043	379.7058	757.4203	379.2138	8
20	2217.9968	1109.5021	2200.9703	1100.9888	2199.9863	1100.4968	E	**647.3359**	324.1716	630.3093	315.6583	**629.3253**	315.1663	7
21	2305.0289	1153.0181	2288.0023	1144.5048	2287.0183	1144.0128	S	**518.2933**	259.6503	501.2667	251.1370	500.2827	250.6450	6
22	2404.0973	1202.5523	2387.0707	1194.0390	2386.0867	1193.5470	V	**431.2613**	216.1343	414.2347	207.6210			5
23	2461.1188	1231.0630	2444.0922	1222.5497	2443.1082	1222.0577	G	**332.1928**	166.6001	315.1663	158.0868			4
24	2532.1559	1266.5816	2515.1293	1258.0683	2514.1453	1257.5763	A	275.1714	138.0893	258.1448	129.5761			3
25	2589.1773	1295.0923	2572.1508	1286.5790	2571.1668	1286.0870	G	204.1343	102.5708	187.1077	94.0575			2
26							K	147.1128	74.0600	130.0863	65.5468			1

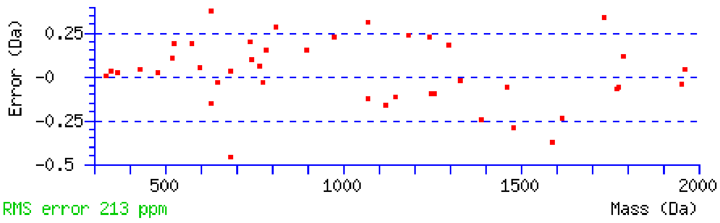

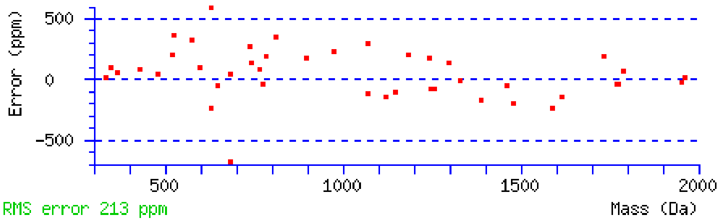

All matches to this query

Score	Mr(calc):	Delta	Sequence
78.0	2734.2756	0.0096	HIDCASVYGNETEIGEALKESVGAGK
35.1	2733.2915	0.9936	HIDCASVYGNETEIGEALKESVGAGK
1.1	2734.2703	0.0149	TPNGLILSHLPNGPTAHFKMSSVR

Spectrum No: 147; Query: 1163; Rank: 1

Peptide View

MS/MS Fragmentation of **ATISPGFTTLYVHQLPNSTYTQAMVETMEELVR**
Found in **IPI00471666**, Tax_Id=10116 Gene_Symbol=MGC94145 Uncharacterized protein C1orf179 homolog

Match to Query 1163: 3727.819662 from(1243.613830,3+)
Title: 091129RatKid_SCX02_15.5703.5703.3.dta
Data file K:\NewmanPaper\Piliang\3SubProteomes\Piliang3SP\mgf5ppm\SCX_3SubProteomes5ppm.mgf

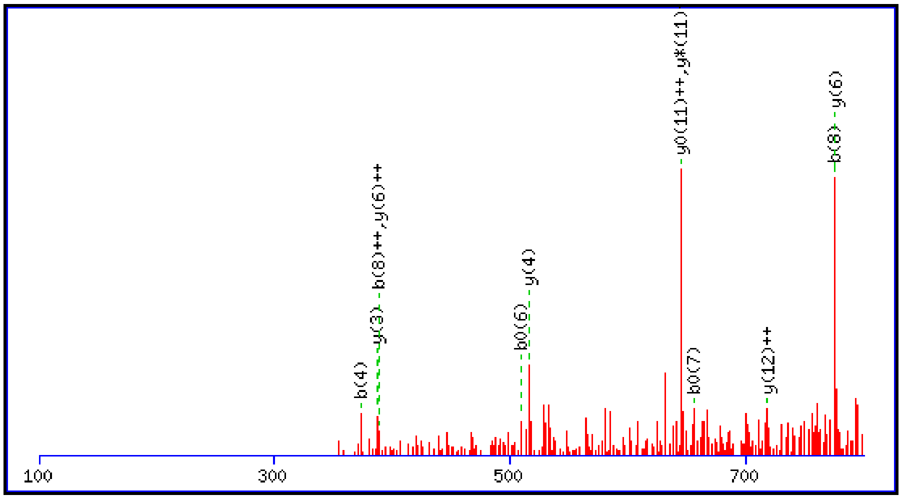

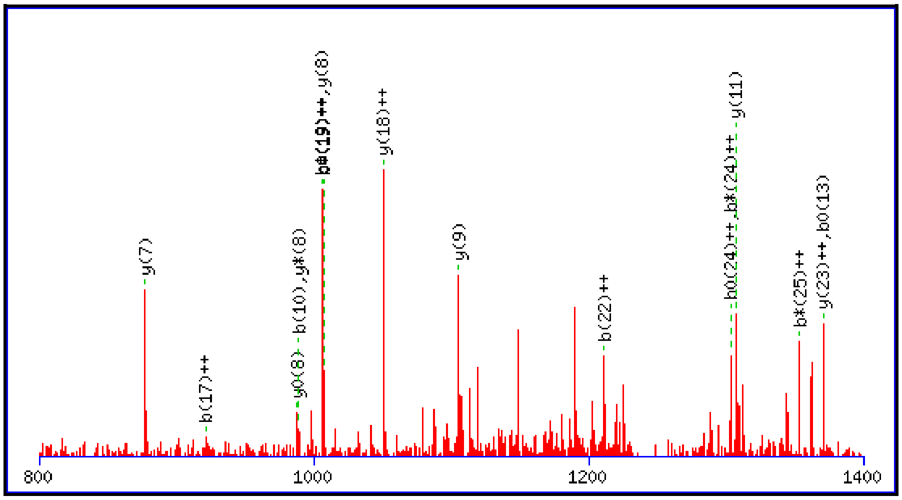

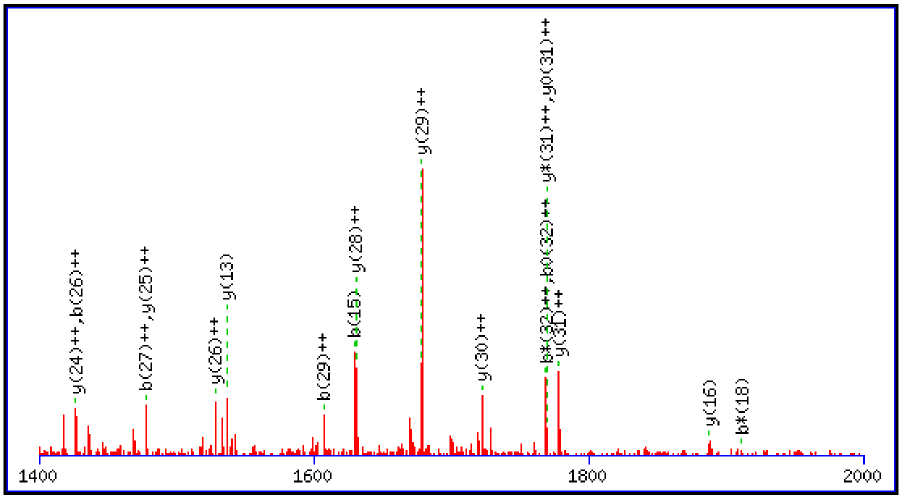

Monoisotopic mass of neutral peptide Mr(calc): 3727.8113
Fixed modifications: Carbamidomethyl (C)
Variable modifications:
N17 : Deamidated_N (N)
Ions Score: 78 **Expect:** 4.6e-006
Matches (Bold Red): 48/350 fragment ions using 72 most intense peaks

#	b	b^{++}	b*	b^{*++}	b^0	b^{0++}	Seq.	y	y^{++}	y*	y^{*++}	y^0	y^{0++}	#
1	72.0444	36.5258					A							33
2	173.0921	87.0497			155.0815	78.0444	T	3657.7815	1829.3944	3640.7550	1820.8811	3639.7709	1820.3891	32
3	286.1761	143.5917			268.1656	134.5864	I	3556.7338	**1778.8706**	3539.7073	**1770.3573**	3538.7233	**1769.8653**	31
4	**373.2082**	187.1077			355.1976	178.1024	S	3443.6498	**1722.3285**	3426.6232	1713.8152	3425.6392	1713.3232	30
5	470.2609	235.6341			452.2504	226.6288	P	3356.6177	**1678.8125**	3339.5912	1670.2992	3338.6072	1669.8072	29
6	527.2824	264.1448			**509.2718**	255.1396	G	3259.5650	**1630.2861**	3242.5384	1621.7729	3241.5544	1621.2808	28
7	674.3508	337.6790			**656.3402**	328.6738	F	3202.5435	1601.7754	3185.5170	1593.2621	3184.5329	1592.7701	27
8	**775.3985**	**388.2029**			757.3879	379.1976	T	3055.4751	**1528.2412**	3038.4485	1519.7279	3037.4645	1519.2359	26
9	876.4462	438.7267			858.4356	429.7214	T	2954.4274	**1477.7173**	2937.4009	1469.2041	2936.4169	1468.7121	25
10	**989.5302**	495.2688			971.5197	486.2635	L	2853.3797	**1427.1935**	2836.3532	1418.6802	2835.3692	1418.1882	24
11	1152.5936	576.8004			1134.5830	567.7951	Y	2740.2957	**1370.6515**	2723.2691	1362.1382	2722.2851	1361.6462	23
12	1251.6620	626.3346			1233.6514	617.3293	V	2577.2323	1289.1198	2560.2058	1280.6065	2559.2218	1280.1145	22
13	1388.7209	694.8641			**1370.7103**	685.8588	H	2478.1639	1239.5856	2461.1374	1231.0723	2460.1534	1230.5803	21
14	1516.7795	758.8934	1499.7529	750.3801	1498.7689	749.8881	Q	2341.1050	1171.0561	2324.0785	1162.5429	2323.0945	1162.0509	20
15	**1629.8635**	815.4354	1612.8370	806.9221	1611.8530	806.4301	L	2213.0464	1107.0269	2196.0199	1098.5136	2195.0359	1098.0216	19
16	1726.9163	863.9618	1709.8897	855.4485	1708.9057	854.9565	P	2099.9624	**1050.4848**	2082.9358	1041.9716	2081.9518	1041.4795	18
17	1841.9432	**921.4752**	1824.9167	912.9620	1823.9327	912.4700	N	2002.9096	1001.9584	1985.8831	993.4452	1984.8991	992.9532	17
18	1928.9753	964.9913	**1911.9487**	956.4780	1910.9647	955.9860	S	**1887.8827**	944.4450	1870.8561	935.9317	1869.8721	935.4397	16
19	2030.0229	1015.5151	2012.9964	**1007.0018**	2012.0124	**1006.5098**	T	1800.8507	900.9290	1783.8241	892.4157	1782.8401	891.9237	15
20	2193.0863	1097.0468	2176.0597	1088.5335	2175.0757	1088.0415	Y	1699.8030	850.4051	1682.7764	841.8918	1681.7924	841.3998	14
21	2294.1339	1147.5706	2277.1074	1139.0573	2276.1234	1138.5653	T	**1536.7396**	768.8735	1519.7131	760.3602	1518.7291	759.8682	13
22	2422.1925	**1211.5999**	2405.1660	1203.0866	2404.1819	1202.5946	Q	1435.6920	**718.3496**	1418.6654	709.8363	1417.6814	709.3443	12
23	2493.2296	1247.1185	2476.2031	1238.6052	2475.2191	1238.1132	A	**1307.6334**	654.3203	1290.6068	**645.8071**	1289.6228	**645.3150**	11
24	2624.2701	1312.6387	2607.2436	**1304.1254**	2606.2595	**1303.6334**	M	1236.5963	618.8018	1219.5697	610.2885	1218.5857	609.7965	10
25	2723.3385	1362.1729	2706.3120	**1353.6596**	2705.3280	1353.1676	V	**1105.5558**	553.2815	1088.5292	544.7683	1087.5452	544.2762	9
26	2852.3811	**1426.6942**	2835.3546	1418.1809	2834.3706	1417.6889	E	**1006.4874**	503.7473	**989.4608**	495.2341	**988.4768**	494.7420	8
27	2953.4288	**1477.2180**	2936.4023	1468.7048	2935.4182	1468.2128	T	**877.4448**	439.2260	860.4182	430.7128	859.4342	430.2207	7
28	3084.4693	1542.7383	3067.4427	1534.2250	3066.4587	1533.7330	M	**776.3971**	**388.7022**	759.3706	380.1889	758.3865	379.6969	6
29	3213.5119	**1607.2596**	3196.4853	1598.7463	3195.5013	1598.2543	E	**645.3566**	323.1819	628.3301	314.6687	627.3461	314.1767	5
30	3342.5545	1671.7809	3325.5279	1663.2676	3324.5439	1662.7756	E	**516.3140**	258.6606	499.2875	250.1474	498.3035	249.6554	4
31	3455.6385	1728.3229	3438.6120	1719.8096	3437.6280	1719.3176	L	**387.2714**	194.1394	370.2449	185.6261			3

32	3554.7069	1777.8571	3537.6804	1769.3438	3536.6964	1768.8518	V	274.1874	137.5973	257.1608	129.0840			2
33							R	175.1190	88.0631	158.0924	79.5498			1

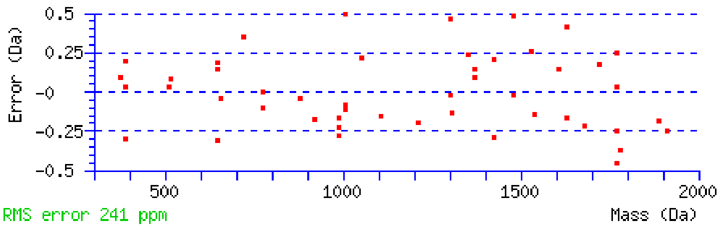

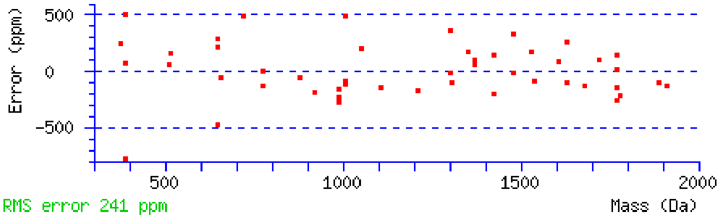

All matches to this query

Score	Mr(calc):	Delta	Sequence
77.9	3727.8113	0.0083	ATISPGFTTLYVHQLPNSTYTQAMVETMEELVR
47.7	3726.8273	0.9923	ATISPGFTTLYVHQLPNSTYTQAMVETMEELVR
5.3	3727.8367	-0.0170	ALSTCSSHLILILFYYTAIVILSVTHLAGRR
3.9	3726.8159	1.0037	LGTVVSVWDAVDMGGSASQDILTSSPLAGPSSHRSDK
3.6	3727.8198	-0.0001	NLTWTLSNLCRNKNPAPPLHAVEQILPTLVR
3.0	3727.8079	0.0117	SLVAGGSASLVAQSITVPIDVVSQHLMMQRKGEK
3.0	3727.8079	0.0117	SLVAGGSASLVAQSITVPIDVVSQHLMMQRKGEK
2.8	3726.8474	0.9723	MAQTAVTNNTAAATVLNQVLSKVAMSQPLFNQLR
1.8	3726.8070	1.0127	CPLAYVVSLLIPSIFLMLVDLGSFYLPPNCR
1.8	3727.7910	0.0287	CPLAYVVSLLIPSIFLMLVDLGSFYLPPNCR

Spectrum No: 148; Query: 783; Rank: 1

Peptide View

MS/MS Fragmentation of **TGEANLTQLYLQEALDFIR**
Found in **IPI00359847**, Tax_Id=10116 Gene_Symbol=Galns N-acetylgalactosamine-6-sulfatase precursor

Match to Query 783: 2195.118788 from(1098.566670,2+)
Title: 091129RatKid_SCX02_16.6376.6376.2.dta
Data file K:\NewmanPaper\Piliang\3SubProteomes\Piliang3SP\mgf5ppm\SCX_3SubProteomes5ppm.mgf

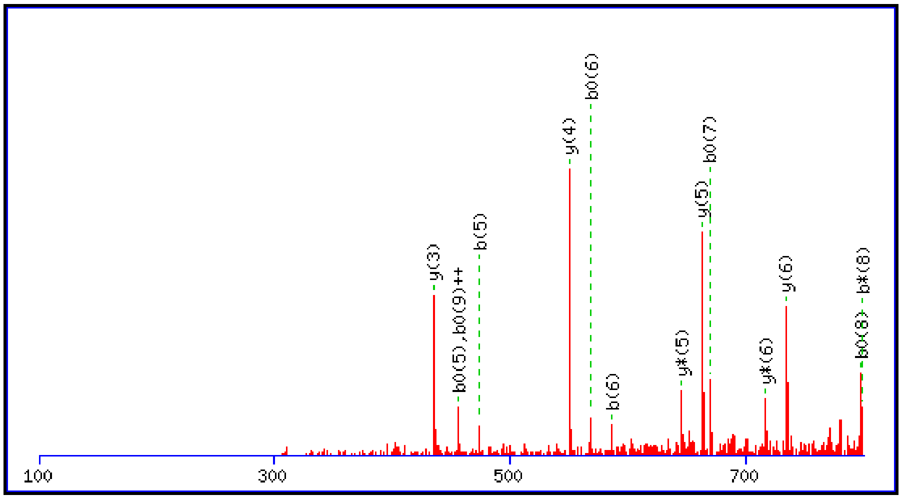

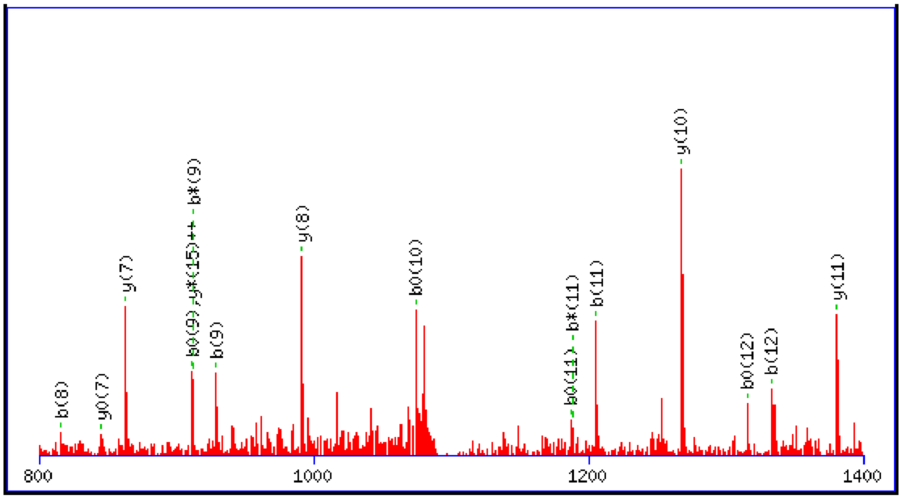

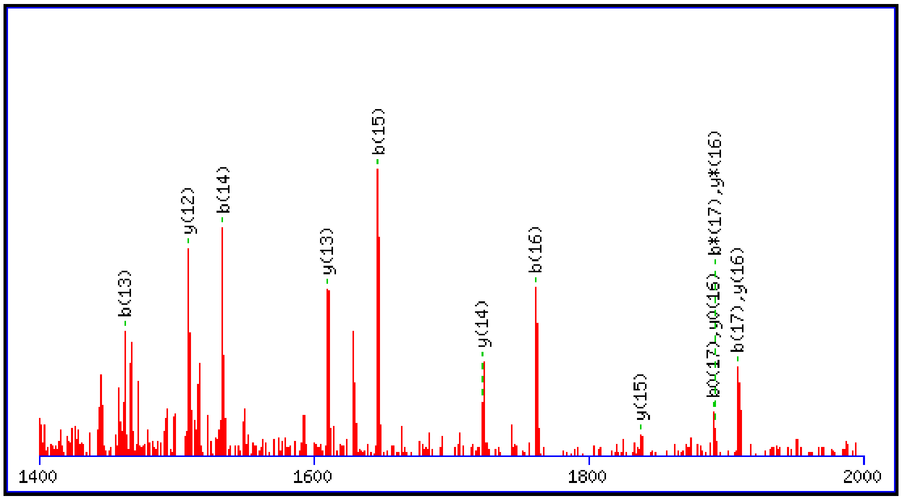

Monoisotopic mass of neutral peptide Mr(calc): 2195.1110
Fixed modifications: Carbamidomethyl (C)
Variable modifications:
N5 : Deamidated_N (N)
Ions Score: 78 **Expect:** 2.9e-006
Matches (Bold Red): 44/202 fragment ions using 73 most intense peaks

#	b	b^{++}	b*	b^{*++}	b^0	b^{0++}	Seq.	y	y^{++}	y*	y^{*++}	y^0	y^{0++}	#
1	102.0550	51.5311			84.0444	42.5258	T							19
2	159.0764	80.0418			141.0659	71.0366	G	2095.0706	1048.0389	2078.0441	1039.5257	2077.0600	1039.0337	18
3	288.1190	144.5631			270.1084	135.5579	E	2038.0491	1019.5282	2021.0226	1011.0149	2020.0386	1010.5229	17
4	359.1561	180.0817			341.1456	171.0764	A	**1909.0066**	955.0069	**1891.9800**	946.4936	**1890.9960**	946.0016	16
5	**474.1831**	237.5952	457.1565	229.0819	**456.1725**	228.5899	N	**1837.9694**	919.4884	1820.9429	**910.9751**	1819.9589	910.4831	15
6	**587.2671**	294.1372	570.2406	285.6239	**569.2566**	285.1319	L	**1722.9425**	861.9749	1705.9160	853.4616	1704.9319	852.9696	14
7	688.3148	344.6610	671.2883	336.1478	**670.3042**	335.6558	T	**1609.8584**	805.4329	1592.8319	796.9196	1591.8479	796.4276	13
8	**816.3734**	408.6903	**799.3468**	400.1771	**798.3628**	399.6850	Q	**1508.8108**	754.9090	1491.7842	746.3957	1490.8002	745.9037	12
9	**929.4574**	465.2324	**912.4309**	456.7191	**911.4469**	**456.2271**	L	**1380.7522**	690.8797	1363.7256	682.3665	1362.7416	681.8744	11
10	1092.5208	546.7640	1075.4942	538.2508	**1074.5102**	537.7587	Y	**1267.6681**	634.3377	1250.6416	625.8244	1249.6576	625.3324	10
11	**1205.6048**	603.3061	**1188.5783**	594.7928	**1187.5943**	594.3008	L	1104.6048	552.8060	1087.5782	544.2928	1086.5942	543.8007	9
12	**1333.6634**	667.3353	1316.6369	658.8221	**1315.6529**	658.3301	Q	**991.5207**	496.2640	974.4942	487.7507	973.5102	487.2587	8
13	**1462.7060**	731.8566	1445.6795	723.3434	1444.6954	722.8514	E	**863.4621**	432.2347	846.4356	423.7214	**845.4516**	423.2294	7
14	**1533.7431**	767.3752	1516.7166	758.8619	1515.7326	758.3699	A	**734.4196**	367.7134	**717.3930**	359.2001	716.4090	358.7081	6
15	**1646.8272**	823.9172	1629.8006	815.4040	1628.8166	814.9119	L	**663.3824**	332.1949	**646.3559**	323.6816	645.3719	323.1896	5
16	**1761.8541**	881.4307	1744.8276	872.9174	1743.8436	872.4254	D	**550.2984**	275.6528	533.2718	267.1396	532.2878	266.6475	4
17	**1908.9225**	954.9649	**1891.8960**	946.4516	**1890.9120**	945.9596	F	**435.2714**	218.1394	418.2449	209.6261			3
18	2022.0066	1011.5069	2004.9801	1002.9937	2003.9960	1002.5017	I	288.2030	144.6051	271.1765	136.0919			2

**19**							**R**	175.1190	88.0631	158.0924	79.5498		**1**

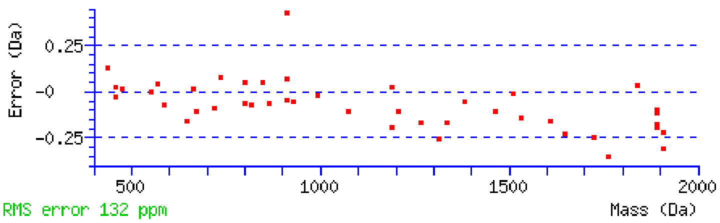

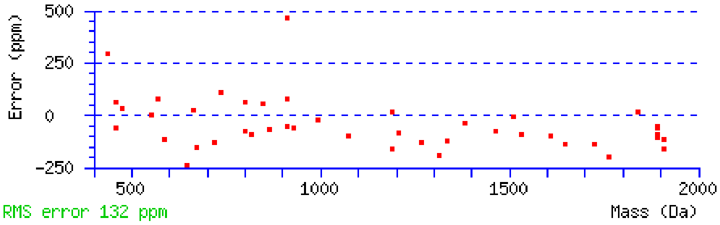

All matches to this query

Score	Mr(calc):	Delta	Sequence
77.6	2195.1110	0.0078	TGEANLTQLYLQEALDFIR
43.1	2194.1270	0.9918	TGEANLTQLYLQEALDFIR
4.4	2195.1395	-0.0207	TLAKVNTFMSETGNILNIVK
0.3	2194.1027	1.0161	TPMGFCDVPRKNLLDFLR

Spectrum No: 149; Query: 79; Rank: 1

Peptide View

MS/MS Fragmentation of **VFGSQNLTTVK**
Found in **IPI00210975**, Tax_Id=10116 Gene_Symbol=Hyou1 Hypoxia up-regulated protein 1 precursor

Match to Query 79: 1193.627828 from(597.821190,2+)
Title: 091129RatKid_SCX02_18.1201.1201.2.dta
Data file K:\NewmanPaper\Piliang\3SubProteomes\Piliang3SP\mgf5ppm\SCX_3SubProteomes5ppm.mgf

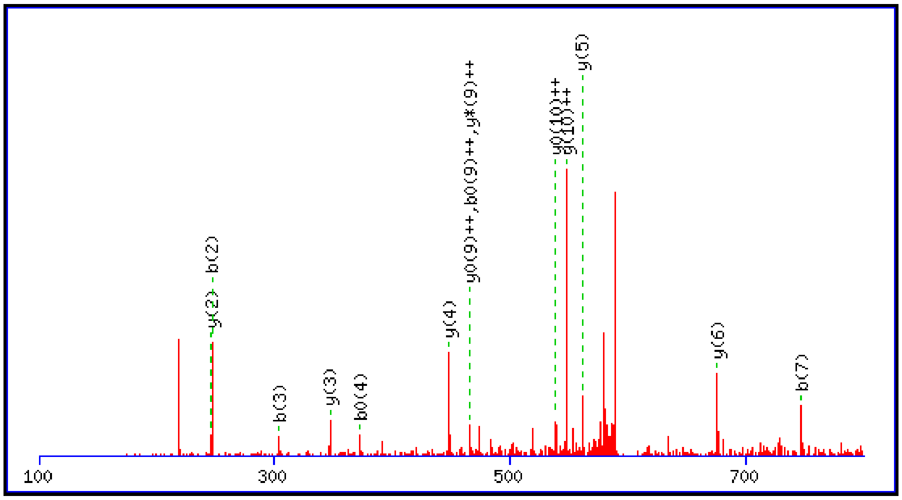

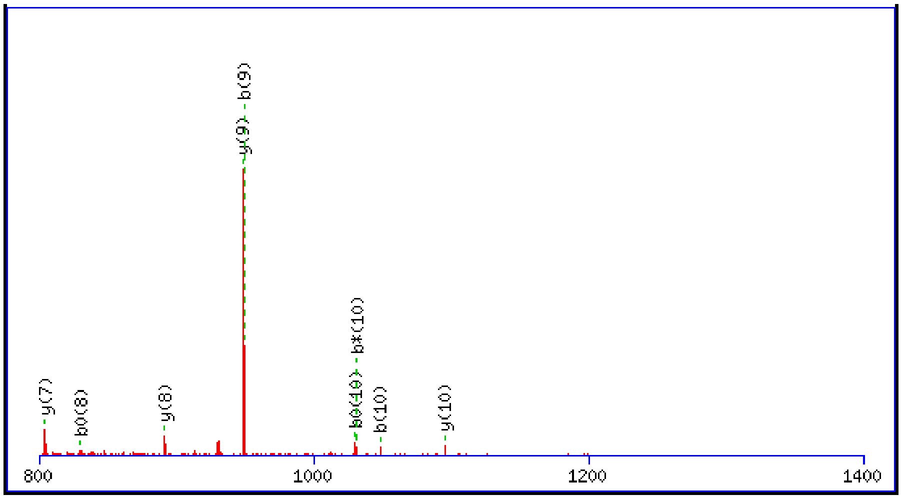

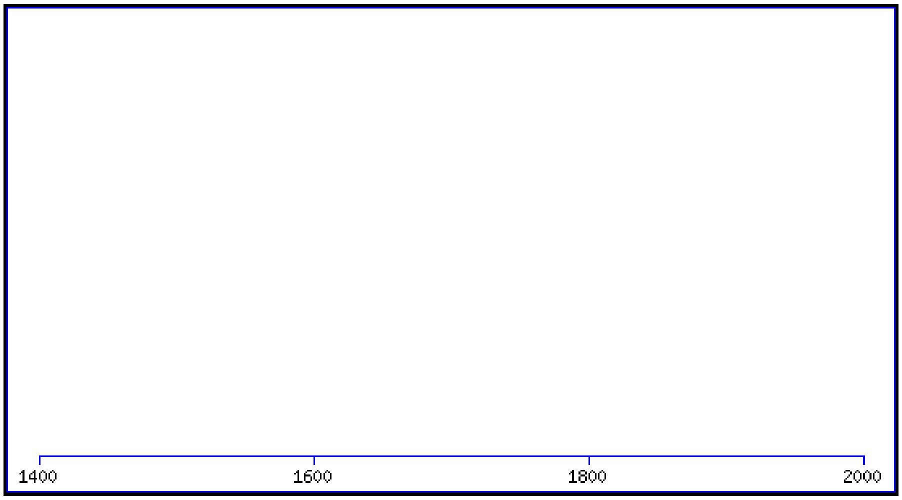

Monoisotopic mass of neutral peptide Mr(calc): 1193.6292
Fixed modifications: Carbamidomethyl (C)
Variable modifications:
N6 : Deamidated_N (N)
Ions Score: 78 **Expect:** 1.6e-006
Matches (Bold Red): 23/102 fragment ions using 34 most intense peaks

#	b	b++	b*	b*++	b0	b0++	Seq.	y	y++	y*	y*++	y0	y0++	#
1	100.0757	50.5415					V							11
2	**247.1441**	124.0757					F	**1095.5681**	**548.2877**	1078.5415	539.7744	1077.5575	**539.2824**	10
3	**304.1656**	152.5864					G	**948.4996**	474.7535	931.4731	**466.2402**	930.4891	**465.7482**	9
4	391.1976	196.1024			**373.1870**	187.0972	S	**891.4782**	446.2427	874.4516	437.7295	873.4676	437.2374	8
5	519.2562	260.1317	502.2296	251.6185	501.2456	251.1264	Q	**804.4462**	402.7267	787.4196	394.2134	786.4356	393.7214	7
6	634.2831	317.6452	617.2566	309.1319	616.2725	308.6399	N	**676.3876**	338.6974	659.3610	330.1842	658.3770	329.6921	6
7	**747.3672**	374.1872	730.3406	365.6740	729.3566	365.1819	L	**561.3606**	281.1840	544.3341	272.6707	543.3501	272.1787	5
8	848.4149	424.7111	831.3883	416.1978	**830.4043**	415.7058	T	**448.2766**	224.6419	431.2500	216.1287	430.2660	215.6366	4
9	**949.4625**	475.2349	932.4360	466.7216	931.4520	**466.2296**	T	**347.2289**	174.1181	330.2023	165.6048	329.2183	165.1128	3
10	**1048.5309**	524.7691	**1031.5044**	516.2558	**1030.5204**	515.7638	V	**246.1812**	123.5942	229.1547	115.0810			2
11							K	147.1128	74.0600	130.0863	65.5468			1

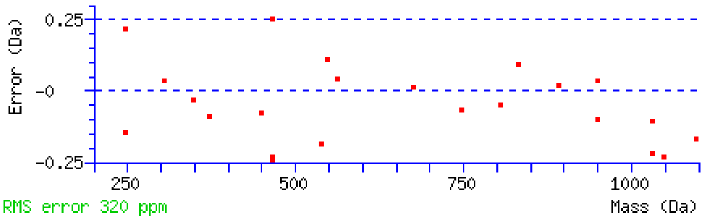

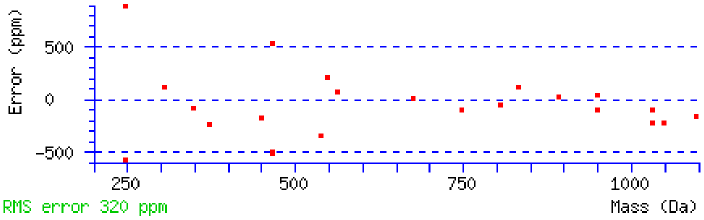

All matches to this query

Score	Mr(calc):	Delta	Sequence
77.5	1193.6292	-0.0014	VFGSQNLTTVK
9.9	1192.6162	1.0116	MPTSFVAPTVK
9.6	1193.6292	-0.0014	ANKAVDFTTVK
7.6	1193.6326	-0.0048	TVMKTGLDSVK
6.6	1193.6227	0.0052	VFKKSSPNCK
4.7	1191.6169	2.0109	MLTNGSLSEIK
3.4	1193.6301	-0.0022	STKIWMAMVK
3.2	1193.6169	0.0109	TRVSNLPTVK
2.8	1193.6326	-0.0047	KMASTSVDLVK
2.4	1193.6169	0.0109	TRVSNLPTVK

Spectrum No: 150; Query: 418; Rank: 1

Peptide View

MS/MS Fragmentation of **NVTELNEPLSNEER**
Found in **IPI00230835**, Tax_Id=10116 Gene_Symbol=Ywhag 14-3-3 protein gamma

Match to Query 418: 1643.767608 from(822.891080,2+)
Title: 091127RatKid_SCX01_14.2311.2311.2.dta
Data file K:\NewmanPaper\Piliang\3SubProteomes\Piliang3SP\mgf5ppm\SCX_3SubProteomes5ppm.mgf

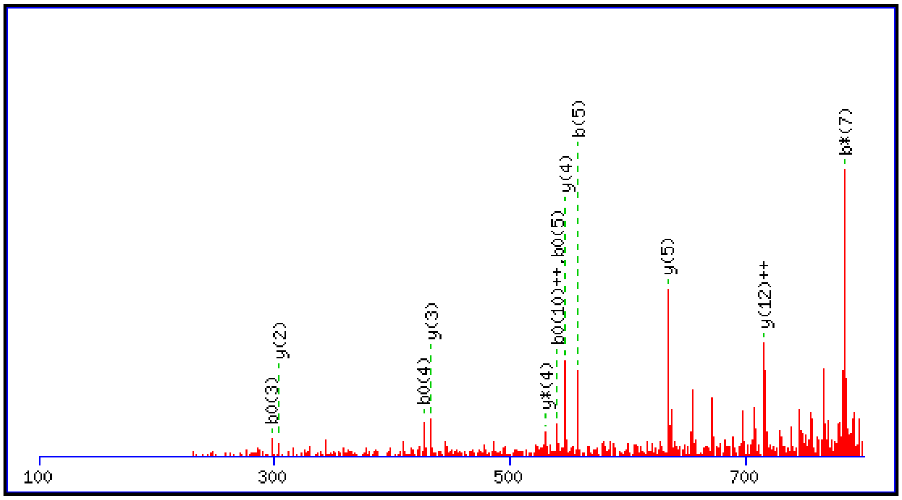

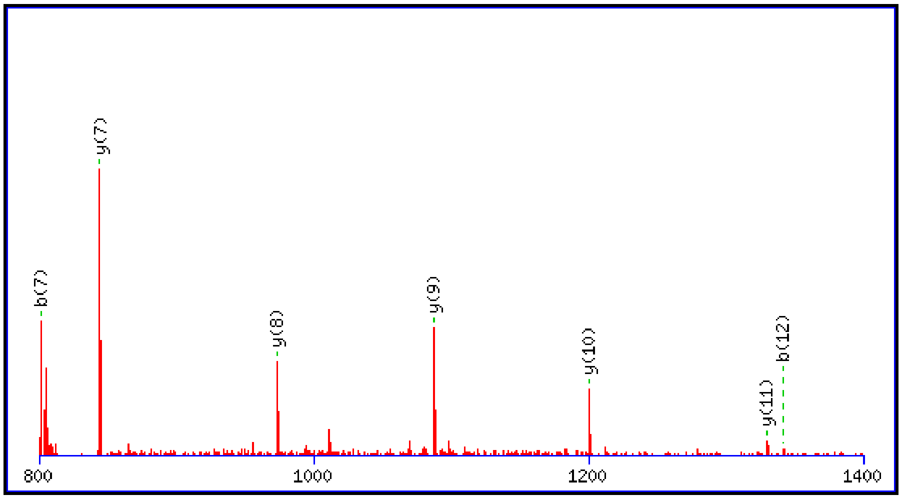

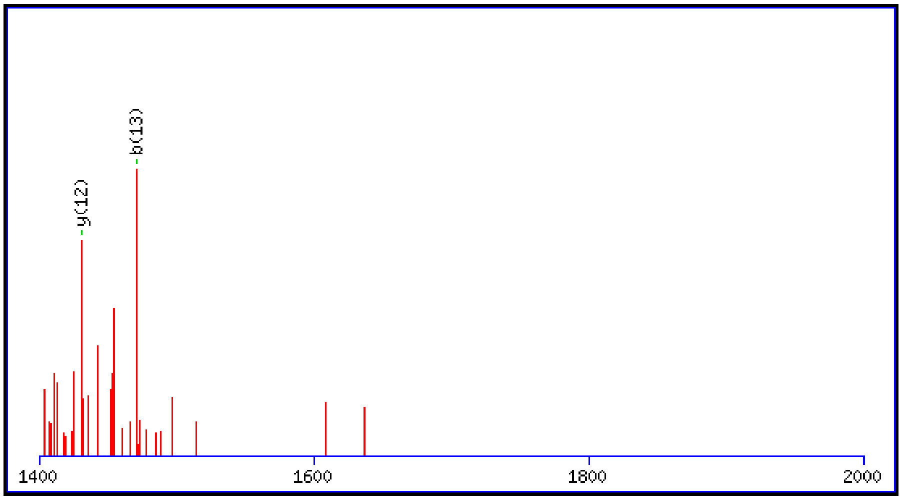

Monoisotopic mass of neutral peptide Mr(calc): 1643.7638
Fixed modifications: Carbamidomethyl (C)
Variable modifications:
N1 : Deamidated_N (N)
Ions Score: 78 **Expect:** 3.3e-006
Matches (Bold Red): 21/150 fragment ions using 32 most intense peaks

#	b	b++	b*	b*++	b0	b0++	Seq.	y	y++	y*	y*++	y0	y0++	#
1	116.0342	58.5207	99.0077	50.0075			N							14
2	215.1026	108.0550	198.0761	99.5417			V	1529.7442	765.3757	1512.7176	756.8625	1511.7336	756.3705	13
3	316.1503	158.5788	299.1238	150.0655	**298.1397**	149.5735	T	**1430.6758**	**715.8415**	1413.6492	707.3283	1412.6652	706.8362	12
4	445.1929	223.1001	428.1663	214.5868	**427.1823**	214.0948	E	**1329.6281**	665.3177	1312.6015	656.8044	1311.6175	656.3124	11
5	**558.2770**	279.6421	541.2504	271.1288	**540.2664**	270.6368	L	**1200.5855**	600.7964	1183.5590	592.2831	1182.5749	591.7911	10
6	672.3199	336.6636	655.2933	328.1503	654.3093	327.6583	N	**1087.5014**	544.2544	1070.4749	535.7411	1069.4909	535.2491	9
7	**801.3625**	401.1849	**784.3359**	392.6716	783.3519	392.1796	E	**973.4585**	487.2329	956.4320	478.7196	955.4479	478.2276	8
8	898.4152	449.7113	881.3887	441.1980	880.4047	440.7060	P	**844.4159**	422.7116	827.3894	414.1983	826.4054	413.7063	7
9	1011.4993	506.2533	994.4728	497.7400	993.4887	497.2480	L	747.3632	374.1852	730.3366	365.6719	729.3526	365.1799	6
10	1098.5313	549.7693	1081.5048	541.2560	1080.5208	**540.7640**	S	**634.2791**	317.6432	617.2525	309.1299	616.2685	308.6379	5
11	1212.5743	606.7908	1195.5477	598.2775	1194.5637	597.7855	N	**547.2471**	274.1272	**530.2205**	265.6139	529.2365	265.1219	4
12	**1341.6169**	671.3121	1324.5903	662.7988	1323.6063	662.3068	E	**433.2041**	217.1057	416.1776	208.5924	415.1936	208.1004	3
13	**1470.6595**	735.8334	1453.6329	727.3201	1452.6489	726.8281	E	**304.1615**	152.5844	287.1350	144.0711	286.1510	143.5791	2
14							R	175.1190	88.0631	158.0924	79.5498			1

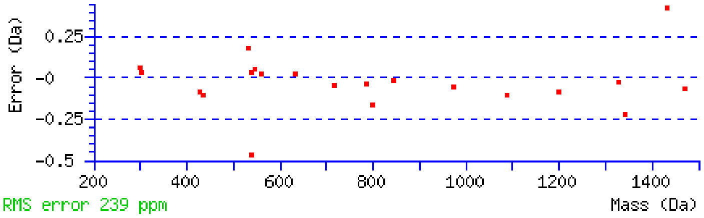

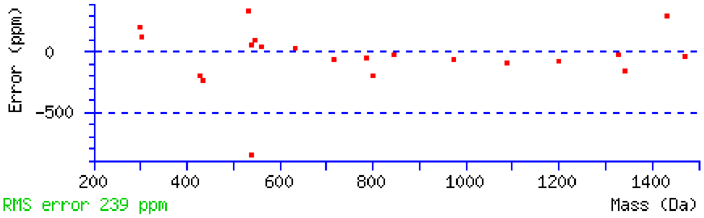

All matches to this query

Score	Mr(calc):	Delta	Sequence
77.5	1643.7638	0.0038	NVTELNEPLSNEER
60.4	1643.7638	0.0038	NVTELNEPLSNEER
56.0	1642.7798	0.9878	NVTELNEPLSNEER
28.7	1643.7638	0.0038	NVTELNEPLSNEER
14.2	1643.7776	-0.0100	GGTKTLMNTIMQLR
7.9	1643.7807	-0.0131	LLESNYNITLVER
7.5	1642.7675	1.0001	NASRESSLKDLSTR

7.5	1642.7675	1.0001	NASRESSLKDLSTR
6.4	1643.7546	0.0130	CHGDRTSESGLRNR
5.4	1643.7742	-0.0066	AAERIMEAIELYR

Spectrum No: 151; Query: 984; Rank: 1

Peptide View

MS/MS Fragmentation of **ALSCLESSWENIEQGGNGSFVYTK**
Found in **IPI00201262**, Tax_Id=10116 Gene_Symbol=LOC297568;Mug2;Mug1 Alpha-1-inhibitor 3 precursor

Match to Query 984: 2676.213368 from(1339.113960,2+)
Title: 091127RatKid_SCX01_02.4397.4397.2.dta
Data file K:\NewmanPaper\Piliang\3SubProteomes\Piliang3SP\mgf5ppm\SCX_3SubProteomes5ppm.mgf

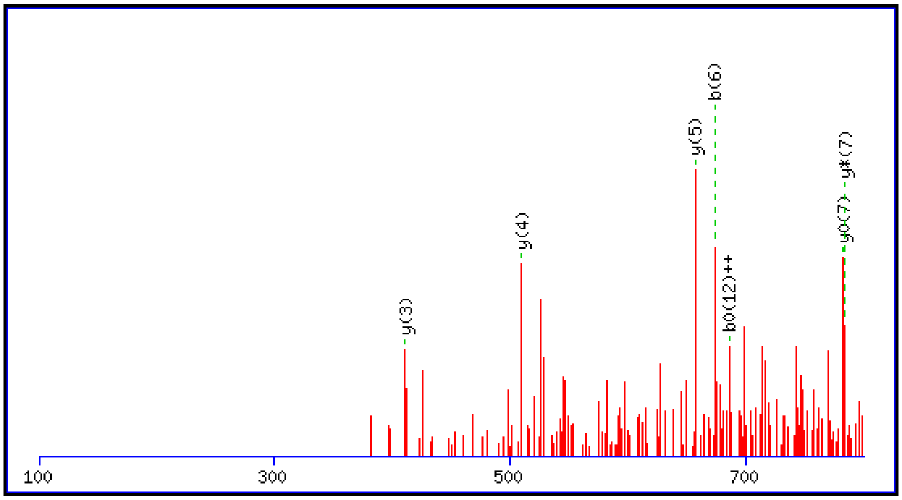

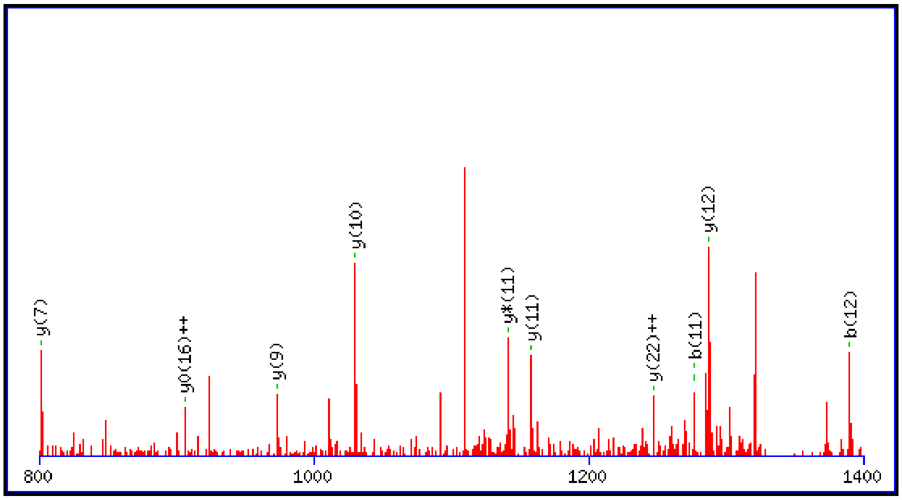

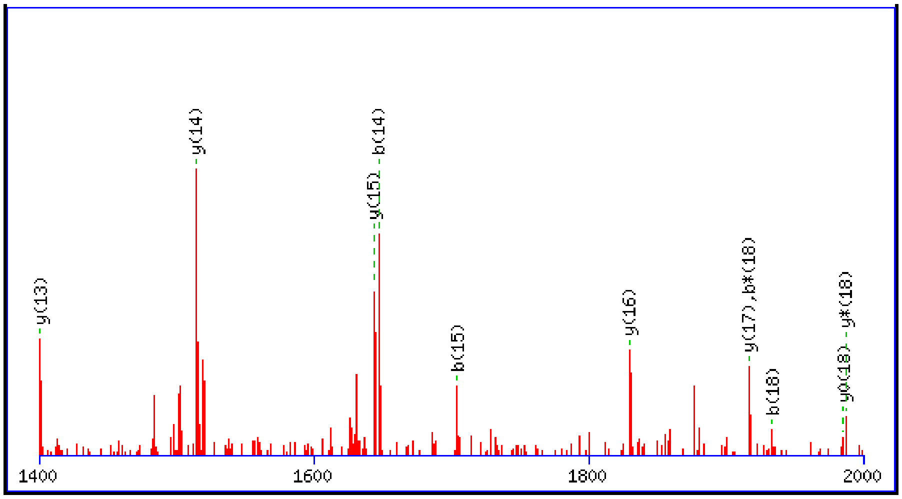

Monoisotopic mass of neutral peptide Mr(calc): 2676.2013
Fixed modifications: Carbamidomethyl (C)
Variable modifications:
N17 : Deamidated_N (N)
Ions Score: 77 **Expect:** 6.4e-006
Matches (Bold Red): 28/250 fragment ions using 41 most intense peaks

#	b	b^{++}	b*	b^{*++}	b^{0}	b^{0++}	Seq.	y	y^{++}	y*	y^{*++}	y^{0}	y^{0++}	#
1	72.0444	36.5258					A							24
2	185.1285	93.0679					L	2606.1715	1303.5894	2589.1450	1295.0761	2588.1610	1294.5841	23
3	272.1605	136.5839			254.1499	127.5786	S	2493.0875	**1247.0474**	2476.0609	1238.5341	2475.0769	1238.0421	22
4	432.1911	216.5992			414.1806	207.5939	C	2406.0554	1203.5314	2389.0289	1195.0181	2388.0449	1194.5261	21
5	545.2752	273.1412			527.2646	264.1360	L	2246.0248	1123.5160	2228.9982	1115.0028	2228.0142	1114.5107	20
6	**674.3178**	337.6625			656.3072	328.6572	E	2132.9407	1066.9740	2115.9142	1058.4607	2114.9301	1057.9687	19
7	761.3498	381.1785			743.3393	372.1733	S	2003.8981	1002.4527	**1986.8716**	993.9394	**1985.8876**	993.4474	18
8	848.3818	424.6946			830.3713	415.6893	S	**1916.8661**	958.9367	1899.8395	950.4234	1898.8555	949.9314	17
9	1034.4612	517.7342			1016.4506	508.7289	W	**1829.8341**	915.4207	1812.8075	906.9074	1811.8235	**906.4154**	16
10	1163.5038	582.2555			1145.4932	573.2502	E	**1643.7548**	822.3810	1626.7282	813.8677	1625.7442	813.3757	15
11	**1277.5467**	639.2770	1260.5201	630.7637	1259.5361	630.2717	N	**1514.7122**	757.8597	1497.6856	749.3464	1496.7016	748.8544	14
12	**1390.6307**	695.8190	1373.6042	687.3057	1372.6202	**686.8137**	I	**1400.6692**	700.8383	1383.6427	692.3250	1382.6587	691.8330	13
13	1519.6733	760.3403	1502.6468	751.8270	1501.6628	751.3350	E	**1287.5852**	644.2962	1270.5586	635.7829	1269.5746	635.2909	12
14	**1647.7319**	824.3696	1630.7054	815.8563	1629.7213	815.3643	Q	**1158.5426**	579.7749	**1141.5160**	571.2617	1140.5320	570.7696	11
15	**1704.7534**	852.8803	1687.7268	844.3671	1686.7428	843.8750	G	**1030.4840**	515.7456	1013.4574	507.2324	1012.4734	506.7404	10
16	1761.7748	881.3911	1744.7483	872.8778	1743.7643	872.3858	G	**973.4625**	487.2349	956.4360	478.7216	955.4520	478.2296	9
17	1876.8018	938.9045	1859.7752	930.3913	1858.7912	929.8992	N	916.4411	458.7242	899.4145	450.2109	898.4305	449.7189	8
18	**1933.8232**	967.4153	**1916.7967**	958.9020	1915.8127	958.4100	G	**801.4141**	401.2107	**784.3876**	392.6974	**783.4036**	392.2054	7
19	2020.8553	1010.9313	2003.8287	1002.4180	2002.8447	1001.9260	S	744.3927	372.7000	727.3661	364.1867	726.3821	363.6947	6
20	2167.9237	1084.4655	2150.8971	1075.9522	2149.9131	1075.4602	F	**657.3606**	329.1840	640.3341	320.6707	639.3501	320.1787	5
21	2266.9921	1133.9997	2249.9655	1125.4864	2248.9815	1124.9944	V	**510.2922**	255.6498	493.2657	247.1365	492.2817	246.6445	4
22	2430.0554	1215.5314	2413.0289	1207.0181	2412.0449	1206.5261	Y	**411.2238**	206.1155	394.1973	197.6023	393.2132	197.1103	3
23	2531.1031	1266.0552	2514.0766	1257.5419	2513.0925	1257.0499	T	248.1605	124.5839	231.1339	116.0706	230.1499	115.5786	2
24							K	147.1128	74.0600	130.0863	65.5468			1

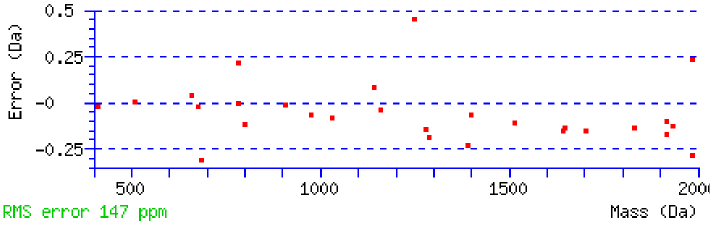

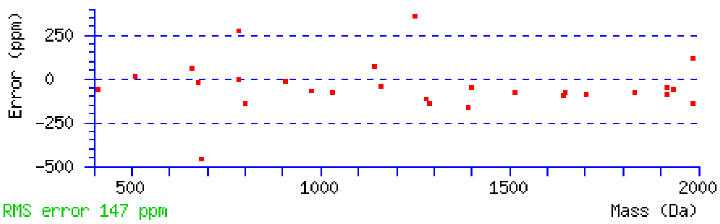

All matches to this query

Score	Mr(calc):	Delta	Sequence
77.3	2676.2013	0.0120	ALSCLESSWENIEQGGNGSFVYTK
34.9	2676.2013	0.0120	ALSCLESSWENIEQGGNGSFVYTK
24.0	2675.2173	0.9960	ALSCLESSWENIEQGGNGSFVYTK
6.4	2676.2288	-0.0154	EKEVMSLLHNLEIKNTEHCQK
2.9	2676.2128	0.0006	NTQQQSWRPLRVSPWVGLRK
2.9	2676.2230	-0.0097	SLEIISTAASHSNSAIRKMENLK
1.3	2676.2288	-0.0155	YSSQLAQMQCLIGSVESQLGEIR
1.3	2676.2288	-0.0155	YSSQLAQMQCLIGSVESQLGEIR
1.3	2676.2288	-0.0155	YSSQLAQMQCLIGSVESQLGEIR

Spectrum No: 152; Query: 286; Rank: 1

Peptide View

MS/MS Fragmentation of **NRTDVEYELDEK**
Found in **IPI00327143**, Tax_Id=10116 Gene_Symbol=Alpl Alkaline phosphatase, tissue-nonspecific isozyme precursor

Match to Query 286: 1510.680288 from(756.347420,2+)
Title: 091127RatKid_SCX01_22.1320.1320.2.dta
Data file K:\NewmanPaper\Piliang\3SubProteomes\Piliang3SP\mgf5ppm\SCX_3SubProteomes5ppm.mgf

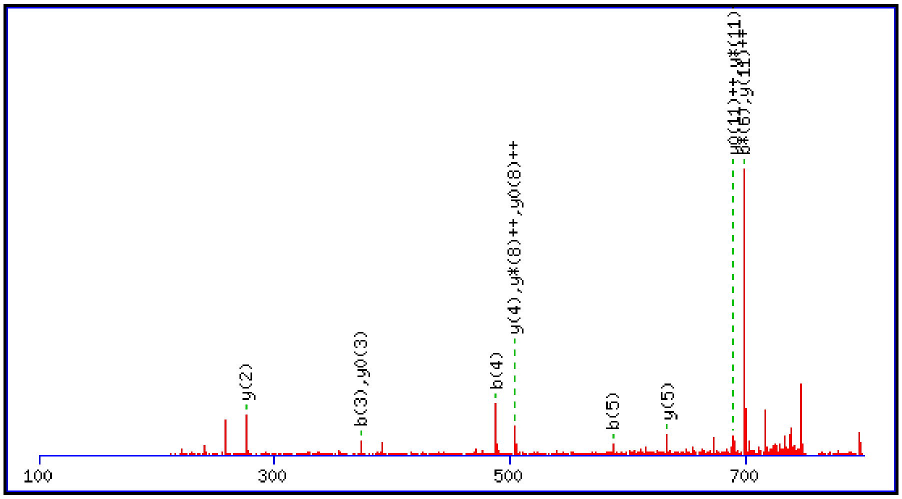

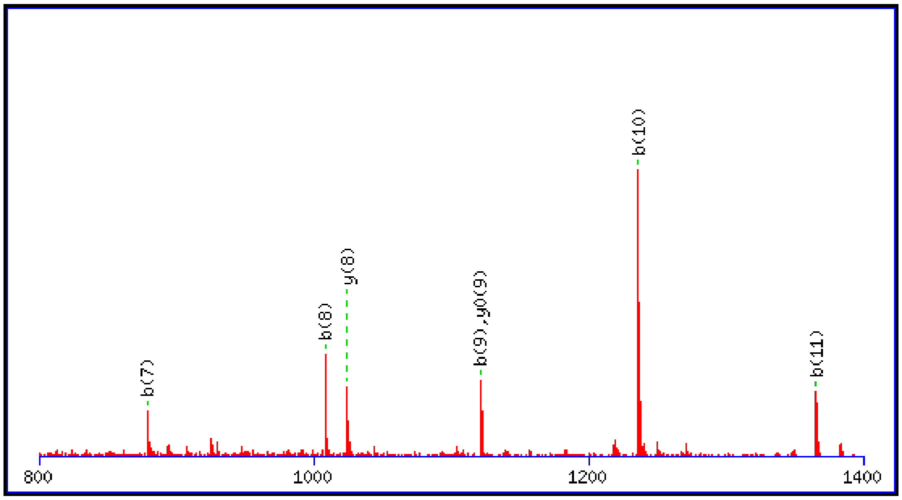

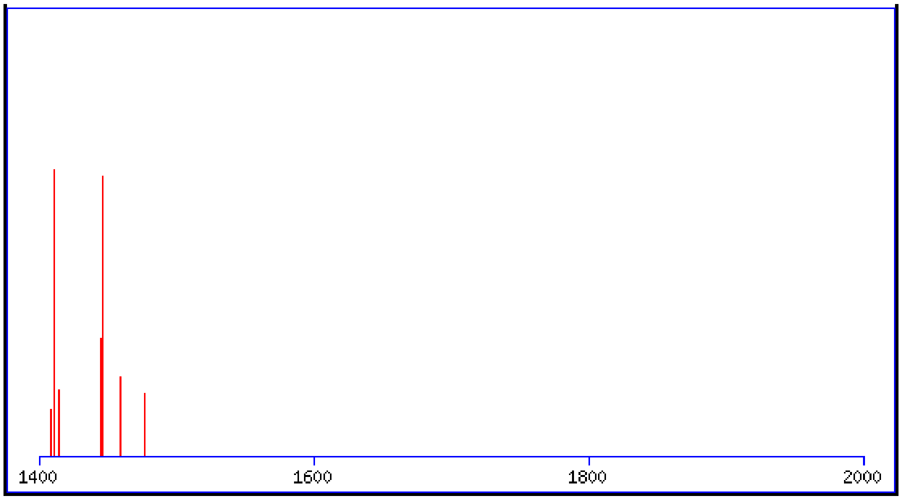

Monoisotopic mass of neutral peptide Mr(calc): 1510.6787
Fixed modifications: Carbamidomethyl (C)
Variable modifications:
N1 : Deamidated_N (N)
Ions Score: 77 **Expect:** 2.8e-006
Matches (Bold Red): 20/126 fragment ions using 26 most intense peaks

#	b	b^{++}	b*	b^{*++}	b^0	b^{0++}	Seq.	y	y^{++}	y*	y^{*++}	y^0	y^{0++}	#
1	116.0342	58.5207	99.0077	50.0075			N							12
2	272.1353	136.5713	255.1088	128.0580			R	1396.6591	**698.8332**	1379.6325	**690.3199**	1378.6485	**689.8279**	11
3	**373.1830**	187.0951	356.1565	178.5819	355.1724	178.0899	T	1240.5580	620.7826	1223.5314	612.2693	1222.5474	611.7773	10
4	**488.2099**	244.6086	471.1834	236.0953	470.1994	235.6033	D	1139.5103	570.2588	1122.4837	561.7455	**1121.4997**	561.2535	9
5	**587.2784**	294.1428	570.2518	285.6295	569.2678	285.1375	V	**1024.4833**	512.7453	1007.4568	**504.2320**	1006.4728	**503.7400**	8
6	716.3210	358.6641	**699.2944**	350.1508	698.3104	349.6588	E	925.4149	463.2111	908.3884	454.6978	907.4044	454.2058	7
7	**879.3843**	440.1958	862.3577	431.6825	861.3737	431.1905	Y	796.3723	398.6898	779.3458	390.1765	778.3618	389.6845	6
8	**1008.4269**	504.7171	991.4003	496.2038	990.4163	495.7118	E	**633.3090**	317.1581	616.2824	308.6449	615.2984	308.1529	5
9	**1121.5109**	561.2591	1104.4844	552.7458	1103.5004	552.2538	L	**504.2664**	252.6368	487.2399	244.1236	486.2558	243.6316	4
10	**1236.5379**	618.7726	1219.5113	610.2593	1218.5273	609.7673	D	391.1823	196.0948	374.1558	187.5815	**373.1718**	187.0895	3
11	**1365.5805**	683.2939	1348.5539	674.7806	1347.5699	674.2886	E	**276.1554**	138.5813	259.1288	130.0681	258.1448	129.5761	2
12							K	147.1128	74.0600	130.0863	65.5468			1

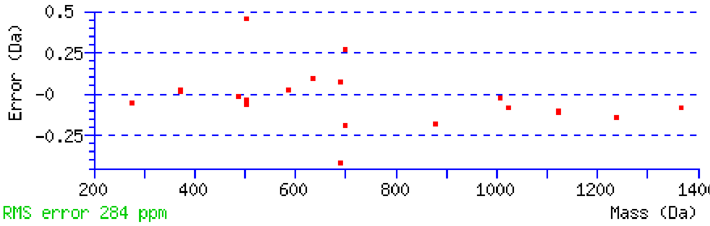

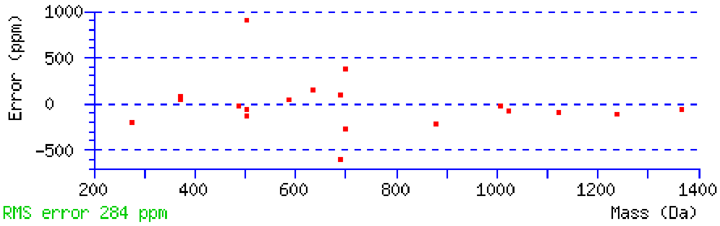

All matches to this query

Score	Mr(calc):	Delta	Sequence
77.3	1510.6787	0.0016	NRTDVEYELDEK
16.9	1510.6778	0.0024	DYEMKFTKIEK
16.6	1510.6778	0.0024	DKMYNLYIVEK
16.6	1510.6778	0.0024	DKMYNLYIVEK
14.7	1510.6777	0.0026	KTGGSGERNATPEK
12.8	1510.6745	0.0058	FFSVFKGNINEK
12.8	1510.6916	-0.0113	TIETQAVNALDEK
12.8	1510.6916	-0.0113	TIETQAVNALDEK
11.3	1510.6851	-0.0048	NCPASVLAXKVTR

11.3	1510.6851	-0.0048	NCPASVLAXKVTR

Spectrum No: 153; Query: 161; Rank: 1

Peptide View

MS/MS Fragmentation of **NATTDALTSVLTK**
Found in **IPI00199445**, Tax_Id=10116 Gene_Symbol=Ank3 ankyrin 3, epithelial isoform 2

Match to Query 161: 1334.695428 from(668.354990,2+)
Title: 091127RatKid_SCX01_13.3364.3364.2.dta
Data file K:\NewmanPaper\Piliang\3SubProteomes\Piliang3SP\mgf5ppm\SCX_3SubProteomes5ppm.mgf

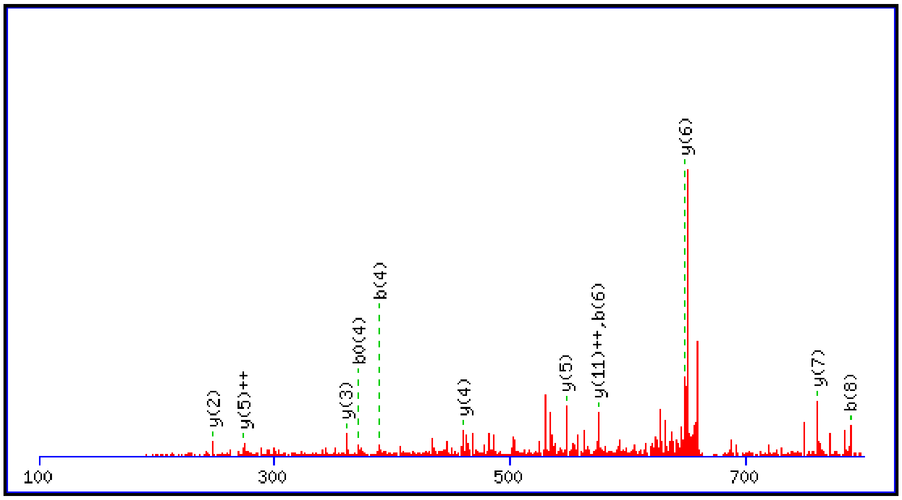

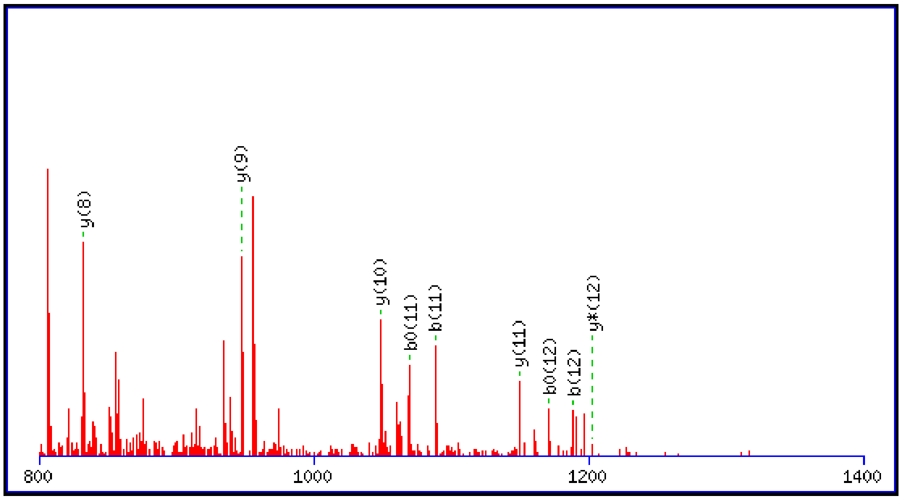

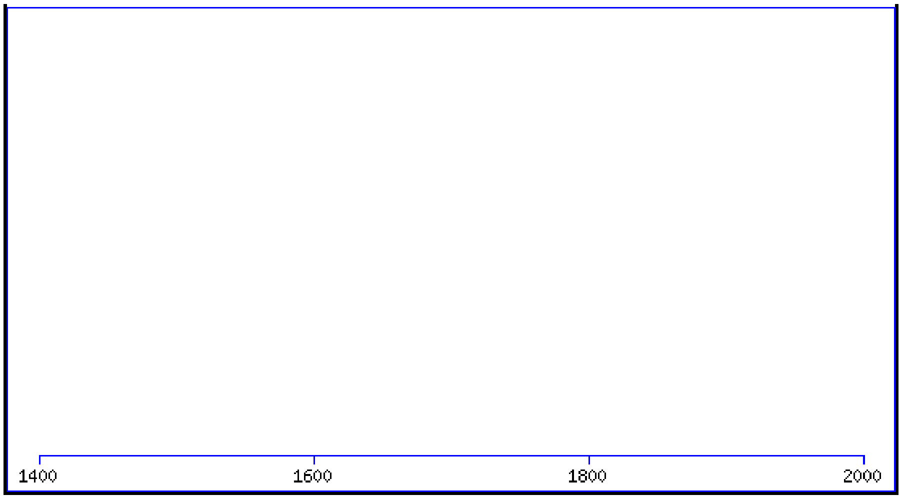

Monoisotopic mass of neutral peptide Mr(calc): 1334.6929
Fixed modifications: Carbamidomethyl (C)
Variable modifications:
N1 : Deamidated_N (N)
Ions Score: 77 **Expect:** 2.5e-006
Matches (Bold Red): 21/138 fragment ions using 42 most intense peaks

#	b	b^{++}	b*	b^{*++}	b^0	b^{0++}	Seq.	y	y^{++}	y*	y^{*++}	y^0	y^{0++}	#
1	116.0342	58.5207	99.0077	50.0075			N							13
2	187.0713	94.0393	170.0448	85.5260			A	1220.6733	610.8403	**1203.6467**	602.3270	1202.6627	601.8350	12
3	288.1190	144.5631	271.0925	136.0499	270.1084	135.5579	T	**1149.6361**	**575.3217**	1132.6096	566.8084	1131.6256	566.3164	11
4	**389.1667**	195.0870	372.1401	186.5737	**371.1561**	186.0817	T	**1048.5885**	524.7979	1031.5619	516.2846	1030.5779	515.7926	10
5	504.1936	252.6005	487.1671	244.0872	486.1831	243.5952	D	**947.5408**	474.2740	930.5142	465.7608	929.5302	465.2688	9
6	**575.2307**	288.1190	558.2042	279.6057	557.2202	279.1137	A	**832.5138**	416.7606	815.4873	408.2473	814.5033	407.7553	8
7	688.3148	344.6610	671.2883	336.1478	670.3042	335.6558	L	**761.4767**	381.2420	744.4502	372.7287	743.4662	372.2367	7
8	**789.3625**	395.1849	772.3359	386.6716	771.3519	386.1796	T	**648.3927**	324.7000	631.3661	316.1867	630.3821	315.6947	6
9	876.3945	438.7009	859.3680	430.1876	858.3839	429.6956	S	**547.3450**	**274.1761**	530.3184	265.6629	529.3344	265.1709	5
10	975.4629	488.2351	958.4364	479.7218	957.4524	479.2298	V	**460.3130**	230.6601	443.2864	222.1468	442.3024	221.6548	4
11	**1088.5470**	544.7771	1071.5204	536.2639	**1070.5364**	535.7719	L	**361.2445**	181.1259	344.2180	172.6126	343.2340	172.1206	3
12	**1189.5947**	595.3010	1172.5681	586.7877	**1171.5841**	586.2957	T	**248.1605**	124.5839	231.1339	116.0706	230.1499	115.5786	2
13							K	147.1128	74.0600	130.0863	65.5468			1

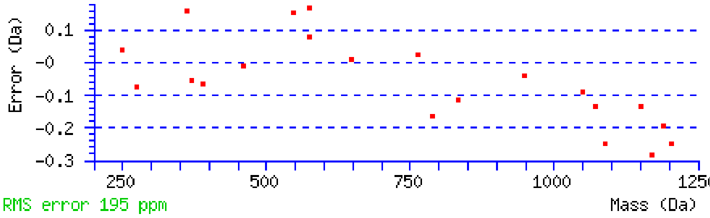

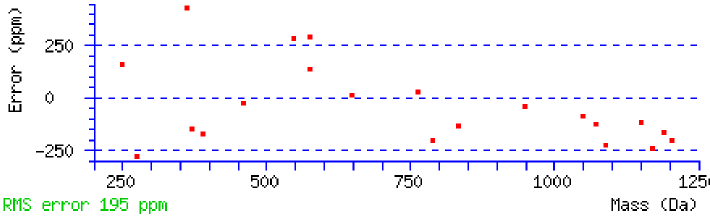

All matches to this query

Score	Mr(calc):	Delta	Sequence
77.0	1334.6929	0.0025	NATTDALTSVLTK
9.1	1334.7041	-0.0087	DTRIDSISSLTK
8.0	1334.6976	-0.0022	QMLLRTESSVR
7.3	1333.6911	1.0043	CTVGEITDALKK
3.6	1334.7041	-0.0087	SSTIVLRTDSEK
3.6	1334.6830	0.0124	LFNLSKEDDVR
2.5	1334.6942	0.0012	ERSLSFQEALR

Spectrum No: 154; Query: 1044; Rank: 1

Peptide View

MS/MS Fragmentation of **ENGTVTAANASTLNDGAAAVVLMTAEAAQR**
Found in **IPI00324302**, Tax_Id=10116 Gene_Symbol=Acat1 Acetyl-CoA acetyltransferase, mitochondrial precursor

Match to Query 1044: 2917.413222 from(973.478350,3+)
Title: 091127RatKid_SCX01_14.4974.4974.3.dta
Data file K:\NewmanPaper\Piliang\3SubProteomes\Piliang3SP\mgf5ppm\SCX_3SubProteomes5ppm.mgf

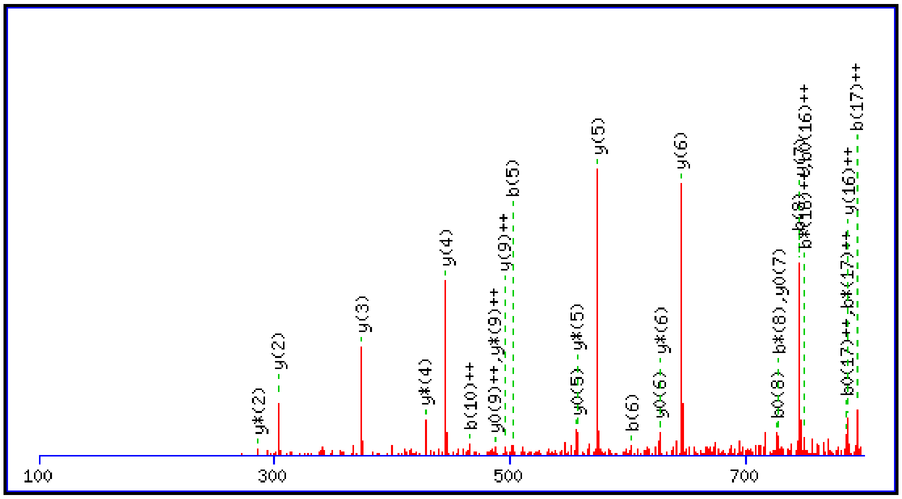

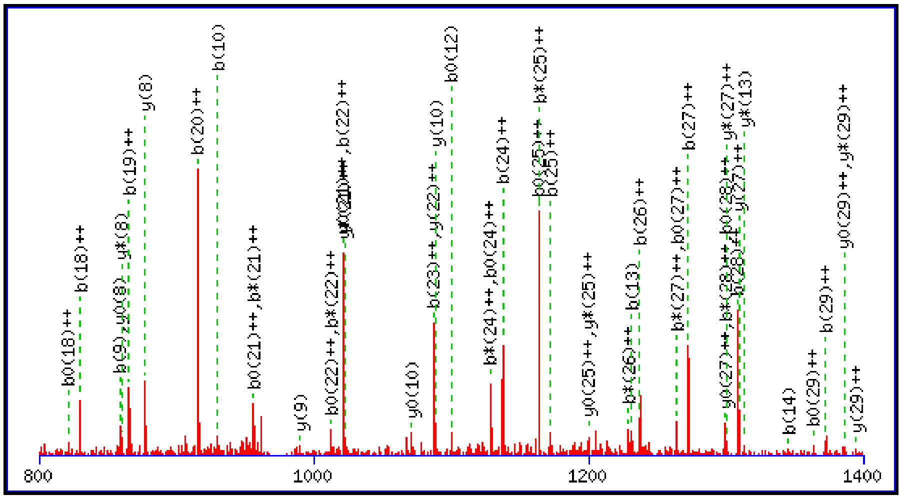

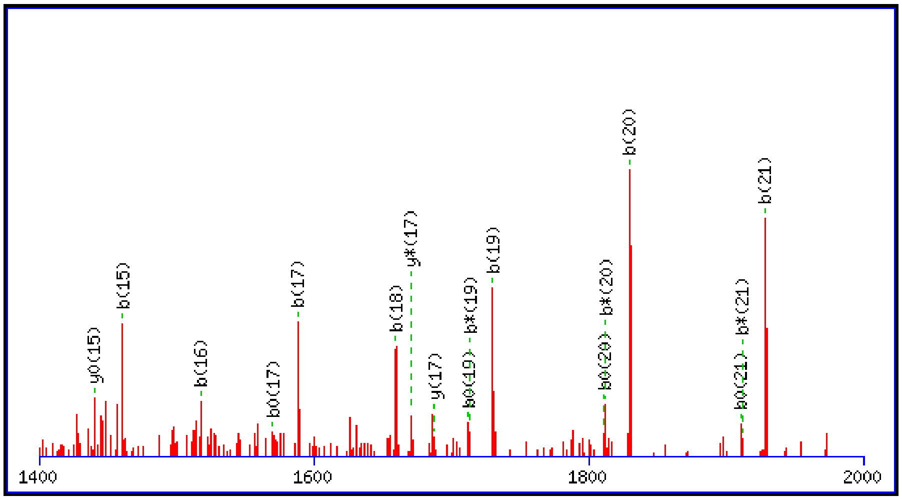

Monoisotopic mass of neutral peptide Mr(calc): 2917.4087

Fixed modifications: Carbamidomethyl (C)
Variable modifications:
N2 : Deamidated_N (N)
Ions Score: 77 **Expect:** 5.8e-006
Matches (Bold Red): 94/338 fragment ions using 156 most intense peaks

#	b	b^{++}	b*	b^{*++}	b^{0}	b^{0++}	Seq.	y	y^{++}	y*	y^{*++}	y^{0}	y^{0++}	#
1	130.0499	65.5286			112.0393	56.5233	E							30
2	245.0768	123.0420	228.0503	114.5288	227.0662	114.0368	N	2789.3734	**1395.1903**	2772.3469	**1386.6771**	2771.3628	**1386.1851**	29
3	302.0983	151.5528	285.0717	143.0395	284.0877	142.5475	G	2674.3465	1337.6769	2657.3199	1329.1636	2656.3359	1328.6716	28
4	403.1459	202.0766	386.1194	193.5633	385.1354	193.0713	T	2617.3250	**1309.1661**	2600.2985	**1300.6529**	2599.3144	**1300.1609**	27
5	**502.2144**	251.6108	485.1878	243.0975	484.2038	242.6055	V	2516.2773	1258.6423	2499.2508	1250.1290	2498.2668	1249.6370	26
6	**603.2620**	302.1347	586.2355	293.6214	585.2515	293.1294	T	2417.2089	1209.1081	2400.1824	**1200.5948**	2399.1984	**1200.1028**	25
7	674.2992	337.6532	657.2726	329.1399	656.2886	328.6479	A	2316.1612	1158.5843	2299.1347	1150.0710	2298.1507	1149.5790	24
8	**745.3363**	373.1718	**728.3097**	364.6585	**727.3257**	364.1665	A	2245.1241	1123.0657	2228.0976	1114.5524	2227.1136	1114.0604	23
9	**859.3792**	430.1932	842.3526	421.6800	841.3686	421.1880	N	2174.0870	**1087.5471**	2157.0605	1079.0339	2156.0764	1078.5419	22
10	**930.4163**	**465.7118**	913.3898	457.1985	912.4057	456.7065	A	2060.0441	1030.5257	2043.0175	**1022.0124**	2042.0335	**1021.5204**	21
11	1017.4483	509.2278	1000.4218	500.7145	999.4378	500.2225	S	1989.0070	995.0071	1971.9804	986.4938	1970.9964	986.0018	20
12	1118.4960	559.7516	1101.4695	551.2384	**1100.4855**	550.7464	T	1901.9749	951.4911	1884.9484	942.9778	1883.9644	942.4858	19
13	**1231.5801**	616.2937	1214.5535	607.7804	1213.5695	607.2884	L	1800.9273	900.9673	1783.9007	892.4540	1782.9167	891.9620	18
14	**1345.6230**	673.3151	1328.5965	664.8019	1327.6124	664.3099	N	**1687.8432**	844.4252	**1670.8166**	835.9120	1669.8326	835.4200	17
15	**1460.6500**	730.8286	1443.6234	722.3153	1442.6394	721.8233	D	1573.8003	**787.4038**	1556.7737	778.8905	1555.7897	778.3985	16
16	**1517.6714**	759.3393	1500.6449	**750.8261**	1499.6608	**750.3341**	G	1458.7733	729.8903	1441.7468	721.3770	**1440.7628**	720.8850	15
17	**1588.7085**	**794.8579**	1571.6820	**786.3446**	**1570.6980**	**785.8526**	A	1401.7519	701.3796	1384.7253	692.8663	1383.7413	692.3743	14
18	**1659.7456**	**830.3765**	1642.7191	821.8632	1641.7351	**821.3712**	A	1330.7147	665.8610	**1313.6882**	657.3477	1312.7042	656.8557	13
19	**1730.7828**	**865.8950**	**1713.7562**	857.3817	**1712.7722**	856.8897	A	1259.6776	630.3425	1242.6511	621.8292	1241.6671	621.3372	12
20	**1829.8512**	**915.4292**	**1812.8246**	906.9159	**1811.8406**	906.4239	V	1188.6405	594.8239	1171.6140	586.3106	1170.6300	585.8186	11
21	**1928.9196**	964.9634	**1911.8930**	**956.4502**	**1910.9090**	**955.9581**	V	**1089.5721**	545.2897	1072.5456	536.7764	**1071.5615**	536.2844	10
22	2042.0036	**1021.5055**	2024.9771	**1012.9922**	2023.9931	**1012.5002**	L	**990.5037**	**495.7555**	973.4771	**487.2422**	972.4931	**486.7502**	9
23	2173.0441	**1087.0257**	2156.0176	1078.5124	2155.0336	1078.0204	M	**877.4196**	439.2135	**860.3931**	430.7002	**859.4091**	430.2082	8
24	2274.0918	**1137.5495**	2257.0653	**1129.0363**	2256.0812	**1128.5443**	T	**746.3791**	373.6932	729.3526	365.1799	**728.3686**	364.6879	7
25	2345.1289	**1173.0681**	2328.1024	**1164.5548**	2327.1184	**1164.0628**	A	**645.3315**	323.1694	**628.3049**	314.6561	**627.3209**	314.1641	6
26	2474.1715	**1237.5894**	2457.1450	**1229.0761**	2456.1610	1228.5841	E	**574.2944**	287.6508	**557.2678**	279.1375	**556.2838**	278.6455	5
27	2545.2086	**1273.1080**	2528.1821	**1264.5947**	2527.1981	**1264.1027**	A	**445.2518**	223.1295	**428.2252**	214.6162			4
28	2616.2457	**1308.6265**	2599.2192	**1300.1132**	2598.2352	**1299.6212**	A	**374.2146**	187.6110	357.1881	179.0977			3
29	2744.3043	**1372.6558**	2727.2778	1364.1425	2726.2938	**1363.6505**	Q	**303.1775**	152.0924	**286.1510**	143.5791			2
30							R	175.1190	88.0631	158.0924	79.5498			1

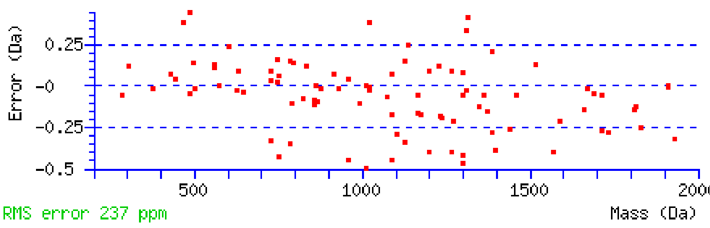

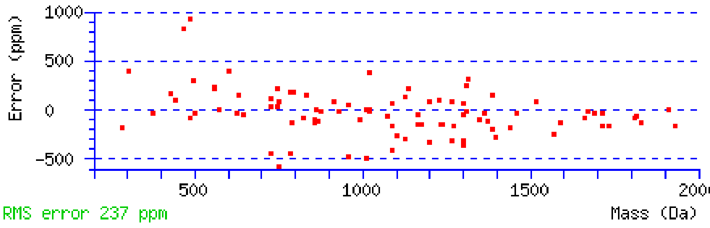

All matches to this query

Score	Mr(calc):	Delta	Sequence
76.9	2917.4087	0.0045	ENGTVTAANASTLNDGAAAVVLMTAEAAQR
64.9	2917.4087	0.0045	ENGTVTAANASTLNDGAAAVVLMTAEAAQR
54.3	2917.4087	0.0045	ENGTVTAANASTLNDGAAAVVLMTAEAAQR
22.8	2916.4247	0.9885	ENGTVTAANASTLNDGAAAVVLMTAEAAQR
8.1	2917.3901	0.0231	TSGGDSNKPTGSDSNKPTSKVPSNTETPK

6.0	2915.3922	2.0210	VEERTPYIVVALQECERMNILTR
5.8	2917.4157	-0.0025	FGLGLAVAGGMVNSALYNVDARNRAVGK
5.5	2917.4157	-0.0025	FGLGLAVAGGMVNSALYNVDARNRAVGK
5.4	2917.4157	-0.0025	FGLGLAVAGGMVNSALYNVDARNRAVGK
5.4	2917.4157	-0.0025	FGLGLAVAGGMVNSALYNVDARNRAVGK

Spectrum No: 155; Query: 1095; Rank: 1

Peptide View

MS/MS Fragmentation of **GVDVSQVAWQSQGDTPCSCCIVNNSNGSR**
Found in **IPI00211989**, Tax_Id=10116 Gene_Symbol=Khk Isoform C of Ketohexokinase

Match to Query 1095: 3182.352072 from(1061.791300,3+)
Title: 091127RatKid_SCX01_17.2588.2588.3.dta
Data file K:\NewmanPaper\Piliang\3SubProteomes\Piliang3SP\mgf5ppm\SCX_3SubProteomes5ppm.mgf

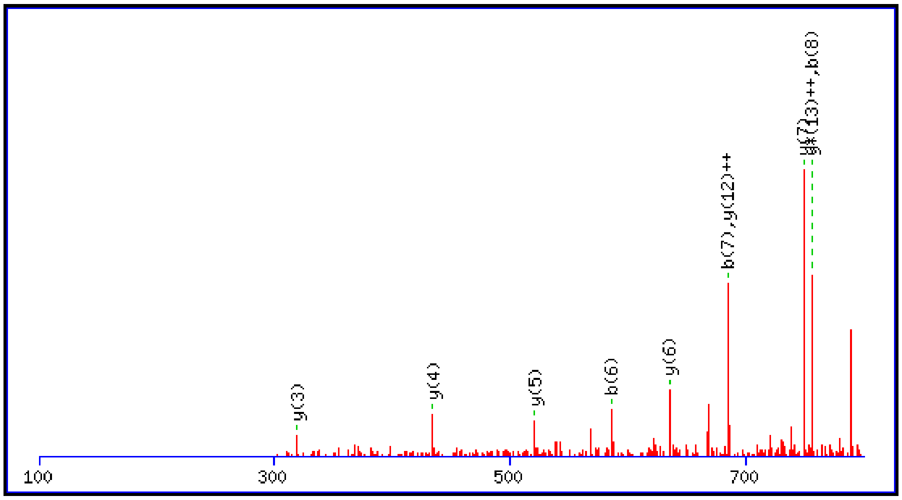

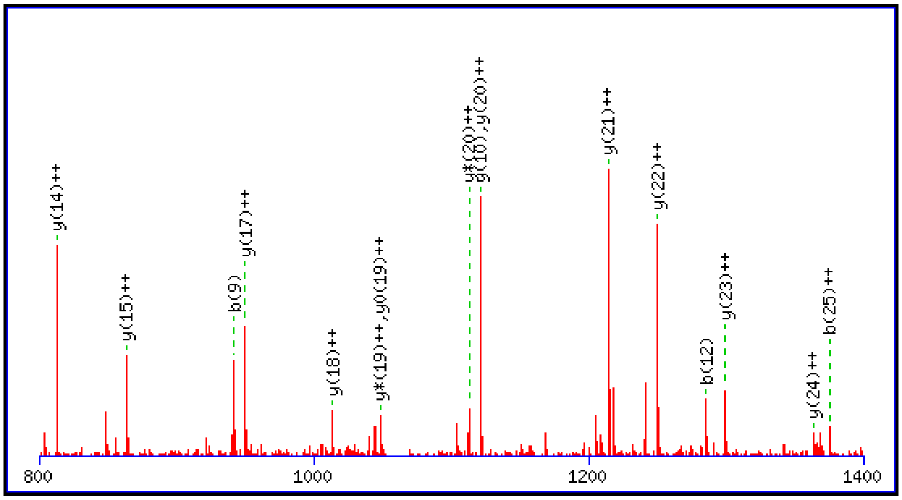

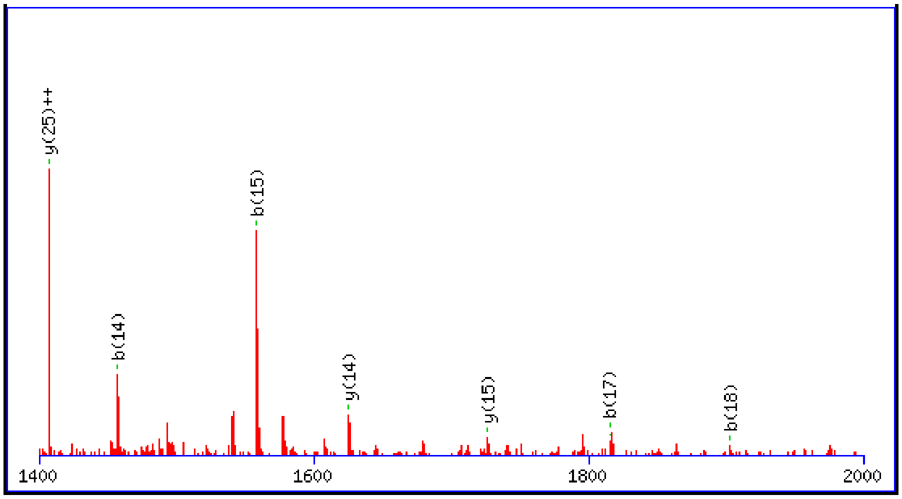

Monoisotopic mass of neutral peptide Mr(calc): 3182.3452
Fixed modifications: Carbamidomethyl (C)
Variable modifications:
N26 : Deamidated_N (N)
Ions Score: 77 **Expect:** 8.9e-006
Matches (Bold Red): 33/320 fragment ions using 40 most intense peaks

#	b	b^{++}	b*	b^{*++}	b^{0}	b^{0++}	Seq.	y	y^{++}	y*	y^{*++}	y^{0}	y^{0++}	#
1	58.0287	29.5180					G							29
2	157.0972	79.0522					V	3126.3310	1563.6691	3109.3044	1555.1558	3108.3204	1554.6638	28
3	272.1241	136.5657			254.1135	127.5604	D	3027.2626	1514.1349	3010.2360	1505.6216	3009.2520	1505.1296	27
4	371.1925	186.0999			353.1819	177.0946	V	2912.2356	1456.6214	2895.2091	1448.1082	2894.2250	1447.6162	26
5	458.2245	229.6159			440.2140	220.6106	S	2813.1672	**1407.0872**	2796.1406	1398.5740	2795.1566	1398.0820	25
6	**586.2831**	293.6452	569.2566	285.1319	568.2726	284.6399	Q	2726.1352	**1363.5712**	2709.1086	1355.0579	2708.1246	1354.5659	24
7	**685.3515**	343.1794	668.3250	334.6661	667.3410	334.1741	V	2598.0766	**1299.5419**	2581.0500	1291.0287	2580.0660	1290.5367	23
8	**756.3886**	378.6980	739.3621	370.1847	738.3781	369.6927	A	2499.0082	**1250.0077**	2481.9816	1241.4945	2480.9976	1241.0024	22
9	**942.4680**	471.7376	925.4414	463.2243	924.4574	462.7323	W	2427.9711	**1214.4892**	2410.9445	1205.9759	2409.9605	1205.4839	21
10	1070.5265	535.7669	1053.5000	527.2536	1052.5160	526.7616	Q	2241.8917	**1121.4495**	2224.8652	**1112.9362**	2223.8812	1112.4442	20
11	1157.5586	579.2829	1140.5320	570.7696	1139.5480	570.2776	S	2113.8332	1057.4202	2096.8066	**1048.9069**	2095.8226	**1048.4149**	19
12	**1285.6171**	643.3122	1268.5906	634.7989	1267.6066	634.3069	Q	2026.8011	**1013.9042**	2009.7746	1005.3909	2008.7906	1004.8989	18
13	1342.6386	671.8229	1325.6121	663.3097	1324.6280	662.8177	G	1898.7426	**949.8749**	1881.7160	941.3616	1880.7320	940.8696	17
14	**1457.6655**	729.3364	1440.6390	720.8231	1439.6550	720.3311	D	1841.7211	921.3642	1824.6946	912.8509	1823.7105	912.3589	16
15	**1558.7132**	779.8603	1541.6867	771.3470	1540.7027	770.8550	T	**1726.6942**	**863.8507**	1709.6676	855.3374	1708.6836	854.8454	15
16	1655.7660	828.3866	1638.7394	819.8734	1637.7554	819.3814	P	**1625.6465**	**813.3269**	1608.6199	804.8136	1607.6359	804.3216	14
17	**1815.7966**	908.4020	1798.7701	899.8887	1797.7861	899.3967	C	1528.5937	764.8005	1511.5672	**756.2872**	1510.5831	755.7952	13
18	**1902.8287**	951.9180	1885.8021	943.4047	1884.8181	942.9127	S	1368.5631	**684.7852**	1351.5365	676.2719	1350.5525	675.7799	12
19	2062.8593	1031.9333	2045.8328	1023.4200	2044.8488	1022.9280	C	1281.5310	641.2692	1264.5045	632.7559	1263.5205	632.2639	11
20	2222.8900	1111.9486	2205.8634	1103.4353	2204.8794	1102.9433	C	**1121.5004**	561.2538	1104.4738	552.7406	1103.4898	552.2485	10
21	2335.9740	1168.4907	2318.9475	1159.9774	2317.9635	1159.4854	I	961.4697	481.2385	944.4432	472.7252	943.4592	472.2332	9
22	2435.0424	1218.0249	2418.0159	1209.5116	2417.0319	1209.0196	V	848.3857	424.6965	831.3591	416.1832	830.3751	415.6912	8
23	2549.0854	1275.0463	2532.0588	1266.5330	2531.0748	1266.0410	N	**749.3173**	375.1623	732.2907	366.6490	731.3067	366.1570	7
24	2663.1283	1332.0678	2646.1017	1323.5545	2645.1177	1323.0625	N	**635.2743**	318.1408	618.2478	309.6275	617.2638	309.1355	6
25	2750.1603	**1375.5838**	2733.1338	1367.0705	2732.1498	1366.5785	S	**521.2314**	261.1193	504.2049	252.6061	503.2208	252.1141	5
26	2865.1873	1433.0973	2848.1607	1424.5840	2847.1767	1424.0920	N	**434.1994**	217.6033	417.1728	209.0901	416.1888	208.5980	4
27	2922.2087	1461.6080	2905.1822	1453.0947	2904.1982	1452.6027	G	**319.1724**	160.0899	302.1459	151.5766	301.1619	151.0846	3
28	3009.2408	1505.1240	2992.2142	1496.6107	2991.2302	1496.1187	S	262.1510	131.5791	245.1244	123.0659	244.1404	122.5738	2
29							R	175.1190	88.0631	158.0924	79.5498			1

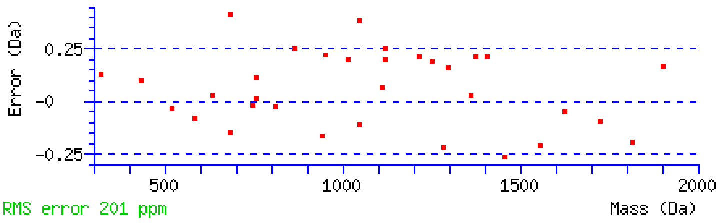

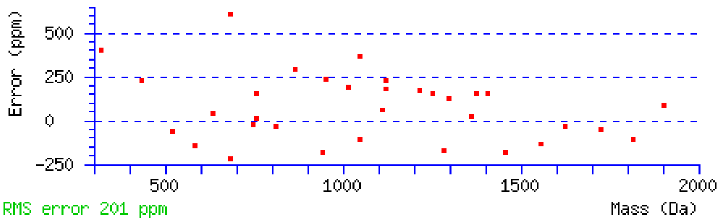

All matches to this query

Score	Mr(calc):	Delta	Sequence
76.8	3182.3452	0.0069	GVDVSQVAWQSQGDTPCSCCIVNNSNGSR
61.4	3182.3452	0.0069	GVDVSQVAWQSQGDTPCSCCIVNNSNGSR
57.4	3182.3452	0.0069	GVDVSQVAWQSQGDTPCSCCIVNNSNGSR
15.6	3181.3612	0.9909	GVDVSQVAWQSQGDTPCSCCIVNNSNGSR
4.8	3180.3416	2.0105	VTSMQAVINETPLPIDLYRYDDVSGK
4.8	3180.3416	2.0105	VTSMQAVINETPLPIDLYRYDDVSGK
4.6	3182.3584	-0.0063	SHHANSPTAGAAKSSPAAKPGSTPSRPSSAK
4.5	3182.3572	-0.0051	AGGYSKATGAMAGAAGGSSAGGSGGASGSGPSGLGSGSK
2.7	3181.3577	0.9944	VGPYGSGDSAPLNEAMAVLQHHDAVTGTAR
2.4	3180.3299	2.0221	FYSSEPNNLREELTRYLFVLQLK

Spectrum No: 156; Query: 837; Rank: 1

Peptide View

MS/MS Fragmentation of **VGPVDPSLNTTYVFFDTFFK**
Found in **IPI00464518**, Tax_Id=10116 Gene_Symbol=Hexb Beta-hexosaminidase beta chain precursor

Match to Query 837: 2294.123028 from(1148.068790,2+)
Title: 091127RatKid_SCX01_15.6755.6755.2.dta
Data file K:\NewmanPaper\Piliang\3SubProteomes\Piliang3SP\mgf5ppm\SCX_3SubProteomes5ppm.mgf

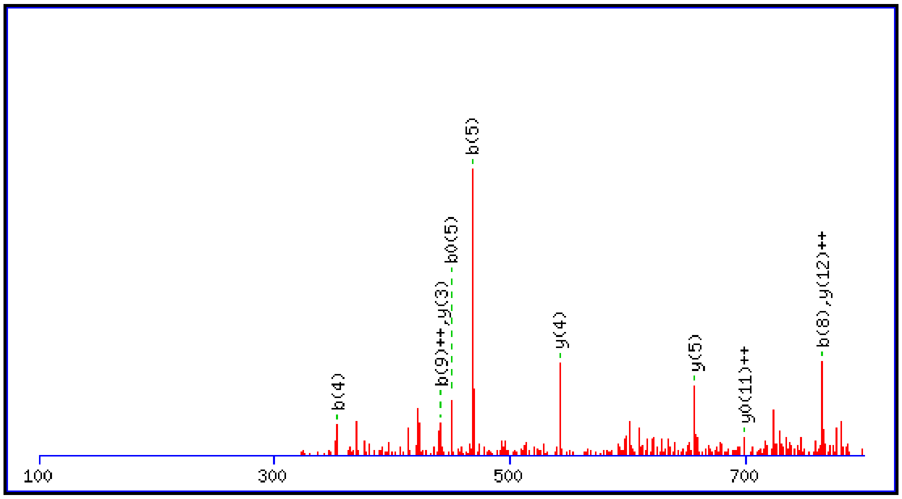

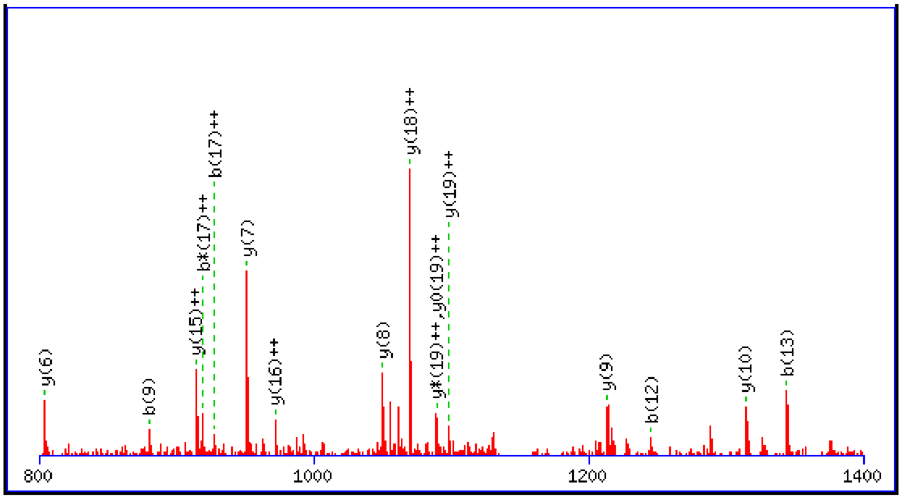

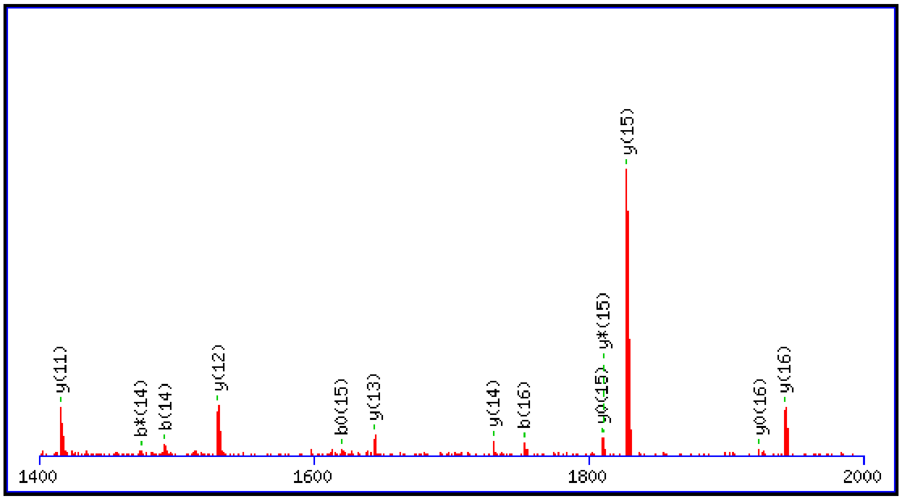

Monoisotopic mass of neutral peptide Mr(calc): 2294.1147
Fixed modifications: Carbamidomethyl (C)
Variable modifications:
N9 : Deamidated_N (N)
Ions Score: 77 **Expect:** 4.8e-006
Matches (Bold Red): 39/198 fragment ions using 72 most intense peaks

#	b	b^{++}	b*	b^{*++}	b^{0}	b^{0++}	Seq.	y	y^{++}	y*	y^{*++}	y^{0}	y^{0++}	#
1	100.0757	50.5415					V							20
2	157.0972	79.0522					G	2196.0536	**1098.5304**	2179.0270	**1090.0171**	2178.0430	**1089.5251**	19
3	254.1499	127.5786					P	2139.0321	**1070.0197**	2122.0055	1061.5064	2121.0215	1061.0144	18
4	**353.2183**	177.1128					V	2041.9793	1021.4933	2024.9528	1012.9800	2023.9688	1012.4880	17
5	**468.2453**	234.6263			**450.2347**	225.6210	D	**1942.9109**	**971.9591**	1925.8844	963.4458	**1924.9004**	962.9538	16
6	565.2980	283.1527			547.2875	274.1474	P	**1827.8840**	**914.4456**	**1810.8574**	905.9324	**1809.8734**	905.4403	15
7	652.3301	326.6687			634.3195	317.6634	S	**1730.8312**	865.9192	1713.8047	857.4060	1712.8206	856.9140	14
8	**765.4141**	383.2107			747.4036	374.2054	L	**1643.7992**	822.4032	1626.7726	813.8900	1625.7886	813.3979	13
9	**880.4411**	**440.7242**	863.4145	432.2109	862.4305	431.7189	N	**1530.7151**	**765.8612**	1513.6886	757.3479	1512.7046	756.8559	12
10	981.4887	491.2480	964.4622	482.7347	963.4782	482.2427	T	**1415.6882**	708.3477	1398.6616	699.8345	1397.6776	**699.3424**	11
11	1082.5364	541.7719	1065.5099	533.2586	1064.5259	532.7666	T	**1314.6405**	657.8239	1297.6140	649.3106	1296.6299	648.8186	10
12	**1245.5998**	623.3035	1228.5732	614.7902	1227.5892	614.2982	Y	**1213.5928**	607.3001	1196.5663	598.7868	1195.5823	598.2948	9
13	**1344.6682**	672.8377	1327.6416	664.3244	1326.6576	663.8324	V	**1050.5295**	525.7684	1033.5029	517.2551	1032.5189	516.7631	8
14	**1491.7366**	746.3719	**1474.7100**	737.8587	1473.7260	737.3666	F	**951.4611**	476.2342	934.4345	467.7209	933.4505	467.2289	7
15	1638.8050	819.9061	1621.7784	811.3929	**1620.7944**	810.9009	F	**804.3927**	402.7000	787.3661	394.1867	786.3821	393.6947	6
16	**1753.8319**	877.4196	1736.8054	868.9063	1735.8214	868.4143	D	**657.3243**	329.1658	640.2977	320.6525	639.3137	320.1605	5
17	1854.8796	**927.9434**	1837.8531	**919.4302**	1836.8691	918.9382	T	**542.2973**	271.6523	525.2708	263.1390	524.2867	262.6470	4
18	2001.9480	1001.4777	1984.9215	992.9644	1983.9375	992.4724	F	**441.2496**	221.1285	424.2231	212.6152			3

**19**	2149.0164	1075.0119	2131.9899	1066.4986	2131.0059	1066.0066	**F**	294.1812	147.5942	277.1547	139.0810			**2**
**20**							**K**	147.1128	74.0600	130.0863	65.5468			**1**

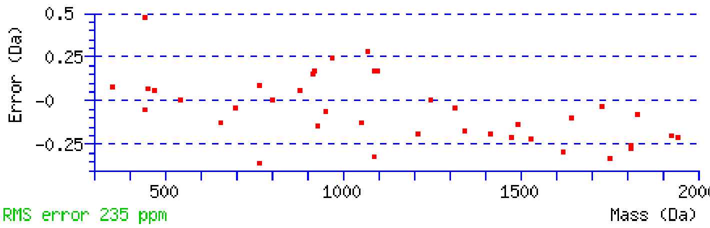

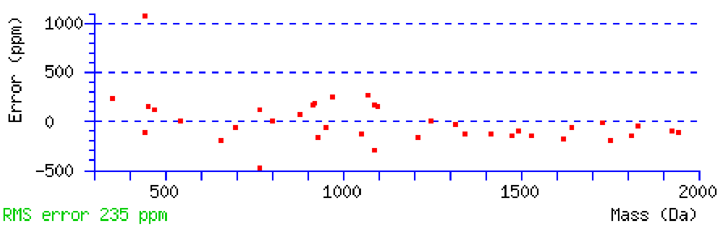

All matches to this query

Score	Mr(calc):	Delta	Sequence
76.8	2294.1147	0.0083	VGPVDPSLNTTYVFFDTFFK
18.8	2293.1307	0.9923	VGPVDPSLNTTYVFFDTFFK

Spectrum No: 157; Query: 209; Rank: 1

Peptide View

MS/MS Fragmentation of **SFSLISGSINQTR**
Found in **IPI00372786**, Tax_Id=10116 Gene_Symbol=Nid2 similar to nidogen 2 isoform 2

Match to Query 209: 1409.717628 from(705.866090,2+)
Title: 091129RatKid_SCX02_17.2327.2327.2.dta
Data file K:\NewmanPaper\Piliang\3SubProteomes\Piliang3SP\mgf5ppm\SCX_3SubProteomes5ppm.mgf

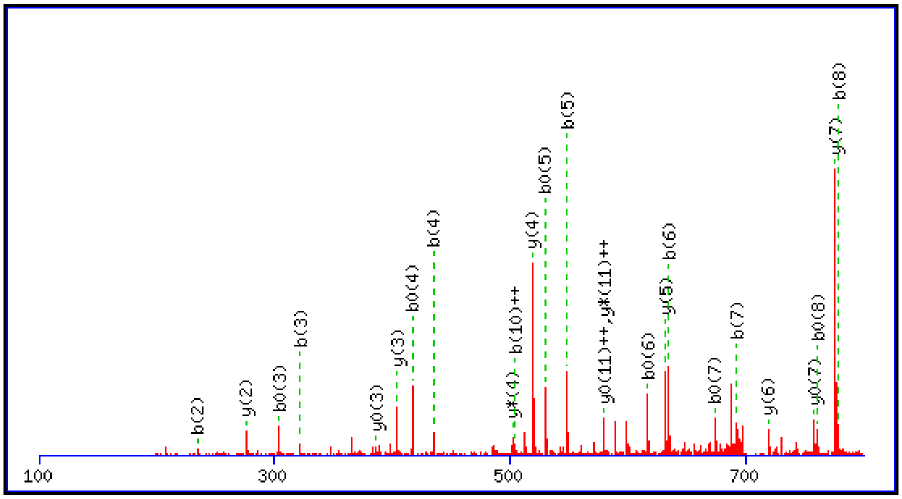

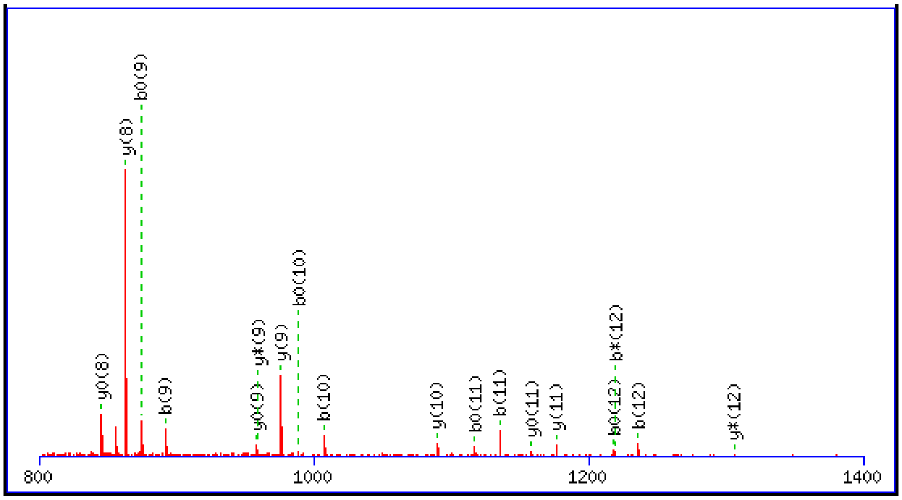

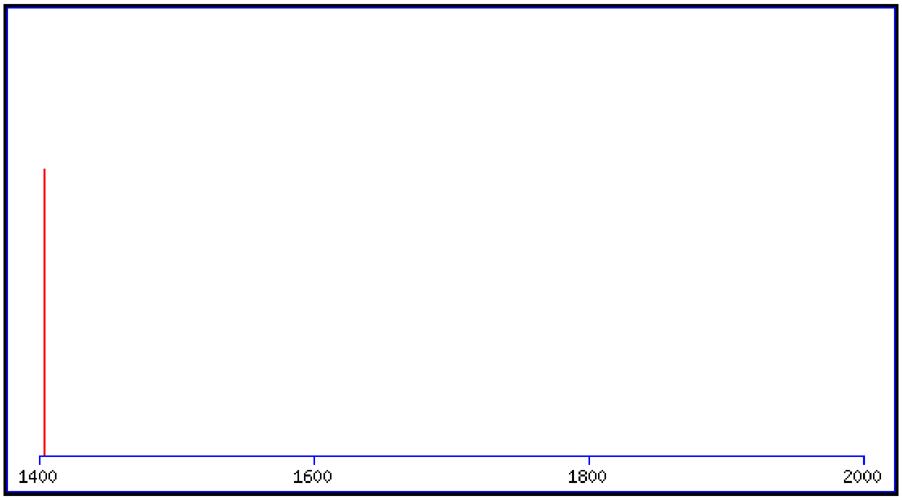

Monoisotopic mass of neutral peptide Mr(calc): 1409.7150
Fixed modifications: Carbamidomethyl (C)
Variable modifications:
N10 : Deamidated_N (N)
Ions Score: 77 **Expect:** 2.8e-006
Matches (Bold Red): 43/124 fragment ions using 84 most intense peaks

#	b	b^{++}	b*	b^{*++}	b^{0}	b^{0++}	Seq.	y	y^{++}	y*	y^{*++}	y^{0}	y^{0++}	#
1	88.0393	44.5233			70.0287	35.5180	S							13
2	**235.1077**	118.0575			217.0972	109.0522	F	1323.6903	662.3488	**1306.6637**	653.8355	1305.6797	653.3435	12
3	**322.1397**	161.5735			**304.1292**	152.5682	S	**1176.6219**	588.8146	1159.5953	**580.3013**	**1158.6113**	**579.8093**	11
4	**435.2238**	218.1155			**417.2132**	209.1103	L	**1089.5899**	545.2986	1072.5633	536.7853	1071.5793	536.2933	10
5	**548.3079**	274.6576			**530.2973**	265.6523	I	**976.5058**	488.7565	**959.4792**	480.2433	**958.4952**	479.7513	9
6	**635.3399**	318.1736			**617.3293**	309.1683	S	**863.4217**	432.2145	846.3952	423.7012	**845.4112**	423.2092	8
7	**692.3614**	346.6843			**674.3508**	337.6790	G	**776.3897**	388.6985	759.3632	380.1852	**758.3791**	379.6932	7
8	**779.3934**	390.2003			**761.3828**	381.1951	S	**719.3682**	360.1878	702.3417	351.6745	701.3577	351.1825	6
9	**892.4775**	446.7424			**874.4669**	437.7371	I	**632.3362**	316.6717	615.3097	308.1585	614.3256	307.6665	5
10	**1007.5044**	**504.2558**	990.4778	495.7426	**989.4938**	495.2506	N	**519.2521**	260.1297	**502.2256**	251.6164	501.2416	251.1244	4
11	**1135.5630**	568.2851	1118.5364	559.7718	**1117.5524**	559.2798	Q	**404.2252**	202.6162	387.1987	194.1030	**386.2146**	193.6110	3
12	**1236.6107**	618.8090	**1219.5841**	610.2957	**1218.6001**	609.8037	T	**276.1666**	138.5870	259.1401	130.0737	258.1561	129.5817	2
13							R	175.1190	88.0631	158.0924	79.5498			1

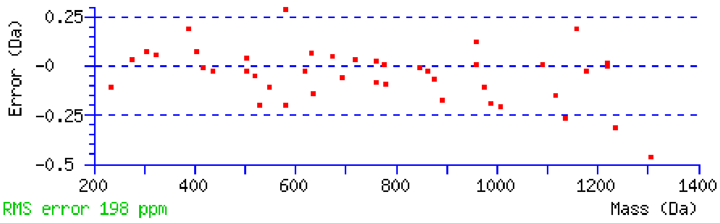

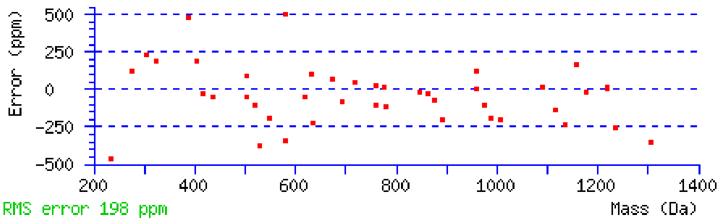

All matches to this query

Score	Mr(calc):	Delta	Sequence
76.7	1409.7150	0.0026	SFSLISGSINQTR
7.6	1409.7224	-0.0048	NEASALLYLLMR
2.5	1409.7263	-0.0087	SVSQETFRISTR
2.1	1409.7303	-0.0127	GLSNFYPDLTKR
2.0	1409.7085	0.0091	MSSLPKPDGHGIR
1.3	1409.7159	0.0017	KFMEKLDACIR
0.2	1408.7054	1.0122	NAMTLMNLDVKK
0.1	1407.6994	2.0183	ISRYIQEQNEK

Spectrum No: 158; Query: 100; Rank: 1

Peptide View

MS/MS Fragmentation of **ELAVAANETAAR**
Found in **IPI00363534**, Tax_Id=10116 Gene_Symbol=Lama1 laminin, alpha 1

Match to Query 100: 1215.609488 from(608.812020,2+)
Title: 091127RatKid_SCX01_18.975.975.2.dta
Data file K:\NewmanPaper\Piliang\3SubProteomes\Piliang3SP\mgf5ppm\SCX_3SubProteomes5ppm.mgf

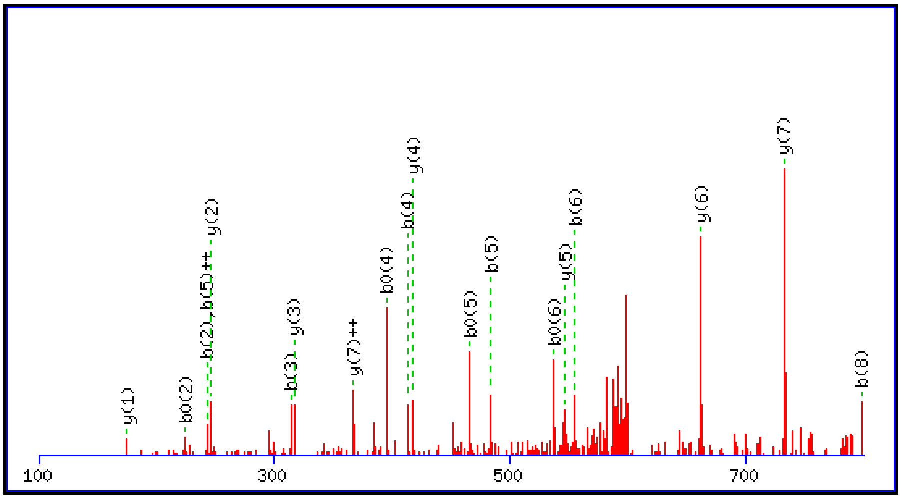

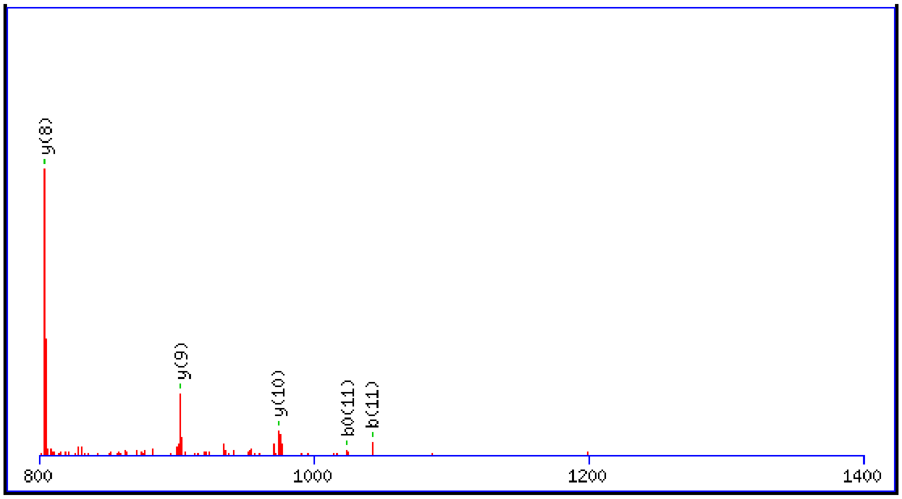

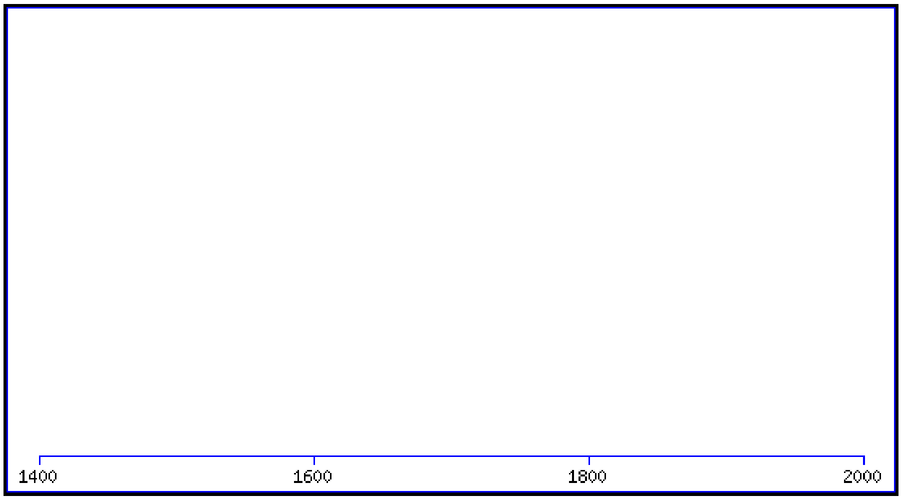

Monoisotopic mass of neutral peptide Mr(calc): 1215.6095
Fixed modifications: Carbamidomethyl (C)
Variable modifications:
N7 : Deamidated_N (N)
Ions Score: 76 **Expect:** 3e-006
Matches (Bold Red): 24/114 fragment ions using 41 most intense peaks

#	b	b++	b*	b*++	b0	b0++	Seq.	y	y++	y*	y*++	y0	y0++	#
1	130.0499	65.5286			112.0393	56.5233	E							12
2	**243.1339**	122.0706			**225.1234**	113.0653	L	1087.5742	544.2907	1070.5477	535.7775	1069.5636	535.2855	11
3	**314.1710**	157.5892			296.1605	148.5839	A	**974.4901**	487.7487	957.4636	479.2354	956.4796	478.7434	10
4	**413.2395**	207.1234			**395.2289**	198.1181	V	**903.4530**	452.2302	886.4265	443.7169	885.4425	443.2249	9
5	**484.2766**	**242.6419**			**466.2660**	233.6366	A	**804.3846**	402.6959	787.3581	394.1827	786.3741	393.6907	8
6	**555.3137**	278.1605			**537.3031**	269.1552	A	**733.3475**	**367.1774**	716.3210	358.6641	715.3369	358.1721	7
7	670.3406	335.6740	653.3141	327.1607	652.3301	326.6687	N	**662.3104**	331.6588	645.2838	323.1456	644.2998	322.6535	6
8	**799.3832**	400.1952	782.3567	391.6820	781.3727	391.1900	E	**547.2835**	274.1454	530.2569	265.6321	529.2729	265.1401	5
9	900.4309	450.7191	883.4043	442.2058	882.4203	441.7138	T	**418.2409**	209.6241	401.2143	201.1108	400.2303	200.6188	4
10	971.4680	486.2376	954.4415	477.7244	953.4574	477.2324	A	**317.1932**	159.1002	300.1666	150.5870			3
11	**1042.5051**	521.7562	1025.4786	513.2429	**1024.4946**	512.7509	A	**246.1561**	123.5817	229.1295	115.0684			2
12							R	**175.1190**	88.0631	158.0924	79.5498			1

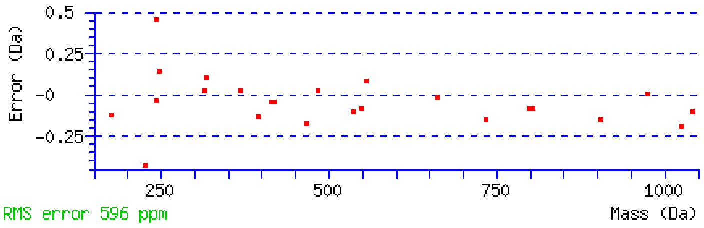

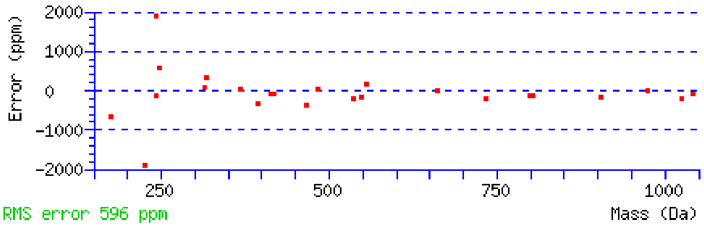

All matches to this query

Score	Mr(calc):	Delta	Sequence
76.3	1215.6095	0.0000	ELAVAANETAAR
5.8	1215.6013	0.0082	NGITPLHVASK
4.6	1215.6095	-0.0000	LEGNTVGVEAAR
4.2	1215.6125	-0.0030	SKVIRSGAYR
3.8	1214.6077	1.0017	TAPAAAVSPMQR
3.7	1215.5983	0.0112	ALGTNPTNAEVK
3.6	1215.6030	0.0065	GGQVVNLMNQR
2.3	1213.6081	2.0014	TKHQTKVHR
1.9	1213.6051	2.0044	QNHTSSISIAR
1.7	1213.5979	2.0116	VTQSNFAVGYK

Spectrum No: 159; Query: 329; Rank: 1

Peptide View

MS/MS Fragmentation of **LEDPTVLQVVNVTK**
Found in **IPI00360957**, Tax_Id=10116 Gene_Symbol=Slc10a5 Sodium/bile acid cotransporter 5 precursor

Match to Query 329: 1554.854088 from(778.434320,2+)
Title: 091127RatKid_SCX01_02.3553.3553.2.dta
Data file K:\NewmanPaper\Piliang\3SubProteomes\Piliang3SP\mgf5ppm\SCX_3SubProteomes5ppm.mgf

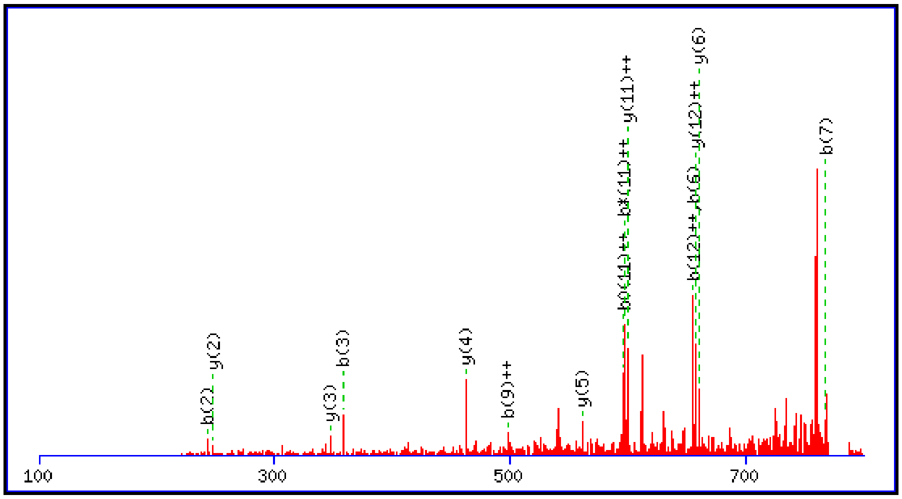

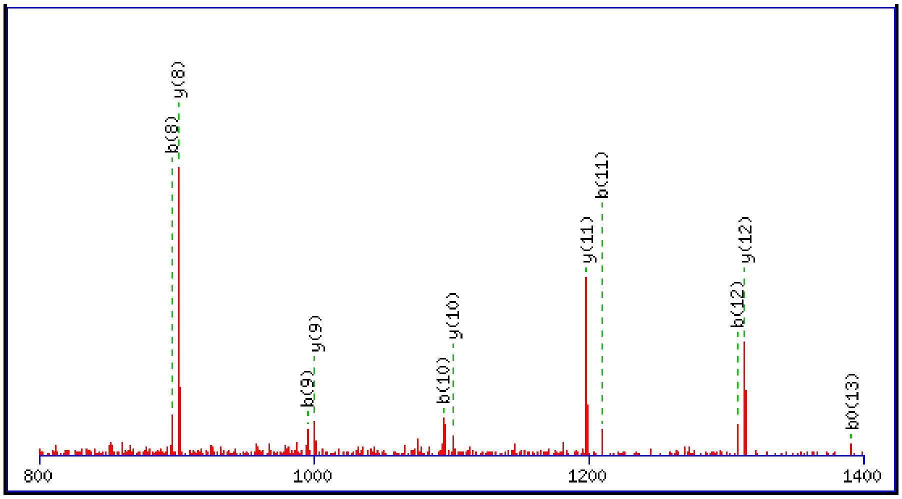

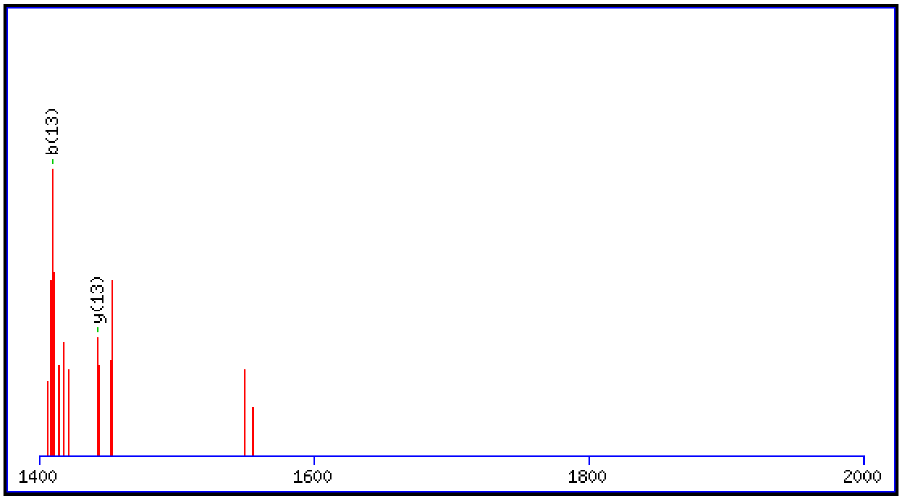

Monoisotopic mass of neutral peptide Mr(calc): 1554.8505
Fixed modifications: Carbamidomethyl (C)
Variable modifications:
N11 : Deamidated_N (N)
Ions Score: 76 **Expect:** 1.7e-006
Matches (Bold Red): 28/138 fragment ions using 49 most intense peaks

#	b	b^{++}	b*	b^{*++}	b^0	b^{0++}	Seq.	y	y^{++}	y*	y^{*++}	y^0	y^{0++}	#
1	114.0913	57.5493					L							14
2	**243.1339**	122.0706			225.1234	113.0653	E	**1442.7737**	721.8905	1425.7471	713.3772	1424.7631	712.8852	13
3	**358.1609**	179.5841			340.1503	170.5788	D	**1313.7311**	**657.3692**	1296.7046	648.8559	1295.7205	648.3639	12
4	455.2136	228.1105			437.2031	219.1052	P	**1198.7042**	**599.8557**	1181.6776	591.3424	1180.6936	590.8504	11
5	556.2613	278.6343			538.2508	269.6290	T	**1101.6514**	551.3293	1084.6248	542.8161	1083.6408	542.3241	10
6	**655.3297**	328.1685			637.3192	319.1632	V	**1000.6037**	500.8055	983.5772	492.2922	982.5932	491.8002	9
7	**768.4138**	384.7105			750.4032	375.7053	L	**901.5353**	451.2713	884.5088	442.7580	883.5247	442.2660	8
8	**896.4724**	448.7398	879.4458	440.2266	878.4618	439.7345	Q	788.4512	394.7293	771.4247	386.2160	770.4407	385.7240	7
9	**995.5408**	**498.2740**	978.5142	489.7608	977.5302	489.2688	V	**660.3927**	330.7000	643.3661	322.1867	642.3821	321.6947	6
10	**1094.6092**	547.8082	1077.5827	539.2950	1076.5986	538.8030	V	**561.3242**	281.1658	544.2977	272.6525	543.3137	272.1605	5
11	**1209.6361**	605.3217	1192.6096	**596.8084**	1191.6256	**596.3164**	N	**462.2558**	231.6316	445.2293	223.1183	444.2453	222.6263	4
12	**1308.7046**	**654.8559**	1291.6780	646.3426	1290.6940	645.8506	V	**347.2289**	174.1181	330.2023	165.6048	329.2183	165.1128	3
13	**1409.7522**	705.3798	1392.7257	696.8665	**1391.7417**	696.3745	T	**248.1605**	124.5839	231.1339	116.0706	230.1499	115.5786	2
14							K	147.1128	74.0600	130.0863	65.5468			1

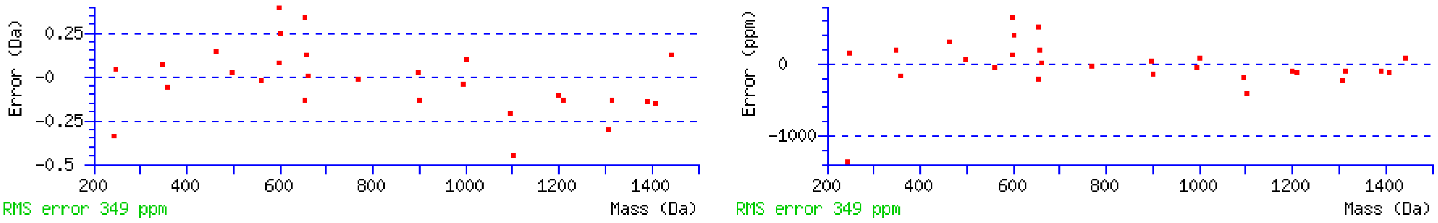

All matches to this query

Score	Mr(calc):	Delta	Sequence
76.2	1554.8505	0.0036	LEDPTVLQVVNVTK
6.1	1554.8513	0.0028	ILMPMASVVAVAEPK
4.2	1554.8518	0.0023	DSKITDHFVRIPK
2.1	1554.8406	0.0135	SLQXRFISVSNLK

Spectrum No: 160; Query: 1089; Rank: 1

Peptide View

MS/MS Fragmentation of **DSSDEANCTTLCSQKEFECGSGECILR**
Found in **IPI00205325**, Tax_Id=10116 Gene_Symbol=Lrp2 Low-density lipoprotein receptor-related protein 2 precursor

Match to Query 1089: 3153.273042 from(1052.098290,3+)
Title: 091127RatKid_SCX01_13.2603.2603.3.dta
Data file K:\NewmanPaper\Piliang\3SubProteomes\Piliang3SP\mgf5ppm\SCX_3SubProteomes5ppm.mgf

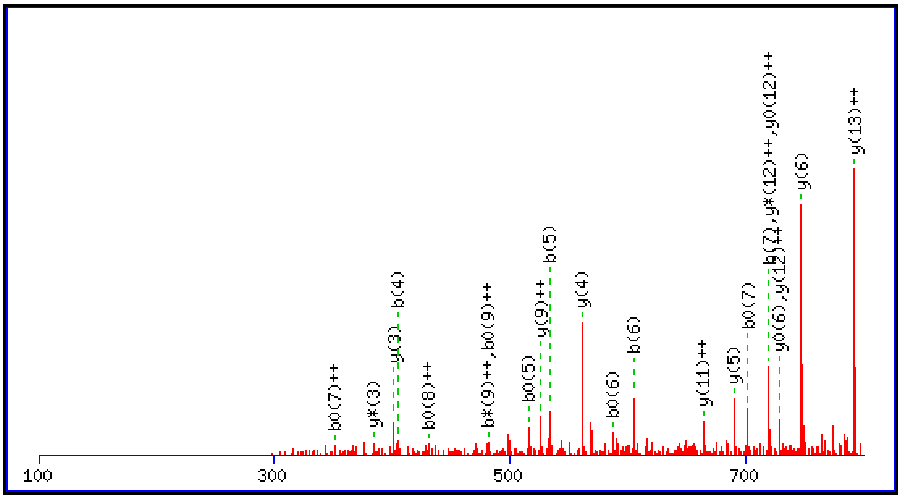

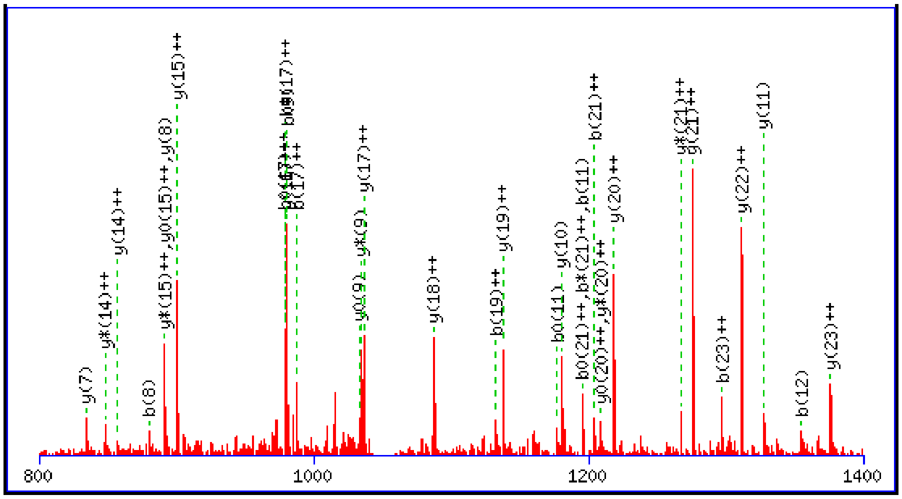

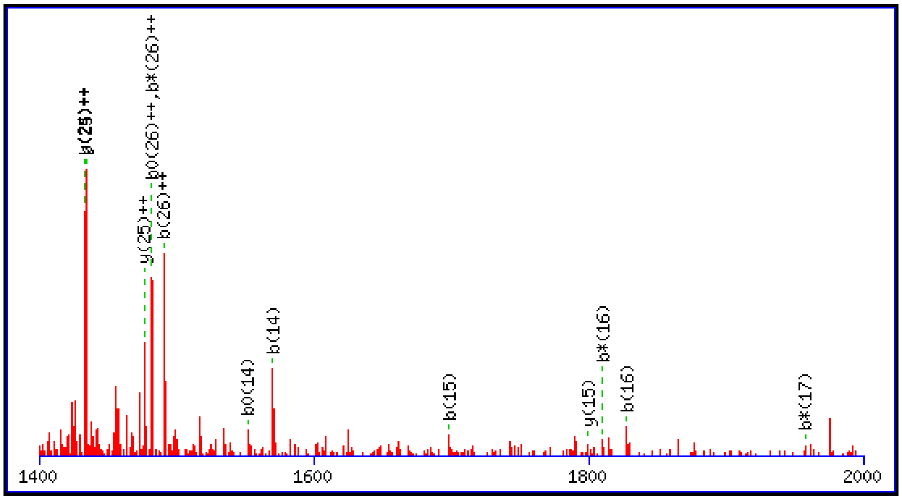

Monoisotopic mass of neutral peptide Mr(calc): 3153.2631
Fixed modifications: Carbamidomethyl (C)
Variable modifications:
N7 : Deamidated_N (N)
Ions Score: 76 **Expect:** 8.9e-006
Matches (Bold Red): 71/292 fragment ions using 140 most intense peaks

#	b	b^{++}	b*	b^{*++}	b^0	b^{0++}	Seq.	y	y^{++}	y*	y^{*++}	y^0	y^{0++}	#
1	116.0342	58.5207			98.0237	49.5155	D							27
2	203.0662	102.0368			185.0557	93.0315	S	3039.2435	1520.1254	3022.2169	1511.6121	3021.2329	1511.1201	26
3	290.0983	145.5528			272.0877	136.5475	S	2952.2114	**1476.6094**	2935.1849	1468.0961	2934.2009	1467.6041	25
4	**405.1252**	203.0662			387.1147	194.0610	D	2865.1794	**1433.0933**	2848.1529	1424.5801	2847.1688	1424.0881	24
5	**534.1678**	267.5875			**516.1572**	258.5823	E	2750.1525	**1375.5799**	2733.1259	1367.0666	2732.1419	1366.5746	23
6	**605.2049**	303.1061			**587.1944**	294.1008	A	2621.1099	**1311.0586**	2604.0833	1302.5453	2603.0993	1302.0533	22
7	**720.2319**	360.6196	703.2053	352.1063	**702.2213**	**351.6143**	N	2550.0728	**1275.5400**	2533.0462	**1267.0267**	2532.0622	1266.5347	21
8	**880.2625**	440.6349	863.2360	432.1216	862.2519	**431.6296**	C	2435.0458	**1218.0265**	2418.0193	**1209.5133**	2417.0353	**1209.0213**	20
9	**981.3102**	491.1587	964.2836	**482.6455**	963.2996	**482.1535**	T	2275.0152	**1138.0112**	2257.9886	1129.4979	2257.0046	1129.0059	19
10	1082.3579	541.6826	1065.3313	533.1693	1064.3473	532.6773	T	2173.9675	**1087.4874**	2156.9409	1078.9741	2155.9569	1078.4821	18
11	**1195.4419**	598.2246	1178.4154	589.7113	**1177.4314**	589.2193	L	2072.9198	**1036.9635**	2055.8933	1028.4503	2054.9092	1027.9583	17
12	**1355.4726**	678.2399	1338.4460	669.7267	1337.4620	669.2346	C	1959.8357	**980.4215**	1942.8092	971.9082	1941.8252	971.4162	16
13	1442.5046	721.7559	1425.4781	713.2427	1424.4940	712.7507	S	**1799.8051**	**900.4062**	1782.7785	**891.8929**	1781.7945	**891.4009**	15
14	**1570.5632**	785.7852	1553.5366	777.2720	**1552.5526**	776.7799	Q	1712.7731	**856.8902**	1695.7465	**848.3769**	1694.7625	847.8849	14
15	**1698.6582**	849.8327	1681.6316	841.3194	1680.6476	840.8274	K	1584.7145	**792.8609**	1567.6879	784.3476	1566.7039	783.8556	13
16	**1827.7007**	914.3540	**1810.6742**	905.8407	1809.6902	905.3487	E	1456.6195	**728.8134**	1439.5930	**720.3001**	1438.6090	**719.8081**	12
17	1974.7692	**987.8882**	**1957.7426**	**979.3749**	1956.7586	**978.8829**	F	**1327.5769**	**664.2921**	1310.5504	655.7788	1309.5664	655.2868	11
18	2103.8117	1052.4095	2086.7852	1043.8962	2085.8012	1043.4042	E	**1180.5085**	590.7579	1163.4820	582.2446	1162.4980	581.7526	10

19	2263.8424	**1132.4248**	2246.8158	1123.9116	2245.8318	1123.4196	C	1051.4659	**526.2366**	**1034.4394**	517.7233	**1033.4554**	517.2313	9
20	2320.8639	1160.9356	2303.8373	1152.4223	2302.8533	1151.9303	G	**891.4353**	446.2213	874.4087	437.7080	873.4247	437.2160	8
21	2407.8959	**1204.4516**	2390.8693	**1195.9383**	2389.8853	**1195.4463**	S	**834.4138**	417.7105	817.3873	409.1973	816.4032	408.7053	7
22	2464.9174	1232.9623	2447.8908	1224.4490	2446.9068	1223.9570	G	**747.3818**	374.1945	730.3552	365.6813	**729.3712**	365.1892	6
23	2593.9599	**1297.4836**	2576.9334	1288.9703	2575.9494	1288.4783	E	**690.3603**	345.6838	673.3338	337.1705	672.3498	336.6785	5
24	2753.9906	1377.4989	2736.9640	1368.9857	2735.9800	1368.4937	C	**561.3177**	281.1625	544.2912	272.6492			4
25	2867.0747	**1434.0410**	2850.0481	1425.5277	2849.0641	1425.0357	I	**401.2871**	201.1472	**384.2605**	192.6339			3
26	2980.1587	**1490.5830**	2963.1322	**1482.0697**	2962.1482	**1481.5777**	L	288.2030	144.6051	271.1765	136.0919			2
27							R	175.1190	88.0631	158.0924	79.5498			1

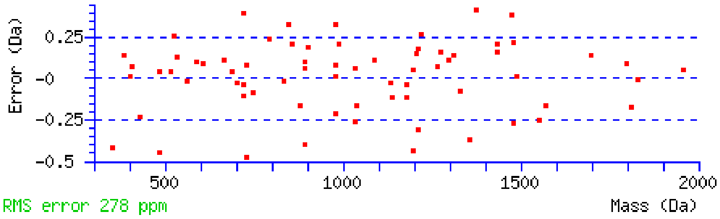

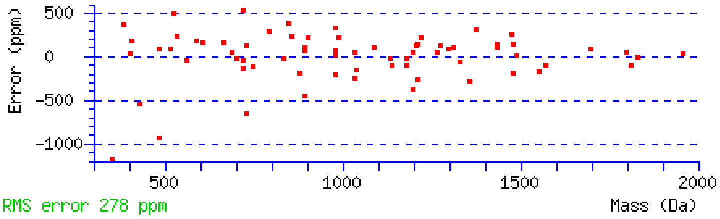

All matches to this query

Score	Mr(calc):	Delta	Sequence
76.0	3153.2631	0.0099	DSSDEANCTTLCSQKEFECGSGECILR
32.1	3152.2791	0.9939	DSSDEANCTTLCSQKEFECGSGECILR
5.1	3153.2776	-0.0046	LEHLSVSHNNLTTLHGELSSLPSLR
4.2	3153.2776	-0.0046	LEHLSVSHNNLTTLHGELSSLPSLR
4.1	3152.2433	1.0298	GPANIKWGDAGDEYVMESTGIFNTMEK
2.6	3151.2790	1.9941	RFQYEEEVKANPHNYDAWFDYLR
1.0	3151.2405	2.0325	YDYLMTLHGVVNESTVCLMGHERR
1.0	3151.2405	2.0325	YDYLMTLHGVVNESTVCLMGHERR
0.7	3152.2433	1.0298	GPANIKWGDAGDEYVMESTGIFNTMEK
0.7	3152.2433	1.0298	GPANIKWGDAGDEYVMESTGIFNTMEK

Spectrum No: 161; Query: 303; Rank: 1

Peptide View

MS/MS Fragmentation of **EDNEGVFNGSWGGR**
Found in **IPI00208917**, Tax_Id=10116 Gene_Symbol=Aldh7a1 similar to aldehyde dehydrogenase family 7, member A1

Match to Query 303: 1523.629848 from(762.822200,2+)
Title: 091129RatKid_SCX02_23.1862.1862.2.dta
Data file K:\NewmanPaper\Piliang\3SubProteomes\Piliang3SP\mgf5ppm\SCX_3SubProteomes5ppm.mgf

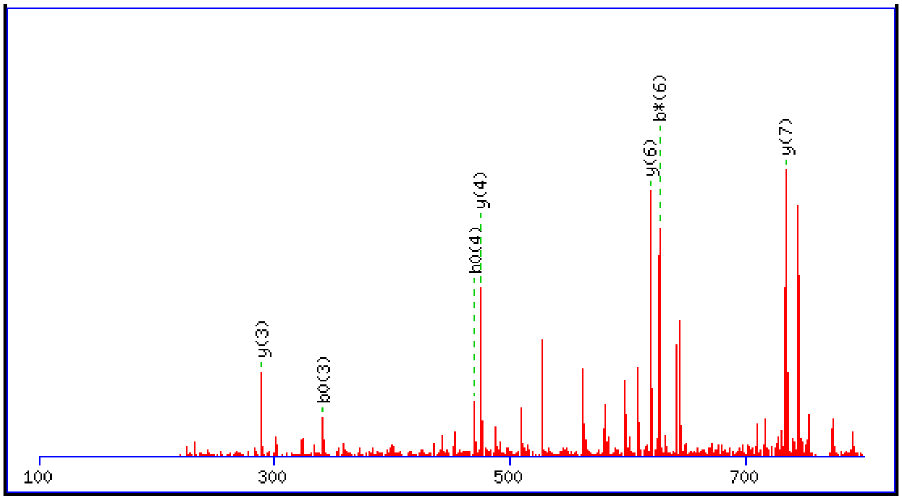

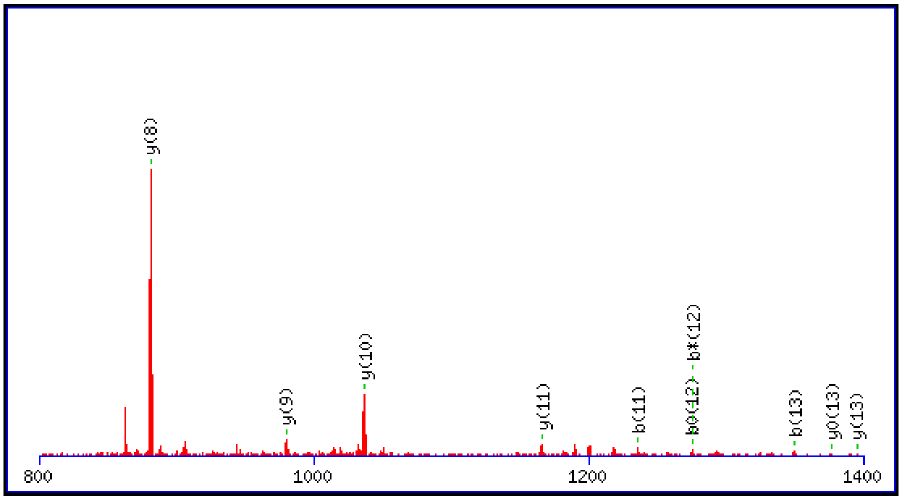

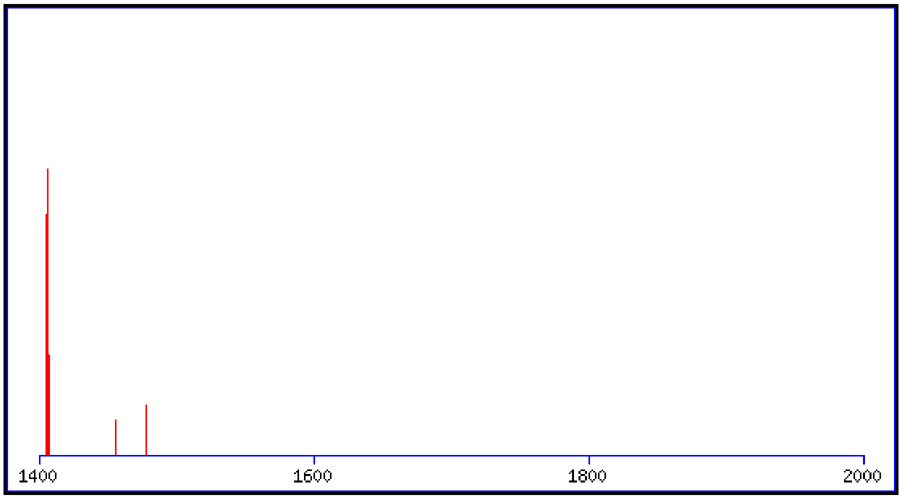

Monoisotopic mass of neutral peptide Mr(calc): 1523.6277
Fixed modifications: Carbamidomethyl (C)
Variable modifications:
N8 : Deamidated_N (N)
Ions Score: 76 **Expect:** 3.4e-006
Matches (Bold Red): 17/144 fragment ions using 33 most intense peaks

#	b	b^{++}	b*	b^{*++}	b^0	b^{0++}	Seq.	y	y^{++}	y*	y^{*++}	y^0	y^{0++}	#
1	130.0499	65.5286			112.0393	56.5233	E							14
2	245.0768	123.0420			227.0662	114.0368	D	**1395.5924**	698.2998	1378.5658	689.7866	**1377.5818**	689.2945	13
3	359.1197	180.0635	342.0932	171.5502	**341.1092**	171.0582	N	1280.5654	640.7864	1263.5389	632.2731	1262.5549	631.7811	12
4	488.1623	244.5848	471.1358	236.0715	**470.1518**	235.5795	E	**1166.5225**	583.7649	1149.4960	575.2516	1148.5119	574.7596	11
5	545.1838	273.0955	528.1572	264.5823	527.1732	264.0903	G	**1037.4799**	519.2436	1020.4534	510.7303	1019.4693	510.2383	10

**6**	644.2522	322.6297	**627.2257**	314.1165	626.2416	313.6245	**V**	**980.4584**	490.7329	963.4319	482.2196	962.4479	481.7276	**9**
**7**	791.3206	396.1640	774.2941	387.6507	773.3101	387.1587	**F**	**881.3900**	441.1987	864.3635	432.6854	863.3795	432.1934	**8**
**8**	906.3476	453.6774	889.3210	445.1641	888.3370	444.6721	**N**	**734.3216**	367.6644	717.2951	359.1512	716.3111	358.6592	**7**
**9**	963.3690	482.1882	946.3425	473.6749	945.3585	473.1829	**G**	**619.2947**	310.1510	602.2681	301.6377	601.2841	301.1457	**6**
**10**	1050.4011	525.7042	1033.3745	517.1909	1032.3905	516.6989	**S**	562.2732	281.6402	545.2467	273.1270	544.2627	272.6350	**5**
**11**	**1236.4804**	618.7438	1219.4538	610.2305	1218.4698	609.7385	**W**	**475.2412**	238.1242	458.2146	229.6110			**4**
**12**	1293.5018	647.2546	**1276.4753**	638.7413	**1275.4913**	638.2493	**G**	**289.1619**	145.0846	272.1353	136.5713			**3**
**13**	**1350.5233**	675.7653	1333.4967	667.2520	1332.5127	666.7600	**G**	232.1404	116.5738	215.1139	108.0606			**2**
**14**							**R**	175.1190	88.0631	158.0924	79.5498			**1**

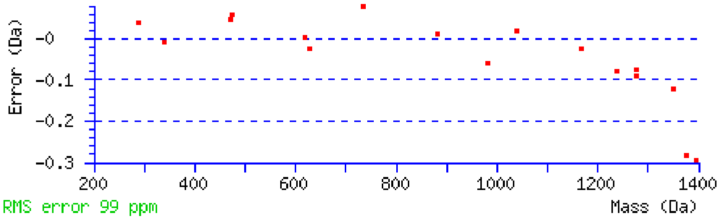

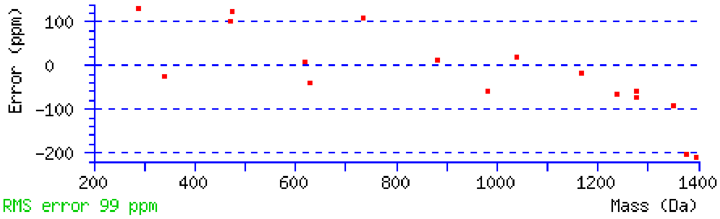

All matches to this query

Score	Mr(calc):	Delta	Sequence
76.0	1523.6277	0.0021	EDNEGVFNGSWGGR
50.0	1523.6277	0.0021	EDNEGVFNGSWGGR
7.2	1523.6171	0.0128	YTRNGQLATPSR
6.3	1523.6323	-0.0025	SFTIWNAGKQGR
6.0	1522.6413	0.9886	NGKEVDSSHEKGR
6.0	1522.6413	0.9886	NGKEVDSSHEKGR
4.5	1523.6228	0.0070	MTSPWGHSADLAR
4.3	1522.6371	0.9927	GVLFQVHTGYSR
4.2	1522.6218	1.0080	SINFTPRSTSPR
4.2	1522.6218	1.0080	SINFTPRSTSPR

Spectrum No: 162; Query: 172; Rank: 1

Peptide View

MS/MS Fragmentation of **GVAVTNTSQLGFR**
Found in **IPI00192845**, Tax_Id=10116 Gene_Symbol=P2rx4 40 kDa protein

Match to Query 172: 1349.697708 from(675.856130,2+)
Title: 091129RatKid_SCX02_12.1602.1602.2.dta
Data file K:\NewmanPaper\Piliang\3SubProteomes\Piliang3SP\mgf5ppm\SCX_3SubProteomes5ppm.mgf

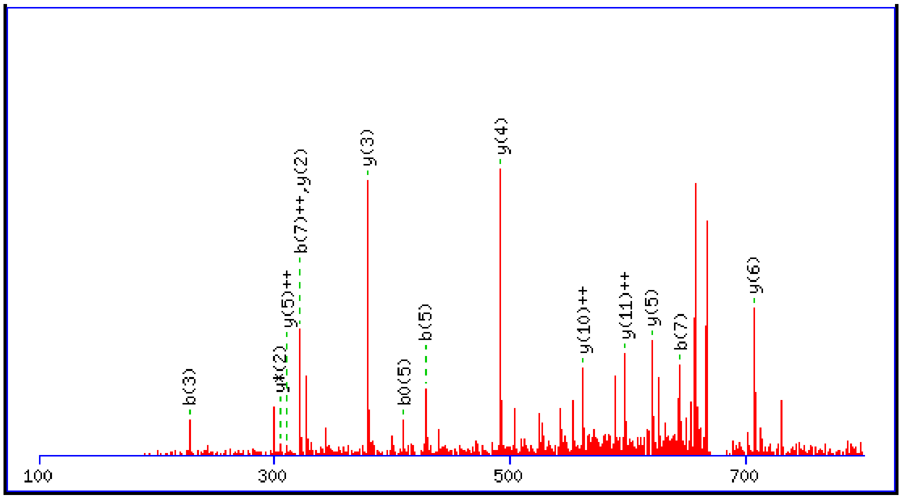

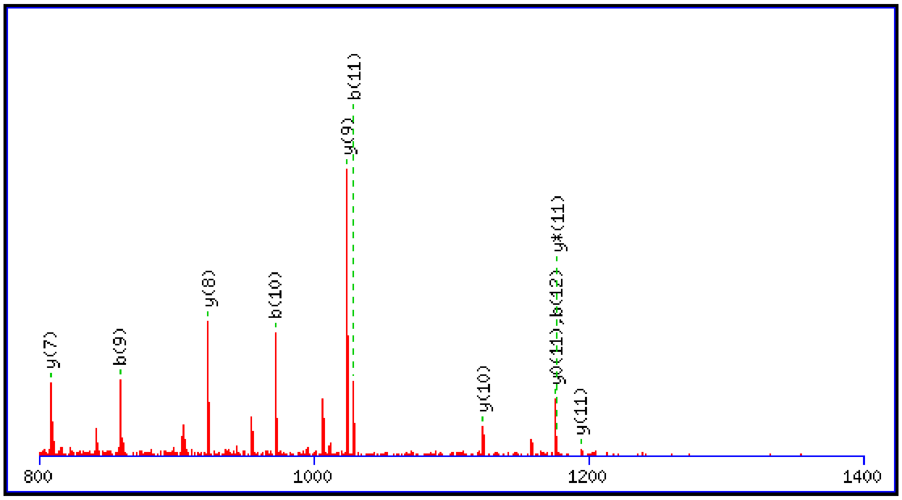

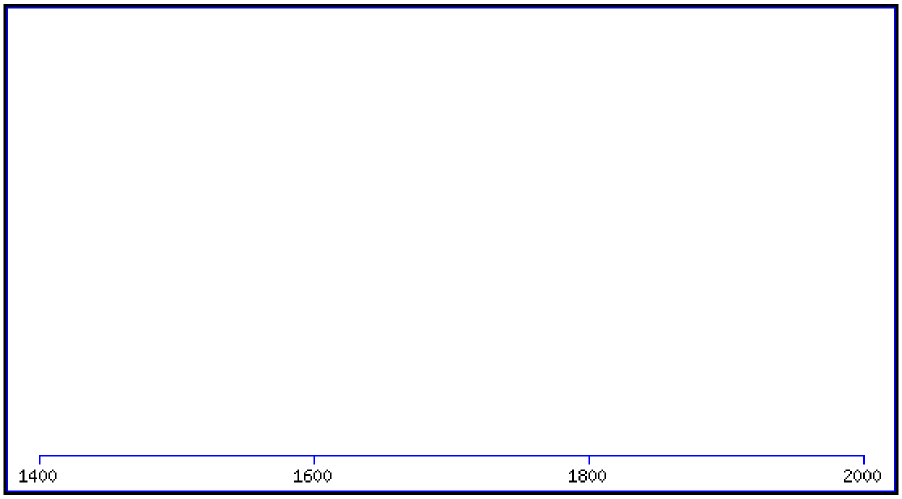

Monoisotopic mass of neutral peptide Mr(calc): 1349.6939
Fixed modifications: Carbamidomethyl (C)
Variable modifications:
N6 : Deamidated_N (N)
Ions Score: 76 **Expect:** 3.1e-006
Matches (Bold Red): 25/116 fragment ions using 40 most intense peaks

#	b	b^{++}	b*	b^{*++}	b^0	b^{0++}	Seq.	y	y^{++}	y*	y^{*++}	y^0	y^{0++}	#
1	58.0287	29.5180					G							13
2	157.0972	79.0522					V	1293.6797	647.3435	1276.6532	638.8302	1275.6692	638.3382	12
3	**228.1343**	114.5708					A	**1194.6113**	**597.8093**	**1177.5848**	589.2960	**1176.6008**	588.8040	11
4	327.2027	164.1050					V	**1123.5742**	**562.2907**	1106.5477	553.7775	1105.5636	553.2855	10
5	**428.2504**	214.6288			**410.2398**	205.6235	T	**1024.5058**	512.7565	1007.4792	504.2433	1006.4952	503.7513	9

6	543.2773	272.1423	526.2507	263.6290	525.2667	263.1370	N	923.4581	462.2327	906.4316	453.7194	905.4476	453.2274	8
7	644.3250	322.6661	627.2984	314.1529	626.3144	313.6608	T	808.4312	404.7192	791.4046	396.2060	790.4206	395.7139	7
8	731.3570	366.1821	714.3305	357.6689	713.3464	357.1769	S	707.3835	354.1954	690.3570	345.6821	689.3729	345.1901	6
9	859.4156	430.2114	842.3890	421.6982	841.4050	421.2061	Q	620.3515	310.6794	603.3249	302.1661			5
10	972.4996	486.7535	955.4731	478.2402	954.4891	477.7482	L	492.2929	246.6501	475.2663	238.1368			4
11	1029.5211	515.2642	1012.4946	506.7509	1011.5105	506.2589	G	379.2088	190.1081	362.1823	181.5948			3
12	1176.5895	588.7984	1159.5630	580.2851	1158.5790	579.7931	F	322.1874	161.5973	305.1608	153.0840			2
13							R	175.1190	88.0631	158.0924	79.5498			1

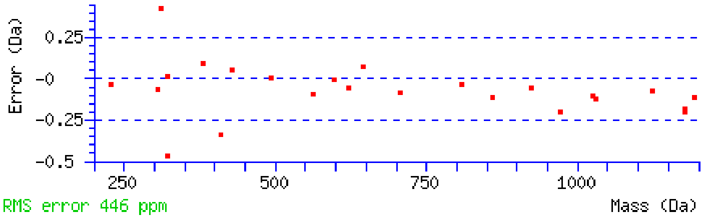

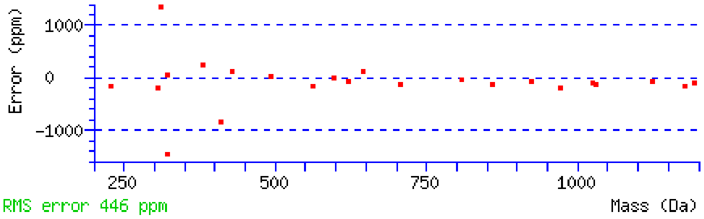

All matches to this query

Score	Mr(calc):	Delta	Sequence
75.9	1349.6939	0.0038	GVAVTNTSQLGFR
10.8	1349.6912	0.0065	RAHQLRPNDSR
8.8	1349.6969	0.0008	RTGLLHALSFR
6.6	1349.7068	-0.0091	NLSISVSGLRPK
5.3	1348.7017	0.9960	KTKHVLPSGFR
3.8	1349.6874	0.0103	VKVGVNGFGCTGR
3.2	1349.6929	0.0048	AGVVSRLERQR
3.1	1349.7068	-0.0091	NLSISVSGLRPK
0.7	1349.6952	0.0025	RASNAFKTWNR

Spectrum No: 163; Query: 719; Rank: 1

Peptide View

MS/MS Fragmentation of **VFTAQTQPHEEQLNGTFR**
Found in **IPI00365922**, Tax_Id=10116 Gene_Symbol=LOC362795 LOC362795 protein

Match to Query 719: 2102.997928 from(1052.506240,2+)
Title: 091129RatKid_SCX02_25.1685.1685.2.dta
Data file K:\NewmanPaper\Piliang\3SubProteomes\Piliang3SP\mgf5ppm\SCX_3SubProteomes5ppm.mgf

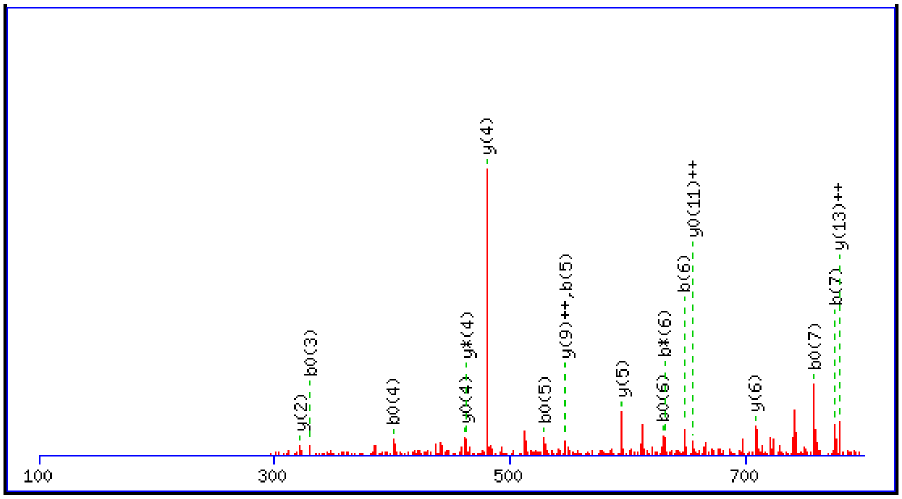

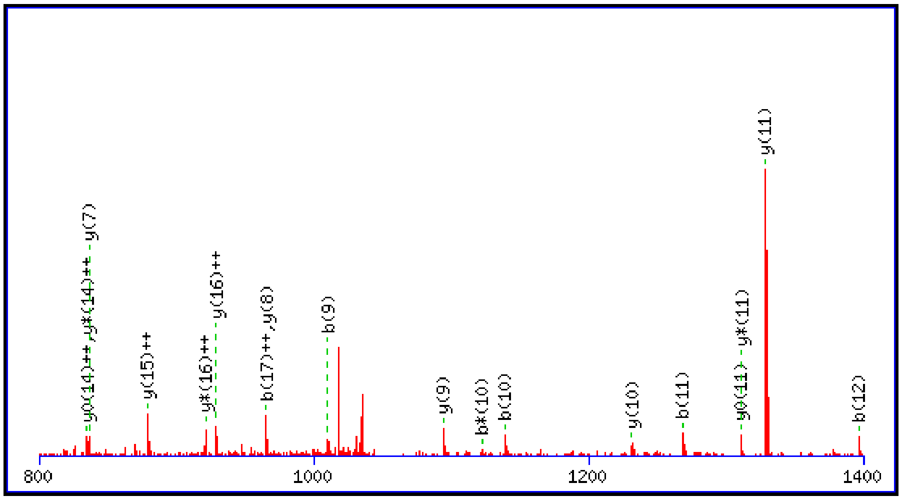

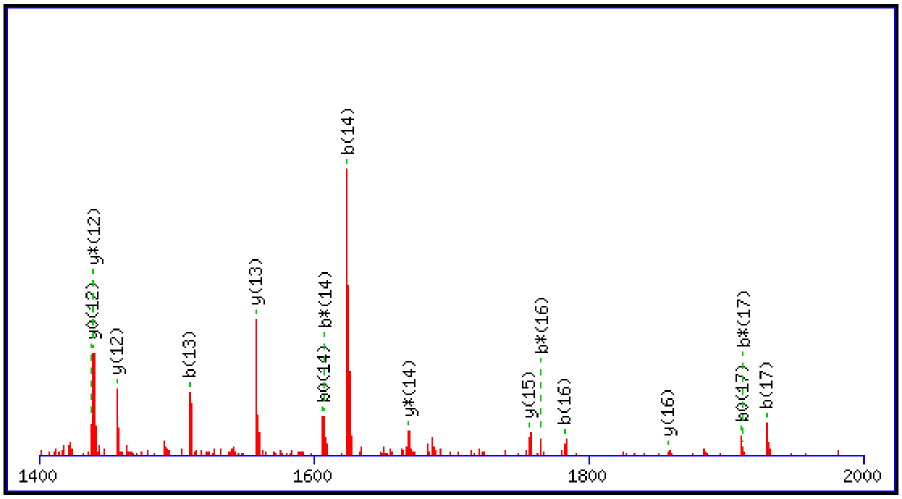

Monoisotopic mass of neutral peptide Mr(calc): 2103.0021
Fixed modifications: Carbamidomethyl (C)
Variable modifications:
N14 : Deamidated_N (N)
Ions Score: 76 **Expect:** 6.6e-006
Matches (Bold Red): 52/188 fragment ions using 93 most intense peaks

#	b	b^{++}	b*	b^{*++}	b^0	b^{0++}	Seq.	y	y^{++}	y*	y^{*++}	y^0	y^{0++}	#
1	100.0757	50.5415					V							18
2	247.1441	124.0757					F	2004.9410	1002.9741	1987.9144	994.4609	1986.9304	993.9689	17
3	348.1918	174.5995			**330.1812**	165.5942	T	**1857.8726**	**929.4399**	1840.8460	**920.9267**	1839.8620	920.4346	16
4	419.2289	210.1181			**401.2183**	201.1128	A	**1756.8249**	**878.9161**	1739.7983	870.4028	1738.8143	869.9108	15
5	**547.2875**	274.1474	530.2609	265.6341	**529.2769**	265.1421	Q	1685.7878	843.3975	**1668.7612**	**834.8843**	1667.7772	**834.3922**	14

**6**	**648.3352**	324.6712	**631.3086**	316.1579	**630.3246**	315.6659	**T**	**1557.7292**	**779.3682**	1540.7027	770.8550	1539.7186	770.3630	**13**
**7**	**776.3937**	388.7005	759.3672	380.1872	**758.3832**	379.6952	**Q**	**1456.6815**	728.8444	**1439.6550**	720.3311	**1438.6710**	719.8391	**12**
**8**	873.4465	437.2269	856.4199	428.7136	855.4359	428.2216	**P**	**1328.6229**	664.8151	**1311.5964**	656.3018	**1310.6124**	**655.8098**	**11**
**9**	**1010.5054**	505.7563	993.4789	497.2431	992.4948	496.7511	**H**	**1231.5702**	616.2887	1214.5436	607.7755	1213.5596	607.2834	**10**
**10**	**1139.5480**	570.2776	**1122.5215**	561.7644	1121.5374	561.2724	**E**	**1094.5113**	**547.7593**	1077.4847	539.2460	1076.5007	538.7540	**9**
**11**	**1268.5906**	634.7989	1251.5640	626.2857	1250.5800	625.7937	**E**	**965.4687**	483.2380	948.4421	474.7247	947.4581	474.2327	**8**
**12**	**1396.6492**	698.8282	1379.6226	690.3149	1378.6386	689.8229	**Q**	**836.4261**	418.7167	819.3995	410.2034	818.4155	409.7114	**7**
**13**	**1509.7332**	755.3703	1492.7067	746.8570	1491.7227	746.3650	**L**	**708.3675**	354.6874	691.3410	346.1741	690.3569	345.6821	**6**
**14**	**1624.7602**	812.8837	**1607.7336**	804.3704	**1606.7496**	803.8784	**N**	**595.2834**	298.1454	578.2569	289.6321	577.2729	289.1401	**5**
**15**	1681.7816	841.3945	1664.7551	832.8812	1663.7711	832.3892	**G**	**480.2565**	240.6319	**463.2300**	232.1186	**462.2459**	231.6266	**4**
**16**	**1782.8293**	891.9183	**1765.8028**	883.4050	1764.8187	882.9130	**T**	423.2350	212.1212	406.2085	203.6079	405.2245	203.1159	**3**
**17**	**1929.8977**	**965.4525**	**1912.8712**	956.9392	**1911.8872**	956.4472	**F**	**322.1874**	161.5973	305.1608	153.0840			**2**
**18**							**R**	175.1190	88.0631	158.0924	79.5498			**1**

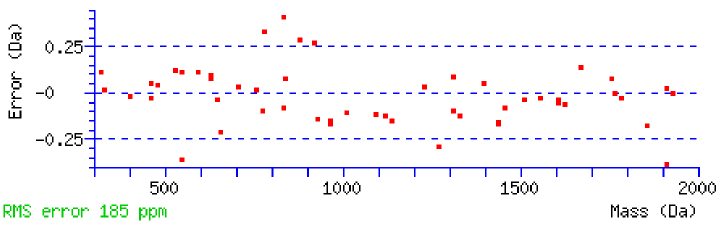

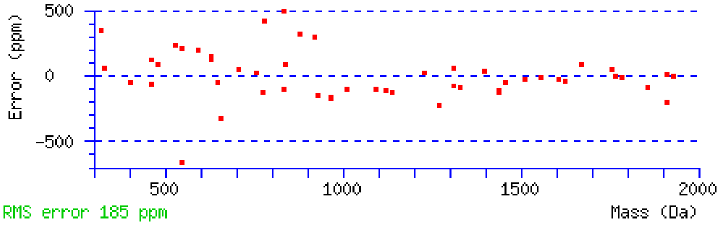

All matches to this query

Score	Mr(calc):	Delta	Sequence
75.8	2103.0021	-0.0042	VFTAQTQPHEEQLNGTFR
4.3	2103.0176	-0.0197	ITNANELKFLDEFLLNK
3.3	2103.0167	-0.0188	VFVNCNTSIRHLDGSVER
2.8	2103.0167	-0.0188	VFVNCNTSIRHLDGSVER
1.8	2102.9803	0.0176	INRDRLQYYSANQMSAR
0.3	2101.9958	1.0021	SMKVQALRDDSTMGYMAAK

Spectrum No: 164; Query: 294; Rank: 1

Peptide View

MS/MS Fragmentation of **FINDYVSNQTQGK**
Found in **IPI00200593**, Tax_Id=10116 Gene_Symbol=Serpina3k Serine protease inhibitor A3K precursor

Match to Query 294: 1513.704348 from(757.859450,2+)
Title: 091129RatKid_SCX02_06.1149.1149.2.dta
Data file K:\NewmanPaper\Piliang\3SubProteomes\Piliang3SP\mgf5ppm\SCX_3SubProteomes5ppm.mgf

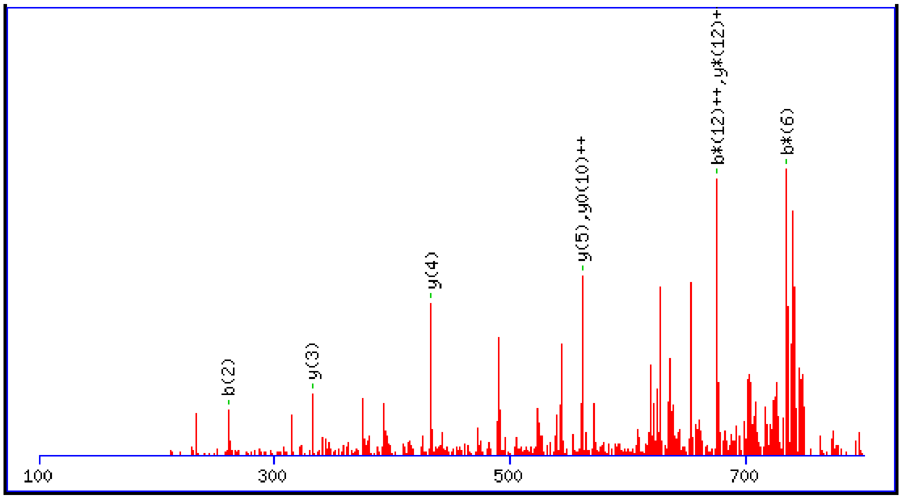

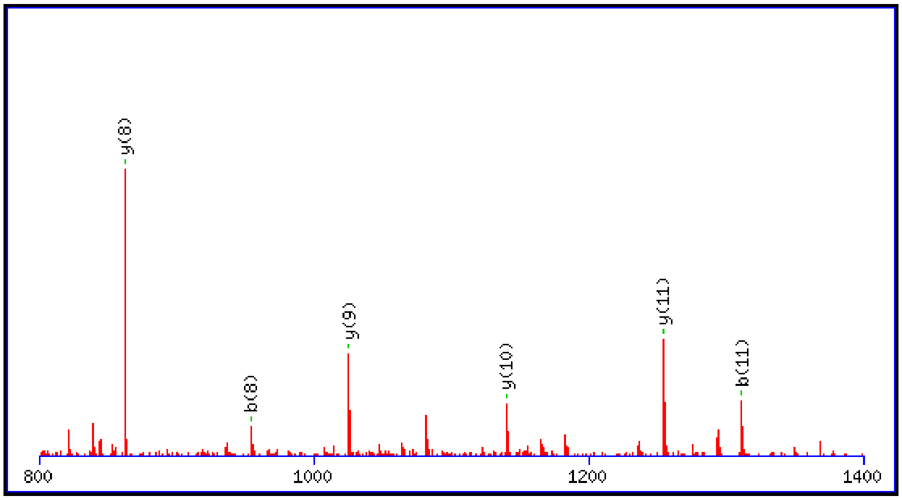

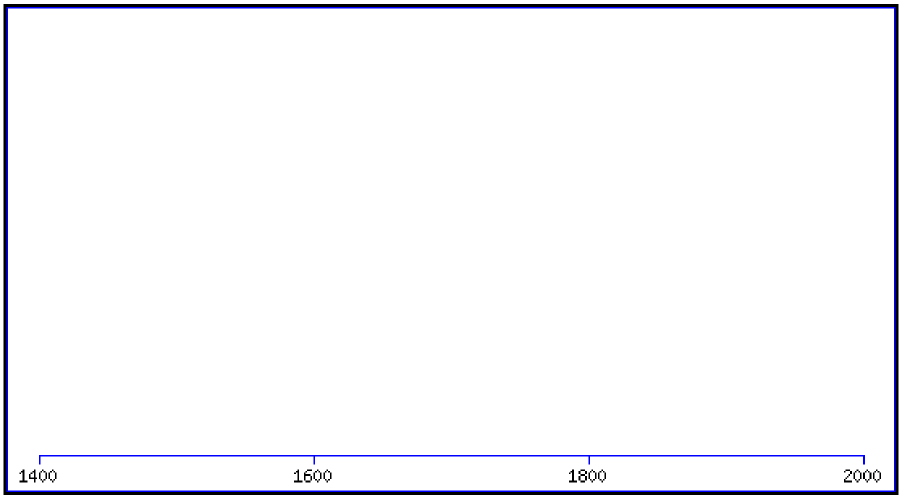

Monoisotopic mass of neutral peptide Mr(calc): 1513.7049
Fixed modifications: Carbamidomethyl (C)
Variable modifications:
N8 : Deamidated_N (N)
Ions Score: 76 **Expect:** 4.3e-006
Matches (Bold Red): 16/128 fragment ions using 15 most intense peaks

#	b	b^{++}	b*	b^{*++}	b^0	b^{0++}	Seq.	y	y^{++}	y*	y^{*++}	y^0	y^{0++}	#
1	148.0757	74.5415					F							13
2	**261.1598**	131.0835					I	1367.6437	684.3255	1350.6172	**675.8122**	1349.6332	675.3202	12
3	375.2027	188.1050	358.1761	179.5917			N	**1254.5597**	627.7835	1237.5331	619.2702	1236.5491	618.7782	11
4	490.2296	245.6185	473.2031	237.1052	472.2191	236.6132	D	**1140.5168**	570.7620	1123.4902	562.2487	1122.5062	**561.7567**	10
5	653.2930	327.1501	636.2664	318.6368	635.2824	318.1448	Y	**1025.4898**	513.2485	1008.4633	504.7353	1007.4792	504.2433	9

6	752.3614	376.6843	735.3348	368.1710	734.3508	367.6790	V	862.4265	431.7169	845.3999	423.2036	844.4159	422.7116	8
7	839.3934	420.2003	822.3668	411.6871	821.3828	411.1951	S	763.3581	382.1827	746.3315	373.6694	745.3475	373.1774	7
8	954.4203	477.7138	937.3938	469.2005	936.4098	468.7085	N	676.3260	338.6667	659.2995	330.1534	658.3155	329.6614	6
9	1082.4789	541.7431	1065.4524	533.2298	1064.4683	532.7378	Q	561.2991	281.1532	544.2726	272.6399	543.2885	272.1479	5
10	1183.5266	592.2669	1166.5000	583.7537	1165.5160	583.2616	T	433.2405	217.1239	416.2140	208.6106	415.2300	208.1186	4
11	1311.5852	656.2962	1294.5586	647.7829	1293.5746	647.2909	Q	332.1928	166.6001	315.1663	158.0868			3
12	1368.6066	684.8070	1351.5801	676.2937	1350.5961	675.8017	G	204.1343	102.5708	187.1077	94.0575			2
13							K	147.1128	74.0600	130.0863	65.5468			1

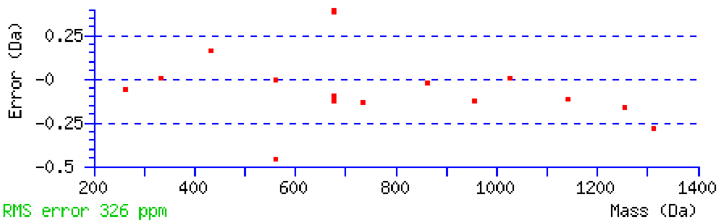

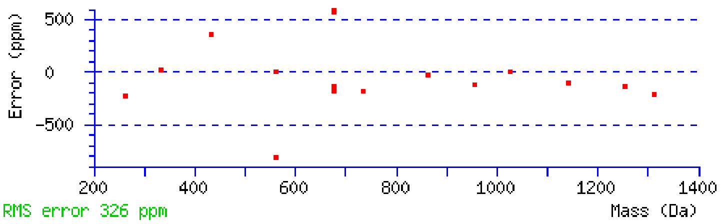

All matches to this query

Score	Mr(calc):	Delta	Sequence
75.7	1513.7049	-0.0005	FINDYVSNQTQGK
21.9	1513.7049	-0.0005	FINDYVSNQTQGK
12.7	1511.7072	1.9971	TETTKDMGRCLGK
10.6	1512.7031	1.0012	WMVAGNADSPVPPR
9.9	1513.7153	-0.0109	FLNWIKTTMHK
9.9	1513.7153	-0.0109	FLNWIKTTMHK
6.3	1513.7125	-0.0081	QMLMLRMIMDGK
5.6	1513.6936	0.0107	DLDDYKEFNDLK
4.3	1513.7078	-0.0035	NVYHIRNYLXK
4.1	1513.7057	-0.0014	AMRMADFWLTEK

Spectrum No: 165; Query: 267; Rank: 1

Peptide View

MS/MS Fragmentation of **LINDYVSNQTQGK**
Found in **IPI00200591**, Tax_Id=10116 Gene_Symbol=LOC299282 Serine protease inhibitor A3L precursor

Match to Query 267: 1479.724068 from(740.869310,2+)
Title: 091129RatKid_SCX02_03.1090.1090.2.dta
Data file K:\NewmanPaper\Piliang\3SubProteomes\Piliang3SP\mgf5ppm\SCX_3SubProteomes5ppm.mgf

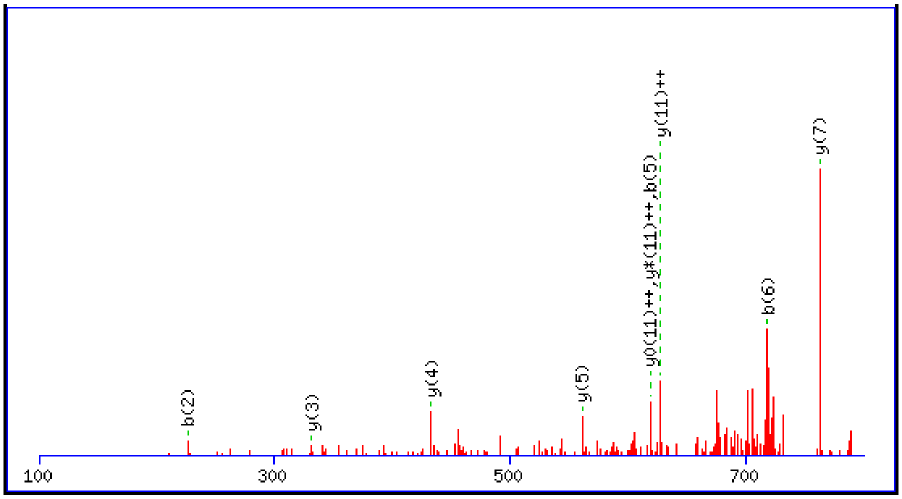

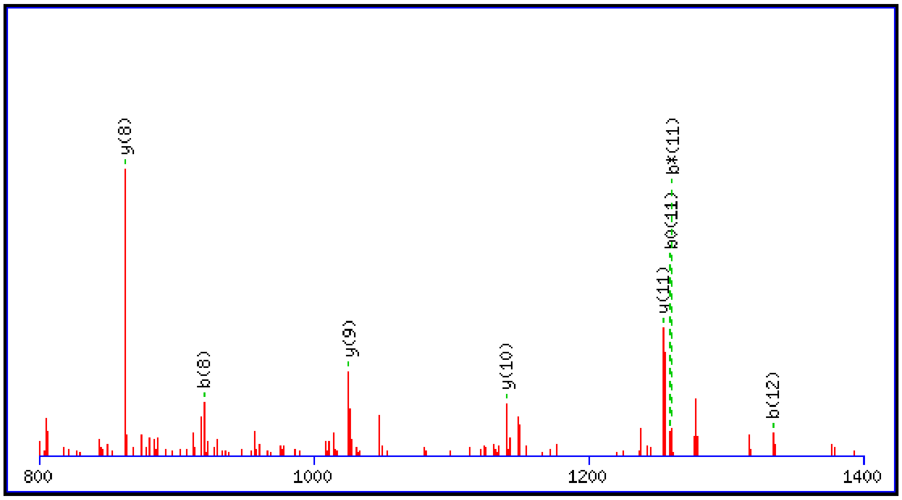

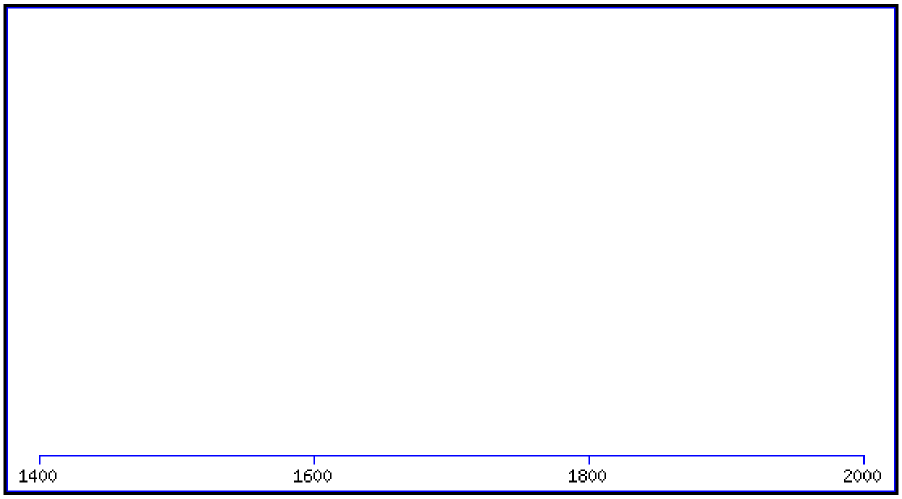

Monoisotopic mass of neutral peptide Mr(calc): 1479.7205
Fixed modifications: Carbamidomethyl (C)
Variable modifications:
N8 : Deamidated_N (N)
Ions Score: 76 **Expect:** 4e-006
Matches (Bold Red): 18/128 fragment ions using 20 most intense peaks

#	b	b^{++}	b*	b^{*++}	b^0	b^{0++}	Seq.	y	y^{++}	y*	y^{*++}	y^0	y^{0++}	#
1	114.0913	57.5493					L							13
2	**227.1754**	114.0913					I	1367.6437	684.3255	1350.6172	675.8122	1349.6332	675.3202	12
3	341.2183	171.1128	324.1918	162.5995			N	**1254.5597**	**627.7835**	1237.5331	**619.2702**	1236.5491	**618.7782**	11
4	456.2453	228.6263	439.2187	220.1130	438.2347	219.6210	D	**1140.5168**	570.7620	1123.4902	562.2487	1122.5062	561.7567	10
5	**619.3086**	310.1579	602.2821	301.6447	601.2980	301.1527	Y	**1025.4898**	513.2485	1008.4633	504.7353	1007.4792	504.2433	9

6	**718.3770**	359.6921	701.3505	351.1789	700.3665	350.6869	V	**862.4265**	431.7169	845.3999	423.2036	844.4159	422.7116	8
7	805.4090	403.2082	788.3825	394.6949	787.3985	394.2029	S	**763.3581**	382.1827	746.3315	373.6694	745.3475	373.1774	7
8	**920.4360**	460.7216	903.4094	452.2084	902.4254	451.7163	N	676.3260	338.6667	659.2995	330.1534	658.3155	329.6614	6
9	1048.4946	524.7509	1031.4680	516.2376	1030.4840	515.7456	Q	**561.2991**	281.1532	544.2726	272.6399	543.2885	272.1479	5
10	1149.5422	575.2748	1132.5157	566.7615	1131.5317	566.2695	T	**433.2405**	217.1239	416.2140	208.6106	415.2300	208.1186	4
11	1277.6008	639.3040	**1260.5743**	630.7908	**1259.5903**	630.2988	Q	**332.1928**	166.6001	315.1663	158.0868			3
12	**1334.6223**	667.8148	1317.5957	659.3015	1316.6117	658.8095	G	204.1343	102.5708	187.1077	94.0575			2
13							K	147.1128	74.0600	130.0863	65.5468			1

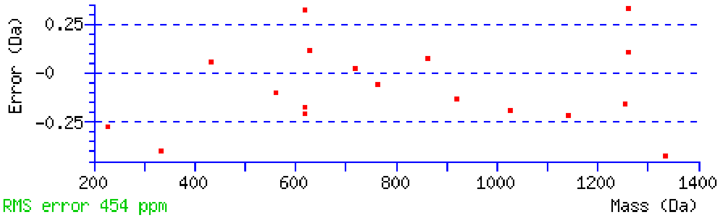

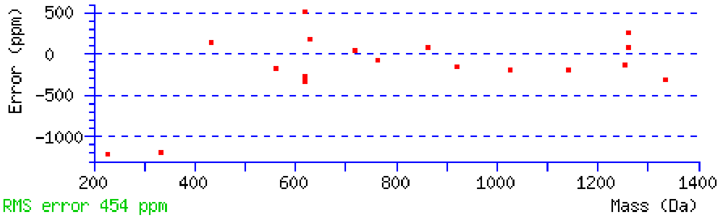

All matches to this query

Score	Mr(calc):	Delta	Sequence
75.6	1479.7205	0.0035	LINDYVSNQTQGK
19.2	1479.7205	0.0035	LINDYVSNQTQGK
1.8	1479.7309	-0.0068	LLSQGLAWPKCK
1.8	1479.7334	-0.0093	KNPDSLELIRSK

Spectrum No: 166; Query: 922; Rank: 1

Peptide View

MS/MS Fragmentation of **IISANGCKVDNSSLTGESEPQTR**
Found in **IPI00326305**, Tax_Id=10116 Gene_Symbol=Atp1a1 Sodium/potassium-transporting ATPase subunit alpha-1 precursor

Match to Query 922: 2463.160872 from(822.060900,3+)
Title: 091129RatKid_SCX02_26.1379.1379.3.dta
Data file K:\NewmanPaper\Piliang\3SubProteomes\Piliang3SP\mgf5ppm\SCX_3SubProteomes5ppm.mgf

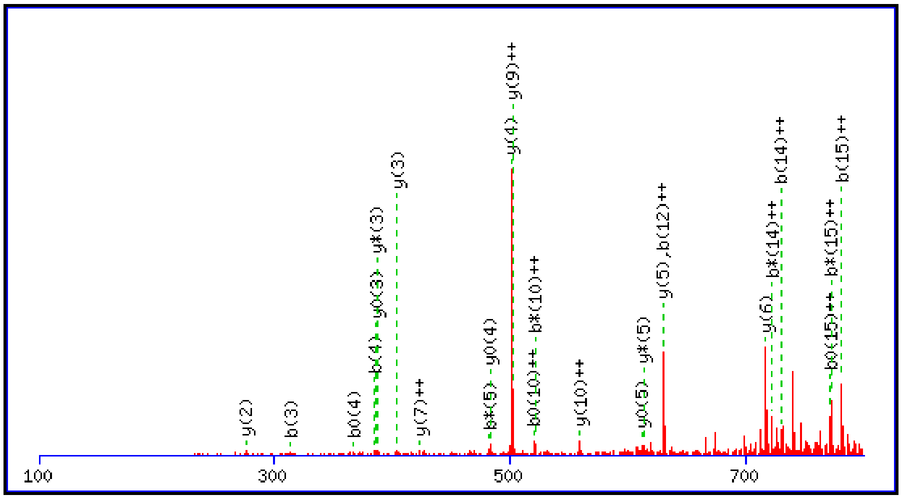

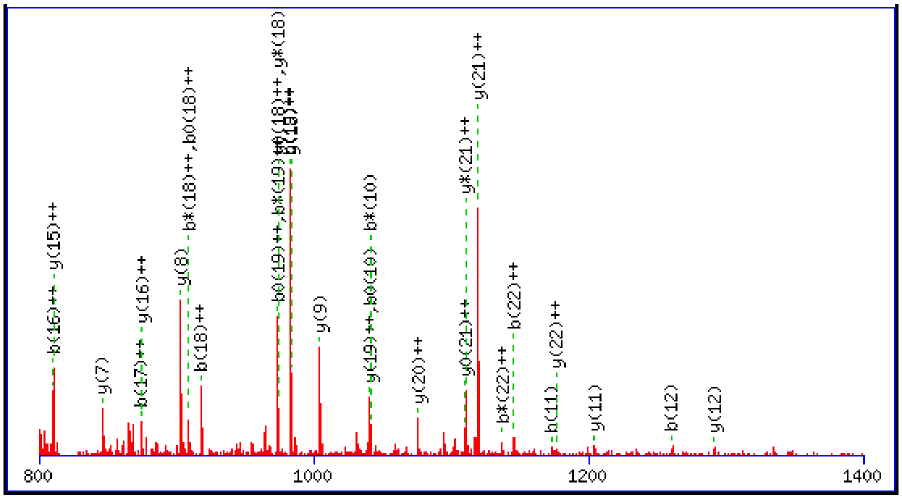

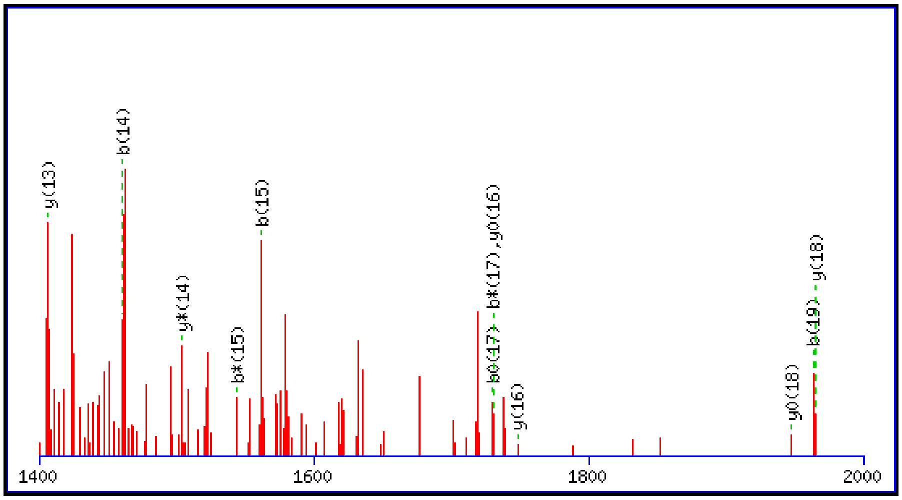

Monoisotopic mass of neutral peptide Mr(calc): 2463.1547
Fixed modifications: Carbamidomethyl (C)
Variable modifications:
N11 : Deamidated_N (N)
Ions Score: 76 **Expect:** 7.8e-006
Matches (Bold Red): 67/250 fragment ions using 118 most intense peaks

#	b	b^{++}	b*	b^{*++}	b^0	b^{0++}	Seq.	y	y^{++}	y*	y^{*++}	y^0	y^{0++}	#
1	114.0913	57.5493					I							23
2	227.1754	114.0913					I	2351.0780	**1176.0426**	2334.0514	1167.5293	2333.0674	1167.0373	22
3	**314.2074**	157.6074			296.1969	148.6021	S	2237.9939	**1119.5006**	2220.9673	**1110.9873**	2219.9833	**1110.4953**	21
4	**385.2445**	193.1259			**367.2340**	184.1206	A	2150.9619	**1075.9846**	2133.9353	1067.4713	2132.9513	1066.9793	20
5	499.2875	250.1474	**482.2609**	241.6341	481.2769	241.1421	N	2079.9247	**1040.4660**	2062.8982	1031.9527	2061.9142	1031.4607	19
6	556.3089	278.6581	539.2824	270.1448	538.2984	269.6528	G	**1965.8818**	**983.4445**	1948.8553	**974.9313**	**1947.8713**	**974.4393**	18
7	716.3396	358.6734	699.3130	350.1602	698.3290	349.6681	C	1908.8604	954.9338	1891.8338	946.4205	1890.8498	945.9285	17
8	844.4345	422.7209	827.4080	414.2076	826.4240	413.7156	K	**1748.8297**	**874.9185**	1731.8032	866.4052	**1730.8191**	865.9132	16
9	943.5030	472.2551	926.4764	463.7418	925.4924	463.2498	V	1620.7347	**810.8710**	1603.7082	802.3577	1602.7242	801.8657	15
10	1058.5299	529.7686	**1041.5034**	**521.2553**	**1040.5193**	**520.7633**	D	1521.6663	761.3368	**1504.6398**	752.8235	1503.6558	752.3315	14
11	**1173.5568**	587.2821	1156.5303	578.7688	1155.5463	578.2768	N	**1406.6394**	703.8233	1389.6128	695.3101	1388.6288	694.8180	13
12	**1260.5889**	**630.7981**	1243.5623	622.2848	1242.5783	621.7928	S	**1291.6125**	646.3099	1274.5859	637.7966	1273.6019	637.3046	12
13	1347.6209	674.3141	1330.5943	665.8008	1329.6103	665.3088	S	**1204.5804**	602.7938	1187.5539	594.2806	1186.5699	593.7886	11
14	**1460.7050**	**730.8561**	1443.6784	**722.3428**	1442.6944	721.8508	L	1117.5484	**559.2778**	1100.5218	550.7646	1099.5378	550.2726	10
15	**1561.7526**	**781.3800**	**1544.7261**	**772.8667**	1543.7421	**772.3747**	T	**1004.4643**	**502.7358**	987.4378	494.2225	986.4538	493.7305	9
16	1618.7741	**809.8907**	1601.7476	801.3774	1600.7635	800.8854	G	**903.4167**	452.2120	886.3901	443.6987	885.4061	443.2067	8
17	1747.8167	**874.4120**	**1730.7901**	865.8987	**1729.8061**	865.4067	E	**846.3952**	**423.7012**	829.3686	415.1880	828.3846	414.6959	7
18	1834.8487	**917.9280**	1817.8222	**909.4147**	1816.8382	**908.9227**	S	**717.3526**	359.1799	700.3260	350.6667	699.3420	350.1747	6

**19**	**1963.8913**	**982.4493**	1946.8648	**973.9360**	1945.8808	**973.4440**	**E**	**630.3206**	315.6639	**613.2940**	307.1506	**612.3100**	306.6586	**5**
**20**	2060.9441	1030.9757	2043.9175	1022.4624	2042.9335	1021.9704	**P**	**501.2780**	251.1426	484.2514	242.6293	**483.2674**	242.1373	**4**
**21**	2189.0027	1095.0050	2171.9761	1086.4917	2170.9921	1085.9997	**Q**	**404.2252**	202.6162	**387.1987**	194.1030	**386.2146**	193.6110	**3**
**22**	2290.0503	**1145.5288**	2273.0238	**1137.0155**	2272.0398	1136.5235	**T**	**276.1666**	138.5870	259.1401	130.0737	258.1561	129.5817	**2**
**23**							**R**	175.1190	88.0631	158.0924	79.5498			**1**

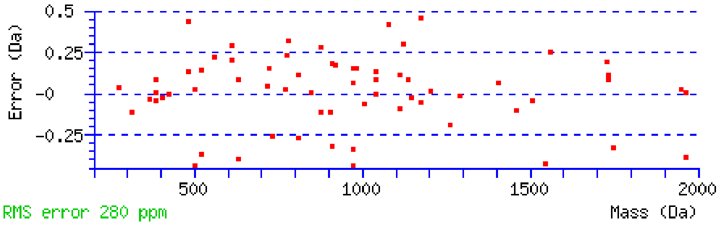

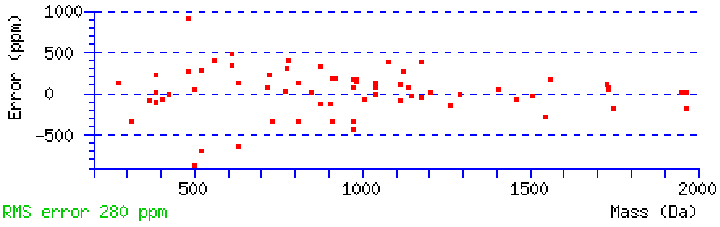

All matches to this query

Score	Mr(calc):	Delta	Sequence
75.6	2463.1547	0.0061	IISANGCKVDNSSLTGESEPQTR
54.9	2463.1547	0.0061	IISANGCKVDNSSLTGESEPQTR
32.3	2462.1707	0.9902	IISANGCKVDNSSLTGESEPQTR
5.3	2462.1715	0.9894	MKKPIGILSPGVALGTAGGAMSSK
5.3	2462.1715	0.9894	MKKPIGILSPGVALGTAGGAMSSK
3.7	2462.1794	0.9815	TGKKPLAHYSSLVRVLGSDMK
3.7	2462.1794	0.9815	TGKKPLAHYSSLVRVLGSDMK
0.6	2463.1382	0.0227	HHLLEQSADMATALLAGEKLR
0.3	2462.1335	1.0274	RNVQALTHNQVVDMLIECPK
0.1	2463.1712	-0.0103	SLDHPNIIKLFHVIESRER

Spectrum No: 167; Query: 84; Rank: 1

Peptide View

MS/MS Fragmentation of **LPVTAVNTTMR**
Found in **IPI00197684**, Tax_Id=10116 Gene_Symbol=Xpnpep2 Membrane-bound aminopeptidase P

Match to Query 84: 1202.633428 from(602.323990,2+)
Title: 091129RatKid_SCX02_12.1213.1213.2.dta
Data file K:\NewmanPaper\Piliang\3SubProteomes\Piliang3SP\mgf5ppm\SCX_3SubProteomes5ppm.mgf

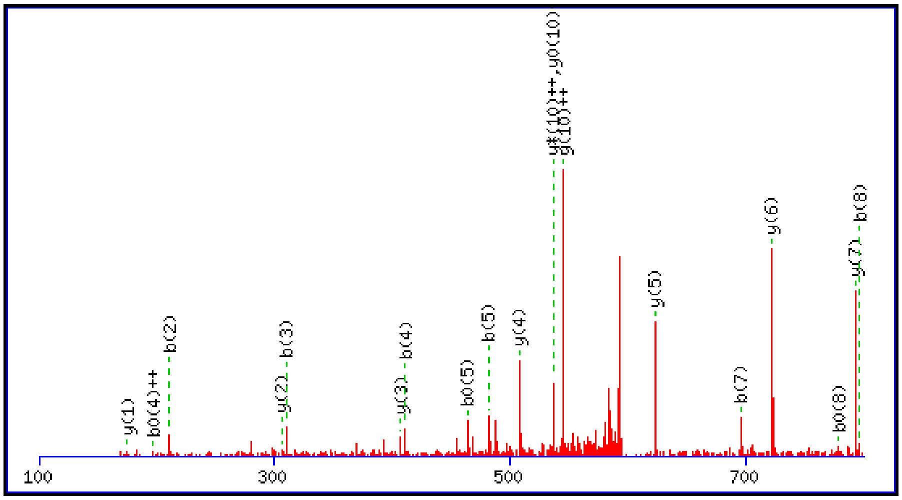

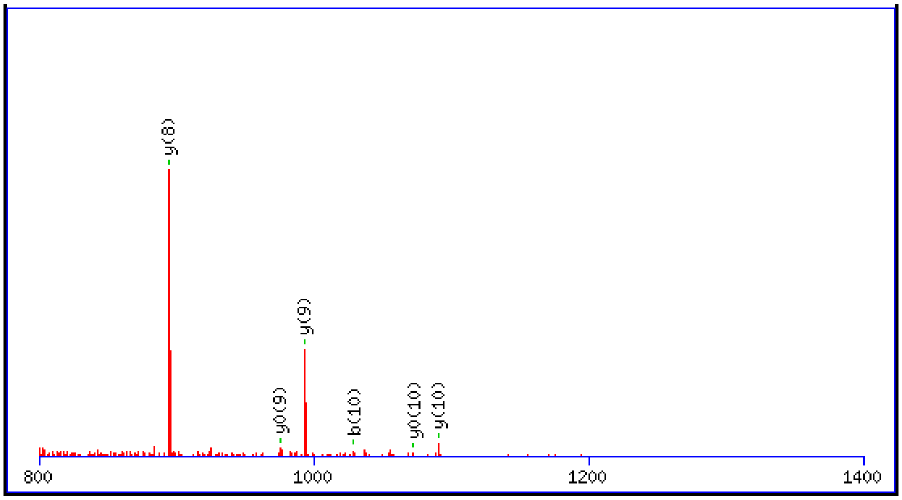

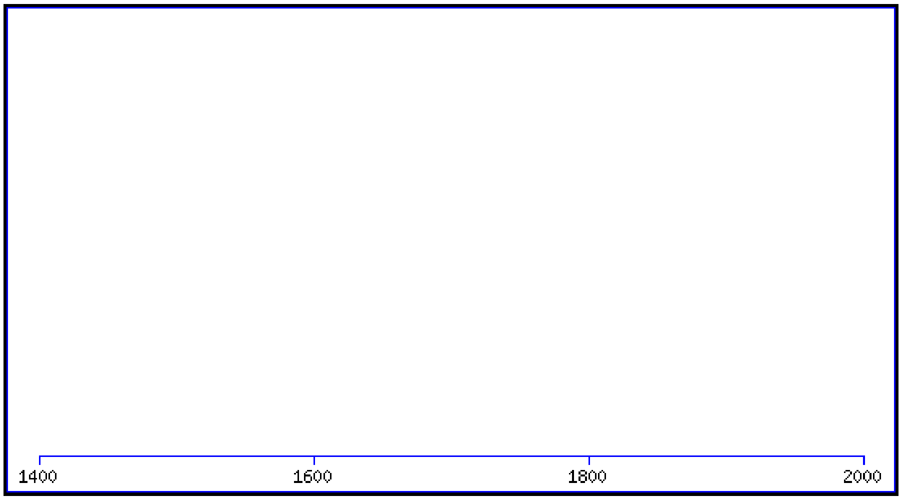

Monoisotopic mass of neutral peptide Mr(calc): 1202.6329
Fixed modifications: Carbamidomethyl (C)
Variable modifications:
N7 : Deamidated_N (N)
Ions Score: 76 **Expect:** 3.4e-006
Matches (Bold Red): 25/98 fragment ions using 51 most intense peaks

#	b	b^{++}	b*	b^{*++}	b^0	b^{0++}	Seq.	y	y^{++}	y*	y^{*++}	y^0	y^{0++}	#
1	114.0913	57.5493					L							11
2	**211.1441**	106.0757					P	**1090.5561**	**545.7817**	1073.5296	**537.2684**	**1072.5456**	**536.7764**	10
3	**310.2125**	155.6099					V	**993.5034**	497.2553	976.4768	488.7420	**975.4928**	488.2500	9
4	**411.2602**	206.1337			393.2496	**197.1285**	T	**894.4349**	447.7211	877.4084	439.2078	876.4244	438.7158	8
5	**482.2973**	241.6523			**464.2867**	232.6470	A	**793.3873**	397.1973	776.3607	388.6840	775.3767	388.1920	7
6	581.3657	291.1865			563.3552	282.1812	V	**722.3501**	361.6787	705.3236	353.1654	704.3396	352.6734	6
7	**696.3927**	348.7000	679.3661	340.1867	678.3821	339.6947	N	**623.2817**	312.1445	606.2552	303.6312	605.2712	303.1392	5
8	**797.4403**	399.2238	780.4138	390.7105	**779.4298**	390.2185	T	**508.2548**	254.6310	491.2282	246.1178	490.2442	245.6258	4
9	898.4880	449.7476	881.4615	441.2344	880.4775	440.7424	T	**407.2071**	204.1072	390.1806	195.5939	389.1966	195.1019	3
10	**1029.5285**	515.2679	1012.5020	506.7546	1011.5179	506.2626	M	**306.1594**	153.5834	289.1329	145.0701			2
11							R	**175.1190**	88.0631	158.0924	79.5498			1

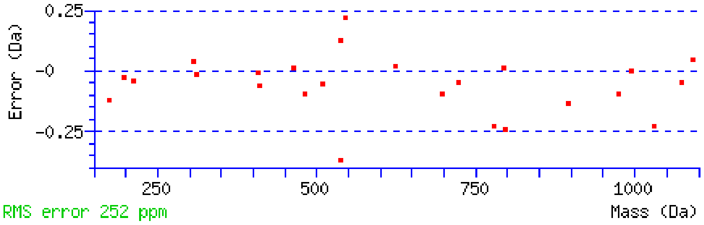

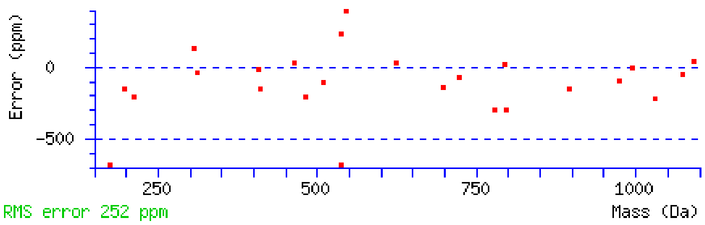

All matches to this query

Score	Mr(calc):	Delta	Sequence
75.5	1202.6329	0.0005	LPVTAVNTTMR
3.4	1202.6441	-0.0107	LQQQMAILSR

Spectrum No: 168; Query: 60; Rank: 1

Peptide View

MS/MS Fragmentation of **LNASIADLQSK**
Found in **IPI00190577**, Tax_Id=10116 Gene_Symbol=Lama5 similar to Laminin alpha-5 chain precursor

Match to Query 60: 1159.610628 from(580.812590,2+)
Title: 091127RatKid_SCX01_12.1811.1811.2.dta
Data file K:\NewmanPaper\Piliang\3SubProteomes\Piliang3SP\mgf5ppm\SCX_3SubProteomes5ppm.mgf

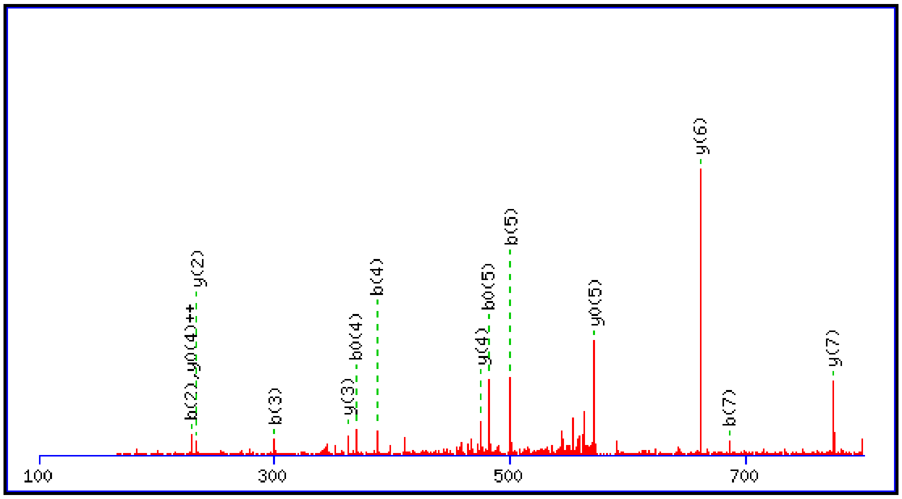

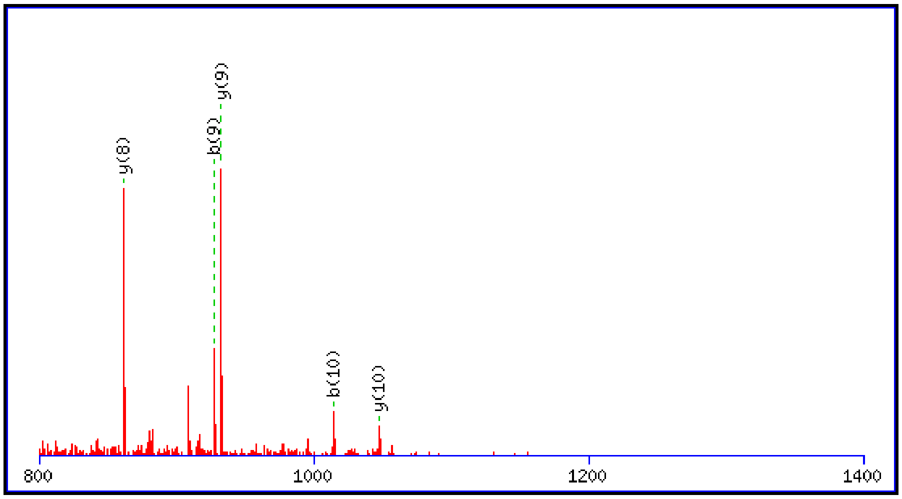

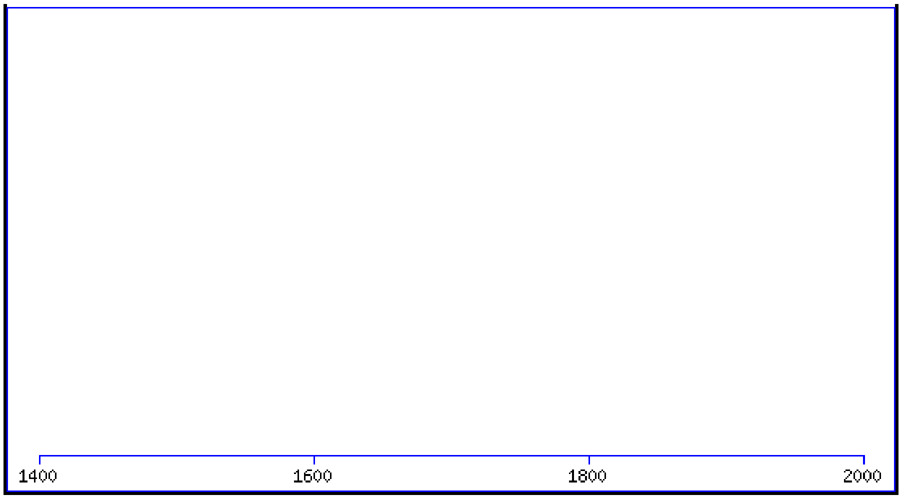

Monoisotopic mass of neutral peptide Mr(calc): 1159.6084
Fixed modifications: Carbamidomethyl (C)
Variable modifications:
N2 : Deamidated_N (N)
Ions Score: 75 **Expect:** 3.4e-006
Matches (Bold Red): 19/110 fragment ions using 26 most intense peaks

#	b	b^{++}	b*	b^{*++}	b^0	b^{0++}	Seq.	y	y^{++}	y*	y^{*++}	y^0	y^{0++}	#
1	114.0913	57.5493					L							11
2	**229.1183**	115.0628	212.0917	106.5495			N	**1047.5317**	524.2695	1030.5051	515.7562	1029.5211	515.2642	10
3	**300.1554**	150.5813	283.1288	142.0681			A	**932.5047**	466.7560	915.4782	458.2427	914.4942	457.7507	9
4	**387.1874**	194.0973	370.1609	185.5841	**369.1769**	185.0921	S	**861.4676**	431.2374	844.4411	422.7242	843.4571	422.2322	8
5	**500.2715**	250.6394	483.2449	242.1261	**482.2609**	241.6341	I	**774.4356**	387.7214	757.4090	379.2082	756.4250	378.7162	7
6	571.3086	286.1579	554.2820	277.6447	553.2980	277.1527	A	**661.3515**	331.1794	644.3250	322.6661	643.3410	322.1741	6
7	**686.3355**	343.6714	669.3090	335.1581	668.3250	334.6661	D	590.3144	295.6608	573.2879	287.1476	**572.3039**	286.6556	5
8	799.4196	400.2134	782.3931	391.7002	781.4090	391.2082	L	**475.2875**	238.1474	458.2609	229.6341	457.2769	**229.1421**	4
9	**927.4782**	464.2427	910.4516	455.7295	909.4676	455.2374	Q	**362.2034**	181.6053	345.1769	173.0921	344.1928	172.6001	3
10	**1014.5102**	507.7587	997.4837	499.2455	996.4996	498.7535	S	**234.1448**	117.5761	217.1183	109.0628	216.1343	108.5708	2
11							K	147.1128	74.0600	130.0863	65.5468			1

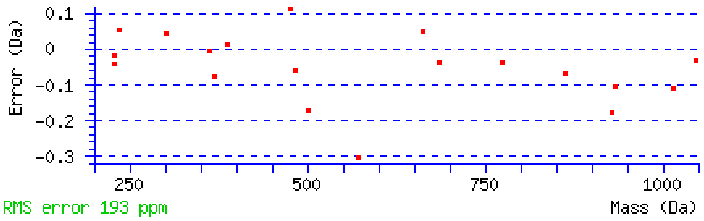

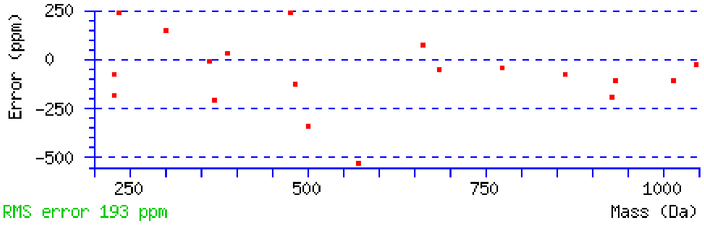

All matches to this query

Score	Mr(calc):	Delta	Sequence
75.5	1159.6084	0.0022	LNASIADLQSK
22.7	1159.6084	0.0022	LEGSLEQEKK
10.8	1159.6210	-0.0104	ERTIWSRGR
9.8	1159.6197	-0.0090	KVSTSSALNPR
8.0	1159.6019	0.0087	LDSAVQMAGLR
7.5	1159.6019	0.0087	GNLTKHMKSK
7.0	1159.6115	-0.0008	LHVSSGLLVR
6.0	1159.6115	-0.0008	LHVSSGLLVR
5.2	1159.6084	0.0022	GESAILVENTK
4.8	1159.6084	0.0022	LEQELKDSAK

Spectrum No: 169; Query: 196; Rank: 1

Peptide View

MS/MS Fragmentation of **NFTDVHPDYGAR**
Found in **IPI00231742**, Tax_Id=10116 Gene_Symbol=Cat Catalase

Match to Query 196: 1391.612528 from(696.813540,2+)
Title: 091129RatKid_SCX02_26.1638.1638.2.dta
Data file K:\NewmanPaper\Piliang\3SubProteomes\Piliang3SP\mgf5ppm\SCX_3SubProteomes5ppm.mgf

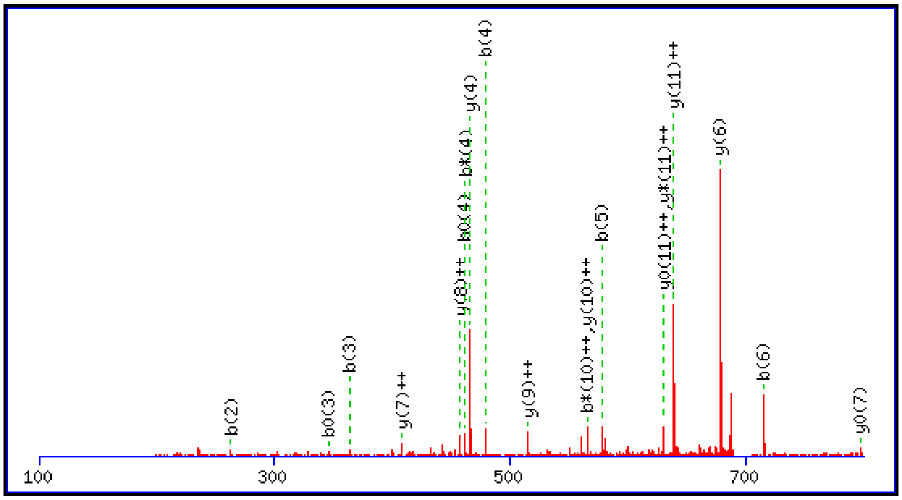

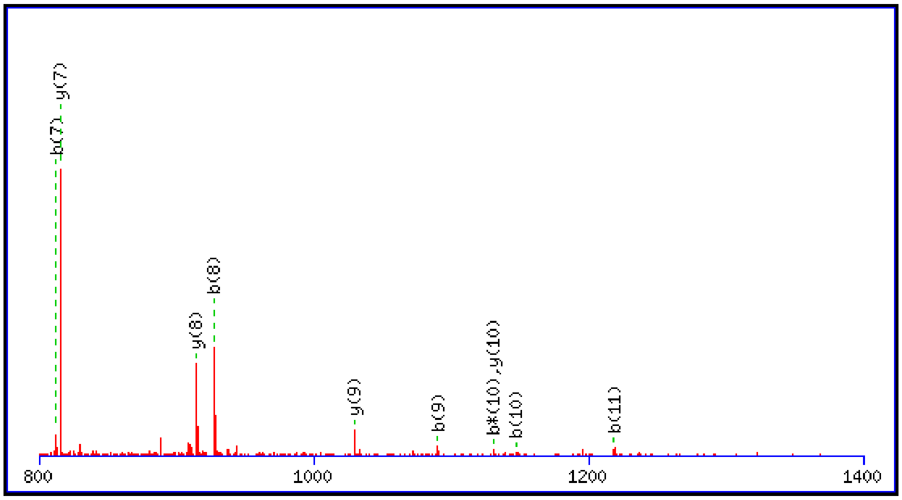

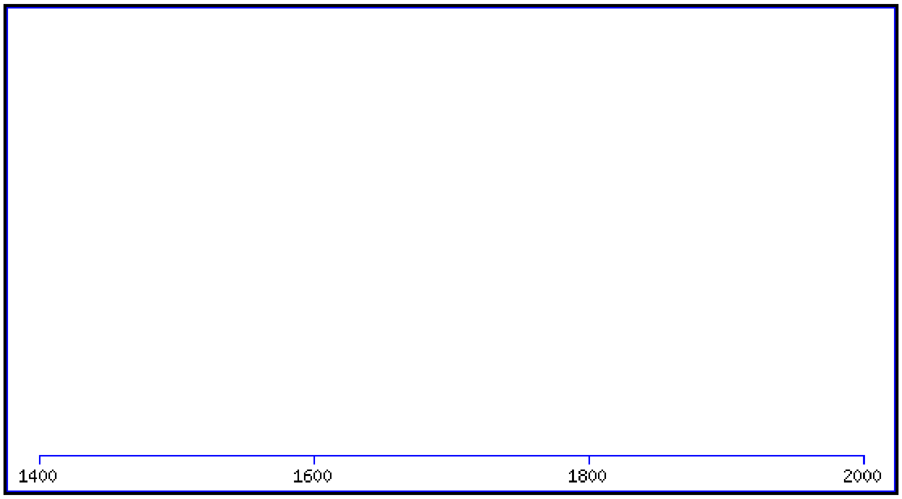

Monoisotopic mass of neutral peptide Mr(calc): 1391.6106
Fixed modifications: Carbamidomethyl (C)
Variable modifications:
N1 : Deamidated_N (N)
Ions Score: 75 **Expect:** 4.4e-006
Matches (Bold Red): 29/120 fragment ions using 44 most intense peaks

#	b	b^{++}	b*	b^{*++}	b^{0}	b^{0++}	Seq.	y	y^{++}	y*	y^{*++}	y^{0}	y^{0++}	#
1	116.0342	58.5207	99.0077	50.0075			N							12
2	**263.1026**	132.0550	246.0761	123.5417			F	1277.5909	**639.2991**	1260.5644	**630.7858**	1259.5804	**630.2938**	11
3	**364.1503**	182.5788	347.1238	174.0655	**346.1397**	173.5735	T	**1130.5225**	**565.7649**	1113.4960	557.2516	1112.5119	556.7596	10
4	**479.1772**	240.0923	**462.1507**	231.5790	**461.1667**	231.0870	D	**1029.4748**	**515.2411**	1012.4483	506.7278	1011.4643	506.2358	9
5	**578.2457**	289.6265	561.2191	281.1132	560.2351	280.6212	V	**914.4479**	**457.7276**	897.4213	449.2143	896.4373	448.7223	8
6	**715.3046**	358.1559	698.2780	349.6427	697.2940	349.1506	H	**815.3795**	**408.1934**	798.3529	399.6801	**797.3689**	399.1881	7
7	**812.3573**	406.6823	795.3308	398.1690	794.3468	397.6770	P	**678.3206**	339.6639	661.2940	331.1506	660.3100	330.6586	6
8	**927.3843**	464.1958	910.3577	455.6825	909.3737	455.1905	D	581.2678	291.1375	564.2413	282.6243	563.2572	282.1323	5
9	**1090.4476**	545.7274	1073.4211	537.2142	1072.4370	536.7222	Y	**466.2409**	233.6241	449.2143	225.1108			4
10	**1147.4691**	574.2382	**1130.4425**	**565.7249**	1129.4585	565.2329	G	303.1775	152.0924	286.1510	143.5791			3
11	**1218.5062**	609.7567	1201.4796	601.2435	1200.4956	600.7514	A	246.1561	123.5817	229.1295	115.0684			2
12							R	175.1190	88.0631	158.0924	79.5498			1

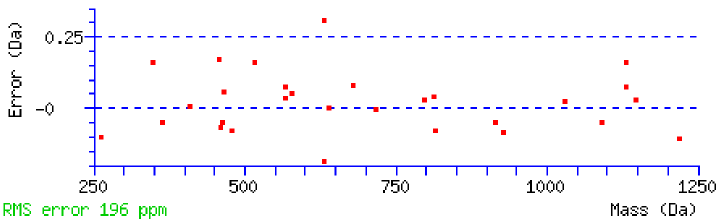

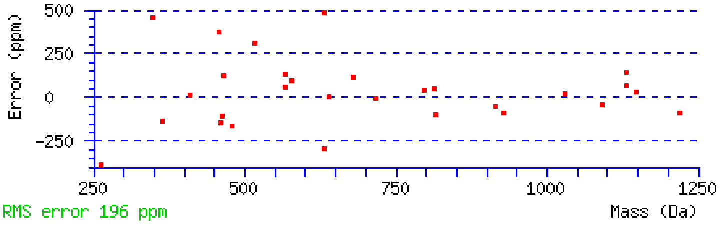

All matches to this query

Score	Mr(calc):	Delta	Sequence
75.2	1391.6106	0.0019	NFTDVHPDYGAR
15.1	1391.5999	0.0126	IYSPVRSPSAR
8.9	1391.6122	0.0003	SPVPPEEAPFSR
5.9	1391.6156	-0.0031	AMVSQISTQGFK
5.7	1391.6156	-0.0031	MAFITSIETQR
5.4	1391.6024	0.0102	THFTFNDFKR
4.5	1391.6153	-0.0028	RYHDWGAMNAR
4.0	1391.6122	0.0003	SSFIDDAFALAR
4.0	1391.6122	0.0003	SSFIDDAFALAR
3.4	1390.6130	0.9996	TDEDVPSAPPRK

Spectrum No: 170; Query: 288; Rank: 1

Peptide View

MS/MS Fragmentation of **TIVYLNGSAQECR**
Found in **IPI00204348**, Tax_Id=10116 Gene_Symbol=Nt5e 5'-nucleotidase precursor

Match to Query 288: 1510.710328 from(756.362440,2+)
Title: 091127RatKid_SCX01_18.1602.1602.2.dta
Data file K:\NewmanPaper\Piliang\3SubProteomes\Piliang3SP\mgf5ppm\SCX_3SubProteomes5ppm.mgf

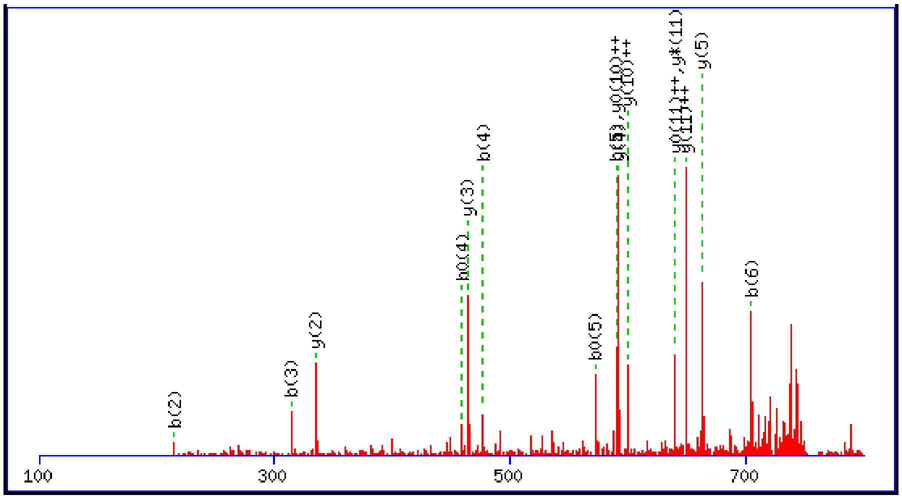

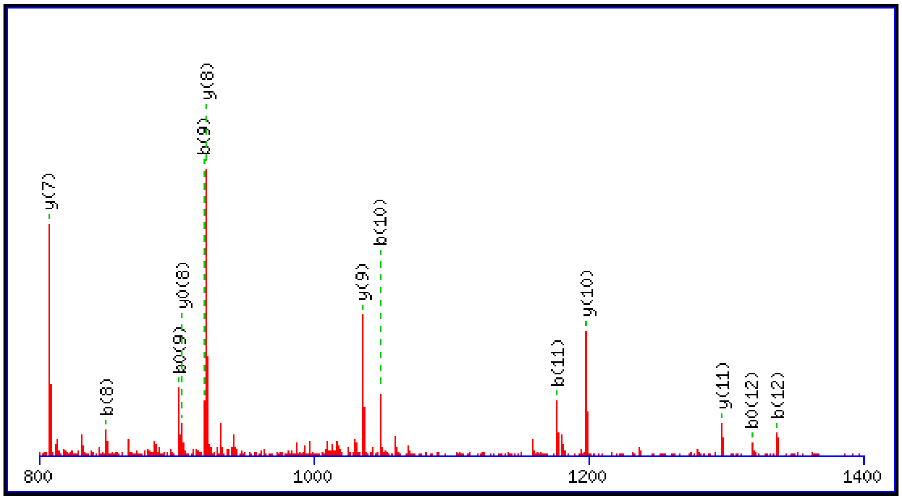

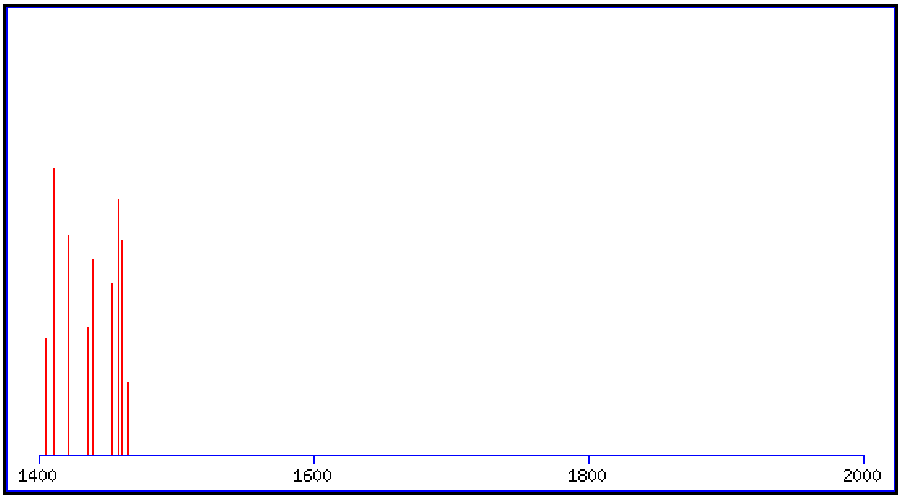

Monoisotopic mass of neutral peptide Mr(calc): 1510.7086
Fixed modifications: Carbamidomethyl (C)
Variable modifications:
N6 : Deamidated_N (N)
Ions Score: 75 **Expect:** 5.1e-006
Matches (Bold Red): 29/130 fragment ions using 48 most intense peaks

#	b	b^{++}	b*	b^{*++}	b^0	b^{0++}	Seq.	y	y^{++}	y*	y^{*++}	y^0	y^{0++}	#
1	102.0550	51.5311			84.0444	42.5258	T							13
2	**215.1390**	108.0731			197.1285	99.0679	I	1410.6682	705.8377	1393.6416	697.3245	1392.6576	696.8324	12
3	**314.2074**	157.6074			296.1969	148.6021	V	**1297.5841**	**649.2957**	1280.5576	**640.7824**	1279.5736	**640.2904**	11
4	**477.2708**	239.1390			**459.2602**	230.1337	Y	**1198.5157**	**599.7615**	1181.4892	591.2482	1180.5051	**590.7562**	10
5	**590.3548**	295.6811			**572.3443**	286.6758	L	**1035.4524**	518.2298	1018.4258	509.7166	1017.4418	509.2245	9

**6**	**705.3818**	353.1945	688.3552	344.6812	687.3712	344.1892	**N**	**922.3683**	461.6878	905.3418	453.1745	**904.3577**	452.6825	**8**
**7**	762.4032	381.7053	745.3767	373.1920	744.3927	372.7000	**G**	**807.3414**	404.1743	790.3148	395.6611	789.3308	395.1690	**7**
**8**	**849.4353**	425.2213	832.4087	416.7080	831.4247	416.2160	**S**	750.3199	375.6636	733.2934	367.1503	732.3093	366.6583	**6**
**9**	**920.4724**	460.7398	903.4458	452.2265	**902.4618**	451.7345	**A**	**663.2879**	332.1476	646.2613	323.6343	645.2773	323.1423	**5**
**10**	**1048.5309**	524.7691	1031.5044	516.2558	1030.5204	515.7638	**Q**	**592.2508**	296.6290	575.2242	288.1157	574.2402	287.6237	**4**
**11**	**1177.5735**	589.2904	1160.5470	580.7771	1159.5630	580.2851	**E**	**464.1922**	232.5997	447.1656	224.0865	446.1816	223.5945	**3**
**12**	**1337.6042**	669.3057	1320.5776	660.7925	**1319.5936**	660.3004	**C**	**335.1496**	168.0784	318.1231	159.5652			**2**
**13**							**R**	175.1190	88.0631	158.0924	79.5498			**1**

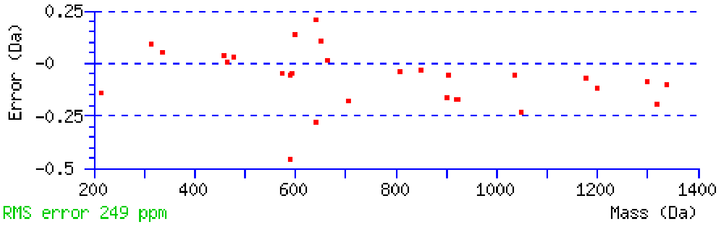

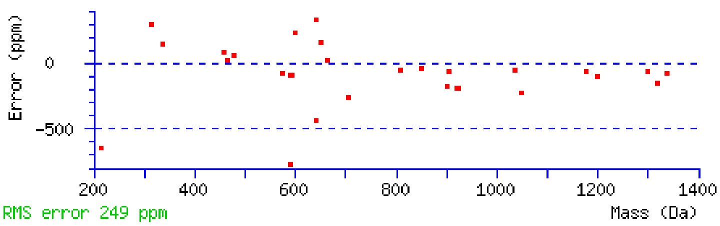

All matches to this query

Score	Mr(calc):	Delta	Sequence
75.2	1510.7086	0.0018	TIVYLNGSAQECR
3.7	1510.6990	0.0114	ITSPLMEPSSIEK
1.3	1510.7086	0.0018	EMEALAPPGQEAPR

Spectrum No: 171; Query: 753; Rank: 1

Peptide View

MS/MS Fragmentation of **EDAQINTTIGSVAAQDPDAAR**
Found in **IPI00215357**, Tax_Id=10116 Gene_Symbol=Cdh6 Cadherin-6 precursor

Match to Query 753: 2144.006868 from(1073.010710,2+)
Title: 091127RatKid_SCX01_12.2364.2364.2.dta
Data file K:\NewmanPaper\Piliang\3SubProteomes\Piliang3SP\mgf5ppm\SCX_3SubProteomes5ppm.mgf

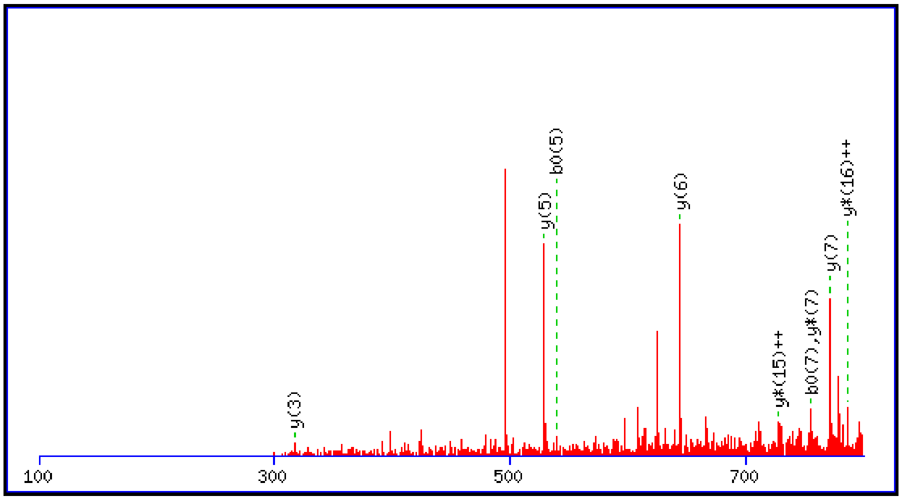

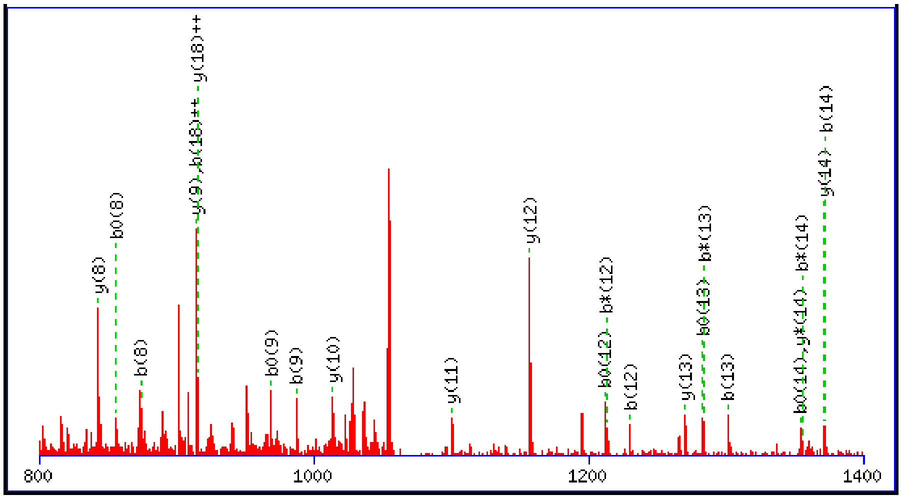

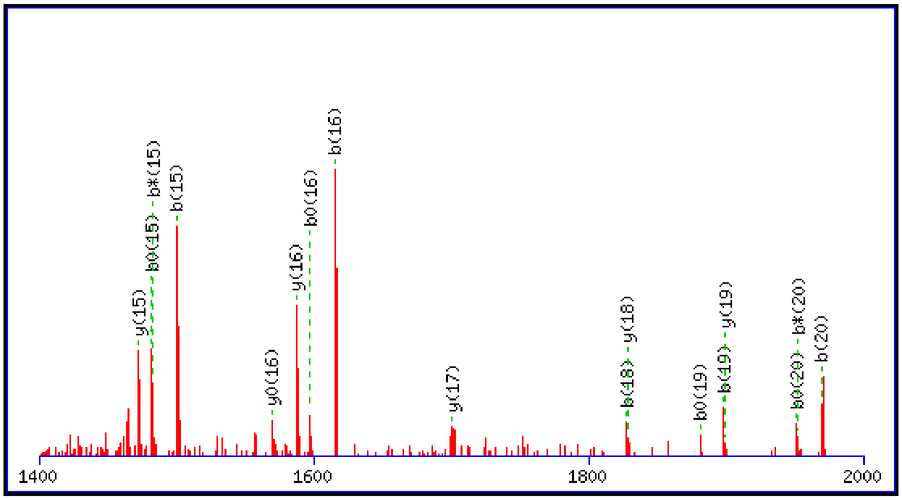

Monoisotopic mass of neutral peptide Mr(calc): 2143.0029
Fixed modifications: Carbamidomethyl (C)
Variable modifications:
N6 : Deamidated_N (N)
Ions Score: 75 **Expect:** 8.1e-006
Matches (Bold Red): 49/228 fragment ions using 122 most intense peaks

#	b	b^{++}	b*	b^{*++}	b^0	b^{0++}	Seq.	y	y^{++}	y*	y^{*++}	y^0	y^{0++}	#
1	130.0499	65.5286			112.0393	56.5233	E							21
2	245.0768	123.0420			227.0662	114.0368	D	2014.9676	1007.9874	1997.9411	999.4742	1996.9570	998.9822	20
3	316.1139	158.5606			298.1034	149.5553	A	**1899.9407**	950.4740	1882.9141	941.9607	1881.9301	941.4687	19
4	444.1725	222.5899	427.1460	214.0766	426.1619	213.5846	Q	**1828.9035**	**914.9554**	1811.8770	906.4421	1810.8930	905.9501	18
5	557.2566	279.1319	540.2300	270.6186	**539.2460**	270.1266	I	**1700.8450**	850.9261	1683.8184	842.4128	1682.8344	841.9208	17
6	672.2835	336.6454	655.2570	328.1321	654.2729	327.6401	N	**1587.7609**	794.3841	1570.7344	**785.8708**	**1569.7503**	785.3788	16
7	773.3312	387.1692	756.3046	378.6560	**755.3206**	378.1639	T	**1472.7340**	736.8706	1455.7074	**728.3573**	1454.7234	727.8653	15
8	**874.3789**	437.6931	857.3523	429.1798	**856.3683**	428.6878	T	**1371.6863**	686.3468	**1354.6597**	677.8335	1353.6757	677.3415	14
9	**987.4629**	494.2351	970.4364	485.7218	**969.4524**	485.2298	I	**1270.6386**	635.8229	1253.6121	627.3097	1252.6280	626.8177	13
10	1044.4844	522.7458	1027.4578	514.2326	1026.4738	513.7406	G	**1157.5545**	579.2809	1140.5280	570.7676	1139.5440	570.2756	12
11	1131.5164	566.2618	1114.4899	557.7486	1113.5059	557.2566	S	**1100.5331**	550.7702	1083.5065	542.2569	1082.5225	541.7649	11
12	**1230.5848**	615.7961	**1213.5583**	607.2828	**1212.5743**	606.7908	V	**1013.5011**	507.2542	996.4745	498.7409	995.4905	498.2489	10
13	**1301.6219**	651.3146	**1284.5954**	642.8013	**1283.6114**	642.3093	A	**914.4326**	457.7200	897.4061	449.2067	896.4221	448.7147	9
14	**1372.6591**	686.8332	**1355.6325**	678.3199	**1354.6485**	677.8279	A	**843.3955**	422.2014	826.3690	413.6881	825.3850	413.1961	8
15	**1500.7176**	750.8625	**1483.6911**	742.3492	**1482.7071**	741.8572	Q	**772.3584**	386.6828	**755.3319**	378.1696	754.3478	377.6776	7
16	**1615.7446**	808.3759	1598.7180	799.8627	**1597.7340**	799.3706	D	**644.2998**	322.6536	627.2733	314.1403	626.2893	313.6483	6
17	1712.7973	856.9023	1695.7708	848.3890	1694.7868	847.8970	P	**529.2729**	265.1401	512.2463	256.6268	511.2623	256.1348	5
18	**1827.8243**	**914.4158**	1810.7977	905.9025	1809.8137	905.4105	D	432.2201	216.6137	415.1936	208.1004	414.2096	207.6084	4

19	1898.8614	949.9343	1881.8349	941.4211	1880.8508	940.9291	A	317.1932	159.1002	300.1666	150.5870			3
20	1969.8985	985.4529	1952.8720	976.9396	1951.8880	976.4476	A	246.1561	123.5817	229.1295	115.0684			2
21							R	175.1190	88.0631	158.0924	79.5498			1

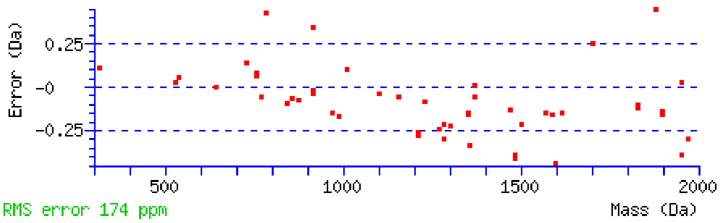

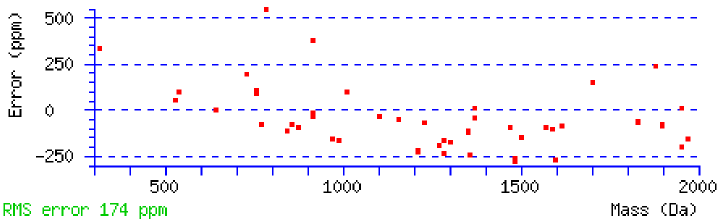

All matches to this query

Score	Mr(calc):	Delta	Sequence
75.1	2143.0029	1.0040	EDAQINTTIGSVAAQDPDAAR
55.7	2142.0189	1.9880	EDAQINTTIGSVAAQDPDAAR
10.5	2143.0038	1.0031	MAAATPTQTPAPEASGLGMDAR
8.7	2142.9946	1.0123	NIRDSLNDYEAKLQDLR
7.8	2143.9886	0.0183	EESVQAKELVVDSSSSGVSK
7.8	2143.9886	0.0183	EESVQAKELVVDSSSSGVSK
5.7	2142.9946	1.0123	NIRDSLNDYEAKLQDLR
4.9	2142.9946	1.0123	NIRDSLNDYEAKLQDLR
4.9	2142.9946	1.0123	NIRDSLNDYEAKLQDLR
4.1	2143.0089	0.9980	LRSSGLRGGGEAALGAWLGR

Spectrum No: 172; Query: 773; Rank: 1

Peptide View

MS/MS Fragmentation of **RSGPSDEGEEEMEDGTVTNGS**
Found in **IPI00192279**, Tax_Id=10116 Gene_Symbol=- 28 kDa protein

Match to Query 773: 2182.845048 from(1092.429800,2+)
Title: 091127RatKid_SCX01_23.1057.1057.2.dta
Data file K:\NewmanPaper\Piliang\3SubProteomes\Piliang3SP\mgf5ppm\SCX_3SubProteomes5ppm.mgf

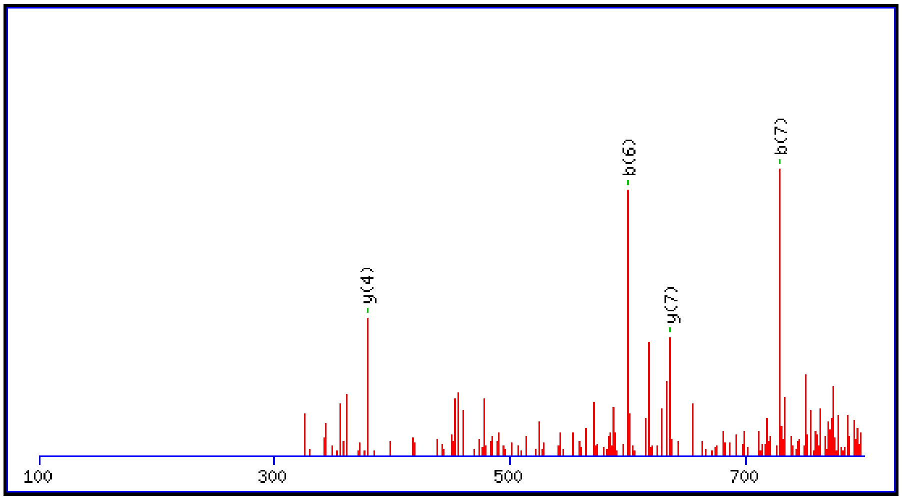

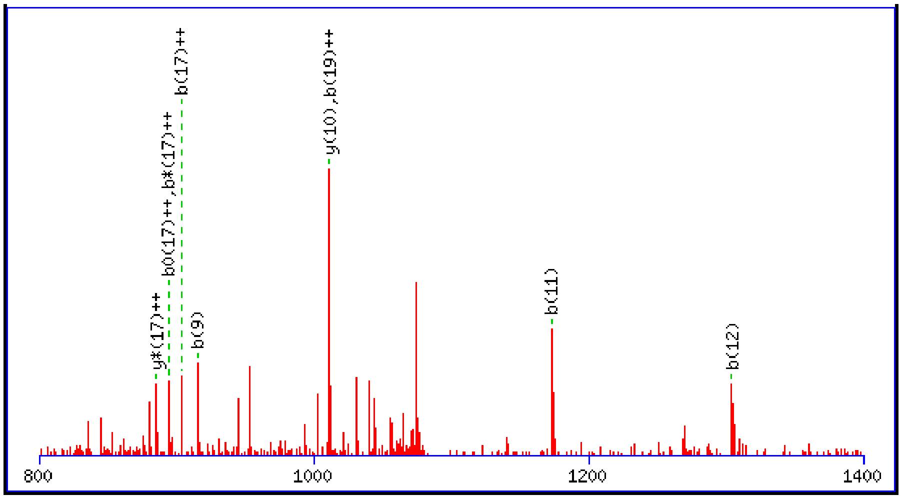

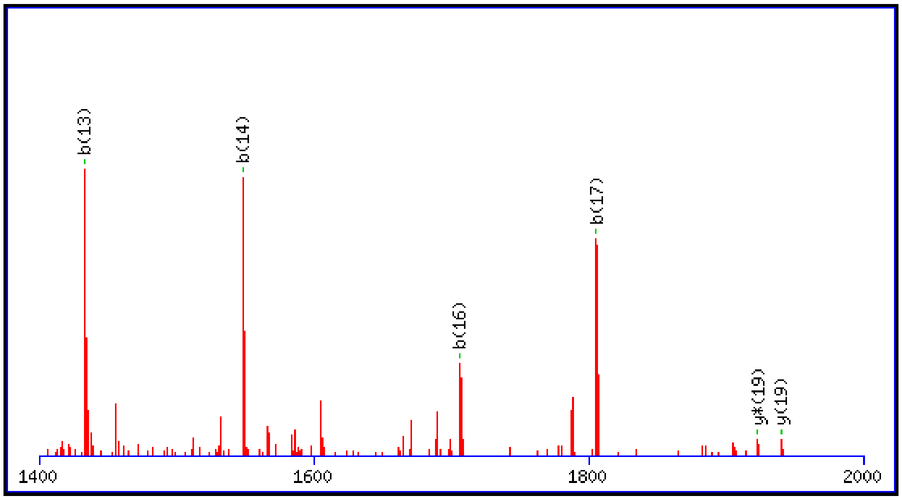

Monoisotopic mass of neutral peptide Mr(calc): 2182.8444
Fixed modifications: Carbamidomethyl (C)
Variable modifications:
N19 : Deamidated_N (N)
Ions Score: 75 **Expect:** 5.7e-006
Matches (Bold Red): 19/234 fragment ions using 26 most intense peaks

#	b	b^{++}	b*	b^{*++}	b^0	b^{0++}	Seq.	y	y^{++}	y*	y^{*++}	y^0	y^{0++}	#
1	157.1084	79.0578	140.0818	70.5446			R							21
2	244.1404	122.5738	227.1139	114.0606	226.1298	113.5686	S	2027.7506	1014.3789	2010.7240	1005.8657	2009.7400	1005.3737	20
3	301.1619	151.0846	284.1353	142.5713	283.1513	142.0793	G	**1940.7186**	970.8629	**1923.6920**	962.3496	1922.7080	961.8576	19
4	398.2146	199.6110	381.1881	191.0977	380.2041	190.6057	P	1883.6971	942.3522	1866.6705	933.8389	1865.6865	933.3469	18
5	485.2467	243.1270	468.2201	234.6137	467.2361	234.1217	S	1786.6443	893.8258	1769.6178	**885.3125**	1768.6338	884.8205	17
6	**600.2736**	300.6404	583.2471	292.1272	582.2630	291.6352	D	1699.6123	850.3098	1682.5858	841.7965	1681.6017	841.3045	16
7	**729.3162**	365.1617	712.2897	356.6485	711.3056	356.1565	E	1584.5854	792.7963	1567.5588	784.2830	1566.5748	783.7910	15
8	786.3377	393.6725	769.3111	385.1592	768.3271	384.6672	G	1455.5428	728.2750	1438.5162	719.7617	1437.5322	719.2697	14
9	**915.3803**	458.1938	898.3537	449.6805	897.3697	449.1885	E	1398.5213	699.7643	1381.4948	691.2510	1380.5107	690.7590	13
10	1044.4229	522.7151	1027.3963	514.2018	1026.4123	513.7098	E	1269.4787	635.2430	1252.4522	626.7297	1251.4681	626.2377	12
11	**1173.4654**	587.2364	1156.4389	578.7231	1155.4549	578.2311	E	1140.4361	570.7217	1123.4096	562.2084	1122.4256	561.7164	11
12	**1304.5059**	652.7566	1287.4794	644.2433	1286.4954	643.7513	M	**1011.3935**	506.2004	994.3670	497.6871	993.3830	497.1951	10
13	**1433.5485**	717.2779	1416.5220	708.7646	1415.5380	708.2726	E	880.3530	440.6802	863.3265	432.1669	862.3425	431.6749	9
14	**1548.5755**	774.7914	1531.5489	766.2781	1530.5649	765.7861	D	751.3104	376.1589	734.2839	367.6456	733.2999	367.1536	8
15	1605.5969	803.3021	1588.5704	794.7888	1587.5864	794.2968	G	**636.2835**	318.6454	619.2570	310.1321	618.2729	309.6401	7
16	**1706.6446**	853.8259	1689.6181	845.3127	1688.6340	844.8207	T	579.2620	290.1347	562.2355	281.6214	561.2515	281.1294	6
17	**1805.7130**	**903.3602**	1788.6865	**894.8469**	1787.7025	**894.3549**	V	478.2144	239.6108	461.1878	231.0975	460.2038	230.6055	5
18	1906.7607	953.8840	1889.7342	945.3707	1888.7501	944.8787	T	**379.1459**	190.0766	362.1194	181.5633	361.1354	181.0713	4

**19**	2021.7876	**1011.3975**	2004.7611	1002.8842	2003.7771	1002.3922	**N**	278.0983	139.5528	261.0717	131.0395	260.0877	130.5475	**3**
**20**	2078.8091	1039.9082	2061.7826	1031.3949	2060.7985	1030.9029	**G**	163.0713	82.0393			145.0608	73.0340	**2**
**21**							**S**	106.0499	53.5286			88.0393	44.5233	**1**

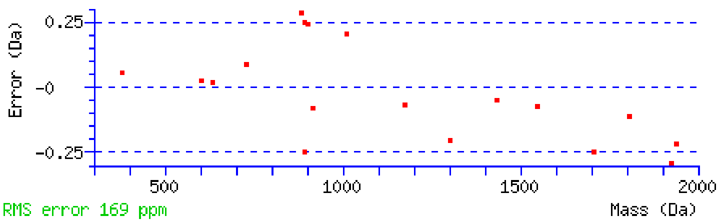

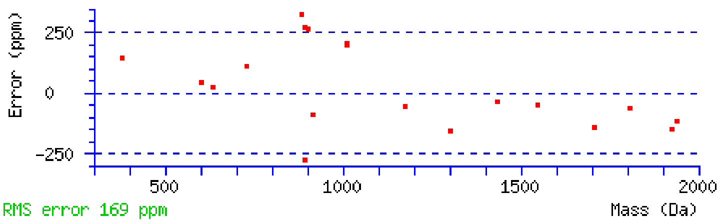

All matches to this query

Score	Mr(calc):	Delta	Sequence
75.0	2182.8444	0.0006	RSGPSDEGEEEMEDGTVTNGS
55.5	2181.8604	0.9846	RSGPSDEGEEEMEDGTVTNGS
2.7	2181.8212	1.0238	SYSTNRSNSNSTSSISSSR
1.8	2181.8212	1.0238	SYSTNRSNSNSTSSISSSR
1.8	2181.8212	1.0238	SYSTNRSNSNSTSSISSSR
1.8	2181.8212	1.0238	SYSTNRSNSNSTSSISSSR
1.5	2180.8353	2.0098	KLDKDDDEDDEDDQDNEK
0.5	2182.8522	-0.0072	YVAICNPLSYTIAMSPK
0.5	2182.8522	-0.0072	YVAICNPLSYTIAMSPK
0.5	2182.8522	-0.0072	YVAICNPLSYTIAMSPK

Spectrum No: 173; Query: 1062; Rank: 1

Peptide View

MS/MS Fragmentation of **VCNCSTVGSLSSQCNINTGQCSCHPK**
Found in **IPI00361301**, Tax_Id=10116 Gene_Symbol=Lama2_predicted similar to Laminin alpha-2 chain precursor

Match to Query 1062: 2955.210612 from(986.077480,3+)
Title: 091129RatKid_SCX02_24.1157.1157.3.dta
Data file K:\NewmanPaper\Piliang\3SubProteomes\Piliang3SP\mgf5ppm\SCX_3SubProteomes5ppm.mgf

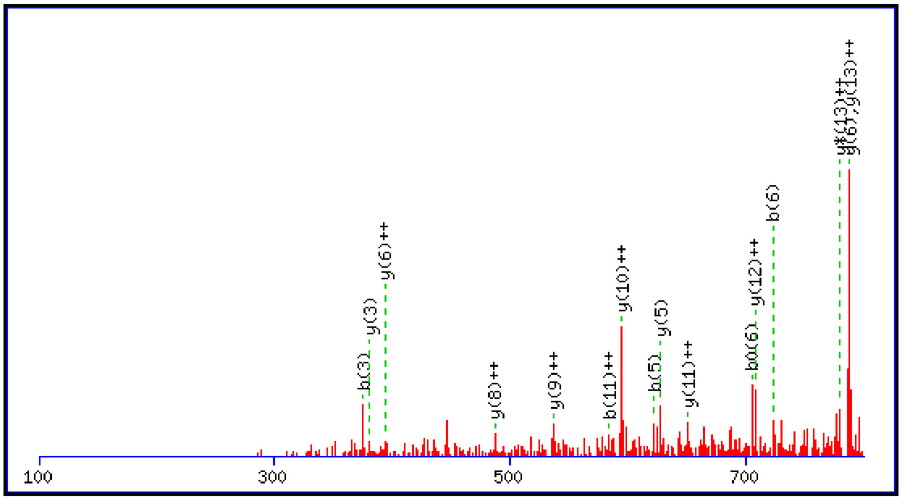

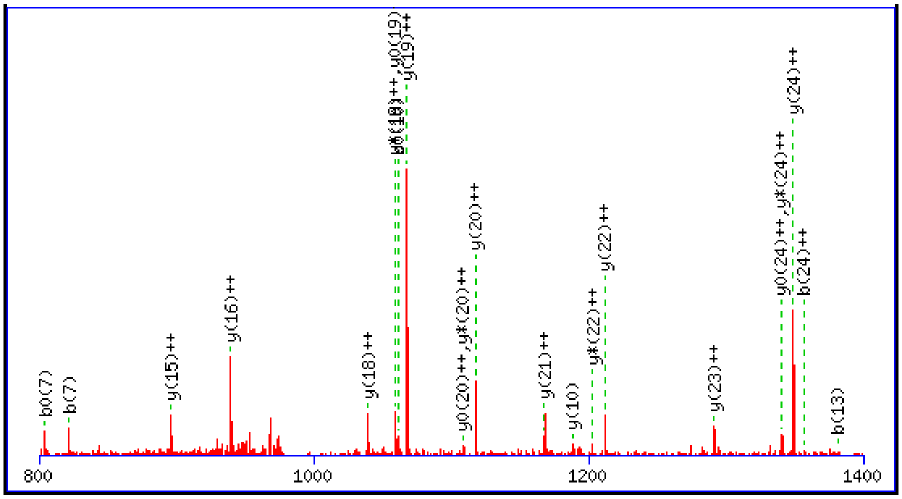

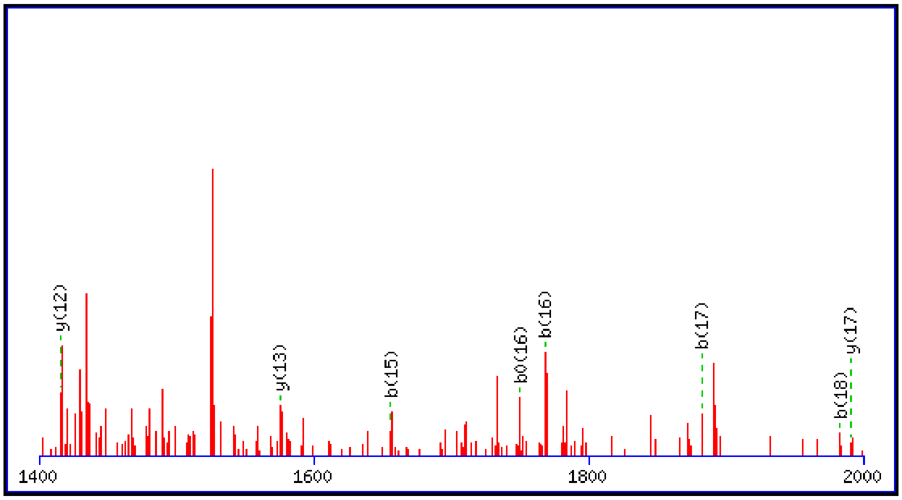

Monoisotopic mass of neutral peptide Mr(calc): 2955.2038
Fixed modifications: Carbamidomethyl (C)
Variable modifications:
N3 : Deamidated_N (N)
Ions Score: 75 **Expect:** 1.1e-005
Matches (Bold Red): 46/280 fragment ions using 97 most intense peaks

#	b	b^{++}	b*	b*$^{++}$	b^{0}	b^{0++}	Seq.	y	y^{++}	y*	y*$^{++}$	y^{0}	y^{0++}	#
1	100.0757	50.5415					V							26
2	260.1063	130.5568					C	2857.1426	1429.0750	2840.1161	1420.5617	2839.1321	1420.0697	25
3	**375.1333**	188.0703	358.1067	179.5570			N	2697.1120	**1349.0596**	2680.0854	**1340.5464**	2679.1014	**1340.0544**	24
4	535.1639	268.0856	518.1374	259.5723			C	2582.0851	**1291.5462**	2565.0585	1283.0329	2564.0745	1282.5409	23
5	**622.1960**	311.6016	605.1694	303.0883	604.1854	302.5963	S	2422.0544	**1211.5308**	2405.0279	**1203.0176**	2404.0438	1202.5256	22
6	**723.2436**	362.1255	706.2171	353.6122	**705.2331**	353.1202	T	2335.0224	**1168.0148**	2317.9958	1159.5016	2317.0118	1159.0095	21
7	**822.3120**	411.6597	805.2855	403.1464	**804.3015**	402.6544	V	2233.9747	**1117.4910**	2216.9481	**1108.9777**	2215.9641	**1108.4857**	20
8	879.3335	440.1704	862.3070	431.6571	861.3229	431.1651	G	2134.9063	**1067.9568**	2117.8797	**1059.4435**	2116.8957	**1058.9515**	19
9	966.3655	483.6864	949.3390	475.1731	948.3550	474.6811	S	2077.8848	**1039.4460**	2060.8583	1030.9328	2059.8743	1030.4408	18
10	1079.4496	540.2284	1062.4231	531.7152	**1061.4390**	531.2232	L	**1990.8528**	995.9300	1973.8262	987.4168	1972.8422	986.9248	17
11	1166.4816	**583.7445**	1149.4551	575.2312	1148.4711	574.7392	S	1877.7687	**939.3880**	1860.7422	930.8747	1859.7582	930.3827	16
12	1253.5137	627.2605	1236.4871	618.7472	1235.5031	618.2552	S	1790.7367	**895.8720**	1773.7102	887.3587	1772.7261	886.8667	15
13	**1381.5722**	691.2898	1364.5457	682.7765	1363.5617	682.2845	Q	1703.7047	852.3560	1686.6781	843.8427	1685.6941	843.3507	14
14	1541.6029	771.3051	1524.5763	762.7918	1523.5923	762.2998	C	**1575.6461**	**788.3267**	1558.6195	**779.8134**	1557.6355	779.3214	13
15	**1655.6458**	828.3265	1638.6193	819.8133	1637.6352	819.3213	N	**1415.6154**	**708.3114**	1398.5889	699.7981	1397.6049	699.3061	12
16	**1768.7299**	884.8686	1751.7033	876.3553	**1750.7193**	875.8633	I	1301.5725	**651.2899**	1284.5460	642.7766	1283.5620	642.2846	11
17	**1882.7728**	941.8900	1865.7463	933.3768	1864.7622	932.8848	N	**1188.4885**	**594.7479**	1171.4619	586.2346	1170.4779	585.7426	10
18	**1983.8205**	992.4139	1966.7939	983.9006	1965.8099	983.4086	T	1074.4455	**537.7264**	1057.4190	529.2131	1056.4350	528.7211	9

**19**	2040.8419	1020.9246	2023.8154	1012.4113	2022.8314	1011.9193	**G**	973.3978	**487.2026**	956.3713	478.6893	955.3873	478.1973	**8**
**20**	2168.9005	1084.9539	2151.8740	1076.4406	2150.8900	1075.9486	**Q**	916.3764	458.6918	899.3498	450.1786	898.3658	449.6865	**7**
**21**	2328.9312	1164.9692	2311.9046	1156.4559	2310.9206	1155.9639	**C**	**788.3178**	**394.6625**	771.2913	386.1493	770.3072	385.6573	**6**
**22**	2415.9632	1208.4852	2398.9367	1199.9720	2397.9526	1199.4800	**S**	**628.2872**	314.6472	611.2606	306.1339	610.2766	305.6419	**5**
**23**	2575.9938	1288.5006	2558.9673	1279.9873	2557.9833	1279.4953	**C**	541.2551	271.1312	524.2286	262.6179			**4**
**24**	2713.0528	**1357.0300**	2696.0262	1348.5167	2695.0422	1348.0247	**H**	**381.2245**	191.1159	364.1979	182.6026			**3**
**25**	2810.1055	1405.5564	2793.0790	1397.0431	2792.0950	1396.5511	**P**	244.1656	122.5864	227.1390	114.0731			**2**
**26**							**K**	147.1128	74.0600	130.0863	65.5468			**1**

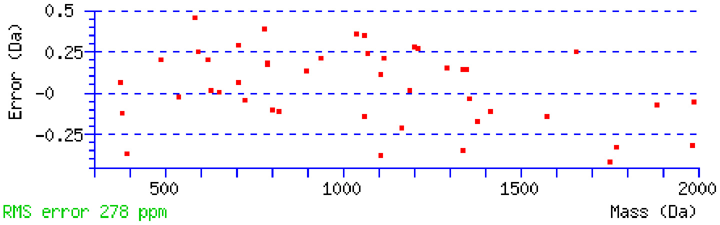

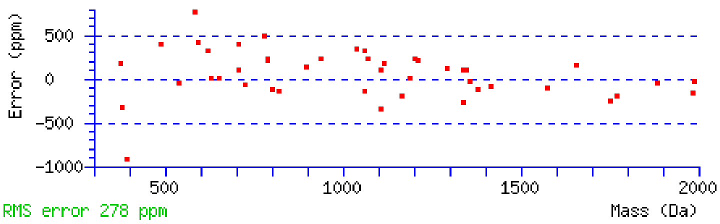

All matches to this query

Score	Mr(calc):	Delta	Sequence
74.9	2955.2038	0.0068	VCNCSTVGSLSSQCNINTGQCSCHPK
70.9	2955.2038	0.0068	VCNCSTVGSLSSQCNINTGQCSCHPK
54.0	2955.2038	0.0068	VCNCSTVGSLSSQCNINTGQCSCHPK
49.5	2954.2198	0.9908	VCNCSTVGSLSSQCNINTGQCSCHPK
6.0	2955.2007	0.0099	SNIGYGSGLGSGIGGFASDIGPLLNRR
4.1	2954.2316	0.9790	SQCTPLFMNAYTMRGAGAVIHTHSK
4.1	2954.2316	0.9790	SQCTPLFMNAYTMRGAGAVIHTHSK
4.1	2954.2316	0.9790	SQCTPLFMNAYTMRGAGAVIHTHSK
4.0	2954.2316	0.9790	SQCTPLFMNAYTMRGAGAVIHTHSK
4.0	2954.2316	0.9790	SQCTPLFMNAYTMRGAGAVIHTHSK

Spectrum No: 174; Query: 827; Rank: 1

Peptide View

MS/MS Fragmentation of **LIFDGVDTVAEILFNNVTIGK**
Found in **IPI00388355**, Tax_Id=10116 Gene_Symbol=Manba Isoform 1 of Beta-mannosidase precursor

Match to Query 827: 2278.218268 from(1140.116410,2+)
Title: 091127RatKid_SCX01_15.7139.7139.2.dta
Data file K:\NewmanPaper\Piliang\3SubProteomes\Piliang3SP\mgf5ppm\SCX_3SubProteomes5ppm.mgf

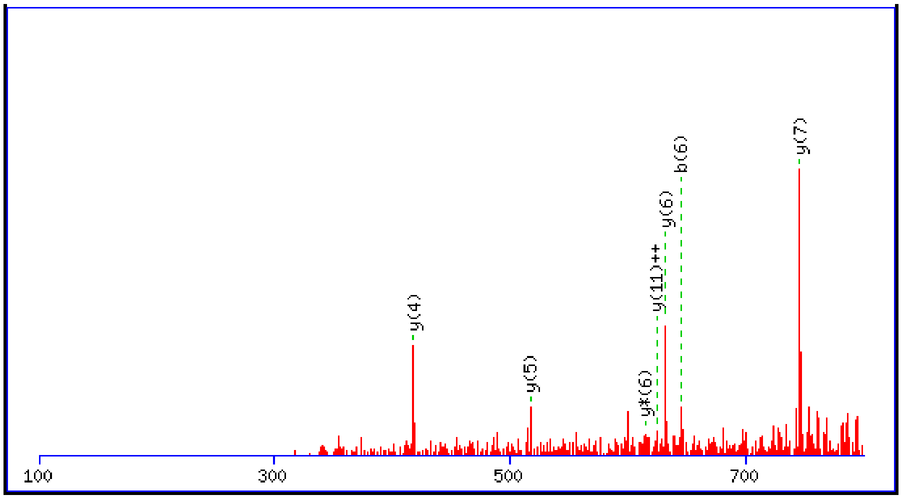

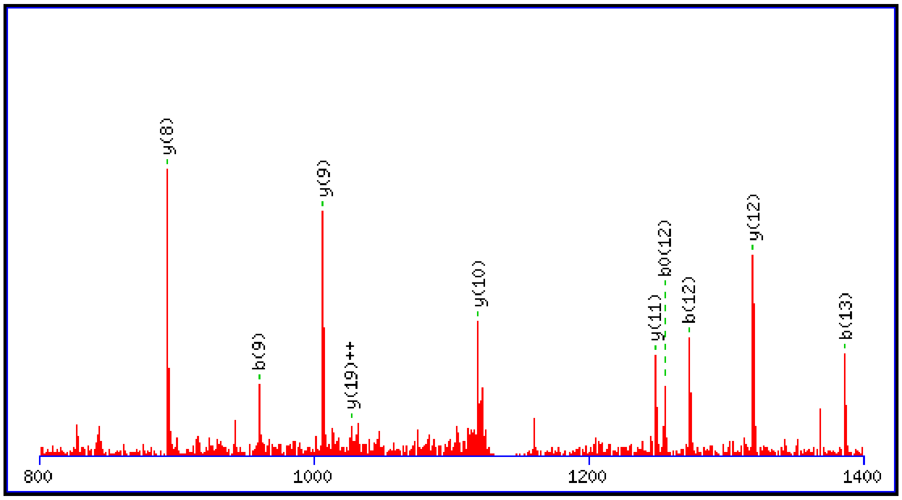

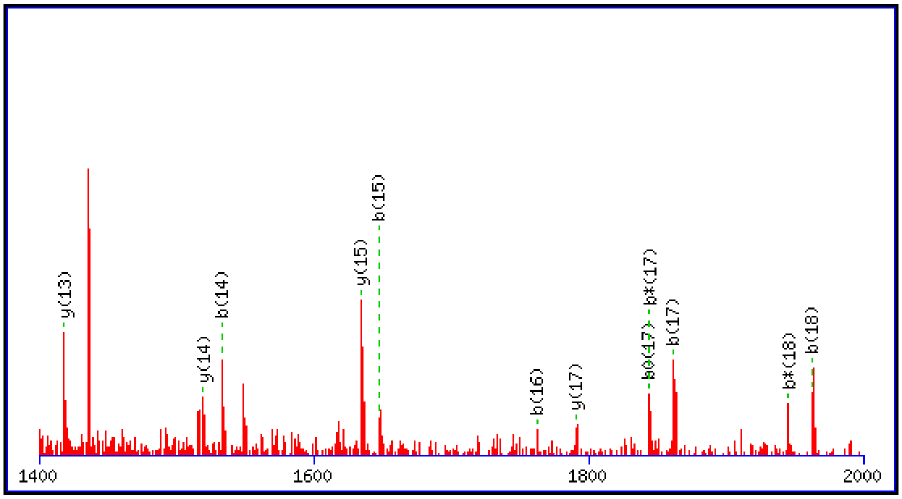

Monoisotopic mass of neutral peptide Mr(calc): 2278.2097
Fixed modifications: Carbamidomethyl (C)
Variable modifications:
N16 : Deamidated_N (N)
Ions Score: 75 **Expect:** 2.4e-006
Matches (Bold Red): 29/200 fragment ions using 58 most intense peaks

#	b	b^{++}	b*	b^{*++}	b^0	b^{0++}	Seq.	y	y^{++}	y*	y^{*++}	y^0	y^{0++}	#
1	114.0913	57.5493					L							21
2	227.1754	114.0913					I	2166.1329	1083.5701	2149.1063	1075.0568	2148.1223	1074.5648	20
3	374.2438	187.6255					F	2053.0488	**1027.0280**	2036.0223	1018.5148	2035.0382	1018.0228	19
4	489.2708	245.1390			471.2602	236.1337	D	1905.9804	953.4938	1888.9538	944.9806	1887.9698	944.4886	18
5	546.2922	273.6498			528.2817	264.6445	G	**1790.9535**	895.9804	1773.9269	887.4671	1772.9429	886.9751	17

**6**	**645.3606**	323.1840			627.3501	314.1787	**V**	1733.9320	867.4696	1716.9054	858.9564	1715.9214	858.4643	**16**
**7**	760.3876	380.6974			742.3770	371.6921	**D**	**1634.8636**	817.9354	1617.8370	809.4222	1616.8530	808.9301	**15**
**8**	861.4353	431.2213			843.4247	422.2160	**T**	**1519.8366**	760.4220	1502.8101	751.9087	1501.8261	751.4167	**14**
**9**	**960.5037**	480.7555			942.4931	471.7502	**V**	**1418.7890**	709.8981	1401.7624	701.3848	1400.7784	700.8928	**13**
**10**	1031.5408	516.2740			1013.5302	507.2687	**A**	**1319.7205**	660.3639	1302.6940	651.8506	1301.7100	651.3586	**12**
**11**	1160.5834	580.7953			1142.5728	571.7900	**E**	**1248.6834**	**624.8453**	1231.6569	616.3321	1230.6729	615.8401	**11**
**12**	**1273.6674**	637.3374			**1255.6569**	628.3321	**I**	**1119.6408**	560.3241	1102.6143	551.8108	1101.6303	551.3188	**10**
**13**	**1386.7515**	693.8794			1368.7409	684.8741	**L**	**1006.5568**	503.7820	989.5302	495.2687	988.5462	494.7767	**9**
**14**	**1533.8199**	767.4136			1515.8094	758.4083	**F**	**893.4727**	447.2400	876.4462	438.7267	875.4621	438.2347	**8**
**15**	**1647.8629**	824.4351	1630.8363	815.9218	1629.8523	815.4298	**N**	**746.4043**	373.7058	729.3777	365.1925	728.3937	364.7005	**7**
**16**	**1762.8898**	881.9485	1745.8632	873.4353	1744.8792	872.9432	**N**	**632.3614**	316.6843	**615.3348**	308.1710	614.3508	307.6790	**6**
**17**	**1861.9582**	931.4827	**1844.9317**	922.9695	**1843.9476**	922.4775	**V**	**517.3344**	259.1709	500.3079	250.6576	499.3239	250.1656	**5**
**18**	**1963.0059**	982.0066	**1945.9793**	973.4933	1944.9953	973.0013	**T**	**418.2660**	209.6366	401.2395	201.1234	400.2554	200.6314	**4**
**19**	2076.0899	1038.5486	2059.0634	1030.0353	2058.0794	1029.5433	**I**	317.2183	159.1128	300.1918	150.5995			**3**
**20**	2133.1114	1067.0593	2116.0849	1058.5461	2115.1008	1058.0541	**G**	204.1343	102.5708	187.1077	94.0575			**2**
**21**							**K**	147.1128	74.0600	130.0863	65.5468			**1**

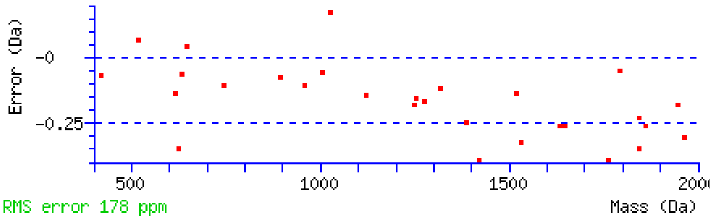

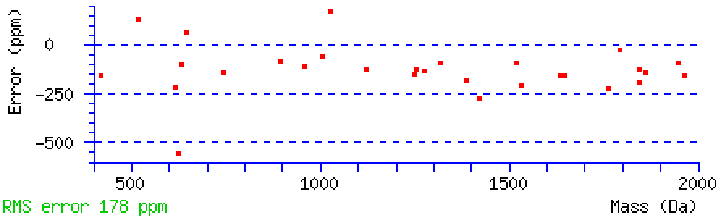

All matches to this query

Score	Mr(calc):	Delta	Sequence
74.8	2278.2097	0.0086	LIFDGVDTVAEILFNNVTIGK
62.6	2278.2097	0.0086	LIFDGVDTVAEILFNNVTIGK
9.0	2277.2257	0.9926	LIFDGVDTVAEILFNNVTIGK
0.1	2276.1953	2.0229	TFGAPRPYNSSAPSPFALAVVK

Spectrum No: 175; Query: 297; Rank: 1

Peptide View

MS/MS Fragmentation of **LGALNSSLLLLEDR**
Found in **IPI00199867**, Tax_Id=10116 Gene_Symbol=Emilin1_predicted elastin microfibril interfacer 1

Match to Query 297: 1513.837748 from(757.926150,2+)
Title: 091129RatKid_SCX02_16.4016.4016.2.dta
Data file K:\NewmanPaper\Piliang\3SubProteomes\Piliang3SP\mgf5ppm\SCX_3SubProteomes5ppm.mgf

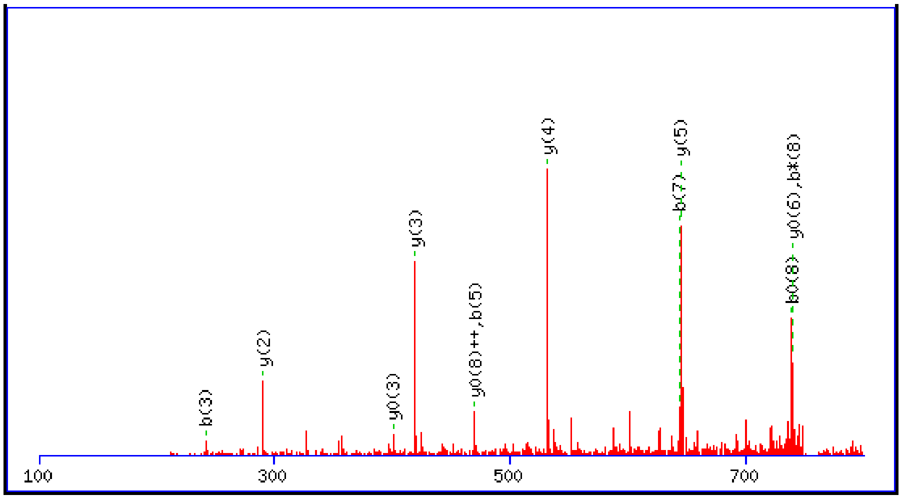

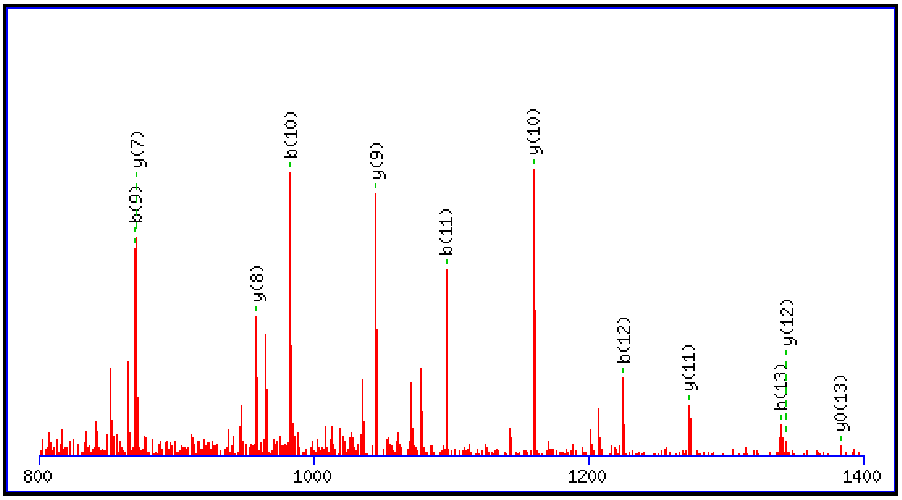

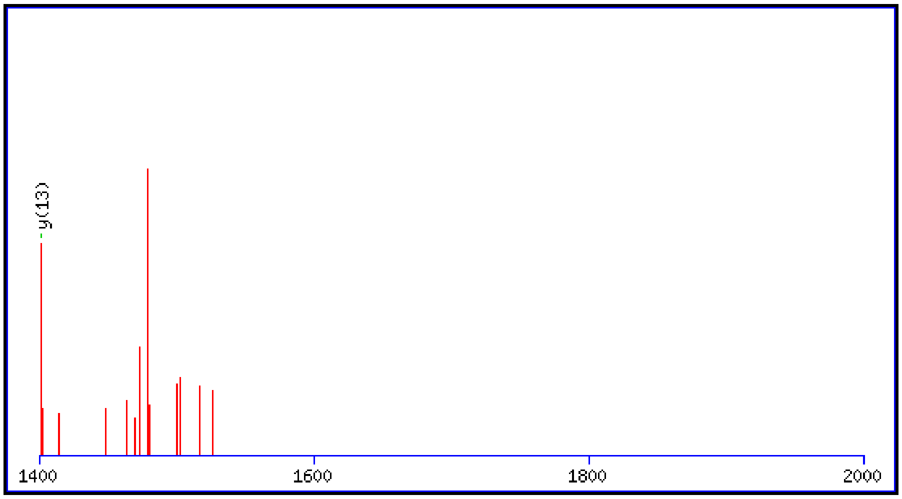

Monoisotopic mass of neutral peptide Mr(calc): 1513.8351
Fixed modifications: Carbamidomethyl (C)
Variable modifications:
N5 : Deamidated_N (N)
Ions Score: 75 **Expect:** 2e-006
Matches (Bold Red): 25/136 fragment ions using 33 most intense peaks

#	b	b^{++}	b*	b^{*++}	b^{0}	b^{0++}	Seq.	y	y^{++}	y*	y^{*++}	y^{0}	y^{0++}	#
1	114.0913	57.5493					L							14
2	171.1128	86.0600					G	**1401.7584**	701.3828	1384.7318	692.8696	**1383.7478**	692.3775	13
3	**242.1499**	121.5786					A	**1344.7369**	672.8721	1327.7104	664.3588	1326.7263	663.8668	12
4	355.2340	178.1206					L	**1273.6998**	637.3535	1256.6733	628.8403	1255.6892	628.3483	11
5	**470.2609**	235.6341	453.2344	227.1208			N	**1160.6157**	580.8115	1143.5892	572.2982	1142.6052	571.8062	10

**6**	557.2929	279.1501	540.2664	270.6368	539.2824	270.1448	**S**	**1045.5888**	523.2980	1028.5623	514.7848	1027.5782	514.2928	**9**
**7**	**644.3250**	322.6661	627.2984	314.1529	626.3144	313.6608	**S**	**958.5568**	479.7820	941.5302	471.2687	940.5462	**470.7767**	**8**
**8**	757.4090	379.2082	**740.3825**	370.6949	**739.3985**	370.2029	**L**	**871.5247**	436.2660	854.4982	427.7527	853.5142	427.2607	**7**
**9**	**870.4931**	435.7502	853.4666	427.2369	852.4825	426.7449	**L**	758.4407	379.7240	741.4141	371.2107	**740.4301**	370.7187	**6**
**10**	**983.5772**	492.2922	966.5506	483.7789	965.5666	483.2869	**L**	**645.3566**	323.1819	628.3301	314.6687	627.3461	314.1767	**5**
**11**	**1096.6612**	548.8343	1079.6347	540.3210	1078.6507	539.8290	**L**	**532.2726**	266.6399	515.2460	258.1266	514.2620	257.6346	**4**
**12**	**1225.7038**	613.3555	1208.6773	604.8423	1207.6933	604.3503	**E**	**419.1885**	210.0979	402.1619	201.5846	**401.1779**	201.0926	**3**
**13**	**1340.7308**	670.8690	1323.7042	662.3557	1322.7202	661.8637	**D**	**290.1459**	145.5766	273.1193	137.0633	272.1353	136.5713	**2**
**14**							**R**	175.1190	88.0631	158.0924	79.5498			**1**

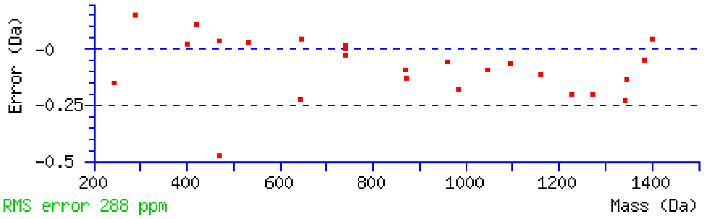

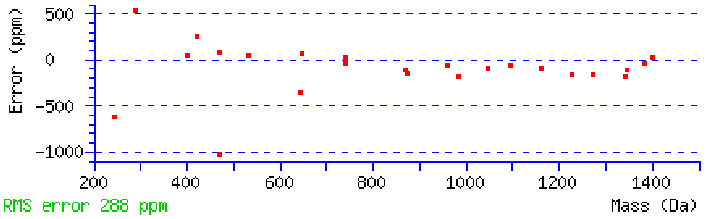

All matches to this query

Score	Mr(calc):	Delta	Sequence
74.8	1513.8351	0.0026	LGALNSSLLLLEDR
6.2	1513.8324	0.0053	KNLISSXLQRGQR
4.4	1512.8412	0.9965	REALVKGDPPIYR
3.7	1512.8372	1.0005	IQDLENRLTKQR
3.3	1513.8253	0.0125	KVLGDLIFNQPDR
3.0	1513.8351	0.0026	LQKVSENALPSSLK
2.8	1512.8484	0.9893	KNLISSXLQRGQR
2.1	1513.8398	-0.0021	LTALQRNGGAILMR

Spectrum No: 176; Query: 649; Rank: 1

Peptide View

MS/MS Fragmentation of **EINISICPASQTSEHFR**
Found in **IPI00400747**, Tax_Id=10116 Gene_Symbol=Man2b1 Mannosidase 2, alpha B1

Match to Query 649: 1988.930188 from(995.472370,2+)
Title: 091127RatKid_SCX01_26.2568.2568.2.dta
Data file K:\NewmanPaper\Piliang\3SubProteomes\Piliang3SP\mgf5ppm\SCX_3SubProteomes5ppm.mgf

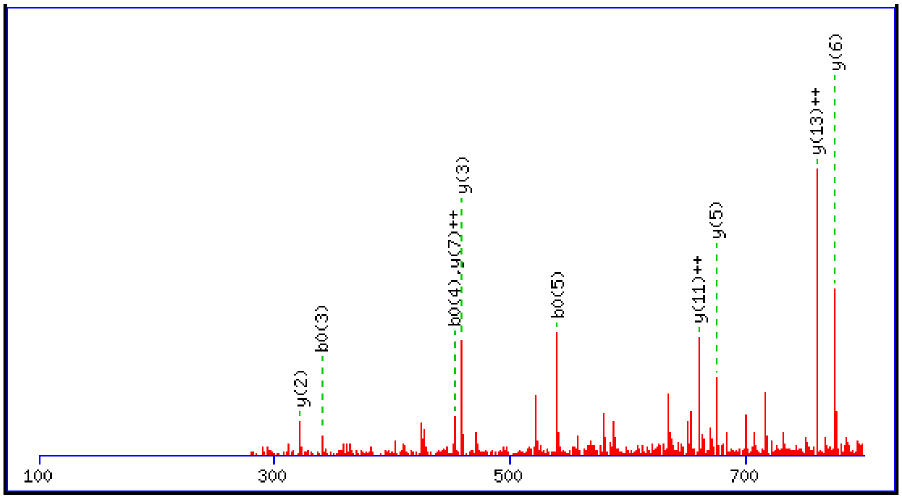

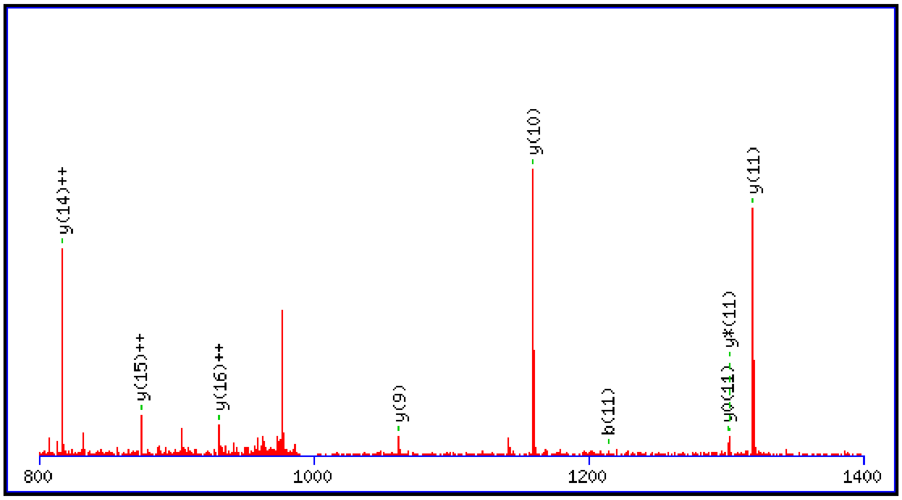

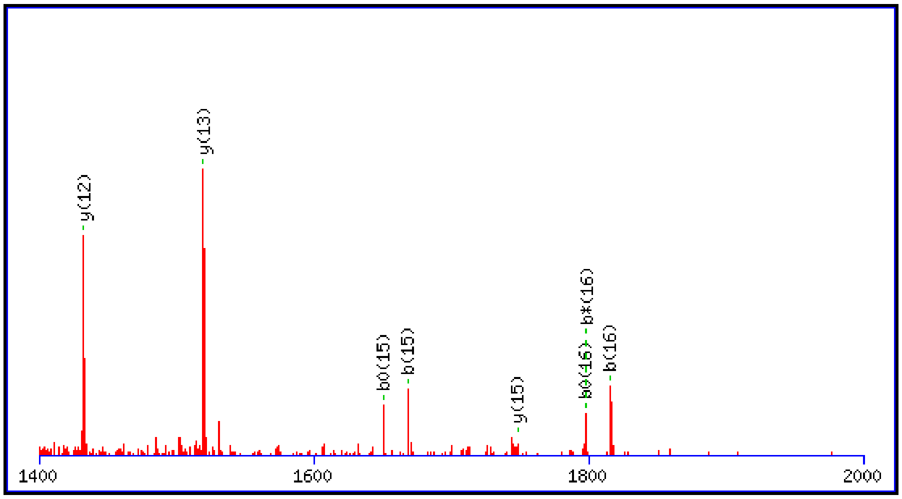

Monoisotopic mass of neutral peptide Mr(calc): 1988.9262
Fixed modifications: Carbamidomethyl (C)
Variable modifications:
N3 : Deamidated_N (N)
Ions Score: 74 **Expect:** 8.8e-006
Matches (Bold Red): 27/182 fragment ions using 51 most intense peaks

#	b	b^{++}	b*	b^{*++}	b^0	b^{0++}	Seq.	y	y^{++}	y*	y^{*++}	y^0	y^{0++}	#
1	130.0499	65.5286			112.0393	56.5233	E							17
2	243.1339	122.0706			225.1234	113.0653	I	1860.8909	**930.9491**	1843.8643	922.4358	1842.8803	921.9438	16
3	358.1609	179.5841	341.1343	171.0708	**340.1503**	170.5788	N	**1747.8068**	**874.4070**	1730.7803	865.8938	1729.7962	865.4018	15
4	471.2449	236.1261	454.2184	227.6128	**453.2344**	227.1208	I	1632.7799	**816.8936**	1615.7533	808.3803	1614.7693	807.8883	14
5	558.2770	279.6421	541.2504	271.1288	**540.2664**	270.6368	S	**1519.6958**	**760.3515**	1502.6693	751.8383	1501.6852	751.3463	13

**6**	671.3610	336.1842	654.3345	327.6709	653.3505	327.1789	**I**	**1432.6638**	716.8355	1415.6372	708.3223	1414.6532	707.8302	**12**
**7**	831.3917	416.1995	814.3651	407.6862	813.3811	407.1942	**C**	**1319.5797**	**660.2935**	**1302.5532**	651.7802	**1301.5691**	651.2882	**11**
**8**	928.4444	464.7259	911.4179	456.2126	910.4339	455.7206	**P**	**1159.5491**	580.2782	1142.5225	571.7649	1141.5385	571.2729	**10**
**9**	999.4816	500.2444	982.4550	491.7311	981.4710	491.2391	**A**	**1062.4963**	531.7518	1045.4697	523.2385	1044.4857	522.7465	**9**
**10**	1086.5136	543.7604	1069.4870	535.2472	1068.5030	534.7551	**S**	991.4592	496.2332	974.4326	487.7200	973.4486	487.2279	**8**
**11**	**1214.5722**	607.7897	1197.5456	599.2764	1196.5616	598.7844	**Q**	904.4272	**452.7172**	887.4006	444.2039	886.4166	443.7119	**7**
**12**	1315.6198	658.3136	1298.5933	649.8003	1297.6093	649.3083	**T**	**776.3686**	388.6879	759.3420	380.1747	758.3580	379.6826	**6**
**13**	1402.6519	701.8296	1385.6253	693.3163	1384.6413	692.8243	**S**	**675.3209**	338.1641	658.2944	329.6508	657.3103	329.1588	**5**
**14**	1531.6945	766.3509	1514.6679	757.8376	1513.6839	757.3456	**E**	588.2889	294.6481	571.2623	286.1348	570.2783	285.6428	**4**
**15**	**1668.7534**	834.8803	1651.7268	826.3670	**1650.7428**	825.8750	**H**	**459.2463**	230.1268	442.2197	221.6135			**3**
**16**	**1815.8218**	908.4145	**1798.7952**	899.9013	**1797.8112**	899.4092	**F**	**322.1874**	161.5973	305.1608	153.0840			**2**
**17**							**R**	175.1190	88.0631	158.0924	79.5498			**1**

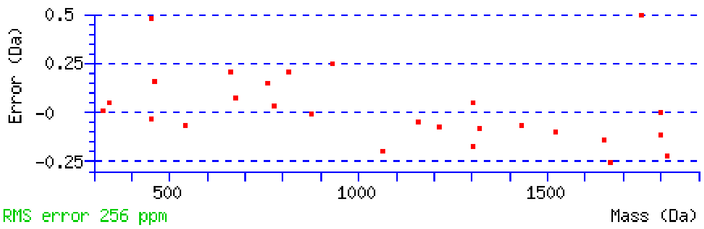

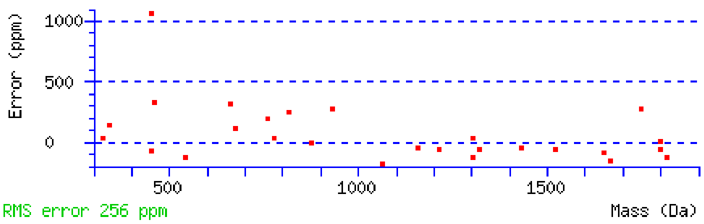

All matches to this query

Score	Mr(calc):	Delta	Sequence
74.5	1988.9262	0.0040	EINISICPASQTSEHFR
46.1	1987.9422	0.9880	EINISICPASQTSEHFR
6.3	1988.9318	-0.0016	KNNLFPPCSYLNSLLK
5.5	1986.9220	2.0082	EKLKMEQENTAILTDK
4.5	1986.9234	2.0068	AYGRGIESTGNMVKQPAK
4.3	1987.9108	1.0194	DMDPTKICTGKGTVTLR
4.2	1988.9318	-0.0016	KNNLFPPCSYLNSLLK
3.9	1986.9346	1.9955	TPNGSLIPLHQGCKQTR
3.0	1986.9048	2.0254	TGYGEINGNAGEREISLK
3.0	1988.9445	-0.0143	NLATSLAELRTASQKAR

Spectrum No: 177; Query: 174; Rank: 1

Peptide View

MS/MS Fragmentation of **NDSFTACIELGK**
Found in **IPI00373419**, Tax_Id=10116 Gene_Symbol=Spnb2 Non-erythrocyte beta-spectrin

Match to Query 174: 1354.608268 from(678.311410,2+)
Title: 091127RatKid_SCX01_14.3036.3036.2.dta
Data file K:\NewmanPaper\Piliang\3SubProteomes\Piliang3SP\mgf5ppm\SCX_3SubProteomes5ppm.mgf

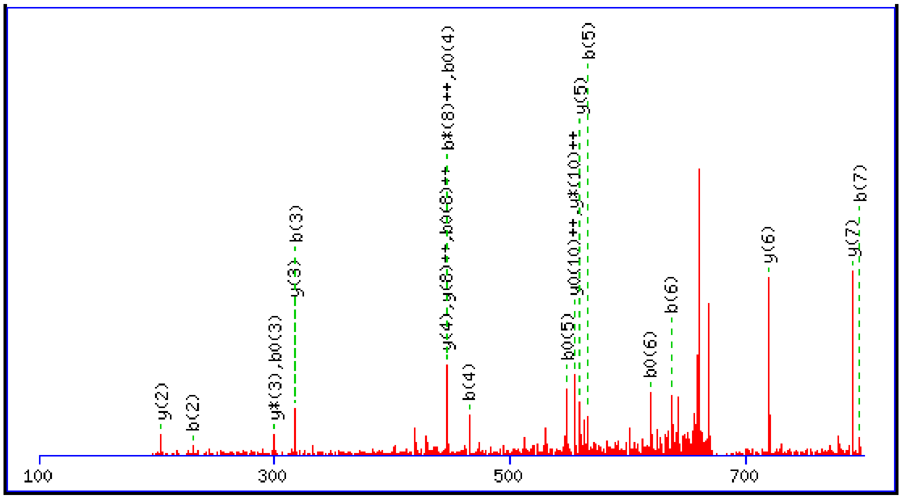

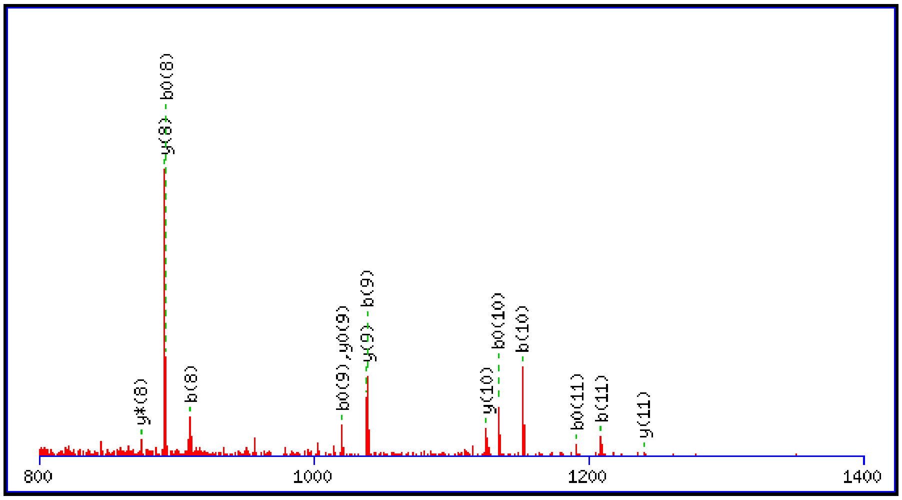

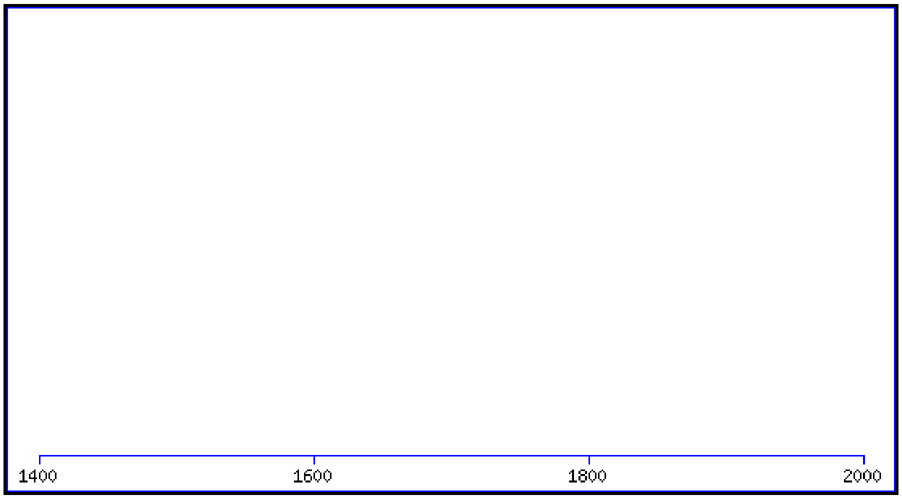

Monoisotopic mass of neutral peptide Mr(calc): 1354.6075
Fixed modifications: Carbamidomethyl (C)
Variable modifications:
N1 : Deamidated_N (N)
Ions Score: 74 **Expect:** 5.3e-006
Matches (Bold Red): 36/124 fragment ions using 55 most intense peaks

#	b	b++	b*	b*++	b0	b0++	Seq.	y	y++	y*	y*++	y0	y0++	#
1	116.0342	58.5207	99.0077	50.0075			N							12
2	**231.0612**	116.0342	214.0346	107.5209	213.0506	107.0289	D	**1240.5878**	620.7975	1223.5613	612.2843	1222.5773	611.7923	11
3	**318.0932**	159.5502	301.0666	151.0370	**300.0826**	150.5449	S	**1125.5609**	563.2841	1108.5343	**554.7708**	1107.5503	**554.2788**	10
4	**465.1616**	233.0844	448.1350	224.5712	**447.1510**	224.0792	F	**1038.5288**	519.7681	1021.5023	511.2548	**1020.5183**	510.7628	9
5	**566.2093**	283.6083	549.1827	275.0950	**548.1987**	274.6030	T	**891.4604**	**446.2339**	**874.4339**	437.7206	873.4499	437.2286	8

**6**	**637.2464**	319.1268	620.2198	310.6136	**619.2358**	310.1216	**A**	**790.4128**	395.7100	773.3862	387.1967	772.4022	386.7047	**7**
**7**	**797.2770**	399.1422	780.2505	390.6289	779.2665	390.1369	**C**	**719.3756**	360.1915	702.3491	351.6782	701.3651	351.1862	**6**
**8**	**910.3611**	455.6842	893.3346	**447.1709**	**892.3505**	**446.6789**	**I**	**559.3450**	280.1761	542.3184	271.6629	541.3344	271.1708	**5**
**9**	**1039.4037**	520.2055	1022.3771	511.6922	**1021.3931**	511.2002	**E**	**446.2609**	223.6341	429.2344	215.1208	428.2504	214.6288	**4**
**10**	**1152.4878**	576.7475	1135.4612	568.2342	**1134.4772**	567.7422	**L**	**317.2183**	159.1128	**300.1918**	150.5995			**3**
**11**	**1209.5092**	605.2582	1192.4827	596.7450	**1191.4987**	596.2530	**G**	**204.1343**	102.5708	187.1077	94.0575			**2**
**12**							**K**	147.1128	74.0600	130.0863	65.5468			**1**

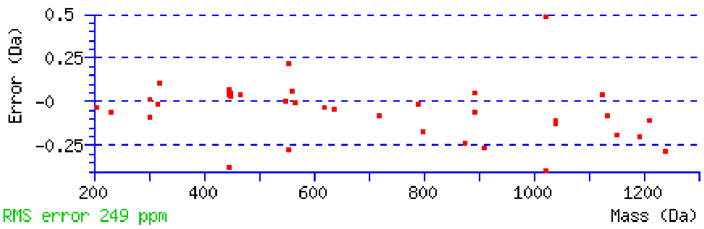

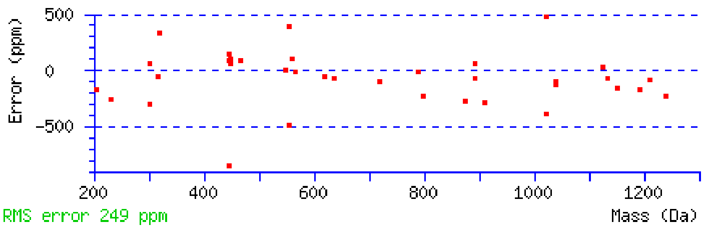

All matches to this query

Score	Mr(calc):	Delta	Sequence
74.4	1354.6075	0.0008	NDSFTACIELGK
9.1	1354.6109	-0.0026	QMGMTDDLSTIK
6.5	1353.6048	1.0035	NSDEIISEEYR
6.0	1354.6074	0.0008	EKFNQNMEGLK
5.4	1354.6048	0.0035	GMGRGDGFDSRGK
4.4	1354.5976	0.0107	SGNIMFHSFGNK
3.7	1353.6048	1.0035	DSIQQSFSNEAK
3.2	1354.6075	0.0008	MAQDVATNVNYK
3.1	1354.6203	-0.0121	MSPGGSALDLALK
3.0	1354.6010	0.0073	SDNQLLCAFCK

Spectrum No: 178; Query: 1154; Rank: 1

Peptide View

MS/MS Fragmentation of **VFSTDINGLNTQEILNVSVDTPENLAVDWINNK**
Found in **IPI00205325**, Tax_Id=10116 Gene_Symbol=Lrp2 Low-density lipoprotein receptor-related protein 2 precursor

Match to Query 1154: 3672.826332 from(1225.282720,3+)
Title: 091129RatKid_SCX02_12.5559.5559.3.dta
Data file K:\NewmanPaper\Piliang\3SubProteomes\Piliang3SP\mgf5ppm\SCX_3SubProteomes5ppm.mgf

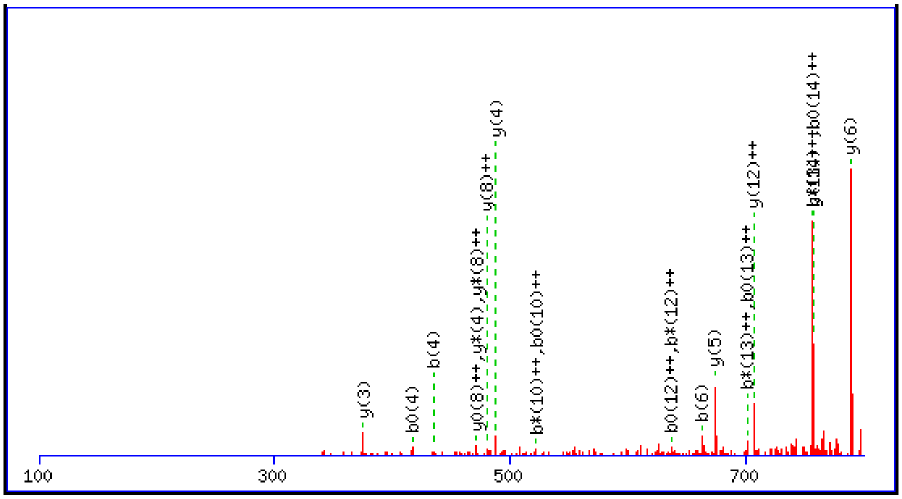

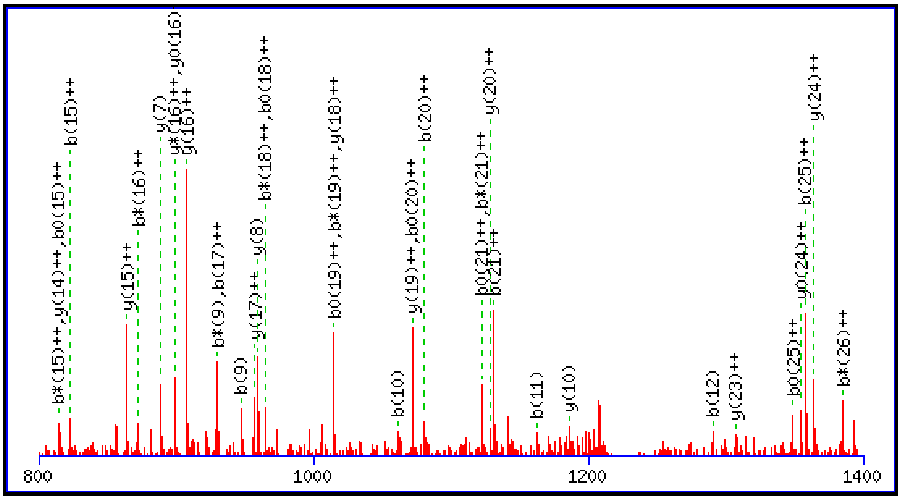

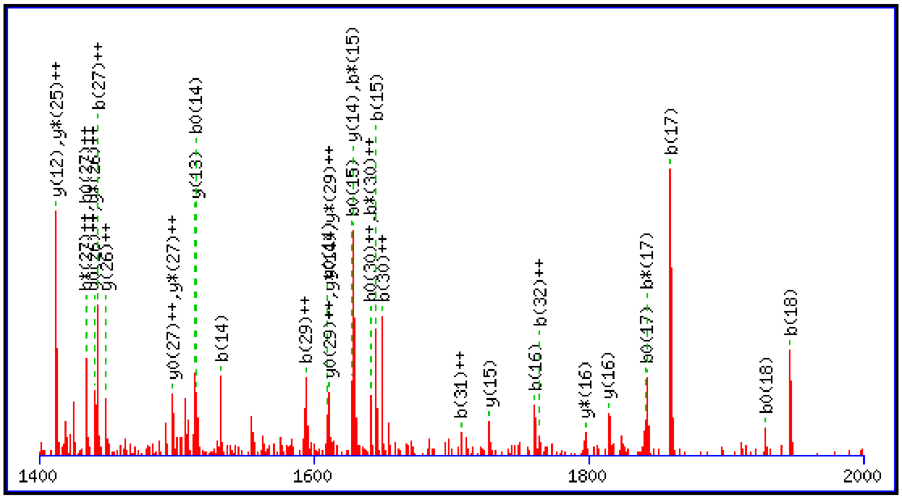

Monoisotopic mass of neutral peptide Mr(calc): 3672.8159
Fixed modifications: Carbamidomethyl (C)
Variable modifications:
N16 : Deamidated_N (N)
Ions Score: 74 **Expect:** 8.1e-006
Matches (Bold Red): 94/358 fragment ions using 125 most intense peaks

#	b	b++	b*	b*++	b0	b0++	Seq.	y	y++	y*	y*++	y0	y0++	#
1	100.0757	50.5415					V							33
2	247.1441	124.0757					F	3574.7548	1787.8810	3557.7282	1779.3677	3556.7442	1778.8757	32
3	334.1761	167.5917			316.1656	158.5864	S	3427.6863	1714.3468	3410.6598	1705.8335	3409.6758	1705.3415	31
4	**435.2238**	218.1155			**417.2132**	209.1103	T	3340.6543	1670.8308	3323.6278	1662.3175	3322.6437	1661.8255	30
5	550.2508	275.6290			532.2402	266.6237	D	3239.6066	1620.3070	3222.5801	**1611.7937**	3221.5961	**1611.3017**	29

6	**663.3348**	332.1710			645.3243	323.1658	I	3124.5797	1562.7935	3107.5531	1554.2802	3106.5691	1553.7882	28
7	777.3777	389.1925	760.3512	380.6792	759.3672	380.1872	N	3011.4956	1506.2515	2994.4691	**1497.7382**	2993.4851	**1497.2462**	27
8	834.3992	417.7032	817.3727	409.1900	816.3886	408.6980	G	2897.4527	**1449.2300**	2880.4262	**1440.7167**	2879.4421	**1440.2247**	26
9	**947.4833**	474.2453	**930.4567**	465.7320	929.4727	465.2400	L	2840.4312	1420.7193	2823.4047	**1412.2060**	2822.4207	1411.7140	25
10	**1061.5262**	531.2667	1044.4997	**522.7535**	1043.5156	**522.2615**	N	2727.3472	**1364.1772**	2710.3206	1355.6639	2709.3366	**1355.1719**	24
11	**1162.5739**	581.7906	1145.5473	573.2773	1144.5633	572.7853	T	2613.3042	**1307.1558**	2596.2777	1298.6425	2595.2937	1298.1505	23
12	**1290.6325**	645.8199	1273.6059	**637.3066**	1272.6219	**636.8146**	Q	2512.2566	1256.6319	2495.2300	1248.1186	2494.2460	1247.6266	22
13	1419.6751	710.3412	1402.6485	**701.8279**	1401.6645	**701.3359**	E	2384.1980	1192.6026	2367.1714	1184.0894	2366.1874	1183.5973	21
14	**1532.7591**	766.8832	1515.7326	**758.3699**	**1514.7485**	**757.8779**	I	2255.1554	**1128.0813**	2238.1288	1119.5681	2237.1448	1119.0761	20
15	**1645.8432**	**823.4252**	**1628.8166**	**814.9120**	**1627.8326**	**814.4199**	L	2142.0713	**1071.5393**	2125.0448	1063.0260	2124.0608	1062.5340	19
16	**1760.8701**	880.9387	1743.8436	**872.4254**	1742.8595	871.9334	N	2028.9873	**1014.9973**	2011.9607	1006.4840	2010.9767	1005.9920	18
17	**1859.9385**	**930.4729**	**1842.9120**	921.9596	**1841.9280**	921.4676	V	1913.9603	**957.4838**	1896.9338	948.9705	1895.9498	948.4785	17
18	**1946.9706**	973.9889	1929.9440	**965.4756**	**1928.9600**	**964.9836**	S	**1814.8919**	**907.9496**	**1797.8654**	**899.4363**	1796.8814	**898.9443**	16
19	2046.0390	1023.5231	2029.0124	**1015.0098**	2028.0284	**1014.5178**	V	**1727.8599**	**864.4336**	1710.8333	855.9203	1709.8493	855.4283	15
20	2161.0659	**1081.0366**	2144.0394	1072.5233	2143.0553	**1072.0313**	D	**1628.7915**	**814.8994**	**1611.7649**	806.3861	**1610.7809**	805.8941	14
21	2262.1136	**1131.5604**	2245.0870	**1123.0472**	2244.1030	**1122.5552**	T	**1513.7645**	**757.3859**	1496.7380	748.8726	1495.7540	748.3806	13
22	2359.1664	1180.0868	2342.1398	1171.5735	2341.1558	1171.0815	P	**1412.7169**	**706.8621**	1395.6903	698.3488	1394.7063	697.8568	12
23	2488.2089	1244.6081	2471.1824	1236.0948	2470.1984	1235.6028	E	1315.6641	658.3357	1298.6375	649.8224	1297.6535	649.3304	11
24	2602.2519	1301.6296	2585.2253	1293.1163	2584.2413	1292.6243	N	**1186.6215**	593.8144	1169.5949	585.3011	1168.6109	584.8091	10
25	2715.3359	**1358.1716**	2698.3094	1349.6583	2697.3254	**1349.1663**	L	1072.5786	536.7929	1055.5520	528.2796	1054.5680	527.7876	9
26	2786.3731	1393.6902	2769.3465	**1385.1769**	2768.3625	1384.6849	A	**959.4945**	**480.2509**	942.4680	**471.7376**	941.4839	**471.2456**	8
27	2885.4415	**1443.2244**	2868.4149	**1434.7111**	2867.4309	**1434.2191**	V	**888.4574**	444.7323	871.4308	436.2191	870.4468	435.7271	7
28	3000.4684	1500.7378	2983.4419	1492.2246	2982.4578	1491.7326	D	**789.3890**	395.1981	772.3624	386.6849	771.3784	386.1928	6
29	3186.5477	**1593.7775**	3169.5212	1585.2642	3168.5372	1584.7722	W	**674.3620**	337.6847	657.3355	329.1714			5
30	3299.6318	**1650.3195**	3282.6052	**1641.8063**	3281.6212	**1641.3142**	I	**488.2827**	244.6450	**471.2562**	236.1317			4
31	3413.6747	**1707.3410**	3396.6482	1698.8277	3395.6642	1698.3357	N	**375.1987**	188.1030	358.1721	179.5897			3
32	3527.7176	**1764.3625**	3510.6911	1755.8492	3509.7071	1755.3572	N	261.1557	131.0815	244.1292	122.5682			2
33							K	147.1128	74.0600	130.0863	65.5468			1

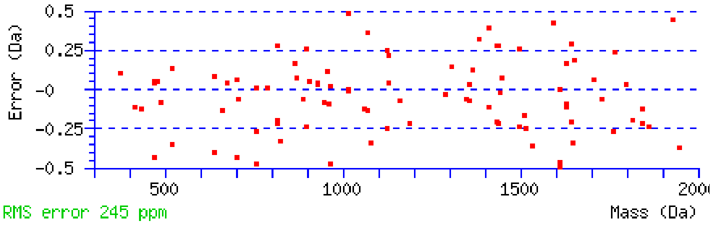

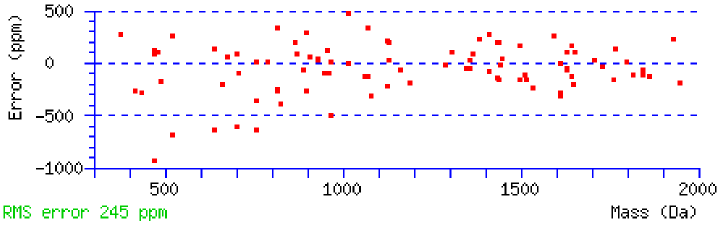

All matches to this query

Score	Mr(calc):	Delta	Sequence
74.4	3672.8159	0.0104	VFSTDINGLNTQEILNVSVDTPENLAVDWINNK
57.5	3672.8159	0.0104	VFSTDINGLNTQEILNVSVDTPENLAVDWINNK
52.1	3672.8159	0.0104	VFSTDINGLNTQEILNVSVDTPENLAVDWINNK
51.2	3671.8319	0.9945	VFSTDINGLNTQEILNVSVDTPENLAVDWINNK
50.6	3672.8159	0.0104	VFSTDINGLNTQEILNVSVDTPENLAVDWINNK
46.0	3672.8159	0.0104	VFSTDINGLNTQEILNVSVDTPENLAVDWINNK
45.3	3672.8159	0.0104	VFSTDINGLNTQEILNVSVDTPENLAVDWINNK
8.9	3671.8243	1.0020	LWSTLCSGLGQSQNLLASPKRPHSPTTGITQTR
7.9	3671.8243	1.0020	LWSTLCSGLGQSQNLLASPKRPHSPTTGITQTR
5.1	3671.8243	1.0020	LWSTLCSGLGQSQNLLASPKRPHSPTTGITQTR

Spectrum No: 179; Query: 1011; Rank: 1

Peptide View

MS/MS Fragmentation of **HTGPGILSMANAGPNTNGSQFFICTAK**
Found in **IPI00364439**, Tax_Id=10116 Gene_Symbol=RGD1559682_predicted similar to Peptidyl-prolyl cis-trans isomerase A

Match to Query 1011: 2792.317268 from(1397.165910,2+)
Title: 091127RatKid_SCX01_27.4173.4173.2.dta
Data file K:\NewmanPaper\Piliang\3SubProteomes\Piliang3SP\mgf5ppm\SCX_3SubProteomes5ppm.mgf

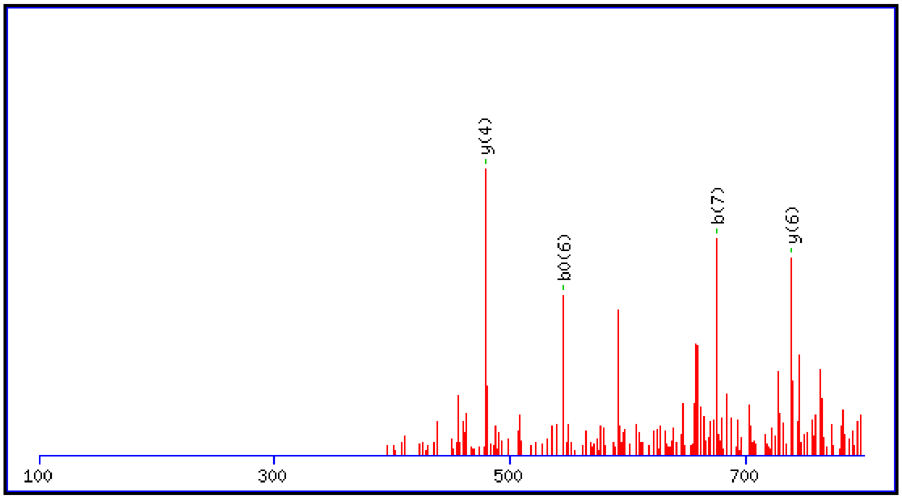

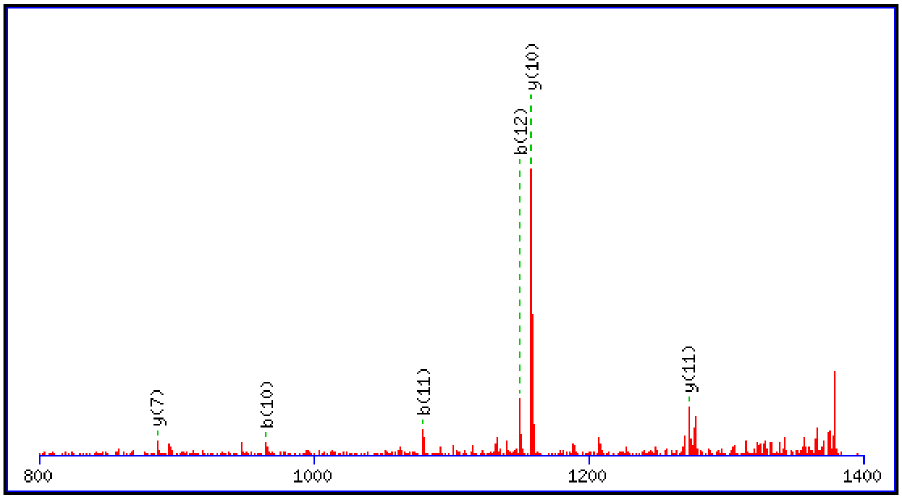

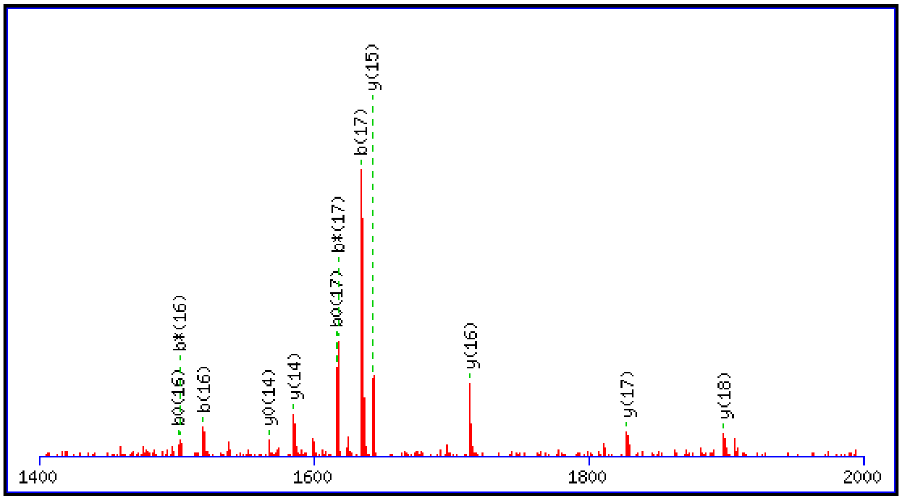

Monoisotopic mass of neutral peptide Mr(calc): 2791.3058
Fixed modifications: Carbamidomethyl (C)

Variable modifications:
N17 : Deamidated_N (N)
Ions Score: 74 **Expect:** 1.2e-005
Matches (Bold Red): 22/286 fragment ions using 34 most intense peaks

#	b	b^{++}	b*	b^{*++}	b^0	b^{0++}	Seq.	y	y^{++}	y*	y^{*++}	y^0	y^{0++}	#
1	138.0662	69.5367					H							27
2	239.1139	120.0606			221.1033	111.0553	T	2655.2541	1328.1307	2638.2276	1319.6174	2637.2436	1319.1254	26
3	296.1353	148.5713			278.1248	139.5660	G	2554.2065	1277.6069	2537.1799	1269.0936	2536.1959	1268.6016	25
4	393.1881	197.0977			375.1775	188.0924	P	2497.1850	1249.0961	2480.1585	1240.5829	2479.1744	1240.0909	24
5	450.2096	225.6084			432.1990	216.6031	G	2400.1322	1200.5698	2383.1057	1192.0565	2382.1217	1191.5645	23
6	563.2936	282.1504			**545.2831**	273.1452	I	2343.1108	1172.0590	2326.0842	1163.5458	2325.1002	1163.0537	22
7	**676.3777**	338.6925			658.3671	329.6872	L	2230.0267	1115.5170	2213.0002	1107.0037	2212.0161	1106.5117	21
8	763.4097	382.2085			745.3991	373.2032	S	2116.9426	1058.9750	2099.9161	1050.4617	2098.9321	1049.9697	20
9	894.4502	447.7287			876.4396	438.7235	M	2029.9106	1015.4589	2012.8841	1006.9457	2011.9001	1006.4537	19
10	**965.4873**	483.2473			947.4767	474.2420	A	**1898.8701**	949.9387	1881.8436	941.4254	1880.8596	940.9334	18
11	**1079.5302**	540.2688	1062.5037	531.7555	1061.5197	531.2635	N	**1827.8330**	914.4201	1810.8065	905.9069	1809.8225	905.4149	17
12	**1150.5674**	575.7873	1133.5408	567.2740	1132.5568	566.7820	A	**1713.7901**	857.3987	1696.7635	848.8854	1695.7795	848.3934	16
13	1207.5888	604.2980	1190.5623	595.7848	1189.5783	595.2928	G	**1642.7530**	821.8801	1625.7264	813.3669	1624.7424	812.8748	15
14	1304.6416	652.8244	1287.6150	644.3112	1286.6310	643.8191	P	**1585.7315**	793.3694	1568.7050	784.8561	**1567.7209**	784.3641	14
15	1418.6845	709.8459	1401.6580	701.3326	1400.6739	700.8406	N	1488.6787	744.8430	1471.6522	736.3297	1470.6682	735.8377	13
16	**1519.7322**	760.3697	**1502.7056**	751.8565	**1501.7216**	751.3644	T	1374.6358	687.8215	1357.6093	679.3083	1356.6253	678.8163	12
17	**1634.7591**	817.8832	**1617.7326**	809.3699	**1616.7486**	808.8779	N	**1273.5881**	637.2977	1256.5616	628.7844	1255.5776	628.2924	11
18	1691.7806	846.3939	1674.7540	837.8807	1673.7700	837.3886	G	**1158.5612**	579.7842	1141.5347	571.2710	1140.5506	570.7790	10
19	1778.8126	889.9099	1761.7861	881.3967	1760.8021	880.9047	S	1101.5397	551.2735	1084.5132	542.7602	1083.5292	542.2682	9
20	1906.8712	953.9392	1889.8446	945.4260	1888.8606	944.9340	Q	1014.5077	507.7575	997.4812	499.2442	996.4972	498.7522	8
21	2053.9396	1027.4734	2036.9131	1018.9602	2035.9290	1018.4682	F	**886.4491**	443.7282	869.4226	435.2149	868.4386	434.7229	7
22	2201.0080	1101.0076	2183.9815	1092.4944	2182.9975	1092.0024	F	**739.3807**	370.1940	722.3542	361.6807	721.3702	361.1887	6
23	2314.0921	1157.5497	2297.0655	1149.0364	2296.0815	1148.5444	I	592.3123	296.6598	575.2858	288.1465	574.3017	287.6545	5
24	2474.1227	1237.5650	2457.0962	1229.0517	2456.1122	1228.5597	C	**479.2282**	240.1178	462.2017	231.6045	461.2177	231.1125	4
25	2575.1704	1288.0888	2558.1439	1279.5756	2557.1598	1279.0836	T	319.1976	160.1024	302.1710	151.5892	301.1870	151.0972	3
26	2646.2075	1323.6074	2629.1810	1315.0941	2628.1970	1314.6021	A	218.1499	109.5786	201.1234	101.0653			2
27							K	147.1128	74.0600	130.0863	65.5468			1

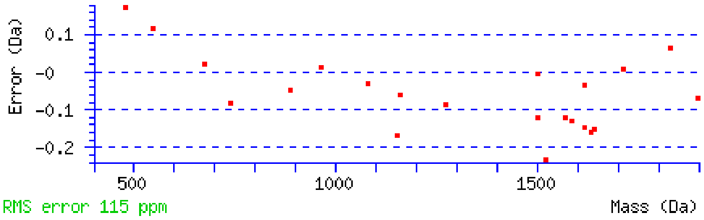

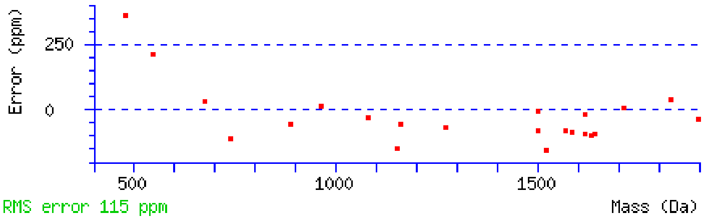

All matches to this query

Score	Mr(calc):	Delta	Sequence
74.3	2791.3058	1.0115	HTGPGILSMANAGPNTNGSQFFICTAK
58.8	2791.3058	1.0115	HTGPGILSMANAGPNTNGSQFFICTAK
52.8	2792.2898	0.0275	HTGPGILSMANAGPNTNGSQFFICTAK
48.8	2792.2898	0.0275	HTGPGILSMANAGPNTNGSQFFICTAK
39.0	2792.2898	0.0275	HTGPGILSMANAGPNTNGSQFFICTAK
32.3	2791.3058	1.0115	HTGPGILSMANAGPNTNGSQFFICTAK
23.9	2790.3218	1.9955	HTGPGILSMANAGPNTNGSQFFICTAK
4.5	2792.2898	0.0274	HTGPGILSMANVGPNTNGSGFFICTDK
4.4	2791.3058	1.0115	HTGPGILSMANVGPNTNGSGFFICTDK

4.0	2792.2898	0.0274	HTGPGILSMANVGPNTNGSGFFICTDK

Spectrum No: 180; Query: 1073; Rank: 1

Peptide View

MS/MS Fragmentation of **AGVLADDHLIEVNGENVENASHEEVVEK**
Found in **IPI00200998**, Tax_Id=10116 Gene_Symbol=Pdzk1 PDZ domain-containing protein 1

Match to Query 1073: 3016.437822 from(1006.486550,3+)
Title: 091129RatKid_SCX02_32.3344.3344.3.dta
Data file K:\NewmanPaper\Piliang\3SubProteomes\Piliang3SP\mgf5ppm\SCX_3SubProteomes5ppm.mgf

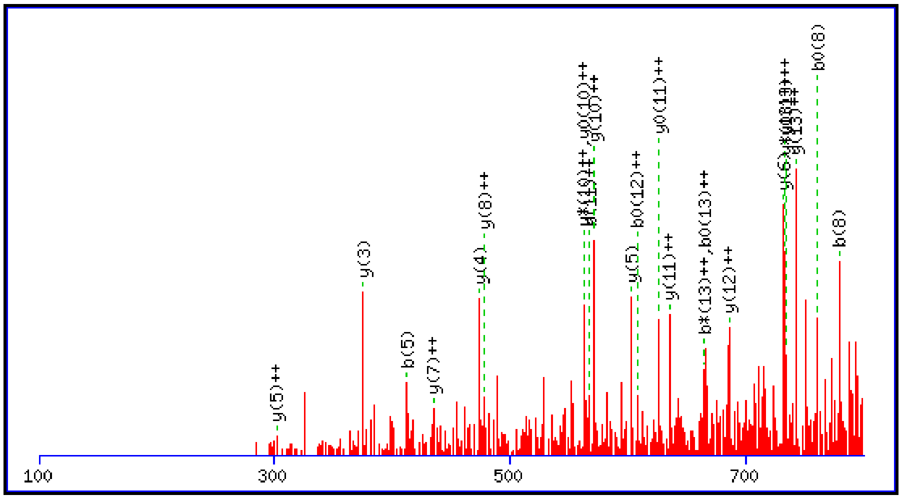

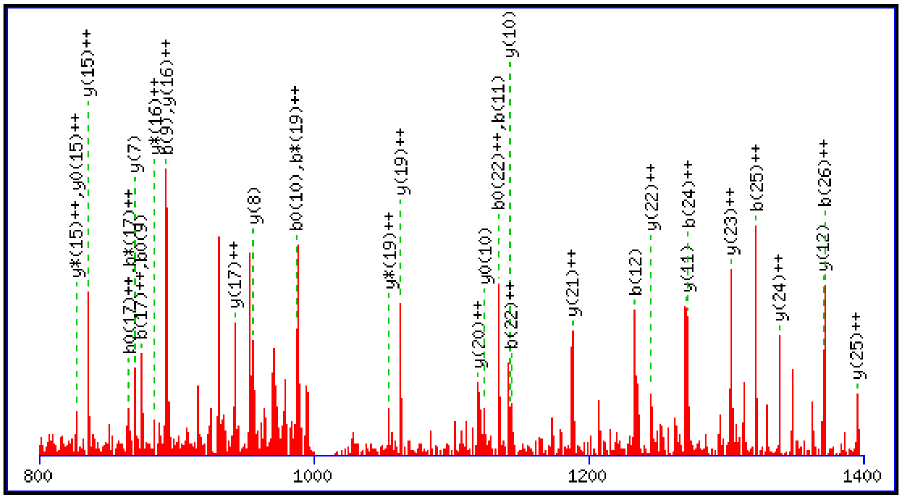

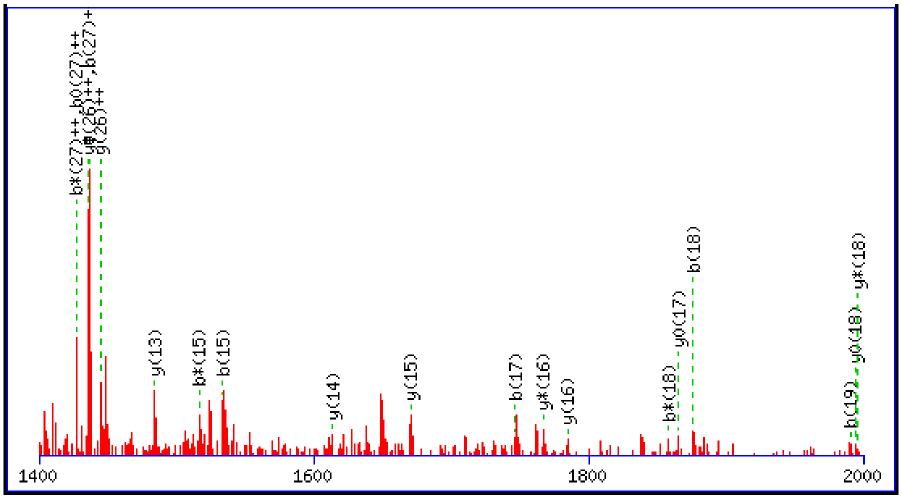

Monoisotopic mass of neutral peptide Mr(calc): 3016.4261
Fixed modifications: Carbamidomethyl (C)
Variable modifications:
N19 : Deamidated_N (N)
Ions Score: 74 **Expect:** 1.2e-005
Matches (Bold Red): 77/288 fragment ions using 162 most intense peaks

#	b	b^{++}	b*	b^{*++}	b^{0}	b^{0++}	Seq.	y	y^{++}	y*	y^{*++}	y^{0}	y^{0++}	#
1	72.0444	36.5258					A							28
2	129.0659	65.0366					G	2946.3963	1473.7018	2929.3698	1465.1885	2928.3857	1464.6965	27
3	228.1343	114.5708					V	2889.3748	**1445.1911**	2872.3483	**1436.6778**	2871.3643	**1436.1858**	26
4	341.2183	171.1128					L	2790.3064	**1395.6569**	2773.2799	1387.1436	2772.2959	1386.6516	25
5	**412.2554**	206.6314					A	2677.2224	**1339.1148**	2660.1958	1330.6015	2659.2118	1330.1095	24
6	527.2824	264.1448			509.2718	255.1396	D	2606.1853	**1303.5963**	2589.1587	1295.0830	2588.1747	1294.5910	23
7	642.3093	321.6583			624.2988	312.6530	D	2491.1583	**1246.0828**	2474.1318	1237.5695	2473.1477	1237.0775	22
8	**779.3682**	390.1878			**761.3577**	381.1825	H	2376.1314	**1188.5693**	2359.1048	1180.0560	2358.1208	1179.5640	21
9	**892.4523**	446.7298			**874.4417**	437.7245	L	2239.0725	**1120.0399**	2222.0459	1111.5266	2221.0619	1111.0346	20
10	1005.5364	503.2718			**987.5258**	494.2665	I	2125.9884	**1063.4978**	2108.9618	**1054.9846**	2107.9778	1054.4926	19
11	**1134.5790**	**567.7931**			1116.5684	558.7878	E	2012.9043	1006.9558	**1995.8778**	998.4425	**1994.8938**	997.9505	18
12	**1233.6474**	617.3273			1215.6368	**608.3220**	V	1883.8617	**942.4345**	1866.8352	933.9212	**1865.8512**	933.4292	17
13	1347.6903	674.3488	1330.6638	**665.8355**	1329.6797	**665.3435**	N	**1784.7933**	**892.9003**	**1767.7668**	**884.3870**	1766.7828	883.8950	16
14	1404.7118	702.8595	1387.6852	694.3462	1386.7012	693.8542	G	**1670.7504**	**835.8788**	1653.7238	**827.3656**	1652.7398	**826.8736**	15
15	**1533.7544**	767.3808	**1516.7278**	758.8675	1515.7438	758.3755	E	**1613.7289**	807.3681	1596.7024	798.8548	1595.7184	798.3628	14
16	1647.7973	824.4023	1630.7707	815.8890	1629.7867	815.3970	N	**1484.6863**	**742.8468**	1467.6598	**734.3335**	1466.6758	**733.8415**	13
17	**1746.8657**	**873.9365**	1729.8392	**865.4232**	1728.8551	**864.9312**	V	**1370.6434**	**685.8253**	1353.6169	677.3121	1352.6328	676.8201	12
18	**1875.9083**	938.4578	**1858.8817**	929.9445	1857.8977	929.4525	E	**1271.5750**	**636.2911**	1254.5484	627.7779	1253.5644	**627.2859**	11
19	**1990.9352**	995.9713	1973.9087	**987.4580**	1972.9247	986.9660	N	**1142.5324**	**571.7698**	1125.5059	**563.2566**	**1124.5218**	**562.7646**	10
20	2061.9723	1031.4898	2044.9458	1022.9765	2043.9618	1022.4845	A	1027.5055	514.2564	1010.4789	505.7431	1009.4949	505.2511	9
21	2149.0044	1075.0058	2131.9778	1066.4926	2130.9938	1066.0005	S	**956.4684**	**478.7378**	939.4418	470.2245	938.4578	469.7325	8
22	2286.0633	**1143.5353**	2269.0367	1135.0220	2268.0527	**1134.5300**	H	**869.4363**	**435.2218**	852.4098	426.7085	851.4258	426.2165	7
23	2415.1059	1208.0566	2398.0793	1199.5433	2397.0953	1199.0513	E	**732.3774**	366.6923	715.3509	358.1791	714.3668	357.6871	6
24	2544.1485	**1272.5779**	2527.1219	1264.0646	2526.1379	1263.5726	E	**603.3348**	**302.1710**	586.3083	293.6578	585.3243	293.1658	5
25	2643.2169	**1322.1121**	2626.1903	1313.5988	2625.2063	1313.1068	V	**474.2922**	237.6498	457.2657	229.1365	456.2817	228.6445	4
26	2742.2853	**1371.6463**	2725.2588	1363.1330	2724.2747	1362.6410	V	**375.2238**	188.1155	358.1973	179.6023	357.2132	179.1103	3
27	2871.3279	**1436.1676**	2854.3013	**1427.6543**	2853.3173	**1427.1623**	E	276.1554	138.5813	259.1288	130.0681	258.1448	129.5761	2
28							K	147.1128	74.0600	130.0863	65.5468			1

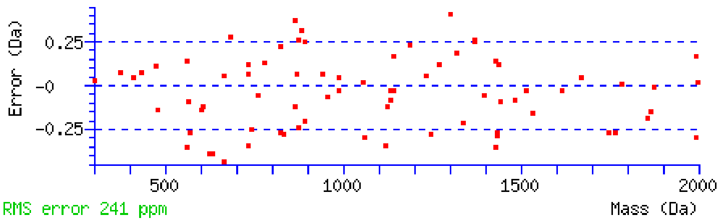

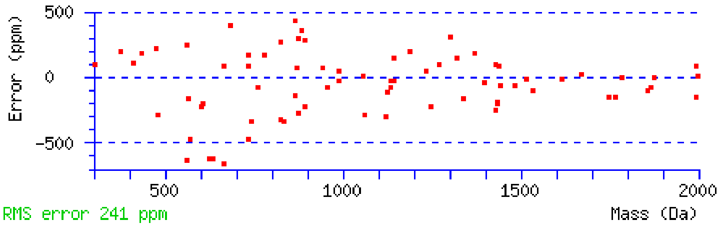

All matches to this query

Score	Mr(calc):	Delta	Sequence
74.3	3016.4261	0.0117	AGVLADDHLIEVNGENVENASHEEVVEK
73.7	3016.4261	0.0117	AGVLADDHLIEVNGENVENASHEEVVEK
73.7	3016.4261	0.0117	AGVLADDHLIEVNGENVENASHEEVVEK
55.3	3015.4421	0.9957	AGVLADDHLIEVNGENVENASHEEVVEK
3.2	3016.4605	-0.0227	VILLIPKSVNIPSNQQSLLGLHSVK
2.9	3014.4209	2.0169	VAEGVPTTEHLLTGLMEGQEYSFRVR
1.1	3016.4513	-0.0135	LCTFFGLTMTVFGLSSLLVASAMAVER
0.8	3015.4500	0.9878	LCVQMTTGAYIAGNIHSIIHTGFLFR

Spectrum No: 181; Query: 580; Rank: 1

Peptide View

MS/MS Fragmentation of **EGTEISHVLLCNVTQR**
Found in **IPI00191929**, Tax_Id=10116 Gene_Symbol=Tmem27 Collectrin precursor

Match to Query 580: 1855.912688 from(928.963620,2+)
Title: 091129RatKid_SCX02_14.2790.2790.2.dta
Data file K:\NewmanPaper\Piliang\3SubProteomes\Piliang3SP\mgf5ppm\SCX_3SubProteomes5ppm.mgf

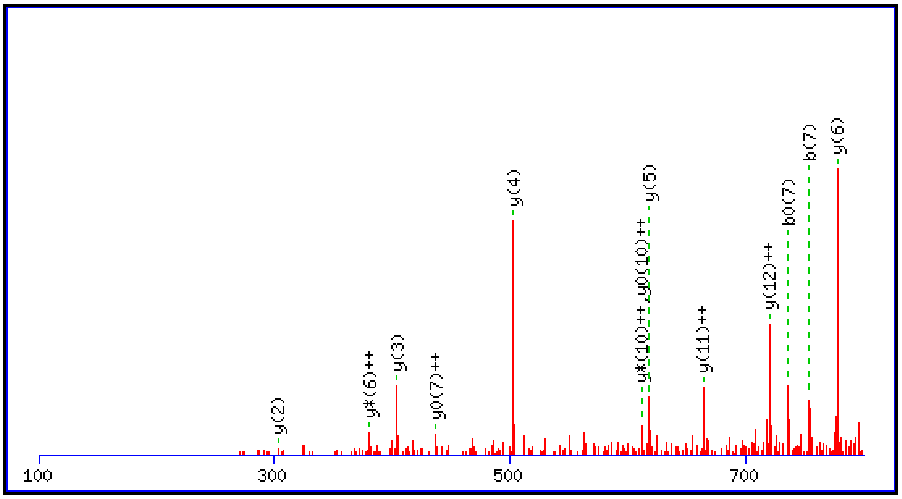

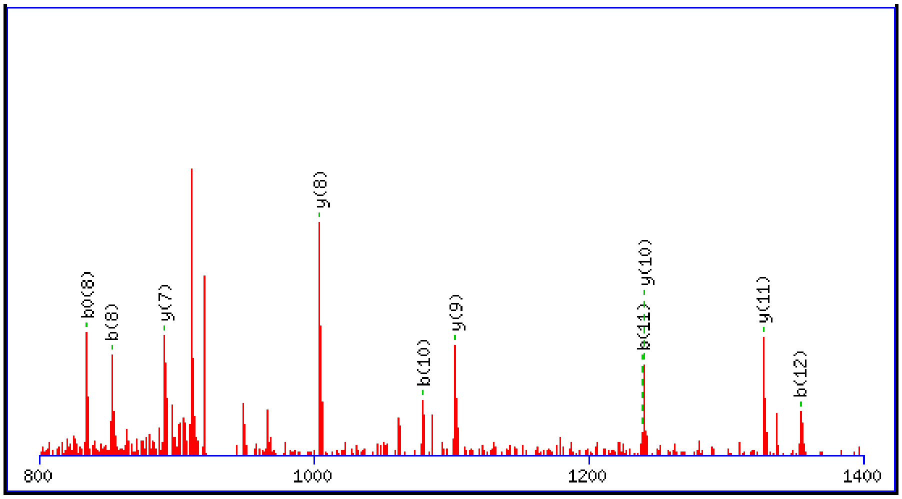

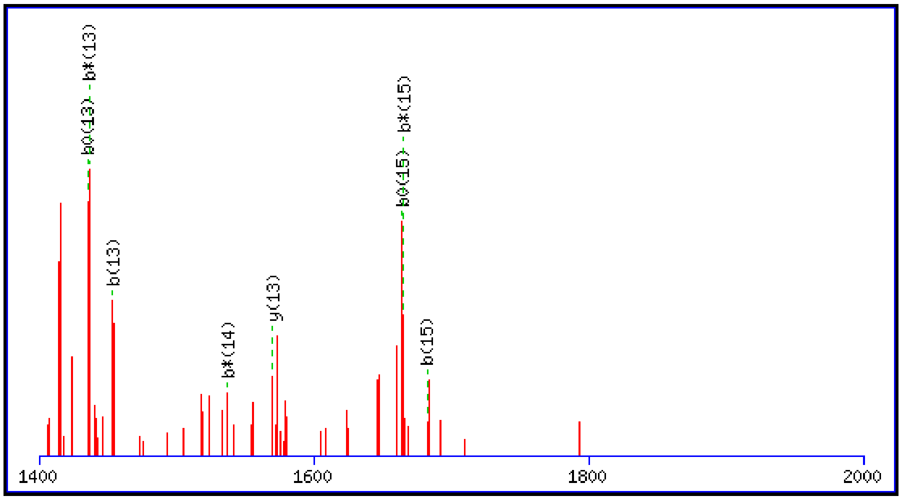

Monoisotopic mass of neutral peptide Mr(calc): 1855.9098
Fixed modifications: Carbamidomethyl (C)
Variable modifications:
N12 : Deamidated_N (N)
Ions Score: 74 **Expect:** 6.7e-006
Matches (Bold Red): 31/154 fragment ions using 51 most intense peaks

#	b	b^{++}	b*	b^{*++}	b^{0}	b^{0++}	Seq.	y	y^{++}	y*	y^{*++}	y^{0}	y^{0++}	#
1	130.0499	65.5286			112.0393	56.5233	E							16
2	187.0713	94.0393			169.0608	85.0340	G	1727.8745	864.4409	1710.8479	855.9276	1709.8639	855.4356	15
3	288.1190	144.5631			270.1084	135.5579	T	1670.8530	835.9302	1653.8265	827.4169	1652.8425	826.9249	14
4	417.1616	209.0844			399.1510	200.0792	E	**1569.8053**	785.4063	1552.7788	776.8930	1551.7948	776.4010	13
5	530.2457	265.6265			512.2351	256.6212	I	1440.7628	**720.8850**	1423.7362	712.3717	1422.7522	711.8797	12
6	617.2777	309.1425			599.2671	300.1372	S	**1327.6787**	**664.3430**	1310.6521	655.8297	1309.6681	655.3377	11
7	**754.3366**	377.6719			**736.3260**	368.6667	H	**1240.6467**	620.8270	1223.6201	**612.3137**	1222.6361	**611.8217**	10
8	**853.4050**	427.2061			**835.3945**	418.2009	V	**1103.5878**	552.2975	1086.5612	543.7842	1085.5772	543.2922	9
9	966.4891	483.7482			948.4785	474.7429	L	**1004.5193**	502.7633	987.4928	494.2500	986.5088	493.7580	8
10	**1079.5732**	540.2902			1061.5626	531.2849	L	**891.4353**	446.2213	874.4087	437.7080	873.4247	**437.2160**	7
11	**1239.6038**	620.3055			1221.5932	611.3003	C	**778.3512**	389.6792	761.3247	**381.1660**	760.3406	380.6740	6
12	**1354.6307**	677.8190	1337.6042	669.3057	1336.6202	668.8137	N	**618.3206**	309.6639	601.2940	301.1506	600.3100	300.6586	5
13	**1453.6991**	727.3532	**1436.6726**	718.8399	**1435.6886**	718.3479	V	**503.2936**	252.1504	486.2671	243.6372	485.2831	243.1452	4
14	1554.7468	777.8771	**1537.7203**	769.3638	1536.7363	768.8718	T	**404.2252**	202.6162	387.1987	194.1030	386.2146	193.6110	3
15	**1682.8054**	841.9063	**1665.7789**	833.3931	**1664.7948**	832.9011	Q	**303.1775**	152.0924	286.1510	143.5791			2
16							R	175.1190	88.0631	158.0924	79.5498			1

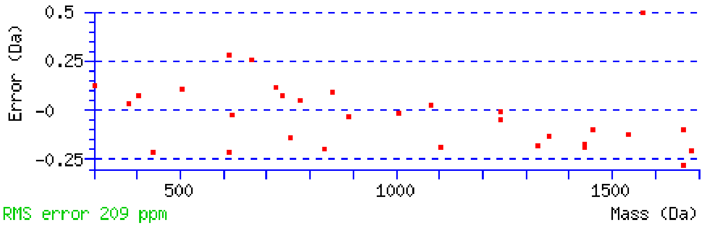

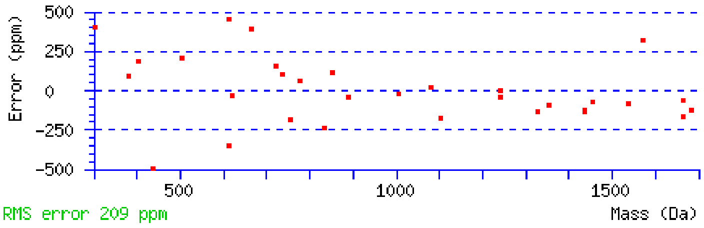

All matches to this query

Score	Mr(calc):	Delta	Sequence
74.3	1855.9098	0.0029	EGTEISHVLLCNVTQR
9.1	1855.8969	0.0158	DLVQDPSLLGGTISAYK
8.8	1854.9258	0.9869	EGTEISHVLLCNVTQR
5.4	1855.8969	0.0158	DLVQDPSLLGGTISAYK
5.3	1855.9207	-0.0080	ARDPHSGHFVALKSVR
3.1	1854.9264	0.9863	ATAGDIAHYYRDYVIK
2.0	1855.9267	-0.0140	AVFIKVPRTNMENLK
1.9	1854.9146	0.9981	ATKNVTGQAALFQGSSMK

Spectrum No: 182; Query: 874; Rank: 1

Peptide View

MS/MS Fragmentation of **TEAGAFEYVADPTFENFTGGVK**
Found in **IPI00370450**, Tax_Id=10116 Gene_Symbol=Plxnb2 plexin B2

Match to Query 874: 2350.073528 from(1176.044040,2+)
Title: 091129RatKid_SCX02_14.4099.4099.2.dta
Data file K:\NewmanPaper\Piliang\3SubProteomes\Piliang3SP\mgf5ppm\SCX_3SubProteomes5ppm.mgf

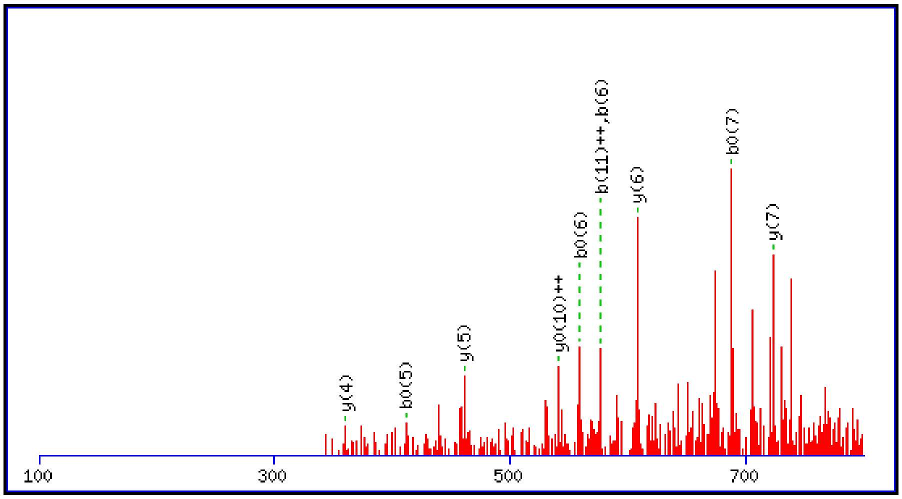

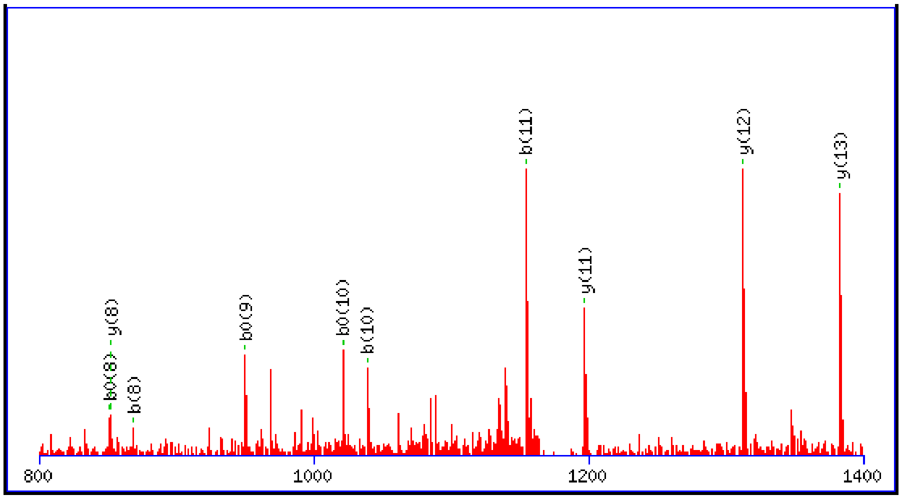

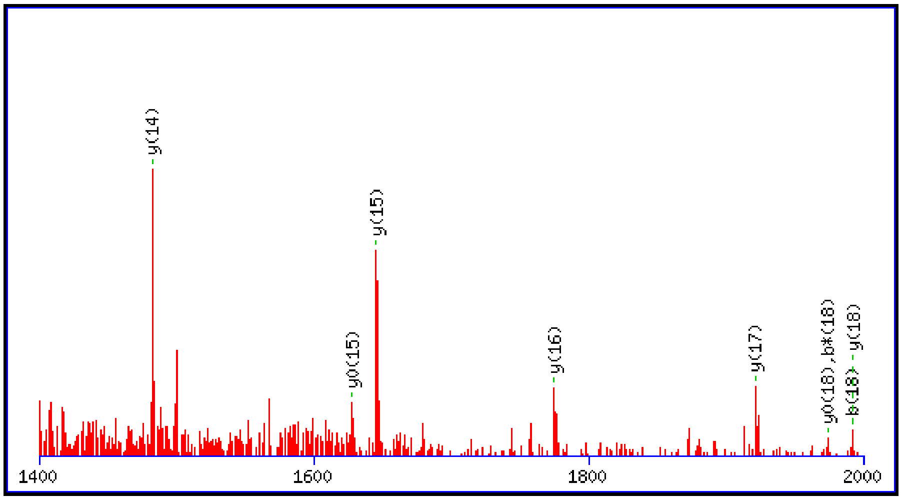

Monoisotopic mass of neutral peptide Mr(calc): 2350.0641
Fixed modifications: Carbamidomethyl (C)
Variable modifications:
N16 : Deamidated_N (N)
Ions Score: 74 **Expect:** 1.1e-005
Matches (Bold Red): 29/214 fragment ions using 52 most intense peaks

#	b	b^{++}	b*	b^{*++}	b^{0}	b^{0++}	Seq.	y	y^{++}	y*	y^{*++}	y^{0}	y^{0++}	#
1	102.0550	51.5311			84.0444	42.5258	T							22
2	231.0975	116.0524			213.0870	107.0471	E	2250.0237	1125.5155	2232.9972	1117.0022	2232.0132	1116.5102	21
3	302.1347	151.5710			284.1241	142.5657	A	2120.9811	1060.9942	2103.9546	1052.4809	2102.9706	1051.9889	20
4	359.1561	180.0817			341.1456	171.0764	G	2049.9440	1025.4756	2032.9175	1016.9624	2031.9334	1016.4704	19
5	430.1932	215.6003			**412.1827**	206.5950	A	**1992.9225**	996.9649	1975.8960	988.4516	**1974.9120**	987.9596	18
6	**577.2617**	289.1345			**559.2511**	280.1292	F	**1921.8854**	961.4464	1904.8589	952.9331	1903.8749	952.4411	17
7	706.3042	353.6558			**688.2937**	344.6505	E	**1774.8170**	887.9121	1757.7905	879.3989	1756.8065	878.9069	16
8	**869.3676**	435.1874			**851.3570**	426.1821	Y	**1645.7744**	823.3909	1628.7479	814.8776	**1627.7639**	814.3856	15
9	968.4360	484.7216			**950.4254**	475.7164	V	**1482.7111**	741.8592	1465.6845	733.3459	1464.7005	732.8539	14
10	**1039.4731**	520.2402			**1021.4625**	511.2349	A	**1383.6427**	692.3250	1366.6161	683.8117	1365.6321	683.3197	13
11	**1154.5000**	**577.7537**			1136.4895	568.7484	D	**1312.6056**	656.8064	1295.5790	648.2931	1294.5950	647.8011	12
12	1251.5528	626.2800			1233.5422	617.2748	P	**1197.5786**	599.2930	1180.5521	590.7797	1179.5681	590.2877	11
13	1352.6005	676.8039			1334.5899	667.7986	T	1100.5259	550.7666	1083.4993	542.2533	1082.5153	**541.7613**	10
14	1499.6689	750.3381			1481.6583	741.3328	F	999.4782	500.2427	982.4516	491.7295	981.4676	491.2374	9
15	1628.7115	814.8594			1610.7009	805.8541	E	**852.4098**	426.7085	835.3832	418.1952	834.3992	417.7032	8
16	1743.7384	872.3729	1726.7119	863.8596	1725.7279	863.3676	N	**723.3672**	362.1872	706.3406	353.6740	705.3566	353.1819	7
17	1890.8068	945.9071	1873.7803	937.3938	1872.7963	936.9018	F	**608.3402**	304.6738	591.3137	296.1605	590.3297	295.6685	6
18	**1991.8545**	996.4309	**1974.8280**	987.9176	1973.8440	987.4256	T	**461.2718**	231.1396	444.2453	222.6263	443.2613	222.1343	5

**19**	2048.8760	1024.9416	2031.8494	1016.4284	2030.8654	1015.9364	**G**	**360.2241**	180.6157	343.1976	172.1024			**4**
**20**	2105.8975	1053.4524	2088.8709	1044.9391	2087.8869	1044.4471	**G**	303.2027	152.1050	286.1761	143.5917			**3**
**21**	2204.9659	1102.9866	2187.9393	1094.4733	2186.9553	1093.9813	**V**	246.1812	123.5942	229.1547	115.0810			**2**
**22**							**K**	147.1128	74.0600	130.0863	65.5468			**1**

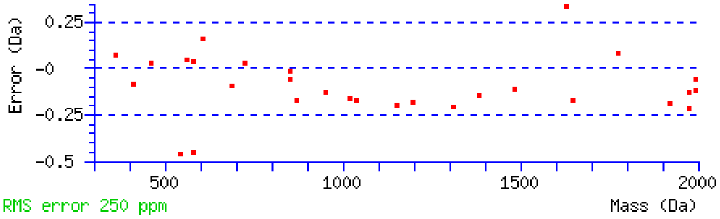

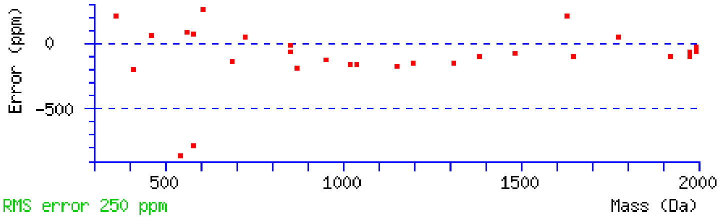

All matches to this query

Score	Mr(calc):	Delta	Sequence
74.3	2350.0641	0.0094	TEAGAFEYVADPTFENFTGGVK
20.6	2349.0801	0.9934	TEAGAFEYVADPTFENFTGGVK
1.4	2348.0896	1.9839	EEAIASPNEKQDAKQAVPESK

Spectrum No: 183; Query: 600; Rank: 1

Peptide View

MS/MS Fragmentation of **NATNVEQSFMTMAAEIK**
Found in **IPI00382300**, Tax_Id=10116 Gene_Symbol=Rab1 Ac2-048

Match to Query 600: 1884.864968 from(943.439760,2+)
Title: 091127RatKid_SCX01_12.5256.5256.2.dta
Data file K:\NewmanPaper\Piliang\3SubProteomes\Piliang3SP\mgf5ppm\SCX_3SubProteomes5ppm.mgf

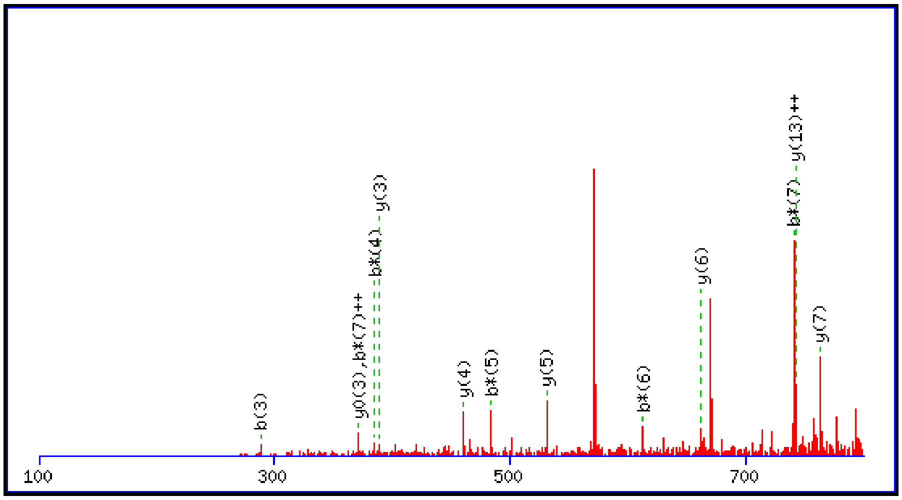

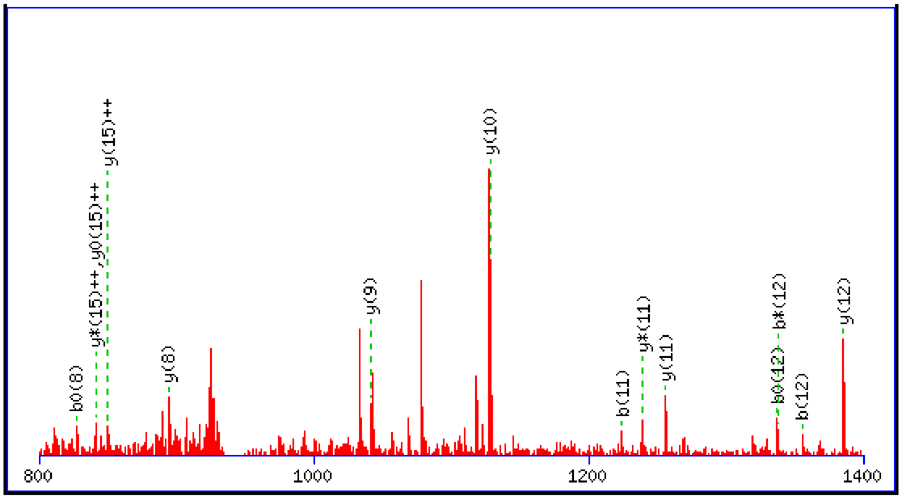

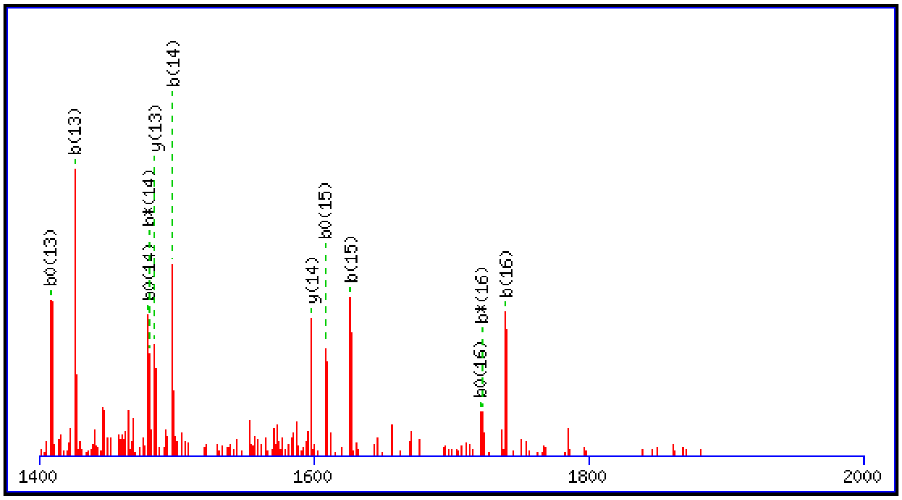

Monoisotopic mass of neutral peptide Mr(calc): 1884.8597
Fixed modifications: Carbamidomethyl (C)
Variable modifications:
N1 : Deamidated_N (N)
Ions Score: 74 **Expect:** 9e-006
Matches (Bold Red): 39/184 fragment ions using 68 most intense peaks

#	b	b++	b*	b*++	b0	b0++	Seq.	y	y++	y*	y*++	y0	y0++	#
1	116.0342	58.5207	99.0077	50.0075			N							17
2	187.0713	94.0393	170.0448	85.5260			A	1770.8401	885.9237	1753.8135	877.4104	1752.8295	876.9184	16
3	**288.1190**	144.5631	271.0925	136.0499	270.1084	135.5579	T	1699.8030	**850.4051**	1682.7764	**841.8918**	1681.7924	**841.3998**	15
4	402.1619	201.5846	**385.1354**	193.0713	384.1514	192.5793	N	**1598.7553**	799.8813	1581.7287	791.3680	1580.7447	790.8760	14
5	501.2303	251.1188	**484.2038**	242.6055	483.2198	242.1135	V	**1484.7124**	**742.8598**	1467.6858	734.3465	1466.7018	733.8545	13
6	630.2729	315.6401	**613.2464**	307.1268	612.2624	306.6348	E	**1385.6440**	693.3256	1368.6174	684.8123	1367.6334	684.3203	12
7	758.3315	379.6694	**741.3050**	**371.1561**	740.3210	370.6641	Q	**1256.6014**	628.8043	**1239.5748**	620.2910	1238.5908	619.7990	11
8	845.3635	423.1854	828.3370	414.6721	**827.3530**	414.1801	S	**1128.5428**	564.7750	1111.5162	556.2618	1110.5322	555.7697	10
9	992.4320	496.7196	975.4054	488.2063	974.4214	487.7143	F	**1041.5108**	521.2590	1024.4842	512.7457	1023.5002	512.2537	9
10	1123.4724	562.2399	1106.4459	553.7266	1105.4619	553.2346	M	**894.4423**	447.7248	877.4158	439.2115	876.4318	438.7195	8
11	**1224.5201**	612.7637	1207.4936	604.2504	1206.5096	603.7584	T	**763.4019**	382.2046	746.3753	373.6913	745.3913	373.1993	7
12	**1355.5606**	678.2839	**1338.5341**	669.7707	**1337.5500**	669.2787	M	**662.3542**	331.6807	645.3276	323.1675	644.3436	322.6754	6
13	**1426.5977**	713.8025	1409.5712	705.2892	**1408.5872**	704.7972	A	**531.3137**	266.1605	514.2871	257.6472	513.3031	257.1552	5
14	**1497.6348**	749.3211	**1480.6083**	740.8078	**1479.6243**	740.3158	A	**460.2766**	230.6419	443.2500	222.1287	442.2660	221.6366	4
15	**1626.6774**	813.8424	1609.6509	805.3291	**1608.6669**	804.8371	E	**389.2395**	195.1234	372.2129	186.6101	**371.2289**	186.1181	3
16	**1739.7615**	870.3844	**1722.7349**	861.8711	**1721.7509**	861.3791	I	260.1969	130.6021	243.1703	122.0888			2
17							K	147.1128	74.0600	130.0863	65.5468			1

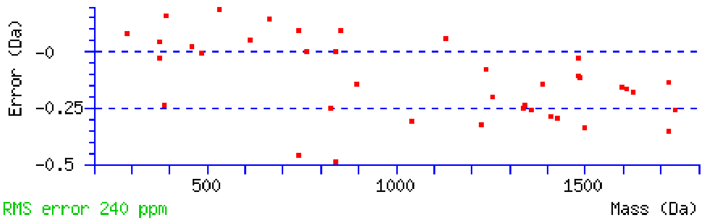

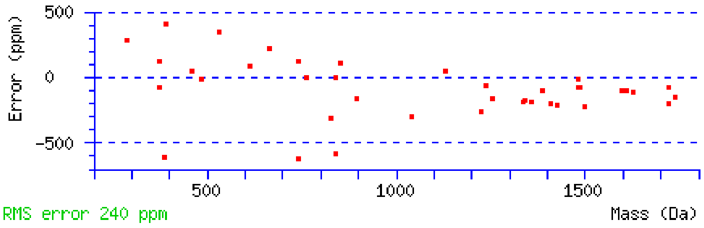

All matches to this query

Score	Mr(calc):	Delta	Sequence
74.2	1884.8597	0.0053	NATNVEQSFMTMAAEIK
61.2	1884.8597	0.0053	NATNVEQSFMTMAAEIK
53.4	1884.8597	0.0053	NATNVEQAFMTMAAEIK
43.6	1884.8597	0.0053	NATNVEQAFMTMAAEIK
36.9	1883.8757	0.9893	NATNVEQSFMTMAAEIK
34.6	1884.8597	0.0053	NATNVEQAFMTMAAEIK
26.8	1884.8597	0.0053	NATNVEQAFMTMAAEIK
19.9	1883.8757	0.9893	NATNVEQAFMTMAAEIK
8.7	1884.8764	-0.0114	MQSNNAKAVSARAEAIK
8.7	1884.8764	-0.0114	MQSNNAKAVSARAEAIK

Spectrum No: 184; Query: 408; Rank: 1

Peptide View

MS/MS Fragmentation of **LEAVQFFNVTTLQK**
Found in **IPI00207480**, Tax_Id=10116 Gene_Symbol=RGD1565180_predicted hypothetical protein LOC363158

Match to Query 408: 1637.869288 from(819.941920,2+)
Title: 091127RatKid_SCX01_16.4461.4461.2.dta
Data file K:\NewmanPaper\Piliang\3SubProteomes\Piliang3SP\mgf5ppm\SCX_3SubProteomes5ppm.mgf

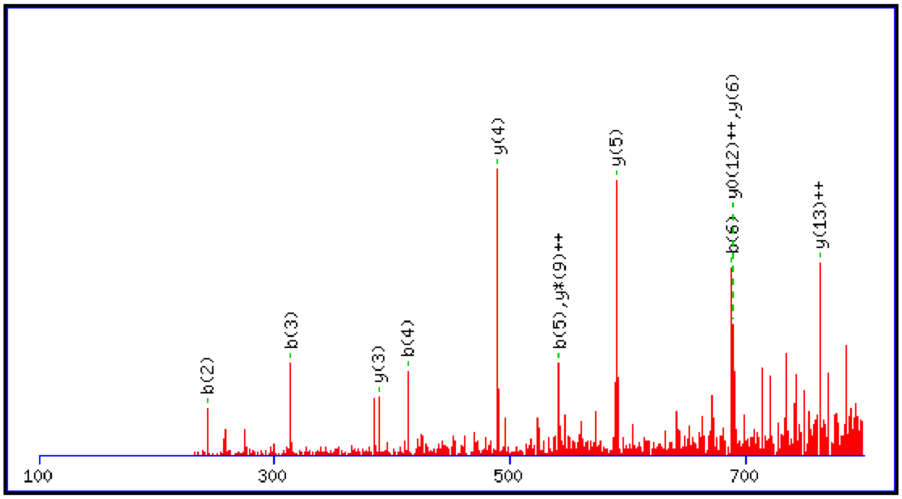

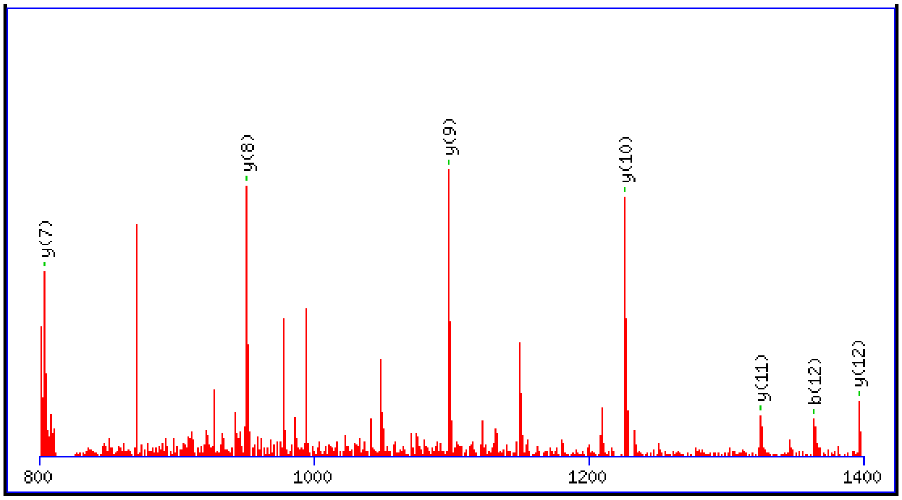

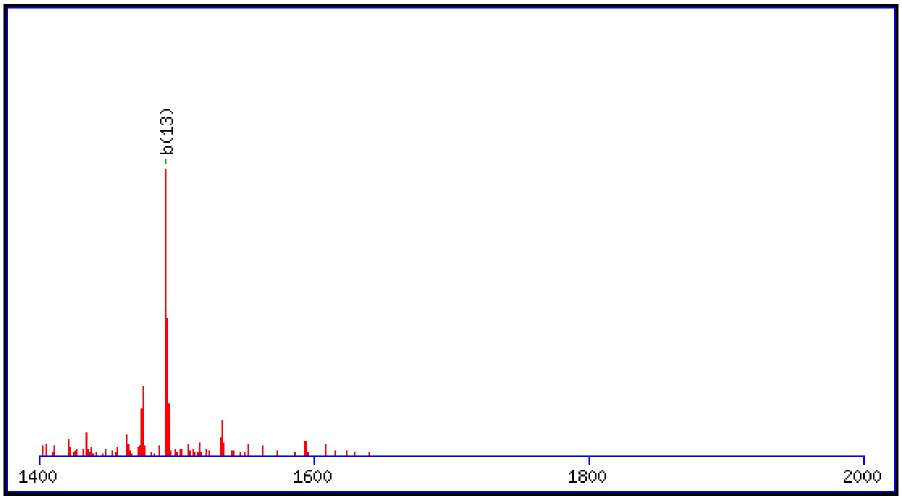

Monoisotopic mass of neutral peptide Mr(calc): 1637.8665
Fixed modifications: Carbamidomethyl (C)
Variable modifications:
N8 : Deamidated_N (N)
Ions Score: 74 **Expect:** 3.7e-006
Matches (Bold Red): 20/140 fragment ions using 31 most intense peaks

#	b	b^{++}	b*	b^{*++}	b^{0}	b^{0++}	Seq.	y	y^{++}	y*	y^{*++}	y^{0}	y^{0++}	#
1	114.0913	57.5493					L							14
2	**243.1339**	122.0706			225.1234	113.0653	E	1525.7897	**763.3985**	1508.7631	754.8852	1507.7791	754.3932	13
3	**314.1710**	157.5892			296.1605	148.5839	A	**1396.7471**	698.8772	1379.7205	690.3639	1378.7365	**689.8719**	12
4	**413.2395**	207.1234			395.2289	198.1181	V	**1325.7100**	663.3586	1308.6834	654.8454	1307.6994	654.3533	11
5	**541.2980**	271.1527	524.2715	262.6394	523.2875	262.1474	Q	**1226.6416**	613.8244	1209.6150	605.3111	1208.6310	604.8191	10
6	**688.3665**	344.6869	671.3399	336.1736	670.3559	335.6816	F	**1098.5830**	549.7951	1081.5564	**541.2819**	1080.5724	540.7898	9
7	835.4349	418.2211	818.4083	409.7078	817.4243	409.2158	F	**951.5146**	476.2609	934.4880	467.7476	933.5040	467.2556	8
8	950.4618	475.7345	933.4353	467.2213	932.4512	466.7293	N	**804.4462**	402.7267	787.4196	394.2134	786.4356	393.7214	7
9	1049.5302	525.2687	1032.5037	516.7555	1031.5197	516.2635	V	**689.4192**	345.2132	672.3927	336.7000	671.4087	336.2080	6
10	1150.5779	575.7926	1133.5513	567.2793	1132.5673	566.7873	T	**590.3508**	295.6790	573.3243	287.1658	572.3402	286.6738	5
11	1251.6256	626.3164	1234.5990	617.8032	1233.6150	617.3111	T	**489.3031**	245.1552	472.2766	236.6419	471.2926	236.1499	4
12	**1364.7096**	682.8585	1347.6831	674.3452	1346.6991	673.8532	L	**388.2554**	194.6314	371.2289	186.1181			3
13	**1492.7682**	746.8877	1475.7417	738.3745	1474.7577	737.8825	Q	275.1714	138.0893	258.1448	129.5761			2
14							K	147.1128	74.0600	130.0863	65.5468			1

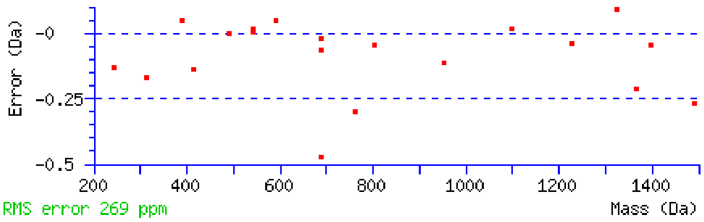

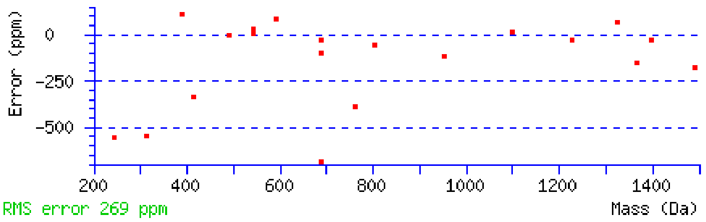

All matches to this query

Score	Mr(calc):	Delta	Sequence
74.1	1637.8665	0.0028	LEAVQFFNVTTLQK
17.9	1637.8671	0.0022	SSRPLMKAPNKEHK
7.6	1635.8733	1.9960	TLPARLDPHFLNNK
7.0	1635.8733	1.9960	TLPARLDPHFLNNK
1.7	1635.8498	2.0195	FVIRTPRIQNSPK
0.4	1636.8549	1.0144	LLSAVLRVSEVESR

Spectrum No: 185; Query: 340; Rank: 1

Peptide View

MS/MS Fragmentation of **VVNVSELYGTPCTK**
Found in **IPI00206668**, Tax_Id=10116 Gene_Symbol=Entpd1 Ectonucleoside triphosphate diphosphohydrolase 1

Match to Query 340: 1566.763128 from(784.388840,2+)
Title: 091127RatKid_SCX01_16.2494.2494.2.dta
Data file K:\NewmanPaper\Piliang\3SubProteomes\Piliang3SP\mgf5ppm\SCX_3SubProteomes5ppm.mgf

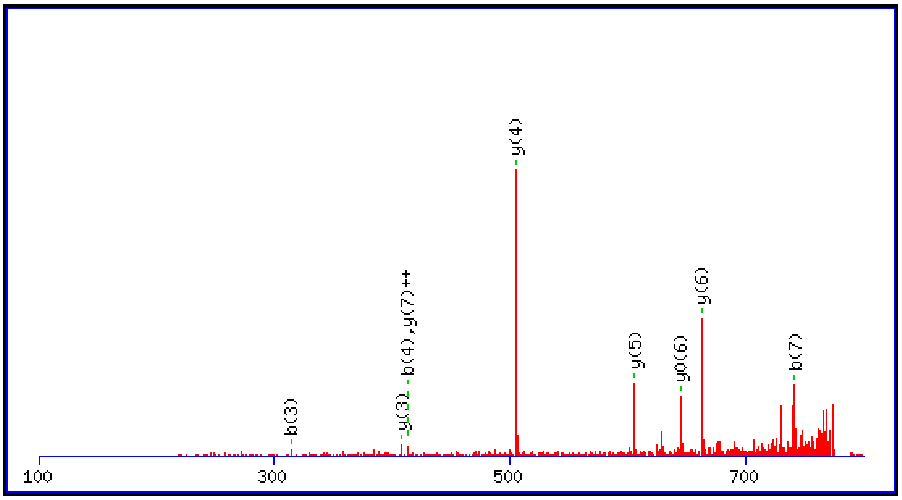

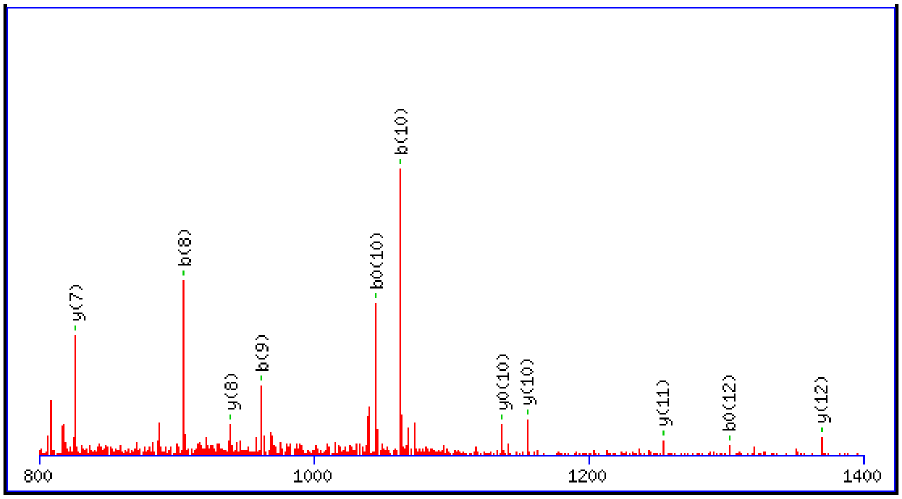

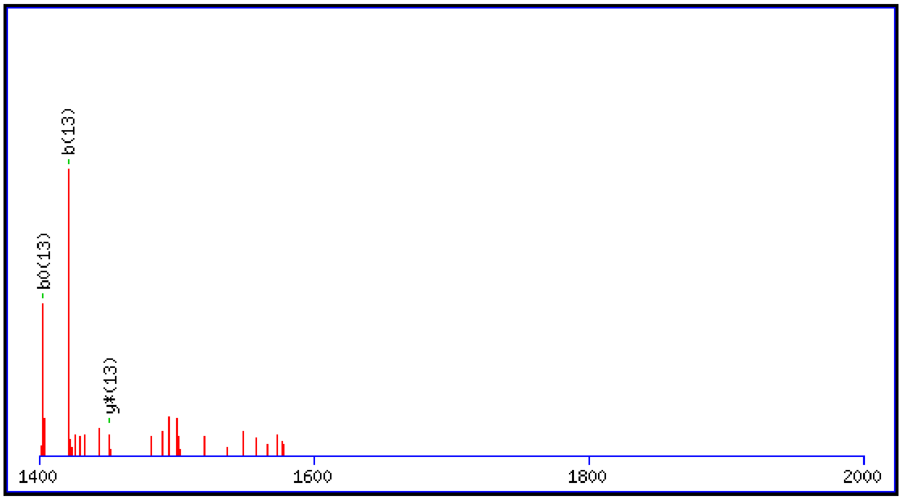

Monoisotopic mass of neutral peptide Mr(calc): 1566.7600
Fixed modifications: Carbamidomethyl (C)
Variable modifications:
N3 : Deamidated_N (N)
Ions Score: 74 **Expect:** 6.8e-006
Matches (Bold Red): 23/142 fragment ions using 40 most intense peaks

#	b	b^{++}	b*	b^{*++}	b^0	b^{0++}	Seq.	y	y^{++}	y*	y^{*++}	y^0	y^{0++}	#
1	100.0757	50.5415					V							14
2	199.1441	100.0757					V	1468.6988	734.8530	**1451.6723**	726.3398	1450.6883	725.8478	13
3	**314.1710**	157.5892	297.1445	149.0759			N	**1369.6304**	685.3188	1352.6039	676.8056	1351.6198	676.3136	12
4	**413.2395**	207.1234	396.2129	198.6101			V	**1254.6035**	627.8054	1237.5769	619.2921	1236.5929	618.8001	11
5	500.2715	250.6394	483.2449	242.1261	482.2609	241.6341	S	**1155.5351**	578.2712	1138.5085	569.7579	**1137.5245**	569.2659	10
6	629.3141	315.1607	612.2875	306.6474	611.3035	306.1554	E	1068.5030	534.7552	1051.4765	526.2419	1050.4925	525.7499	9
7	**742.3981**	371.7027	725.3716	363.1894	724.3876	362.6974	L	**939.4604**	470.2339	922.4339	461.7206	921.4499	461.2286	8
8	**905.4615**	453.2344	888.4349	444.7211	887.4509	444.2291	Y	**826.3764**	**413.6918**	809.3498	405.1785	808.3658	404.6865	7
9	**962.4829**	481.7451	945.4564	473.2318	944.4724	472.7398	G	**663.3130**	332.1602	646.2865	323.6469	**645.3025**	323.1549	6
10	**1063.5306**	532.2689	1046.5041	523.7557	**1045.5200**	523.2637	T	**606.2916**	303.6494	589.2650	295.1362	588.2810	294.6441	5
11	1160.5834	580.7953	1143.5568	572.2821	1142.5728	571.7900	P	**505.2439**	253.1256	488.2173	244.6123	487.2333	244.1203	4
12	1320.6140	660.8107	1303.5875	652.2974	**1302.6035**	651.8054	C	**408.1911**	204.5992	391.1646	196.0859	390.1806	195.5939	3
13	**1421.6617**	711.3345	1404.6352	702.8212	**1403.6511**	702.3292	T	248.1605	124.5839	231.1339	116.0706	230.1499	115.5786	2
14							K	147.1128	74.0600	130.0863	65.5468			1

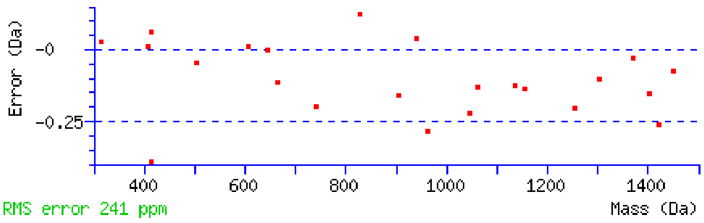

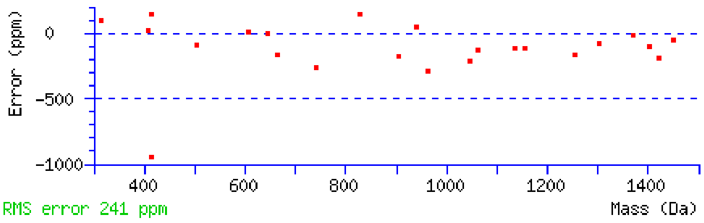

All matches to this query

Score	Mr(calc):	Delta	Sequence
74.0	1566.7600	0.0032	VVNVSELYGTPCTK
7.4	1566.7599	0.0032	YVELTEGVECIQK
2.0	1566.7712	-0.0080	ILYNTSQECLAVR

Spectrum No: 186; Query: 516; Rank: 1

Peptide View

MS/MS Fragmentation of **FTCNETTNVIIIHSK**
Found in **IPI00230862**, Tax_Id=10116 Gene_Symbol=Anpep Aminopeptidase N

Match to Query 516: 1776.876288 from(889.445420,2+)
Title: 091127RatKid_SCX01_26.1900.1900.2.dta
Data file K:\NewmanPaper\Piliang\3SubProteomes\Piliang3SP\mgf5ppm\SCX_3SubProteomes5ppm.mgf

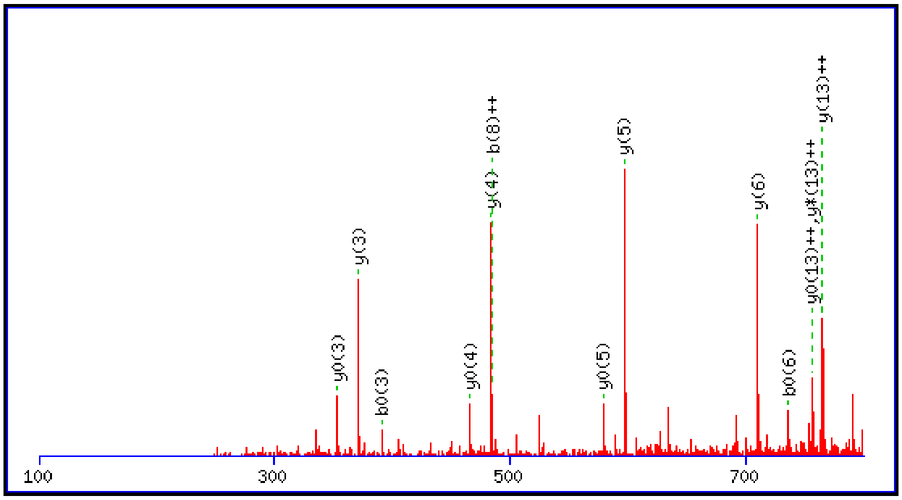

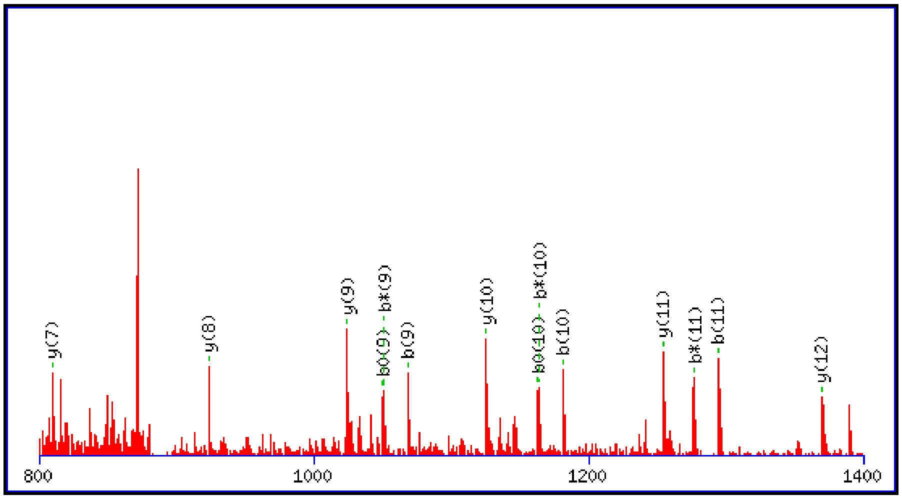

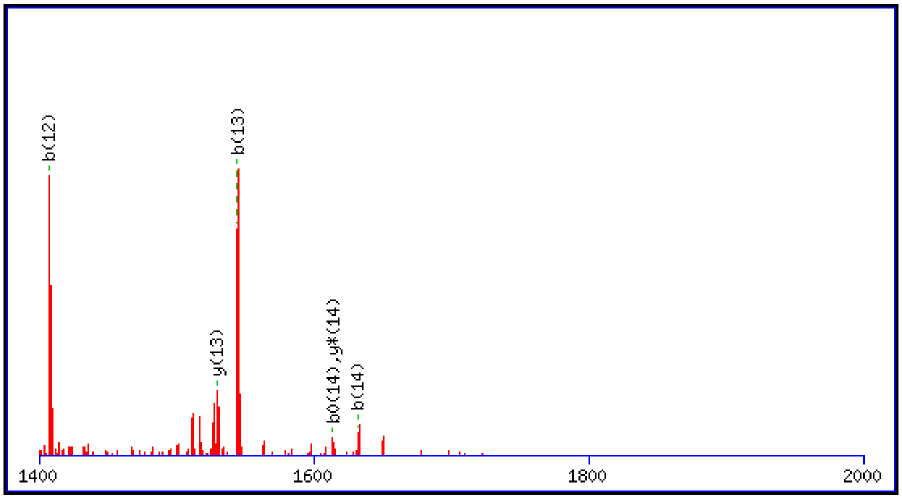

Monoisotopic mass of neutral peptide Mr(calc): 1776.8716
Fixed modifications: Carbamidomethyl (C)
Variable modifications:
N4 : Deamidated_N (N)
Ions Score: 74 **Expect:** 7.1e-006
Matches (Bold Red): 33/158 fragment ions using 50 most intense peaks

#	b	b++	b*	b*++	b0	b0++	Seq.	y	y++	y*	y*++	y0	y0++	#
1	148.0757	74.5415					F							15
2	249.1234	125.0653			231.1128	116.0600	T	1630.8105	815.9089	**1613.7839**	807.3956	1612.7999	806.9036	14
3	409.1540	205.0806			**391.1435**	196.0754	C	**1529.7628**	**765.3850**	1512.7363	**756.8718**	1511.7522	**756.3798**	13
4	524.1810	262.5941	507.1544	254.0808	506.1704	253.5888	N	**1369.7322**	685.3697	1352.7056	676.8564	1351.7216	676.3644	12
5	653.2235	327.1154	636.1970	318.6021	635.2130	318.1101	E	**1254.7052**	627.8563	1237.6787	619.3430	1236.6947	618.8510	11
6	754.2712	377.6393	737.2447	369.1260	**736.2607**	368.6340	T	**1125.6626**	563.3350	1108.6361	554.8217	1107.6521	554.3297	10
7	855.3189	428.1631	838.2924	419.6498	837.3083	419.1578	T	**1024.6150**	512.8111	1007.5884	504.2978	1006.6044	503.8058	9
8	969.3618	**485.1846**	952.3353	476.6713	951.3513	476.1793	N	**923.5673**	462.2873	906.5407	453.7740	905.5567	453.2820	8
9	**1068.4302**	534.7188	**1051.4037**	526.2055	**1050.4197**	525.7135	V	**809.5243**	405.2658	792.4978	396.7525	791.5138	396.2605	7
10	**1181.5143**	591.2608	**1164.4878**	582.7475	**1163.5037**	582.2555	I	**710.4559**	355.7316	693.4294	347.2183	692.4454	346.7263	6
11	**1294.5984**	647.8028	**1277.5718**	639.2896	1276.5878	638.7975	I	**597.3719**	299.1896	580.3453	290.6763	**579.3613**	290.1843	5
12	**1407.6824**	704.3449	1390.6559	695.8316	1389.6719	695.3396	I	**484.2878**	242.6475	467.2613	234.1343	**466.2772**	233.6423	4
13	**1544.7413**	772.8743	1527.7148	764.3610	1526.7308	763.8690	H	**371.2037**	186.1055	354.1772	177.5922	**353.1932**	177.1002	3
14	**1631.7734**	816.3903	1614.7468	807.8771	**1613.7628**	807.3850	S	234.1448	117.5761	217.1183	109.0628	216.1343	108.5708	2
15							K	147.1128	74.0600	130.0863	65.5468			1

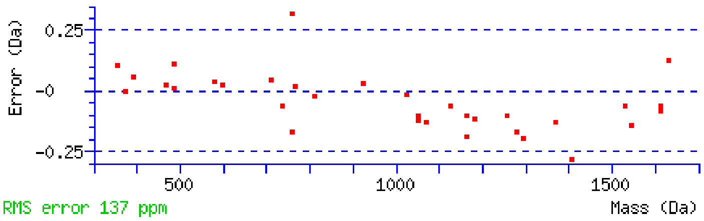

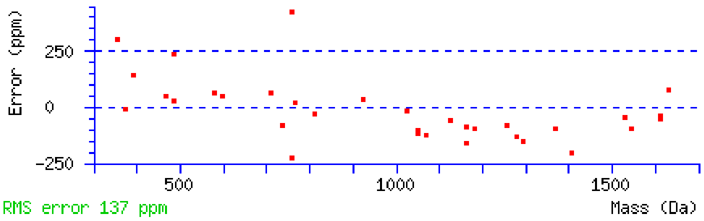

All matches to this query

Score	Mr(calc):	Delta	Sequence
73.9	1776.8716	0.0047	FTCNETTNVIIIHSK
49.9	1776.8716	0.0047	FTCNETTNVIIIHSK
35.0	1775.8876	0.9887	FTCNETTNVIIIHSK
9.8	1776.8675	0.0088	QIKNLEMERNAESSK
9.1	1776.8675	0.0088	QIKNLEMERNAESSK
4.6	1776.8712	0.0051	FPSGIKHLANYVHSK

4.2	1776.8811	-0.0048	KIELEDALALGPSWR
3.7	1774.8655	2.0108	DGVIVSNSLAAFAYIR
3.3	1776.8934	-0.0171	FVFGSESVKSIFSSEK
2.3	1775.8777	0.9986	LAWLIYLVGTVVGGR

Spectrum No: 187; Query: 957; Rank: 1

Peptide View

MS/MS Fragmentation of **LANASLYLAEWQNITKNFSEDR**
Found in **IPI00211616**, Tax_Id=10116 Gene_Symbol=Slc3a2 4F2 cell-surface antigen heavy chain

Match to Query 957: 2585.234922 from(862.752250,3+)
Title: 091129RatKid_SCX02_14.5208.5208.3.dta
Data file K:\NewmanPaper\Piliang\3SubProteomes\Piliang3SP\mgf5ppm\SCX_3SubProteomes5ppm.mgf

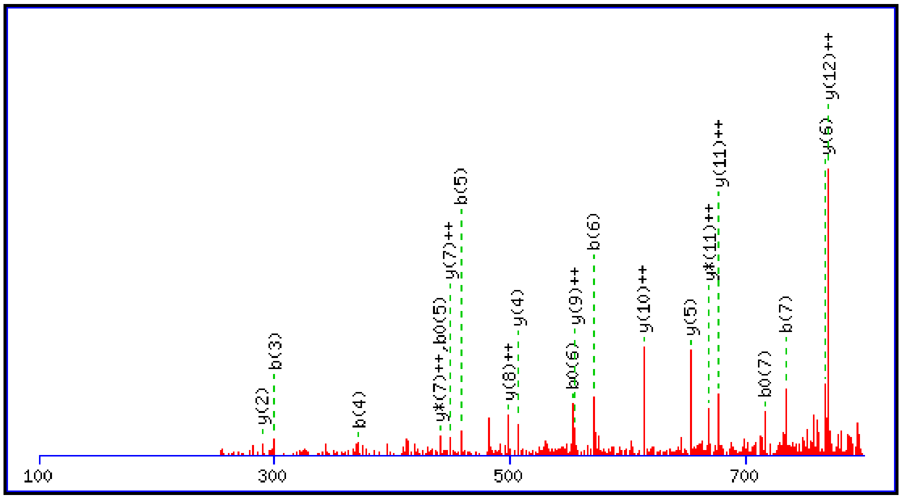

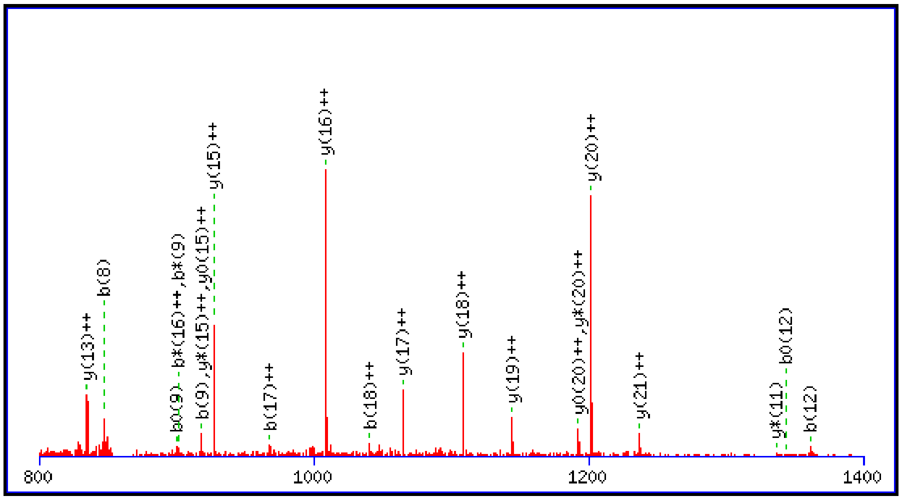

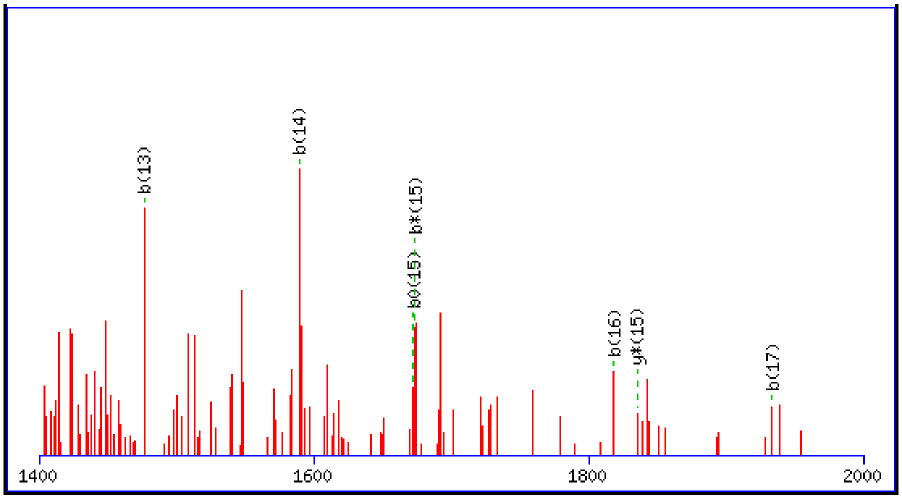

Monoisotopic mass of neutral peptide Mr(calc): 2585.2285
Fixed modifications: Carbamidomethyl (C)
Variable modifications:
N3 : Deamidated_N (N)
N13 : Deamidated_N (N)
N17 : Deamidated_N (N)
Ions Score: 74 **Expect:** 1.2e-005
Matches (Bold Red): 49/238 fragment ions using 102 most intense peaks

#	b	b^{++}	b*	b^{*++}	b^{0}	b^{0++}	Seq.	y	y^{++}	y*	y^{*++}	y^{0}	y^{0++}	#
1	114.0913	57.5493					L							22
2	185.1285	93.0679					A	2473.1518	**1237.0795**	2456.1252	1228.5662	2455.1412	1228.0742	21
3	**300.1554**	150.5813	283.1288	142.0681			N	2402.1146	**1201.5610**	2385.0881	**1193.0477**	2384.1041	**1192.5557**	20
4	**371.1925**	186.0999	354.1660	177.5866			A	2287.0877	**1144.0475**	2270.0612	1135.5342	2269.0771	1135.0422	19
5	**458.2245**	229.6159	441.1980	221.1026	**440.2140**	220.6106	S	2216.0506	**1108.5289**	2199.0240	1100.0157	2198.0400	1099.5237	18
6	**571.3086**	286.1579	554.2820	277.6447	**553.2980**	277.1527	L	2129.0186	**1065.0129**	2111.9920	1056.4996	2111.0080	1056.0076	17
7	**734.3719**	367.6896	717.3454	359.1763	**716.3614**	358.6843	Y	2015.9345	**1008.4709**	1998.9079	999.9576	1997.9239	999.4656	16
8	**847.4560**	424.2316	830.4294	415.7184	829.4454	415.2264	L	1852.8712	**926.9392**	**1835.8446**	**918.4259**	1834.8606	**917.9339**	15
9	**918.4931**	459.7502	**901.4666**	451.2369	**900.4825**	450.7449	A	1739.7871	870.3972	1722.7606	861.8839	1721.7765	861.3919	14
10	1047.5357	524.2715	1030.5091	515.7582	1029.5251	515.2662	E	1668.7500	**834.8786**	1651.7234	826.3654	1650.7394	825.8734	13
11	1233.6150	617.3111	1216.5885	608.7979	1215.6044	608.3059	W	1539.7074	**770.3573**	1522.6808	761.8441	1521.6968	761.3521	12
12	**1361.6736**	681.3404	1344.6470	672.8272	**1343.6630**	672.3351	Q	1353.6281	**677.3177**	**1336.6015**	**668.8044**	1335.6175	668.3124	11
13	**1476.7005**	738.8539	1459.6740	730.3406	1458.6900	729.8486	N	1225.5695	**613.2884**	1208.5430	604.7751	1207.5589	604.2831	10
14	**1589.7846**	795.3959	1572.7580	786.8827	1571.7740	786.3906	I	1110.5426	**555.7749**	1093.5160	547.2616	1092.5320	546.7696	9
15	1690.8323	845.9198	**1673.8057**	837.4065	**1672.8217**	836.9145	T	997.4585	**499.2329**	980.4320	490.7196	979.4479	490.2276	8
16	**1818.9272**	909.9673	1801.9007	**901.4540**	1800.9167	900.9620	K	896.4108	**448.7091**	879.3843	**440.1958**	878.4003	439.7038	7
17	**1933.9542**	**967.4807**	1916.9276	958.9674	1915.9436	958.4754	N	**768.3159**	384.6616	751.2893	376.1483	750.3053	375.6563	6
18	2081.0226	**1041.0149**	2063.9960	1032.5017	2063.0120	1032.0096	F	**653.2889**	327.1481	636.2624	318.6348	635.2784	318.1428	5
19	2168.0546	1084.5309	2151.0281	1076.0177	2150.0440	1075.5257	S	**506.2205**	253.6139	489.1940	245.1006	488.2100	244.6086	4
20	2297.0972	1149.0522	2280.0707	1140.5390	2279.0866	1140.0470	E	419.1885	210.0979	402.1619	201.5846	401.1779	201.0926	3
21	2412.1241	1206.5657	2395.0976	1198.0524	2394.1136	1197.5604	D	**290.1459**	145.5766	273.1193	137.0633	272.1353	136.5713	2
22							R	175.1190	88.0631	158.0924	79.5498			1

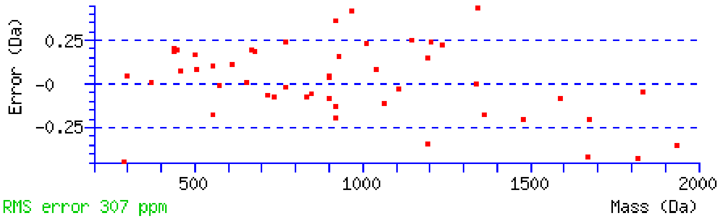

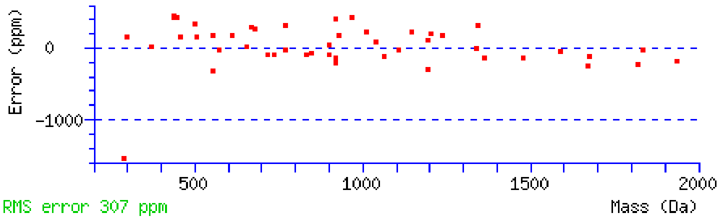

All matches to this query

Score	Mr(calc):	Delta	Sequence
73.8	2585.2285	0.0064	LANASLYLAEWQNITKNFSEDR
35.8	2584.2445	0.9904	LANASLYLAEWQNITKNFSEDR
28.5	2584.2445	0.9904	LANASLYLAEWQNITKNFSEDR
13.6	2584.2445	0.9904	LANASLYLAEWQNITKNFSEDR
7.3	2585.2156	0.0193	RTLNSDKTLLIQNTDLCESQR
7.0	2584.2315	1.0034	WLACQSMDNQILIFGAQNRFR
6.0	2585.2156	0.0193	RTLNSDKTLLIQNTDLCESQR
3.5	2584.2316	1.0033	RTLNSDKTLLIQNTDLCESQR
3.5	2584.2316	1.0033	RTLNSDKTLLIQNTDLCESQR
3.3	2584.2331	1.0018	LRMAFWSQQLQCQPSLLGVNK

Spectrum No: 188; Query: 1036; Rank: 1

Peptide View

MS/MS Fragmentation of **IEAVFPAEFFEVLSSSQNGSYHHVR**
Found in **IPI00195875**, Tax_Id=10116 Gene_Symbol=Pom210 Nuclear pore membrane glycoprotein 210 precursor

Match to Query 1036: 2851.371456 from(713.850140,4+)
Title: 091127RatKid_SCX01_31.5912.5912.4.dta
Data file K:\NewmanPaper\Piliang\3SubProteomes\Piliang3SP\mgf5ppm\SCX_3SubProteomes5ppm.mgf

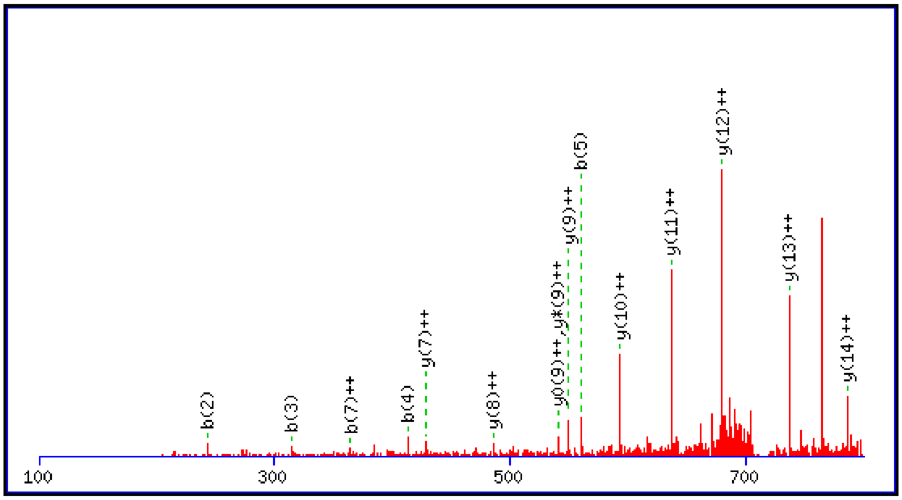

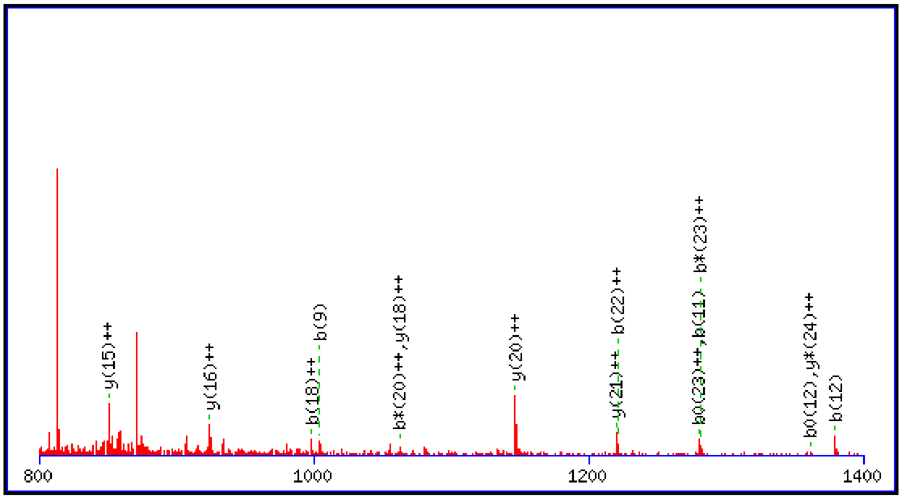

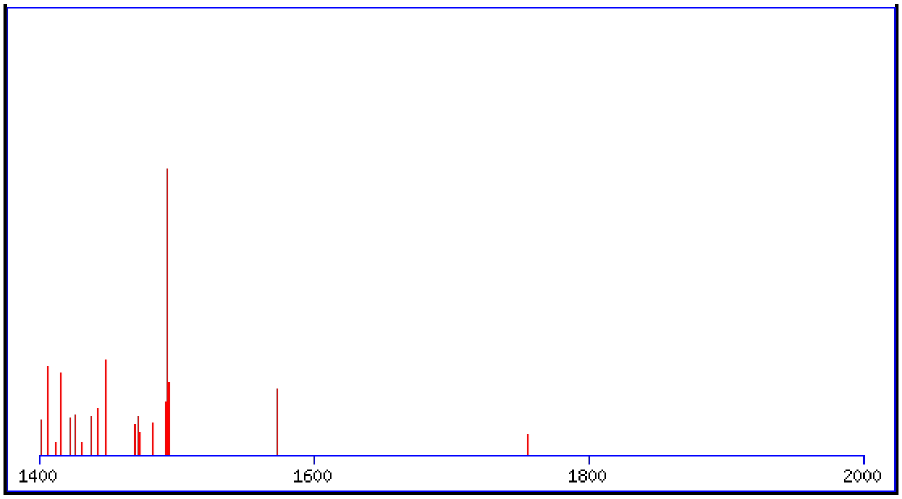

Monoisotopic mass of neutral peptide Mr(calc): 2850.3613
Fixed modifications: Carbamidomethyl (C)
Variable modifications:
N18 : Deamidated_N (N)
Ions Score: 74 **Expect:** 1.3e-005
Matches (Bold Red): 30/244 fragment ions using 52 most intense peaks

#	b	b^{++}	b*	b^{*++}	b^0	b^{0++}	Seq.	y	y^{++}	y*	y^{*++}	y^0	y^{0++}	#
1	114.0913	57.5493					I							25
2	**243.1339**	122.0706			225.1234	113.0653	E	2738.2845	1369.6459	2721.2580	**1361.1326**	2720.2739	1360.6406	24
3	**314.1710**	157.5892			296.1605	148.5839	A	2609.2419	1305.1246	2592.2154	1296.6113	2591.2314	1296.1193	23
4	**413.2395**	207.1234			395.2289	198.1181	V	2538.2048	1269.6060	2521.1783	1261.0928	2520.1942	1260.6008	22
5	**560.3079**	280.6576			542.2973	271.6523	F	2439.1364	**1220.0718**	2422.1098	1211.5586	2421.1258	1211.0666	21
6	657.3606	329.1840			639.3501	320.1787	P	2292.0680	**1146.5376**	2275.0414	1138.0244	2274.0574	1137.5323	20
7	728.3978	**364.7025**			710.3872	355.6972	A	2195.0152	1098.0112	2177.9887	1089.4980	2177.0046	1089.0060	19
8	857.4403	429.2238			839.4298	420.2185	E	2123.9781	**1062.4927**	2106.9516	1053.9794	2105.9675	1053.4874	18
9	**1004.5088**	502.7580			986.4982	493.7527	F	1994.9355	997.9714	1977.9090	989.4581	1976.9249	988.9661	17
10	1151.5772	576.2922			1133.5666	567.2869	F	1847.8671	**924.4372**	1830.8405	915.9239	1829.8565	915.4319	16
11	**1280.6198**	640.8135			1262.6092	631.8082	E	1700.7987	**850.9030**	1683.7721	842.3897	1682.7881	841.8977	15
12	**1379.6882**	690.3477			**1361.6776**	681.3424	V	1571.7561	**786.3817**	1554.7295	777.8684	1553.7455	777.3764	14
13	1492.7722	746.8898			1474.7617	737.8845	L	1472.6877	**736.8475**	1455.6611	728.3342	1454.6771	727.8422	13
14	1579.8043	790.4058			1561.7937	781.4005	S	1359.6036	**680.3054**	1342.5771	671.7922	1341.5930	671.3002	12
15	1666.8363	833.9218			1648.8257	824.9165	S	1272.5716	**636.7894**	1255.5450	628.2762	1254.5610	627.7841	11
16	1753.8683	877.4378			1735.8578	868.4325	S	1185.5396	**593.2734**	1168.5130	584.7601	1167.5290	584.2681	10
17	1881.9269	941.4671	1864.9004	932.9538	1863.9163	932.4618	Q	1098.5075	**549.7574**	1081.4810	**541.2441**	1080.4970	**540.7521**	9
18	1996.9538	**998.9806**	1979.9273	990.4673	1978.9433	989.9753	N	970.4489	**485.7281**	953.4224	477.2148	952.4384	476.7228	8
19	2053.9753	1027.4913	2036.9488	1018.9780	2035.9647	1018.4860	G	855.4220	**428.2146**	838.3955	419.7014	837.4114	419.2094	7
20	2141.0073	1071.0073	2123.9808	**1062.4940**	2122.9968	1062.0020	S	798.4005	399.7039	781.3740	391.1906	780.3900	390.6986	6
21	2304.0707	1152.5390	2287.0441	1144.0257	2286.0601	1143.5337	Y	711.3685	356.1879	694.3420	347.6746			5
22	2441.1296	**1221.0684**	2424.1030	1212.5552	2423.1190	1212.0631	H	548.3052	274.6562	531.2786	266.1430			4
23	2578.1885	1289.5979	2561.1619	**1281.0846**	2560.1779	**1280.5926**	H	411.2463	206.1268	394.2197	197.6135			3
24	2677.2569	1339.1321	2660.2304	1330.6188	2659.2463	1330.1268	V	274.1874	137.5973	257.1608	129.0840			2
25							R	175.1190	88.0631	158.0924	79.5498			1

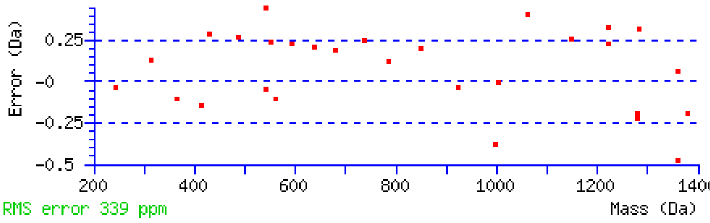

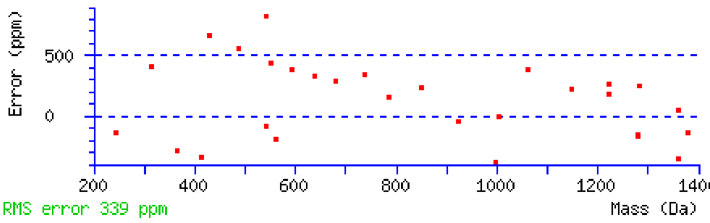

All matches to this query

Score	Mr(calc):	Delta	Sequence
73.5	2850.3613	1.0102	IEAVFPAEFFEVLSSSQNGSYHHVR
9.0	2849.3855	1.9860	ELSRLAGSSRSPGAEAQGLGVVMASPSR
8.3	2849.3855	1.9860	ELSRLAGSSRSPGAEAQGLGVVMASPSR
8.3	2850.3446	1.0269	MSMDVTFLGTGAAYPSPTRGASAVVLR
8.0	2849.3773	1.9942	IEAVFPAEFFEVLSSSQNGSYHHVR
7.8	2850.3446	1.0269	MSMDVTFLGTGAAYPSPTRGASAVVLR
6.6	2849.3855	1.9860	ELSRLAGSSRSPGAEAQGLGVVMASPSR
5.5	2849.3855	1.9860	ELSRLAGSSRSPGAEAQGLGVVMASPSR
4.5	2851.3562	0.0152	LSVYMEAGKVTTSVSSDAGGSVTSITPK
3.6	2851.3558	0.0156	RRFTITDSDQLPGYSVGTNILPIK

Spectrum No: 189; Query: 492; Rank: 1

Peptide View

MS/MS Fragmentation of **MESDLANTTAGFTAALK**
Found in **IPI00392894**, Tax_Id=10116 Gene_Symbol=Mgam_predicted 89 kDa protein

Match to Query 492: 1740.827588 from(871.421070,2+)
Title: 091129RatKid_SCX02_12.2957.2957.2.dta
Data file K:\NewmanPaper\Piliang\3SubProteomes\Piliang3SP\mgf5ppm\SCX_3SubProteomes5ppm.mgf

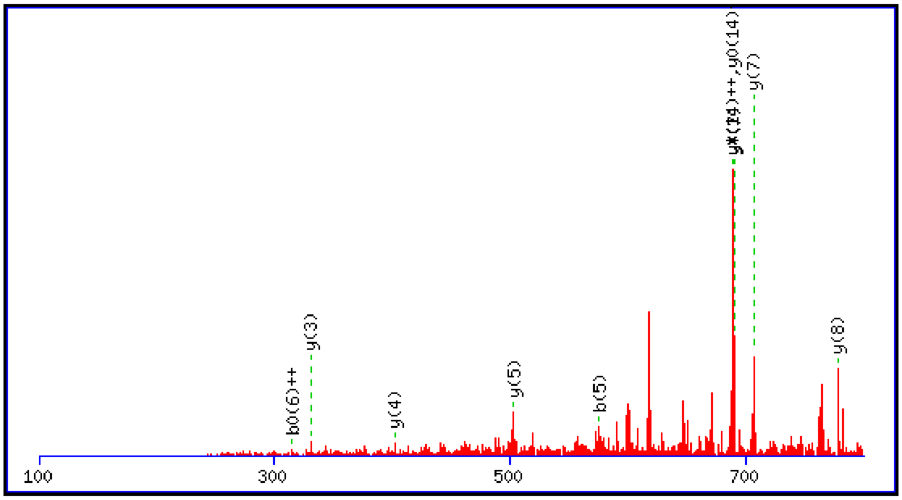

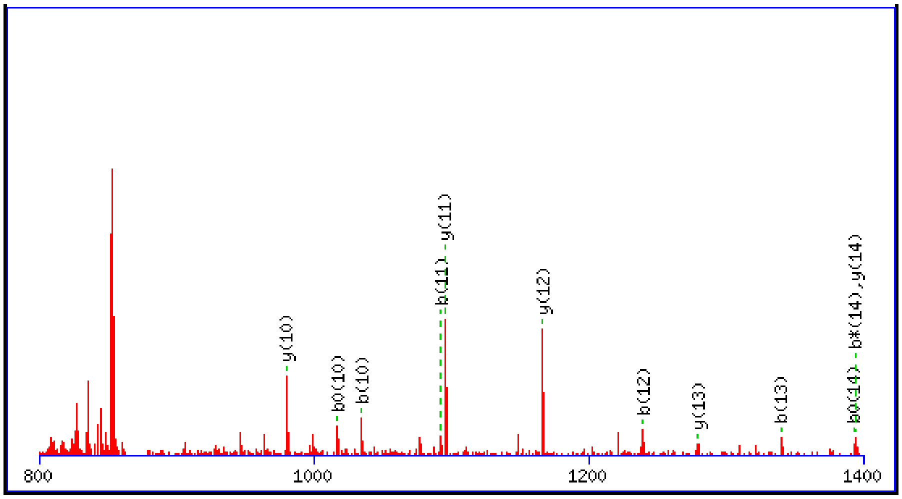

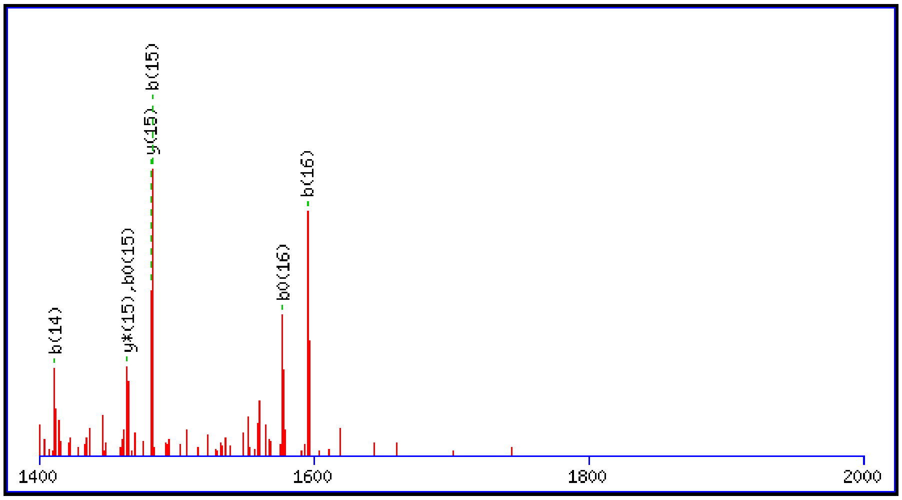

Monoisotopic mass of neutral peptide Mr(calc): 1740.8240
Fixed modifications: Carbamidomethyl (C)
Variable modifications:
N7 : Deamidated_N (N)
Ions Score: 73 **Expect:** 8.5e-006
Matches (Bold Red): 30/170 fragment ions using 57 most intense peaks

#	b	b^{++}	b*	b*$^{++}$	b^{0}	b^{0++}	Seq.	y	y^{++}	y*	y*$^{++}$	y^{0}	y^{0++}	#
1	132.0478	66.5275					M							17
2	261.0904	131.0488			243.0798	122.0435	E	1610.7908	805.8990	1593.7643	797.3858	1592.7802	796.8938	16
3	348.1224	174.5648			330.1118	165.5595	S	**1481.7482**	741.3777	**1464.7217**	732.8645	1463.7376	732.3725	15
4	463.1493	232.0783			445.1388	223.0730	D	**1394.7162**	697.8617	1377.6896	**689.3485**	1376.7056	**688.8564**	14
5	**576.2334**	288.6203			558.2228	279.6151	L	**1279.6892**	640.3483	1262.6627	631.8350	1261.6787	631.3430	13
6	647.2705	324.1389			629.2599	**315.1336**	A	**1166.6052**	583.8062	1149.5786	575.2930	1148.5946	574.8009	12
7	762.2974	381.6524	745.2709	373.1391	744.2869	372.6471	N	**1095.5681**	548.2877	1078.5415	539.7744	1077.5575	539.2824	11
8	863.3451	432.1762	846.3186	423.6629	845.3346	423.1709	T	**980.5411**	490.7742	963.5146	482.2609	962.5306	481.7689	10
9	964.3928	482.7000	947.3662	474.1868	946.3822	473.6948	T	879.4934	440.2504	862.4669	431.7371	861.4829	431.2451	9
10	**1035.4299**	518.2186	1018.4034	509.7053	**1017.4193**	509.2133	A	**778.4458**	389.7265	761.4192	381.2132	760.4352	380.7212	8
11	**1092.4514**	546.7293	1075.4248	538.2161	1074.4408	537.7240	G	**707.4087**	354.2080	**690.3821**	345.6947	**689.3981**	345.2027	7
12	**1239.5198**	620.2635	1222.4932	611.7503	1221.5092	611.2582	F	650.3872	325.6972	633.3606	317.1840	632.3766	316.6920	6
13	**1340.5675**	670.7874	1323.5409	662.2741	1322.5569	661.7821	T	**503.3188**	252.1630	486.2922	243.6498	485.3082	243.1577	5
14	**1411.6046**	706.3059	**1394.5780**	697.7927	**1393.5940**	697.3006	A	**402.2711**	201.6392	385.2445	193.1259			4
15	**1482.6417**	741.8245	1465.6151	733.3112	**1464.6311**	732.8192	A	**331.2340**	166.1206	314.2074	157.6074			3
16	**1595.7258**	798.3665	1578.6992	789.8532	**1577.7152**	789.3612	L	260.1969	130.6021	243.1703	122.0888			2
17							K	147.1128	74.0600	130.0863	65.5468			1

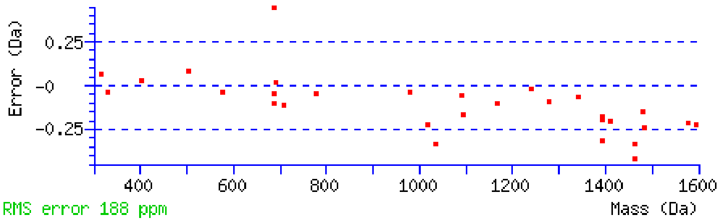

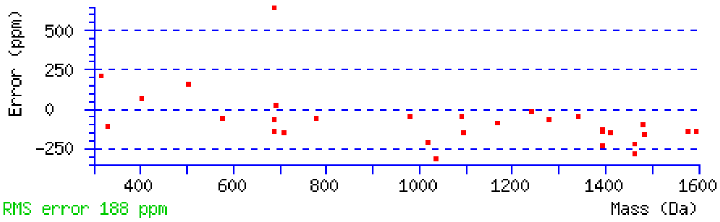

All matches to this query

Score	Mr(calc):	Delta	Sequence
73.4	1740.8240	0.0036	MESDLANTTAGFTAALK
16.9	1739.8400	0.9876	MESDLANTTAGFTAALK
6.7	1740.8270	0.0006	LKVTHQAESNLYMK
6.1	1740.8191	0.0085	CLNELGKTVPMALAK
5.0	1740.8295	-0.0019	ETEIESSRLNTLAAK
4.6	1740.8308	-0.0032	RRQSVGDFVNGALNK
4.5	1740.8308	-0.0032	RRQSVGDFVNGALNK
3.9	1740.8196	0.0080	RSPSAATSSHYLGSLK
3.8	1740.8124	0.0152	FTGDFLLSRYDSLK
2.8	1740.8230	0.0046	MVTGDNINTARAIAAK

Spectrum No: 190; Query: 160; Rank: 1

Peptide View

MS/MS Fragmentation of **LGAINSTLSNESK**
Found in **IPI00765277**, Tax_Id=10116 Gene_Symbol=RGD1563548_predicted similar to endoplasmic oxidoreductase 1 beta

Match to Query 160: 1334.659368 from(668.336960,2+)
Title: 091127RatKid_SCX01_16.1614.1614.2.dta
Data file K:\NewmanPaper\Piliang\3SubProteomes\Piliang3SP\mgf5ppm\SCX_3SubProteomes5ppm.mgf

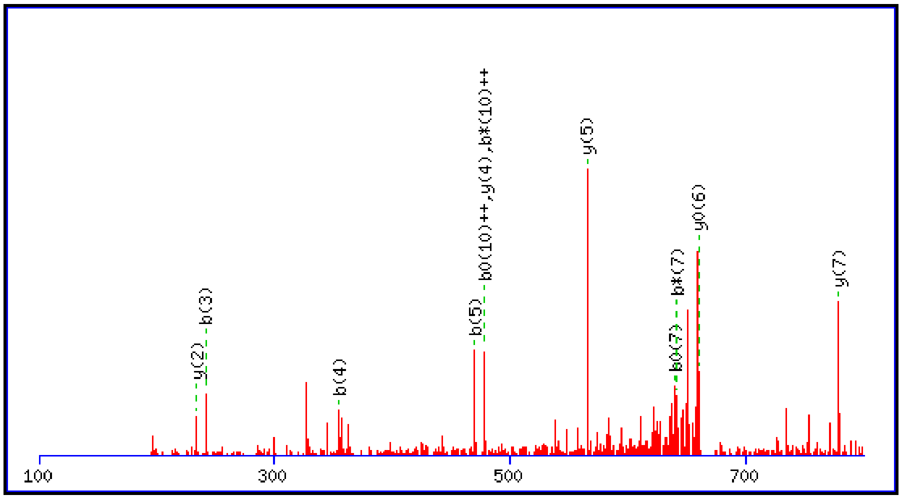

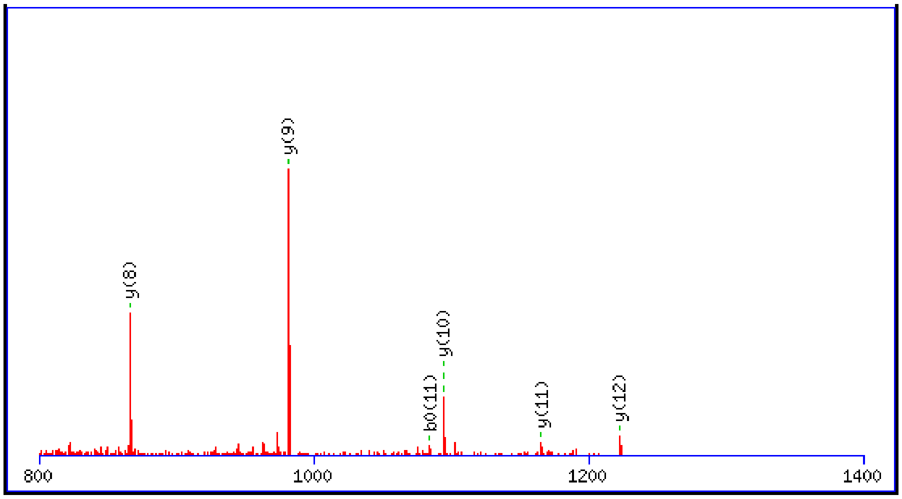

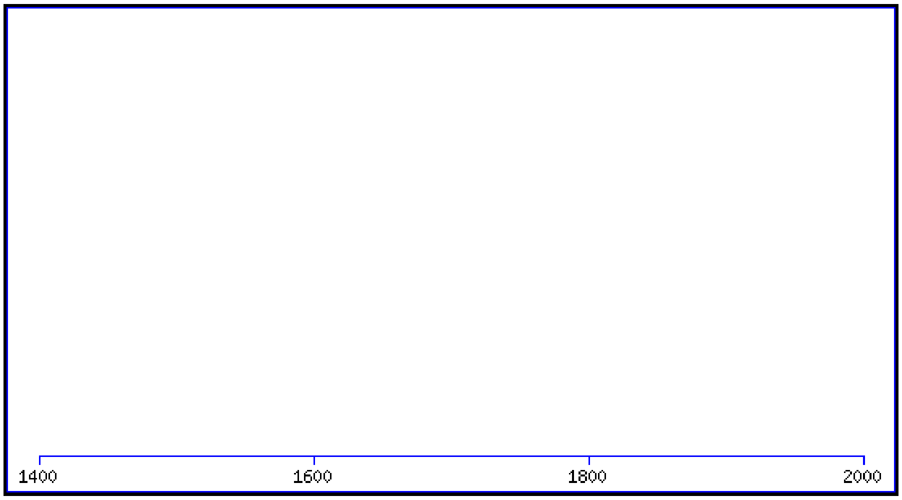

Monoisotopic mass of neutral peptide Mr(calc): 1334.6565
Fixed modifications: Carbamidomethyl (C)
Variable modifications:
N5 : Deamidated_N (N)
N10 : Deamidated_N (N)
Ions Score: 73 **Expect:** 6.4e-006
Matches (Bold Red): 18/124 fragment ions using 25 most intense peaks

#	b	b^{++}	b*	b^{*++}	b^{0}	b^{0++}	Seq.	y	y^{++}	y*	y^{*++}	y^{0}	y^{0++}	#
1	114.0913	57.5493					L							13
2	171.1128	86.0600					G	**1222.5797**	611.7935	1205.5532	603.2802	1204.5692	602.7882	12
3	**242.1499**	121.5786					A	**1165.5583**	583.2828	1148.5317	574.7695	1147.5477	574.2775	11
4	**355.2340**	178.1206					I	**1094.5212**	547.7642	1077.4946	539.2509	1076.5106	538.7589	10
5	**470.2609**	235.6341	453.2344	227.1208			N	**981.4371**	491.2222	964.4105	482.7089	963.4265	482.2169	9
6	557.2929	279.1501	540.2664	270.6368	539.2824	270.1448	S	**866.4102**	433.7087	849.3836	425.1954	848.3996	424.7034	8
7	658.3406	329.6740	**641.3141**	321.1607	**640.3301**	320.6687	T	**779.3781**	390.1927	762.3516	381.6794	761.3676	381.1874	7
8	771.4247	386.2160	754.3981	377.7027	753.4141	377.2107	L	678.3305	339.6689	661.3039	331.1556	**660.3199**	330.6636	6
9	858.4567	429.7320	841.4302	421.2187	840.4462	420.7267	S	**565.2464**	283.1268	548.2198	274.6136	547.2358	274.1216	5
10	973.4837	487.2455	956.4571	**478.7322**	955.4731	**478.2402**	N	**478.2144**	239.6108	461.1878	231.0975	460.2038	230.6055	4
11	1102.5262	551.7668	1085.4997	543.2535	**1084.5157**	542.7615	E	363.1874	182.0974	346.1609	173.5841	345.1769	173.0921	3
12	1189.5583	595.2828	1172.5317	586.7695	1171.5477	586.2775	S	**234.1448**	117.5761	217.1183	109.0628	216.1343	108.5708	2
13							K	147.1128	74.0600	130.0863	65.5468			1

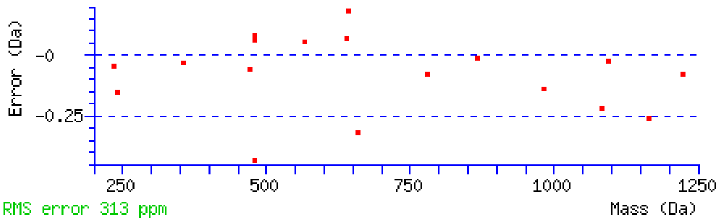

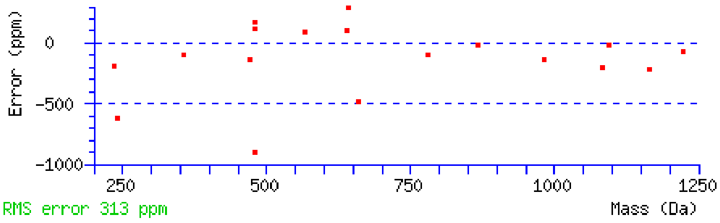

All matches to this query

Score	Mr(calc):	Delta	Sequence
73.0	1334.6565	0.0029	LGAINSTLSNESK
9.3	1334.6707	-0.0113	LKNLKANSPNR
8.3	1334.6595	-0.0001	IIQDSNKVNPK
3.5	1334.6707	-0.0113	NKEPLGATIRR
1.7	1334.6595	-0.0001	AVINTVEPSAVR

Spectrum No: 191; Query: 263; Rank: 1

Peptide View

MS/MS Fragmentation of **VSNMTLPASAEVLK**
Found in **IPI00206336**, Tax_Id=10116 Gene_Symbol=Lamp1 Lysosome-associated membrane glycoprotein 1 precursor

Match to Query 263: 1475.755608 from(738.885080,2+)
Title: 091127RatKid_SCX01_16.2205.2205.2.dta
Data file K:\NewmanPaper\Piliang\3SubProteomes\Piliang3SP\mgf5ppm\SCX_3SubProteomes5ppm.mgf

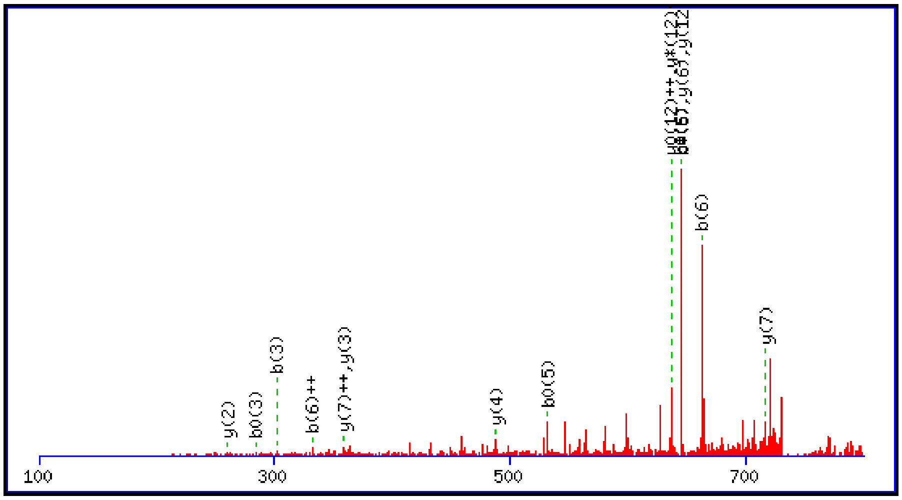

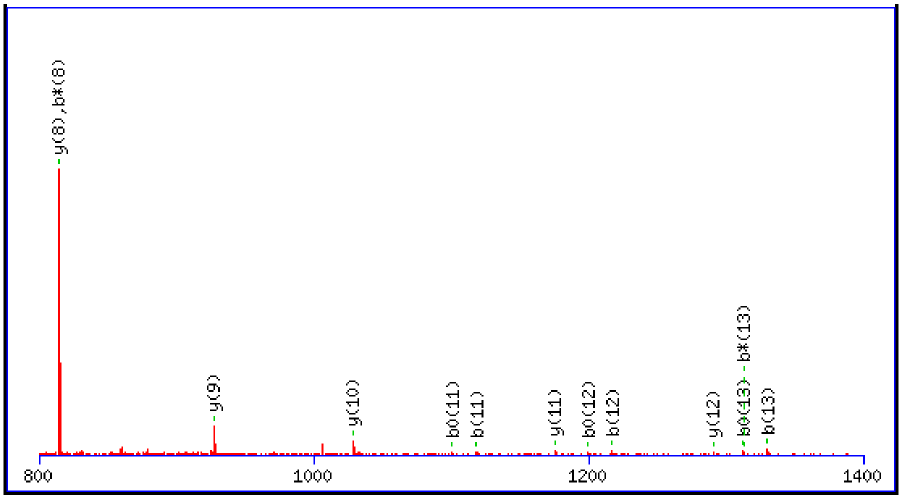

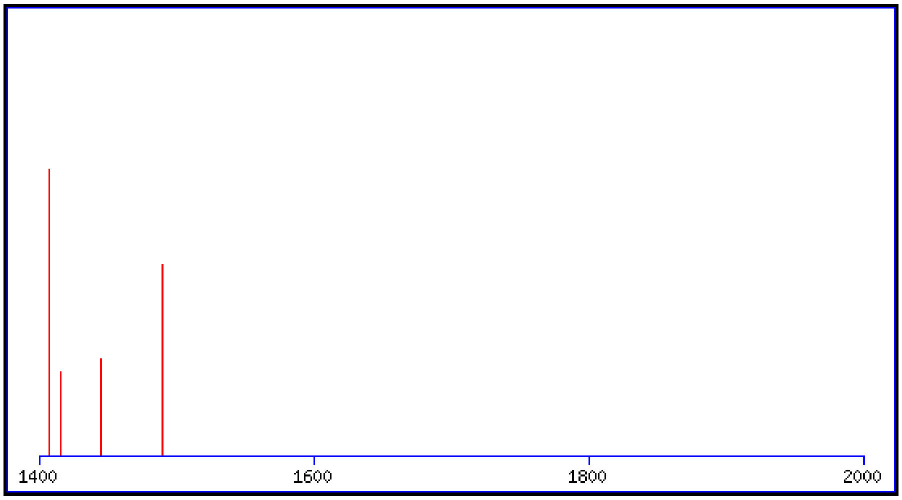

Monoisotopic mass of neutral peptide Mr(calc): 1475.7541
Fixed modifications: Carbamidomethyl (C)
Variable modifications:
N3 : Deamidated_N (N)
M4 : Oxidation (M)
Ions Score: 73 **Expect:** 7e-006
Matches (Bold Red): 29/144 fragment ions using 55 most intense peaks

#	b	b^{++}	b*	b^{*++}	b^0	b^{0++}	Seq.	y	y^{++}	y*	y^{*++}	y^0	y^{0++}	#
1	100.0757	50.5415					V							14
2	187.1077	94.0575			169.0972	85.0522	S	1377.6930	689.3501	1360.6665	680.8369	1359.6824	680.3449	13
3	**302.1347**	151.5710	285.1081	143.0577	**284.1241**	142.5657	N	**1290.6610**	**645.8341**	1273.6344	**637.3209**	1272.6504	**636.8288**	12
4	449.1701	225.0887	432.1435	216.5754	431.1595	216.0834	M	**1175.6340**	588.3207	1158.6075	579.8074	1157.6235	579.3154	11
5	550.2177	275.6125	533.1912	267.0992	**532.2072**	266.6072	T	**1028.5986**	514.8030	1011.5721	506.2897	1010.5881	505.7977	10
6	**663.3018**	**332.1545**	**646.2753**	323.6413	**645.2912**	323.1493	L	**927.5510**	464.2791	910.5244	455.7658	909.5404	455.2738	9
7	760.3546	380.6809	743.3280	372.1676	742.3440	371.6756	P	**814.4669**	407.7371	797.4403	399.2238	796.4563	398.7318	8
8	831.3917	416.1995	**814.3651**	407.6862	813.3811	407.1942	A	**717.4141**	**359.2107**	700.3876	350.6974	699.4036	350.2054	7
9	918.4237	459.7155	901.3972	451.2022	900.4131	450.7102	S	**646.3770**	323.6921	629.3505	315.1789	628.3665	314.6869	6
10	989.4608	495.2340	972.4343	486.7208	971.4503	486.2288	A	559.3450	280.1761	542.3184	271.6629	541.3344	271.1709	5
11	**1118.5034**	559.7553	1101.4769	551.2421	**1100.4928**	550.7501	E	**488.3079**	244.6576	471.2813	236.1443	470.2973	235.6523	4
12	**1217.5718**	609.2896	1200.5453	600.7763	**1199.5613**	600.2843	V	**359.2653**	180.1363	342.2387	171.6230			3
13	**1330.6559**	665.8316	**1313.6293**	657.3183	**1312.6453**	656.8263	L	**260.1969**	130.6021	243.1703	122.0888			2
14							K	147.1128	74.0600	130.0863	65.5468			1

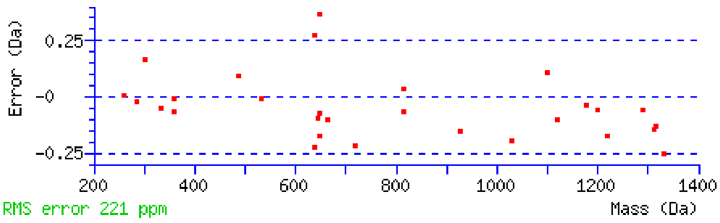

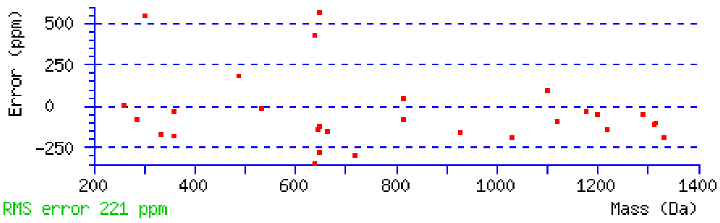

All matches to this query

Score	Mr(calc):	Delta	Sequence
73.0	1475.7541	0.0015	VSNMTLPASAEVLK
12.4	1475.7468	0.0089	GDSGDTLAATQSLLK
11.8	1474.7640	0.9916	SAASAAEGRARFPGK
9.5	1475.7620	-0.0064	GDFDNLSGKGKPLK
7.9	1475.7541	0.0015	MDLVLELKNNASK
7.2	1475.7442	0.0114	TSITALAWSPNGMK
5.0	1474.7456	1.0100	WYPVSTPTPNKGK
5.0	1475.7467	0.0089	SVDSRNTLNDLLK
3.4	1475.7425	0.0131	AFLSPTFTSGKLK
2.1	1473.7497	2.0059	MAPKLSDSVEELR

Spectrum No: 192; Query: 1047; Rank: 1

Peptide View

MS/MS Fragmentation of **ACIENGTSCIDICGEPQFLELMHVK**
Found in **IPI00372804**, Tax_Id=10116 Gene_Symbol=Sccpdh Probable saccharopine dehydrogenase

Match to Query 1047: 2921.314122 from(974.778650,3+)
Title: 091127RatKid_SCX01_24.5309.5309.3.dta
Data file K:\NewmanPaper\Piliang\3SubProteomes\Piliang3SP\mgf5ppm\SCX_3SubProteomes5ppm.mgf

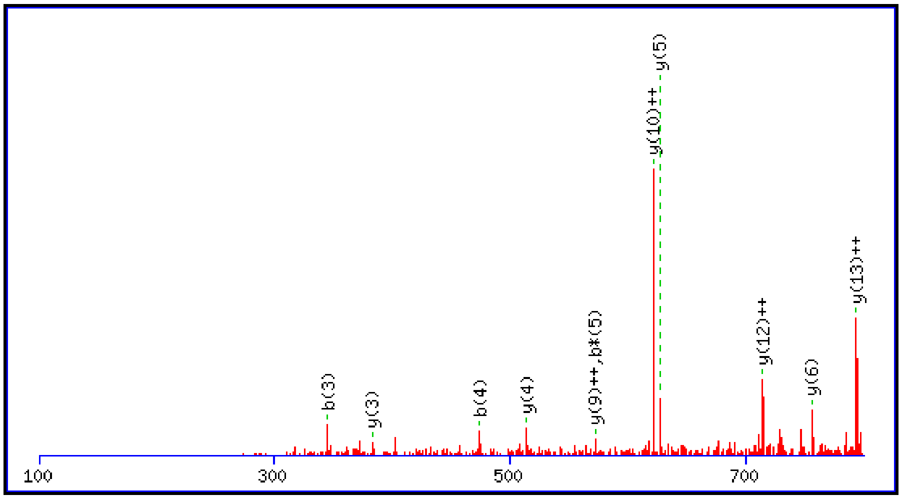

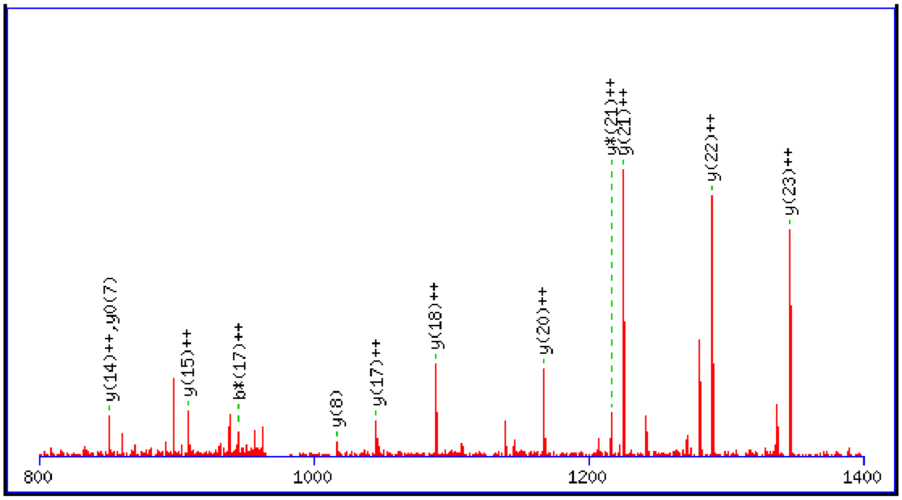

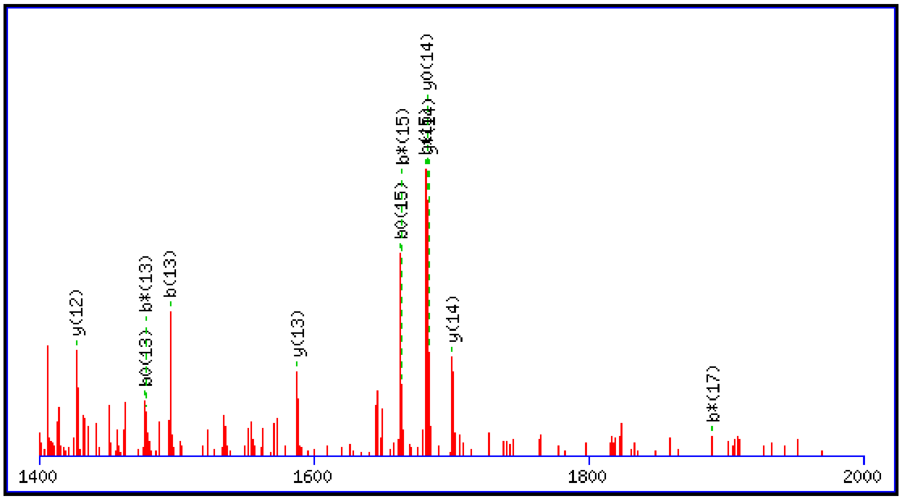

Monoisotopic mass of neutral peptide Mr(calc): 2921.3068
Fixed modifications: Carbamidomethyl (C)
Variable modifications:
N5 : Deamidated_N (N)
Ions Score: 73 **Expect:** 2e-005
Matches (Bold Red): 35/264 fragment ions using 63 most intense peaks

#	b	b^{++}	b*	b^{*++}	b^0	b^{0++}	Seq.	y	y^{++}	y*	y^{*++}	y^0	y^{0++}	#
1	72.0444	36.5258					A							25
2	232.0750	116.5412					C	2851.2769	1426.1421	2834.2504	1417.6288	2833.2664	1417.1368	24
3	**345.1591**	173.0832					I	2691.2463	**1346.1268**	2674.2197	1337.6135	2673.2357	1337.1215	23
4	**474.2017**	237.6045			456.1911	228.5992	E	2578.1622	**1289.5847**	2561.1357	1281.0715	2560.1517	1280.5795	22
5	589.2286	295.1180	**572.2021**	286.6047	571.2181	286.1127	N	2449.1196	**1225.0635**	2432.0931	**1216.5502**	2431.1091	1216.0582	21
6	646.2501	323.6287	629.2235	315.1154	628.2395	314.6234	G	2334.0927	**1167.5500**	2317.0661	1159.0367	2316.0821	1158.5447	20
7	747.2978	374.1525	730.2712	365.6393	729.2872	365.1472	T	2277.0712	1139.0393	2260.0447	1130.5260	2259.0607	1130.0340	19
8	834.3298	417.6685	817.3033	409.1553	816.3192	408.6633	S	2176.0235	**1088.5154**	2158.9970	1080.0021	2158.0130	1079.5101	18
9	994.3605	497.6839	977.3339	489.1706	976.3499	488.6786	C	2088.9915	**1044.9994**	2071.9650	1036.4861	2070.9810	1035.9941	17
10	1107.4445	554.2259	1090.4180	545.7126	1089.4340	545.2206	I	1928.9609	964.9841	1911.9343	956.4708	1910.9503	955.9788	16
11	1222.4715	611.7394	1205.4449	603.2261	1204.4609	602.7341	D	1815.8768	**908.4420**	1798.8503	899.9288	1797.8662	899.4368	15
12	1335.5555	668.2814	1318.5290	659.7681	1317.5450	659.2761	I	**1700.8499**	**850.9286**	**1683.8233**	842.4153	**1682.8393**	841.9233	14
13	**1495.5862**	748.2967	**1478.5596**	739.7834	**1477.5756**	739.2914	C	**1587.7658**	**794.3865**	1570.7392	785.8733	1569.7552	785.3813	13
14	1552.6076	776.8075	1535.5811	768.2942	1534.5971	767.8022	G	**1427.7351**	**714.3712**	1410.7086	705.8579	1409.7246	705.3659	12
15	**1681.6502**	841.3288	**1664.6237**	832.8155	**1663.6397**	832.3235	E	1370.7137	685.8605	1353.6871	677.3472	1352.7031	676.8552	11
16	1778.7030	889.8551	1761.6764	881.3419	1760.6924	880.8499	P	1241.6711	**621.3392**	1224.6445	612.8259	1223.6605	612.3339	10
17	1906.7616	953.8844	**1889.7350**	**945.3711**	1888.7510	944.8791	Q	1144.6183	**572.8128**	1127.5918	564.2995	1126.6078	563.8075	9
18	2053.8300	1027.4186	2036.8034	1018.9054	2035.8194	1018.4133	F	**1016.5598**	508.7835	999.5332	500.2702	998.5492	499.7782	8

**19**	2166.9140	1083.9607	2149.8875	1075.4474	2148.9035	1074.9554	**L**	869.4913	435.2493	852.4648	426.7360	**851.4808**	426.2440	**7**
**20**	2295.9566	1148.4820	2278.9301	1139.9687	2277.9461	1139.4767	**E**	**756.4073**	378.7073	739.3807	370.1940	738.3967	369.7020	**6**
**21**	2409.0407	1205.0240	2392.0142	1196.5107	2391.0301	1196.0187	**L**	**627.3647**	314.1860	610.3381	305.6727			**5**
**22**	2540.0812	1270.5442	2523.0546	1262.0310	2522.0706	1261.5390	**M**	**514.2806**	257.6439	497.2541	249.1307			**4**
**23**	2677.1401	1339.0737	2660.1136	1330.5604	2659.1295	1330.0684	**H**	**383.2401**	192.1237	366.2136	183.6104			**3**
**24**	2776.2085	1388.6079	2759.1820	1380.0946	2758.1980	1379.6026	**V**	246.1812	123.5942	229.1547	115.0810			**2**
**25**							**K**	147.1128	74.0600	130.0863	65.5468			**1**

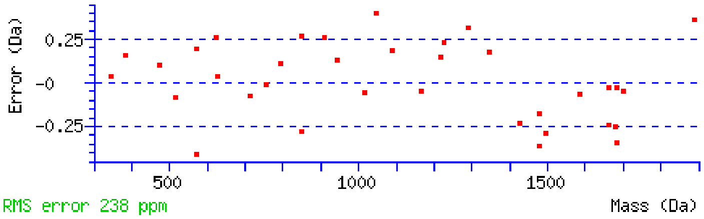

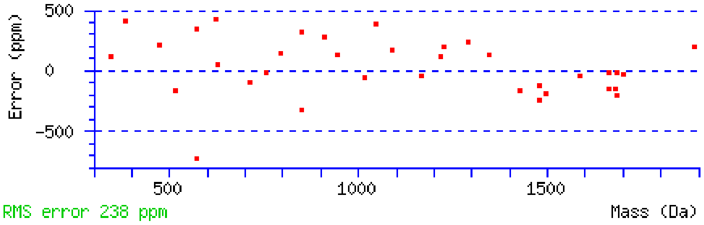

All matches to this query

Score	Mr(calc):	Delta	Sequence
73.0	2921.3068	0.0074	ACIENGTSCIDICGEPQFLELMHVK
31.2	2920.3228	0.9914	ACIENGTSCIDICGEPQFLELMHVK
8.2	2921.2952	0.0189	QESELCGNCTEKQPVSLLLSSLLR
6.9	2921.3122	0.0019	CPSDPEMLKSSVSSPLHPTMRINLN
5.6	2919.2949	2.0193	SHWSMDLLQSGPLVKDMNTKSLEK
5.4	2921.3195	-0.0053	GGDTFSQMLGFPTPEPTLNTNFVNLK
4.1	2920.3215	0.9927	MNNSLDYLAYPVIVSNHRQSTNFR
4.0	2919.3160	1.9981	MPNILTSNLKIINEDTNCISPTQK
4.0	2919.3160	1.9981	MPNILTSNLKIINEDTNCISPTQK
3.6	2919.3160	1.9981	MAHEMTGTQIVTERLVALLESGTEK

Spectrum No: 193; Query: 943; Rank: 1

Peptide View

MS/MS Fragmentation of **NGSYCVLQMDQSYKPDENEVR**
Found in **IPI00393333**, Tax_Id=10116 Gene_Symbol=Atic Bifunctional purine biosynthesis protein PURH

Match to Query 943: 2532.090732 from(845.037520,3+)
Title: 091129RatKid_SCX02_21.2631.2631.3.dta
Data file K:\NewmanPaper\Piliang\3SubProteomes\Piliang3SP\mgf5ppm\SCX_3SubProteomes5ppm.mgf

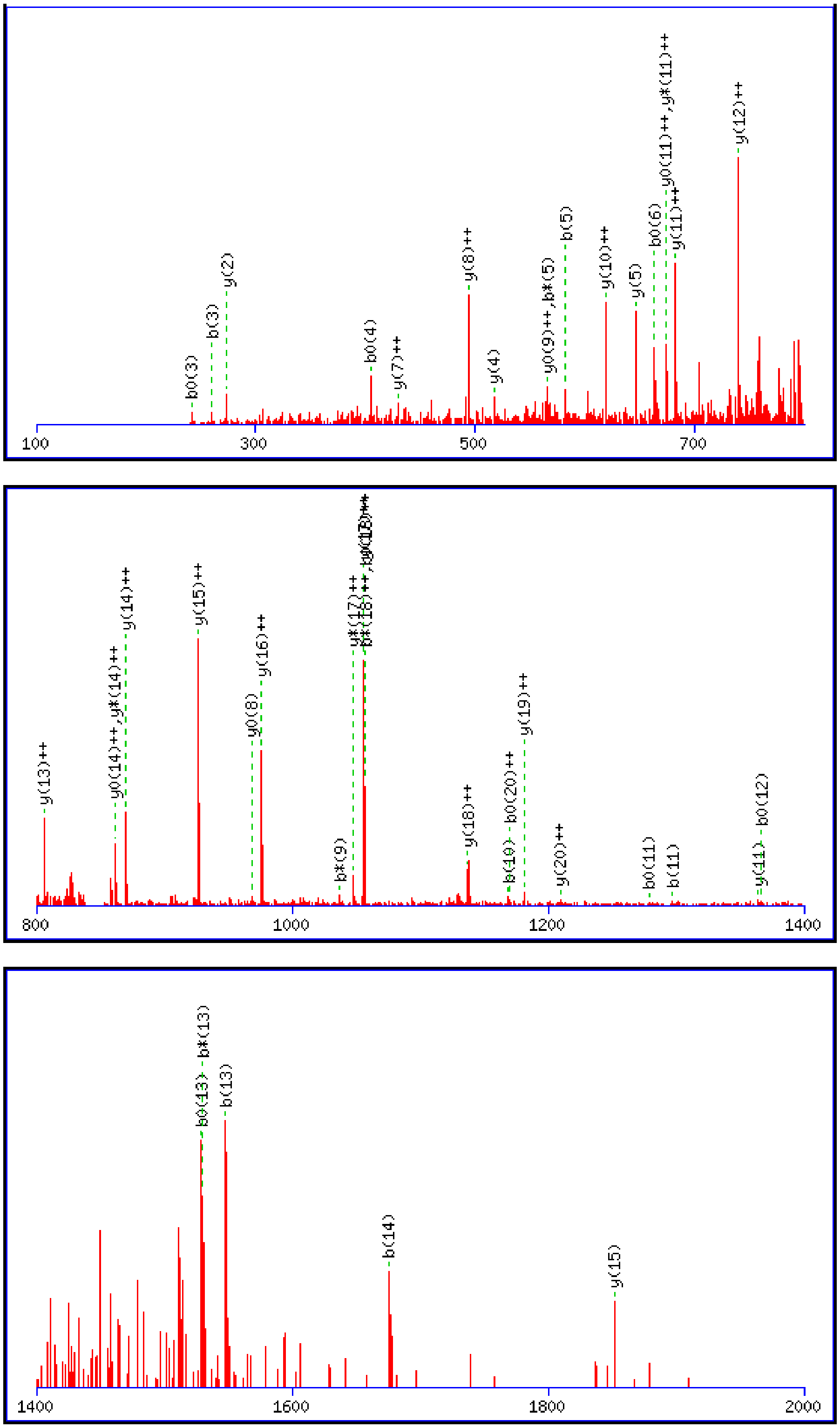

Monoisotopic mass of neutral peptide Mr(calc): 2532.0897
Fixed modifications: Carbamidomethyl (C)
Variable modifications:
N1 : Deamidated_N (N)
Ions Score: 73 **Expect:** 1.7e-005
Matches (Bold Red): 43/232 fragment ions using 72 most intense peaks

#	b	b^{++}	b*	b^{*++}	b^0	b^{0++}	Seq.	y	y^{++}	y*	y^{*++}	y^0	y^{0++}	#
1	116.0342	58.5207	99.0077	50.0075			N							21
2	173.0557	87.0315	156.0291	78.5182			G	2418.0700	**1209.5387**	2401.0435	1201.0254	2400.0595	1200.5334	20
3	**260.0877**	130.5475	243.0612	122.0342	**242.0771**	121.5422	S	2361.0486	**1181.0279**	2344.0220	1172.5147	2343.0380	1172.0226	19
4	423.1510	212.0792	406.1245	203.5659	**405.1405**	203.0739	Y	2274.0165	**1137.5119**	2256.9900	1128.9986	2256.0060	1128.5066	18
5	**583.1817**	292.0945	**566.1551**	283.5812	565.1711	283.0892	C	2110.9532	**1055.9802**	2093.9267	**1047.4670**	2092.9427	1046.9750	17

6	682.2501	341.6287	665.2235	333.1154	**664.2395**	332.6234	V	1950.9226	**975.9649**	1933.8960	967.4516	1932.9120	966.9596	16
7	795.3342	398.1707	778.3076	389.6574	777.3236	389.1654	L	**1851.8542**	**926.4307**	1834.8276	917.9174	1833.8436	917.4254	15
8	923.3927	462.2000	906.3662	453.6867	905.3822	453.1947	Q	1738.7701	**869.8887**	1721.7435	**861.3754**	1720.7595	**860.8834**	14
9	1054.4332	527.7202	**1037.4067**	519.2070	1036.4227	518.7150	M	1610.7115	**805.8594**	1593.6850	797.3461	1592.7009	796.8541	13
10	**1169.4602**	585.2337	1152.4336	576.7204	1151.4496	576.2284	D	1479.6710	**740.3392**	1462.6445	731.8259	1461.6605	731.3339	12
11	**1297.5187**	649.2630	1280.4922	640.7497	**1279.5082**	640.2577	Q	**1364.6441**	**682.8257**	1347.6175	**674.3124**	1346.6335	**673.8204**	11
12	1384.5508	692.7790	1367.5242	684.2657	**1366.5402**	683.7737	S	1236.5855	**618.7964**	1219.5590	610.2831	1218.5749	609.7911	10
13	**1547.6141**	774.3107	**1530.5876**	765.7974	**1529.6035**	765.3054	Y	1149.5535	575.2804	1132.5269	566.7671	1131.5429	**566.2751**	9
14	**1675.7091**	838.3582	1658.6825	829.8449	1657.6985	829.3529	K	986.4901	**493.7487**	969.4636	485.2354	**968.4796**	484.7434	8
15	1772.7618	886.8846	1755.7353	878.3713	1754.7513	877.8793	P	858.3952	**429.7012**	841.3686	421.1880	840.3846	420.6959	7
16	1887.7888	944.3980	1870.7622	935.8847	1869.7782	935.3927	D	761.3424	381.1748	744.3159	372.6616	743.3319	372.1696	6
17	2016.8314	1008.9193	1999.8048	1000.4060	1998.8208	999.9140	E	**646.3155**	323.6614	629.2889	315.1481	628.3049	314.6561	5
18	2130.8743	1065.9408	2113.8477	**1057.4275**	2112.8637	**1056.9355**	N	**517.2729**	259.1401	500.2463	250.6268	499.2623	250.1348	4
19	2259.9169	1130.4621	2242.8903	1121.9488	2241.9063	1121.4568	E	403.2300	202.1186	386.2034	193.6053	385.2194	193.1133	3
20	2358.9853	1179.9963	2341.9587	1171.4830	2340.9747	**1170.9910**	V	**274.1874**	137.5973	257.1608	129.0840			2
21							R	175.1190	88.0631	158.0924	79.5498			1

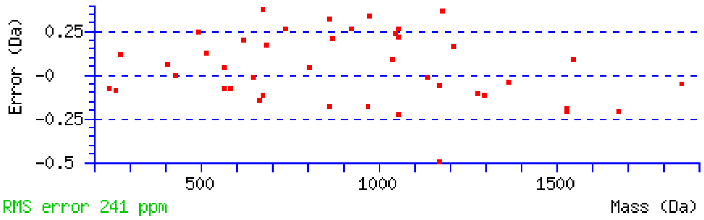

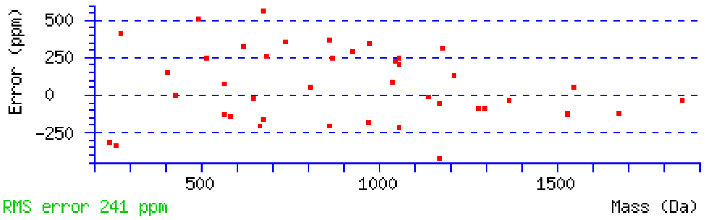

All matches to this query

Score	Mr(calc):	Delta	Sequence
72.9	2532.0897	0.0011	NGSYCVLQMDQSYKPDENEVR
49.7	2531.1057	0.9851	NGSYCVLQMDQSYKPDENEVR
49.1	2532.0897	0.0011	NGSYCVLQMDQSYKPDENEVR
5.1	2531.0860	1.0047	VSHVCSQTNGSSAQQASAPPAPDR
4.6	2531.0860	1.0047	VSHVCSQTNGSSAQQASAPPAPDR
4.3	2531.0860	1.0047	VSHVCSQTNGSSAQQASAPPAPDR
4.2	2531.0860	1.0047	VSHVCSQTNGSSAQQASAPPAPDR
1.9	2531.1029	0.9878	GLEPPVASGPTAGAGMPGGGGHAAALR
1.8	2531.0860	1.0047	VSHVCSQTNGSSAQQASAPPAPDR
1.6	2532.0700	0.0207	VSHVCSQTNGSSAQQASAPPAPDR

Spectrum No: 194; Query: 899; Rank: 1

Peptide View

MS/MS Fragmentation of **YRAEYAVANDTGFVDIPQQEK**
Found in **IPI00326070**, Tax_Id=10116 Gene_Symbol=Ctsl Cathepsin L1 precursor

Match to Query 899: 2414.140308 from(1208.077430,2+)
Title: 091127RatKid_SCX01_27.2893.2893.2.dta
Data file K:\NewmanPaper\Piliang\3SubProteomes\Piliang3SP\mgf5ppm\SCX_3SubProteomes5ppm.mgf

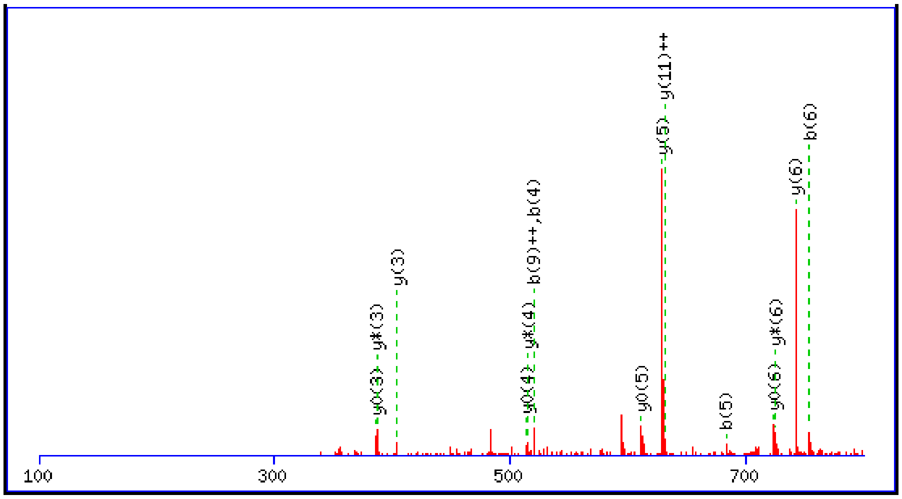

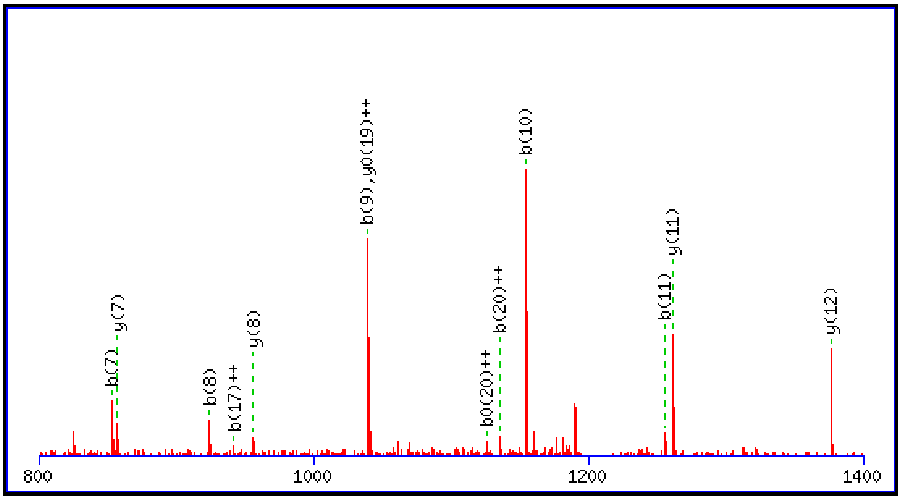

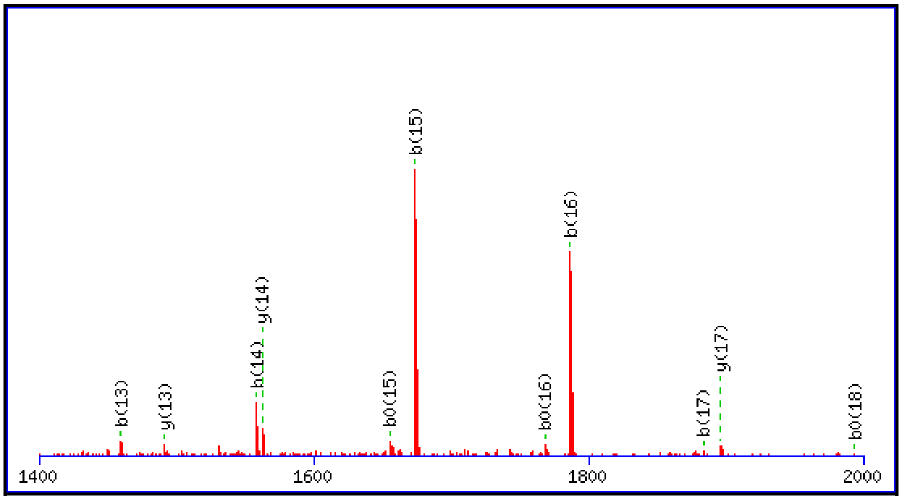

Monoisotopic mass of neutral peptide Mr(calc): 2414.1390
Fixed modifications: Carbamidomethyl (C)
Variable modifications:
N9 : Deamidated_N (N)
Ions Score: 73 **Expect:** 1.4e-005
Matches (Bold Red): 39/230 fragment ions using 72 most intense peaks

#	b	b^{++}	b*	b^{*++}	b^{0}	b^{0++}	Seq.	y	y^{++}	y*	y^{*++}	y^{0}	y^{0++}	#
1	164.0706	82.5389					Y							21
2	320.1717	160.5895	303.1452	152.0762			R	2252.0830	1126.5451	2235.0564	1118.0318	2234.0724	1117.5398	20
3	391.2088	196.1081	374.1823	187.5948			A	2095.9819	1048.4946	2078.9553	1039.9813	2077.9713	**1039.4893**	19
4	**520.2514**	260.6293	503.2249	252.1161	502.2409	251.6241	E	2024.9447	1012.9760	2007.9182	1004.4627	2006.9342	1003.9707	18
5	**683.3148**	342.1610	666.2882	333.6477	665.3042	333.1557	Y	**1895.9021**	948.4547	1878.8756	939.9414	1877.8916	939.4494	17

6	**754.3519**	377.6796	737.3253	369.1663	736.3413	368.6743	A	1732.8388	866.9230	1715.8123	858.4098	1714.8283	857.9178	16
7	**853.4203**	427.2138	836.3937	418.7005	835.4097	418.2085	V	1661.8017	831.4045	1644.7752	822.8912	1643.7911	822.3992	15
8	**924.4574**	462.7323	907.4308	454.2191	906.4468	453.7271	A	**1562.7333**	781.8703	1545.7067	773.3570	1544.7227	772.8650	14
9	**1039.4843**	**520.2458**	1022.4578	511.7325	1021.4738	511.2405	N	**1491.6962**	746.3517	1474.6696	737.8385	1473.6856	737.3464	13
10	**1154.5113**	577.7593	1137.4847	569.2460	1136.5007	568.7540	D	**1376.6692**	688.8383	1359.6427	680.3250	1358.6587	679.8330	12
11	**1255.5590**	628.2831	1238.5324	619.7698	1237.5484	619.2778	T	**1261.6423**	**631.3248**	1244.6157	622.8115	1243.6317	622.3195	11
12	1312.5804	656.7938	1295.5539	648.2806	1294.5699	647.7886	G	1160.5946	580.8009	1143.5681	572.2877	1142.5841	571.7957	10
13	**1459.6488**	730.3281	1442.6223	721.8148	1441.6383	721.3228	F	1103.5732	552.2902	1086.5466	543.7769	1085.5626	543.2849	9
14	**1558.7172**	779.8623	1541.6907	771.3490	1540.7067	770.8570	V	**956.5047**	478.7560	939.4782	470.2427	938.4942	469.7507	8
15	**1673.7442**	837.3757	1656.7176	828.8625	**1655.7336**	828.3704	D	**857.4363**	429.2218	840.4098	420.7085	839.4258	420.2165	7
16	**1786.8283**	893.9178	1769.8017	885.4045	**1768.8177**	884.9125	I	**742.4094**	371.7083	**725.3828**	363.1951	**724.3988**	362.7030	6
17	**1883.8810**	**942.4441**	1866.8545	933.9309	1865.8705	933.4389	P	**629.3253**	315.1663	612.2988	306.6530	**611.3148**	306.1610	5
18	2011.9396	1006.4734	1994.9130	997.9602	**1993.9290**	997.4682	Q	532.2726	266.6399	**515.2460**	258.1266	**514.2620**	257.6346	4
19	2139.9982	1070.5027	2122.9716	1061.9894	2121.9876	1061.4974	Q	**404.2140**	202.6106	**387.1874**	194.0974	**386.2034**	193.6053	3
20	2269.0408	**1135.0240**	2252.0142	1126.5107	2251.0302	**1126.0187**	E	276.1554	138.5813	259.1288	130.0681	258.1448	129.5761	2
21							K	147.1128	74.0600	130.0863	65.5468			1

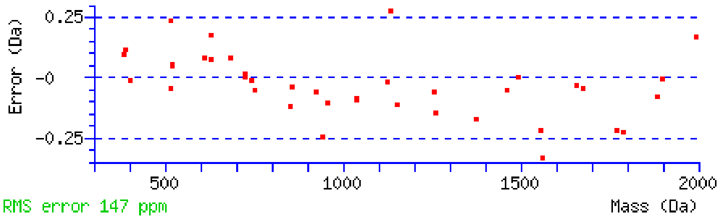

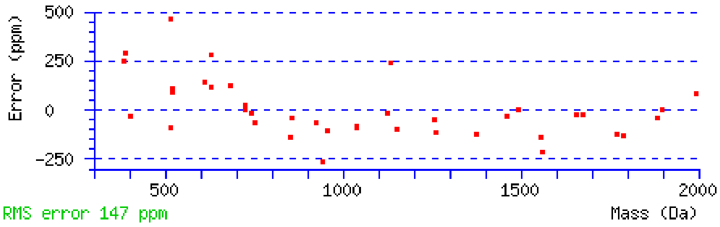

All matches to this query

Score	Mr(calc):	Delta	Sequence
73.0	2414.1390	0.0013	YRAEYAVANDTGFVDIPQQEK
25.0	2413.1550	0.9853	YRAEYAVANDTGFVDIPQQEK
9.9	2414.1341	0.0062	TPGDLAWNFLMKVQALDSTAR
9.9	2414.1341	0.0062	TPGDLAWNFLMKVQALDSTAR
5.5	2414.1586	-0.0183	MAQLANIGELLSMLDSSTLGVR
5.5	2414.1586	-0.0183	MAQLANIGELLSMLDSSTLGVR
5.5	2414.1586	-0.0183	MAQLANIGELLSMLDSSTLGVR
5.5	2414.1586	-0.0183	MAQLANIGELLSMLDSSTLGVR
5.0	2414.1375	0.0028	CQPRLMYPAKLSINIDGETK
4.7	2413.1501	0.9902	TPGDLAWNFLMKVQALDSTAR

Spectrum No: 195; Query: 423; Rank: 1

Peptide View

MS/MS Fragmentation of **EGITLNANLSGDGFLK**
Found in **IPI00327398**, Tax_Id=10116 Gene_Symbol=Enpep Isoform 1 of Glutamyl aminopeptidase

Match to Query 423: 1648.837368 from(825.425960,2+)
Title: 091129RatKid_SCX02_12.3395.3395.2.dta
Data file K:\NewmanPaper\Piliang\3SubProteomes\Piliang3SP\mgf5ppm\SCX_3SubProteomes5ppm.mgf

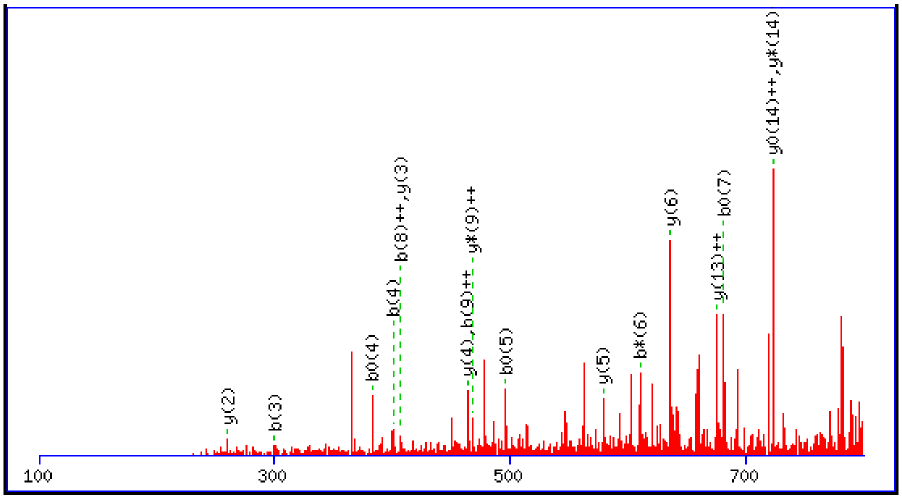

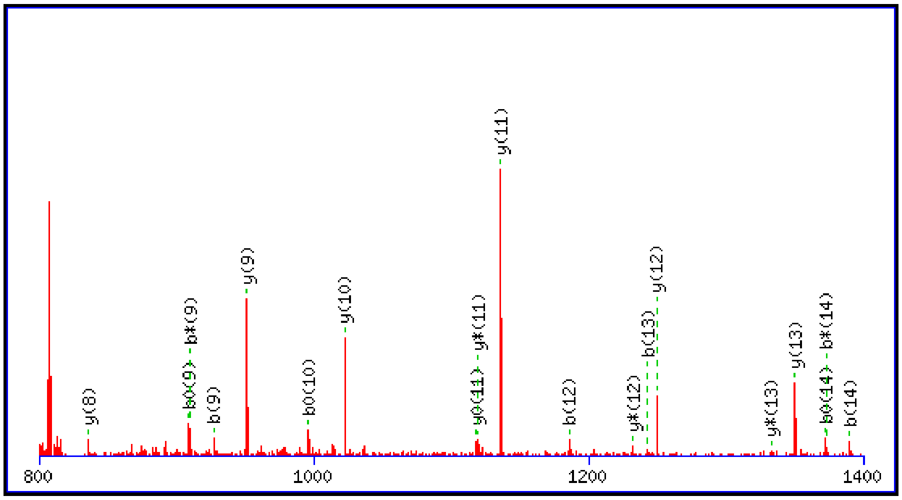

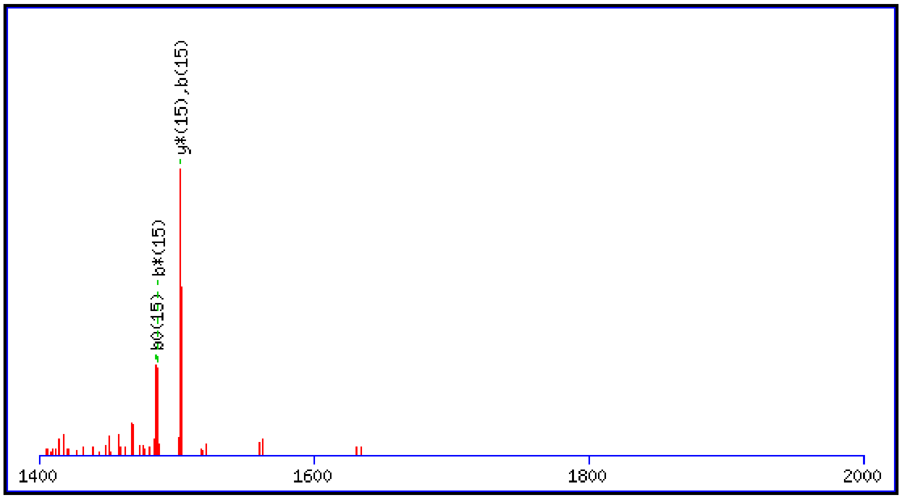

Monoisotopic mass of neutral peptide Mr(calc): 1648.8308
Fixed modifications: Carbamidomethyl (C)
Variable modifications:
N8 : Deamidated_N (N)
Ions Score: 73 **Expect:** 8.4e-006
Matches (Bold Red): 41/162 fragment ions using 74 most intense peaks

#	b	b^{++}	b*	b^{*++}	b^0	b^{0++}	Seq.	y	y^{++}	y*	y^{*++}	y^0	y^{0++}	#
1	130.0499	65.5286			112.0393	56.5233	E							16
2	187.0713	94.0393			169.0608	85.0340	G	1520.7955	760.9014	**1503.7689**	752.3881	1502.7849	751.8961	15
3	**300.1554**	150.5813			282.1448	141.5761	I	1463.7740	732.3907	1446.7475	**723.8774**	1445.7635	**723.3854**	14
4	**401.2031**	201.1052			**383.1925**	192.0999	T	**1350.6900**	**675.8486**	**1333.6634**	667.3353	1332.6794	666.8433	13
5	514.2871	257.6472			**496.2766**	248.6419	L	**1249.6423**	625.3248	**1232.6157**	616.8115	1231.6317	616.3195	12

6	628.3301	314.6687	**611.3035**	306.1554	610.3195	305.6634	N	**1136.5582**	568.7827	**1119.5317**	560.2695	**1118.5477**	559.7775	11
7	699.3672	350.1872	682.3406	341.6740	**681.3566**	341.1819	A	**1022.5153**	511.7613	1005.4887	503.2480	1004.5047	502.7560	10
8	814.3941	**407.7007**	797.3676	399.1874	796.3836	398.6954	N	**951.4782**	476.2427	934.4516	**467.7295**	933.4676	467.2374	9
9	**927.4782**	**464.2427**	**910.4516**	455.7295	**909.4676**	455.2374	L	**836.4512**	418.7293	819.4247	410.2160	818.4407	409.7240	8
10	1014.5102	507.7587	997.4837	499.2455	**996.4996**	498.7535	S	**723.3672**	362.1872	706.3406	353.6740	705.3566	353.1819	7
11	1071.5317	536.2695	1054.5051	527.7562	1053.5211	527.2642	G	**636.3352**	318.6712	619.3086	310.1579	618.3246	309.6659	6
12	**1186.5586**	593.7829	1169.5321	585.2697	1168.5481	584.7777	D	**579.3137**	290.1605	562.2871	281.6472	561.3031	281.1552	5
13	**1243.5801**	622.2937	1226.5535	613.7804	1225.5695	613.2884	G	**464.2867**	232.6470	447.2602	224.1337			4
14	**1390.6485**	695.8279	**1373.6219**	687.3146	**1372.6379**	686.8226	F	**407.2653**	204.1363	390.2387	195.6230			3
15	**1503.7326**	752.3699	**1486.7060**	743.8566	**1485.7220**	743.3646	L	**260.1969**	130.6021	243.1703	122.0888			2
16							K	147.1128	74.0600	130.0863	65.5468			1

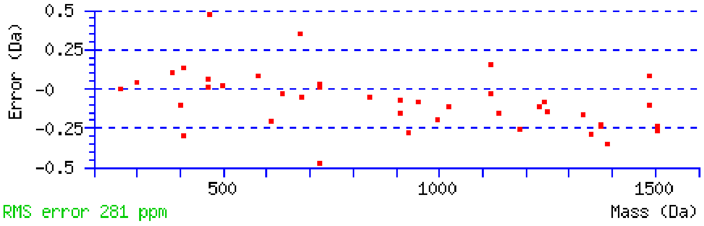

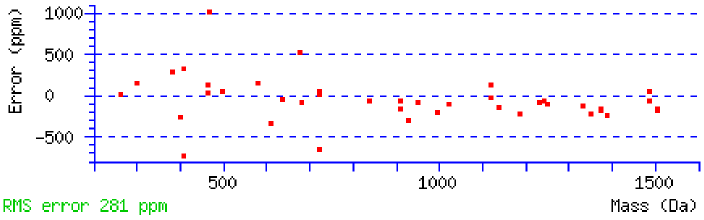

All matches to this query

Score	Mr(calc):	Delta	Sequence
72.9	1648.8308	0.0066	EGITLNANLSGDGFLK
47.7	1648.8308	0.0066	EGITLNANLSGDGFLK
10.9	1647.8468	0.9906	EGITLNANLSGDGFLK
4.4	1648.8532	-0.0159	EAPDPARVGENPRIK
3.0	1648.8356	0.0018	SRGSFGMQVVSVGGPGK
1.9	1648.8378	-0.0004	YGVISPRFYVQLK
0.9	1648.8494	-0.0120	YDMIAEDLQAVLLR
0.7	1646.8168	2.0206	STEIGKLLSSYLEK

Spectrum No: 196; Query: 1071; Rank: 1

Peptide View

MS/MS Fragmentation of **CISIDKVCDGVPDCPEGDDENNVTSGR**
Found in **IPI00205325**, Tax_Id=10116 Gene_Symbol=Lrp2 Low-density lipoprotein receptor-related protein 2 precursor

Match to Query 1071: 3008.238582 from(1003.753470,3+)
Title: 091129RatKid_SCX02_22.2285.2285.3.dta
Data file K:\NewmanPaper\Piliang\3SubProteomes\Piliang3SP\mgf5ppm\SCX_3SubProteomes5ppm.mgf

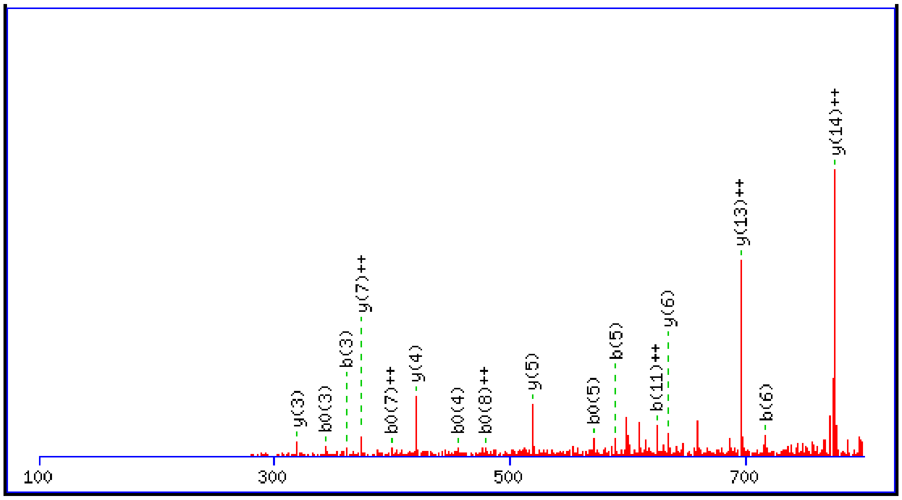

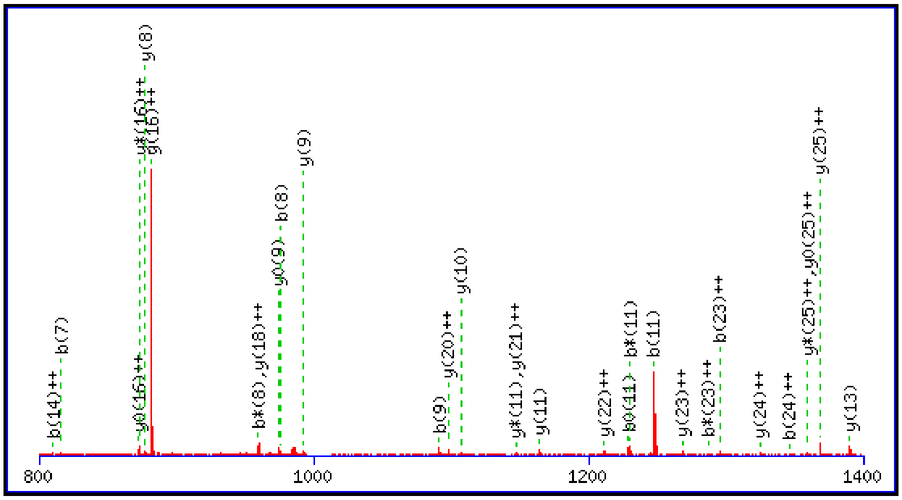

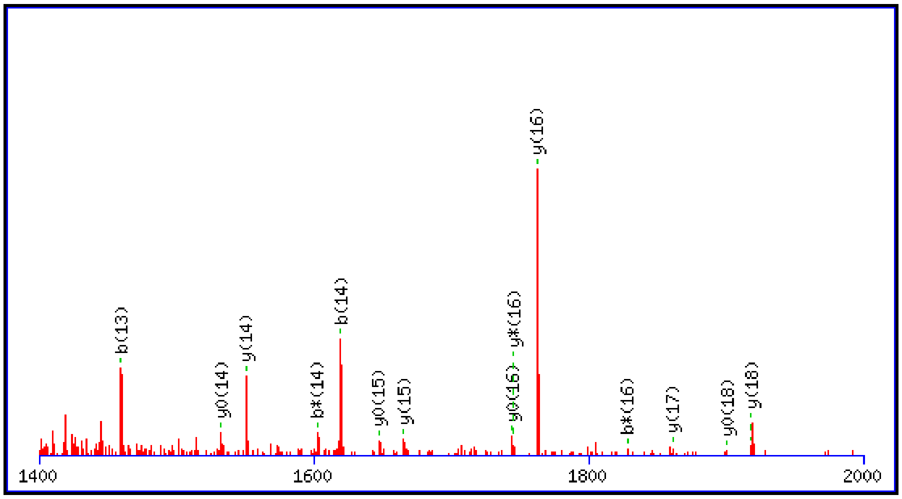

Monoisotopic mass of neutral peptide Mr(calc): 3008.2434
Fixed modifications: Carbamidomethyl (C)
Variable modifications:
N22 : Deamidated_N (N)
Ions Score: 73 **Expect:** 1.8e-005
Matches (Bold Red): 60/294 fragment ions using 126 most intense peaks

#	b	b^{++}	b*	b^{*++}	b^0	b^{0++}	Seq.	y	y^{++}	y*	y^{*++}	y^0	y^{0++}	#
1	161.0379	81.0226					C							27
2	274.1220	137.5646					I	2849.2200	1425.1136	2832.1935	1416.6004	2831.2094	1416.1084	26
3	**361.1540**	181.0806			**343.1435**	172.0754	S	2736.1359	**1368.5716**	2719.1094	**1360.0583**	2718.1254	**1359.5663**	25
4	474.2381	237.6227			**456.2275**	228.6174	I	2649.1039	**1325.0556**	2632.0774	1316.5423	2631.0933	1316.0503	24
5	**589.2650**	295.1362			**571.2545**	286.1309	D	2536.0198	**1268.5136**	2518.9933	1260.0003	2518.0093	1259.5083	23

6	**717.3600**	359.1836	700.3334	350.6704	699.3494	350.1783	K	2420.9929	**1211.0001**	2403.9664	1202.4868	2402.9823	1201.9948	22
7	**816.4284**	408.7178	799.4019	400.2046	798.4178	**399.7126**	V	2292.8979	**1146.9526**	2275.8714	1138.4393	2274.8874	1137.9473	21
8	**976.4591**	488.7332	**959.4325**	480.2199	958.4485	**479.7279**	C	2193.8295	**1097.4184**	2176.8030	1088.9051	2175.8190	1088.4131	20
9	**1091.4860**	546.2466	1074.4594	537.7334	1073.4754	537.2414	D	2033.7989	1017.4031	2016.7723	1008.8898	2015.7883	1008.3978	19
10	1148.5075	574.7574	1131.4809	566.2441	1130.4969	565.7521	G	**1918.7719**	**959.8896**	1901.7454	951.3763	**1900.7614**	950.8843	18
11	**1247.5759**	**624.2916**	**1230.5493**	615.7783	**1229.5653**	615.2863	V	**1861.7505**	931.3789	1844.7239	922.8656	1843.7399	922.3736	17
12	1344.6286	672.8180	1327.6021	664.3047	1326.6181	663.8127	P	**1762.6821**	**881.8447**	**1745.6555**	**873.3314**	**1744.6715**	**872.8394**	16
13	**1459.6556**	730.3314	1442.6290	721.8182	1441.6450	721.3261	D	**1665.6293**	833.3183	1648.6027	824.8050	**1647.6187**	824.3130	15
14	**1619.6862**	**810.3468**	**1602.6597**	801.8335	1601.6757	801.3415	C	**1550.6023**	**775.8048**	1533.5758	767.2915	**1532.5918**	766.7995	14
15	1716.7390	858.8731	1699.7124	850.3599	1698.7284	849.8679	P	**1390.5717**	**695.7895**	1373.5452	687.2762	1372.5611	686.7842	13
16	1845.7816	923.3944	**1828.7550**	914.8812	1827.7710	914.3891	E	1293.5189	647.2631	1276.4924	638.7498	1275.5084	638.2578	12
17	1902.8030	951.9052	1885.7765	943.3919	1884.7925	942.8999	G	**1164.4763**	582.7418	**1147.4498**	574.2285	1146.4658	573.7365	11
18	2017.8300	1009.4186	2000.8034	1000.9054	1999.8194	1000.4134	D	**1107.4549**	554.2311	1090.4283	545.7178	1089.4443	545.2258	10
19	2132.8569	1066.9321	2115.8304	1058.4188	2114.8464	1057.9268	D	**992.4279**	496.7176	975.4014	488.2043	**974.4174**	487.7123	9
20	2261.8995	1131.4534	2244.8730	1122.9401	2243.8890	1122.4481	E	**877.4010**	439.2041	860.3744	430.6909	859.3904	430.1989	8
21	2375.9425	1188.4749	2358.9159	1179.9616	2357.9319	1179.4696	N	748.3584	**374.6828**	731.3319	366.1696	730.3478	365.6776	7
22	2490.9694	1245.9883	2473.9428	1237.4751	2472.9588	1236.9831	N	**634.3155**	317.6614	617.2889	309.1481	616.3049	308.6561	6
23	2590.0378	**1295.5225**	2573.0113	**1287.0093**	2572.0272	1286.5173	V	**519.2885**	260.1479	502.2620	251.6346	501.2780	251.1426	5
24	2691.0855	**1346.0464**	2674.0589	1337.5331	2673.0749	1337.0411	T	**420.2201**	210.6137	403.1936	202.1004	402.2096	201.6084	4
25	2778.1175	1389.5624	2761.0910	1381.0491	2760.1069	1380.5571	S	**319.1724**	160.0899	302.1459	151.5766	301.1619	151.0846	3
26	2835.1390	1418.0731	2818.1124	1409.5599	2817.1284	1409.0678	G	232.1404	116.5738	215.1139	108.0606			2
27							R	175.1190	88.0631	158.0924	79.5498			1

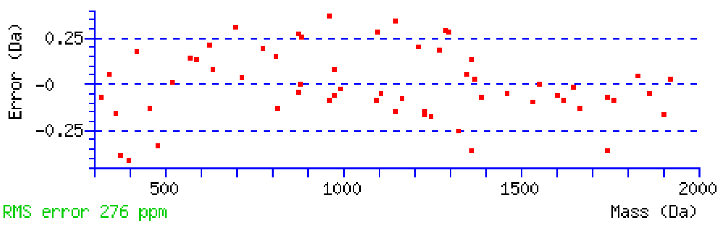

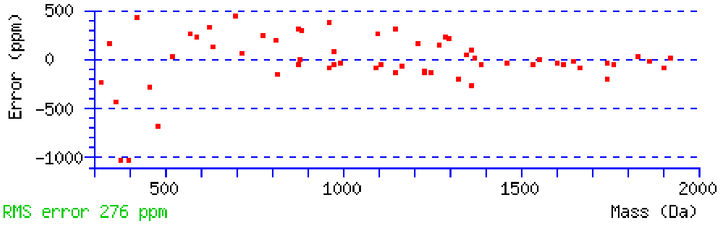

All matches to this query

Score	Mr(calc):	Delta	Sequence
72.8	3008.2434	-0.0048	CISIDKVCDGVPDCPEGDDENNVTSGR
65.7	3008.2434	-0.0048	CISIDKVCDGVPDCPEGDDENNVTSGR
7.1	3008.2260	0.0126	SRDDSQLNGDPSALLNPSKECEPYR
7.1	3008.2260	0.0126	SRDDSQLNGDPSALLNPSKECEPYR
7.1	3008.2260	0.0126	SRDDSQLNGDPSALLNPSKECEPYR
6.9	3007.2169	1.0217	RPKPHCFNCGSEEHQMKECPMPR
6.3	3007.2594	0.9792	CISIDKVCDGVPDCPEGDDENNVTSGR
6.0	3008.2347	0.0039	ALLGKHCDIPTNGCGSERWNSTFAR
5.2	3008.2349	0.0036	MMPEGPSFPVCSTFVQELFQAQYR
5.2	3008.2349	0.0036	MMPEGPSFPVCSTFVQELFQAQYR

Spectrum No: 197; Query: 389; Rank: 1

Peptide View

MS/MS Fragmentation of **KLINDYVSNQTQGK**
Found in **IPI00200591**, Tax_Id=10116 Gene_Symbol=LOC299282 Serine protease inhibitor A3L precursor

Match to Query 389: 1607.813648 from(804.914100,2+)
Title: 091129RatKid_SCX02_13.821.821.2.dta
Data file K:\NewmanPaper\Piliang\3SubProteomes\Piliang3SP\mgf5ppm\SCX_3SubProteomes5ppm.mgf

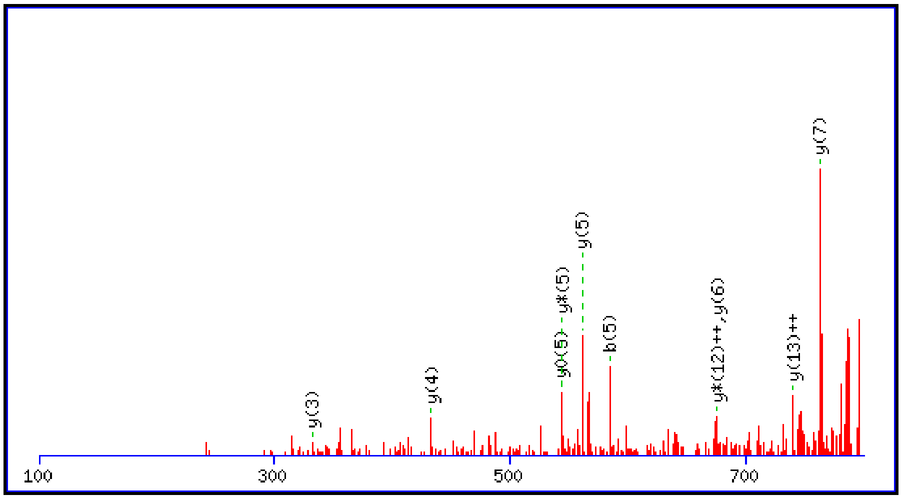

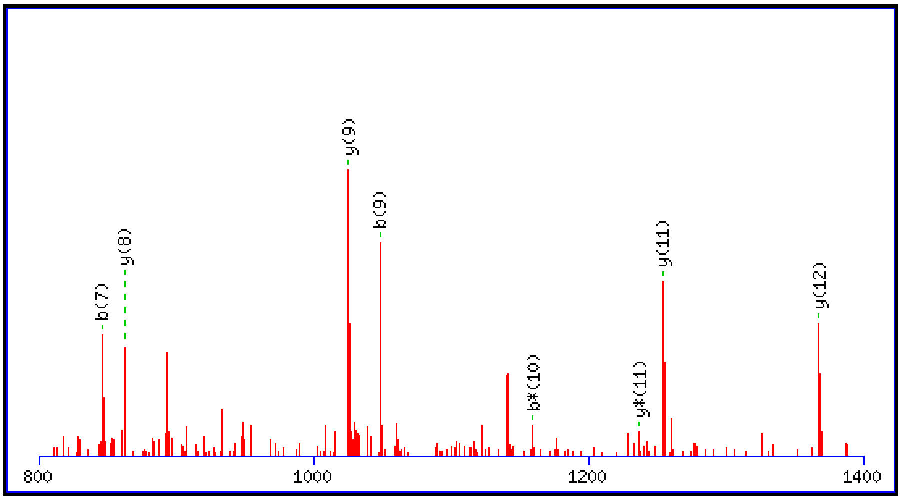

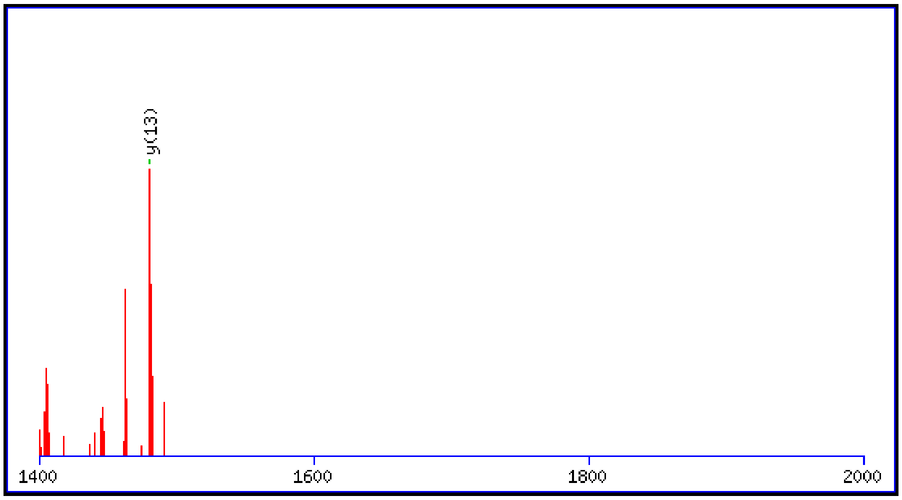

Monoisotopic mass of neutral peptide Mr(calc): 1607.8155
Fixed modifications: Carbamidomethyl (C)
Variable modifications:
N9 : Deamidated_N (N)
Ions Score: 73 **Expect:** 7.8e-006
Matches (Bold Red): 19/142 fragment ions using 28 most intense peaks

#	b	b^{++}	b*	b^{*++}	b^0	b^{0++}	Seq.	y	y^{++}	y*	y^{*++}	y^0	y^{0++}	#
1	129.1022	65.0548	112.0757	56.5415			K							14
2	242.1863	121.5968	225.1598	113.0835			L	**1480.7278**	**740.8675**	1463.7013	732.3543	1462.7172	731.8623	13

3	355.2704	178.1388	338.2438	169.6255			I	**1367.6437**	684.3255	1350.6172	**675.8122**	1349.6332	675.3202	12
4	469.3133	235.1603	452.2867	226.6470			N	**1254.5597**	627.7835	**1237.5331**	619.2702	1236.5491	618.7782	11
5	**584.3402**	292.6738	567.3137	284.1605	566.3297	283.6685	D	1140.5168	570.7620	1123.4902	562.2487	1122.5062	561.7567	10
6	747.4036	374.2054	730.3770	365.6921	729.3930	365.2001	Y	**1025.4898**	513.2485	1008.4633	504.7353	1007.4792	504.2433	9
7	**846.4720**	423.7396	829.4454	415.2264	828.4614	414.7343	V	**862.4265**	431.7169	845.3999	423.2036	844.4159	422.7116	8
8	933.5040	467.2556	916.4775	458.7424	915.4934	458.2504	S	**763.3581**	382.1827	746.3315	373.6694	745.3475	373.1774	7
9	**1048.5309**	524.7691	1031.5044	516.2558	1030.5204	515.7638	N	**676.3260**	338.6667	659.2995	330.1534	658.3155	329.6614	6
10	1176.5895	588.7984	**1159.5630**	580.2851	1158.5790	579.7931	Q	**561.2991**	281.1532	**544.2726**	272.6399	**543.2885**	272.1479	5
11	1277.6372	639.3222	1260.6107	630.8090	1259.6266	630.3170	T	**433.2405**	217.1239	416.2140	208.6106	415.2300	208.1186	4
12	1405.6958	703.3515	1388.6692	694.8383	1387.6852	694.3462	Q	**332.1928**	166.6001	315.1663	158.0868			3
13	1462.7172	731.8623	1445.6907	723.3490	1444.7067	722.8570	G	204.1343	102.5708	187.1077	94.0575			2
14							K	147.1128	74.0600	130.0863	65.5468			1

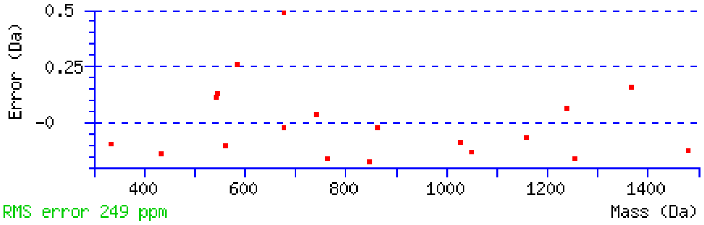

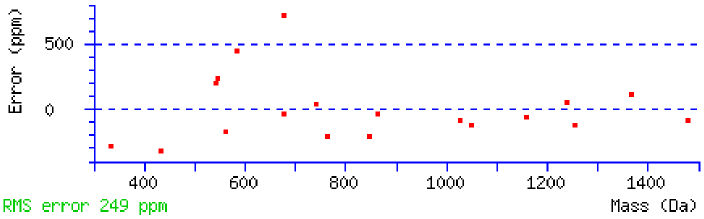

All matches to this query

Score	Mr(calc):	Delta	Sequence
72.7	1607.8155	-0.0018	KLINDYVSNQTQGK
33.5	1607.8155	-0.0018	KLINDYVSNQTQGK
8.2	1607.8158	-0.0021	QLIRSSPAAHHRR
8.2	1607.8158	-0.0021	QLIRSSPAAHHRR
5.8	1607.7977	0.0159	KQQAKFDECVLDK
5.0	1607.8072	0.0064	QILDGVNYLHTKK
4.2	1607.8276	-0.0139	KLVENGGMFVCKAR
3.6	1607.8130	0.0007	LGALNCTAFSIQWK
3.5	1607.8072	0.0064	QLLAPGNSAGAFLIR
3.4	1607.8283	-0.0147	KLQTLDLRDNALK

Spectrum No: 198; Query: 954; Rank: 1

Peptide View

MS/MS Fragmentation of **GTDPSLSPSPAAALPAPGTELSYLNR**
Found in **IPI00192504**, Tax_Id=10116 Gene_Symbol=Mrc2 Macrophage mannose receptor 2 precursor

Match to Query 954: 2582.289568 from(1292.152060,2+)
Title: 091127RatKid_SCX01_14.3906.3906.2.dta
Data file K:\NewmanPaper\Piliang\3SubProteomes\Piliang3SP\mgf5ppm\SCX_3SubProteomes5ppm.mgf

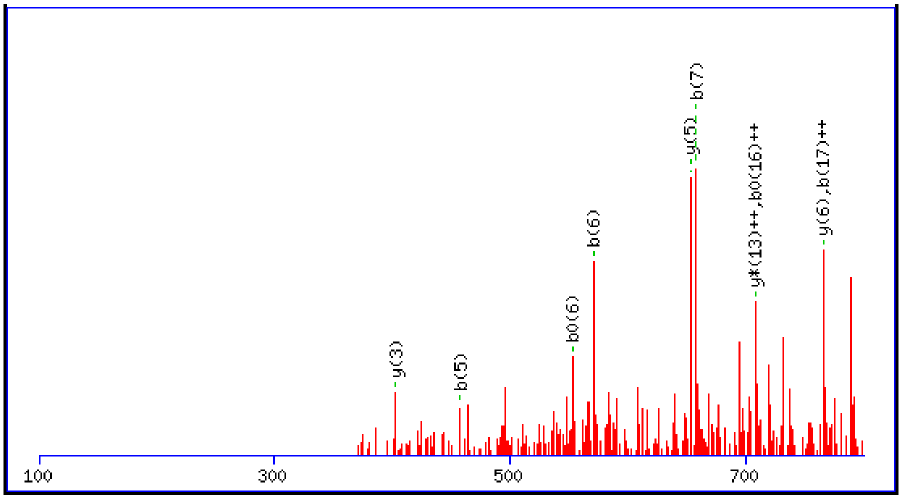

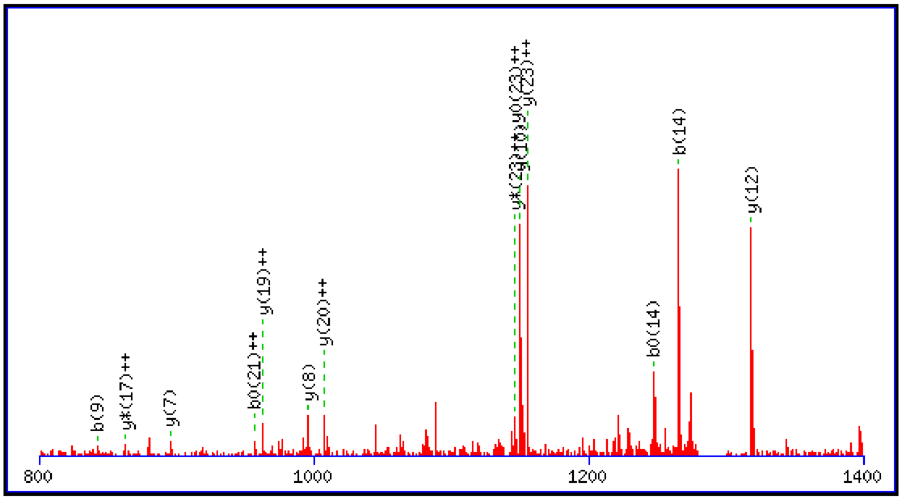

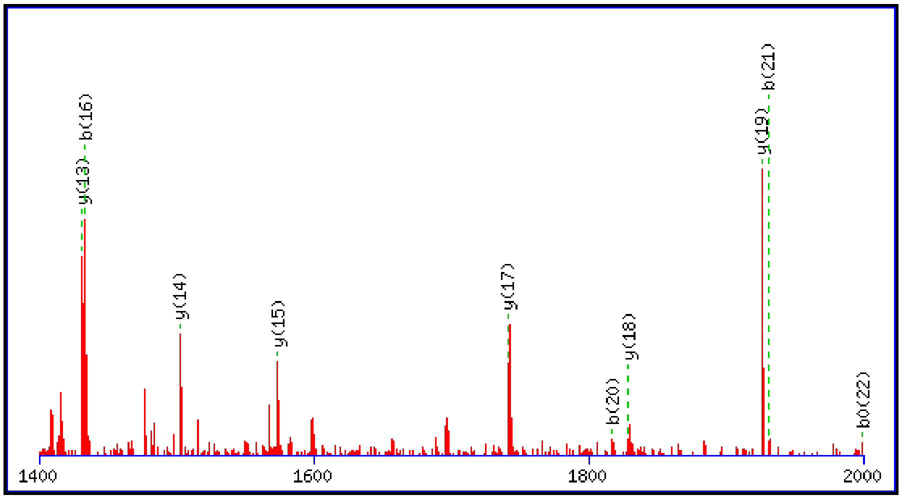

Monoisotopic mass of neutral peptide Mr(calc): 2582.2864
Fixed modifications: Carbamidomethyl (C)
Variable modifications:
N25 : Deamidated_N (N)
Ions Score: 73 **Expect:** 1.1e-005
Matches (Bold Red): 34/242 fragment ions using 58 most intense peaks

#	b	b^{++}	b*	b^{*++}	b^0	b^{0++}	Seq.	y	y^{++}	y*	y^{*++}	y^0	y^{0++}	#
1	58.0287	29.5180					G							26
2	159.0764	80.0418			141.0659	71.0366	T	2526.2722	1263.6397	2509.2457	1255.1265	2508.2617	1254.6345	25
3	274.1034	137.5553			256.0928	128.5500	D	2425.2245	1213.1159	2408.1980	1204.6026	2407.2140	1204.1106	24
4	371.1561	186.0817			353.1456	177.0764	P	2310.1976	**1155.6024**	2293.1710	**1147.0892**	2292.1870	**1146.5972**	23
5	**458.1882**	229.5977			440.1776	220.5924	S	2213.1448	1107.0761	2196.1183	1098.5628	2195.1343	1098.0708	22

6	571.2722	286.1397			553.2617	277.1345	L	2126.1128	1063.5600	2109.0863	1055.0468	2108.1022	1054.5548	21
7	658.3042	329.6558			640.2937	320.6505	S	2013.0287	1007.0180	1996.0022	998.5047	1995.0182	998.0127	20
8	755.3570	378.1821			737.3464	369.1769	P	1925.9967	963.5020	1908.9702	954.9887	1907.9861	954.4967	19
9	842.3890	421.6982			824.3785	412.6929	S	1828.9439	914.9756	1811.9174	906.4623	1810.9334	905.9703	18
10	939.4418	470.2245			921.4312	461.2193	P	1741.9119	871.4596	1724.8854	862.9463	1723.9014	862.4543	17
11	1010.4789	505.7431			992.4684	496.7378	A	1644.8592	822.9332	1627.8326	814.4199	1626.8486	813.9279	16
12	1081.5160	541.2617			1063.5055	532.2564	A	1573.8220	787.4147	1556.7955	778.9014	1555.8115	778.4094	15
13	1152.5531	576.7802			1134.5426	567.7749	A	1502.7849	751.8961	1485.7584	743.3828	1484.7744	742.8908	14
14	1265.6372	633.3222			1247.6266	624.3170	L	1431.7478	716.3775	1414.7213	707.8643	1413.7372	707.3723	13
15	1362.6900	681.8486			1344.6794	672.8433	P	1318.6638	659.8355	1301.6372	651.3222	1300.6532	650.8302	12
16	1433.7271	717.3672			1415.7165	708.3619	A	1221.6110	611.3091	1204.5844	602.7959	1203.6004	602.3038	11
17	1530.7798	765.8936			1512.7693	756.8883	P	1150.5739	575.7906	1133.5473	567.2773	1132.5633	566.7853	10
18	1587.8013	794.4043			1569.7907	785.3990	G	1053.5211	527.2642	1036.4946	518.7509	1035.5105	518.2589	9
19	1688.8490	844.9281			1670.8384	835.9229	T	996.4996	498.7535	979.4731	490.2402	978.4891	489.7482	8
20	1817.8916	909.4494			1799.8810	900.4441	E	895.4520	448.2296	878.4254	439.7163	877.4414	439.2243	7
21	1930.9756	965.9915			1912.9651	956.9862	L	766.4094	383.7083	749.3828	375.1950	748.3988	374.7030	6
22	2018.0077	1009.5075			1999.9971	1000.5022	S	653.3253	327.1663	636.2988	318.6530	635.3147	318.1610	5
23	2181.0710	1091.0391			2163.0604	1082.0339	Y	566.2933	283.6503	549.2667	275.1370			4
24	2294.1551	1147.5812			2276.1445	1138.5759	L	403.2300	202.1186	386.2034	193.6053			3
25	2409.1820	1205.0946	2392.1555	1196.5814	2391.1714	1196.0894	N	290.1459	145.5766	273.1193	137.0633			2
26							R	175.1190	88.0631	158.0924	79.5498			1

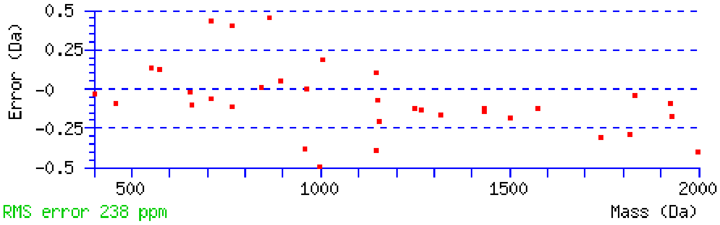

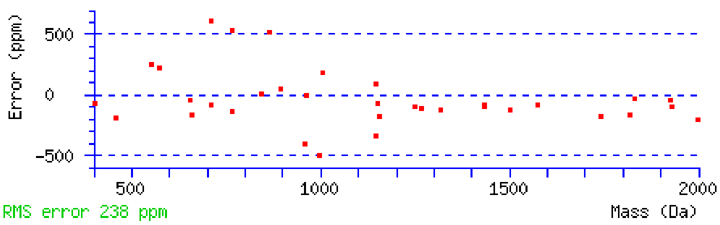

All matches to this query

Score	Mr(calc):	Delta	Sequence
72.7	2582.2864	0.0032	GTDPSLSPSPAAALPAPGTELSYLNR
11.4	2581.3024	0.9872	GTDPSLSPSPAAALPAPGTELSYLNR

Spectrum No: 199; Query: 315; Rank: 1

Peptide View

MS/MS Fragmentation of **IYGGILNLSEITNK**
Found in **IPI00203384**, Tax_Id=10116 Gene_Symbol=Klkb1 Plasma kallikrein precursor

Match to Query 315: 1534.826928 from(768.420740,2+)
Title: 091129RatKid_SCX02_11.3187.3187.2.dta
Data file K:\NewmanPaper\Piliang\3SubProteomes\Piliang3SP\mgf5ppm\SCX_3SubProteomes5ppm.mgf

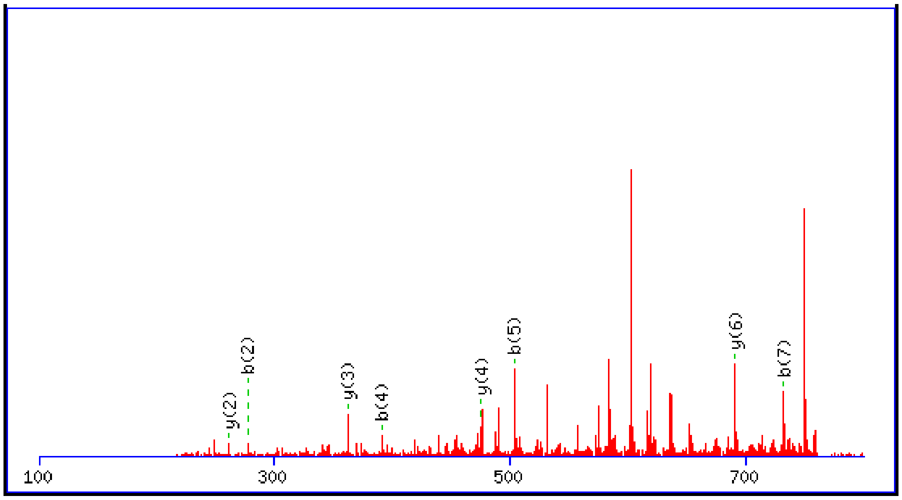

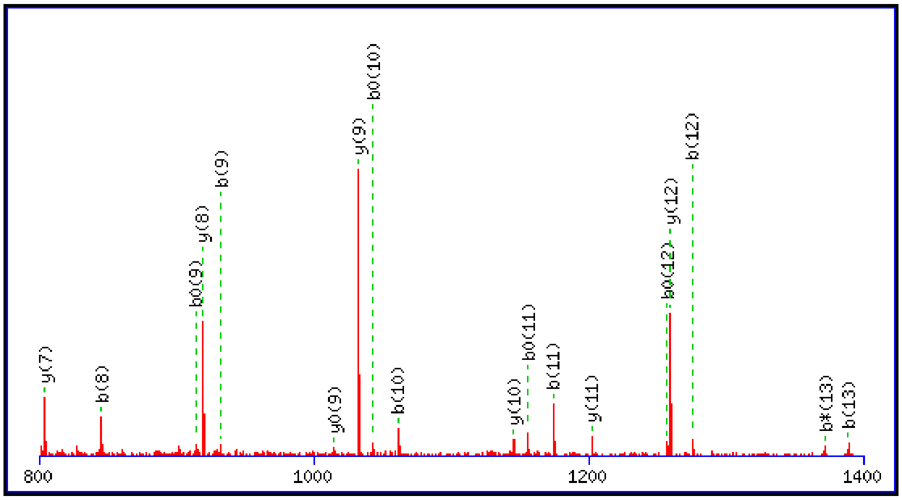

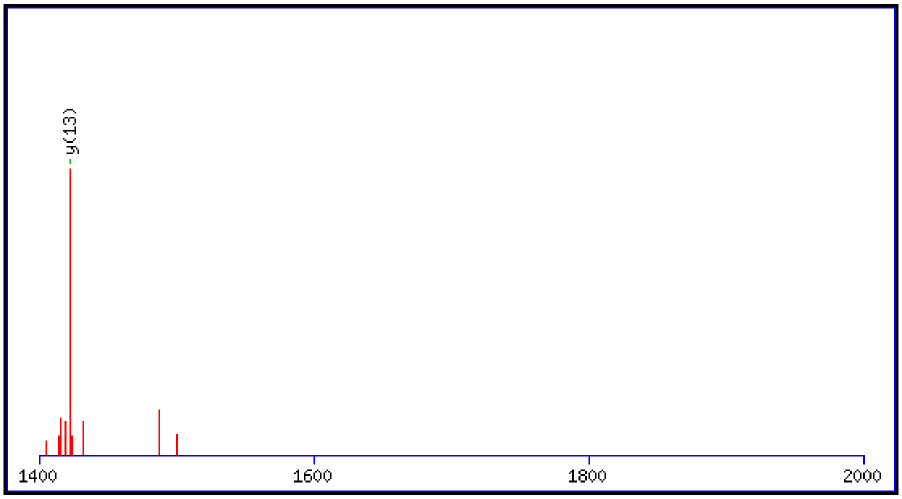

Monoisotopic mass of neutral peptide Mr(calc): 1534.8242
Fixed modifications: Carbamidomethyl (C)
Variable modifications:
N7 : Deamidated_N (N)
Ions Score: 73 **Expect:** 4.8e-006
Matches (Bold Red): 27/124 fragment ions using 62 most intense peaks

#	b	b^{++}	b*	b^{*++}	b^{0}	b^{0++}	Seq.	y	y^{++}	y*	y^{*++}	y^{0}	y^{0++}	#
1	114.0913	57.5493					I							14
2	**277.1547**	139.0810					Y	**1422.7475**	711.8774	1405.7209	703.3641	1404.7369	702.8721	13
3	334.1761	167.5917					G	**1259.6842**	630.3457	1242.6576	621.8324	1241.6736	621.3404	12
4	**391.1976**	196.1024					G	**1202.6627**	601.8350	1185.6361	593.3217	1184.6521	592.8297	11
5	**504.2817**	252.6445					I	**1145.6412**	573.3242	1128.6147	564.8110	1127.6307	564.3190	10

**6**	617.3657	309.1865					**L**	**1032.5572**	516.7822	1015.5306	508.2689	**1014.5466**	507.7769	**9**
**7**	**732.3927**	366.7000	715.3661	358.1867			**N**	**919.4731**	460.2402	902.4465	451.7269	901.4625	451.2349	**8**
**8**	**845.4767**	423.2420	828.4502	414.7287			**L**	**804.4462**	402.7267	787.4196	394.2134	786.4356	393.7214	**7**
**9**	**932.5088**	466.7580	915.4822	458.2447	**914.4982**	457.7527	**S**	**691.3621**	346.1847	674.3355	337.6714	673.3515	337.1794	**6**
**10**	**1061.5513**	531.2793	1044.5248	522.7660	**1043.5408**	522.2740	**E**	604.3301	302.6687	587.3035	294.1554	586.3195	293.6634	**5**
**11**	**1174.6354**	587.8213	1157.6089	579.3081	**1156.6248**	578.8161	**I**	**475.2875**	238.1474	458.2609	229.6341	457.2769	229.1421	**4**
**12**	**1275.6831**	638.3452	1258.6565	629.8319	**1257.6725**	629.3399	**T**	**362.2034**	181.6053	345.1769	173.0921	344.1928	172.6001	**3**
**13**	**1389.7260**	695.3666	**1372.6995**	686.8534	1371.7155	686.3614	**N**	**261.1557**	131.0815	244.1292	122.5682			**2**
**14**							**K**	147.1128	74.0600	130.0863	65.5468			**1**

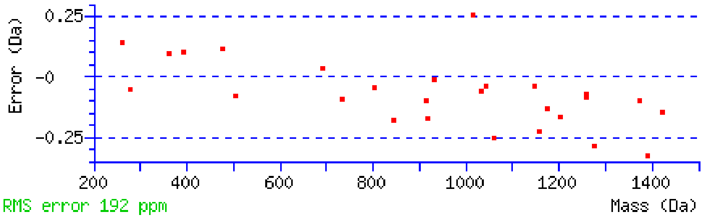

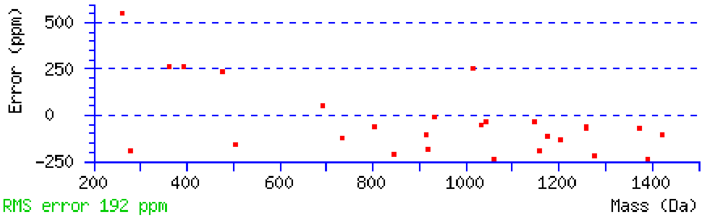

All matches to this query

Score	Mr(calc):	Delta	Sequence
72.6	1534.8242	0.0027	IYGGILNLSEITNK
31.7	1534.8242	0.0027	IYGGILNLSEITNK
18.2	1534.8355	-0.0085	KFNLDATELSIRK
16.5	1534.8242	0.0027	LLNYLLDNLSGTAK
7.3	1533.8151	1.0119	YNLKPQTSEKQAK
5.8	1533.8225	1.0044	LTSTVMLWLQTNK
5.2	1534.8218	0.0052	NFTAFPSLPGLIMK
4.8	1534.8251	0.0018	YLMVPALQGALTMK
4.7	1534.8355	-0.0086	FLSQESGVAQTLKK
4.6	1534.8251	0.0018	TLMLKNVMLAWAK

Spectrum No: 200; Query: 310; Rank: 1

Peptide View

MS/MS Fragmentation of **ILSGGALQLWNVTR**
Found in **IPI00205668**, Tax_Id=10116 Gene_Symbol=Nphs1 Isoform 1 of Nephrin precursor

Match to Query 310: 1527.844708 from(764.929630,2+)
Title: 091127RatKid_SCX01_02.4111.4111.2.dta
Data file K:\NewmanPaper\Piliang\3SubProteomes\Piliang3SP\mgf5ppm\SCX_3SubProteomes5ppm.mgf

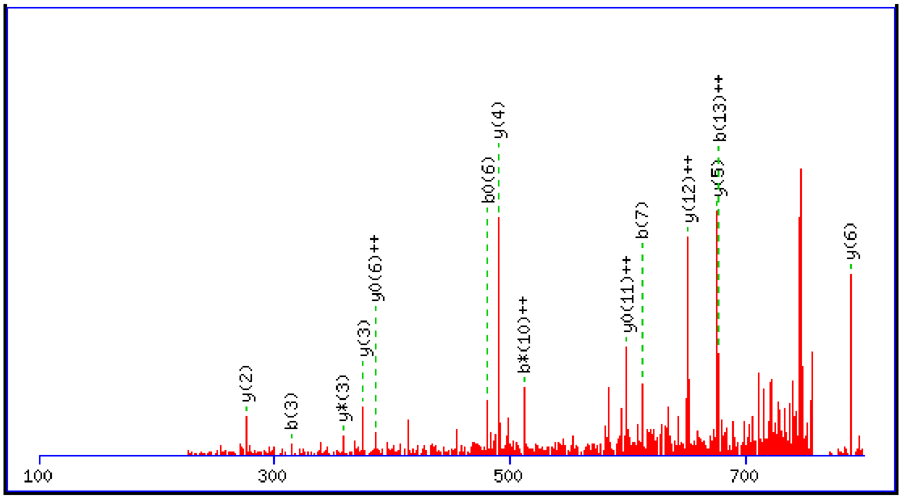

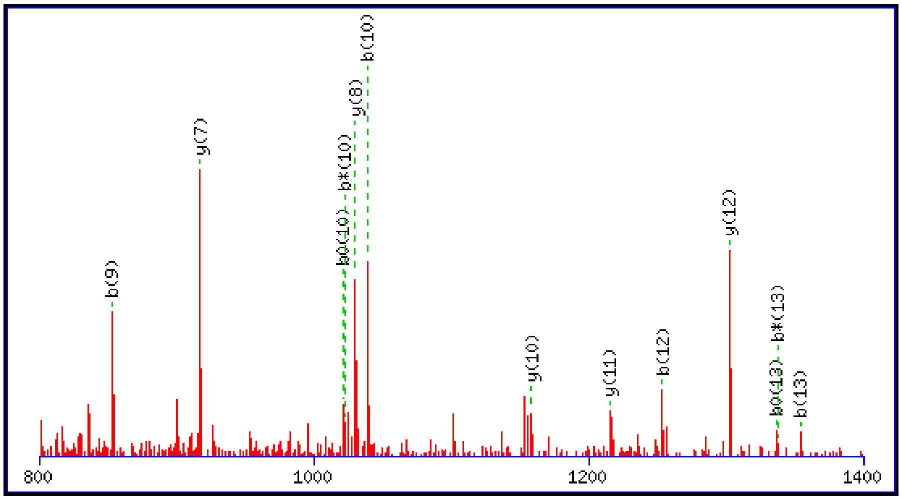

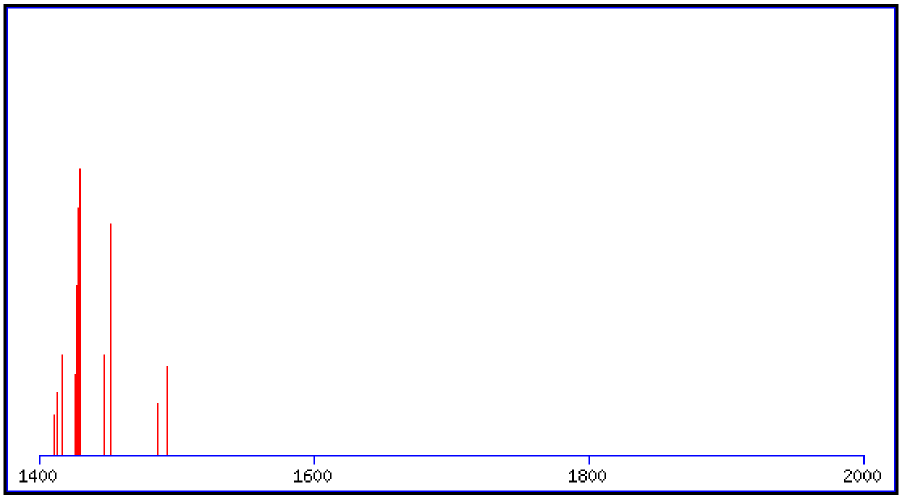

Monoisotopic mass of neutral peptide Mr(calc): 1527.8409
Fixed modifications: Carbamidomethyl (C)
Variable modifications:
N11 : Deamidated_N (N)
Ions Score: 72 **Expect:** 3.5e-006
Matches (Bold Red): 27/136 fragment ions using 44 most intense peaks

#	b	b++	b*	b*++	b0	b0++	Seq.	y	y++	y*	y*++	y0	y0++	#
1	114.0913	57.5493					I							14
2	227.1754	114.0913					L	1415.7641	708.3857	1398.7376	699.8724	1397.7536	699.3804	13
3	**314.2074**	157.6074			296.1969	148.6021	S	**1302.6801**	**651.8437**	1285.6535	643.3304	1284.6695	642.8384	12
4	371.2289	186.1181			353.2183	177.1128	G	**1215.6480**	608.3277	1198.6215	599.8144	1197.6375	**599.3224**	11
5	428.2504	214.6288			410.2398	205.6235	G	**1158.6266**	579.8169	1141.6000	571.3037	1140.6160	570.8116	10

**6**	499.2875	250.1474			**481.2769**	241.1421	**A**	1101.6051	551.3062	1084.5786	542.7929	1083.5945	542.3009	**9**
**7**	**612.3715**	306.6894			594.3610	297.6841	**L**	**1030.5680**	515.7876	1013.5415	507.2744	1012.5574	506.7824	**8**
**8**	740.4301	370.7187	723.4036	362.2054	722.4196	361.7134	**Q**	**917.4839**	459.2456	900.4574	450.7323	899.4734	450.2403	**7**
**9**	**853.5142**	427.2607	836.4876	418.7475	835.5036	418.2554	**L**	**789.4254**	395.2163	772.3988	386.7030	771.4148	**386.2110**	**6**
**10**	**1039.5935**	520.3004	**1022.5669**	**511.7871**	**1021.5829**	511.2951	**W**	**676.3413**	338.6743	659.3147	330.1610	658.3307	329.6690	**5**
**11**	1154.6204	577.8139	1137.5939	569.3006	1136.6099	568.8086	**N**	**490.2620**	245.6346	473.2354	237.1214	472.2514	236.6293	**4**
**12**	**1253.6888**	627.3481	1236.6623	618.8348	1235.6783	618.3428	**V**	**375.2350**	188.1212	**358.2085**	179.6079	357.2245	179.1159	**3**
**13**	**1354.7365**	**677.8719**	**1337.7100**	669.3586	**1336.7260**	668.8666	**T**	**276.1666**	138.5870	259.1401	130.0737	258.1561	129.5817	**2**
**14**							**R**	175.1190	88.0631	158.0924	79.5498			**1**

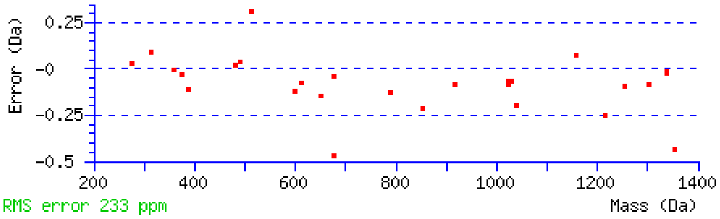

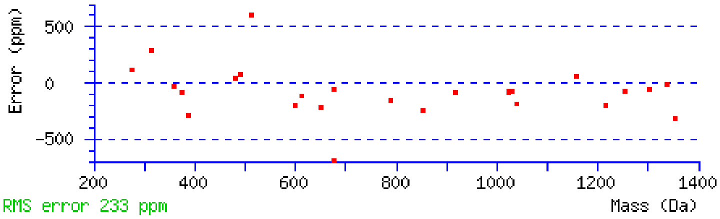

All matches to this query

Score	Mr(calc):	Delta	Sequence
72.4	1527.8409	0.0038	ILSGGALQLWNVTR
5.3	1527.8562	-0.0115	QFLTSFHIALVPR
4.1	1527.8382	0.0065	SSTLTLHHRIHAR
3.8	1527.8369	0.0078	QRLQGELQSLTQK

Mascot: http://www.matrixscience.com/

Mascot Search Results

Results Generated by a Script Modified from Mascot Pepitde View
by Newman Sze, School of Biological Sciences, Nanyang Technological University

---

Spectrum No: 201; Query: 1183; Rank: 1

Peptide View

MS/MS Fragmentation of **ALAGLVYNSSGMEPCFDIYQMYQSCADPTGCGTGSNAR**
Found in **IPI00230946**, Tax_Id=10116 Gene_Symbol=Dpp7 Dipeptidyl-peptidase 2 precursor

Match to Query 1183: 4166.738562 from(1389.920130,3+)
Title: 091127RatKid_SCX01_13.4674.4674.3.dta
Data file K:\NewmanPaper\Piliang\3SubProteomes\Piliang3SP\mgf5ppm\SCX_3SubProteomes5ppm.mgf

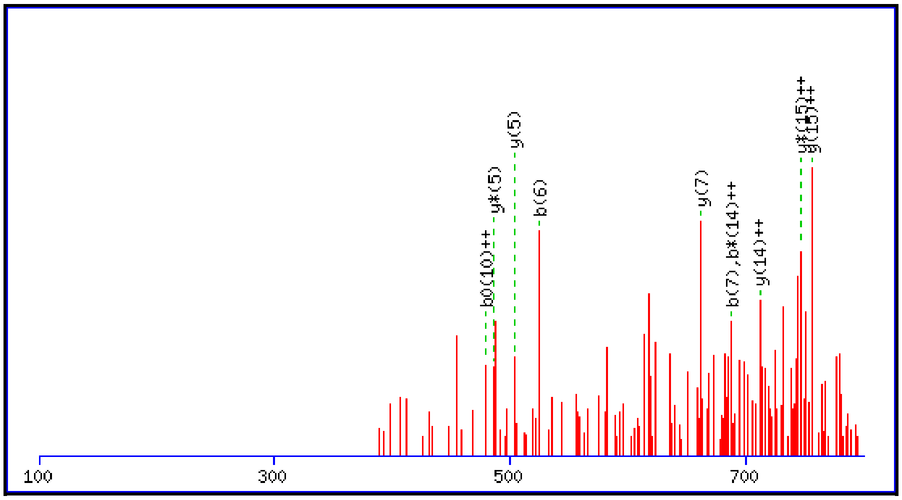

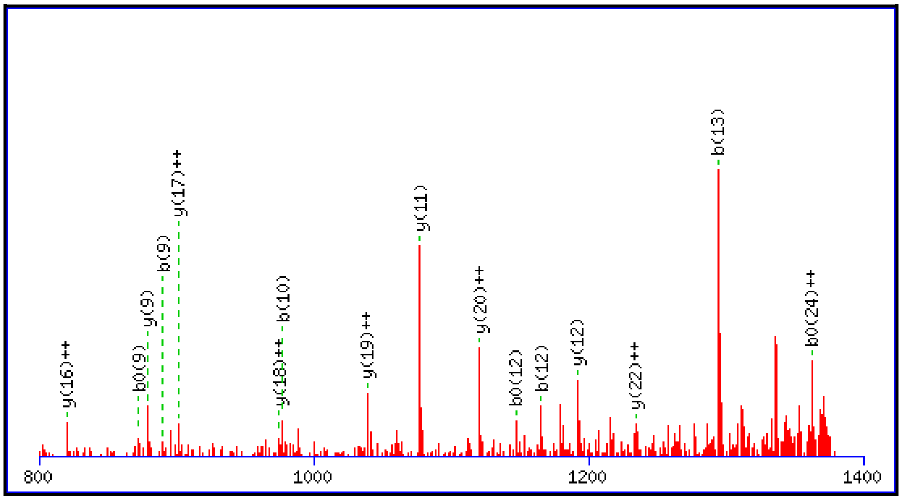

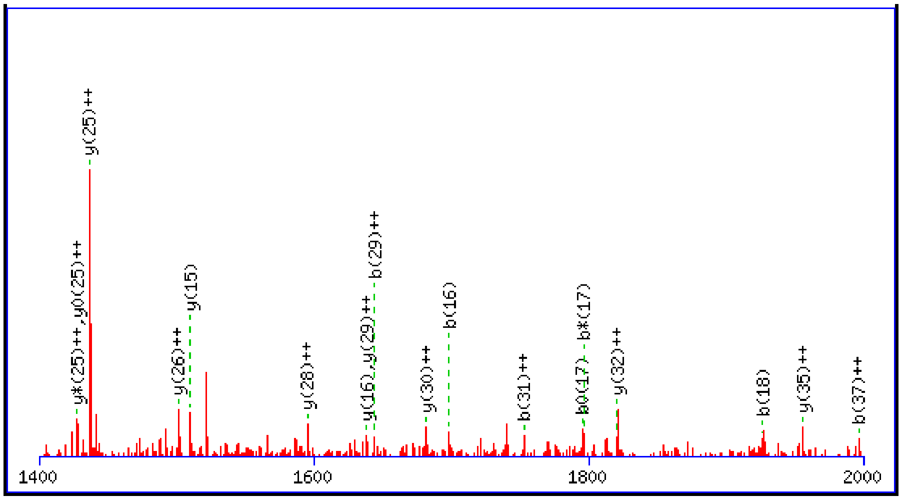

Monoisotopic mass of neutral peptide Mr(calc): 4164.7268
Fixed modifications: Carbamidomethyl (C)
Variable modifications:
N8 : Deamidated_N (N)
M21 : Oxidation (M)
Ions Score: 72 **Expect:** 2.8e-005
Matches (Bold Red): 44/408 fragment ions using 75 most intense peaks

#	b	b^{++}	b*	b^{*++}	b^0	b^{0++}	Seq.	y	y^{++}	y*	y^{*++}	y^0	y^{0++}	#
1	72.0444	36.5258					A							38
2	185.1285	93.0679					L	4094.6970	2047.8521	4077.6704	2039.3388	4076.6864	2038.8468	37
3	256.1656	128.5864					A	3981.6129	1991.3101	3964.5863	1982.7968	3963.6023	1982.3048	36
4	313.1870	157.0972					G	3910.5758	**1955.7915**	3893.5492	1947.2783	3892.5652	1946.7862	35
5	426.2711	213.6392					L	3853.5543	1927.2808	3836.5278	1918.7675	3835.5437	1918.2755	34
6	**525.3395**	263.1734					V	3740.4702	1870.7388	3723.4437	1862.2255	3722.4597	1861.7335	33
7	**688.4028**	344.7051					Y	3641.4018	**1821.2046**	3624.3753	1812.6913	3623.3913	1812.1993	32
8	803.4298	402.2185	786.4032	393.7053			N	3478.3385	1739.6729	3461.3120	1731.1596	3460.3279	1730.6676	31
9	**890.4618**	445.7345	873.4353	437.2213	**872.4512**	436.7293	S	3363.3116	**1682.1594**	3346.2850	1673.6461	3345.3010	1673.1541	30
10	**977.4938**	489.2506	960.4673	480.7373	959.4833	**480.2453**	S	3276.2795	**1638.6434**	3259.2530	1630.1301	3258.2690	1629.6381	29
11	1034.5153	517.7613	1017.4887	509.2480	1016.5047	508.7560	G	3189.2475	**1595.1274**	3172.2210	1586.6141	3171.2369	1586.1221	28
12	**1165.5558**	583.2815	1148.5292	574.7683	**1147.5452**	574.2762	M	3132.2260	1566.6167	3115.1995	1558.1034	3114.2155	1557.6114	27
13	**1294.5984**	647.8028	1277.5718	639.2896	1276.5878	638.7975	E	3001.1856	**1501.0964**	2984.1590	1492.5831	2983.1750	1492.0911	26
14	1391.6511	696.3292	1374.6246	**687.8159**	1373.6406	687.3239	P	2872.1430	**1436.5751**	2855.1164	**1428.0618**	2854.1324	**1427.5698**	25
15	1551.6818	776.3445	1534.6552	767.8313	1533.6712	767.3392	C	2775.0902	1388.0487	2758.0637	1379.5355	2757.0796	1379.0435	24
16	**1698.7502**	849.8787	1681.7237	841.3655	1680.7396	840.8735	F	2615.0596	1308.0334	2598.0330	1299.5201	2597.0490	1299.0281	23
17	1813.7771	907.3922	**1796.7506**	898.8789	**1795.7666**	898.3869	D	2467.9911	**1234.4992**	2450.9646	1225.9859	2449.9806	1225.4939	22
18	**1926.8612**	963.9342	1909.8347	955.4210	1908.8506	954.9290	I	2352.9642	1176.9857	2335.9377	1168.4725	2334.9536	1167.9805	21
19	2089.9245	1045.4659	2072.8980	1036.9526	2071.9140	1036.4606	Y	2239.8801	**1120.4437**	2222.8536	1111.9304	2221.8696	1111.4384	20
20	2217.9831	1109.4952	2200.9566	1100.9819	2199.9725	1100.4899	Q	2076.8168	**1038.9120**	2059.7903	1030.3988	2058.8062	1029.9068	19
21	2365.0185	1183.0129	2347.9920	1174.4996	2347.0080	1174.0076	M	1948.7582	**974.8828**	1931.7317	966.3695	1930.7477	965.8775	18
22	2528.0818	1264.5446	2511.0553	1256.0313	2510.0713	1255.5393	Y	1801.7228	**901.3651**	1784.6963	892.8518	1783.7123	892.3598	17
23	2656.1404	1328.5739	2639.1139	1320.0606	2638.1299	1319.5686	Q	**1638.6595**	**819.8334**	1621.6329	811.3201	1620.6489	810.8281	16
24	2743.1725	1372.0899	2726.1459	1363.5766	2725.1619	**1363.0846**	S	**1510.6009**	**755.8041**	1493.5744	**747.2908**	1492.5904	746.7988	15
25	2903.2031	1452.1052	2886.1766	1443.5919	2885.1925	1443.0999	C	1423.5689	**712.2881**	1406.5423	703.7748	1405.5583	703.2828	14
26	2974.2402	1487.6237	2957.2137	1479.1105	2956.2297	1478.6185	A	1263.5382	632.2728	1246.5117	623.7595	1245.5277	623.2675	13
27	3089.2672	1545.1372	3072.2406	1536.6239	3071.2566	1536.1319	D	**1192.5011**	596.7542	1175.4746	588.2409	1174.4906	587.7489	12
28	3186.3199	1593.6636	3169.2934	1585.1503	3168.3094	1584.6583	P	**1077.4742**	539.2407	1060.4476	530.7275	1059.4636	530.2354	11
29	3287.3676	**1644.1874**	3270.3411	1635.6742	3269.3570	1635.1822	T	980.4214	490.7143	963.3949	482.2011	962.4109	481.7091	10
30	3344.3891	1672.6982	3327.3625	1664.1849	3326.3785	1663.6929	G	**879.3737**	440.1905	862.3472	431.6772	861.3632	431.1852	9
31	3504.4197	**1752.7135**	3487.3932	1744.2002	3486.4092	1743.7082	C	822.3523	411.6798	805.3257	403.1665	804.3417	402.6745	8

**32**	3561.4412	1781.2242	3544.4146	1772.7110	3543.4306	1772.2189	**G**	**662.3216**	331.6645	645.2951	323.1512	644.3111	322.6592	**7**
**33**	3662.4889	1831.7481	3645.4623	1823.2348	3644.4783	1822.7428	**T**	605.3002	303.1537	588.2736	294.6404	587.2896	294.1484	**6**
**34**	3719.5103	1860.2588	3702.4838	1851.7455	3701.4998	1851.2535	**G**	**504.2525**	252.6299	**487.2259**	244.1166	486.2419	243.6246	**5**
**35**	3806.5423	1903.7748	3789.5158	1895.2615	3788.5318	1894.7695	**S**	447.2310	224.1191	430.2045	215.6059	429.2205	215.1139	**4**
**36**	3920.5853	1960.7963	3903.5587	1952.2830	3902.5747	1951.7910	**N**	360.1990	180.6031	343.1724	172.0899			**3**
**37**	3991.6224	**1996.3148**	3974.5958	1987.8016	3973.6118	1987.3096	**A**	246.1561	123.5817	229.1295	115.0684			**2**
**38**							**R**	175.1190	88.0631	158.0924	79.5498			**1**

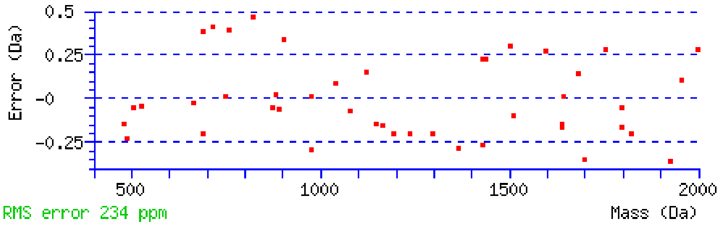

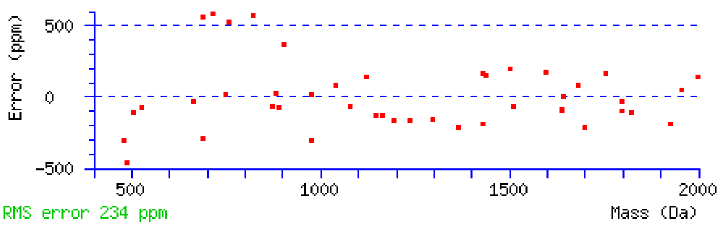

All matches to this query

Score	Mr(calc):	Delta	Sequence
72.3	4164.7268	2.0118	ALAGLVYNSSGMEPCFDIYQMYQSCADPTGCGTGSNAR
70.4	4165.7108	1.0278	ALAGLVYNSSGMEPCFDIYQMYQSCADPTGCGTGSNAR
33.4	4164.7268	2.0118	ALAGLVYNSSGMEPCFDIYQMYQSCADPTGCGTGSNAR
29.3	4164.7268	2.0118	ALAGLVYNSSGMEPCFDIYQMYQSCADPTGCGTGSNAR
27.1	4165.7108	1.0278	ALAGLVYNSSGMEPCFDIYQMYQSCADPTGCGTGSNAR
8.2	4164.7268	2.0118	ALAGLVYNSSGMEPCFDIYQMYQSCADPTGCGTGSNAR
7.2	4164.7027	2.0359	ALSTSGEDTLSDSDDEDDEEEDEEEEIDVVTVEKRR
4.7	4166.7317	0.0068	EQLVYSLWNMEKGFEVTYMTEPIDEYCTQQFK
4.0	4165.7305	1.0081	FPTCPLIPVFLGSEVLSECRSADGAVHTVERSCR
2.5	4166.7240	0.0146	CPQPASIGSDEVTGPCETEESIDVSAQGSDSSTTKMSSR

Spectrum No: 202; Query: 71; Rank: 1

Peptide View

MS/MS Fragmentation of **ISSNETEIMR**
Found in **IPI00370427**, Tax_Id=10116 Gene_Symbol=Tinag Tubulointerstitial nephritis antigen

Match to Query 71: 1179.546148 from(590.780350,2+)
Title: 091127RatKid_SCX01_13.1010.1010.2.dta
Data file K:\NewmanPaper\Piliang\3SubProteomes\Piliang3SP\mgf5ppm\SCX_3SubProteomes5ppm.mgf

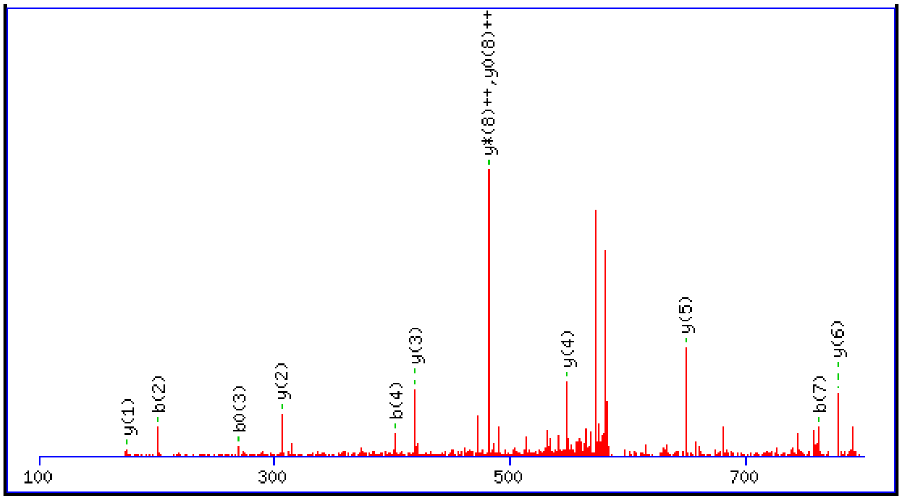

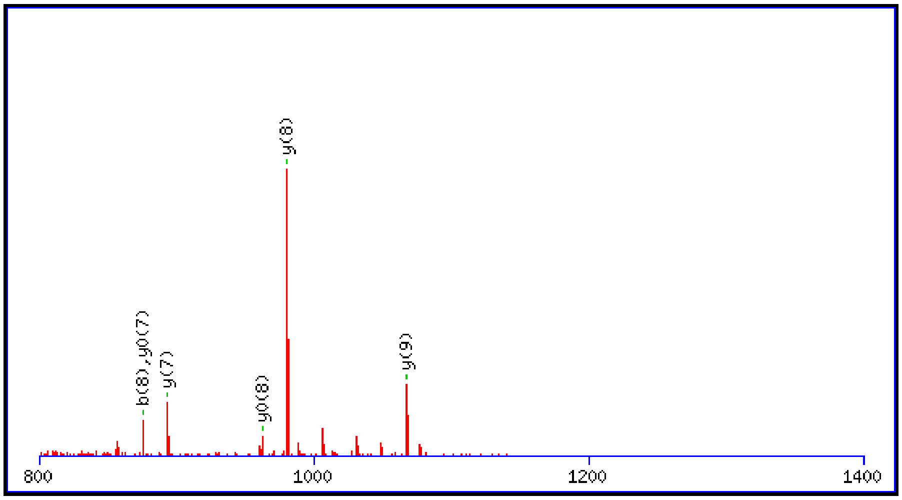

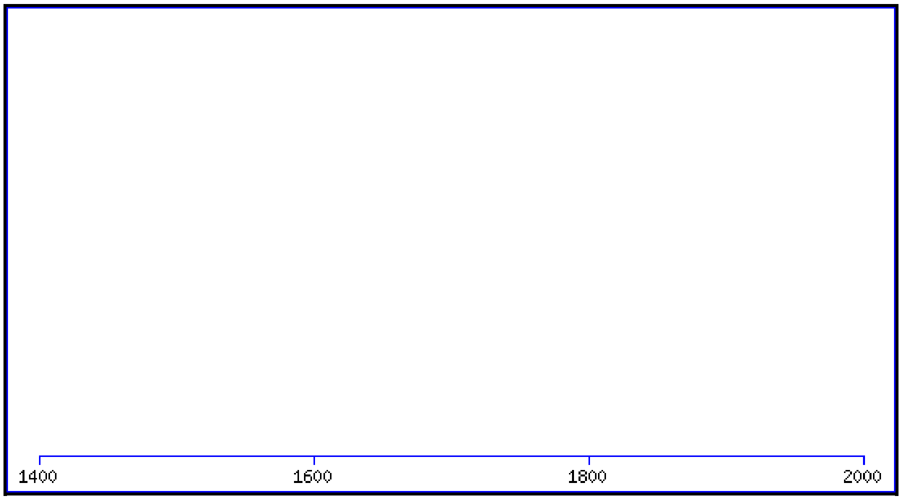

Monoisotopic mass of neutral peptide Mr(calc): 1179.5441
Fixed modifications: Carbamidomethyl (C)
Variable modifications:
N4 : Deamidated_N (N)
Ions Score: 72 **Expect:** 6.9e-006
Matches (Bold Red): 18/94 fragment ions using 32 most intense peaks

#	b	b^{++}	b*	b^{*++}	b^{0}	b^{0++}	Seq.	y	y^{++}	y*	y^{*++}	y^{0}	y^{0++}	#
1	114.0913	57.5493					I							10
2	**201.1234**	101.0653			183.1128	92.0600	S	**1067.4674**	534.2373	1050.4408	525.7240	1049.4568	525.2320	9
3	288.1554	144.5813			**270.1448**	135.5761	S	**980.4353**	490.7213	963.4088	**482.2080**	**962.4248**	**481.7160**	8
4	**403.1823**	202.0948	386.1558	193.5815	385.1718	193.0895	N	**893.4033**	447.2053	876.3768	438.6920	**875.3927**	438.2000	7
5	532.2249	266.6161	515.1984	258.1028	514.2144	257.6108	E	**778.3764**	389.6918	761.3498	381.1785	760.3658	380.6865	6

6	633.2726	317.1399	616.2461	308.6267	615.2620	308.1347	T	649.3338	325.1705	632.3072	316.6573	631.3232	316.1652	5
7	762.3152	381.6612	745.2886	373.1480	744.3046	372.6560	E	548.2861	274.6467	531.2595	266.1334	530.2755	265.6414	4
8	875.3993	438.2033	858.3727	429.6900	857.3887	429.1980	I	419.2435	210.1254	402.2170	201.6121			3
9	1006.4397	503.7235	989.4132	495.2102	988.4292	494.7182	M	306.1594	153.5834	289.1329	145.0701			2
10							R	175.1190	88.0631	158.0924	79.5498			1

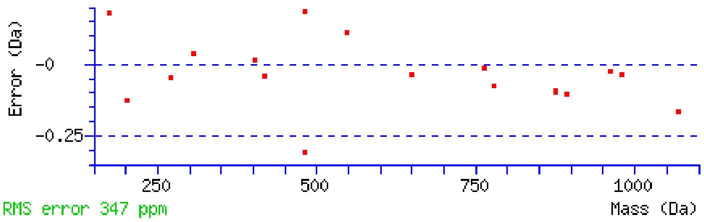

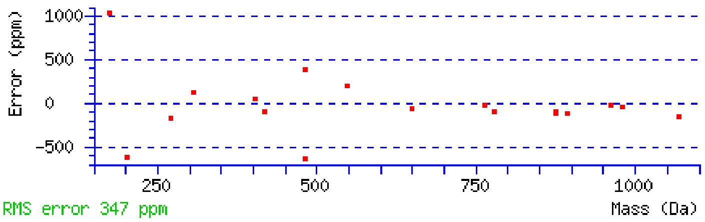

All matches to this query

Score	Mr(calc):	Delta	Sequence
72.3	1179.5441	0.0020	ISSNETEIMR
12.8	1179.5520	-0.0059	TVPSYNQTNR
12.7	1179.5560	-0.0099	LWGLYEEDR
8.7	1179.5537	-0.0075	LSPTPLGNSSK
8.3	1179.5554	-0.0092	ISSGAKDMAER
7.1	1179.5520	-0.0059	AFSDQNTLQR
7.1	1177.5471	1.9990	MAPTEMVLDR
6.3	1179.5536	-0.0075	LPLSSEGELR
5.9	1179.5520	-0.0059	TVPSYNQTNR
4.3	1178.5332	1.0129	KASPDPELSR

Spectrum No: 203; Query: 192; Rank: 1

Peptide View

MS/MS Fragmentation of **SAVFTVENGSTIR**
Found in **IPI00214373**, Tax_Id=10116 Gene_Symbol=Aadat Kynurenine/alpha-aminoadipate aminotransferase mitochondrial precursor

Match to Query 192: 1380.689228 from(691.351890,2+)
Title: 091129RatKid_SCX02_21.1728.1728.2.dta
Data file K:\NewmanPaper\Piliang\3SubProteomes\Piliang3SP\mgf5ppm\SCX_3SubProteomes5ppm.mgf

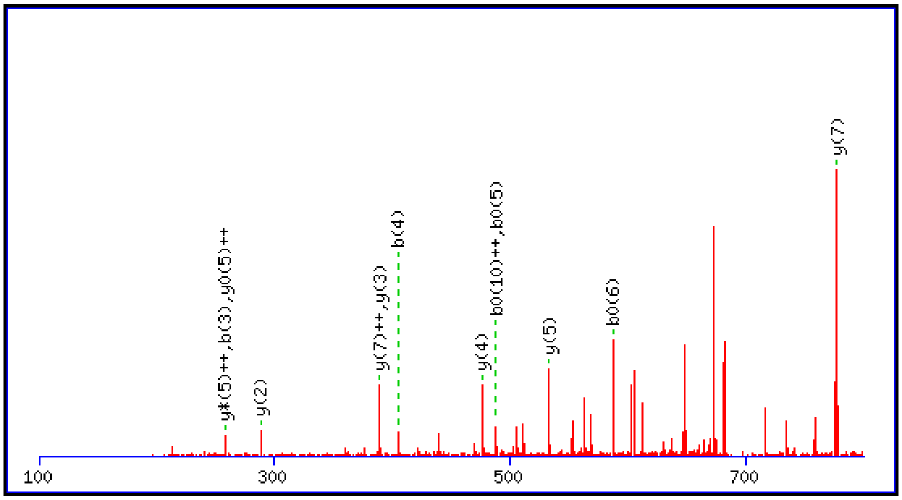

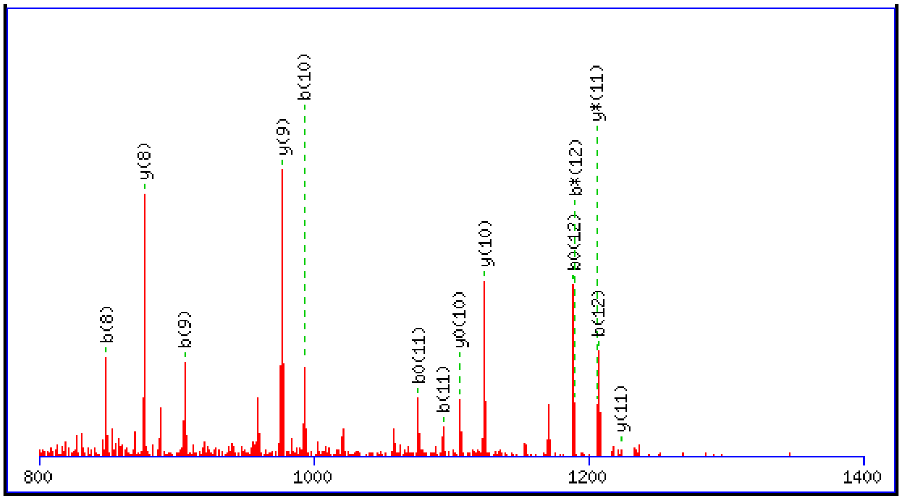

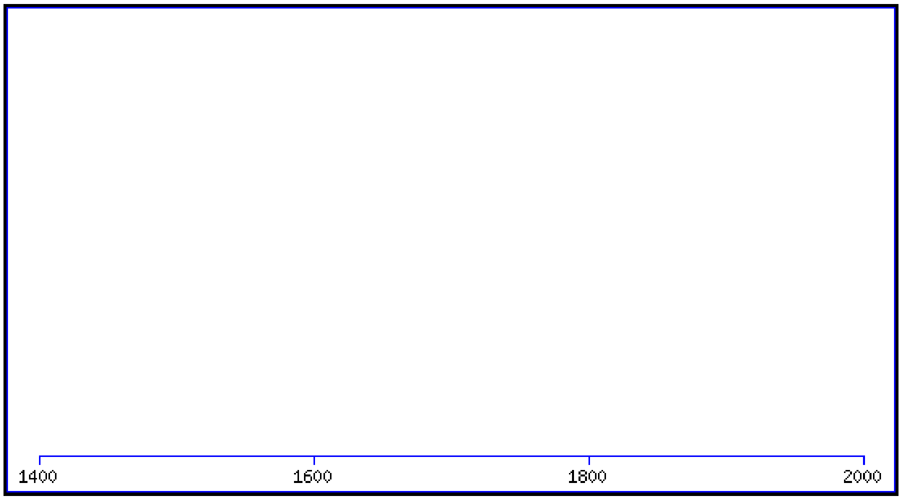

Monoisotopic mass of neutral peptide Mr(calc): 1380.6885
Fixed modifications: Carbamidomethyl (C)
Variable modifications:
N8 : Deamidated_N (N)
Ions Score: 72 **Expect:** 8.9e-006
Matches (Bold Red): 27/126 fragment ions using 50 most intense peaks

#	b	b^{++}	b*	b^{*++}	b^0	b^{0++}	Seq.	y	y^{++}	y*	y^{*++}	y^0	y^{0++}	#
1	88.0393	44.5233			70.0287	35.5180	S							13
2	159.0764	80.0418			141.0659	71.0366	A	1294.6638	647.8355	1277.6372	639.3222	1276.6532	638.8302	12
3	**258.1448**	129.5761			240.1343	120.5708	V	**1223.6266**	612.3170	**1206.6001**	603.8037	1205.6161	603.3117	11
4	**405.2132**	203.1103			387.2027	194.1050	F	**1124.5582**	562.7827	1107.5317	554.2695	**1106.5477**	553.7775	10
5	506.2609	253.6341			**488.2504**	244.6288	T	**977.4898**	489.2485	960.4633	480.7353	959.4792	480.2433	9
6	605.3293	303.1683			**587.3188**	294.1630	V	**876.4421**	438.7247	859.4156	430.2114	858.4316	429.7194	8
7	734.3719	367.6896			716.3614	358.6843	E	**777.3737**	**389.1905**	760.3472	380.6772	759.3632	380.1852	7
8	**849.3989**	425.2031	832.3723	416.6898	831.3883	416.1978	N	648.3311	324.6692	631.3046	316.1559	630.3206	315.6639	6
9	**906.4203**	453.7138	889.3938	445.2005	888.4098	444.7085	G	**533.3042**	267.1557	516.2776	**258.6425**	515.2936	**258.1504**	5
10	**993.4524**	497.2298	976.4258	488.7165	975.4418	**488.2245**	S	**476.2827**	238.6450	459.2562	230.1317	458.2722	229.6397	4
11	**1094.5000**	547.7537	1077.4735	539.2404	**1076.4895**	538.7484	T	**389.2507**	195.1290	372.2241	186.6157	371.2401	186.1237	3
12	**1207.5841**	604.2957	**1190.5576**	595.7824	**1189.5735**	595.2904	I	**288.2030**	144.6051	271.1765	136.0919			2
13							R	175.1190	88.0631	158.0924	79.5498			1

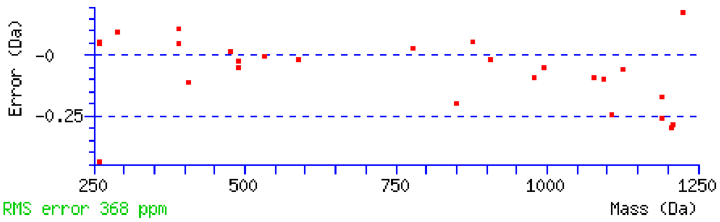

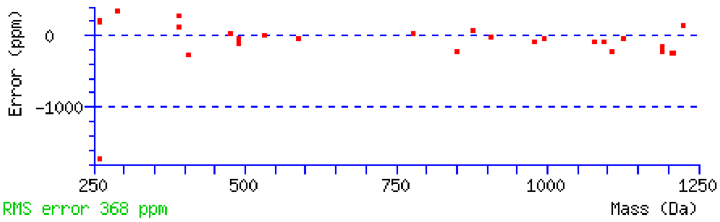

All matches to this query

Score	Mr(calc):	Delta	Sequence
72.2	1380.6885	0.0007	SAVFTVENGSTIR
17.1	1380.6820	0.0073	RTFKEGMDELR
13.0	1379.6980	0.9912	GVTFAGDVGRMVR
12.6	1380.6997	-0.0105	EKFQSRTSEAAK
8.5	1380.6867	0.0026	QERHMIMHKR
7.1	1380.6915	-0.0022	TGRLPEAAFLAR
6.8	1380.6820	0.0073	DNSFRICVLTR
6.5	1379.6793	1.0099	LLEVXHAEADGGR
6.2	1379.6980	0.9913	EQMAISGGFIRR
6.0	1379.6753	1.0139	SRSGTSSGAGGSITR

Spectrum No: 204; Query: 273; Rank: 1

Peptide View

MS/MS Fragmentation of **EVMSLLQPLNITK**
Found in **IPI00388265**, Tax_Id=10116 Gene_Symbol=Pgcp 48 kDa protein

Match to Query 273: 1485.813968 from(743.914260,2+)
Title: 091127RatKid_SCX01_17.4096.4096.2.dta
Data file K:\NewmanPaper\Piliang\3SubProteomes\Piliang3SP\mgf5ppm\SCX_3SubProteomes5ppm.mgf

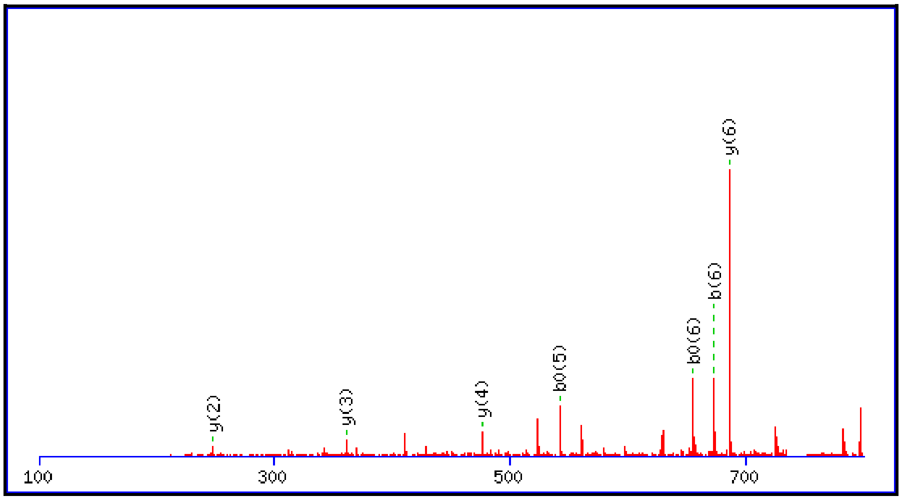

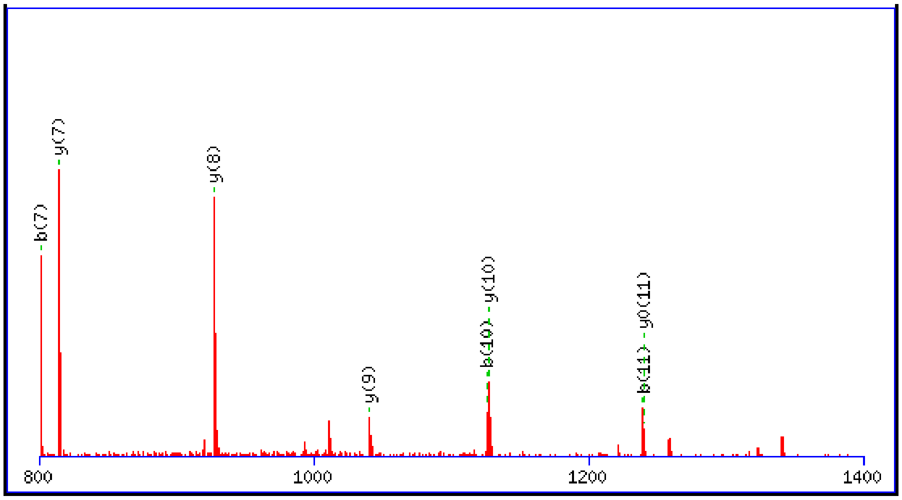

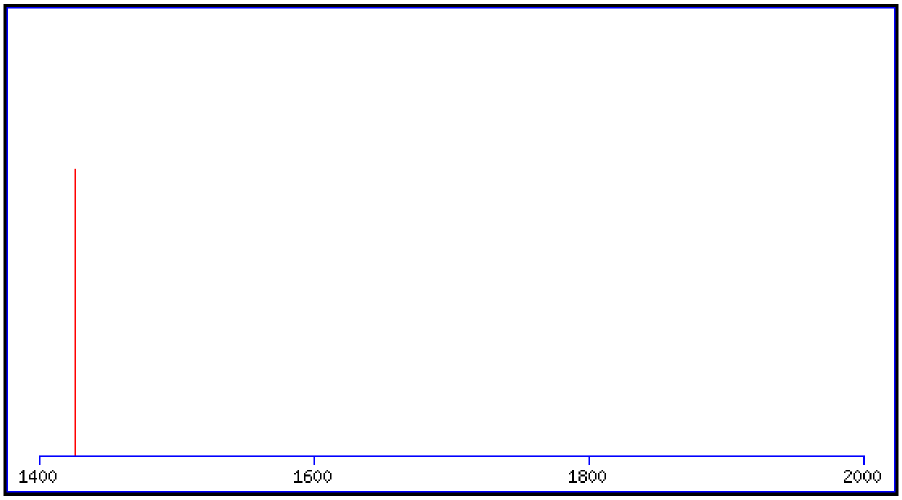

Monoisotopic mass of neutral peptide Mr(calc): 1485.8112
Fixed modifications: Carbamidomethyl (C)
Variable modifications:
N10 : Deamidated_N (N)
Ions Score: 72 **Expect:** 4.7e-006
Matches (Bold Red): 15/130 fragment ions using 21 most intense peaks

#	b	b^{++}	b*	b*$^{++}$	b^{0}	b^{0++}	Seq.	y	y^{++}	y*	y*$^{++}$	y^{0}	y^{0++}	#
1	130.0499	65.5286			112.0393	56.5233	E							13
2	229.1183	115.0628			211.1077	106.0575	V	1357.7759	679.3916	1340.7494	670.8783	1339.7654	670.3863	12
3	360.1588	180.5830			342.1482	171.5777	M	1258.7075	629.8574	1241.6810	621.3441	**1240.6970**	620.8521	11
4	447.1908	224.0990			429.1802	215.0938	S	**1127.6670**	564.3372	1110.6405	555.8239	1109.6565	555.3319	10
5	560.2749	280.6411			**542.2643**	271.6358	L	**1040.6350**	520.8211	1023.6085	512.3079	1022.6245	511.8159	9
6	**673.3589**	337.1831			**655.3484**	328.1778	L	**927.5510**	464.2791	910.5244	455.7658	909.5404	455.2738	8
7	**801.4175**	401.2124	784.3910	392.6991	783.4069	392.2071	Q	**814.4669**	407.7371	797.4403	399.2238	796.4563	398.7318	7
8	898.4703	449.7388	881.4437	441.2255	880.4597	440.7335	P	**686.4083**	343.7078	669.3818	335.1945	668.3977	334.7025	6
9	1011.5543	506.2808	994.5278	497.7675	993.5438	497.2755	L	589.3555	295.1814	572.3290	286.6681	571.3450	286.1761	5
10	**1126.5813**	563.7943	1109.5547	555.2810	1108.5707	554.7890	N	**476.2715**	238.6394	459.2449	230.1261	458.2609	229.6341	4
11	**1239.6653**	620.3363	1222.6388	611.8230	1221.6548	611.3310	I	**361.2445**	181.1259	344.2180	172.6126	343.2340	172.1206	3
12	1340.7130	670.8601	1323.6865	662.3469	1322.7024	661.8549	T	**248.1605**	124.5839	231.1339	116.0706	230.1499	115.5786	2
13							K	147.1128	74.0600	130.0863	65.5468			1

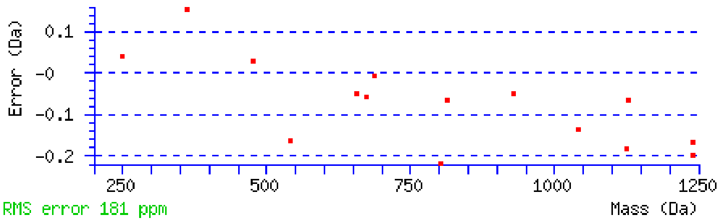

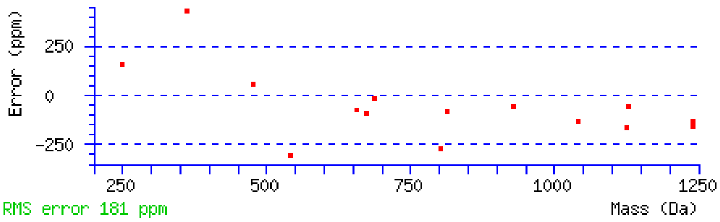

All matches to this query

Score	Mr(calc):	Delta	Sequence
72.2	1485.8112	0.0027	EVMSLLQPLNITK
17.2	1485.8151	-0.0011	DILSRTKEGVGSPK
14.5	1483.8085	2.0055	MLKSKLNVLTLK
11.0	1485.8191	-0.0051	EFNPTKPLLSVNK
8.7	1485.8112	0.0027	DLLSKMLVIDPDK
7.9	1483.8035	2.0105	INSNKSVAFLYTK
6.9	1485.8052	0.0088	YPANRAKGKPDAAK
6.3	1484.8239	0.9901	NPLNVEPYSLIVK
6.2	1485.8224	-0.0085	VEMALEALRNVIK
5.8	1485.8191	-0.0051	VKWGAEKETPTLK

Spectrum No: 205; Query: 224; Rank: 1

Peptide View

MS/MS Fragmentation of **AIPTLVSDGNITVK**
Found in **IPI00212933**, Tax_Id=10116 Gene_Symbol=Slc5a1 Sodium/glucose cotransporter 1

Match to Query 224: 1427.787448 from(714.901000,2+)
Title: 091129RatKid_SCX02_02.2344.2344.2.dta
Data file K:\NewmanPaper\Piliang\3SubProteomes\Piliang3SP\mgf5ppm\SCX_3SubProteomes5ppm.mgf

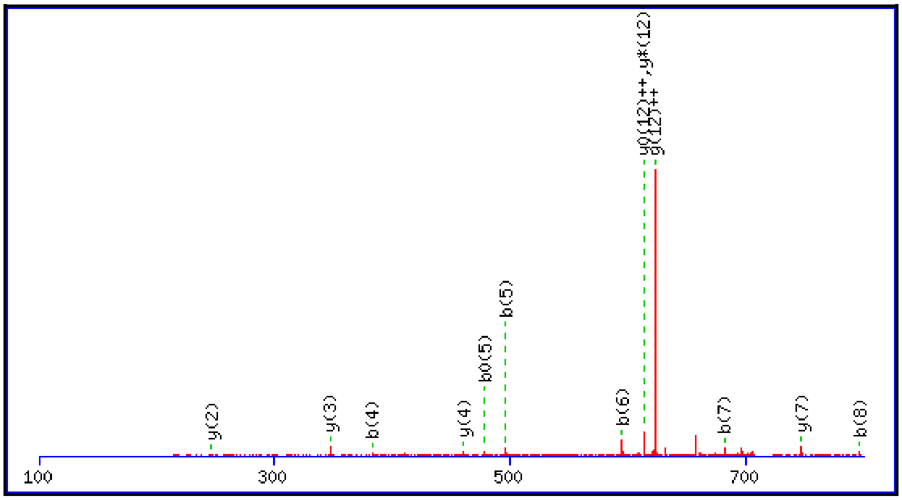

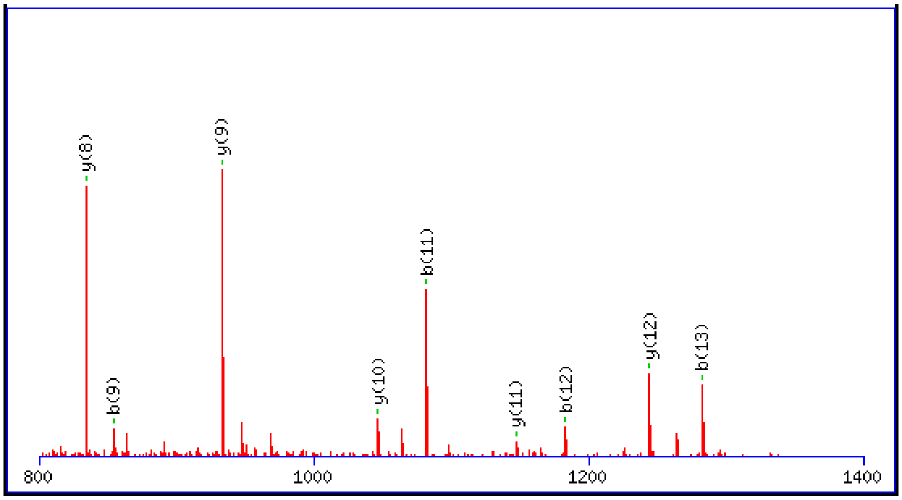

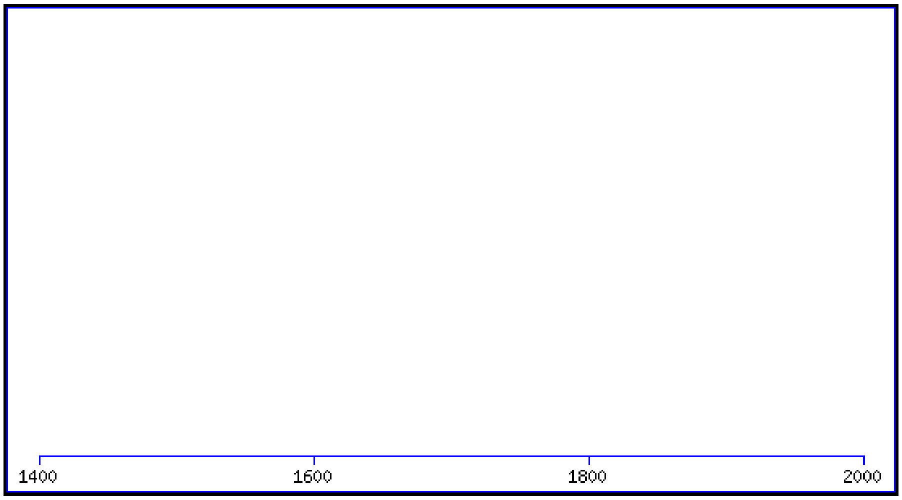

Monoisotopic mass of neutral peptide Mr(calc): 1427.7872
Fixed modifications: Carbamidomethyl (C)
Variable modifications:
N10 : Deamidated_N (N)
Ions Score: 72 **Expect:** 4.9e-006
Matches (Bold Red): 23/128 fragment ions using 41 most intense peaks

#	b	b++	b*	b*++	b0	b0++	Seq.	y	y++	y*	y*++	y0	y0++	#
1	72.0444	36.5258					A							14
2	185.1285	93.0679					I	1357.7573	679.3823	1340.7308	670.8690	1339.7468	670.3770	13
3	282.1812	141.5942					P	**1244.6733**	**622.8403**	1227.6467	**614.3270**	1226.6627	**613.8350**	12
4	**383.2289**	192.1181			365.2183	183.1128	T	**1147.6205**	574.3139	1130.5939	565.8006	1129.6099	565.3086	11
5	**496.3130**	248.6601			**478.3024**	239.6548	L	**1046.5728**	523.7900	1029.5463	515.2768	1028.5622	514.7848	10
6	**595.3814**	298.1943			577.3708	289.1890	V	**933.4887**	467.2480	916.4622	458.7347	915.4782	458.2427	9
7	**682.4134**	341.7103			664.4028	332.7051	S	**834.4203**	417.7138	817.3938	409.2005	816.4098	408.7085	8
8	**797.4403**	399.2238			779.4298	390.2185	D	**747.3883**	374.1978	730.3618	365.6845	729.3777	365.1925	7
9	**854.4618**	427.7345			836.4512	418.7293	G	632.3614	316.6843	615.3348	308.1710	**614.3508**	307.6790	6
10	969.4887	485.2480	952.4622	476.7347	951.4782	476.2427	N	575.3399	288.1736	558.3133	279.6603	557.3293	279.1683	5
11	**1082.5728**	541.7900	1065.5463	533.2768	1064.5622	532.7848	I	**460.3130**	230.6601	443.2864	222.1468	442.3024	221.6548	4
12	**1183.6205**	592.3139	1166.5939	583.8006	1165.6099	583.3086	T	**347.2289**	174.1181	330.2023	165.6048	329.2183	165.1128	3
13	**1282.6889**	641.8481	1265.6624	633.3348	1264.6783	632.8428	V	**246.1812**	123.5942	229.1547	115.0810			2
14							K	147.1128	74.0600	130.0863	65.5468			1

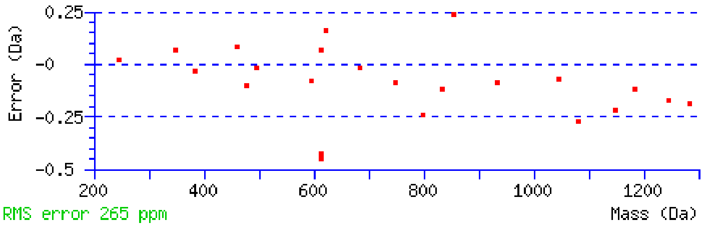

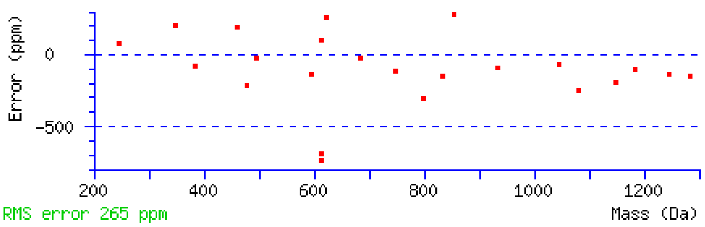

All matches to this query

Score	Mr(calc):	Delta	Sequence
72.0	1427.7872	0.0003	AIPTLVSDGNITVK
9.4	1426.7836	1.0038	KVPTIILSVSYK
6.8	1427.7959	-0.0084	VLKELGFCVHVK
6.2	1425.7827	2.0047	TVIEYKTTKTSR
3.5	1426.7836	1.0038	KVPTIILSVSYK
2.4	1427.7844	0.0030	ALRTENRNLLAR
2.4	1427.7806	0.0069	LAEIMNRKEPVK
2.3	1425.7745	2.0130	LQLGTPPPLLAAR
2.1	1426.7780	1.0095	NVIAPKGQEGSISK
2.0	1425.7728	2.0146	SIAASRFAPHLEK

Spectrum No: 206; Query: 891; Rank: 1

Peptide View

MS/MS Fragmentation of **LDPPCTNTTAPSTYLNNPYVR**
Found in **IPI00464785**, Tax_Id=10116 Gene_Symbol=Ppgb Protective protein for beta-galactosidase

Match to Query 891: 2394.119608 from(1198.067080,2+)
Title: 091127RatKid_SCX01_16.3246.3246.2.dta
Data file K:\NewmanPaper\Piliang\3SubProteomes\Piliang3SP\mgf5ppm\SCX_3SubProteomes5ppm.mgf

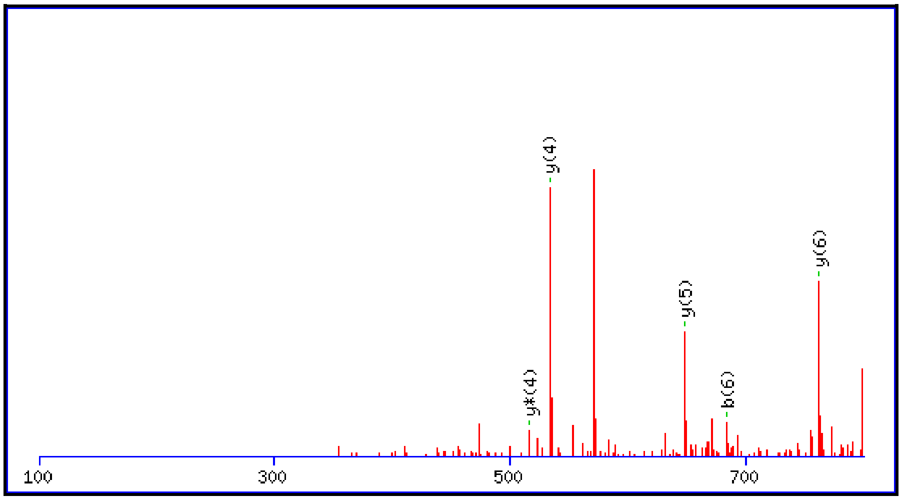

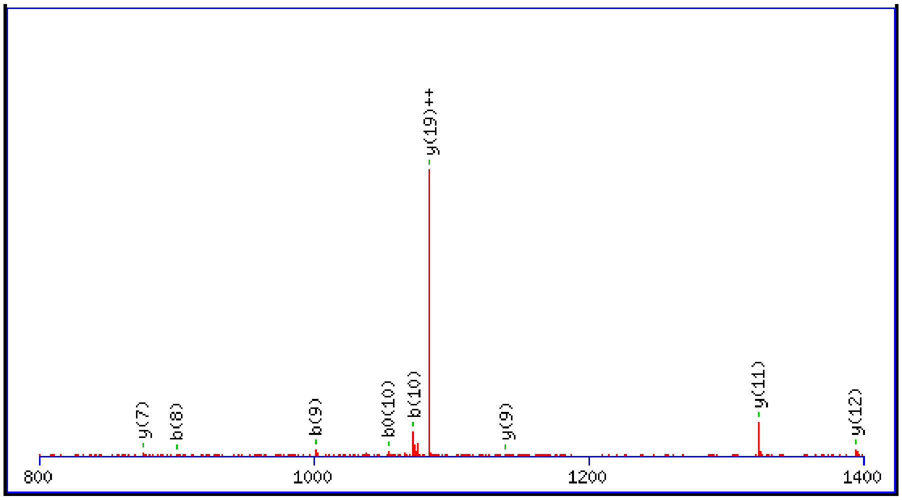

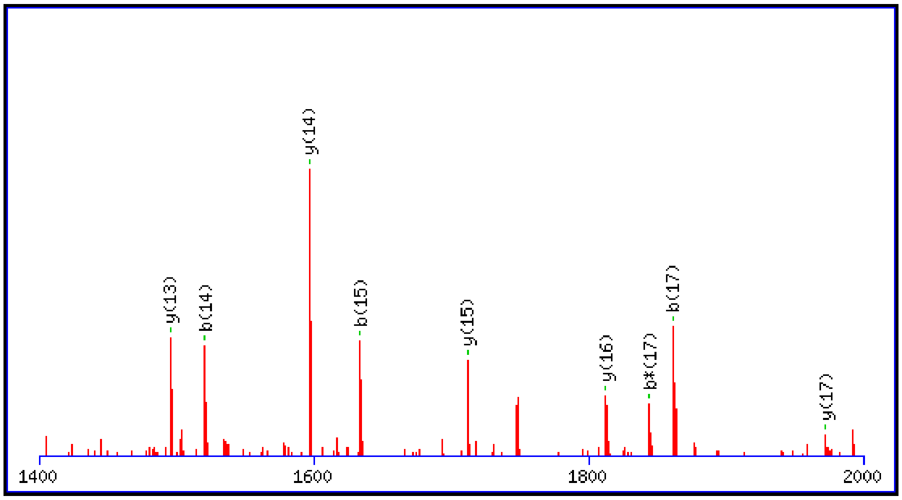

Monoisotopic mass of neutral peptide Mr(calc): 2394.1162
Fixed modifications: Carbamidomethyl (C)
Variable modifications:
N7 : Deamidated_N (N)
Ions Score: 72 **Expect:** 1.8e-005
Matches (Bold Red): 23/210 fragment ions using 41 most intense peaks

#	b	b^{++}	b*	b^{*++}	b^0	b^{0++}	Seq.	y	y^{++}	y*	y^{*++}	y^0	y^{0++}	#
1	114.0913	57.5493					L							21
2	229.1183	115.0628			211.1077	106.0575	D	2282.0394	1141.5233	2265.0128	1133.0101	2264.0288	1132.5180	20
3	326.1710	163.5892			308.1605	154.5839	P	2167.0124	**1084.0099**	2149.9859	1075.4966	2149.0019	1075.0046	19
4	423.2238	212.1155			405.2132	203.1103	P	2069.9597	1035.4835	2052.9331	1026.9702	2051.9491	1026.4782	18
5	583.2545	292.1309			565.2439	283.1256	C	**1972.9069**	986.9571	1955.8804	978.4438	1954.8963	977.9518	17
6	**684.3021**	342.6547			666.2916	333.6494	T	**1812.8763**	906.9418	1795.8497	898.4285	1794.8657	897.9365	16
7	799.3291	400.1682	782.3025	391.6549	781.3185	391.1629	N	**1711.8286**	856.4179	1694.8020	847.9047	1693.8180	847.4126	15
8	**900.3768**	450.6920	883.3502	442.1787	882.3662	441.6867	T	**1596.8016**	798.9045	1579.7751	790.3912	1578.7911	789.8992	14
9	**1001.4244**	501.2159	984.3979	492.7026	983.4139	492.2106	T	**1495.7540**	748.3806	1478.7274	739.8673	1477.7434	739.3753	13
10	**1072.4615**	536.7344	1055.4350	528.2211	**1054.4510**	527.7291	A	**1394.7063**	697.8568	1377.6797	689.3435	1376.6957	688.8515	12
11	1169.5143	585.2608	1152.4878	576.7475	1151.5037	576.2555	P	**1323.6692**	662.3382	1306.6426	653.8250	1305.6586	653.3329	11
12	1256.5463	628.7768	1239.5198	620.2635	1238.5358	619.7715	S	1226.6164	613.8118	1209.5899	605.2986	1208.6058	604.8066	10
13	1357.5940	679.3006	1340.5675	670.7874	1339.5835	670.2954	T	**1139.5844**	570.2958	1122.5578	561.7826	1121.5738	561.2905	9
14	**1520.6573**	760.8323	1503.6308	752.3190	1502.6468	751.8270	Y	1038.5367	519.7720	1021.5102	511.2587			8
15	**1633.7414**	817.3743	1616.7149	808.8611	1615.7308	808.3691	L	**875.4734**	438.2403	858.4468	429.7271			7
16	1747.7843	874.3958	1730.7578	865.8825	1729.7738	865.3905	N	**762.3893**	381.6983	745.3628	373.1850			6
17	**1861.8273**	931.4173	**1844.8007**	922.9040	1843.8167	922.4120	N	**648.3464**	324.6768	631.3198	316.1636			5
18	1958.8800	979.9437	1941.8535	971.4304	1940.8695	970.9384	P	**534.3035**	267.6554	**517.2769**	259.1421			4

**19**	2121.9434	1061.4753	2104.9168	1052.9620	2103.9328	1052.4700	**Y**	437.2507	219.1290	420.2241	210.6157			**3**
**20**	2221.0118	1111.0095	2203.9852	1102.4962	2203.0012	1102.0042	**V**	274.1874	137.5973	257.1608	129.0840			**2**
**21**							**R**	175.1190	88.0631	158.0924	79.5498			**1**

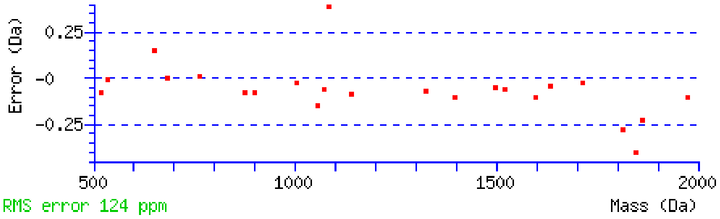

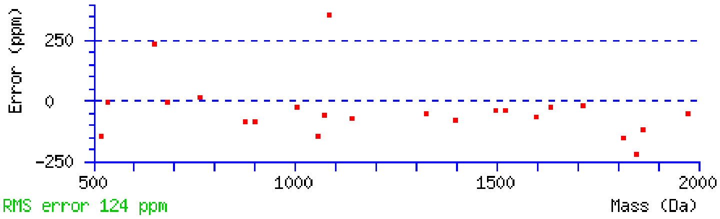

All matches to this query

Score	Mr(calc):	Delta	Sequence
71.9	2394.1162	0.0034	LDPPCTNTTAPSTYLNNPYVR
43.0	2394.1162	0.0034	LDPPCTNTTAPSTYLNNPYVR
31.0	2394.1162	0.0034	LDPPCTNTTAPSTYLNNPYVR
25.1	2393.1322	0.9875	LDPPCTNTTAPSTYLNNPYVR
5.6	2394.1104	0.0092	EFQENIIKQVEAHKEESQK
5.1	2393.1363	0.9833	VQLSSTAIVDVTVTDVNDSPPR
5.1	2393.1363	0.9833	VQLSSTAIVDVTVTDVNDSPPR
4.7	2394.1121	0.0075	AHETNLLIATSVVEEGVDIPK
4.7	2394.1121	0.0075	AHETNLLIATSVVEEGVDIPK
4.7	2394.1121	0.0075	AHETNLLIATSVVEEGVDIPK

Spectrum No: 207; Query: 757; Rank: 1

Peptide View

MS/MS Fragmentation of **SLTFNESYQDVSEIVYGAK**
Found in **IPI00372372**, Tax_Id=10116 Gene_Symbol=Serpinc1 Serine (Or cysteine) peptidase inhibitor, clade C (Antithrombin), member 1

Match to Query 757: 2150.009548 from(1076.012050,2+)
Title: 091127RatKid_SCX01_02.4738.4738.2.dta
Data file K:\NewmanPaper\Piliang\3SubProteomes\Piliang3SP\mgf5ppm\SCX_3SubProteomes5ppm.mgf

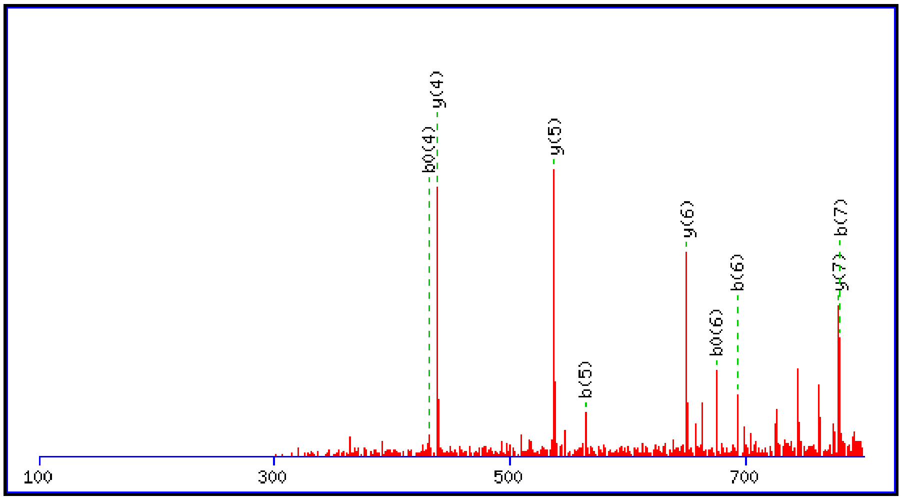

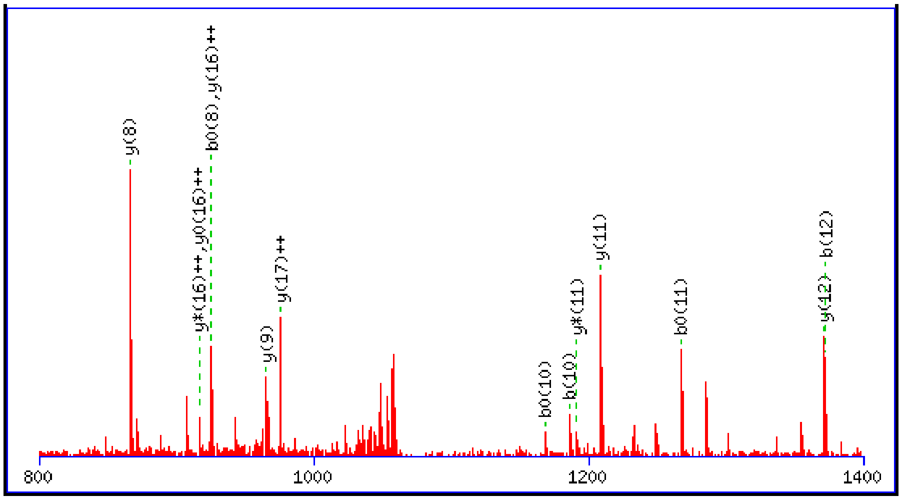

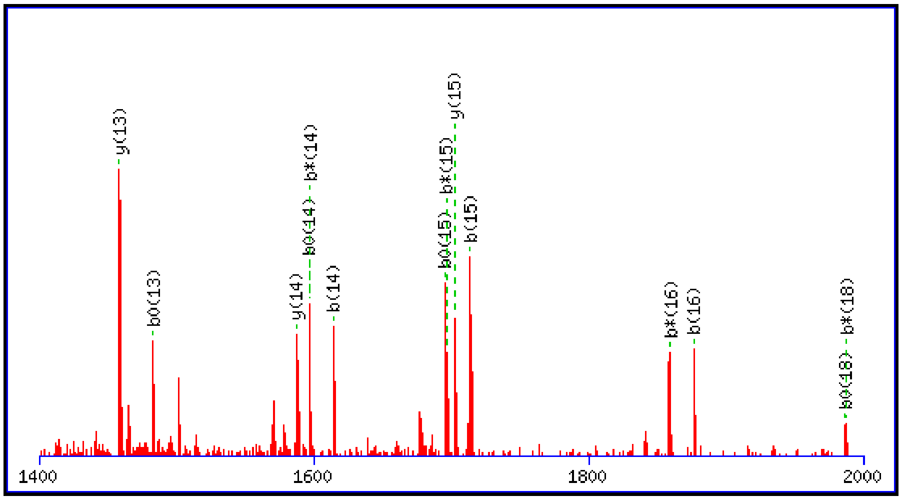

Monoisotopic mass of neutral peptide Mr(calc): 2150.0055
Fixed modifications: Carbamidomethyl (C)
Variable modifications:
N5 : Deamidated_N (N)
Ions Score: 72 **Expect:** 1.7e-005
Matches (Bold Red): 37/196 fragment ions using 55 most intense peaks

#	b	b^{++}	b*	b^{*++}	b^{0}	b^{0++}	Seq.	y	y^{++}	y*	y^{*++}	y^{0}	y^{0++}	#
1	88.0393	44.5233			70.0287	35.5180	S							19
2	201.1234	101.0653			183.1128	92.0600	L	2063.9808	1032.4940	2046.9542	1023.9808	2045.9702	1023.4887	18
3	302.1710	151.5892			284.1605	142.5839	T	1950.8967	**975.9520**	1933.8702	967.4387	1932.8862	966.9467	17
4	449.2395	225.1234			**431.2289**	216.1181	F	1849.8490	**925.4282**	1832.8225	**916.9149**	1831.8385	**916.4229**	16
5	**564.2664**	282.6368	547.2398	274.1236	546.2558	273.6316	N	**1702.7806**	851.8940	1685.7541	843.3807	1684.7701	842.8887	15
6	**693.3090**	347.1581	676.2824	338.6449	**675.2984**	338.1529	E	**1587.7537**	794.3805	1570.7271	785.8672	1569.7431	785.3752	14
7	**780.3410**	390.6741	763.3145	382.1609	762.3305	381.6689	S	**1458.7111**	729.8592	1441.6846	721.3459	1440.7005	720.8539	13
8	943.4043	472.2058	926.3778	463.6925	**925.3938**	463.2005	Y	**1371.6791**	686.3432	1354.6525	677.8299	1353.6685	677.3379	12
9	1071.4629	536.2351	1054.4364	527.7218	1053.4524	527.2298	Q	**1208.6157**	604.8115	**1191.5892**	596.2982	1190.6052	595.8062	11
10	**1186.4899**	593.7486	1169.4633	585.2353	**1168.4793**	584.7433	D	1080.5572	540.7822	1063.5306	532.2689	1062.5466	531.7769	10
11	1285.5583	643.2828	1268.5317	634.7695	**1267.5477**	634.2775	V	**965.5302**	483.2688	948.5037	474.7555	947.5197	474.2635	9
12	**1372.5903**	686.7988	1355.5638	678.2855	1354.5797	677.7935	S	**866.4618**	433.7345	849.4353	425.2213	848.4512	424.7293	8
13	1501.6329	751.3201	1484.6064	742.8068	**1483.6223**	742.3148	E	**779.4298**	390.2185	762.4032	381.7053	761.4192	381.2132	7
14	**1614.7170**	807.8621	**1597.6904**	799.3488	**1596.7064**	798.8568	I	**650.3872**	325.6972	633.3606	317.1840			6
15	**1713.7854**	857.3963	**1696.7588**	848.8831	**1695.7748**	848.3910	V	**537.3031**	269.1552	520.2766	260.6419			5
16	**1876.8487**	938.9280	**1859.8222**	930.4147	1858.8381	929.9227	Y	**438.2347**	219.6210	421.2082	211.1077			4
17	1933.8702	967.4387	1916.8436	958.9255	1915.8596	958.4334	G	275.1714	138.0893	258.1448	129.5761			3
18	2004.9073	1002.9573	**1987.8807**	994.4440	**1986.8967**	993.9520	A	218.1499	109.5786	201.1234	101.0653			2

19						K	147.1128	74.0600	130.0863	65.5468			1

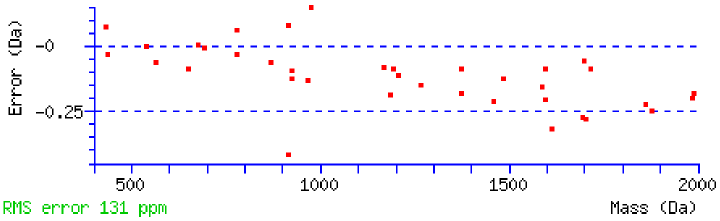

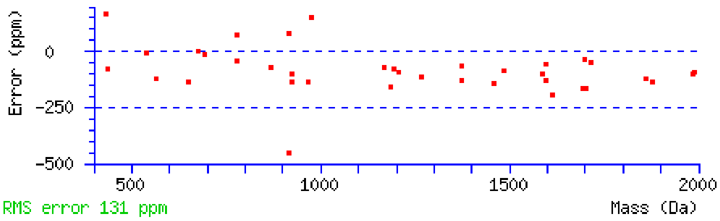

All matches to this query

Score	Mr(calc):	Delta	Sequence
71.8	2150.0055	0.0040	SLTFNESYQDVSEIVYGAK
46.4	2149.0215	0.9880	SLTFNESYQDVSEIVYGAK

Spectrum No: 208; Query: 204; Rank: 1

Peptide View

MS/MS Fragmentation of **SWPAVGNCTAALR**
Found in **IPI00195516**, Tax_Id=10116 Gene_Symbol=Hpx Hemopexin precursor

Match to Query 204: 1402.668308 from(702.341430,2+)
Title: 091127RatKid_SCX01_10.2264.2264.2.dta
Data file K:\NewmanPaper\Piliang\3SubProteomes\Piliang3SP\mgf5ppm\SCX_3SubProteomes5ppm.mgf

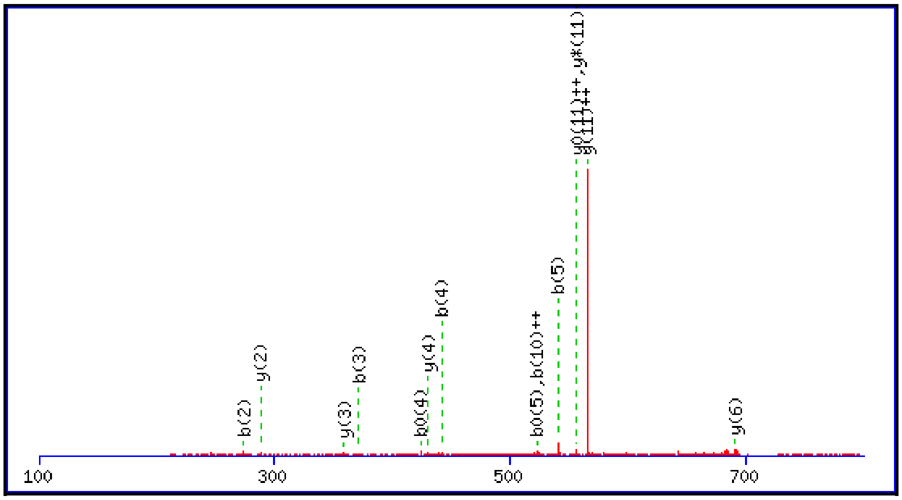

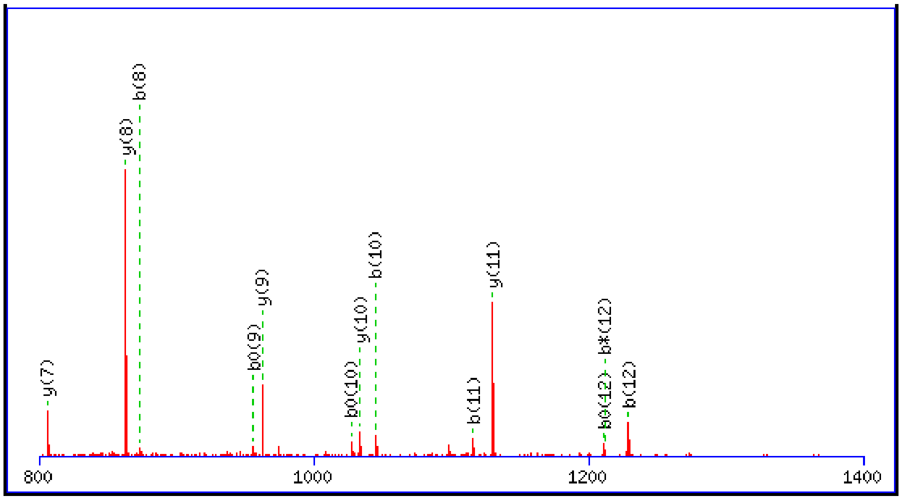

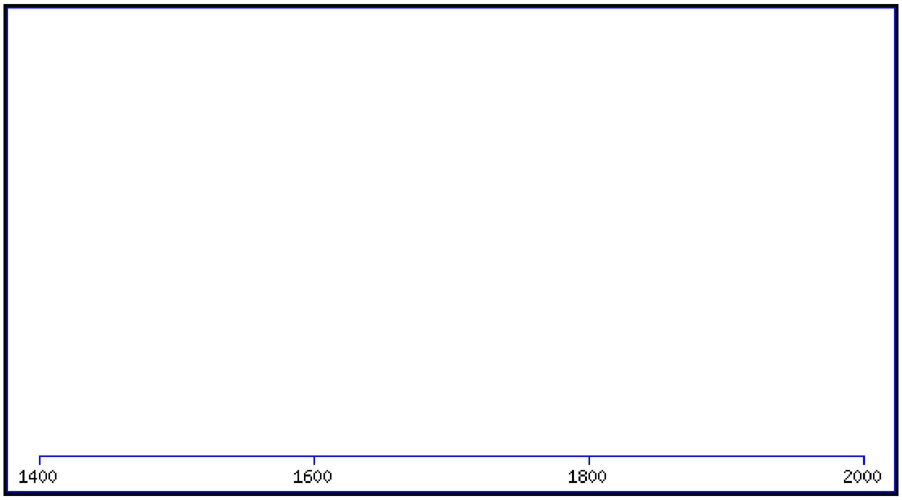

Monoisotopic mass of neutral peptide Mr(calc): 1402.6663
Fixed modifications: Carbamidomethyl (C)
Variable modifications:
N7 : Deamidated_N (N)
Ions Score: 72 **Expect:** 9.8e-006
Matches (Bold Red): 27/124 fragment ions using 46 most intense peaks

#	b	b^{++}	b*	b^{*++}	b^0	b^{0++}	Seq.	y	y^{++}	y*	y^{*++}	y^0	y^{0++}	#
1	88.0393	44.5233			70.0287	35.5180	S							13
2	**274.1186**	137.5629			256.1081	128.5577	W	1316.6416	658.8244	1299.6150	650.3112	1298.6310	649.8191	12
3	**371.1714**	186.0893			353.1608	177.0840	P	**1130.5623**	**565.7848**	1113.5357	**557.2715**	1112.5517	**556.7795**	11
4	**442.2085**	221.6079			**424.1979**	212.6026	A	**1033.5095**	517.2584	1016.4830	508.7451	1015.4989	508.2531	10
5	**541.2769**	271.1421			**523.2663**	262.1368	V	**962.4724**	481.7398	945.4458	473.2266	944.4618	472.7345	9
6	598.2984	299.6528			580.2878	290.6475	G	**863.4040**	432.2056	846.3774	423.6923	845.3934	423.2003	8
7	713.3253	357.1663	696.2988	348.6530	695.3147	348.1610	N	**806.3825**	403.6949	789.3560	395.1816	788.3719	394.6896	7
8	**873.3560**	437.1816	856.3294	428.6683	855.3454	428.1763	C	**691.3556**	346.1814	674.3290	337.6681	673.3450	337.1761	6
9	974.4036	487.7055	957.3771	479.1922	**956.3931**	478.7002	T	531.3249	266.1661	514.2984	257.6528	513.3144	257.1608	5
10	**1045.4408**	**523.2240**	1028.4142	514.7107	**1027.4302**	514.2187	A	**430.2772**	215.6423	413.2507	207.1290			4
11	**1116.4779**	558.7426	1099.4513	550.2293	1098.4673	549.7373	A	**359.2401**	180.1237	342.2136	171.6104			3
12	**1229.5619**	615.2846	**1212.5354**	606.7713	**1211.5514**	606.2793	L	**288.2030**	144.6051	271.1765	136.0919			2
13							R	175.1190	88.0631	158.0924	79.5498			1

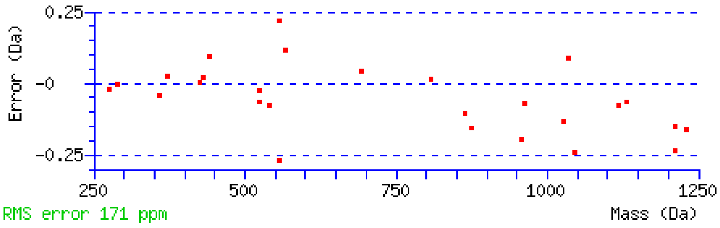

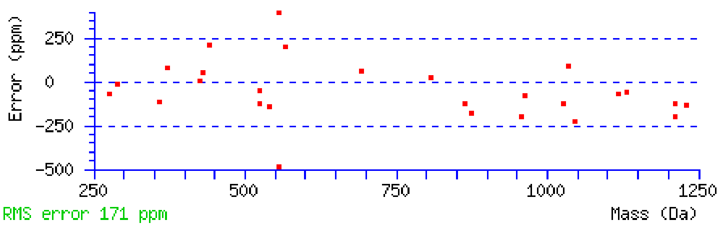

All matches to this query

Score	Mr(calc):	Delta	Sequence
71.8	1402.6663	0.0020	SWPAVGNCTAALR
13.3	1402.6688	-0.0005	SGENAANIASELAR
13.3	1401.6766	0.9917	GGATERPLTPAPR
13.3	1401.6766	0.9917	GGATERPLTPAPR
13.0	1401.6766	0.9917	QLQASPGIGAGAPR
11.9	1400.6523	2.0160	XIDSLIGPPPPR
10.8	1402.6792	-0.0109	VMLGNFKSRVR
10.7	1401.6765	0.9918	ANYLQKSRSVR
9.3	1402.6688	-0.0005	SGENAANIASELAR
9.0	1401.6653	1.0030	SGKQYSIEAALR

Spectrum No: 209; Query: 1118; Rank: 1

Peptide View

MS/MS Fragmentation of **RGVDVSQVAWQSQGDTPCSCCIVNNSNGSR**
Found in **IPI00211989**, Tax_Id=10116 Gene_Symbol=Khk Isoform C of Ketohexokinase

Match to Query 1118: 3338.455482 from(1113.825770,3+)
Title: 091129RatKid_SCX02_29.2276.2276.3.dta
Data file K:\NewmanPaper\Piliang\3SubProteomes\Piliang3SP\mgf5ppm\SCX_3SubProteomes5ppm.mgf

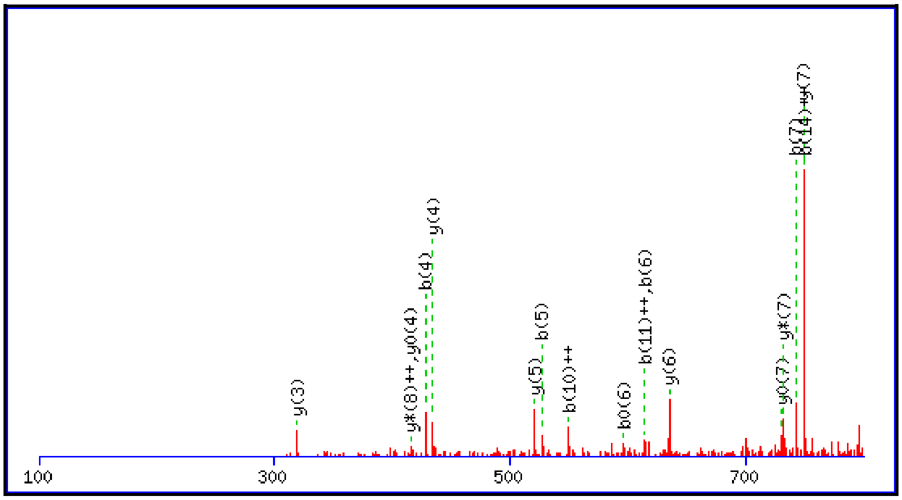

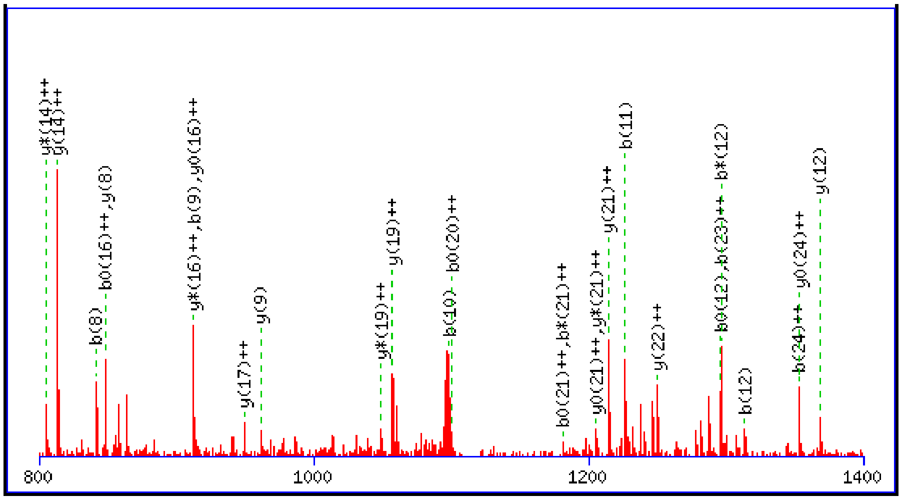

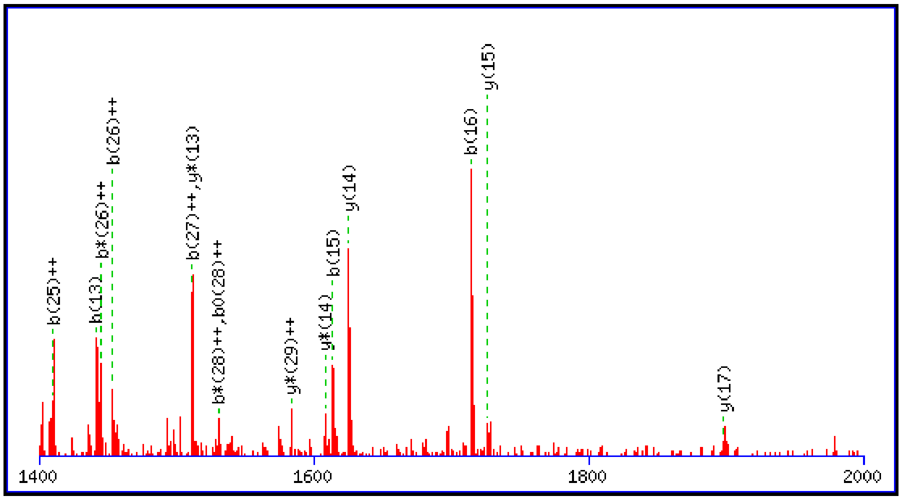

Monoisotopic mass of neutral peptide Mr(calc): 3338.4463
Fixed modifications: Carbamidomethyl (C)
Variable modifications:
N27 : Deamidated_N (N)
Ions Score: 72 **Expect:** 3.1e-005
Matches (Bold Red): 60/340 fragment ions using 93 most intense peaks

#	b	b^{++}	b*	b^{*++}	b^0	b^{0++}	Seq.	y	y^{++}	y*	y^{*++}	y^0	y^{0++}	#
1	157.1084	79.0578	140.0818	70.5446			R							30
2	214.1299	107.5686	197.1033	99.0553			G	3183.3524	1592.1799	3166.3259	**1583.6666**	3165.3419	1583.1746	29
3	313.1983	157.1028	296.1717	148.5895			V	3126.3310	1563.6691	3109.3044	1555.1558	3108.3204	1554.6638	28
4	**428.2252**	214.6162	411.1987	206.1030	410.2146	205.6110	D	3027.2626	1514.1349	3010.2360	1505.6216	3009.2520	1505.1296	27
5	**527.2936**	264.1504	510.2671	255.6372	509.2831	255.1452	V	2912.2356	1456.6214	2895.2091	1448.1082	2894.2250	1447.6162	26
6	**614.3257**	307.6665	597.2991	299.1532	**596.3151**	298.6612	S	2813.1672	1407.0872	2796.1406	1398.5740	2795.1566	1398.0820	25
7	**742.3842**	371.6958	725.3577	363.1825	724.3737	362.6905	Q	2726.1352	1363.5712	2709.1086	1355.0579	2708.1246	**1354.5659**	24
8	**841.4526**	421.2300	824.4261	412.7167	823.4421	412.2247	V	2598.0766	1299.5419	2581.0500	1291.0287	2580.0660	1290.5367	23
9	**912.4898**	456.7485	895.4632	448.2352	894.4792	447.7432	A	2499.0082	**1250.0077**	2481.9816	1241.4945	2480.9976	1241.0024	22
10	**1098.5691**	**549.7882**	1081.5425	541.2749	1080.5585	540.7829	W	2427.9711	**1214.4892**	2410.9445	**1205.9759**	2409.9605	**1205.4839**	21
11	**1226.6276**	**613.8175**	1209.6011	605.3042	1208.6171	604.8122	Q	2241.8917	1121.4495	2224.8652	1112.9362	2223.8812	1112.4442	20
12	**1313.6597**	657.3335	**1296.6331**	648.8202	**1295.6491**	648.3282	S	2113.8332	**1057.4202**	2096.8066	**1048.9069**	2095.8226	1048.4149	19
13	**1441.7183**	721.3628	1424.6917	712.8495	1423.7077	712.3575	Q	2026.8011	1013.9042	2009.7746	1005.3909	2008.7906	1004.8989	18
14	1498.7397	**749.8735**	1481.7132	741.3602	1480.7292	740.8682	G	**1898.7426**	**949.8749**	1881.7160	941.3616	1880.7320	940.8696	17
15	**1613.7667**	807.3870	1596.7401	798.8737	1595.7561	798.3817	D	1841.7211	921.3642	1824.6946	**912.8509**	1823.7105	**912.3589**	16
16	**1714.8143**	857.9108	1697.7878	849.3975	1696.8038	**848.9055**	T	**1726.6942**	863.8507	1709.6676	855.3374	1708.6836	854.8454	15
17	1811.8671	906.4372	1794.8406	897.9239	1793.8565	897.4319	P	**1625.6465**	**813.3269**	**1608.6199**	**804.8136**	1607.6359	804.3216	14
18	1971.8978	986.4525	1954.8712	977.9392	1953.8872	977.4472	C	1528.5937	764.8005	**1511.5672**	756.2872	1510.5831	755.7952	13

**19**	2058.9298	1029.9685	2041.9032	1021.4553	2040.9192	1020.9632	**S**	**1368.5631**	684.7852	1351.5365	676.2719	1350.5525	675.7799	**12**
**20**	2218.9604	1109.9839	2201.9339	1101.4706	2200.9499	**1100.9786**	**C**	1281.5310	641.2692	1264.5045	632.7559	1263.5205	632.2639	**11**
**21**	2378.9911	1189.9992	2361.9645	**1181.4859**	2360.9805	**1180.9939**	**C**	1121.5004	561.2538	1104.4738	552.7406	1103.4898	552.2485	**10**
**22**	2492.0751	1246.5412	2475.0486	1238.0279	2474.0646	1237.5359	**I**	**961.4697**	481.2385	944.4432	472.7252	943.4592	472.2332	**9**
**23**	2591.1436	**1296.0754**	2574.1170	1287.5621	2573.1330	1287.0701	**V**	**848.3857**	424.6965	831.3591	**416.1832**	830.3751	415.6912	**8**
**24**	2705.1865	**1353.0969**	2688.1599	1344.5836	2687.1759	1344.0916	**N**	**749.3173**	375.1623	**732.2907**	366.6490	**731.3067**	366.1570	**7**
**25**	2819.2294	**1410.1183**	2802.2029	1401.6051	2801.2188	1401.1131	**N**	**635.2743**	318.1408	618.2478	309.6275	617.2638	309.1355	**6**
**26**	2906.2614	**1453.6344**	2889.2349	**1445.1211**	2888.2509	1444.6291	**S**	**521.2314**	261.1193	504.2049	252.6061	503.2208	252.1141	**5**
**27**	3021.2884	**1511.1478**	3004.2618	1502.6346	3003.2778	1502.1425	**N**	**434.1994**	217.6033	417.1728	209.0901	**416.1888**	208.5980	**4**
**28**	3078.3098	1539.6586	3061.2833	**1531.1453**	3060.2993	**1530.6533**	**G**	**319.1724**	160.0899	302.1459	151.5766	301.1619	151.0846	**3**
**29**	3165.3419	1583.1746	3148.3153	1574.6613	3147.3313	1574.1693	**S**	262.1510	131.5791	245.1244	123.0659	244.1404	122.5738	**2**
**30**							**R**	175.1190	88.0631	158.0924	79.5498			**1**

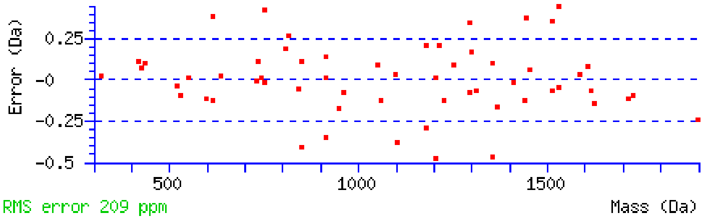

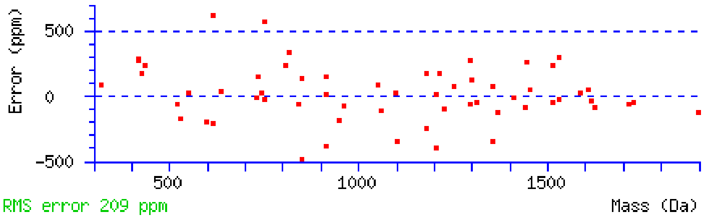

All matches to this query

Score	Mr(calc):	Delta	Sequence
71.7	3338.4463	0.0092	RGVDVSQVAWQSQGDTPCSCCIVNNSNGSR
58.1	3338.4463	0.0092	RGVDVSQVAWQSQGDTPCSCCIVNNSNGSR
58.0	3338.4463	0.0092	RGVDVSQVAWQSQGDTPCSCCIVNNSNGSR
27.6	3337.4623	0.9932	RGVDVSQVAWQSQGDTPCSCCIVNNSNGSR
17.7	3338.4351	0.0204	LSWAFNLYDLNKDGCITKEEMLDIMK
11.6	3338.4534	0.0021	ALYTLSDGHPVTNLADLQNGGQYVAAGFER
11.6	3338.4534	0.0021	ALYTLSDGHPVTNLADLQNGGQYVAAGFER
11.6	3338.4534	0.0021	ALYTLSDGHPVTNLADLQNGGQYVAAGFER
7.6	3336.4579	1.9976	VALQDLQTNSKIAALLPYFVYVVSGVK
5.8	3337.4693	0.9861	ALYTLSDGHPVTNLADLQNGGQYVAAGFER

Spectrum No: 210; Query: 400; Rank: 1

Peptide View

MS/MS Fragmentation of **ANTTQPGIIEGGQVLK**
Found in **IPI00764984**, Tax_Id=10116 Gene_Symbol=Itgav_predicted 106 kDa protein

Match to Query 400: 1625.865868 from(813.940210,2+)
Title: 091127RatKid_SCX01_16.2173.2173.2.dta
Data file K:\NewmanPaper\Piliang\3SubProteomes\Piliang3SP\mgf5ppm\SCX_3SubProteomes5ppm.mgf

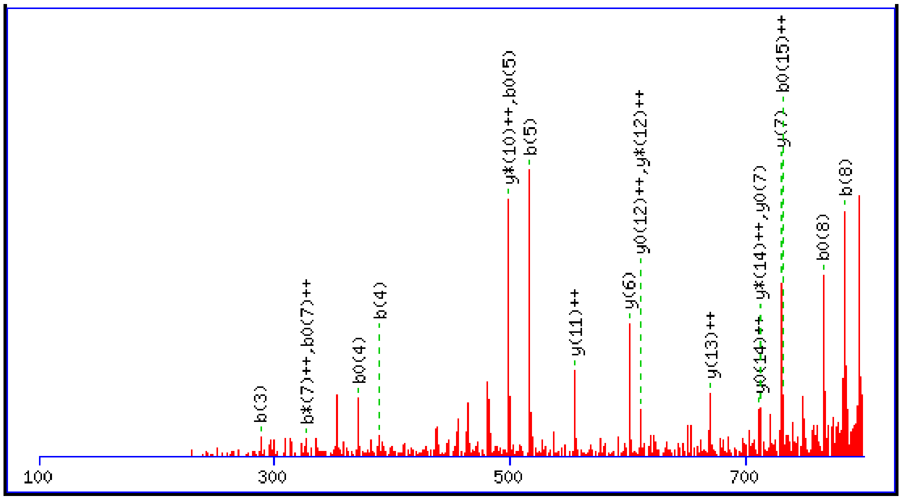

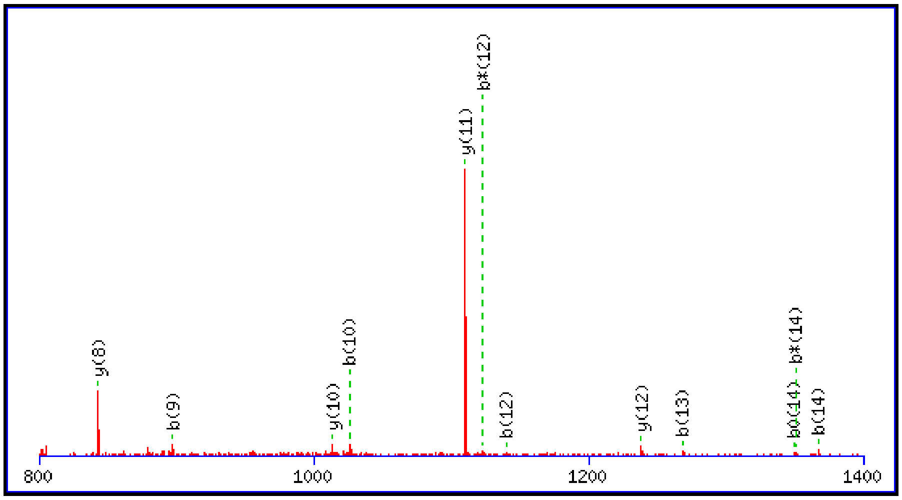

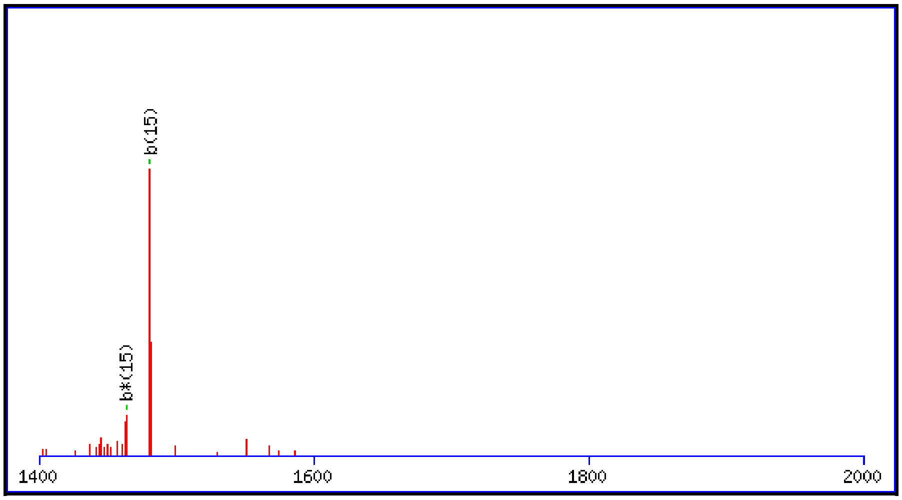

Monoisotopic mass of neutral peptide Mr(calc): 1625.8624
Fixed modifications: Carbamidomethyl (C)
Variable modifications:
N2 : Deamidated_N (N)
Ions Score: 72 **Expect:** 6.8e-006
Matches (Bold Red): 34/162 fragment ions using 53 most intense peaks

#	b	b^{++}	b*	b^{*++}	b^0	b^{0++}	Seq.	y	y^{++}	y*	y^{*++}	y^0	y^{0++}	#
1	72.0444	36.5258					A							16
2	187.0713	94.0393	170.0448	85.5260			N	1555.8326	778.4199	1538.8061	769.9067	1537.8220	769.4147	15
3	**288.1190**	144.5631	271.0925	136.0499	270.1084	135.5579	T	1440.8057	720.9065	1423.7791	**712.3932**	1422.7951	**711.9012**	14
4	**389.1667**	195.0870	372.1401	186.5737	**371.1561**	186.0817	T	1339.7580	**670.3826**	1322.7314	661.8694	1321.7474	661.3774	13
5	**517.2253**	259.1163	500.1987	250.6030	**499.2147**	250.1110	Q	**1238.7103**	619.8588	1221.6838	**611.3455**	1220.6998	**610.8535**	12

**6**	614.2780	307.6427	597.2515	299.1294	596.2675	298.6374	**P**	**1110.6517**	**555.8295**	1093.6252	547.3162	1092.6412	546.8242	**11**
**7**	671.2995	336.1534	654.2729	**327.6401**	653.2889	**327.1481**	**G**	**1013.5990**	507.3031	996.5724	**498.7899**	995.5884	498.2978	**10**
**8**	**784.3836**	392.6954	767.3570	384.1821	**766.3730**	383.6901	**I**	956.5775	478.7924	939.5510	470.2791	938.5669	469.7871	**9**
**9**	**897.4676**	449.2374	880.4411	440.7242	879.4571	440.2322	**I**	**843.4934**	422.2504	826.4669	413.7371	825.4829	413.2451	**8**
**10**	**1026.5102**	513.7587	1009.4837	505.2455	1008.4996	504.7535	**E**	**730.4094**	365.7083	713.3828	357.1951	**712.3988**	356.7030	**7**
**11**	1083.5317	542.2695	1066.5051	533.7562	1065.5211	533.2642	**G**	**601.3668**	301.1870	584.3402	292.6738			**6**
**12**	**1140.5531**	570.7802	**1123.5266**	562.2669	1122.5426	561.7749	**G**	544.3453	272.6763	527.3188	264.1630			**5**
**13**	**1268.6117**	634.8095	1251.5852	626.2962	1250.6012	625.8042	**Q**	487.3239	244.1656	470.2973	235.6523			**4**
**14**	**1367.6801**	684.3437	**1350.6536**	675.8304	**1349.6696**	675.3384	**V**	359.2653	180.1363	342.2387	171.6230			**3**
**15**	**1480.7642**	740.8857	**1463.7376**	732.3725	1462.7536	**731.8805**	**L**	260.1969	130.6021	243.1703	122.0888			**2**
**16**							**K**	147.1128	74.0600	130.0863	65.5468			**1**

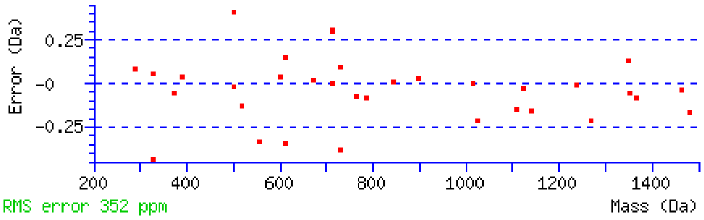

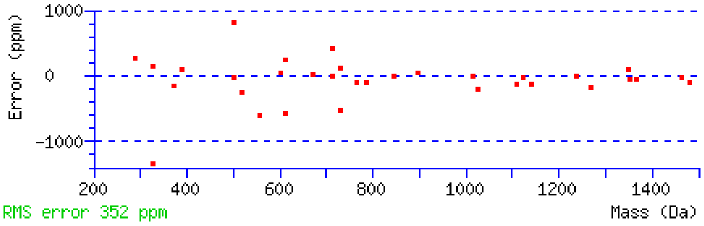

All matches to this query

Score	Mr(calc):	Delta	Sequence
71.6	1625.8624	0.0034	ANTTQPGIIEGGQVLK
15.0	1624.8784	0.9874	ANTTQPGIIEGGQVLK
14.4	1625.8575	0.0083	TRILVTSKMEQLK
14.4	1625.8575	0.0083	TRILVTSKMEQLK
12.8	1625.8624	0.0035	DINTKLPQAQQELK
7.9	1625.8575	0.0083	TRILVTSKMEQLK
5.4	1625.8616	0.0043	IYVVQPKAMKIEK
5.3	1625.8624	0.0034	SLDEGAPQDTPKLKK
4.0	1625.8542	0.0117	KSGKLVFLGLDNAGK
3.4	1625.8599	0.0059	FSVLNQIFCKNKK

Spectrum No: 211; Query: 729; Rank: 1

Peptide View

MS/MS Fragmentation of **FQYNDKTFLNLMDLLNK**
Found in **IPI00327991**, Tax_Id=10116 Gene_Symbol=Cyp2c23 Cytochrome P450 2C23

Match to Query 729: 2116.069308 from(1059.041930,2+)
Title: 091129RatKid_SCX02_29.6065.6065.2.dta
Data file K:\NewmanPaper\Piliang\3SubProteomes\Piliang3SP\mgf5ppm\SCX_3SubProteomes5ppm.mgf

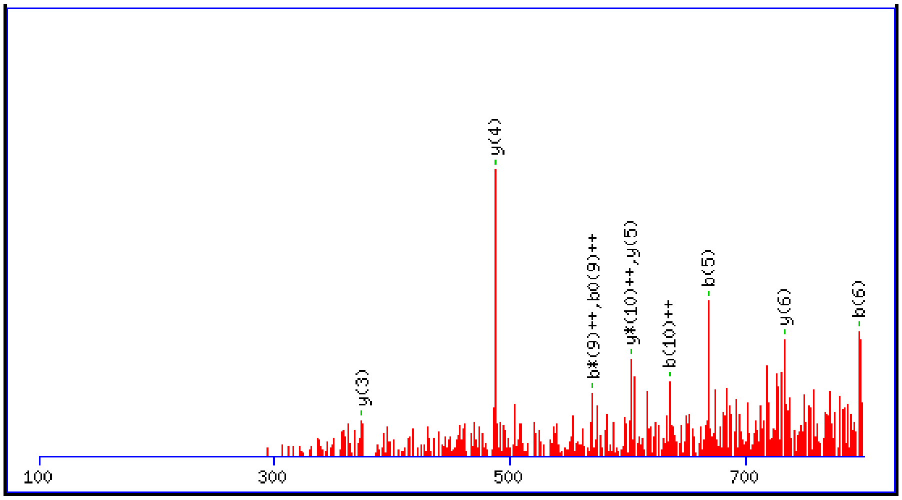

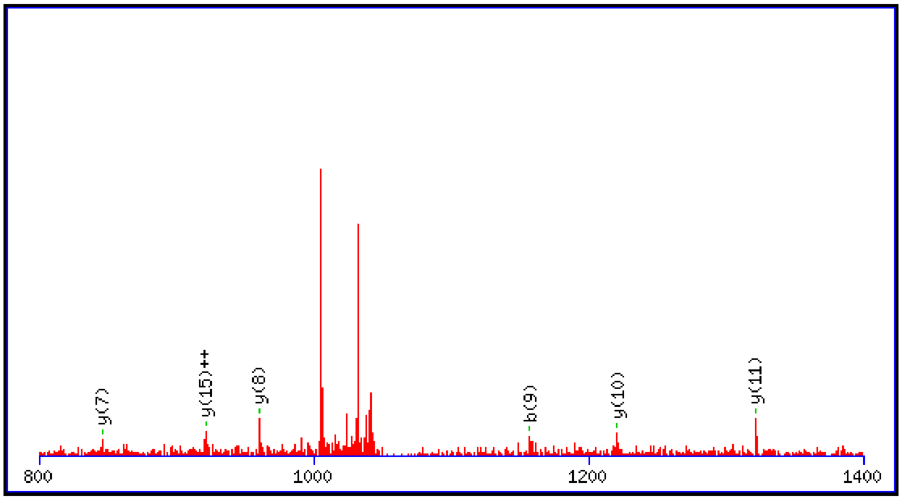

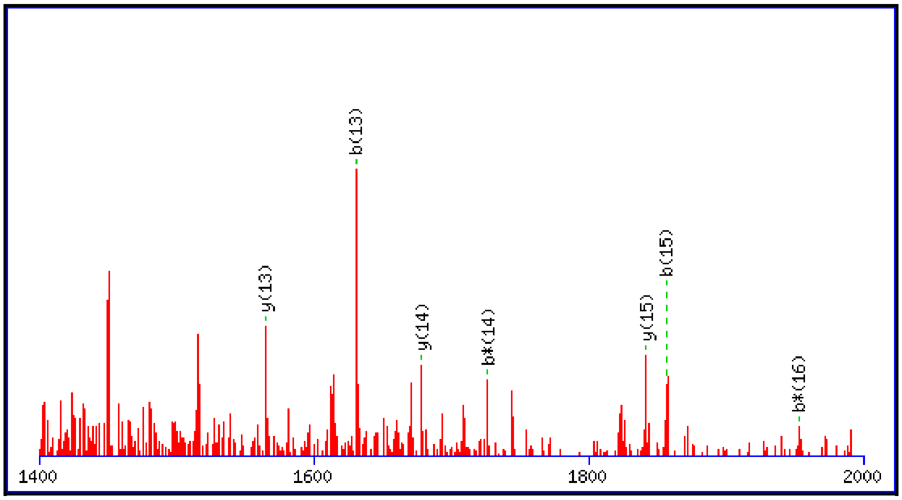

Monoisotopic mass of neutral peptide Mr(calc): 2116.0663
Fixed modifications: Carbamidomethyl (C)
Ions Score: 72 **Expect:** 1.1e-005
Matches (Bold Red): 23/174 fragment ions using 43 most intense peaks

#	b	b^{++}	b*	b^{*++}	b^0	b^{0++}	Seq.	y	y^{++}	y*	y^{*++}	y^0	y^{0++}	#
1	148.0757	74.5415					F							17
2	276.1343	138.5708	259.1077	130.0575			Q	1970.0052	985.5062	1952.9786	976.9930	1951.9946	976.5009	16
3	439.1976	220.1024	422.1710	211.5892			Y	**1841.9466**	**921.4769**	1824.9200	912.9637	1823.9360	912.4717	15
4	553.2405	277.1239	536.2140	268.6106			N	**1678.8833**	839.9453	1661.8567	831.4320	1660.8727	830.9400	14
5	**668.2675**	334.6374	651.2409	326.1241	650.2569	325.6321	D	**1564.8403**	782.9238	1547.8138	774.4105	1546.8298	773.9185	13
6	**796.3624**	398.6849	779.3359	390.1716	778.3519	389.6796	K	1449.8134	725.4103	1432.7868	716.8971	1431.8028	716.4051	12

**7**	897.4101	449.2087	880.3836	440.6954	879.3995	440.2034	**T**	**1321.7184**	661.3629	1304.6919	652.8496	1303.7079	652.3576	**11**
**8**	1044.4785	522.7429	1027.4520	514.2296	1026.4680	513.7376	**F**	**1220.6708**	610.8390	1203.6442	**602.3257**	1202.6602	601.8337	**10**
**9**	**1157.5626**	579.2849	1140.5360	**570.7717**	1139.5520	**570.2796**	**L**	1073.6023	537.3048	1056.5758	528.7915	1055.5918	528.2995	**9**
**10**	1271.6055	**636.3064**	1254.5790	627.7931	1253.5949	627.3011	**N**	**960.5183**	480.7628	943.4917	472.2495	942.5077	471.7575	**8**
**11**	1384.6896	692.8484	1367.6630	684.3352	1366.6790	683.8431	**L**	**846.4754**	423.7413	829.4488	415.2280	828.4648	414.7360	**7**
**12**	1515.7301	758.3687	1498.7035	749.8554	1497.7195	749.3634	**M**	**733.3913**	367.1993	716.3647	358.6860	715.3807	358.1940	**6**
**13**	**1630.7570**	815.8821	1613.7305	807.3689	1612.7464	806.8769	**D**	**602.3508**	301.6790	585.3243	293.1658	584.3402	292.6738	**5**
**14**	1743.8411	872.4242	**1726.8145**	863.9109	1725.8305	863.4189	**L**	**487.3239**	244.1656	470.2973	235.6523			**4**
**15**	**1856.9251**	928.9662	1839.8986	920.4529	1838.9146	919.9609	**L**	**374.2398**	187.6235	357.2132	179.1103			**3**
**16**	1970.9681	985.9877	**1953.9415**	977.4744	1952.9575	976.9824	**N**	261.1557	131.0815	244.1292	122.5682			**2**
**17**							**K**	147.1128	74.0600	130.0863	65.5468			**1**

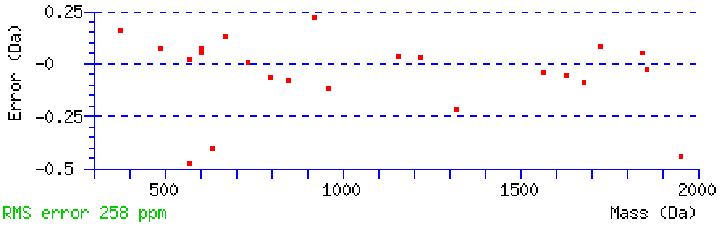

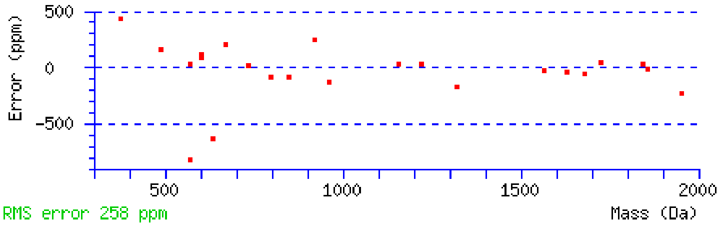

All matches to this query

Score	Mr(calc):	Delta	Sequence
71.5	2116.0663	0.0030	FQYNDKTFLNLMDLLNK
71.5	2116.0663	0.0030	FQYNNKTFLNLMDLLNK
56.0	2116.0663	0.0030	FQYNNKTFLNLMDLLNK
50.2	2116.0663	0.0030	FQYNNKTFLNLMDLLNK
24.4	2115.0823	0.9870	FQYNNKTFLNLMDLLNK
8.5	2116.0663	0.0030	FQYNNKTFLNLMDLLNK
2.5	2116.0623	0.0070	STSPTHYSLLPSLQMDALR

Spectrum No: 212; Query: 311; Rank: 1

Peptide View

MS/MS Fragmentation of **AVAQGNLSSADVQAAK**
Found in **IPI00188924**, Tax_Id=10116 Gene_Symbol=Uqcrc2 Cytochrome b-c1 complex subunit 2, mitochondrial precursor

Match to Query 311: 1529.765708 from(765.890130,2+)
Title: 091127RatKid_SCX01_20.1223.1223.2.dta
Data file K:\NewmanPaper\Piliang\3SubProteomes\Piliang3SP\mgf5ppm\SCX_3SubProteomes5ppm.mgf

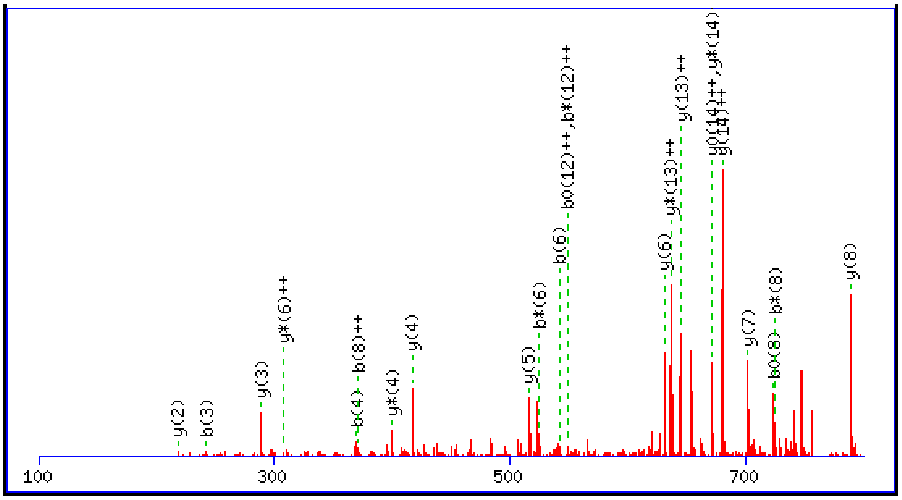

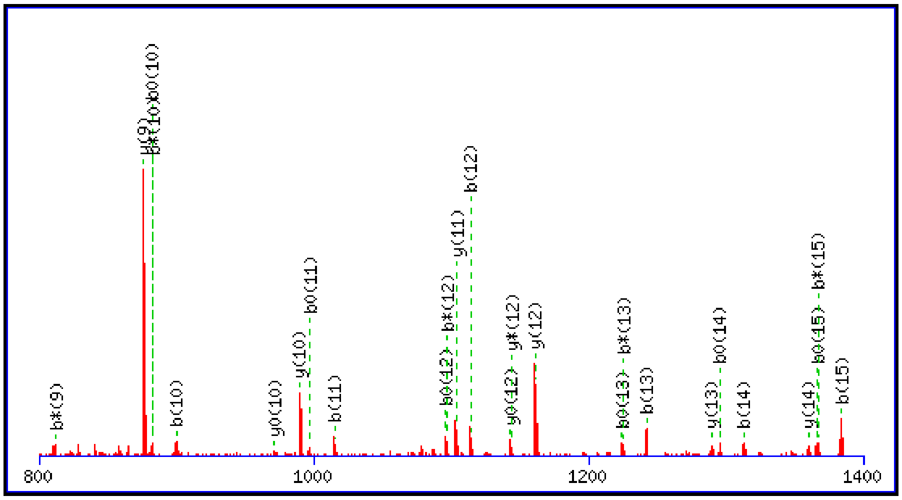

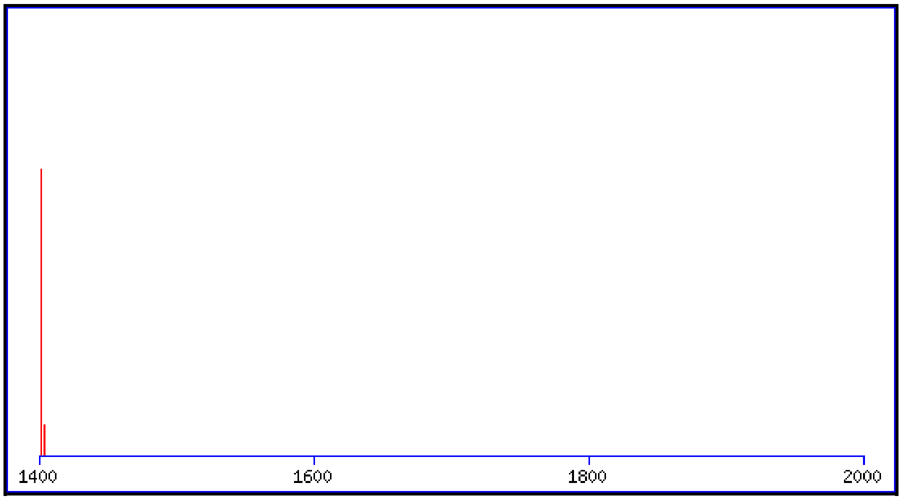

Monoisotopic mass of neutral peptide Mr(calc): 1529.7685
Fixed modifications: Carbamidomethyl (C)
Variable modifications:
N6 : Deamidated_N (N)
Ions Score: 72 **Expect:** 1e-005
Matches (Bold Red): 49/150 fragment ions using 102 most intense peaks

#	b	b++	b*	b*++	b0	b0++	Seq.	y	y++	y*	y*++	y0	y0++	#
1	72.0444	36.5258					A							16
2	171.1128	86.0600					V	1459.7387	730.3730	1442.7122	721.8597	1441.7281	721.3677	15
3	**242.1499**	121.5786					A	**1360.6703**	**680.8388**	1343.6437	**672.3255**	1342.6597	**671.8335**	14
4	**370.2085**	185.6079	353.1819	177.0946			Q	**1289.6332**	**645.3202**	1272.6066	**636.8070**	1271.6226	636.3149	13
5	427.2300	214.1186	410.2034	205.6053			G	**1161.5746**	581.2909	**1144.5481**	572.7777	**1143.5640**	572.2857	12

6	542.2569	271.6321	525.2303	263.1188			N	1104.5531	552.7802	1087.5266	544.2669	1086.5426	543.7749	11
7	655.3410	328.1741	638.3144	319.6608			L	989.5262	495.2667	972.4997	486.7535	971.5156	486.2615	10
8	742.3730	371.6901	725.3464	363.1769	724.3624	362.6848	S	876.4421	438.7247	859.4156	430.2114	858.4316	429.7194	9
9	829.4050	415.2061	812.3785	406.6929	811.3945	406.2009	S	789.4101	395.2087	772.3836	386.6954	771.3995	386.2034	8
10	900.4421	450.7247	883.4156	442.2114	882.4316	441.7194	A	702.3781	351.6927	685.3515	343.1794	684.3675	342.6874	7
11	1015.4691	508.2382	998.4425	499.7249	997.4585	499.2329	D	631.3410	316.1741	614.3144	307.6608	613.3304	307.1688	6
12	1114.5375	557.7724	1097.5109	549.2591	1096.5269	548.7671	V	516.3140	258.6607	499.2875	250.1474			5
13	1242.5961	621.8017	1225.5695	613.2884	1224.5855	612.7964	Q	417.2456	209.1264	400.2191	200.6132			4
14	1313.6332	657.3202	1296.6066	648.8070	1295.6226	648.3149	A	289.1870	145.0972	272.1605	136.5839			3
15	1384.6703	692.8388	1367.6437	684.3255	1366.6597	683.8335	A	218.1499	109.5786	201.1234	101.0653			2
16							K	147.1128	74.0600	130.0863	65.5468			1

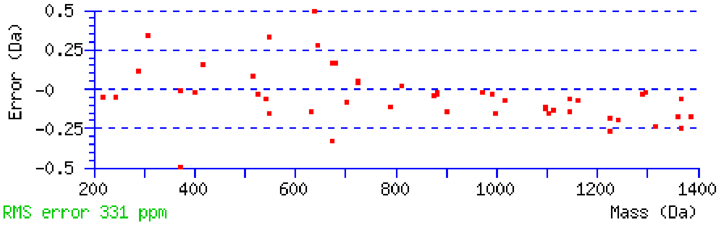

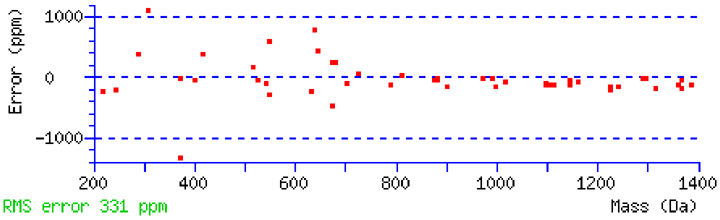

All matches to this query

Score	Mr(calc):	Delta	Sequence
71.5	1529.7685	-0.0028	AVAQGNLSSADVQAAK
1.6	1529.7531	0.0126	LAWFLVTSANLSK
0.2	1527.7698	1.9959	TIINESLNFKIR

Spectrum No: 213; Query: 543; Rank: 1

Peptide View

MS/MS Fragmentation of **NETLGGTCLNVGCIPSK**
Found in **IPI00365545**, Tax_Id=10116 Gene_Symbol=Dld Dihydrolipoyl dehydrogenase, mitochondrial precursor

Match to Query 543: 1819.840728 from(910.927640,2+)
Title: 091129RatKid_SCX02_13.2212.2212.2.dta
Data file K:\NewmanPaper\Piliang\3SubProteomes\Piliang3SP\mgf5ppm\SCX_3SubProteomes5ppm.mgf

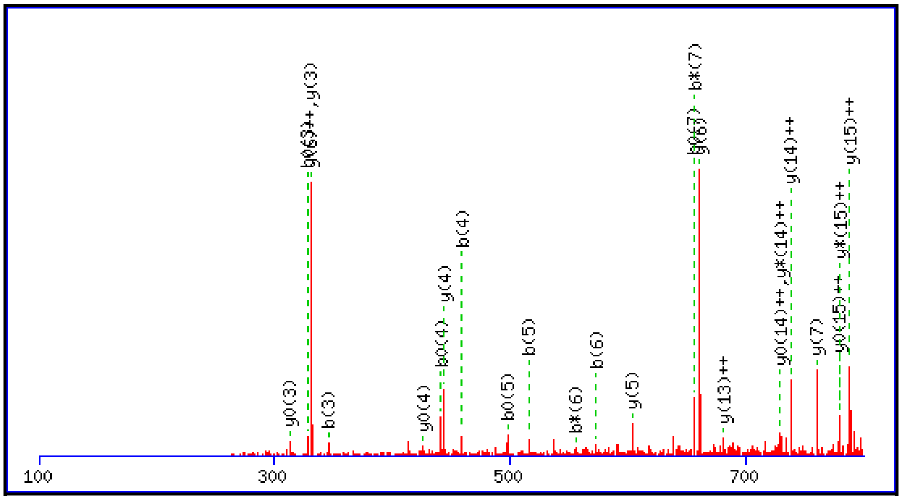

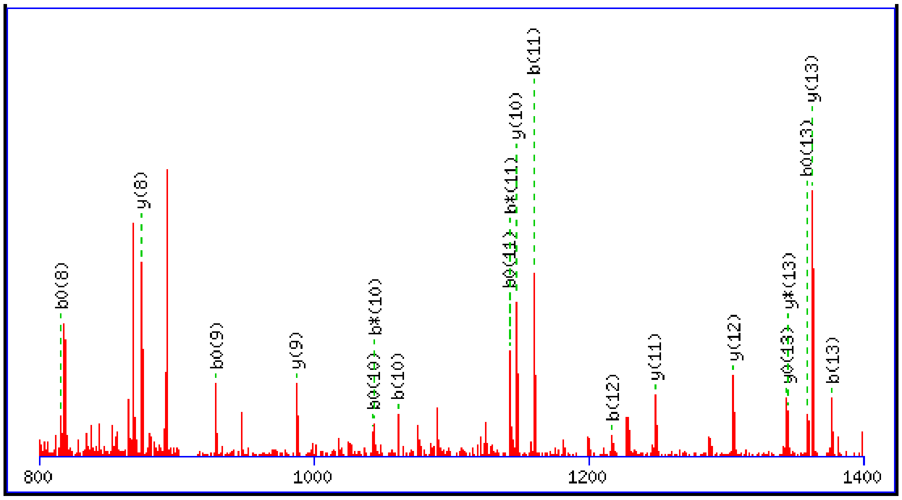

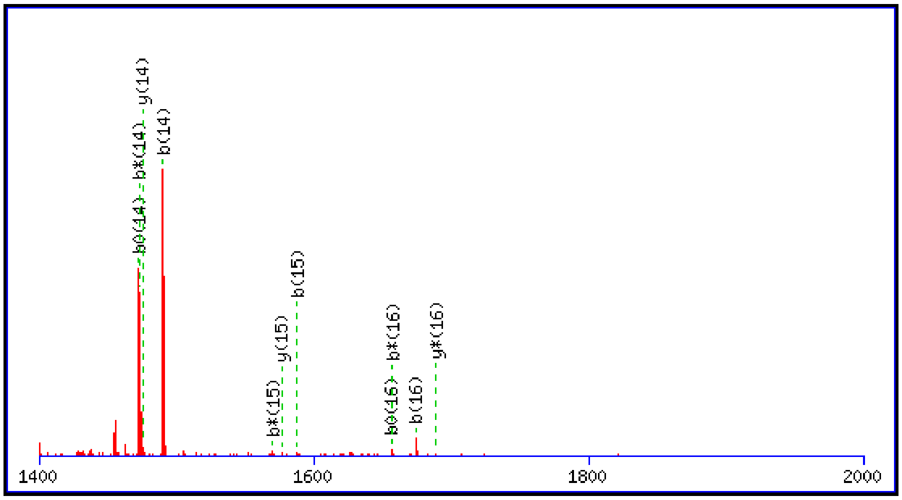

Monoisotopic mass of neutral peptide Mr(calc): 1819.8444
Fixed modifications: Carbamidomethyl (C)
Variable modifications:
N1 : Deamidated_N (N)
Ions Score: 71 **Expect:** 1.6e-005
Matches (Bold Red): 55/188 fragment ions using 108 most intense peaks

#	b	b^{++}	b^{*}	b^{*++}	b^{0}	b^{0++}	Seq.	y	y^{++}	y^{*}	y^{*++}	y^{0}	y^{0++}	#
1	116.0342	58.5207	99.0077	50.0075			N							17
2	245.0768	123.0420	228.0503	114.5288	227.0662	114.0368	E	1705.8248	853.4160	**1688.7982**	844.9027	1687.8142	844.4107	16
3	**346.1245**	173.5659	329.0979	165.0526	**328.1139**	164.5606	T	**1576.7822**	**788.8947**	1559.7556	**780.3815**	1558.7716	**779.8894**	15
4	**459.2085**	230.1079	442.1820	221.5946	**441.1980**	221.1026	L	**1475.7345**	**738.3709**	1458.7079	**729.8576**	1457.7239	**729.3656**	14
5	**516.2300**	258.6186	499.2035	250.1054	**498.2194**	249.6134	G	**1362.6504**	**681.8289**	**1345.6239**	673.3156	**1344.6399**	672.8236	13
6	**573.2515**	287.1294	**556.2249**	278.6161	555.2409	278.1241	G	**1305.6290**	653.3181	1288.6024	644.8048	1287.6184	644.3128	12
7	674.2992	337.6532	**657.2726**	329.1399	**656.2886**	328.6479	T	**1248.6075**	624.8074	1231.5810	616.2941	1230.5969	615.8021	11
8	834.3298	417.6685	817.3033	409.1553	**816.3192**	408.6633	C	**1147.5598**	574.2836	1130.5333	565.7703	1129.5493	565.2783	10
9	947.4139	474.2106	930.3873	465.6973	**929.4033**	465.2053	L	**987.5292**	494.2682	970.5026	485.7550	969.5186	485.2629	9
10	**1061.4568**	531.2320	**1044.4302**	522.7188	**1043.4462**	522.2268	N	**874.4451**	437.7262	857.4186	429.2129	856.4345	428.7209	8
11	**1160.5252**	580.7662	**1143.4987**	572.2530	**1142.5146**	571.7610	V	**760.4022**	380.7047	743.3756	372.1915	742.3916	371.6994	7
12	**1217.5467**	609.2770	1200.5201	600.7637	1199.5361	600.2717	G	**661.3338**	**331.1705**	644.3072	322.6573	643.3232	322.1652	6
13	**1377.5773**	689.2923	1360.5508	680.7790	**1359.5668**	680.2870	C	**604.3123**	302.6598	587.2858	294.1465	586.3017	293.6545	5
14	**1490.6614**	745.8343	**1473.6348**	737.3211	**1472.6508**	736.8290	I	**444.2817**	222.6445	427.2551	214.1312	**426.2711**	213.6392	4
15	**1587.7142**	794.3607	**1570.6876**	785.8474	1569.7036	785.3554	P	**331.1976**	166.1024	314.1710	157.5892	**313.1870**	157.0972	3
16	**1674.7462**	837.8767	**1657.7196**	829.3635	**1656.7356**	828.8714	S	234.1448	117.5761	217.1183	109.0628	216.1343	108.5708	2
17							K	147.1128	74.0600	130.0863	65.5468			1

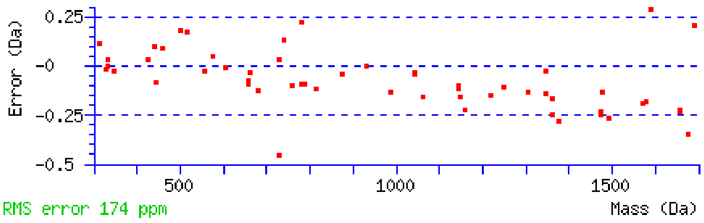

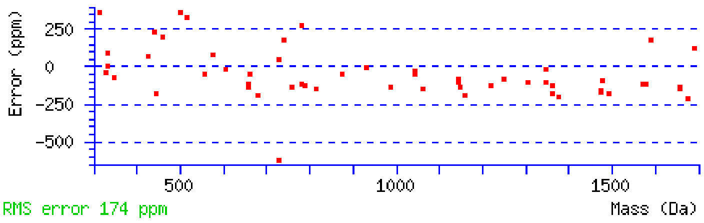

All matches to this query

Score	Mr(calc):	Delta	Sequence
71.5	1819.8444	-0.0037	NETLGGTCLNVGCIPSK
51.4	1819.8444	-0.0037	NETLGGTCLNVGCIPSK
9.6	1819.8370	0.0037	LAAVQSSCNNGGTNDIAK
9.6	1819.8370	0.0037	LAAVQSSCNNGGTNDIAK
5.5	1819.8230	0.0177	KLQTLQSENSSLRR
2.8	1819.8298	0.0109	SGDSMAIYFTSGTTGTPK
2.6	1819.8383	0.0025	TRPQNGSIILYNRK
2.6	1819.8383	0.0025	TRPQNGSIILYNRK
1.9	1819.8482	-0.0075	GSQSVQKLVTSSQALK
1.9	1819.8482	-0.0075	GSQSVQKLVTSSQALK

Spectrum No: 214; Query: 90; Rank: 1

Peptide View

MS/MS Fragmentation of **QTAEYAANITK**
Found in **IPI00327398**, Tax_Id=10116 Gene_Symbol=Enpep Isoform 1 of Glutamyl aminopeptidase

Match to Query 90: 1209.587428 from(605.800990,2+)
Title: 091127RatKid_SCX01_18.1030.1030.2.dta
Data file K:\NewmanPaper\Piliang\3SubProteomes\Piliang3SP\mgf5ppm\SCX_3SubProteomes5ppm.mgf

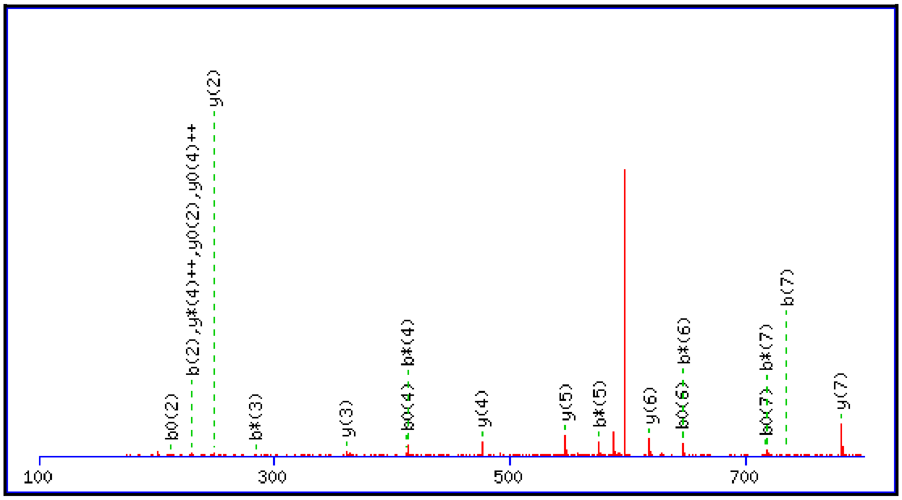

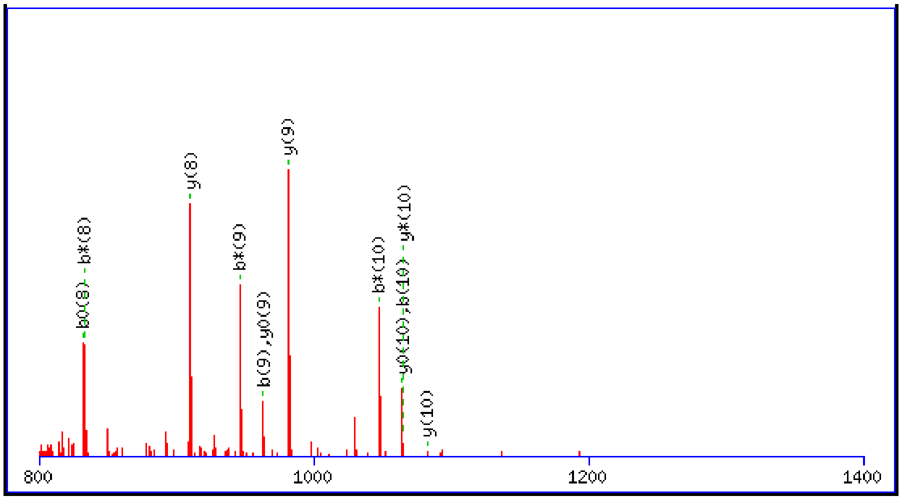

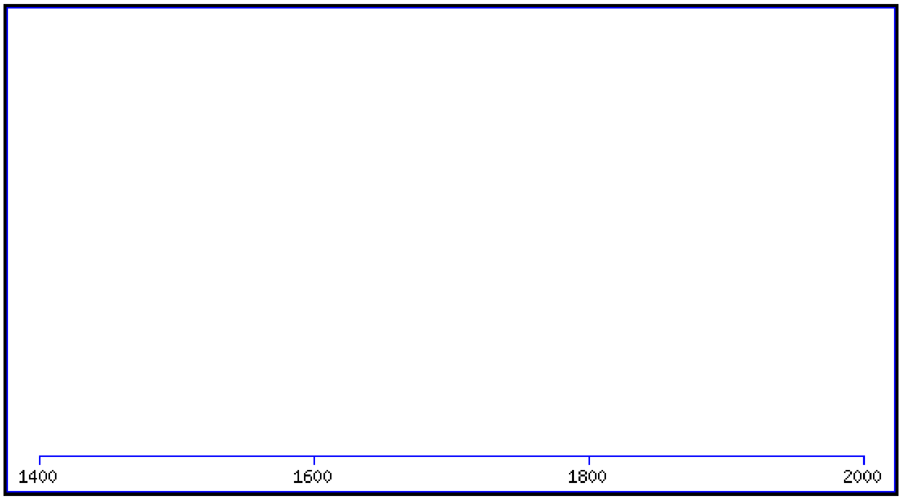

Monoisotopic mass of neutral peptide Mr(calc): 1209.5877
Fixed modifications: Carbamidomethyl (C)
Variable modifications:
N8 : Deamidated_N (N)
Ions Score: 71 **Expect:** 1.2e-005
Matches (Bold Red): 32/116 fragment ions using 47 most intense peaks

#	b	b^{++}	b*	b^{*++}	b^{0}	b^{0++}	Seq.	y	y^{++}	y*	y^{*++}	y^{0}	y^{0++}	#
1	129.0659	65.0366	112.0393	56.5233			Q							11
2	**230.1135**	115.5604	213.0870	107.0471	**212.1030**	106.5551	T	**1082.5364**	541.7719	**1065.5099**	533.2586	**1064.5259**	532.7666	10
3	301.1506	151.0790	**284.1241**	142.5657	283.1401	142.0737	A	**981.4887**	491.2480	964.4622	482.7347	**963.4782**	482.2427	9
4	430.1932	215.6003	**413.1667**	207.0870	**412.1827**	206.5950	E	**910.4516**	455.7295	893.4251	447.2162	892.4411	446.7242	8
5	593.2566	297.1319	**576.2300**	288.6186	575.2460	288.1266	Y	**781.4090**	391.2082	764.3825	382.6949	763.3985	382.2029	7
6	664.2937	332.6505	**647.2671**	324.1372	**646.2831**	323.6452	A	**618.3457**	309.6765	601.3192	301.1632	600.3351	300.6712	6
7	**735.3308**	368.1690	**718.3042**	359.6558	**717.3202**	359.1638	A	**547.3086**	274.1579	530.2820	265.6447	529.2980	265.1527	5
8	850.3577	425.6825	**833.3312**	417.1692	**832.3472**	416.6772	N	**476.2715**	238.6394	459.2449	**230.1261**	458.2609	**229.6341**	4
9	**963.4418**	482.2245	**946.4152**	473.7113	945.4312	473.2193	I	**361.2445**	181.1259	344.2180	172.6126	343.2340	172.1206	3
10	**1064.4895**	532.7484	**1047.4629**	524.2351	1046.4789	523.7431	T	**248.1605**	124.5839	231.1339	116.0706	**230.1499**	115.5786	2
11							K	147.1128	74.0600	130.0863	65.5468			1

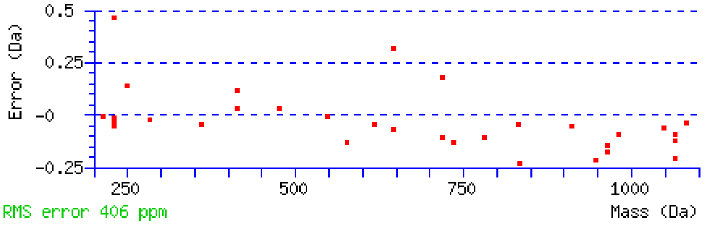

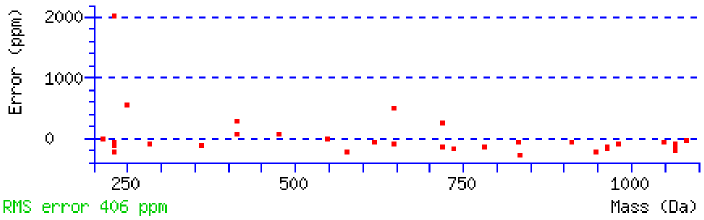

All matches to this query

Score	Mr(calc):	Delta	Sequence
71.2	1209.5877	-0.0003	QTAEYAANITK
12.0	1208.5826	1.0048	DTVLAYWADR
8.6	1209.5989	-0.0115	ASAEFQTNSKK
6.6	1207.5710	2.0164	EAEATAKRPR
6.4	1208.5781	1.0093	MVEVCVESIK
6.3	1209.5951	-0.0077	VELNMFISEK
3.8	1207.5710	2.0164	DGTGPSGLRLR
3.5	1208.5955	0.9920	DILSVQIXR
3.4	1208.5802	1.0072	NVNTGELAAIK
3.4	1208.5914	0.9960	SETPVTIARR

Spectrum No: 215; Query: 386; Rank: 1

Peptide View

MS/MS Fragmentation of **VTINNWVANKTEGR**
Found in **IPI00372372**, Tax_Id=10116 Gene_Symbol=Serpinc1 Serine (Or cysteine) peptidase inhibitor, clade C (Antithrombin), member 1

Match to Query 386: 1601.817548 from(801.916050,2+)
Title: 091127RatKid_SCX01_13.1895.1895.2.dta
Data file K:\NewmanPaper\Piliang\3SubProteomes\Piliang3SP\mgf5ppm\SCX_3SubProteomes5ppm.mgf

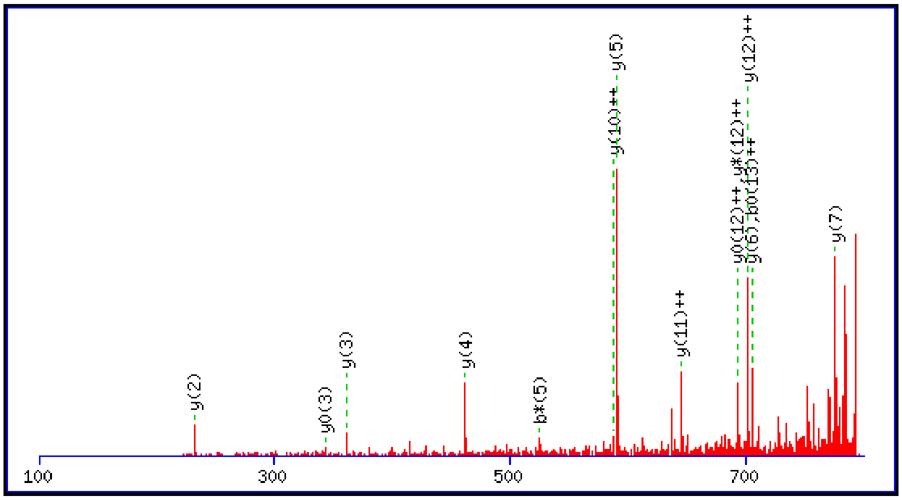

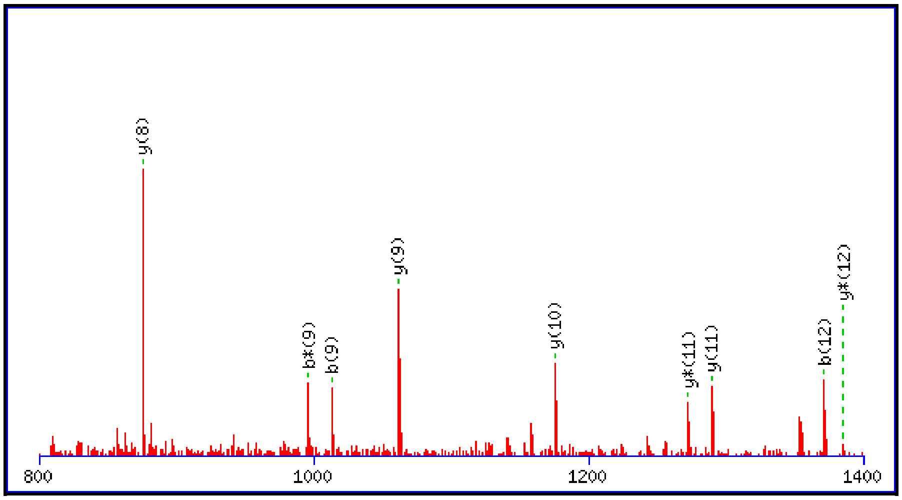

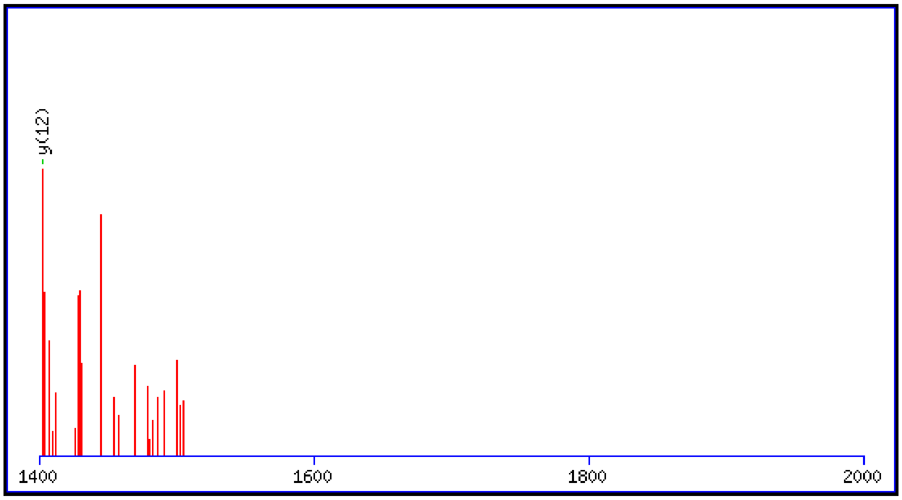

Monoisotopic mass of neutral peptide Mr(calc): 1601.8161
Fixed modifications: Carbamidomethyl (C)
Variable modifications:
N9 : Deamidated_N (N)
Ions Score: 71 **Expect:** 1.1e-005
Matches (Bold Red): 24/144 fragment ions using 35 most intense peaks

#	b	b^{++}	b*	b^{*++}	b^0	b^{0++}	Seq.	y	y^{++}	y*	y^{*++}	y^0	y^{0++}	#
1	100.0757	50.5415					V							14
2	201.1234	101.0653			183.1128	92.0600	T	1503.7550	752.3812	1486.7285	743.8679	1485.7445	743.3759	13
3	314.2074	157.6074			296.1969	148.6021	I	**1402.7073**	**701.8573**	**1385.6808**	**693.3440**	1384.6968	**692.8520**	12
4	428.2504	214.6288	411.2238	206.1155	410.2398	205.6235	N	**1289.6233**	**645.3153**	**1272.5967**	636.8020	1271.6127	636.3100	11
5	542.2933	271.6503	**525.2667**	263.1370	524.2827	262.6450	N	**1175.5804**	**588.2938**	1158.5538	579.7805	1157.5698	579.2885	10
6	728.3726	364.6899	711.3461	356.1767	710.3620	355.6847	W	**1061.5374**	531.2724	1044.5109	522.7591	1043.5269	522.2671	9
7	827.4410	414.2241	810.4145	405.7109	809.4304	405.2189	V	**875.4581**	438.2327	858.4316	429.7194	857.4475	429.2274	8
8	898.4781	449.7427	881.4516	441.2294	880.4676	440.7374	A	**776.3897**	388.6985	759.3632	380.1852	758.3791	379.6932	7
9	**1013.5051**	507.2562	**996.4785**	498.7429	995.4945	498.2509	N	**705.3526**	353.1799	688.3260	344.6667	687.3420	344.1746	6
10	1141.6000	571.3037	1124.5735	562.7904	1123.5895	562.2984	K	**590.3257**	295.6665	573.2991	287.1532	572.3151	286.6612	5
11	1242.6477	621.8275	1225.6212	613.3142	1224.6371	612.8222	T	**462.2307**	231.6190	445.2041	223.1057	444.2201	222.6137	4
12	**1371.6903**	686.3488	1354.6638	677.8355	1353.6797	677.3435	E	**361.1830**	181.0951	344.1565	172.5819	**343.1724**	172.0899	3
13	1428.7118	714.8595	1411.6852	706.3462	1410.7012	**705.8542**	G	**232.1404**	116.5738	215.1139	108.0606			2
14							R	175.1190	88.0631	158.0924	79.5498			1

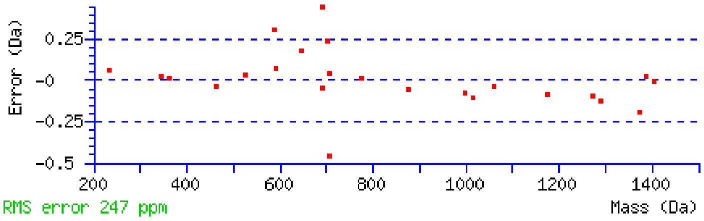

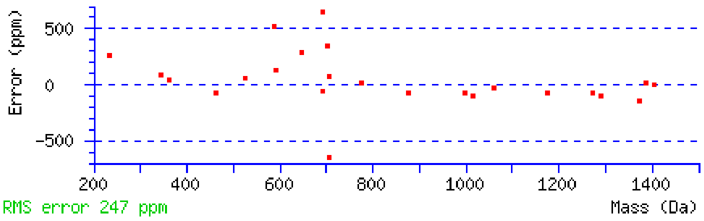

All matches to this query

Score	Mr(calc):	Delta	Sequence
71.0	1601.8161	0.0014	VTINNWVANKTEGR
33.2	1601.8161	0.0014	VTINNWVANKTEGR
21.8	1601.8161	0.0014	VTINNWVANKTEGR
6.7	1601.8151	0.0024	AASLLGGRTGPGGPRR
5.0	1601.8106	0.0070	VKITVNYPPYISK
2.3	1601.8039	0.0137	SHRLQESLGAVLGR
2.3	1601.8331	-0.0155	VTFNKSFLRPVSK

1.1	1601.8137	0.0039	CPVFVVRWTPEGR
0.6	1600.8281	0.9894	RVGISGDNSGGNLKAR
0.5	1601.8151	0.0024	AASLLGGRTGPGGPRR

Spectrum No: 216; Query: 742; Rank: 1

Peptide View

MS/MS Fragmentation of **EGNCSVQSGLTWQDCDFK**
Found in **IPI00327182**, Tax_Id=10116 Gene_Symbol=Kng1 T-kininogen 1 precursor

Match to Query 742: 2131.873948 from(1066.944250,2+)
Title: 091127RatKid_SCX01_02.3050.3050.2.dta
Data file K:\NewmanPaper\Piliang\3SubProteomes\Piliang3SP\mgf5ppm\SCX_3SubProteomes5ppm.mgf

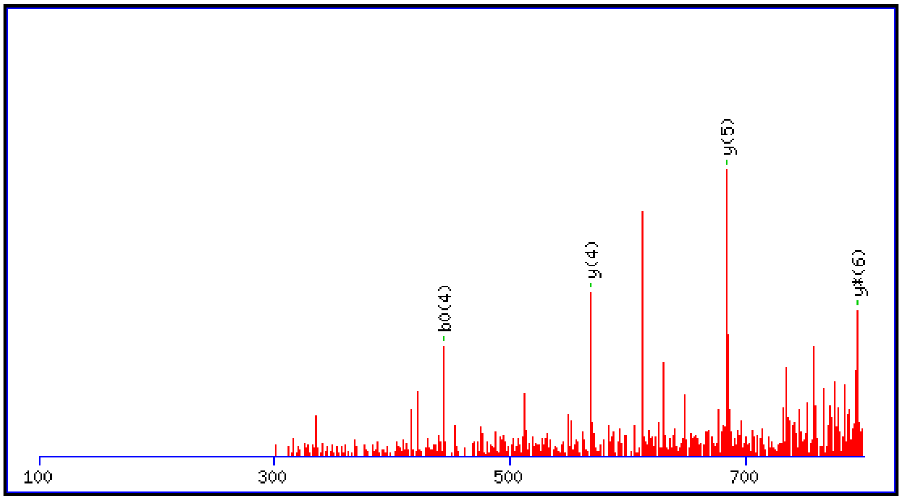

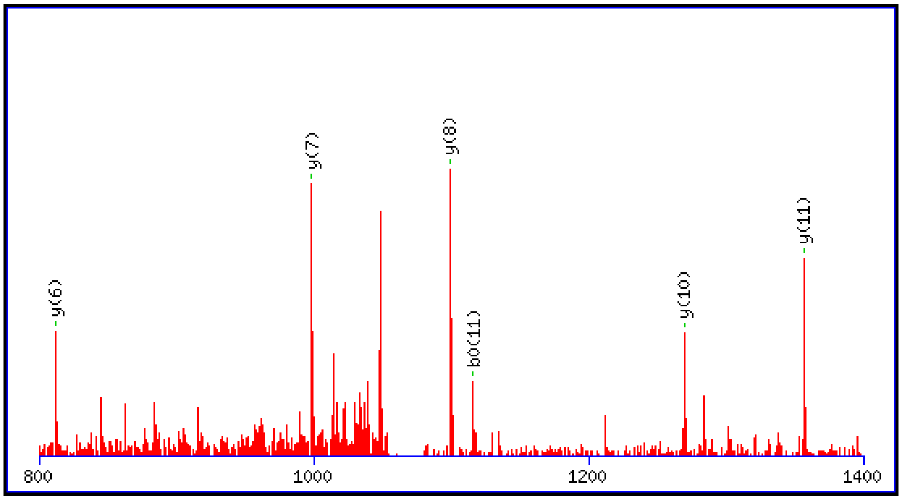

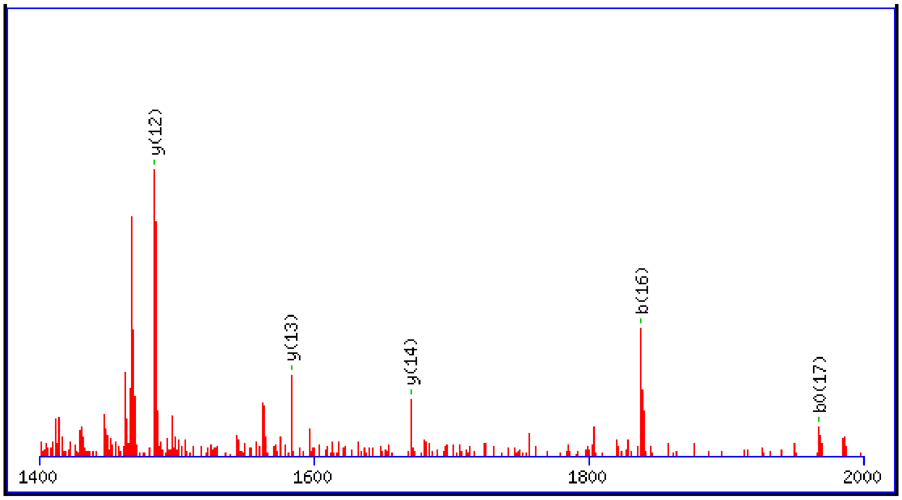

Monoisotopic mass of neutral peptide Mr(calc): 2130.8623
Fixed modifications: Carbamidomethyl (C)
Variable modifications:
N3 : Deamidated_N (N)
Ions Score: 71 **Expect:** 1.7e-005
Matches (Bold Red): 15/196 fragment ions using 27 most intense peaks

#	b	b^{++}	b*	b^{*++}	b^{0}	b^{0++}	Seq.	y	y^{++}	y*	y^{*++}	y^{0}	y^{0++}	#
1	130.0499	65.5286			112.0393	56.5233	E							18
2	187.0713	94.0393			169.0608	85.0340	G	2002.8269	1001.9171	1985.8004	993.4038	1984.8164	992.9118	17
3	302.0983	151.5528	285.0717	143.0395	284.0877	142.5475	N	1945.8055	973.4064	1928.7789	964.8931	1927.7949	964.4011	16
4	462.1289	231.5681	445.1024	223.0548	**444.1184**	222.5628	C	1830.7785	915.8929	1813.7520	907.3796	1812.7680	906.8876	15
5	549.1609	275.0841	532.1344	266.5708	531.1504	266.0788	S	**1670.7479**	835.8776	1653.7214	827.3643	1652.7373	826.8723	14
6	648.2294	324.6183	631.2028	316.1050	630.2188	315.6130	V	**1583.7159**	792.3616	1566.6893	783.8483	1565.7053	783.3563	13
7	776.2879	388.6476	759.2614	380.1343	758.2774	379.6423	Q	**1484.6475**	742.8274	1467.6209	734.3141	1466.6369	733.8221	12
8	863.3200	432.1636	846.2934	423.6503	845.3094	423.1583	S	**1356.5889**	678.7981	1339.5623	670.2848	1338.5783	669.7928	11
9	920.3414	460.6744	903.3149	452.1611	902.3309	451.6691	G	**1269.5569**	635.2821	1252.5303	626.7688	1251.5463	626.2768	10
10	1033.4255	517.2164	1016.3989	508.7031	1015.4149	508.2111	L	1212.5354	606.7713	1195.5088	598.2581	1194.5248	597.7660	9
11	1134.4732	567.7402	1117.4466	559.2270	**1116.4626**	558.7349	T	**1099.4513**	550.2293	1082.4248	541.7160	1081.4408	541.2240	8
12	1320.5525	660.7799	1303.5259	652.2666	1302.5419	651.7746	W	**998.4036**	499.7055	981.3771	491.1922	980.3931	490.7002	7
13	1448.6111	724.8092	1431.5845	716.2959	1430.6005	715.8039	Q	**812.3243**	406.6658	**795.2978**	398.1525	794.3138	397.6605	6
14	1563.6380	782.3226	1546.6115	773.8094	1545.6274	773.3174	D	**684.2658**	342.6365	667.2392	334.1232	666.2552	333.6312	5
15	1723.6687	862.3380	1706.6421	853.8247	1705.6581	853.3327	C	**569.2388**	285.1230	552.2123	276.6098	551.2282	276.1178	4
16	**1838.6956**	919.8514	1821.6691	911.3382	1820.6850	910.8462	D	409.2082	205.1077	392.1816	196.5944	391.1976	196.1024	3
17	1985.7640	993.3856	1968.7375	984.8724	**1967.7534**	984.3804	F	294.1812	147.5942	277.1547	139.0810			2
18							K	147.1128	74.0600	130.0863	65.5468			1

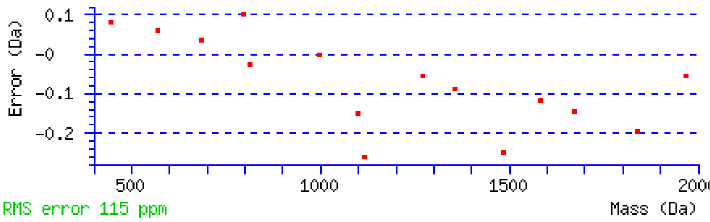

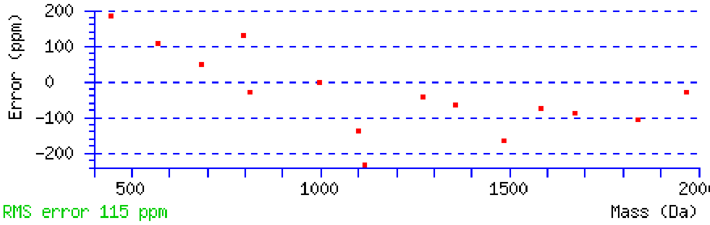

All matches to this query

Score	Mr(calc):	Delta	Sequence
71.0	2130.8623	1.0117	EGNCSVQSGLTWQDCDFK
70.5	2129.8783	1.9957	EGNCSVQSGLTWQDCDFK
5.0	2131.8564	0.0176	FTSRILNHSTSVMRNK

4.1	2131.8769	-0.0030	MEAGDTFPATEESSPPKSR
3.0	2130.8724	1.0016	FTSRILNHSTSVMRNK
2.6	2131.8564	0.0176	FTSRILNHSTSVMRNK
2.0	2131.8564	0.0176	FTSRILNHSTSVMRNK
1.3	2131.8591	0.0149	LSSISKESLTYASMTFK

Spectrum No: 217; Query: 444; Rank: 1

Peptide View

MS/MS Fragmentation of **FSLVNDSEGWLCIK**
Found in **IPI00471800**, Tax_Id=10116 Gene_Symbol=Cdh16 Cadherin 16

Match to Query 444: 1668.790508 from(835.402530,2+)
Title: 091129RatKid_SCX02_11.4190.4190.2.dta
Data file K:\NewmanPaper\Piliang\3SubProteomes\Piliang3SP\mgf5ppm\SCX_3SubProteomes5ppm.mgf

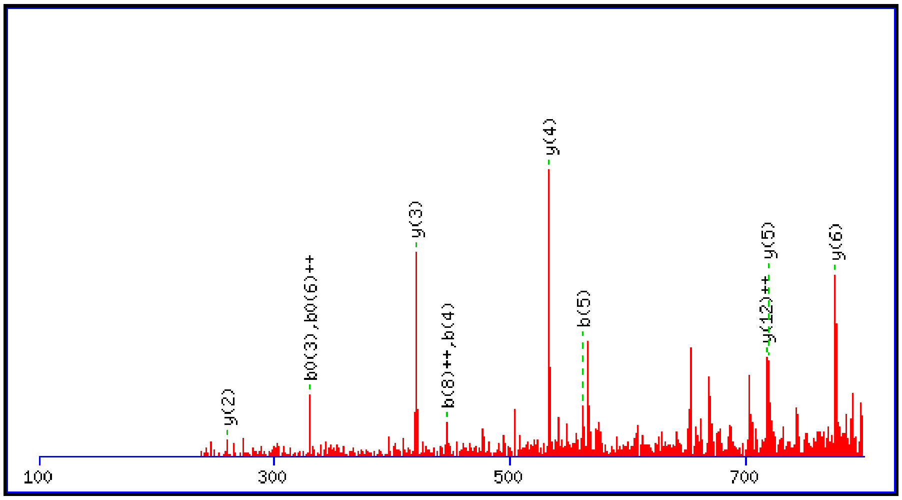

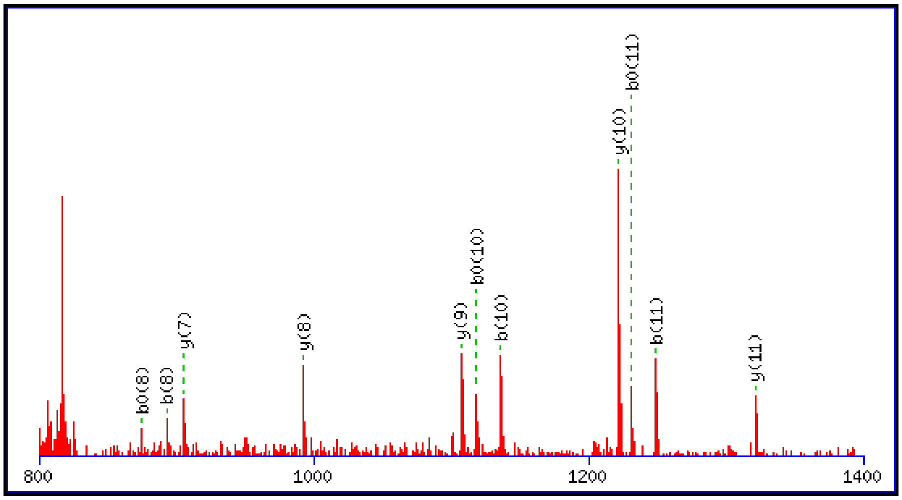

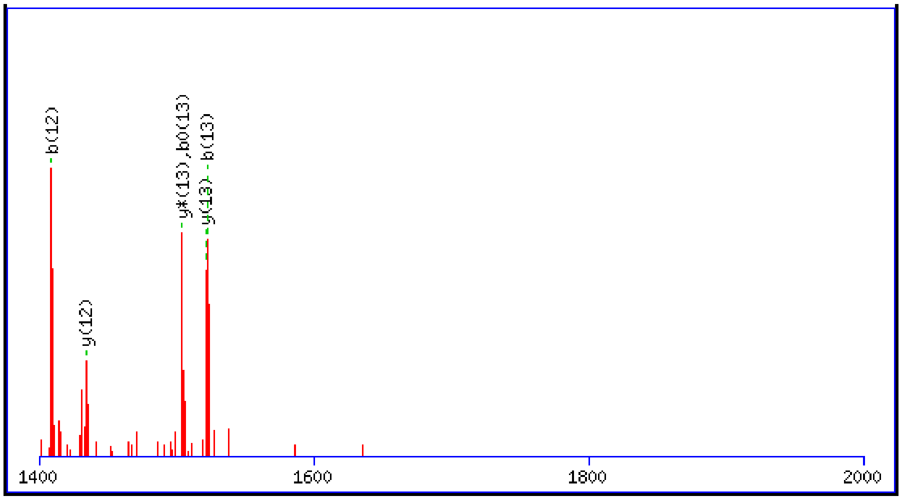

Monoisotopic mass of neutral peptide Mr(calc): 1667.7865
Fixed modifications: Carbamidomethyl (C)
Variable modifications:
N5 : Deamidated_N (N)
Ions Score: 71 **Expect:** 1.5e-005
Matches (Bold Red): 28/134 fragment ions using 61 most intense peaks

#	b	b^{++}	b*	b^{*++}	b^{0}	b^{0++}	Seq.	y	y^{++}	y*	y^{*++}	y^{0}	y^{0++}	#
1	148.0757	74.5415					F							14
2	235.1077	118.0575			217.0972	109.0522	S	**1521.7254**	761.3663	**1504.6988**	752.8530	1503.7148	752.3610	13
3	348.1918	174.5995			**330.1812**	165.5942	L	**1434.6933**	**717.8503**	1417.6668	709.3370	1416.6828	708.8450	12
4	**447.2602**	224.1337			429.2496	215.1285	V	**1321.6093**	661.3083	1304.5827	652.7950	1303.5987	652.3030	11
5	**562.2871**	281.6472	545.2606	273.1339	544.2766	272.6419	N	**1222.5409**	611.7741	1205.5143	603.2608	1204.5303	602.7688	10
6	677.3141	339.1607	660.2875	330.6474	659.3035	**330.1554**	D	**1107.5139**	554.2606	1090.4874	545.7473	1089.5034	545.2553	9
7	764.3461	382.6767	747.3196	374.1634	746.3355	373.6714	S	**992.4870**	496.7471	975.4604	488.2339	974.4764	487.7418	8
8	**893.3887**	**447.1980**	876.3621	438.6847	**875.3781**	438.1927	E	**905.4550**	453.2311	888.4284	444.7178	887.4444	444.2258	7
9	950.4102	475.7087	933.3836	467.1954	932.3996	466.7034	G	**776.4124**	388.7098	759.3858	380.1965			6
10	**1136.4895**	568.7484	1119.4629	560.2351	**1118.4789**	559.7431	W	**719.3909**	360.1991	702.3643	351.6858			5
11	**1249.5735**	625.2904	1232.5470	616.7771	**1231.5630**	616.2851	L	**533.3116**	267.1594	516.2850	258.6462			4
12	**1409.6042**	705.3057	1392.5776	696.7925	1391.5936	696.3004	C	**420.2275**	210.6174	403.2010	202.1041			3
13	**1522.6882**	761.8478	1505.6617	753.3345	**1504.6777**	752.8425	I	**260.1969**	130.6021	243.1703	122.0888			2
14							K	147.1128	74.0600	130.0863	65.5468			1

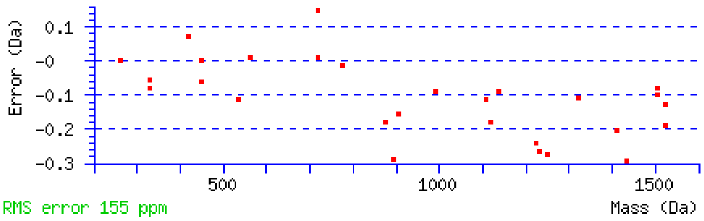

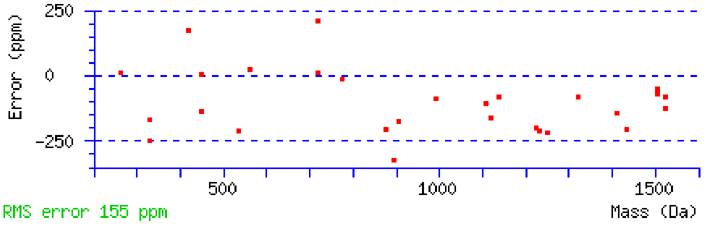

All matches to this query

Score	Mr(calc):	Delta	Sequence
71.0	1667.7865	1.0040	FSLVNDSEGWLCIK
8.6	1667.7878	1.0027	SNAFFPRWDQCLK
6.8	1666.7953	1.9952	EMDCSVLKRLMNR
4.8	1666.7832	2.0073	SLAMLGSSEDNTALSR
3.1	1667.7712	1.0193	FGIEDTLMEENVQK
2.2	1668.8018	-0.0113	SPRISETSAVCVRK
2.0	1667.7858	1.0047	EEANEMLTTMSRLK

1.3	1668.8051	-0.0146	FNALLTTYEYIIK
0.9	1667.7858	1.0047	EEANEMLTTMSRLK
0.3	1667.7792	1.0113	SSGKLMLLQKMLK

Spectrum No: 218; Query: 1003; Rank: 1

Peptide View

MS/MS Fragmentation of **NASTDNSVITVFTGVGDYYSSGNDLR**
Found in **IPI00362963**, Tax_Id=10116 Gene_Symbol=RGD1310224 Similar to RIKEN cDNA 1810022C23

Match to Query 1003: 2752.258228 from(1377.136390,2+)
Title: 091127RatKid_SCX01_12.4947.4947.2.dta
Data file K:\NewmanPaper\Piliang\3SubProteomes\Piliang3SP\mgf5ppm\SCX_3SubProteomes5ppm.mgf

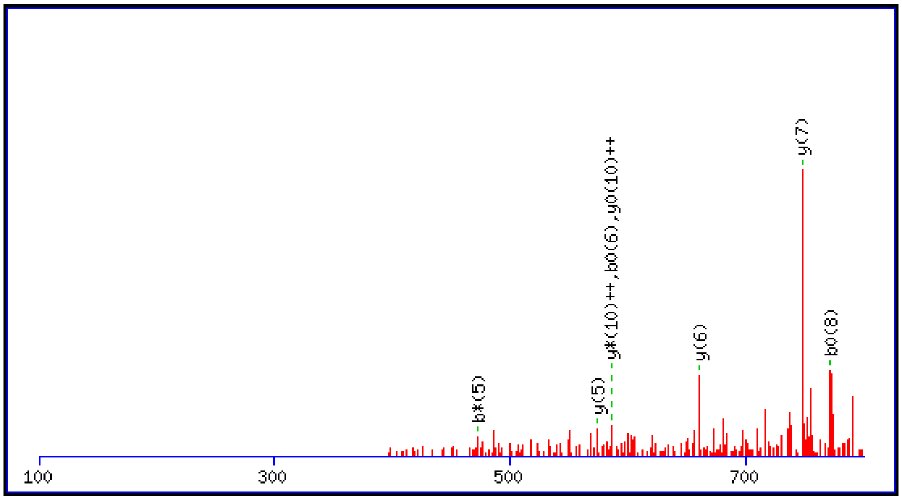

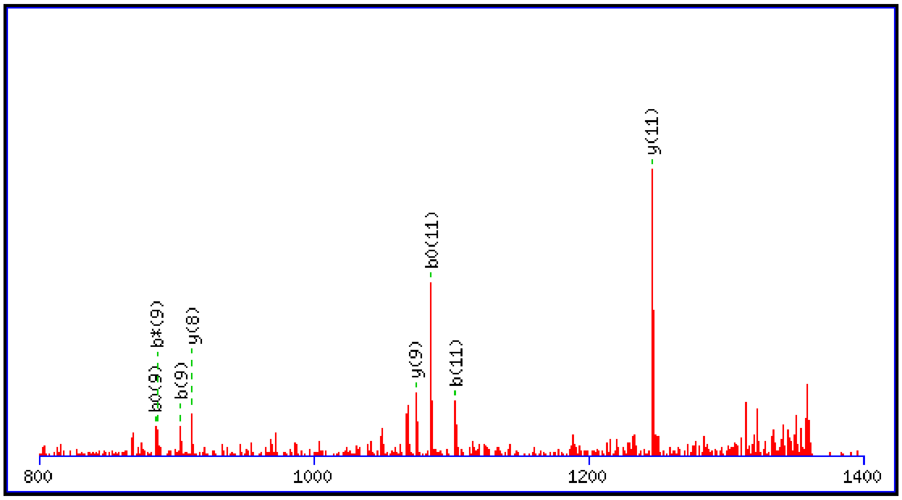

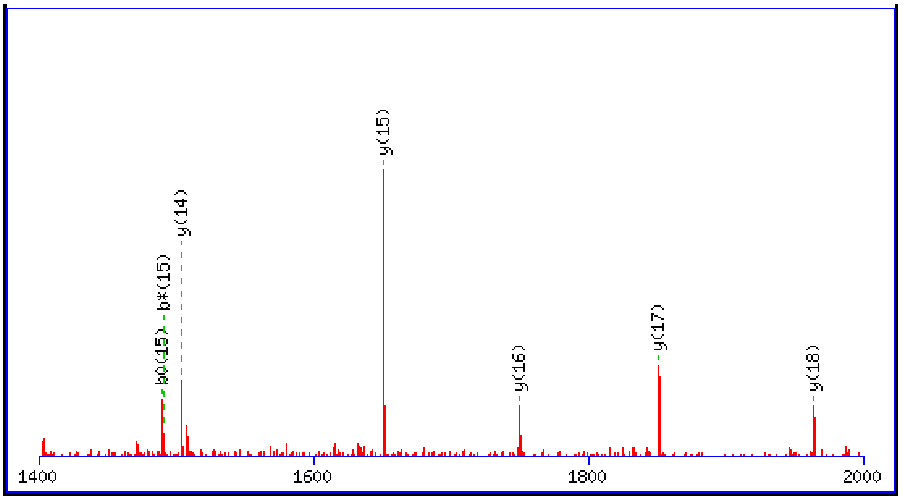

Monoisotopic mass of neutral peptide Mr(calc): 2752.2464
Fixed modifications: Carbamidomethyl (C)
Variable modifications:
N1 : Deamidated_N (N)
Ions Score: 71 **Expect:** 2.7e-005
Matches (Bold Red): 23/292 fragment ions using 37 most intense peaks

#	b	b^{++}	b*	b^{*++}	b^0	b^{0++}	Seq.	y	y^{++}	y*	y^{*++}	y^0	y^{0++}	#
1	116.0342	58.5207	99.0077	50.0075			N							26
2	187.0713	94.0393	170.0448	85.5260			A	2638.2267	1319.6170	2621.2002	1311.1037	2620.2162	1310.6117	25
3	274.1034	137.5553	257.0768	129.0420	256.0928	128.5500	S	2567.1896	1284.0984	2550.1631	1275.5852	2549.1791	1275.0932	24
4	375.1510	188.0792	358.1245	179.5659	357.1405	179.0739	T	2480.1576	1240.5824	2463.1310	1232.0692	2462.1470	1231.5772	23
5	490.1780	245.5926	**473.1514**	237.0794	472.1674	236.5873	D	2379.1099	1190.0586	2362.0834	1181.5453	2361.0993	1181.0533	22
6	604.2209	302.6141	587.1944	294.1008	**586.2103**	293.6088	N	2264.0830	1132.5451	2247.0564	1124.0318	2246.0724	1123.5398	21
7	691.2529	346.1301	674.2264	337.6168	673.2424	337.1248	S	2150.0400	1075.5237	2133.0135	1067.0104	2132.0295	1066.5184	20
8	790.3213	395.6643	773.2948	387.1510	**772.3108**	386.6590	V	2063.0080	1032.0076	2045.9815	1023.4944	2044.9974	1023.0024	19
9	**903.4054**	452.2063	**886.3789**	443.6931	**885.3948**	443.2011	I	**1963.9396**	982.4734	1946.9131	973.9602	1945.9290	973.4682	18
10	1004.4531	502.7302	987.4265	494.2169	986.4425	493.7249	T	**1850.8555**	925.9314	1833.8290	917.4181	1832.8450	916.9261	17
11	**1103.5215**	552.2644	1086.4950	543.7511	**1085.5109**	543.2591	V	**1749.8079**	875.4076	1732.7813	866.8943	1731.7973	866.4023	16
12	1250.5899	625.7986	1233.5634	617.2853	1232.5794	616.7933	F	**1650.7394**	825.8734	1633.7129	817.3601	1632.7289	816.8681	15
13	1351.6376	676.3224	1334.6110	667.8092	1333.6270	667.3172	T	**1503.6710**	752.3392	1486.6445	743.8259	1485.6605	743.3339	14
14	1408.6591	704.8332	1391.6325	696.3199	1390.6485	695.8279	G	1402.6233	701.8153	1385.5968	693.3020	1384.6128	692.8100	13
15	1507.7275	754.3674	**1490.7009**	745.8541	**1489.7169**	745.3621	V	1345.6019	673.3046	1328.5753	664.7913	1327.5913	664.2993	12
16	1564.7489	782.8781	1547.7224	774.3648	1546.7384	773.8728	G	**1246.5335**	623.7704	1229.5069	615.2571	1228.5229	614.7651	11
17	1679.7759	840.3916	1662.7493	831.8783	1661.7653	831.3863	D	1189.5120	595.2596	1172.4855	**586.7464**	1171.5014	**586.2544**	10
18	1842.8392	921.9232	1825.8127	913.4100	1824.8286	912.9180	Y	**1074.4851**	537.7462	1057.4585	529.2329	1056.4745	528.7409	9
19	2005.9025	1003.4549	1988.8760	994.9416	1987.8920	994.4496	Y	**911.4217**	456.2145	894.3952	447.7012	893.4112	447.2092	8
20	2092.9346	1046.9709	2075.9080	1038.4576	2074.9240	1037.9656	S	**748.3584**	374.6828	731.3319	366.1696	730.3478	365.6776	7
21	2179.9666	1090.4869	2162.9400	1081.9737	2161.9560	1081.4817	S	**661.3264**	331.1668	644.2998	322.6536	643.3158	322.1615	6
22	2236.9881	1118.9977	2219.9615	1110.4844	2218.9775	1109.9924	G	**574.2943**	287.6508	557.2678	279.1375	556.2838	278.6455	5
23	2351.0310	1176.0191	2334.0044	1167.5059	2333.0204	1167.0138	N	517.2729	259.1401	500.2463	250.6268	499.2623	250.1348	4
24	2466.0579	1233.5326	2449.0314	1225.0193	2448.0474	1224.5273	D	403.2300	202.1186	386.2034	193.6053	385.2194	193.1133	3
25	2579.1420	1290.0746	2562.1154	1281.5614	2561.1314	1281.0694	L	288.2030	144.6051	271.1765	136.0919			2
26							R	175.1190	88.0631	158.0924	79.5498			1

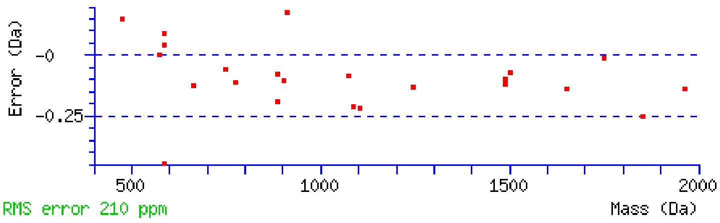

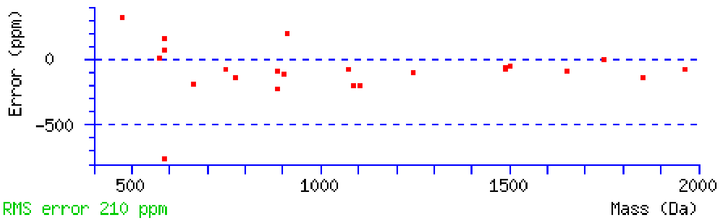

All matches to this query

Score	Mr(calc):	Delta	Sequence
71.0	2752.2464	0.0118	NASTDNSVITVFTGVGDYYSSGNDLR
68.6	2752.2464	0.0118	NASTDNSVITVFTGVGDYYSSGNDLR
54.6	2751.2624	0.9958	NASTDNSVITVFTGVGDYYSSGNDLR
5.2	2751.2683	0.9899	VTHDSIGMVEGLMTMVYAITATRK
5.2	2751.2683	0.9899	VTHDSIGMVEGLMTMVYAITATRK
4.7	2750.2687	1.9895	GQNYLLSLLVGLSFKNESNSQDNK
4.7	2750.2687	1.9895	GQNYLLSLLVGLSFKNESNSQDNK
4.6	2752.2464	0.0118	NASTDNSVITVFTGVGDYYSSGNDLR
4.3	2751.2683	0.9899	VTHDSIGMVEGLMTMVYAITATRK
2.7	2751.2527	1.0055	GQNYLLSLLVGLSFKNESNSQDNK

Spectrum No: 219; Query: 10; Rank: 1

Peptide View

MS/MS Fragmentation of **TPGNASLSLR**
Found in **IPI00193010**, Tax_Id=10116 Gene_Symbol=Slc23a3_predicted solute carrier family 23 (nucleobase transporters), member 3

Match to Query 10: 1015.530348 from(508.772450,2+)
Title: 091127RatKid_SCX01_02.1215.1215.2.dta
Data file K:\NewmanPaper\Piliang\3SubProteomes\Piliang3SP\mgf5ppm\SCX_3SubProteomes5ppm.mgf

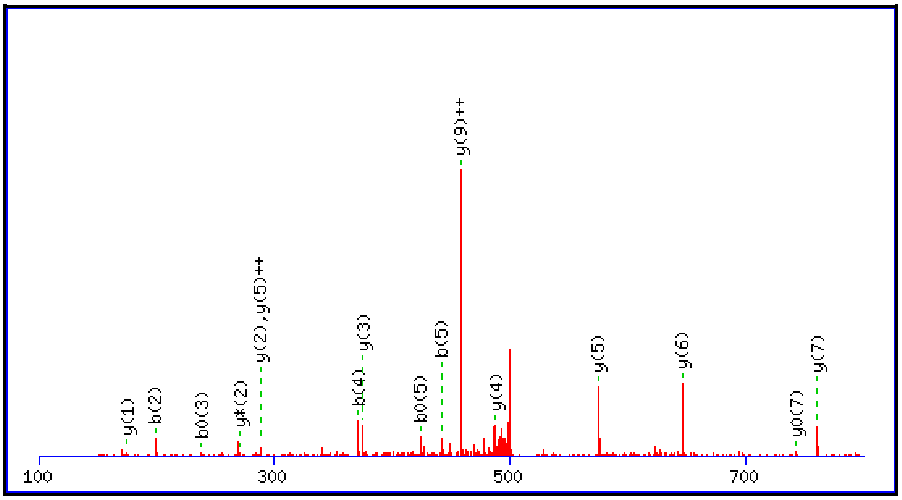

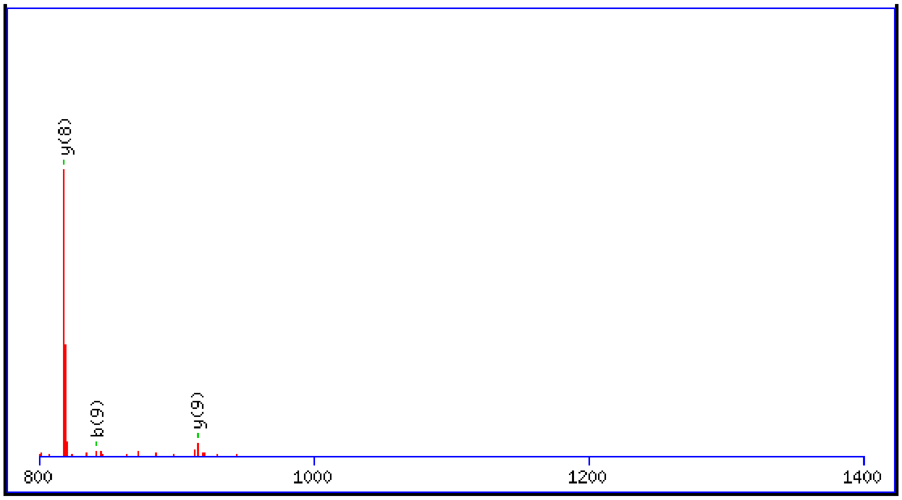

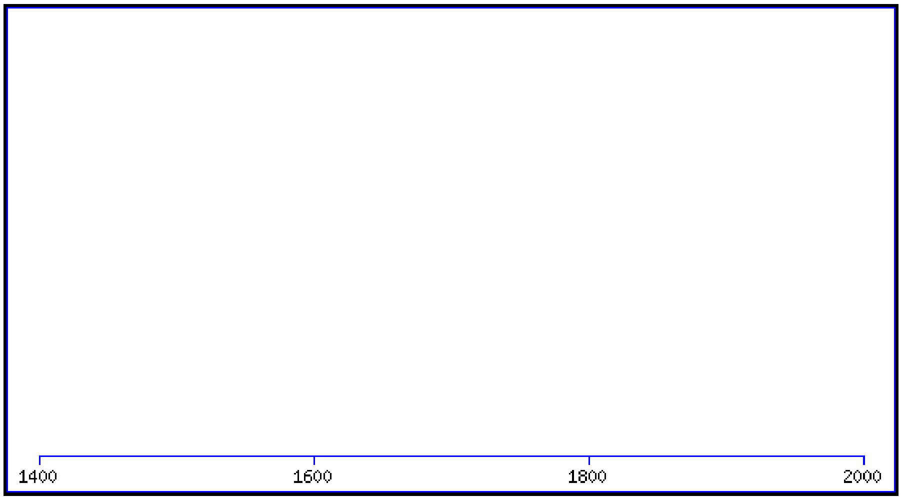

Monoisotopic mass of neutral peptide Mr(calc): 1015.5298
Fixed modifications: Carbamidomethyl (C)
Variable modifications:
N4 : Deamidated_N (N)
Ions Score: 71 **Expect:** 6.7e-006
Matches (Bold Red): 19/98 fragment ions using 35 most intense peaks

#	b	b^{++}	b*	b^{*++}	b^0	b^{0++}	Seq.	y	y^{++}	y*	y^{*++}	y^0	y^{0++}	#
1	102.0550	51.5311			84.0444	42.5258	T							10
2	**199.1077**	100.0575			181.0972	91.0522	P	**915.4894**	**458.2483**	898.4629	449.7351	897.4788	449.2431	9
3	256.1292	128.5682			**238.1186**	119.5629	G	**818.4367**	409.7220	801.4101	401.2087	800.4261	400.7167	8
4	**371.1561**	186.0817	354.1296	177.5684	353.1456	177.0764	N	**761.4152**	381.2112	744.3886	372.6980	**743.4046**	372.2059	7
5	**442.1932**	221.6003	425.1667	213.0870	**424.1827**	212.5950	A	**646.3883**	323.6978	629.3617	315.1845	628.3777	314.6925	6
6	529.2253	265.1163	512.1987	256.6030	511.2147	256.1110	S	**575.3511**	**288.1792**	558.3246	279.6659	557.3406	279.1739	5
7	642.3093	321.6583	625.2828	313.1450	624.2988	312.6530	L	**488.3191**	244.6632	471.2926	236.1499	470.3085	235.6579	4
8	729.3414	365.1743	712.3148	356.6610	711.3308	356.1690	S	**375.2350**	188.1212	358.2085	179.6079	357.2245	179.1159	3
9	**842.4254**	421.7163	825.3989	413.2031	824.4149	412.7111	L	**288.2030**	144.6051	**271.1765**	136.0919			2
10							R	**175.1190**	88.0631	158.0924	79.5498			1

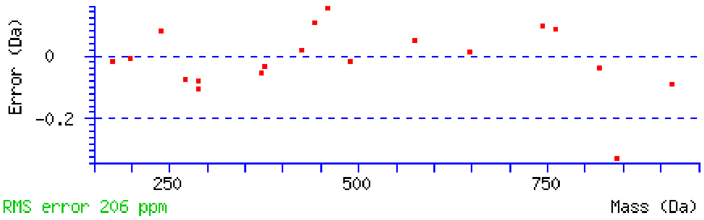

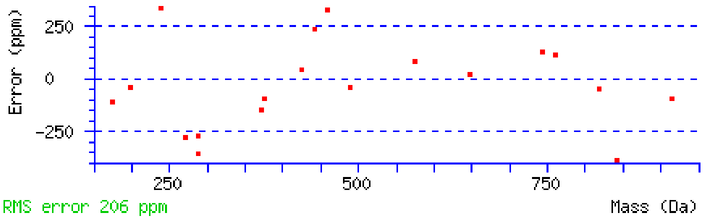

All matches to this query

Score	Mr(calc):	Delta	Sequence
70.8	1015.5298	0.0005	TPGNASLSLR
11.4	1013.5328	1.9975	VTAPCTPLR
6.2	1013.5142	2.0162	VPTTGAPESR
5.6	1013.5254	2.0050	VATAREDPR
4.5	1015.5298	0.0006	LKEAGNINR
4.0	1014.5233	1.0070	NPDLSILSGV
3.7	1015.5233	0.0071	CPLAGTNKR
3.2	1015.5298	0.0006	EQLANLATR
2.3	1013.5328	1.9975	VPVLGCVDR
2.3	1013.5311	1.9993	LFTLTALR

Spectrum No: 220; Query: 377; Rank: 1

Peptide View

MS/MS Fragmentation of **CSGDGLDSDGNQTLR**
Found in **IPI00364764**, Tax_Id=10116 Gene_Symbol=RGD1559717_predicted similar to R17.3

Match to Query 377: 1594.655248 from(798.334900,2+)
Title: 091127RatKid_SCX01_13.839.839.2.dta
Data file K:\NewmanPaper\Piliang\3SubProteomes\Piliang3SP\mgf5ppm\SCX_3SubProteomes5ppm.mgf

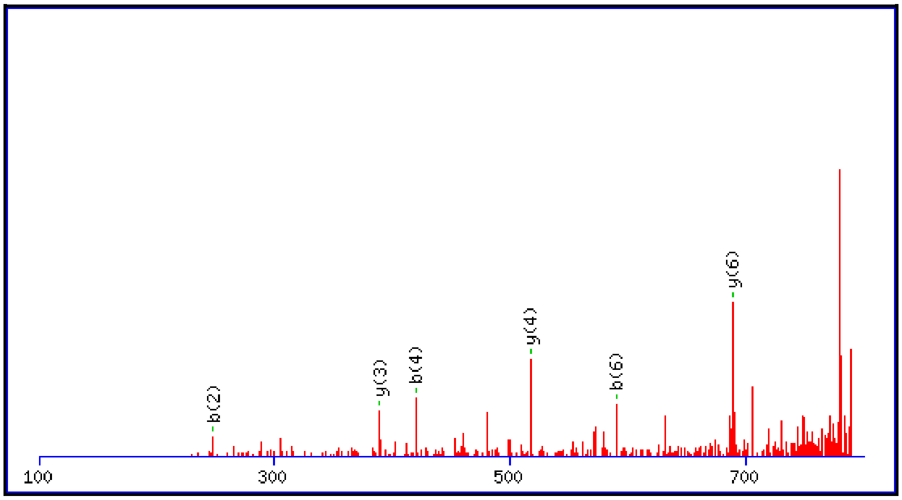

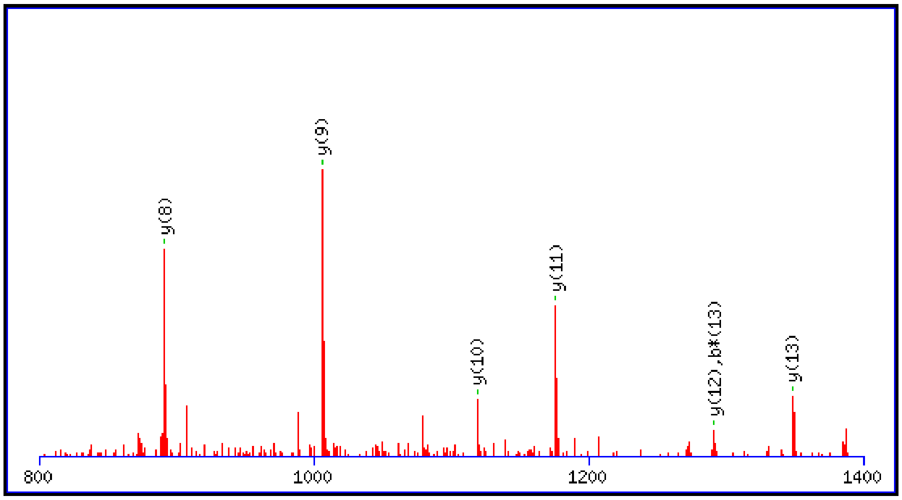

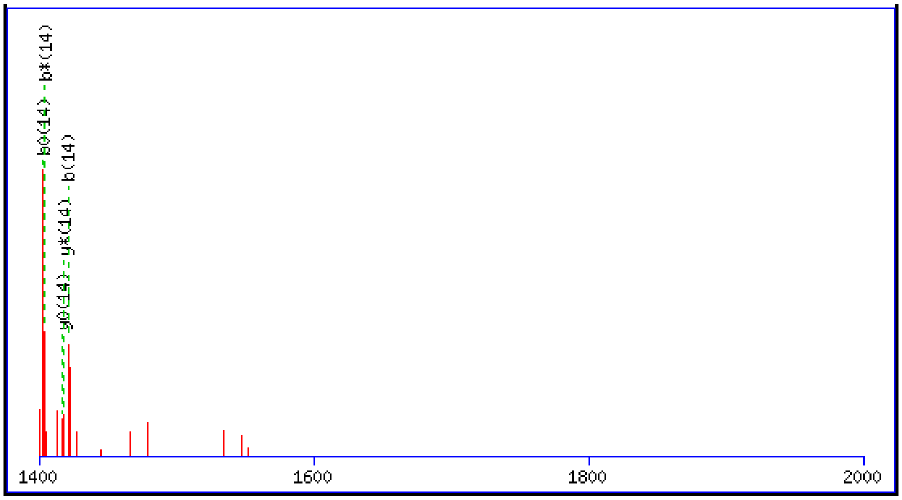

Monoisotopic mass of neutral peptide Mr(calc): 1594.6529
Fixed modifications: Carbamidomethyl (C)
Variable modifications:
N11 : Deamidated_N (N)
Ions Score: 71 **Expect:** 1.2e-005
Matches (Bold Red): 18/142 fragment ions using 29 most intense peaks

#	b	b^{++}	b*	b^{*++}	b^{0}	b^{0++}	Seq.	y	y^{++}	y*	y^{*++}	y^{0}	y^{0++}	#
1	161.0379	81.0226					C							15
2	**248.0700**	124.5386			230.0594	115.5333	S	1435.6295	718.3184	**1418.6030**	709.8051	**1417.6190**	709.3131	14
3	305.0914	153.0493			287.0809	144.0441	G	**1348.5975**	674.8024	1331.5710	666.2891	1330.5870	665.7971	13
4	**420.1184**	210.5628			402.1078	201.5575	D	**1291.5761**	646.2917	1274.5495	637.7784	1273.5655	637.2864	12
5	477.1398	239.0735			459.1293	230.0683	G	**1176.5491**	588.7782	1159.5226	580.2649	1158.5385	579.7729	11
6	**590.2239**	295.6156			572.2133	286.6103	L	**1119.5277**	560.2675	1102.5011	551.7542	1101.5171	551.2622	10
7	705.2508	353.1291			687.2403	344.1238	D	**1006.4436**	503.7254	989.4170	495.2122	988.4330	494.7201	9
8	792.2829	396.6451			774.2723	387.6398	S	**891.4166**	446.2120	874.3901	437.6987	873.4061	437.2067	8
9	907.3098	454.1585			889.2992	445.1533	D	804.3846	402.6959	787.3581	394.1827	786.3741	393.6907	7
10	964.3313	482.6693			946.3207	473.6640	G	**689.3577**	345.1825	672.3311	336.6692	671.3471	336.1772	6
11	1079.3582	540.1827	1062.3317	531.6695	1061.3476	531.1775	N	632.3362	316.6717	615.3097	308.1585	614.3256	307.6665	5
12	1207.4168	604.2120	1190.3902	595.6988	1189.4062	595.2067	Q	**517.3093**	259.1583	500.2827	250.6450	499.2987	250.1530	4
13	1308.4645	654.7359	**1291.4379**	646.2226	1290.4539	645.7306	T	**389.2507**	195.1290	372.2241	186.6157	371.2401	186.1237	3
14	**1421.5485**	711.2779	**1404.5220**	702.7646	**1403.5380**	702.2726	L	288.2030	144.6051	271.1765	136.0919			2
15							R	175.1190	88.0631	158.0924	79.5498			1

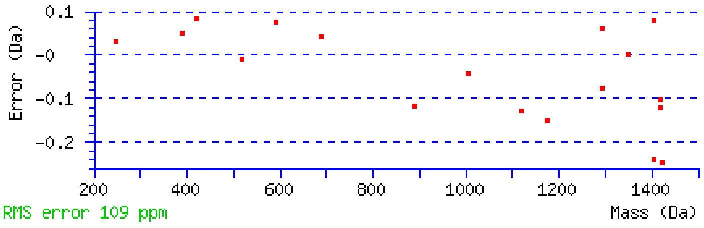

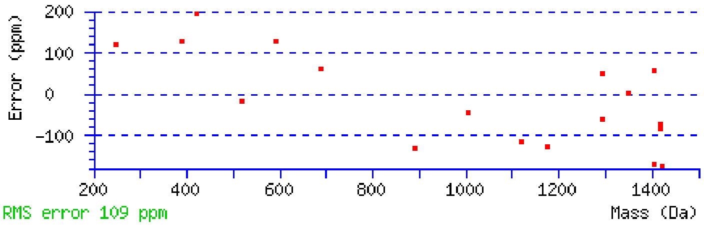

All matches to this query

Score	Mr(calc):	Delta	Sequence
70.5	1594.6529	0.0023	CSGDGLDSDGNQTLR
5.6	1594.6529	0.0024	EGSTLGLTISGGTDK
1.0	1594.6503	0.0049	LTIMYSHINGASK
1.0	1594.6503	0.0049	LTIMYSHINGASK
1.0	1594.6503	0.0049	LTIMYSHINGASK
0.7	1592.6484	2.0068	NTGDLSDQTLITR

Spectrum No: 221; Query: 684; Rank: 1

Peptide View

MS/MS Fragmentation of **KLHINYNNLTESVGPLPK**
Found in **IPI00206403**, Tax_Id=10116 Gene_Symbol=Lum Lumican precursor

Match to Query 684: 2037.091628 from(1019.553090,2+)
Title: 091127RatKid_SCX01_33.3261.3261.2.dta
Data file K:\NewmanPaper\Piliang\3SubProteomes\Piliang3SP\mgf5ppm\SCX_3SubProteomes5ppm.mgf

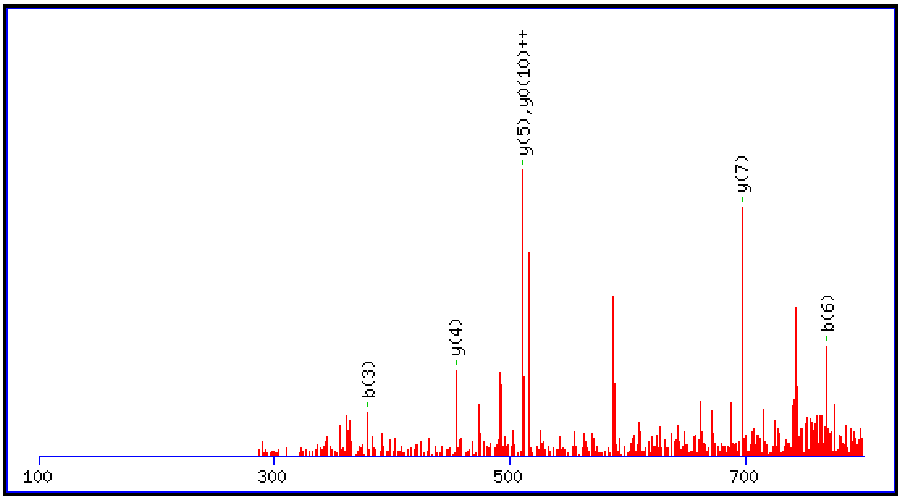

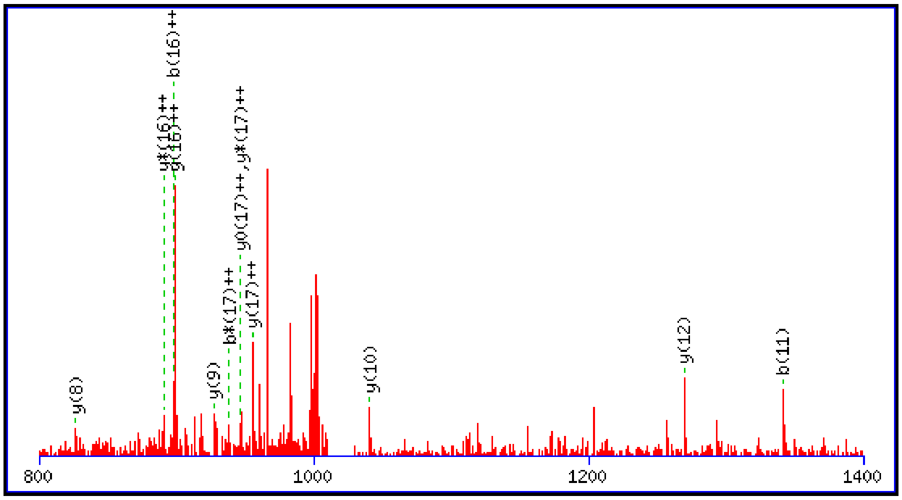

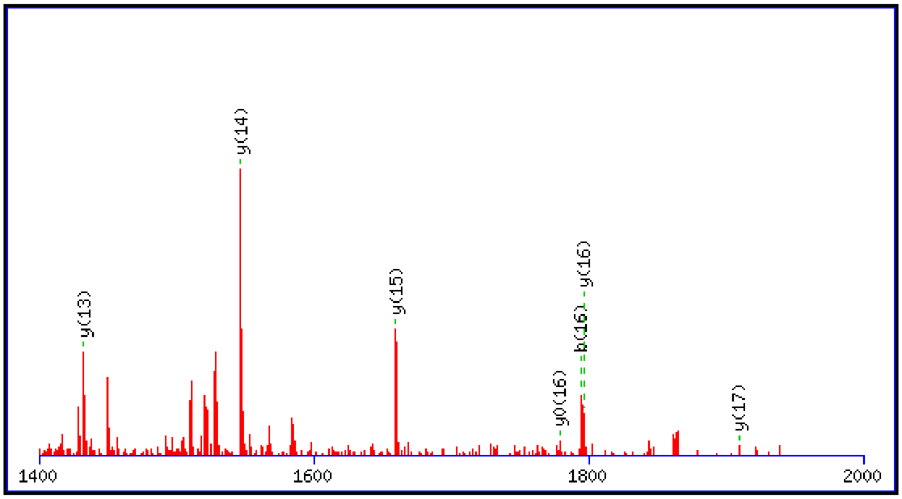

Monoisotopic mass of neutral peptide Mr(calc): 2037.0894
Fixed modifications: Carbamidomethyl (C)
Variable modifications:
N8 : Deamidated_N (N)
Ions Score: 70 **Expect:** 6.9e-006
Matches (Bold Red): 26/174 fragment ions using 49 most intense peaks

#	b	b^{++}	b^{*}	b^{*++}	b^{0}	b^{0++}	Seq.	y	y^{++}	y^{*}	y^{*++}	y^{0}	y^{0++}	#
1	129.1022	65.0548	112.0757	56.5415			K							18
2	242.1863	121.5968	225.1598	113.0835			L	**1910.0018**	**955.5045**	1892.9752	**946.9913**	1891.9912	**946.4993**	17
3	**379.2452**	190.1262	362.2187	181.6130			H	**1796.9177**	**898.9625**	1779.8912	**890.4492**	**1778.9072**	889.9572	16
4	492.3293	246.6683	475.3027	238.1550			I	**1659.8588**	830.4330	1642.8323	821.9198	1641.8483	821.4278	15
5	606.3722	303.6897	589.3457	295.1765			N	**1546.7748**	773.8910	1529.7482	765.3777	1528.7642	764.8857	14
6	**769.4355**	385.2214	752.4090	376.7081			Y	**1432.7318**	716.8696	1415.7053	708.3563	1414.7213	707.8643	13
7	883.4785	442.2429	866.4519	433.7296			N	**1269.6685**	635.3379	1252.6420	626.8246	1251.6579	626.3326	12
8	998.5054	499.7563	981.4788	491.2431			N	1155.6256	578.3164	1138.5990	569.8032	1137.6150	569.3111	11
9	1111.5895	556.2984	1094.5629	547.7851			L	**1040.5986**	520.8030	1023.5721	512.2897	1022.5881	**511.7977**	10
10	1212.6371	606.8222	1195.6106	598.3089	1194.6266	597.8169	T	**927.5146**	464.2609	910.4880	455.7477	909.5040	455.2556	9
11	**1341.6797**	671.3435	1324.6532	662.8302	1323.6692	662.3382	E	**826.4669**	413.7371	809.4403	405.2238	808.4563	404.7318	8
12	1428.7118	714.8595	1411.6852	706.3462	1410.7012	705.8542	S	**697.4243**	349.2158	680.3978	340.7025	679.4137	340.2105	7
13	1527.7802	764.3937	1510.7536	755.8805	1509.7696	755.3884	V	610.3923	305.6998	593.3657	297.1865			6
14	1584.8016	792.9045	1567.7751	784.3912	1566.7911	783.8992	G	**511.3239**	256.1656	494.2973	247.6523			5
15	1681.8544	841.4308	1664.8279	832.9176	1663.8438	832.4256	P	**454.3024**	227.6548	437.2758	219.1416			4
16	**1794.9385**	**897.9729**	1777.9119	889.4596	1776.9279	888.9676	L	357.2496	179.1285	340.2231	170.6152			3
17	1891.9912	**946.4993**	1874.9647	**937.9860**	1873.9807	937.4940	P	244.1656	122.5864	227.1390	114.0731			2
18							K	147.1128	74.0600	130.0863	65.5468			1

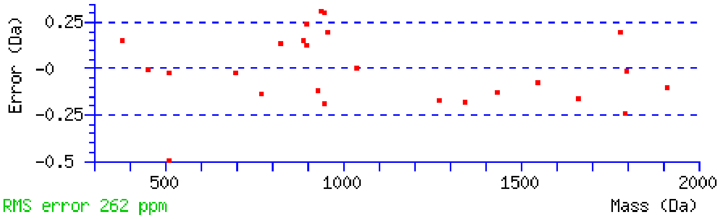

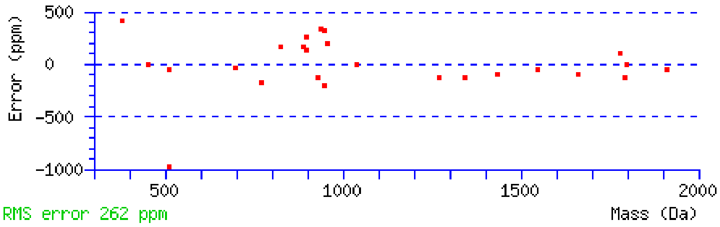

All matches to this query

Score	Mr(calc):	Delta	Sequence
70.5	2037.0894	0.0022	KLHINYNNLTESVGPLPK
70.5	2037.0894	0.0022	KLHINYNNLTESVGPLPK
41.7	2037.0894	0.0022	KLHINYNNLTESVGPLPK
6.1	2036.1054	0.9862	KLHINYNNLTESVGPLPK

Spectrum No: 222; Query: 393; Rank: 1

Peptide View

MS/MS Fragmentation of **FENQTCFPLPDSR**
Found in **IPI00190776**, Tax_Id=10116 Gene_Symbol=Cspg2 Isoform V0 of Versican core protein precursor (Fragment)

Match to Query 393: 1610.706508 from(806.360530,2+)
Title: 091127RatKid_SCX01_16.2727.2727.2.dta
Data file K:\NewmanPaper\Piliang\3SubProteomes\Piliang3SP\mgf5ppm\SCX_3SubProteomes5ppm.mgf

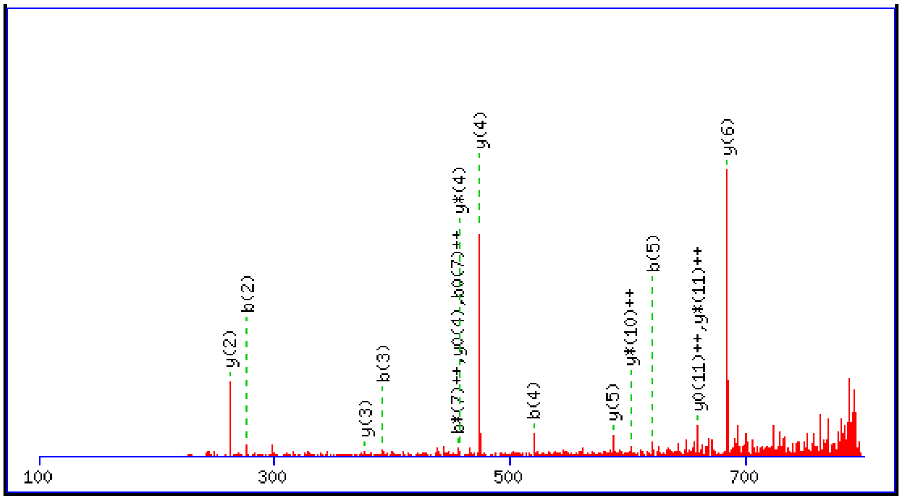

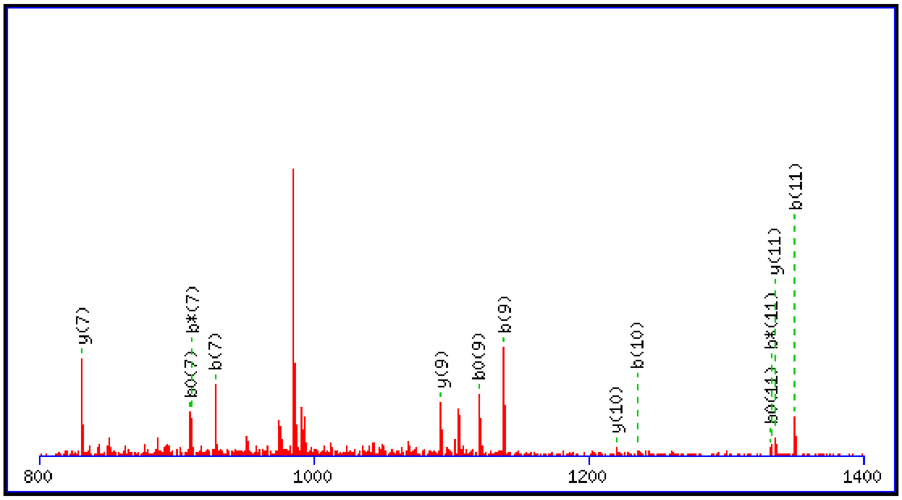

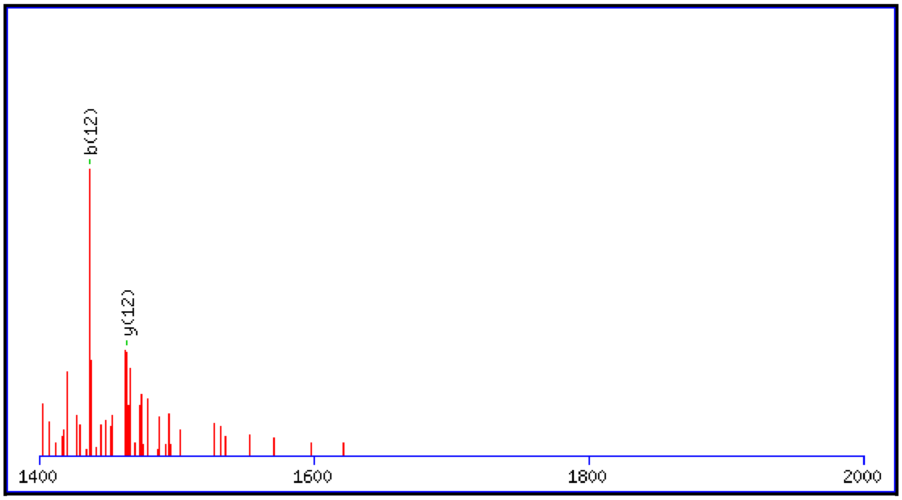

Monoisotopic mass of neutral peptide Mr(calc): 1610.7035
Fixed modifications: Carbamidomethyl (C)
Variable modifications:
N3 : Deamidated_N (N)
Ions Score: 70 **Expect:** 1.7e-005
Matches (Bold Red): 31/136 fragment ions using 57 most intense peaks

#	b	b^{++}	b*	b^{*++}	b^0	b^{0++}	Seq.	y	y^{++}	y*	y^{*++}	y^0	y^{0++}	#
1	148.0757	74.5415					F							13
2	**277.1183**	139.0628			259.1077	130.0575	E	**1464.6424**	732.8248	1447.6158	724.3115	1446.6318	723.8195	12
3	**392.1452**	196.5762	375.1187	188.0630	374.1347	187.5710	N	**1335.5998**	668.3035	1318.5732	**659.7902**	1317.5892	**659.2982**	11
4	**520.2038**	260.6055	503.1772	252.0923	502.1932	251.6003	Q	**1220.5728**	610.7901	1203.5463	**602.2768**	1202.5623	601.7848	10
5	**621.2515**	311.1294	604.2249	302.6161	603.2409	302.1241	T	**1092.5143**	546.7608	1075.4877	538.2475	1074.5037	537.7555	9

**6**	781.2821	391.1447	764.2556	382.6314	763.2716	382.1394	**C**	991.4666	496.2369	974.4400	487.7237	973.4560	487.2316	**8**
**7**	**928.3505**	464.6789	**911.3240**	**456.1656**	**910.3400**	**455.6736**	**F**	**831.4359**	416.2216	814.4094	407.7083	813.4254	407.2163	**7**
**8**	1025.4033	513.2053	1008.3768	504.6920	1007.3927	504.2000	**P**	**684.3675**	342.6874	667.3410	334.1741	666.3569	333.6821	**6**
**9**	**1138.4874**	569.7473	1121.4608	561.2340	**1120.4768**	560.7420	**L**	**587.3148**	294.1610	570.2882	285.6477	569.3042	285.1557	**5**
**10**	**1235.5401**	618.2737	1218.5136	609.7604	1217.5296	609.2684	**P**	**474.2307**	237.6190	**457.2041**	229.1057	**456.2201**	228.6137	**4**
**11**	**1350.5671**	675.7872	**1333.5405**	667.2739	**1332.5565**	666.7819	**D**	**377.1779**	189.0926	360.1514	180.5793	359.1674	180.0873	**3**
**12**	**1437.5991**	719.3032	1420.5726	710.7899	1419.5885	710.2979	**S**	**262.1510**	131.5791	245.1244	123.0659	244.1404	122.5738	**2**
**13**							**R**	175.1190	88.0631	158.0924	79.5498			**1**

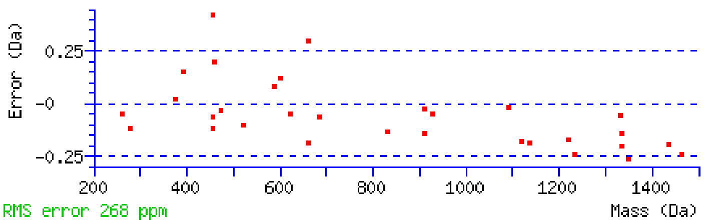

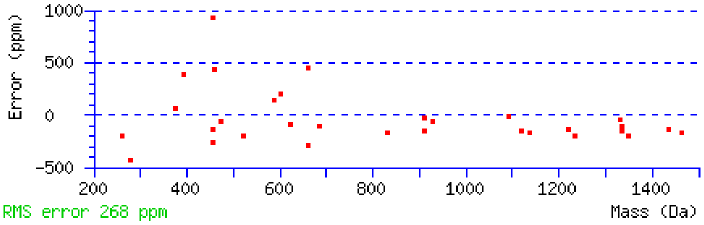

All matches to this query

Score	Mr(calc):	Delta	Sequence
70.2	1610.7035	0.0030	FENQTCFPLPDSR
11.3	1610.7028	0.0037	EMDQTMAANAQKNK
10.8	1610.7189	-0.0123	ENKVDPTLSSGLNR
8.0	1610.7141	-0.0076	SMPSASSCSTRTPSR
6.9	1608.6871	2.0194	TSAIEVMLLETSR
6.9	1608.6871	2.0194	TSAIEVMLLETSR
4.9	1610.7049	0.0016	RSASPPPAASSSSSSR
4.9	1610.7049	0.0016	RSASPPPAASSSSSSR
1.9	1610.7123	-0.0058	LSSLLTIGLIGER
1.6	1610.7049	0.0016	RSASPPPAASSSSSSR

Spectrum No: 223; Query: 121; Rank: 1

Peptide View

MS/MS Fragmentation of **LANTSMFNNSK**
Found in **IPI00206254**, Tax_Id=10116 Gene_Symbol=Ggt1 Gamma-glutamyltranspeptidase 1 precursor

Match to Query 121: 1243.539688 from(622.777120,2+)
Title: 091129RatKid_SCX02_02.943.943.2.dta
Data file K:\NewmanPaper\Piliang\3SubProteomes\Piliang3SP\mgf5ppm\SCX_3SubProteomes5ppm.mgf

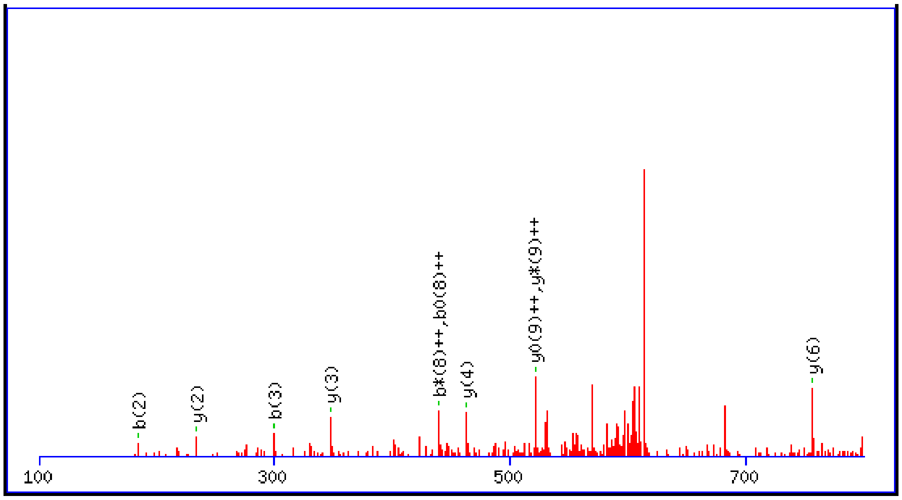

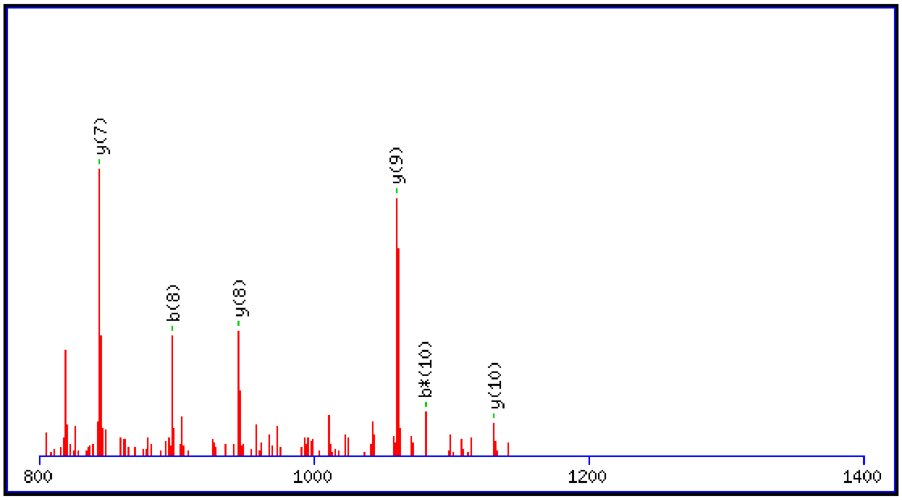

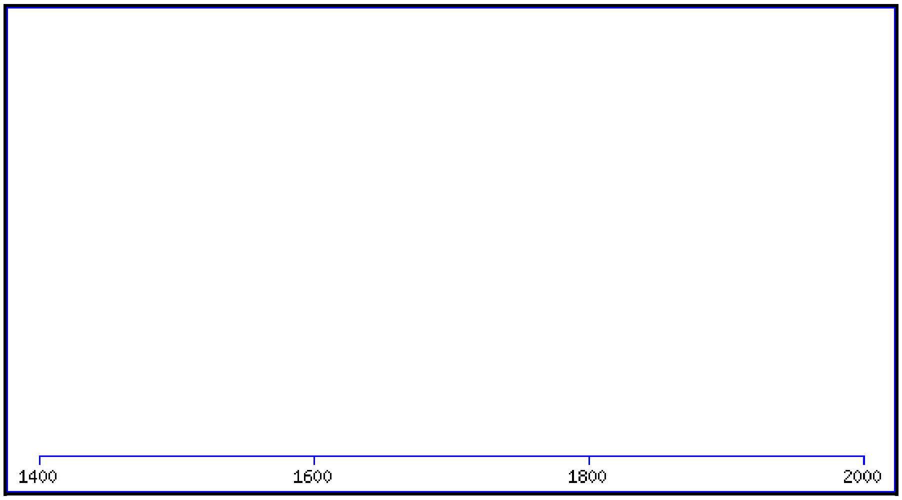

Monoisotopic mass of neutral peptide Mr(calc): 1243.5390
Fixed modifications: Carbamidomethyl (C)
Variable modifications:
N3 : Deamidated_N (N)
M6 : Oxidation (M)
N8 : Deamidated_N (N)
Ions Score: 70 **Expect:** 1.1e-005
Matches (Bold Red): 16/108 fragment ions using 20 most intense peaks

#	b	b^{++}	b*	b^{*++}	b^0	b^{0++}	Seq.	y	y^{++}	y*	y^{*++}	y^0	y^{0++}	#
**1**	114.0913	57.5493					**L**							**11**
**2**	**185.1285**	93.0679					**A**	**1131.4623**	566.2348	1114.4357	557.7215	1113.4517	557.2295	**10**
**3**	**300.1554**	150.5813	283.1288	142.0681			**N**	**1060.4252**	530.7162	1043.3986	**522.2029**	1042.4146	**521.7109**	**9**
**4**	401.2031	201.1052	384.1765	192.5919	383.1925	192.0999	**T**	**945.3982**	473.2027	928.3717	464.6895	927.3877	464.1975	**8**

**5**	488.2351	244.6212	471.2085	236.1079	470.2245	235.6159	**S**	**844.3505**	422.6789	827.3240	414.1656	826.3400	413.6736	**7**
**6**	635.2705	318.1389	618.2440	309.6256	617.2599	309.1336	**M**	**757.3185**	379.1629	740.2920	370.6496	739.3079	370.1576	**6**
**7**	782.3389	391.6731	765.3124	383.1598	764.3284	382.6678	**F**	610.2831	305.6452	593.2566	297.1319	592.2725	296.6399	**5**
**8**	**897.3659**	449.1866	880.3393	**440.6733**	879.3553	**440.1813**	**N**	**463.2147**	232.1110	446.1881	223.5977	445.2041	223.1057	**4**
**9**	1011.4088	506.2080	994.3822	497.6948	993.3982	497.2027	**N**	**348.1878**	174.5975	331.1612	166.0842	330.1772	165.5922	**3**
**10**	1098.4408	549.7240	**1081.4143**	541.2108	1080.4302	540.7188	**S**	**234.1448**	117.5761	217.1183	109.0628	216.1343	108.5708	**2**
**11**							**K**	147.1128	74.0600	130.0863	65.5468			**1**

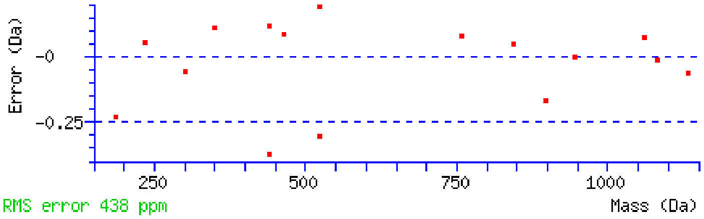

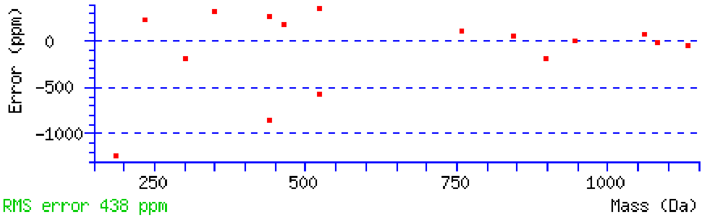

All matches to this query

Score	Mr(calc):	Delta	Sequence
70.0	1243.5390	0.0007	LANTSMFNNSK
54.7	1243.5390	0.0007	LANTSMFNNSK
26.5	1243.5390	0.0007	LANTSMFNNSK
3.5	1241.5289	2.0108	TVAGENQSQTK

Spectrum No: 224; Query: 425; Rank: 1

Peptide View

MS/MS Fragmentation of **NNVSLLQSCIDLFK**
Found in **IPI00373144**, Tax_Id=10116 Gene_Symbol=Snx5_predicted sorting nexin 5

Match to Query 425: 1650.831108 from(826.422830,2+)
Title: 091127RatKid_SCX01_13.5090.5090.2.dta
Data file K:\NewmanPaper\Piliang\3SubProteomes\Piliang3SP\mgf5ppm\SCX_3SubProteomes5ppm.mgf

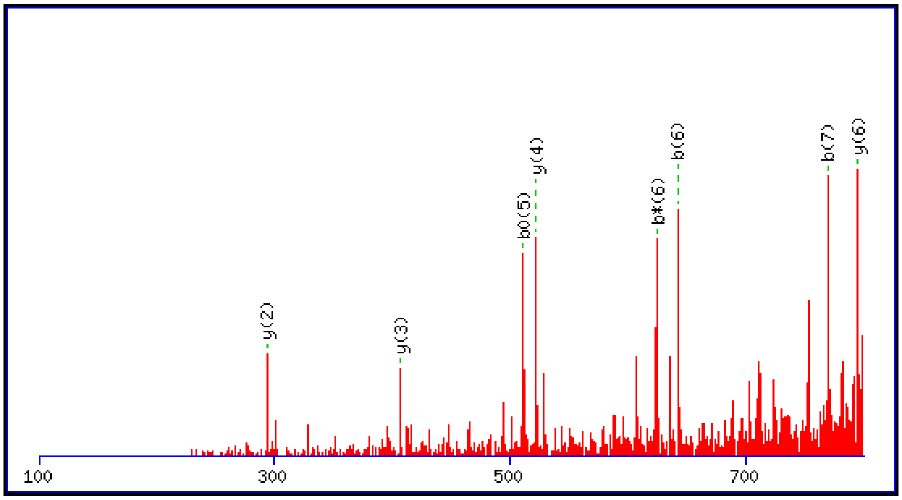

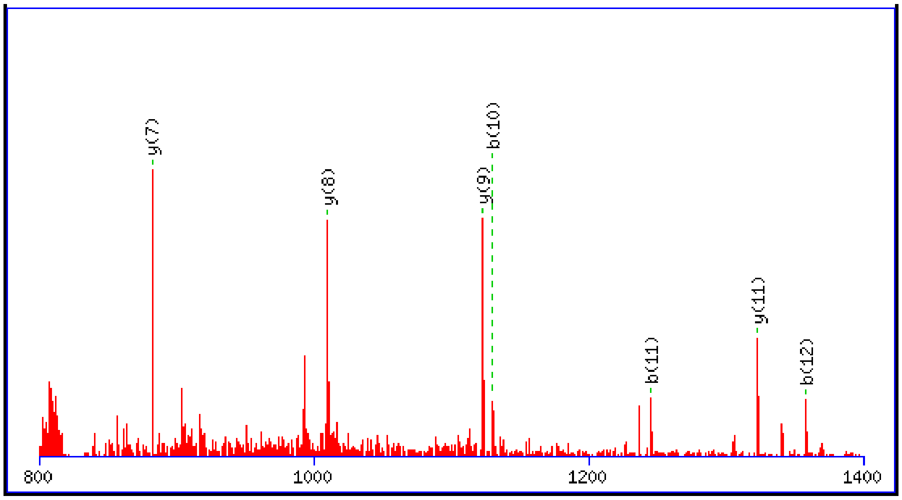

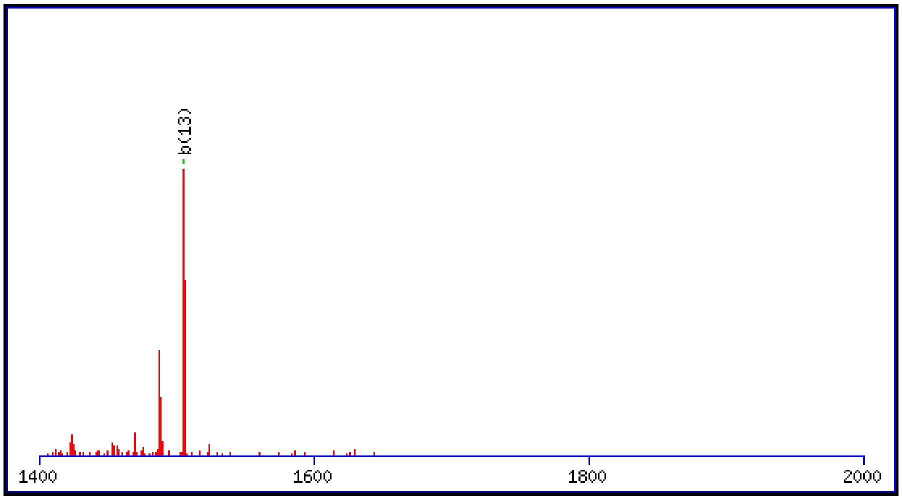

Monoisotopic mass of neutral peptide Mr(calc): 1650.8287
Fixed modifications: Carbamidomethyl (C)
Variable modifications:
N2 : Deamidated_N (N)
Ions Score: 70 **Expect:** 1.6e-005
Matches (Bold Red): 16/144 fragment ions using 22 most intense peaks

#	b	b++	b*	b*++	b0	b0++	Seq.	y	y++	y*	y*++	y0	y0++	#
1	115.0502	58.0287	98.0237	49.5155			N							14
2	230.0771	115.5422	213.0506	107.0289			N	1537.7931	769.4002	1520.7665	760.8869	1519.7825	760.3949	13
3	329.1456	165.0764	312.1190	156.5631			V	1422.7661	711.8867	1405.7396	703.3734	1404.7555	702.8814	12
4	416.1776	208.5924	399.1510	200.0792	398.1670	199.5871	S	**1323.6977**	662.3525	1306.6712	653.8392	1305.6871	653.3472	11
5	529.2616	265.1345	512.2351	256.6212	**511.2511**	256.1292	L	1236.6657	618.8365	1219.6391	610.3232	1218.6551	609.8312	10
6	**642.3457**	321.6765	**625.3192**	313.1632	624.3351	312.6712	L	**1123.5816**	562.2944	1106.5551	553.7812	1105.5710	553.2892	9
7	**770.4043**	385.7058	753.3777	377.1925	752.3937	376.7005	Q	**1010.4975**	505.7524	993.4710	497.2391	992.4870	496.7471	8
8	857.4363	429.2218	840.4098	420.7085	839.4257	420.2165	S	**882.4390**	441.7231	865.4124	433.2098	864.4284	432.7178	7
9	1017.4670	509.2371	1000.4404	500.7238	999.4564	500.2318	C	**795.4069**	398.2071	778.3804	389.6938	777.3964	389.2018	6
10	**1130.5510**	565.7792	1113.5245	557.2659	1112.5405	556.7739	I	635.3763	318.1918	618.3497	309.6785	617.3657	309.1865	5
11	**1245.5780**	623.2926	1228.5514	614.7793	1227.5674	614.2873	D	**522.2922**	261.6498	505.2657	253.1365	504.2817	252.6445	4
12	**1358.6620**	679.8347	1341.6355	671.3214	1340.6515	670.8294	L	**407.2653**	204.1363	390.2387	195.6230			3
13	**1505.7304**	753.3689	1488.7039	744.8556	1487.7199	744.3636	F	**294.1812**	147.5942	277.1547	139.0810			2
14							K	147.1128	74.0600	130.0863	65.5468			1

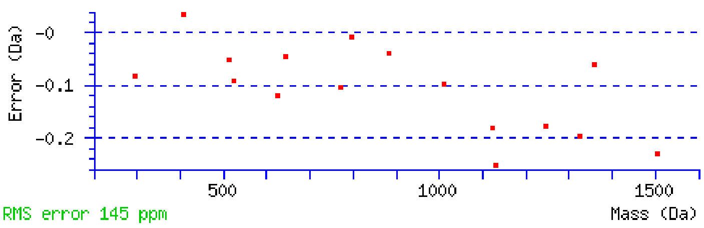

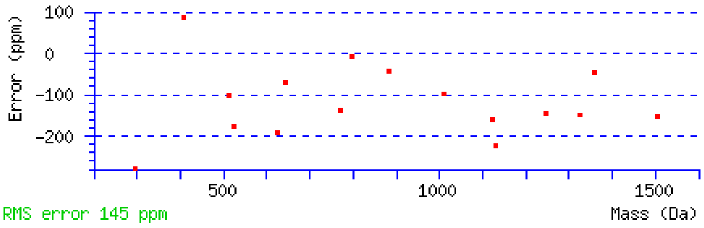

All matches to this query

Score	Mr(calc):	Delta	Sequence
70.1	1650.8287	0.0024	NNVSLLQSCIDLFK
70.1	1650.8287	0.0024	NNVSLLQSCIDLFK
6.0	1650.8213	0.0098	EEDPPSKVQNPQGVK
5.7	1649.8116	1.0195	DIVDRAVKEMSMEK
4.8	1650.8310	0.0001	FFIDTSIILFLNK
4.4	1650.8262	0.0049	HMVFFGGAVLADIMK
3.1	1650.8262	0.0049	KFNFPTVALHSMMK
3.0	1650.8246	0.0065	QSIIVAASSRSEMEK
1.5	1649.8178	1.0133	FAQSILQSLDALHK
1.2	1649.8138	1.0173	DGQDRPLTKINSVK

Spectrum No: 225; Query: 392; Rank: 1

Peptide View

MS/MS Fragmentation of **LHNQLLPNTTTVEK**
Found in **IPI00206254**, Tax_Id=10116 Gene_Symbol=Ggt1 Gamma-glutamyltranspeptidase 1 precursor

Match to Query 392: 1607.857528 from(804.936040,2+)
Title: 091127RatKid_SCX01_28.1670.1670.2.dta
Data file K:\NewmanPaper\Piliang\3SubProteomes\Piliang3SP\mgf5ppm\SCX_3SubProteomes5ppm.mgf

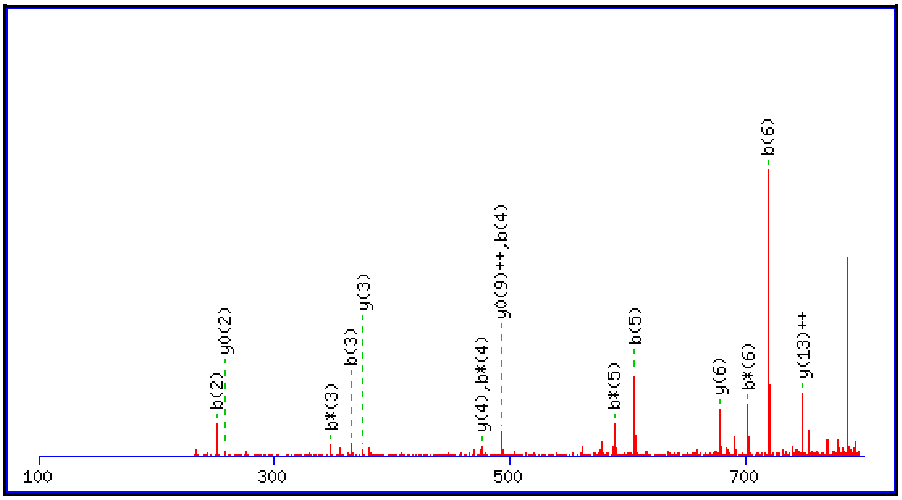

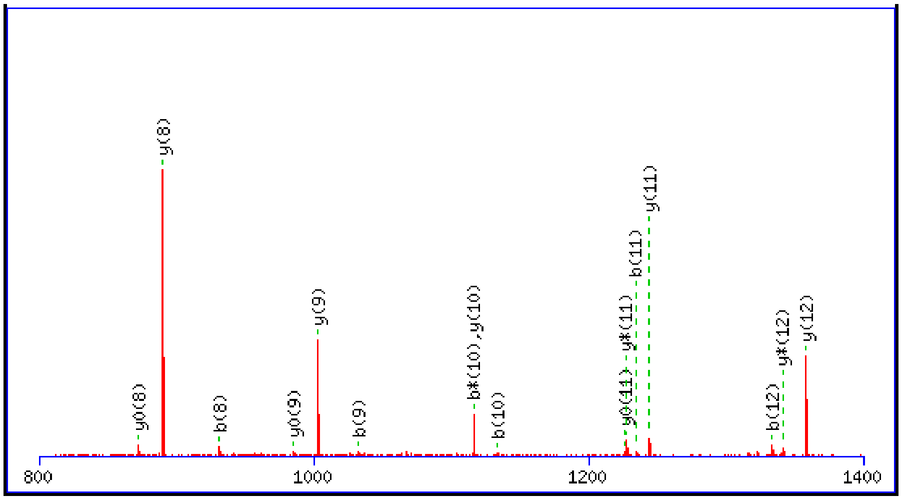

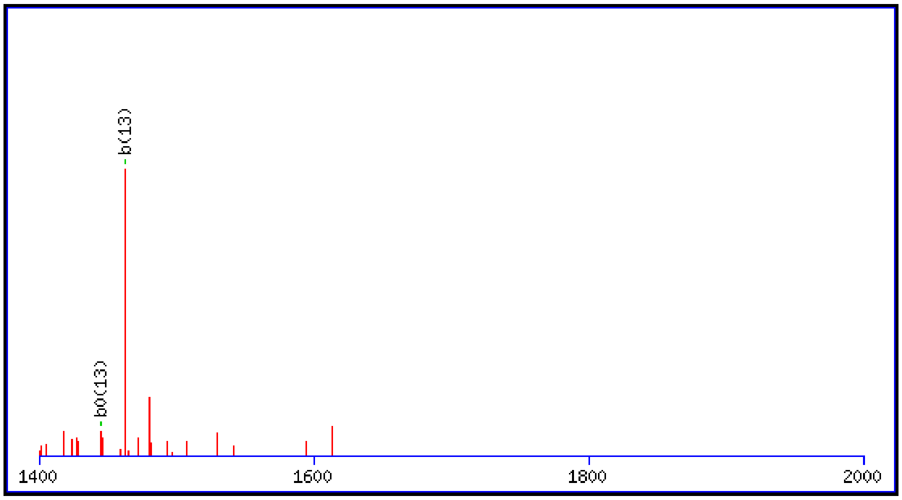

Monoisotopic mass of neutral peptide Mr(calc): 1607.8519
Fixed modifications: Carbamidomethyl (C)
Variable modifications:
N8 : Deamidated_N (N)
Ions Score: 70 **Expect:** 1e-005
Matches (Bold Red): 33/134 fragment ions using 65 most intense peaks

#	b	b^{++}	b*	b^{*++}	b^{0}	b^{0++}	Seq.	y	y^{++}	y*	y^{*++}	y^{0}	y^{0++}	#
1	114.0913	57.5493					L							14
2	**251.1503**	126.0788					H	1495.7751	**748.3912**	1478.7485	739.8779	1477.7645	739.3859	13
3	**365.1932**	183.1002	**348.1666**	174.5870			N	**1358.7162**	679.8617	**1341.6896**	671.3485	1340.7056	670.8564	12
4	**493.2518**	247.1295	**476.2252**	238.6162			Q	**1244.6733**	622.8403	**1227.6467**	614.3270	**1226.6627**	613.8350	11
5	**606.3358**	303.6715	**589.3093**	295.1583			L	**1116.6147**	558.8110	1099.5881	550.2977	1098.6041	549.8057	10
6	**719.4199**	360.2136	**702.3933**	351.7003			L	**1003.5306**	502.2689	986.5041	493.7557	**985.5200**	**493.2637**	9
7	816.4726	408.7400	799.4461	400.2267			P	**890.4465**	445.7269	873.4200	437.2136	**872.4360**	436.7216	8
8	**931.4996**	466.2534	914.4730	457.7402			N	793.3938	397.2005	776.3672	388.6873	775.3832	388.1952	7
9	**1032.5473**	516.7773	1015.5207	508.2640	1014.5367	507.7720	T	**678.3668**	339.6871	661.3403	331.1738	660.3563	330.6818	6
10	**1133.5949**	567.3011	**1116.5684**	558.7878	1115.5844	558.2958	T	577.3192	289.1632	560.2926	280.6499	559.3086	280.1579	5
11	**1234.6426**	617.8249	1217.6161	609.3117	1216.6321	608.8197	T	**476.2715**	238.6394	459.2449	230.1261	458.2609	229.6341	4
12	**1333.7110**	667.3592	1316.6845	658.8459	1315.7005	658.3539	V	**375.2238**	188.1155	358.1973	179.6023	357.2132	179.1103	3
13	**1462.7536**	731.8805	1445.7271	723.3672	**1444.7431**	722.8752	E	276.1554	138.5813	259.1288	130.0681	**258.1448**	129.5761	2
14							K	147.1128	74.0600	130.0863	65.5468			1

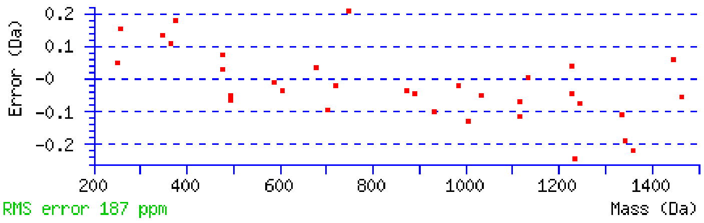

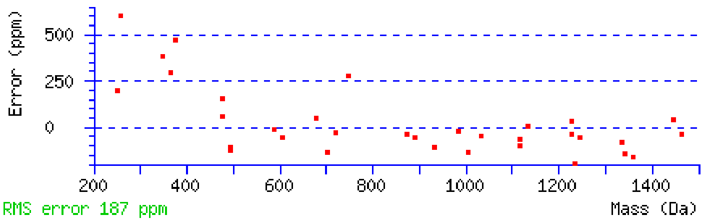

All matches to this query

Score	Mr(calc):	Delta	Sequence
69.9	1607.8519	0.0057	LHNQLLPNTTTVEK
46.4	1607.8519	0.0057	LHNQLLPNTTTVEK
16.1	1606.8679	0.9897	LHNQLLPNTTTVEK
10.7	1607.8592	-0.0017	EISLYIPRMLTEK
10.6	1607.8420	0.0156	HLHKVFQENNITK
10.6	1607.8420	0.0156	HLHKVFQENNITK
8.4	1607.8705	-0.0129	YSKPGLSMRLLESK
6.7	1607.8519	0.0056	IKDEHPGLSIGDVTK
6.4	1607.8572	0.0003	LHNNLLTWWGNLK
5.4	1607.8645	-0.0069	VKHAGGHSPQVNHLK

Spectrum No: 226; Query: 25; Rank: 1

Peptide View

MS/MS Fragmentation of **NATDAVGVVLK**
Found in **IPI00372400**, Tax_Id=10116 Gene_Symbol=Dap3 Death associated protein 3

Match to Query 25: 1086.593068 from(544.303810,2+)
Title: 091127RatKid_SCX01_14.2490.2490.2.dta
Data file K:\NewmanPaper\Piliang\3SubProteomes\Piliang3SP\mgf5ppm\SCX_3SubProteomes5ppm.mgf

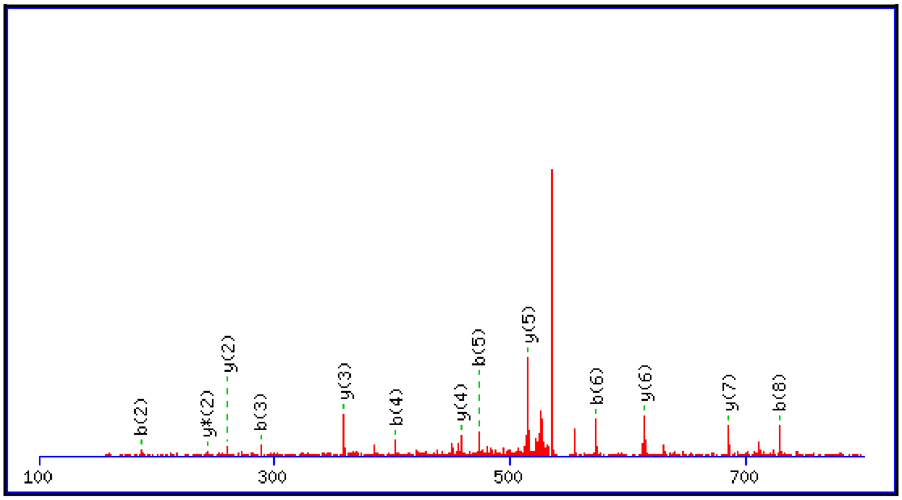

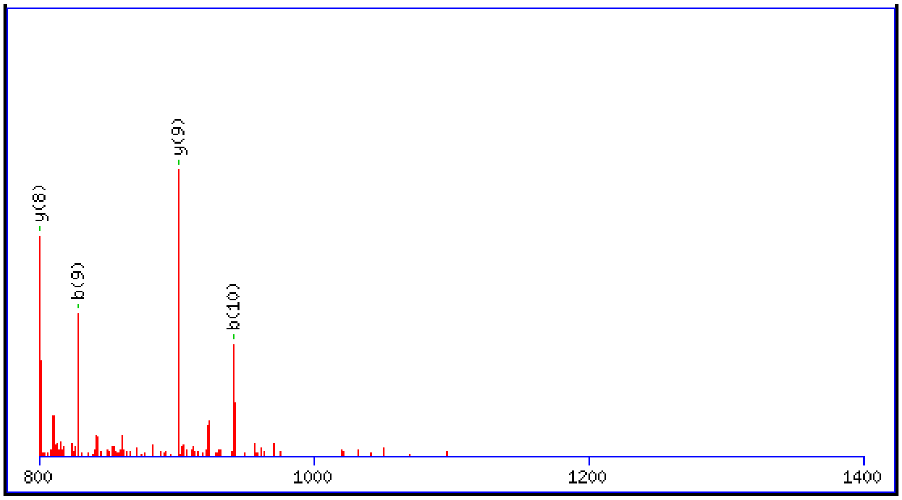

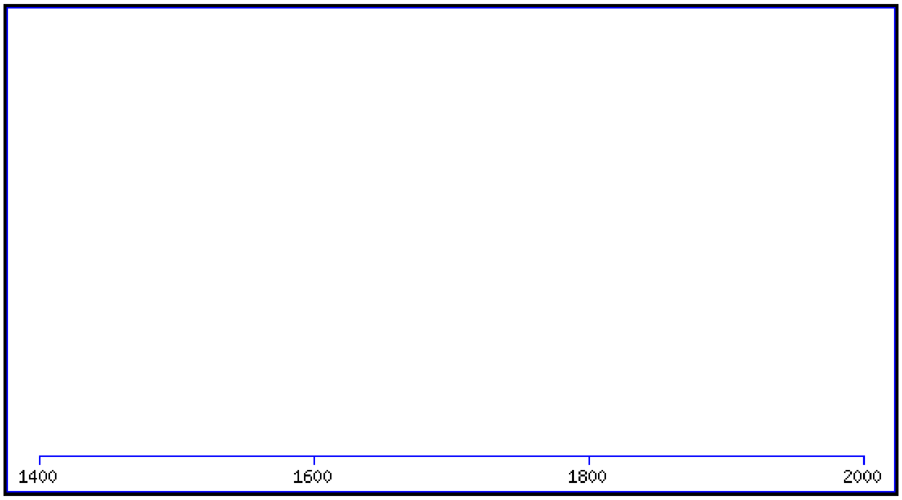

Monoisotopic mass of neutral peptide Mr(calc): 1086.5921
Fixed modifications: Carbamidomethyl (C)
Variable modifications:
N1 : Deamidated_N (N)
Ions Score: 70 **Expect:** 1e-005
Matches (Bold Red): 17/102 fragment ions using 36 most intense peaks

#	b	b^{++}	b^{*}	b^{*++}	b^{0}	b^{0++}	Seq.	y	y^{++}	y^{*}	y^{*++}	y^{0}	y^{0++}	#
1	116.0342	58.5207	99.0077	50.0075			N							11
2	**187.0713**	94.0393	170.0448	85.5260			A	972.5724	486.7899	955.5459	478.2766	954.5619	477.7846	10
3	**288.1190**	144.5631	271.0925	136.0499	270.1084	135.5579	T	**901.5353**	451.2713	884.5088	442.7580	883.5247	442.2660	9
4	**403.1459**	202.0766	386.1194	193.5633	385.1354	193.0713	D	**800.4876**	400.7475	783.4611	392.2342	782.4771	391.7422	8
5	**474.1831**	237.5952	457.1565	229.0819	456.1725	228.5899	A	**685.4607**	343.2340	668.4341	334.7207			7
6	**573.2515**	287.1294	556.2249	278.6161	555.2409	278.1241	V	**614.4236**	307.7154	597.3970	299.2022			6
7	630.2729	315.6401	613.2464	307.1268	612.2624	306.6348	G	**515.3552**	258.1812	498.3286	249.6679			5
8	**729.3414**	365.1743	712.3148	356.6610	711.3308	356.1690	V	**458.3337**	229.6705	441.3071	221.1572			4
9	**828.4098**	414.7085	811.3832	406.1952	810.3992	405.7032	V	**359.2653**	180.1363	342.2387	171.6230			3
10	**941.4938**	471.2506	924.4673	462.7373	923.4833	462.2453	L	**260.1969**	130.6021	**243.1703**	122.0888			2
11							K	147.1128	74.0600	130.0863	65.5468			1

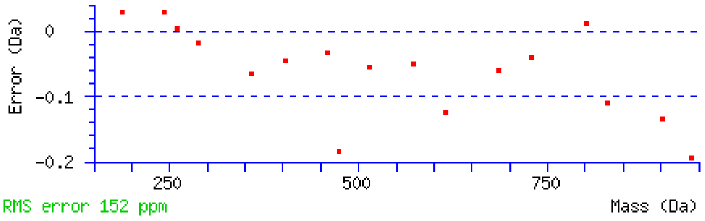

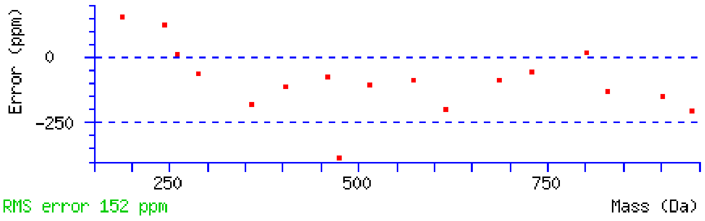

All matches to this query

Score	Mr(calc):	Delta	Sequence
69.8	1086.5921	0.0010	NATDAVGVVLK
23.5	1086.5855	0.0075	SPGAMERVLK
16.4	1086.6033	-0.0102	NKEIKNAIR
16.4	1086.6033	-0.0102	NKELKDALR
15.3	1086.6033	-0.0102	ADTEKVVGLR
14.4	1086.5968	-0.0037	NQMVRVALR
12.1	1086.5856	0.0075	VMQNQQVKL
11.5	1086.5921	0.0010	ELSKDSPALK
11.1	1086.6008	-0.0077	KMPSLGWLR
11.2	1086.6033	-0.0102	SDRLQVEIK

Spectrum No: 227; Query: 1182; Rank: 1

Peptide View

MS/MS Fragmentation of **ALAGLVYNSSGMEPCFDIYQMYQSCADPTGCGTGSNAR**
Found in **IPI00230946**, Tax_Id=10116 Gene_Symbol=Dpp7 Dipeptidyl-peptidase 2 precursor

Match to Query 1182: 4165.745772 from(1389.589200,3+)
Title: 091127RatKid_SCX01_13.4829.4829.3.dta
Data file K:\NewmanPaper\Piliang\3SubProteomes\Piliang3SP\mgf5ppm\SCX_3SubProteomes5ppm.mgf

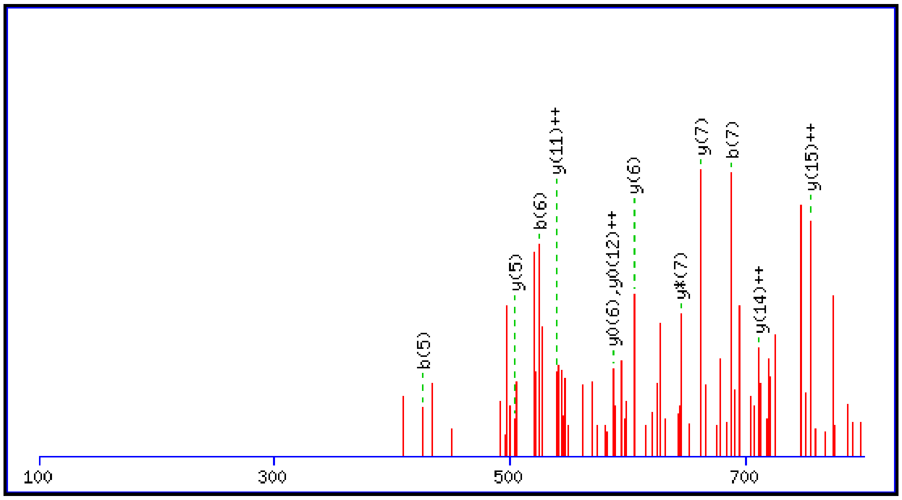

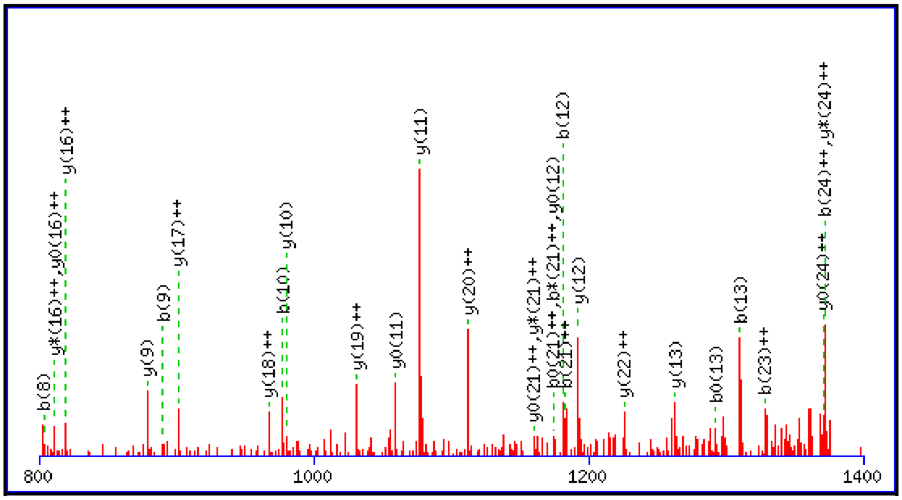

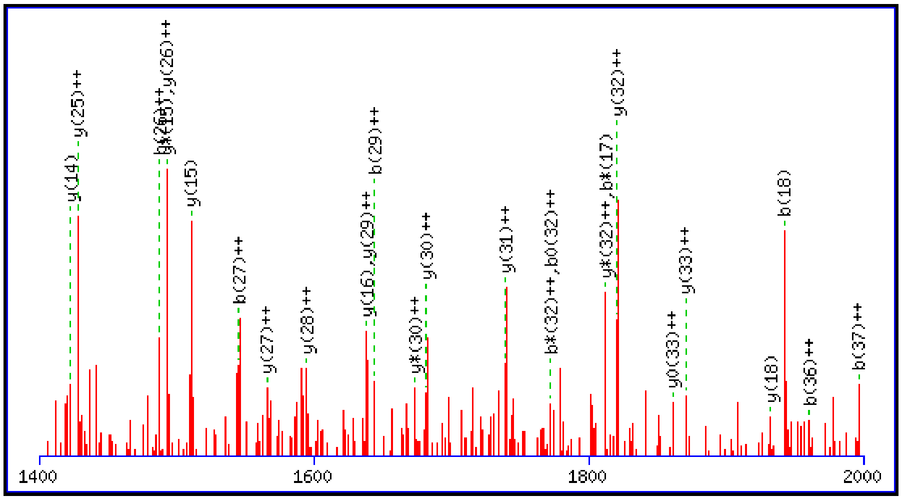

Monoisotopic mass of neutral peptide Mr(calc): 4164.7268
Fixed modifications: Carbamidomethyl (C)
Variable modifications:
N8 : Deamidated_N (N)
M12 : Oxidation (M)
Ions Score: 70 **Expect:** 5.1e-005
Matches (Bold Red): 68/408 fragment ions using 145 most intense peaks

#	b	b^{++}	b*	b^{*++}	b^0	b^{0++}	Seq.	y	y^{++}	y*	y^{*++}	y^0	y^{0++}	#
1	72.0444	36.5258					A							38
2	185.1285	93.0679					L	4094.6970	2047.8521	4077.6704	2039.3388	4076.6864	2038.8468	37
3	256.1656	128.5864					A	3981.6129	1991.3101	3964.5863	1982.7968	3963.6023	1982.3048	36
4	313.1870	157.0972					G	3910.5758	1955.7915	3893.5492	1947.2783	3892.5652	1946.7862	35
5	**426.2711**	213.6392					L	3853.5543	1927.2808	3836.5278	1918.7675	3835.5437	1918.2755	34
6	**525.3395**	263.1734					V	3740.4702	**1870.7388**	3723.4437	1862.2255	3722.4597	**1861.7335**	33
7	**688.4028**	344.7051					Y	3641.4018	**1821.2046**	3624.3753	**1812.6913**	3623.3913	1812.1993	32
8	**803.4298**	402.2185	786.4032	393.7053			N	3478.3385	**1739.6729**	3461.3120	1731.1596	3460.3279	1730.6676	31
9	**890.4618**	445.7345	873.4353	437.2213	872.4512	436.7293	S	3363.3116	**1682.1594**	3346.2850	**1673.6461**	3345.3010	1673.1541	30
10	**977.4938**	489.2506	960.4673	480.7373	959.4833	480.2453	S	3276.2795	**1638.6434**	3259.2530	1630.1301	3258.2690	1629.6381	29
11	1034.5153	517.7613	1017.4887	509.2480	1016.5047	508.7560	G	3189.2475	**1595.1274**	3172.2210	1586.6141	3171.2369	1586.1221	28
12	**1181.5507**	591.2790	1164.5242	582.7657	1163.5401	582.2737	M	3132.2260	**1566.6167**	3115.1995	1558.1034	3114.2155	1557.6114	27
13	**1310.5933**	655.8003	1293.5667	647.2870	**1292.5827**	646.7950	E	2985.1906	**1493.0990**	2968.1641	1484.5857	2967.1801	1484.0937	26
14	1407.6461	704.3267	1390.6195	695.8134	1389.6355	695.3214	P	2856.1481	**1428.5777**	2839.1215	1420.0644	2838.1375	1419.5724	25
15	1567.6767	784.3420	1550.6502	775.8287	1549.6661	775.3367	C	2759.0953	1380.0513	2742.0687	**1371.5380**	2741.0847	**1371.0460**	24
16	1714.7451	857.8762	1697.7186	849.3629	1696.7346	848.8709	F	2599.0646	1300.0360	2582.0381	1291.5227	2581.0541	1291.0307	23
17	1829.7721	915.3897	**1812.7455**	906.8764	1811.7615	906.3844	D	2451.9962	**1226.5017**	2434.9697	1217.9885	2433.9857	1217.4965	22
18	**1942.8561**	971.9317	1925.8296	963.4184	1924.8456	962.9264	I	2336.9693	1168.9883	2319.9427	**1160.4750**	2318.9587	**1159.9830**	21
19	2105.9195	1053.4634	2088.8929	1044.9501	2087.9089	1044.4581	Y	2223.8852	**1112.4462**	2206.8587	1103.9330	2205.8747	1103.4410	20
20	2233.9780	1117.4927	2216.9515	1108.9794	2215.9675	1108.4874	Q	2060.8219	**1030.9146**	2043.7953	1022.4013	2042.8113	1021.9093	19
21	2365.0185	**1183.0129**	2347.9920	**1174.4996**	2347.0080	**1174.0076**	M	**1932.7633**	**966.8853**	1915.7368	958.3720	1914.7527	957.8800	18
22	2528.0818	1264.5446	2511.0553	1256.0313	2510.0713	1255.5393	Y	1801.7228	**901.3651**	1784.6963	892.8518	1783.7123	892.3598	17
23	2656.1404	**1328.5739**	2639.1139	1320.0606	2638.1299	1319.5686	Q	**1638.6595**	**819.8334**	1621.6329	**811.3201**	1620.6489	**810.8281**	16
24	2743.1725	**1372.0899**	2726.1459	1363.5766	2725.1619	1363.0846	S	**1510.6009**	**755.8041**	**1493.5744**	747.2908	1492.5904	746.7988	15
25	2903.2031	1452.1052	2886.1766	1443.5919	2885.1925	1443.0999	C	**1423.5689**	**712.2881**	1406.5423	703.7748	1405.5583	703.2828	14
26	2974.2402	**1487.6237**	2957.2137	1479.1105	2956.2297	1478.6185	A	**1263.5382**	632.2728	1246.5117	623.7595	1245.5277	623.2675	13
27	3089.2672	**1545.1372**	3072.2406	1536.6239	3071.2566	1536.1319	D	**1192.5011**	596.7542	1175.4746	588.2409	**1174.4906**	**587.7489**	12
28	3186.3199	1593.6636	3169.2934	1585.1503	3168.3094	1584.6583	P	**1077.4742**	**539.2407**	1060.4476	530.7275	**1059.4636**	530.2354	11
29	3287.3676	**1644.1874**	3270.3411	1635.6742	3269.3570	1635.1822	T	**980.4214**	490.7143	963.3949	482.2011	962.4109	481.7091	10
30	3344.3891	1672.6982	3327.3625	1664.1849	3326.3785	1663.6929	G	**879.3737**	440.1905	862.3472	431.6772	861.3632	431.1852	9
31	3504.4197	1752.7135	3487.3932	1744.2002	3486.4091	1743.7082	C	822.3523	411.6798	805.3257	403.1665	804.3417	402.6745	8

**32**	3561.4412	1781.2242	3544.4146	**1772.7110**	3543.4306	**1772.2189**	**G**	**662.3216**	331.6645	**645.2951**	323.1512	644.3111	322.6592	**7**
**33**	3662.4889	1831.7481	3645.4623	1823.2348	3644.4783	1822.7428	**T**	**605.3002**	303.1537	588.2736	294.6404	**587.2896**	294.1484	**6**
**34**	3719.5103	1860.2588	3702.4838	1851.7455	3701.4998	1851.2535	**G**	**504.2525**	252.6299	487.2259	244.1166	486.2419	243.6246	**5**
**35**	3806.5423	1903.7748	3789.5158	1895.2615	3788.5318	1894.7695	**S**	447.2310	224.1191	430.2045	215.6059	429.2205	215.1139	**4**
**36**	3920.5853	**1960.7963**	3903.5587	1952.2830	3902.5747	1951.7910	**N**	360.1990	180.6031	343.1724	172.0899			**3**
**37**	3991.6224	**1996.3148**	3974.5958	1987.8016	3973.6118	1987.3096	**A**	246.1561	123.5817	229.1295	115.0684			**2**
**38**							**R**	175.1190	88.0631	158.0924	79.5498			**1**

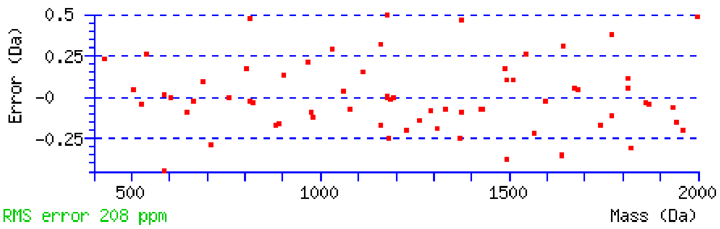

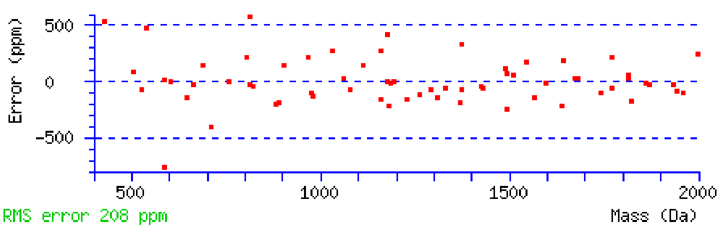

All matches to this query

Score	Mr(calc):	Delta	Sequence
69.8	4164.7268	1.0190	ALAGLVYNSSGMEPCFDIYQMYQSCADPTGCGTGSNAR
64.8	4163.7428	2.0030	ALAGLVYNSSGMEPCFDIYQMYQSCADPTGCGTGSNAR
33.9	4165.7108	0.0350	ALAGLVYNSSGMEPCFDIYQMYQSCADPTGCGTGSNAR
33.2	4164.7268	1.0190	ALAGLVYNSSGMEPCFDIYQMYQSCADPTGCGTGSNAR
32.0	4163.7428	2.0030	ALAGLVYNSSGMEPCFDIYQMYQSCADPTGCGTGSNAR
29.7	4164.7268	1.0190	ALAGLVYNSSGMEPCFDIYQMYQSCADPTGCGTGSNAR
14.1	4165.7108	0.0350	ALAGLVYNSSGMEPCFDIYQMYQSCADPTGCGTGSNAR
14.0	4164.7268	1.0190	ALAGLVYNSSGMEPCFDIYQMYQSCADPTGCGTGSNAR
7.6	4165.7242	0.0215	CLENIATFMEALPMDSPSSLWTTISNQFQTFFAK
6.3	4163.7406	2.0052	RDTAGASGGVAAPLPQKVPPTTAVEATVGTGGNSSAQSTR

Spectrum No: 228; Query: 592; Rank: 1

Peptide View

MS/MS Fragmentation of **TSLQPMVSALNISLGGTGK**
Found in **IPI00198021**, Tax_Id=10116 Gene_Symbol=Umod Uromodulin precursor

Match to Query 592: 1873.990788 from(938.002670,2+)
Title: 091129RatKid_SCX02_12.3782.3782.2.dta
Data file K:\NewmanPaper\Piliang\3SubProteomes\Piliang3SP\mgf5ppm\SCX_3SubProteomes5ppm.mgf

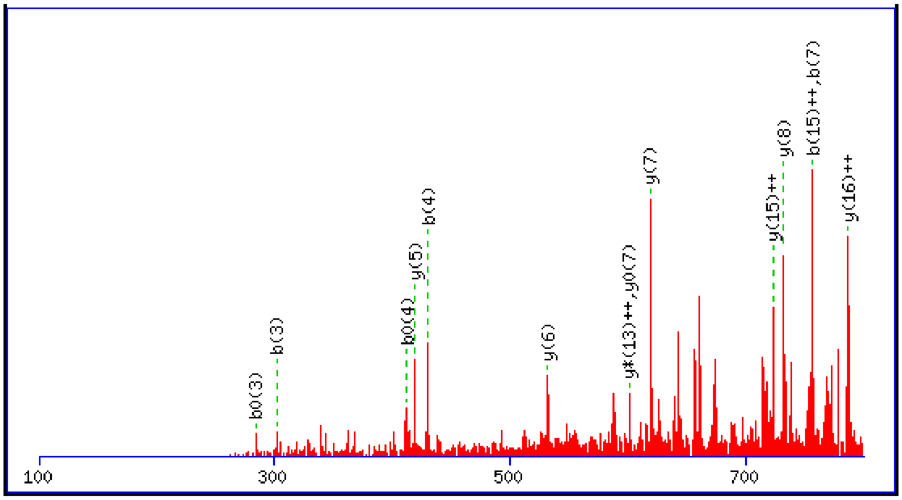

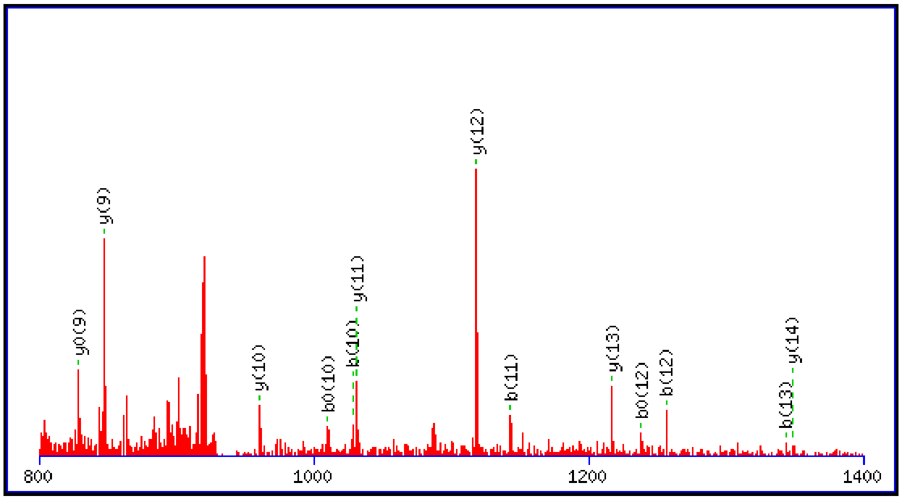

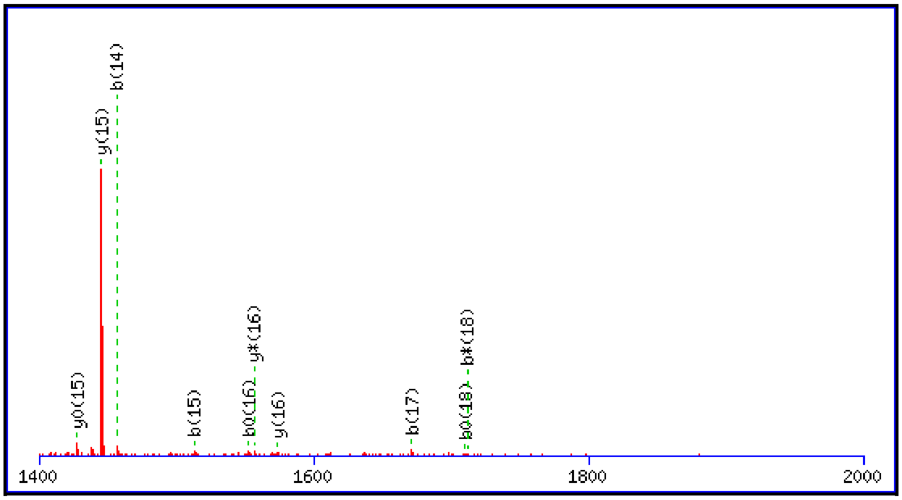

Monoisotopic mass of neutral peptide Mr(calc): 1873.9819
Fixed modifications: Carbamidomethyl (C)
Variable modifications:
N11 : Deamidated_N (N)
Ions Score: 70 **Expect:** 1.3e-005
Matches (Bold Red): 37/206 fragment ions using 59 most intense peaks

#	b	b^{++}	b*	b^{*++}	b^0	b^{0++}	Seq.	y	y^{++}	y*	y^{*++}	y^0	y^{0++}	#
1	102.0550	51.5311			84.0444	42.5258	T							19
2	189.0870	95.0471			171.0764	86.0418	S	1773.9415	887.4744	1756.9150	878.9611	1755.9309	878.4691	18
3	**302.1710**	151.5892			**284.1605**	142.5839	L	1686.9095	843.9584	1669.8829	835.4451	1668.8989	834.9531	17
4	**430.2296**	215.6185	413.2031	207.1052	**412.2191**	206.6132	Q	**1573.8254**	**787.4163**	**1556.7989**	778.9031	1555.8148	778.4111	16
5	527.2824	264.1448	510.2558	255.6316	509.2718	255.1396	P	**1445.7668**	**723.3871**	1428.7403	714.8738	**1427.7563**	714.3818	15

6	658.3229	329.6651	641.2963	321.1518	640.3123	320.6598	M	**1348.7141**	674.8607	1331.6875	666.3474	1330.7035	665.8554	14
7	**757.3913**	379.1993	740.3647	370.6860	739.3807	370.1940	V	**1217.6736**	609.3404	1200.6470	**600.8272**	1199.6630	600.3351	13
8	844.4233	422.7153	827.3968	414.2020	826.4128	413.7100	S	**1118.6052**	559.8062	1101.5786	551.2929	1100.5946	550.8009	12
9	915.4604	458.2339	898.4339	449.7206	897.4499	449.2286	A	**1031.5731**	516.2902	1014.5466	507.7769	1013.5626	507.2849	11
10	**1028.5445**	514.7759	1011.5179	506.2626	**1010.5339**	505.7706	L	**960.5360**	480.7717	943.5095	472.2584	942.5255	471.7664	10
11	**1143.5714**	572.2894	1126.5449	563.7761	1125.5609	563.2841	N	**847.4520**	424.2296	830.4254	415.7163	**829.4414**	415.2243	9
12	**1256.6555**	628.8314	1239.6289	620.3181	**1238.6449**	619.8261	I	**732.4250**	366.7162	715.3985	358.2029	714.4145	357.7109	8
13	**1343.6875**	672.3474	1326.6610	663.8341	1325.6770	663.3421	S	**619.3410**	310.1741	602.3144	301.6608	**601.3304**	301.1688	7
14	**1456.7716**	728.8894	1439.7450	720.3762	1438.7610	719.8841	L	**532.3089**	266.6581	515.2824	258.1448	514.2984	257.6528	6
15	**1513.7930**	**757.4002**	1496.7665	748.8869	1495.7825	748.3949	G	**419.2249**	210.1161	402.1983	201.6028	401.2143	201.1108	5
16	1570.8145	785.9109	1553.7880	777.3976	**1552.8039**	776.9056	G	362.2034	181.6053	345.1769	173.0921	344.1928	172.6001	4
17	**1671.8622**	836.4347	1654.8356	827.9215	1653.8516	827.4295	T	305.1819	153.0946	288.1554	144.5813	287.1714	144.0893	3
18	1728.8837	864.9455	**1711.8571**	856.4322	**1710.8731**	855.9402	G	204.1343	102.5708	187.1077	94.0575			2
19							K	147.1128	74.0600	130.0863	65.5468			1

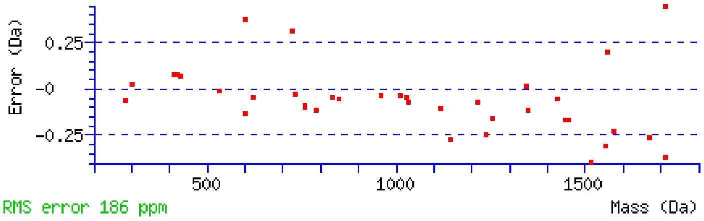

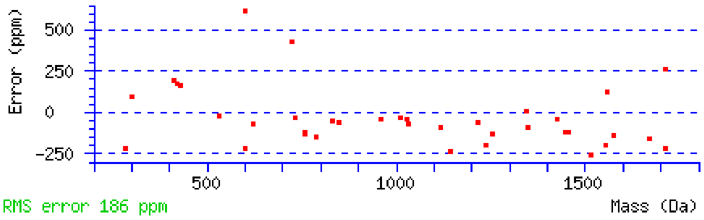

All matches to this query

Score	Mr(calc):	Delta	Sequence
69.7	1873.9819	0.0089	TSLQPMVSALNISLGGTGK
18.1	1872.9979	0.9929	TSLQPMVSALNISLGGTGK
2.2	1871.9870	2.0038	AELLLHVTIAAQSGLTR

Spectrum No: 229; Query: 339; Rank: 1

Peptide View

MS/MS Fragmentation of **TCNPETFPLNESR**
Found in **IPI00366496**, Tax_Id=10116 Gene_Symbol=Slc44a2_predicted similar to CTL2 gene

Match to Query 339: 1564.687808 from(783.351180,2+)
Title: 091129RatKid_SCX02_12.1394.1394.2.dta
Data file K:\NewmanPaper\Piliang\3SubProteomes\Piliang3SP\mgf5ppm\SCX_3SubProteomes5ppm.mgf

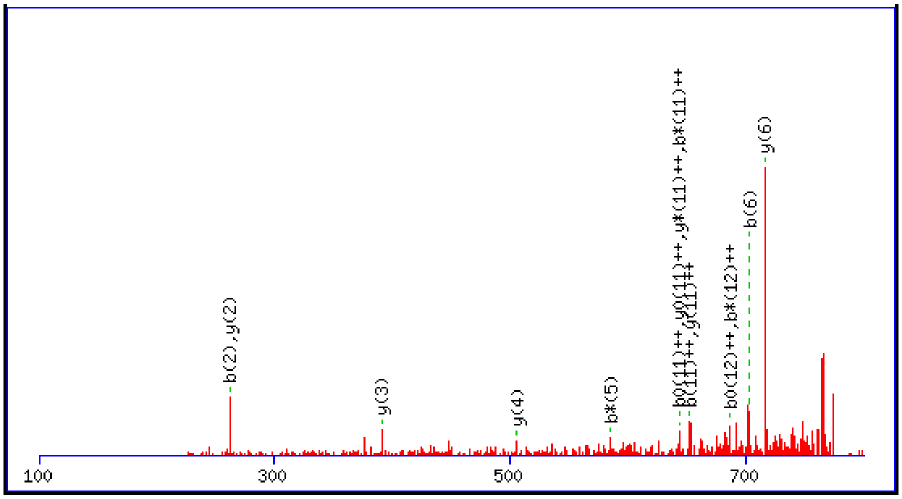

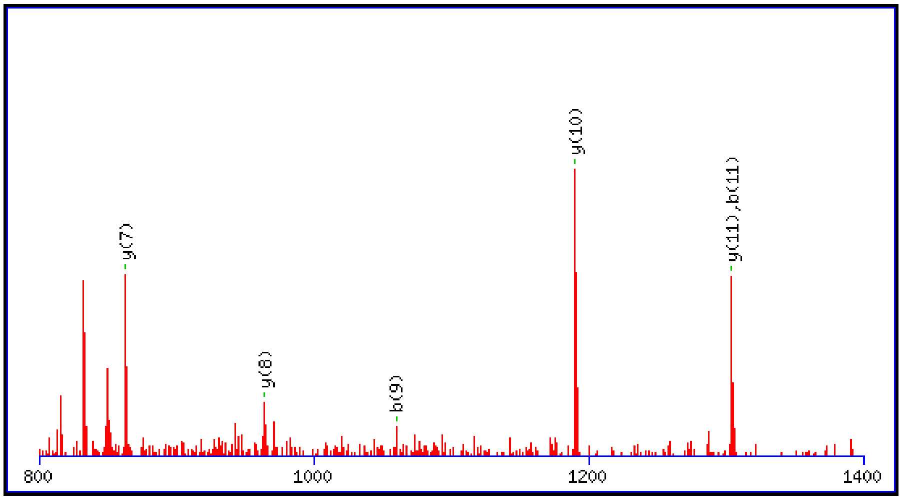

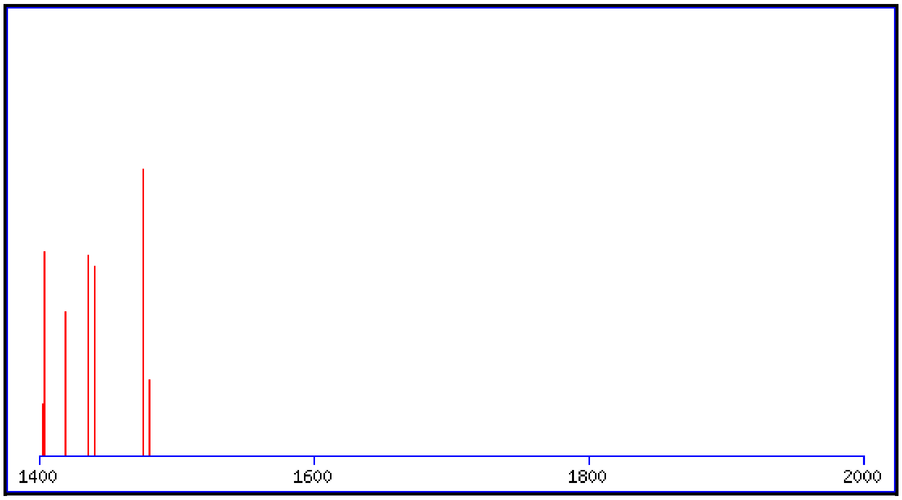

Monoisotopic mass of neutral peptide Mr(calc): 1564.6828
Fixed modifications: Carbamidomethyl (C)
Variable modifications:
N10 : Deamidated_N (N)
Ions Score: 70 **Expect:** 1.7e-005
Matches (Bold Red): 21/138 fragment ions using 30 most intense peaks

#	b	b^{++}	b*	b^{*++}	b^0	b^{0++}	Seq.	y	y^{++}	y*	y^{*++}	y^0	y^{0++}	#
1	102.0550	51.5311			84.0444	42.5258	T							13
2	**262.0856**	131.5464			244.0750	122.5412	C	1464.6424	732.8248	1447.6158	724.3115	1446.6318	723.8195	12
3	376.1285	188.5679	359.1020	180.0546	358.1180	179.5626	N	**1304.6117**	**652.8095**	1287.5852	**644.2962**	1286.6011	**643.8042**	11
4	473.1813	237.0943	456.1547	228.5810	455.1707	228.0890	P	**1190.5688**	595.7880	1173.5422	587.2748	1172.5582	586.7827	10
5	602.2239	301.6156	**585.1973**	293.1023	584.2133	292.6103	E	1093.5160	547.2616	1076.4895	538.7484	1075.5055	538.2564	9

**6**	**703.2716**	352.1394	686.2450	343.6261	685.2610	343.1341	**T**	**964.4734**	482.7404	947.4469	474.2271	946.4629	473.7351	**8**
**7**	850.3400	425.6736	833.3134	417.1604	832.3294	416.6683	**F**	**863.4258**	432.2165	846.3992	423.7032	845.4152	423.2112	**7**
**8**	947.3927	474.2000	930.3662	465.6867	929.3822	465.1947	**P**	**716.3573**	358.6823	699.3308	350.1690	698.3468	349.6770	**6**
**9**	**1060.4768**	530.7420	1043.4503	522.2288	1042.4662	521.7368	**L**	619.3046	310.1559	602.2780	301.6426	601.2940	301.1506	**5**
**10**	1175.5037	588.2555	1158.4772	579.7422	1157.4932	579.2502	**N**	**506.2205**	253.6139	489.1940	245.1006	488.2099	244.6086	**4**
**11**	**1304.5463**	**652.7768**	1287.5198	**644.2635**	1286.5358	**643.7715**	**E**	**391.1936**	196.1004	374.1670	187.5871	373.1830	187.0951	**3**
**12**	1391.5784	696.2928	1374.5518	**687.7795**	1373.5678	**687.2875**	**S**	**262.1510**	131.5791	245.1244	123.0659	244.1404	122.5738	**2**
**13**							**R**	175.1190	88.0631	158.0924	79.5498			**1**

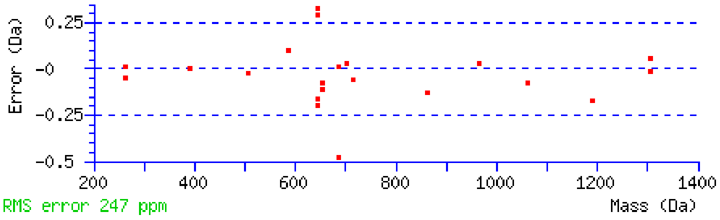

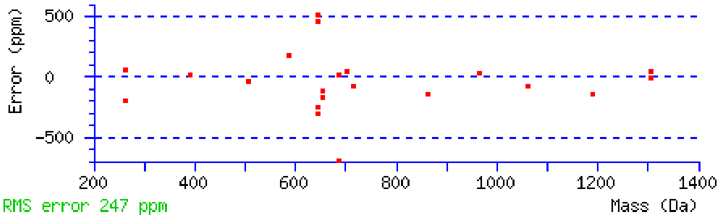

All matches to this query

Score	Mr(calc):	Delta	Sequence
69.7	1564.6828	0.0051	TCNPETFPLNESR
14.9	1563.6987	0.9891	TCNPETFPLNESR
7.3	1564.6995	-0.0117	LHTNREGAASVSSR
6.4	1564.6810	0.0068	ASGKFYPESKENK
5.9	1564.7005	-0.0127	ASDSSWKGQLNEDK
5.7	1564.6828	0.0051	TCNPETFPLNESR
5.3	1564.6861	0.0017	NMEETRTPSQPMK
4.3	1563.6769	1.0109	LMEGNRTENPCSR
4.2	1564.6896	-0.0018	KEHHPSHGASRDK
2.7	1564.6974	-0.0095	MNQSQRMAPVGSDK

Spectrum No: 230; Query: 636; Rank: 1

Peptide View

MS/MS Fragmentation of **FSPNLNISESVIPAIIEK**
Found in **IPI00195148**, Tax_Id=10116 Gene_Symbol=Es2 Liver carboxylesterase 1 precursor

Match to Query 636: 1971.058408 from(986.536480,2+)
Title: 091129RatKid_SCX02_11.3947.3947.2.dta
Data file K:\NewmanPaper\Piliang\3SubProteomes\Piliang3SP\mgf5ppm\SCX_3SubProteomes5ppm.mgf

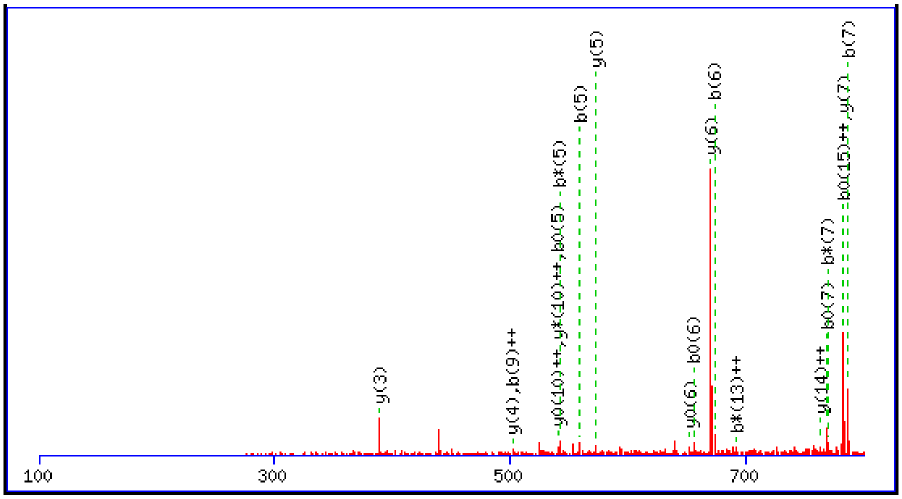

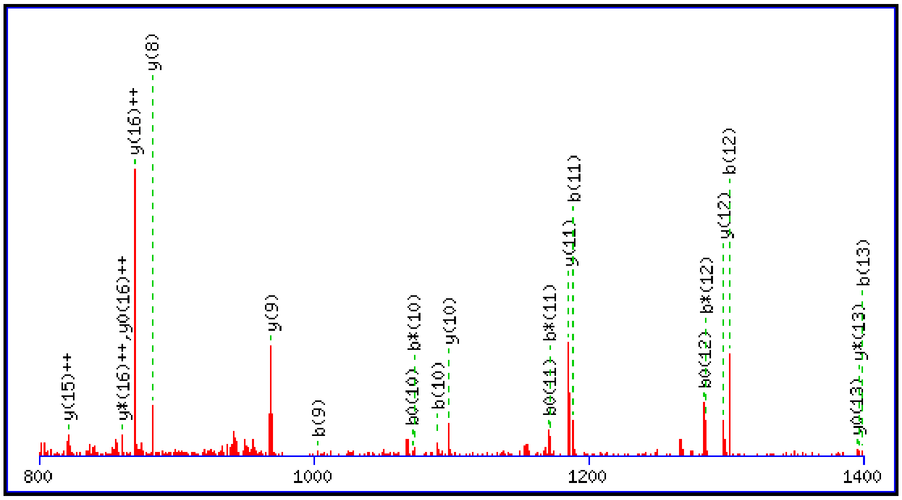

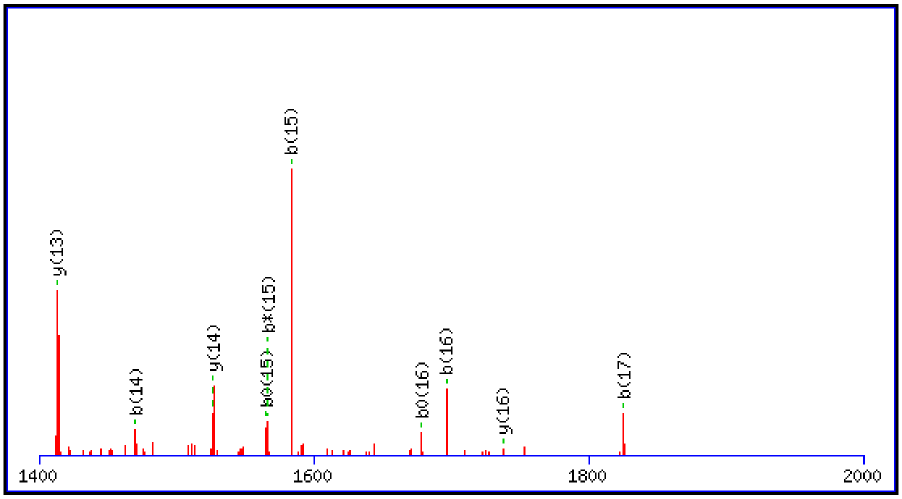

Monoisotopic mass of neutral peptide Mr(calc): 1971.0564
Fixed modifications: Carbamidomethyl (C)
Variable modifications:
N6 : Deamidated_N (N)
Ions Score: 70 **Expect:** 1.2e-005
Matches (Bold Red): 52/194 fragment ions using 94 most intense peaks

#	b	b++	b*	b*++	b0	b0++	Seq.	y	y++	y*	y*++	y0	y0++	#
1	148.0757	74.5415					F							18
2	235.1077	118.0575			217.0972	109.0522	S	1824.9953	913.0013	1807.9688	904.4880	1806.9847	903.9960	17
3	332.1605	166.5839			314.1499	157.5786	P	**1737.9633**	**869.4853**	1720.9367	**860.9720**	1719.9527	**860.4800**	16
4	446.2034	223.6053	429.1769	215.0921	428.1928	214.6001	N	1640.9105	**820.9589**	1623.8840	812.4456	1622.9000	811.9536	15
5	**559.2875**	280.1474	**542.2609**	271.6341	**541.2769**	271.1421	L	**1526.8676**	**763.9374**	1509.8410	755.4242	1508.8570	754.9322	14

6	674.3144	337.6608	657.2879	329.1476	656.3038	328.6556	N	1413.7835	707.3954	1396.7570	698.8821	1395.7730	698.3901	13
7	787.3985	394.2029	770.3719	385.6896	769.3879	385.1976	I	1298.7566	649.8819	1281.7300	641.3687	1280.7460	640.8767	12
8	874.4305	437.7189	857.4040	429.2056	856.4199	428.7136	S	1185.6725	593.3399	1168.6460	584.8266	1167.6620	584.3346	11
9	1003.4731	502.2402	986.4465	493.7269	985.4625	493.2349	E	1098.6405	549.8239	1081.6140	541.3106	1080.6299	540.8186	10
10	1090.5051	545.7562	1073.4786	537.2429	1072.4946	536.7509	S	969.5979	485.3026	952.5714	476.7893	951.5873	476.2973	9
11	1189.5735	595.2904	1172.5470	586.7771	1171.5630	586.2851	V	882.5659	441.7866	865.5393	433.2733	864.5553	432.7813	8
12	1302.6576	651.8324	1285.6311	643.3192	1284.6470	642.8272	I	783.4975	392.2524	766.4709	383.7391	765.4869	383.2471	7
13	1399.7104	700.3588	1382.6838	691.8455	1381.6998	691.3535	P	670.4134	335.7103	653.3869	327.1971	652.4028	326.7051	6
14	1470.7475	735.8774	1453.7209	727.3641	1452.7369	726.8721	A	573.3606	287.1840	556.3341	278.6707	555.3501	278.1787	5
15	1583.8315	792.4194	1566.8050	783.9061	1565.8210	783.4141	I	502.3235	251.6654	485.2970	243.1521	484.3130	242.6601	4
16	1696.9156	848.9614	1679.8891	840.4482	1678.9050	839.9562	I	389.2395	195.1234	372.2129	186.6101	371.2289	186.1181	3
17	1825.9582	913.4827	1808.9317	904.9695	1807.9476	904.4775	E	276.1554	138.5813	259.1288	130.0681	258.1448	129.5761	2
18							K	147.1128	74.0600	130.0863	65.5468			1

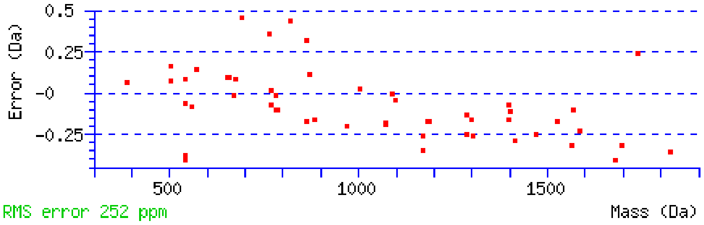

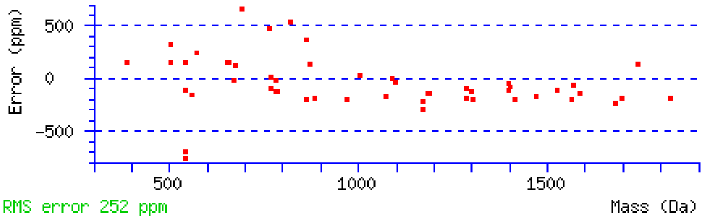

All matches to this query

Score	Mr(calc):	Delta	Sequence
69.7	1971.0564	0.0020	FSPNLNISESVIPAIIEK
51.1	1971.0564	0.0020	FSPNLNISESVIPAIIEK
18.2	1971.0636	-0.0052	KEQVETSPQASLKTGLQK
15.9	1970.0724	0.9860	FSPNLNISESVIPAIIEK
7.9	1971.0554	0.0030	LHKILGDKVNNTAVIEK
6.7	1971.0398	0.0186	SELSTHQRLHLGKNSHK
5.5	1971.0571	0.0013	MDRSRVLEQLIPELTR
5.4	1971.0707	-0.0123	TLNVKPLVTHRFPLEK
5.0	1971.0710	-0.0126	SPADIVKNLKESMAVLEK
3.6	1971.0524	0.0060	GSLEEAEAERLLLIATEK

Spectrum No: 231; Query: 27; Rank: 1

Peptide View

MS/MS Fragmentation of **IANISMEVGR**
Found in **IPI00363534**, Tax_Id=10116 Gene_Symbol=Lama1 laminin, alpha 1

Match to Query 27: 1089.549948 from(545.782250,2+)
Title: 091127RatKid_SCX01_12.1794.1794.2.dta
Data file K:\NewmanPaper\Piliang\3SubProteomes\Piliang3SP\mgf5ppm\SCX_3SubProteomes5ppm.mgf

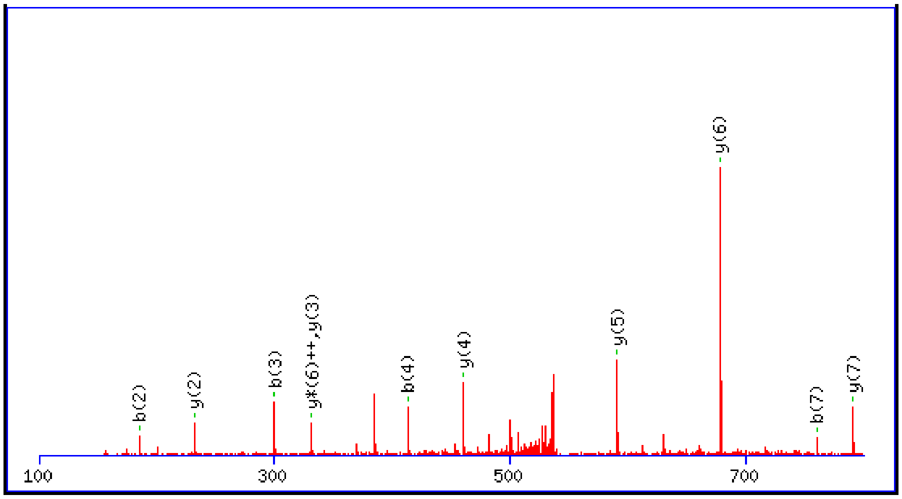

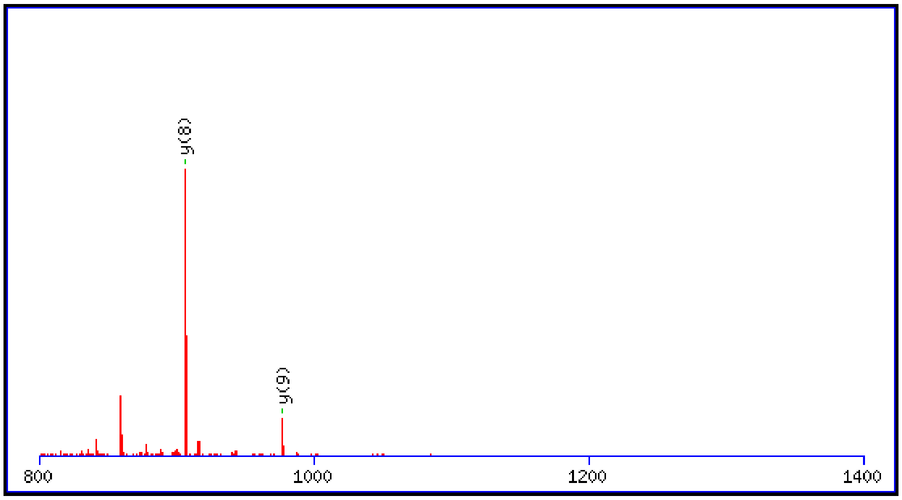

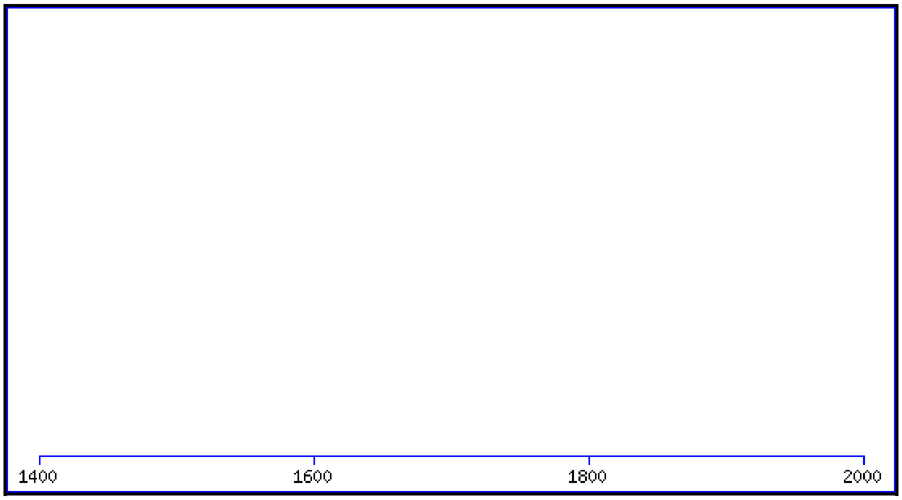

Monoisotopic mass of neutral peptide Mr(calc): 1089.5488
Fixed modifications: Carbamidomethyl (C)
Variable modifications:
N3 : Deamidated_N (N)
Ions Score: 70 **Expect:** 1.3e-005
Matches (Bold Red): 13/90 fragment ions using 28 most intense peaks

#	b	b^{++}	b*	b^{*++}	b^{0}	b^{0++}	Seq.	y	y^{++}	y*	y^{*++}	y^{0}	y^{0++}	#
1	114.0913	57.5493					I							10
2	**185.1285**	93.0679					A	**977.4721**	489.2397	960.4455	480.7264	959.4615	480.2344	9
3	**300.1554**	150.5813	283.1288	142.0681			N	**906.4349**	453.7211	889.4084	445.2078	888.4244	444.7158	8
4	**413.2395**	207.1234	396.2129	198.6101			I	**791.4080**	396.2076	774.3815	387.6944	773.3974	387.2024	7
5	500.2715	250.6394	483.2449	242.1261	482.2609	241.6341	S	**678.3239**	339.6656	661.2974	**331.1523**	660.3134	330.6603	6

**6**	631.3120	316.1596	614.2854	307.6463	613.3014	307.1543	**M**	**591.2919**	296.1496	574.2654	287.6363	573.2813	287.1443	**5**
**7**	**760.3546**	380.6809	743.3280	372.1676	742.3440	371.6756	**E**	**460.2514**	230.6293	443.2249	222.1161	442.2409	221.6241	**4**
**8**	859.4230	430.2151	842.3964	421.7019	841.4124	421.2098	**V**	**331.2088**	166.1081	314.1823	157.5948			**3**
**9**	916.4444	458.7259	899.4179	450.2126	898.4339	449.7206	**G**	**232.1404**	116.5738	215.1139	108.0606			**2**
**10**							**R**	175.1190	88.0631	158.0924	79.5498			**1**

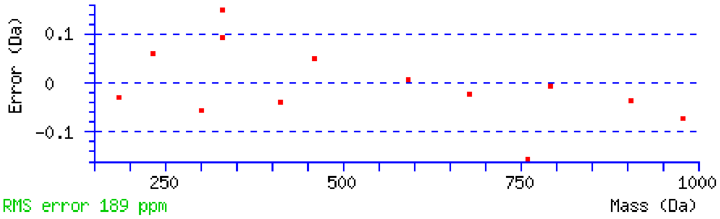

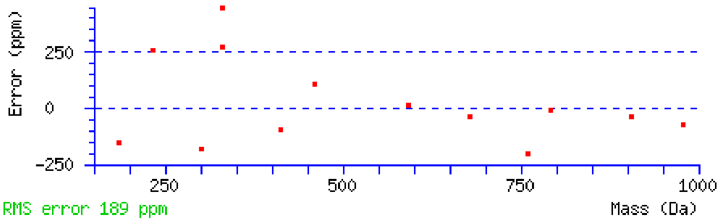

All matches to this query

Score	Mr(calc):	Delta	Sequence
69.7	1089.5488	0.0011	IANISMEVGR
13.3	1089.5567	-0.0067	IGKYESHTR
11.5	1089.5488	0.0011	AIDLENIMR
10.5	1089.5528	-0.0029	IALNMDWVK
9.4	1089.5600	-0.0101	LANREAIMR
7.6	1088.5462	1.0037	ETVVKEDGGR
7.4	1089.5488	0.0011	EALVAMGIDR
2.4	1088.5379	1.0120	ALLLHTEGR
2.2	1089.5461	0.0038	RGNTMAERR
1.8	1089.5455	0.0045	ESAPFVLNGR

Spectrum No: 232; Query: 706; Rank: 1

Peptide View

MS/MS Fragmentation of **IDCYPDEHGVSEANCTAR**
Found in **IPI00193894**, Tax_Id=10116 Gene_Symbol=Mgam_predicted 198 kDa protein

Match to Query 706: 2093.846328 from(1047.930440,2+)
Title: 091127RatKid_SCX01_24.1128.1128.2.dta
Data file K:\NewmanPaper\Piliang\3SubProteomes\Piliang3SP\mgf5ppm\SCX_3SubProteomes5ppm.mgf

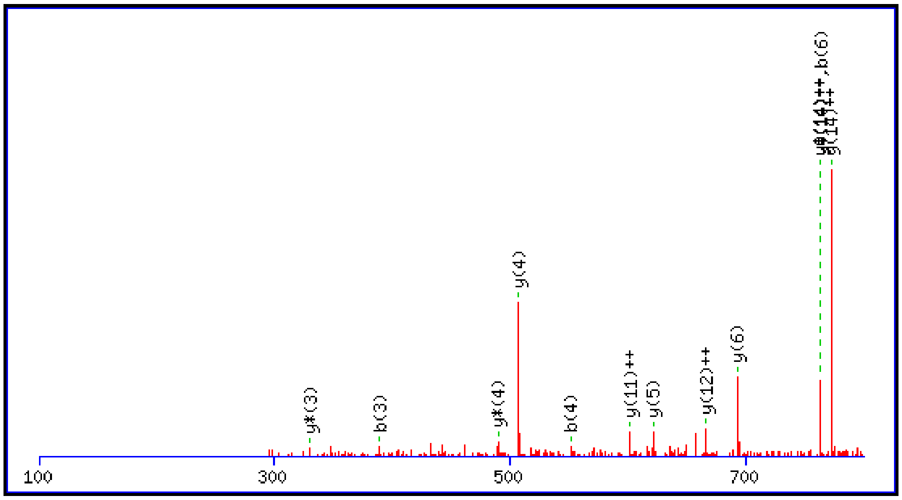

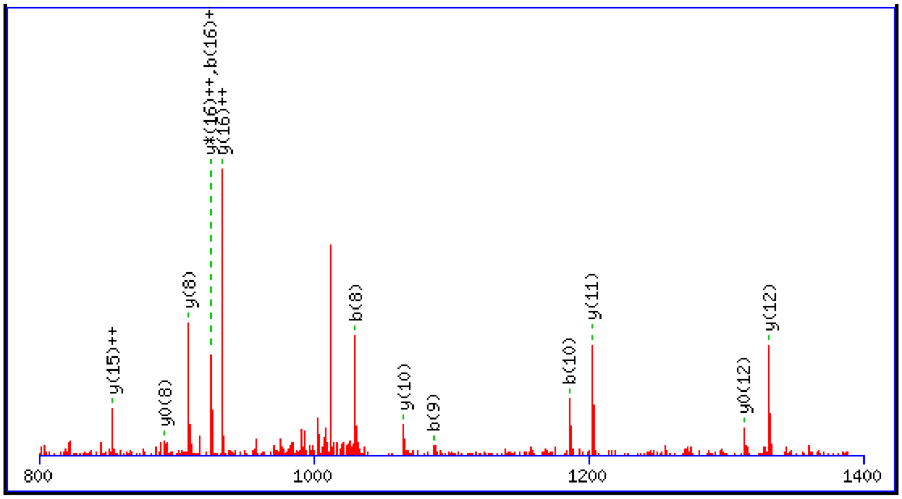

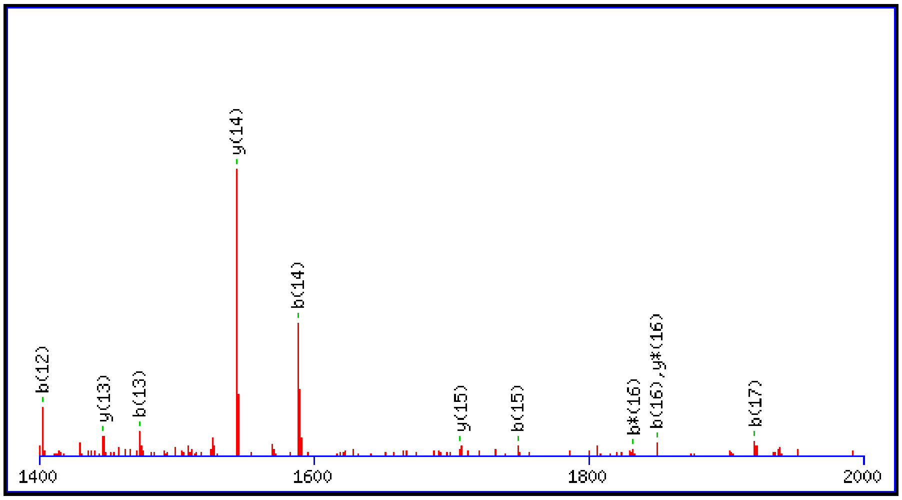

Monoisotopic mass of neutral peptide Mr(calc): 2093.8419
Fixed modifications: Carbamidomethyl (C)
Variable modifications:
N14 : Deamidated_N (N)
Ions Score: 70 **Expect:** 2.2e-005
Matches (Bold Red): 38/172 fragment ions using 60 most intense peaks

#	b	b^{++}	b*	b^{*++}	b^0	b^{0++}	Seq.	y	y^{++}	y*	y^{*++}	y^0	y^{0++}	#
1	114.0913	57.5493					I							18
2	229.1183	115.0628			211.1077	106.0575	D	1981.7651	991.3862	1964.7385	982.8729	1963.7545	982.3809	17
3	**389.1489**	195.0781			371.1384	186.0728	C	1866.7381	**933.8727**	**1849.7116**	**925.3594**	1848.7276	**924.8674**	16
4	**552.2123**	276.6098			534.2017	267.6045	Y	**1706.7075**	**853.8574**	1689.6809	845.3441	1688.6969	844.8521	15
5	649.2650	325.1362			631.2545	316.1309	P	**1543.6442**	**772.3257**	1526.6176	**763.8124**	1525.6336	**763.3204**	14
6	**764.2920**	382.6496			746.2814	373.6443	D	**1446.5914**	723.7993	1429.5648	715.2861	1428.5808	714.7941	13
7	893.3346	447.1709			875.3240	438.1656	E	**1331.5644**	**666.2859**	1314.5379	657.7726	**1313.5539**	657.2806	12
8	**1030.3935**	515.7004			1012.3829	506.6951	H	**1202.5219**	**601.7646**	1185.4953	593.2513	1184.5113	592.7593	11
9	**1087.4149**	544.2111			1069.4044	535.2058	G	**1065.4629**	533.2351	1048.4364	524.7218	1047.4524	524.2298	10
10	**1186.4834**	593.7453			1168.4728	584.7400	V	1008.4415	504.7244	991.4149	496.2111	990.4309	495.7191	9
11	1273.5154	637.2613			1255.5048	628.2560	S	**909.3731**	455.1902	892.3465	446.6769	**891.3625**	446.1849	8
12	**1402.5580**	701.7826			1384.5474	692.7773	E	822.3410	411.6742	805.3145	403.1609	804.3305	402.6689	7
13	**1473.5951**	737.3012			1455.5845	728.2959	A	**693.2984**	347.1529	676.2719	338.6396	675.2879	338.1476	6
14	**1588.6220**	794.8146	1571.5955	786.3014	1570.6115	785.8094	N	**622.2613**	311.6343	605.2348	303.1210	604.2508	302.6290	5
15	**1748.6527**	874.8300	1731.6261	866.3167	1730.6421	865.8247	C	**507.2344**	254.1208	**490.2078**	245.6076	489.2238	245.1156	4
16	**1849.7004**	**925.3538**	**1832.6738**	916.8405	1831.6898	916.3485	T	347.2037	174.1055	**330.1772**	165.5922	329.1932	165.1002	3
17	**1920.7375**	960.8724	1903.7109	952.3591	1902.7269	951.8671	A	246.1561	123.5817	229.1295	115.0684			2
18							R	175.1190	88.0631	158.0924	79.5498			1

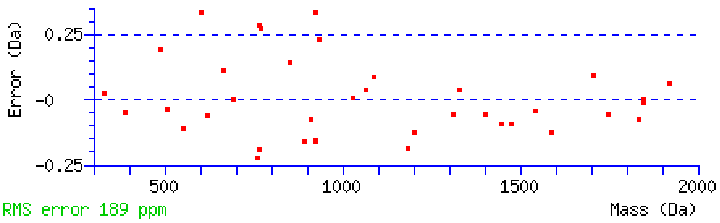

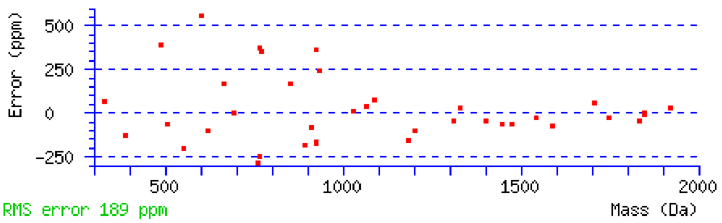

All matches to this query

Score	Mr(calc):	Delta	Sequence
69.6	2093.8419	0.0045	IDCYPDEHGVSEANCTAR
5.6	2093.8490	-0.0027	STSACNRVLNGAVPTDGSK
2.7	2093.8486	-0.0022	TAEPPSPPASRAAYRQR
1.9	2092.8578	0.9885	IDCYPDEHGVSEANCTAR
1.5	2092.8373	1.0090	NIRSNYRDMHTHMVK
0.5	2091.8577	1.9886	KDHSVNKMCASSCDFVK
0.3	2092.8408	1.0056	TYIETSIGEGANLASLSK
0.3	2092.8408	1.0056	TYIETSIGEGANLASLSK

Spectrum No: 233; Query: 569; Rank: 1

Peptide View

MS/MS Fragmentation of **TRCNTTQGNEVTSILR**
Found in **IPI00327143**, Tax_Id=10116 Gene_Symbol=Alpl Alkaline phosphatase, tissue-nonspecific isozyme precursor

Match to Query 569: 1849.895442 from(617.639090,3+)
Title: 091127RatKid_SCX01_12.1659.1659.3.dta
Data file K:\NewmanPaper\Piliang\3SubProteomes\Piliang3SP\mgf5ppm\SCX_3SubProteomes5ppm.mgf

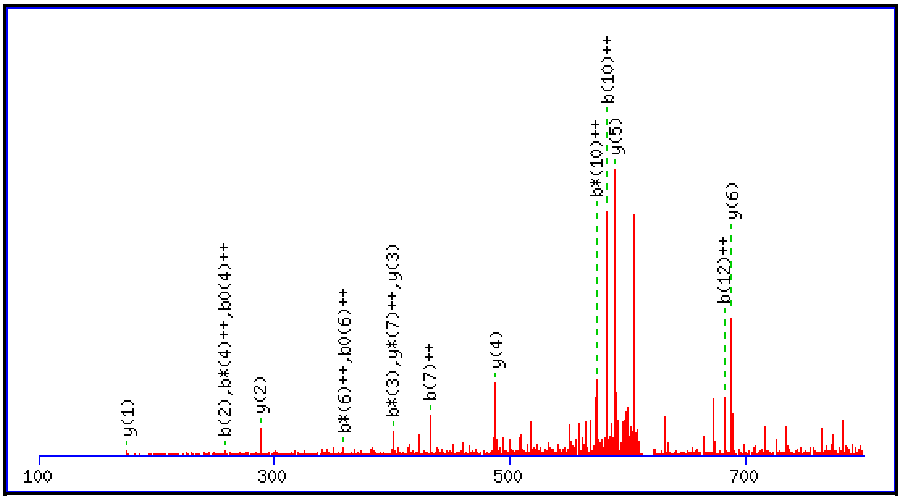

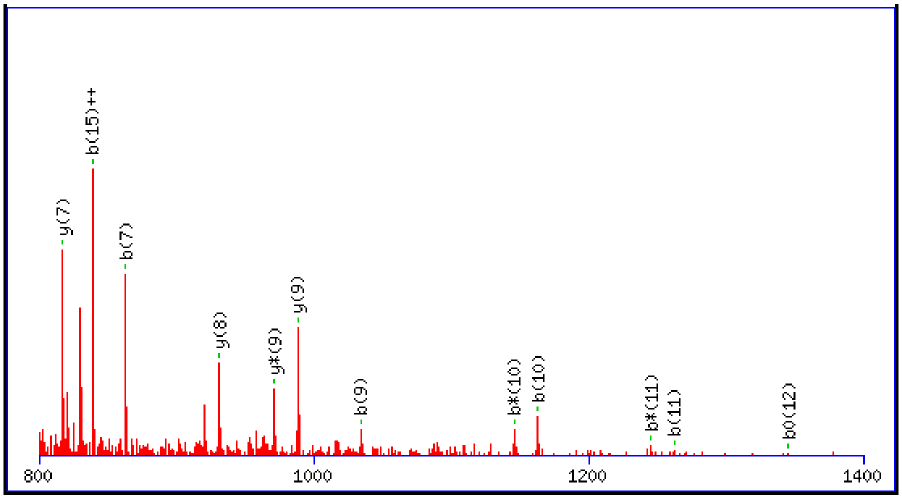

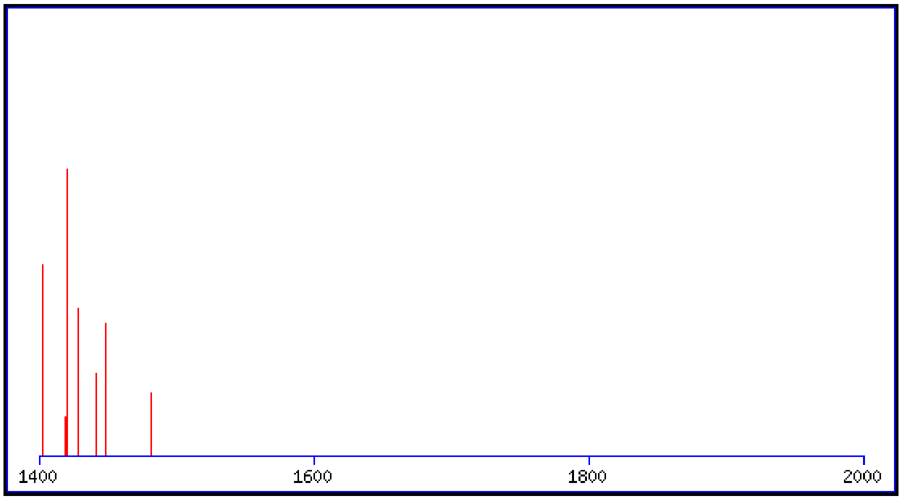

Monoisotopic mass of neutral peptide Mr(calc): 1849.8952
Fixed modifications: Carbamidomethyl (C)
Variable modifications:
N4 : Deamidated_N (N)
Ions Score: 70 **Expect:** 2.8e-005
Matches (Bold Red): 29/172 fragment ions using 42 most intense peaks

#	b	b^{++}	b*	b^{*++}	b^0	b^{0++}	Seq.	y	y^{++}	y*	y^{*++}	y^0	y^{0++}	#
1	102.0550	51.5311			84.0444	42.5258	T							16
2	**258.1561**	129.5817	241.1295	121.0684	240.1455	120.5764	R	1749.8548	875.4310	1732.8283	866.9178	1731.8443	866.4258	15
3	418.1867	209.5970	**401.1602**	201.0837	400.1762	200.5917	C	1593.7537	797.3805	1576.7272	788.8672	1575.7431	788.3752	14
4	533.2137	267.1105	516.1871	**258.5972**	515.2031	**258.1052**	N	1433.7231	717.3652	1416.6965	708.8519	1415.7125	708.3599	13
5	634.2613	317.6343	617.2348	309.1210	616.2508	308.6290	T	1318.6961	659.8517	1301.6696	651.3384	1300.6856	650.8464	12
6	735.3090	368.1581	718.2825	**359.6449**	717.2984	**359.1529**	T	1217.6484	609.3279	1200.6219	600.8146	1199.6379	600.3226	11
7	**863.3676**	**432.1874**	846.3410	423.6742	845.3570	423.1821	Q	1116.6008	558.8040	1099.5742	550.2907	1098.5902	549.7987	10
8	920.3891	460.6982	903.3625	452.1849	902.3785	451.6929	G	**988.5422**	494.7747	**971.5156**	486.2615	970.5316	485.7694	9
9	**1034.4320**	517.7196	1017.4054	509.2064	1016.4214	508.7143	N	**931.5207**	466.2640	914.4942	457.7507	913.5102	457.2587	8
10	**1163.4746**	**582.2409**	**1146.4480**	**573.7276**	1145.4640	573.2356	E	**817.4778**	409.2425	800.4512	**400.7293**	799.4672	400.2373	7
11	**1262.5430**	631.7751	**1245.5164**	623.2619	1244.5324	622.7698	V	**688.4352**	344.7212	671.4087	336.2080	670.4246	335.7160	6
12	1363.5907	**682.2990**	1346.5641	673.7857	**1345.5801**	673.2937	T	**589.3668**	295.1870	572.3402	286.6738	571.3562	286.1817	5
13	1450.6227	725.8150	1433.5961	717.3017	1432.6121	716.8097	S	**488.3191**	244.6632	471.2926	236.1499	470.3085	235.6579	4
14	1563.7068	782.3570	1546.6802	773.8437	1545.6962	773.3517	I	**401.2871**	201.1472	384.2605	192.6339			3
15	1676.7908	**838.8990**	1659.7643	830.3858	1658.7803	829.8938	L	**288.2030**	144.6051	271.1765	136.0919			2
16							R	**175.1190**	88.0631	158.0924	79.5498			1

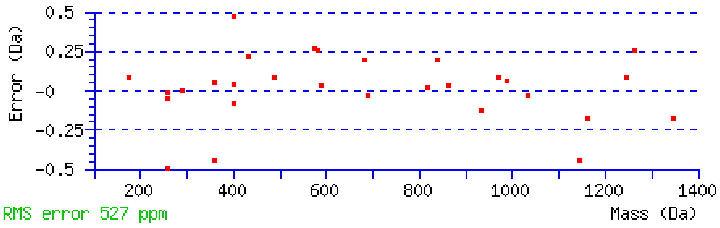

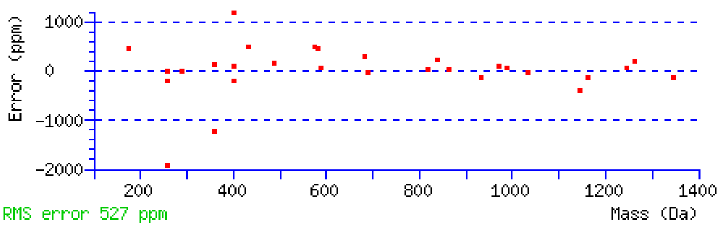

All matches to this query

Score	Mr(calc):	Delta	Sequence
69.5	1849.8952	0.0002	TRCNTTQGNEVTSILR
53.5	1849.8952	0.0002	TRCNTTQGNEVTSILR
8.9	1849.8840	0.0114	DQQNMANGSTVVVTLTR
7.3	1847.8931	2.0024	IYSNAGEKSFDRLLR
7.0	1847.8931	2.0024	IYSNAGEKSFDRLLR
7.0	1848.8780	1.0175	VQSFCNGFLKGMLLR
6.3	1847.8869	2.0086	IKTVDISMILSEAIR
6.3	1849.8950	0.0004	VLVFPMEGSHWLSIR
5.2	1848.9000	0.9954	DQQNMANGSTVVVTLTR
4.7	1847.8778	2.0176	HDLSGKVVNIDTNSLR

Spectrum No: 234; Query: 406; Rank: 1

Peptide View

MS/MS Fragmentation of **VIQQIAMGDSEAQNK**
Found in **IPI00200859**, Tax_Id=10116 Gene_Symbol=Slc34a2 Sodium-dependent phosphate transport protein 2B

Match to Query 406: 1631.784588 from(816.899570,2+)
Title: 091127RatKid_SCX01_02.1456.1456.2.dta
Data file K:\NewmanPaper\Piliang\3SubProteomes\Piliang3SP\mgf5ppm\SCX_3SubProteomes5ppm.mgf

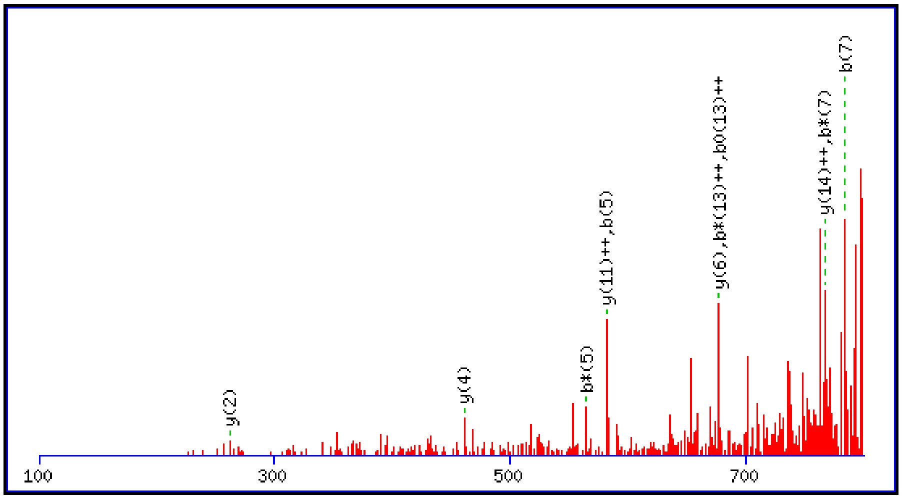

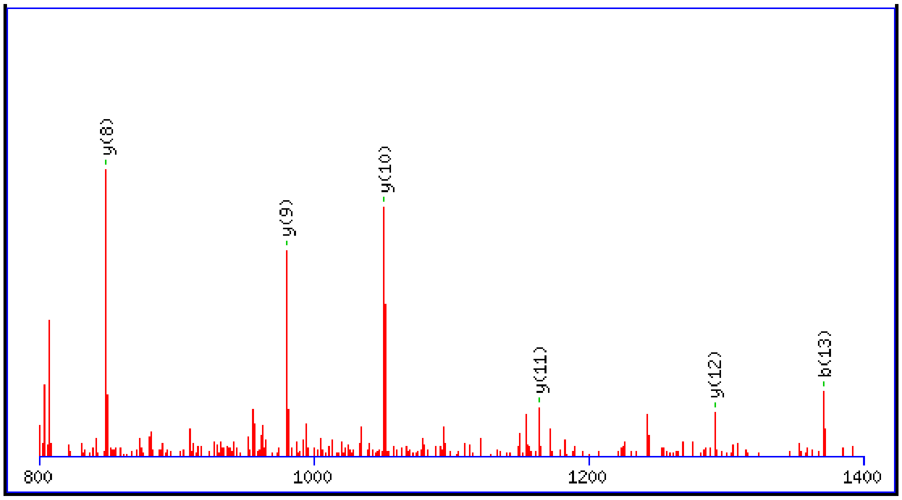

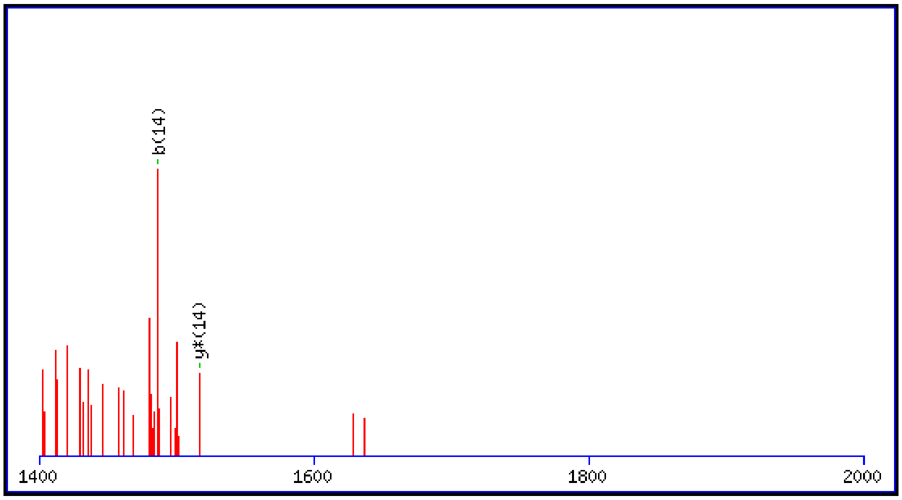

Monoisotopic mass of neutral peptide Mr(calc): 1631.7825
Fixed modifications: Carbamidomethyl (C)
Variable modifications:
N14 : Deamidated_N (N)
Ions Score: 69 **Expect:** 2.3e-005
Matches (Bold Red): 19/140 fragment ions using 27 most intense peaks

#	b	b^{++}	b*	b^{*++}	b^{0}	b^{0++}	Seq.	y	y^{++}	y*	y^{*++}	y^{0}	y^{0++}	#
1	100.0757	50.5415					V							15
2	213.1598	107.0835					I	1533.7213	**767.3643**	**1516.6948**	758.8510	1515.7108	758.3590	14
3	341.2183	171.1128	324.1918	162.5995			Q	1420.6373	710.8223	1403.6107	702.3090	1402.6267	701.8170	13
4	469.2769	235.1421	452.2504	226.6288			Q	**1292.5787**	646.7930	1275.5522	638.2797	1274.5681	637.7877	12
5	**582.3610**	291.6841	**565.3344**	283.1709			I	**1164.5201**	**582.7637**	1147.4936	574.2504	1146.5096	573.7584	11
6	653.3981	327.2027	636.3715	318.6894			A	**1051.4361**	526.2217	1034.4095	517.7084	1033.4255	517.2164	10
7	**784.4386**	392.7229	**767.4120**	384.2097			M	**980.3989**	490.7031	963.3724	482.1898	962.3884	481.6978	9
8	841.4600	421.2337	824.4335	412.7204			G	**849.3585**	425.1829	832.3319	416.6696	831.3479	416.1776	8
9	956.4870	478.7471	939.4604	470.2339	938.4764	469.7418	D	792.3370	396.6721	775.3104	388.1589	774.3264	387.6669	7
10	1043.5190	522.2631	1026.4925	513.7499	1025.5084	513.2579	S	**677.3101**	339.1587	660.2835	330.6454	659.2995	330.1534	6
11	1172.5616	586.7844	1155.5351	578.2712	1154.5510	577.7792	E	590.2780	295.6427	573.2515	287.1294	572.2675	286.6374	5
12	1243.5987	622.3030	1226.5722	613.7897	1225.5882	613.2977	A	**461.2354**	231.1214	444.2089	222.6081			4
13	**1371.6573**	686.3323	1354.6307	**677.8190**	1353.6467	**677.3270**	Q	390.1983	195.6028	373.1718	187.0895			3
14	**1486.6842**	743.8458	1469.6577	735.3325	1468.6737	734.8405	N	**262.1397**	131.5735	245.1132	123.0602			2
15							K	147.1128	74.0600	130.0863	65.5468			1

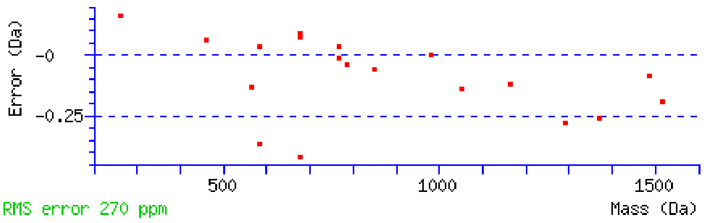

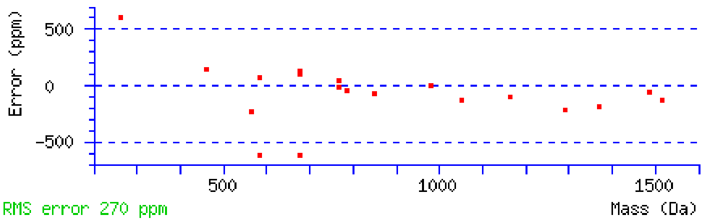

All matches to this query

Score	Mr(calc):	Delta	Sequence
69.5	1631.7825	0.0021	VIQQIAMGDSEAQNK
8.2	1631.7994	-0.0148	MATTGLPPLLPAENK
8.2	1631.7994	-0.0148	MATTGLPPLLPAENK
8.2	1631.7871	-0.0025	RLAQILSACILSK
7.8	1631.7831	0.0015	FLSLFGGIYENESR
7.2	1631.7807	0.0038	TTQLGSKAILYDDK
7.2	1631.7807	0.0038	TTQLGSKAILYDDK
6.5	1631.7824	0.0021	KPLEGVKNNMDENK
6.1	1631.7807	0.0038	TTQLGSKAILYDDK
1.9	1631.7960	-0.0114	YGAPDKALYTLVNK

Spectrum No: 235; Query: 103; Rank: 1

Peptide View

MS/MS Fragmentation of **LDFIVLNETR**
Found in **IPI00208422**, Tax_Id=10116 Gene_Symbol=Dpp4 Dipeptidyl peptidase 4

Match to Query 103: 1219.645968 from(610.830260,2+)
Title: 091129RatKid_SCX02_18.3147.3147.2.dta
Data file K:\NewmanPaper\Piliang\3SubProteomes\Piliang3SP\mgf5ppm\SCX_3SubProteomes5ppm.mgf

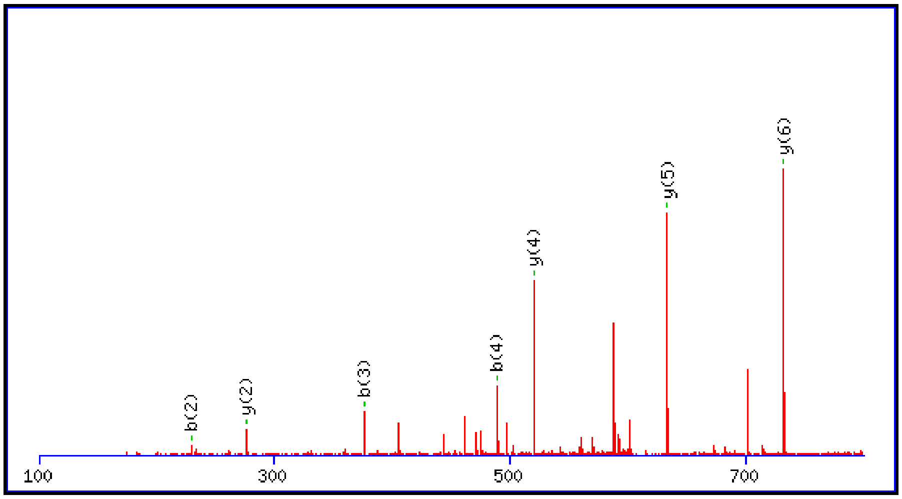

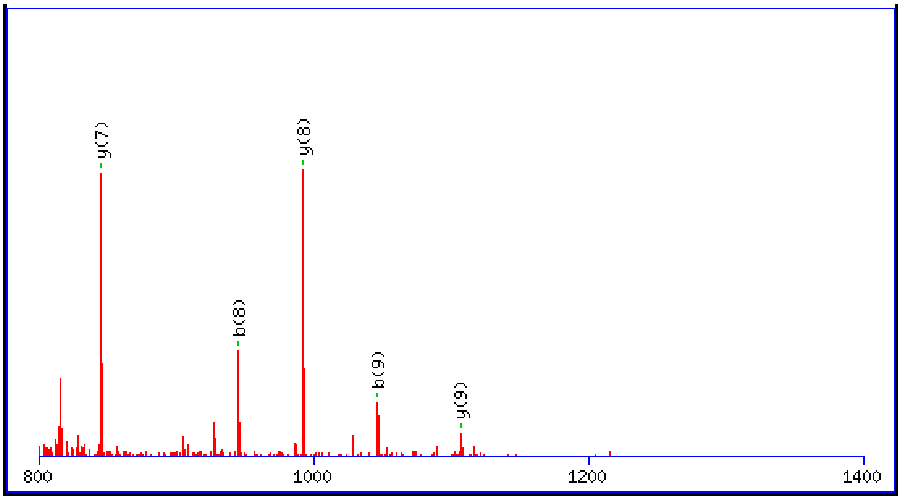

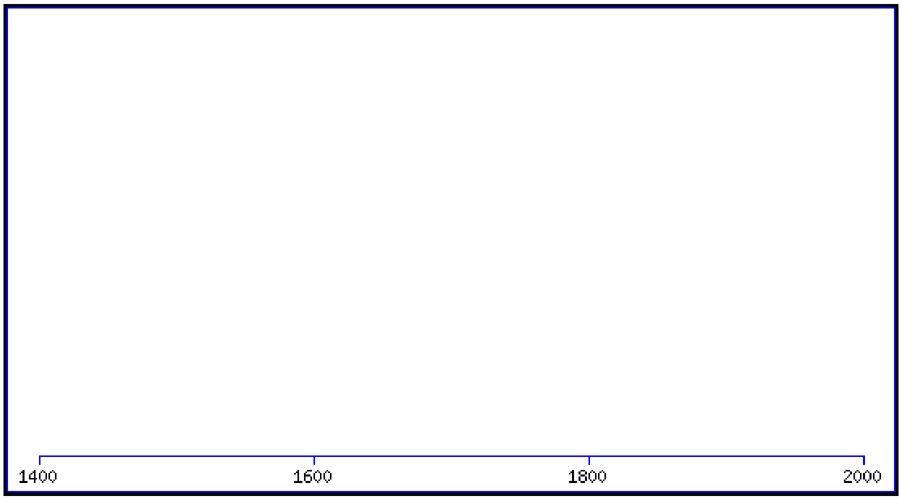

Monoisotopic mass of neutral peptide Mr(calc): 1219.6448
Fixed modifications: Carbamidomethyl (C)
Variable modifications:
N7 : Deamidated_N (N)
Ions Score: 69 **Expect:** 1.5e-005
Matches (Bold Red): 12/92 fragment ions using 18 most intense peaks

#	b	b++	b*	b*++	b0	b0++	Seq.	y	y++	y*	y*++	y0	y0++	#
1	114.0913	57.5493					L							10
2	**229.1183**	115.0628			211.1077	106.0575	D	**1107.5681**	554.2877	1090.5415	545.7744	1089.5575	545.2824	9
3	**376.1867**	188.5970			358.1761	179.5917	F	**992.5411**	496.7742	975.5146	488.2609	974.5306	487.7689	8
4	**489.2708**	245.1390			471.2602	236.1337	I	**845.4727**	423.2400	828.4462	414.7267	827.4621	414.2347	7
5	588.3392	294.6732			570.3286	285.6679	V	**732.3886**	366.6980	715.3621	358.1847	714.3781	357.6927	6
6	701.4232	351.2153			683.4127	342.2100	L	**633.3202**	317.1638	616.2937	308.6505	615.3097	308.1585	5
7	816.4502	408.7287	799.4236	400.2155	798.4396	399.7234	N	**520.2362**	260.6217	503.2096	252.1084	502.2256	251.6164	4
8	**945.4928**	473.2500	928.4662	464.7367	927.4822	464.2447	E	405.2092	203.1082	388.1827	194.5950	387.1987	194.1030	3
9	**1046.5404**	523.7739	1029.5139	515.2606	1028.5299	514.7686	T	**276.1666**	138.5870	259.1401	130.0737	258.1561	129.5817	2
10							R	175.1190	88.0631	158.0924	79.5498			1

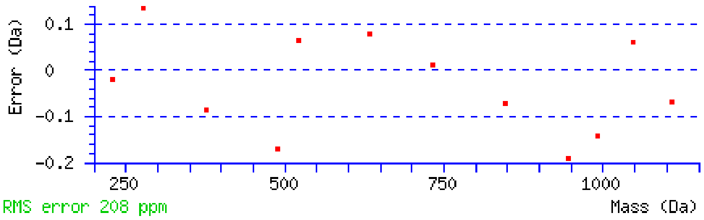

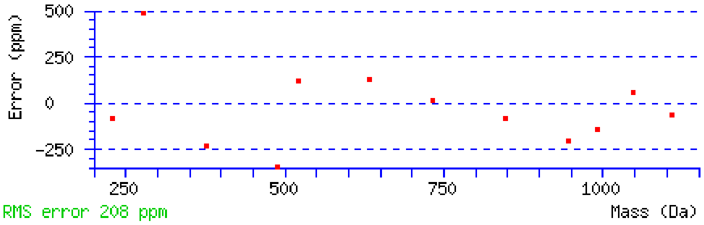

All matches to this query

Score	Mr(calc):	Delta	Sequence
69.3	1219.6448	0.0011	LDFIVLNETR
18.7	1219.6449	0.0011	VQSGVVINFEK
14.1	1219.6482	-0.0022	NLEIKKSMEK
10.7	1219.6343	0.0117	NKDMKQTVTR
6.9	1219.6343	0.0117	ALMRVSGXETR
5.5	1218.6431	1.0029	YQPVVDLVMR
5.0	1218.6456	1.0004	TSSVLEGSALQK
5.0	1218.6316	1.0143	LSNKQTSGASAR
3.6	1218.6357	1.0103	NFQIEVQKGR
2.7	1218.6390	1.0069	LMVERLSAER

Spectrum No: 236; Query: 715; Rank: 1

Peptide View

MS/MS Fragmentation of **IYHPNINSNGSICLDILR**
Found in **IPI00192159**, Tax_Id=10116 Gene_Symbol=Ube2d3 Ubiquitin-conjugating enzyme E2 D3

Match to Query 715: 2099.054188 from(1050.534370,2+)
Title: 091129RatKid_SCX02_29.3590.3590.2.dta
Data file K:\NewmanPaper\Piliang\3SubProteomes\Piliang3SP\mgf5ppm\SCX_3SubProteomes5ppm.mgf

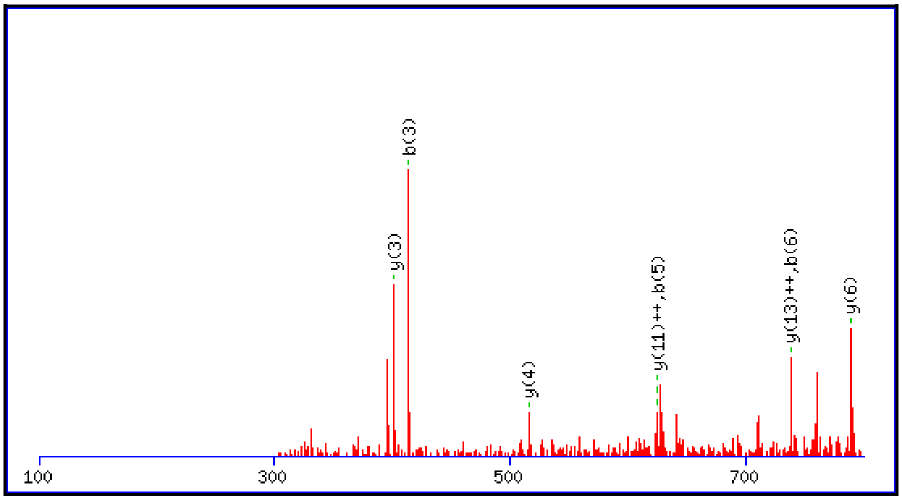

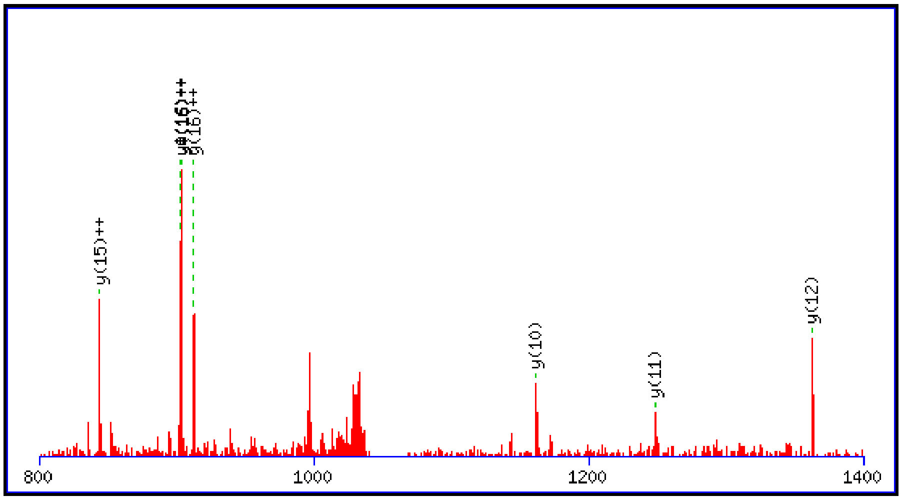

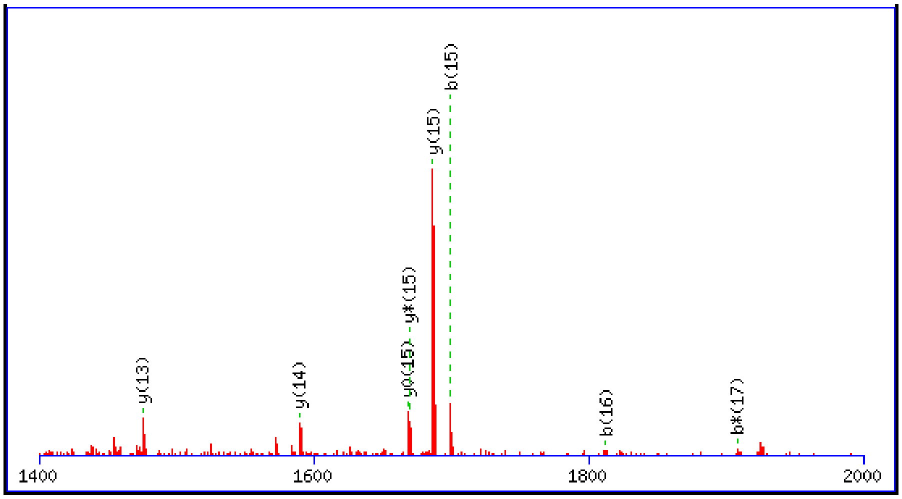

Monoisotopic mass of neutral peptide Mr(calc): 2099.0469
Fixed modifications: Carbamidomethyl (C)
Variable modifications:
N9 : Deamidated_N (N)
Ions Score: 69 **Expect:** 2.2e-005
Matches (Bold Red): 23/176 fragment ions using 36 most intense peaks

#	b	b^{++}	b*	b^{*++}	b^0	b^{0++}	Seq.	y	y^{++}	y*	y^{*++}	y^0	y^{0++}	#
1	114.0913	57.5493					I							18
2	277.1547	139.0810					Y	1986.9702	993.9887	1969.9436	985.4755	1968.9596	984.9834	17
3	**414.2136**	207.6104					H	1823.9068	**912.4571**	1806.8803	**903.9438**	1805.8963	**903.4518**	16
4	511.2663	256.1368					P	**1686.8479**	**843.9276**	**1669.8214**	835.4143	**1668.8374**	834.9223	15
5	**625.3093**	313.1583	608.2827	304.6450			N	**1589.7952**	795.4012	1572.7686	786.8880	1571.7846	786.3959	14
6	**738.3933**	369.7003	721.3668	361.1870			I	**1475.7522**	**738.3798**	1458.7257	729.8665	1457.7417	729.3745	13
7	852.4363	426.7218	835.4097	418.2085			N	**1362.6682**	681.8377	1345.6416	673.3245	1344.6576	672.8324	12
8	939.4683	470.2378	922.4417	461.7245	921.4577	461.2325	S	**1248.6253**	**624.8163**	1231.5987	616.3030	1230.6147	615.8110	11
9	1054.4952	527.7513	1037.4687	519.2380	1036.4847	518.7460	N	**1161.5932**	581.3003	1144.5667	572.7870	1143.5827	572.2950	10
10	1111.5167	556.2620	1094.4901	547.7487	1093.5061	547.2567	G	1046.5663	523.7868	1029.5397	515.2735	1028.5557	514.7815	9
11	1198.5487	599.7780	1181.5222	591.2647	1180.5382	590.7727	S	989.5448	495.2761	972.5183	486.7628	971.5343	486.2708	8
12	1311.6328	656.3200	1294.6062	647.8068	1293.6222	647.3147	I	902.5128	451.7600	885.4863	443.2468	884.5022	442.7548	7
13	1471.6634	736.3354	1454.6369	727.8221	1453.6529	727.3301	C	**789.4287**	395.2180	772.4022	386.7047	771.4182	386.2127	6
14	1584.7475	792.8774	1567.7209	784.3641	1566.7369	783.8721	L	629.3981	315.2027	612.3715	306.6894	611.3875	306.1974	5
15	**1699.7744**	850.3909	1682.7479	841.8776	1681.7639	841.3856	D	**516.3140**	258.6606	499.2875	250.1474	498.3035	249.6554	4
16	**1812.8585**	906.9329	1795.8320	898.4196	1794.8479	897.9276	I	**401.2871**	201.1472	384.2605	192.6339			3
17	1925.9426	963.4749	**1908.9160**	954.9616	1907.9320	954.4696	L	288.2030	144.6051	271.1765	136.0919			2
18							R	175.1190	88.0631	158.0924	79.5498			1

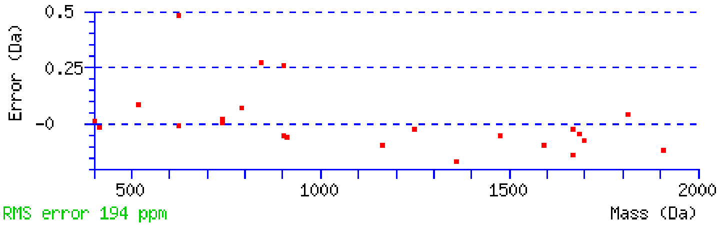

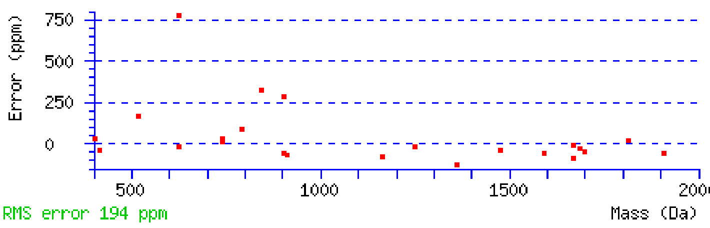

All matches to this query

Score	Mr(calc):	Delta	Sequence
69.0	2099.0469	0.0073	IYHPNINSNGSICLDILR
41.7	2099.0469	0.0073	IYHPNINSNGSICLDILR
20.3	2099.0469	0.0073	IYHPNINSNGSICLDILR

12.2	2098.0629	0.9913	IYHPNINSNGSICLDILR
7.8	2099.0526	0.0016	LWIIMEYLGGGSALDLLR
6.4	2099.0526	0.0016	LWIIMEYLGGGSALDLLR
4.3	2098.0388	1.0154	TQSLPTEPPTFPPTIPPAK
3.2	2099.0663	-0.0121	RKINLNISYEINEVSVK
0.5	2099.0663	-0.0121	RKINLNISYEINEVSVK
0.3	2098.0316	1.0226	DQGLSIMVSGKLDAVMKAR

Spectrum No: 237; Query: 1086; Rank: 1

Peptide View

MS/MS Fragmentation of **EGTYSLPSSNFTVPDLELPSWLSTGNYR**
Found in **IPI00470260**, Tax_Id=10116 Gene_Symbol=Gm2a GM2 ganglioside activator protein

Match to Query 1086: 3130.482972 from(1044.501600,3+)
Title: 091127RatKid_SCX01_14.6013.6013.3.dta
Data file K:\NewmanPaper\Piliang\3SubProteomes\Piliang3SP\mgf5ppm\SCX_3SubProteomes5ppm.mgf

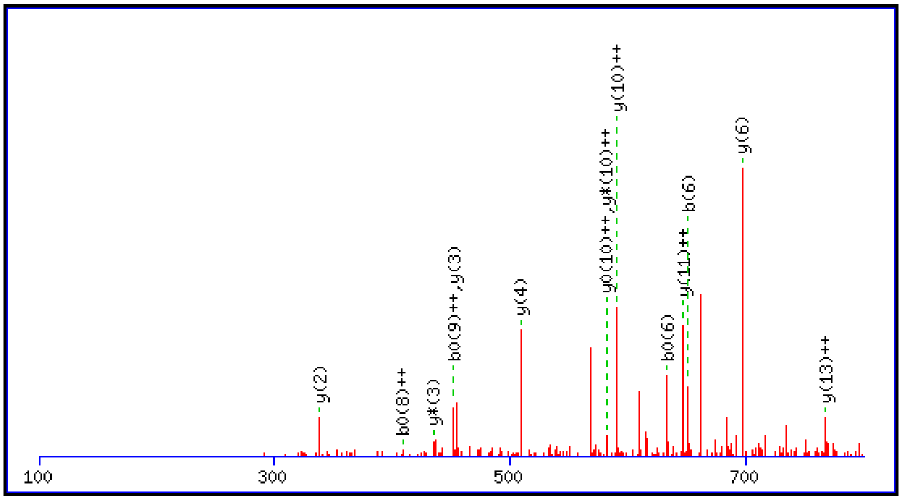

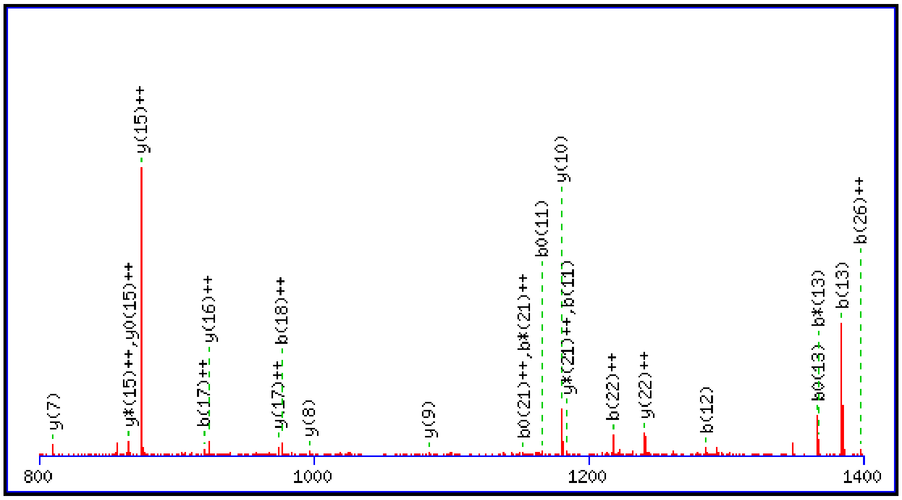

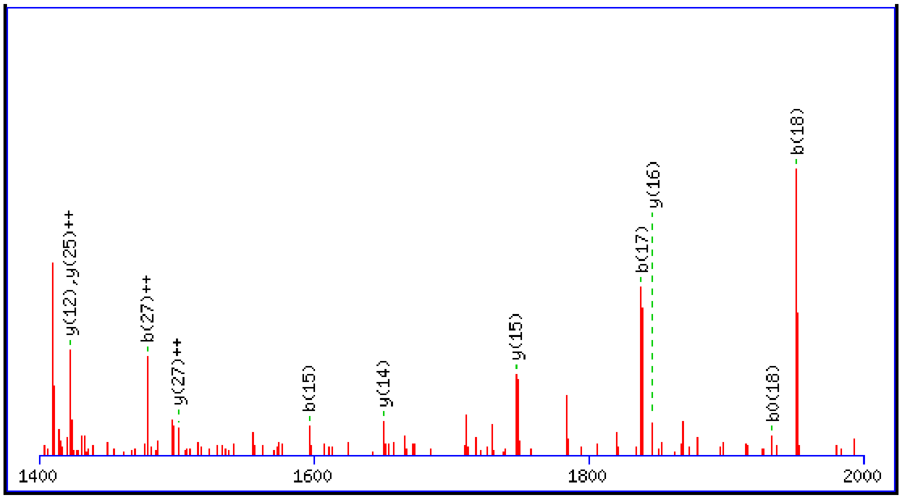

Monoisotopic mass of neutral peptide Mr(calc): 3130.4771
Fixed modifications: Carbamidomethyl (C)
Variable modifications:
N10 : Deamidated_N (N)
Ions Score: 69 **Expect:** 4.9e-005
Matches (Bold Red): 48/298 fragment ions using 76 most intense peaks

#	b	b++	b*	b*++	b0	b0++	Seq.	y	y++	y*	y*++	y0	y0++	#
1	130.0499	65.5286			112.0393	56.5233	E							28
2	187.0713	94.0393			169.0608	85.0340	G	3002.4418	**1501.7245**	2985.4153	1493.2113	2984.4312	1492.7193	27
3	288.1190	144.5631			270.1084	135.5579	T	2945.4203	1473.2138	2928.3938	1464.7005	2927.4098	1464.2085	26
4	451.1823	226.0948			433.1718	217.0895	Y	2844.3727	**1422.6900**	2827.3461	1414.1767	2826.3621	1413.6847	25
5	538.2144	269.6108			520.2038	260.6055	S	2681.3093	1341.1583	2664.2828	1332.6450	2663.2988	1332.1530	24
6	**651.2984**	326.1529			**633.2879**	317.1476	L	2594.2773	1297.6423	2577.2508	1289.1290	2576.2667	1288.6370	23
7	748.3512	374.6792			730.3406	365.6740	P	2481.1932	**1241.1003**	2464.1667	1232.5870	2463.1827	1232.0950	22
8	835.3832	418.1953			817.3727	**409.1900**	S	2384.1405	1192.5739	2367.1139	**1184.0606**	2366.1299	1183.5686	21
9	922.4153	461.7113			904.4047	**452.7060**	S	2297.1084	1149.0579	2280.0819	1140.5446	2279.0979	1140.0526	20
10	1037.4422	519.2247	1020.4156	510.7115	1019.4316	510.2195	N	2210.0764	1105.5418	2193.0499	1097.0286	2192.0659	1096.5366	19
11	**1184.5106**	592.7589	1167.4841	584.2457	**1166.5000**	583.7537	F	2095.0495	1048.0284	2078.0229	1039.5151	2077.0389	1039.0231	18
12	**1285.5583**	643.2828	1268.5317	634.7695	1267.5477	634.2775	T	1947.9811	**974.4942**	1930.9545	965.9809	1929.9705	965.4889	17
13	**1384.6267**	692.8170	**1367.6001**	684.3037	**1366.6161**	683.8117	V	**1846.9334**	**923.9703**	1829.9068	915.4571	1828.9228	914.9651	16
14	1481.6795	741.3434	1464.6529	732.8301	1463.6689	732.3381	P	**1747.8650**	**874.4361**	1730.8384	**865.9229**	1729.8544	**865.4308**	15
15	**1596.7064**	798.8568	1579.6799	790.3436	1578.6958	789.8516	D	**1650.8122**	825.9097	1633.7857	817.3965	1632.8016	816.9045	14
16	1709.7905	855.3989	1692.7639	846.8856	1691.7799	846.3936	L	1535.7853	**768.3963**	1518.7587	759.8830	1517.7747	759.3910	13
17	**1838.8331**	**919.9202**	1821.8065	911.4069	1820.8225	910.9149	E	**1422.7012**	711.8542	1405.6747	703.3410	1404.6906	702.8490	12
18	**1951.9171**	**976.4622**	1934.8906	967.9489	**1933.9066**	967.4569	L	1293.6586	**647.3329**	1276.6321	638.8197	1275.6480	638.3277	11
19	2048.9699	1024.9886	2031.9433	1016.4753	2030.9593	1015.9833	P	**1180.5745**	**590.7909**	1163.5480	**582.2776**	1162.5640	**581.7856**	10
20	2136.0019	1068.5046	2118.9754	1059.9913	2117.9914	1059.4993	S	**1083.5218**	542.2645	1066.4952	533.7513	1065.5112	533.2592	9
21	2322.0812	1161.5443	2305.0547	**1153.0310**	2304.0707	**1152.5390**	W	**996.4898**	498.7485	979.4632	490.2352	978.4792	489.7432	8
22	2435.1653	**1218.0863**	2418.1387	1209.5730	2417.1547	1209.0810	L	**810.4104**	405.7089	793.3839	397.1956	792.3999	396.7036	7
23	2522.1973	1261.6023	2505.1708	1253.0890	2504.1868	1252.5970	S	**697.3264**	349.1668	680.2998	340.6536	679.3158	340.1615	6
24	2623.2450	1312.1261	2606.2185	1303.6129	2605.2344	1303.1209	T	610.2944	305.6508	593.2678	297.1375	592.2838	296.6455	5
25	2680.2665	1340.6369	2663.2399	1332.1236	2662.2559	1331.6316	G	**509.2467**	255.1270	492.2201	246.6137			4
26	2794.3094	**1397.6583**	2777.2828	1389.1451	2776.2988	1388.6531	N	**452.2252**	226.6162	**435.1987**	218.1030			3
27	2957.3727	**1479.1900**	2940.3462	1470.6767	2939.3622	1470.1847	Y	**338.1823**	169.5948	321.1557	161.0815			2
28							R	175.1190	88.0631	158.0924	79.5498			1

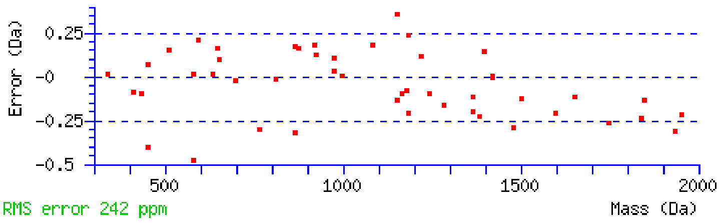

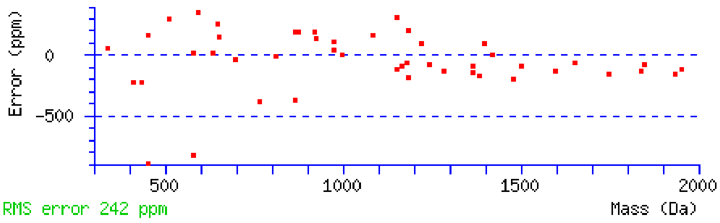

All matches to this query

Score	Mr(calc):	Delta	Sequence
68.9	3130.4771	0.0059	EGTYSLPSSNFTVPDLELPSWLSTGNYR
43.3	3129.4931	0.9899	EGTYSLPSSNFTVPDLELPSWLSTGNYR
14.3	3130.4771	0.0059	EGTYSLPSSNFTVPDLELPSWLSTGNYR
9.2	3128.4654	2.0175	LELDGGQMKLTVNLGAAGKGPETLFAGHK
8.2	3128.4849	1.9981	NRNICTRPSTLSFIPPINESTALSSSGK
7.2	3129.4689	1.0141	NRNICTRPSTLSFIPPINESTALSSSGK
7.1	3130.4695	0.0135	TMSAESVHYNALLDLINREQNRWYK
7.0	3130.4695	0.0135	TMSAESVHYNALLDLINREQNRWYK
6.1	3130.5061	-0.0231	GSLNLTQLQQASRGVNQATAAVVASTISGK
5.1	3129.5106	0.9724	LVRIYTKPKGQLPDYTSPVVLPYSR

Spectrum No: 238; Query: 526; Rank: 1

Peptide View

MS/MS Fragmentation of **ATEPCLDGWIYNSTR**
Found in **IPI00207702**, Tax_Id=10116 Gene_Symbol=Slc22a8 Solute carrier family 22 member 8

Match to Query 526: 1782.792248 from(892.403400,2+)
Title: 091127RatKid_SCX01_11.3021.3021.2.dta
Data file K:\NewmanPaper\Piliang\3SubProteomes\Piliang3SP\mgf5ppm\SCX_3SubProteomes5ppm.mgf

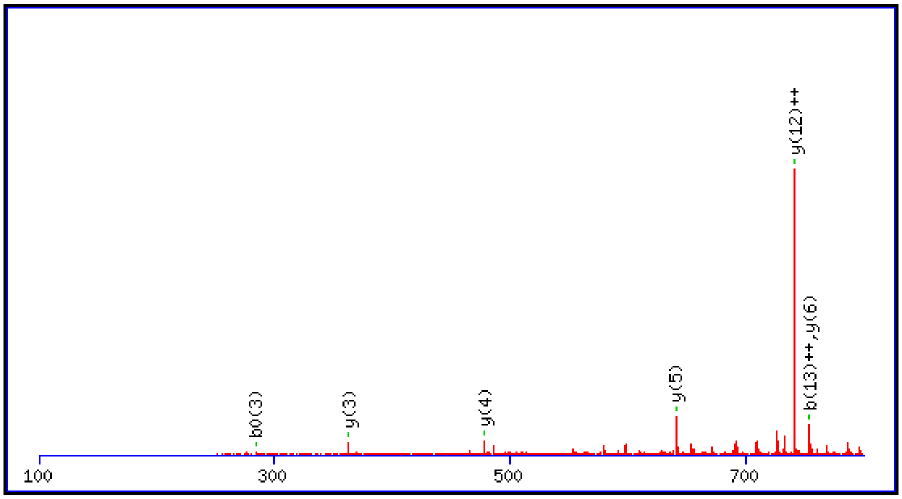

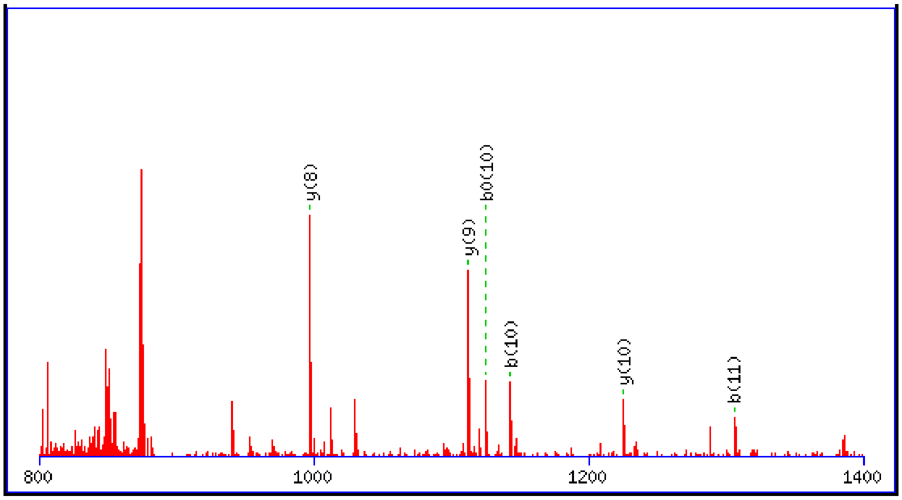

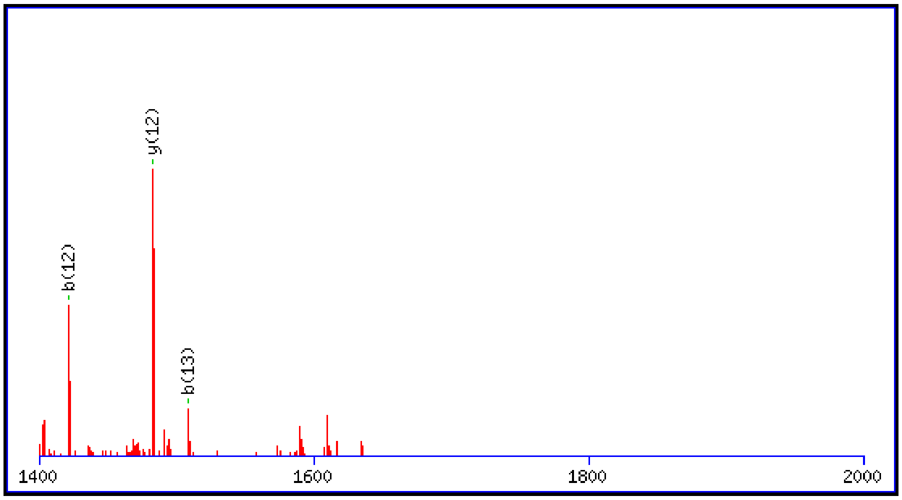

Monoisotopic mass of neutral peptide Mr(calc): 1782.7883
Fixed modifications: Carbamidomethyl (C)
Variable modifications:
N12 : Deamidated_N (N)
Ions Score: 69 **Expect:** 2.5e-005
Matches (Bold Red): 16/142 fragment ions using 19 most intense peaks

#	b	b^{++}	b*	b^{*++}	b^0	b^{0++}	Seq.	y	y^{++}	y*	y^{*++}	y^0	y^{0++}	#
1	72.0444	36.5258					A							15
2	173.0921	87.0497			155.0815	78.0444	T	1712.7585	856.8829	1695.7319	848.3696	1694.7479	847.8776	14
3	302.1347	151.5710			**284.1241**	142.5657	E	1611.7108	806.3590	1594.6842	797.8458	1593.7002	797.3537	13
4	399.1874	200.0974			381.1769	191.0921	P	**1482.6682**	**741.8377**	1465.6416	733.3245	1464.6576	732.8324	12
5	559.2181	280.1127			541.2075	271.1074	C	1385.6154	693.3113	1368.5889	684.7981	1367.6049	684.3061	11
6	672.3021	336.6547			654.2916	327.6494	L	**1225.5848**	613.2960	1208.5582	604.7827	1207.5742	604.2907	10
7	787.3291	394.1682			769.3185	385.1629	D	**1112.5007**	556.7540	1095.4742	548.2407	1094.4901	547.7487	9
8	844.3505	422.6789			826.3400	413.6736	G	**997.4738**	499.2405	980.4472	490.7272	979.4632	490.2352	8
9	1030.4299	515.7186			1012.4193	506.7133	W	940.4523	470.7298	923.4258	462.2165	922.4417	461.7245	7
10	**1143.5139**	572.2606			**1125.5034**	563.2553	I	**754.3730**	377.6901	737.3464	369.1769	736.3624	368.6848	6
11	**1306.5773**	653.7923			1288.5667	644.7870	Y	**641.2889**	321.1481	624.2624	312.6348	623.2784	312.1428	5
12	**1421.6042**	711.3057	1404.5776	702.7925	1403.5936	702.3004	N	**478.2256**	239.6164	461.1990	231.1032	460.2150	230.6112	4
13	**1508.6362**	**754.8217**	1491.6097	746.3085	1490.6257	745.8165	S	**363.1987**	182.1030	346.1721	173.5897	345.1881	173.0977	3
14	1609.6839	805.3456	1592.6573	796.8323	1591.6733	796.3403	T	276.1666	138.5870	259.1401	130.0737	258.1561	129.5817	2
15							R	175.1190	88.0631	158.0924	79.5498			1

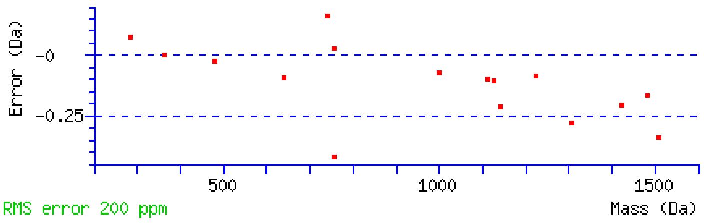

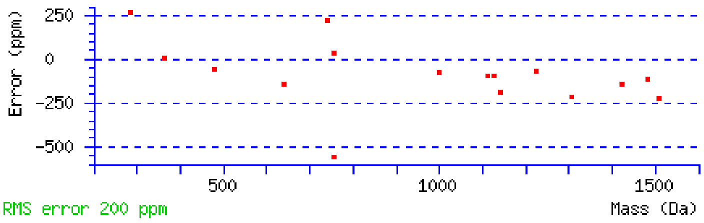

All matches to this query

Score	Mr(calc):	Delta	Sequence
68.8	1782.7883	0.0040	ATEPCLDGWIYNSTR
11.9	1781.7729	1.0193	DVMNGEQTGLLMLHK
3.5	1782.8036	-0.0114	EKGPNNTANSSEVKVK
3.5	1782.8036	-0.0114	EKGPNNTANSSEVKVK
3.1	1782.8050	-0.0127	SNTRYYTGTQSRIR
2.3	1780.8033	1.9890	EEAEGPEPKGLSPPHK
1.1	1782.7898	0.0025	TSIVQAAAGGGTGGGSNNGK
1.1	1782.7898	0.0025	TSIVQAAAGGGTGGGSNNGK
1.0	1782.8036	-0.0114	EKGPNNTANSSEVKVK
1.0	1782.8036	-0.0114	EKGPNNTANSSEVKVK

Spectrum No: 239; Query: 251; Rank: 1

Peptide View

MS/MS Fragmentation of **VSNMTLPASAEVLK**
Found in **IPI00206336**, Tax_Id=10116 Gene_Symbol=Lamp1 Lysosome-associated membrane glycoprotein 1 precursor

Match to Query 251: 1459.760768 from(730.887660,2+)
Title: 091127RatKid_SCX01_15.2412.2412.2.dta
Data file K:\NewmanPaper\Piliang\3SubProteomes\Piliang3SP\mgf5ppm\SCX_3SubProteomes5ppm.mgf

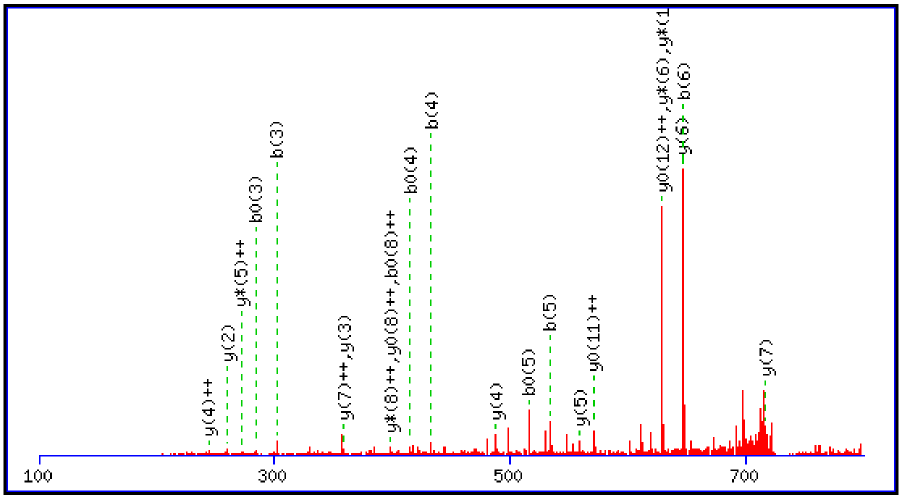

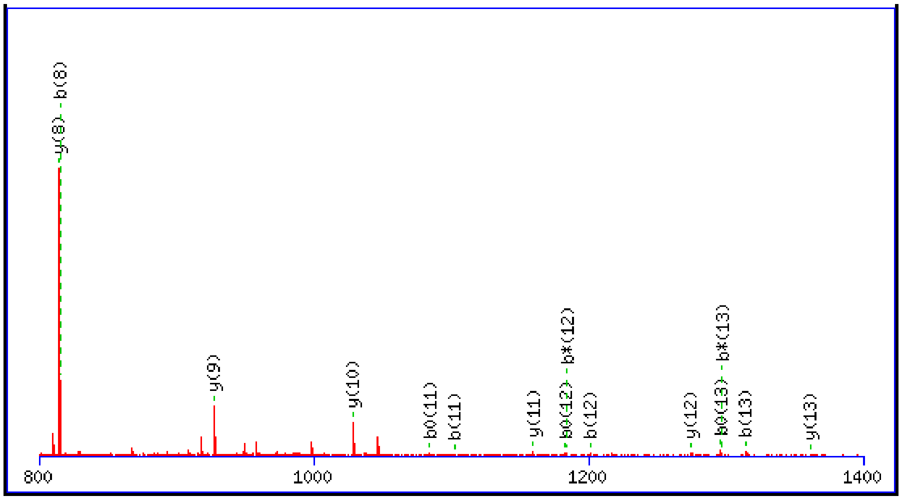

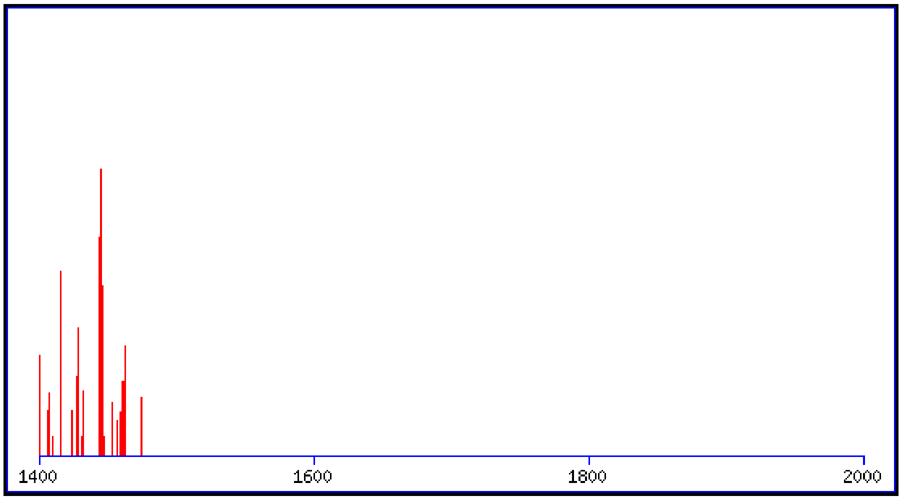

Monoisotopic mass of neutral peptide Mr(calc): 1459.7592
Fixed modifications: Carbamidomethyl (C)
Variable modifications:
N3 : Deamidated_N (N)
Ions Score: 69 **Expect:** 1.6e-005
Matches (Bold Red): 39/144 fragment ions using 81 most intense peaks

#	b	b^{++}	b*	b^{*++}	b^0	b^{0++}	Seq.	y	y^{++}	y*	y^{*++}	y^0	y^{0++}	#
1	100.0757	50.5415					V							14
2	187.1077	94.0575			169.0972	85.0522	S	**1361.6981**	681.3527	1344.6715	672.8394	1343.6875	672.3474	13
3	**302.1347**	151.5710	285.1081	143.0577	**284.1241**	142.5657	N	**1274.6661**	637.8367	1257.6395	**629.3234**	1256.6555	**628.8314**	12
4	**433.1751**	217.0912	416.1486	208.5779	**415.1646**	208.0859	M	**1159.6391**	580.3232	1142.6126	571.8099	1141.6286	**571.3179**	11
5	**534.2228**	267.6150	517.1963	259.1018	**516.2123**	258.6098	T	**1028.5986**	514.8030	1011.5721	506.2897	1010.5881	505.7977	10
6	**647.3069**	324.1571	630.2803	315.6438	**629.2963**	315.1518	L	**927.5510**	464.2791	910.5244	455.7658	909.5404	455.2738	9
7	744.3596	372.6835	727.3331	364.1702	726.3491	363.6782	P	**814.4669**	407.7371	797.4403	**399.2238**	796.4563	**398.7318**	8
8	**815.3968**	408.2020	798.3702	399.6887	797.3862	**399.1967**	A	**717.4141**	**359.2107**	700.3876	350.6974	699.4036	350.2054	7
9	902.4288	451.7180	885.4022	443.2048	884.4182	442.7127	S	**646.3770**	323.6921	**629.3505**	315.1789	628.3665	314.6869	6
10	973.4659	487.2366	956.4394	478.7233	955.4553	478.2313	A	**559.3450**	280.1761	542.3184	**271.6629**	541.3344	271.1709	5
11	**1102.5085**	551.7579	1085.4819	543.2446	**1084.4979**	542.7526	E	**488.3079**	**244.6576**	471.2813	236.1443	470.2973	235.6523	4
12	**1201.5769**	601.2921	**1184.5504**	592.7788	**1183.5663**	592.2868	V	**359.2653**	180.1363	342.2387	171.6230			3
13	**1314.6610**	657.8341	**1297.6344**	649.3209	**1296.6504**	648.8288	L	**260.1969**	130.6021	243.1703	122.0888			2
14							K	147.1128	74.0600	130.0863	65.5468			1

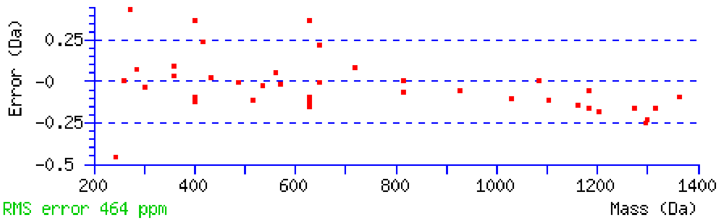

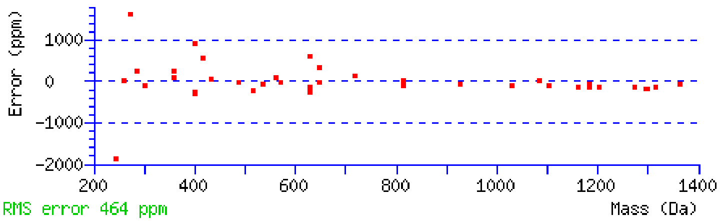

All matches to this query

Score	Mr(calc):	Delta	Sequence
68.7	1459.7592	0.0016	VSNMTLPASAEVLK
12.4	1459.7606	0.0002	KSCGXIPGKQAWK
10.9	1459.7671	-0.0063	WLGTGNKGSNALLK
9.9	1459.7476	0.0131	FTLGKVWITTSK
9.9	1459.7476	0.0131	FTLGKVWITTSK
3.9	1459.7518	0.0090	KLNESLKQQNEK
3.6	1459.7476	0.0131	FTLGKVWITTSK
2.2	1459.7622	-0.0014	YLIVMTKVRGGK
1.4	1459.7745	-0.0137	VEQMFQILLDPK
1.3	1459.7592	0.0015	DAVEEIVCVSVLK

Spectrum No: 240; Query: 1008; Rank: 1

Peptide View

MS/MS Fragmentation of **NCHYPTCDQLTCANGACYNTSQR**
Found in **IPI00205325**, Tax_Id=10116 Gene_Symbol=Lrp2 Low-density lipoprotein receptor-related protein 2 precursor

Match to Query 1008: 2791.084842 from(931.368890,3+)
Title: 091127RatKid_SCX01_22.1516.1516.3.dta
Data file K:\NewmanPaper\Piliang\3SubProteomes\Piliang3SP\mgf5ppm\SCX_3SubProteomes5ppm.mgf

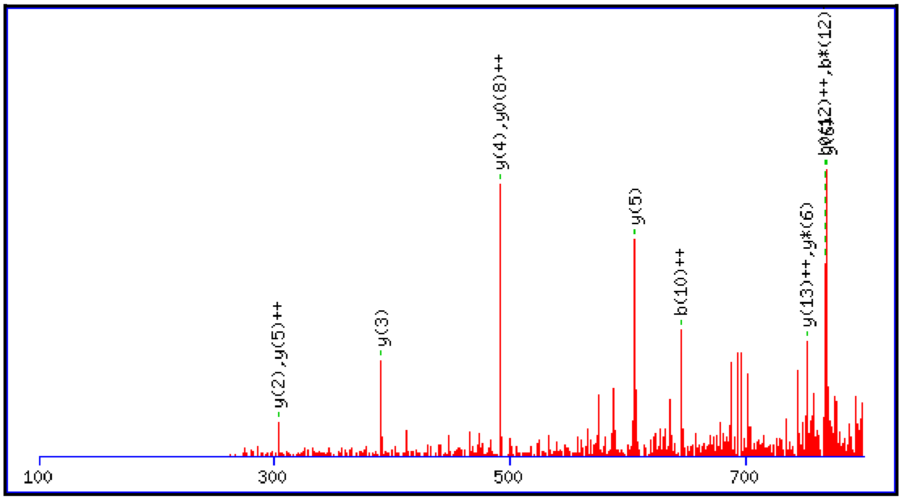

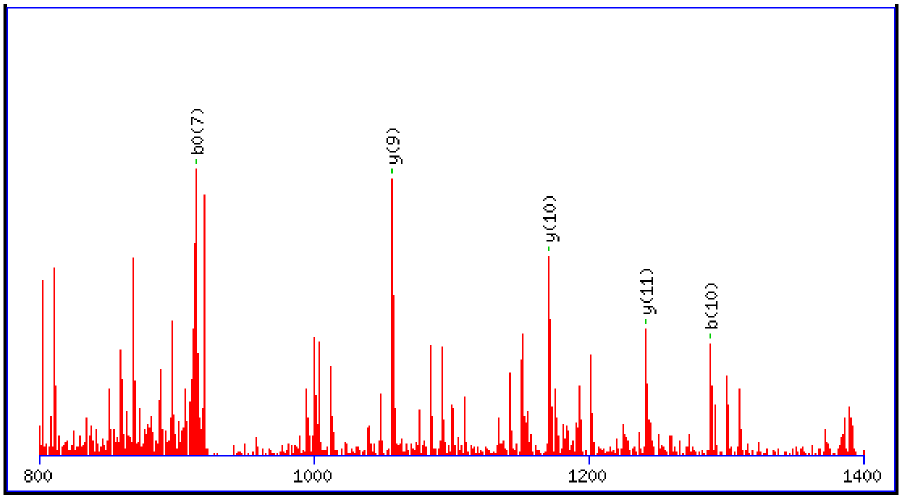

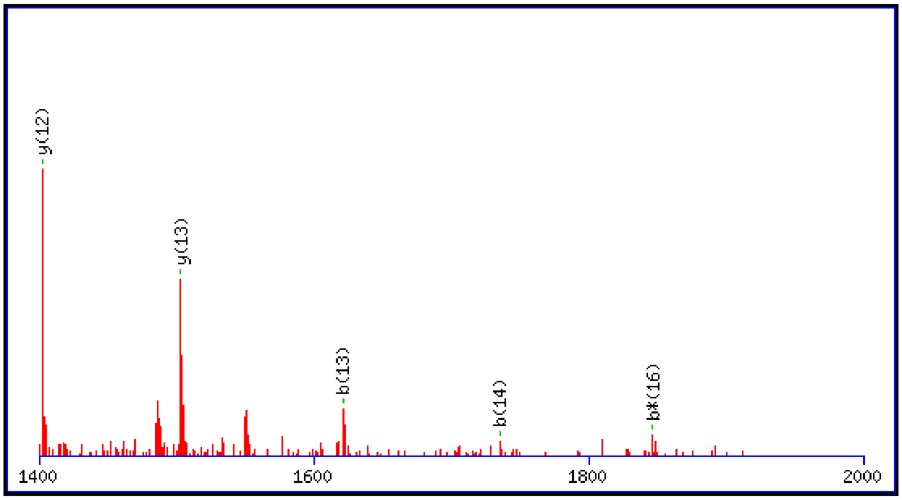

Monoisotopic mass of neutral peptide Mr(calc): 2791.0843
Fixed modifications: Carbamidomethyl (C)
Variable modifications:
N19 : Deamidated_N (N)
Ions Score: 69 **Expect:** 3.7e-005
Matches (Bold Red): 22/250 fragment ions using 27 most intense peaks

#	b	b^{++}	b*	b*$^{++}$	b^{0}	b^{0++}	Seq.	y	y^{++}	y*	y*$^{++}$	y^{0}	y^{0++}	#
1	115.0502	58.0287	98.0237	49.5155			N							23
2	275.0809	138.0441	258.0543	129.5308			C	2678.0487	1339.5280	2661.0221	1331.0147	2660.0381	1330.5227	22
3	412.1398	206.5735	395.1132	198.0602			H	2518.0180	1259.5126	2500.9915	1250.9994	2500.0075	1250.5074	21
4	575.2031	288.1052	558.1765	279.5919			Y	2380.9591	1190.9832	2363.9326	1182.4699	2362.9485	1181.9779	20
5	672.2559	336.6316	655.2293	328.1183			P	2217.8958	1109.4515	2200.8692	1100.9383	2199.8852	1100.4462	19
6	773.3035	387.1554	756.2770	378.6421	755.2930	378.1501	T	2120.8430	1060.9251	2103.8165	1052.4119	2102.8324	1051.9199	18
7	933.3342	467.1707	916.3076	458.6575	**915.3236**	458.1654	C	2019.7953	1010.4013	2002.7688	1001.8880	2001.7848	1001.3960	17
8	1048.3611	524.6842	1031.3346	516.1709	1030.3506	515.6789	D	1859.7647	930.3860	1842.7381	921.8727	1841.7541	921.3807	16
9	1176.4197	588.7135	1159.3932	580.2002	1158.4091	579.7082	Q	1744.7377	872.8725	1727.7112	864.3592	1726.7272	863.8672	15
10	**1289.5038**	**645.2555**	1272.4772	636.7422	1271.4932	636.2502	L	1616.6792	808.8432	1599.6526	800.3299	1598.6686	799.8379	14
11	1390.5514	695.7794	1373.5249	687.2661	1372.5409	686.7741	T	**1503.5951**	**752.3012**	1486.5685	743.7879	1485.5845	743.2959	13
12	1550.5821	775.7947	1533.5555	**767.2814**	1532.5715	**766.7894**	C	**1402.5474**	701.7773	1385.5209	693.2641	1384.5369	692.7721	12
13	**1621.6192**	811.3132	1604.5927	802.8000	1603.6086	802.3080	A	**1242.5168**	621.7620	1225.4902	613.2487	1224.5062	612.7567	11
14	**1735.6621**	868.3347	1718.6356	859.8214	1717.6516	859.3294	N	**1171.4797**	586.2435	1154.4531	577.7302	1153.4691	577.2382	10
15	1792.6836	896.8454	1775.6571	888.3322	1774.6730	887.8402	G	**1057.4367**	529.2220	1040.4102	520.7087	1039.4262	520.2167	9
16	1863.7207	932.3640	**1846.6942**	923.8507	1845.7102	923.3587	A	1000.4153	500.7113	983.3887	492.1980	982.4047	**491.7060**	8
17	2023.7514	1012.3793	2006.7248	1003.8660	2005.7408	1003.3740	C	929.3782	465.1927	912.3516	456.6794	911.3676	456.1874	7
18	2186.8147	1093.9110	2169.7881	1085.3977	2168.8041	1084.9057	Y	**769.3475**	385.1774	**752.3210**	376.6641	751.3369	376.1721	6

**19**	2301.8416	1151.4245	2284.8151	1142.9112	2283.8311	1142.4192	**N**	**606.2842**	**303.6457**	589.2576	295.1324	588.2736	294.6404	**5**
**20**	2402.8893	1201.9483	2385.8628	1193.4350	2384.8787	1192.9430	**T**	**491.2572**	246.1323	474.2307	237.6190	473.2467	237.1270	**4**
**21**	2489.9213	1245.4643	2472.8948	1236.9510	2471.9108	1236.4590	**S**	**390.2096**	195.6084	373.1830	187.0951	372.1990	186.6031	**3**
**22**	2617.9799	1309.4936	2600.9534	1300.9803	2599.9694	1300.4883	**Q**	**303.1775**	152.0924	286.1510	143.5791			**2**
**23**							**R**	175.1190	88.0631	158.0924	79.5498			**1**

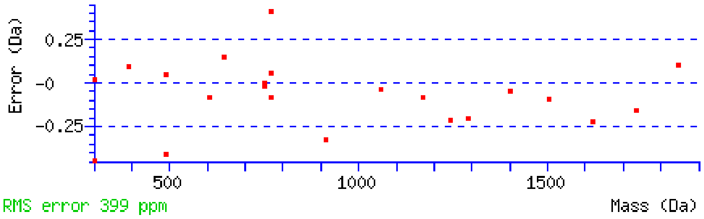

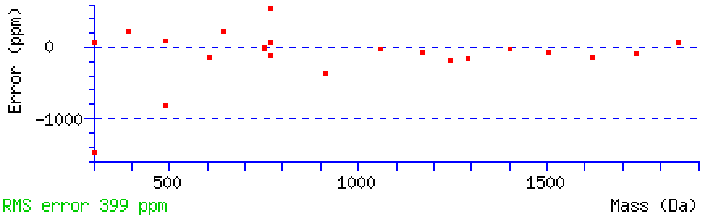

All matches to this query

Score	Mr(calc):	Delta	Sequence
68.7	2791.0843	0.0005	NCHYPTCDQLTCANGACYNTSQR
48.3	2791.0843	0.0005	NCHYPTCDQLTCANGACYNTSQR
24.2	2790.1003	0.9845	NCHYPTCDQLTCANGACYNTSQR
19.2	2791.0843	0.0005	NCHYPTCDQLTCANGACYNTSQR
2.9	2791.0817	0.0031	DCIMHGYMSKMGNPFLTQWQR
2.9	2791.0817	0.0031	DCIMHGYMSKMGNPFLTQWQR
1.1	2790.0542	1.0306	DDASQVSSTSNDVSSSDFEEGPSRK
1.1	2790.0542	1.0306	DDASQVSSTSNDVSSSDFEEGPSRK
0.8	2791.0817	0.0031	DCIMHGYMSKMGNPFLTQWQR
0.8	2791.0817	0.0031	DCIMHGYMSKMGNPFLTQWQR

Spectrum No: 241; Query: 549; Rank: 1

Peptide View

MS/MS Fragmentation of **GTFTDCALANMTQQIR**
Found in **IPI00372839**, Tax_Id=10116 Gene_Symbol=Col6a2 similar to procollagen, type VI, alpha 2

Match to Query 549: 1827.832208 from(914.923380,2+)
Title: 091127RatKid_SCX01_12.3466.3466.2.dta
Data file K:\NewmanPaper\Piliang\3SubProteomes\Piliang3SP\mgf5ppm\SCX_3SubProteomes5ppm.mgf

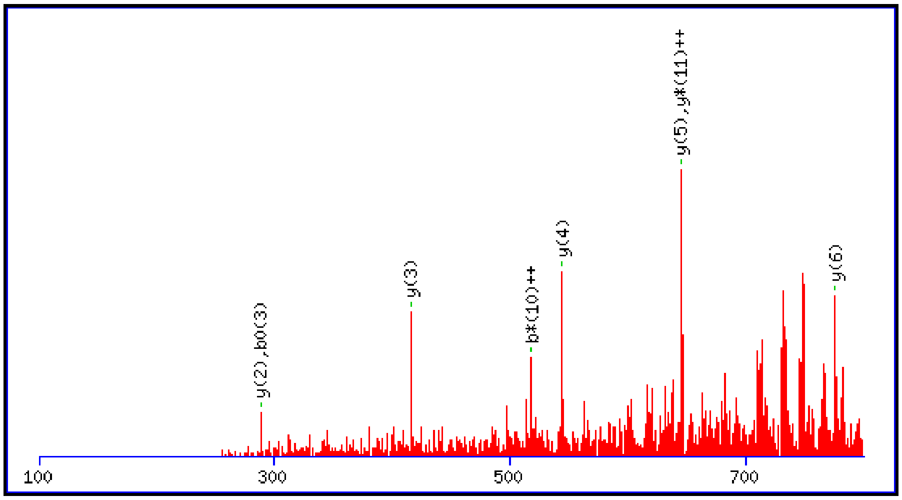

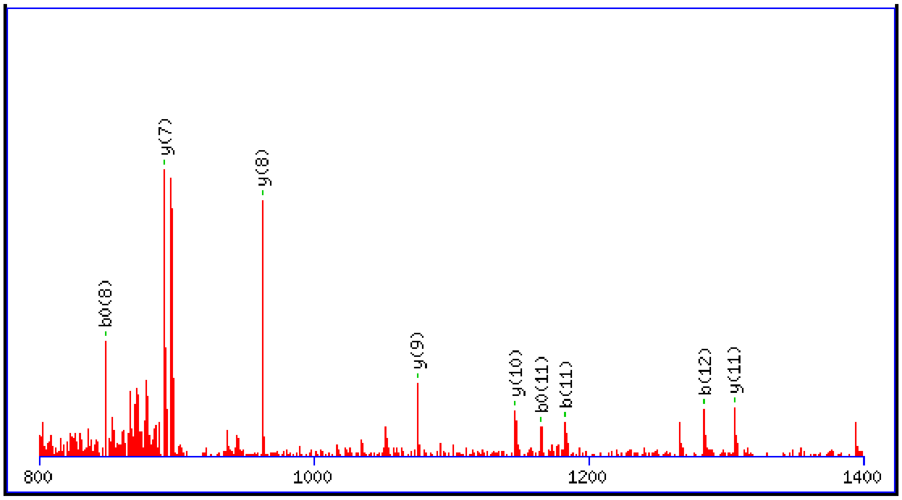

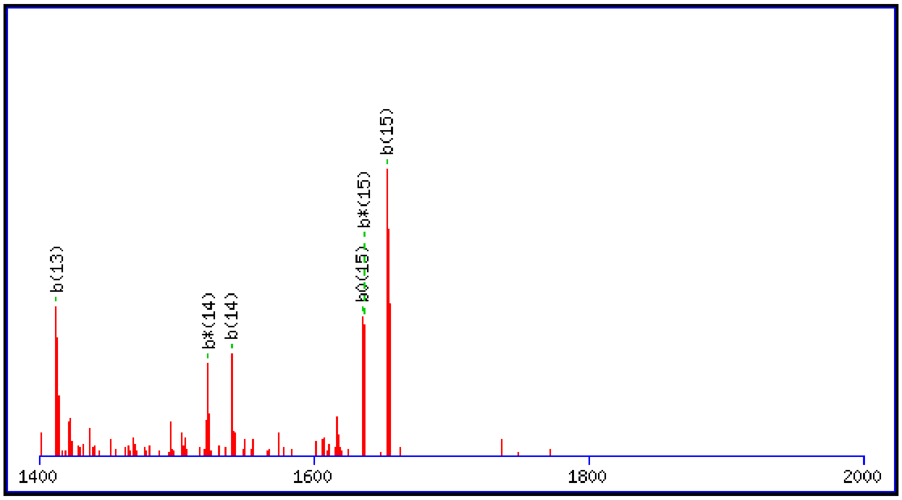

Monoisotopic mass of neutral peptide Mr(calc): 1826.8291
Fixed modifications: Carbamidomethyl (C)
Variable modifications:
N10 : Deamidated_N (N)
Ions Score: 69 **Expect:** 3e-005
Matches (Bold Red): 23/152 fragment ions using 38 most intense peaks

#	b	b++	b*	b*++	b0	b0++	Seq.	y	y++	y*	y*++	y0	y0++	#
1	58.0287	29.5180					G							16
2	159.0764	80.0418			141.0659	71.0366	T	1770.8149	885.9111	1753.7884	877.3978	1752.8044	876.9058	15
3	306.1448	153.5761			**288.1343**	144.5708	F	1669.7672	835.3873	1652.7407	826.8740	1651.7567	826.3820	14
4	407.1925	204.0999			389.1819	195.0946	T	1522.6988	761.8531	1505.6723	753.3398	1504.6883	752.8478	13
5	522.2195	261.6134			504.2089	252.6081	D	1421.6512	711.3292	1404.6246	702.8159	1403.6406	702.3239	12
6	682.2501	341.6287			664.2395	332.6234	C	**1306.6242**	653.8157	1289.5977	**645.3025**	1288.6136	644.8105	11
7	753.2872	377.1472			735.2767	368.1420	A	**1146.5936**	573.8004	1129.5670	565.2871	1128.5830	564.7951	10
8	866.3713	433.6893			**848.3607**	424.6840	L	**1075.5565**	538.2819	1058.5299	529.7686	1057.5459	529.2766	9
9	937.4084	469.2078			919.3978	460.2026	A	**962.4724**	481.7398	945.4458	473.2266	944.4618	472.7345	8
10	1052.4353	526.7213	1035.4088	**518.2080**	1034.4248	517.7160	N	**891.4353**	446.2213	874.4087	437.7080	873.4247	437.2160	7
11	**1183.4758**	592.2415	1166.4493	583.7283	**1165.4653**	583.2363	M	**776.4083**	388.7078	759.3818	380.1945	758.3978	379.7025	6
12	**1284.5235**	642.7654	1267.4969	634.2521	1266.5129	633.7601	T	**645.3679**	323.1876	628.3413	314.6743	627.3573	314.1823	5
13	**1412.5821**	706.7947	1395.5555	698.2814	1394.5715	697.7894	Q	**544.3202**	272.6637	527.2936	264.1504			4
14	**1540.6407**	770.8240	**1523.6141**	762.3107	1522.6301	761.8187	Q	**416.2616**	208.6344	399.2350	200.1212			3
15	**1653.7247**	827.3660	**1636.6982**	818.8527	**1635.7142**	818.3607	I	**288.2030**	144.6051	271.1765	136.0919			2
16							R	175.1190	88.0631	158.0924	79.5498			1

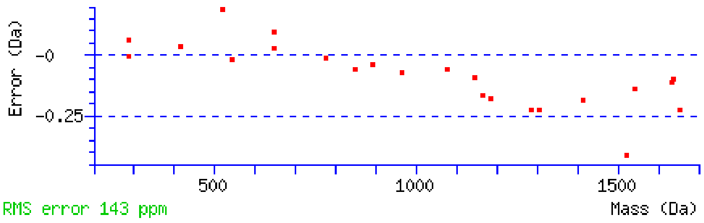

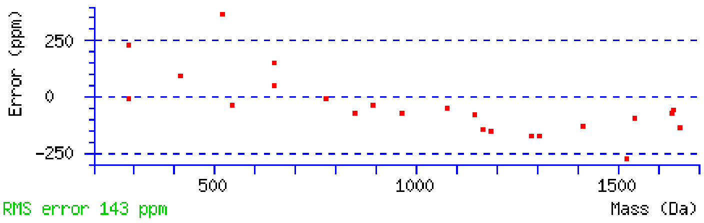

All matches to this query

Score	Mr(calc):	Delta	Sequence
68.5	1826.8291	1.0031	GTFTDCALANMTQQIR
8.4	1827.8383	-0.0061	CTEGDFGVPLTMSSLSK
7.3	1827.8284	0.0038	TLWMPSSTSXWSMLR
5.4	1825.8083	2.0239	ERQHYAWLCSQLR
4.4	1826.8240	1.0082	NYGKFLEEYTSQLR
2.9	1826.8234	1.0088	NGQEEMAGVVSTQLIR
2.9	1826.8234	1.0088	NGQEEMAGVVSTQLIR
1.5	1826.8240	1.0082	NYGKFLEEYTSQLR
1.5	1826.8240	1.0082	NYGKFLEEYTSQLR
1.5	1826.8240	1.0082	NYGKFLEEYTSQLR

Spectrum No: 242; Query: 1006; Rank: 1

Peptide View

MS/MS Fragmentation of **SENGLEFTSSGSANTETTKVNGSLETK**
Found in **IPI00421874**, Tax_Id=10116 Gene_Symbol=Vdac1 Voltage-dependent anion-selective channel protein 1

Match to Query 1006: 2788.293492 from(930.438440,3+)
Title: 091129RatKid_SCX02_25.1763.1763.3.dta
Data file K:\NewmanPaper\Piliang\3SubProteomes\Piliang3SP\mgf5ppm\SCX_3SubProteomes5ppm.mgf

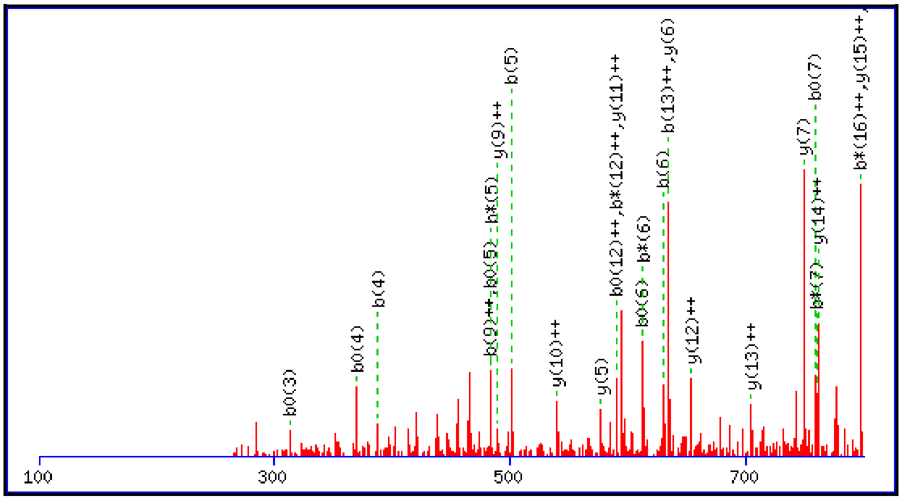

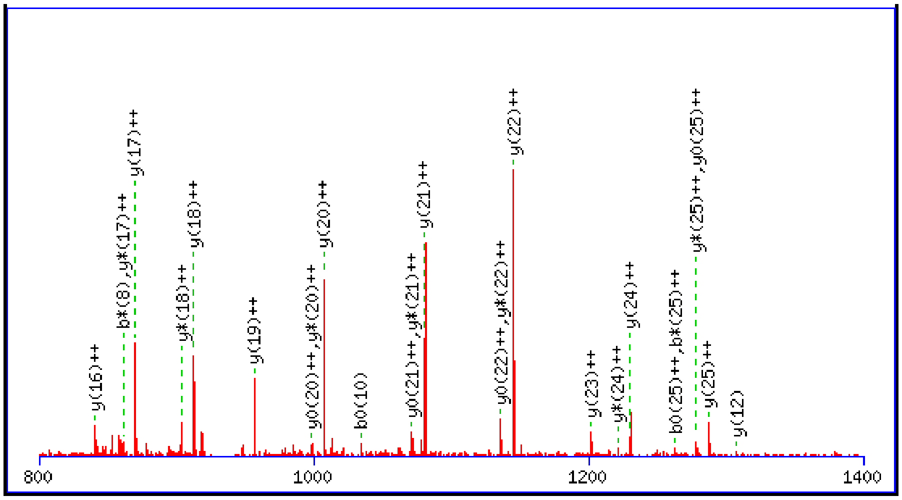

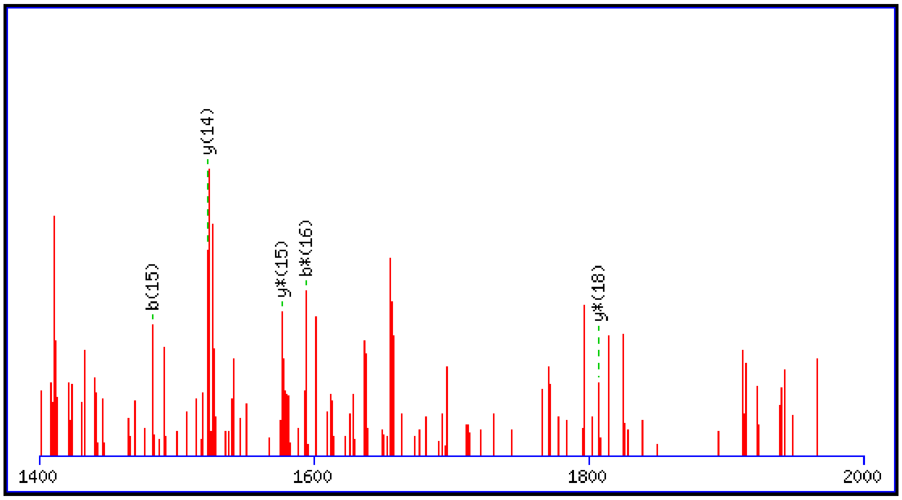

Monoisotopic mass of neutral peptide Mr(calc): 2788.2886
Fixed modifications: Carbamidomethyl (C)
Variable modifications:
N21 : Deamidated_N (N)
Ions Score: 68 **Expect:** 5.4e-005
Matches (Bold Red): 58/306 fragment ions using 126 most intense peaks

#	b	b^{++}	b*	b^{*++}	b^0	b^{0++}	Seq.	y	y^{++}	y*	y^{*++}	y^0	y^{0++}	#
1	88.0393	44.5233			70.0287	35.5180	S							27
2	217.0819	109.0446			199.0713	100.0393	E	2702.2639	1351.6356	2685.2373	1343.1223	2684.2533	1342.6303	26
3	331.1248	166.0660	314.0983	157.5528	**313.1143**	157.0608	N	2573.2213	**1287.1143**	2556.1948	**1278.6010**	2555.2107	**1278.1090**	25
4	**388.1463**	194.5768	371.1197	186.0635	**370.1357**	185.5715	G	2459.1784	**1230.0928**	2442.1518	**1221.5796**	2441.1678	1221.0875	24
5	**501.2304**	251.1188	**484.2038**	242.6055	**483.2198**	242.1135	L	2402.1569	**1201.5821**	2385.1304	1193.0688	2384.1463	1192.5768	23
6	**630.2729**	315.6401	**613.2464**	307.1268	**612.2624**	306.6348	E	2289.0728	**1145.0401**	2272.0463	**1136.5268**	2271.0623	**1136.0348**	22
7	777.3414	389.1743	**760.3148**	380.6610	**759.3308**	380.1690	F	2160.0303	**1080.5188**	2143.0037	**1072.0055**	2142.0197	**1071.5135**	21
8	878.3890	439.6982	**861.3625**	431.1849	860.3785	430.6929	T	2012.9618	**1006.9846**	1995.9353	**998.4713**	1994.9513	**997.9793**	20
9	965.4211	**483.2142**	948.3945	474.7009	947.4105	474.2089	S	1911.9142	**956.4607**	1894.8876	947.9474	1893.9036	947.4554	19
10	1052.4531	526.7302	1035.4265	518.2169	**1034.4425**	517.7249	S	1824.8821	**912.9447**	**1807.8556**	**904.4314**	1806.8716	903.9394	18
11	1109.4746	555.2409	1092.4480	546.7276	1091.4640	546.2356	G	1737.8501	**869.4287**	1720.8236	**860.9154**	1719.8395	860.4234	17
12	1196.5066	598.7569	1179.4800	**590.2437**	1178.4960	**589.7516**	S	1680.8286	**840.9180**	1663.8021	832.4047	1662.8181	831.9127	16
13	1267.5437	**634.2755**	1250.5172	625.7622	1249.5331	625.2702	A	1593.7966	**797.4019**	**1576.7701**	788.8887	1575.7861	788.3967	15
14	1381.5866	691.2970	1364.5601	682.7837	1363.5761	682.2917	N	**1522.7595**	**761.8834**	1505.7330	753.3701	1504.7489	752.8781	14
15	**1482.6343**	741.8208	1465.6078	733.3075	1464.6237	732.8155	T	1408.7166	**704.8619**	1391.6900	696.3487	1390.7060	695.8566	13
16	1611.6769	806.3421	**1594.6503**	**797.8288**	1593.6663	**797.3368**	E	**1307.6689**	**654.3381**	1290.6423	645.8248	1289.6583	645.3328	12
17	1712.7246	856.8659	1695.6980	848.3527	1694.7140	847.8606	T	1178.6263	**589.8168**	1161.5998	581.3035	1160.6157	580.8115	11
18	1813.7723	907.3898	1796.7457	898.8765	1795.7617	898.3845	T	1077.5786	**539.2930**	1060.5521	530.7797	1059.5681	530.2877	10

**19**	1941.8672	971.4372	1924.8407	962.9240	1923.8567	962.4320	**K**	976.5309	**488.7691**	959.5044	480.2558	958.5204	479.7638	**9**
**20**	2040.9356	1020.9715	2023.9091	1012.4582	2022.9251	1011.9662	**V**	848.4360	424.7216	831.4094	416.2084	830.4254	415.7163	**8**
**21**	2155.9626	1078.4849	2138.9360	1069.9716	2137.9520	1069.4796	**N**	**749.3676**	375.1874	732.3410	366.6741	731.3570	366.1821	**7**
**22**	2212.9840	1106.9957	2195.9575	1098.4824	2194.9735	1097.9904	**G**	**634.3406**	317.6740	617.3141	309.1607	616.3301	308.6687	**6**
**23**	2300.0161	1150.5117	2282.9895	1141.9984	2282.0055	1141.5064	**S**	**577.3192**	289.1632	560.2926	280.6499	559.3086	280.1579	**5**
**24**	2413.1001	1207.0537	2396.0736	1198.5404	2395.0896	1198.0484	**L**	490.2871	245.6472	473.2606	237.1339	472.2766	236.6419	**4**
**25**	2542.1427	1271.5750	2525.1162	**1263.0617**	2524.1322	**1262.5697**	**E**	377.2031	189.1052	360.1765	180.5919	359.1925	180.0999	**3**
**26**	2643.1904	1322.0988	2626.1638	1313.5856	2625.1798	1313.0936	**T**	248.1605	124.5839	231.1339	116.0706	230.1499	115.5786	**2**
**27**							**K**	147.1128	74.0600	130.0863	65.5468			**1**

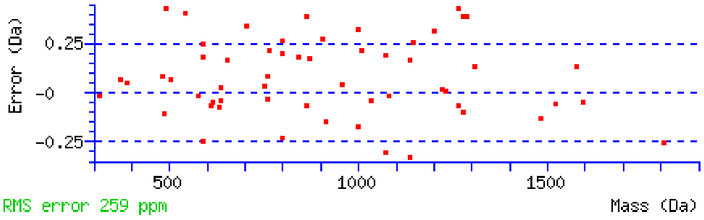

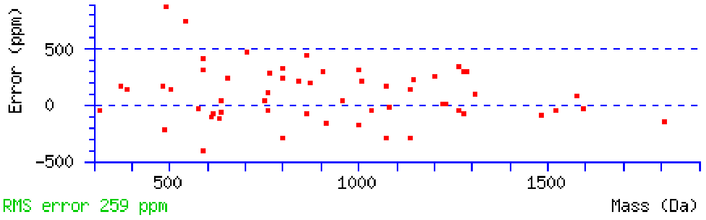

All matches to this query

Score	Mr(calc):	Delta	Sequence
68.5	2788.2886	0.0049	SENGLEFTSSGSANTETTKVNGSLETK
42.8	2788.2886	0.0049	SENGLEFTSSGSANTETTKVNGSLETK
5.8	2786.2734	2.0201	LTAHPAPSPEDLALSTGTIKWLTK
4.9	2788.3112	-0.0177	QSRPRPPLSAPWTQGGPWELRGR
3.1	2786.3024	1.9910	GFLSYLEEIQSVRRHTNSTSEPR
1.4	2786.2800	2.0135	INGDKTDYLTVDQLVSFLNEHQR
1.3	2788.2925	0.0010	HSGKYVCMVQTGVDSVSSAAELIVR
1.1	2786.2677	2.0258	NLEDQITLVRQKHDEEVLQYR
0.9	2787.3027	0.9908	TNLAQMTDANGNIQQRTVLPKLVS
0.9	2788.2867	0.0067	TNLAQMTDANGNIQQRTVLPKLVS

Spectrum No: 243; Query: 453; Rank: 1

Peptide View

MS/MS Fragmentation of **TYCSSELVSNCTQK**
Found in **IPI00212725**, Tax_Id=10116 Gene_Symbol=Cd164 Endolyn precursor

Match to Query 453: 1676.706608 from(839.360580,2+)
Title: 091127RatKid_SCX01_02.1118.1118.2.dta
Data file K:\NewmanPaper\Piliang\3SubProteomes\Piliang3SP\mgf5ppm\SCX_3SubProteomes5ppm.mgf

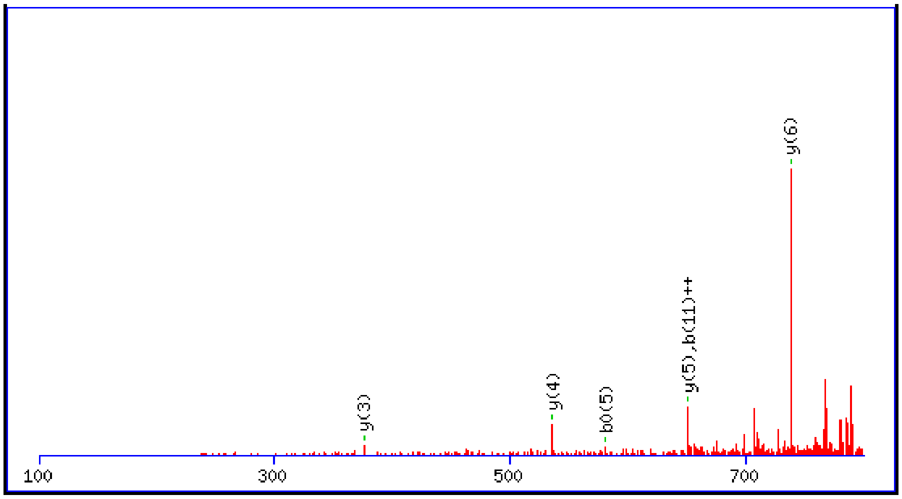

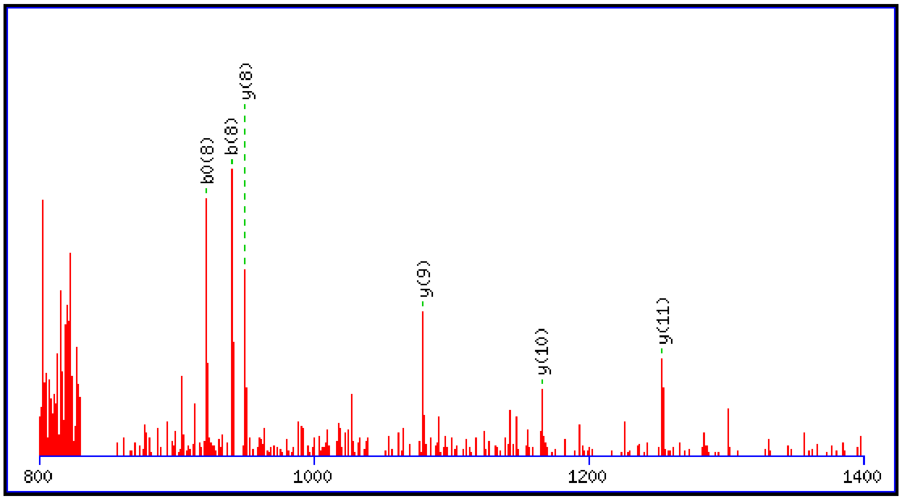

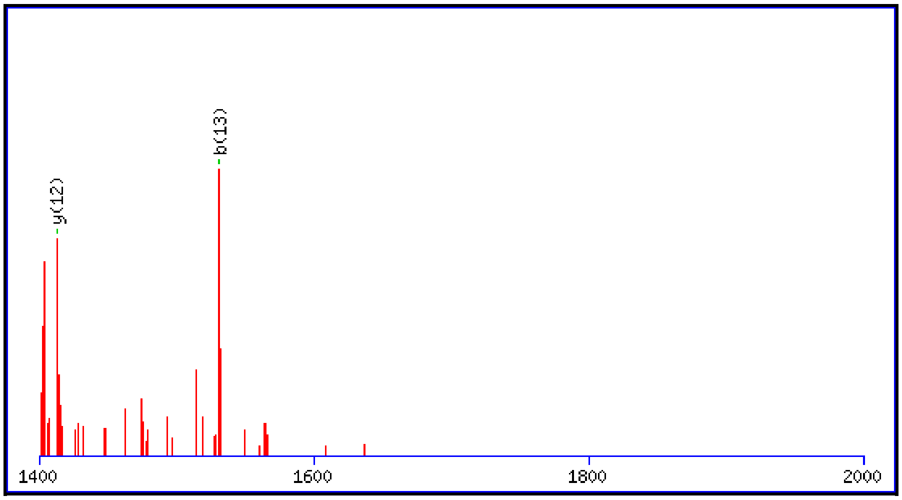

Monoisotopic mass of neutral peptide Mr(calc): 1676.7022
Fixed modifications: Carbamidomethyl (C)
Variable modifications:
N10 : Deamidated_N (N)
Ions Score: 68 **Expect:** 2.6e-005
Matches (Bold Red): 14/134 fragment ions using 23 most intense peaks

#	b	b^{++}	b*	b^{*++}	b^{0}	b^{0++}	Seq.	y	y^{++}	y*	y^{*++}	y^{0}	y^{0++}	#
1	102.0550	51.5311			84.0444	42.5258	T							14
2	265.1183	133.0628			247.1077	124.0575	Y	1576.6618	788.8345	1559.6352	780.3213	1558.6512	779.8292	13
3	425.1489	213.0781			407.1384	204.0728	C	**1413.5985**	707.3029	1396.5719	698.7896	1395.5879	698.2976	12
4	512.1810	256.5941			494.1704	247.5888	S	**1253.5678**	627.2875	1236.5413	618.7743	1235.5572	618.2823	11
5	599.2130	300.1101			**581.2024**	291.1049	S	**1166.5358**	583.7715	1149.5092	575.2583	1148.5252	574.7662	10

**6**	728.2556	364.6314			710.2450	355.6261	**E**	**1079.5037**	540.2555	1062.4772	531.7422	1061.4932	531.2502	**9**
**7**	841.3396	421.1735			823.3291	412.1682	**L**	**950.4612**	475.7342	933.4346	467.2209	932.4506	466.7289	**8**
**8**	**940.4081**	470.7077			**922.3975**	461.7024	**V**	837.3771	419.1922	820.3505	410.6789	819.3665	410.1869	**7**
**9**	1027.4401	514.2237			1009.4295	505.2184	**S**	**738.3087**	369.6580	721.2821	361.1447	720.2981	360.6527	**6**
**10**	1142.4670	571.7372	1125.4405	563.2239	1124.4565	562.7319	**N**	**651.2766**	326.1420	634.2501	317.6287	633.2661	317.1367	**5**
**11**	1302.4977	**651.7525**	1285.4711	643.2392	1284.4871	642.7472	**C**	**536.2497**	268.6285	519.2232	260.1152	518.2391	259.6232	**4**
**12**	1403.5454	702.2763	1386.5188	693.7630	1385.5348	693.2710	**T**	**376.2191**	188.6132	359.1925	180.0999	358.2085	179.6079	**3**
**13**	**1531.6039**	766.3056	1514.5774	757.7923	1513.5934	757.3003	**Q**	275.1714	138.0893	258.1448	129.5761			**2**
**14**							**K**	147.1128	74.0600	130.0863	65.5468			**1**

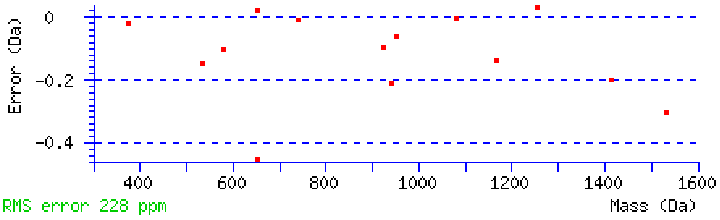

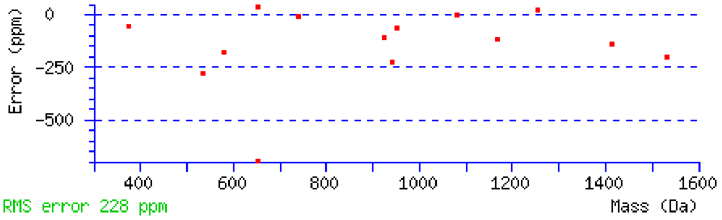

All matches to this query

Score	Mr(calc):	Delta	Sequence
68.4	1676.7022	0.0044	TYCSSELVSNCTQK
11.5	1676.7117	-0.0051	MLNFGASLQQASEGK
2.2	1675.7164	0.9902	SGNSLPLSNSTMFIQ
2.2	1675.7164	0.9902	SGNSLPLSNSTMFIQ
2.0	1676.7124	-0.0057	DSSVDFQFSYFKK
2.0	1676.7124	-0.0057	DSSVDFQFSYFKK
1.9	1675.6967	1.0099	VKAGNANSKTGEPSR
0.8	1676.6906	0.0161	QMTAQHYVTEFNK

Spectrum No: 244; Query: 276; Rank: 1

Peptide View

MS/MS Fragmentation of **FNSANEDNVTQVR**
Found in **IPI00231742**, Tax_Id=10116 Gene_Symbol=Cat Catalase

Match to Query 276: 1493.671268 from(747.842910,2+)
Title: 091129RatKid_SCX02_23.732.732.2.dta
Data file K:\NewmanPaper\Piliang\3SubProteomes\Piliang3SP\mgf5ppm\SCX_3SubProteomes5ppm.mgf

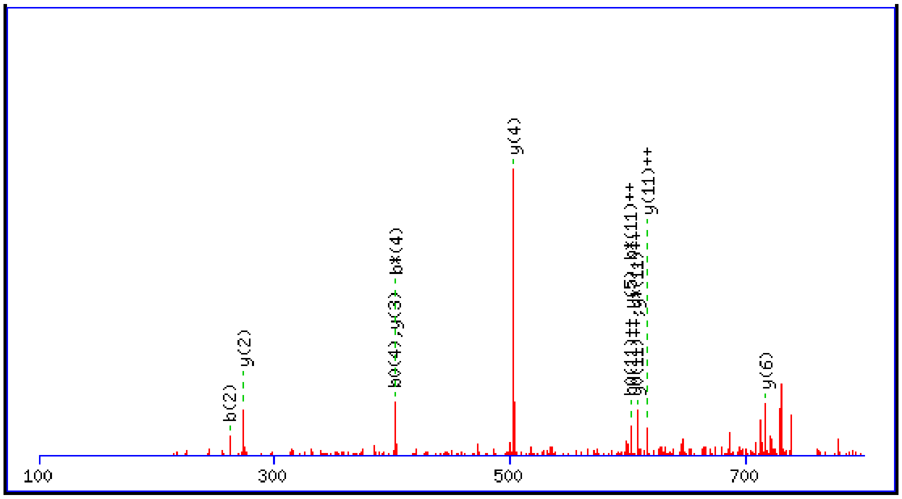

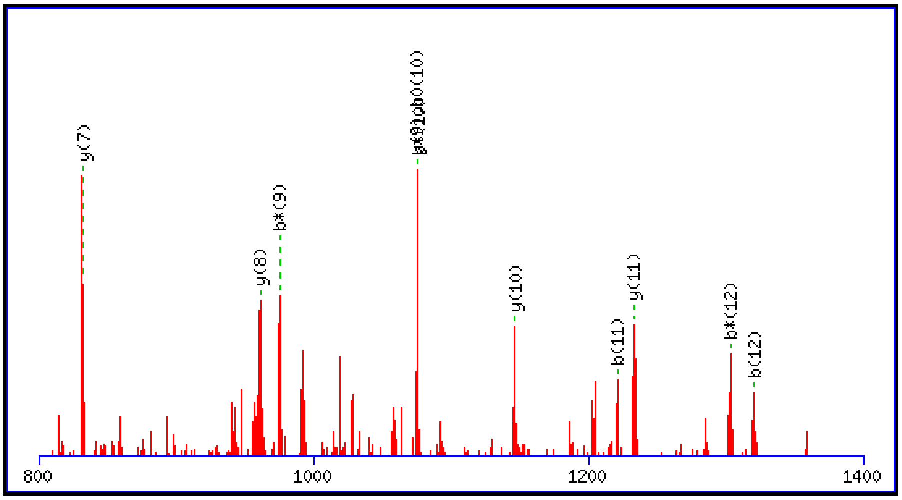

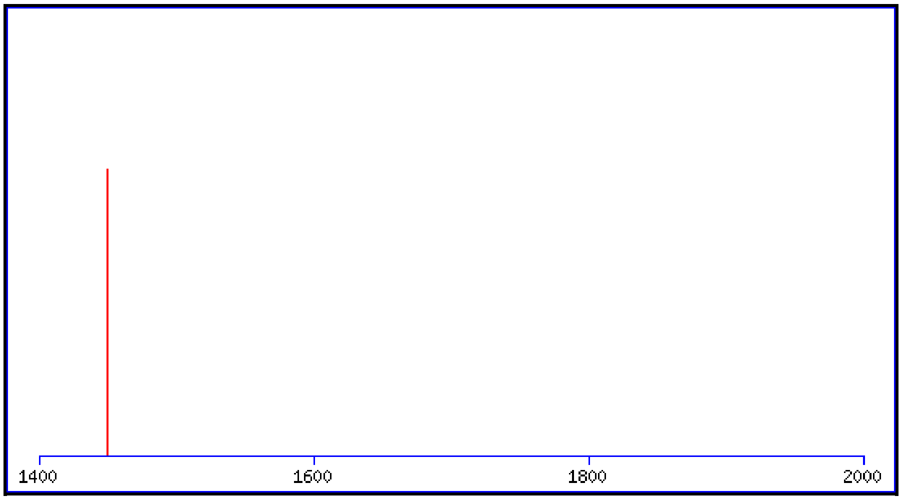

Monoisotopic mass of neutral peptide Mr(calc): 1493.6746
Fixed modifications: Carbamidomethyl (C)
Variable modifications:
N8 : Deamidated_N (N)
Ions Score: 68 **Expect:** 2.3e-005
Matches (Bold Red): 24/132 fragment ions using 35 most intense peaks

#	b	b^{++}	b*	b^{*++}	b^0	b^{0++}	Seq.	y	y^{++}	y*	y^{*++}	y^0	y^{0++}	#
1	148.0757	74.5415					F							13
2	**262.1186**	131.5629	245.0921	123.0497			N	1347.6135	674.3104	1330.5870	665.7971	1329.6029	665.3051	12
3	349.1506	175.0790	332.1241	166.5657	331.1401	166.0737	S	**1233.5706**	**617.2889**	1216.5440	**608.7757**	1215.5600	**608.2836**	11
4	420.1878	210.5975	**403.1612**	202.0842	**402.1772**	201.5922	A	**1146.5386**	573.7729	1129.5120	565.2596	1128.5280	564.7676	10
5	534.2307	267.6190	517.2041	259.1057	516.2201	258.6137	N	**1075.5014**	538.2544	1058.4749	529.7411	1057.4909	529.2491	9

6	663.2733	332.1403	646.2467	323.6270	645.2627	323.1350	E	961.4585	481.2329	944.4320	472.7196	943.4479	472.2276	8
7	778.3002	389.6537	761.2737	381.1405	760.2897	380.6485	D	832.4159	416.7116	815.3894	408.1983	814.4054	407.7063	7
8	893.3272	447.1672	876.3006	438.6539	875.3166	438.1619	N	717.3890	359.1981	700.3624	350.6849	699.3784	350.1928	6
9	992.3956	496.7014	975.3690	488.1881	974.3850	487.6961	V	602.3620	301.6847	585.3355	293.1714	584.3515	292.6794	5
10	1093.4433	547.2253	1076.4167	538.7120	1075.4327	538.2200	T	503.2936	252.1504	486.2671	243.6372	485.2831	243.1452	4
11	1221.5018	611.2546	1204.4753	602.7413	1203.4913	602.2493	Q	402.2459	201.6266	385.2194	193.1133			3
12	1320.5702	660.7888	1303.5437	652.2755	1302.5597	651.7835	V	274.1874	137.5973	257.1608	129.0840			2
13							R	175.1190	88.0631	158.0924	79.5498			1

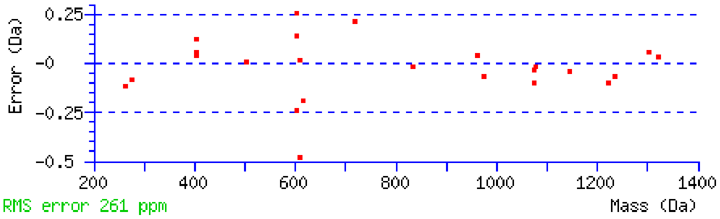

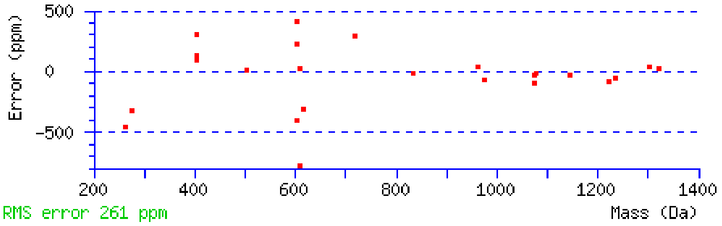

All matches to this query

Score	Mr(calc):	Delta	Sequence
68.3	1493.6746	-0.0034	FNSANEDNVTQVR
63.8	1493.6746	-0.0034	FNSANEDNVTQVR
55.5	1493.6746	-0.0034	FNSANEDNVTQVR
13.8	1493.6602	0.0110	MGNLSSMGSPSPSSR
11.7	1493.6818	-0.0106	ESRGGNTRESASSR
10.5	1493.6585	0.0128	NEVEASHLIMVR
8.1	1493.6667	0.0045	SGGMSNELNNIISR
7.5	1492.6827	0.9885	SGGMSNELNNIISR
7.3	1492.6819	0.9894	MELNMKKFTVR
5.5	1493.6738	-0.0025	YQPISYKICSR

Spectrum No: 245; Query: 824; Rank: 1

Peptide View

MS/MS Fragmentation of **NGSCGVSYIAQEPGNYEVSIK**
Found in **IPI00373752**, Tax_Id=10116 Gene_Symbol=Flnb_predicted filamin, beta

Match to Query 824: 2272.035868 from(1137.025210,2+)
Title: 091129RatKid_SCX02_13.2485.2485.2.dta
Data file K:\NewmanPaper\Piliang\3SubProteomes\Piliang3SP\mgf5ppm\SCX_3SubProteomes5ppm.mgf

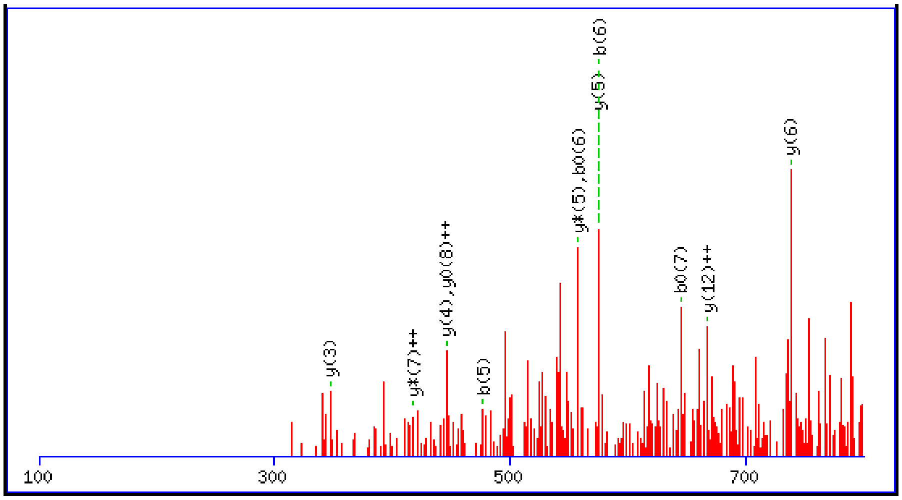

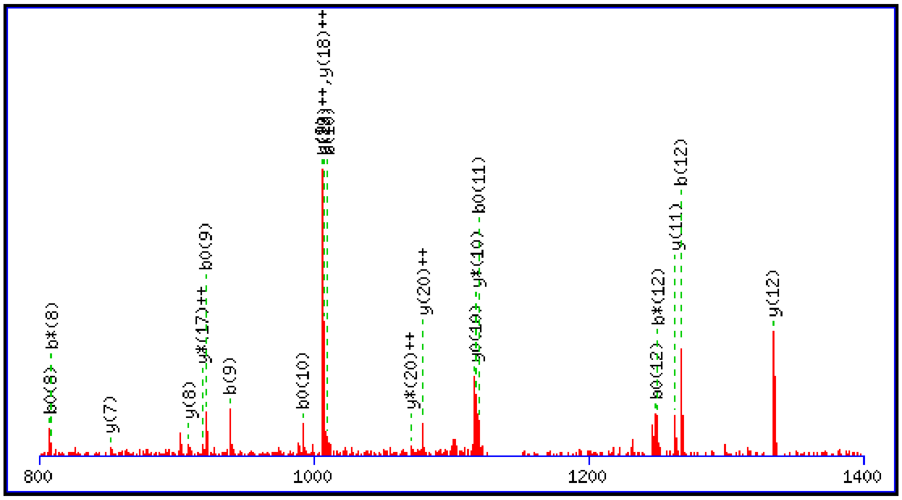

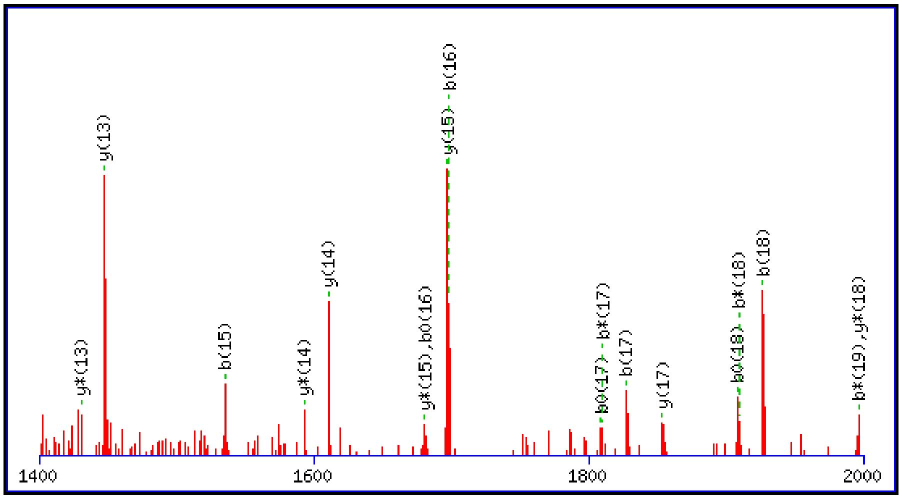

Monoisotopic mass of neutral peptide Mr(calc): 2272.0318
Fixed modifications: Carbamidomethyl (C)
Variable modifications:
N1 : Deamidated_N (N)
Ions Score: 68 **Expect:** 4.2e-005
Matches (Bold Red): 52/232 fragment ions using 98 most intense peaks

#	b	b^{++}	b*	b^{*++}	b^0	b^{0++}	Seq.	y	y^{++}	y*	y^{*++}	y^0	y^{0++}	#
1	116.0342	58.5207	99.0077	50.0075			N							21
2	173.0557	87.0315	156.0291	78.5182			G	2158.0121	**1079.5097**	2140.9856	**1070.9964**	2140.0015	1070.5044	20
3	260.0877	130.5475	243.0612	122.0342	242.0771	121.5422	S	2100.9906	1050.9990	2083.9641	1042.4857	2082.9801	1041.9937	19
4	420.1184	210.5628	403.0918	202.0495	402.1078	201.5575	C	2013.9586	**1007.4829**	**1996.9321**	998.9697	1995.9481	998.4777	18
5	**477.1398**	239.0735	460.1133	230.5603	459.1293	230.0683	G	**1853.9280**	927.4676	1836.9014	**918.9543**	1835.9174	918.4623	17

6	**576.2082**	288.6078	559.1817	280.0945	**558.1977**	279.6025	V	1796.9065	898.9569	1779.8800	890.4436	1778.8959	889.9516	16
7	663.2403	332.1238	646.2137	323.6105	**645.2297**	323.1185	S	**1697.8381**	849.4227	**1680.8115**	840.9094	1679.8275	840.4174	15
8	826.3036	413.6554	**809.2770**	405.1422	**808.2930**	404.6501	Y	**1610.8061**	805.9067	**1593.7795**	797.3934	1592.7955	796.9014	14
9	**939.3877**	470.1975	922.3611	461.6842	**921.3771**	461.1922	I	**1447.7427**	724.3750	**1430.7162**	715.8617	1429.7322	715.3697	13
10	**1010.4248**	505.7160	993.3982	497.2027	**992.4142**	496.7107	A	**1334.6587**	**667.8330**	1317.6321	659.3197	1316.6481	658.8277	12
11	1138.4833	569.7453	1121.4568	561.2320	**1120.4728**	560.7400	Q	**1263.6216**	632.3144	1246.5950	623.8011	1245.6110	623.3091	11
12	**1267.5259**	634.2666	**1250.4994**	625.7533	**1249.5154**	625.2613	E	1135.5630	568.2851	**1118.5364**	559.7719	**1117.5524**	559.2798	10
13	1364.5787	682.7930	1347.5522	674.2797	1346.5681	673.7877	P	**1006.5204**	503.7638	989.4938	495.2506	988.5098	494.7585	9
14	1421.6002	711.3037	1404.5736	702.7904	1403.5896	702.2984	G	**909.4676**	455.2374	892.4411	446.7242	891.4571	**446.2322**	8
15	**1535.6431**	768.3252	1518.6165	759.8119	1517.6325	759.3199	N	**852.4462**	426.7267	835.4196	**418.2134**	834.4356	417.7214	7
16	**1698.7064**	849.8568	1681.6799	841.3436	**1680.6959**	840.8516	Y	**738.4032**	369.7053	721.3767	361.1920	720.3927	360.7000	6
17	**1827.7490**	914.3781	**1810.7225**	905.8649	**1809.7384**	905.3729	E	**575.3399**	288.1736	**558.3134**	279.6603	557.3293	279.1683	5
18	**1926.8174**	963.9124	**1909.7909**	955.3991	**1908.8069**	954.9071	V	**446.2973**	223.6523	429.2708	215.1390	428.2867	214.6470	4
19	2013.8495	**1007.4284**	**1996.8229**	998.9151	1995.8389	998.4231	S	**347.2289**	174.1181	330.2023	165.6048	329.2183	165.1128	3
20	2126.9335	1063.9704	2109.9070	1055.4571	2108.9230	1054.9651	I	260.1969	130.6021	243.1703	122.0888			2
21							K	147.1128	74.0600	130.0863	65.5468			1

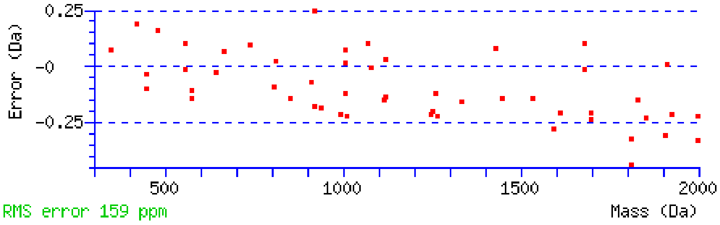

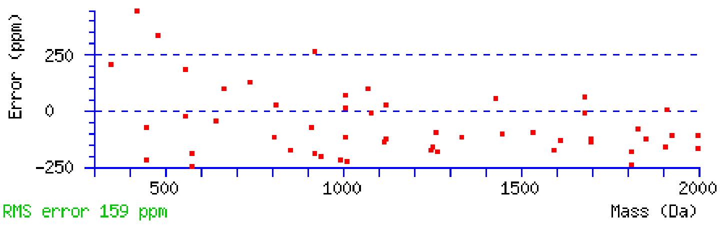

All matches to this query

Score	Mr(calc):	Delta	Sequence
68.3	2272.0318	0.0041	NGSCGVSYIAQEPGNYEVSIK
42.3	2271.0477	0.9881	NGSCGVSYIAQEPGNYEVSIK
36.5	2272.0318	0.0041	NGSCGVSYIAQEPGNYEVSIK
10.3	2272.0470	-0.0111	MEWIGHISYSGSTSYNPSLK
1.9	2272.0501	-0.0143	IGNPLSGTSTPAVRIASVTDR
1.9	2272.0501	-0.0143	IGNPLSGTSTPAVRIASVTDR
1.6	2272.0501	-0.0143	IGNPLSGTSTPAVRIASVTDR
1.6	2272.0501	-0.0143	IGNPLSGTSTPAVRIASVTDR
1.6	2272.0501	-0.0143	IGNPLSGTSTPAVRIASVTDR
1.6	2272.0501	-0.0143	IGNPLSGTSTPAVRIASVTDR

Spectrum No: 246; Query: 519; Rank: 1

Peptide View

MS/MS Fragmentation of **SYIFIYDGNKNSTTR**
Found in **IPI00470238**, Tax_Id=10116 Gene_Symbol=Cd47 Isoform 2 of Leukocyte surface antigen CD47 precursor

Match to Query 519: 1778.844488 from(890.429520,2+)
Title: 091129RatKid_SCX02_12.1619.1619.2.dta
Data file K:\NewmanPaper\Piliang\3SubProteomes\Piliang3SP\mgf5ppm\SCX_3SubProteomes5ppm.mgf

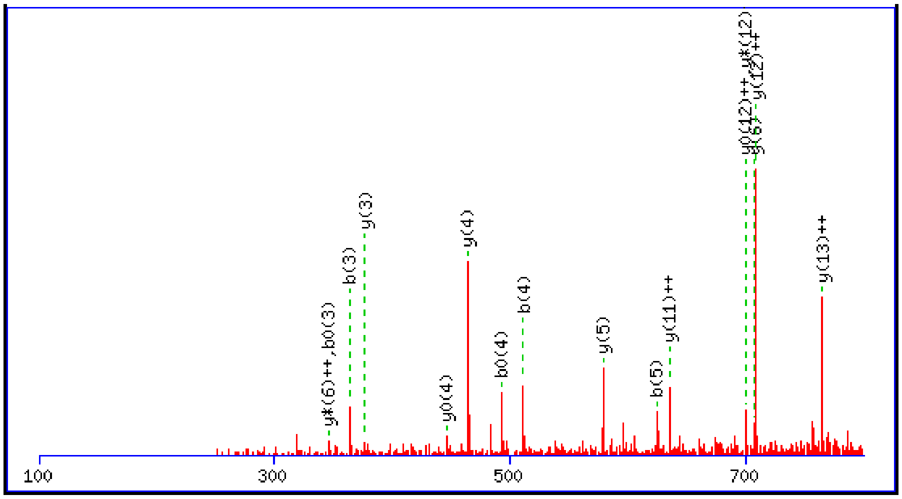

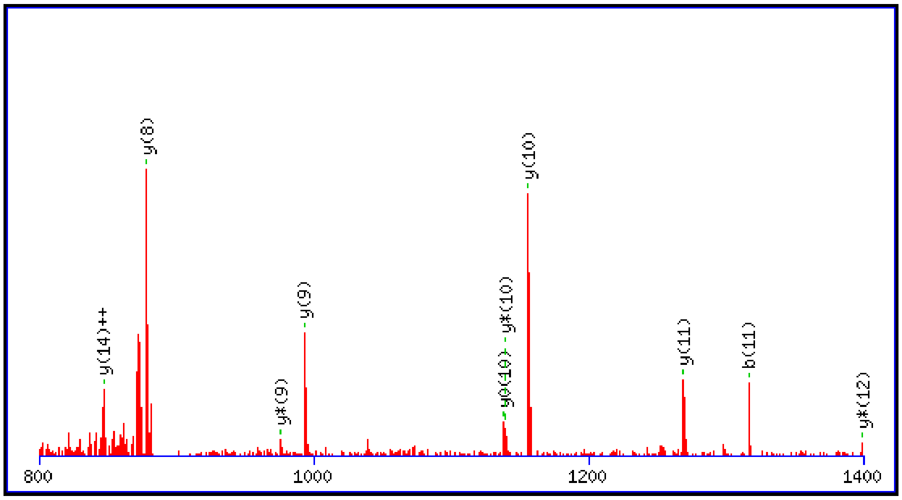

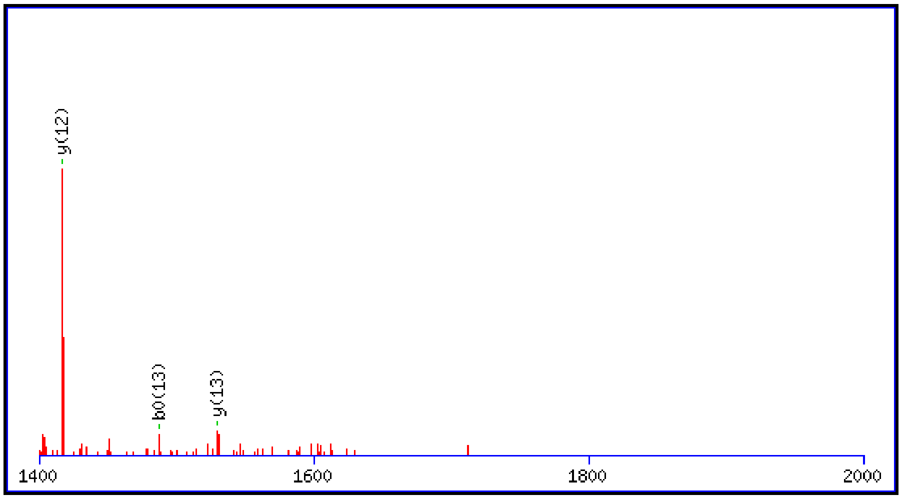

Monoisotopic mass of neutral peptide Mr(calc): 1778.8475
Fixed modifications: Carbamidomethyl (C)
Variable modifications:
N11 : Deamidated_N (N)
Ions Score: 68 **Expect:** 3.2e-005
Matches (Bold Red): 29/150 fragment ions using 49 most intense peaks

#	b	b^{++}	b*	b^{*++}	b^0	b^{0++}	Seq.	y	y^{++}	y*	y^{*++}	y^0	y^{0++}	#
**1**	88.0393	44.5233			70.0287	35.5180	**S**							**15**
**2**	251.1026	126.0550			233.0921	117.0497	**Y**	1692.8228	**846.9150**	1675.7962	838.4017	1674.8122	837.9097	**14**
**3**	**364.1867**	182.5970			**346.1761**	173.5917	**I**	**1529.7594**	**765.3834**	1512.7329	756.8701	1511.7489	756.3781	**13**
**4**	**511.2551**	256.1312			**493.2445**	247.1259	**F**	**1416.6754**	**708.8413**	**1399.6488**	**700.3281**	1398.6648	**699.8360**	**12**
**5**	**624.3392**	312.6732			606.3286	303.6679	**I**	**1269.6070**	**635.3071**	1252.5804	626.7938	1251.5964	626.3018	**11**

**6**	787.4025	394.2049			769.3919	385.1996	**Y**	**1156.5229**	578.7651	**1139.4964**	570.2518	**1138.5123**	569.7598	**10**
**7**	902.4294	451.7184			884.4189	442.7131	**D**	**993.4596**	497.2334	**976.4330**	488.7201	975.4490	488.2281	**9**
**8**	959.4509	480.2291			941.4403	471.2238	**G**	**878.4326**	439.7200	861.4061	431.2067	860.4221	430.7147	**8**
**9**	1073.4938	537.2506	1056.4673	528.7373	1055.4833	528.2453	**N**	821.4112	411.2092	804.3846	402.6959	803.4006	402.2039	**7**
**10**	1201.5888	601.2980	1184.5623	592.7848	1183.5782	592.2928	**K**	**707.3682**	354.1878	690.3417	**345.6745**	689.3577	345.1825	**6**
**11**	**1316.6157**	658.8115	1299.5892	650.2982	1298.6052	649.8062	**N**	**579.2733**	290.1403	562.2467	281.6270	561.2627	281.1350	**5**
**12**	1403.6478	702.3275	1386.6212	693.8142	1385.6372	693.3222	**S**	**464.2463**	232.6268	447.2198	224.1135	**446.2358**	223.6215	**4**
**13**	1504.6954	752.8514	1487.6689	744.3381	**1486.6849**	743.8461	**T**	**377.2143**	189.1108	360.1878	180.5975	359.2037	180.1055	**3**
**14**	1605.7431	803.3752	1588.7166	794.8619	1587.7326	794.3699	**T**	276.1666	138.5870	259.1401	130.0737	258.1561	129.5817	**2**
**15**							**R**	175.1190	88.0631	158.0924	79.5498			**1**

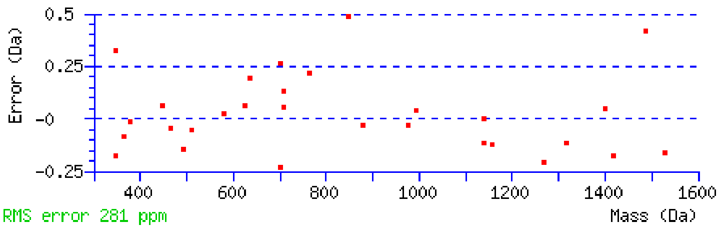

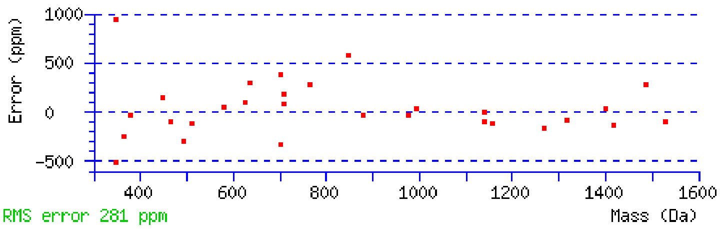

All matches to this query

Score	Mr(calc):	Delta	Sequence
68.0	1778.8475	-0.0030	SYIFIYDGNKNSTTR
50.4	1778.8475	-0.0030	SYIFIYDGNKNSTTR
5.6	1776.8263	2.0182	MTLLMASSTAQSRLR
5.6	1776.8263	2.0182	MTLLMASSTAQSRLR
5.6	1776.8263	2.0182	MTLLMASSTAQSRLR
5.6	1776.8263	2.0182	MTLLMASSTAQSRLR
4.4	1777.8264	1.0181	TLTLLGEVLKSSDSR
4.4	1777.8264	1.0181	TLTLLGEVLKSSDSR
0.8	1777.8288	1.0157	YLNVREAVLGGYDTK

Spectrum No: 247; Query: 656; Rank: 1

Peptide View

MS/MS Fragmentation of **VNASTTDPNSTVEQSALTR**
Found in **IPI00365542**, Tax_Id=10116 Gene_Symbol=Lamb1_predicted laminin, beta 1

Match to Query 656: 1990.951988 from(996.483270,2+)
Title: 091127RatKid_SCX01_11.1009.1009.2.dta
Data file K:\NewmanPaper\Piliang\3SubProteomes\Piliang3SP\mgf5ppm\SCX_3SubProteomes5ppm.mgf

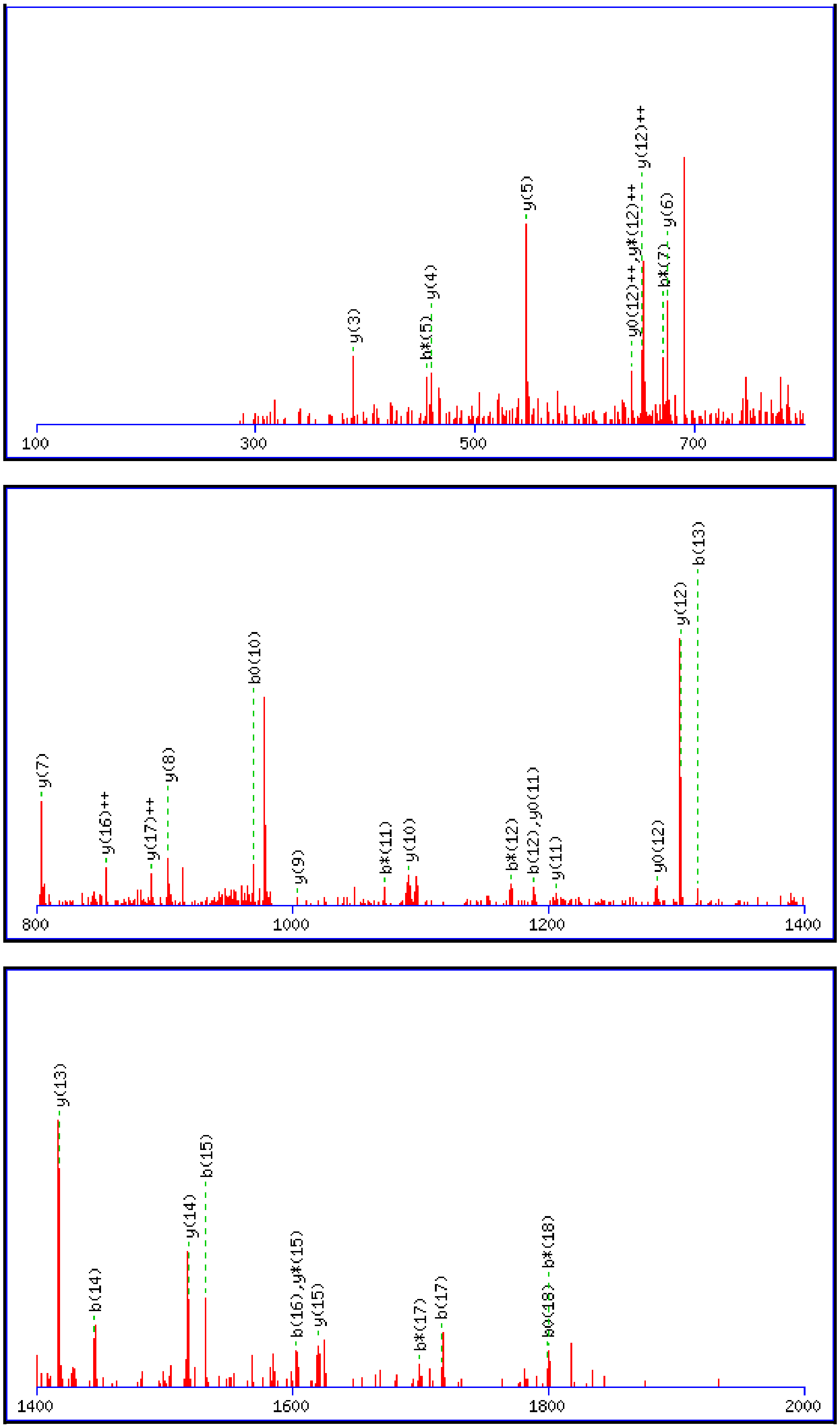

Monoisotopic mass of neutral peptide Mr(calc): 1990.9443
Fixed modifications: Carbamidomethyl (C)
Variable modifications:
N9 : Deamidated_N (N)
Ions Score: 68 **Expect:** 4e-005
Matches (Bold Red): 35/206 fragment ions using 71 most intense peaks

#	b	b^{++}	b*	b^{*++}	b^0	b^{0++}	Seq.	y	y^{++}	y*	y^{*++}	y^0	y^{0++}	#
1	100.0757	50.5415					V							19
2	214.1186	107.5629	197.0921	99.0497			N	1892.8832	946.9452	1875.8567	938.4320	1874.8726	937.9400	18
3	285.1557	143.0815	268.1292	134.5682			A	1778.8403	**889.9238**	1761.8137	881.4105	1760.8297	880.9185	17
4	372.1878	186.5975	355.1612	178.0842	354.1772	177.5922	S	1707.8032	**854.4052**	1690.7766	845.8919	1689.7926	845.3999	16
5	473.2354	237.1214	**456.2089**	228.6081	455.2249	228.1161	T	**1620.7711**	810.8892	**1603.7446**	802.3759	1602.7606	801.8839	15

**6**	574.2831	287.6452	557.2566	279.1319	556.2726	278.6399	**T**	**1519.7235**	760.3654	1502.6969	751.8521	1501.7129	751.3601	**14**
**7**	689.3101	345.1587	**672.2835**	336.6454	671.2995	336.1534	**D**	**1418.6758**	709.8415	1401.6492	701.3282	1400.6652	700.8362	**13**
**8**	786.3628	393.6851	769.3363	385.1718	768.3523	384.6798	**P**	**1303.6488**	**652.3281**	1286.6223	**643.8148**	**1285.6383**	**643.3228**	**12**
**9**	901.3898	451.1985	884.3632	442.6852	883.3792	442.1932	**N**	**1206.5961**	603.8017	1189.5695	595.2884	**1188.5855**	594.7964	**11**
**10**	988.4218	494.7145	971.3952	486.2013	**970.4112**	485.7092	**S**	**1091.5691**	546.2882	1074.5426	537.7749	1073.5586	537.2829	**10**
**11**	1089.4695	545.2384	**1072.4429**	536.7251	1071.4589	536.2331	**T**	**1004.5371**	502.7722	987.5106	494.2589	986.5265	493.7669	**9**
**12**	**1188.5379**	594.7726	**1171.5113**	586.2593	1170.5273	585.7673	**V**	**903.4894**	452.2483	886.4629	443.7351	885.4789	443.2431	**8**
**13**	**1317.5805**	659.2939	1300.5539	650.7806	1299.5699	650.2886	**E**	**804.4210**	402.7141	787.3945	394.2009	786.4104	393.7089	**7**
**14**	**1445.6391**	723.3232	1428.6125	714.8099	1427.6285	714.3179	**Q**	**675.3784**	338.1928	658.3519	329.6796	657.3679	329.1876	**6**
**15**	**1532.6711**	766.8392	1515.6445	758.3259	1514.6605	757.8339	**S**	**547.3198**	274.1636	530.2933	265.6503	529.3093	265.1583	**5**
**16**	**1603.7082**	802.3577	1586.6816	793.8445	1585.6976	793.3525	**A**	**460.2878**	230.6475	443.2613	222.1343	442.2772	221.6423	**4**
**17**	**1716.7923**	858.8998	**1699.7657**	850.3865	1698.7817	849.8945	**L**	**389.2507**	195.1290	372.2241	186.6157	371.2401	186.1237	**3**
**18**	1817.8399	909.4236	**1800.8134**	900.9103	**1799.8294**	900.4183	**T**	276.1666	138.5870	259.1401	130.0737	258.1561	129.5817	**2**
**19**							**R**	175.1190	88.0631	158.0924	79.5498			**1**

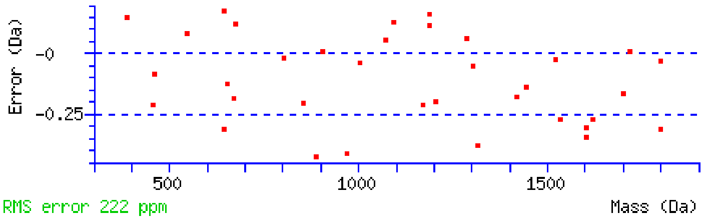

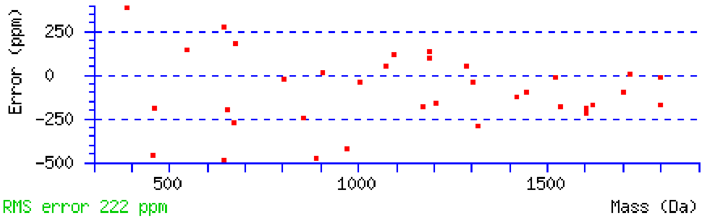

All matches to this query

Score	Mr(calc):	Delta	Sequence
67.7	1990.9443	0.0077	VNASTTDPNSTVEQSALTR
56.9	1990.9443	0.0077	VNASTTDPNSTVEQSALTR
39.4	1989.9603	0.9917	VNASTTDPNSTVEQSALTR
7.5	1988.9456	2.0064	SPSTYNALESGLTSLTIR
4.3	1988.9456	2.0064	SPSTYNALESGLTSLTIR
2.7	1989.9455	1.0064	GETALHMAARAGQVEVVR
2.4	1990.9401	0.0119	SLSTWQPTLLPNNTGPGK
1.6	1988.9456	2.0064	SPSTYNALESGLTSLTIR
1.4	1990.9492	0.0028	CDICDKTFNYKSLLSK
1.4	1988.9456	2.0064	SPSTYNALESGLTSLTIR

Spectrum No: 248; Query: 202; Rank: 1

Peptide View

MS/MS Fragmentation of **QELNDSLQVAER**
Found in **IPI00198667**, Tax_Id=10116 Gene_Symbol=Clu Clusterin precursor

Match to Query 202: 1401.679488 from(701.847020,2+)
Title: 091129RatKid_SCX02_02.1176.1176.2.dta
Data file K:\NewmanPaper\Piliang\3SubProteomes\Piliang3SP\mgf5ppm\SCX_3SubProteomes5ppm.mgf

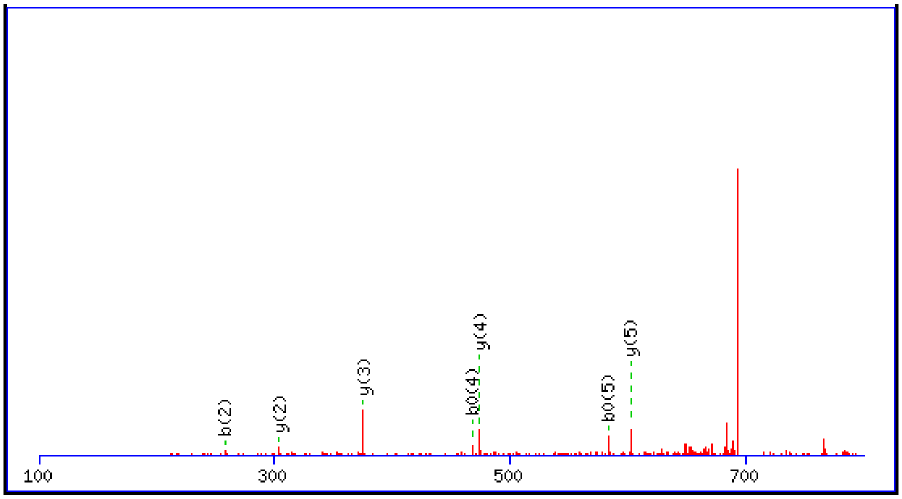

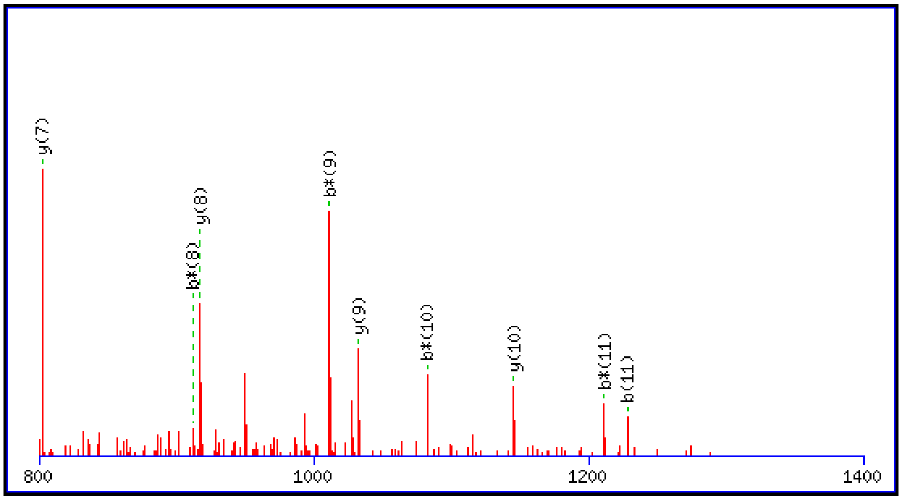

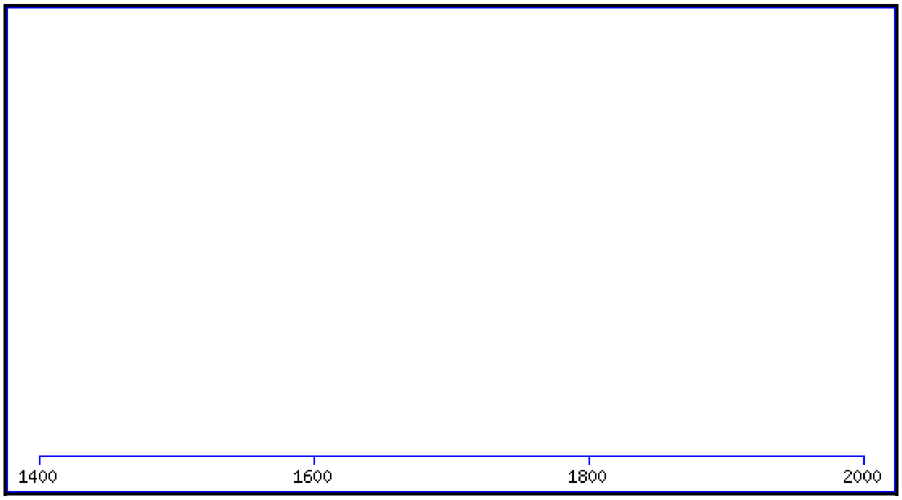

Monoisotopic mass of neutral peptide Mr(calc): 1401.6736
Fixed modifications: Carbamidomethyl (C)
Variable modifications:
N4 : Deamidated_N (N)
Ions Score: 67 **Expect:** 2.7e-005
Matches (Bold Red): 16/128 fragment ions using 23 most intense peaks

#	b	b++	b*	b*++	b0	b0++	Seq.	y	y++	y*	y*++	y0	y0++	#
1	129.0659	65.0366	112.0393	56.5233			Q							12
2	**258.1084**	129.5579	241.0819	121.0446	240.0979	120.5526	E	1274.6223	637.8148	1257.5957	629.3015	1256.6117	628.8095	11
3	371.1925	186.0999	354.1660	177.5866	353.1819	177.0946	L	**1145.5797**	573.2935	1128.5531	564.7802	1127.5691	564.2882	10
4	486.2194	243.6134	469.1929	235.1001	**468.2089**	234.6081	N	**1032.4956**	516.7514	1015.4691	508.2382	1014.4851	507.7462	9
5	601.2464	301.1268	584.2198	292.6136	**583.2358**	292.1216	D	**917.4687**	459.2380	900.4421	450.7247	899.4581	450.2327	8

**6**	688.2784	344.6428	671.2519	336.1296	670.2679	335.6376	**S**	**802.4417**	401.7245	785.4152	393.2112	784.4312	392.7192	**7**
**7**	801.3625	401.1849	784.3359	392.6716	783.3519	392.1796	**L**	715.4097	358.2085	698.3832	349.6952	697.3991	349.2032	**6**
**8**	929.4211	465.2142	**912.3945**	456.7009	911.4105	456.2089	**Q**	**602.3257**	301.6665	585.2991	293.1532	584.3151	292.6612	**5**
**9**	1028.4895	514.7484	**1011.4629**	506.2351	1010.4789	505.7431	**V**	**474.2671**	237.6372	457.2405	229.1239	456.2565	228.6319	**4**
**10**	1099.5266	550.2669	**1082.5000**	541.7537	1081.5160	541.2616	**A**	**375.1987**	188.1030	358.1721	179.5897	357.1881	179.0977	**3**
**11**	**1228.5692**	614.7882	**1211.5426**	606.2750	1210.5586	605.7829	**E**	**304.1615**	152.5844	287.1350	144.0711	286.1510	143.5791	**2**
**12**							**R**	175.1190	88.0631	158.0924	79.5498			**1**

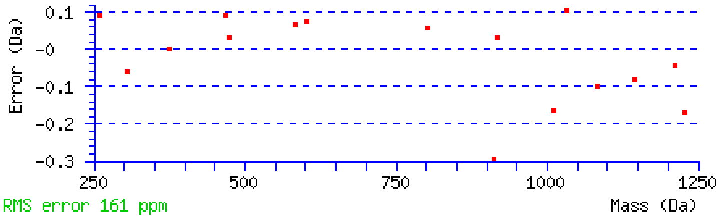

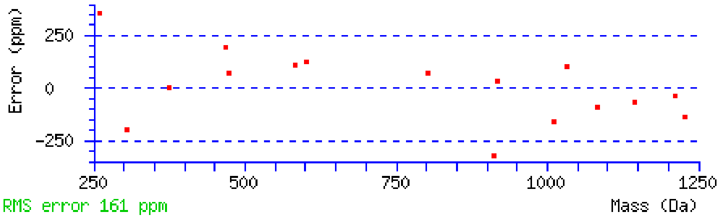

All matches to this query

Score	Mr(calc):	Delta	Sequence
67.3	1401.6736	0.0059	QELNDSLQVAER
29.6	1400.6895	0.9899	QELNDSLQVAER
11.5	1401.6727	0.0068	VFQLISLMDTR
11.2	1401.6922	-0.0127	QAEAMALLAEAER
10.3	1401.6727	0.0067	VCSFVKQVVEK
10.0	1400.6700	1.0094	NYILTNNLLSR
10.0	1400.6748	1.0047	HLGHMGSKVSLR
9.8	1401.6904	-0.0109	NFSAAKSLLNKK
7.2	1400.6691	1.0104	TACHSRSRAEAR
5.5	1401.6895	-0.0100	AMTAGSPGDRGARR

Spectrum No: 249; Query: 1148; Rank: 1

Peptide View

MS/MS Fragmentation of **VFNGSGKPIDRGPVVLAEDFLDIMGQPINPQCR**
Found in **IPI00199305**, Tax_Id=10116 Gene_Symbol=Atp6v1b2 Vacuolar ATP synthase subunit B, brain isoform

Match to Query 1148: 3640.827312 from(1214.616380,3+)
Title: 091129RatKid_SCX02_29.7171.7171.3.dta
Data file K:\NewmanPaper\Piliang\3SubProteomes\Piliang3SP\mgf5ppm\SCX_3SubProteomes5ppm.mgf

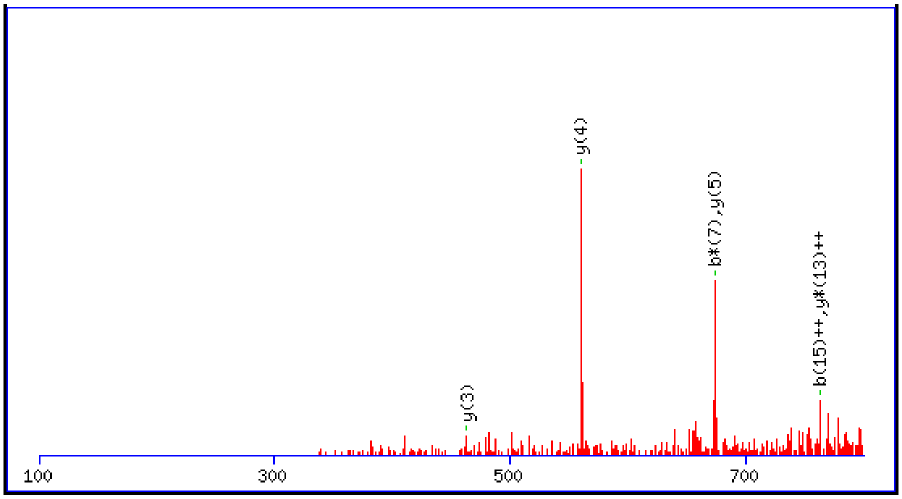

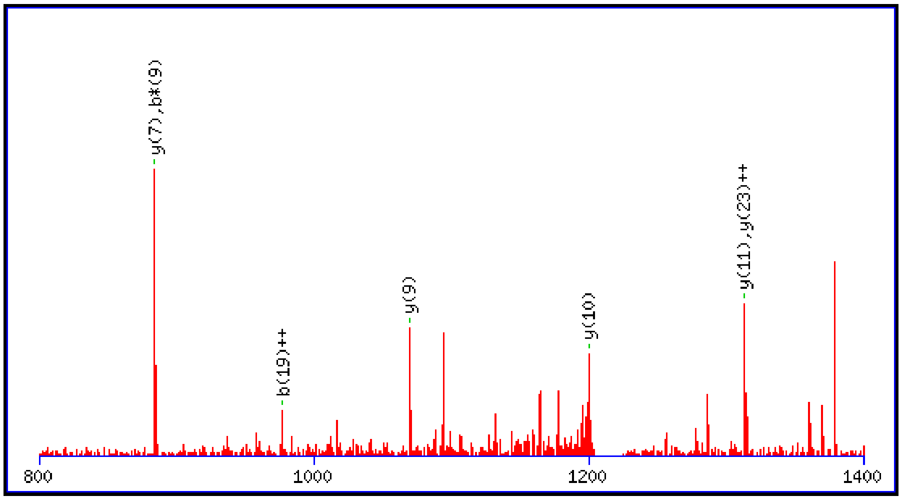

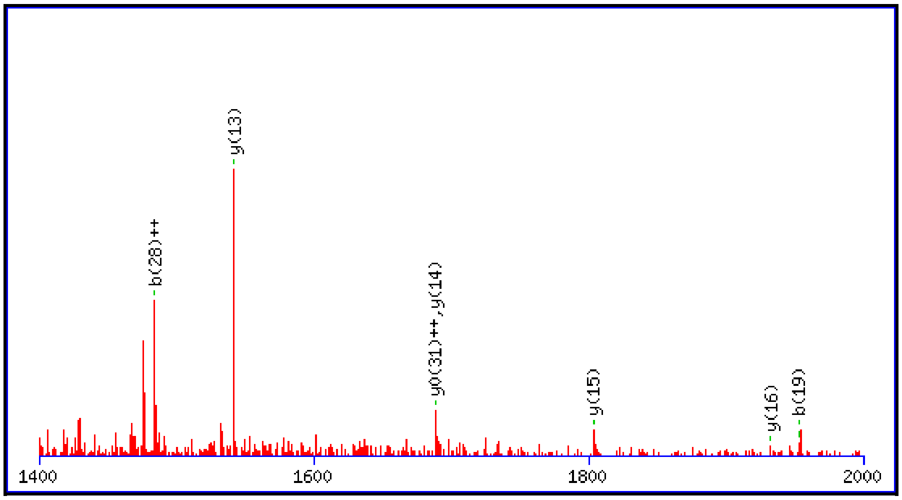

Monoisotopic mass of neutral peptide Mr(calc): 3639.8178
Fixed modifications: Carbamidomethyl (C)
Variable modifications:
N3 : Deamidated_N (N)
Ions Score: 67 **Expect:** 3.5e-005
Matches (Bold Red): 20/350 fragment ions using 22 most intense peaks

#	b	b^{++}	b*	b^{*++}	b^0	b^{0++}	Seq.	y	y^{++}	y*	y^{*++}	y^0	y^{0++}	#
1	100.0757	50.5415					V							33
2	247.1441	124.0757					F	3541.7566	1771.3820	3524.7301	1762.8687	3523.7461	1762.3767	32
3	362.1710	181.5892	345.1445	173.0759			N	3394.6882	1697.8477	3377.6617	1689.3345	3376.6777	**1688.8425**	31
4	419.1925	210.0999	402.1660	201.5866			G	3279.6613	1640.3343	3262.6347	1631.8210	3261.6507	1631.3290	30
5	506.2245	253.6159	489.1980	245.1026	488.2140	244.6106	S	3222.6398	1611.8235	3205.6133	1603.3103	3204.6293	1602.8183	29

6	563.2460	282.1266	546.2194	273.6134	545.2354	273.1214	G	3135.6078	1568.3075	3118.5812	1559.7943	3117.5972	1559.3022	28
7	691.3410	346.1741	**674.3144**	337.6608	673.3304	337.1688	K	3078.5863	1539.7968	3061.5598	1531.2835	3060.5758	1530.7915	27
8	788.3937	394.7005	771.3672	386.1872	770.3832	385.6952	P	2950.4914	1475.7493	2933.4648	1467.2360	2932.4808	1466.7440	26
9	901.4778	451.2425	**884.4512**	442.7293	883.4672	442.2372	I	2853.4386	1427.2229	2836.4120	1418.7097	2835.4280	1418.2177	25
10	1016.5047	508.7560	999.4782	500.2427	998.4942	499.7507	D	2740.3545	1370.6809	2723.3280	1362.1676	2722.3440	1361.6756	24
11	1172.6058	586.8066	1155.5793	578.2933	1154.5953	577.8013	R	2625.3276	**1313.1674**	2608.3010	1304.6542	2607.3170	1304.1622	23
12	1229.6273	615.3173	1212.6008	606.8040	1211.6167	606.3120	G	2469.2265	1235.1169	2452.1999	1226.6036	2451.2159	1226.1116	22
13	1326.6801	663.8437	1309.6535	655.3304	1308.6695	654.8384	P	2412.2050	1206.6061	2395.1785	1198.0929	2394.1945	1197.6009	21
14	1425.7485	713.3779	1408.7219	704.8646	1407.7379	704.3726	V	2315.1523	1158.0798	2298.1257	1149.5665	2297.1417	1149.0745	20
15	1524.8169	**762.9121**	1507.7903	754.3988	1506.8063	753.9068	V	2216.0838	1108.5456	2199.0573	1100.0323	2198.0733	1099.5403	19
16	1637.9010	819.4541	1620.8744	810.9408	1619.8904	810.4488	L	2117.0154	1059.0113	2099.9889	1050.4981	2099.0049	1050.0061	18
17	1708.9381	854.9727	1691.9115	846.4594	1690.9275	845.9674	A	2003.9314	1002.4693	1986.9048	993.9560	1985.9208	993.4640	17
18	1837.9807	919.4940	1820.9541	910.9807	1819.9701	910.4887	E	**1932.8942**	966.9508	1915.8677	958.4375	1914.8837	957.9455	16
19	**1953.0076**	**977.0074**	1935.9811	968.4942	1934.9970	968.0022	D	**1803.8517**	902.4295	1786.8251	893.9162	1785.8411	893.4242	15
20	2100.0760	1050.5417	2083.0495	1042.0284	2082.0655	1041.5364	F	**1688.8247**	844.9160	1671.7982	836.4027	1670.8141	835.9107	14
21	2213.1601	1107.0837	2196.1335	1098.5704	2195.1495	1098.0784	L	**1541.7563**	771.3818	1524.7297	**762.8685**	1523.7457	762.3765	13
22	2328.1870	1164.5972	2311.1605	1156.0839	2310.1765	1155.5919	D	1428.6722	714.8398	1411.6457	706.3265	1410.6617	705.8345	12
23	2441.2711	1221.1392	2424.2445	1212.6259	2423.2605	1212.1339	I	**1313.6453**	657.3263	1296.6187	648.8130			11
24	2572.3116	1286.6594	2555.2850	1278.1462	2554.3010	1277.6541	M	**1200.5612**	600.7843	1183.5347	592.2710			10
25	2629.3330	1315.1702	2612.3065	1306.6569	2611.3225	1306.1649	G	**1069.5207**	535.2640	1052.4942	526.7507			9
26	2757.3916	1379.1994	2740.3651	1370.6862	2739.3811	1370.1942	Q	1012.4993	506.7533	995.4727	498.2400			8
27	2854.4444	1427.7258	2837.4178	1419.2126	2836.4338	1418.7205	P	**884.4407**	442.7240	867.4141	434.2107			7
28	2967.5285	**1484.2679**	2950.5019	1475.7546	2949.5179	1475.2626	I	787.3879	394.1976	770.3614	385.6843			6
29	3081.5714	1541.2893	3064.5448	1532.7761	3063.5608	1532.2840	N	**674.3039**	337.6556	657.2773	329.1423			5
30	3178.6241	1589.8157	3161.5976	1581.3024	3160.6136	1580.8104	P	**560.2609**	280.6341	543.2344	272.1208			4
31	3306.6827	1653.8450	3289.6562	1645.3317	3288.6722	1644.8397	Q	**463.2082**	232.1077	446.1816	223.5945			3
32	3466.7134	1733.8603	3449.6868	1725.3470	3448.7028	1724.8550	C	335.1496	168.0784	318.1231	159.5652			2
33							R	175.1190	88.0631	158.0924	79.5498			1

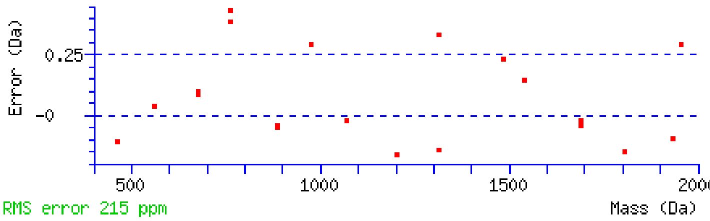

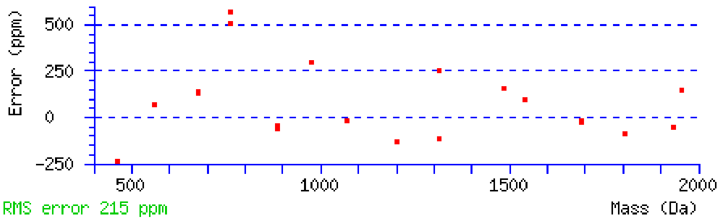

All matches to this query

Score	Mr(calc):	Delta	Sequence
67.3	3639.8178	1.0095	VFNGSGKPIDRGPVVLAEDFLDIMGQPINPQCR
59.5	3638.8338	1.9935	VFNGSGKPIDRGPVVLAEDFLDIMGQPINPQCR
19.8	3640.8018	0.0255	VFNGSGKPIDRGPVVLAEDFLDIMGQPINPQCR
12.0	3639.8178	1.0095	VFNGSGKPIDRGPVVLAEDFLDIMGQPINPQCR
6.7	3639.8519	0.9754	LLNSVLNTLIMISVIVVMTIFLVVLYKYR
5.7	3639.8519	0.9754	LLNSVLNTLIMISVIVVMTIFLVVLYKYR
0.8	3640.8359	-0.0086	LLNSVLNTLIMISVIVVMTIFLVVLYKYR
0.8	3640.8249	0.0024	IFLVSNLSPNRYDFPMLVTTWEHDLPAHRR
0.8	3638.8151	2.0122	TLPFTLRTAASINGAEVEVQSWLRMSPGFSSNR
0.7	3639.8519	0.9754	LLNSVLNTLIMISVIVVMTIFLVVLYKYR

Spectrum No: 250; Query: 710; Rank: 1

Peptide View

MS/MS Fragmentation of **LAWGTTASAQNHTEPQLDR**
Found in **IPI00214328**, Tax_Id=10116 Gene_Symbol=Naglt1 Sodium-dependent glucose transporter 1

Match to Query 710: 2095.998908 from(1049.006730,2+)
Title: 091127RatKid_SCX01_12.1783.1783.2.dta
Data file K:\NewmanPaper\Piliang\3SubProteomes\Piliang3SP\mgf5ppm\SCX_3SubProteomes5ppm.mgf

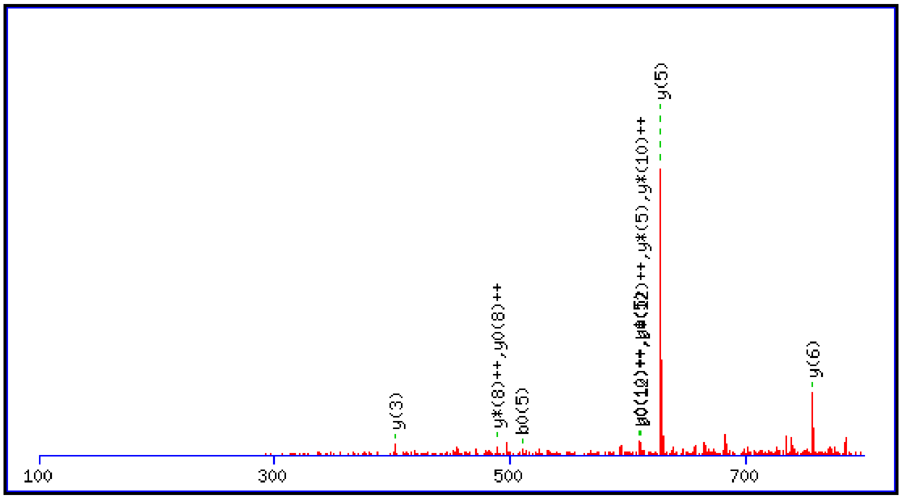

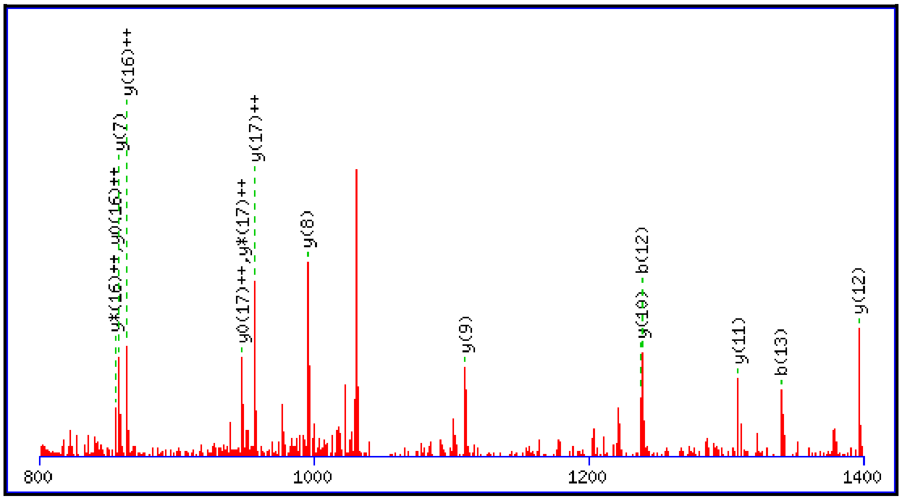

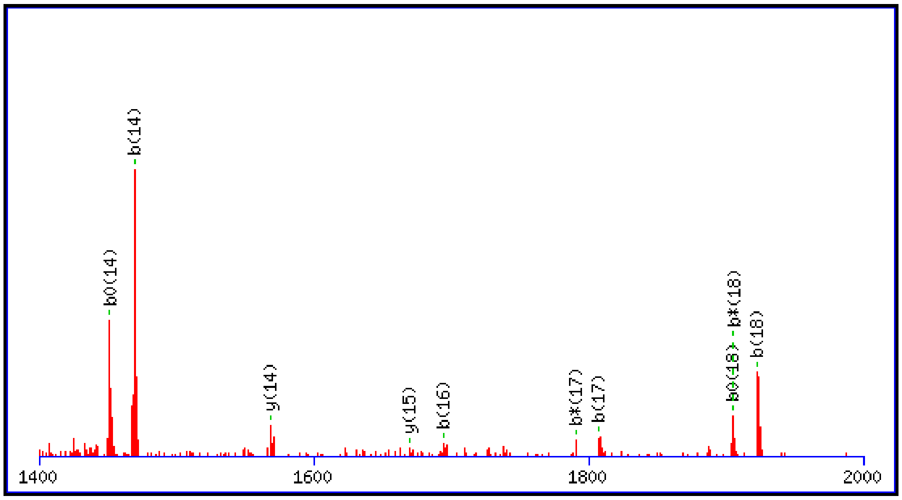

Monoisotopic mass of neutral peptide Mr(calc): 2095.9923
Fixed modifications: Carbamidomethyl (C)

Variable modifications:
N11 : Deamidated_N (N)
Ions Score: 67 **Expect:** 4.7e-005
Matches (Bold Red): 36/188 fragment ions using 45 most intense peaks

#	b	b++	b*	b*++	b0	b0++	Seq.	y	y++	y*	y*++	y0	y0++	#
1	114.0913	57.5493					L							19
2	185.1285	93.0679					A	1983.9155	992.4614	1966.8890	983.9481	1965.9049	983.4561	18
3	371.2078	186.1075					W	1912.8784	**956.9428**	1895.8518	**948.4296**	1894.8678	**947.9376**	17
4	428.2292	214.6183					G	1726.7991	**863.9032**	1709.7725	**855.3899**	1708.7885	**854.8979**	16
5	529.2769	265.1421			**511.2663**	256.1368	T	**1669.7776**	835.3924	1652.7511	826.8792	1651.7670	826.3872	15
6	630.3246	315.6659			612.3140	306.6607	T	**1568.7299**	784.8686	1551.7034	776.3553	1550.7194	775.8633	14
7	701.3617	351.1845			683.3511	342.1792	A	1467.6823	734.3448	1450.6557	725.8315	1449.6717	725.3395	13
8	788.3937	394.7005			770.3832	385.6952	S	**1396.6451**	698.8262	1379.6186	690.3129	1378.6346	689.8209	12
9	859.4308	430.2191			841.4203	421.2138	A	**1309.6131**	655.3102	1292.5866	646.7969	1291.6025	646.3049	11
10	987.4894	494.2483	970.4629	485.7351	969.4789	485.2431	Q	**1238.5760**	619.7916	1221.5495	**611.2784**	1220.5654	**610.7864**	10
11	1102.5164	551.7618	1085.4898	543.2485	1084.5058	542.7565	N	**1110.5174**	555.7623	1093.4909	547.2491	1092.5069	546.7571	9
12	**1239.5753**	620.2913	1222.5487	**611.7780**	1221.5647	**611.2860**	H	**995.4905**	498.2489	978.4639	**489.7356**	977.4799	**489.2436**	8
13	**1340.6229**	670.8151	1323.5964	662.3018	1322.6124	661.8098	T	**858.4316**	429.7194	841.4050	421.2062	840.4210	420.7141	7
14	**1469.6655**	735.3364	1452.6390	726.8231	**1451.6550**	726.3311	E	**757.3839**	379.1956	740.3573	370.6823	739.3733	370.1903	6
15	1566.7183	783.8628	1549.6918	775.3495	1548.7077	774.8575	P	**628.3413**	314.6743	**611.3148**	306.1610	**610.3307**	305.6690	5
16	**1694.7769**	847.8921	1677.7503	839.3788	1676.7663	838.8868	Q	531.2885	266.1479	514.2620	257.6346	513.2780	257.1426	4
17	**1807.8609**	904.4341	**1790.8344**	895.9208	1789.8504	895.4288	L	**403.2300**	202.1186	386.2034	193.6053	385.2194	193.1133	3
18	**1922.8879**	961.9476	**1905.8613**	953.4343	**1904.8773**	952.9423	D	290.1459	145.5766	273.1193	137.0633	272.1353	136.5713	2
19							R	175.1190	88.0631	158.0924	79.5498			1

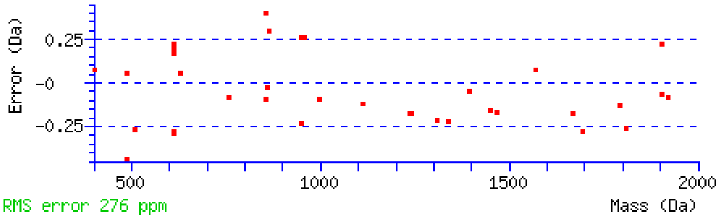

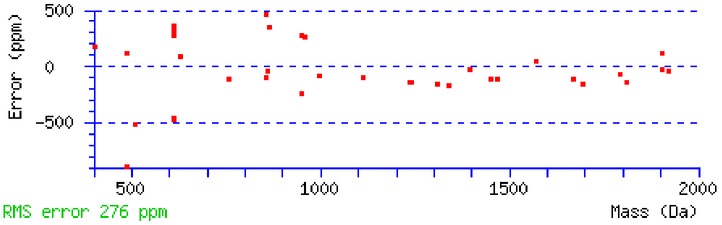

All matches to this query

Score	Mr(calc):	Delta	Sequence
67.2	2095.9923	0.0066	LAWGTTASAQNHTEPQLDR
9.6	2095.9922	0.0067	NYLXENKTHTETLQNPR
7.7	2095.9810	0.0179	SSIENYYHLETERALDR
5.1	2095.9886	0.0103	KQMEAMDMEVMEARLIR
4.8	2095.9922	0.0067	NYLXENKTHTETLQNPR
4.5	2095.0083	0.9906	LAWGTTASAQNHTEPQLDR
4.5	2096.0060	-0.0071	RLMNVLVPYTCQTHIR
4.2	2095.9886	0.0103	KQMEAMDMEVMEARLIR
4.2	2095.9886	0.0103	KQMEAMDMEVMEARLIR
3.0	2095.9827	0.0162	IWNLSENSTSGPTQLVLR

Spectrum No: 251; Query: 353; Rank: 1

Peptide View

MS/MS Fragmentation of **MIENGSLSFLPTLR**

Found in **IPI00191090**, Tax_Id=10116 Gene_Symbol=Bgn Biglycan precursor

Match to Query 353: 1577.815828 from(789.915190,2+)
Title: 091129RatKid_SCX02_16.3785.3785.2.dta
Data file K:\NewmanPaper\Piliang\3SubProteomes\Piliang3SP\mgf5ppm\SCX_3SubProteomes5ppm.mgf

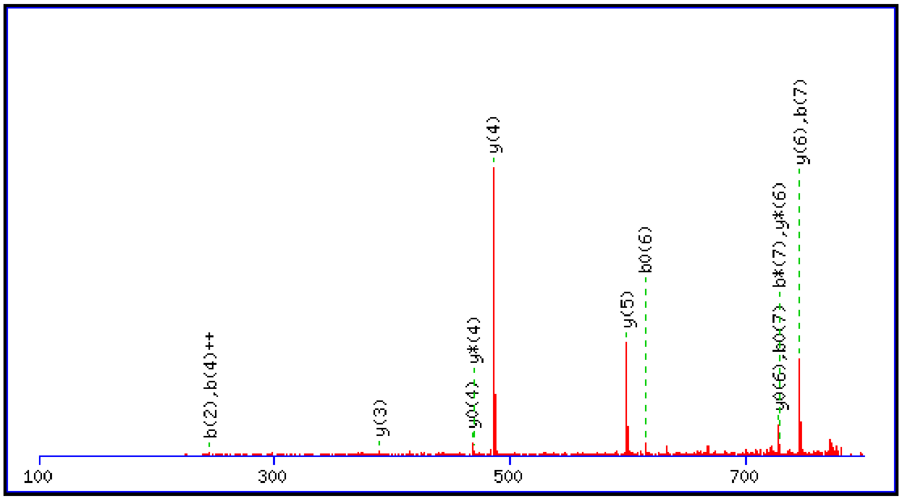

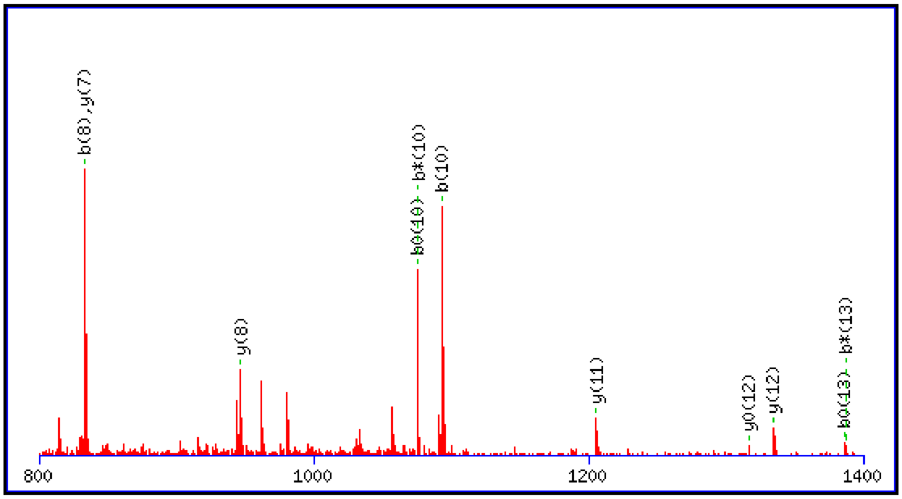

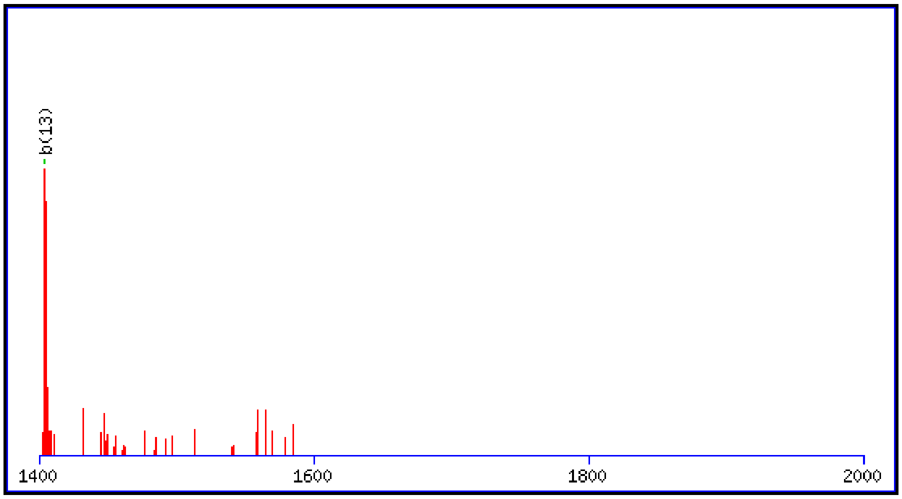

Monoisotopic mass of neutral peptide Mr(calc): 1577.8123
Fixed modifications: Carbamidomethyl (C)
Variable modifications:
N4 : Deamidated_N (N)
Ions Score: 67 **Expect:** 2.5e-005
Matches (Bold Red): 26/142 fragment ions using 37 most intense peaks

#	b	b^{++}	b*	b^{*++}	b^0	b^{0++}	Seq.	y	y^{++}	y*	y^{*++}	y^0	y^{0++}	#
1	132.0478	66.5275					M							14

**2**	**245.1318**	123.0696					**I**	1447.7791	724.3932	1430.7526	715.8799	1429.7685	715.3879	**13**
**3**	374.1744	187.5908			356.1639	178.5856	**E**	**1334.6951**	667.8512	1317.6685	659.3379	**1316.6845**	658.8459	**12**
**4**	489.2014	**245.1043**	472.1748	236.5910	471.1908	236.0990	**N**	**1205.6525**	603.3299	1188.6259	594.8166	1187.6419	594.3246	**11**
**5**	546.2228	273.6150	529.1963	265.1018	528.2123	264.6098	**G**	1090.6255	545.8164	1073.5990	537.3031	1072.6150	536.8111	**10**
**6**	633.2548	317.1311	616.2283	308.6178	**615.2443**	308.1258	**S**	1033.6041	517.3057	1016.5775	508.7924	1015.5935	508.3004	**9**
**7**	**746.3389**	373.6731	**729.3124**	365.1598	**728.3283**	364.6678	**L**	**946.5720**	473.7897	929.5455	465.2764	928.5615	464.7844	**8**
**8**	**833.3709**	417.1891	816.3444	408.6758	815.3604	408.1838	**S**	**833.4880**	417.2476	816.4614	408.7343	815.4774	408.2423	**7**
**9**	980.4394	490.7233	963.4128	482.2100	962.4288	481.7180	**F**	**746.4559**	373.7316	**729.4294**	365.2183	**728.4454**	364.7263	**6**
**10**	**1093.5234**	547.2653	**1076.4969**	538.7521	**1075.5129**	538.2601	**L**	**599.3875**	300.1974	582.3610	291.6841	581.3770	291.1921	**5**
**11**	1190.5762	595.7917	1173.5496	587.2785	1172.5656	586.7864	**P**	**486.3035**	243.6554	**469.2769**	235.1421	**468.2929**	234.6501	**4**
**12**	1291.6239	646.3156	1274.5973	637.8023	1273.6133	637.3103	**T**	**389.2507**	195.1290	372.2241	186.6157	371.2401	186.1237	**3**
**13**	**1404.7079**	702.8576	**1387.6814**	694.3443	**1386.6974**	693.8523	**L**	288.2030	144.6051	271.1765	136.0919			**2**
**14**							**R**	175.1190	88.0631	158.0924	79.5498			**1**

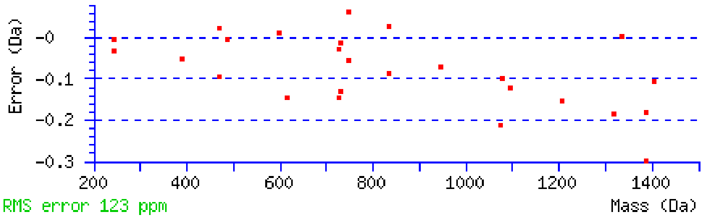

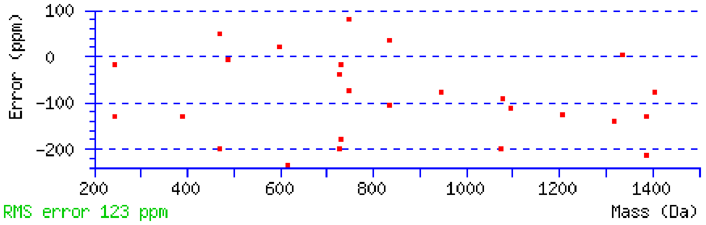

All matches to this query

Score	Mr(calc):	Delta	Sequence
67.1	1577.8123	0.0035	MIENGSLSFLPTLR
7.2	1577.8161	-0.0003	IEELNRYRDTLR
7.0	1577.8300	-0.0142	VKYSNSQATLEIPK
6.3	1577.8300	-0.0142	KNELEQLKETFAK
5.4	1577.8202	-0.0043	VGNLTPHLHNIFSK
5.3	1575.8186	1.9972	MKLELNTMQLPMK
4.8	1577.8235	-0.0077	DFCQNIASRLLLK
4.2	1576.8026	1.0132	MKLELNTMQLPMK
4.1	1577.8300	-0.0142	KNEYLLSLLAEER
2.4	1577.8083	0.0076	QELDLNSSMRLKK

Spectrum No: 252; Query: 1143; Rank: 1

Peptide View

MS/MS Fragmentation of **TVVQHSPNLIFSGIEEIQKDSCESNQNITCR**
Found in **IPI00324585**, Tax_Id=10116 Gene_Symbol=Itga1 Integrin alpha-1 precursor

Match to Query 1143: 3603.694992 from(1202.238940,3+)
Title: 091129RatKid_SCX02_29.4061.4061.3.dta
Data file K:\NewmanPaper\Piliang\3SubProteomes\Piliang3SP\mgf5ppm\SCX_3SubProteomes5ppm.mgf

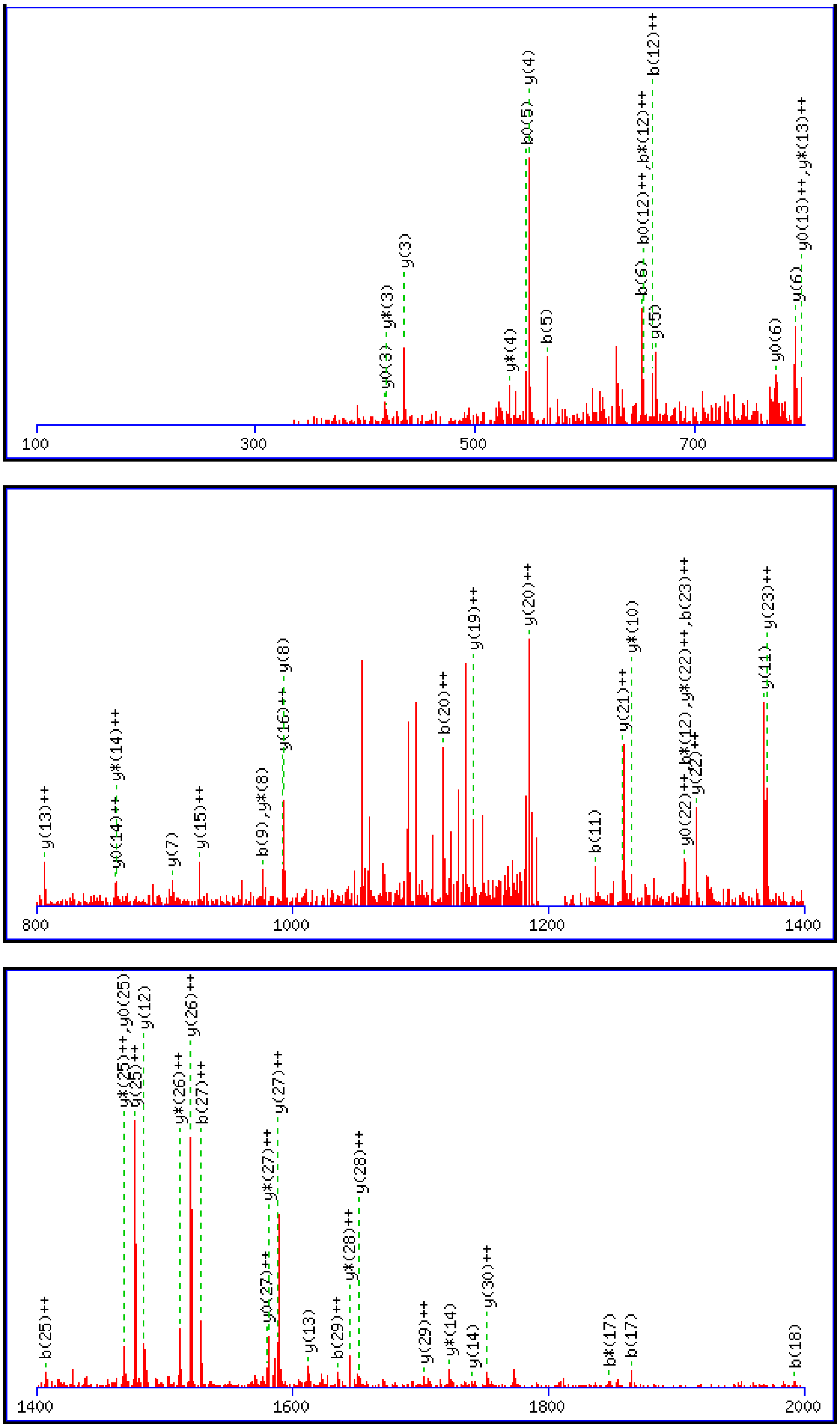

Monoisotopic mass of neutral peptide Mr(calc): 3603.6933
Fixed modifications: Carbamidomethyl (C)
Variable modifications:
N27 : Deamidated_N (N)
Ions Score: 67 **Expect:** 8.2e-005
Matches (Bold Red): 60/350 fragment ions using 130 most intense peaks

#	b	b^{++}	b*	b^{*++}	b^0	b^{0++}	Seq.	y	y^{++}	y*	y^{*++}	y^0	y^{0++}	#
1	102.0550	51.5311			84.0444	42.5258	T							31
2	201.1234	101.0653			183.1128	92.0600	V	3503.6530	**1752.3301**	3486.6264	1743.8168	3485.6424	1743.3248	30
3	300.1918	150.5995			282.1812	141.5942	V	3404.5845	**1702.7959**	3387.5580	1694.2826	3386.5740	1693.7906	29
4	428.2504	214.6288	411.2238	206.1155	410.2398	205.6235	Q	3305.5161	**1653.2617**	3288.4896	**1644.7484**	3287.5056	1644.2564	28
5	**565.3093**	283.1583	548.2827	274.6450	**547.2987**	274.1530	H	3177.4575	**1589.2324**	3160.4310	**1580.7191**	3159.4470	**1580.2271**	27

6	652.3413	326.6743	635.3148	318.1610	634.3307	317.6690	S	3040.3986	1520.7030	3023.3721	1512.1897	3022.3881	1511.6977	26
7	749.3941	375.2007	732.3675	366.6874	731.3835	366.1954	P	2953.3666	1477.1869	2936.3401	1468.6737	2935.3560	1468.1817	25
8	863.4370	432.2221	846.4104	423.7089	845.4264	423.2169	N	2856.3138	1428.6606	2839.2873	1420.1473	2838.3033	1419.6553	24
9	976.5211	488.7642	959.4945	480.2509	958.5105	479.7589	L	2742.2709	1371.6391	2725.2444	1363.1258	2724.2603	1362.6338	23
10	1089.6051	545.3062	1072.5786	536.7929	1071.5946	536.3009	I	2629.1869	1315.0971	2612.1603	1306.5838	2611.1763	1306.0918	22
11	1236.6735	618.8404	1219.6470	610.3271	1218.6630	609.8351	F	2516.1028	1258.5550	2499.0762	1250.0418	2498.0922	1249.5497	21
12	1323.7056	662.3564	1306.6790	653.8431	1305.6950	653.3511	S	2369.0344	1185.0208	2352.0078	1176.5075	2351.0238	1176.0155	20
13	1380.7270	690.8672	1363.7005	682.3539	1362.7165	681.8619	G	2282.0023	1141.5048	2264.9758	1132.9915	2263.9918	1132.4995	19
14	1493.8111	747.4092	1476.7845	738.8959	1475.8005	738.4039	I	2224.9809	1112.9941	2207.9543	1104.4808	2206.9703	1103.9888	18
15	1622.8537	811.9305	1605.8271	803.4172	1604.8431	802.9252	E	2111.8968	1056.4520	2094.8703	1047.9388	2093.8863	1047.4468	17
16	1751.8963	876.4518	1734.8697	867.9385	1733.8857	867.4465	E	1982.8542	991.9307	1965.8277	983.4175	1964.8437	982.9255	16
17	1864.9803	932.9938	1847.9538	924.4805	1846.9698	923.9885	I	1853.8116	927.4095	1836.7851	918.8962	1835.8011	918.4042	15
18	1993.0389	997.0231	1976.0124	988.5098	1975.0284	988.0178	Q	1740.7276	870.8674	1723.7010	862.3541	1722.7170	861.8621	14
19	2121.1339	1061.0706	2104.1073	1052.5573	2103.1233	1052.0653	K	1612.6690	806.8381	1595.6424	798.3249	1594.6584	797.8328	13
20	2236.1608	1118.5840	2219.1343	1110.0708	2218.1503	1109.5788	D	1484.5740	742.7907	1467.5475	734.2774	1466.5635	733.7854	12
21	2323.1929	1162.1001	2306.1663	1153.5868	2305.1823	1153.0948	S	1369.5471	685.2772	1352.5205	676.7639	1351.5365	676.2719	11
22	2483.2235	1242.1154	2466.1970	1233.6021	2465.2129	1233.1101	C	1282.5151	641.7612	1265.4885	633.2479	1264.5045	632.7559	10
23	2612.2661	1306.6367	2595.2395	1298.1234	2594.2555	1297.6314	E	1122.4844	561.7458	1105.4579	553.2326	1104.4738	552.7406	9
24	2699.2981	1350.1527	2682.2716	1341.6394	2681.2876	1341.1474	S	993.4418	497.2245	976.4153	488.7113	975.4312	488.2193	8
25	2813.3410	1407.1742	2796.3145	1398.6609	2795.3305	1398.1689	N	906.4098	453.7085	889.3832	445.1953	888.3992	444.7032	7
26	2941.3996	1471.2035	2924.3731	1462.6902	2923.3891	1462.1982	Q	792.3669	396.6871	775.3403	388.1738	774.3563	387.6818	6
27	3056.4266	1528.7169	3039.4000	1520.2036	3038.4160	1519.7116	N	664.3083	332.6578	647.2817	324.1445	646.2977	323.6525	5
28	3169.5106	1585.2590	3152.4841	1576.7457	3151.5001	1576.2537	I	549.2813	275.1443	532.2548	266.6310	531.2708	266.1390	4
29	3270.5583	1635.7828	3253.5318	1627.2695	3252.5477	1626.7775	T	436.1973	218.6023	419.1707	210.0890	418.1867	209.5970	3
30	3430.5890	1715.7981	3413.5624	1707.2848	3412.5784	1706.7928	C	335.1496	168.0784	318.1231	159.5652			2
31							R	175.1190	88.0631	158.0924	79.5498			1

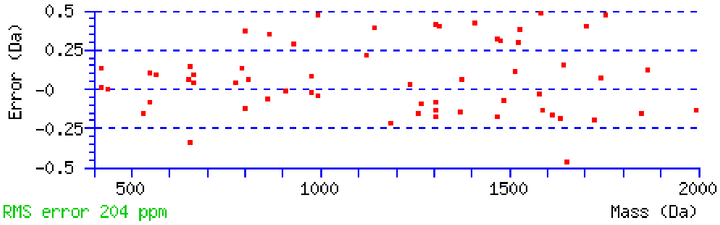

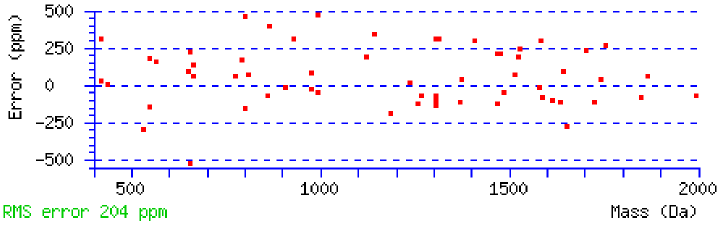

All matches to this query

Score	Mr(calc):	Delta	Sequence
66.9	3603.6933	0.0016	TVVQHSPNLIFSGIEEIQKDSCESNQNITCR
60.6	3603.6933	0.0016	TVVQHSPNLIFSGIEEIQKDSCESNQNITCR
22.5	3603.6933	0.0016	TVVQHSPNLIFSGIEEIQKDSCESNQNITCR
5.6	3601.6886	2.0064	SHQFFVVKEIMAAYPAGHSVIIILSNSKFK
3.8	3602.7093	0.9857	TVVQHSPNLIFSGIEEIQKDSCESNQNITCR
1.4	3602.6899	1.0051	GCQDITSVFLNVERMTPQTQKELETAWGLR
1.3	3601.6928	2.0022	GGGTAPEKPDPAQKPPYSYVALIAMAIRESAEK
1.3	3601.6928	2.0022	GGGTAPEKPDPAQKPPYSYVALIAMAIRESAEK
1.3	3601.6928	2.0022	GGGTAPEKPDPAQKPPYSYVALIAMAIRESAEK
0.9	3601.6796	2.0154	YAVVLNATWLVNSAAHLYGYRPYDKNISSR

Spectrum No: 253; Query: 282; Rank: 1

Peptide View

MS/MS Fragmentation of **TVANLSGCEQVDSK**
Found in **IPI00557299**, Tax_Id=10116 Gene_Symbol=LOC498940 Similar to Carboxylesterase 2

Match to Query 282: 1507.686188 from(754.850370,2+)
Title: 091127RatKid_SCX01_12.1125.1125.2.dta
Data file K:\NewmanPaper\Piliang\3SubProteomes\Piliang3SP\mgf5ppm\SCX_3SubProteomes5ppm.mgf

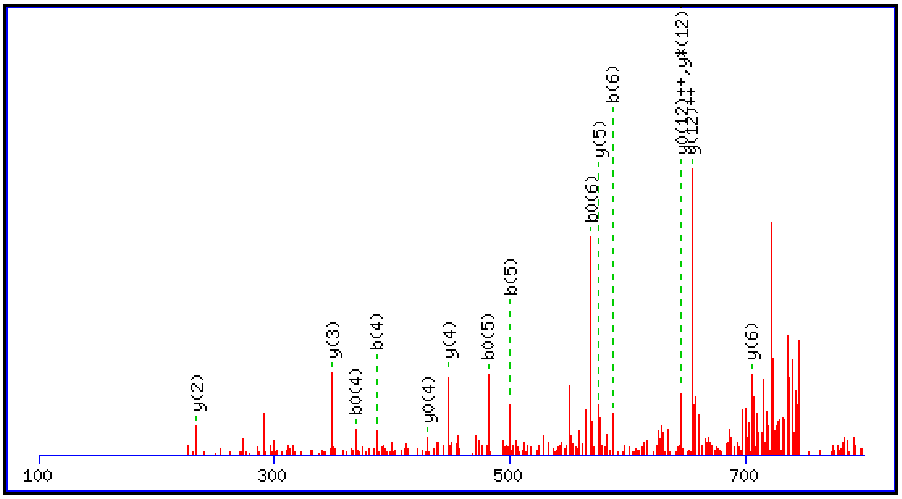

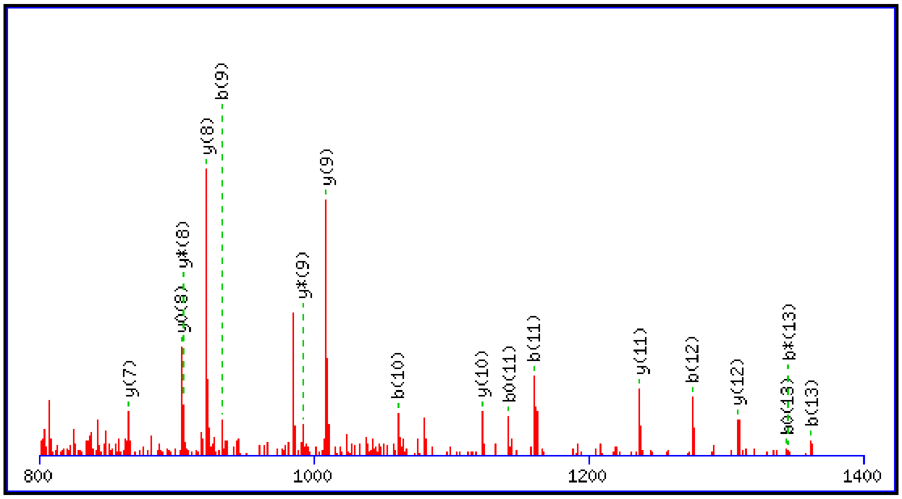

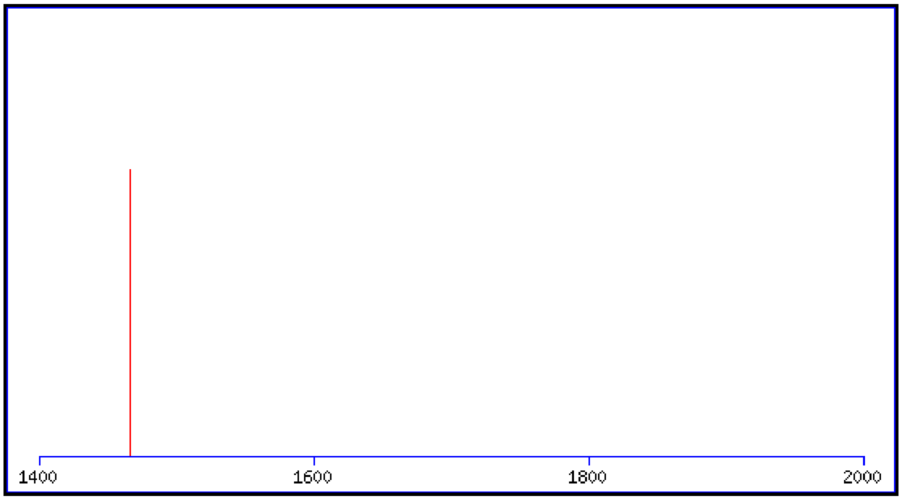

Monoisotopic mass of neutral peptide Mr(calc): 1507.6824
Fixed modifications: Carbamidomethyl (C)
Variable modifications:
N4 : Deamidated_N (N)
Ions Score: 67 **Expect:** 3.3e-005

Matches (Bold Red): 32/148 fragment ions using 68 most intense peaks

#	b	b^{++}	b*	b^{*++}	b^{0}	b^{0++}	Seq.	y	y^{++}	y*	y^{*++}	y^{0}	y^{0++}	#
1	102.0550	51.5311			84.0444	42.5258	T							14
2	201.1234	101.0653			183.1128	92.0600	V	1407.6420	704.3247	1390.6155	695.8114	1389.6315	695.3194	13
3	272.1605	136.5839			254.1499	127.5786	A	**1308.5736**	**654.7904**	1291.5471	**646.2772**	1290.5631	**645.7852**	12
4	**387.1874**	194.0973	370.1609	185.5841	**369.1769**	185.0921	N	**1237.5365**	619.2719	1220.5100	610.7586	1219.5259	610.2666	11
5	**500.2715**	250.6394	483.2449	242.1261	**482.2609**	241.6341	L	**1122.5096**	561.7584	1105.4830	553.2451	1104.4990	552.7531	10
6	**587.3035**	294.1554	570.2770	285.6421	**569.2929**	285.1501	S	**1009.4255**	505.2164	**992.3990**	496.7031	991.4149	496.2111	9
7	644.3250	322.6661	627.2984	314.1529	626.3144	313.6608	G	**922.3935**	461.7004	**905.3669**	453.1871	**904.3829**	452.6951	8
8	804.3556	402.6815	787.3291	394.1682	786.3451	393.6762	C	**865.3720**	433.1896	848.3455	424.6764	847.3614	424.1844	7
9	**933.3982**	467.2027	916.3717	458.6895	915.3877	458.1975	E	**705.3414**	353.1743	688.3148	344.6610	687.3308	344.1690	6
10	**1061.4568**	531.2320	1044.4302	522.7188	1043.4462	522.2268	Q	**576.2988**	288.6530	559.2722	280.1397	558.2882	279.6477	5
11	**1160.5252**	580.7662	1143.4987	572.2530	**1142.5146**	571.7610	V	**448.2402**	224.6237	431.2136	216.1105	**430.2296**	215.6185	4
12	**1275.5522**	638.2797	1258.5256	629.7664	1257.5416	629.2744	D	**349.1718**	175.0895	332.1452	166.5763	331.1612	166.0842	3
13	**1362.5842**	681.7957	**1345.5576**	673.2825	**1344.5736**	672.7904	S	**234.1448**	117.5761	217.1183	109.0628	216.1343	108.5708	2
14							K	147.1128	74.0600	130.0863	65.5468			1

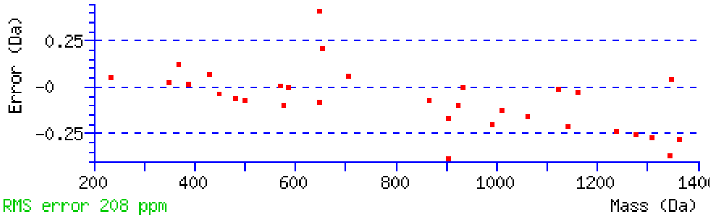

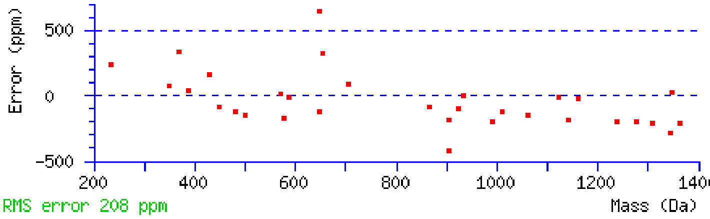

All matches to this query

Score	Mr(calc):	Delta	Sequence
66.9	1507.6824	0.0038	TVANLSGCEQVDSK
0.2	1507.6936	-0.0074	SVIATSTKVDISK

Spectrum No: 254; Query: 307; Rank: 1

Peptide View

MS/MS Fragmentation of **ENGVFEEISNSSGR**
Found in **IPI00326260**, Tax_Id=10116 Gene_Symbol=Sdfr1 Isoform 2 of Neuroplastin precursor

Match to Query 307: 1525.668048 from(763.841300,2+)
Title: 091129RatKid_SCX02_02.1880.1880.2.dta
Data file K:\NewmanPaper\Piliang\3SubProteomes\Piliang3SP\mgf5ppm\SCX_3SubProteomes5ppm.mgf

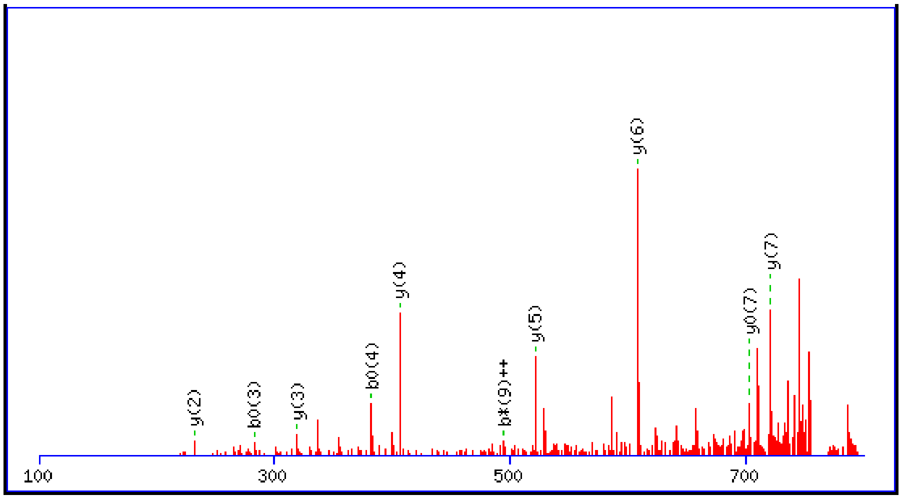

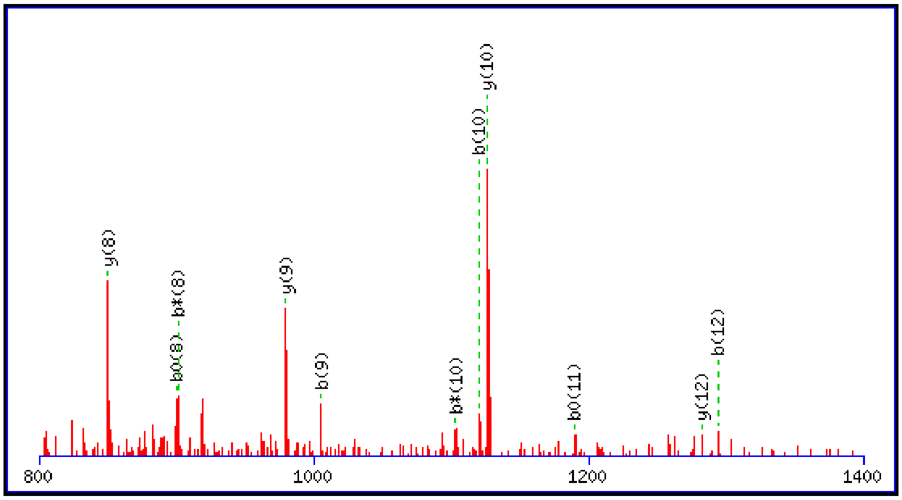

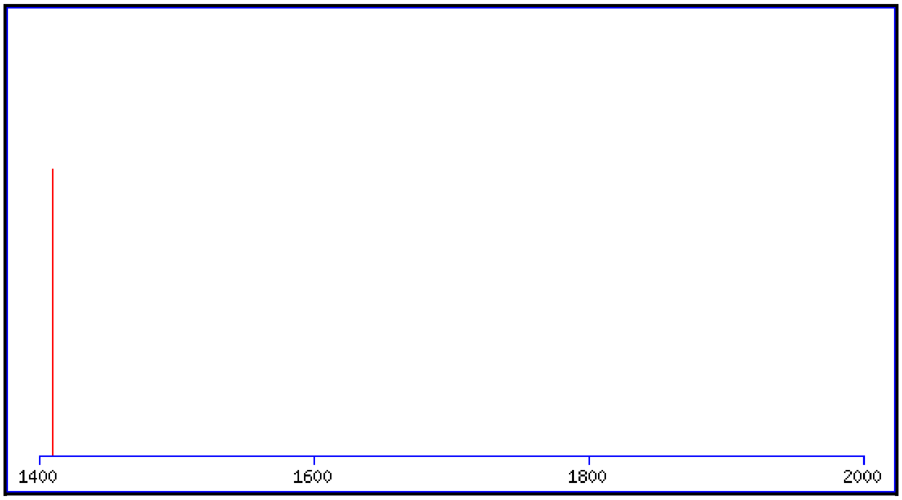

Monoisotopic mass of neutral peptide Mr(calc): 1524.6692
Fixed modifications: Carbamidomethyl (C)
Variable modifications:
N10 : Deamidated_N (N)
Ions Score: 67 **Expect:** 3.1e-005
Matches (Bold Red): 21/150 fragment ions using 43 most intense peaks

#	b	b^{++}	b*	b^{*++}	b^0	b^{0++}	Seq.	y	y^{++}	y*	y^{*++}	y^0	y^{0++}	#
1	130.0499	65.5286			112.0393	56.5233	E							14
2	244.0928	122.5500	227.0662	114.0368	226.0822	113.5448	N	1396.6339	698.8206	1379.6074	690.3073	1378.6233	689.8153	13
3	301.1143	151.0608	284.0877	142.5475	**283.1037**	142.0555	G	**1282.5910**	641.7991	1265.5644	633.2859	1264.5804	632.7938	12
4	400.1827	200.5950	383.1561	192.0817	**382.1721**	191.5897	V	1225.5695	613.2884	1208.5430	604.7751	1207.5589	604.2831	11
5	547.2511	274.1292	530.2245	265.6159	529.2405	265.1239	F	**1126.5011**	563.7542	1109.4746	555.2409	1108.4905	554.7489	10

**6**	676.2937	338.6505	659.2671	330.1372	658.2831	329.6452	**E**	**979.4327**	490.2200	962.4061	481.7067	961.4221	481.2147	**9**
**7**	805.3363	403.1718	788.3097	394.6585	787.3257	394.1665	**E**	**850.3901**	425.6987	833.3635	417.1854	832.3795	416.6934	**8**
**8**	918.4203	459.7138	**901.3938**	451.2005	**900.4098**	450.7085	**I**	**721.3475**	361.1774	704.3210	352.6641	**703.3369**	352.1721	**7**
**9**	**1005.4524**	503.2298	988.4258	**494.7165**	987.4418	494.2245	**S**	**608.2634**	304.6354	591.2369	296.1221	590.2529	295.6301	**6**
**10**	**1120.4793**	560.7433	**1103.4528**	552.2300	1102.4687	551.7380	**N**	**521.2314**	261.1193	504.2049	252.6061	503.2208	252.1141	**5**
**11**	1207.5113	604.2593	1190.4848	595.7460	**1189.5008**	595.2540	**S**	**406.2045**	203.6059	389.1779	195.0926	388.1939	194.6006	**4**
**12**	**1294.5434**	647.7753	1277.5168	639.2620	1276.5328	638.7700	**S**	**319.1724**	160.0899	302.1459	151.5766	301.1619	151.0846	**3**
**13**	1351.5648	676.2860	1334.5383	667.7728	1333.5543	667.2808	**G**	**232.1404**	116.5738	215.1139	108.0606			**2**
**14**							**R**	175.1190	88.0631	158.0924	79.5498			**1**

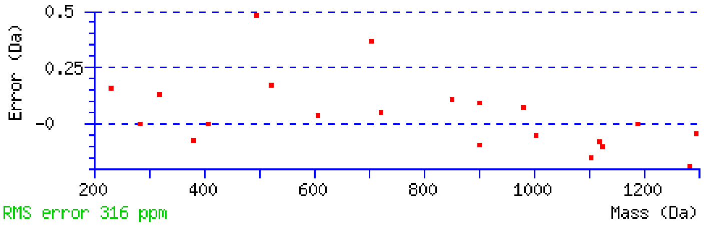

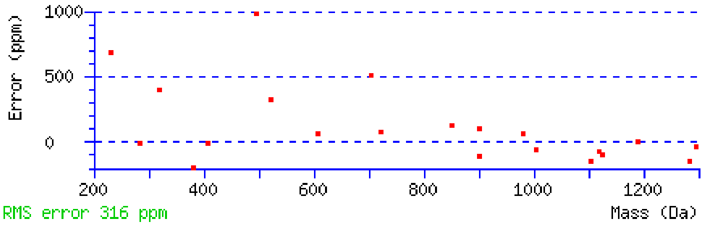

All matches to this query

Score	Mr(calc):	Delta	Sequence
66.8	1524.6692	0.9988	ENGVFEEISNSSGR
62.3	1525.6532	0.0148	ENGVFEEISNSSGR
5.8	1524.6756	0.9925	THSTTSQAIGVGMR
0.5	1525.6540	0.0140	MSYLLLPLDSSK
0.4	1524.6692	0.9988	ENGVFEEISNSSGR

Spectrum No: 255; Query: 365; Rank: 1

Peptide View

MS/MS Fragmentation of **FNLTETPETEIHR**
Found in **IPI00211075**, Tax_Id=10116 Gene_Symbol=Serpina3n Serine protease inhibitor A3N precursor

Match to Query 365: 1586.760988 from(794.387770,2+)
Title: 091127RatKid_SCX01_12.2028.2028.2.dta
Data file K:\NewmanPaper\Piliang\3SubProteomes\Piliang3SP\mgf5ppm\SCX_3SubProteomes5ppm.mgf

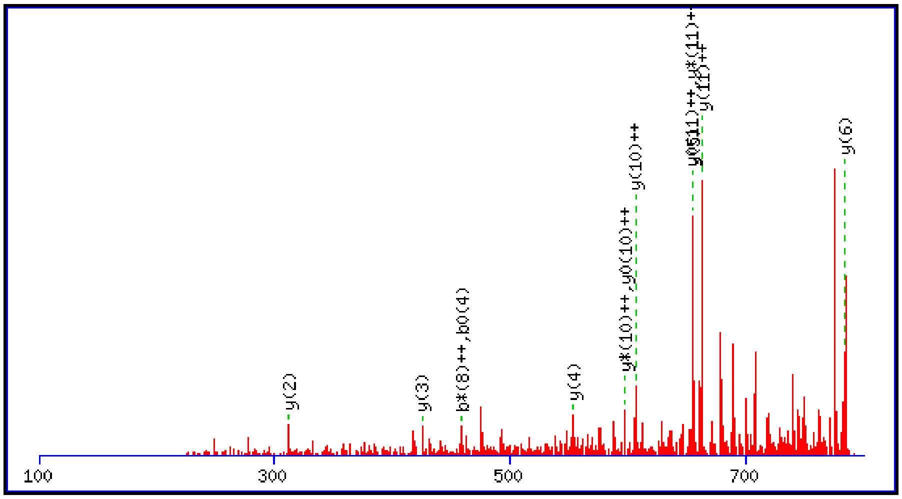

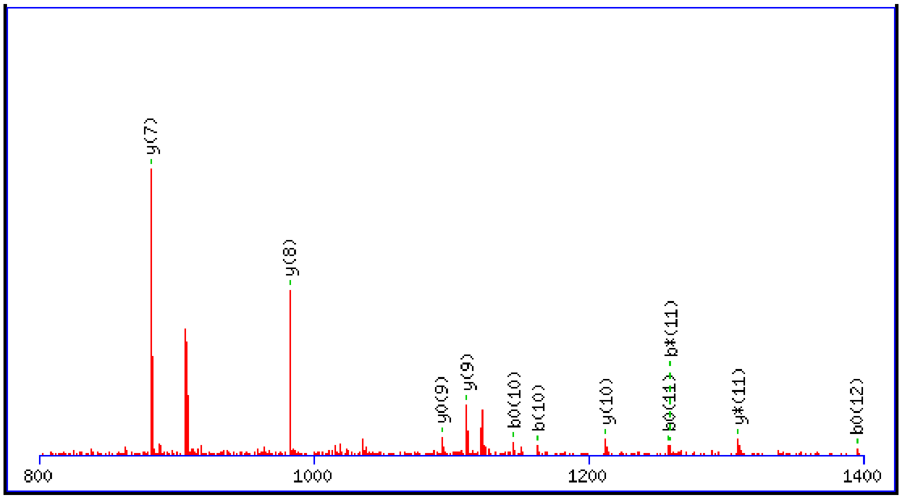

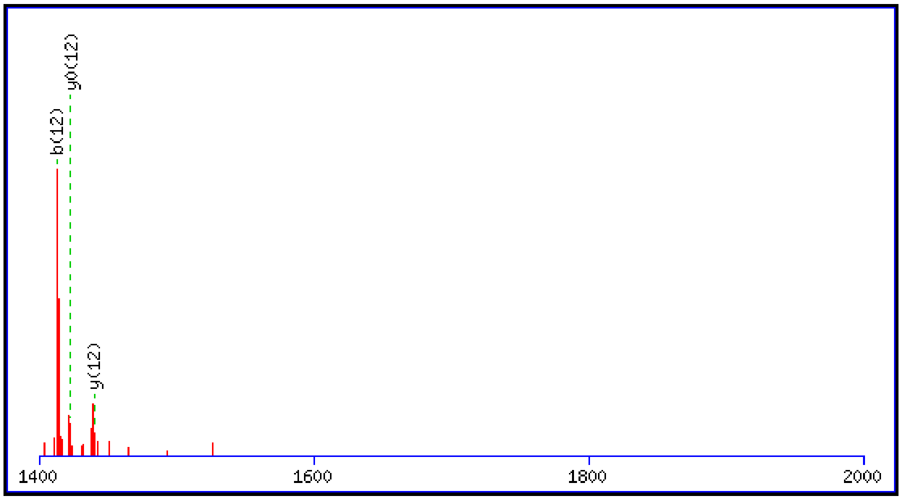

Monoisotopic mass of neutral peptide Mr(calc): 1586.7576
Fixed modifications: Carbamidomethyl (C)
Variable modifications:
N2 : Deamidated_N (N)
Ions Score: 67 **Expect:** 3.7e-005
Matches (Bold Red): 27/130 fragment ions using 49 most intense peaks

#	b	b^{++}	b*	b^{*++}	b^0	b^{0++}	Seq.	y	y^{++}	y*	y^{*++}	y^0	y^{0++}	#
1	148.0757	74.5415					F							13
2	263.1026	132.0550	246.0761	123.5417			N	**1440.6965**	720.8519	1423.6700	712.3386	**1422.6859**	711.8466	12
3	376.1867	188.5970	359.1601	180.0837			L	1325.6696	**663.3384**	**1308.6430**	**654.8251**	1307.6590	**654.3331**	11
4	477.2344	239.1208	460.2078	230.6075	**459.2238**	230.1155	T	**1212.5855**	**606.7964**	1195.5590	**598.2831**	1194.5749	**597.7911**	10
5	606.2770	303.6421	589.2504	295.1288	588.2664	294.6368	E	**1111.5378**	556.2726	1094.5113	547.7593	**1093.5273**	547.2673	9
6	707.3246	354.1660	690.2981	345.6527	689.3141	345.1607	T	**982.4952**	491.7513	965.4687	483.2380	964.4847	482.7460	8
7	804.3774	402.6923	787.3509	394.1791	786.3668	393.6871	P	**881.4476**	441.2274	864.4210	432.7141	863.4370	432.2221	7
8	933.4200	467.2136	916.3934	**458.7004**	915.4094	458.2084	E	**784.3948**	392.7010	767.3682	384.1878	766.3842	383.6958	6
9	1034.4677	517.7375	1017.4411	509.2242	1016.4571	508.7322	T	**655.3522**	328.1797	638.3257	319.6665	637.3416	319.1745	5
10	**1163.5103**	582.2588	1146.4837	573.7455	**1145.4997**	573.2535	E	**554.3045**	277.6559	537.2780	269.1426	536.2940	268.6506	4
11	1276.5943	638.8008	**1259.5678**	630.2875	**1258.5838**	629.7955	I	**425.2619**	213.1346	408.2354	204.6213			3
12	**1413.6532**	707.3303	1396.6267	698.8170	**1395.6427**	698.3250	H	**312.1779**	156.5926	295.1513	148.0793			2
13							R	175.1190	88.0631	158.0924	79.5498			1

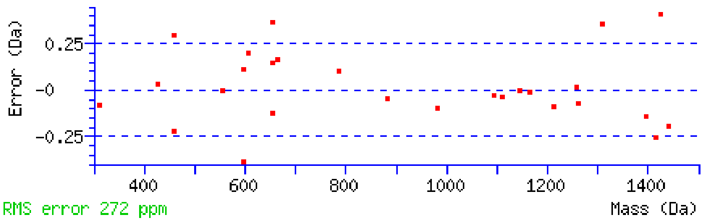

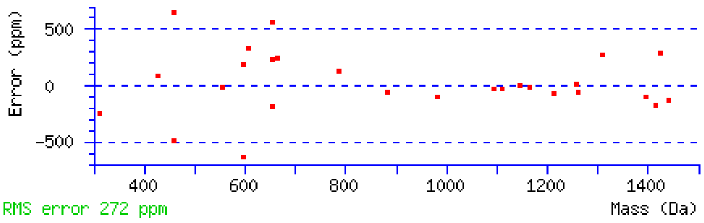

All matches to this query

Score	Mr(calc):	Delta	Sequence
66.7	1586.7576	0.0034	FNLTETPETEIHR
7.8	1585.7535	1.0075	MVKVSIGLGEGTGSR
6.6	1585.7436	1.0174	FVQQCIRTVEAR
5.5	1584.7508	2.0102	NESSLRKSEQLSK
5.3	1585.7654	0.9956	TPLYTRVATWGNK
5.3	1586.7494	0.0116	TPLYTRVATWGNK
4.8	1584.7396	2.0214	IAQDGVSTSATTNLK
4.8	1584.7396	2.0214	IAQDGVSTSATTNLK
4.1	1584.7677	1.9932	VPTERSAVLVNLK
3.4	1584.7396	2.0214	IAQDGVSTSATTNLK

Spectrum No: 256; Query: 678; Rank: 1

Peptide View

MS/MS Fragmentation of **YQTSSAINGSVIWDRPTK**
Found in **IPI00210872**, Tax_Id=10116 Gene_Symbol=Mep1a Meprin A subunit alpha precursor

Match to Query 678: 2023.009468 from(1012.512010,2+)
Title: 091129RatKid_SCX02_12.1828.1828.2.dta
Data file K:\NewmanPaper\Piliang\3SubProteomes\Piliang3SP\mgf5ppm\SCX_3SubProteomes5ppm.mgf

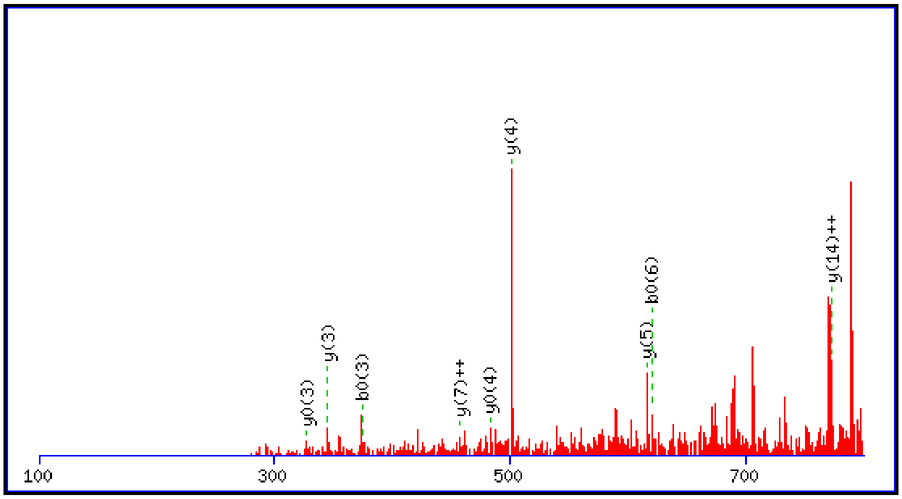

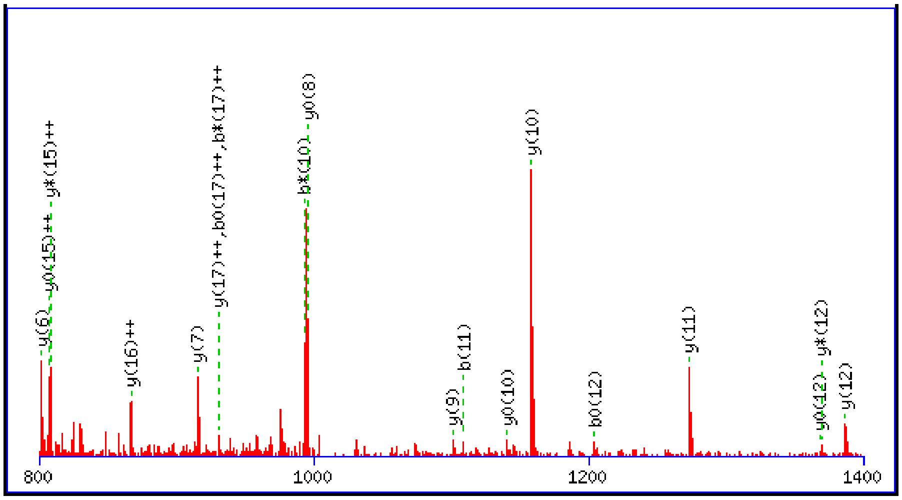

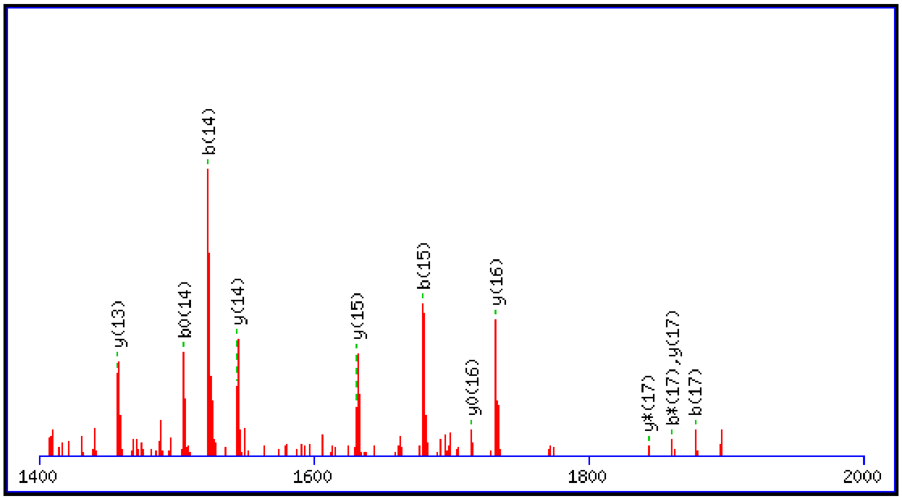

Monoisotopic mass of neutral peptide Mr(calc): 2023.0011
Fixed modifications: Carbamidomethyl (C)
Variable modifications:
N8 : Deamidated_N (N)
Ions Score: 67 **Expect:** 4.4e-005
Matches (Bold Red): 40/196 fragment ions using 102 most intense peaks

#	b	b++	b*	b*++	b0	b0++	Seq.	y	y++	y*	y*++	y0	y0++	#
1	164.0706	82.5389					Y							18
2	292.1292	146.5682	275.1026	138.0550			Q	**1860.9450**	**930.9761**	**1843.9185**	922.4629	1842.9344	921.9709	17
3	393.1769	197.0921	376.1503	188.5788	**375.1663**	188.0868	T	**1732.8864**	**866.9469**	1715.8599	858.4336	**1714.8759**	857.9416	16
4	480.2089	240.6081	463.1823	232.0948	462.1983	231.6028	S	**1631.8388**	816.4230	1614.8122	**807.9097**	1613.8282	**807.4177**	15
5	567.2409	284.1241	550.2144	275.6108	549.2304	275.1188	S	**1544.8067**	**772.9070**	1527.7802	764.3937	1526.7962	763.9017	14
6	638.2780	319.6427	621.2515	311.1294	**620.2675**	310.6374	A	**1457.7747**	729.3910	1440.7481	720.8777	1439.7641	720.3857	13
7	751.3621	376.1847	734.3355	367.6714	733.3515	367.1794	I	**1386.7376**	693.8724	**1369.7110**	685.3592	**1368.7270**	684.8671	12
8	866.3890	433.6982	849.3625	425.1849	848.3785	424.6929	N	**1273.6535**	637.3304	1256.6270	628.8171	1255.6430	628.3251	11
9	923.4105	462.2089	906.3839	453.6956	905.3999	453.2036	G	**1158.6266**	579.8169	1141.6000	571.3037	**1140.6160**	570.8116	10
10	1010.4425	505.7249	**993.4160**	497.2116	992.4320	496.7196	S	**1101.6051**	551.3062	1084.5786	542.7929	1083.5946	542.3009	9
11	**1109.5109**	555.2591	1092.4844	546.7458	1091.5004	546.2538	V	1014.5731	507.7902	997.5465	499.2769	**996.5625**	498.7849	8
12	1222.5950	611.8011	1205.5685	603.2879	**1204.5844**	602.7959	I	**915.5047**	**458.2560**	898.4781	449.7427	897.4941	449.2507	7
13	1408.6743	704.8408	1391.6478	696.3275	1390.6637	695.8355	W	**802.4206**	401.7139	785.3941	393.2007	784.4100	392.7087	6
14	**1523.7013**	762.3543	1506.6747	753.8410	**1505.6907**	753.3490	D	**616.3413**	308.6743	599.3148	300.1610	598.3307	299.6690	5
15	**1679.8024**	840.4048	1662.7758	831.8915	1661.7918	831.3995	R	**501.3144**	251.1608	484.2878	242.6475	**483.3038**	242.1555	4
16	1776.8551	888.9312	1759.8286	880.4179	1758.8446	879.9259	P	**345.2132**	173.1103	328.1867	164.5970	**327.2027**	164.1050	3
17	**1877.9028**	939.4550	**1860.8763**	**930.9418**	1859.8922	**930.4498**	T	248.1605	124.5839	231.1339	116.0706	230.1499	115.5786	2
18							K	147.1128	74.0600	130.0863	65.5468			1

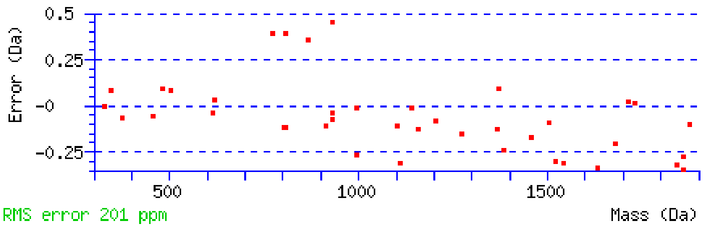

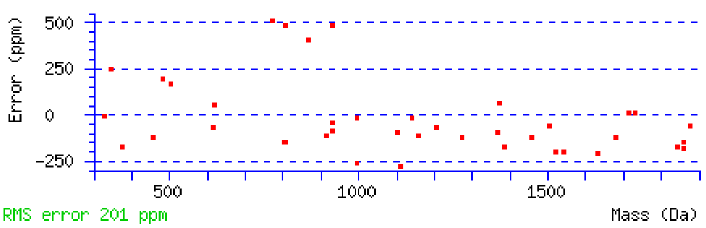

All matches to this query

Score	Mr(calc):	Delta	Sequence
66.7	2023.0011	0.0084	YQTSSAINGSVIWDRPTK
8.0	2022.0170	0.9924	YQTSSAINGSVIWDRPTK
7.8	2023.0044	0.0051	NLKLGSSFPRGDLSQMEK
2.6	2023.0255	-0.0161	AGITSAMATRTSLKDEDLK
2.5	2023.0173	-0.0078	QNTKVTIKGAKPCVEAAK
0.1	2023.0156	-0.0061	NGLHLSEHIRNEIKSMK

Spectrum No: 257; Query: 1184; Rank: 1

Peptide View

MS/MS Fragmentation of **SNTGGQAFPQCVFDHWQILPGDPFDNSSRPSQVVAETR**
Found in **IPI00203214**, Tax_Id=10116 Gene_Symbol=Eef2 Elongation factor 2

Match to Query 1184: 4244.981412 from(1416.001080,3+)
Title: 091129RatKid_SCX02_29.5827.5827.3.dta
Data file K:\NewmanPaper\Piliang\3SubProteomes\Piliang3SP\mgf5ppm\SCX_3SubProteomes5ppm.mgf

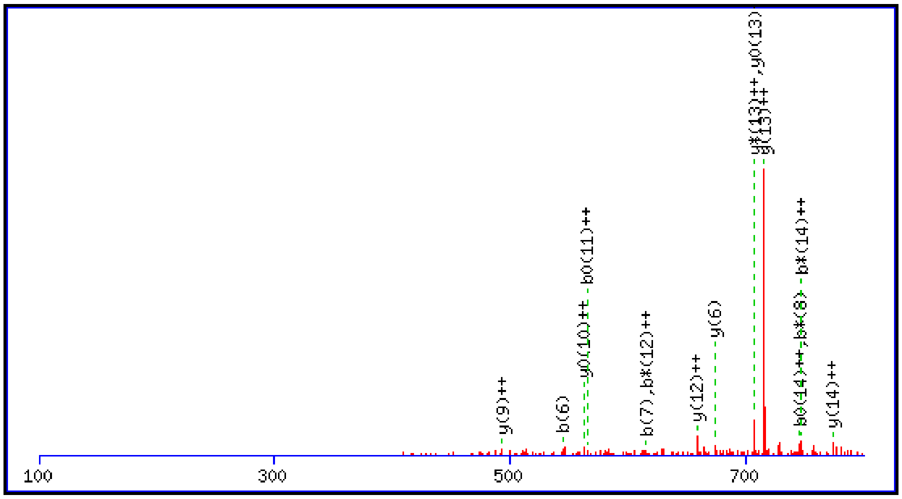

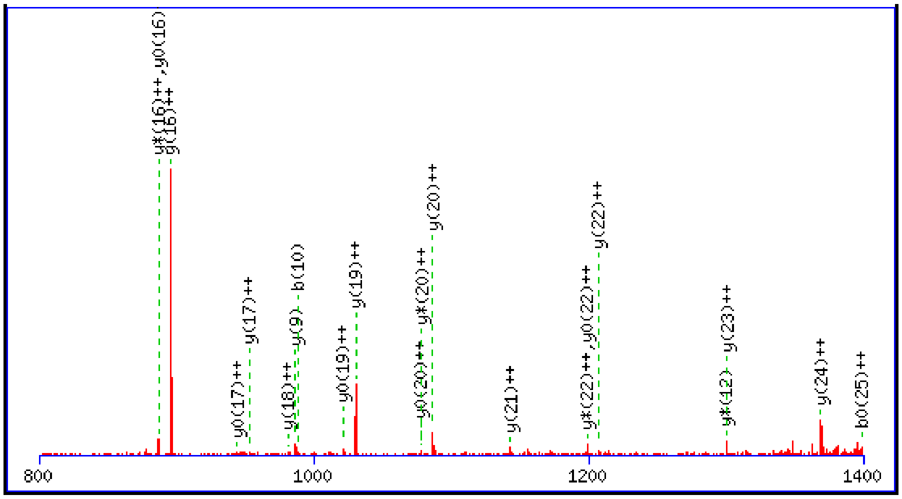

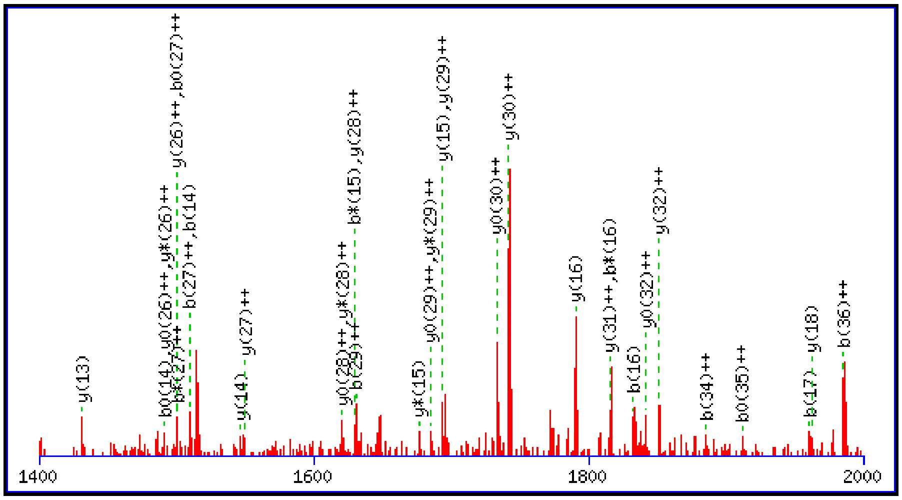

Monoisotopic mass of neutral peptide Mr(calc): 4244.9611
Fixed modifications: Carbamidomethyl (C)
Variable modifications:
N26 : Deamidated_N (N)
Ions Score: 67 **Expect:** 0.0001
Matches (Bold Red): 70/440 fragment ions using 149 most intense peaks

#	b	b^{++}	b*	b^{*++}	b^0	b^{0++}	Seq.	y	y^{++}	y*	y^{*++}	y^0	y^{0++}	#
1	88.0393	44.5233			70.0287	35.5180	S							38
2	202.0822	101.5448	185.0557	93.0315	184.0717	92.5395	N	4158.9363	2079.9718	4141.9097	2071.4585	4140.9257	2070.9665	37
3	303.1299	152.0686	286.1034	143.5553	285.1193	143.0633	T	4044.8933	2022.9503	4027.8668	2014.4370	4026.8828	2013.9450	36
4	360.1514	180.5793	343.1248	172.0661	342.1408	171.5740	G	3943.8457	1972.4265	3926.8191	1963.9132	3925.8351	1963.4212	35
5	417.1728	209.0901	400.1463	200.5768	399.1623	200.0848	G	3886.8242	1943.9157	3869.7976	1935.4025	3868.8136	1934.9105	34
6	**545.2314**	273.1193	528.2049	264.6061	527.2209	264.1141	Q	3829.8027	1915.4050	3812.7762	1906.8917	3811.7922	1906.3997	33
7	**616.2685**	308.6379	599.2420	300.1246	598.2580	299.6326	A	3701.7442	**1851.3757**	3684.7176	1842.8624	3683.7336	**1842.3704**	32
8	763.3369	382.1721	**746.3104**	373.6588	745.3264	373.1668	F	3630.7070	**1815.8572**	3613.6805	1807.3439	3612.6965	1806.8519	31
9	860.3897	430.6985	843.3632	422.1852	842.3791	421.6932	P	3483.6386	**1742.3229**	3466.6121	1733.8097	3465.6281	**1733.3177**	30
10	**988.4483**	494.7278	971.4217	486.2145	970.4377	485.7225	Q	3386.5859	**1693.7966**	3369.5593	**1685.2833**	3368.5753	**1684.7913**	29
11	1148.4789	574.7431	1131.4524	566.2298	1130.4684	**565.7378**	C	3258.5273	**1629.7673**	3241.5007	**1621.2540**	3240.5167	**1620.7620**	28
12	1247.5473	624.2773	1230.5208	**615.7640**	1229.5368	615.2720	V	3098.4966	**1549.7520**	3081.4701	1541.2387	3080.4861	1540.7467	27
13	1394.6158	697.8115	1377.5892	689.2982	1376.6052	688.8062	F	2999.4282	**1500.2177**	2982.4017	**1491.7045**	2981.4177	**1491.2125**	26
14	**1509.6427**	755.3250	1492.6162	**746.8117**	**1491.6321**	**746.3197**	D	2852.3598	1426.6835	2835.3333	1418.1703	2834.3492	1417.6783	25
15	1646.7016	823.8544	**1629.6751**	815.3412	1628.6911	814.8492	H	2737.3329	**1369.1701**	2720.3063	1360.6568	2719.3223	1360.1648	24
16	**1832.7809**	916.8941	**1815.7544**	908.3808	1814.7704	907.8888	W	2600.2739	**1300.6406**	2583.2474	1292.1273	2582.2634	1291.6353	23
17	**1960.8395**	980.9234	1943.8130	972.4101	1942.8289	971.9181	Q	2414.1946	**1207.6010**	2397.1681	**1199.0877**	2396.1841	**1198.5957**	22
18	2073.9236	1037.4654	2056.8970	1028.9521	2055.9130	1028.4601	I	2286.1361	**1143.5717**	2269.1095	1135.0584	2268.1255	1134.5664	21

19	2187.0076	1094.0075	2169.9811	1085.4942	2168.9971	1085.0022	L	2173.0520	**1087.0296**	2156.0254	**1078.5164**	2155.0414	**1078.0244**	20
20	2284.0604	1142.5338	2267.0339	1134.0206	2266.0498	1133.5286	P	2059.9679	**1030.4876**	2042.9414	1021.9743	2041.9574	**1021.4823**	19
21	2341.0819	1171.0446	2324.0553	1162.5313	2323.0713	1162.0393	G	**1962.9152**	**981.9612**	1945.8886	973.4479	1944.9046	972.9559	18
22	2456.1088	1228.5580	2439.0823	1220.0448	2438.0982	1219.5528	D	1905.8937	**953.4505**	1888.8672	944.9372	1887.8831	**944.4452**	17
23	2553.1616	1277.0844	2536.1350	1268.5711	2535.1510	1268.0791	P	**1790.8668**	**895.9370**	1773.8402	**887.4237**	1772.8562	**886.9317**	16
24	2700.2300	1350.6186	2683.2034	1342.1054	2682.2194	1341.6133	F	**1693.8140**	847.4106	**1676.7874**	838.8974	1675.8034	838.4054	15
25	2815.2569	1408.1321	2798.2304	1399.6188	2797.2464	**1399.1268**	D	**1546.7456**	**773.8764**	1529.7190	765.3632	1528.7350	764.8711	14
26	2930.2839	1465.6456	2913.2573	1457.1323	2912.2733	1456.6403	N	**1431.7186**	**716.3630**	1414.6921	**707.8497**	1413.7081	**707.3577**	13
27	3017.3159	**1509.1616**	3000.2893	**1500.6483**	2999.3053	**1500.1563**	S	1316.6917	**658.8495**	**1299.6652**	650.3362	1298.6811	649.8442	12
28	3104.3479	1552.6776	3087.3214	1544.1643	3086.3374	1543.6723	S	1229.6597	615.3335	1212.6331	606.8202	1211.6491	606.3282	11
29	3260.4490	**1630.7282**	3243.4225	1622.2149	3242.4385	1621.7229	R	1142.6276	571.8175	1125.6011	563.3042	1124.6171	**562.8122**	10
30	3357.5018	1679.2545	3340.4752	1670.7413	3339.4912	1670.2493	P	**986.5265**	**493.7669**	969.5000	485.2536	968.5160	484.7616	9
31	3444.5338	1722.7705	3427.5073	1714.2573	3426.5233	1713.7653	S	889.4738	445.2405	872.4472	436.7272	871.4632	436.2352	8
32	3572.5924	1786.7998	3555.5659	1778.2866	3554.5818	1777.7946	Q	802.4417	401.7245	785.4152	393.2112	784.4312	392.7192	7
33	3671.6608	1836.3340	3654.6343	1827.8208	3653.6503	1827.3288	V	**674.3832**	337.6952	657.3566	329.1819	656.3726	328.6899	6
34	3770.7292	**1885.8683**	3753.7027	1877.3550	3752.7187	1876.8630	V	575.3148	288.1610	558.2882	279.6477	557.3042	279.1557	5
35	3841.7663	1921.3868	3824.7398	1912.8735	3823.7558	**1912.3815**	A	476.2463	238.6268	459.2198	230.1135	458.2358	229.6215	4
36	3970.8089	**1985.9081**	3953.7824	1977.3948	3952.7984	1976.9028	E	405.2092	203.1082	388.1827	194.5950	387.1987	194.1030	3
37	4071.8566	2036.4319	4054.8301	2027.9187	4053.8461	2027.4267	T	276.1666	138.5870	259.1401	130.0737	258.1561	129.5817	2
38							R	175.1190	88.0631	158.0924	79.5498			1

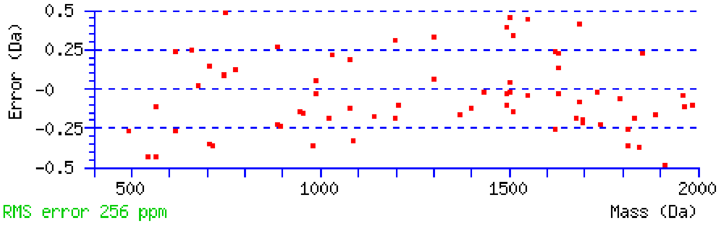

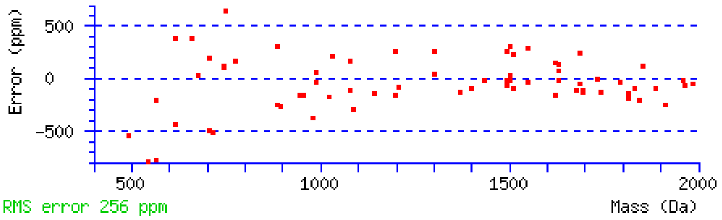

All matches to this query

Score	Mr(calc):	Delta	Sequence
66.6	4244.9611	0.0204	SNTGGQAFPQCVFDHWQILPGDPFDNSSRPSQVVAETR
60.1	4244.9611	0.0204	SNTGGQAFPQCVFDHWQILPGDPFDNSSRPSQVVAETR
46.8	4243.9771	1.0044	SNTGGQAFPQCVFDHWQILPGDPFDNSSRPSQVVAETR
6.5	4242.9810	2.0004	RGDFTEDTTHNRPVYTTSQVGGLITYVLREILEMR
6.3	4242.9810	2.0004	RGDFTEDTTHNRPVYTTSQVGGLITYVLREILEMR
6.3	4242.9810	2.0004	RGDFTEDTTHNRPVYTTSQVGGLITYVLREILEMR
6.3	4242.9810	2.0004	RGDFTEDTTHNRPVYTTSQVGGLITYVLREILEMR
5.6	4243.9929	0.9885	MFTGQPGHQELSGPPHLPHLPNPSPGQGLQHLSPNRPK
4.7	4243.9488	1.0326	TFLNDAYNWEFLIWETALLLFLLRLASLGSETNK
4.7	4243.9488	1.0326	TFLNDAYNWEFLIWETALLLFLLRLASLGSETNK

Spectrum No: 258; Query: 836; Rank: 1

Peptide View

MS/MS Fragmentation of **VCDGVPDCPEGDDENNVTSGR**
Found in **IPI00205325**, Tax_Id=10116 Gene_Symbol=Lrp2 Low-density lipoprotein receptor-related protein 2 precursor

Match to Query 836: 2291.895008 from(1146.954780,2+)
Title: 091127RatKid_SCX01_12.1449.1449.2.dta

Data file K:\NewmanPaper\Piliang\3SubProteomes\Piliang3SP\mgf5ppm\SCX_3SubProteomes5ppm.mgf

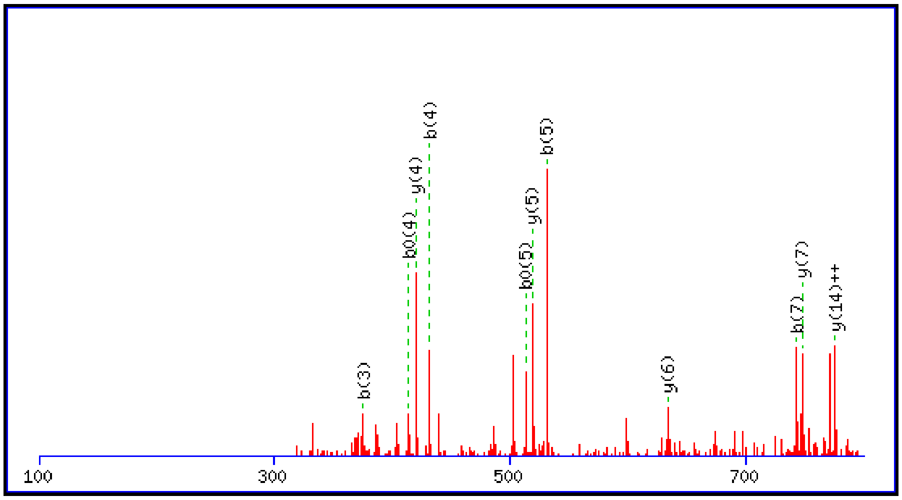

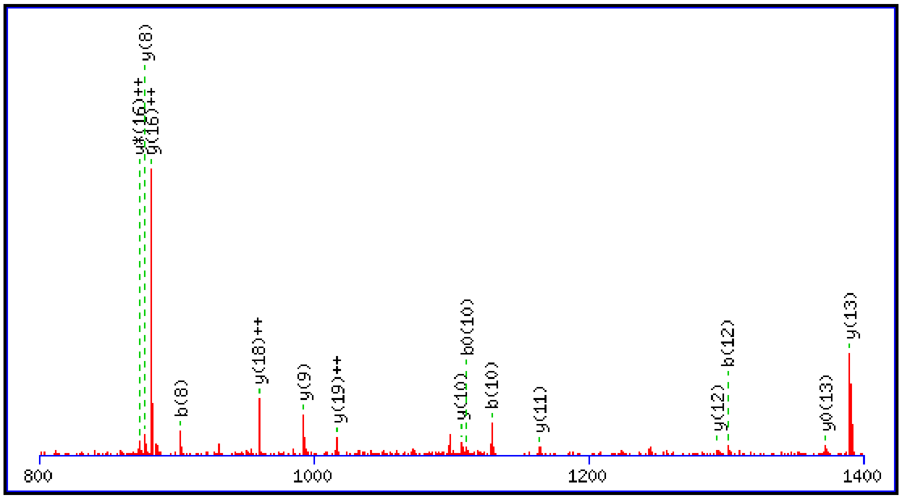

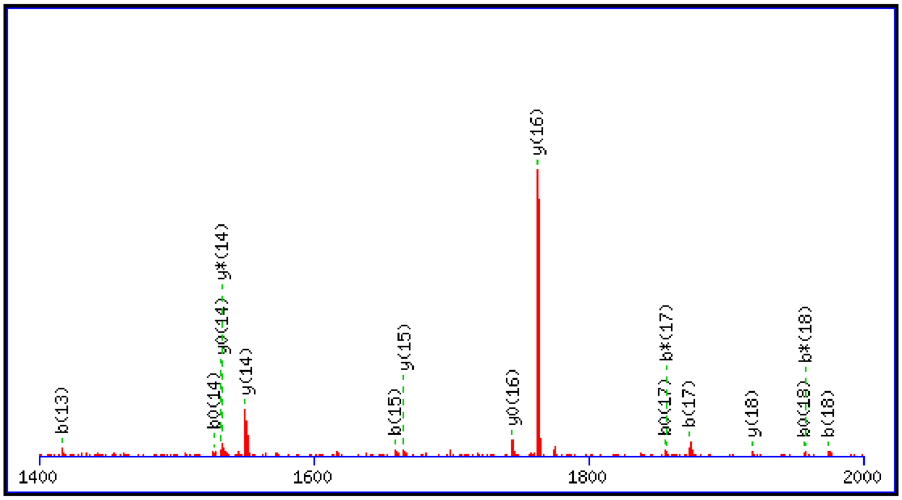

Monoisotopic mass of neutral peptide Mr(calc): 2291.8907
Fixed modifications: Carbamidomethyl (C)
Variable modifications:
N16 : Deamidated_N (N)
Ions Score: 66 **Expect:** 4.1e-005
Matches (Bold Red): 42/204 fragment ions using 93 most intense peaks

#	b	b^{++}	b*	b^{*++}	b^0	b^{0++}	Seq.	y	y^{++}	y*	y^{*++}	y^0	y^{0++}	#
1	100.0757	50.5415					V							21
2	260.1063	130.5568					C	2193.8295	1097.4184	2176.8030	1088.9051	2175.8190	1088.4131	20
3	**375.1333**	188.0703			357.1227	179.0650	D	2033.7989	**1017.4031**	2016.7723	1008.8898	2015.7883	1008.3978	19
4	**432.1547**	216.5810			**414.1442**	207.5757	G	**1918.7719**	**959.8896**	1901.7454	951.3763	1900.7614	950.8843	18

5	**531.2232**	266.1152			**513.2126**	257.1099	V	1861.7505	931.3789	1844.7239	922.8656	1843.7399	922.3736	17
6	628.2759	314.6416			610.2654	305.6363	P	**1762.6821**	**881.8447**	1745.6555	**873.3314**	**1744.6715**	872.8394	16
7	**743.3029**	372.1551			725.2923	363.1498	D	**1665.6293**	833.3183	1648.6027	824.8050	1647.6187	824.3130	15
8	**903.3335**	452.1704			885.3230	443.1651	C	**1550.6023**	**775.8048**	**1533.5758**	767.2915	**1532.5918**	766.7995	14
9	1000.3863	500.6968			982.3757	491.6915	P	**1390.5717**	695.7895	1373.5452	687.2762	**1372.5611**	686.7842	13
10	**1129.4289**	565.2181			**1111.4183**	556.2128	E	**1293.5189**	647.2631	1276.4924	638.7498	1275.5084	638.2578	12
11	1186.4503	593.7288			1168.4398	584.7235	G	**1164.4763**	582.7418	1147.4498	574.2285	1146.4658	573.7365	11
12	**1301.4773**	651.2423			1283.4667	642.2370	D	**1107.4549**	554.2311	1090.4283	545.7178	1089.4443	545.2258	10
13	**1416.5042**	708.7557			1398.4937	699.7505	D	**992.4279**	496.7176	975.4014	488.2043	974.4174	487.7123	9
14	1545.5468	773.2770			**1527.5363**	764.2718	E	**877.4010**	439.2041	860.3744	430.6909	859.3904	430.1989	8
15	**1659.5897**	830.2985	1642.5632	821.7852	1641.5792	821.2932	N	**748.3584**	374.6828	731.3319	366.1696	730.3478	365.6776	7
16	1774.6167	887.8120	1757.5901	879.2987	1756.6061	878.8067	N	**634.3155**	317.6614	617.2889	309.1481	616.3049	308.6561	6
17	**1873.6851**	937.3462	**1856.6585**	928.8329	**1855.6745**	928.3409	V	**519.2885**	260.1479	502.2620	251.6346	501.2780	251.1426	5
18	**1974.7328**	987.8700	**1957.7062**	979.3567	**1956.7222**	978.8647	T	**420.2201**	210.6137	403.1936	202.1004	402.2096	201.6084	4
19	2061.7648	1031.3860	2044.7383	1022.8728	2043.7542	1022.3808	S	319.1724	160.0899	302.1459	151.5766	301.1619	151.0846	3
20	2118.7863	1059.8968	2101.7597	1051.3835	2100.7757	1050.8915	G	232.1404	116.5738	215.1139	108.0606			2
21							R	175.1190	88.0631	158.0924	79.5498			1

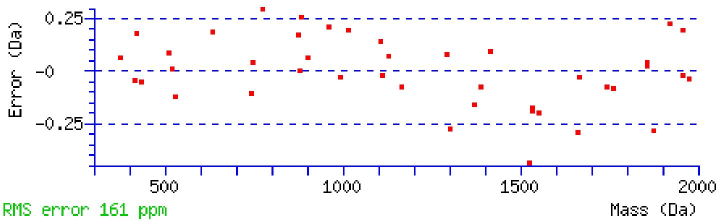

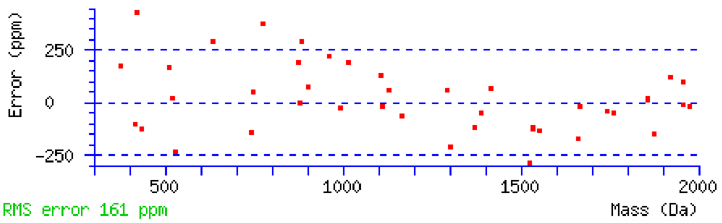

All matches to this query

Score	Mr(calc):	Delta	Sequence
66.5	2291.8907	0.0043	VCDGVPDCPEGDDENNVTSGR
64.6	2291.8907	0.0043	VCDGVPDCPEGDDENNVTSGR
13.1	2290.9067	0.9883	VCDGVPDCPEGDDENNVTSGR
4.7	2291.8955	-0.0005	MYGKTLSSMIMGDTSGYYK
4.3	2291.8955	-0.0005	MYGKTLSSMIMGDTSGYYK
4.2	2290.9123	0.9827	WQMMKQXDQELNNPTSDK
4.2	2290.9123	0.9827	WQMMKQXDQELNNPTSDK
4.2	2290.9123	0.9827	WQMMKQXDQELNNPTSDK
4.2	2290.9123	0.9827	WQMMKQXDQELNNPTSDK
1.4	2291.9074	-0.0124	LSGNGTDSXNGRNGGSTAGRER

Spectrum No: 259; Query: 405; Rank: 1

Peptide View

MS/MS Fragmentation of **WYSAGLASNSSWFR**
Found in **IPI00203102**, Tax_Id=10116 Gene_Symbol=Ptgds Prostaglandin-H2 D-isomerase precursor

Match to Query 405: 1631.740248 from(816.877400,2+)
Title: 091127RatKid_SCX01_13.4075.4075.2.dta
Data file K:\NewmanPaper\Piliang\3SubProteomes\Piliang3SP\mgf5ppm\SCX_3SubProteomes5ppm.mgf

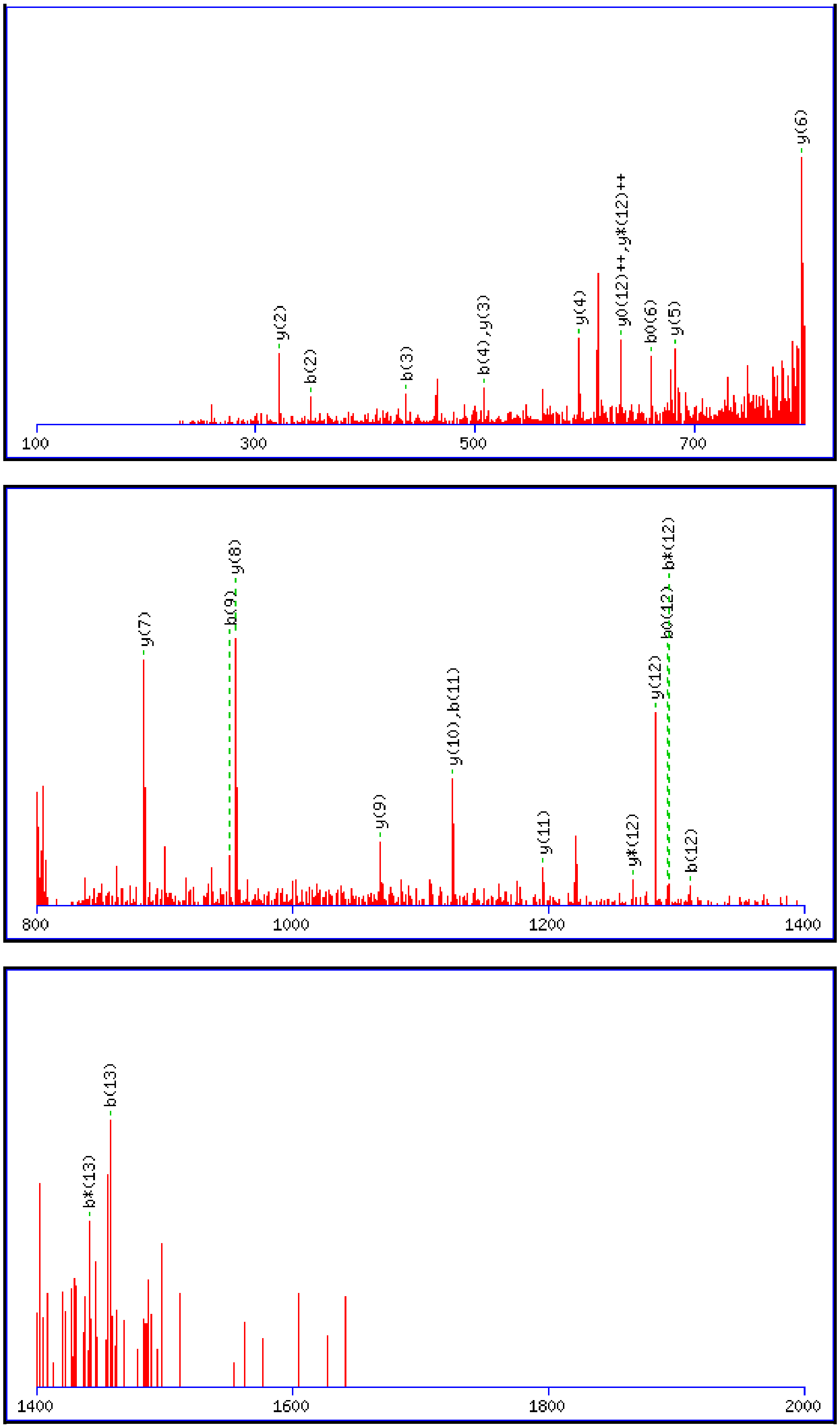

Monoisotopic mass of neutral peptide Mr(calc): 1631.7368
Fixed modifications: Carbamidomethyl (C)
Variable modifications:
N9 : Deamidated_N (N)
Ions Score: 66 **Expect:** 4.2e-005
Matches (Bold Red): 25/130 fragment ions using 59 most intense peaks

#	b	b^{++}	b*	b^{*++}	b^0	b^{0++}	Seq.	y	y^{++}	y*	y^{*++}	y^0	y^{0++}	#
1	187.0866	94.0469					W							14
2	**350.1499**	175.5786					Y	1446.6648	723.8360	1429.6383	715.3228	1428.6542	714.8308	13
3	**437.1819**	219.0946			419.1714	210.0893	S	**1283.6015**	642.3044	**1266.5749**	**633.7911**	1265.5909	**633.2991**	12
4	**508.2191**	254.6132			490.2085	245.6079	A	**1196.5695**	598.7884	1179.5429	590.2751	1178.5589	589.7831	11
5	565.2405	283.1239			547.2300	274.1186	G	**1125.5323**	563.2698	1108.5058	554.7565	1107.5218	554.2645	10

**6**	678.3246	339.6659			**660.3140**	330.6606	**L**	**1068.5109**	534.7591	1051.4843	526.2458	1050.5003	525.7538	**9**
**7**	749.3617	375.1845			731.3511	366.1792	**A**	**955.4268**	478.2170	938.4003	469.7038	937.4162	469.2118	**8**
**8**	836.3937	418.7005			818.3832	409.6952	**S**	**884.3897**	442.6985	867.3632	434.1852	866.3791	433.6932	**7**
**9**	**951.4207**	476.2140	934.3941	467.7007	933.4101	467.2087	**N**	**797.3577**	399.1825	780.3311	390.6692	779.3471	390.1772	**6**
**10**	1038.4527	519.7300	1021.4261	511.2167	1020.4421	510.7247	**S**	**682.3307**	341.6690	665.3042	333.1557	664.3202	332.6637	**5**
**11**	**1125.4847**	563.2460	1108.4582	554.7327	1107.4742	554.2407	**S**	**595.2987**	298.1530	578.2722	289.6397	577.2881	289.1477	**4**
**12**	**1311.5640**	656.2857	**1294.5375**	647.7724	**1293.5535**	647.2804	**W**	**508.2667**	254.6370	491.2401	246.1237			**3**
**13**	**1458.6324**	729.8199	**1441.6059**	721.3066	1440.6219	720.8146	**F**	**322.1874**	161.5973	305.1608	153.0840			**2**
**14**							**R**	175.1190	88.0631	158.0924	79.5498			**1**

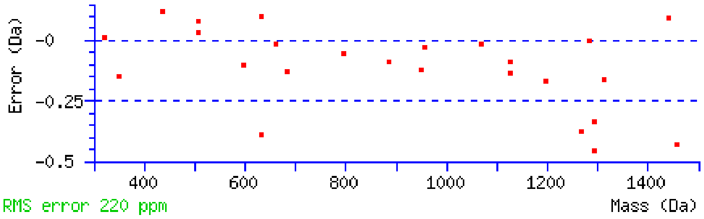

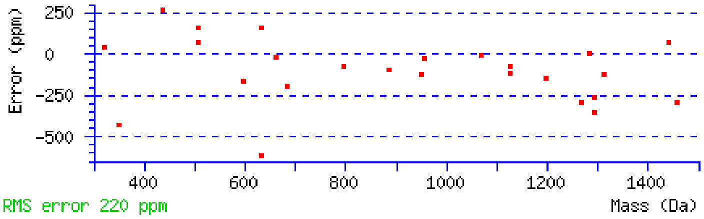

All matches to this query

Score	Mr(calc):	Delta	Sequence
66.4	1631.7368	0.0034	WYSAGLASNSSWFR
4.9	1630.7528	0.9874	WYSAGLASNSSWFR

Spectrum No: 260; Query: 277; Rank: 1

Peptide View

MS/MS Fragmentation of **EVMSLLQPLNITK**
Found in **IPI00388265**, Tax_Id=10116 Gene_Symbol=Pgcp 48 kDa protein

Match to Query 277: 1501.806688 from(751.910620,2+)
Title: 091129RatKid_SCX02_16.2959.2959.2.dta
Data file K:\NewmanPaper\Piliang\3SubProteomes\Piliang3SP\mgf5ppm\SCX_3SubProteomes5ppm.mgf

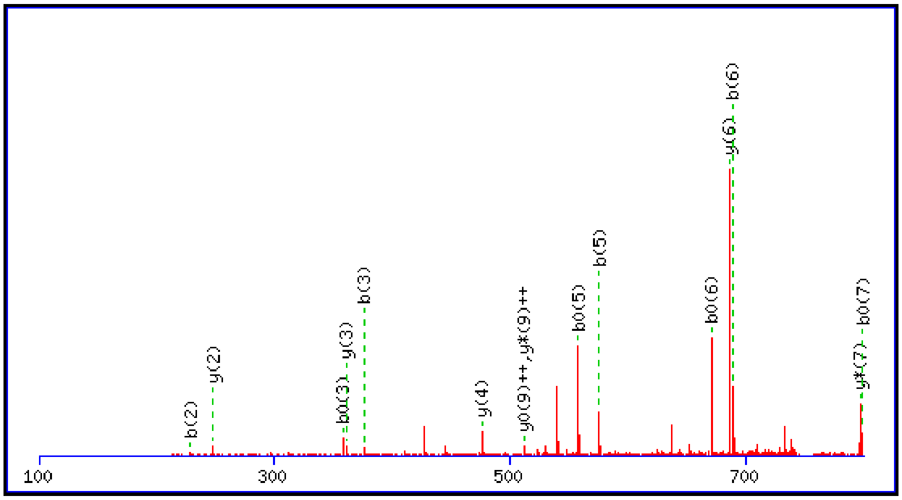

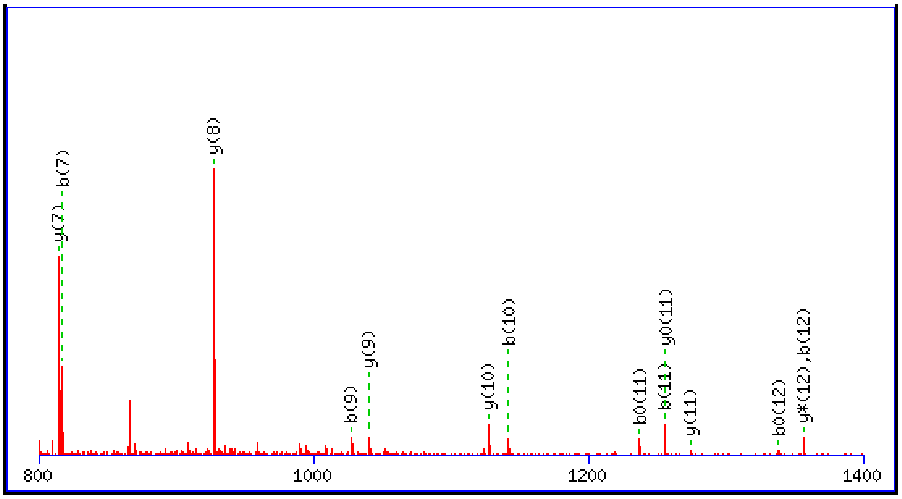

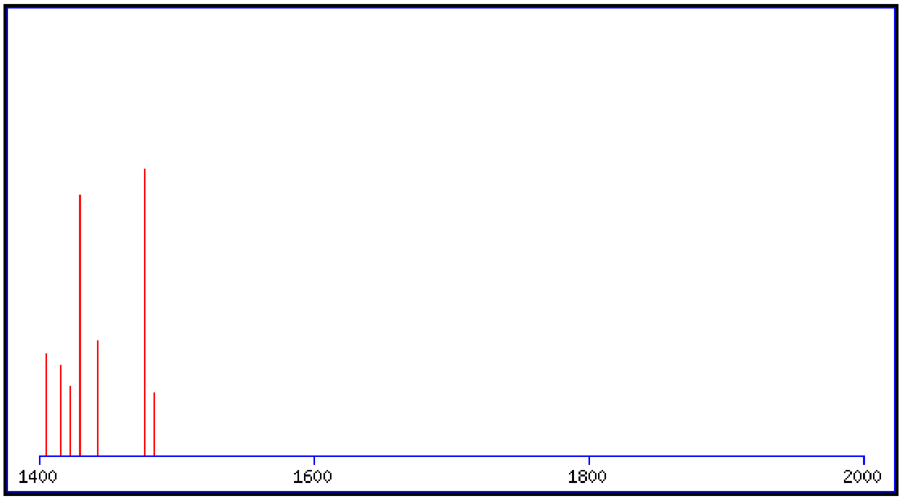

Monoisotopic mass of neutral peptide Mr(calc): 1501.8062
Fixed modifications: Carbamidomethyl (C)
Variable modifications:
M3 : Oxidation (M)
N10 : Deamidated_N (N)
Ions Score: 66 **Expect:** 2.1e-005
Matches (Bold Red): 29/130 fragment ions using 43 most intense peaks

#	b	b^{++}	b*	b^{*++}	b^0	b^{0++}	Seq.	y	y^{++}	y*	y^{*++}	y^0	y^{0++}	#
1	130.0499	65.5286			112.0393	56.5233	E							13
2	**229.1183**	115.0628			211.1077	106.0575	V	1373.7709	687.3891	**1356.7443**	678.8758	1355.7603	678.3838	12
3	**376.1537**	188.5805			**358.1431**	179.5752	M	**1274.7024**	637.8549	1257.6759	629.3416	**1256.6919**	628.8496	11
4	463.1857	232.0965			445.1752	223.0912	S	**1127.6670**	564.3372	1110.6405	555.8239	1109.6565	555.3319	10
5	**576.2698**	288.6385			**558.2592**	279.6332	L	**1040.6350**	520.8211	1023.6085	**512.3079**	1022.6245	**511.8159**	9
6	**689.3538**	345.1806			**671.3433**	336.1753	L	**927.5510**	464.2791	910.5244	455.7658	909.5404	455.2738	8
7	**817.4124**	409.2098	800.3859	400.6966	**799.4019**	400.2046	Q	**814.4669**	407.7371	**797.4403**	399.2238	796.4563	398.7318	7
8	914.4652	457.7362	897.4386	449.2230	896.4546	448.7309	P	**686.4083**	343.7078	669.3818	335.1945	668.3977	334.7025	6
9	**1027.5492**	514.2783	1010.5227	505.7650	1009.5387	505.2730	L	589.3555	295.1814	572.3290	286.6681	571.3450	286.1761	5
10	**1142.5762**	571.7917	1125.5496	563.2785	1124.5656	562.7864	N	**476.2715**	238.6394	459.2449	230.1261	458.2609	229.6341	4
11	**1255.6602**	628.3338	1238.6337	619.8205	**1237.6497**	619.3285	I	**361.2445**	181.1259	344.2180	172.6126	343.2340	172.1206	3
12	**1356.7079**	678.8576	1339.6814	670.3443	**1338.6974**	669.8523	T	**248.1605**	124.5839	231.1339	116.0706	230.1499	115.5786	2
13							K	147.1128	74.0600	130.0863	65.5468			1

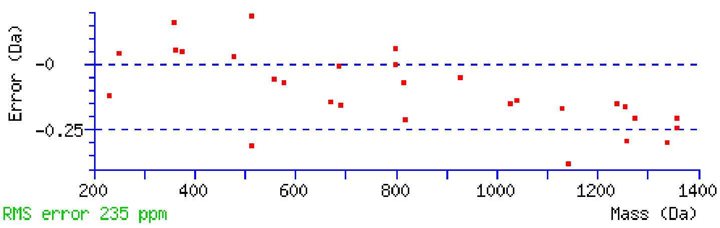

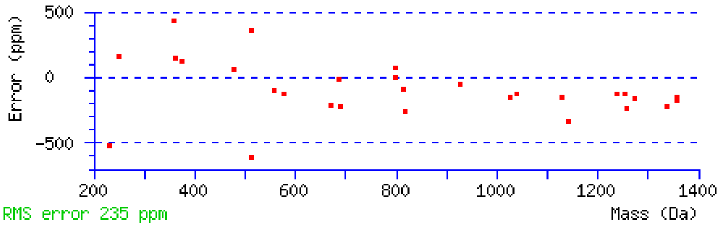

All matches to this query

Score	Mr(calc):	Delta	Sequence
66.2	1501.8062	0.0005	EVMSLLQPLNITK
10.2	1501.8174	-0.0107	MPILSKLSQDINK
8.1	1501.7988	0.0079	LKIDTDKENAEVK
4.6	1501.8174	-0.0107	VEMALEALRNVIK
3.9	1499.7943	2.0123	QESEPDKLTIGKR
3.4	1501.7987	0.0080	ELKSLDQAINDKK
2.7	1500.8082	0.9985	KCSPVVKELQASR
2.6	1501.8035	0.0032	RQMLERIQNSVK
1.8	1501.7923	0.0144	SLPDVRLEGCKTK
1.1	1500.7896	1.0171	ELSVEEQIKRNR

Spectrum No: 261; Query: 863; Rank: 1

Peptide View

MS/MS Fragmentation of **LDNASAFQGAVISPHYDSLLVK**
Found in **IPI00210435**, Tax_Id=10116 Gene_Symbol=Pc Pyruvate carboxylase, mitochondrial precursor

Match to Query 863: 2345.193312 from(782.738380,3+)
Title: 091129RatKid_SCX02_24.3961.3961.3.dta
Data file K:\NewmanPaper\Piliang\3SubProteomes\Piliang3SP\mgf5ppm\SCX_3SubProteomes5ppm.mgf

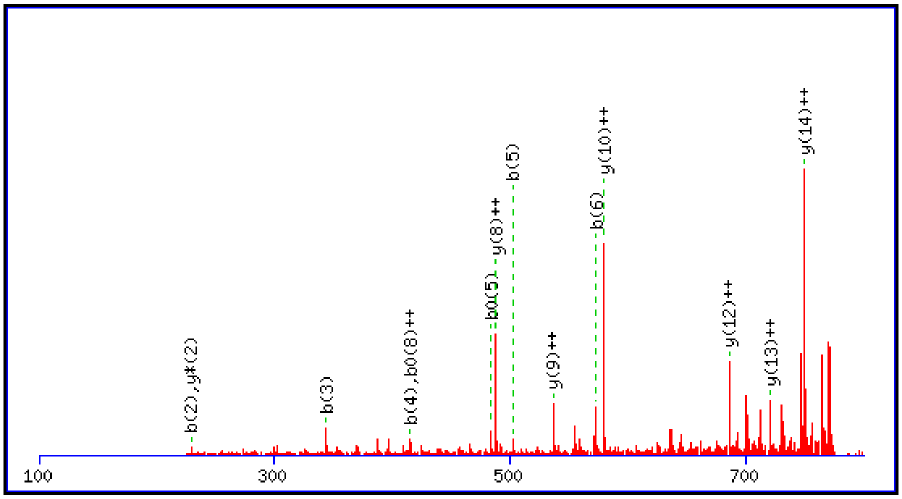

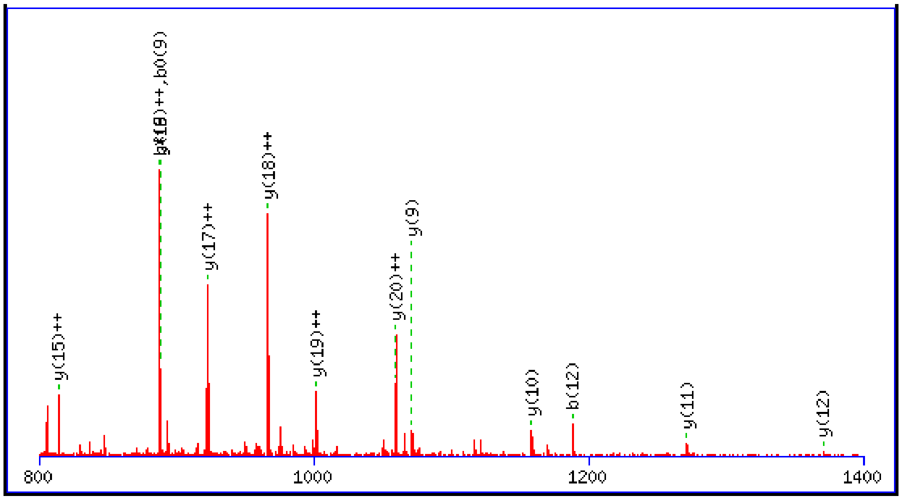

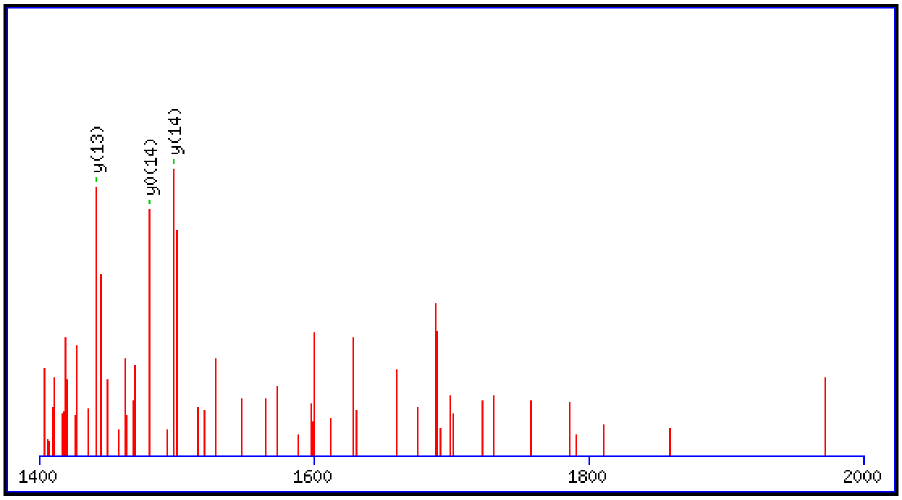

Monoisotopic mass of neutral peptide Mr(calc): 2345.1903
Fixed modifications: Carbamidomethyl (C)
Variable modifications:
N3 : Deamidated_N (N)
Ions Score: 66 **Expect:** 3.7e-005
Matches (Bold Red): 30/238 fragment ions using 57 most intense peaks

#	b	b^{++}	b*	b^{*++}	b^{0}	b^{0++}	Seq.	y	y^{++}	y*	y^{*++}	y^{0}	y^{0++}	#
1	114.0913	57.5493					L							22
2	**229.1183**	115.0628			211.1077	106.0575	D	2233.1135	1117.0604	2216.0870	1108.5471	2215.1030	1108.0551	21
3	**344.1452**	172.5762	327.1187	164.0630	326.1347	163.5710	N	2118.0866	**1059.5469**	2101.0600	1051.0337	2100.0760	1050.5417	20
4	**415.1823**	208.0948	398.1558	199.5815	397.1718	199.0895	A	2003.0597	**1002.0335**	1986.0331	993.5202	1985.0491	993.0282	19
5	**502.2144**	251.6108	485.1878	243.0975	**484.2038**	242.6055	S	1932.0225	**966.5149**	1914.9960	958.0016	1914.0120	957.5096	18
6	**573.2515**	287.1294	556.2249	278.6161	555.2409	278.1241	A	1844.9905	**922.9989**	1827.9640	914.4856	1826.9799	913.9936	17
7	720.3199	360.6636	703.2933	352.1503	702.3093	351.6583	F	1773.9534	**887.4803**	1756.9268	878.9671	1755.9428	878.4751	16
8	848.3785	424.6929	831.3519	416.1796	830.3679	**415.6876**	Q	1626.8850	**813.9461**	1609.8584	805.4329	1608.8744	804.9408	15
9	905.3999	453.2036	**888.3734**	444.6903	**887.3894**	444.1983	G	**1498.8264**	**749.9168**	1481.7999	741.4036	**1480.8158**	740.9116	14
10	976.4370	488.7222	959.4105	480.2089	958.4265	479.7169	A	**1441.8049**	**721.4061**	1424.7784	712.8928	1423.7944	712.4008	13
11	1075.5055	538.2564	1058.4789	529.7431	1057.4949	529.2511	V	**1370.7678**	**685.8876**	1353.7413	677.3743	1352.7573	676.8823	12
12	**1188.5895**	594.7984	1171.5630	586.2851	1170.5790	585.7931	I	**1271.6994**	636.3533	1254.6729	627.8401	1253.6888	627.3481	11
13	1275.6216	638.3144	1258.5950	629.8011	1257.6110	629.3091	S	**1158.6154**	**579.8113**	1141.5888	571.2980	1140.6048	570.8060	10
14	1372.6743	686.8408	1355.6478	678.3275	1354.6637	677.8355	P	**1071.5833**	**536.2953**	1054.5568	527.7820	1053.5728	527.2900	9
15	1509.7332	755.3703	1492.7067	746.8570	1491.7227	746.3650	H	974.5306	**487.7689**	957.5040	479.2556	956.5200	478.7636	8
16	1672.7966	836.9019	1655.7700	828.3886	1654.7860	827.8966	Y	837.4716	419.2395	820.4451	410.7262	819.4611	410.2342	7
17	1787.8235	894.4154	1770.7970	885.9021	1769.8129	885.4101	D	674.4083	337.7078	657.3818	329.1945	656.3978	328.7025	6
18	1874.8555	937.9314	1857.8290	929.4181	1856.8450	928.9261	S	559.3814	280.1943	542.3548	271.6811	541.3708	271.1890	5

**19**	1987.9396	994.4734	1970.9130	985.9602	1969.9290	985.4682	**L**	472.3493	236.6783	455.3228	228.1650			**4**
**20**	2101.0237	1051.0155	2083.9971	1042.5022	2083.0131	1042.0102	**L**	359.2653	180.1363	342.2387	171.6230			**3**
**21**	2200.0921	1100.5497	2183.0655	1092.0364	2182.0815	1091.5444	**V**	246.1812	123.5942	**229.1547**	115.0810			**2**
**22**							**K**	147.1128	74.0600	130.0863	65.5468			**1**

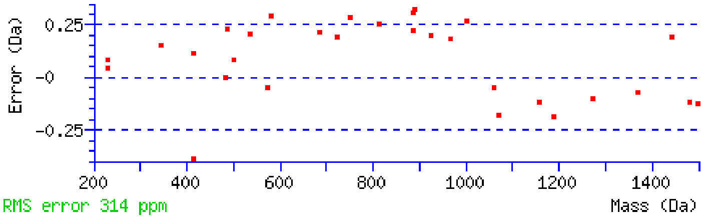

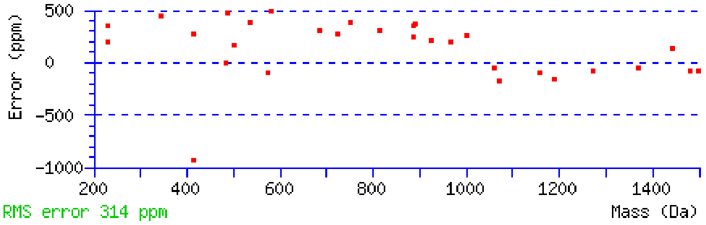

All matches to this query

Score	Mr(calc):	Delta	Sequence
66.2	2345.1903	0.0030	LDNASAFQGAVISPHYDSLLVK
60.9	2344.2063	0.9870	LDNASAFQGAVISPHYDSLLVK
7.0	2344.2014	0.9919	RVIISAPSADVPIFLMGVNHK
5.7	2344.2014	0.9919	RVIISAPSADVPIFLMGVNHK
3.8	2343.2008	1.9925	KVSSKEEISLSTMELSGIVVK
2.9	2343.2008	1.9925	KVSSKEEISLSTMELSGIVVK
1.2	2343.1715	2.0218	SSQTMVLRPANSPSHVFLPMK
1.0	2345.1896	0.0037	NLNGVPMVTKSIKTANQQQQK
0.9	2343.2008	1.9925	KVSSKEEISLSTMELSGIVVK
0.5	2344.1725	1.0209	NIVSGVLFGTGLWVAVIMTMR

Spectrum No: 262; Query: 366; Rank: 1

Peptide View

MS/MS Fragmentation of **HQAFEAELSANQSR**
Found in **IPI00209258**, Tax_Id=10116 Gene_Symbol=Spna2 Spectrin alpha chain, brain

Match to Query 366: 1587.728568 from(794.871560,2+)
Title: 091129RatKid_SCX02_35.1186.1186.2.dta
Data file K:\NewmanPaper\Piliang\3SubProteomes\Piliang3SP\mgf5ppm\SCX_3SubProteomes5ppm.mgf

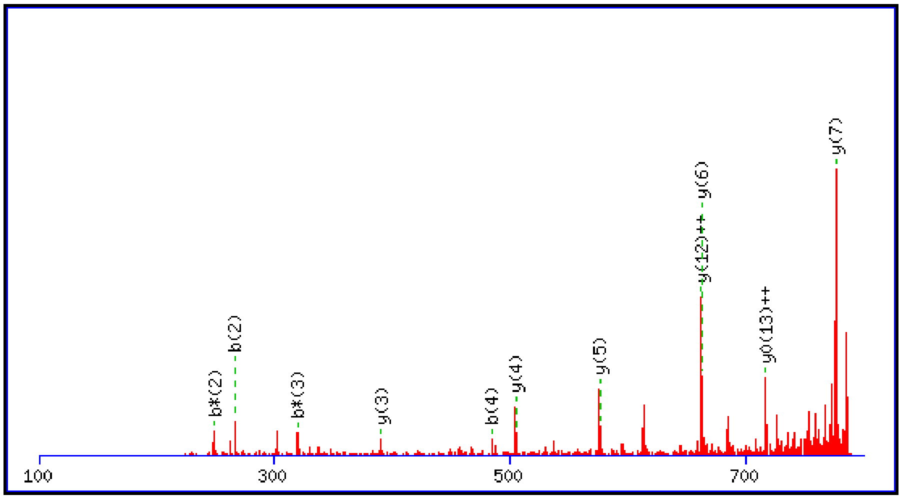

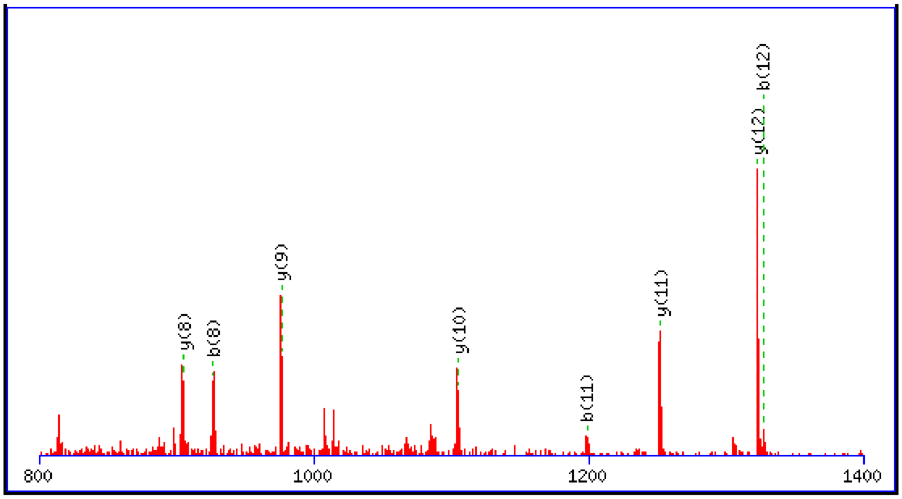

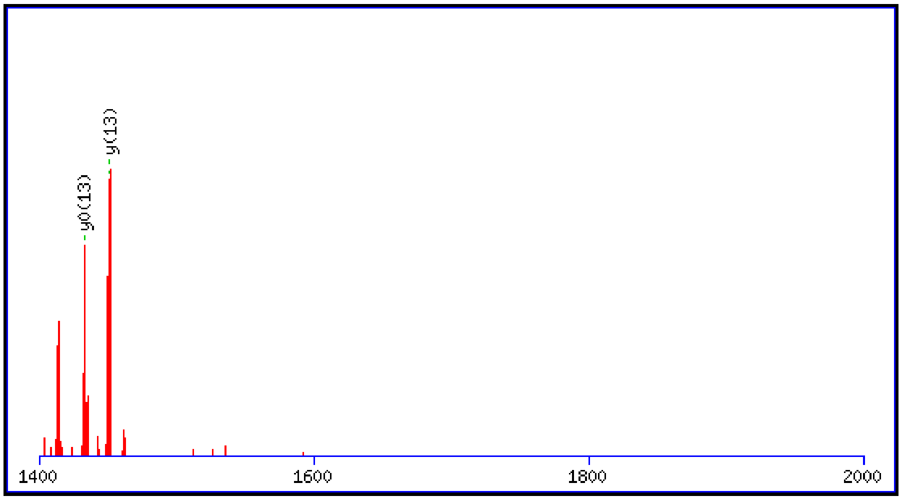

Monoisotopic mass of neutral peptide Mr(calc): 1587.7277
Fixed modifications: Carbamidomethyl (C)
Variable modifications:
N11 : Deamidated_N (N)
Ions Score: 66 **Expect:** 4.6e-005
Matches (Bold Red): 21/144 fragment ions using 58 most intense peaks

#	b	b^{++}	b*	b^{*++}	b^0	b^{0++}	Seq.	y	y^{++}	y*	y^{*++}	y^0	y^{0++}	#
1	138.0662	69.5367					H							14
2	**266.1248**	133.5660	**249.0982**	125.0527			Q	**1451.6761**	726.3417	1434.6496	717.8284	**1433.6655**	**717.3364**	13
3	337.1619	169.0846	**320.1353**	160.5713			A	**1323.6175**	**662.3124**	1306.5910	653.7991	1305.6070	653.3071	12
4	**484.2303**	242.6188	467.2037	234.1055			F	**1252.5804**	626.7938	1235.5539	618.2806	1234.5698	617.7886	11
5	613.2729	307.1401	596.2463	298.6268	595.2623	298.1348	E	**1105.5120**	553.2596	1088.4855	544.7464	1087.5014	544.2544	10
6	684.3100	342.6586	667.2835	334.1454	666.2994	333.6534	A	**976.4694**	488.7383	959.4429	480.2251	958.4588	479.7331	9
7	813.3526	407.1799	796.3260	398.6667	795.3420	398.1747	E	**905.4323**	453.2198	888.4057	444.7065	887.4217	444.2145	8
8	**926.4367**	463.7220	909.4101	455.2087	908.4261	454.7167	L	**776.3897**	388.6985	759.3632	380.1852	758.3791	379.6932	7
9	1013.4687	507.2380	996.4421	498.7247	995.4581	498.2327	S	**663.3056**	332.1565	646.2791	323.6432	645.2951	323.1512	6
10	1084.5058	542.7565	1067.4793	534.2433	1066.4952	533.7513	A	**576.2736**	288.6404	559.2471	280.1272	558.2630	279.6352	5
11	**1199.5327**	600.2700	1182.5062	591.7567	1181.5222	591.2647	N	**505.2365**	253.1219	488.2099	244.6086	487.2259	244.1166	4
12	**1327.5913**	664.2993	1310.5648	655.7860	1309.5807	655.2940	Q	**390.2096**	195.6084	373.1830	187.0951	372.1990	186.6031	3
13	1414.6233	707.8153	1397.5968	699.3020	1396.6128	698.8100	S	262.1510	131.5791	245.1244	123.0659	244.1404	122.5738	2
14							R	175.1190	88.0631	158.0924	79.5498			1

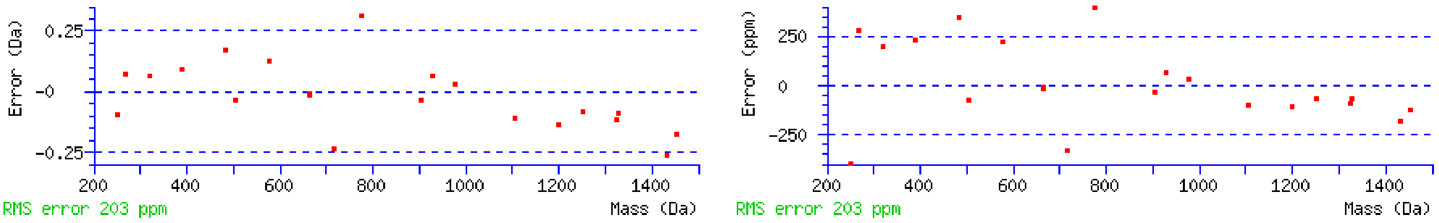

All matches to this query

Score	Mr(calc):	Delta	Sequence
66.1	1587.7277	0.0009	HQAFEAELSANQSR
2.7	1587.7423	-0.0138	MSERAADDVRGEPR

Spectrum No: 263; Query: 463; Rank: 1

Peptide View

MS/MS Fragmentation of **CDQCEENYFYNR**
Found in **IPI00363849**, Tax_Id=10116 Gene_Symbol=Lamc1 similar to Laminin gamma-1 chain precursor

Match to Query 463: 1697.610228 from(849.812390,2+)
Title: 091127RatKid_SCX01_13.1729.1729.2.dta
Data file K:\NewmanPaper\Piliang\3SubProteomes\Piliang3SP\mgf5ppm\SCX_3SubProteomes5ppm.mgf

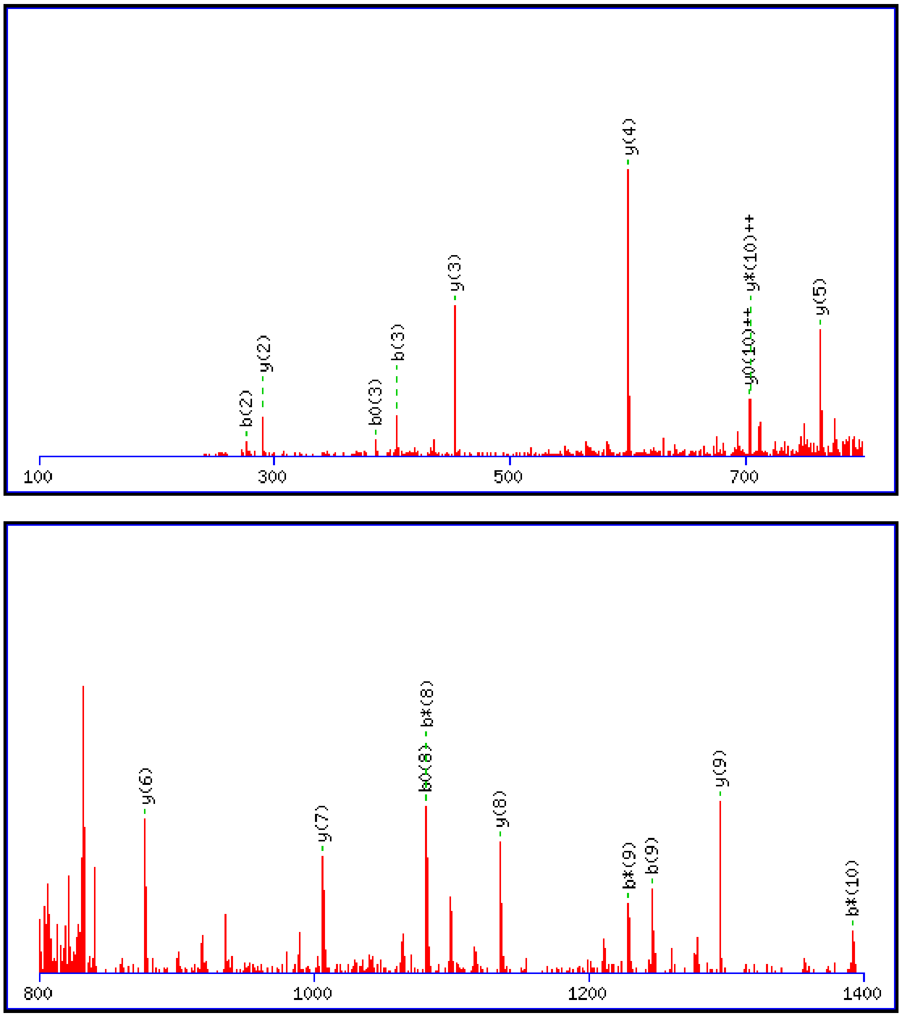

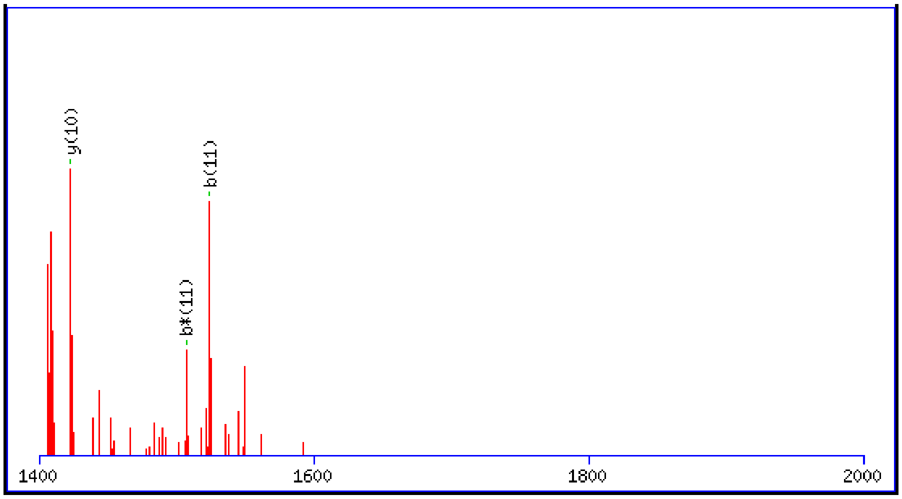

Monoisotopic mass of neutral peptide Mr(calc): 1697.6086
Fixed modifications: Carbamidomethyl (C)
Variable modifications:
N11 : Deamidated_N (N)
Ions Score: 66 **Expect:** 1.7e-005
Matches (Bold Red): 21/114 fragment ions using 32 most intense peaks

#	b	b++	b*	b*++	b0	b0++	Seq.	y	y++	y*	y*++	y0	y0++	#
1	161.0379	81.0226					C							12
2	**276.0649**	138.5361			258.0543	129.5308	D	1538.5852	769.7963	1521.5587	761.2830	1520.5747	760.7910	11
3	**404.1234**	202.5654	387.0969	194.0521	**386.1129**	193.5601	Q	**1423.5583**	712.2828	1406.5318	**703.7695**	1405.5477	**703.2775**	10
4	564.1541	282.5807	547.1275	274.0674	546.1435	273.5754	C	**1295.4997**	648.2535	1278.4732	639.7402	1277.4892	639.2482	9
5	693.1967	347.1020	676.1701	338.5887	675.1861	338.0967	E	**1135.4691**	568.2382	1118.4425	559.7249	1117.4585	559.2329	8
6	822.2393	411.6233	805.2127	403.1100	804.2287	402.6180	E	**1006.4265**	503.7169	989.3999	495.2036	988.4159	494.7116	7
7	936.2822	468.6447	919.2557	460.1315	918.2716	459.6395	N	**877.3839**	439.1956	860.3573	430.6823			6
8	1099.3455	550.1764	**1082.3190**	541.6631	**1081.3350**	541.1711	Y	**763.3410**	382.1741	746.3144	373.6608			5
9	**1246.4140**	623.7106	**1229.3874**	615.1973	1228.4034	614.7053	F	**600.2776**	300.6425	583.2511	292.1292			4
10	1409.4773	705.2423	**1392.4507**	696.7290	1391.4667	696.2370	Y	**453.2092**	227.1082	436.1827	218.5950			3
11	**1524.5042**	762.7557	**1507.4777**	754.2425	1506.4937	753.7505	N	**290.1459**	145.5766	273.1193	137.0633			2
12							R	175.1190	88.0631	158.0924	79.5498			1

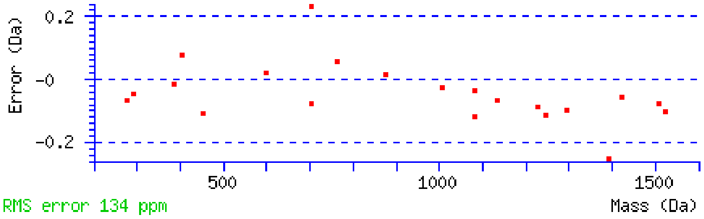

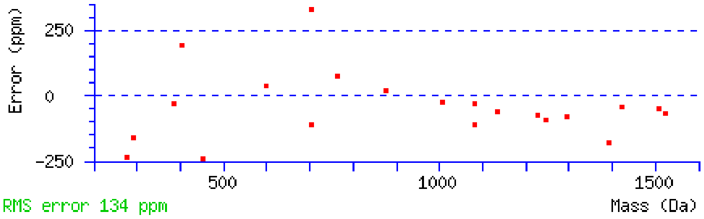

All matches to this query

Score	Mr(calc):	Delta	Sequence
66.0	1697.6086	0.0016	CDQCEENYFYNR
28.6	1697.6086	0.0016	CDQCEENYFYNR

Spectrum No: 264; Query: 1096; Rank: 1

Peptide View

MS/MS Fragmentation of **GVDVSQVAWQSQGDTPCSCCIVNNSNGSR**
Found in **IPI00211989**, Tax_Id=10116 Gene_Symbol=Khk Isoform C of Ketohexokinase

Match to Query 1096: 3183.335712 from(1062.119180,3+)
Title: 091127RatKid_SCX01_17.2799.2799.3.dta
Data file K:\NewmanPaper\Piliang\3SubProteomes\Piliang3SP\mgf5ppm\SCX_3SubProteomes5ppm.mgf

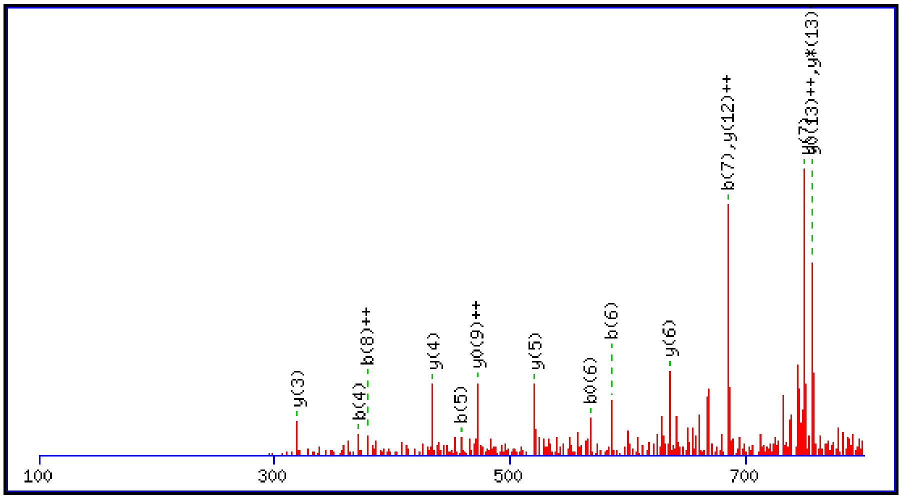

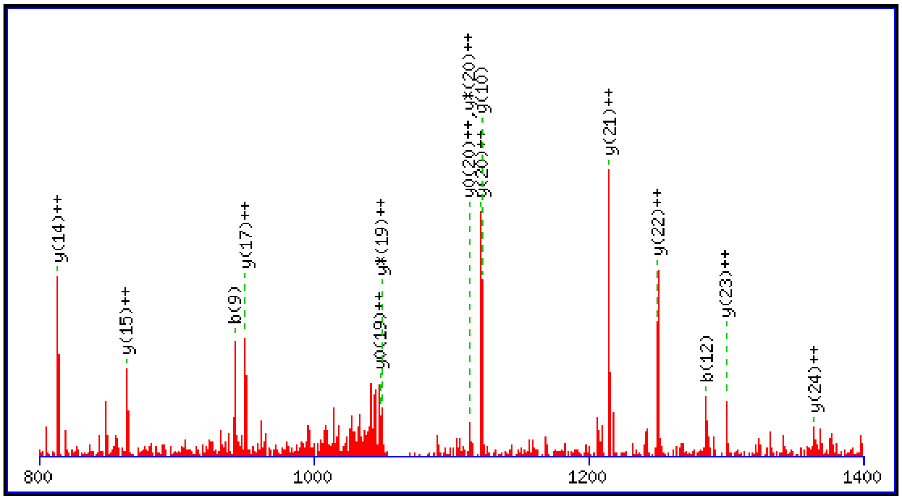

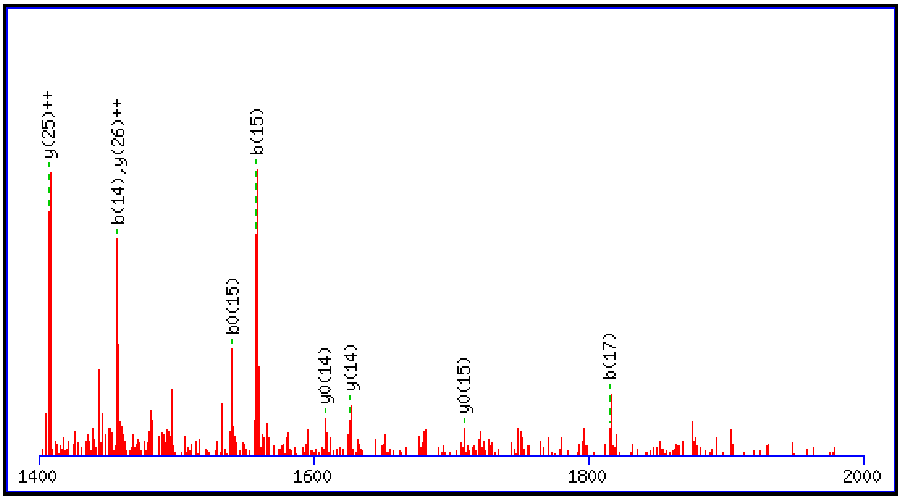

Monoisotopic mass of neutral peptide Mr(calc): 3183.3292
Fixed modifications: Carbamidomethyl (C)
Variable modifications:
N23 : Deamidated_N (N)
N26 : Deamidated_N (N)
Ions Score: 66 **Expect:** 0.00011
Matches (Bold Red): 40/320 fragment ions using 58 most intense peaks

#	b	b^{++}	b*	b^{*++}	b^0	b^{0++}	Seq.	y	y^{++}	y*	y^{*++}	y^0	y^{0++}	#
1	58.0287	29.5180					G							29

2	157.0972	79.0522					V	3127.3150	1564.1611	3110.2884	1555.6479	3109.3044	1555.1558	28
3	272.1241	136.5657			254.1135	127.5604	D	3028.2466	1514.6269	3011.2200	1506.1136	3010.2360	1505.6216	27
4	**371.1925**	186.0999			353.1819	177.0946	V	2913.2196	**1457.1134**	2896.1931	1448.6002	2895.2091	1448.1082	26
5	**458.2245**	229.6159			440.2140	220.6106	S	2814.1512	**1407.5792**	2797.1247	1399.0660	2796.1406	1398.5740	25
6	**586.2831**	293.6452	569.2566	285.1319	**568.2726**	284.6399	Q	2727.1192	**1364.0632**	2710.0926	1355.5500	2709.1086	1355.0579	24
7	**685.3515**	343.1794	668.3250	334.6661	667.3410	334.1741	V	2599.0606	**1300.0339**	2582.0340	1291.5207	2581.0500	1291.0287	23
8	**756.3886**	**378.6980**	739.3621	370.1847	738.3781	369.6927	A	2499.9922	**1250.4997**	2482.9656	1241.9865	2481.9816	1241.4944	22
9	**942.4680**	471.7376	925.4414	463.2243	924.4574	462.7323	W	2428.9551	**1214.9812**	2411.9285	1206.4679	2410.9445	1205.9759	21
10	1070.5265	535.7669	1053.5000	527.2536	1052.5160	526.7616	Q	2242.8758	**1121.9415**	2225.8492	**1113.4282**	2224.8652	**1112.9362**	20
11	1157.5586	579.2829	1140.5320	570.7696	1139.5480	570.2776	S	2114.8172	1057.9122	2097.7906	**1049.3990**	2096.8066	**1048.9069**	19
12	**1285.6171**	643.3122	1268.5906	634.7989	1267.6066	634.3069	Q	2027.7852	1014.3962	2010.7586	1005.8829	2009.7746	1005.3909	18
13	1342.6386	671.8229	1325.6121	663.3097	1324.6280	662.8177	G	1899.7266	**950.3669**	1882.7000	941.8537	1881.7160	941.3616	17
14	**1457.6655**	729.3364	1440.6390	720.8231	1439.6550	720.3311	D	1842.7051	921.8562	1825.6786	913.3429	1824.6945	912.8509	16
15	**1558.7132**	779.8603	1541.6867	771.3470	**1540.7027**	770.8550	T	1727.6782	**864.3427**	1710.6516	855.8294	**1709.6676**	855.3374	15
16	1655.7660	828.3866	1638.7394	819.8734	1637.7554	819.3814	P	**1626.6305**	**813.8189**	1609.6039	805.3056	**1608.6199**	804.8136	14
17	**1815.7966**	908.4020	1798.7701	899.8887	1797.7861	899.3967	C	1529.5777	765.2925	1512.5512	**756.7792**	1511.5672	**756.2872**	13
18	1902.8287	951.9180	1885.8021	943.4047	1884.8181	942.9127	S	1369.5471	**685.2772**	1352.5205	676.7639	1351.5365	676.2719	12
19	2062.8593	1031.9333	2045.8328	1023.4200	2044.8488	1022.9280	C	1282.5150	641.7612	1265.4885	633.2479	1264.5045	632.7559	11
20	2222.8900	1111.9486	2205.8634	1103.4353	2204.8794	1102.9433	C	**1122.4844**	561.7458	1105.4578	553.2326	1104.4738	552.7406	10
21	2335.9740	1168.4907	2318.9475	1159.9774	2317.9635	1159.4854	I	962.4537	481.7305	945.4272	473.2172	944.4432	**472.7252**	9
22	2435.0424	1218.0249	2418.0159	1209.5116	2417.0319	1209.0196	V	849.3697	425.1885	832.3431	416.6752	831.3591	416.1832	8
23	2550.0694	1275.5383	2533.0428	1267.0251	2532.0588	1266.5330	N	**750.3013**	375.6543	733.2747	367.1410	732.2907	366.6490	7
24	2664.1123	1332.5598	2647.0858	1324.0465	2646.1017	1323.5545	N	**635.2743**	318.1408	618.2478	309.6275	617.2638	309.1355	6
25	2751.1443	1376.0758	2734.1178	1367.5625	2733.1338	1367.0705	S	**521.2314**	261.1193	504.2049	252.6061	503.2208	252.1141	5
26	2866.1713	1433.5893	2849.1447	1425.0760	2848.1607	1424.5840	N	**434.1994**	217.6033	417.1728	209.0901	416.1888	208.5980	4
27	2923.1927	1462.1000	2906.1662	1453.5867	2905.1822	1453.0947	G	**319.1724**	160.0899	302.1459	151.5766	301.1619	151.0846	3
28	3010.2248	1505.6160	2993.1982	1497.1027	2992.2142	1496.6107	S	262.1510	131.5791	245.1244	123.0659	244.1404	122.5738	2
29							R	175.1190	88.0631	158.0924	79.5498			1

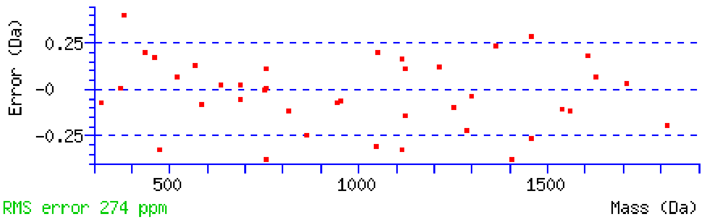

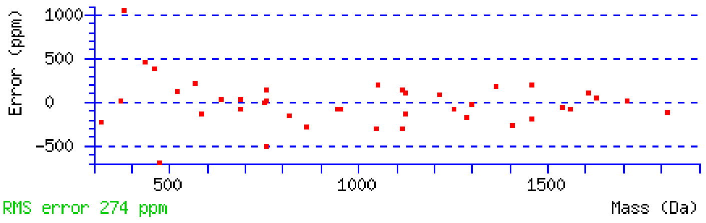

All matches to this query

Score	Mr(calc):	Delta	Sequence
65.8	3183.3292	0.0065	GVDVSQVAWQSQGDTPCSCCIVNNSNGSR
60.9	3183.3292	0.0065	GVDVSQVAWQSQGDTPCSCCIVNNSNGSR
51.0	3183.3292	0.0065	GVDVSQVAWQSQGDTPCSCCIVNNSNGSR
40.1	3182.3452	0.9905	GVDVSQVAWQSQGDTPCSCCIVNNSNGSR
29.9	3182.3452	0.9905	GVDVSQVAWQSQGDTPCSCCIVNNSNGSR
25.4	3182.3452	0.9905	GVDVSQVAWQSQGDTPCSCCIVNNSNGSR
4.3	3183.3265	0.0092	LAWDAAEGTQKEDTQEEPSGEEDGREAR
2.2	3183.3426	-0.0069	VATVHVKNGSMGTFESQLSLNFYTNAK
2.0	3182.3404	0.9953	DLLNKVDSDTLCTEEIFPNEGAGTDLR
1.4	3182.3035	1.0322	CSFCGGHGAQSLASSRLEPCWSRLGSR

Spectrum No: 265; Query: 588; Rank: 1

Peptide View

MS/MS Fragmentation of **TLFPNYFNGSEIVIAGK**
Found in **IPI00763060**, Tax_Id=10116 Gene_Symbol=LOC684327 similar to inter-alpha (globulin) inhibitor H5

Match to Query 588: 1869.955168 from(935.984860,2+)
Title: 091129RatKid_SCX02_16.4050.4050.2.dta
Data file K:\NewmanPaper\Piliang\3SubProteomes\Piliang3SP\mgf5ppm\SCX_3SubProteomes5ppm.mgf

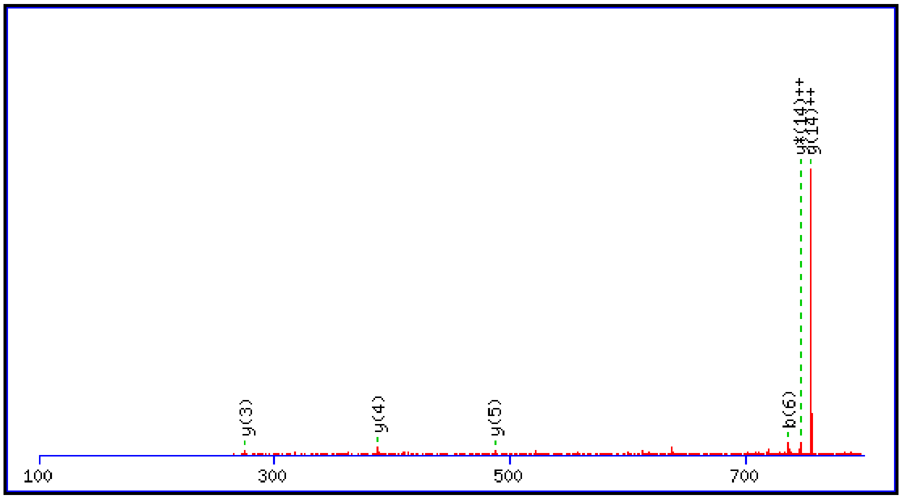

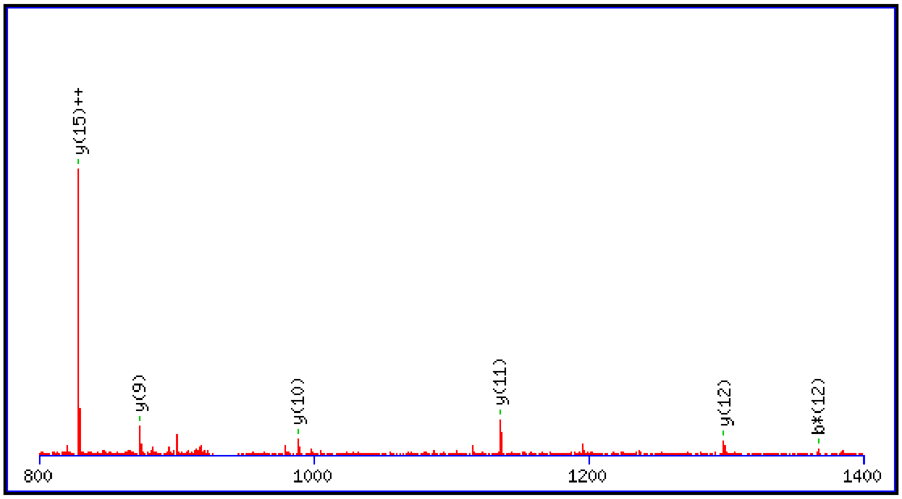

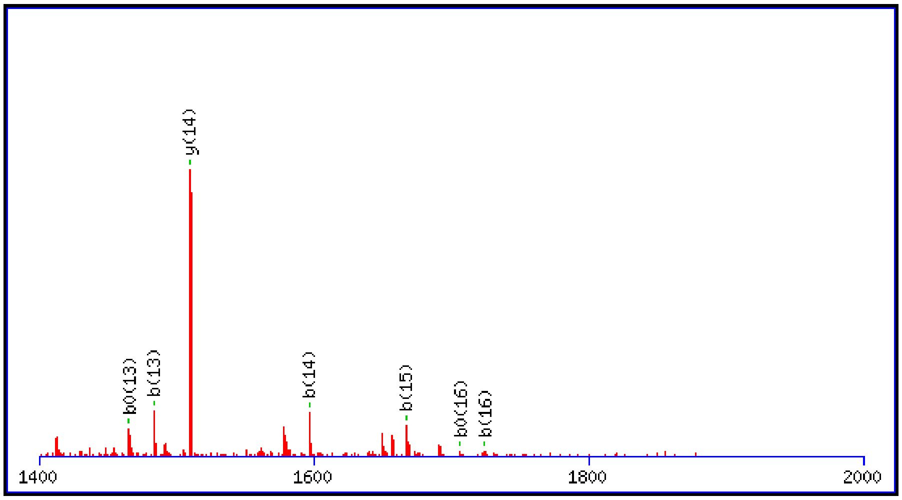

Monoisotopic mass of neutral peptide Mr(calc): 1869.9512
Fixed modifications: Carbamidomethyl (C)

Variable modifications:
N8 : Deamidated_N (N)
Ions Score: 66 **Expect:** 3.8e-005
Matches (Bold Red): 19/172 fragment ions using 35 most intense peaks

#	b	b^{++}	b*	b^{*++}	b^0	b^{0++}	Seq.	y	y^{++}	y*	y^{*++}	y^0	y^{0++}	#
1	102.0550	51.5311			84.0444	42.5258	T							17
2	215.1390	108.0731			197.1285	99.0679	L	1769.9109	885.4591	1752.8843	876.9458	1751.9003	876.4538	16
3	362.2074	181.6074			344.1969	172.6021	F	1656.8268	**828.9170**	1639.8002	820.4038	1638.8162	819.9118	15
4	459.2602	230.1337			441.2496	221.1285	P	**1509.7584**	**755.3828**	1492.7318	**746.8696**	1491.7478	746.3775	14
5	573.3031	287.1552	556.2766	278.6419	555.2926	278.1499	N	1412.7056	706.8564	1395.6791	698.3432	1394.6950	697.8512	13
6	**736.3665**	368.6869	719.3399	360.1736	718.3559	359.6816	Y	**1298.6627**	649.8350	1281.6361	641.3217	1280.6521	640.8297	12
7	883.4349	442.2211	866.4083	433.7078	865.4243	433.2158	F	**1135.5994**	568.3033	1118.5728	559.7900	1117.5888	559.2980	11
8	998.4618	499.7345	981.4353	491.2213	980.4512	490.7293	N	**988.5309**	494.7691	971.5044	486.2558	970.5204	485.7638	10
9	1055.4833	528.2453	1038.4567	519.7320	1037.4727	519.2400	G	**873.5040**	437.2556	856.4775	428.7424	855.4934	428.2504	9
10	1142.5153	571.7613	1125.4887	563.2480	1124.5047	562.7560	S	816.4825	408.7449	799.4560	400.2316	798.4720	399.7396	8
11	1271.5579	636.2826	1254.5313	627.7693	1253.5473	627.2773	E	729.4505	365.2289	712.4240	356.7156	711.4400	356.2236	7
12	1384.6420	692.8246	**1367.6154**	684.3113	1366.6314	683.8193	I	600.4079	300.7076	583.3814	292.1943			6
13	**1483.7104**	742.3588	1466.6838	733.8455	**1465.6998**	733.3535	V	**487.3239**	244.1656	470.2973	235.6523			5
14	**1596.7944**	798.9009	1579.7679	790.3876	1578.7839	789.8956	I	**388.2554**	194.6314	371.2289	186.1181			4
15	**1667.8315**	834.4194	1650.8050	825.9061	1649.8210	825.4141	A	**275.1714**	138.0893	258.1448	129.5761			3
16	**1724.8530**	862.9301	1707.8265	854.4169	**1706.8424**	853.9249	G	204.1343	102.5708	187.1077	94.0575			2
17							K	147.1128	74.0600	130.0863	65.5468			1

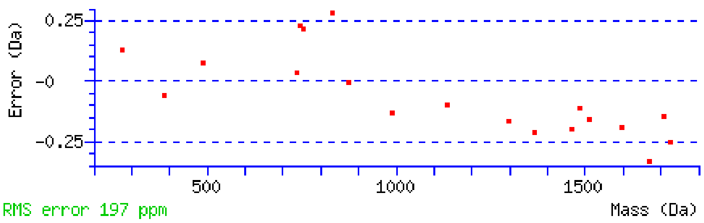

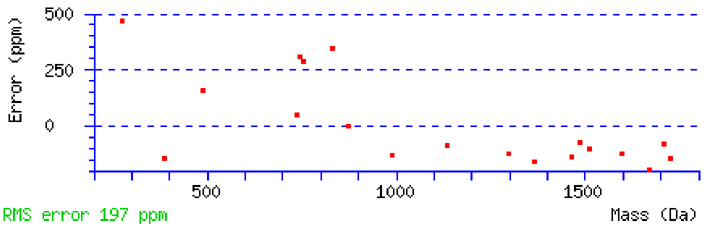

All matches to this query

Score	Mr(calc):	Delta	Sequence
65.8	1869.9512	0.0039	TLFPNYFNGSEIVIAGK
27.2	1869.9512	0.0039	TLFPNYFNGSEIVIAGK
7.3	1869.9618	-0.0067	MQDEKLQDKINQPVGK
7.2	1868.9437	1.0114	VLFASEEIPASPFRVK
5.5	1869.9648	-0.0097	RGLVMVKPGSIQPHQK
3.4	1868.9437	1.0114	VLFASEEIPASPFRVK
2.6	1867.9543	2.0008	SSTSEGLLLKTDSLLPK
1.6	1869.9601	-0.0049	NLVDPITHDLIISLAR
1.4	1869.9724	-0.0172	VVVTEVKEEDAFYSKK
1.4	1867.9316	2.0236	GFEESEPLTGFLTTGKR

Spectrum No: 266; Query: 92; Rank: 1

Peptide View

MS/MS Fragmentation of **STNSSLTLLFK**
Found in **IPI00196620**, Tax_Id=10116 Gene_Symbol=Cubn Cubilin precursor

Match to Query 92: 1210.645488 from(606.330020,2+)
Title: 091129RatKid_SCX02_17.2969.2969.2.dta
Data file K:\NewmanPaper\Piliang\3SubProteomes\Piliang3SP\mgf5ppm\SCX_3SubProteomes5ppm.mgf

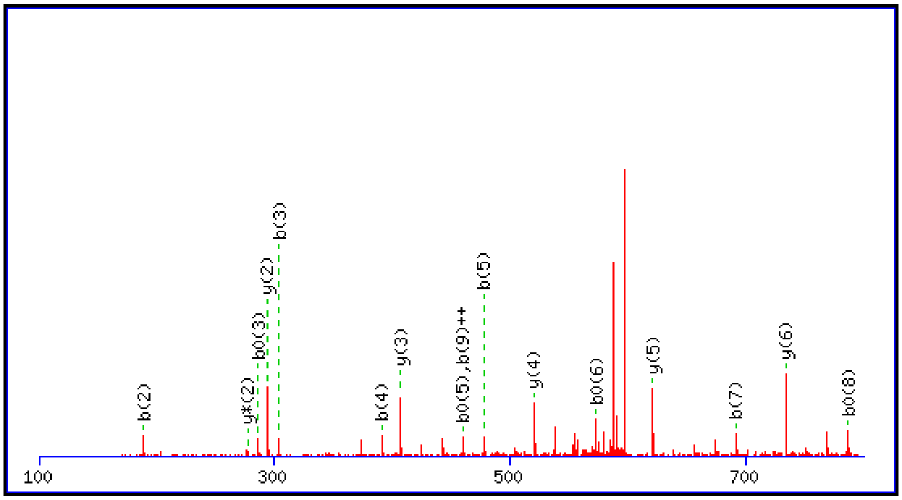

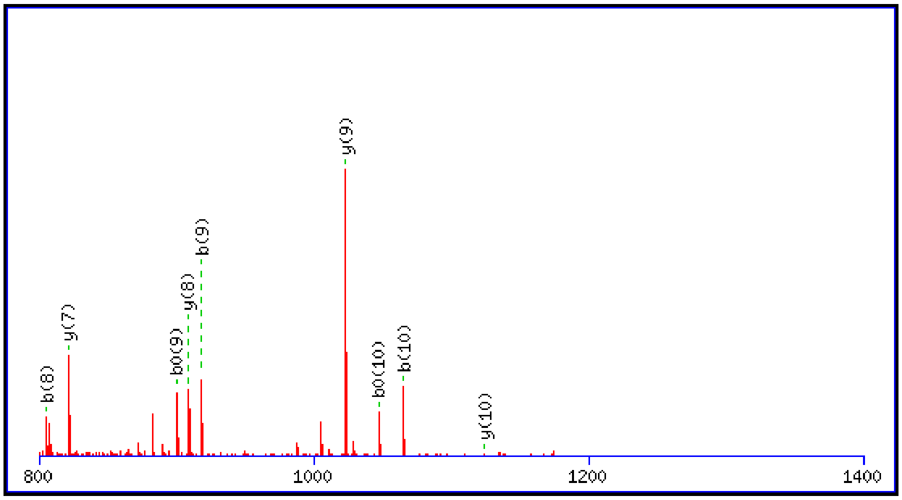

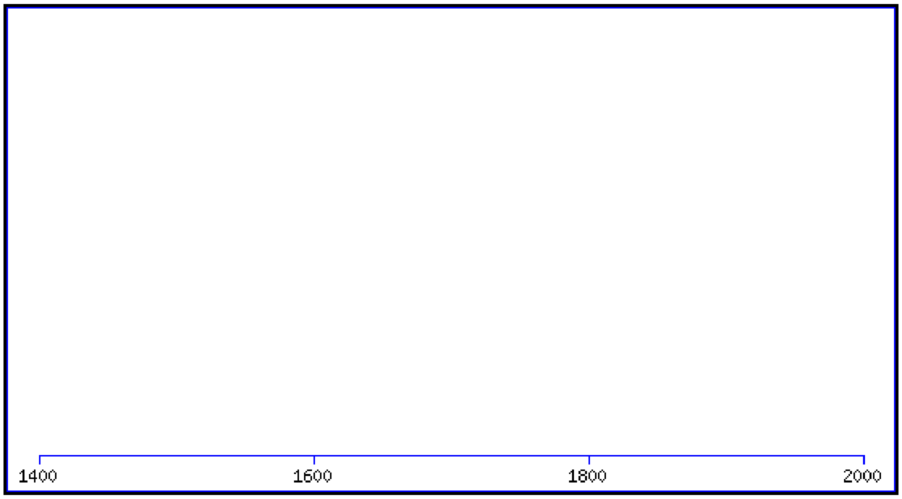

Monoisotopic mass of neutral peptide Mr(calc): 1210.6445
Fixed modifications: Carbamidomethyl (C)
Variable modifications:
N3 : Deamidated_N (N)
Ions Score: 66 **Expect:** 2.3e-005
Matches (Bold Red): 25/108 fragment ions using 51 most intense peaks

#	b	b^{++}	b*	b^{*++}	b^0	b^{0++}	Seq.	y	y^{++}	y*	y^{*++}	y^0	y^{0++}	#
1	88.0393	44.5233			70.0287	35.5180	S							11
2	**189.0870**	95.0471			171.0764	86.0418	T	**1124.6198**	562.8135	1107.5932	554.3002	1106.6092	553.8082	10

3	**304.1139**	152.5606	287.0874	144.0473	**286.1034**	143.5553	N	**1023.5721**	512.2897	1006.5455	503.7764	1005.5615	503.2844	9
4	**391.1459**	196.0766	374.1194	187.5633	373.1354	187.0713	S	**908.5451**	454.7762	891.5186	446.2629	890.5346	445.7709	8
5	**478.1780**	239.5926	461.1514	231.0794	**460.1674**	230.5873	S	**821.5131**	411.2602	804.4866	402.7469	803.5026	402.2549	7
6	591.2620	296.1347	574.2355	287.6214	**573.2515**	287.1294	L	**734.4811**	367.7442	717.4545	359.2309	716.4705	358.7389	6
7	**692.3097**	346.6585	675.2832	338.1452	674.2992	337.6532	T	**621.3970**	311.2022	604.3705	302.6889	603.3865	302.1969	5
8	**805.3938**	403.2005	788.3672	394.6873	**787.3832**	394.1952	L	**520.3493**	260.6783	503.3228	252.1650			4
9	**918.4778**	**459.7426**	901.4513	451.2293	**900.4673**	450.7373	L	**407.2653**	204.1363	390.2387	195.6230			3
10	**1065.5463**	533.2768	1048.5197	524.7635	**1047.5357**	524.2715	F	**294.1812**	147.5942	**277.1547**	139.0810			2
11							K	147.1128	74.0600	130.0863	65.5468			1

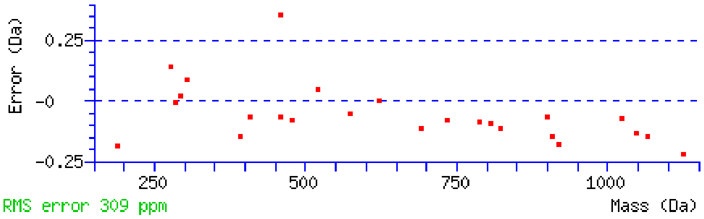

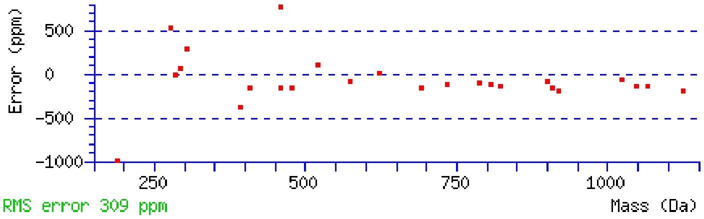

All matches to this query

Score	Mr(calc):	Delta	Sequence
65.7	1210.6445	0.0010	STNSSLTLLFK
9.0	1210.6492	-0.0037	FNSRTKAIMK
8.0	1210.6346	0.0109	RFSELGFEVK
7.0	1210.6532	-0.0078	QCVAFKALFK
6.4	1210.6454	0.0001	YLVLANMLMK
5.2	1210.6557	-0.0102	TSVVLNHLAEK
4.7	1210.6380	0.0075	SAPSVPPQGLMK
3.9	1210.6530	-0.0076	RNHETLGTRK
3.7	1210.6363	0.0092	ITGAPAIIFTK
1.8	1209.6370	1.0085	AGIKSTIAIEK

Spectrum No: 267; Query: 1015; Rank: 1

Peptide View

MS/MS Fragmentation of **LVIYLEHNLEKNSTKEEILAALEK**
Found in **IPI00195160**, Tax_Id=10116 Gene_Symbol=Psap Sulfated glycoprotein 1 precursor

Match to Query 1015: 2797.516152 from(933.512660,3+)
Title: 091129RatKid_SCX02_36.4698.4698.3.dta
Data file K:\NewmanPaper\Piliang\3SubProteomes\Piliang3SP\mgf5ppm\SCX_3SubProteomes5ppm.mgf

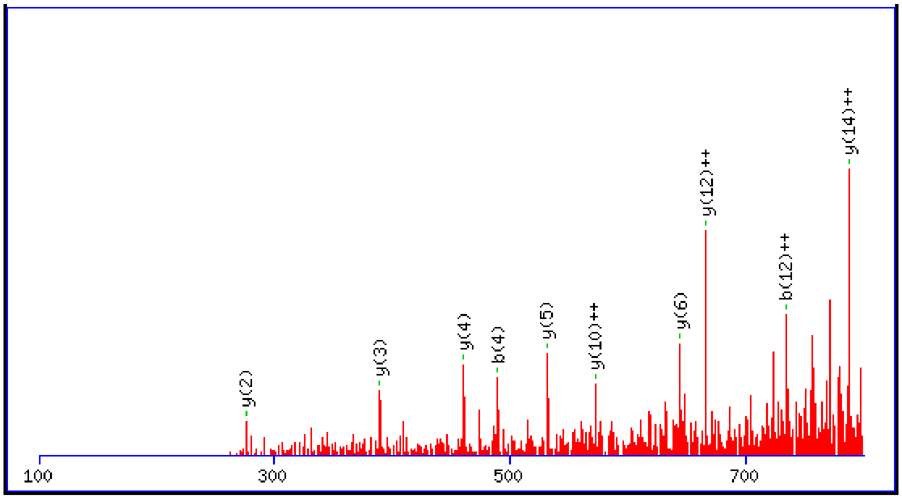

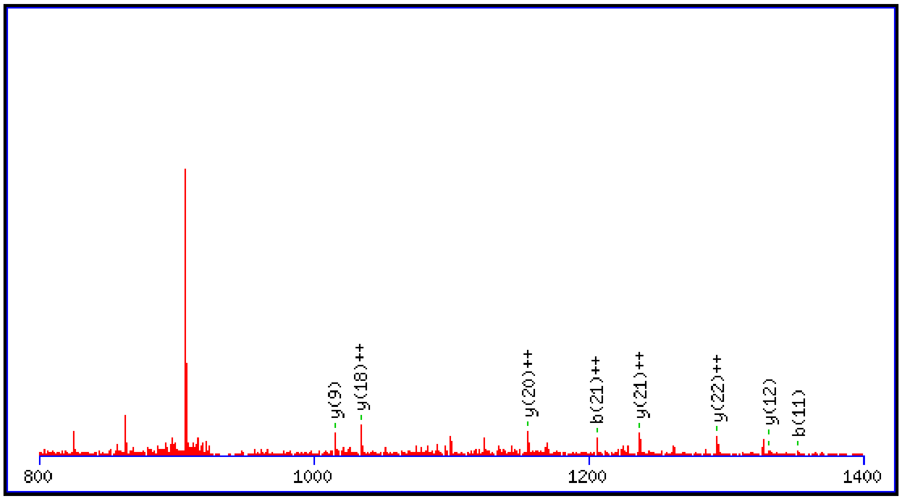

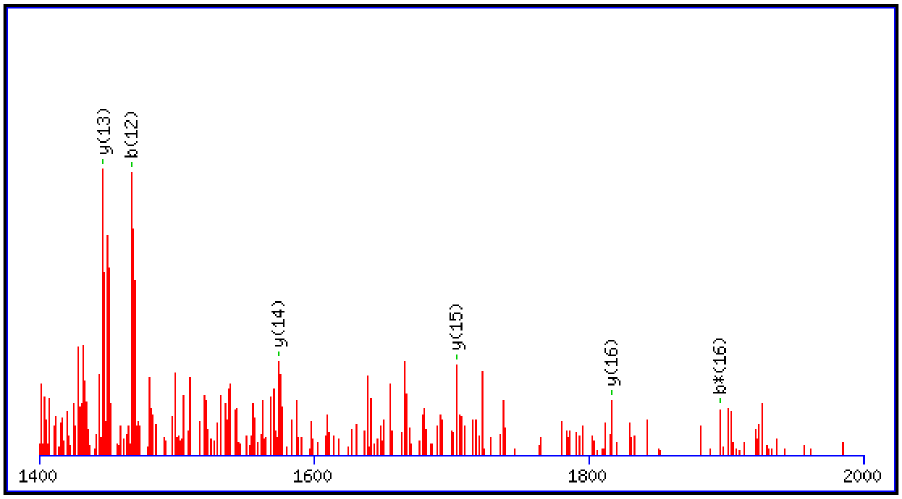

Monoisotopic mass of neutral peptide Mr(calc): 2797.5112
Fixed modifications: Carbamidomethyl (C)
Variable modifications:
N12 : Deamidated_N (N)
Ions Score: 66 **Expect:** 1.3e-005
Matches (Bold Red): 24/250 fragment ions using 47 most intense peaks

#	b	b^{++}	b*	b^{*++}	b^0	b^{0++}	Seq.	y	y^{++}	y*	y^{*++}	y^0	y^{0++}	#
1	114.0913	57.5493					L							24
2	213.1598	107.0835					V	2685.4345	1343.2209	2668.4080	1334.7076	2667.4240	1334.2156	23
3	326.2438	163.6255					I	2586.3661	**1293.6867**	2569.3396	1285.1734	2568.3556	1284.6814	22
4	**489.3071**	245.1572					Y	2473.2821	**1237.1447**	2456.2555	1228.6314	2455.2715	1228.1394	21
5	602.3912	301.6992					L	2310.2187	**1155.6130**	2293.1922	1147.0997	2292.2082	1146.6077	20

**6**	731.4338	366.2205			713.4232	357.2153	**E**	2197.1347	1099.0710	2180.1081	1090.5577	2179.1241	1090.0657	**19**
**7**	868.4927	434.7500			850.4822	425.7447	**H**	2068.0921	**1034.5497**	2051.0655	1026.0364	2050.0815	1025.5444	**18**
**8**	982.5356	491.7715	965.5091	483.2582	964.5251	482.7662	**N**	1931.0332	966.0202	1914.0066	957.5069	1913.0226	957.0149	**17**
**9**	1095.6197	548.3135	1078.5932	539.8002	1077.6091	539.3082	**L**	**1816.9902**	908.9988	1799.9637	900.4855	1798.9797	899.9935	**16**
**10**	1224.6623	612.8348	1207.6358	604.3215	1206.6517	603.8295	**E**	**1703.9062**	852.4567	1686.8796	843.9434	1685.8956	843.4514	**15**
**11**	**1352.7573**	676.8823	1335.7307	668.3690	1334.7467	667.8770	**K**	**1574.8636**	**787.9354**	1557.8370	779.4221	1556.8530	778.9301	**14**
**12**	**1467.7842**	**734.3957**	1450.7576	725.8825	1449.7736	725.3905	**N**	**1446.7686**	723.8879	1429.7421	715.3747	1428.7580	714.8827	**13**
**13**	1554.8162	777.9118	1537.7897	769.3985	1536.8057	768.9065	**S**	**1331.7417**	**666.3745**	1314.7151	657.8612	1313.7311	657.3692	**12**
**14**	1655.8639	828.4356	1638.8374	819.9223	1637.8533	819.4303	**T**	1244.7096	622.8585	1227.6831	614.3452	1226.6991	613.8532	**11**
**15**	1783.9589	892.4831	1766.9323	883.9698	1765.9483	883.4778	**K**	1143.6620	**572.3346**	1126.6354	563.8213	1125.6514	563.3293	**10**
**16**	1913.0015	957.0044	**1895.9749**	948.4911	1894.9909	947.9991	**E**	**1015.5670**	508.2871	998.5405	499.7739	997.5564	499.2819	**9**
**17**	2042.0441	1021.5257	2025.0175	1013.0124	2024.0335	1012.5204	**E**	886.5244	443.7658	869.4979	435.2526	868.5138	434.7606	**8**
**18**	2155.1281	1078.0677	2138.1016	1069.5544	2137.1176	1069.0624	**I**	757.4818	379.2445	740.4553	370.7313	739.4713	370.2393	**7**
**19**	2268.2122	1134.6097	2251.1856	1126.0965	2250.2016	1125.6044	**L**	**644.3978**	322.7025	627.3712	314.1892	626.3872	313.6972	**6**
**20**	2339.2493	1170.1283	2322.2227	1161.6150	2321.2387	1161.1230	**A**	**531.3137**	266.1605	514.2871	257.6472	513.3031	257.1552	**5**
**21**	2410.2864	**1205.6468**	2393.2599	1197.1336	2392.2758	1196.6416	**A**	**460.2766**	230.6419	443.2500	222.1287	442.2660	221.6366	**4**
**22**	2523.3705	1262.1889	2506.3439	1253.6756	2505.3599	1253.1836	**L**	**389.2395**	195.1234	372.2129	186.6101	371.2289	186.1181	**3**
**23**	2652.4131	1326.7102	2635.3865	1318.1969	2634.4025	1317.7049	**E**	**276.1554**	138.5813	259.1288	130.0681	258.1448	129.5761	**2**
**24**							**K**	147.1128	74.0600	130.0863	65.5468			**1**

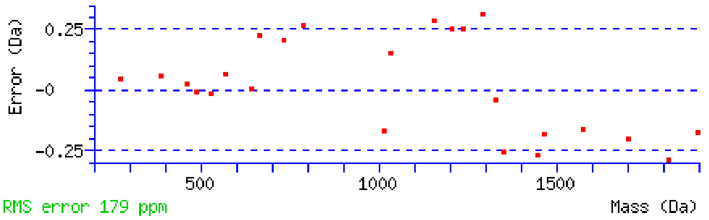

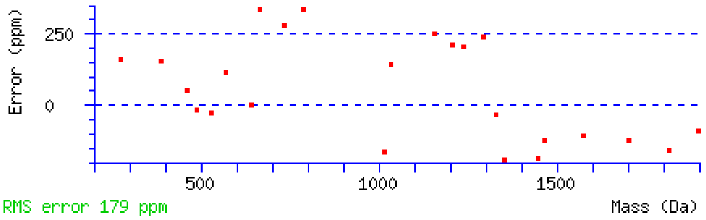

All matches to this query

Score	Mr(calc):	Delta	Sequence
65.6	2797.5112	0.0049	LVIYLEHNLEKNSTKEEILAALEK
28.5	2797.5112	0.0049	LVIYLEHNLEKNSTKEEILAALEK
3.8	2796.5262	0.9899	ATVLSTLRGLGLRPALLESAVEVHSK
3.8	2796.5262	0.9899	ATVLSTLRGLGLRPALLESAVEVHSK
2.1	2796.5262	0.9899	ATVLSTLRGLGLRPALLESAVEVHSK
1.4	2796.4882	1.0280	GHLSRPEAQSLSPYTTSANRAKLLAK
0.9	2797.5104	0.0057	LISVLYTVLTPILNPLIYCLRNK
0.8	2797.5282	-0.0121	DGVSTAILLLLLAVISLSLTFTSWGK
0.5	2797.5282	-0.0121	DGVSTAILLLLLAVISLSLTFTSWGK
0.5	2797.5282	-0.0121	DGVSTAILLLLLAVISLSLTFTSWGK

Spectrum No: 268; Query: 53; Rank: 1

Peptide View

MS/MS Fragmentation of **ALNVTLSSMGR**
Found in **IPI00213036**, Tax_Id=10116 Gene_Symbol=C4a Complement C4 precursor

Match to Query 53: 1148.587508 from(575.301030,2+)
Title: 091127RatKid_SCX01_12.2184.2184.2.dta
Data file K:\NewmanPaper\Piliang\3SubProteomes\Piliang3SP\mgf5ppm\SCX_3SubProteomes5ppm.mgf

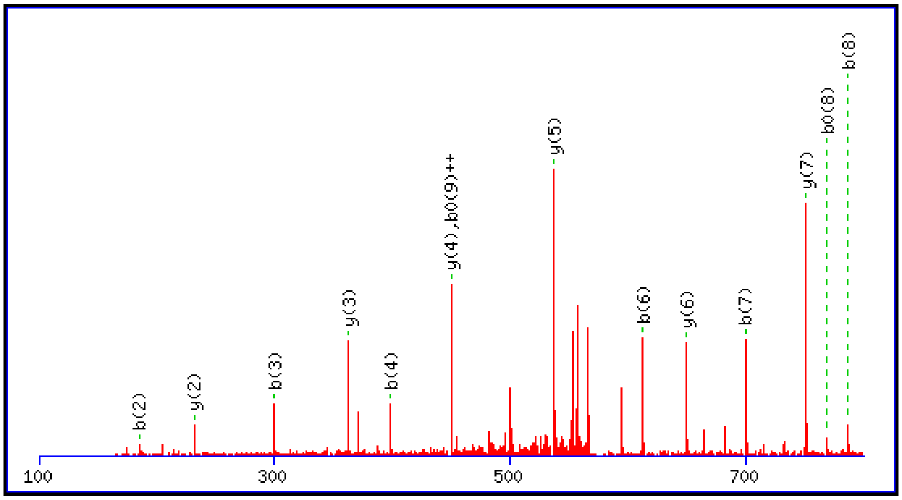

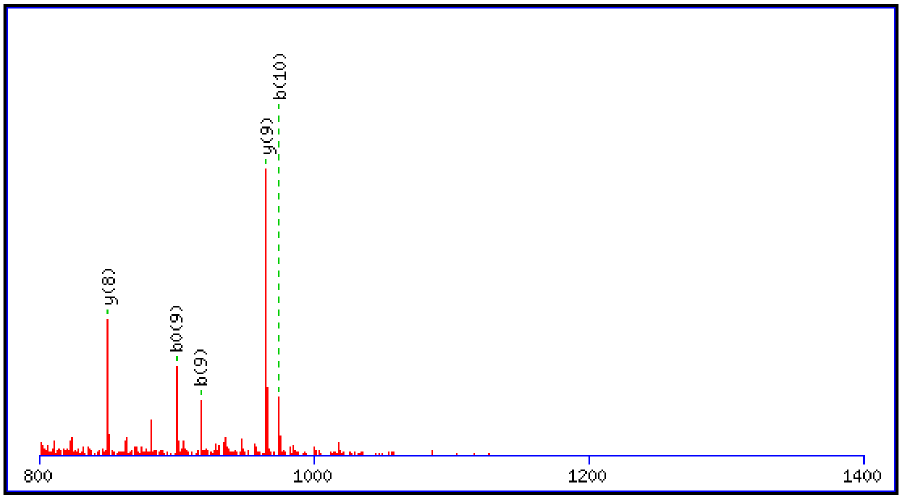

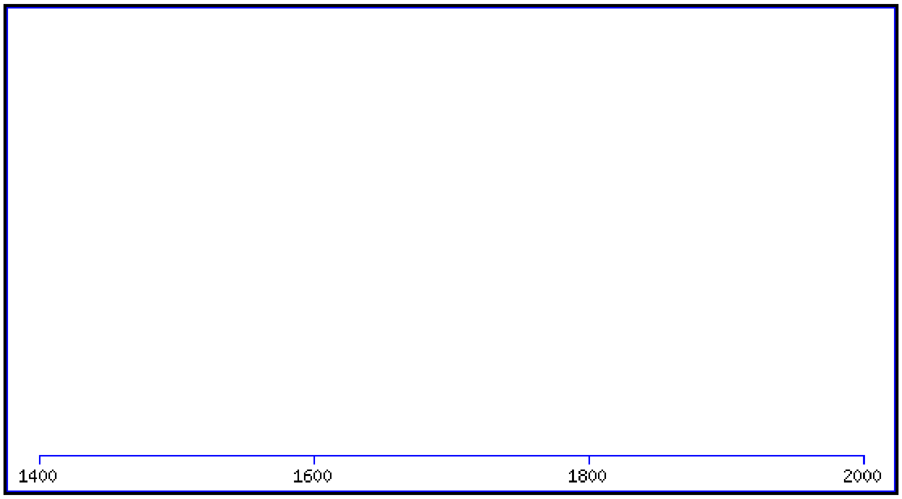

Monoisotopic mass of neutral peptide Mr(calc): 1148.5859
Fixed modifications: Carbamidomethyl (C)
Variable modifications:
N3 : Deamidated_N (N)
Ions Score: 66 **Expect:** 4.2e-005
Matches (Bold Red): 19/102 fragment ions using 40 most intense peaks

#	b	b^{++}	b*	b^{*++}	b^{0}	b^{0++}	Seq.	y	y^{++}	y*	y^{*++}	y^{0}	y^{0++}	#
1	72.0444	36.5258					A							11
2	**185.1285**	93.0679					L	1078.5561	539.7817	1061.5296	531.2684	1060.5455	530.7764	10
3	**300.1554**	150.5813	283.1288	142.0681			N	**965.4721**	483.2397	948.4455	474.7264	947.4615	474.2344	9
4	**399.2238**	200.1155	382.1973	191.6023			V	**850.4451**	425.7262	833.4186	417.2129	832.4345	416.7209	8
5	500.2715	250.6394	483.2449	242.1261	482.2609	241.6341	T	**751.3767**	376.1920	734.3502	367.6787	733.3661	367.1867	7

**6**	**613.3555**	307.1814	596.3290	298.6681	595.3450	298.1761	**L**	**650.3290**	325.6681	633.3025	317.1549	632.3185	316.6629	**6**
**7**	**700.3876**	350.6974	683.3610	342.1842	682.3770	341.6921	**S**	**537.2450**	269.1261	520.2184	260.6128	519.2344	260.1208	**5**
**8**	**787.4196**	394.2134	770.3931	385.7002	**769.4090**	385.2082	**S**	**450.2129**	225.6101	433.1864	217.0968	432.2024	216.6048	**4**
**9**	**918.4601**	459.7337	901.4335	451.2204	**900.4495**	**450.7284**	**M**	**363.1809**	182.0941	346.1544	173.5808			**3**
**10**	**975.4816**	488.2444	958.4550	479.7311	957.4710	479.2391	**G**	**232.1404**	116.5738	215.1139	108.0606			**2**
**11**							**R**	175.1190	88.0631	158.0924	79.5498			**1**

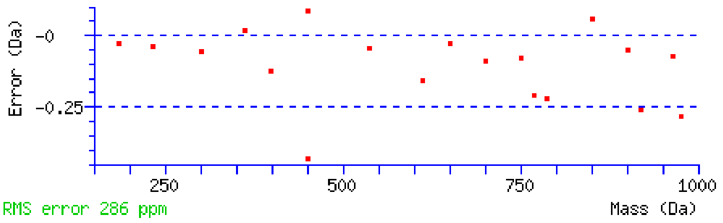

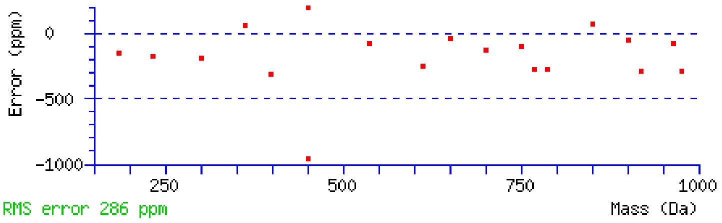

All matches to this query

Score	Mr(calc):	Delta	Sequence
65.5	1148.5859	0.0016	ALNVTLSSMGR
12.1	1148.5938	-0.0063	ALDPAEQHLR
10.1	1148.5760	0.0115	MASPLRFDGR
9.2	1147.5750	1.0125	SVISEHIKR
8.9	1147.5833	1.0042	VRELNSSNTK
6.2	1148.5839	0.0036	ALDRHWHSK
6.2	1148.5799	0.0076	HSATRPHSTR
5.3	1148.5826	0.0049	SDTFPSGIIGR
5.0	1148.5859	0.0016	VNREIVSGMK
4.3	1147.5751	1.0125	ALSGQLPAVGR

Spectrum No: 269; Query: 40; Rank: 1

Peptide View

MS/MS Fragmentation of **LEVLLANVSR**
Found in **IPI00199867**, Tax_Id=10116 Gene_Symbol=Emilin1_predicted elastin microfibril interfacer 1

Match to Query 40: 1113.643028 from(557.828790,2+)
Title: 091127RatKid_SCX01_02.3027.3027.2.dta
Data file K:\NewmanPaper\Piliang\3SubProteomes\Piliang3SP\mgf5ppm\SCX_3SubProteomes5ppm.mgf

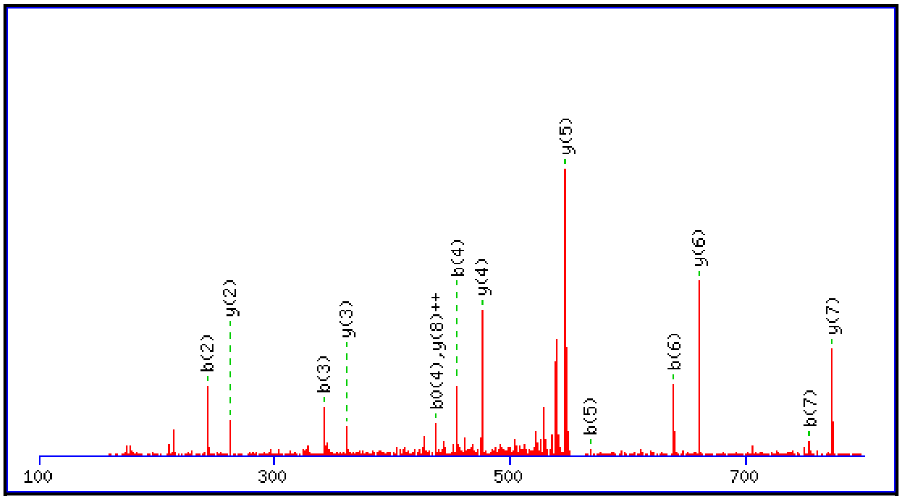

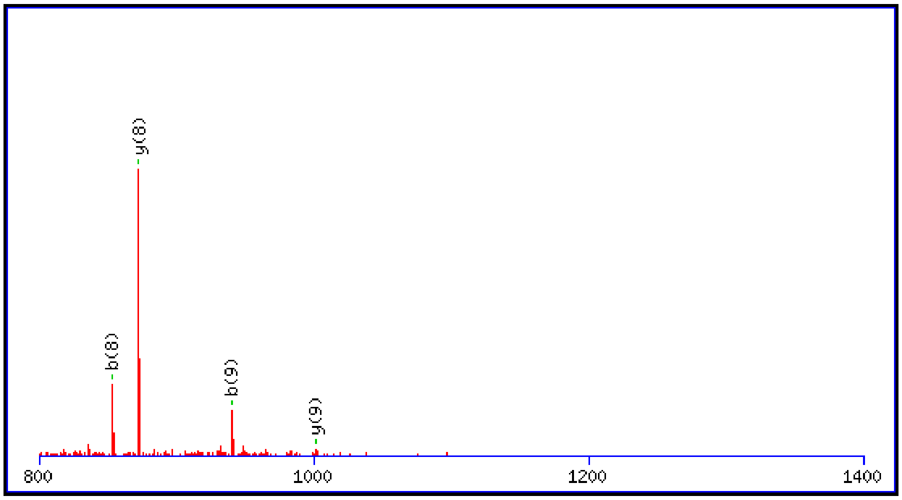

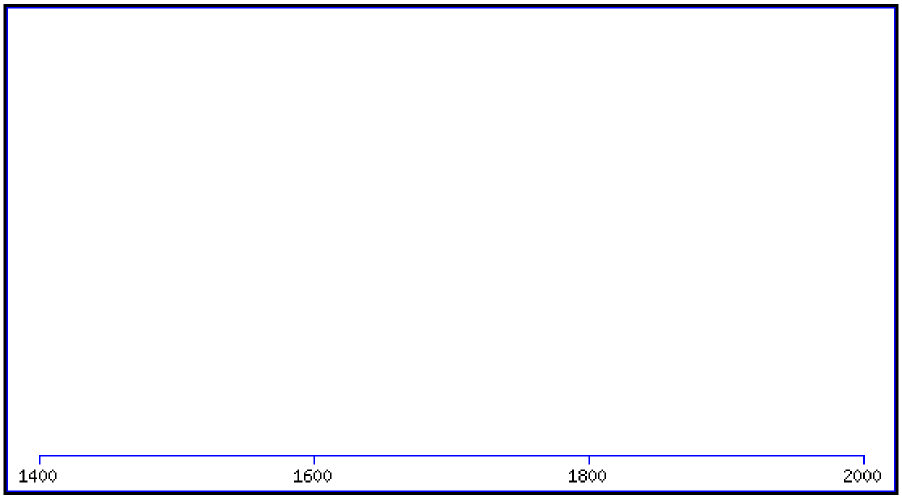

Monoisotopic mass of neutral peptide Mr(calc): 1113.6393
Fixed modifications: Carbamidomethyl (C)
Variable modifications:
N7 : Deamidated_N (N)
Ions Score: 65 **Expect:** 1.7e-005
Matches (Bold Red): 18/92 fragment ions using 35 most intense peaks

#	b	b^{++}	b*	b^{*++}	b^0	b^{0++}	Seq.	y	y^{++}	y*	y^{*++}	y^0	y^{0++}	#
1	114.0913	57.5493					L							10
2	**243.1339**	122.0706			225.1234	113.0653	E	**1001.5626**	501.2849	984.5360	492.7717	983.5520	492.2796	9
3	**342.2023**	171.6048			324.1918	162.5995	V	**872.5200**	**436.7636**	855.4934	428.2504	854.5094	427.7583	8
4	**455.2864**	228.1468			**437.2758**	219.1416	L	**773.4516**	387.2294	756.4250	378.7161	755.4410	378.2241	7
5	**568.3705**	284.6889			550.3599	275.6836	L	**660.3675**	330.6874	643.3410	322.1741	642.3569	321.6821	6
6	**639.4076**	320.2074			621.3970	311.2022	A	**547.2834**	274.1454	530.2569	265.6321	529.2729	265.1401	5
7	**754.4345**	377.7209	737.4080	369.2076	736.4240	368.7156	N	**476.2463**	238.6268	459.2198	230.1135	458.2358	229.6215	4
8	**853.5029**	427.2551	836.4764	418.7418	835.4924	418.2498	V	**361.2194**	181.1133	344.1928	172.6001	343.2088	172.1081	3
9	**940.5350**	470.7711	923.5084	462.2578	922.5244	461.7658	S	**262.1510**	131.5791	245.1244	123.0659	244.1404	122.5738	2
10							R	175.1190	88.0631	158.0924	79.5498			1

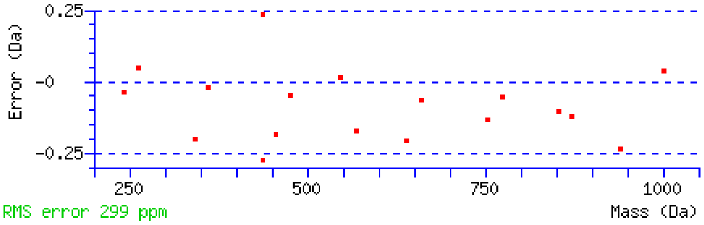

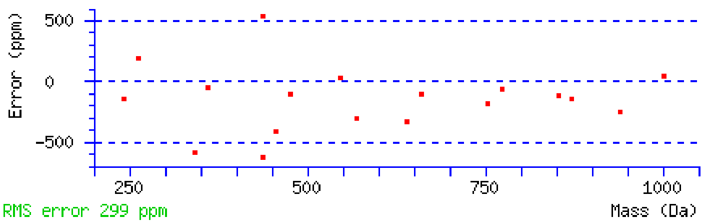

All matches to this query

Score	Mr(calc):	Delta	Sequence
65.3	1113.6393	0.0037	LEVLLANVSR
13.6	1113.6393	0.0037	DILLISQNAK
9.9	1113.6506	-0.0075	NIVKLAEATR
9.8	1113.6393	0.0037	LINLVGESLR
5.7	1113.6393	0.0037	VIELENRLK
5.2	1113.6393	0.0037	LLNLNLERK
4.5	1113.6367	0.0064	LPSSRRGVSR
2.7	1112.6414	1.0016	NQVIKAERR
2.1	1113.6506	-0.0075	LAALNGLSLSR
1.5	1113.6506	-0.0075	LQLQKESLR

Spectrum No: 270; Query: 259; Rank: 1

Peptide View

MS/MS Fragmentation of **CNASSQFLCSSGR**
Found in **IPI00369995**, Tax_Id=10116 Gene_Symbol=Lrp1 similar to low density lipoprotein receptor-related protein 1

Match to Query 259: 1473.600348 from(737.807450,2+)
Title: 091127RatKid_SCX01_13.1257.1257.2.dta
Data file K:\NewmanPaper\Piliang\3SubProteomes\Piliang3SP\mgf5ppm\SCX_3SubProteomes5ppm.mgf

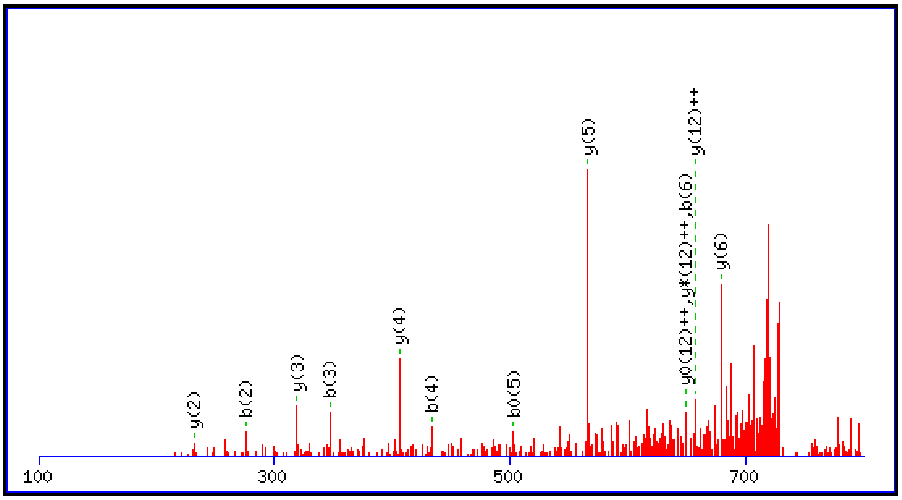

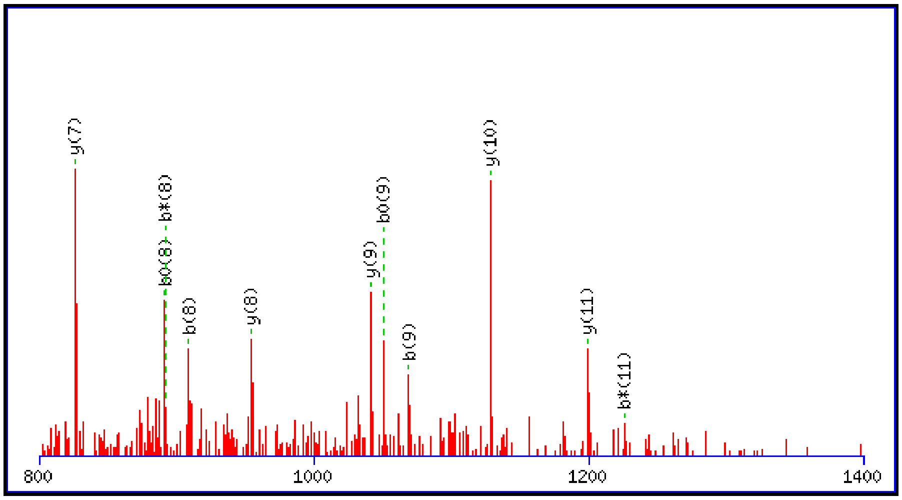

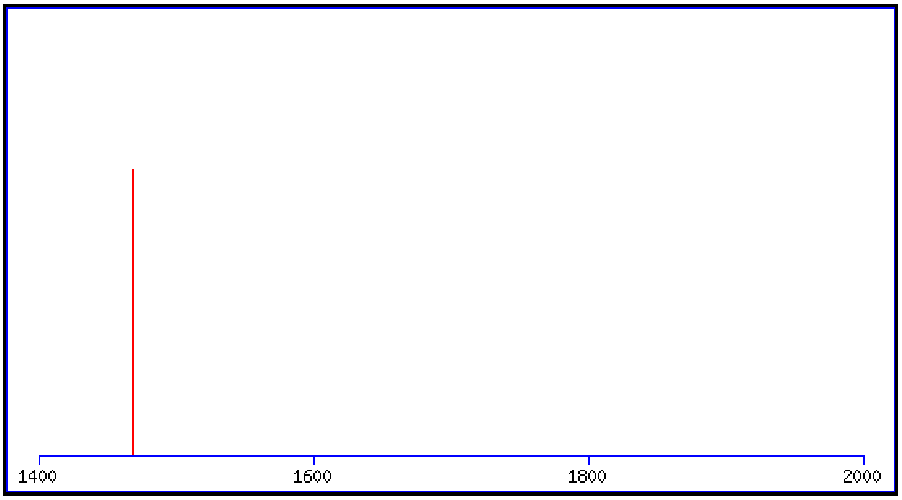

Monoisotopic mass of neutral peptide Mr(calc): 1473.5977
Fixed modifications: Carbamidomethyl (C)
Variable modifications:
N2 : Deamidated_N (N)
Ions Score: 65 **Expect:** 3.7e-005
Matches (Bold Red): 24/132 fragment ions using 54 most intense peaks

#	b	b^{++}	b*	b^{*++}	b^{0}	b^{0++}	Seq.	y	y^{++}	y*	y^{*++}	y^{0}	y^{0++}	#
1	161.0379	81.0226					C							13
2	**276.0649**	138.5361	259.0383	130.0228			N	1314.5743	**657.7908**	1297.5477	**649.2775**	1296.5637	**648.7855**	12
3	**347.1020**	174.0546	330.0754	165.5414			A	**1199.5473**	600.2773	1182.5208	591.7640	1181.5368	591.2720	11
4	**434.1340**	217.5706	417.1075	209.0574	416.1234	208.5654	S	**1128.5102**	564.7588	1111.4837	556.2455	1110.4997	555.7535	10
5	521.1660	261.0867	504.1395	252.5734	**503.1555**	252.0814	S	**1041.4782**	521.2427	1024.4517	512.7295	1023.4676	512.2375	9
6	**649.2246**	325.1159	632.1981	316.6027	631.2140	316.1107	Q	**954.4462**	477.7267	937.4196	469.2135	936.4356	468.7214	8
7	796.2930	398.6501	779.2665	390.1369	778.2825	389.6449	F	**826.3876**	413.6974	809.3610	405.1842	808.3770	404.6922	7
8	**909.3771**	455.1922	**892.3505**	446.6789	**891.3665**	446.1869	L	**679.3192**	340.1632	662.2926	331.6500	661.3086	331.1579	6
9	**1069.4077**	535.2075	1052.3812	526.6942	**1051.3972**	526.2022	C	**566.2351**	283.6212	549.2086	275.1079	548.2246	274.6159	5
10	1156.4398	578.7235	1139.4132	570.2102	1138.4292	569.7182	S	**406.2045**	203.6059	389.1779	195.0926	388.1939	194.6006	4
11	1243.4718	622.2395	**1226.4452**	613.7263	1225.4612	613.2343	S	**319.1724**	160.0899	302.1459	151.5766	301.1619	151.0846	3
12	1300.4933	650.7503	1283.4667	642.2370	1282.4827	641.7450	G	**232.1404**	116.5738	215.1139	108.0606			2
13							R	175.1190	88.0631	158.0924	79.5498			1

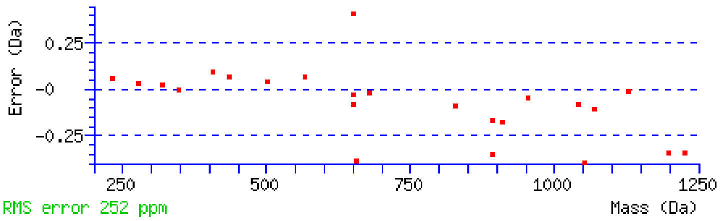

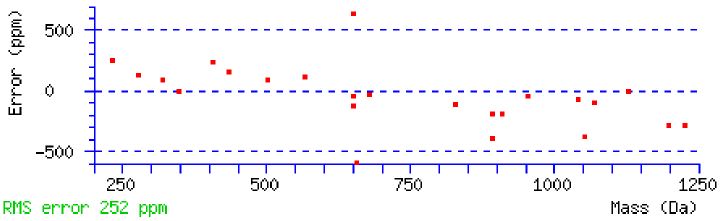

All matches to this query

Score	Mr(calc):	Delta	Sequence
65.2	1473.5977	0.0027	CNASSQFLCSSGR
10.3	1473.5976	0.0027	NCYLAMNSRGDR
7.1	1471.5811	2.0192	GEGEEDHESPSSGR
5.6	1473.5875	0.0129	TSPHSRSRSSGR
5.6	1473.5875	0.0129	TSPHSRSRSSGR
3.9	1473.5875	0.0129	TSPHSRSRSSGR
3.9	1473.5875	0.0129	TSPHSRSRSSGR
2.5	1473.5959	0.0044	NSPLPNCTYATR

2.5	1473.5875	0.0129	TSPHSRSRSSGR
0.9	1473.6014	-0.0011	TREAGPSLPSSGR

Spectrum No: 271; Query: 52; Rank: 1

Peptide View

MS/MS Fragmentation of **GINASSTAWAR**
Found in **IPI00190577**, Tax_Id=10116 Gene_Symbol=Lama5 similar to Laminin alpha-5 chain precursor

Match to Query 52: 1133.548288 from(567.781420,2+)
Title: 091127RatKid_SCX01_13.1387.1387.2.dta
Data file K:\NewmanPaper\Piliang\3SubProteomes\Piliang3SP\mgf5ppm\SCX_3SubProteomes5ppm.mgf

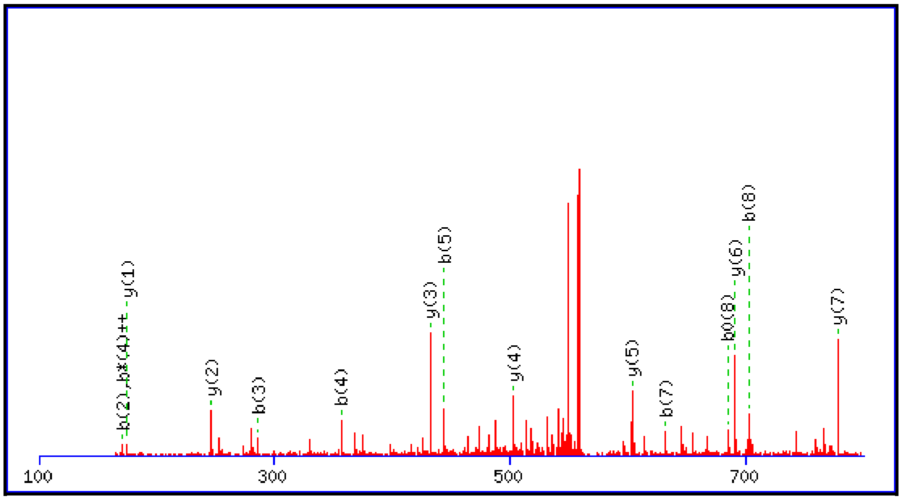

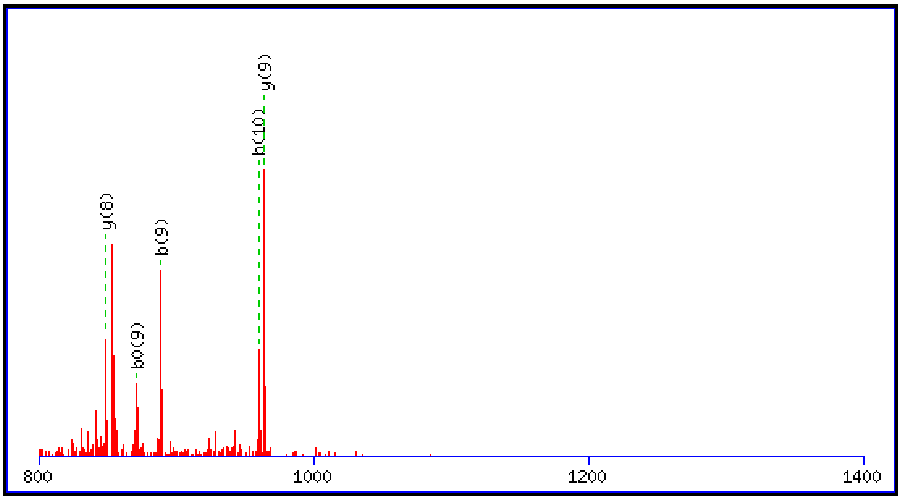

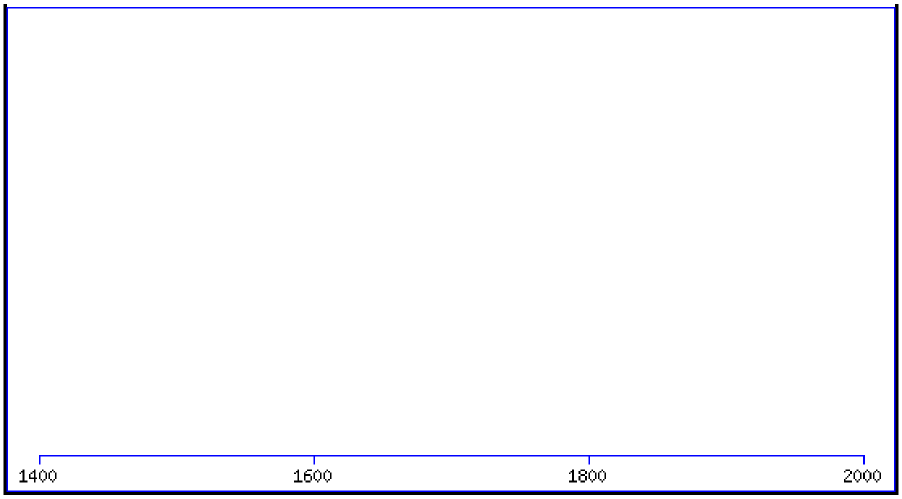

Monoisotopic mass of neutral peptide Mr(calc): 1133.5465
Fixed modifications: Carbamidomethyl (C)
Variable modifications:
N3 : Deamidated_N (N)
Ions Score: 65 **Expect:** 4.2e-005
Matches (Bold Red): 20/100 fragment ions using 44 most intense peaks

#	b	b^{++}	b*	b^{*++}	b^0	b^{0++}	Seq.	y	y^{++}	y*	y^{*++}	y^0	y^{0++}	#
1	58.0287	29.5180					G							11
2	**171.1128**	86.0600					I	1077.5323	539.2698	1060.5058	530.7565	1059.5218	530.2645	10
3	**286.1397**	143.5735	269.1132	135.0602			N	**964.4483**	482.7278	947.4217	474.2145	946.4377	473.7225	9
4	**357.1769**	179.0921	340.1503	**170.5788**			A	**849.4213**	425.2143	832.3948	416.7010	831.4108	416.2090	8
5	**444.2089**	222.6081	427.1823	214.0948	426.1983	213.6028	S	**778.3842**	389.6958	761.3577	381.1825	760.3737	380.6905	7
6	531.2409	266.1241	514.2144	257.6108	513.2303	257.1188	S	**691.3522**	346.1797	674.3257	337.6665	673.3416	337.1745	6
7	**632.2886**	316.6479	615.2620	308.1347	614.2780	307.6427	T	**604.3202**	302.6637	587.2936	294.1504	586.3096	293.6584	5
8	**703.3257**	352.1665	686.2992	343.6532	**685.3151**	343.1612	A	**503.2725**	252.1399	486.2459	243.6266			4
9	**889.4050**	445.2061	872.3785	436.6929	**871.3945**	436.2009	W	**432.2354**	216.6213	415.2088	208.1081			3
10	**960.4421**	480.7247	943.4156	472.2114	942.4316	471.7194	A	**246.1561**	123.5817	229.1295	115.0684			2
11							R	**175.1190**	88.0631	158.0924	79.5498			1

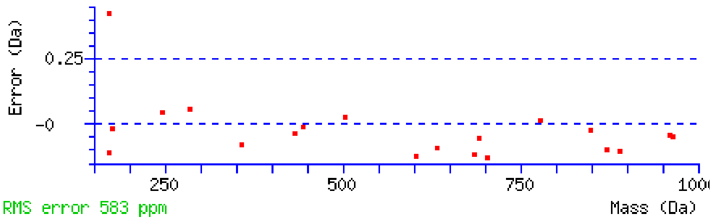

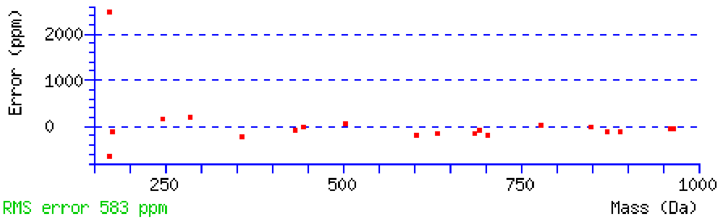

All matches to this query

Score	Mr(calc):	Delta	Sequence
64.7	1133.5465	0.0018	GINASSTAWAR
10.7	1133.5499	-0.0016	AVMNLSSADAR
8.5	1133.5425	0.0058	NSGVNSSGEKR
8.4	1132.5472	1.0011	NKLSSEGNER
7.1	1131.5381	2.0102	GSERGRDEAR
6.8	1133.5564	-0.0081	ASESKEAVGEK
6.8	1131.5520	1.9963	TAEGQALSEAR
6.6	1133.5495	-0.0012	GLYYRRAR
6.6	1133.5495	-0.0012	GLYYRRAR
5.4	1133.5564	-0.0081	GIAQTSSEDVK

Spectrum No: 272; Query: 631; Rank: 1

Peptide View

MS/MS Fragmentation of **QGAIVAVTGDGVNDSPALKK**
Found in **IPI00205693**, Tax_Id=10116 Gene_Symbol=Atp1a2 Sodium/potassium-transporting ATPase subunit alpha-2 precursor

Match to Query 631: 1940.020782 from(647.680870,3+)
Title: 091129RatKid_SCX02_27.1735.1735.3.dta
Data file K:\NewmanPaper\Piliang\3SubProteomes\Piliang3SP\mgf5ppm\SCX_3SubProteomes5ppm.mgf

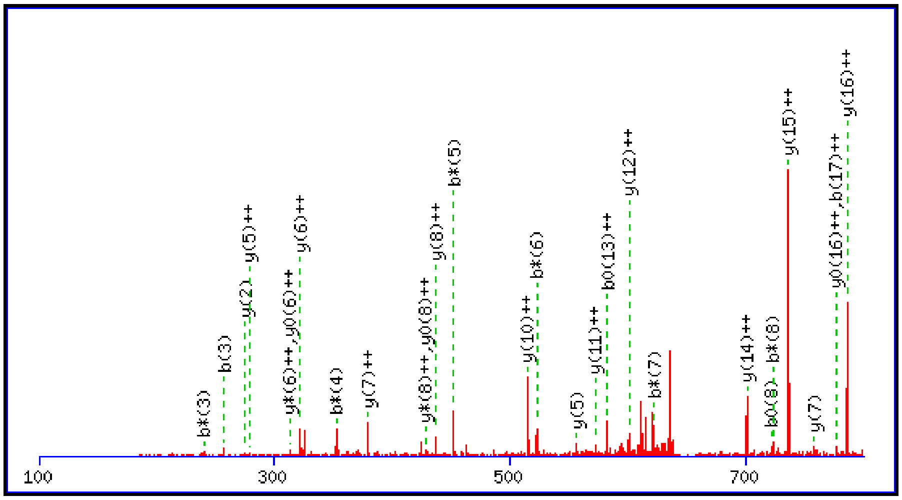

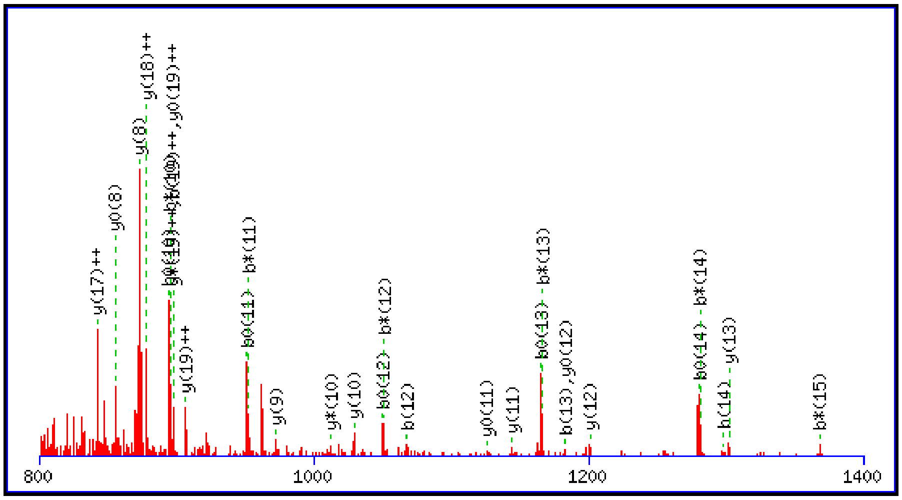

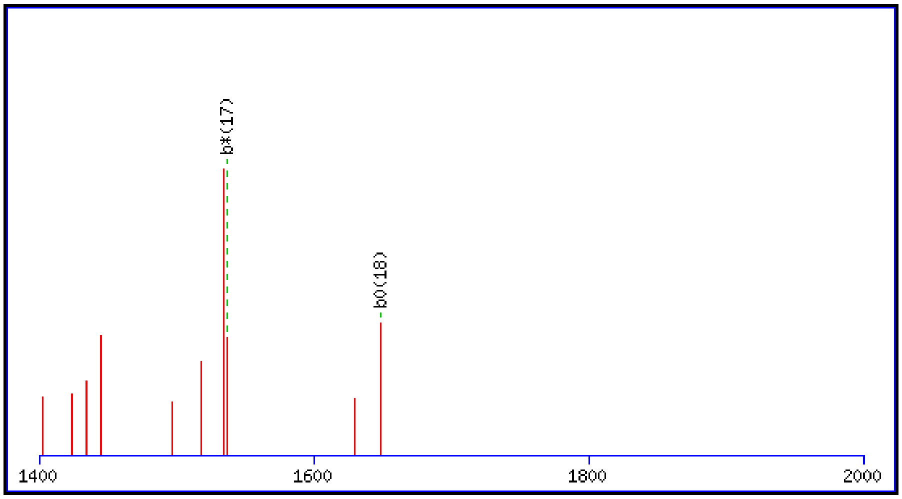

Monoisotopic mass of neutral peptide Mr(calc): 1940.0215
Fixed modifications: Carbamidomethyl (C)
Variable modifications:
N13 : Deamidated_N (N)
Ions Score: 65 **Expect:** 3.8e-005
Matches (Bold Red): 60/204 fragment ions using 109 most intense peaks

#	b	b^{++}	b*	b^{*++}	b^0	b^{0++}	Seq.	y	y^{++}	y*	y^{*++}	y^0	y^{0++}	#
1	129.0659	65.0366	112.0393	56.5233			Q							20
2	186.0873	93.5473	169.0608	85.0340			G	1812.9702	**906.9887**	1795.9436	**898.4754**	1794.9596	**897.9834**	19
3	**257.1244**	129.0659	**240.0979**	120.5526			A	1755.9487	**878.4780**	1738.9222	869.9647	1737.9381	869.4727	18
4	370.2085	185.6079	**353.1819**	177.0946			I	1684.9116	**842.9594**	1667.8850	834.4462	1666.9010	833.9541	17
5	469.2769	235.1421	**452.2504**	226.6288			V	1571.8275	**786.4174**	1554.8010	777.9041	1553.8170	**777.4121**	16
6	540.3140	270.6607	**523.2875**	262.1474			A	1472.7591	**736.8832**	1455.7326	728.3699	1454.7485	727.8779	15
7	639.3824	320.1949	**622.3559**	311.6816			V	1401.7220	**701.3646**	1384.6954	692.8514	1383.7114	692.3594	14
8	740.4301	370.7187	**723.4036**	362.2054	**722.4196**	361.7134	T	**1302.6536**	651.8304	1285.6270	643.3172	1284.6430	642.8251	13
9	797.4516	399.2294	780.4250	390.7162	779.4410	390.2241	G	**1201.6059**	**601.3066**	1184.5794	592.7933	**1183.5953**	592.3013	12
10	912.4785	456.7429	**895.4520**	448.2296	**894.4680**	447.7376	D	**1144.5844**	**572.7959**	1127.5579	564.2826	**1126.5739**	563.7906	11
11	969.5000	485.2536	**952.4734**	476.7404	**951.4894**	476.2483	G	**1029.5575**	**515.2824**	**1012.5309**	506.7691	1011.5469	506.2771	10
12	**1068.5684**	534.7878	**1051.5419**	526.2746	**1050.5578**	525.7826	V	**972.5360**	486.7717	955.5095	478.2584	954.5255	477.7664	9
13	**1183.5953**	592.3013	**1166.5688**	583.7880	**1165.5848**	**583.2960**	N	**873.4676**	**437.2374**	856.4411	**428.7242**	**855.4571**	**428.2322**	8
14	**1298.6223**	649.8148	**1281.5957**	641.3015	**1280.6117**	640.8095	D	**758.4407**	**379.7240**	741.4141	371.2107	740.4301	370.7187	7
15	1385.6543	693.3308	**1368.6278**	684.8175	1367.6437	684.3255	S	643.4137	**322.2105**	626.3872	**313.6972**	625.4032	**313.2052**	6
16	1482.7071	741.8572	1465.6805	733.3439	1464.6965	732.8519	P	**556.3817**	**278.6945**	539.3552	270.1812			5
17	1553.7442	**777.3757**	**1536.7176**	768.8625	1535.7336	768.3704	A	459.3289	230.1681	442.3024	221.6548			4
18	1666.8283	833.9178	1649.8017	825.4045	**1648.8177**	824.9125	L	388.2918	194.6496	371.2653	186.1363			3
19	1794.9232	**897.9652**	1777.8967	889.4520	1776.9126	888.9600	K	**275.2078**	138.1075	258.1812	129.5942			2
20							K	147.1128	74.0600	130.0863	65.5468			1

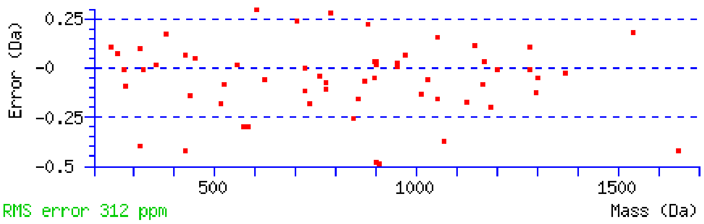

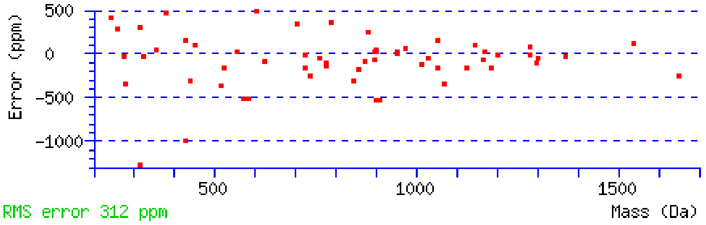

All matches to this query

Score	Mr(calc):	Delta	Sequence
64.7	1940.0215	-0.0007	QGAIVAVTGDGVNDSPALKK

Spectrum No: 273; Query: 403; Rank: 1

Peptide View

MS/MS Fragmentation of **LATIANFSALGLEPGR**
Found in **IPI00199585**, Tax_Id=10116 Gene_Symbol=Slc22a5 Organic cation/carnitine transporter 2

Match to Query 403: 1629.873252 from(544.298360,3+)
Title: 091129RatKid_SCX02_02.3586.3586.3.dta
Data file K:\NewmanPaper\Piliang\3SubProteomes\Piliang3SP\mgf5ppm\SCX_3SubProteomes5ppm.mgf

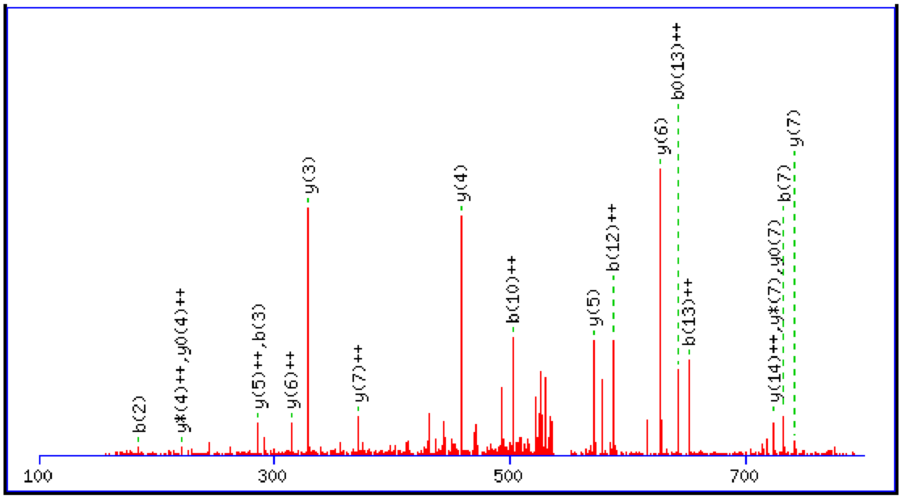

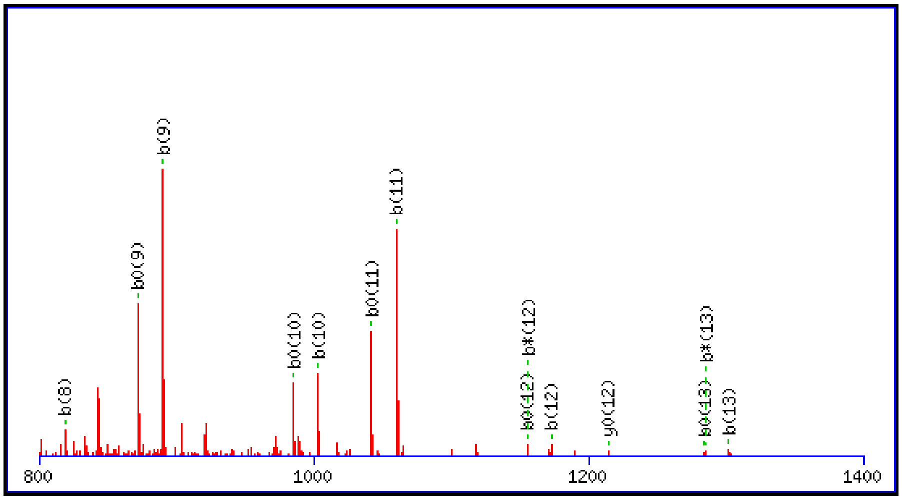

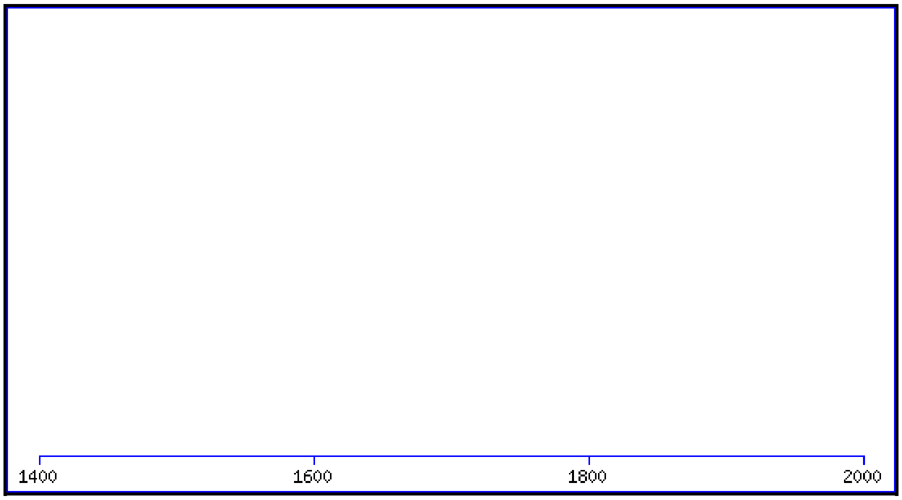

Monoisotopic mass of neutral peptide Mr(calc): 1629.8726
Fixed modifications: Carbamidomethyl (C)
Variable modifications:
N6 : Deamidated_N (N)
Ions Score: 65 **Expect:** 3.8e-005
Matches (Bold Red): 34/160 fragment ions using 41 most intense peaks

#	b	b++	b*	b*++	b0	b0++	Seq.	y	y++	y*	y*++	y0	y0++	#
1	114.0913	57.5493					L							16
2	**185.1285**	93.0679					A	1517.7958	759.4016	1500.7693	750.8883	1499.7853	750.3963	15
3	**286.1761**	143.5917			268.1656	134.5864	T	1446.7587	**723.8830**	1429.7322	715.3697	1428.7481	714.8777	14
4	399.2602	200.1337			381.2496	191.1285	I	1345.7110	673.3592	1328.6845	664.8459	1327.7005	664.3539	13
5	470.2973	235.6523			452.2867	226.6470	A	1232.6270	616.8171	1215.6004	608.3038	**1214.6164**	607.8118	12

6	585.3242	293.1658	568.2977	284.6525	567.3137	284.1605	N	1161.5899	581.2986	1144.5633	572.7853	1143.5793	572.2933	11
7	**732.3927**	366.7000	715.3661	358.1867	714.3821	357.6947	F	1046.5629	523.7851	1029.5364	515.2718	1028.5524	514.7798	10
8	**819.4247**	410.2160	802.3981	401.7027	801.4141	401.2107	S	899.4945	450.2509	882.4680	441.7376	881.4839	441.2456	9
9	**890.4618**	445.7345	873.4353	437.2213	**872.4512**	436.7293	A	812.4625	406.7349	795.4359	398.2216	794.4519	397.7296	8
10	**1003.5459**	**502.2766**	986.5193	493.7633	**985.5353**	493.2713	L	**741.4254**	**371.2163**	**724.3988**	362.7030	**723.4148**	362.2110	7
11	**1060.5673**	530.7873	1043.5408	522.2740	**1042.5568**	521.7820	G	**628.3413**	**314.6743**	611.3148	306.1610	610.3307	305.6690	6
12	**1173.6514**	**587.3293**	**1156.6248**	578.8161	**1155.6408**	578.3241	L	**571.3198**	**286.1636**	554.2933	277.6503	553.3093	277.1583	5
13	**1302.6940**	**651.8506**	**1285.6674**	643.3374	**1284.6834**	**642.8453**	E	**458.2358**	229.6215	441.2092	**221.1082**	440.2252	**220.6162**	4
14	1399.7468	700.3770	1382.7202	691.8637	1381.7362	691.3717	P	**329.1932**	165.1002	312.1666	156.5870			3
15	1456.7682	728.8877	1439.7417	720.3745	1438.7577	719.8825	G	232.1404	116.5738	215.1139	108.0606			2
16							R	175.1190	88.0631	158.0924	79.5498			1

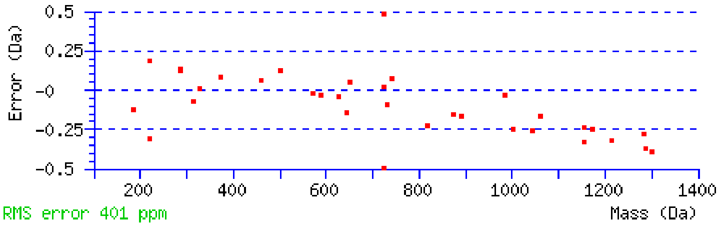

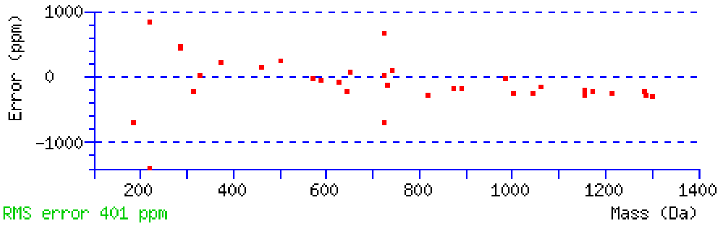

All matches to this query

Score	Mr(calc):	Delta	Sequence
64.5	1629.8726	0.0007	LATIANFSALGLEPGR
9.6	1629.8630	0.0102	YLTLLSDVLVVSTK
5.7	1629.8798	-0.0065	ERNGTLISISQQKR
4.5	1629.8630	0.0102	YLTLLSDVLVVSTK
3.4	1629.8587	0.0146	VGGNIEVLGFNARQR
2.9	1629.8603	0.0129	RKPDIVTPNGLNVK
1.6	1627.8586	2.0147	LTIYTTLVGLLNAR
0.7	1628.8733	0.9999	ETEDLRKVALEAQK

Spectrum No: 274; Query: 70; Rank: 1

Peptide View

MS/MS Fragmentation of **IVDVNLTSEGK**
Found in **IPI00192796**, Tax_Id=10116 Gene_Symbol=RGD1564625_predicted similar to Transmembrane 9 superfamily protein member 3 precursor

Match to Query 70: 1174.614108 from(588.314330,2+)
Title: 091129RatKid_SCX02_02.1050.1050.2.dta
Data file K:\NewmanPaper\Piliang\3SubProteomes\Piliang3SP\mgf5ppm\SCX_3SubProteomes5ppm.mgf

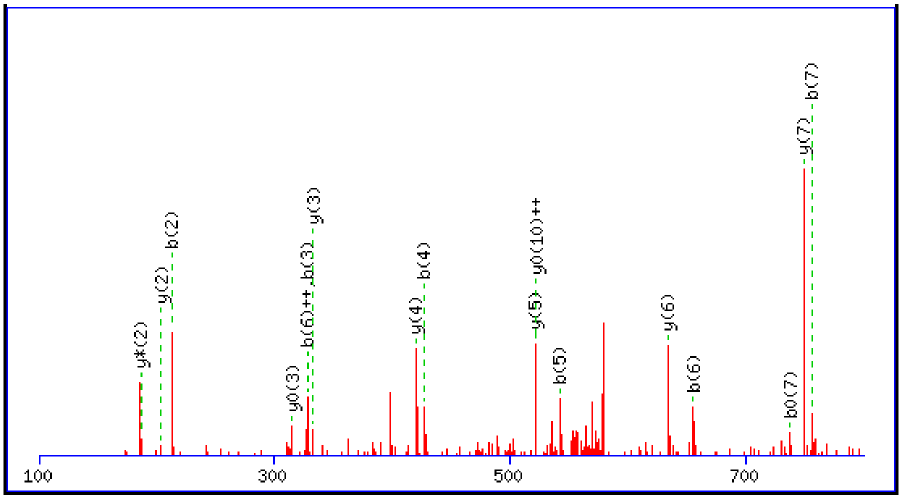

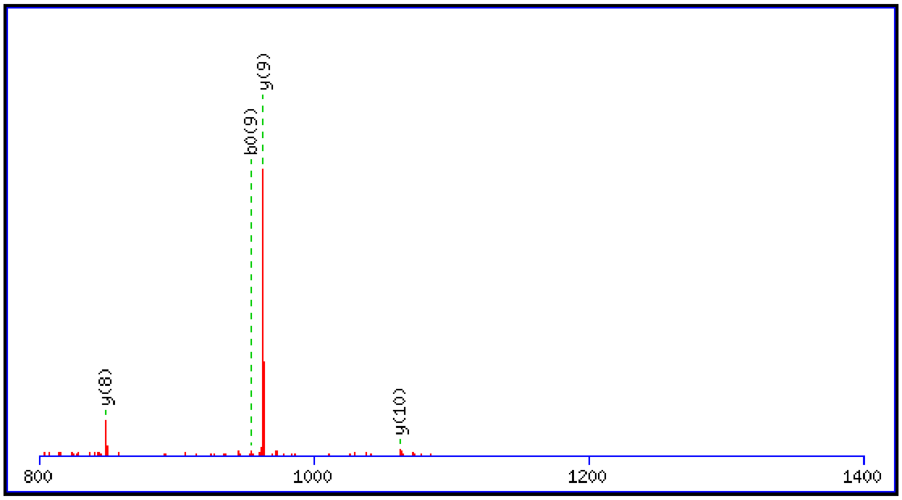

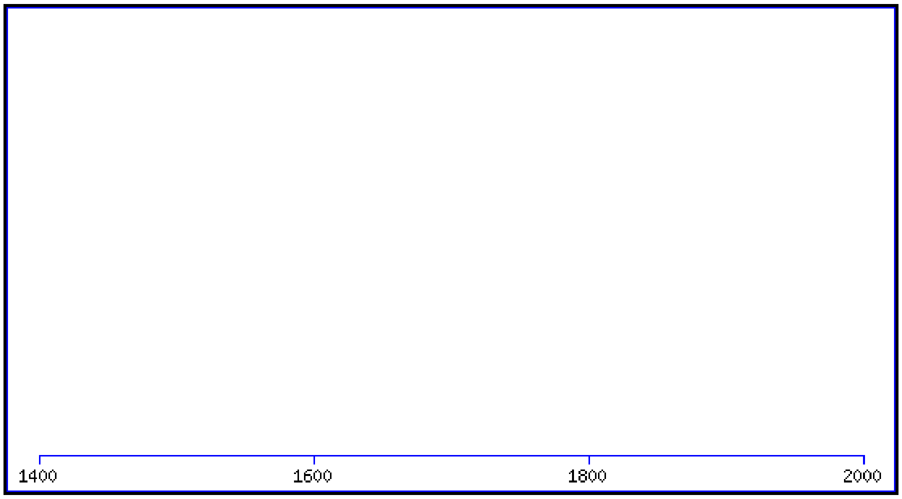

Monoisotopic mass of neutral peptide Mr(calc): 1174.6081
Fixed modifications: Carbamidomethyl (C)
Variable modifications:
N5 : Deamidated_N (N)
Ions Score: 64 **Expect:** 3.8e-005
Matches (Bold Red): 21/104 fragment ions using 44 most intense peaks

#	b	b^{++}	b*	b^{*++}	b^0	b^{0++}	Seq.	y	y^{++}	y*	y^{*++}	y^0	y^{0++}	#
1	114.0913	57.5493					I							11
2	**213.1598**	107.0835					V	**1062.5313**	531.7693	1045.5048	523.2560	1044.5208	**522.7640**	10
3	**328.1867**	164.5970			310.1761	155.5917	D	**963.4629**	482.2351	946.4364	473.7218	945.4524	473.2298	9
4	**427.2551**	214.1312			409.2445	205.1259	V	**848.4360**	424.7216	831.4094	416.2084	830.4254	415.7163	8
5	**542.2820**	271.6447	525.2555	263.1314	524.2715	262.6394	N	**749.3676**	375.1874	732.3410	366.6741	731.3570	366.1821	7

6	**655.3661**	**328.1867**	638.3396	319.6734	637.3555	319.1814	L	**634.3406**	317.6740	617.3141	309.1607	616.3301	308.6687	6
7	**756.4138**	378.7105	739.3872	370.1973	**738.4032**	369.7053	T	**521.2566**	261.1319	504.2300	252.6186	503.2460	252.1266	5
8	843.4458	422.2265	826.4193	413.7133	825.4353	413.2213	S	**420.2089**	210.6081	403.1823	202.0948	402.1983	201.6028	4
9	972.4884	486.7478	955.4619	478.2346	**954.4778**	477.7426	E	**333.1769**	167.0921	316.1503	158.5788	**315.1663**	158.0868	3
10	1029.5099	515.2586	1012.4833	506.7453	1011.4993	506.2533	G	**204.1343**	102.5708	**187.1077**	94.0575			2
11							K	147.1128	74.0600	130.0863	65.5468			1

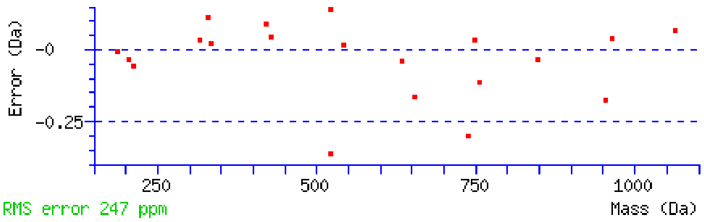

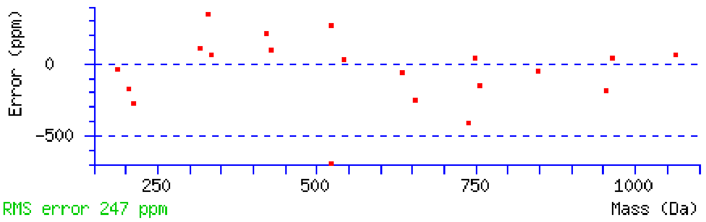

All matches to this query

Score	Mr(calc):	Delta	Sequence
64.5	1174.6081	0.0060	IVDVNLTSEGK
5.1	1174.6111	0.0030	VLEQHKLTK
3.0	1174.6127	0.0014	IALKKTITK
1.6	1174.6223	-0.0082	DLVKHRLSK
1.6	1173.6158	0.9983	LAEIAHSLLK
1.4	1174.6223	-0.0082	LVSERKVHK

Spectrum No: 275; Query: 988; Rank: 1

Peptide View

MS/MS Fragmentation of **YIAENGTDPINNQPLSEEQLIDIK**
Found in **IPI00204772**, Tax_Id=10116 Gene_Symbol=Prpf19 Pre-mRNA-processing factor 19

Match to Query 988: 2714.333948 from(1358.174250,2+)
Title: 091129RatKid_SCX02_15.3097.3097.2.dta
Data file K:\NewmanPaper\Piliang\3SubProteomes\Piliang3SP\mgf5ppm\SCX_3SubProteomes5ppm.mgf

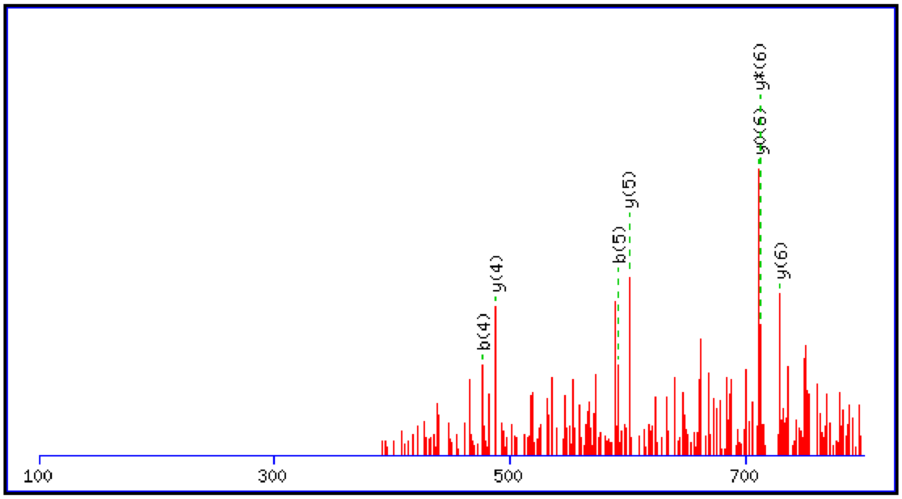

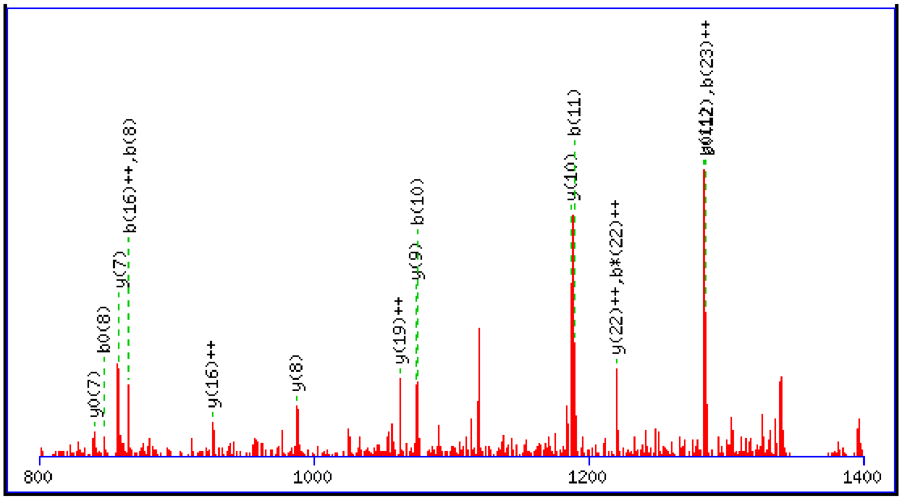

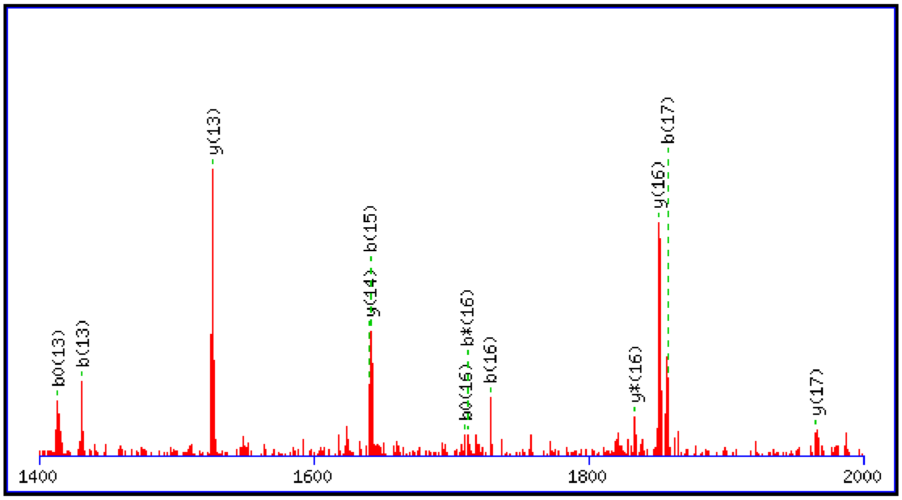

Monoisotopic mass of neutral peptide Mr(calc): 2714.3286
Fixed modifications: Carbamidomethyl (C)
Variable modifications:
N5 : Deamidated_N (N)
Ions Score: 64 **Expect:** 8.6e-005
Matches (Bold Red): 36/258 fragment ions using 66 most intense peaks

#	b	b^{++}	b*	b^{*++}	b^{0}	b^{0++}	Seq.	y	y^{++}	y*	y^{*++}	y^{0}	y^{0++}	#
1	164.0706	82.5389					Y							24
2	277.1547	139.0810					I	2552.2726	1276.6399	2535.2461	1268.1267	2534.2620	1267.6347	23
3	348.1918	174.5995					A	2439.1885	**1220.0979**	2422.1620	1211.5846	2421.1780	1211.0926	22
4	**477.2344**	239.1208			459.2238	230.1155	E	2368.1514	1184.5794	2351.1249	1176.0661	2350.1409	1175.5741	21
5	**592.2613**	296.6343	575.2348	288.1210	574.2507	287.6290	N	2239.1088	1120.0581	2222.0823	1111.5448	2221.0983	1111.0528	20
6	649.2828	325.1450	632.2562	316.6318	631.2722	316.1397	G	2124.0819	**1062.5446**	2107.0554	1054.0313	2106.0713	1053.5393	19
7	750.3305	375.6689	733.3039	367.1556	732.3199	366.6636	T	2067.0604	1034.0339	2050.0339	1025.5206	2049.0499	1025.0286	18
8	**865.3574**	433.1823	848.3308	424.6691	**847.3468**	424.1771	D	**1966.0128**	983.5100	1948.9862	974.9967	1948.0022	974.5047	17
9	962.4102	481.7087	945.3836	473.1954	944.3996	472.7034	P	**1850.9858**	**925.9965**	**1833.9593**	917.4833	1832.9753	916.9913	16
10	**1075.4942**	538.2508	1058.4677	529.7375	1057.4837	529.2455	I	1753.9331	877.4702	1736.9065	868.9569	1735.9225	868.4649	15
11	**1189.5372**	595.2722	1172.5106	586.7589	1171.5266	586.2669	N	**1640.8490**	820.9281	1623.8224	812.4149	1622.8384	811.9229	14
12	1303.5801	652.2937	1286.5535	643.7804	**1285.5695**	643.2884	N	**1526.8061**	763.9067	1509.7795	755.3934	1508.7955	754.9014	13
13	**1431.6387**	716.3230	1414.6121	707.8097	**1413.6281**	707.3177	Q	1412.7631	706.8852	1395.7366	698.3719	1394.7526	697.8799	12
14	1528.6914	764.8493	1511.6649	756.3361	1510.6809	755.8441	P	**1284.7046**	642.8559	1267.6780	634.3426	1266.6940	633.8506	11
15	**1641.7755**	821.3914	1624.7489	812.8781	1623.7649	812.3861	L	**1187.6518**	594.3295	1170.6252	585.8163	1169.6412	585.3243	10
16	**1728.8075**	**864.9074**	**1711.7810**	856.3941	**1710.7969**	855.9021	S	**1074.5677**	537.7875	1057.5412	529.2742	1056.5572	528.7822	9
17	**1857.8501**	929.4287	1840.8236	920.9154	1839.8395	920.4234	E	**987.5357**	494.2715	970.5092	485.7582	969.5251	485.2662	8
18	1986.8927	993.9500	1969.8662	985.4367	1968.8821	984.9447	E	**858.4931**	429.7502	841.4666	421.2369	**840.4825**	420.7449	7

**19**	2114.9513	1057.9793	2097.9247	1049.4660	2096.9407	1048.9740	**Q**	**729.4505**	365.2289	**712.4240**	356.7156	**711.4400**	356.2236	**6**
**20**	2228.0353	1114.5213	2211.0088	1106.0080	2210.0248	1105.5160	**L**	**601.3919**	301.1996	584.3654	292.6863	583.3814	292.1943	**5**
**21**	2341.1194	1171.0633	2324.0929	1162.5501	2323.1088	1162.0581	**I**	**488.3079**	244.6576	471.2813	236.1443	470.2973	235.6523	**4**
**22**	2456.1463	1228.5768	2439.1198	**1220.0635**	2438.1358	1219.5715	**D**	375.2238	188.1155	358.1973	179.6023	357.2132	179.1103	**3**
**23**	2569.2304	**1285.1188**	2552.2039	1276.6056	2551.2198	1276.1136	**I**	260.1969	130.6021	243.1703	122.0888			**2**
**24**							**K**	147.1128	74.0600	130.0863	65.5468			**1**

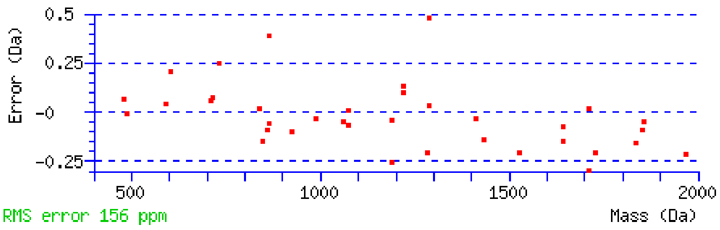

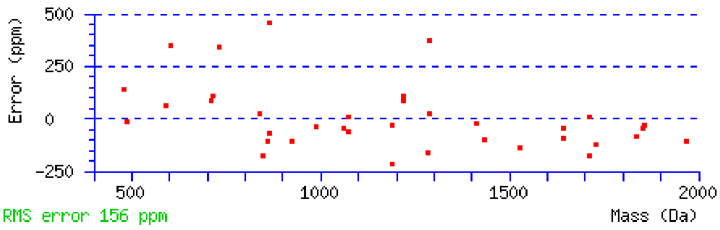

All matches to this query

Score	Mr(calc):	Delta	Sequence
64.4	2714.3286	0.0053	YIAENGTDPINNQPLSEEQLIDIK
57.9	2714.3286	0.0053	YIAENGTDPINNQPLSEEQLIDIK
55.9	2714.3286	0.0053	YIAENGTDPINNQPLSEEQLIDIK
54.6	2713.3446	0.9893	YIAENGTDPINNQPLSEEQLIDIK
5.5	2714.3229	0.0110	KMLSLSYTVVTPMLNPIIYSLR
2.3	2712.3380	1.9959	ICLLTCLVVLPGTVTSETHPGTHK
0.8	2714.3229	0.0110	KMLSLSYTVVTPMLNPIIYSLR
0.7	2712.3002	2.0337	LEMNTLNVMLGTLNLALVAEQESK

Spectrum No: 276; Query: 596; Rank: 1

Peptide View

MS/MS Fragmentation of **TLPLPLSTEFCVQQNK**
Found in **IPI00565969**, Tax_Id=10116 Gene_Symbol=RGD1311652_predicted 56 kDa protein

Match to Query 596: 1874.954248 from(938.484400,2+)
Title: 091129RatKid_SCX02_02.3175.3175.2.dta
Data file K:\NewmanPaper\Piliang\3SubProteomes\Piliang3SP\mgf5ppm\SCX_3SubProteomes5ppm.mgf

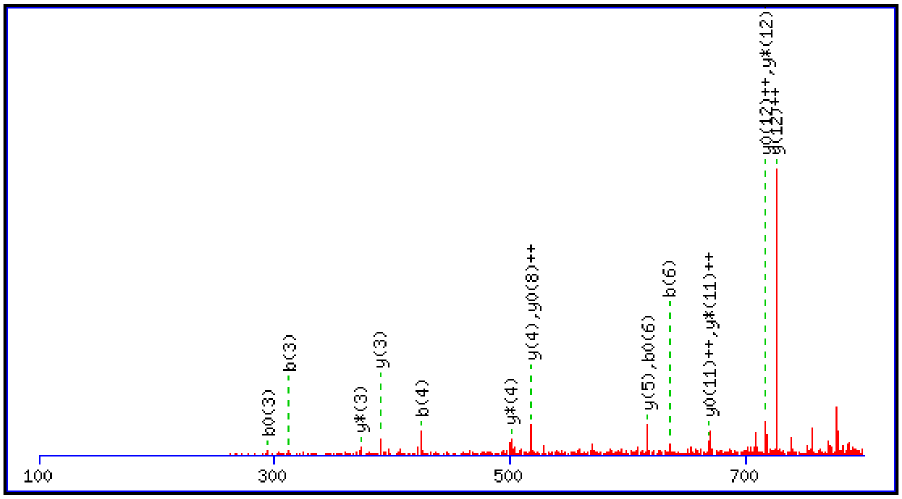

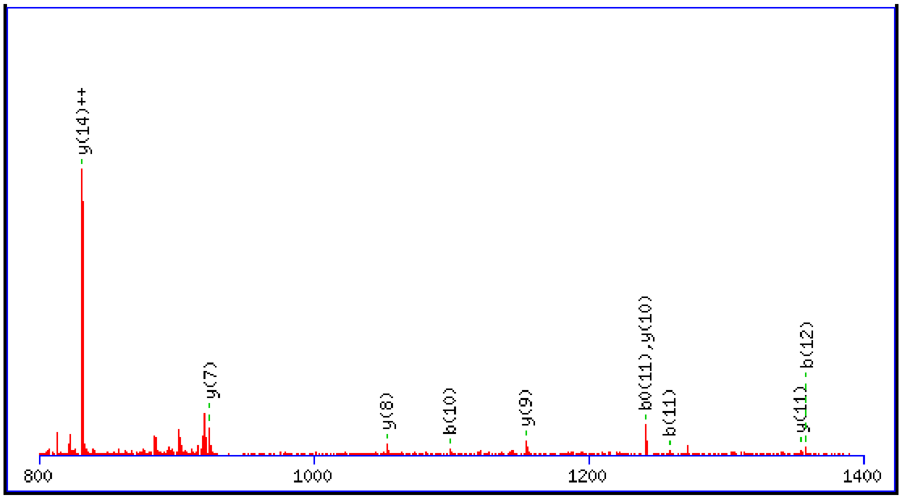

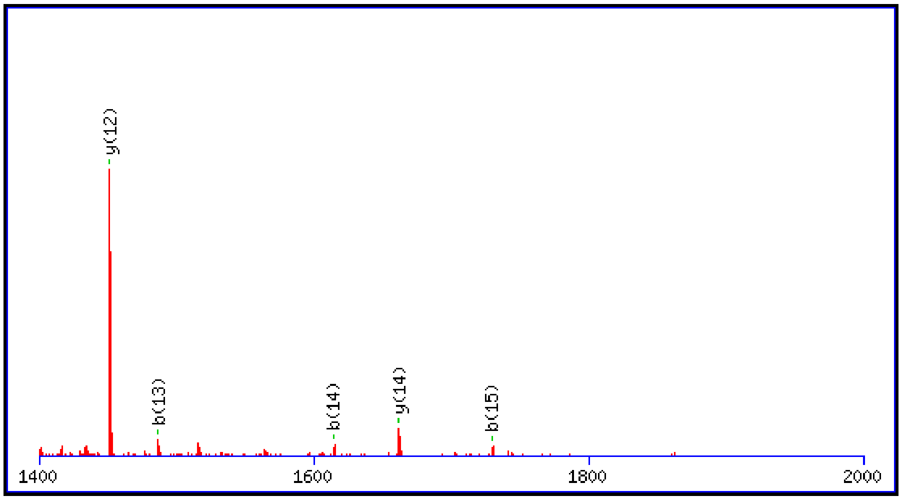

Monoisotopic mass of neutral peptide Mr(calc): 1874.9448
Fixed modifications: Carbamidomethyl (C)
Variable modifications:
N15 : Deamidated_N (N)
Ions Score: 64 **Expect:** 5.7e-005
Matches (Bold Red): 31/142 fragment ions using 63 most intense peaks

#	b	b^{++}	b*	b^{*++}	b^{0}	b^{0++}	Seq.	y	y^{++}	y*	y^{*++}	y^{0}	y^{0++}	#
1	102.0550	51.5311			84.0444	42.5258	T							16
2	215.1390	108.0731			197.1285	99.0679	L	1774.9044	887.9558	1757.8778	879.4426	1756.8938	878.9506	15
3	**312.1918**	156.5995			**294.1812**	147.5942	P	**1661.8203**	**831.4138**	1644.7938	822.9005	1643.8098	822.4085	14
4	**425.2758**	213.1416			407.2653	204.1363	L	1564.7676	782.8874	1547.7410	774.3741	1546.7570	773.8821	13
5	522.3286	261.6679			504.3180	252.6627	P	**1451.6835**	**726.3454**	1434.6570	**717.8321**	1433.6729	**717.3401**	12
6	**635.4127**	318.2100			**617.4021**	309.2047	L	**1354.6307**	677.8190	1337.6042	**669.3057**	1336.6202	**668.8137**	11
7	722.4447	361.7260			704.4341	352.7207	S	**1241.5467**	621.2770	1224.5201	612.7637	1223.5361	612.2717	10
8	823.4924	412.2498			805.4818	403.2445	T	**1154.5146**	577.7610	1137.4881	569.2477	1136.5041	568.7557	9
9	952.5350	476.7711			934.5244	467.7658	E	**1053.4670**	527.2371	1036.4404	518.7238	1035.4564	**518.2318**	8
10	**1099.6034**	550.3053			1081.5928	541.3001	F	**924.4244**	462.7158	907.3978	454.2026			7
11	**1259.6340**	630.3207			**1241.6235**	621.3154	C	777.3560	389.1816	760.3294	380.6683			6
12	**1358.7025**	679.8549			1340.6919	670.8496	V	**617.3253**	309.1663	600.2988	300.6530			5
13	**1486.7610**	743.8842	1469.7345	735.3709	1468.7505	734.8789	Q	**518.2569**	259.6321	**501.2303**	251.1188			4
14	**1614.8196**	807.9134	1597.7931	799.4002	1596.8090	798.9082	Q	**390.1983**	195.6028	**373.1718**	187.0895			3
15	**1729.8465**	865.4269	1712.8200	856.9136	1711.8360	856.4216	N	262.1397	131.5735	245.1132	123.0602			2
16							K	147.1128	74.0600	130.0863	65.5468			1

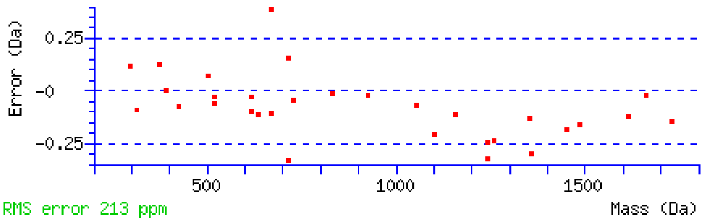

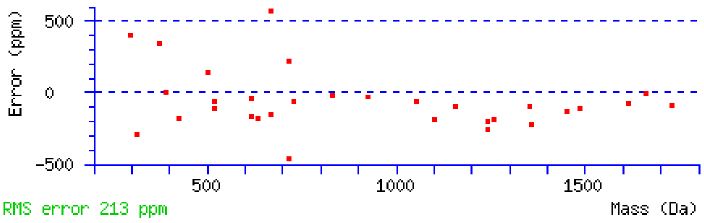

All matches to this query

Score	Mr(calc):	Delta	Sequence
64.3	1874.9448	0.0095	TLPLPLSTEFCVQQNK
19.3	1873.9608	0.9935	TLPLPLSTEFCVQQNK
3.4	1873.9356	1.0186	GRPIRITPDFSPENMK
2.6	1873.9339	1.0204	SAVPLHLIYPSVENVR
2.6	1873.9339	1.0204	SAVPLHLIYPSVENVR
0.2	1874.9369	0.0174	TPIIVMITNIEEMNEK

Spectrum No: 277; Query: 945; Rank: 1

Peptide View

MS/MS Fragmentation of **DSLNCSCLALQTSGQEEQVNQR**
Found in **IPI00326462**, Tax_Id=10116 Gene_Symbol=Enpp3 Ectonucleotide pyrophosphatase/phosphodiesterase family member 3

Match to Query 945: 2537.115132 from(846.712320,3+)
Title: 091127RatKid_SCX01_02.2441.2441.3.dta
Data file K:\NewmanPaper\Piliang\3SubProteomes\Piliang3SP\mgf5ppm\SCX_3SubProteomes5ppm.mgf

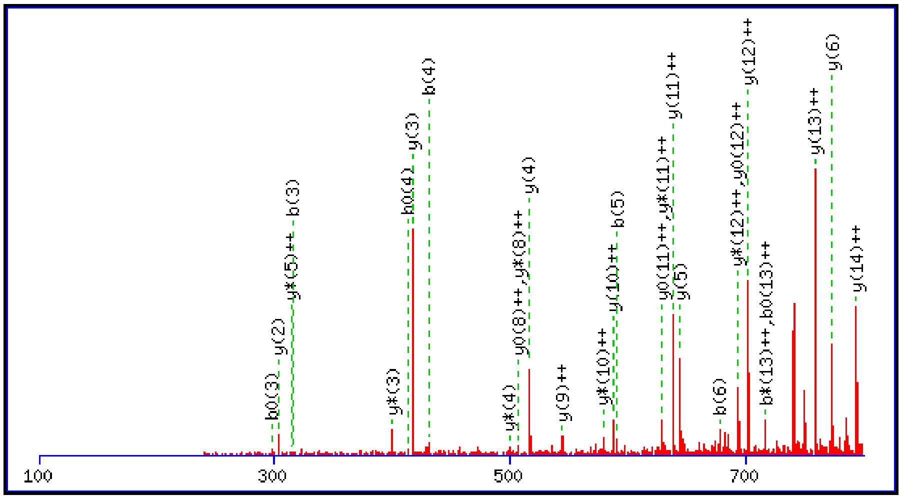

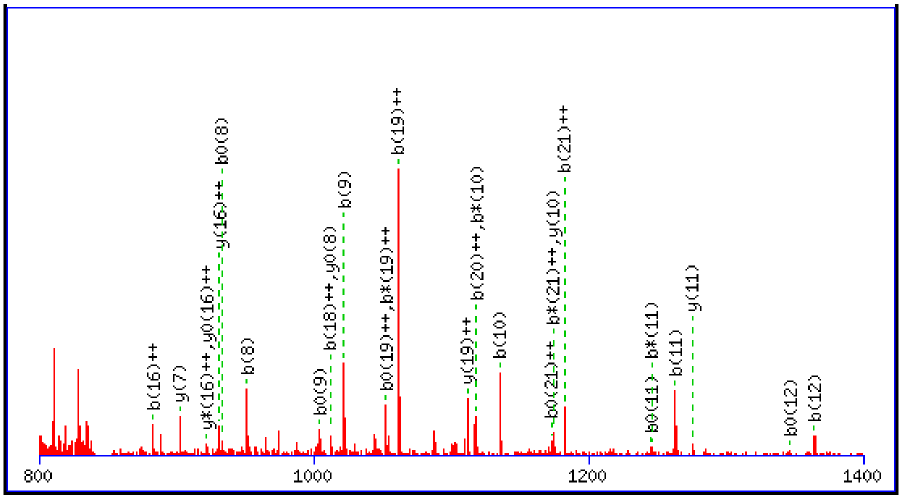

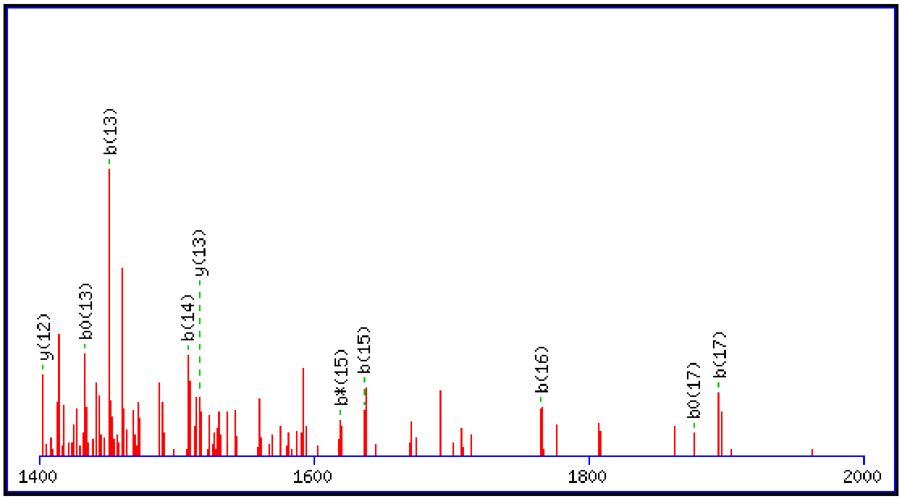

Monoisotopic mass of neutral peptide Mr(calc): 2537.1122
Fixed modifications: Carbamidomethyl (C)
Variable modifications:
N4 : Deamidated_N (N)
Ions Score: 64 **Expect:** 0.00012
Matches (Bold Red): 67/236 fragment ions using 122 most intense peaks

#	b	b^{++}	b*	b^{*++}	b^0	b^{0++}	Seq.	y	y^{++}	y*	y^{*++}	y^0	y^{0++}	#
1	116.0342	58.5207			98.0237	49.5155	D							22
2	203.0662	102.0368			185.0557	93.0315	S	2423.0926	1212.0499	2406.0660	1203.5366	2405.0820	1203.0446	21
3	**316.1503**	158.5788			**298.1397**	149.5735	L	2336.0605	1168.5339	2319.0340	1160.0206	2318.0500	1159.5286	20
4	**431.1772**	216.0923	414.1507	207.5790	**413.1667**	207.0870	N	2222.9765	**1111.9919**	2205.9499	1103.4786	2204.9659	1102.9866	19
5	**591.2079**	296.1076	574.1813	287.5943	573.1973	287.1023	C	2107.9495	1054.4784	2090.9230	1045.9651	2089.9390	1045.4731	18
6	**678.2399**	339.6236	661.2134	331.1103	660.2294	330.6183	S	1947.9189	974.4631	1930.8923	965.9498	1929.9083	965.4578	17
7	838.2706	419.6389	821.2440	411.1257	820.2600	410.6336	C	1860.8869	**930.9471**	1843.8603	**922.4338**	1842.8763	**921.9418**	16
8	**951.3546**	476.1810	934.3281	467.6677	**933.3441**	467.1757	L	1700.8562	850.9317	1683.8297	842.4185	1682.8456	841.9265	15
9	**1022.3918**	511.6995	1005.3652	503.1862	**1004.3812**	502.6942	A	1587.7721	**794.3897**	1570.7456	785.8764	1569.7616	785.3844	14
10	**1135.4758**	568.2415	**1118.4493**	559.7283	1117.4653	559.2363	L	**1516.7350**	**758.8712**	1499.7085	750.3579	1498.7245	749.8659	13
11	**1263.5344**	632.2708	**1246.5078**	623.7576	**1245.5238**	623.2656	Q	**1403.6510**	**702.3291**	1386.6244	**693.8158**	1385.6404	**693.3238**	12
12	**1364.5821**	682.7947	1347.5555	674.2814	**1346.5715**	673.7894	T	**1275.5924**	**638.2998**	1258.5658	**629.7866**	1257.5818	**629.2945**	11
13	**1451.6141**	726.3107	1434.5876	**717.7974**	**1433.6035**	**717.3054**	S	**1174.5447**	**587.7760**	1157.5182	**579.2627**	1156.5341	578.7707	10
14	**1508.6356**	754.8214	1491.6090	746.3081	1490.6250	745.8161	G	1087.5127	**544.2600**	1070.4861	535.7467	1069.5021	535.2547	9
15	**1636.6941**	818.8507	**1619.6676**	810.3374	1618.6836	809.8454	Q	1030.4912	515.7492	1013.4647	**507.2360**	**1012.4806**	**506.7440**	8
16	**1765.7367**	**883.3720**	1748.7102	874.8587	1747.7262	874.3667	E	**902.4326**	451.7200	885.4061	443.2067	884.4221	442.7147	7
17	**1894.7793**	947.8933	1877.7528	939.3800	**1876.7688**	938.8880	E	**773.3900**	387.1987	756.3635	378.6854	755.3795	378.1934	6
18	2022.8379	**1011.9226**	2005.8114	1003.4093	2004.8273	1002.9173	Q	**644.3474**	322.6774	627.3209	**314.1641**			5

**19**	2121.9063	**1061.4568**	2104.8798	**1052.9435**	2103.8958	**1052.4515**	**V**	**516.2889**	258.6481	**499.2623**	250.1348			**4**
**20**	2235.9492	**1118.4783**	2218.9227	1109.9650	2217.9387	1109.4730	**N**	**417.2205**	209.1139	**400.1939**	200.6006			**3**
**21**	2364.0078	**1182.5076**	2346.9813	**1173.9943**	2345.9973	**1173.5023**	**Q**	**303.1775**	152.0924	286.1510	143.5791			**2**
**22**							**R**	175.1190	88.0631	158.0924	79.5498			**1**

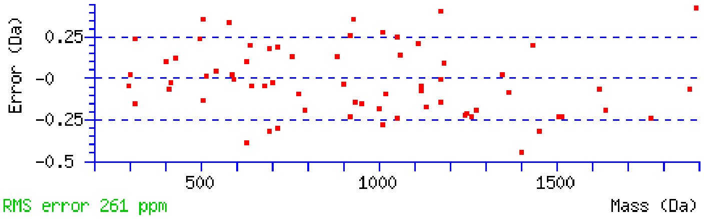

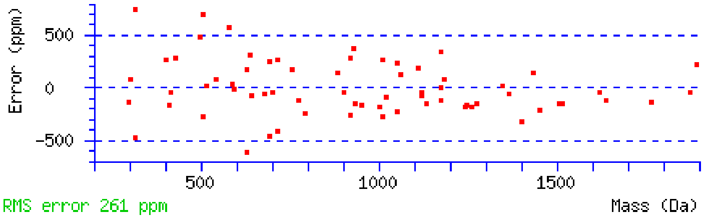

All matches to this query

Score	Mr(calc):	Delta	Sequence
64.1	2537.1122	0.0029	DSLNCSCLALQTSGQEEQVNQR
17.1	2536.1282	0.9869	DSLNCSCLALQTSGQEEQVNQR
10.0	2537.1122	0.0029	DSLNCSCLALQTSGQEEQVNQR
4.5	2537.0975	0.0176	ANTTGEPEEVSGALSLPSASAYVK
4.3	2537.1234	-0.0083	TLENGVNVTVSQINTMLGGRTR
3.9	2537.1234	-0.0083	TLENGVNVTVSQINTMLGGRTR
3.5	2537.1173	-0.0022	DPERAGLASSLLAGGTGHGDRGGAR
3.5	2537.1173	-0.0022	DPERAGLASSLLAGGTGHGDRGGAR
3.4	2537.1314	-0.0163	TFLDNLVNFEKLHMIADTVR
3.4	2537.1234	-0.0083	TLENGVNVTVSQINTMLGGRTR

Spectrum No: 278; Query: 1027; Rank: 1

Peptide View

MS/MS Fragmentation of **HVGPGVLSMANAGPNTNGSQFFICTIK**
Found in **IPI00201489**, Tax_Id=10116 Gene_Symbol=Ppif Peptidyl-prolyl cis-trans isomerase, mitochondrial precursor

Match to Query 1027: 2817.371262 from(940.131030,3+)
Title: 091127RatKid_SCX01_27.4544.4544.3.dta
Data file K:\NewmanPaper\Piliang\3SubProteomes\Piliang3SP\mgf5ppm\SCX_3SubProteomes5ppm.mgf

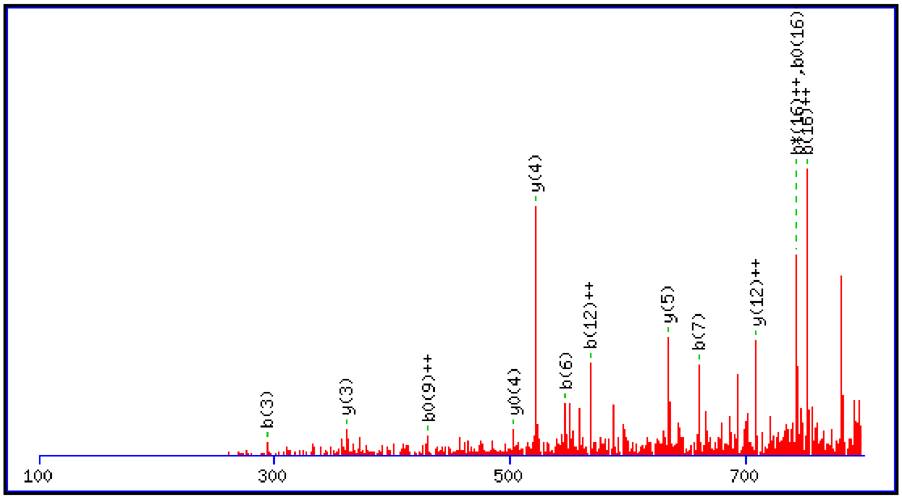

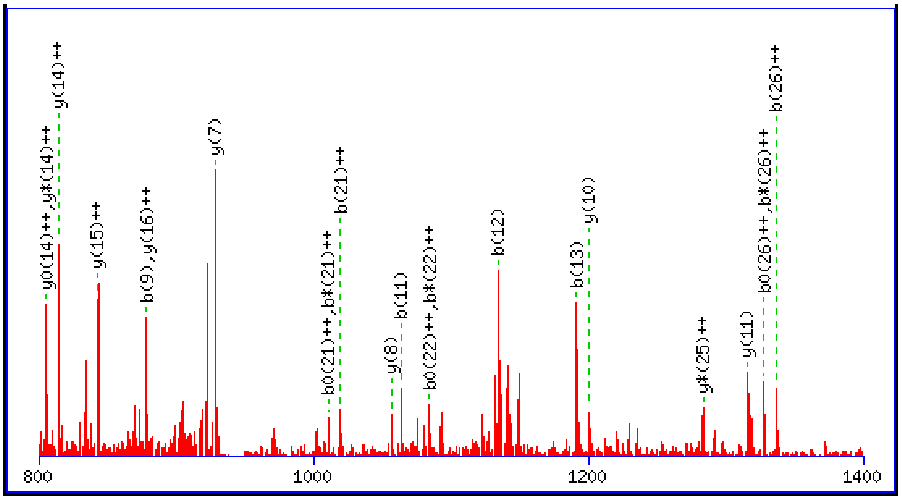

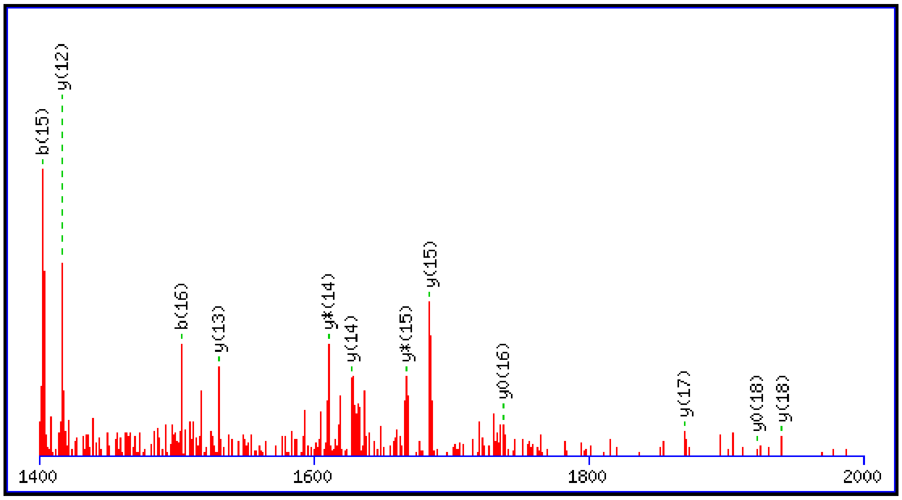

Monoisotopic mass of neutral peptide Mr(calc): 2817.3578
Fixed modifications: Carbamidomethyl (C)
Variable modifications:
N17 : Deamidated_N (N)
Ions Score: 64 **Expect:** 0.00011
Matches (Bold Red): 47/274 fragment ions using 76 most intense peaks

#	b	b^{++}	b*	b^{*++}	b^{0}	b^{0++}	Seq.	y	y^{++}	y*	y^{*++}	y^{0}	y^{0++}	#
1	138.0662	69.5367					H							27
2	237.1346	119.0709					V	2681.3062	1341.1567	2664.2796	1332.6435	2663.2956	1332.1514	26
3	**294.1561**	147.5817					G	2582.2378	1291.6225	2565.2112	**1283.1092**	2564.2272	1282.6172	25
4	391.2088	196.1081					P	2525.2163	1263.1118	2508.1898	1254.5985	2507.2057	1254.1065	24
5	448.2303	224.6188					G	2428.1635	1214.5854	2411.1370	1206.0721	2410.1530	1205.5801	23
6	**547.2987**	274.1530					V	2371.1421	1186.0747	2354.1155	1177.5614	2353.1315	1177.0694	22
7	**660.3828**	330.6950					L	2272.0737	1136.5405	2255.0471	1128.0272	2254.0631	1127.5352	21
8	747.4148	374.2110			729.4042	365.2058	S	2158.9896	1079.9984	2141.9630	1071.4852	2140.9790	1070.9932	20
9	**878.4553**	439.7313			860.4447	**430.7260**	M	2071.9576	1036.4824	2054.9310	1027.9691	2053.9470	1027.4771	19
10	949.4924	475.2498			931.4818	466.2446	A	**1940.9171**	970.9622	1923.8905	962.4489	**1922.9065**	961.9569	18
11	**1063.5353**	532.2713	1046.5088	523.7580	1045.5248	523.2660	N	**1869.8800**	935.4436	1852.8534	926.9303	1851.8694	926.4383	17
12	**1134.5724**	**567.7899**	1117.5459	559.2766	1116.5619	558.7846	A	1755.8370	**878.4222**	1738.8105	869.9089	**1737.8265**	869.4169	16
13	**1191.5939**	596.3006	1174.5674	587.7873	1173.5833	587.2953	G	**1684.7999**	**842.9036**	**1667.7734**	834.3903	1666.7894	833.8983	15
14	1288.6467	644.8270	1271.6201	636.3137	1270.6361	635.8217	P	**1627.7785**	**814.3929**	**1610.7519**	**805.8796**	1609.7679	**805.3876**	14
15	**1402.6896**	701.8484	1385.6630	693.3352	1384.6790	692.8432	N	**1530.7257**	765.8665	1513.6992	757.3532	1512.7151	756.8612	13
16	**1503.7373**	**752.3723**	1486.7107	**743.8590**	1485.7267	**743.3670**	T	**1416.6828**	**708.8450**	1399.6562	700.3318	1398.6722	699.8397	12
17	1618.7642	809.8857	1601.7377	801.3725	1600.7536	800.8805	N	**1315.6351**	658.3212	1298.6085	649.8079	1297.6245	649.3159	11
18	1675.7857	838.3965	1658.7591	829.8832	1657.7751	829.3912	G	**1200.6082**	600.8077	1183.5816	592.2944	1182.5976	591.8024	10

**19**	1762.8177	881.9125	1745.7912	873.3992	1744.8071	872.9072	**S**	1143.5867	572.2970	1126.5601	563.7837	1125.5761	563.2917	**9**
**20**	1890.8763	945.9418	1873.8497	937.4285	1872.8657	936.9365	**Q**	**1056.5547**	528.7810	1039.5281	520.2677	1038.5441	519.7757	**8**
**21**	2037.9447	**1019.4760**	2020.9181	**1010.9627**	2019.9341	**1010.4707**	**F**	**928.4961**	464.7517	911.4695	456.2384	910.4855	455.7464	**7**
**22**	2185.0131	1093.0102	2167.9866	**1084.4969**	2167.0025	**1084.0049**	**F**	781.4277	391.2175	764.4011	382.7042	763.4171	382.2122	**6**
**23**	2298.0972	1149.5522	2281.0706	1141.0389	2280.0866	1140.5469	**I**	**634.3593**	317.6833	617.3327	309.1700	616.3487	308.6780	**5**
**24**	2458.1278	1229.5675	2441.1013	1221.0543	2440.1173	1220.5623	**C**	**521.2752**	261.1412	504.2486	252.6280	**503.2646**	252.1360	**4**
**25**	2559.1755	1280.0914	2542.1490	1271.5781	2541.1649	1271.0861	**T**	**361.2445**	181.1259	344.2180	172.6126	343.2340	172.1206	**3**
**26**	2672.2596	**1336.6334**	2655.2330	**1328.1201**	2654.2490	**1327.6281**	**I**	260.1969	130.6021	243.1703	122.0888			**2**
**27**							**K**	147.1128	74.0600	130.0863	65.5468			**1**

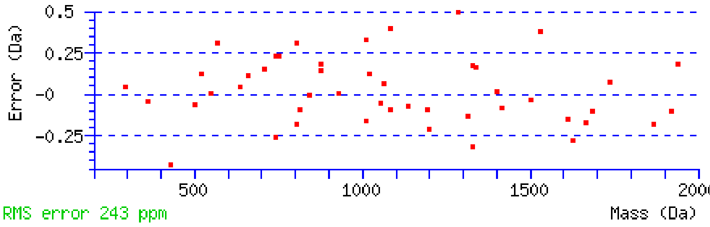

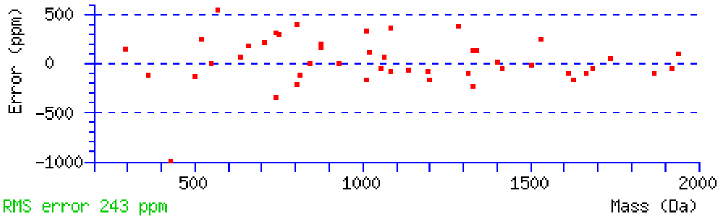

All matches to this query

Score	Mr(calc):	Delta	Sequence
64.1	2817.3578	0.0134	HVGPGVLSMANAGPNTNGSQFFICTIK
55.6	2817.3578	0.0134	HVGPGVLSMANAGPNTNGSQFFICTIK
33.5	2817.3578	0.0134	HVGPGVLSMANAGPNTNGSQFFICTIK
27.4	2816.3738	0.9974	HVGPGVLSMANAGPNTNGSQFFICTIK
1.2	2816.3812	0.9901	TVCMQTKPKVNVSSYIHFMLNFR
1.2	2815.3495	2.0218	KPPVVITWMSLDFYTKVLKSCK
0.8	2817.3747	-0.0035	GTSVIYIVKIPVKDHCIFFCEGR
0.8	2817.3747	-0.0035	GTSVIYIVKIPVKDHCIFFCEGR
0.8	2817.3747	-0.0035	GTSVIYIVKIPVKDHCIFFCEGR

Spectrum No: 279; Query: 896; Rank: 1

Peptide View

MS/MS Fragmentation of **ASFNITLIHPNNLTALSNMLPK**
Found in **IPI00230862**, Tax_Id=10116 Gene_Symbol=Anpep Aminopeptidase N

Match to Query 896: 2410.264328 from(1206.139440,2+)
Title: 091127RatKid_SCX01_19.6859.6859.2.dta
Data file K:\NewmanPaper\Piliang\3SubProteomes\Piliang3SP\mgf5ppm\SCX_3SubProteomes5ppm.mgf

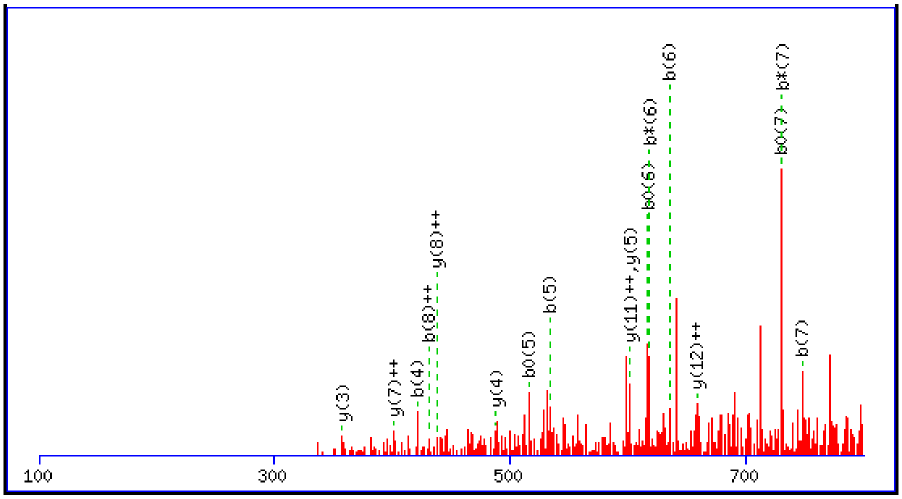

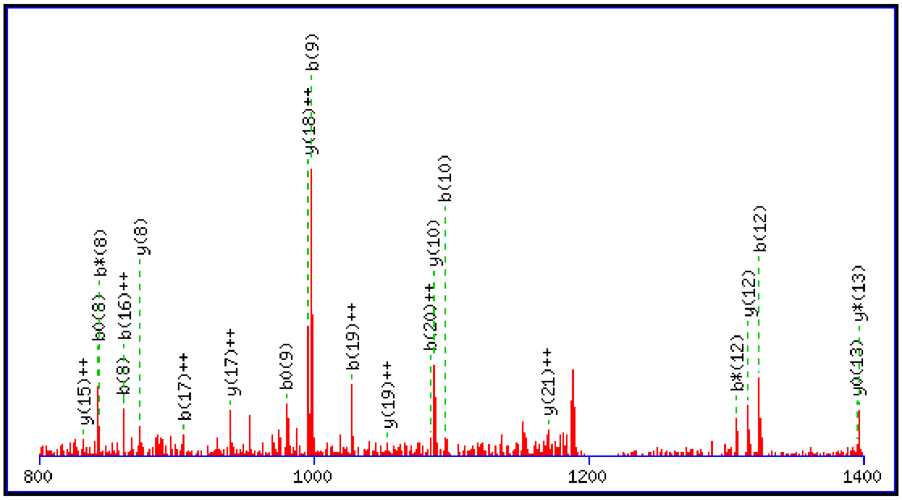

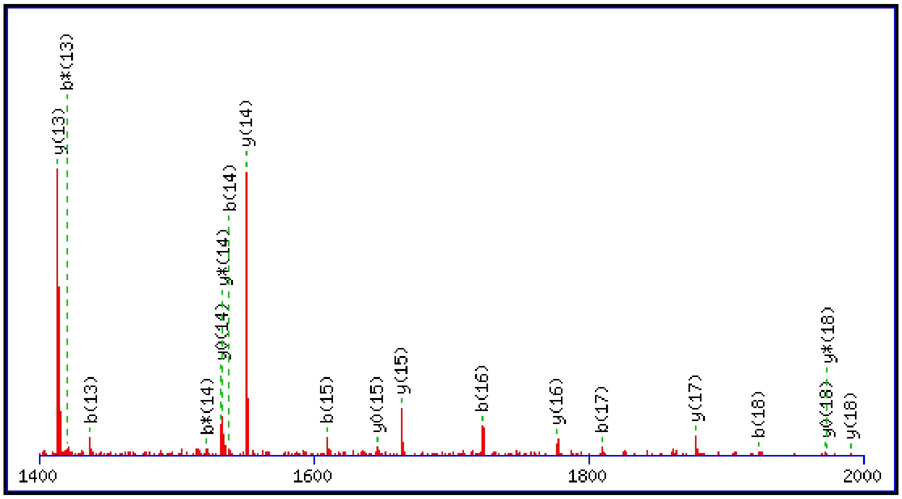

Monoisotopic mass of neutral peptide Mr(calc): 2410.2566
Fixed modifications: Carbamidomethyl (C)
Variable modifications:
N4 : Deamidated_N (N)
N12 : Deamidated_N (N)
Ions Score: 64 **Expect:** 5e-005
Matches (Bold Red): 58/234 fragment ions using 134 most intense peaks

#	b	b^{++}	b*	b^{*++}	b^0	b^{0++}	Seq.	y	y^{++}	y*	y^{*++}	y^0	y^{0++}	#
1	72.0444	36.5258					A							22
2	159.0764	80.0418			141.0659	71.0366	S	2340.2268	**1170.6170**	2323.2002	1162.1038	2322.2162	1161.6117	21
3	306.1448	153.5761			288.1343	144.5708	F	2253.1948	1127.1010	2236.1682	1118.5877	2235.1842	1118.0957	20
4	**421.1718**	211.0895	404.1452	202.5762	403.1612	202.0842	N	2106.1263	**1053.5668**	2089.0998	1045.0535	2088.1158	1044.5615	19

**5**	**534.2558**	267.6316	517.2293	259.1183	**516.2453**	258.6263	**I**	**1991.0994**	**996.0533**	**1974.0729**	987.5401	**1973.0888**	987.0481	**18**
**6**	**635.3035**	318.1554	**618.2770**	309.6421	**617.2929**	309.1501	**T**	**1878.0153**	**939.5113**	1860.9888	930.9980	1860.0048	930.5060	**17**
**7**	**748.3876**	374.6974	**731.3610**	366.1842	**730.3770**	365.6921	**L**	**1776.9677**	888.9875	1759.9411	880.4742	1758.9571	879.9822	**16**
**8**	**861.4716**	**431.2395**	**844.4451**	422.7262	**843.4611**	422.2342	**I**	**1663.8836**	**832.4454**	1646.8570	823.9322	**1645.8730**	823.4402	**15**
**9**	**998.5306**	499.7689	981.5040	491.2556	**980.5200**	490.7636	**H**	**1550.7995**	775.9034	**1533.7730**	767.3901	**1532.7890**	766.8981	**14**
**10**	**1095.5833**	548.2953	1078.5568	539.7820	1077.5728	539.2900	**P**	**1413.7406**	707.3739	**1396.7141**	698.8607	**1395.7301**	698.3687	**13**
**11**	1209.6262	605.3168	1192.5997	596.8035	1191.6157	596.3115	**N**	**1316.6879**	**658.8476**	1299.6613	650.3343	1298.6773	649.8423	**12**
**12**	**1324.6532**	662.8302	**1307.6266**	654.3170	1306.6426	653.8249	**N**	1202.6449	**601.8261**	1185.6184	593.3128	1184.6344	592.8208	**11**
**13**	**1437.7372**	719.3723	**1420.7107**	710.8590	1419.7267	710.3670	**L**	**1087.6180**	544.3126	1070.5914	535.7994	1069.6074	535.3074	**10**
**14**	**1538.7849**	769.8961	**1521.7584**	761.3828	1520.7744	760.8908	**T**	974.5339	487.7706	957.5074	479.2573	956.5234	478.7653	**9**
**15**	**1609.8220**	805.4147	1592.7955	796.9014	1591.8115	796.4094	**A**	**873.4863**	**437.2468**	856.4597	428.7335	855.4757	428.2415	**8**
**16**	**1722.9061**	**861.9567**	1705.8795	853.4434	1704.8955	852.9514	**L**	802.4491	**401.7282**	785.4226	393.2149	784.4386	392.7229	**7**
**17**	**1809.9381**	**905.4727**	1792.9116	896.9594	1791.9276	896.4674	**S**	689.3651	345.1862	672.3385	336.6729	671.3545	336.1809	**6**
**18**	**1923.9811**	962.4942	1906.9545	953.9809	1905.9705	953.4889	**N**	**602.3330**	301.6702	585.3065	293.1569			**5**
**19**	2055.0215	**1028.0144**	2037.9950	1019.5011	2037.0110	1019.0091	**M**	**488.2901**	244.6487	471.2636	236.1354			**4**
**20**	2168.1056	**1084.5564**	2151.0791	1076.0432	2150.0950	1075.5512	**L**	**357.2496**	179.1285	340.2231	170.6152			**3**
**21**	2265.1584	1133.0828	2248.1318	1124.5695	2247.1478	1124.0775	**P**	244.1656	122.5864	227.1390	114.0731			**2**
**22**							**K**	147.1128	74.0600	130.0863	65.5468			**1**

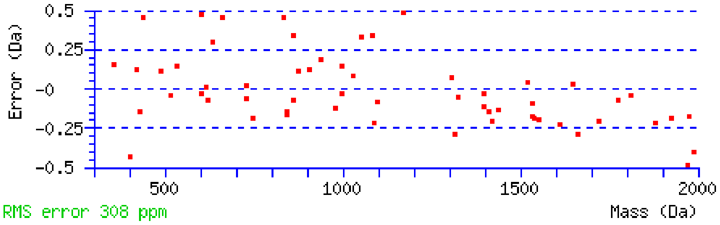

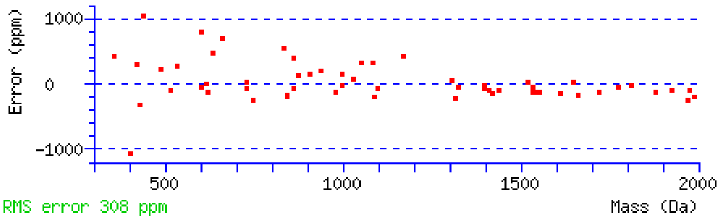

All matches to this query

Score	Mr(calc):	Delta	Sequence
64.0	2410.2566	0.0078	ASFNITLIHPNNLTALSNMLPK
64.0	2410.2566	0.0078	ASFNITLIHPNNLTALSNMLPK
39.1	2410.2566	0.0078	ASFNITLIHPNNLTALSNMLPK
29.0	2410.2566	0.0078	ASFNITLIHPNNLTALSNMLPK
22.1	2409.2726	0.9918	ASFNITLIHPNNLTALSNMLPK
22.1	2409.2726	0.9918	ASFNITLIHPNNLTALSNMLPK
14.2	2410.2566	0.0078	ASFNITLIHPNNLTALSNMLPK
14.2	2410.2566	0.0078	ASFNITLIHPNNLTALSNMLPK
11.9	2409.2726	0.9918	ASFNITLIHPNNLTALSNMLPK
6.3	2409.2726	0.9918	ASFNITLIHPNNLTALSNMLPK

Spectrum No: 280; Query: 437; Rank: 1

Peptide View

MS/MS Fragmentation of **TEIQVNCLSAPGENK**
Found in **IPI00471762**, Tax_Id=10116 Gene_Symbol=Sypl Synaptophysin-like protein

Match to Query 437: 1659.780328 from(830.897440,2+)
Title: 091127RatKid_SCX01_02.1965.1965.2.dta
Data file K:\NewmanPaper\Piliang\3SubProteomes\Piliang3SP\mgf5ppm\SCX_3SubProteomes5ppm.mgf

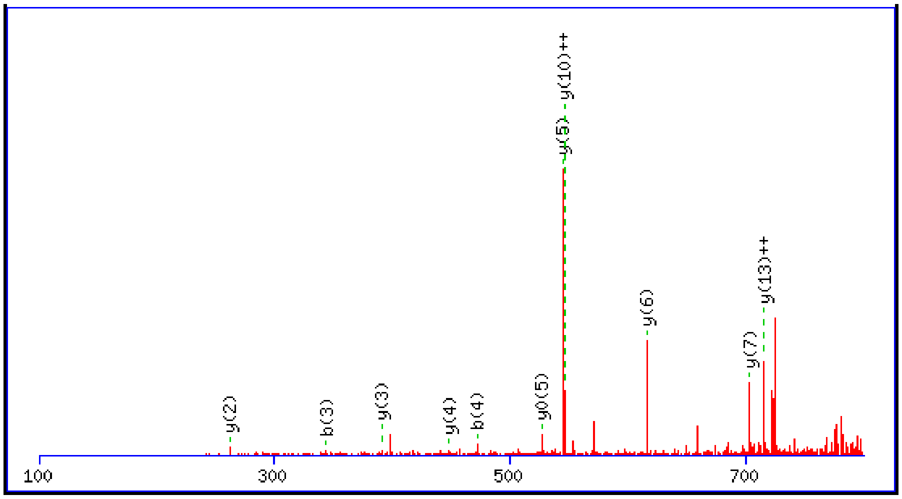

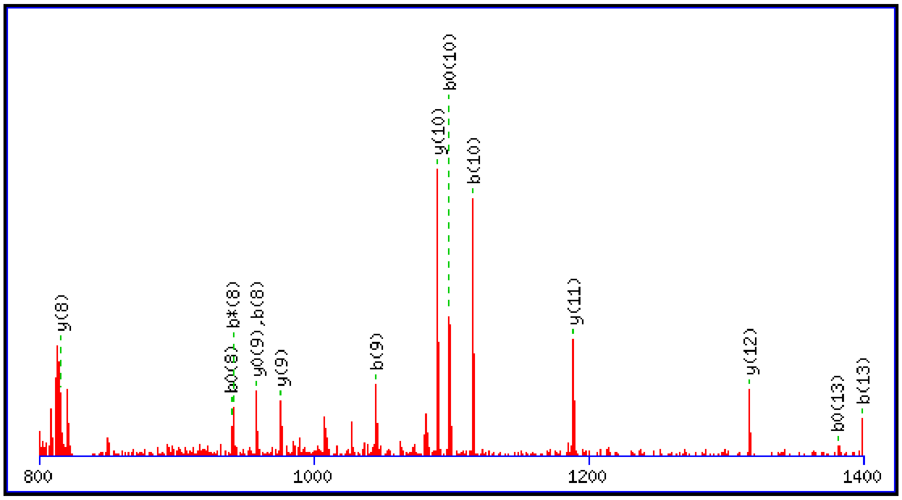

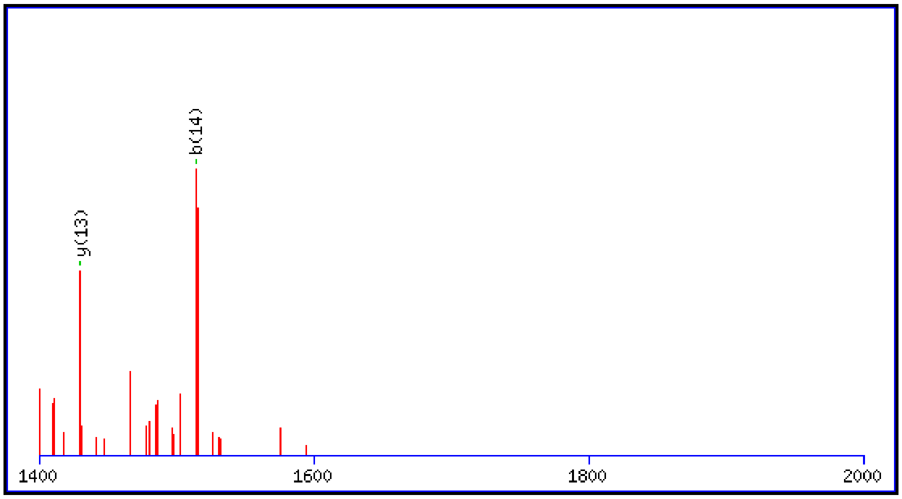

Monoisotopic mass of neutral peptide Mr(calc): 1659.7774
Fixed modifications: Carbamidomethyl (C)
Variable modifications:
N14 : Deamidated_N (N)
Ions Score: 64 **Expect:** 7.7e-005
Matches (Bold Red): 27/158 fragment ions using 52 most intense peaks

#	b	b++	b*	b*++	b0	b0++	Seq.	y	y++	y*	y*++	y0	y0++	#
1	102.0550	51.5311			84.0444	42.5258	T							15
2	231.0975	116.0524			213.0870	107.0471	E	1559.7370	780.3721	1542.7104	771.8589	1541.7264	771.3669	14
3	**344.1816**	172.5944			326.1710	163.5892	I	**1430.6944**	**715.8508**	1413.6678	707.3376	1412.6838	706.8456	13
4	**472.2402**	236.6237	455.2136	228.1105	454.2296	227.6185	Q	**1317.6103**	659.3088	1300.5838	650.7955	1299.5998	650.3035	12
5	571.3086	286.1579	554.2821	277.6447	553.2980	277.1527	V	**1189.5518**	595.2795	1172.5252	586.7662	1171.5412	586.2742	11

6	685.3515	343.1794	668.3250	334.6661	667.3410	334.1741	N	**1090.4833**	**545.7453**	1073.4568	537.2320	1072.4728	536.7400	10
7	845.3822	423.1947	828.3556	414.6815	827.3716	414.1894	C	**976.4404**	488.7238	959.4139	480.2106	**958.4299**	479.7186	9
8	**958.4662**	479.7368	**941.4397**	471.2235	**940.4557**	470.7315	L	**816.4098**	408.7085	799.3832	400.1952	798.3992	399.7032	8
9	**1045.4983**	523.2528	1028.4717	514.7395	1027.4877	514.2475	S	**703.3257**	352.1665	686.2992	343.6532	685.3151	343.1612	7
10	**1116.5354**	558.7713	1099.5088	550.2581	**1098.5248**	549.7660	A	**616.2937**	308.6505	599.2671	300.1372	598.2831	299.6452	6
11	1213.5882	607.2977	1196.5616	598.7844	1195.5776	598.2924	P	**545.2566**	273.1319	528.2300	264.6186	**527.2460**	264.1266	5
12	1270.6096	635.8084	1253.5831	627.2952	1252.5990	626.8032	G	**448.2038**	224.6055	431.1772	216.0923	430.1932	215.6003	4
13	**1399.6522**	700.3297	1382.6257	691.8165	**1381.6416**	691.3245	E	**391.1823**	196.0948	374.1558	187.5815	373.1718	187.0895	3
14	**1514.6791**	757.8432	1497.6526	749.3299	1496.6686	748.8379	N	**262.1397**	131.5735	245.1132	123.0602			2
15							K	147.1128	74.0600	130.0863	65.5468			1

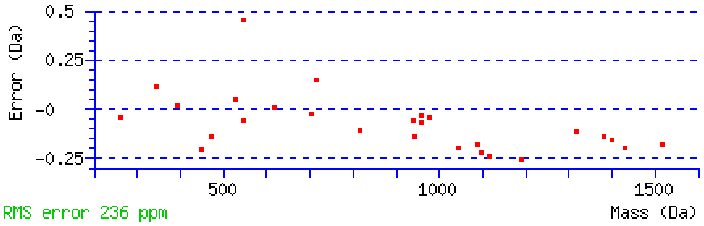

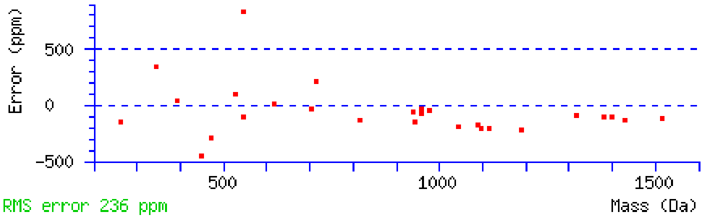

All matches to this query

Score	Mr(calc):	Delta	Sequence
63.9	1659.7774	0.0030	TEIQVNCLSAPGENK
19.0	1658.7934	0.9870	TEIQVNCLSAPGENK
18.9	1659.7774	0.0030	TEIQVNCLSAPGENK
5.6	1659.7960	-0.0157	QHSSSNMVIMLIGNK
5.3	1659.7926	-0.0123	MYSFNKIGRSEPSK
5.0	1659.7757	0.0047	NEDYVAVSNLTKVK
4.2	1659.7757	0.0047	QLKTVEDYQTLNK
4.2	1659.7885	-0.0082	LVDAGSDALSLKLAK
2.6	1658.7739	1.0064	YNVSSTPPLQSMKK
1.7	1659.7869	-0.0066	LFTDASSRSIGALNK

Spectrum No: 281; Query: 245; Rank: 1

Peptide View

MS/MS Fragmentation of **NAIANASTLAEVER**
Found in **IPI00192279**, Tax_Id=10116 Gene_Symbol=- 28 kDa protein

Match to Query 245: 1458.733008 from(730.373780,2+)
Title: 091127RatKid_SCX01_14.2787.2787.2.dta
Data file K:\NewmanPaper\Piliang\3SubProteomes\Piliang3SP\mgf5ppm\SCX_3SubProteomes5ppm.mgf

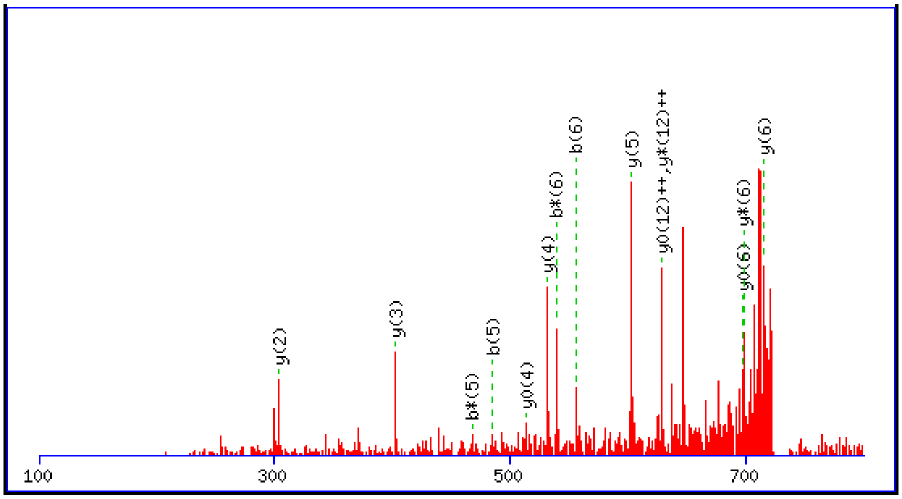

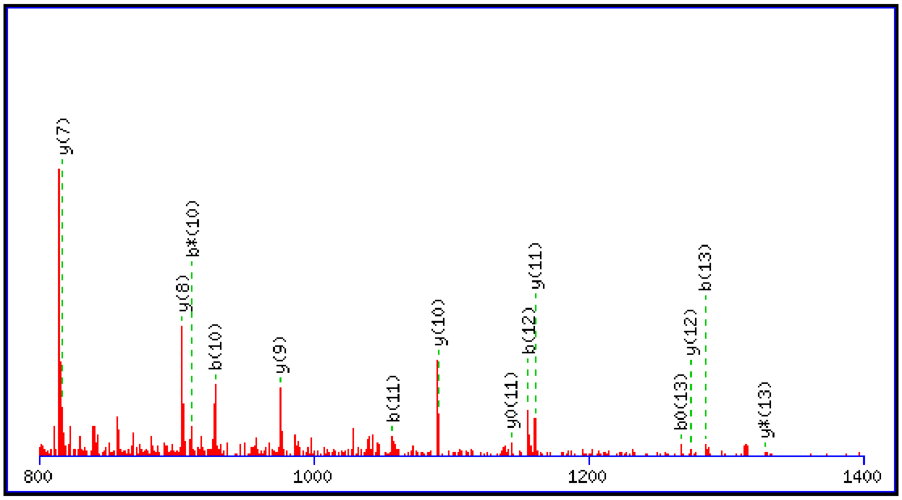

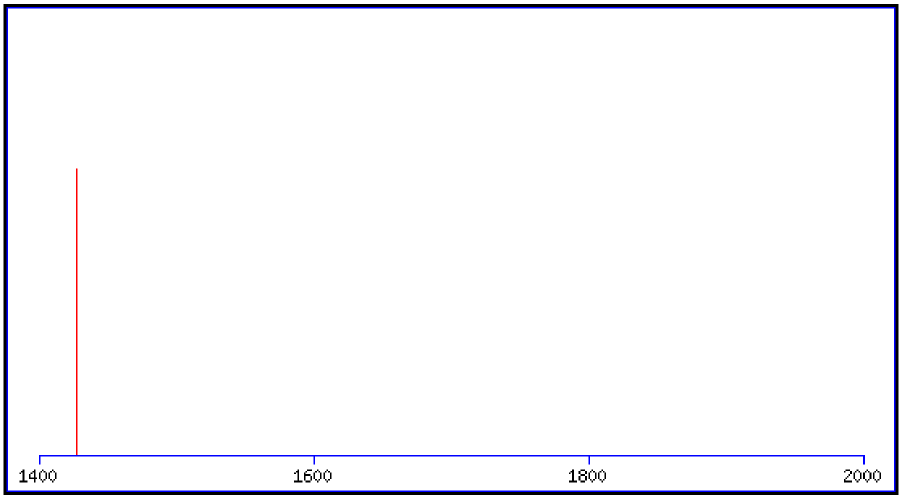

Monoisotopic mass of neutral peptide Mr(calc): 1458.7314
Fixed modifications: Carbamidomethyl (C)
Variable modifications:
N5 : Deamidated_N (N)
Ions Score: 64 **Expect:** 5.3e-005
Matches (Bold Red): 28/142 fragment ions using 68 most intense peaks

#	b	b++	b*	b*++	b0	b0++	Seq.	y	y++	y*	y*++	y0	y0++	#
1	115.0502	58.0287	98.0237	49.5155			N							14
2	186.0873	93.5473	169.0608	85.0340			A	1345.6958	673.3515	**1328.6692**	664.8383	1327.6852	664.3462	13
3	299.1714	150.0893	282.1448	141.5761			I	**1274.6587**	637.8330	1257.6321	**629.3197**	1256.6481	**628.8277**	12
4	370.2085	185.6079	353.1819	177.0946			A	**1161.5746**	581.2909	1144.5481	572.7777	**1143.5640**	572.2857	11
5	**485.2354**	243.1214	**468.2089**	234.6081			N	**1090.5375**	545.7724	1073.5109	537.2591	1072.5269	536.7671	10

**6**	**556.2725**	278.6399	**539.2460**	270.1266			**A**	**975.5106**	488.2589	958.4840	479.7456	957.5000	479.2536	**9**
**7**	643.3046	322.1559	626.2780	313.6427	625.2940	313.1506	**S**	**904.4734**	452.7404	887.4469	444.2271	886.4629	443.7351	**8**
**8**	744.3523	372.6798	727.3257	364.1665	726.3417	363.6745	**T**	**817.4414**	409.2243	800.4149	400.7111	799.4308	400.2191	**7**
**9**	857.4363	429.2218	840.4098	420.7085	839.4258	420.2165	**L**	**716.3937**	358.7005	**699.3672**	350.1872	**698.3832**	349.6952	**6**
**10**	**928.4734**	464.7404	**911.4469**	456.2271	910.4629	455.7351	**A**	**603.3097**	302.1585	586.2831	293.6452	585.2991	293.1532	**5**
**11**	**1057.5160**	529.2616	1040.4895	520.7484	1039.5055	520.2564	**E**	**532.2726**	266.6399	515.2460	258.1266	**514.2620**	257.6346	**4**
**12**	**1156.5844**	578.7959	1139.5579	570.2826	1138.5739	569.7906	**V**	**403.2300**	202.1186	386.2034	193.6053	385.2194	193.1133	**3**
**13**	**1285.6270**	643.3172	1268.6005	634.8039	**1267.6165**	634.3119	**E**	**304.1615**	152.5844	287.1350	144.0711	286.1510	143.5791	**2**
**14**							**R**	175.1190	88.0631	158.0924	79.5498			**1**

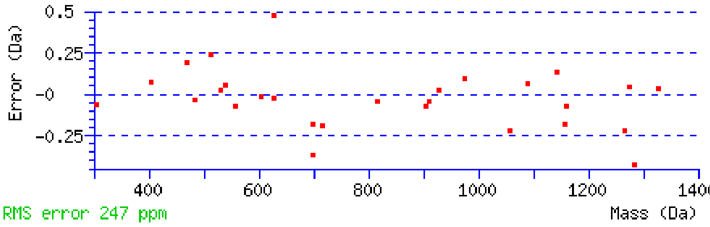

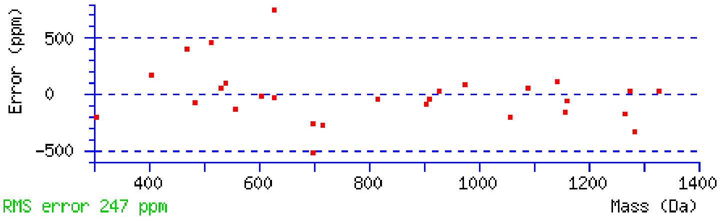

All matches to this query

Score	Mr(calc):	Delta	Sequence
63.7	1458.7314	0.0016	NAIANASTLAEVER
55.8	1458.7314	0.0016	NAIANASTLAEVER
10.8	1458.7361	-0.0031	SDTVTRPGPAMRR
6.1	1458.7314	0.0016	QGLVSKLDGEEER
3.8	1458.7466	-0.0136	NLAANRPPNTYVK
3.5	1458.7426	-0.0096	ALQTERNDLNKR
3.4	1458.7314	0.0016	QLEEESKAELQR
1.7	1458.7289	0.0041	WIADLPSMQLNR
1.5	1458.7256	0.0075	QYPEQGPQLPFR
1.0	1458.7314	0.0016	TVQQVNQEKQEK

Spectrum No: 282; Query: 333; Rank: 1

Peptide View

MS/MS Fragmentation of **QIDYILGQYNTTK**
Found in **IPI00325312**, Tax_Id=10116 Gene_Symbol=Prom1 prominin 1 isoform 1

Match to Query 333: 1556.775668 from(779.395110,2+)
Title: 091127RatKid_SCX01_12.2672.2672.2.dta
Data file K:\NewmanPaper\Piliang\3SubProteomes\Piliang3SP\mgf5ppm\SCX_3SubProteomes5ppm.mgf

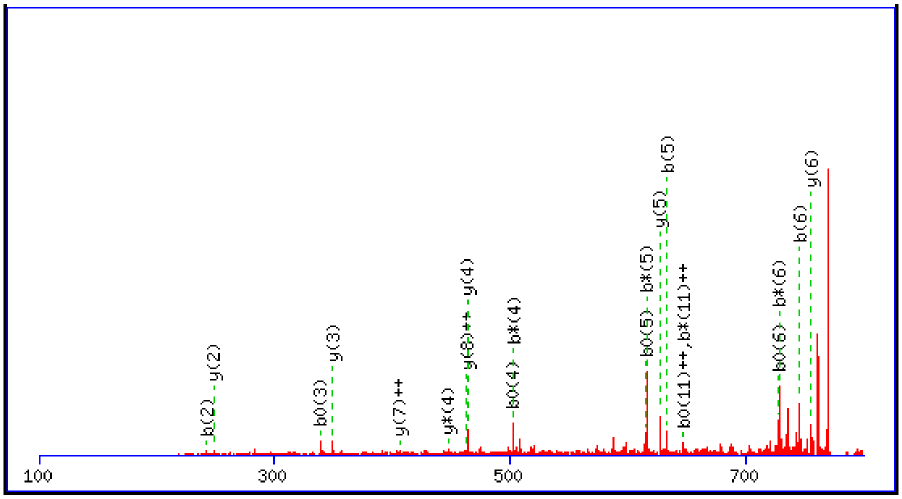

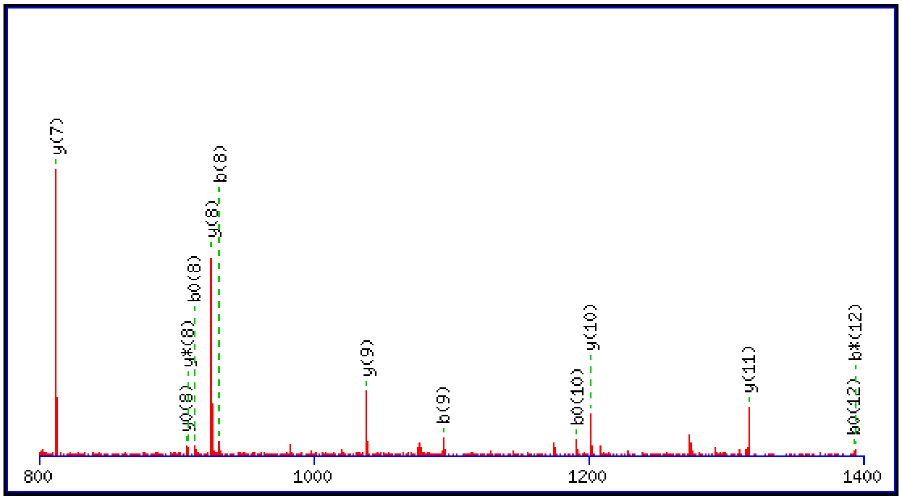

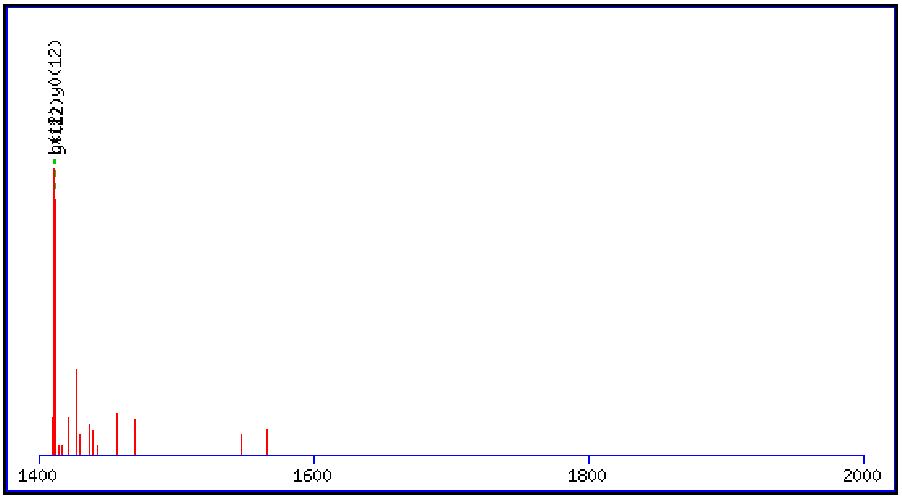

Monoisotopic mass of neutral peptide Mr(calc): 1556.7722
Fixed modifications: Carbamidomethyl (C)
Variable modifications:
N10 : Deamidated_N (N)
Ions Score: 64 **Expect:** 6.8e-005
Matches (Bold Red): 36/138 fragment ions using 60 most intense peaks

#	b	b^{++}	b*	b^{*++}	b^0	b^{0++}	Seq.	y	y^{++}	y*	y^{*++}	y^0	y^{0++}	#
1	129.0659	65.0366	112.0393	56.5233			Q							13
2	**242.1499**	121.5786	225.1234	113.0653			I	1429.7209	715.3641	**1412.6944**	706.8508	**1411.7104**	706.3588	12
3	357.1769	179.0921	340.1503	170.5788	**339.1663**	170.0868	D	**1316.6369**	658.8221	1299.6103	650.3088	1298.6263	649.8168	11
4	520.2402	260.6237	**503.2136**	252.1105	**502.2296**	251.6185	Y	**1201.6099**	601.3086	1184.5834	592.7953	1183.5994	592.3033	10
5	**633.3243**	317.1658	**616.2977**	308.6525	**615.3137**	308.1605	I	**1038.5466**	519.7769	1021.5200	511.2637	1020.5360	510.7717	9

**6**	**746.4083**	373.7078	**729.3818**	365.1945	**728.3978**	364.7025	**L**	**925.4625**	**463.2349**	**908.4360**	454.7216	**907.4520**	454.2296	**8**
**7**	803.4298	402.2185	786.4032	393.7053	785.4192	393.2132	**G**	**812.3785**	**406.6929**	795.3519	398.1796	794.3679	397.6876	**7**
**8**	**931.4884**	466.2478	914.4618	457.7345	**913.4778**	457.2425	**Q**	**755.3570**	378.1821	738.3305	369.6689	737.3464	369.1769	**6**
**9**	**1094.5517**	547.7795	1077.5251	539.2662	1076.5411	538.7742	**Y**	**627.2984**	314.1529	610.2719	305.6396	609.2879	305.1476	**5**
**10**	1209.5786	605.2930	1192.5521	596.7797	**1191.5681**	596.2877	**N**	**464.2351**	232.6212	**447.2085**	224.1079	446.2245	223.6159	**4**
**11**	1310.6263	655.8168	1293.5998	**647.3035**	1292.6157	**646.8115**	**T**	**349.2082**	175.1077	332.1816	166.5944	331.1976	166.1024	**3**
**12**	**1411.6740**	706.3406	**1394.6474**	697.8274	**1393.6634**	697.3353	**T**	**248.1605**	124.5839	231.1339	116.0706	230.1499	115.5786	**2**
**13**							**K**	147.1128	74.0600	130.0863	65.5468			**1**

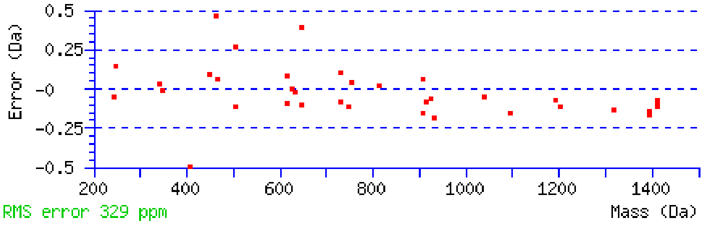

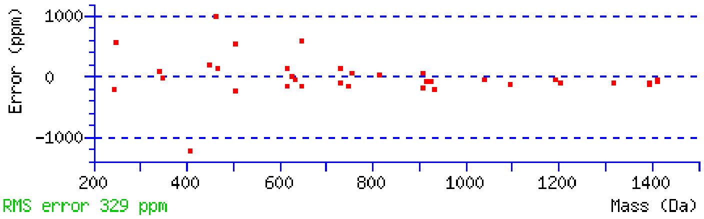

All matches to this query

Score	Mr(calc):	Delta	Sequence
63.7	1556.7722	0.0035	QIDYILGQYNTTK
8.0	1556.7712	0.0045	DSRGHLGLVANPLK
5.5	1556.7633	0.0123	MPASKTTKVQTASK
5.3	1556.7738	0.0018	ELLNIDLIKDYK
3.3	1556.7712	0.0045	IKYDAIARQGQSK
1.6	1556.7786	-0.0029	GPLAVLTCIATHPK
0.8	1555.7599	1.0157	NCGVLVHCLAGISR
0.6	1554.7799	1.9957	VVMMIRPNPNTFH
0.2	1556.7868	-0.0111	EMENKETLKGLHK

Spectrum No: 283; Query: 336; Rank: 1

Peptide View

MS/MS Fragmentation of **DGNCTSPQALCNAR**
Found in **IPI00205325**, Tax_Id=10116 Gene_Symbol=Lrp2 Low-density lipoprotein receptor-related protein 2 precursor

Match to Query 336: 1563.637768 from(782.826160,2+)
Title: 091129RatKid_SCX02_16.630.630.2.dta
Data file K:\NewmanPaper\Piliang\3SubProteomes\Piliang3SP\mgf5ppm\SCX_3SubProteomes5ppm.mgf

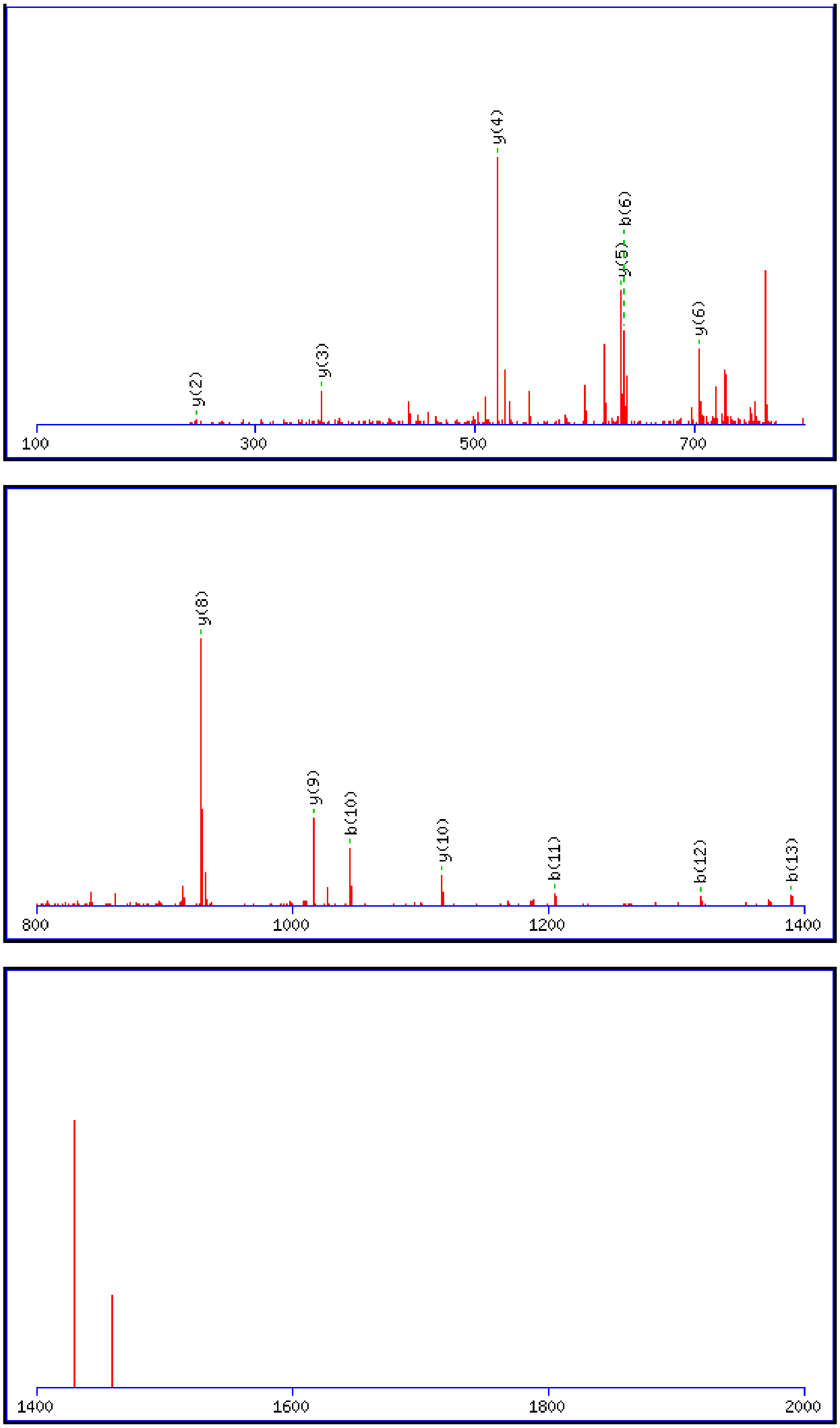

Monoisotopic mass of neutral peptide Mr(calc): 1563.6406
Fixed modifications: Carbamidomethyl (C)
Variable modifications:
N3 : Deamidated_N (N)
Ions Score: 64 **Expect:** 5.3e-005
Matches (Bold Red): 13/136 fragment ions using 20 most intense peaks

#	b	b^{++}	b*	b^{*++}	b^{0}	b^{0++}	Seq.	y	y^{++}	y*	y^{*++}	y^{0}	y^{0++}	#
1	116.0342	58.5207			98.0237	49.5155	D							14
2	173.0557	87.0315			155.0451	78.0262	G	1449.6209	725.3141	1432.5944	716.8008	1431.6104	716.3088	13
3	288.0826	144.5449	271.0561	136.0317	270.0721	135.5397	N	1392.5995	696.8034	1375.5729	688.2901	1374.5889	687.7981	12
4	448.1133	224.5603	431.0867	216.0470	430.1027	215.5550	C	1277.5725	639.2899	1260.5460	630.7766	1259.5620	630.2846	11
5	549.1609	275.0841	532.1344	266.5708	531.1504	266.0788	T	**1117.5419**	559.2746	1100.5153	550.7613	1099.5313	550.2693	10

**6**	**636.1930**	318.6001	619.1664	310.0869	618.1824	309.5948	**S**	**1016.4942**	508.7507	999.4676	500.2375	998.4836	499.7455	**9**
**7**	733.2457	367.1265	716.2192	358.6132	715.2352	358.1212	**P**	**929.4622**	465.2347	912.4356	456.7214			**8**
**8**	861.3043	431.1558	844.2778	422.6425	843.2938	422.1505	**Q**	832.4094	416.7083	815.3828	408.1951			**7**
**9**	932.3414	466.6744	915.3149	458.1611	914.3309	457.6691	**A**	**704.3508**	352.6790	687.3243	344.1658			**6**
**10**	**1045.4255**	523.2164	1028.3989	514.7031	1027.4149	514.2111	**L**	**633.3137**	317.1605	616.2872	308.6472			**5**
**11**	**1205.4561**	603.2317	1188.4296	594.7184	1187.4456	594.2264	**C**	**520.2296**	260.6185	503.2031	252.1052			**4**
**12**	**1319.4991**	660.2532	1302.4725	651.7399	1301.4885	651.2479	**N**	**360.1990**	180.6031	343.1724	172.0899			**3**
**13**	**1390.5362**	695.7717	1373.5096	687.2585	1372.5256	686.7664	**A**	**246.1561**	123.5817	229.1295	115.0684			**2**
**14**							**R**	175.1190	88.0631	158.0924	79.5498			**1**

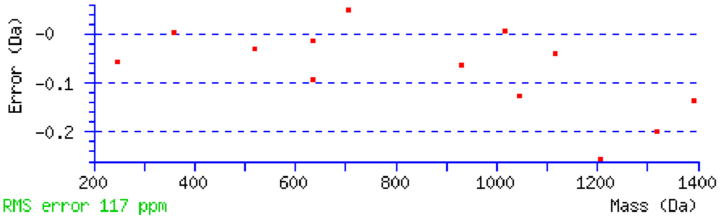

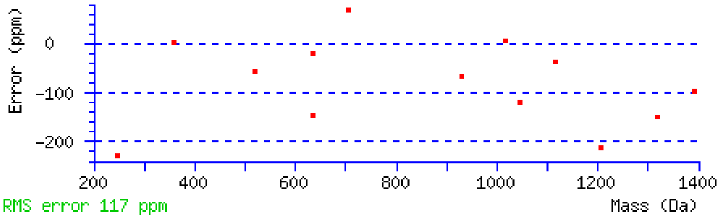

All matches to this query

Score	Mr(calc):	Delta	Sequence
63.6	1563.6406	-0.0028	DGNCTSPQALCNAR
16.8	1563.6283	0.0095	MRSESQRMSNAR
16.8	1563.6283	0.0095	MRSESQRMSNAR
10.2	1561.6418	1.9959	DKPYACDICGKR
8.8	1563.6517	-0.0140	SPGSESKVLGCKR
6.1	1563.6406	-0.0028	DGNCTSPQALCNAR
5.5	1563.6429	-0.0051	DMNDYPPVFSKR
5.4	1562.6469	0.9908	MERSLELANMTR
3.9	1563.6517	-0.0140	SPGSESKVLGCKR
3.3	1563.6355	0.0023	ASSFYGSASSNHLR

Spectrum No: 284; Query: 167; Rank: 1

Peptide View

MS/MS Fragmentation of **LLNLTNDGHSVR**
Found in **IPI00366665**, Tax_Id=10116 Gene_Symbol=Car12 Membrane-bound carbonic anhydrase 12

Match to Query 167: 1338.689628 from(670.352090,2+)
Title: 091127RatKid_SCX01_15.1516.1516.2.dta
Data file K:\NewmanPaper\Piliang\3SubProteomes\Piliang3SP\mgf5ppm\SCX_3SubProteomes5ppm.mgf

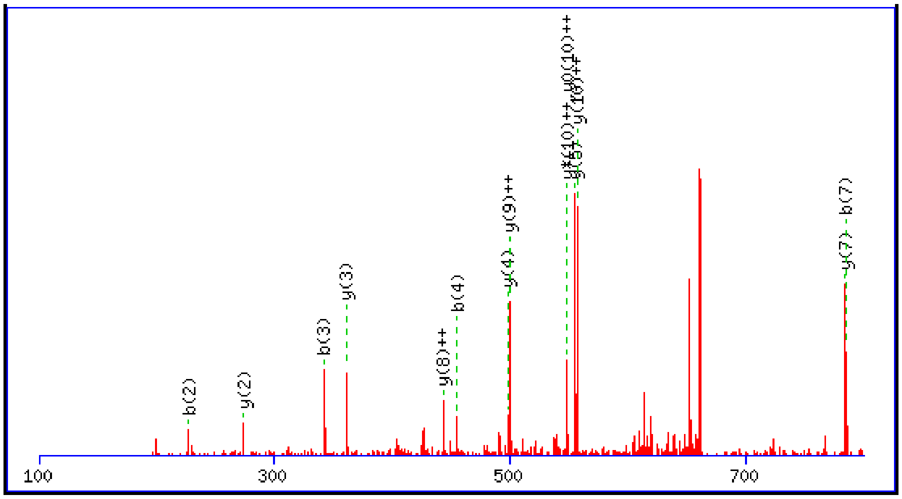

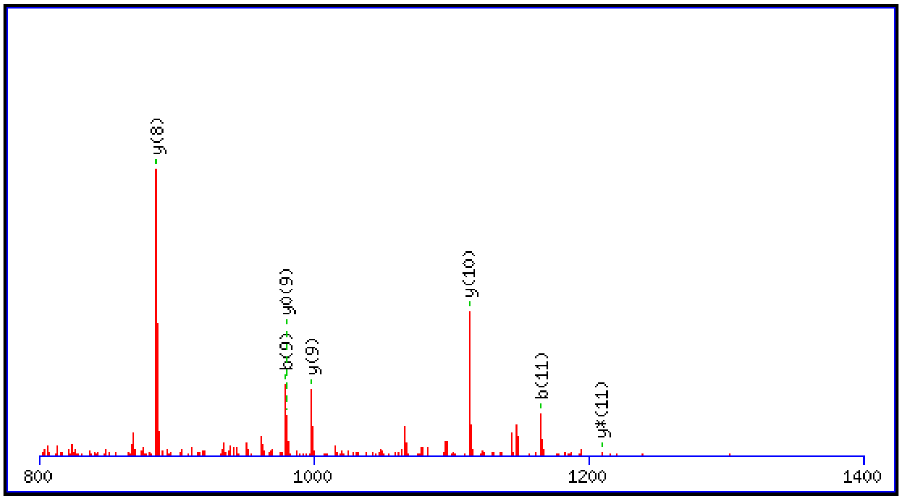

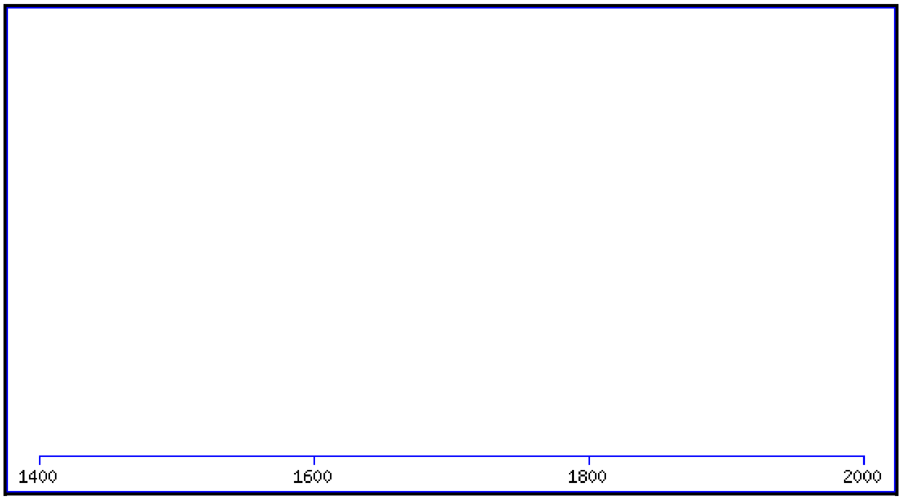

Monoisotopic mass of neutral peptide Mr(calc): 1338.6892
Fixed modifications: Carbamidomethyl (C)
Variable modifications:
N3 : Deamidated_N (N)
Ions Score: 64 **Expect:** 6e-005
Matches (Bold Red): 21/116 fragment ions using 30 most intense peaks

#	b	b^{++}	b*	b^{*++}	b^0	b^{0++}	Seq.	y	y^{++}	y*	y^{*++}	y^0	y^{0++}	#
1	114.0913	57.5493					L							12
2	**227.1754**	114.0913					L	1226.6124	613.8098	**1209.5858**	605.2966	1208.6018	604.8045	11
3	**342.2023**	171.6048	325.1758	163.0915			N	**1113.5283**	**557.2678**	1096.5018	**548.7545**	1095.5178	**548.2625**	10
4	**455.2864**	228.1468	438.2599	219.6336			L	**998.5014**	**499.7543**	981.4748	491.2411	**980.4908**	490.7490	9
5	556.3341	278.6707	539.3075	270.1574	538.3235	269.6654	T	**885.4173**	**443.2123**	868.3908	434.6990	867.4068	434.2070	8

**6**	670.3770	335.6921	653.3505	327.1789	652.3664	326.6869	**N**	**784.3696**	392.6885	767.3431	384.1752	766.3591	383.6832	**7**
**7**	**785.4040**	393.2056	768.3774	384.6923	767.3934	384.2003	**D**	670.3267	335.6670	653.3002	327.1537	652.3161	326.6617	**6**
**8**	842.4254	421.7163	825.3989	413.2031	824.4149	412.7111	**G**	**555.2998**	278.1535	538.2732	269.6402	537.2892	269.1482	**5**
**9**	**979.4843**	490.2458	962.4578	481.7325	961.4738	481.2405	**H**	**498.2783**	249.6428	481.2518	241.1295	480.2677	240.6375	**4**
**10**	1066.5164	533.7618	1049.4898	525.2485	1048.5058	524.7565	**S**	**361.2194**	181.1133	344.1928	172.6001	343.2088	172.1081	**3**
**11**	**1165.5848**	583.2960	1148.5582	574.7827	1147.5742	574.2907	**V**	**274.1874**	137.5973	257.1608	129.0840			**2**
**12**							**R**	175.1190	88.0631	158.0924	79.5498			**1**

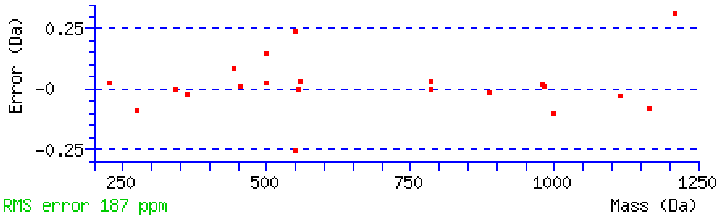

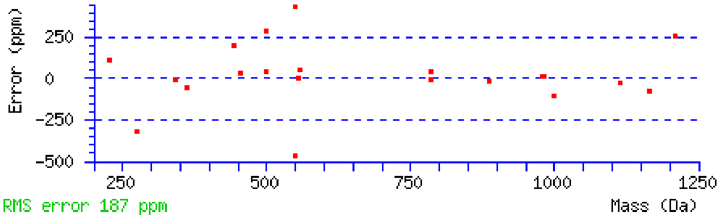

All matches to this query

Score	Mr(calc):	Delta	Sequence
63.5	1338.6892	0.0005	LLNLTNDGHSVR
49.3	1338.6892	0.0005	LLNLTNDGHSVR
10.1	1338.6769	0.0128	IRETARASLSR
9.5	1338.6769	0.0127	LLGNSSRTRVR
9.1	1338.7020	-0.0124	RTLKGSIELSR
8.8	1338.6769	0.0127	LLGNSSRTRVR
8.7	1338.6769	0.0127	LLGNSSRTRVR
7.3	1338.7020	-0.0124	SLRTGSALLSVR
7.2	1338.6809	0.0087	FGGLPSRAVTVR
6.2	1338.6932	-0.0035	LLFSSEKSSWR

Spectrum No: 285; Query: 1074; Rank: 1

Peptide View

MS/MS Fragmentation of **TKSENGLEFTSSGSANTETTKVNGSLETK**
Found in **IPI00421874**, Tax_Id=10116 Gene_Symbol=Vdac1 Voltage-dependent anion-selective channel protein 1

Match to Query 1074: 3017.429712 from(1006.817180,3+)
Title: 091129RatKid_SCX02_34.1808.1808.3.dta
Data file K:\NewmanPaper\Piliang\3SubProteomes\Piliang3SP\mgf5ppm\SCX_3SubProteomes5ppm.mgf

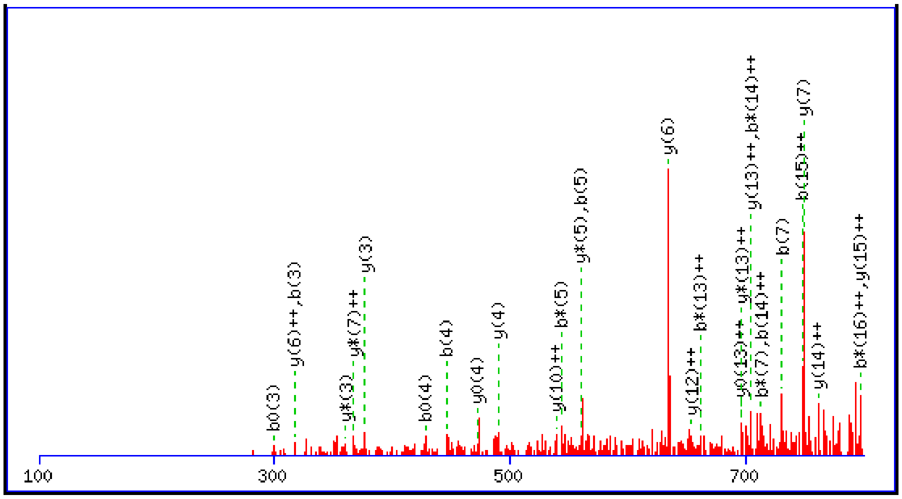

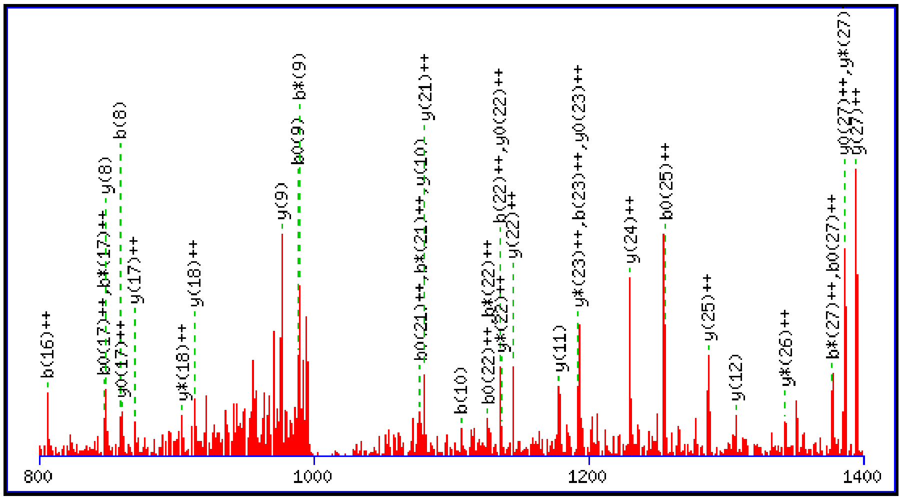

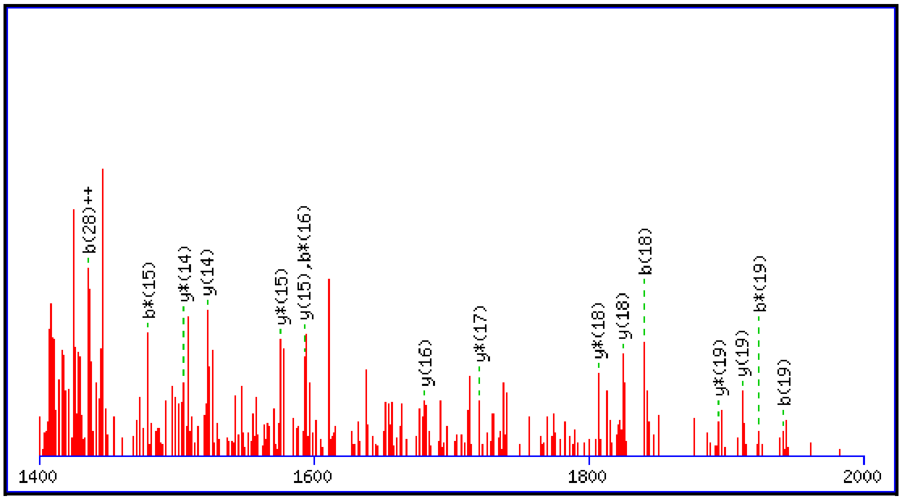

Monoisotopic mass of neutral peptide Mr(calc): 3017.4313
Fixed modifications: Carbamidomethyl (C)
Variable modifications:
N23 : Deamidated_N (N)
Ions Score: 64 **Expect:** 0.00015
Matches (Bold Red): 83/332 fragment ions using 162 most intense peaks

#	b	b++	b*	b*++	b0	b0++	Seq.	y	y++	y*	y*++	y0	y0++	#
1	102.0550	51.5311			84.0444	42.5258	T							29
2	230.1499	115.5786	213.1234	107.0653	212.1394	106.5733	K	2917.3909	1459.1991	2900.3643	1450.6858	2899.3803	1450.1938	28
3	**317.1819**	159.0946	300.1554	150.5813	**299.1714**	150.0893	S	2789.2959	**1395.1516**	2772.2694	**1386.6383**	2771.2854	**1386.1463**	27
4	**446.2245**	223.6159	429.1980	215.1026	**428.2140**	214.6106	E	2702.2639	1351.6356	2685.2373	**1343.1223**	2684.2533	1342.6303	26
5	**560.2675**	280.6374	**543.2409**	272.1241	542.2569	271.6321	N	2573.2213	**1287.1143**	2556.1948	1278.6010	2555.2107	1278.1090	25

6	617.2889	309.1481	600.2624	300.6348	599.2784	300.1428	G	2459.1784	**1230.0928**	2442.1518	1221.5796	2441.1678	1221.0875	24
7	**730.3730**	365.6901	**713.3464**	357.1769	712.3624	356.6849	L	2402.1569	1201.5821	2385.1304	**1193.0688**	2384.1463	**1192.5768**	23
8	**859.4156**	430.2114	842.3890	421.6982	841.4050	421.2061	E	2289.0728	**1145.0401**	2272.0463	**1136.5268**	2271.0623	**1136.0348**	22
9	1006.4840	503.7456	**989.4575**	495.2324	**988.4734**	494.7404	F	2160.0303	**1080.5188**	2143.0037	1072.0055	2142.0197	1071.5135	21
10	**1107.5317**	554.2695	1090.5051	545.7562	1089.5211	545.2642	T	2012.9618	1006.9846	1995.9353	998.4713	1994.9513	997.9793	20
11	1194.5637	597.7855	1177.5372	589.2722	1176.5531	588.7802	S	**1911.9142**	956.4607	**1894.8876**	947.9474	1893.9036	947.4554	19
12	1281.5957	641.3015	1264.5692	632.7882	1263.5852	632.2962	S	**1824.8821**	**912.9447**	**1807.8556**	**904.4314**	1806.8716	903.9394	18
13	1338.6172	669.8122	1321.5907	**661.2990**	1320.6066	660.8070	G	1737.8501	**869.4287**	**1720.8236**	860.9154	1719.8395	**860.4234**	17
14	1425.6492	**713.3283**	1408.6227	**704.8150**	1407.6387	704.3230	S	**1680.8286**	840.9180	1663.8021	832.4047	1662.8181	831.9127	16
15	1496.6863	**748.8468**	**1479.6598**	740.3335	1478.6758	739.8415	A	**1593.7966**	**797.4019**	**1576.7701**	788.8887	1575.7861	788.3967	15
16	1610.7293	**805.8683**	**1593.7027**	**797.3550**	1592.7187	796.8630	N	**1522.7595**	**761.8834**	**1505.7330**	753.3701	1504.7489	752.8781	14
17	1711.7769	856.3921	1694.7504	**847.8788**	1693.7664	**847.3868**	T	1408.7166	**704.8619**	1391.6900	**696.3487**	1390.7060	**695.8566**	13
18	**1840.8195**	920.9134	1823.7930	912.4001	1822.8090	911.9081	E	**1307.6689**	**654.3381**	1290.6423	645.8248	1289.6583	645.3328	12
19	**1941.8672**	971.4372	**1924.8407**	962.9240	1923.8567	962.4320	T	**1178.6263**	589.8168	1161.5998	581.3035	1160.6157	580.8115	11
20	2042.9149	1021.9611	2025.8884	1013.4478	2024.9043	1012.9558	T	**1077.5786**	**539.2929**	1060.5521	530.7797	1059.5681	530.2877	10
21	2171.0099	1086.0086	2153.9833	**1077.4953**	2152.9993	**1077.0033**	K	**976.5309**	488.7691	959.5044	480.2558	958.5204	479.7638	9
22	2270.0783	**1135.5428**	2253.0517	**1127.0295**	2252.0677	**1126.5375**	V	**848.4360**	424.7216	831.4094	416.2084	830.4254	415.7163	8
23	2385.1052	**1193.0562**	2368.0787	1184.5430	2367.0946	1184.0510	N	**749.3676**	375.1874	732.3410	**366.6741**	731.3570	366.1821	7
24	2442.1267	1221.5670	2425.1001	1213.0537	2424.1161	1212.5617	G	**634.3406**	**317.6740**	617.3141	309.1607	616.3301	308.6687	6
25	2529.1587	1265.0830	2512.1322	1256.5697	2511.1481	**1256.0777**	S	577.3192	289.1632	**560.2926**	280.6499	559.3086	280.1579	5
26	2642.2428	1321.6250	2625.2162	1313.1117	2624.2322	1312.6197	L	**490.2871**	245.6472	473.2606	237.1339	**472.2766**	236.6419	4
27	2771.2854	**1386.1463**	2754.2588	**1377.6330**	2753.2748	**1377.1410**	E	**377.2031**	189.1052	**360.1765**	180.5919	359.1925	180.0999	3
28	2872.3330	**1436.6702**	2855.3065	1428.1569	2854.3225	1427.6649	T	248.1605	124.5839	231.1339	116.0706	230.1499	115.5786	2
29							K	147.1128	74.0600	130.0863	65.5468			1

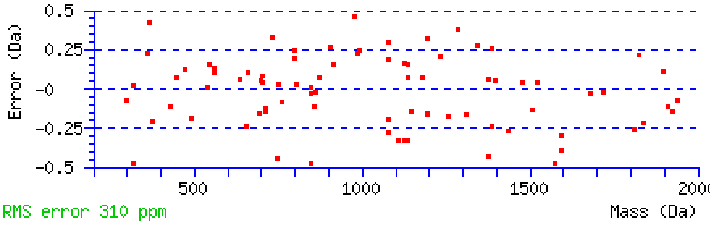

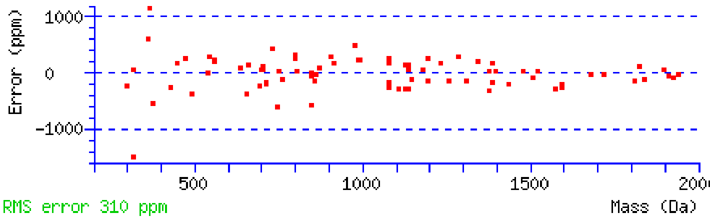

All matches to this query

Score	Mr(calc):	Delta	Sequence
63.5	3017.4313	-0.0016	TKSENGLEFTSSGSANTETTKVNGSLETK
48.1	3017.4313	-0.0016	TKSENGLEFTSSGSANTETTKVNGSLETK
21.8	3017.4313	-0.0016	TKSENGLEFTSSGSANTETTKVNGSLETK
17.7	3016.4473	0.9824	TKSENGLEFTSSGSANTETTKVNGSLETK
3.0	3015.4297	2.0000	SYLNAHGSFWVAESGGQVVGIVAALPVK
1.8	3016.4137	1.0160	SYLNAHGSFWVAESGGQVVGIVAALPVK
1.5	3016.4057	1.0240	MPYRTXDLTISINVLYNYLEANPK
1.5	3016.4057	1.0240	MPYRTXDLTISINVLYNYLEANPK
1.5	3016.4057	1.0240	MPYRTXDLTISINVLYNYLEANPK
1.5	3016.4057	1.0240	MPYRTXDLTISINVLYNYLEANPK

Spectrum No: 286; Query: 1018; Rank: 1

Peptide View

MS/MS Fragmentation of **CFANTVNLSFPSAQSLPASDTHLTVK**
Found in **IPI00326140**, Tax_Id=10116 Gene_Symbol=Pzp Alpha-1-macroglobulin precursor

Match to Query 1018: 2805.375192 from(936.132340,3+)
Title: 091127RatKid_SCX01_13.3826.3826.3.dta
Data file K:\NewmanPaper\Piliang\3SubProteomes\Piliang3SP\mgf5ppm\SCX_3SubProteomes5ppm.mgf

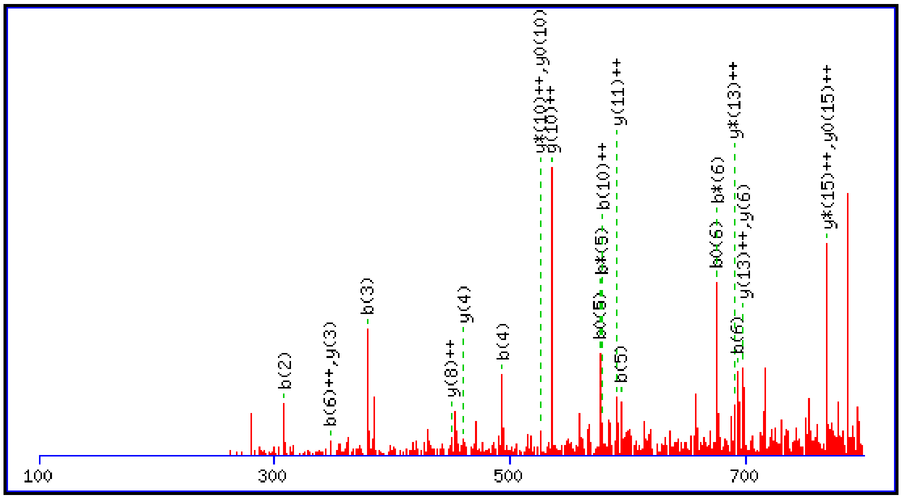

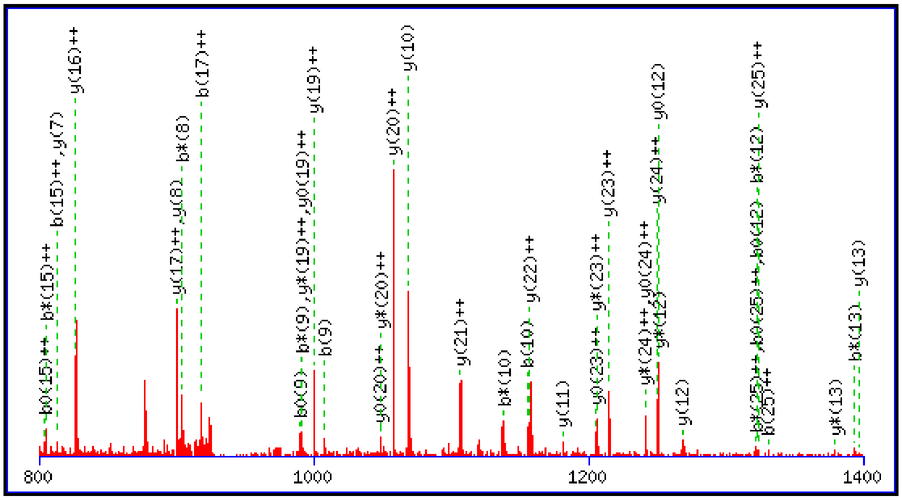

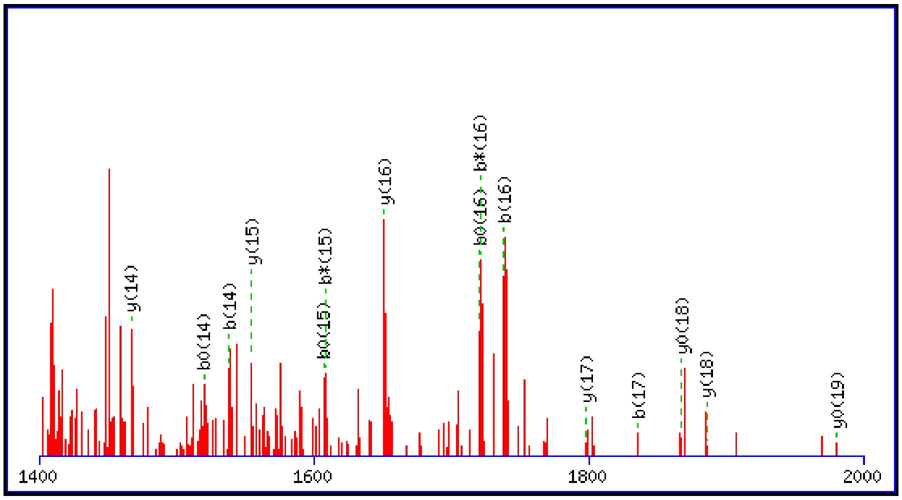

Monoisotopic mass of neutral peptide Mr(calc): 2805.3643
Fixed modifications: Carbamidomethyl (C)
Variable modifications:
N7 : Deamidated_N (N)
Ions Score: 63 **Expect:** 0.00012
Matches (Bold Red): 80/282 fragment ions using 154 most intense peaks

#	b	b^{++}	b*	b^{*++}	b^0	b^{0++}	Seq.	y	y^{++}	y*	y^{*++}	y^0	y^{0++}	#

1	161.0379	81.0226					C							26
2	**308.1063**	154.5568					F	2646.3410	**1323.6741**	2629.3144	1315.1608	2628.3304	1314.6688	25
3	**379.1435**	190.0754					A	2499.2726	**1250.1399**	2482.2460	**1241.6266**	2481.2620	**1241.1346**	24
4	**493.1864**	247.0968	476.1598	238.5836			N	2428.2354	**1214.6214**	2411.2089	**1206.1081**	2410.2249	**1205.6161**	23
5	**594.2341**	297.6207	**577.2075**	289.1074	**576.2235**	288.6154	T	2314.1925	**1157.5999**	2297.1660	1149.0866	2296.1819	1148.5946	22
6	**693.3025**	**347.1549**	**676.2759**	338.6416	**675.2919**	338.1496	V	2213.1448	**1107.0761**	2196.1183	1098.5628	2195.1343	1098.0708	21
7	808.3294	404.6683	791.3029	396.1551	790.3188	395.6631	N	2114.0764	**1057.5418**	2097.0499	**1049.0286**	2096.0659	**1048.5366**	20
8	921.4135	461.2104	**904.3869**	452.6971	903.4029	452.2051	L	1999.0495	**1000.0284**	1982.0229	**991.5151**	**1981.0389**	**991.0231**	19
9	**1008.4455**	504.7264	**991.4190**	496.2131	**990.4349**	495.7211	S	**1885.9654**	943.4863	1868.9389	934.9731	**1867.9549**	934.4811	18
10	**1155.5139**	**578.2606**	**1138.4874**	569.7473	1137.5034	569.2553	F	**1798.9334**	**899.9703**	1781.9068	891.4571	1780.9228	890.9651	17
11	1252.5667	626.7870	1235.5401	618.2737	1234.5561	617.7817	P	**1651.8650**	**826.4361**	1634.8384	817.9229	1633.8544	817.4308	16
12	1339.5987	670.3030	**1322.5722**	661.7897	**1321.5881**	661.2977	S	**1554.8122**	777.9097	1537.7857	**769.3965**	1536.8016	**768.9045**	15
13	1410.6358	705.8215	**1393.6093**	697.3083	1392.6253	696.8163	A	**1467.7802**	734.3937	1450.7536	725.8805	1449.7696	725.3884	14
14	**1538.6944**	769.8508	1521.6679	761.3376	**1520.6838**	760.8456	Q	**1396.7431**	**698.8752**	**1379.7165**	**690.3619**	1378.7325	689.8699	13
15	1625.7264	**813.3669**	**1608.6999**	**804.8536**	**1607.7159**	**804.3616**	S	**1268.6845**	634.8459	**1251.6579**	626.3326	**1250.6739**	625.8406	12
16	**1738.8105**	869.9089	**1721.7839**	861.3956	**1720.7999**	860.9036	L	**1181.6525**	**591.3299**	1164.6259	582.8166	1163.6419	582.3246	11
17	**1835.8633**	**918.4353**	1818.8367	909.9220	1817.8527	909.4300	P	**1068.5684**	**534.7878**	1051.5419	**526.2746**	1050.5578	**525.7826**	10
18	1906.9004	953.9538	1889.8738	945.4405	1888.8898	944.9485	A	971.5156	486.2615	954.4891	477.7482	953.5051	477.2562	9
19	1993.9324	997.4698	1976.9058	988.9566	1975.9218	988.4646	S	**900.4785**	**450.7429**	883.4520	442.2296	882.4680	441.7376	8
20	2108.9593	1054.9833	2091.9328	1046.4700	2090.9488	1045.9780	D	**813.4465**	407.2269	796.4199	398.7136	795.4359	398.2216	7
21	2210.0070	1105.5071	2192.9805	1096.9939	2191.9965	1096.5019	T	**698.4196**	349.7134	681.3930	341.2001	680.4090	340.7081	6
22	2347.0659	1174.0366	2330.0394	1165.5233	2329.0554	1165.0313	H	597.3719	299.1896	580.3453	290.6763	579.3613	290.1843	5
23	2460.1500	1230.5786	2443.1234	1222.0654	2442.1394	1221.5734	L	**460.3130**	230.6601	443.2864	222.1468	442.3024	221.6548	4
24	2561.1977	1281.1025	2544.1711	1272.5892	2543.1871	1272.0972	T	**347.2289**	174.1181	330.2023	165.6048	329.2183	165.1128	3
25	2660.2661	**1330.6367**	2643.2395	**1322.1234**	2642.2555	**1321.6314**	V	246.1812	123.5942	229.1547	115.0810			2
26							K	147.1128	74.0600	130.0863	65.5468			1

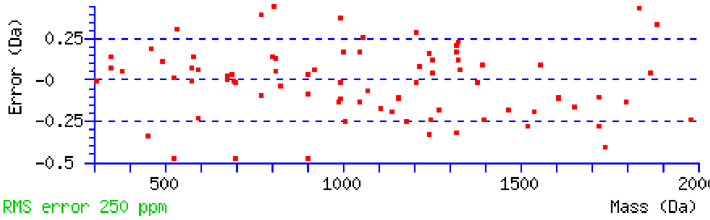

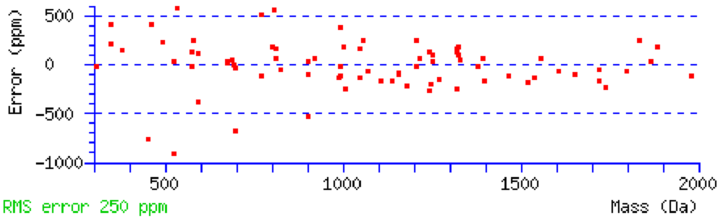

All matches to this query

Score	Mr(calc):	Delta	Sequence
63.3	2805.3643	0.0108	CFANTVNLSFPSAQSLPASDTHLTVK
57.7	2805.3643	0.0108	CFANTVNLSFPSAQSLPASDTHLTVK
43.9	2804.3803	0.9949	CFANTVNLSFPSAQSLPASDTHLTVK
5.1	2805.3578	0.0174	MSSGNAKIGHPAPSFKATAVMPDGQFK
0.3	2803.3802	1.9950	KHILQVSTFQMTILMLFNNREK
0.3	2803.3802	1.9950	KHILQVSTFQMTILMLFNNREK

Spectrum No: 287; Query: 1056; Rank: 1

Peptide View

MS/MS Fragmentation of **LLEFDSTNVSEGTQPPGKPYPPYSLAK**
Found in **IPI00196226**, Tax_Id=10116 Gene_Symbol=RGD1303130 Uncharacterized protein C1orf85 homolog precursor

Match to Query 1056: 2936.466222 from(979.829350,3+)
Title: 091129RatKid_SCX02_15.2508.2508.3.dta
Data file K:\NewmanPaper\Piliang\3SubProteomes\Piliang3SP\mgf5ppm\SCX_3SubProteomes5ppm.mgf

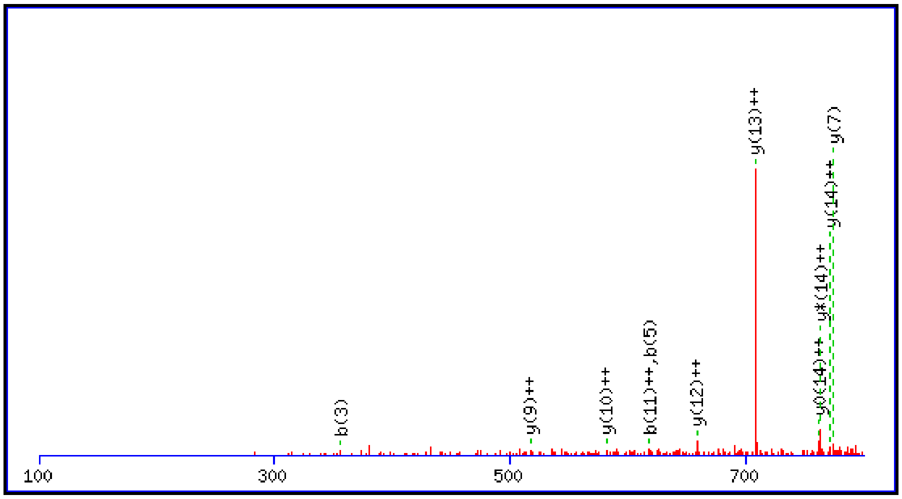

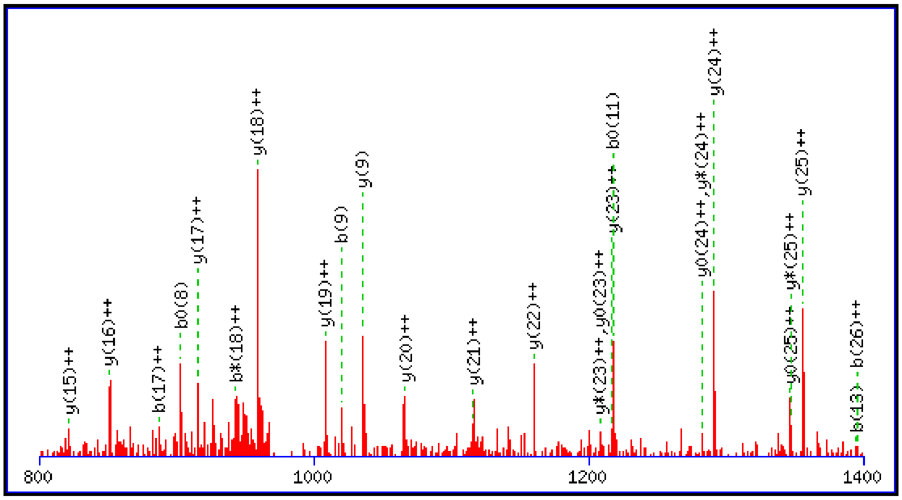

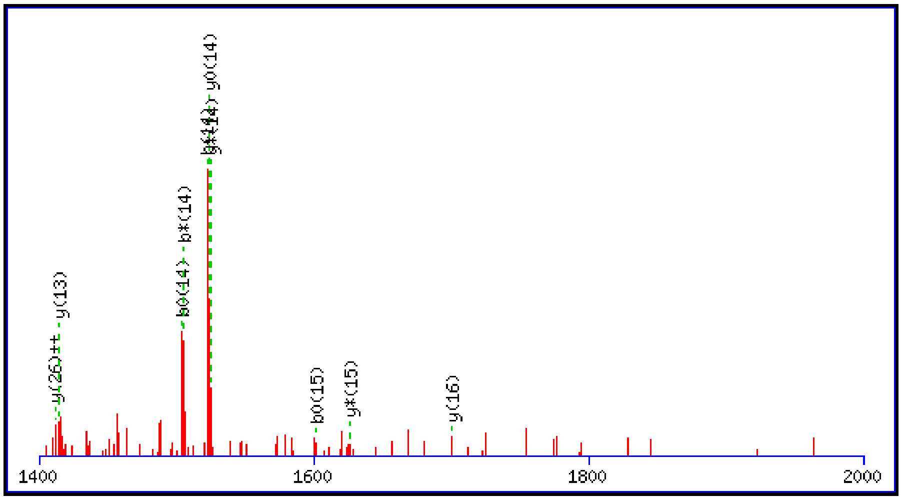

Monoisotopic mass of neutral peptide Mr(calc): 2935.4491
Fixed modifications: Carbamidomethyl (C)
Variable modifications:
N8 : Deamidated_N (N)
Ions Score: 63 **Expect:** 9.8e-005
Matches (Bold Red): 46/288 fragment ions using 133 most intense peaks

#	b	b^{++}	b*	b^{*++}	b^0	b^{0++}	Seq.	y	y^{++}	y*	y^{*++}	y^0	y^{0++}	#
1	114.0913	57.5493					L							27
2	227.1754	114.0913					L	2823.3723	**1412.1898**	2806.3458	1403.6765	2805.3618	1403.1845	26

3	**356.2180**	178.6126			338.2074	169.6074	E	2710.2883	**1355.6478**	2693.2617	**1347.1345**	2692.2777	**1346.6425**	25
4	503.2864	252.1468			485.2758	243.1416	F	2581.2457	**1291.1265**	2564.2191	**1282.6132**	2563.2351	**1282.1212**	24
5	**618.3134**	309.6603			600.3028	300.6550	D	2434.1773	**1217.5923**	2417.1507	**1209.0790**	2416.1667	**1208.5870**	23
6	705.3454	353.1763			687.3348	344.1710	S	2319.1503	**1160.0788**	2302.1238	1151.5655	2301.1397	1151.0735	22
7	806.3931	403.7002			788.3825	394.6949	T	2232.1183	**1116.5628**	2215.0917	1108.0495	2214.1077	1107.5575	21
8	921.4200	461.2136	904.3934	452.7004	**903.4094**	452.2084	N	2131.0706	**1066.0389**	2114.0441	1057.5257	2113.0600	1057.0337	20
9	**1020.4884**	510.7478	1003.4619	502.2346	1002.4778	501.7426	V	2016.0437	**1008.5255**	1999.0171	1000.0122	1998.0331	999.5202	19
10	1107.5204	554.2639	1090.4939	545.7506	1089.5099	545.2586	S	1916.9753	**958.9913**	1899.9487	950.4780	1898.9647	949.9860	18
11	1236.5630	**618.7852**	1219.5365	610.2719	**1218.5525**	609.7799	E	1829.9432	**915.4753**	1812.9167	906.9620	1811.9327	906.4700	17
12	1293.5845	647.2959	1276.5579	638.7826	1275.5739	638.2906	G	**1700.9006**	**850.9540**	1683.8741	842.4407	1682.8901	841.9487	16
13	**1394.6322**	697.8197	1377.6056	689.3065	1376.6216	688.8144	T	1643.8792	**822.4432**	**1626.8526**	813.9299	1625.8686	813.4379	15
14	**1522.6908**	761.8490	**1505.6642**	753.3357	**1504.6802**	752.8437	Q	1542.8315	**771.9194**	**1525.8049**	**763.4061**	**1524.8209**	**762.9141**	14
15	1619.7435	810.3754	1602.7170	801.8621	**1601.7330**	801.3701	P	**1414.7729**	**707.8901**	1397.7464	699.3768	1396.7624	698.8848	13
16	1716.7963	858.9018	1699.7697	850.3885	1698.7857	849.8965	P	1317.7202	**659.3637**	1300.6936	650.8504	1299.7096	650.3584	12
17	1773.8177	**887.4125**	1756.7912	878.8992	1755.8072	878.4072	G	1220.6674	610.8373	1203.6408	602.3241	1202.6568	601.8320	11
18	1901.9127	951.4600	1884.8862	**942.9467**	1883.9021	942.4547	K	1163.6459	**582.3266**	1146.6194	573.8133	1145.6354	573.3213	10
19	1998.9655	999.9864	1981.9389	991.4731	1980.9549	990.9811	P	**1035.5510**	**518.2791**	1018.5244	509.7658	1017.5404	509.2738	9
20	2162.0288	1081.5180	2145.0023	1073.0048	2144.0182	1072.5128	Y	938.4982	469.7527	921.4716	461.2395	920.4876	460.7475	8
21	2259.0816	1130.0444	2242.0550	1121.5311	2241.0710	1121.0391	P	**775.4349**	388.2211	758.4083	379.7078	757.4243	379.2158	7
22	2356.1343	1178.5708	2339.1078	1170.0575	2338.1238	1169.5655	P	678.3821	339.6947	661.3556	331.1814	660.3715	330.6894	6
23	2519.1977	1260.1025	2502.1711	1251.5892	2501.1871	1251.0972	Y	581.3293	291.1683	564.3028	282.6550	563.3188	282.1630	5
24	2606.2297	1303.6185	2589.2031	1295.1052	2588.2191	1294.6132	S	418.2660	209.6366	401.2395	201.1234	400.2554	200.6314	4
25	2719.3137	1360.1605	2702.2872	1351.6472	2701.3032	1351.1552	L	331.2340	166.1206	314.2074	157.6074			3
26	2790.3509	**1395.6791**	2773.3243	1387.1658	2772.3403	1386.6738	A	218.1499	109.5786	201.1234	101.0653			2
27							K	147.1128	74.0600	130.0863	65.5468			1

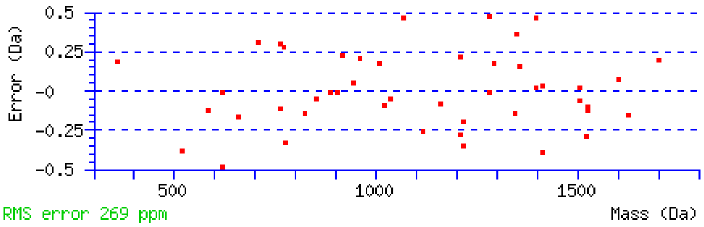

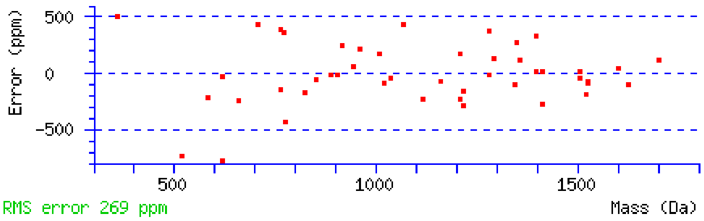

All matches to this query

Score	Mr(calc):	Delta	Sequence
63.3	2935.4491	1.0171	LLEFDSTNVSEGTQPPGKPYPPYSLAK
30.3	2934.4651	2.0011	LLEFDSTNVSEGTQPPGKPYPPYSLAK
2.5	2934.4691	1.9971	SGGLPLVLSMLTRNNFLPNADMETRR
2.5	2934.4691	1.9971	SGGLPLVLSMLTRNNFLPNADMETRR
0.9	2935.4643	1.0019	LLEPLIIQVTTLVNCPQNPSNRKK
0.9	2935.4643	1.0019	LLEPLIIQVTTLVNCPQNPSNRKK
0.9	2935.4643	1.0019	LLEPLIIQVTTLVNCPQNPSNRKK
0.6	2935.4643	1.0019	LLEPLIIQVTTLVNCPQNPSNRKK
0.6	2935.4643	1.0019	LLEPLIIQVTTLVNCPQNPSNRKK
0.6	2935.4643	1.0019	LLEPLIIQVTTLVNCPQNPSNRKK

Spectrum No: 288; Query: 265; Rank: 1

Peptide View

MS/MS Fragmentation of **VEAIGSLANTTMEK**
Found in **IPI00193397**, Tax_Id=10116 Gene_Symbol=Slc34a1 Sodium-dependent phosphate transport protein 2A

Match to Query 265: 1479.713828 from(740.864190,2+)
Title: 091127RatKid_SCX01_01.1155.1155.2.dta
Data file K:\NewmanPaper\Piliang\3SubProteomes\Piliang3SP\mgf5ppm\SCX_3SubProteomes5ppm.mgf

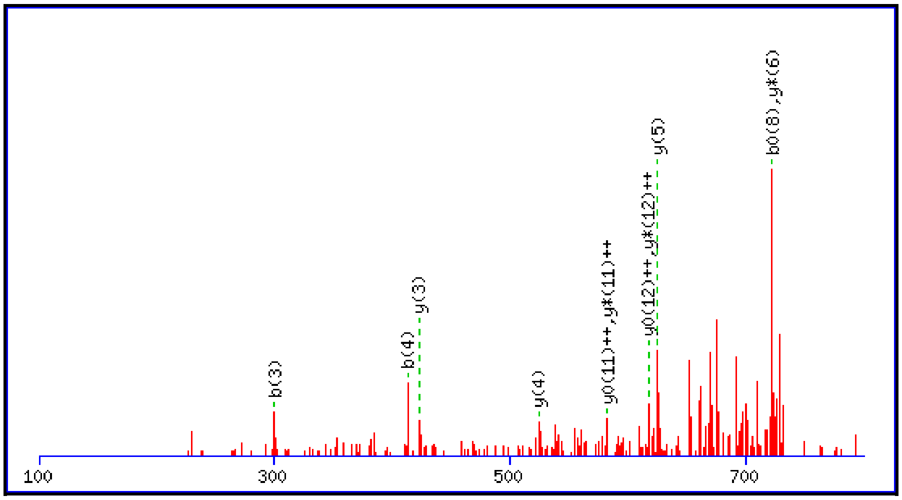

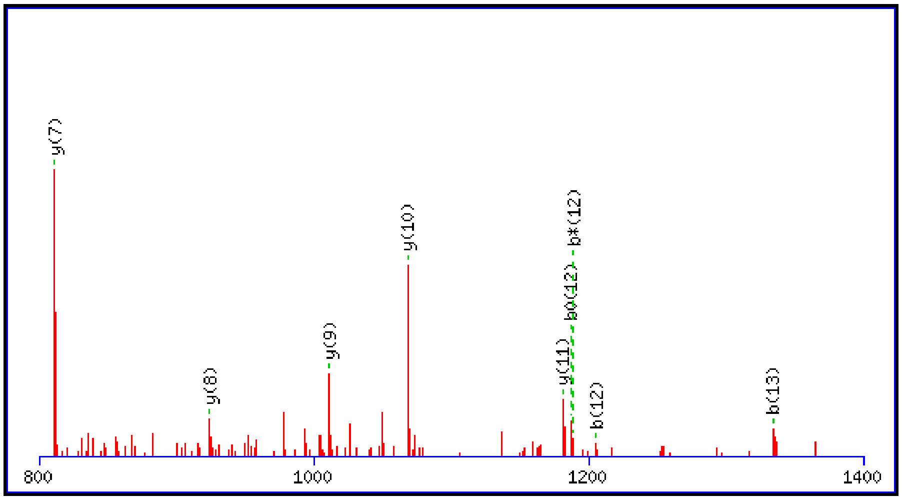

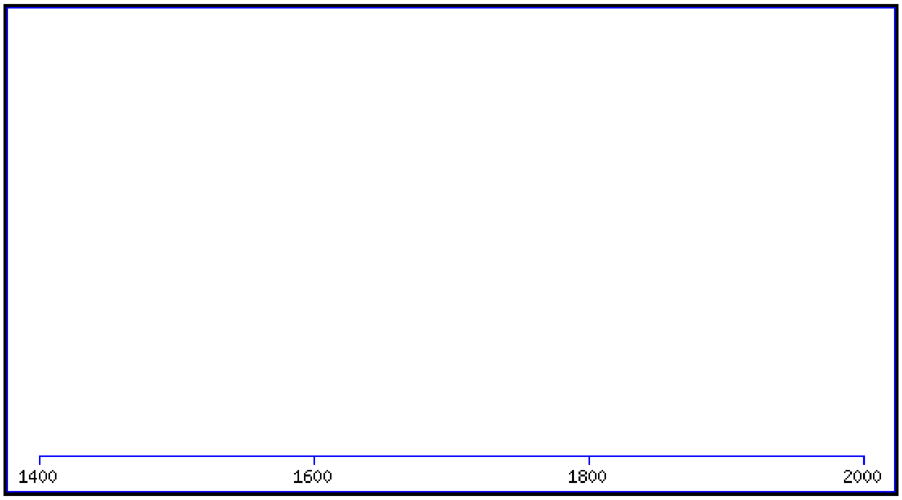

Monoisotopic mass of neutral peptide Mr(calc): 1479.7126
Fixed modifications: Carbamidomethyl (C)
Variable modifications:
N9 : Deamidated_N (N)
M12 : Oxidation (M)
Ions Score: 63 **Expect:** 7.5e-005

Matches (Bold Red): 20/136 fragment ions using 23 most intense peaks

#	b	b^{++}	b*	b^{*++}	b^0	b^{0++}	Seq.	y	y^{++}	y*	y^{*++}	y^0	y^{0++}	#
1	100.0757	50.5415					V							14
2	229.1183	115.0628			211.1077	106.0575	E	1381.6515	691.3294	1364.6250	682.8161	1363.6410	682.3241	13
3	**300.1554**	150.5813			282.1448	141.5761	A	1252.6089	626.8081	1235.5824	**618.2948**	1234.5984	**617.8028**	12
4	**413.2395**	207.1234			395.2289	198.1181	I	**1181.5718**	591.2896	1164.5453	**582.7763**	1163.5613	**582.2843**	11
5	470.2609	235.6341			452.2504	226.6288	G	**1068.4878**	534.7475	1051.4612	526.2342	1050.4772	525.7422	10
6	557.2930	279.1501			539.2824	270.1448	S	**1011.4663**	506.2368	994.4398	497.7235	993.4557	497.2315	9
7	670.3770	335.6921			652.3665	326.6869	L	**924.4343**	462.7208	907.4077	454.2075	906.4237	453.7155	8
8	741.4141	371.2107			**723.4036**	362.2054	A	**811.3502**	406.1787	794.3237	397.6655	793.3396	397.1735	7
9	856.4411	428.7242	839.4145	420.2109	838.4305	419.7189	N	740.3131	370.6602	**723.2865**	362.1469	722.3025	361.6549	6
10	957.4887	479.2480	940.4622	470.7347	939.4782	470.2427	T	**625.2862**	313.1467	608.2596	304.6334	607.2756	304.1414	5
11	1058.5364	529.7719	1041.5099	521.2586	1040.5259	520.7666	T	**524.2385**	262.6229	507.2119	254.1096	506.2279	253.6176	4
12	**1205.5718**	603.2896	**1188.5453**	594.7763	**1187.5613**	594.2843	M	**423.1908**	212.0990	406.1643	203.5858	405.1802	203.0938	3
13	**1334.6144**	667.8108	1317.5879	659.2976	1316.6039	658.8056	E	276.1554	138.5813	259.1288	130.0681	258.1448	129.5761	2
14							K	147.1128	74.0600	130.0863	65.5468			1

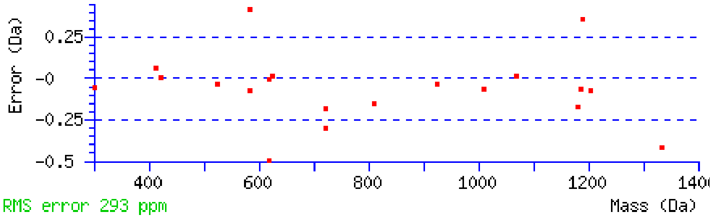

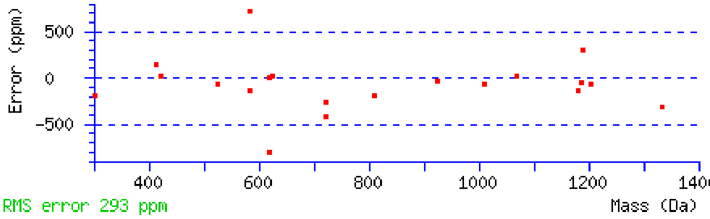

All matches to this query

Score	Mr(calc):	Delta	Sequence
63.1	1479.7126	0.0012	VEAIGSLANTTMEK
8.0	1479.7269	-0.0131	GNQLCGLISGIIR
4.3	1479.7195	-0.0057	LLEGIRGASGGTGGR
3.1	1479.7140	-0.0001	NPKKSVNNQYCK
3.0	1479.7222	-0.0083	DKSLADILDPSVK
3.0	1479.7010	0.0128	XVEQPSEVIVSK
2.7	1478.7130	1.0008	GAAQNIIPASTGATK
2.6	1479.7195	-0.0057	GKPVAVTSNRGTGR
2.3	1478.7105	1.0033	LIGKGAFSRCYK
1.9	1479.7221	-0.0083	ALNQSNTPLLSLK

Spectrum No: 289; Query: 1067; Rank: 1

Peptide View

MS/MS Fragmentation of **LQAALDNEAGGRPAMEPGNGSLDLGGDAAGR**
Found in **IPI00210090**, Tax_Id=10116 Gene_Symbol=Hnrpu SP120

Match to Query 1067: 2997.394632 from(1000.138820,3+)
Title: 091127RatKid_SCX01_23.2140.2140.3.dta
Data file K:\NewmanPaper\Piliang\3SubProteomes\Piliang3SP\mgf5ppm\SCX_3SubProteomes5ppm.mgf

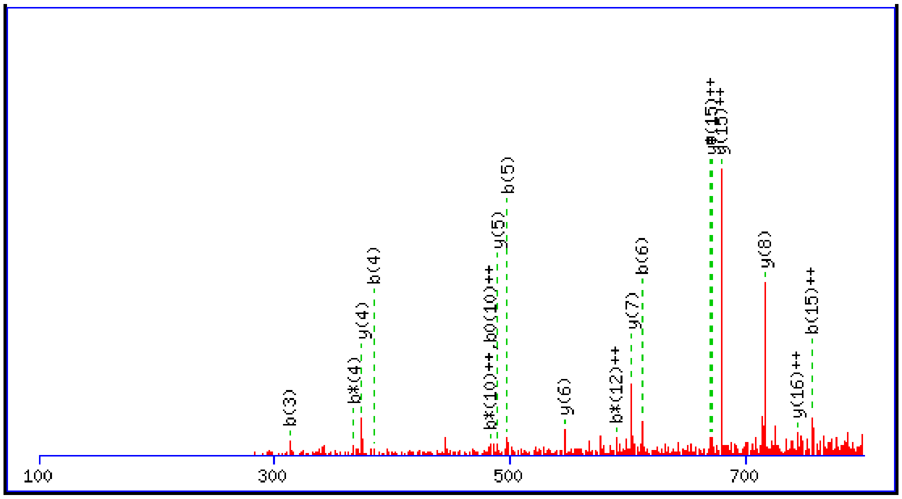

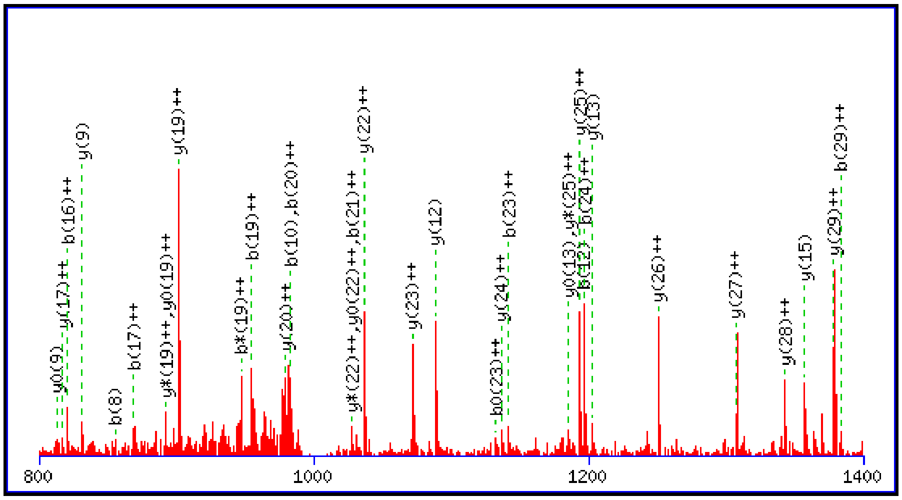

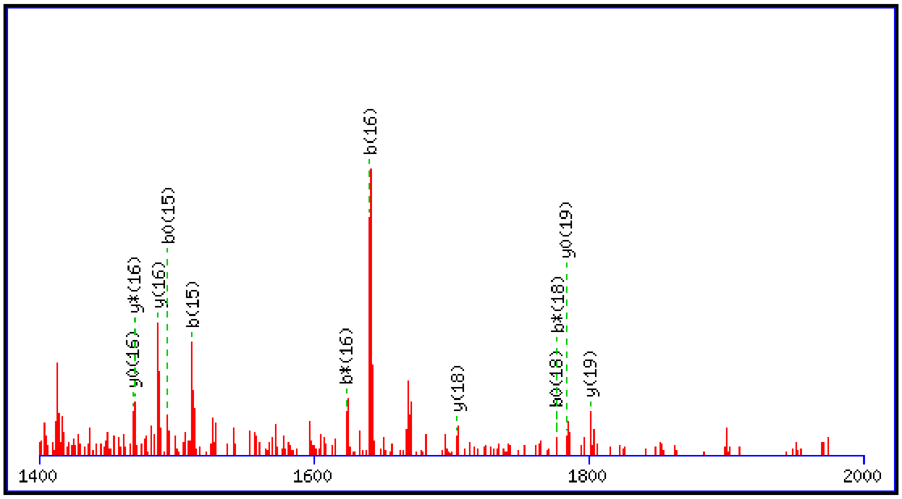

Monoisotopic mass of neutral peptide Mr(calc): 2996.3894
Fixed modifications: Carbamidomethyl (C)
Variable modifications:
M15 : Oxidation (M)
N19 : Deamidated_N (N)
Ions Score: 63 **Expect:** 0.00019
Matches (Bold Red): 65/340 fragment ions using 142 most intense peaks

#	b	b^{++}	b*	b^{*++}	b^0	b^{0++}	Seq.	y	y^{++}	y*	y^{*++}	y^0	y^{0++}	#
1	114.0913	57.5493					L							31
2	242.1499	121.5786	225.1234	113.0653			Q	2884.3126	1442.6599	2867.2861	1434.1467	2866.3020	1433.6547	30
3	**313.1870**	157.0972	296.1605	148.5839			A	2756.2540	**1378.6307**	2739.2275	1370.1174	2738.2435	1369.6254	29
4	**384.2241**	192.6157	**367.1976**	184.1024			A	2685.2169	**1343.1121**	2668.1904	1334.5988	2667.2063	1334.1068	28

**5**	**497.3082**	249.1577	480.2817	240.6445			**L**	2614.1798	**1307.5935**	2597.1532	1299.0803	2596.1692	1298.5883	**27**
**6**	**612.3352**	306.6712	595.3086	298.1579	594.3246	297.6659	**D**	2501.0957	**1251.0515**	2484.0692	1242.5382	2483.0852	1242.0462	**26**
**7**	726.3781	363.6927	709.3515	355.1794	708.3675	354.6874	**N**	2386.0688	**1193.5380**	2369.0422	**1185.0248**	2368.0582	1184.5328	**25**
**8**	**855.4207**	428.2140	838.3941	419.7007	837.4101	419.2087	**E**	2272.0259	**1136.5166**	2254.9993	1128.0033	2254.0153	1127.5113	**24**
**9**	926.4578	463.7325	909.4312	455.2193	908.4472	454.7272	**A**	2142.9833	**1071.9953**	2125.9567	1063.4820	2124.9727	1062.9900	**23**
**10**	**983.4793**	492.2433	966.4527	**483.7300**	965.4687	**483.2380**	**G**	2071.9462	**1036.4767**	2054.9196	**1027.9634**	2053.9356	**1027.4714**	**22**
**11**	1040.5007	520.7540	1023.4742	512.2407	1022.4901	511.7487	**G**	2014.9247	1007.9660	1997.8981	999.4527	1996.9141	998.9607	**21**
**12**	**1196.6018**	598.8046	1179.5753	**590.2913**	1178.5913	589.7993	**R**	1957.9032	**979.4553**	1940.8767	970.9420	1939.8927	970.4500	**20**
**13**	1293.6546	647.3309	1276.6280	638.8177	1275.6440	638.3257	**P**	**1801.8021**	**901.4047**	1784.7756	**892.8914**	**1783.7916**	**892.3994**	**19**
**14**	1364.6917	682.8495	1347.6652	674.3362	1346.6811	673.8442	**A**	**1704.7494**	852.8783	1687.7228	844.3650	1686.7388	843.8730	**18**
**15**	**1511.7271**	**756.3672**	1494.7006	747.8539	**1493.7165**	747.3619	**M**	1633.7122	**817.3598**	1616.6857	808.8465	1615.7017	808.3545	**17**
**16**	**1640.7697**	**820.8885**	**1623.7432**	812.3752	1622.7591	811.8832	**E**	**1486.6768**	**743.8421**	**1469.6503**	735.3288	**1468.6663**	734.8368	**16**
**17**	1737.8225	**869.4149**	1720.7959	860.9016	1719.8119	860.4096	**P**	**1357.6342**	**679.3208**	1340.6077	**670.8075**	1339.6237	**670.3155**	**15**
**18**	1794.8439	897.9256	**1777.8174**	889.4123	**1776.8334**	888.9203	**G**	1260.5815	630.7944	1243.5549	622.2811	1242.5709	621.7891	**14**
**19**	1909.8709	**955.4391**	1892.8443	**946.9258**	1891.8603	946.4338	**N**	**1203.5600**	602.2836	1186.5335	593.7704	**1185.5494**	593.2784	**13**
**20**	1966.8923	**983.9498**	1949.8658	975.4365	1948.8818	974.9445	**G**	**1088.5331**	544.7702	1071.5065	536.2569	1070.5225	535.7649	**12**
**21**	2053.9244	**1027.4658**	2036.8978	1018.9525	2035.9138	1018.4605	**S**	1031.5116	516.2594	1014.4851	507.7462	1013.5010	507.2542	**11**
**22**	2167.0084	1084.0078	2149.9819	1075.4946	2148.9979	1075.0026	**L**	944.4796	472.7434	927.4530	464.2302	926.4690	463.7381	**10**
**23**	2282.0354	**1141.5213**	2265.0088	1133.0080	2264.0248	**1132.5160**	**D**	**831.3955**	416.2014	814.3690	407.6881	**813.3850**	407.1961	**9**
**24**	2395.1194	**1198.0634**	2378.0929	1189.5501	2377.1089	1189.0581	**L**	**716.3686**	358.6879	699.3420	350.1747	698.3580	349.6826	**8**
**25**	2452.1409	1226.5741	2435.1143	1218.0608	2434.1303	1217.5688	**G**	**603.2845**	302.1459	586.2580	293.6326	585.2740	293.1406	**7**
**26**	2509.1624	1255.0848	2492.1358	1246.5715	2491.1518	1246.0795	**G**	**546.2631**	273.6352	529.2365	265.1219	528.2525	264.6299	**6**
**27**	2624.1893	1312.5983	2607.1627	1304.0850	2606.1787	1303.5930	**D**	**489.2416**	245.1244	472.2150	236.6112	471.2310	236.1191	**5**
**28**	2695.2264	1348.1168	2678.1999	1339.6036	2677.2158	1339.1116	**A**	**374.2146**	187.6110	357.1881	179.0977			**4**
**29**	2766.2635	**1383.6354**	2749.2370	1375.1221	2748.2530	1374.6301	**A**	303.1775	152.0924	286.1510	143.5791			**3**
**30**	2823.2850	1412.1461	2806.2584	1403.6329	2805.2744	1403.1409	**G**	232.1404	116.5738	215.1139	108.0606			**2**
**31**							**R**	175.1190	88.0631	158.0924	79.5498			**1**

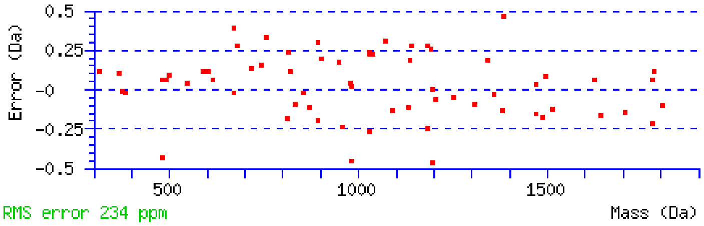

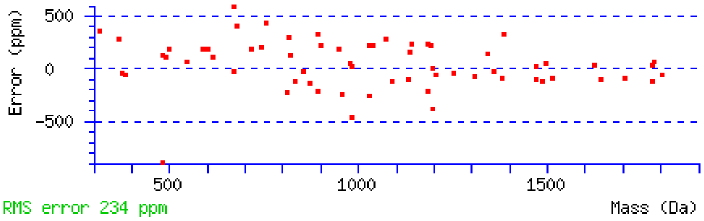

All matches to this query

Score	Mr(calc):	Delta	Sequence
63.0	2996.3894	1.0053	LQAALDNEAGGRPAMEPGNGSLDLGGDAAGR
61.8	2997.3734	0.0213	LQAALDNEAGGRPAMEPGNGSLDLGGDAAGR
25.9	2996.3894	1.0053	LQAALDNEAGGRPAMEPGNGSLDLGGDAAGR
17.7	2995.4054	1.9893	LQAALDNEAGGRPAMEPGNGSLDLGGDAAGR
4.3	2997.4039	-0.0092	AFSWASVTSKNLPPSGTVSSSGIPPHVK
1.1	2995.3973	1.9973	ALLSGYFMQIARDVDGSGNYLMLTHK

Spectrum No: 290; Query: 843; Rank: 1

Peptide View

MS/MS Fragmentation of **NATYSYVLDDPDPDDGFNYK**

Found in **IPI00776882**, Tax_Id=10116 Gene_Symbol=Calu calumenin isoform a

Match to Query 843: 2308.972548 from(1155.493550,2+)
Title: 091127RatKid_SCX01_15.3238.3238.2.dta
Data file K:\NewmanPaper\Piliang\3SubProteomes\Piliang3SP\mgf5ppm\SCX_3SubProteomes5ppm.mgf

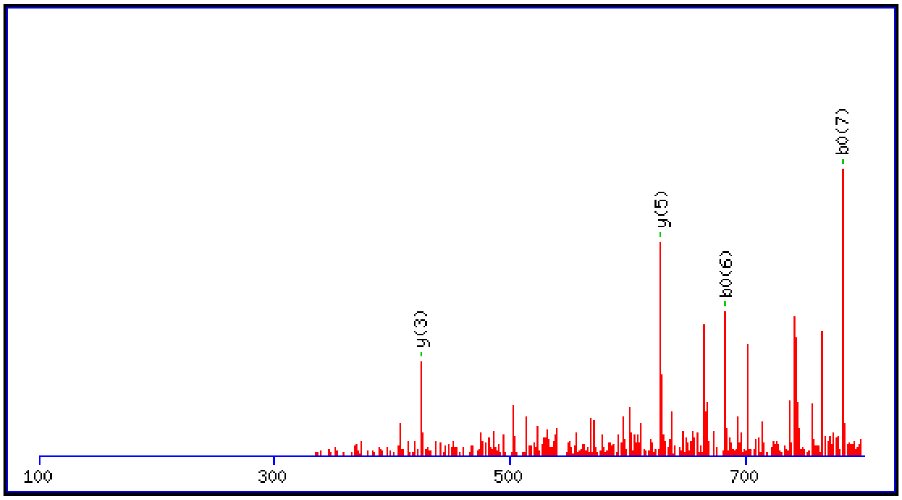

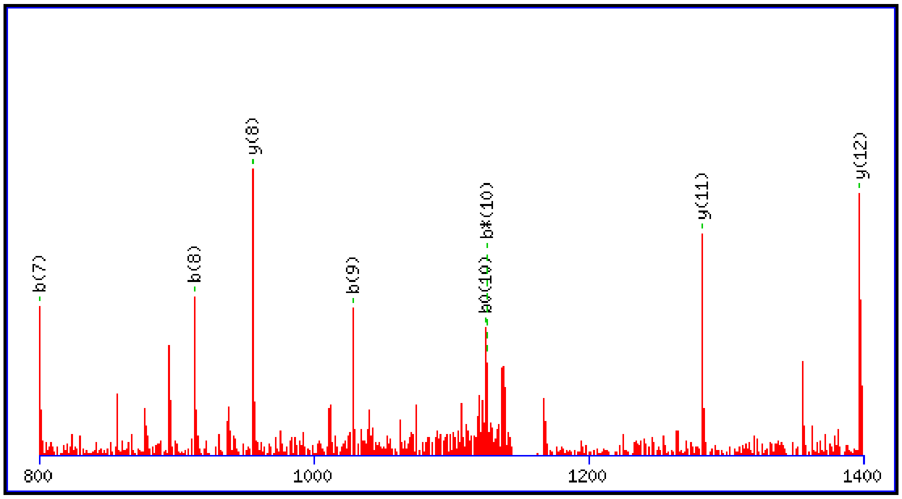

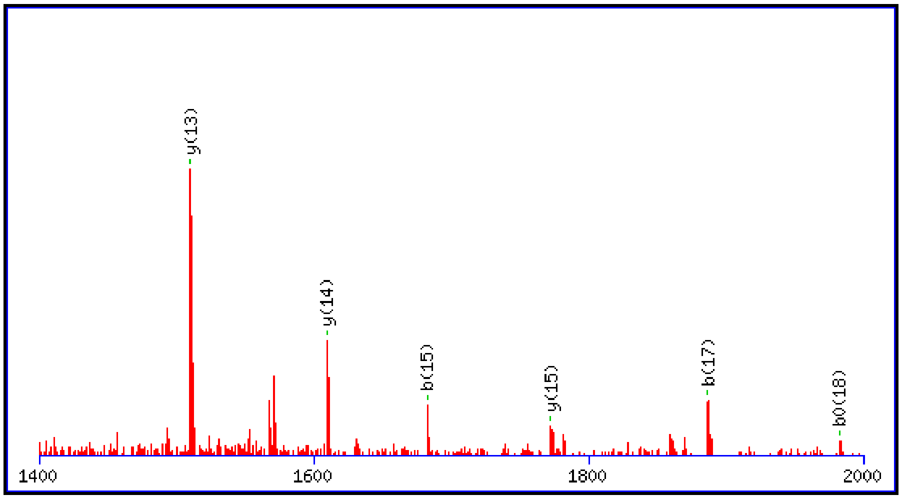

Monoisotopic mass of neutral peptide Mr(calc): 2308.9648
Fixed modifications: Carbamidomethyl (C)
Variable modifications:
N1 : Deamidated_N (N)
Ions Score: 63 **Expect:** 0.00014
Matches (Bold Red): 18/214 fragment ions using 26 most intense peaks

#	b	b++	b*	b*++	b0	b0++	Seq.	y	y++	y*	y*++	y0	y0++	#
1	116.0342	58.5207	99.0077	50.0075			N							20

2	187.0713	94.0393	170.0448	85.5260			A	2194.9451	1097.9762	2177.9186	1089.4629	2176.9346	1088.9709	19
3	288.1190	144.5631	271.0925	136.0499	270.1084	135.5579	T	2123.9080	1062.4576	2106.8815	1053.9444	2105.8975	1053.4524	18
4	451.1823	226.0948	434.1558	217.5815	433.1718	217.0895	Y	2022.8603	1011.9338	2005.8338	1003.4205	2004.8498	1002.9285	17
5	538.2144	269.6108	521.1878	261.0975	520.2038	260.6055	S	1859.7970	930.4021	1842.7705	921.8889	1841.7865	921.3969	16
6	701.2777	351.1425	684.2511	342.6292	**683.2671**	342.1372	Y	**1772.7650**	886.8861	1755.7384	878.3729	1754.7544	877.8808	15
7	**800.3461**	400.6767	783.3196	392.1634	**782.3355**	391.6714	V	**1609.7017**	805.3545	1592.6751	796.8412	1591.6911	796.3492	14
8	**913.4302**	457.2187	896.4036	448.7054	895.4196	448.2134	L	**1510.6332**	755.8203	1493.6067	747.3070	1492.6227	746.8150	13
9	**1028.4571**	514.7322	1011.4306	506.2189	1010.4465	505.7269	D	**1397.5492**	699.2782	1380.5226	690.7650	1379.5386	690.2729	12
10	1143.4841	572.2457	**1126.4575**	563.7324	**1125.4735**	563.2404	D	**1282.5222**	641.7648	1265.4957	633.2515	1264.5117	632.7595	11
11	1240.5368	620.7720	1223.5103	612.2588	1222.5263	611.7668	P	1167.4953	584.2513	1150.4687	575.7380	1149.4847	575.2460	10
12	1355.5638	678.2855	1338.5372	669.7722	1337.5532	669.2802	D	1070.4425	535.7249	1053.4160	527.2116	1052.4320	526.7196	9
13	1452.6165	726.8119	1435.5900	718.2986	1434.6060	717.8066	P	**955.4156**	478.2114	938.3890	469.6982	937.4050	469.2061	8
14	1567.6435	784.3254	1550.6169	775.8121	1549.6329	775.3201	D	858.3628	429.6851	841.3363	421.1718	840.3523	420.6798	7
15	**1682.6704**	841.8388	1665.6439	833.3256	1664.6598	832.8336	D	743.3359	372.1716	726.3093	363.6583	725.3253	363.1663	6
16	1739.6919	870.3496	1722.6653	861.8363	1721.6813	861.3443	G	**628.3089**	314.6581	611.2824	306.1448			5
17	**1886.7603**	943.8838	1869.7337	935.3705	1868.7497	934.8785	F	571.2875	286.1474	554.2609	277.6341			4
18	2000.8032	1000.9052	1983.7767	992.3920	**1982.7927**	991.9000	N	**424.2191**	212.6132	407.1925	204.0999			3
19	2163.8665	1082.4369	2146.8400	1073.9236	2145.8560	1073.4316	Y	310.1761	155.5917	293.1496	147.0784			2
20							K	147.1128	74.0600	130.0863	65.5468			1

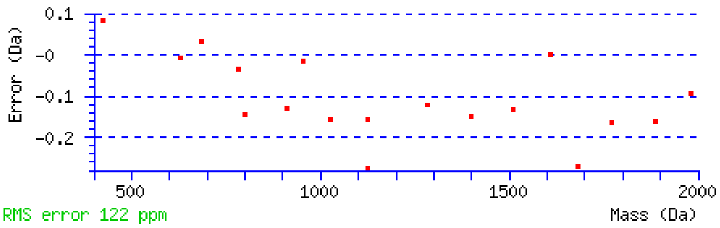

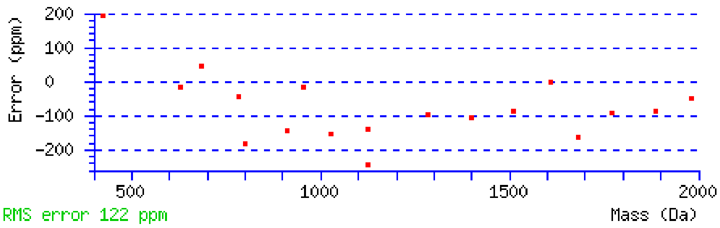

All matches to this query

Score	Mr(calc):	Delta	Sequence
63.0	2308.9648	0.0077	NATYSYVLDDPDPDDGFNYK
39.0	2307.9808	0.9918	NATYSYVLDDPDPDDGFNYK
33.2	2308.9648	0.0077	NVTYGTYLDDPDPDDGFNYK
18.6	2307.9808	0.9917	NVTYGTYLDDPDPDDGFNYK
7.8	2308.9648	0.0077	NATYSYVLDDPDPDDGFNYK
5.8	2307.9695	1.0031	LANCTSSNTLQEVNLEVQK
4.4	2307.9695	1.0031	LANCTSSNTLQEVNLEVQK
2.1	2308.9825	-0.0099	SGELRKEINSNTEQLQSSK
2.1	2308.9825	-0.0099	SGELRKEINSNTEQLQSSK
2.1	2308.9825	-0.0099	SGELRKEINSNTEQLQSSK

Spectrum No: 291; Query: 530; Rank: 1

Peptide View

MS/MS Fragmentation of **LDNTSMSTVIDGVVLPK**
Found in **IPI00195148**, Tax_Id=10116 Gene_Symbol=Es2 Liver carboxylesterase 1 precursor

Match to Query 530: 1788.922308 from(895.468430,2+)
Title: 091127RatKid_SCX01_02.4140.4140.2.dta
Data file K:\NewmanPaper\Piliang\3SubProteomes\Piliang3SP\mgf5ppm\SCX_3SubProteomes5ppm.mgf

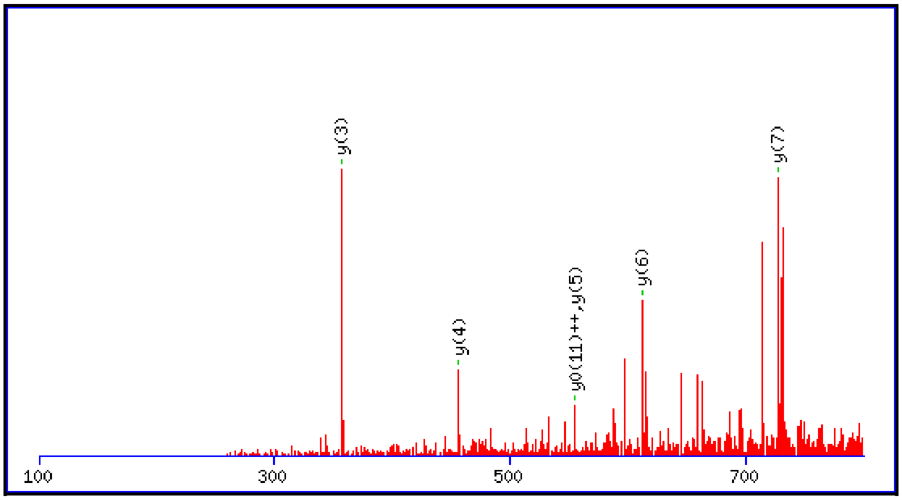

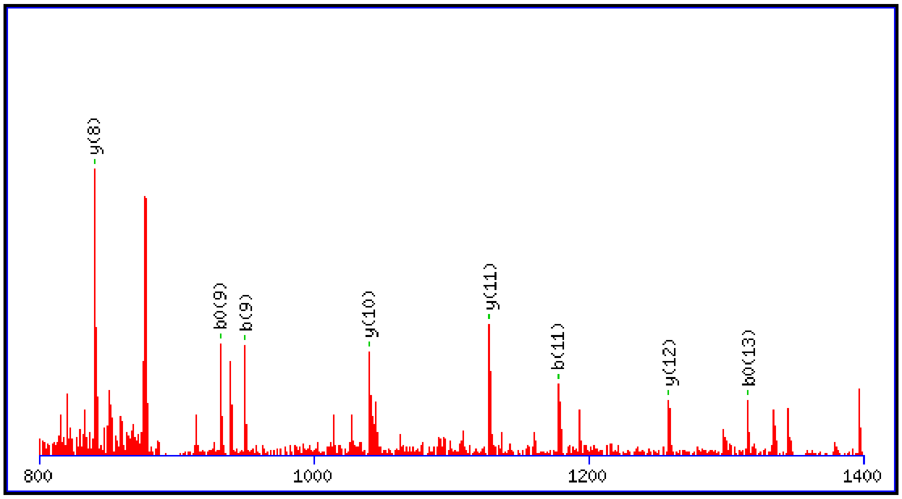

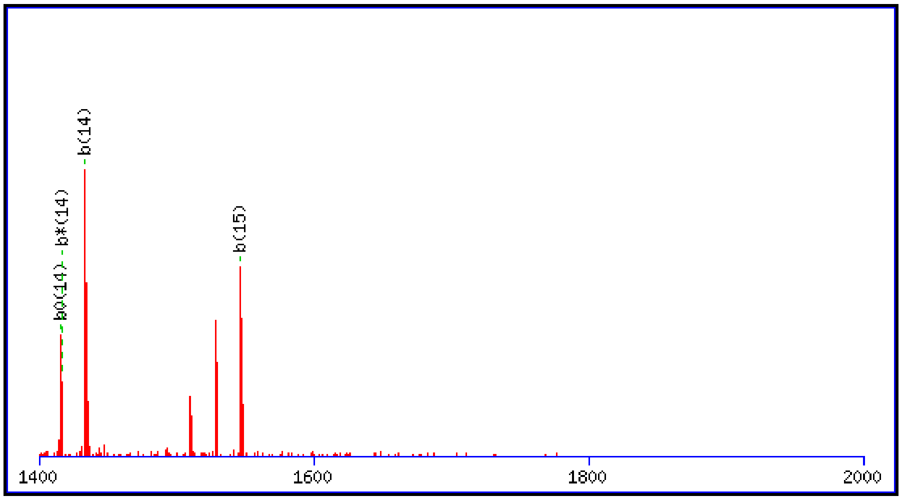

Monoisotopic mass of neutral peptide Mr(calc): 1788.9179
Fixed modifications: Carbamidomethyl (C)
Variable modifications:
N3 : Deamidated_N (N)
Ions Score: 63 **Expect:** 7.3e-005
Matches (Bold Red): 18/174 fragment ions using 31 most intense peaks

#	b	b^{++}	b*	b^{*++}	b^0	b^{0++}	Seq.	y	y^{++}	y*	y^{*++}	y^0	y^{0++}	#
1	114.0913	57.5493					L							17
2	229.1183	115.0628			211.1077	106.0575	D	1676.8411	838.9242	1659.8146	830.4109	1658.8306	829.9189	16
3	344.1452	172.5762	327.1187	164.0630	326.1347	163.5710	N	1561.8142	781.4107	1544.7876	772.8975	1543.8036	772.4054	15
4	445.1929	223.1001	428.1663	214.5868	427.1823	214.0948	T	1446.7872	723.8973	1429.7607	715.3840	1428.7767	714.8920	14
5	532.2249	266.6161	515.1984	258.1028	514.2144	257.6108	S	1345.7396	673.3734	1328.7130	664.8601	1327.7290	664.3681	13

**6**	663.2654	332.1363	646.2389	323.6231	645.2548	323.1311	**M**	**1258.7075**	629.8574	1241.6810	621.3441	1240.6970	620.8521	**12**
**7**	750.2974	375.6524	733.2709	367.1391	732.2869	366.6471	**S**	**1127.6671**	564.3372	1110.6405	555.8239	1109.6565	**555.3319**	**11**
**8**	851.3451	426.1762	834.3186	417.6629	833.3346	417.1709	**T**	**1040.6350**	520.8212	1023.6085	512.3079	1022.6245	511.8159	**10**
**9**	**950.4135**	475.7104	933.3870	467.1971	**932.4030**	466.7051	**V**	939.5873	470.2973	922.5608	461.7840	921.5768	461.2920	**9**
**10**	1063.4976	532.2524	1046.4710	523.7392	1045.4870	523.2472	**I**	**840.5189**	420.7631	823.4924	412.2498	822.5084	411.7578	**8**
**11**	**1178.5245**	589.7659	1161.4980	581.2526	1160.5140	580.7606	**D**	**727.4349**	364.2211	710.4083	355.7078	709.4243	355.2158	**7**
**12**	1235.5460	618.2766	1218.5195	609.7634	1217.5354	609.2714	**G**	**612.4079**	306.7076	595.3814	298.1943			**6**
**13**	1334.6144	667.8108	1317.5879	659.2976	**1316.6039**	658.8056	**V**	**555.3865**	278.1969	538.3599	269.6836			**5**
**14**	**1433.6828**	717.3451	**1416.6563**	708.8318	**1415.6723**	708.3398	**V**	**456.3180**	228.6627	439.2915	220.1494			**4**
**15**	**1546.7669**	773.8871	1529.7403	765.3738	1528.7563	764.8818	**L**	**357.2496**	179.1285	340.2231	170.6152			**3**
**16**	1643.8197	822.4135	1626.7931	813.9002	1625.8091	813.4082	**P**	244.1656	122.5864	227.1390	114.0731			**2**
**17**							**K**	147.1128	74.0600	130.0863	65.5468			**1**

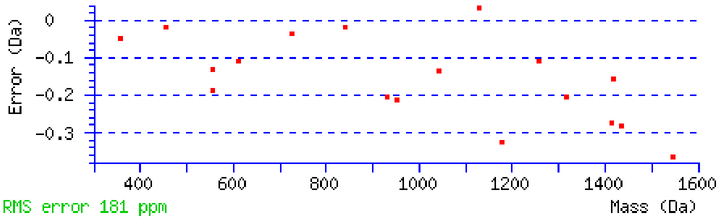

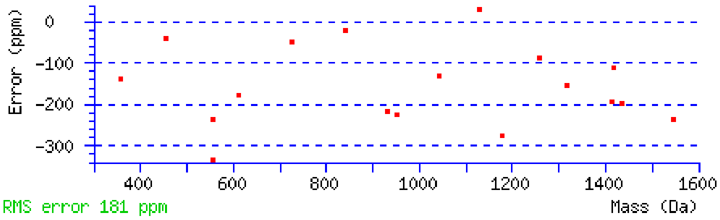

All matches to this query

Score	Mr(calc):	Delta	Sequence
63.0	1788.9179	0.0044	LDNTSMSTVIDGVVLPK
43.9	1787.9339	0.9884	LDNTSMSTVIDGVVLPK
0.8	1788.9291	-0.0068	DQLALAIGTLGENMSLK

Spectrum No: 292; Query: 78; Rank: 1

Peptide View

MS/MS Fragmentation of **DALQNMSSSLK**
Found in **IPI00325312**, Tax_Id=10116 Gene_Symbol=Prom1 prominin 1 isoform 1

Match to Query 78: 1193.560528 from(597.787540,2+)
Title: 091127RatKid_SCX01_12.2217.2217.2.dta
Data file K:\NewmanPaper\Piliang\3SubProteomes\Piliang3SP\mgf5ppm\SCX_3SubProteomes5ppm.mgf

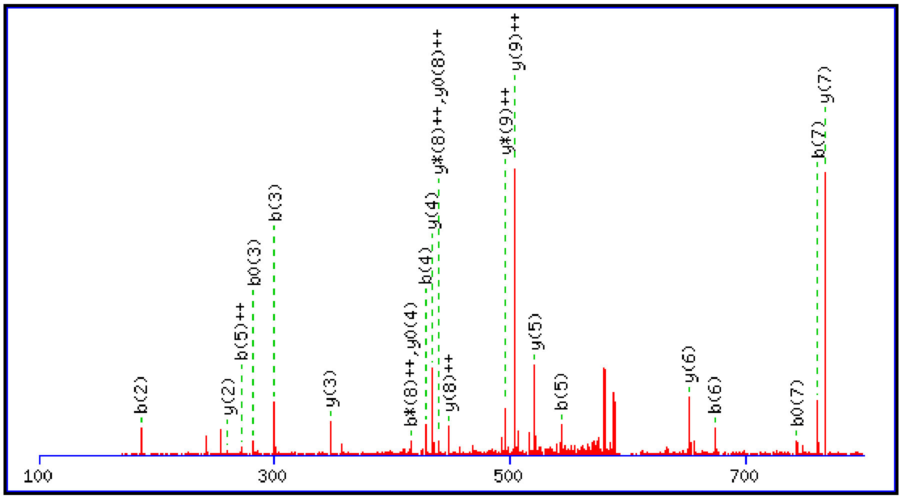

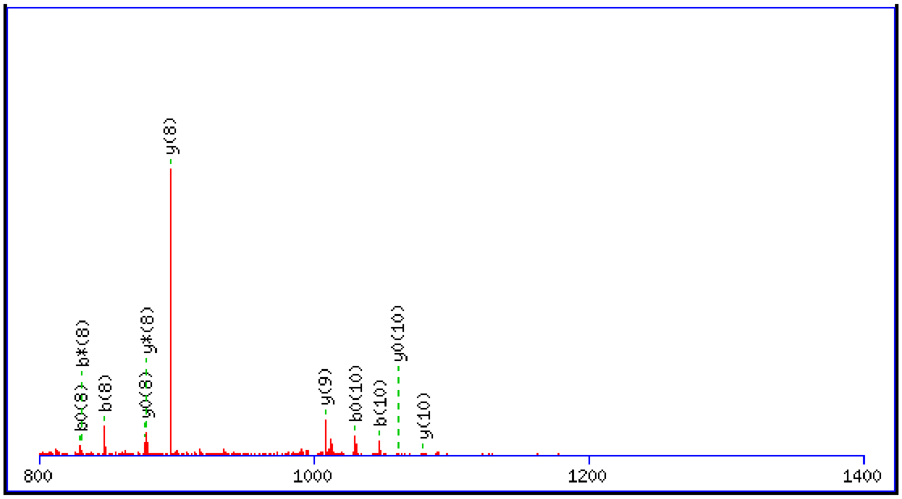

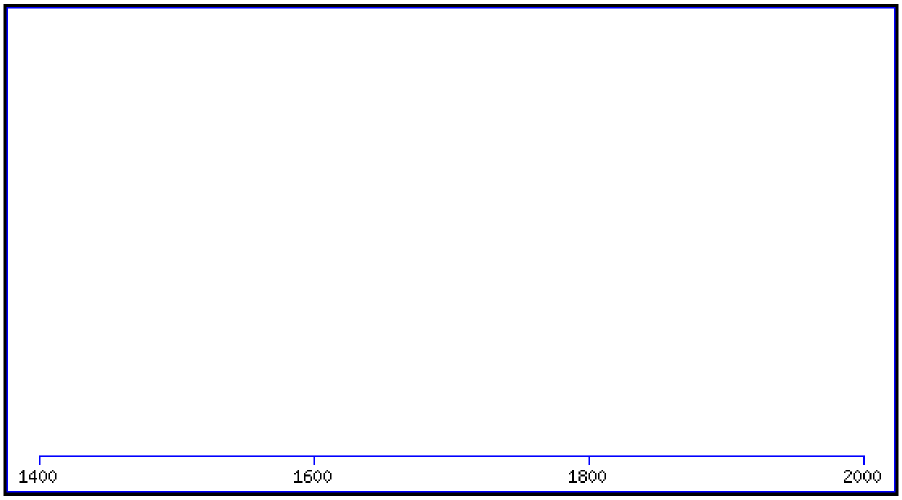

Monoisotopic mass of neutral peptide Mr(calc): 1193.5598
Fixed modifications: Carbamidomethyl (C)
Variable modifications:
N5 : Deamidated_N (N)
Ions Score: 63 **Expect:** 5.8e-005
Matches (Bold Red): 33/110 fragment ions using 55 most intense peaks

#	b	b++	b*	b*++	b0	b0++	Seq.	y	y++	y*	y*++	y0	y0++	#
1	116.0342	58.5207			98.0237	49.5155	D							11
2	**187.0713**	94.0393			169.0608	85.0340	A	**1079.5401**	540.2737	1062.5136	531.7604	**1061.5296**	531.2684	10
3	**300.1554**	150.5813			**282.1448**	141.5761	L	**1008.5030**	**504.7551**	991.4765	**496.2419**	990.4924	495.7499	9
4	**428.2140**	214.6106	411.1874	206.0974	410.2034	205.6053	Q	**895.4190**	**448.2131**	**878.3924**	**439.6998**	**877.4084**	**439.2078**	8
5	**543.2409**	**272.1241**	526.2144	263.6108	525.2303	263.1188	N	**767.3604**	384.1838	750.3338	375.6705	749.3498	375.1785	7
6	**674.2814**	337.6443	657.2548	329.1311	656.2708	328.6391	M	**652.3334**	326.6704	635.3069	318.1571	634.3229	317.6651	6
7	**761.3134**	381.1604	744.2869	372.6471	**743.3029**	372.1551	S	**521.2930**	261.1501	504.2664	252.6368	503.2824	252.1448	5
8	**848.3455**	424.6764	**831.3189**	**416.1631**	**830.3349**	415.6711	S	**434.2609**	217.6341	417.2344	209.1208	**416.2504**	208.6288	4
9	935.3775	468.1924	918.3509	459.6791	917.3669	459.1871	S	**347.2289**	174.1181	330.2023	165.6048	329.2183	165.1128	3
10	**1048.4615**	524.7344	1031.4350	516.2211	**1030.4510**	515.7291	L	**260.1969**	130.6021	243.1703	122.0888			2
11							K	147.1128	74.0600	130.0863	65.5468			1

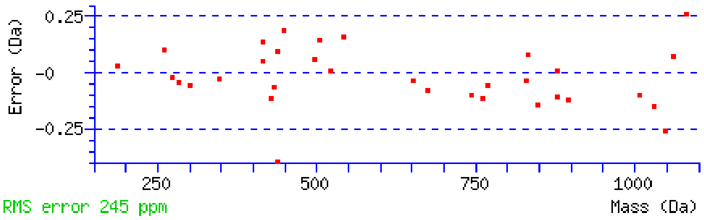

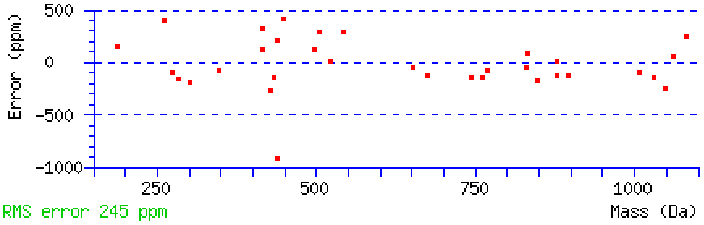

All matches to this query

Score	Mr(calc):	Delta	Sequence
62.9	1193.5598	0.0008	DALQNMSSSLK
9.7	1193.5532	0.0073	NAEMKNAMRK
6.4	1193.5693	-0.0088	EAVKTLGGDPK
6.3	1193.5598	0.0008	NLEDKCSSLK
3.3	1193.5598	0.0008	DQNASIMASLK
3.2	1193.5645	-0.0040	EGLSKCGRCK
1.2	1191.5627	1.9978	IAMMEESLPR
1.2	1193.5668	-0.0063	YIAICHPIK
0.9	1193.5693	-0.0088	GEVGATTALAPK
0.9	1193.5693	-0.0088	GEVGATTALAPK

Spectrum No: 293; Query: 194; Rank: 1

Peptide View

MS/MS Fragmentation of **SENSSLTLSSSNR**
Found in **IPI00324585**, Tax_Id=10116 Gene_Symbol=Itga1 Integrin alpha-1 precursor

Match to Query 194: 1381.634848 from(691.824700,2+)
Title: 091127RatKid_SCX01_20.923.923.2.dta
Data file K:\NewmanPaper\Piliang\3SubProteomes\Piliang3SP\mgf5ppm\SCX_3SubProteomes5ppm.mgf

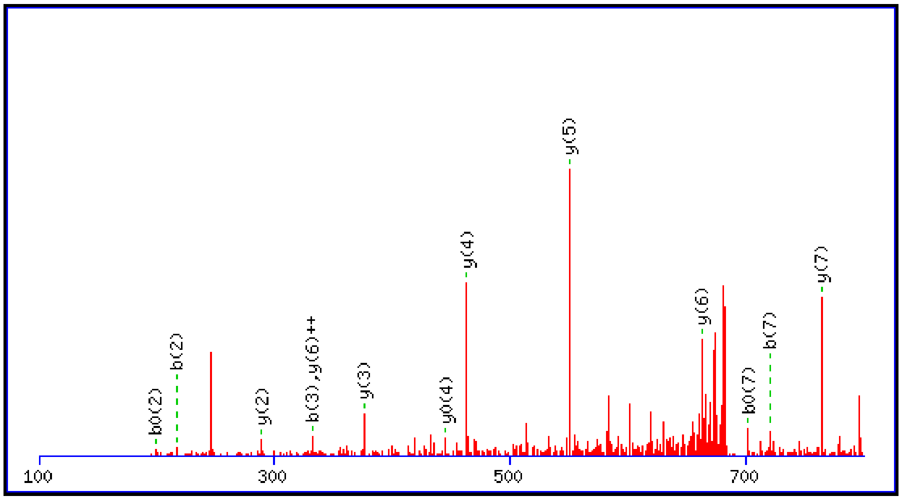

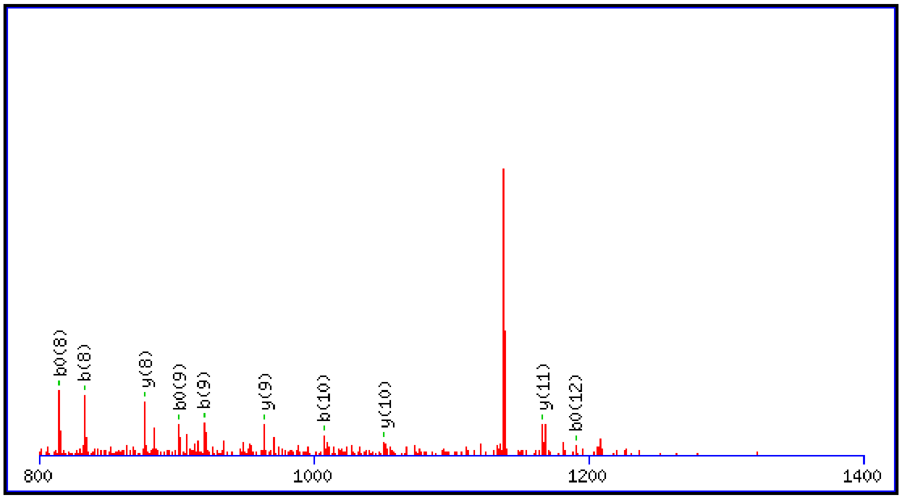

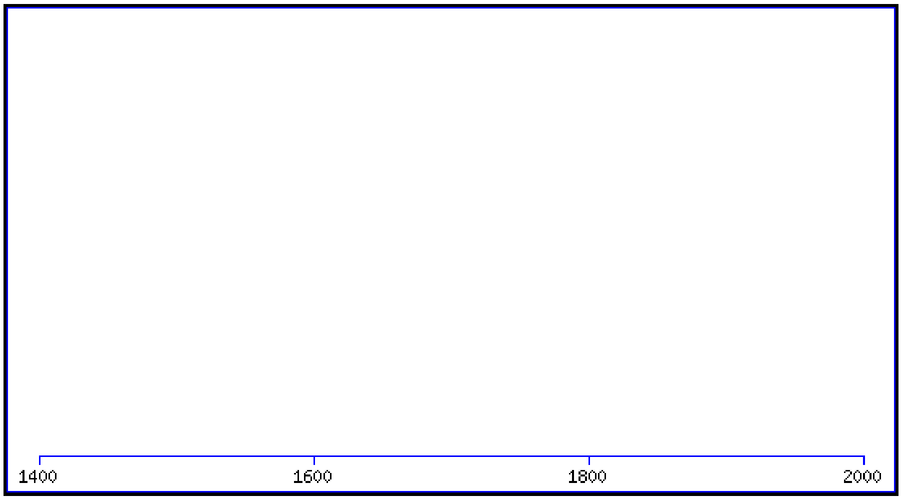

Monoisotopic mass of neutral peptide Mr(calc): 1381.6321
Fixed modifications: Carbamidomethyl (C)
Variable modifications:
N3 : Deamidated_N (N)
Ions Score: 63 **Expect:** 7.5e-005
Matches (Bold Red): 23/136 fragment ions using 54 most intense peaks

#	b	b^{++}	b*	b^{*++}	b^0	b^{0++}	Seq.	y	y^{++}	y*	y^{*++}	y^0	y^{0++}	#
1	88.0393	44.5233			70.0287	35.5180	S							13
2	**217.0819**	109.0446			**199.0713**	100.0393	E	1295.6074	648.3073	1278.5808	639.7940	1277.5968	639.3020	12
3	**332.1088**	166.5581	315.0823	158.0448	314.0983	157.5528	N	**1166.5648**	583.7860	1149.5382	575.2727	1148.5542	574.7807	11
4	419.1409	210.0741	402.1143	201.5608	401.1303	201.0688	S	**1051.5378**	526.2726	1034.5113	517.7593	1033.5273	517.2673	10
5	506.1729	253.5901	489.1463	245.0768	488.1623	244.5848	S	**964.5058**	482.7565	947.4792	474.2433	946.4952	473.7513	9
6	619.2570	310.1321	602.2304	301.6188	601.2464	301.1268	L	**877.4738**	439.2405	860.4472	430.7272	859.4632	430.2352	8
7	**720.3046**	360.6560	703.2781	352.1427	**702.2941**	351.6507	T	**764.3897**	382.6985	747.3632	374.1852	746.3791	373.6932	7
8	**833.3887**	417.1980	816.3621	408.6847	**815.3781**	408.1927	L	**663.3420**	**332.1747**	646.3155	323.6614	645.3315	323.1694	6
9	**920.4207**	460.7140	903.3942	452.2007	**902.4102**	451.7087	S	**550.2580**	275.6326	533.2314	267.1193	532.2474	266.6273	5
10	**1007.4528**	504.2300	990.4262	495.7167	989.4422	495.2247	S	**463.2259**	232.1166	446.1994	223.6033	**445.2154**	223.1113	4
11	1094.4848	547.7460	1077.4582	539.2328	1076.4742	538.7407	S	**376.1939**	188.6006	359.1674	180.0873	358.1833	179.5953	3
12	1208.5277	604.7675	1191.5012	596.2542	**1190.5171**	595.7622	N	**289.1619**	145.0846	272.1353	136.5713			2
13							R	175.1190	88.0631	158.0924	79.5498			1

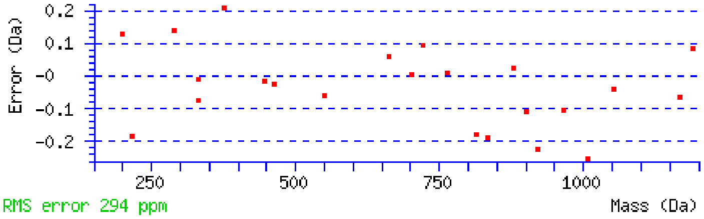

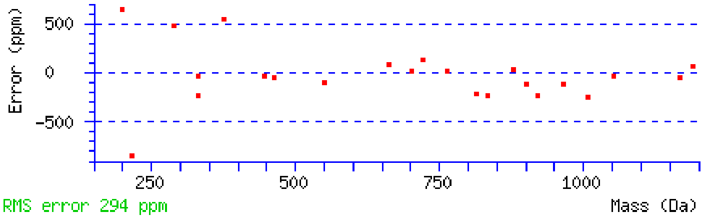

All matches to this query

Score	Mr(calc):	Delta	Sequence
62.7	1381.6321	0.0028	SENSSLTLSSSNR
6.8	1380.6415	0.9933	SRNSMRVSGSNGK
2.4	1380.6286	1.0062	QDVIQDVEISR
2.4	1380.6400	0.9948	SIIYYMVTSPK
0.1	1381.6288	0.0061	NXFHSVMPIMK
0.0	1381.6378	-0.0029	LVPEELTESASK

Spectrum No: 294; Query: 177; Rank: 1

Peptide View

MS/MS Fragmentation of **LFIVGSNSSSSTR**
Found in **IPI00191738**, Tax_Id=10116 Gene_Symbol=Gclm Glutamate--cysteine ligase regulatory subunit

Match to Query 177: 1354.674588 from(678.344570,2+)
Title: 091127RatKid_SCX01_20.2065.2065.2.dta
Data file K:\NewmanPaper\Piliang\3SubProteomes\Piliang3SP\mgf5ppm\SCX_3SubProteomes5ppm.mgf

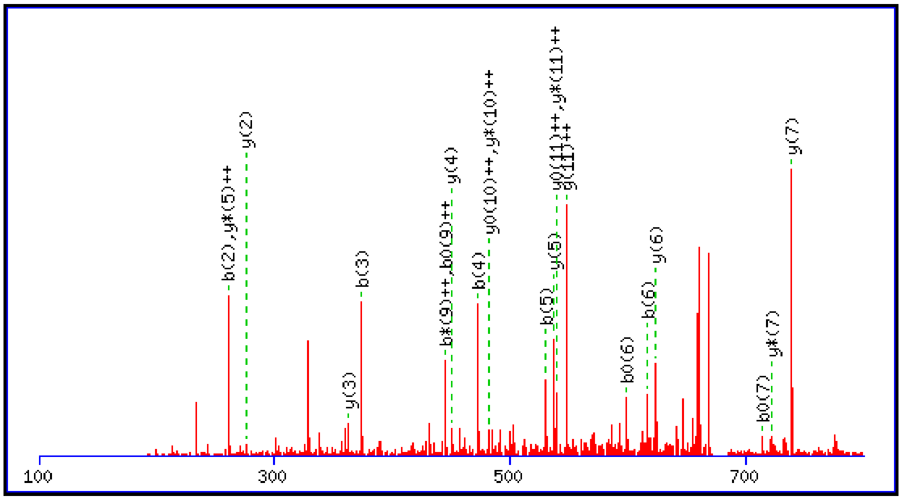

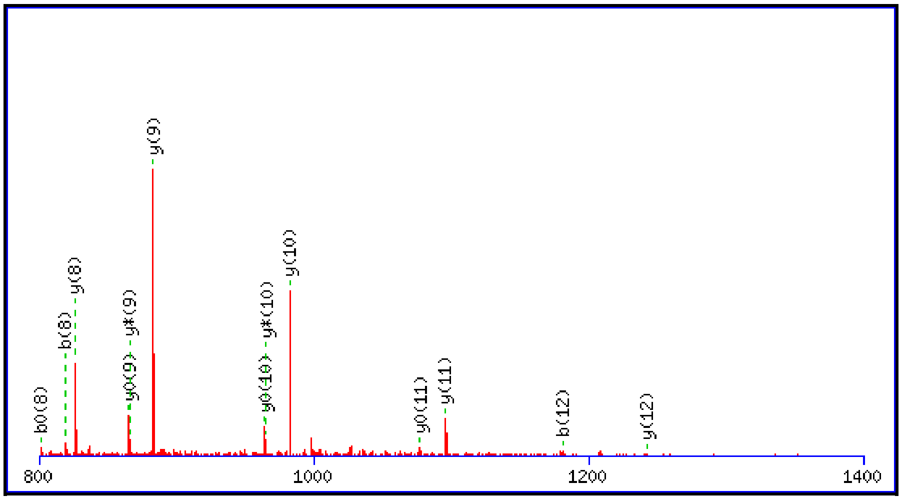

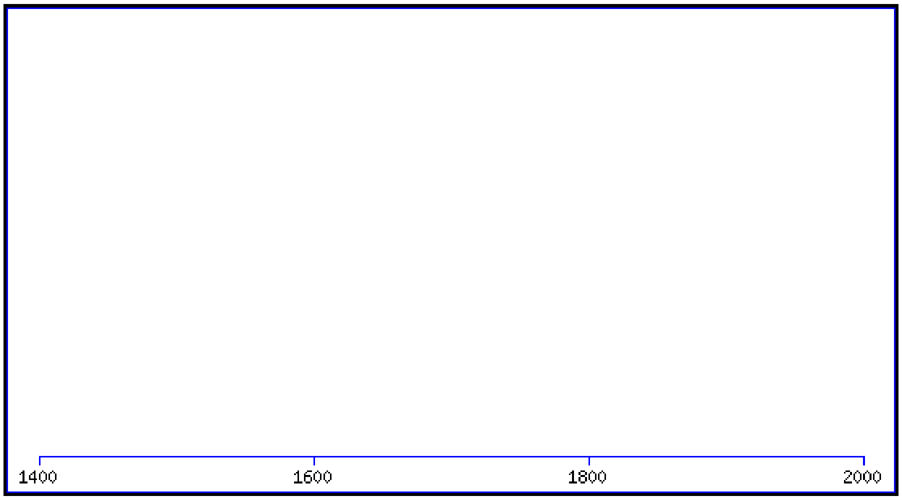

Monoisotopic mass of neutral peptide Mr(calc): 1354.6729
Fixed modifications: Carbamidomethyl (C)

```
Variable modifications:
N7     : Deamidated_N (N)
Ions Score: 63  Expect: 7.4e-005
Matches (Bold Red): 35/120 fragment ions using 73 most intense peaks
```

#	b	b^{++}	b*	b^{*++}	b^0	b^{0++}	Seq.	y	y^{++}	y*	y^{*++}	y^0	y^{0++}	#
1	114.0913	57.5493					L							13
2	**261.1598**	131.0835					F	**1242.5961**	621.8017	1225.5695	613.2884	1224.5855	612.7964	12
3	**374.2438**	187.6255					I	**1095.5276**	**548.2675**	1078.5011	**539.7542**	**1077.5171**	**539.2622**	11
4	**473.3122**	237.1598					V	**982.4436**	491.7254	**965.4170**	**483.2122**	**964.4330**	**482.7201**	10
5	**530.3337**	265.6705					G	**883.3752**	442.1912	**866.3486**	433.6779	**865.3646**	433.1859	9
6	**617.3657**	309.1865			**599.3552**	300.1812	S	**826.3537**	413.6805	809.3272	405.1672	808.3431	404.6752	8
7	732.3927	366.7000	715.3661	358.1867	**714.3821**	357.6947	N	**739.3217**	370.1645	**722.2951**	361.6512	721.3111	361.1592	7
8	**819.4247**	410.2160	802.3981	401.7027	**801.4141**	401.2107	S	**624.2947**	312.6510	607.2682	304.1377	606.2842	303.6457	6
9	906.4567	453.7320	889.4302	**445.2187**	888.4462	**444.7267**	S	**537.2627**	269.1350	520.2362	**260.6217**	519.2521	260.1297	5
10	993.4887	497.2480	976.4622	488.7347	975.4782	488.2427	S	**450.2307**	225.6190	433.2041	217.1057	432.2201	216.6137	4
11	1080.5208	540.7640	1063.4942	532.2507	1062.5102	531.7587	S	**363.1987**	182.1030	346.1721	173.5897	345.1881	173.0977	3
12	**1181.5685**	591.2879	1164.5419	582.7746	1163.5579	582.2826	T	**276.1666**	138.5870	259.1401	130.0737	258.1561	129.5817	2
13							R	175.1190	88.0631	158.0924	79.5498			1

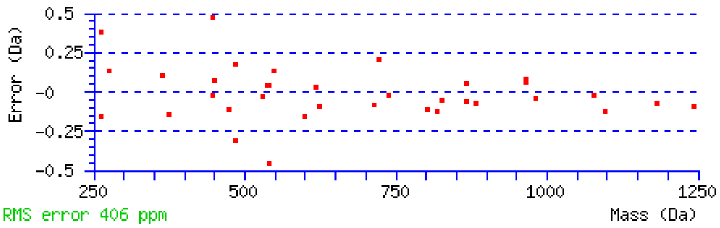

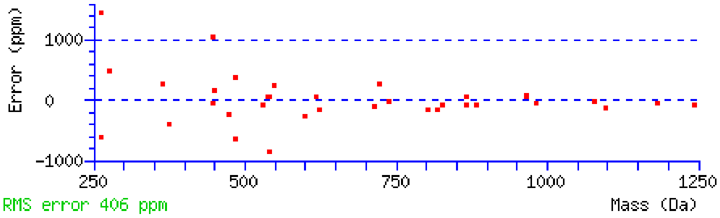

All matches to this query

Score	Mr(calc):	Delta	Sequence
62.6	1354.6729	0.0017	LFIVGSNSSSSTR
18.4	1353.6653	1.0093	QSQDLSRSLLK
16.0	1354.6625	0.0121	MILSTISWMGGK
15.9	1353.6806	0.9940	HFISSSRSLLK
15.9	1353.6806	0.9940	HFISSSRSLLK
15.9	1353.6806	0.9940	HFISSSRSLLK
15.9	1353.6653	1.0093	QSQDLSRSLLK
15.4	1353.6776	0.9970	DSSTGANSYVIIK
14.3	1353.6632	1.0114	MMNTDLSRILK
10.2	1354.6679	0.0066	MLIRVANNISK

Spectrum No: 295; Query: 198; Rank: 1

Peptide View

MS/MS Fragmentation of **AANYTSSLNLPDK**
Found in **IPI00366011**, Tax_Id=10116 Gene_Symbol=Sema4d_predicted similar to semaphorin 4D

Match to Query 198: 1393.676068 from(697.845310,2+)
Title: 091127RatKid_SCX01_12.1942.1942.2.dta
Data file K:\NewmanPaper\Piliang\3SubProteomes\Piliang3SP\mgf5ppm\SCX_3SubProteomes5ppm.mgf

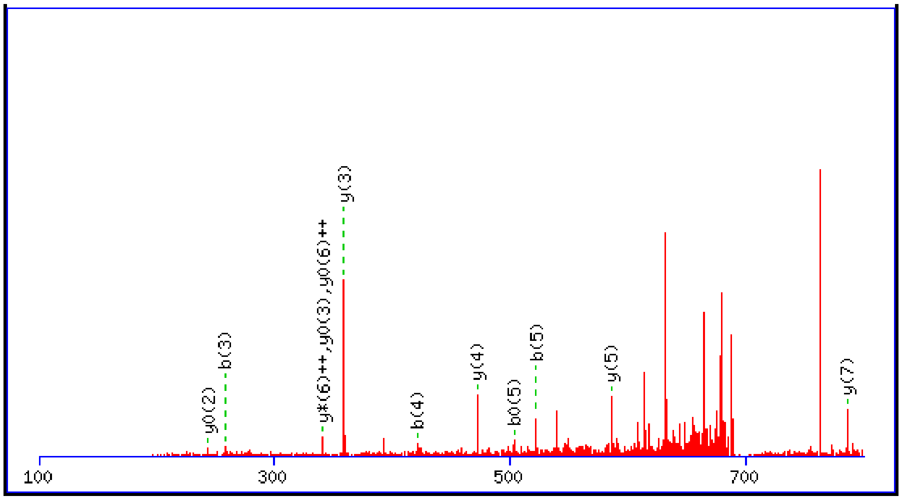

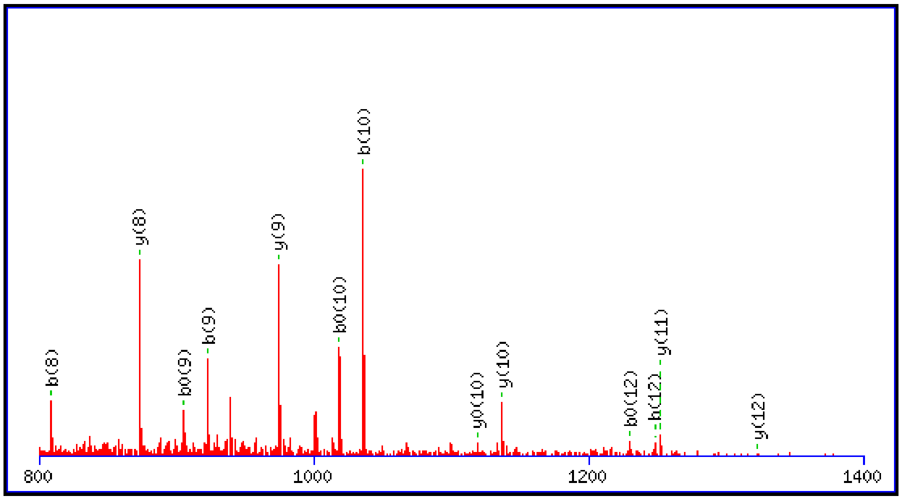

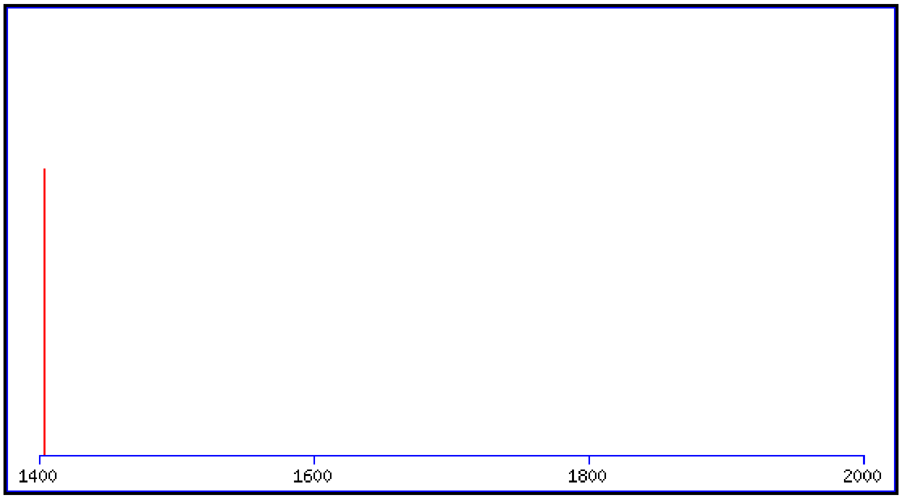

Monoisotopic mass of neutral peptide Mr(calc): 1393.6725
Fixed modifications: Carbamidomethyl (C)
Variable modifications:
N3 : Deamidated_N (N)
Ions Score: 63 **Expect:** 8e-005
Matches (Bold Red): 25/130 fragment ions using 49 most intense peaks

#	b	b++	b*	b*++	b0	b0++	Seq.	y	y++	y*	y*++	y0	y0++	#
1	72.0444	36.5258					A							13
2	143.0815	72.0444					A	**1323.6427**	662.3250	1306.6161	653.8117	1305.6321	653.3197	12
3	**258.1084**	129.5579	241.0819	121.0446			N	**1252.6056**	626.8064	1235.5790	618.2931	1234.5950	617.8011	11
4	**421.1718**	211.0895	404.1452	202.5762			Y	**1137.5786**	569.2930	1120.5521	560.7797	**1119.5681**	560.2877	10
5	**522.2194**	261.6134	505.1929	253.1001	**504.2089**	252.6081	T	**974.5153**	487.7613	957.4888	479.2480	956.5047	478.7560	9

6	609.2515	305.1294	592.2249	296.6161	591.2409	296.1241	S	**873.4676**	437.2374	856.4411	428.7242	855.4571	428.2322	8
7	696.2835	348.6454	679.2570	340.1321	678.2729	339.6401	S	**786.4356**	393.7214	769.4090	385.2082	768.4250	384.7162	7
8	**809.3676**	405.1874	792.3410	396.6741	791.3570	396.1821	L	699.4036	350.2054	682.3770	**341.6921**	681.3930	**341.2001**	6
9	**923.4105**	462.2089	906.3839	453.6956	**905.3999**	453.2036	N	**586.3195**	293.6634	569.2930	285.1501	568.3089	284.6581	5
10	**1036.4946**	518.7509	1019.4680	510.2376	**1018.4840**	509.7456	L	**472.2766**	236.6419	455.2500	228.1287	454.2660	227.6366	4
11	1133.5473	567.2773	1116.5208	558.7640	1115.5368	558.2720	P	**359.1925**	180.0999	342.1660	171.5866	**341.1819**	171.0946	3
12	**1248.5743**	624.7908	1231.5477	616.2775	**1230.5637**	615.7855	D	262.1397	131.5735	245.1132	123.0602	**244.1292**	122.5682	2
13							K	147.1128	74.0600	130.0863	65.5468			1

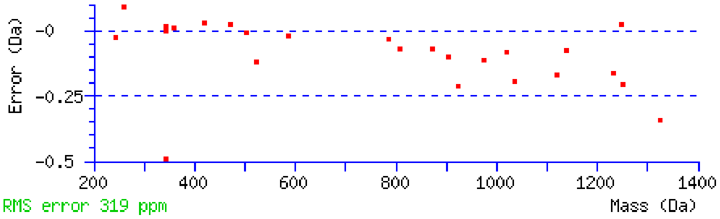

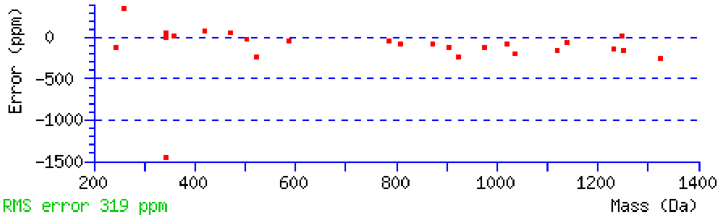

All matches to this query

Score	Mr(calc):	Delta	Sequence
62.6	1393.6725	0.0036	AANYTSSLNLPDK
35.8	1393.6725	0.0036	AANYTSSLNLPDK
8.3	1393.6715	0.0046	VQSKSAQARDPK
5.7	1393.6854	-0.0093	EKTLETVPLER
3.6	1393.6715	0.0046	VQSKSAQARDPK
2.5	1393.6621	0.0139	LETLETWMMPK
2.5	1393.6621	0.0139	LETLETWMMPK
1.9	1391.6615	2.0145	ENKMTWAPRSR
1.3	1391.6558	2.0202	SGNIHATKTLDR

Spectrum No: 296; Query: 477; Rank: 1

Peptide View

MS/MS Fragmentation of **VLPFNVTDYCQLVR**
Found in **IPI00204006**, Tax_Id=10116 Gene_Symbol=Fbn1 Fibrillin-1

Match to Query 477: 1723.865008 from(862.939780,2+)
Title: 091127RatKid_SCX01_16.4644.4644.2.dta
Data file K:\NewmanPaper\Piliang\3SubProteomes\Piliang3SP\mgf5ppm\SCX_3SubProteomes5ppm.mgf

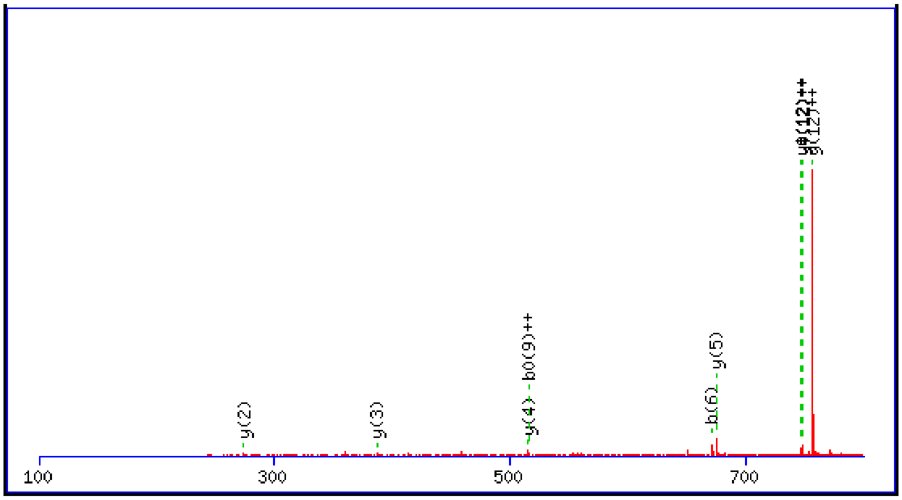

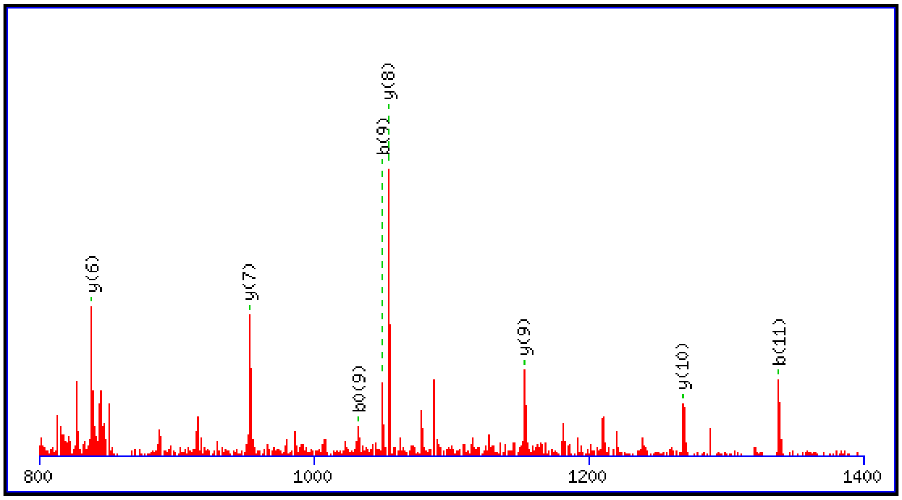

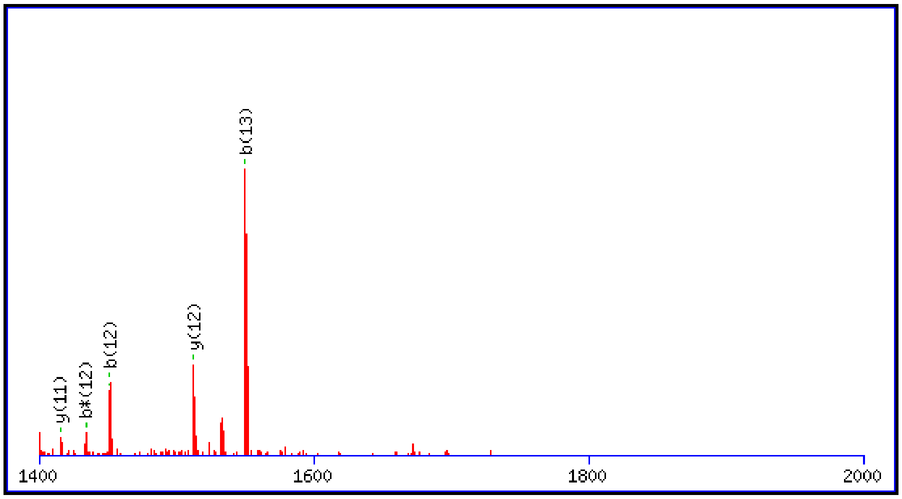

Monoisotopic mass of neutral peptide Mr(calc): 1723.8604
Fixed modifications: Carbamidomethyl (C)
Variable modifications:
N5 : Deamidated_N (N)
Ions Score: 63 **Expect:** 9.6e-005
Matches (Bold Red): 22/124 fragment ions using 61 most intense peaks

#	b	b^{++}	b*	b^{*++}	b^0	b^{0++}	Seq.	y	y^{++}	y*	y^{*++}	y^0	y^{0++}	#
1	100.0757	50.5415					V							14
2	213.1598	107.0835					L	1625.7992	813.4032	1608.7727	804.8900	1607.7886	804.3980	13
3	310.2125	155.6099					P	**1512.7151**	**756.8612**	1495.6886	**748.3479**	1494.7046	**747.8559**	12
4	457.2809	229.1441					F	**1415.6624**	708.3348	1398.6358	699.8215	1397.6518	699.3295	11
5	572.3079	286.6576	555.2813	278.1443			N	**1268.5940**	634.8006	1251.5674	626.2873	1250.5834	625.7953	10

**6**	**671.3763**	336.1918	654.3497	327.6785			**V**	**1153.5670**	577.2871	1136.5405	568.7739	1135.5565	568.2819	**9**
**7**	772.4240	386.7156	755.3974	378.2023	754.4134	377.7103	**T**	**1054.4986**	527.7529	1037.4721	519.2397	1036.4880	518.7477	**8**
**8**	887.4509	444.2291	870.4244	435.7158	869.4403	435.2238	**D**	**953.4509**	477.2291	936.4244	468.7158	935.4404	468.2238	**7**
**9**	**1050.5142**	525.7608	1033.4877	517.2475	**1032.5037**	**516.7555**	**Y**	**838.4240**	419.7156	821.3974	411.2024			**6**
**10**	1210.5449	605.7761	1193.5183	597.2628	1192.5343	596.7708	**C**	**675.3607**	338.1840	658.3341	329.6707			**5**
**11**	**1338.6035**	669.8054	1321.5769	661.2921	1320.5929	660.8001	**Q**	**515.3300**	258.1686	498.3035	249.6554			**4**
**12**	**1451.6875**	726.3474	**1434.6610**	717.8341	1433.6770	717.3421	**L**	**387.2714**	194.1394	370.2449	185.6261			**3**
**13**	**1550.7559**	775.8816	1533.7294	767.3683	1532.7454	766.8763	**V**	**274.1874**	137.5973	257.1608	129.0840			**2**
**14**							**R**	175.1190	88.0631	158.0924	79.5498			**1**

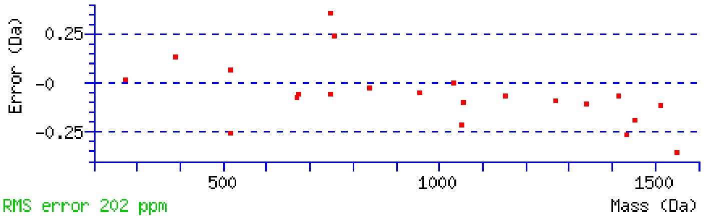

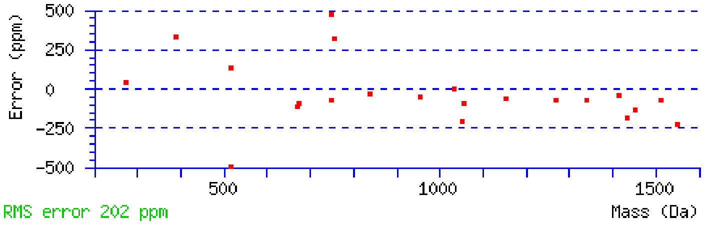

All matches to this query

Score	Mr(calc):	Delta	Sequence
62.6	1723.8604	0.0047	VLPFNVTDYCQLVR
21.6	1722.8763	0.9887	VLPFNVTDYCQLVR
10.8	1723.8514	0.0136	LCLSPLTNLCGKLR
10.2	1723.8514	0.0136	LCLSPLTNLCGKLR
7.8	1723.8645	0.0005	VISQTNLITTVTPEK
7.6	1723.8770	-0.0120	IYKNPSLRAQGQIR
6.6	1723.8570	0.0080	VIFHPPNPFPSEASGK
5.5	1721.8559	2.0091	WVVNAVNCSLFSAVR
4.2	1723.8645	0.0005	VISQTNLITTVTPEK
3.1	1721.8713	1.9937	IQAKATANSLQLEVR

Spectrum No: 297; Query: 16; Rank: 1

Peptide View

MS/MS Fragmentation of **NLSTFAVDGK**
Found in **IPI00231028**, Tax_Id=10116 Gene_Symbol=RGD1303003 ES1 protein homolog, mitochondrial precursor

Match to Query 16: 1051.519268 from(526.766910,2+)
Title: 091127RatKid_SCX01_16.2550.2550.2.dta
Data file K:\NewmanPaper\Piliang\3SubProteomes\Piliang3SP\mgf5ppm\SCX_3SubProteomes5ppm.mgf

Mascot Search Results: Peptide View

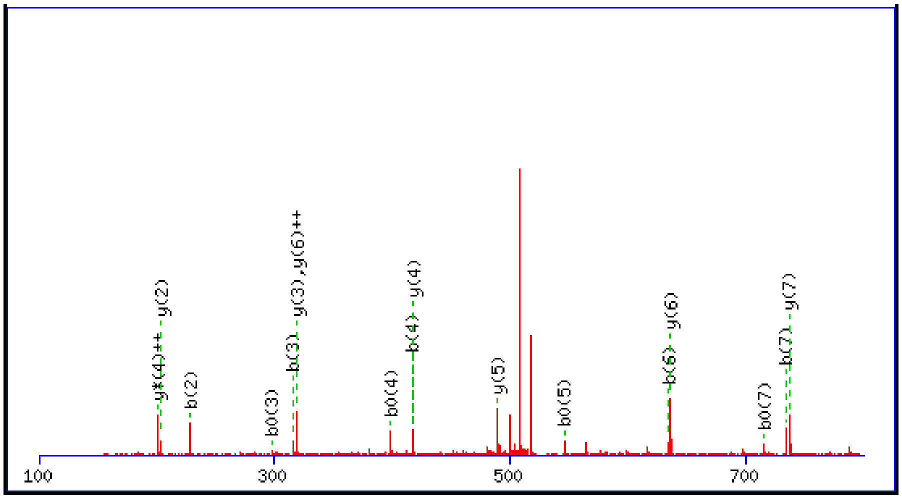

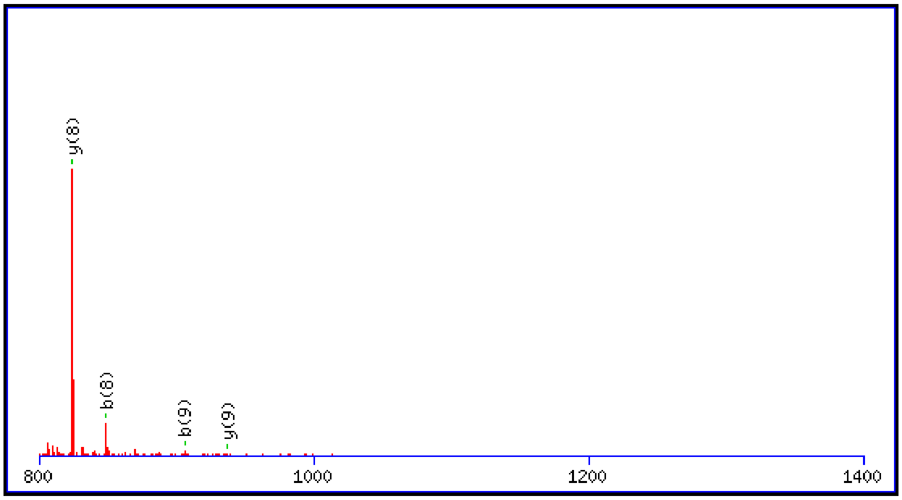

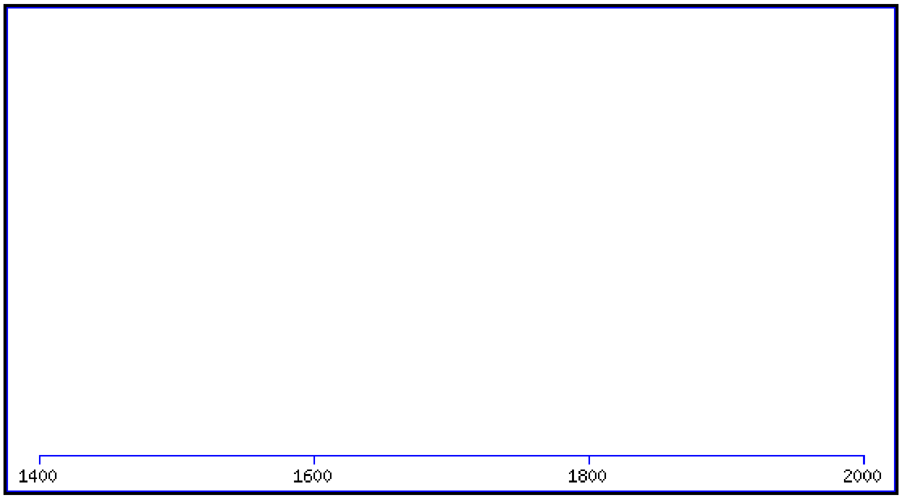

Monoisotopic mass of neutral peptide Mr(calc): 1051.5186
Fixed modifications: Carbamidomethyl (C)
Variable modifications:
N1 : Deamidated_N (N)
Ions Score: 62 **Expect:** 5.7e-005
Matches (Bold Red): 21/100 fragment ions using 31 most intense peaks

#	b	b^{++}	b*	b^{*++}	b^0	b^{0++}	Seq.	y	y^{++}	y*	y^{*++}	y^0	y^{0++}	#
1	116.0342	58.5207	99.0077	50.0075			N							10
2	**229.1183**	115.0628	212.0917	106.5495			L	**937.4989**	469.2531	920.4724	460.7398	919.4884	460.2478	9
3	**316.1503**	158.5788	299.1238	150.0655	**298.1397**	149.5735	S	**824.4149**	412.7111	807.3883	404.1978	806.4043	403.7058	8
4	**417.1980**	209.1026	400.1714	200.5894	**399.1874**	200.0973	T	**737.3828**	369.1951	720.3563	360.6818	719.3723	360.1898	7
5	564.2664	282.6368	547.2398	274.1236	**546.2558**	273.6316	F	**636.3352**	**318.6712**	619.3086	310.1579	618.3246	309.6659	6

6	635.3035	318.1554	618.2770	309.6421	617.2929	309.1501	A	489.2667	245.1370	472.2402	236.6237	471.2562	236.1317	5
7	734.3719	367.6896	717.3454	359.1763	716.3614	358.6843	V	418.2296	209.6185	401.2031	201.1052	400.2191	200.6132	4
8	849.3989	425.2031	832.3723	416.6898	831.3883	416.1978	D	319.1612	160.0842	302.1347	151.5710	301.1506	151.0790	3
9	906.4203	453.7138	889.3938	445.2005	888.4098	444.7085	G	204.1343	102.5708	187.1077	94.0575			2
10							K	147.1128	74.0600	130.0863	65.5468			1

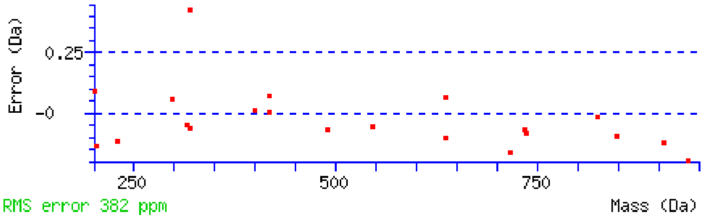

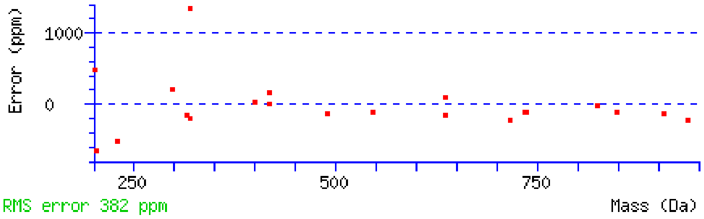

All matches to this query

Score	Mr(calc):	Delta	Sequence
62.3	1051.5186	0.0007	NLSTFAVDGK
6.5	1051.5186	0.0007	XISNTFLDK
6.5	1051.5186	0.0007	XISNTFLDK
6.4	1051.5216	-0.0023	AILTVHYR
5.3	1051.5146	0.0047	SSISSKSGDGK
5.2	1049.5158	2.0034	TATPSPVLGK
2.8	1050.5094	1.0099	NQASHPPATK
1.1	1051.5273	-0.0080	MWVFSNLR
0.9	1051.5186	0.0007	VEVEQSYAK
0.9	1050.5223	0.9970	LSVNSRAPK

Spectrum No: 298; Query: 173; Rank: 1

Peptide View

MS/MS Fragmentation of **YQTLNCSVNVR**
Found in **IPI00205166**, Tax_Id=10116 Gene_Symbol=Itga6 similar to integrin alpha 6 isoform 2

Match to Query 173: 1353.635228 from(677.824890,2+)
Title: 091127RatKid_SCX01_13.1327.1327.2.dta
Data file K:\NewmanPaper\Piliang\3SubProteomes\Piliang3SP\mgf5ppm\SCX_3SubProteomes5ppm.mgf

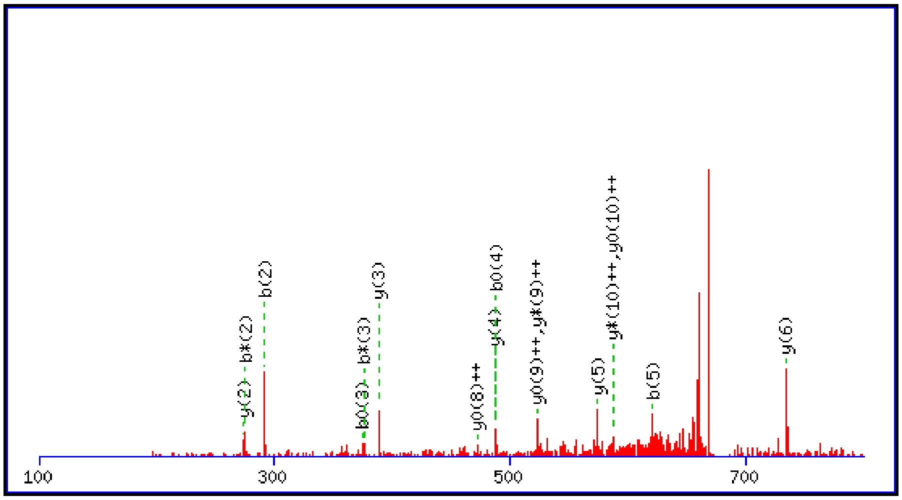

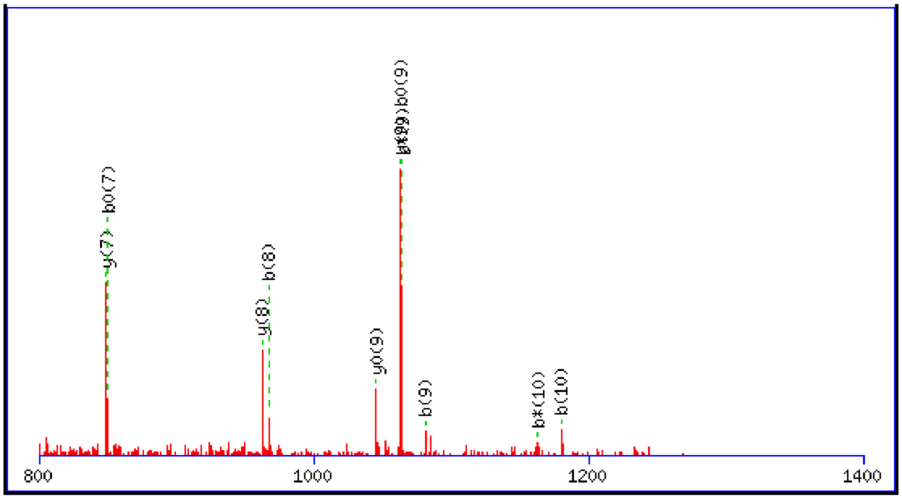

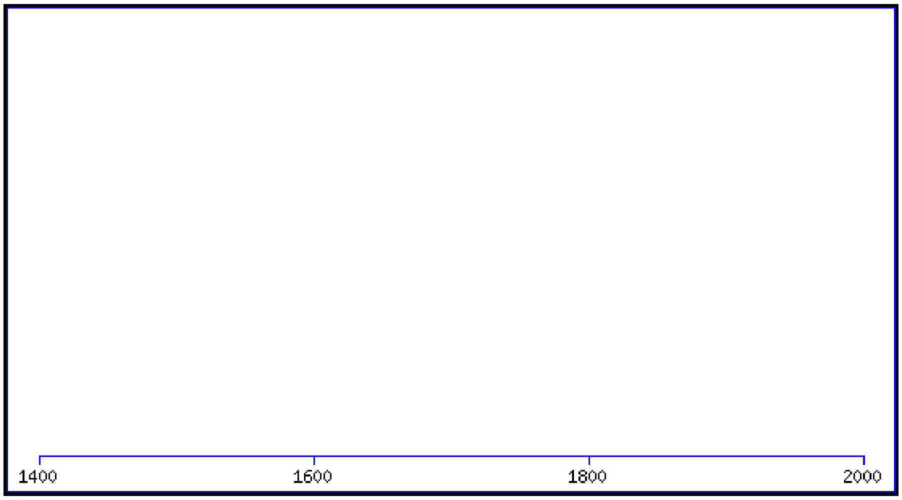

Monoisotopic mass of neutral peptide Mr(calc): 1353.6347
Fixed modifications: Carbamidomethyl (C)
Variable modifications:
N5 : Deamidated_N (N)
Ions Score: 62 **Expect:** 0.0001
Matches (Bold Red): 27/106 fragment ions using 51 most intense peaks

#	b	b^{++}	b*	b^{*++}	b^0	b^{0++}	Seq.	y	y^{++}	y*	y^{*++}	y^0	y^{0++}	#
1	164.0706	82.5389					Y							11
2	**292.1292**	146.5682	**275.1026**	138.0550			Q	1191.5786	596.2930	1174.5521	**587.7797**	1173.5681	**587.2877**	10
3	393.1769	197.0921	**376.1503**	188.5788	**375.1663**	188.0868	T	**1063.5201**	532.2637	1046.4935	**523.7504**	**1045.5095**	**523.2584**	9
4	506.2609	253.6341	489.2344	245.1208	**488.2504**	244.6288	L	**962.4724**	481.7398	945.4458	473.2266	944.4618	**472.7345**	8
5	**621.2879**	311.1476	604.2613	302.6343	603.2773	302.1423	N	**849.3883**	425.1978	832.3618	416.6845	831.3778	416.1925	7
6	781.3185	391.1629	764.2920	382.6496	763.3079	382.1576	C	**734.3614**	367.6843	717.3348	359.1711	716.3508	358.6790	6
7	868.3505	434.6789	851.3240	426.1656	**850.3400**	425.6736	S	**574.3307**	287.6690	557.3042	279.1557	556.3202	278.6637	5
8	**967.4190**	484.2131	950.3924	475.6998	949.4084	475.2078	V	**487.2987**	244.1530	470.2722	235.6397			4
9	**1081.4619**	541.2346	**1064.4353**	532.7213	**1063.4513**	532.2293	N	**388.2303**	194.6188	371.2037	186.1055			3
10	**1180.5303**	590.7688	**1163.5037**	582.2555	1162.5197	581.7635	V	**274.1874**	137.5973	257.1608	129.0840			2
11							R	175.1190	88.0631	158.0924	79.5498			1

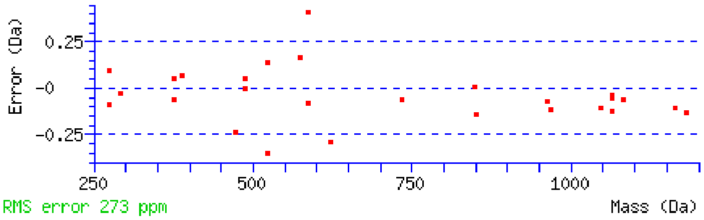

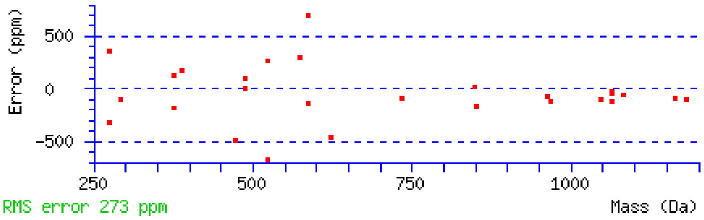

All matches to this query

Score	Mr(calc):	Delta	Sequence
62.2	1353.6347	0.0005	YQTLNCSVNVR
34.3	1353.6347	0.0005	YQTLNCSVNVR
15.6	1352.6282	1.0070	EVYLDCALNQK
8.7	1353.6476	-0.0123	LNLTKVVNGCR
7.7	1353.6347	0.0006	VKDDNYNLAMR
6.8	1353.6476	-0.0124	MAATTGSGVKVPR
5.3	1353.6264	0.0088	MVNPWRLSVR
4.8	1353.6377	-0.0025	GSHLGYMVRVR
4.8	1353.6486	-0.0133	YNILGTNAIMDK
4.6	1352.6316	1.0037	MATSDLMSELQK

Spectrum No: 299; Query: 679; Rank: 1

Peptide View

MS/MS Fragmentation of **DEYPSVLMYLNATQPER**
Found in **IPI00207180**, Tax_Id=10116 Gene_Symbol=Slc6a18 Sodium- and chloride-dependent transporter XTRP2

Match to Query 679: 2025.941648 from(1013.978100,2+)
Title: 091127RatKid_SCX01_02.4600.4600.2.dta
Data file K:\NewmanPaper\Piliang\3SubProteomes\Piliang3SP\mgf5ppm\SCX_3SubProteomes5ppm.mgf

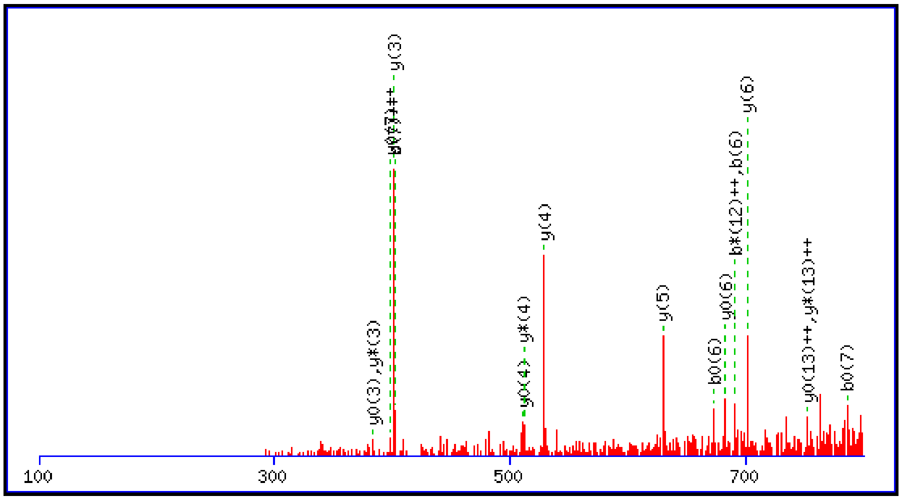

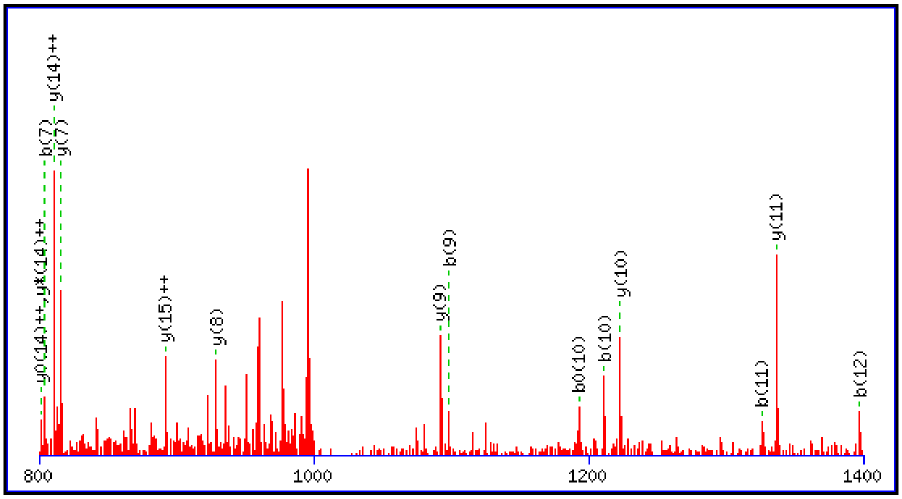

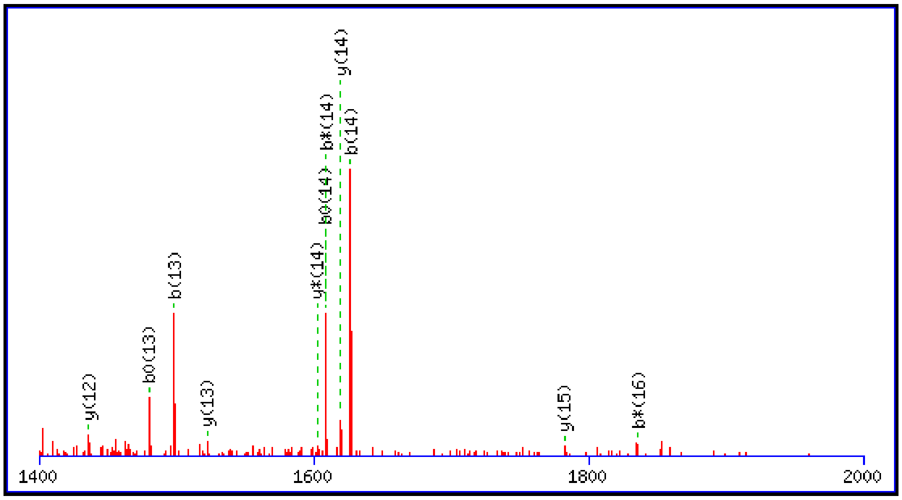

Monoisotopic mass of neutral peptide Mr(calc): 2025.9353
Fixed modifications: Carbamidomethyl (C)
Variable modifications:
N11 : Deamidated_N (N)
Ions Score: 62 **Expect:** 0.00015
Matches (Bold Red): 43/170 fragment ions using 90 most intense peaks

#	b	b^{++}	b^{*}	b^{*++}	b^{0}	b^{0++}	Seq.	y	y^{++}	y^{*}	y^{*++}	y^{0}	y^{0++}	#
1	116.0342	58.5207			98.0237	49.5155	D							17
2	245.0768	123.0420			227.0662	114.0368	E	1911.9157	956.4615	1894.8891	947.9482	1893.9051	947.4562	16
3	408.1401	204.5737			390.1296	195.5684	Y	**1782.8731**	**891.9402**	1765.8465	883.4269	1764.8625	882.9349	15
4	505.1929	253.1001			487.1823	244.0948	P	**1619.8098**	**810.4085**	**1602.7832**	**801.8952**	1601.7992	**801.4032**	14
5	592.2249	296.6161			574.2144	287.6108	S	**1522.7570**	761.8821	1505.7305	**753.3689**	1504.7464	**752.8769**	13
6	**691.2933**	346.1503			**673.2828**	337.1450	V	**1435.7250**	718.3661	1418.6984	709.8528	1417.7144	709.3608	12
7	**804.3774**	**402.6923**			**786.3668**	393.6871	L	**1336.6566**	668.8319	1319.6300	660.3186	1318.6460	659.8266	11
8	935.4179	468.2126			917.4073	459.2073	M	**1223.5725**	612.2899	1206.5459	603.7766	1205.5619	603.2846	10
9	**1098.4812**	549.7443			1080.4707	540.7390	Y	**1092.5320**	546.7696	1075.5055	538.2564	1074.5214	537.7644	9
10	**1211.5653**	606.2863			**1193.5547**	597.2810	L	**929.4687**	465.2380	912.4421	456.7247	911.4581	456.2327	8
11	**1326.5922**	663.7998	1309.5657	655.2865	1308.5817	654.7945	N	**816.3846**	408.6959	799.3581	400.1827	798.3741	**399.6907**	7
12	**1397.6293**	699.3183	1380.6028	**690.8050**	1379.6188	690.3130	A	**701.3577**	351.1825	684.3311	342.6692	**683.3471**	342.1772	6
13	**1498.6770**	749.8421	1481.6505	741.3289	**1480.6665**	740.8369	T	**630.3206**	315.6639	613.2940	307.1506	612.3100	306.6586	5
14	**1626.7356**	813.8714	**1609.7090**	805.3582	**1608.7250**	804.8662	Q	**529.2729**	265.1401	**512.2463**	256.6268	**511.2623**	256.1348	4
15	1723.7884	862.3978	1706.7618	853.8845	1705.7778	853.3925	P	**401.2143**	201.1108	**384.1878**	192.5975	**383.2037**	192.1055	3
16	1852.8310	926.9191	**1835.8044**	918.4058	1834.8204	917.9138	E	304.1615	152.5844	287.1350	144.0711	286.1510	143.5791	2
17							R	175.1190	88.0631	158.0924	79.5498			1

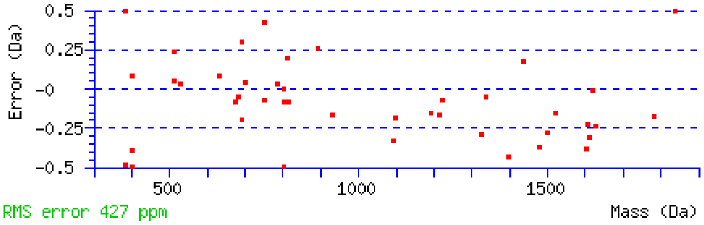

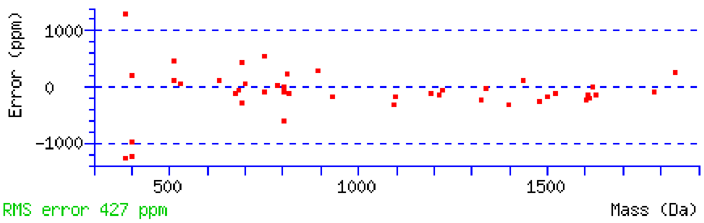

All matches to this query

Score	Mr(calc):	Delta	Sequence
62.1	2025.9353	0.0063	DEYPSVLMYLNATQPER
7.9	2024.9513	0.9903	DEYPSVLMYLNATQPER
3.5	2025.9368	0.0048	RTTSPSSDTDLLDRPASK
2.0	2025.9288	0.0129	DVLLTDFLPTMAVYIR

2.0	2025.9269	0.0147	QIDNTGSSTYRPPPRTR
1.3	2025.9269	0.0147	QIDNTGSSTYRPPPRTR
1.3	2025.9269	0.0147	QIDNTGSSTYRPPPRTR

Spectrum No: 300; Query: 1092; Rank: 1

Peptide View

MS/MS Fragmentation of **VNYEAETWDWIAETLSSNHMNFSSADR**
Found in **IPI00327398**, Tax_Id=10116 Gene_Symbol=Enpep Isoform 1 of Glutamyl aminopeptidase

Match to Query 1092: 3173.375052 from(1058.798960,3+)
Title: 091127RatKid_SCX01_20.7907.7907.3.dta
Data file K:\NewmanPaper\Piliang\3SubProteomes\Piliang3SP\mgf5ppm\SCX_3SubProteomes5ppm.mgf

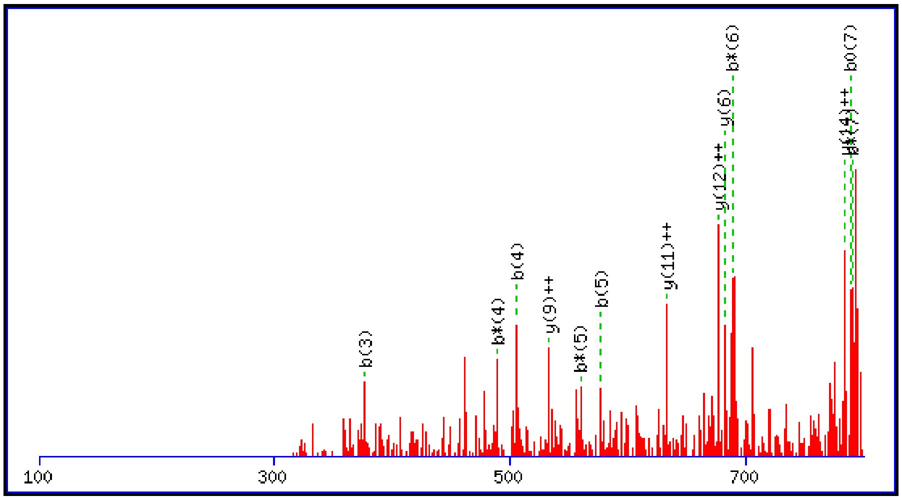

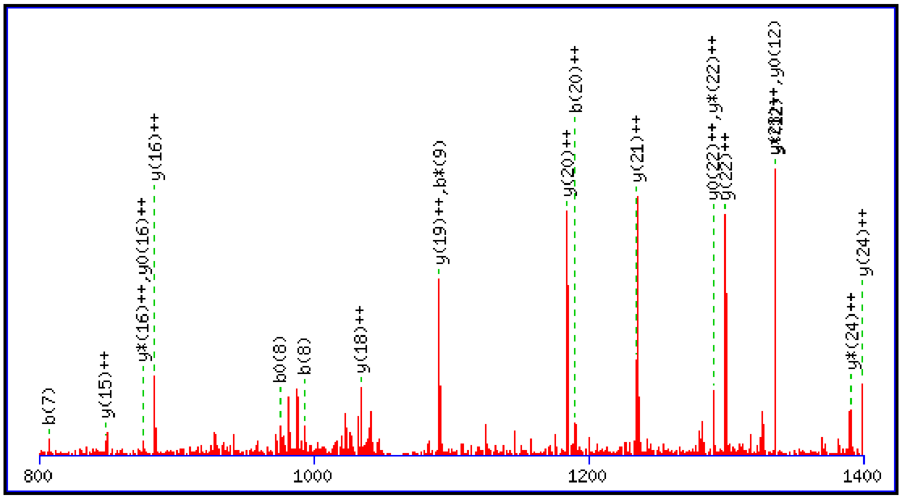

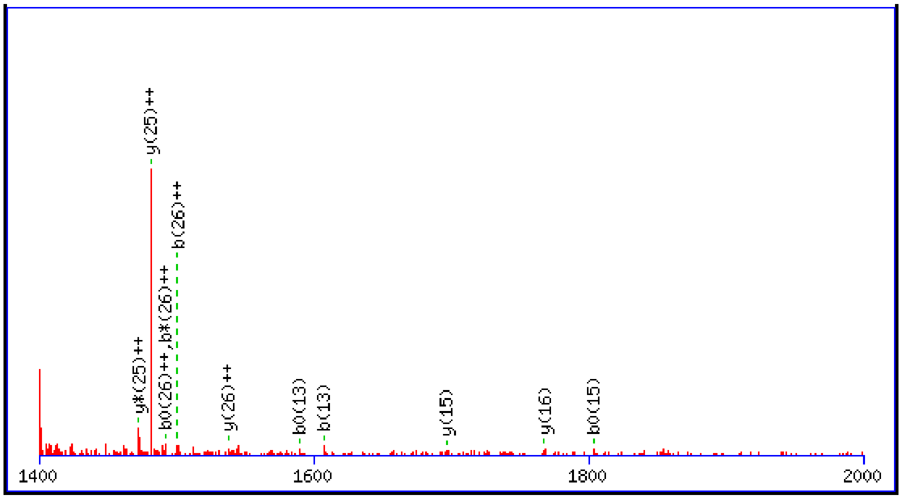

Monoisotopic mass of neutral peptide Mr(calc): 3173.3672
Fixed modifications: Carbamidomethyl (C)
Variable modifications:
N21 : Deamidated_N (N)
Ions Score: 62 **Expect:** 0.00029
Matches (Bold Red): 45/302 fragment ions using 94 most intense peaks

#	b	b^{++}	b*	b*$^{++}$	b^{0}	b^{0++}	Seq.	y	y^{++}	y*	y*$^{++}$	y^{0}	y^{0++}	#
1	100.0757	50.5415					V							27
2	214.1186	107.5629	197.0921	99.0497			N	3075.3061	**1538.1567**	3058.2796	1529.6434	3057.2955	1529.1514	26
3	**377.1819**	189.0946	360.1554	180.5813			Y	2961.2632	**1481.1352**	2944.2366	**1472.6220**	2943.2526	1472.1299	25
4	**506.2245**	253.6159	**489.1980**	245.1026	488.2140	244.6106	E	2798.1999	**1399.6036**	2781.1733	**1391.0903**	2780.1893	1390.5983	24
5	**577.2617**	289.1345	**560.2351**	280.6212	559.2511	280.1292	A	2669.1573	**1335.0823**	2652.1307	1326.5690	2651.1467	1326.0770	23
6	706.3042	353.6558	**689.2777**	345.1425	688.2937	344.6505	E	2598.1201	**1299.5637**	2581.0936	**1291.0504**	2580.1096	**1290.5584**	22
7	**807.3519**	404.1796	**790.3254**	395.6663	**789.3414**	395.1743	T	2469.0776	**1235.0424**	2452.0510	1226.5291	2451.0670	1226.0371	21
8	**993.4312**	497.2193	976.4047	488.7060	**975.4207**	488.2140	W	2368.0299	**1184.5186**	2351.0033	1176.0053	2350.0193	1175.5133	20
9	1108.4582	554.7327	**1091.4316**	546.2195	1090.4476	545.7274	D	2181.9506	**1091.4789**	2164.9240	1082.9656	2163.9400	1082.4736	19
10	1294.5375	647.7724	1277.5109	639.2591	1276.5269	638.7671	W	2066.9236	**1033.9654**	2049.8971	1025.4522	2048.9131	1024.9602	18
11	1407.6216	704.3144	1390.5950	695.8011	1389.6110	695.3091	I	1880.8443	940.9258	1863.8178	932.4125	1862.8337	931.9205	17
12	1478.6587	739.8330	1461.6321	731.3197	1460.6481	730.8277	A	**1767.7602**	**884.3838**	1750.7337	**875.8705**	1749.7497	**875.3785**	16
13	**1607.7013**	804.3543	1590.6747	795.8410	**1589.6907**	795.3490	E	**1696.7231**	**848.8652**	1679.6966	840.3519	1678.7126	839.8599	15
14	1708.7489	854.8781	1691.7224	846.3648	1690.7384	845.8728	T	1567.6805	**784.3439**	1550.6540	775.8306	1549.6700	775.3386	14
15	1821.8330	911.4201	1804.8065	902.9069	**1803.8224**	902.4149	L	1466.6329	733.8201	1449.6063	725.3068	1448.6223	724.8148	13
16	1908.8650	954.9362	1891.8385	946.4229	1890.8545	945.9309	S	1353.5488	**677.2780**	**1336.5222**	668.7648	**1335.5382**	668.2728	12
17	1995.8971	998.4522	1978.8705	989.9389	1977.8865	989.4469	S	1266.5168	**633.7620**	1249.4902	625.2487	1248.5062	624.7567	11
18	2109.9400	1055.4736	2092.9134	1046.9604	2091.9294	1046.4684	N	1179.4847	590.2460	1162.4582	581.7327	1161.4742	581.2407	10
19	2246.9989	1124.0031	2229.9724	1115.4898	2228.9883	1114.9978	H	1065.4418	**533.2245**	1048.4153	524.7113	1047.4312	524.2193	9
20	2378.0394	**1189.5233**	2361.0128	1181.0101	2360.0288	1180.5180	M	928.3829	464.6951	911.3564	456.1818	910.3723	455.6898	8
21	2493.0663	1247.0368	2476.0398	1238.5235	2475.0558	1238.0315	N	797.3424	399.1748	780.3159	390.6616	779.3318	390.1696	7
22	2640.1347	1320.5710	2623.1082	1312.0577	2622.1242	1311.5657	F	**682.3155**	341.6614	665.2889	333.1481	664.3049	332.6561	6
23	2727.1668	1364.0870	2710.1402	1355.5737	2709.1562	1355.0817	S	535.2471	268.1272	518.2205	259.6139	517.2365	259.1219	5
24	2814.1988	1407.6030	2797.1722	1399.0898	2796.1882	1398.5978	S	448.2150	224.6112	431.1885	216.0979	430.2045	215.6059	4
25	2885.2359	1443.1216	2868.2094	1434.6083	2867.2253	1434.1163	A	361.1830	181.0951	344.1565	172.5819	343.1724	172.0899	3
26	3000.2629	**1500.6351**	2983.2363	**1492.1218**	2982.2523	**1491.6298**	D	290.1459	145.5766	273.1193	137.0633	272.1353	136.5713	2
27							R	175.1190	88.0631	158.0924	79.5498			1

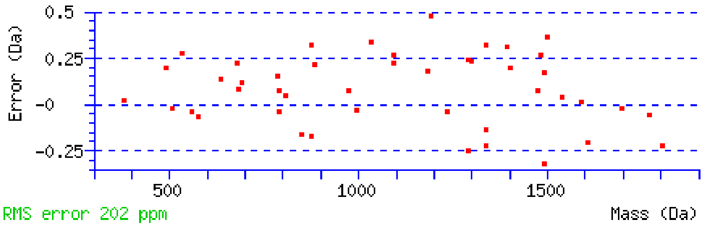

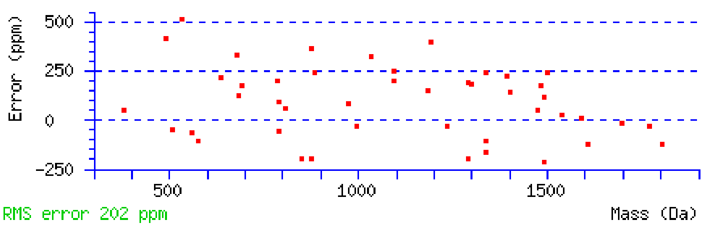

All matches to this query

Score	Mr(calc):	Delta	Sequence
61.8	3173.3672	0.0078	VNYEAETWDWIAETLSSNHMNFSSADR
53.0	3173.3672	0.0078	VNYEAETWDWIAETLSSNHMNFSSADR
8.4	3172.3577	1.0174	KSASQDTSENGMENPGLELMEVGNLEQGK
8.0	3172.3577	1.0174	KSASQDTSENGMENPGLELMEVGNLEQGK
4.4	3173.3877	-0.0126	LVQLSVMHMMLTQHSLFLPTMLTSK
3.4	3173.3811	-0.0060	TLLNTQENMALSLANVSGRLSGMANAFR
1.8	3172.3404	1.0346	CSSAKAFHTEISSSENNTLTSSNTYNSR
1.6	3172.3742	1.0008	LQPSNTQHPELKITAWPMGTQDAELK
1.4	3172.3404	1.0346	CSSAKAFHTEISSSENNTLTSSNTYNSR
1.4	3172.3404	1.0346	CSSAKAFHTEISSSENNTLTSSNTYNSR

Spectrum No: 301; Query: 535; Rank: 1

Peptide View

MS/MS Fragmentation of **ANEGAIYPDNTTDFQR**
Found in **IPI00325847**, Tax_Id=10116 Gene_Symbol=Cp GPI-anchored ceruloplasmin

Match to Query 535: 1811.790128 from(906.902340,2+)
Title: 091129RatKid_SCX02_12.1421.1421.2.dta
Data file K:\NewmanPaper\Piliang\3SubProteomes\Piliang3SP\mgf5ppm\SCX_3SubProteomes5ppm.mgf

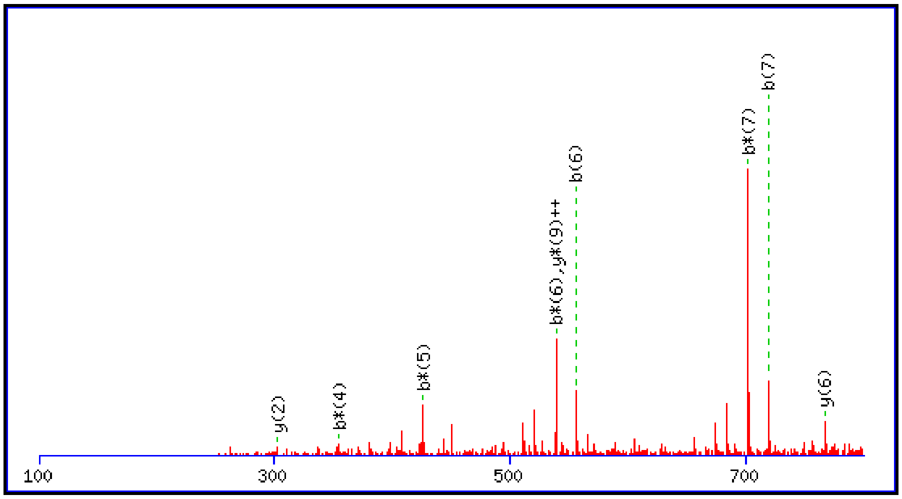

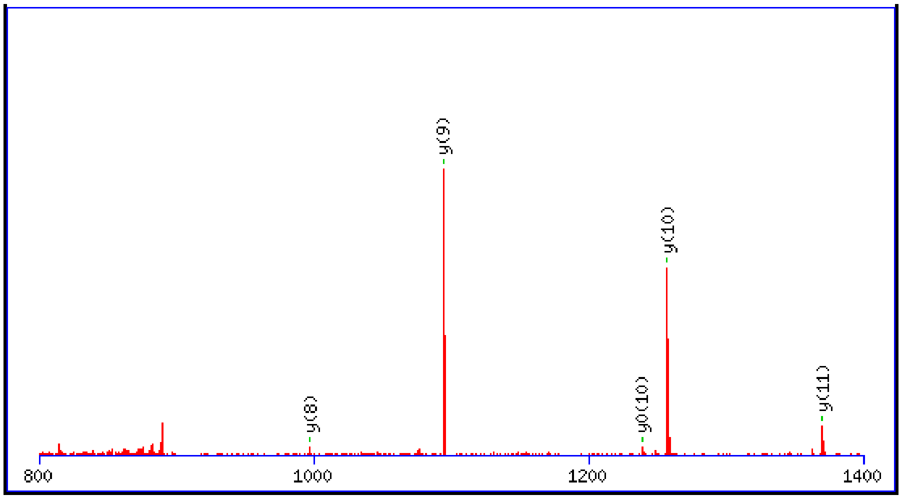

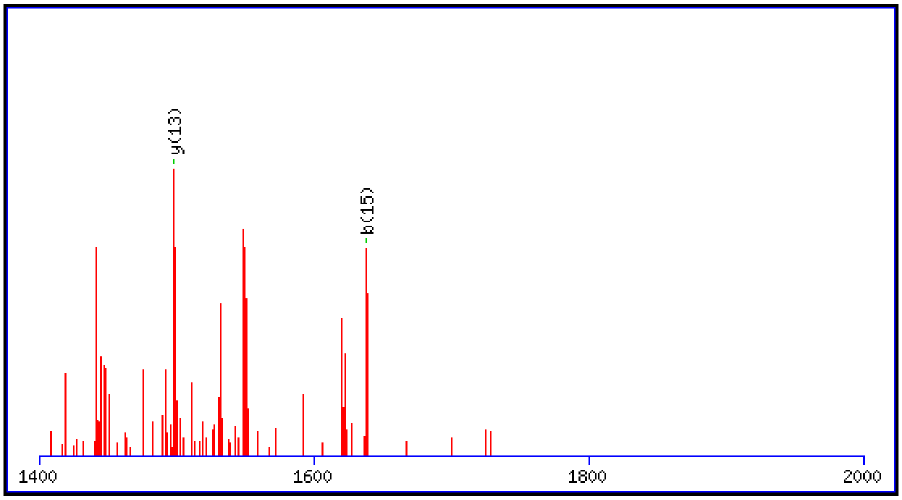

Monoisotopic mass of neutral peptide Mr(calc): 1811.7962
Fixed modifications: Carbamidomethyl (C)
Variable modifications:
N10 : Deamidated_N (N)
Ions Score: 62 **Expect:** 0.00016
Matches (Bold Red): 16/168 fragment ions using 35 most intense peaks

#	b	b^{++}	b*	b^{*++}	b^{0}	b^{0++}	Seq.	y	y^{++}	y*	y^{*++}	y^{0}	y^{0++}	#
1	72.0444	36.5258					A							16
2	186.0873	93.5473	169.0608	85.0340			N	1741.7664	871.3868	1724.7398	862.8736	1723.7558	862.3815	15
3	315.1299	158.0686	298.1034	149.5553	297.1193	149.0633	E	1627.7235	814.3654	1610.6969	805.8521	1609.7129	805.3601	14
4	372.1514	186.5793	**355.1248**	178.0661	354.1408	177.5740	G	**1498.6809**	749.8441	1481.6543	741.3308	1480.6703	740.8388	13
5	443.1885	222.0979	**426.1619**	213.5846	425.1779	213.0926	A	1441.6594	721.3333	1424.6328	712.8201	1423.6488	712.3281	12
6	**556.2726**	278.6399	**539.2460**	270.1266	538.2620	269.6346	I	**1370.6223**	685.8148	1353.5957	677.3015	1352.6117	676.8095	11
7	**719.3359**	360.1716	**702.3093**	351.6583	701.3253	351.1663	Y	**1257.5382**	629.2727	1240.5117	620.7595	**1239.5277**	620.2675	10
8	816.3886	408.6980	799.3621	400.1847	798.3781	399.6927	P	**1094.4749**	547.7411	1077.4483	**539.2278**	1076.4643	538.7358	9
9	931.4156	466.2114	914.3890	457.6982	913.4050	457.2061	D	**997.4221**	499.2147	980.3956	490.7014	979.4116	490.2094	8
10	1046.4425	523.7249	1029.4160	515.2116	1028.4320	514.7196	N	882.3952	441.7012	865.3686	433.1880	864.3846	432.6959	7
11	1147.4902	574.2487	1130.4637	565.7355	1129.4796	565.2435	T	**767.3682**	384.1878	750.3417	375.6745	749.3577	375.1825	6
12	1248.5379	624.7726	1231.5113	616.2593	1230.5273	615.7673	T	666.3206	333.6639	649.2940	325.1506	648.3100	324.6586	5
13	1363.5648	682.2861	1346.5383	673.7728	1345.5543	673.2808	D	565.2729	283.1401	548.2463	274.6268	547.2623	274.1348	4
14	1510.6332	755.8203	1493.6067	747.3070	1492.6227	746.8150	F	450.2459	225.6266	433.2194	217.1133			3
15	**1638.6918**	819.8495	1621.6653	811.3363	1620.6813	810.8443	Q	**303.1775**	152.0924	286.1510	143.5791			2
16							R	175.1190	88.0631	158.0924	79.5498			1

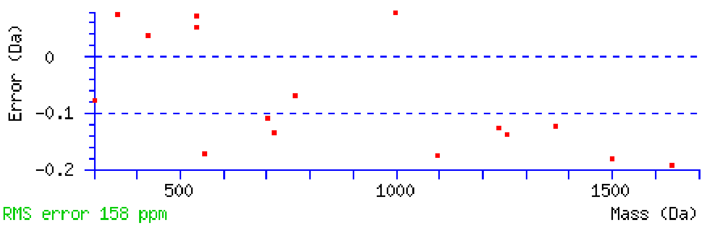

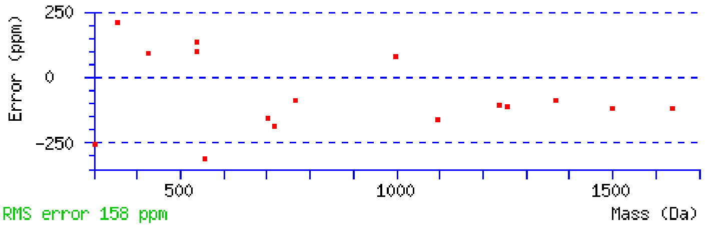

All matches to this query

Score	Mr(calc):	Delta	Sequence
61.9	1811.7962	-0.0061	ANEGAIYPDNTTDFQR
17.2	1811.8050	-0.0149	NLSKITNNNLNDSQR
17.2	1811.8050	-0.0149	NLSKITNNNLNDSQR
17.2	1811.8050	-0.0149	NLSKITNNNLNDSQR
17.2	1811.8050	-0.0149	NLSKITNNNLNDSQR
17.2	1811.8050	-0.0149	NLSKITNNNLNDSQR
17.2	1811.8050	-0.0149	NLSKITNNNLNDSQR
7.8	1811.7962	-0.0061	ANEGAIYPDNTTDFQR
7.0	1811.7873	0.0029	SLEENNAELECRIR
6.1	1811.7963	-0.0062	ILNVTTEMLKMATR

Spectrum No: 302; Query: 346; Rank: 1

Peptide View

MS/MS Fragmentation of **GVFITNETGQPLIGK**
Found in **IPI00400579**, Tax_Id=10116 Gene_Symbol=Gaa Lysosomal alpha-glucosidase precursor

Match to Query 346: 1573.839088 from(787.926820,2+)
Title: 091127RatKid_SCX01_17.2859.2859.2.dta
Data file K:\NewmanPaper\Piliang\3SubProteomes\Piliang3SP\mgf5ppm\SCX_3SubProteomes5ppm.mgf

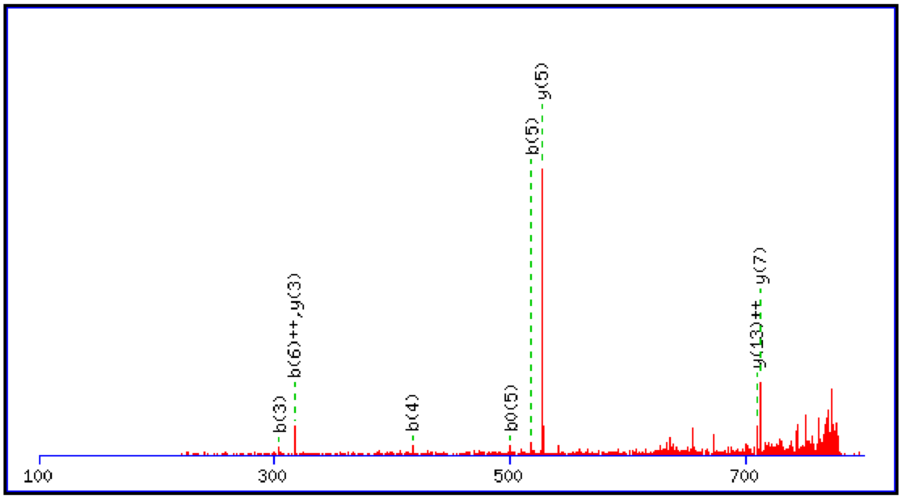

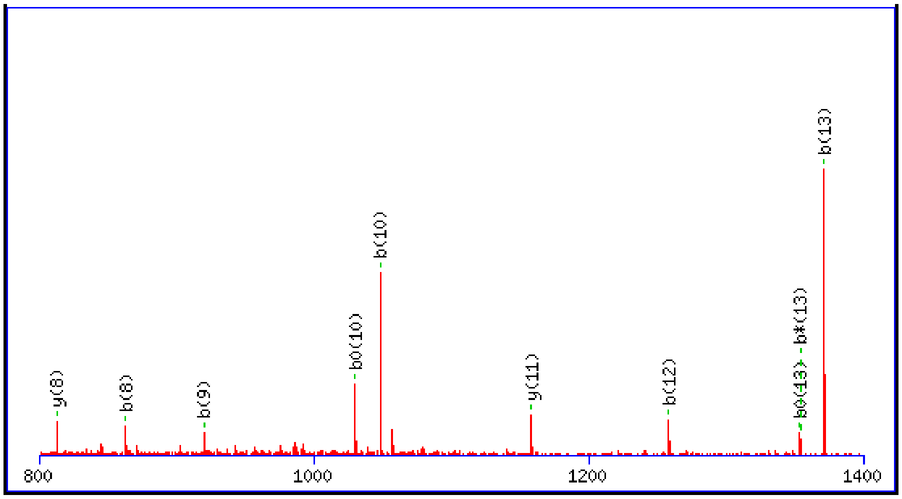

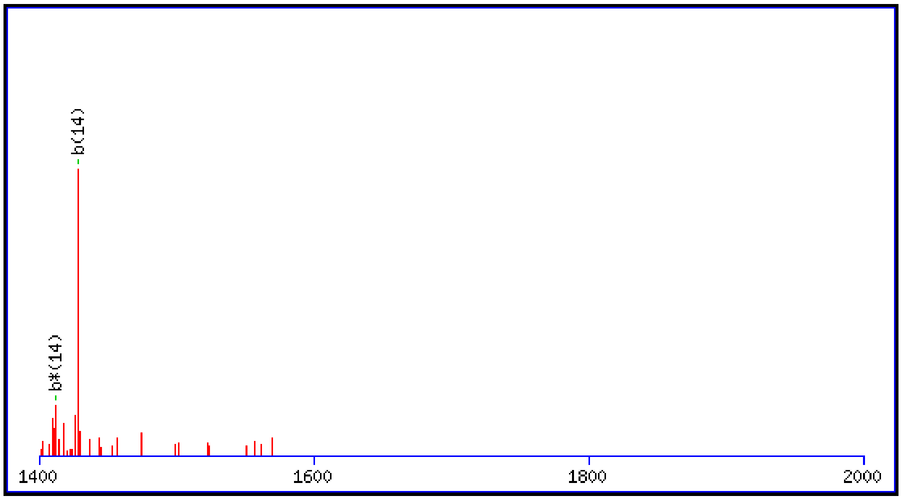

Monoisotopic mass of neutral peptide Mr(calc): 1573.8352
Fixed modifications: Carbamidomethyl (C)
Variable modifications:
N6 : Deamidated_N (N)
Ions Score: 62 **Expect:** 6.8e-005
Matches (Bold Red): 21/136 fragment ions using 38 most intense peaks

#	b	b^{++}	b*	b^{*++}	b^0	b^{0++}	Seq.	y	y^{++}	y*	y^{*++}	y^0	y^{0++}	#
1	58.0287	29.5180					G							15
2	157.0972	79.0522					V	1517.8210	759.4141	1500.7944	750.9009	1499.8104	750.4088	14
3	**304.1656**	152.5864					F	1418.7526	**709.8799**	1401.7260	701.3666	1400.7420	700.8746	13
4	**417.2496**	209.1285					I	1271.6842	636.3457	1254.6576	627.8324	1253.6736	627.3404	12
5	**518.2973**	259.6523			**500.2867**	250.6470	T	**1158.6001**	579.8037	1141.5735	571.2904	1140.5895	570.7984	11
6	633.3242	**317.1658**	616.2977	308.6525	615.3137	308.1605	N	1057.5524	529.2798	1040.5259	520.7666	1039.5418	520.2746	10
7	762.3668	381.6871	745.3403	373.1738	744.3563	372.6818	E	942.5255	471.7664	925.4989	463.2531	924.5149	462.7611	9
8	**863.4145**	432.2109	846.3880	423.6976	845.4040	423.2056	T	**813.4829**	407.2451	796.4563	398.7318	795.4723	398.2398	8
9	**920.4360**	460.7216	903.4094	452.2084	902.4254	451.7163	G	**712.4352**	356.7212	695.4087	348.2080			7
10	**1048.4946**	524.7509	1031.4680	516.2376	**1030.4840**	515.7456	Q	655.4137	328.2105	638.3872	319.6972			6
11	1145.5473	573.2773	1128.5208	564.7640	1127.5368	564.2720	P	**527.3552**	264.1812	510.3286	255.6679			5
12	**1258.6314**	629.8193	1241.6048	621.3061	1240.6208	620.8141	L	430.3024	215.6548	413.2758	207.1416			4
13	**1371.7155**	686.3614	**1354.6889**	677.8481	**1353.7049**	677.3561	I	**317.2183**	159.1128	300.1918	150.5995			3
14	**1428.7369**	714.8721	**1411.7104**	706.3588	1410.7264	705.8668	G	204.1343	102.5708	187.1077	94.0575			2
15							K	147.1128	74.0600	130.0863	65.5468			1

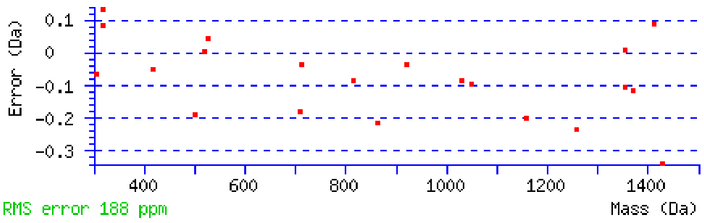

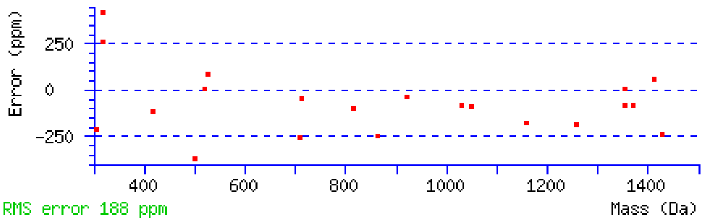

All matches to this query

Score	Mr(calc):	Delta	Sequence
61.8	1573.8352	0.0039	GVFITNETGQPLIGK
24.0	1572.8512	0.9879	GVFITNETGQPLIGK
2.9	1573.8497	-0.0107	EMVRSKLPNSVLGK
2.3	1573.8423	-0.0033	SLDESTLRKQIER
1.6	1573.8327	0.0064	NMKLQPVPWFIGK
1.0	1572.8260	1.0131	YVDDIEKGHTIKR

Spectrum No: 303; Query: 975; Rank: 1

Peptide View

MS/MS Fragmentation of **KENSSEICSNNGECVCGQCVCR**
Found in **IPI00191681**, Tax_Id=10116 Gene_Symbol=Itgb1 Integrin beta-1 precursor

Match to Query 975: 2647.021032 from(883.347620,3+)
Title: 091127RatKid_SCX01_22.999.999.3.dta
Data file K:\NewmanPaper\Piliang\3SubProteomes\Piliang3SP\mgf5ppm\SCX_3SubProteomes5ppm.mgf

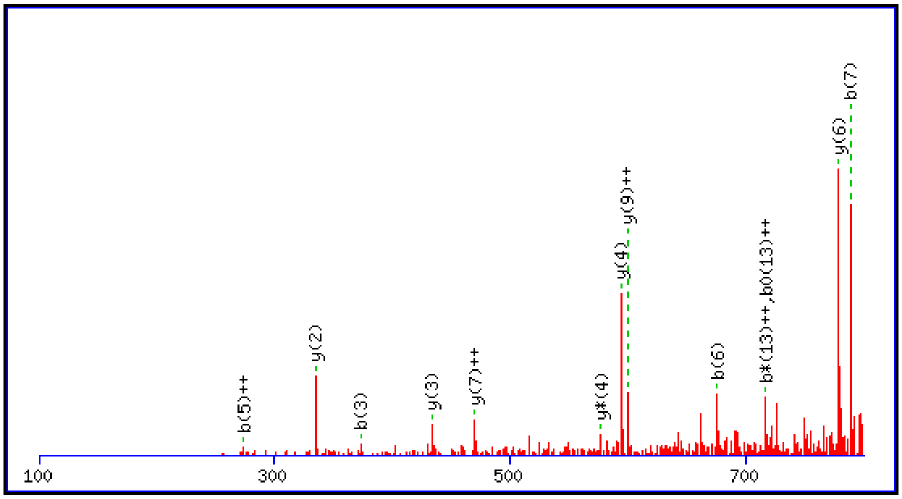

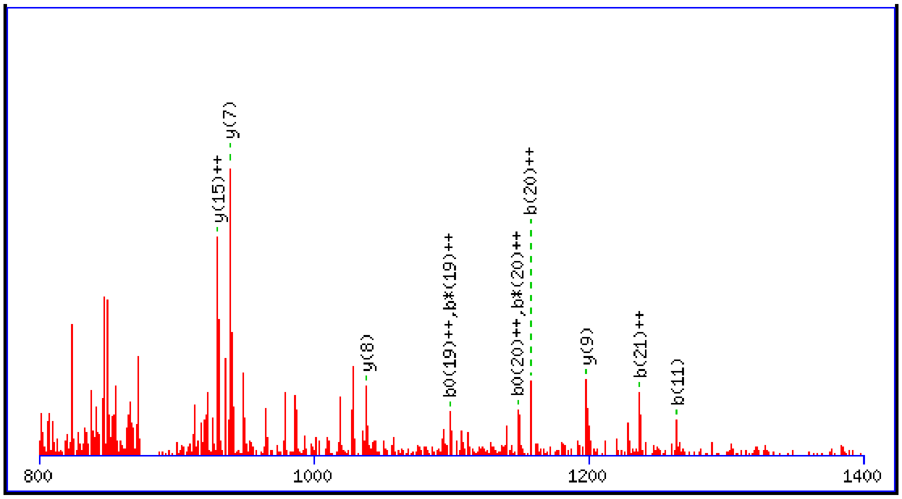

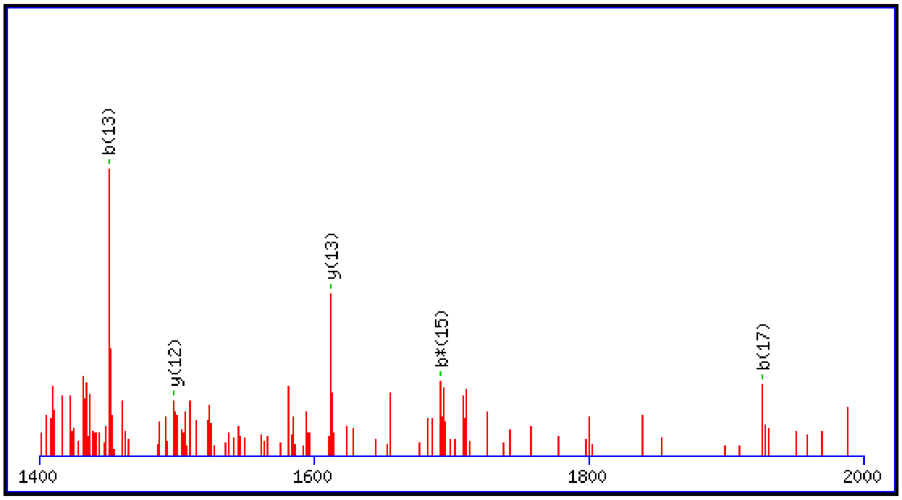

Monoisotopic mass of neutral peptide Mr(calc): 2647.0189
Fixed modifications: Carbamidomethyl (C)
Variable modifications:
N3 : Deamidated_N (N)
Ions Score: 62 **Expect:** 0.00014
Matches (Bold Red): 29/232 fragment ions using 48 most intense peaks

#	b	b^{++}	b*	b^{*++}	b^0	b^{0++}	Seq.	y	y^{++}	y*	y^{*++}	y^0	y^{0++}	#
1	129.1022	65.0548	112.0757	56.5415			K							22
2	258.1448	129.5761	241.1183	121.0628	240.1343	120.5708	E	2519.9312	1260.4693	2502.9047	1251.9560	2501.9207	1251.4640	21
3	**373.1718**	187.0895	356.1452	178.5762	355.1612	178.0842	N	2390.8887	1195.9480	2373.8621	1187.4347	2372.8781	1186.9427	20
4	460.2038	230.6055	443.1772	222.0923	442.1932	221.6003	S	2275.8617	1138.4345	2258.8352	1129.9212	2257.8512	1129.4292	19
5	547.2358	**274.1216**	530.2093	265.6083	529.2253	265.1163	S	2188.8297	1094.9185	2171.8031	1086.4052	2170.8191	1085.9132	18
6	**676.2784**	338.6428	659.2519	330.1296	658.2679	329.6376	E	2101.7977	1051.4025	2084.7711	1042.8892	2083.7871	1042.3972	17
7	**789.3625**	395.1849	772.3359	386.6716	771.3519	386.1796	I	1972.7551	986.8812	1955.7285	978.3679	1954.7445	977.8759	16
8	949.3931	475.2002	932.3666	466.6869	931.3826	466.1949	C	1859.6710	**930.3391**	1842.6445	921.8259	1841.6604	921.3339	15
9	1036.4252	518.7162	1019.3986	510.2029	1018.4146	509.7109	S	1699.6404	850.3238	1682.6138	841.8105	1681.6298	841.3185	14
10	1150.4681	575.7377	1133.4415	567.2244	1132.4575	566.7324	N	**1612.6083**	806.8078	1595.5818	798.2945	1594.5978	797.8025	13
11	**1264.5110**	632.7591	1247.4845	624.2459	1246.5004	623.7539	N	**1498.5654**	749.7863	1481.5389	741.2731	1480.5548	740.7811	12
12	1321.5325	661.2699	1304.5059	652.7566	1303.5219	652.2646	G	1384.5225	692.7649	1367.4959	684.2516	1366.5119	683.7596	11
13	**1450.5751**	725.7912	1433.5485	**717.2779**	1432.5645	**716.7859**	E	1327.5010	664.2541	1310.4745	655.7409	1309.4904	655.2489	10
14	1610.6057	805.8065	1593.5792	797.2932	1592.5952	796.8012	C	**1198.4584**	**599.7328**	1181.4319	591.2196			9
15	1709.6741	855.3407	**1692.6476**	846.8274	1691.6636	846.3354	V	**1038.4278**	519.7175	1021.4012	511.2042			8
16	1869.7048	935.3560	1852.6782	926.8428	1851.6942	926.3507	C	**939.3594**	**470.1833**	922.3328	461.6700			7
17	**1926.7262**	963.8668	1909.6997	955.3535	1908.7157	954.8615	G	**779.3287**	390.1680	762.3022	381.6547			6
18	2054.7848	1027.8960	2037.7583	1019.3828	2036.7743	1018.8908	Q	722.3072	361.6573	705.2807	353.1440			5

19	2214.8155	1107.9114	2197.7889	**1099.3981**	2196.8049	**1098.9061**	C	**594.2487**	297.6280	**577.2221**	289.1147			4
20	2313.8839	**1157.4456**	2296.8573	**1148.9323**	2295.8733	**1148.4403**	V	**434.2180**	217.6126	417.1915	209.0994			3
21	2473.9145	**1237.4609**	2456.8880	1228.9476	2455.9040	1228.4556	C	**335.1496**	168.0784	318.1231	159.5652			2
22							R	175.1190	88.0631	158.0924	79.5498			1

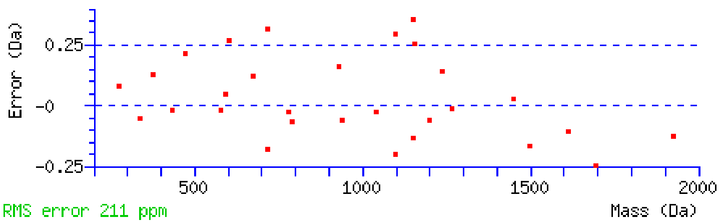

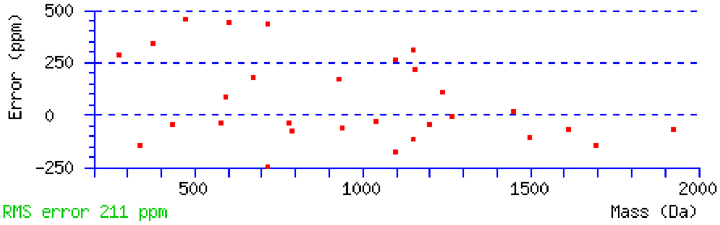

All matches to this query

Score	Mr(calc):	Delta	Sequence
61.8	2647.0189	0.0021	KENSSEICSNNGECVCGQCVCR
32.7	2647.0189	0.0021	KENSSEICSNNGECVCGQCVCR
28.4	2646.0349	0.9861	KENSSEICSNNGECVCGQCVCR
26.4	2647.0189	0.0021	KENSSEICSNNGECVCGQCVCR
3.7	2647.0411	-0.0201	DSQYKSSDQLSWNTRDQLCR
3.7	2647.0411	-0.0201	DSQYKSSDQLSWNTRDQLCR
0.4	2646.0008	1.0203	YYNTKMTIEDVLSPGTCVCR
0.4	2646.0008	1.0203	YYNTKMTIEDVLSPGTCVCR
0.1	2645.0295	1.9915	TNHSGFLFGFGDGGGGPTQTMLDR

Spectrum No: 304; Query: 1158; Rank: 1

Peptide View

MS/MS Fragmentation of **SYLDDGPLQHYIAVSSPTNTTYVVQYALANLTGK**
Found in **IPI00192495**, Tax_Id=10116 Gene_Symbol=Ncstn Isoform 1 of Nicastrin precursor

Match to Query 1158: 3700.810482 from(1234.610770,3+)
Title: 091129RatKid_SCX02_18.6910.6910.3.dta
Data file K:\NewmanPaper\Piliang\3SubProteomes\Piliang3SP\mgf5ppm\SCX_3SubProteomes5ppm.mgf

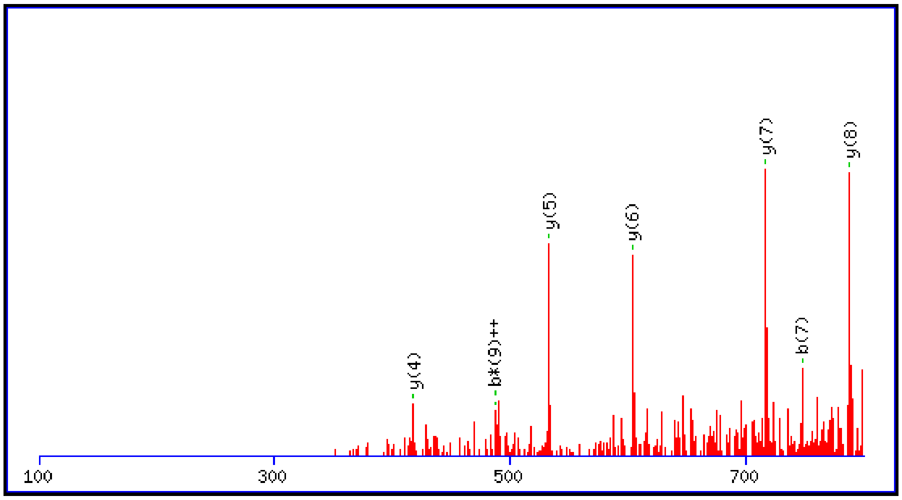

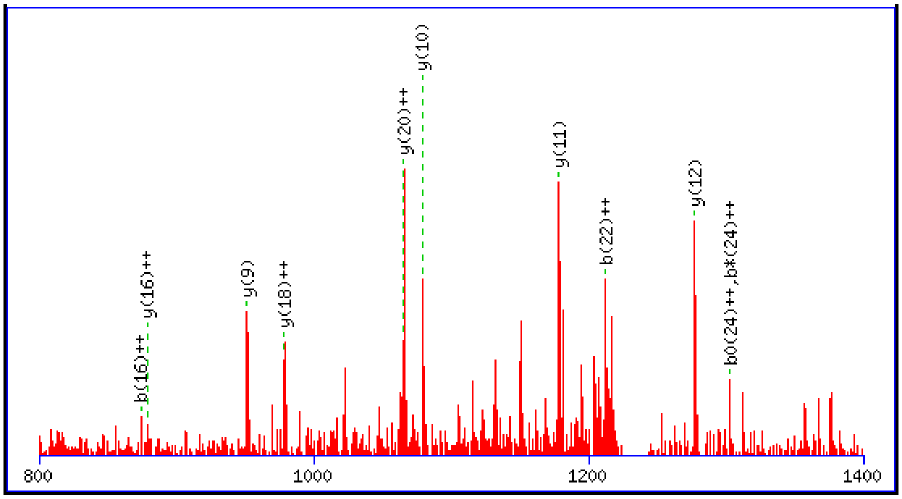

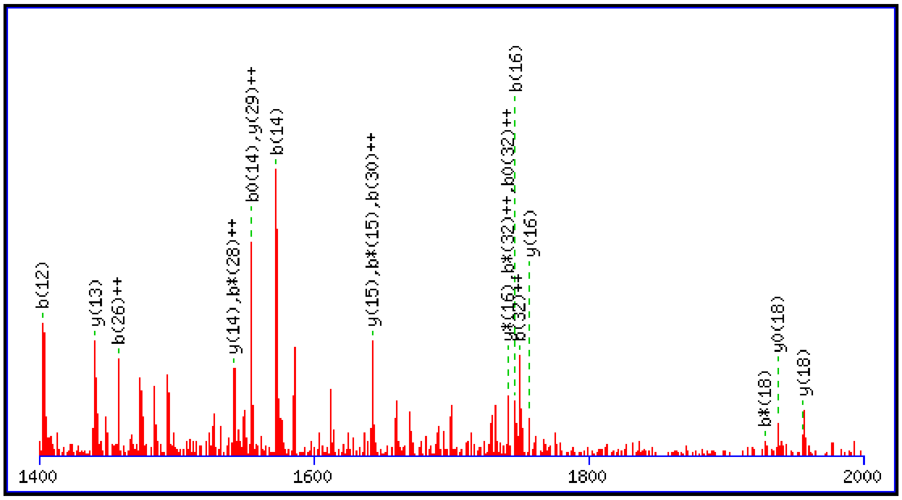

Monoisotopic mass of neutral peptide Mr(calc): 3700.8148
Fixed modifications: Carbamidomethyl (C)
Variable modifications:
N19 : Deamidated_N (N)
N30 : Deamidated_N (N)
Ions Score: 62 **Expect:** 0.00018
Matches (Bold Red): 38/376 fragment ions using 62 most intense peaks

#	b	b^{++}	b*	b^{*++}	b^{0}	b^{0++}	Seq.	y	y^{++}	y*	y^{*++}	y^{0}	y^{0++}	#
1	88.0393	44.5233			70.0287	35.5180	S							34
2	251.1026	126.0550			233.0921	117.0497	Y	3614.7901	1807.8987	3597.7635	1799.3854	3596.7795	1798.8934	33
3	364.1867	182.5970			346.1761	173.5917	L	3451.7267	1726.3670	3434.7002	1717.8537	3433.7162	1717.3617	32
4	479.2136	240.1105			461.2031	231.1052	D	3338.6427	1669.8250	3321.6161	1661.3117	3320.6321	1660.8197	31
5	594.2406	297.6239			576.2300	288.6186	D	3223.6157	1612.3115	3206.5892	1603.7982	3205.6052	1603.3062	30
6	651.2620	326.1347			633.2515	317.1294	G	3108.5888	**1554.7980**	3091.5622	1546.2848	3090.5782	1545.7928	29
7	**748.3148**	374.6610			730.3042	365.6558	P	3051.5673	1526.2873	3034.5408	1517.7740	3033.5568	1517.2820	28
8	861.3989	431.2031			843.3883	422.1978	L	2954.5146	1477.7609	2937.4880	1469.2476	2936.5040	1468.7556	27
9	989.4575	495.2324	972.4309	**486.7191**	971.4469	486.2271	Q	2841.4305	1421.2189	2824.4040	1412.7056	2823.4199	1412.2136	26
10	1126.5164	563.7618	1109.4898	555.2485	1108.5058	554.7565	H	2713.3719	1357.1896	2696.3454	1348.6763	2695.3614	1348.1843	25
11	1289.5797	645.2935	1272.5531	636.7802	1271.5691	636.2882	Y	2576.3130	1288.6601	2559.2865	1280.1469	2558.3025	1279.6549	24
12	**1402.6638**	701.8355	1385.6372	693.3222	1384.6532	692.8302	I	2413.2497	1207.1285	2396.2231	1198.6152	2395.2391	1198.1232	23
13	1473.7009	737.3541	1456.6743	728.8408	1455.6903	728.3488	A	2300.1656	1150.5864	2283.1391	1142.0732	2282.1551	1141.5812	22
14	**1572.7693**	786.8883	1555.7427	778.3750	**1554.7587**	777.8830	V	2229.1285	1115.0679	2212.1020	1106.5546	2211.1179	1106.0626	21
15	1659.8013	830.4043	**1642.7748**	821.8910	1641.7907	821.3990	S	2130.0601	**1065.5337**	2113.0335	1057.0204	2112.0495	1056.5284	20
16	**1746.8333**	**873.9203**	1729.8068	865.4070	1728.8228	864.9150	S	2043.0281	1022.0177	2026.0015	1013.5044	2025.0175	1013.0124	19
17	1843.8861	922.4467	1826.8596	913.9334	1825.8755	913.4414	P	**1955.9960**	**978.5017**	1938.9695	969.9884	**1937.9855**	969.4964	18
18	1944.9338	972.9705	**1927.9072**	964.4573	1926.9232	963.9652	T	1858.9433	929.9753	1841.9167	921.4620	1840.9327	920.9700	17

**19**	2059.9607	1030.4840	2042.9342	1021.9707	2041.9502	1021.4787	**N**	**1757.8956**	**879.4514**	**1740.8690**	870.9382	1739.8850	870.4462	**16**
**20**	2161.0084	1081.0078	2143.9819	1072.4946	2142.9978	1072.0026	**T**	**1642.8687**	821.9380	1625.8421	813.4247	1624.8581	812.9327	**15**
**21**	2262.0561	1131.5317	2245.0295	1123.0184	2244.0455	1122.5264	**T**	**1541.8210**	771.4141	1524.7944	762.9009	1523.8104	762.4088	**14**
**22**	2425.1194	**1213.0633**	2408.0929	1204.5501	2407.1088	1204.0581	**Y**	**1440.7733**	720.8903	1423.7468	712.3770	1422.7627	711.8850	**13**
**23**	2524.1878	1262.5975	2507.1613	1254.0843	2506.1773	1253.5923	**V**	**1277.7100**	639.3586	1260.6834	630.8454	1259.6994	630.3533	**12**
**24**	2623.2562	1312.1318	2606.2297	**1303.6185**	2605.2457	**1303.1265**	**V**	**1178.6416**	589.8244	1161.6150	581.3111	1160.6310	580.8191	**11**
**25**	2751.3148	1376.1610	2734.2883	1367.6478	2733.3042	1367.1558	**Q**	**1079.5731**	540.2902	1062.5466	531.7769	1061.5626	531.2849	**10**
**26**	2914.3781	**1457.6927**	2897.3516	1449.1794	2896.3676	1448.6874	**Y**	**951.5146**	476.2609	934.4880	467.7476	933.5040	467.2556	**9**
**27**	2985.4153	1493.2113	2968.3887	1484.6980	2967.4047	1484.2060	**A**	**788.4512**	394.7293	771.4247	386.2160	770.4407	385.7240	**8**
**28**	3098.4993	1549.7533	3081.4728	**1541.2400**	3080.4888	1540.7480	**L**	**717.4141**	359.2107	700.3876	350.6974	699.4036	350.2054	**7**
**29**	3169.5364	1585.2719	3152.5099	1576.7586	3151.5259	1576.2666	**A**	**604.3301**	302.6687	587.3035	294.1554	586.3195	293.6634	**6**
**30**	3284.5634	**1642.7853**	3267.5368	1634.2720	3266.5528	1633.7800	**N**	**533.2929**	267.1501	516.2664	258.6368	515.2824	258.1448	**5**
**31**	3397.6474	1699.3274	3380.6209	1690.8141	3379.6369	1690.3221	**L**	**418.2660**	209.6366	401.2395	201.1234	400.2554	200.6314	**4**
**32**	3498.6951	**1749.8512**	3481.6686	**1741.3379**	3480.6845	**1740.8459**	**T**	305.1819	153.0946	288.1554	144.5813	287.1714	144.0893	**3**
**33**	3555.7166	1778.3619	3538.6900	1769.8487	3537.7060	1769.3566	**G**	204.1343	102.5708	187.1077	94.0575			**2**
**34**							**K**	147.1128	74.0600	130.0863	65.5468			**1**

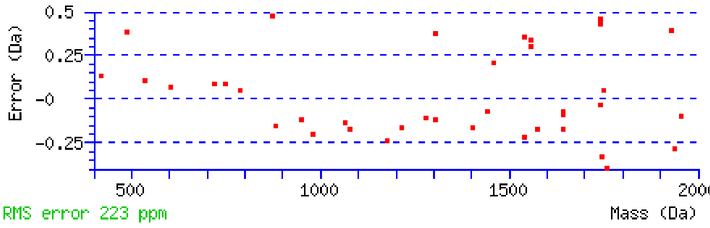

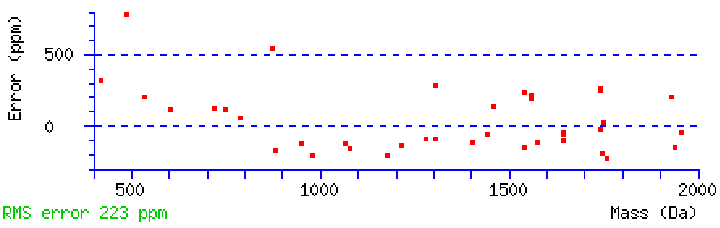

All matches to this query

Score	Mr(calc):	Delta	Sequence
61.7	3700.8148	-0.0043	SYLDDGPLQHYIAVSSPTNTTYVVQYALANLTGK
39.6	3699.8308	0.9797	SYLDDGPLQHYIAVSSPTNTTYVVQYALANLTGK
5.1	3700.8138	-0.0033	FEDGDFILKRTGPGLLSVANAGPNTNGSGVFIGTAK
5.1	3700.8138	-0.0033	FEDGDFILKRTGPGLLSVANAGPNTNGSGVFIGTAK
4.2	3699.8308	0.9797	SYLDDGPLQHYIAVSSPTNTTYVVQYALANLTGK
2.5	3698.8266	1.9839	IKTNVINPAYAAELGQIENSLHMGYSALEIKSK
2.5	3698.8266	1.9839	IKTNVINPAYAAELGQIENSLHMGYSALEIKSK
2.4	3700.7946	0.0159	IKTNVINPAYAAELGQIENSLHMGYSALEIKSK
1.9	3699.8106	0.9999	IKTNVINPAYAAELGQIENSLHMGYSALEIKSK
1.9	3699.8106	0.9999	IKTNVINPAYAAELGQIENSLHMGYSALEIKSK

Spectrum No: 305; Query: 797; Rank: 1

Peptide View

MS/MS Fragmentation of **VAANFSTPVISTSGSSDPGQER**
Found in **IPI00391338**, Tax_Id=10116 Gene_Symbol=RGD1562791_predicted similar to ICOS ligand precursor

Match to Query 797: 2207.036468 from(1104.525510,2+)
Title: 091127RatKid_SCX01_02.2501.2501.2.dta
Data file K:\NewmanPaper\Piliang\3SubProteomes\Piliang3SP\mgf5ppm\SCX_3SubProteomes5ppm.mgf

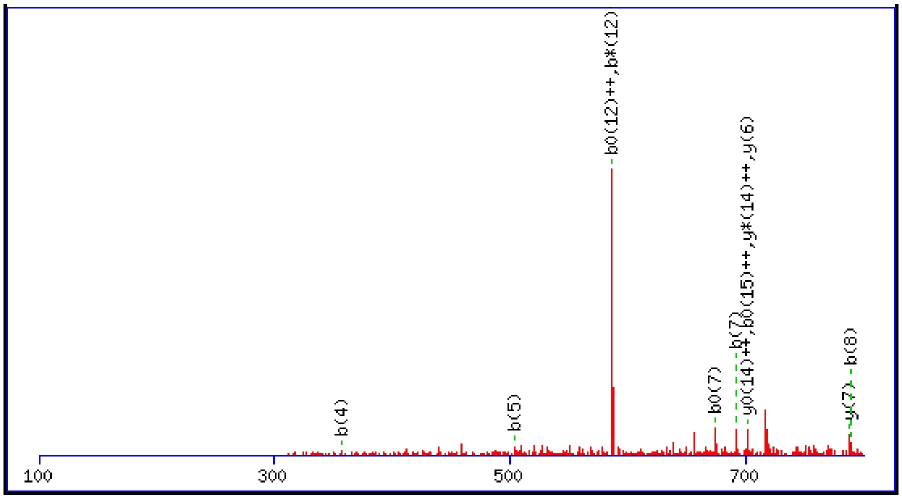

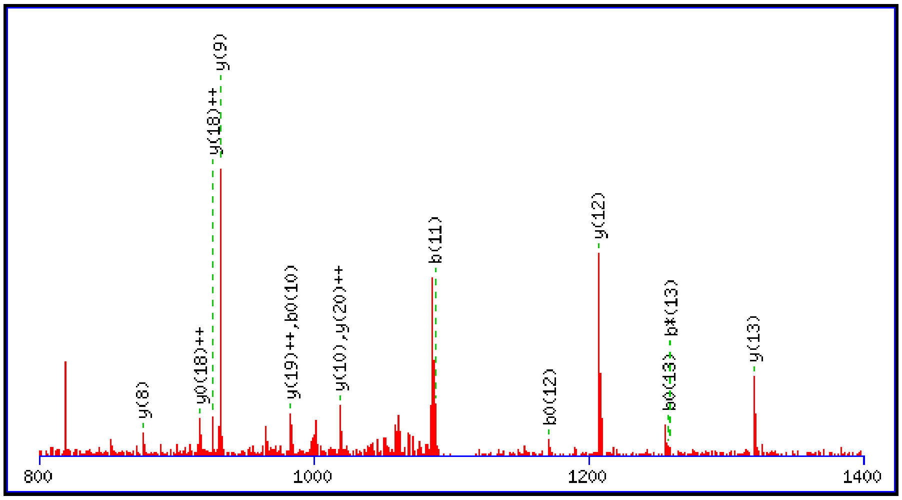

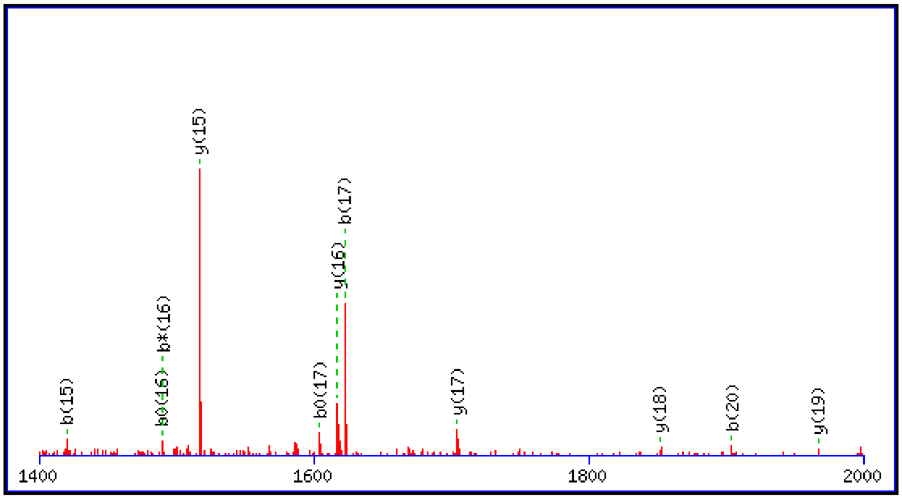

Monoisotopic mass of neutral peptide Mr(calc): 2207.0342
Fixed modifications: Carbamidomethyl (C)
Variable modifications:
N4 : Deamidated_N (N)
Ions Score: 62 **Expect:** 0.00019
Matches (Bold Red): 38/234 fragment ions using 79 most intense peaks

#	b	b++	b*	b*++	b0	b0++	Seq.	y	y++	y*	y*++	y0	y0++	#
1	100.0757	50.5415					V							22
2	171.1128	86.0600					A	2108.9731	1054.9902	2091.9465	1046.4769	2090.9625	1045.9849	21
3	242.1499	121.5786					A	2037.9360	**1019.4716**	2020.9094	1010.9583	2019.9254	1010.4663	20
4	**357.1769**	179.0921	340.1503	170.5788			N	**1966.8988**	**983.9531**	1949.8723	975.4398	1948.8883	974.9478	19
5	**504.2453**	252.6263	487.2187	244.1130			F	**1851.8719**	**926.4396**	1834.8454	917.9263	1833.8613	**917.4343**	18

**6**	591.2773	296.1423	574.2507	287.6290	573.2667	287.1370	**S**	**1704.8035**	852.9054	1687.7769	844.3921	1686.7929	843.9001	**17**
**7**	**692.3250**	346.6661	675.2984	338.1529	**674.3144**	337.6608	**T**	**1617.7715**	809.3894	1600.7449	800.8761	1599.7609	800.3841	**16**
**8**	**789.3777**	395.1925	772.3512	386.6792	771.3672	386.1872	**P**	**1516.7238**	758.8655	1499.6972	750.3523	1498.7132	749.8603	**15**
**9**	888.4462	444.7267	871.4196	436.2134	870.4356	435.7214	**V**	1419.6710	710.3392	1402.6445	**701.8259**	1401.6605	**701.3339**	**14**
**10**	1001.5302	501.2687	984.5037	492.7555	**983.5197**	492.2635	**I**	**1320.6026**	660.8049	1303.5761	652.2917	1302.5920	651.7997	**13**
**11**	**1088.5622**	544.7848	1071.5357	536.2715	1070.5517	535.7795	**S**	**1207.5185**	604.2629	1190.4920	595.7496	1189.5080	595.2576	**12**
**12**	1189.6099	595.3086	1172.5834	**586.7953**	**1171.5994**	**586.3033**	**T**	1120.4865	560.7469	1103.4600	552.2336	1102.4760	551.7416	**11**
**13**	1276.6420	638.8246	**1259.6154**	630.3113	**1258.6314**	629.8193	**S**	**1019.4388**	510.2231	1002.4123	501.7098	1001.4283	501.2178	**10**
**14**	1333.6634	667.3353	1316.6369	658.8221	1315.6529	658.3301	**G**	**932.4068**	466.7070	915.3803	458.1938	914.3962	457.7018	**9**
**15**	**1420.6954**	710.8514	1403.6689	702.3381	1402.6849	**701.8461**	**S**	**875.3854**	438.1963	858.3588	429.6830	857.3748	429.1910	**8**
**16**	1507.7275	754.3674	**1490.7009**	745.8541	**1489.7169**	745.3621	**S**	**788.3533**	394.6803	771.3268	386.1670	770.3428	385.6750	**7**
**17**	**1622.7544**	811.8808	1605.7279	803.3676	**1604.7439**	802.8756	**D**	**701.3213**	351.1643	684.2947	342.6510	683.3107	342.1590	**6**
**18**	1719.8072	860.4072	1702.7806	851.8940	1701.7966	851.4019	**P**	**586.2944**	293.6508	569.2678	285.1375	568.2838	284.6455	**5**
**19**	1776.8286	888.9180	1759.8021	880.4047	1758.8181	879.9127	**G**	489.2416	245.1244	472.2150	236.6112	471.2310	236.1191	**4**
**20**	**1904.8872**	952.9472	1887.8607	944.4340	1886.8767	943.9420	**Q**	432.2201	216.6137	415.1936	208.1004	414.2096	207.6084	**3**
**21**	2033.9298	1017.4685	2016.9033	1008.9553	2015.9192	1008.4633	**E**	304.1615	152.5844	287.1350	144.0711	286.1510	143.5791	**2**
**22**							**R**	175.1190	88.0631	158.0924	79.5498			**1**

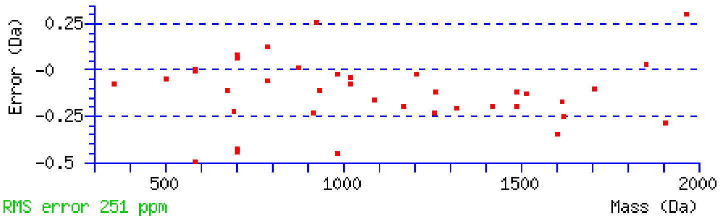

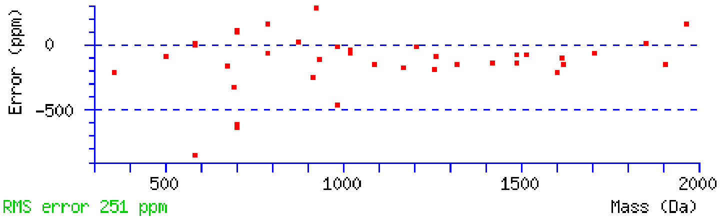

All matches to this query

Score	Mr(calc):	Delta	Sequence
61.6	2207.0342	0.0022	VAANFSTPVISTSGSSDPGQER
36.4	2206.0502	0.9863	VAANFSTPVISTSGSSDPGQER
6.6	2207.0574	-0.0210	YKSLMVSRGAPKPSSLAQK
6.6	2207.0574	-0.0210	YKSLMVSRGAPKPSSLAQK
4.7	2207.0581	-0.0217	YFLGLVDLTTVYGFRKR
4.7	2207.0581	-0.0217	YFLGLVDLTTVYGFRKR
4.7	2207.0581	-0.0217	YFLGLVDLTTVYGFRKR
3.0	2207.0574	-0.0210	YKSLMVSRGAPKPSSLAQK
3.0	2207.0574	-0.0210	YKSLMVSRGAPKPSSLAQK
0.6	2205.0363	2.0001	HGFNVGQTMFSLLMTGKLK

Spectrum No: 306; Query: 133; Rank: 1

Peptide View

MS/MS Fragmentation of **NDSQLVVEAYK**
Found in **IPI00201083**, Tax_Id=10116 Gene_Symbol=Fnbp1 Isoform 2 of Formin-binding protein 1

Match to Query 133: 1265.614008 from(633.814280,2+)
Title: 091127RatKid_SCX01_14.2259.2259.2.dta
Data file K:\NewmanPaper\Piliang\3SubProteomes\Piliang3SP\mgf5ppm\SCX_3SubProteomes5ppm.mgf

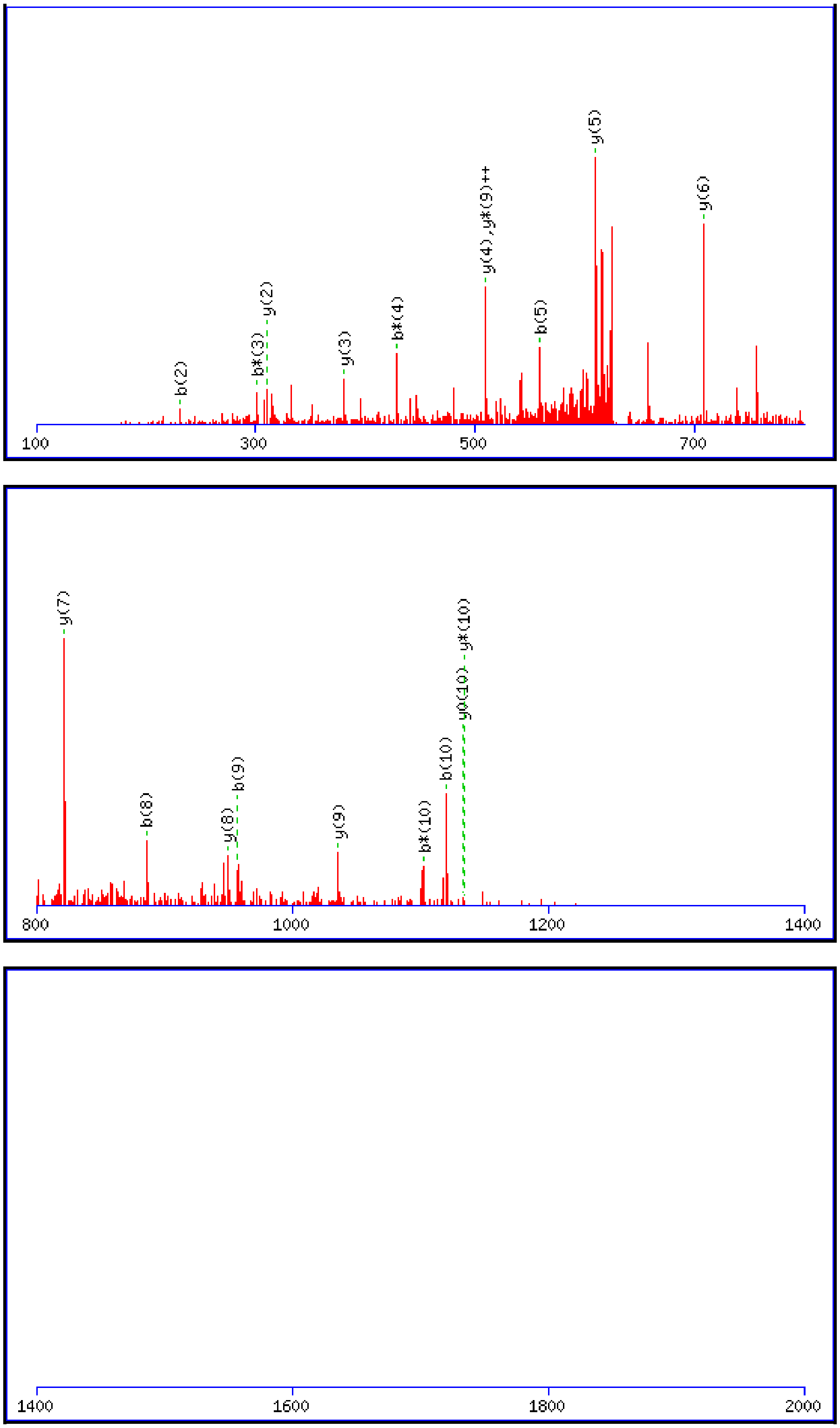

Monoisotopic mass of neutral peptide Mr(calc): 1265.6139
Fixed modifications: Carbamidomethyl (C)
Variable modifications:
N1 : Deamidated_N (N)
Ions Score: 62 **Expect:** 9.7e-005
Matches (Bold Red): 19/112 fragment ions using 34 most intense peaks

#	b	b^{++}	b*	b^{*++}	b^{0}	b^{0++}	Seq.	y	y^{++}	y*	y^{*++}	y^{0}	y^{0++}	#
1	116.0342	58.5207	99.0077	50.0075			N							11
2	**231.0612**	116.0342	214.0346	107.5209	213.0506	107.0289	D	1151.5943	576.3008	**1134.5677**	567.7875	**1133.5837**	567.2955	10
3	318.0932	159.5502	**301.0666**	151.0370	300.0826	150.5449	S	**1036.5673**	518.7873	1019.5408	**510.2740**	1018.5568	509.7820	9
4	446.1518	223.5795	**429.1252**	215.0662	428.1412	214.5742	Q	**949.5353**	475.2713	932.5088	466.7580	931.5247	466.2660	8
5	**559.2358**	280.1216	542.2093	271.6083	541.2253	271.1163	L	**821.4767**	411.2420	804.4502	402.7287	803.4662	402.2367	7

**6**	658.3042	329.6558	641.2777	321.1425	640.2937	320.6505	**V**	**708.3927**	354.7000	691.3661	346.1867	690.3821	345.6947	**6**
**7**	757.3727	379.1900	740.3461	370.6767	739.3621	370.1847	**V**	**609.3243**	305.1658	592.2977	296.6525	591.3137	296.1605	**5**
**8**	**886.4152**	443.7113	869.3887	435.1980	868.4047	434.7060	**E**	**510.2558**	255.6316	493.2293	247.1183	492.2453	246.6263	**4**
**9**	**957.4524**	479.2298	940.4258	470.7165	939.4418	470.2245	**A**	**381.2132**	191.1103	364.1867	182.5970			**3**
**10**	**1120.5157**	560.7615	**1103.4891**	552.2482	1102.5051	551.7562	**Y**	**310.1761**	155.5917	293.1496	147.0784			**2**
**11**							**K**	147.1128	74.0600	130.0863	65.5468			**1**

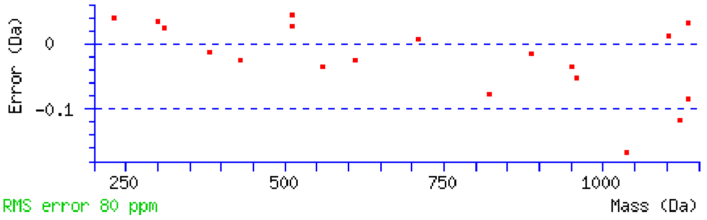

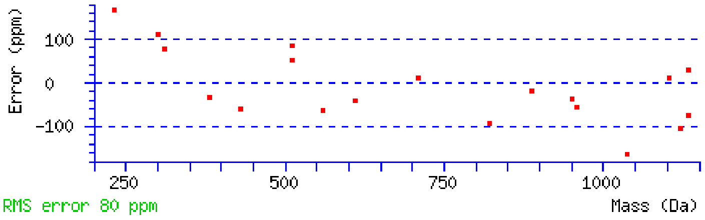

All matches to this query

Score	Mr(calc):	Delta	Sequence
61.6	1265.6139	0.0001	NDSQLVVEAYK
12.0	1265.6016	0.0124	SIQALNLQGNK
10.8	1265.6182	-0.0042	AGDALMVMIAMK
10.1	1265.6182	-0.0042	AGDALMVMIAMK
6.2	1265.6173	-0.0033	NSNKLMTISEK
5.4	1265.6081	0.0059	NPPPFPGEPWK
4.7	1265.6186	-0.0046	AMLNNSGPRYK
4.5	1265.6140	0.0001	SDVSGLSDPYVK
3.8	1265.6016	0.0124	AQTKAEATAPAK
3.3	1265.6091	0.0050	MAXPLVPSSQK

Spectrum No: 307; Query: 790; Rank: 1

Peptide View

MS/MS Fragmentation of **EGNSALINCSAFGIPDLEYK**
Found in **IPI00188728**, Tax_Id=10116 Gene_Symbol=Mfap3l Microfibrillar-associated protein 3-like precursor

Match to Query 790: 2200.034708 from(1101.024630,2+)
Title: 091127RatKid_SCX01_02.4412.4412.2.dta
Data file K:\NewmanPaper\Piliang\3SubProteomes\Piliang3SP\mgf5ppm\SCX_3SubProteomes5ppm.mgf

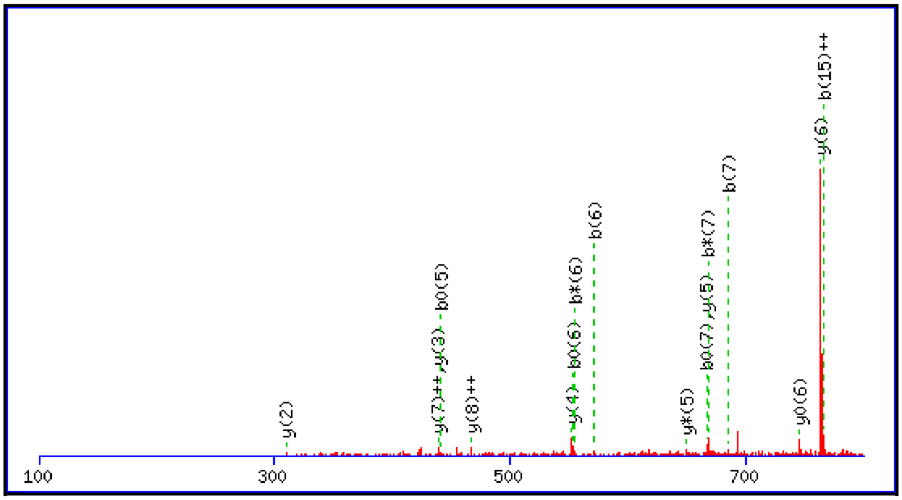

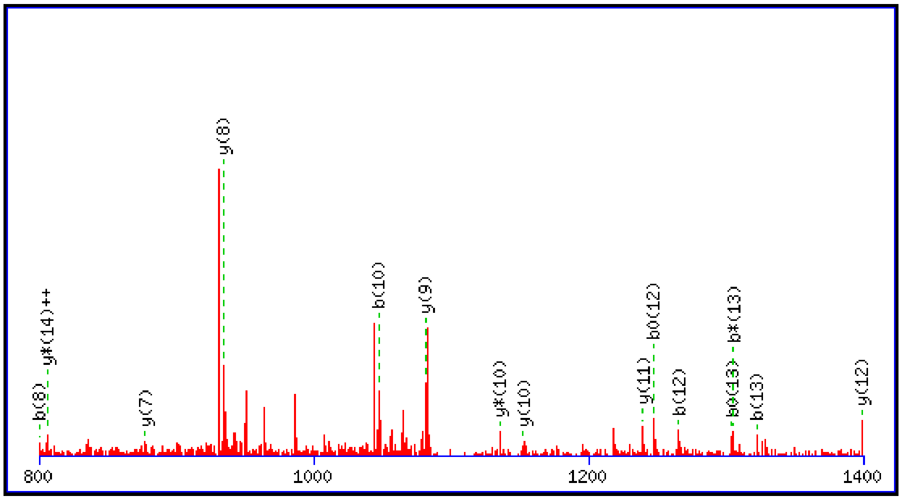

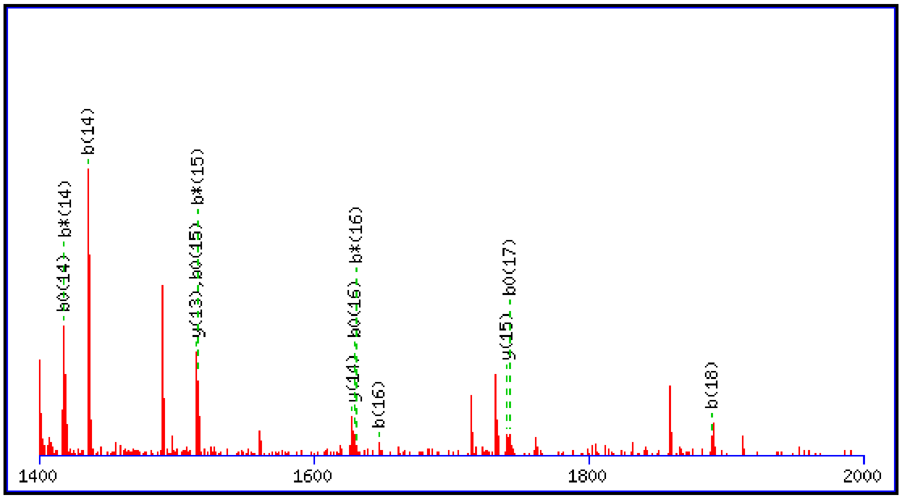

Monoisotopic mass of neutral peptide Mr(calc): 2198.0201
Fixed modifications: Carbamidomethyl (C)
Variable modifications:
N8 : Deamidated_N (N)
Ions Score: 62 **Expect:** 0.00018
Matches (Bold Red): 45/220 fragment ions using 106 most intense peaks

#	b	b^{++}	b*	b^{*++}	b^0	b^{0++}	Seq.	y	y^{++}	y*	y^{*++}	y^0	y^{0++}	#
1	130.0499	65.5286			112.0393	56.5233	E							20
2	187.0713	94.0393			169.0608	85.0340	G	2069.9848	1035.4961	2052.9583	1026.9828	2051.9743	1026.4908	19
3	301.1143	151.0608	284.0877	142.5475	283.1037	142.0555	N	2012.9634	1006.9853	1995.9368	998.4720	1994.9528	997.9800	18
4	388.1463	194.5768	371.1197	186.0635	370.1357	185.5715	S	1898.9204	949.9639	1881.8939	941.4506	1880.9099	940.9586	17
5	459.1834	230.0953	442.1569	221.5821	**441.1728**	221.0901	A	1811.8884	906.4478	1794.8619	897.9346	1793.8778	897.4426	16
6	**572.2675**	286.6374	**555.2409**	278.1241	**554.2569**	277.6321	L	**1740.8513**	870.9293	1723.8247	862.4160	1722.8407	861.9240	15
7	**685.3515**	343.1794	**668.3250**	334.6661	**667.3410**	334.1741	I	**1627.7672**	814.3873	1610.7407	**805.8740**	1609.7567	805.3820	14
8	**800.3785**	400.6929	783.3519	392.1796	782.3679	391.6876	N	**1514.6832**	757.8452	1497.6566	749.3319	1496.6726	748.8399	13
9	960.4091	480.7082	943.3826	472.1949	942.3986	471.7029	C	**1399.6562**	700.3318	1382.6297	691.8185	1381.6457	691.3265	12
10	**1047.4411**	524.2242	1030.4146	515.7109	1029.4306	515.2189	S	**1239.6256**	620.3164	1222.5990	611.8032	1221.6150	611.3111	11
11	1118.4783	559.7428	1101.4517	551.2295	1100.4677	550.7375	A	**1152.5936**	576.8004	**1135.5670**	568.2871	1134.5830	567.7951	10
12	**1265.5467**	633.2770	1248.5201	624.7637	**1247.5361**	624.2717	F	**1081.5564**	541.2819	1064.5299	532.7686	1063.5459	532.2766	9
13	**1322.5681**	661.7877	**1305.5416**	653.2744	**1304.5576**	652.7824	G	**934.4880**	**467.7477**	917.4615	459.2344	916.4775	458.7424	8
14	**1435.6522**	718.3297	**1418.6257**	709.8165	**1417.6416**	709.3245	I	**877.4666**	**439.2369**	860.4400	430.7236	859.4560	430.2316	7
15	1532.7050	**766.8561**	**1515.6784**	758.3428	**1514.6944**	757.8508	P	**764.3825**	382.6949	747.3559	374.1816	**746.3719**	373.6896	6
16	**1647.7319**	824.3696	**1630.7054**	815.8563	**1629.7213**	815.3643	D	**667.3297**	334.1685	**650.3032**	325.6552	649.3192	325.1632	5
17	1760.8160	880.9116	1743.7894	872.3983	**1742.8054**	871.9063	L	**552.3028**	276.6550	535.2762	268.1418	534.2922	267.6498	4
18	**1889.8586**	945.4329	1872.8320	936.9196	1871.8480	936.4276	E	**439.2187**	220.1130	422.1922	211.5997	421.2082	211.1077	3

**19**	2052.9219	1026.9646	2035.8953	1018.4513	2034.9113	1017.9593	**Y**	**310.1761**	155.5917	293.1496	147.0784			**2**
**20**							**K**	147.1128	74.0600	130.0863	65.5468			**1**

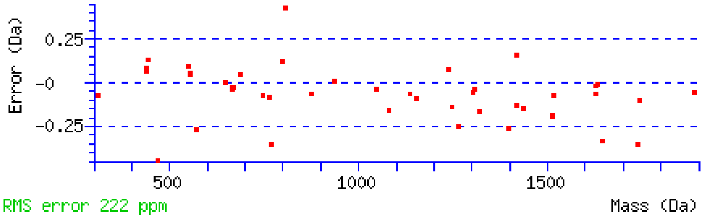

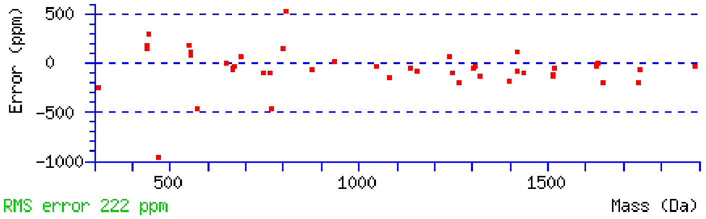

All matches to this query

Score	Mr(calc):	Delta	Sequence
61.6	2198.0201	2.0146	EGNSALINCSAFGIPDLEYK
39.7	2198.0201	2.0146	EGNSALINCSAFGIPDLEYK
3.8	2198.0320	2.0028	ATGIRKGISPMNRPPLSDK
3.3	2199.0126	1.0221	FTSLRPLLGGIPESGGPDAR
3.3	2199.0126	1.0221	FTSLRPLLGGIPESGGPDAR
0.5	2199.0321	1.0026	SSRGSIPLATQQLEEGFGSR
0.4	2199.0321	1.0026	SSRGSIPLATQQLEEGFGSR

Spectrum No: 308; Query: 1077; Rank: 1

Peptide View

MS/MS Fragmentation of **ILQEYQVQYTPQGDSDDGKENQTVHK**
Found in **IPI00199465**, Tax_Id=10116 Gene_Symbol=Gls Glutaminase kidney isoform, mitochondrial precursor

Match to Query 1077: 3020.400432 from(1007.807420,3+)
Title: 091127RatKid_SCX01_33.1862.1862.3.dta
Data file K:\NewmanPaper\Piliang\3SubProteomes\Piliang3SP\mgf5ppm\SCX_3SubProteomes5ppm.mgf

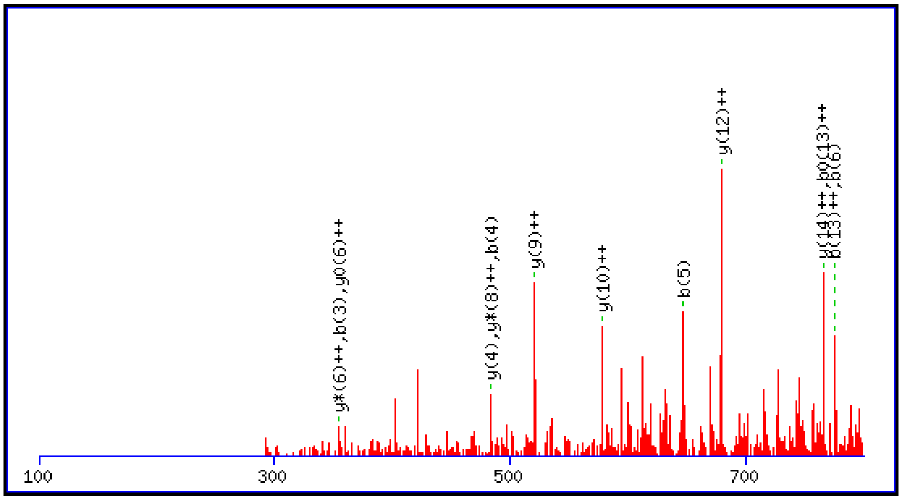

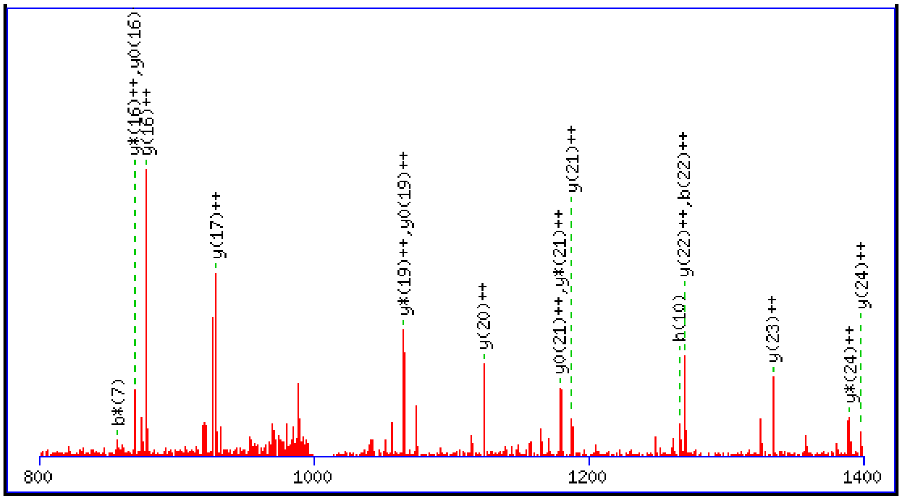

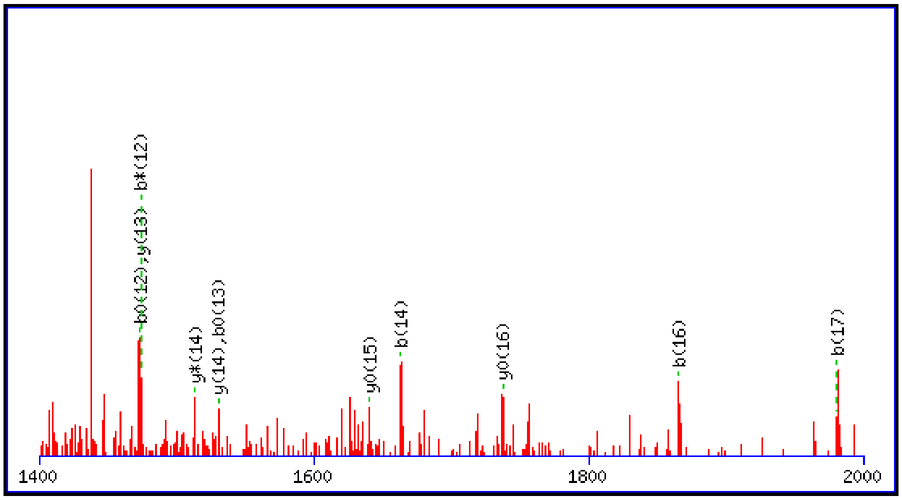

Monoisotopic mass of neutral peptide Mr(calc): 3020.3999
Fixed modifications: Carbamidomethyl (C)
Variable modifications:
N21 : Deamidated_N (N)
Ions Score: 62 **Expect:** 0.00027
Matches (Bold Red): 42/284 fragment ions using 47 most intense peaks

#	b	b^{++}	b*	b^{*++}	b^{0}	b^{0++}	Seq.	y	y^{++}	y*	y^{*++}	y^{0}	y^{0++}	#
1	114.0913	57.5493					I							26
2	227.1754	114.0913					L	2908.3231	1454.6652	2891.2966	1446.1519	2890.3126	1445.6599	25
3	**355.2340**	178.1206	338.2074	169.6074			Q	2795.2391	**1398.1232**	2778.2125	**1389.6099**	2777.2285	1389.1179	24
4	**484.2766**	242.6419	467.2500	234.1287	466.2660	233.6366	E	2667.1805	**1334.0939**	2650.1540	1325.5806	2649.1699	1325.0886	23
5	**647.3399**	324.1736	630.3134	315.6603	629.3293	315.1683	Y	2538.1379	**1269.5726**	2521.1114	1261.0593	2520.1273	1260.5673	22
6	**775.3985**	388.2029	758.3719	379.6896	757.3879	379.1976	Q	2375.0746	**1188.0409**	2358.0480	**1179.5277**	2357.0640	**1179.0356**	21
7	874.4669	437.7371	**857.4403**	429.2238	856.4563	428.7318	V	2247.0160	**1124.0116**	2229.9895	1115.4984	2229.0054	1115.0064	20
8	1002.5255	501.7664	985.4989	493.2531	984.5149	492.7611	Q	2147.9476	1074.4774	2130.9210	**1065.9642**	2129.9370	**1065.4722**	19
9	1165.5888	583.2980	1148.5623	574.7848	1147.5782	574.2928	Y	2019.8890	1010.4481	2002.8625	1001.9349	2001.8784	1001.4429	18
10	**1266.6365**	633.8219	1249.6099	625.3086	1248.6259	624.8166	T	1856.8257	**928.9165**	1839.7991	920.4032	1838.8151	919.9112	17
11	1363.6892	682.3483	1346.6627	673.8350	1345.6787	673.3430	P	1755.7780	**878.3926**	1738.7515	**869.8794**	**1737.7674**	**869.3874**	16
12	1491.7478	746.3776	**1474.7213**	737.8643	**1473.7373**	737.3723	Q	1658.7252	829.8663	1641.6987	821.3530	**1640.7147**	820.8610	15
13	1548.7693	**774.8883**	1531.7427	766.3750	**1530.7587**	**765.8830**	G	**1530.6667**	**765.8370**	**1513.6401**	757.3237	1512.6561	756.8317	14
14	**1663.7962**	832.4018	1646.7697	823.8885	1645.7857	823.3965	D	**1473.6452**	737.3262	1456.6187	728.8130	1455.6346	728.3210	13
15	1750.8283	875.9178	1733.8017	867.4045	1732.8177	866.9125	S	1358.6183	**679.8128**	1341.5917	671.2995	1340.6077	670.8075	12
16	**1865.8552**	933.4312	1848.8287	924.9180	1847.8446	924.4260	D	1271.5862	636.2968	1254.5597	627.7835	1253.5757	627.2915	11
17	**1980.8821**	990.9447	1963.8556	982.4314	1962.8716	981.9394	D	1156.5593	**578.7833**	1139.5327	570.2700	1138.5487	569.7780	10
18	2037.9036	1019.4554	2020.8771	1010.9422	2019.8930	1010.4502	G	1041.5323	**521.2698**	1024.5058	512.7565	1023.5218	512.2645	9

19	2165.9986	1083.5029	2148.9720	1074.9896	2147.9880	1074.4976	K	984.5109	492.7591	967.4843	**484.2458**	966.5003	483.7538	8
20	2295.0412	1148.0242	2278.0146	1139.5109	2277.0306	1139.0189	E	856.4159	428.7116	839.3894	420.1983	838.4054	419.7063	7
21	2410.0681	1205.5377	2393.0416	1197.0244	2392.0575	1196.5324	N	727.3733	364.1903	710.3468	**355.6770**	709.3628	**355.1850**	6
22	2538.1267	**1269.5670**	2521.1001	1261.0537	2520.1161	1260.5617	Q	612.3464	306.6768	595.3198	298.1636	594.3358	297.6715	5
23	2639.1744	1320.0908	2622.1478	1311.5775	2621.1638	1311.0855	T	**484.2878**	242.6475	467.2613	234.1343	466.2772	233.6423	4
24	2738.2428	1369.6250	2721.2162	1361.1117	2720.2322	1360.6197	V	383.2401	192.1237	366.2136	183.6104			3
25	2875.3017	1438.1545	2858.2751	1429.6412	2857.2911	1429.1492	H	284.1717	142.5895	267.1452	134.0762			2
26							K	147.1128	74.0600	130.0863	65.5468			1

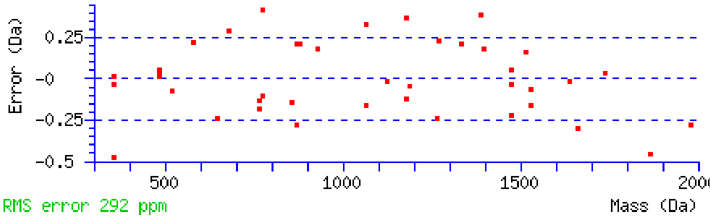

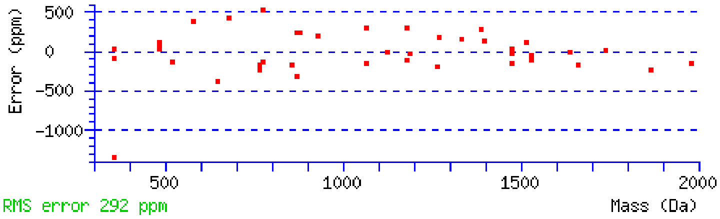

All matches to this query

Score	Mr(calc):	Delta	Sequence
61.5	3020.3999	0.0005	ILQEYQVQYTPQGDSDDGKENQTVHK
25.8	3019.4159	0.9845	ILQEYQVQYTPQGDSDDGKENQTVHK
8.0	3019.3974	1.0030	SSTSRDKVVKPAMNQNEVQEIIGVTK
8.0	3019.3974	1.0030	SSTSRDKVVKPAMNQNEVQEIIGVTK
8.0	3019.3974	1.0030	SSTSRDKVVKPAMNQNEVQEIIGVTK
8.0	3019.3974	1.0030	SSTSRDKVVKPAMNQNEVQEIIGVTK
4.8	3020.4288	-0.0283	KHQQRSIIGFLQAGNQGLSPTGCTADR
4.8	3020.4288	-0.0283	KHQQRSIIGFLQAGNQGLSPTGCTADR
4.8	3020.4288	-0.0283	KHQQRSIIGFLQAGNQGLSPTGCTADR
3.1	3020.4134	-0.0130	QKQRRPSKPNIAQELSMGQNETENR

Spectrum No: 309; Query: 1133; Rank: 1

Peptide View

MS/MS Fragmentation of **IGCFALSEPGNGSDAGAASTTAREEGDSWVLNGTK**
Found in **IPI00231359**, Tax_Id=10116 Gene_Symbol=Acads Acetyl-Coenzyme A dehydrogenase, short chain

Match to Query 1133: 3526.602492 from(1176.541440,3+)
Title: 091127RatKid_SCX01_22.4313.4313.3.dta
Data file K:\NewmanPaper\Piliang\3SubProteomes\Piliang3SP\mgf5ppm\SCX_3SubProteomes5ppm.mgf

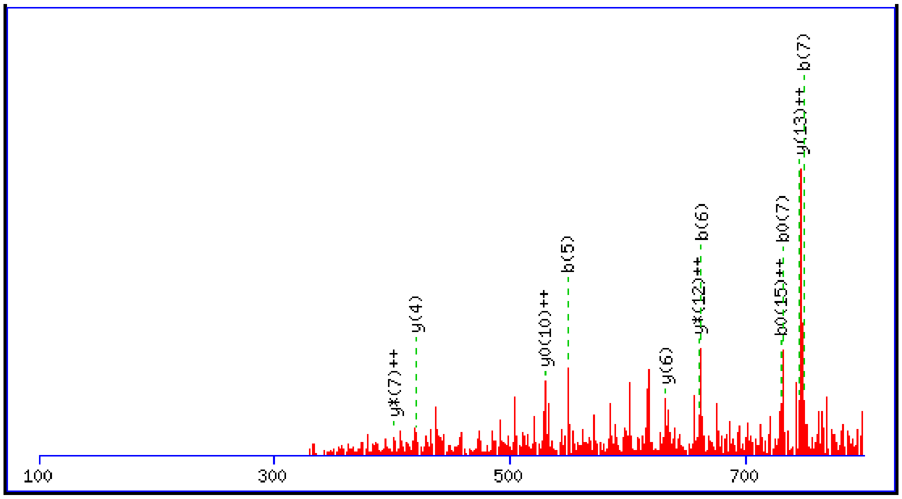

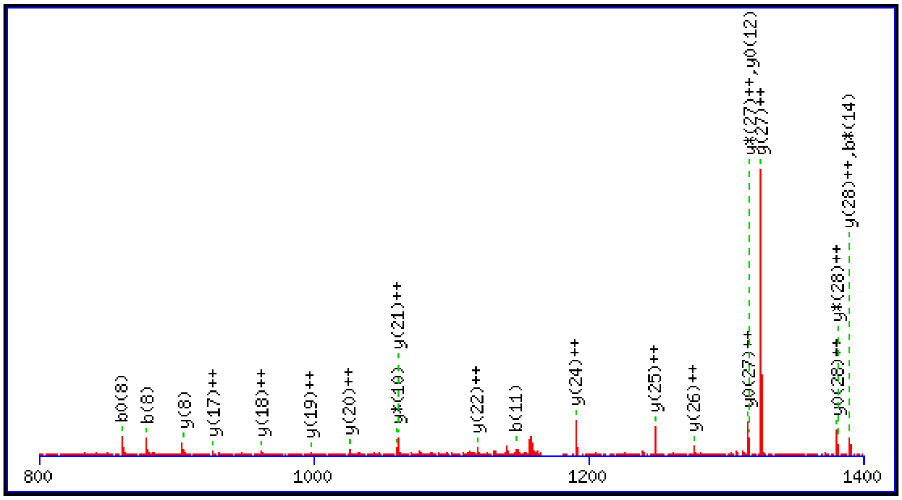

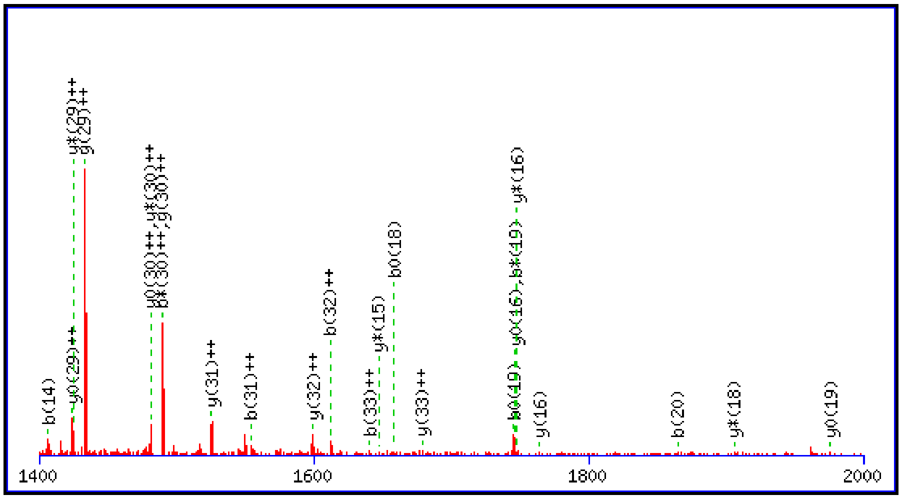

Monoisotopic mass of neutral peptide Mr(calc): 3526.5794
Fixed modifications: Carbamidomethyl (C)
Variable modifications:
N11 : Deamidated_N (N)
N32 : Deamidated_N (N)
Ions Score: 62 **Expect:** 0.00033
Matches (Bold Red): 57/374 fragment ions using 128 most intense peaks

#	b	b^{++}	b*	b^{*++}	b^{0}	b^{0++}	Seq.	y	y^{++}	y*	y^{*++}	y^{0}	y^{0++}	#
1	114.0913	57.5493					I							35
2	171.1128	86.0600					G	3414.5026	1707.7550	3397.4761	1699.2417	3396.4921	1698.7497	34
3	331.1435	166.0754					C	3357.4812	**1679.2442**	3340.4546	1670.7309	3339.4706	1670.2389	33
4	478.2119	239.6096					F	3197.4505	**1599.2289**	3180.4240	1590.7156	3179.4400	1590.2236	32

5	**549.2490**	275.1281					A	3050.3821	**1525.6947**	3033.3556	1517.1814	3032.3715	1516.6894	31
6	**662.3330**	331.6702					L	2979.3450	**1490.1761**	2962.3184	**1481.6629**	2961.3344	**1481.1709**	30
7	**749.3651**	375.1862			**731.3545**	366.1809	S	2866.2609	**1433.6341**	2849.2344	**1425.1208**	2848.2504	**1424.6288**	29
8	**878.4077**	439.7075			**860.3971**	430.7022	E	2779.2289	**1390.1181**	2762.2024	**1381.6048**	2761.2183	**1381.1128**	28
9	975.4604	488.2339			957.4499	479.2286	P	2650.1863	**1325.5968**	2633.1598	**1317.0835**	2632.1757	**1316.5915**	27
10	1032.4819	516.7446			1014.4713	507.7393	G	2553.1335	**1277.0704**	2536.1070	1268.5571	2535.1230	1268.0651	26
11	**1147.5088**	574.2581	1130.4823	565.7448	1129.4983	565.2528	N	2496.1121	**1248.5597**	2479.0855	1240.0464	2478.1015	1239.5544	25
12	1204.5303	602.7688	1187.5037	594.2555	1186.5197	593.7635	G	2381.0851	**1191.0462**	2364.0586	1182.5329	2363.0746	1182.0409	24
13	1291.5623	646.2848	1274.5358	637.7715	1273.5518	637.2795	S	2324.0637	1162.5355	2307.0371	1154.0222	2306.0531	1153.5302	23
14	**1406.5893**	703.7983	**1389.5627**	695.2850	1388.5787	694.7930	D	2237.0317	**1119.0195**	2220.0051	1110.5062	2219.0211	1110.0142	22
15	1477.6264	739.3168	1460.5998	730.8036	1459.6158	**730.3115**	A	2122.0047	**1061.5060**	2104.9782	1052.9927	2103.9941	1052.5007	21
16	1534.6478	767.8276	1517.6213	759.3143	1516.6373	758.8223	G	2050.9676	**1025.9874**	2033.9410	1017.4742	2032.9570	1016.9822	20
17	1605.6850	803.3461	1588.6584	794.8328	1587.6744	794.3408	A	1993.9461	**997.4767**	1976.9196	988.9634	**1975.9356**	988.4714	19
18	1676.7221	838.8647	1659.6955	830.3514	**1658.7115**	829.8594	A	1922.9090	**961.9581**	**1905.8825**	953.4449	1904.8985	952.9529	18
19	1763.7541	882.3807	**1746.7276**	873.8674	**1745.7435**	873.3754	S	1851.8719	**926.4396**	1834.8454	917.9263	1833.8613	917.4343	17
20	**1864.8018**	932.9045	1847.7752	924.3913	1846.7912	923.8992	T	**1764.8399**	882.9236	**1747.8133**	874.4103	**1746.8293**	873.9183	16
21	1965.8495	983.4284	1948.8229	974.9151	1947.8389	974.4231	T	1663.7922	832.3997	**1646.7657**	823.8865	1645.7816	823.3945	15
22	2036.8866	1018.9469	2019.8600	1010.4336	2018.8760	1009.9416	A	1562.7445	781.8759	1545.7180	773.3626	1544.7340	772.8706	14
23	2192.9877	1096.9975	2175.9611	1088.4842	2174.9771	1087.9922	R	1491.7074	**746.3573**	1474.6809	737.8441	1473.6968	737.3521	13
24	2322.0303	1161.5188	2305.0037	1153.0055	2304.0197	1152.5135	E	1335.6063	668.3068	1318.5797	**659.7935**	**1317.5957**	659.3015	12
25	2451.0729	1226.0401	2434.0463	1217.5268	2433.0623	1217.0348	E	1206.5637	603.7855	1189.5372	595.2722	1188.5531	594.7802	11
26	2508.0943	1254.5508	2491.0678	1246.0375	2490.0838	1245.5455	G	1077.5211	539.2642	**1060.4946**	530.7509	1059.5105	**530.2589**	10
27	2623.1213	1312.0643	2606.0947	1303.5510	2605.1107	1303.0590	D	1020.4996	510.7535	1003.4731	502.2402	1002.4891	501.7482	9
28	2710.1533	1355.5803	2693.1268	1347.0670	2692.1427	1346.5750	S	**905.4727**	453.2400	888.4462	444.7267	887.4621	444.2347	8
29	2896.2326	1448.6199	2879.2061	1440.1067	2878.2221	1439.6147	W	818.4407	409.7240	801.4141	**401.2107**	800.4301	400.7187	7
30	2995.3010	1498.1542	2978.2745	**1489.6409**	2977.2905	1489.1489	V	**632.3614**	316.6843	615.3348	308.1710	614.3508	307.6790	6
31	3108.3851	**1554.6962**	3091.3585	1546.1829	3090.3745	1545.6909	L	533.2929	267.1501	516.2664	258.6368	515.2824	258.1448	5
32	3223.4120	**1612.2097**	3206.3855	1603.6964	3205.4015	1603.2044	N	**420.2089**	210.6081	403.1823	202.0948	402.1983	201.6028	4
33	3280.4335	**1640.7204**	3263.4069	1632.2071	3262.4229	1631.7151	G	305.1819	153.0946	288.1554	144.5813	287.1714	144.0893	3
34	3381.4812	1691.2442	3364.4546	1682.7309	3363.4706	1682.2389	T	248.1605	124.5839	231.1339	116.0706	230.1499	115.5786	2
35							K	147.1128	74.0600	130.0863	65.5468			1

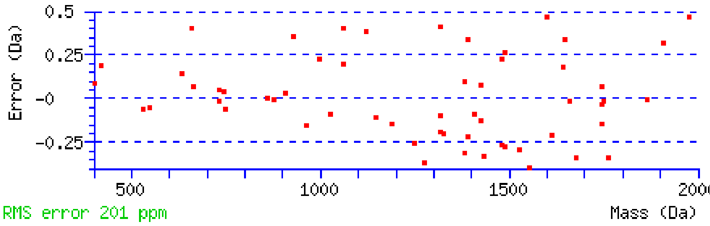

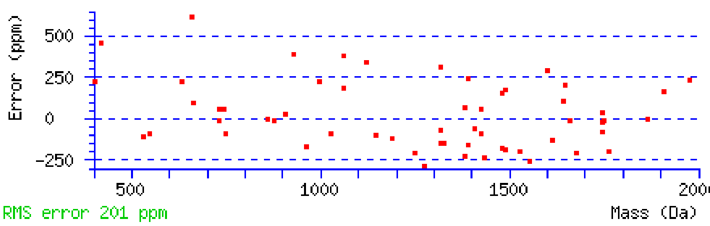

All matches to this query

Score	Mr(calc):	Delta	Sequence
61.5	3526.5794	0.0231	IGCFALSEPGNGSDAGAASTTAREEGDSWVLNGTK
56.3	3525.5954	1.0071	IGCFALSEPGNGSDAGAASTTAREEGDSWVLNGTK
40.4	3525.5954	1.0071	IGCFALSEPGNGSDAGAASTTAREEGDSWVLNGTK
6.8	3526.5997	0.0028	AWGAMMTEADKVSELHQEVKNSLLNEDLEK
6.6	3526.6178	-0.0153	YLPLFMIQXNLSSLLESLRLVSLSWTR
5.9	3525.5734	1.0291	NSQSFFSGLFGGSSKIEEACEIYARAANMFK
5.7	3524.6244	1.9781	MSKSESMFHNQSSVETLFVSPAFKAVSPLNK
5.2	3526.6170	-0.0145	LITHGLGGEGYLNFMGNEFGHPEWLDFPRK
5.0	3524.6244	1.9781	MSKSESMFHNQSSVETLFVSPAFKAVSPLNK

2.9	3526.5997	0.0028	AWGAMMTEADKVSELHQEVKNSLLNEDLEK

Spectrum No: 310; Query: 887; Rank: 1

Peptide View

MS/MS Fragmentation of **VLTLANFTTKDEGDYMCELR**
Found in **IPI00188956**, Tax_Id=10116 Gene_Symbol=Thy1 Thy-1 membrane glycoprotein precursor

Match to Query 887: 2376.097842 from(793.039890,3+)
Title: 091129RatKid_SCX02_22.4830.4830.3.dta
Data file K:\NewmanPaper\Piliang\3SubProteomes\Piliang3SP\mgf5ppm\SCX_3SubProteomes5ppm.mgf

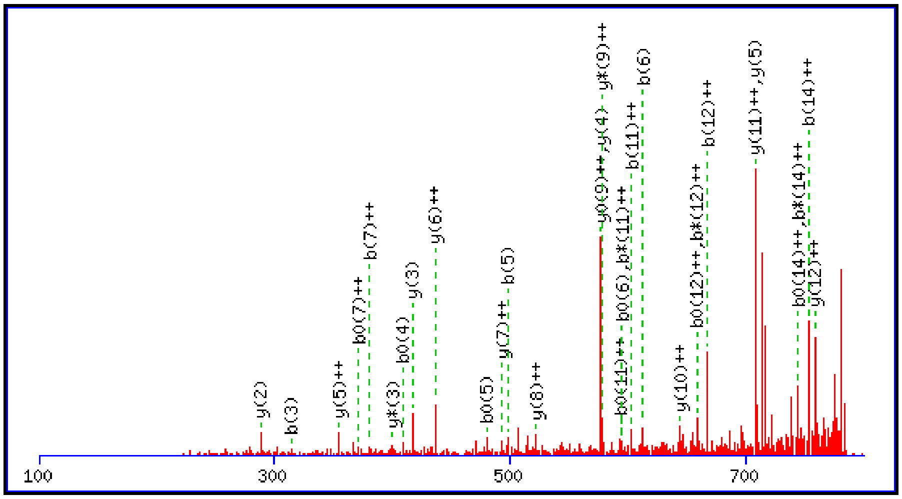

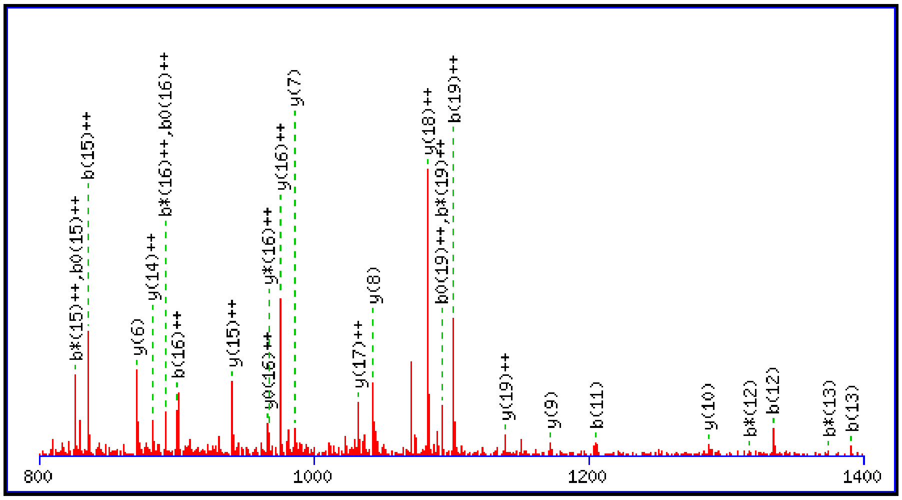

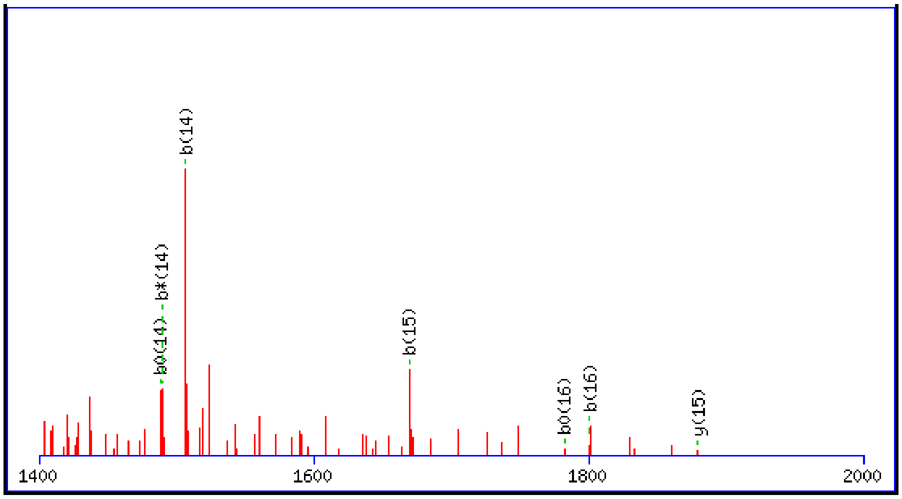

Monoisotopic mass of neutral peptide Mr(calc): 2376.0977
Fixed modifications: Carbamidomethyl (C)
Variable modifications:
N6 : Deamidated_N (N)
Ions Score: 61 **Expect:** 0.00022
Matches (Bold Red): 65/210 fragment ions using 124 most intense peaks

#	b	b^{++}	b^{*}	b^{*++}	b^{0}	b^{0++}	Seq.	y	y^{++}	y^{*}	y^{*++}	y^{0}	y^{0++}	#
1	100.0757	50.5415					V							20
2	213.1598	107.0835					L	2278.0366	**1139.5219**	2261.0101	1131.0087	2260.0260	1130.5167	19
3	**314.2074**	157.6074			296.1969	148.6021	T	2164.9525	**1082.9799**	2147.9260	1074.4666	2146.9420	1073.9746	18
4	427.2915	214.1494			**409.2809**	205.1441	L	2063.9049	**1032.4561**	2046.8783	1023.9428	2045.8943	1023.4508	17
5	**498.3286**	249.6679			**480.3180**	240.6627	A	1950.8208	**975.9140**	1933.7943	**967.4008**	1932.8102	**966.9088**	16
6	**613.3555**	307.1814	596.3290	298.6681	**595.3450**	298.1761	N	**1879.7837**	**940.3955**	1862.7571	931.8822	1861.7731	931.3902	15
7	760.4240	**380.7156**	743.3974	372.2023	742.4134	**371.7103**	F	1764.7568	**882.8820**	1747.7302	874.3687	1746.7462	873.8767	14
8	861.4716	431.2395	844.4451	422.7262	843.4611	422.2342	T	1617.6883	809.3478	1600.6618	800.8345	1599.6778	800.3425	13
9	962.5193	481.7633	945.4928	473.2500	944.5088	472.7580	T	1516.6407	**758.8240**	1499.6141	750.3107	1498.6301	749.8187	12
10	1090.6143	545.8108	1073.5877	537.2975	1072.6037	536.8055	K	1415.5930	**708.3001**	1398.5664	699.7869	1397.5824	699.2948	11
11	**1205.6412**	**603.3243**	1188.6147	**594.8110**	1187.6307	**594.3190**	D	**1287.4980**	**644.2526**	1270.4715	635.7394	1269.4875	635.2474	10
12	**1334.6838**	**667.8455**	**1317.6573**	**659.3323**	1316.6733	**658.8403**	E	**1172.4711**	586.7392	1155.4445	**578.2259**	1154.4605	**577.7339**	9
13	**1391.7053**	696.3563	**1374.6787**	687.8430	1373.6947	687.3510	G	**1043.4285**	**522.2179**	1026.4019	513.7046	1025.4179	513.2126	8
14	**1506.7322**	**753.8698**	**1489.7057**	**745.3565**	**1488.7217**	**744.8645**	D	**986.4070**	**493.7071**	969.3805	485.1939	968.3965	484.7019	7
15	**1669.7956**	**835.4014**	1652.7690	**826.8881**	1651.7850	**826.3961**	Y	**871.3801**	**436.1937**	854.3535	427.6804	853.3695	427.1884	6
16	**1800.8360**	**900.9217**	1783.8095	**892.4084**	**1782.8255**	**891.9164**	M	**708.3167**	**354.6620**	691.2902	346.1487	690.3062	345.6567	5
17	1960.8667	980.9370	1943.8401	972.4237	1942.8561	971.9317	C	**577.2763**	289.1418	560.2497	280.6285	559.2657	280.1365	4
18	2089.9093	1045.4583	2072.8827	1036.9450	2071.8987	1036.4530	E	**417.2456**	209.1264	**400.2191**	200.6132	399.2350	200.1212	3
19	2202.9933	**1102.0003**	2185.9668	**1093.4870**	2184.9828	**1092.9950**	L	**288.2030**	144.6051	271.1765	136.0919			2
20							R	175.1190	88.0631	158.0924	79.5498			1

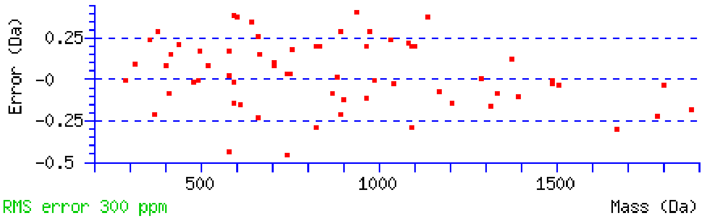

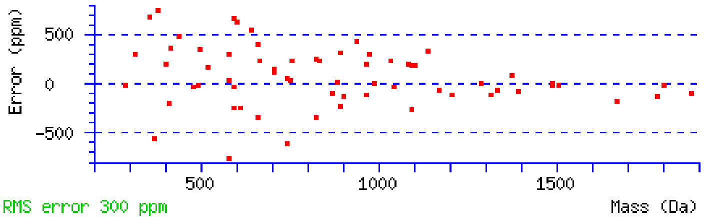

All matches to this query

Score	Mr(calc):	Delta	Sequence
61.2	2376.0977	0.0001	VLTLANFTTKDEGDYMCELR

19.8	2375.1137	0.9841	VLTLANFTTKDEGDYMCELR
3.9	2375.1175	0.9804	SYDYRVSLKTAISTTLELR
3.9	2375.1175	0.9804	SYDYRVSLKTAISTTLELR
3.9	2375.1175	0.9804	SYDYRVSLKTAISTTLELR
3.9	2375.1175	0.9804	SYDYRVSLKTAISTTLELR
3.9	2375.1175	0.9804	SYDYRVSLKTAISTTLELR
3.9	2375.1175	0.9804	SYDYRVSLKTAISTTLELR
3.6	2376.1049	-0.0071	XLLDMDTGVTEGGLNVTLTIR
0.7	2375.1175	0.9804	SYDYRVSLKTAISTTLELR

Spectrum No: 311; Query: 1130; Rank: 1

Peptide View

MS/MS Fragmentation of **SNSTAGELDALQAEAGSLDKTVNELAEQLEFIK**
Found in **IPI00365542**, Tax_Id=10116 Gene_Symbol=Lamb1_predicted laminin, beta 1

Match to Query 1130: 3491.733222 from(1164.918350,3+)
Title: 091129RatKid_SCX02_19.6985.6985.3.dta
Data file K:\NewmanPaper\Piliang\3SubProteomes\Piliang3SP\mgf5ppm\SCX_3SubProteomes5ppm.mgf

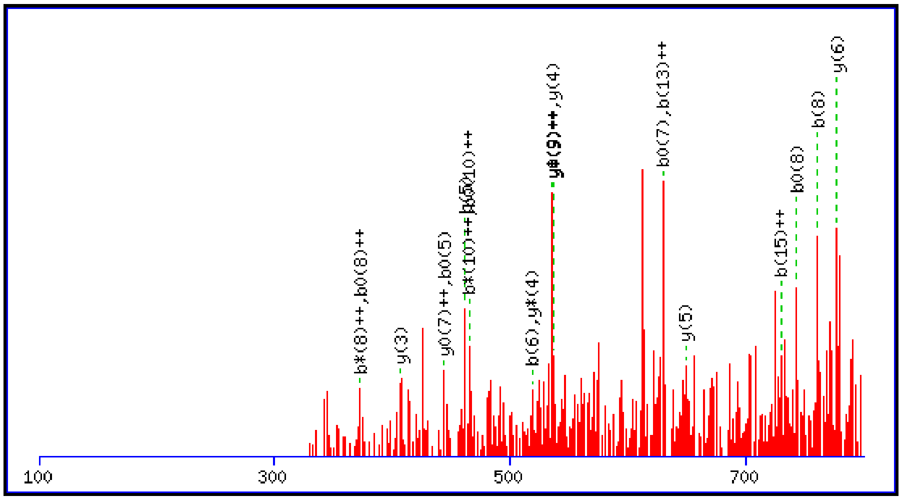

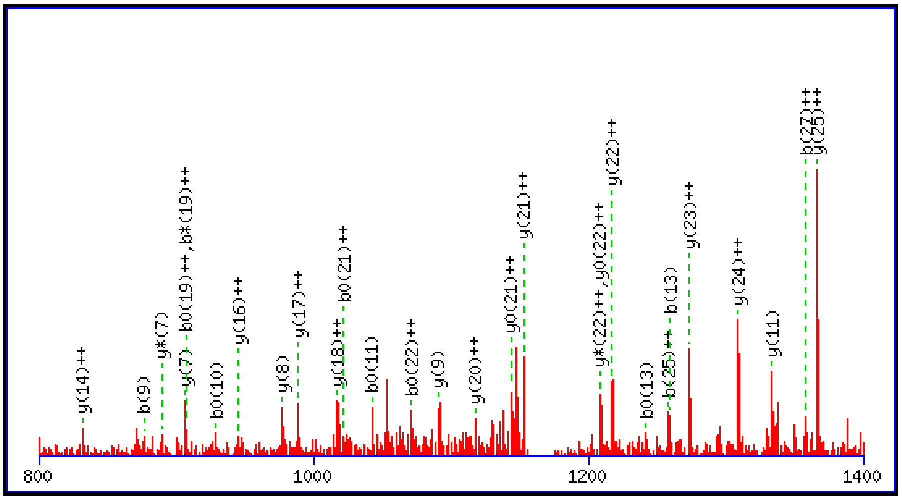

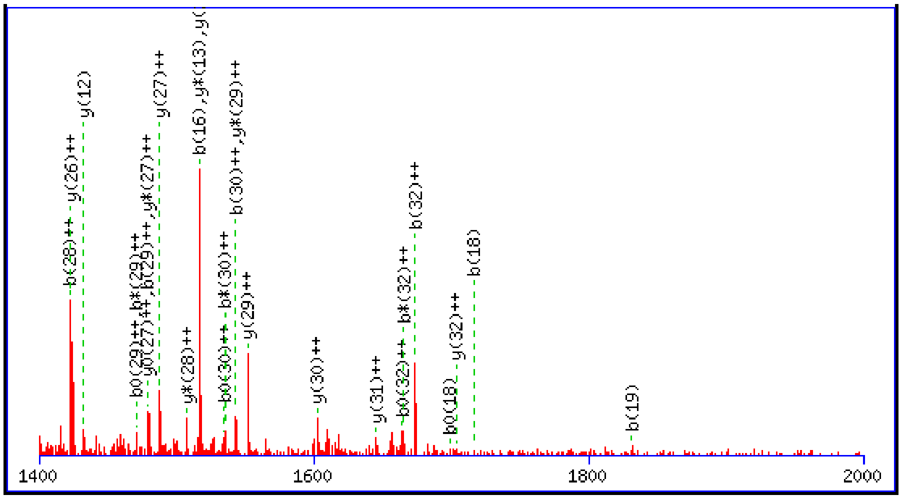

Monoisotopic mass of neutral peptide Mr(calc): 3491.7155
Fixed modifications: Carbamidomethyl (C)
Variable modifications:
N2 : Deamidated_N (N)
Ions Score: 61 **Expect:** 0.00018
Matches (Bold Red): 76/376 fragment ions using 181 most intense peaks

#	b	b^{++}	b*	b^{*++}	b^{0}	b^{0++}	Seq.	y	y^{++}	y*	y^{*++}	y^{0}	y^{0++}	#
1	88.0393	44.5233			70.0287	35.5180	S							33
2	203.0662	102.0368	186.0397	93.5235	185.0557	93.0315	N	3405.6908	**1703.3490**	3388.6642	1694.8357	3387.6802	1694.3437	32
3	290.0983	145.5528	273.0717	137.0395	272.0877	136.5475	S	3290.6638	**1645.8356**	3273.6373	1637.3223	3272.6533	1636.8303	31
4	391.1459	196.0766	374.1194	187.5633	373.1354	187.0713	T	3203.6318	**1602.3195**	3186.6052	1593.8063	3185.6212	1593.3143	30
5	**462.1831**	231.5952	445.1565	223.0819	**444.1725**	222.5899	A	3102.5841	**1551.7957**	3085.5576	**1543.2824**	3084.5736	1542.7904	29
6	**519.2045**	260.1059	502.1780	251.5926	501.1940	251.1006	G	3031.5470	**1516.2771**	3014.5205	**1507.7639**	3013.5364	1507.2719	28
7	648.2471	324.6272	631.2206	316.1139	**630.2366**	315.6219	E	2974.5255	**1487.7664**	2957.4990	**1479.2531**	2956.5150	**1478.7611**	27
8	**761.3312**	381.1692	744.3046	**372.6560**	**743.3206**	**372.1639**	L	2845.4829	**1423.2451**	2828.4564	1414.7318	2827.4724	1414.2398	26
9	**876.3581**	438.6827	859.3316	430.1694	858.3476	429.6774	D	2732.3989	**1366.7031**	2715.3723	1358.1898	2714.3883	1357.6978	25
10	947.3952	474.2013	930.3687	**465.6880**	**929.3847**	**465.1960**	A	2617.3719	**1309.1896**	2600.3454	1300.6763	2599.3614	1300.1843	24
11	1060.4793	530.7433	1043.4528	522.2300	**1042.4687**	521.7380	L	2546.3348	**1273.6711**	2529.3083	1265.1578	2528.3243	1264.6658	23
12	1188.5379	594.7726	1171.5113	586.2593	1170.5273	585.7673	Q	2433.2508	**1217.1290**	2416.2242	**1208.6157**	2415.2402	**1208.1237**	22
13	**1259.5750**	**630.2911**	1242.5484	621.7779	**1241.5644**	621.2859	A	2305.1922	**1153.0997**	2288.1656	1144.5865	2287.1816	**1144.0944**	21
14	1388.6176	694.8124	1371.5910	686.2992	1370.6070	685.8071	E	2234.1551	**1117.5812**	2217.1285	1109.0679	2216.1445	1108.5759	20
15	1459.6547	**730.3310**	1442.6282	721.8177	1441.6441	721.3257	A	2105.1125	1053.0599	2088.0859	1044.5466	2087.1019	1044.0546	19
16	**1516.6762**	758.8417	1499.6496	750.3284	1498.6656	749.8364	G	2034.0754	**1017.5413**	2017.0488	1009.0280	2016.0648	1008.5360	18
17	1603.7082	802.3577	1586.6816	793.8445	1585.6976	793.3525	S	1977.0539	**989.0306**	1960.0274	980.5173	1959.0433	980.0253	17
18	**1716.7923**	858.8998	1699.7657	850.3865	**1698.7817**	849.8945	L	1890.0219	**945.5146**	1872.9953	937.0013	1872.0113	936.5093	16
19	**1831.8192**	916.4132	1814.7927	**907.9000**	1813.8086	**907.4080**	D	1776.9378	888.9725	1759.9113	880.4593	1758.9272	879.9673	15
20	1959.9142	980.4607	1942.8876	971.9474	1941.9036	971.4554	K	1661.9109	**831.4591**	1644.8843	822.9458	1643.9003	822.4538	14
21	2060.9618	1030.9846	2043.9353	1022.4713	2042.9513	**1021.9793**	T	1533.8159	767.4116	**1516.7894**	758.8983	1515.8053	758.4063	13
22	2160.0303	1080.5188	2143.0037	1072.0055	2142.0197	**1071.5135**	V	**1432.7682**	716.8877	1415.7417	708.3745	1414.7577	707.8825	12
23	2274.0732	1137.5402	2257.0466	1129.0270	2256.0626	1128.5349	N	**1333.6998**	667.3535	1316.6733	658.8403	1315.6892	658.3483	11
24	2403.1158	1202.0615	2386.0892	1193.5483	2385.1052	1193.0562	E	1219.6569	610.3321	1202.6303	601.8188	1201.6463	601.3268	10
25	2516.1998	**1258.6036**	2499.1733	1250.0903	2498.1893	1249.5983	L	**1090.6143**	545.8108	1073.5877	**537.2975**	1072.6037	**536.8055**	9
26	2587.2370	1294.1221	2570.2104	1285.6088	2569.2264	1285.1168	A	**977.5302**	489.2688	960.5037	480.7555	959.5197	480.2635	8
27	2716.2795	**1358.6434**	2699.2530	1350.1301	2698.2690	1349.6381	E	**906.4931**	453.7502	**889.4666**	445.2369	888.4825	**444.7449**	7
28	2844.3381	**1422.6727**	2827.3116	1414.1594	2826.3276	1413.6674	Q	**777.4505**	389.2289	760.4240	380.7156	759.4400	380.2236	6
29	2957.4222	**1479.2147**	2940.3956	**1470.7015**	2939.4116	**1470.2095**	L	**649.3919**	325.1996	632.3654	316.6863	631.3814	316.1943	5
30	3086.4648	**1543.7360**	3069.4382	**1535.2228**	3068.4542	**1534.7307**	E	**536.3079**	268.6576	**519.2813**	260.1443	518.2973	259.6523	4
31	3233.5332	1617.2702	3216.5066	1608.7570	3215.5226	1608.2650	F	**407.2653**	204.1363	390.2387	195.6230			3
32	3346.6173	**1673.8123**	3329.5907	**1665.2990**	3328.6067	**1664.8070**	I	260.1969	130.6021	243.1703	122.0888			2

33							K	147.1128	74.0600	130.0863	65.5468			1

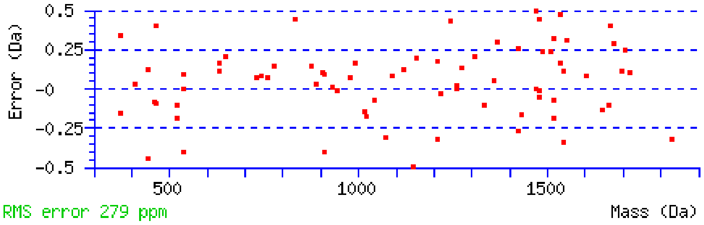

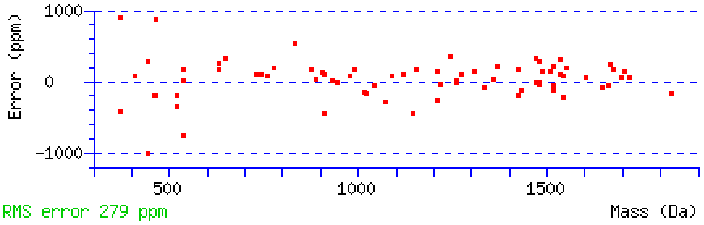

All matches to this query

Score	Mr(calc):	Delta	Sequence
61.1	3491.7155	0.0178	SNSTAGELDALQAEAGSLDKTVNELAEQLEFIK
49.7	3491.7155	0.0178	SNSTAGELDALQAEAGSLDKTVNELAEQLEFIK
41.2	3490.7314	1.0018	SNSTAGELDALQAEAGSLDKTVNELAEQLEFIK
9.2	3491.7639	-0.0307	QIGENETDERFQDVLMSLAKAVANAAAMLVLK
1.5	3491.7295	0.0037	SHFLYLAPEIQLPFSPLTSTEFELIRRK
1.5	3491.7295	0.0037	SHFLYLAPEIQLPFSPLTSTEFELIRRK
1.5	3491.7295	0.0037	SHFLYLAPEIQLPFSPLTSTEFELIRRK
1.2	3491.7524	-0.0192	VIHYNFGIVEGLMTTVHVITATQKTVDGPSGK
1.0	3491.7345	-0.0013	MSIEIILCHKQFHVWFLVFASYPHTMNK
0.9	3489.7382	1.9950	TILLLMTFFVVFYILGTVIFHSRTKFK

Spectrum No: 312; Query: 528; Rank: 1

Peptide View

MS/MS Fragmentation of **LSALDNLLNHSSIFLK**
Found in **IPI00210975**, Tax_Id=10116 Gene_Symbol=Hyou1 Hypoxia up-regulated protein 1 precursor

Match to Query 528: 1784.971588 from(893.493070,2+)
Title: 091127RatKid_SCX01_25.5657.5657.2.dta
Data file K:\NewmanPaper\Piliang\3SubProteomes\Piliang3SP\mgf5ppm\SCX_3SubProteomes5ppm.mgf

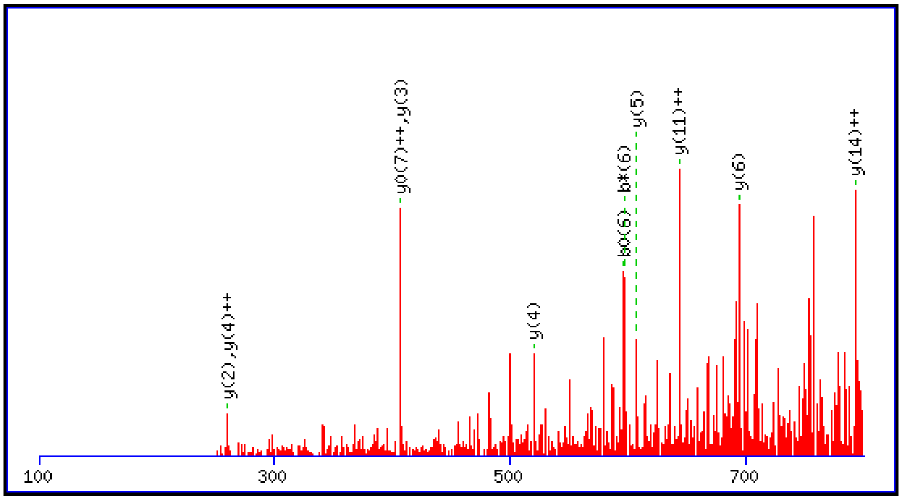

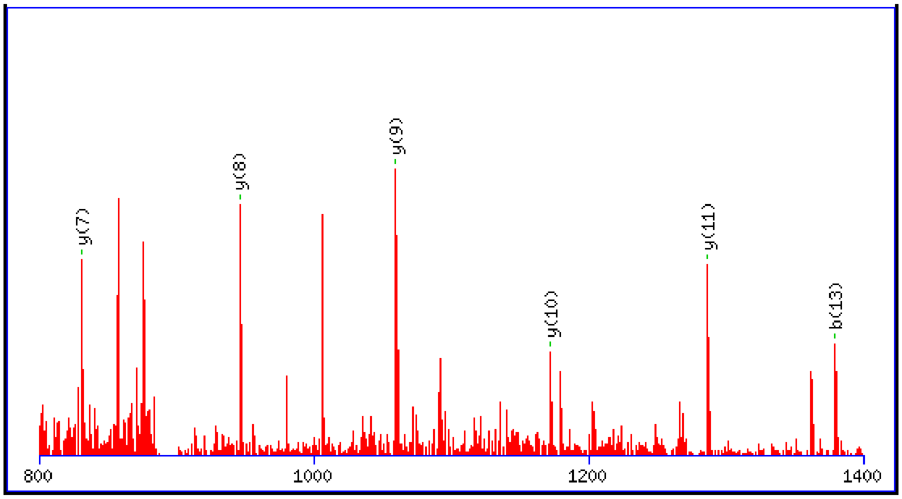

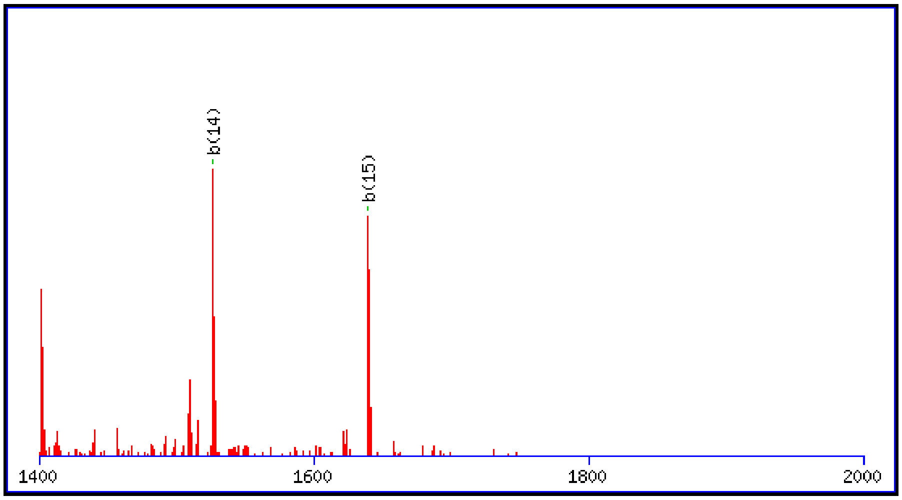

Monoisotopic mass of neutral peptide Mr(calc): 1784.9672
Fixed modifications: Carbamidomethyl (C)
Variable modifications:
N9 : Deamidated_N (N)
Ions Score: 61 **Expect:** 6e-005
Matches (Bold Red): 19/160 fragment ions using 26 most intense peaks

#	b	b++	b*	b*++	b0	b0++	Seq.	y	y++	y*	y*++	y0	y0++	#
1	114.0913	57.5493					L							16
2	201.1234	101.0653			183.1128	92.0600	S	1672.8905	836.9489	1655.8639	828.4356	1654.8799	827.9436	15
3	272.1605	136.5839			254.1499	127.5786	A	1585.8584	**793.4329**	1568.8319	784.9196	1567.8479	784.4276	14
4	385.2445	193.1259			367.2340	184.1206	L	1514.8213	757.9143	1497.7948	749.4010	1496.8107	748.9090	13
5	500.2715	250.6394			482.2609	241.6341	D	1401.7372	701.3723	1384.7107	692.8590	1383.7267	692.3670	12
6	614.3144	307.6608	**597.2879**	299.1476	**596.3039**	298.6556	N	**1286.7103**	**643.8588**	1269.6838	635.3455	1268.6997	634.8535	11
7	727.3985	364.2029	710.3719	355.6896	709.3879	355.1976	L	**1172.6674**	586.8373	1155.6408	578.3241	1154.6568	577.8320	10
8	840.4825	420.7449	823.4560	412.2316	822.4720	411.7396	L	**1059.5833**	530.2953	1042.5568	521.7820	1041.5727	521.2900	9
9	955.5095	478.2584	938.4829	469.7451	937.4989	469.2531	N	**946.4993**	473.7533	929.4727	465.2400	928.4887	464.7480	8
10	1092.5684	546.7878	1075.5418	538.2746	1074.5578	537.7826	H	**831.4723**	416.2398	814.4458	407.7265	813.4617	**407.2345**	7
11	1179.6004	590.3038	1162.5739	581.7906	1161.5899	581.2986	S	**694.4134**	347.7103	677.3869	339.1971	676.4028	338.7051	6
12	1266.6324	633.8199	1249.6059	625.3066	1248.6219	624.8146	S	**607.3814**	304.1943	590.3548	295.6811	589.3708	295.1890	5
13	**1379.7165**	690.3619	1362.6900	681.8486	1361.7059	681.3566	I	**520.3493**	**260.6783**	503.3228	252.1650			4
14	**1526.7849**	763.8961	1509.7584	755.3828	1508.7744	754.8908	F	**407.2653**	204.1363	390.2387	195.6230			3
15	**1639.8690**	820.4381	1622.8424	811.9249	1621.8584	811.4329	L	**260.1969**	130.6021	243.1703	122.0888			2
16							K	147.1128	74.0600	130.0863	65.5468			1

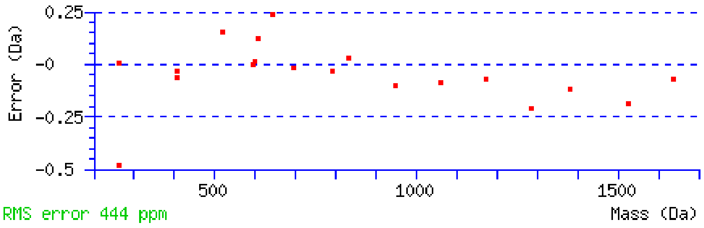

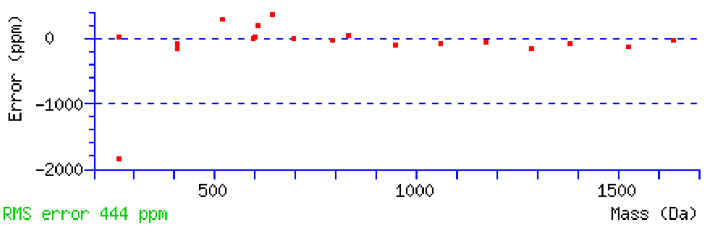

All matches to this query

Score	Mr(calc):	Delta	Sequence
61.2	1784.9672	0.0044	LSALDNLLNHSSIFLK
32.7	1784.9672	0.0044	LSALDNLLNHSSIFLK
9.6	1783.9832	0.9884	LSALDNLLNHSSIFLK
3.7	1784.9567	0.0149	AITGRIPDSLKNCVNK
3.2	1784.9585	0.0131	YPLMVKLNLLLXLK
2.0	1784.9632	0.0084	EELNKELVQASERLK

Spectrum No: 313; Query: 374; Rank: 1

Peptide View

MS/MS Fragmentation of **YLNETQQLTENIK**
Found in **IPI00564327**, Tax_Id=10116 Gene_Symbol=Mug2 Murinoglobulin-2 precursor

Match to Query 374: 1593.791008 from(797.902780,2+)
Title: 091127RatKid_SCX01_11.1748.1748.2.dta
Data file K:\NewmanPaper\Piliang\3SubProteomes\Piliang3SP\mgf5ppm\SCX_3SubProteomes5ppm.mgf

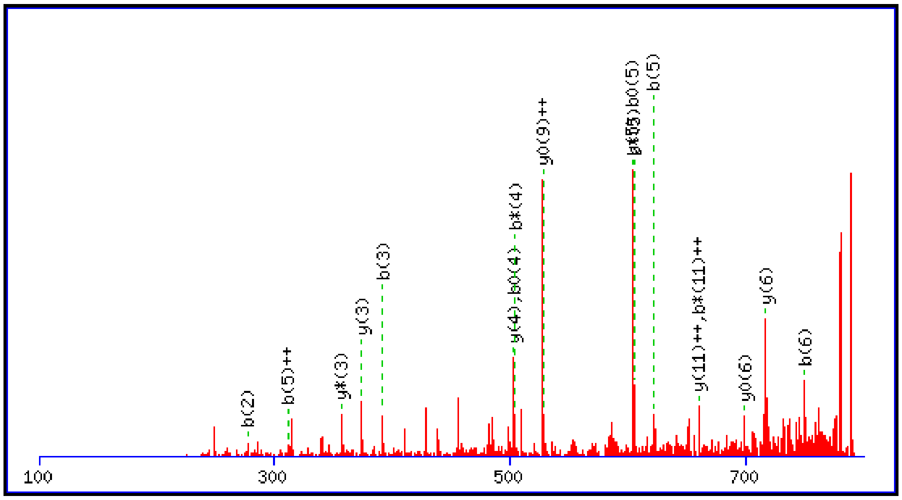

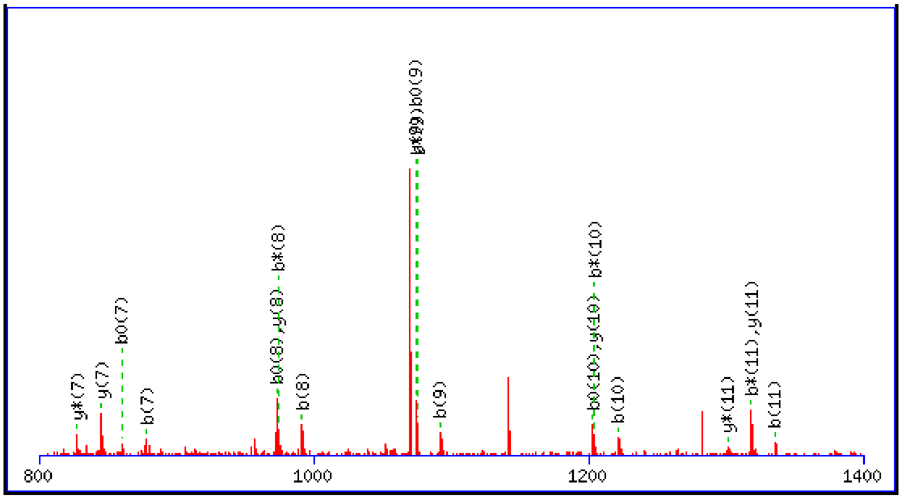

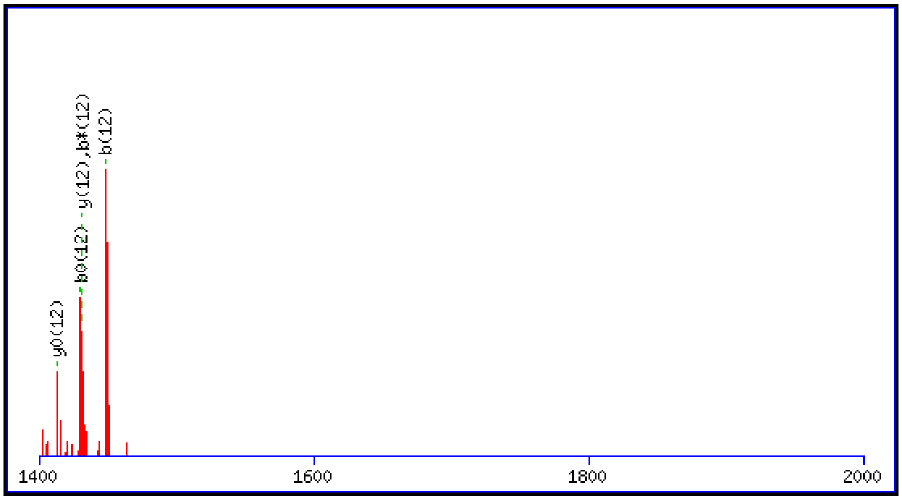

Monoisotopic mass of neutral peptide Mr(calc): 1593.7886
Fixed modifications: Carbamidomethyl (C)
Variable modifications:
N3 : Deamidated_N (N)
Ions Score: 61 **Expect:** 0.00012
Matches (Bold Red): 43/128 fragment ions using 65 most intense peaks

#	b	b^{++}	b*	b^{*++}	b^0	b^{0++}	Seq.	y	y^{++}	y*	y^{*++}	y^0	y^{0++}	#
1	164.0706	82.5389					Y							13
2	**277.1547**	139.0810					L	**1431.7326**	716.3699	1414.7060	707.8566	**1413.7220**	707.3646	12
3	**392.1816**	196.5944	375.1551	188.0812			N	**1318.6485**	**659.8279**	**1301.6219**	651.3146	1300.6379	650.8226	11
4	521.2242	261.1157	**504.1976**	252.6025	**503.2136**	252.1105	E	**1203.6216**	602.3144	1186.5950	593.8011	1185.6110	593.3091	10
5	**622.2719**	**311.6396**	**605.2453**	303.1263	**604.2613**	302.6343	T	**1074.5790**	537.7931	1057.5524	529.2798	1056.5684	**528.7878**	9
6	**750.3305**	375.6689	733.3039	367.1556	732.3199	366.6636	Q	**973.5313**	487.2693	956.5047	478.7560	955.5207	478.2640	8
7	**878.3890**	439.6982	861.3625	431.1849	**860.3785**	430.6929	Q	**845.4727**	423.2400	**828.4462**	414.7267	827.4621	414.2347	7
8	**991.4731**	496.2402	**974.4465**	487.7269	**973.4625**	487.2349	L	**717.4141**	359.2107	700.3876	350.6974	**699.4036**	350.2054	6
9	**1092.5208**	546.7640	**1075.4942**	538.2508	**1074.5102**	537.7587	T	**604.3301**	302.6687	587.3035	294.1554	586.3195	293.6634	5
10	**1221.5634**	611.2853	**1204.5368**	602.7720	**1203.5528**	602.2800	E	**503.2824**	252.1448	486.2558	243.6316	485.2718	243.1395	4
11	**1335.6063**	668.3068	**1318.5797**	**659.7935**	1317.5957	659.3015	N	**374.2398**	187.6235	**357.2132**	179.1103			3
12	**1448.6904**	724.8488	**1431.6638**	716.3355	**1430.6798**	715.8435	I	260.1969	130.6021	243.1703	122.0888			2
13							K	147.1128	74.0600	130.0863	65.5468			1

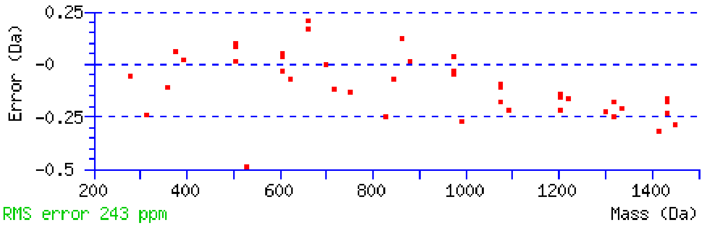

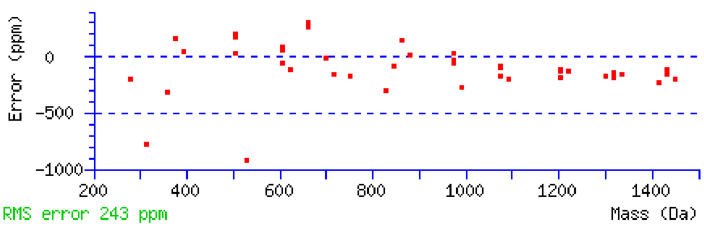

All matches to this query

Score	Mr(calc):	Delta	Sequence
61.1	1593.7886	0.0024	YLNETQQLTENIK
36.2	1593.7886	0.0024	YLNETQQLTENIK
18.7	1592.7728	1.0182	AILGANRTLTYIK
11.8	1593.7763	0.0147	EQARISLLNDGGLK
7.0	1593.7928	-0.0018	IICTTQMLENMIK
5.0	1593.7803	0.0107	SLFPEGNPAKVNLK
3.5	1593.7817	0.0093	YIPNRGPLDKWR
3.4	1592.7728	1.0182	AILGANRTLTYIK
2.9	1593.7956	-0.0046	LNINKFNFKTFK
1.6	1591.7929	1.9981	RWNFIQNGAIMNK

Spectrum No: 314; Query: 903; Rank: 1

Peptide View

MS/MS Fragmentation of **ASFNITLIHPNNLTALSNMLPK**
Found in **IPI00230862**, Tax_Id=10116 Gene_Symbol=Anpep Aminopeptidase N

Match to Query 903: 2426.255202 from(809.759010,3+)
Title: 091127RatKid_SCX01_15.4597.4597.3.dta
Data file K:\NewmanPaper\Piliang\3SubProteomes\Piliang3SP\mgf5ppm\SCX_3SubProteomes5ppm.mgf

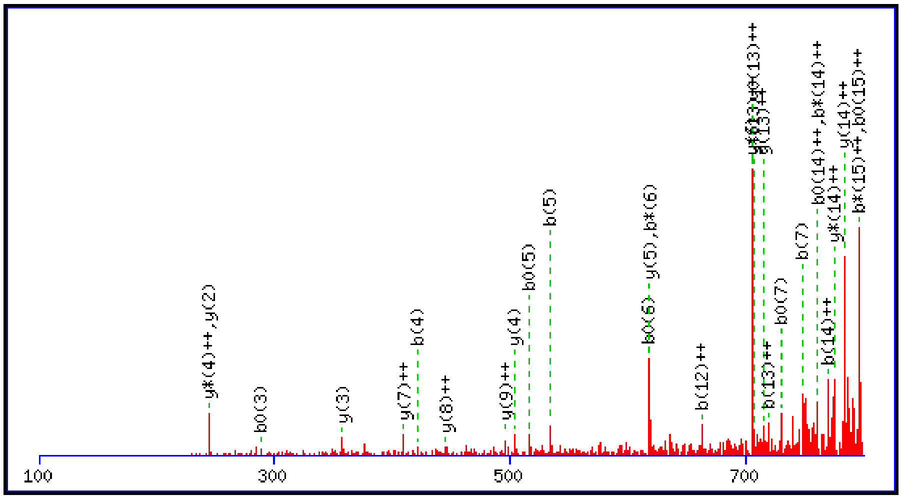

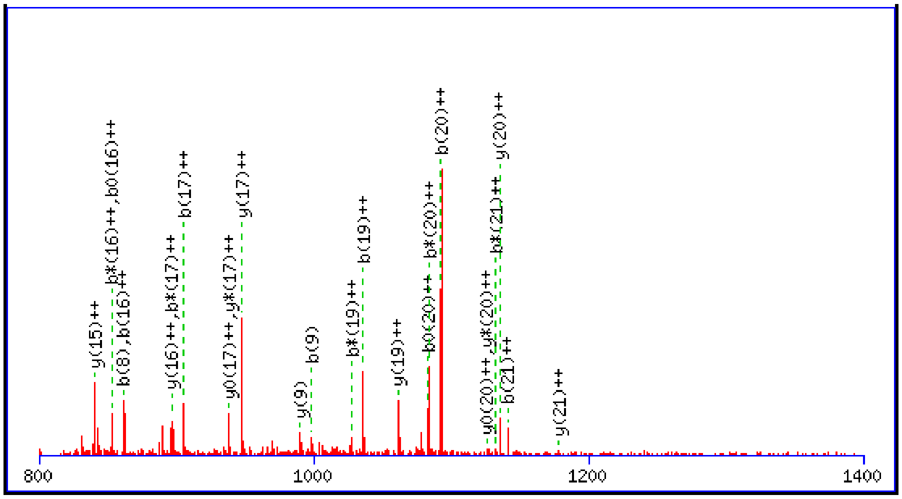

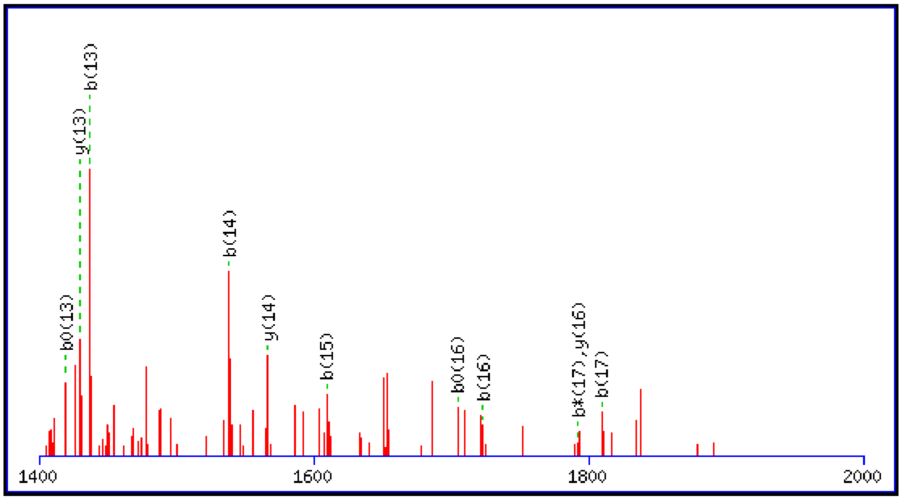

Monoisotopic mass of neutral peptide Mr(calc): 2426.2515
Fixed modifications: Carbamidomethyl (C)
Variable modifications:
N4 : Deamidated_N (N)
N12 : Deamidated_N (N)
M19 : Oxidation (M)
Ions Score: 61 **Expect:** 0.0001
Matches (Bold Red): 65/234 fragment ions using 108 most intense peaks

#	b	b++	b*	b*++	b0	b0++	Seq.	y	y++	y*	y*++	y0	y0++	#
1	72.0444	36.5258					A							22
2	159.0764	80.0418			141.0659	71.0366	S	2356.2217	**1178.6145**	2339.1952	1170.1012	2338.2111	1169.6092	21
3	306.1448	153.5761			**288.1343**	144.5708	F	2269.1897	**1135.0985**	2252.1631	**1126.5852**	2251.1791	**1126.0932**	20
4	**421.1718**	211.0895	404.1452	202.5762	403.1612	202.0842	N	2122.1213	**1061.5643**	2105.0947	1053.0510	2104.1107	1052.5590	19
5	**534.2558**	267.6316	517.2293	259.1183	**516.2453**	258.6263	I	2007.0943	1004.0508	1990.0678	995.5375	1989.0838	995.0455	18
6	635.3035	318.1554	**618.2770**	309.6421	**617.2929**	309.1501	T	1894.0103	**947.5088**	1876.9837	**938.9955**	1875.9997	**938.5035**	17
7	**748.3876**	374.6974	731.3610	366.1842	**730.3770**	365.6921	L	**1792.9626**	**896.9849**	1775.9360	888.4717	1774.9520	887.9796	16
8	**861.4716**	431.2395	844.4451	422.7262	843.4611	422.2342	I	1679.8785	**840.4429**	1662.8520	831.9296	1661.8680	831.4376	15
9	**998.5306**	499.7689	981.5040	491.2556	980.5200	490.7636	H	**1566.7945**	**783.9009**	1549.7679	**775.3876**	1548.7839	774.8956	14
10	1095.5833	548.2953	1078.5568	539.7820	1077.5728	539.2900	P	**1429.7355**	**715.3714**	1412.7090	**706.8581**	1411.7250	**706.3661**	13
11	1209.6262	605.3168	1192.5997	596.8035	1191.6157	596.3115	N	1332.6828	666.8450	1315.6562	658.3318	1314.6722	657.8397	12
12	1324.6532	**662.8302**	1307.6266	654.3170	1306.6426	653.8249	N	1218.6398	609.8236	1201.6133	601.3103	1200.6293	600.8183	11
13	**1437.7372**	**719.3723**	1420.7107	710.8590	**1419.7267**	710.3670	L	1103.6129	552.3101	1086.5864	543.7968	1085.6023	543.3048	10
14	**1538.7849**	**769.8961**	1521.7584	**761.3828**	1520.7744	**760.8908**	T	**990.5288**	**495.7681**	973.5023	487.2548	972.5183	486.7628	9
15	**1609.8220**	805.4147	1592.7955	**796.9014**	1591.8115	**796.4094**	A	889.4812	**445.2442**	872.4546	436.7309	871.4706	436.2389	8
16	**1722.9061**	**861.9567**	1705.8795	**853.4434**	**1704.8955**	**852.9514**	L	818.4441	**409.7257**	801.4175	401.2124	800.4335	400.7204	7
17	**1809.9381**	**905.4727**	**1792.9116**	**896.9594**	1791.9276	896.4674	S	**705.3600**	353.1836	688.3334	344.6704	687.3494	344.1784	6

18	1923.9811	962.4942	1906.9545	953.9809	1905.9705	953.4889	N	**618.3280**	309.6676	601.3014	301.1543			5
19	2071.0165	**1036.0119**	2053.9899	**1027.4986**	2053.0059	1027.0066	M	**504.2850**	252.6462	487.2585	**244.1329**			4
20	2184.1005	**1092.5539**	2167.0740	**1084.0406**	2166.0900	**1083.5486**	L	**357.2496**	179.1285	340.2231	170.6152			3
21	2281.1533	**1141.0803**	2264.1267	**1132.5670**	2263.1427	1132.0750	P	**244.1656**	122.5864	227.1390	114.0731			2
22							K	147.1128	74.0600	130.0863	65.5468			1

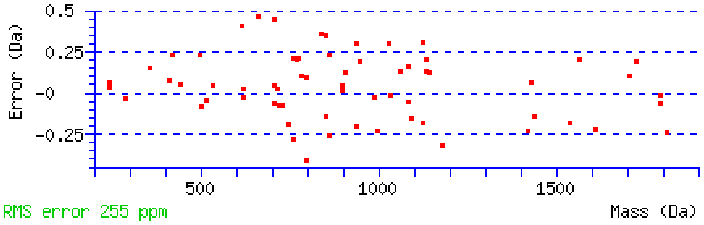

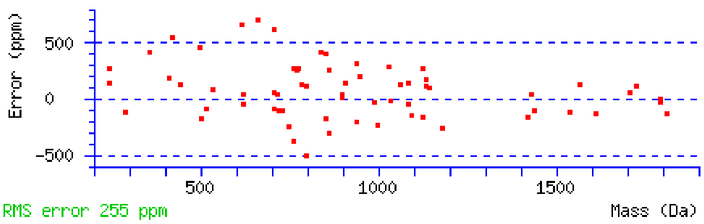

All matches to this query

Score	Mr(calc):	Delta	Sequence
61.0	2426.2515	0.0037	ASFNITLIHPNNLTALSNMLPK
61.0	2426.2515	0.0037	ASFNITLIHPNNLTALSNMLPK
58.4	2426.2515	0.0037	ASFNITLIHPNNLTALSNMLPK
52.6	2426.2515	0.0037	ASFNITLIHPNNLTALSNMLPK
34.3	2426.2515	0.0037	ASFNITLIHPNNLTALSNMLPK
34.3	2426.2515	0.0037	ASFNITLIHPNNLTALSNMLPK
26.8	2425.2675	0.9877	ASFNITLIHPNNLTALSNMLPK
26.8	2425.2675	0.9877	ASFNITLIHPNNLTALSNMLPK
21.3	2425.2675	0.9877	ASFNITLIHPNNLTALSNMLPK
18.4	2425.2675	0.9877	ASFNITLIHPNNLTALSNMLPK

Spectrum No: 315; Query: 819; Rank: 1

Peptide View

MS/MS Fragmentation of **ETQSPAPLQNSSGDPAEEESPV**
Found in **IPI00214787**, Tax_Id=10116 Gene_Symbol=Slc16a1 Monocarboxylate transporter 1

Match to Query 819: 2268.990188 from(1135.502370,2+)
Title: 091127RatKid_SCX01_03.2132.2132.2.dta
Data file K:\NewmanPaper\Piliang\3SubProteomes\Piliang3SP\mgf5ppm\SCX_3SubProteomes5ppm.mgf

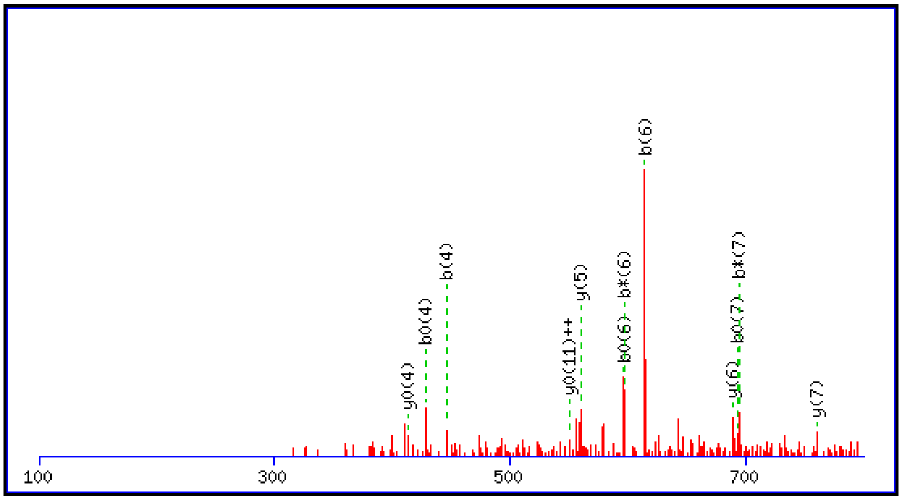

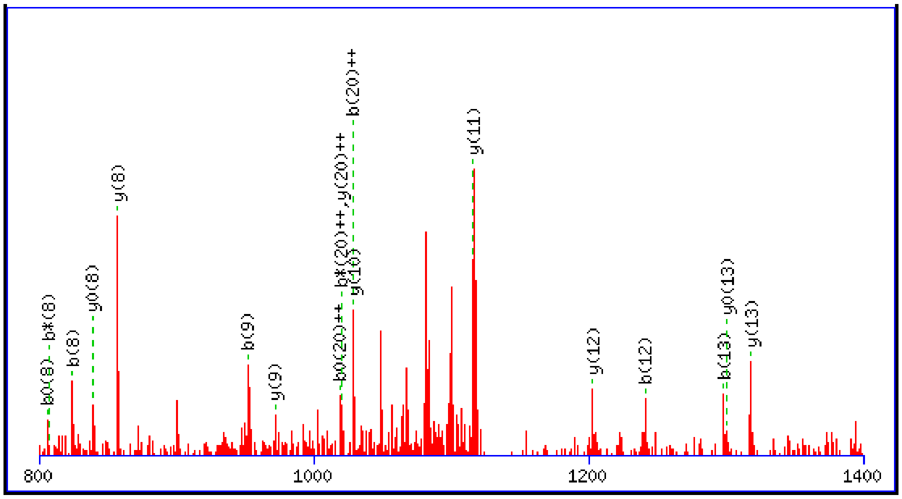

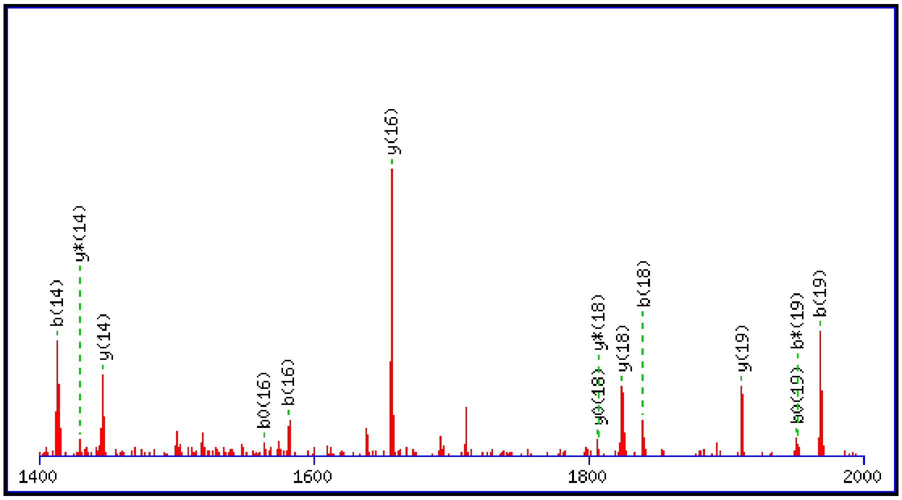

Monoisotopic mass of neutral peptide Mr(calc): 2268.9870
Fixed modifications: Carbamidomethyl (C)
Variable modifications:
N10 : Deamidated_N (N)
Ions Score: 61 **Expect:** 0.00024
Matches (Bold Red): 44/220 fragment ions using 97 most intense peaks

#	b	b^{++}	b*	b^{*++}	b^{0}	b^{0++}	Seq.	y	y^{++}	y*	y^{*++}	y^{0}	y^{0++}	#
1	130.0499	65.5286			112.0393	56.5233	E							22
2	231.0975	116.0524			213.0870	107.0471	T	2140.9517	1070.9795	2123.9251	1062.4662	2122.9411	1061.9742	21
3	359.1561	180.0817	342.1296	171.5684	341.1456	171.0764	Q	2039.9040	**1020.4556**	2022.8774	1011.9424	2021.8934	1011.4504	20
4	**446.1882**	223.5977	429.1616	215.0844	**428.1776**	214.5924	S	**1911.8454**	956.4263	1894.8189	947.9131	1893.8349	947.4211	19
5	543.2409	272.1241	526.2144	263.6108	525.2304	263.1188	P	**1824.8134**	912.9103	**1807.7868**	904.3971	**1806.8028**	903.9050	18
6	**614.2780**	307.6427	**597.2515**	299.1294	**596.2675**	298.6374	A	1727.7606	864.3839	1710.7341	855.8707	1709.7501	855.3787	17
7	711.3308	356.1690	**694.3042**	347.6558	**693.3202**	347.1638	P	**1656.7235**	828.8654	1639.6970	820.3521	1638.7129	819.8601	16
8	**824.4149**	412.7111	**807.3883**	404.1978	**806.4043**	403.7058	L	1559.6707	780.3390	1542.6442	771.8257	1541.6602	771.3337	15
9	**952.4734**	476.7404	935.4469	468.2271	934.4629	467.7351	Q	**1446.5867**	723.7970	**1429.5601**	715.2837	1428.5761	714.7917	14
10	1067.5004	534.2538	1050.4738	525.7406	1049.4898	525.2485	N	**1318.5281**	659.7677	1301.5016	651.2544	**1300.5175**	650.7624	13
11	1154.5324	577.7698	1137.5059	569.2566	1136.5218	568.7646	S	**1203.5012**	602.2542			1185.4906	593.2489	12
12	**1241.5644**	621.2859	1224.5379	612.7726	1223.5539	612.2806	S	**1116.4691**	558.7382			1098.4586	**549.7329**	11
13	**1298.5859**	649.7966	1281.5593	641.2833	1280.5753	640.7913	G	**1029.4371**	515.2222			1011.4265	506.2169	10
14	**1413.6128**	707.3101	1396.5863	698.7968	1395.6023	698.3048	D	**972.4156**	486.7115			954.4051	477.7062	9
15	1510.6656	755.8364	1493.6391	747.3232	1492.6550	746.8312	P	**857.3887**	429.1980			**839.3781**	420.1927	8
16	**1581.7027**	791.3550	1564.6762	782.8417	**1563.6922**	782.3497	A	**760.3359**	380.6716			742.3254	371.6663	7
17	1710.7453	855.8763	1693.7188	847.3630	1692.7347	846.8710	E	**689.2988**	345.1531			671.2883	336.1478	6
18	**1839.7879**	920.3976	1822.7614	911.8843	1821.7773	911.3923	E	**560.2562**	280.6318			542.2457	271.6265	5

**19**	**1968.8305**	984.9189	**1951.8039**	976.4056	**1950.8199**	975.9136	**E**	431.2136	216.1105			**413.2031**	207.1052	**4**
**20**	2055.8625	**1028.4349**	2038.8360	**1019.9216**	2037.8520	**1019.4296**	**S**	302.1710	151.5892			284.1605	142.5839	**3**
**21**	2152.9153	1076.9613	2135.8887	1068.4480	2134.9047	1067.9560	**P**	215.1390	108.0731					**2**
**22**							**V**	118.0863	59.5468					**1**

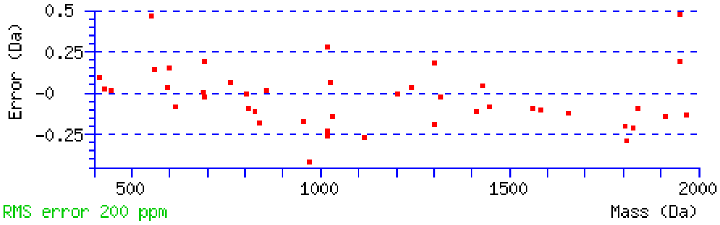

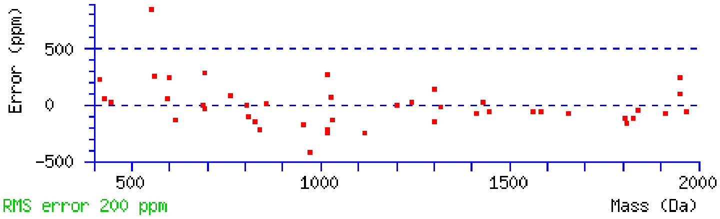

All matches to this query

Score	Mr(calc):	Delta	Sequence
60.8	2268.9870	0.0032	ETQSPAPLQNSSGDPAEEESPV
35.5	2268.0030	0.9872	ETQSPAPLQNSSGDPAEEESPV
3.0	2268.9864	0.0038	ERHHSMLQFQVPSSSLAR

Spectrum No: 316; Query: 112; Rank: 1

Peptide View

MS/MS Fragmentation of **NGTGVLILSPTR**
Found in **IPI00367078**, Tax_Id=10116 Gene_Symbol=Ddx18 DEAD (Asp-Glu-Ala-Asp) box polypeptide 18

Match to Query 112: 1227.683428 from(614.848990,2+)
Title: 091127RatKid_SCX01_15.2470.2470.2.dta
Data file K:\NewmanPaper\Piliang\3SubProteomes\Piliang3SP\mgf5ppm\SCX_3SubProteomes5ppm.mgf

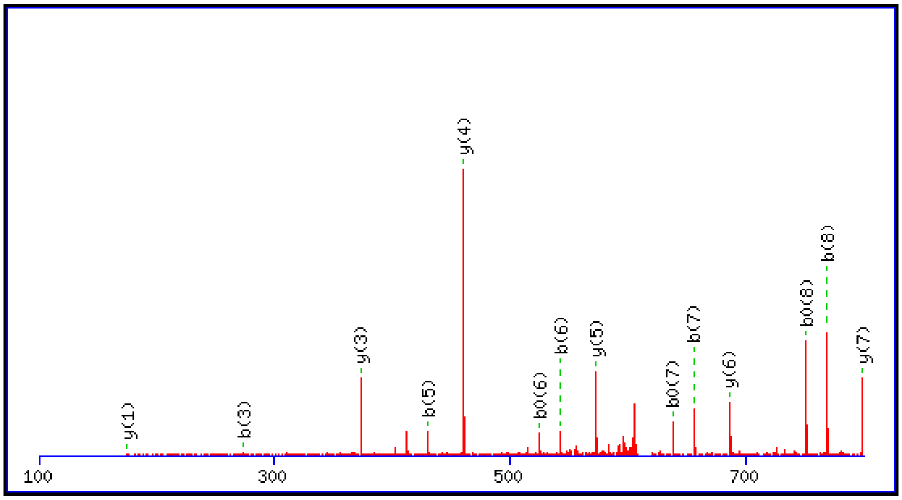

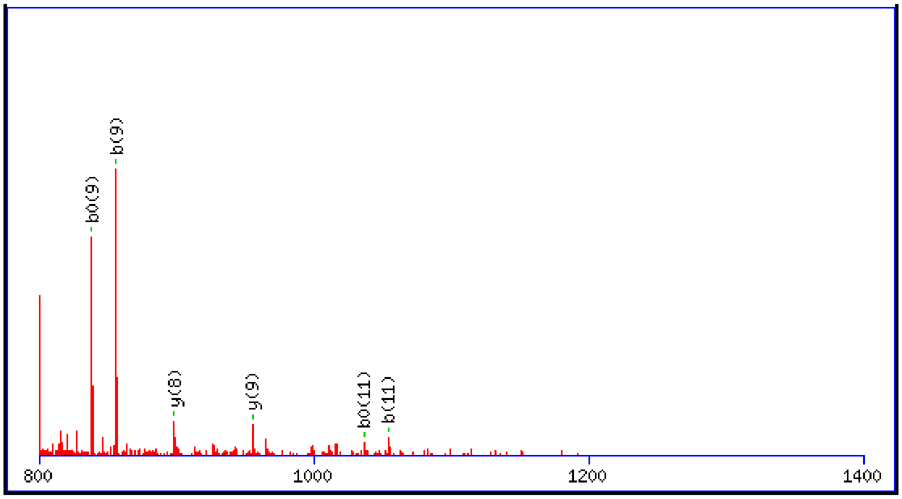

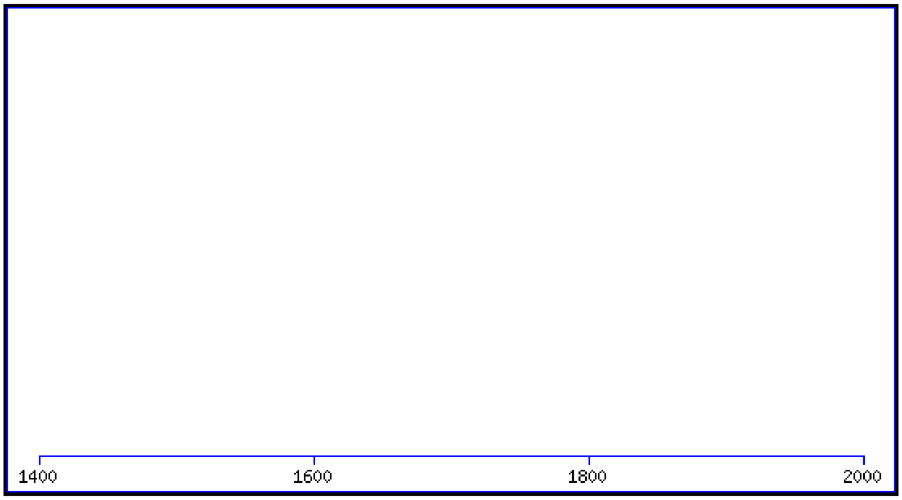

Monoisotopic mass of neutral peptide Mr(calc): 1227.6823
Fixed modifications: Carbamidomethyl (C)
Variable modifications:
N1 : Deamidated_N (N)
Ions Score: 61 **Expect:** 5.5e-005
Matches (Bold Red): 20/126 fragment ions using 36 most intense peaks

#	b	b^{++}	b*	b^{*++}	b^{0}	b^{0++}	Seq.	y	y^{++}	y*	y^{*++}	y^{0}	y^{0++}	#
1	116.0342	58.5207	99.0077	50.0075			N							12
2	173.0557	87.0315	156.0291	78.5182			G	1113.6626	557.3350	1096.6361	548.8217	1095.6521	548.3297	11
3	**274.1034**	137.5553	257.0768	129.0420	256.0928	128.5500	T	1056.6412	528.8242	1039.6146	520.3109	1038.6306	519.8189	10
4	331.1248	166.0660	314.0983	157.5528	313.1143	157.0608	G	**955.5935**	478.3004	938.5669	469.7871	937.5829	469.2951	9
5	**430.1932**	215.6003	413.1667	207.0870	412.1827	206.5950	V	**898.5720**	449.7897	881.5455	441.2764	880.5615	440.7844	8
6	**543.2773**	272.1423	526.2507	263.6290	**525.2667**	263.1370	L	**799.5036**	400.2554	782.4771	391.7422	781.4930	391.2502	7
7	**656.3614**	328.6843	639.3348	320.1710	**638.3508**	319.6790	I	**686.4196**	343.7134	669.3930	335.2001	668.4090	334.7081	6
8	**769.4454**	385.2264	752.4189	376.7131	**751.4349**	376.2211	L	**573.3355**	287.1714	556.3089	278.6581	555.3249	278.1661	5
9	**856.4775**	428.7424	839.4509	420.2291	**838.4669**	419.7371	S	**460.2514**	230.6293	443.2249	222.1161	442.2409	221.6241	4
10	953.5302	477.2687	936.5037	468.7555	935.5197	468.2635	P	**373.2194**	187.1133	356.1928	178.6001	355.2088	178.1081	3
11	**1054.5779**	527.7926	1037.5513	519.2793	**1036.5673**	518.7873	T	276.1666	138.5870	259.1401	130.0737	258.1561	129.5817	2
12							R	**175.1190**	88.0631	158.0924	79.5498			1

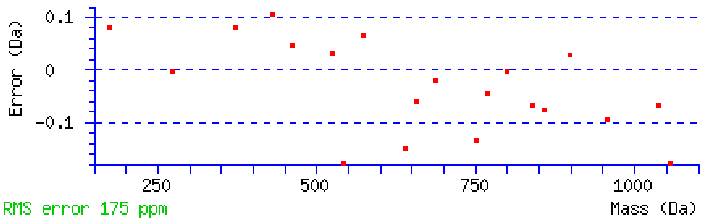

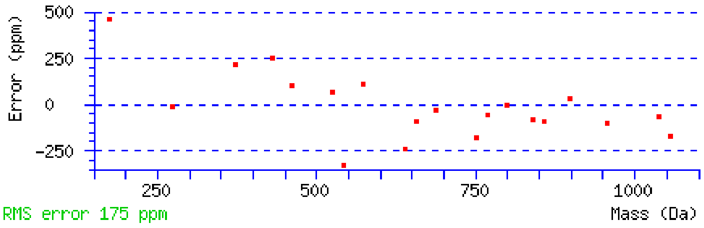

All matches to this query

Score	Mr(calc):	Delta	Sequence
60.7	1227.6823	0.0011	NGTGVLILSPTR
9.9	1227.6935	-0.0101	VGADITVLRER
9.0	1226.6758	1.0077	ELPENALSILK
8.9	1227.6724	0.0111	HYLEIELRR
8.5	1225.6853	1.9982	KPPLVMTSPTR
6.0	1227.6935	-0.0101	ITDDQLRVLR
5.9	1226.6771	1.0063	WQNIELAVVR
5.9	1225.6779	2.0055	VDTVRVVEGPR
5.0	1226.6731	1.0103	LDARTGALSAPR
3.1	1226.6843	0.9991	ERELQLQRR

Spectrum No: 317; Query: 462; Rank: 1

Peptide View

MS/MS Fragmentation of **NSSYFVEWIPNNVK**
Found in **IPI00195673**, Tax_Id=10116 Gene_Symbol=Tubb6 Tubulin, beta 6

Match to Query 462: 1696.814928 from(849.414740,2+)
Title: 091129RatKid_SCX02_13.4012.4012.2.dta
Data file K:\NewmanPaper\Piliang\3SubProteomes\Piliang3SP\mgf5ppm\SCX_3SubProteomes5ppm.mgf

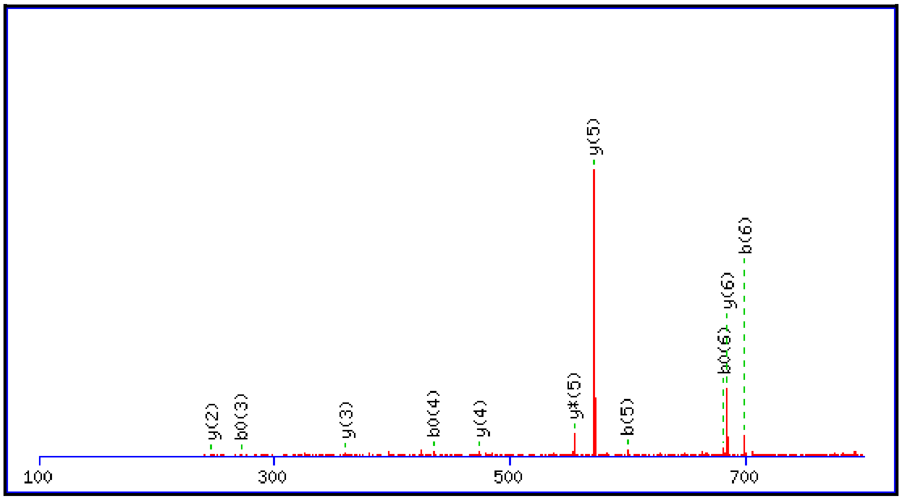

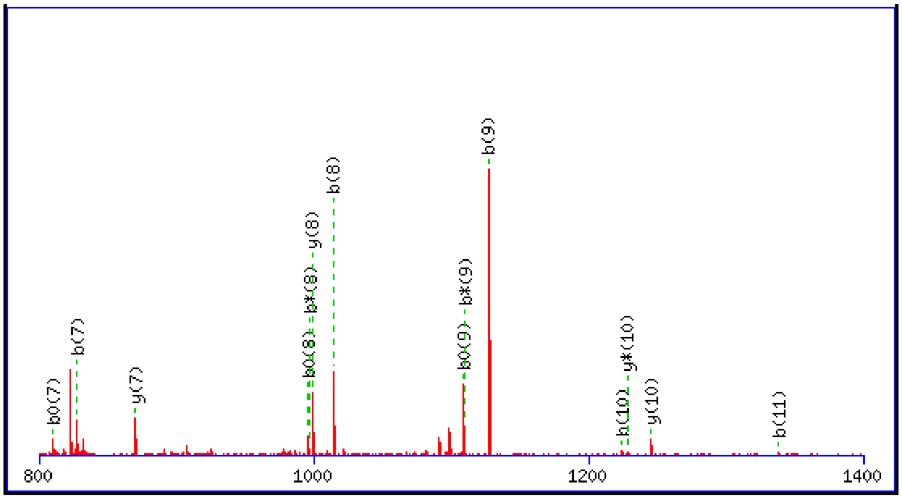

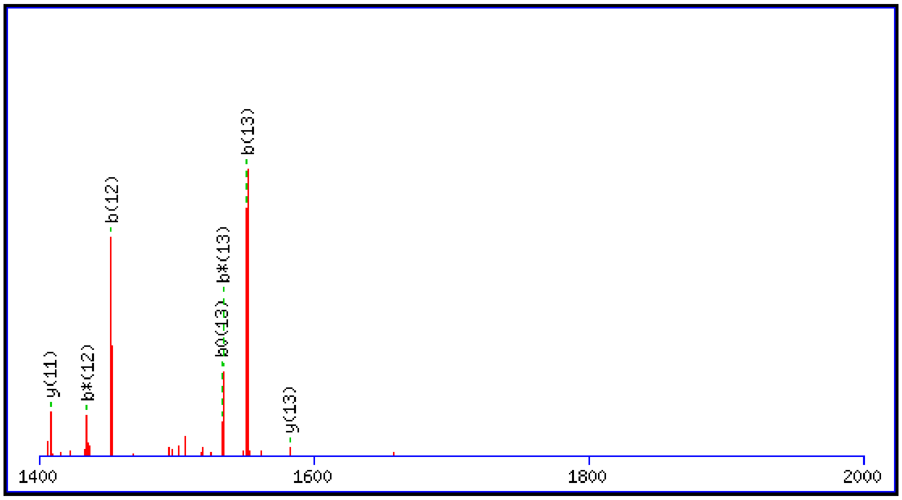

Monoisotopic mass of neutral peptide Mr(calc): 1696.8097
Fixed modifications: Carbamidomethyl (C)
Variable modifications:
N1 : Deamidated_N (N)
Ions Score: 61 **Expect:** 0.00016
Matches (Bold Red): 32/140 fragment ions using 62 most intense peaks

#	b	b^{++}	b*	b^{*++}	b^0	b^{0++}	Seq.	y	y^{++}	y*	y^{*++}	y^0	y^{0++}	#
1	116.0342	58.5207	99.0077	50.0075			N							14
2	203.0662	102.0368	186.0397	93.5235	185.0557	93.0315	S	**1582.7900**	791.8986	1565.7635	783.3854	1564.7795	782.8934	13
3	290.0983	145.5528	273.0717	137.0395	**272.0877**	136.5475	S	1495.7580	748.3826	1478.7314	739.8694	1477.7474	739.3774	12
4	453.1616	227.0844	436.1350	218.5712	**435.1510**	218.0792	Y	**1408.7260**	704.8666	1391.6994	696.3533	1390.7154	695.8613	11
5	**600.2300**	300.6186	583.2035	292.1054	582.2194	291.6134	F	**1245.6626**	623.3350	**1228.6361**	614.8217	1227.6521	614.3297	10
6	**699.2984**	350.1529	682.2719	341.6396	**681.2879**	341.1476	V	1098.5942	549.8007	1081.5677	541.2875	1080.5837	540.7955	9
7	**828.3410**	414.6741	811.3145	406.1609	**810.3305**	405.6689	E	**999.5258**	500.2665	982.4993	491.7533	981.5152	491.2613	8
8	**1014.4203**	507.7138	**997.3938**	499.2005	**996.4098**	498.7085	W	**870.4832**	435.7452	853.4567	427.2320			7
9	**1127.5044**	564.2558	**1110.4778**	555.7426	**1109.4938**	555.2506	I	**684.4039**	342.7056	667.3774	334.1923			6
10	**1224.5572**	612.7822	1207.5306	604.2689	1206.5466	603.7769	P	**571.3198**	286.1636	**554.2933**	277.6503			5
11	**1338.6001**	669.8037	1321.5735	661.2904	1320.5895	660.7984	N	**474.2671**	237.6372	457.2405	229.1239			4
12	**1452.6430**	726.8251	**1435.6165**	718.3119	1434.6324	717.8199	N	**360.2241**	180.6157	343.1976	172.1024			3
13	**1551.7114**	776.3594	**1534.6849**	767.8461	**1533.7009**	767.3541	V	**246.1812**	123.5942	229.1547	115.0810			2
14							K	147.1128	74.0600	130.0863	65.5468			1

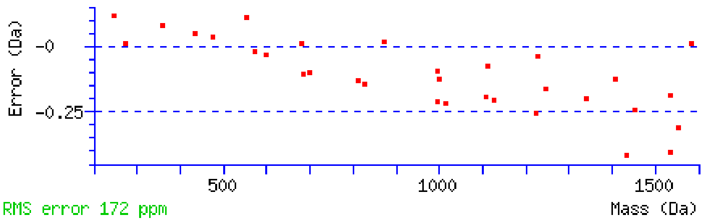

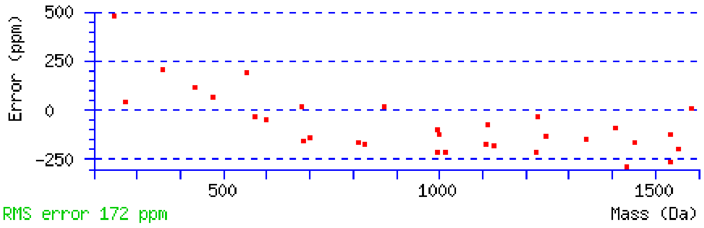

All matches to this query

Score	Mr(calc):	Delta	Sequence
60.6	1696.8097	0.0053	NSSYFVEWIPNNVK
28.9	1695.8257	0.9893	NSSYFVEWIPNNVK
16.9	1696.8097	0.0053	NSSYFVEWIPNNVK
16.1	1696.8259	-0.0110	KLCVFSPSQPLDVK
15.0	1696.8259	-0.0110	KLCVFSPSQPLDVK
10.7	1696.8308	-0.0159	YQDEYGVSSVIPNVK
6.5	1695.8262	0.9887	YARILAFLQSKAR
5.5	1696.8317	-0.0168	IIAHTYCEFMAVVK
3.9	1696.8233	-0.0083	MQRRGALFSVPGGGGK
3.0	1696.8281	-0.0132	QGNAVTLGDYYQGRR

Spectrum No: 318; Query: 503; Rank: 1

Peptide View

MS/MS Fragmentation of **GAVSILCNSSGNCQCK**
Found in **IPI00357926**, Tax_Id=10116 Gene_Symbol=Egfl5_predicted EGF-like-domain, multiple 5

Match to Query 503: 1754.744588 from(878.379570,2+)
Title: 091129RatKid_SCX02_13.1015.1015.2.dta
Data file K:\NewmanPaper\Piliang\3SubProteomes\Piliang3SP\mgf5ppm\SCX_3SubProteomes5ppm.mgf

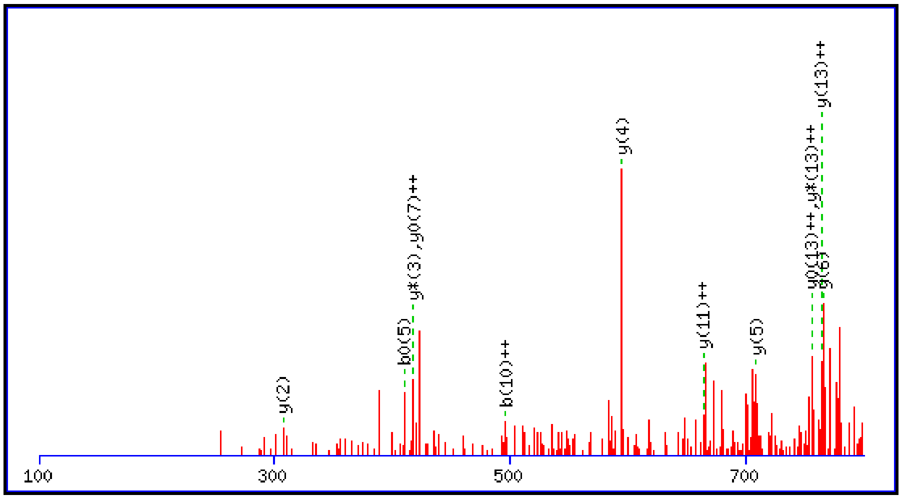

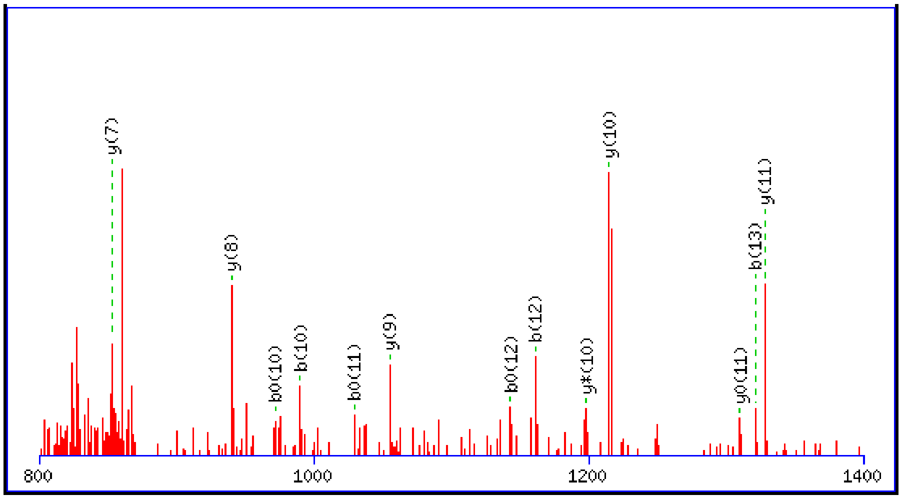

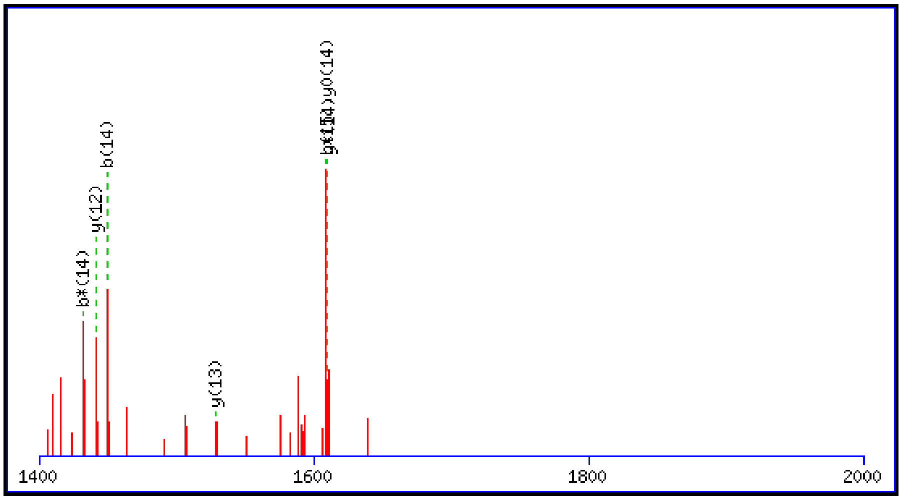

Monoisotopic mass of neutral peptide Mr(calc): 1754.7386
Fixed modifications: Carbamidomethyl (C)
Variable modifications:
N8 : Deamidated_N (N)
Ions Score: 61 **Expect:** 0.00015
Matches (Bold Red): 32/148 fragment ions using 62 most intense peaks

#	b	b^{++}	b*	b^{*++}	b^{0}	b^{0++}	Seq.	y	y^{++}	y*	y^{*++}	y^{0}	y^{0++}	#
1	58.0287	29.5180					G							16
2	129.0659	65.0366					A	1698.7244	849.8658	1681.6978	841.3526	1680.7138	840.8606	15
3	228.1343	114.5708					V	1627.6873	814.3473	**1610.6607**	805.8340	**1609.6767**	805.3420	14
4	315.1663	158.0868			297.1557	149.0815	S	**1528.6189**	**764.8131**	1511.5923	**756.2998**	1510.6083	**755.8078**	13
5	428.2504	214.6288			**410.2398**	205.6235	I	**1441.5868**	721.2971	1424.5603	712.7838	1423.5763	712.2918	12
6	541.3344	271.1709			523.3239	262.1656	L	**1328.5028**	**664.7550**	1311.4762	656.2418	**1310.4922**	655.7497	11
7	701.3651	351.1862			683.3545	342.1809	C	**1215.4187**	608.2130	**1198.3922**	599.6997	1197.4081	599.2077	10
8	816.3920	408.6996	799.3655	400.1864	798.3814	399.6944	N	**1055.3881**	528.1977	1038.3615	519.6844	1037.3775	519.1924	9
9	903.4240	452.2157	886.3975	443.7024	885.4135	443.2104	S	**940.3611**	470.6842	923.3346	462.1709	922.3506	461.6789	8
10	**990.4561**	**495.7317**	973.4295	487.2184	**972.4455**	486.7264	S	**853.3291**	427.1682	836.3025	418.6549	835.3185	**418.1629**	7
11	1047.4775	524.2424	1030.4510	515.7291	**1029.4670**	515.2371	G	**766.2971**	383.6522	749.2705	375.1389			6
12	**1161.5205**	581.2639	1144.4939	572.7506	**1143.5099**	572.2586	N	**709.2756**	355.1414	692.2491	346.6282			5
13	**1321.5511**	661.2792	1304.5246	652.7659	1303.5405	652.2739	C	**595.2327**	298.1200	578.2061	289.6067			4
14	**1449.6097**	725.3085	**1432.5831**	716.7952	1431.5991	716.3032	Q	435.2020	218.1047	**418.1755**	209.5914			3
15	**1609.6403**	805.3238	1592.6138	796.8105	1591.6298	796.3185	C	**307.1435**	154.0754	290.1169	145.5621			2
16							K	147.1128	74.0600	130.0863	65.5468			1

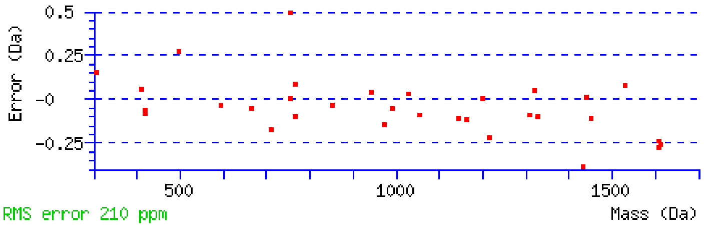

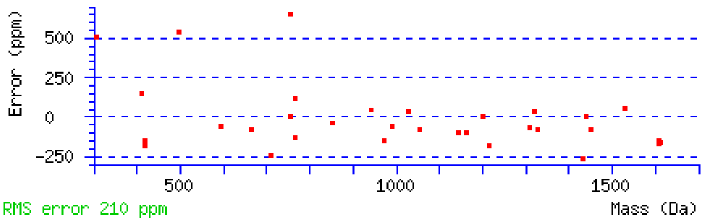

All matches to this query

Score	Mr(calc):	Delta	Sequence
60.6	1754.7386	0.0060	GAVSILCNSSGNCQCK
27.4	1754.7386	0.0060	GAVSILCNSSGNCQCK
5.6	1754.7390	0.0056	RGIAGSSGSSVSNFLR
5.6	1754.7390	0.0056	RGIAGSSGSSVSNFLR
2.2	1753.7546	0.9900	GAVSILCNSSGNCQCK
0.4	1753.7399	1.0047	TYLECNEPKLSLK
0.0	1753.7399	1.0047	TYLECNEPKLSLK
0.0	1754.7326	0.0120	MISFIKSFCKYR

Spectrum No: 319; Query: 462; Rank: 1

Peptide View

MS/MS Fragmentation of **NSSYFVEWIPNNVK**
Found in **IPI00195673**, Tax_Id=10116 Gene_Symbol=Tubb6 Tubulin, beta 6

Match to Query 462: 1696.814928 from(849.414740,2+)
Title: 091129RatKid_SCX02_13.4012.4012.2.dta
Data file K:\NewmanPaper\Piliang\3SubProteomes\Piliang3SP\mgf5ppm\SCX_3SubProteomes5ppm.mgf

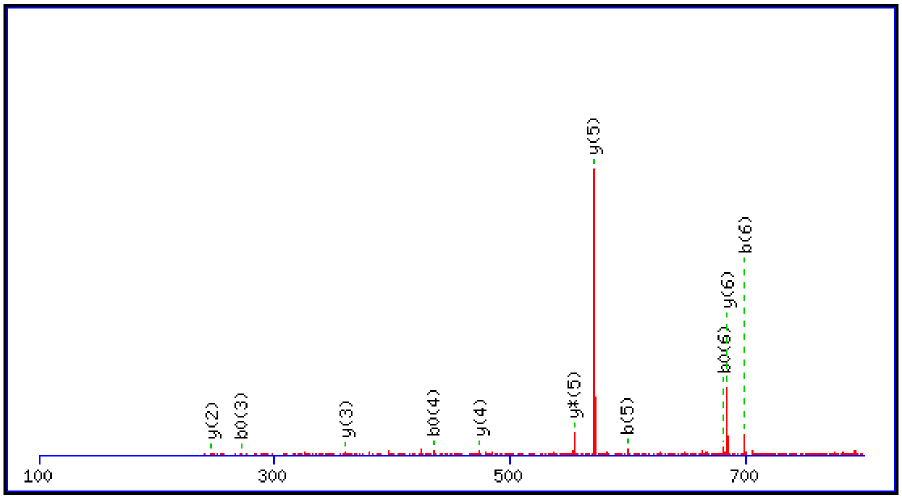

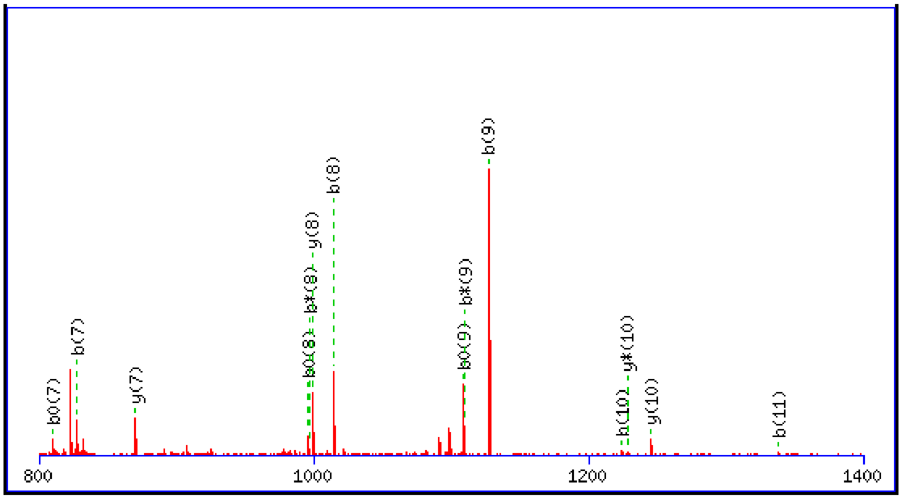

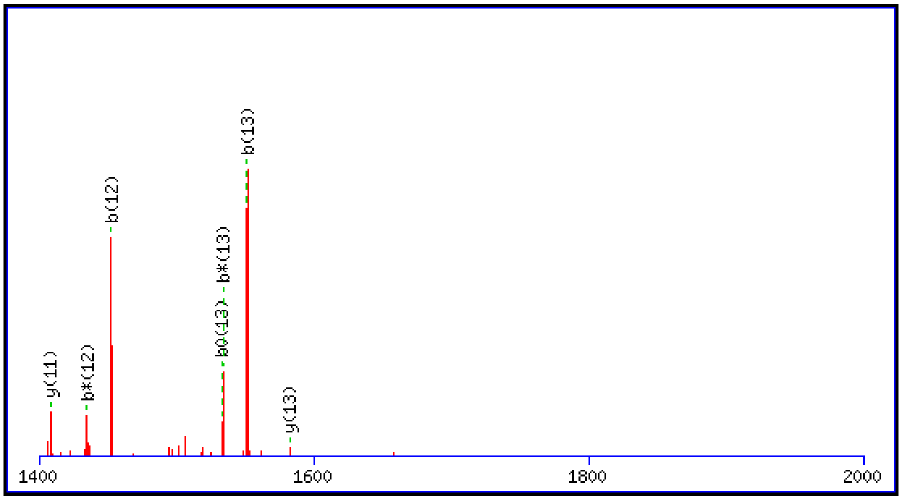

Monoisotopic mass of neutral peptide Mr(calc): 1696.8097
Fixed modifications: Carbamidomethyl (C)
Variable modifications:
N1 : Deamidated_N (N)
Ions Score: 61 **Expect:** 0.00016
Matches (Bold Red): 32/140 fragment ions using 62 most intense peaks

#	b	b^{++}	b*	b^{*++}	b^0	b^{0++}	Seq.	y	y^{++}	y*	y^{*++}	y^0	y^{0++}	#
1	116.0342	58.5207	99.0077	50.0075			N							14
2	203.0662	102.0368	186.0397	93.5235	185.0557	93.0315	S	**1582.7900**	791.8986	1565.7635	783.3854	1564.7795	782.8934	13
3	290.0983	145.5528	273.0717	137.0395	**272.0877**	136.5475	S	1495.7580	748.3826	1478.7314	739.8694	1477.7474	739.3774	12
4	453.1616	227.0844	436.1350	218.5712	**435.1510**	218.0792	Y	**1408.7260**	704.8666	1391.6994	696.3533	1390.7154	695.8613	11
5	**600.2300**	300.6186	583.2035	292.1054	582.2194	291.6134	F	**1245.6626**	623.3350	**1228.6361**	614.8217	1227.6521	614.3297	10
6	**699.2984**	350.1529	682.2719	341.6396	**681.2879**	341.1476	V	1098.5942	549.8007	1081.5677	541.2875	1080.5837	540.7955	9
7	**828.3410**	414.6741	811.3145	406.1609	**810.3305**	405.6689	E	**999.5258**	500.2665	982.4993	491.7533	981.5152	491.2613	8
8	**1014.4203**	507.7138	**997.3938**	499.2005	**996.4098**	498.7085	W	**870.4832**	435.7452	853.4567	427.2320			7
9	**1127.5044**	564.2558	**1110.4778**	555.7426	**1109.4938**	555.2506	I	**684.4039**	342.7056	667.3774	334.1923			6
10	**1224.5572**	612.7822	1207.5306	604.2689	1206.5466	603.7769	P	**571.3198**	286.1636	**554.2933**	277.6503			5
11	**1338.6001**	669.8037	1321.5735	661.2904	1320.5895	660.7984	N	**474.2671**	237.6372	457.2405	229.1239			4
12	**1452.6430**	726.8251	**1435.6165**	718.3119	1434.6324	717.8199	N	**360.2241**	180.6157	343.1976	172.1024			3
13	**1551.7114**	776.3594	**1534.6849**	767.8461	**1533.7009**	767.3541	V	**246.1812**	123.5942	229.1547	115.0810			2
14							K	147.1128	74.0600	130.0863	65.5468			1

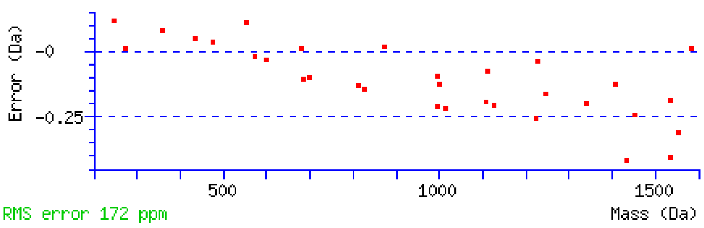

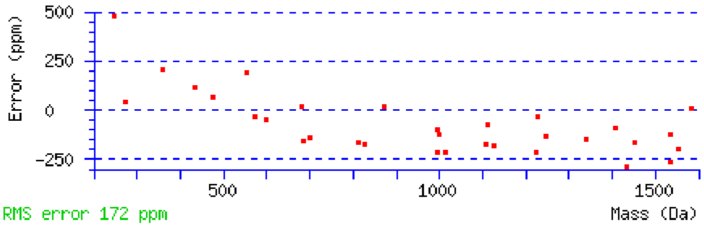

All matches to this query

Score	Mr(calc):	Delta	Sequence
60.6	1696.8097	0.0053	NSSYFVEWIPNNVK
28.9	1695.8257	0.9893	NSSYFVEWIPNNVK
16.9	1696.8097	0.0053	NSSYFVEWIPNNVK
16.1	1696.8259	-0.0110	KLCVFSPSQPLDVK
15.0	1696.8259	-0.0110	KLCVFSPSQPLDVK
10.7	1696.8308	-0.0159	YQDEYGVSSVIPNVK
6.5	1695.8262	0.9887	YARILAFLQSKAR
5.5	1696.8317	-0.0168	IIAHTYCEFMAVVK
3.9	1696.8233	-0.0083	MQRRGALFSVPGGGGK
3.0	1696.8281	-0.0132	QGNAVTLGDYYQGRR

Spectrum No: 320; Query: 1126; Rank: 1

Peptide View

MS/MS Fragmentation of **MYVTNDTEVAENNYQALKDFYHLFPEYK**
Found in **IPI00464785**, Tax_Id=10116 Gene_Symbol=Ppgb Protective protein for beta-galactosidase

Match to Query 1126: 3442.582782 from(1148.534870,3+)
Title: 091127RatKid_SCX01_29.5808.5808.3.dta
Data file K:\NewmanPaper\Piliang\3SubProteomes\Piliang3SP\mgf5ppm\SCX_3SubProteomes5ppm.mgf

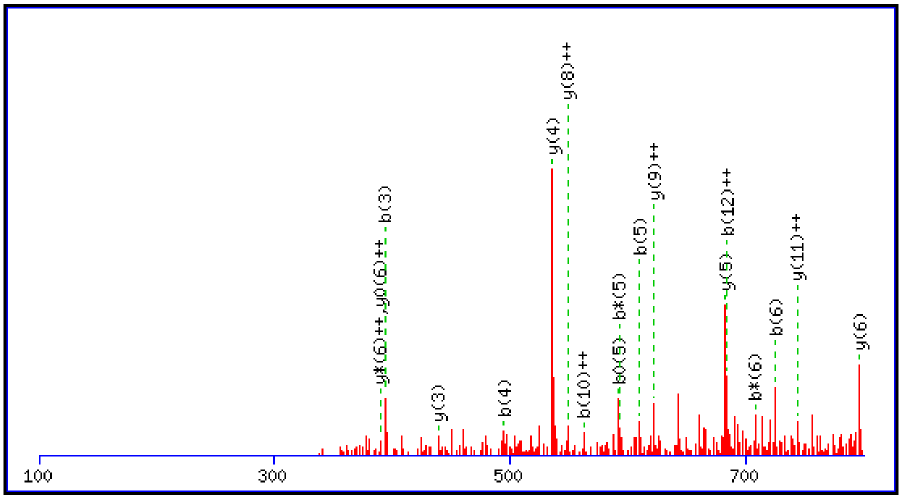

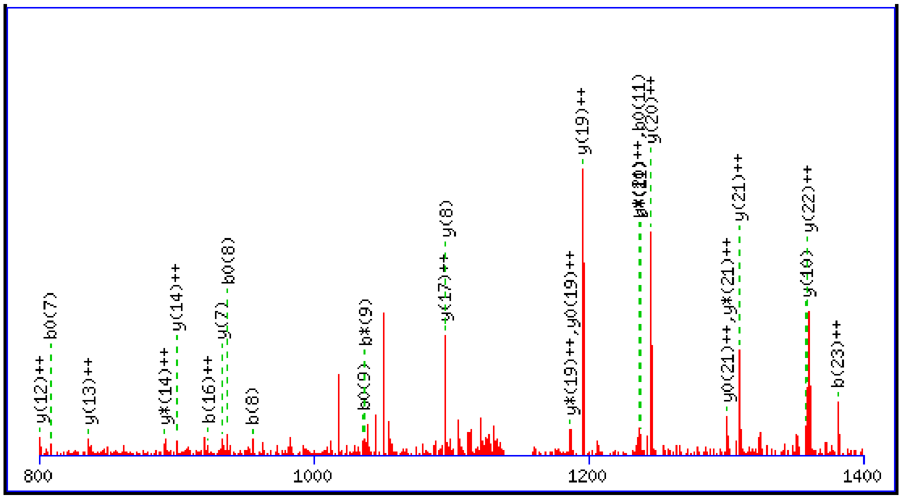

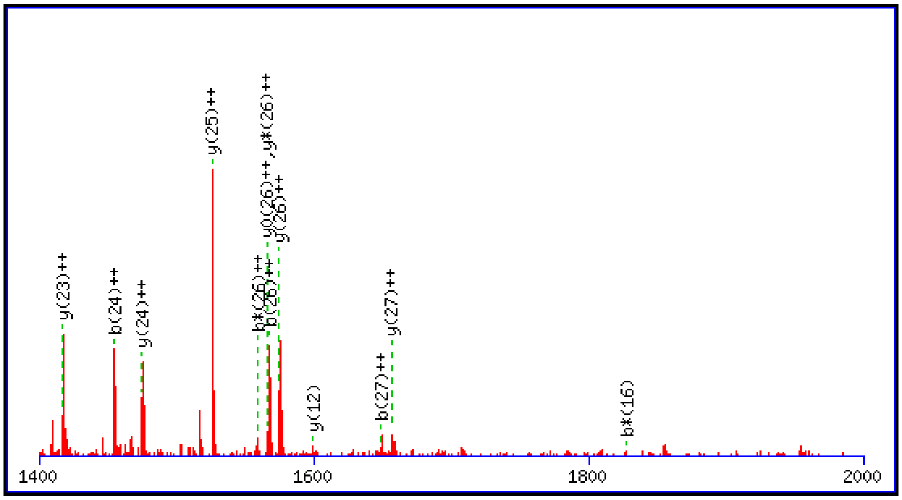

Monoisotopic mass of neutral peptide Mr(calc): 3442.5703
Fixed modifications: Carbamidomethyl (C)
Variable modifications:
N5 : Deamidated_N (N)
Ions Score: 61 **Expect:** 0.00036
Matches (Bold Red): 57/306 fragment ions using 128 most intense peaks

#	b	b++	b*	b*++	b0	b0++	Seq.	y	y++	y*	y*++	y0	y0++	#
1	132.0478	66.5275					M							28
2	295.1111	148.0592					Y	3312.5372	**1656.7722**	3295.5106	1648.2589	3294.5266	1647.7669	27
3	**394.1795**	197.5934					V	3149.4738	**1575.2406**	3132.4473	**1566.7273**	3131.4633	**1566.2353**	26
4	**495.2272**	248.1172			477.2166	239.1119	T	3050.4054	**1525.7063**	3033.3789	1517.1931	3032.3949	1516.7011	25
5	**610.2541**	305.6307	**593.2276**	297.1174	**592.2436**	296.6254	N	2949.3577	**1475.1825**	2932.3312	1466.6692	2931.3472	1466.1772	24
6	**725.2811**	363.1442	**708.2545**	354.6309	707.2705	354.1389	D	2834.3308	**1417.6690**	2817.3043	1409.1558	2816.3202	1408.6638	23
7	826.3287	413.6680	809.3022	405.1547	**808.3182**	404.6627	T	2719.3039	**1360.1556**	2702.2773	1351.6423	2701.2933	1351.1503	22
8	**955.3713**	478.1893	938.3448	469.6760	**937.3608**	469.1840	E	2618.2562	**1309.6317**	2601.2296	**1301.1185**	2600.2456	**1300.6264**	21
9	1054.4397	527.7235	**1037.4132**	519.2102	**1036.4292**	518.7182	V	2489.2136	**1245.1104**	2472.1870	**1236.5972**	2471.2030	1236.1051	20
10	1125.4769	**563.2421**	1108.4503	554.7288	1107.4663	554.2368	A	2390.1452	**1195.5762**	2373.1186	**1187.0630**	2372.1346	**1186.5709**	19
11	1254.5195	627.7634	**1237.4929**	619.2501	**1236.5089**	618.7581	E	2319.1081	1160.0577	2302.0815	1151.5444	2301.0975	1151.0524	18
12	1368.5624	**684.7848**	1351.5358	676.2716	1350.5518	675.7795	N	2190.0655	**1095.5364**	2173.0389	1087.0231	2172.0549	1086.5311	17
13	1482.6053	741.8063	1465.5788	733.2930	1464.5947	732.8010	N	2076.0225	1038.5149	2058.9960	1030.0016	2058.0120	1029.5096	16
14	1645.6686	823.3380	1628.6421	814.8247	1627.6581	814.3327	Y	1961.9796	981.4934	1944.9531	972.9802	1943.9690	972.4882	15
15	1773.7272	887.3672	1756.7007	878.8540	1755.7167	878.3620	Q	1798.9163	**899.9618**	1781.8897	**891.4485**	1780.9057	890.9565	14
16	1844.7643	**922.8858**	**1827.7378**	914.3725	1826.7538	913.8805	A	1670.8577	**835.9325**	1653.8312	827.4192	1652.8471	826.9272	13
17	1957.8484	979.4278	1940.8218	970.9146	1939.8378	970.4226	L	**1599.8206**	**800.4139**	1582.7940	791.9007	1581.8100	791.4087	12
18	2085.9434	1043.4753	2068.9168	1034.9620	2067.9328	1034.4700	K	1486.7365	**743.8719**	1469.7100	735.3586	1468.7260	734.8666	11

**19**	2200.9703	1100.9888	2183.9438	1092.4755	2182.9597	1091.9835	**D**	**1358.6416**	679.8244	1341.6150	671.3111	1340.6310	670.8191	**10**
**20**	2348.0387	1174.5230	2331.0122	1166.0097	2330.0281	1165.5177	**F**	1243.6146	**622.3109**	1226.5881	613.7977	1225.6041	613.3057	**9**
**21**	2511.1020	1256.0547	2494.0755	1247.5414	2493.0915	1247.0494	**Y**	**1096.5462**	**548.7767**	1079.5197	540.2635	1078.5356	539.7715	**8**
**22**	2648.1610	1324.5841	2631.1344	1316.0708	2630.1504	1315.5788	**H**	**933.4829**	467.2451	916.4563	458.7318	915.4723	458.2398	**7**
**23**	2761.2450	**1381.1261**	2744.2185	1372.6129	2743.2345	1372.1209	**L**	**796.4240**	398.7156	779.3974	**390.2023**	778.4134	**389.7103**	**6**
**24**	2908.3134	**1454.6604**	2891.2869	1446.1471	2890.3029	1445.6551	**F**	**683.3399**	342.1736	666.3134	333.6603	665.3293	333.1683	**5**
**25**	3005.3662	1503.1867	2988.3396	1494.6735	2987.3556	1494.1815	**P**	**536.2715**	268.6394	519.2449	260.1261	518.2609	259.6341	**4**
**26**	3134.4088	**1567.7080**	3117.3822	**1559.1948**	3116.3982	1558.7028	**E**	**439.2187**	220.1130	422.1922	211.5997	421.2082	211.1077	**3**
**27**	3297.4721	**1649.2397**	3280.4456	1640.7264	3279.4616	1640.2344	**Y**	310.1761	155.5917	293.1496	147.0784			**2**
**28**							**K**	147.1128	74.0600	130.0863	65.5468			**1**

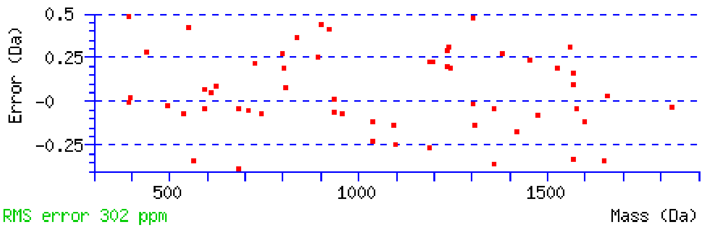

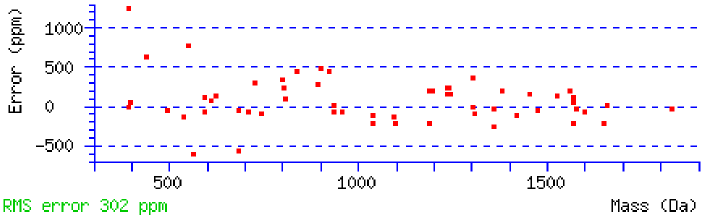

All matches to this query

Score	Mr(calc):	Delta	Sequence
60.6	3442.5703	0.0125	MYVTNDTEVAENNYQALKDFYHLFPEYK
47.5	3442.5703	0.0125	MYVTNDTEVAENNYQALKDFYHLFPEYK
47.5	3442.5703	0.0125	MYVTNDTEVAENNYQALKDFYHLFPEYK
28.6	3441.5863	0.9965	MYVTNDTEVAENNYQALKDFYHLFPEYK
3.8	3440.5612	2.0216	KAQFLATKTGGTLEDQIIEANPAMEAFGNAK
3.8	3440.5612	2.0216	KAQFLATKTGGTLEDQIIEANPAMEAFGNAK
1.5	3440.5854	1.9974	AQIXLSSSHTACKSSGVIPLCAGSQESKSER
1.5	3440.5854	1.9974	AQIXLSSSHTACKSSGVIPLCAGSQESKSER
1.5	3440.5854	1.9974	AQIXLSSSHTACKSSGVIPLCAGSQESKSER
1.0	3440.5951	1.9877	HGSQAGNQMISVFYTVVIPMLNPLIYSLR

Spectrum No: 321; Query: 375; Rank: 1

Peptide View

MS/MS Fragmentation of **MIENGSLSFLPTLR**
Found in **IPI00191090**, Tax_Id=10116 Gene_Symbol=Bgn Biglycan precursor

Match to Query 375: 1593.809768 from(797.912160,2+)
Title: 091129RatKid_SCX02_16.3593.3593.2.dta
Data file K:\NewmanPaper\Piliang\3SubProteomes\Piliang3SP\mgf5ppm\SCX_3SubProteomes5ppm.mgf

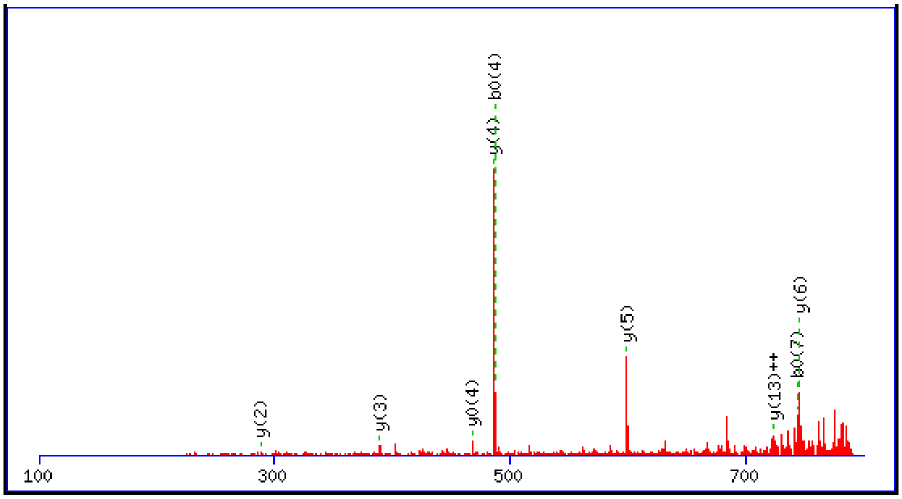

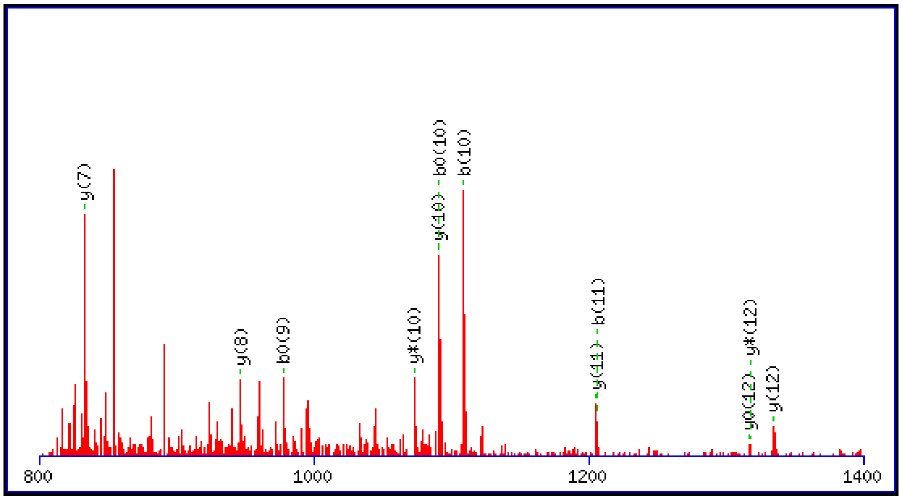

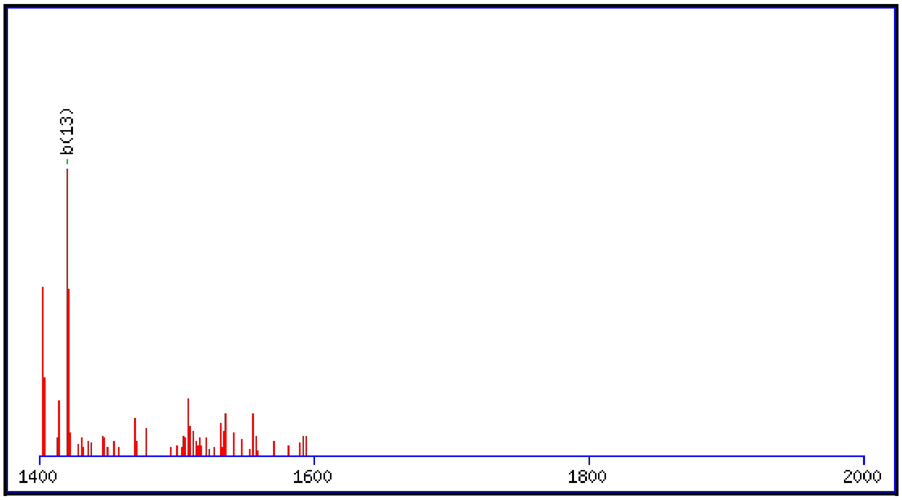

Monoisotopic mass of neutral peptide Mr(calc): 1593.8072
Fixed modifications: Carbamidomethyl (C)
Variable modifications:
M1 : Oxidation (M)
N4 : Deamidated_N (N)
Ions Score: 61 **Expect:** 0.00012
Matches (Bold Red): 22/142 fragment ions using 39 most intense peaks

#	b	b^{++}	b*	b^{*++}	b^0	b^{0++}	Seq.	y	y^{++}	y*	y^{*++}	y^0	y^{0++}	#
1	148.0427	74.5250					M							14
2	261.1267	131.0670					I	1447.7791	**724.3932**	1430.7526	715.8799	1429.7685	715.3879	13
3	390.1693	195.5883			372.1588	186.5830	E	**1334.6951**	667.8512	**1317.6685**	659.3379	**1316.6845**	658.8459	12
4	505.1963	253.1018	488.1697	244.5885	**487.1857**	244.0965	N	**1205.6525**	603.3299	1188.6259	594.8166	1187.6419	594.3246	11

**5**	562.2177	281.6125	545.1912	273.0992	544.2072	272.6072	**G**	**1090.6255**	545.8164	**1073.5990**	537.3031	1072.6150	536.8111	**10**
**6**	649.2498	325.1285	632.2232	316.6152	631.2392	316.1232	**S**	1033.6041	517.3057	1016.5775	508.7924	1015.5935	508.3004	**9**
**7**	762.3338	381.6706	745.3073	373.1573	**744.3233**	372.6653	**L**	**946.5720**	473.7897	929.5455	465.2764	928.5615	464.7844	**8**
**8**	849.3659	425.1866	832.3393	416.6733	831.3553	416.1813	**S**	**833.4880**	417.2476	816.4614	408.7343	815.4774	408.2423	**7**
**9**	996.4343	498.7208	979.4077	490.2075	**978.4237**	489.7155	**F**	**746.4559**	373.7316	729.4294	365.2183	728.4454	364.7263	**6**
**10**	**1109.5183**	555.2628	1092.4918	546.7495	**1091.5078**	546.2575	**L**	**599.3875**	300.1974	582.3610	291.6841	581.3770	291.1921	**5**
**11**	**1206.5711**	603.7892	1189.5446	595.2759	1188.5605	594.7839	**P**	**486.3035**	243.6554	469.2769	235.1421	**468.2929**	234.6501	**4**
**12**	1307.6188	654.3130	1290.5922	645.7998	1289.6082	645.3077	**T**	**389.2507**	195.1290	372.2241	186.6157	371.2401	186.1237	**3**
**13**	**1420.7028**	710.8551	1403.6763	702.3418	1402.6923	701.8498	**L**	**288.2030**	144.6051	271.1765	136.0919			**2**
**14**							**R**	175.1190	88.0631	158.0924	79.5498			**1**

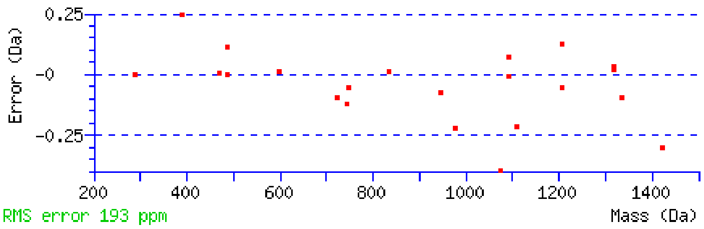

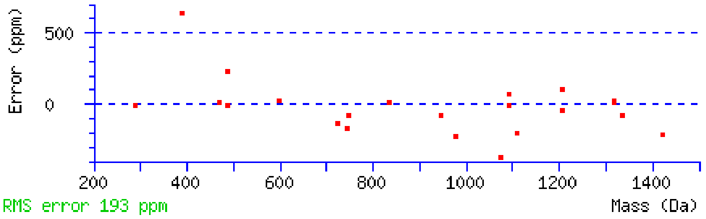

All matches to this query

Score	Mr(calc):	Delta	Sequence
60.5	1593.8072	0.0025	MIENGSLSFLPTLR
11.5	1593.8055	0.0043	FKEVIPISDPELK
10.1	1593.8223	-0.0125	SLRELHLQDNNVR
9.0	1591.8174	1.9924	RSQNLMAMQELKK
6.5	1593.8223	-0.0125	SLRELHLQDNNVR
5.6	1593.8223	-0.0125	RISELEHENAQLR
5.1	1593.8218	-0.0121	NQMNLLTMDVKKK
3.3	1593.8106	-0.0008	LMQDKEEMIGKLK
2.5	1591.7945	2.0152	GNYRFMKLLLTR
1.6	1593.8028	0.0069	TAQGQRLVSQWIK

Spectrum No: 322; Query: 1021; Rank: 1

Peptide View

MS/MS Fragmentation of **MCQLYPETFECVTNSSDILQAFR**
Found in **IPI00327697**, Tax_Id=10116 Gene_Symbol=Dpep1 Dipeptidase 1 precursor

Match to Query 1021: 2810.242128 from(1406.128340,2+)
Title: 091129RatKid_SCX02_11.4613.4613.2.dta
Data file K:\NewmanPaper\Piliang\3SubProteomes\Piliang3SP\mgf5ppm\SCX_3SubProteomes5ppm.mgf

Mascot Search Results: Peptide View

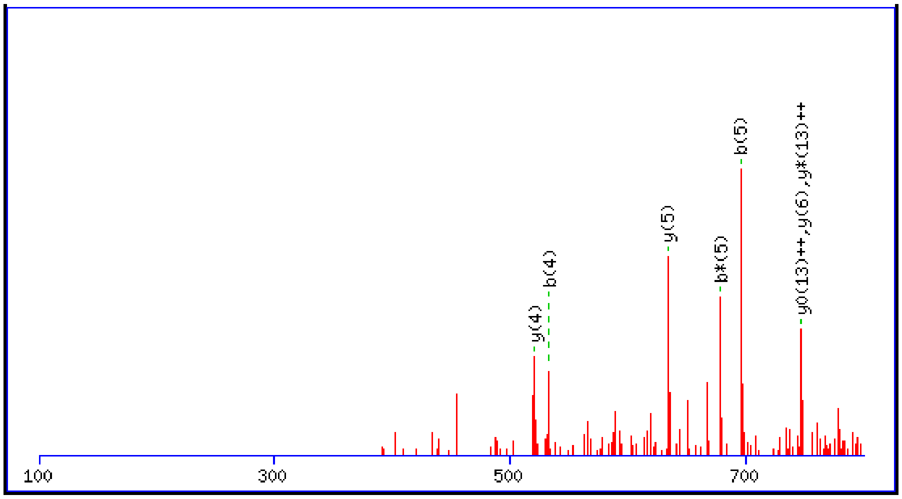

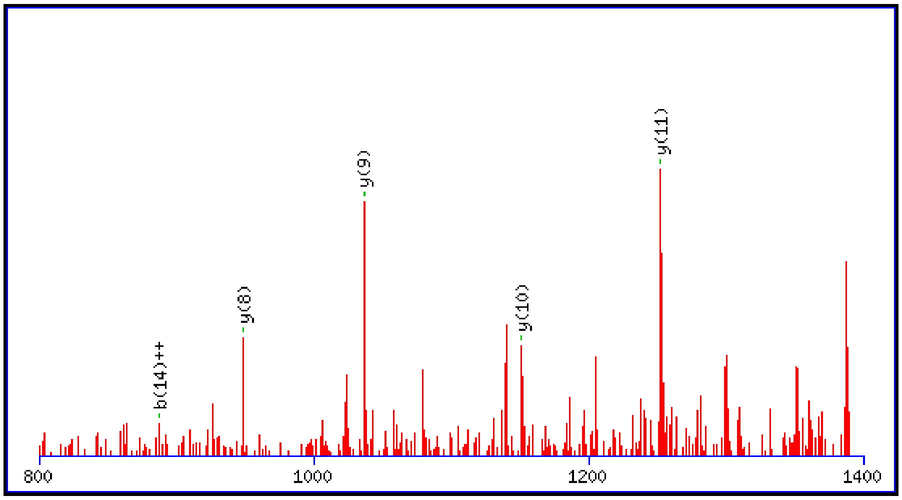

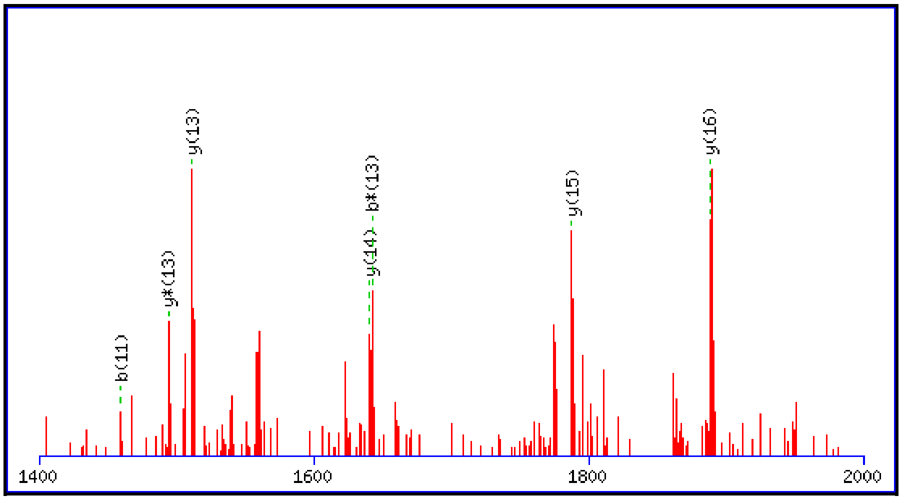

Monoisotopic mass of neutral peptide Mr(calc): 2809.2397
Fixed modifications: Carbamidomethyl (C)
Variable modifications:
N14 : Deamidated_N (N)
Ions Score: 60 **Expect:** 0.00033
Matches (Bold Red): 20/236 fragment ions using 41 most intense peaks

#	b	b^{++}	b*	b^{*++}	b^0	b^{0++}	Seq.	y	y^{++}	y*	y^{*++}	y^0	y^{0++}	#
1	132.0478	66.5275					M							23
2	292.0784	146.5428					C	2679.2065	1340.1069	2662.1800	1331.5936	2661.1960	1331.1016	22
3	420.1370	210.5721	403.1104	202.0589			Q	2519.1759	1260.0916	2502.1493	1251.5783	2501.1653	1251.0863	21
4	**533.2211**	267.1142	516.1945	258.6009			L	2391.1173	1196.0623	2374.0907	1187.5490	2373.1067	1187.0570	20
5	**696.2844**	348.6458	**679.2578**	340.1326			Y	2278.0332	1139.5203	2261.0067	1131.0070	2260.0227	1130.5150	19

**6**	793.3371	397.1722	776.3106	388.6589			**P**	2114.9699	1057.9886	2097.9434	1049.4753	2096.9593	1048.9833	**18**
**7**	922.3797	461.6935	905.3532	453.1802	904.3692	452.6882	**E**	2017.9171	1009.4622	2000.8906	1000.9489	1999.9066	1000.4569	**17**
**8**	1023.4274	512.2173	1006.4009	503.7041	1005.4169	503.2121	**T**	**1888.8745**	944.9409	1871.8480	936.4276	1870.8640	935.9356	**16**
**9**	1170.4958	585.7516	1153.4693	577.2383	1152.4853	576.7463	**F**	**1787.8269**	894.4171	1770.8003	885.9038	1769.8163	885.4118	**15**
**10**	1299.5384	650.2729	1282.5119	641.7596	1281.5279	641.2676	**E**	**1640.7585**	820.8829	1623.7319	812.3696	1622.7479	811.8776	**14**
**11**	**1459.5691**	730.2882	1442.5425	721.7749	1441.5585	721.2829	**C**	**1511.7159**	756.3616	**1494.6893**	**747.8483**	1493.7053	**747.3563**	**13**
**12**	1558.6375	779.8224	1541.6109	771.3091	1540.6269	770.8171	**V**	1351.6852	676.3462	1334.6587	667.8330	1333.6746	667.3410	**12**
**13**	1659.6852	830.3462	**1642.6586**	821.8329	1641.6746	821.3409	**T**	**1252.6168**	626.8120	1235.5903	618.2988	1234.6062	617.8068	**11**
**14**	1774.7121	**887.8597**	1757.6856	879.3464	1756.7015	878.8544	**N**	**1151.5691**	576.2882	1134.5426	567.7749	1133.5586	567.2829	**10**
**15**	1861.7441	931.3757	1844.7176	922.8624	1843.7336	922.3704	**S**	**1036.5422**	518.7747	1019.5156	510.2615	1018.5316	509.7694	**9**
**16**	1948.7762	974.8917	1931.7496	966.3784	1930.7656	965.8864	**S**	**949.5102**	475.2587	932.4836	466.7454	931.4996	466.2534	**8**
**17**	2063.8031	1032.4052	2046.7766	1023.8919	2045.7925	1023.3999	**D**	862.4781	431.7427	845.4516	423.2294	844.4676	422.7374	**7**
**18**	2176.8872	1088.9472	2159.8606	1080.4339	2158.8766	1079.9419	**I**	**747.4512**	374.2292	730.4246	365.7160			**6**
**19**	2289.9712	1145.4893	2272.9447	1136.9760	2271.9607	1136.4840	**L**	**634.3671**	317.6872	617.3406	309.1739			**5**
**20**	2418.0298	1209.5185	2401.0033	1201.0053	2400.0192	1200.5133	**Q**	**521.2831**	261.1452	504.2565	252.6319			**4**
**21**	2489.0669	1245.0371	2472.0404	1236.5238	2471.0564	1236.0318	**A**	393.2245	197.1159	376.1979	188.6026			**3**
**22**	2636.1353	1318.5713	2619.1088	1310.0580	2618.1248	1309.5660	**F**	322.1874	161.5973	305.1608	153.0840			**2**
**23**							**R**	175.1190	88.0631	158.0924	79.5498			**1**

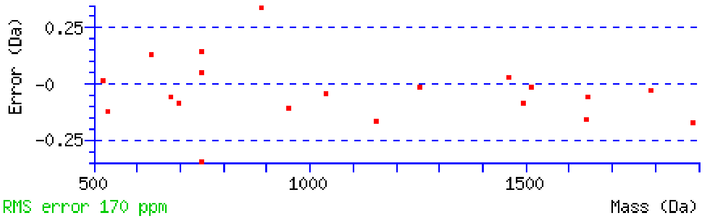

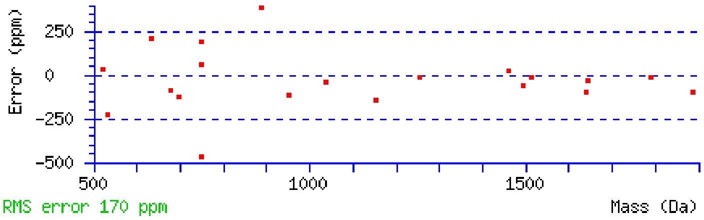

All matches to this query

Score	Mr(calc):	Delta	Sequence
60.4	2809.2397	1.0024	MCQLYPETFECVTNSSDILQAFR
18.8	2808.2557	1.9864	MCQLYPETFECVTNSSDILQAFR

Spectrum No: 323; Query: 1131; Rank: 1

Peptide View

MS/MS Fragmentation of **TVFQKENGTVTAANASTLNDGAAAVVLMTAEAAQR**
Found in **IPI00324302**, Tax_Id=10116 Gene_Symbol=Acat1 Acetyl-CoA acetyltransferase, mitochondrial precursor

Match to Query 1131: 3520.759272 from(1174.593700,3+)
Title: 091127RatKid_SCX01_22.5885.5885.3.dta
Data file K:\NewmanPaper\Piliang\3SubProteomes\Piliang3SP\mgf5ppm\SCX_3SubProteomes5ppm.mgf

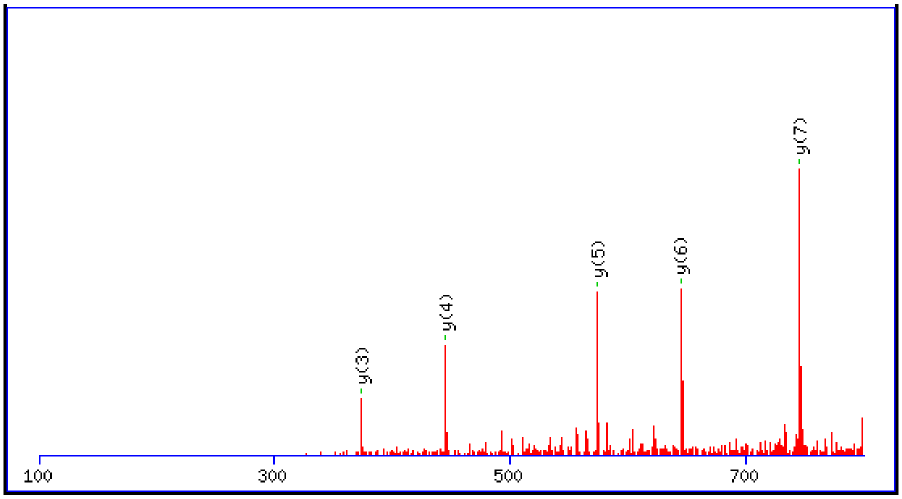

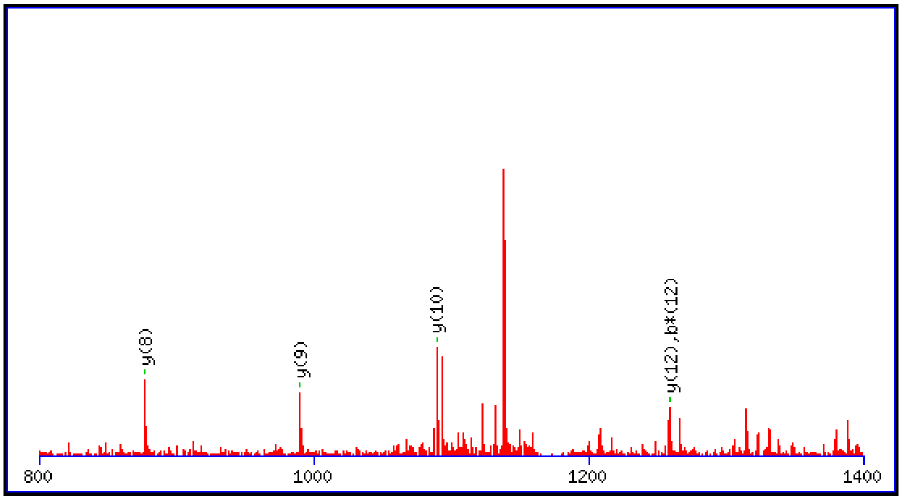

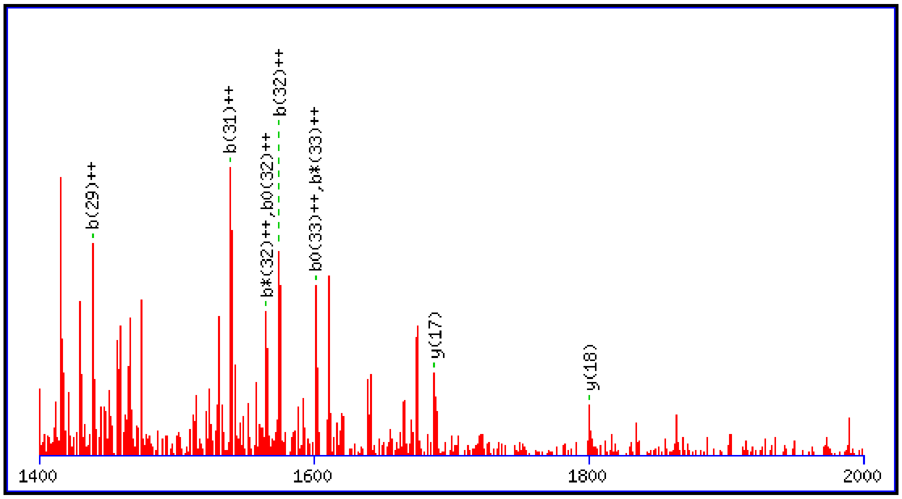

Monoisotopic mass of neutral peptide Mr(calc): 3520.7467
Fixed modifications: Carbamidomethyl (C)
Variable modifications:
N14 : Deamidated_N (N)
Ions Score: 60 **Expect:** 0.0002
Matches (Bold Red): 19/394 fragment ions using 26 most intense peaks

#	b	b++	b*	b*++	b0	b0++	Seq.	y	y++	y*	y*++	y0	y0++	#
1	102.0550	51.5311			84.0444	42.5258	T							35
2	201.1234	101.0653			183.1128	92.0600	V	3420.7064	1710.8568	3403.6798	1702.3435	3402.6958	1701.8515	34
3	348.1918	174.5995			330.1812	165.5942	F	3321.6380	1661.3226	3304.6114	1652.8093	3303.6274	1652.3173	33
4	476.2504	238.6288	459.2238	230.1155	458.2398	229.6235	Q	3174.5695	1587.7884	3157.5430	1579.2751	3156.5590	1578.7831	32
5	604.3453	302.6763	587.3188	294.1630	586.3348	293.6710	K	3046.5110	1523.7591	3029.4844	1515.2458	3028.5004	1514.7538	31

6	733.3879	367.1976	716.3614	358.6843	715.3774	358.1923	E	2918.4160	1459.7116	2901.3895	1451.1984	2900.4054	1450.7064	30
7	847.4308	424.2191	830.4043	415.7058	829.4203	415.2138	N	2789.3734	1395.1903	2772.3469	1386.6771	2771.3628	1386.1851	29
8	904.4523	452.7298	887.4258	444.2165	886.4417	443.7245	G	2675.3305	1338.1689	2658.3039	1329.6556	2657.3199	1329.1636	28
9	1005.5000	503.2536	988.4734	494.7404	987.4894	494.2483	T	2618.3090	1309.6581	2601.2825	1301.1449	2600.2985	1300.6529	27
10	1104.5684	552.7878	1087.5419	544.2746	1086.5578	543.7826	V	2517.2613	1259.1343	2500.2348	1250.6210	2499.2508	1250.1290	26
11	1205.6161	603.3117	1188.5895	594.7984	1187.6055	594.3064	T	2418.1929	1209.6001	2401.1664	1201.0868	2400.1824	1200.5948	25
12	1276.6532	638.8302	**1259.6266**	630.3170	1258.6426	629.8250	A	2317.1452	1159.0763	2300.1187	1150.5630	2299.1347	1150.0710	24
13	1347.6903	674.3488	1330.6638	665.8355	1329.6797	665.3435	A	2246.1081	1123.5577	2229.0816	1115.0444	2228.0976	1114.5524	23
14	1462.7172	731.8623	1445.6907	723.3490	1444.7067	722.8570	N	2175.0710	1088.0391	2158.0445	1079.5259	2157.0605	1079.0339	22
15	1533.7544	767.3808	1516.7278	758.8675	1515.7438	758.3755	A	2060.0441	1030.5257	2043.0175	1022.0124	2042.0335	1021.5204	21
16	1620.7864	810.8968	1603.7598	802.3836	1602.7758	801.8915	S	1989.0070	995.0071	1971.9804	986.4938	1970.9964	986.0018	20
17	1721.8341	861.4207	1704.8075	852.9074	1703.8235	852.4154	T	1901.9749	951.4911	1884.9484	942.9778	1883.9644	942.4858	19
18	1834.9181	917.9627	1817.8916	909.4494	1816.9076	908.9574	L	**1800.9273**	900.9673	1783.9007	892.4540	1782.9167	891.9620	18
19	1948.9611	974.9842	1931.9345	966.4709	1930.9505	965.9789	N	**1687.8432**	844.4252	1670.8166	835.9120	1669.8326	835.4200	17
20	2063.9880	1032.4976	2046.9615	1023.9844	2045.9774	1023.4924	D	1573.8003	787.4038	1556.7737	778.8905	1555.7897	778.3985	16
21	2121.0095	1061.0084	2103.9829	1052.4951	2102.9989	1052.0031	G	1458.7733	729.8903	1441.7468	721.3770	1440.7628	720.8850	15
22	2192.0466	1096.5269	2175.0200	1088.0137	2174.0360	1087.5216	A	1401.7519	701.3796	1384.7253	692.8663	1383.7413	692.3743	14
23	2263.0837	1132.0455	2246.0571	1123.5322	2245.0731	1123.0402	A	1330.7147	665.8610	1313.6882	657.3477	1312.7042	656.8557	13
24	2334.1208	1167.5640	2317.0943	1159.0508	2316.1102	1158.5588	A	**1259.6776**	630.3425	1242.6511	621.8292	1241.6671	621.3372	12
25	2433.1892	1217.0982	2416.1627	1208.5850	2415.1787	1208.0930	V	1188.6405	594.8239	1171.6140	586.3106	1170.6300	585.8186	11
26	2532.2576	1266.6325	2515.2311	1258.1192	2514.2471	1257.6272	V	**1089.5721**	545.2897	1072.5456	536.7764	1071.5615	536.2844	10
27	2645.3417	1323.1745	2628.3151	1314.6612	2627.3311	1314.1692	L	**990.5037**	495.7555	973.4771	487.2422	972.4931	486.7502	9
28	2776.3822	1388.6947	2759.3556	1380.1815	2758.3716	1379.6894	M	**877.4196**	439.2135	860.3931	430.7002	859.4091	430.2082	8
29	2877.4299	**1439.2186**	2860.4033	1430.7053	2859.4193	1430.2133	T	**746.3791**	373.6932	729.3526	365.1799	728.3686	364.6879	7
30	2948.4670	1474.7371	2931.4404	1466.2239	2930.4564	1465.7318	A	**645.3315**	323.1694	628.3049	314.6561	627.3209	314.1641	6
31	3077.5096	**1539.2584**	3060.4830	1530.7451	3059.4990	1530.2531	E	**574.2944**	287.6508	557.2678	279.1375	556.2838	278.6455	5
32	3148.5467	**1574.7770**	3131.5201	**1566.2637**	3130.5361	**1565.7717**	A	**445.2518**	223.1295	428.2252	214.6162			4
33	3219.5838	1610.2955	3202.5572	**1601.7823**	3201.5732	**1601.2903**	A	**374.2146**	187.6110	357.1881	179.0977			3
34	3347.6424	1674.3248	3330.6158	1665.8116	3329.6318	1665.3195	Q	303.1775	152.0924	286.1510	143.5791			2
35							R	175.1190	88.0631	158.0924	79.5498			1

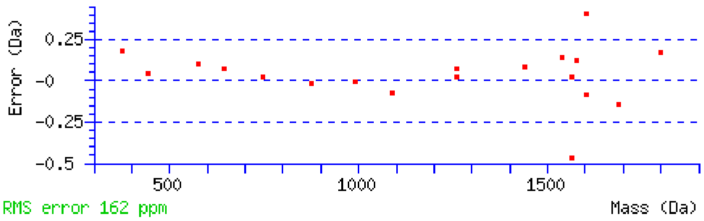

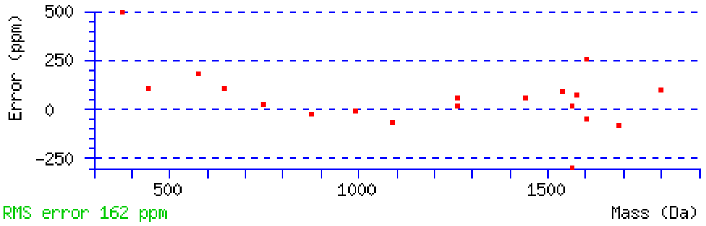

All matches to this query

Score	Mr(calc):	Delta	Sequence
60.3	3520.7467	0.0125	TVFQKENGTVTAANASTLNDGAAAVVLMTAEAAQR
60.3	3520.7467	0.0125	TVFQKENGTVTAANASTLNDGAAAVVLMTAEAAQR
52.6	3519.7627	0.9965	TVFQKENGTVTAANASTLNDGAAAVVLMTAEAAQR
48.0	3520.7467	0.0125	TVFQKENGTVTAANASTLNDGAAAVVLMTAEAAQR
6.1	3519.7462	1.0130	DSPKHPQLVKNSLCAAPRPTALGLLPNSGPPK
4.3	3520.7729	-0.0136	SRQSQPVNVGLVDWISLAYQHYAIAVRNTR
4.3	3520.7729	-0.0136	SRQSQPVNVGLVDWISLAYQHYAIAVRNTR
0.2	3519.7253	1.0340	THYFLLVMLFFLFSQMELGAGILTSLGRR
0.2	3519.7253	1.0340	THYFLLVMLFFLFSQMELGAGILTSLGRR

Spectrum No: 324; Query: 1113; Rank: 1

Peptide View

MS/MS Fragmentation of **SPLGPHNQTAEQLQTLEQQSISLQQDTER**
Found in **IPI00190577**, Tax_Id=10116 Gene_Symbol=Lama5 similar to Laminin alpha-5 chain precursor

Match to Query 1113: 3276.593262 from(1093.205030,3+)
Title: 091129RatKid_SCX02_14.3379.3379.3.dta
Data file K:\NewmanPaper\Piliang\3SubProteomes\Piliang3SP\mgf5ppm\SCX_3SubProteomes5ppm.mgf

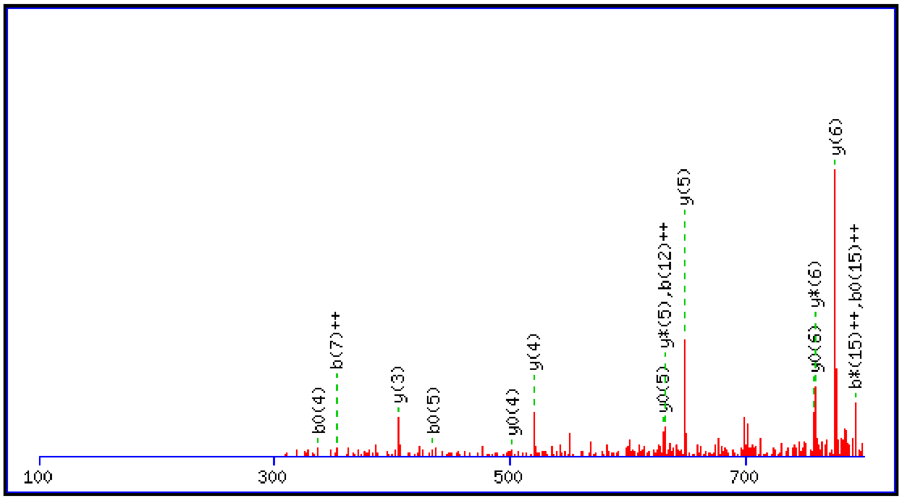

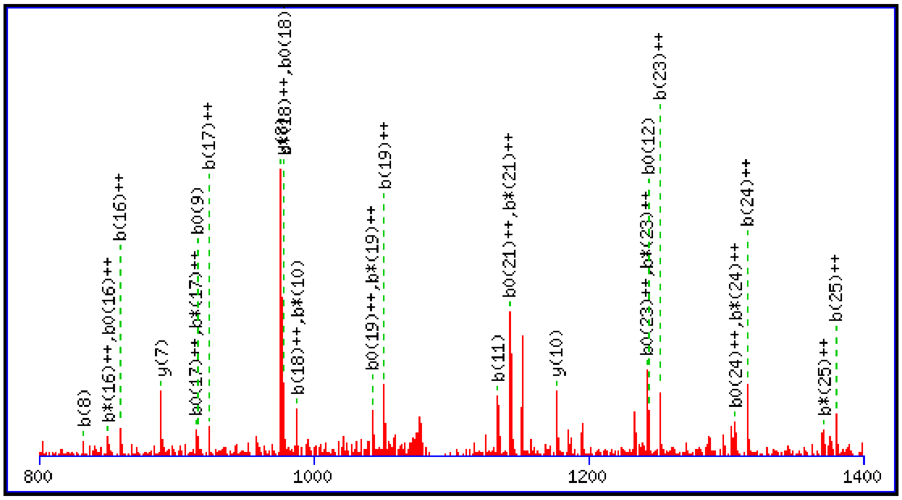

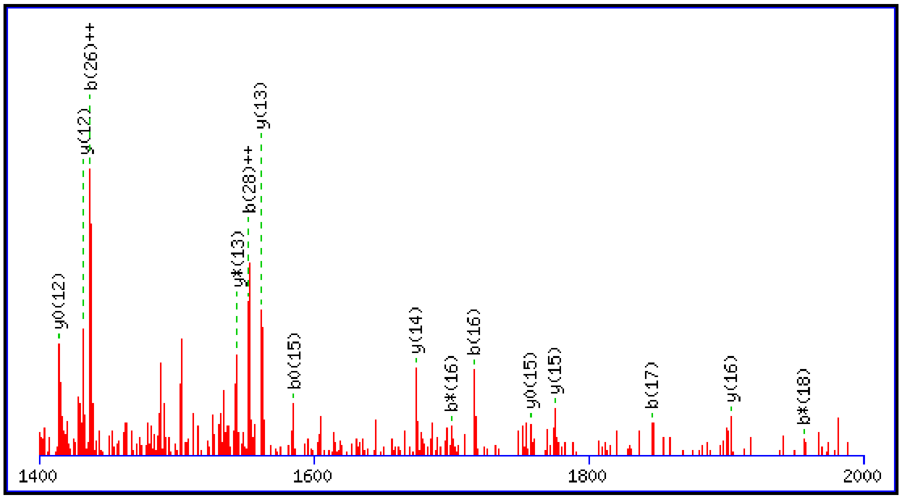

Monoisotopic mass of neutral peptide Mr(calc): 3276.5858
Fixed modifications: Carbamidomethyl (C)
Variable modifications:
N7 : Deamidated_N (N)
Ions Score: 60 **Expect:** 0.00031
Matches (Bold Red): 60/322 fragment ions using 90 most intense peaks

#	b	b^{++}	b*	b^{*++}	b^{0}	b^{0++}	Seq.	y	y^{++}	y*	y^{*++}	y^{0}	y^{0++}	#
1	88.0393	44.5233			70.0287	35.5180	S							29
2	185.0921	93.0497			167.0815	84.0444	P	3190.5611	1595.7842	3173.5345	1587.2709	3172.5505	1586.7789	28
3	298.1761	149.5917			280.1656	140.5864	L	3093.5083	1547.2578	3076.4818	1538.7445	3075.4978	1538.2525	27
4	355.1976	178.1024			**337.1870**	169.0972	G	2980.4243	1490.7158	2963.3977	1482.2025	2962.4137	1481.7105	26
5	452.2504	226.6288			**434.2398**	217.6235	P	2923.4028	1462.2050	2906.3762	1453.6918	2905.3922	1453.1998	25
6	589.3093	295.1583			571.2987	286.1530	H	2826.3500	1413.6787	2809.3235	1405.1654	2808.3395	1404.6734	24
7	704.3362	**352.6717**	687.3097	344.1585	686.3256	343.6665	N	2689.2911	1345.1492	2672.2646	1336.6359	2671.2806	1336.1439	23
8	**832.3948**	416.7010	815.3682	408.1878	814.3842	407.6957	Q	2574.2642	1287.6357	2557.2376	1279.1225	2556.2536	1278.6304	22
9	933.4425	467.2249	916.4159	458.7116	**915.4319**	458.2196	T	2446.2056	1223.6064	2429.1791	1215.0932	2428.1950	1214.6012	21
10	1004.4796	502.7434	**987.4530**	494.2302	986.4690	493.7381	A	2345.1579	1173.0826	2328.1314	1164.5693	2327.1474	1164.0773	20
11	**1133.5222**	567.2647	1116.4956	558.7514	1115.5116	558.2594	E	2274.1208	1137.5640	2257.0943	1129.0508	2256.1102	1128.5588	19
12	1261.5807	**631.2940**	1244.5542	622.7807	**1243.5702**	622.2887	Q	2145.0782	1073.0427	2128.0517	1064.5295	2127.0677	1064.0375	18
13	1374.6648	687.8360	1357.6383	679.3228	1356.6542	678.8308	L	2017.0196	1009.0135	1999.9931	1000.5002	1999.0091	1000.0082	17
14	1502.7234	751.8653	1485.6968	743.3521	1484.7128	742.8601	Q	**1903.9356**	952.4714	1886.9090	943.9582	1885.9250	943.4661	16
15	1603.7711	802.3892	1586.7445	**793.8759**	**1585.7605**	**793.3839**	T	**1775.8770**	888.4421	1758.8505	879.9289	**1757.8664**	879.4369	15
16	**1716.8551**	**858.9312**	**1699.8286**	**850.4179**	1698.8446	**849.9259**	L	**1674.8293**	837.9183	1657.8028	829.4050	1656.8188	828.9130	14
17	**1845.8977**	**923.4525**	1828.8712	**914.9392**	1827.8872	**914.4472**	E	**1561.7453**	781.3763	**1544.7187**	772.8630	1543.7347	772.3710	13
18	1973.9563	**987.4818**	**1956.9298**	**978.9685**	1955.9457	**978.4765**	Q	**1432.7027**	716.8550	1415.6761	708.3417	**1414.6921**	707.8497	12
19	2102.0149	**1051.5111**	2084.9883	**1042.9978**	2084.0043	**1042.5058**	Q	1304.6441	652.8257	1287.6175	644.3124	1286.6335	643.8204	11
20	2189.0469	1095.0271	2172.0204	1086.5138	2171.0363	1086.0218	S	**1176.5855**	588.7964	1159.5590	580.2831	1158.5749	579.7911	10
21	2302.1310	1151.5691	2285.1044	**1143.0559**	2284.1204	**1142.5638**	I	1089.5535	545.2804	1072.5269	536.7671	1071.5429	536.2751	9
22	2389.1630	1195.0851	2372.1365	1186.5719	2371.1524	1186.0799	S	**976.4694**	488.7383	959.4429	480.2251	958.4589	479.7331	8
23	2502.2471	**1251.6272**	2485.2205	**1243.1139**	2484.2365	**1242.6219**	L	**889.4374**	445.2223	872.4108	436.7091	871.4268	436.2170	7
24	2630.3056	**1315.6565**	2613.2791	**1307.1432**	2612.2951	**1306.6512**	Q	**776.3533**	388.6803	**759.3268**	380.1670	**758.3428**	379.6750	6
25	2758.3642	**1379.6858**	2741.3377	**1371.1725**	2740.3537	1370.6805	Q	**648.2947**	324.6510	**631.2682**	316.1377	**630.2842**	315.6457	5
26	2873.3912	**1437.1992**	2856.3646	1428.6859	2855.3806	1428.1939	D	**520.2362**	260.6217	503.2096	252.1084	**502.2256**	251.6164	4
27	2974.4388	1487.7231	2957.4123	1479.2098	2956.4283	1478.7178	T	**405.2092**	203.1082	388.1827	194.5950	387.1987	194.1030	3
28	3103.4814	**1552.2444**	3086.4549	1543.7311	3085.4709	1543.2391	E	304.1615	152.5844	287.1350	144.0711	286.1510	143.5791	2
29							R	175.1190	88.0631	158.0924	79.5498			1

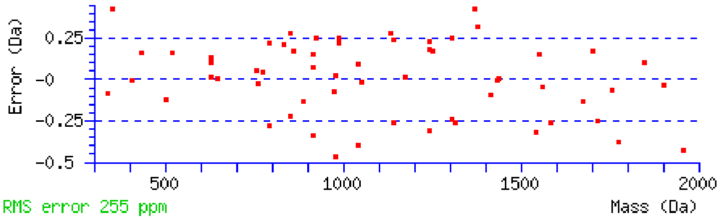

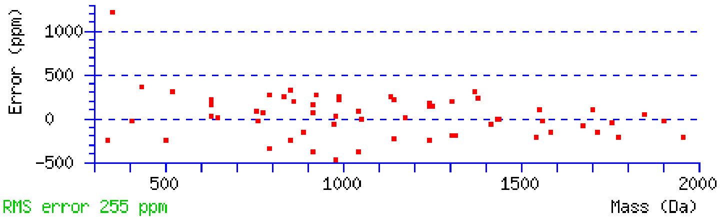

All matches to this query

Score	Mr(calc):	Delta	Sequence
60.0	3276.5858	0.0074	SPLGPHNQTAEQLQTLEQQSISLQQDTER
39.3	3275.6018	0.9915	SPLGPHNQTAEQLQTLEQQSISLQQDTER
2.9	3275.5630	1.0302	FEQRTFSVIKIFPDLSNNDMLLFIVK
2.6	3276.5808	0.0125	YLQKSSISLAAVLPGWLPPPPTPGSPCAGR
2.6	3276.5808	0.0125	YLQKSSISLAAVLPGWLPPPPTPGSPCAGR

2.6	3276.5808	0.0125	YLQKSSISLAAVLPGWLPPPPTPGSPCAGR
1.5	3275.5951	0.9981	REHLQEGVNINNSLLALINILNTLANAR
1.5	3276.5791	0.0141	REHLQEGVNINNSLLALINILNTLANAR
1.5	3276.5791	0.0141	REHLQEGVNINNSLLALINILNTLANAR
1.3	3274.5790	2.0143	FEQRTFSVIKIFPDLSNNDMLLFIVK

Spectrum No: 325; Query: 1145; Rank: 1

Peptide View

MS/MS Fragmentation of **LLYISPENFQIDNSPNSTAGPSCSLLLEAFRR**
Found in **IPI00464518**, Tax_Id=10116 Gene_Symbol=Hexb Beta-hexosaminidase beta chain precursor

Match to Query 1145: 3609.793632 from(1204.271820,3+)
Title: 091127RatKid_SCX01_21.7242.7242.3.dta
Data file K:\NewmanPaper\Piliang\3SubProteomes\Piliang3SP\mgf5ppm\SCX_3SubProteomes5ppm.mgf

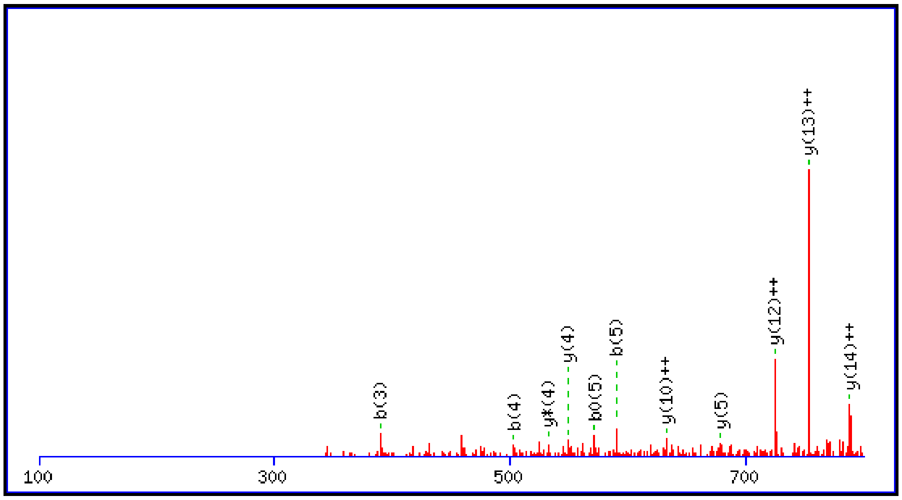

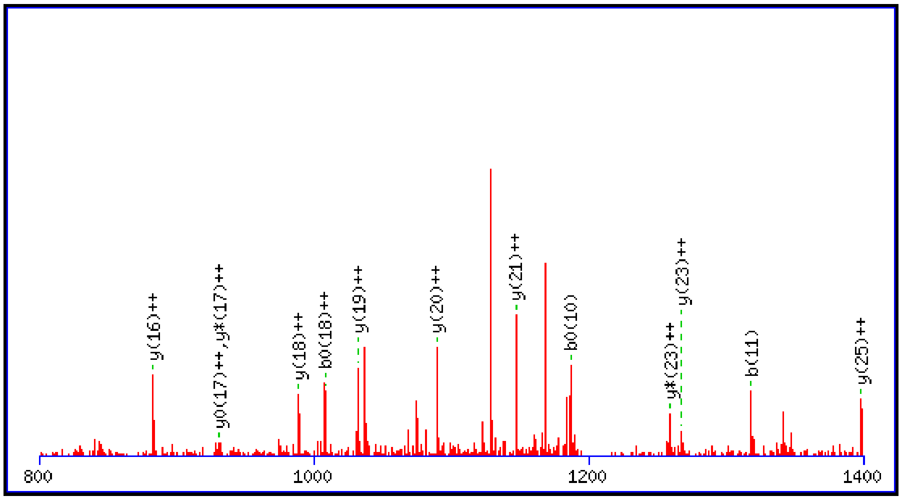

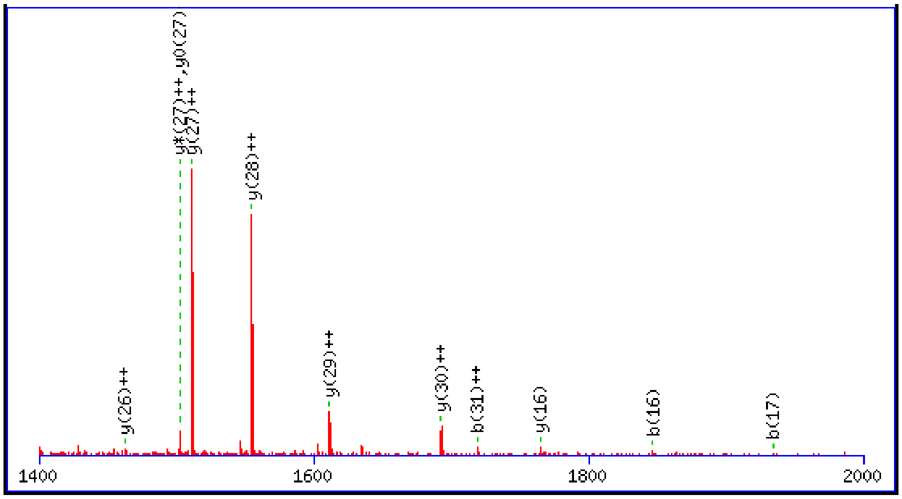

Monoisotopic mass of neutral peptide Mr(calc): 3609.7773
Fixed modifications: Carbamidomethyl (C)
Variable modifications:
N16 : Deamidated_N (N)
Ions Score: 60 **Expect:** 0.00027
Matches (Bold Red): 35/342 fragment ions using 88 most intense peaks

#	b	b^{++}	b*	b^{*++}	b^0	b^{0++}	Seq.	y	y^{++}	y*	y^{*++}	y^0	y^{0++}	#
1	114.0913	57.5493					L							32
2	227.1754	114.0913					L	3497.7006	1749.3539	3480.6740	1740.8406	3479.6900	1740.3486	31
3	**390.2387**	195.6230					Y	3384.6165	**1692.8119**	3367.5899	1684.2986	3366.6059	1683.8066	30
4	**503.3228**	252.1650					I	3221.5532	**1611.2802**	3204.5266	1602.7669	3203.5426	1602.2749	29
5	**590.3548**	295.6811			**572.3443**	286.6758	S	3108.4691	**1554.7382**	3091.4425	1546.2249	3090.4585	1545.7329	28
6	687.4076	344.2074			669.3970	335.2022	P	3021.4371	**1511.2222**	3004.4105	**1502.7089**	3003.4265	**1502.2169**	27
7	816.4502	408.7287			798.4396	399.7234	E	2924.3843	**1462.6958**	2907.3578	1454.1825	2906.3737	1453.6905	26
8	930.4931	465.7502	913.4666	457.2369	912.4825	456.7449	N	2795.3417	**1398.1745**	2778.3152	1389.6612	2777.3311	1389.1692	25
9	1077.5615	539.2844	1060.5350	530.7711	1059.5510	530.2791	F	2681.2988	1341.1530	2664.2722	1332.6398	2663.2882	1332.1477	24
10	1205.6201	603.3137	1188.5936	594.8004	**1187.6095**	594.3084	Q	2534.2304	**1267.6188**	2517.2038	**1259.1055**	2516.2198	1258.6135	23
11	**1318.7042**	659.8557	1301.6776	651.3424	1300.6936	650.8504	I	2406.1718	1203.5895	2389.1452	1195.0763	2388.1612	1194.5843	22
12	1433.7311	717.3692	1416.7046	708.8559	1415.7205	708.3639	D	2293.0877	**1147.0475**	2276.0612	1138.5342	2275.0772	1138.0422	21
13	1547.7740	774.3907	1530.7475	765.8774	1529.7635	765.3854	N	2178.0608	**1089.5340**	2161.0342	1081.0208	2160.0502	1080.5287	20
14	1634.8061	817.9067	1617.7795	809.3934	1616.7955	808.9014	S	2064.0179	**1032.5126**	2046.9913	1023.9993	2046.0073	1023.5073	19
15	1731.8588	866.4331	1714.8323	857.9198	1713.8483	857.4278	P	1976.9858	**988.9966**	1959.9593	980.4833	1958.9753	979.9913	18
16	**1846.8858**	923.9465	1829.8592	915.4332	1828.8752	914.9412	N	1879.9331	940.4702	1862.9065	**931.9569**	1861.9225	**931.4649**	17
17	**1933.9178**	967.4625	1916.8912	958.9493	1915.9072	958.4573	S	**1764.9061**	**882.9567**	1747.8796	874.4434	1746.8956	873.9514	16
18	2034.9655	1017.9864	2017.9389	1009.4731	2016.9549	**1008.9811**	T	1677.8741	839.4407	1660.8476	830.9274	1659.8635	830.4354	15
19	2106.0026	1053.5049	2088.9760	1044.9917	2087.9920	1044.4996	A	1576.8264	**788.9168**	1559.7999	780.4036	1558.8159	779.9116	14
20	2163.0240	1082.0157	2145.9975	1073.5024	2145.0135	1073.0104	G	1505.7893	**753.3983**	1488.7628	744.8850	1487.7787	744.3930	13
21	2260.0768	1130.5420	2243.0503	1122.0288	2242.0662	1121.5368	P	1448.7678	**724.8876**	1431.7413	716.3743	1430.7573	715.8823	12
22	2347.1088	1174.0581	2330.0823	1165.5448	2329.0983	1165.0528	S	1351.7151	676.3612	1334.6885	667.8479	1333.7045	667.3559	11
23	2507.1395	1254.0734	2490.1129	1245.5601	2489.1289	1245.0681	C	1264.6831	**632.8452**	1247.6565	624.3319	1246.6725	623.8399	10
24	2594.1715	1297.5894	2577.1450	1289.0761	2576.1610	1288.5841	S	1104.6524	552.8298	1087.6259	544.3166	1086.6418	543.8246	9
25	2707.2556	1354.1314	2690.2290	1345.6182	2689.2450	1345.1261	L	1017.6204	509.3138	1000.5938	500.8006	999.6098	500.3085	8
26	2820.3396	1410.6735	2803.3131	1402.1602	2802.3291	1401.6682	L	904.5363	452.7718	887.5098	444.2585	886.5257	443.7665	7
27	2933.4237	1467.2155	2916.3972	1458.7022	2915.4131	1458.2102	L	791.4522	396.2298	774.4257	387.7165	773.4417	387.2245	6
28	3062.4663	1531.7368	3045.4398	1523.2235	3044.4557	1522.7315	E	**678.3682**	339.6877	661.3416	331.1745	660.3576	330.6824	5
29	3133.5034	1567.2553	3116.4769	1558.7421	3115.4929	1558.2501	A	**549.3256**	275.1664	**532.2990**	266.6532			4
30	3280.5718	1640.7896	3263.5453	1632.2763	3262.5613	1631.7843	F	478.2885	239.6479	461.2619	231.1346			3
31	3436.6729	**1718.8401**	3419.6464	1710.3268	3418.6624	1709.8348	R	331.2201	166.1137	314.1935	157.6004			2
32							R	175.1190	88.0631	158.0924	79.5498			1

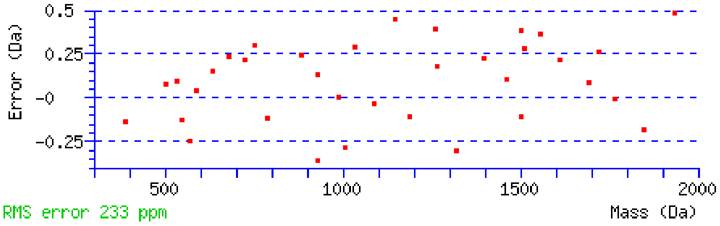

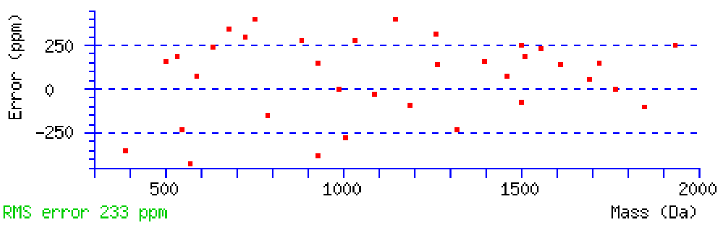

All matches to this query

Score	Mr(calc):	Delta	Sequence
60.0	3609.7773	0.0163	LLYISPENFQIDNSPNSTAGPSCSLLLEAFRR
52.8	3609.7773	0.0163	LLYISPENFQIDNSPNSTAGPSCSLLLEAFRR
34.8	3609.7773	0.0163	LLYISPENFQIDNSPNSTAGPSCSLLLEAFRR
5.2	3608.7933	1.0003	LLYISPENFQIDNSPNSTAGPSCSLLLEAFRR
5.2	3609.7919	0.0018	AVEHINNTIAPALVSKKFNVVQQDVVQLMVK
5.2	3609.7919	0.0018	AVEHINNTIAPALVSKKFNVVQQDVVQLMVK
4.3	3609.7956	-0.0020	NTHIFISWIIAQSVTAVAGLTSYPFDMVRHR
2.9	3608.7714	1.0222	DVMLNAAFALAADISSKSPVAVQGSKINLIYSR
2.8	3608.7765	1.0171	LRQALTNCDDILIKQNQTLAELQNNMMLVK
2.2	3609.7597	0.0339	VLQSPATTVLKTLNDRSSIVMGEPISQSSSNSSP

Spectrum No: 326; Query: 1121; Rank: 1

Peptide View

MS/MS Fragmentation of **LHNPPTVNYSPNEGQMDLCITSGCQDGLCK**
Found in **IPI00214373**, Tax_Id=10116 Gene_Symbol=Aadat Kynurenine/alpha-aminoadipate aminotransferase mitochondrial precursor

Match to Query 1121: 3406.494102 from(1136.505310,3+)
Title: 091129RatKid_SCX02_26.5223.5223.3.dta
Data file K:\NewmanPaper\Piliang\3SubProteomes\Piliang3SP\mgf5ppm\SCX_3SubProteomes5ppm.mgf

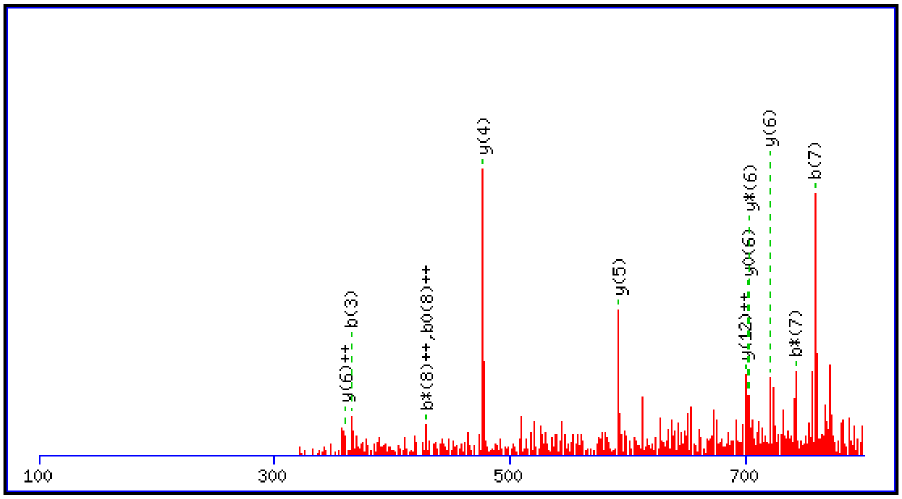

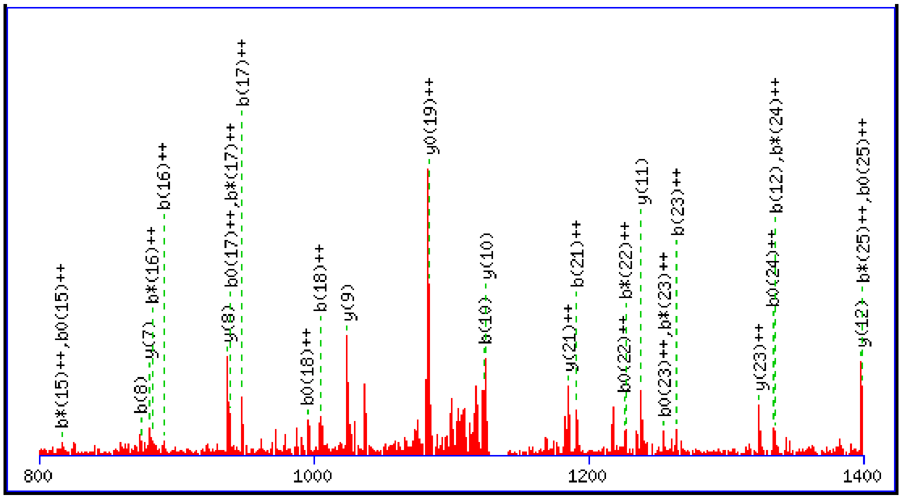

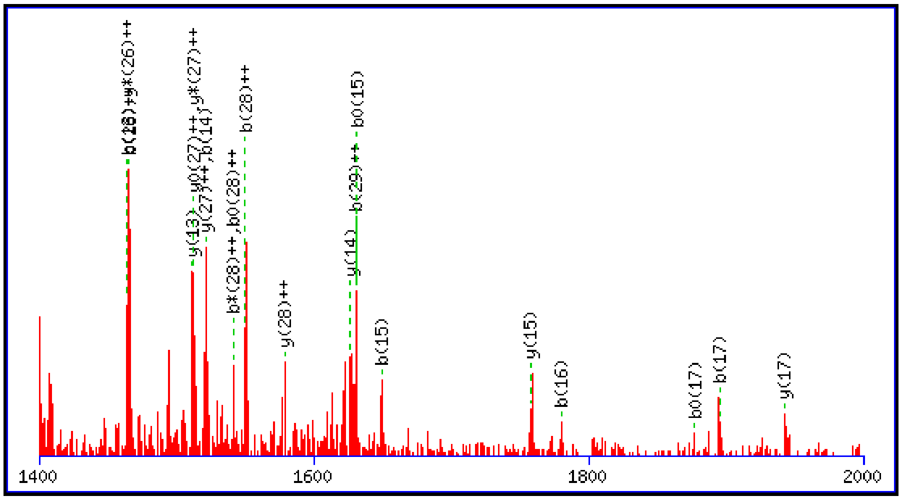

Monoisotopic mass of neutral peptide Mr(calc): 3405.4734
Fixed modifications: Carbamidomethyl (C)
Variable modifications:
N8 : Deamidated_N (N)
Ions Score: 60 **Expect:** 0.00049
Matches (Bold Red): 64/326 fragment ions using 106 most intense peaks

#	b	b^{++}	b*	b^{*++}	b^0	b^{0++}	Seq.	y	y^{++}	y*	y^{*++}	y^0	y^{0++}	#
1	114.0913	57.5493					L							30
2	251.1503	126.0788					H	3293.3966	1647.2019	3276.3701	1638.6887	3275.3860	1638.1967	29
3	**365.1932**	183.1002	348.1666	174.5870			N	3156.3377	**1578.6725**	3139.3111	1570.1592	3138.3271	1569.6672	28
4	462.2459	231.6266	445.2194	223.1133			P	3042.2948	**1521.6510**	3025.2682	**1513.1377**	3024.2842	**1512.6457**	27
5	559.2987	280.1530	542.2722	271.6397			P	2945.2420	1473.1246	2928.2155	**1464.6114**	2927.2314	1464.1194	26
6	660.3464	330.6768	643.3198	322.1636	642.3358	321.6715	T	2848.1892	1424.5983	2831.1627	1416.0850	2830.1787	1415.5930	25
7	**759.4148**	380.2110	**742.3883**	371.6978	741.4042	371.2058	V	2747.1416	1374.0744	2730.1150	1365.5611	2729.1310	1365.0691	24
8	**874.4417**	437.7245	857.4152	**429.2112**	856.4312	**428.7192**	N	2648.0731	**1324.5402**	2631.0466	1316.0269	2630.0626	1315.5349	23
9	1037.5051	519.2562	1020.4785	510.7429	1019.4945	510.2509	Y	2533.0462	1267.0267	2516.0197	1258.5135	2515.0356	1258.0215	22
10	**1124.5371**	562.7722	1107.5105	554.2589	1106.5265	553.7669	S	2369.9829	**1185.4951**	2352.9563	1176.9818	2351.9723	1176.4898	21
11	1221.5899	611.2986	1204.5633	602.7853	1203.5793	602.2933	P	2282.9509	1141.9791	2265.9243	1133.4658	2264.9403	1132.9738	20
12	**1335.6328**	668.3200	1318.6062	659.8068	1317.6222	659.3147	N	2185.8981	1093.4527	2168.8715	1084.9394	2167.8875	**1084.4474**	19
13	**1464.6754**	732.8413	1447.6488	724.3281	1446.6648	723.8360	E	2071.8552	1036.4312	2054.8286	1027.9179	2053.8446	1027.4259	18
14	**1521.6968**	761.3521	1504.6703	752.8388	1503.6863	752.3468	G	**1942.8126**	971.9099	1925.7860	963.3966	1924.8020	962.9046	17
15	**1649.7554**	825.3813	1632.7289	**816.8681**	**1631.7449**	**816.3761**	Q	1885.7911	943.3992	1868.7646	934.8859	1867.7805	934.3939	16
16	**1780.7959**	**890.9016**	1763.7694	**882.3883**	1762.7853	881.8963	M	**1757.7325**	879.3699	1740.7060	870.8566	1739.7220	870.3646	15
17	**1895.8228**	**948.4151**	1878.7963	**939.9018**	**1877.8123**	**939.4098**	D	**1626.6920**	813.8497	1609.6655	805.3364	1608.6815	804.8444	14
18	2008.9069	**1004.9571**	1991.8804	996.4438	1990.8963	**995.9518**	L	**1511.6651**	756.3362	1494.6385	747.8229	1493.6545	747.3309	13

19	2168.9376	1084.9724	2151.9110	1076.4591	2150.9270	1075.9671	C	1398.5810	699.7942	1381.5545	691.2809	1380.5705	690.7889	12
20	2282.0216	1141.5144	2264.9951	1133.0012	2264.0111	1132.5092	I	1238.5504	619.7788	1221.5238	611.2656	1220.5398	610.7735	11
21	2383.0693	1192.0383	2366.0428	1183.5250	2365.0587	1183.0330	T	1125.4663	563.2368	1108.4398	554.7235	1107.4558	554.2315	10
22	2470.1013	1235.5543	2453.0748	1227.0410	2452.0908	1226.5490	S	1024.4186	512.7130	1007.3921	504.1997	1006.4081	503.7077	9
23	2527.1228	1264.0650	2510.0962	1255.5518	2509.1122	1255.0598	G	937.3866	469.1969	920.3601	460.6837	919.3760	460.1917	8
24	2687.1534	1344.0804	2670.1269	1335.5671	2669.1429	1335.0751	C	880.3652	440.6862	863.3386	432.1729	862.3546	431.6809	7
25	2815.2120	1408.1096	2798.1855	1399.5964	2797.2015	1399.1044	Q	720.3345	360.6709	703.3080	352.1576	702.3239	351.6656	6
26	2930.2390	1465.6231	2913.2124	1457.1098	2912.2284	1456.6178	D	592.2759	296.6416	575.2494	288.1283	574.2654	287.6363	5
27	2987.2604	1494.1339	2970.2339	1485.6206	2969.2499	1485.1286	G	477.2490	239.1281	460.2224	230.6149			4
28	3100.3445	1550.6759	3083.3179	1542.1626	3082.3339	1541.6706	L	420.2275	210.6174	403.2010	202.1041			3
29	3260.3751	1630.6912	3243.3486	1622.1779	3242.3646	1621.6859	C	307.1435	154.0754	290.1169	145.5621			2
30							K	147.1128	74.0600	130.0863	65.5468			1

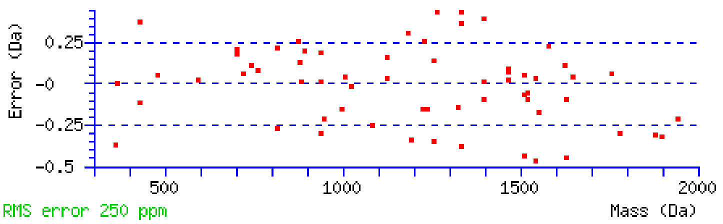

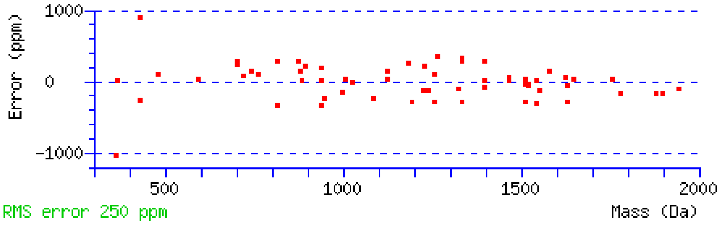

All matches to this query

Score	Mr(calc):	Delta	Sequence
59.9	3405.4734	1.0207	LHNPPTVNYSPNEGQMDLCITSGCQDGLCK
59.7	3405.4734	1.0207	LHNPPTVNYSPNEGQMDLCITSGCQDGLCK
57.5	3405.4734	1.0207	LHNPPTVNYSPNEGQMDLCITSGCQDGLCK
56.9	3404.4894	2.0047	LHNPPTVNYSPNEGQMDLCITSGCQDGLCK
4.5	3404.5045	1.9896	MLAPASPVLMNGGQSPPAFQDALQAAMQYPK
2.8	3406.5259	-0.0318	SGTATIQITVVDTNDNAPVFTQPEYHISVK
2.2	3406.5233	-0.0292	KFEFVEPMLSFFQGMFTFYHQGHELSK
2.0	3405.5118	0.9823	MGSWKSGQSYLAAGLLQNQVAVVTGGATGIGK
1.9	3404.4808	2.0133	LQAGTVFINTYNKTDVAAPFGGFKQSGFGK
1.9	3404.4808	2.0133	LQAGTVFINTYNKTDVAAPFGGFKQSGFGK

Spectrum No: 327; Query: 1171; Rank: 1

Peptide View

MS/MS Fragmentation of **YIEQFKDTLNTSVATSIAGFFAEPIQGVNGVVQYPK**
Found in **IPI00213584**, Tax_Id=10116 Gene_Symbol=Agxt2 Alanine--glyoxylate aminotransferase 2, mitochondrial precursor

Match to Query 1171: 3932.004912 from(1311.675580,3+)
Title: 091129RatKid_SCX02_21.7864.7864.3.dta
Data file K:\NewmanPaper\Piliang\3SubProteomes\Piliang3SP\mgf5ppm\SCX_3SubProteomes5ppm.mgf

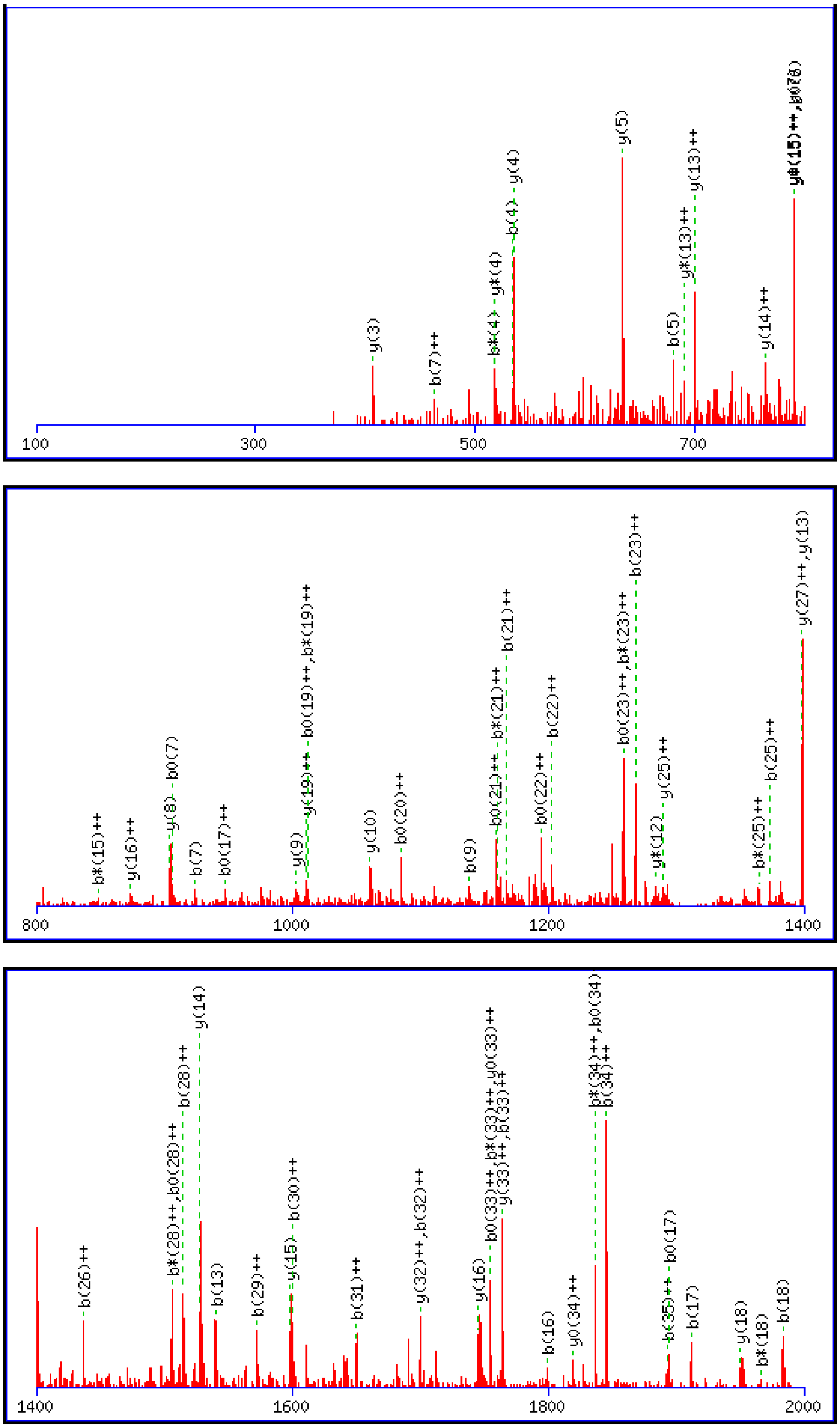

Monoisotopic mass of neutral peptide Mr(calc): 3931.9884
Fixed modifications: Carbamidomethyl (C)
Variable modifications:
N10 : Deamidated_N (N)
Ions Score: 60 **Expect:** 0.00014
Matches (Bold Red): 71/384 fragment ions using 124 most intense peaks

#	b	b^{++}	b*	b^{*++}	b^0	b^{0++}	Seq.	y	y^{++}	y*	y^{*++}	y^0	y^{0++}	#
1	164.0706	82.5389					Y							36
2	277.1547	139.0810					I	3769.9323	1885.4698	3752.9058	1876.9565	3751.9218	1876.4645	35
3	406.1973	203.6023			388.1867	194.5970	E	3656.8483	1828.9278	3639.8217	1820.4145	3638.8377	**1819.9225**	34
4	**534.2558**	267.6316	**517.2293**	259.1183	516.2453	258.6263	Q	3527.8057	**1764.4065**	3510.7791	1755.8932	3509.7951	**1755.4012**	33
5	**681.3243**	341.1658	664.2977	332.6525	663.3137	332.1605	F	3399.7471	**1700.3772**	3382.7205	1691.8639	3381.7365	1691.3719	32

6	809.4192	405.2132	792.3927	396.7000	**791.4087**	396.2080	K	3252.6787	1626.8430	3235.6521	1618.3297	3234.6681	1617.8377	31
7	**924.4462**	**462.7267**	907.4196	454.2134	**906.4356**	453.7214	D	3124.5837	1562.7955	3107.5572	1554.2822	3106.5732	1553.7902	30
8	1025.4938	513.2506	1008.4673	504.7373	1007.4833	504.2453	T	3009.5568	1505.2820	2992.5302	1496.7688	2991.5462	1496.2767	29
9	**1138.5779**	569.7926	1121.5514	561.2793	1120.5673	560.7873	L	2908.5091	1454.7582	2891.4825	1446.2449	2890.4985	1445.7529	28
10	1253.6048	627.3061	1236.5783	618.7928	1235.5943	618.3008	N	2795.4250	**1398.2162**	2778.3985	1389.7029	2777.4145	1389.2109	27
11	1354.6525	677.8299	1337.6260	669.3166	1336.6420	668.8246	T	2680.3981	1340.7027	2663.3715	1332.1894	2662.3875	1331.6974	26
12	1441.6845	721.3459	1424.6580	712.8326	1423.6740	712.3406	S	2579.3504	**1290.1788**	2562.3239	1281.6656	2561.3399	1281.1736	25
13	**1540.7530**	770.8801	1523.7264	762.3668	1522.7424	761.8748	V	2492.3184	1246.6628	2475.2918	1238.1496	2474.3078	1237.6576	24
14	1611.7901	806.3987	1594.7635	797.8854	1593.7795	797.3934	A	2393.2500	1197.1286	2376.2234	1188.6154	2375.2394	1188.1233	23
15	1712.8378	856.9225	1695.8112	**848.4092**	1694.8272	847.9172	T	2322.2129	1161.6101	2305.1863	1153.0968	2304.2023	1152.6048	22
16	**1799.8698**	900.4385	1782.8432	891.9253	1781.8592	891.4332	S	2221.1652	1111.0862	2204.1386	1102.5730	2203.1546	1102.0809	21
17	**1912.9538**	956.9806	1895.9273	948.4673	**1894.9433**	**947.9753**	I	2134.1332	1067.5702	2117.1066	1059.0569	2116.1226	1058.5649	20
18	**1983.9910**	992.4991	**1966.9644**	983.9858	1965.9804	983.4938	A	2021.0491	**1011.0282**	2004.0225	1002.5149	2003.0385	1002.0229	19
19	2041.0124	1021.0098	2023.9859	**1012.4966**	2023.0019	**1012.0046**	G	**1950.0120**	975.5096	1932.9854	966.9964	1932.0014	966.5043	18
20	2188.0808	1094.5441	2171.0543	1086.0308	2170.0703	**1085.5388**	F	1892.9905	946.9989	1875.9640	938.4856	1874.9799	937.9936	17
21	2335.1493	**1168.0783**	2318.1227	**1159.5650**	2317.1387	**1159.0730**	F	**1745.9221**	**873.4647**	1728.8956	864.9514	1727.9115	864.4594	16
22	2406.1864	**1203.5968**	2389.1598	1195.0835	2388.1758	**1194.5915**	A	**1598.8537**	799.9305	1581.8271	**791.4172**	1580.8431	**790.9252**	15
23	2535.2290	**1268.1181**	2518.2024	**1259.6048**	2517.2184	**1259.1128**	E	**1527.8166**	**764.4119**	1510.7900	755.8986	1509.8060	755.4066	14
24	2632.2817	1316.6445	2615.2552	1308.1312	2614.2712	1307.6392	P	**1398.7740**	**699.8906**	1381.7474	**691.3774**			13
25	2745.3658	**1373.1865**	2728.3392	**1364.6733**	2727.3552	1364.1812	I	1301.7212	651.3642	**1284.6947**	642.8510			12
26	2873.4244	**1437.2158**	2856.3978	1428.7025	2855.4138	1428.2105	Q	1188.6371	594.8222	1171.6106	586.3089			11
27	2930.4458	1465.7266	2913.4193	1457.2133	2912.4353	1456.7213	G	**1060.5786**	530.7929	1043.5520	522.2796			10
28	3029.5142	**1515.2608**	3012.4877	**1506.7475**	3011.5037	**1506.2555**	V	**1003.5571**	502.2822	986.5306	493.7689			9
29	3143.5572	**1572.2822**	3126.5306	1563.7689	3125.5466	1563.2769	N	**904.4887**	452.7480	887.4621	444.2347			8
30	3200.5786	**1600.7930**	3183.5521	1592.2797	3182.5681	1591.7877	G	**790.4458**	395.7265	773.4192	387.2132			7
31	3299.6470	**1650.3272**	3282.6205	1641.8139	3281.6365	1641.3219	V	733.4243	367.2158	716.3978	358.7025			6
32	3398.7155	**1699.8614**	3381.6889	1691.3481	3380.7049	1690.8561	V	**634.3559**	317.6816	617.3293	309.1683			5
33	3526.7740	**1763.8907**	3509.7475	**1755.3774**	3508.7635	**1754.8854**	Q	**535.2875**	268.1474	**518.2609**	259.6341			4
34	3689.8374	**1845.4223**	3672.8108	**1836.9090**	3671.8268	**1836.4170**	Y	**407.2289**	204.1181	390.2023	195.6048			3
35	3786.8901	**1893.9487**	3769.8636	1885.4354	3768.8796	1884.9434	P	244.1656	122.5864	227.1390	114.0731			2
36							K	147.1128	74.0600	130.0863	65.5468			1

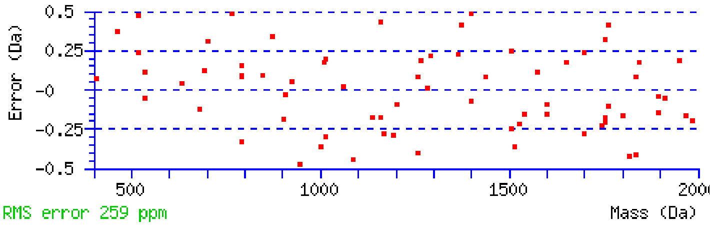

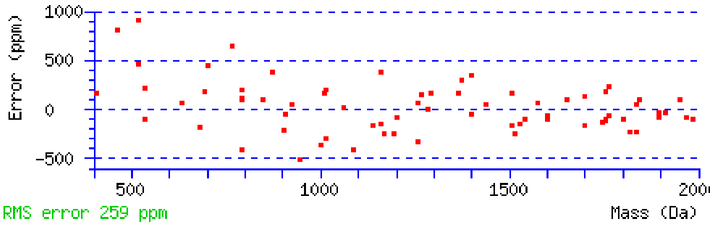

All matches to this query

Score	Mr(calc):	Delta	Sequence
59.9	3931.9884	0.0165	YIEQFKDTLNTSVATSIAGFFAEPIQGVNGVVQYPK
58.2	3931.9884	0.0165	YIEQFKDTLNTSVATSIAGFFAEPIQGVNGVVQYPK
46.9	3931.0044	1.0005	YIEQFKDTLNTSVATSIAGFFAEPIQGVNGVVQYPK
9.3	3932.0039	0.0010	NSVMELIANGTLSILEEENHIKDVLSRIVVEMIK
9.3	3932.0039	0.0010	NSVMELIANGTLSILEEENHIKDVLSRIVVEMIK
6.8	3932.0039	0.0010	NSVMELIANGTLSILEEENHIKDVLSRIVVEMIK

Spectrum No: 328; Query: 219; Rank: 1

Peptide View

MS/MS Fragmentation of **VIPVCNASLPAQR**
Found in **IPI00192504**, Tax_Id=10116 Gene_Symbol=Mrc2 Macrophage mannose receptor 2 precursor

Match to Query 219: 1424.748268 from(713.381410,2+)
Title: 091129RatKid_SCX02_12.1255.1255.2.dta
Data file K:\NewmanPaper\Piliang\3SubProteomes\Piliang3SP\mgf5ppm\SCX_3SubProteomes5ppm.mgf

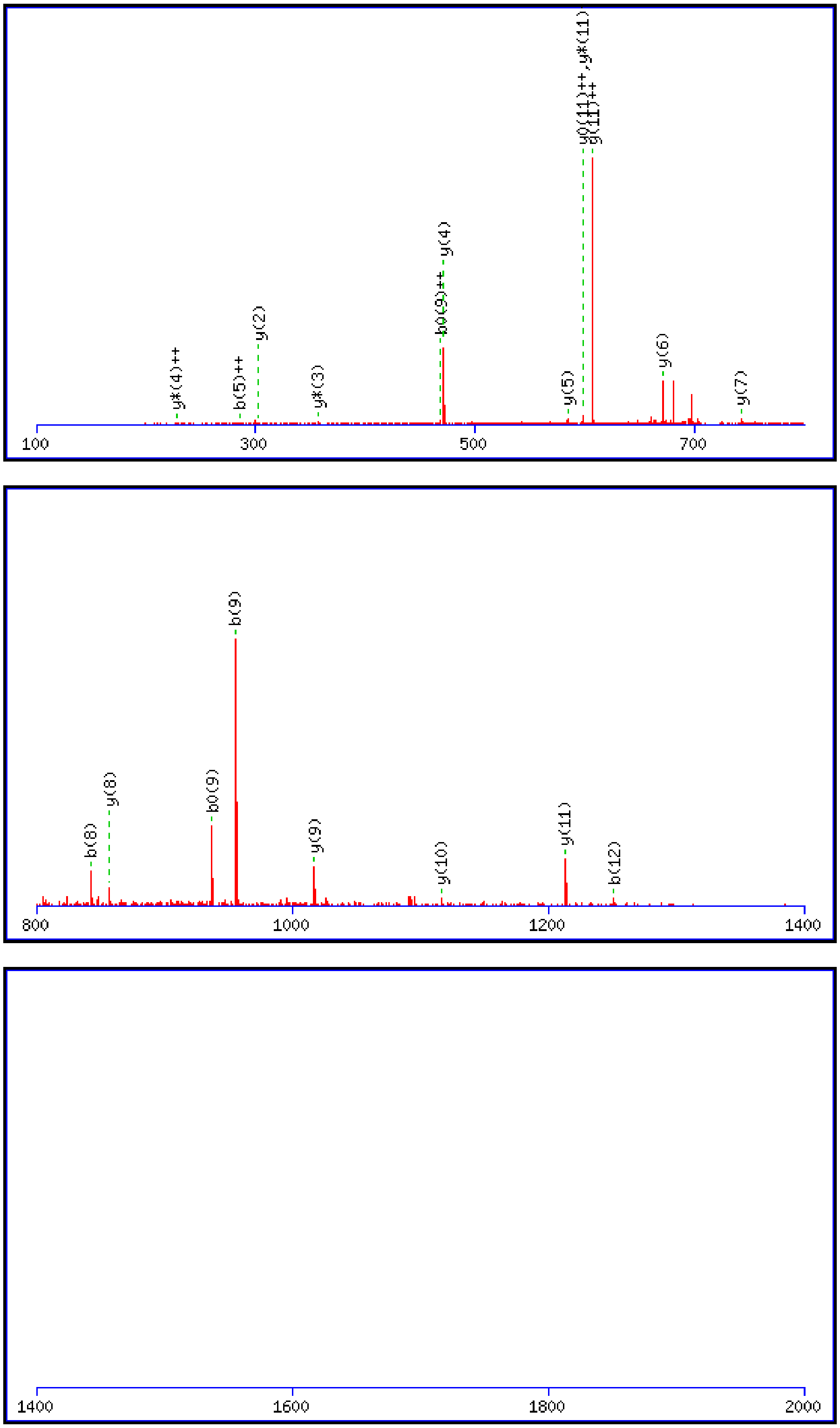

Monoisotopic mass of neutral peptide Mr(calc): 1424.7446
Fixed modifications: Carbamidomethyl (C)
Variable modifications:

N6 : Deamidated_N (N)
Ions Score: 60 **Expect:** 9.9e-005
Matches (Bold Red): 20/110 fragment ions using 50 most intense peaks

#	b	b^{++}	b*	b^{*++}	b^{0}	b^{0++}	Seq.	y	y^{++}	y*	y^{*++}	y^{0}	y^{0++}	#
1	100.0757	50.5415					V							13
2	213.1598	107.0835					I	1326.6834	663.8454	1309.6569	655.3321	1308.6729	654.8401	12
3	310.2125	155.6099					P	**1213.5994**	**607.3033**	1196.5728	**598.7901**	1195.5888	**598.2980**	11
4	409.2809	205.1441					V	**1116.5466**	558.7769	1099.5201	550.2637	1098.5360	549.7717	10
5	569.3116	**285.1594**					C	**1017.4782**	509.2427	1000.4516	500.7295	999.4676	500.2375	9
6	684.3385	342.6729	667.3120	334.1596			N	**857.4475**	429.2274	840.4210	420.7141	839.4370	420.2221	8
7	755.3756	378.1915	738.3491	369.6782			A	**742.4206**	371.7139	725.3941	363.2007	724.4100	362.7087	7
8	**842.4077**	421.7075	825.3811	413.1942	824.3971	412.7022	S	**671.3835**	336.1954	654.3570	327.6821	653.3729	327.1901	6
9	**955.4917**	478.2495	938.4652	469.7362	**937.4812**	**469.2442**	L	**584.3515**	292.6794	567.3249	284.1661			5
10	1052.5445	526.7759	1035.5179	518.2626	1034.5339	517.7706	P	**471.2674**	236.1373	454.2409	**227.6241**			4
11	1123.5816	562.2944	1106.5551	553.7812	1105.5710	553.2892	A	374.2146	187.6110	**357.1881**	179.0977			3
12	**1251.6402**	626.3237	1234.6136	617.8105	1233.6296	617.3184	Q	**303.1775**	152.0924	286.1510	143.5791			2
13							R	175.1190	88.0631	158.0924	79.5498			1

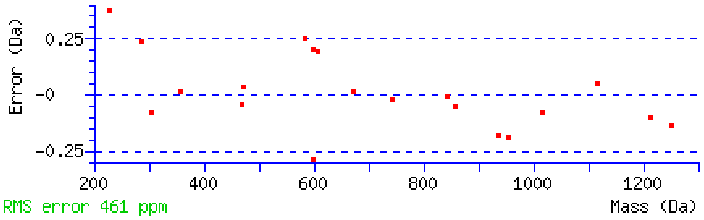

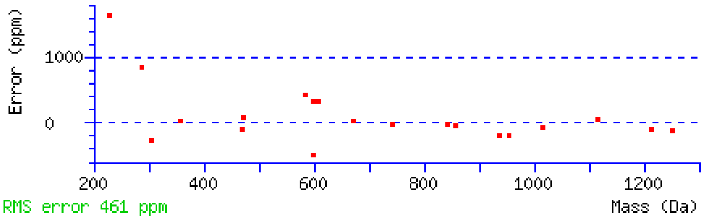

All matches to this query

Score	Mr(calc):	Delta	Sequence
59.8	1424.7446	0.0037	VIPVCNASLPAQR
12.5	1423.7320	1.0163	NPPVLYHNSRAR
8.4	1424.7558	-0.0075	NSRLLMSSFSKR
7.8	1423.7323	1.0159	LGSKVITDPSLSK
7.8	1423.7323	1.0159	LGSKVITDPSLSK
7.8	1423.7323	1.0159	LGSKVITDPSLSK
4.0	1424.7388	0.0095	LSKVSTAEKINR
3.2	1422.7306	2.0177	MSGDLPKQVKLK
2.8	1424.7581	-0.0098	LPPHILWATGLK
2.1	1424.7412	0.0071	NPIHKIQEGDFK

Spectrum No: 329; Query: 134; Rank: 1

Peptide View

MS/MS Fragmentation of **FNDTNVVHTVK**
Found in **IPI00327469**, Tax_Id=10116 Gene_Symbol=Ahsg Alpha-2-HS-glycoprotein precursor

Match to Query 134: 1273.626568 from(637.820560,2+)
Title: 091129RatKid_SCX02_14.739.739.2.dta
Data file K:\NewmanPaper\Piliang\3SubProteomes\Piliang3SP\mgf5ppm\SCX_3SubProteomes5ppm.mgf

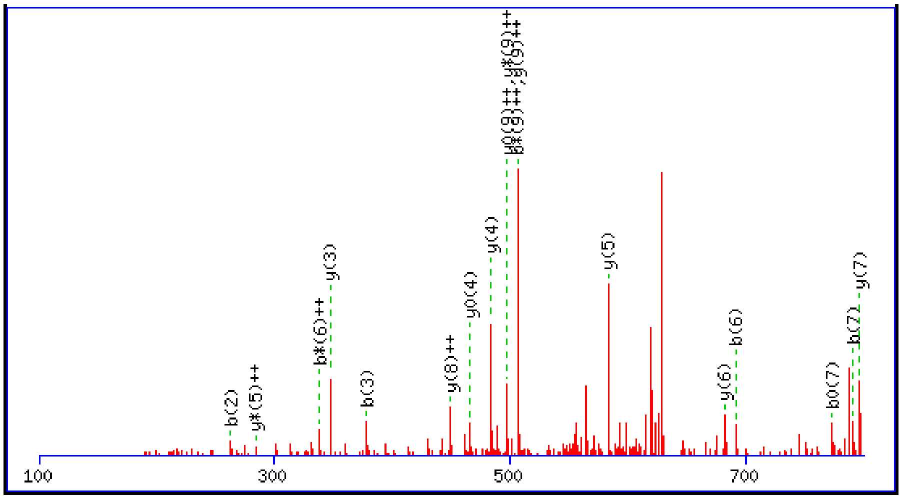

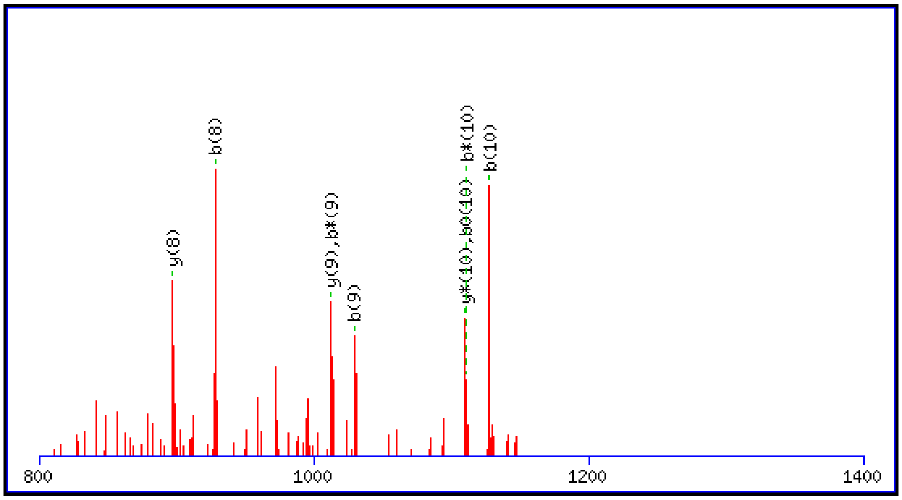

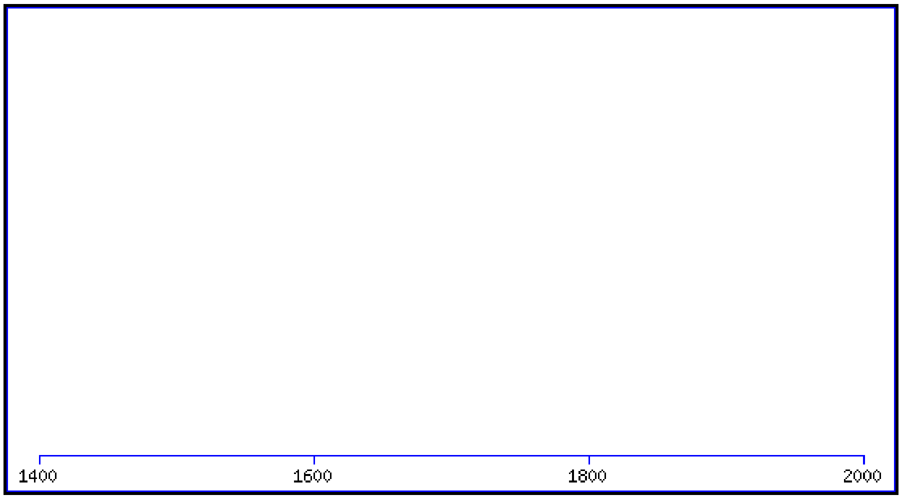

Monoisotopic mass of neutral peptide Mr(calc): 1273.6303
Fixed modifications: Carbamidomethyl (C)
Variable modifications:
N2 : Deamidated_N (N)
Ions Score: 60 **Expect:** 0.00014
Matches (Bold Red): 27/110 fragment ions using 34 most intense peaks

#	b	b^{++}	b*	b^{*++}	b^0	b^{0++}	Seq.	y	y^{++}	y*	y^{*++}	y^0	y^{0++}	#
1	148.0757	74.5415					F							11
2	**263.1026**	132.0550	246.0761	123.5417			N	1127.5691	564.2882	**1110.5426**	555.7749	1109.5586	555.2829	10
3	**378.1296**	189.5684	361.1030	181.0551	360.1190	180.5631	D	**1012.5422**	**506.7747**	995.5156	**498.2615**	994.5316	**497.7694**	9
4	479.1772	240.0923	462.1507	231.5790	461.1667	231.0870	T	**897.5152**	**449.2613**	880.4887	440.7480	879.5047	440.2560	8
5	593.2202	297.1137	576.1936	288.6005	575.2096	288.1084	N	**796.4676**	398.7374	779.4410	390.2241	778.4570	389.7321	7

6	692.2886	346.6479	675.2620	338.1347	674.2780	337.6427	V	682.4246	341.7160	665.3981	333.2027	664.4141	332.7107	6
7	791.3570	396.1821	774.3305	387.6689	773.3464	387.1769	V	583.3562	292.1817	566.3297	283.6685	565.3457	283.1765	5
8	928.4159	464.7116	911.3894	456.1983	910.4053	455.7063	H	484.2878	242.6475	467.2613	234.1343	466.2772	233.6423	4
9	1029.4636	515.2354	1012.4370	506.7222	1011.4530	506.2302	T	347.2289	174.1181	330.2023	165.6048	329.2183	165.1128	3
10	1128.5320	564.7696	1111.5055	556.2564	1110.5214	555.7644	V	246.1812	123.5942	229.1547	115.0810			2
11							K	147.1128	74.0600	130.0863	65.5468			1

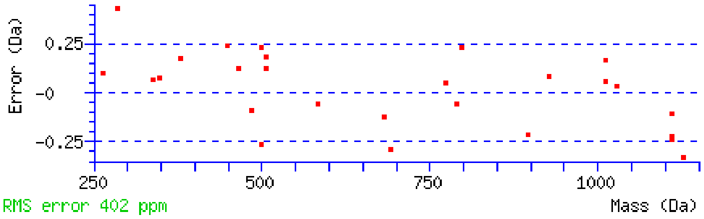

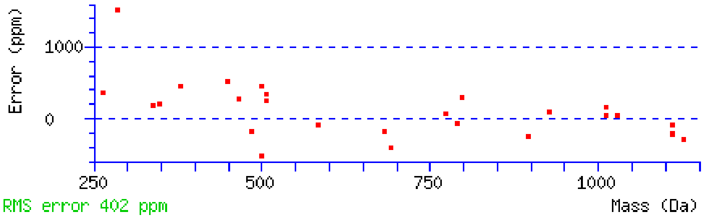

All matches to this query

Score	Mr(calc):	Delta	Sequence
59.8	1273.6303	-0.0037	FNDTNVVHTVK
40.7	1273.6303	-0.0037	FNDTNVVHTVK
9.8	1272.6115	1.0151	ANKAVDFTTVK
9.7	1273.6319	-0.0053	SLTSLVLYGNK
9.6	1273.6319	-0.0053	SLTSLVLYGNK
8.9	1273.6180	0.0086	QLGGKSHGVSPK
8.3	1273.6336	-0.0070	TALPNMDREVK
8.3	1272.6115	1.0151	ANKAVDFTTVK
7.4	1273.6263	0.0003	VNLGVDSQGGTAR
7.2	1273.6150	0.0116	QEAENGALDISK

Spectrum No: 330; Query: 1032; Rank: 1

Peptide View

MS/MS Fragmentation of **HVCPVENWSYNDSCSPDPAEQGGPK**
Found in **IPI00214915**, Tax_Id=10116 Gene_Symbol=Tm6p1 Transmembrane protein 150 precursor

Match to Query 1032: 2830.165872 from(944.395900,3+)
Title: 091127RatKid_SCX01_12.1927.1927.3.dta
Data file K:\NewmanPaper\Piliang\3SubProteomes\Piliang3SP\mgf5ppm\SCX_3SubProteomes5ppm.mgf

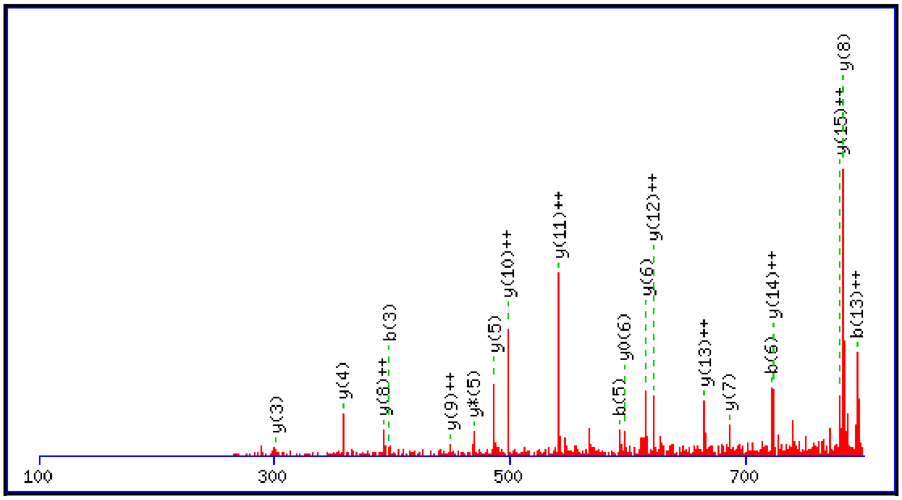

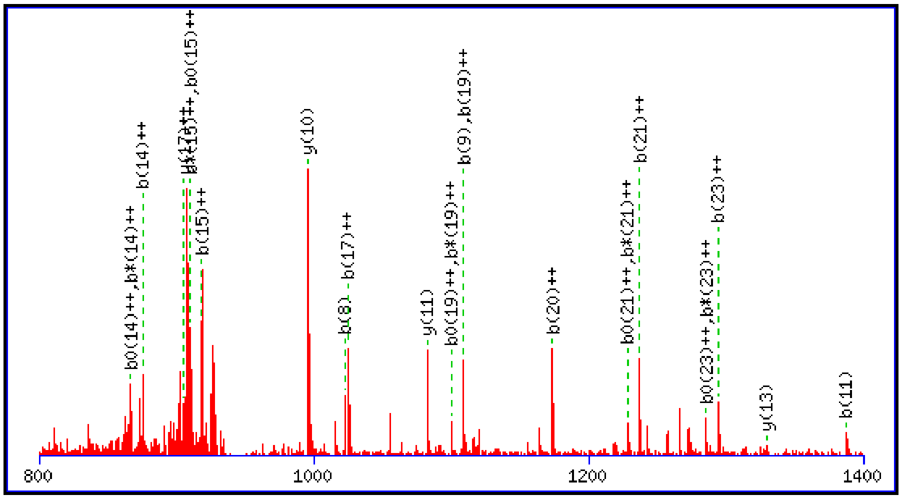

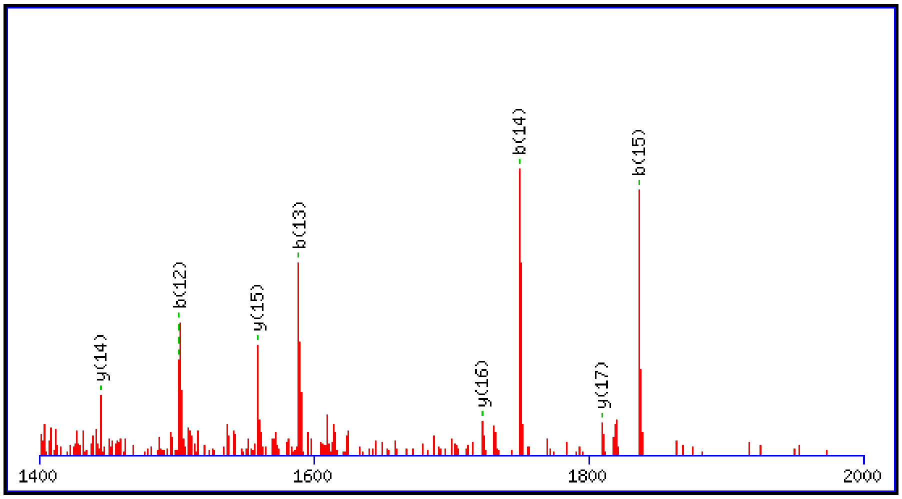

Monoisotopic mass of neutral peptide Mr(calc): 2830.1599
Fixed modifications: Carbamidomethyl (C)
Variable modifications:
N11 : Deamidated_N (N)
Ions Score: 60 **Expect:** 0.00035
Matches (Bold Red): 52/256 fragment ions using 104 most intense peaks

#	b	b^{++}	b*	b^{*++}	b^0	b^{0++}	Seq.	y	y^{++}	y*	y^{*++}	y^0	y^{0++}	#
1	138.0662	69.5367					H							25
2	237.1346	119.0709					V	2694.1083	1347.5578	2677.0817	1339.0445	2676.0977	1338.5525	24
3	**397.1653**	199.0863					C	2595.0399	1298.0236	2578.0133	1289.5103	2577.0293	1289.0183	23
4	494.2180	247.6126					P	2435.0092	1218.0082	2417.9827	1209.4950	2416.9986	1209.0030	22
5	**593.2864**	297.1469					V	2337.9564	1169.4819	2320.9299	1160.9686	2319.9459	1160.4766	21
6	**722.3290**	361.6681			704.3185	352.6629	E	2238.8880	1119.9477	2221.8615	1111.4344	2220.8775	1110.9424	20
7	836.3719	418.6896	819.3454	410.1763	818.3614	409.6843	N	2109.8454	1055.4264	2092.8189	1046.9131	2091.8349	1046.4211	19
8	**1022.4513**	511.7293	1005.4247	503.2160	1004.4407	502.7240	W	1995.8025	998.4049	1978.7760	989.8916	1977.7919	989.3996	18
9	**1109.4833**	555.2453	1092.4567	546.7320	1091.4727	546.2400	S	**1809.7232**	**905.3652**	1792.6966	896.8520	1791.7126	896.3600	17
10	1272.5466	636.7769	1255.5201	628.2637	1254.5361	627.7717	Y	**1722.6912**	861.8492	1705.6646	853.3359	1704.6806	852.8439	16
11	**1387.5736**	694.2904	1370.5470	685.7771	1369.5630	685.2851	N	**1559.6278**	**780.3176**	1542.6013	771.8043	1541.6173	771.3123	15
12	**1502.6005**	751.8039	1485.5739	743.2906	1484.5899	742.7986	D	**1444.6009**	**722.8041**	1427.5744	714.2908	1426.5903	713.7988	14
13	**1589.6325**	**795.3199**	1572.6060	786.8066	1571.6220	786.3146	S	**1329.5740**	**665.2906**	1312.5474	656.7773	1311.5634	656.2853	13
14	**1749.6632**	**875.3352**	1732.6366	**866.8220**	1731.6526	**866.3299**	C	1242.5419	**621.7746**	1225.5154	613.2613	1224.5314	612.7693	12
15	**1836.6952**	**918.8512**	1819.6687	**910.3380**	1818.6846	**909.8460**	S	**1082.5113**	**541.7593**	1065.4847	533.2460	1064.5007	532.7540	11
16	1933.7480	967.3776	1916.7214	958.8643	1915.7374	958.3723	P	**995.4793**	**498.2433**	978.4527	489.7300	977.4687	489.2380	10
17	2048.7749	**1024.8911**	2031.7484	1016.3778	2030.7643	1015.8858	D	898.4265	**449.7169**	881.3999	441.2036	880.4159	440.7116	9
18	2145.8277	1073.4175	2128.8011	1064.9042	2127.8171	1064.4122	P	**783.3995**	**392.2034**	766.3730	383.6901	765.3890	383.1981	8

**19**	2216.8648	**1108.9360**	2199.8382	**1100.4228**	2198.8542	**1099.9307**	**A**	**686.3468**	343.6770	669.3202	335.1638	668.3362	334.6717	**7**
**20**	2345.9074	**1173.4573**	2328.8808	1164.9441	2327.8968	1164.4520	**E**	**615.3097**	308.1585	598.2831	299.6452	**597.2991**	299.1532	**6**
**21**	2473.9660	**1237.4866**	2456.9394	**1228.9733**	2455.9554	**1228.4813**	**Q**	**486.2671**	243.6372	**469.2405**	235.1239			**5**
**22**	2530.9874	1265.9973	2513.9609	1257.4841	2512.9769	1256.9921	**G**	**358.2085**	179.6079	341.1819	171.0946			**4**
**23**	2588.0089	**1294.5081**	2570.9823	**1285.9948**	2569.9983	**1285.5028**	**G**	**301.1870**	151.0972	284.1605	142.5839			**3**
**24**	2685.0617	1343.0345	2668.0351	1334.5212	2667.0511	1334.0292	**P**	244.1656	122.5864	227.1390	114.0731			**2**
**25**							**K**	147.1128	74.0600	130.0863	65.5468			**1**

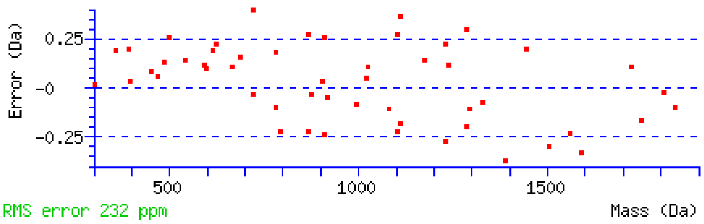

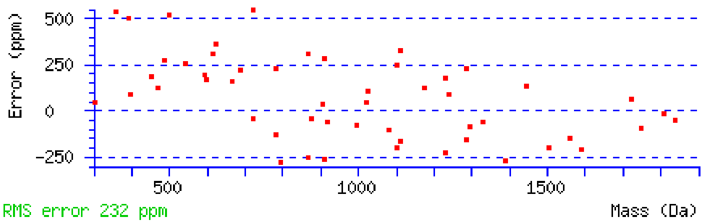

All matches to this query

Score	Mr(calc):	Delta	Sequence
59.7	2830.1599	0.0060	HVCPVENWSYNDSCSPDPAEQGGPK
40.8	2830.1599	0.0060	HVCPVENWSYNDSCSPDPAEQGGPK
31.1	2829.1759	0.9900	HVCPVENWSYNDSCSPDPAEQGGPK
3.4	2828.1783	1.9876	CGNMSDGEGGSLFVRNASADVTFSIR
3.0	2830.1687	-0.0028	GRSPSDISSDVSIQMTQNYPPAIK
2.9	2829.1483	1.0176	ELDSSPGLKYAHTQCSLQAQTQK
1.9	2828.1838	1.9821	MSGDQTAQDPGSSLGELDQHNVVKR
1.9	2828.1838	1.9821	MSGDQTAQDPGSSLGELDQHNVVKR
1.5	2830.1687	-0.0028	GRSPSDISSDVSIQMTQNYPPAIK
0.7	2830.1752	-0.0093	KEVQENLVNIPEGTQRNTEYVK

Spectrum No: 331; Query: 949; Rank: 1

Peptide View

MS/MS Fragmentation of **DLNISLDTDAPLSVGKDYGVSAYR**
Found in **IPI00326990**, Tax_Id=10116 Gene_Symbol=Slc15a2 Oligopeptide transporter, kidney isoform

Match to Query 949: 2569.266972 from(857.429600,3+)
Title: 091129RatKid_SCX02_12.3373.3373.3.dta
Data file K:\NewmanPaper\Piliang\3SubProteomes\Piliang3SP\mgf5ppm\SCX_3SubProteomes5ppm.mgf

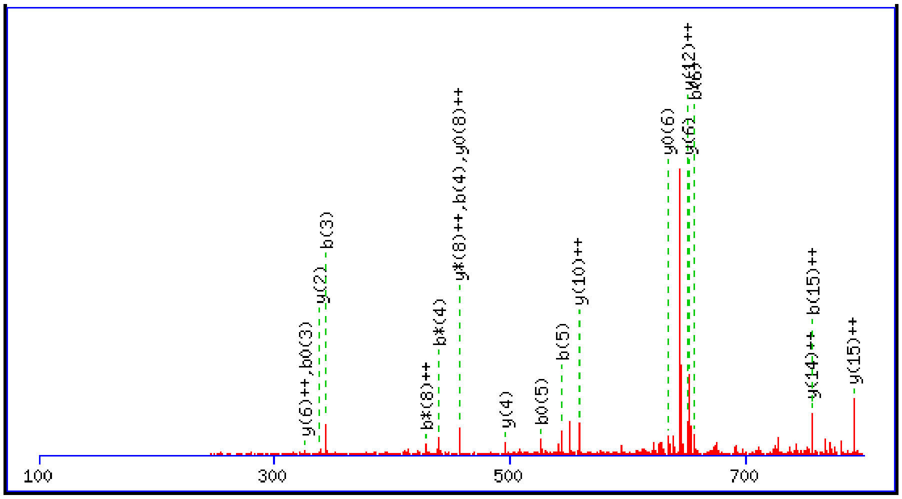

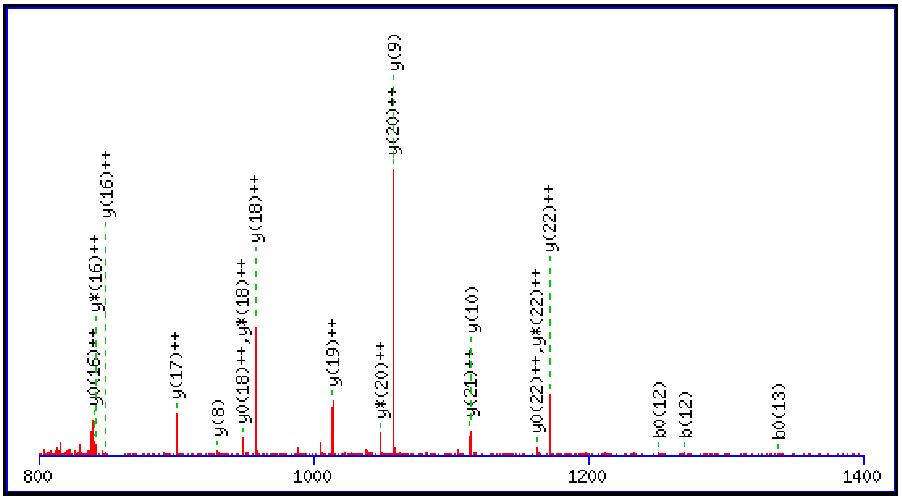

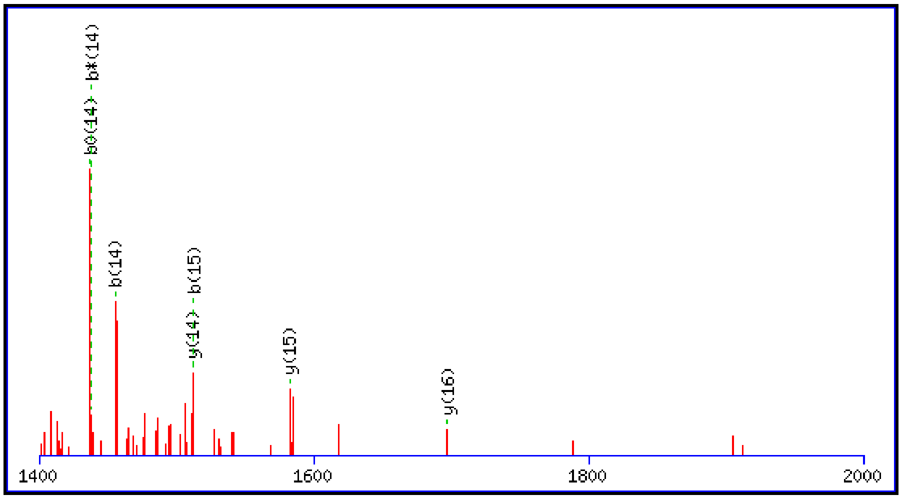

Monoisotopic mass of neutral peptide Mr(calc): 2569.2548
Fixed modifications: Carbamidomethyl (C)
Variable modifications:
N3 : Deamidated_N (N)
Ions Score: 60 **Expect:** 0.00026
Matches (Bold Red): 47/266 fragment ions using 87 most intense peaks

#	b	b++	b*	b*++	b0	b0++	Seq.	y	y++	y*	y*++	y0	y0++	#
1	116.0342	58.5207			98.0237	49.5155	D							24
2	229.1183	115.0628			211.1077	106.0575	L	2455.2351	1228.1212	2438.2086	1219.6079	2437.2245	1219.1159	23
3	**344.1452**	172.5762	327.1187	164.0630	**326.1347**	163.5710	N	2342.1510	**1171.5792**	2325.1245	**1163.0659**	2324.1405	**1162.5739**	22
4	**457.2293**	229.1183	**440.2027**	220.6050	439.2187	220.1130	I	2227.1241	**1114.0657**	2210.0976	1105.5524	2209.1135	1105.0604	21
5	**544.2613**	272.6343	527.2348	264.1210	**526.2507**	263.6290	S	2114.0400	**1057.5237**	2097.0135	**1049.0104**	2096.0295	1048.5184	20

6	**657.3454**	329.1763	640.3188	320.6631	639.3348	320.1710	L	2027.0080	**1014.0076**	2009.9815	1005.4944	2008.9974	1005.0024	19
7	772.3723	386.6898	755.3458	378.1765	754.3618	377.6845	D	1913.9239	**957.4656**	1896.8974	**948.9523**	1895.9134	**948.4603**	18
8	873.4200	437.2136	856.3934	**428.7004**	855.4094	428.2084	T	1798.8970	**899.9521**	1781.8705	891.4389	1780.8864	890.9469	17
9	988.4469	494.7271	971.4204	486.2138	970.4364	485.7218	D	**1697.8493**	**849.4283**	1680.8228	**840.9150**	1679.8388	**840.4230**	16
10	1059.4841	530.2457	1042.4575	521.7324	1041.4735	521.2404	A	**1582.8224**	**791.9148**	1565.7958	783.4016	1564.8118	782.9095	15
11	1156.5368	578.7720	1139.5103	570.2588	1138.5263	569.7668	P	**1511.7853**	**756.3963**	1494.7587	747.8830	1493.7747	747.3910	14
12	**1269.6209**	635.3141	1252.5943	626.8008	**1251.6103**	626.3088	L	1414.7325	707.8699	1397.7060	699.3566	1396.7219	698.8646	13
13	1356.6529	678.8301	1339.6264	670.3168	**1338.6423**	669.8248	S	1301.6484	**651.3279**	1284.6219	642.8146	1283.6379	642.3226	12
14	**1455.7213**	728.3643	**1438.6948**	719.8510	**1437.7108**	719.3590	V	1214.6164	607.8118	1197.5899	599.2986	1196.6058	598.8066	11
15	**1512.7428**	**756.8750**	1495.7162	748.3618	1494.7322	747.8697	G	**1115.5480**	**558.2776**	1098.5215	549.7644	1097.5374	549.2724	10
16	1640.8378	820.9225	1623.8112	812.4092	1622.8272	811.9172	K	**1058.5265**	529.7669	1041.5000	521.2536	1040.5160	520.7616	9
17	1755.8647	878.4360	1738.8381	869.9227	1737.8541	869.4307	D	**930.4316**	465.7194	913.4050	**457.2062**	912.4210	**456.7141**	8
18	1918.9280	959.9676	1901.9015	951.4544	1900.9175	950.9624	Y	815.4046	408.2060	798.3781	399.6927	797.3941	399.2007	7
19	1975.9495	988.4784	1958.9229	979.9651	1957.9389	979.4731	G	**652.3413**	**326.6743**	635.3148	318.1610	**634.3307**	317.6690	6
20	2075.0179	1038.0126	2057.9914	1029.4993	2057.0073	1029.0073	V	595.3198	298.1636	578.2933	289.6503	577.3093	289.1583	5
21	2162.0499	1081.5286	2145.0234	1073.0153	2144.0394	1072.5233	S	**496.2514**	248.6293	479.2249	240.1161	478.2409	239.6241	4
22	2233.0870	1117.0472	2216.0605	1108.5339	2215.0765	1108.0419	A	409.2194	205.1133	392.1928	196.6001			3
23	2396.1504	1198.5788	2379.1238	1190.0655	2378.1398	1189.5735	Y	**338.1823**	169.5948	321.1557	161.0815			2
24							R	175.1190	88.0631	158.0924	79.5498			1

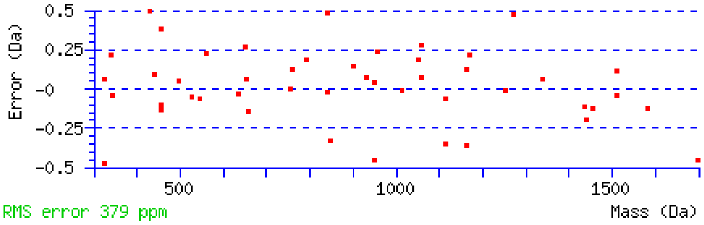

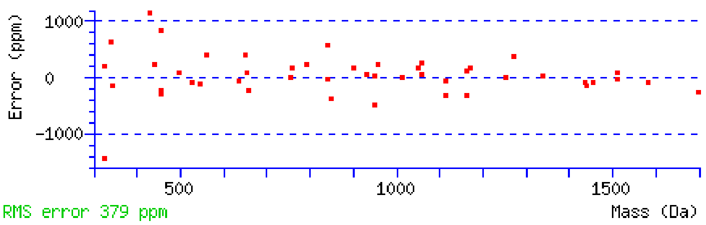

All matches to this query

Score	Mr(calc):	Delta	Sequence
59.7	2569.2548	0.0122	DLNISLDTDAPLSVGKDYGVSAYR
32.8	2568.2708	0.9962	DLNISLDTDAPLSVGKDYGVSAYR
4.8	2568.2676	0.9993	ALEGMCSGGGVTANAPAAAPGGVTLVPK
2.9	2569.2516	0.0153	ALEGMCSGGGVTANAPAAAPGGVTLVPK
2.3	2567.2424	2.0246	KPMVGRKVCILGDCSGVVGDGGVK
2.0	2568.2430	1.0239	TVFVSGLPPAPALSPPSFDLAGLR
2.0	2568.2585	1.0085	NIDRTDLQTITNDPRLVFTVR
1.9	2568.2672	0.9998	IIITNTNCWSPRKAQSRPAFK
1.9	2569.2636	0.0034	RKLTSGSLSEDLGSAGGSGEVILEK
1.8	2568.2430	1.0239	TVFVSGLPPAPALSPPSFDLAGLR

Spectrum No: 332; Query: 258; Rank: 1

Peptide View

MS/MS Fragmentation of **SFSGLLDCGNCSR**
Found in **IPI00391769**, Tax_Id=10116 Gene_Symbol=Cacna2d1 Voltage-gated calcium channel alpha2/delta-1 subunit

Match to Query 258: 1472.604108 from(737.309330,2+)
Title: 091127RatKid_SCX01_13.2231.2231.2.dta
Data file K:\NewmanPaper\Piliang\3SubProteomes\Piliang3SP\mgf5ppm\SCX_3SubProteomes5ppm.mgf

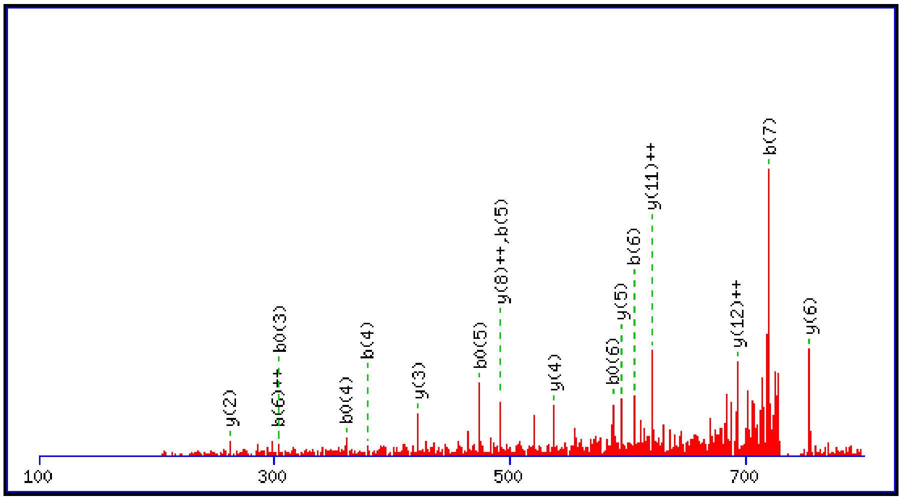

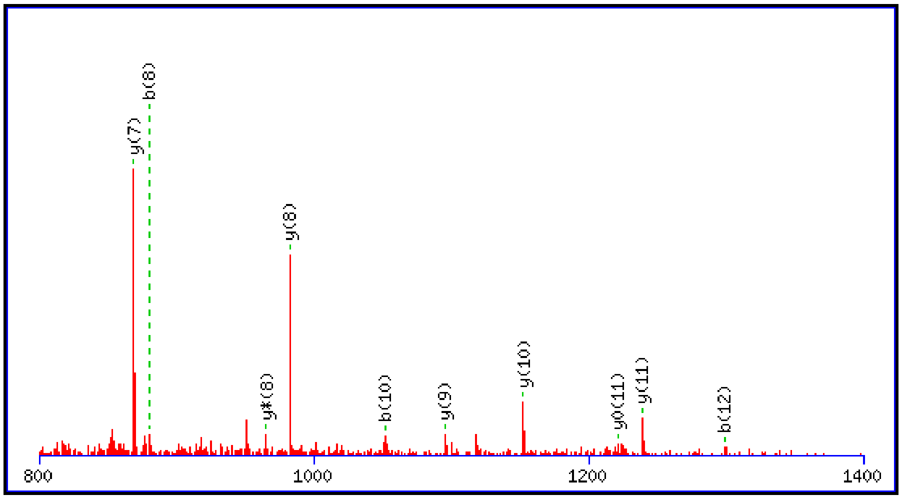

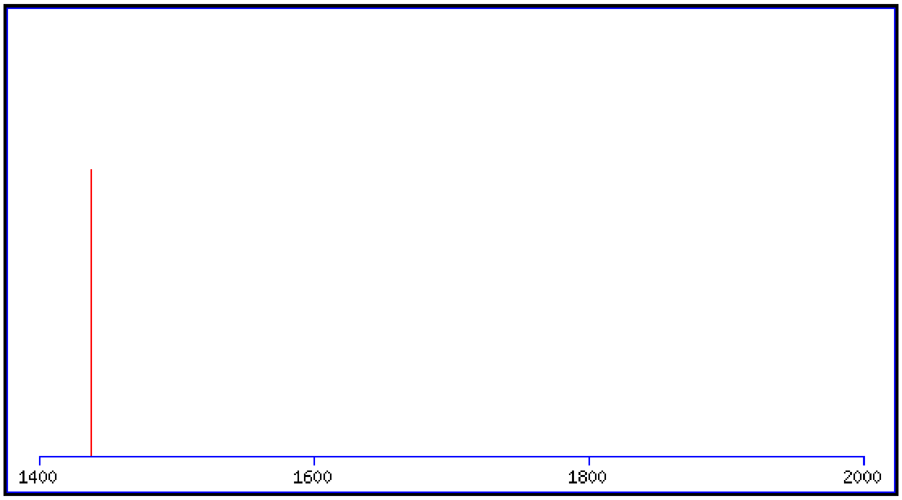

Monoisotopic mass of neutral peptide Mr(calc): 1472.6024
Fixed modifications: Carbamidomethyl (C)
Variable modifications:
N10 : Deamidated_N (N)
Ions Score: 60 **Expect:** 0.00013
Matches (Bold Red): 27/124 fragment ions using 56 most intense peaks

#	b	b^{++}	b*	b^{*++}	b^{0}	b^{0++}	Seq.	y	y^{++}	y*	y^{*++}	y^{0}	y^{0++}	#
1	88.0393	44.5233			70.0287	35.5180	S							13
2	235.1077	118.0575			217.0972	109.0522	F	1386.5777	**693.7925**	1369.5511	685.2792	1368.5671	684.7872	12
3	322.1397	161.5735			**304.1292**	152.5682	S	**1239.5092**	**620.2583**	1222.4827	611.7450	**1221.4987**	611.2530	11
4	**379.1612**	190.0842			**361.1506**	181.0790	G	**1152.4772**	576.7422	1135.4507	568.2290	1134.4666	567.7370	10
5	**492.2453**	246.6263			**474.2347**	237.6210	L	**1095.4557**	548.2315	1078.4292	539.7182	1077.4452	539.2262	9

6	605.3293	303.1683			587.3188	294.1630	L	982.3717	491.6895	965.3451	483.1762	964.3611	482.6842	8
7	720.3563	360.6818			702.3457	351.6765	D	869.2876	435.1474	852.2611	426.6342	851.2771	426.1422	7
8	880.3869	440.6971			862.3764	431.6918	C	754.2607	377.6340	737.2341	369.1207	736.2501	368.6287	6
9	937.4084	469.2078			919.3978	460.2026	G	594.2300	297.6187	577.2035	289.1054	576.2195	288.6134	5
10	1052.4353	526.7213	1035.4088	518.2080	1034.4248	517.7160	N	537.2086	269.1079	520.1820	260.5946	519.1980	260.1026	4
11	1212.4660	606.7366	1195.4394	598.2234	1194.4554	597.7313	C	422.1816	211.5945	405.1551	203.0812	404.1711	202.5892	3
12	1299.4980	650.2526	1282.4715	641.7394	1281.4874	641.2474	S	262.1510	131.5791	245.1244	123.0659	244.1404	122.5738	2
13							R	175.1190	88.0631	158.0924	79.5498			1

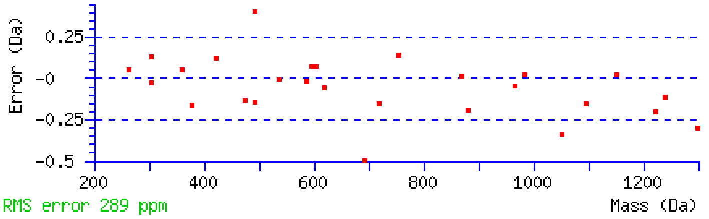

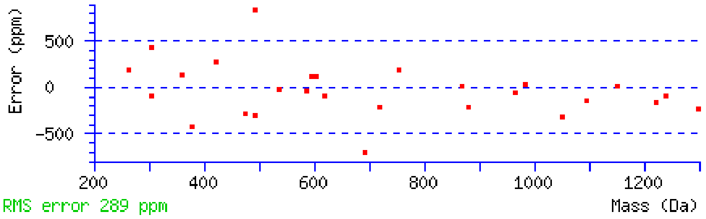

All matches to this query

Score	Mr(calc):	Delta	Sequence
59.6	1472.6024	0.0017	SFSGLLDCGNCSR
5.1	1472.6007	0.0034	MTYEGDPIPAQR
4.9	1472.6003	0.0038	RSSIWQFFSR
2.4	1472.6007	0.0034	MTYEGDPIPAQR
0.6	1470.6062	1.9979	DVSEDTGVPMVSR
0.3	1471.6109	0.9932	RHSELSELNVK

Spectrum No: 333; Query: 1123; Rank: 1

Peptide View

MS/MS Fragmentation of **LTFFNSTLNTSGLSTQGEALPIPGAHRPGVVTK**
Found in **IPI00196725**, Tax_Id=10116 Gene_Symbol=Fthfd 10-formyltetrahydrofolate dehydrogenase

Match to Query 1123: 3410.800182 from(1137.940670,3+)
Title: 091129RatKid_SCX02_30.4769.4769.3.dta
Data file K:\NewmanPaper\Piliang\3SubProteomes\Piliang3SP\mgf5ppm\SCX_3SubProteomes5ppm.mgf

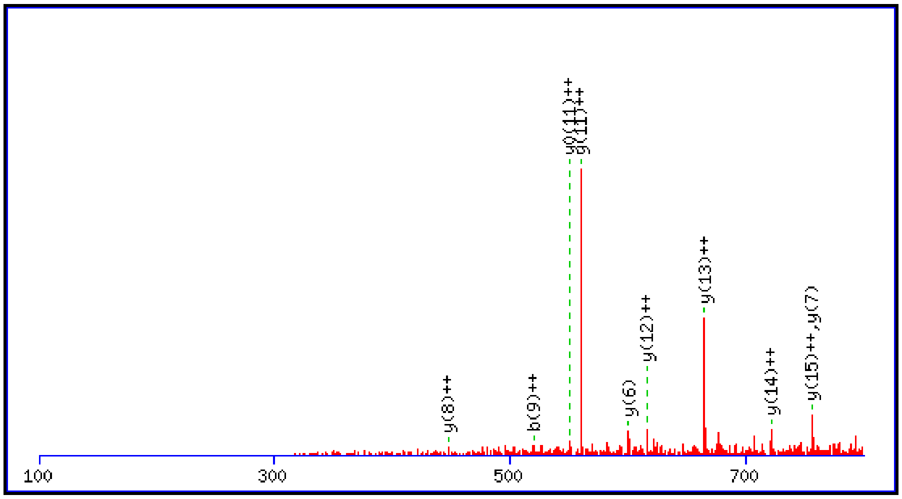

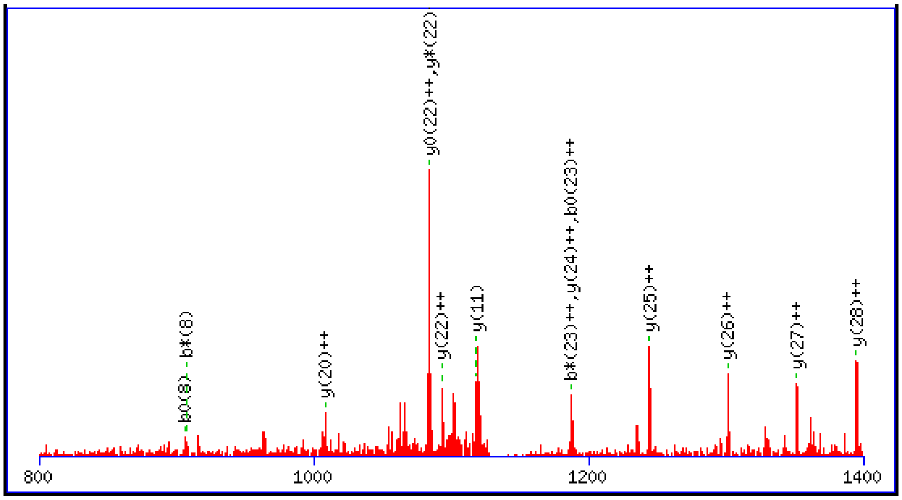

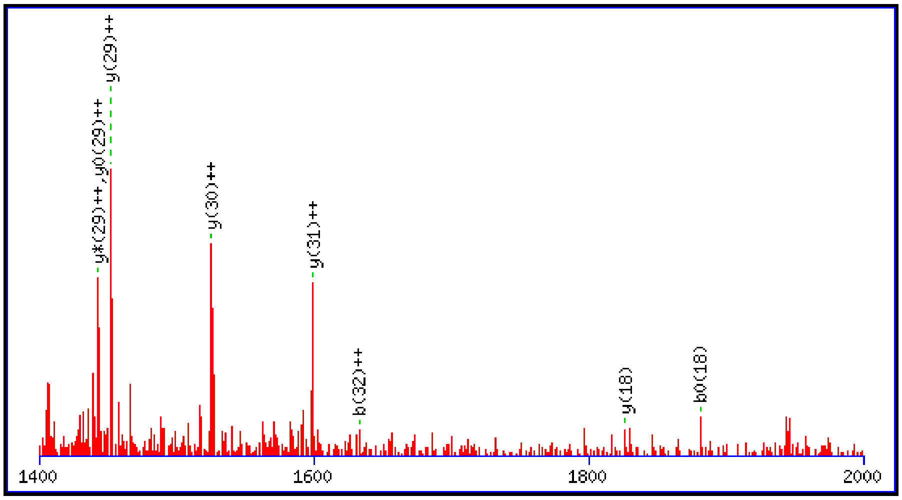

Monoisotopic mass of neutral peptide Mr(calc): 3410.7834
Fixed modifications: Carbamidomethyl (C)
Variable modifications:
N9 : Deamidated_N (N)
Ions Score: 60 **Expect:** 7.9e-005
Matches (Bold Red): 32/372 fragment ions using 76 most intense peaks

#	b	b^{++}	b*	b^{*++}	b^0	b^{0++}	Seq.	y	y^{++}	y*	y^{*++}	y^0	y^{0++}	#
1	114.0913	57.5493					L							33
2	215.1390	108.0731			197.1285	99.0679	T	3298.7066	1649.8570	3281.6801	1641.3437	3280.6961	1640.8517	32
3	362.2074	181.6074			344.1969	172.6021	F	3197.6589	**1599.3331**	3180.6324	1590.8198	3179.6484	1590.3278	31
4	509.2758	255.1416			491.2653	246.1363	F	3050.5905	**1525.7989**	3033.5640	1517.2856	3032.5800	1516.7936	30
5	623.3188	312.1630	606.2922	303.6498	605.3082	303.1577	N	2903.5221	**1452.2647**	2886.4956	**1443.7514**	2885.5116	**1443.2594**	29
6	710.3508	355.6790	693.3243	347.1658	692.3402	346.6738	S	2789.4792	**1395.2432**	2772.4526	1386.7300	2771.4686	1386.2380	28
7	811.3985	406.2029	794.3719	397.6896	793.3879	397.1976	T	2702.4472	**1351.7272**	2685.4206	1343.2139	2684.4366	1342.7219	27
8	924.4825	462.7449	**907.4560**	454.2316	**906.4720**	453.7396	L	2601.3995	**1301.2034**	2584.3729	1292.6901	2583.3889	1292.1981	26
9	1039.5095	**520.2584**	1022.4829	511.7451	1021.4989	511.2531	N	2488.3154	**1244.6613**	2471.2889	1236.1481	2470.3049	1235.6561	25
10	1140.5572	570.7822	1123.5306	562.2689	1122.5466	561.7769	T	2373.2885	**1187.1479**	2356.2619	1178.6346	2355.2779	1178.1426	24
11	1227.5892	614.2982	1210.5626	605.7850	1209.5786	605.2929	S	2272.2408	1136.6240	2255.2143	1128.1108	2254.2302	1127.6188	23
12	1284.6107	642.8090	1267.5841	634.2957	1266.6001	633.8037	G	2185.2088	**1093.1080**	2168.1822	**1084.5948**	2167.1982	**1084.1027**	22
13	1397.6947	699.3510	1380.6682	690.8377	1379.6842	690.3457	L	2128.1873	1064.5973	2111.1608	1056.0840	2110.1768	1055.5920	21
14	1484.7267	742.8670	1467.7002	734.3537	1466.7162	733.8617	S	2015.1033	**1008.0553**	1998.0767	999.5420	1997.0927	999.0500	20
15	1585.7744	793.3908	1568.7479	784.8776	1567.7639	784.3856	T	1928.0712	964.5392	1911.0447	956.0260	1910.0607	955.5340	19
16	1713.8330	857.4201	1696.8065	848.9069	1695.8224	848.4149	Q	**1827.0235**	914.0154	1809.9970	905.5021	1809.0130	905.0101	18
17	1770.8545	885.9309	1753.8279	877.4176	1752.8439	876.9256	G	1698.9650	849.9861	1681.9384	841.4728	1680.9544	840.9808	17
18	1899.8971	950.4522	1882.8705	941.9389	**1881.8865**	941.4469	E	1641.9435	821.4754	1624.9170	812.9621	1623.9329	812.4701	16

**19**	1970.9342	985.9707	1953.9076	977.4574	1952.9236	976.9654	**A**	1512.9009	**756.9541**	1495.8744	748.4408	1494.8903	747.9488	**15**
**20**	2084.0182	1042.5128	2066.9917	1033.9995	2066.0077	1033.5075	**L**	1441.8638	**721.4355**	1424.8372	712.9223	1423.8532	712.4303	**14**
**21**	2181.0710	1091.0391	2164.0445	1082.5259	2163.0604	1082.0339	**P**	1328.7797	**664.8935**	1311.7532	656.3802	1310.7692	655.8882	**13**
**22**	2294.1551	1147.5812	2277.1285	1139.0679	2276.1445	1138.5759	**I**	1231.7270	**616.3671**	1214.7004	607.8538	1213.7164	607.3618	**12**
**23**	2391.2078	1196.1076	2374.1813	**1187.5943**	2373.1973	**1187.1023**	**P**	**1118.6429**	**559.8251**	1101.6164	551.3118	1100.6323	**550.8198**	**11**
**24**	2448.2293	1224.6183	2431.2027	1216.1050	2430.2187	1215.6130	**G**	1021.5901	511.2987	1004.5636	502.7854	1003.5796	502.2934	**10**
**25**	2519.2664	1260.1368	2502.2399	1251.6236	2501.2558	1251.1316	**A**	964.5687	482.7880	947.5421	474.2747	946.5581	473.7827	**9**
**26**	2656.3253	1328.6663	2639.2988	1320.1530	2638.3148	1319.6610	**H**	893.5316	**447.2694**	876.5050	438.7561	875.5210	438.2641	**8**
**27**	2812.4264	1406.7169	2795.3999	1398.2036	2794.4159	1397.7116	**R**	**756.4726**	378.7400	739.4461	370.2267	738.4621	369.7347	**7**
**28**	2909.4792	1455.2432	2892.4526	1446.7300	2891.4686	1446.2380	**P**	**600.3715**	300.6894	583.3450	292.1761	582.3610	291.6841	**6**
**29**	2966.5007	1483.7540	2949.4741	1475.2407	2948.4901	1474.7487	**G**	503.3188	252.1630	486.2922	243.6498	485.3082	243.1577	**5**
**30**	3065.5691	1533.2882	3048.5425	1524.7749	3047.5585	1524.2829	**V**	446.2973	223.6523	429.2708	215.1390	428.2867	214.6470	**4**
**31**	3164.6375	1582.8224	3147.6109	1574.3091	3146.6269	1573.8171	**V**	347.2289	174.1181	330.2023	165.6048	329.2183	165.1128	**3**
**32**	3265.6852	**1633.3462**	3248.6586	1624.8329	3247.6746	1624.3409	**T**	248.1605	124.5839	231.1339	116.0706	230.1499	115.5786	**2**
**33**							**K**	147.1128	74.0600	130.0863	65.5468			**1**

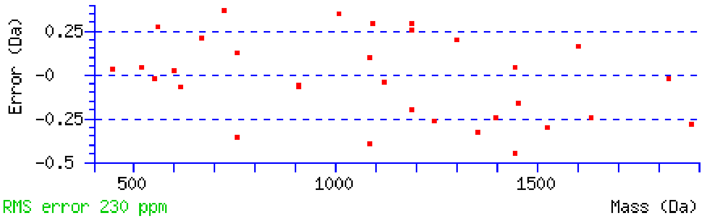

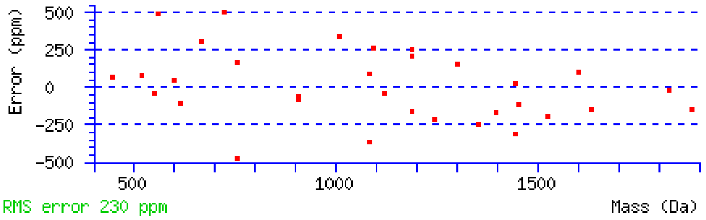

All matches to this query

Score	Mr(calc):	Delta	Sequence
59.6	3410.7834	0.0168	LTFFNSTLNTSGLSTQGEALPIPGAHRPGVVTK
48.7	3410.7834	0.0168	LTFFNSTLNTSGLSTQGEALPIPGAHRPGVVTK
45.3	3409.7994	1.0008	LTFFNSTLNTSGLSTQGEALPIPGAHRPGVVTK
0.7	3410.8124	-0.0122	SYENVDTSSQGSTISVDSQEDQLDS

Spectrum No: 334; Query: 342; Rank: 1

Peptide View

MS/MS Fragmentation of **GVPGAIVNVSSQASQR**
Found in **IPI00200794**, Tax_Id=10116 Gene_Symbol=Dcxr L-xylulose reductase

Match to Query 342: 1569.807268 from(785.910910,2+)
Title: 091129RatKid_SCX02_20.1663.1663.2.dta
Data file K:\NewmanPaper\Piliang\3SubProteomes\Piliang3SP\mgf5ppm\SCX_3SubProteomes5ppm.mgf

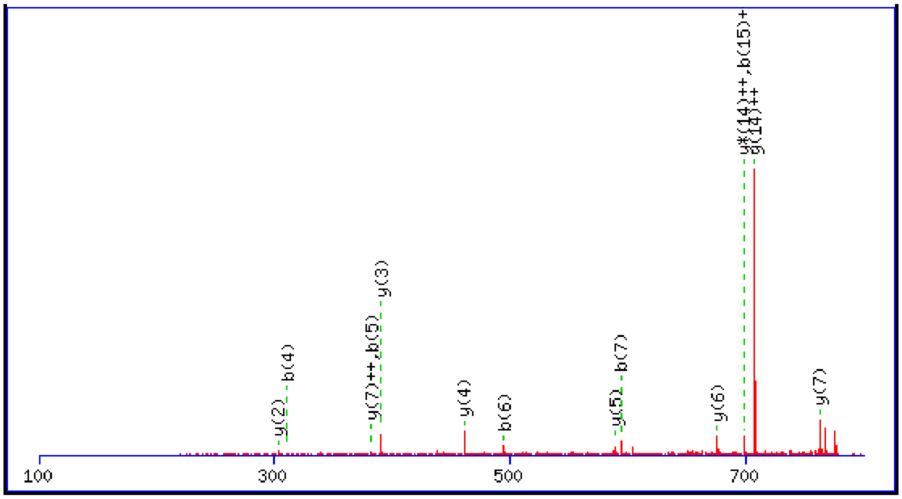

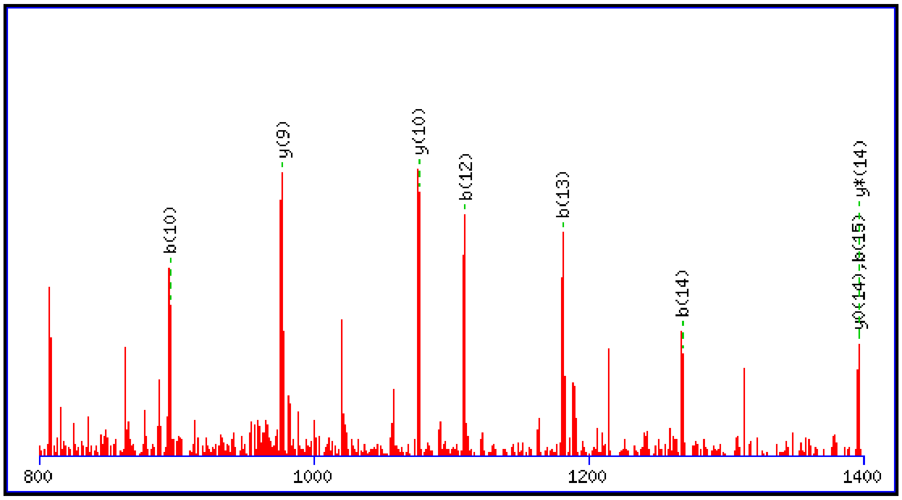

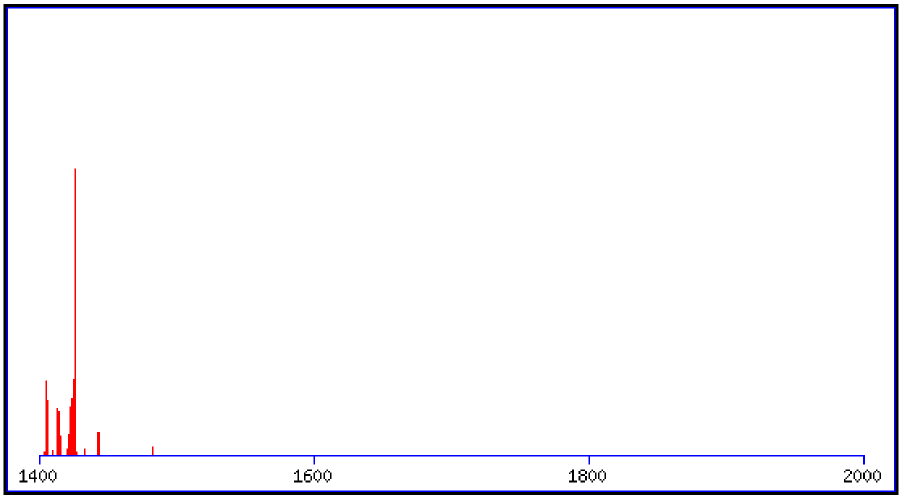

Monoisotopic mass of neutral peptide Mr(calc): 1569.8111
Fixed modifications: Carbamidomethyl (C)
Variable modifications:
N8 : Deamidated_N (N)
Ions Score: 59 **Expect:** 0.00014
Matches (Bold Red): 24/144 fragment ions using 34 most intense peaks

#	b	b^{++}	b*	b^{*++}	b^{0}	b^{0++}	Seq.	y	y^{++}	y*	y^{*++}	y^{0}	y^{0++}	#
1	58.0287	29.5180					G							16
2	157.0972	79.0522					V	1513.7969	757.4021	1496.7703	748.8888	1495.7863	748.3968	15
3	254.1499	127.5786					P	1414.7285	**707.8679**	**1397.7019**	**699.3546**	**1396.7179**	**698.8626**	14
4	**311.1714**	156.0893					G	1317.6757	659.3415	1300.6492	650.8282	1299.6651	650.3362	13
5	**382.2085**	191.6079					A	1260.6542	630.8308	1243.6277	622.3175	1242.6437	621.8255	12

**6**	**495.2926**	248.1499					**I**	1189.6171	595.3122	1172.5906	586.7989	1171.6066	586.3069	**11**
**7**	**594.3610**	297.6841					**V**	**1076.5331**	538.7702	1059.5065	530.2569	1058.5225	529.7649	**10**
**8**	709.3879	355.1976	692.3614	346.6843			**N**	**977.4647**	489.2360	960.4381	480.7227	959.4541	480.2307	**9**
**9**	808.4563	404.7318	791.4298	396.2185			**V**	862.4377	431.7225	845.4112	423.2092	844.4272	422.7172	**8**
**10**	**895.4884**	448.2478	878.4618	439.7345	877.4778	439.2425	**S**	**763.3693**	**382.1883**	746.3428	373.6750	745.3587	373.1830	**7**
**11**	982.5204	491.7638	965.4938	483.2506	964.5098	482.7585	**S**	**676.3373**	338.6723	659.3107	330.1590	658.3267	329.6670	**6**
**12**	**1110.5790**	555.7931	1093.5524	547.2798	1092.5684	546.7878	**Q**	**589.3052**	295.1563	572.2787	286.6430	571.2947	286.1510	**5**
**13**	**1181.6161**	591.3117	1164.5895	582.7984	1163.6055	582.3064	**A**	**461.2467**	231.1270	444.2201	222.6137	443.2361	222.1217	**4**
**14**	**1268.6481**	634.8277	1251.6216	626.3144	1250.6375	625.8224	**S**	**390.2096**	195.6084	373.1830	187.0951	372.1990	186.6031	**3**
**15**	**1396.7067**	**698.8570**	1379.6801	690.3437	1378.6961	689.8517	**Q**	**303.1775**	152.0924	286.1510	143.5791			**2**
**16**							**R**	175.1190	88.0631	158.0924	79.5498			**1**

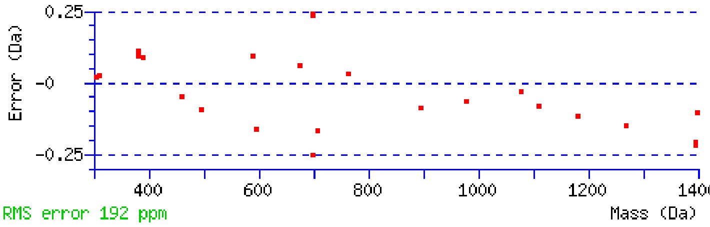

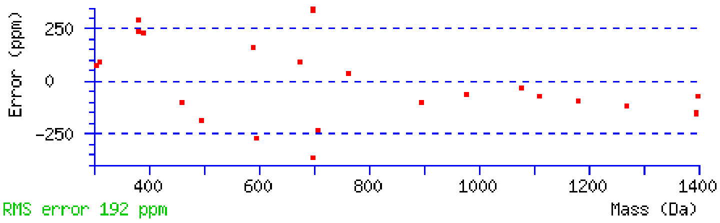

All matches to this query

Score	Mr(calc):	Delta	Sequence
59.5	1569.8111	-0.0038	GVPGAIVNVSSQASQR
17.6	1569.8028	0.0045	RFIGSIVKEASQR
8.2	1568.8055	1.0018	TIMDQSISLFMRK
7.6	1568.8086	0.9986	DGYVDISLLVSFNK
5.8	1569.8151	-0.0078	DPDRSLPGGTLAFPK
4.8	1568.7923	1.0150	KSPSAISSGTLASKR
4.8	1568.7923	1.0150	KSPSAISSGTLASKR
4.6	1568.7923	1.0150	KSPSAISSGTLASKR
4.4	1568.7923	1.0150	KSPSAISSGTLASKR
4.4	1568.7898	1.0175	IMNHSSILRKYK

Spectrum No: 335; Query: 1020; Rank: 1

Peptide View

MS/MS Fragmentation of **HTGPGILSMANAGPNTNGSQFFICTAK**
Found in **IPI00364439**, Tax_Id=10116 Gene_Symbol=RGD1559682_predicted similar to Peptidyl-prolyl cis-trans isomerase A

Match to Query 1020: 2807.305272 from(936.775700,3+)
Title: 091127RatKid_SCX01_27.3385.3385.3.dta
Data file K:\NewmanPaper\Piliang\3SubProteomes\Piliang3SP\mgf5ppm\SCX_3SubProteomes5ppm.mgf

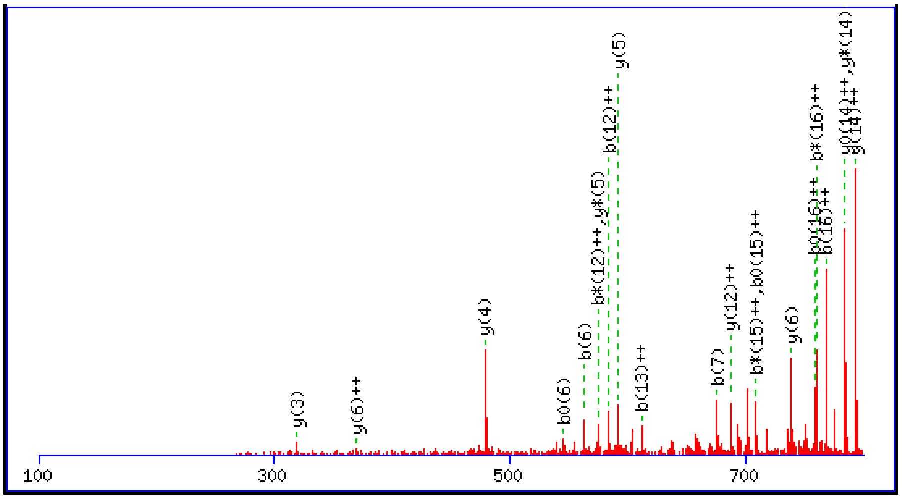

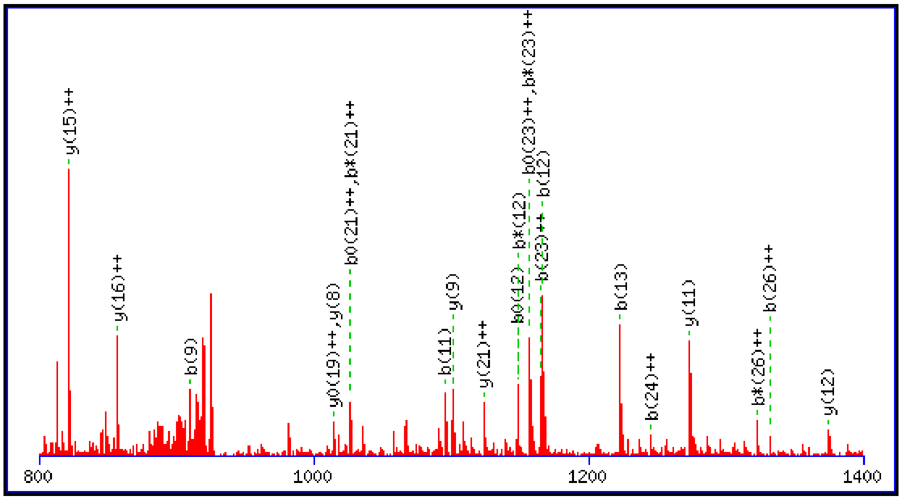

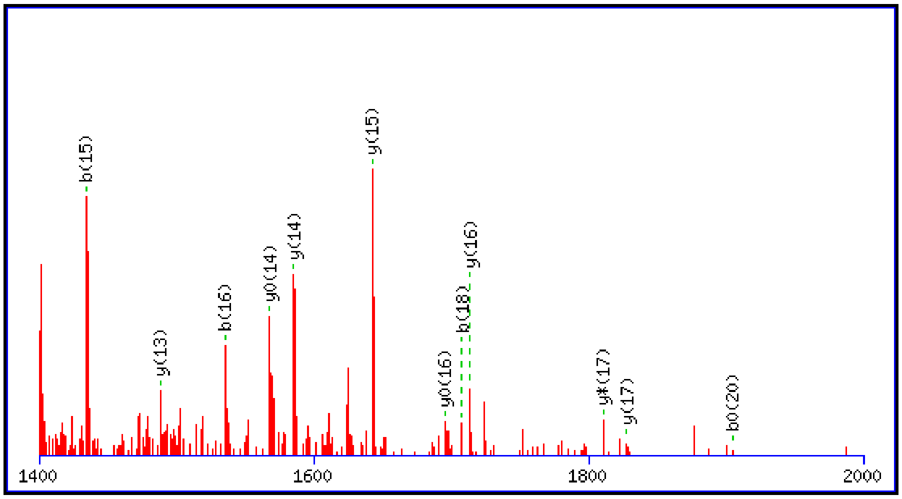

Monoisotopic mass of neutral peptide Mr(calc): 2807.3007
Fixed modifications: Carbamidomethyl (C)
Variable modifications:
M9 : Oxidation (M)
N17 : Deamidated_N (N)
Ions Score: 60 **Expect:** 0.00039
Matches (Bold Red): 55/286 fragment ions using 85 most intense peaks

#	b	b^{++}	b*	b^{*++}	b^0	b^{0++}	Seq.	y	y^{++}	y*	y^{*++}	y^0	y^{0++}	#
1	138.0662	69.5367					H							27
2	239.1139	120.0606			221.1033	111.0553	T	2671.2491	1336.1282	2654.2225	1327.6149	2653.2385	1327.1229	26
3	296.1353	148.5713			278.1248	139.5660	G	2570.2014	1285.6043	2553.1748	1277.0911	2552.1908	1276.5990	25
4	393.1881	197.0977			375.1775	188.0924	P	2513.1799	1257.0936	2496.1534	1248.5803	2495.1694	1248.0883	24

5	450.2096	225.6084			432.1990	216.6031	G	2416.1272	1208.5672	2399.1006	1200.0539	2398.1166	1199.5619	23
6	**563.2936**	282.1504			**545.2831**	273.1452	I	2359.1057	1180.0565	2342.0791	1171.5432	2341.0951	1171.0512	22
7	**676.3777**	338.6925			658.3671	329.6872	L	2246.0216	**1123.5145**	2228.9951	1115.0012	2228.0111	1114.5092	21
8	763.4097	382.2085			745.3991	373.2032	S	2132.9376	1066.9724	2115.9110	1058.4591	2114.9270	1057.9671	20
9	**910.4451**	455.7262			892.4346	446.7209	M	2045.9055	1023.4564	2028.8790	1014.9431	2027.8950	**1014.4511**	19
10	981.4822	491.2448			963.4717	482.2395	A	1898.8701	949.9387	1881.8436	941.4254	1880.8596	940.9334	18
11	**1095.5252**	548.2662	1078.4986	539.7529	1077.5146	539.2609	N	**1827.8330**	914.4201	**1810.8065**	905.9069	1809.8225	905.4149	17
12	**1166.5623**	**583.7848**	**1149.5357**	**575.2715**	**1148.5517**	574.7795	A	**1713.7901**	**857.3987**	1696.7635	848.8854	**1695.7795**	848.3934	16
13	**1223.5837**	**612.2955**	1206.5572	603.7822	1205.5732	603.2902	G	**1642.7530**	**821.8801**	1625.7264	813.3669	1624.7424	812.8748	15
14	1320.6365	660.8219	1303.6100	652.3086	1302.6259	651.8166	P	**1585.7315**	**793.3694**	1568.7050	**784.8561**	**1567.7209**	**784.3641**	14
15	**1434.6794**	717.8434	1417.6529	**709.3301**	1416.6689	**708.8381**	N	**1488.6787**	744.8430	1471.6522	736.3297	1470.6682	735.8377	13
16	**1535.7271**	**768.3672**	1518.7006	**759.8539**	1517.7165	**759.3619**	T	**1374.6358**	**687.8215**	1357.6093	679.3083	1356.6253	678.8163	12
17	1650.7540	825.8807	1633.7275	817.3674	1632.7435	816.8754	N	**1273.5881**	637.2977	1256.5616	628.7844	1255.5776	628.2924	11
18	**1707.7755**	854.3914	1690.7490	845.8781	1689.7649	845.3861	G	1158.5612	579.7842	1141.5347	571.2710	1140.5506	570.7790	10
19	1794.8075	897.9074	1777.7810	889.3941	1776.7970	888.9021	S	**1101.5397**	551.2735	1084.5132	542.7602	1083.5292	542.2682	9
20	1922.8661	961.9367	1905.8396	953.4234	**1904.8555**	952.9314	Q	**1014.5077**	507.7575	997.4812	499.2442	996.4972	498.7522	8
21	2069.9345	1035.4709	2052.9080	**1026.9576**	2051.9240	**1026.4656**	F	886.4491	443.7282	869.4226	435.2149	868.4386	434.7229	7
22	2217.0029	1109.0051	2199.9764	1100.4918	2198.9924	1099.9998	F	**739.3807**	**370.1940**	722.3542	361.6807	721.3702	361.1887	6
23	2330.0870	**1165.5471**	2313.0605	**1157.0339**	2312.0764	**1156.5419**	I	**592.3123**	296.6598	**575.2858**	288.1465	574.3017	287.6545	5
24	2490.1177	**1245.5625**	2473.0911	1237.0492	2472.1071	1236.5572	C	**479.2282**	240.1178	462.2017	231.6045	461.2177	231.1125	4
25	2591.1653	1296.0863	2574.1388	1287.5730	2573.1548	1287.0810	T	**319.1976**	160.1024	302.1710	151.5892	301.1870	151.0972	3
26	2662.2024	**1331.6049**	2645.1759	**1323.0916**	2644.1919	1322.5996	A	218.1499	109.5786	201.1234	101.0653			2
27							K	147.1128	74.0600	130.0863	65.5468			1

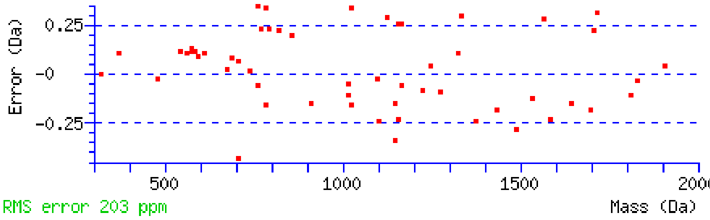

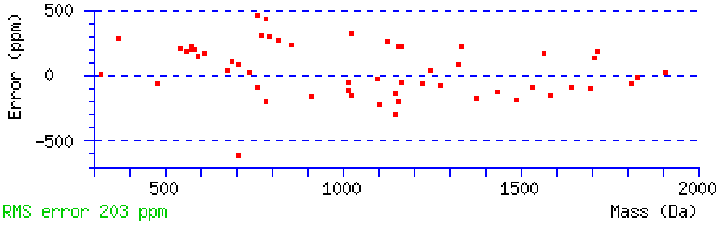

All matches to this query

Score	Mr(calc):	Delta	Sequence
59.5	2807.3007	0.0046	HTGPGILSMANAGPNTNGSQFFICTAK
51.7	2807.3007	0.0046	HTGPGILSMANAGPNTNGSQFFICTAK
31.1	2807.3007	0.0046	HTGPGILSMANAGPNTNGSQFFICTAK
19.4	2806.3167	0.9886	HTGPGILSMANAGPNTNGSQFFICTAK
1.2	2805.3095	1.9957	QMQNINPLTMCLNGVIDAAVNGGVSR
0.4	2807.3038	0.0015	QSHGKSLEWIGEIYPNSGATNFNEK
0.4	2807.2915	0.0137	AKSFEGNNNYDTPELRAFTPLSTR

Spectrum No: 336; Query: 612; Rank: 1

Peptide View

MS/MS Fragmentation of **DAFTNVSSPDAGATLPNDK**
Found in **IPI00369140**, Tax_Id=10116 Gene_Symbol=RGD1309019_predicted 38 kDa protein

Match to Query 612: 1919.881348 from(960.947950,2+)
Title: 091127RatKid_SCX01_02.2510.2510.2.dta

Data file K:\NewmanPaper\Piliang\3SubProteomes\Piliang3SP\mgf5ppm\SCX_3SubProteomes5ppm.mgf

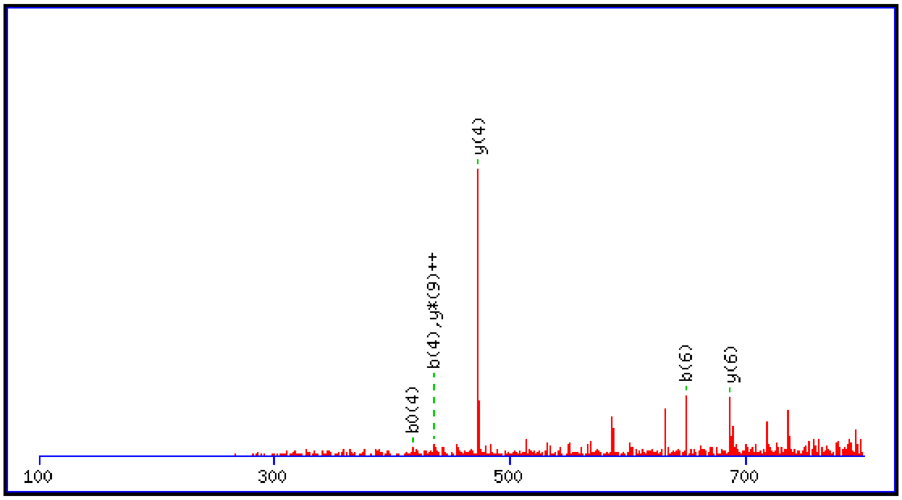

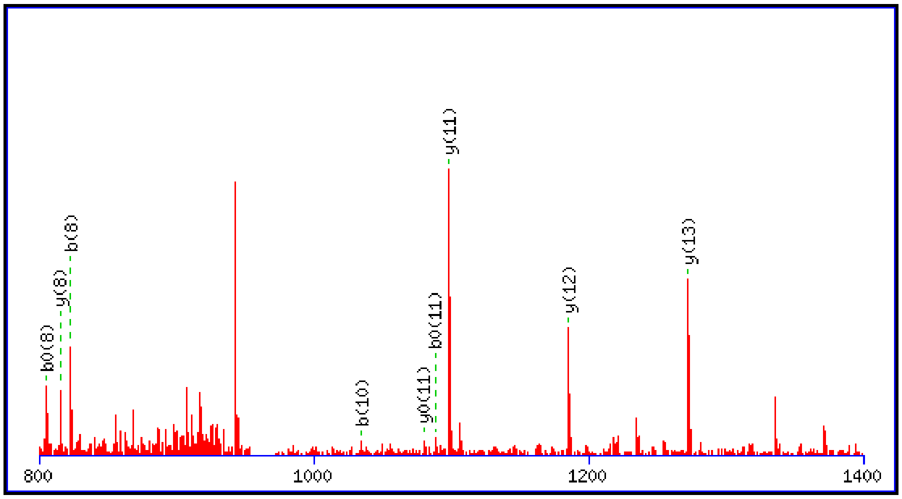

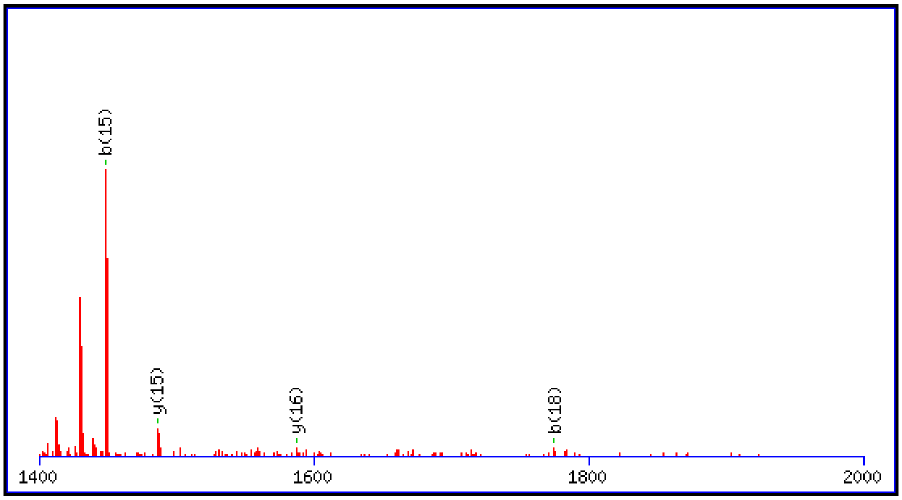

Monoisotopic mass of neutral peptide Mr(calc): 1919.8749
Fixed modifications: Carbamidomethyl (C)
Variable modifications:
N5 : Deamidated_N (N)
Ions Score: 59 **Expect:** 0.00026
Matches (Bold Red): 19/206 fragment ions using 42 most intense peaks

#	b	b^{++}	b*	b^{*++}	b^0	b^{0++}	Seq.	y	y^{++}	y*	y^{*++}	y^0	y^{0++}	#
1	116.0342	58.5207			98.0237	49.5155	D							19
2	187.0713	94.0393			169.0608	85.0340	A	1805.8552	903.4312	1788.8286	894.9180	1787.8446	894.4260	18
3	334.1397	167.5735			316.1292	158.5682	F	1734.8181	867.9127	1717.7915	859.3994	1716.8075	858.9074	17
4	**435.1874**	218.0974			**417.1769**	209.0921	T	**1587.7497**	794.3785	1570.7231	785.8652	1569.7391	785.3732	16

**5**	550.2144	275.6108	533.1878	267.0975	532.2038	266.6055	**N**	**1486.7020**	743.8546	1469.6754	735.3414	1468.6914	734.8493	**15**
**6**	**649.2828**	325.1450	632.2562	316.6318	631.2722	316.1397	**V**	1371.6751	686.3412	1354.6485	677.8279	1353.6645	677.3359	**14**
**7**	736.3148	368.6610	719.2883	360.1478	718.3042	359.6558	**S**	**1272.6066**	636.8070	1255.5801	628.2937	1254.5961	627.8017	**13**
**8**	**823.3468**	412.1771	806.3203	403.6638	**805.3363**	403.1718	**S**	**1185.5746**	593.2909	1168.5481	584.7777	1167.5640	584.2857	**12**
**9**	920.3996	460.7034	903.3730	452.1902	902.3890	451.6982	**P**	**1098.5426**	549.7749	1081.5160	541.2617	**1080.5320**	540.7696	**11**
**10**	**1035.4265**	518.2169	1018.4000	509.7036	1017.4160	509.2116	**D**	1001.4898	501.2485	984.4633	492.7353	983.4793	492.2433	**10**
**11**	1106.4637	553.7355	1089.4371	545.2222	**1088.4531**	544.7302	**A**	886.4629	443.7351	869.4363	**435.2218**	868.4523	434.7298	**9**
**12**	1163.4851	582.2462	1146.4586	573.7329	1145.4746	573.2409	**G**	**815.4258**	408.2165	798.3992	399.7032	797.4152	399.2112	**8**
**13**	1234.5222	617.7648	1217.4957	609.2515	1216.5117	608.7595	**A**	758.4043	379.7058	741.3777	371.1925	740.3937	370.7005	**7**
**14**	1335.5699	668.2886	1318.5434	659.7753	1317.5593	659.2833	**T**	**687.3672**	344.1872	670.3406	335.6740	669.3566	335.1819	**6**
**15**	**1448.6540**	724.8306	1431.6274	716.3174	1430.6434	715.8253	**L**	586.3195	293.6634	569.2930	285.1501	568.3089	284.6581	**5**
**16**	1545.7067	773.3570	1528.6802	764.8437	1527.6962	764.3517	**P**	**473.2354**	237.1214	456.2089	228.6081	455.2249	228.1161	**4**
**17**	1659.7497	830.3785	1642.7231	821.8652	1641.7391	821.3732	**N**	376.1827	188.5950	359.1561	180.0817	358.1721	179.5897	**3**
**18**	**1774.7766**	887.8919	1757.7501	879.3787	1756.7660	878.8867	**D**	262.1397	131.5735	245.1132	123.0602	244.1292	122.5682	**2**
**19**							**K**	147.1128	74.0600	130.0863	65.5468			**1**

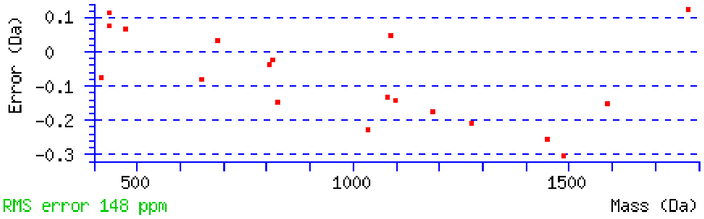

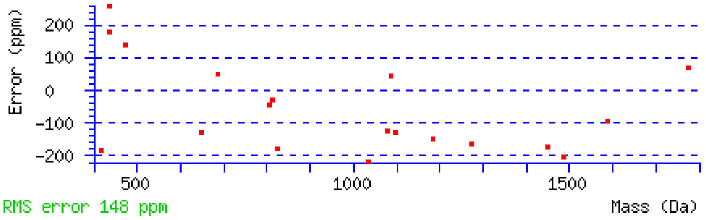

All matches to this query

Score	Mr(calc):	Delta	Sequence
59.3	1919.8749	0.0065	DAFTNVSSPDAGATLPNDK
18.0	1919.8749	0.0065	DAFTNVSSPDAGATLPNDK
13.9	1918.8909	0.9905	DAFTNVSSPDAGATLPNDK
3.7	1919.8973	-0.0160	NQDDADRAQKAFEAVDK
0.3	1919.8856	-0.0042	QEAPNIDTCLEMEILK
0.1	1919.8965	-0.0151	LHEITHTGEKPYKCK
0.1	1919.8965	-0.0151	LHEITHTGEKPYKCK

Spectrum No: 337; Query: 647; Rank: 1

Peptide View

MS/MS Fragmentation of **TVTNAVVTVPAYFNDSQR**
Found in **IPI00208205**, Tax_Id=10116 Gene_Symbol=Hspa8 Heat shock cognate 71 kDa protein

Match to Query 647: 1981.982908 from(991.998730,2+)
Title: 091129RatKid_SCX02_17.2657.2657.2.dta
Data file K:\NewmanPaper\Piliang\3SubProteomes\Piliang3SP\mgf5ppm\SCX_3SubProteomes5ppm.mgf

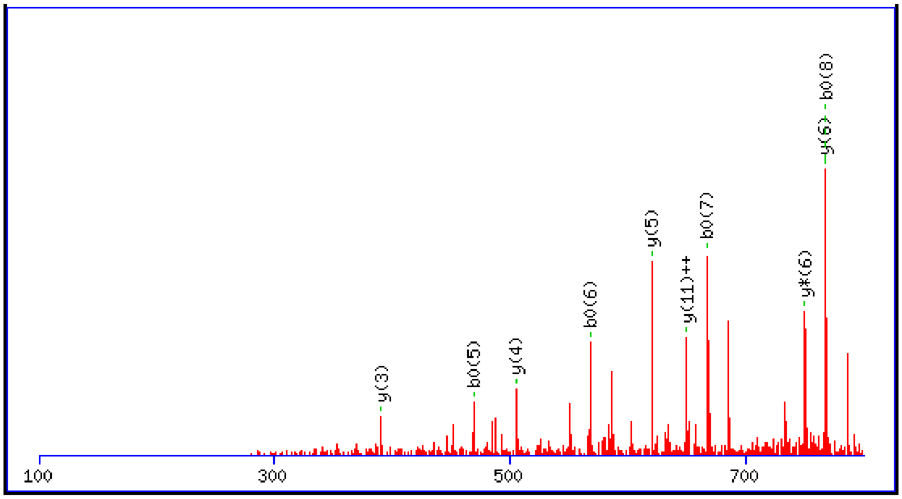

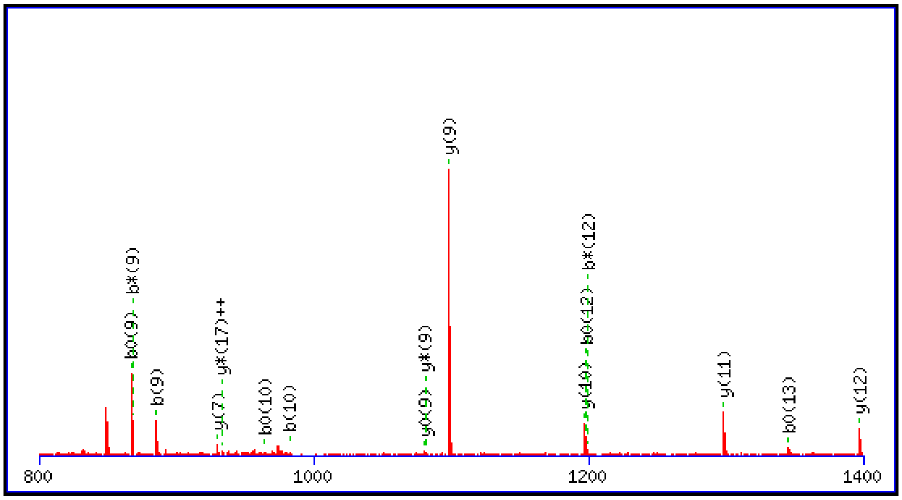

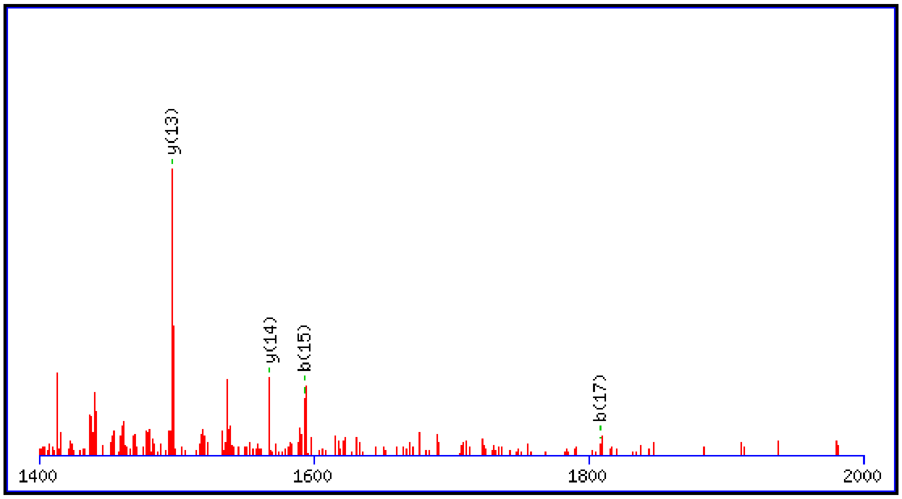

Monoisotopic mass of neutral peptide Mr(calc): 1981.9745
Fixed modifications: Carbamidomethyl (C)
Variable modifications:
N14 : Deamidated_N (N)
Ions Score: 59 **Expect:** 0.00023
Matches (Bold Red): 30/194 fragment ions using 78 most intense peaks

#	b	b++	b*	b*++	b0	b0++	Seq.	y	y++	y*	y*++	y0	y0++	#
1	102.0550	51.5311			84.0444	42.5258	T							18
2	201.1234	101.0653			183.1128	92.0600	V	1881.9341	941.4707	1864.9076	**932.9574**	1863.9235	932.4654	17
3	302.1710	151.5892			284.1605	142.5839	T	1782.8657	891.9365	1765.8392	883.4232	1764.8551	882.9312	16
4	416.2140	208.6106	399.1874	200.0974	398.2034	199.6053	N	1681.8180	841.4126	1664.7915	832.8994	1663.8075	832.4074	15
5	487.2511	244.1292	470.2245	235.6159	**469.2405**	235.1239	A	**1567.7751**	784.3912	1550.7485	775.8779	1549.7645	775.3859	14

**6**	586.3195	293.6634	569.2930	285.1501	**568.3089**	284.6581	**V**	**1496.7380**	748.8726	1479.7114	740.3594	1478.7274	739.8673	**13**
**7**	685.3879	343.1976	668.3614	334.6843	**667.3774**	334.1923	**V**	**1397.6696**	699.3384	1380.6430	690.8251	1379.6590	690.3331	**12**
**8**	786.4356	393.7214	769.4090	385.2082	**768.4250**	384.7162	**T**	**1298.6012**	**649.8042**	1281.5746	641.2909	1280.5906	640.7989	**11**
**9**	**885.5040**	443.2556	**868.4775**	434.7424	**867.4934**	434.2504	**V**	**1197.5535**	599.2804	1180.5269	590.7671	1179.5429	590.2751	**10**
**10**	**982.5568**	491.7820	965.5302	483.2688	**964.5462**	482.7767	**P**	**1098.4851**	549.7462	**1081.4585**	541.2329	**1080.4745**	540.7409	**9**
**11**	1053.5939	527.3006	1036.5673	518.7873	1035.5833	518.2953	**A**	1001.4323	501.2198	984.4057	492.7065	983.4217	492.2145	**8**
**12**	1216.6572	608.8322	**1199.6307**	600.3190	**1198.6467**	599.8270	**Y**	**930.3952**	465.7012	913.3686	457.1880	912.3846	456.6959	**7**
**13**	1363.7256	682.3665	1346.6991	673.8532	**1345.7151**	673.3612	**F**	**767.3319**	384.1696	**750.3053**	375.6563	749.3213	375.1643	**6**
**14**	1478.7526	739.8799	1461.7260	731.3666	1460.7420	730.8746	**N**	**620.2634**	310.6354	603.2369	302.1221	602.2529	301.6301	**5**
**15**	**1593.7795**	797.3934	1576.7530	788.8801	1575.7689	788.3881	**D**	**505.2365**	253.1219	488.2100	244.6086	487.2259	244.1166	**4**
**16**	1680.8115	840.9094	1663.7850	832.3961	1662.8010	831.9041	**S**	**390.2096**	195.6084	373.1830	187.0951	372.1990	186.6031	**3**
**17**	**1808.8701**	904.9387	1791.8436	896.4254	1790.8596	895.9334	**Q**	303.1775	152.0924	286.1510	143.5791			**2**
**18**							**R**	175.1190	88.0631	158.0924	79.5498			**1**

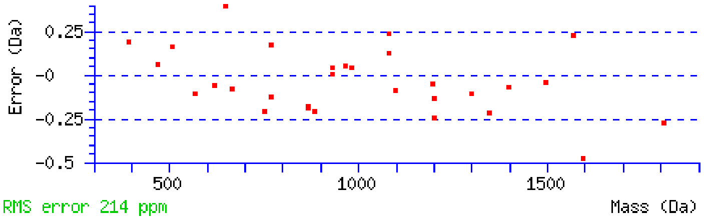

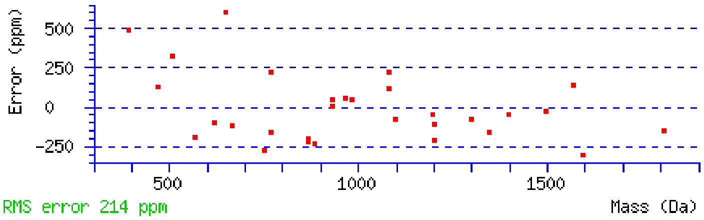

All matches to this query

Score	Mr(calc):	Delta	Sequence
59.2	1981.9745	0.0084	TVTNAVVTVPAYFNDSQR
9.8	1980.9905	0.9924	TVTNAVVTVPAYFNDSQR
7.9	1979.9717	2.0112	EEQVVQVWSKKNIGEK
6.8	1981.9874	-0.0045	WSLISLSNNTGKNVELK
5.7	1981.9656	0.0173	RKSMEASLTIVPANETR
5.3	1981.9874	-0.0045	WSLISLSNNTGKNVELK
4.5	1980.9955	0.9874	CLIVAQKSVTNTTPLEK
3.1	1981.9874	-0.0045	WSLISLSNNTGKNVELK
3.0	1981.9874	-0.0045	WSLISLSNNTGKNVELK
2.9	1981.9930	-0.0101	EADGGGVGRGKDISTITGHR

Spectrum No: 338; Query: 57; Rank: 1

Peptide View

MS/MS Fragmentation of **NQTLVQDNVK**
Found in **IPI00327202**, Tax_Id=10116 Gene_Symbol=Aqp1 Aquaporin-1

Match to Query 57: 1158.589148 from(580.301850,2+)
Title: 091127RatKid_SCX01_16.1026.1026.2.dta
Data file K:\NewmanPaper\Piliang\3SubProteomes\Piliang3SP\mgf5ppm\SCX_3SubProteomes5ppm.mgf

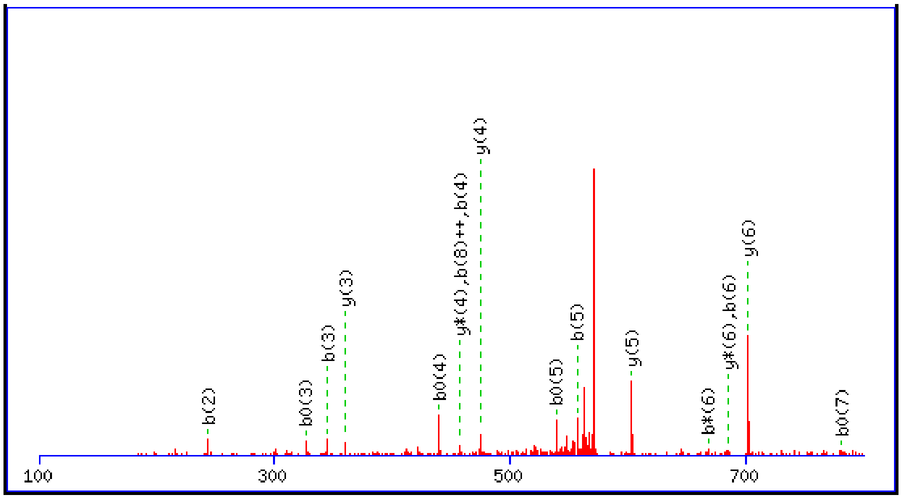

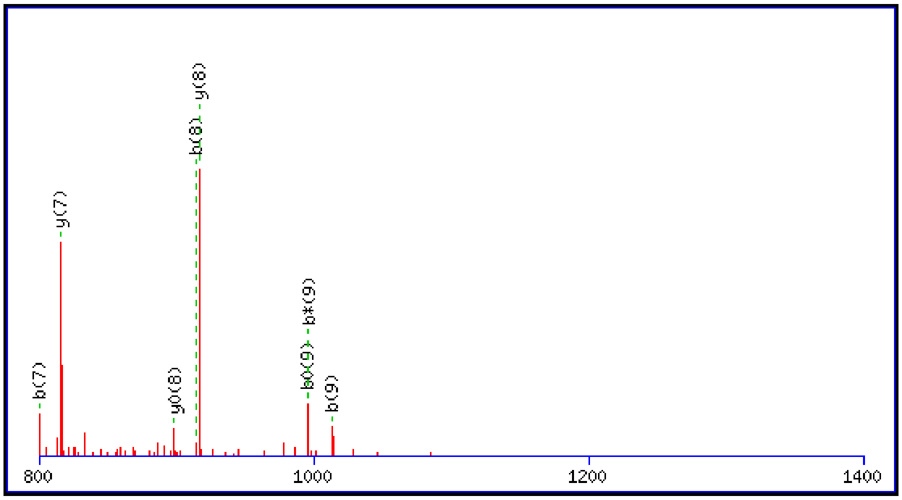

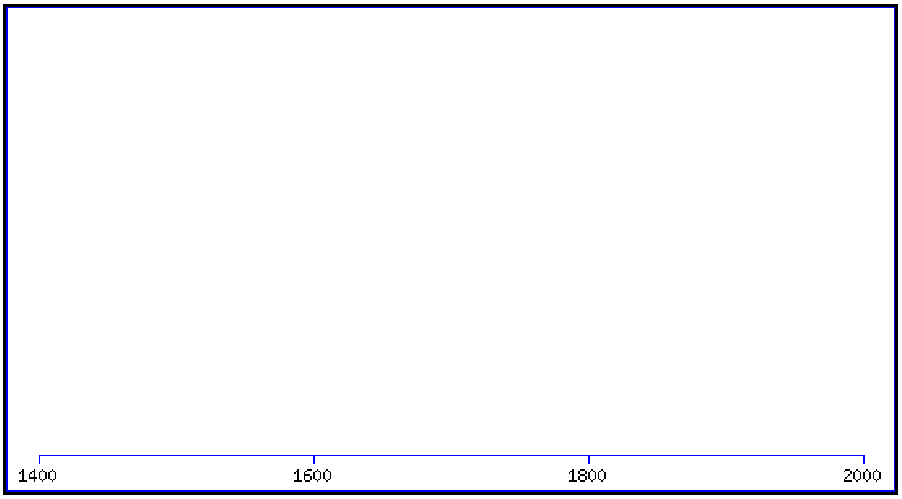

Monoisotopic mass of neutral peptide Mr(calc): 1158.5881
Fixed modifications: Carbamidomethyl (C)
Variable modifications:
N1 : Deamidated_N (N)
Ions Score: 59 **Expect:** 0.00015
Matches (Bold Red): 25/98 fragment ions using 38 most intense peaks

#	b	b^{++}	b*	b^{*++}	b^0	b^{0++}	Seq.	y	y^{++}	y*	y^{*++}	y^0	y^{0++}	#
1	116.0342	58.5207	99.0077	50.0075			N							10
2	**244.0928**	122.5500	227.0662	114.0368			Q	1044.5684	522.7878	1027.5419	514.2746	1026.5578	513.7826	9
3	**345.1405**	173.0739	328.1139	164.5606	**327.1299**	164.0686	T	**916.5098**	458.7585	899.4833	450.2453	**898.4993**	449.7533	8
4	**458.2245**	229.6159	441.1980	221.1026	**440.2140**	220.6106	L	**815.4621**	408.2347	798.4356	399.7214	797.4516	399.2294	7
5	**557.2929**	279.1501	540.2664	270.6368	**539.2824**	270.1448	V	**702.3781**	351.6927	**685.3515**	343.1794	684.3675	342.6874	6

**6**	**685.3515**	343.1794	**668.3250**	334.6661	667.3410	334.1741	**Q**	**603.3097**	302.1585	586.2831	293.6452	585.2991	293.1532	**5**
**7**	**800.3785**	400.6929	783.3519	392.1796	**782.3679**	391.6876	**D**	**475.2511**	238.1292	**458.2245**	229.6159	457.2405	229.1239	**4**
**8**	**914.4214**	**457.7143**	897.3948	449.2011	896.4108	448.7091	**N**	**360.2241**	180.6157	343.1976	172.1024			**3**
**9**	**1013.4898**	507.2485	**996.4633**	498.7353	**995.4792**	498.2433	**V**	246.1812	123.5942	229.1547	115.0810			**2**
**10**							**K**	147.1128	74.0600	130.0863	65.5468			**1**

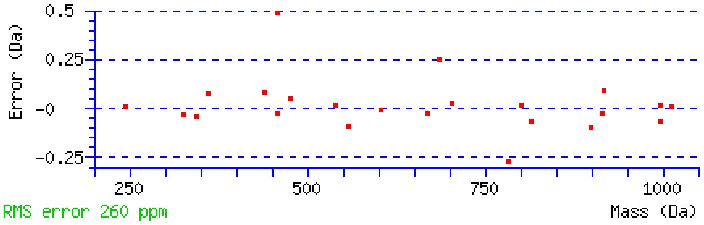

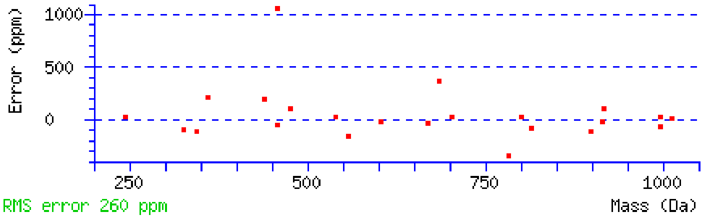

All matches to this query

Score	Mr(calc):	Delta	Sequence
58.9	1158.5881	0.0011	NQTLVQDNVK
23.0	1158.5993	-0.0101	RSSGSKSEPPK
20.5	1158.5880	0.0011	KAGENDSITPK
12.4	1158.5881	0.0011	NQTLVQDNVK
11.9	1158.5993	-0.0101	QNGAVITNKGR
10.8	1157.5928	0.9964	GSQALLESEPK
10.5	1158.5880	0.0011	NLSEALGDNVK
9.3	1157.5928	0.9963	QDTEPAALVSK
9.2	1157.5751	1.0141	DQTCTPIPVK
7.5	1158.5798	0.0094	ENHLKTLPK

Spectrum No: 339; Query: 322; Rank: 1

Peptide View

MS/MS Fragmentation of **DLLTNQSDFSGNTK**
Found in **IPI00210824**, Tax_Id=10116 Gene_Symbol=Serpina6 Corticosteroid-binding globulin precursor

Match to Query 322: 1539.709628 from(770.862090,2+)
Title: 091127RatKid_SCX01_12.2023.2023.2.dta
Data file K:\NewmanPaper\Piliang\3SubProteomes\Piliang3SP\mgf5ppm\SCX_3SubProteomes5ppm.mgf

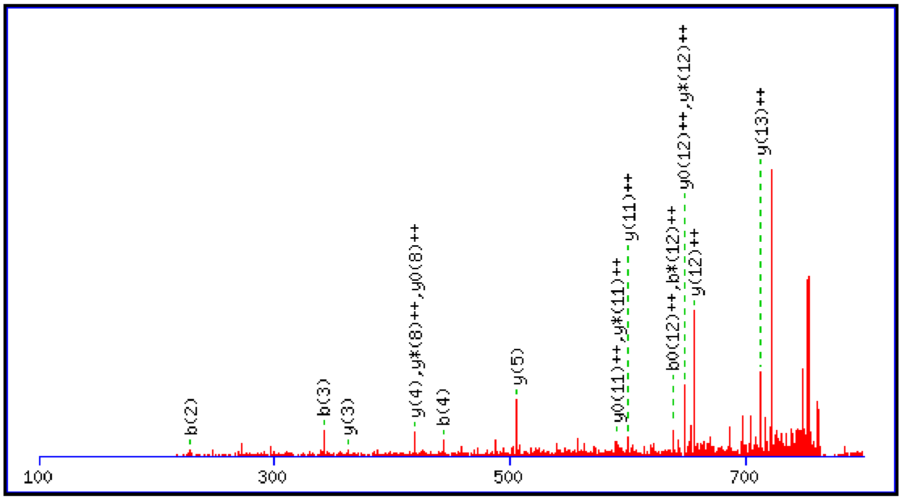

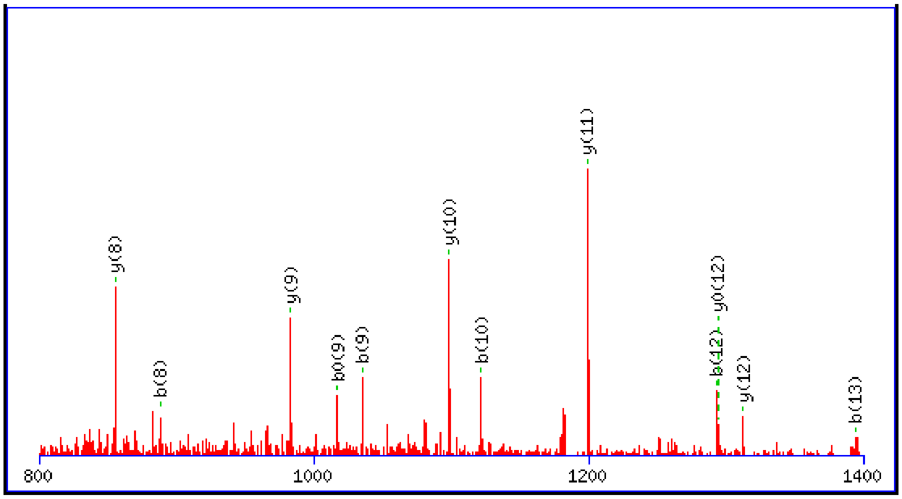

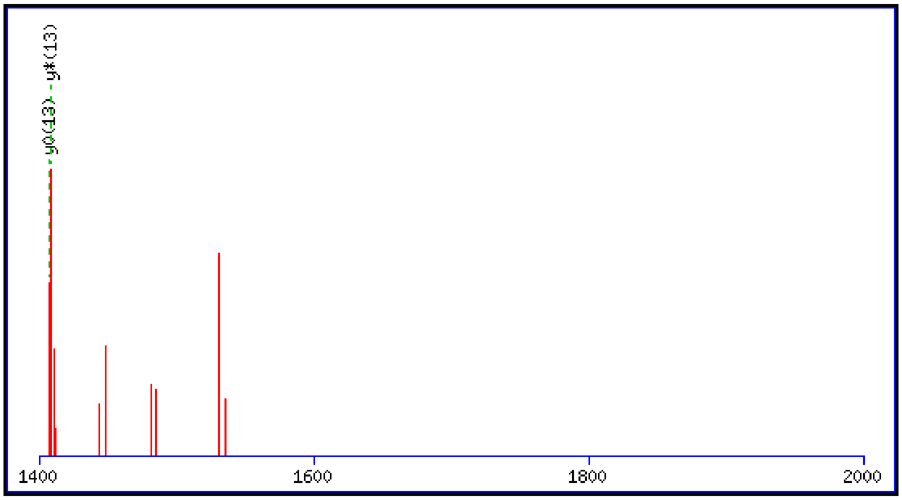

Monoisotopic mass of neutral peptide Mr(calc): 1539.7053
Fixed modifications: Carbamidomethyl (C)
Variable modifications:
N5 : Deamidated_N (N)
Ions Score: 59 **Expect:** 0.00023
Matches (Bold Red): 31/146 fragment ions using 45 most intense peaks

#	b	b^{++}	b*	b^{*++}	b^0	b^{0++}	Seq.	y	y^{++}	y*	y^{*++}	y^0	y^{0++}	#
1	116.0342	58.5207			98.0237	49.5155	D							14
2	**229.1183**	115.0628			211.1077	106.0575	L	1425.6856	**713.3464**	**1408.6591**	704.8332	**1407.6750**	704.3412	13
3	**342.2023**	171.6048			324.1918	162.5995	L	**1312.6015**	**656.8044**	1295.5750	**648.2911**	**1294.5910**	**647.7991**	12
4	**443.2500**	222.1287			425.2395	213.1234	T	**1199.5175**	**600.2624**	1182.4909	**591.7491**	1181.5069	**591.2571**	11
5	558.2770	279.6421	541.2504	271.1288	540.2664	270.6368	N	**1098.4698**	549.7385	1081.4433	541.2253	1080.4592	540.7333	10
6	686.3355	343.6714	669.3090	335.1581	668.3250	334.6661	Q	**983.4429**	492.2251	966.4163	483.7118	965.4323	483.2198	9
7	773.3676	387.1874	756.3410	378.6741	755.3570	378.1821	S	**855.3843**	428.1958	838.3577	**419.6825**	837.3737	**419.1905**	8
8	**888.3945**	444.7009	871.3680	436.1876	870.3839	435.6956	D	768.3523	384.6798	751.3257	376.1665	750.3417	375.6745	7
9	**1035.4629**	518.2351	1018.4364	509.7218	**1017.4524**	509.2298	F	653.3253	327.1663	636.2988	318.6530	635.3148	318.1610	6
10	**1122.4950**	561.7511	1105.4684	553.2378	1104.4844	552.7458	S	**506.2569**	253.6321	489.2304	245.1188	488.2463	244.6268	5
11	1179.5164	590.2618	1162.4899	581.7486	1161.5059	581.2566	G	**419.2249**	210.1161	402.1983	201.6028	401.2143	201.1108	4
12	**1293.5593**	647.2833	1276.5328	**638.7700**	1275.5488	**638.2780**	N	**362.2034**	181.6053	345.1769	173.0921	344.1928	172.6001	3
13	**1394.6070**	697.8071	1377.5805	689.2939	1376.5965	688.8019	T	248.1605	124.5839	231.1339	116.0706	230.1499	115.5786	2
14							K	147.1128	74.0600	130.0863	65.5468			1

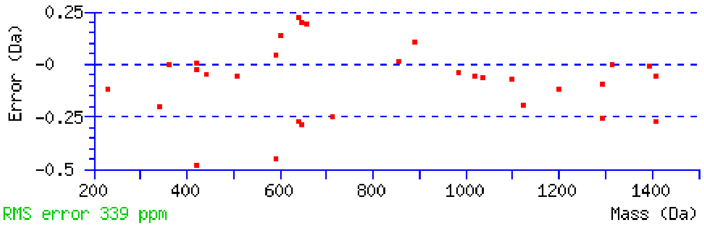

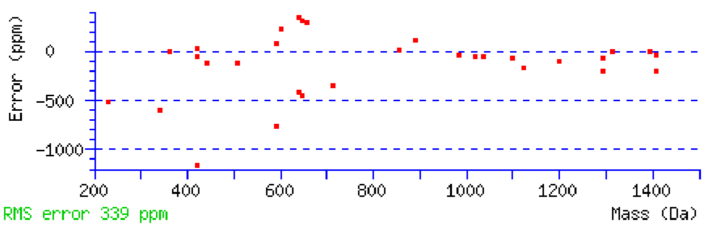

All matches to this query

Score	Mr(calc):	Delta	Sequence
58.8	1539.7053	0.0044	DLLTNQSDFSGNTK
23.3	1538.7213	0.9884	DLLTNQSDFSGNTK
22.8	1539.7053	0.0044	DLLTNQSDFSGNTK
6.6	1539.7069	0.0027	DIINNTSLAELEK
6.6	1539.7069	0.0027	DIINNTSLAELEK
6.0	1539.7083	0.0014	GVGQETLLWNTSR
6.0	1539.7165	-0.0068	NKYSVEENNTGRK
6.0	1539.7165	-0.0068	NKYSVEENNTGRK
6.0	1539.7235	-0.0139	INNKWTWVGTNK
5.6	1539.7043	0.0054	EVLTGSRGDXSLGGR

Spectrum No: 340; Query: 246; Rank: 1

Peptide View

MS/MS Fragmentation of **VWVNSSDGNALIGK**
Found in **IPI00193894**, Tax_Id=10116 Gene_Symbol=Mgam_predicted 198 kDa protein

Match to Query 246: 1459.733328 from(730.873940,2+)
Title: 091127RatKid_SCX01_17.2255.2255.2.dta
Data file K:\NewmanPaper\Piliang\3SubProteomes\Piliang3SP\mgf5ppm\SCX_3SubProteomes5ppm.mgf

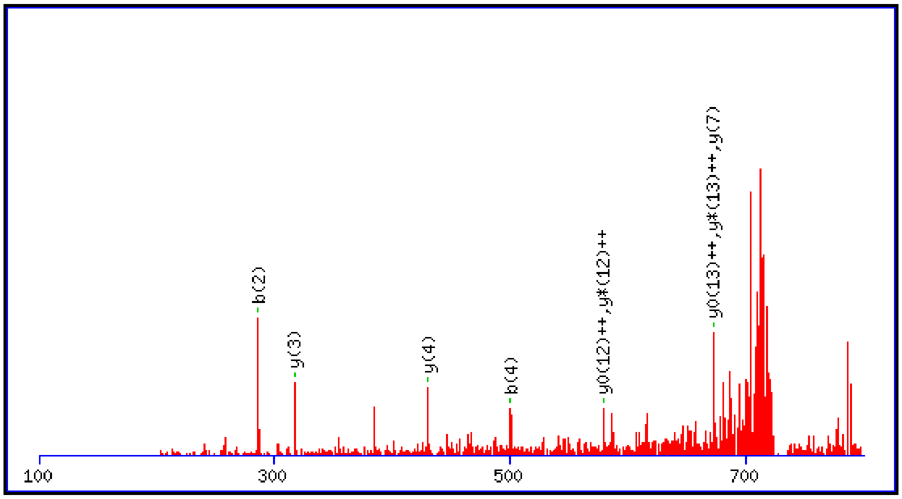

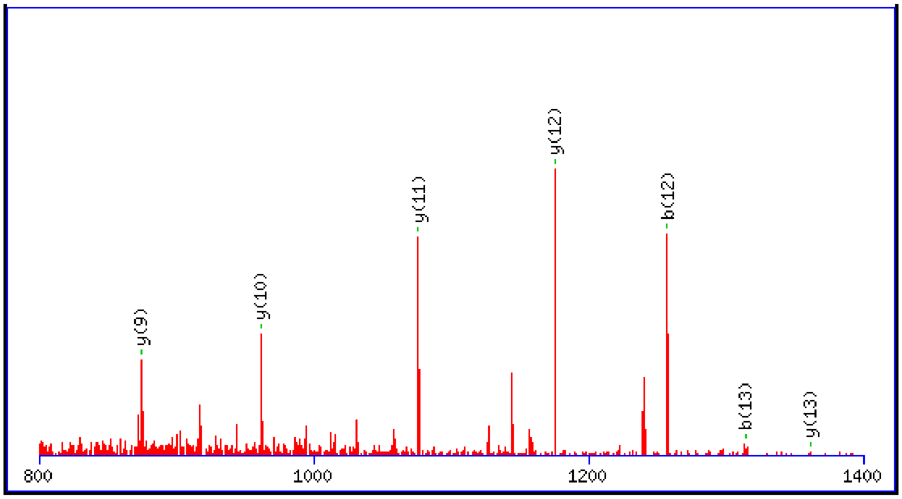

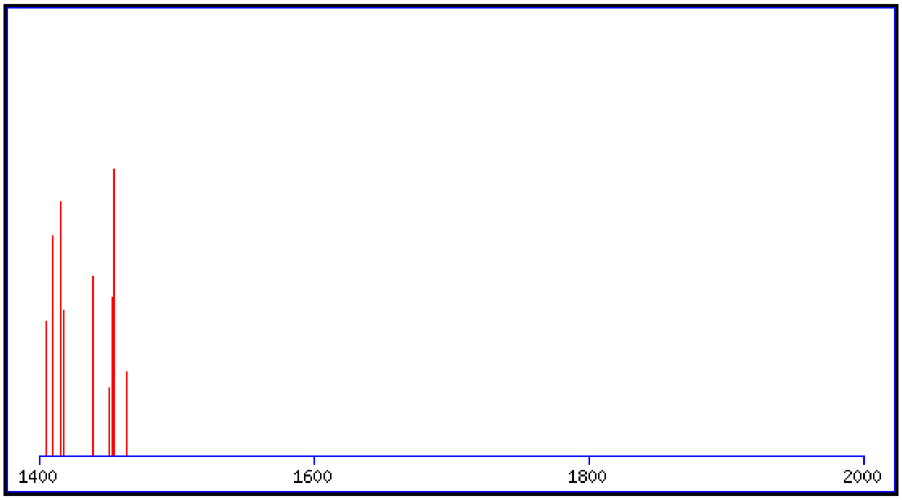

Monoisotopic mass of neutral peptide Mr(calc): 1459.7307
Fixed modifications: Carbamidomethyl (C)
Variable modifications:
N4 : Deamidated_N (N)
Ions Score: 59 **Expect:** 0.00019
Matches (Bold Red): 16/128 fragment ions using 26 most intense peaks

#	b	b++	b*	b*++	b0	b0++	Seq.	y	y++	y*	y*++	y0	y0++	#
1	100.0757	50.5415					V							14
2	**286.1550**	143.5811					W	**1361.6696**	681.3384	1344.6430	**672.8251**	1343.6590	**672.3331**	13
3	385.2234	193.1153					V	**1175.5902**	588.2988	1158.5637	**579.7855**	1157.5797	**579.2935**	12
4	**500.2504**	250.6288	483.2238	242.1155			N	**1076.5218**	538.7646	1059.4953	530.2513	1058.5113	529.7593	11
5	587.2824	294.1448	570.2558	285.6316	569.2718	285.1395	S	**961.4949**	481.2511	944.4684	472.7378	943.4843	472.2458	10
6	674.3144	337.6608	657.2879	329.1476	656.3038	328.6556	S	**874.4629**	437.7351	857.4363	429.2218	856.4523	428.7298	9
7	789.3414	395.1743	772.3148	386.6610	771.3308	386.1690	D	787.4308	394.2191	770.4043	385.7058	769.4203	385.2138	8
8	846.3628	423.6850	829.3363	415.1718	828.3523	414.6798	G	**672.4039**	336.7056	655.3774	328.1923			7
9	960.4057	480.7065	943.3792	472.1932	942.3952	471.7012	N	615.3824	308.1949	598.3559	299.6816			6
10	1031.4429	516.2251	1014.4163	507.7118	1013.4323	507.2198	A	501.3395	251.1734	484.3130	242.6601			5
11	1144.5269	572.7671	1127.5004	564.2538	1126.5164	563.7618	L	**430.3024**	215.6548	413.2758	207.1416			4
12	**1257.6110**	629.3091	1240.5844	620.7959	1239.6004	620.3038	I	**317.2183**	159.1128	300.1918	150.5995			3
13	**1314.6324**	657.8199	1297.6059	649.3066	1296.6219	648.8146	G	204.1343	102.5708	187.1077	94.0575			2
14							K	147.1128	74.0600	130.0863	65.5468			1

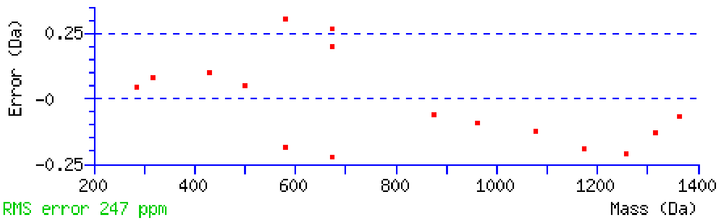

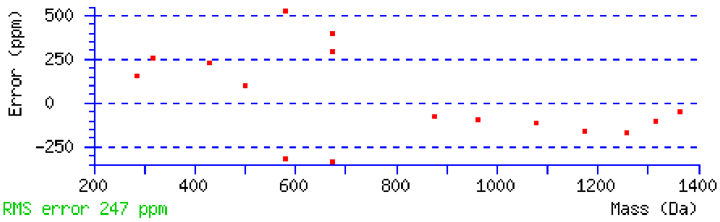

All matches to this query

Score	Mr(calc):	Delta	Sequence
58.7	1459.7307	0.0026	VWVNSSDGNALIGK
33.4	1459.7307	0.0026	VWVNSSDGNALIGK
8.3	1459.7201	0.0133	IASLSRSVVALGK
4.5	1458.7388	0.9945	NSLLKDAMAPGTPK
3.1	1459.7225	0.0109	AITAFTAGFVNIR
2.8	1459.7200	0.0133	KNLAKSQTLLGK
1.7	1458.7249	1.0084	TTLGSKVVNSCHR
1.1	1458.7202	1.0132	KEANDLLSSPQEK

Spectrum No: 341; Query: 804; Rank: 1

Peptide View

MS/MS Fragmentation of **SCATSQEDFLIYSLNNSLR**
Found in **IPI00205325**, Tax_Id=10116 Gene_Symbol=Lrp2 Low-density lipoprotein receptor-related protein 2 precursor

Match to Query 804: 2218.026388 from(1110.020470,2+)
Title: 091127RatKid_SCX01_17.5002.5002.2.dta
Data file K:\NewmanPaper\Piliang\3SubProteomes\Piliang3SP\mgf5ppm\SCX_3SubProteomes5ppm.mgf

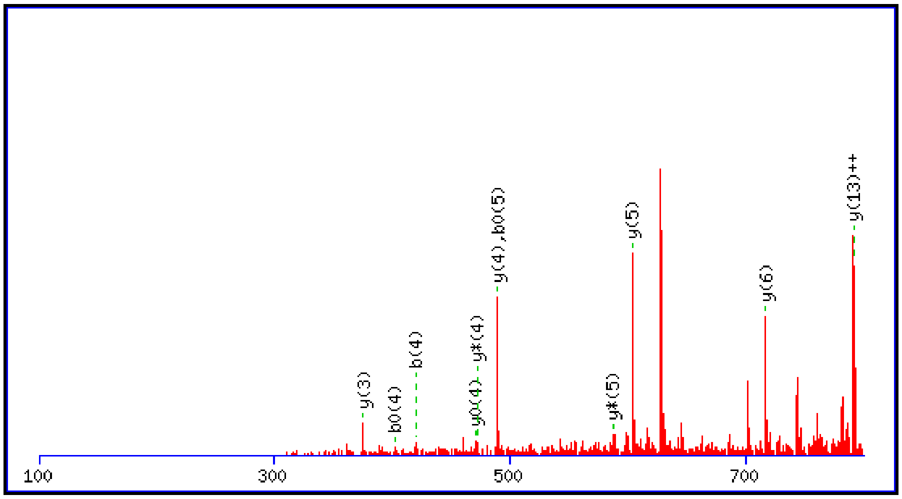

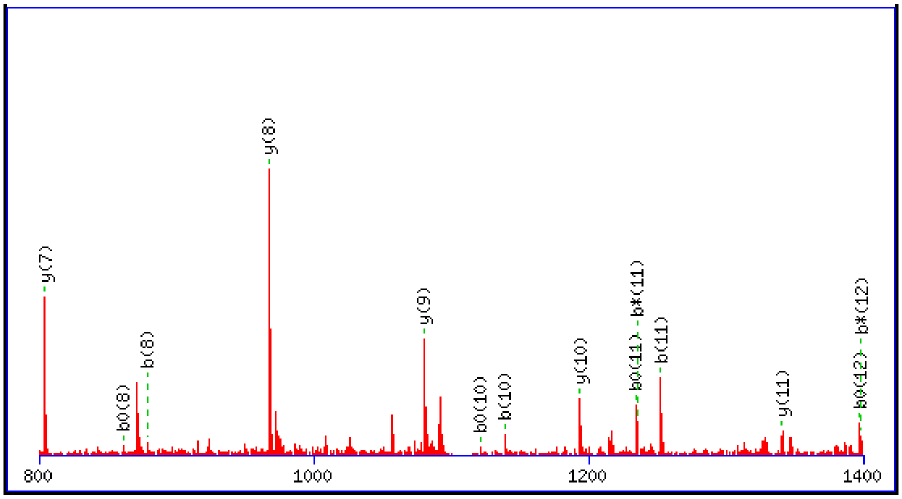

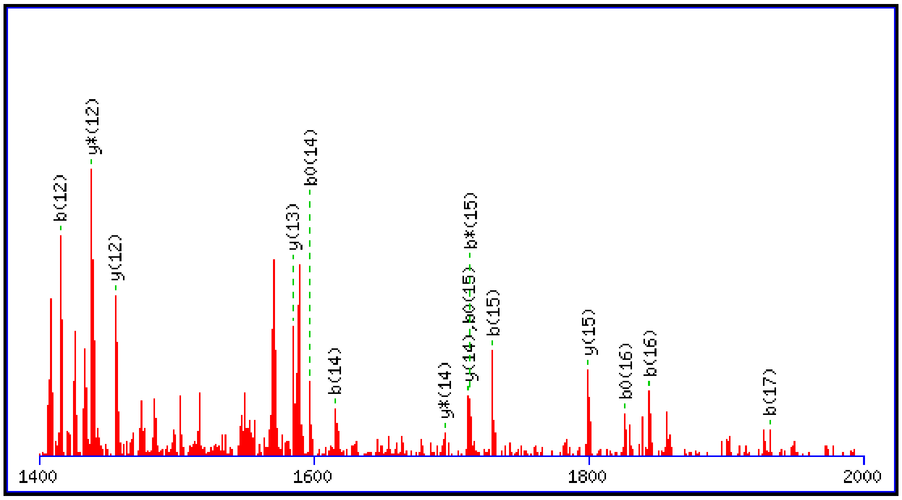

Monoisotopic mass of neutral peptide Mr(calc): 2218.0212
Fixed modifications: Carbamidomethyl (C)
Variable modifications:
N15 : Deamidated_N (N)
Ions Score: 59 **Expect:** 0.00039
Matches (Bold Red): 40/202 fragment ions using 94 most intense peaks

#	b	b^{++}	b*	b^{*++}	b^{0}	b^{0++}	Seq.	y	y^{++}	y*	y^{*++}	y^{0}	y^{0++}	#
1	88.0393	44.5233			70.0287	35.5180	S							19
2	248.0700	124.5386			230.0594	115.5333	C	2131.9965	1066.5019	2114.9699	1057.9886	2113.9859	1057.4966	18
3	319.1071	160.0572			301.0965	151.0519	A	1971.9658	986.4865	1954.9393	977.9733	1953.9552	977.4813	17
4	**420.1547**	210.5810			**402.1442**	201.5757	T	1900.9287	950.9680	1883.9021	942.4547	1882.9181	941.9627	16
5	507.1868	254.0970			**489.1762**	245.0917	S	**1799.8810**	900.4441	1782.8545	891.9309	1781.8704	891.4389	15
6	635.2454	318.1263	618.2188	309.6130	617.2348	309.1210	Q	**1712.8490**	856.9281	**1695.8224**	848.4149	1694.8384	847.9228	14
7	764.2879	382.6476	747.2614	374.1343	746.2774	373.6423	E	**1584.7904**	**792.8988**	1567.7639	784.3856	1566.7798	783.8936	13
8	**879.3149**	440.1611	862.2883	431.6478	**861.3043**	431.1558	D	**1455.7478**	728.3775	**1438.7213**	719.8643	1437.7372	719.3723	12
9	1026.3833	513.6953	1009.3568	505.1820	1008.3727	504.6900	F	**1340.7209**	670.8641	1323.6943	662.3508	1322.7103	661.8588	11
10	**1139.4674**	570.2373	1122.4408	561.7240	**1121.4568**	561.2320	L	**1193.6525**	597.3299	1176.6259	588.8166	1175.6419	588.3246	10
11	**1252.5514**	626.7794	**1235.5249**	618.2661	**1234.5409**	617.7741	I	**1080.5684**	540.7878	1063.5418	532.2746	1062.5578	531.7826	9
12	**1415.6148**	708.3110	**1398.5882**	699.7977	**1397.6042**	699.3057	Y	**967.4843**	484.2458	950.4578	475.7325	949.4738	475.2405	8
13	1502.6468	751.8270	1485.6202	743.3138	1484.6362	742.8217	S	**804.4210**	402.7141	787.3945	394.2009	786.4104	393.7089	7
14	**1615.7309**	808.3691	1598.7043	799.8558	**1597.7203**	799.3638	L	**717.3890**	359.1981	700.3624	350.6848	699.3784	350.1928	6
15	**1730.7578**	865.8825	**1713.7312**	857.3693	**1712.7472**	856.8772	N	**604.3049**	302.6561	**587.2784**	294.1428	586.2943	293.6508	5
16	**1844.8007**	922.9040	1827.7742	914.3907	**1826.7901**	913.8987	N	**489.2780**	245.1426	**472.2514**	236.6293	**471.2674**	236.1373	4
17	**1931.8327**	966.4200	1914.8062	957.9067	1913.8222	957.4147	S	**375.2350**	188.1212	358.2085	179.6079	357.2245	179.1159	3
18	2044.9168	1022.9620	2027.8903	1014.4488	2026.9062	1013.9568	L	288.2030	144.6051	271.1765	136.0919			2

19							R	175.1190	88.0631	158.0924	79.5498		1

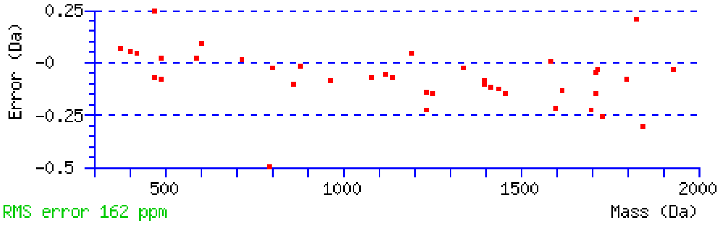

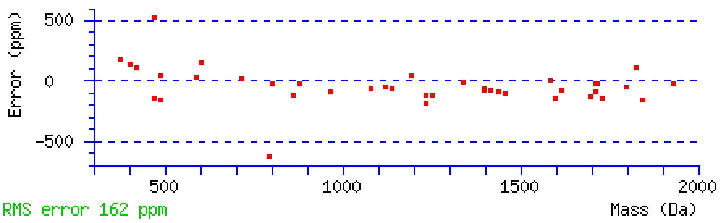

All matches to this query

Score	Mr(calc):	Delta	Sequence
58.6	2218.0212	0.0052	SCATSQEDFLIYSLNNSLR
56.2	2218.0212	0.0052	SCATSQEDFLIYSLNNSLR
8.9	2217.0191	1.0073	TKGPSRRPSLISSDSNNLK
8.9	2217.0191	1.0073	TKGPSRRPSLISSDSNNLK
8.5	2217.0191	1.0073	TKGPSRRPSLISSDSNNLK
8.5	2217.0191	1.0073	TKGPSRRPSLISSDSNNLK
5.4	2218.0449	-0.0185	WSTASAYLRPALSRPNLR

Spectrum No: 342; Query: 1081; Rank: 1

Peptide View

MS/MS Fragmentation of **LVGGPVAGGDPNQTIQGQYCDICIAANSNK**
Found in **IPI00190577**, Tax_Id=10116 Gene_Symbol=Lama5 similar to Laminin alpha-5 chain precursor

Match to Query 1081: 3117.459342 from(1040.160390,3+)
Title: 091127RatKid_SCX01_14.3242.3242.3.dta
Data file K:\NewmanPaper\Piliang\3SubProteomes\Piliang3SP\mgf5ppm\SCX_3SubProteomes5ppm.mgf

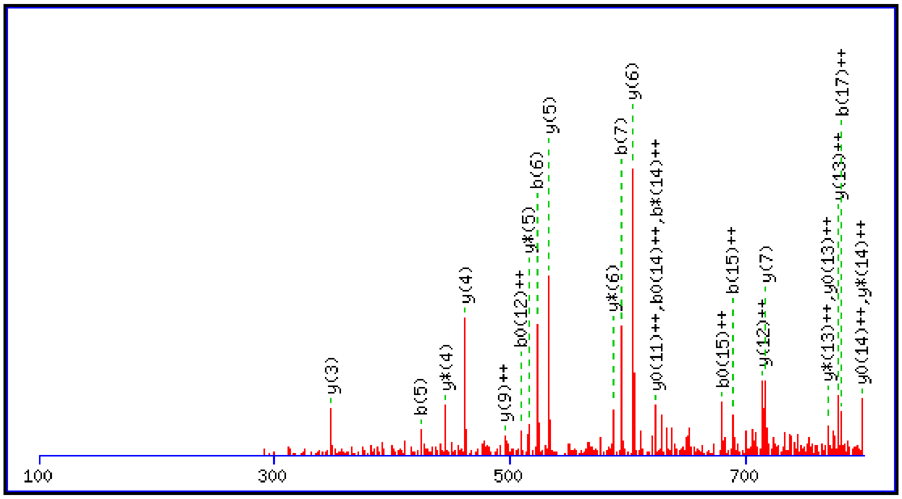

Mascot Search Results: Peptide View

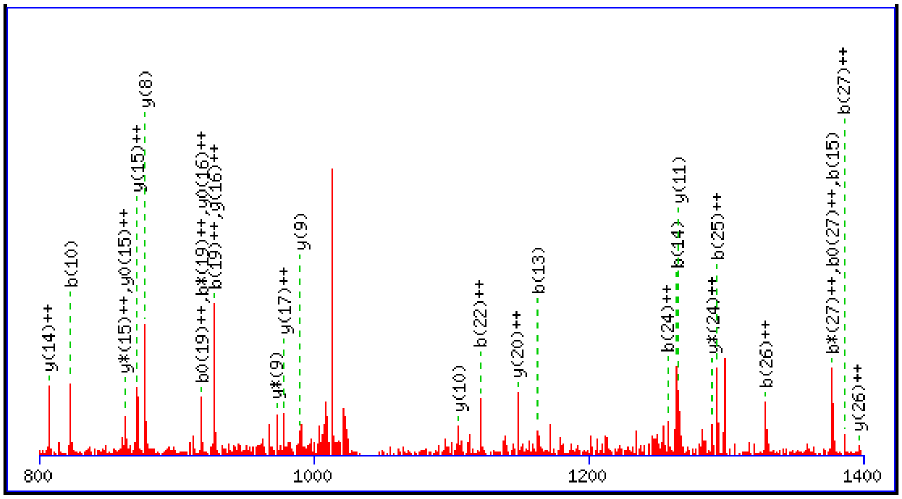

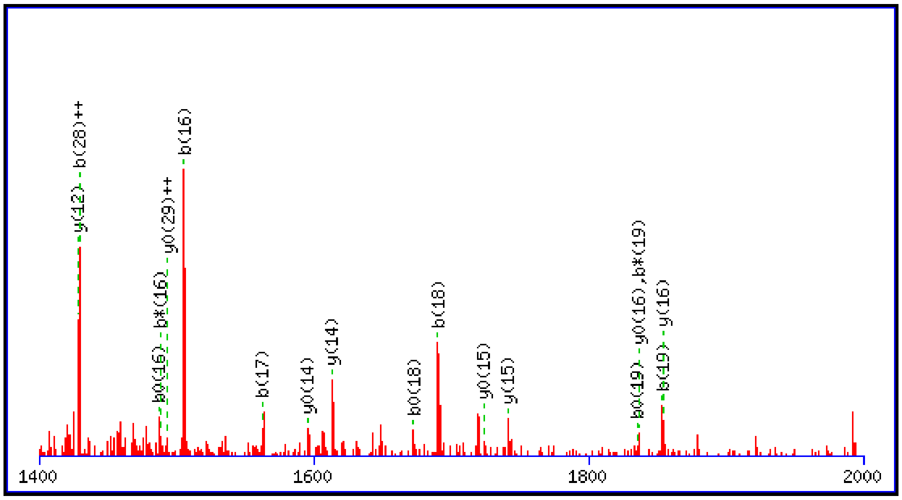

Monoisotopic mass of neutral peptide Mr(calc): 3117.4496
Fixed modifications: Carbamidomethyl (C)
Variable modifications:
N12 : Deamidated_N (N)
Ions Score: 59 **Expect:** 0.00052
Matches (Bold Red): 72/304 fragment ions using 128 most intense peaks

#	b	b^{++}	b*	b^{*++}	b^{0}	b^{0++}	Seq.	y	y^{++}	y*	y^{*++}	y^{0}	y^{0++}	#
1	114.0913	57.5493					L							30
2	213.1598	107.0835					V	3005.3728	1503.1900	2988.3462	1494.6767	2987.3622	**1494.1847**	29
3	270.1812	135.5942					G	2906.3043	1453.6558	2889.2778	1445.1425	2888.2938	1444.6505	28
4	327.2027	164.1050					G	2849.2829	1425.1451	2832.2563	1416.6318	2831.2723	1416.1398	27
5	**424.2554**	212.6314					P	2792.2614	**1396.6343**	2775.2349	1388.1211	2774.2509	1387.6291	26
6	**523.3239**	262.1656					V	2695.2087	1348.1080	2678.1821	1339.5947	2677.1981	1339.1027	25
7	**594.3610**	297.6841					A	2596.1402	1298.5738	2579.1137	**1290.0605**	2578.1297	1289.5685	24
8	651.3824	326.1949					G	2525.1031	1263.0552	2508.0766	1254.5419	2507.0926	1254.0499	23
9	708.4039	354.7056					G	2468.0817	1234.5445	2451.0551	1226.0312	2450.0711	1225.5392	22
10	**823.4308**	412.2191			805.4203	403.2138	D	2411.0602	1206.0337	2394.0336	1197.5205	2393.0496	1197.0285	21
11	920.4836	460.7454			902.4730	451.7402	P	2296.0333	**1148.5203**	2279.0067	1140.0070	2278.0227	1139.5150	20
12	1035.5105	518.2589	1018.4840	509.7456	1017.5000	**509.2536**	N	2198.9805	1099.9939	2181.9539	1091.4806	2180.9699	1090.9886	19
13	**1163.5691**	582.2882	1146.5426	573.7749	1145.5586	573.2829	Q	2083.9536	1042.4804	2066.9270	1033.9671	2065.9430	1033.4751	18
14	**1264.6168**	632.8120	1247.5903	**624.2988**	1246.6062	**623.8068**	T	1955.8950	**978.4511**	1938.8684	969.9379	1937.8844	969.4458	17
15	**1377.7009**	**689.3541**	1360.6743	680.8408	1359.6903	**680.3488**	I	**1854.8473**	**927.9273**	1837.8207	919.4140	**1836.8367**	**918.9220**	16
16	**1505.7594**	753.3834	**1488.7329**	744.8701	**1487.7489**	744.3781	Q	**1741.7632**	**871.3853**	1724.7367	**862.8720**	**1723.7527**	**862.3800**	15
17	**1562.7809**	**781.8941**	1545.7544	773.3808	1544.7703	772.8888	G	**1613.7047**	**807.3560**	1596.6781	**798.8427**	**1595.6941**	**798.3507**	14
18	**1690.8395**	845.9234	1673.8129	837.4101	**1672.8289**	836.9181	Q	1556.6832	**778.8452**	1539.6566	**770.3320**	1538.6726	**769.8400**	13

**19**	**1853.9028**	**927.4550**	**1836.8763**	**918.9418**	**1835.8923**	**918.4498**	**Y**	**1428.6246**	**714.8159**	1411.5981	706.3027	1410.6140	705.8107	**12**
**20**	2013.9335	1007.4704	1996.9069	998.9571	1995.9229	998.4651	**C**	**1265.5613**	633.2843	1248.5347	624.7710	1247.5507	**624.2790**	**11**
**21**	2128.9604	1064.9838	2111.9339	1056.4706	2110.9498	1055.9786	**D**	**1105.5306**	553.2690	1088.5041	544.7557	1087.5201	544.2637	**10**
**22**	2242.0445	**1121.5259**	2225.0179	1113.0126	2224.0339	1112.5206	**I**	**990.5037**	**495.7555**	**973.4771**	487.2422	972.4931	486.7502	**9**
**23**	2402.0751	1201.5412	2385.0486	1193.0279	2384.0646	1192.5359	**C**	**877.4196**	439.2135	860.3931	430.7002	859.4091	430.2082	**8**
**24**	2515.1592	**1258.0832**	2498.1326	1249.5700	2497.1486	1249.0779	**I**	**717.3890**	359.1981	700.3624	350.6849	699.3784	350.1928	**7**
**25**	2586.1963	**1293.6018**	2569.1697	1285.0885	2568.1857	1284.5965	**A**	**604.3049**	302.6561	**587.2784**	294.1428	586.2943	293.6508	**6**
**26**	2657.2334	**1329.1203**	2640.2069	1320.6071	2639.2228	1320.1151	**A**	**533.2678**	267.1375	**516.2413**	258.6243	515.2572	258.1323	**5**
**27**	2771.2763	**1386.1418**	2754.2498	**1377.6285**	2753.2658	**1377.1365**	**N**	**462.2307**	231.6190	**445.2041**	223.1057	444.2201	222.6137	**4**
**28**	2858.3084	**1429.6578**	2841.2818	1421.1445	2840.2978	1420.6525	**S**	**348.1878**	174.5975	331.1612	166.0842	330.1772	165.5922	**3**
**29**	2972.3513	1486.6793	2955.3247	1478.1660	2954.3407	1477.6740	**N**	261.1557	131.0815	244.1292	122.5682			**2**
**30**							**K**	147.1128	74.0600	130.0863	65.5468			**1**

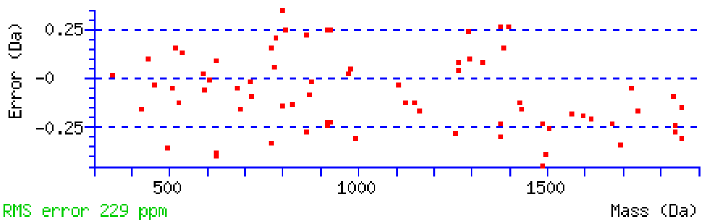

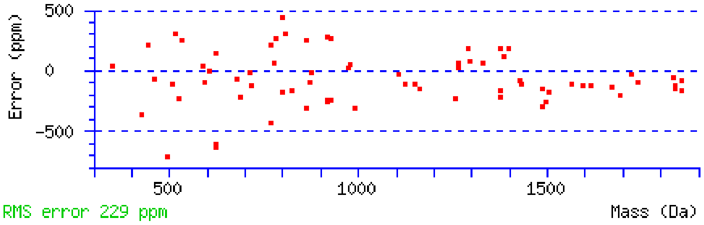

All matches to this query

Score	Mr(calc):	Delta	Sequence
58.6	3117.4496	0.0098	LVGGPVAGGDPNQTIQGQYCDICIAANSNK
37.6	3116.4655	0.9938	LVGGPVAGGDPNQTIQGQYCDICIAANSNK
32.8	3117.4496	0.0098	LVGGPVAGGDPNQTIQGQYCDICIAANSNK
32.8	3117.4496	0.0098	LVGGPVAGGDPNQTIQGQYCDICIAANSNK
6.2	3117.4630	-0.0037	WMQKNGGYIWIQSSATIAINAKNANEK
4.6	3117.4630	-0.0037	WMQKNGGYIWIQSSATIAINAKNANEK
3.2	3117.4630	-0.0037	WMQKNGGYIWIQSSATIAINAKNANEK
2.9	3116.4321	1.0273	VALTPSMRLLFETHHLQTATPATVSR
2.2	3117.4702	-0.0109	TLAIDVGKTGGSTLHVVGASHVETFLIR

Spectrum No: 343; Query: 507; Rank: 1

Peptide View

MS/MS Fragmentation of **NSSASNPQDSVGSLCSR**
Found in **IPI00363651**, Tax_Id=10116 Gene_Symbol=Pxn 64 kDa protein

Match to Query 507: 1765.755868 from(883.885210,2+)
Title: 091127RatKid_SCX01_15.1409.1409.2.dta
Data file K:\NewmanPaper\Piliang\3SubProteomes\Piliang3SP\mgf5ppm\SCX_3SubProteomes5ppm.mgf

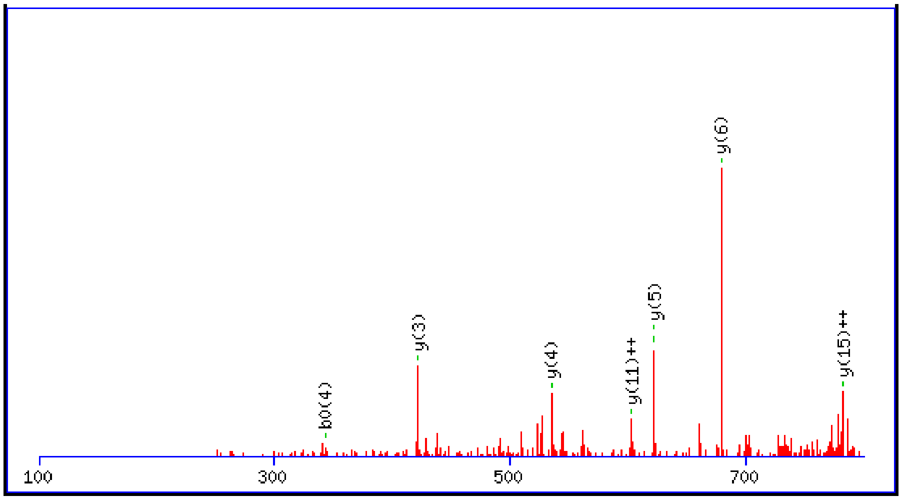

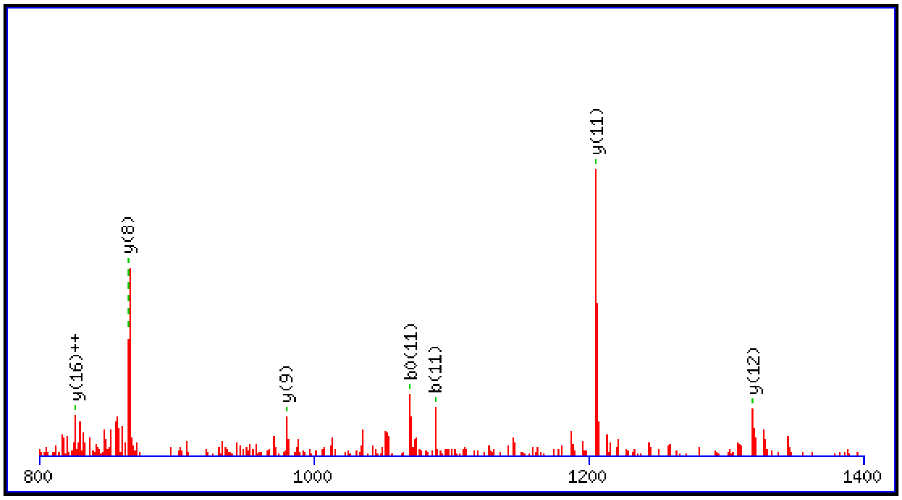

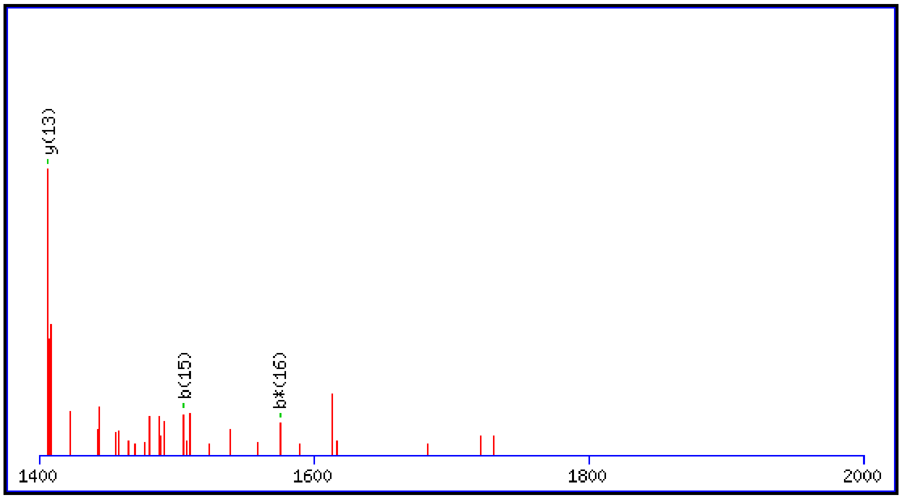

Monoisotopic mass of neutral peptide Mr(calc): 1765.7537
Fixed modifications: Carbamidomethyl (C)
Variable modifications:
N1 : Deamidated_N (N)
Ions Score: 59 **Expect:** 0.00026
Matches (Bold Red): 17/188 fragment ions using 37 most intense peaks

#	b	b^{++}	b*	b^{*++}	b^0	b^{0++}	Seq.	y	y^{++}	y*	y^{*++}	y^0	y^{0++}	#
1	116.0342	58.5207	99.0077	50.0075			N							17
2	203.0662	102.0368	186.0397	93.5235	185.0557	93.0315	S	1651.7340	**826.3707**	1634.7075	817.8574	1633.7235	817.3654	16
3	290.0983	145.5528	273.0717	137.0395	272.0877	136.5475	S	1564.7020	**782.8546**	1547.6755	774.3414	1546.6914	773.8494	15
4	361.1354	181.0713	344.1088	172.5581	**343.1248**	172.0660	A	1477.6700	739.3386	1460.6434	730.8254	1459.6594	730.3333	14
5	448.1674	224.5873	431.1409	216.0741	430.1568	215.5821	S	**1406.6329**	703.8201	1389.6063	695.3068	1388.6223	694.8148	13

6	562.2103	281.6088	545.1838	273.0955	544.1998	272.6035	N	1319.6008	660.3041	1302.5743	651.7908	1301.5903	651.2988	12
7	659.2631	330.1352	642.2366	321.6219	641.2525	321.1299	P	1205.5579	603.2826	1188.5314	594.7693	1187.5473	594.2773	11
8	787.3217	394.1645	770.2951	385.6512	769.3111	385.1592	Q	1108.5051	554.7562	1091.4786	546.2429	1090.4946	545.7509	10
9	902.3486	451.6779	885.3221	443.1647	884.3381	442.6727	D	980.4466	490.7269	963.4200	482.2136	962.4360	481.7216	9
10	989.3806	495.1940	972.3541	486.6807	971.3701	486.1887	S	865.4196	433.2135	848.3931	424.7002	847.4091	424.2082	8
11	1088.4491	544.7282	1071.4225	536.2149	1070.4385	535.7229	V	778.3876	389.6974	761.3610	381.1842	760.3770	380.6922	7
12	1145.4705	573.2389	1128.4440	564.7256	1127.4600	564.2336	G	679.3192	340.1632	662.2926	331.6500	661.3086	331.1579	6
13	1232.5026	616.7549	1215.4760	608.2416	1214.4920	607.7496	S	622.2977	311.6525	605.2712	303.1392	604.2872	302.6472	5
14	1345.5866	673.2969	1328.5601	664.7837	1327.5761	664.2917	L	535.2657	268.1365	518.2391	259.6232	517.2551	259.1312	4
15	1505.6173	753.3123	1488.5907	744.7990	1487.6067	744.3070	C	422.1816	211.5945	405.1551	203.0812	404.1711	202.5892	3
16	1592.6493	796.8283	1575.6227	788.3150	1574.6387	787.8230	S	262.1510	131.5791	245.1244	123.0659	244.1404	122.5738	2
17							R	175.1190	88.0631	158.0924	79.5498			1

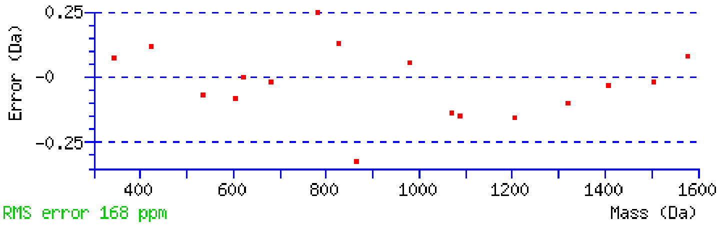

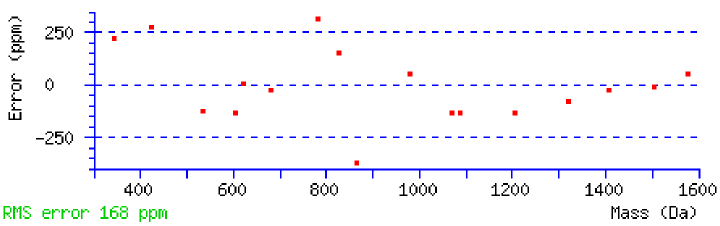

All matches to this query

Score	Mr(calc):	Delta	Sequence
58.6	1765.7537	0.0022	NSSASNPQDSVGSLCSR
58.3	1765.7537	0.0022	NSSASNPQDSVGSLCSR
48.9	1764.7697	0.9862	NSSASNPQDSVGSLCSR
8.4	1763.7606	1.9952	MAVDVTEYHLSVIK
8.2	1764.7597	0.9962	GGAVSARYVLADEAAR
0.8	1765.7439	0.0120	SELFIFFTSLMQK

Spectrum No: 344; Query: 148; Rank: 1

Peptide View

MS/MS Fragmentation of **TVVVDNETVATR**
Found in **IPI00211023**, Tax_Id=10116 Gene_Symbol=Slc12a4 Solute carrier family 12 member 4

Match to Query 148: 1303.663428 from(652.838990,2+)
Title: 091127RatKid_SCX01_02.1101.1101.2.dta
Data file K:\NewmanPaper\Piliang\3SubProteomes\Piliang3SP\mgf5ppm\SCX_3SubProteomes5ppm.mgf

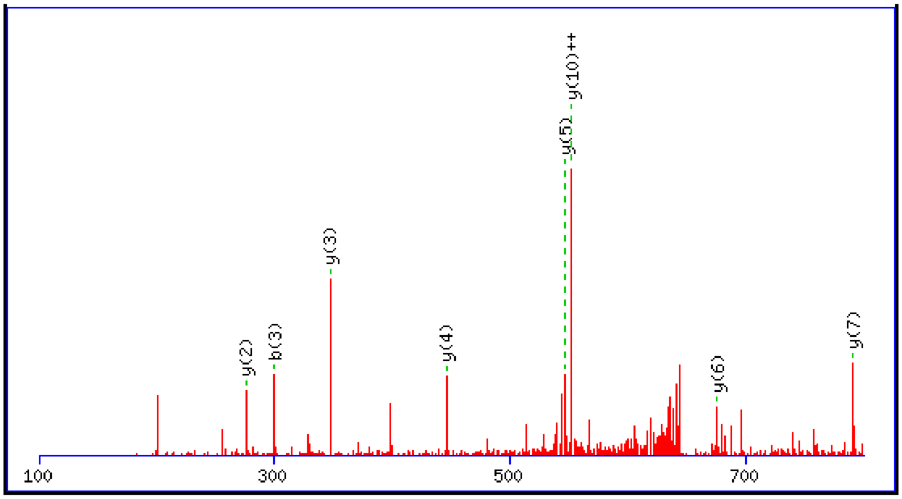

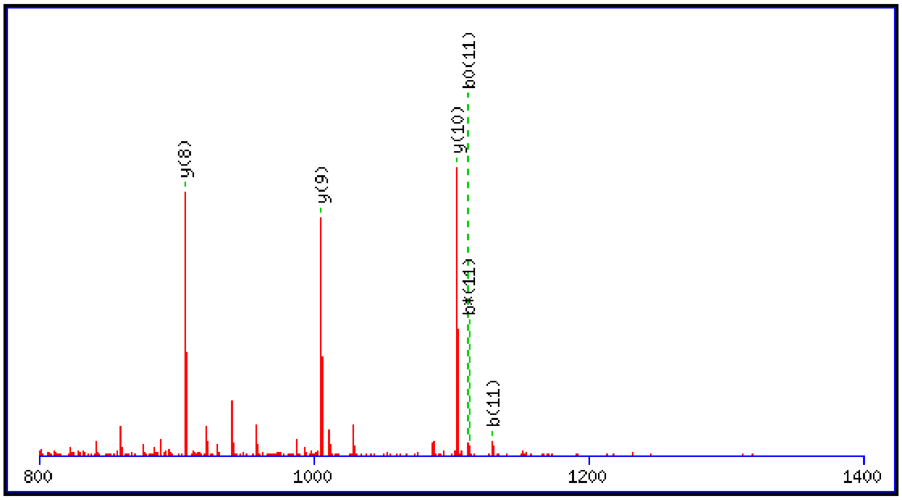

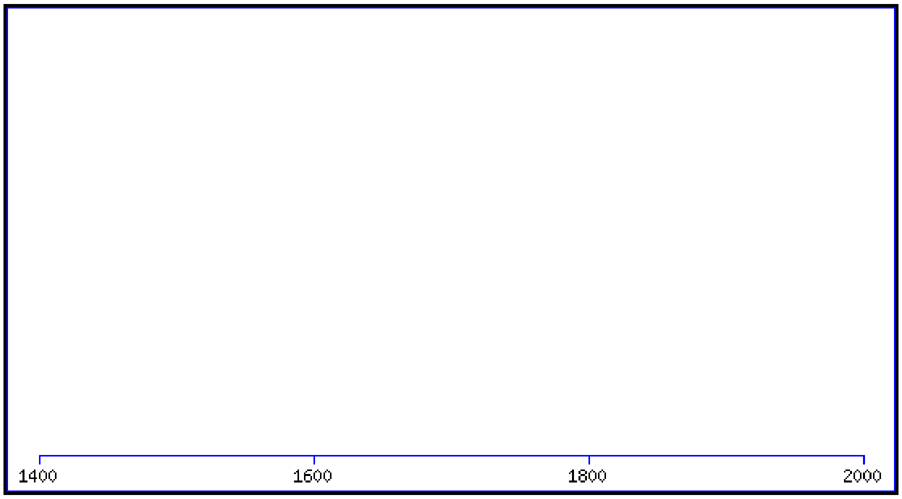

Monoisotopic mass of neutral peptide Mr(calc): 1303.6620
Fixed modifications: Carbamidomethyl (C)
Variable modifications:
N6 : Deamidated_N (N)
Ions Score: 58 **Expect:** 0.0002
Matches (Bold Red): 14/120 fragment ions using 24 most intense peaks

#	b	b^{++}	b*	b^{*++}	b^0	b^{0++}	Seq.	y	y^{++}	y*	y^{*++}	y^0	y^{0++}	#
**1**	102.0550	51.5311			84.0444	42.5258	**T**							**12**
**2**	201.1234	101.0653			183.1128	92.0600	**V**	1203.6216	602.3144	1186.5950	593.8011	1185.6110	593.3091	**11**
**3**	**300.1918**	150.5995			282.1812	141.5942	**V**	**1104.5531**	**552.7802**	1087.5266	544.2669	1086.5426	543.7749	**10**
**4**	399.2602	200.1337			381.2496	191.1285	**V**	**1005.4847**	503.2460	988.4582	494.7327	987.4742	494.2407	**9**
**5**	514.2871	257.6472			496.2766	248.6419	**D**	**906.4163**	453.7118	889.3898	445.1985	888.4057	444.7065	**8**

**6**	629.3141	315.1607	612.2875	306.6474	611.3035	306.1554	**N**	**791.3894**	396.1983	774.3628	387.6850	773.3788	387.1930	**7**
**7**	758.3567	379.6820	741.3301	371.1687	740.3461	370.6767	**E**	**676.3624**	338.6849	659.3359	330.1716	658.3519	329.6796	**6**
**8**	859.4043	430.2058	842.3778	421.6925	841.3938	421.2005	**T**	**547.3198**	274.1636	530.2933	265.6503	529.3093	265.1583	**5**
**9**	958.4728	479.7400	941.4462	471.2267	940.4622	470.7347	**V**	**446.2722**	223.6397	429.2456	215.1264	428.2616	214.6344	**4**
**10**	1029.5099	515.2586	1012.4833	506.7453	1011.4993	506.2533	**A**	**347.2037**	174.1055	330.1772	165.5922	329.1932	165.1002	**3**
**11**	**1130.5576**	565.7824	**1113.5310**	557.2691	**1112.5470**	556.7771	**T**	**276.1666**	138.5870	259.1401	130.0737	258.1561	129.5817	**2**
**12**							**R**	175.1190	88.0631	158.0924	79.5498			**1**

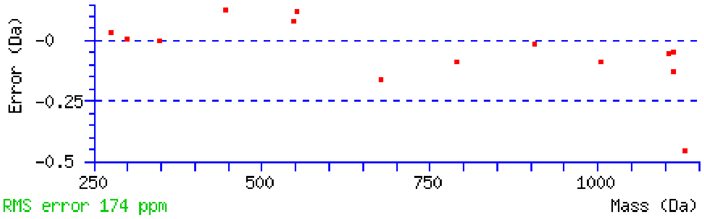

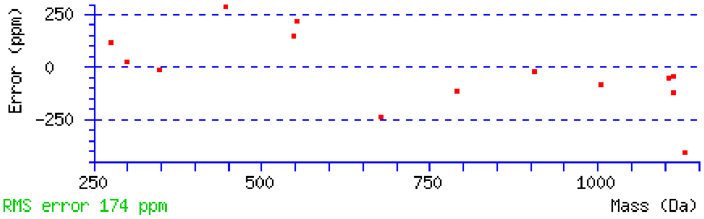

All matches to this query

Score	Mr(calc):	Delta	Sequence
58.4	1303.6620	0.0015	TVVVDNETVATR
9.6	1302.6472	1.0162	TLVTSSFLVEK
9.6	1302.6472	1.0162	TLVTSSFLVEK
8.3	1303.6667	-0.0032	LKSRNCVVDGR
6.0	1303.6537	0.0097	TTPKHIVTEAK
5.1	1302.6472	1.0162	TLVTSSFLVEK
5.0	1303.6619	0.0015	DVKGTQESLAEK
4.4	1303.6537	0.0097	TTPKHIVTEAK
3.6	1301.6503	2.0131	TPNPTASEFIPK
2.7	1302.6568	1.0066	AERSVDXIAEK

Spectrum No: 345; Query: 929; Rank: 1

Peptide View

MS/MS Fragmentation of **KMIAQHCVDANNTFCFEHPR**
Found in **IPI00205325**, Tax_Id=10116 Gene_Symbol=Lrp2 Low-density lipoprotein receptor-related protein 2 precursor

Match to Query 929: 2475.096462 from(826.039430,3+)
Title: 091127RatKid_SCX01_38.1493.1493.3.dta
Data file K:\NewmanPaper\Piliang\3SubProteomes\Piliang3SP\mgf5ppm\SCX_3SubProteomes5ppm.mgf

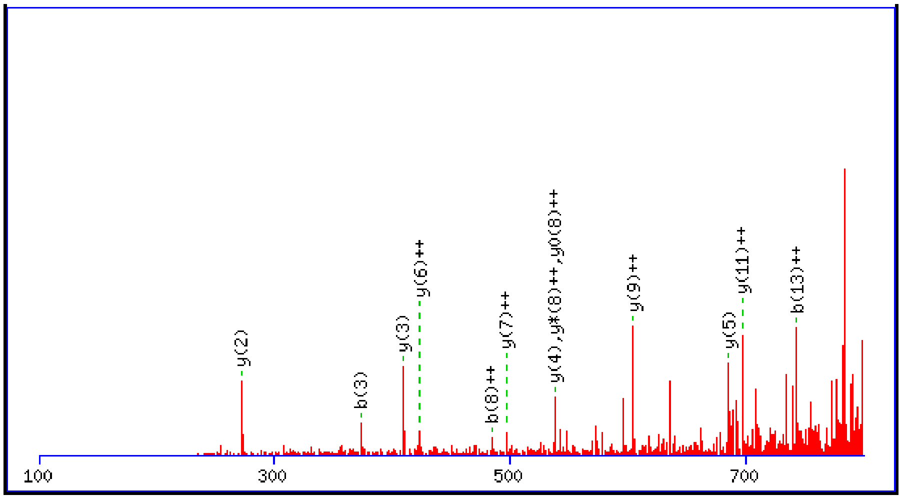

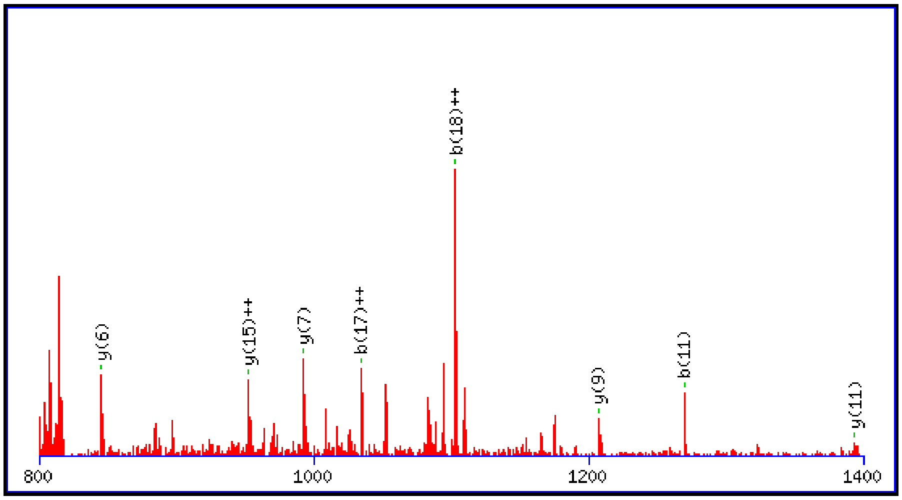

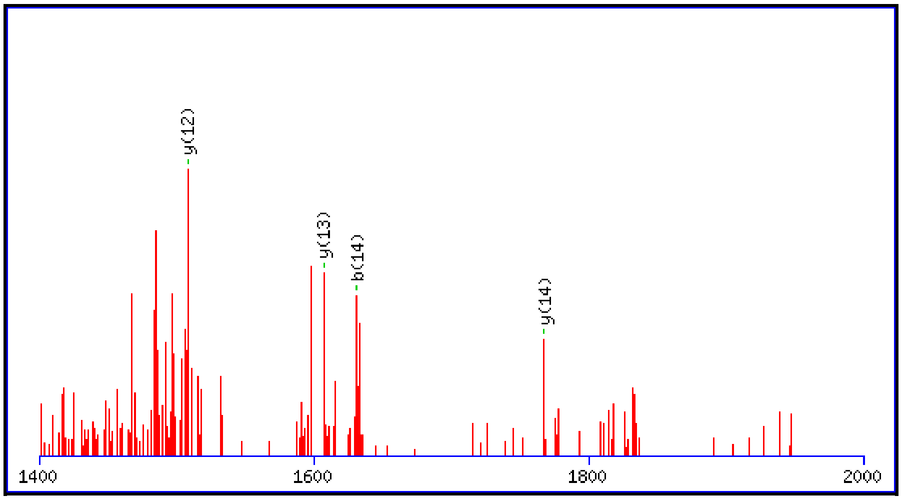

Monoisotopic mass of neutral peptide Mr(calc): 2475.0882
Fixed modifications: Carbamidomethyl (C)
Variable modifications:
N11 : Deamidated_N (N)
Ions Score: 58 **Expect:** 0.00046
Matches (Bold Red): 25/206 fragment ions using 51 most intense peaks

#	b	b++	b*	b*++	b0	b0++	Seq.	y	y++	y*	y*++	y0	y0++	#
1	129.1022	65.0548	112.0757	56.5415			K							20
2	260.1427	130.5750	243.1162	122.0617			M	2348.0005	1174.5039	2330.9740	1165.9906	2329.9899	1165.4986	19
3	**373.2268**	187.1170	356.2002	178.6038			I	2216.9600	1108.9837	2199.9335	1100.4704	2198.9495	1099.9784	18
4	444.2639	222.6356	427.2374	214.1223			A	2103.8760	1052.4416	2086.8494	1043.9283	2085.8654	1043.4363	17
5	572.3225	286.6649	555.2959	278.1516			Q	2032.8389	1016.9231	2015.8123	1008.4098	2014.8283	1007.9178	16

**6**	709.3814	355.1943	692.3548	346.6811			**H**	1904.7803	**952.8938**	1887.7537	944.3805	1886.7697	943.8885	**15**
**7**	869.4120	435.2097	852.3855	426.6964			**C**	**1767.7214**	884.3643	1750.6948	875.8510	1749.7108	875.3590	**14**
**8**	968.4805	**484.7439**	951.4539	476.2306			**V**	**1607.6907**	804.3490	1590.6642	795.8357	1589.6801	795.3437	**13**
**9**	1083.5074	542.2573	1066.4808	533.7441	1065.4968	533.2521	**D**	**1508.6223**	754.8148	1491.5957	746.3015	1490.6117	745.8095	**12**
**10**	1154.5445	577.7759	1137.5180	569.2626	1136.5339	568.7706	**A**	**1393.5954**	**697.3013**	1376.5688	688.7880	1375.5848	688.2960	**11**
**11**	**1269.5714**	635.2894	1252.5449	626.7761	1251.5609	626.2841	**N**	1322.5582	661.7828	1305.5317	653.2695	1304.5477	652.7775	**10**
**12**	1383.6144	692.3108	1366.5878	683.7976	1365.6038	683.3055	**N**	**1207.5313**	**604.2693**	1190.5048	595.7560	1189.5207	595.2640	**9**
**13**	1484.6621	**742.8347**	1467.6355	734.3214	1466.6515	733.8294	**T**	1093.4884	547.2478	1076.4618	**538.7346**	1075.4778	**538.2425**	**8**
**14**	**1631.7305**	816.3689	1614.7039	807.8556	1613.7199	807.3636	**F**	**992.4407**	**496.7240**	975.4141	488.2107	974.4301	487.7187	**7**
**15**	1791.7611	896.3842	1774.7346	887.8709	1773.7506	887.3789	**C**	**845.3723**	**423.1898**	828.3457	414.6765	827.3617	414.1845	**6**
**16**	1938.8295	969.9184	1921.8030	961.4051	1920.8190	960.9131	**F**	**685.3416**	343.1745	668.3151	334.6612	667.3311	334.1692	**5**
**17**	2067.8721	**1034.4397**	2050.8456	1025.9264	2049.8616	1025.4344	**E**	**538.2732**	269.6402	521.2467	261.1270	520.2627	260.6350	**4**
**18**	2204.9310	**1102.9692**	2187.9045	1094.4559	2186.9205	1093.9639	**H**	**409.2306**	205.1190	392.2041	196.6057			**3**
**19**	2301.9838	1151.4955	2284.9573	1142.9823	2283.9732	1142.4903	**P**	**272.1717**	136.5895	255.1452	128.0762			**2**
**20**							**R**	175.1190	88.0631	158.0924	79.5498			**1**

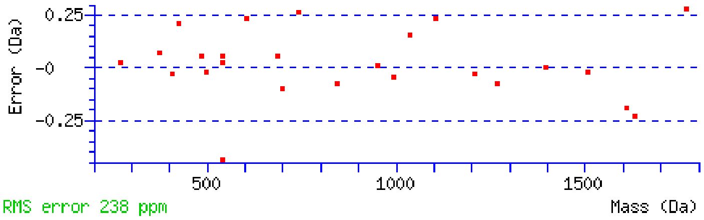

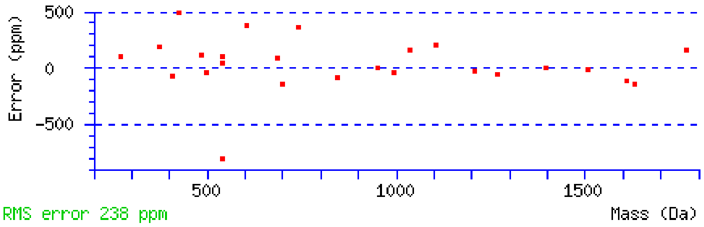

All matches to this query

Score	Mr(calc):	Delta	Sequence
58.4	2475.0882	0.0083	KMIAQHCVDANNTFCFEHPR
51.9	2475.0882	0.0083	KMIAQHCVDANNTFCFEHPR
13.4	2474.1042	0.9923	KMIAQHCVDANNTFCFEHPR
3.3	2475.0737	0.0227	ASNQGNSQPQQKYTVMSGTPEK
2.4	2475.1043	-0.0078	KQASNLETAIADAEQRGDSALK

Spectrum No: 346; Query: 335; Rank: 1

Peptide View

MS/MS Fragmentation of **ITDIENGTFANIPR**
Found in **IPI00365784**, Tax_Id=10116 Gene_Symbol=LOC306805 Similar to asporin

Match to Query 335: 1560.785988 from(781.400270,2+)
Title: 091127RatKid_SCX01_12.2714.2714.2.dta
Data file K:\NewmanPaper\Piliang\3SubProteomes\Piliang3SP\mgf5ppm\SCX_3SubProteomes5ppm.mgf

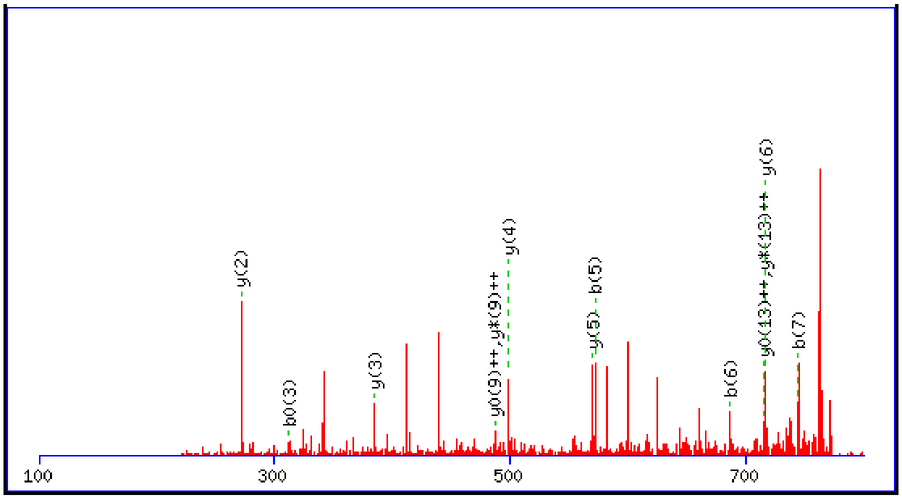

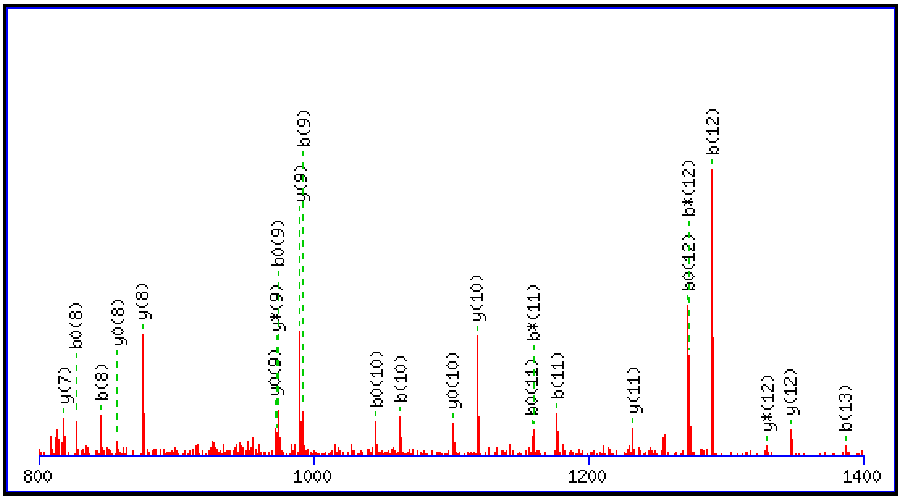

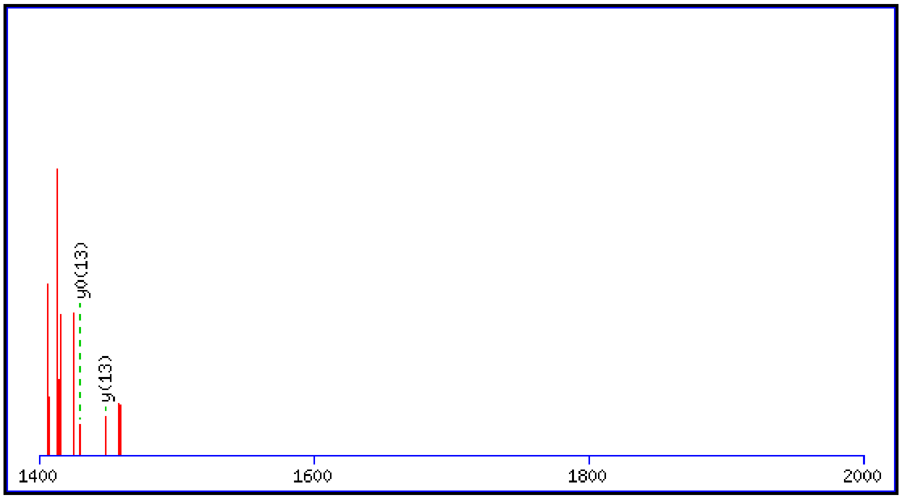

Monoisotopic mass of neutral peptide Mr(calc): 1560.7784
Fixed modifications: Carbamidomethyl (C)
Variable modifications:
N6 : Deamidated_N (N)
Ions Score: 58 **Expect:** 0.00023
Matches (Bold Red): 39/132 fragment ions using 86 most intense peaks

#	b	b++	b*	b*++	b0	b0++	Seq.	y	y++	y*	y*++	y0	y0++	#
1	114.0913	57.5493					I							14
2	215.1390	108.0731			197.1285	99.0679	T	**1448.7016**	724.8544	1431.6750	**716.3412**	**1430.6910**	**715.8492**	13
3	330.1660	165.5866			**312.1554**	156.5813	D	**1347.6539**	674.3306	**1330.6274**	665.8173	1329.6433	665.3253	12
4	443.2500	222.1287			425.2395	213.1234	I	**1232.6270**	616.8171	1215.6004	608.3038	1214.6164	607.8118	11
5	**572.2926**	286.6499			554.2821	277.6447	E	**1119.5429**	560.2751	1102.5164	551.7618	**1101.5323**	551.2698	10

**6**	**687.3196**	344.1634	670.2930	335.6501	669.3090	335.1581	**N**	**990.5003**	495.7538	**973.4738**	**487.2405**	**972.4897**	**486.7485**	**9**
**7**	**744.3410**	372.6741	727.3145	364.1609	726.3305	363.6689	**G**	**875.4734**	438.2403	858.4468	429.7271	**857.4628**	429.2350	**8**
**8**	**845.3887**	423.1980	828.3621	414.6847	**827.3781**	414.1927	**T**	**818.4519**	409.7296	801.4254	401.2163	800.4413	400.7243	**7**
**9**	**992.4571**	496.7322	975.4306	488.2189	**974.4465**	487.7269	**F**	**717.4042**	359.2058	700.3777	350.6925			**6**
**10**	**1063.4942**	532.2508	1046.4677	523.7375	**1045.4837**	523.2455	**A**	**570.3358**	285.6715	553.3093	277.1583			**5**
**11**	**1177.5372**	589.2722	**1160.5106**	580.7589	**1159.5266**	580.2669	**N**	**499.2987**	250.1530	482.2722	241.6397			**4**
**12**	**1290.6212**	645.8142	**1273.5947**	637.3010	**1272.6107**	636.8090	**I**	**385.2558**	193.1315	368.2292	184.6183			**3**
**13**	**1387.6740**	694.3406	1370.6474	685.8274	1369.6634	685.3353	**P**	**272.1717**	136.5895	255.1452	128.0762			**2**
**14**							**R**	175.1190	88.0631	158.0924	79.5498			**1**

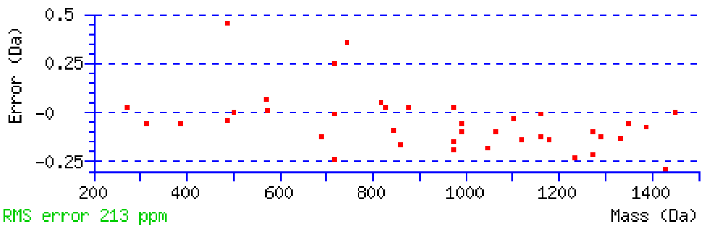

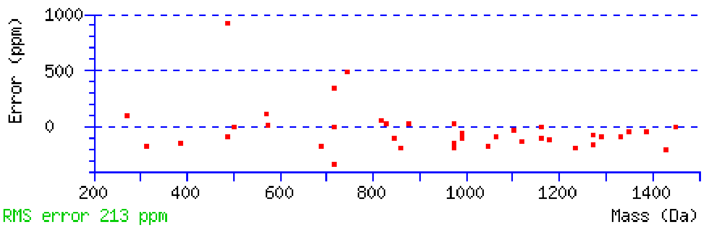

All matches to this query

Score	Mr(calc):	Delta	Sequence
58.3	1560.7784	0.0076	ITDIENGTFANIPR
37.9	1560.7784	0.0076	ITDIENGTFANIPR
7.2	1560.7864	-0.0004	DRARTQMMAEKPK
5.7	1559.7944	0.9916	ITDIENGTFANIPR
3.2	1560.7896	-0.0036	LEYRVPTDTQSPR
2.4	1560.7930	-0.0070	KLAGVMKDGVNSANR
0.5	1559.7820	1.0039	RRANANQPLLTPK

Spectrum No: 347; Query: 260; Rank: 1

Peptide View

MS/MS Fragmentation of **VLTNQENPYQNR**
Found in **IPI00215296**, Tax_Id=10116 Gene_Symbol=C1qa Complement C1q subcomponent subunit A precursor

Match to Query 260: 1475.697648 from(738.856100,2+)
Title: 091129RatKid_SCX02_13.532.532.2.dta
Data file K:\NewmanPaper\Piliang\3SubProteomes\Piliang3SP\mgf5ppm\SCX_3SubProteomes5ppm.mgf

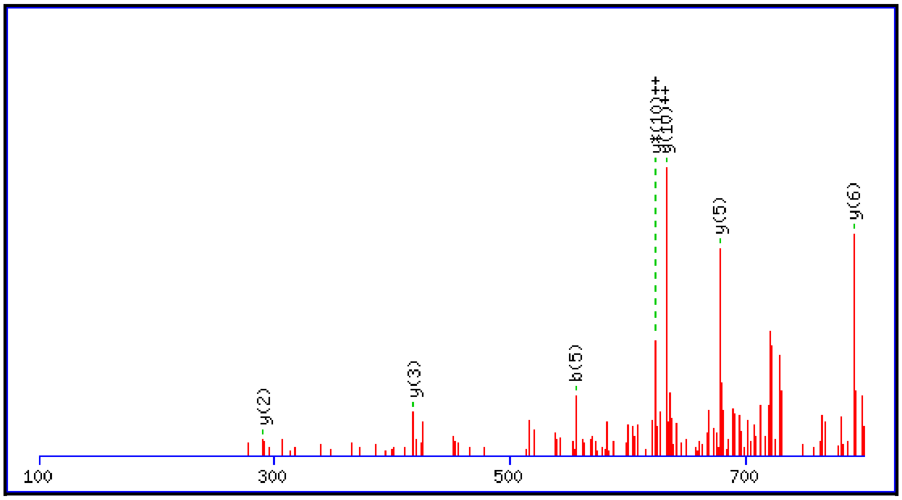

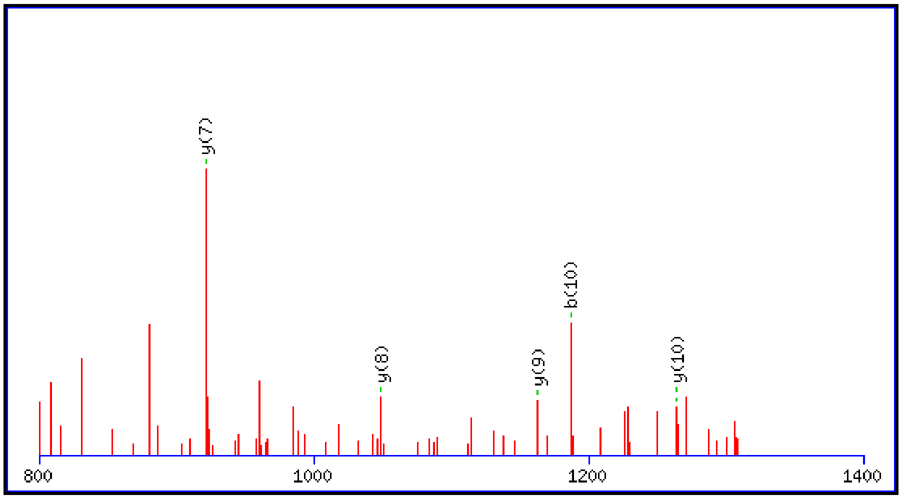

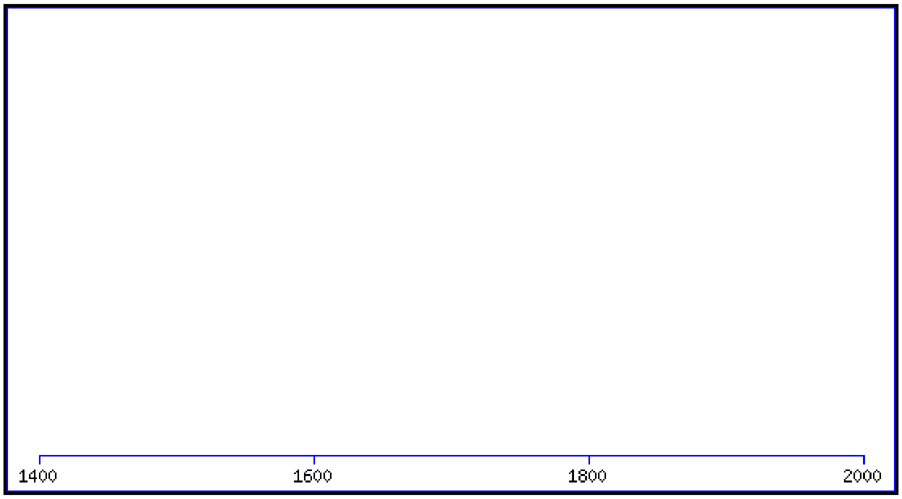

Monoisotopic mass of neutral peptide Mr(calc): 1475.7004
Fixed modifications: Carbamidomethyl (C)
Variable modifications:
N11 : Deamidated_N (N)
Ions Score: 58 **Expect:** 0.00023
Matches (Bold Red): 12/110 fragment ions using 18 most intense peaks

#	b	b++	b*	b*++	b0	b0++	Seq.	y	y++	y*	y*++	y0	y0++	#
1	100.0757	50.5415					V							12
2	213.1598	107.0835					L	1377.6393	689.3233	1360.6128	680.8100	1359.6288	680.3180	11
3	314.2074	157.6074			296.1969	148.6021	T	**1264.5553**	**632.7813**	1247.5287	**624.2680**	1246.5447	623.7760	10
4	428.2504	214.6288	411.2238	206.1155	410.2398	205.6235	N	**1163.5076**	582.2574	1146.4810	573.7442	1145.4970	573.2521	9
5	**556.3089**	278.6581	539.2824	270.1448	538.2984	269.6528	Q	**1049.4647**	525.2360	1032.4381	516.7227	1031.4541	516.2307	8
6	685.3515	343.1794	668.3250	334.6661	667.3410	334.1741	E	**921.4061**	461.2067	904.3795	452.6934	903.3955	452.2014	7
7	799.3945	400.2009	782.3679	391.6876	781.3839	391.1956	N	**792.3635**	396.6854	775.3369	388.1721			6
8	896.4472	448.7272	879.4207	440.2140	878.4367	439.7220	P	**678.3206**	339.6639	661.2940	331.1506			5
9	1059.5106	530.2589	1042.4840	521.7456	1041.5000	521.2536	Y	581.2678	291.1375	564.2412	282.6243			4
10	**1187.5691**	594.2882	1170.5426	585.7749	1169.5586	585.2829	Q	**418.2045**	209.6059	401.1779	201.0926			3
11	1302.5961	651.8017	1285.5695	643.2884	1284.5855	642.7964	N	**290.1459**	145.5766	273.1193	137.0633			2
12							R	175.1190	88.0631	158.0924	79.5498			1

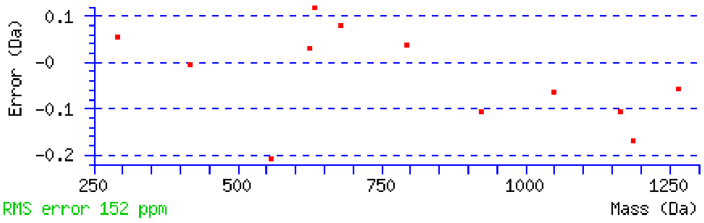

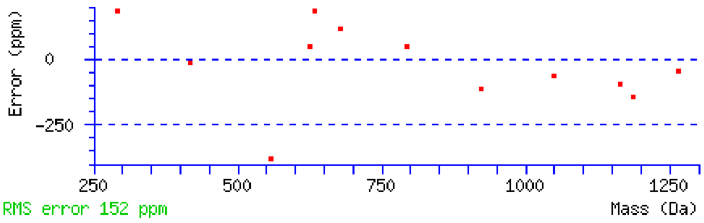

All matches to this query

Score	Mr(calc):	Delta	Sequence
58.2	1475.7004	-0.0028	VLTNQENPYQNR
18.9	1475.7004	-0.0028	VLTNQENPYQNR
10.2	1474.7041	0.9935	AEQKEQRHTLR

Spectrum No: 348; Query: 583; Rank: 1

Peptide View

MS/MS Fragmentation of **GQPIPNKNLTVQVNSVR**
Found in **IPI00326140**, Tax_Id=10116 Gene_Symbol=Pzp Alpha-1-macroglobulin precursor

Match to Query 583: 1864.015212 from(622.345680,3+)
Title: 091129RatKid_SCX02_17.1191.1191.3.dta
Data file K:\NewmanPaper\Piliang\3SubProteomes\Piliang3SP\mgf5ppm\SCX_3SubProteomes5ppm.mgf

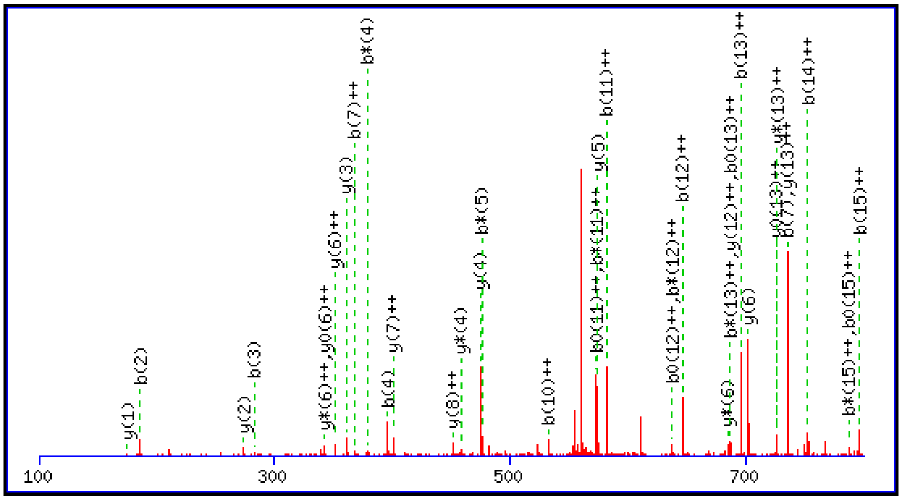

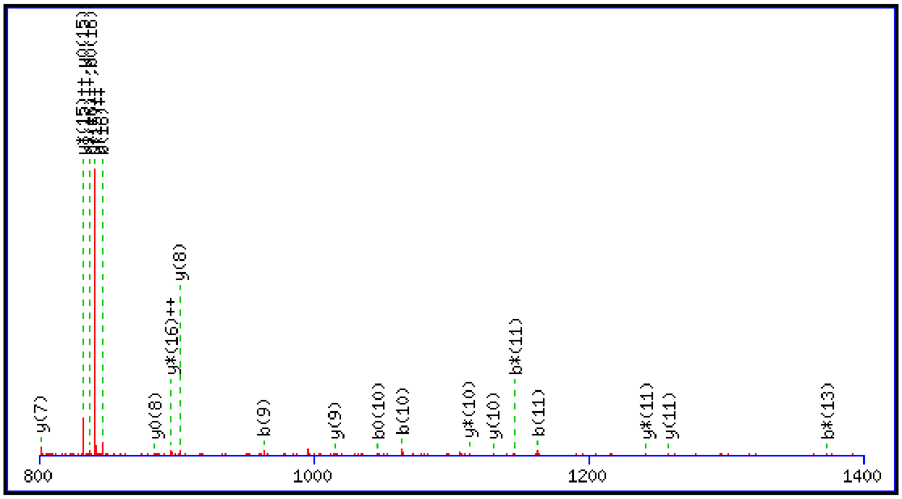

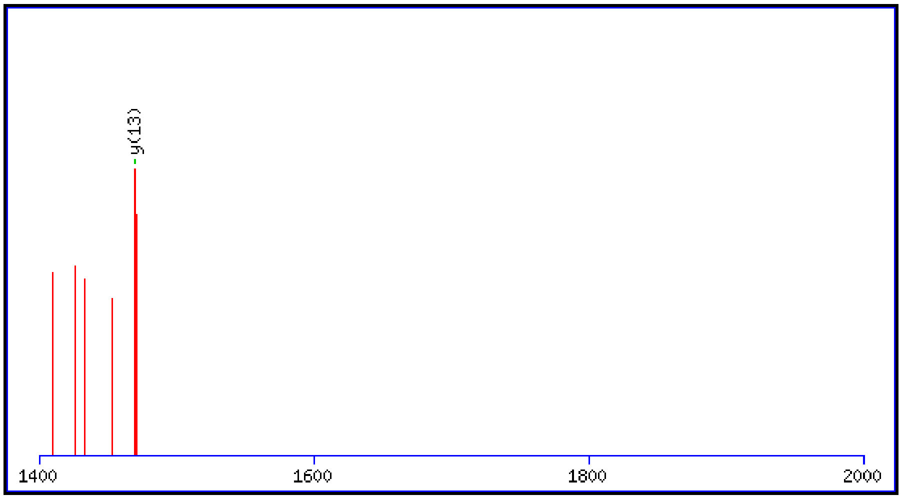

Monoisotopic mass of neutral peptide Mr(calc): 1864.0166
Fixed modifications: Carbamidomethyl (C)
Variable modifications:
N8 : Deamidated_N (N)
Ions Score: 58 **Expect:** 9.3e-005
Matches (Bold Red): 60/168 fragment ions using 112 most intense peaks

#	b	b^{++}	b*	b^{*++}	b^{0}	b^{0++}	Seq.	y	y^{++}	y*	y^{*++}	y^{0}	y^{0++}	#
1	58.0287	29.5180					G							17
2	**186.0873**	93.5473	169.0608	85.0340			Q	1808.0025	904.5049	1790.9759	**895.9916**	1789.9919	895.4996	16
3	**283.1401**	142.0737	266.1135	133.5604			P	1679.9439	**840.4756**	1662.9173	**831.9623**	1661.9333	**831.4703**	15
4	**396.2241**	198.6157	**379.1976**	190.1024			I	1582.8911	791.9492	1565.8646	783.4359	1564.8806	782.9439	14
5	493.2769	247.1421	**476.2504**	238.6288			P	**1469.8071**	**735.4072**	1452.7805	**726.8939**	1451.7965	**726.4019**	13
6	607.3198	304.1636	590.2933	295.6503			N	1372.7543	**686.8808**	1355.7277	678.3675	1354.7437	677.8755	12
7	**735.4148**	**368.2110**	718.3883	359.6978			K	**1258.7114**	629.8593	**1241.6848**	621.3460	1240.7008	620.8540	11
8	850.4417	425.7245	833.4152	417.2112			N	**1130.6164**	565.8118	**1113.5899**	557.2986	1112.6058	556.8066	10
9	**963.5258**	482.2665	946.4993	473.7533			L	**1015.5895**	508.2984	998.5629	499.7851	997.5789	499.2931	9
10	**1064.5735**	**532.7904**	1047.5469	524.2771	**1046.5629**	523.7851	T	**902.5054**	**451.7563**	885.4789	443.2431	**884.4948**	442.7511	8
11	**1163.6419**	**582.3246**	**1146.6153**	**573.8113**	1145.6313	**573.3193**	V	**801.4577**	**401.2325**	784.4312	392.7192	783.4472	392.2272	7
12	1291.7005	**646.3539**	1274.6739	**637.8406**	1273.6899	**637.3486**	Q	**702.3893**	**351.6983**	**685.3628**	**343.1850**	684.3787	**342.6930**	6
13	1390.7689	**695.8881**	**1373.7423**	**687.3748**	1372.7583	**686.8828**	V	**574.3307**	287.6690	557.3042	279.1557	556.3202	278.6637	5
14	1504.8118	**752.9095**	1487.7853	744.3963	1486.8012	743.9043	N	**475.2623**	238.1348	**458.2358**	229.6215	457.2518	229.1295	4
15	1591.8438	**796.4256**	1574.8173	**787.9123**	1573.8333	**787.4203**	S	**361.2194**	181.1133	344.1928	172.6001	343.2088	172.1081	3
16	1690.9123	**845.9598**	1673.8857	**837.4465**	1672.9017	**836.9545**	V	**274.1874**	137.5973	257.1608	129.0840			2
17							R	**175.1190**	88.0631	158.0924	79.5498			1

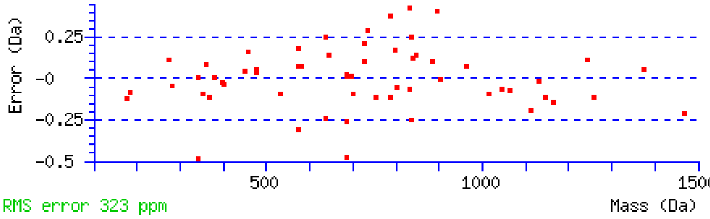

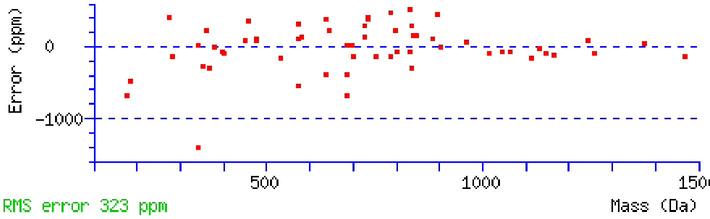

All matches to this query

Score	Mr(calc):	Delta	Sequence
58.2	1864.0166	-0.0014	GQPIPNKNLTVQVNSVR
39.6	1864.0166	-0.0014	GQPIPNKNLTVQVNSVR
38.8	1864.0166	-0.0014	GQPIPNKNLTVQVNSVR
4.0	1864.0029	0.0123	MKPNTLVIGYKKNWR

2.0	1864.0305	-0.0153	APLLPSQVNLEDKGALAK
1.3	1864.0213	-0.0061	AIASHPSAALMALRRGSR
1.0	1864.0045	0.0107	NAIKKLMNHILLFTK
0.4	1864.0029	0.0123	MKPNTLVIGYKKNWR
0.3	1862.9962	1.0190	VPESLARSLRGEEPAPR
0.2	1864.0005	0.0147	LASVNLRKLIDCILR

Spectrum No: 349; Query: 1084; Rank: 1

Peptide View

MS/MS Fragmentation of **SIFGEDALANVSIEKPVHQGPDAAVTGHIR**
Found in **IPI00211812**, Tax_Id=10116 Gene_Symbol=Copb1 Coatomer subunit beta

Match to Query 1084: 3129.606732 from(1044.209520,3+)
Title: 091127RatKid_SCX01_37.3507.3507.3.dta
Data file K:\NewmanPaper\Piliang\3SubProteomes\Piliang3SP\mgf5ppm\SCX_3SubProteomes5ppm.mgf

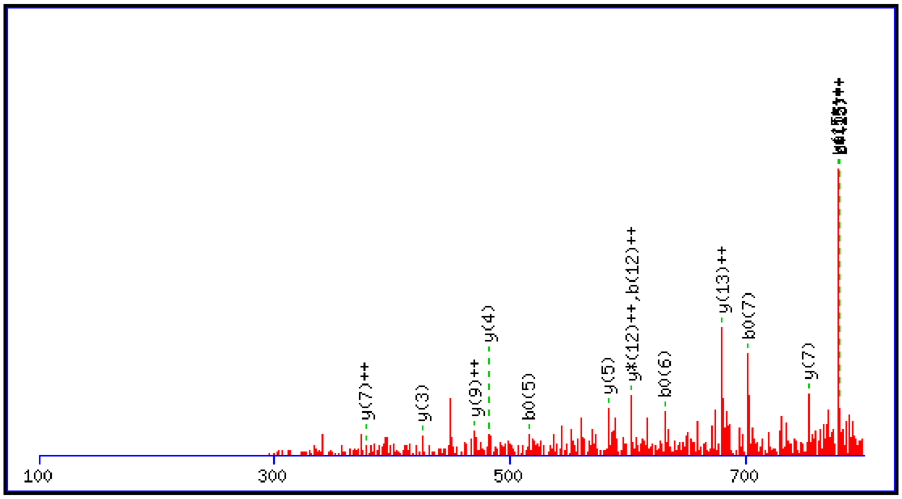

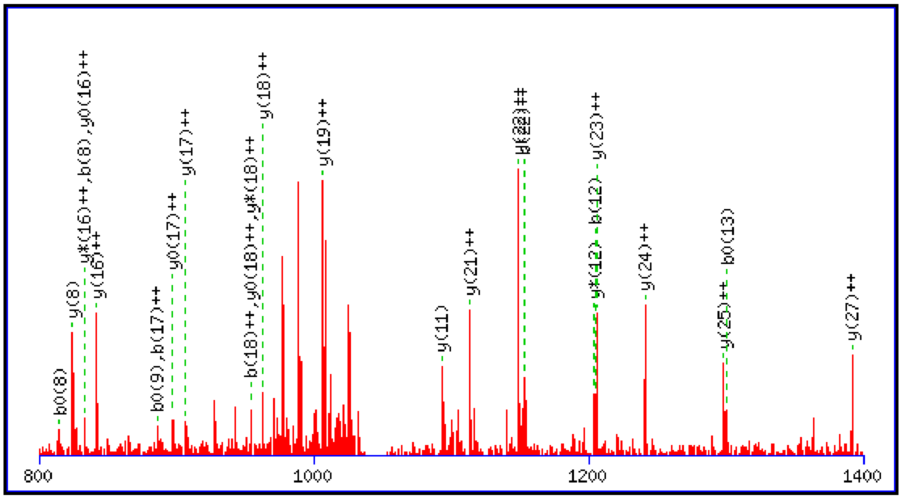

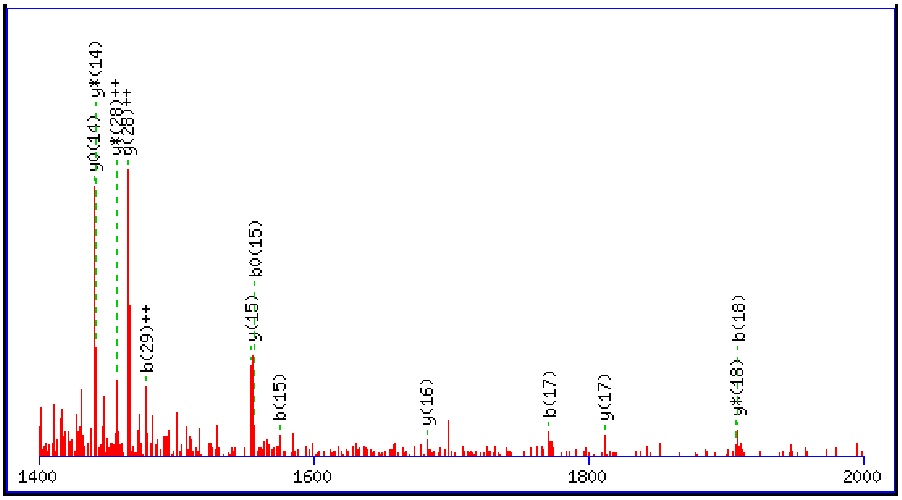

Monoisotopic mass of neutral peptide Mr(calc): 3128.5891
Fixed modifications: Carbamidomethyl (C)
Variable modifications:
N10 : Deamidated_N (N)
Ions Score: 58 **Expect:** 0.00022
Matches (Bold Red): 54/322 fragment ions using 121 most intense peaks

#	b	b++	b*	b*++	b0	b0++	Seq.	y	y++	y*	y*++	y0	y0++	#
1	88.0393	44.5233			70.0287	35.5180	S							30
2	201.1234	101.0653			183.1128	92.0600	I	3042.5643	1521.7858	3025.5378	1513.2725	3024.5538	1512.7805	29
3	348.1918	174.5995			330.1812	165.5942	F	2929.4803	**1465.2438**	2912.4537	**1456.7305**	2911.4697	1456.2385	28
4	405.2132	203.1103			387.2027	194.1050	G	2782.4118	**1391.7096**	2765.3853	1383.1963	2764.4013	1382.7043	27
5	534.2558	267.6316			**516.2453**	258.6263	E	2725.3904	1363.1988	2708.3638	1354.6856	2707.3798	1354.1935	26
6	649.2828	325.1450			**631.2722**	316.1397	D	2596.3478	**1298.6775**	2579.3212	1290.1643	2578.3372	1289.6722	25
7	720.3199	360.6636			**702.3093**	351.6583	A	2481.3208	**1241.1641**	2464.2943	1232.6508	2463.3103	1232.1588	24
8	**833.4040**	417.2056			**815.3934**	408.2003	L	2410.2837	**1205.6455**	2393.2572	1197.1322	2392.2732	1196.6402	23
9	904.4411	452.7242			**886.4305**	443.7189	A	2297.1997	**1149.1035**	2280.1731	1140.5902	2279.1891	1140.0982	22
10	1019.4680	510.2376	1002.4415	501.7244	1001.4574	501.2324	N	2226.1625	**1113.5849**	2209.1360	1105.0716	2208.1520	1104.5796	21
11	1118.5364	559.7718	1101.5099	551.2586	1100.5259	550.7666	V	2111.1356	1056.0714	2094.1091	1047.5582	2093.1250	1047.0662	20
12	**1205.5685**	**603.2879**	1188.5419	594.7746	1187.5579	594.2826	S	2012.0672	**1006.5372**	1995.0407	998.0240	1994.0566	997.5320	19
13	1318.6525	659.8299	1301.6260	651.3166	**1300.6420**	650.8246	I	1925.0352	**963.0212**	**1908.0086**	**954.5079**	1907.0246	**954.0159**	18
14	1447.6951	724.3512	1430.6686	715.8379	1429.6845	715.3459	E	**1811.9511**	**906.4792**	1794.9246	897.9659	1793.9405	**897.4739**	17
15	**1575.7901**	788.3987	1558.7635	**779.8854**	**1557.7795**	**779.3934**	K	**1682.9085**	**841.9579**	1665.8820	**833.4446**	1664.8979	**832.9526**	16
16	1672.8428	836.9251	1655.8163	828.4118	1654.8323	827.9198	P	**1554.8136**	**777.9104**	1537.7870	769.3971	1536.8030	768.9051	15
17	**1771.9113**	**886.4593**	1754.8847	877.9460	1753.9007	877.4540	V	1457.7608	729.3840	**1440.7342**	720.8708	**1439.7502**	720.3787	14
18	**1908.9702**	**954.9887**	1891.9436	946.4754	1890.9596	945.9834	H	1358.6924	**679.8498**	1341.6658	671.3366	1340.6818	670.8445	13
19	2037.0287	1019.0180	2020.0022	1010.5047	2019.0182	1010.0127	Q	1221.6335	611.3204	**1204.6069**	**602.8071**	1203.6229	602.3151	12
20	2094.0502	1047.5287	2077.0237	1039.0155	2076.0396	1038.5235	G	**1093.5749**	547.2911	1076.5483	538.7778	1075.5643	538.2858	11
21	2191.1030	1096.0551	2174.0764	1087.5418	2173.0924	1087.0498	P	1036.5534	518.7803	1019.5269	510.2671	1018.5429	509.7751	10
22	2306.1299	**1153.5686**	2289.1034	1145.0553	2288.1193	1144.5633	D	939.5007	**470.2540**	922.4741	461.7407	921.4901	461.2487	9
23	2377.1670	1189.0872	2360.1405	1180.5739	2359.1565	1180.0819	A	**824.4737**	412.7405	807.4472	404.2272	806.4631	403.7352	8
24	2448.2041	1224.6057	2431.1776	1216.0924	2430.1936	1215.6004	A	**753.4366**	**377.2219**	736.4100	368.7087	735.4260	368.2167	7
25	2547.2726	1274.1399	2530.2460	1265.6266	2529.2620	1265.1346	V	682.3995	341.7034	665.3729	333.1901	664.3889	332.6981	6
26	2648.3202	1324.6638	2631.2937	1316.1505	2630.3097	1315.6585	T	**583.3311**	292.1692	566.3045	283.6559	565.3205	283.1639	5
27	2705.3417	1353.1745	2688.3151	1344.6612	2687.3311	1344.1692	G	**482.2834**	241.6453	465.2568	233.1321			4
28	2842.4006	1421.7039	2825.3741	1413.1907	2824.3900	1412.6987	H	**425.2619**	213.1346	408.2354	204.6213			3
29	2955.4847	**1478.2460**	2938.4581	1469.7327	2937.4741	1469.2407	I	288.2030	144.6051	271.1765	136.0919			2
30							R	175.1190	88.0631	158.0924	79.5498			1

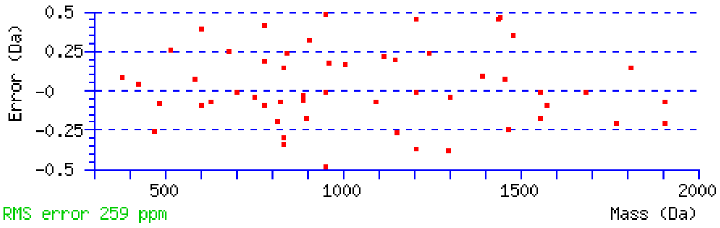

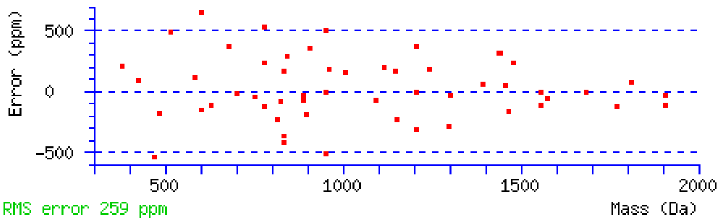

All matches to this query

Score	Mr(calc):	Delta	Sequence
58.2	3128.5891	1.0177	SIFGEDALANVSIEKPVHQGPDAAVTGHIR
43.8	3127.6051	2.0017	SIFGEDALANVSIEKPVHQGPDAAVTGHIR

Spectrum No: 350; Query: 721; Rank: 1

Peptide View

MS/MS Fragmentation of **VNNSSLIGLGYTQTLKPGIK**
Found in **IPI00365813**, Tax_Id=10116 Gene_Symbol=RGD1565338_predicted similar to Voltage-dependent anion-selective channel protein 1

Match to Query 721: 2103.161868 from(1052.588210,2+)
Title: 091127RatKid_SCX01_27.3340.3340.2.dta
Data file K:\NewmanPaper\Piliang\3SubProteomes\Piliang3SP\mgf5ppm\SCX_3SubProteomes5ppm.mgf

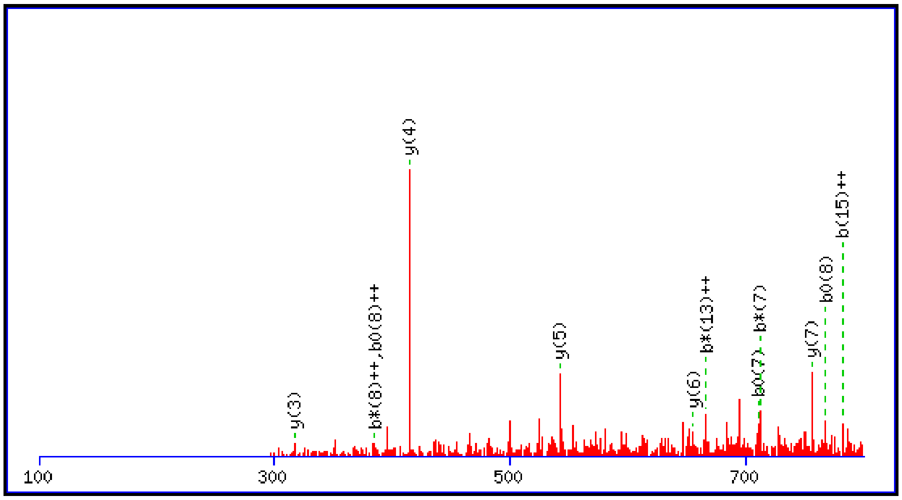

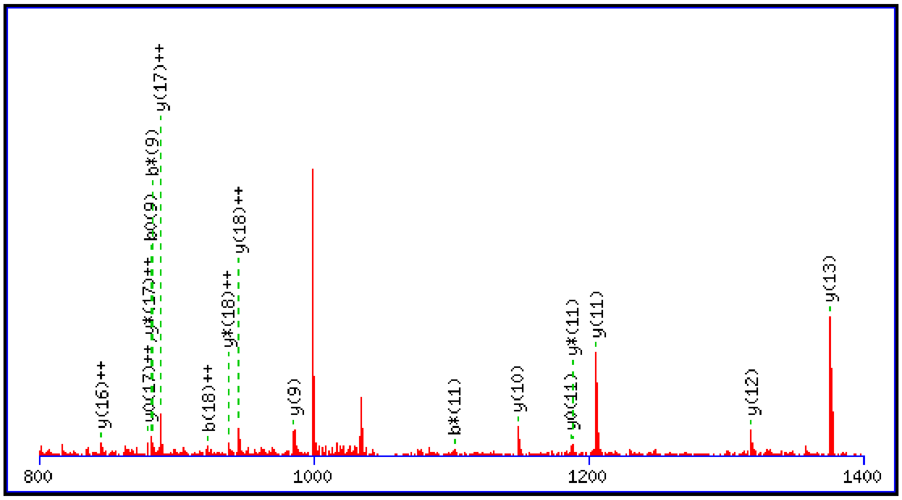

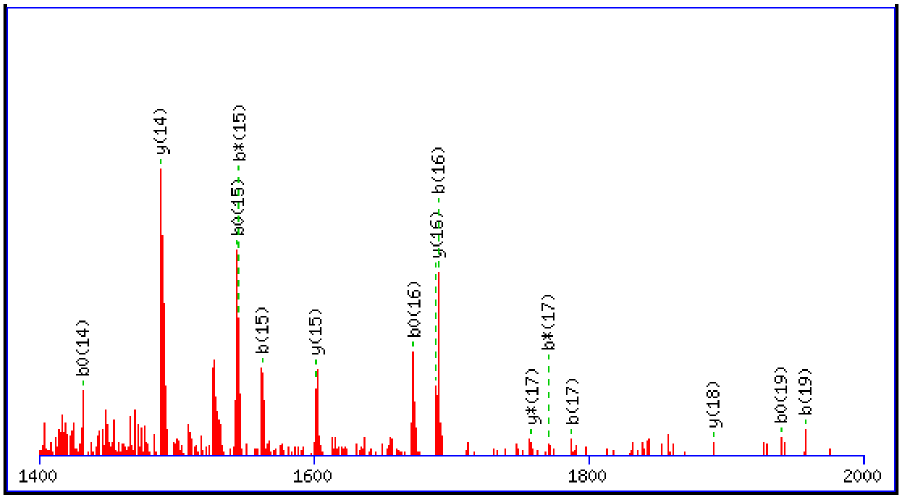

Monoisotopic mass of neutral peptide Mr(calc): 2103.1575
Fixed modifications: Carbamidomethyl (C)
Variable modifications:
N3 : Deamidated_N (N)
Ions Score: 58 **Expect:** 7.4e-005
Matches (Bold Red): 44/208 fragment ions using 112 most intense peaks

#	b	b^{++}	b*	b^{*++}	b^{0}	b^{0++}	Seq.	y	y^{++}	y*	y^{*++}	y^{0}	y^{0++}	#
1	100.0757	50.5415					V							20
2	214.1186	107.5629	197.0921	99.0497			N	2005.0964	1003.0519	1988.0699	994.5386	1987.0859	994.0466	19
3	329.1456	165.0764	312.1190	156.5631			N	**1891.0535**	**946.0304**	1874.0270	**937.5171**	1873.0429	937.0251	18
4	416.1776	208.5924	399.1510	200.0792	398.1670	199.5871	S	1776.0266	**888.5169**	**1759.0000**	**880.0036**	1758.0160	**879.5116**	17
5	503.2096	252.1084	486.1831	243.5952	485.1990	243.1032	S	**1688.9945**	**845.0009**	1671.9680	836.4876	1670.9840	835.9956	16
6	616.2937	308.6505	599.2671	300.1372	598.2831	299.6452	L	**1601.9625**	801.4849	1584.9360	792.9716	1583.9519	792.4796	15
7	729.3777	365.1925	**712.3512**	356.6792	**711.3672**	356.1872	I	**1488.8784**	744.9429	1471.8519	736.4296	1470.8679	735.9376	14
8	786.3992	393.7032	769.3727	**385.1900**	**768.3886**	**384.6980**	G	**1375.7944**	688.4008	1358.7678	679.8876	1357.7838	679.3955	13
9	899.4833	450.2453	**882.4567**	441.7320	**881.4727**	441.2400	L	**1318.7729**	659.8901	1301.7464	651.3768	1300.7624	650.8848	12
10	956.5047	478.7560	939.4782	470.2427	938.4942	469.7507	G	**1205.6889**	603.3481	**1188.6623**	594.8348	**1187.6783**	594.3428	11
11	1119.5681	560.2877	**1102.5415**	551.7744	1101.5575	551.2824	Y	**1148.6674**	574.8373	1131.6408	566.3241	1130.6568	565.8320	10
12	1220.6157	610.8115	1203.5892	602.2982	1202.6052	601.8062	T	**985.6041**	493.3057	968.5775	484.7924	967.5935	484.3004	9
13	1348.6743	674.8408	1331.6478	**666.3275**	1330.6637	665.8355	Q	884.5564	442.7818	867.5298	434.2686	866.5458	433.7765	8
14	1449.7220	725.3646	1432.6954	716.8514	**1431.7114**	716.3594	T	**756.4978**	378.7525	739.4713	370.2393	738.4872	369.7473	7
15	**1562.8061**	**781.9067**	**1545.7795**	773.3934	**1544.7955**	772.9014	L	**655.4501**	328.2287	638.4236	319.7154			6
16	**1690.9010**	845.9541	1673.8745	837.4409	**1672.8905**	836.9489	K	**542.3661**	271.6867	525.3395	263.1734			5
17	**1787.9538**	894.4805	**1770.9272**	885.9673	1769.9432	885.4752	P	**414.2711**	207.6392	397.2445	199.1259			4
18	1844.9752	**922.9913**	1827.9487	914.4780	1826.9647	913.9860	G	**317.2183**	159.1128	300.1918	150.5995			3
19	**1958.0593**	979.5333	1941.0328	971.0200	**1940.0487**	970.5280	I	260.1969	130.6021	243.1703	122.0888			2
20							K	147.1128	74.0600	130.0863	65.5468			1

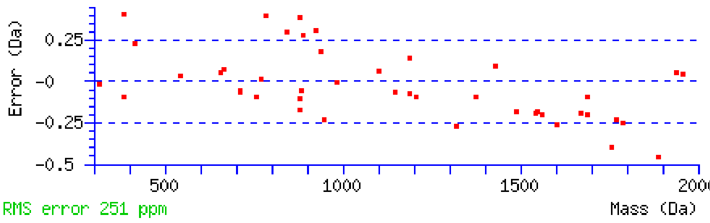

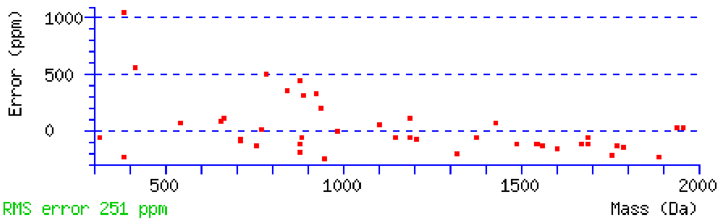

All matches to this query

Score	Mr(calc):	Delta	Sequence
58.1	2103.1575	0.0043	VNNSSLIGLGYTQTLKPGIK

47.9	2103.1575	0.0043	VNNSSLIGLGYTQTLKPGIK
43.0	2102.1735	0.9883	VNNSSLIGLGYTQTLKPGIK
5.5	2102.1484	1.0135	VNNSSLIGVGYTQTLRPGVK

Spectrum No: 351; Query: 137; Rank: 1

Peptide View

MS/MS Fragmentation of **GLFTNGSCADIK**
Found in **IPI00190846**, Tax_Id=10116 Gene_Symbol=Casr Extracellular calcium-sensing receptor precursor

Match to Query 137: 1282.591908 from(642.303230,2+)
Title: 091129RatKid_SCX02_13.1565.1565.2.dta
Data file K:\NewmanPaper\Piliang\3SubProteomes\Piliang3SP\mgf5ppm\SCX_3SubProteomes5ppm.mgf

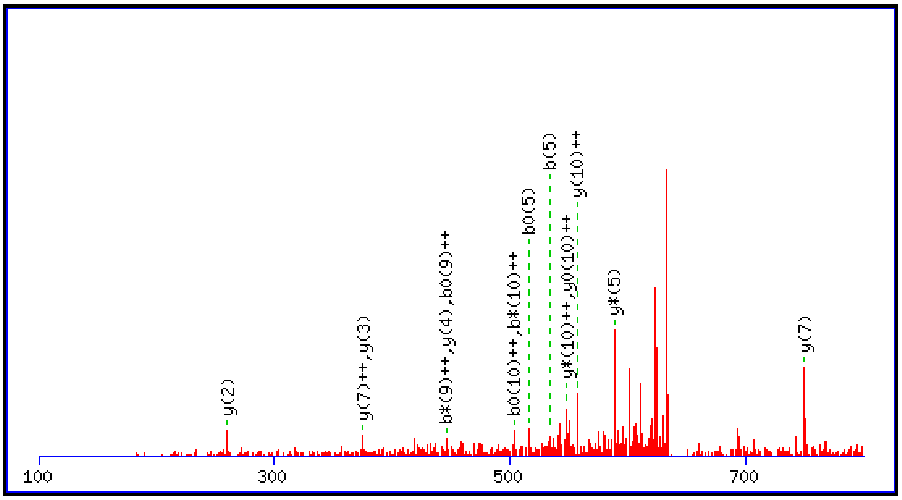

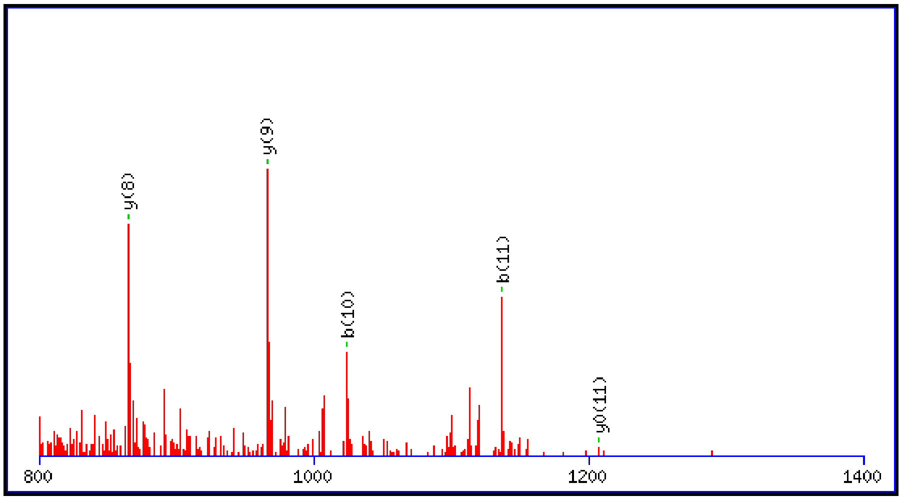

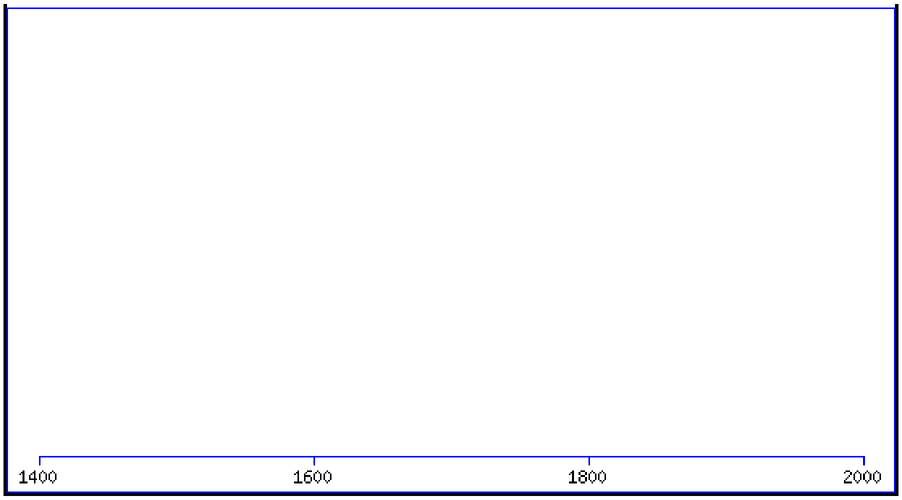

Monoisotopic mass of neutral peptide Mr(calc): 1282.5863
Fixed modifications: Carbamidomethyl (C)
Variable modifications:
N5 : Deamidated_N (N)
Ions Score: 58 **Expect:** 0.00025
Matches (Bold Red): 20/114 fragment ions using 32 most intense peaks

#	b	b^{++}	b^{*}	b^{*++}	b^{0}	b^{0++}	Seq.	y	y^{++}	y^{*}	y^{*++}	y^{0}	y^{0++}	#
1	58.0287	29.5180					G							12
2	171.1128	86.0600					L	1226.5722	613.7897	1209.5456	605.2764	**1208.5616**	604.7844	11
3	318.1812	159.5942					F	1113.4881	**557.2477**	1096.4615	**548.7344**	1095.4775	**548.2424**	10
4	419.2289	210.1181			401.2183	201.1128	T	**966.4197**	483.7135	949.3931	475.2002	948.4091	474.7082	9
5	**534.2558**	267.6316	517.2293	259.1183	**516.2453**	258.6263	N	**865.3720**	433.1896	848.3455	424.6764	847.3614	424.1844	8
6	591.2773	296.1423	574.2507	287.6290	573.2667	287.1370	G	**750.3451**	**375.6762**	733.3185	367.1629	732.3345	366.6709	7
7	678.3093	339.6583	661.2828	331.1450	660.2988	330.6530	S	693.3236	347.1654	676.2971	338.6522	675.3130	338.1602	6
8	838.3400	419.6736	821.3134	411.1604	820.3294	410.6683	C	606.2916	303.6494	**589.2650**	295.1362	588.2810	294.6441	5
9	909.3771	455.1922	892.3505	**446.6789**	891.3665	**446.1869**	A	**446.2609**	223.6341	429.2344	215.1208	428.2504	214.6288	4
10	**1024.4040**	512.7057	1007.3775	**504.1924**	1006.3935	**503.7004**	D	**375.2238**	188.1155	358.1973	179.6023	357.2132	179.1103	3
11	**1137.4881**	569.2477	1120.4615	560.7344	1119.4775	560.2424	I	**260.1969**	130.6021	243.1703	122.0888			2
12							K	147.1128	74.0600	130.0863	65.5468			1

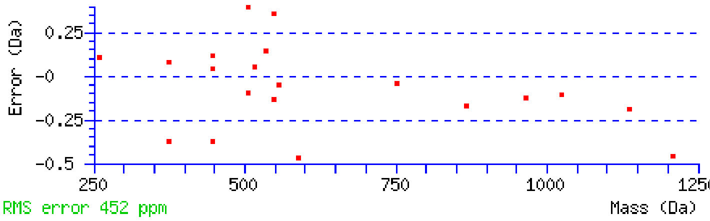

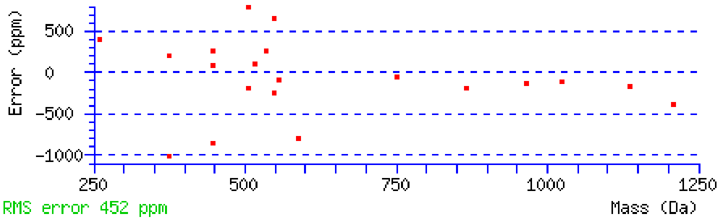

All matches to this query

Score	Mr(calc):	Delta	Sequence
58.0	1282.5863	0.0056	GLFTNGSCADIK
17.8	1282.5976	-0.0057	ASTFQNTCNLK
16.9	1282.5798	0.0121	VCDGCFRELK
9.0	1282.6041	-0.0122	DNISYGTNSALK
8.5	1282.6016	-0.0097	GSWACSIFDLK
7.9	1282.5992	-0.0073	MVKQASEGPLK
7.9	1282.5992	-0.0073	VMKQASEGPLK
7.7	1280.5735	2.0184	QQNSRGRGGGGK
7.2	1280.5836	2.0083	CPGQITLGDLK

6.8	1282.5863	0.0056	RNFELMEAEK

Spectrum No: 352; Query: 986; Rank: 1

Peptide View

MS/MS Fragmentation of **HNNDTQHIWESDSNEFSVIADPR**
Found in **IPI00365985**, Tax_Id=10116 Gene_Symbol=Tra1_predicted 93 kDa protein

Match to Query 986: 2711.188812 from(904.736880,3+)
Title: 091127RatKid_SCX01_35.3213.3213.3.dta
Data file K:\NewmanPaper\Piliang\3SubProteomes\Piliang3SP\mgf5ppm\SCX_3SubProteomes5ppm.mgf

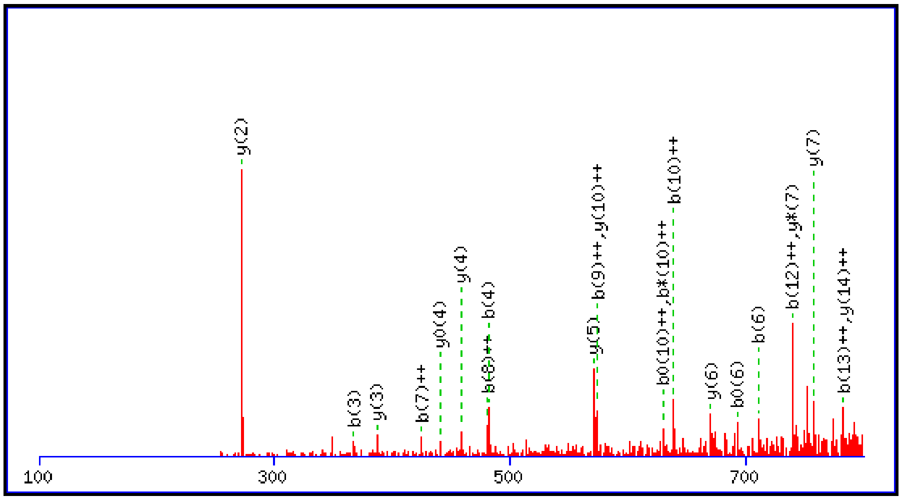

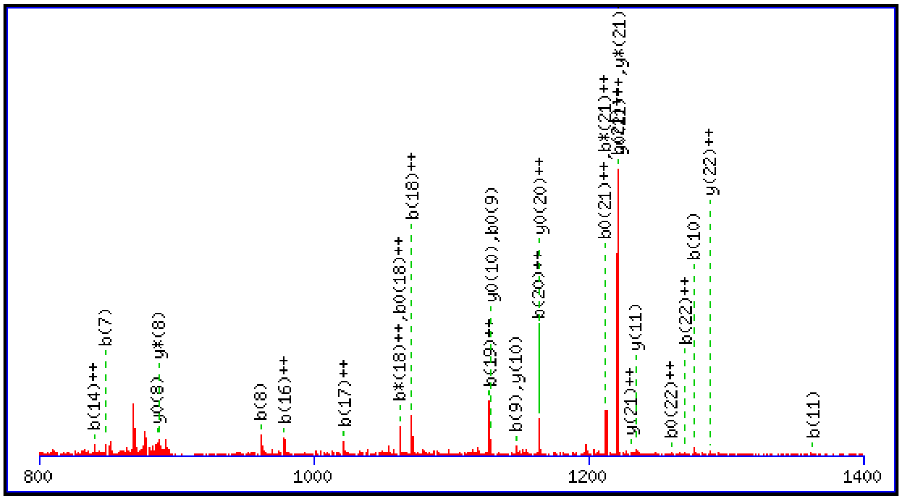

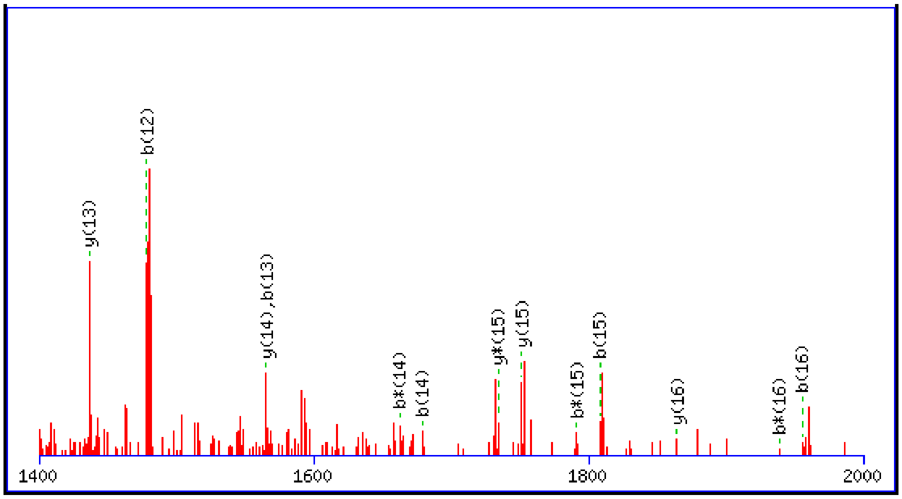

Monoisotopic mass of neutral peptide Mr(calc): 2711.1848
Fixed modifications: Carbamidomethyl (C)
Variable modifications:
N3 : Deamidated_N (N)
Ions Score: 58 **Expect:** 0.00056
Matches (Bold Red): 64/252 fragment ions using 152 most intense peaks

#	b	b^{++}	b*	b^{*++}	b^0	b^{0++}	Seq.	y	y^{++}	y*	y^{*++}	y^0	y^{0++}	#
1	138.0662	69.5367					H							23
2	252.1091	126.5582	235.0826	118.0449			N	2575.1332	**1288.0702**	2558.1066	1279.5569	2557.1226	1279.0649	22
3	**367.1361**	184.0717	350.1095	175.5584			N	2461.0902	**1231.0488**	2444.0637	**1222.5355**	2443.0797	**1222.0435**	21
4	**482.1630**	241.5851	465.1364	233.0719	464.1524	232.5799	D	2346.0633	1173.5353	2329.0367	1165.0220	2328.0527	**1164.5300**	20
5	583.2107	292.1090	566.1841	283.5957	565.2001	283.1037	T	2231.0364	1116.0218	2214.0098	1107.5085	2213.0258	1107.0165	19
6	**711.2693**	356.1383	694.2427	347.6250	**693.2587**	347.1330	Q	2129.9887	1065.4980	2112.9621	1056.9847	2111.9781	1056.4927	18
7	**848.3282**	**424.6677**	831.3016	416.1544	830.3176	415.6624	H	2001.9301	1001.4687	1984.9035	992.9554	1983.9195	992.4634	17
8	**961.4122**	**481.2098**	944.3857	472.6965	943.4017	472.2045	I	**1864.8712**	932.9392	1847.8446	924.4260	1846.8606	923.9339	16
9	**1147.4915**	**574.2494**	1130.4650	565.7361	**1129.4810**	565.2441	W	**1751.7871**	876.3972	**1734.7606**	867.8839	1733.7766	867.3919	15
10	**1276.5341**	**638.7707**	1259.5076	**630.2574**	1258.5236	**629.7654**	E	**1565.7078**	**783.3575**	1548.6813	774.8443	1547.6972	774.3523	14
11	**1363.5662**	682.2867	1346.5396	673.7734	1345.5556	673.2814	S	**1436.6652**	718.8362	1419.6387	710.3230	1418.6546	709.8310	13
12	**1478.5931**	**739.8002**	1461.5666	731.2869	1460.5825	730.7949	D	1349.6332	675.3202	1332.6066	666.8070	1331.6226	666.3149	12
13	**1565.6251**	**783.3162**	1548.5986	774.8029	1547.6146	774.3109	S	**1234.6062**	617.8068	1217.5797	609.2935	1216.5957	608.8015	11
14	**1679.6681**	**840.3377**	**1662.6415**	831.8244	1661.6575	831.3324	N	**1147.5742**	**574.2907**	1130.5477	565.7775	**1129.5636**	565.2855	10
15	**1808.7107**	904.8590	**1791.6841**	896.3457	1790.7001	895.8537	E	1033.5313	517.2693	1016.5047	508.7560	1015.5207	508.2640	9
16	**1955.7791**	**978.3932**	**1938.7525**	969.8799	1937.7685	969.3879	F	904.4887	452.7480	**887.4621**	444.2347	**886.4781**	443.7427	8
17	2042.8111	**1021.9092**	2025.7845	1013.3959	2024.8005	1012.9039	S	**757.4203**	379.2138	**740.3937**	370.7005	739.4097	370.2085	7
18	2141.8795	**1071.4434**	2124.8530	**1062.9301**	2123.8689	**1062.4381**	V	**670.3883**	335.6978	653.3617	327.1845	652.3777	326.6925	6
19	2254.9636	**1127.9854**	2237.9370	1119.4721	2236.9530	1118.9801	I	**571.3198**	286.1636	554.2933	277.6503	553.3093	277.1583	5
20	2326.0007	**1163.5040**	2308.9741	1154.9907	2307.9901	1154.4987	A	**458.2358**	229.6215	441.2092	221.1083	**440.2252**	220.6162	4
21	2441.0276	**1221.0175**	2424.0011	**1212.5042**	2423.0171	**1212.0122**	D	**387.1987**	194.1030	370.1721	185.5897	369.1881	185.0977	3
22	2538.0804	**1269.5438**	2521.0538	1261.0306	2520.0698	**1260.5386**	P	**272.1717**	136.5895	255.1452	128.0762			2
23							R	175.1190	88.0631	158.0924	79.5498			1

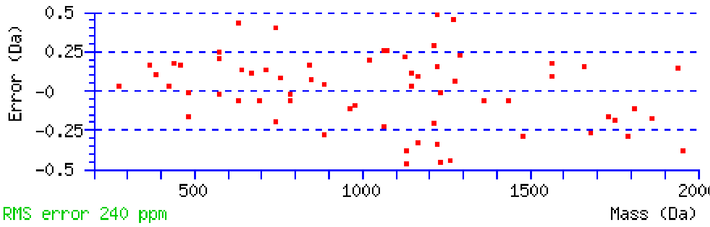

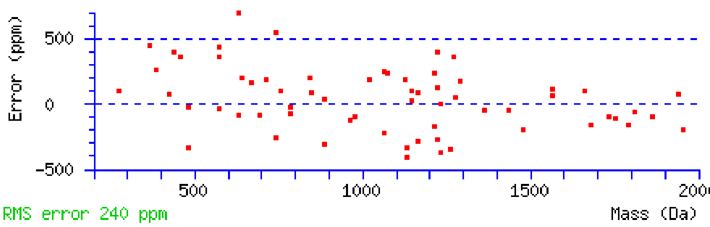

All matches to this query

Score	Mr(calc):	Delta	Sequence
58.0	2711.1848	0.0040	HNNDTQHIWESDSNEFSVIADPR
58.0	2711.1848	0.0040	HNNDTQHIWESDSNEFSVIADPR
18.8	2711.1848	0.0040	HNNDTQHIWESDSNEFSVIADPR
16.1	2710.2008	0.9880	HNNDTQHIWESDSNEFSVIADPR
3.6	2711.2043	-0.0155	SELEGSMLSVLSAASTASRLLPPR
3.0	2711.1742	0.0146	WSSEEPKSSTSFTTQRRPSTPR
2.4	2711.1694	0.0194	LKYSILEQSPSPPMLFTMHPR
1.9	2711.1911	-0.0023	SFPTLQSLTESRFPGPRDALXR
1.5	2711.1898	-0.0010	DLWITQTCSSGQTSNIPPSSEAPR
1.5	2711.1898	-0.0010	DLWITQTCSSGQTSNIPPSSEAPR

Spectrum No: 353; Query: 582; Rank: 1

Peptide View

MS/MS Fragmentation of **MAATFIGNSTAIQELFK**
Found in **IPI00765366**, Tax_Id=10116 Gene_Symbol=Tubb4 tubulin, beta 4

Match to Query 582: 1857.919308 from(929.966930,2+)
Title: 091127RatKid_SCX01_18.5663.5663.2.dta
Data file K:\NewmanPaper\Piliang\3SubProteomes\Piliang3SP\mgf5ppm\SCX_3SubProteomes5ppm.mgf

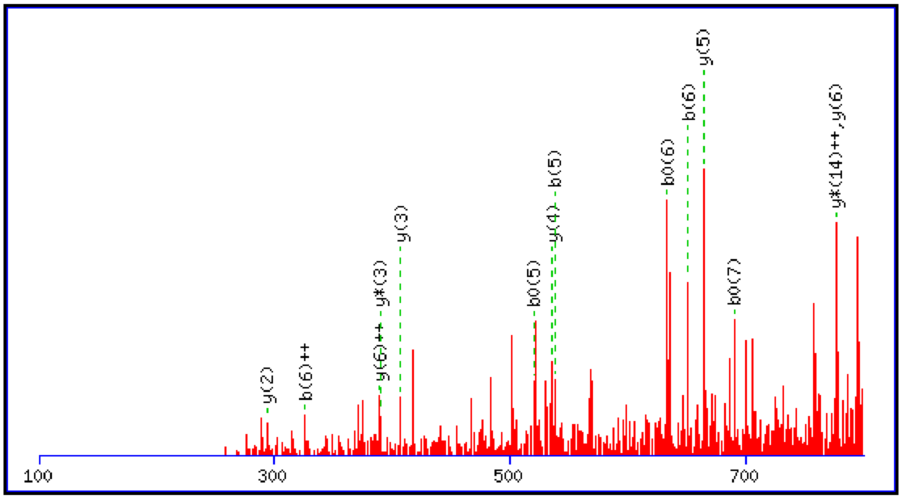

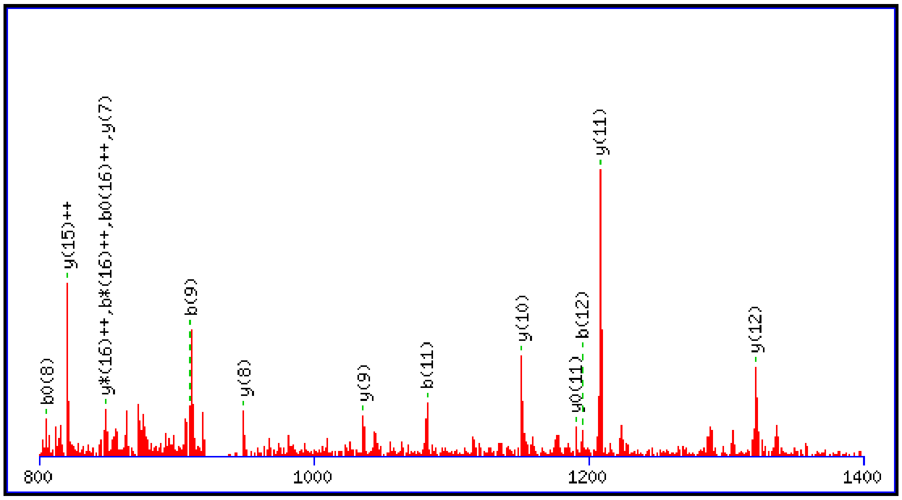

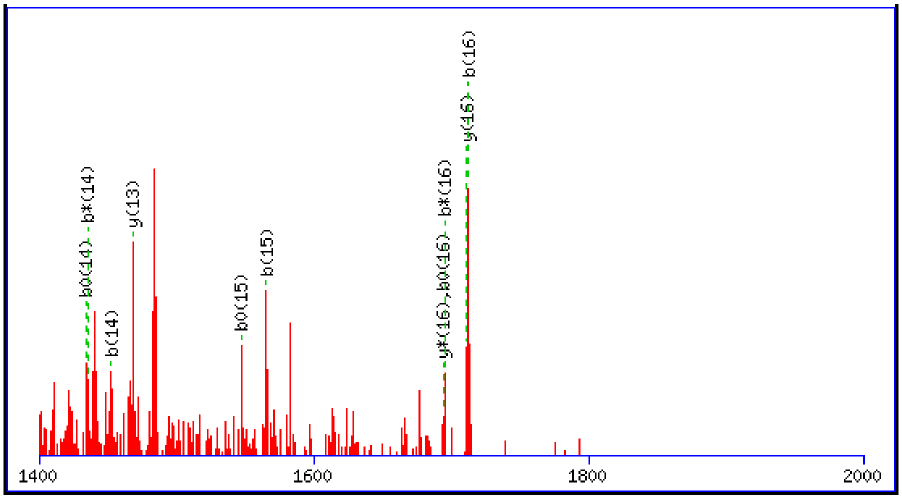

Monoisotopic mass of neutral peptide Mr(calc): 1857.9182
Fixed modifications: Carbamidomethyl (C)
Variable modifications:
M1 : Oxidation (M)
N8 : Deamidated_N (N)
Ions Score: 58 **Expect:** 0.00031
Matches (Bold Red): 40/166 fragment ions using 98 most intense peaks

#	b	b^{++}	b*	b^{*++}	b^0	b^{0++}	Seq.	y	y^{++}	y*	y^{*++}	y^0	y^{0++}	#
1	148.0427	74.5250					M							17
2	219.0798	110.0435					A	**1711.8901**	856.4487	**1694.8636**	**847.9354**	1693.8796	847.4434	16
3	290.1169	145.5621					A	1640.8530	**820.9301**	1623.8265	812.4169	1622.8424	811.9249	15
4	391.1646	196.0859			373.1540	187.0806	T	1569.8159	785.4116	1552.7893	**776.8983**	1551.8053	776.4063	14
5	**538.2330**	269.6201			**520.2224**	260.6149	F	**1468.7682**	734.8877	1451.7417	726.3745	1450.7577	725.8825	13
6	**651.3171**	**326.1622**			**633.3065**	317.1569	I	**1321.6998**	661.3535	1304.6733	652.8403	1303.6892	652.3483	12
7	708.3385	354.6729			**690.3280**	345.6676	G	**1208.6157**	604.8115	1191.5892	596.2982	**1190.6052**	595.8062	11
8	823.3655	412.1864	806.3389	403.6731	**805.3549**	403.1811	N	**1151.5943**	576.3008	1134.5677	567.7875	1133.5837	567.2955	10
9	**910.3975**	455.7024	893.3709	447.1891	892.3869	446.6971	S	**1036.5673**	518.7873	1019.5408	510.2740	1018.5568	509.7820	9
10	1011.4452	506.2262	994.4186	497.7129	993.4346	497.2209	T	**949.5353**	475.2713	932.5088	466.7580	931.5247	466.2660	8
11	**1082.4823**	541.7448	1065.4557	533.2315	1064.4717	532.7395	A	**848.4876**	424.7475	831.4611	416.2342	830.4771	415.7422	7
12	**1195.5664**	598.2868	1178.5398	589.7735	1177.5558	589.2815	I	**777.4505**	**389.2289**	760.4240	380.7156	759.4400	380.2236	6
13	1323.6249	662.3161	1306.5984	653.8028	1305.6144	653.3108	Q	**664.3665**	332.6869	647.3399	324.1736	646.3559	323.6816	5
14	**1452.6675**	726.8374	**1435.6410**	718.3241	**1434.6570**	717.8321	E	**536.3079**	268.6576	519.2813	260.1443	518.2973	259.6523	4
15	**1565.7516**	783.3794	1548.7250	774.8662	**1547.7410**	774.3741	L	**407.2653**	204.1363	**390.2387**	195.6230			3
16	**1712.8200**	856.9136	**1695.7935**	**848.4004**	**1694.8094**	**847.9084**	F	**294.1812**	147.5942	277.1547	139.0810			2
17							K	147.1128	74.0600	130.0863	65.5468			1

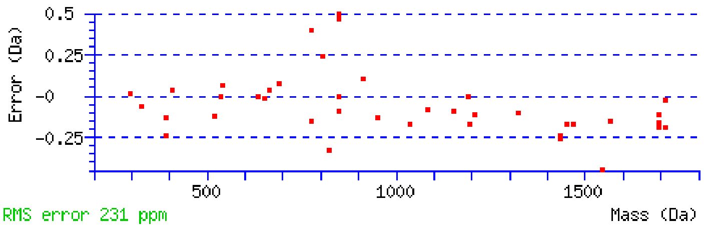

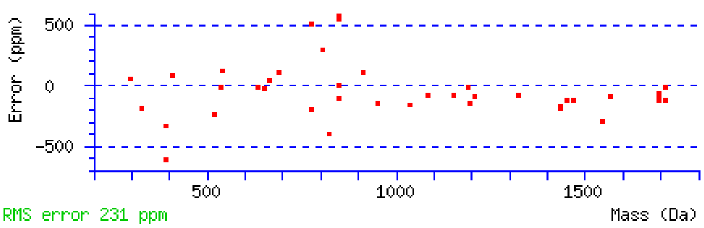

All matches to this query

Score	Mr(calc):	Delta	Sequence
58.0	1857.9182	0.0011	MAATFIGNSTAIQELFK
51.2	1857.9182	0.0011	MSATFIGNSTAIQELFK
49.2	1857.9182	0.0011	MASTFIGNSTAIQELFK
32.4	1856.9342	0.9851	MSATFIGNSTAIQELFK

31.6	1856.9342	0.9851	MAATFIGNSTAIQELFK
30.9	1856.9342	0.9851	MASTFIGNSTAIQELFK
28.8	1857.9148	0.0045	AYSEALAAFGNGALFVEK
11.8	1856.9308	0.9885	AYSEALAAFGNGALFVEK
10.9	1857.9365	-0.0172	MFRPLINLSHIYFK
5.3	1857.9253	-0.0060	LSLKDQLIAQSLLEK

Spectrum No: 354; Query: 961; Rank: 1

Peptide View

MS/MS Fragmentation of **EGTVTAGNASGMSDGAGVVIIASEDAVK**
Found in **IPI00201413**, Tax_Id=10116 Gene_Symbol=Acaa2 3-ketoacyl-CoA thiolase, mitochondrial

Match to Query 961: 2607.242682 from(870.088170,3+)
Title: 091129RatKid_SCX02_15.3158.3158.3.dta
Data file K:\NewmanPaper\Piliang\3SubProteomes\Piliang3SP\mgf5ppm\SCX_3SubProteomes5ppm.mgf

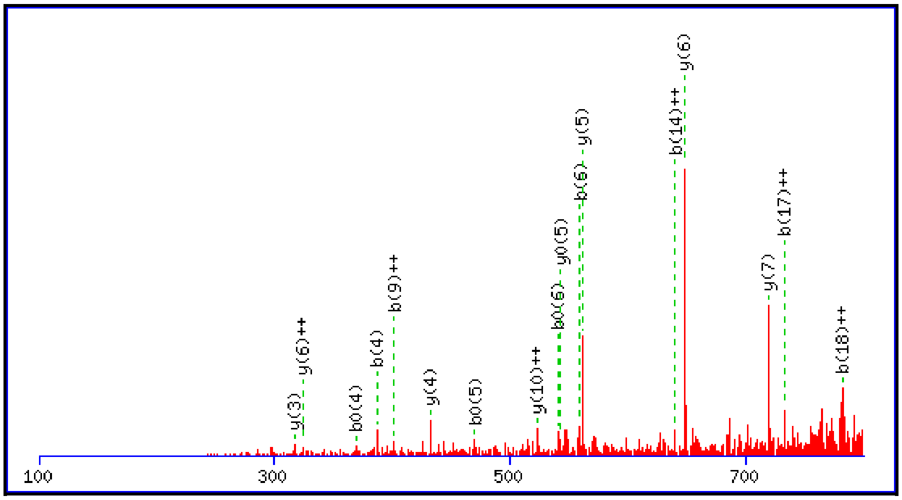

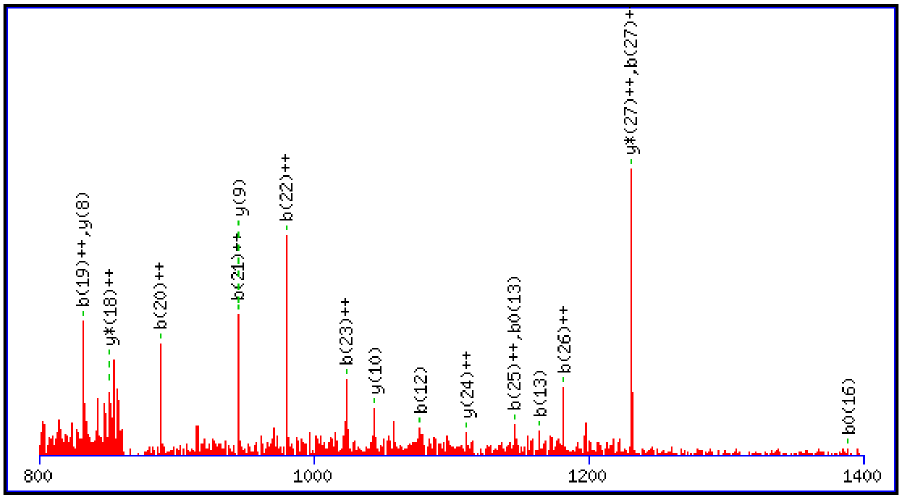

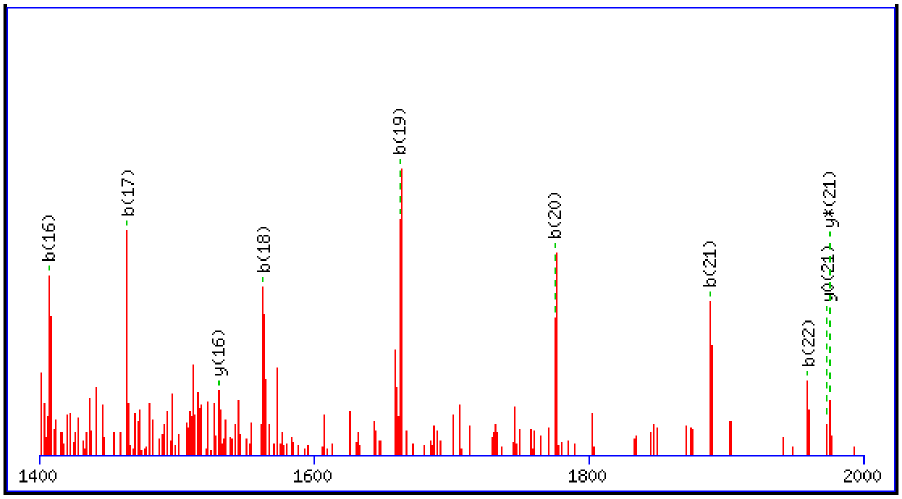

Monoisotopic mass of neutral peptide Mr(calc): 2606.2381
Fixed modifications: Carbamidomethyl (C)
Variable modifications:
N8 : Deamidated_N (N)
Ions Score: 58 **Expect:** 0.00048
Matches (Bold Red): 45/304 fragment ions using 80 most intense peaks

#	b	b^{++}	b*	b^{*++}	b^{0}	b^{0++}	Seq.	y	y^{++}	y*	y^{*++}	y^{0}	y^{0++}	#
1	130.0499	65.5286			112.0393	56.5233	E							28
2	187.0713	94.0393			169.0608	85.0340	G	2478.2028	1239.6050	2461.1763	**1231.0918**	2460.1923	1230.5998	27
3	288.1190	144.5631			270.1084	135.5579	T	2421.1814	1211.0943	2404.1548	1202.5810	2403.1708	1202.0890	26
4	**387.1874**	194.0974			**369.1769**	185.0921	V	2320.1337	1160.5705	2303.1071	1152.0572	2302.1231	1151.5652	25
5	488.2351	244.6212			**470.2245**	235.6159	T	2221.0653	**1111.0363**	2204.0387	1102.5230	2203.0547	1102.0310	24
6	**559.2722**	280.1397			**541.2617**	271.1345	A	2120.0176	1060.5124	2102.9910	1051.9992	2102.0070	1051.5071	23
7	616.2937	308.6505			598.2831	299.6452	G	2048.9805	1024.9939	2031.9539	1016.4806	2030.9699	1015.9886	22
8	731.3206	366.1639	714.2941	357.6507	713.3101	357.1587	N	1991.9590	996.4831	**1974.9325**	987.9699	**1973.9484**	987.4779	21
9	802.3577	**401.6825**	785.3312	393.1692	784.3472	392.6772	A	1876.9321	938.9697	1859.9055	930.4564	1858.9215	929.9644	20
10	889.3898	445.1985	872.3632	436.6852	871.3792	436.1932	S	1805.8950	903.4511	1788.8684	894.9378	1787.8844	894.4458	19
11	946.4112	473.7093	929.3847	465.1960	928.4007	464.7040	G	1718.8629	859.9351	1701.8364	**851.4218**	1700.8524	850.9298	18
12	**1077.4517**	539.2295	1060.4252	530.7162	1059.4411	530.2242	M	1661.8415	831.4244	1644.8149	822.9111	1643.8309	822.4191	17
13	**1164.4837**	582.7455	1147.4572	574.2322	**1146.4732**	573.7402	S	**1530.8010**	765.9041	1513.7744	757.3909	1512.7904	756.8988	16
14	1279.5107	**640.2590**	1262.4841	631.7457	1261.5001	631.2537	D	1443.7690	722.3881	1426.7424	713.8748	1425.7584	713.3828	15
15	1336.5321	668.7697	1319.5056	660.2564	1318.5216	659.7644	G	1328.7420	664.8746	1311.7155	656.3614	1310.7314	655.8694	14
16	**1407.5693**	704.2883	1390.5427	695.7750	**1389.5587**	695.2830	A	1271.7205	636.3639	1254.6940	627.8506	1253.7100	627.3586	13
17	**1464.5907**	**732.7990**	1447.5642	724.2857	1446.5802	723.7937	G	1200.6834	600.8454	1183.6569	592.3321	1182.6729	591.8401	12
18	**1563.6591**	**782.3332**	1546.6326	773.8199	1545.6486	773.3279	V	1143.6620	572.3346	1126.6354	563.8213	1125.6514	563.3293	11
19	**1662.7276**	**831.8674**	1645.7010	823.3541	1644.7170	822.8621	V	**1044.5936**	**522.8004**	1027.5670	514.2871	1026.5830	513.7951	10
20	**1775.8116**	**888.4094**	1758.7851	879.8962	1757.8011	879.4042	I	**945.5251**	473.2662	928.4986	464.7529	927.5146	464.2609	9
21	**1888.8957**	**944.9515**	1871.8691	936.4382	1870.8851	935.9462	I	**832.4411**	416.7242	815.4145	408.2109	814.4305	407.7189	8
22	**1959.9328**	**980.4700**	1942.9062	971.9568	1941.9222	971.4648	A	**719.3570**	360.1821	702.3305	351.6689	701.3464	351.1769	7
23	2046.9648	**1023.9860**	2029.9383	1015.4728	2028.9543	1014.9808	S	**648.3199**	**324.6636**	631.2933	316.1503	630.3093	315.6583	6
24	2176.0074	1088.5073	2158.9809	1079.9941	2157.9968	1079.5021	E	**561.2879**	281.1476	544.2613	272.6343	**543.2773**	272.1423	5
25	2291.0344	**1146.0208**	2274.0078	1137.5075	2273.0238	1137.0155	D	**432.2453**	216.6263	415.2187	208.1130	414.2347	207.6210	4
26	2362.0715	**1181.5394**	2345.0449	1173.0261	2344.0609	1172.5341	A	**317.2183**	159.1128	300.1918	150.5995			3
27	2461.1399	**1231.0736**	2444.1133	1222.5603	2443.1293	1222.0683	V	246.1812	123.5942	229.1547	115.0810			2
28							K	147.1128	74.0600	130.0863	65.5468			1

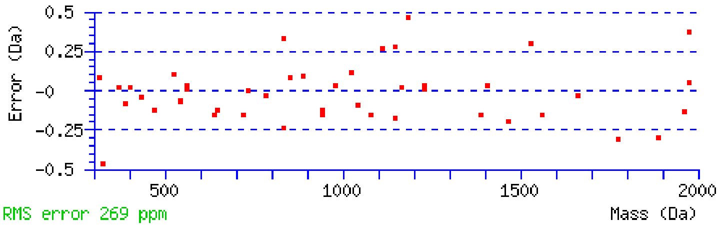

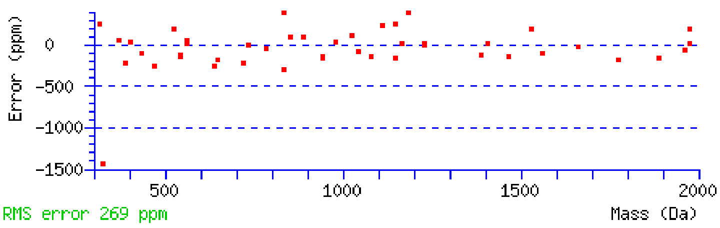

All matches to this query

Score	Mr(calc):	Delta	Sequence
58.0	2606.2381	1.0045	EGTVTAGNASGMSDGAGVVIIASEDAVK
24.1	2605.2541	1.9885	EGTVTAGNASGMSDGAGVVIIASEDAVK
12.0	2607.2234	0.0193	TSKDANKSLPSAALYGIPEISTGK
10.6	2607.2234	0.0193	TSKDANKSLPSAALYGIPEISTGK
10.6	2607.2234	0.0193	TSKDANKSLPSAALYGIPEISTGK
7.3	2607.2234	0.0193	TSKDANKSLPSAALYGIPEISTGK
5.6	2606.2353	1.0074	LHCNQTPNFPGSTEPLLFIVSR
5.0	2607.2193	0.0234	YRSLLQPSKFGGTICSGDIWDK
4.6	2607.2234	0.0193	TSKDANKSLPSAALYGIPEISTGK
4.6	2607.2234	0.0193	TSKDANKSLPSAALYGIPEISTGK

Spectrum No: 355; Query: 37; Rank: 1

Peptide View

MS/MS Fragmentation of **SAEGTFFINK**
Found in **IPI00394353**, Tax_Id=10116 Gene_Symbol=Hexa Beta-hexosaminidase alpha chain precursor

Match to Query 37: 1113.534728 from(557.774640,2+)
Title: 091127RatKid_SCX01_19.2212.2212.2.dta
Data file K:\NewmanPaper\Piliang\3SubProteomes\Piliang3SP\mgf5ppm\SCX_3SubProteomes5ppm.mgf

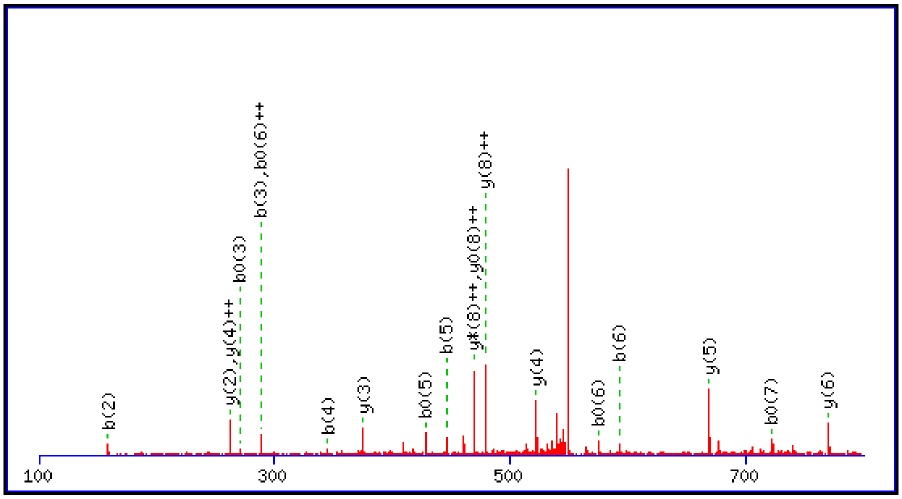

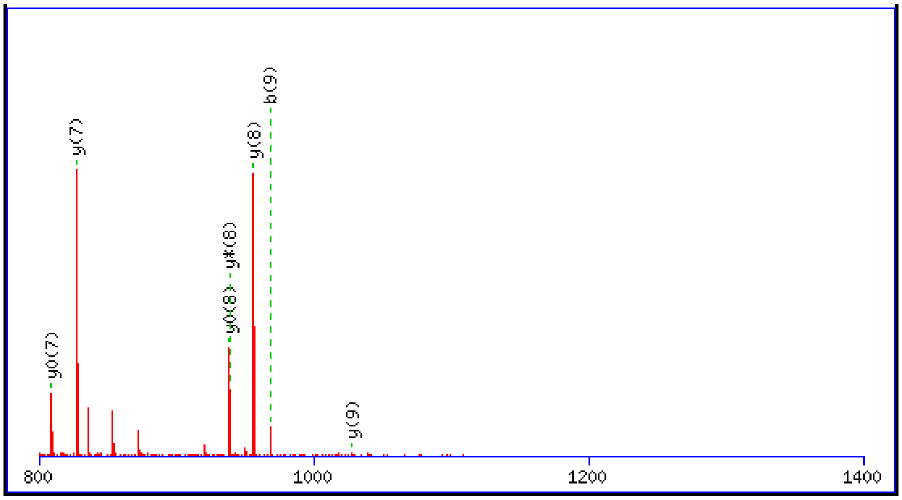

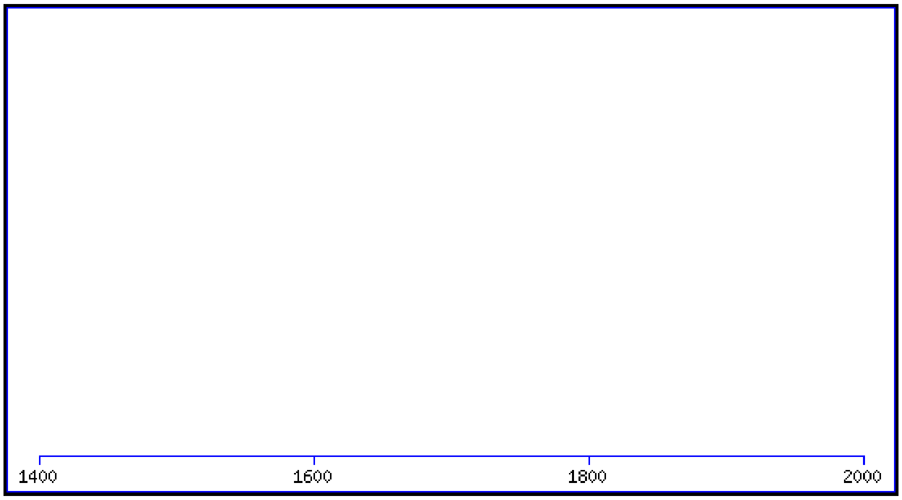

Monoisotopic mass of neutral peptide Mr(calc): 1113.5342
Fixed modifications: Carbamidomethyl (C)
Variable modifications:
N9 : Deamidated_N (N)
Ions Score: 58 **Expect:** 0.00018
Matches (Bold Red): 26/82 fragment ions using 42 most intense peaks

#	b	b^{++}	b*	b^{*++}	b^{0}	b^{0++}	Seq.	y	y^{++}	y*	y^{*++}	y^{0}	y^{0++}	#
1	88.0393	44.5233			70.0287	35.5180	S							10
2	**159.0764**	80.0418			141.0659	71.0366	A	**1027.5095**	514.2584	1010.4829	505.7451	1009.4989	505.2531	9
3	**288.1190**	144.5631			**270.1084**	135.5579	E	**956.4724**	**478.7398**	**939.4458**	**470.2265**	**938.4618**	**469.7345**	8
4	**345.1405**	173.0739			327.1299	164.0686	G	**827.4298**	414.2185	810.4032	405.7053	**809.4192**	405.2132	7
5	**446.1882**	223.5977			**428.1776**	214.5924	T	**770.4083**	385.7078	753.3818	377.1945	752.3977	376.7025	6
6	**593.2566**	297.1319			**575.2460**	**288.1266**	F	**669.3606**	335.1840	652.3341	326.6707			5
7	740.3250	370.6661			**722.3144**	361.6608	F	**522.2922**	**261.6497**	505.2657	253.1365			4
8	853.4090	427.2082			835.3985	418.2029	I	**375.2238**	188.1155	358.1973	179.6023			3
9	**968.4360**	484.7216	951.4094	476.2084	950.4254	475.7163	N	**262.1397**	131.5735	245.1132	123.0602			2
10							K	147.1128	74.0600	130.0863	65.5468			1

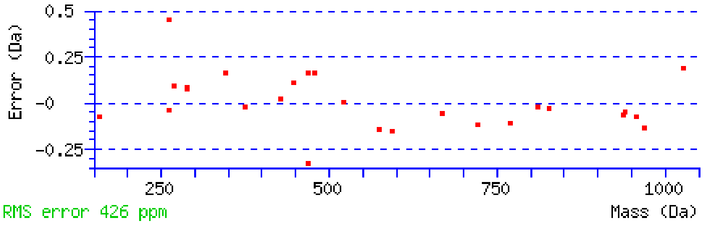

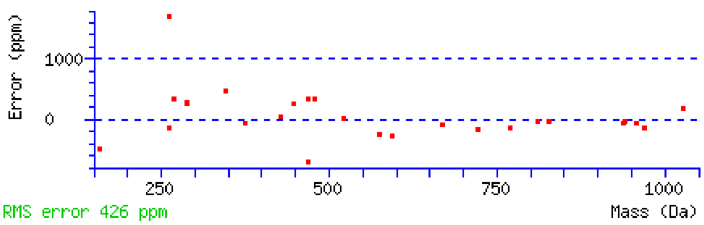

All matches to this query

Score	Mr(calc):	Delta	Sequence
57.9	1113.5342	0.0005	SAEGTFFINK
19.9	1113.5253	0.0094	GMVTGLTLSR
17.6	1111.5379	1.9969	REFMMRSR
14.5	1113.5253	0.0094	TGXTRLLNK
10.4	1113.5253	0.0094	GMVTGLTLSR
7.9	1113.5431	-0.0083	KIETTSSLR
7.4	1113.5431	-0.0083	KIETTSSLR
5.3	1112.5274	1.0074	RMRLXSSR
5.0	1113.5365	-0.0018	NKKMSATVR
5.0	1113.5431	-0.0083	KIETTSSLR

Spectrum No: 356; Query: 323; Rank: 1

Peptide View

MS/MS Fragmentation of **DDILNGSHPVSFDK**
Found in **IPI00362014**, Tax_Id=10116 Gene_Symbol=Tln1 Tln1 protein

Match to Query 323: 1543.716548 from(772.865550,2+)
Title: 091127RatKid_SCX01_28.2189.2189.2.dta
Data file K:\NewmanPaper\Piliang\3SubProteomes\Piliang3SP\mgf5ppm\SCX_3SubProteomes5ppm.mgf

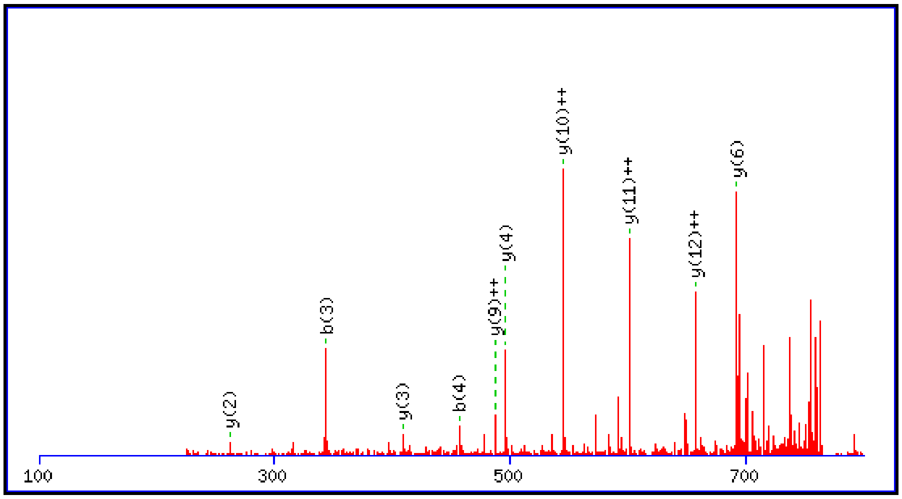

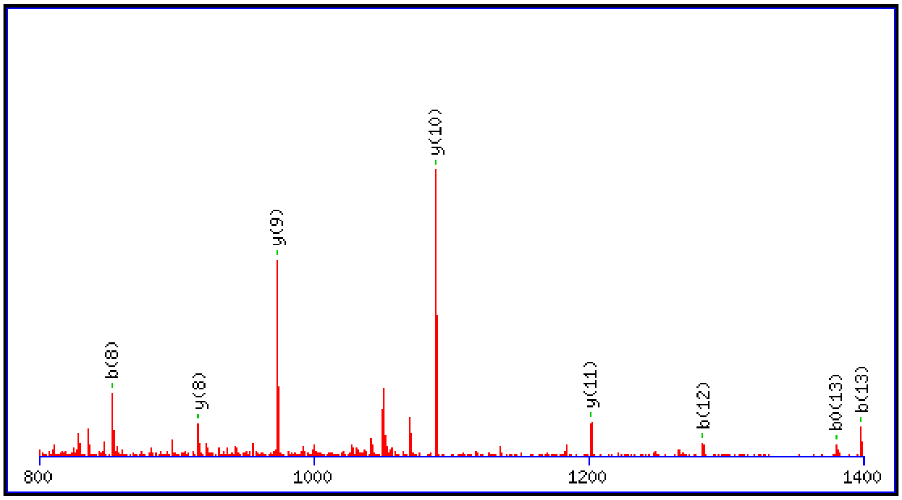

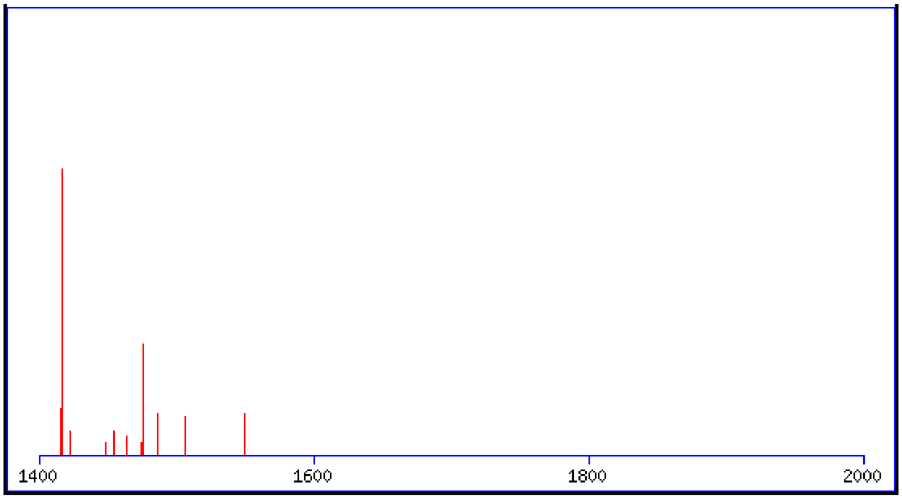

Monoisotopic mass of neutral peptide Mr(calc): 1543.7155
Fixed modifications: Carbamidomethyl (C)
Variable modifications:
N5 : Deamidated_N (N)
Ions Score: 58 **Expect:** 0.00029
Matches (Bold Red): 18/146 fragment ions using 31 most intense peaks

#	b	b^{++}	b*	b^{*++}	b^0	b^{0++}	Seq.	y	y^{++}	y*	y^{*++}	y^0	y^{0++}	#
1	116.0342	58.5207			98.0237	49.5155	D							14
2	231.0612	116.0342			213.0506	107.0289	D	1429.6958	715.3515	1412.6692	706.8383	1411.6852	706.3462	13
3	**344.1452**	172.5763			326.1347	163.5710	I	1314.6688	**657.8381**	1297.6423	649.3248	1296.6583	648.8328	12
4	**457.2293**	229.1183			439.2187	220.1130	L	**1201.5848**	**601.2960**	1184.5582	592.7827	1183.5742	592.2907	11
5	572.2562	286.6318	555.2297	278.1185	554.2457	277.6265	N	**1088.5007**	**544.7540**	1071.4742	536.2407	1070.4901	535.7487	10
6	629.2777	315.1425	612.2511	306.6292	611.2671	306.1372	G	**973.4738**	**487.2405**	956.4472	478.7272	955.4632	478.2352	9
7	716.3097	358.6585	699.2832	350.1452	698.2992	349.6532	S	**916.4523**	458.7298	899.4258	450.2165	898.4417	449.7245	8
8	**853.3686**	427.1880	836.3421	418.6747	835.3581	418.1827	H	829.4203	415.2138	812.3937	406.7005	811.4097	406.2085	7
9	950.4214	475.7143	933.3948	467.2011	932.4108	466.7091	P	**692.3614**	346.6843	675.3348	338.1710	674.3508	337.6790	6
10	1049.4898	525.2485	1032.4633	516.7353	1031.4792	516.2433	V	595.3086	298.1579	578.2821	289.6447	577.2980	289.1527	5
11	1136.5218	568.7646	1119.4953	560.2513	1118.5113	559.7593	S	**496.2402**	248.6237	479.2136	240.1105	478.2296	239.6185	4
12	**1283.5903**	642.2988	1266.5637	633.7855	1265.5797	633.2935	F	**409.2082**	205.1077	392.1816	196.5944	391.1976	196.1024	3
13	**1398.6172**	699.8122	1381.5906	691.2990	**1380.6066**	690.8070	D	**262.1397**	131.5735	245.1132	123.0602	244.1292	122.5682	2
14							K	147.1128	74.0600	130.0863	65.5468			1

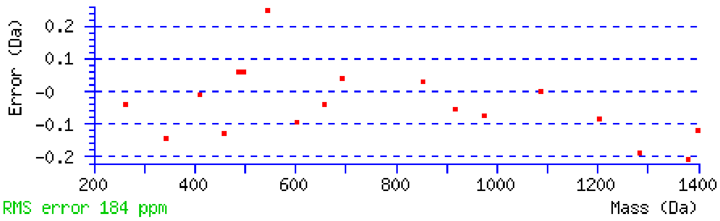

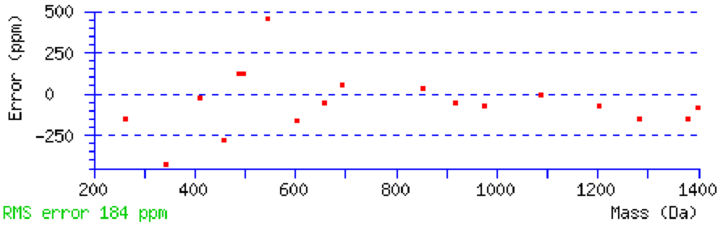

All matches to this query

Score	Mr(calc):	Delta	Sequence
57.7	1543.7155	0.0011	DDILNGSHPVSFDK
11.4	1543.7188	-0.0022	ENPDPVEAELMRK
10.1	1543.7160	0.0005	ITAVPRKVSGDNK
5.0	1543.7106	0.0060	CLTKINYGGDVPK
5.0	1543.7106	0.0060	CLTKINYGGDVPK
4.9	1543.7160	0.0005	SQTKLKSHGASLK
4.0	1543.7160	0.0005	SQTKLKSHGASLK

4.0	1543.7160	0.0005	SQTKLKSHGASLK
3.6	1543.7160	0.0005	SQTKLKSHGASLK
3.5	1543.7188	-0.0022	DRLTYITEGSMNK

Spectrum No: 357; Query: 113; Rank: 1

Peptide View

MS/MS Fragmentation of **LNGTDPEDVIR**
Found in **IPI00421625**, Tax_Id=10116 Gene_Symbol=Mrlcb;RGD1565978_predicted Myosin regulatory light chain 2-B, smooth muscle isoform

Match to Query 113: 1228.594788 from(615.304670,2+)
Title: 091127RatKid_SCX01_04.1761.1761.2.dta
Data file K:\NewmanPaper\Piliang\3SubProteomes\Piliang3SP\mgf5ppm\SCX_3SubProteomes5ppm.mgf

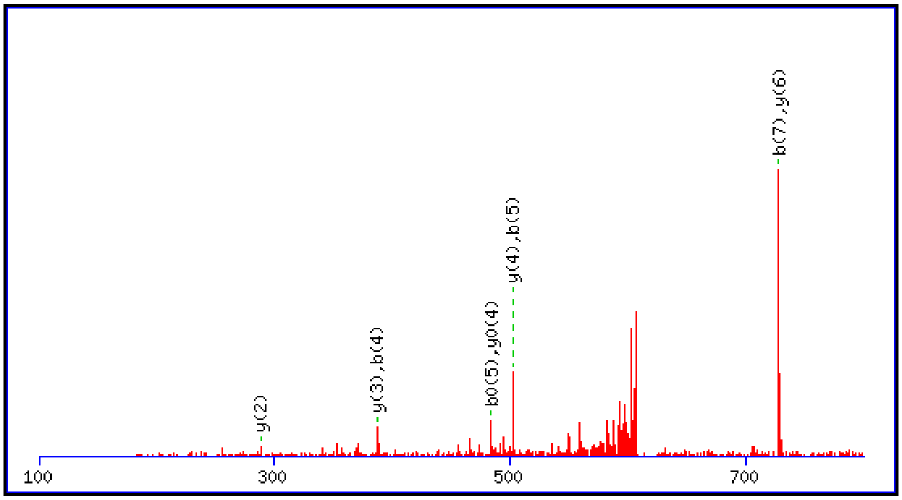

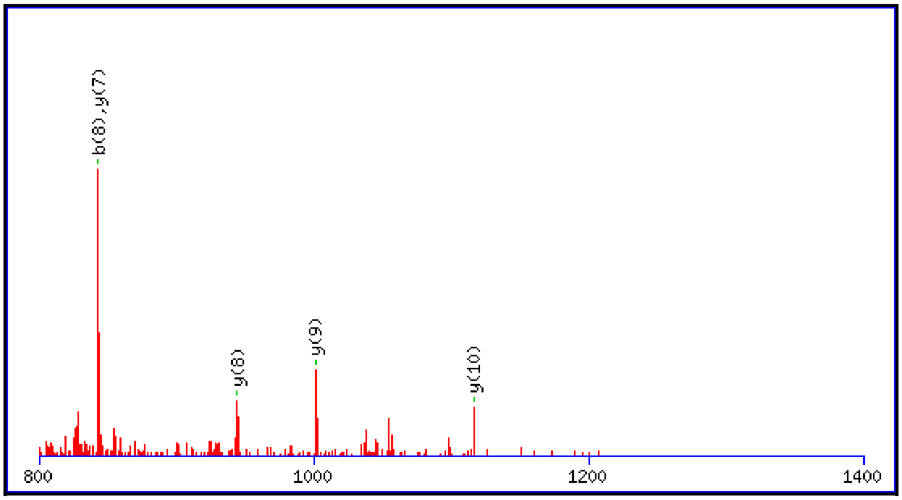

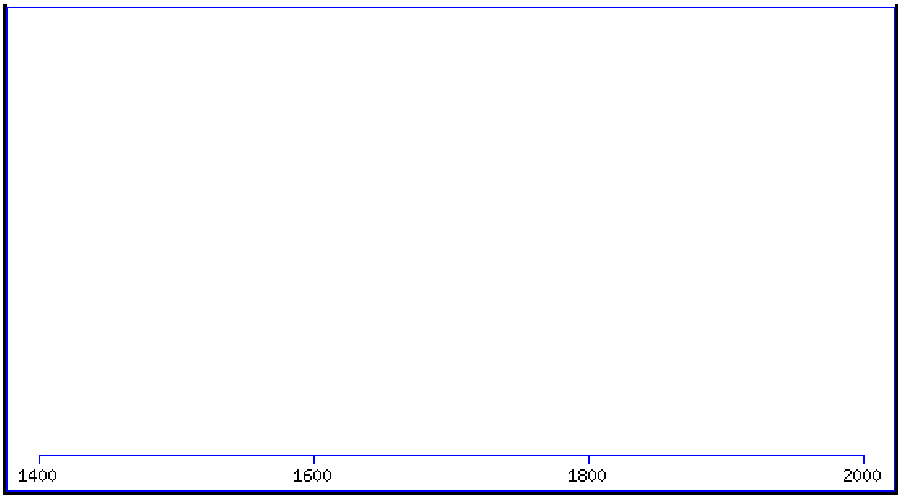

Monoisotopic mass of neutral peptide Mr(calc): 1228.5935
Fixed modifications: Carbamidomethyl (C)
Variable modifications:
N2 : Deamidated_N (N)
Ions Score: 58 **Expect:** 0.00023
Matches (Bold Red): 14/106 fragment ions using 19 most intense peaks

#	b	b^{++}	b*	b^{*++}	b^{0}	b^{0++}	Seq.	y	y^{++}	y*	y^{*++}	y^{0}	y^{0++}	#
1	114.0913	57.5493					L							11
2	229.1183	115.0628	212.0917	106.5495			N	**1116.5168**	558.7620	1099.4902	550.2487	1098.5062	549.7567	10
3	286.1397	143.5735	269.1132	135.0602			G	**1001.4898**	501.2485	984.4633	492.7353	983.4793	492.2433	9
4	**387.1874**	194.0973	370.1609	185.5841	369.1769	185.0921	T	**944.4684**	472.7378	927.4418	464.2245	926.4578	463.7325	8
5	**502.2144**	251.6108	485.1878	243.0975	**484.2038**	242.6055	D	**843.4207**	422.2140	826.3941	413.7007	825.4101	413.2087	7
6	599.2671	300.1372	582.2406	291.6239	581.2566	291.1319	P	**728.3937**	364.7005	711.3672	356.1872	710.3832	355.6952	6
7	**728.3097**	364.6585	711.2832	356.1452	710.2992	355.6532	E	631.3410	316.1741	614.3144	307.6608	613.3304	307.1688	5
8	**843.3367**	422.1720	826.3101	413.6587	825.3261	413.1667	D	**502.2984**	251.6528	485.2718	243.1395	**484.2878**	242.6475	4
9	942.4051	471.7062	925.3785	463.1929	924.3945	462.7009	V	**387.2714**	194.1394	370.2449	185.6261			3
10	1055.4891	528.2482	1038.4626	519.7349	1037.4786	519.2429	I	**288.2030**	144.6051	271.1765	136.0919			2
11							R	175.1190	88.0631	158.0924	79.5498			1

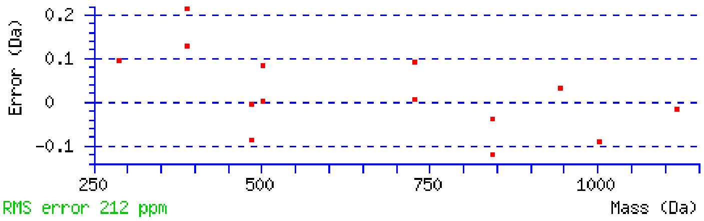

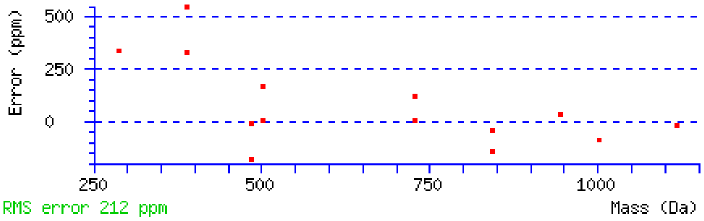

All matches to this query

Score	Mr(calc):	Delta	Sequence
57.5	1228.5935	0.0013	LNGTDPEDVIR
10.2	1228.5908	0.0040	NNKNQENNVR
6.0	1228.5853	0.0095	QELIDFKTR
4.0	1228.5965	-0.0017	LNGKYRLER
3.8	1228.5853	0.0095	SFADLITNLR
3.0	1227.5900	1.0047	VGDTFILNLR
2.5	1228.6039	-0.0091	MQAALLYALR
1.9	1228.5887	0.0061	SVSQLLSMLR
1.6	1228.5965	-0.0017	IRTDAGFTLR
0.1	1228.5999	-0.0051	LASMTQKKAR

Spectrum No: 358; Query: 65; Rank: 1

Peptide View

MS/MS Fragmentation of **VFNTSEDLSR**
Found in **IPI00363534**, Tax_Id=10116 Gene_Symbol=Lama1 laminin, alpha 1

Match to Query 65: 1167.540608 from(584.777580,2+)
Title: 091127RatKid_SCX01_02.1281.1281.2.dta
Data file K:\NewmanPaper\Piliang\3SubProteomes\Piliang3SP\mgf5ppm\SCX_3SubProteomes5ppm.mgf

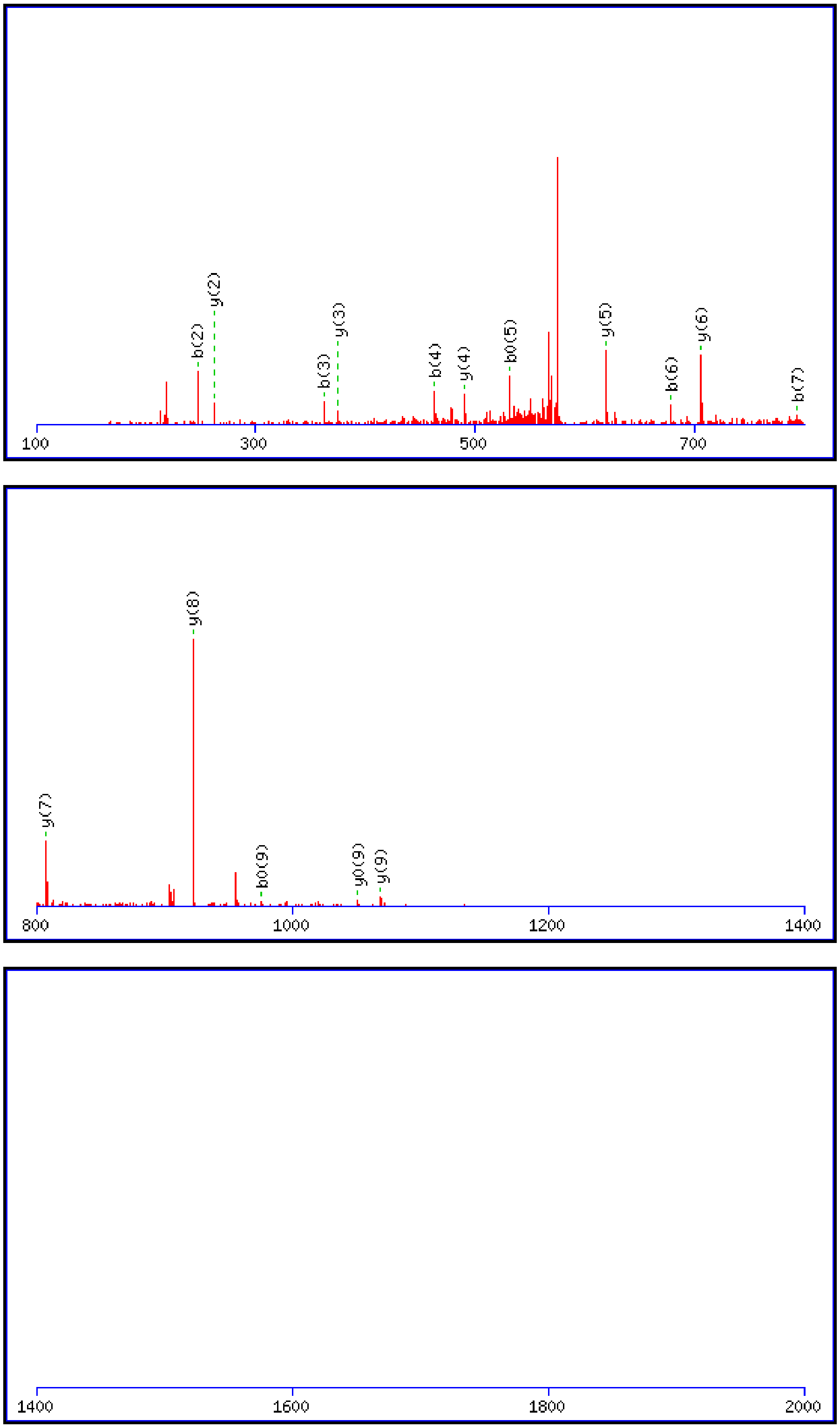

Monoisotopic mass of neutral peptide Mr(calc): 1167.5408
Fixed modifications: Carbamidomethyl (C)
Variable modifications:
N3 : Deamidated_N (N)
Ions Score: 57 **Expect:** 0.00022
Matches (Bold Red): 16/96 fragment ions using 35 most intense peaks

#	b	b^{++}	b*	b^{*++}	b^0	b^{0++}	Seq.	y	y^{++}	y*	y^{*++}	y^0	y^{0++}	#
1	100.0757	50.5415					V							10
2	**247.1441**	124.0757					F	**1069.4796**	535.2435	1052.4531	526.7302	**1051.4691**	526.2382	9
3	**362.1710**	181.5892	345.1445	173.0759			N	**922.4112**	461.7092	905.3847	453.1960	904.4007	452.7040	8
4	**463.2187**	232.1130	446.1922	223.5997	445.2082	223.1077	T	**807.3843**	404.1958	790.3577	395.6825	789.3737	395.1905	7
5	550.2507	275.6290	533.2242	267.1157	**532.2402**	266.6237	S	**706.3366**	353.6719	689.3101	345.1587	688.3260	344.6667	6
6	**679.2933**	340.1503	662.2668	331.6370	661.2828	331.1450	E	**619.3046**	310.1559	602.2780	301.6427	601.2940	301.1506	5
7	**794.3203**	397.6638	777.2937	389.1505	776.3097	388.6585	D	**490.2620**	245.6346	473.2354	237.1214	472.2514	236.6293	4
8	907.4043	454.2058	890.3778	445.6925	889.3938	445.2005	L	**375.2350**	188.1212	358.2085	179.6079	357.2245	179.1159	3
9	994.4364	497.7218	977.4098	489.2086	**976.4258**	488.7165	S	**262.1510**	131.5791	245.1244	123.0659	244.1404	122.5738	2
10							R	175.1190	88.0631	158.0924	79.5498			1

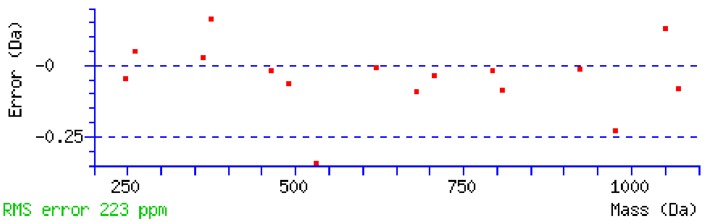

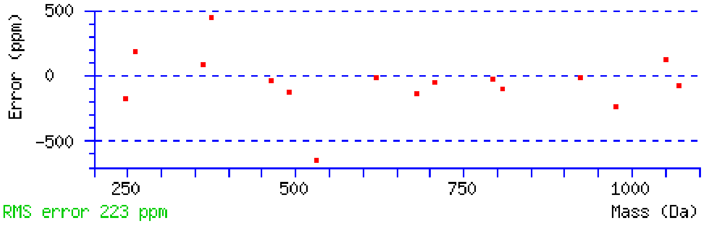

All matches to this query

Score	Mr(calc):	Delta	Sequence
57.5	1167.5408	-0.0002	VFNTSEDLSR
15.8	1165.5285	2.0122	NEDMKSALNK
8.5	1167.5294	0.0113	VMMKLSHSR
8.3	1167.5383	0.0023	MVQFHFTNK
5.7	1165.5380	2.0026	SLPLAQSDQK
5.2	1165.5285	2.0121	NSDLCIASGTK
5.1	1165.5285	2.0122	NKMESDGEKK
3.2	1167.5450	-0.0044	MALLMAEMSR
3.0	1167.5294	0.0113	VMMKLSHSR
1.8	1166.5455	0.9951	NLSFEESNVK

Spectrum No: 359; Query: 1098; Rank: 1

Peptide View

MS/MS Fragmentation of **GETPQGSSEECDLSGSCTERNSSASPGEDR**
Found in **IPI00203529**, Tax_Id=10116 Gene_Symbol=Slc34a3 Sodium-dependent phosphate transport protein 2C

Match to Query 1098: 3186.267912 from(1063.096580,3+)
Title: 091127RatKid_SCX01_12.1019.1019.3.dta
Data file K:\NewmanPaper\Piliang\3SubProteomes\Piliang3SP\mgf5ppm\SCX_3SubProteomes5ppm.mgf

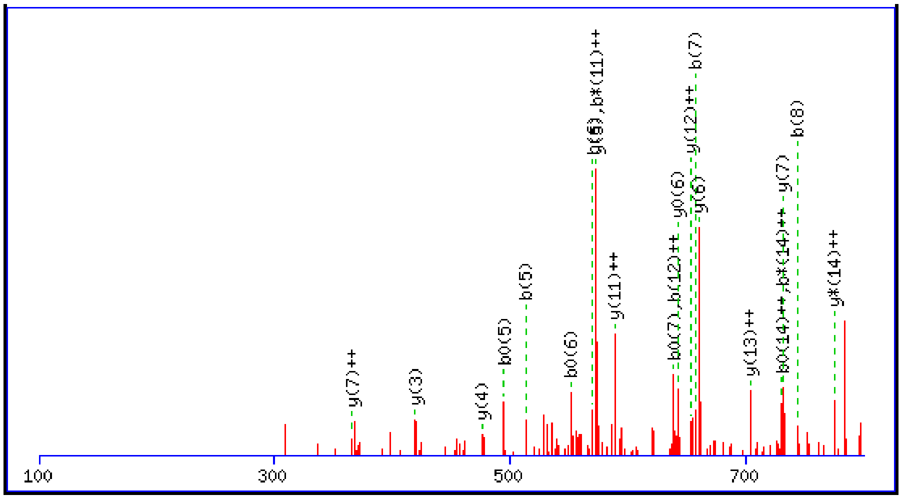

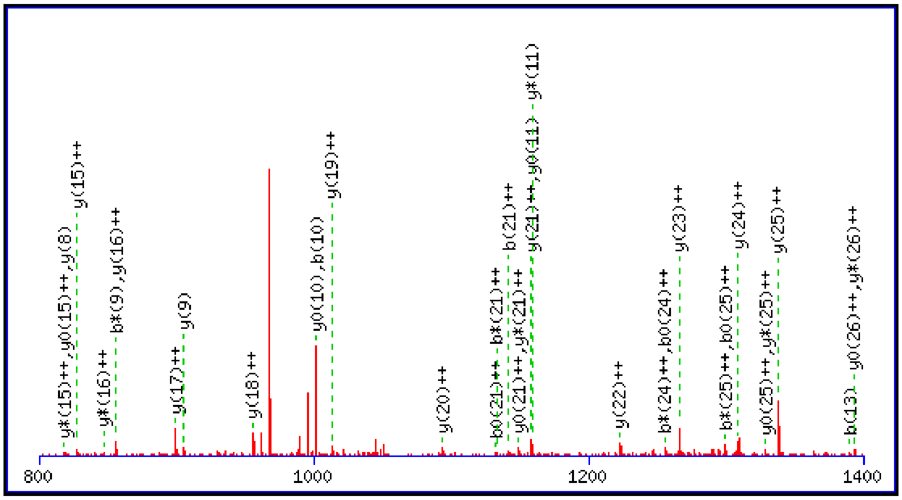

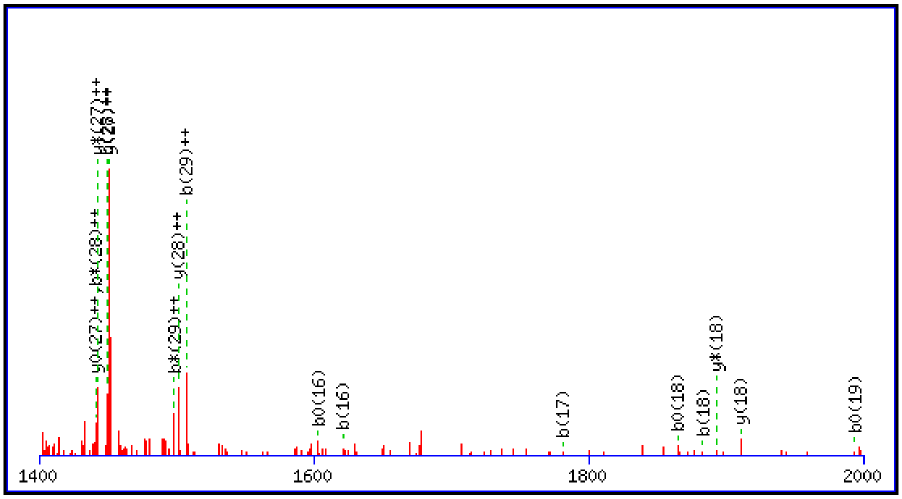

Monoisotopic mass of neutral peptide Mr(calc): 3186.2586
Fixed modifications: Carbamidomethyl (C)
Variable modifications:
N21 : Deamidated_N (N)
Ions Score: 57 **Expect:** 0.00058
Matches (Bold Red): 73/336 fragment ions using 170 most intense peaks

#	b	b^{++}	b*	b^{*++}	b^{0}	b^{0++}	Seq.	y	y^{++}	y*	y^{*++}	y^{0}	y^{0++}	#
1	58.0287	29.5180					G							30
2	187.0713	94.0393			169.0608	85.0340	E	3130.2444	1565.6258	3113.2178	1557.1126	3112.2338	1556.6205	29
3	288.1190	144.5631			270.1084	135.5579	T	3001.2018	**1501.1045**	2984.1752	1492.5913	2983.1912	1492.0992	28
4	385.1718	193.0895			367.1612	184.0842	P	2900.1541	**1450.5807**	2883.1276	**1442.0674**	2882.1435	**1441.5754**	27
5	**513.2304**	257.1188	496.2038	248.6055	**495.2198**	248.1135	Q	2803.1013	1402.0543	2786.0748	**1393.5410**	2785.0908	**1393.0490**	26

6	570.2518	285.6295	553.2253	277.1163	552.2413	276.6243	G	2675.0428	1338.0250	2658.0162	1329.5117	2657.0322	1329.0197	25
7	657.2838	329.1456	640.2573	320.6323	639.2733	320.1403	S	2618.0213	1309.5143	2600.9948	1301.0010	2600.0107	1300.5090	24
8	744.3159	372.6616	727.2893	364.1483	726.3053	363.6563	S	2530.9893	1265.9983	2513.9627	1257.4850	2512.9787	1256.9930	23
9	873.3585	437.1829	856.3319	428.6696	855.3479	428.1776	E	2443.9572	1222.4823	2426.9307	1213.9690	2425.9467	1213.4770	22
10	1002.4011	501.7042	985.3745	493.1909	984.3905	492.6989	E	2314.9147	1157.9610	2297.8881	1149.4477	2296.9041	1148.9557	21
11	1162.4317	581.7195	1145.4052	573.2062	1144.4211	572.7142	C	2185.8721	1093.4397	2168.8455	1084.9264	2167.8615	1084.4344	20
12	1277.4587	639.2330	1260.4321	630.7197	1259.4481	630.2277	D	2025.8414	1013.4243	2008.8149	1004.9111	2007.8308	1004.4191	19
13	1390.5427	695.7750	1373.5162	687.2617	1372.5322	686.7697	L	1910.8145	955.9109	1893.7879	947.3976	1892.8039	946.9056	18
14	1477.5747	739.2910	1460.5482	730.7777	1459.5642	730.2857	S	1797.7304	899.3688	1780.7039	890.8556	1779.7198	890.3636	17
15	1534.5962	767.8017	1517.5697	759.2885	1516.5856	758.7965	G	1710.6984	855.8528	1693.6718	847.3396	1692.6878	846.8475	16
16	1621.6282	811.3178	1604.6017	802.8045	1603.6177	802.3125	S	1653.6769	827.3421	1636.6504	818.8288	1635.6663	818.3368	15
17	1781.6589	891.3331	1764.6323	882.8198	1763.6483	882.3278	C	1566.6449	783.8261	1549.6183	775.3128	1548.6343	774.8208	14
18	1882.7066	941.8569	1865.6800	933.3436	1864.6960	932.8516	T	1406.6142	703.8108	1389.5877	695.2975	1388.6037	694.8055	13
19	2011.7492	1006.3782	1994.7226	997.8649	1993.7386	997.3729	E	1305.5666	653.2869	1288.5400	644.7736	1287.5560	644.2816	12
20	2167.8503	1084.4288	2150.8237	1075.9155	2149.8397	1075.4235	R	1176.5240	588.7656	1159.4974	580.2523	1158.5134	579.7603	11
21	2282.8772	1141.9422	2265.8507	1133.4290	2264.8666	1132.9370	N	1020.4229	510.7151	1003.3963	502.2018	1002.4123	501.7098	10
22	2369.9092	1185.4583	2352.8827	1176.9450	2351.8987	1176.4530	S	905.3959	453.2016	888.3694	444.6883	887.3853	444.1963	9
23	2456.9413	1228.9743	2439.9147	1220.4610	2438.9307	1219.9690	S	818.3639	409.6856	801.3373	401.1723	800.3533	400.6803	8
24	2527.9784	1264.4928	2510.9518	1255.9796	2509.9678	1255.4875	A	731.3319	366.1696	714.3053	357.6563	713.3213	357.1643	7
25	2615.0104	1308.0088	2597.9839	1299.4956	2596.9998	1299.0036	S	660.2947	330.6510	643.2682	322.1377	642.2842	321.6457	6
26	2712.0632	1356.5352	2695.0366	1348.0219	2694.0526	1347.5299	P	573.2627	287.1350	556.2362	278.6217	555.2522	278.1297	5
27	2769.0846	1385.0460	2752.0581	1376.5327	2751.0741	1376.0407	G	476.2100	238.6086	459.1834	230.0953	458.1994	229.6033	4
28	2898.1272	1449.5672	2881.1007	1441.0540	2880.1167	1440.5620	E	419.1885	210.0979	402.1619	201.5846	401.1779	201.0926	3
29	3013.1542	1507.0807	2996.1276	1498.5674	2995.1436	1498.0754	D	290.1459	145.5766	273.1193	137.0633	272.1353	136.5713	2
30							R	175.1190	88.0631	158.0924	79.5498			1

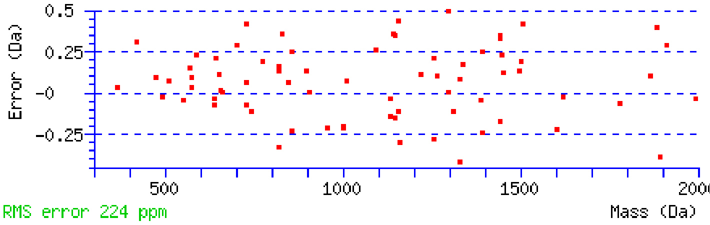

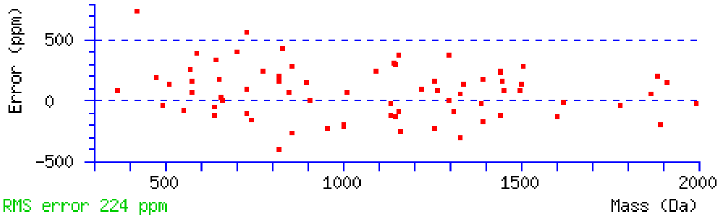

All matches to this query

Score	Mr(calc):	Delta	Sequence
57.4	3186.2586	0.0094	GETPQGSSEECDLSGSCTERNSSASPGEDR
4.4	3184.2799	1.9880	MYFAFNPKLCVSEIYRMEEVTGTK
4.4	3184.2799	1.9880	MYFAFNPKLCVSEIYRMEEVTGTK
4.4	3184.2799	1.9880	MYFAFNPKLCVSEIYRMEEVTGTK
4.4	3184.2799	1.9880	MYFAFNPKLCVSEIYRMEEVTGTK
4.3	3185.2746	0.9934	GETPQGSSEECDLSGSCTERNSSASPGEDR
2.4	3186.2641	0.0038	DLSSSFSRHTAPLTGHPSSHLQRLR
2.4	3186.2641	0.0038	DLSSSFSRHTAPLTGHPSSHLQRLR
1.5	3185.2531	1.0148	TNINMQSNPVSMMHQQARPTTTQRR
1.4	3186.2378	0.0301	GAVDSGPLHRSLTSPFSLVLYDACPK

Spectrum No: 360; Query: 82; Rank: 1

Peptide View

MS/MS Fragmentation of **NQSPVLEPVGR**
Found in **IPI00192458**, Tax_Id=10116 Gene_Symbol=RGD1563860_predicted similar to ribosomal protein S6 kinase polypeptide 3

Match to Query 82: 1195.621188 from(598.817870,2+)
Title: 091127RatKid_SCX01_16.1586.1586.2.dta
Data file K:\NewmanPaper\Piliang\3SubProteomes\Piliang3SP\mgf5ppm\SCX_3SubProteomes5ppm.mgf

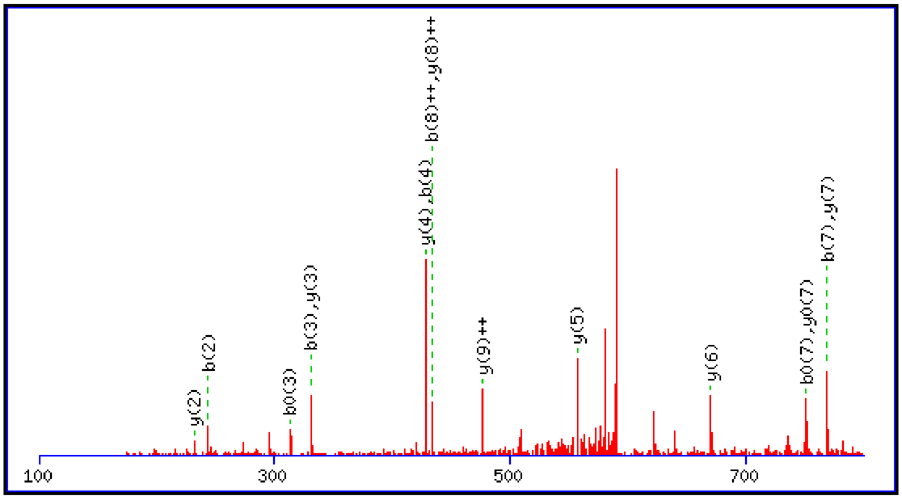

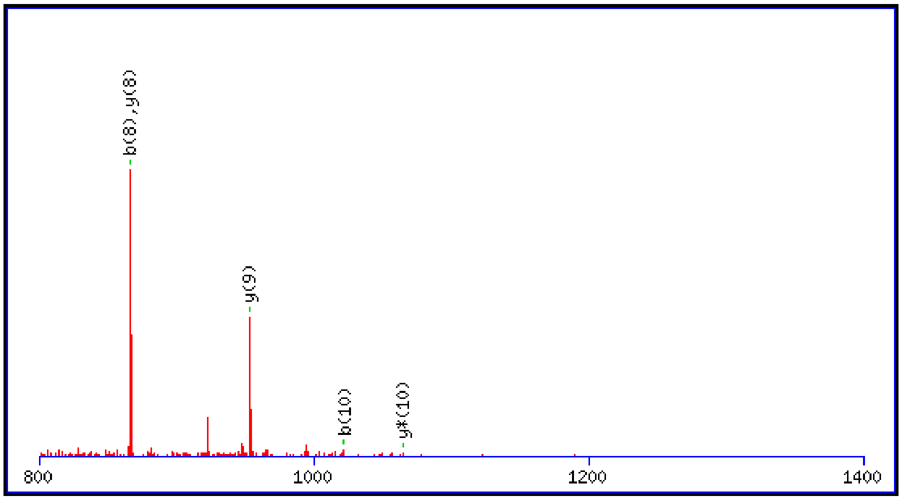

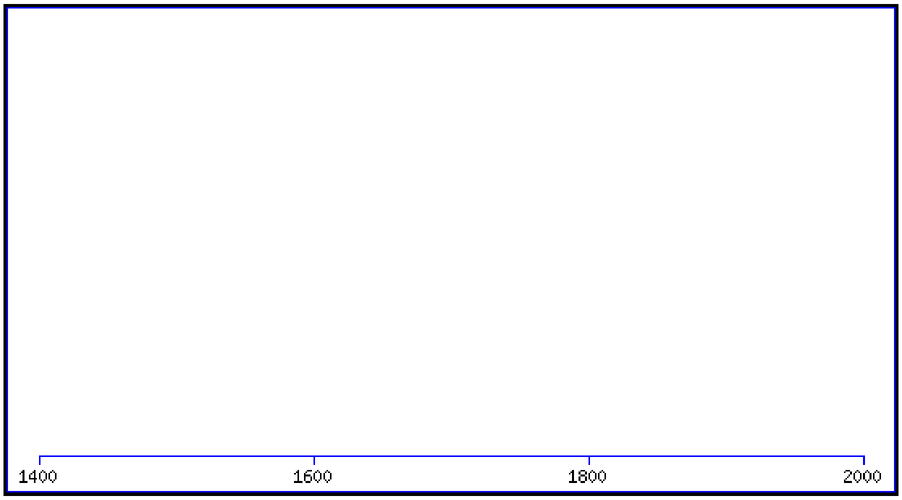

Monoisotopic mass of neutral peptide Mr(calc): 1195.6197
Fixed modifications: Carbamidomethyl (C)
Variable modifications:
N1 : Deamidated_N (N)
Ions Score: 57 **Expect:** 0.0002
Matches (Bold Red): 21/108 fragment ions using 28 most intense peaks

#	b	b++	b*	b*++	b0	b0++	Seq.	y	y++	y*	y*++	y0	y0++	#
1	116.0342	58.5207	99.0077	50.0075			N							11
2	**244.0928**	122.5500	227.0662	114.0368			Q	1081.6000	541.3037	**1064.5735**	532.7904	1063.5895	532.2984	10
3	**331.1248**	166.0660	314.0983	157.5528	**313.1143**	157.0608	S	**953.5415**	**477.2744**	936.5149	468.7611	935.5309	468.2691	9
4	**428.1776**	214.5924	411.1510	206.0792	410.1670	205.5871	P	**866.5094**	**433.7584**	849.4829	425.2451	848.4989	424.7531	8
5	527.2460	264.1266	510.2194	255.6134	509.2354	255.1214	V	**769.4567**	385.2320	752.4301	376.7187	**751.4461**	376.2267	7
6	640.3301	320.6687	623.3035	312.1554	622.3195	311.6634	L	**670.3883**	335.6978	653.3617	327.1845	652.3777	326.6925	6
7	**769.3727**	385.1900	752.3461	376.6767	**751.3621**	376.1847	E	**557.3042**	279.1557	540.2776	270.6425	539.2936	270.1504	5
8	**866.4254**	**433.7163**	849.3989	425.2031	848.4149	424.7111	P	**428.2616**	214.6344	411.2350	206.1212			4
9	965.4938	483.2506	948.4673	474.7373	947.4833	474.2453	V	**331.2088**	166.1081	314.1823	157.5948			3
10	**1022.5153**	511.7613	1005.4887	503.2480	1004.5047	502.7560	G	**232.1404**	116.5738	215.1139	108.0606			2
11							R	175.1190	88.0631	158.0924	79.5498			1

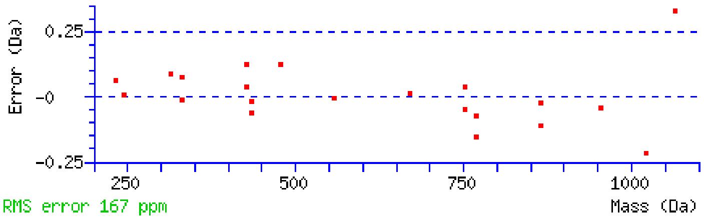

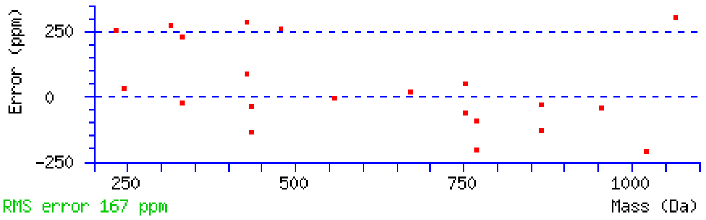

All matches to this query

Score	Mr(calc):	Delta	Sequence
57.3	1195.6197	0.0015	NQSPVLEPVGR
11.4	1194.6217	0.9995	NQSNLAVHRR
9.3	1195.6132	0.0080	AGDISKMFRR
8.2	1195.6186	0.0026	SRLARGSTIR
8.2	1195.6186	0.0026	SRLARGSTIR
7.5	1195.6260	-0.0048	MALQLTKRR
7.4	1195.6309	-0.0097	DGAVPLPESRR
7.3	1195.6322	-0.0111	XWRSLEHRR
6.5	1194.6217	0.9995	NQSNLAVHRR
6.4	1193.6169	2.0043	TEVVKAPKSR

Spectrum No: 361; Query: 47; Rank: 1

Peptide View

MS/MS Fragmentation of **ITNYTVVYR**
Found in **IPI00231988**, Tax_Id=10116 Gene_Symbol=Ptprf Leukocyte common antigen-related phosphatase precursor

Match to Query 47: 1128.582048 from(565.298300,2+)
Title: 091127RatKid_SCX01_13.1609.1609.2.dta
Data file K:\NewmanPaper\Piliang\3SubProteomes\Piliang3SP\mgf5ppm\SCX_3SubProteomes5ppm.mgf

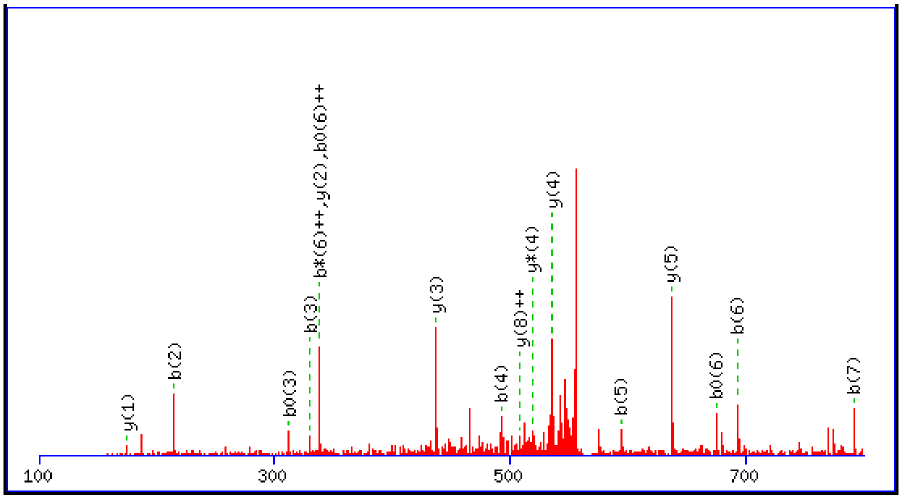

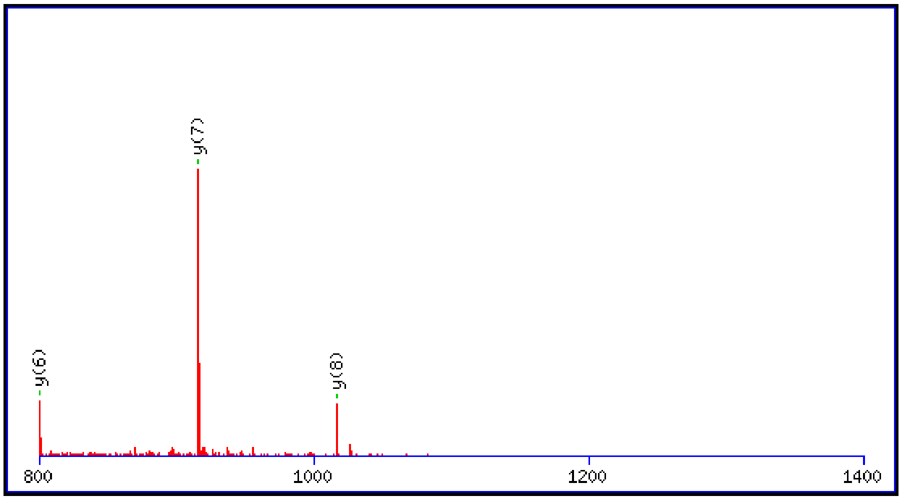

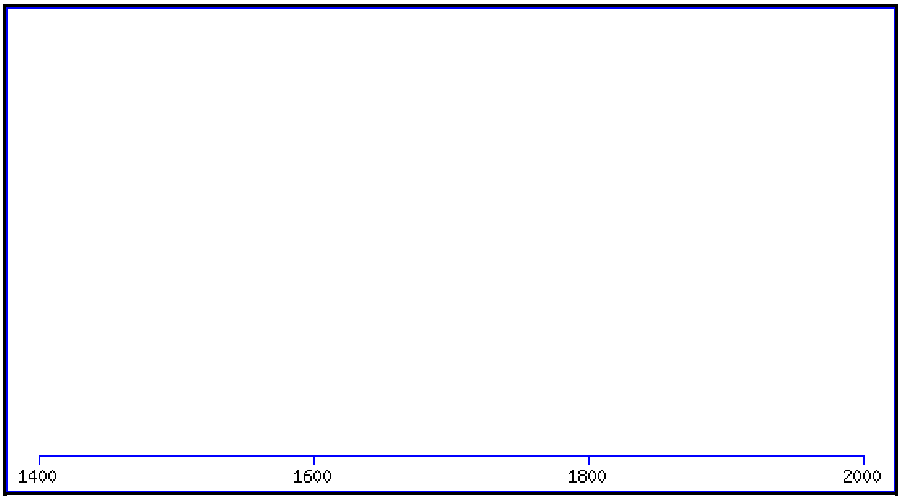

Monoisotopic mass of neutral peptide Mr(calc): 1128.5815
Fixed modifications: Carbamidomethyl (C)
Variable modifications:
N3 : Deamidated_N (N)
Ions Score: 57 **Expect:** 0.00017
Matches (Bold Red): 20/82 fragment ions using 48 most intense peaks

#	b	b^{++}	b*	b^{*++}	b^0	b^{0++}	Seq.	y	y^{++}	y*	y^{*++}	y^0	y^{0++}	#
1	114.0913	57.5493					I							9
2	**215.1390**	108.0731			197.1285	99.0679	T	**1016.5047**	**508.7560**	999.4782	500.2427	998.4942	499.7507	8
3	**330.1660**	165.5866	313.1394	157.0733	**312.1554**	156.5813	N	**915.4571**	458.2322	898.4305	449.7189	897.4465	449.2269	7
4	**493.2293**	247.1183	476.2027	238.6050	475.2187	238.1130	Y	**800.4301**	400.7187	783.4036	392.2054	782.4196	391.7134	6
5	**594.2770**	297.6421	577.2504	289.1288	576.2664	288.6368	T	**637.3668**	319.1870	620.3402	310.6738	619.3562	310.1817	5

6	693.3454	347.1763	676.3188	338.6631	675.3348	338.1710	V	536.3191	268.6632	519.2926	260.1499			4
7	792.4138	396.7105	775.3872	388.1973	774.4032	387.7053	V	437.2507	219.1290	420.2241	210.6157			3
8	955.4771	478.2422	938.4506	469.7289	937.4666	469.2369	Y	338.1823	169.5948	321.1557	161.0815			2
9							R	175.1190	88.0631	158.0924	79.5498			1

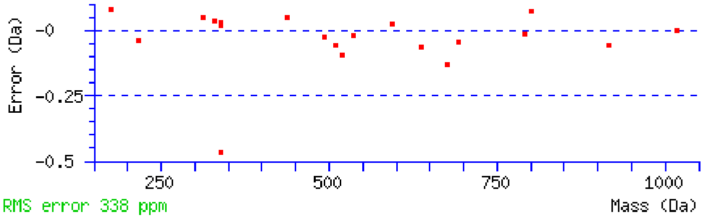

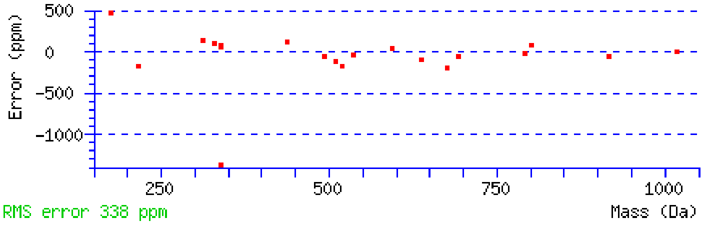

All matches to this query

Score	Mr(calc):	Delta	Sequence
57.3	1128.5815	0.0005	ITNYTVVYR
8.6	1128.5805	0.0016	LRSHPAQLK
5.3	1128.5927	-0.0107	LNLTLDFHR
4.8	1128.5710	0.0111	MTSLGSQLHR
3.2	1128.5887	-0.0067	SLHLDKSSSR
2.1	1128.5750	0.0070	LFVHGMPGSGK
0.5	1128.5815	0.0005	LTGPPPSFEGK
0.5	1128.5710	0.0111	LTSSHLGMQR
0.2	1126.5731	2.0090	DPDVAREAVR
0.2	1126.5675	2.0145	LVDPLYSIK

Spectrum No: 362; Query: 789; Rank: 1

Peptide View

MS/MS Fragmentation of **DLRNEIQVNTSQIPDTVTR**
Found in **IPI00211648**, Tax_Id=10116 Gene_Symbol=Slc3a1 Neutral and basic amino acid transport protein rBAT

Match to Query 789: 2199.115392 from(734.045740,3+)
Title: 091129RatKid_SCX02_14.3043.3043.3.dta
Data file K:\NewmanPaper\Piliang\3SubProteomes\Piliang3SP\mgf5ppm\SCX_3SubProteomes5ppm.mgf

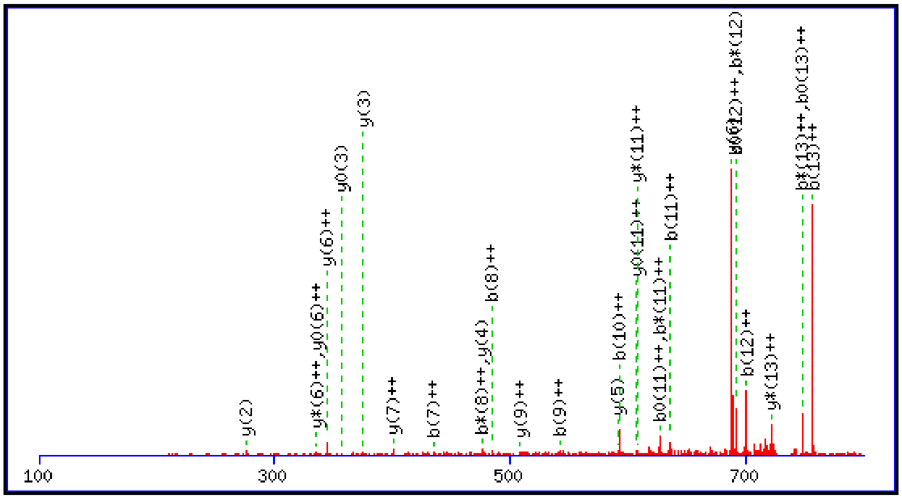

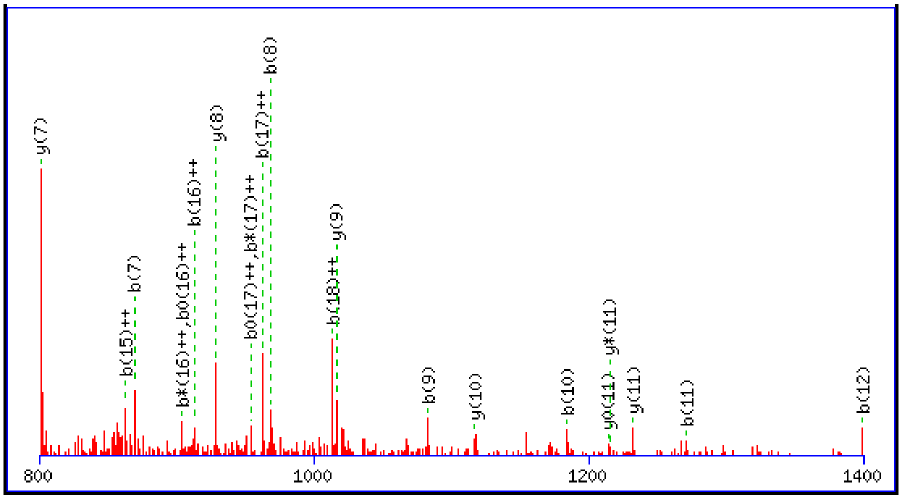

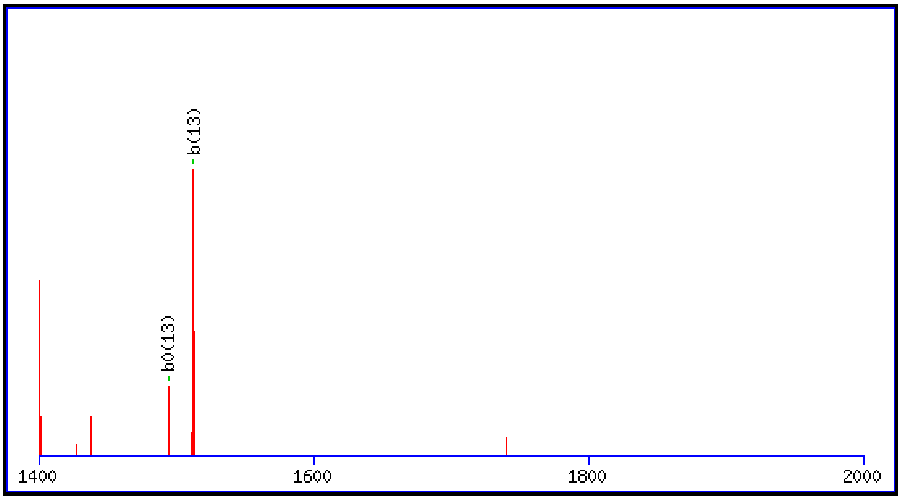

Monoisotopic mass of neutral peptide Mr(calc): 2199.1131
Fixed modifications: Carbamidomethyl (C)
Variable modifications:
N9 : Deamidated_N (N)
Ions Score: 57 **Expect:** 0.00031
Matches (Bold Red): 51/210 fragment ions using 84 most intense peaks

#	b	b^{++}	b*	b^{*++}	b^0	b^{0++}	Seq.	y	y^{++}	y*	y^{*++}	y^0	y^{0++}	#
1	116.0342	58.5207			98.0237	49.5155	D							19
2	229.1183	115.0628			211.1077	106.0575	L	2085.0935	1043.0504	2068.0669	1034.5371	2067.0829	1034.0451	18
3	385.2194	193.1133	368.1928	184.6001	367.2088	184.1081	R	1972.0094	986.5083	1954.9829	977.9951	1953.9988	977.5031	17
4	499.2623	250.1348	482.2358	241.6215	481.2518	241.1295	N	1815.9083	908.4578	1798.8817	899.9445	1797.8977	899.4525	16
5	628.3049	314.6561	611.2784	306.1428	610.2943	305.6508	E	1701.8654	851.4363	1684.8388	842.9230	1683.8548	842.4310	15
6	741.3890	371.1981	724.3624	362.6849	723.3784	362.1928	I	1572.8228	786.9150	1555.7962	778.4017	1554.8122	777.9097	14
7	**869.4476**	**435.2274**	852.4210	426.7141	851.4370	426.2221	Q	1459.7387	730.3730	1442.7122	**721.8597**	1441.7281	721.3677	13
8	**968.5160**	**484.7616**	951.4894	**476.2483**	950.5054	475.7563	V	1331.6801	666.3437	1314.6536	657.8304	1313.6696	657.3384	12
9	**1083.5429**	**542.2751**	1066.5164	533.7618	1065.5323	533.2698	N	**1232.6117**	616.8095	**1215.5852**	**608.2962**	**1214.6012**	**607.8042**	11
10	**1184.5906**	**592.7989**	1167.5640	584.2857	1166.5800	583.7936	T	**1117.5848**	559.2960	1100.5582	550.7828	1099.5742	550.2907	10
11	**1271.6226**	**636.3149**	1254.5961	**627.8017**	1253.6120	**627.3097**	S	**1016.5371**	**508.7722**	999.5106	500.2589	998.5265	499.7669	9
12	**1399.6812**	**700.3442**	1382.6546	**691.8310**	1381.6706	**691.3390**	Q	**929.5051**	465.2562	912.4785	456.7429	911.4945	456.2509	8
13	**1512.7653**	**756.8863**	1495.7387	**748.3730**	**1494.7547**	**747.8810**	I	**801.4465**	**401.2269**	784.4199	392.7136	783.4359	392.2216	7
14	1609.8180	805.4126	1592.7915	796.8994	1591.8075	796.4074	P	**688.3624**	**344.6849**	671.3359	**336.1716**	670.3519	**335.6796**	6
15	1724.8450	**862.9261**	1707.8184	854.4128	1706.8344	853.9208	D	**591.3097**	296.1585	574.2831	287.6452	573.2991	287.1532	5
16	1825.8926	**913.4500**	1808.8661	**904.9367**	1807.8821	**904.4447**	T	**476.2827**	238.6450	459.2562	230.1317	458.2722	229.6397	4
17	1924.9611	**962.9842**	1907.9345	**954.4709**	1906.9505	**953.9789**	V	**375.2350**	188.1212	358.2085	179.6079	**357.2245**	179.1159	3
18	2026.0087	**1013.5080**	2008.9822	1004.9947	2007.9982	1004.5027	T	**276.1666**	138.5870	259.1401	130.0737	258.1561	129.5817	2

**19**							**R**	175.1190	88.0631	158.0924	79.5498		**1**

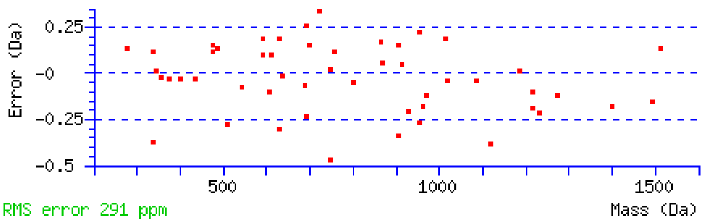

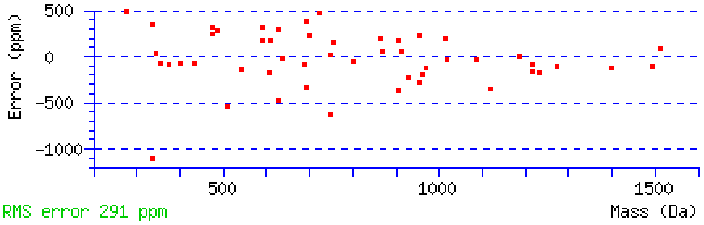

All matches to this query

Score	Mr(calc):	Delta	Sequence
57.3	2199.1131	0.0023	DLRNEIQVNTSQIPDTVTR
50.2	2199.1131	0.0023	DLRNEIQVNTSQIPDTVTR
30.0	2198.1291	0.9863	DLRNEIQVNTSQIPDTVTR
10.0	2197.1174	1.9980	VLDLTRNALRSLPPGLFR
9.9	2199.0936	0.0218	YNIDSLSPGRKVSISEVQK
7.5	2199.1024	0.0130	VSFSGLPLMPARRVAPSYR
7.5	2199.1024	0.0130	VSFSGLPLMPARRVAPSYR
7.2	2199.0936	0.0218	YNIDSLSPGRKVSISEVQK
5.7	2199.1106	0.0048	ESQQQPGMYXKRLTSLVR
5.6	2199.0936	0.0218	YNIDSLSPGRKVSISEVQK

Spectrum No: 363; Query: 480; Rank: 1

Peptide View

MS/MS Fragmentation of **SCVTITDAFPQNMSR**
Found in **IPI00370450**, Tax_Id=10116 Gene_Symbol=Plxnb2 plexin B2

Match to Query 480: 1726.769328 from(864.391940,2+)
Title: 091127RatKid_SCX01_11.2287.2287.2.dta
Data file K:\NewmanPaper\Piliang\3SubProteomes\Piliang3SP\mgf5ppm\SCX_3SubProteomes5ppm.mgf

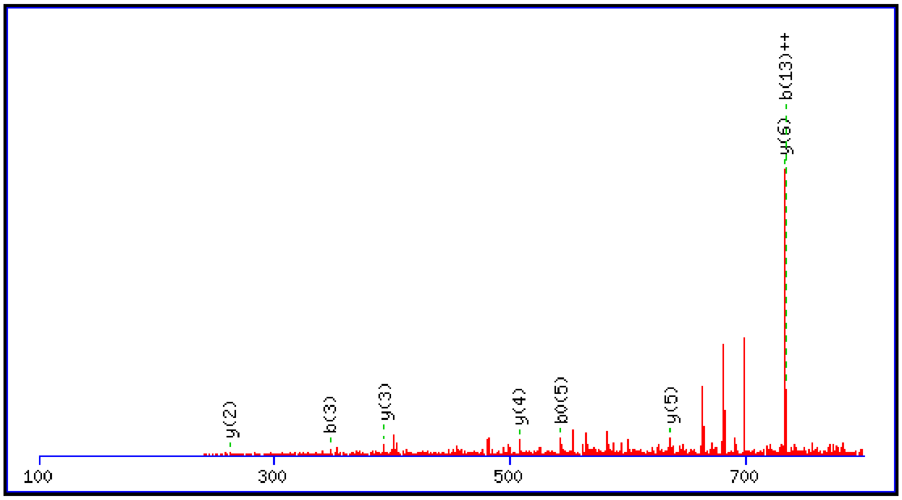

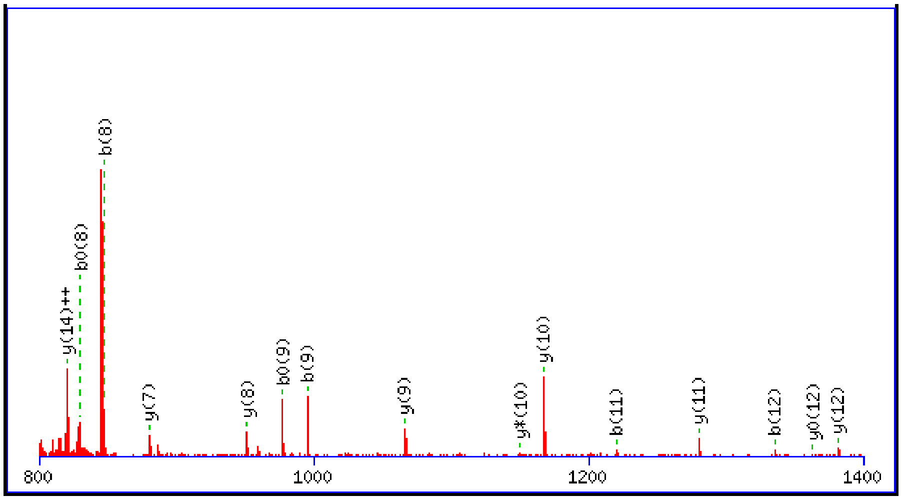

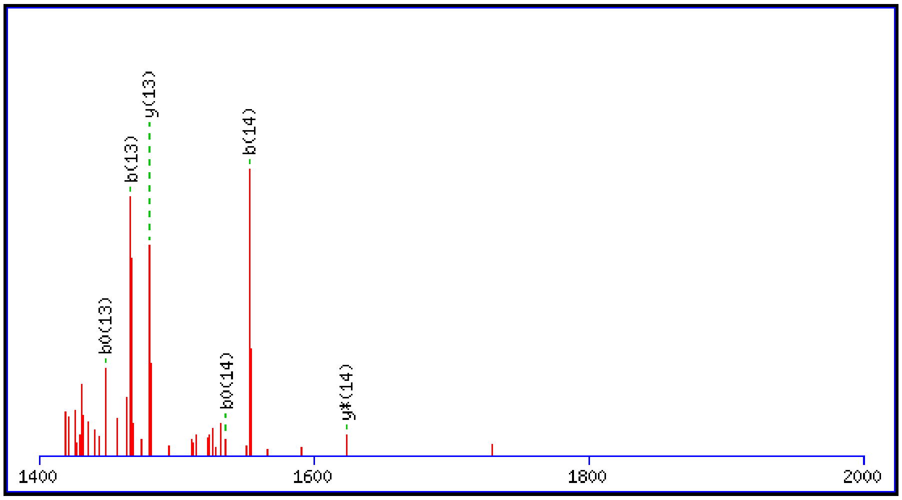

Monoisotopic mass of neutral peptide Mr(calc): 1726.7655
Fixed modifications: Carbamidomethyl (C)
Variable modifications:
N12 : Deamidated_N (N)
Ions Score: 57 **Expect:** 0.00038
Matches (Bold Red): 29/146 fragment ions using 81 most intense peaks

#	b	b^{++}	b*	b^{*++}	b^{0}	b^{0++}	Seq.	y	y^{++}	y*	y^{*++}	y^{0}	y^{0++}	#
1	88.0393	44.5233			70.0287	35.5180	S							15
2	248.0700	124.5386			230.0594	115.5333	C	1640.7407	**820.8740**	**1623.7142**	812.3607	1622.7301	811.8687	14
3	**347.1384**	174.0728			329.1278	165.0675	V	**1480.7100**	740.8587	1463.6835	732.3454	1462.6995	731.8534	13
4	448.1860	224.5967			430.1755	215.5914	T	**1381.6416**	691.3245	1364.6151	682.8112	**1363.6311**	682.3192	12
5	561.2701	281.1387			**543.2595**	272.1334	I	**1280.5940**	640.8006	1263.5674	632.2873	1262.5834	631.7953	11
6	662.3178	331.6625			644.3072	322.6573	T	**1167.5099**	584.2586	**1150.4833**	575.7453	1149.4993	575.2533	10
7	777.3447	389.1760			759.3342	380.1707	D	**1066.4622**	533.7347	1049.4357	525.2215	1048.4516	524.7295	9
8	**848.3818**	424.6946			**830.3713**	415.6893	A	**951.4353**	476.2213	934.4087	467.7080	933.4247	467.2160	8
9	**995.4503**	498.2288			**977.4397**	489.2235	F	**880.3982**	440.7027	863.3716	432.1894	862.3876	431.6974	7
10	1092.5030	546.7551			1074.4925	537.7499	P	**733.3297**	367.1685	716.3032	358.6552	715.3192	358.1632	6
11	**1220.5616**	610.7844	1203.5351	602.2712	1202.5510	601.7792	Q	**636.2770**	318.6421	619.2504	310.1289	618.2664	309.6368	5
12	**1335.5885**	668.2979	1318.5620	659.7846	1317.5780	659.2926	N	**508.2184**	254.6128	491.1919	246.0996	490.2078	245.6076	4
13	**1466.6290**	**733.8181**	1449.6025	725.3049	**1448.6185**	724.8129	M	**393.1915**	197.0994	376.1649	188.5861	375.1809	188.0941	3
14	**1553.6611**	777.3342	1536.6345	768.8209	**1535.6505**	768.3289	S	**262.1510**	131.5791	245.1244	123.0659	244.1404	122.5738	2
15							R	175.1190	88.0631	158.0924	79.5498			1

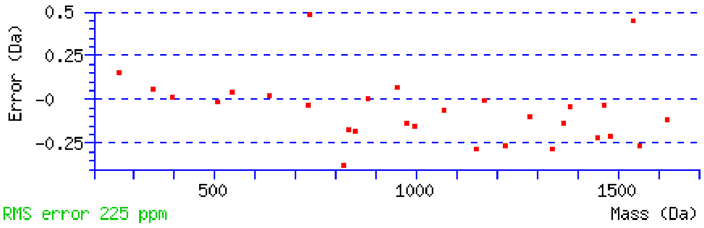

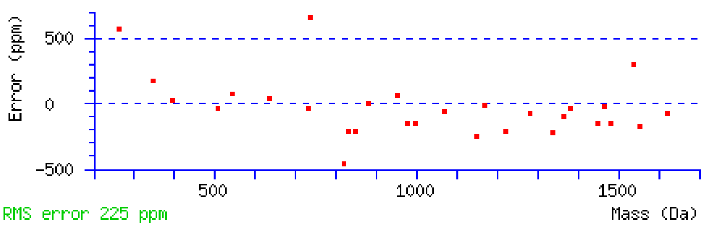

All matches to this query

Score	Mr(calc):	Delta	Sequence
57.3	1726.7655	0.0039	SCVTITDAFPQNMSR
7.9	1724.7535	2.0158	SLKPESGGNHIADLK
4.0	1726.7862	-0.0169	TGLGVIRMWADEGSR
3.6	1724.7778	1.9916	RSAPSGPVRSSSGMGGR
2.9	1726.7655	0.0039	TCVFEKENDPSVMR
2.0	1726.7727	-0.0033	ASAKCGTGGKQMEGXSR
1.7	1725.7739	0.9954	SGSLPSLHDIIKGNK
1.5	1726.7804	-0.0111	SQPARLENPKATVR
0.9	1724.7771	1.9923	SLYESFVSSSDRLR
0.9	1724.7771	1.9923	SLYESFVSSSDRLR

Spectrum No: 364; Query: 131; Rank: 1

Peptide View

MS/MS Fragmentation of **MTLSQDNSTLR**
Found in **IPI00199607**, Tax_Id=10116 Gene_Symbol=Ceacam1_v4;Ceacam1_v1;Ceacam1;Ceacam1_v3 carcinoembryonic antigen-related cell adhesion molecule isoform 2

Match to Query 131: 1265.590068 from(633.802310,2+)
Title: 091129RatKid_SCX02_13.841.841.2.dta
Data file K:\NewmanPaper\Piliang\3SubProteomes\Piliang3SP\mgf5ppm\SCX_3SubProteomes5ppm.mgf

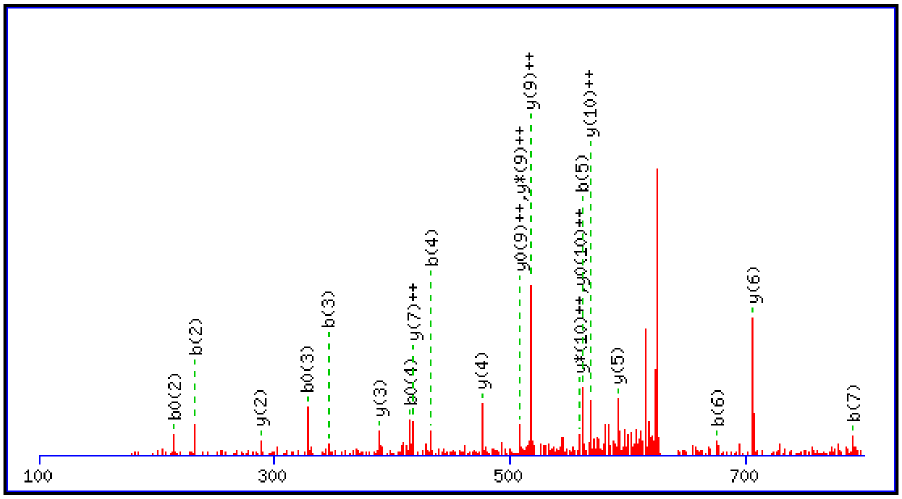

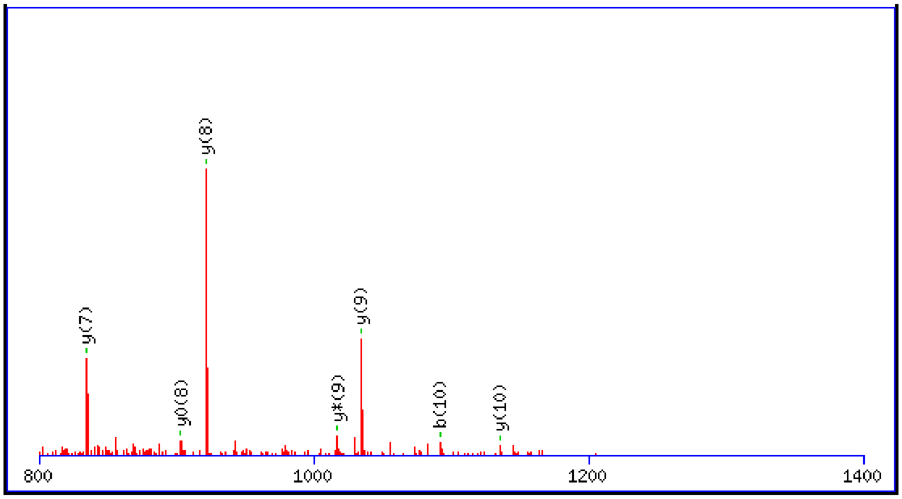

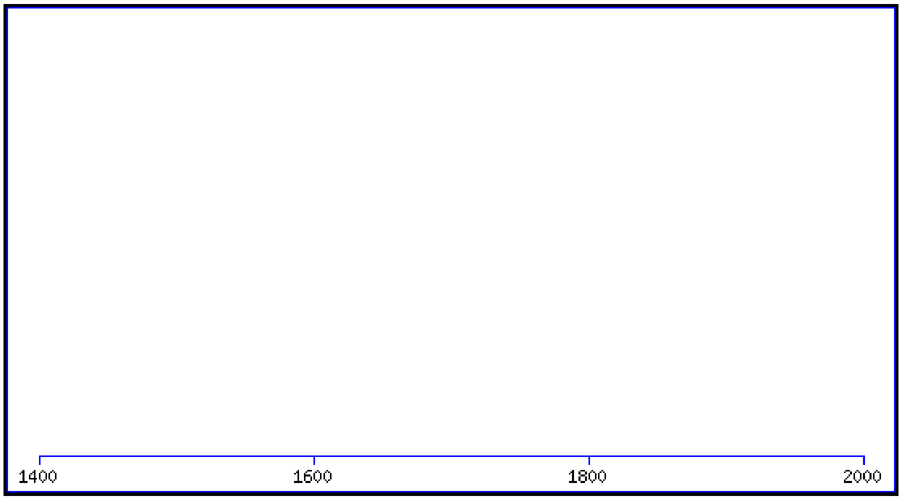

Monoisotopic mass of neutral peptide Mr(calc): 1265.5921
Fixed modifications: Carbamidomethyl (C)
Variable modifications:
N7 : Deamidated_N (N)
Ions Score: 57 **Expect:** 0.00025
Matches (Bold Red): 28/106 fragment ions using 61 most intense peaks

#	b	b^{++}	b*	b^{*++}	b^0	b^{0++}	Seq.	y	y^{++}	y*	y^{*++}	y^0	y^{0++}	#
1	132.0478	66.5275					M							11
2	**233.0954**	117.0514			**215.0849**	108.0461	T	**1135.5590**	**568.2831**	1118.5324	**559.7698**	1117.5484	**559.2778**	10
3	**346.1795**	173.5934			**328.1689**	164.5881	L	**1034.5113**	**517.7593**	**1017.4847**	**509.2460**	1016.5007	**508.7540**	9
4	**433.2115**	217.1094			**415.2010**	208.1041	S	**921.4272**	461.2172	904.4007	452.7040	**903.4166**	452.2120	8
5	**561.2701**	281.1387	544.2436	272.6254	543.2595	272.1334	Q	**834.3952**	**417.7012**	817.3686	409.1880	816.3846	408.6959	7
6	**676.2971**	338.6522	659.2705	330.1389	658.2865	329.6469	D	**706.3366**	353.6719	689.3101	345.1587	688.3260	344.6667	6
7	**791.3240**	396.1656	774.2974	387.6524	773.3134	387.1603	N	**591.3097**	296.1585	574.2831	287.6452	573.2991	287.1532	5
8	878.3560	439.6816	861.3295	431.1684	860.3455	430.6764	S	**476.2827**	238.6450	459.2562	230.1317	458.2722	229.6397	4
9	979.4037	490.2055	962.3771	481.6922	961.3931	481.2002	T	**389.2507**	195.1290	372.2241	186.6157	371.2401	186.1237	3
10	**1092.4878**	546.7475	1075.4612	538.2342	1074.4772	537.7422	L	**288.2030**	144.6051	271.1765	136.0919			2
11							R	175.1190	88.0631	158.0924	79.5498			1

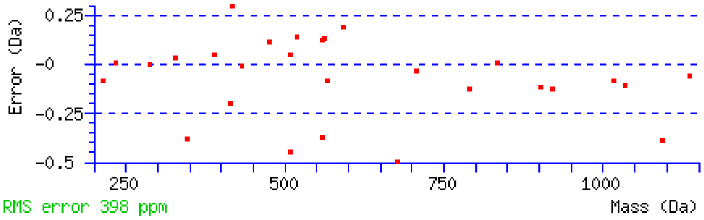

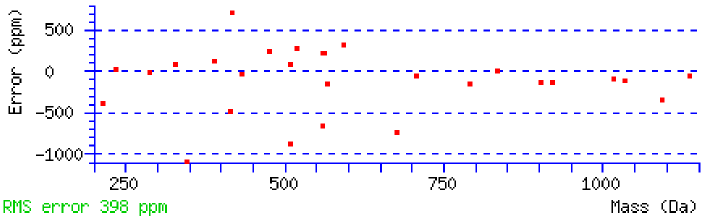

All matches to this query

Score	Mr(calc):	Delta	Sequence
57.0	1265.5921	-0.0021	MTLSQDNSTLR
9.8	1265.5856	0.0045	TGMREEMSLGR
8.4	1265.5922	-0.0021	VKDDMTEVTGR
4.5	1264.5813	1.0088	TKDSPTPLGGGR
1.9	1265.6000	-0.0099	EPPRPEQEER
0.6	1265.6017	-0.0116	QEQLELTLGR

Spectrum No: 365; Query: 735; Rank: 1

Peptide View

MS/MS Fragmentation of **LTNQTMGFSFAVEQDIPVK**
Found in **IPI00326140**, Tax_Id=10116 Gene_Symbol=Pzp Alpha-1-macroglobulin precursor

Match to Query 735: 2125.044728 from(1063.529640,2+)
Title: 091127RatKid_SCX01_02.4366.4366.2.dta
Data file K:\NewmanPaper\Piliang\3SubProteomes\Piliang3SP\mgf5ppm\SCX_3SubProteomes5ppm.mgf

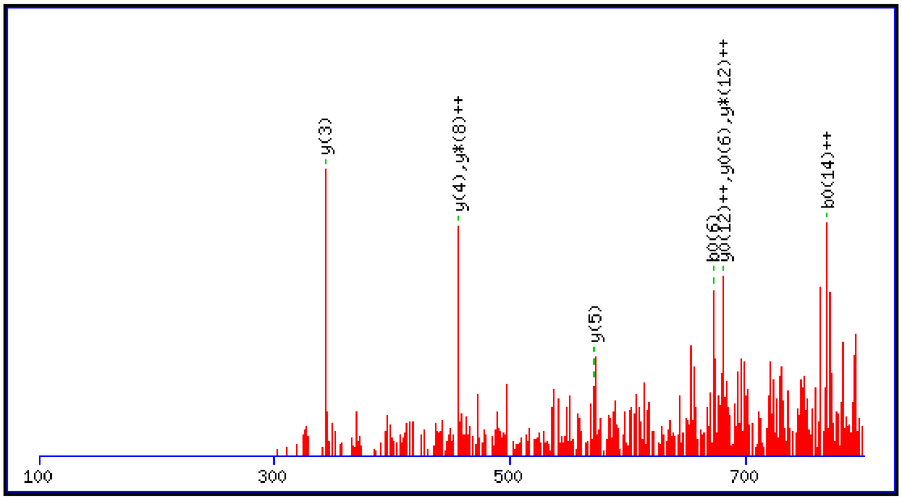

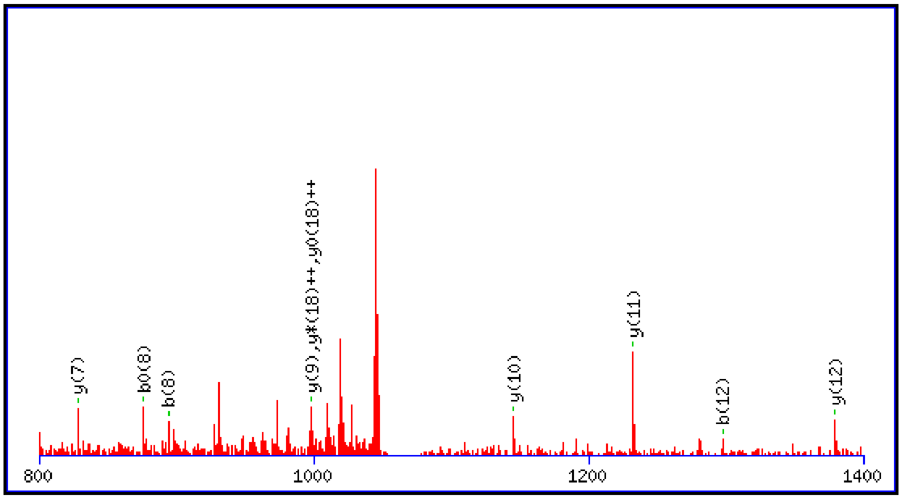

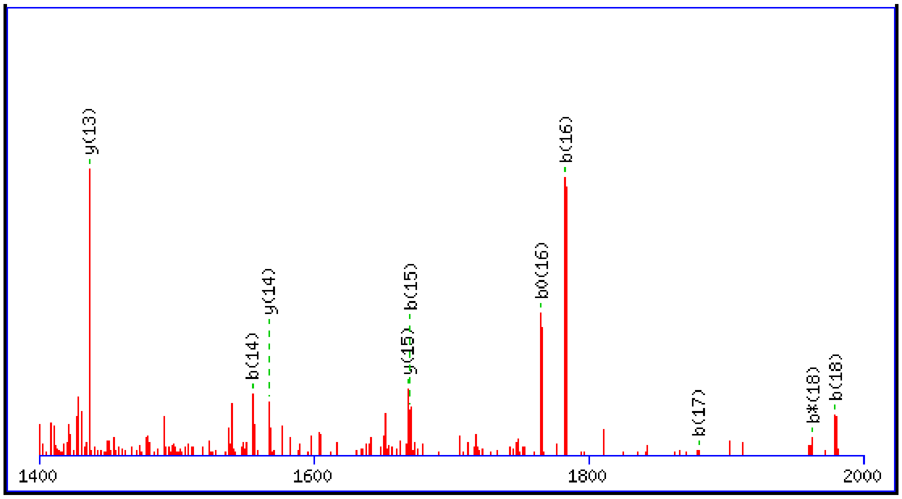

Monoisotopic mass of neutral peptide Mr(calc): 2125.0402
Fixed modifications: Carbamidomethyl (C)
Variable modifications:
N3 : Deamidated_N (N)
Ions Score: 57 **Expect:** 0.00044
Matches (Bold Red): 29/202 fragment ions using 57 most intense peaks

#	b	b^{++}	b*	b^{*++}	b^0	b^{0++}	Seq.	y	y^{++}	y*	y^{*++}	y^0	y^{0++}	#
1	114.0913	57.5493					L							19
2	215.1390	108.0731			197.1285	99.0679	T	2012.9634	1006.9853	1995.9368	**998.4720**	1994.9528	**997.9800**	18
3	330.1660	165.5866	313.1394	157.0733	312.1554	156.5813	N	1911.9157	956.4615	1894.8891	947.9482	1893.9051	947.4562	17
4	458.2245	229.6159	441.1980	221.1026	440.2140	220.6106	Q	1796.8888	898.9480	1779.8622	890.4347	1778.8782	889.9427	16
5	559.2722	280.1397	542.2457	271.6265	541.2616	271.1345	T	**1668.8302**	834.9187	1651.8036	826.4054	1650.8196	825.9134	15
6	690.3127	345.6600	673.2861	337.1467	**672.3021**	336.6547	M	**1567.7825**	784.3949	1550.7559	775.8816	1549.7719	775.3896	14
7	747.3342	374.1707	730.3076	365.6574	729.3236	365.1654	G	**1436.7420**	718.8746	1419.7155	710.3614	1418.7314	709.8694	13
8	**894.4026**	447.7049	877.3760	439.1917	**876.3920**	438.6996	F	**1379.7205**	690.3639	1362.6940	**681.8506**	1361.7100	**681.3586**	12
9	981.4346	491.2209	964.4081	482.7077	963.4240	482.2157	S	**1232.6521**	616.8297	1215.6256	608.3164	1214.6416	607.8244	11
10	1128.5030	564.7551	1111.4765	556.2419	1110.4925	555.7499	F	**1145.6201**	573.3137	1128.5936	564.8004	1127.6095	564.3084	10
11	1199.5401	600.2737	1182.5136	591.7604	1181.5296	591.2684	A	**998.5517**	499.7795	981.5251	491.2662	980.5411	490.7742	9
12	**1298.6085**	649.8079	1281.5820	641.2946	1280.5980	640.8026	V	927.5146	464.2609	910.4880	**455.7477**	909.5040	455.2556	8
13	1427.6511	714.3292	1410.6246	705.8159	1409.6406	705.3239	E	**828.4462**	414.7267	811.4196	406.2134	810.4356	405.7214	7
14	**1555.7097**	778.3585	1538.6832	769.8452	1537.6992	**769.3532**	Q	699.4036	350.2054	682.3770	341.6921	**681.3930**	341.2001	6
15	**1670.7367**	835.8720	1653.7101	827.3587	1652.7261	826.8667	D	**571.3450**	286.1761	554.3184	277.6629	553.3344	277.1709	5
16	**1783.8207**	892.4140	1766.7942	883.9007	**1765.8102**	883.4087	I	**456.3180**	228.6627	439.2915	220.1494			4
17	**1880.8735**	940.9404	1863.8469	932.4271	1862.8629	931.9351	P	**343.2340**	172.1206	326.2074	163.6074			3
18	**1979.9419**	990.4746	**1962.9154**	981.9613	1961.9313	981.4693	V	246.1812	123.5942	229.1547	115.0810			2
19							K	147.1128	74.0600	130.0863	65.5468			1

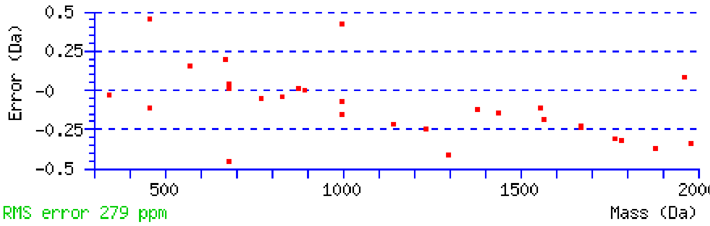

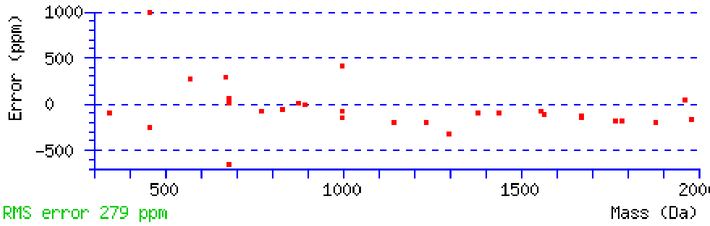

All matches to this query

Score	Mr(calc):	Delta	Sequence
57.0	2125.0402	0.0046	LTNQTMGFSFAVEQDIPVK
39.6	2124.0562	0.9886	LTNQTMGFSFAVEQDIPVK

7.6	2125.0448	-0.0001	RMGQAIAQYNMDQFIPVK
6.2	2125.0448	-0.0001	RMGQAIAQYNMDQFIPVK
5.2	2123.0402	2.0046	LASALLPLSGRHRTTSQR
5.2	2123.0402	2.0046	LASALLPLSGRHRTTSQR
4.6	2125.0643	-0.0195	VMLVKVGEGEAGQWSVKTK
0.4	2123.0241	2.0206	YFEGPAFSFLXGAIGLGRK
0.3	2125.0530	-0.0083	LLKSCFETLASNPEIPVK

Spectrum No: 366; Query: 866; Rank: 1

Peptide View

MS/MS Fragmentation of **MIAQHCVDANNTFCFEHPR**
Found in **IPI00205325**, Tax_Id=10116 Gene_Symbol=Lrp2 Low-density lipoprotein receptor-related protein 2 precursor

Match to Query 866: 2346.996708 from(1174.505630,2+)
Title: 091127RatKid_SCX01_32.2110.2110.2.dta
Data file K:\NewmanPaper\Piliang\3SubProteomes\Piliang3SP\mgf5ppm\SCX_3SubProteomes5ppm.mgf

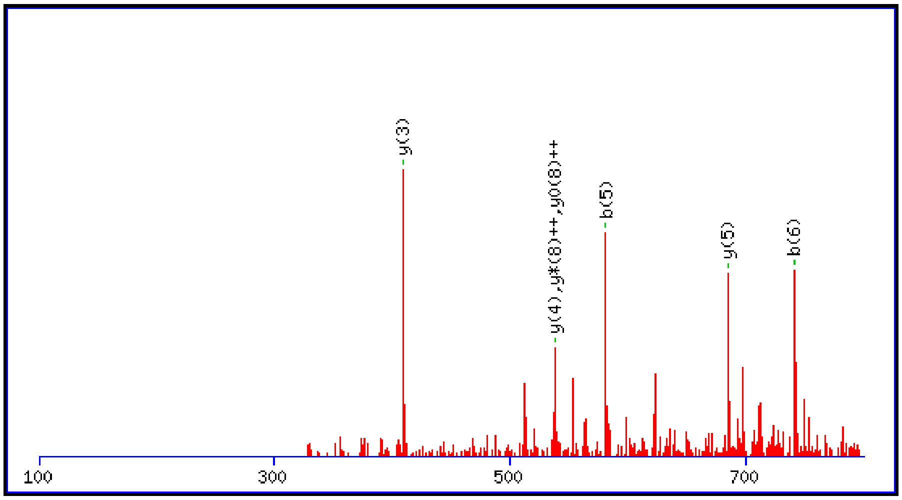

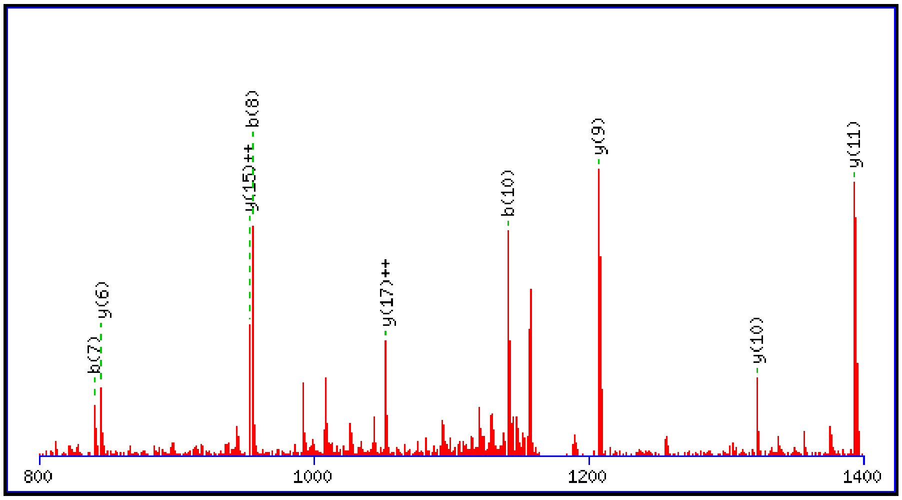

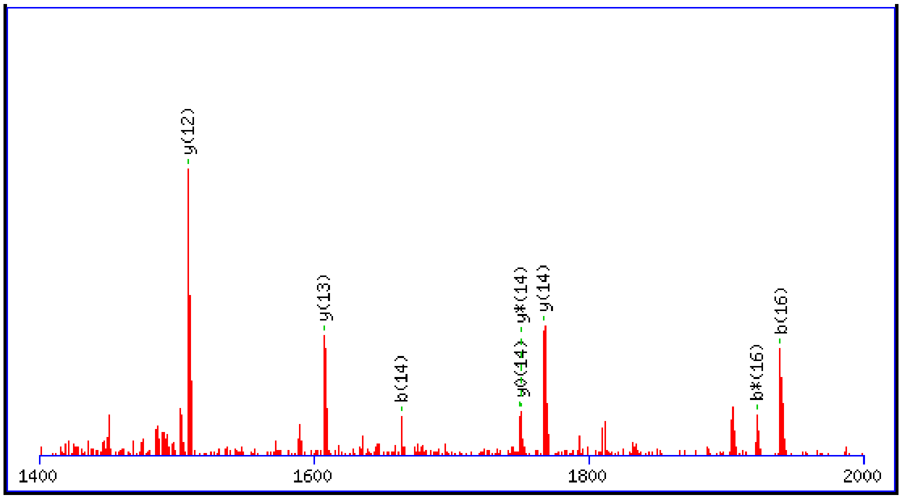

Monoisotopic mass of neutral peptide Mr(calc): 2346.9932
Fixed modifications: Carbamidomethyl (C)
Variable modifications:
N10 : Deamidated_N (N)
Ions Score: 57 **Expect:** 0.00054
Matches (Bold Red): 24/190 fragment ions using 41 most intense peaks

#	b	b^{++}	b^{*}	b^{*++}	b^{0}	b^{0++}	Seq.	y	y^{++}	y^{*}	y^{*++}	y^{0}	y^{0++}	#
1	132.0478	66.5275					M							19
2	245.1318	123.0696					I	2216.9600	1108.9837	2199.9335	1100.4704	2198.9495	1099.9784	18
3	316.1689	158.5881					A	2103.8760	**1052.4416**	2086.8494	1043.9283	2085.8654	1043.4363	17
4	444.2275	222.6174	427.2010	214.1041			Q	2032.8389	1016.9231	2015.8123	1008.4098	2014.8283	1007.9178	16
5	**581.2864**	291.1469	564.2599	282.6336			H	1904.7803	**952.8938**	1887.7537	944.3805	1886.7697	943.8885	15
6	**741.3171**	371.1622	724.2905	362.6489			C	**1767.7214**	884.3643	**1750.6948**	875.8510	**1749.7108**	875.3590	14
7	**840.3855**	420.6964	823.3589	412.1831			V	**1607.6907**	804.3490	1590.6642	795.8357	1589.6801	795.3437	13
8	**955.4124**	478.2099	938.3859	469.6966	937.4019	469.2046	D	**1508.6223**	754.8148	1491.5957	746.3015	1490.6117	745.8095	12
9	1026.4495	513.7284	1009.4230	505.2151	1008.4390	504.7231	A	**1393.5954**	697.3013	1376.5688	688.7880	1375.5848	688.2960	11
10	**1141.4765**	571.2419	1124.4499	562.7286	1123.4659	562.2366	N	**1322.5582**	661.7828	1305.5317	653.2695	1304.5477	652.7775	10
11	1255.5194	628.2633	1238.4929	619.7501	1237.5088	619.2581	N	**1207.5313**	604.2693	1190.5048	595.7560	1189.5207	595.2640	9
12	1356.5671	678.7872	1339.5405	670.2739	1338.5565	669.7819	T	1093.4884	547.2478	1076.4618	**538.7346**	1075.4778	**538.2425**	8
13	1503.6355	752.3214	1486.6090	743.8081	1485.6249	743.3161	F	992.4407	496.7240	975.4141	488.2107	974.4301	487.7187	7
14	**1663.6662**	832.3367	1646.6396	823.8234	1645.6556	823.3314	C	**845.3723**	423.1898	828.3457	414.6765	827.3617	414.1845	6
15	1810.7346	905.8709	1793.7080	897.3576	1792.7240	896.8656	F	**685.3416**	343.1745	668.3151	334.6612	667.3311	334.1692	5
16	**1939.7772**	970.3922	**1922.7506**	961.8789	1921.7666	961.3869	E	**538.2732**	269.6402	521.2467	261.1270	520.2627	260.6350	4
17	2076.8361	1038.9217	2059.8095	1030.4084	2058.8255	1029.9164	H	**409.2306**	205.1190	392.2041	196.6057			3
18	2173.8888	1087.4481	2156.8623	1078.9348	2155.8783	1078.4428	P	272.1717	136.5895	255.1452	128.0762			2
19							R	175.1190	88.0631	158.0924	79.5498			1

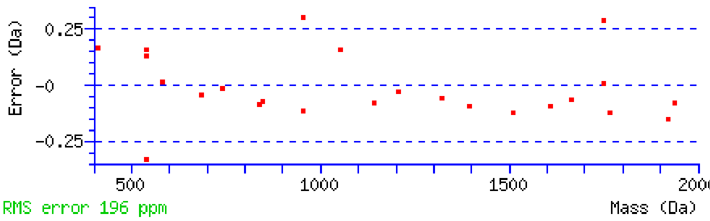

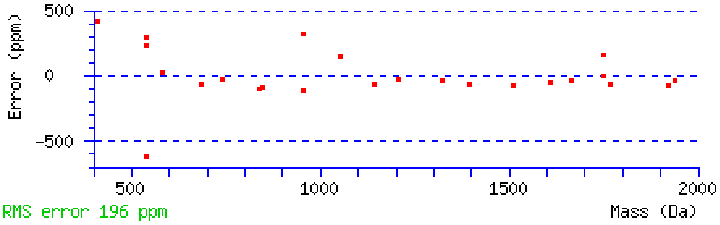

All matches to this query

Score	Mr(calc):	Delta	Sequence
57.0	2346.9932	0.0035	MIAQHCVDANNTFCFEHPR
45.0	2346.9932	0.0035	MIAQHCVDANNTFCFEHPR

11.6	2346.0092	0.9875	MIAQHCVDANNTFCFEHPR
3.3	2345.0068	1.9899	EAKSNSFISIPKMEVVTPK
0.7	2346.9739	0.0228	CSSPESMADRLLLTTPTHR
0.7	2346.9739	0.0228	CSSPESMADRLLLTTPTHR

Spectrum No: 367; Query: 1125; Rank: 1

Peptide View

MS/MS Fragmentation of **EHNFNIYNDSVSDSLNVPYDYTSVMHYSK**
Found in **IPI00204808**, Tax_Id=10116 Gene_Symbol=Mep1b Meprin A subunit beta precursor

Match to Query 1125: 3438.507702 from(1147.176510,3+)
Title: 091127RatKid_SCX01_31.4381.4381.3.dta
Data file K:\NewmanPaper\Piliang\3SubProteomes\Piliang3SP\mgf5ppm\SCX_3SubProteomes5ppm.mgf

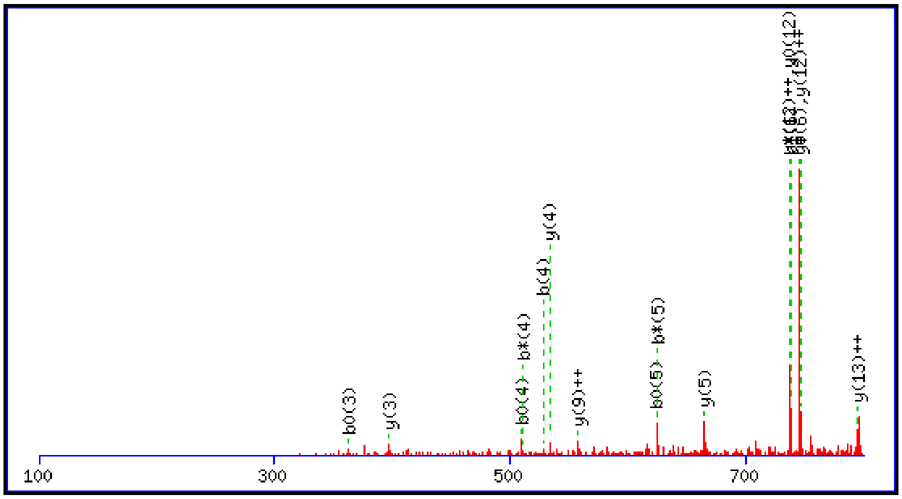

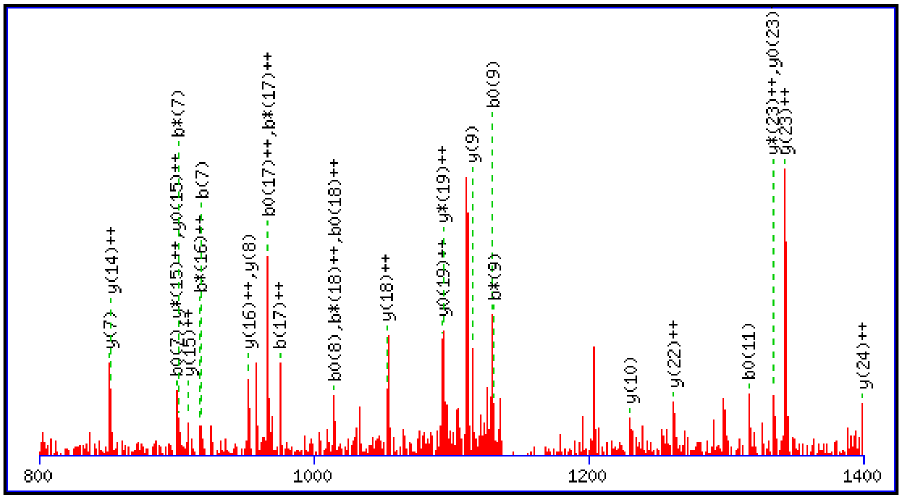

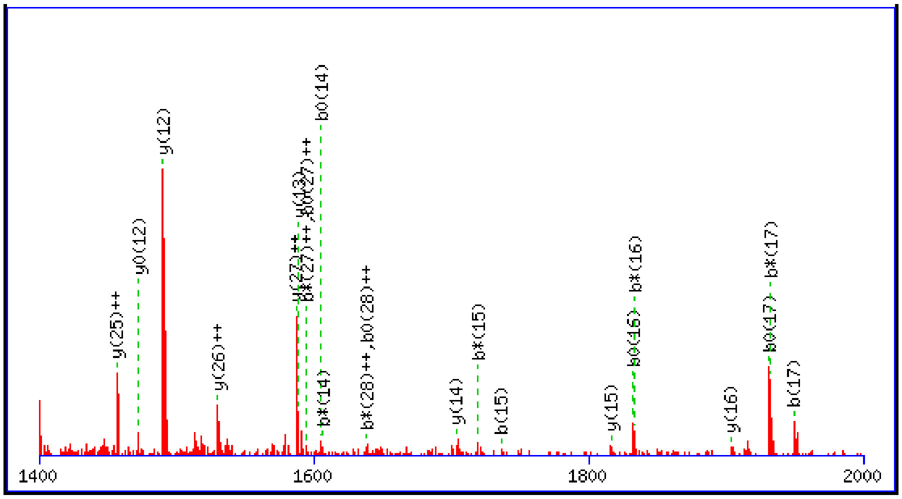

Monoisotopic mass of neutral peptide Mr(calc): 3438.4986
Fixed modifications: Carbamidomethyl (C)
Variable modifications:
N8 : Deamidated_N (N)
Ions Score: 57 **Expect:** 0.001
Matches (Bold Red): 71/330 fragment ions using 126 most intense peaks

#	b	b^{++}	b*	b^{*++}	b^0	b^{0++}	Seq.	y	y^{++}	y*	y^{*++}	y^0	y^{0++}	#
1	130.0499	65.5286			112.0393	56.5233	E							29
2	267.1088	134.0580			249.0982	125.0527	H	3310.4633	1655.7353	3293.4368	1647.2220	3292.4528	1646.7300	28
3	381.1517	191.0795	364.1252	182.5662	**363.1411**	182.0742	N	3173.4044	**1587.2059**	3156.3779	1578.6926	3155.3939	1578.2006	27
4	**528.2201**	264.6137	**511.1936**	256.1004	**510.2096**	255.6084	F	3059.3615	**1530.1844**	3042.3350	1521.6711	3041.3509	1521.1791	26
5	642.2630	321.6352	**625.2365**	313.1219	**624.2525**	312.6299	N	2912.2931	**1456.6502**	2895.2665	1448.1369	2894.2825	1447.6449	25
6	755.3471	378.1772	**738.3206**	369.6639	**737.3365**	369.1719	I	2798.2502	**1399.6287**	2781.2236	1391.1154	2780.2396	1390.6234	24
7	**918.4104**	459.7089	**901.3839**	451.1956	**900.3999**	450.7036	Y	2685.1661	**1343.0867**	2668.1395	**1334.5734**	2667.1555	**1334.0814**	23
8	1033.4374	517.2223	1016.4108	508.7091	**1015.4268**	508.2170	N	2522.1028	**1261.5550**	2505.0762	1253.0417	2504.0922	1252.5497	22
9	1148.4643	574.7358	**1131.4378**	566.2225	**1130.4538**	565.7305	D	2407.0758	1204.0416	2390.0493	1195.5283	2389.0653	1195.0363	21
10	1235.4963	618.2518	1218.4698	609.7385	1217.4858	609.2465	S	2292.0489	1146.5281	2275.0223	1138.0148	2274.0383	1137.5228	20
11	**1334.5648**	667.7860	1317.5382	659.2727	**1316.5542**	658.7807	V	2205.0169	1103.0121	2187.9903	**1094.4988**	2187.0063	**1094.0068**	19
12	1421.5968	711.3020	1404.5702	702.7888	1403.5862	702.2968	S	2105.9484	**1053.4779**	2088.9219	1044.9646	2087.9379	1044.4726	18
13	1536.6237	768.8155	1519.5972	760.3022	1518.6132	759.8102	D	2018.9164	1009.9618	2001.8899	1001.4486	2000.9059	1000.9566	17
14	1623.6558	812.3315	**1606.6292**	803.8182	**1605.6452**	803.3262	S	**1903.8895**	**952.4484**	1886.8629	943.9351	1885.8789	943.4431	16
15	**1736.7398**	868.8736	**1719.7133**	860.3603	1718.7293	859.8683	L	**1816.8574**	**908.9324**	1799.8309	**900.4191**	1798.8469	**899.9271**	15
16	1850.7828	925.8950	**1833.7562**	**917.3817**	**1832.7722**	916.8897	N	**1703.7734**	**852.3903**	1686.7468	843.8771	1685.7628	843.3850	14
17	**1949.8512**	**975.4292**	**1932.8246**	**966.9159**	**1931.8406**	**966.4239**	V	**1589.7305**	**795.3689**	1572.7039	786.8556	1571.7199	786.3636	13
18	2046.9039	1023.9556	2029.8774	**1015.4423**	2028.8934	**1014.9503**	P	**1490.6620**	**745.8347**	1473.6355	**737.3214**	**1472.6515**	**736.8294**	12
19	2209.9673	1105.4873	2192.9407	1096.9740	2191.9567	1096.4820	Y	1393.6093	697.3083	1376.5827	688.7950	1375.5987	688.3030	11
20	2324.9942	1163.0007	2307.9677	1154.4875	2306.9836	1153.9955	D	**1230.5460**	615.7766	1213.5194	607.2633	1212.5354	606.7713	10
21	2488.0575	1244.5324	2471.0310	1236.0191	2470.0470	1235.5271	Y	**1115.5190**	**558.2631**	1098.4925	549.7499	1097.5084	549.2579	9
22	2589.1052	1295.0562	2572.0787	1286.5430	2571.0946	1286.0510	T	**952.4557**	476.7315	935.4291	468.2182	934.4451	467.7262	8
23	2676.1372	1338.5723	2659.1107	1330.0590	2658.1267	1329.5670	S	**851.4080**	426.2076	834.3815	417.6944	833.3974	417.2024	7
24	2775.2057	1388.1065	2758.1791	1379.5932	2757.1951	1379.1012	V	764.3760	382.6916	**747.3494**	374.1783	**746.3654**	373.6863	6
25	2906.2461	1453.6267	2889.2196	1445.1134	2888.2356	1444.6214	M	**665.3076**	333.1574	648.2810	324.6441	647.2970	324.1521	5
26	3043.3050	1522.1562	3026.2785	1513.6429	3025.2945	1513.1509	H	**534.2671**	267.6372	517.2405	259.1239	516.2565	258.6319	4
27	3206.3684	1603.6878	3189.3418	**1595.1746**	3188.3578	**1594.6825**	Y	**397.2082**	199.1077	380.1816	190.5944	379.1976	190.1024	3
28	3293.4004	1647.2038	3276.3739	**1638.6906**	3275.3898	**1638.1986**	S	234.1448	117.5761	217.1183	109.0628	216.1343	108.5708	2
29							K	147.1128	74.0600	130.0863	65.5468			1

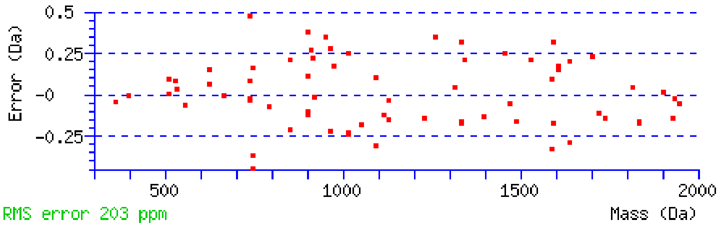

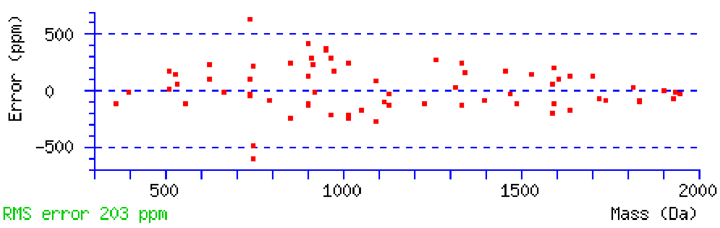

All matches to this query

Score	Mr(calc):	Delta	Sequence
56.8	3438.4986	0.0091	EHNFNIYNDSVSDSLNVPYDYTSVMHYSK
50.0	3438.4986	0.0091	EHNFNIYNDSVSDSLNVPYDYTSVMHYSK
43.2	3438.4986	0.0091	EHNFNIYNDSVSDSLNVPYDYTSVMHYSK
32.2	3438.4986	0.0091	EHNFNIYNDSVSDSLNVPYDYTSVMHYSK
18.4	3437.5146	0.9931	EHNFNIYNDSVSDSLNVPYDYTSVMHYSK
9.1	3437.5284	0.9793	NSPGLLVSPGNLNKNIQAKSPPPMNLGMNNR
6.9	3436.5120	1.9957	YLTVAYACVPKNILTAVDPAVANLNPSLK
6.3	3437.5229	0.9848	FPLTTESAMKKIEVNNTLVFIVDVMDNK
5.3	3437.5346	0.9731	ELTLWINDKLLTSPDISYDEARNLHNK
5.2	3437.4715	1.0363	EEERYSGYGSAVNPDAGFHINGTGLDLRPR

Spectrum No: 368; Query: 668; Rank: 1

Peptide View

MS/MS Fragmentation of **ATDDIIVNWVNGTLSEAGK**
Found in **IPI00210234**, Tax_Id=10116 Gene_Symbol=Pls3 Plastin-3

Match to Query 668: 2002.988248 from(1002.501400,2+)
Title: 091129RatKid_SCX02_17.3560.3560.2.dta
Data file K:\NewmanPaper\Piliang\3SubProteomes\Piliang3SP\mgf5ppm\SCX_3SubProteomes5ppm.mgf

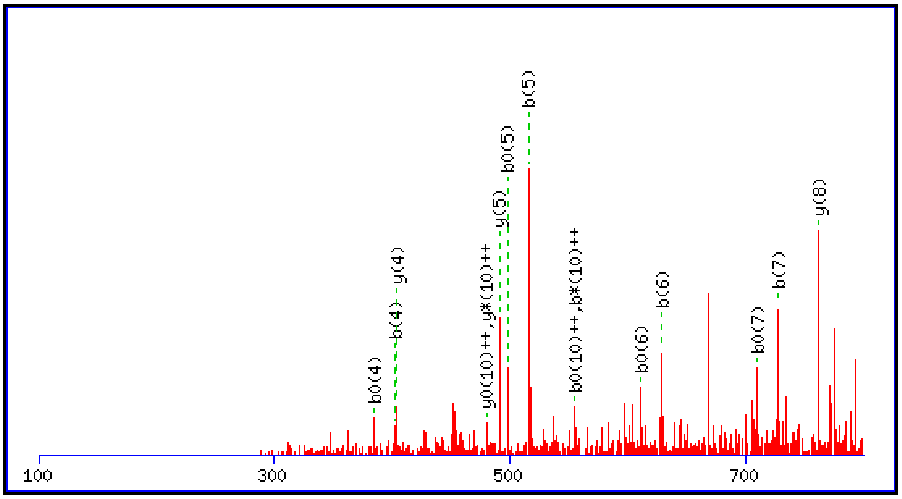

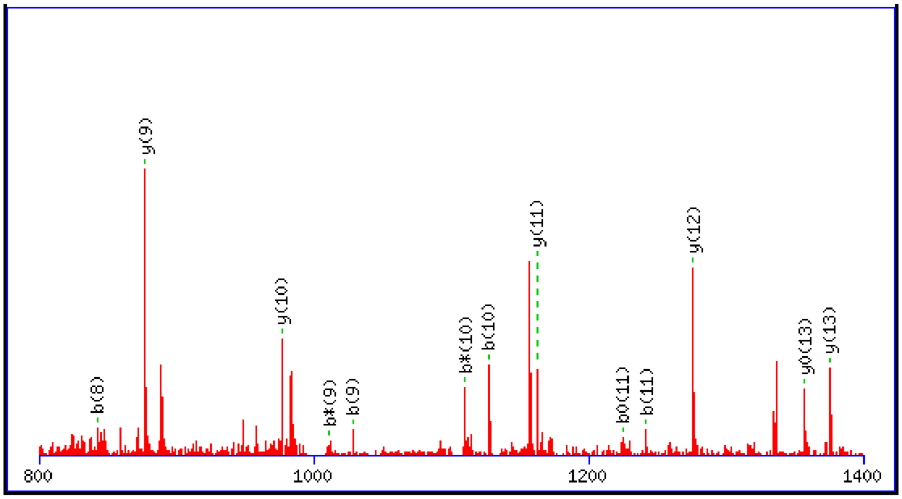

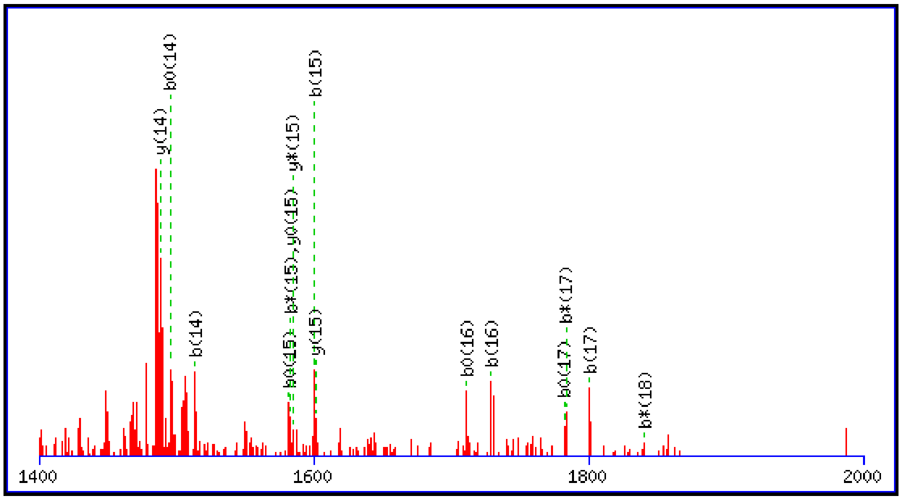

Monoisotopic mass of neutral peptide Mr(calc): 2002.9847
Fixed modifications: Carbamidomethyl (C)
Variable modifications:
N11 : Deamidated_N (N)
Ions Score: 57 **Expect:** 0.00046
Matches (Bold Red): 43/194 fragment ions using 92 most intense peaks

#	b	b^{++}	b*	b^{*++}	b^{0}	b^{0++}	Seq.	y	y^{++}	y*	y^{*++}	y^{0}	y^{0++}	#
1	72.0444	36.5258					A							19
2	173.0921	87.0497			155.0815	78.0444	T	1932.9549	966.9811	1915.9284	958.4678	1914.9443	957.9758	18
3	288.1190	144.5631			270.1084	135.5579	D	1831.9072	916.4573	1814.8807	907.9440	1813.8967	907.4520	17
4	**403.1460**	202.0766			**385.1354**	193.0713	D	1716.8803	858.9438	1699.8537	850.4305	1698.8697	849.9385	16
5	**516.2300**	258.6186			**498.2195**	249.6134	I	**1601.8533**	801.4303	**1584.8268**	792.9170	**1583.8428**	792.4250	15
6	**629.3141**	315.1607			**611.3035**	306.1554	I	**1488.7693**	744.8883	1471.7427	736.3750	1470.7587	735.8830	14
7	**728.3825**	364.6949			**710.3719**	355.6896	V	**1375.6852**	688.3462	1358.6587	679.8330	**1357.6746**	679.3410	13
8	**842.4254**	421.7164	825.3989	413.2031	824.4149	412.7111	N	**1276.6168**	638.8120	1259.5903	630.2988	1258.6062	629.8068	12
9	**1028.5047**	514.7560	**1011.4782**	506.2427	1010.4942	505.7507	W	**1162.5739**	581.7906	1145.5473	573.2773	1144.5633	572.7853	11
10	**1127.5732**	564.2902	**1110.5466**	**555.7769**	1109.5626	**555.2849**	V	**976.4946**	488.7509	959.4680	**480.2376**	958.4840	**479.7456**	10
11	**1242.6001**	621.8037	1225.5735	613.2904	**1224.5895**	612.7984	N	**877.4261**	439.2167	860.3996	430.7034	859.4156	430.2114	9
12	1299.6216	650.3144	1282.5950	641.8011	1281.6110	641.3091	G	**762.3992**	381.7032	745.3727	373.1900	744.3886	372.6980	8
13	1400.6692	700.8383	1383.6427	692.3250	1382.6587	691.8330	T	705.3777	353.1925	688.3512	344.6792	687.3672	344.1872	7
14	**1513.7533**	757.3803	1496.7267	748.8670	**1495.7427**	748.3750	L	604.3301	302.6687	587.3035	294.1554	586.3195	293.6634	6
15	**1600.7853**	800.8963	**1583.7588**	792.3830	**1582.7748**	791.8910	S	**491.2460**	246.1266	474.2195	237.6134	473.2354	237.1214	5
16	**1729.8279**	865.4176	1712.8014	856.9043	**1711.8174**	856.4123	E	**404.2140**	202.6106	387.1874	194.0974	386.2034	193.6053	4
17	**1800.8650**	900.9362	**1783.8385**	892.4229	**1782.8545**	891.9309	A	275.1714	138.0893	258.1448	129.5761			3
18	1857.8865	929.4469	**1840.8599**	920.9336	1839.8759	920.4416	G	204.1343	102.5708	187.1077	94.0575			2

19							K	147.1128	74.0600	130.0863	65.5468			1

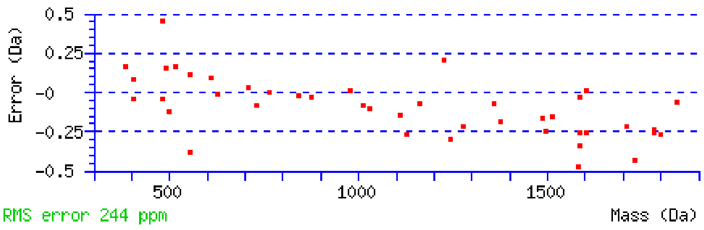

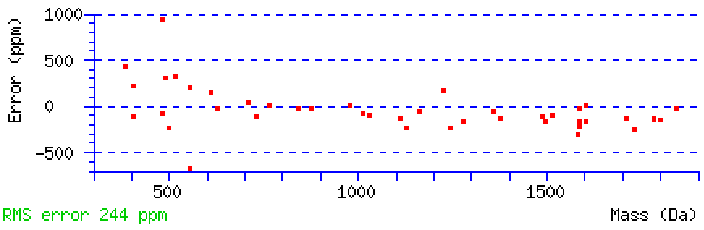

All matches to this query

Score	Mr(calc):	Delta	Sequence
56.8	2002.9847	0.0035	ATDDIIVNWVNGTLSEAGK
33.5	2002.9847	0.0035	ATDDIIVNWVNGTLSEAGK
20.7	2002.0007	0.9875	ATDDIIVNWVNGTLSEAGK
3.4	2003.0000	-0.0118	FTFFADVLSVDNREKSK
1.7	2001.0014	1.9869	LSNSNLLEELEELGRER
1.4	2001.0014	1.9869	LSNSNLLEELEELGRER
0.7	2000.9819	2.0063	AKLKQNTSEQFETVAVK
0.0	2000.9642	2.0241	QMKLENVSVALEFLER

Spectrum No: 369; Query: 915; Rank: 1

Peptide View

MS/MS Fragmentation of **VATNPQGLVGTENTTSEMDPSHR**
Found in **IPI00358014**, Tax_Id=10116 Gene_Symbol=Btd Biotinidase precursor

Match to Query 915: 2441.125062 from(814.715630,3+)
Title: 091127RatKid_SCX01_24.1938.1938.3.dta
Data file K:\NewmanPaper\Piliang\3SubProteomes\Piliang3SP\mgf5ppm\SCX_3SubProteomes5ppm.mgf

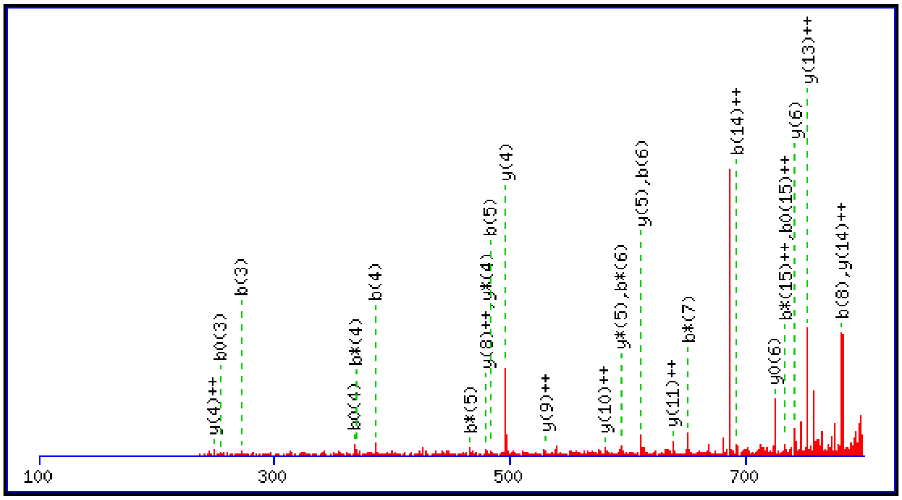

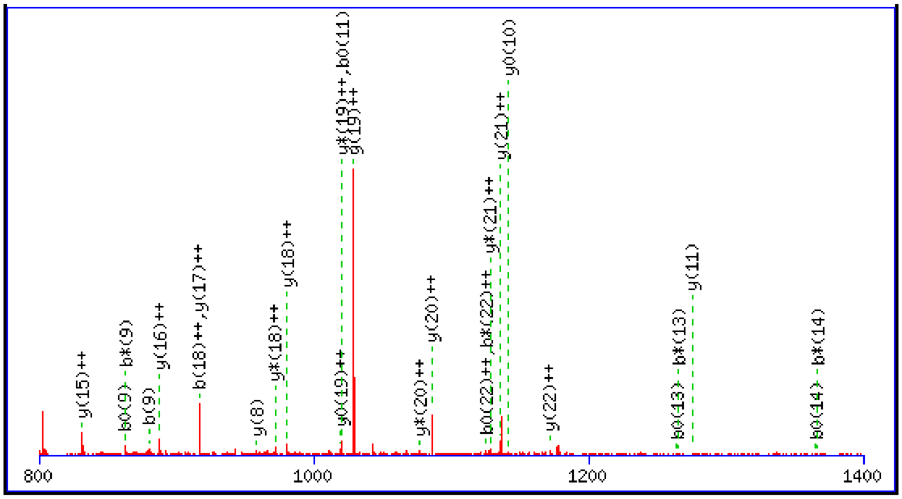

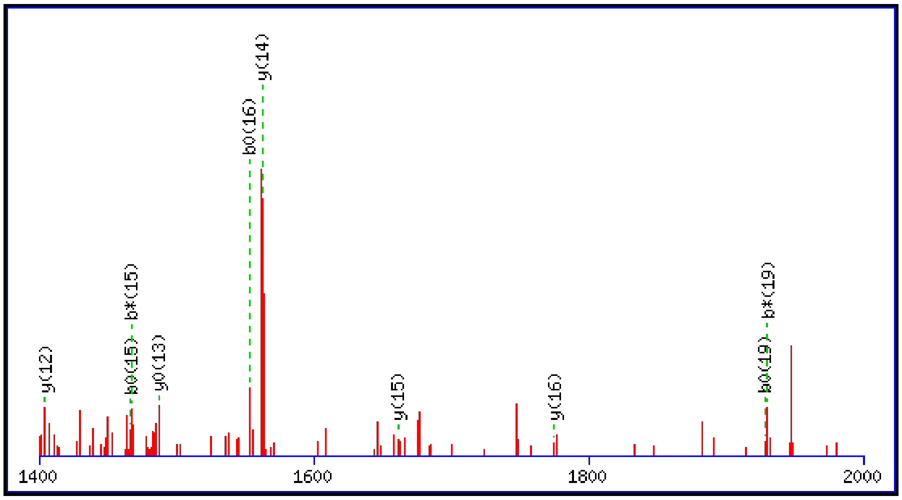

Monoisotopic mass of neutral peptide Mr(calc): 2441.1129
Fixed modifications: Carbamidomethyl (C)
Variable modifications:
N13 : Deamidated_N (N)
Ions Score: 57 **Expect:** 0.00066
Matches (Bold Red): 64/250 fragment ions using 154 most intense peaks

#	b	b^{++}	b*	b^{*++}	b^{0}	b^{0++}	Seq.	y	y^{++}	y*	y^{*++}	y^{0}	y^{0++}	#
1	100.0757	50.5415					V							23
2	171.1128	86.0600					A	2343.0517	**1172.0295**	2326.0252	1163.5162	2325.0412	1163.0242	22
3	**272.1605**	136.5839			**254.1499**	127.5786	T	2272.0146	**1136.5110**	2254.9881	**1127.9977**	2254.0041	1127.5057	21
4	**386.2034**	193.6053	**369.1769**	185.0921	**368.1928**	184.6001	N	2170.9669	**1085.9871**	2153.9404	**1077.4738**	2152.9564	1076.9818	20
5	**483.2562**	242.1317	**466.2296**	233.6185	465.2456	233.1264	P	2056.9240	**1028.9656**	2039.8975	**1020.4524**	2038.9135	**1019.9604**	19
6	**611.3148**	306.1610	**594.2882**	297.6477	593.3042	297.1557	Q	1959.8713	**980.4393**	1942.8447	**971.9260**	1941.8607	971.4340	18
7	668.3362	334.6717	**651.3097**	326.1585	650.3257	325.6665	G	1831.8127	**916.4100**	1814.7861	907.8967	1813.8021	907.4047	17
8	**781.4203**	391.2138	764.3937	382.7005	763.4097	382.2085	L	**1774.7912**	**887.8992**	1757.7647	879.3860	1756.7806	878.8940	16
9	**880.4887**	440.7480	**863.4621**	432.2347	**862.4781**	431.7427	V	**1661.7071**	**831.3572**	1644.6806	822.8439	1643.6966	822.3519	15
10	937.5102	469.2587	920.4836	460.7454	919.4996	460.2534	G	**1562.6387**	**781.8230**	1545.6122	773.3097	1544.6282	772.8177	14
11	1038.5578	519.7826	1021.5313	511.2693	**1020.5473**	510.7773	T	1505.6173	**753.3123**	1488.5907	744.7990	**1487.6067**	744.3070	13
12	1167.6004	584.3039	1150.5739	575.7906	1149.5899	575.2986	E	**1404.5696**	702.7884	1387.5430	694.2752	1386.5590	693.7832	12
13	1282.6274	641.8173	**1265.6008**	633.3040	**1264.6168**	632.8120	N	**1275.5270**	**638.2671**	1258.5005	629.7539	1257.5164	629.2619	11
14	1383.6750	**692.3412**	**1366.6485**	683.8279	**1365.6645**	683.3359	T	1160.5001	**580.7537**	1143.4735	572.2404	**1142.4895**	571.7484	10
15	1484.7227	742.8650	**1467.6962**	**734.3517**	**1466.7122**	**733.8597**	T	1059.4524	**530.2298**	1042.4258	521.7166	1041.4418	521.2245	9
16	1571.7548	786.3810	1554.7282	777.8677	**1553.7442**	777.3757	S	**958.4047**	**479.7060**	941.3782	471.1927	940.3941	470.7007	8
17	1700.7973	850.9023	1683.7708	842.3890	1682.7868	841.8970	E	871.3727	436.1900	854.3461	427.6767	853.3621	427.1847	7
18	1831.8378	**916.4226**	1814.8113	907.9093	1813.8273	907.4173	M	**742.3301**	371.6687	725.3035	363.1554	**724.3195**	362.6634	6

**19**	1946.8648	973.9360	**1929.8382**	965.4228	**1928.8542**	964.9307	**D**	**611.2896**	306.1484	**594.2630**	297.6352	593.2790	297.1432	**5**
**20**	2043.9175	1022.4624	2026.8910	1013.9491	2025.9070	1013.4571	**P**	**496.2627**	**248.6350**	**479.2361**	240.1217	478.2521	239.6297	**4**
**21**	2130.9496	1065.9784	2113.9230	1057.4651	2112.9390	1056.9731	**S**	399.2099	200.1086	382.1833	191.5953	381.1993	191.1033	**3**
**22**	2268.0085	1134.5079	2250.9819	**1125.9946**	2249.9979	**1125.5026**	**H**	312.1779	156.5926	295.1513	148.0793			**2**
**23**							**R**	175.1190	88.0631	158.0924	79.5498			**1**

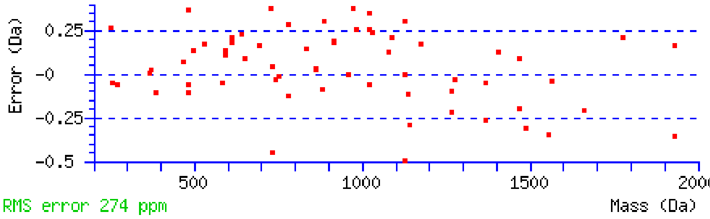

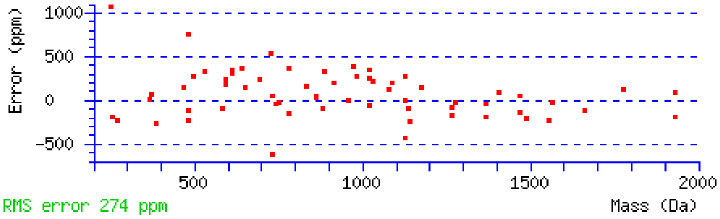

All matches to this query

Score	Mr(calc):	Delta	Sequence
56.6	2441.1129	0.0122	VATNPQGLVGTENTTSEMDPSHR
11.1	2441.1129	0.0122	VATNPQGLVGTENTTSEMDPSHR
9.4	2439.1365	1.9886	STNIIASFKNLVPQQATVIR
5.9	2440.1289	0.9962	VATNPQGLVGTENTTSEMDPSHR
4.9	2439.1365	1.9886	STNIIASFKNLVPQQATVIR
4.9	2440.1205	1.0045	STNIIASFKNLVPQQATVIR
4.3	2440.0993	1.0258	SGSAEPTNNHANGTQNKPRQPR
4.0	2440.1440	0.9810	DGIPLDPGSPRLEFLLEGSLR
4.0	2440.1205	1.0045	STNIIASFKNLVPQQATVIR
2.7	2441.1045	0.0205	STNIIASFKNLVPQQATVIR

Spectrum No: 370; Query: 96; Rank: 1

Peptide View

MS/MS Fragmentation of **VYVYAVNQTR**
Found in **IPI00324585**, Tax_Id=10116 Gene_Symbol=Itga1 Integrin alpha-1 precursor

Match to Query 96: 1212.617988 from(607.316270,2+)
Title: 091129RatKid_SCX02_13.954.954.2.dta
Data file K:\NewmanPaper\Piliang\3SubProteomes\Piliang3SP\mgf5ppm\SCX_3SubProteomes5ppm.mgf

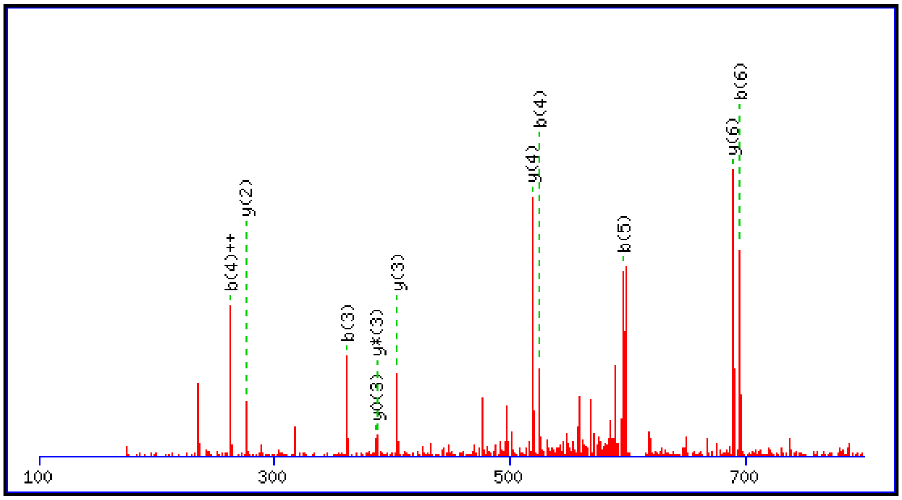

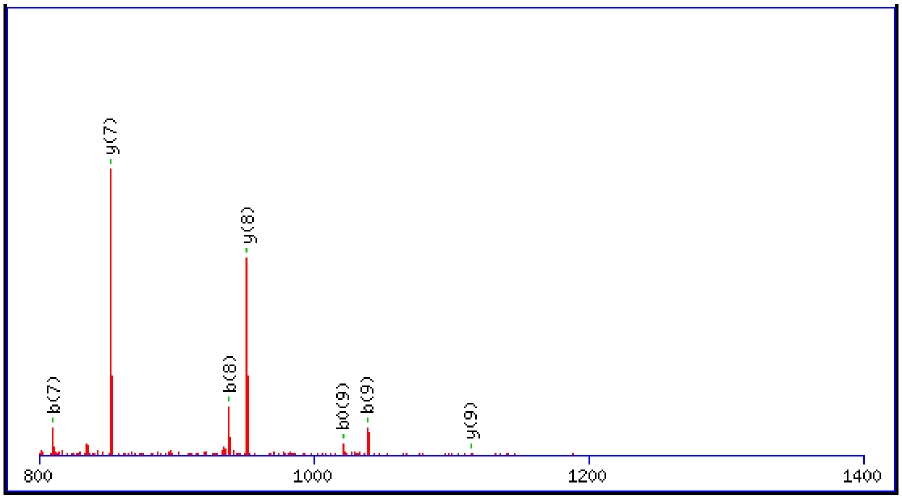

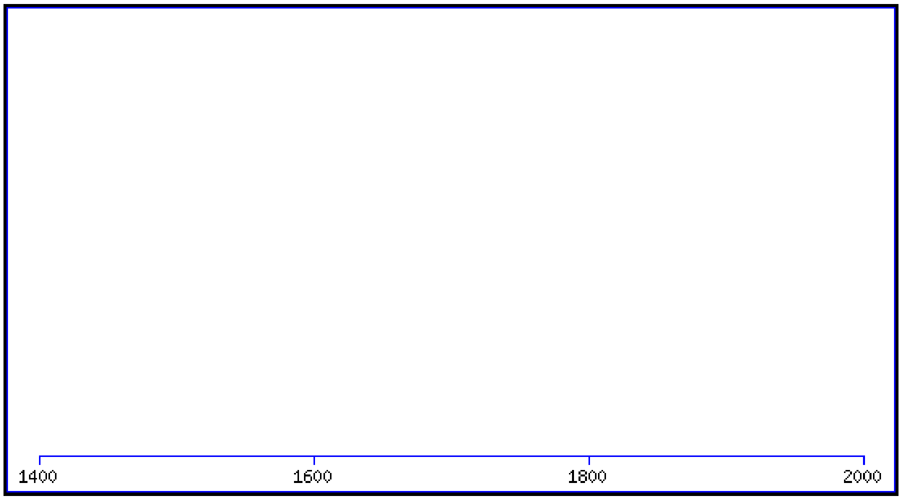

Monoisotopic mass of neutral peptide Mr(calc): 1212.6139
Fixed modifications: Carbamidomethyl (C)
Variable modifications:
N7 : Deamidated_N (N)
Ions Score: 56 **Expect:** 0.00022
Matches (Bold Red): 19/78 fragment ions using 41 most intense peaks

#	b	b^{++}	b*	b^{*++}	b^0	b^{0++}	Seq.	y	y^{++}	y*	y^{*++}	y^0	y^{0++}	#
1	100.0757	50.5415					V							10
2	**263.1390**	132.0731					Y	**1114.5527**	557.7800	1097.5262	549.2667	1096.5422	548.7747	9
3	**362.2074**	181.6074					V	**951.4894**	476.2483	934.4629	467.7351	933.4789	467.2431	8
4	**525.2708**	**263.1390**					Y	**852.4210**	426.7141	835.3945	418.2009	834.4104	417.7089	7
5	**596.3079**	298.6576					A	**689.3577**	345.1825	672.3311	336.6692	671.3471	336.1772	6
6	**695.3763**	348.1918					V	618.3206	309.6639	601.2940	301.1506	600.3100	300.6586	5
7	**810.4032**	405.7053	793.3767	397.1920			N	**519.2521**	260.1297	502.2256	251.6164	501.2416	251.1244	4
8	**938.4618**	469.7345	921.4353	461.2213			Q	**404.2252**	202.6162	**387.1987**	194.1030	**386.2146**	193.6110	3
9	**1039.5095**	520.2584	1022.4829	511.7451	**1021.4989**	511.2531	T	**276.1666**	138.5870	259.1401	130.0737	258.1561	129.5817	2
10							R	175.1190	88.0631	158.0924	79.5498			1

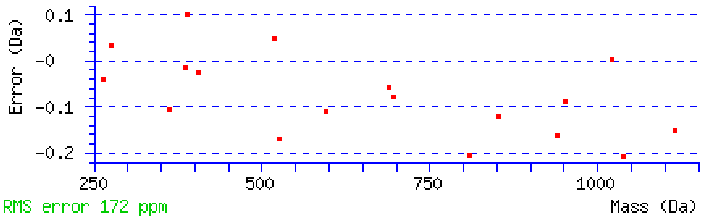

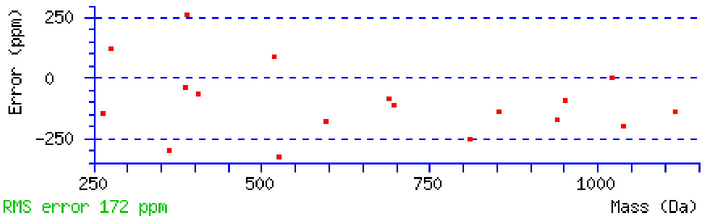

All matches to this query

Score	Mr(calc):	Delta	Sequence
56.3	1212.6139	0.0041	VYVYAVNQTR
21.3	1212.6268	-0.0088	VYVNGLISLR
6.8	1212.6073	0.0106	KFQYQLACR
5.1	1212.6251	-0.0071	KFSVEVHDPR
4.5	1212.6139	0.0041	LGEPQAGLWNK
4.3	1212.6145	0.0034	RCAHXEIKSR
3.9	1212.6098	0.0082	QAPPLSTANASR
2.9	1212.6210	-0.0031	SHLKTKNNNR
2.6	1210.6032	2.0148	MASIVPLKEK
2.3	1212.6139	0.0041	DYTGALAVFTR

Spectrum No: 371; Query: 947; Rank: 1

Peptide View

MS/MS Fragmentation of **RIYYSDFSNQTINSMAEDGSNR**
Found in **IPI00205325**, Tax_Id=10116 Gene_Symbol=Lrp2 Low-density lipoprotein receptor-related protein 2 precursor

Match to Query 947: 2568.122292 from(857.048040,3+)
Title: 091127RatKid_SCX01_23.2904.2904.3.dta
Data file K:\NewmanPaper\Piliang\3SubProteomes\Piliang3SP\mgf5ppm\SCX_3SubProteomes5ppm.mgf

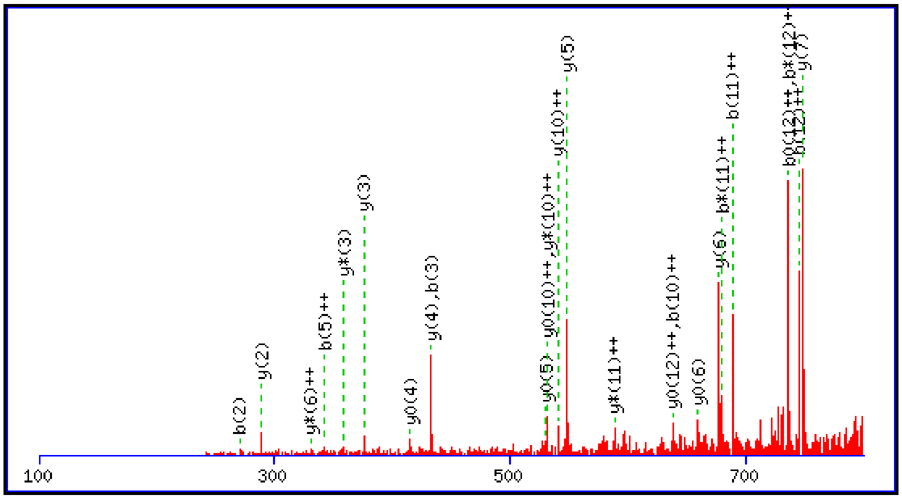

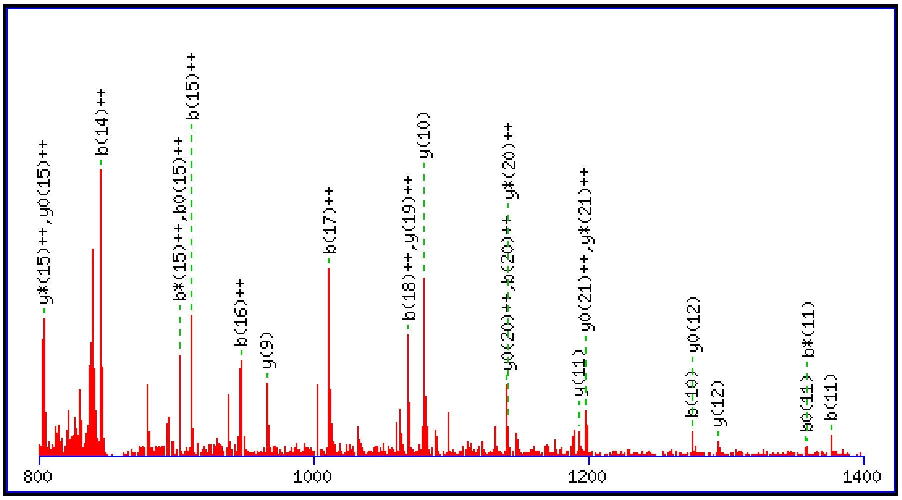

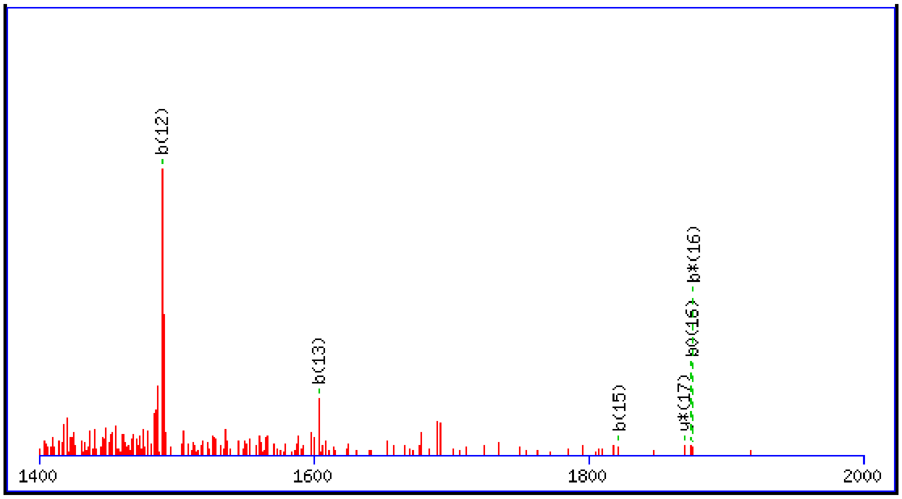

Monoisotopic mass of neutral peptide Mr(calc): 2568.1187
Fixed modifications: Carbamidomethyl (C)
Variable modifications:
N9 : Deamidated_N (N)
Ions Score: 56 **Expect:** 0.00084
Matches (Bold Red): 56/240 fragment ions using 80 most intense peaks

#	b	b^{++}	b*	b^{*++}	b^0	b^{0++}	Seq.	y	y^{++}	y*	y^{*++}	y^0	y^{0++}	#
1	157.1084	79.0578	140.0818	70.5446			R							22
2	**270.1925**	135.5999	253.1659	127.0866			I	2413.0249	1207.0161	2395.9983	**1198.5028**	2395.0143	**1198.0108**	21
3	**433.2558**	217.1315	416.2292	208.6183			Y	2299.9408	1150.4740	2282.9142	**1141.9608**	2281.9302	**1141.4687**	20
4	596.3191	298.6632	579.2926	290.1499			Y	2136.8775	**1068.9424**	2119.8509	1060.4291	2118.8669	1059.9371	19
5	683.3511	**342.1792**	666.3246	333.6659	665.3406	333.1739	S	1973.8141	987.4107	1956.7876	978.8974	1955.8036	978.4054	18
6	798.3781	399.6927	781.3515	391.1794	780.3675	390.6874	D	1886.7821	943.8947	**1869.7556**	935.3814	1868.7715	934.8894	17
7	945.4465	473.2269	928.4199	464.7136	927.4359	464.2216	F	1771.7552	886.3812	1754.7286	877.8679	1753.7446	877.3759	16
8	1032.4785	516.7429	1015.4520	508.2296	1014.4680	507.7376	S	1624.6867	812.8470	1607.6602	**804.3337**	1606.6762	**803.8417**	15
9	1147.5055	574.2564	1130.4789	565.7431	1129.4949	565.2511	N	1537.6547	769.3310	1520.6282	760.8177	1519.6442	760.3257	14
10	**1275.5640**	**638.2857**	1258.5375	629.7724	1257.5535	629.2804	Q	1422.6278	711.8175	1405.6012	703.3043	1404.6172	702.8122	13
11	**1376.6117**	**688.8095**	**1359.5852**	**680.2962**	**1358.6012**	679.8042	T	**1294.5692**	647.7882	1277.5427	639.2750	**1276.5586**	**638.7830**	12
12	**1489.6958**	**745.3515**	1472.6692	**736.8383**	1471.6852	**736.3462**	I	**1193.5215**	597.2644	1176.4950	**588.7511**	1175.5110	588.2591	11
13	**1603.7387**	802.2730	1586.7122	793.8597	1585.7281	793.3677	N	**1080.4375**	**540.7224**	1063.4109	**532.2091**	1062.4269	**531.7171**	10
14	1690.7707	**845.8890**	1673.7442	837.3757	1672.7602	836.8837	S	**966.3945**	483.7009	949.3680	475.1876	948.3840	474.6956	9
15	**1821.8112**	**911.4092**	1804.7847	**902.8960**	1803.8007	**902.4040**	M	879.3625	440.1849	862.3360	431.6716	861.3519	431.1796	8
16	1892.8483	**946.9278**	**1875.8218**	938.4145	**1874.8378**	937.9225	A	**748.3220**	374.6646	731.2955	366.1514	730.3115	365.6594	7
17	2021.8909	**1011.4491**	2004.8644	1002.9358	2003.8804	1002.4438	E	**677.2849**	339.1461	660.2584	**330.6328**	**659.2743**	330.1408	6
18	2136.9179	**1068.9626**	2119.8913	1060.4493	2118.9073	1059.9573	D	**548.2423**	274.6248	531.2158	266.1115	**530.2317**	265.6195	5
19	2193.9393	1097.4733	2176.9128	1088.9600	2175.9288	1088.4680	G	**433.2154**	217.1113	416.1888	208.5980	**415.2048**	208.1060	4
20	2280.9714	**1140.9893**	2263.9448	1132.4760	2262.9608	1131.9840	S	**376.1939**	188.6006	**359.1674**	180.0873	358.1833	179.5953	3
21	2395.0143	**1198.0108**	2377.9877	1189.4975	2377.0037	1189.0055	N	**289.1619**	145.0846	272.1353	136.5713			2
22							R	175.1190	88.0631	158.0924	79.5498			1

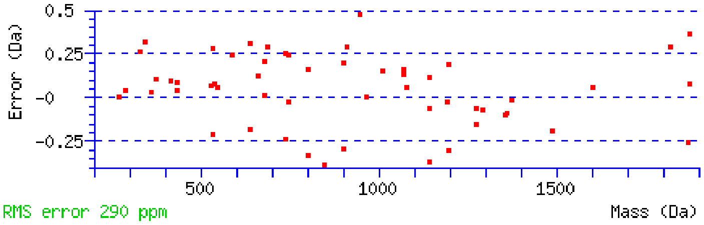

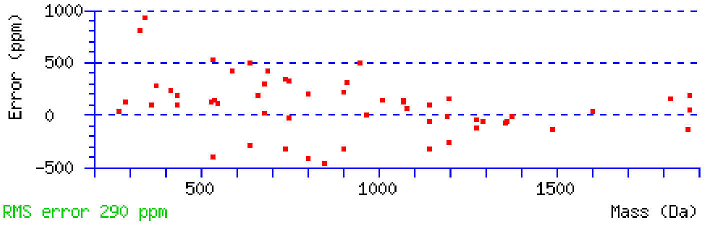

All matches to this query

Score	Mr(calc):	Delta	Sequence
56.2	2568.1187	0.0036	RIYYSDFSNQTINSMAEDGSNR
35.6	2568.1187	0.0036	RIYYSDFSNQTINSMAEDGSNR
31.9	2567.1346	0.9876	RIYYSDFSNQTINSMAEDGSNR
8.2	2566.1353	1.9869	LSGEGVGPVNISVVTNSLSSGYGGR
7.5	2568.1258	-0.0035	KGSVVNVNPTNTRAHSETPEIR
5.8	2566.1353	1.9869	LSGEGVGPVNISVVTNSLSSGYGGR
5.4	2567.1418	0.9805	KGSVVNVNPTNTRAHSETPEIR
5.4	2567.1418	0.9805	KGSVVNVNPTNTRAHSETPEIR
5.4	2566.1353	1.9869	LSGEGVGPVNISVVTNSLSSGYGGR
4.7	2566.1353	1.9869	LSGEGVGPVNISVVTNSLSSGYGGR

Spectrum No: 372; Query: 44; Rank: 1

Peptide View

MS/MS Fragmentation of **VWDTAAALNR**
Found in **IPI00363776**, Tax_Id=10116 Gene_Symbol=Ttyh3_predicted tweety homolog 3

Match to Query 44: 1116.556908 from(559.285730,2+)
Title: 091127RatKid_SCX01_13.1898.1898.2.dta
Data file K:\NewmanPaper\Piliang\3SubProteomes\Piliang3SP\mgf5ppm\SCX_3SubProteomes5ppm.mgf

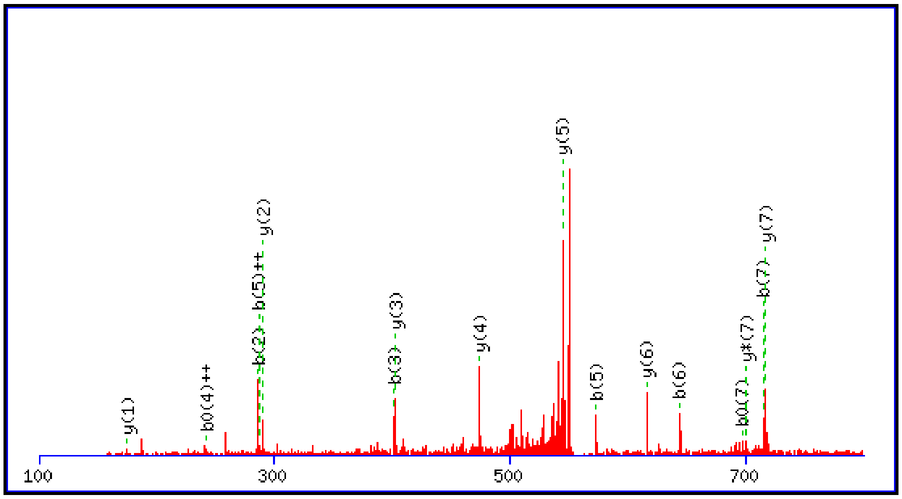

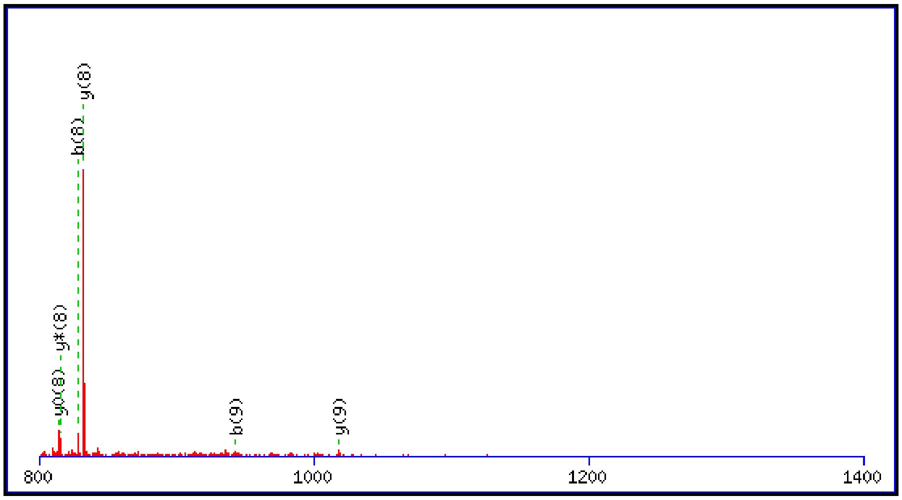

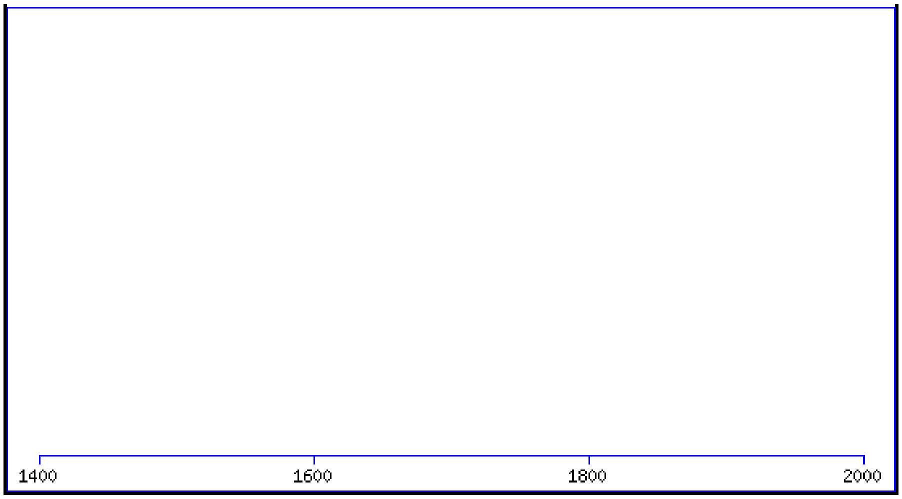

Monoisotopic mass of neutral peptide Mr(calc): 1116.5564
Fixed modifications: Carbamidomethyl (C)
Variable modifications:
N9 : Deamidated_N (N)
Ions Score: 56 **Expect:** 0.00026
Matches (Bold Red): 22/76 fragment ions using 55 most intense peaks

#	b	b^{++}	b*	b^{*++}	b^0	b^{0++}	Seq.	y	y^{++}	y*	y^{*++}	y^0	y^{0++}	#
1	100.0757	50.5415					V							10
2	**286.1550**	143.5811					W	**1018.4952**	509.7513	1001.4687	501.2380	1000.4847	500.7460	9
3	**401.1819**	201.0946			383.1714	192.0893	D	**832.4159**	416.7116	**815.3894**	408.1983	**814.4054**	407.7063	8
4	502.2296	251.6185			484.2191	**242.6132**	T	**717.3890**	359.1981	**700.3624**	350.6849	699.3784	350.1928	7
5	**573.2667**	**287.1370**			555.2562	278.1317	A	**616.3413**	308.6743	599.3147	300.1610			6
6	**644.3039**	322.6556			626.2933	313.6503	A	**545.3042**	273.1557	528.2776	264.6425			5
7	**715.3410**	358.1741			**697.3304**	349.1688	A	**474.2671**	237.6372	457.2405	229.1239			4
8	**828.4250**	414.7162			810.4145	405.7109	L	**403.2300**	202.1186	386.2034	193.6053			3
9	**943.4520**	472.2296	926.4254	463.7163	925.4414	463.2243	N	**290.1459**	145.5766	273.1193	137.0633			2
10							R	**175.1190**	88.0631	158.0924	79.5498			1

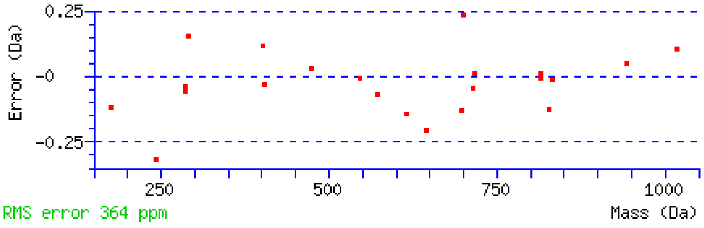

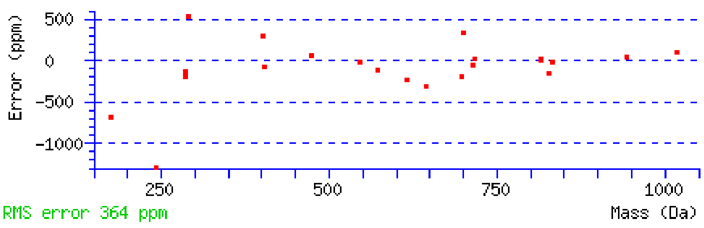

All matches to this query

Score	Mr(calc):	Delta	Sequence
56.2	1116.5564	0.0006	VWDTAAALNR
13.8	1116.5570	-0.0001	ERRMANNAR
10.0	1116.5523	0.0046	RQTEEAIDR
9.6	1115.5529	1.0040	HFTTFKKK
9.6	1115.5529	1.0040	HFTTFKKK
9.3	1116.5597	-0.0028	VANLCGINQK
7.3	1116.5676	-0.0107	WVRKEEDR
6.1	1116.5676	-0.0107	VGETYHQRK
4.8	1116.5458	0.0111	MRPNAKNNR
4.8	1116.5458	0.0111	MRPNAKNNR

Spectrum No: 373; Query: 34; Rank: 1

Peptide View

MS/MS Fragmentation of **AAENFTLLVK**
Found in **IPI00324987**, Tax_Id=10116 Gene_Symbol=P2rx4 P2X purinoceptor 4

Match to Query 34: 1105.603808 from(553.809180,2+)
Title: 091127RatKid_SCX01_12.2731.2731.2.dta
Data file K:\NewmanPaper\Piliang\3SubProteomes\Piliang3SP\mgf5ppm\SCX_3SubProteomes5ppm.mgf

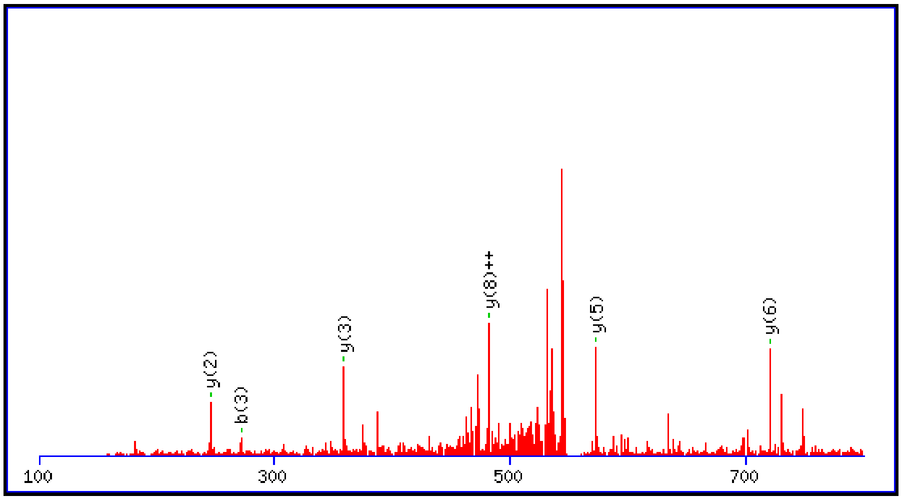

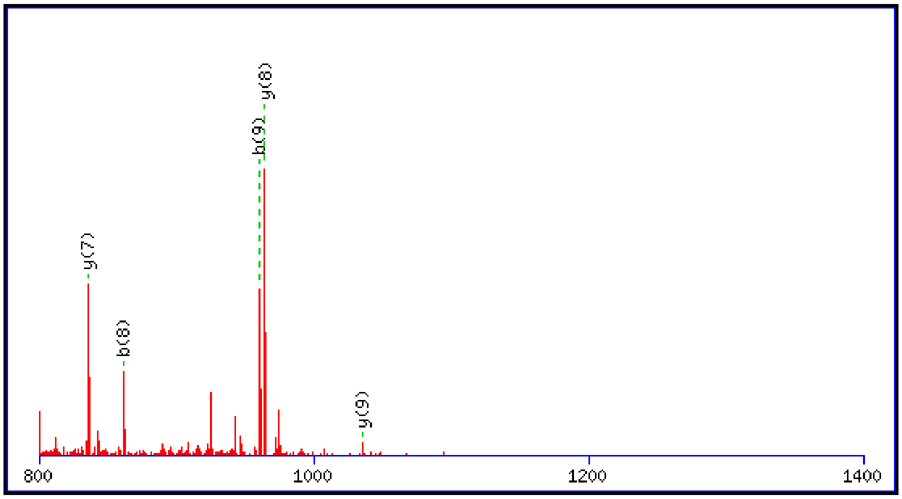

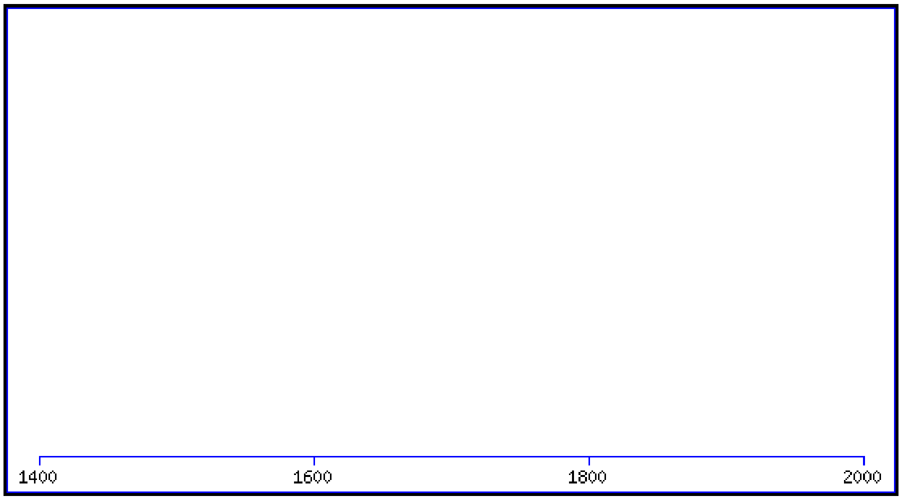

Monoisotopic mass of neutral peptide Mr(calc): 1105.6019

Fixed modifications: Carbamidomethyl (C)
Variable modifications:
N4 : Deamidated_N (N)
Ions Score: 56 **Expect:** 0.00019
Matches (Bold Red): 11/90 fragment ions using 28 most intense peaks

#	b	b^{++}	b*	b^{*++}	b^{0}	b^{0++}	Seq.	y	y^{++}	y*	y^{*++}	y^{0}	y^{0++}	#
1	72.0444	36.5258					A							10
2	143.0815	72.0444					A	**1035.5721**	518.2897	1018.5455	509.7764	1017.5615	509.2844	9
3	**272.1241**	136.5657			254.1135	127.5604	E	**964.5350**	**482.7711**	947.5084	474.2578	946.5244	473.7658	8
4	387.1510	194.0792	370.1245	185.5659	369.1405	185.0739	N	**835.4924**	418.2498	818.4658	409.7366	817.4818	409.2445	7
5	534.2194	267.6134	517.1929	259.1001	516.2089	258.6081	F	**720.4654**	360.7364	703.4389	352.2231	702.4549	351.7311	6
6	635.2671	318.1372	618.2406	309.6239	617.2566	309.1319	T	**573.3970**	287.2022	556.3705	278.6889	555.3865	278.1969	5
7	748.3512	374.6792	731.3246	366.1660	730.3406	365.6740	L	472.3493	236.6783	455.3228	228.1650			4
8	**861.4353**	431.2213	844.4087	422.7080	843.4247	422.2160	L	**359.2653**	180.1363	342.2387	171.6230			3
9	**960.5037**	480.7555	943.4771	472.2422	942.4931	471.7502	V	**246.1812**	123.5942	229.1547	115.0810			2
10							K	147.1128	74.0600	130.0863	65.5468			1

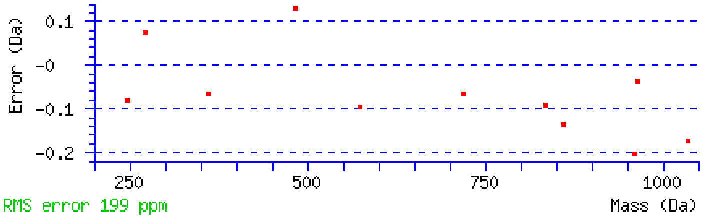

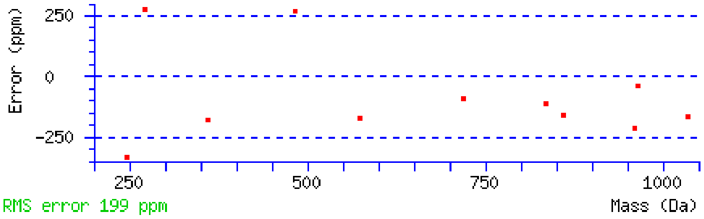

All matches to this query

Score	Mr(calc):	Delta	Sequence
56.0	1105.6019	0.0019	AAENFTLLVK
15.0	1105.6131	-0.0093	TGISKPYNVK
7.9	1103.5975	2.0063	GVSETVFLPR
7.7	1103.5863	2.0175	DPGTFDLLVK
6.8	1105.6009	0.0029	ARLKTNPVK
6.0	1105.5954	0.0084	MFLNLLSPR
4.7	1105.6131	-0.0093	TFKEVANAVK
4.5	1105.5979	0.0059	KETNSKATVK
3.6	1105.6148	-0.0110	TIPLKDVLK
3.5	1105.6131	-0.0093	RDLSFLLDK

Spectrum No: 374; Query: 384; Rank: 1

Peptide View

MS/MS Fragmentation of **NNVTPDMMEEMYK**
Found in **IPI00230914**, Tax_Id=10116 Gene_Symbol=Rpl5 60S ribosomal protein L5

Match to Query 384: 1601.643528 from(801.829040,2+)
Title: 091129RatKid_SCX02_14.2943.2943.2.dta
Data file K:\NewmanPaper\Piliang\3SubProteomes\Piliang3SP\mgf5ppm\SCX_3SubProteomes5ppm.mgf

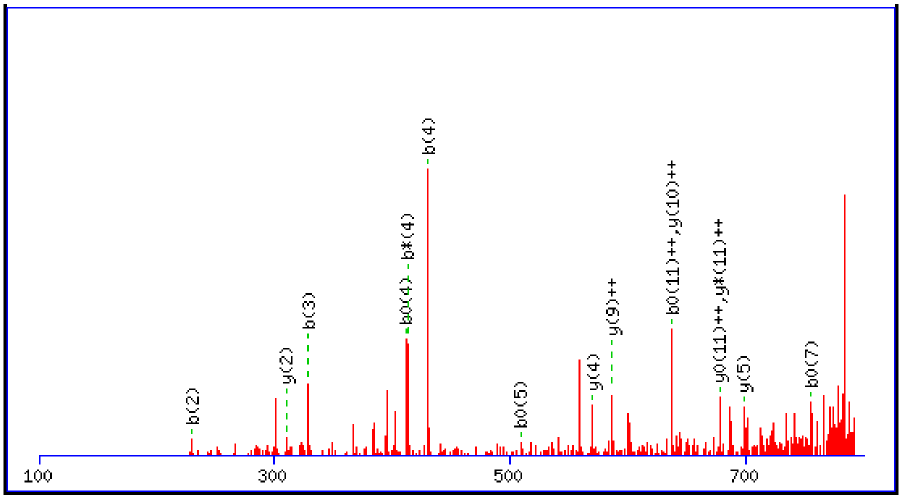

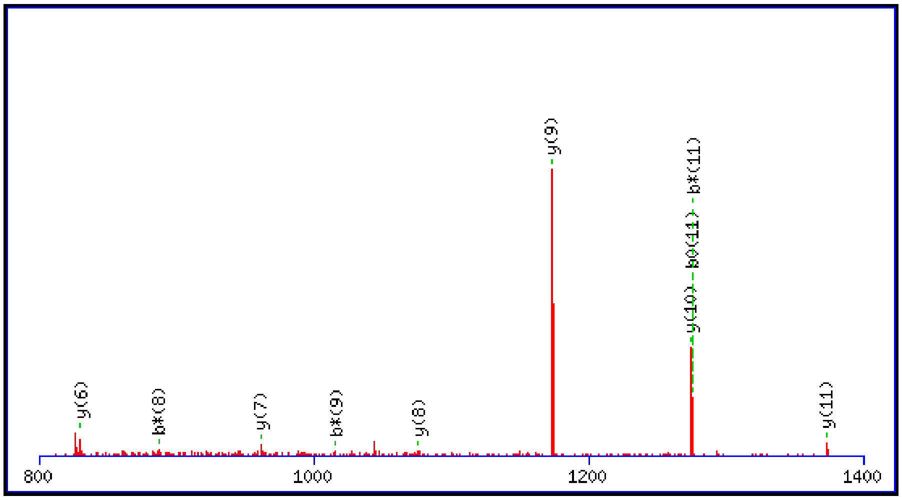

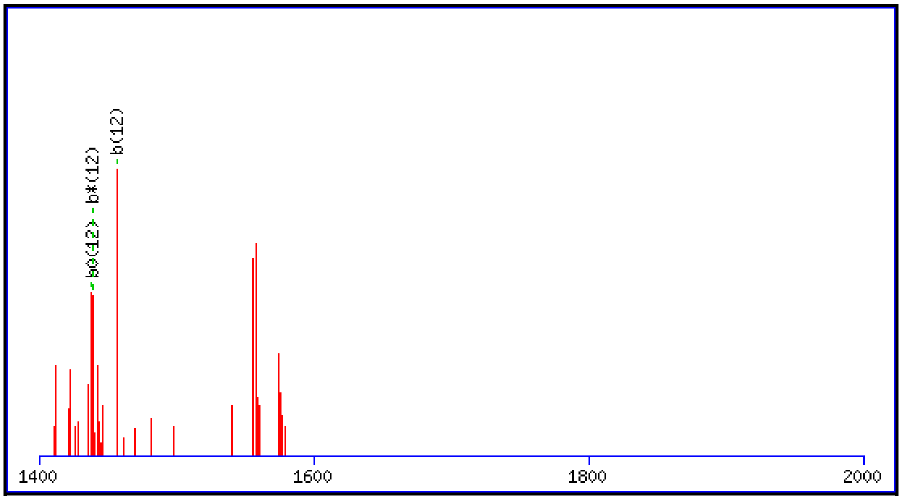

Monoisotopic mass of neutral peptide Mr(calc): 1601.6411
Fixed modifications: Carbamidomethyl (C)
Variable modifications:
N2 : Deamidated_N (N)
Ions Score: 56 **Expect:** 0.0003
Matches (Bold Red): 28/132 fragment ions using 48 most intense peaks

#	b	b^{++}	b*	b^{*++}	b^{0}	b^{0++}	Seq.	y	y^{++}	y*	y^{*++}	y^{0}	y^{0++}	#
1	115.0502	58.0287	98.0237	49.5155			N							13
2	**230.0771**	115.5422	213.0506	107.0289			N	1488.6055	744.8064	1471.5790	736.2931	1470.5949	735.8011	12
3	**329.1456**	165.0764	312.1190	156.5631			V	**1373.5786**	687.2929	1356.5520	**678.7797**	1355.5680	**678.2876**	11
4	**430.1932**	215.6003	**413.1667**	207.0870	**412.1827**	206.5950	T	**1274.5102**	**637.7587**	1257.4836	629.2454	1256.4996	628.7534	10
5	527.2460	264.1266	510.2194	255.6134	**509.2354**	255.1214	P	**1173.4625**	**587.2349**	1156.4359	578.7216	1155.4519	578.2296	9

**6**	642.2729	321.6401	625.2464	313.1268	624.2624	312.6348	**D**	**1076.4097**	538.7085	1059.3832	530.1952	1058.3992	529.7032	**8**
**7**	773.3134	387.1604	756.2869	378.6471	**755.3029**	378.1551	**M**	**961.3828**	481.1950	944.3562	472.6818	943.3722	472.1897	**7**
**8**	904.3539	452.6806	**887.3274**	444.1673	886.3433	443.6753	**M**	**830.3423**	415.6748	813.3157	407.1615	812.3317	406.6695	**6**
**9**	1033.3965	517.2019	**1016.3700**	508.6886	1015.3859	508.1966	**E**	**699.3018**	350.1545	682.2753	341.6413	681.2912	341.1493	**5**
**10**	1162.4391	581.7232	1145.4125	573.2099	1144.4285	572.7179	**E**	**570.2592**	285.6332	553.2327	277.1200	552.2486	276.6280	**4**
**11**	1293.4796	647.2434	**1276.4530**	638.7302	**1275.4690**	**638.2381**	**M**	441.2166	221.1119	424.1901	212.5987			**3**
**12**	**1456.5429**	728.7751	**1439.5164**	720.2618	**1438.5323**	719.7698	**Y**	**310.1761**	155.5917	293.1496	147.0784			**2**
**13**							**K**	147.1128	74.0600	130.0863	65.5468			**1**

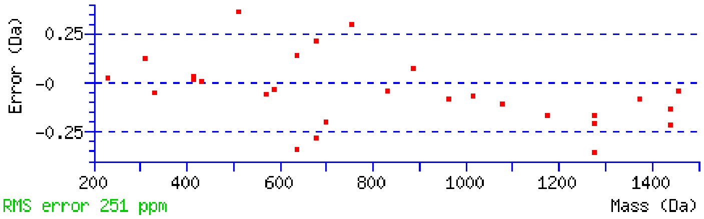

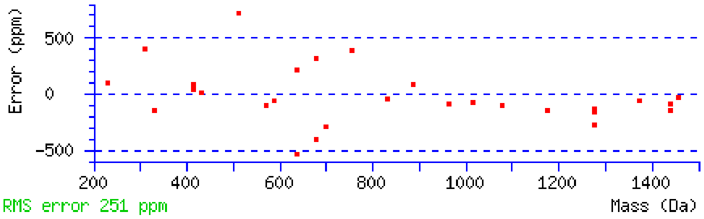

All matches to this query

Score	Mr(calc):	Delta	Sequence
55.9	1601.6411	0.0024	NNVTPDMMEEMYK
55.9	1601.6411	0.0024	NNVTPDMMEEMYK
8.8	1601.6480	-0.0045	CSCREGWVGNGIK
4.0	1600.6440	0.9995	LPMSSTGDNSELDR
3.7	1601.6490	-0.0055	HYEDNCISYCIK
2.9	1600.6245	1.0191	LNAMNISETRYK
2.6	1600.6436	0.9999	GDFTRLEYERR
2.6	1600.6283	1.0152	QTTRASESYKDR
2.2	1601.6528	-0.0093	TGDRVTFGVFESK
2.0	1601.6334	0.0101	GTFHWSGNEQMTK

Spectrum No: 375; Query: 1105; Rank: 1

Peptide View

MS/MS Fragmentation of **MTVAHMWFDNQIHEADTTENQSGVSFDK**
Found in **IPI00326305**, Tax_Id=10116 Gene_Symbol=Atp1a1 Sodium/potassium-transporting ATPase subunit alpha-1 precursor

Match to Query 1105: 3238.406712 from(1080.476180,3+)
Title: 091127RatKid_SCX01_32.4411.4411.3.dta
Data file K:\NewmanPaper\Piliang\3SubProteomes\Piliang3SP\mgf5ppm\SCX_3SubProteomes5ppm.mgf

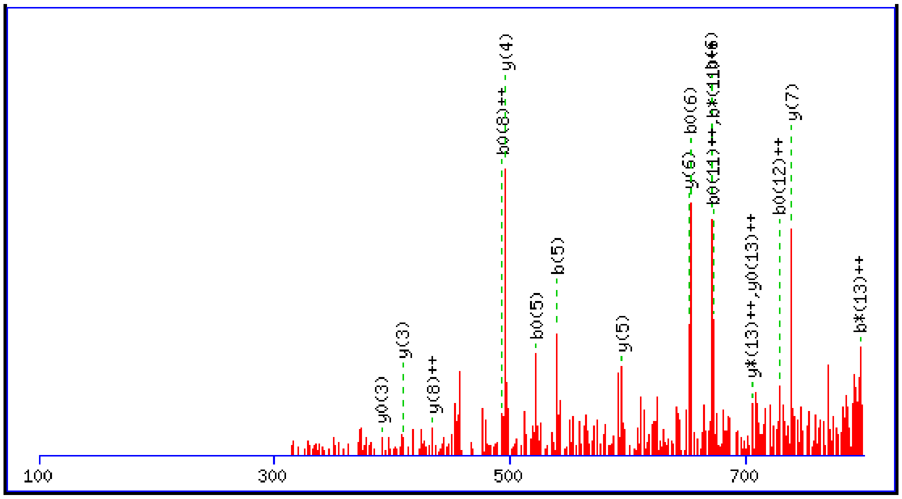

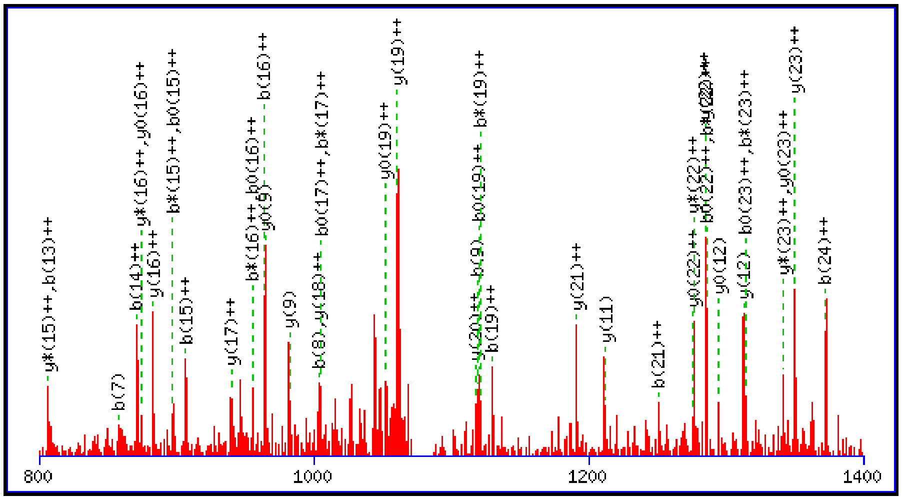

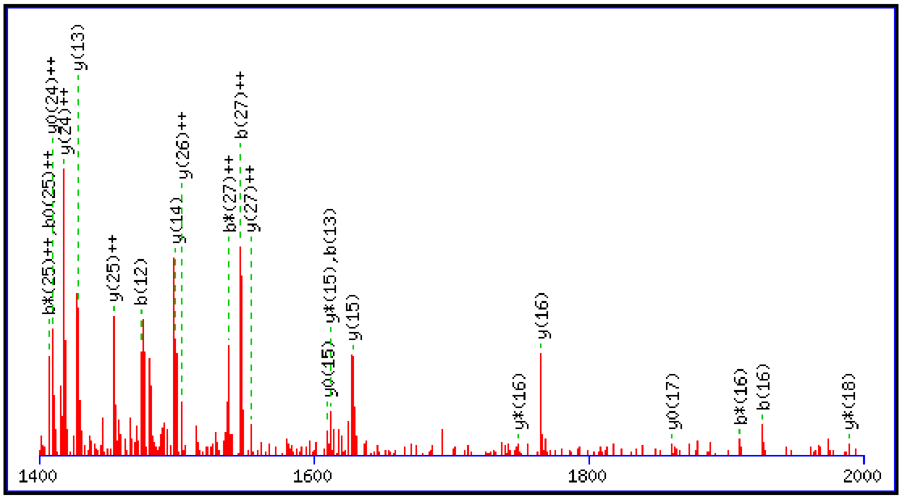

Monoisotopic mass of neutral peptide Mr(calc): 3238.3972
Fixed modifications: Carbamidomethyl (C)
Variable modifications:
N20 : Deamidated_N (N)
Ions Score: 56 **Expect:** 0.0011
Matches (Bold Red): 83/302 fragment ions using 178 most intense peaks

#	b	b^{++}	b*	b^{*++}	b^0	b^{0++}	Seq.	y	y^{++}	y*	y^{*++}	y^0	y^{0++}	#
1	132.0478	66.5275					M							28
2	233.0954	117.0514			215.0849	108.0461	T	3108.3640	**1554.6856**	3091.3374	1546.1723	3090.3534	1545.6803	27
3	332.1639	166.5856			314.1533	157.5803	V	3007.3163	**1504.1618**	2990.2897	1495.6485	2989.3057	1495.1565	26
4	403.2010	202.1041			385.1904	193.0988	A	2908.2479	**1454.6276**	2891.2213	1446.1143	2890.2373	1445.6223	25
5	**540.2599**	270.6336			**522.2493**	261.6283	H	2837.2108	**1419.1090**	2820.1842	1410.5957	2819.2002	**1410.1037**	24

6	671.3004	336.1538			653.2898	327.1485	M	2700.1518	1350.5796	2683.1253	1342.0663	2682.1413	1341.5743	23
7	857.3797	429.1935			839.3691	420.1882	W	2569.1114	1285.0593	2552.0848	1276.5460	2551.1008	1276.0540	22
8	1004.4481	502.7277			986.4375	493.7224	F	2383.0320	1192.0197	2366.0055	1183.5064	2365.0215	1183.0144	21
9	1119.4750	560.2412			1101.4645	551.2359	D	2235.9636	1118.4855	2218.9371	1109.9722	2217.9531	1109.4802	20
10	1233.5180	617.2626	1216.4914	608.7493	1215.5074	608.2573	N	2120.9367	1060.9720	2103.9101	1052.4587	2102.9261	1051.9667	19
11	1361.5765	681.2919	1344.5500	672.7786	1343.5660	672.2866	Q	2006.8938	1003.9505	1989.8672	995.4372	1988.8832	994.9452	18
12	1474.6606	737.8339	1457.6341	729.3207	1456.6500	728.8287	I	1878.8352	939.9212	1861.8086	931.4080	1860.8246	930.9159	17
13	1611.7195	806.3634	1594.6930	797.8501	1593.7090	797.3581	H	1765.7511	883.3792	1748.7246	874.8659	1747.7406	874.3739	16
14	1740.7621	870.8847	1723.7356	862.3714	1722.7515	861.8794	E	1628.6922	814.8497	1611.6657	806.3365	1610.6816	805.8445	15
15	1811.7992	906.4032	1794.7727	897.8900	1793.7887	897.3980	A	1499.6496	750.3284	1482.6231	741.8152	1481.6391	741.3232	14
16	1926.8262	963.9167	1909.7996	955.4034	1908.8156	954.9114	D	1428.6125	714.8099	1411.5860	706.2966	1410.6019	705.8046	13
17	2027.8738	1014.4406	2010.8473	1005.9273	2009.8633	1005.4353	T	1313.5856	657.2964	1296.5590	648.7831	1295.5750	648.2911	12
18	2128.9215	1064.9644	2111.8950	1056.4511	2110.9110	1055.9591	T	1212.5379	606.7726	1195.5113	598.2593	1194.5273	597.7673	11
19	2257.9641	1129.4857	2240.9376	1120.9724	2239.9536	1120.4804	E	1111.4902	556.2487	1094.4637	547.7355	1093.4796	547.2435	10
20	2372.9911	1186.9992	2355.9645	1178.4859	2354.9805	1177.9939	N	982.4476	491.7274	965.4211	483.2142	964.4370	482.7222	9
21	2501.0496	1251.0285	2484.0231	1242.5152	2483.0391	1242.0232	Q	867.4207	434.2140	850.3941	425.7007	849.4101	425.2087	8
22	2588.0817	1294.5445	2571.0551	1286.0312	2570.0711	1285.5392	S	739.3621	370.1847	722.3355	361.6714	721.3515	361.1794	7
23	2645.1031	1323.0552	2628.0766	1314.5419	2627.0926	1314.0499	G	652.3301	326.6687	635.3035	318.1554	634.3195	317.6634	6
24	2744.1715	1372.5894	2727.1450	1364.0761	2726.1610	1363.5841	V	595.3086	298.1579	578.2821	289.6447	577.2980	289.1527	5
25	2831.2036	1416.1054	2814.1770	1407.5921	2813.1930	1407.1001	S	496.2402	248.6237	479.2136	240.1105	478.2296	239.6184	4
26	2978.2720	1489.6396	2961.2454	1481.1264	2960.2614	1480.6343	F	409.2082	205.1077	392.1816	196.5944	391.1976	196.1024	3
27	3093.2989	1547.1531	3076.2724	1538.6398	3075.2884	1538.1478	D	262.1397	131.5735	245.1132	123.0602	244.1292	122.5682	2
28							K	147.1128	74.0600	130.0863	65.5468			1

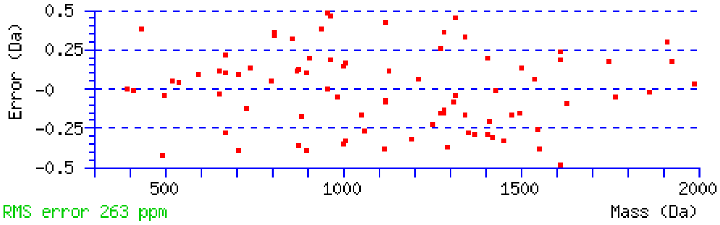

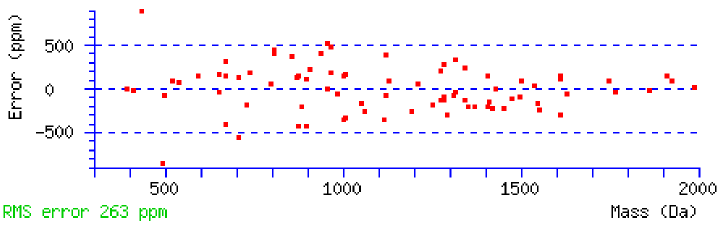

All matches to this query

Score	Mr(calc):	Delta	Sequence
55.8	3238.3972	0.0095	MTVAHMWFDNQIHEADTTENQSGVSFDK
54.4	3238.3972	0.0095	MTVAHMWFDNQIHEADTTENQSGVSFDK
51.7	3237.4132	0.9935	MTVAHMWFDNQIHEADTTENQSGVSFDK
8.5	3238.4229	-0.0162	QFGMMQSQIGYDILAEDTITALLHNNR
4.9	3237.4063	1.0004	KRSGSLPVLGTLQVSFSHSQVSQYGQK
1.9	3237.4063	1.0004	KRSGSLPVLGTLQVSFSHSQVSQYGQK
1.7	3238.4328	-0.0261	AENIMLLELDMPSRTTRDYEGPAISPK
1.7	3238.4328	-0.0261	AENIMLLELDMPSRTTRDYEGPAISPK
1.7	3238.4328	-0.0261	AENIMLLELDMPSRTTRDYEGPAISPK
0.8	3238.4093	-0.0026	ESGAADKLEAAHAPAESAMMENVDQAMTRK

Spectrum No: 376; Query: 215; Rank: 1

Peptide View

MS/MS Fragmentation of **FQLLNFSSSELK**

Found in **IPI00464765**, Tax_Id=10116 Gene_Symbol=Igsf4a Immunoglobulin superfamily, member 4A

Match to Query 215: 1412.720228 from(707.367390,2+)
Title: 091127RatKid_SCX01_02.4260.4260.2.dta
Data file K:\NewmanPaper\Piliang\3SubProteomes\Piliang3SP\mgf5ppm\SCX_3SubProteomes5ppm.mgf

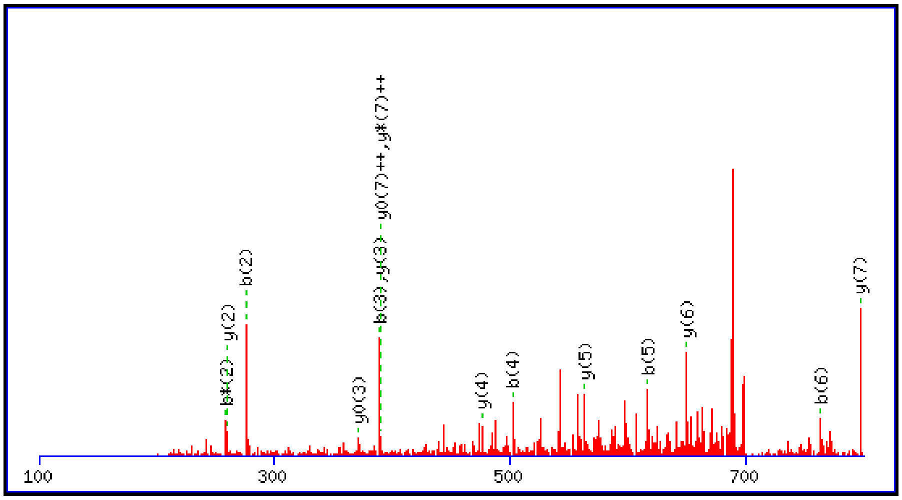

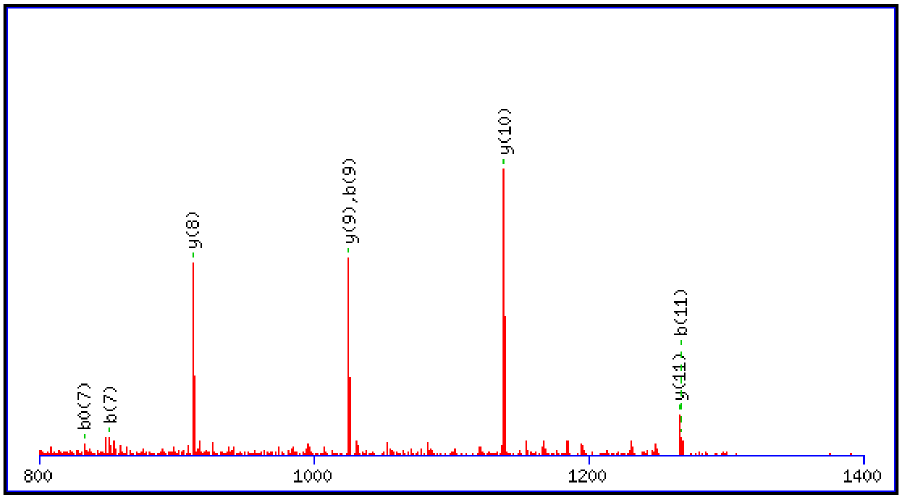

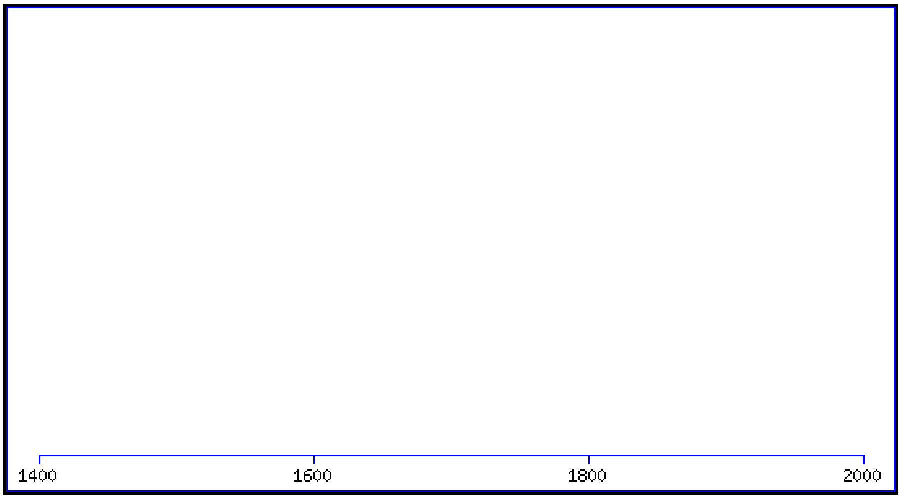

Monoisotopic mass of neutral peptide Mr(calc): 1412.7187
Fixed modifications: Carbamidomethyl (C)
Variable modifications:
N5 : Deamidated_N (N)
Ions Score: 56 **Expect:** 0.00032
Matches (Bold Red): 23/114 fragment ions using 58 most intense peaks

#	b	b^{++}	b*	b^{*++}	b^0	b^{0++}	Seq.	y	y^{++}	y*	y^{*++}	y^0	y^{0++}	#
1	148.0757	74.5415					F							12

**2**	**276.1343**	138.5708	**259.1077**	130.0575			**Q**	**1266.6576**	633.8324	1249.6311	625.3192	1248.6470	624.8272	**11**
**3**	**389.2183**	195.1128	372.1918	186.5995			**L**	**1138.5990**	569.8031	1121.5725	561.2899	1120.5885	560.7979	**10**
**4**	**502.3024**	251.6548	485.2758	243.1416			**L**	**1025.5150**	513.2611	1008.4884	504.7478	1007.5044	504.2558	**9**
**5**	**617.3293**	309.1683	600.3028	300.6550			**N**	**912.4309**	456.7191	895.4043	448.2058	894.4203	447.7138	**8**
**6**	**764.3977**	382.7025	747.3712	374.1892			**F**	**797.4040**	399.2056	780.3774	**390.6923**	779.3934	**390.2003**	**7**
**7**	**851.4298**	426.2185	834.4032	417.7053	**833.4192**	417.2132	**S**	**650.3355**	325.6714	633.3090	317.1581	632.3250	316.6661	**6**
**8**	938.4618	469.7345	921.4353	461.2213	920.4512	460.7293	**S**	**563.3035**	282.1554	546.2770	273.6421	545.2930	273.1501	**5**
**9**	**1025.4938**	513.2506	1008.4673	504.7373	1007.4833	504.2453	**S**	**476.2715**	238.6394	459.2449	230.1261	458.2609	229.6341	**4**
**10**	1154.5364	577.7718	1137.5099	569.2586	1136.5259	568.7666	**E**	**389.2395**	195.1234	372.2129	186.6101	**371.2289**	186.1181	**3**
**11**	**1267.6205**	634.3139	1250.5939	625.8006	1249.6099	625.3086	**L**	**260.1969**	130.6021	243.1703	122.0888			**2**
**12**							**K**	147.1128	74.0600	130.0863	65.5468			**1**

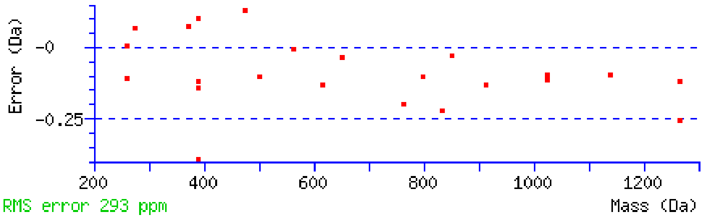

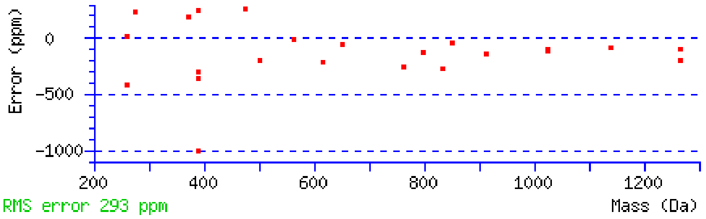

All matches to this query

Score	Mr(calc):	Delta	Sequence
55.8	1412.7187	0.0015	FQLLNFSSSELK
25.4	1411.7307	0.9896	NDPNQLNNLIKK
25.4	1411.7307	0.9896	NDPNQLNNLIKK
25.4	1411.7307	0.9896	NDPNQLNNLIKK
22.3	1411.7152	1.0050	YYYVKNIIKK
22.3	1411.7152	1.0050	YYYVKNIIKK
22.3	1411.7152	1.0050	YYYVKNIIKK
20.2	1412.7156	0.0047	CMLLAHADLIEK
18.2	1412.7177	0.0025	KFNATERVLQK
15.8	1412.7147	0.0056	NDPNQLNNLIKK

Spectrum No: 377; Query: 319; Rank: 1

Peptide View

MS/MS Fragmentation of **IKLDNYSTQELGR**
Found in **IPI00204348**, Tax_Id=10116 Gene_Symbol=Nt5e 5'-nucleotidase precursor

Match to Query 319: 1536.782868 from(769.398710,2+)
Title: 091127RatKid_SCX01_22.1923.1923.2.dta
Data file K:\NewmanPaper\Piliang\3SubProteomes\Piliang3SP\mgf5ppm\SCX_3SubProteomes5ppm.mgf

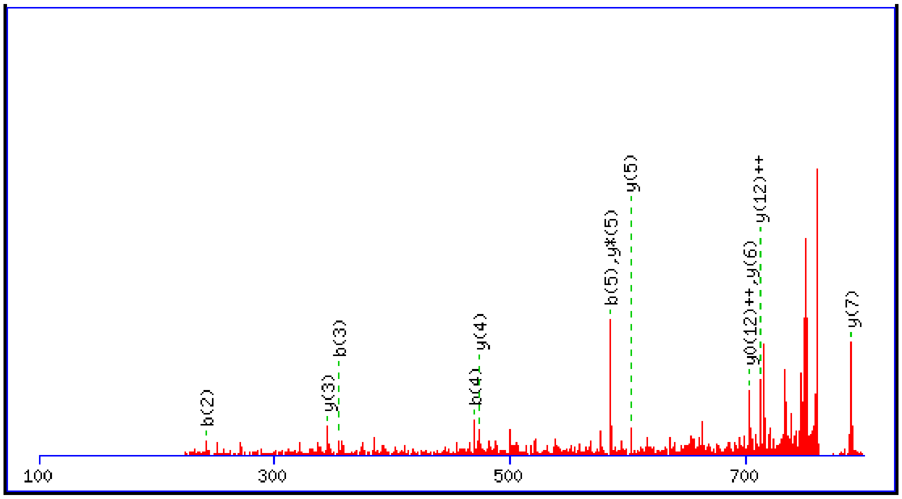

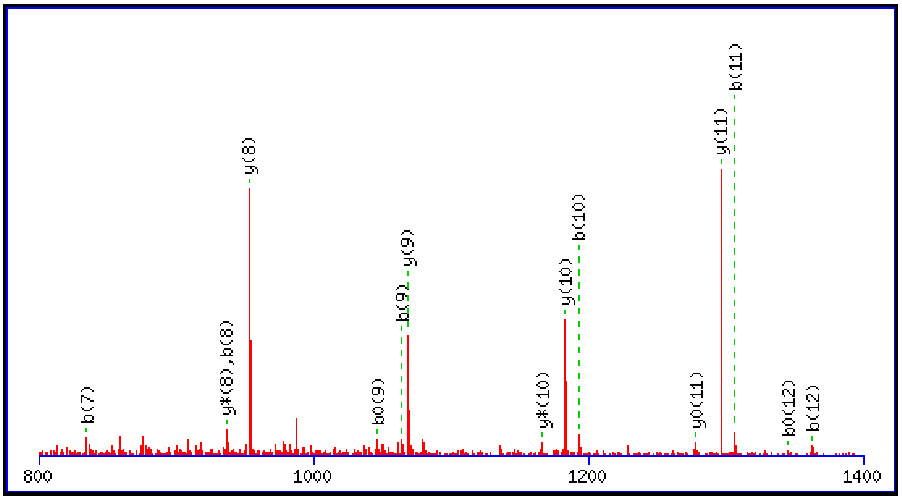

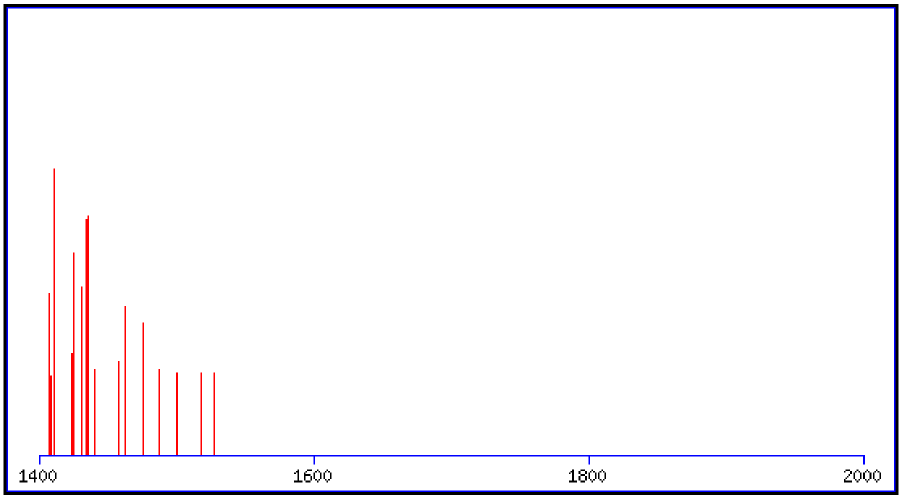

Monoisotopic mass of neutral peptide Mr(calc): 1536.7784
Fixed modifications: Carbamidomethyl (C)
Variable modifications:
N5 : Deamidated_N (N)
Ions Score: 56 **Expect:** 0.00037
Matches (Bold Red): 27/130 fragment ions using 69 most intense peaks

#	b	b^{++}	b*	b^{*++}	b^{0}	b^{0++}	Seq.	y	y^{++}	y*	y^{*++}	y^{0}	y^{0++}	#
1	114.0913	57.5493					I							13
2	**242.1863**	121.5968	225.1598	113.0835			K	1424.7016	**712.8544**	1407.6750	704.3412	1406.6910	**703.8492**	12
3	**355.2704**	178.1388	338.2438	169.6255			L	**1296.6066**	648.8070	1279.5801	640.2937	**1278.5961**	639.8017	11
4	**470.2973**	235.6523	453.2708	227.1390	452.2867	226.6470	D	**1183.5226**	592.2649	**1166.4960**	583.7516	1165.5120	583.2596	10
5	**585.3242**	293.1658	568.2977	284.6525	567.3137	284.1605	N	**1068.4956**	534.7514	1051.4691	526.2382	1050.4851	525.7462	9

6	748.3876	374.6974	731.3610	366.1842	730.3770	365.6921	Y	**953.4687**	477.2380	**936.4421**	468.7247	935.4581	468.2327	8
7	**835.4196**	418.2134	818.3931	409.7002	817.4090	409.2082	S	**790.4054**	395.7063	773.3788	387.1930	772.3948	386.7010	7
8	**936.4673**	468.7373	919.4407	460.2240	918.4567	459.7320	T	**703.3733**	352.1903	686.3468	343.6770	685.3628	343.1850	6
9	**1064.5259**	532.7666	1047.4993	524.2533	**1046.5153**	523.7613	Q	**602.3257**	301.6665	**585.2991**	293.1532	584.3151	292.6612	5
10	**1193.5685**	597.2879	1176.5419	588.7746	1175.5579	588.2826	E	**474.2671**	237.6372	457.2405	229.1239	456.2565	228.6319	4
11	**1306.6525**	653.8299	1289.6260	645.3166	1288.6420	644.8246	L	**345.2245**	173.1159	328.1979	164.6026			3
12	**1363.6740**	682.3406	1346.6474	673.8274	**1345.6634**	673.3353	G	232.1404	116.5738	215.1139	108.0606			2
13							R	175.1190	88.0631	158.0924	79.5498			1

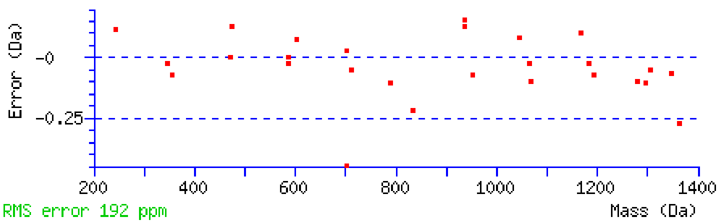

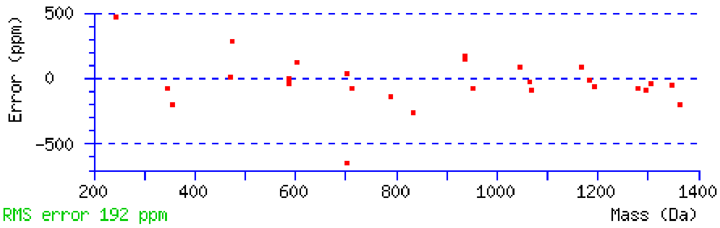

All matches to this query

Score	Mr(calc):	Delta	Sequence
55.8	1536.7784	0.0045	IKLDNYSTQELGR
12.5	1535.7943	0.9885	IKLDNYSTQELGR
5.9	1536.7814	0.0015	KLYVSNLGIGHTR
5.9	1536.7814	0.0015	KLYVSNLGIGHTR
3.8	1536.7719	0.0110	EEPIIVHCSAGIGR
3.7	1536.7752	0.0077	IIKNTAMMREAAR
0.9	1536.7757	0.0072	RSGYGVSQRSNGLR

Spectrum No: 378; Query: 840; Rank: 1

Peptide View

MS/MS Fragmentation of **VTNLTQEQCQDPSKVPNESK**
Found in **IPI00192495**, Tax_Id=10116 Gene_Symbol=Ncstn Isoform 1 of Nicastrin precursor

Match to Query 840: 2302.081812 from(768.367880,3+)
Title: 091129RatKid_SCX02_12.580.580.3.dta
Data file K:\NewmanPaper\Piliang\3SubProteomes\Piliang3SP\mgf5ppm\SCX_3SubProteomes5ppm.mgf

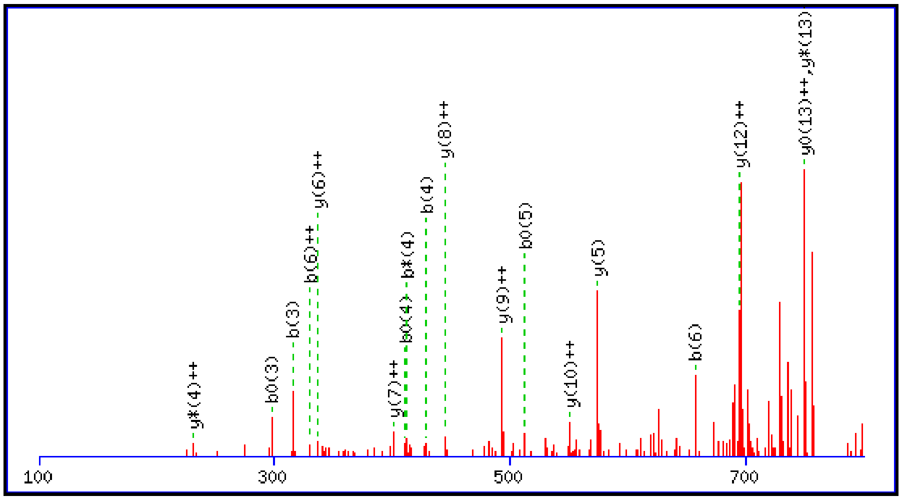

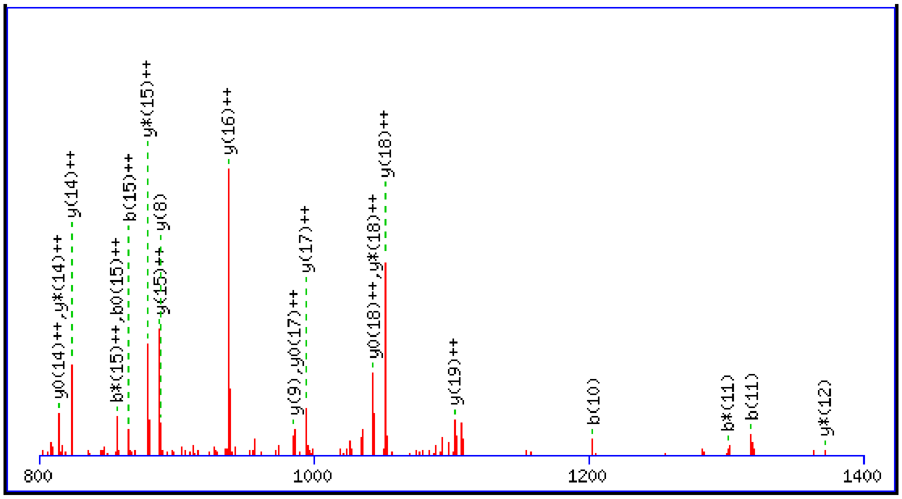

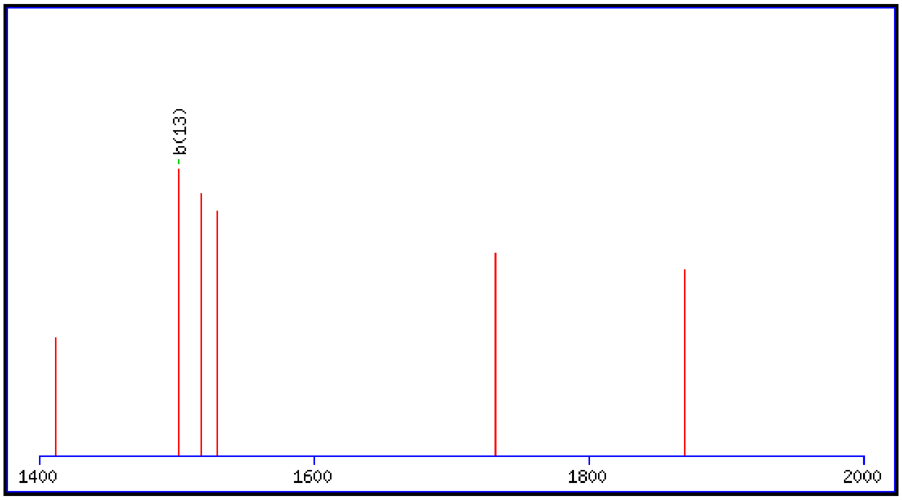

Monoisotopic mass of neutral peptide Mr(calc): 2302.0747
Fixed modifications: Carbamidomethyl (C)
Variable modifications:
N3 : Deamidated_N (N)
Ions Score: 56 **Expect:** 0.00076
Matches (Bold Red): 40/220 fragment ions using 73 most intense peaks

#	b	b^{++}	b*	b^{*++}	b^{0}	b^{0++}	Seq.	y	y^{++}	y*	y^{*++}	y^{0}	y^{0++}	#
1	100.0757	50.5415					V							20
2	201.1234	101.0653			183.1128	92.0600	T	2204.0136	**1102.5104**	2186.9870	1093.9971	2186.0030	1093.5051	19
3	**316.1503**	158.5788	299.1238	150.0655	**298.1397**	149.5735	N	2102.9659	**1051.9866**	2085.9393	**1043.4733**	2084.9553	**1042.9813**	18
4	**429.2344**	215.1208	**412.2078**	206.6075	**411.2238**	206.1155	L	1987.9389	**994.4731**	1970.9124	985.9598	1969.9284	**985.4678**	17
5	530.2820	265.6447	513.2555	257.1314	**512.2715**	256.6394	T	1874.8549	**937.9311**	1857.8283	929.4178	1856.8443	928.9258	16
6	**658.3406**	**329.6740**	641.3141	321.1607	640.3301	320.6687	Q	1773.8072	**887.4072**	1756.7807	**878.8940**	1755.7966	878.4020	15
7	787.3832	394.1952	770.3567	385.6820	769.3727	385.1900	E	1645.7486	**823.3780**	1628.7221	**814.8647**	1627.7381	**814.3727**	14
8	915.4418	458.2245	898.4152	449.7113	897.4312	449.2193	Q	1516.7060	758.8567	1499.6795	**750.3434**	1498.6955	**749.8514**	13
9	1075.4724	538.2399	1058.4459	529.7266	1057.4619	529.2346	C	1388.6475	**694.8274**	**1371.6209**	686.3141	1370.6369	685.8221	12
10	**1203.5310**	602.2692	1186.5045	593.7559	1185.5205	593.2639	Q	1228.6168	614.8120	1211.5903	606.2988	1210.6062	605.8068	11
11	**1318.5580**	659.7826	**1301.5314**	651.2693	1300.5474	650.7773	D	1100.5582	**550.7828**	1083.5317	542.2695	1082.5477	541.7775	10
12	1415.6107	708.3090	1398.5842	699.7957	1397.6002	699.3037	P	**985.5313**	**493.2693**	968.5047	484.7560	967.5207	484.2640	9
13	**1502.6428**	751.8250	1485.6162	743.3117	1484.6322	742.8197	S	**888.4785**	**444.7429**	871.4520	436.2296	870.4680	435.7376	8
14	1630.7377	815.8725	1613.7112	807.3592	1612.7272	806.8672	K	801.4465	**401.2269**	784.4199	392.7136	783.4359	392.2216	7
15	1729.8061	**865.4067**	1712.7796	**856.8934**	1711.7956	**856.4014**	V	673.3515	**337.1794**	656.3250	328.6661	655.3410	328.1741	6
16	1826.8589	913.9331	1809.8324	905.4198	1808.8483	904.9278	P	**574.2831**	287.6452	557.2566	279.1319	556.2726	278.6399	5
17	1940.9018	970.9546	1923.8753	962.4413	1922.8913	961.9493	N	477.2304	239.1188	460.2038	**230.6055**	459.2198	230.1135	4
18	2069.9444	1035.4758	2052.9179	1026.9626	2051.9339	1026.4706	E	363.1874	182.0974	346.1609	173.5841	345.1769	173.0921	3

19	2156.9764	1078.9919	2139.9499	1070.4786	2138.9659	1069.9866	S	234.1448	117.5761	217.1183	109.0628	216.1343	108.5708	2
20							K	147.1128	74.0600	130.0863	65.5468			1

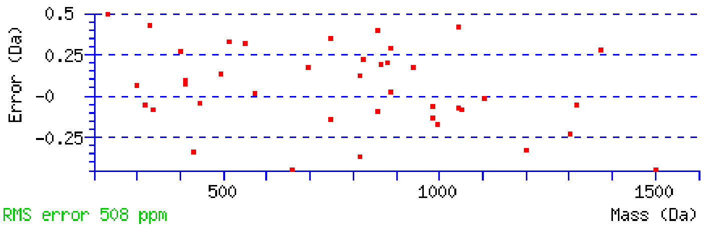

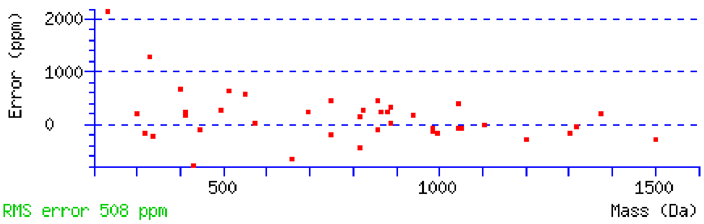

All matches to this query

Score	Mr(calc):	Delta	Sequence
55.7	2302.0747	0.0071	VTNLTQEQCQDPSKVPNESK
40.0	2301.0907	0.9911	VTNLTQEQCQDPSKVPNESK
24.1	2302.0747	0.0071	VTNLTQEQCQDPSKVPNESK
5.9	2300.0715	2.0103	LLGIAAGVGAGVPWGSRETSSR
5.9	2300.0715	2.0103	LLGIAAGVGAGVPWGSRETSSR
5.6	2300.0531	2.0287	LSYIIAFSKDEVVDVTWR
4.9	2300.0937	1.9881	NVLVTLYERDEGNNLLTEK
4.9	2300.0937	1.9881	NVLVTLYERDEGNNLLTEK
4.5	2302.0956	-0.0138	TLWEFKTCDGETPLTLAIK
4.4	2300.0715	2.0103	LLGIAAGVGAGVPWGSRETSSR

Spectrum No: 379; Query: 673; Rank: 1

Peptide View

MS/MS Fragmentation of **VFADGAPHYVAFYSNVTR**
Found in **IPI00190577**, Tax_Id=10116 Gene_Symbol=Lama5 similar to Laminin alpha-5 chain precursor

Match to Query 673: 2014.964232 from(672.662020,3+)
Title: 091127RatKid_SCX01_14.3102.3102.3.dta
Data file K:\NewmanPaper\Piliang\3SubProteomes\Piliang3SP\mgf5ppm\SCX_3SubProteomes5ppm.mgf

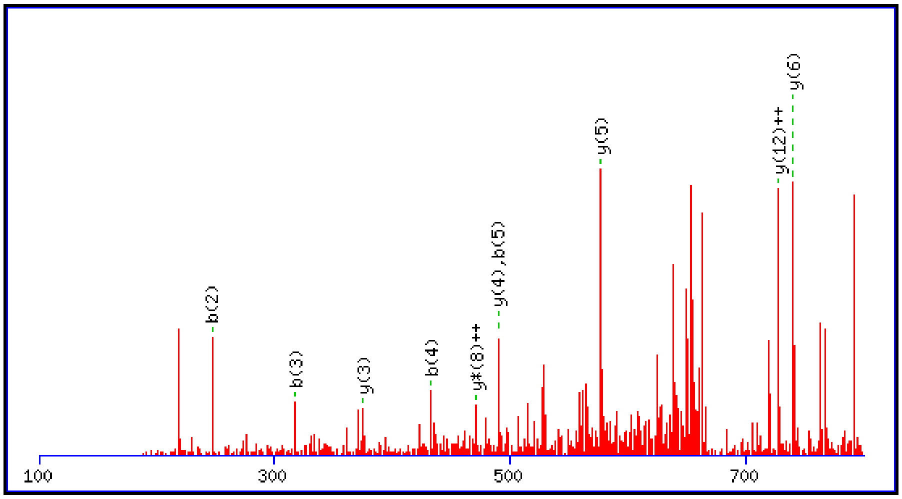

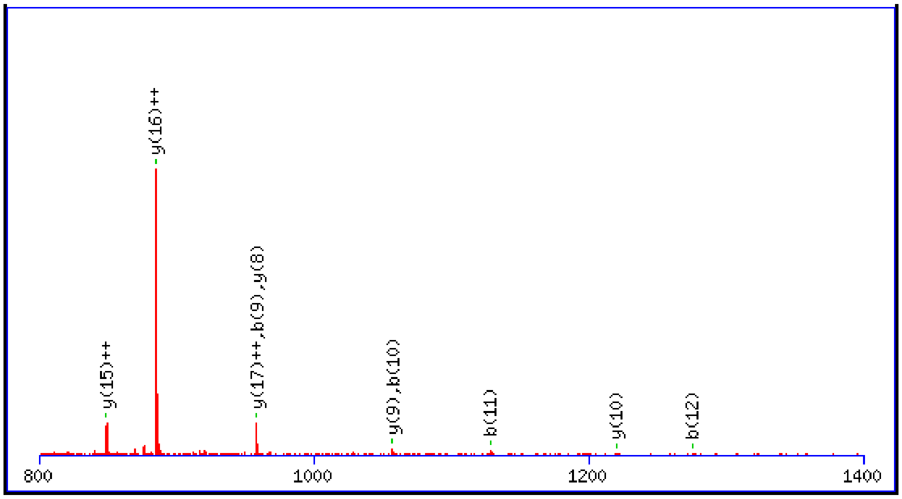

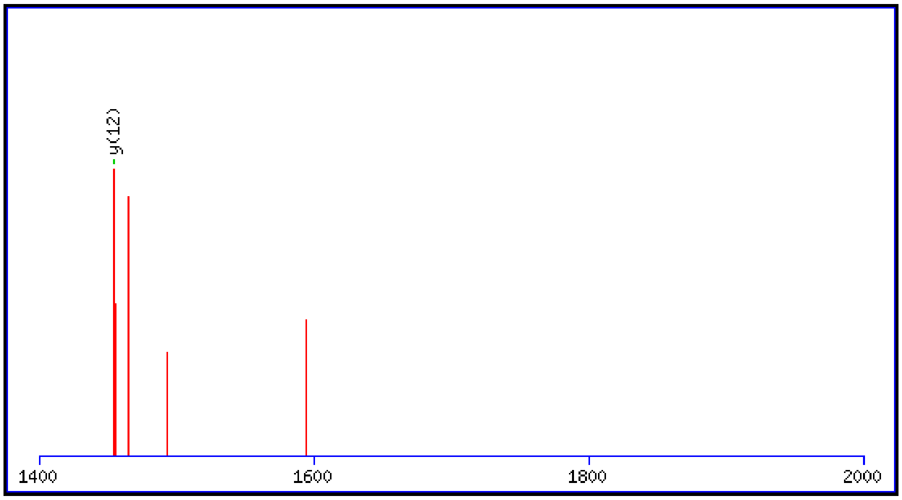

Monoisotopic mass of neutral peptide Mr(calc): 2013.9585
Fixed modifications: Carbamidomethyl (C)
Variable modifications:
N15 : Deamidated_N (N)
Ions Score: 56 **Expect:** 0.00064
Matches (Bold Red): 21/168 fragment ions using 35 most intense peaks

#	b	b^{++}	b*	b^{*++}	b^0	b^{0++}	Seq.	y	y^{++}	y*	y^{*++}	y^0	y^{0++}	#
1	100.0757	50.5415					V							18
2	**247.1441**	124.0757					F	1915.8973	**958.4523**	1898.8708	949.9390	1897.8868	949.4470	17
3	**318.1812**	159.5942					A	1768.8289	**884.9181**	1751.8024	876.4048	1750.8184	875.9128	16
4	**433.2082**	217.1077			415.1976	208.1024	D	1697.7918	**849.3995**	1680.7653	840.8863	1679.7812	840.3943	15
5	**490.2296**	245.6185			472.2191	236.6132	G	1582.7649	791.8861	1565.7383	783.3728	1564.7543	782.8808	14
6	561.2667	281.1370			543.2562	272.1317	A	1525.7434	763.3753	1508.7169	754.8621	1507.7328	754.3701	13
7	658.3195	329.6634			640.3089	320.6581	P	**1454.7063**	**727.8568**	1437.6797	719.3435	1436.6957	718.8515	12
8	795.3784	398.1928			777.3678	389.1876	H	1357.6535	679.3304	1340.6270	670.8171	1339.6430	670.3251	11
9	**958.4417**	479.7245			940.4312	470.7192	Y	**1220.5946**	610.8009	1203.5681	602.2877	1202.5840	601.7957	10
10	**1057.5102**	529.2587			1039.4996	520.2534	V	**1057.5313**	529.2693	1040.5047	520.7560	1039.5207	520.2640	9
11	**1128.5473**	564.7773			1110.5367	555.7720	A	**958.4629**	479.7351	941.4363	**471.2218**	940.4523	470.7298	8
12	**1275.6157**	638.3115			1257.6051	629.3062	F	887.4258	444.2165	870.3992	435.7032	869.4152	435.2112	7
13	1438.6790	719.8431			1420.6685	710.8379	Y	**740.3573**	370.6823	723.3308	362.1690	722.3468	361.6770	6
14	1525.7110	763.3592			1507.7005	754.3539	S	**577.2940**	289.1506	560.2675	280.6374	559.2834	280.1454	5
15	1640.7380	820.8726	1623.7114	812.3594	1622.7274	811.8673	N	**490.2620**	245.6346	473.2354	237.1214	472.2514	236.6293	4
16	1739.8064	870.4068	1722.7798	861.8936	1721.7958	861.4016	V	**375.2350**	188.1212	358.2085	179.6079	357.2245	179.1159	3
17	1840.8541	920.9307	1823.8275	912.4174	1822.8435	911.9254	T	276.1666	138.5870	259.1401	130.0737	258.1561	129.5817	2
18							R	175.1190	88.0631	158.0924	79.5498			1

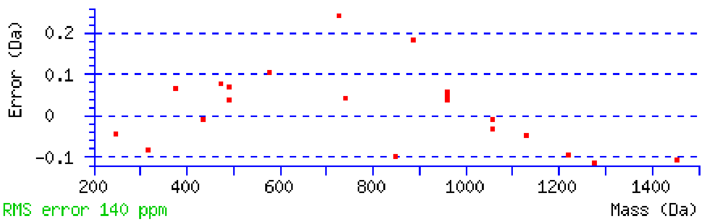

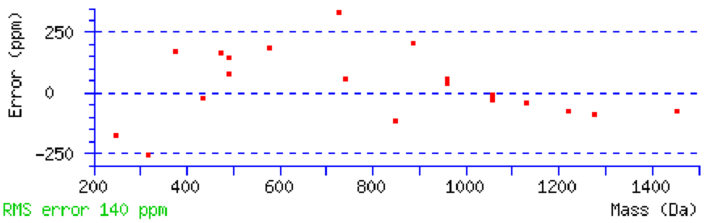

All matches to this query

Score	Mr(calc):	Delta	Sequence
55.5	2013.9585	1.0058	VFADGAPHYVAFYSNVTR
13.6	2012.9745	1.9898	VFADGAPHYVAFYSNVTR
7.1	2013.9489	1.0153	MIDMLAANSGRVTNLRR
6.8	2012.9754	1.9888	HIANRMLPPEDAIPKSK
6.6	2012.9489	2.0154	MDQVMQFVQPSWQFVK
5.0	2012.9489	2.0154	MDQVMQFVQPSWQFVK
4.7	2012.9642	2.0001	WMNGSILNTNLLRITGK
4.0	2012.9642	2.0000	LGVKEVRTAIEEMEWK
3.7	2014.9587	0.0055	AFVTQDIQLYHNLVMK
3.6	2012.9642	2.0001	WMNGSILNTNLLRITGK

Spectrum No: 380; Query: 232; Rank: 1

Peptide View

MS/MS Fragmentation of **DITNLINNTFIR**
Found in **IPI00373408**, Tax_Id=10116 Gene_Symbol=Sort1 Sortilin precursor

Match to Query 232: 1433.755248 from(717.884900,2+)
Title: 091129RatKid_SCX02_11.3971.3971.2.dta
Data file K:\NewmanPaper\Piliang\3SubProteomes\Piliang3SP\mgf5ppm\SCX_3SubProteomes5ppm.mgf

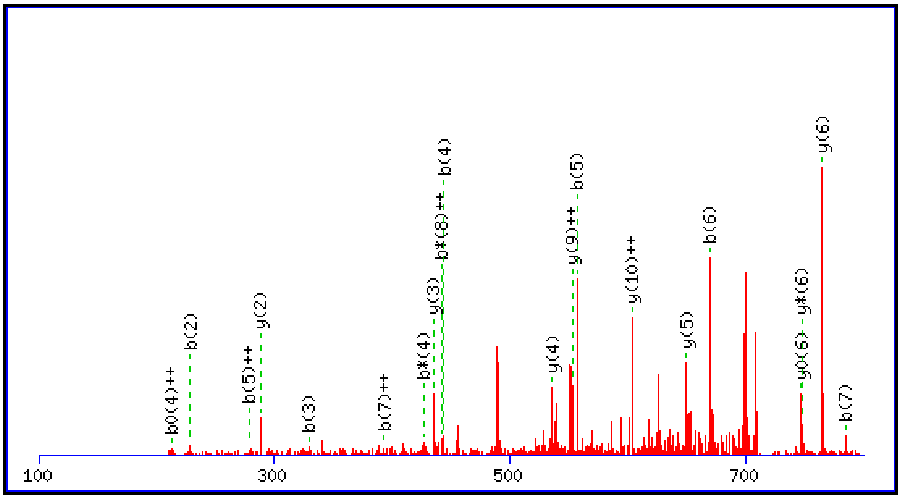

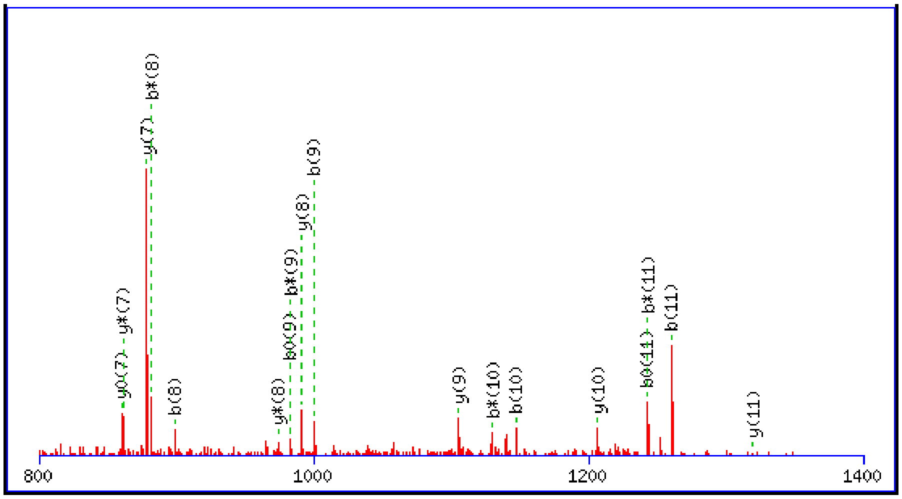

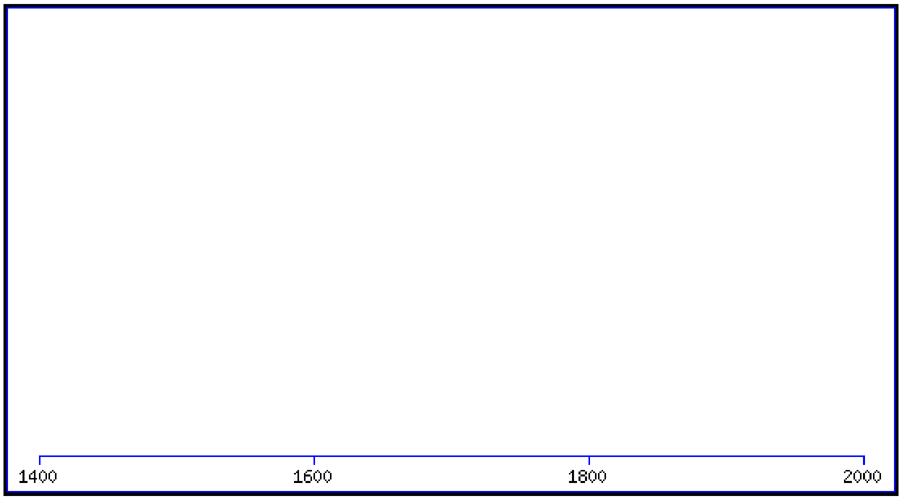

Monoisotopic mass of neutral peptide Mr(calc): 1433.7514
Fixed modifications: Carbamidomethyl (C)
Variable modifications:
N7 : Deamidated_N (N)
Ions Score: 56 **Expect:** 0.00031
Matches (Bold Red): 38/120 fragment ions using 78 most intense peaks

#	b	b^{++}	b*	b^{*++}	b^0	b^{0++}	Seq.	y	y^{++}	y*	y^{*++}	y^0	y^{0++}	#
1	116.0342	58.5207			98.0237	49.5155	D							12
2	**229.1183**	115.0628			211.1077	106.0575	I	**1319.7318**	660.3695	1302.7052	651.8562	1301.7212	651.3642	11
3	**330.1660**	165.5866			312.1554	156.5813	T	**1206.6477**	**603.8275**	1189.6212	595.3142	1188.6371	594.8222	10
4	**444.2089**	222.6081	**427.1823**	214.0948	426.1983	**213.6028**	N	**1105.6000**	**553.3037**	1088.5735	544.7904	1087.5895	544.2984	9
5	**557.2930**	**279.1501**	540.2664	270.6368	539.2824	270.1448	L	**991.5571**	496.2822	**974.5306**	487.7689	973.5465	487.2769	8
6	**670.3770**	335.6921	653.3505	327.1789	652.3665	326.6869	I	**878.4730**	439.7402	**861.4465**	431.2269	**860.4625**	430.7349	7
7	**785.4040**	**393.2056**	768.3774	384.6923	767.3934	384.2003	N	**765.3890**	383.1981	**748.3624**	374.6848	**747.3784**	374.1928	6
8	**899.4469**	450.2271	**882.4203**	**441.7138**	881.4363	441.2218	N	**650.3620**	325.6847	633.3355	317.1714	632.3515	316.6794	5
9	**1000.4946**	500.7509	**983.4680**	492.2376	**982.4840**	491.7456	T	**536.3191**	268.6632	519.2926	260.1499	518.3085	259.6579	4
10	**1147.5630**	574.2851	**1130.5364**	565.7719	1129.5524	565.2798	F	**435.2714**	218.1394	418.2449	209.6261			3
11	**1260.6470**	630.8272	**1243.6205**	622.3139	**1242.6365**	621.8219	I	**288.2030**	144.6051	271.1765	136.0919			2
12							R	175.1190	88.0631	158.0924	79.5498			1

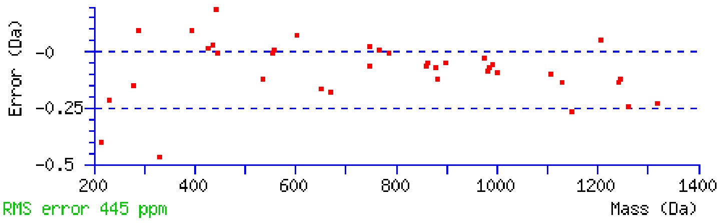

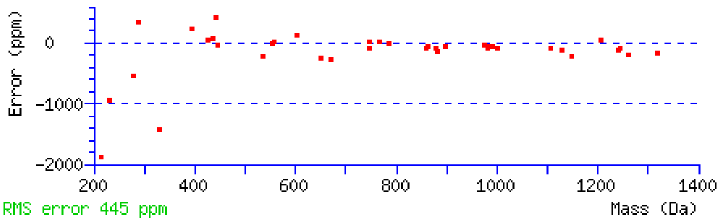

All matches to this query

Score	Mr(calc):	Delta	Sequence
55.5	1433.7514	0.0038	DITNLINNTFIR
44.8	1433.7514	0.0038	DITNLINNTFIR
30.9	1433.7514	0.0038	DITNLINNTFIR
13.2	1433.7626	-0.0074	GEKELLFNKNSR
12.3	1433.7626	-0.0074	DLFIDGRSKNIR
9.0	1432.7595	0.9957	QSINIMELTLQK
7.3	1433.7474	0.0079	SSLSNDISEKAKR
3.2	1433.7578	-0.0025	MSKIRGLPPAIR
2.3	1433.7514	0.0038	LNSIKNSFLGSPR
2.3	1433.7448	0.0104	TITVILAFKLR

Spectrum No: 381; Query: 814; Rank: 1

Peptide View

MS/MS Fragmentation of **EDSCQLNYSEGPCLGMQQK**
Found in **IPI00210900**, Tax_Id=10116 Gene_Symbol=Ambp AMBP protein precursor

Match to Query 814: 2243.920928 from(1122.967740,2+)
Title: 091129RatKid_SCX02_02.1746.1746.2.dta
Data file K:\NewmanPaper\Piliang\3SubProteomes\Piliang3SP\mgf5ppm\SCX_3SubProteomes5ppm.mgf

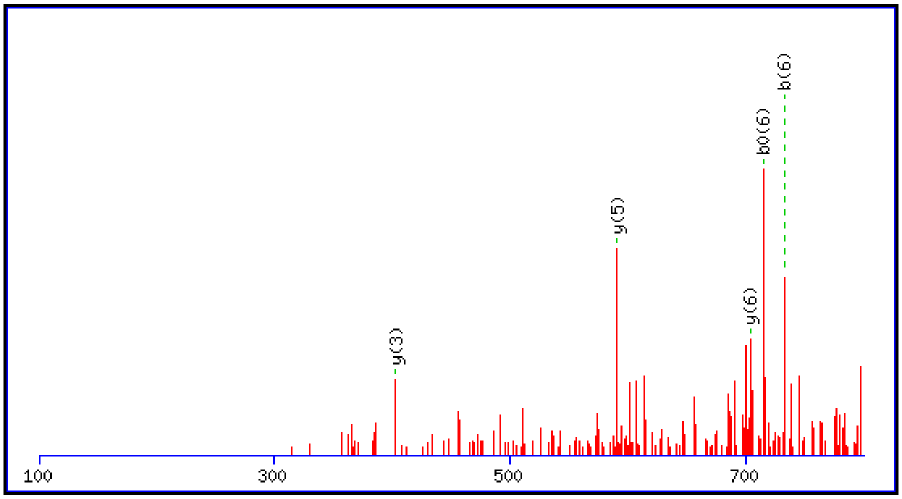

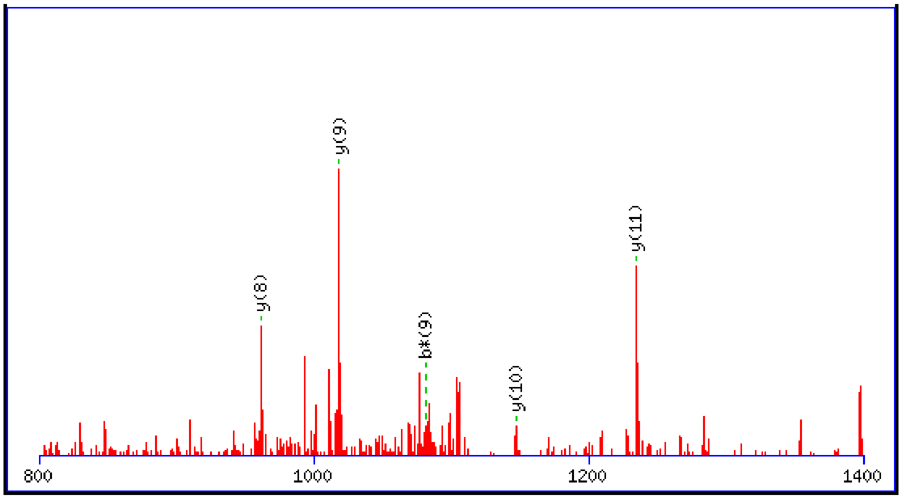

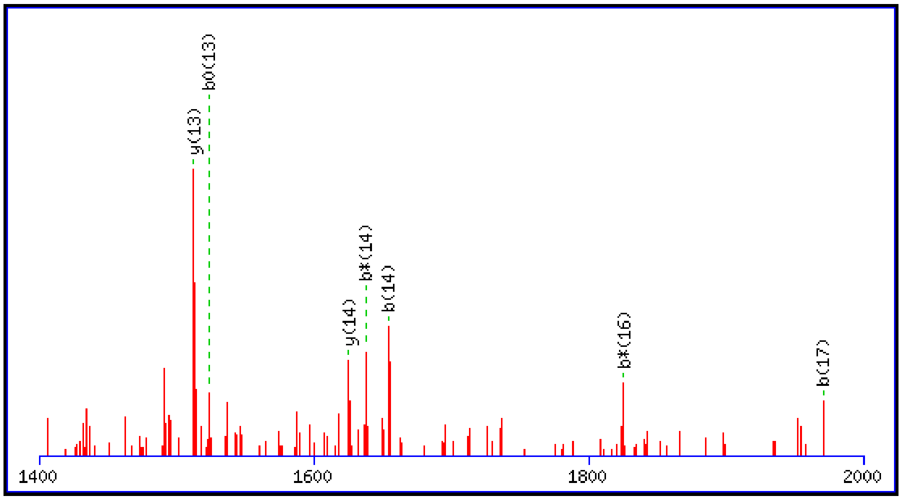

Monoisotopic mass of neutral peptide Mr(calc): 2243.9133
Fixed modifications: Carbamidomethyl (C)
Variable modifications:
N7 : Deamidated_N (N)
Ions Score: 55 **Expect:** 0.00065
Matches (Bold Red): 17/190 fragment ions using 40 most intense peaks

#	b	b^{++}	b*	b^{*++}	b^0	b^{0++}	Seq.	y	y^{++}	y*	y^{*++}	y^0	y^{0++}	#
1	130.0499	65.5286			112.0393	56.5233	E							19
2	245.0768	123.0420			227.0662	114.0368	D	2115.8780	1058.4426	2098.8514	1049.9294	2097.8674	1049.4374	18
3	332.1088	166.5581			314.0983	157.5528	S	2000.8511	1000.9292	1983.8245	992.4159	1982.8405	991.9239	17
4	492.1395	246.5734			474.1289	237.5681	C	1913.8190	957.4132	1896.7925	948.8999	1895.8085	948.4079	16
5	620.1981	310.6027	603.1715	302.0894	602.1875	301.5974	Q	1753.7884	877.3978	1736.7618	868.8846	1735.7778	868.3925	15
6	**733.2821**	367.1447	716.2556	358.6314	**715.2716**	358.1394	L	**1625.7298**	813.3685	1608.7033	804.8553	1607.7192	804.3633	14
7	848.3091	424.6582	831.2825	416.1449	830.2985	415.6529	N	**1512.6457**	756.8265	1495.6192	748.3132	1494.6352	747.8212	13
8	1011.3724	506.1898	994.3458	497.6766	993.3618	497.1846	Y	1397.6188	699.3130	1380.5923	690.7998	1379.6082	690.3078	12
9	1098.4044	549.7059	**1081.3779**	541.1926	1080.3939	540.7006	S	**1234.5555**	617.7814	1217.5289	609.2681	1216.5449	608.7761	11
10	1227.4470	614.2271	1210.4205	605.7139	1209.4365	605.2219	E	**1147.5234**	574.2654	1130.4969	565.7521	1129.5129	565.2601	10
11	1284.4685	642.7379	1267.4419	634.2246	1266.4579	633.7326	G	**1018.4809**	509.7441	1001.4543	501.2308			9
12	1381.5212	691.2643	1364.4947	682.7510	1363.5107	682.2590	P	**961.4594**	481.2333	944.4328	472.7201			8
13	1541.5519	771.2796	1524.5253	762.7663	**1523.5413**	762.2743	C	864.4066	432.7069	847.3801	424.1937			7
14	**1654.6360**	827.8216	**1637.6094**	819.3083	1636.6254	818.8163	L	**704.3760**	352.6916	687.3494	344.1784			6
15	1711.6574	856.3323	1694.6309	847.8191	1693.6469	847.3271	G	**591.2919**	296.1496	574.2654	287.6363			5
16	1842.6979	921.8526	**1825.6714**	913.3393	1824.6873	912.8473	M	534.2704	267.6389	517.2439	259.1256			4
17	**1970.7565**	985.8819	1953.7299	977.3686	1952.7459	976.8766	Q	**403.2300**	202.1186	386.2034	193.6053			3
18	2098.8151	1049.9112	2081.7885	1041.3979	2080.8045	1040.9059	Q	275.1714	138.0893	258.1448	129.5761			2

19							K	147.1128	74.0600	130.0863	65.5468			1

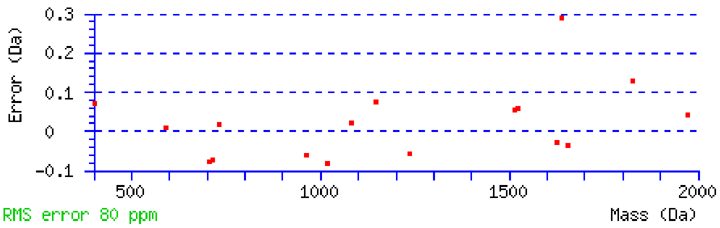

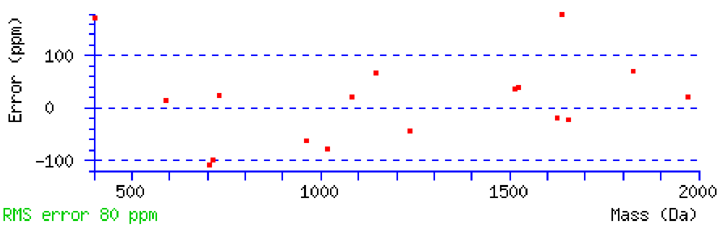

All matches to this query

Score	Mr(calc):	Delta	Sequence
55.3	2243.9133	0.0076	EDSCQLNYSEGPCLGMQQK
28.3	2242.9293	0.9916	EDSCQLNYSEGPCLGMQQK

Spectrum No: 382; Query: 1101; Rank: 1

Peptide View

MS/MS Fragmentation of **FDEFFSQGCAPGYKKNSTLCDLCIGPAK**
Found in **IPI00196656**, Tax_Id=10116 Gene_Symbol=Srprb Ba1-667

Match to Query 1101: 3211.454832 from(1071.492220,3+)
Title: 091127RatKid_SCX01_20.3845.3845.3.dta
Data file K:\NewmanPaper\Piliang\3SubProteomes\Piliang3SP\mgf5ppm\SCX_3SubProteomes5ppm.mgf

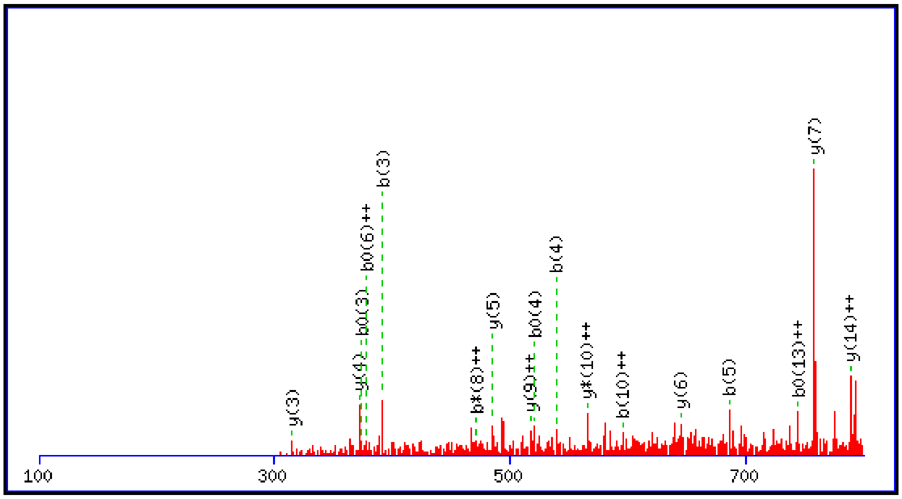

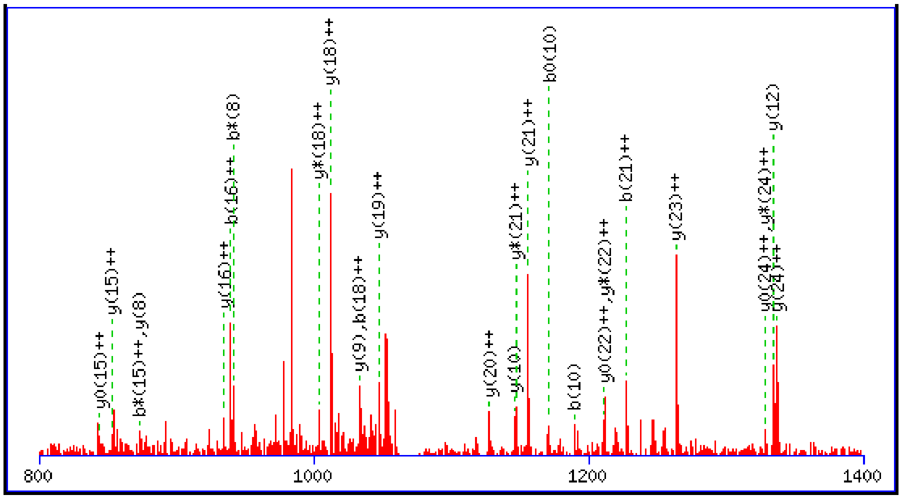

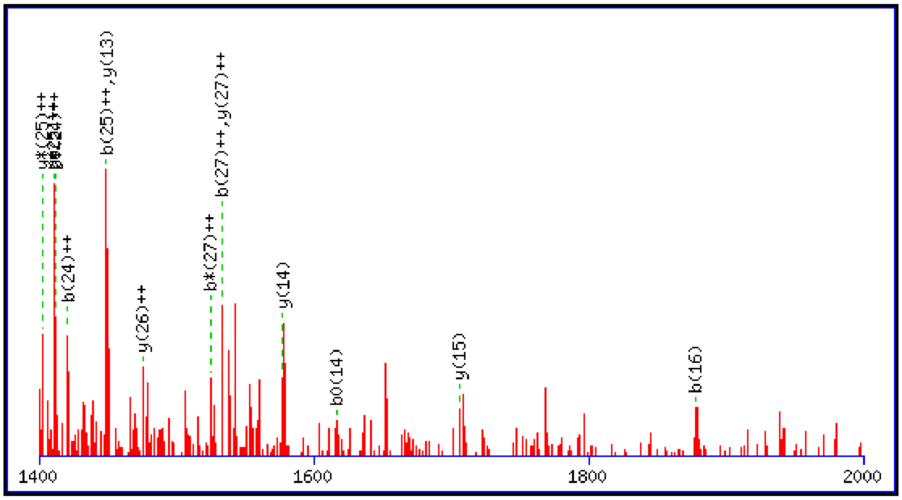

Monoisotopic mass of neutral peptide Mr(calc): 3210.4460
Fixed modifications: Carbamidomethyl (C)
Variable modifications:
N16 : Deamidated_N (N)
Ions Score: 55 **Expect:** 0.0013
Matches (Bold Red): 58/296 fragment ions using 141 most intense peaks

#	b	b^{++}	b*	b^{*++}	b^{0}	b^{0++}	Seq.	y	y^{++}	y*	y^{*++}	y^{0}	y^{0++}	#
1	148.0757	74.5415					F							28
2	263.1026	132.0550			245.0921	123.0497	D	3064.3849	**1532.6961**	3047.3584	1524.1828	3046.3743	1523.6908	27
3	**392.1452**	196.5763			**374.1347**	187.5710	E	2949.3580	**1475.1826**	2932.3314	1466.6693	2931.3474	1466.1773	26
4	**539.2136**	270.1105			**521.2031**	261.1052	F	2820.3154	**1410.6613**	2803.2888	**1402.1480**	2802.3048	1401.6560	25
5	**686.2821**	343.6447			668.2715	334.6394	F	2673.2470	**1337.1271**	2656.2204	**1328.6138**	2655.2364	**1328.1218**	24
6	773.3141	387.1607			755.3035	**378.1554**	S	2526.1785	**1263.5929**	2509.1520	1255.0796	2508.1680	1254.5876	23
7	901.3727	451.1900	884.3461	442.6767	883.3621	442.1847	Q	2439.1465	1220.0769	2422.1200	**1211.5636**	2421.1359	**1211.0716**	22
8	958.3941	479.7007	**941.3676**	**471.1874**	940.3836	470.6954	G	2311.0879	**1156.0476**	2294.0614	**1147.5343**	2293.0774	1147.0423	21
9	1118.4248	559.7160	1101.3982	551.2028	1100.4142	550.7107	C	2254.0665	**1127.5369**	2237.0399	1119.0236	2236.0559	1118.5316	20
10	**1189.4619**	**595.2346**	1172.4353	586.7213	**1171.4513**	586.2293	A	2094.0358	**1047.5215**	2077.0093	1039.0083	2076.0253	1038.5163	19
11	1286.5147	643.7610	1269.4881	635.2477	1268.5041	634.7557	P	2022.9987	**1012.0030**	2005.9722	**1003.4897**	2004.9881	1002.9977	18
12	1343.5361	672.2717	1326.5096	663.7584	1325.5255	663.2664	G	1925.9459	963.4766	1908.9194	954.9633	1907.9354	954.4713	17
13	1506.5994	753.8034	1489.5729	745.2901	1488.5889	**744.7981**	Y	1868.9245	**934.9659**	1851.8979	926.4526	1850.9139	925.9606	16
14	1634.6944	817.8508	1617.6679	809.3376	**1616.6838**	808.8456	K	**1705.8611**	**853.4342**	1688.8346	844.9209	1687.8506	**844.4289**	15
15	1762.7894	881.8983	1745.7628	**873.3850**	1744.7788	872.8930	K	**1577.7662**	**789.3867**	1560.7396	780.8735	1559.7556	780.3814	14
16	**1877.8163**	**939.4118**	1860.7898	930.8985	1859.8057	930.4065	N	**1449.6712**	725.3392	1432.6447	716.8260	1431.6607	716.3340	13
17	1964.8483	982.9278	1947.8218	974.4145	1946.8378	973.9225	S	**1334.6443**	667.8258	1317.6177	659.3125	1316.6337	658.8205	12
18	2065.8960	**1033.4516**	2048.8695	1024.9384	2047.8854	1024.4464	T	1247.6123	624.3098	1230.5857	615.7965	1229.6017	615.3045	11

**19**	2178.9801	1089.9937	2161.9535	1081.4804	2160.9695	1080.9884	**L**	**1146.5646**	573.7859	1129.5380	**565.2727**	1128.5540	564.7806	**10**
**20**	2339.0107	1170.0090	2321.9842	1161.4957	2321.0002	1161.0037	**C**	**1033.4805**	**517.2439**	1016.4540	508.7306	1015.4699	508.2386	**9**
**21**	2454.0377	**1227.5225**	2437.0111	1219.0092	2436.0271	1218.5172	**D**	**873.4499**	437.2286	856.4233	428.7153	855.4393	428.2233	**8**
**22**	2567.1217	1284.0645	2550.0952	1275.5512	2549.1112	1275.0592	**L**	**758.4229**	379.7151	741.3964	371.2018			**7**
**23**	2727.1524	1364.0798	2710.1258	1355.5666	2709.1418	1355.0745	**C**	**645.3389**	323.1731	628.3123	314.6598			**6**
**24**	2840.2364	**1420.6219**	2823.2099	**1412.1086**	2822.2259	**1411.6166**	**I**	**485.3082**	243.1577	468.2817	234.6445			**5**
**25**	2897.2579	**1449.1326**	2880.2314	1440.6193	2879.2473	1440.1273	**G**	**372.2241**	186.6157	355.1976	178.1024			**4**
**26**	2994.3107	1497.6590	2977.2841	1489.1457	2976.3001	1488.6537	**P**	**315.2027**	158.1050	298.1761	149.5917			**3**
**27**	3065.3478	**1533.1775**	3048.3212	**1524.6643**	3047.3372	1524.1722	**A**	218.1499	109.5786	201.1234	101.0653			**2**
**28**							**K**	147.1128	74.0600	130.0863	65.5468			**1**

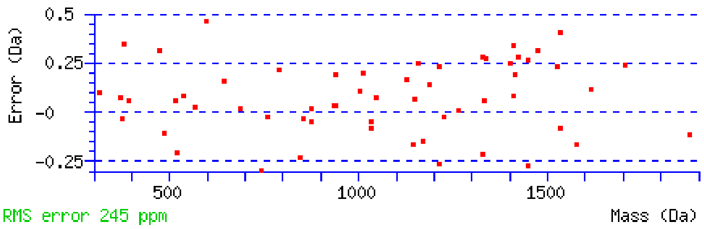

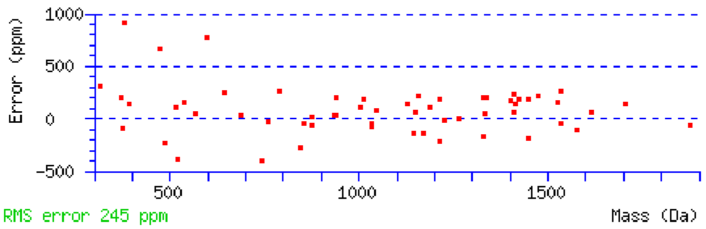

All matches to this query

Score	Mr(calc):	Delta	Sequence
55.3	3210.4460	1.0088	FDEFFSQGCAPGYKKNSTLCDLCIGPAK
7.6	3209.4345	2.0203	LQDLFTIWTEESLMCVGCGAESSRRGK
7.0	3209.4620	1.9928	FDEFFSQGCAPGYKKNSTLCDLCIGPAK
2.4	3209.4661	1.9887	MLMELSAGAANFTDQKEFSLQDIEVLGR
2.4	3209.4661	1.9887	MLMELSAGAANFTDQKEFSLQDIEVLGR
2.3	3209.4345	2.0203	LQDLFTIWTEESLMCVGCGAESSRRGK
1.8	3209.4541	2.0008	EDEFNLTALALENSSEFISLSTDGVVDSK
1.6	3210.4509	1.0039	GAEDDLNTVAAGTMTGMLYKCTGGLRGIAR
1.6	3210.4509	1.0039	GAEDDLNTVAAGTMTGMLYKCTGGLRGIAR
1.6	3210.4509	1.0039	GAEDDLNTVAAGTMTGMLYKCTGGLRGIAR

Spectrum No: 383; Query: 410; Rank: 1

Peptide View

MS/MS Fragmentation of **CLVPHTVNLSSAWR**
Found in **IPI00199585**, Tax_Id=10116 Gene_Symbol=Slc22a5 Organic cation/carnitine transporter 2

Match to Query 410: 1639.815822 from(547.612550,3+)
Title: 091127RatKid_SCX01_13.2990.2990.3.dta
Data file K:\NewmanPaper\Piliang\3SubProteomes\Piliang3SP\mgf5ppm\SCX_3SubProteomes5ppm.mgf

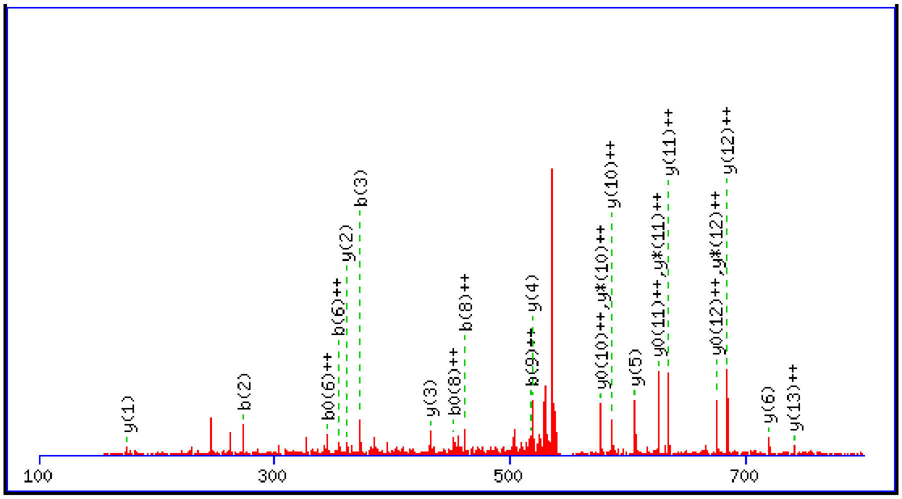

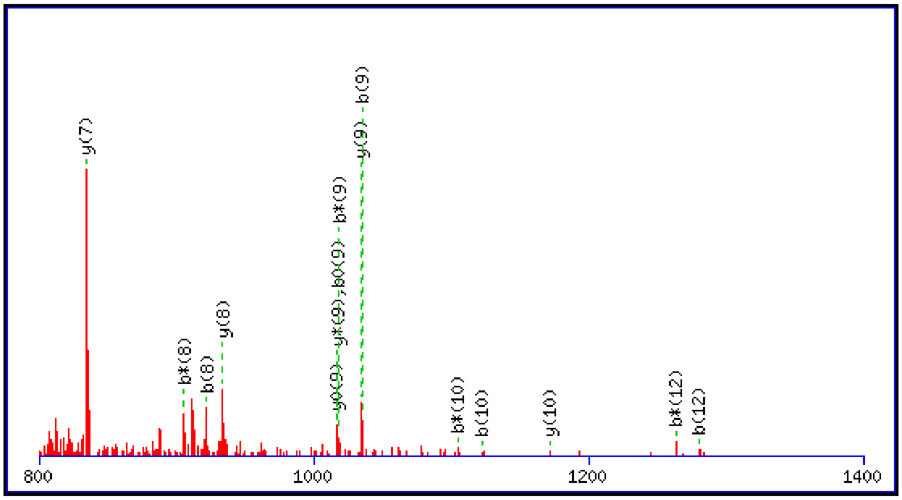

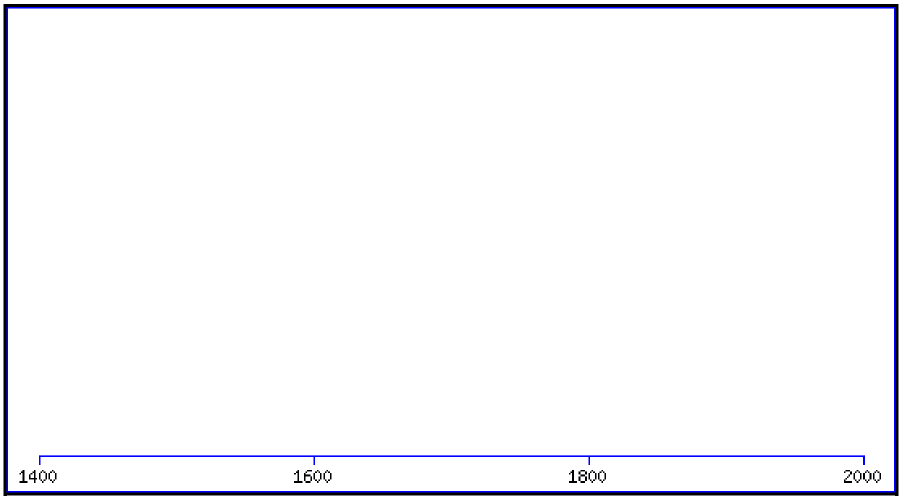

Monoisotopic mass of neutral peptide Mr(calc): 1639.8141
Fixed modifications: Carbamidomethyl (C)
Variable modifications:
N8 : Deamidated_N (N)
Ions Score: 55 **Expect:** 0.00049
Matches (Bold Red): 38/126 fragment ions using 68 most intense peaks

#	b	b^{++}	b*	b^{*++}	b^0	b^{0++}	Seq.	y	y^{++}	y*	y^{*++}	y^0	y^{0++}	#
1	161.0379	81.0226					C							14
2	**274.1220**	137.5646					L	1480.7907	**740.8990**	1463.7641	732.3857	1462.7801	731.8937	13
3	**373.1904**	187.0988					V	1367.7066	**684.3569**	1350.6801	**675.8437**	1349.6961	**675.3517**	12
4	470.2432	235.6252					P	1268.6382	**634.8227**	1251.6117	**626.3095**	1250.6276	**625.8175**	11
5	607.3021	304.1547					H	**1171.5854**	**586.2964**	1154.5589	**577.7831**	1153.5749	**577.2911**	10

**6**	708.3498	**354.6785**			690.3392	**345.6732**	**T**	**1034.5265**	517.7669	**1017.5000**	509.2536	**1016.5160**	508.7616	**9**
**7**	807.4182	404.2127			789.4076	395.2074	**V**	**933.4788**	467.2431	916.4523	458.7298	915.4683	458.2378	**8**
**8**	**922.4451**	**461.7262**	**905.4186**	453.2129	904.4345	**452.7209**	**N**	**834.4104**	417.7089	817.3839	409.1956	816.3999	408.7036	**7**
**9**	**1035.5292**	**518.2682**	**1018.5026**	509.7549	**1017.5186**	509.2629	**L**	**719.3835**	360.1954	702.3570	351.6821	701.3729	351.1901	**6**
**10**	**1122.5612**	561.7842	**1105.5347**	553.2710	1104.5506	552.7790	**S**	**606.2994**	303.6534	589.2729	295.1401	588.2889	294.6481	**5**
**11**	1209.5932	605.3003	1192.5667	596.7870	1191.5827	596.2950	**S**	**519.2674**	260.1373	502.2409	251.6241	501.2568	251.1321	**4**
**12**	**1280.6303**	640.8188	**1263.6038**	632.3055	1262.6198	631.8135	**A**	**432.2354**	216.6213	415.2088	208.1081			**3**
**13**	1466.7097	733.8585	1449.6831	725.3452	1448.6991	724.8532	**W**	**361.1983**	181.1028	344.1717	172.5895			**2**
**14**							**R**	**175.1190**	88.0631	158.0924	79.5498			**1**

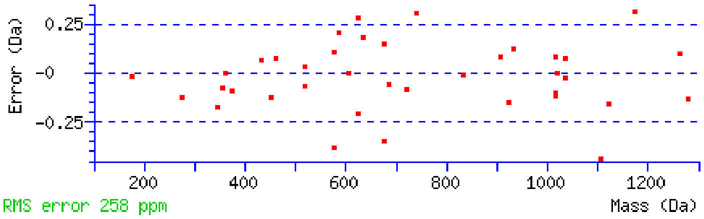

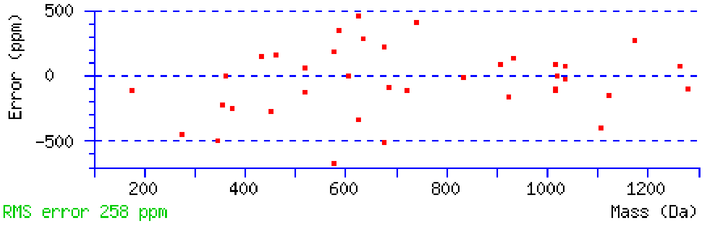

All matches to this query

Score	Mr(calc):	Delta	Sequence
55.2	1639.8141	0.0018	CLVPHTVNLSSAWR
5.6	1639.8239	-0.0081	VKGVAYTNTTMLGER
5.2	1638.8277	0.9882	MTGRGKTKPLNTVR
4.5	1638.8032	1.0127	SQGVVLARYGPAWR
4.5	1637.8039	2.0119	SFSQSSILIQHRR
3.7	1639.8164	-0.0006	AVLTGLFGFFAYVR
3.5	1639.8043	0.0115	QGVRQSVSEKTSVR
3.4	1638.8114	1.0044	GFPGADGVAGPKGPAGER
3.4	1639.8286	-0.0128	LTQQHLLAMACQAR
3.4	1639.8004	0.0154	TCKDGATELLLLAR

Spectrum No: 384; Query: 741; Rank: 1

Peptide View

MS/MS Fragmentation of **DCSTNPPRLPVTAVNTTMR**
Found in **IPI00197684**, Tax_Id=10116 Gene_Symbol=Xpnpep2 Membrane-bound aminopeptidase P

Match to Query 741: 2130.022482 from(711.014770,3+)
Title: 091129RatKid_SCX02_23.1767.1767.3.dta
Data file K:\NewmanPaper\Piliang\3SubProteomes\Piliang3SP\mgf5ppm\SCX_3SubProteomes5ppm.mgf

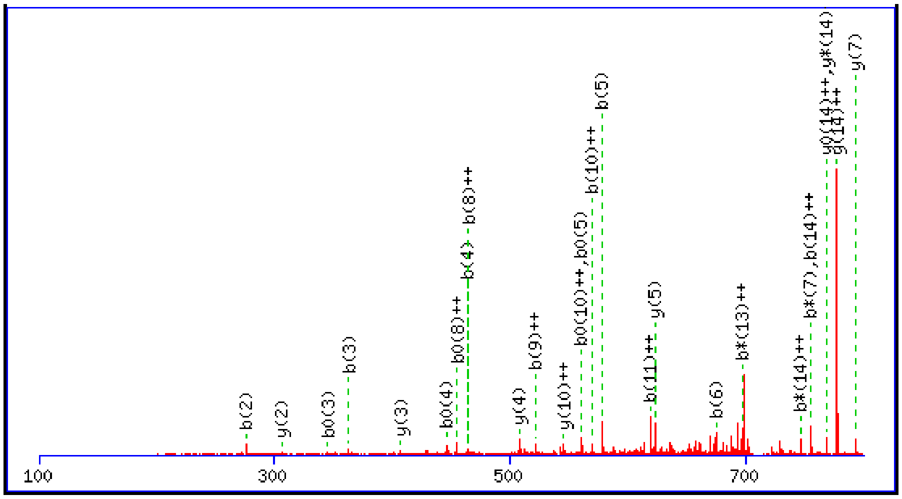

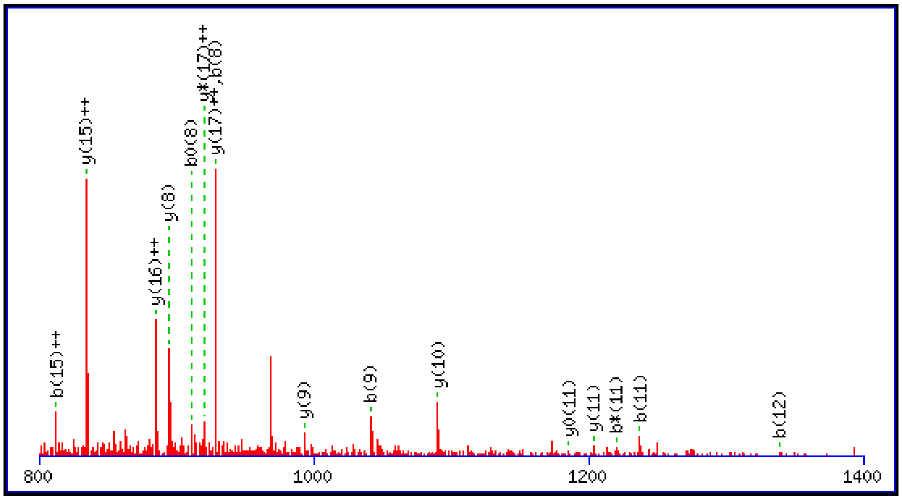

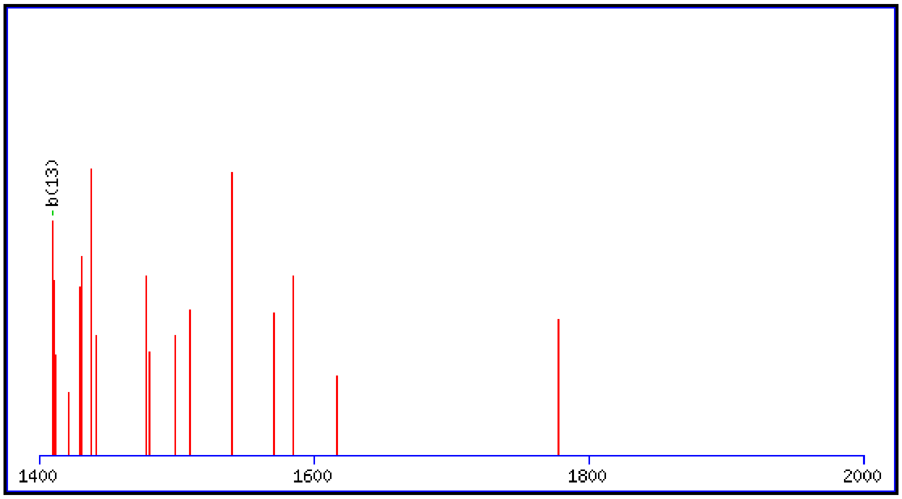

Monoisotopic mass of neutral peptide Mr(calc): 2130.0198
Fixed modifications: Carbamidomethyl (C)
Variable modifications:
N15 : Deamidated_N (N)
Ions Score: 55 **Expect:** 0.00071
Matches (Bold Red): 44/204 fragment ions using 90 most intense peaks

#	b	b^{++}	b*	b^{*++}	b^0	b^{0++}	Seq.	y	y^{++}	y*	y^{*++}	y^0	y^{0++}	#
1	116.0342	58.5207			98.0237	49.5155	D							19
2	**276.0649**	138.5361			258.0543	129.5308	C	2016.0001	1008.5037	1998.9736	999.9904	1997.9895	999.4984	18
3	**363.0969**	182.0521			**345.0863**	173.0468	S	1855.9695	**928.4884**	1838.9429	**919.9751**	1837.9589	919.4831	17
4	**464.1446**	232.5759			**446.1340**	223.5706	T	1768.9374	**884.9724**	1751.9109	876.4591	1750.9269	875.9671	16
5	**578.1875**	289.5974	561.1610	281.0841	**560.1769**	280.5921	N	1667.8897	**834.4485**	1650.8632	825.9352	1649.8792	825.4432	15

**6**	**675.2403**	338.1238	658.2137	329.6105	657.2297	329.1185	**P**	1553.8468	**777.4270**	1536.8203	**768.9138**	1535.8363	**768.4218**	**14**
**7**	772.2930	386.6502	**755.2665**	378.1369	754.2825	377.6449	**P**	1456.7941	728.9007	1439.7675	720.3874	1438.7835	719.8954	**13**
**8**	**928.3941**	**464.7007**	911.3676	456.1874	**910.3836**	**455.6954**	**R**	1359.7413	680.3743	1342.7147	671.8610	1341.7307	671.3690	**12**
**9**	**1041.4782**	**521.2427**	1024.4517	512.7295	1023.4676	512.2375	**L**	**1203.6402**	602.3237	1186.6136	593.8105	**1185.6296**	593.3184	**11**
**10**	1138.5310	**569.7691**	1121.5044	561.2558	1120.5204	**560.7638**	**P**	**1090.5561**	**545.7817**	1073.5296	537.2684	1072.5456	536.7764	**10**
**11**	**1237.5994**	**619.3033**	**1220.5728**	610.7901	1219.5888	610.2980	**V**	**993.5034**	497.2553	976.4768	488.7420	975.4928	488.2500	**9**
**12**	**1338.6471**	669.8272	1321.6205	661.3139	1320.6365	660.8219	**T**	**894.4349**	447.7211	877.4084	439.2078	876.4244	438.7158	**8**
**13**	**1409.6842**	705.3457	1392.6576	**696.8325**	1391.6736	696.3404	**A**	**793.3873**	397.1973	776.3607	388.6840	775.3767	388.1920	**7**
**14**	1508.7526	**754.8799**	1491.7260	**746.3667**	1490.7420	745.8747	**V**	722.3501	361.6787	705.3236	353.1654	704.3396	352.6734	**6**
**15**	1623.7795	**812.3934**	1606.7530	803.8801	1605.7690	803.3881	**N**	**623.2817**	312.1445	606.2552	303.6312	605.2712	303.1392	**5**
**16**	1724.8272	862.9172	1707.8007	854.4040	1706.8166	853.9120	**T**	**508.2548**	254.6310	491.2282	246.1178	490.2442	245.6258	**4**
**17**	1825.8749	913.4411	1808.8483	904.9278	1807.8643	904.4358	**T**	**407.2071**	204.1072	390.1806	195.5939	389.1966	195.1019	**3**
**18**	1956.9154	978.9613	1939.8888	970.4480	1938.9048	969.9560	**M**	**306.1594**	153.5834	289.1329	145.0701			**2**
**19**							**R**	175.1190	88.0631	158.0924	79.5498			**1**

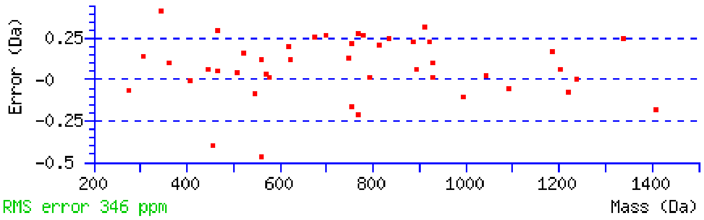

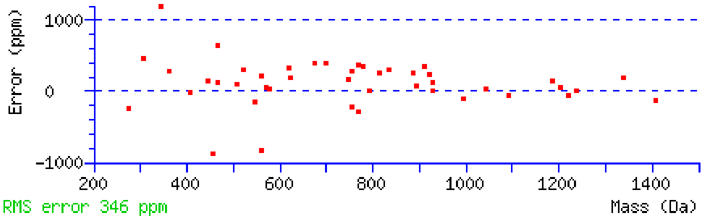

All matches to this query

Score	Mr(calc):	Delta	Sequence
55.1	2130.0198	0.0027	DCSTNPPRLPVTAVNTTMR
28.6	2129.0358	0.9867	DCSTNPPRLPVTAVNTTMR
26.0	2130.0198	0.0027	DCSTNPPRLPVTAVNTTMR
2.6	2129.0201	1.0024	LSYNGRSSVVQCRPLTGR
2.2	2130.0320	-0.0095	IDFSDIMVLGDINTKPKK
2.1	2130.0309	-0.0084	IRVNSVNPTVVLTDMGKK
2.1	2130.0309	-0.0084	IRVNSVNPTVVLTDMGKK
2.1	2130.0309	-0.0084	IRVNSVNPTVVLTDMGKK
1.8	2128.0130	2.0095	DAEVVVVFHEETLVTFSK
1.6	2129.0295	0.9930	HHSTIVYHWMKWLRR

Spectrum No: 385; Query: 358; Rank: 1

Peptide View

MS/MS Fragmentation of **EAGNITTDGYEILGK**
Found in **IPI00189714**, Tax_Id=10116 Gene_Symbol=Col12a1 similar to Collagen alpha-1(XII) chain precursor

Match to Query 358: 1580.761968 from(791.388260,2+)
Title: 091127RatKid_SCX01_12.2706.2706.2.dta
Data file K:\NewmanPaper\Piliang\3SubProteomes\Piliang3SP\mgf5ppm\SCX_3SubProteomes5ppm.mgf

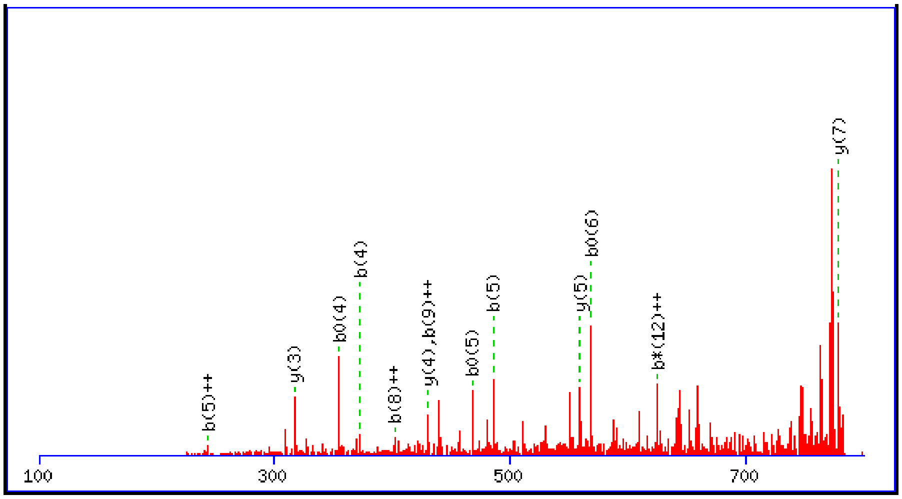

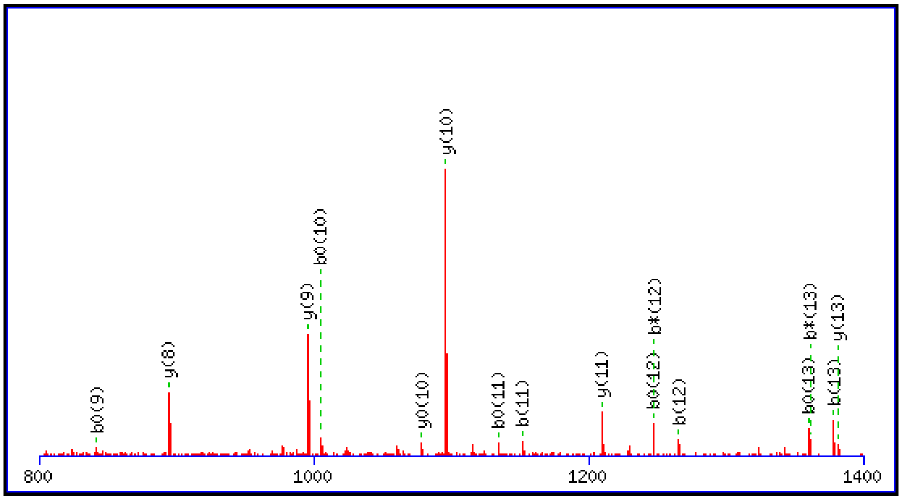

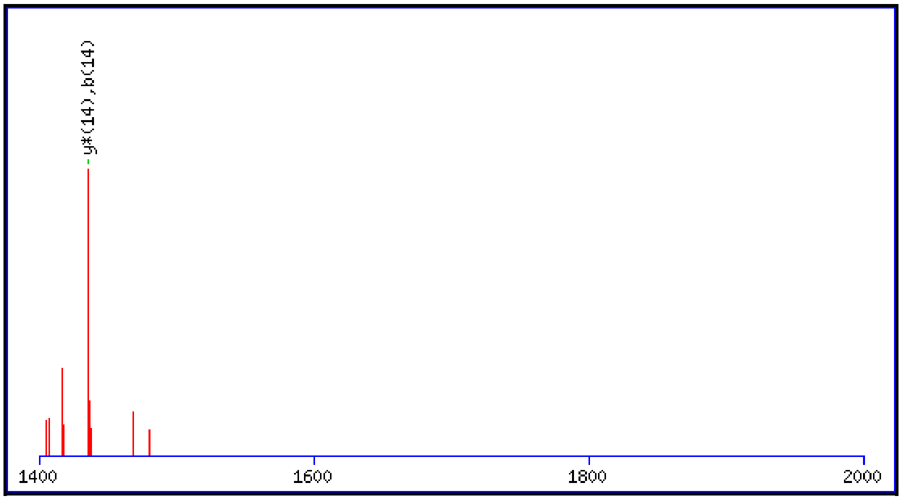

Monoisotopic mass of neutral peptide Mr(calc): 1580.7570
Fixed modifications: Carbamidomethyl (C)
Variable modifications:
N4 : Deamidated_N (N)
Ions Score: 55 **Expect:** 0.00049
Matches (Bold Red): 31/154 fragment ions using 50 most intense peaks

#	b	b^{++}	b*	b^{*++}	b^0	b^{0++}	Seq.	y	y^{++}	y*	y^{*++}	y^0	y^{0++}	#
1	130.0499	65.5286			112.0393	56.5233	E							15
2	201.0870	101.0471			183.0764	92.0418	A	1452.7217	726.8645	**1435.6951**	718.3512	1434.7111	717.8592	14
3	258.1084	129.5579			240.0979	120.5526	G	**1381.6845**	691.3459	1364.6580	682.8326	1363.6740	682.3406	13
4	**373.1354**	187.0713	356.1088	178.5581	**355.1248**	178.0660	N	1324.6631	662.8352	1307.6365	654.3219	1306.6525	653.8299	12
5	**486.2194**	**243.6134**	469.1929	235.1001	**468.2089**	234.6081	I	**1209.6361**	605.3217	1192.6096	596.8084	1191.6256	596.3164	11

6	587.2671	294.1372	570.2406	285.6239	**569.2566**	285.1319	T	**1096.5521**	548.7797	1079.5255	540.2664	**1078.5415**	539.7744	10
7	688.3148	344.6610	671.2883	336.1478	670.3042	335.6558	T	**995.5044**	498.2558	978.4779	489.7426	977.4938	489.2506	9
8	803.3417	**402.1745**	786.3152	393.6612	785.3312	393.1692	D	**894.4567**	447.7320	877.4302	439.2187	876.4462	438.7267	8
9	860.3632	**430.6852**	843.3367	422.1720	**842.3526**	421.6800	G	**779.4298**	390.2185	762.4032	381.7053	761.4192	381.2132	7
10	1023.4265	512.2169	1006.4000	503.7036	**1005.4160**	503.2116	Y	722.4083	361.7078	705.3818	353.1945	704.3978	352.7025	6
11	**1152.4691**	576.7382	1135.4426	568.2249	**1134.4586**	567.7329	E	**559.3450**	280.1761	542.3184	271.6629	541.3344	271.1708	5
12	**1265.5532**	633.2802	**1248.5266**	**624.7670**	**1247.5426**	624.2750	I	**430.3024**	215.6548	413.2758	207.1416			4
13	**1378.6373**	689.8223	**1361.6107**	681.3090	**1360.6267**	680.8170	L	**317.2183**	159.1128	300.1918	150.5995			3
14	**1435.6587**	718.3330	1418.6322	709.8197	1417.6482	709.3277	G	204.1343	102.5708	187.1077	94.0575			2
15							K	147.1128	74.0600	130.0863	65.5468			1

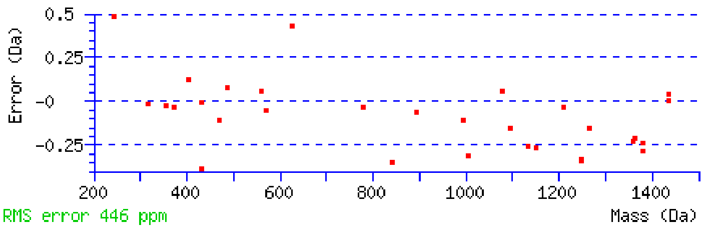

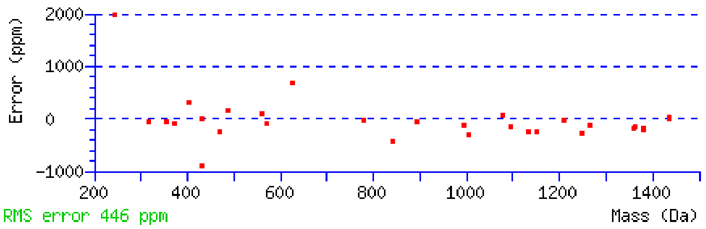

All matches to this query

Score	Mr(calc):	Delta	Sequence
55.2	1580.7570	0.0050	EAGNITTDGYEILGK
18.7	1579.7729	0.9890	EAGNITTDGYEILGK
10.0	1580.7575	0.0045	GHFYMHVGSLLLK
6.3	1580.7682	-0.0062	ERVPENPSNALPEK
4.8	1580.7521	0.0099	TDLPCDAAIATILK
3.5	1580.7534	0.0085	GTLLXWSINCPGK
2.9	1580.7521	0.0099	MPQLLNSILNVNK
2.9	1580.7521	0.0099	MPQLLNSILNVNK
2.9	1580.7521	0.0099	MPQLLNSILNVNK
2.9	1580.7633	-0.0014	TKAQGPALDLICSK

Spectrum No: 386; Query: 1024; Rank: 1

Peptide View

MS/MS Fragmentation of **ENYAELLDDGFLKNITAQICIDKK**
Found in **IPI00371853**, Tax_Id=10116 Gene_Symbol=Col6a1_predicted similar to Collagen alpha-1(VI) chain precursor

Match to Query 1024: 2812.412082 from(938.477970,3+)
Title: 091127RatKid_SCX01_31.6276.6276.3.dta
Data file K:\NewmanPaper\Piliang\3SubProteomes\Piliang3SP\mgf5ppm\SCX_3SubProteomes5ppm.mgf

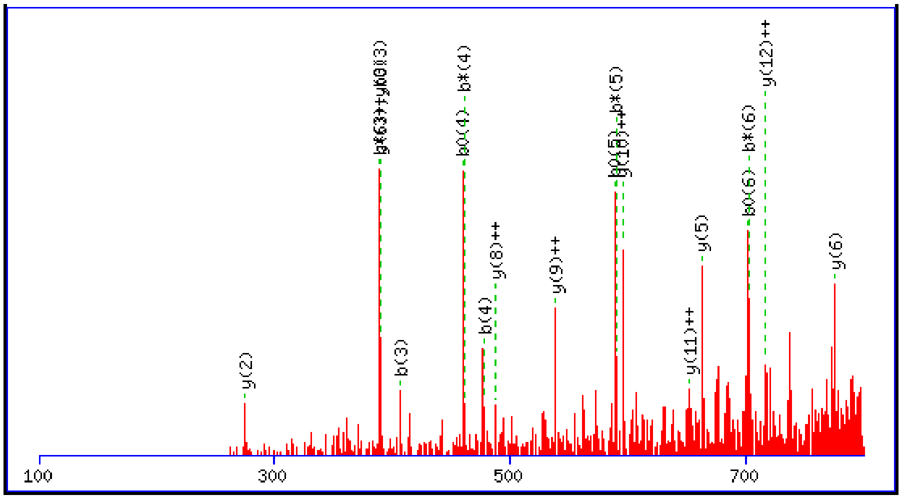

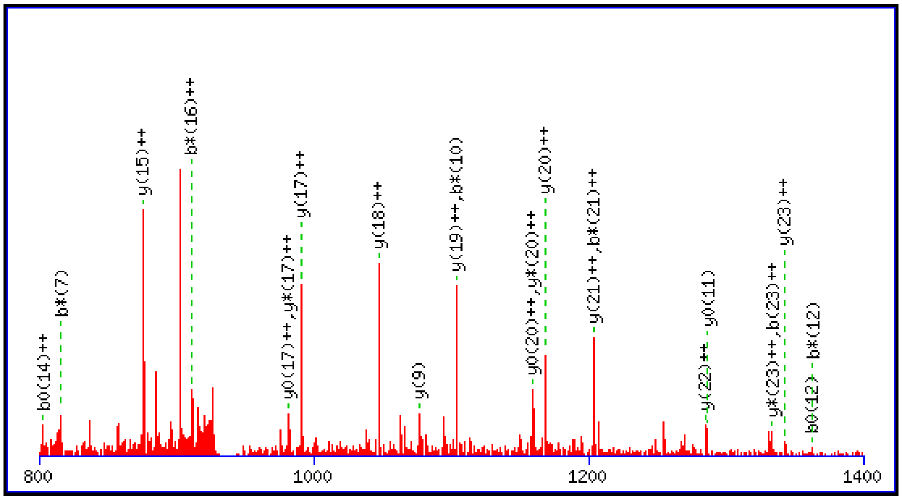

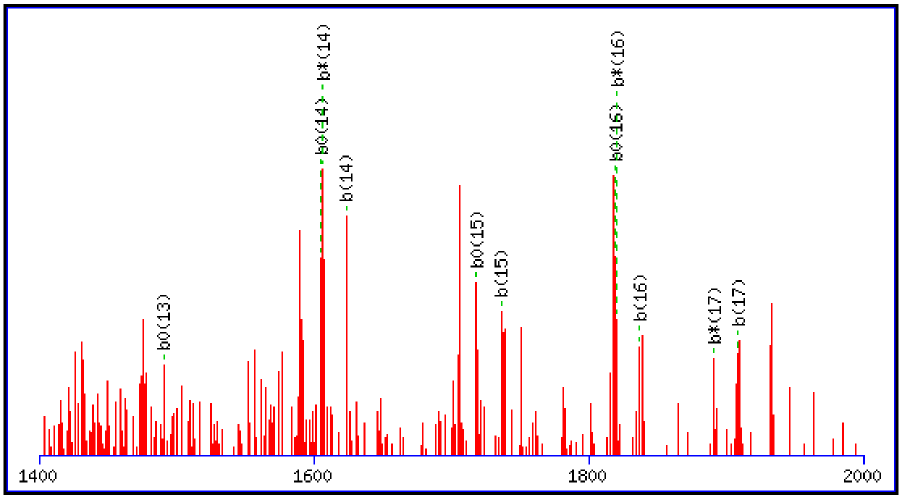

Monoisotopic mass of neutral peptide Mr(calc): 2811.4000
Fixed modifications: Carbamidomethyl (C)
Variable modifications:
N14 : Deamidated_N (N)
Ions Score: 55 **Expect:** 0.00064
Matches (Bold Red): 54/270 fragment ions using 98 most intense peaks

#	b	b^{++}	b*	b^{*++}	b^0	b^{0++}	Seq.	y	y^{++}	y*	y^{*++}	y^0	y^{0++}	#
1	130.0499	65.5286			112.0393	56.5233	E							24
2	244.0928	122.5500	227.0662	114.0368	226.0822	113.5448	N	2683.3647	**1342.1860**	2666.3382	**1333.6727**	2665.3542	1333.1807	23
3	**407.1561**	204.0817	**390.1296**	195.5684	**389.1456**	195.0764	Y	2569.3218	**1285.1645**	2552.2953	1276.6513	2551.3112	1276.1593	22
4	**478.1932**	239.6003	**461.1667**	231.0870	**460.1827**	230.5950	A	2406.2585	**1203.6329**	2389.2319	1195.1196	2388.2479	1194.6276	21
5	607.2358	304.1216	**590.2093**	295.6083	**589.2253**	295.1163	E	2335.2214	**1168.1143**	2318.1948	**1159.6010**	2317.2108	**1159.1090**	20

6	720.3199	360.6636	703.2933	352.1503	702.3093	351.6583	L	2206.1788	1103.5930	2189.1522	1095.0798	2188.1682	1094.5877	19
7	833.4040	417.2056	816.3774	408.6923	815.3934	408.2003	L	2093.0947	1047.0510	2076.0682	1038.5377	2075.0841	1038.0457	18
8	948.4309	474.7191	931.4044	466.2058	930.4203	465.7138	D	1980.0106	990.5090	1962.9841	981.9957	1962.0001	981.5037	17
9	1063.4578	532.2326	1046.4313	523.7193	1045.4473	523.2273	D	1864.9837	932.9955	1847.9572	924.4822	1846.9731	923.9902	16
10	1120.4793	560.7433	1103.4528	552.2300	1102.4687	551.7380	G	1749.9568	875.4820	1732.9302	866.9687	1731.9462	866.4767	15
11	1267.5477	634.2775	1250.5212	625.7642	1249.5372	625.2722	F	1692.9353	846.9713	1675.9088	838.4580	1674.9247	837.9660	14
12	1380.6318	690.8195	1363.6052	682.3063	1362.6212	681.8142	L	1545.8669	773.4371	1528.8403	764.9238	1527.8563	764.4318	13
13	1508.7268	754.8670	1491.7002	746.3537	1490.7162	745.8617	K	1432.7828	716.8950	1415.7563	708.3818	1414.7723	707.8898	12
14	1623.7537	812.3805	1606.7271	803.8672	1605.7431	803.3752	N	1304.6879	652.8476	1287.6613	644.3343	1286.6773	643.8423	11
15	1736.8378	868.9225	1719.8112	860.4092	1718.8272	859.9172	I	1189.6609	595.3341	1172.6344	586.8208	1171.6504	586.3288	10
16	1837.8854	919.4464	1820.8589	910.9331	1819.8749	910.4411	T	1076.5769	538.7921	1059.5503	530.2788	1058.5663	529.7868	9
17	1908.9225	954.9649	1891.8960	946.4516	1890.9120	945.9596	A	975.5292	488.2682	958.5026	479.7550	957.5186	479.2629	8
18	2036.9811	1018.9942	2019.9546	1010.4809	2018.9706	1009.9889	Q	904.4921	452.7497	887.4655	444.2364	886.4815	443.7444	7
19	2150.0652	1075.5362	2133.0386	1067.0230	2132.0546	1066.5309	I	776.4335	388.7204	759.4069	380.2071	758.4229	379.7151	6
20	2310.0958	1155.5516	2293.0693	1147.0383	2292.0853	1146.5463	C	663.3494	332.1783	646.3229	323.6651	645.3389	323.1731	5
21	2423.1799	1212.0936	2406.1533	1203.5803	2405.1693	1203.0883	I	503.3188	252.1630	486.2922	243.6498	485.3082	243.1577	4
22	2538.2068	1269.6071	2521.1803	1261.0938	2520.1963	1260.6018	D	390.2347	195.6210	373.2082	187.1077	372.2241	186.6157	3
23	2666.3018	1333.6545	2649.2753	1325.1413	2648.2912	1324.6493	K	275.2078	138.1075	258.1812	129.5942			2
24							K	147.1128	74.0600	130.0863	65.5468			1

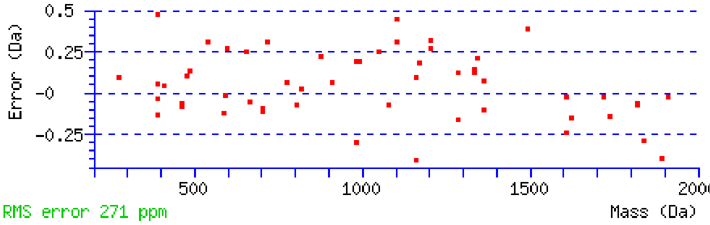

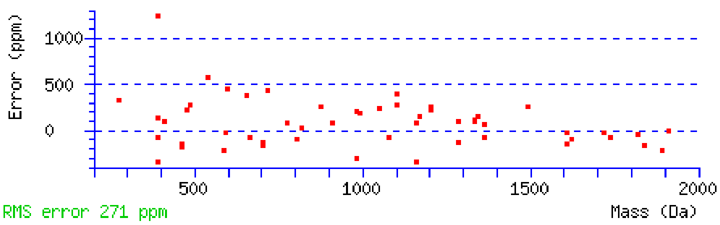

All matches to this query

Score	Mr(calc):	Delta	Sequence
55.1	2811.4000	1.0121	ENYAELLDDGFLKNITAQICIDKK
43.8	2812.3840	0.0281	ENYAELLDDGFLKNITAQICIDKK
24.0	2810.4160	1.9961	ENYAELLDDGFLKNITAQICIDKK
17.6	2812.3928	0.0193	NMILHFYGTIIGLYLCPSGNNSTVK
16.0	2812.3928	0.0193	NMILHFYGTIIGLYLCPSGNNSTVK
12.4	2811.4000	1.0121	ENYAELLDDGFLKNITAQICIDKK
3.4	2812.3926	0.0195	IEHQVLCEGHGKQASPQLQPDVSQK
2.0	2811.4088	1.0033	NMILHFYGTIIGLYLCPSGNNSTVK
1.5	2810.3982	2.0139	NKITSVLYSVLMPTLNPLIYTLR
1.5	2810.3982	2.0139	NKITSVLYSVLMPTLNPLIYTLR

Spectrum No: 387; Query: 448; Rank: 1

Peptide View

MS/MS Fragmentation of **GGGGPWANGSALALCQR**
Found in **IPI00370827**, Tax_Id=10116 Gene_Symbol=Mcoln1_predicted mucolipin 1

Match to Query 448: 1671.776648 from(836.895600,2+)
Title: 091127RatKid_SCX01_02.3049.3049.2.dta
Data file K:\NewmanPaper\Piliang\3SubProteomes\Piliang3SP\mgf5ppm\SCX_3SubProteomes5ppm.mgf

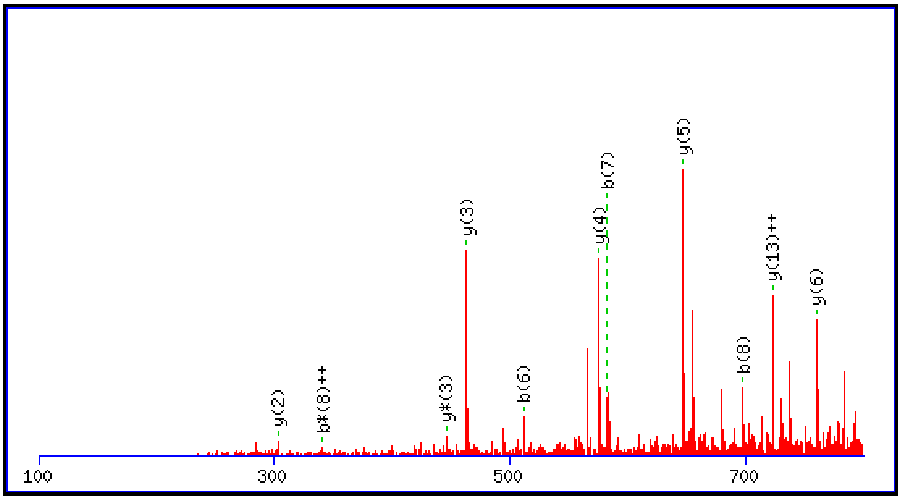

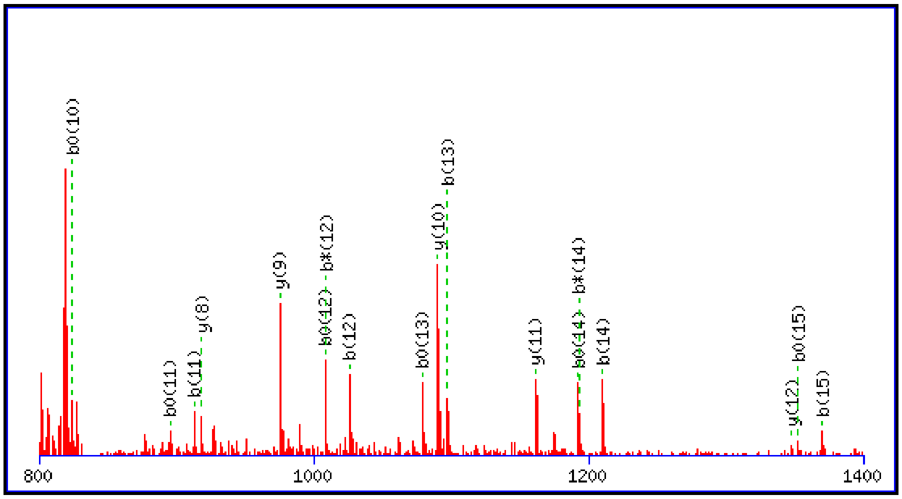

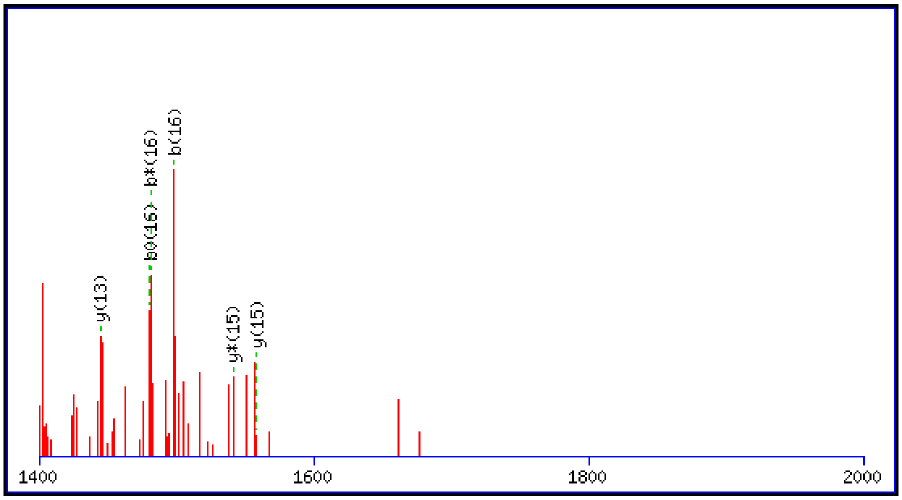

Monoisotopic mass of neutral peptide Mr(calc): 1671.7787
Fixed modifications: Carbamidomethyl (C)
Variable modifications:
N8 : Deamidated_N (N)
Ions Score: 54 **Expect:** 0.00063
Matches (Bold Red): 35/146 fragment ions using 91 most intense peaks

#	b	b^{++}	b*	b^{*++}	b^0	b^{0++}	Seq.	y	y^{++}	y*	y^{*++}	y^0	y^{0++}	#
1	58.0287	29.5180					G							17
2	115.0502	58.0287					G	1615.7645	808.3859	1598.7380	799.8726	1597.7540	799.3806	16
3	172.0717	86.5395					G	**1558.7431**	779.8752	**1541.7165**	771.3619	1540.7325	770.8699	15
4	229.0931	115.0502					G	1501.7216	751.3644	1484.6951	742.8512	1483.7111	742.3592	14
5	326.1459	163.5766					P	**1444.7002**	**722.8537**	1427.6736	714.3404	1426.6896	713.8484	13

**6**	**512.2252**	256.6162					**W**	**1347.6474**	674.3273	1330.6208	665.8141	1329.6368	665.3221	**12**
**7**	**583.2623**	292.1348					**A**	**1161.5681**	581.2877	1144.5415	572.7744	1143.5575	572.2824	**11**
**8**	**698.2893**	349.6483	681.2627	**341.1350**			**N**	**1090.5310**	545.7691	1073.5044	537.2558	1072.5204	536.7638	**10**
**9**	755.3107	378.1590	738.2842	369.6457			**G**	**975.5040**	488.2557	958.4775	479.7424	957.4935	479.2504	**9**
**10**	842.3428	421.6750	825.3162	413.1617	**824.3322**	412.6697	**S**	**918.4826**	459.7449	901.4560	451.2316	900.4720	450.7396	**8**
**11**	**913.3799**	457.1936	896.3533	448.6803	**895.3693**	448.1883	**A**	831.4505	416.2289	814.4240	407.7156			**7**
**12**	**1026.4639**	513.7356	**1009.4374**	505.2223	**1008.4534**	504.7303	**L**	**760.4134**	380.7103	743.3869	372.1971			**6**
**13**	**1097.5010**	549.2542	1080.4745	540.7409	**1079.4905**	540.2489	**A**	**647.3294**	324.1683	630.3028	315.6550			**5**
**14**	**1210.5851**	605.7962	**1193.5586**	597.2829	**1192.5745**	596.7909	**L**	**576.2922**	288.6498	559.2657	280.1365			**4**
**15**	**1370.6158**	685.8115	1353.5892	677.2982	**1352.6052**	676.8062	**C**	**463.2082**	232.1077	**446.1816**	223.5945			**3**
**16**	**1498.6743**	749.8408	**1481.6478**	741.3275	**1480.6638**	740.8355	**Q**	**303.1775**	152.0924	286.1510	143.5791			**2**
**17**							**R**	175.1190	88.0631	158.0924	79.5498			**1**

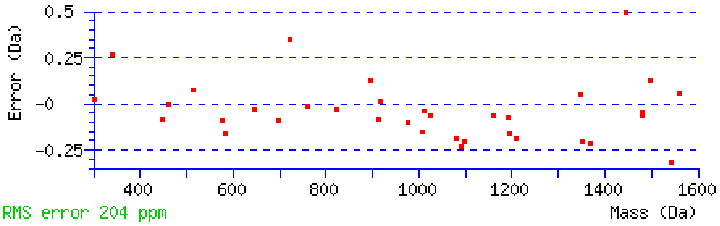

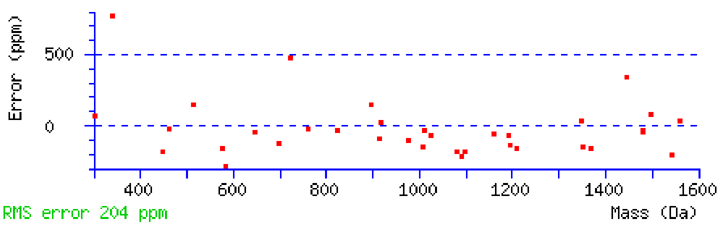

All matches to this query

Score	Mr(calc):	Delta	Sequence
54.5	1671.7787	-0.0021	GGGGPWANGSALALCQR
4.2	1671.7882	-0.0116	GRSVGKGWLSFNQR
4.2	1671.7886	-0.0120	LGPGQSEVAEELCQR
3.2	1671.7691	0.0075	SSQCAEPKYKPLGK
2.9	1671.7882	-0.0116	GRSVGKGWLSFNQR
1.7	1671.7626	0.0140	MPNVFQNLVSCKR

Spectrum No: 388; Query: 15; Rank: 1

Peptide View

MS/MS Fragmentation of **LLNNLTSIK**
Found in **IPI00363849**, Tax_Id=10116 Gene_Symbol=Lamc1 similar to Laminin gamma-1 chain precursor

Match to Query 15: 1015.593668 from(508.804110,2+)
Title: 091129RatKid_SCX02_13.1737.1737.2.dta
Data file K:\NewmanPaper\Piliang\3SubProteomes\Piliang3SP\mgf5ppm\SCX_3SubProteomes5ppm.mgf

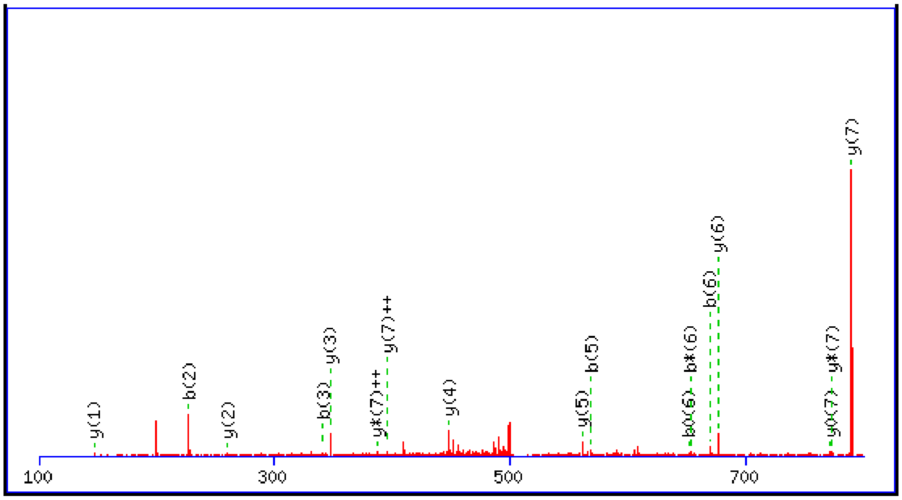

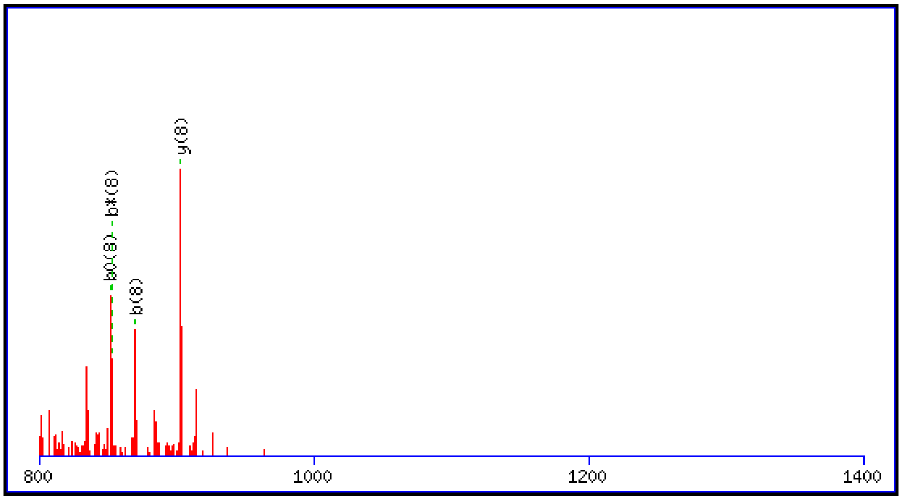

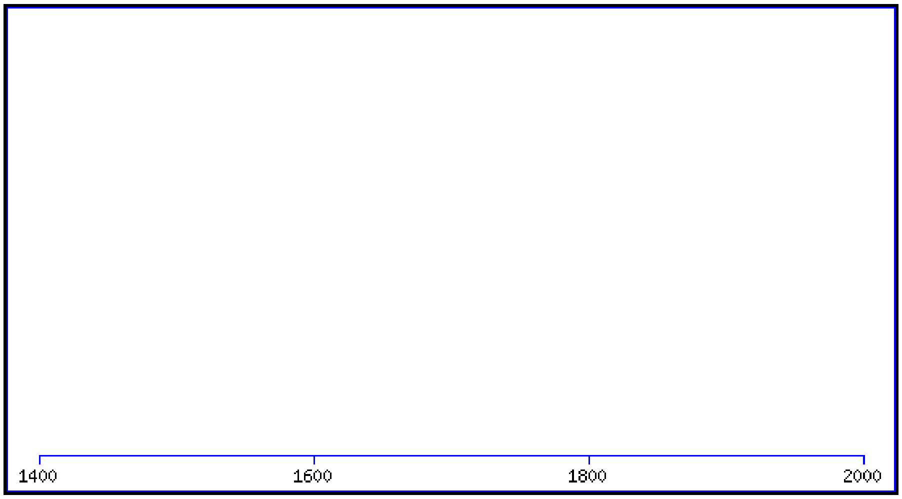

Monoisotopic mass of neutral peptide Mr(calc): 1015.5913
Fixed modifications: Carbamidomethyl (C)
Variable modifications:
N4 : Deamidated_N (N)
Ions Score: 54 **Expect:** 0.00024
Matches (Bold Red): 21/78 fragment ions using 48 most intense peaks

#	b	b++	b*	b*++	b0	b0++	Seq.	y	y++	y*	y*++	y0	y0++	#
1	114.0913	57.5493					L							9
2	**227.1754**	114.0913					L	**903.5146**	452.2609	886.4880	443.7476	885.5040	443.2556	8
3	**341.2183**	171.1128	324.1918	162.5995			N	**790.4305**	**395.7189**	**773.4040**	**387.2056**	**772.4199**	386.7136	7
4	456.2453	228.6263	439.2187	220.1130			N	**676.3876**	338.6974	659.3610	330.1842	658.3770	329.6921	6
5	**569.3293**	285.1683	552.3028	276.6550			L	**561.3606**	281.1840	544.3341	272.6707	543.3501	272.1787	5

6	670.3770	335.6921	653.3505	327.1789	652.3664	326.6869	T	448.2766	224.6419	431.2500	216.1287	430.2660	215.6366	4
7	757.4090	379.2082	740.3825	370.6949	739.3985	370.2029	S	347.2289	174.1181	330.2023	165.6048	329.2183	165.1128	3
8	870.4931	435.7502	853.4666	427.2369	852.4825	426.7449	I	260.1969	130.6021	243.1703	122.0888			2
9							K	147.1128	74.0600	130.0863	65.5468			1

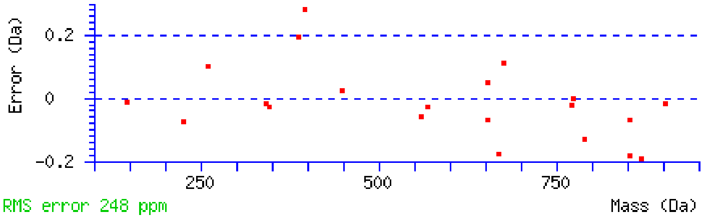

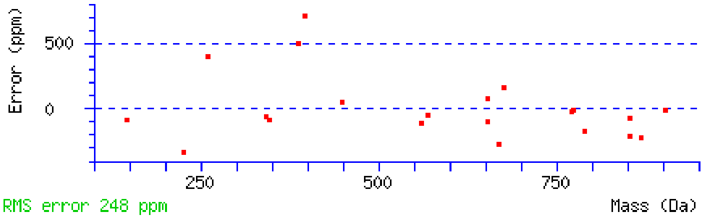

All matches to this query

Score	Mr(calc):	Delta	Sequence
54.5	1015.5913	0.0023	LLNNLTSIK
40.8	1015.5913	0.0023	LLNNLTSIK
20.9	1015.6026	-0.0089	LLLSSREAK
16.6	1015.5913	0.0023	LNILDTLSK
11.1	1015.6026	-0.0089	LIAKSNGKGK
10.4	1015.5913	0.0023	LILDSLSQK
8.4	1015.5927	0.0010	ILQYARPR
7.3	1015.6026	-0.0089	IITTAVDKR
5.7	1015.5848	0.0089	ILQRLNMK
5.3	1015.6026	-0.0089	LDRALSLTK

Spectrum No: 389; Query: 355; Rank: 1

Peptide View

MS/MS Fragmentation of **VYGQNINNETWSR**
Found in **IPI00361557**, Tax_Id=10116 Gene_Symbol=Cnnm2 94 kDa protein

Match to Query 355: 1580.725348 from(791.369950,2+)
Title: 091127RatKid_SCX01_13.1743.1743.2.dta
Data file K:\NewmanPaper\Piliang\3SubProteomes\Piliang3SP\mgf5ppm\SCX_3SubProteomes5ppm.mgf

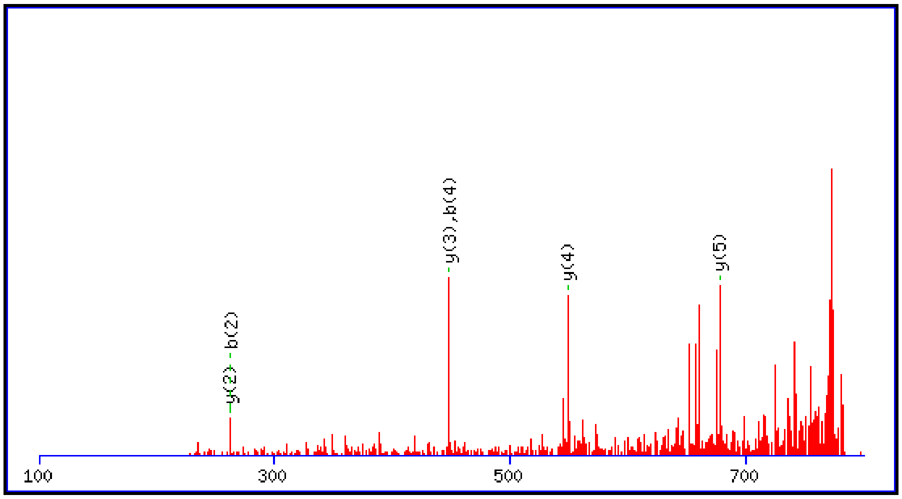

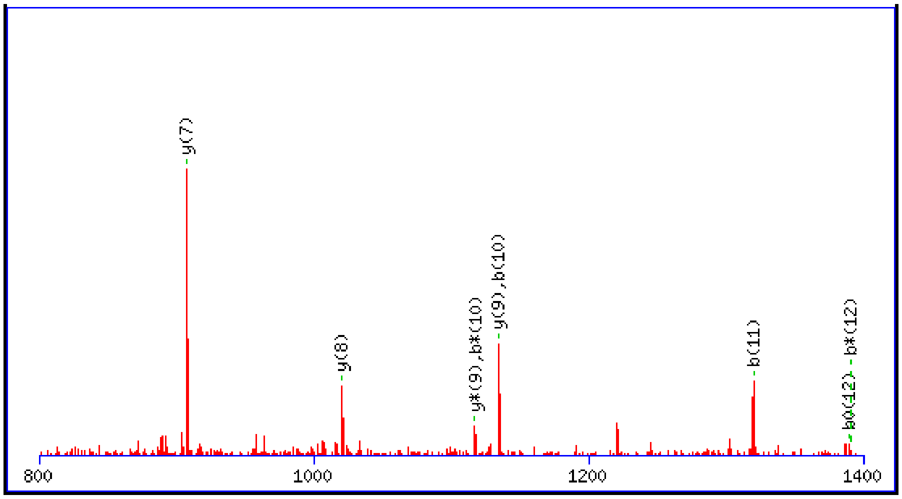

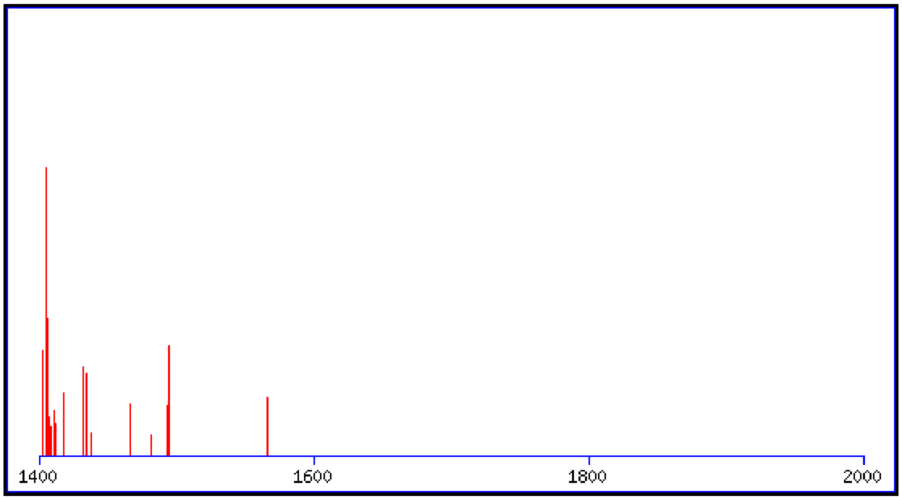

Monoisotopic mass of neutral peptide Mr(calc): 1580.7219
Fixed modifications: Carbamidomethyl (C)
Variable modifications:
N8 : Deamidated_N (N)
Ions Score: 54 **Expect:** 0.00068
Matches (Bold Red): 15/120 fragment ions using 20 most intense peaks

#	b	b^{++}	b*	b^{*++}	b^0	b^{0++}	Seq.	y	y^{++}	y*	y^{*++}	y^0	y^{0++}	#
1	100.0757	50.5415					V							13
2	**263.1390**	132.0731					Y	1482.6608	741.8340	1465.6342	733.3208	1464.6502	732.8288	12
3	320.1605	160.5839					G	1319.5975	660.3024	1302.5709	651.7891	1301.5869	651.2971	11
4	**448.2191**	224.6132	431.1925	216.0999			Q	1262.5760	631.7916	1245.5494	623.2784	1244.5654	622.7864	10
5	562.2620	281.6346	545.2354	273.1214			N	**1134.5174**	567.7623	**1117.4909**	559.2491	1116.5069	558.7571	9
6	675.3461	338.1767	658.3195	329.6634			I	**1020.4745**	510.7409	1003.4479	502.2276	1002.4639	501.7356	8
7	789.3890	395.1981	772.3624	386.6849			N	**907.3904**	454.1989	890.3639	445.6856	889.3799	445.1936	7
8	904.4159	452.7116	887.3894	444.1983			N	793.3475	397.1774	776.3210	388.6641	775.3369	388.1721	6
9	1033.4585	517.2329	1016.4320	508.7196	1015.4479	508.2276	E	**678.3206**	339.6639	661.2940	331.1506	660.3100	330.6586	5
10	**1134.5062**	567.7567	**1117.4796**	559.2435	1116.4956	558.7514	T	**549.2780**	275.1426	532.2514	266.6293	531.2674	266.1373	4
11	**1320.5855**	660.7964	1303.5590	652.2831	1302.5749	651.7911	W	**448.2303**	224.6188	431.2037	216.1055	430.2197	215.6135	3
12	1407.6175	704.3124	**1390.5910**	695.7991	**1389.6070**	695.3071	S	**262.1510**	131.5791	245.1244	123.0659	244.1404	122.5738	2
13							R	175.1190	88.0631	158.0924	79.5498			1

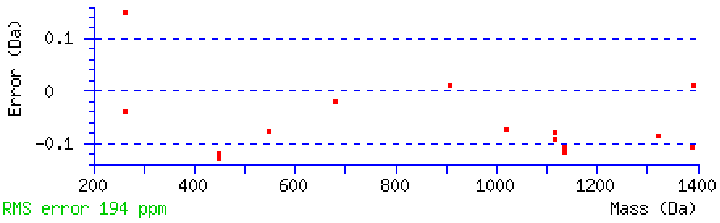

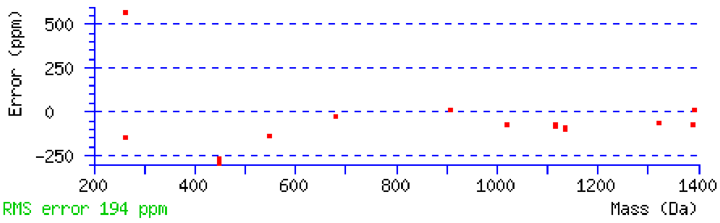

All matches to this query

Score	Mr(calc):	Delta	Sequence
54.3	1580.7219	0.0034	VYGQNINNETWSR
54.3	1580.7219	0.0034	VYGQNINNETWSR
26.3	1580.7219	0.0034	VYGQNINNETWSR
9.1	1580.7154	0.0100	MNNWESRSLSWR
7.5	1580.7174	0.0079	SLDQVTDMMIANSR
5.9	1579.7256	0.9997	APGPTRFDGRPSSR
5.7	1579.7240	1.0014	THHRAHYETEGSR
5.0	1579.7201	1.0053	KVLSVAVYNHYK
4.5	1580.7269	-0.0016	SYLMKLSNXSSSR
4.5	1580.7269	-0.0016	SYLMKLSNXSSSR

Spectrum No: 390; Query: 754; Rank: 1

Peptide View

MS/MS Fragmentation of **DCSTNPPRLPVTAVNTTMR**
Found in **IPI00197684**, Tax_Id=10116 Gene_Symbol=Xpnpep2 Membrane-bound aminopeptidase P

Match to Query 754: 2146.017222 from(716.346350,3+)
Title: 091127RatKid_SCX01_15.1803.1803.3.dta
Data file K:\NewmanPaper\Piliang\3SubProteomes\Piliang3SP\mgf5ppm\SCX_3SubProteomes5ppm.mgf

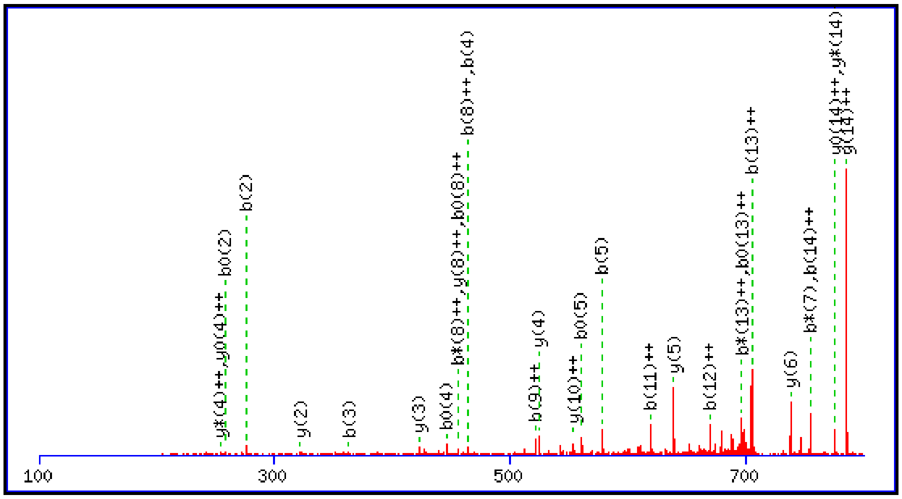

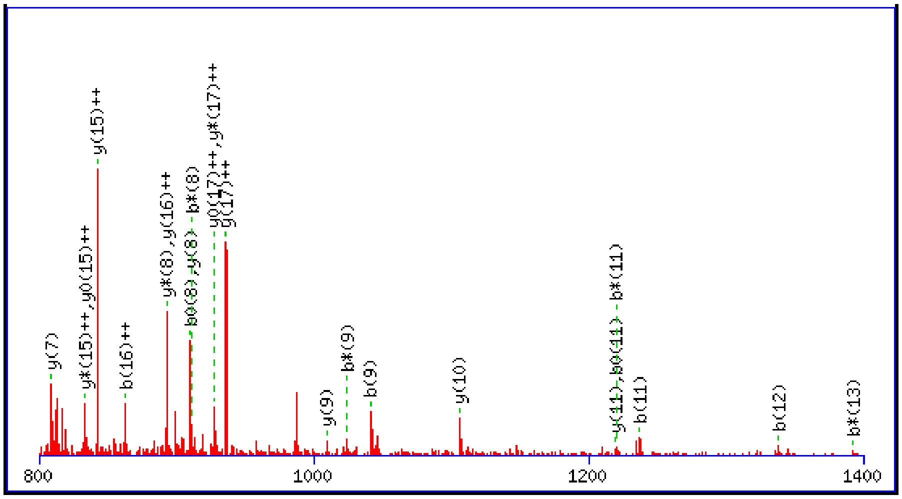

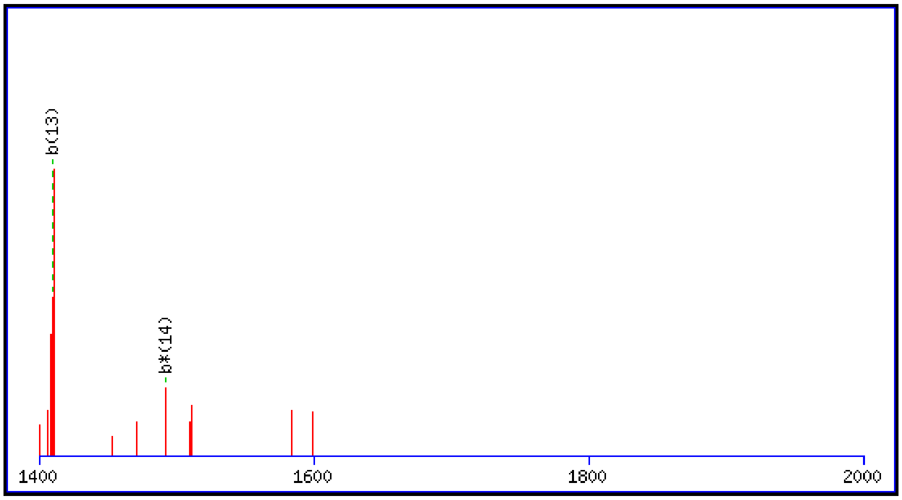

Monoisotopic mass of neutral peptide Mr(calc): 2146.0147
Fixed modifications: Carbamidomethyl (C)
Variable modifications:
N15 : Deamidated_N (N)
M18 : Oxidation (M)
Ions Score: 54 **Expect:** 0.00096
Matches (Bold Red): 55/204 fragment ions using 83 most intense peaks

#	b	b^{++}	b*	b^{*++}	b^{0}	b^{0++}	Seq.	y	y^{++}	y*	y^{*++}	y^{0}	y^{0++}	#
1	116.0342	58.5207			98.0237	49.5155	D							19
2	**276.0649**	138.5361			**258.0543**	129.5308	C	2031.9950	1016.5011	2014.9685	1007.9879	2013.9845	1007.4959	18
3	**363.0969**	182.0521			345.0863	173.0468	S	1871.9644	**936.4858**	1854.9378	**927.9725**	1853.9538	**927.4805**	17
4	**464.1446**	232.5759			**446.1340**	223.5706	T	1784.9323	**892.9698**	1767.9058	884.4565	1766.9218	883.9645	16
5	**578.1875**	289.5974	561.1610	281.0841	**560.1769**	280.5921	N	1683.8847	**842.4460**	1666.8581	**833.9327**	1665.8741	**833.4407**	15
6	675.2403	338.1238	658.2137	329.6105	657.2297	329.1185	P	1569.8417	**785.4245**	1552.8152	**776.9112**	1551.8312	**776.4192**	14
7	772.2930	386.6502	**755.2665**	378.1369	754.2825	377.6449	P	1472.7890	736.8981	1455.7624	728.3849	1454.7784	727.8928	13
8	928.3941	**464.7007**	**911.3676**	**456.1874**	**910.3836**	**455.6954**	R	1375.7362	688.3717	1358.7097	679.8585	1357.7256	679.3665	12
9	**1041.4782**	**521.2427**	**1024.4517**	512.7295	1023.4676	512.2375	L	**1219.6351**	610.3212	1202.6086	601.8079	1201.6245	601.3159	11
10	1138.5310	569.7691	1121.5044	561.2558	1120.5204	560.7638	P	**1106.5510**	**553.7792**	1089.5245	545.2659	1088.5405	544.7739	10
11	**1237.5994**	**619.3033**	**1220.5728**	610.7901	**1219.5888**	610.2980	V	**1009.4983**	505.2528	992.4717	496.7395	991.4877	496.2475	9
12	**1338.6471**	**669.8272**	1321.6205	661.3139	1320.6365	660.8219	T	**910.4299**	**455.7186**	**893.4033**	447.2053	892.4193	446.7133	8
13	**1409.6842**	**705.3457**	**1392.6576**	**696.8325**	1391.6736	**696.3404**	A	**809.3822**	405.1947	792.3556	396.6815	791.3716	396.1894	7
14	1508.7526	**754.8799**	**1491.7260**	746.3667	1490.7420	745.8747	V	**738.3451**	369.6762	721.3185	361.1629	720.3345	360.6709	6
15	1623.7795	812.3934	1606.7530	803.8801	1605.7690	803.3881	N	**639.2767**	320.1420	622.2501	311.6287	621.2661	311.1367	5
16	1724.8272	**862.9172**	1707.8007	854.4040	1706.8166	853.9120	T	**524.2497**	262.6285	507.2232	**254.1152**	506.2391	**253.6232**	4
17	1825.8749	913.4411	1808.8483	904.9278	1807.8643	904.4358	T	**423.2020**	212.1047	406.1755	203.5914	405.1915	203.0994	3
18	1972.9103	986.9588	1955.8837	978.4455	1954.8997	977.9535	M	**322.1544**	161.5808	305.1278	153.0675			2

19						R	175.1190	88.0631	158.0924	79.5498			1

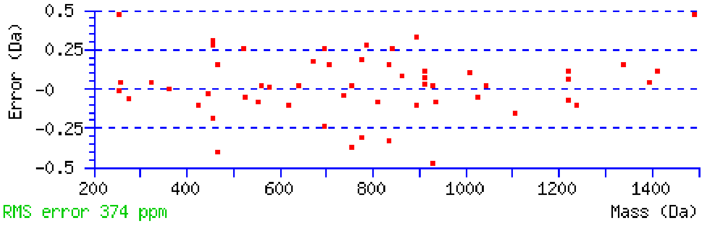

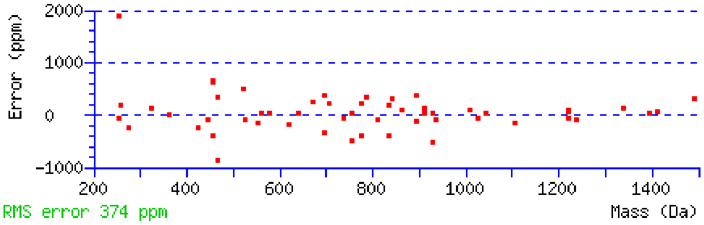

All matches to this query

Score	Mr(calc):	Delta	Sequence
54.3	2146.0147	0.0025	DCSTNPPRLPVTAVNTTMR
32.0	2146.0147	0.0025	DCSTNPPRLPVTAVNTTMR
27.6	2145.0307	0.9865	DCSTNPPRLPVTAVNTTMR
4.7	2143.9901	2.0271	YTDFKMFSDEILLSLAR
4.7	2143.9901	2.0271	YTDFKMFSDEILLSLAR
4.6	2144.0051	2.0121	KKPGAGNANFALDRQNTVY
4.6	2144.0051	2.0121	KKPGAGNANFALDRQNTVY
3.4	2146.0106	0.0066	TITDFDNEFQAFLLSEEK
2.9	2145.0143	1.0029	SLSLFSNVSQSHGIYIGEK
2.1	2144.0110	2.0062	SSRPASASSSTKDITGAVQSK

Spectrum No: 391; Query: 1177; Rank: 1

Peptide View

MS/MS Fragmentation of **NGSITQIPILTMPNDDITHPIPDLTGYITEGQIYVDR**
Found in **IPI00199305**, Tax_Id=10116 Gene_Symbol=Atp6v1b2 Vacuolar ATP synthase subunit B, brain isoform

Match to Query 1177: 4127.044632 from(1376.688820,3+)
Title: 091127RatKid_SCX01_15.6062.6062.3.dta
Data file K:\NewmanPaper\Piliang\3SubProteomes\Piliang3SP\mgf5ppm\SCX_3SubProteomes5ppm.mgf

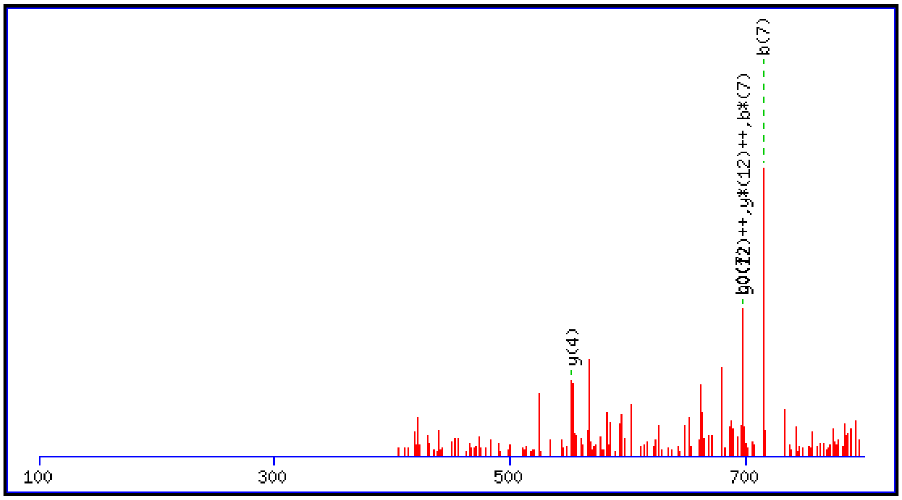

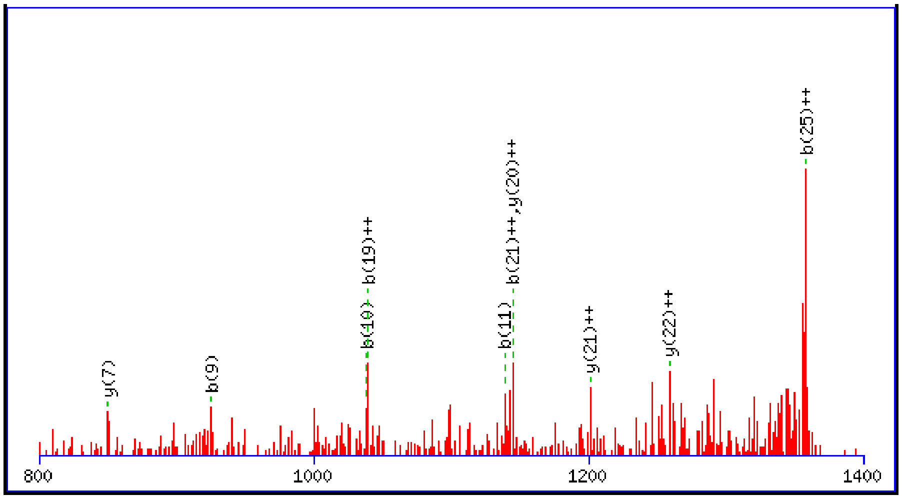

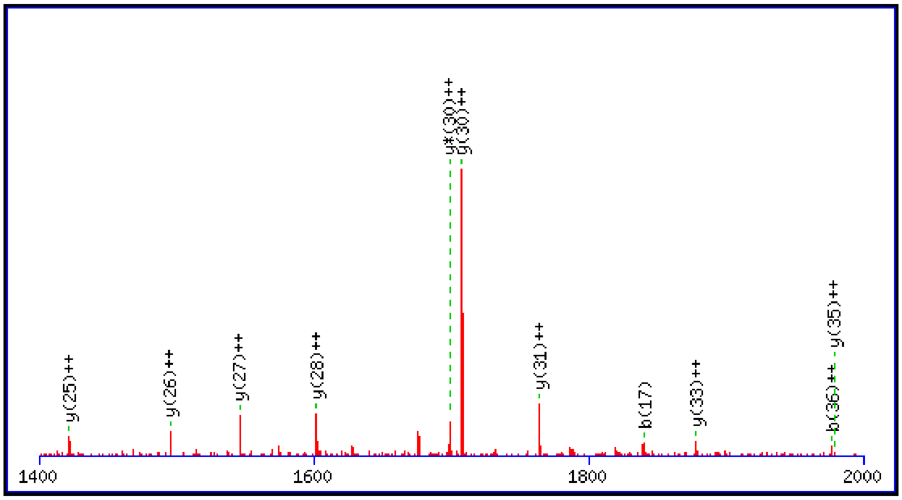

Monoisotopic mass of neutral peptide Mr(calc): 4127.0409
Fixed modifications: Carbamidomethyl (C)
Variable modifications:
N1 : Deamidated_N (N)
M12 : Oxidation (M)
Ions Score: 54 **Expect:** 0.00087
Matches (Bold Red): 27/426 fragment ions using 39 most intense peaks

#	b	b^{++}	b*	b^{*++}	b^0	b^{0++}	Seq.	y	y^{++}	y*	y^{*++}	y^0	y^{0++}	#
1	116.0342	58.5207	99.0077	50.0075			N							37
2	173.0557	87.0315	156.0291	78.5182			G	4013.0212	2007.0143	3995.9947	1998.5010	3995.0107	1998.0090	36
3	260.0877	130.5475	243.0612	122.0342	242.0771	121.5422	S	3955.9998	**1978.5035**	3938.9732	1969.9902	3937.9892	1969.4982	35
4	373.1718	187.0895	356.1452	178.5762	355.1612	178.0842	I	3868.9677	1934.9875	3851.9412	1926.4742	3850.9572	1925.9822	34
5	474.2194	237.6134	457.1929	229.1001	456.2089	228.6081	T	3755.8837	**1878.4455**	3738.8571	1869.9322	3737.8731	1869.4402	33
6	602.2780	301.6427	585.2515	293.1294	584.2675	292.6374	Q	3654.8360	1827.9216	3637.8095	1819.4084	3636.8254	1818.9164	32
7	**715.3621**	358.1847	**698.3355**	349.6714	**697.3515**	349.1794	I	3526.7774	**1763.8923**	3509.7509	1755.3791	3508.7669	1754.8871	31
8	812.4149	406.7111	795.3883	398.1978	794.4043	397.7058	P	3413.6934	**1707.3503**	3396.6668	**1698.8370**	3395.6828	1698.3450	30
9	**925.4989**	463.2531	908.4724	454.7398	907.4884	454.2478	I	3316.6406	1658.8239	3299.6140	1650.3107	3298.6300	1649.8187	29
10	**1038.5830**	519.7951	1021.5564	511.2819	1020.5724	510.7898	L	3203.5565	**1602.2819**	3186.5300	1593.7686	3185.5460	1593.2766	28
11	**1139.6307**	570.3190	1122.6041	561.8057	1121.6201	561.3137	T	3090.4725	**1545.7399**	3073.4459	1537.2266	3072.4619	1536.7346	27
12	1286.6661	643.8367	1269.6395	635.3234	1268.6555	634.8314	M	2989.4248	**1495.2160**	2972.3982	1486.7028	2971.4142	1486.2107	26
13	1383.7188	692.3631	1366.6923	683.8498	1365.7083	683.3578	P	2842.3894	**1421.6983**	2825.3628	1413.1851	2824.3788	1412.6930	25
14	1497.7618	749.3845	1480.7352	740.8712	1479.7512	740.3792	N	2745.3366	1373.1719	2728.3101	1364.6587	2727.3261	1364.1667	24
15	1612.7887	806.8980	1595.7621	798.3847	1594.7781	797.8927	D	2631.2937	1316.1505	2614.2671	1307.6372	2613.2831	1307.1452	23
16	1727.8156	864.4115	1710.7891	855.8982	1709.8051	855.4062	D	2516.2667	**1258.6370**	2499.2402	1250.1237	2498.2562	1249.6317	22
17	**1840.8997**	920.9535	1823.8732	912.4402	1822.8891	911.9482	I	2401.2398	**1201.1235**	2384.2133	1192.6103	2383.2292	1192.1183	21
18	1941.9474	971.4773	1924.9208	962.9641	1923.9368	962.4720	T	2288.1557	**1144.5815**	2271.1292	1136.0682	2270.1452	1135.5762	20

19	2079.0063	1040.0068	2061.9797	1031.4935	2060.9957	1031.0015	H	2187.1081	1094.0577	2170.0815	1085.5444	2169.0975	1085.0524	19
20	2176.0591	1088.5332	2159.0325	1080.0199	2158.0485	1079.5279	P	2050.0492	1025.5282	2033.0226	1017.0149	2032.0386	1016.5229	18
21	2289.1431	1145.0752	2272.1166	1136.5619	2271.1326	1136.0699	I	1952.9964	977.0018	1935.9698	968.4886	1934.9858	967.9965	17
22	2386.1959	1193.6016	2369.1693	1185.0883	2368.1853	1184.5963	P	1839.9123	920.4598	1822.8858	911.9465	1821.9018	911.4545	16
23	2501.2228	1251.1151	2484.1963	1242.6018	2483.2123	1242.1098	D	1742.8596	871.9334	1725.8330	863.4201	1724.8490	862.9281	15
24	2614.3069	1307.6571	2597.2803	1299.1438	2596.2963	1298.6518	L	1627.8326	814.4199	1610.8061	805.9067	1609.8221	805.4147	14
25	2715.3546	1358.1809	2698.3280	1349.6677	2697.3440	1349.1756	T	1514.7486	757.8779	1497.7220	749.3646	1496.7380	748.8726	13
26	2772.3760	1386.6917	2755.3495	1378.1784	2754.3655	1377.6864	G	1413.7009	707.3541	1396.6743	698.8408	1395.6903	698.3488	12
27	2935.4394	1468.2233	2918.4128	1459.7100	2917.4288	1459.2180	Y	1356.6794	678.8433	1339.6529	670.3301	1338.6688	669.8381	11
28	3048.5234	1524.7654	3031.4969	1516.2521	3030.5129	1515.7601	I	1193.6161	597.3117	1176.5895	588.7984	1175.6055	588.3064	10
29	3149.5711	1575.2892	3132.5446	1566.7759	3131.5605	1566.2839	T	1080.5320	540.7696	1063.5055	532.2564	1062.5215	531.7644	9
30	3278.6137	1639.8105	3261.5872	1631.2972	3260.6031	1630.8052	E	979.4843	490.2458	962.4578	481.7325	961.4738	481.2405	8
31	3335.6352	1668.3212	3318.6086	1659.8079	3317.6246	1659.3159	G	850.4417	425.7245	833.4152	417.2112	832.4312	416.7192	7
32	3463.6937	1732.3505	3446.6672	1723.8372	3445.6832	1723.3452	Q	793.4203	397.2138	776.3937	388.7005	775.4097	388.2085	6
33	3576.7778	1788.8925	3559.7513	1780.3793	3558.7672	1779.8873	I	665.3617	333.1845	648.3352	324.6712	647.3511	324.1792	5
34	3739.8411	1870.4242	3722.8146	1861.9109	3721.8306	1861.4189	Y	552.2776	276.6425	535.2511	268.1292	534.2671	267.6372	4
35	3838.9096	1919.9584	3821.8830	1911.4451	3820.8990	1910.9531	V	389.2143	195.1108	372.1878	186.5975	371.2037	186.1055	3
36	3953.9365	1977.4719	3936.9099	1968.9586	3935.9259	1968.4666	D	290.1459	145.5766	273.1193	137.0633	272.1353	136.5713	2
37							R	175.1190	88.0631	158.0924	79.5498			1

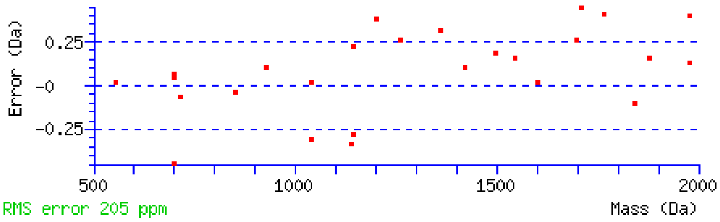

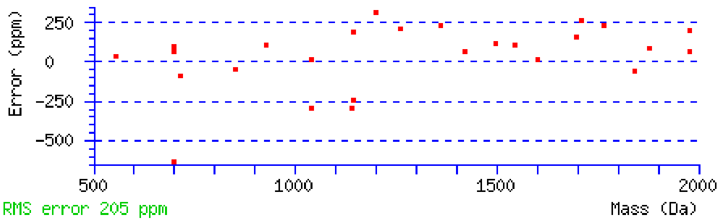

All matches to this query

Score	Mr(calc):	Delta	Sequence
54.3	4127.0409	0.0037	NGSITQIPILTMPNDDITHPIPDLTGYITEGQIYVDR
43.1	4127.0409	0.0037	NGSITQIPILTMPNDDITHPIPDLTGYITEGQIYVDR
42.1	4126.0569	0.9877	NGSITQIPILTMPNDDITHPIPDLTGYITEGQIYVDR
5.1	4126.0016	1.0430	ISTILFGAAYTCLEAATGRALLRQHMDVSGEENPLNK
4.5	4126.0016	1.0430	ISTILFGAAYTCLEAATGRALLRQHMDVSGEENPLNK
4.5	4127.0627	-0.0181	VALVYPNNDPVMFMVAFYGCLLAEVIPVPIEVPLTR
4.5	4127.0627	-0.0181	VALVYPNNDPVMFMVAFYGCLLAEVIPVPIEVPLTR
4.0	4126.0500	0.9946	TGDVLFPLNQSEVITSVGSWQTALMXHQLSKWRVR
4.0	4126.0500	0.9946	TGDVLFPLNQSEVITSVGSWQTALMXHQLSKWRVR
4.0	4126.0500	0.9946	TGDVLFPLNQSEVITSVGSWQTALMXHQLSKWRVR

Spectrum No: 392; Query: 270; Rank: 1

Peptide View

MS/MS Fragmentation of **NVNCSIEESFQR**
Found in **IPI00205374**, Tax_Id=10116 Gene_Symbol=Hmgcl Hydroxymethylglutaryl-CoA lyase, mitochondrial precursor

Match to Query 270: 1482.641068 from(742.327810,2+)
Title: 091129RatKid_SCX02_23.1582.1582.2.dta
Data file K:\NewmanPaper\Piliang\3SubProteomes\Piliang3SP\mgf5ppm\SCX_3SubProteomes5ppm.mgf

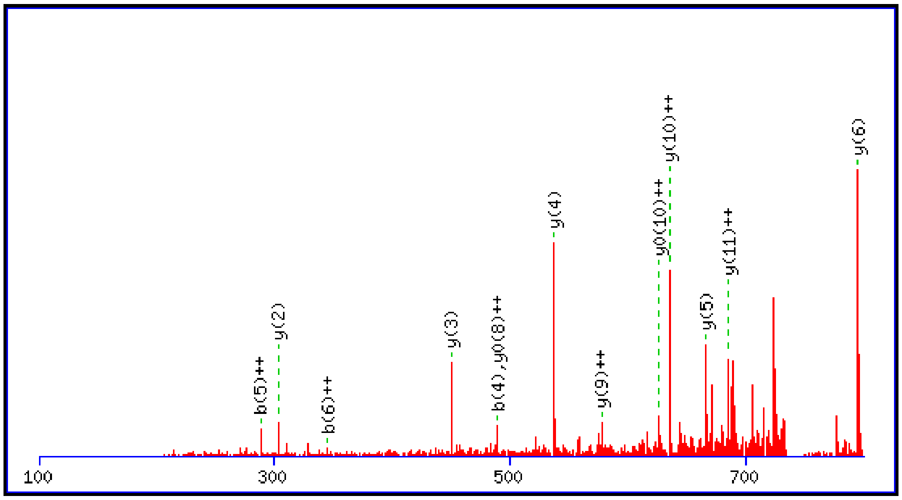

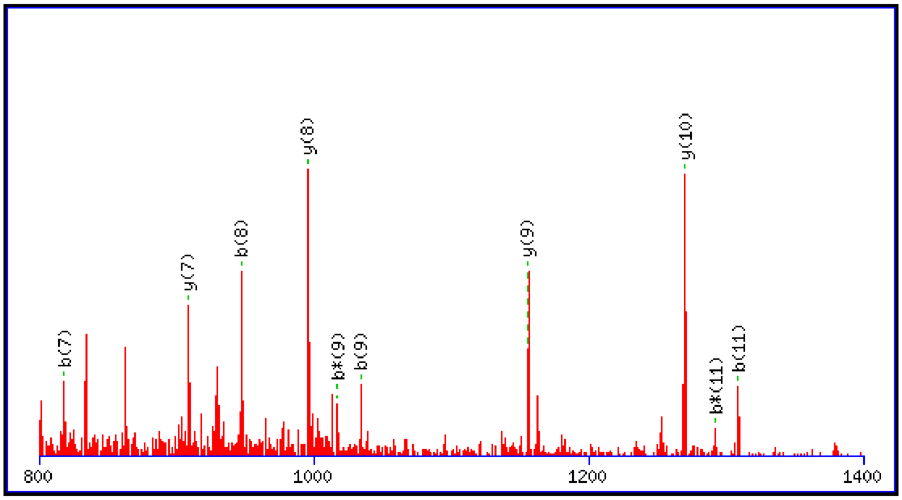

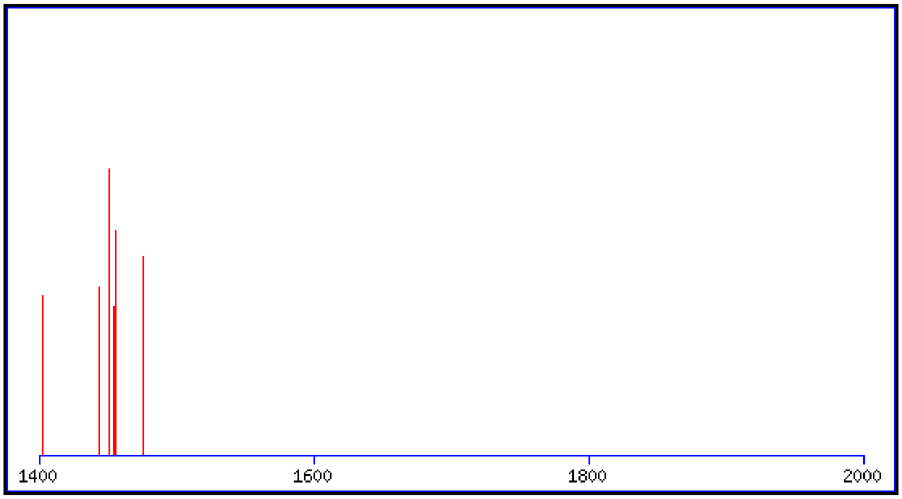

Monoisotopic mass of neutral peptide Mr(calc): 1482.6409
Fixed modifications: Carbamidomethyl (C)
Variable modifications:
N3 : Deamidated_N (N)
Ions Score: 54 **Expect:** 0.00055
Matches (Bold Red): 23/118 fragment ions using 49 most intense peaks

#	b	b^{++}	b*	b^{*++}	b^0	b^{0++}	Seq.	y	y^{++}	y*	y^{*++}	y^0	y^{0++}	#
1	115.0502	58.0287	98.0237	49.5155			N							12
2	214.1186	107.5629	197.0921	99.0497			V	1369.6052	**685.3063**	1352.5787	676.7930	1351.5947	676.3010	11
3	329.1456	165.0764	312.1190	156.5631			N	**1270.5368**	**635.7721**	1253.5103	627.2588	1252.5263	**626.7668**	10
4	**489.1762**	245.0917	472.1497	236.5785			C	**1155.5099**	**578.2586**	1138.4834	569.7453	1137.4993	569.2533	9
5	576.2082	**288.6078**	559.1817	280.0945	558.1977	279.6025	S	**995.4793**	498.2433	978.4527	489.7300	977.4687	**489.2380**	8

**6**	689.2923	**345.1498**	672.2657	336.6365	671.2817	336.1445	**I**	**908.4472**	454.7272	891.4207	446.2140	890.4367	445.7220	**7**
**7**	**818.3349**	409.6711	801.3083	401.1578	800.3243	400.6658	**E**	**795.3632**	398.1852	778.3366	389.6719	777.3526	389.1799	**6**
**8**	**947.3775**	474.1924	930.3509	465.6791	929.3669	465.1871	**E**	**666.3206**	333.6639	649.2940	325.1506	648.3100	324.6586	**5**
**9**	**1034.4095**	517.7084	**1017.3830**	509.1951	1016.3989	508.7031	**S**	**537.2780**	269.1426	520.2514	260.6293	519.2674	260.1373	**4**
**10**	1181.4779	591.2426	1164.4514	582.7293	1163.4674	582.2373	**F**	**450.2459**	225.6266	433.2194	217.1133			**3**
**11**	**1309.5365**	655.2719	**1292.5100**	646.7586	1291.5259	646.2666	**Q**	**303.1775**	152.0924	286.1510	143.5791			**2**
**12**							**R**	175.1190	88.0631	158.0924	79.5498			**1**

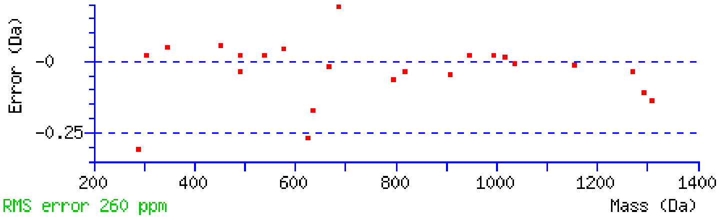

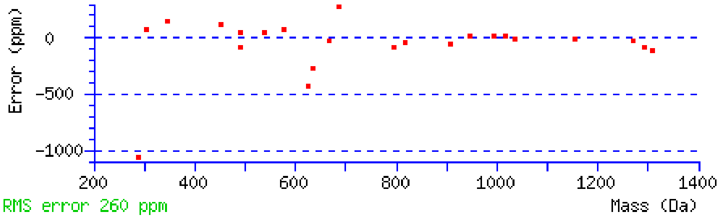

All matches to this query

Score	Mr(calc):	Delta	Sequence
54.2	1482.6409	0.0002	NVNCSIEESFQR
52.1	1482.6409	0.0002	NVNCSIEESFQR
13.1	1482.6398	0.0013	LRTSPALKTTR
8.0	1482.6398	0.0013	LRTSPALKTTR
4.9	1482.6269	0.0142	AVTSISGPGEHLR
0.2	1480.6447	1.9964	LSQSSPDNITDPK
0.1	1482.6399	0.0012	SPSVSPCRRSDR
0.1	1482.6399	0.0012	SPSVSPCRRSDR
0.1	1482.6399	0.0012	SPSVSPCRRSDR

Spectrum No: 393; Query: 236; Rank: 1

Peptide View

MS/MS Fragmentation of **NSTLCDLCIGPAK**
Found in **IPI00196656**, Tax_Id=10116 Gene_Symbol=Srprb Ba1-667

Match to Query 236: 1448.664928 from(725.339740,2+)
Title: 091129RatKid_SCX02_12.1904.1904.2.dta
Data file K:\NewmanPaper\Piliang\3SubProteomes\Piliang3SP\mgf5ppm\SCX_3SubProteomes5ppm.mgf

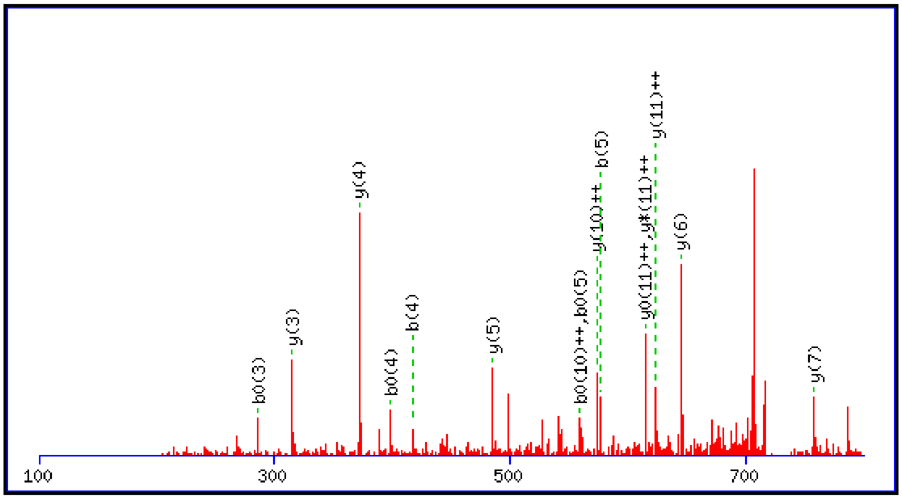

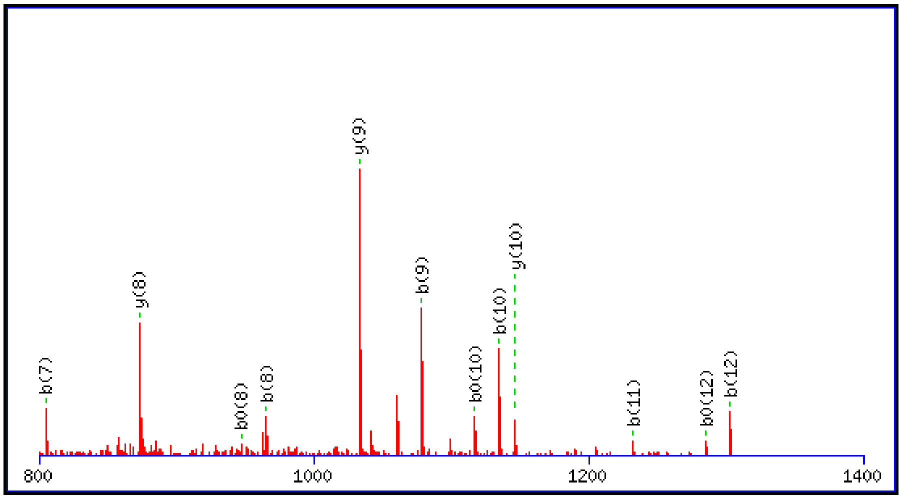

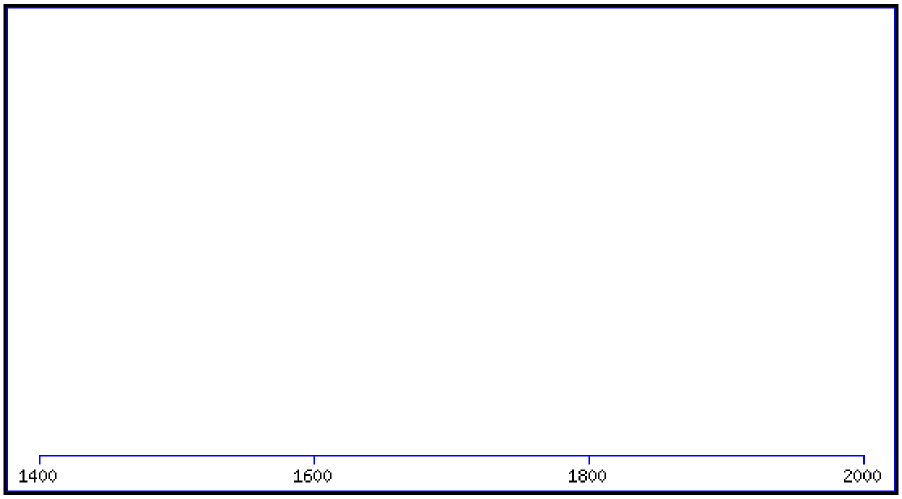

Monoisotopic mass of neutral peptide Mr(calc): 1448.6639
Fixed modifications: Carbamidomethyl (C)
Variable modifications:
N1 : Deamidated_N (N)
Ions Score: 54 **Expect:** 0.00055
Matches (Bold Red): 27/128 fragment ions using 46 most intense peaks

#	b	b^{++}	b^{*}	b^{*++}	b^{0}	b^{0++}	Seq.	y	y^{++}	y^{*}	y^{*++}	y^{0}	y^{0++}	#
1	116.0342	58.5207	99.0077	50.0075			N							13
2	203.0662	102.0368	186.0397	93.5235	185.0557	93.0315	S	1334.6443	667.8258	1317.6177	659.3125	1316.6337	658.8205	12
3	304.1139	152.5606	287.0874	144.0473	**286.1034**	143.5553	T	1247.6123	**624.3098**	1230.5857	**615.7965**	1229.6017	**615.3045**	11
4	**417.1980**	209.1026	400.1714	200.5894	**399.1874**	200.0973	L	**1146.5646**	**573.7859**	1129.5380	565.2727	1128.5540	564.7806	10
5	**577.2286**	289.1180	560.2021	280.6047	**559.2181**	280.1127	C	**1033.4805**	517.2439	1016.4540	508.7306	1015.4699	508.2386	9
6	692.2556	346.6314	675.2290	338.1182	674.2450	337.6261	D	**873.4499**	437.2286	856.4233	428.7153	855.4393	428.2233	8
7	**805.3396**	403.1735	788.3131	394.6602	787.3291	394.1682	L	**758.4229**	379.7151	741.3964	371.2018			7
8	**965.3703**	483.1888	948.3437	474.6755	**947.3597**	474.1835	C	**645.3389**	323.1731	628.3123	314.6598			6
9	**1078.4544**	539.7308	1061.4278	531.2175	1060.4438	530.7255	I	**485.3082**	243.1577	468.2817	234.6445			5
10	**1135.4758**	568.2415	1118.4493	559.7283	**1117.4653**	**559.2363**	G	**372.2241**	186.6157	355.1976	178.1024			4
11	**1232.5286**	616.7679	1215.5020	608.2547	1214.5180	607.7626	P	**315.2027**	158.1050	298.1761	149.5917			3
12	**1303.5657**	652.2865	1286.5391	643.7732	**1285.5551**	643.2812	A	218.1499	109.5786	201.1234	101.0653			2
13							K	147.1128	74.0600	130.0863	65.5468			1

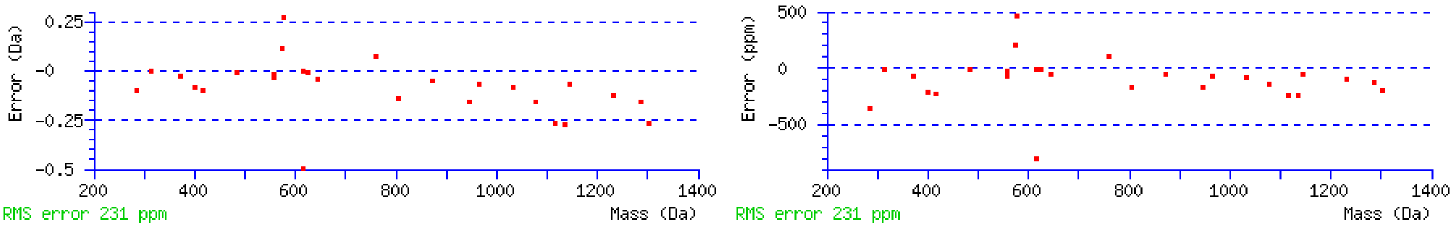

All matches to this query

Score	Mr(calc):	Delta	Sequence
54.2	1448.6639	0.0010	NSTLCDLCIGPAK
7.3	1448.6538	0.0112	ASRTTGGATALXK
6.6	1448.6538	0.0112	ASRTTGGATALXK
6.1	1448.6622	0.0027	NLGSLDVTIMYK
5.9	1448.6622	0.0027	NLGSLDVTIMYK
5.8	1448.6574	0.0075	SXMTPMRSVPMK
5.4	1448.6639	0.0010	QDGAVAMEMQPLK
4.8	1446.6690	1.9959	LATLCSNLYRR
4.3	1448.6538	0.0112	ASRTTGGATALXK
3.1	1448.6557	0.0093	EQMFRLLMASK

Spectrum No: 394; Query: 749; Rank: 1

Peptide View

MS/MS Fragmentation of **VFNTSEDLSRVNATVQETK**
Found in **IPI00363534**, Tax_Id=10116 Gene_Symbol=Lama1 laminin, alpha 1

Match to Query 749: 2139.034212 from(714.018680,3+)
Title: 091129RatKid_SCX02_16.2305.2305.3.dta
Data file K:\NewmanPaper\Piliang\3SubProteomes\Piliang3SP\mgf5ppm\SCX_3SubProteomes5ppm.mgf

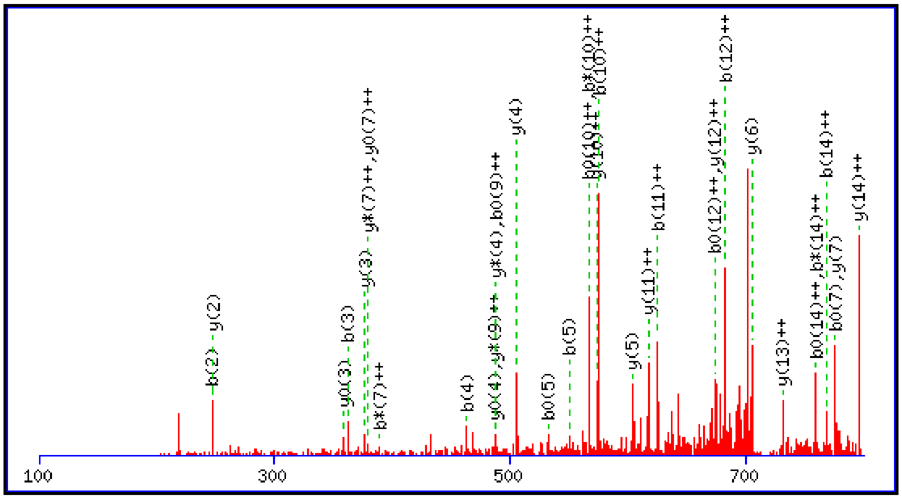

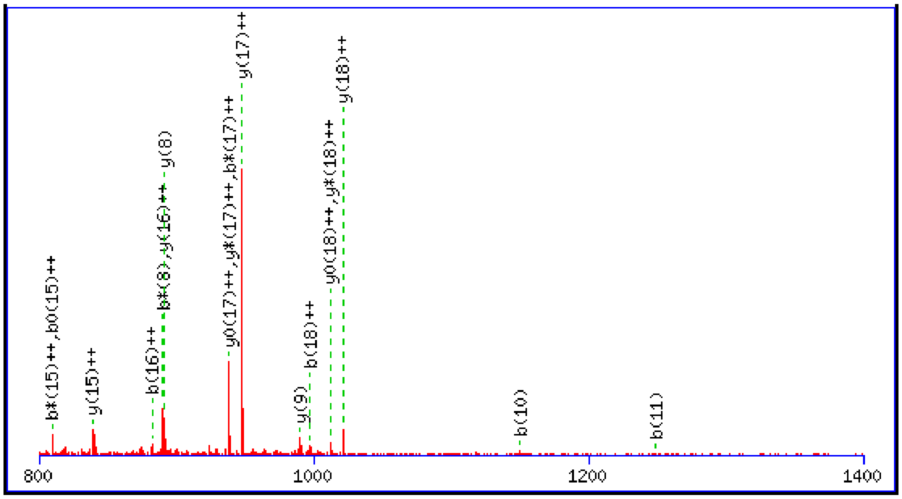

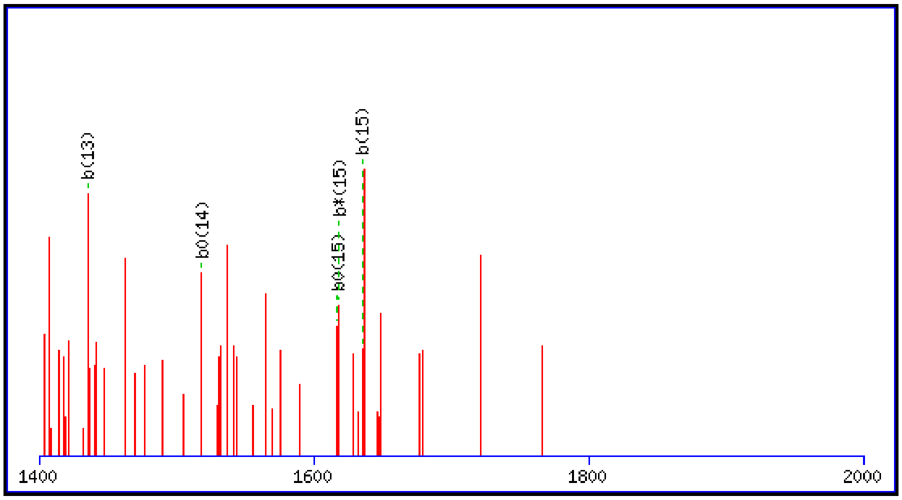

Monoisotopic mass of neutral peptide Mr(calc): 2139.0331
Fixed modifications: Carbamidomethyl (C)
Variable modifications:
N3 : Deamidated_N (N)
N12 : Deamidated_N (N)
Ions Score: 54 **Expect:** 0.00093
Matches (Bold Red): 57/204 fragment ions using 123 most intense peaks

#	b	b++	b*	b*++	b0	b0++	Seq.	y	y++	y*	y*++	y0	y0++	#
1	100.0757	50.5415					V							19
2	**247.1441**	124.0757					F	2040.9720	**1020.9896**	2023.9455	**1012.4764**	2022.9614	**1011.9844**	18
3	**362.1710**	181.5892	345.1445	173.0759			N	1893.9036	**947.4554**	1876.8770	**938.9422**	1875.8930	**938.4502**	17
4	**463.2187**	232.1130	446.1922	223.5997	445.2082	223.1077	T	1778.8767	**889.9420**	1761.8501	881.4287	1760.8661	880.9367	16
5	**550.2507**	275.6290	533.2242	267.1157	**532.2402**	266.6237	S	1677.8290	**839.4181**	1660.8024	830.9049	1659.8184	830.4128	15
6	679.2933	340.1503	662.2668	331.6370	661.2828	331.1450	E	1590.7969	**795.9021**	1573.7704	787.3888	1572.7864	786.8968	14
7	794.3203	397.6638	777.2937	**389.1505**	**776.3097**	388.6585	D	1461.7544	**731.3808**	1444.7278	722.8675	1443.7438	722.3755	13
8	907.4043	454.2058	**890.3778**	445.6925	889.3938	445.2005	L	1346.7274	**673.8673**	1329.7009	665.3541	1328.7168	664.8621	12
9	994.4364	497.7218	977.4098	489.2086	976.4258	**488.7165**	S	1233.6434	**617.3253**	1216.6168	608.8120	1215.6328	608.3200	11
10	**1150.5375**	**575.7724**	1133.5109	**567.2591**	1132.5269	**566.7671**	R	1146.6113	**573.8093**	1129.5848	565.2960	1128.6008	564.8040	10
11	**1249.6059**	**625.3066**	1232.5794	616.7933	1231.5953	616.3013	V	**990.5102**	495.7587	973.4837	**487.2455**	972.4996	486.7535	9
12	1364.6328	**682.8201**	1347.6063	674.3068	1346.6223	**673.8148**	N	**891.4418**	446.2245	874.4152	437.7113	873.4312	437.2193	8
13	**1435.6700**	718.3386	1418.6434	709.8253	1417.6594	709.3333	A	**776.4149**	388.7111	759.3883	**380.1978**	758.4043	**379.7058**	7
14	1536.7176	**768.8625**	1519.6911	**760.3492**	**1518.7071**	**759.8572**	T	**705.3777**	353.1925	688.3512	344.6792	687.3672	344.1872	6
15	**1635.7860**	818.3967	**1618.7595**	**809.8834**	**1617.7755**	**809.3914**	V	**604.3301**	302.6687	587.3035	294.1554	586.3195	293.6634	5
16	1763.8446	**882.4259**	1746.8181	873.9127	1745.8341	873.4207	Q	**505.2617**	253.1345	**488.2351**	244.6212	**487.2511**	244.1292	4
17	1892.8872	946.9472	1875.8607	**938.4340**	1874.8766	937.9420	E	**377.2031**	189.1052	360.1765	180.5919	**359.1925**	180.0999	3
18	1993.9349	**997.4711**	1976.9083	988.9578	1975.9243	988.4658	T	**248.1605**	124.5839	231.1339	116.0706	230.1499	115.5786	2

19							K	147.1128	74.0600	130.0863	65.5468			1

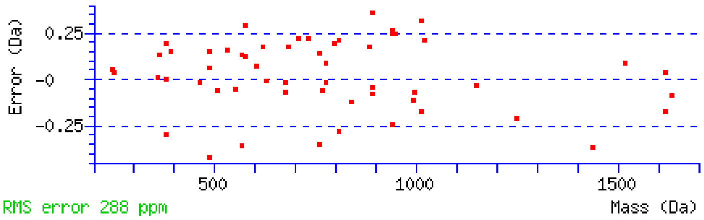

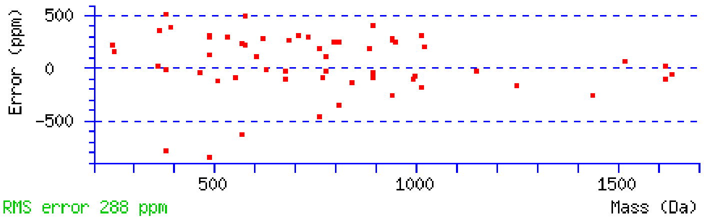

All matches to this query

Score	Mr(calc):	Delta	Sequence
54.1	2139.0331	0.0011	VFNTSEDLSRVNATVQETK
20.5	2138.0491	0.9851	VFNTSEDLSRVNATVQETK
19.2	2138.0491	0.9851	VFNTSEDLSRVNATVQETK
15.4	2137.0093	2.0250	DKNFSTLQTVLSATGGGAYK
15.4	2137.0093	2.0250	DKNFSTLQTVLSATGGGAYK
6.3	2137.0093	2.0250	DKNFSTLQTVLSATGGGAYK
4.4	2138.0392	0.9950	QDGAPEPGSGLWADVGKDIXR
3.1	2137.0093	2.0250	DKNFSTLQTVLSATGGGAYK
2.7	2137.0126	2.0216	ATEALKAEEKPVDVSAMPR
2.6	2139.0469	-0.0127	MVNEPRSGMLVLVIQAASK

Spectrum No: 395; Query: 129; Rank: 1

Peptide View

MS/MS Fragmentation of **AVLVNNITTGEK**
Found in **IPI00764009**, Tax_Id=10116 Gene_Symbol=LOC682869 similar to Golgi phosphoprotein 2 (Golgi membrane protein GP73) isoform 2

Match to Query 129: 1258.675628 from(630.345090,2+)
Title: 091129RatKid_SCX02_02.1079.1079.2.dta
Data file K:\NewmanPaper\Piliang\3SubProteomes\Piliang3SP\mgf5ppm\SCX_3SubProteomes5ppm.mgf

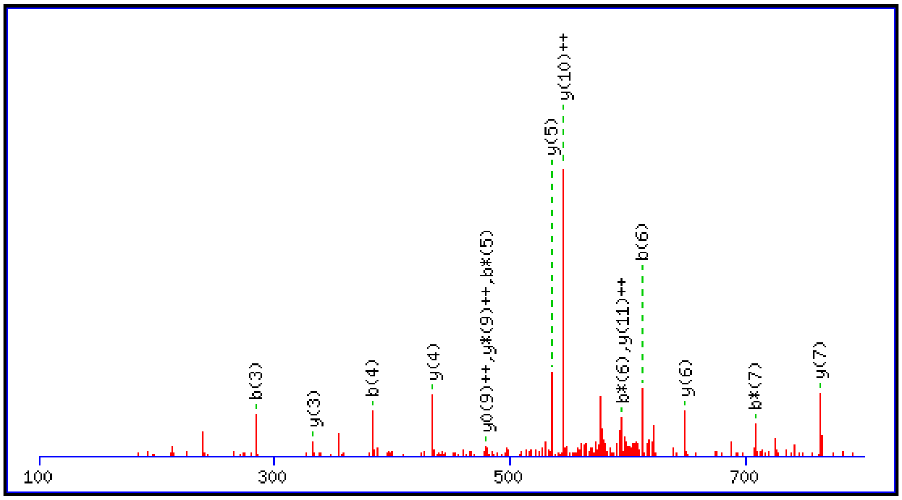

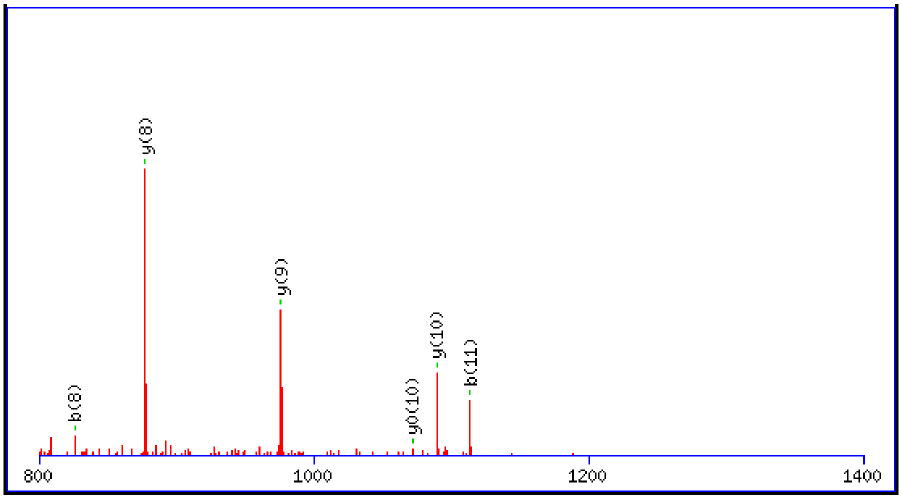

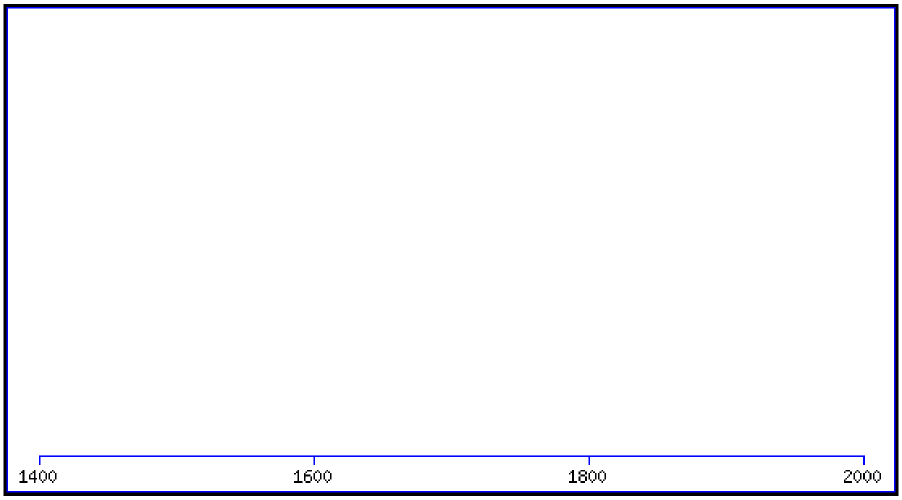

Monoisotopic mass of neutral peptide Mr(calc): 1258.6769
Fixed modifications: Carbamidomethyl (C)
Variable modifications:
N6 : Deamidated_N (N)
Ions Score: 54 **Expect:** 0.00037
Matches (Bold Red): 21/108 fragment ions using 35 most intense peaks

#	b	b++	b*	b*++	b0	b0++	Seq.	y	y++	y*	y*++	y0	y0++	#
1	72.0444	36.5258					A							12
2	171.1128	86.0600					V	1188.6470	**594.8272**	1171.6205	586.3139	1170.6365	585.8219	11
3	**284.1969**	142.6021					L	**1089.5786**	**545.2930**	1072.5521	536.7797	**1071.5681**	536.2877	10
4	**383.2653**	192.1363					V	**976.4946**	488.7509	959.4680	**480.2376**	958.4840	**479.7456**	9
5	497.3082	249.1577	**480.2817**	240.6445			N	**877.4261**	439.2167	860.3996	430.7034	859.4156	430.2114	8
6	**612.3351**	306.6712	**595.3086**	298.1579			N	**763.3832**	382.1952	746.3567	373.6820	745.3727	373.1900	7
7	725.4192	363.2132	**708.3927**	354.7000			I	**648.3563**	324.6818	631.3297	316.1685	630.3457	315.6765	6
8	**826.4669**	413.7371	809.4403	405.2238	808.4563	404.7318	T	**535.2722**	268.1397	518.2457	259.6265	517.2617	259.1345	5
9	927.5146	464.2609	910.4880	455.7476	909.5040	455.2556	T	**434.2245**	217.6159	417.1980	209.1026	416.2140	208.6106	4
10	984.5360	492.7717	967.5095	484.2584	966.5255	483.7664	G	**333.1769**	167.0921	316.1503	158.5788	315.1663	158.0868	3
11	**1113.5786**	557.2930	1096.5521	548.7797	1095.5681	548.2877	E	276.1554	138.5813	259.1288	130.0681	258.1448	129.5761	2
12							K	147.1128	74.0600	130.0863	65.5468			1

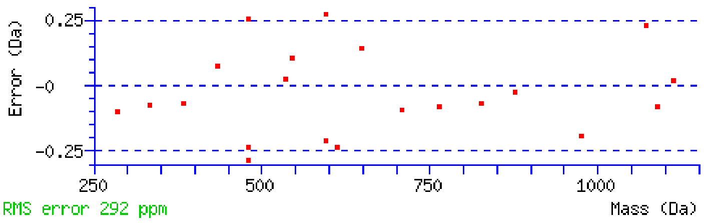

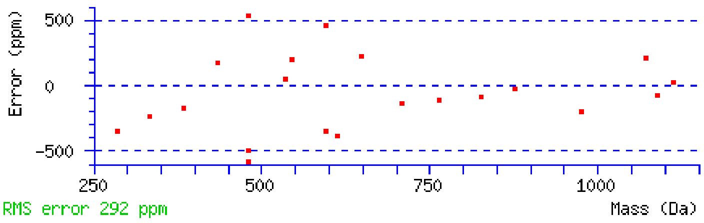

All matches to this query

Score	Mr(calc):	Delta	Sequence
54.1	1258.6769	-0.0012	AVLVNNITTGEK
41.9	1258.6769	-0.0012	AVLVNNITTGEK
6.9	1258.6703	0.0053	IIRNNELMQK
4.5	1258.6659	0.0097	ALGTRLRQHK
2.5	1256.6612	2.0144	VPAINVNDSVTK
0.1	1258.6703	0.0053	IIRNNELMQK

Spectrum No: 396; Query: 163; Rank: 1

Peptide View

MS/MS Fragmentation of **AYYNCSCIER**
Found in **IPI00393407**, Tax_Id=10116 Gene_Symbol=Slco4c1 Kidney-specific organic anion transporter

Match to Query 163: 1335.523708 from(668.769130,2+)
Title: 091127RatKid_SCX01_03.1270.1270.2.dta
Data file K:\NewmanPaper\Piliang\3SubProteomes\Piliang3SP\mgf5ppm\SCX_3SubProteomes5ppm.mgf

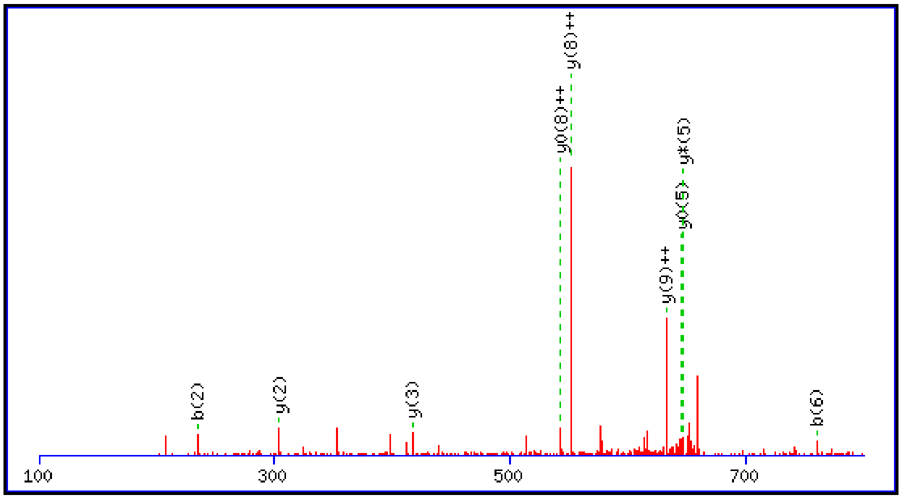

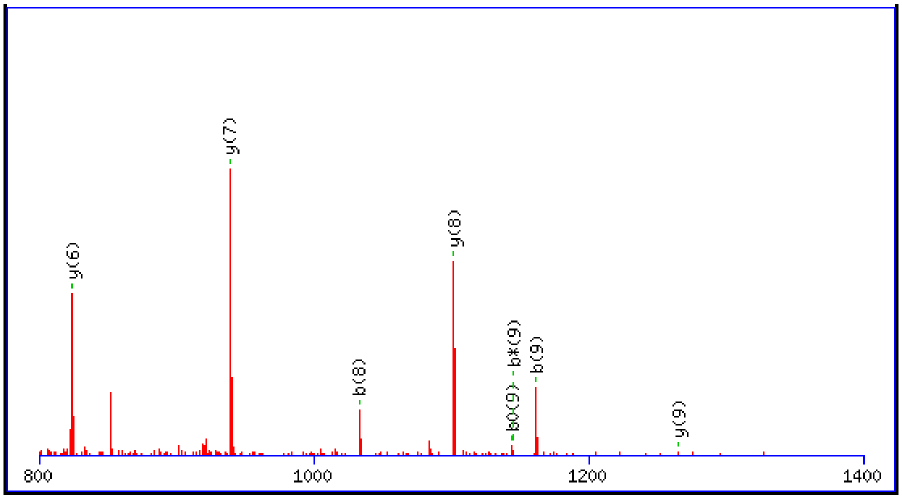

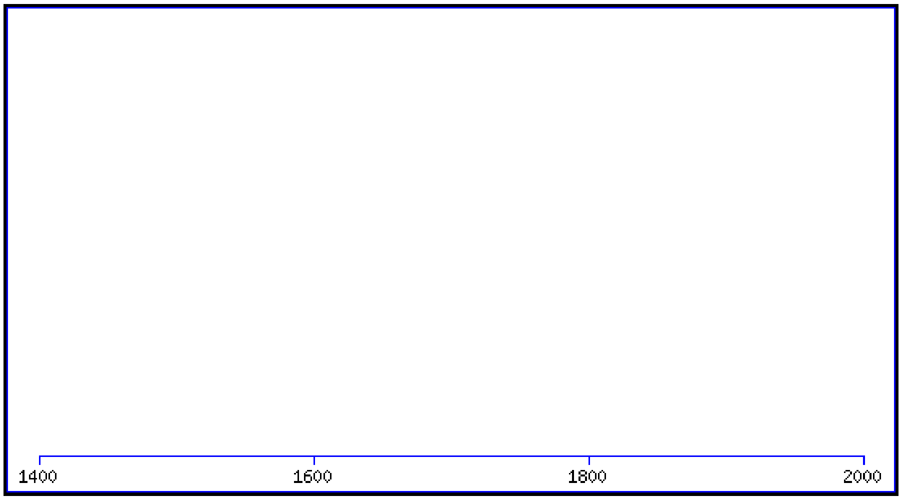

Monoisotopic mass of neutral peptide Mr(calc): 1335.5223
Fixed modifications: Carbamidomethyl (C)
Variable modifications:
N4 : Deamidated_N (N)
Ions Score: 54 **Expect:** 0.00035
Matches (Bold Red): 17/90 fragment ions using 31 most intense peaks

#	b	b^{++}	b*	b^{*++}	b^0	b^{0++}	Seq.	y	y^{++}	y*	y^{*++}	y^0	y^{0++}	#
1	72.0444	36.5258					A							10
2	**235.1077**	118.0575					Y	**1265.4925**	**633.2499**	1248.4660	624.7366	1247.4820	624.2446	9
3	398.1710	199.5892					Y	**1102.4292**	**551.7182**	1085.4027	543.2050	1084.4186	**542.7130**	8
4	513.1980	257.1026	496.1714	248.5894			N	**939.3659**	470.1866	922.3393	461.6733	921.3553	461.1813	7
5	673.2286	337.1180	656.2021	328.6047			C	**824.3389**	412.6731	807.3124	404.1598	806.3284	403.6678	6
6	**760.2607**	380.6340	743.2341	372.1207	742.2501	371.6287	S	664.3083	332.6578	**647.2817**	324.1445	**646.2977**	323.6525	5
7	920.2913	460.6493	903.2648	452.1360	902.2807	451.6440	C	577.2763	289.1418	560.2497	280.6285	559.2657	280.1365	4
8	**1033.3754**	517.1913	1016.3488	508.6781	1015.3648	508.1860	I	**417.2456**	209.1264	400.2191	200.6132	399.2350	200.1212	3
9	**1162.4180**	581.7126	**1145.3914**	573.1993	**1144.4074**	572.7073	E	**304.1615**	152.5844	287.1350	144.0711	286.1510	143.5791	2
10							R	175.1190	88.0631	158.0924	79.5498			1

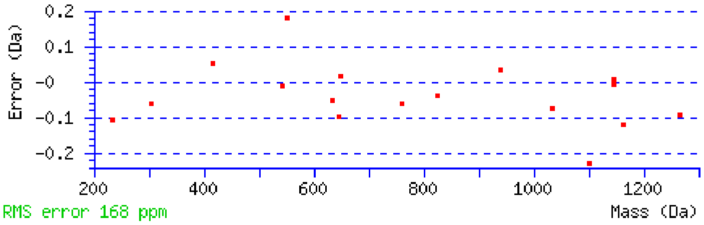

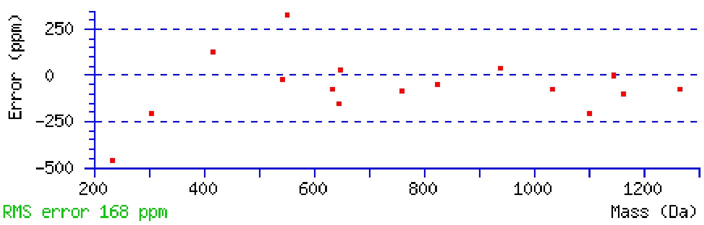

All matches to this query

Score	Mr(calc):	Delta	Sequence
54.1	1335.5223	0.0014	AYYNCSCIER
3.2	1335.5221	0.0016	ANVKTEQSSGR
2.4	1335.5301	-0.0064	AYLTYFRDK
1.6	1333.5179	2.0058	SHPXTVMNLK
1.4	1335.5278	-0.0041	ESTQGFRMER
1.0	1335.5312	-0.0075	STMKDTGGCXR
1.0	1335.5312	-0.0075	STMKDTGGCXR

Spectrum No: 397; Query: 234; Rank: 1

Peptide View

MS/MS Fragmentation of **SLNLTEDVYKPR**
Found in **IPI00199448**, Tax_Id=10116 Gene_Symbol=Lgals3bp Mama

Match to Query 234: 1434.737648 from(718.376100,2+)
Title: 091127RatKid_SCX01_12.1967.1967.2.dta
Data file K:\NewmanPaper\Piliang\3SubProteomes\Piliang3SP\mgf5ppm\SCX_3SubProteomes5ppm.mgf

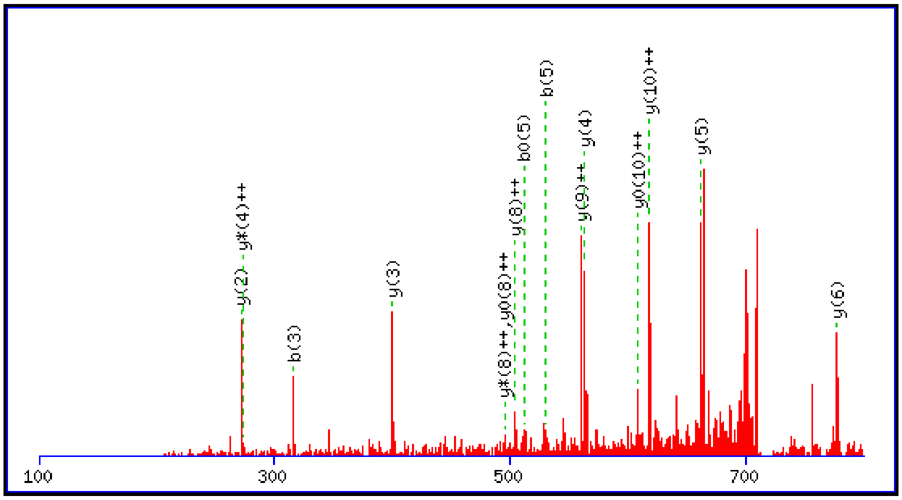

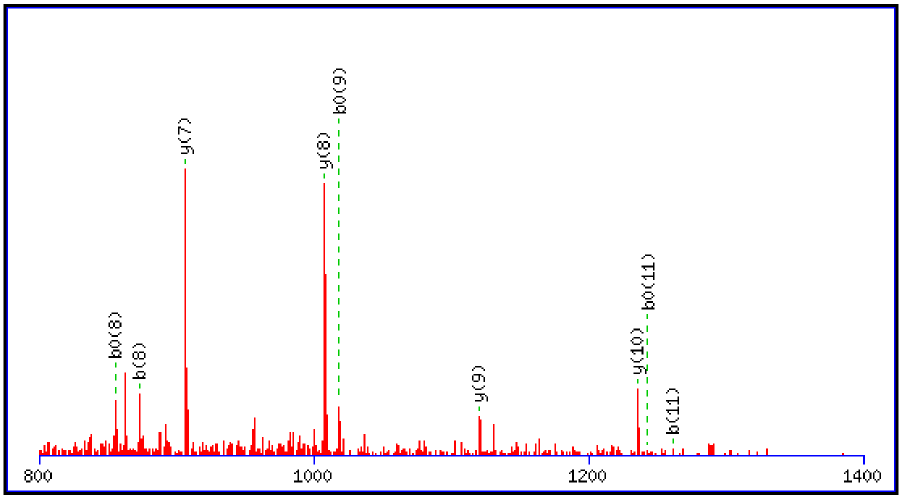

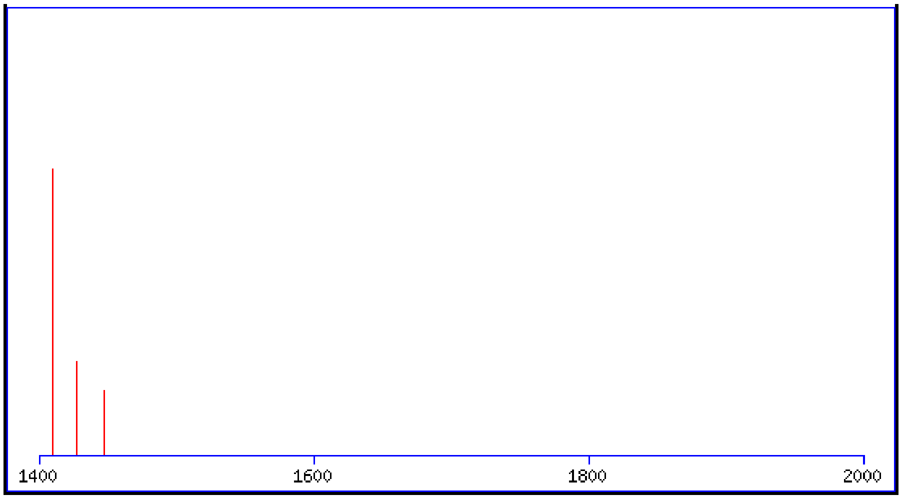

Monoisotopic mass of neutral peptide Mr(calc): 1434.7354
Fixed modifications: Carbamidomethyl (C)
Variable modifications:
N3 : Deamidated_N (N)
Ions Score: 54 **Expect:** 0.00049
Matches (Bold Red): 24/118 fragment ions using 55 most intense peaks

#	b	b^{++}	b*	b^{*++}	b^{0}	b^{0++}	Seq.	y	y^{++}	y*	y^{*++}	y^{0}	y^{0++}	#
1	88.0393	44.5233			70.0287	35.5180	S							12
2	201.1234	101.0653			183.1128	92.0600	L	1348.7107	674.8590	1331.6842	666.3457	1330.7001	665.8537	11
3	**316.1503**	158.5788	299.1238	150.0655	298.1397	149.5735	N	**1235.6266**	**618.3170**	1218.6001	609.8037	1217.6161	**609.3117**	10
4	429.2344	215.1208	412.2078	206.6075	411.2238	206.1155	L	**1120.5997**	**560.8035**	1103.5732	552.2902	1102.5891	551.7982	9
5	**530.2820**	265.6447	513.2555	257.1314	**512.2715**	256.6394	T	**1007.5156**	**504.2615**	990.4891	**495.7482**	989.5051	**495.2562**	8
6	659.3246	330.1660	642.2981	321.6527	641.3141	321.1607	E	**906.4680**	453.7376	889.4414	445.2243	888.4574	444.7323	7
7	774.3516	387.6794	757.3250	379.1662	756.3410	378.6741	D	**777.4254**	389.2163	760.3988	380.7030	759.4148	380.2110	6
8	**873.4200**	437.2136	856.3934	428.7004	**855.4094**	428.2084	V	**662.3984**	331.7028	645.3719	323.1896			5
9	1036.4833	518.7453	1019.4568	510.2320	**1018.4728**	509.7400	Y	**563.3300**	282.1686	546.3035	**273.6554**			4
10	1164.5783	582.7928	1147.5517	574.2795	1146.5677	573.7875	K	**400.2667**	200.6370	383.2401	192.1237			3
11	**1261.6311**	631.3192	1244.6045	622.8059	**1243.6205**	622.3139	P	**272.1717**	136.5895	255.1452	128.0762			2
12							R	175.1190	88.0631	158.0924	79.5498			1

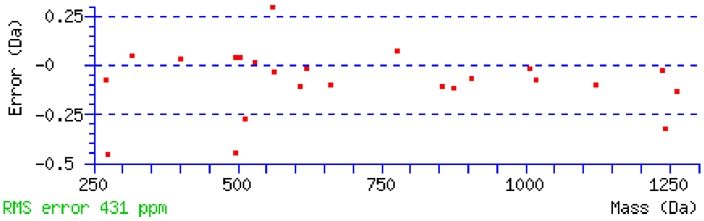

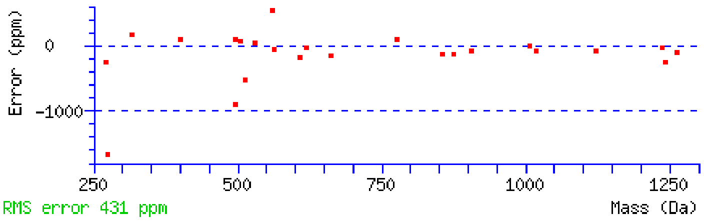

All matches to this query

Score	Mr(calc):	Delta	Sequence
53.9	1434.7354	0.0022	SLNLTEDVYKPR
7.0	1434.7394	-0.0018	WELTKYPELEK
7.0	1433.7238	1.0139	WKLTKMDGNWR
6.1	1434.7467	-0.0090	LSDINQSVKFQR
5.5	1432.7439	1.9937	VEVTPTVPRISR
3.8	1434.7500	-0.0124	SINEMLLSRLSR
1.2	1432.7439	1.9937	VEVTPTVPRISR

Spectrum No: 398; Query: 465; Rank: 1

Peptide View

MS/MS Fragmentation of **DNATEEEILHYLEK**
Found in **IPI00195160**, Tax_Id=10116 Gene_Symbol=Psap Sulfated glycoprotein 1 precursor

Match to Query 465: 1703.790648 from(852.902600,2+)
Title: 091129RatKid_SCX02_25.4347.4347.2.dta
Data file K:\NewmanPaper\Piliang\3SubProteomes\Piliang3SP\mgf5ppm\SCX_3SubProteomes5ppm.mgf

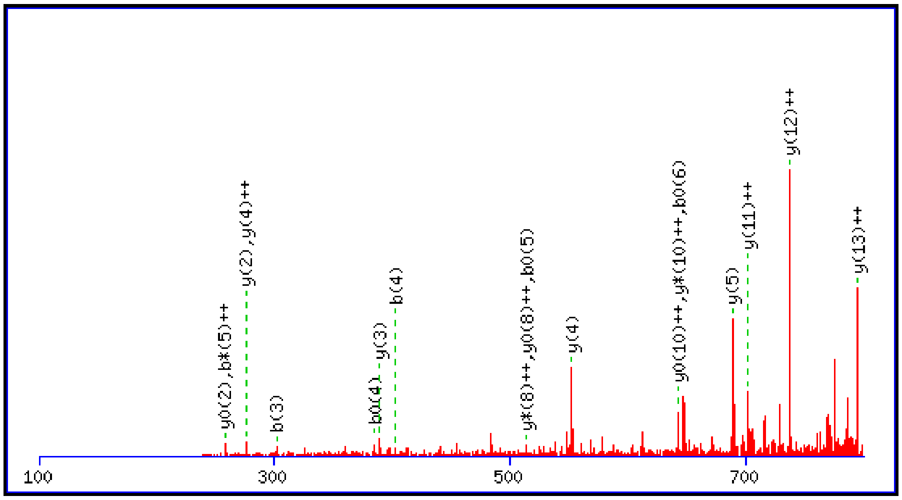

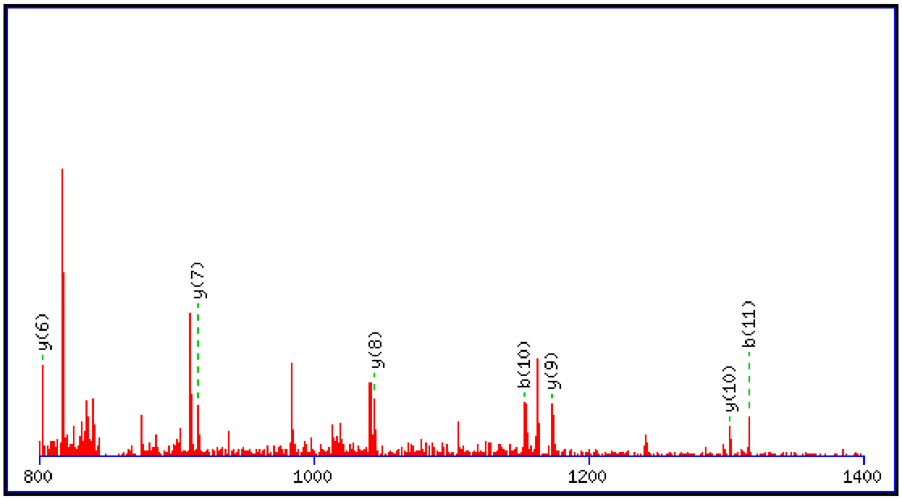

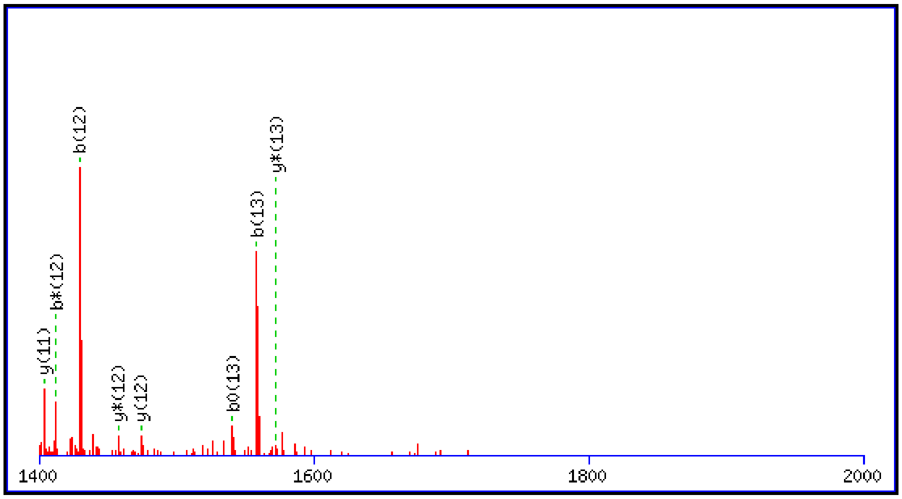

Monoisotopic mass of neutral peptide Mr(calc): 1703.7890
Fixed modifications: Carbamidomethyl (C)
Variable modifications:

N2 : Deamidated_N (N)
Ions Score: 54 **Expect:** 0.0008
Matches (Bold Red): 34/152 fragment ions using 77 most intense peaks

#	b	b^{++}	b*	b^{*++}	b^0	b^{0++}	Seq.	y	y^{++}	y*	y^{*++}	y^0	y^{0++}	#
1	116.0342	58.5207			98.0237	49.5155	D							14
2	231.0612	116.0342	214.0346	107.5209	213.0506	107.0289	N	1589.7693	**795.3883**	**1572.7428**	786.8750	1571.7588	786.3830	13
3	**302.0983**	151.5528	285.0717	143.0395	284.0877	142.5475	A	**1474.7424**	**737.8748**	**1457.7159**	729.3616	1456.7318	728.8696	12
4	**403.1459**	202.0766	386.1194	193.5633	**385.1354**	193.0713	T	**1403.7053**	**702.3563**	1386.6787	693.8430	1385.6947	693.3510	11
5	532.1885	266.5979	515.1620	**258.0846**	**514.1780**	257.5926	E	**1302.6576**	651.8324	1285.6311	**643.3192**	1284.6470	**642.8272**	10
6	661.2311	331.1192	644.2046	322.6059	**643.2206**	322.1139	E	**1173.6150**	587.3111	1156.5885	578.7979	1155.6045	578.3059	9
7	790.2737	395.6405	773.2472	387.1272	772.2632	386.6352	E	**1044.5724**	522.7898	1027.5459	**514.2766**	1026.5619	**513.7846**	8
8	903.3578	452.1825	886.3312	443.6693	885.3472	443.1773	I	**915.5298**	458.2686	898.5033	449.7553	897.5193	449.2633	7
9	1016.4419	508.7246	999.4153	500.2113	998.4313	499.7193	L	**802.4458**	401.7265	785.4192	393.2132	784.4352	392.7212	6
10	**1153.5008**	577.2540	1136.4742	568.7407	1135.4902	568.2487	H	**689.3617**	345.1845	672.3352	336.6712	671.3511	336.1792	5
11	**1316.5641**	658.7857	1299.5375	650.2724	1298.5535	649.7804	Y	**552.3028**	**276.6550**	535.2762	268.1418	534.2922	267.6498	4
12	**1429.6482**	715.3277	**1412.6216**	706.8144	1411.6376	706.3224	L	**389.2395**	195.1234	372.2129	186.6101	371.2289	186.1181	3
13	**1558.6908**	779.8490	1541.6642	771.3357	**1540.6802**	770.8437	E	**276.1554**	138.5813	259.1288	130.0681	**258.1448**	129.5761	2
14							K	147.1128	74.0600	130.0863	65.5468			1

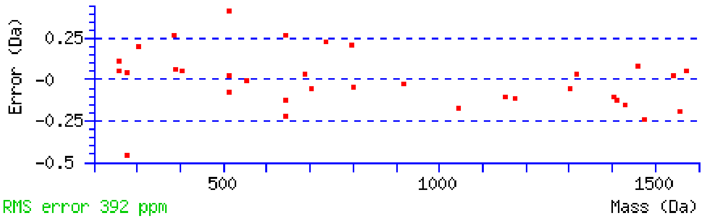

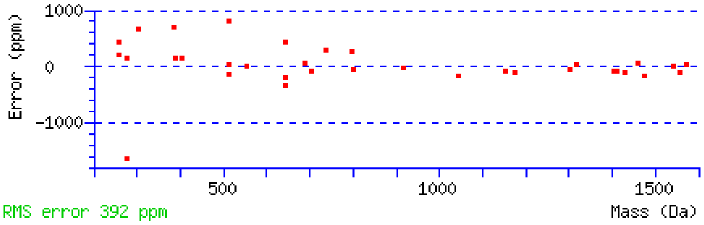

All matches to this query

Score	Mr(calc):	Delta	Sequence
53.9	1703.7890	0.0017	DNATEEEILHYLEK
6.1	1703.7987	-0.0081	KTSVADSMLRMLEK
6.1	1703.7987	-0.0081	KTSVADSMLRMLEK
5.0	1703.8011	-0.0104	YIVRNTMNCYLEK
5.0	1703.8011	-0.0104	YIVRNTMNCYLEK
1.6	1702.7974	0.9933	MASNHKSAAPRPISR

Spectrum No: 399; Query: 243; Rank: 1

Peptide View

MS/MS Fragmentation of **SVLENSTSYEEAK**
Found in **IPI00421601**, Tax_Id=10116 Gene_Symbol=Asah1 N-acylsphingosine amidohydrolase 1

Match to Query 243: 1456.656908 from(729.335730,2+)
Title: 091129RatKid_SCX02_18.776.776.2.dta
Data file K:\NewmanPaper\Piliang\3SubProteomes\Piliang3SP\mgf5ppm\SCX_3SubProteomes5ppm.mgf

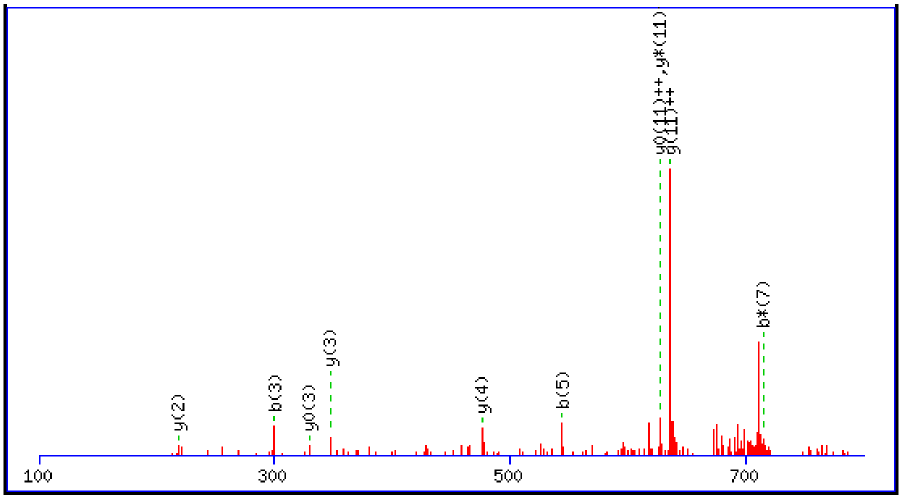

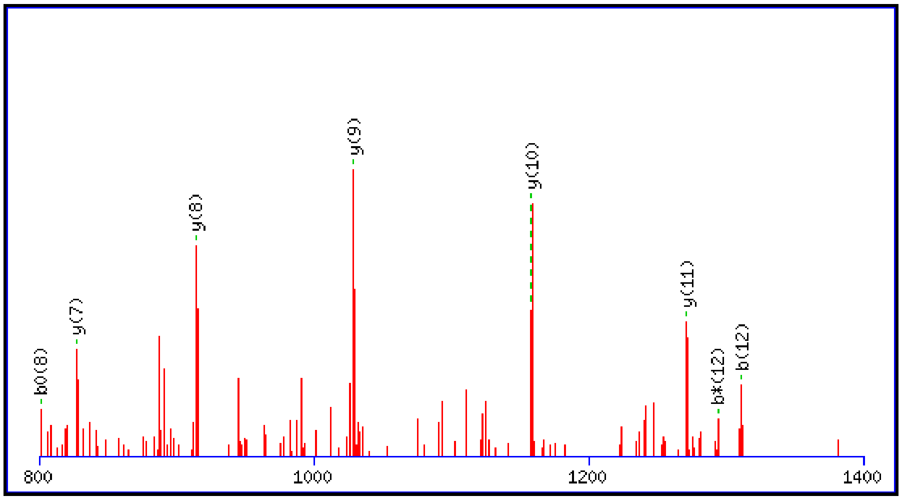

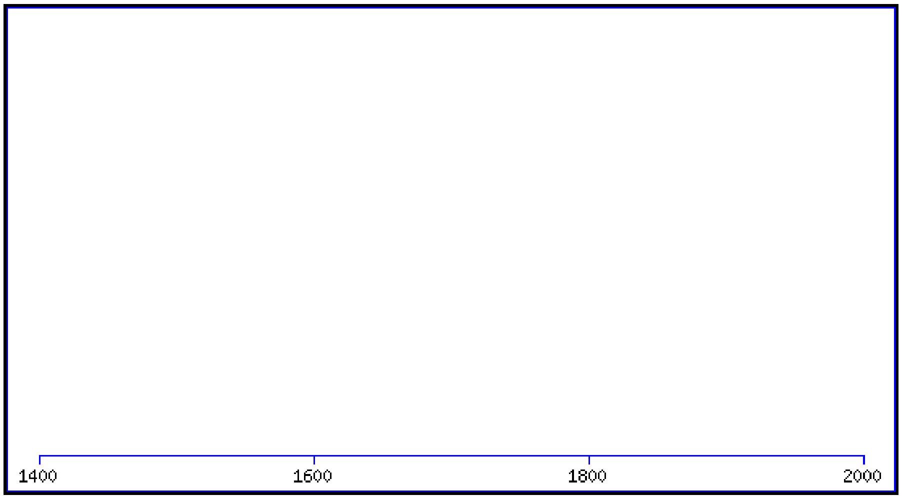

Monoisotopic mass of neutral peptide Mr(calc): 1456.6569
Fixed modifications: Carbamidomethyl (C)
Variable modifications:
N5 : Deamidated_N (N)
Ions Score: 54 **Expect:** 0.00067
Matches (Bold Red): 18/132 fragment ions using 33 most intense peaks

#	b	b^{++}	b*	b^{*++}	b^{0}	b^{0++}	Seq.	y	y^{++}	y*	y^{*++}	y^{0}	y^{0++}	#
1	88.0393	44.5233			70.0287	35.5180	S							13
2	187.1077	94.0575			169.0972	85.0522	V	1370.6322	685.8197	1353.6056	677.3065	1352.6216	676.8144	12
3	**300.1918**	150.5995			282.1812	141.5942	L	**1271.5638**	**636.2855**	1254.5372	**627.7722**	1253.5532	**627.2802**	11
4	429.2344	215.1208			411.2238	206.1155	E	**1158.4797**	579.7435	1141.4531	571.2302	1140.4691	570.7382	10
5	**544.2613**	272.6343	527.2348	264.1210	526.2507	263.6290	N	**1029.4371**	515.2222	1012.4106	506.7089	1011.4265	506.2169	9

6	631.2933	316.1503	614.2668	307.6370	613.2828	307.1450	S	914.4102	457.7087	897.3836	449.1954	896.3996	448.7034	8
7	732.3410	366.6741	715.3145	358.1609	714.3305	357.6689	T	827.3781	414.1927	810.3516	405.6794	809.3676	405.1874	7
8	819.3730	410.1902	802.3465	401.6769	801.3625	401.1849	S	726.3305	363.6689	709.3039	355.1556	708.3199	354.6636	6
9	982.4364	491.7218	965.4098	483.2086	964.4258	482.7165	Y	639.2984	320.1529	622.2719	311.6396	621.2879	311.1476	5
10	1111.4790	556.2431	1094.4524	547.7298	1093.4684	547.2378	E	476.2351	238.6212	459.2086	230.1079	458.2245	229.6159	4
11	1240.5216	620.7644	1223.4950	612.2511	1222.5110	611.7591	E	347.1925	174.0999	330.1660	165.5866	329.1819	165.0946	3
12	1311.5587	656.2830	1294.5321	647.7697	1293.5481	647.2777	A	218.1499	109.5786	201.1234	101.0653			2
13							K	147.1128	74.0600	130.0863	65.5468			1

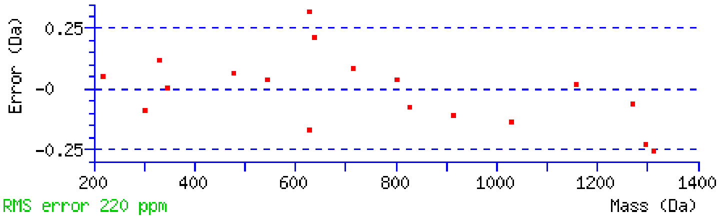

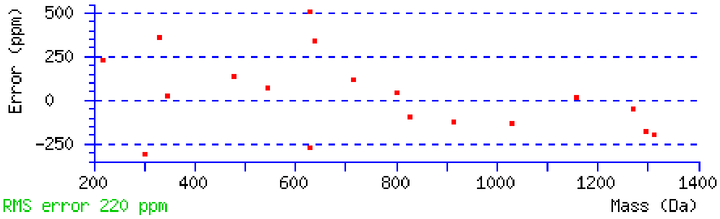

All matches to this query

Score	Mr(calc):	Delta	Sequence
53.8	1456.6569	0.0000	SVLENSTSYEEAK
16.3	1456.6438	0.0131	SLLMVTSGYKAK
16.3	1456.6438	0.0131	SLLMVTSGYKAK
5.0	1456.6447	0.0123	VSLTNVSISDEGR
3.9	1455.6494	1.0075	SPSSLSANITSSPK
3.9	1455.6494	1.0075	SPSSLSANITSSPK
3.9	1455.6494	1.0075	SPSSLSANITSSPK
3.9	1456.6632	-0.0063	SLAEMLISKNNR
3.0	1454.6364	2.0205	VTLNDVPCEVTK
2.7	1456.6503	0.0066	AMKEYEEEERK

Spectrum No: 400; Query: 280; Rank: 1

Peptide View

MS/MS Fragmentation of **SQHDKFNVSLTVK**
Found in **IPI00324585**, Tax_Id=10116 Gene_Symbol=Itga1 Integrin alpha-1 precursor

Match to Query 280: 1502.774028 from(752.394290,2+)
Title: 091129RatKid_SCX02_37.1399.1399.2.dta
Data file K:\NewmanPaper\Piliang\3SubProteomes\Piliang3SP\mgf5ppm\SCX_3SubProteomes5ppm.mgf

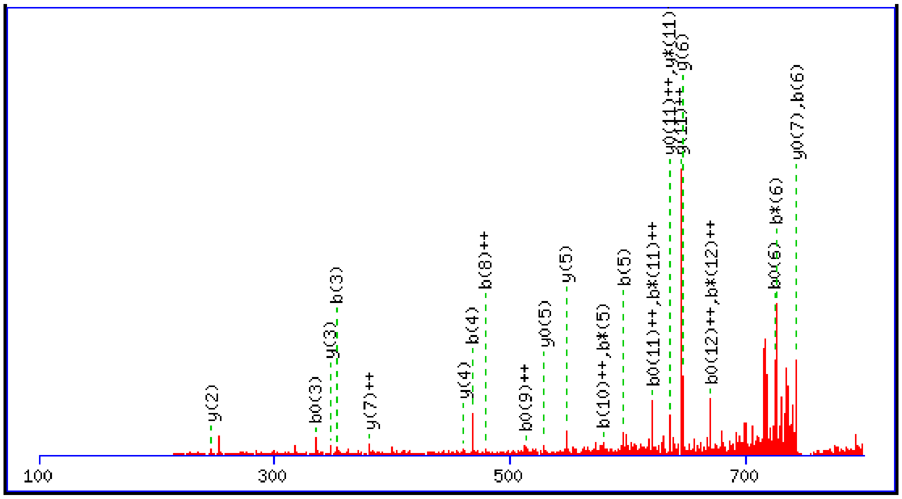

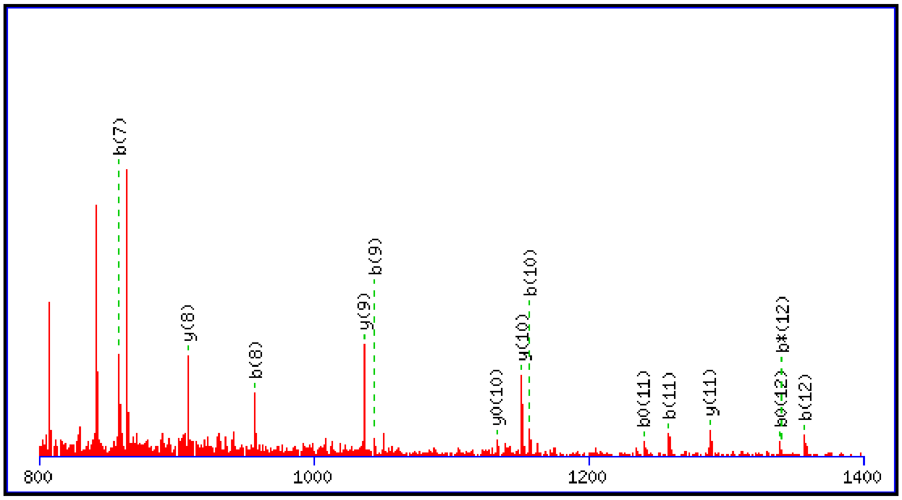

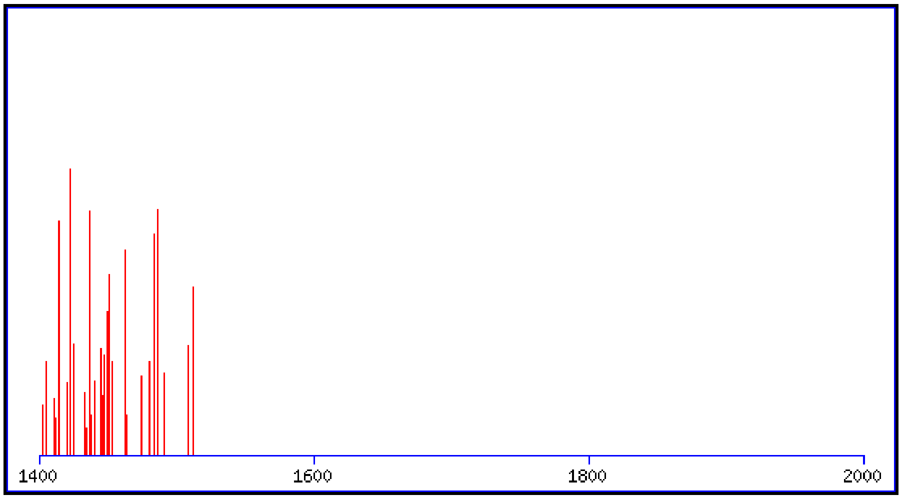

Monoisotopic mass of neutral peptide Mr(calc): 1502.7729
Fixed modifications: Carbamidomethyl (C)
Variable modifications:
N7 : Deamidated_N (N)
Ions Score: 54 **Expect:** 0.00056
Matches (Bold Red): 40/138 fragment ions using 75 most intense peaks

#	b	b^{++}	b*	b^{*++}	b^0	b^{0++}	Seq.	y	y^{++}	y*	y^{*++}	y^0	y^{0++}	#
1	88.0393	44.5233			70.0287	35.5180	S							13
2	216.0979	108.5526	199.0713	100.0393	198.0873	99.5473	Q	1416.7481	708.8777	1399.7216	700.3644	1398.7376	699.8724	12
3	**353.1568**	177.0820	336.1302	168.5688	**335.1462**	168.0768	H	**1288.6896**	**644.8484**	1271.6630	**636.3351**	1270.6790	**635.8431**	11
4	**468.1837**	234.5955	451.1572	226.0822	450.1732	225.5902	D	**1151.6307**	576.3190	1134.6041	567.8057	**1133.6201**	567.3137	10
5	**596.2787**	298.6430	**579.2522**	290.1297	578.2681	289.6377	K	**1036.6037**	518.8055	1019.5772	510.2922	1018.5932	509.8002	9

6	743.3471	372.1772	726.3206	363.6639	725.3365	363.1719	F	908.5088	454.7580	891.4822	446.2447	890.4982	445.7527	8
7	858.3741	429.6907	841.3475	421.1774	840.3635	420.6854	N	761.4403	381.2238	744.4138	372.7105	743.4298	372.2185	7
8	957.4425	479.2249	940.4159	470.7116	939.4319	470.2196	V	646.4134	323.7103	629.3869	315.1971	628.4028	314.7051	6
9	1044.4745	522.7409	1027.4479	514.2276	1026.4639	513.7356	S	547.3450	274.1761	530.3184	265.6629	529.3344	265.1709	5
10	1157.5586	579.2829	1140.5320	570.7696	1139.5480	570.2776	L	460.3130	230.6601	443.2864	222.1468	442.3024	221.6548	4
11	1258.6062	629.8068	1241.5797	621.2935	1240.5957	620.8015	T	347.2289	174.1181	330.2023	165.6048	329.2183	165.1128	3
12	1357.6746	679.3410	1340.6481	670.8277	1339.6641	670.3357	V	246.1812	123.5942	229.1547	115.0810			2
13							K	147.1128	74.0600	130.0863	65.5468			1

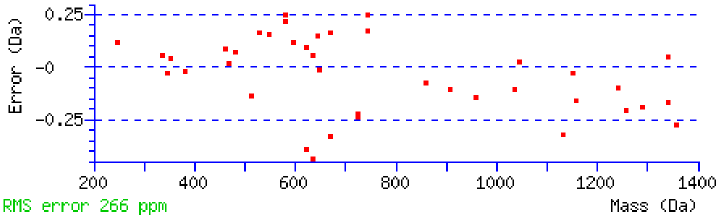

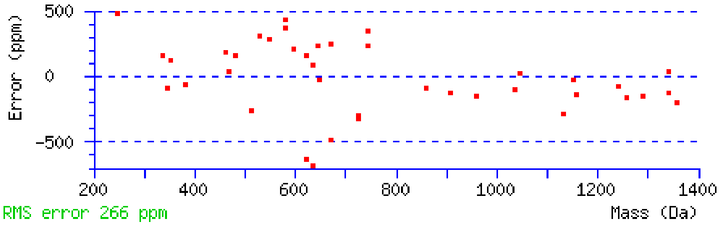

All matches to this query

Score	Mr(calc):	Delta	Sequence
53.7	1502.7729	0.0011	SQHDKFNVSLTVK
2.7	1502.7769	-0.0029	FHLDPVNGYITVK
2.3	1502.7729	0.0011	SKHQTFLSEAEVK

Mascot: http://www.matrixscience.com/

{MATRIX SCIENCE} Mascot Search Results

Results Generated by a Script Modified from Mascot Pepitde View
by Newman Sze, School of Biological Sciences, Nanyang Technological University

---

Spectrum No: 401; Query: 320; Rank: 1

Peptide View

MS/MS Fragmentation of **LLQTAEHNISGAER**
Found in **IPI00207123**, Tax_Id=10116 Gene_Symbol=Tnc Tenascin (Fragment)

Match to Query 320: 1538.773708 from(770.394130,2+)
Title: 091127RatKid_SCX01_12.1390.1390.2.dta
Data file K:\NewmanPaper\Piliang\3SubProteomes\Piliang3SP\mgf5ppm\SCX_3SubProteomes5ppm.mgf

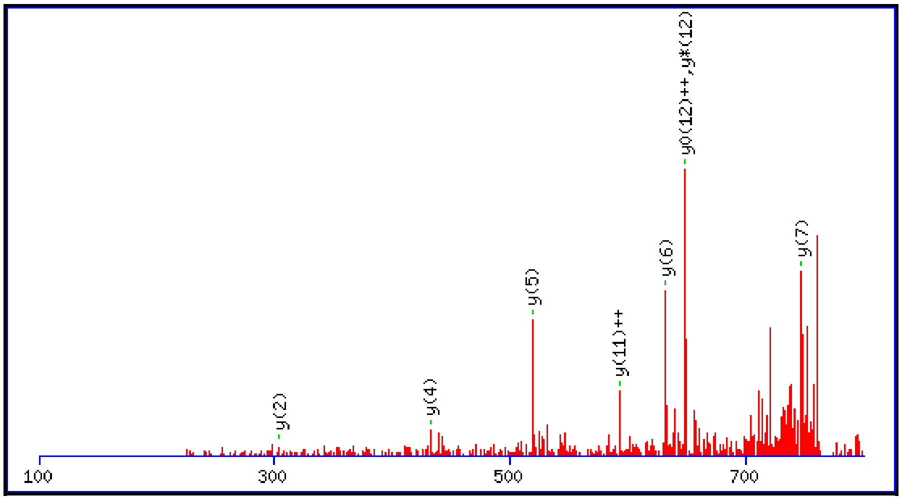

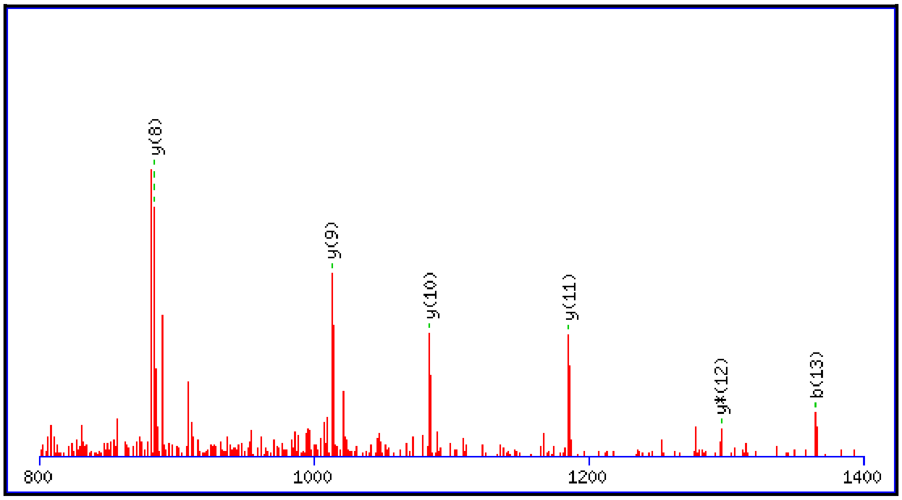

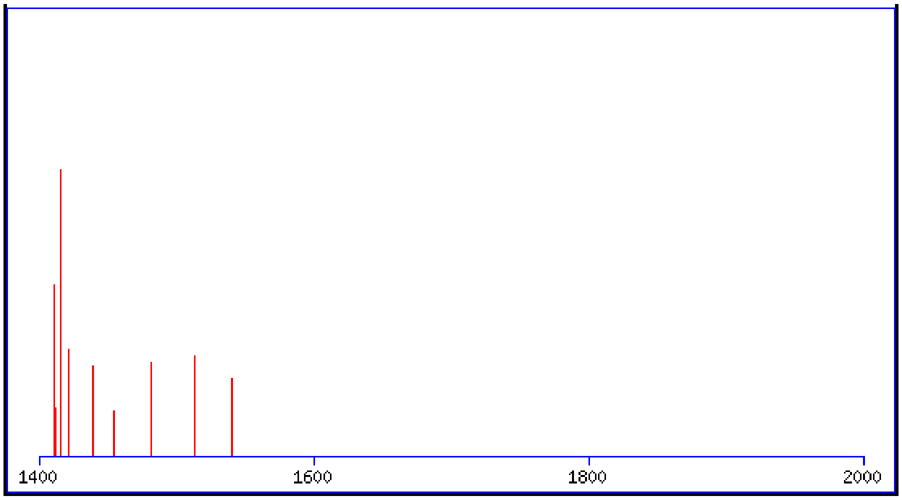

Monoisotopic mass of neutral peptide Mr(calc): 1538.7688
Fixed modifications: Carbamidomethyl (C)
Variable modifications:
N8 : Deamidated_N (N)
Ions Score: 54 **Expect:** 0.0007
Matches (Bold Red): 14/144 fragment ions using 32 most intense peaks

#	b	b^{++}	b*	b*$^{++}$	b^{0}	b^{0++}	Seq.	y	y^{++}	y*	y*$^{++}$	y^{0}	y^{0++}	#
1	114.0913	57.5493					L							14
2	227.1754	114.0913					L	1426.6921	713.8497	1409.6655	705.3364	1408.6815	704.8444	13
3	355.2340	178.1206	338.2074	169.6074			Q	1313.6080	657.3077	**1296.5815**	**648.7944**	1295.5975	**648.3024**	12
4	456.2817	228.6445	439.2551	220.1312	438.2711	219.6392	T	**1185.5494**	**593.2784**	1168.5229	584.7651	1167.5389	584.2731	11
5	527.3188	264.1630	510.2922	255.6498	509.3082	255.1577	A	**1084.5018**	542.7545	1067.4752	534.2412	1066.4912	533.7492	10
6	656.3614	328.6843	639.3348	320.1710	638.3508	319.6790	E	**1013.4647**	507.2360	996.4381	498.7227	995.4541	498.2307	9
7	793.4203	397.2138	776.3937	388.7005	775.4097	388.2085	H	**884.4221**	442.7147	867.3955	434.2014	866.4115	433.7094	8
8	908.4472	454.7272	891.4207	446.2140	890.4367	445.7220	N	**747.3632**	374.1852	730.3366	365.6719	729.3526	365.1799	7
9	1021.5313	511.2693	1004.5047	502.7560	1003.5207	502.2640	I	**632.3362**	316.6717	615.3097	308.1585	614.3256	307.6665	6
10	1108.5633	554.7853	1091.5368	546.2720	1090.5527	545.7800	S	**519.2522**	260.1297	502.2256	251.6164	501.2416	251.1244	5
11	1165.5848	583.2960	1148.5582	574.7827	1147.5742	574.2907	G	**432.2201**	216.6137	415.1936	208.1004	414.2096	207.6084	4
12	1236.6219	618.8146	1219.5953	610.3013	1218.6113	609.8093	A	375.1987	188.1030	358.1721	179.5897	357.1881	179.0977	3
13	**1365.6645**	683.3359	1348.6379	674.8226	1347.6539	674.3306	E	**304.1615**	152.5844	287.1350	144.0711	286.1510	143.5791	2
14							R	175.1190	88.0631	158.0924	79.5498			1

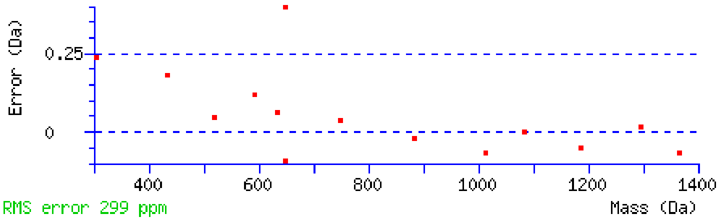

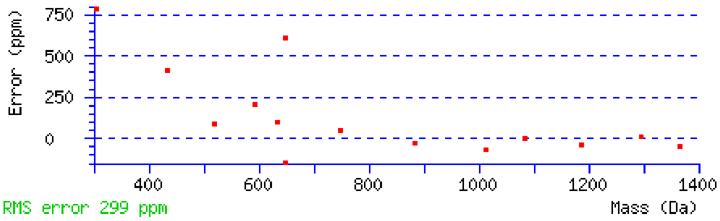

All matches to this query

Score	Mr(calc):	Delta	Sequence
53.6	1538.7688	0.0049	LLQTAEHNISGAER
3.4	1538.7705	0.0032	VSKVSLVDLAGSER
1.6	1537.7637	1.0100	GANGAPGIAGAPGFPGAR
0.5	1537.7848	0.9889	LLQTAEHNISGAER

Spectrum No: 402; Query: 1034; Rank: 1

Peptide View

MS/MS Fragmentation of **IDSEGISQNHTGQVASADASGFAGVVGHK**
Found in **IPI00326948**, Tax_Id=10116 Gene_Symbol=Hsd17b4 Hsd17b4 protein

Match to Query 1034: 2839.350282 from(947.457370,3+)
Title: 091129RatKid_SCX02_31.2462.2462.3.dta
Data file K:\NewmanPaper\Piliang\3SubProteomes\Piliang3SP\mgf5ppm\SCX_3SubProteomes5ppm.mgf

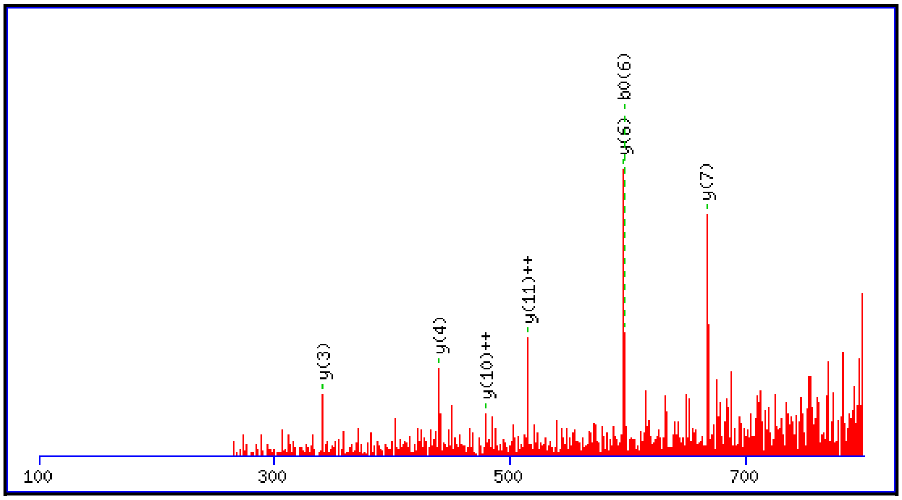

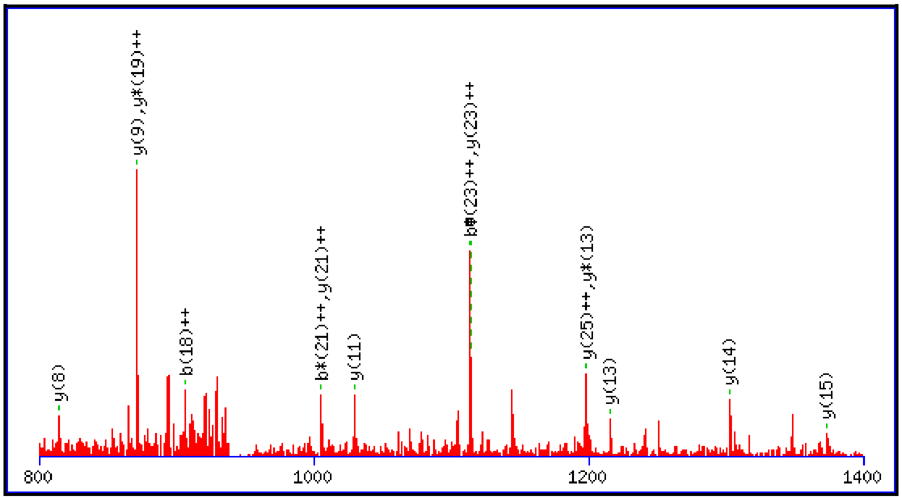

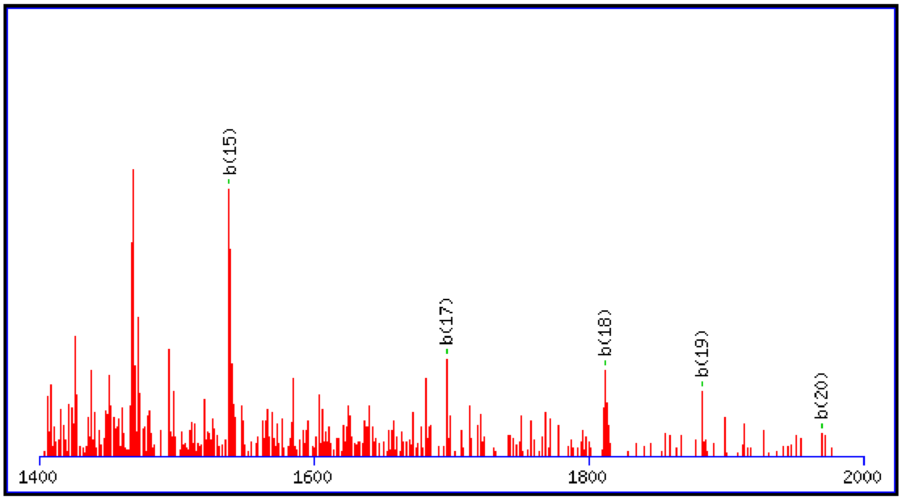

Monoisotopic mass of neutral peptide Mr(calc): 2839.3373
Fixed modifications: Carbamidomethyl (C)
Variable modifications:
N9 : Deamidated_N (N)
Ions Score: 54 **Expect:** 0.0015

Matches (Bold Red): 27/302 fragment ions using 48 most intense peaks

#	b	b^{++}	b*	b^{*++}	b^{0}	b^{0++}	Seq.	y	y^{++}	y*	y^{*++}	y^{0}	y^{0++}	#
1	114.0913	57.5493					I							29
2	229.1183	115.0628			211.1077	106.0575	D	2727.2605	1364.1339	2710.2339	1355.6206	2709.2499	1355.1286	28
3	316.1503	158.5788			298.1397	149.5735	S	2612.2335	1306.6204	2595.2070	1298.1071	2594.2230	1297.6151	27
4	445.1929	223.1001			427.1823	214.0948	E	2525.2015	1263.1044	2508.1750	1254.5911	2507.1909	1254.0991	26
5	502.2144	251.6108			484.2038	242.6055	G	2396.1589	**1198.5831**	2379.1324	1190.0698	2378.1484	1189.5778	25
6	615.2984	308.1529			**597.2879**	299.1476	I	2339.1375	1170.0724	2322.1109	1161.5591	2321.1269	1161.0671	24
7	702.3305	351.6689			684.3199	342.6636	S	2226.0534	**1113.5303**	2209.0268	1105.0171	2208.0428	1104.5251	23
8	830.3890	415.6982	813.3625	407.1849	812.3785	406.6929	Q	2139.0214	1070.0143	2121.9948	1061.5010	2121.0108	1061.0090	22
9	945.4160	473.2116	928.3894	464.6984	927.4054	464.2063	N	2010.9628	**1005.9850**	1993.9362	997.4718	1992.9522	996.9797	21
10	1082.4749	541.7411	1065.4483	533.2278	1064.4643	532.7358	H	1895.9359	948.4716	1878.9093	939.9583	1877.9253	939.4663	20
11	1183.5226	592.2649	1166.4960	583.7516	1165.5120	583.2596	T	1758.8769	879.9421	1741.8504	**871.4288**	1740.8664	870.9368	19
12	1240.5440	620.7757	1223.5175	612.2624	1222.5335	611.7704	G	1657.8293	829.4183	1640.8027	820.9050	1639.8187	820.4130	18
13	1368.6026	684.8049	1351.5761	676.2917	1350.5920	675.7997	Q	1600.8078	800.9075	1583.7812	792.3943	1582.7972	791.9023	17
14	1467.6710	734.3391	1450.6445	725.8259	1449.6605	725.3339	V	1472.7492	736.8782	1455.7227	728.3650	1454.7387	727.8730	16
15	**1538.7081**	769.8577	1521.6816	761.3444	1520.6976	760.8524	A	**1373.6808**	687.3440	1356.6543	678.8308	1355.6702	678.3388	15
16	1625.7402	813.3737	1608.7136	804.8604	1607.7296	804.3684	S	**1302.6437**	651.8255	1285.6171	643.3122	1284.6331	642.8202	14
17	**1696.7773**	848.8923	1679.7507	840.3790	1678.7667	839.8870	A	**1215.6117**	608.3095	**1198.5851**	599.7962	1197.6011	599.3042	13
18	**1811.8042**	**906.4057**	1794.7777	897.8925	1793.7937	897.4005	D	1144.5745	572.7909	1127.5480	564.2776	1126.5640	563.7856	12
19	**1882.8413**	941.9243	1865.8148	933.4110	1864.8308	932.9190	A	**1029.5476**	**515.2774**	1012.5211	506.7642	1011.5370	506.2722	11
20	**1969.8734**	985.4403	1952.8468	976.9270	1951.8628	976.4350	S	958.5105	**479.7589**	941.4839	471.2456	940.4999	470.7536	10
21	2026.8948	1013.9511	2009.8683	**1005.4378**	2008.8843	1004.9458	G	**871.4785**	436.2429	854.4519	427.7296			9
22	2173.9632	1087.4853	2156.9367	1078.9720	2155.9527	1078.4800	F	**814.4570**	407.7321	797.4305	399.2189			8
23	2245.0004	1123.0038	2227.9738	**1114.4905**	2226.9898	**1113.9985**	A	**667.3886**	334.1979	650.3620	325.6847			7
24	2302.0218	1151.5145	2284.9953	1143.0013	2284.0113	1142.5093	G	**596.3515**	298.6794	579.3249	290.1661			6
25	2401.0902	1201.0488	2384.0637	1192.5355	2383.0797	1192.0435	V	539.3300	270.1686	522.3035	261.6554			5
26	2500.1586	1250.5830	2483.1321	1242.0697	2482.1481	1241.5777	V	**440.2616**	220.6344	423.2350	212.1212			4
27	2557.1801	1279.0937	2540.1536	1270.5804	2539.1695	1270.0884	G	**341.1932**	171.1002	324.1666	162.5870			3
28	2694.2390	1347.6231	2677.2125	1339.1099	2676.2285	1338.6179	H	284.1717	142.5895	267.1452	134.0762			2
29							K	147.1128	74.0600	130.0863	65.5468			1

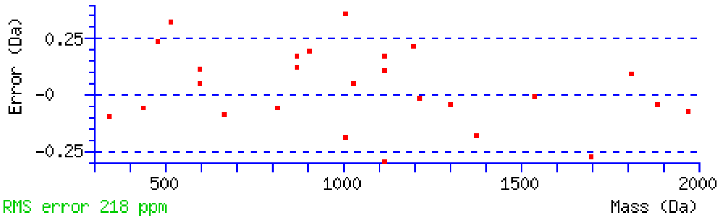

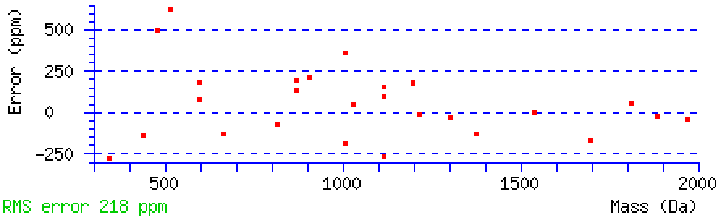

All matches to this query

Score	Mr(calc):	Delta	Sequence
53.5	2839.3373	0.0130	IDSEGISQNHTGQVASADASGFAGVVGHK
36.4	2838.3533	0.9970	IDSEGISQNHTGQVASADASGFAGVVGHK
5.4	2839.3479	0.0024	VTISTMTSADLLSALRAVINEPSYK
5.4	2839.3479	0.0024	VTISTMTSADLLSALRAVINEPSYK
5.4	2839.3479	0.0024	VTISTMTSADLLSALRAVINEPSYK
3.1	2839.3479	0.0024	VTISTMTSADLLSALRAVINEPSYK
1.8	2839.3479	0.0024	VTISTMTSADLLSALRAVINEPSYK
1.1	2839.3479	0.0024	VTISTMTSADLLSALRAVINEPSYK

1.0	2837.3323	2.0180	MNLPTFSSDLILIKSVLSQETDAR
1.0	2837.3323	2.0180	MNLPTFSSDLILIKSVLSQETDAR

Spectrum No: 403; Query: 577; Rank: 1

Peptide View

MS/MS Fragmentation of **SVEFTSCNDTVVIPCK**
Found in **IPI00470238**, Tax_Id=10116 Gene_Symbol=Cd47 Isoform 2 of Leukocyte surface antigen CD47 precursor

Match to Query 577: 1855.836688 from(928.925620,2+)
Title: 091127RatKid_SCX01_17.2454.2454.2.dta
Data file K:\NewmanPaper\Piliang\3SubProteomes\Piliang3SP\mgf5ppm\SCX_3SubProteomes5ppm.mgf

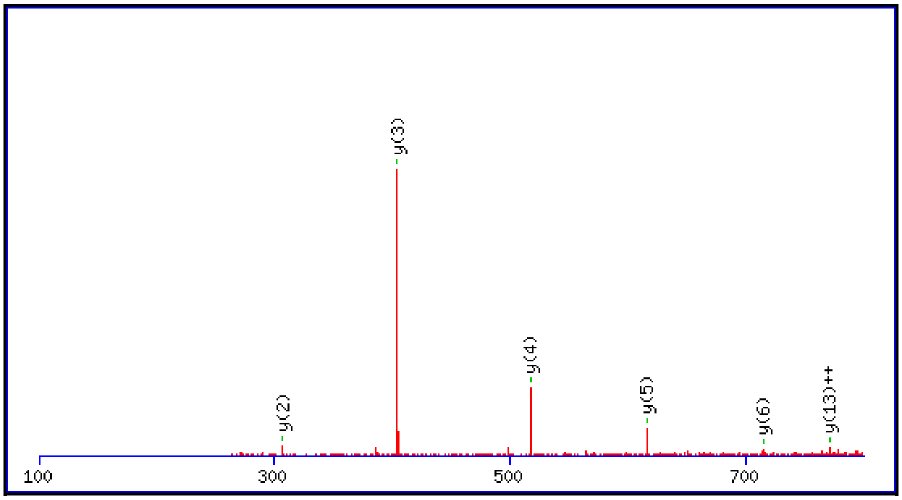

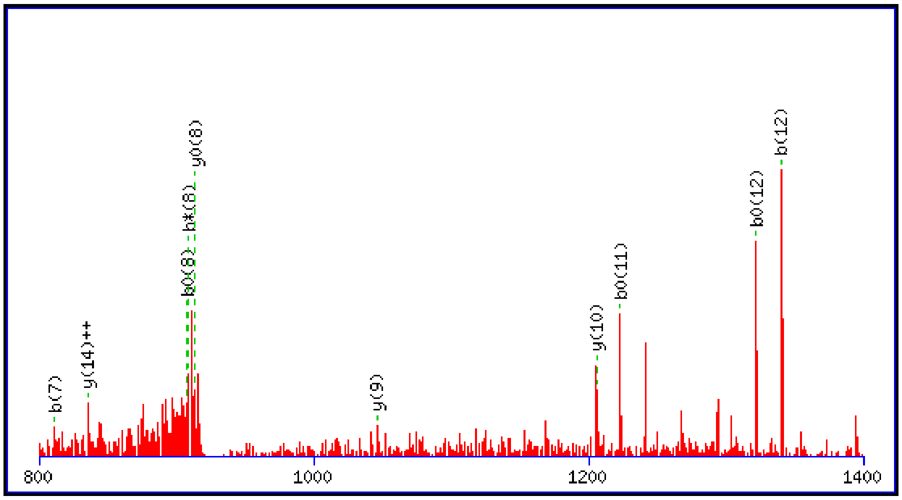

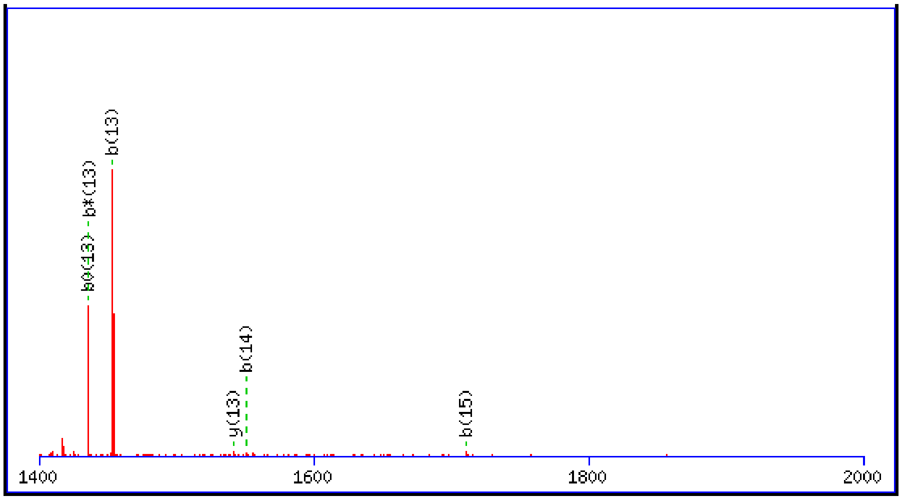

Monoisotopic mass of neutral peptide Mr(calc): 1855.8332
Fixed modifications: Carbamidomethyl (C)
Variable modifications:
N8 : Deamidated_N (N)
Ions Score: 54 **Expect:** 0.00099
Matches (Bold Red): 22/154 fragment ions using 36 most intense peaks

#	b	b^{++}	b*	b^{*++}	b^0	b^{0++}	Seq.	y	y^{++}	y*	y^{*++}	y^0	y^{0++}	#
1	88.0393	44.5233			70.0287	35.5180	S							16
2	187.1077	94.0575			169.0972	85.0522	V	1769.8084	885.4079	1752.7819	876.8946	1751.7979	876.4026	15
3	316.1503	158.5788			298.1397	149.5735	E	1670.7400	**835.8737**	1653.7135	827.3604	1652.7295	826.8684	14
4	463.2187	232.1130			445.2082	223.1077	F	**1541.6974**	**771.3524**	1524.6709	762.8391	1523.6869	762.3471	13
5	564.2664	282.6368			546.2558	273.6316	T	1394.6290	697.8181	1377.6025	689.3049	1376.6185	688.8129	12
6	651.2984	326.1529			633.2879	317.1476	S	1293.5813	647.2943	1276.5548	638.7810	1275.5708	638.2890	11
7	**811.3291**	406.1682			793.3185	397.1629	C	**1206.5493**	603.7783	1189.5228	595.2650	1188.5388	594.7730	10
8	926.3560	463.6816	**909.3295**	455.1684	**908.3455**	454.6764	N	**1046.5187**	523.7630	1029.4921	515.2497	1028.5081	514.7577	9
9	1041.3830	521.1951	1024.3564	512.6818	1023.3724	512.1898	D	931.4917	466.2495	914.4652	457.7362	**913.4812**	457.2442	8
10	1142.4306	571.7190	1125.4041	563.2057	1124.4201	562.7137	T	816.4648	408.7360	799.4382	400.2228	798.4542	399.7307	7
11	1241.4991	621.2532	1224.4725	612.7399	**1223.4885**	612.2479	V	**715.4171**	358.2122	698.3906	349.6989			6
12	**1340.5675**	670.7874	1323.5409	662.2741	**1322.5569**	661.7821	V	**616.3487**	308.6780	599.3221	300.1647			5
13	**1453.6515**	727.3294	**1436.6250**	718.8161	**1435.6410**	718.3241	I	**517.2803**	259.1438	500.2537	250.6305			4
14	**1550.7043**	775.8558	1533.6777	767.3425	1532.6937	766.8505	P	**404.1962**	202.6017	387.1697	194.0885			3
15	**1710.7349**	855.8711	1693.7084	847.3578	1692.7244	846.8658	C	**307.1435**	154.0754	290.1169	145.5621			2
16							K	147.1128	74.0600	130.0863	65.5468			1

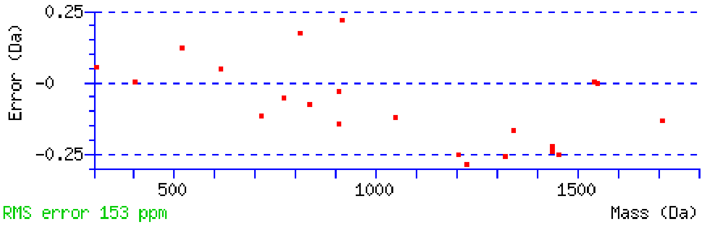

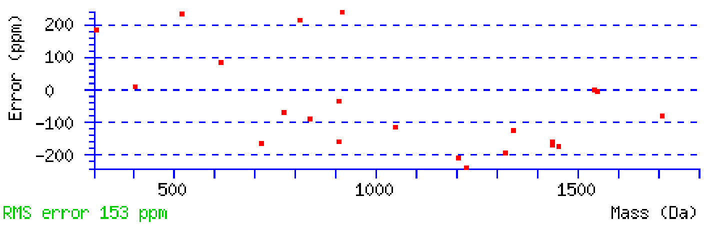

All matches to this query

Score	Mr(calc):	Delta	Sequence
53.5	1855.8332	0.0035	SVEFTSCNDTVVIPCK
14.9	1855.8386	-0.0019	SKPMKEDNNLSLQEK
14.9	1855.8386	-0.0019	SKPMKEDNNLSLQEK
14.8	1854.8492	0.9875	SVEFTSCNDTVVIPCK
9.3	1855.8288	0.0079	CRSLAEEYGLHTVNK

9.2	1855.8288	0.0079	CRSLAEEYGLHTVNK
9.0	1855.8230	0.0137	SRRYGASSSVELSLGK
9.0	1855.8230	0.0137	SRRYGASSSVELSLGK
9.0	1855.8230	0.0137	SRRYGASSSVELSLGK
9.0	1855.8230	0.0137	SRRYGASSSVELSLGK

Spectrum No: 404; Query: 604; Rank: 1

Peptide View

MS/MS Fragmentation of **AADPPAENSSAPEAEQGGAE**
Found in **IPI00551815**, Tax_Id=10116 Gene_Symbol=Ybx1-ps3 Ybx1 protein

Match to Query 604: 1897.780108 from(949.897330,2+)
Title: 091127RatKid_SCX01_04.835.835.2.dta
Data file K:\NewmanPaper\Piliang\3SubProteomes\Piliang3SP\mgf5ppm\SCX_3SubProteomes5ppm.mgf

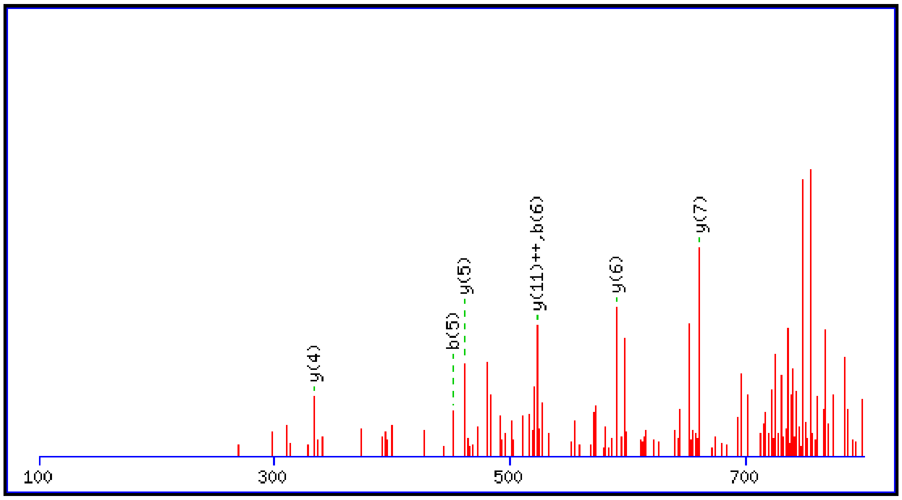

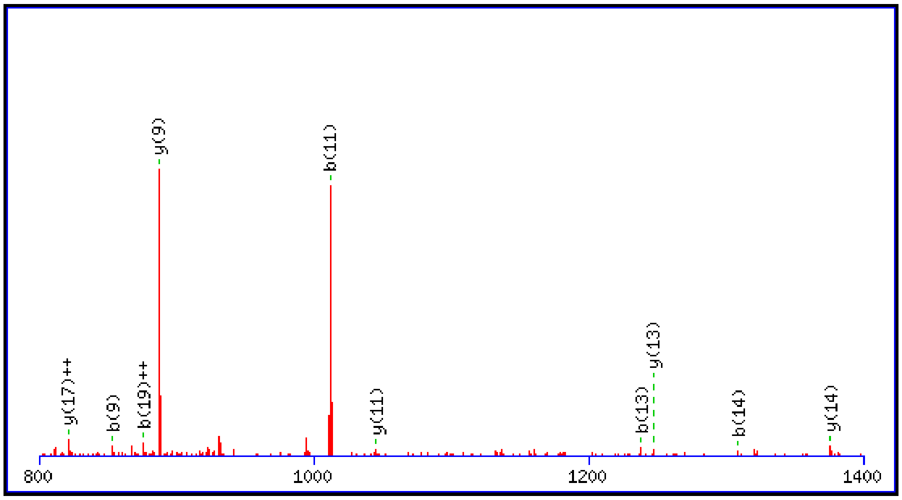

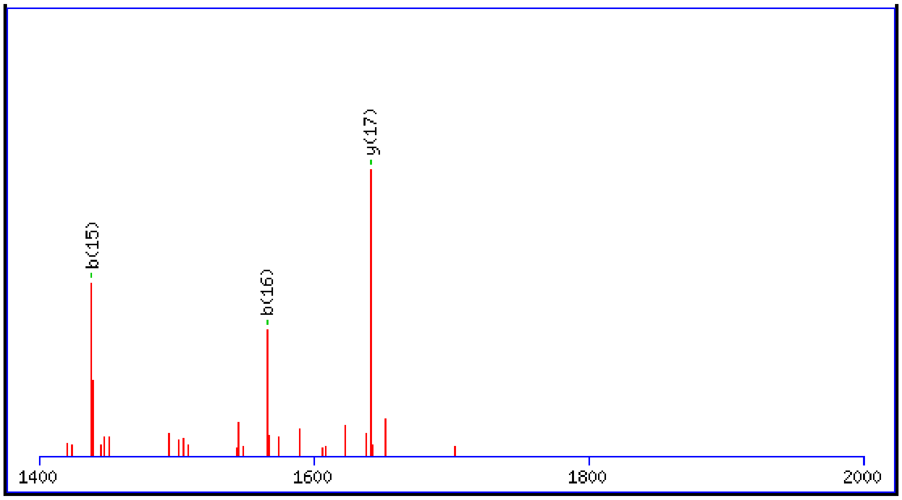

Monoisotopic mass of neutral peptide Mr(calc): 1897.7813
Fixed modifications: Carbamidomethyl (C)
Variable modifications:
N8 : Deamidated_N (N)
Ions Score: 54 **Expect:** 0.00078
Matches (Bold Red): 20/202 fragment ions using 34 most intense peaks

#	b	b++	b*	b*++	b0	b0++	Seq.	y	y++	y*	y*++	y0	y0++	#
1	72.0444	36.5258					A							20
2	143.0815	72.0444					A	1827.7515	914.3794	1810.7250	905.8661	1809.7410	905.3741	19
3	258.1084	129.5579			240.0979	120.5526	D	1756.7144	878.8608	1739.6879	870.3476	1738.7038	869.8556	18
4	355.1612	178.0842			337.1506	169.0790	P	**1641.6875**	**821.3474**	1624.6609	812.8341	1623.6769	812.3421	17
5	**452.2140**	226.6106			434.2034	217.6053	P	1544.6347	772.8210	1527.6081	764.3077	1526.6241	763.8157	16
6	**523.2511**	262.1292			505.2405	253.1239	A	1447.5819	724.2946	1430.5554	715.7813	1429.5714	715.2893	15
7	652.2937	326.6505			634.2831	317.6452	E	**1376.5448**	688.7760	1359.5183	680.2628	1358.5343	679.7708	14
8	767.3206	384.1639	750.2941	375.6507	749.3101	375.1587	N	**1247.5022**	624.2547	1230.4757	615.7415	1229.4917	615.2495	13
9	**854.3526**	427.6800	837.3261	419.1667	836.3421	418.6747	S	1132.4753	566.7413	1115.4487	558.2280	1114.4647	557.7360	12
10	941.3847	471.1960	924.3581	462.6827	923.3741	462.1907	S	**1045.4433**	**523.2253**	1028.4167	514.7120	1027.4327	514.2200	11
11	**1012.4218**	506.7145	995.3952	498.2013	994.4112	497.7092	A	958.4112	479.7093	941.3847	471.1960	940.4007	470.7040	10
12	1109.4746	555.2409	1092.4480	546.7276	1091.4640	546.2356	P	**887.3741**	444.1907	870.3476	435.6774	869.3636	435.1854	9
13	**1238.5171**	619.7622	1221.4906	611.2489	1220.5066	610.7569	E	790.3214	395.6643	773.2948	387.1510	772.3108	386.6590	8
14	**1309.5543**	655.2808	1292.5277	646.7675	1291.5437	646.2755	A	**661.2788**	331.1430	644.2522	322.6297	643.2682	322.1377	7
15	**1438.5969**	719.8021	1421.5703	711.2888	1420.5863	710.7968	E	**590.2416**	295.6245	573.2151	287.1112	572.2311	286.6192	6
16	**1566.6554**	783.8314	1549.6289	775.3181	1548.6449	774.8261	Q	**461.1991**	231.1032	444.1725	222.5899	443.1885	222.0979	5
17	1623.6769	812.3421	1606.6503	803.8288	1605.6663	803.3368	G	**333.1405**	167.0739			315.1299	158.0686	4
18	1680.6984	840.8528	1663.6718	832.3395	1662.6878	831.8475	G	276.1190	138.5631			258.1084	129.5579	3
19	1751.7355	**876.3714**	1734.7089	867.8581	1733.7249	867.3661	A	219.0975	110.0524			201.0870	101.0471	2
20							E	148.0604	74.5339			130.0499	65.5286	1

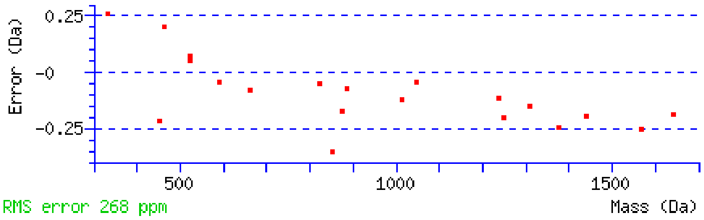

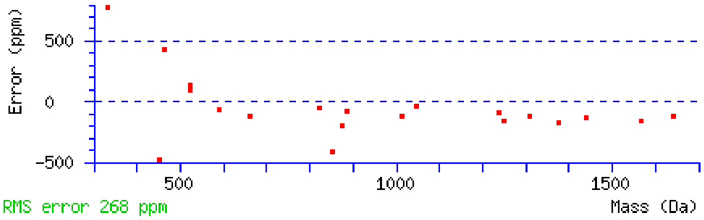

All matches to this query

Score	Mr(calc):	Delta	Sequence
53.5	1897.7813	-0.0012	AADPPAENSSAPEAEQGGAE

2.9	1897.7884	-0.0083	EKVFTSDSDASGWAYR
2.9	1897.7884	-0.0083	EKVFTSDSDASGWAYR
1.9	1897.7794	0.0007	LMTAQNVASYRTPDR
1.7	1897.7794	0.0007	LMTAQNVASYRTPDR
1.4	1896.7760	1.0041	SWSVHCEPCSERRK
1.4	1895.7850	1.9951	MDQVGDSTVVGTSRLR
0.8	1897.7830	-0.0029	KTGMLMEYMLSKYK

Spectrum No: 405; Query: 206; Rank: 1

Peptide View

MS/MS Fragmentation of **WGEVDLIGNCSR**
Found in **IPI00204451**, Tax_Id=10116 Gene_Symbol=Cfi Complement factor I precursor

Match to Query 206: 1405.633548 from(703.824050,2+)
Title: 091127RatKid_SCX01_12.2715.2715.2.dta
Data file K:\NewmanPaper\Piliang\3SubProteomes\Piliang3SP\mgf5ppm\SCX_3SubProteomes5ppm.mgf

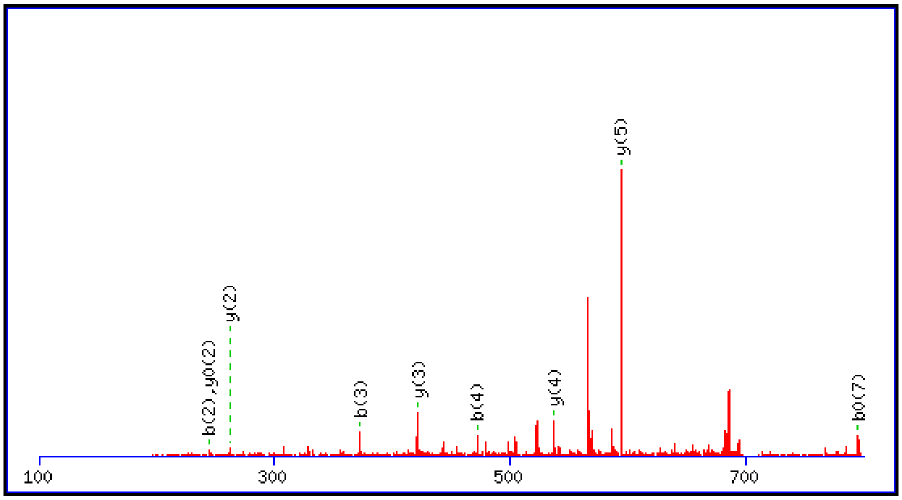

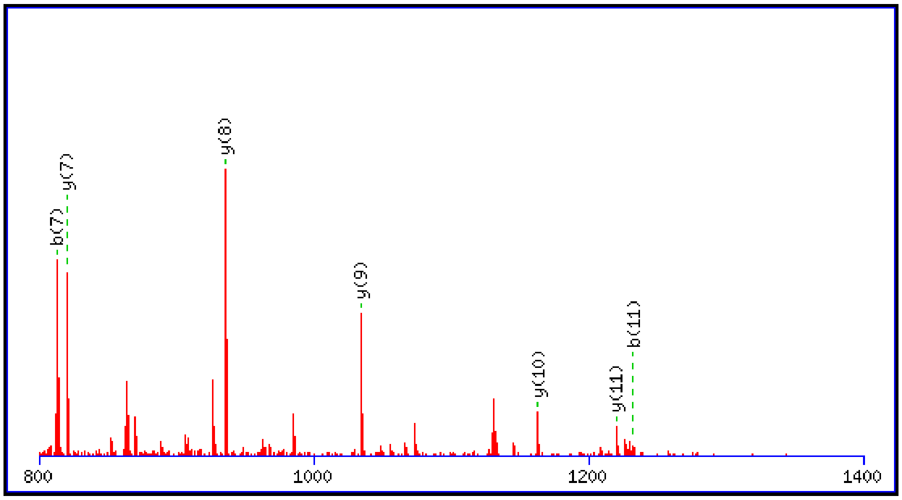

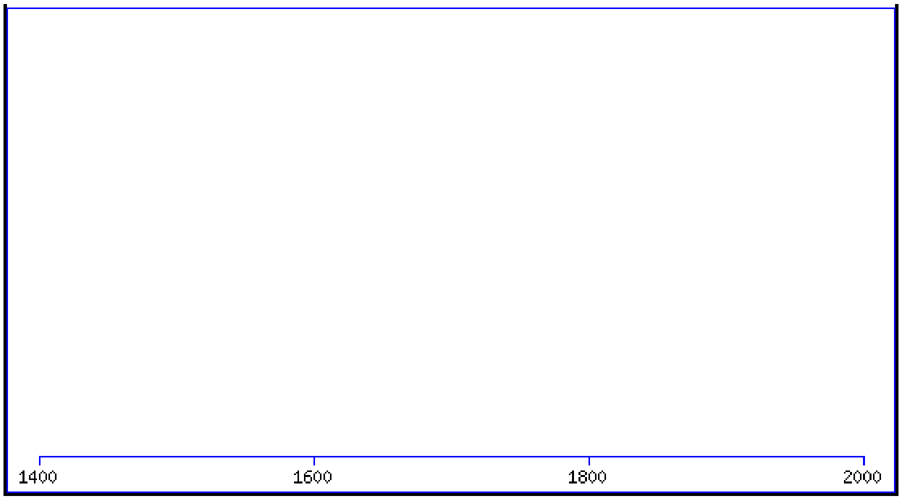

Monoisotopic mass of neutral peptide Mr(calc): 1405.6296
Fixed modifications: Carbamidomethyl (C)
Variable modifications:
N9 : Deamidated_N (N)
Ions Score: 53 **Expect:** 0.00071
Matches (Bold Red): 16/110 fragment ions using 41 most intense peaks

#	b	b++	b*	b*++	b0	b0++	Seq.	y	y++	y*	y*++	y0	y0++	#
1	187.0866	94.0469					W							12
2	**244.1081**	122.5577					G	**1220.5576**	610.7824	1203.5310	602.2691	1202.5470	601.7771	11
3	**373.1506**	187.0790			355.1401	178.0737	E	**1163.5361**	582.2717	1146.5096	573.7584	1145.5255	573.2664	10
4	**472.2191**	236.6132			454.2085	227.6079	V	**1034.4935**	517.7504	1017.4670	509.2371	1016.4829	508.7451	9
5	587.2460	294.1266			569.2354	285.1214	D	**935.4251**	468.2162	918.3986	459.7029	917.4145	459.2109	8
6	700.3301	350.6687			682.3195	341.6634	L	**820.3982**	410.7027	803.3716	402.1894	802.3876	401.6974	7
7	**813.4141**	407.2107			**795.4036**	398.2054	I	707.3141	354.1607	690.2875	345.6474	689.3035	345.1554	6
8	870.4356	435.7214			852.4250	426.7162	G	**594.2300**	297.6187	577.2035	289.1054	576.2195	288.6134	5
9	985.4625	493.2349	968.4360	484.7216	967.4520	484.2296	N	**537.2086**	269.1079	520.1820	260.5946	519.1980	260.1026	4
10	1145.4932	573.2502	1128.4666	564.7370	1127.4826	564.2449	C	**422.1816**	211.5945	405.1551	203.0812	404.1711	202.5892	3
11	**1232.5252**	616.7662	1215.4987	608.2530	1214.5146	607.7610	S	**262.1510**	131.5791	245.1244	123.0659	**244.1404**	122.5738	2
12							R	175.1190	88.0631	158.0924	79.5498			1

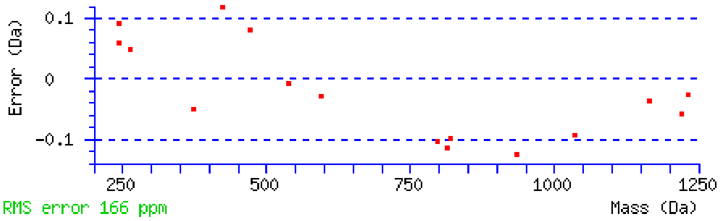

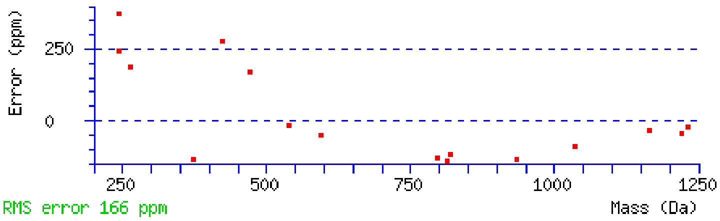

All matches to this query

Score	Mr(calc):	Delta	Sequence
53.4	1405.6296	0.0039	WGEVDLIGNCSR
3.7	1405.6321	0.0014	TGSNEESTEVPTR
1.1	1405.6398	-0.0062	KPRNGKSHCSR
0.3	1405.6449	-0.0113	SMVHHLYFNDK

Spectrum No: 406; Query: 958; Rank: 1

Peptide View

MS/MS Fragmentation of **QPNATNPCGSNNGGCAQVCVLSHR**
Found in **IPI00205325**, Tax_Id=10116 Gene_Symbol=Lrp2 Low-density lipoprotein receptor-related protein 2 precursor

Match to Query 958: 2598.116442 from(867.046090,3+)
Title: 091127RatKid_SCX01_24.1348.1348.3.dta
Data file K:\NewmanPaper\Piliang\3SubProteomes\Piliang3SP\mgf5ppm\SCX_3SubProteomes5ppm.mgf

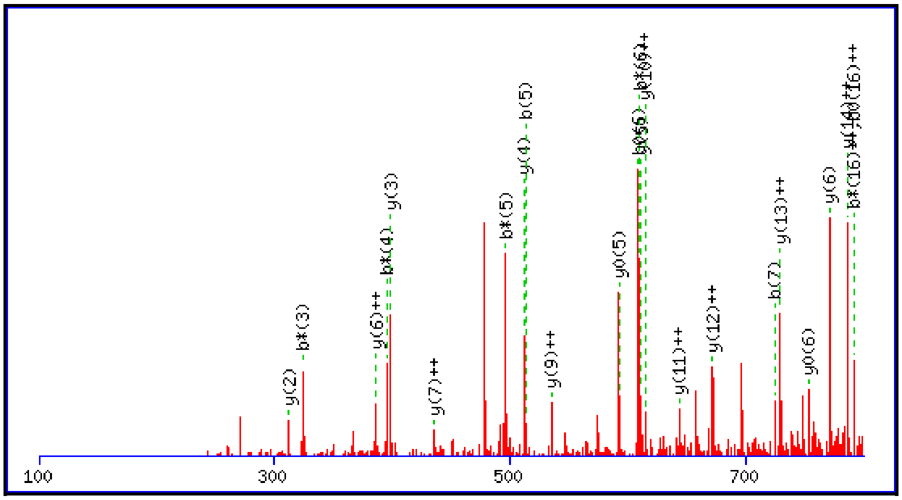

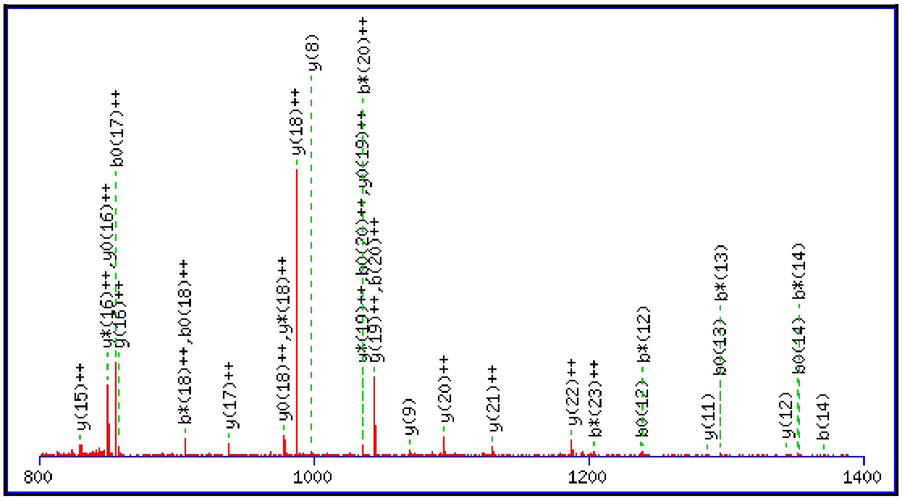

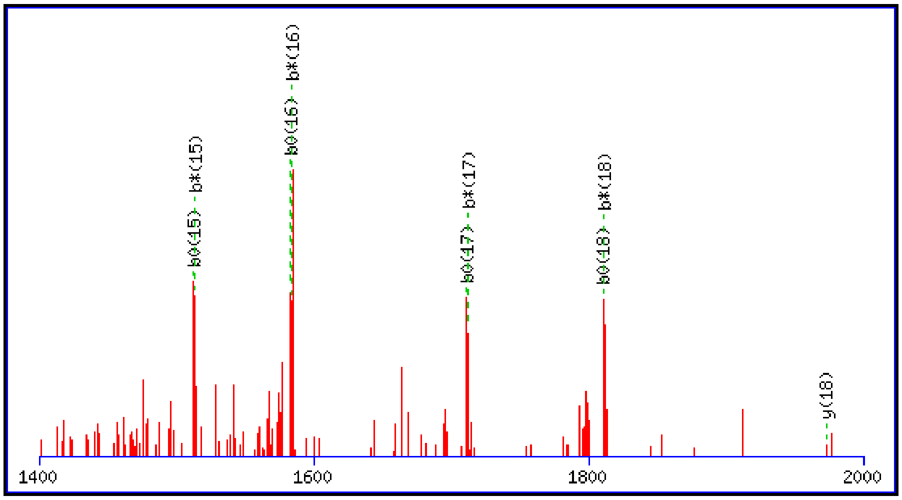

Monoisotopic mass of neutral peptide Mr(calc): 2598.1122
Fixed modifications: Carbamidomethyl (C)
Variable modifications:
N3 : Deamidated_N (N)
Ions Score: 53 **Expect:** 0.0017
Matches (Bold Red): 65/264 fragment ions using 122 most intense peaks

#	b	++	b*	++	0	0++	Seq.	y	++	y*	++	0	0++	#

		b		b*	b	b			y		y*	y	y	
1	129.0659	65.0366	112.0393	56.5233			Q							24
2	226.1186	113.5629	209.0921	105.0497			P	2471.0609	1236.0341	2454.0343	1227.5208	2453.0503	1227.0288	23
3	341.1456	171.0764	**324.1190**	162.5631			N	2374.0081	**1187.5077**	2356.9816	1178.9944	2355.9976	1178.5024	22
4	412.1827	206.5950	**395.1561**	198.0817			A	2258.9812	**1129.9942**	2241.9546	1121.4810	2240.9706	1120.9889	21
5	**513.2303**	257.1188	**496.2038**	248.6055	495.2198	248.1135	T	2187.9441	**1094.4757**	2170.9175	1085.9624	2169.9335	1085.4704	20
6	627.2733	314.1403	**610.2467**	305.6270	**609.2627**	305.1350	N	2086.8964	**1043.9518**	2069.8698	**1035.4386**	2068.8858	**1034.9465**	19
7	**724.3260**	362.6667	707.2995	354.1534	706.3155	353.6614	P	**1972.8535**	**986.9304**	1955.8269	**978.4171**	1954.8429	**977.9251**	18
8	884.3567	442.6820	867.3301	434.1687	866.3461	433.6767	C	1875.8007	**938.4040**	1858.7741	929.8907	1857.7901	929.3987	17
9	941.3782	471.1927	924.3516	462.6794	923.3676	462.1874	G	1715.7700	**858.3887**	1698.7435	**849.8754**	1697.7595	**849.3834**	16
10	1028.4102	514.7087	1011.3836	506.1955	1010.3996	505.7034	S	1658.7486	**829.8779**	1641.7220	821.3647	1640.7380	820.8726	15
11	1142.4531	571.7302	1125.4266	563.2169	1124.4425	562.7249	N	1571.7166	**786.3619**	1554.6900	777.8486	1553.7060	777.3566	14
12	1256.4960	628.7517	**1239.4695**	620.2384	**1238.4855**	619.7464	N	1457.6736	**729.3405**	1440.6471	720.8272	1439.6631	720.3352	13
13	1313.5175	657.2624	**1296.4909**	648.7491	**1295.5069**	648.2571	G	**1343.6307**	**672.3190**	1326.6042	663.8057	1325.6201	663.3137	12
14	**1370.5390**	685.7731	**1353.5124**	677.2598	**1352.5284**	676.7678	G	**1286.6092**	**643.8083**	1269.5827	635.2950	1268.5987	634.8030	11
15	1530.5696	765.7884	**1513.5431**	757.2752	**1512.5590**	756.7832	C	1229.5878	**615.2975**	1212.5612	606.7843	1211.5772	606.2922	10
16	1601.6067	801.3070	**1584.5802**	**792.7937**	**1583.5962**	**792.3017**	A	**1069.5571**	**535.2822**	1052.5306	526.7689	1051.5466	526.2769	9
17	1729.6653	865.3363	**1712.6388**	856.8230	**1711.6547**	**856.3310**	Q	**998.5200**	499.7636	981.4935	491.2504	980.5094	490.7584	8
18	1828.7337	914.8705	**1811.7072**	**906.3572**	**1810.7232**	**905.8652**	V	870.4614	**435.7344**	853.4349	427.2211	852.4509	426.7291	7
19	1988.7644	994.8858	1971.7378	986.3725	1970.7538	985.8805	C	**771.3930**	**386.2001**	754.3665	377.6869	**753.3825**	377.1949	6
20	2087.8328	**1044.4200**	2070.8062	**1035.9068**	2069.8222	**1035.4147**	V	**611.3624**	306.1848	594.3358	297.6715	**593.3518**	297.1795	5
21	2200.9168	1100.9621	2183.8903	1092.4488	2182.9063	1091.9568	L	**512.2940**	256.6506	495.2674	248.1373	494.2834	247.6453	4
22	2287.9489	1144.4781	2270.9223	1135.9648	2269.9383	1135.4728	S	**399.2099**	200.1086	382.1833	191.5953	381.1993	191.1033	3
23	2425.0078	1213.0075	2407.9812	**1204.4943**	2406.9972	1204.0022	H	**312.1779**	156.5926	295.1513	148.0793			2
24							R	175.1190	88.0631	158.0924	79.5498			1

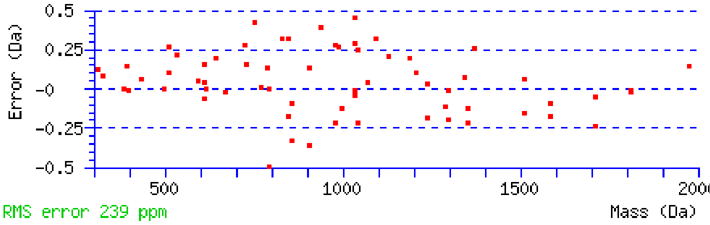

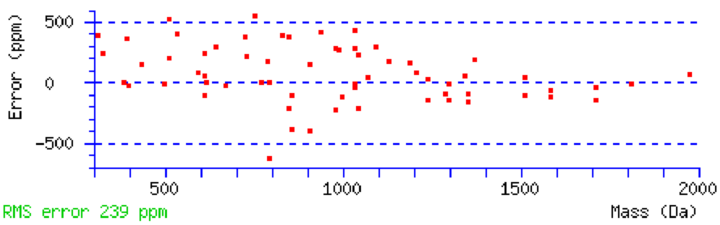

All matches to this query

Score	Mr(calc):	Delta	Sequence
53.2	2598.1122	0.0042	QPNATNPCGSNNGGCAQVCVLSHR
46.4	2598.1122	0.0042	QPNATNPCGSNNGGCAQVCVLSHR
43.6	2598.1122	0.0042	QPNATNPCGSNNGGCAQVCVLSHR
43.6	2598.1122	0.0042	QPNATNPCGSNNGGCAQVCVLSHR
38.6	2597.1282	0.9883	QPNATNPCGSNNGGCAQVCVLSHR
5.0	2598.1355	-0.0191	AMSIPTSPTRISFHSIKQTAAV
1.6	2598.1355	-0.0191	AMSIPTSPTRISFHSIKQTAAV
1.5	2596.1339	1.9826	VESDIKRFFENLNPMGNSMEK
1.5	2596.1249	1.9916	IWLLPVFPSSCLMNNHCPCR
1.5	2596.1249	1.9916	IWLLPVFPSSCLMNNHCPCR

Spectrum No: 407; Query: 116; Rank: 1

Peptide View

MS/MS Fragmentation of **FINYNQTVSR**
Found in **IPI00359123**, Tax_Id=10116 Gene_Symbol=RGD1310427_predicted hypothetical protein LOC362643

Match to Query 116: 1241.605408 from(621.809980,2+)
Title: 091127RatKid_SCX01_19.1383.1383.2.dta
Data file K:\NewmanPaper\Piliang\3SubProteomes\Piliang3SP\mgf5ppm\SCX_3SubProteomes5ppm.mgf

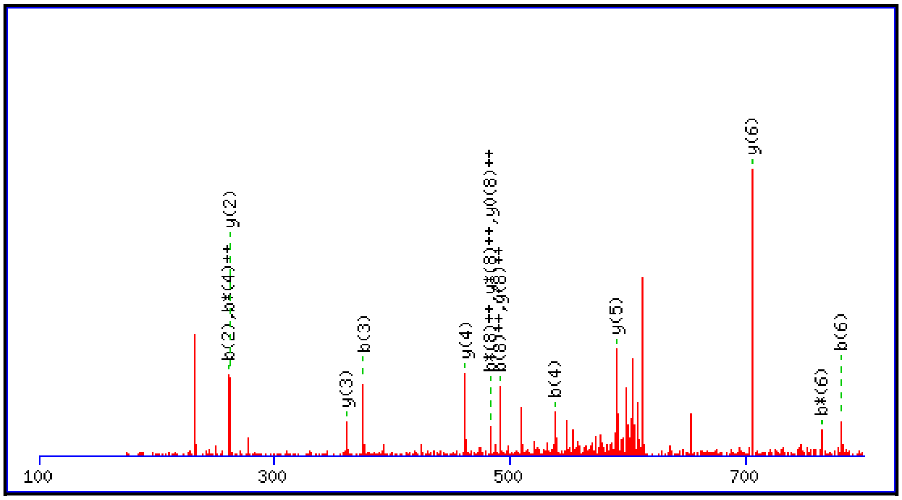

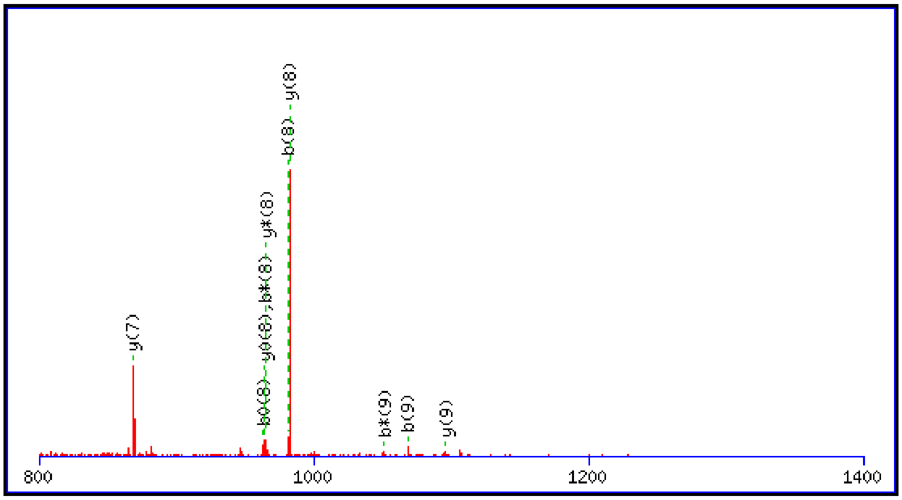

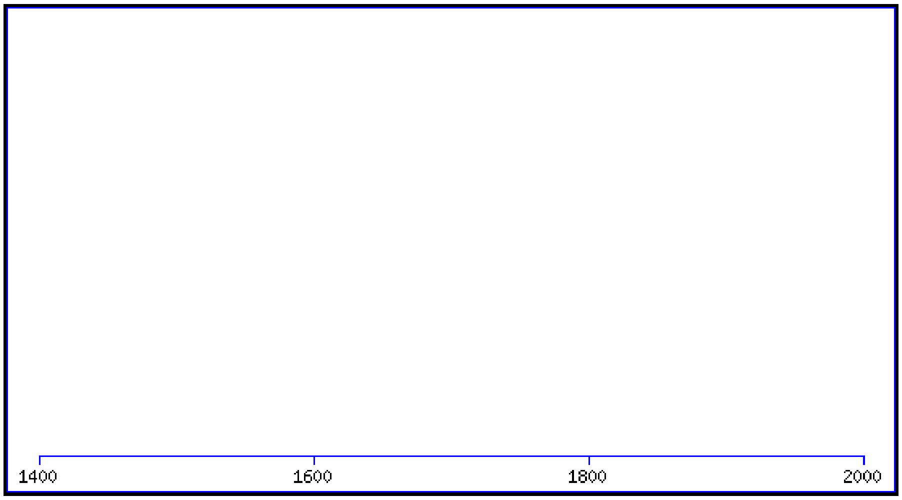

Monoisotopic mass of neutral peptide Mr(calc): 1241.6040
Fixed modifications: Carbamidomethyl (C)
Variable modifications:
N5 : Deamidated_N (N)
Ions Score: 53 **Expect:** 0.00059
Matches (Bold Red): 26/90 fragment ions using 43 most intense peaks

#	b	b^{++}	b^{*}	b^{*++}	b^{0}	b^{0++}	Seq.	y	y^{++}	y^{*}	y^{*++}	y^{0}	y^{0++}	#
1	148.0757	74.5415					F							10
2	**261.1598**	131.0835					I	**1095.5429**	548.2751	1078.5164	539.7618	1077.5323	539.2698	9
3	**375.2027**	188.1050	358.1761	179.5917			N	**982.4588**	**491.7331**	**965.4323**	**483.2198**	**964.4483**	**482.7278**	8
4	**538.2660**	269.6366	521.2395	**261.1234**			Y	**868.4159**	434.7116	851.3894	426.1983	850.4054	425.7063	7
5	653.2929	327.1501	636.2664	318.6368			N	**705.3526**	353.1799	688.3260	344.6667	687.3420	344.1746	6
6	**781.3515**	391.1794	**764.3250**	382.6661			Q	**590.3257**	295.6665	573.2991	287.1532	572.3151	286.6612	5
7	882.3992	441.7032	865.3727	433.1900	864.3886	432.6980	T	**462.2671**	231.6372	445.2405	223.1239	444.2565	222.6319	4
8	**981.4676**	**491.2374**	**964.4411**	**482.7242**	**963.4571**	482.2322	V	**361.2194**	181.1133	344.1928	172.6001	343.2088	172.1081	3
9	**1068.4996**	534.7535	**1051.4731**	526.2402	1050.4891	525.7482	S	**262.1510**	131.5791	245.1244	123.0659	244.1404	122.5738	2
10							R	175.1190	88.0631	158.0924	79.5498			1

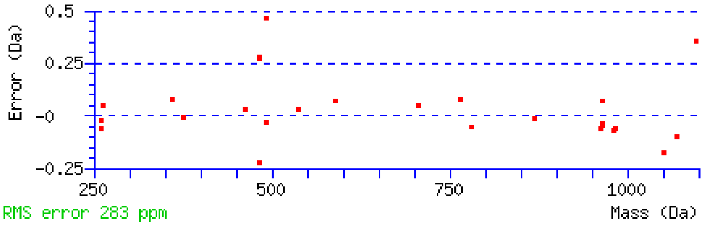

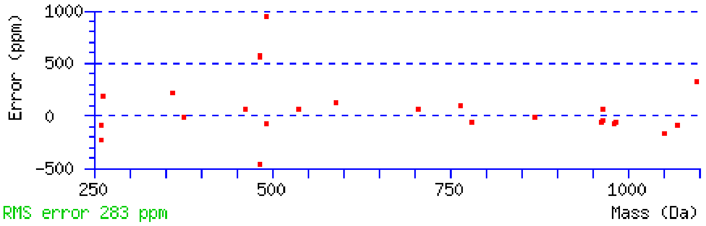

All matches to this query

Score	Mr(calc):	Delta	Sequence
53.0	1241.6040	0.0014	FINYNQTVSR
31.2	1241.6040	0.0014	FINYNQTVSR
10.1	1240.6064	0.9990	DSNKSQKLLK
9.8	1240.6064	0.9990	DSNKSQKLLK
9.4	1240.6009	1.0045	MLISIFSNGNK
9.4	1240.6009	1.0045	MLISLFSNGNK
8.5	1240.5999	1.0055	CSLTKTPARK
8.2	1240.6138	0.9916	MTSATSPIILK
8.2	1240.6138	0.9916	MTSATSPIILK
8.2	1240.6138	0.9916	MTSATSPIILK

Spectrum No: 408; Query: 154; Rank: 1

Peptide View

MS/MS Fragmentation of **SPCNSSVLQDGR**
Found in **IPI00368484**, Tax_Id=10116 Gene_Symbol=- 46 kDa protein

Match to Query 154: 1319.578608 from(660.796580,2+)
Title: 091127RatKid_SCX01_02.795.795.2.dta
Data file K:\NewmanPaper\Piliang\3SubProteomes\Piliang3SP\mgf5ppm\SCX_3SubProteomes5ppm.mgf

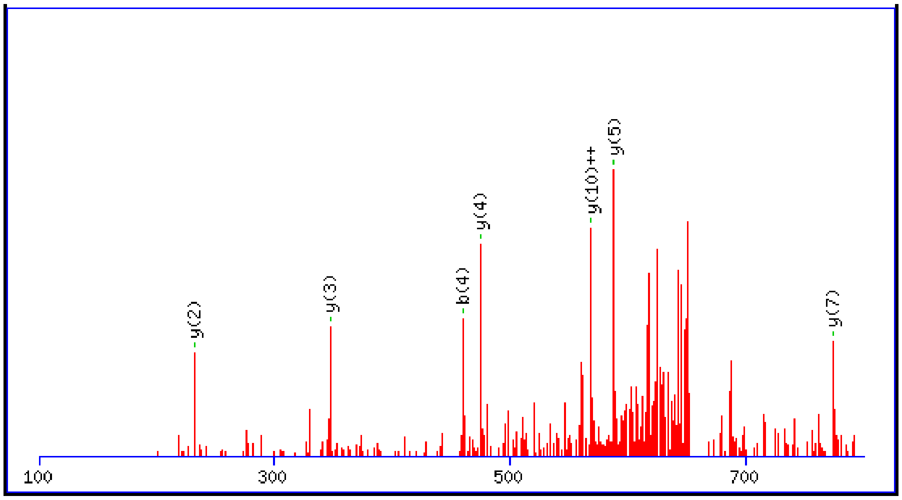

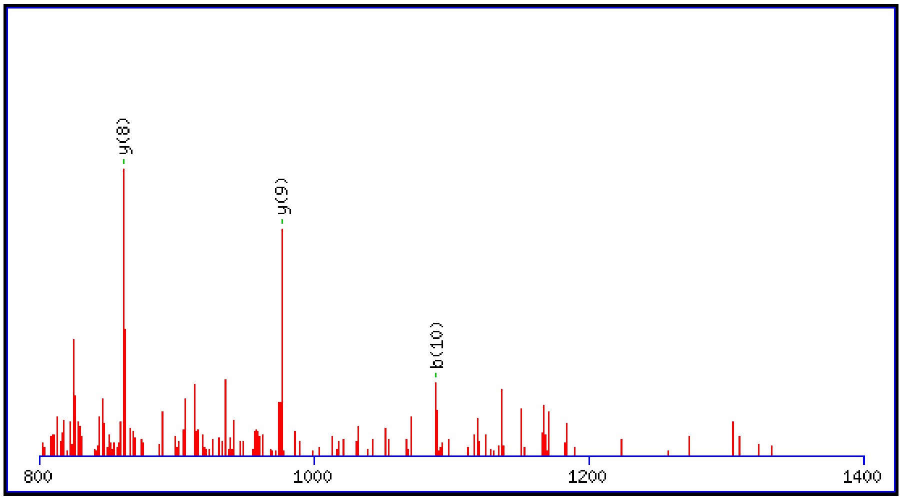

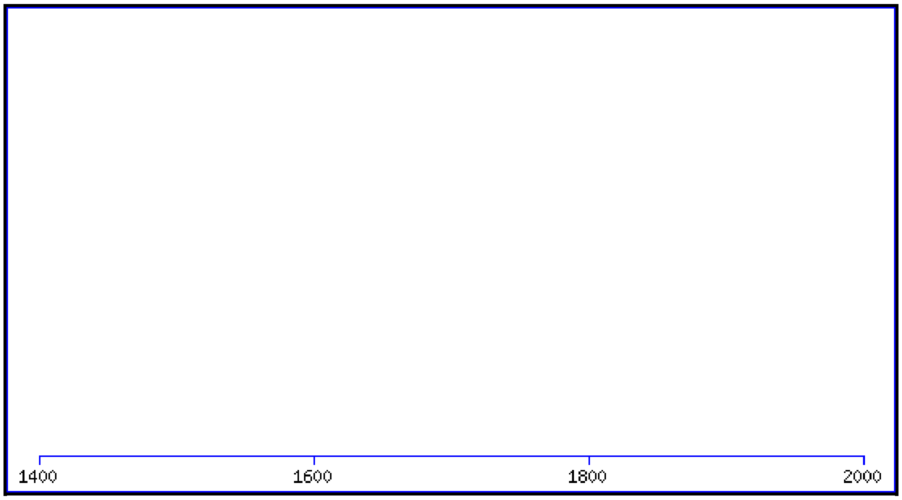

Monoisotopic mass of neutral peptide Mr(calc): 1319.5776
Fixed modifications: Carbamidomethyl (C)
Variable modifications:
N4 : Deamidated_N (N)
Ions Score: 53 **Expect:** 0.00069
Matches (Bold Red): 10/122 fragment ions using 20 most intense peaks

#	b	b^{++}	b*	b^{*++}	b^0	b^{0++}	Seq.	y	y^{++}	y*	y^{*++}	y^0	y^{0++}	#
1	88.0393	44.5233			70.0287	35.5180	S							12
2	185.0921	93.0497			167.0815	84.0444	P	1233.5528	617.2800	1216.5263	608.7668	1215.5423	608.2748	11
3	345.1227	173.0650			327.1122	164.0597	C	1136.5001	**568.7537**	1119.4735	560.2404	1118.4895	559.7484	10
4	**460.1497**	230.5785	443.1231	222.0652	442.1391	221.5732	N	**976.4694**	488.7383	959.4429	480.2251	958.4588	479.7331	9
5	547.1817	274.0945	530.1551	265.5812	529.1711	265.0892	S	**861.4425**	431.2249	844.4159	422.7116	843.4319	422.2196	8

**6**	634.2137	317.6105	617.1872	309.0972	616.2031	308.6052	**S**	**774.4104**	387.7089	757.3839	379.1956	756.3999	378.7036	**7**
**7**	733.2821	367.1447	716.2556	358.6314	715.2716	358.1394	**V**	687.3784	344.1928	670.3519	335.6796	669.3678	335.1876	**6**
**8**	846.3662	423.6867	829.3396	415.1735	828.3556	414.6814	**L**	**588.3100**	294.6586	571.2835	286.1454	570.2994	285.6534	**5**
**9**	974.4248	487.7160	957.3982	479.2027	956.4142	478.7107	**Q**	**475.2259**	238.1166	458.1994	229.6033	457.2154	229.1113	**4**
**10**	**1089.4517**	545.2295	1072.4252	536.7162	1071.4411	536.2242	**D**	**347.1674**	174.0873	330.1408	165.5740	329.1568	165.0820	**3**
**11**	1146.4732	573.7402	1129.4466	565.2269	1128.4626	564.7349	**G**	**232.1404**	116.5738	215.1139	108.0606			**2**
**12**							**R**	175.1190	88.0631	158.0924	79.5498			**1**

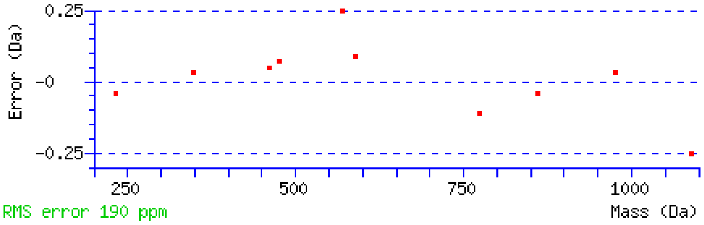

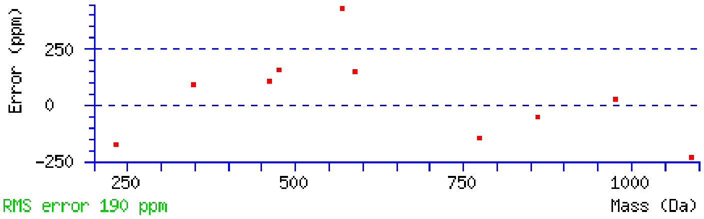

All matches to this query

Score	Mr(calc):	Delta	Sequence
52.8	1319.5776	0.0010	SPCNSSVLQDGR
4.7	1319.5748	0.0038	RSSLEIRSGR
4.5	1319.5887	-0.0101	TVRESLQSIK
4.1	1317.5796	1.9990	EKEAGEEAENGR
3.0	1319.5841	-0.0055	DLDDTSVVEDGR
2.1	1318.5628	1.0158	ACNSVFTALEK

Spectrum No: 409; Query: 868; Rank: 1

Peptide View

MS/MS Fragmentation of **VPFNPNDTFESEFYLDEKR**
Found in **IPI00200591**, Tax_Id=10116 Gene_Symbol=LOC299282 Serine protease inhibitor A3L precursor

Match to Query 868: 2347.074042 from(783.365290,3+)
Title: 091129RatKid_SCX02_12.3102.3102.3.dta
Data file K:\NewmanPaper\Piliang\3SubProteomes\Piliang3SP\mgf5ppm\SCX_3SubProteomes5ppm.mgf

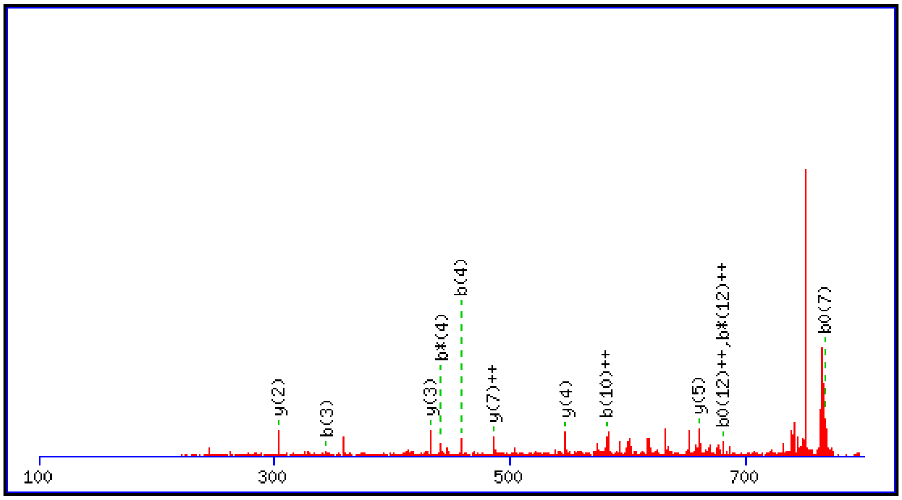

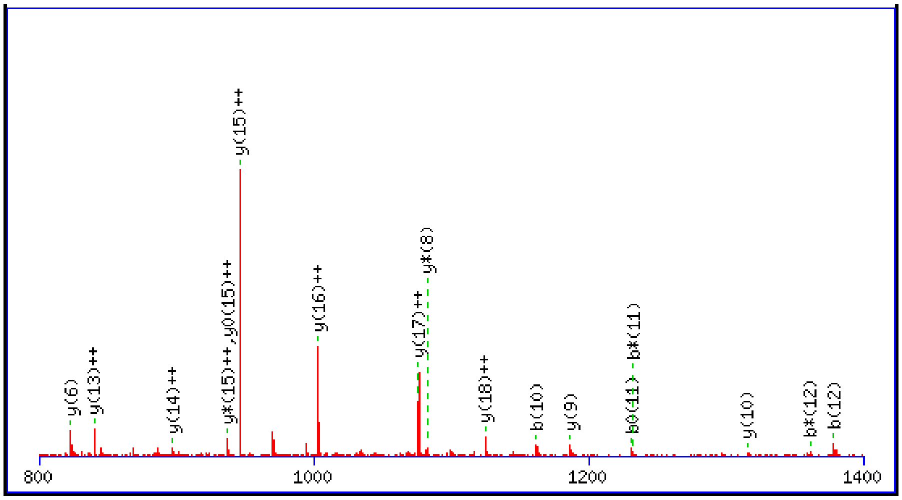

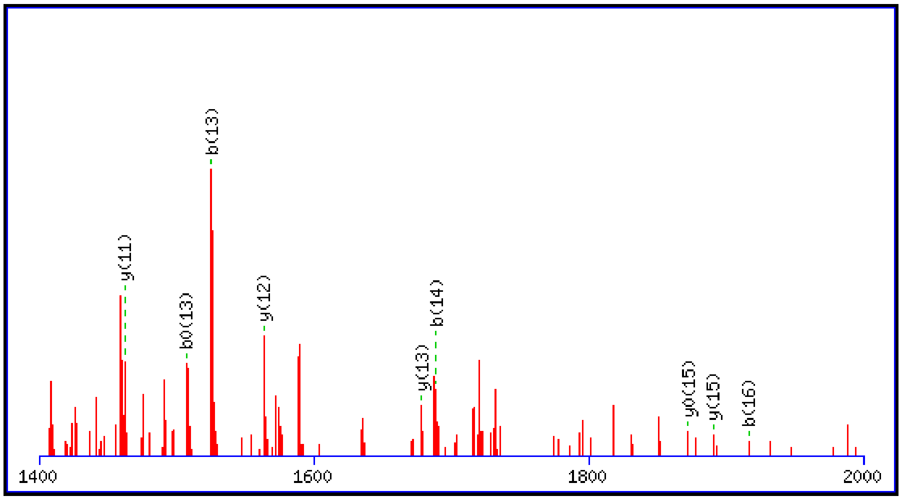

Monoisotopic mass of neutral peptide Mr(calc): 2347.0644
Fixed modifications: Carbamidomethyl (C)
Variable modifications:
N6 : Deamidated_N (N)
Ions Score: 53 **Expect:** 0.0019
Matches (Bold Red): 38/194 fragment ions using 83 most intense peaks

#	b	b^{++}	b*	b^{*++}	b^0	b^{0++}	Seq.	y	y^{++}	y*	y^{*++}	y^0	y^{0++}	#
1	100.0757	50.5415					V							19
2	197.1285	99.0679					P	2249.0033	**1125.0053**	2231.9768	1116.4920	2230.9927	1116.0000	18
3	**344.1969**	172.6021					F	2151.9505	**1076.4789**	2134.9240	1067.9656	2133.9400	1067.4736	17
4	**458.2398**	229.6235	**441.2132**	221.1103			N	2004.8821	**1002.9447**	1987.8556	994.4314	1986.8716	993.9394	16
5	555.2926	278.1499	538.2660	269.6366			P	**1890.8392**	**945.9232**	1873.8127	**937.4100**	**1872.8286**	**936.9180**	15
6	670.3195	335.6634	653.2929	327.1501			N	1793.7864	**897.3969**	1776.7599	888.8836	1775.7759	888.3916	14
7	785.3464	393.1769	768.3199	384.6636	**767.3359**	384.1716	D	**1678.7595**	**839.8834**	1661.7330	831.3701	1660.7489	830.8781	13
8	886.3941	443.7007	869.3676	435.1874	868.3836	434.6954	T	**1563.7326**	782.3699	1546.7060	773.8566	1545.7220	773.3646	12
9	1033.4625	517.2349	1016.4360	508.7216	1015.4520	508.2296	F	**1462.6849**	731.8461	1445.6583	723.3328	1444.6743	722.8408	11
10	**1162.5051**	**581.7562**	1145.4786	573.2429	1144.4946	572.7509	E	**1315.6165**	658.3119	1298.5899	649.7986	1297.6059	649.3066	10
11	1249.5372	625.2722	**1232.5106**	616.7589	**1231.5266**	616.2669	S	**1186.5739**	593.7906	1169.5473	585.2773	1168.5633	584.7853	9
12	**1378.5797**	689.7935	**1361.5532**	**681.2802**	1360.5692	**680.7882**	E	1099.5419	550.2746	**1082.5153**	541.7613	1081.5313	541.2693	8
13	**1525.6482**	763.3277	1508.6216	754.8144	**1507.6376**	754.3224	F	970.4993	**485.7533**	953.4727	477.2400	952.4887	476.7480	7
14	**1688.7115**	844.8594	1671.6849	836.3461	1670.7009	835.8541	Y	**823.4308**	412.2191	806.4043	403.7058	805.4203	403.2138	6
15	1801.7956	901.4014	1784.7690	892.8881	1783.7850	892.3961	L	**660.3675**	330.6874	643.3410	322.1741	642.3569	321.6821	5
16	**1916.8225**	958.9149	1899.7959	950.4016	1898.8119	949.9096	D	**547.2835**	274.1454	530.2569	265.6321	529.2729	265.1401	4
17	2045.8651	1023.4362	2028.8385	1014.9229	2027.8545	1014.4309	E	**432.2565**	216.6319	415.2300	208.1186	414.2459	207.6266	3
18	2173.9601	1087.4837	2156.9335	1078.9704	2155.9495	1078.4784	K	**303.2139**	152.1106	286.1874	143.5973			2

19							R	175.1190	88.0631	158.0924	79.5498			1

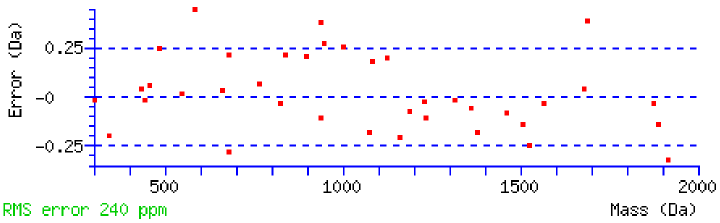

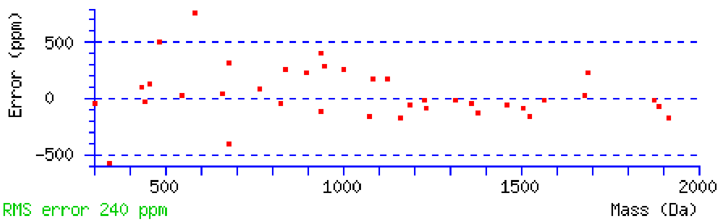

All matches to this query

Score	Mr(calc):	Delta	Sequence
52.7	2347.0644	0.0096	VPFNPNDTFESEFYLDEKR
33.1	2347.0644	0.0096	VPFNPNDTFESEFYLDEKR
29.0	2346.0804	0.9936	VPFNPNDTFESEFYLDEKR
5.6	2346.0748	0.9992	SSPLRASSLTIMATFLGLLI
5.0	2347.0515	0.0225	EQTGFRVEADQMTILSPSSR
5.0	2347.0515	0.0225	EQTGFRVEADQMTILSPSSR
4.6	2347.0515	0.0225	EQTGFRVEADQMTILSPSSR
3.0	2346.0713	1.0027	ETQASLREQRAAHEEDLQR
2.5	2346.0748	0.9992	SSPLRASSLTIMATFLGLLI
2.0	2347.0666	0.0074	SSLNSHVTKSTDSRQHSGSPR

Spectrum No: 410; Query: 22; Rank: 1

Peptide View

MS/MS Fragmentation of **INVTEVYNK**
Found in **IPI00371761**, Tax_Id=10116 Gene_Symbol=Enpp4_predicted ectonucleotide pyrophosphatase/phosphodiesterase 4

Match to Query 22: 1079.549068 from(540.781810,2+)
Title: 091129RatKid_SCX02_02.957.957.2.dta
Data file K:\NewmanPaper\Piliang\3SubProteomes\Piliang3SP\mgf5ppm\SCX_3SubProteomes5ppm.mgf

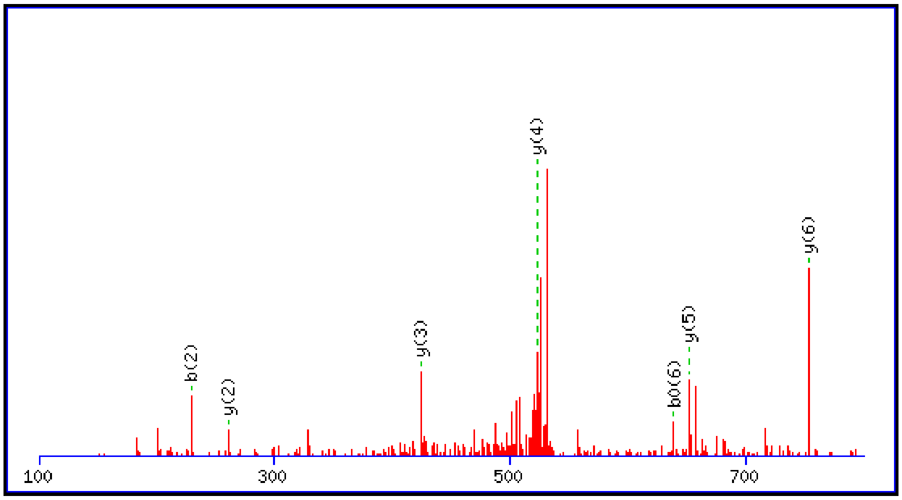

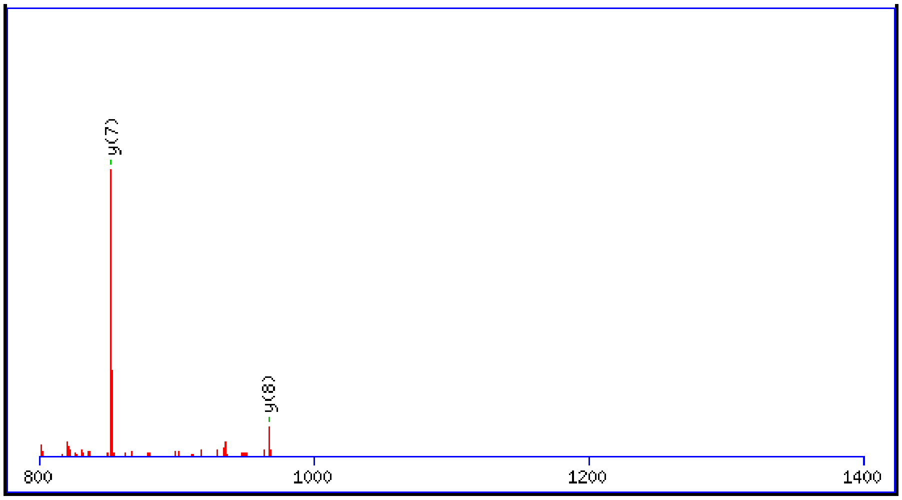

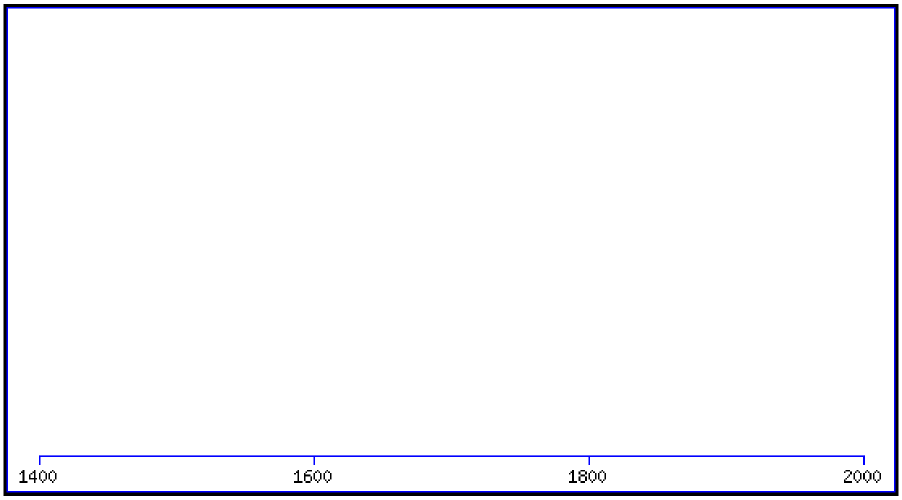

Monoisotopic mass of neutral peptide Mr(calc): 1079.5499
Fixed modifications: Carbamidomethyl (C)
Variable modifications:
N2 : Deamidated_N (N)
Ions Score: 53 **Expect:** 0.00056
Matches (Bold Red): 9/80 fragment ions using 12 most intense peaks

#	b	b^{++}	b*	b^{*++}	b^0	b^{0++}	Seq.	y	y^{++}	y*	y^{*++}	y^0	y^{0++}	#
1	114.0913	57.5493					I							9
2	**229.1183**	115.0628	212.0917	106.5495			N	**967.4731**	484.2402	950.4465	475.7269	949.4625	475.2349	8
3	328.1867	164.5970	311.1601	156.0837			V	**852.4462**	426.7267	835.4196	418.2134	834.4356	417.7214	7
4	429.2344	215.1208	412.2078	206.6075	411.2238	206.1155	T	**753.3777**	377.1925	736.3512	368.6792	735.3672	368.1872	6
5	558.2770	279.6421	541.2504	271.1288	540.2664	270.6368	E	**652.3301**	326.6687	635.3035	318.1554	634.3195	317.6634	5
6	657.3454	329.1763	640.3188	320.6631	**639.3348**	320.1710	V	**523.2875**	262.1474	506.2609	253.6341			4
7	820.4087	410.7080	803.3822	402.1947	802.3981	401.7027	Y	**424.2191**	212.6132	407.1925	204.0999			3
8	934.4516	467.7295	917.4251	459.2162	916.4411	458.7242	N	**261.1557**	131.0815	244.1292	122.5682			2
9							K	147.1128	74.0600	130.0863	65.5468			1

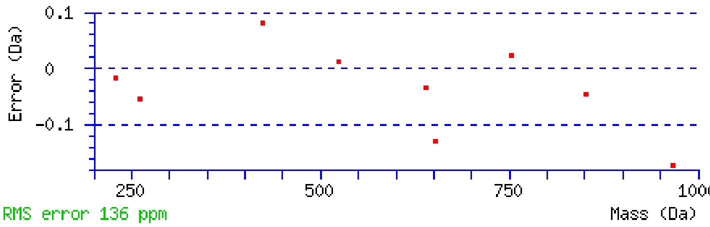

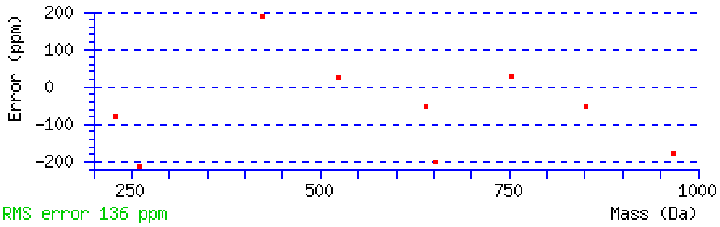

All matches to this query

Score	Mr(calc):	Delta	Sequence
52.6	1079.5499	-0.0008	INVTEVYNK
14.1	1079.5488	0.0002	INVAVTRAR
12.1	1079.5488	0.0002	EVVQLTRR
10.7	1077.5511	1.9979	ILVTYYVK
10.7	1077.5511	1.9979	ILVTYYVK
9.8	1079.5499	-0.0008	INVTEVYNK
8.9	1079.5433	0.0057	IPMGKNYNK
6.9	1078.5536	0.9955	KVPSLRDGK
5.3	1079.5499	-0.0008	NLISQETFK
4.0	1079.5499	-0.0008	DLVSXYQEK

Spectrum No: 411; Query: 534; Rank: 1

Peptide View

MS/MS Fragmentation of **NVNRNISSLSEIFVGR**
Found in **IPI00214328**, Tax_Id=10116 Gene_Symbol=Naglt1 Sodium-dependent glucose transporter 1

Match to Query 534: 1804.948242 from(602.656690,3+)
Title: 091129RatKid_SCX02_12.3219.3219.3.dta
Data file K:\NewmanPaper\Piliang\3SubProteomes\Piliang3SP\mgf5ppm\SCX_3SubProteomes5ppm.mgf

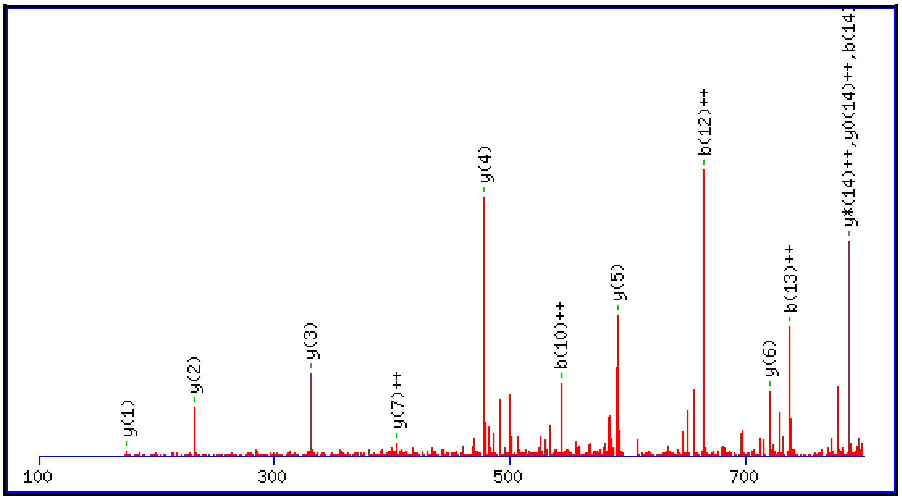

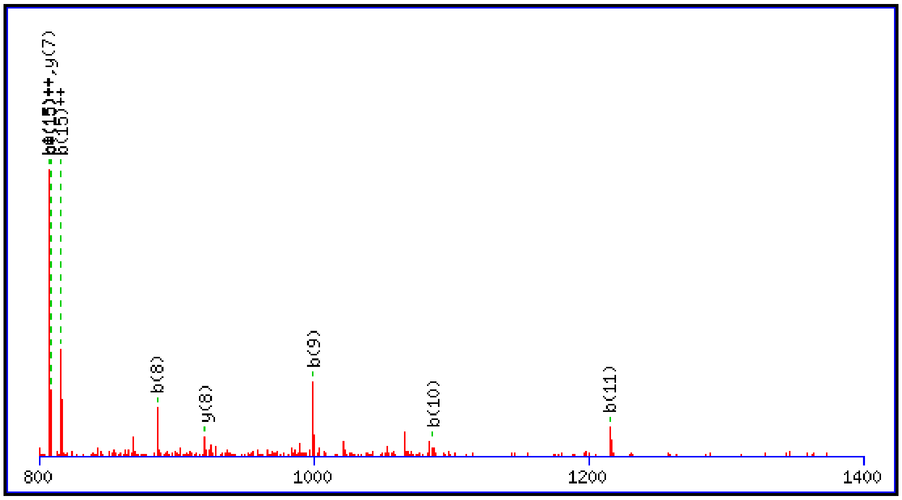

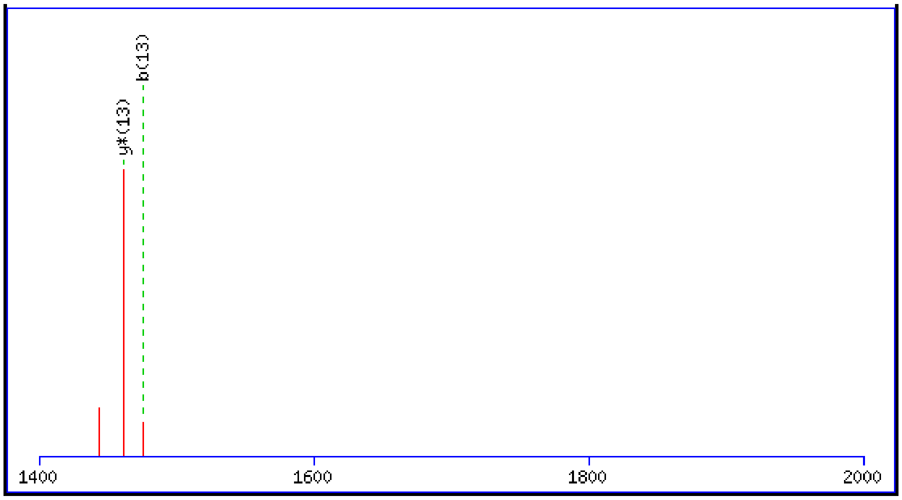

Monoisotopic mass of neutral peptide Mr(calc): 1804.9431
Fixed modifications: Carbamidomethyl (C)
Variable modifications:
N5 : Deamidated_N (N)
Ions Score: 53 **Expect:** 0.00063
Matches (Bold Red): 24/158 fragment ions using 31 most intense peaks

#	b	b++	b*	b*++	b0	b0++	Seq.	y	y++	y*	y*++	y0	y0++	#
1	115.0502	58.0287	98.0237	49.5155			N							16
2	214.1186	107.5629	197.0921	99.0497			V	1691.9075	846.4574	1674.8810	837.9441	1673.8969	837.4521	15
3	328.1615	164.5844	311.1350	156.0711			N	1592.8391	796.9232	1575.8125	**788.4099**	1574.8285	**787.9179**	14
4	484.2627	242.6350	467.2361	234.1217			R	1478.7962	739.9017	**1461.7696**	731.3884	1460.7856	730.8964	13
5	599.2896	300.1484	582.2630	291.6352			N	1322.6950	661.8512	1305.6685	653.3379	1304.6845	652.8459	12
6	712.3737	356.6905	695.3471	348.1772			I	1207.6681	604.3377	1190.6416	595.8244	1189.6575	595.3324	11
7	799.4057	400.2065	782.3791	391.6932	781.3951	391.2012	S	1094.5840	547.7957	1077.5575	539.2824	1076.5735	538.7904	10
8	**886.4377**	443.7225	869.4112	435.2092	868.4271	434.7172	S	1007.5520	504.2796	990.5255	495.7664	989.5415	495.2744	9
9	**999.5218**	500.2645	982.4952	491.7513	981.5112	491.2592	L	**920.5200**	460.7636	903.4934	452.2504	902.5094	451.7584	8
10	**1086.5538**	**543.7805**	1069.5273	535.2673	1068.5432	534.7753	S	**807.4359**	**404.2216**	790.4094	395.7083	789.4254	395.2163	7
11	**1215.5964**	608.3018	1198.5698	599.7886	1197.5858	599.2966	E	**720.4039**	360.7056	703.3774	352.1923	702.3933	351.7003	6
12	1328.6805	**664.8439**	1311.6539	656.3306	1310.6699	655.8386	I	**591.3613**	296.1843	574.3348	287.6710			5
13	**1475.7489**	**738.3781**	1458.7223	729.8648	1457.7383	729.3728	F	**478.2772**	239.6423	461.2507	231.1290			4
14	1574.8173	**787.9123**	1557.7907	779.3990	1556.8067	778.9070	V	**331.2088**	166.1081	314.1823	157.5948			3
15	1631.8388	**816.4230**	1614.8122	**807.9097**	1613.8282	**807.4177**	G	**232.1404**	116.5738	215.1139	108.0606			2
16							R	**175.1190**	88.0631	158.0924	79.5498			1

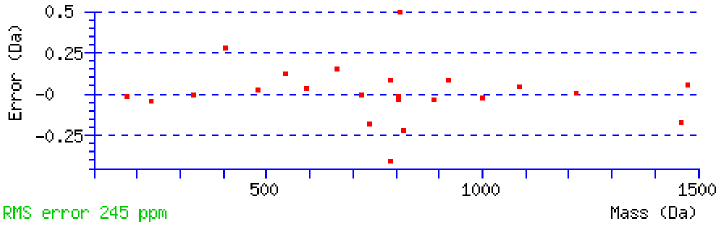

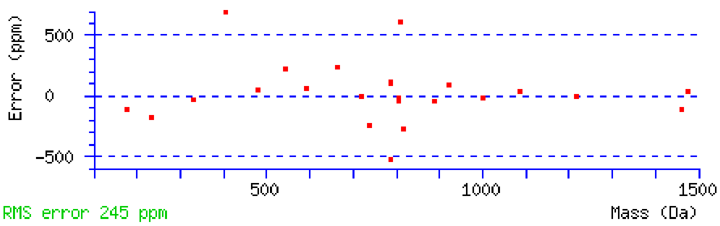

All matches to this query

Score	Mr(calc):	Delta	Sequence
52.5	1804.9431	0.0051	NVNRNISSLSEIFVGR
50.4	1804.9431	0.0051	NVNRNISSLSEIFVGR
50.4	1804.9431	0.0051	NVNRNISSLSEIFVGR
32.7	1803.9591	0.9891	NVNRNISSLSEIFVGR
14.5	1804.9319	0.0164	NLSFEESNVKLLAANR

11.8	1804.9319	0.0164	NQSFTDLLAELRAAQK
10.0	1802.9447	2.0035	EEAEAKMLILSQNIAK
9.2	1804.9349	0.0134	VIPRRSPLSYPAVNR
8.0	1804.9618	-0.0135	CVQSNIVLLTQAFRR
7.9	1804.9319	0.0164	NLSFEESNVKLLAANR

Spectrum No: 412; Query: 852; Rank: 1

Peptide View

MS/MS Fragmentation of **ELINALQINNTAVGHALVLPAR**
Found in **IPI00565677**, Tax_Id=10116 Gene_Symbol=Col6a3_predicted 289 kDa protein

Match to Query 852: 2327.302812 from(776.774880,3+)
Title: 091129RatKid_SCX02_22.4868.4868.3.dta
Data file K:\NewmanPaper\Piliang\3SubProteomes\Piliang3SP\mgf5ppm\SCX_3SubProteomes5ppm.mgf

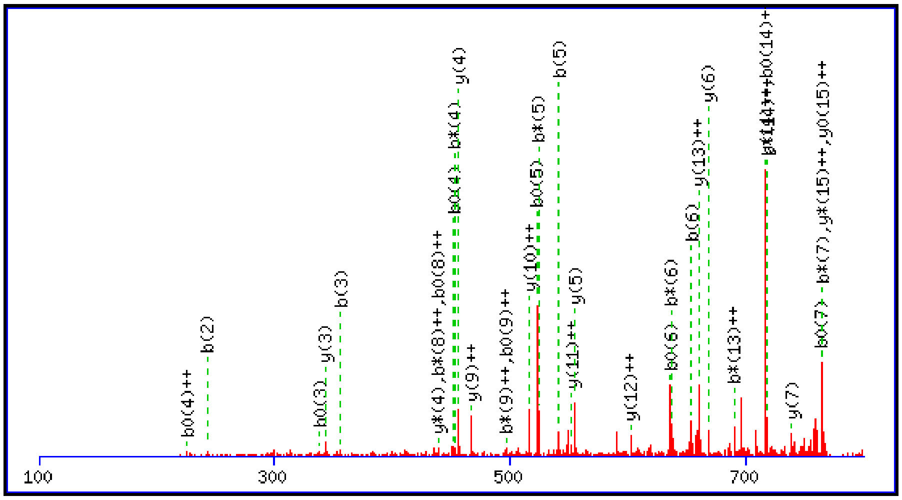

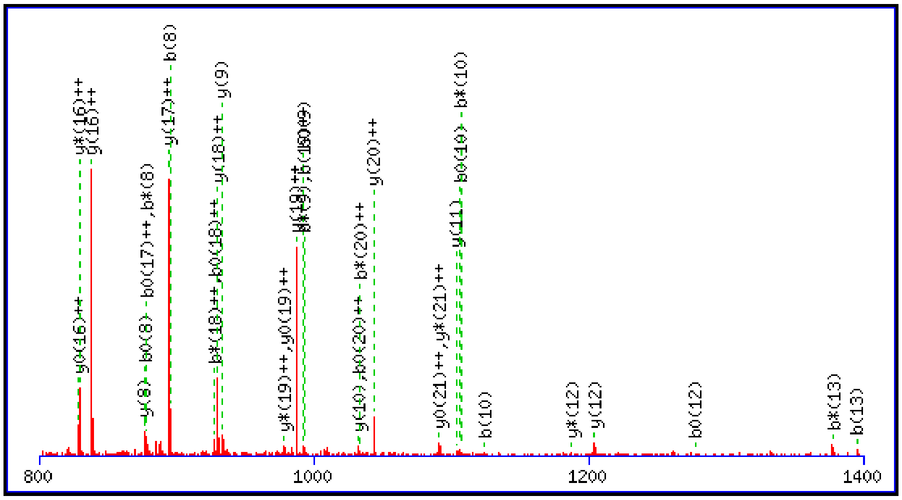

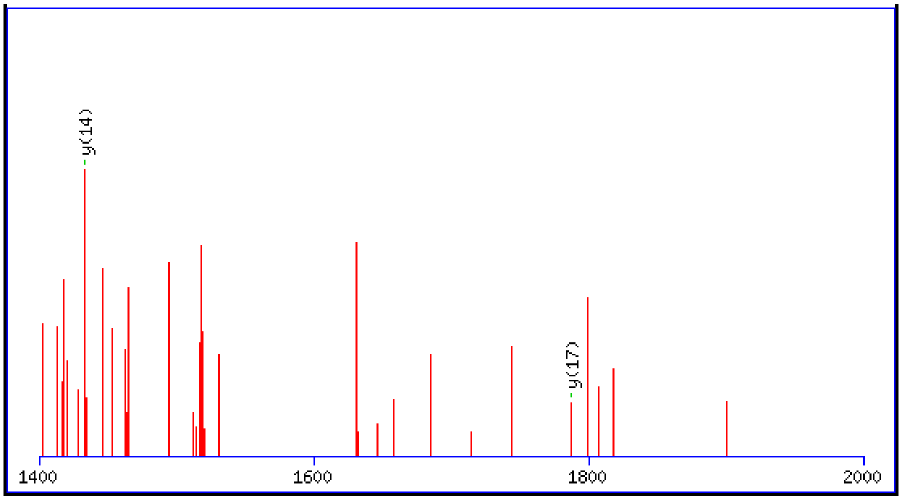

Monoisotopic mass of neutral peptide Mr(calc): 2327.2961
Fixed modifications: Carbamidomethyl (C)
Variable modifications:
N9 : Deamidated_N (N)
Ions Score: 52 **Expect:** 0.00016
Matches (Bold Red): 71/224 fragment ions using 151 most intense peaks

#	b	b++	b*	b*++	b0	b0++	Seq.	y	y++	y*	y*++	y0	y0++	#
1	130.0499	65.5286			112.0393	56.5233	E							22
2	**243.1339**	122.0706			225.1234	113.0653	L	2199.2608	1100.1340	2182.2343	**1091.6208**	2181.2502	**1091.1288**	21
3	**356.2180**	178.6126			**338.2074**	169.6074	I	2086.1767	**1043.5920**	2069.1502	1035.0787	2068.1662	1034.5867	20
4	470.2609	235.6341	**453.2344**	227.1208	**452.2504**	**226.6288**	N	1973.0927	**987.0500**	1956.0661	**978.5367**	1955.0821	**978.0447**	19
5	**541.2980**	271.1527	**524.2715**	262.6394	**523.2875**	262.1474	A	1859.0498	**930.0285**	1842.0232	921.5152	1841.0392	921.0232	18
6	**654.3821**	327.6947	**637.3556**	319.1814	**636.3715**	318.6894	L	**1788.0126**	**894.5100**	1770.9861	885.9967	1770.0021	885.5047	17
7	782.4407	391.7240	**765.4141**	383.2107	**764.4301**	382.7187	Q	1674.9286	**837.9679**	1657.9020	**829.4547**	1656.9180	**828.9626**	16
8	**895.5247**	448.2660	**878.4982**	**439.7527**	**877.5142**	**439.2607**	I	1546.8700	773.9386	1529.8434	**765.4254**	1528.8594	**764.9334**	15
9	1010.5517	505.7795	**993.5251**	**497.2662**	**992.5411**	**496.7742**	N	**1433.7859**	**717.3966**	1416.7594	708.8833	1415.7754	708.3913	14
10	**1124.5946**	562.8009	**1107.5681**	554.2877	**1106.5840**	553.7957	N	1318.7590	**659.8831**	1301.7324	651.3699	1300.7484	650.8779	13
11	1225.6423	613.3248	1208.6157	604.8115	1207.6317	604.3195	T	**1204.7161**	**602.8617**	**1187.6895**	594.3484	1186.7055	593.8564	12
12	1296.6794	648.8433	1279.6529	640.3301	**1278.6688**	639.8381	A	**1103.6684**	**552.3378**	1086.6418	543.8246			11
13	**1395.7478**	698.3775	**1378.7213**	**689.8643**	1377.7372	689.3723	V	**1032.6313**	**516.8193**	1015.6047	508.3060			10
14	1452.7693	726.8883	1435.7427	**718.3750**	1434.7587	**717.8830**	G	**933.5629**	**467.2851**	916.5363	458.7718			9
15	1589.8282	795.4177	1572.8016	786.9045	1571.8176	786.4125	H	**876.5414**	438.7743	859.5148	430.2611			8
16	1660.8653	830.9363	1643.8388	822.4230	1642.8547	821.9310	A	**739.4825**	370.2449	722.4559	361.7316			7
17	1773.9494	887.4783	1756.9228	878.9650	1755.9388	**878.4730**	L	**668.4454**	334.7263	651.4188	326.2130			6
18	1873.0178	937.0125	1855.9912	**928.4993**	1855.0072	**928.0072**	V	**555.3613**	278.1843	538.3348	269.6710			5
19	1986.1018	**993.5546**	1969.0753	985.0413	1968.0913	984.5493	L	**456.2929**	228.6501	**439.2663**	220.1368			4
20	2083.1546	1042.0809	2066.1281	**1033.5677**	2065.1440	**1033.0757**	P	**343.2088**	172.1081	326.1823	163.5948			3
21	2154.1917	1077.5995	2137.1652	1069.0862	2136.1812	1068.5942	A	246.1561	123.5817	229.1295	115.0684			2
22							R	175.1190	88.0631	158.0924	79.5498			1

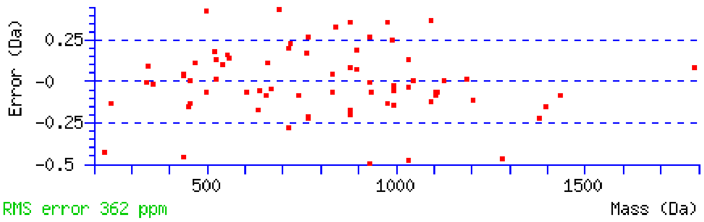

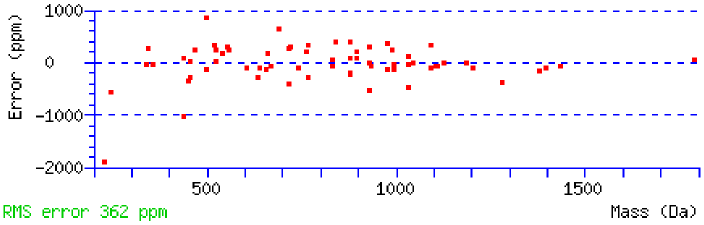

All matches to this query

Score	Mr(calc):	Delta	Sequence
52.4	2327.2961	0.0067	ELINALQINNTAVGHALVLPAR
51.8	2327.2961	0.0067	ELINALQINNTAVGHALVLPAR
22.6	2327.2961	0.0067	ELINALQINNTAVGHALVLPAR
20.1	2326.3121	0.9907	ELINALQINNTAVGHALVLPAR
2.2	2327.2974	0.0054	EILQGLAHLHAHQVIHRDIK
1.1	2327.2896	0.0132	RRGLGLCGTALVSYLISLHNK
0.3	2325.2818	2.0210	NLTPGVLPPAHAIGRTRYVHR
0.0	2326.2998	1.0030	QLLLAGLRGAARRPTNLAQVK

Spectrum No: 413; Query: 63; Rank: 1

Peptide View

MS/MS Fragmentation of **NTTGVTEEALK**
Found in **IPI00209258**, Tax_Id=10116 Gene_Symbol=Spna2 Spectrin alpha chain, brain

Match to Query 63: 1162.570408 from(582.292480,2+)
Title: 091129RatKid_SCX02_15.723.723.2.dta
Data file K:\NewmanPaper\Piliang\3SubProteomes\Piliang3SP\mgf5ppm\SCX_3SubProteomes5ppm.mgf

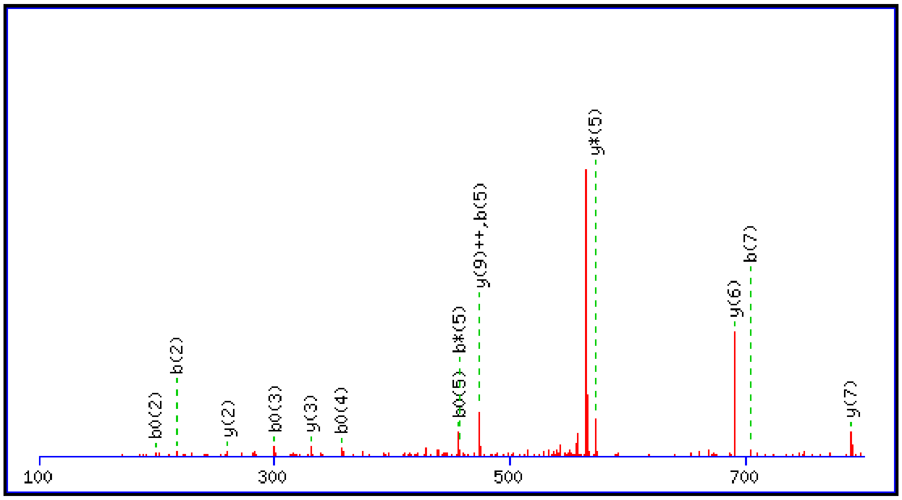

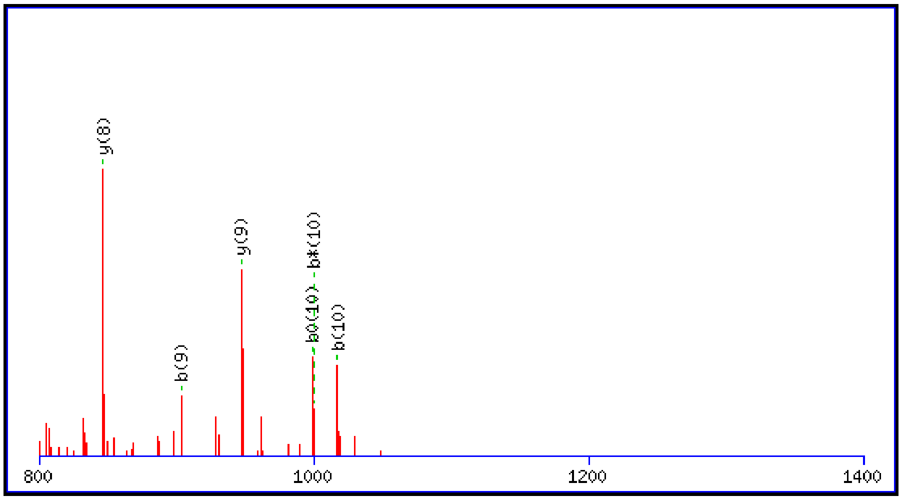

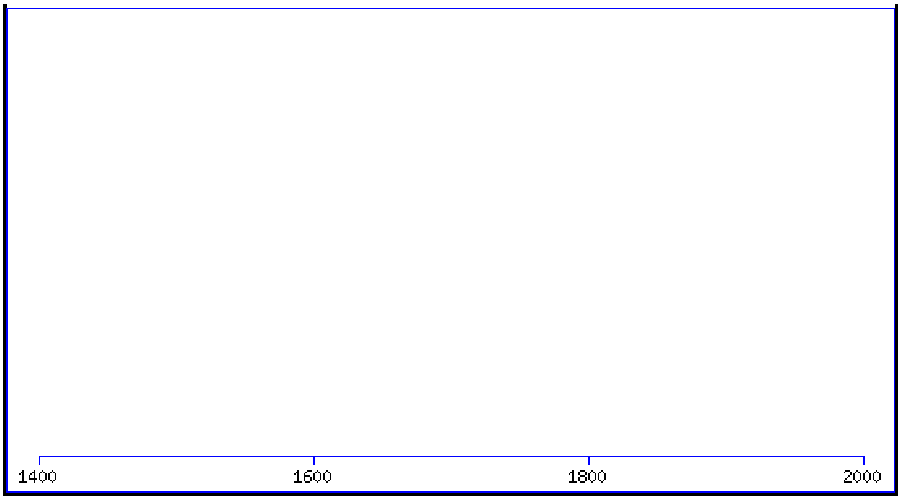

Monoisotopic mass of neutral peptide Mr(calc): 1162.5717
Fixed modifications: Carbamidomethyl (C)
Variable modifications:
N1 : Deamidated_N (N)
Ions Score: 52 **Expect:** 0.0007
Matches (Bold Red): 20/112 fragment ions using 28 most intense peaks

#	b	b^{++}	b^{*}	b^{*++}	b^{0}	b^{0++}	Seq.	y	y^{++}	y^{*}	y^{*++}	y^{0}	y^{0++}	#
1	116.0342	58.5207	99.0077	50.0075			N							11
2	**217.0819**	109.0446	200.0553	100.5313	**199.0713**	100.0393	T	1048.5521	524.7797	1031.5255	516.2664	1030.5415	515.7744	10
3	318.1296	159.5684	301.1030	151.0551	**300.1190**	150.5631	T	**947.5044**	**474.2558**	930.4779	465.7426	929.4938	465.2506	9
4	375.1510	188.0792	358.1245	179.5659	**357.1405**	179.0739	G	**846.4567**	423.7320	829.4302	415.2187	828.4462	414.7267	8
5	**474.2194**	237.6134	**457.1929**	229.1001	**456.2089**	228.6081	V	**789.4353**	395.2213	772.4087	386.7080	771.4247	386.2160	7
6	575.2671	288.1372	558.2406	279.6239	557.2566	279.1319	T	**690.3668**	345.6871	673.3403	337.1738	672.3563	336.6818	6
7	**704.3097**	352.6585	687.2832	344.1452	686.2992	343.6532	E	589.3192	295.1632	**572.2926**	286.6499	571.3086	286.1579	5
8	833.3523	417.1798	816.3258	408.6665	815.3417	408.1745	E	460.2766	230.6419	443.2500	222.1287	442.2660	221.6366	4
9	**904.3894**	452.6984	887.3629	444.1851	886.3789	443.6931	A	**331.2340**	166.1206	314.2074	157.6074			3
10	**1017.4735**	509.2404	**1000.4469**	500.7271	**999.4629**	500.2351	L	**260.1969**	130.6021	243.1703	122.0888			2
11							K	147.1128	74.0600	130.0863	65.5468			1

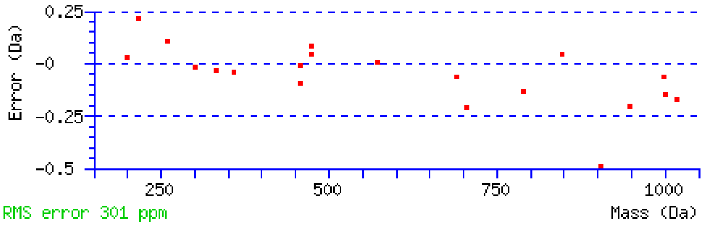

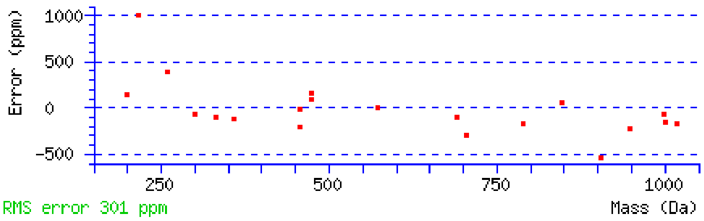

All matches to this query

Score	Mr(calc):	Delta	Sequence
52.4	1162.5717	-0.0013	NTTGVTEEALK
13.9	1162.5726	-0.0022	MEPSKMAPLK
9.5	1162.5635	0.0069	DVVPTQSPLK
9.5	1162.5787	-0.0083	SYXNFLALK
9.5	1162.5787	-0.0083	SYXNFLALK
8.7	1162.5764	-0.0060	DRVGGSMNSIK
8.4	1161.5778	0.9926	NSFQPVNSLR
8.0	1162.5805	-0.0101	TGQSVYMHLK
7.9	1162.5652	0.0052	TNAACAALDKK
7.4	1162.5635	0.0069	DVVPTQSPLK

Spectrum No: 414; Query: 884; Rank: 1

Peptide View

MS/MS Fragmentation of **VYINYYDMNAANVGWNGSTFA**
Found in **IPI00230907**, Tax_Id=10116 Gene_Symbol=Mif Macrophage migration inhibitory factor

Match to Query 884: 2370.031808 from(1186.023180,2+)
Title: 091129RatKid_SCX02_05.5426.5426.2.dta
Data file K:\NewmanPaper\Piliang\3SubProteomes\Piliang3SP\mgf5ppm\SCX_3SubProteomes5ppm.mgf

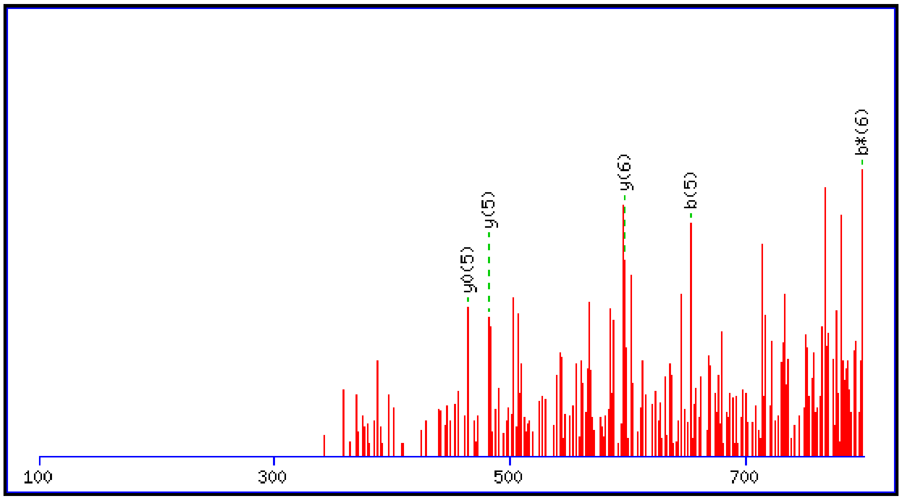

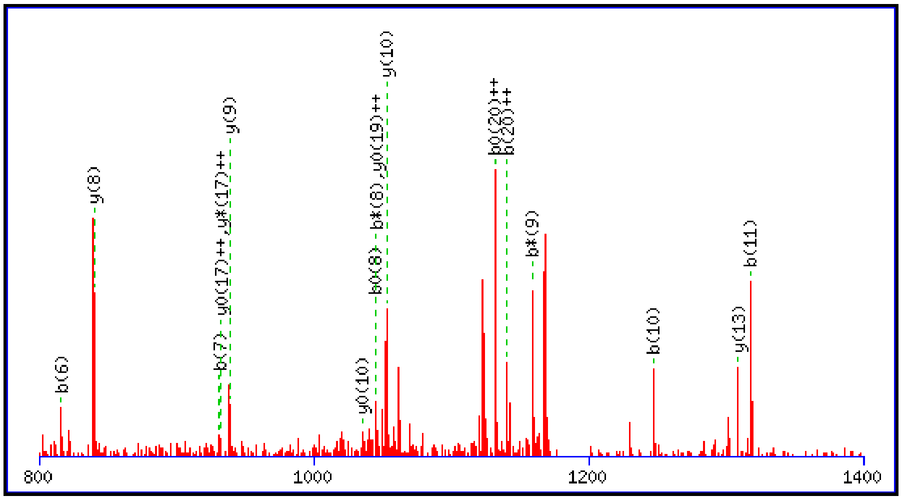

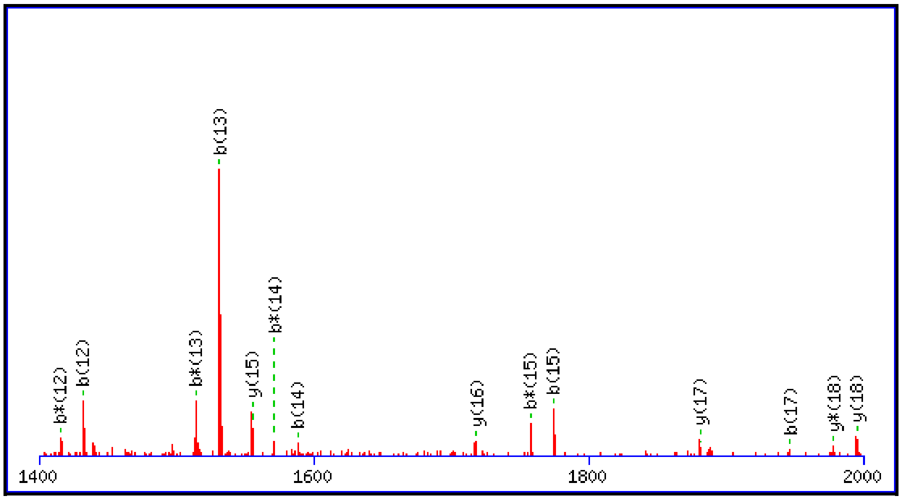

Monoisotopic mass of neutral peptide Mr(calc): 2370.0263
Fixed modifications: Carbamidomethyl (C)
Variable modifications:
N16 : Deamidated_N (N)
Ions Score: 52 **Expect:** 0.0018
Matches (Bold Red): 36/208 fragment ions using 83 most intense peaks

#	b	b^{++}	b*	b^{*++}	b^{0}	b^{0++}	Seq.	y	y^{++}	y*	y^{*++}	y^{0}	y^{0++}	#
1	100.0757	50.5415					V							21
2	263.1390	132.0731					Y	2271.9652	1136.4862	2254.9386	1127.9729	2253.9546	1127.4809	20
3	376.2231	188.6152					I	2108.9018	1054.9546	2091.8753	1046.4413	2090.8913	**1045.9493**	19
4	490.2660	245.6366	473.2395	237.1234			N	**1995.8178**	998.4125	**1978.7912**	989.8992	1977.8072	989.4072	18
5	**653.3293**	327.1683	636.3028	318.6550			Y	**1881.7748**	941.3911	1864.7483	**932.8778**	1863.7643	**932.3858**	17
6	**816.3927**	408.7000	**799.3661**	400.1867			Y	**1718.7115**	859.8594	1701.6850	851.3461	1700.7009	850.8541	16
7	**931.4196**	466.2134	914.3931	457.7002	913.4090	457.2082	D	**1555.6482**	778.3277	1538.6216	769.8145	1537.6376	769.3224	15
8	1062.4601	531.7337	**1045.4335**	523.2204	**1044.4495**	522.7284	M	1440.6212	720.8143	1423.5947	712.3010	1422.6107	711.8090	14
9	1176.5030	588.7551	**1159.4765**	580.2419	1158.4925	579.7499	N	**1309.5807**	655.2940	1292.5542	646.7807	1291.5702	646.2887	13
10	**1247.5401**	624.2737	1230.5136	615.7604	1229.5296	615.2684	A	1195.5378	598.2725	1178.5113	589.7593	1177.5273	589.2673	12
11	**1318.5773**	659.7923	1301.5507	651.2790	1300.5667	650.7870	A	1124.5007	562.7540	1107.4742	554.2407	1106.4901	553.7487	11
12	**1432.6202**	716.8137	**1415.5936**	708.3005	1414.6096	707.8084	N	**1053.4636**	527.2354	1036.4370	518.7222	**1035.4530**	518.2302	10
13	**1531.6886**	766.3479	**1514.6620**	757.8347	1513.6780	757.3427	V	**939.4207**	470.2140	922.3941	461.7007	921.4101	461.2087	9
14	**1588.7101**	794.8587	**1571.6835**	786.3454	1570.6995	785.8534	G	**840.3523**	420.6798	823.3257	412.1665	822.3417	411.6745	8
15	**1774.7894**	887.8983	**1757.7628**	879.3850	1756.7788	878.8930	W	783.3308	392.1690	766.3042	383.6558	765.3202	383.1638	7
16	1889.8163	945.4118	1872.7898	936.8985	1871.8057	936.4065	N	**597.2515**	299.1294	580.2249	290.6161	579.2409	290.1241	6
17	**1946.8378**	973.9225	1929.8112	965.4092	1928.8272	964.9172	G	**482.2245**	241.6159			**464.2140**	232.6106	5
18	2033.8698	1017.4385	2016.8432	1008.9253	2015.8592	1008.4333	S	425.2031	213.1052			407.1925	204.0999	4
19	2134.9175	1067.9624	2117.8909	1059.4491	2116.9069	1058.9571	T	338.1710	169.5892			320.1605	160.5839	3
20	2281.9859	**1141.4966**	2264.9593	1132.9833	2263.9753	**1132.4913**	F	237.1234	119.0653					2
21							A	90.0550	45.5311					1

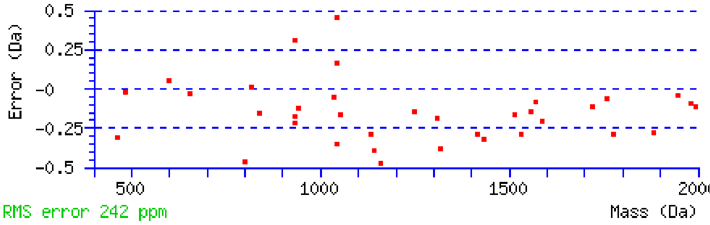

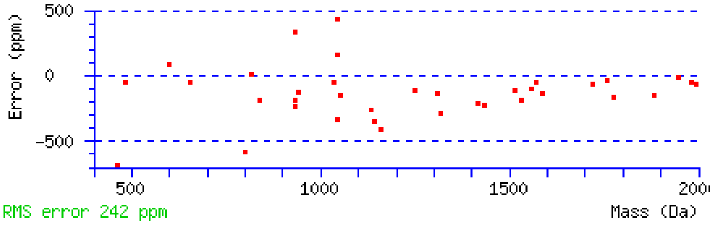

All matches to this query

Score	Mr(calc):	Delta	Sequence
52.4	2370.0263	0.0055	VYINYYDMNAANVGWNGSTFA
51.7	2369.0423	0.9895	VYINYYDMNAANVGWNGSTFA
48.4	2370.0263	0.0055	VYINYYDMNAANVGWNGSTFA
47.5	2370.0263	0.0055	VYINYYDMNAANVGWNGSTFA
44.6	2370.0263	0.0055	VYINYYDMNAANVGWNGSTFA
2.7	2370.0265	0.0053	FGEAPMHFCEVMLKDMADSR
0.4	2370.0327	-0.0009	VSAENRAGIGPPSDASNPVLMK

Spectrum No: 415; Query: 435; Rank: 1

Peptide View

MS/MS Fragmentation of **NDSPCGTTIGPILASR**
Found in **IPI00358059**, Tax_Id=10116 Gene_Symbol=Dnpep Aspartyl aminopeptidase

Match to Query 435: 1658.795368 from(830.404960,2+)
Title: 091129RatKid_SCX02_14.2568.2568.2.dta
Data file K:\NewmanPaper\Piliang\3SubProteomes\Piliang3SP\mgf5ppm\SCX_3SubProteomes5ppm.mgf

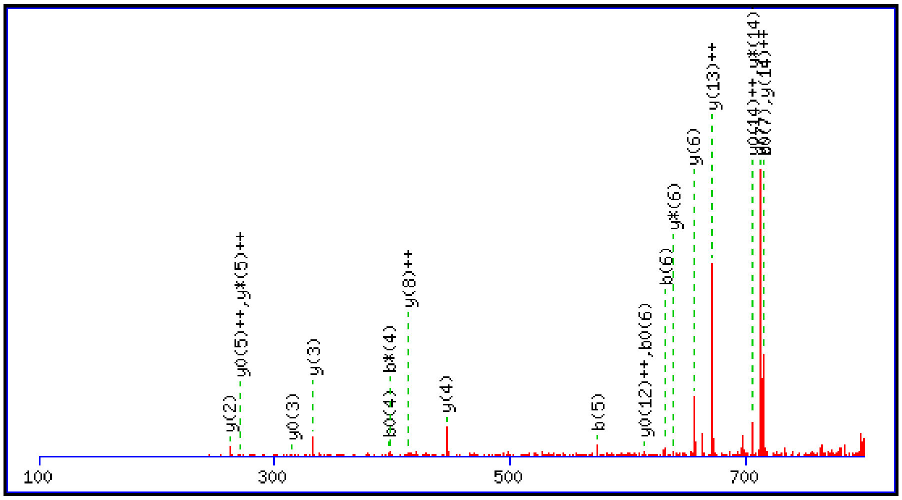

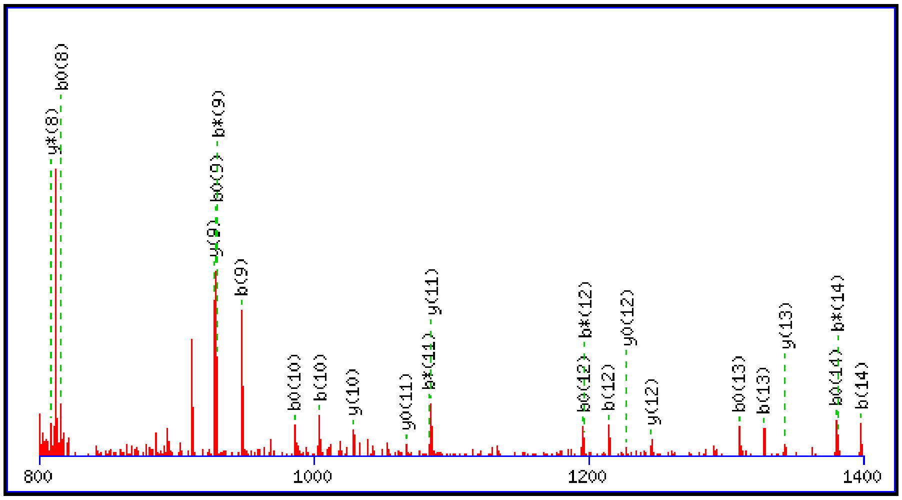

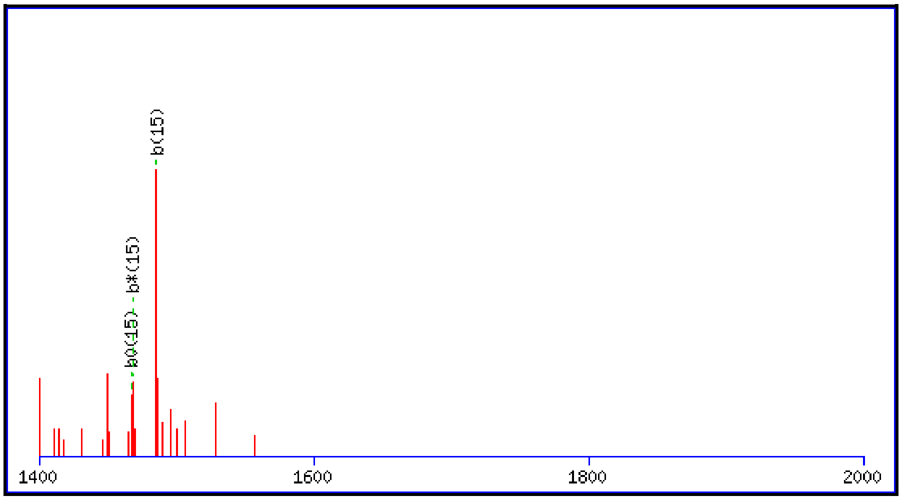

Monoisotopic mass of neutral peptide Mr(calc): 1658.7934
Fixed modifications: Carbamidomethyl (C)
Variable modifications:
N1 : Deamidated_N (N)
Ions Score: 52 **Expect:** 0.0011
Matches (Bold Red): 47/176 fragment ions using 83 most intense peaks

#	b	b^{++}	b*	b^{*++}	b^0	b^{0++}	Seq.	y	y^{++}	y*	y^{*++}	y^0	y^{0++}	#

1	116.0342	58.5207	99.0077	50.0075			N							16
2	231.0612	116.0342	214.0346	107.5209	213.0506	107.0289	D	1544.7737	772.8905	1527.7472	764.3772	1526.7632	763.8852	15
3	318.0932	159.5502	301.0666	151.0370	300.0826	150.5449	S	1429.7468	**715.3770**	1412.7202	**706.8638**	1411.7362	**706.3717**	14
4	415.1459	208.0766	**398.1194**	199.5633	**397.1354**	199.0713	P	**1342.7147**	**671.8610**	1325.6882	663.3477	1324.7042	662.8557	13
5	**575.1766**	288.0919	558.1500	279.5787	557.1660	279.0867	C	**1245.6620**	623.3346	1228.6354	614.8214	**1227.6514**	**614.3293**	12
6	**632.1981**	316.6027	615.1715	308.0894	**614.1875**	307.5974	G	**1085.6313**	543.3193	1068.6048	534.8060	**1067.6208**	534.3140	11
7	733.2457	367.1265	716.2192	358.6132	**715.2352**	358.1212	T	**1028.6099**	514.8086	1011.5833	506.2953	1010.5993	505.8033	10
8	834.2934	417.6503	817.2669	409.1371	**816.2829**	408.6451	T	**927.5622**	464.2847	910.5356	455.7715	909.5516	455.2795	9
9	**947.3775**	474.1924	**930.3509**	465.6791	**929.3669**	465.1871	I	826.5145	**413.7609**	**809.4880**	405.2476	808.5039	404.7556	8
10	**1004.3989**	502.7031	987.3724	494.1898	**986.3884**	493.6978	G	**713.4304**	357.2189	696.4039	348.7056	695.4199	348.2136	7
11	1101.4517	551.2295	**1084.4252**	542.7162	1083.4411	542.2242	P	**656.4090**	328.7081	**639.3824**	320.1949	638.3984	319.7028	6
12	**1214.5358**	607.7715	**1197.5092**	599.2583	**1196.5252**	598.7662	I	559.3562	280.1817	542.3297	**271.6685**	541.3457	**271.1765**	5
13	**1327.6198**	664.3136	1310.5933	655.8003	**1309.6093**	655.3083	L	**446.2722**	223.6397	429.2456	215.1264	428.2616	214.6344	4
14	**1398.6570**	699.8321	**1381.6304**	691.3188	**1380.6464**	690.8268	A	**333.1881**	167.0977	316.1615	158.5844	**315.1775**	158.0924	3
15	**1485.6890**	743.3481	**1468.6624**	734.8349	**1467.6784**	734.3428	S	**262.1510**	131.5791	245.1244	123.0659	244.1404	122.5738	2
16							R	175.1190	88.0631	158.0924	79.5498			1

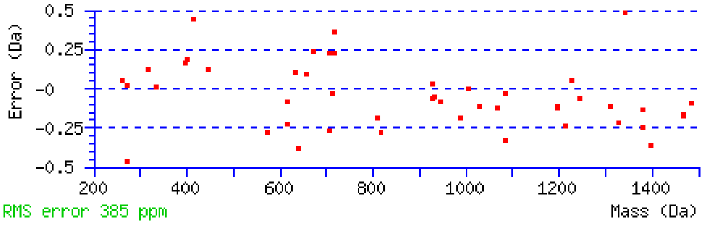

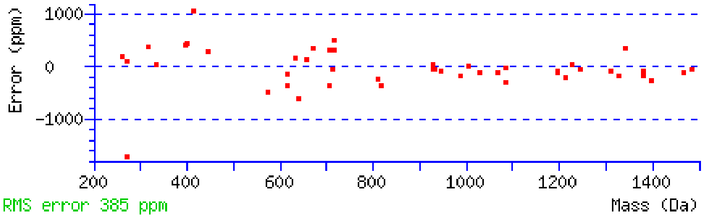

All matches to this query

Score	Mr(calc):	Delta	Sequence
52.3	1658.7934	0.0020	NDSPCGTTIGPILASR
7.2	1658.7885	0.0069	MLNSLLKGQVMSSR
6.3	1658.8103	-0.0149	MKTFVIGIGGVTNGGK
4.5	1656.7971	1.9983	KSNLESSQKSDLLK
4.5	1656.7971	1.9983	KSNLESSQKSDLLK
2.6	1656.7980	1.9974	CMALSTAVLVGEAKK
1.7	1656.7980	1.9974	CMALSTAVLVGEAKK
1.7	1657.7787	1.0167	EGAKMPTSFVAPTVK
1.5	1658.8111	-0.0158	SSTPSTVLSSSQPEPR
1.2	1658.7885	0.0069	MLNSLLKGQVMSSR

Spectrum No: 416; Query: 886; Rank: 1

Peptide View

MS/MS Fragmentation of **KEDSCQLNYSEGPCLGMQQK**
Found in **IPI00210900**, Tax_Id=10116 Gene_Symbol=Ambp AMBP protein precursor

Match to Query 886: 2372.016162 from(791.679330,3+)
Title: 091129RatKid_SCX02_13.1380.1380.3.dta
Data file K:\NewmanPaper\Piliang\3SubProteomes\Piliang3SP\mgf5ppm\SCX_3SubProteomes5ppm.mgf

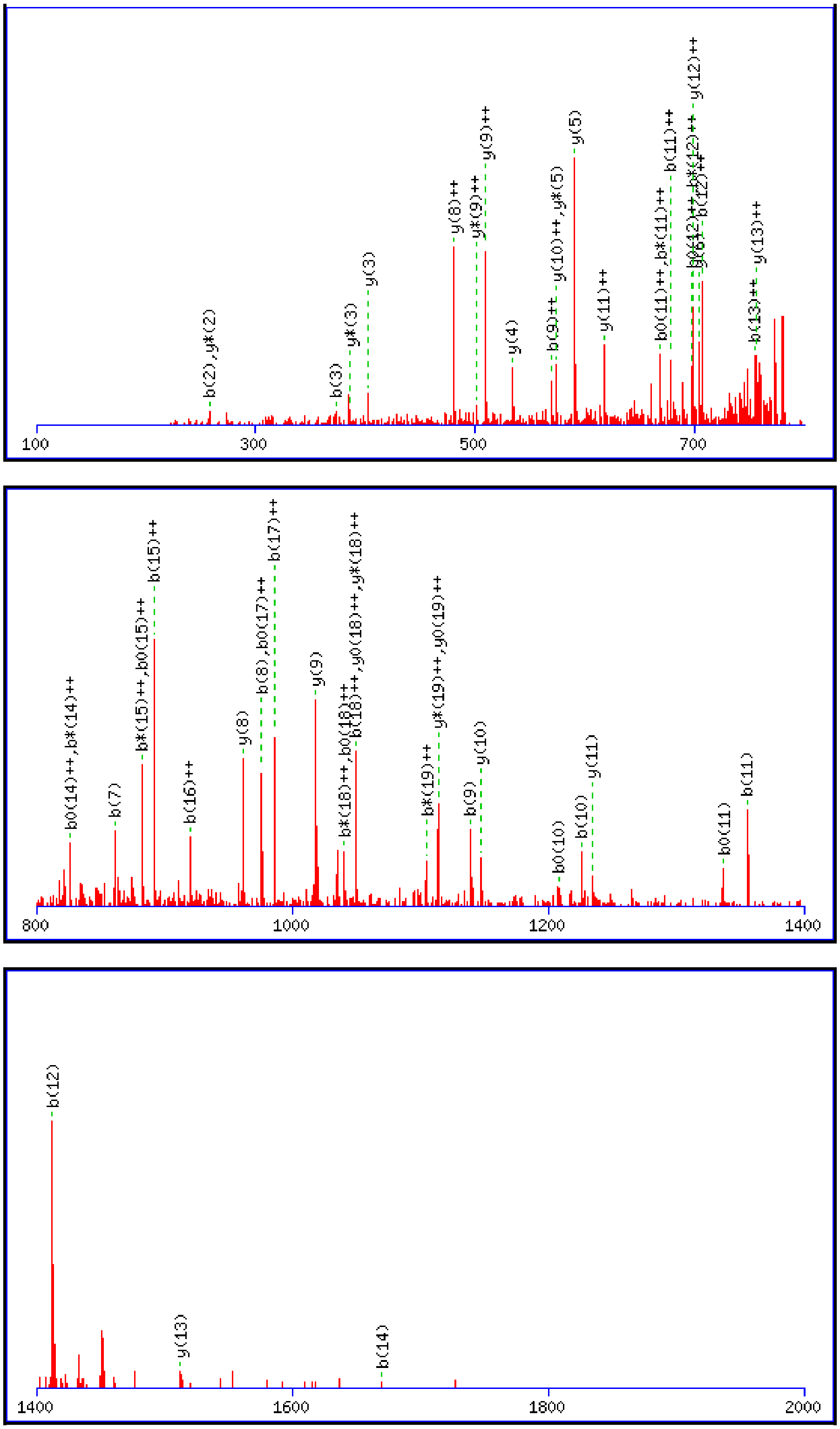

Monoisotopic mass of neutral peptide Mr(calc): 2372.0083
Fixed modifications: Carbamidomethyl (C)
Variable modifications:
N8 : Deamidated_N (N)
Ions Score: 52 **Expect:** 0.0021
Matches (Bold Red): 55/208 fragment ions using 91 most intense peaks

#	b	b++	b*	b*++	b0	b0++	Seq.	y	y++	y*	y*++	y0	y0++	#
1	129.1022	65.0548	112.0757	56.5415			K							20
2	**258.1448**	129.5761	241.1183	121.0628	240.1343	120.5708	E	2244.9206	1122.9639	2227.8940	**1114.4507**	2226.9100	**1113.9587**	19
3	**373.1718**	187.0895	356.1452	178.5763	355.1612	178.0842	D	2115.8780	1058.4426	2098.8514	**1049.9294**	2097.8674	**1049.4374**	18
4	460.2038	230.6055	443.1773	222.0923	442.1932	221.6003	S	2000.8511	1000.9292	1983.8245	992.4159	1982.8405	991.9239	17
5	620.2345	310.6209	603.2079	302.1076	602.2239	301.6156	C	1913.8190	957.4132	1896.7925	948.8999	1895.8085	948.4079	16

**6**	748.2930	374.6502	731.2665	366.1369	730.2825	365.6449	**Q**	1753.7884	877.3978	1736.7618	868.8846	1735.7778	868.3925	**15**
**7**	**861.3771**	431.1922	844.3505	422.6789	843.3665	422.1869	**L**	1625.7298	813.3685	1608.7033	804.8553	1607.7192	804.3633	**14**
**8**	**976.4040**	488.7057	959.3775	480.1924	958.3935	479.7004	**N**	**1512.6457**	**756.8265**	1495.6192	748.3132	1494.6352	747.8212	**13**
**9**	**1139.4674**	**570.2373**	1122.4408	561.7240	1121.4568	561.2320	**Y**	1397.6188	**699.3130**	1380.5923	690.7998	1379.6082	690.3078	**12**
**10**	**1226.4994**	613.7533	1209.4728	605.2401	**1208.4888**	604.7480	**S**	**1234.5555**	**617.7814**	1217.5289	609.2681	1216.5449	608.7761	**11**
**11**	**1355.5420**	**678.2746**	1338.5154	**669.7614**	**1337.5314**	**669.2693**	**E**	**1147.5234**	**574.2654**	1130.4969	565.7521	1129.5129	565.2601	**10**
**12**	**1412.5634**	**706.7854**	1395.5369	**698.2721**	1394.5529	**697.7801**	**G**	**1018.4809**	**509.7441**	1001.4543	**501.2308**			**9**
**13**	1509.6162	**755.3117**	1492.5897	746.7985	1491.6056	746.3065	**P**	**961.4594**	**481.2333**	944.4328	472.7201			**8**
**14**	**1669.6469**	835.3271	1652.6203	**826.8138**	1651.6363	**826.3218**	**C**	864.4066	432.7069	847.3801	424.1937			**7**
**15**	1782.7309	**891.8691**	1765.7044	**883.3558**	1764.7204	**882.8638**	**L**	**704.3760**	352.6916	687.3494	344.1784			**6**
**16**	1839.7524	**920.3798**	1822.7258	911.8666	1821.7418	911.3745	**G**	**591.2919**	296.1496	**574.2654**	287.6363			**5**
**17**	1970.7929	**985.9001**	1953.7663	977.3868	1952.7823	**976.8948**	**M**	**534.2704**	267.6389	517.2439	259.1256			**4**
**18**	2098.8514	**1049.9294**	2081.8249	**1041.4161**	2080.8409	**1040.9241**	**Q**	**403.2300**	202.1186	**386.2034**	193.6053			**3**
**19**	2226.9100	**1113.9587**	2209.8835	**1105.4454**	2208.8995	1104.9534	**Q**	275.1714	138.0893	**258.1448**	129.5761			**2**
**20**							**K**	147.1128	74.0600	130.0863	65.5468			**1**

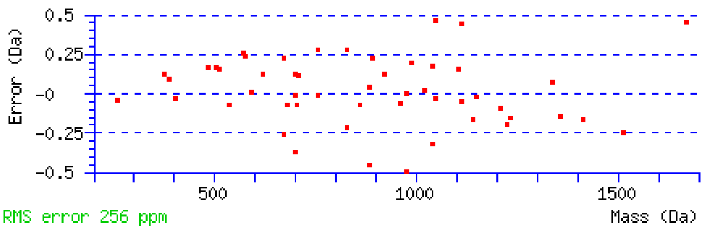

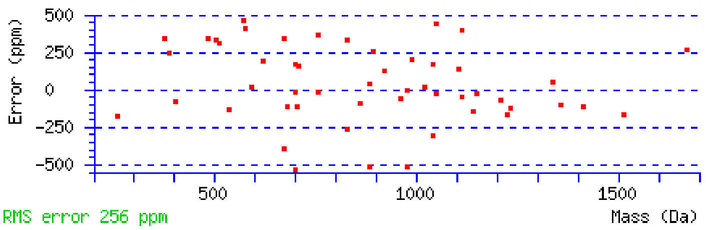

All matches to this query

Score	Mr(calc):	Delta	Sequence
51.8	2372.0083	0.0079	KEDSCQLNYSEGPCLGMQQK
20.9	2371.0242	0.9919	KEDSCQLNYSEGPCLGMQQK
10.8	2372.0169	-0.0008	QQGFLTDDLVSQEENGQQQK
3.0	2371.0014	1.0148	YAACNAVGQMATDFAPGFQKK
2.4	2372.0235	-0.0074	DMVECMYGRNYQQGSYIVK
2.3	2371.9983	0.0179	MGSEVTVGPEFFCRNLLQK
2.0	2371.9934	0.0228	RTPENLEEPSATPNSSRTPK
2.0	2371.9934	0.0228	RTPENLEEPSATPNSSRTPK
0.3	2370.9990	1.0172	ENMTLQPKSFGSKCQLSEK
0.2	2371.9943	0.0219	FCLGLLSNVNRNATVEMTR

Spectrum No: 417; Query: 3; Rank: 1

Peptide View

MS/MS Fragmentation of **VNVTNEFK**
Found in **IPI00196620**, Tax_Id=10116 Gene_Symbol=Cubn Cubilin precursor

Match to Query 3: 950.470248 from(476.242400,2+)
Title: 091127RatKid_SCX01_18.1262.1262.2.dta
Data file K:\NewmanPaper\Piliang\3SubProteomes\Piliang3SP\mgf5ppm\SCX_3SubProteomes5ppm.mgf

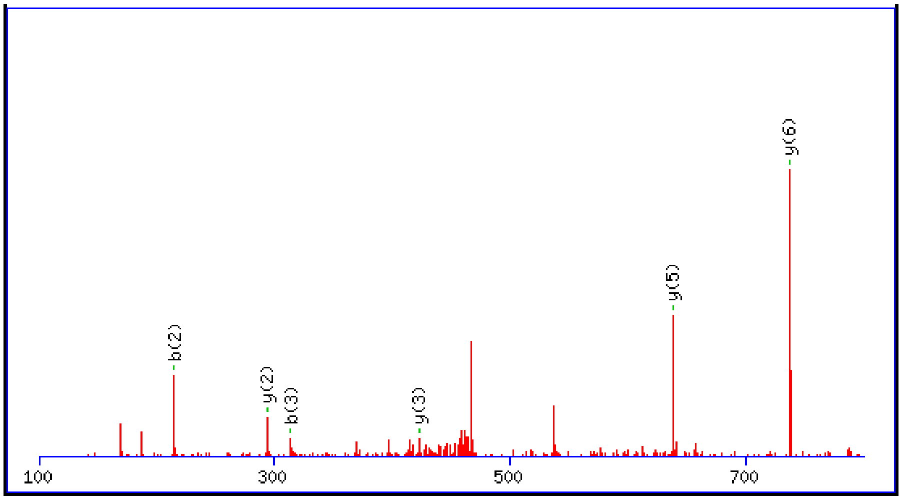

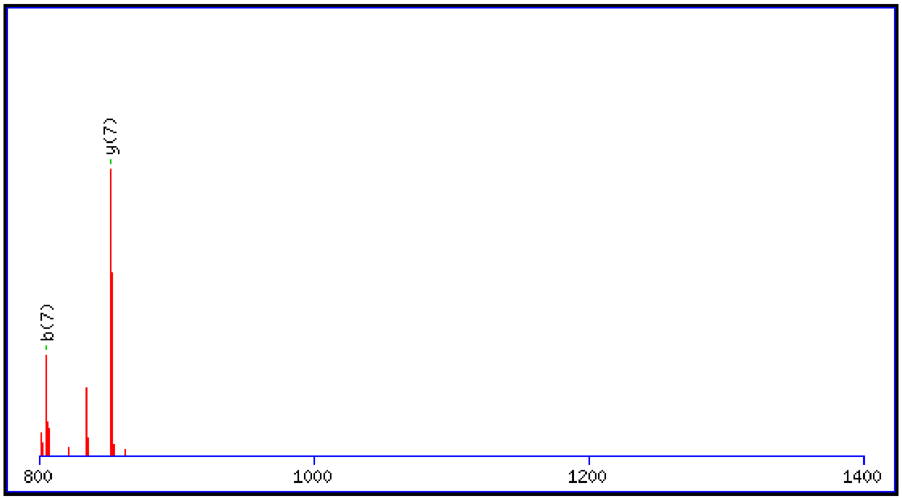

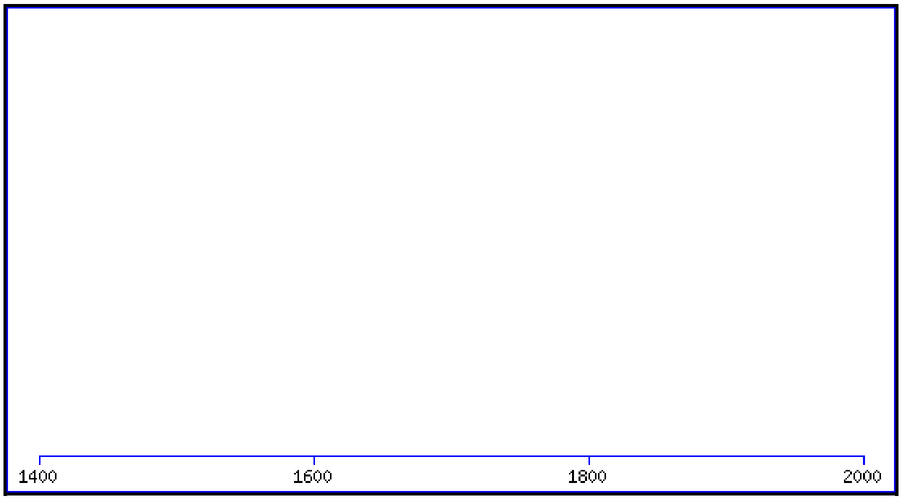

Monoisotopic mass of neutral peptide Mr(calc): 950.4709
Fixed modifications: Carbamidomethyl (C)
Variable modifications:
N2 : Deamidated_N (N)
Ions Score: 52 **Expect:** 0.00065
Matches (Bold Red): 8/72 fragment ions using 14 most intense peaks

#	b	b++	b*	b*++	b0	b0++	Seq.	y	y++	y*	y*++	y0	y0++	#
1	100.0757	50.5415					V							8
2	**215.1026**	108.0550	198.0761	99.5417			N	**852.4098**	426.7085	835.3832	418.1952	834.3992	417.7032	7
3	**314.1710**	157.5892	297.1445	149.0759			V	**737.3828**	369.1951	720.3563	360.6818	719.3723	360.1898	6
4	415.2187	208.1130	398.1922	199.5997	397.2082	199.1077	T	**638.3144**	319.6608	621.2879	311.1476	620.3039	310.6556	5
5	529.2616	265.1345	512.2351	256.6212	511.2511	256.1292	N	537.2667	269.1370	520.2402	260.6237	519.2562	260.1317	4

**6**	658.3042	329.6558	641.2777	321.1425	640.2937	320.6505	**E**	**423.2238**	212.1155	406.1973	203.6023	405.2132	203.1103	**3**
**7**	**805.3727**	403.1900	788.3461	394.6767	787.3621	394.1847	**F**	**294.1812**	147.5942	277.1547	139.0810			**2**
**8**							**K**	147.1128	74.0600	130.0863	65.5468			**1**

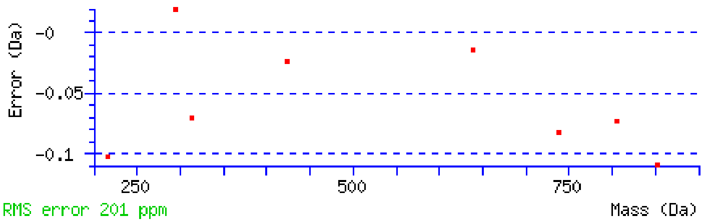

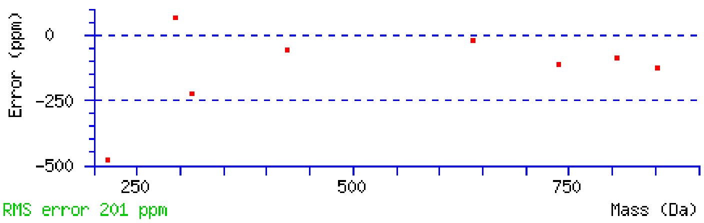

All matches to this query

Score	Mr(calc):	Delta	Sequence
51.5	950.4709	-0.0007	VNVTNEFK
25.5	950.4709	-0.0007	VNVTNEFK
11.8	950.4743	-0.0040	LTAVEGSMK
10.7	950.4644	0.0059	WIDRSMK
6.1	950.4709	-0.0007	NVQFTVDK
2.8	950.4709	-0.0007	VSQTPEYK
2.5	950.4709	-0.0006	ISLDFENNK

Spectrum No: 418; Query: 155; Rank: 1

Peptide View

MS/MS Fragmentation of **ICGNETLFPIR**
Found in **IPI00196620**, Tax_Id=10116 Gene_Symbol=Cubn Cubilin precursor

Match to Query 155: 1319.655028 from(660.834790,2+)
Title: 091127RatKid_SCX01_12.2859.2859.2.dta
Data file K:\NewmanPaper\Piliang\3SubProteomes\Piliang3SP\mgf5ppm\SCX_3SubProteomes5ppm.mgf

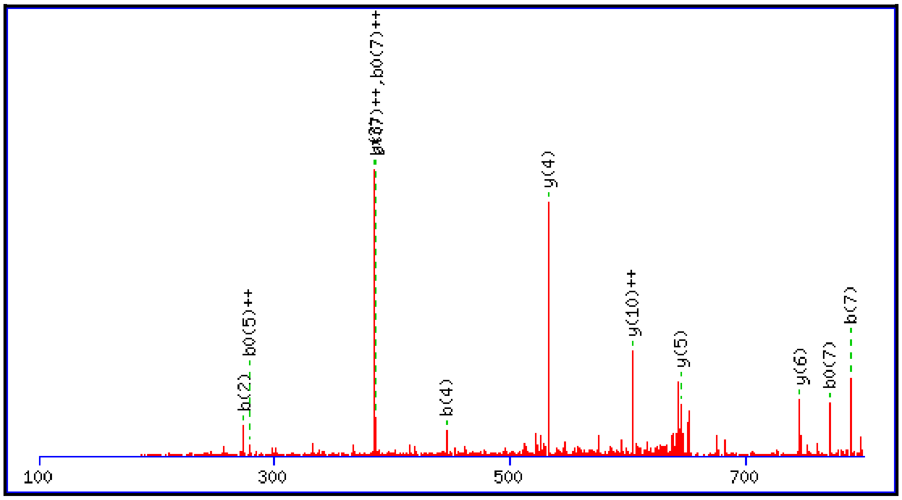

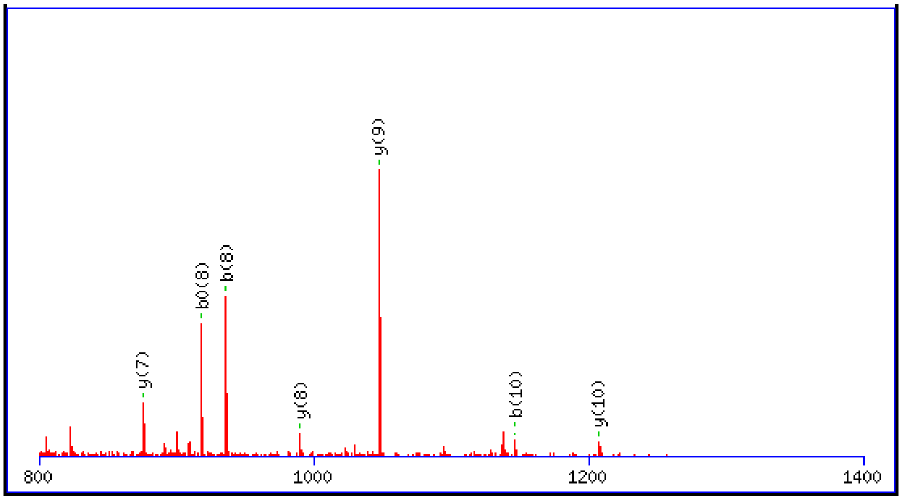

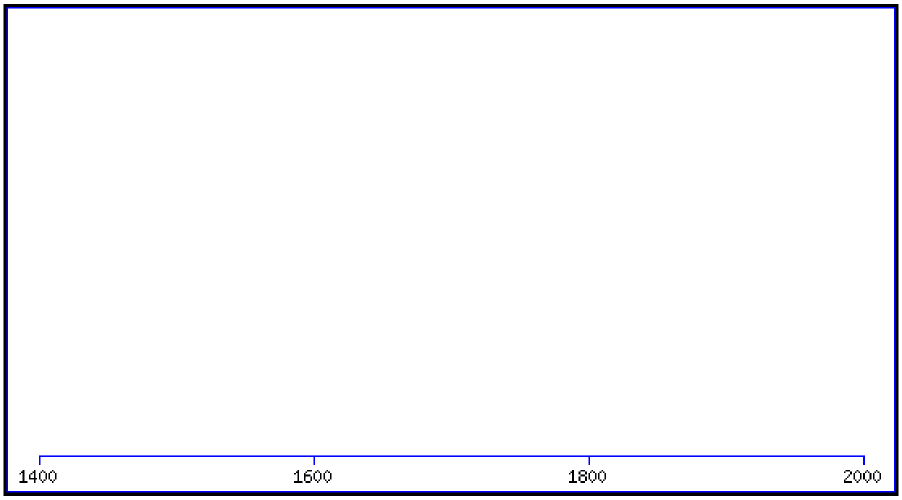

Monoisotopic mass of neutral peptide Mr(calc): 1319.6544
Fixed modifications: Carbamidomethyl (C)
Variable modifications:
N4 : Deamidated_N (N)
Ions Score: 51 **Expect:** 0.00087
Matches (Bold Red): 19/96 fragment ions using 35 most intense peaks

#	b	b^{++}	b*	b^{*++}	b^0	b^{0++}	Seq.	y	y^{++}	y*	y^{*++}	y^0	y^{0++}	#
1	114.0913	57.5493					I							11
2	**274.1220**	137.5646					C	**1207.5776**	**604.2924**	1190.5510	595.7792	1189.5670	595.2871	10
3	331.1435	166.0754					G	**1047.5469**	524.2771	1030.5204	515.7638	1029.5364	515.2718	9
4	**446.1704**	223.5888	429.1438	215.0756			N	**990.5255**	495.7664	973.4989	487.2531	972.5149	486.7611	8
5	575.2130	288.1101	558.1864	279.5969	557.2024	**279.1048**	E	**875.4985**	438.2529	858.4720	429.7396	857.4880	429.2476	7
6	676.2607	338.6340	659.2341	330.1207	658.2501	329.6287	T	**746.4559**	373.7316	729.4294	365.2183	728.4454	364.7263	6
7	**789.3447**	395.1760	772.3182	**386.6627**	**771.3342**	**386.1707**	L	**645.4083**	323.2078	628.3817	314.6945			5
8	**936.4131**	468.7102	919.3866	460.1969	**918.4026**	459.7049	F	**532.3242**	266.6657	515.2976	258.1525			4
9	1033.4659	517.2366	1016.4394	508.7233	1015.4553	508.2313	P	**385.2558**	193.1315	368.2292	184.6183			3
10	**1146.5500**	573.7786	1129.5234	565.2653	1128.5394	564.7733	I	288.2030	144.6051	271.1765	136.0919			2
11							R	175.1190	88.0631	158.0924	79.5498			1

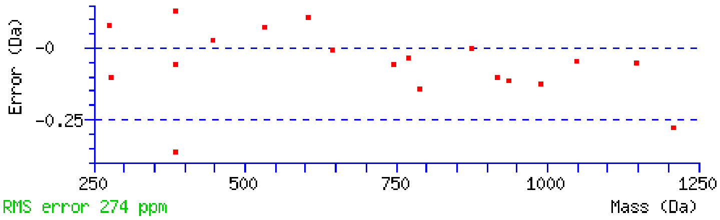

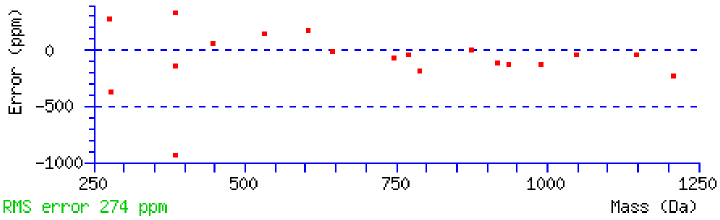

All matches to this query

Score	Mr(calc):	Delta	Sequence
51.3	1319.6544	0.0007	ICGNETLFPIR
13.3	1319.6622	-0.0072	GKQAFEGAPYPR
5.2	1319.6470	0.0081	NSSAGNFIAPSVR
5.2	1319.6470	0.0081	NSSAGNFIAPSVR
3.1	1319.6616	-0.0065	QMSVRGSEGITR
2.7	1319.6656	-0.0105	MFPLKEENRR
2.2	1319.6503	0.0047	NASEQVKMLQR
1.2	1318.6629	0.9921	RNFSNSIDLPR
0.1	1319.6656	-0.0106	GEFPTLQNKMR

Spectrum No: 419; Query: 67; Rank: 1

Peptide View

MS/MS Fragmentation of **INSSGPVNISGK**
Found in **IPI00209975**, Tax_Id=10116 Gene_Symbol=C4bpb C4b-binding protein beta chain precursor

Match to Query 67: 1173.589088 from(587.801820,2+)
Title: 091127RatKid_SCX01_02.1046.1046.2.dta
Data file K:\NewmanPaper\Piliang\3SubProteomes\Piliang3SP\mgf5ppm\SCX_3SubProteomes5ppm.mgf

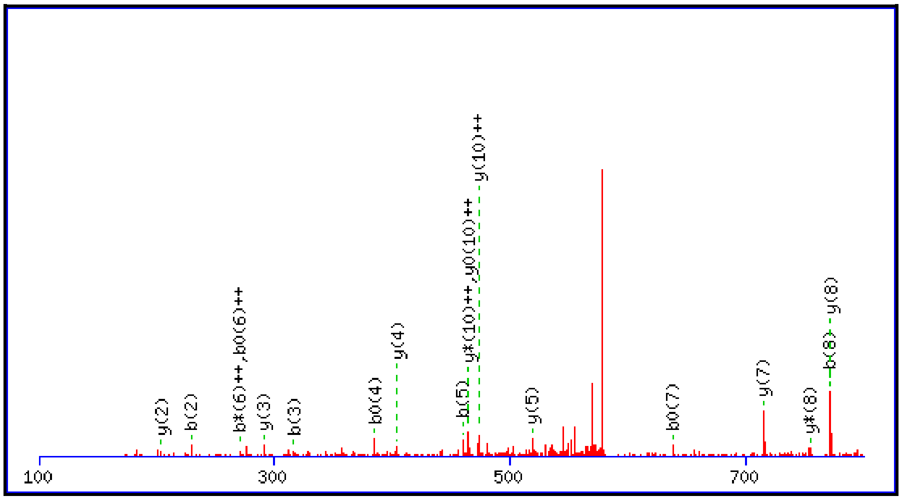

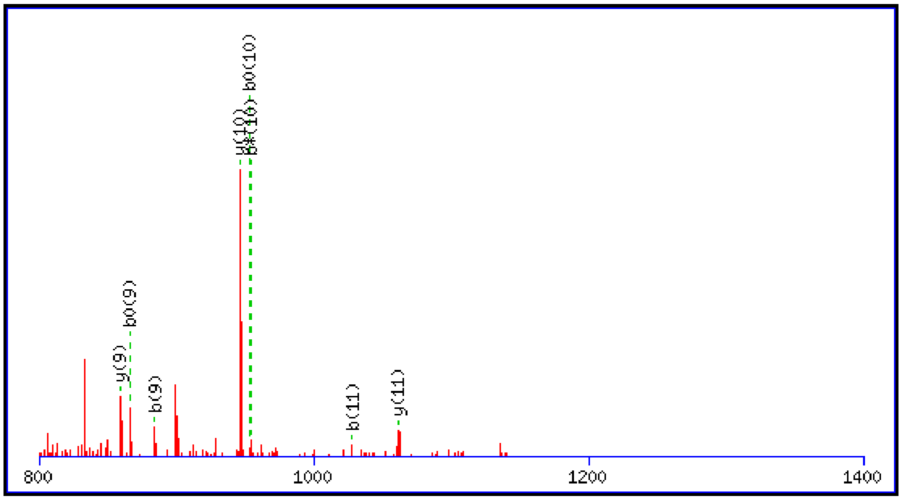

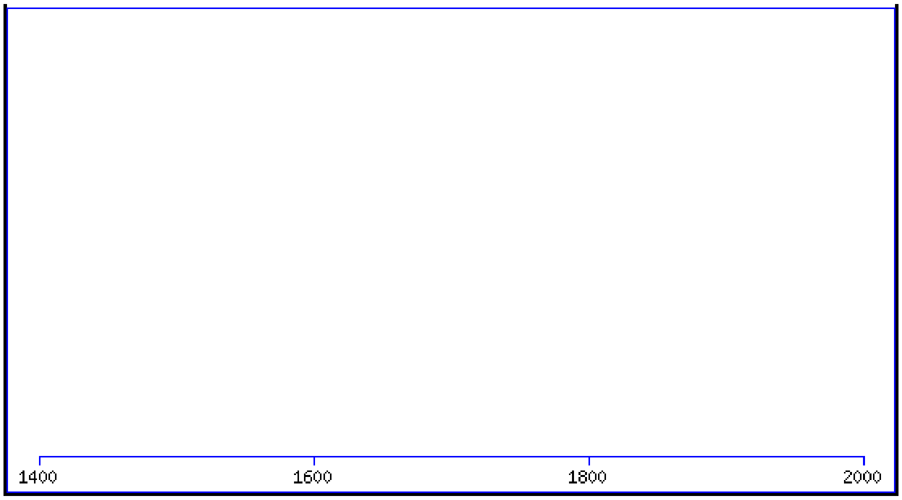

Monoisotopic mass of neutral peptide Mr(calc): 1173.5877
Fixed modifications: Carbamidomethyl (C)
Variable modifications:
N2 : Deamidated_N (N)
N8 : Deamidated_N (N)
Ions Score: 51 **Expect:** 0.00086
Matches (Bold Red): 26/122 fragment ions using 57 most intense peaks

#	b	b^{++}	b^{*}	b^{*++}	b^{0}	b^{0++}	Seq.	y	y^{++}	y^{*}	y^{*++}	y^{0}	y^{0++}	#
1	114.0913	57.5493					I							12
2	**229.1183**	115.0628	212.0917	106.5495			N	**1061.5109**	531.2591	1044.4844	522.7458	1043.5004	522.2538	11
3	**316.1503**	158.5788	299.1238	150.0655	298.1397	149.5735	S	**946.4840**	**473.7456**	929.4574	**465.2324**	928.4734	**464.7404**	10
4	403.1823	202.0948	386.1558	193.5815	**385.1718**	193.0895	S	**859.4520**	430.2296	842.4254	421.7163	841.4414	421.2243	9
5	**460.2038**	230.6055	443.1772	222.0923	442.1932	221.6003	G	**772.4199**	386.7136	**755.3934**	378.2003	754.4094	377.7083	8
6	557.2566	279.1319	540.2300	**270.6186**	539.2460	**270.1266**	P	**715.3985**	358.2029	698.3719	349.6896	697.3879	349.1976	7
7	656.3250	328.6661	639.2984	320.1529	**638.3144**	319.6608	V	618.3457	309.6765	601.3192	301.1632	600.3351	300.6712	6
8	**771.3519**	386.1796	754.3254	377.6663	753.3413	377.1743	N	**519.2773**	260.1423	502.2507	251.6290	501.2667	251.1370	5
9	**884.4360**	442.7216	867.4094	434.2084	**866.4254**	433.7163	I	**404.2504**	202.6288	387.2238	194.1155	386.2398	193.6235	4
10	971.4680	486.2376	**954.4415**	477.7244	**953.4574**	477.2324	S	**291.1663**	146.0868	274.1397	137.5735	273.1557	137.0815	3
11	**1028.4895**	514.7484	1011.4629	506.2351	1010.4789	505.7431	G	**204.1343**	102.5708	187.1077	94.0575			2
12							K	147.1128	74.0600	130.0863	65.5468			1

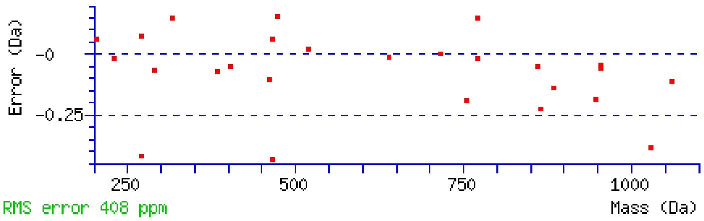

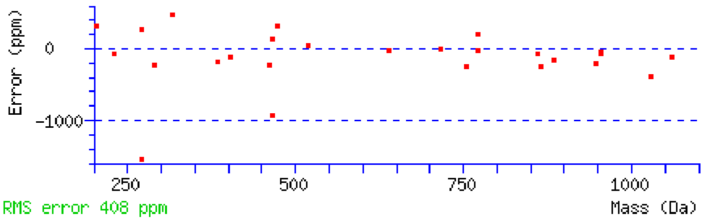

All matches to this query

Score	Mr(calc):	Delta	Sequence
51.3	1173.5877	0.0014	INSSGPVNISGK
16.1	1173.5989	-0.0098	AERSVDXIAEK
11.1	1173.5989	-0.0098	AESRAEAALEK
8.0	1173.5778	0.0113	NINSHFANKK
7.1	1173.5990	-0.0099	VDVTGDKGEVR
7.0	1173.5812	0.0079	EDASVKCLPR
5.9	1173.5989	-0.0098	AENRVVETEK
5.5	1173.5890	0.0001	ARNEWNKEK
5.4	1173.5877	0.0014	AGIDSVLNAGEK

5.3	1173.5989	-0.0098	DLIKENNATR

Spectrum No: 420; Query: 1060; Rank: 1

Peptide View

MS/MS Fragmentation of **VCNGSNKVETTTECVQTDSQTDSQAGK**
Found in **IPI00395293**, Tax_Id=10116 Gene_Symbol=Pde8a cAMP-specific cyclic nucleotide phosphodiesterase PDE8

Match to Query 1060: 2944.269012 from(982.430280,3+)
Title: 091127RatKid_SCX01_27.1049.1049.3.dta
Data file K:\NewmanPaper\Piliang\3SubProteomes\Piliang3SP\mgf5ppm\SCX_3SubProteomes5ppm.mgf

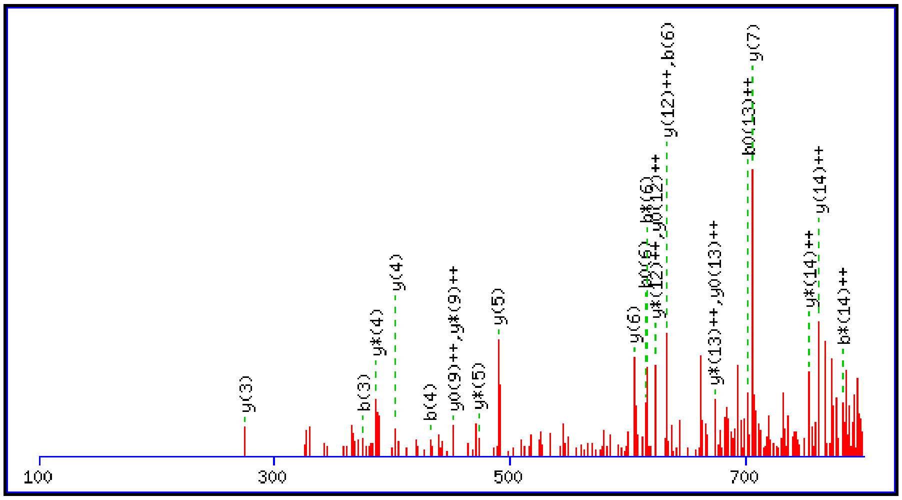

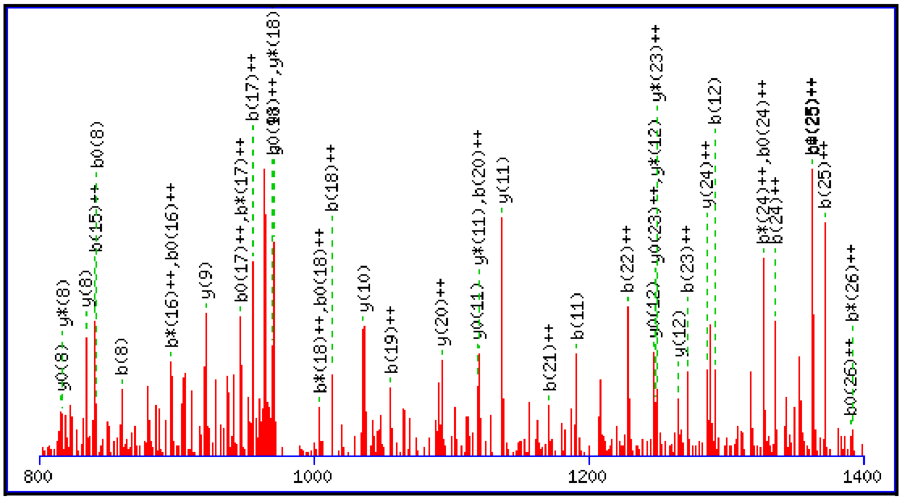

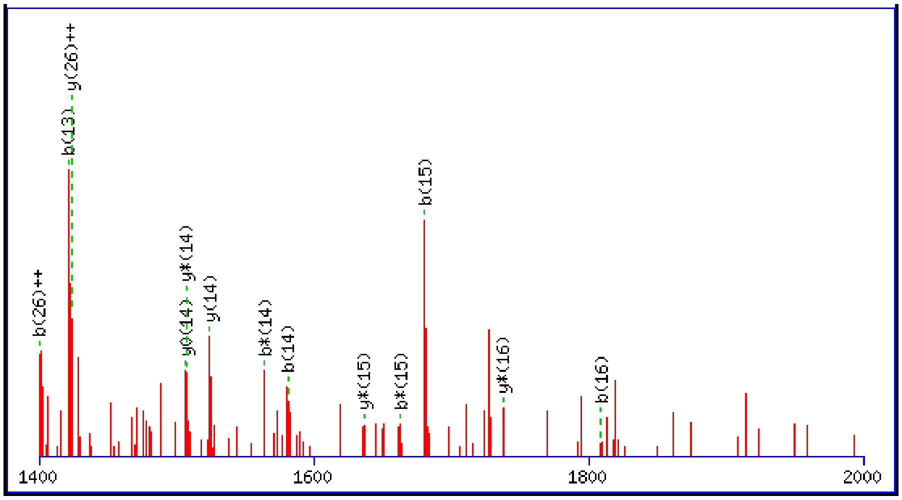

Monoisotopic mass of neutral peptide Mr(calc): 2944.2663
Fixed modifications: Carbamidomethyl (C)
Variable modifications:
N3 : Deamidated_N (N)
Ions Score: 51 **Expect:** 0.003
Matches (Bold Red): 80/292 fragment ions using 175 most intense peaks

#	b	b^{++}	b^{*}	b^{*++}	b^{0}	b^{0++}	Seq.	y	y^{++}	y^{*}	y^{*++}	y^{0}	y^{0++}	#
1	100.0757	50.5415					V							27
2	260.1063	130.5568					C	2846.2051	**1423.6062**	2829.1785	1415.0929	2828.1945	1414.6009	26
3	**375.1333**	188.0703	358.1067	179.5570			N	2686.1744	1343.5909	2669.1479	1335.0776	2668.1639	1334.5856	25
4	**432.1547**	216.5810	415.1282	208.0677			G	2571.1475	**1286.0774**	2554.1209	1277.5641	2553.1369	1277.0721	24
5	519.1868	260.0970	502.1602	251.5837	501.1762	251.0917	S	2514.1260	1257.5667	2497.0995	**1249.0534**	2496.1155	**1248.5614**	23
6	**633.2297**	317.1185	**616.2031**	308.6052	**615.2191**	308.1132	N	2427.0940	1214.0506	2410.0675	1205.5374	2409.0834	1205.0454	22
7	761.3247	381.1660	744.2981	372.6527	743.3141	372.1607	K	2313.0511	1157.0292	2296.0245	1148.5159	2295.0405	1148.0239	21
8	**860.3931**	430.7002	843.3665	422.1869	**842.3825**	421.6949	V	2184.9561	**1092.9817**	2167.9296	1084.4684	2166.9456	1083.9764	20
9	989.4357	495.2215	972.4091	486.7082	**971.4251**	486.2162	E	2085.8877	1043.4475	2068.8612	1034.9342	2067.8771	1034.4422	19
10	1090.4833	545.7453	1073.4568	537.2320	1072.4728	536.7400	T	1956.8451	978.9262	1939.8186	**970.4129**	1938.8345	**969.9209**	18
11	**1191.5310**	596.2691	1174.5045	587.7559	1173.5205	587.2639	T	1855.7974	928.4024	1838.7709	919.8891	1837.7869	919.3971	17
12	**1292.5787**	646.7930	1275.5522	638.2797	1274.5681	637.7877	T	1754.7498	877.8785	**1737.7232**	869.3652	1736.7392	868.8732	16
13	**1421.6213**	711.3143	1404.5947	702.8010	1403.6107	**702.3090**	E	1653.7021	827.3547	**1636.6755**	818.8414	1635.6915	818.3494	15
14	**1581.6519**	791.3296	**1564.6254**	**782.8163**	1563.6414	782.3243	C	**1524.6595**	**762.8334**	**1507.6329**	**754.3201**	**1506.6489**	753.8281	14
15	**1680.7204**	**840.8638**	**1663.6938**	832.3505	1662.7098	831.8585	V	1364.6288	682.8181	1347.6023	**674.3048**	1346.6183	**673.8128**	13
16	**1808.7789**	904.8931	1791.7524	**896.3798**	1790.7684	**895.8878**	Q	**1265.5604**	**633.2838**	**1248.5339**	**624.7706**	**1247.5499**	**624.2786**	12
17	1909.8266	**955.4169**	1892.8001	**946.9037**	1891.8160	**946.4117**	T	**1137.5018**	569.2546	**1120.4753**	560.7413	**1119.4913**	560.2493	11
18	2024.8536	**1012.9304**	2007.8270	**1004.4171**	2006.8430	**1003.9251**	D	**1036.4542**	518.7307	1019.4276	510.2174	1018.4436	509.7254	10
19	2111.8856	**1056.4464**	2094.8590	1047.9332	2093.8750	1047.4411	S	**921.4272**	461.2172	904.4007	**452.7040**	903.4167	**452.2120**	9
20	2239.9442	**1120.4757**	2222.9176	1111.9624	2221.9336	1111.4704	Q	**834.3952**	417.7012	**817.3686**	409.1880	**816.3846**	408.6959	8
21	2340.9918	**1170.9996**	2323.9653	1162.4863	2322.9813	1161.9943	T	**706.3366**	353.6719	689.3101	345.1587	688.3260	344.6667	7
22	2456.0188	**1228.5130**	2438.9922	1219.9998	2438.0082	1219.5077	D	**605.2889**	303.1481	588.2624	294.6348	587.2784	294.1428	6
23	2543.0508	**1272.0290**	2526.0243	1263.5158	2525.0402	1263.0238	S	**490.2620**	245.6346	**473.2354**	237.1214	472.2514	236.6293	5
24	2671.1094	**1336.0583**	2654.0828	**1327.5451**	2653.0988	**1327.0531**	Q	**403.2300**	202.1186	**386.2034**	193.6053			4
25	2742.1465	**1371.5769**	2725.1200	**1363.0636**	2724.1359	**1362.5716**	A	**275.1714**	138.0893	258.1448	129.5761			3
26	2799.1680	**1400.0876**	2782.1414	**1391.5743**	2781.1574	**1391.0823**	G	204.1343	102.5708	187.1077	94.0575			2
27							K	147.1128	74.0600	130.0863	65.5468			1

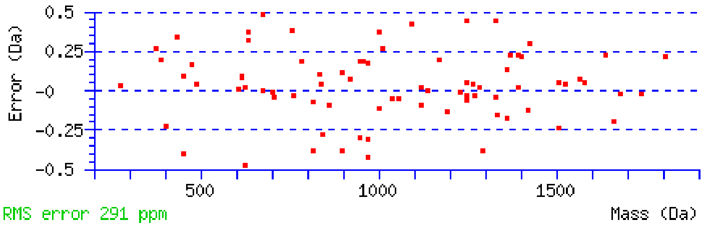

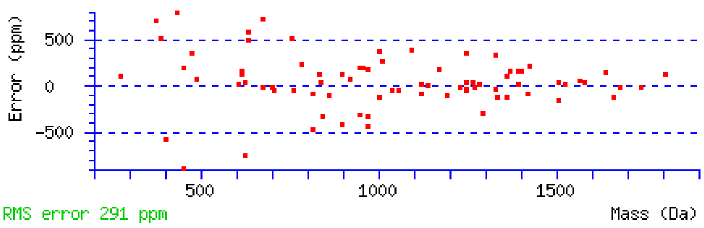

All matches to this query

Score	Mr(calc):	Delta	Sequence
51.2	2944.2663	0.0028	VCNGSNKVETTTECVQTDSQTDSQAGK
43.4	2944.2663	0.0028	VCNGSNKVETTTECVQTDSQTDSQAGK
13.9	2943.2822	0.9868	VCNGSNKVETTTECVQTDSQTDSQAGK
4.0	2944.2542	0.0148	VYFQSPPGAAGEGPGGADDDGPVRRQGK
2.7	2944.2979	-0.0289	KSPAVLTGPSWSASAHIGTLLQLYK

Spectrum No: 421; Query: 128; Rank: 1

Peptide View

MS/MS Fragmentation of **ATYNISLIHPK**
Found in **IPI00327398**, Tax_Id=10116 Gene_Symbol=Enpep Isoform 1 of Glutamyl aminopeptidase

Match to Query 128: 1256.677122 from(419.899650,3+)
Title: 091127RatKid_SCX01_28.2106.2106.3.dta
Data file K:\NewmanPaper\Piliang\3SubProteomes\Piliang3SP\mgf5ppm\SCX_3SubProteomes5ppm.mgf

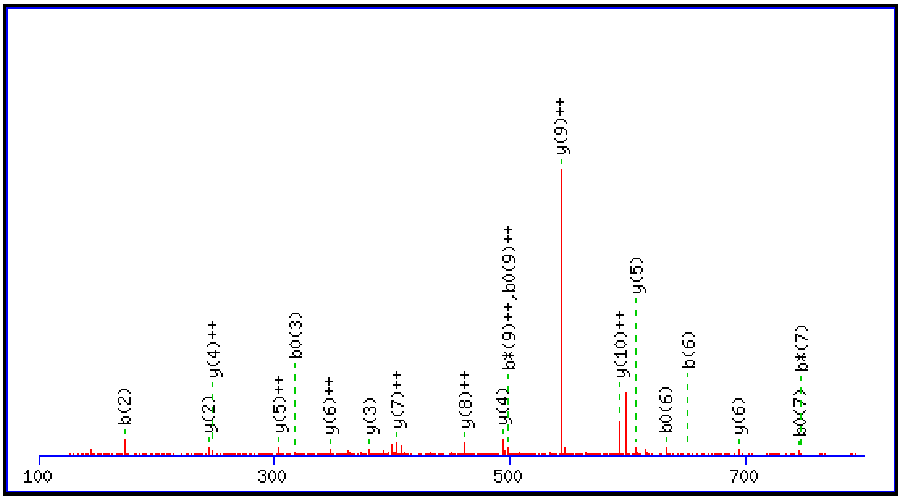

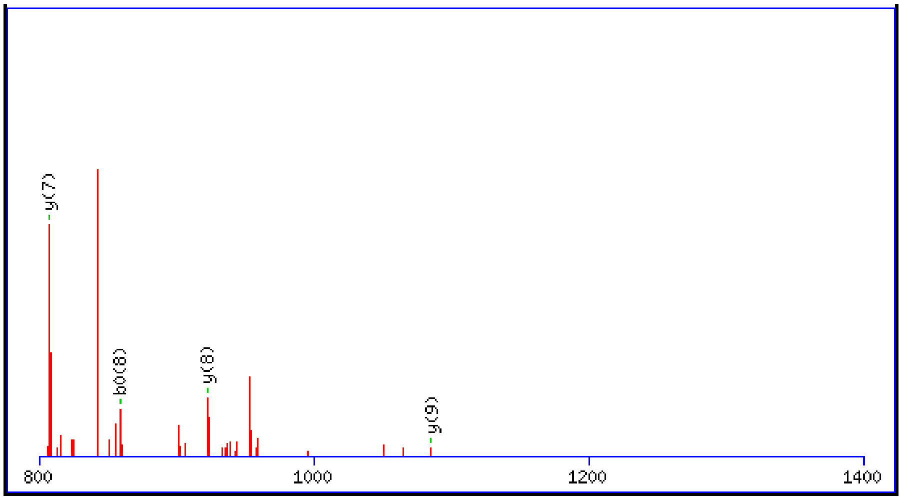

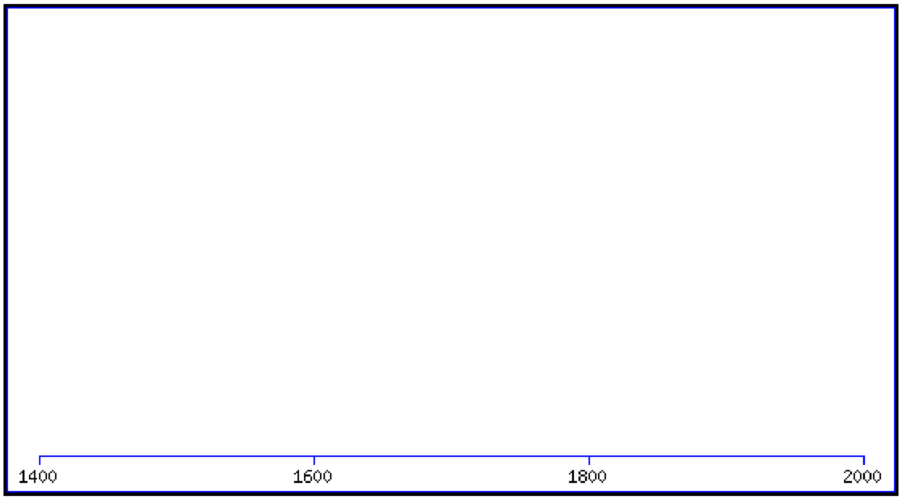

Monoisotopic mass of neutral peptide Mr(calc): 1256.6765
Fixed modifications: Carbamidomethyl (C)
Variable modifications:
N4 : Deamidated_N (N)
Ions Score: 51 **Expect:** 0.0006
Matches (Bold Red): 24/102 fragment ions using 42 most intense peaks

#	b	b^{++}	b*	b^{*++}	b^0	b^{0++}	Seq.	y	y^{++}	y*	y^{*++}	y^0	y^{0++}	#
1	72.0444	36.5258					A							11
2	**173.0921**	87.0497			155.0815	78.0444	T	1186.6466	**593.8270**	1169.6201	585.3137	1168.6361	584.8217	10
3	336.1554	168.5813			**318.1448**	159.5761	Y	**1085.5990**	**543.3031**	1068.5724	534.7898	1067.5884	534.2978	9
4	451.1823	226.0948	434.1558	217.5815	433.1718	217.0895	N	**922.5356**	**461.7715**	905.5091	453.2582	904.5251	452.7662	8
5	564.2664	282.6368	547.2398	274.1236	546.2558	273.6316	I	**807.5087**	**404.2580**	790.4822	395.7447	789.4981	395.2527	7
6	**651.2984**	326.1529	634.2719	317.6396	**633.2879**	317.1476	S	**694.4246**	**347.7160**	677.3981	339.2027	676.4141	338.7107	6
7	764.3825	382.6949	**747.3559**	374.1816	**746.3719**	373.6896	L	**607.3926**	**304.1999**	590.3661	295.6867			5
8	877.4666	439.2369	860.4400	430.7236	**859.4560**	430.2316	I	**494.3085**	**247.6579**	477.2820	239.1446			4
9	1014.5255	507.7664	997.4989	**499.2531**	996.5149	**498.7611**	H	**381.2245**	191.1159	364.1979	182.6026			3
10	1111.5782	556.2928	1094.5517	547.7795	1093.5677	547.2875	P	**244.1656**	122.5864	227.1390	114.0731			2
11							K	147.1128	74.0600	130.0863	65.5468			1

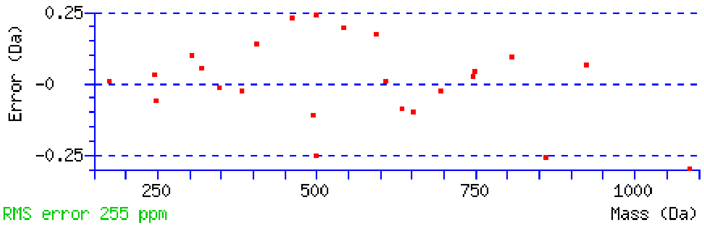

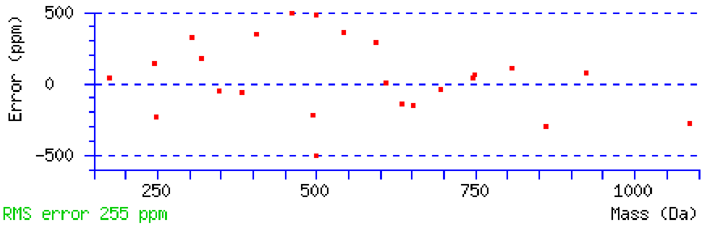

All matches to this query

Score	Mr(calc):	Delta	Sequence
51.2	1256.6765	0.0007	ATYNISLIHPK
7.8	1255.6772	0.9999	AVINTVEPSAVR
3.1	1254.6761	2.0010	AVYSWFLTLR
2.9	1255.6633	1.0138	GILVQADRDNR
2.7	1256.6798	-0.0027	DGVPMLIADAKK
2.5	1254.6584	2.0187	AVDTKLSSVKK
2.5	1254.6584	2.0187	AVDTKLSSVKK
2.5	1254.6584	2.0187	AVDTKLSSVKK
2.5	1254.6598	2.0173	AVTQAVFRRK
1.5	1254.6819	1.9952	AVLIPKDDQEK

Spectrum No: 422; Query: 532; Rank: 1

Peptide View

MS/MS Fragmentation of **NGGLGHMNITLLSDLTK**
Found in **IPI00208215**, Tax_Id=10116 Gene_Symbol=Prdx3 Thioredoxin-dependent peroxide reductase, mitochondrial precursor

Match to Query 532: 1799.911388 from(900.962970,2+)
Title: 091127RatKid_SCX01_22.4320.4320.2.dta
Data file K:\NewmanPaper\Piliang\3SubProteomes\Piliang3SP\mgf5ppm\SCX_3SubProteomes5ppm.mgf

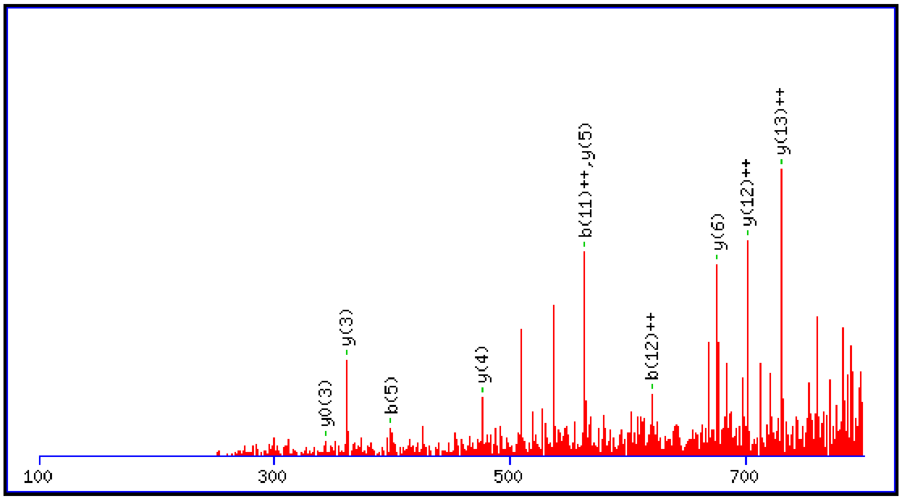

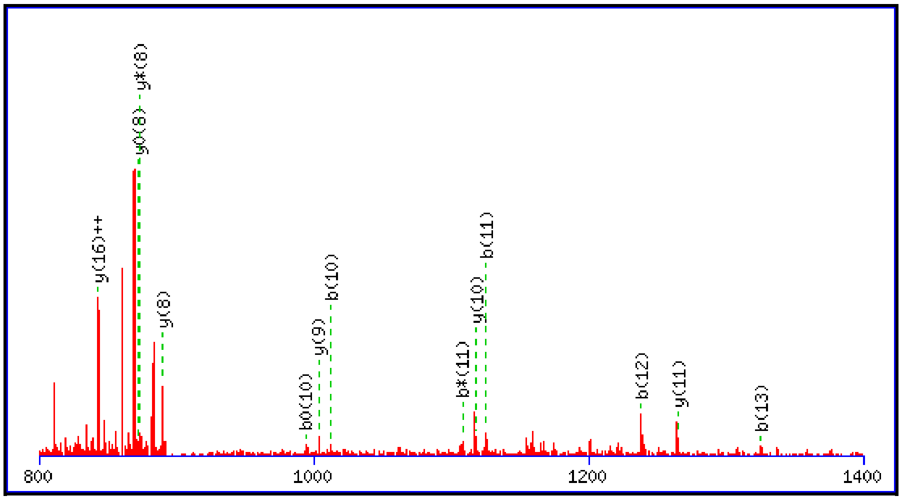

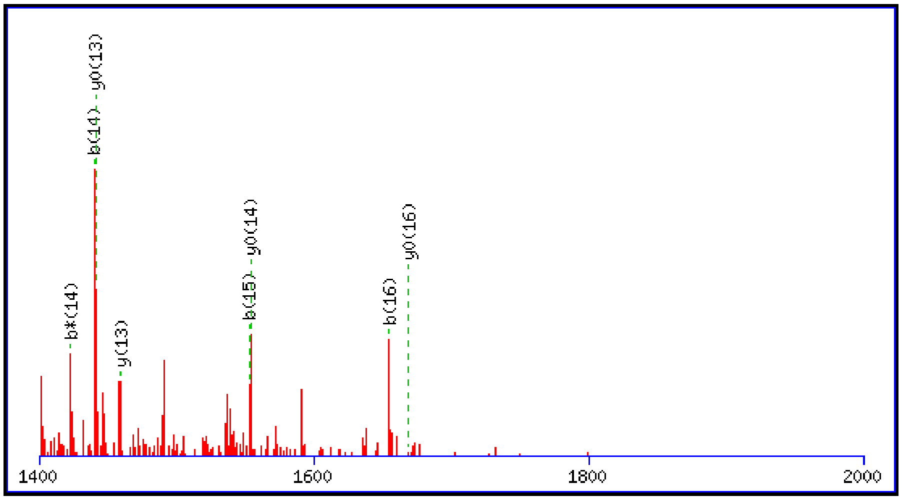

Monoisotopic mass of neutral peptide Mr(calc): 1799.9087
Fixed modifications: Carbamidomethyl (C)
Variable modifications:
M7 : Oxidation (M)
N8 : Deamidated_N (N)
Ions Score: 51 **Expect:** 0.0012
Matches (Bold Red): 31/172 fragment ions using 69 most intense peaks

#	b	b^{++}	b*	b^{*++}	b^{0}	b^{0++}	Seq.	y	y^{++}	y*	y^{*++}	y^{0}	y^{0++}	#
1	115.0502	58.0287	98.0237	49.5155			N							17
2	172.0717	86.5395	155.0451	78.0262			G	1686.8731	**843.9402**	1669.8465	835.4269	**1668.8625**	834.9349	16
3	229.0931	115.0502	212.0666	106.5369			G	1629.8516	815.4295	1612.8251	806.9162	1611.8411	806.4242	15
4	342.1772	171.5922	325.1506	163.0790			L	1572.8302	786.9187	1555.8036	778.4054	**1554.8196**	777.9134	14
5	**399.1987**	200.1030	382.1721	191.5897			G	**1459.7461**	**730.3767**	1442.7196	721.8634	**1441.7355**	721.3714	13
6	536.2576	268.6324	519.2310	260.1191			H	1402.7246	**701.8660**	1385.6981	693.3527	1384.7141	692.8607	12
7	683.2930	342.1501	666.2664	333.6369			M	**1265.6657**	633.3365	1248.6392	624.8232	1247.6552	624.3312	11
8	798.3199	399.6636	781.2934	391.1503			N	**1118.6303**	559.8188	1101.6038	551.3055	1100.6198	550.8135	10
9	911.4040	456.2056	894.3774	447.6924			I	**1003.6034**	502.3053	986.5768	493.7921	985.5928	493.3001	9
10	**1012.4517**	506.7295	995.4251	498.2162	**994.4411**	497.7242	T	**890.5193**	445.7633	**873.4928**	437.2500	**872.5088**	436.7580	8
11	**1125.5357**	**563.2715**	**1108.5092**	554.7582	1107.5252	554.2662	L	789.4716	395.2395	772.4451	386.7262	771.4611	386.2342	7
12	**1238.6198**	**619.8135**	1221.5932	611.3003	1220.6092	610.8082	L	**676.3876**	338.6974	659.3610	330.1842	658.3770	329.6921	6
13	**1325.6518**	663.3295	1308.6253	654.8163	1307.6412	654.3243	S	**563.3035**	282.1554	546.2770	273.6421	545.2930	273.1501	5
14	**1440.6788**	720.8430	**1423.6522**	712.3297	1422.6682	711.8377	D	**476.2715**	238.6394	459.2449	230.1261	458.2609	229.6341	4
15	**1553.7628**	777.3850	1536.7363	768.8718	1535.7523	768.3798	L	**361.2445**	181.1259	344.2180	172.6126	**343.2340**	172.1206	3
16	**1654.8105**	827.9089	1637.7839	819.3956	1636.7999	818.9036	T	248.1605	124.5839	231.1339	116.0706	230.1499	115.5786	2
17							K	147.1128	74.0600	130.0863	65.5468			1

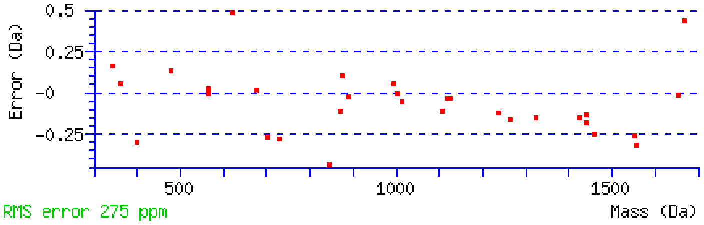

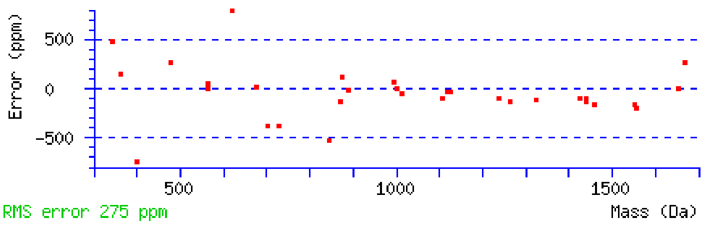

All matches to this query

Score	Mr(calc):	Delta	Sequence
51.2	1799.9087	0.0027	NGGLGHMNITLLSDLTK
47.6	1799.9087	0.0027	NGGLGHMNITLLSDLTK
23.7	1798.9247	0.9867	NGGLGHMNITLLSDLTK
5.2	1799.9247	-0.0133	DSLFVLAGFKWQDFK

Spectrum No: 423; Query: 698; Rank: 1

Peptide View

MS/MS Fragmentation of **VMELEKEIANATTKPEDR**
Found in **IPI00231789**, Tax_Id=10116 Gene_Symbol=Mme Neprilysin

Match to Query 698: 2074.029342 from(692.350390,3+)
Title: 091127RatKid_SCX01_33.3101.3101.3.dta
Data file K:\NewmanPaper\Piliang\3SubProteomes\Piliang3SP\mgf5ppm\SCX_3SubProteomes5ppm.mgf

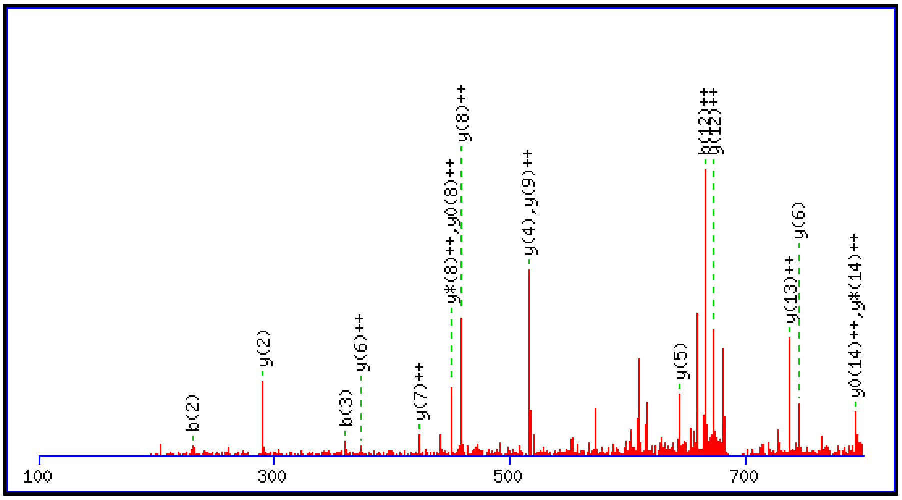

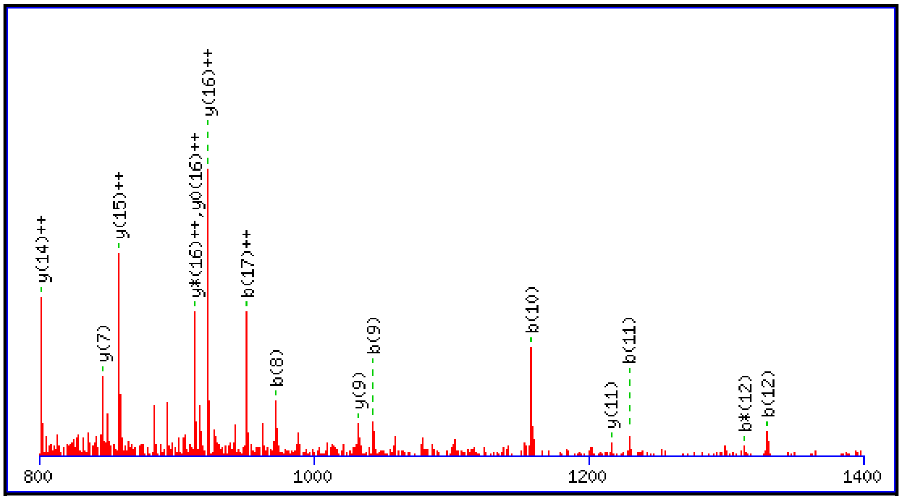

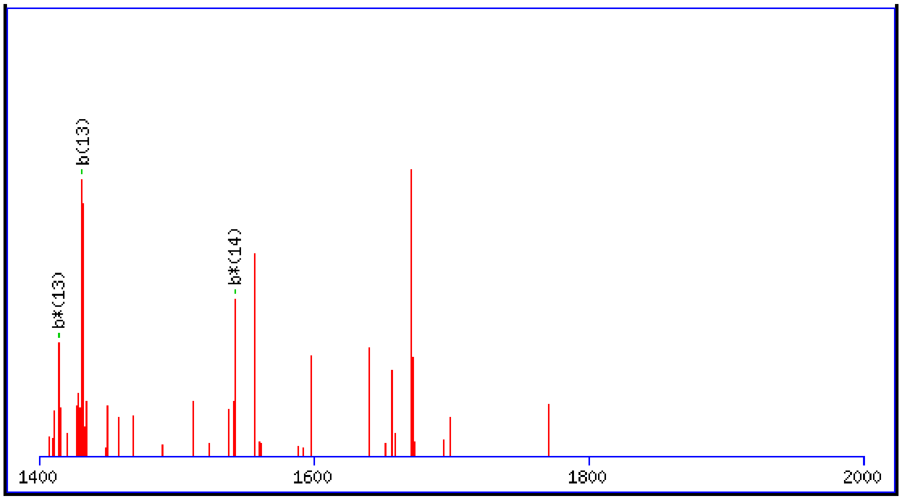

Monoisotopic mass of neutral peptide Mr(calc): 2074.0252
Fixed modifications: Carbamidomethyl (C)
Variable modifications:
N10 : Deamidated_N (N)
Ions Score: 51 **Expect:** 0.0015
Matches (Bold Red): 35/188 fragment ions using 53 most intense peaks

#	b	b^{++}	b*	b*$^{++}$	b^{0}	b^{0++}	Seq.	y	y^{++}	y*	y*$^{++}$	y^{0}	y^{0++}	#
1	100.0757	50.5415					V							18
2	**231.1162**	116.0617					M	1975.9641	988.4857	1958.9375	979.9724	1957.9535	979.4804	17
3	**360.1588**	180.5830			342.1482	171.5777	E	1844.9236	**922.9654**	1827.8971	**914.4522**	1826.9130	**913.9602**	16
4	473.2428	237.1251			455.2323	228.1198	L	1715.8810	**858.4441**	1698.8545	849.9309	1697.8704	849.4389	15
5	602.2854	301.6464			584.2749	292.6411	E	1602.7970	**801.9021**	1585.7704	**793.3888**	1584.7864	**792.8968**	14
6	730.3804	365.6938	713.3538	357.1806	712.3698	356.6885	K	1473.7544	**737.3808**	1456.7278	728.8675	1455.7438	728.3755	13
7	859.4230	430.2151	842.3964	421.7019	841.4124	421.2098	E	1345.6594	**673.3333**	1328.6328	664.8201	1327.6488	664.3281	12
8	**972.5070**	486.7572	955.4805	478.2439	954.4965	477.7519	I	**1216.6168**	608.8120	1199.5903	600.2988	1198.6062	599.8068	11
9	**1043.5442**	522.2757	1026.5176	513.7624	1025.5336	513.2704	A	1103.5327	552.2700	1086.5062	543.7567	1085.5222	543.2647	10
10	**1158.5711**	579.7892	1141.5445	571.2759	1140.5605	570.7839	N	**1032.4956**	**516.7514**	1015.4691	508.2382	1014.4851	507.7462	9
11	**1229.6082**	615.3077	1212.5817	606.7945	1211.5976	606.3025	A	917.4687	**459.2380**	900.4421	**450.7247**	899.4581	**450.2327**	8
12	**1330.6559**	**665.8316**	**1313.6293**	657.3183	1312.6453	656.8263	T	**846.4316**	**423.7194**	829.4050	415.2062	828.4210	414.7141	7
13	**1431.7036**	716.3554	**1414.6770**	707.8421	1413.6930	707.3501	T	**745.3839**	**373.1956**	728.3573	364.6823	727.3733	364.1903	6
14	1559.7985	780.4029	**1542.7720**	771.8896	1541.7880	771.3976	K	**644.3362**	322.6717	627.3097	314.1585	626.3257	313.6665	5
15	1656.8513	828.9293	1639.8247	820.4160	1638.8407	819.9240	P	**516.2413**	258.6243	499.2147	250.1110	498.2307	249.6190	4
16	1785.8939	893.4506	1768.8673	884.9373	1767.8833	884.4453	E	419.1885	210.0979	402.1619	201.5846	401.1779	201.0926	3
17	1900.9208	**950.9641**	1883.8943	942.4508	1882.9103	941.9588	D	**290.1459**	145.5766	273.1193	137.0633	272.1353	136.5713	2
18							R	175.1190	88.0631	158.0924	79.5498			1

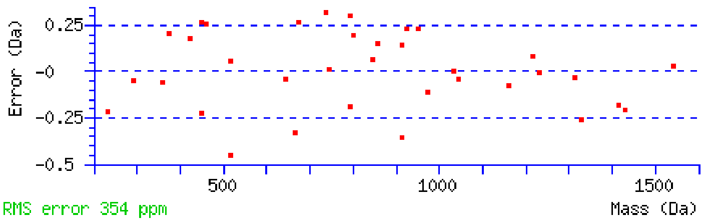

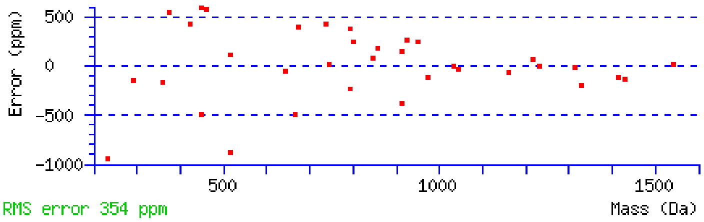

All matches to this query

Score	Mr(calc):	Delta	Sequence
51.1	2074.0252	0.0042	VMELEKEIANATTKPEDR
14.1	2073.0412	0.9882	VMELEKEIANATTKPEDR
9.4	2074.0113	0.0181	CQALERKNSATPSELNEK

7.2	2074.0322	-0.0029	YDLLCLEGLARGLQVFK
4.6	2074.0405	-0.0112	DGGNPAFSAEVLVPITVMNK
4.0	2074.0266	0.0028	MGQTLPTWLREDLQNTR
3.5	2072.0238	2.0056	YINQVGSTCLALRGVRGK
1.5	2072.0199	2.0094	SWVQCGISMKILNSLKK
1.3	2072.0327	1.9967	EVQLEKAGHFSWKDVNGK
0.9	2074.0143	0.0150	VPGLANGVKCGSPRQLQGR

Spectrum No: 424; Query: 996; Rank: 1

Peptide View

MS/MS Fragmentation of **NGTNLDWATAETLALGSLLAQGFNVR**
Found in **IPI00364925**, Tax_Id=10116 Gene_Symbol=Dhtkd1 Probable 2-oxoglutarate dehydrogenase E1 component DHKTD1, mitochondrial precursor

Match to Query 996: 2732.383392 from(911.801740,3+)
Title: 091127RatKid_SCX01_12.6645.6645.3.dta
Data file K:\NewmanPaper\Piliang\3SubProteomes\Piliang3SP\mgf5ppm\SCX_3SubProteomes5ppm.mgf

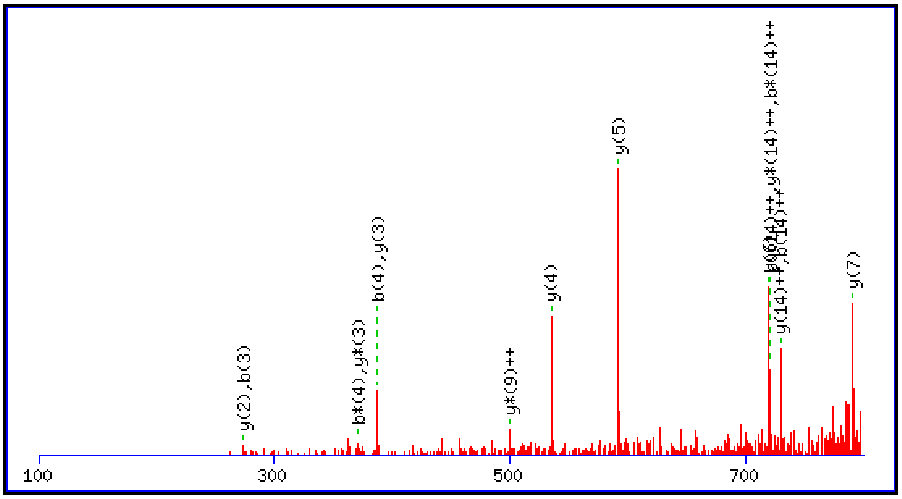

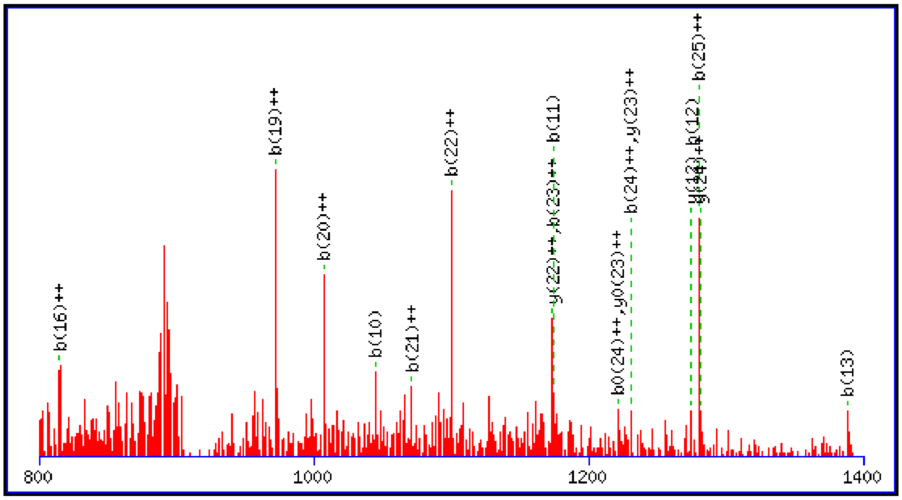

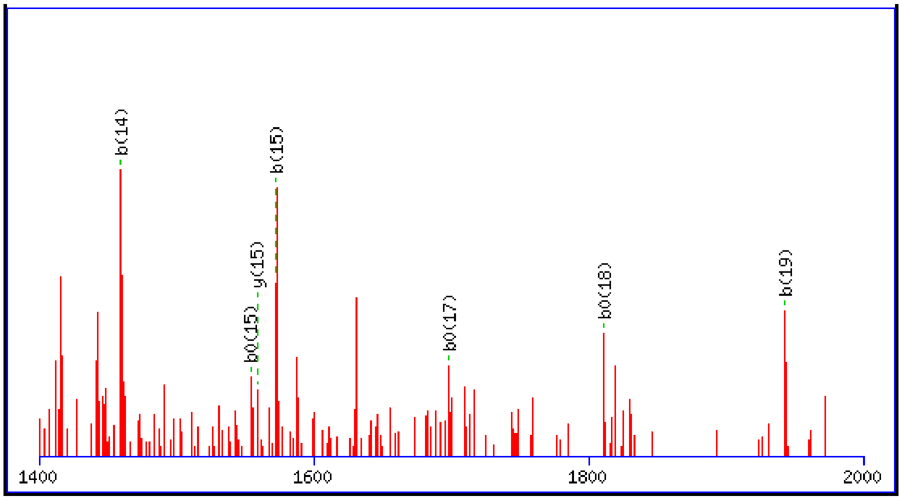

Monoisotopic mass of neutral peptide Mr(calc): 2732.3769
Fixed modifications: Carbamidomethyl (C)
Variable modifications:
N1 : Deamidated_N (N)
Ions Score: 51 **Expect:** 0.0014
Matches (Bold Red): 41/278 fragment ions using 64 most intense peaks

#	b	b^{++}	b*	b^{*++}	b^{0}	b^{0++}	Seq.	y	y^{++}	y*	y^{*++}	y^{0}	y^{0++}	#
1	116.0342	58.5207	99.0077	50.0075			N							26
2	173.0557	87.0315	156.0291	78.5182			G	2618.3573	1309.6823	2601.3307	1301.1690	2600.3467	1300.6770	25
3	**274.1034**	137.5553	257.0768	129.0420	256.0928	128.5500	T	2561.3358	**1281.1716**	2544.3093	1272.6583	2543.3253	1272.1663	24
4	**388.1463**	194.5768	**371.1197**	186.0635	370.1357	185.5715	N	2460.2882	**1230.6477**	2443.2616	1222.1344	2442.2776	**1221.6424**	23
5	501.2303	251.1188	484.2038	242.6055	483.2198	242.1135	L	2346.2452	**1173.6262**	2329.2187	1165.1130	2328.2347	1164.6210	22
6	616.2573	308.6323	599.2307	300.1190	598.2467	299.6270	D	2233.1612	1117.0842	2216.1346	1108.5709	2215.1506	1108.0789	21
7	802.3366	401.6719	785.3101	393.1587	784.3260	392.6667	W	2118.1342	1059.5707	2101.1077	1051.0575	2100.1237	1050.5655	20
8	873.3737	437.1905	856.3472	428.6772	855.3632	428.1852	A	1932.0549	966.5311	1915.0284	958.0178	1914.0443	957.5258	19
9	974.4214	487.7143	957.3948	479.2011	956.4108	478.7091	T	1861.0178	931.0125	1843.9912	922.4993	1843.0072	922.0073	18
10	**1045.4585**	523.2329	1028.4320	514.7196	1027.4479	514.2276	A	1759.9701	880.4887	1742.9436	871.9754	1741.9595	871.4834	17
11	**1174.5011**	587.7542	1157.4746	579.2409	1156.4905	578.7489	E	1688.9330	844.9701	1671.9064	836.4569	1670.9224	835.9649	16
12	**1275.5488**	638.2780	1258.5222	629.7648	1257.5382	629.2727	T	**1559.8904**	780.4488	1542.8639	771.9356	1541.8798	771.4436	15
13	**1388.6328**	694.8201	1371.6063	686.3068	1370.6223	685.8148	L	1458.8427	**729.9250**	1441.8162	**721.4117**	1440.8322	720.9197	14
14	**1459.6700**	**730.3386**	1442.6434	**721.8253**	1441.6594	**721.3333**	A	1345.7587	673.3830	1328.7321	664.8697	1327.7481	664.3777	13
15	**1572.7540**	786.8806	1555.7275	778.3674	**1554.7435**	777.8754	L	**1274.7215**	637.8644	1257.6950	629.3511	1256.7110	628.8591	12
16	1629.7755	**815.3914**	1612.7489	806.8781	1611.7649	806.3861	G	1161.6375	581.3224	1144.6109	572.8091	1143.6269	572.3171	11
17	1716.8075	858.9074	1699.7810	850.3941	**1698.7969**	849.9021	S	1104.6160	552.8116	1087.5895	544.2984	1086.6055	543.8064	10
18	1829.8916	915.4494	1812.8650	906.9362	**1811.8810**	906.4441	L	1017.5840	509.2956	1000.5574	**500.7824**			9
19	**1942.9756**	**971.9915**	1925.9491	963.4782	1924.9651	962.9862	L	904.4999	452.7536	887.4734	444.2403			8
20	2014.0128	**1007.5100**	1996.9862	998.9967	1996.0022	998.5047	A	**791.4159**	396.2116	774.3893	387.6983			7
21	2142.0713	**1071.5393**	2125.0448	1063.0260	2124.0608	1062.5340	Q	**720.3787**	360.6930	703.3522	352.1797			6
22	2199.0928	**1100.0500**	2182.0662	1091.5368	2181.0822	1091.0448	G	**592.3202**	296.6637	575.2936	288.1504			5
23	2346.1612	**1173.5842**	2329.1347	1165.0710	2328.1506	1164.5790	F	**535.2987**	268.1530	518.2722	259.6397			4
24	2460.2041	**1230.6057**	2443.1776	1222.0924	2442.1936	**1221.6004**	N	**388.2303**	194.6188	**371.2037**	186.1055			3
25	2559.2726	**1280.1399**	2542.2460	1271.6266	2541.2620	1271.1346	V	**274.1874**	137.5973	257.1608	129.0840			2
26							R	175.1190	88.0631	158.0924	79.5498			1

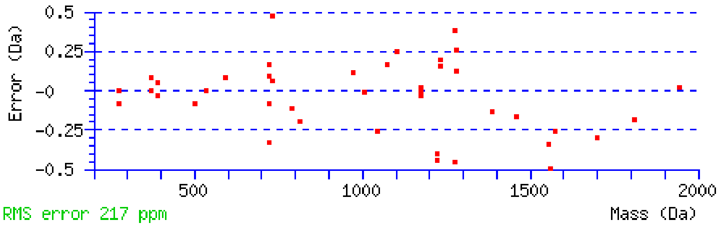

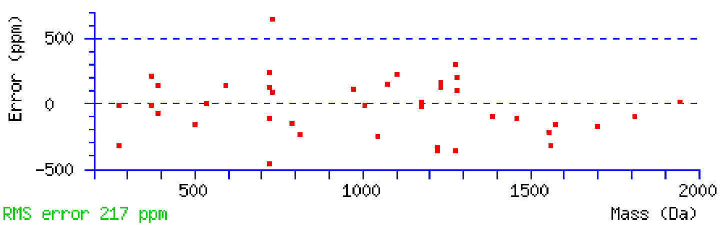

All matches to this query

Score	Mr(calc):	Delta	Sequence
51.1	2732.3769	0.0065	NGTNLDWATAETLALGSLLAQGFNVR
45.2	2732.3769	0.0065	NGTNLDWATAETLALGSLLAQGFNVR
15.7	2731.3929	0.9905	NGTNLDWATAETLALGSLLAQGFNVR
5.5	2732.3769	0.0065	NGTNLDWATAETLALGSLLAQGFNVR
5.4	2732.3816	0.0018	GESLWLNLRNRISQQMDGLAPYR
4.0	2732.3608	0.0226	ASLSALTMNTIVFNKLGGAVIFDDR
0.8	2730.3653	2.0181	YSHFPDFSYSASSKIIISDVPSRK

Spectrum No: 425; Query: 61; Rank: 1

Peptide View

MS/MS Fragmentation of **LNVTSPDLFR**
Found in **IPI00190577**, Tax_Id=10116 Gene_Symbol=Lama5 similar to Laminin alpha-5 chain precursor

Match to Query 61: 1161.601928 from(581.808240,2+)
Title: 091127RatKid_SCX01_12.2786.2786.2.dta
Data file K:\NewmanPaper\Piliang\3SubProteomes\Piliang3SP\mgf5ppm\SCX_3SubProteomes5ppm.mgf

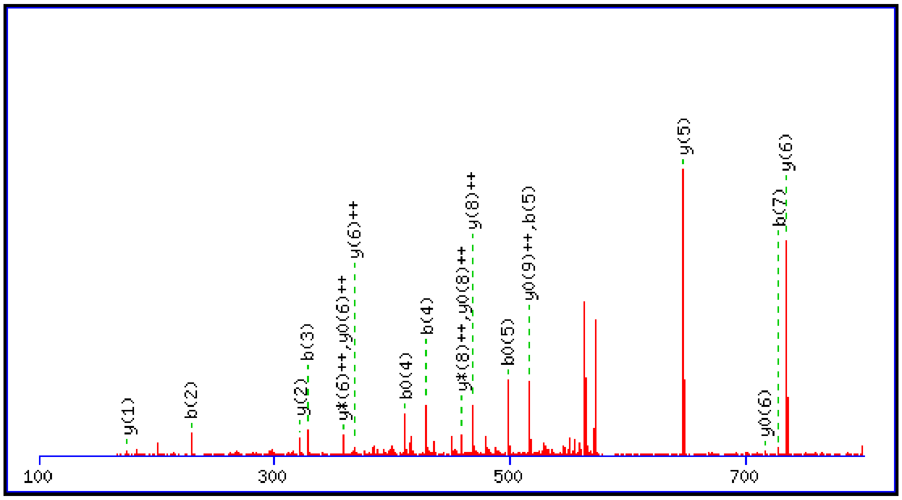

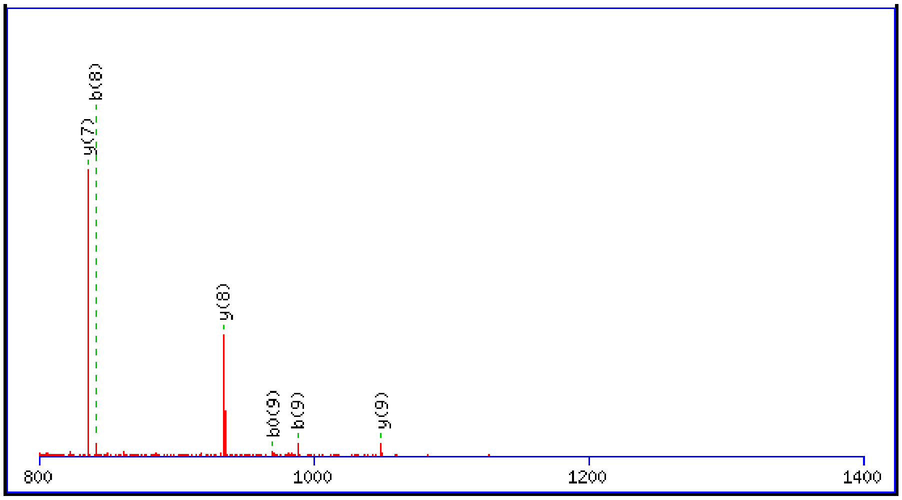

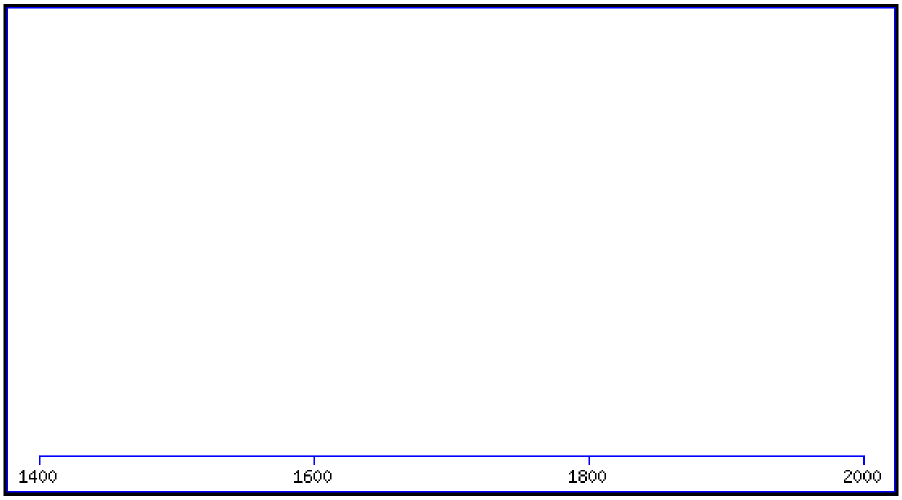

Monoisotopic mass of neutral peptide Mr(calc): 1161.6030
Fixed modifications: Carbamidomethyl (C)
Variable modifications:
N2 : Deamidated_N (N)
Ions Score: 51 **Expect:** 0.00084
Matches (Bold Red): 25/94 fragment ions using 51 most intense peaks

#	b	b++	b*	b*++	b0	b0++	Seq.	y	y++	y*	y*++	y0	y0++	#
1	114.0913	57.5493					L							10
2	**229.1183**	115.0628	212.0917	106.5495			N	**1049.5262**	525.2667	1032.4996	516.7535	1031.5156	**516.2615**	9
3	**328.1867**	164.5970	311.1601	156.0837			V	**934.4993**	**467.7533**	917.4727	**459.2400**	916.4887	**458.7480**	8
4	**429.2344**	215.1208	412.2078	206.6075	**411.2238**	206.1155	T	**835.4308**	418.2191	818.4043	409.7058	817.4203	409.2138	7
5	**516.2664**	258.6368	499.2398	250.1236	**498.2558**	249.6316	S	**734.3832**	**367.6952**	717.3566	**359.1819**	**716.3726**	**358.6899**	6
6	613.3192	307.1632	596.2926	298.6499	595.3086	298.1579	P	**647.3511**	324.1792	630.3246	315.6659	629.3406	315.1739	5
7	**728.3461**	364.6767	711.3196	356.1634	710.3355	355.6714	D	550.2984	275.6528	533.2718	267.1396	532.2878	266.6475	4
8	**841.4302**	421.2187	824.4036	412.7054	823.4196	412.2134	L	435.2714	218.1394	418.2449	209.6261			3
9	**988.4986**	494.7529	971.4720	486.2397	**970.4880**	485.7476	F	**322.1874**	161.5973	305.1608	153.0840			2
10							R	**175.1190**	88.0631	158.0924	79.5498			1

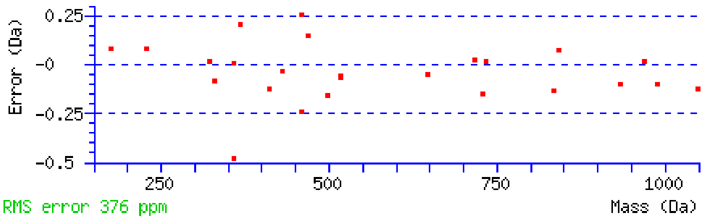

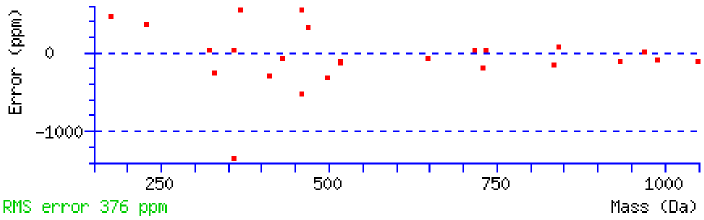

All matches to this query

Score	Mr(calc):	Delta	Sequence
50.9	1161.6030	-0.0011	LNVTSPDLFR
8.3	1161.5964	0.0055	IMDRFNLPR
8.2	1160.5972	1.0047	VELSTGMPRR
8.2	1161.5998	0.0021	ICXTQKLMR
6.7	1161.6030	-0.0010	LPAFLEETSR
6.3	1161.6064	-0.0044	VELSQLCVSK
4.8	1161.5990	0.0030	LSSNAVSQVTR
4.4	1161.5990	0.0030	QTETLTSNLR
4.2	1161.5964	0.0055	WNKLADMLR
4.1	1161.5924	0.0095	VQKCKEGTGR

Spectrum No: 426; Query: 1124; Rank: 1

Peptide View

MS/MS Fragmentation of **AFQFVETHGEVCPANWTPESPTIKPSPTASK**
Found in **IPI00208215**, Tax_Id=10116 Gene_Symbol=Prdx3 Thioredoxin-dependent peroxide reductase, mitochondrial precursor

Match to Query 1124: 3413.640192 from(1138.887340,3+)
Title: 091127RatKid_SCX01_30.3346.3346.3.dta
Data file K:\NewmanPaper\Piliang\3SubProteomes\Piliang3SP\mgf5ppm\SCX_3SubProteomes5ppm.mgf

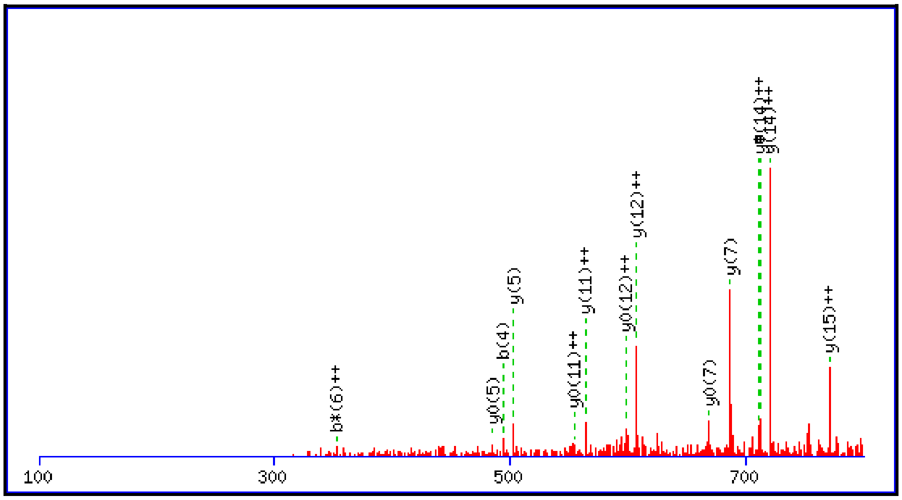

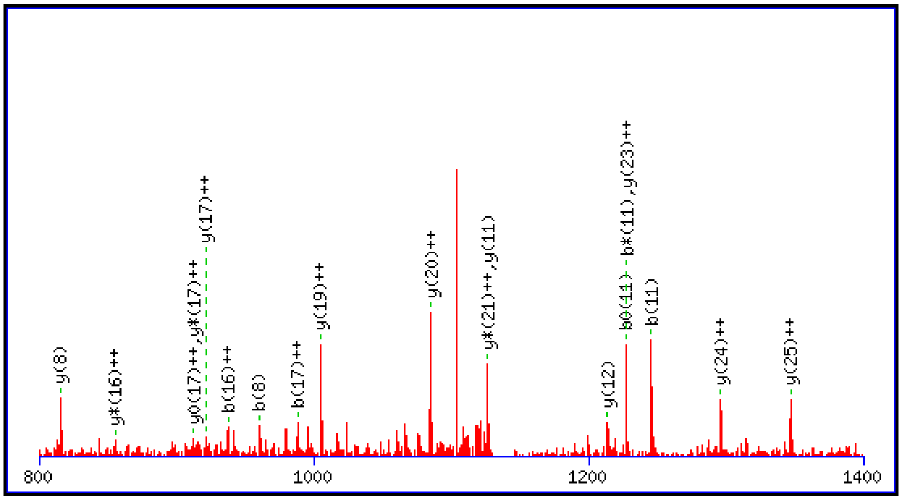

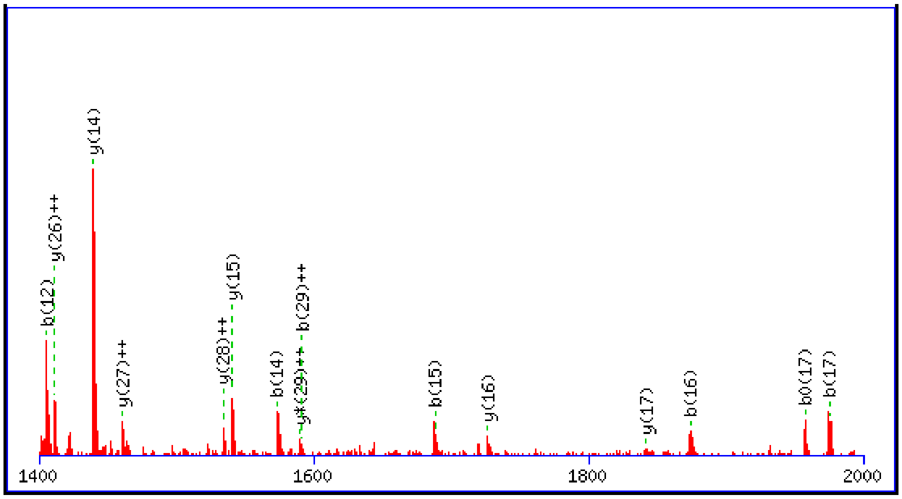

Monoisotopic mass of neutral peptide Mr(calc): 3413.6238
Fixed modifications: Carbamidomethyl (C)
Variable modifications:
N15 : Deamidated_N (N)
Ions Score: 51 **Expect:** 0.0027
Matches (Bold Red): 48/344 fragment ions using 102 most intense peaks

#	b	b^{++}	b*	b^{*++}	b^0	b^{0++}	Seq.	y	y^{++}	y*	y^{*++}	y^0	y^{0++}	#
1	72.0444	36.5258					A							31
2	219.1128	110.0600					F	3343.5940	1672.3006	3326.5674	1663.7873	3325.5834	1663.2953	30
3	347.1714	174.0893	330.1448	165.5761			Q	3196.5256	1598.7664	3179.4990	**1590.2531**	3178.5150	1589.7611	29
4	**494.2398**	247.6235	477.2132	239.1103			F	3068.4670	**1534.7371**	3051.4404	1526.2239	3050.4564	1525.7318	28
5	593.3082	297.1577	576.2817	288.6445			V	2921.3986	**1461.2029**	2904.3720	1452.6896	2903.3880	1452.1976	27
6	722.3508	361.6790	705.3243	**353.1658**	704.3402	352.6738	E	2822.3301	**1411.6687**	2805.3036	1403.1554	2804.3196	1402.6634	26
7	823.3985	412.2029	806.3719	403.6896	805.3879	403.1976	T	2693.2876	**1347.1474**	2676.2610	1338.6341	2675.2770	1338.1421	25
8	**960.4574**	480.7323	943.4308	472.2191	942.4468	471.7271	H	2592.2399	**1296.6236**	2575.2133	1288.1103	2574.2293	1287.6183	24
9	1017.4789	509.2431	1000.4523	500.7298	999.4683	500.2378	G	2455.1810	**1228.0941**	2438.1544	1219.5808	2437.1704	1219.0888	23
10	1146.5215	573.7644	1129.4949	565.2511	1128.5109	564.7591	E	2398.1595	1199.5834	2381.1329	1191.0701	2380.1489	1190.5781	22
11	**1245.5899**	623.2986	**1228.5633**	614.7853	**1227.5793**	614.2933	V	2269.1169	1135.0621	2252.0904	**1126.5488**	2251.1063	1126.0568	21
12	**1405.6205**	703.3139	1388.5940	694.8006	1387.6099	694.3086	C	2170.0485	**1085.5279**	2153.0219	1077.0146	2152.0379	1076.5226	20
13	1502.6733	751.8403	1485.6467	743.3270	1484.6627	742.8350	P	2010.0178	**1005.5126**	1992.9913	996.9993	1992.0073	996.5073	19
14	**1573.7104**	787.3588	1556.6838	778.8456	1555.6998	778.3536	A	1912.9651	956.9862	1895.9385	948.4729	1894.9545	947.9809	18
15	**1688.7373**	844.8723	1671.7108	836.3590	1670.7268	835.8670	N	**1841.9280**	**921.4676**	1824.9014	**912.9543**	1823.9174	**912.4623**	17
16	**1874.8166**	**937.9120**	1857.7901	929.3987	1856.8061	928.9067	W	**1726.9010**	863.9542	1709.8745	**855.4409**	1708.8905	854.9489	16
17	**1975.8643**	**988.4358**	1958.8378	979.9225	**1957.8538**	979.4305	T	**1540.8217**	**770.9145**	1523.7952	762.4012	1522.8112	761.9092	15
18	2072.9171	1036.9622	2055.8905	1028.4489	2054.9065	1027.9569	P	**1439.7740**	**720.3907**	1422.7475	**711.8774**	1421.7635	**711.3854**	14
19	2201.9597	1101.4835	2184.9331	1092.9702	2183.9491	1092.4782	E	1342.7213	671.8643	1325.6947	663.3510	1324.7107	662.8590	13
20	2288.9917	1144.9995	2271.9652	1136.4862	2270.9811	1135.9942	S	**1213.6787**	**607.3430**	1196.6521	598.8297	1195.6681	**598.3377**	12
21	2386.0445	1193.5259	2369.0179	1185.0126	2368.0339	1184.5206	P	**1126.6467**	**563.8270**	1109.6201	555.3137	1108.6361	**554.8217**	11
22	2487.0921	1244.0497	2470.0656	1235.5364	2469.0816	1235.0444	T	1029.5939	515.3006	1012.5673	506.7873	1011.5833	506.2953	10
23	2600.1762	1300.5917	2583.1497	1292.0785	2582.1656	1291.5865	I	928.5462	464.7767	911.5197	456.2635	910.5356	455.7715	9
24	2728.2712	1364.6392	2711.2446	1356.1260	2710.2606	1355.6339	K	**815.4621**	408.2347	798.4356	399.7214	797.4516	399.2294	8
25	2825.3239	1413.1656	2808.2974	1404.6523	2807.3134	1404.1603	P	**687.3672**	344.1872	670.3406	335.6740	**669.3566**	335.1819	7
26	2912.3560	1456.6816	2895.3294	1448.1683	2894.3454	1447.6763	S	590.3144	295.6608	573.2879	287.1476	572.3039	286.6556	6
27	3009.4087	1505.2080	2992.3822	1496.6947	2991.3982	1496.2027	P	**503.2824**	252.1448	486.2558	243.6316	**485.2718**	243.1396	5
28	3110.4564	1555.7318	3093.4299	1547.2186	3092.4458	1546.7266	T	406.2296	203.6185	389.2031	195.1052	388.2191	194.6132	4
29	3181.4935	**1591.2504**	3164.4670	1582.7371	3163.4830	1582.2451	A	305.1819	153.0946	288.1554	144.5813	287.1714	144.0893	3
30	3268.5256	1634.7664	3251.4990	1626.2531	3250.5150	1625.7611	S	234.1448	117.5761	217.1183	109.0628	216.1343	108.5708	2
31							K	147.1128	74.0600	130.0863	65.5468			1

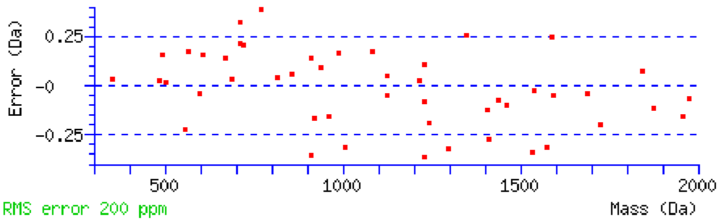

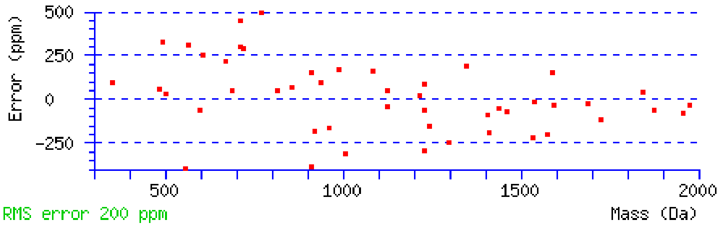

All matches to this query

Score	Mr(calc):	Delta	Sequence
50.9	3413.6238	0.0164	AFQFVETHGEVCPANWTPESPTIKPSPTASK
42.4	3412.6398	1.0004	AFQFVETHGEVCPANWTPESPTIKPSPTASK
2.0	3411.6074	2.0328	EKMKSLHIEILDDNLPEGPEEFVLAITR
1.1	3413.6310	0.0092	LRLDVAITGEPPPTATWLKGDEVFTVSEGR
1.1	3413.6310	0.0092	LRLDVAITGEPPPTATWLKGDEVFTVSEGR
1.1	3413.6561	-0.0159	NPILLPNVTLGYNIHNAGSIESETVLSFLK
1.1	3413.6561	-0.0159	NPILLPNVTLGYNIHNAGSIESETVLSFLK
0.8	3413.6343	0.0059	SNNITDSLANRTLIVTTILEEPYVMYRK
0.8	3413.6561	-0.0159	NPILLPNVTLGYNIHNAGSIESETVLSFLK
0.8	3413.6561	-0.0159	NPILLPNVTLGYNIHNAGSIESETVLSFLK

Spectrum No: 427; Query: 875; Rank: 1

Peptide View

MS/MS Fragmentation of **EIDRDQLMTVLANVTHLLIR**
Found in **IPI00363534**, Tax_Id=10116 Gene_Symbol=Lama1 laminin, alpha 1

Match to Query 875: 2350.270856 from(588.574990,4+)
Title: 091127RatKid_SCX01_31.7626.7626.4.dta
Data file K:\NewmanPaper\Piliang\3SubProteomes\Piliang3SP\mgf5ppm\SCX_3SubProteomes5ppm.mgf

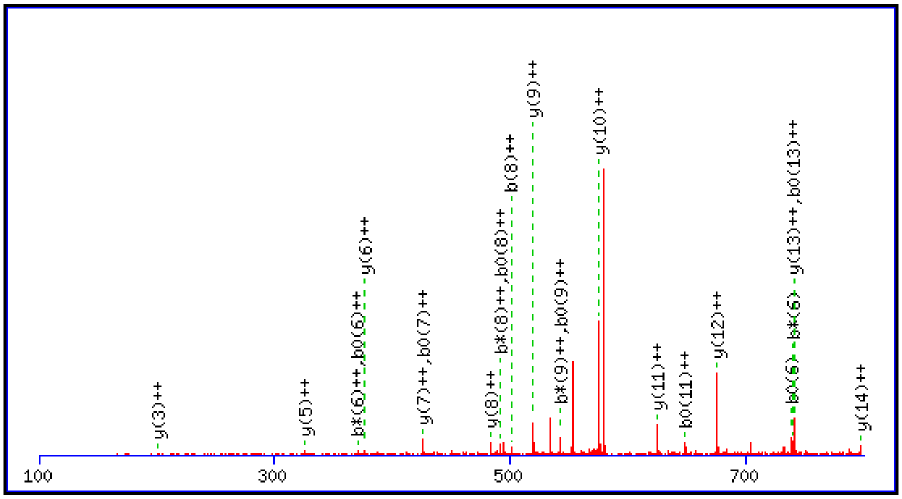

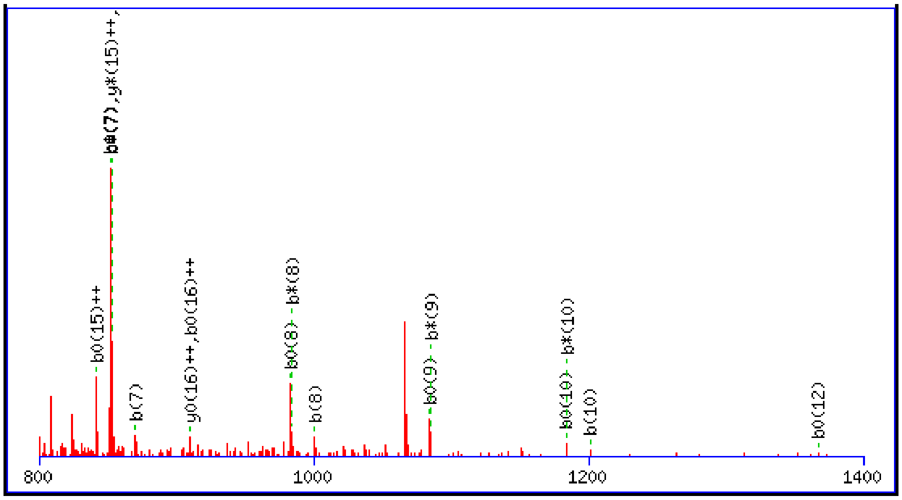

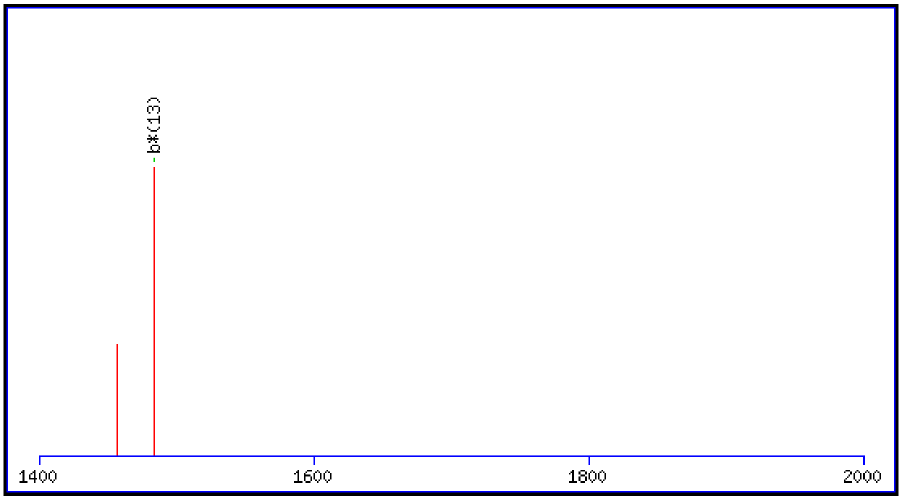

Monoisotopic mass of neutral peptide Mr(calc): 2350.2678
Fixed modifications: Carbamidomethyl (C)
Variable modifications:
N13 : Deamidated_N (N)
Ions Score: 50 **Expect:** 0.00055
Matches (Bold Red): 41/212 fragment ions using 57 most intense peaks

#	b	b++	b*	b*++	b0	b0++	Seq.	y	y++	y*	y*++	y0	y0++	#
1	130.0499	65.5286			112.0393	56.5233	E							20
2	243.1339	122.0706			225.1234	113.0653	I	2222.2325	1111.6199	2205.2060	1103.1066	2204.2220	1102.6146	19
3	358.1609	179.5841			340.1503	170.5788	D	2109.1485	1055.0779	2092.1219	1046.5646	2091.1379	1046.0726	18
4	514.2620	257.6346	497.2354	249.1214	496.2514	248.6293	R	1994.1215	997.5644	1977.0950	989.0511	1976.1110	988.5591	17
5	629.2889	315.1481	612.2624	306.6348	611.2784	306.1428	D	1838.0204	919.5139	1820.9939	911.0006	1820.0099	**910.5086**	16
6	757.3475	379.1774	**740.3210**	**370.6641**	**739.3369**	**370.1721**	Q	1722.9935	862.0004	1705.9669	**853.4871**	1704.9829	**852.9951**	15
7	**870.4316**	435.7194	**853.4050**	427.2061	**852.4210**	**426.7141**	L	1594.9349	**797.9711**	1577.9084	789.4578	1576.9243	788.9658	14
8	**1001.4721**	**501.2397**	**984.4455**	**492.7264**	**983.4615**	**492.2344**	M	1481.8508	**741.4291**	1464.8243	732.9158	1463.8403	732.4238	13
9	1102.5197	551.7635	**1085.4932**	**543.2502**	**1084.5092**	**542.7582**	T	1350.8104	**675.9088**	1333.7838	667.3955	1332.7998	666.9035	12
10	**1201.5882**	601.2977	**1184.5616**	592.7844	**1183.5776**	592.2924	V	1249.7627	**625.3850**	1232.7361	616.8717	1231.7521	616.3797	11
11	1314.6722	657.8397	1297.6457	649.3265	1296.6616	**648.8345**	L	1150.6943	**575.8508**	1133.6677	567.3375	1132.6837	566.8455	10
12	1385.7093	693.3583	1368.6828	684.8450	**1367.6988**	684.3530	A	1037.6102	**519.3087**	1020.5836	510.7955	1019.5996	510.3035	9
13	1500.7363	750.8718	**1483.7097**	742.3585	1482.7257	**741.8665**	N	966.5731	**483.7902**	949.5465	475.2769	948.5625	474.7849	8
14	1599.8047	800.4060	1582.7781	791.8927	1581.7941	791.4007	V	851.5461	**426.2767**	834.5196	417.7634	833.5356	417.2714	7
15	1700.8524	850.9298	1683.8258	842.4165	1682.8418	**841.9245**	T	752.4777	**376.7425**	735.4512	368.2292	734.4672	367.7372	6
16	1837.9113	919.4593	1820.8847	910.9460	1819.9007	**910.4540**	H	651.4301	**326.2187**	634.4035	317.7054			5
17	1950.9953	976.0013	1933.9688	967.4880	1932.9848	966.9960	L	514.3711	257.6892	497.3446	249.1759			4
18	2064.0794	1032.5433	2047.0528	1024.0301	2046.0688	1023.5381	L	401.2871	**201.1472**	384.2605	192.6339			3

19	2177.1635	1089.0854	2160.1369	1080.5721	2159.1529	1080.0801	I	288.2030	144.6051	271.1765	136.0919			2
20							R	175.1190	88.0631	158.0924	79.5498			1

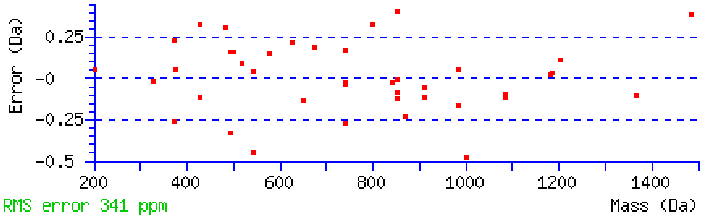

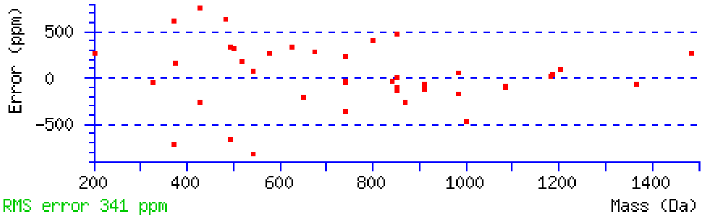

All matches to this query

Score	Mr(calc):	Delta	Sequence
50.5	2350.2678	0.0030	EIDRDQLMTVLANVTHLLIR
6.9	2350.2583	0.0126	AIKGVVVMDSALEALSGSLLIGK
6.6	2349.2838	0.9870	EIDRDQLMTVLANVTHLLIR
1.7	2350.2583	0.0126	AIKGVVVMDSALEALSGSLLIGK
1.0	2348.2488	2.0220	ATPASTPDTRALVADFVGYKLR
0.1	2350.2661	0.0047	ALTLLRIITVGSVASFEAPNAK
0.1	2350.2661	0.0047	ALTLLRIITVGSVASFEAPNAK

Spectrum No: 428; Query: 972; Rank: 1

Peptide View

MS/MS Fragmentation of **DPAVGNISSTCYQPRPDSYHLLR**
Found in **IPI00201120**, Tax_Id=10116 Gene_Symbol=Slc5a2 Low affinity Na-dependent glucose transporter SGLT2 delta e trans

Match to Query 972: 2646.248232 from(883.090020,3+)
Title: 091129RatKid_SCX02_14.2428.2428.3.dta
Data file K:\NewmanPaper\Piliang\3SubProteomes\Piliang3SP\mgf5ppm\SCX_3SubProteomes5ppm.mgf

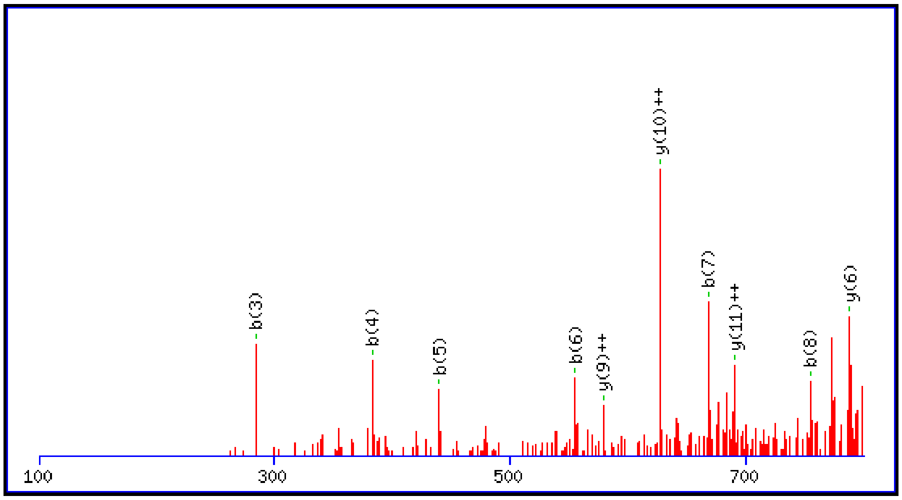

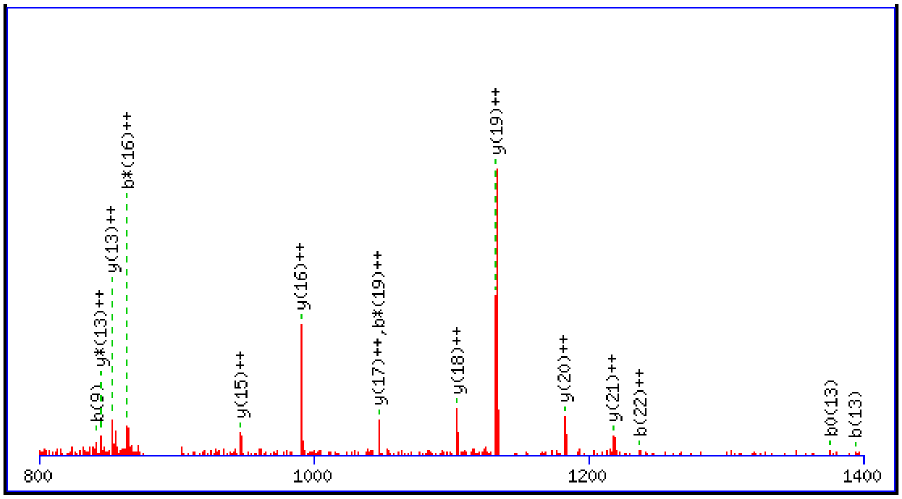

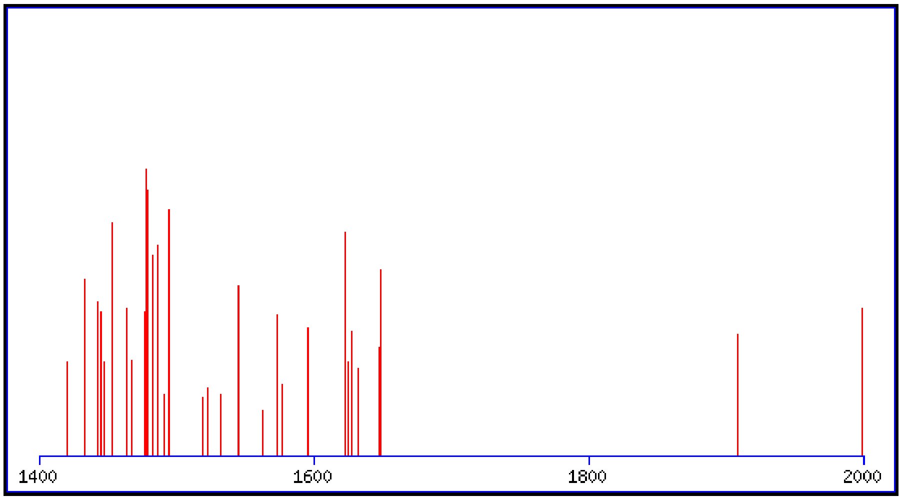

Monoisotopic mass of neutral peptide Mr(calc): 2646.2496
Fixed modifications: Carbamidomethyl (C)
Variable modifications:
N6 : Deamidated_N (N)
Ions Score: 51 **Expect:** 0.0028
Matches (Bold Red): 25/244 fragment ions using 55 most intense peaks

#	b	b^{++}	b*	b^{*++}	b^0	b^{0++}	Seq.	y	y^{++}	y*	y^{*++}	y^0	y^{0++}	#
1	116.0342	58.5207			98.0237	49.5155	D							23
2	213.0870	107.0471			195.0764	98.0418	P	2532.2300	1266.6186	2515.2034	1258.1054	2514.2194	1257.6133	22
3	**284.1241**	142.5657			266.1135	133.5604	A	2435.1772	**1218.0922**	2418.1507	1209.5790	2417.1666	1209.0870	21
4	**383.1925**	192.0999			365.1819	183.0946	V	2364.1401	**1182.5737**	2347.1136	1174.0604	2346.1295	1173.5684	20
5	**440.2140**	220.6106			422.2034	211.6053	G	2265.0717	**1133.0395**	2248.0451	1124.5262	2247.0611	1124.0342	19
6	**555.2409**	278.1241	538.2144	269.6108	537.2303	269.1188	N	2208.0502	**1104.5287**	2191.0237	1096.0155	2190.0397	1095.5235	18
7	**668.3250**	334.6661	651.2984	326.1529	650.3144	325.6608	I	2093.0233	**1047.0153**	2075.9967	1038.5020	2075.0127	1038.0100	17
8	**755.3570**	378.1821	738.3305	369.6689	737.3464	369.1769	S	1979.9392	**990.4732**	1962.9127	981.9600	1961.9287	981.4680	16
9	**842.3890**	421.6982	825.3625	413.1849	824.3785	412.6929	S	1892.9072	**946.9572**	1875.8806	938.4440	1874.8966	937.9520	15
10	943.4367	472.2220	926.4102	463.7087	925.4261	463.2167	T	1805.8752	903.4412	1788.8486	894.9279	1787.8646	894.4359	14
11	1103.4674	552.2373	1086.4408	543.7240	1085.4568	543.2320	C	1704.8275	**852.9174**	1687.8009	**844.4041**	1686.8169	843.9121	13
12	1266.5307	633.7690	1249.5041	625.2557	1248.5201	624.7637	Y	1544.7968	772.9021	1527.7703	764.3888	1526.7863	763.8968	12
13	**1394.5893**	697.7983	1377.5627	689.2850	**1376.5787**	688.7930	Q	1381.7335	**691.3704**	1364.7070	682.8571	1363.7229	682.3651	11
14	1491.6420	746.3247	1474.6155	737.8114	1473.6315	737.3194	P	1253.6749	**627.3411**	1236.6484	618.8278	1235.6644	618.3358	10
15	1647.7431	824.3752	1630.7166	815.8619	1629.7326	815.3699	R	1156.6222	**578.8147**	1139.5956	570.3014	1138.6116	569.8094	9
16	1744.7959	872.9016	1727.7694	**864.3883**	1726.7853	863.8963	P	1000.5211	500.7642	983.4945	492.2509	982.5105	491.7589	8
17	1859.8228	930.4151	1842.7963	921.9018	1841.8123	921.4098	D	903.4683	452.2378	886.4417	443.7245	885.4577	443.2325	7
18	1946.8549	973.9311	1929.8283	965.4178	1928.8443	964.9258	S	**788.4413**	394.7243	771.4148	386.2110	770.4308	385.7190	6

**19**	2109.9182	1055.4627	2092.8917	**1046.9495**	2091.9076	1046.4575	**Y**	701.4093	351.2083	684.3828	342.6950			**5**
**20**	2246.9771	1123.9922	2229.9506	1115.4789	2228.9666	1114.9869	**H**	538.3460	269.6766	521.3194	261.1634			**4**
**21**	2360.0612	1180.5342	2343.0346	1172.0210	2342.0506	1171.5289	**L**	401.2871	201.1472	384.2605	192.6339			**3**
**22**	2473.1452	**1237.0763**	2456.1187	1228.5630	2455.1347	1228.0710	**L**	288.2030	144.6051	271.1765	136.0919			**2**
**23**							**R**	175.1190	88.0631	158.0924	79.5498			**1**

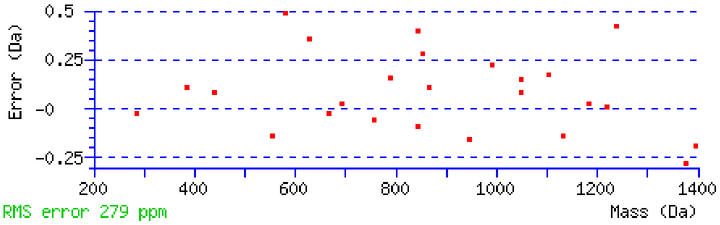

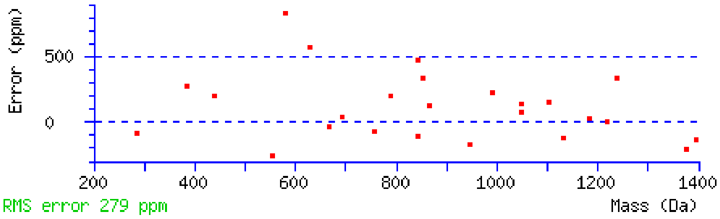

All matches to this query

Score	Mr(calc):	Delta	Sequence
50.5	2646.2496	-0.0014	DPAVGNISSTCYQPRPDSYHLLR
24.4	2645.2656	0.9826	DPAVGNISSTCYQPRPDSYHLLR
6.2	2645.2577	0.9905	LVYLLTDKTGTLTQNEMVFKR
4.8	2646.2479	0.0003	SITRSYYRNSVGGFLVFDITNR
2.2	2646.2417	0.0065	LVYLLTDKTGTLTQNEMVFKR
2.0	2645.2691	0.9792	MTSVFYTLIIPMLNPLIYSLR
1.9	2646.2468	0.0014	KYPEREQHLSSAAHAPLRPATK
1.5	2646.2287	0.0195	ALNLSLEYNFVTALTSLVVIPK
1.4	2646.2543	-0.0061	VQRFHSIHNMNPGPLPPCTDRR
1.1	2646.2686	-0.0203	WLLRATQQFPSPPSHWFFGHK

Spectrum No: 429; Query: 627; Rank: 1

Peptide View

MS/MS Fragmentation of **ISIEMNGTLEDQLSHLK**
Found in **IPI00213463**, Tax_Id=10116 Gene_Symbol=Actn4 Alpha-actinin-4

Match to Query 627: 1927.959368 from(964.986960,2+)
Title: 091129RatKid_SCX02_26.4425.4425.2.dta
Data file K:\NewmanPaper\Piliang\3SubProteomes\Piliang3SP\mgf5ppm\SCX_3SubProteomes5ppm.mgf

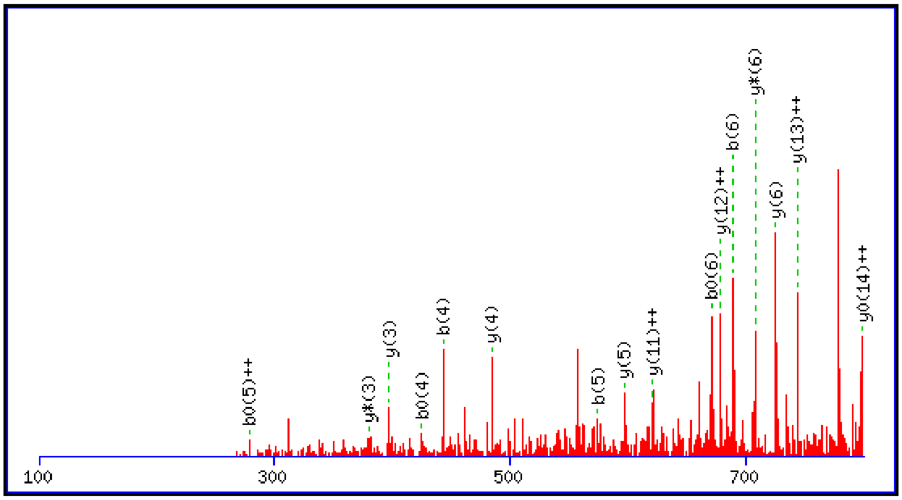

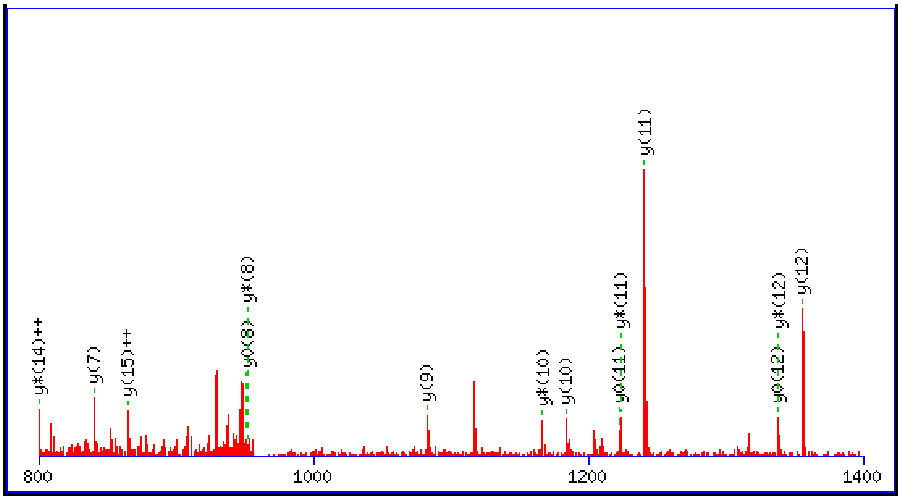

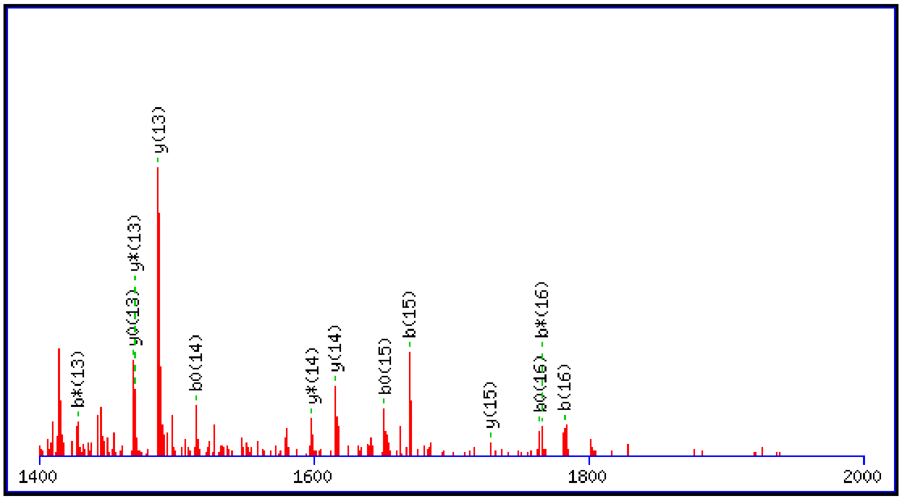

Monoisotopic mass of neutral peptide Mr(calc): 1927.9561
Fixed modifications: Carbamidomethyl (C)
Variable modifications:
N6 : Deamidated_N (N)
Ions Score: 50 **Expect:** 0.0017
Matches (Bold Red): 43/174 fragment ions using 112 most intense peaks

#	b	b^{++}	b*	b^{*++}	b^0	b^{0++}	Seq.	y	y^{++}	y*	y^{*++}	y^0	y^{0++}	#
1	114.0913	57.5493					I							17
2	201.1234	101.0653			183.1128	92.0600	S	1815.8793	908.4433	1798.8528	899.9300	1797.8687	899.4380	16
3	314.2074	157.6074			296.1969	148.6021	I	**1728.8473**	**864.9273**	1711.8207	856.4140	1710.8367	855.9220	15
4	**443.2500**	222.1287			**425.2395**	213.1234	E	**1615.7632**	808.3852	**1598.7367**	**799.8720**	1597.7526	**799.3800**	14
5	**574.2905**	287.6489			556.2799	**278.6436**	M	**1486.7206**	**743.8639**	**1469.6941**	735.3507	**1468.7100**	734.8587	13
6	**689.3174**	345.1624	672.2909	336.6491	**671.3069**	336.1571	N	**1355.6801**	**678.3437**	**1338.6536**	669.8304	**1337.6696**	669.3384	12
7	746.3389	373.6731	729.3124	365.1598	728.3283	364.6678	G	**1240.6532**	**620.8302**	**1223.6266**	612.3170	**1222.6426**	611.8250	11
8	847.3866	424.1969	830.3600	415.6837	829.3760	415.1917	T	**1183.6317**	592.3195	**1166.6052**	583.8062	1165.6212	583.3142	10
9	960.4707	480.7390	943.4441	472.2257	942.4601	471.7337	L	**1082.5841**	541.7957	1065.5575	533.2824	1064.5735	532.7904	9
10	1089.5132	545.2603	1072.4867	536.7470	1071.5027	536.2550	E	969.5000	485.2536	**952.4734**	476.7404	**951.4894**	476.2483	8
11	1204.5402	602.7737	1187.5136	594.2605	1186.5296	593.7685	D	**840.4574**	420.7323	823.4308	412.2191	822.4468	411.7271	7
12	1332.5988	666.8030	1315.5722	658.2897	1314.5882	657.7977	Q	**725.4304**	363.2189	**708.4039**	354.7056	707.4199	354.2136	6
13	1445.6828	723.3451	**1428.6563**	714.8318	1427.6723	714.3398	L	**597.3719**	299.1896	580.3453	290.6763	579.3613	290.1843	5
14	1532.7149	766.8611	1515.6883	758.3478	**1514.7043**	757.8558	S	**484.2878**	242.6475	467.2613	234.1343	466.2772	233.6423	4
15	**1669.7738**	835.3905	1652.7472	826.8772	**1651.7632**	826.3852	H	**397.2558**	199.1315	**380.2292**	190.6183			3
16	**1782.8578**	891.9326	**1765.8313**	883.4193	**1764.8473**	882.9273	L	260.1969	130.6021	243.1703	122.0888			2
17							K	147.1128	74.0600	130.0863	65.5468			1

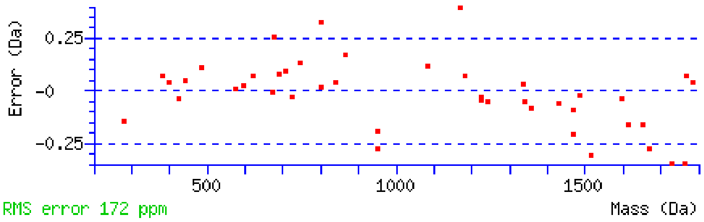

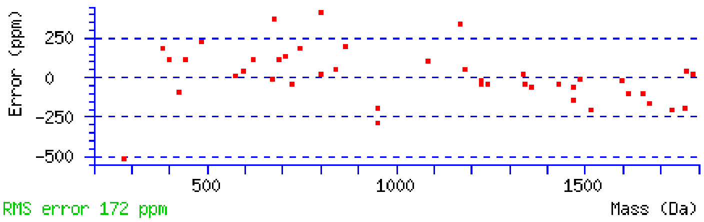

All matches to this query

Score	Mr(calc):	Delta	Sequence
50.3	1927.9561	0.0033	ISIEMNGTLEDQLSHLK
7.2	1926.9720	0.9873	ISIEMNGTLEDQLSHLK
6.0	1927.9639	-0.0046	AGQSVYSFVATEGSREIK
2.9	1927.9785	-0.0191	KMQANNSKAVSAHTEAIK
2.4	1927.9599	-0.0005	QAEEQVEAARKDLDQAK

Spectrum No: 430; Query: 1050; Rank: 1

Peptide View

MS/MS Fragmentation of **VWPDYPNITVDPSLDWDSQVQQYR**
Found in **IPI00191437**, Tax_Id=10116 Gene_Symbol=- 64 kDa protein

Match to Query 1050: 2922.368248 from(1462.191400,2+)
Title: 091127RatKid_SCX01_12.4769.4769.2.dta
Data file K:\NewmanPaper\Piliang\3SubProteomes\Piliang3SP\mgf5ppm\SCX_3SubProteomes5ppm.mgf

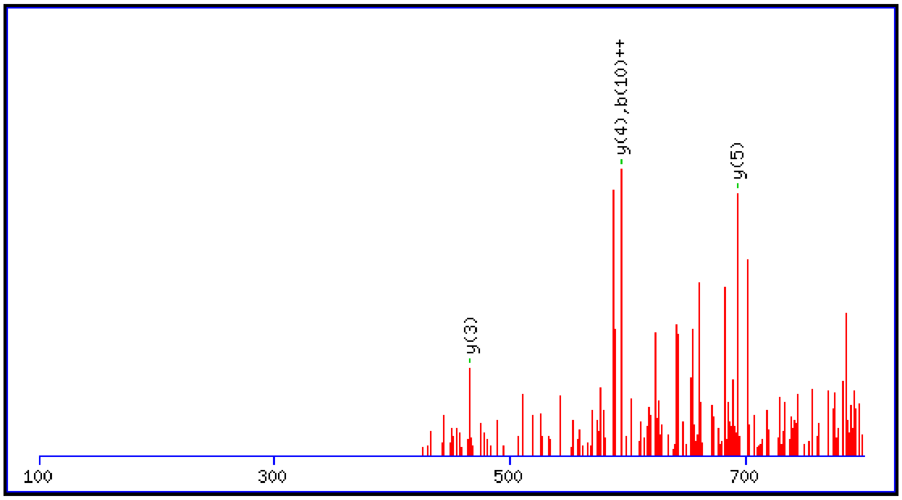

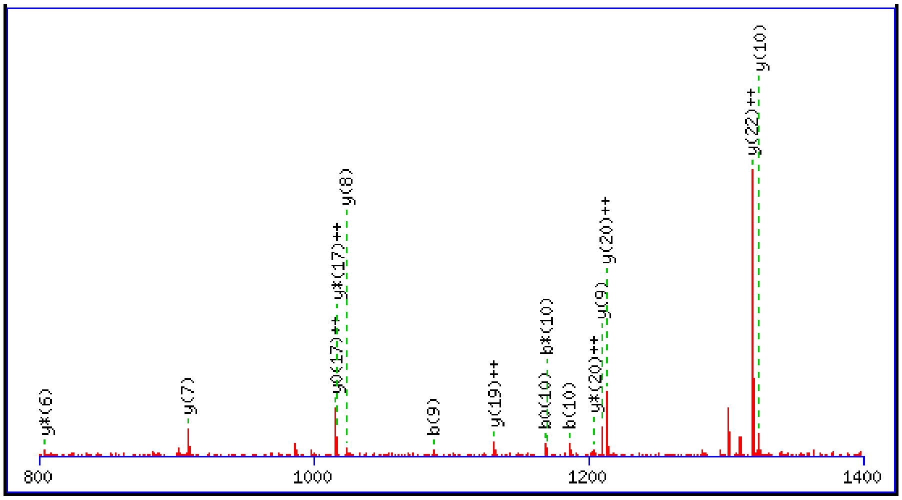

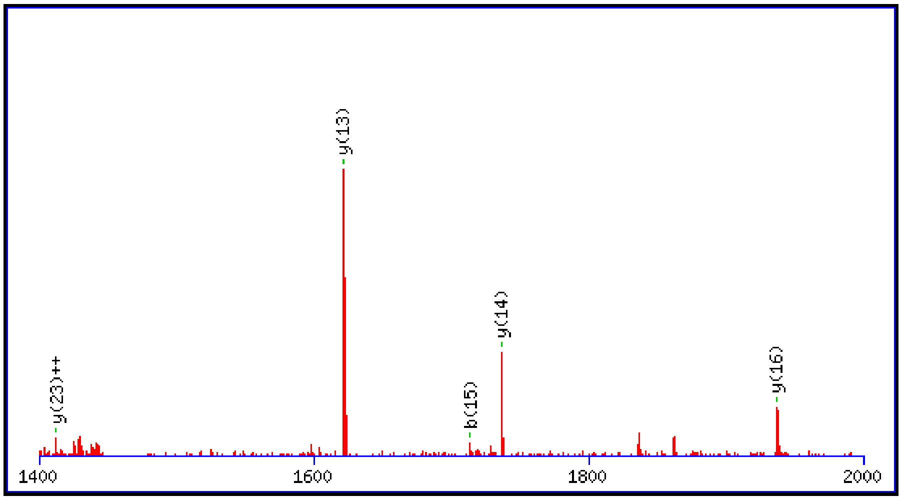

Monoisotopic mass of neutral peptide Mr(calc): 2921.3508
Fixed modifications: Carbamidomethyl (C)
Variable modifications:
N7 : Deamidated_N (N)
Ions Score: 50 **Expect:** 0.0036
Matches (Bold Red): 24/246 fragment ions using 35 most intense peaks

#	b	b^{++}	b*	b^{*++}	b^0	b^{0++}	Seq.	y	y^{++}	y*	y^{*++}	y^0	y^{0++}	#
1	100.0757	50.5415					V							24
2	286.1550	143.5811					W	2823.2897	**1412.1485**	2806.2631	1403.6352	2805.2791	1403.1432	23
3	383.2078	192.1075					P	2637.2103	**1319.1088**	2620.1838	1310.5955	2619.1998	1310.1035	22
4	498.2347	249.6210			480.2241	240.6157	D	2540.1576	1270.5824	2523.1310	1262.0692	2522.1470	1261.5771	21
5	661.2980	331.1527			643.2875	322.1474	Y	2425.1306	**1213.0690**	2408.1041	**1204.5557**	2407.1201	1204.0637	20
6	758.3508	379.6790			740.3402	370.6738	P	2262.0673	**1131.5373**	2245.0408	1123.0240	2244.0567	1122.5320	19
7	873.3777	437.1925	856.3512	428.6792	855.3672	428.1872	N	2165.0145	1083.0109	2147.9880	1074.4976	2147.0040	1074.0056	18
8	986.4618	493.7345	969.4353	485.2213	968.4512	484.7293	I	2049.9876	1025.4974	2032.9611	**1016.9842**	2031.9770	**1016.4922**	17
9	**1087.5095**	544.2584	1070.4829	535.7451	1069.4989	535.2531	T	**1936.9035**	968.9554	1919.8770	960.4421	1918.8930	959.9501	16
10	**1186.5779**	**593.7926**	**1169.5513**	585.2793	**1168.5673**	584.7873	V	1835.8559	918.4316	1818.8293	909.9183	1817.8453	909.4263	15
11	1301.6048	651.3061	1284.5783	642.7928	1283.5943	642.3008	D	**1736.7875**	868.8974	1719.7609	860.3841	1718.7769	859.8921	14
12	1398.6576	699.8324	1381.6311	691.3192	1380.6470	690.8272	P	**1621.7605**	811.3839	1604.7340	802.8706	1603.7499	802.3786	13
13	1485.6896	743.3485	1468.6631	734.8352	1467.6791	734.3432	S	1524.7077	762.8575	1507.6812	754.3442	1506.6972	753.8522	12
14	1598.7737	799.8905	1581.7471	791.3772	1580.7631	790.8852	L	1437.6757	719.3415	1420.6492	710.8282	1419.6652	710.3362	11
15	**1713.8006**	857.4040	1696.7741	848.8907	1695.7901	848.3987	D	**1324.5917**	662.7995	1307.5651	654.2862	1306.5811	653.7942	10
16	1899.8800	950.4436	1882.8534	941.9303	1881.8694	941.4383	W	**1209.5647**	605.2860	1192.5382	596.7727	1191.5541	596.2807	9
17	2014.9069	1007.9571	1997.8803	999.4438	1996.8963	998.9518	D	**1023.4854**	512.2463	1006.4589	503.7331	1005.4748	503.2411	8
18	2101.9389	1051.4731	2084.9124	1042.9598	2083.9284	1042.4678	S	**908.4585**	454.7329	891.4319	446.2196	890.4479	445.7276	7

**19**	2229.9975	1115.5024	2212.9710	1106.9891	2211.9869	1106.4971	**Q**	821.4264	411.2169	**804.3999**	402.7036			**6**
**20**	2329.0659	1165.0366	2312.0394	1156.5233	2311.0553	1156.0313	**V**	**693.3679**	347.1876	676.3413	338.6743			**5**
**21**	2457.1245	1229.0659	2440.0979	1220.5526	2439.1139	1220.0606	**Q**	**594.2994**	297.6534	577.2729	289.1401			**4**
**22**	2585.1831	1293.0952	2568.1565	1284.5819	2567.1725	1284.0899	**Q**	**466.2409**	233.6241	449.2143	225.1108			**3**
**23**	2748.2464	1374.6268	2731.2199	1366.1136	2730.2358	1365.6216	**Y**	338.1823	169.5948	321.1557	161.0815			**2**
**24**							**R**	175.1190	88.0631	158.0924	79.5498			**1**

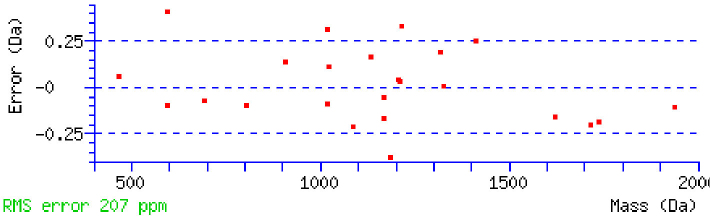

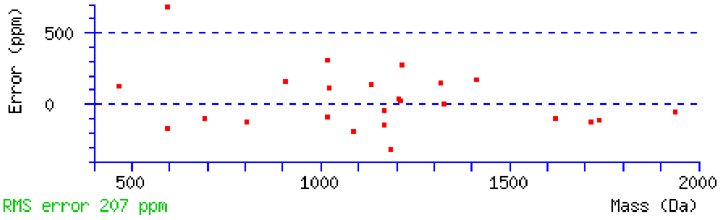

All matches to this query

Score	Mr(calc):	Delta	Sequence
50.2	2921.3508	1.0174	VWPDYPNITVDPSLDWDSQVQQYR
32.5	2920.3668	2.0014	VWPDYPNITVDPSLDWDSQVQQYR
5.2	2921.3766	0.9917	EFPFPTTITAVRTNVANLSDAALWK
2.1	2920.3708	1.9975	LWLRGQGCSVTPALSTDPSVLGKYR
2.1	2920.3708	1.9975	LWLRGQGCSVTPALSTDPSVLGKYR
0.8	2922.3678	0.0004	VDADMNARPHKDEELWNEREPVTR
0.8	2921.3766	0.9917	EFPFPTTITAVRTNVANLSDAALWK
0.8	2921.3766	0.9917	EFPFPTTITAVRTNVANLSDAALWK
0.6	2921.3614	1.0069	HFRGVPMEDSHNIQTHTNSLDLLDK
0.6	2922.3860	-0.0178	ERVRIDMHADGTQDLMMMIIGAPFK

Spectrum No: 431; Query: 968; Rank: 1

Peptide View

MS/MS Fragmentation of **LVIILDPAISNNSLSSNPYGPYDR**
Found in **IPI00193894**, Tax_Id=10116 Gene_Symbol=Mgam_predicted 198 kDa protein

Match to Query 968: 2618.329812 from(873.783880,3+)
Title: 091127RatKid_SCX01_13.4656.4656.3.dta
Data file K:\NewmanPaper\Piliang\3SubProteomes\Piliang3SP\mgf5ppm\SCX_3SubProteomes5ppm.mgf

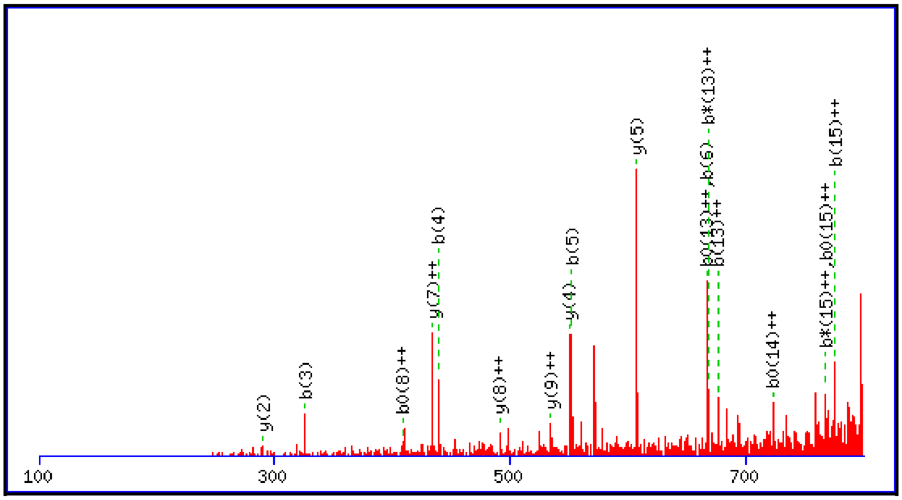

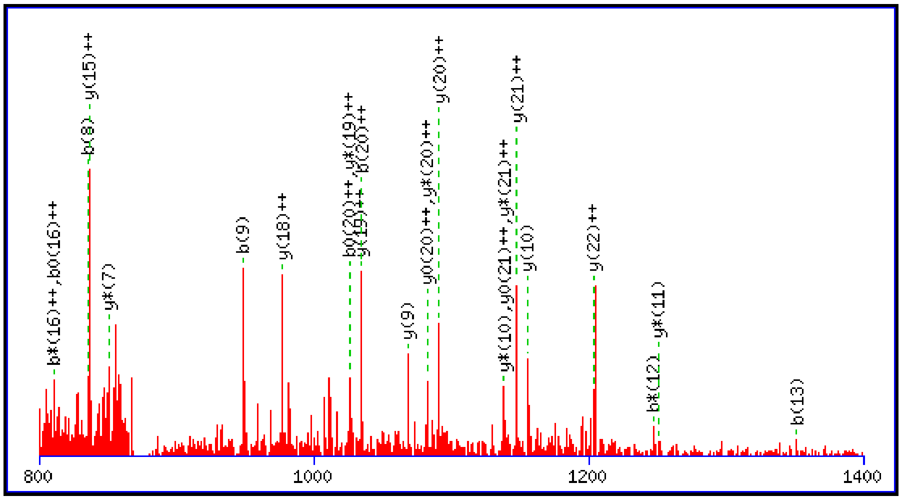

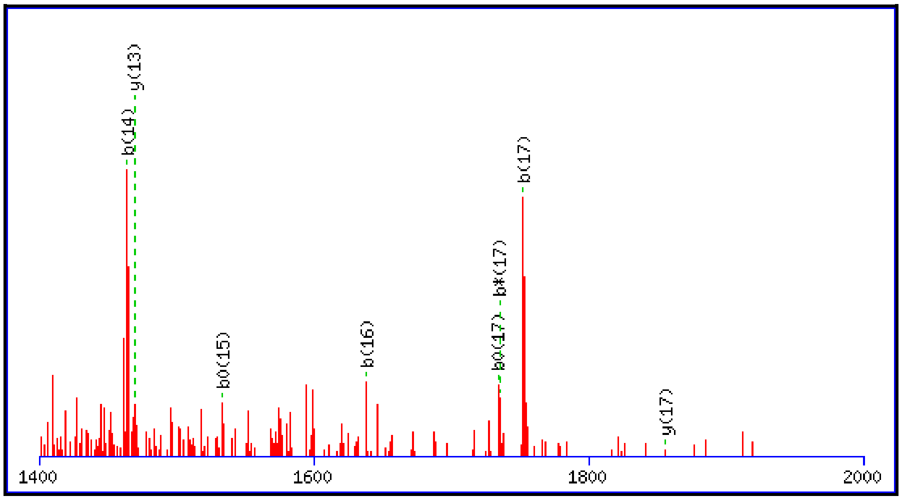

Monoisotopic mass of neutral peptide Mr(calc): 2618.3228
Fixed modifications: Carbamidomethyl (C)
Variable modifications:
N11 : Deamidated_N (N)
Ions Score: 50 **Expect:** 0.0017
Matches (Bold Red): 50/244 fragment ions using 90 most intense peaks

#	b	b^{++}	b*	b^{*++}	b^0	b^{0++}	Seq.	y	y^{++}	y*	y^{*++}	y^0	y^{0++}	#
1	114.0913	57.5493					L							24
2	213.1598	107.0835					V	2506.2460	1253.6266	2489.2195	1245.1134	2488.2354	1244.6214	23
3	**326.2438**	163.6255					I	2407.1776	**1204.0924**	2390.1510	1195.5792	2389.1670	1195.0871	22
4	**439.3279**	220.1676					I	2294.0935	**1147.5504**	2277.0670	**1139.0371**	2276.0830	**1138.5451**	21
5	**552.4119**	276.7096					L	2181.0095	**1091.0084**	2163.9829	**1082.4951**	2162.9989	**1082.0031**	20
6	**667.4389**	334.2231			649.4283	325.2178	D	2067.9254	**1034.4663**	2050.8988	**1025.9531**	2049.9148	1025.4611	19
7	764.4917	382.7495			746.4811	373.7442	P	1952.8985	**976.9529**	1935.8719	968.4396	1934.8879	967.9476	18
8	**835.5288**	418.2680			817.5182	**409.2627**	A	**1855.8457**	928.4265	1838.8191	919.9132	1837.8351	919.4212	17
9	**948.6128**	474.8101			930.6023	465.8048	I	1784.8086	892.9079	1767.7820	884.3947	1766.7980	883.9026	16
10	1035.6449	518.3261			1017.6343	509.3208	S	1671.7245	**836.3659**	1654.6980	827.8526	1653.7139	827.3606	15
11	1150.6718	575.8395	1133.6452	567.3263	1132.6612	566.8343	N	1584.6925	792.8499	1567.6659	784.3366	1566.6819	783.8446	14
12	1264.7147	632.8610	**1247.6882**	624.3477	1246.7042	623.8557	N	**1469.6655**	735.3364	1452.6390	726.8231	1451.6550	726.3311	13
13	**1351.7468**	**676.3770**	1334.7202	**667.8637**	1333.7362	**667.3717**	S	1355.6226	678.3149	1338.5961	669.8017	1337.6121	669.3097	12
14	**1464.8308**	732.9190	1447.8043	724.4058	1446.8202	**723.9138**	L	1268.5906	634.7989	**1251.5640**	626.2857	1250.5800	625.7937	11
15	1551.8628	**776.4351**	1534.8363	**767.9218**	**1533.8523**	**767.4298**	S	**1155.5065**	578.2569	**1138.4800**	569.7436	1137.4960	569.2516	10
16	**1638.8949**	819.9511	1621.8683	**811.4378**	1620.8843	**810.9458**	S	**1068.4745**	**534.7409**	1051.4480	526.2276	1050.4639	525.7356	9
17	**1752.9378**	876.9725	**1735.9112**	868.4593	**1734.9272**	867.9673	N	981.4425	**491.2249**	964.4159	482.7116	963.4319	482.2196	8
18	1849.9906	925.4989	1832.9640	916.9856	1831.9800	916.4936	P	867.3995	**434.2034**	**850.3730**	425.6901	849.3890	425.1981	7

**19**	2013.0539	1007.0306	1996.0273	998.5173	1995.0433	998.0253	**Y**	770.3468	385.6770	753.3202	377.1638	752.3362	376.6717	**6**
**20**	2070.0754	**1035.5413**	2053.0488	1027.0280	2052.0648	**1026.5360**	**G**	**607.2835**	304.1454	590.2569	295.6321	589.2729	295.1401	**5**
**21**	2167.1281	1084.0677	2150.1016	1075.5544	2149.1176	1075.0624	**P**	**550.2620**	275.6346	533.2354	267.1214	532.2514	266.6293	**4**
**22**	2330.1914	1165.5994	2313.1649	1157.0861	2312.1809	1156.5941	**Y**	453.2092	227.1082	436.1827	218.5950	435.1987	218.1030	**3**
**23**	2445.2184	1223.1128	2428.1918	1214.5996	2427.2078	1214.1076	**D**	**290.1459**	145.5766	273.1193	137.0633	272.1353	136.5713	**2**
**24**							**R**	175.1190	88.0631	158.0924	79.5498			**1**

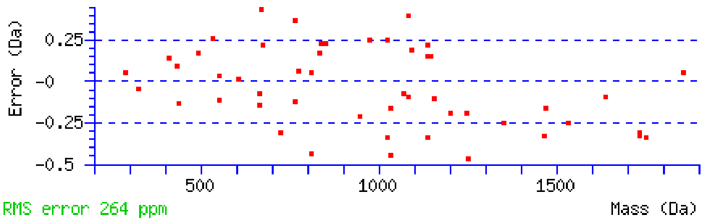

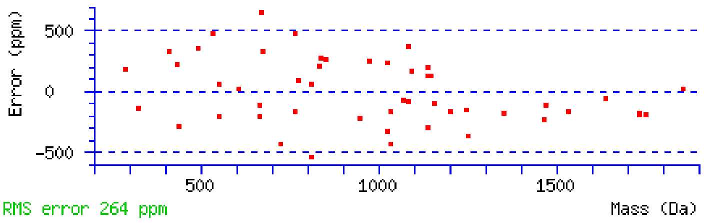

All matches to this query

Score	Mr(calc):	Delta	Sequence
50.2	2618.3228	0.0071	LVIILDPAISNNSLSSNPYGPYDR
49.7	2618.3228	0.0071	LVIILDPAISNNSLSSNPYGPYDR
30.3	2618.3228	0.0071	LVIILDPAISNNSLSSNPYGPYDR
21.1	2617.3387	0.9911	LVIILDPAISNNSLSSNPYGPYDR
1.5	2618.3551	-0.0253	QNLSQQLDVKLVPSSYSEAVASVR

Spectrum No: 432; Query: 75; Rank: 1

Peptide View

MS/MS Fragmentation of **EKIEENGSMR**
Found in **IPI00191711**, Tax_Id=10116 Gene_Symbol=LOC259246;LOC298116;LOC259245 Major urinary protein precursor

Match to Query 75: 1192.539088 from(597.276820,2+)
Title: 091127RatKid_SCX01_34.221.221.2.dta
Data file K:\NewmanPaper\Piliang\3SubProteomes\Piliang3SP\mgf5ppm\SCX_3SubProteomes5ppm.mgf

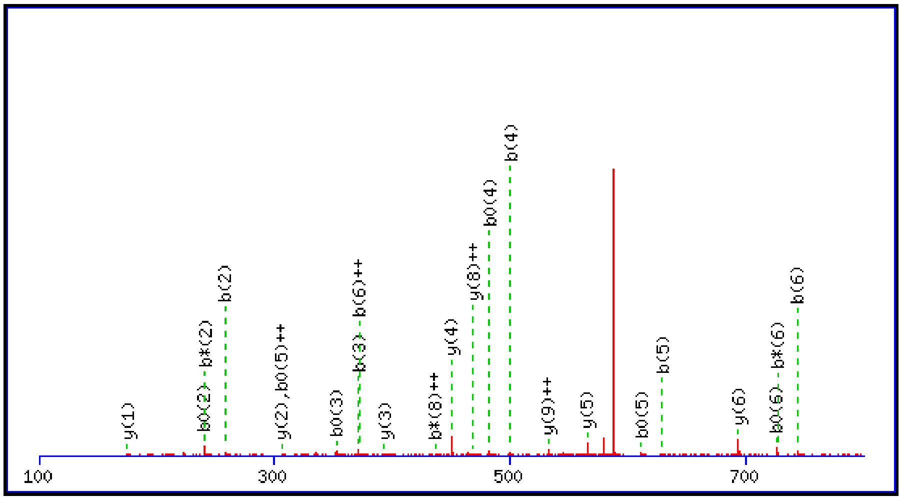

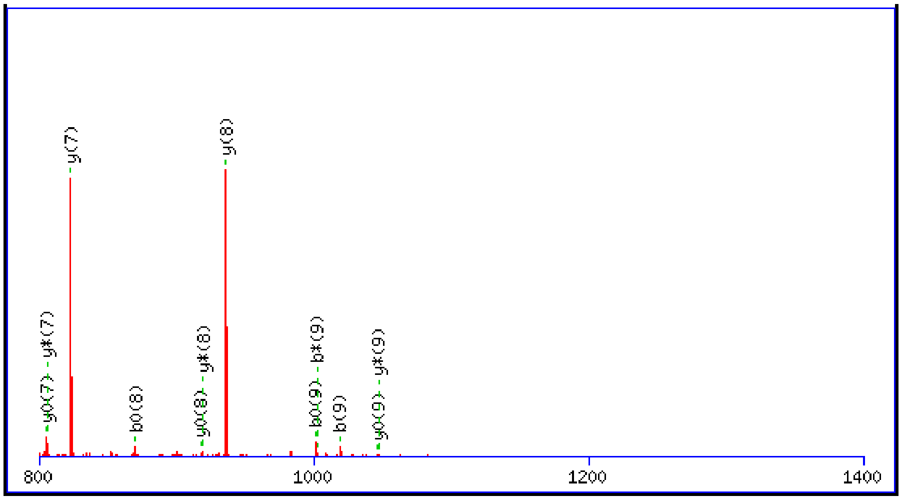

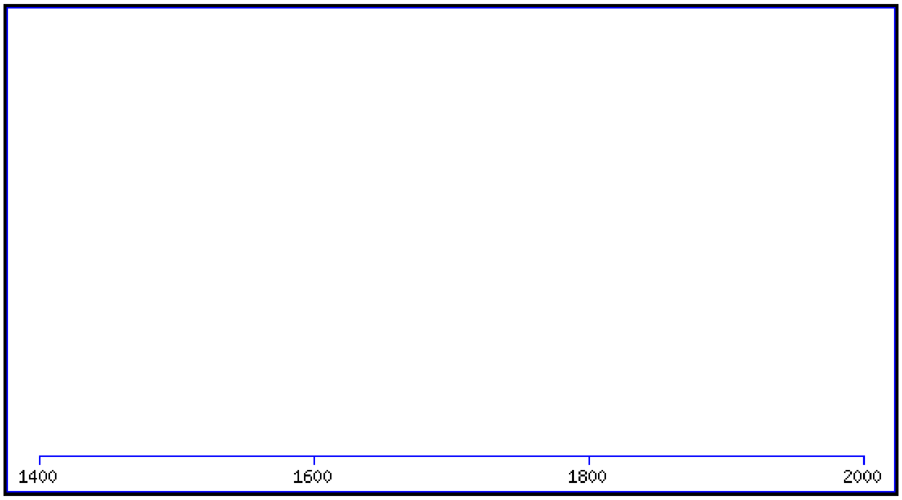

Monoisotopic mass of neutral peptide Mr(calc): 1192.5394
Fixed modifications: Carbamidomethyl (C)
Variable modifications:
N6 : Deamidated_N (N)
Ions Score: 50 **Expect:** 0.0011
Matches (Bold Red): 35/102 fragment ions using 63 most intense peaks

#	b	b^{++}	b*	b^{*++}	b^0	b^{0++}	Seq.	y	y^{++}	y*	y^{*++}	y^0	y^{0++}	#
1	130.0499	65.5286			112.0393	56.5233	E							10
2	**258.1448**	129.5761	**241.1183**	121.0628	**240.1343**	120.5708	K	1064.5041	**532.7557**	**1047.4775**	524.2424	**1046.4935**	523.7504	9
3	**371.2289**	186.1181	354.2023	177.6048	**353.2183**	177.1128	I	**936.4091**	**468.7082**	**919.3826**	460.1949	**918.3986**	459.7029	8
4	**500.2715**	250.6394	483.2449	242.1261	**482.2609**	241.6341	E	**823.3251**	412.1662	**806.2985**	403.6529	**805.3145**	403.1609	7
5	**629.3141**	315.1607	612.2875	306.6474	**611.3035**	**306.1554**	E	**694.2825**	347.6449	677.2559	339.1316	676.2719	338.6396	6
6	**744.3410**	**372.6741**	**727.3145**	364.1609	**726.3305**	363.6689	N	**565.2399**	283.1236	548.2133	274.6103	547.2293	274.1183	5
7	801.3625	401.1849	784.3359	392.6716	783.3519	392.1796	G	**450.2129**	225.6101	433.1864	217.0968	432.2024	216.6048	4
8	888.3945	444.7009	871.3680	**436.1876**	**870.3839**	435.6956	S	**393.1915**	197.0994	376.1649	188.5861	375.1809	188.0941	3
9	**1019.4350**	510.2211	**1002.4084**	501.7079	**1001.4244**	501.2159	M	**306.1594**	153.5834	289.1329	145.0701			2
10							R	**175.1190**	88.0631	158.0924	79.5498			1

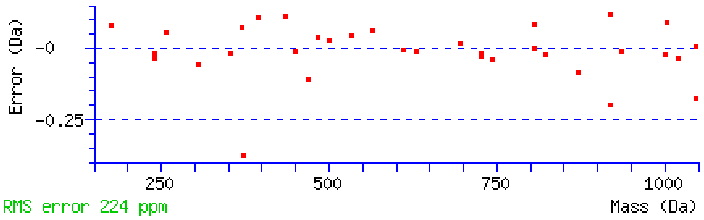

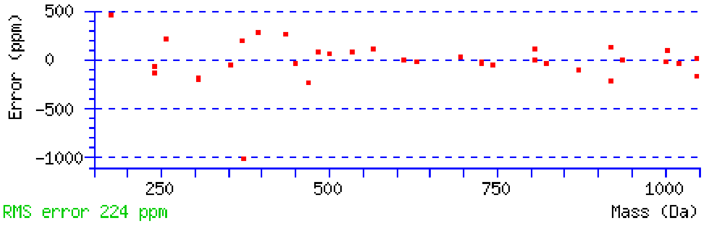

All matches to this query

Score	Mr(calc):	Delta	Sequence
50.1	1192.5394	-0.0003	EKIEENGSMR
27.9	1192.5394	-0.0003	QELDLNSSMR
23.9	1192.5394	-0.0003	IEVANTDGSMR
15.7	1192.5424	-0.0033	GEAMPPLRSR
9.5	1192.5506	-0.0115	KCSPNKDSTR
8.7	1192.5403	-0.0012	LAVSPMCMER
7.3	1192.5278	0.0113	QKDLSGFYR
6.2	1192.5424	-0.0033	CAHXEIKSR
6.2	1192.5424	-0.0033	CAHXEIKSR
4.8	1192.5462	-0.0071	QSHGSTRGKR

Spectrum No: 433; Query: 284; Rank: 1

Peptide View

MS/MS Fragmentation of **NSTTDQVYQAIAAK**
Found in **IPI00203641**, Tax_Id=10116 Gene_Symbol=Snx27 Isoform 2 of Sorting nexin-27

Match to Query 284: 1509.733768 from(755.874160,2+)
Title: 091129RatKid_SCX02_13.2036.2036.2.dta
Data file K:\NewmanPaper\Piliang\3SubProteomes\Piliang3SP\mgf5ppm\SCX_3SubProteomes5ppm.mgf

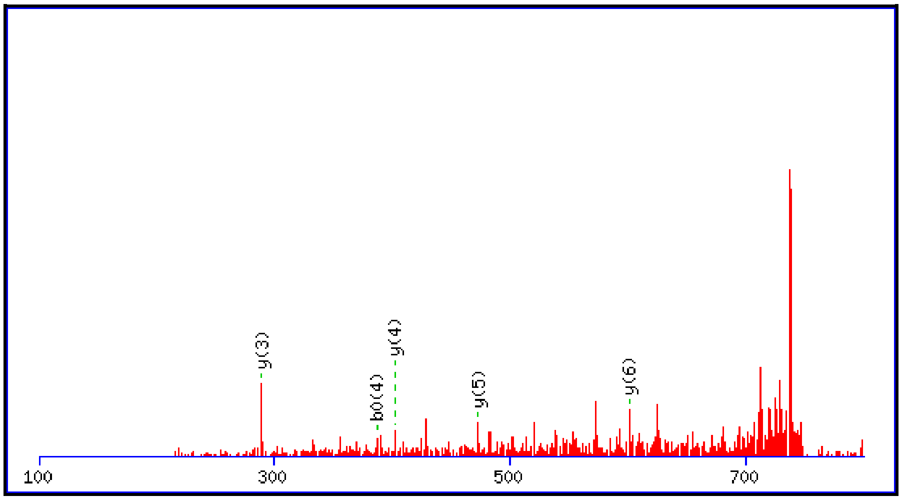

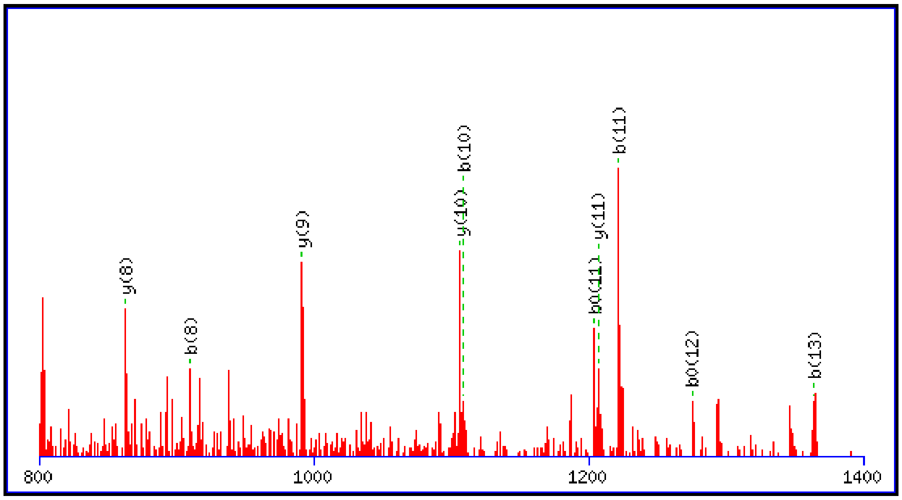

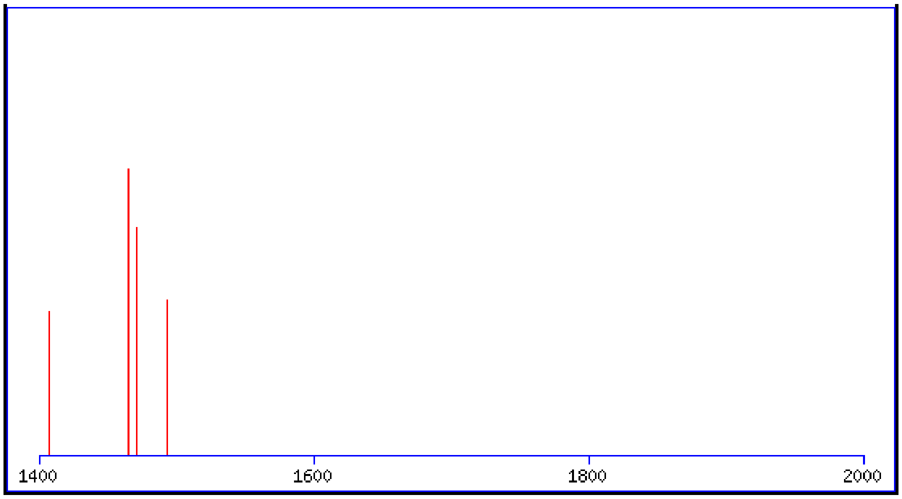

Monoisotopic mass of neutral peptide Mr(calc): 1509.7311
Fixed modifications: Carbamidomethyl (C)
Variable modifications:
N1 : Deamidated_N (N)
Ions Score: 50 **Expect:** 0.0019
Matches (Bold Red): 15/136 fragment ions using 39 most intense peaks

#	b	b^{++}	b^{*}	b^{*++}	b^{0}	b^{0++}	Seq.	y	y^{++}	y^{*}	y^{*++}	y^{0}	y^{0++}	#
1	116.0342	58.5207	99.0077	50.0075			N							14
2	203.0662	102.0368	186.0397	93.5235	185.0557	93.0315	S	1395.7114	698.3594	1378.6849	689.8461	1377.7009	689.3541	13
3	304.1139	152.5606	287.0874	144.0473	286.1034	143.5553	T	1308.6794	654.8433	1291.6529	646.3301	1290.6688	645.8381	12
4	405.1616	203.0844	388.1350	194.5712	**387.1510**	194.0792	T	**1207.6317**	604.3195	1190.6052	595.8062	1189.6212	595.3142	11
5	520.1885	260.5979	503.1620	252.0846	502.1780	251.5926	D	**1106.5841**	553.7957	1089.5575	545.2824	1088.5735	544.7904	10
6	648.2471	324.6272	631.2206	316.1139	630.2366	315.6219	Q	**991.5571**	496.2822	974.5306	487.7689			9
7	747.3155	374.1614	730.2890	365.6481	729.3050	365.1561	V	**863.4985**	432.2529	846.4720	423.7396			8
8	**910.3789**	455.6931	893.3523	447.1798	892.3683	446.6878	Y	764.4301	382.7187	747.4036	374.2054			7
9	1038.4374	519.7224	1021.4109	511.2091	1020.4269	510.7171	Q	**601.3668**	301.1870	584.3402	292.6738			6
10	**1109.4746**	555.2409	1092.4480	546.7276	1091.4640	546.2356	A	**473.3082**	237.1577	456.2817	228.6445			5
11	**1222.5586**	611.7829	1205.5321	603.2697	**1204.5481**	602.7777	I	**402.2711**	201.6392	385.2445	193.1259			4
12	1293.5957	647.3015	1276.5692	638.7882	**1275.5852**	638.2962	A	**289.1870**	145.0972	272.1605	136.5839			3
13	**1364.6328**	682.8201	1347.6063	674.3068	1346.6223	673.8148	A	218.1499	109.5786	201.1234	101.0653			2
14							K	147.1128	74.0600	130.0863	65.5468			1

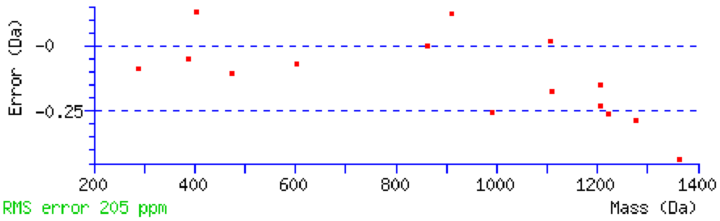

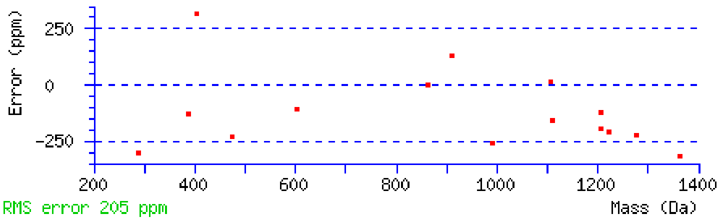

All matches to this query

Score	Mr(calc):	Delta	Sequence
49.8	1509.7311	0.0027	NSTTDQVYQAIAAK
17.9	1509.7374	-0.0037	VMGAVTALSQALNR
17.9	1509.7374	-0.0037	VMGAVTALSQALNR
5.2	1509.7301	0.0037	SSLDSQVLGVRGGR
4.5	1509.7331	0.0007	QRCAHEAGQARAR
4.3	1509.7262	0.0075	SSXTPLGQCVIAAK
4.3	1509.7262	0.0075	SSXTPLGQCVIAAK

3.6	1509.7188	0.0149	ERNTDTLGLQVGK
3.6	1509.7262	0.0075	SSXTPLGQCVIAAK
2.6	1509.7188	0.0150	NESAAEKALQTLR

Spectrum No: 434; Query: 431; Rank: 1

Peptide View

MS/MS Fragmentation of **VGVEAFFPNGTQFSR**
Found in **IPI00204808**, Tax_Id=10116 Gene_Symbol=Mep1b Meprin A subunit beta precursor

Match to Query 431: 1655.798588 from(828.906570,2+)
Title: 091129RatKid_SCX02_17.3368.3368.2.dta
Data file K:\NewmanPaper\Piliang\3SubProteomes\Piliang3SP\mgf5ppm\SCX_3SubProteomes5ppm.mgf

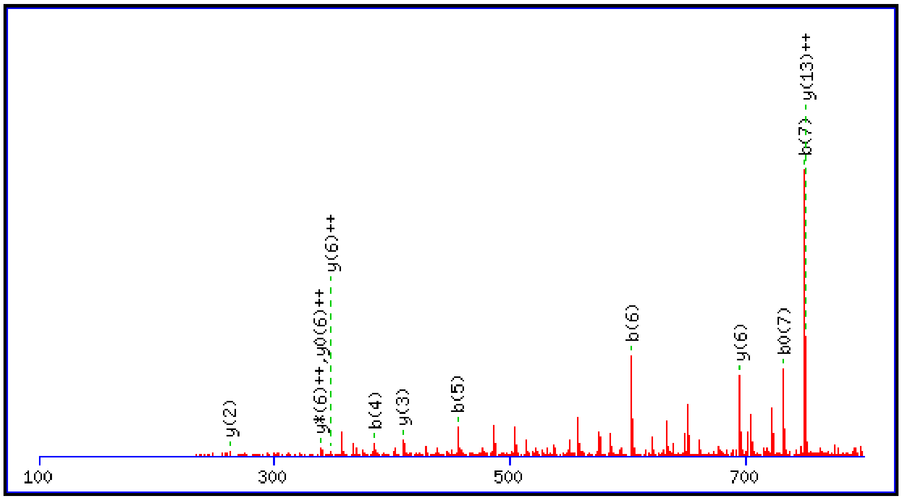

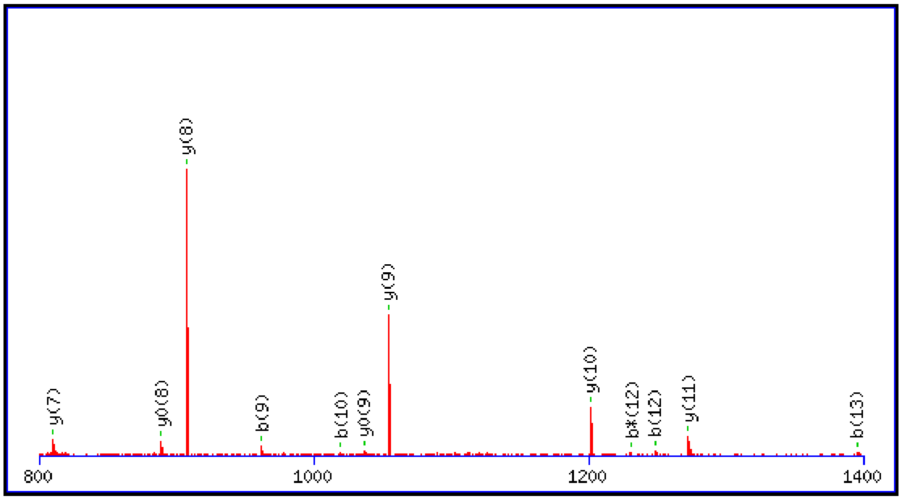

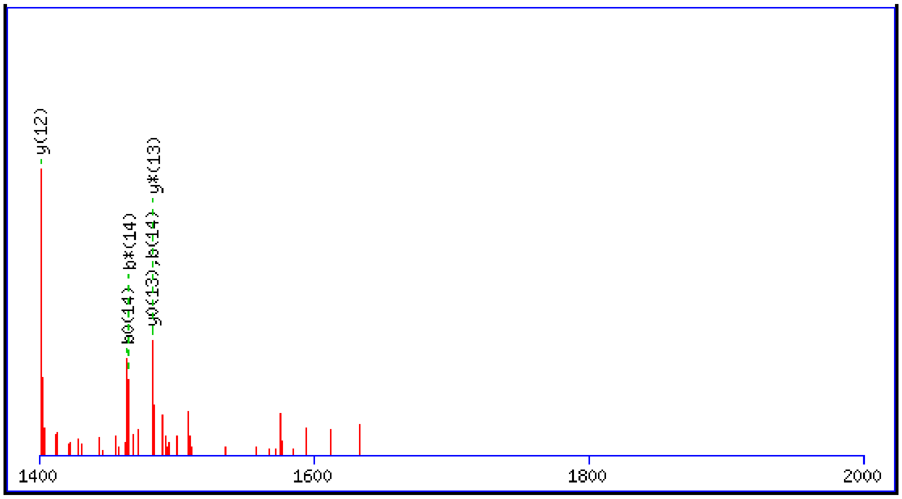

Monoisotopic mass of neutral peptide Mr(calc): 1655.7944
Fixed modifications: Carbamidomethyl (C)
Variable modifications:
N9 : Deamidated_N (N)
Ions Score: 50 **Expect:** 0.002
Matches (Bold Red): 30/144 fragment ions using 61 most intense peaks

#	b	b^{++}	b*	b^{*++}	b^0	b^{0++}	Seq.	y	y^{++}	y*	y^{*++}	y^0	y^{0++}	#
1	100.0757	50.5415					V							15
2	157.0972	79.0522					G	1557.7332	779.3703	1540.7067	770.8570	1539.7227	770.3650	14
3	256.1656	128.5864					V	1500.7118	**750.8595**	**1483.6852**	742.3462	**1482.7012**	741.8542	13
4	**385.2082**	193.1077			367.1976	184.1024	E	**1401.6434**	701.3253	1384.6168	692.8120	1383.6328	692.3200	12
5	**456.2453**	228.6263			438.2347	219.6210	A	**1272.6008**	636.8040	1255.5742	628.2907	1254.5902	627.7987	11
6	**603.3137**	302.1605			585.3031	293.1552	F	**1201.5636**	601.2855	1184.5371	592.7722	1183.5531	592.2802	10
7	**750.3821**	375.6947			**732.3715**	366.6894	F	**1054.4952**	527.7513	1037.4687	519.2380	**1036.4847**	518.7460	9
8	847.4349	424.2211			829.4243	415.2158	P	**907.4268**	454.2170	890.4003	445.7038	**889.4163**	445.2118	8
9	**962.4618**	481.7345	945.4353	473.2213	944.4512	472.7293	N	**810.3741**	405.6907	793.3475	397.1774	792.3635	396.6854	7
10	**1019.4833**	510.2453	1002.4567	501.7320	1001.4727	501.2400	G	**695.3471**	**348.1772**	678.3206	**339.6639**	677.3366	**339.1719**	6
11	1120.5309	560.7691	1103.5044	552.2558	1102.5204	551.7638	T	638.3257	319.6665	621.2991	311.1532	620.3151	310.6612	5
12	**1248.5895**	624.7984	**1231.5630**	616.2851	1230.5790	615.7931	Q	537.2780	269.1426	520.2514	260.6293	519.2674	260.1373	4
13	**1395.6579**	698.3326	1378.6314	689.8193	1377.6474	689.3273	F	**409.2194**	205.1133	392.1928	196.6001	391.2088	196.1081	3
14	**1482.6900**	741.8486	**1465.6634**	733.3353	**1464.6794**	732.8433	S	**262.1510**	131.5791	245.1244	123.0659	244.1404	122.5738	2
15							R	175.1190	88.0631	158.0924	79.5498			1

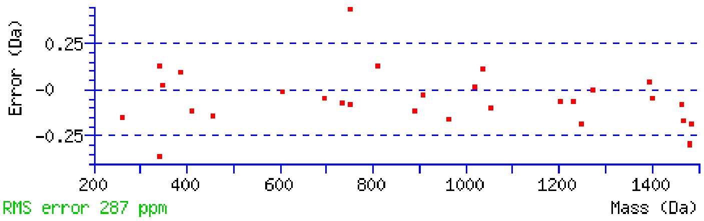

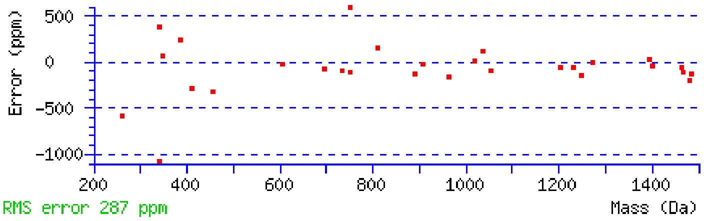

All matches to this query

Score	Mr(calc):	Delta	Sequence
49.7	1655.7944	0.0042	VGVEAFFPNGTQFSR
12.1	1654.8001	0.9985	RDTMLGVSIVLENK
11.7	1654.7941	1.0045	ATSPFRSPVRGASSR
9.3	1655.8106	-0.0120	SPPQLMISKVFSAR
9.3	1655.7821	0.0165	SSFASLFSFRRSGK
9.3	1655.7821	0.0165	SSFASLFSFRRSGK

8.5	1654.8001	0.9985	RDTMLGVSIVLENK
5.7	1655.7821	0.0165	SSFASLFSFRRSGK
5.5	1654.8039	0.9946	ATLAEVATVSSGSARR
5.5	1654.8039	0.9946	ATLAEVATVSSGSARR

Spectrum No: 435; Query: 970; Rank: 1

Peptide View

MS/MS Fragmentation of **LEDLTTPYVRDEELNCTVVELK**
Found in **IPI00211075**, Tax_Id=10116 Gene_Symbol=Serpina3n Serine protease inhibitor A3N precursor

Match to Query 970: 2636.297832 from(879.773220,3+)
Title: 091127RatKid_SCX01_13.3848.3848.3.dta
Data file K:\NewmanPaper\Piliang\3SubProteomes\Piliang3SP\mgf5ppm\SCX_3SubProteomes5ppm.mgf

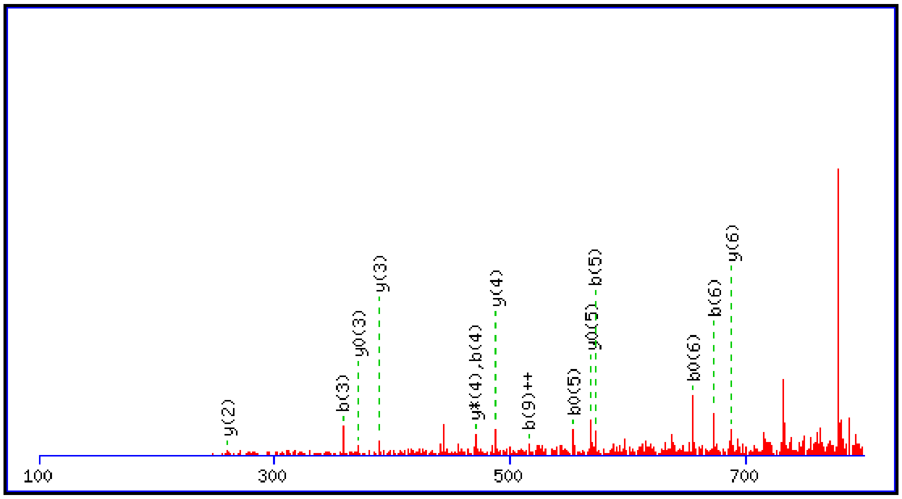

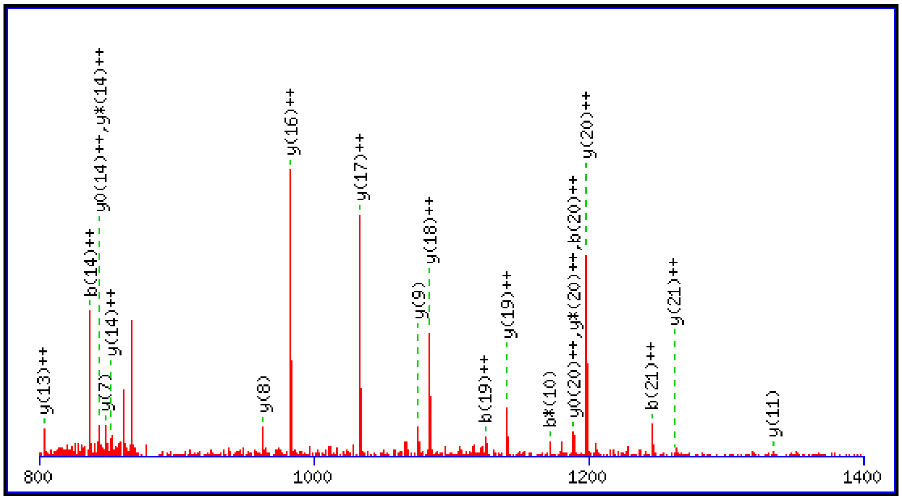

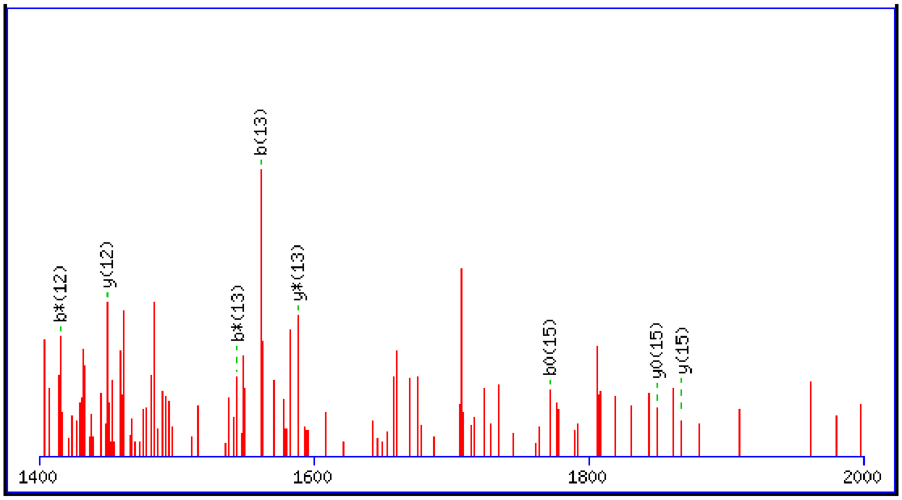

Monoisotopic mass of neutral peptide Mr(calc): 2636.2891
Fixed modifications: Carbamidomethyl (C)
Variable modifications:
N15 : Deamidated_N (N)
Ions Score: 50 **Expect:** 0.0024
Matches (Bold Red): 43/228 fragment ions using 106 most intense peaks

#	b	b^{++}	b*	b^{*++}	b^{0}	b^{0++}	Seq.	y	y^{++}	y*	y^{*++}	y^{0}	y^{0++}	#
1	114.0913	57.5493					L							22
2	243.1339	122.0706			225.1234	113.0653	E	2524.2123	**1262.6098**	2507.1858	1254.0965	2506.2018	1253.6045	21
3	**358.1609**	179.5841			340.1503	170.5788	D	2395.1697	**1198.0885**	2378.1432	**1189.5752**	2377.1592	**1189.0832**	20
4	**471.2449**	236.1261			453.2344	227.1208	L	2280.1428	**1140.5750**	2263.1162	1132.0618	2262.1322	1131.5697	19
5	**572.2926**	286.6499			**554.2821**	277.6447	T	2167.0587	**1084.0330**	2150.0322	1075.5197	2149.0482	1075.0277	18
6	**673.3403**	337.1738			**655.3297**	328.1685	T	2066.0110	**1033.5092**	2048.9845	1024.9959	2048.0005	1024.5039	17
7	770.3931	385.7002			752.3825	376.6949	P	1964.9634	**982.9853**	1947.9368	974.4720	1946.9528	973.9800	16
8	933.4564	467.2318			915.4458	458.2266	Y	**1867.9106**	934.4589	1850.8841	925.9457	**1849.9000**	925.4537	15
9	1032.5248	**516.7660**			1014.5142	507.7608	V	1704.8473	**852.9273**	1687.8207	**844.4140**	1686.8367	**843.9220**	14
10	1188.6259	594.8166	**1171.5994**	586.3033	1170.6154	585.8113	R	1605.7789	**803.3931**	**1588.7523**	794.8798	1587.7683	794.3878	13
11	1303.6529	652.3301	1286.6263	643.8168	1285.6423	643.3248	D	**1449.6777**	725.3425	1432.6512	716.8292	1431.6672	716.3372	12
12	1432.6955	716.8514	**1415.6689**	708.3381	1414.6849	707.8461	E	**1334.6508**	667.8290	1317.6243	659.3158	1316.6402	658.8238	11
13	**1561.7380**	781.3727	**1544.7115**	772.8594	1543.7275	772.3674	E	1205.6082	603.3077	1188.5817	594.7945	1187.5976	594.3025	10
14	1674.8221	**837.9147**	1657.7956	829.4014	1656.8115	828.9094	L	**1076.5656**	538.7864	1059.5391	530.2732	1058.5551	529.7812	9
15	1789.8490	895.4282	1772.8225	886.9149	**1771.8385**	886.4229	N	**963.4816**	482.2444	946.4550	473.7311	945.4710	473.2391	8
16	1949.8797	975.4435	1932.8531	966.9302	1931.8691	966.4382	C	**848.4546**	424.7309	831.4281	416.2177	830.4441	415.7257	7
17	2050.9274	1025.9673	2033.9008	1017.4541	2032.9168	1016.9620	T	**688.4240**	344.7156	671.3974	336.2023	670.4134	335.7103	6
18	2149.9958	1075.5015	2132.9692	1066.9883	2131.9852	1066.4962	V	587.3763	294.1918	570.3497	285.6785	**569.3657**	285.1865	5
19	2249.0642	**1125.0357**	2232.0377	1116.5225	2231.0536	1116.0305	V	**488.3079**	244.6576	**471.2813**	236.1443	470.2973	235.6523	4
20	2378.1068	**1189.5570**	2361.0802	1181.0438	2360.0962	1180.5518	E	**389.2395**	195.1234	372.2129	186.6101	**371.2289**	186.1181	3
21	2491.1909	**1246.0991**	2474.1643	1237.5858	2473.1803	1237.0938	L	**260.1969**	130.6021	243.1703	122.0888			2
22							K	147.1128	74.0600	130.0863	65.5468			1

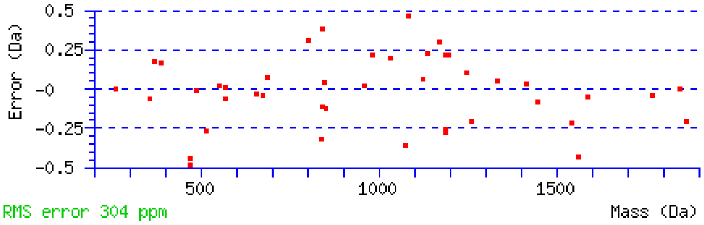

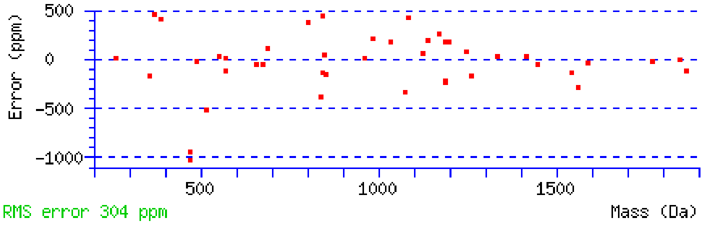

All matches to this query

Score	Mr(calc):	Delta	Sequence
49.6	2636.2891	0.0087	LEDLTTPYVRDEELNCTVVELK
3.8	2636.3210	-0.0232	LEDLASLVYLNESSVLHTLRQR
3.8	2636.3210	-0.0232	LEDLASLVYLNESSVLHTLRQR
1.1	2634.3050	1.9928	STGGAPTFNVTVTMTAKTLVLLMGK
1.0	2636.2764	0.0215	KLNNFNSYLAILSALDSAPIRR
1.0	2636.2764	0.0215	KLNNFNSYLAILSALDSAPIRR
1.0	2636.2764	0.0215	KLNNFNSYLAILSALDSAPIRR
1.0	2636.2764	0.0215	KLNNFNSYLAILSALDSAPIRR
0.9	2635.2924	1.0055	KLNNFNSYLAILSALDSAPIRR
0.9	2635.2924	1.0055	KLNNFNSYLAILSALDSAPIRR

Spectrum No: 436; Query: 1045; Rank: 1

Peptide View

MS/MS Fragmentation of **ENGTVTAANASTLNDGAAAVVLMTAEAAQR**
Found in **IPI00324302**, Tax_Id=10116 Gene_Symbol=Acat1 Acetyl-CoA acetyltransferase, mitochondrial precursor

Match to Query 1045: 2917.413852 from(973.478560,3+)
Title: 091127RatKid_SCX01_16.5518.5518.3.dta
Data file K:\NewmanPaper\Piliang\3SubProteomes\Piliang3SP\mgf5ppm\SCX_3SubProteomes5ppm.mgf

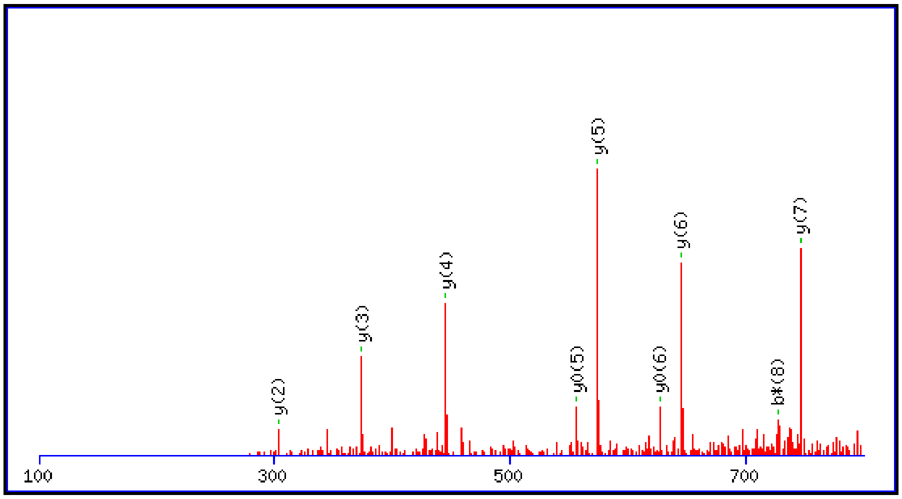

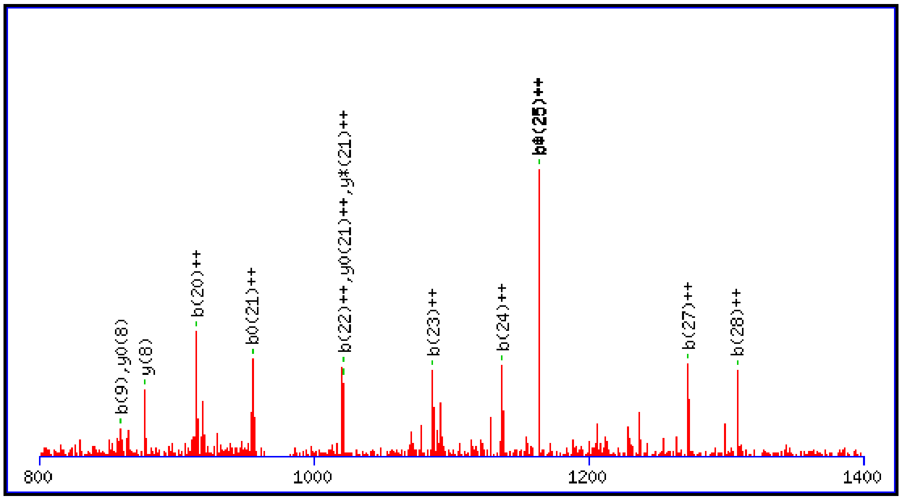

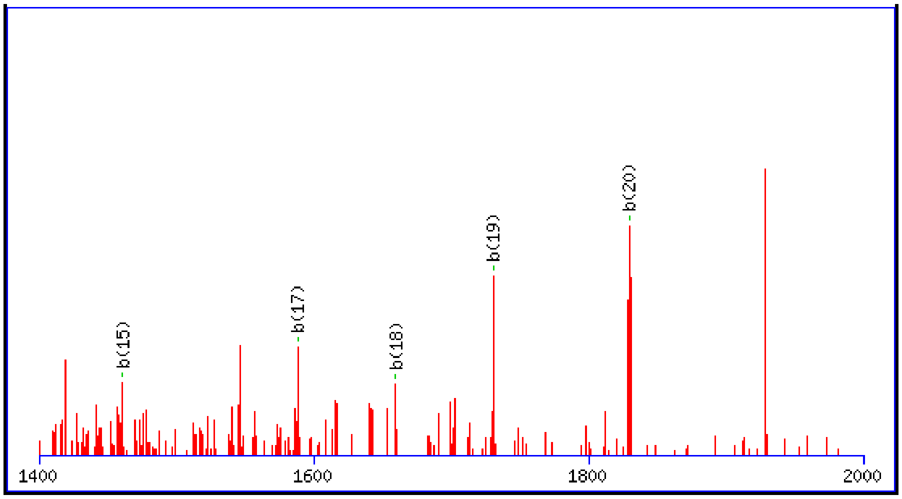

Monoisotopic mass of neutral peptide Mr(calc): 2917.4087
Fixed modifications: Carbamidomethyl (C)
Variable modifications:
N9 : Deamidated_N (N)
Ions Score: 50 **Expect:** 0.0031
Matches (Bold Red): 28/338 fragment ions using 44 most intense peaks

#	b	b^{++}	b*	b^{*++}	b^{0}	b^{0++}	Seq.	y	y^{++}	y*	y^{*++}	y^{0}	y^{0++}	#
1	130.0499	65.5286			112.0393	56.5233	E							30
2	244.0928	122.5500	227.0662	114.0368	226.0822	113.5448	N	2789.3734	1395.1903	2772.3469	1386.6771	2771.3628	1386.1851	29
3	301.1143	151.0608	284.0877	142.5475	283.1037	142.0555	G	2675.3305	1338.1689	2658.3039	1329.6556	2657.3199	1329.1636	28
4	402.1619	201.5846	385.1354	193.0713	384.1514	192.5793	T	2618.3090	1309.6581	2601.2825	1301.1449	2600.2985	1300.6529	27
5	501.2304	251.1188	484.2038	242.6055	483.2198	242.1135	V	2517.2613	1259.1343	2500.2348	1250.6210	2499.2508	1250.1290	26
6	602.2780	301.6427	585.2515	293.1294	584.2675	292.6374	T	2418.1929	1209.6001	2401.1664	1201.0868	2400.1824	1200.5948	25
7	673.3151	337.1612	656.2886	328.6479	655.3046	328.1559	A	2317.1452	1159.0763	2300.1187	1150.5630	2299.1347	1150.0710	24
8	744.3523	372.6798	**727.3257**	364.1665	726.3417	363.6745	A	2246.1081	1123.5577	2229.0816	1115.0444	2228.0976	1114.5524	23
9	**859.3792**	430.1932	842.3526	421.6800	841.3686	421.1880	N	2175.0710	1088.0391	2158.0445	1079.5259	2157.0605	1079.0339	22
10	930.4163	465.7118	913.3898	457.1985	912.4057	456.7065	A	2060.0441	1030.5257	2043.0175	**1022.0124**	2042.0335	**1021.5204**	21
11	1017.4483	509.2278	1000.4218	500.7145	999.4378	500.2225	S	1989.0070	995.0071	1971.9804	986.4938	1970.9964	986.0018	20
12	1118.4960	559.7516	1101.4695	551.2384	1100.4855	550.7464	T	1901.9749	951.4911	1884.9484	942.9778	1883.9644	942.4858	19
13	1231.5801	616.2937	1214.5535	607.7804	1213.5695	607.2884	L	1800.9273	900.9673	1783.9007	892.4540	1782.9167	891.9620	18
14	1345.6230	673.3151	1328.5965	664.8019	1327.6124	664.3099	N	1687.8432	844.4252	1670.8166	835.9120	1669.8326	835.4200	17
15	**1460.6500**	730.8286	1443.6234	722.3153	1442.6394	721.8233	D	1573.8003	787.4038	1556.7737	778.8905	1555.7897	778.3985	16
16	1517.6714	759.3393	1500.6449	750.8261	1499.6608	750.3341	G	1458.7733	729.8903	1441.7468	721.3770	1440.7628	720.8850	15
17	**1588.7085**	794.8579	1571.6820	786.3446	1570.6980	785.8526	A	1401.7519	701.3796	1384.7253	692.8663	1383.7413	692.3743	14
18	**1659.7456**	830.3765	1642.7191	821.8632	1641.7351	821.3712	A	1330.7147	665.8610	1313.6882	657.3477	1312.7042	656.8557	13
19	**1730.7828**	865.8950	1713.7562	857.3817	1712.7722	856.8897	A	1259.6776	630.3425	1242.6511	621.8292	1241.6671	621.3372	12
20	**1829.8512**	**915.4292**	1812.8246	906.9159	1811.8406	906.4239	V	1188.6405	594.8239	1171.6140	586.3106	1170.6300	585.8186	11
21	1928.9196	964.9634	1911.8930	956.4502	1910.9090	**955.9581**	V	1089.5721	545.2897	1072.5456	536.7764	1071.5615	536.2844	10
22	2042.0036	**1021.5055**	2024.9771	1012.9922	2023.9931	1012.5002	L	990.5037	495.7555	973.4771	487.2422	972.4931	486.7502	9
23	2173.0441	**1087.0257**	2156.0176	1078.5124	2155.0336	1078.0204	M	**877.4196**	439.2135	860.3931	430.7002	**859.4091**	430.2082	8
24	2274.0918	**1137.5495**	2257.0653	1129.0363	2256.0812	1128.5443	T	**746.3791**	373.6932	729.3526	365.1799	728.3686	364.6879	7
25	2345.1289	1173.0681	2328.1024	**1164.5548**	2327.1184	**1164.0628**	A	**645.3315**	323.1694	628.3049	314.6561	**627.3209**	314.1641	6
26	2474.1715	1237.5894	2457.1450	1229.0761	2456.1610	1228.5841	E	**574.2944**	287.6508	557.2678	279.1375	**556.2838**	278.6455	5
27	2545.2086	**1273.1080**	2528.1821	1264.5947	2527.1981	1264.1027	A	**445.2518**	223.1295	428.2252	214.6162			4
28	2616.2457	**1308.6265**	2599.2192	1300.1132	2598.2352	1299.6212	A	**374.2146**	187.6110	357.1881	179.0977			3
29	2744.3043	1372.6558	2727.2778	1364.1425	2726.2938	1363.6505	Q	**303.1775**	152.0924	286.1510	143.5791			2
30							R	175.1190	88.0631	158.0924	79.5498			1

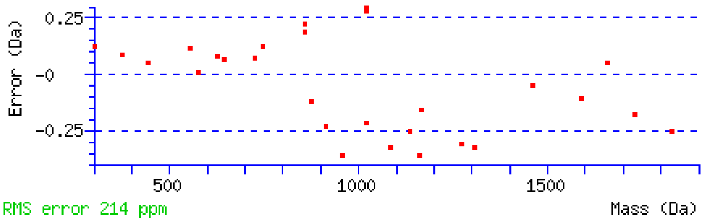

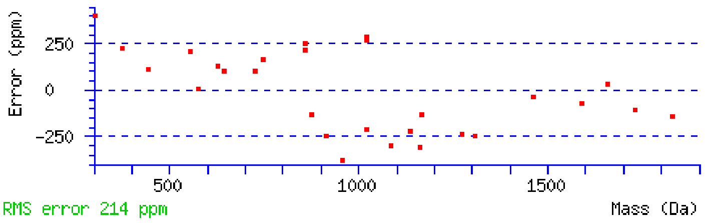

All matches to this query

Score	Mr(calc):	Delta	Sequence
49.6	2917.4087	0.0052	ENGTVTAANASTLNDGAAAVVLMTAEAAQR
49.6	2917.4087	0.0052	ENGTVTAANASTLNDGAAAVVLMTAEAAQR
43.6	2917.4087	0.0052	ENGTVTAANASTLNDGAAAVVLMTAEAAQR
34.2	2916.4247	0.9892	ENGTVTAANASTLNDGAAAVVLMTAEAAQR
10.2	2917.4157	-0.0019	FGLGLAVAGGMVNSALYNVDARNRAVGK
8.9	2915.3922	2.0217	VEERTPYIVVALQECERMNILTR
7.0	2916.4317	0.9821	FGLGLAVAGGMVNSALYNVDARNRAVGK
6.8	2916.4317	0.9821	FGLGLAVAGGMVNSALYNVDARNRAVGK
6.0	2916.4317	0.9821	FGLGLAVAGGMVNSALYNVDARNRAVGK
5.7	2917.4157	-0.0019	FGLGLAVAGGMVNSALYNVDARNRAVGK

Spectrum No: 437; Query: 894; Rank: 1

Peptide View

MS/MS Fragmentation of **EQNYTCLVEHEGLPEPLSQR**
Found in **IPI00393441**, Tax_Id=10116 Gene_Symbol=RT1-CE2 MHC class I protein

Match to Query 894: 2399.111742 from(800.711190,3+)
Title: 091127RatKid_SCX01_14.3152.3152.3.dta
Data file K:\NewmanPaper\Piliang\3SubProteomes\Piliang3SP\mgf5ppm\SCX_3SubProteomes5ppm.mgf

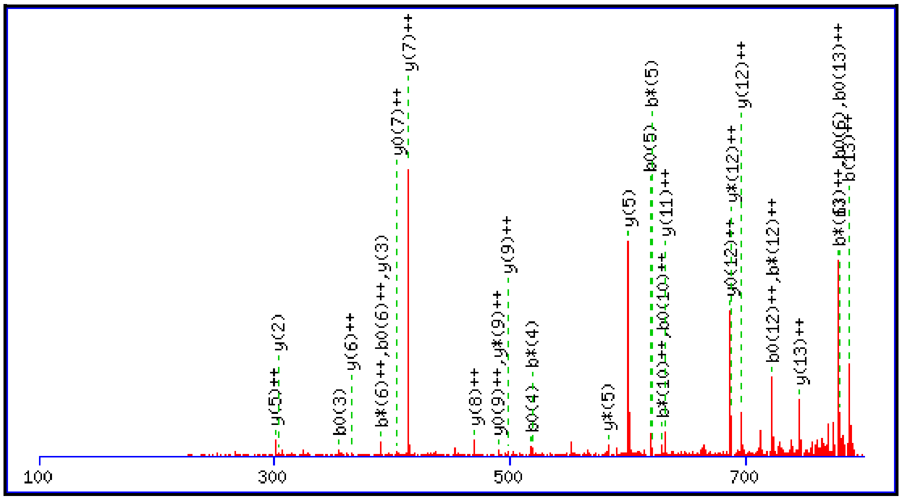

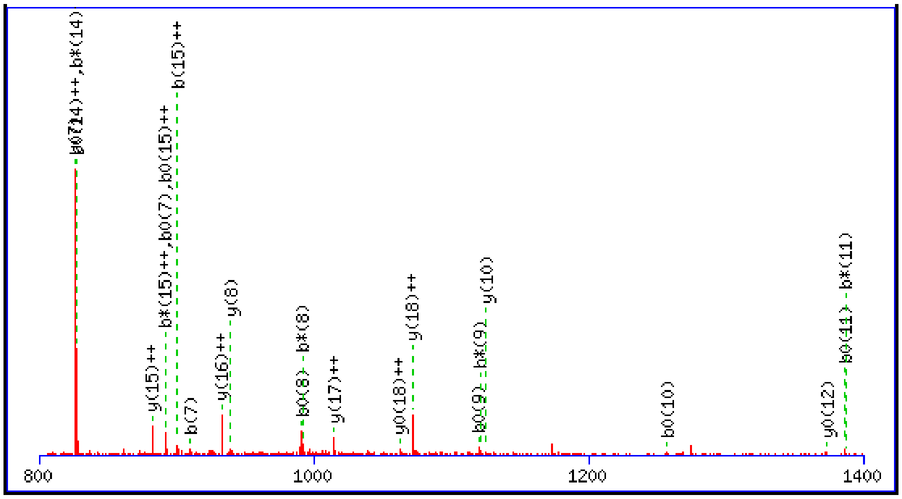

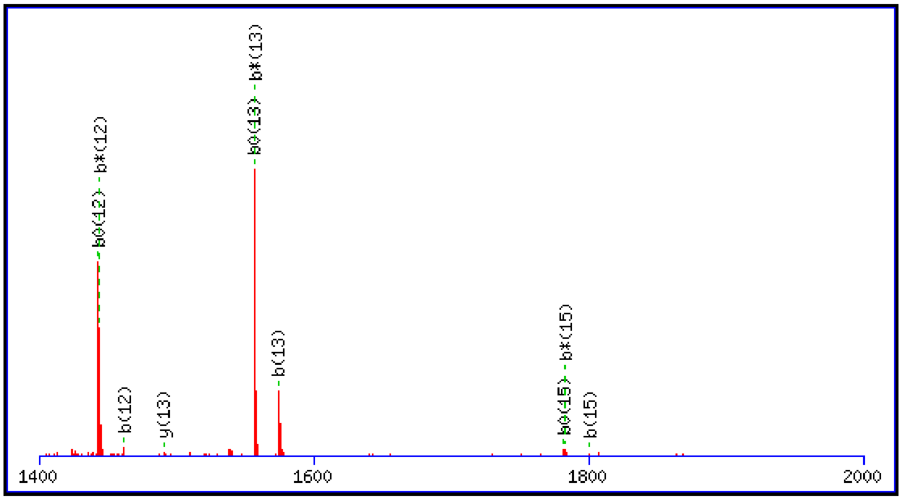

Monoisotopic mass of neutral peptide Mr(calc): 2399.1063
Fixed modifications: Carbamidomethyl (C)
Variable modifications:
N3 : Deamidated_N (N)
Ions Score: 50 **Expect:** 0.0034
Matches (Bold Red): 66/222 fragment ions using 117 most intense peaks

#	b	b^{++}	b*	b^{*++}	b^0	b^{0++}	Seq.	y	y^{++}	y*	y^{*++}	y^0	y^{0++}	#
1	130.0499	65.5286			112.0393	56.5233	E							20
2	258.1084	129.5579	241.0819	121.0446	240.0979	120.5526	Q	2271.0710	1136.0391	2254.0445	1127.5259	2253.0605	1127.0339	19
3	373.1354	187.0713	356.1088	178.5581	**355.1248**	178.0660	N	2143.0124	**1072.0099**	2125.9859	1063.4966	2125.0019	**1063.0046**	18
4	536.1987	268.6030	**519.1722**	260.0897	**518.1881**	259.5977	Y	2027.9855	**1014.4964**	2010.9590	1005.9831	2009.9749	1005.4911	17
5	637.2464	319.1268	**620.2198**	310.6136	**619.2358**	310.1216	T	1864.9222	**932.9647**	1847.8956	924.4515	1846.9116	923.9594	16
6	797.2770	399.1422	**780.2505**	**390.6289**	**779.2665**	**390.1369**	C	1763.8745	**882.4409**	1746.8479	873.9276	1745.8639	873.4356	15
7	**910.3611**	455.6842	893.3346	447.1709	**892.3505**	446.6789	L	1603.8438	802.4256	1586.8173	793.9123	1585.8333	793.4203	14
8	1009.4295	505.2184	**992.4030**	496.7051	**991.4190**	496.2131	V	**1490.7598**	**745.8835**	1473.7332	737.3703	1472.7492	736.8782	13
9	1138.4721	569.7397	**1121.4456**	561.2264	**1120.4615**	560.7344	E	1391.6914	**696.3493**	1374.6648	**687.8360**	**1373.6808**	**687.3440**	12
10	1275.5310	638.2691	1258.5045	**629.7559**	**1257.5205**	**629.2639**	H	1262.6488	**631.8280**	1245.6222	623.3148	1244.6382	622.8227	11
11	1404.5736	702.7904	**1387.5471**	694.2772	**1386.5631**	693.7852	E	**1125.5899**	563.2986	1108.5633	554.7853	1107.5793	554.2933	10
12	**1461.5951**	731.3012	**1444.5685**	**722.7879**	**1443.5845**	**722.2959**	G	996.5473	**498.7773**	979.5207	**490.2640**	978.5367	**489.7720**	9
13	**1574.6791**	**787.8432**	**1557.6526**	**779.3299**	**1556.6686**	**778.8379**	L	**939.5258**	**470.2665**	922.4993	461.7533	921.5152	461.2613	8
14	1671.7319	836.3696	1654.7054	**827.8563**	1653.7213	**827.3643**	P	**826.4417**	**413.7245**	809.4152	405.2112	808.4312	**404.7192**	7
15	**1800.7745**	**900.8909**	**1783.7480**	**892.3776**	**1782.7639**	**891.8856**	E	729.3890	**365.1981**	712.3624	356.6849	711.3784	356.1928	6
16	1897.8273	949.4173	1880.8007	940.9040	1879.8167	940.4120	P	**600.3464**	**300.6768**	**583.3198**	292.1636	582.3358	291.6715	5
17	2010.9113	1005.9593	1993.8848	997.4460	1992.9008	996.9540	L	503.2936	252.1504	486.2671	243.6372	485.2831	243.1452	4
18	2097.9434	1049.4753	2080.9168	1040.9620	2079.9328	1040.4700	S	**390.2096**	195.6084	373.1830	187.0951	372.1990	186.6031	3

19	2226.0019	1113.5046	2208.9754	1104.9913	2207.9914	1104.4993	Q	303.1775	152.0924	286.1510	143.5791			2
20							R	175.1190	88.0631	158.0924	79.5498			1

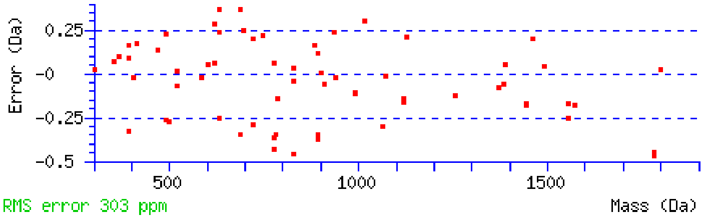

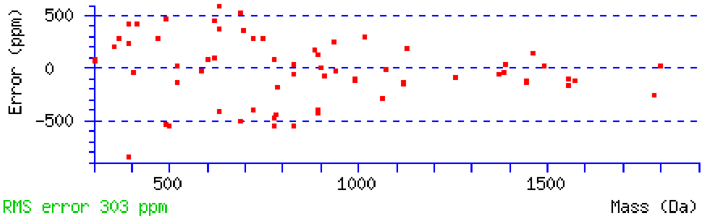

All matches to this query

Score	Mr(calc):	Delta	Sequence
49.5	2399.1063	0.0054	EQNYTCLVEHEGLPEPLSQR
25.2	2398.1223	0.9894	EQNYTCLVEHEGLPEPLSQR
9.4	2399.1298	-0.0180	GTVLXTGGLEAFTALATSAYGEK
8.8	2399.0923	0.0194	ETAGKLILANPASQAHSEFQK
5.9	2397.0993	2.0124	ASCSAVWAAAFLAGLPTLLYR
5.3	2397.0993	2.0124	ASCSAVWAAAFLAGLPTLLYR
3.5	2398.1125	0.9992	SQDSGSDNRINTVSLKEALER
3.4	2398.1157	0.9960	EKLCGYVPVHPDILSLKNR
2.9	2398.1256	0.9861	ATISQFNALTKCVVSTILGSK
2.9	2398.1256	0.9861	ATISQFNALTKCVVSTILGSK

Spectrum No: 438; Query: 443; Rank: 1

Peptide View

MS/MS Fragmentation of **GHVDPANDTFDIDPR**
Found in **IPI00370827**, Tax_Id=10116 Gene_Symbol=Mcoln1_predicted mucolipin 1

Match to Query 443: 1668.740652 from(557.254160,3+)
Title: 091127RatKid_SCX01_12.1870.1870.3.dta
Data file K:\NewmanPaper\Piliang\3SubProteomes\Piliang3SP\mgf5ppm\SCX_3SubProteomes5ppm.mgf

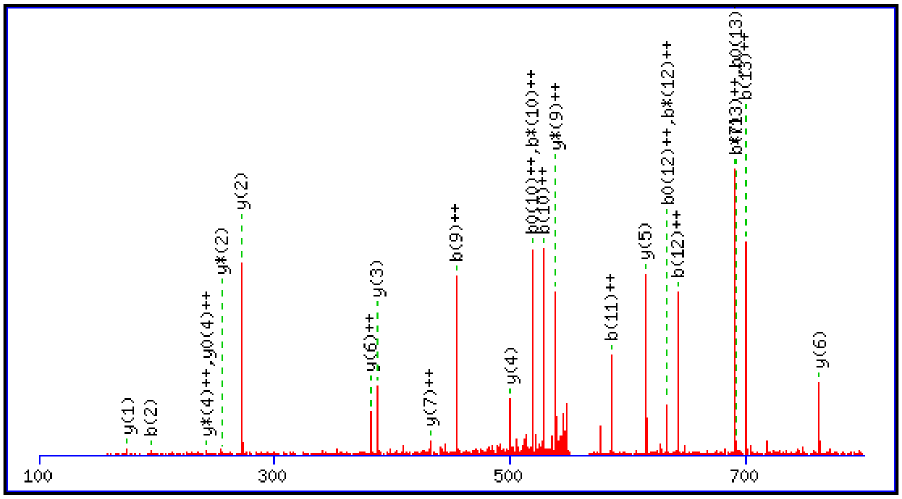

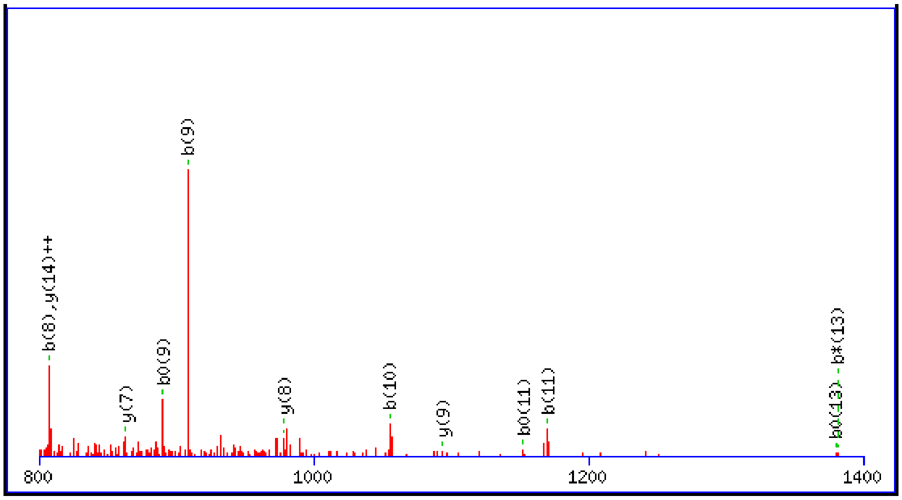

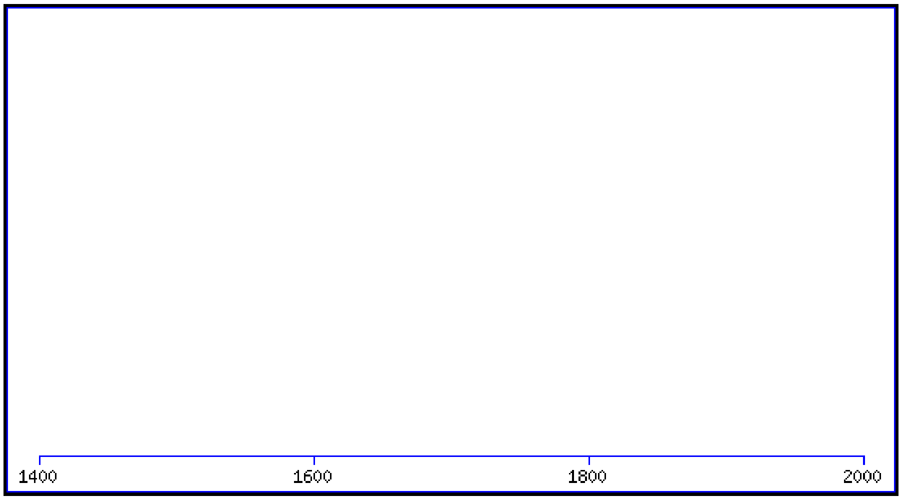

Monoisotopic mass of neutral peptide Mr(calc): 1668.7380
Fixed modifications: Carbamidomethyl (C)
Variable modifications:
N7 : Deamidated_N (N)
Ions Score: 50 **Expect:** 0.0022
Matches (Bold Red): 37/146 fragment ions using 57 most intense peaks

#	b	b^{++}	b*	b^{*++}	b^0	b^{0++}	Seq.	y	y^{++}	y*	y^{*++}	y^0	y^{0++}	#
1	58.0287	29.5180					G							15
2	**195.0877**	98.0475					H	1612.7238	**806.8655**	1595.6972	798.3523	1594.7132	797.8602	14
3	294.1561	147.5817					V	1475.6649	738.3361	1458.6383	729.8228	1457.6543	729.3308	13
4	409.1830	205.0951			391.1724	196.0899	D	1376.5965	688.8019	1359.5699	680.2886	1358.5859	679.7966	12
5	506.2358	253.6215			488.2252	244.6162	P	1261.5695	631.2884	1244.5430	622.7751	1243.5590	622.2831	11
6	577.2729	289.1401			559.2623	280.1348	A	1164.5168	582.7620	1147.4902	574.2487	1146.5062	573.7567	10
7	**692.2998**	346.6535	675.2733	338.1403	674.2893	337.6483	N	**1093.4796**	547.2435	1076.4531	**538.7302**	1075.4691	538.2382	9
8	**807.3268**	404.1670	790.3002	395.6537	789.3162	395.1617	D	**978.4527**	489.7300	961.4262	481.2167	960.4421	480.7247	8
9	**908.3744**	**454.6909**	891.3479	446.1776	**890.3639**	445.6856	T	**863.4258**	**432.2165**	846.3992	423.7032	845.4152	423.2112	7
10	**1055.4429**	**528.2251**	1038.4163	**519.7118**	1037.4323	**519.2198**	F	**762.3781**	**381.6927**	745.3515	373.1794	744.3675	372.6874	6
11	**1170.4698**	**585.7385**	1153.4433	577.2253	**1152.4592**	576.7333	D	**615.3097**	308.1585	598.2831	299.6452	597.2991	299.1532	5
12	1283.5539	**642.2806**	1266.5273	**633.7673**	1265.5433	**633.2753**	I	**500.2827**	250.6450	483.2562	**242.1317**	482.2722	**241.6397**	4
13	1398.5808	**699.7940**	**1381.5543**	**691.2808**	**1380.5702**	**690.7888**	D	**387.1987**	194.1030	370.1721	185.5897	369.1881	185.0977	3
14	1495.6336	748.3204	1478.6070	739.8072	1477.6230	739.3151	P	**272.1717**	136.5895	**255.1452**	128.0762			2
15							R	**175.1190**	88.0631	158.0924	79.5498			1

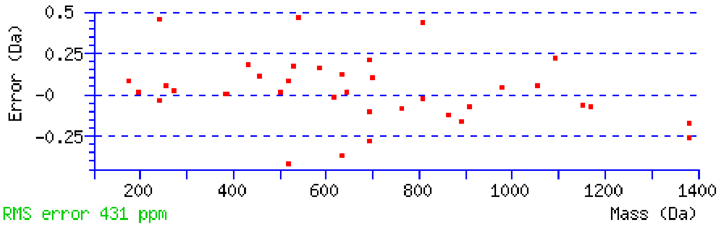

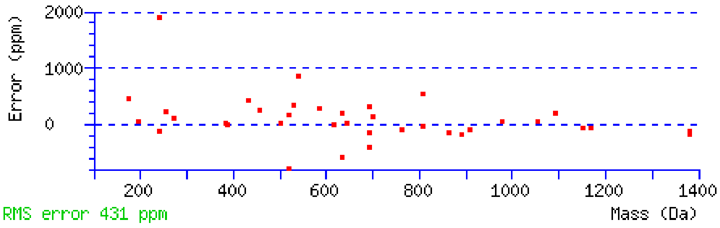

All matches to this query

Score	Mr(calc):	Delta	Sequence
49.5	1668.7380	0.0027	GHVDPANDTFDIDPR
18.6	1667.7540	0.9867	GHVDPANDTFDIDPR
16.4	1668.7257	0.0149	YVSHGATGKGNDQVR
13.0	1668.7290	0.0116	ARSSAACANVLTPDR
11.9	1667.7300	1.0107	MAMPINVSDPDLLR
11.9	1667.7300	1.0107	MAMPINVSDPDLLR
11.6	1668.7525	-0.0119	ELVGTPSPTSGLVPR
11.6	1668.7525	-0.0119	ELVGTPSPTSGLVPR
9.7	1666.7311	2.0095	QQEEAQAVAAASAASR
9.7	1666.7311	2.0095	QQEEAQAVAAASAASR

Spectrum No: 439; Query: 1134; Rank: 1

Peptide View

MS/MS Fragmentation of **IGCFALSEPGNGSDAGAASTTAREEGDSWVLNGTK**
Found in **IPI00231359**, Tax_Id=10116 Gene_Symbol=Acads Acetyl-Coenzyme A dehydrogenase, short chain

Match to Query 1134: 3526.603272 from(1176.541700,3+)
Title: 091129RatKid_SCX02_22.4124.4124.3.dta
Data file K:\NewmanPaper\Piliang\3SubProteomes\Piliang3SP\mgf5ppm\SCX_3SubProteomes5ppm.mgf

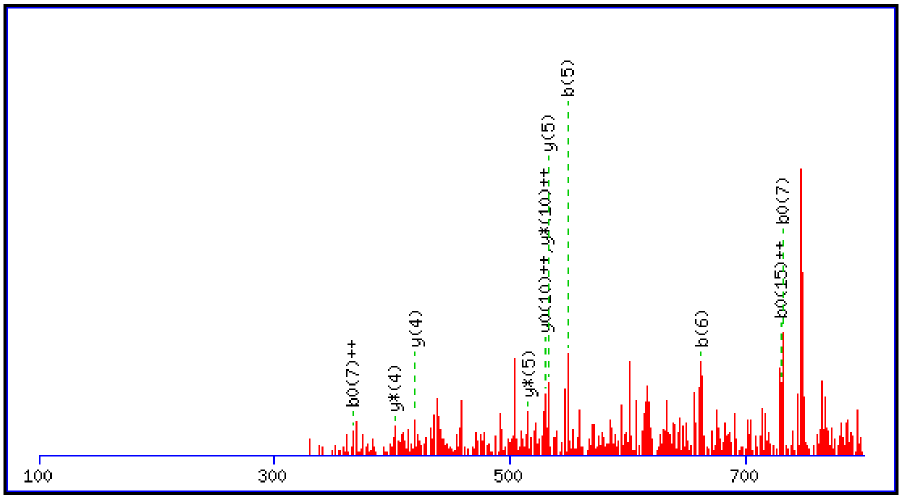

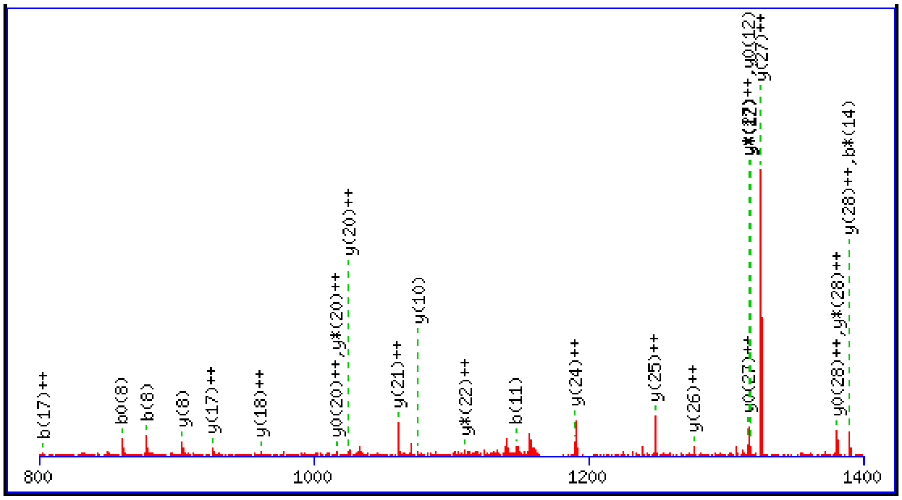

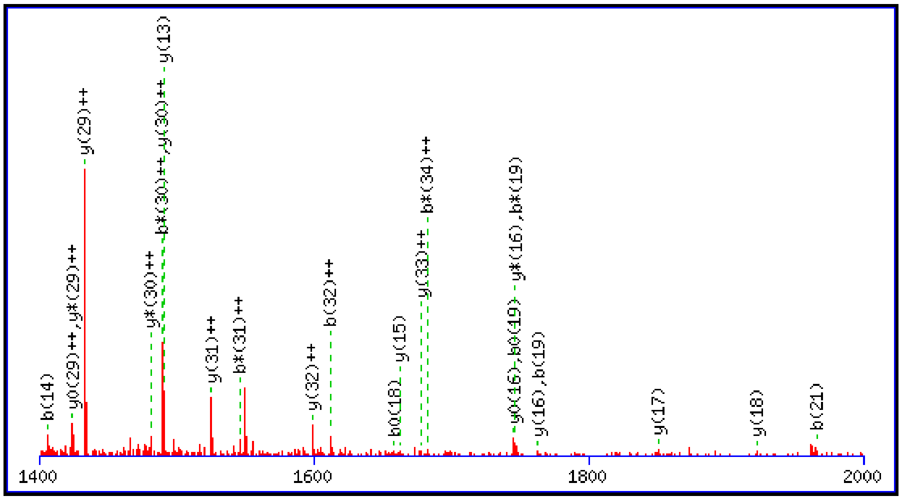

Monoisotopic mass of neutral peptide Mr(calc): 3525.5954
Fixed modifications: Carbamidomethyl (C)
Variable modifications:
N11 : Deamidated_N (N)
Ions Score: 49 **Expect:** 0.0053
Matches (Bold Red): 61/374 fragment ions using 128 most intense peaks

#	b	b^{++}	b*	b^{*++}	b^0	b^{0++}	Seq.	y	y^{++}	y*	y^{*++}	y^0	y^{0++}	#
1	114.0913	57.5493					I							35
2	171.1128	86.0600					G	3413.5186	1707.2630	3396.4921	1698.7497	3395.5081	1698.2577	34
3	331.1435	166.0754					C	3356.4972	**1678.7522**	3339.4706	1670.2389	3338.4866	1669.7469	33
4	478.2119	239.6096					F	3196.4665	**1598.7369**	3179.4400	1590.2236	3178.4559	1589.7316	32
5	**549.2490**	275.1281					A	3049.3981	**1525.2027**	3032.3716	1516.6894	3031.3875	1516.1974	31
6	**662.3330**	331.6702					L	2978.3610	**1489.6841**	2961.3344	**1481.1709**	2960.3504	1480.6788	30
7	749.3651	375.1862			**731.3545**	**366.1809**	S	2865.2769	**1433.1421**	2848.2504	**1424.6288**	2847.2664	**1424.1368**	29
8	**878.4077**	439.7075			**860.3971**	430.7022	E	2778.2449	**1389.6261**	2761.2183	**1381.1128**	2760.2343	**1380.6208**	28
9	975.4604	488.2339			957.4499	479.2286	P	2649.2023	**1325.1048**	2632.1758	**1316.5915**	2631.1917	**1316.0995**	27
10	1032.4819	516.7446			1014.4713	507.7393	G	2552.1495	**1276.5784**	2535.1230	1268.0651	2534.1390	1267.5731	26
11	**1147.5088**	574.2581	1130.4823	565.7448	1129.4983	565.2528	N	2495.1281	**1248.0677**	2478.1015	1239.5544	2477.1175	1239.0624	25
12	1204.5303	602.7688	1187.5037	594.2555	1186.5197	593.7635	G	2380.1011	**1190.5542**	2363.0746	1182.0409	2362.0906	1181.5489	24
13	1291.5623	646.2848	1274.5358	637.7715	1273.5518	637.2795	S	2323.0797	1162.0435	2306.0531	1153.5302	2305.0691	1153.0382	23
14	**1406.5893**	703.7983	**1389.5627**	695.2850	1388.5787	694.7930	D	2236.0476	1118.5275	2219.0211	**1110.0142**	2218.0371	1109.5222	22
15	1477.6264	739.3168	1460.5998	730.8036	1459.6158	**730.3115**	A	2121.0207	**1061.0140**	2103.9942	1052.5007	2103.0101	1052.0087	21
16	1534.6478	767.8276	1517.6213	759.3143	1516.6373	758.8223	G	2049.9836	**1025.4954**	2032.9570	**1016.9822**	2031.9730	**1016.4901**	20
17	1605.6850	**803.3461**	1588.6584	794.8328	1587.6744	794.3408	A	1992.9621	996.9847	1975.9356	988.4714	1974.9516	987.9794	19
18	1676.7221	838.8647	1659.6955	830.3514	**1658.7115**	829.8594	A	**1921.9250**	**961.4661**	1904.8985	952.9529	1903.9144	952.4609	18

**19**	**1763.7541**	882.3807	**1746.7276**	873.8674	**1745.7435**	873.3754	**S**	**1850.8879**	**925.9476**	1833.8613	917.4343	1832.8773	916.9423	**17**
**20**	1864.8018	932.9045	1847.7752	924.3913	1846.7912	923.8992	**T**	**1763.8559**	882.4316	**1746.8293**	873.9183	**1745.8453**	873.4263	**16**
**21**	**1965.8495**	983.4284	1948.8229	974.9151	1947.8389	974.4231	**T**	**1662.8082**	831.9077	1645.7816	823.3945	1644.7976	822.9025	**15**
**22**	2036.8866	1018.9469	2019.8600	1010.4336	2018.8760	1009.9416	**A**	1561.7605	781.3839	1544.7340	772.8706	1543.7499	772.3786	**14**
**23**	2192.9877	1096.9975	2175.9611	1088.4842	2174.9771	1087.9922	**R**	**1490.7234**	745.8653	1473.6968	737.3521	1472.7128	736.8601	**13**
**24**	2322.0303	1161.5188	2305.0037	1153.0055	2304.0197	1152.5135	**E**	1334.6223	667.8148	**1317.5957**	659.3015	**1316.6117**	658.8095	**12**
**25**	2451.0729	1226.0401	2434.0463	1217.5268	2433.0623	1217.0348	**E**	1205.5797	603.2935	1188.5531	594.7802	1187.5691	594.2882	**11**
**26**	2508.0943	1254.5508	2491.0678	1246.0375	2490.0838	1245.5455	**G**	**1076.5371**	538.7722	1059.5106	**530.2589**	1058.5265	**529.7669**	**10**
**27**	2623.1213	1312.0643	2606.0947	1303.5510	2605.1107	1303.0590	**D**	1019.5156	510.2615	1002.4891	501.7482	1001.5051	501.2562	**9**
**28**	2710.1533	1355.5803	2693.1268	1347.0670	2692.1427	1346.5750	**S**	**904.4887**	452.7480	887.4621	444.2347	886.4781	443.7427	**8**
**29**	2896.2326	1448.6199	2879.2061	1440.1067	2878.2221	1439.6147	**W**	817.4567	409.2320	800.4301	400.7187	799.4461	400.2267	**7**
**30**	2995.3010	1498.1542	2978.2745	**1489.6409**	2977.2905	1489.1489	**V**	631.3774	316.1923	614.3508	307.6790	613.3668	307.1870	**6**
**31**	3108.3851	1554.6962	3091.3585	**1546.1829**	3090.3745	1545.6909	**L**	**532.3089**	266.6581	**515.2824**	258.1448	514.2984	257.6528	**5**
**32**	3222.4280	**1611.7176**	3205.4015	1603.2044	3204.4175	1602.7124	**N**	**419.2249**	210.1161	**402.1983**	201.6028	401.2143	201.1108	**4**
**33**	3279.4495	1640.2284	3262.4229	1631.7151	3261.4389	1631.2231	**G**	305.1819	153.0946	288.1554	144.5813	287.1714	144.0893	**3**
**34**	3380.4972	1690.7522	3363.4706	**1682.2389**	3362.4866	1681.7469	**T**	248.1605	124.5839	231.1339	116.0706	230.1499	115.5786	**2**
**35**							**K**	147.1128	74.0600	130.0863	65.5468			**1**

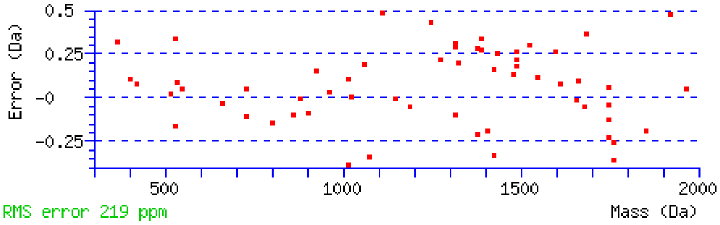

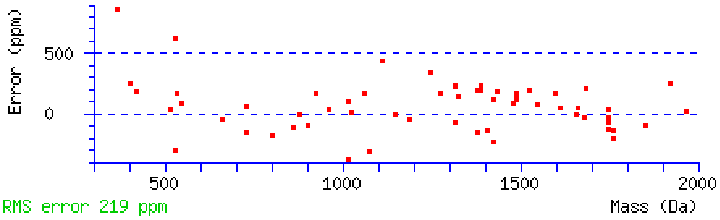

All matches to this query

Score	Mr(calc):	Delta	Sequence
49.5	3525.5954	1.0079	IGCFALSEPGNGSDAGAASTTAREEGDSWVLNGTK
35.7	3525.5954	1.0079	IGCFALSEPGNGSDAGAASTTAREEGDSWVLNGTK
34.8	3526.5794	0.0239	IGCFALSEPGNGSDAGAASTTAREEGDSWVLNGTK
10.2	3525.5734	1.0299	NSQSFFSGLFGGSSKIEEACEIYARAANMFK
9.6	3526.6178	-0.0146	YLPLFMIQXNLSSLLESLRLVSLSWTR
8.3	3524.6114	1.9919	IGCFALSEPGNGSDAGAASTTAREEGDSWVLNGTK
8.0	3526.5775	0.0258	TACHLNPSPDGEAYTLASRPPVRLNDVMLR
7.0	3525.5734	1.0299	NSQSFFSGLFGGSSKIEEACEIYARAANMFK
5.2	3526.5775	0.0258	TACHLNPSPDGEAYTLASRPPVRLNDVMLR
5.2	3526.5775	0.0258	TACHLNPSPDGEAYTLASRPPVRLNDVMLR

Spectrum No: 440; Query: 360; Rank: 1

Peptide View

MS/MS Fragmentation of **YLWSEPQNCSATK**
Found in **IPI00212809**, Tax_Id=10116 Gene_Symbol=Ctsh Cathepsin H precursor

Match to Query 360: 1583.694888 from(792.854720,2+)
Title: 091127RatKid_SCX01_12.2037.2037.2.dta
Data file K:\NewmanPaper\Piliang\3SubProteomes\Piliang3SP\mgf5ppm\SCX_3SubProteomes5ppm.mgf

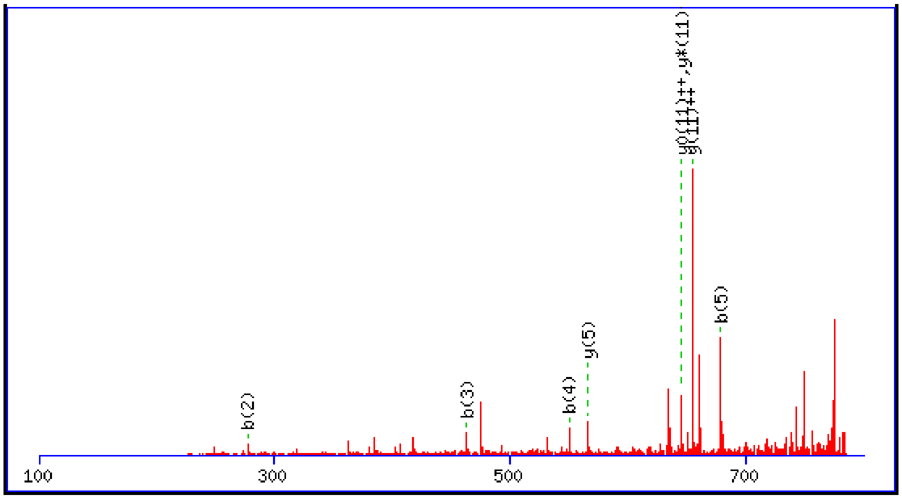

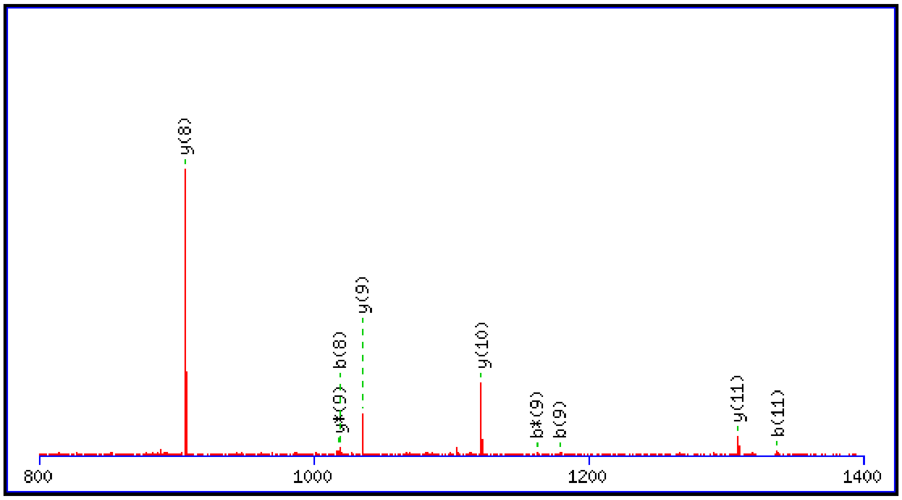

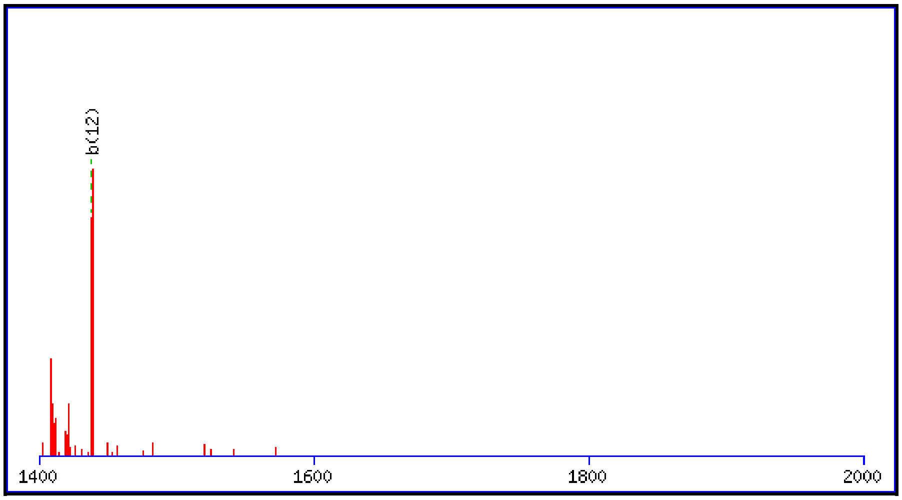

Monoisotopic mass of neutral peptide Mr(calc): 1583.6926
Fixed modifications: Carbamidomethyl (C)
Variable modifications:
N8 : Deamidated_N (N)
Ions Score: 49 **Expect:** 0.0019
Matches (Bold Red): 18/124 fragment ions using 37 most intense peaks

#	b	b^{++}	b*	b^{*++}	b^0	b^{0++}	Seq.	y	y^{++}	y*	y^{*++}	y^0	y^{0++}	#
1	164.0706	82.5389					Y							13
2	**277.1547**	139.0810					L	1421.6366	711.3219	1404.6100	702.8086	1403.6260	702.3166	12
3	**463.2340**	232.1206					W	**1308.5525**	**654.7799**	1291.5259	**646.2666**	1290.5419	**645.7746**	11
4	**550.2660**	275.6366			532.2554	266.6314	S	**1122.4732**	561.7402	1105.4466	553.2270	1104.4626	552.7349	10
5	**679.3086**	340.1579			661.2980	331.1527	E	**1035.4411**	518.2242	**1018.4146**	509.7109	1017.4306	509.2189	9

**6**	776.3614	388.6843			758.3508	379.6790	**P**	**906.3986**	453.7029	889.3720	445.1896	888.3880	444.6976	**8**
**7**	904.4199	452.7136	887.3934	444.2003	886.4094	443.7083	**Q**	809.3458	405.1765	792.3192	396.6633	791.3352	396.1712	**7**
**8**	**1019.4469**	510.2271	1002.4203	501.7138	1001.4363	501.2218	**N**	681.2872	341.1472	664.2607	332.6340	663.2766	332.1420	**6**
**9**	**1179.4775**	590.2424	**1162.4510**	581.7291	1161.4670	581.2371	**C**	**566.2603**	283.6338	549.2337	275.1205	548.2497	274.6285	**5**
**10**	1266.5096	633.7584	1249.4830	625.2451	1248.4990	624.7531	**S**	406.2296	203.6185	389.2031	195.1052	388.2191	194.6132	**4**
**11**	**1337.5467**	669.2770	1320.5201	660.7637	1319.5361	660.2717	**A**	319.1976	160.1024	302.1710	151.5892	301.1870	151.0972	**3**
**12**	**1438.5944**	719.8008	1421.5678	711.2875	1420.5838	710.7955	**T**	248.1605	124.5839	231.1339	116.0706	230.1499	115.5786	**2**
**13**							**K**	147.1128	74.0600	130.0863	65.5468			**1**

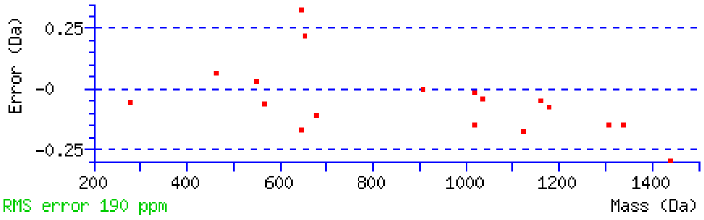

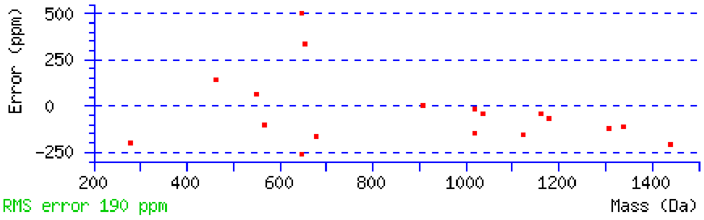

All matches to this query

Score	Mr(calc):	Delta	Sequence
49.2	1583.6926	0.0023	YLWSEPQNCSATK
8.1	1582.6988	0.9961	ARPEGSAEISTETR
7.8	1581.6763	2.0186	GSENGTPMDMKAGVR
4.8	1582.6854	1.0095	ATNMEDMLKEEEK
3.4	1582.7045	0.9904	FVKTDALTSGEKK
2.8	1582.7045	0.9904	FVKTDALTSGEKK
2.2	1581.6786	2.0163	CLYELNFTVQSK
2.0	1583.6942	0.0007	ASNPDLMLWEISK
1.8	1583.6942	0.0006	YLSMDGLQISSFK
1.3	1583.6803	0.0146	LRQSNPEFCPEK

Spectrum No: 441; Query: 49; Rank: 1

Peptide View

MS/MS Fragmentation of **NGTTYEGIFK**
Found in **IPI00366038**, Tax_Id=10116 Gene_Symbol=Atxn2l_predicted similar to ataxin 2-like

Match to Query 49: 1129.527068 from(565.770810,2+)
Title: 091129RatKid_SCX02_16.2199.2199.2.dta
Data file K:\NewmanPaper\Piliang\3SubProteomes\Piliang3SP\mgf5ppm\SCX_3SubProteomes5ppm.mgf

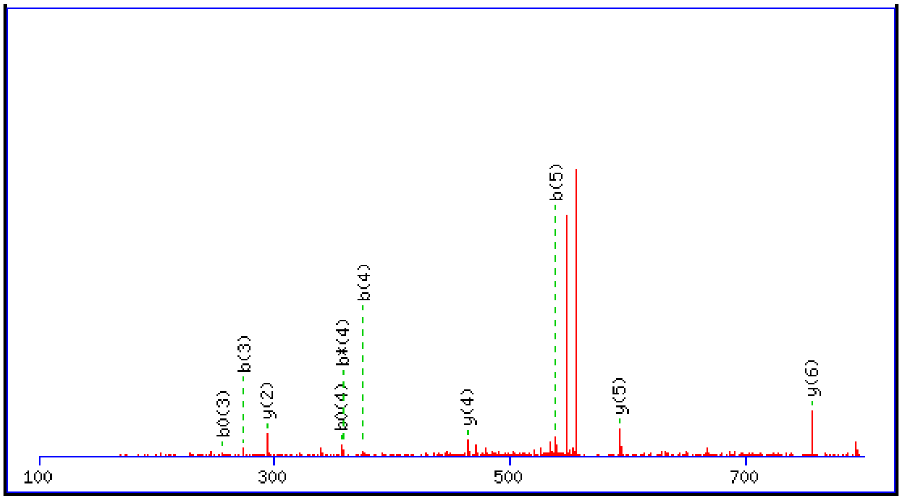

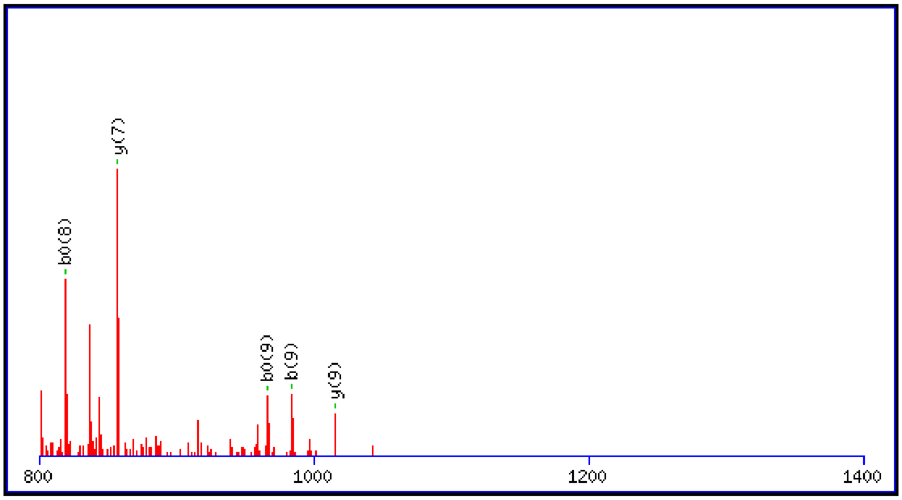

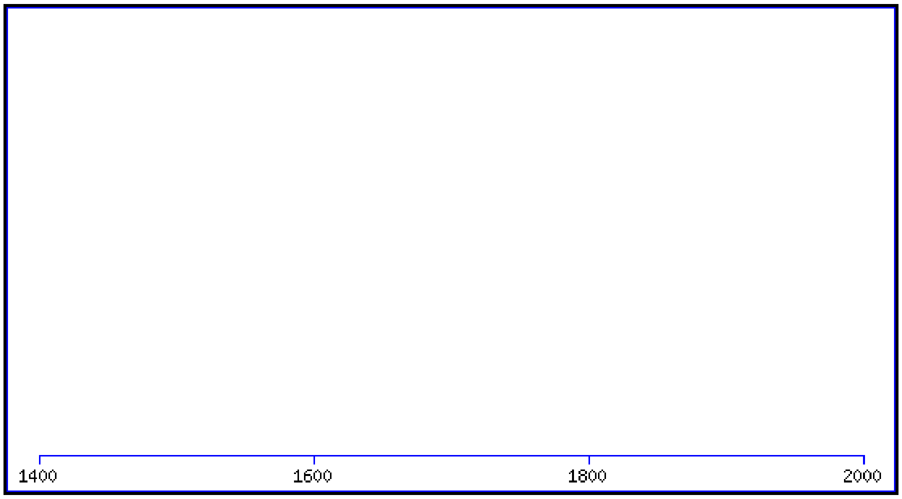

Monoisotopic mass of neutral peptide Mr(calc): 1129.5291
Fixed modifications: Carbamidomethyl (C)
Variable modifications:
N1 : Deamidated_N (N)
Ions Score: 49 **Expect:** 0.0014
Matches (Bold Red): 15/96 fragment ions using 23 most intense peaks

#	b	b^{++}	b*	b^{*++}	b^0	b^{0++}	Seq.	y	y^{++}	y*	y^{*++}	y^0	y^{0++}	#
1	116.0342	58.5207	99.0077	50.0075			N							10
2	173.0557	87.0315	156.0291	78.5182			G	**1015.5095**	508.2584	998.4829	499.7451	997.4989	499.2531	9
3	**274.1034**	137.5553	257.0768	129.0420	**256.0928**	128.5500	T	958.4880	479.7477	941.4615	471.2344	940.4775	470.7424	8
4	**375.1510**	188.0792	**358.1245**	179.5659	**357.1405**	179.0739	T	**857.4403**	429.2238	840.4138	420.7105	839.4298	420.2185	7
5	**538.2144**	269.6108	521.1878	261.0975	520.2038	260.6055	Y	**756.3927**	378.7000	739.3661	370.1867	738.3821	369.6947	6

**6**	667.2570	334.1321	650.2304	325.6188	649.2464	325.1268	**E**	**593.3293**	297.1683	576.3028	288.6550	575.3188	288.1630	**5**
**7**	724.2784	362.6428	707.2519	354.1296	706.2679	353.6376	**G**	**464.2867**	232.6470	447.2602	224.1337			**4**
**8**	837.3625	419.1849	820.3359	410.6716	**819.3519**	410.1796	**I**	407.2653	204.1363	390.2387	195.6230			**3**
**9**	**984.4309**	492.7191	967.4043	484.2058	**966.4203**	483.7138	**F**	**294.1812**	147.5942	277.1547	139.0810			**2**
**10**							**K**	147.1128	74.0600	130.0863	65.5468			**1**

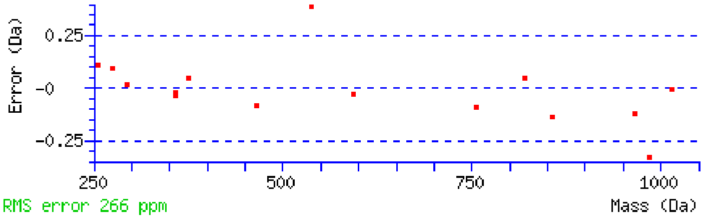

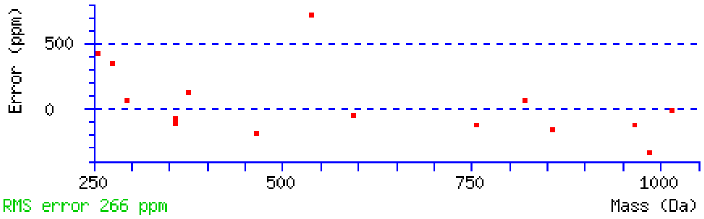

All matches to this query

Score	Mr(calc):	Delta	Sequence
49.0	1129.5291	-0.0021	NGTTYEGIFK
13.4	1128.5250	1.0021	QKSIEMSVK
13.1	1129.5325	-0.0054	NTMKTYVEK
11.5	1129.5298	-0.0027	RVLGSPTLK
11.0	1129.5339	-0.0068	VNWMAHTVR
9.7	1129.5315	-0.0044	NKKMSATVR
7.5	1129.5372	-0.0102	DLVHKACCK
6.6	1129.5168	0.0102	SPSLERAYK
6.6	1129.5168	0.0102	SPSLERAYK
6.5	1129.5169	0.0102	SFIQSNNLK

Spectrum No: 442; Query: 913; Rank: 1

Peptide View

MS/MS Fragmentation of **KQILEGLEFNLTQIPEADIHK**
Found in **IPI00324019**, Tax_Id=10116 Gene_Symbol=Serpina1 Alpha-1-antiproteinase precursor

Match to Query 913: 2436.291976 from(610.080270,4+)
Title: 091129RatKid_SCX02_23.4246.4246.4.dta
Data file K:\NewmanPaper\Piliang\3SubProteomes\Piliang3SP\mgf5ppm\SCX_3SubProteomes5ppm.mgf

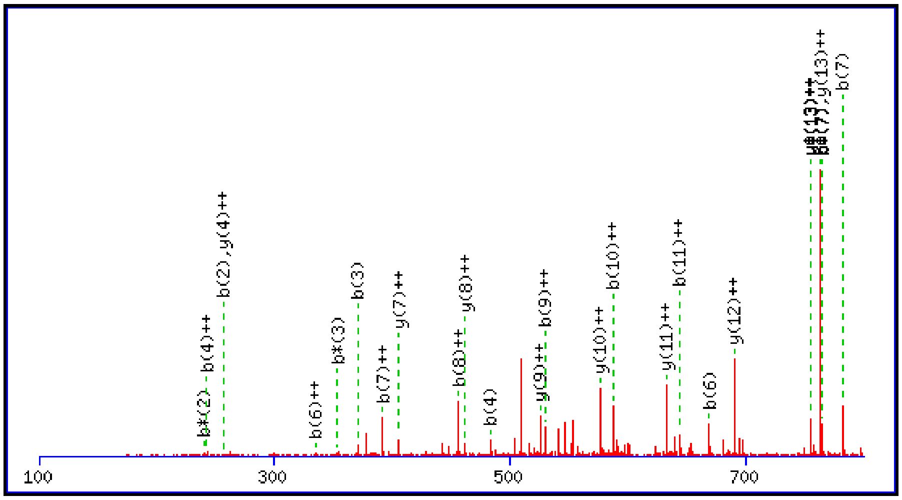

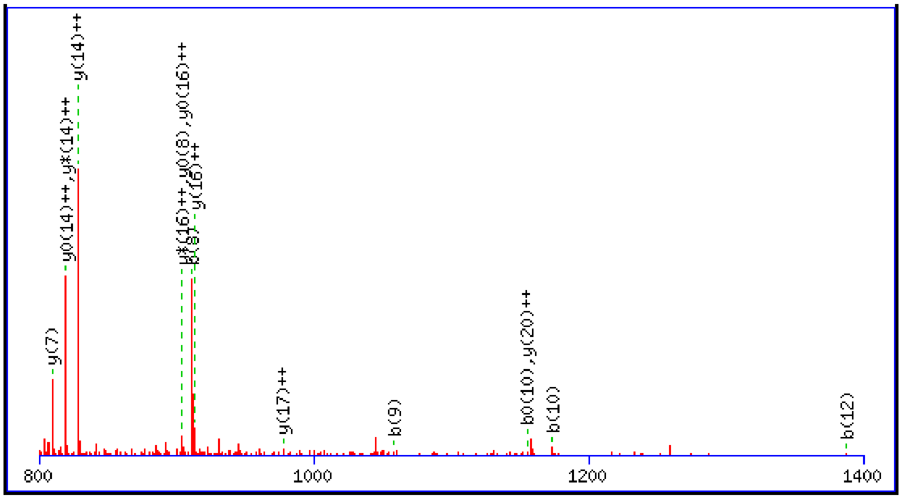

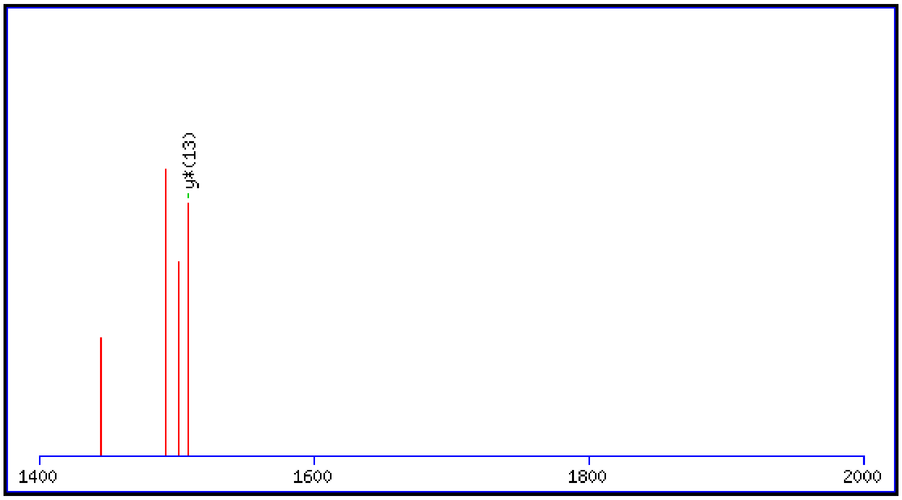

Monoisotopic mass of neutral peptide Mr(calc): 2436.2900
Fixed modifications: Carbamidomethyl (C)
Variable modifications:
N10 : Deamidated_N (N)
Ions Score: 49 **Expect:** 0.0011
Matches (Bold Red): 42/226 fragment ions using 80 most intense peaks

#	b	b^{++}	b*	b^{*++}	b^0	b^{0++}	Seq.	y	y^{++}	y*	y^{*++}	y^0	y^{0++}	#
1	129.1022	65.0548	112.0757	56.5415			K							21
2	**257.1608**	129.0840	**240.1343**	120.5708			Q	2309.2023	**1155.1048**	2292.1758	1146.5915	2291.1918	1146.0995	20
3	**370.2449**	185.6261	**353.2183**	177.1128			I	2181.1438	1091.0755	2164.1172	1082.5622	2163.1332	1082.0702	19
4	**483.3289**	**242.1681**	466.3024	233.6548			L	2068.0597	1034.5335	2051.0332	1026.0202	2050.0491	1025.5282	18
5	612.3715	306.6894	595.3450	298.1761	594.3610	297.6841	E	1954.9756	**977.9915**	1937.9491	969.4782	1936.9651	968.9862	17
6	**669.3930**	**335.2001**	652.3665	326.6869	651.3824	326.1949	G	1825.9330	**913.4702**	1808.9065	**904.9569**	1807.9225	**904.4649**	16
7	**782.4771**	**391.7422**	**765.4505**	383.2289	**764.4665**	382.7369	L	1768.9116	884.9594	1751.8850	876.4462	1750.9010	875.9541	15
8	**911.5197**	**456.2635**	894.4931	447.7502	893.5091	447.2582	E	1655.8275	**828.4174**	1638.8010	**819.9041**	1637.8170	**819.4121**	14
9	**1058.5881**	**529.7977**	1041.5615	521.2844	1040.5775	520.7924	F	1526.7849	**763.8961**	**1509.7584**	**755.3828**	1508.7744	**754.8908**	13
10	**1173.6150**	**587.3111**	1156.5885	578.7979	**1155.6044**	578.3059	N	1379.7165	**690.3619**	1362.6900	681.8486	1361.7059	681.3566	12
11	1286.6991	**643.8532**	1269.6725	635.3399	1268.6885	634.8479	L	1264.6896	**632.8484**	1247.6630	624.3352	1246.6790	623.8431	11
12	**1387.7468**	694.3770	1370.7202	685.8637	1369.7362	685.3717	T	1151.6055	**576.3064**	1134.5790	567.7931	1133.5949	567.3011	10
13	1515.8053	758.4063	1498.7788	749.8930	1497.7948	749.4010	Q	1050.5578	**525.7826**	1033.5313	517.2693	1032.5473	516.7773	9
14	1628.8894	814.9483	1611.8628	806.4351	1610.8788	805.9431	I	922.4993	**461.7533**	905.4727	453.2400	**904.4887**	452.7480	8
15	1725.9422	863.4747	1708.9156	854.9614	1707.9316	854.4694	P	**809.4152**	**405.2112**	792.3886	396.6980	791.4046	396.2060	7
16	1854.9848	927.9960	1837.9582	919.4827	1836.9742	918.9907	E	712.3624	356.6849	695.3359	348.1716	694.3519	347.6796	6
17	1926.0219	963.5146	1908.9953	955.0013	1908.0113	954.5093	A	583.3198	292.1636	566.2933	283.6503	565.3093	283.1583	5
18	2041.0488	1021.0280	2024.0223	1012.5148	2023.0382	1012.0228	D	512.2827	**256.6450**	495.2562	248.1317	494.2722	247.6397	4

**19**	2154.1329	1077.5701	2137.1063	1069.0568	2136.1223	1068.5648	**I**	397.2558	199.1315	380.2292	190.6183			**3**
**20**	2291.1918	1146.0995	2274.1652	1137.5863	2273.1812	1137.0942	**H**	284.1717	142.5895	267.1452	134.0762			**2**
**21**							**K**	147.1128	74.0600	130.0863	65.5468			**1**

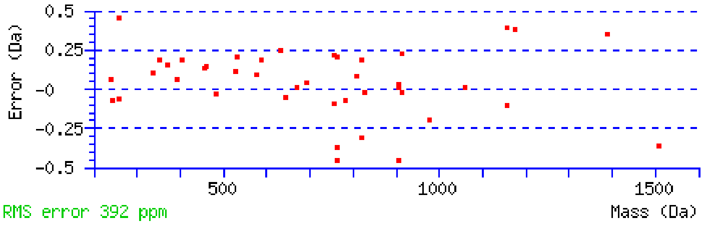

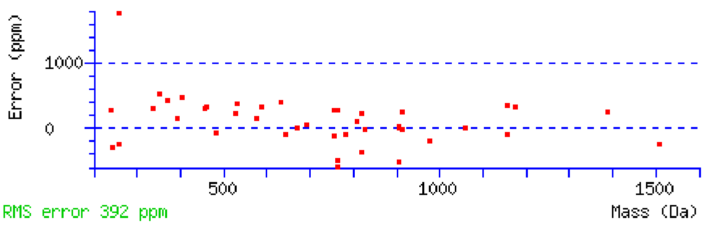

All matches to this query

Score	Mr(calc):	Delta	Sequence
48.8	2436.2900	0.0020	KQILEGLEFNLTQIPEADIHK
27.1	2435.3060	0.9860	KQILEGLEFNLTQIPEADIHK
2.4	2436.3125	-0.0205	NLEELHLPTGDGIHQVAKPIVR
2.3	2434.2814	2.0106	RILQAVEFPFLVRLEYSFK
1.1	2434.2663	2.0256	LRLLVSAGMLLVALSPCLPCR

Spectrum No: 443; Query: 851; Rank: 1

Peptide View

MS/MS Fragmentation of **DRVPDSCCINITVGCGNDFK**
Found in **IPI00231686**, Tax_Id=10116 Gene_Symbol=Cd63 CD63 antigen

Match to Query 851: 2326.999992 from(776.673940,3+)
Title: 091129RatKid_SCX02_14.2464.2464.3.dta
Data file K:\NewmanPaper\Piliang\3SubProteomes\Piliang3SP\mgf5ppm\SCX_3SubProteomes5ppm.mgf

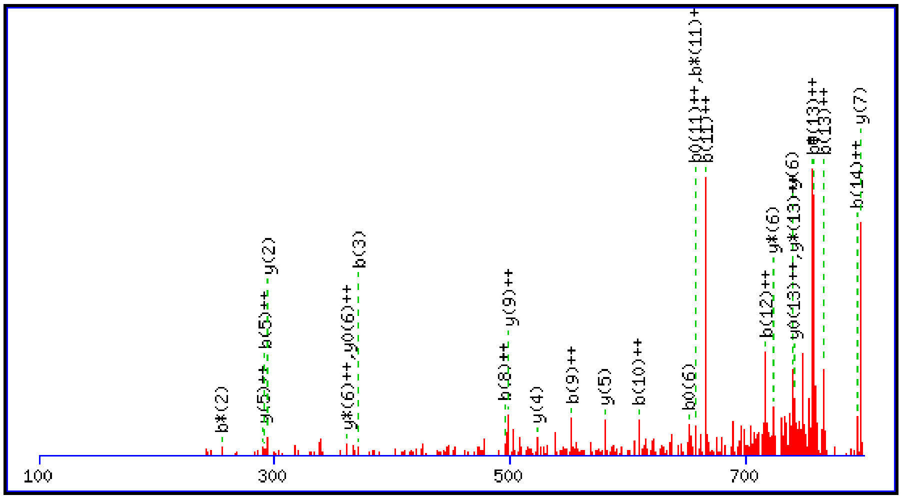

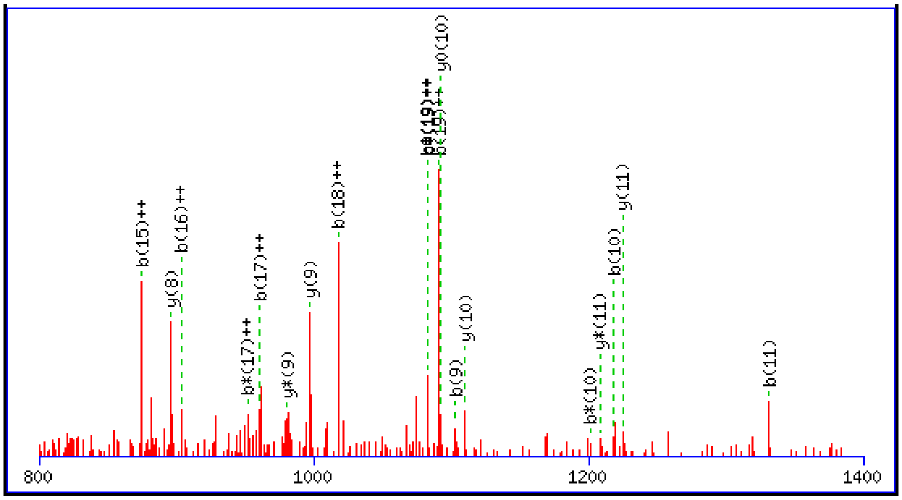

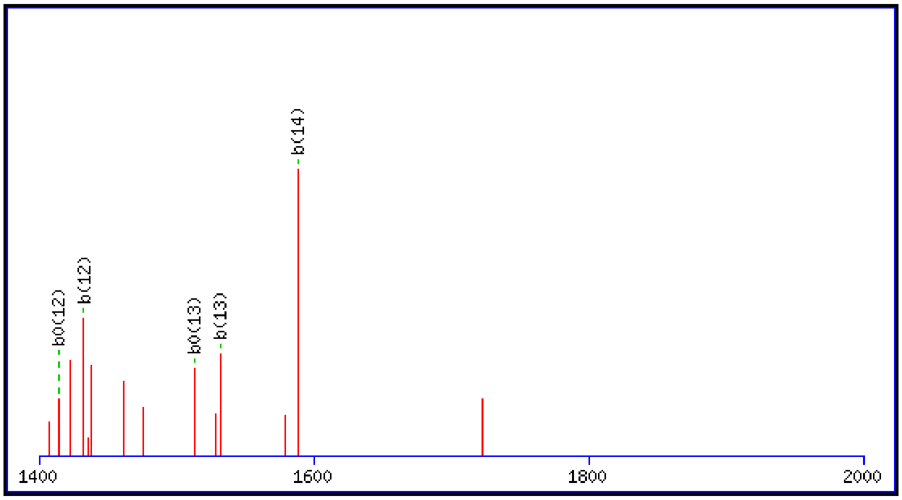

Monoisotopic mass of neutral peptide Mr(calc): 2326.9981
Fixed modifications: Carbamidomethyl (C)
Variable modifications:
N10 : Deamidated_N (N)
Ions Score: 49 **Expect:** 0.0044
Matches (Bold Red): 51/222 fragment ions using 119 most intense peaks

#	b	b^{++}	b^{*}	b^{*++}	b^{0}	b^{0++}	Seq.	y	y^{++}	y^{*}	y^{*++}	y^{0}	y^{0++}	#
1	116.0342	58.5207			98.0237	49.5155	D							20
2	272.1353	136.5713	**255.1088**	128.0580	254.1248	127.5660	R	2212.9784	1106.9928	2195.9518	1098.4796	2194.9678	1097.9875	19
3	**371.2037**	186.1055	354.1772	177.5922	353.1932	177.1002	V	2056.8773	1028.9423	2039.8507	1020.4290	2038.8667	1019.9370	18
4	468.2565	234.6319	451.2300	226.1186	450.2459	225.6266	P	1957.8089	979.4081	1940.7823	970.8948	1939.7983	970.4028	17
5	583.2835	**292.1454**	566.2569	283.6321	565.2729	283.1401	D	1860.7561	930.8817	1843.7295	922.3684	1842.7455	921.8764	16
6	670.3155	335.6614	653.2889	327.1481	**652.3049**	326.6561	S	1745.7291	873.3682	1728.7026	864.8549	1727.7186	864.3629	15
7	830.3461	415.6767	813.3196	407.1634	812.3356	406.6714	C	1658.6971	829.8522	1641.6706	821.3389	1640.6866	820.8469	14
8	990.3768	**495.6920**	973.3502	487.1788	972.3662	486.6867	C	1498.6665	749.8369	1481.6399	**741.3236**	1480.6559	**740.8316**	13
9	**1103.4608**	**552.2341**	1086.4343	543.7208	1085.4503	543.2288	I	1338.6358	669.8215	1321.6093	661.3083	1320.6253	660.8163	12
10	**1218.4878**	**609.7475**	**1201.4612**	601.2343	1200.4772	600.7422	N	**1225.5518**	613.2795	**1208.5252**	604.7662	1207.5412	604.2742	11
11	**1331.5718**	**666.2896**	1314.5453	**657.7763**	1313.5613	**657.2843**	I	**1110.5248**	555.7660	1093.4983	547.2528	**1092.5143**	546.7608	10
12	**1432.6195**	**716.8134**	1415.5930	708.3001	**1414.6090**	707.8081	T	**997.4408**	**499.2240**	**980.4142**	490.7107	979.4302	490.2187	9
13	**1531.6879**	**766.3476**	1514.6614	**757.8343**	**1513.6774**	**757.3423**	V	**896.3931**	448.7002	879.3665	440.1869	878.3825	439.6949	8
14	**1588.7094**	**794.8583**	1571.6828	786.3451	1570.6988	785.8531	G	**797.3247**	399.1660	780.2981	390.6527	779.3141	390.1607	7
15	1748.7400	**874.8737**	1731.7135	866.3604	1730.7295	865.8684	C	**740.3032**	370.6552	**723.2767**	**362.1420**	722.2926	**361.6500**	6
16	1805.7615	**903.3844**	1788.7350	894.8711	1787.7509	894.3791	G	**580.2726**	**290.6399**	563.2460	282.1266	562.2620	281.6346	5
17	1919.8044	**960.4059**	1902.7779	**951.8926**	1901.7939	951.4006	N	**523.2511**	262.1292	506.2245	253.6159	505.2405	253.1239	4
18	2034.8314	**1017.9193**	2017.8048	1009.4061	2016.8208	1008.9140	D	409.2082	205.1077	392.1816	196.5944	391.1976	196.1024	3

19	2181.8998	1091.4535	2164.8732	1082.9403	2163.8892	1082.4483	F	294.1812	147.5942	277.1547	139.0810			2
20							K	147.1128	74.0600	130.0863	65.5468			1

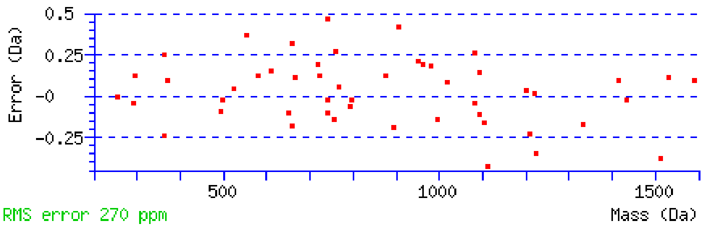

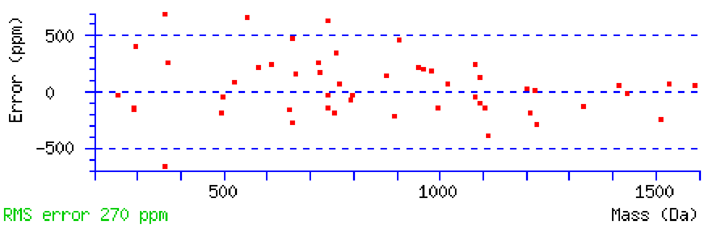

All matches to this query

Score	Mr(calc):	Delta	Sequence
48.8	2326.9981	0.0019	DRVPDSCCINITVGCGNDFK
9.1	2327.0102	-0.0102	KAYEPKLTPEEMMDSSTFK
9.1	2327.0141	-0.0141	IRNLDTTDTGYFQCVATSGK
8.5	2327.0030	-0.0030	MAMAMRQKALGTLGMTTNEK
6.3	2326.0141	0.9859	DRVPDSCCINITVGCGNDFK
6.3	2325.0056	1.9944	SSHEGLQKMQSEGNQVQSVR
6.3	2325.0056	1.9944	SSHEGLQKMQSEGNQVQSVR
4.8	2325.0130	1.9870	VNGTMVTNSSHLEVVKLIK
4.6	2326.9946	0.0054	TNLIVNYLPQNMTQDEFK
4.4	2326.9913	0.0087	GFAFVTFDNHDTVDKVVQK

Spectrum No: 444; Query: 1068; Rank: 1

Peptide View

MS/MS Fragmentation of **NENFSSGPSFETISASGLNAALAHYSPTK**
Found in **IPI00197684**, Tax_Id=10116 Gene_Symbol=Xpnpep2 Membrane-bound aminopeptidase P

Match to Query 1068: 2997.403722 from(1000.141850,3+)
Title: 091129RatKid_SCX02_20.4752.4752.3.dta
Data file K:\NewmanPaper\Piliang\3SubProteomes\Piliang3SP\mgf5ppm\SCX_3SubProteomes5ppm.mgf

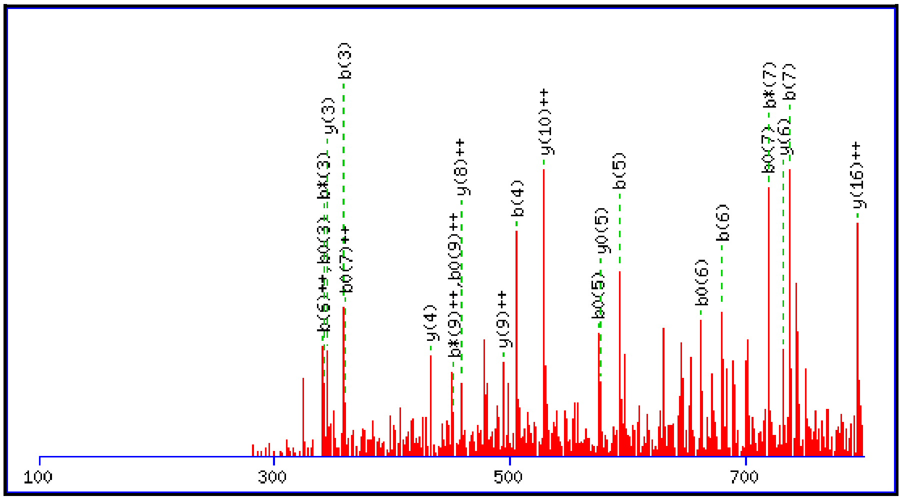

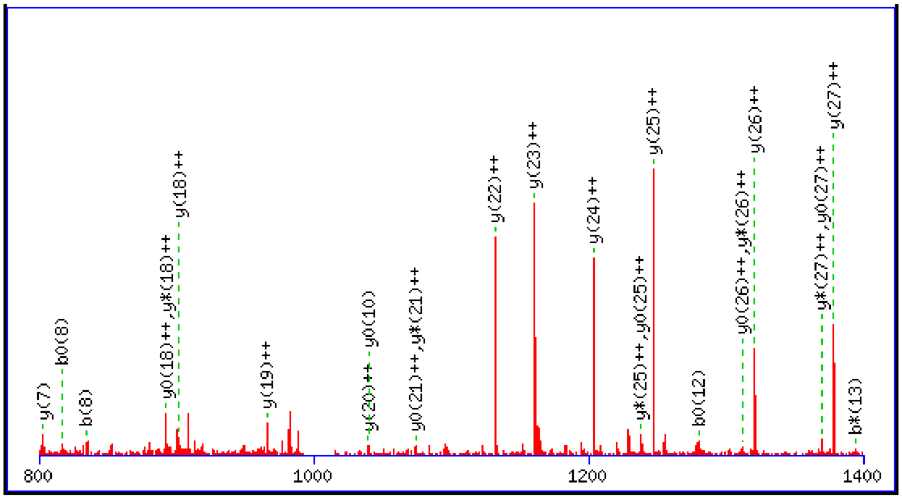

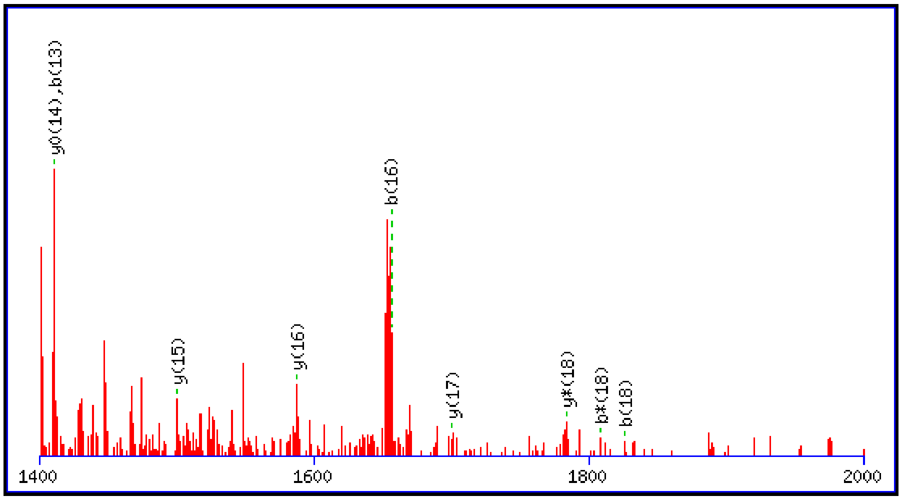

Monoisotopic mass of neutral peptide Mr(calc): 2997.3992
Fixed modifications: Carbamidomethyl (C)
Variable modifications:
N3 : Deamidated_N (N)
Ions Score: 49 **Expect:** 0.0051
Matches (Bold Red): 57/332 fragment ions using 128 most intense peaks

#	b	b^{++}	b*	b^{*++}	b^0	b^{0++}	Seq.	y	y^{++}	y*	y^{*++}	y^0	y^{0++}	#
1	115.0502	58.0287	98.0237	49.5155			N							29
2	244.0928	122.5500	227.0662	114.0368	226.0822	113.5448	E	2884.3636	1442.6854	2867.3370	1434.1721	2866.3530	1433.6801	28
3	**359.1197**	180.0635	**342.0932**	171.5502	**341.1092**	171.0582	N	2755.3210	**1378.1641**	2738.2944	**1369.6508**	2737.3104	**1369.1588**	27
4	**506.1881**	253.5977	489.1616	245.0844	488.1776	244.5924	F	2640.2940	**1320.6506**	2623.2675	**1312.1374**	2622.2835	**1311.6454**	26
5	**593.2202**	297.1137	576.1936	288.6005	**575.2096**	288.1084	S	2493.2256	**1247.1164**	2476.1991	**1238.6032**	2475.2150	**1238.1112**	25
6	**680.2522**	**340.6297**	663.2257	332.1165	**662.2416**	331.6245	S	2406.1936	**1203.6004**	2389.1670	1195.0872	2388.1830	1194.5951	24
7	**737.2737**	369.1405	**720.2471**	360.6272	**719.2631**	**360.1352**	G	2319.1616	**1160.0844**	2302.1350	1151.5711	2301.1510	1151.0791	23
8	**834.3264**	417.6669	817.2999	409.1536	**816.3159**	408.6616	P	2262.1401	**1131.5737**	2245.1135	1123.0604	2244.1295	1122.5684	22
9	921.3585	461.1829	904.3319	**452.6696**	903.3479	**452.1776**	S	2165.0873	1083.0473	2148.0608	**1074.5340**	2147.0768	**1074.0420**	21
10	1068.4269	534.7171	1051.4003	526.2038	1050.4163	525.7118	F	2078.0553	**1039.5313**	2061.0287	1031.0180	2060.0447	1030.5260	20
11	1197.4695	599.2384	1180.4429	590.7251	1179.4589	590.2331	E	1930.9869	**965.9971**	1913.9603	957.4838	1912.9763	956.9918	19
12	1298.5171	649.7622	1281.4906	641.2489	**1280.5066**	640.7569	T	1801.9443	**901.4758**	**1784.9177**	**892.9625**	1783.9337	**892.4705**	18
13	**1411.6012**	706.3042	**1394.5747**	697.7910	1393.5906	697.2990	I	**1700.8966**	850.9519	1683.8701	842.4387	1682.8860	841.9467	17
14	1498.6332	749.8203	1481.6067	741.3070	1480.6227	740.8150	S	**1587.8125**	**794.4099**	1570.7860	785.8966	1569.8020	785.4046	16
15	1569.6703	785.3388	1552.6438	776.8255	1551.6598	776.3335	A	**1500.7805**	750.8939	1483.7540	742.3806	1482.7700	741.8886	15
16	**1656.7024**	828.8548	1639.6758	820.3416	1638.6918	819.8495	S	1429.7434	715.3753	1412.7169	706.8621	**1411.7328**	706.3701	14
17	1713.7238	857.3656	1696.6973	848.8523	1695.7133	848.3603	G	1342.7114	671.8593	1325.6848	663.3461	1324.7008	662.8540	13
18	**1826.8079**	913.9076	**1809.7814**	905.3943	1808.7973	904.9023	L	1285.6899	643.3486	1268.6634	634.8353	1267.6793	634.3433	12

**19**	1940.8508	970.9291	1923.8243	962.4158	1922.8403	961.9238	**N**	1172.6058	586.8066	1155.5793	578.2933	1154.5953	577.8013	**11**
**20**	2011.8879	1006.4476	1994.8614	997.9343	1993.8774	997.4423	**A**	1058.5629	**529.7851**	1041.5364	521.2718	**1040.5524**	520.7798	**10**
**21**	2082.9251	1041.9662	2065.8985	1033.4529	2064.9145	1032.9609	**A**	987.5258	**494.2665**	970.4993	485.7533	969.5152	485.2613	**9**
**22**	2196.0091	1098.5082	2178.9826	1089.9949	2177.9986	1089.5029	**L**	916.4887	**458.7480**	899.4621	450.2347	898.4781	449.7427	**8**
**23**	2267.0462	1134.0268	2250.0197	1125.5135	2249.0357	1125.0215	**A**	**803.4046**	402.2060	786.3781	393.6927	785.3941	393.2007	**7**
**24**	2404.1052	1202.5562	2387.0786	1194.0429	2386.0946	1193.5509	**H**	**732.3675**	366.6874	715.3410	358.1741	714.3570	357.6821	**6**
**25**	2567.1685	1284.0879	2550.1419	1275.5746	2549.1579	1275.0826	**Y**	595.3086	298.1579	578.2821	289.6447	**577.2980**	289.1527	**5**
**26**	2654.2005	1327.6039	2637.1740	1319.0906	2636.1899	1318.5986	**S**	**432.2453**	216.6263	415.2187	208.1130	414.2347	207.6210	**4**
**27**	2751.2533	1376.1303	2734.2267	1367.6170	2733.2427	1367.1250	**P**	**345.2132**	173.1103	328.1867	164.5970	327.2027	164.1050	**3**
**28**	2852.3010	1426.6541	2835.2744	1418.1408	2834.2904	1417.6488	**T**	248.1605	124.5839	231.1339	116.0706	230.1499	115.5786	**2**
**29**							**K**	147.1128	74.0600	130.0863	65.5468			**1**

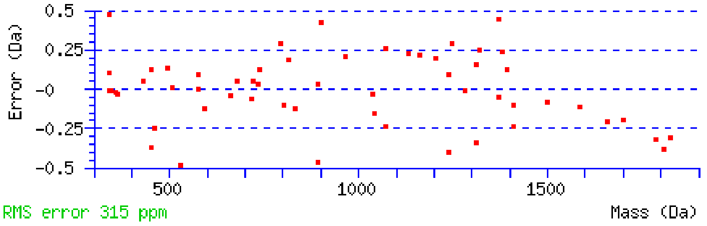

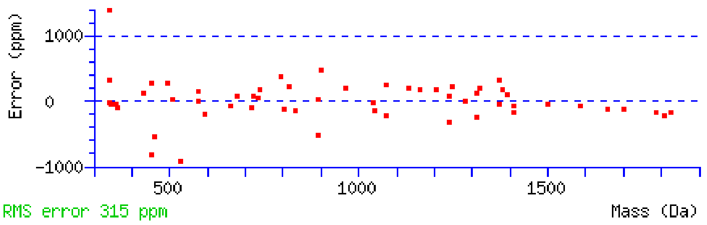

All matches to this query

Score	Mr(calc):	Delta	Sequence
48.7	2997.3992	0.0046	NENFSSGPSFETISASGLNAALAHYSPTK
43.3	2997.3992	0.0046	NENFSSGPSFETISASGLNAALAHYSPTK
35.0	2997.3992	0.0046	NENFSSGPSFETISASGLNAALAHYSPTK
26.2	2996.4152	0.9886	NENFSSGPSFETISASGLNAALAHYSPTK
4.5	2997.4054	-0.0017	VKSLNLIGQGTFSYYAISNATRLLK
1.4	2996.3887	1.0150	KCGKIWGLFDGQTPVFAIMDTEMIK
0.8	2997.4054	-0.0017	VKSLNLIGQGTFSYYAISNATRLLK
0.8	2995.3689	2.0348	IQNSQLQLQLNQDLSILHQEDGSSK
0.8	2996.3949	1.0089	SIILGWAQGKLYQDMSYVFLFSVR
0.8	2996.3949	1.0089	SIILGWAQGKLYQDMSYVFLFSVR

Spectrum No: 445; Query: 140; Rank: 1

Peptide View

MS/MS Fragmentation of **VFQNCSELYK**
Found in **IPI00368780**, Tax_Id=10116 Gene_Symbol=Tmem87a_predicted similar to CG17660-PA

Match to Query 140: 1287.582188 from(644.798370,2+)
Title: 091127RatKid_SCX01_02.1678.1678.2.dta
Data file K:\NewmanPaper\Piliang\3SubProteomes\Piliang3SP\mgf5ppm\SCX_3SubProteomes5ppm.mgf

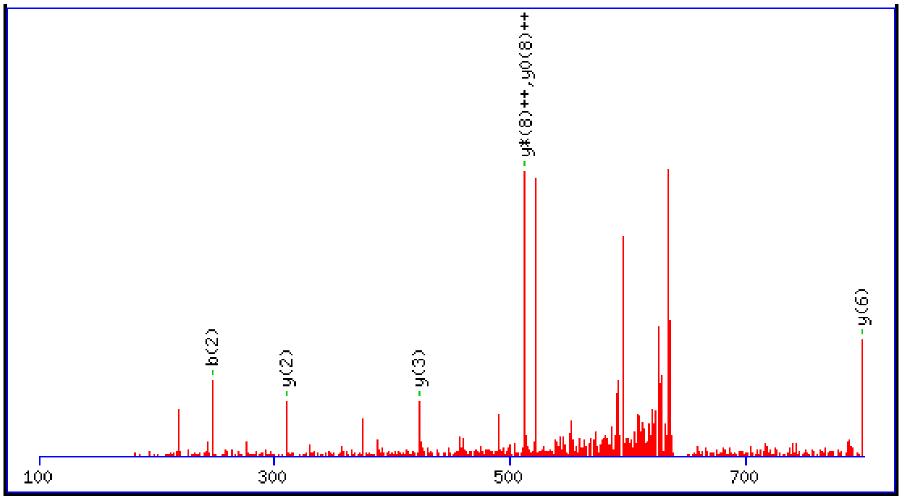

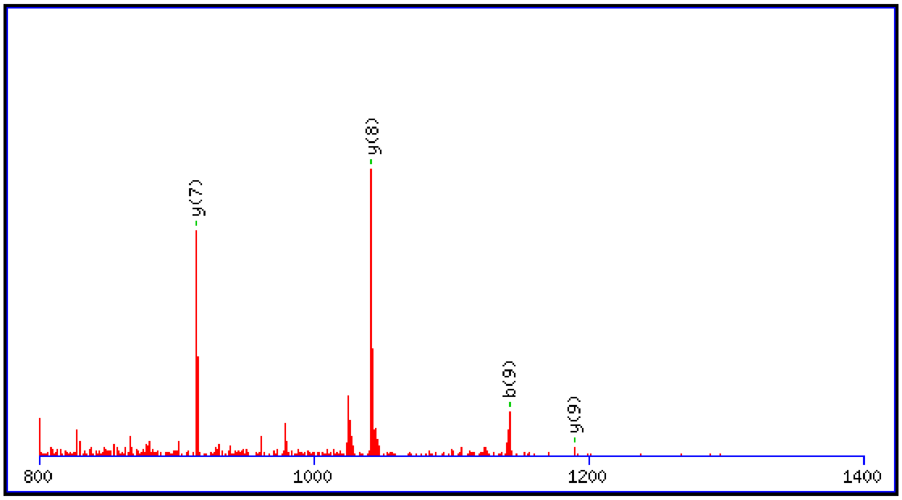

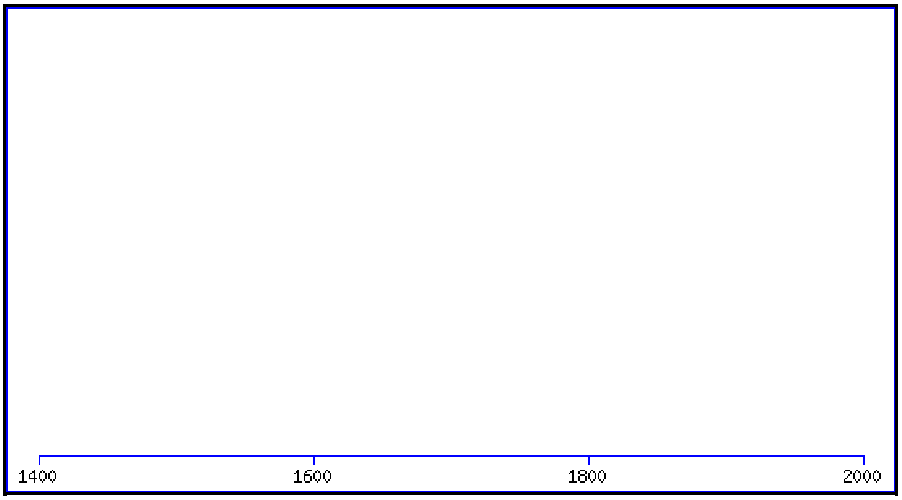

Monoisotopic mass of neutral peptide Mr(calc): 1287.5805
Fixed modifications: Carbamidomethyl (C)
Variable modifications:
N4 : Deamidated_N (N)
Ions Score: 49 **Expect:** 0.0019
Matches (Bold Red): 10/88 fragment ions using 17 most intense peaks

#	b	b^{++}	b*	b^{*++}	b^{0}	b^{0++}	Seq.	y	y^{++}	y*	y^{*++}	y^{0}	y^{0++}	#
1	100.0757	50.5415					V							10
2	**247.1441**	124.0757					F	**1189.5194**	595.2633	1172.4928	586.7501	1171.5088	586.2581	9
3	375.2027	188.1050	358.1761	179.5917			Q	**1042.4510**	521.7291	1025.4244	**513.2159**	1024.4404	**512.7238**	8
4	490.2296	245.6184	473.2031	237.1052			N	**914.3924**	457.6998	897.3659	449.1866	896.3818	448.6946	7
5	650.2603	325.6338	633.2337	317.1205			C	**799.3655**	400.1864	782.3389	391.6731	781.3549	391.1811	6

**6**	737.2923	369.1498	720.2657	360.6365	719.2817	360.1445	**S**	639.3348	320.1710	622.3083	311.6578	621.3243	311.1658	**5**
**7**	866.3349	433.6711	849.3083	425.1578	848.3243	424.6658	**E**	552.3028	276.6550	535.2762	268.1418	534.2922	267.6498	**4**
**8**	979.4190	490.2131	962.3924	481.6998	961.4084	481.2078	**L**	**423.2602**	212.1337	406.2336	203.6205			**3**
**9**	**1142.4823**	571.7448	1125.4557	563.2315	1124.4717	562.7395	**Y**	**310.1761**	155.5917	293.1496	147.0784			**2**
**10**							**K**	147.1128	74.0600	130.0863	65.5468			**1**

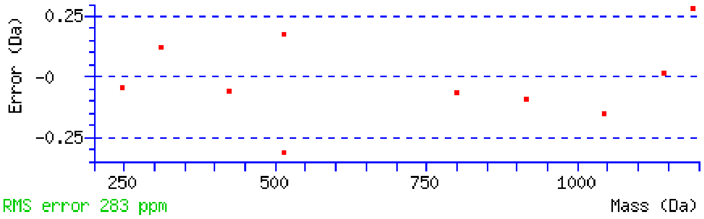

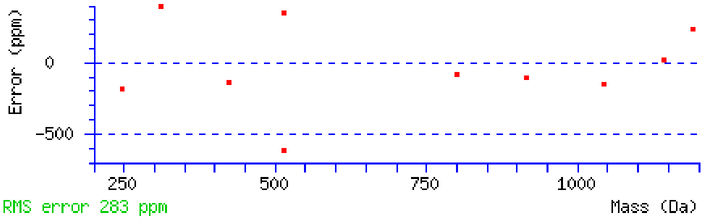

All matches to this query

Score	Mr(calc):	Delta	Sequence
48.6	1287.5805	0.0017	VFQNCSELYK
12.6	1287.5877	-0.0056	MNQDGLTPQQR
11.9	1285.5778	2.0044	FVMTIENPAGK
11.8	1285.5778	2.0044	QITMVPFDQK
10.4	1287.5748	0.0074	FAQIDEKTEK
10.0	1286.5867	0.9955	NISKASRSSEK
9.3	1286.5867	0.9955	NISKASRSSEK
9.1	1285.5876	1.9946	LITLEEEMTK
8.4	1287.5894	-0.0072	EMRSSLDKVK
8.4	1287.5894	-0.0072	EMRSSLDKVK

Spectrum No: 446; Query: 963; Rank: 1

Peptide View

MS/MS Fragmentation of **VIDLWDLAQSANFTEKELESFR**
Found in **IPI00364124**, Tax_Id=10116 Gene_Symbol=Lrpap1 Alpha-2-macroglobulin receptor-associated protein precursor

Match to Query 963: 2611.282542 from(871.434790,3+)
Title: 091129RatKid_SCX02_23.7130.7130.3.dta
Data file K:\NewmanPaper\Piliang\3SubProteomes\Piliang3SP\mgf5ppm\SCX_3SubProteomes5ppm.mgf

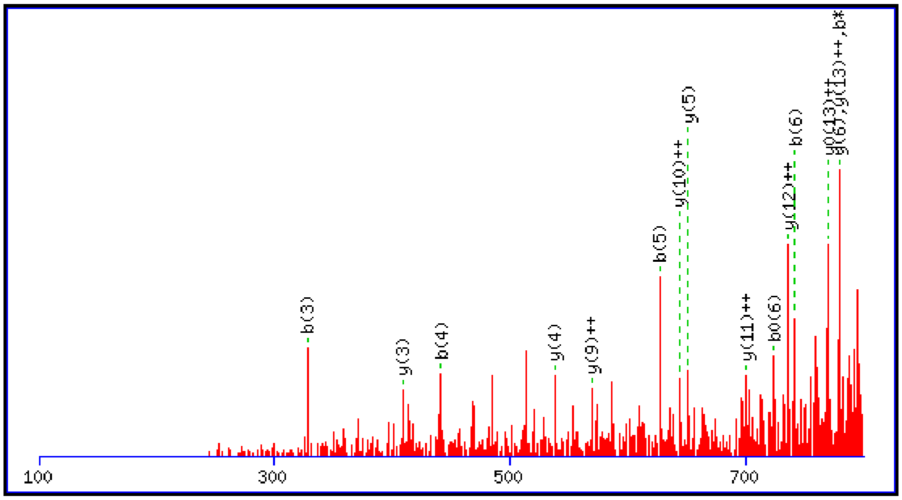

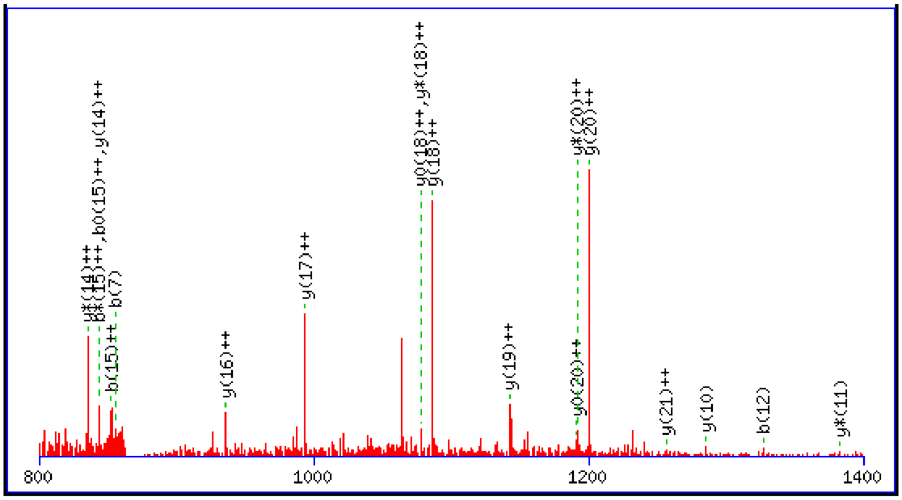

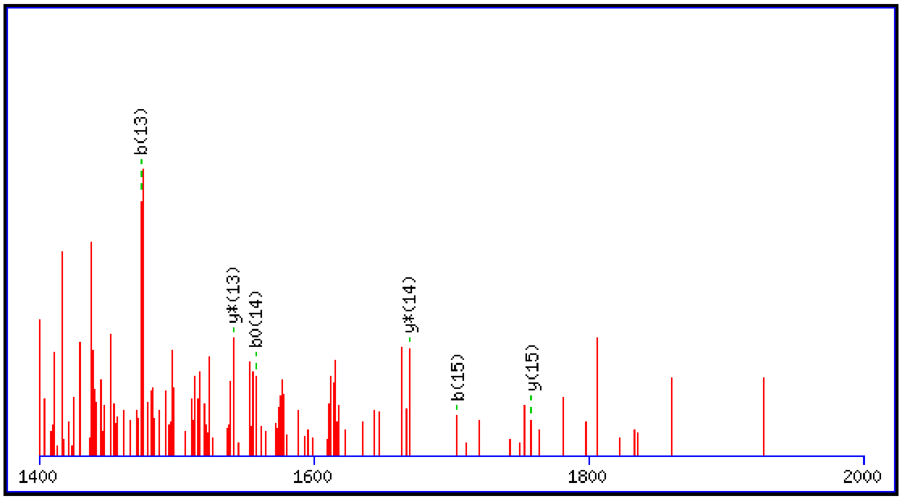

Monoisotopic mass of neutral peptide Mr(calc): 2611.2806
Fixed modifications: Carbamidomethyl (C)
Variable modifications:
N12 : Deamidated_N (N)
Ions Score: 49 **Expect:** 0.0038
Matches (Bold Red): 41/228 fragment ions using 91 most intense peaks

#	b	b++	b*	b*++	b0	b0++	Seq.	y	y++	y*	y*++	y0	y0++	#
1	100.0757	50.5415					V							22
2	213.1598	107.0835					I	2513.2195	**1257.1134**	2496.1929	1248.6001	2495.2089	1248.1081	21
3	**328.1867**	164.5970			310.1761	155.5917	D	2400.1354	**1200.5713**	2383.1088	**1192.0581**	2382.1248	**1191.5661**	20
4	**441.2708**	221.1390			423.2602	212.1337	L	2285.1084	**1143.0579**	2268.0819	1134.5446	2267.0979	1134.0526	19
5	**627.3501**	314.1787			609.3395	305.1734	W	2172.0244	**1086.5158**	2154.9978	**1078.0026**	2154.0138	**1077.5105**	18
6	**742.3770**	371.6921			**724.3665**	362.6869	D	1985.9451	**993.4762**	1968.9185	984.9629	1967.9345	984.4709	17
7	**855.4611**	428.2342			837.4505	419.2289	L	1870.9181	**935.9627**	1853.8916	927.4494	1852.9076	926.9574	16
8	926.4982	463.7527			908.4876	454.7475	A	**1757.8341**	879.4207	1740.8075	870.9074	1739.8235	870.4154	15
9	1054.5568	527.7820	1037.5302	519.2688	1036.5462	518.7767	Q	1686.7969	**843.9021**	**1669.7704**	**835.3888**	1668.7864	834.8968	14
10	1141.5888	571.2980	1124.5623	562.7848	1123.5782	562.2928	S	1558.7384	**779.8728**	**1541.7118**	771.3595	1540.7278	**770.8675**	13
11	1212.6259	606.8166	1195.5994	598.3033	1194.6154	597.8113	A	1471.7063	**736.3568**	1454.6798	727.8435	1453.6958	727.3515	12
12	**1327.6529**	664.3301	1310.6263	655.8168	1309.6423	655.3248	N	1400.6692	**700.8383**	**1383.6427**	692.3250	1382.6587	691.8330	11
13	**1474.7213**	737.8643	1457.6947	729.3510	1456.7107	728.8590	F	**1285.6423**	**643.3248**	1268.6157	634.8115	1267.6317	634.3195	10
14	1575.7689	788.3881	1558.7424	**779.8748**	**1557.7584**	779.3828	T	1138.5739	**569.7906**	1121.5473	561.2773	1120.5633	560.7853	9
15	**1704.8115**	**852.9094**	1687.7850	**844.3961**	1686.8010	**843.9041**	E	1037.5262	519.2667	1020.4997	510.7535	1019.5156	510.2615	8
16	1832.9065	916.9569	1815.8800	908.4436	1814.8959	907.9516	K	908.4836	454.7454	891.4571	446.2322	890.4730	445.7402	7
17	1961.9491	981.4782	1944.9225	972.9649	1943.9385	972.4729	E	**780.3886**	390.6980	763.3621	382.1847	762.3781	381.6927	6
18	2075.0332	1038.0202	2058.0066	1029.5069	2057.0226	1029.0149	L	**651.3461**	326.1767	634.3195	317.6634	633.3355	317.1714	5

**19**	2204.0758	1102.5415	2187.0492	1094.0282	2186.0652	1093.5362	**E**	**538.2620**	269.6346	521.2354	261.1214	520.2514	260.6293	**4**
**20**	2291.1078	1146.0575	2274.0812	1137.5443	2273.0972	1137.0522	**S**	**409.2194**	205.1133	392.1928	196.6001	391.2088	196.1081	**3**
**21**	2438.1762	1219.5917	2421.1496	1211.0785	2420.1656	1210.5865	**F**	322.1874	161.5973	305.1608	153.0840			**2**
**22**							**R**	175.1190	88.0631	158.0924	79.5498			**1**

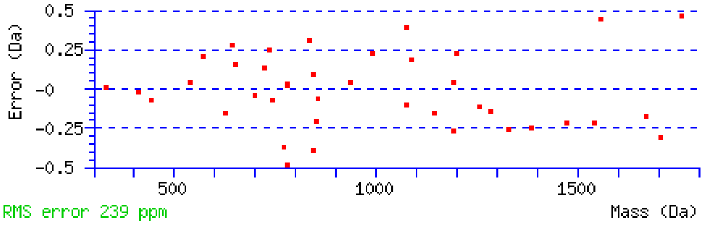

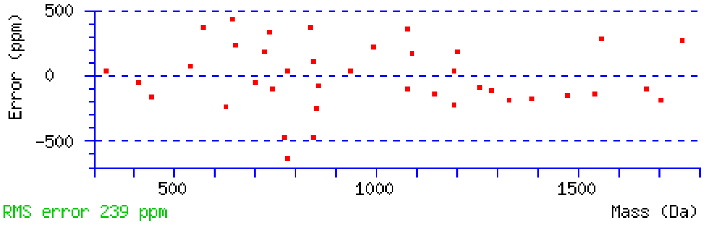

All matches to this query

Score	Mr(calc):	Delta	Sequence
48.6	2611.2806	0.0020	VIDLWDLAQSANFTEKELESFR
14.8	2610.2966	0.9860	VIDLWDLAQSANFTEKELESFR
4.1	2610.2682	1.0143	LLDFCKTNGIILVAYGVLGTQR
2.0	2611.2903	-0.0077	IVDLACLNHFTRVSNMIAKLAK
1.2	2609.2954	1.9871	IPVEANFLYVYSTKPNYYSWR
1.2	2610.2794	1.0031	IPVEANFLYVYSTKPNYYSWR
1.2	2611.2975	-0.0149	NIDGEALVAIESFYPVFTPPPKK
1.2	2611.2975	-0.0149	NIDGEALVAIESFYPVFTPPPKK
1.1	2609.2585	2.0240	NDELTSLAEETRALKDEIDVLR
1.1	2609.2585	2.0240	NDELTSLAEETRALKDEIDVLR

Spectrum No: 447; Query: 934; Rank: 1

Peptide View

MS/MS Fragmentation of **EVAGIPLMDPTVNNWSQIQTFK**
Found in **IPI00204469**, Tax_Id=10116 Gene_Symbol=Sult1c2a Sulfotransferase 1C2A

Match to Query 934: 2488.231648 from(1245.123100,2+)
Title: 091127RatKid_SCX01_16.5496.5496.2.dta
Data file K:\NewmanPaper\Piliang\3SubProteomes\Piliang3SP\mgf5ppm\SCX_3SubProteomes5ppm.mgf

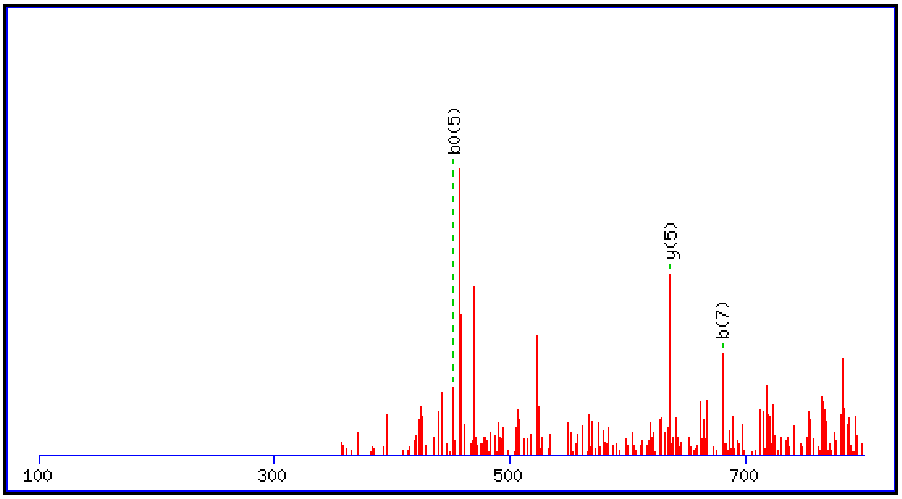

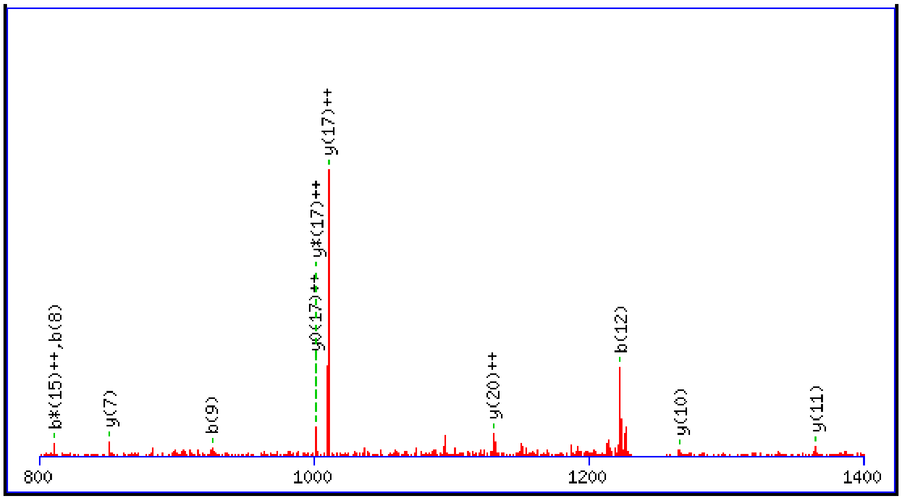

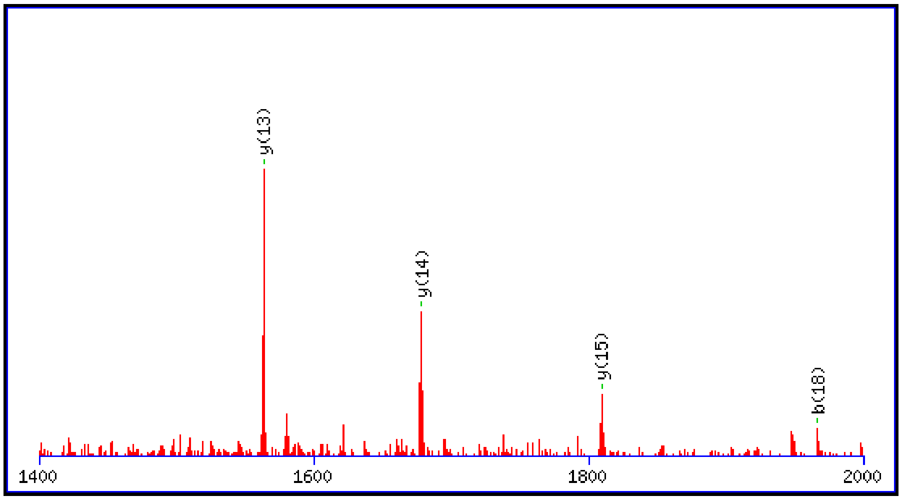

Monoisotopic mass of neutral peptide Mr(calc): 2488.2308
Fixed modifications: Carbamidomethyl (C)
Variable modifications:
N14 : Deamidated_N (N)
Ions Score: 49 **Expect:** 0.0041
Matches (Bold Red): 18/224 fragment ions using 28 most intense peaks

#	b	b^{++}	b*	b^{*++}	b^0	b^{0++}	Seq.	y	y^{++}	y*	y^{*++}	y^0	y^{0++}	#
1	130.0499	65.5286			112.0393	56.5233	E							22
2	229.1183	115.0628			211.1077	106.0575	V	2360.1955	1180.6014	2343.1689	1172.0881	2342.1849	1171.5961	21
3	300.1554	150.5813			282.1448	141.5761	A	2261.1271	**1131.0672**	2244.1005	1122.5539	2243.1165	1122.0619	20
4	357.1769	179.0921			339.1663	170.0868	G	2190.0900	1095.5486	2173.0634	1087.0353	2172.0794	1086.5433	19
5	470.2609	235.6341			**452.2504**	226.6288	I	2133.0685	1067.0379	2116.0419	1058.5246	2115.0579	1058.0326	18
6	567.3137	284.1605			549.3031	275.1552	P	2019.9844	**1010.4959**	2002.9579	**1001.9826**	2001.9739	**1001.4906**	17
7	**680.3978**	340.7025			662.3872	331.6972	L	1922.9317	961.9695	1905.9051	953.4562	1904.9211	952.9642	16
8	**811.4382**	406.2228			793.4277	397.2175	M	**1809.8476**	905.4274	1792.8211	896.9142	1791.8370	896.4222	15
9	**926.4652**	463.7362			908.4546	454.7309	D	**1678.8071**	839.9072	1661.7806	831.3939	1660.7966	830.9019	14
10	1023.5179	512.2626			1005.5074	503.2573	P	**1563.7802**	782.3937	1546.7536	773.8805	1545.7696	773.3884	13
11	1124.5656	562.7865			1106.5551	553.7812	T	1466.7274	733.8673	1449.7009	725.3541	1448.7168	724.8621	12
12	**1223.6340**	612.3207			1205.6235	603.3154	V	**1365.6797**	683.3435	1348.6532	674.8302	1347.6692	674.3382	11
13	1337.6770	669.3421	1320.6504	660.8288	1319.6664	660.3368	N	**1266.6113**	633.8093	1249.5848	625.2960	1248.6008	624.8040	10
14	1452.7039	726.8556	1435.6774	718.3423	1434.6933	717.8503	N	1152.5684	576.7878	1135.5418	568.2746	1134.5578	567.7826	9
15	1638.7832	819.8952	1621.7567	**811.3820**	1620.7726	810.8900	W	1037.5415	519.2744	1020.5149	510.7611	1019.5309	510.2691	8
16	1725.8152	863.4113	1708.7887	854.8980	1707.8047	854.4060	S	**851.4621**	426.2347	834.4356	417.7214	833.4516	417.2294	7
17	1853.8738	927.4405	1836.8473	918.9273	1835.8633	918.4353	Q	764.4301	382.7187	747.4036	374.2054	746.4196	373.7134	6
18	**1966.9579**	983.9826	1949.9313	975.4693	1948.9473	974.9773	I	**636.3715**	318.6894	619.3450	310.1761	618.3610	309.6841	5

19	2095.0165	1048.0119	2077.9899	1039.4986	2077.0059	1039.0066	Q	523.2875	262.1474	506.2609	253.6341	505.2769	253.1421	4
20	2196.0641	1098.5357	2179.0376	1090.0224	2178.0536	1089.5304	T	395.2289	198.1181	378.2023	189.6048	377.2183	189.1128	3
21	2343.1326	1172.0699	2326.1060	1163.5566	2325.1220	1163.0646	F	294.1812	147.5942	277.1547	139.0810			2
22							K	147.1128	74.0600	130.0863	65.5468			1

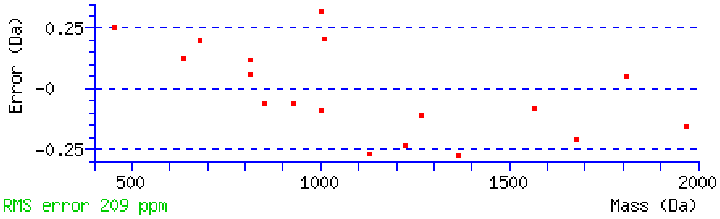

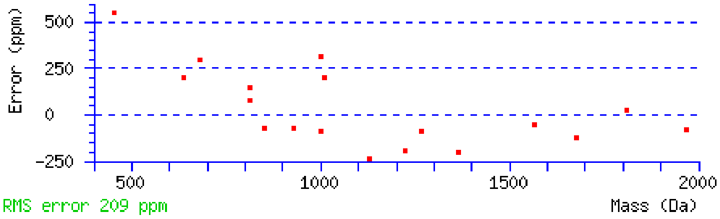

All matches to this query

Score	Mr(calc):	Delta	Sequence
48.5	2488.2308	0.0008	EVAGIPLMDPTVNNWSQIQTFK
48.5	2488.2308	0.0008	EVAGIPLMDPTVNNWSQIQTFK
30.5	2487.2468	0.9849	EVAGIPLMDPTVNNWSQIQTFK
2.4	2488.2168	0.0149	ILSRLILLWYNPVTEEDVR
1.2	2488.2227	0.0090	TPLSIVTVPQSAAKSETVSKSAK
0.4	2487.2491	0.9825	DSLGWLPIQLTPFYPVGYTIK
0.3	2488.2106	0.0210	LNEVEKLLETVISMPCKYSR
0.2	2487.2491	0.9825	DSLGWLPIQLTPFYPVGYTIK

Spectrum No: 448; Query: 186; Rank: 1

Peptide View

MS/MS Fragmentation of **NGTILQLAISPSR**
Found in **IPI00196499**, Tax_Id=10116 Gene_Symbol=Elmo2 similar to Engulfment and cell motility protein 2

Match to Query 186: 1369.757808 from(685.886180,2+)
Title: 091129RatKid_SCX02_14.3450.3450.2.dta
Data file K:\NewmanPaper\Piliang\3SubProteomes\Piliang3SP\mgf5ppm\SCX_3SubProteomes5ppm.mgf

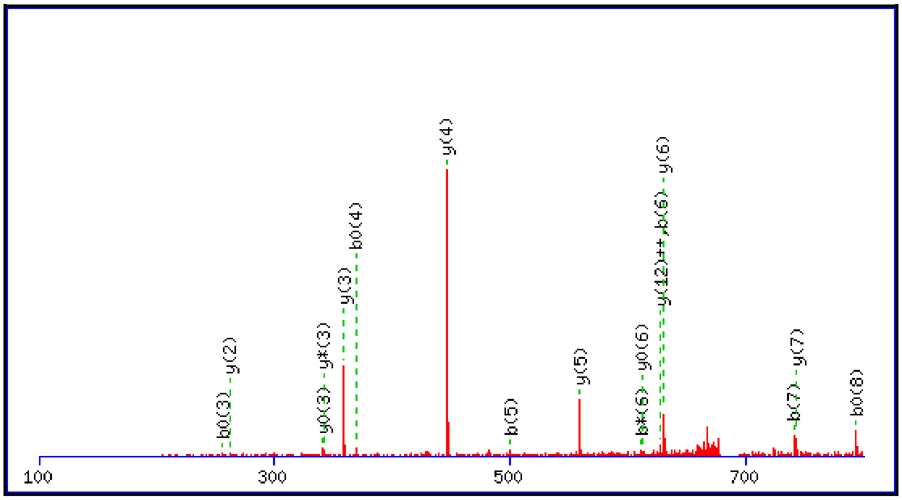

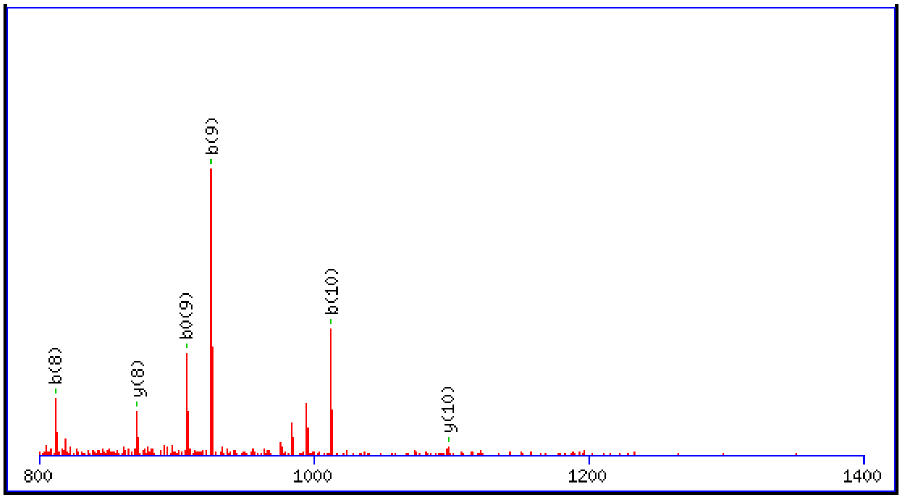

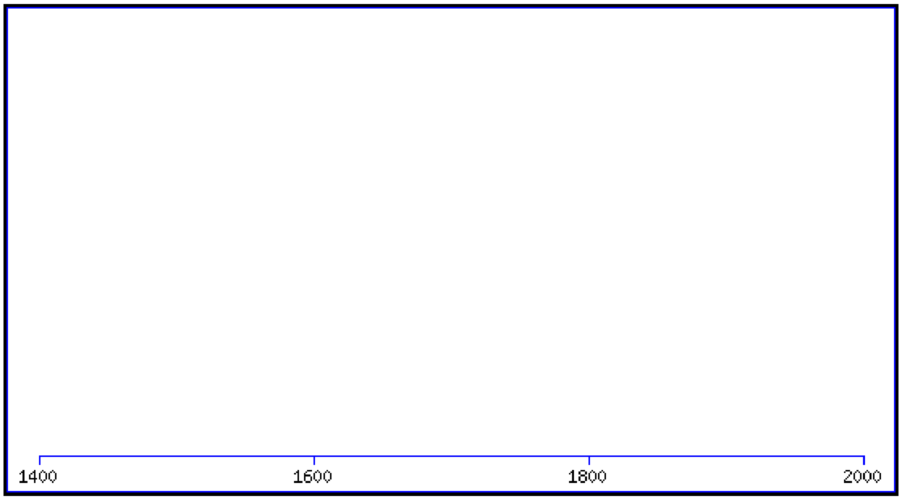

Monoisotopic mass of neutral peptide Mr(calc): 1369.7565
Fixed modifications: Carbamidomethyl (C)
Variable modifications:
N1 : Deamidated_N (N)
Ions Score: 48 **Expect:** 0.00082
Matches (Bold Red): 23/138 fragment ions using 43 most intense peaks

#	b	b++	b*	b*++	b0	b0++	Seq.	y	y++	y*	y*++	y0	y0++	#
1	116.0342	58.5207	99.0077	50.0075			N							13
2	173.0557	87.0315	156.0291	78.5182			G	1255.7369	**628.3721**	1238.7103	619.8588	1237.7263	619.3668	12
3	274.1034	137.5553	257.0768	129.0420	**256.0928**	128.5500	T	1198.7154	599.8613	1181.6889	591.3481	1180.7048	590.8561	11
4	387.1874	194.0973	370.1609	185.5841	**369.1769**	185.0921	I	**1097.6677**	549.3375	1080.6412	540.8242	1079.6572	540.3322	10
5	**500.2715**	250.6394	483.2449	242.1261	482.2609	241.6341	L	984.5837	492.7955	967.5571	484.2822	966.5731	483.7902	9
6	**628.3301**	314.6687	**611.3035**	306.1554	610.3195	305.6634	Q	**871.4996**	436.2534	854.4730	427.7402	853.4890	427.2482	8
7	**741.4141**	371.2107	724.3876	362.6974	723.4036	362.2054	L	**743.4410**	372.2241	726.4145	363.7109	725.4304	363.2189	7
8	**812.4512**	406.7293	795.4247	398.2160	**794.4407**	397.7240	A	**630.3569**	315.6821	613.3304	307.1688	**612.3464**	306.6768	6
9	**925.5353**	463.2713	908.5088	454.7580	**907.5247**	454.2660	I	**559.3198**	280.1636	542.2933	271.6503	541.3093	271.1583	5
10	**1012.5673**	506.7873	995.5408	498.2740	994.5568	497.7820	S	**446.2358**	223.6215	429.2092	215.1082	428.2252	214.6162	4
11	1109.6201	555.3137	1092.5935	546.8004	1091.6095	546.3084	P	**359.2037**	180.1055	**342.1772**	171.5922	**341.1932**	171.1002	3
12	1196.6521	598.8297	1179.6256	590.3164	1178.6416	589.8244	S	**262.1510**	131.5791	245.1244	123.0659	244.1404	122.5738	2
13							R	175.1190	88.0631	158.0924	79.5498			1

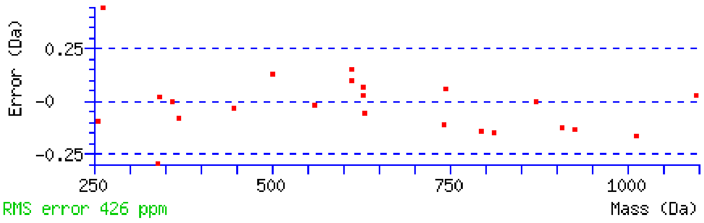

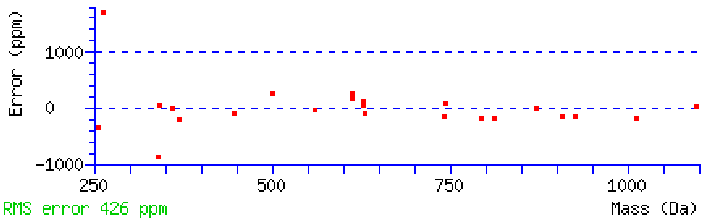

All matches to this query

Score	Mr(calc):	Delta	Sequence
48.5	1369.7565	0.0013	NGTILQLAISPSR
15.6	1369.7677	-0.0099	EIPTKRSLSPSR
6.6	1369.7565	0.0013	VRAPTAESLVAEK
5.0	1369.7453	0.0125	SSGLLASPVLDSPK
4.4	1369.7677	-0.0099	ELDARRADLLAK
4.1	1369.7540	0.0038	GMTRYFKQIVK
3.7	1369.7453	0.0126	LQEAQLELADIK
3.7	1369.7453	0.0125	LINLDKDSIPDK

Spectrum No: 449; Query: 58; Rank: 1

Peptide View

MS/MS Fragmentation of **NGSQIDAVLDK**
Found in **IPI00393036**, Tax_Id=10116 Gene_Symbol=RGD1560364_predicted similar to vacuolar protein sorting 13C protein isoform 1A

Match to Query 58: 1159.574368 from(580.794460,2+)
Title: 091127RatKid_SCX01_15.2058.2058.2.dta
Data file K:\NewmanPaper\Piliang\3SubProteomes\Piliang3SP\mgf5ppm\SCX_3SubProteomes5ppm.mgf

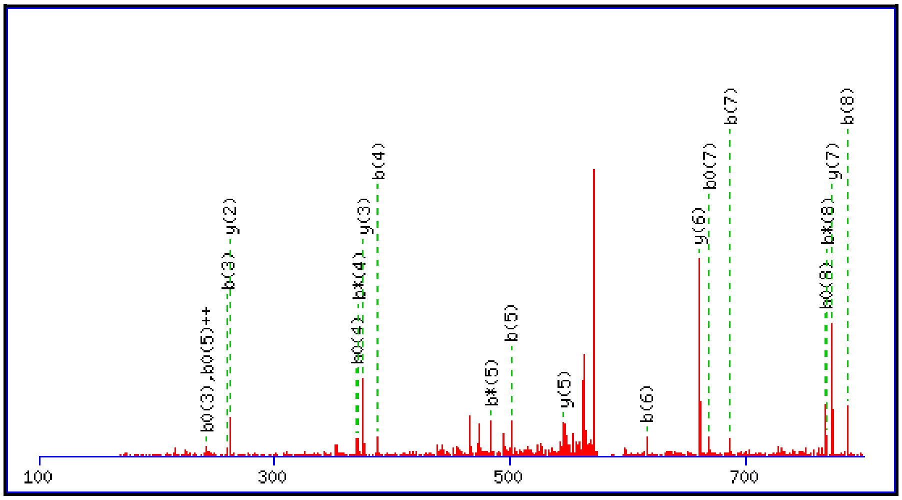

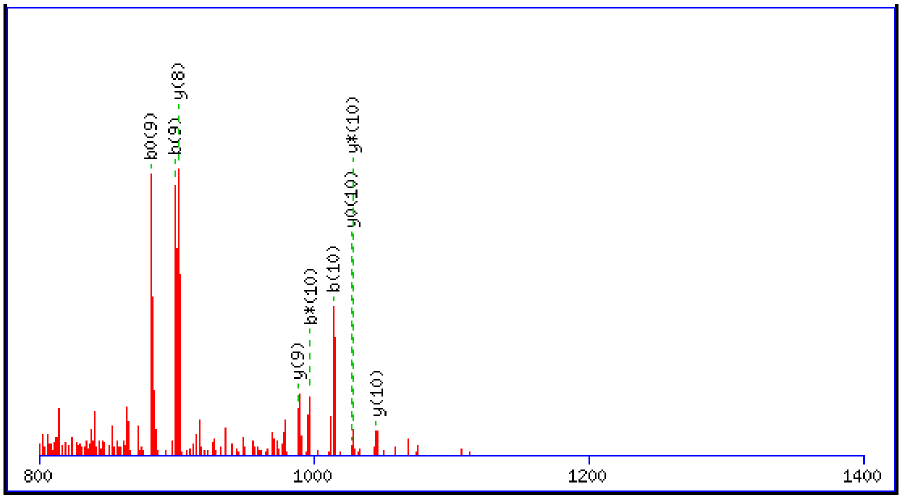

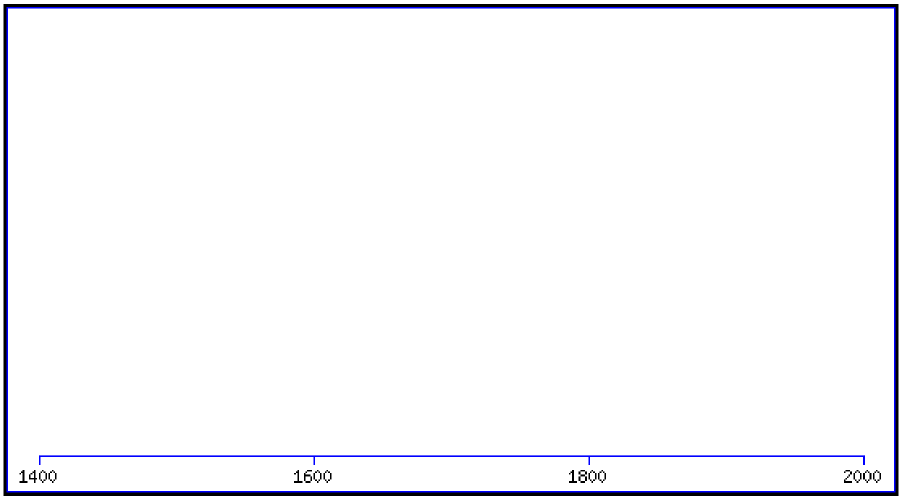

Monoisotopic mass of neutral peptide Mr(calc): 1159.5721
Fixed modifications: Carbamidomethyl (C)
Variable modifications:
N1 : Deamidated_N (N)
Ions Score: 48 **Expect:** 0.0017
Matches (Bold Red): 28/114 fragment ions using 63 most intense peaks

#	b	b++	b*	b*++	b0	b0++	Seq.	y	y++	y*	y*++	y0	y0++	#
1	116.0342	58.5207	99.0077	50.0075			N							11
2	173.0557	87.0315	156.0291	78.5182			G	**1045.5524**	523.2798	**1028.5259**	514.7666	**1027.5419**	514.2746	10
3	**260.0877**	130.5475	243.0612	122.0342	**242.0771**	121.5422	S	**988.5310**	494.7691	971.5044	486.2558	970.5204	485.7638	9
4	**388.1463**	194.5768	**371.1197**	186.0635	**370.1357**	185.5715	Q	**901.4989**	451.2531	884.4724	442.7398	883.4884	442.2478	8
5	**501.2303**	251.1188	**484.2038**	242.6055	483.2198	**242.1135**	I	**773.4403**	387.2238	756.4138	378.7105	755.4298	378.2185	7
6	**616.2573**	308.6323	599.2307	300.1190	598.2467	299.6270	D	**660.3563**	330.6818	643.3297	322.1685	642.3457	321.6765	6
7	**687.2944**	344.1508	670.2679	335.6376	**669.2838**	335.1456	A	**545.3293**	273.1683	528.3028	264.6550	527.3188	264.1630	5
8	**786.3628**	393.6850	**769.3363**	385.1718	**768.3523**	384.6798	V	474.2922	237.6498	457.2657	229.1365	456.2817	228.6445	4
9	**899.4469**	450.2271	882.4203	441.7138	**881.4363**	441.2218	L	**375.2238**	188.1155	358.1973	179.6023	357.2132	179.1103	3
10	**1014.4738**	507.7405	**997.4473**	499.2273	996.4633	498.7353	D	**262.1397**	131.5735	245.1132	123.0602	244.1292	122.5682	2
11							K	147.1128	74.0600	130.0863	65.5468			1

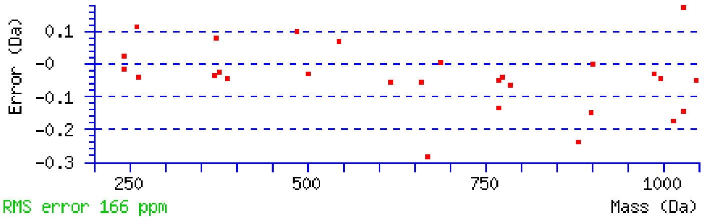

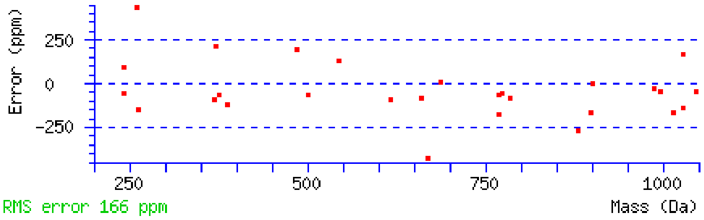

All matches to this query

Score	Mr(calc):	Delta	Sequence
48.4	1159.5721	0.0023	NGSQIDAVLDK
12.1	1159.5721	0.0023	IQEESEGAGLK
11.4	1159.5712	0.0031	TCLLIVFSK
10.2	1159.5678	0.0065	GLTYLWSLK
9.6	1159.5695	0.0048	HLEFMNQLK
7.7	1159.5720	0.0023	QLEAENNTLK
6.8	1159.5720	0.0023	QLEAENNTLK
6.6	1158.5685	1.0058	NFSAAKSLLK
6.4	1159.5825	-0.0081	MKTVFVTVR
6.0	1159.5833	-0.0089	NLGDTRQNLK

Spectrum No: 450; Query: 308; Rank: 1

Peptide View

MS/MS Fragmentation of **LCDYENHFNTSK**
Found in **IPI00326242**, Tax_Id=10116 Gene_Symbol=Clcn5 Chloride channel protein 5

Match to Query 308: 1527.631508 from(764.823030,2+)
Title: 091127RatKid_SCX01_13.965.965.2.dta
Data file K:\NewmanPaper\Piliang\3SubProteomes\Piliang3SP\mgf5ppm\SCX_3SubProteomes5ppm.mgf

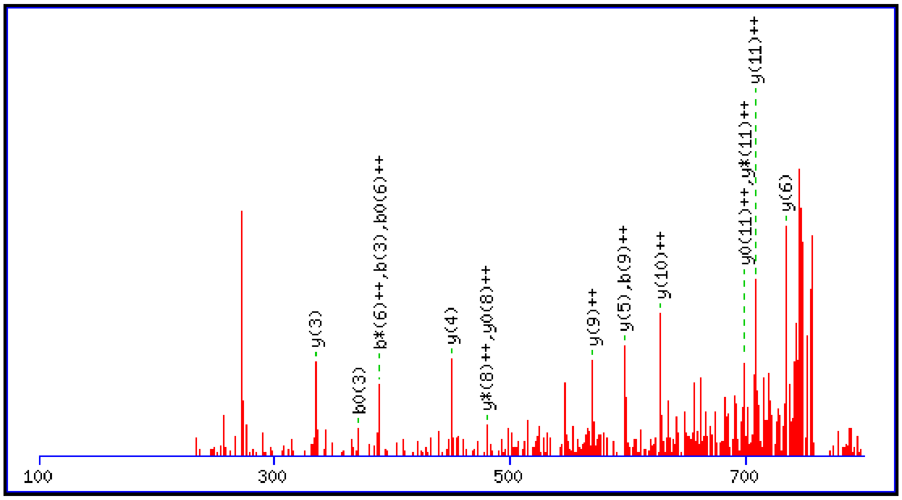

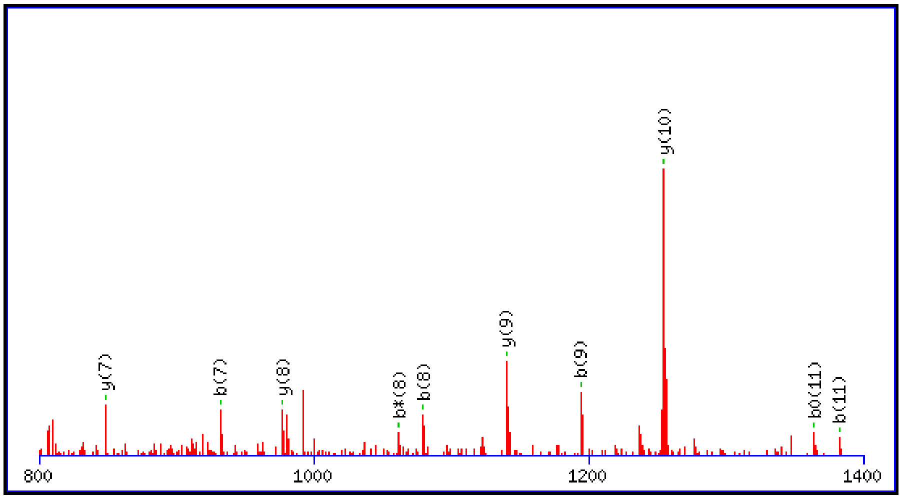

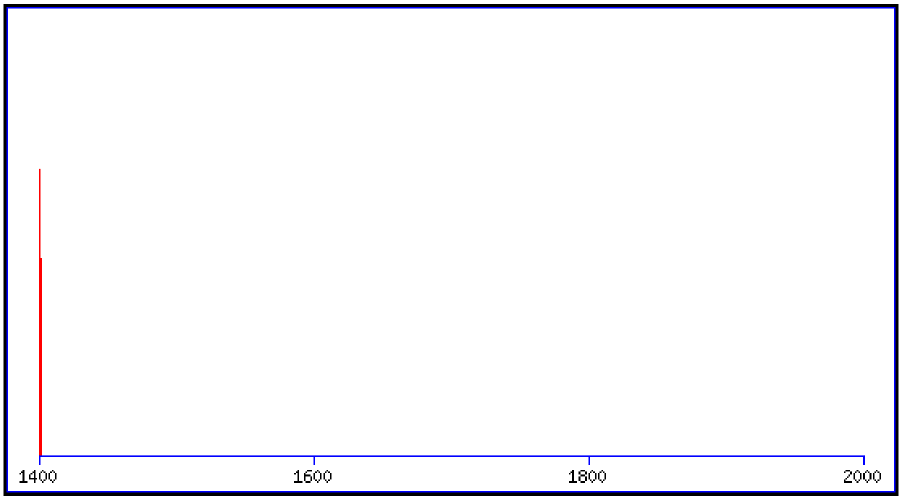

Monoisotopic mass of neutral peptide Mr(calc): 1527.6300
Fixed modifications: Carbamidomethyl (C)
Variable modifications:
N9 : Deamidated_N (N)
Ions Score: 48 **Expect:** 0.0018
Matches (Bold Red): 26/116 fragment ions using 40 most intense peaks

#	b	b^{++}	b*	b^{*++}	b^{0}	b^{0++}	Seq.	y	y^{++}	y*	y^{*++}	y^{0}	y^{0++}	#
1	114.0913	57.5493					L							12
2	274.1220	137.5646					C	1415.5532	**708.2802**	1398.5267	**699.7670**	1397.5426	**699.2750**	11
3	**389.1489**	195.0781			**371.1384**	186.0728	D	**1255.5226**	**628.2649**	1238.4960	619.7516	1237.5120	619.2596	10
4	552.2123	276.6098			534.2017	267.6045	Y	**1140.4956**	**570.7514**	1123.4691	562.2382	1122.4851	561.7462	9
5	681.2549	341.1311			663.2443	332.1258	E	**977.4323**	489.2198	960.4057	**480.7065**	959.4217	**480.2145**	8
6	795.2978	398.1525	778.2712	**389.6393**	777.2872	**389.1472**	N	**848.3897**	424.6985	831.3632	416.1852	830.3791	415.6932	7
7	**932.3567**	466.6820	915.3301	458.1687	914.3461	457.6767	H	**734.3468**	367.6770	717.3202	359.1638	716.3362	358.6717	6
8	**1079.4251**	540.2162	**1062.3986**	531.7029	1061.4145	531.2109	F	**597.2879**	299.1476	580.2613	290.6343	579.2773	290.1423	5
9	**1194.4520**	**597.7297**	1177.4255	589.2164	1176.4415	588.7244	N	**450.2194**	225.6134	433.1929	217.1001	432.2089	216.6081	4
10	1295.4997	648.2535	1278.4732	639.7402	1277.4892	639.2482	T	**335.1925**	168.0999	318.1660	159.5866	317.1819	159.0946	3
11	**1382.5318**	691.7695	1365.5052	683.2562	**1364.5212**	682.7642	S	234.1448	117.5761	217.1183	109.0628	216.1343	108.5708	2
12							K	147.1128	74.0600	130.0863	65.5468			1

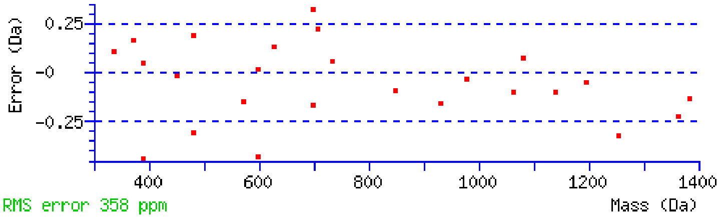

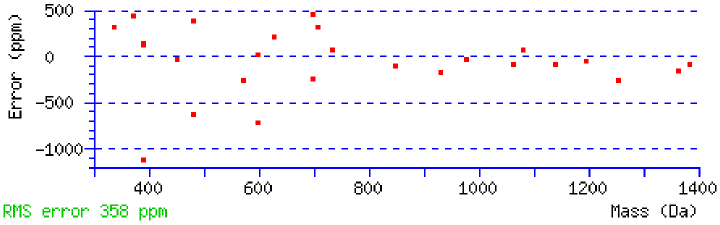

All matches to this query

Score	Mr(calc):	Delta	Sequence
48.4	1527.6300	0.0015	LCDYENHFNTSK
21.0	1527.6300	0.0015	LCDYENHFNTSK
6.7	1525.6300	2.0015	RDDRTHASRPR
3.7	1525.6160	2.0155	MGYTNLSYLNPR
3.0	1526.6250	1.0065	ASSSGNDDDLTIPR
2.6	1526.6151	1.0164	NSSYPETGHGKNR
2.6	1526.6151	1.0164	NSSYPETGHGKNR
2.0	1527.6388	-0.0073	TIQQIKSTIEK
1.9	1527.6330	-0.0015	SLCVWGAQWXDR

1.4	1527.6380	-0.0065	KMGVPYCTIKR

Spectrum No: 451; Query: 106; Rank: 1

Peptide View

MS/MS Fragmentation of **TGNWSALPSCK**
Found in **IPI00195241**, Tax_Id=10116 Gene_Symbol=Apoh Beta-2-glycoprotein 1 precursor

Match to Query 106: 1220.552368 from(611.283460,2+)
Title: 091127RatKid_SCX01_03.1694.1694.2.dta
Data file K:\NewmanPaper\Piliang\3SubProteomes\Piliang3SP\mgf5ppm\SCX_3SubProteomes5ppm.mgf

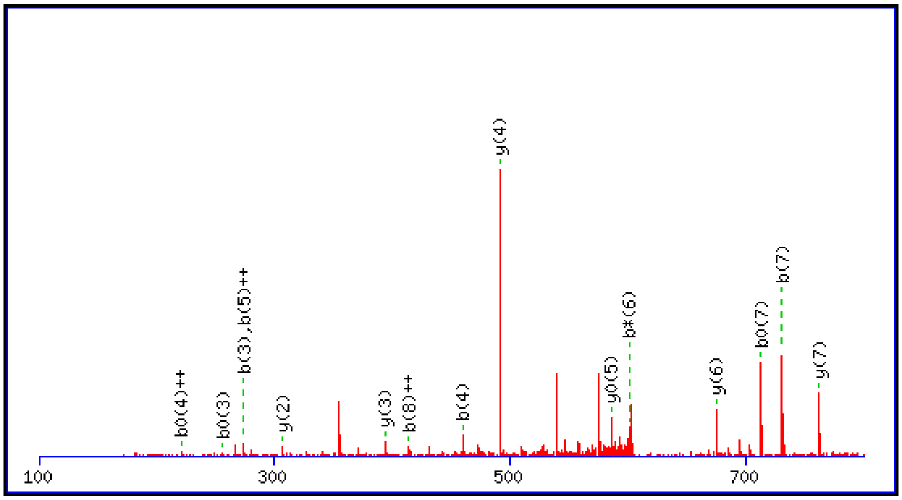

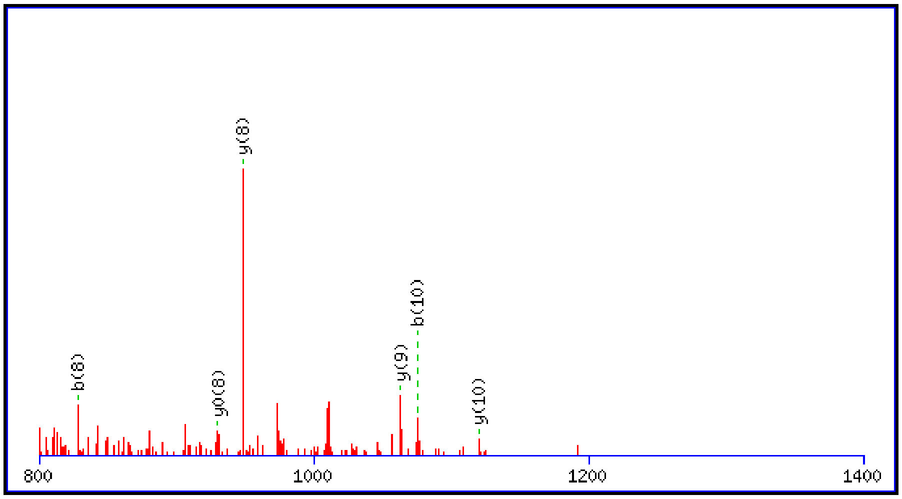

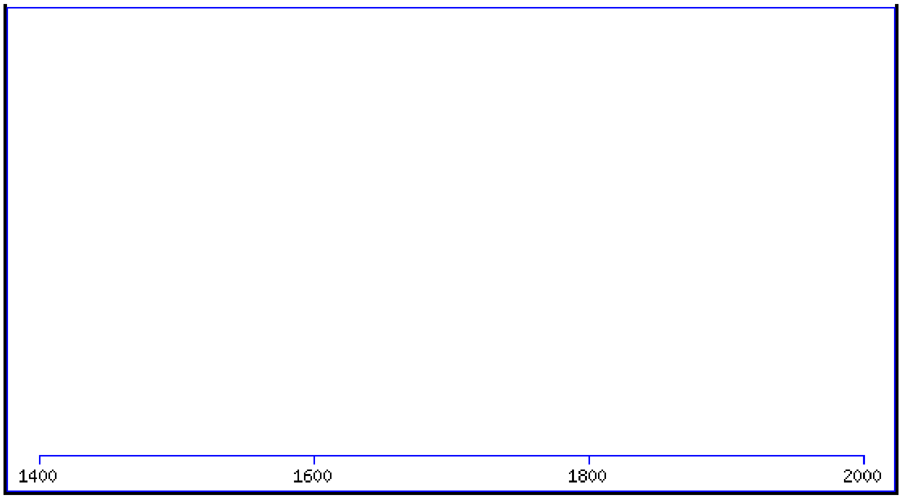

Monoisotopic mass of neutral peptide Mr(calc): 1220.5496
Fixed modifications: Carbamidomethyl (C)
Variable modifications:
N3 : Deamidated_N (N)
Ions Score: 48 **Expect:** 0.0016
Matches (Bold Red): 21/112 fragment ions using 43 most intense peaks

#	b	b^{++}	b*	b*$^{++}$	b^{0}	b^{0++}	Seq.	y	y^{++}	y*	y*$^{++}$	y^{0}	y^{0++}	#
1	102.0550	51.5311			84.0444	42.5258	T							11
2	159.0764	80.0418			141.0659	71.0366	G	**1120.5092**	560.7582	1103.4826	552.2449	1102.4986	551.7529	10
3	**274.1034**	137.5553	257.0768	129.0420	**256.0928**	128.5500	N	**1063.4877**	532.2475	1046.4612	523.7342	1045.4771	523.2422	9
4	**460.1827**	230.5950	443.1561	222.0817	442.1721	**221.5897**	W	**948.4608**	474.7340	931.4342	466.2207	**930.4502**	465.7287	8
5	547.2147	**274.1110**	530.1881	265.5977	529.2041	265.1057	S	**762.3815**	381.6944	745.3549	373.1811	744.3709	372.6891	7
6	618.2518	309.6295	**601.2253**	301.1163	600.2412	300.6243	A	**675.3494**	338.1783	658.3229	329.6651	657.3389	329.1731	6
7	**731.3359**	366.1716	714.3093	357.6583	**713.3253**	357.1663	L	604.3123	302.6598	587.2858	294.1465	**586.3017**	293.6545	5
8	**828.3886**	**414.6980**	811.3621	406.1847	810.3781	405.6927	P	**491.2282**	246.1178	474.2017	237.6045	473.2177	237.1125	4
9	915.4207	458.2140	898.3941	449.7007	897.4101	449.2087	S	**394.1755**	197.5914	377.1489	189.0781	376.1649	188.5861	3
10	**1075.4513**	538.2293	1058.4248	529.7160	1057.4408	529.2240	C	**307.1435**	154.0754	290.1169	145.5621			2
11							K	147.1128	74.0600	130.0863	65.5468			1

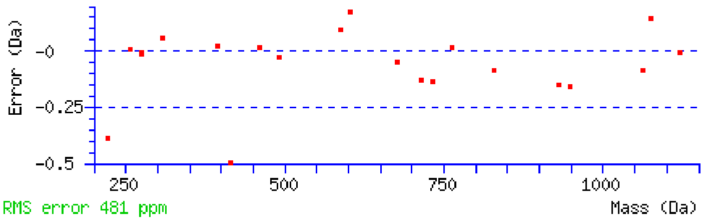

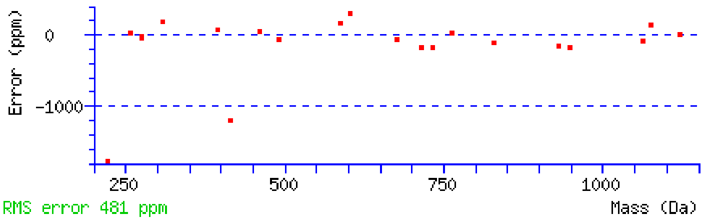

All matches to this query

Score	Mr(calc):	Delta	Sequence
48.4	1220.5496	0.0028	TGNWSALPSCK
0.6	1220.5452	0.0072	HRSPNGGLFR
0.4	1220.5594	-0.0070	LEELNLNSCK

Spectrum No: 452; Query: 32; Rank: 1

Peptide View

MS/MS Fragmentation of **IANISMEVGR**
Found in **IPI00363534**, Tax_Id=10116 Gene_Symbol=Lama1 laminin, alpha 1

Match to Query 32: 1105.545828 from(553.780190,2+)
Title: 091127RatKid_SCX01_13.1091.1091.2.dta
Data file K:\NewmanPaper\Piliang\3SubProteomes\Piliang3SP\mgf5ppm\SCX_3SubProteomes5ppm.mgf

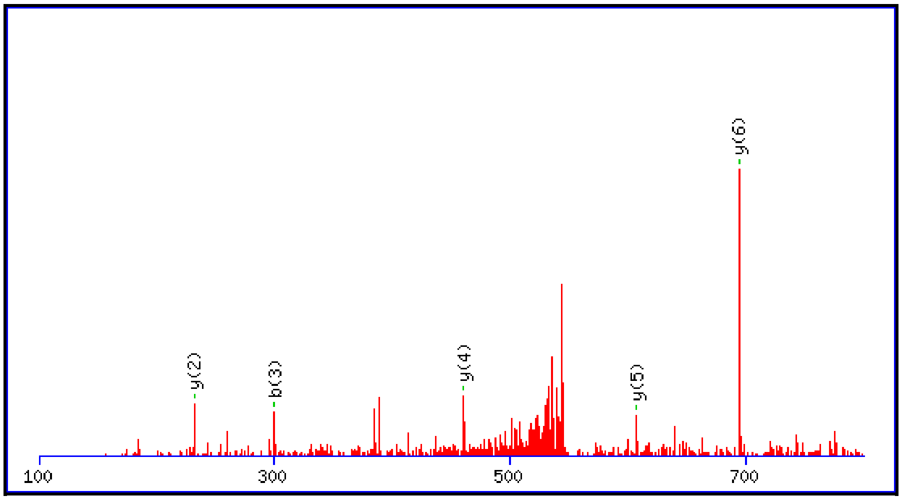

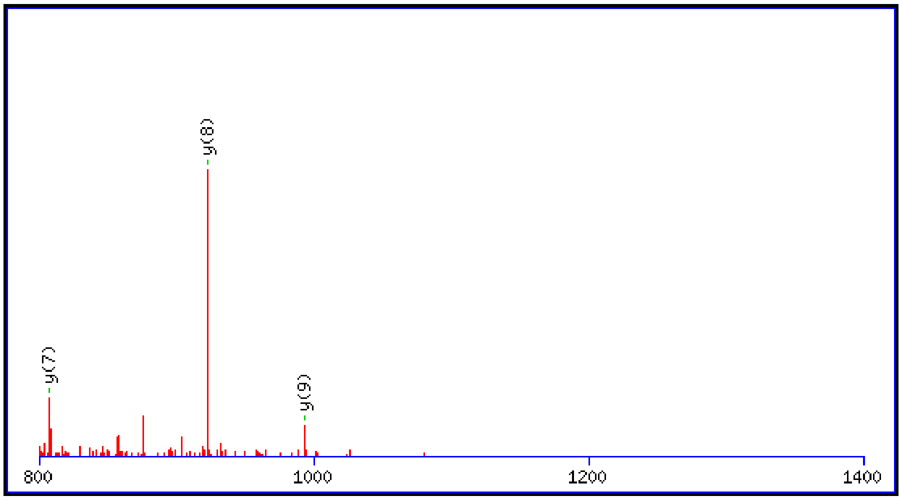

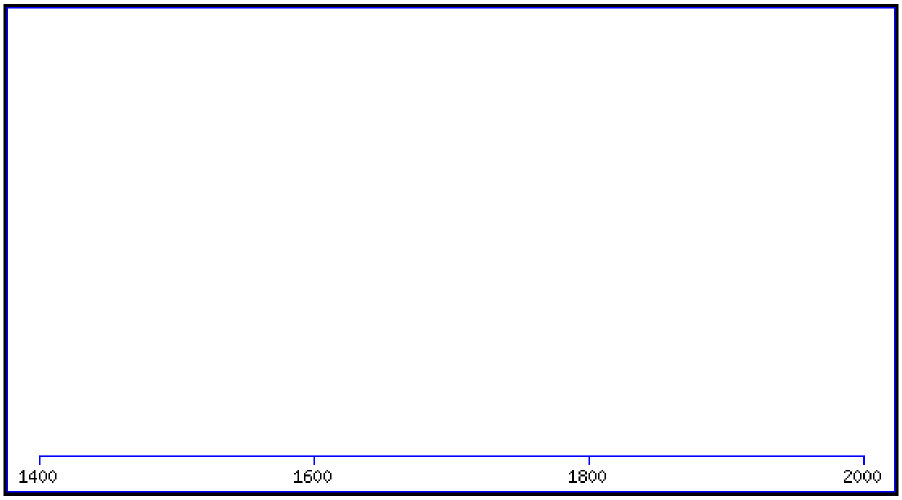

Monoisotopic mass of neutral peptide Mr(calc): 1105.5437
Fixed modifications: Carbamidomethyl (C)
Variable modifications:
N3 : Deamidated_N (N)
M6 : Oxidation (M)
Ions Score: 48 **Expect:** 0.0018
Matches (Bold Red): 8/90 fragment ions using 24 most intense peaks

#	b	b^{++}	b*	b^{*++}	b^0	b^{0++}	Seq.	y	y^{++}	y*	y^{*++}	y^0	y^{0++}	#
1	114.0913	57.5493					I							10

2	185.1285	93.0679					A	**993.4670**	497.2371	976.4404	488.7238	975.4564	488.2318	9
3	**300.1554**	150.5813	283.1288	142.0681			N	**922.4299**	461.7186	905.4033	453.2053	904.4193	452.7133	8
4	413.2395	207.1234	396.2129	198.6101			I	**807.4029**	404.2051	790.3764	395.6918	789.3924	395.1998	7
5	500.2715	250.6394	483.2449	242.1261	482.2609	241.6341	S	**694.3189**	347.6631	677.2923	339.1498	676.3083	338.6578	6
6	647.3069	324.1571	630.2803	315.6438	629.2963	315.1518	M	**607.2868**	304.1471	590.2603	295.6338	589.2763	295.1418	5
7	776.3495	388.6784	759.3229	380.1651	758.3389	379.6731	E	**460.2514**	230.6293	443.2249	222.1161	442.2409	221.6241	4
8	875.4179	438.2126	858.3913	429.6993	857.4073	429.2073	V	331.2088	166.1081	314.1823	157.5948			3
9	932.4394	466.7233	915.4128	458.2100	914.4288	457.7180	G	**232.1404**	116.5738	215.1139	108.0606			2
10							R	175.1190	88.0631	158.0924	79.5498			1

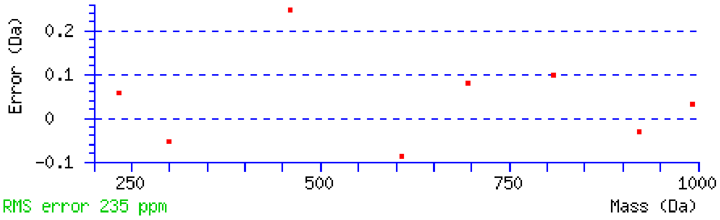

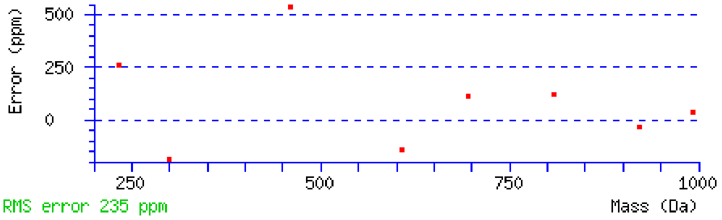

All matches to this query

Score	Mr(calc):	Delta	Sequence
48.1	1105.5437	0.0021	IANISMEVGR
13.7	1105.5404	0.0055	LADAAENFQK
9.9	1105.5478	-0.0019	IALNMDWVK
7.9	1105.5403	0.0055	LANAAGFNAEK
4.3	1103.5376	2.0082	TDIYKKTR
3.5	1104.5485	0.9973	IADLCLGTDK
2.5	1103.5458	2.0000	VERENSELK

Spectrum No: 453; Query: 362; Rank: 1

Peptide View

MS/MS Fragmentation of **EVTPVDLPNCTLVK**
Found in **IPI00231264**, Tax_Id=10116 Gene_Symbol=Pon1 Serum paraoxonase/arylesterase 1

Match to Query 362: 1584.812148 from(793.413350,2+)
Title: 091127RatKid_SCX01_02.3063.3063.2.dta
Data file K:\NewmanPaper\Piliang\3SubProteomes\Piliang3SP\mgf5ppm\SCX_3SubProteomes5ppm.mgf

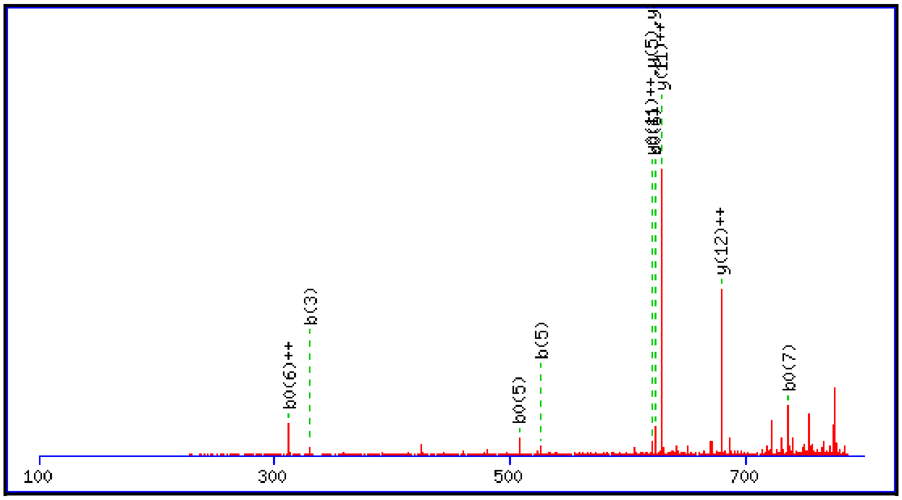

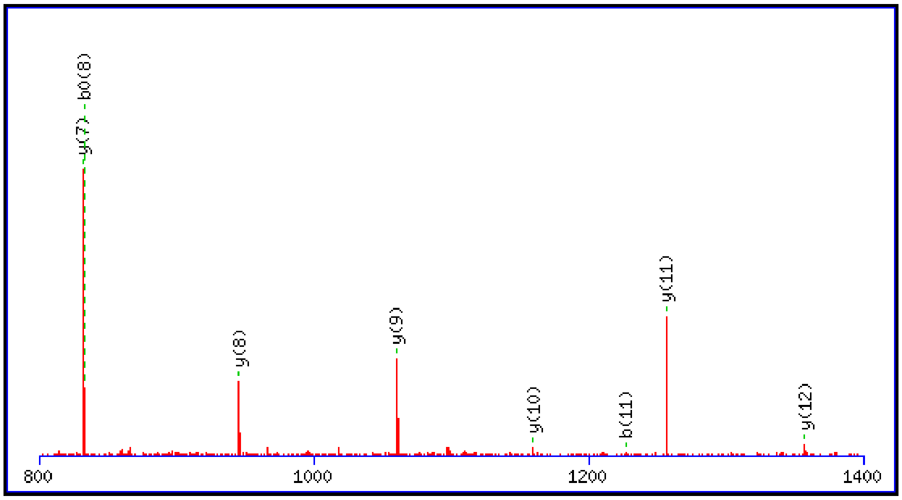

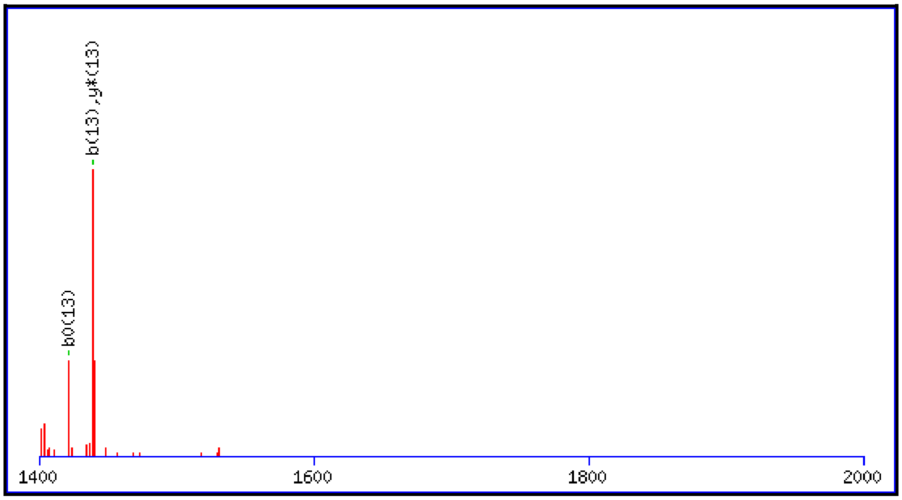

Monoisotopic mass of neutral peptide Mr(calc): 1584.8069
Fixed modifications: Carbamidomethyl (C)
Variable modifications:
N9 : Deamidated_N (N)
Ions Score: 48 **Expect:** 0.002
Matches (Bold Red): 23/134 fragment ions using 31 most intense peaks

#	b	b^{++}	b*	b^{*++}	b^{0}	b^{0++}	Seq.	y	y^{++}	y*	y^{*++}	y^{0}	y^{0++}	#
1	130.0499	65.5286			112.0393	56.5233	E							14
2	229.1183	115.0628			211.1077	106.0575	V	1456.7716	728.8894	**1439.7450**	720.3762	1438.7610	719.8841	13
3	**330.1660**	165.5866			**312.1554**	156.5813	T	**1357.7032**	**679.3552**	1340.6766	670.8420	1339.6926	670.3499	12
4	427.2187	214.1130			409.2082	205.1077	P	**1256.6555**	**628.8314**	1239.6289	**620.3181**	1238.6449	**619.8261**	11
5	**526.2871**	263.6472			**508.2766**	254.6419	V	**1159.6027**	580.3050	1142.5762	571.7917	1141.5922	571.2997	10
6	641.3141	321.1607			**623.3035**	**312.1554**	D	**1060.5343**	530.7708	1043.5078	522.2575	1042.5238	521.7655	9
7	754.3981	377.7027			**736.3876**	368.6974	L	**945.5074**	473.2573	928.4808	464.7441	927.4968	464.2520	8
8	851.4509	426.2291			**833.4403**	417.2238	P	**832.4233**	416.7153	815.3968	408.2020	814.4127	407.7100	7
9	966.4778	483.7426	949.4513	475.2293	948.4673	474.7373	N	735.3705	368.1889	718.3440	359.6756	717.3600	359.1836	6
10	1126.5085	563.7579	1109.4819	555.2446	1108.4979	554.7526	C	**620.3436**	310.6754	603.3171	302.1622	602.3330	301.6702	5
11	**1227.5562**	614.2817	1210.5296	605.7685	1209.5456	605.2764	T	460.3130	230.6601	443.2864	222.1468	442.3024	221.6548	4
12	1340.6402	670.8238	1323.6137	662.3105	1322.6297	661.8185	L	359.2653	180.1363	342.2387	171.6230			3
13	**1439.7087**	720.3580	1422.6821	711.8447	**1421.6981**	711.3527	V	246.1812	123.5942	229.1547	115.0810			2
14							K	147.1128	74.0600	130.0863	65.5468			1

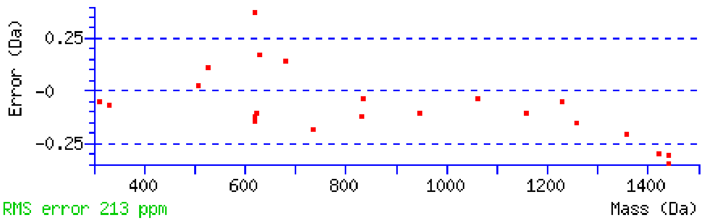

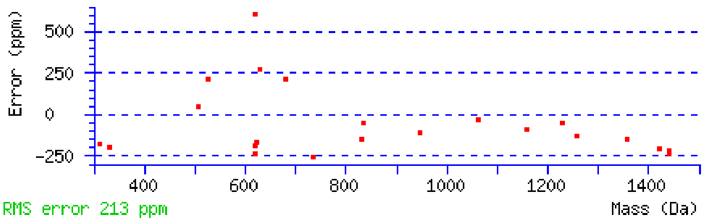

All matches to this query

Score	Mr(calc):	Delta	Sequence
48.0	1584.8069	0.0052	EVTPVDLPNCTLVK
7.1	1584.8148	-0.0026	EVTADSTLAGFHLPK
4.8	1584.8221	-0.0100	LSFLMNKINVFNK
4.4	1584.8154	-0.0033	VKGQRAGGTPNMAAAR
4.3	1584.8154	-0.0033	NSQGPLRGGRMPSTK
4.1	1584.7970	0.0152	YGRVELNMFISEK
3.4	1584.8035	0.0086	IDNQVKTFITYNK
2.1	1583.7984	1.0137	TLNPVFNEQFTFK
1.7	1583.8168	0.9953	KAPPTQTFRGDGGPR
1.5	1584.8137	-0.0016	FTGALALSRNTRAK

Spectrum No: 454; Query: 1106; Rank: 1

Peptide View

MS/MS Fragmentation of **AWSDGSHLMPIVNTSLGWPNGLAIDWSASR**
Found in **IPI00205325**, Tax_Id=10116 Gene_Symbol=Lrp2 Low-density lipoprotein receptor-related protein 2 precursor

Match to Query 1106: 3239.561022 from(1080.860950,3+)
Title: 091129RatKid_SCX02_20.6556.6556.3.dta
Data file K:\NewmanPaper\Piliang\3SubProteomes\Piliang3SP\mgf5ppm\SCX_3SubProteomes5ppm.mgf

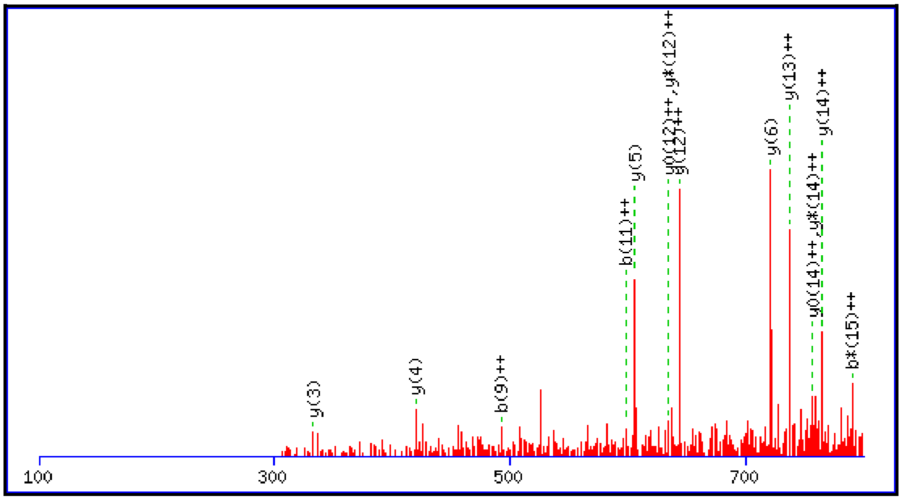

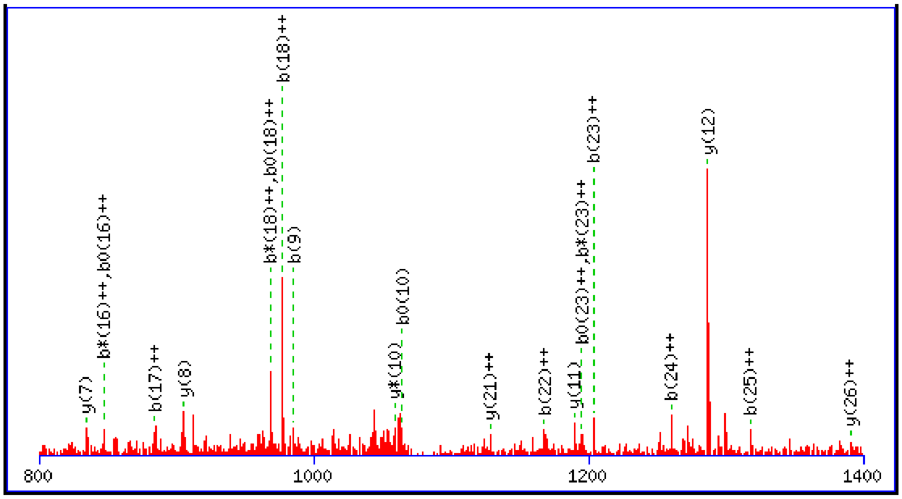

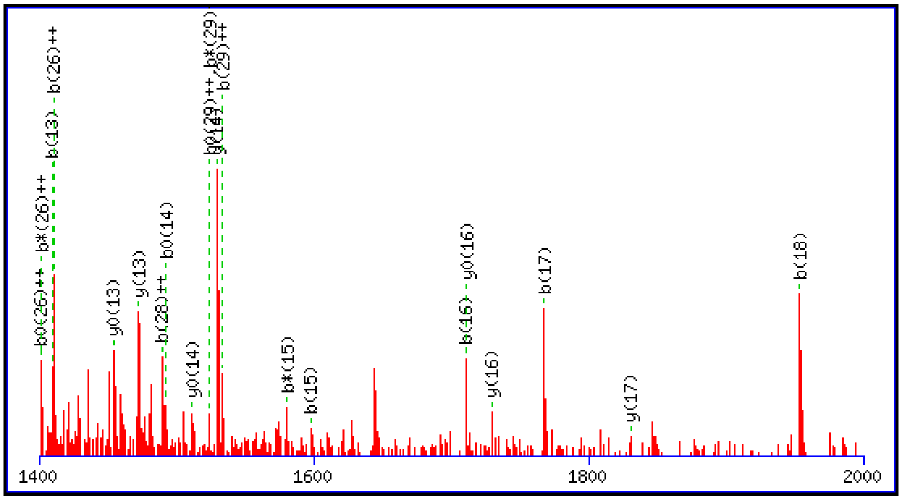

Monoisotopic mass of neutral peptide Mr(calc): 3238.5506
Fixed modifications: Carbamidomethyl (C)
Variable modifications:
N13 : Deamidated_N (N)
Ions Score: 48 **Expect:** 0.005
Matches (Bold Red): 56/318 fragment ions using 109 most intense peaks

#	b	b++	b*	b*++	b0	b0++	Seq.	y	y++	y*	y*++	y0	y0++	#
1	72.0444	36.5258					A							30
2	258.1237	129.5655					W	3168.5207	1584.7640	3151.4942	1576.2507	3150.5102	1575.7587	29
3	345.1557	173.0815			327.1452	164.0762	S	2982.4414	1491.7244	2965.4149	1483.2111	2964.4309	1482.7191	28
4	460.1827	230.5950			442.1721	221.5897	D	2895.4094	1448.2083	2878.3828	1439.6951	2877.3988	1439.2031	27
5	517.2041	259.1057			499.1936	250.1004	G	2780.3825	**1390.6949**	2763.3559	1382.1816	2762.3719	1381.6896	26
6	604.2362	302.6217			586.2256	293.6164	S	2723.3610	1362.1841	2706.3344	1353.6709	2705.3504	1353.1789	25
7	741.2951	371.1512			723.2845	362.1459	H	2636.3290	1318.6681	2619.3024	1310.1548	2618.3184	1309.6628	24
8	854.3791	427.6932			836.3686	418.6879	L	2499.2701	1250.1387	2482.2435	1241.6254	2481.2595	1241.1334	23
9	**985.4196**	**493.2135**			967.4091	484.2082	M	2386.1860	1193.5966	2369.1594	1185.0834	2368.1754	1184.5913	22
10	1082.4724	541.7398			**1064.4618**	532.7346	P	2255.1455	**1128.0764**	2238.1190	1119.5631	2237.1349	1119.0711	21
11	1195.5565	**598.2819**			1177.5459	589.2766	I	2158.0927	1079.5500	2141.0662	1071.0367	2140.0822	1070.5447	20
12	1294.6249	647.8161			1276.6143	638.8108	V	2045.0087	1023.0080	2027.9821	1014.4947	2026.9981	1014.0027	19
13	**1409.6518**	705.3295	1392.6253	696.8163	1391.6412	696.3243	N	1945.9403	973.4738	1928.9137	964.9605	1927.9297	964.4685	18
14	1510.6995	755.8534	1493.6729	747.3401	**1492.6889**	746.8481	T	**1830.9133**	915.9603	1813.8868	907.4470	1812.9028	906.9550	17
15	**1597.7315**	799.3694	**1580.7050**	**790.8561**	1579.7209	790.3641	S	**1729.8656**	865.4365	1712.8391	856.9232	**1711.8551**	856.4312	16
16	**1710.8156**	855.9114	1693.7890	**847.3982**	1692.8050	**846.9061**	L	1642.8336	821.9204	1625.8071	813.4072	1624.8231	812.9152	15
17	**1767.8370**	**884.4222**	1750.8105	875.9089	1749.8265	875.4169	G	**1529.7496**	**765.3784**	1512.7230	**756.8651**	**1511.7390**	**756.3731**	14
18	**1953.9164**	**977.4618**	1936.8898	**968.9485**	1935.9058	**968.4565**	W	**1472.7281**	**736.8677**	1455.7015	728.3544	**1454.7175**	727.8624	13

**19**	2050.9691	1025.9882	2033.9426	1017.4749	2032.9586	1016.9829	**P**	**1286.6488**	**643.8280**	1269.6222	**635.3148**	1268.6382	**634.8227**	**12**
**20**	2165.0120	1083.0097	2147.9855	1074.4964	2147.0015	1074.0044	**N**	**1189.5960**	595.3016	1172.5695	586.7884	1171.5854	586.2964	**11**
**21**	2222.0335	1111.5204	2205.0070	1103.0071	2204.0229	1102.5151	**G**	1075.5531	538.2802	**1058.5265**	529.7669	1057.5425	529.2749	**10**
**22**	2335.1176	**1168.0624**	2318.0910	1159.5491	2317.1070	1159.0571	**L**	1018.5316	509.7694	1001.5051	501.2562	1000.5211	500.7642	**9**
**23**	2406.1547	**1203.5810**	2389.1281	**1195.0677**	2388.1441	**1194.5757**	**A**	**905.4476**	453.2274	888.4210	444.7141	887.4370	444.2221	**8**
**24**	2519.2388	**1260.1230**	2502.2122	1251.6097	2501.2282	1251.1177	**I**	**834.4104**	417.7089	817.3839	409.1956	816.3999	408.7036	**7**
**25**	2634.2657	**1317.6365**	2617.2391	1309.1232	2616.2551	1308.6312	**D**	**721.3264**	361.1668	704.2998	352.6536	703.3158	352.1615	**6**
**26**	2820.3450	**1410.6761**	2803.3185	**1402.1629**	2802.3344	**1401.6709**	**W**	**606.2994**	303.6534	589.2729	295.1401	588.2889	294.6481	**5**
**27**	2907.3770	1454.1922	2890.3505	1445.6789	2889.3665	1445.1869	**S**	**420.2201**	210.6137	403.1936	202.1004	402.2096	201.6084	**4**
**28**	2978.4141	**1489.7107**	2961.3876	1481.1974	2960.4036	1480.7054	**A**	**333.1881**	167.0977	316.1615	158.5844	315.1775	158.0924	**3**
**29**	3065.4462	**1533.2267**	3048.4196	**1524.7135**	3047.4356	**1524.2214**	**S**	262.1510	131.5791	245.1244	123.0659	244.1404	122.5738	**2**
**30**							**R**	175.1190	88.0631	158.0924	79.5498			**1**

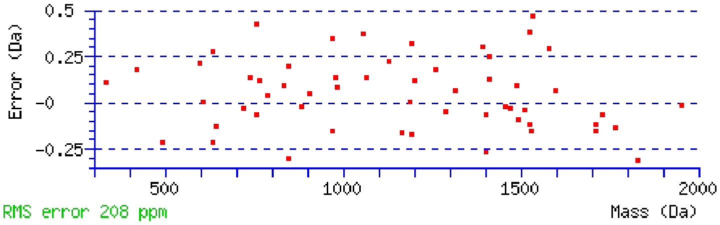

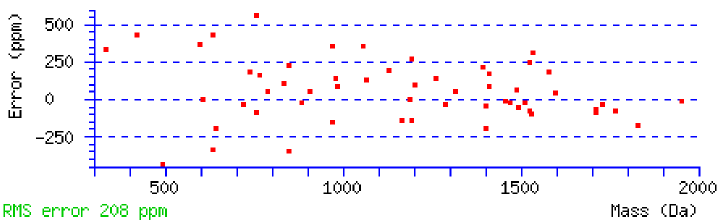

All matches to this query

Score	Mr(calc):	Delta	Sequence
48.0	3238.5506	1.0105	AWSDGSHLMPIVNTSLGWPNGLAIDWSASR
39.9	3239.5346	0.0264	AWSDGSHLMPIVNTSLGWPNGLAIDWSASR
27.2	3237.5666	1.9945	AWSDGSHLMPIVNTSLGWPNGLAIDWSASR
17.4	3238.5506	1.0105	AWSDGSHLMPIVNTSLGWPNGLAIDWSASR
2.9	3239.5751	-0.0141	YAAVYDNIHIVGVLHTITSKITNSESGEK
2.4	3239.5751	-0.0141	YAAVYDNIHIVGVLHTITSKITNSESGEK
1.1	3239.5751	-0.0141	YAAVYDNIHIVGVLHTITSKITNSESGEK
1.1	3239.5751	-0.0141	YAAVYDNIHIVGVLHTITSKITNSESGEK
0.9	3237.5852	1.9758	GTEASEIALPRGSDITVHIKLVSPALPVK
0.8	3238.5304	1.0306	SQEIGVKPLACTSANIWPHGTSLMNYLR

Spectrum No: 455; Query: 1108; Rank: 1

Peptide View

MS/MS Fragmentation of **VFDLHQNMGSVNVSVGCTPAQLQTSGATAQK**
Found in **IPI00190577**, Tax_Id=10116 Gene_Symbol=Lama5 similar to Laminin alpha-5 chain precursor

Match to Query 1108: 3245.542152 from(1082.854660,3+)
Title: 091129RatKid_SCX02_22.3359.3359.3.dta
Data file K:\NewmanPaper\Piliang\3SubProteomes\Piliang3SP\mgf5ppm\SCX_3SubProteomes5ppm.mgf

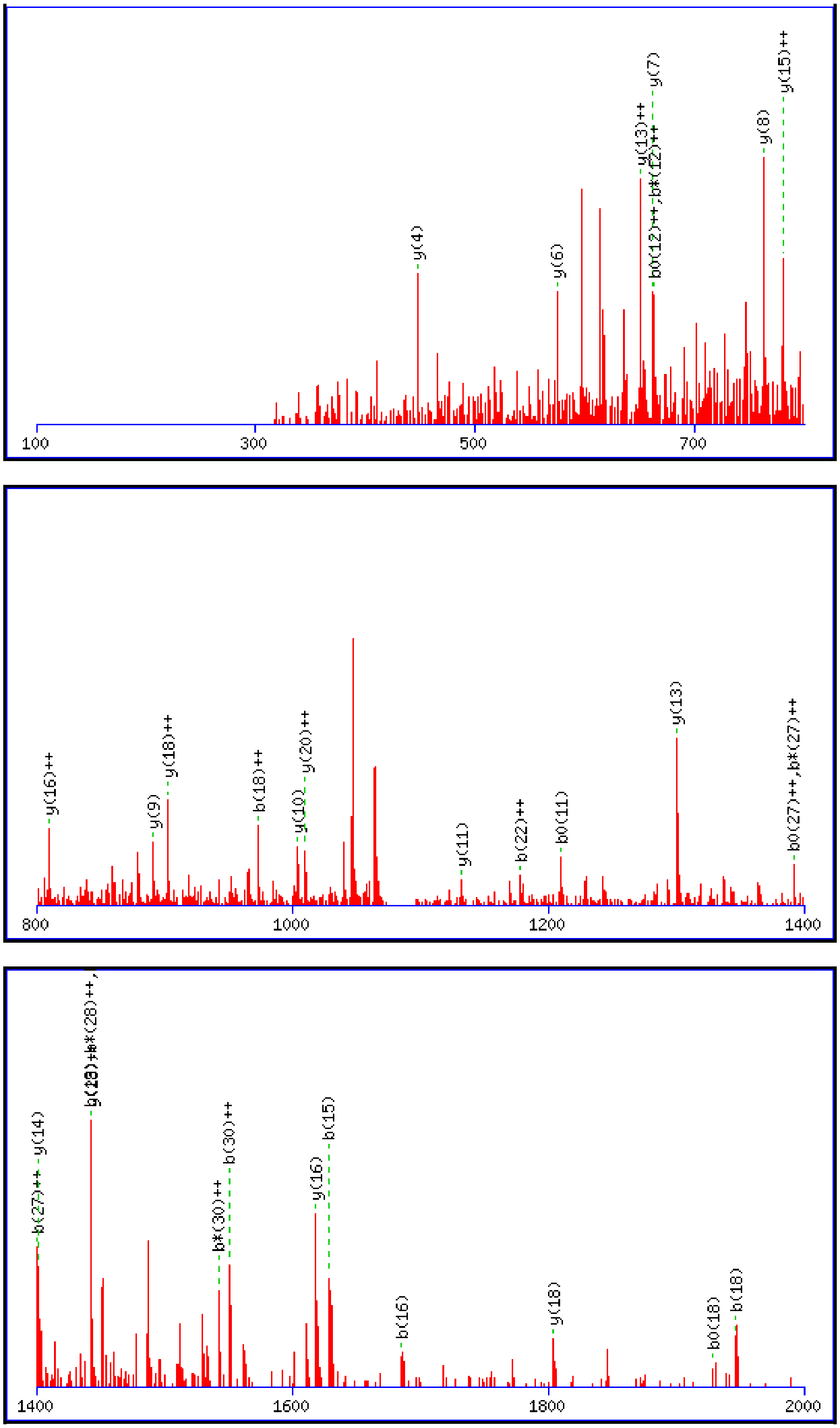

Monoisotopic mass of neutral peptide Mr(calc): 3245.5445
Fixed modifications: Carbamidomethyl (C)
Variable modifications:
N12 : Deamidated_N (N)
Ions Score: 48 **Expect:** 0.0058
Matches (Bold Red): 34/340 fragment ions using 60 most intense peaks

#	b	b^{++}	b*	b^{*++}	b^0	b^{0++}	Seq.	y	y^{++}	y*	y^{*++}	y^0	y^{0++}	#
1	100.0757	50.5415					V							31
2	247.1441	124.0757					F	3147.4834	1574.2453	3130.4568	1565.7320	3129.4728	1565.2400	30
3	362.1710	181.5892			344.1605	172.5839	D	3000.4150	1500.7111	2983.3884	1492.1978	2982.4044	1491.7058	29
4	475.2551	238.1312			457.2445	229.1259	L	2885.3880	**1443.1976**	2868.3615	1434.6844	2867.3774	1434.1924	28
5	612.3140	306.6606			594.3035	297.6554	H	2772.3040	1386.6556	2755.2774	1378.1423	2754.2934	1377.6503	27

6	740.3726	370.6899	723.3461	362.1767	722.3620	361.6847	Q	2635.2450	1318.1262	2618.2185	1309.6129	2617.2345	1309.1209	26
7	854.4155	427.7114	837.3890	419.1981	836.4050	418.7061	N	2507.1865	1254.0969	2490.1599	1245.5836	2489.1759	1245.0916	25
8	985.4560	493.2316	968.4295	484.7184	967.4454	484.2264	M	2393.1435	1197.0754	2376.1170	1188.5621	2375.1330	1188.0701	24
9	1042.4775	521.7424	1025.4509	513.2291	1024.4669	512.7371	G	2262.1030	1131.5552	2245.0765	1123.0419	2244.0925	1122.5499	23
10	1129.5095	565.2584	1112.4830	556.7451	1111.4989	556.2531	S	2205.0816	1103.0444	2188.0550	1094.5312	2187.0710	1094.0391	22
11	1228.5779	614.7926	1211.5514	606.2793	**1210.5674**	605.7873	V	2118.0496	1059.5284	2101.0230	1051.0151	2100.0390	1050.5231	21
12	1343.6049	672.3061	1326.5783	**663.7928**	1325.5943	**663.3008**	N	2018.9811	**1009.9942**	2001.9546	1001.4809	2000.9706	1000.9889	20
13	**1442.6733**	721.8403	1425.6467	713.3270	1424.6627	712.8350	V	1903.9542	952.4807	1886.9277	943.9675	1885.9436	943.4755	19
14	1529.7053	765.3563	1512.6787	756.8430	1511.6947	756.3510	S	**1804.8858**	**902.9465**	1787.8592	894.4333	1786.8752	893.9413	18
15	**1628.7737**	814.8905	1611.7472	806.3772	1610.7631	805.8852	V	1717.8538	859.4305	1700.8272	850.9172	1699.8432	850.4252	17
16	**1685.7952**	843.4012	1668.7686	834.8880	1667.7846	834.3959	G	**1618.7854**	**809.8963**	1601.7588	801.3830	1600.7748	800.8910	16
17	1845.8258	923.4166	1828.7993	914.9033	1827.8153	914.4113	C	1561.7639	**781.3856**	1544.7373	772.8723	1543.7533	772.3803	15
18	**1946.8735**	**973.9404**	1929.8470	965.4271	**1928.8629**	964.9351	T	**1401.7332**	701.3703	1384.7067	692.8570	1383.7227	692.3650	14
19	2043.9263	1022.4668	2026.8997	1013.9535	2025.9157	1013.4615	P	**1300.6856**	**650.8464**	1283.6590	642.3331	1282.6750	641.8411	13
20	2114.9634	1057.9853	2097.9368	1049.4721	2096.9528	1048.9800	A	1203.6328	602.3200	1186.6062	593.8068	1185.6222	593.3148	12
21	2243.0220	1122.0146	2225.9954	1113.5013	2225.0114	1113.0093	Q	**1132.5957**	566.8015	1115.5691	558.2882	1114.5851	557.7962	11
22	2356.1060	**1178.5566**	2339.0795	1170.0434	2338.0955	1169.5514	L	**1004.5371**	502.7722	987.5106	494.2589	986.5265	493.7669	10
23	2484.1646	1242.5859	2467.1381	1234.0727	2466.1540	1233.5807	Q	**891.4530**	446.2302	874.4265	437.7169	873.4425	437.2249	9
24	2585.2123	1293.1098	2568.1857	1284.5965	2567.2017	1284.1045	T	**763.3945**	382.2009	746.3679	373.6876	745.3839	373.1956	8
25	2672.2443	1336.6258	2655.2178	1328.1125	2654.2337	1327.6205	S	**662.3468**	331.6770	645.3202	323.1638	644.3362	322.6717	7
26	2729.2658	1365.1365	2712.2392	1356.6232	2711.2552	1356.1312	G	**575.3148**	288.1610	558.2882	279.6477	557.3042	279.1557	6
27	2800.3029	**1400.6551**	2783.2763	**1392.1418**	2782.2923	**1391.6498**	A	518.2933	259.6503	501.2667	251.1370	500.2827	250.6450	5
28	2901.3506	1451.1789	2884.3240	**1442.6656**	2883.3400	**1442.1736**	T	**447.2562**	224.1317	430.2296	215.6185	429.2456	215.1264	4
29	2972.3877	1486.6975	2955.3611	1478.1842	2954.3771	1477.6922	A	346.2085	173.6079	329.1819	165.0946			3
30	3100.4463	**1550.7268**	3083.4197	**1542.2135**	3082.4357	1541.7215	Q	275.1714	138.0893	258.1448	129.5761			2
31							K	147.1128	74.0600	130.0863	65.5468			1

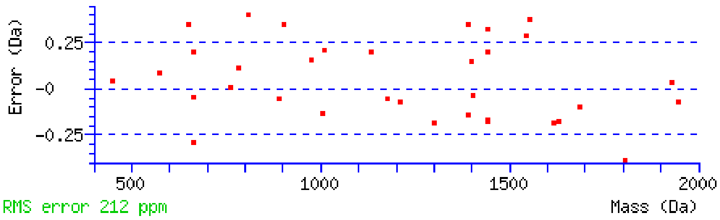

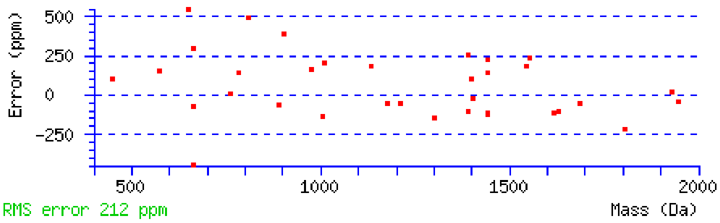

All matches to this query

Score	Mr(calc):	Delta	Sequence
47.9	3245.5445	-0.0024	VFDLHQNMGSVNVSVGCTPAQLQTSGATAQK
44.8	3245.5445	-0.0024	VFDLHQNMGSVNVSVGCTPAQLQTSGATAQK
29.2	3244.5605	0.9816	VFDLHQNMGSVNVSVGCTPAQLQTSGATAQK
3.7	3244.5614	0.9807	YVNGLDQLAFAESQVGEMKLELVELQPK
0.3	3245.5253	0.0169	LISEVIGESLGIPMSVLMGANIASEVAEEK
0.2	3245.5131	0.0290	SSIVEDGVMVQITAENMDSLRQALREMK

Spectrum No: 456; Query: 242; Rank: 1

Peptide View

MS/MS Fragmentation of **GVNCTWHVVVQR**
Found in **IPI00196620**, Tax_Id=10116 Gene_Symbol=Cubn Cubilin precursor

Match to Query 242: 1454.711352 from(485.911060,3+)
Title: 091127RatKid_SCX01_27.1783.1783.3.dta
Data file K:\NewmanPaper\Piliang\3SubProteomes\Piliang3SP\mgf5ppm\SCX_3SubProteomes5ppm.mgf

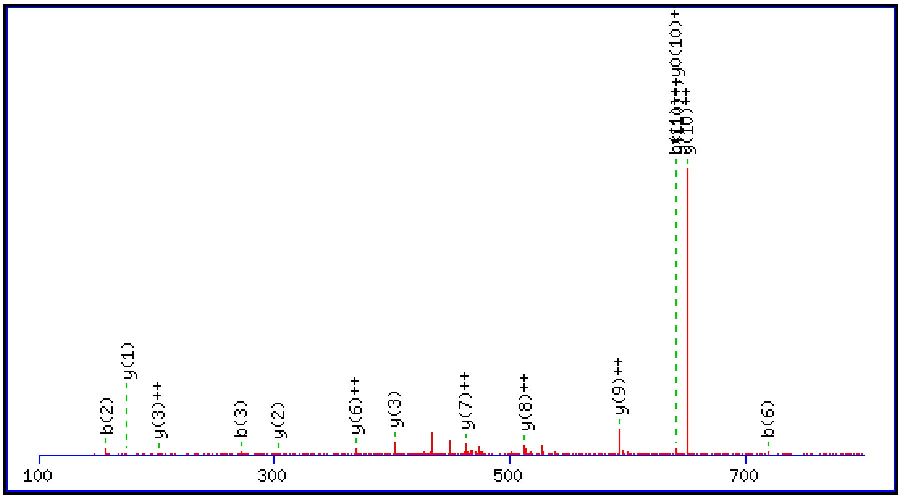

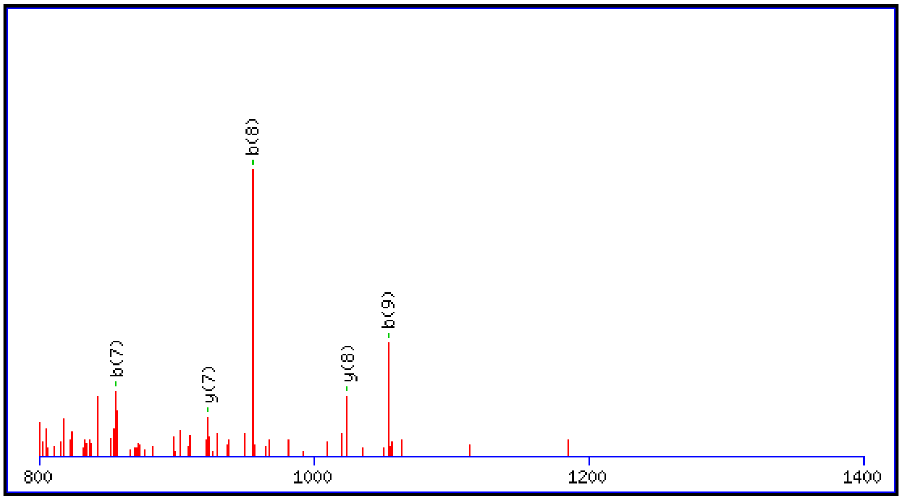

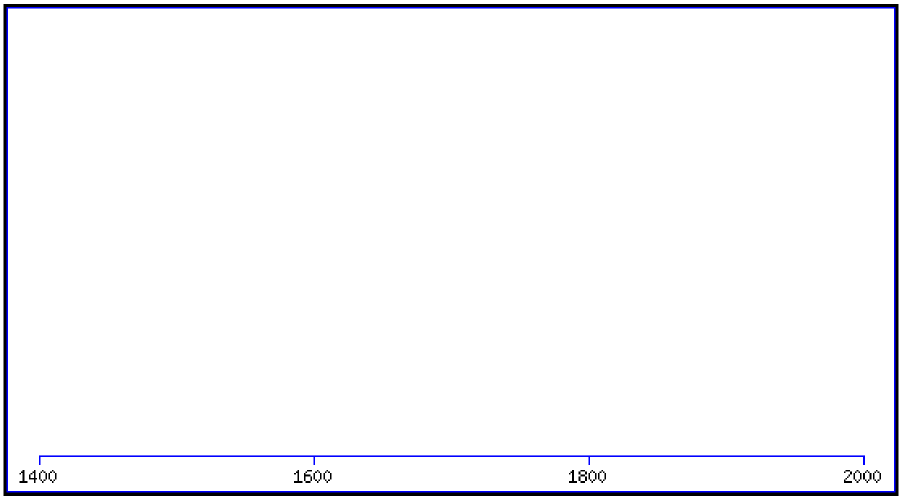

Monoisotopic mass of neutral peptide Mr(calc): 1454.7089
Fixed modifications: Carbamidomethyl (C)
Variable modifications:
N3 : Deamidated_N (N)
Ions Score: 48 **Expect:** 0.0027
Matches (Bold Red): 20/106 fragment ions using 31 most intense peaks

#	b	b^{++}	b*	b^{*++}	b^0	b^{0++}	Seq.	y	y^{++}	y*	y^{*++}	y^0	y^{0++}	#
1	58.0287	29.5180					G							12
2	**157.0972**	79.0522					V	1398.6947	699.8510	1381.6681	691.3377	1380.6841	690.8457	11

3	**272.1241**	136.5657	255.0975	128.0524			N	1299.6263	**650.3168**	1282.5997	**641.8035**	1281.6157	**641.3115**	10
4	432.1547	216.5810	415.1282	208.0677			C	1184.5993	**592.8033**	1167.5728	584.2900	1166.5888	583.7980	9
5	533.2024	267.1048	516.1759	258.5916	515.1919	258.0996	T	**1024.5687**	**512.7880**	1007.5421	504.2747	1006.5581	503.7827	8
6	**719.2817**	360.1445	702.2552	351.6312	701.2712	351.1392	W	**923.5210**	**462.2641**	906.4944	453.7509			7
7	**856.3406**	428.6740	839.3141	420.1607	838.3301	419.6687	H	737.4417	**369.2245**	720.4151	360.7112			6
8	**955.4091**	478.2082	938.3825	469.6949	937.3985	469.2029	V	600.3828	300.6950	583.3562	292.1817			5
9	**1054.4775**	527.7424	1037.4509	519.2291	1036.4669	518.7371	V	501.3144	251.1608	484.2878	242.6475			4
10	1153.5459	577.2766	1136.5193	568.7633	1135.5353	568.2713	V	**402.2459**	**201.6266**	385.2194	193.1133			3
11	1281.6045	**641.3059**	1264.5779	632.7926	1263.5939	632.3006	Q	**303.1775**	152.0924	286.1510	143.5791			2
12							R	**175.1190**	88.0631	158.0924	79.5498			1

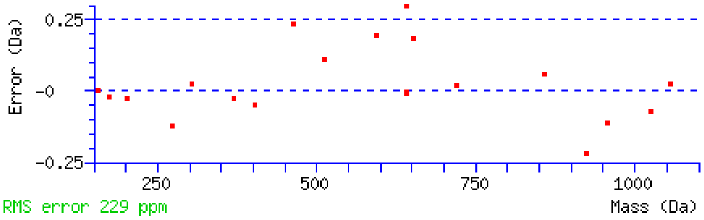

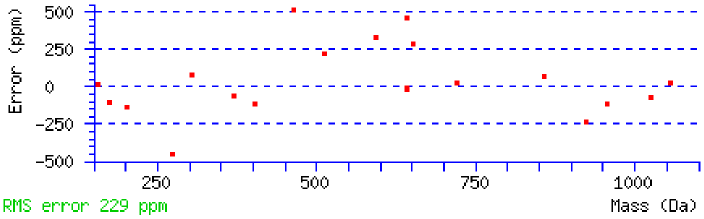

All matches to this query

Score	Mr(calc):	Delta	Sequence
47.7	1454.7089	0.0025	GVNCTWHVVVQR
9.4	1453.7102	1.0011	GPFEGPNYHIAPR
9.4	1453.7000	1.0114	GNTNMVKLLLDR
8.6	1454.7225	-0.0112	RENREAVAQEPR
6.2	1453.7161	0.9952	HSLEVTQEEVQR
3.9	1453.7112	1.0001	RMSLVEEGAVKR
3.7	1452.6919	2.0195	ACIPQSFTVDTSK
3.4	1454.7048	0.0066	GLSLPLSPVRTR
3.3	1453.7000	1.0114	GNTNMVKLLLDR
3.2	1453.7065	1.0048	GDSLDSVEALIKK

Spectrum No: 457; Query: 1005; Rank: 1

Peptide View

MS/MS Fragmentation of **QLITEEKIPNNTQWITWSQEGHK**
Found in **IPI00208422**, Tax_Id=10116 Gene_Symbol=Dpp4 Dipeptidyl peptidase 4

Match to Query 1005: 2780.390892 from(927.804240,3+)
Title: 091127RatKid_SCX01_25.3504.3504.3.dta
Data file K:\NewmanPaper\Piliang\3SubProteomes\Piliang3SP\mgf5ppm\SCX_3SubProteomes5ppm.mgf

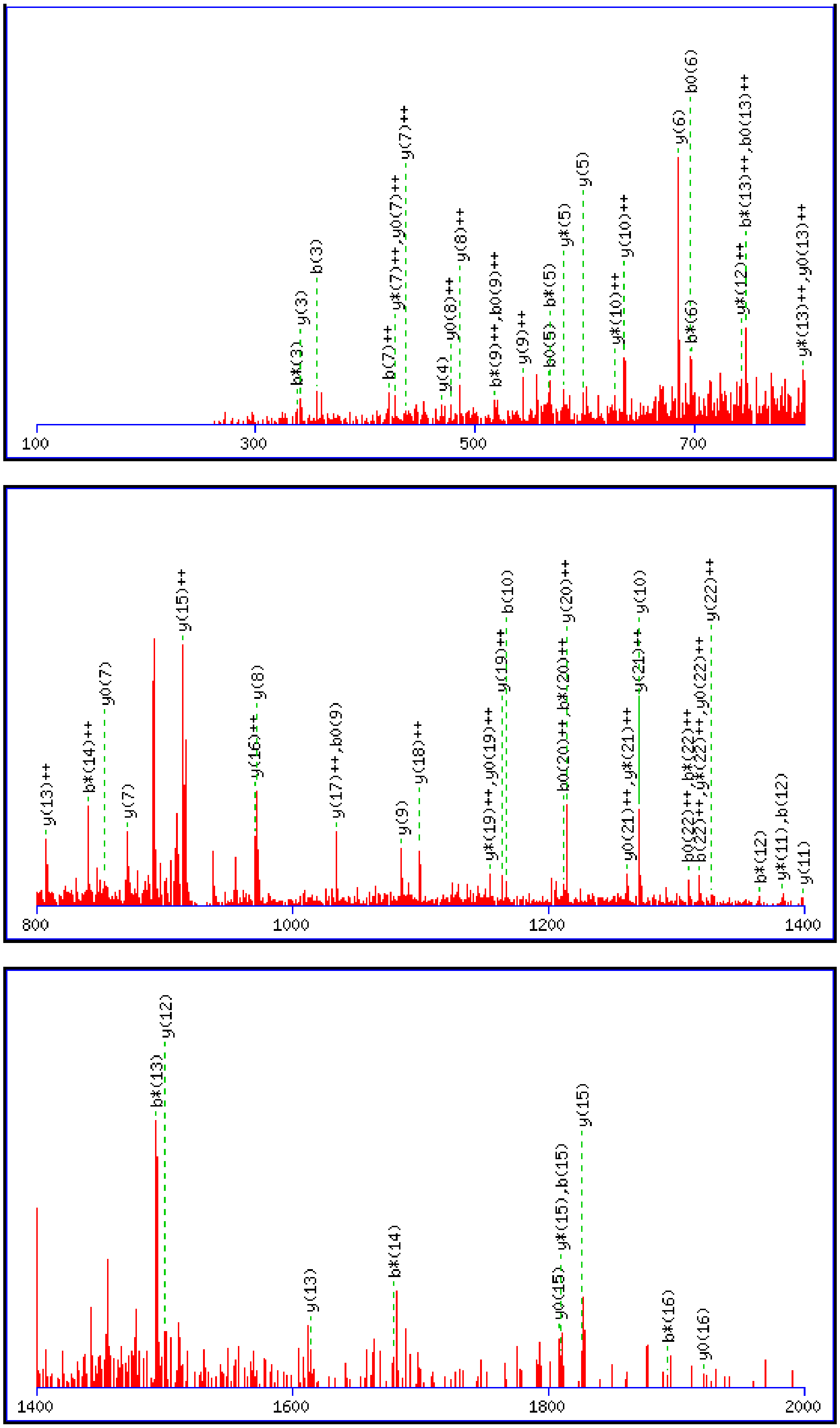

Monoisotopic mass of neutral peptide Mr(calc): 2780.3769
Fixed modifications: Carbamidomethyl (C)
Variable modifications:
N10 : Deamidated_N (N)
Ions Score: 47 **Expect:** 0.0039
Matches (Bold Red): 69/252 fragment ions using 151 most intense peaks

#	b	b^{++}	b*	b^{*++}	b^{0}	b^{0++}	Seq.	y	y^{++}	y*	y^{*++}	y^{0}	y^{0++}	#
1	129.0659	65.0366	112.0393	56.5233			Q							23
2	242.1499	121.5786	225.1234	113.0653			L	2653.3257	**1327.1665**	2636.2991	**1318.6532**	2635.3151	**1318.1612**	22
3	**355.2340**	178.1206	**338.2074**	169.6074			I	2540.2416	**1270.6244**	2523.2150	**1262.1112**	2522.2310	**1261.6191**	21
4	456.2817	228.6445	439.2551	220.1312	438.2711	219.6392	T	2427.1575	**1214.0824**	2410.1310	1205.5691	2409.1470	1205.0771	20
5	585.3243	293.1658	**568.2977**	284.6525	**567.3137**	284.1605	E	2326.1098	**1163.5586**	2309.0833	**1155.0453**	2308.0993	**1154.5533**	19

6	714.3668	357.6871	**697.3403**	349.1738	**696.3563**	348.6818	E	2197.0673	**1099.0373**	2180.0407	1090.5240	2179.0567	1090.0320	18
7	842.4618	**421.7345**	825.4353	413.2213	824.4512	412.7293	K	2068.0247	**1034.5160**	2050.9981	1026.0027	2050.0141	1025.5107	17
8	955.5459	478.2766	938.5193	469.7633	937.5353	469.2713	I	1939.9297	**970.4685**	1922.9031	961.9552	**1921.9191**	961.4632	16
9	1052.5986	526.8030	1035.5721	**518.2897**	**1034.5881**	**517.7977**	P	**1826.8456**	**913.9265**	**1809.8191**	905.4132	**1808.8351**	904.9212	15
10	**1167.6256**	584.3164	1150.5990	575.8032	1149.6150	575.3111	N	1729.7929	865.4001	1712.7663	856.8868	1711.7823	856.3948	14
11	1281.6685	641.3379	1264.6420	632.8246	1263.6579	632.3326	N	**1614.7659**	**807.8866**	1597.7394	**799.3733**	1596.7554	**798.8813**	13
12	**1382.7162**	691.8617	**1365.6896**	683.3485	1364.7056	682.8564	T	**1500.7230**	750.8651	1483.6965	**742.3519**	1482.7124	741.8599	12
13	1510.7748	755.8910	**1493.7482**	**747.3777**	1492.7642	**746.8857**	Q	**1399.6753**	700.3413	**1382.6488**	691.8280	1381.6648	691.3360	11
14	1696.8541	848.9307	**1679.8275**	**840.4174**	1678.8435	839.9254	W	**1271.6167**	**636.3120**	1254.5902	**627.7987**	1253.6062	627.3067	10
15	**1809.9381**	905.4727	1792.9116	896.9594	1791.9276	896.4674	I	**1085.5374**	**543.2724**	1068.5109	534.7591	1067.5269	534.2671	9
16	1910.9858	955.9965	**1893.9593**	947.4833	1892.9752	946.9913	T	**972.4534**	**486.7303**	955.4268	478.2170	954.4428	**477.7250**	8
17	2097.0651	1049.0362	2080.0386	1040.5229	2079.0546	1040.0309	W	**871.4057**	**436.2065**	854.3791	**427.6932**	**853.3951**	**427.2012**	7
18	2184.0972	1092.5522	2167.0706	1084.0389	2166.0866	1083.5469	S	**685.3264**	343.1668	668.2998	334.6536	667.3158	334.1615	6
19	2312.1557	1156.5815	2295.1292	1148.0682	2294.1452	1147.5762	Q	**598.2944**	299.6508	**581.2678**	291.1375	580.2838	290.6455	5
20	2441.1983	1221.1028	2424.1718	**1212.5895**	2423.1878	**1212.0975**	E	**470.2358**	235.6215	453.2092	227.1083	452.2252	226.6162	4
21	2498.2198	1249.6135	2481.1932	1241.1003	2480.2092	1240.6083	G	**341.1932**	171.1002	324.1666	162.5870			3
22	2635.2787	**1318.1430**	2618.2522	**1309.6297**	2617.2681	**1309.1377**	H	284.1717	142.5895	267.1452	134.0762			2
23							K	147.1128	74.0600	130.0863	65.5468			1

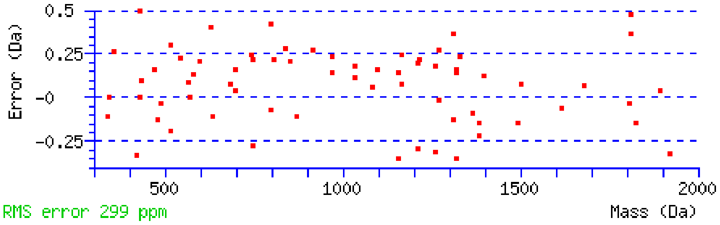

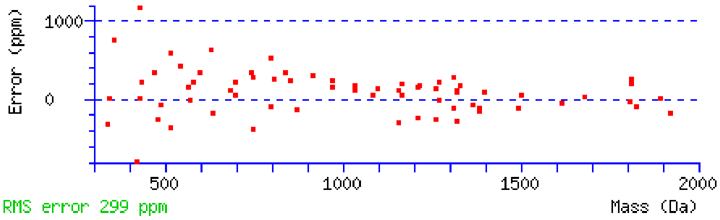

All matches to this query

Score	Mr(calc):	Delta	Sequence
47.4	2780.3769	0.0140	QLITEEKIPNNTQWITWSQEGHK
42.6	2780.3769	0.0140	QLITEEKIPNNTQWITWSQEGHK
10.4	2779.3929	0.9980	QLITEEKIPNNTQWITWSQEGHK

Spectrum No: 458; Query: 942; Rank: 1

Peptide View

MS/MS Fragmentation of **TGDFQLHTNVNNGTEFGGSIYQK**
Found in **IPI00198327**, Tax_Id=10116 Gene_Symbol=Vdac2 Voltage-dependent anion-selective channel protein 2

Match to Query 942: 2527.173102 from(843.398310,3+)
Title: 091129RatKid_SCX02_27.3246.3246.3.dta
Data file K:\NewmanPaper\Piliang\3SubProteomes\Piliang3SP\mgf5ppm\SCX_3SubProteomes5ppm.mgf

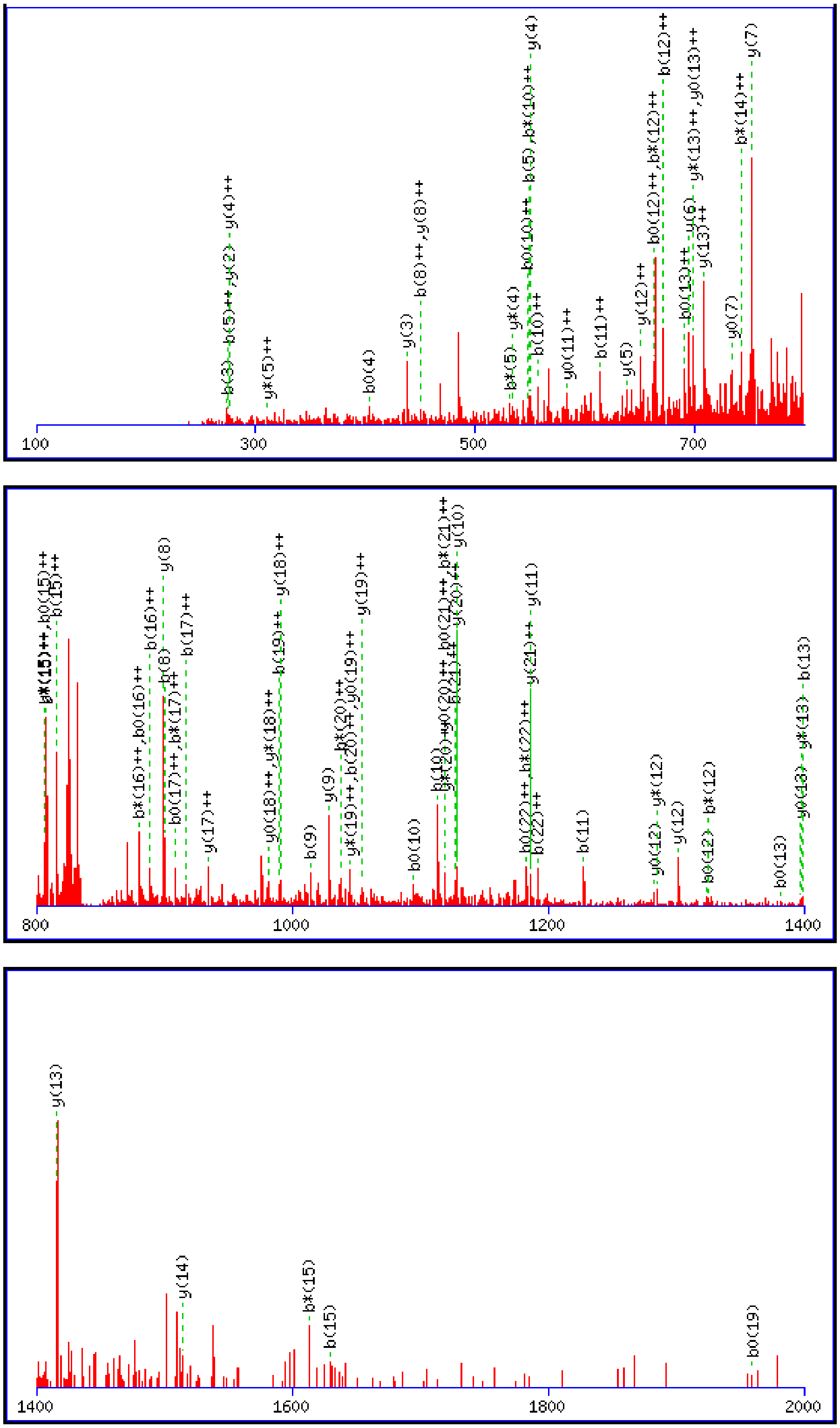

Monoisotopic mass of neutral peptide Mr(calc): 2527.1616
Fixed modifications: Carbamidomethyl (C)
Variable modifications:
N12 : Deamidated_N (N)
Ions Score: 47 **Expect:** 0.0058
Matches (Bold Red): 84/248 fragment ions using 174 most intense peaks

#	b	b++	b*	b*++	b0	b0++	Seq.	y	y++	y*	y*++	y0	y0++	#
1	102.0550	51.5311			84.0444	42.5258	T							23
2	159.0764	80.0418			141.0659	71.0366	G	2427.1211	1214.0642	2410.0946	1205.5509	2409.1106	1205.0589	22
3	**274.1034**	137.5553			256.0928	128.5500	D	2370.0997	**1185.5535**	2353.0731	1177.0402	2352.0891	1176.5482	21
4	421.1718	211.0895			**403.1612**	202.0842	F	2255.0727	**1128.0400**	2238.0462	**1119.5267**	2237.0622	**1119.0347**	20
5	**549.2304**	**275.1188**	**532.2038**	266.6055	531.2198	266.1135	Q	2108.0043	**1054.5058**	2090.9778	**1045.9925**	2089.9938	**1045.5005**	19

6	662.3144	331.6608	645.2879	323.1476	644.3039	322.6556	L	1979.9457	**990.4765**	1962.9192	**981.9632**	1961.9352	**981.4712**	18
7	799.3733	400.1903	782.3468	391.6770	781.3628	391.1850	H	1866.8617	**933.9345**	1849.8351	925.4212	1848.8511	924.9292	17
8	**900.4210**	**450.7141**	883.3945	442.2009	882.4104	441.7089	T	1729.8028	865.4050	1712.7762	856.8917	1711.7922	856.3997	16
9	**1014.4639**	507.7356	997.4374	499.2223	996.4534	498.7303	N	1628.7551	814.8812	1611.7285	**806.3679**	1610.7445	805.8759	15
10	**1113.5324**	**557.2698**	1096.5058	**548.7565**	**1095.5218**	**548.2645**	V	**1514.7122**	757.8597	1497.6856	749.3464	1496.7016	748.8544	14
11	**1227.5753**	**614.2913**	1210.5487	605.7780	1209.5647	605.2860	N	**1415.6437**	**708.3255**	**1398.6172**	**699.8122**	**1397.6332**	**699.3202**	13
12	1342.6022	**671.8047**	**1325.5757**	**663.2915**	**1324.5916**	**662.7995**	N	**1301.6008**	**651.3040**	**1284.5743**	642.7908	**1283.5903**	642.2988	12
13	**1399.6237**	700.3155	1382.5971	691.8022	**1381.6131**	**691.3102**	G	**1186.5739**	593.7906	1169.5473	585.2773	1168.5633	**584.7853**	11
14	1500.6714	750.8393	1483.6448	**742.3260**	1482.6608	741.8340	T	**1129.5524**	565.2798	1112.5259	556.7666	1111.5419	556.2746	10
15	**1629.7139**	**815.3606**	**1612.6874**	**806.8473**	1611.7034	**806.3553**	E	**1028.5047**	514.7560	1011.4782	506.2427	1010.4942	505.7507	9
16	1776.7824	**888.8948**	1759.7558	**880.3815**	1758.7718	**879.8895**	F	**899.4621**	**450.2347**	882.4356	441.7214	881.4516	441.2294	8
17	1833.8038	**917.4056**	1816.7773	**908.8923**	1815.7933	**908.4003**	G	**752.3937**	376.7005	735.3672	368.1872	**734.3832**	367.6952	7
18	1890.8253	945.9163	1873.7987	937.4030	1872.8147	936.9110	G	**695.3723**	348.1898	678.3457	339.6765	677.3617	339.1845	6
19	1977.8573	**989.4323**	1960.8308	980.9190	**1959.8468**	980.4270	S	**638.3508**	319.6790	621.3243	**311.1658**	620.3402	310.6738	5
20	2090.9414	**1045.9743**	2073.9148	**1037.4611**	2072.9308	1036.9690	I	**551.3188**	**276.1630**	**534.2922**	267.6498			4
21	2254.0047	**1127.5060**	2236.9782	**1118.9927**	2235.9941	**1118.5007**	Y	**438.2347**	219.6210	421.2082	211.1077			3
22	2382.0633	**1191.5353**	2365.0367	**1183.0220**	2364.0527	**1182.5300**	Q	**275.1714**	138.0893	258.1448	129.5761			2
23							K	147.1128	74.0600	130.0863	65.5468			1

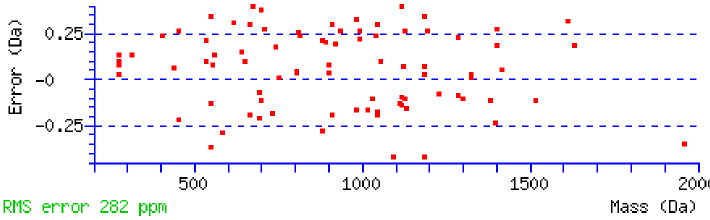

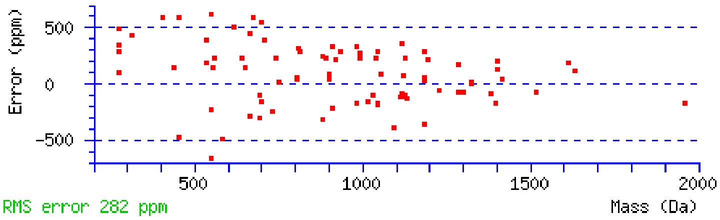

All matches to this query

Score	Mr(calc):	Delta	Sequence
47.3	2527.1616	0.0115	TGDFQLHTNVNNGTEFGGSIYQK
44.7	2527.1616	0.0115	TGDFQLHTNVNNGTEFGGSIYQK
36.2	2527.1616	0.0115	TGDFQLHTNVNNGTEFGGSIYQK
26.1	2526.1776	0.9956	TGDFQLHTNVNNGTEFGGSIYQK
5.1	2527.1665	0.0066	MGQNNSVSEFILLGLTEDPAGQK
2.0	2527.1665	0.0066	MGQNNSVSEFILLGLTEDPAGQK

Spectrum No: 459; Query: 1173; Rank: 1

Peptide View

MS/MS Fragmentation of **ILTNNSQTPILSPQEVVSCSPYAQGCDGGFPYLIAGK**
Found in **IPI00193765**, Tax_Id=10116 Gene_Symbol=Ctsc Dipeptidyl-peptidase 1 precursor

Match to Query 1173: 3981.926896 from(996.489000,4+)
Title: 091129RatKid_SCX02_12.4338.4338.4.dta
Data file K:\NewmanPaper\Piliang\3SubProteomes\Piliang3SP\mgf5ppm\SCX_3SubProteomes5ppm.mgf

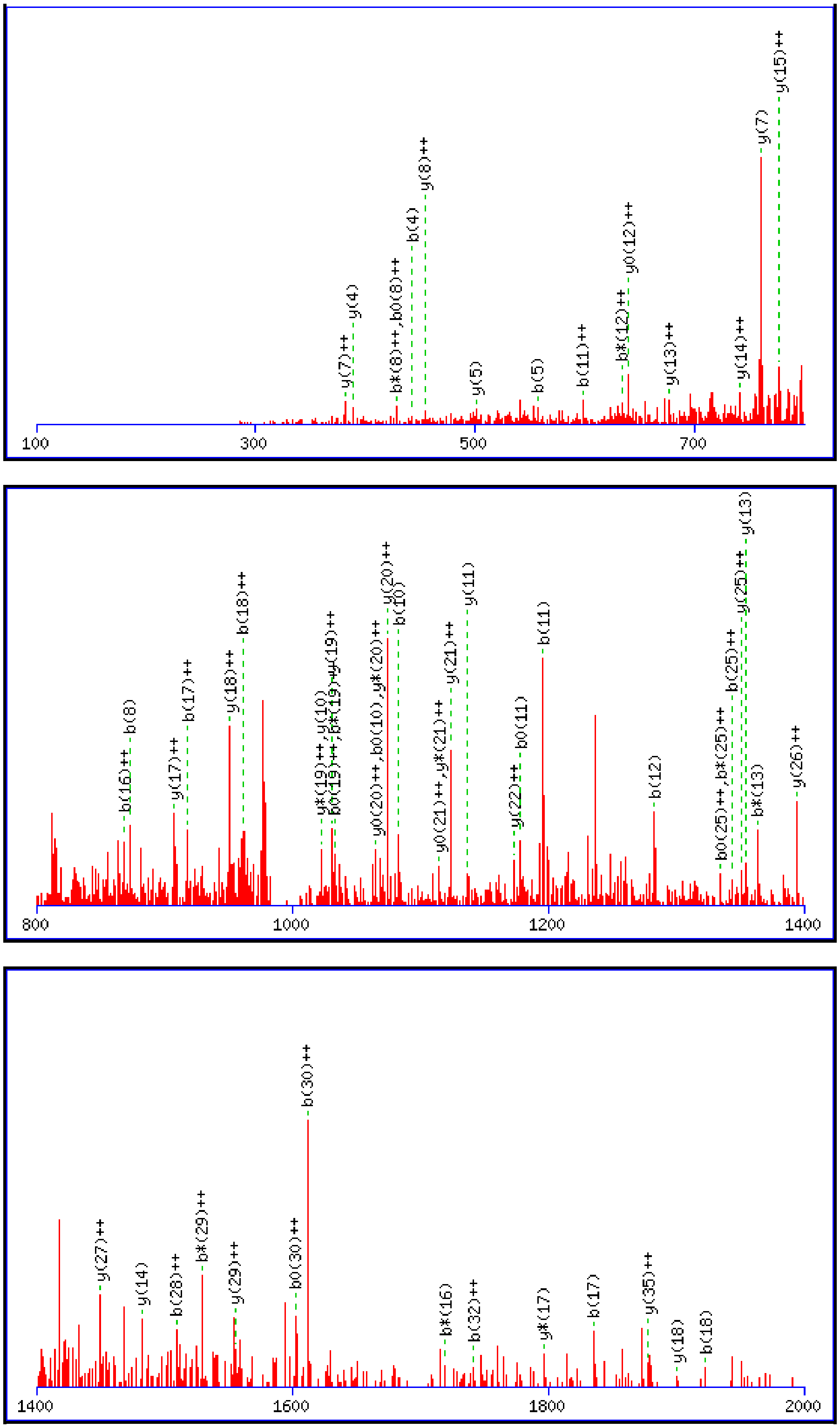

Monoisotopic mass of neutral peptide Mr(calc): 3981.9129
Fixed modifications: Carbamidomethyl (C)
Variable modifications:
N4 : Deamidated_N (N)
Ions Score: 47 **Expect:** 0.0061
Matches (Bold Red): 60/402 fragment ions using 146 most intense peaks

#	b	b^{++}	b*	b^{*++}	b^{0}	b^{0++}	Seq.	y	y^{++}	y*	y^{*++}	y^{0}	y^{0++}	#
1	114.0913	57.5493					I							37
2	227.1754	114.0913					L	3869.8361	1935.4217	3852.8095	1926.9084	3851.8255	1926.4164	36
3	328.2231	164.6152			310.2125	155.6099	T	3756.7520	**1878.8796**	3739.7255	1870.3664	3738.7414	1869.8744	35
4	**443.2500**	222.1286	426.2235	213.6154	425.2395	213.1234	N	3655.7043	1828.3558	3638.6778	1819.8425	3637.6938	1819.3505	34
5	**557.2929**	279.1501	540.2664	270.6368	539.2824	270.1448	N	3540.6774	1770.8423	3523.6508	1762.3291	3522.6668	1761.8370	33

6	644.3250	322.6661	627.2984	314.1529	626.3144	313.6608	S	3426.6345	1713.8209	3409.6079	1705.3076	3408.6239	1704.8156	32
7	772.3836	386.6954	755.3570	378.1821	754.3730	377.6901	Q	3339.6024	1670.3049	3322.5759	1661.7916	3321.5919	1661.2996	31
8	873.4312	437.2193	856.4047	428.7060	855.4207	428.2140	T	3211.5439	1606.2756	3194.5173	1597.7623	3193.5333	1597.2703	30
9	970.4840	485.7456	953.4574	477.2324	952.4734	476.7404	P	3110.4962	1555.7517	3093.4696	1547.2385	3092.4856	1546.7464	29
10	1083.5681	542.2877	1066.5415	533.7744	1065.5575	533.2824	I	3013.4434	1507.2253	2996.4169	1498.7121	2995.4328	1498.2201	28
11	1196.6521	598.8297	1179.6256	590.3164	1178.6416	589.8244	L	2900.3593	1450.6833	2883.3328	1442.1700	2882.3488	1441.6780	27
12	1283.6842	642.3457	1266.6576	633.8324	1265.6736	633.3404	S	2787.2753	1394.1413	2770.2487	1385.6280	2769.2647	1385.1360	26
13	1380.7369	690.8721	1363.7104	682.3588	1362.7263	681.8668	P	2700.2433	1350.6253	2683.2167	1342.1120	2682.2327	1341.6200	25
14	1508.7955	754.9014	1491.7689	746.3881	1490.7849	745.8961	Q	2603.1905	1302.0989	2586.1639	1293.5856	2585.1799	1293.0936	24
15	1637.8381	819.4227	1620.8115	810.9094	1619.8275	810.4174	E	2475.1319	1238.0696	2458.1054	1229.5563	2457.1213	1229.0643	23
16	1736.9065	868.9569	1719.8800	860.4436	1718.8959	859.9516	V	2346.0893	1173.5483	2329.0628	1165.0350	2328.0788	1164.5430	22
17	1835.9749	918.4911	1818.9484	909.9778	1817.9643	909.4858	V	2247.0209	1124.0141	2229.9944	1115.5008	2229.0103	1115.0088	21
18	1923.0069	962.0071	1905.9804	953.4938	1904.9964	953.0018	S	2147.9525	1074.4799	2130.9259	1065.9666	2129.9419	1065.4746	20
19	2083.0376	1042.0224	2066.0110	1033.5092	2065.0270	1033.0172	C	2060.9205	1030.9639	2043.8939	1022.4506	2042.9099	1021.9586	19
20	2170.0696	1085.5384	2153.0431	1077.0252	2152.0591	1076.5332	S	1900.8898	950.9485	1883.8633	942.4353	1882.8792	941.9433	18
21	2267.1224	1134.0648	2250.0958	1125.5516	2249.1118	1125.0595	P	1813.8578	907.4325	1796.8312	898.9193	1795.8472	898.4272	17
22	2430.1857	1215.5965	2413.1592	1207.0832	2412.1751	1206.5912	Y	1716.8050	858.9061	1699.7785	850.3929	1698.7945	849.9009	16
23	2501.2228	1251.1151	2484.1963	1242.6018	2483.2123	1242.1098	A	1553.7417	777.3745	1536.7151	768.8612	1535.7311	768.3692	15
24	2629.2814	1315.1443	2612.2549	1306.6311	2611.2708	1306.1391	Q	1482.7046	741.8559	1465.6780	733.3427	1464.6940	732.8506	14
25	2686.3029	1343.6551	2669.2763	1335.1418	2668.2923	1334.6498	G	1354.6460	677.8266	1337.6195	669.3134	1336.6354	668.8214	13
26	2846.3335	1423.6704	2829.3070	1415.1571	2828.3230	1414.6651	C	1297.6245	649.3159	1280.5980	640.8026	1279.6140	640.3106	12
27	2961.3605	1481.1839	2944.3339	1472.6706	2943.3499	1472.1786	D	1137.5939	569.3006	1120.5673	560.7873	1119.5833	560.2953	11
28	3018.3819	1509.6946	3001.3554	1501.1813	3000.3714	1500.6893	G	1022.5669	511.7871	1005.5404	503.2738			10
29	3075.4034	1538.2053	3058.3768	1529.6921	3057.3928	1529.2000	G	965.5455	483.2764	948.5189	474.7631			9
30	3222.4718	1611.7395	3205.4453	1603.2263	3204.4612	1602.7343	F	908.5240	454.7656	891.4975	446.2524			8
31	3319.5246	1660.2659	3302.4980	1651.7526	3301.5140	1651.2606	P	761.4556	381.2314	744.4291	372.7182			7
32	3482.5879	1741.7976	3465.5613	1733.2843	3464.5773	1732.7923	Y	664.4028	332.7051	647.3763	324.1918			6
33	3595.6720	1798.3396	3578.6454	1789.8263	3577.6614	1789.3343	L	501.3395	251.1734	484.3130	242.6601			5
34	3708.7560	1854.8816	3691.7295	1846.3684	3690.7455	1845.8764	I	388.2554	194.6314	371.2289	186.1181			4
35	3779.7931	1890.4002	3762.7666	1881.8869	3761.7826	1881.3949	A	275.1714	138.0893	258.1448	129.5761			3
36	3836.8146	1918.9109	3819.7881	1910.3977	3818.8040	1909.9057	G	204.1343	102.5708	187.1077	94.0575			2
37							K	147.1128	74.0600	130.0863	65.5468			1

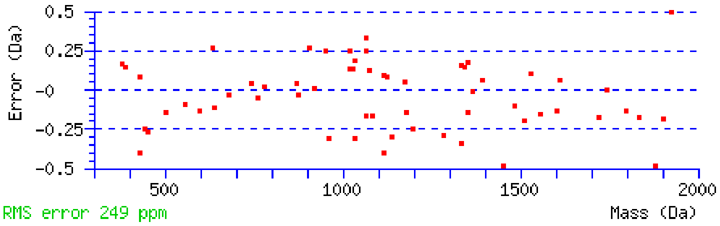

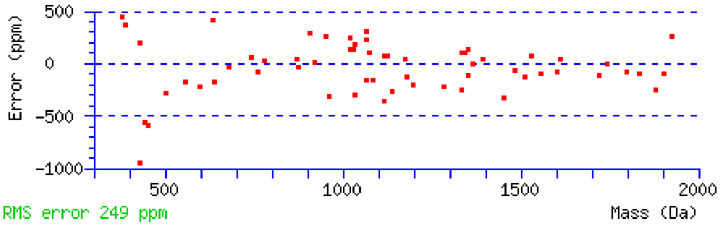

All matches to this query

Score	Mr(calc):	Delta	Sequence
47.0	3981.9129	0.0140	ILTNNSQTPILSPQEVVSCSPYAQGCDGGFPYLIAGK
46.2	3981.9129	0.0140	ILTNNSQTPILSPQEVVSCSPYAQGCDGGFPYLIAGK
28.7	3980.9288	0.9980	ILTNNSQTPILSPQEVVSCSPYAQGCDGGFPYLIAGK
3.8	3980.9504	0.9765	SLNALAPAARYSAGLAVYEGQARPLSDAQNFQVSDNSK
1.6	3981.9126	0.0143	HNSTGQGNVSVSLVPPTKIVEFDLAQQTVIDAKDSK
0.3	3981.9008	0.0261	KTISYSNCVTQMFIFLVLGVTESWLFAIMSIDR
0.3	3981.9008	0.0261	KTISYSNCVTQMFIFLVLGVTESWLFAIMSIDR
0.1	3981.9008	0.0261	KTISYSNCVTQMFIFLVLGVTESWLFAIMSIDR

Spectrum No: 460; Query: 1002; Rank: 1

Peptide View

MS/MS Fragmentation of **STNVAVDSGGGLLKPTVASQNQSLPVAK**
Found in **IPI00194958**, Tax_Id=10116 Gene_Symbol=Picalm Isoform 2 of Phosphatidylinositol-binding clathrin assembly protein

Match to Query 1002: 2738.448462 from(913.823430,3+)
Title: 091129RatKid_SCX02_23.1913.1913.3.dta
Data file K:\NewmanPaper\Piliang\3SubProteomes\Piliang3SP\mgf5ppm\SCX_3SubProteomes5ppm.mgf

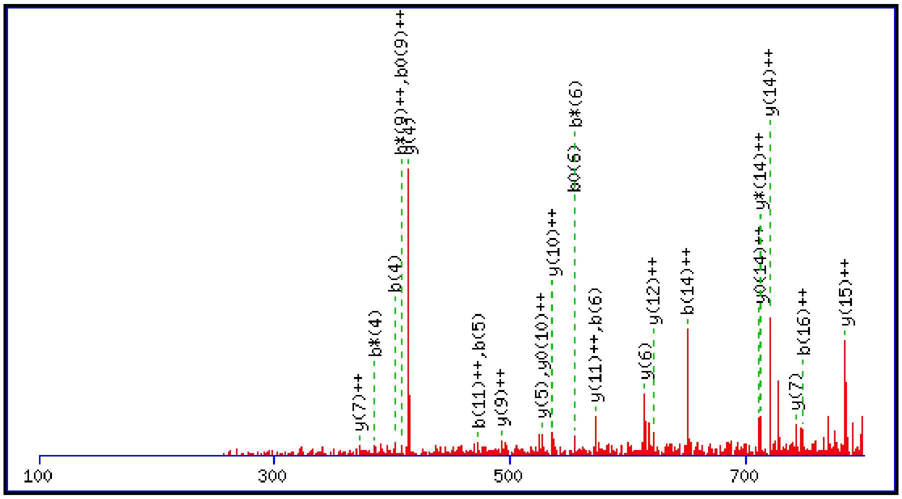

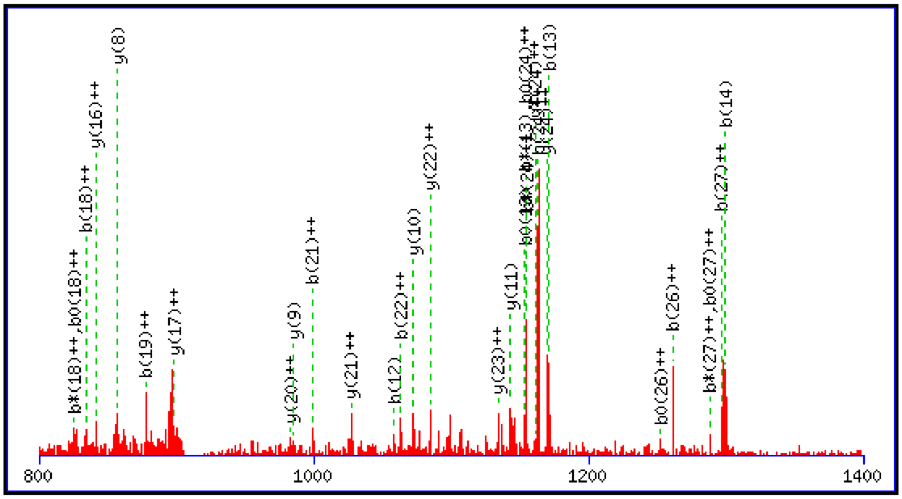

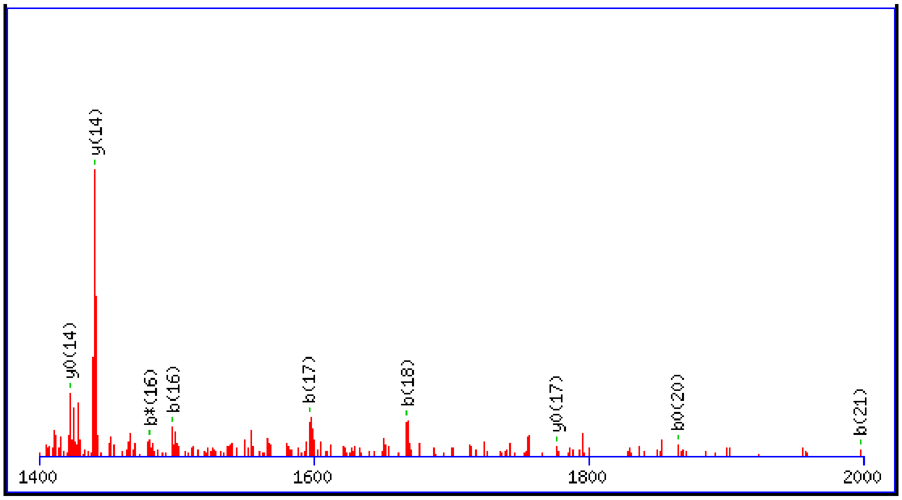

Monoisotopic mass of neutral peptide Mr(calc): 2738.4450
Fixed modifications: Carbamidomethyl (C)
Variable modifications:
N21 : Deamidated_N (N)
Ions Score: 47 **Expect:** 0.002
Matches (Bold Red): 65/310 fragment ions using 147 most intense peaks

#	b	b^{++}	b*	b^{*++}	b^{0}	b^{0++}	Seq.	y	y^{++}	y*	y^{*++}	y^{0}	y^{0++}	#
1	88.0393	44.5233			70.0287	35.5180	S							28
2	189.0870	95.0471			171.0764	86.0418	T	2652.4203	1326.7138	2635.3937	1318.2005	2634.4097	1317.7085	27
3	303.1299	152.0686	286.1034	143.5553	285.1193	143.0633	N	2551.3726	1276.1899	2534.3461	1267.6767	2533.3620	1267.1847	26
4	**402.1983**	201.6028	**385.1718**	193.0895	384.1878	192.5975	V	2437.3297	1219.1685	2420.3031	1210.6552	2419.3191	1210.1632	25
5	**473.2354**	237.1214	456.2089	228.6081	455.2249	228.1161	A	2338.2613	**1169.6343**	2321.2347	**1161.1210**	2320.2507	1160.6290	24
6	**572.3039**	286.6556	**555.2773**	278.1423	**554.2933**	277.6503	V	2267.2241	**1134.1157**	2250.1976	1125.6024	2249.2136	1125.1104	23
7	687.3308	344.1690	670.3042	335.6558	669.3202	335.1638	D	2168.1557	**1084.5815**	2151.1292	1076.0682	2150.1452	1075.5762	22
8	774.3628	387.6850	757.3363	379.1718	756.3523	378.6798	S	2053.1288	**1027.0680**	2036.1022	1018.5548	2035.1182	1018.0628	21
9	831.3843	416.1958	814.3577	**407.6825**	813.3737	**407.1905**	G	1966.0968	**983.5520**	1949.0702	975.0387	1948.0862	974.5467	20
10	888.4058	444.7065	871.3792	436.1932	870.3952	435.7012	G	1909.0753	955.0413	1892.0487	946.5280	1891.0647	946.0360	19
11	945.4272	**473.2172**	928.4007	464.7040	927.4167	464.2120	G	1852.0538	926.5306	1835.0273	918.0173	1834.0433	917.5253	18
12	**1058.5113**	529.7593	1041.4847	521.2460	1040.5007	520.7540	L	1795.0324	**898.0198**	1778.0058	889.5065	**1777.0218**	889.0145	17
13	**1171.5953**	586.3013	**1154.5688**	577.7880	**1153.5848**	577.2960	L	1681.9483	**841.4778**	1664.9218	832.9645	1663.9377	832.4725	16
14	**1299.6903**	**650.3488**	1282.6638	641.8355	1281.6797	641.3435	K	1568.8642	**784.9358**	1551.8377	776.4225	1550.8537	775.9305	15
15	1396.7431	698.8752	1379.7165	690.3619	1378.7325	689.8699	P	**1440.7693**	**720.8883**	1423.7427	**712.3750**	**1422.7587**	**711.8830**	14
16	**1497.7907**	**749.3990**	**1480.7642**	740.8857	1479.7802	740.3937	T	1343.7165	672.3619	1326.6900	663.8486	1325.7060	663.3566	13
17	**1596.8592**	798.9332	1579.8326	790.4199	1578.8486	789.9279	V	1242.6688	**621.8381**	1225.6423	613.3248	1224.6583	612.8328	12
18	**1667.8963**	**834.4518**	1650.8697	**825.9385**	1649.8857	**825.4465**	A	**1143.6004**	**572.3038**	1126.5739	563.7906	1125.5899	563.2986	11
19	1754.9283	**877.9678**	1737.9018	869.4545	1736.9177	868.9625	S	**1072.5633**	**536.7853**	1055.5368	528.2720	1054.5527	**527.7800**	10
20	1882.9869	941.9971	1865.9603	933.4838	**1864.9763**	932.9918	Q	**985.5313**	**493.2693**	968.5047	484.7560	967.5207	484.2640	9
21	**1998.0138**	**999.5105**	1980.9873	990.9973	1980.0033	990.5053	N	**857.4727**	429.2400	840.4462	420.7267	839.4621	420.2347	8
22	2126.0724	**1063.5398**	2109.0458	1055.0266	2108.0618	1054.5346	Q	**742.4458**	**371.7265**	725.4192	363.2132	724.4352	362.7212	7
23	2213.1044	1107.0559	2196.0779	1098.5426	2195.0939	1098.0506	S	**614.3872**	307.6972	597.3606	299.1840	596.3766	298.6919	6
24	2326.1885	**1163.5979**	2309.1619	**1155.0846**	2308.1779	**1154.5926**	L	**527.3552**	264.1812	510.3286	255.6679			5
25	2423.2413	1212.1243	2406.2147	1203.6110	2405.2307	1203.1190	P	**414.2711**	207.6392	397.2445	199.1259			4
26	2522.3097	**1261.6585**	2505.2831	1253.1452	2504.2991	**1252.6532**	V	317.2183	159.1128	300.1918	150.5995			3
27	2593.3468	**1297.1770**	2576.3202	**1288.6638**	2575.3362	**1288.1717**	A	218.1499	109.5786	201.1234	101.0653			2
28							K	147.1128	74.0600	130.0863	65.5468			1

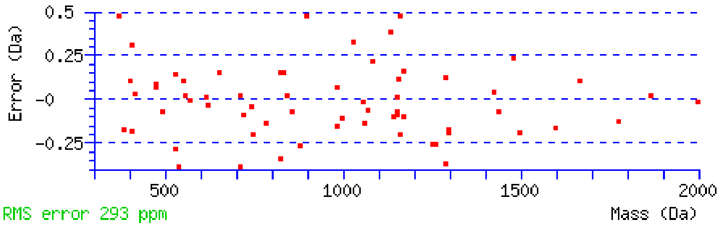

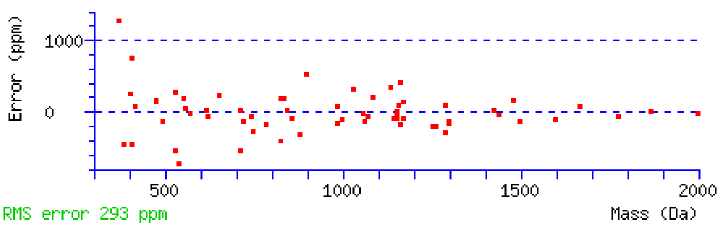

All matches to this query

Score	Mr(calc):	Delta	Sequence
46.5	2738.4450	0.0034	STNVAVDSGGGLLKPTVASQNQSLPVAK
22.5	2738.4450	0.0034	STNVAVDSGGGLLKPTVASQNQSLPVAK
21.9	2737.4610	0.9874	STNVAVDSGGGLLKPTVASQNQSLPVAK
2.5	2738.4506	-0.0021	KGFYLAFQDVGACIALVSVKVYYK
2.0	2737.4316	1.0168	GHLEVVKLLVLTHGADPENYAVRK

Spectrum No: 461; Query: 808; Rank: 1

Peptide View

MS/MS Fragmentation of **FTSPQWGPPFYNGTEANGTR**
Found in **IPI00214158**, Tax_Id=10116 Gene_Symbol=Slc22a6 Renal organic anion transporter 1

Match to Query 808: 2228.994448 from(1115.504500,2+)
Title: 091127RatKid_SCX01_02.3711.3711.2.dta
Data file K:\NewmanPaper\Piliang\3SubProteomes\Piliang3SP\mgf5ppm\SCX_3SubProteomes5ppm.mgf

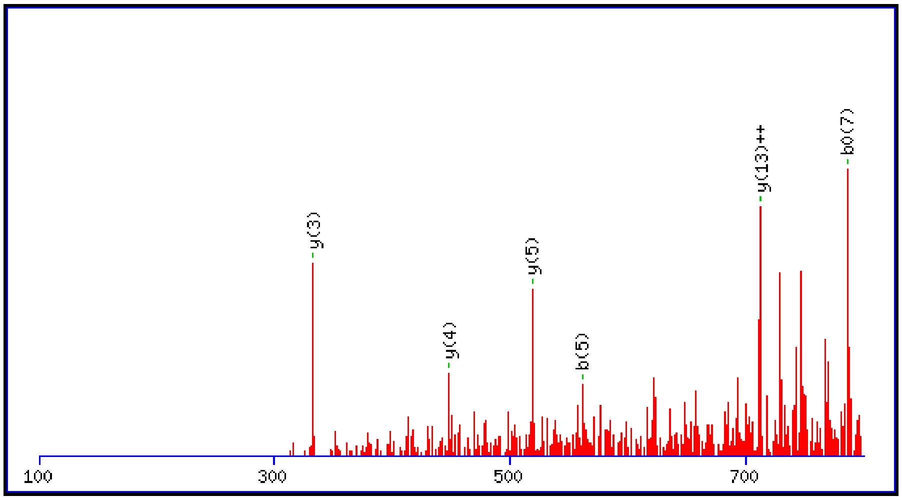

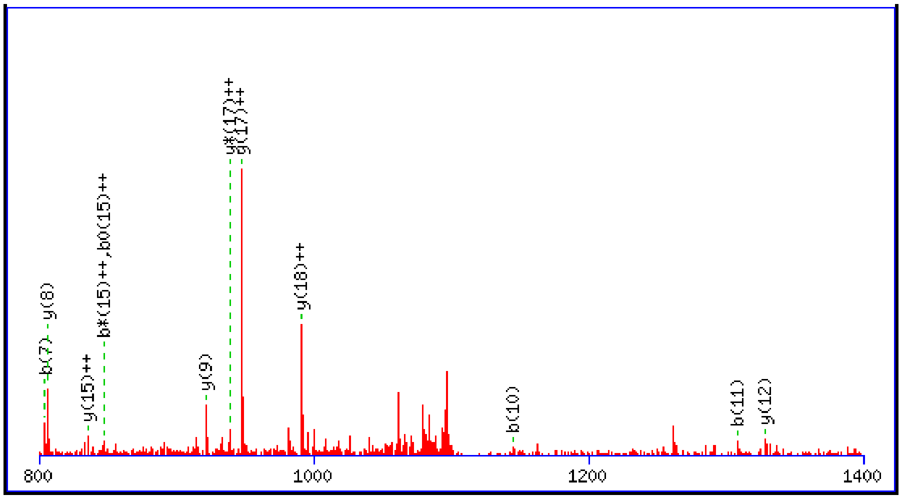

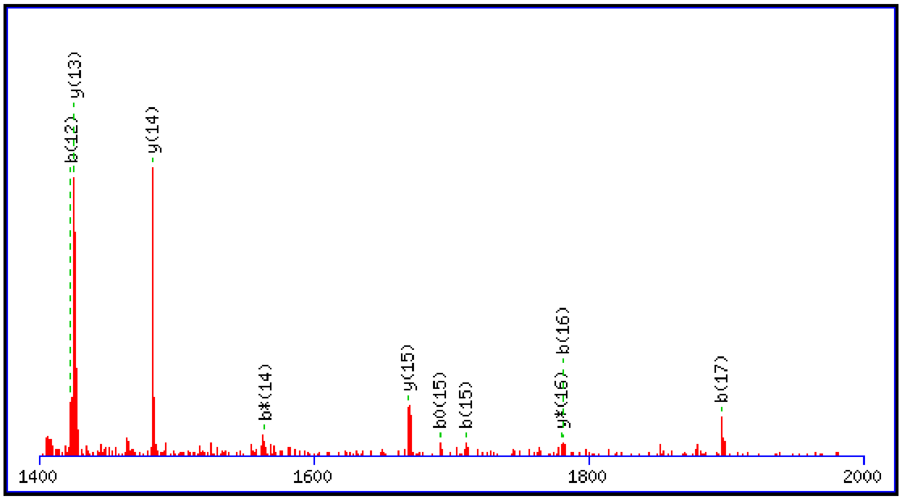

Monoisotopic mass of neutral peptide Mr(calc): 2227.9811
Fixed modifications: Carbamidomethyl (C)
Variable modifications:
N12 : Deamidated_N (N)
N17 : Deamidated_N (N)
Ions Score: 46 **Expect:** 0.0065
Matches (Bold Red): 28/216 fragment ions using 55 most intense peaks

#	b	b^{++}	b*	b^{*++}	b^0	b^{0++}	Seq.	y	y^{++}	y*	y^{*++}	y^0	y^{0++}	#
1	148.0757	74.5415					F							20
2	249.1234	125.0653			231.1128	116.0600	T	2081.9199	1041.4636	2064.8934	1032.9503	2063.9093	1032.4583	19
3	336.1554	168.5813			318.1448	159.5761	S	1980.8722	**990.9398**	1963.8457	982.4265	1962.8617	981.9345	18
4	433.2082	217.1077			415.1976	208.1024	P	1893.8402	**947.4237**	1876.8137	**938.9105**	1875.8296	938.4185	17
5	**561.2667**	281.1370	544.2402	272.6237	543.2562	272.1317	Q	1796.7874	898.8974	**1779.7609**	890.3841	1778.7769	889.8921	16
6	747.3461	374.1767	730.3195	365.6634	729.3355	365.1714	W	**1668.7289**	**834.8681**	1651.7023	826.3548	1650.7183	825.8628	15
7	**804.3675**	402.6874	787.3410	394.1741	**786.3570**	393.6821	G	**1482.6496**	741.8284	1465.6230	733.3151	1464.6390	732.8231	14
8	901.4203	451.2138	884.3937	442.7005	883.4097	442.2085	P	**1425.6281**	**713.3177**	1408.6015	704.8044	1407.6175	704.3124	13
9	998.4730	499.7402	981.4465	491.2269	980.4625	490.7349	P	**1328.5753**	664.7913	1311.5488	656.2780	1310.5648	655.7860	12
10	**1145.5415**	573.2744	1128.5149	564.7611	1127.5309	564.2691	F	1231.5226	616.2649	1214.4960	607.7516	1213.5120	607.2596	11
11	**1308.6048**	654.8060	1291.5782	646.2928	1290.5942	645.8007	Y	1084.4541	542.7307	1067.4276	534.2174	1066.4436	533.7254	10
12	**1423.6317**	712.3195	1406.6052	703.8062	1405.6212	703.3142	N	**921.3908**	461.1990	904.3643	452.6858	903.3803	452.1938	9
13	1480.6532	740.8302	1463.6266	732.3170	1462.6426	731.8249	G	**806.3639**	403.6856	789.3373	395.1723	788.3533	394.6803	8
14	1581.7009	791.3541	**1564.6743**	782.8408	1563.6903	782.3488	T	749.3424	375.1748	732.3159	366.6616	731.3319	366.1696	7
15	**1710.7435**	855.8754	1693.7169	**847.3621**	**1692.7329**	**846.8701**	E	648.2947	324.6510	631.2682	316.1377	630.2842	315.6457	6
16	**1781.7806**	891.3939	1764.7540	882.8807	1763.7700	882.3886	A	**519.2521**	260.1297	502.2256	251.6164	501.2416	251.1244	5
17	**1896.8075**	948.9074	1879.7810	940.3941	1878.7969	939.9021	N	**448.2150**	224.6112	431.1885	216.0979	430.2045	215.6059	4
18	1953.8290	977.4181	1936.8024	968.9049	1935.8184	968.4128	G	**333.1881**	167.0977	316.1615	158.5844	315.1775	158.0924	3

**19**	2054.8767	1027.9420	2037.8501	1019.4287	2036.8661	1018.9367	**T**	276.1666	138.5870	259.1401	130.0737	258.1561	129.5817	**2**
**20**							**R**	175.1190	88.0631	158.0924	79.5498			**1**

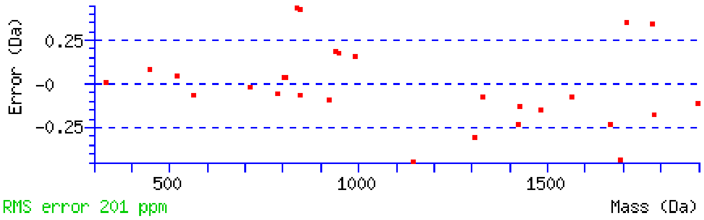

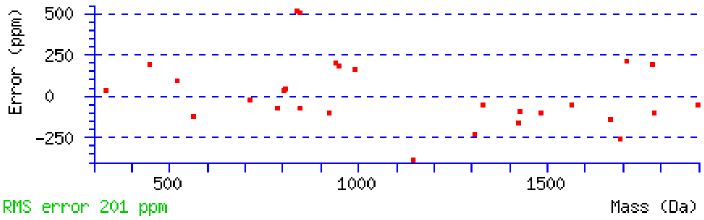

All matches to this query

Score	Mr(calc):	Delta	Sequence
46.4	2227.9811	1.0134	FTSPQWGPPFYNGTEANGTR
9.2	2226.9971	1.9974	FTSPQWGPPFYNGTEANGTR
8.5	2226.9971	1.9974	FTSPQWGPPFYNGTEANGTR
6.6	2228.9910	0.0034	SRSASQERSLDEGVSIDGEK
3.4	2228.9910	0.0034	SRSASQERSLDEGVSIDGEK
1.7	2228.9910	0.0034	SRSASQERSLDEGVSIDGEK
1.5	2228.9910	0.0034	SRSASQERSLDEGVSIDGEK
1.2	2226.9729	2.0216	VYQQFNETTSMGESRARK
0.8	2226.9729	2.0216	VYQQFNETTSMGESRARK
0.8	2226.9729	2.0216	VYQQFNETTSMGESRARK

Spectrum No: 462; Query: 524; Rank: 1

Peptide View

MS/MS Fragmentation of **VSVNGTVVVEEPVGQLR**
Found in **IPI00369646**, Tax_Id=10116 Gene_Symbol=Pfas_predicted phosphoribosylformylglycinamidine synthase

Match to Query 524: 1781.957888 from(891.986220,2+)
Title: 091127RatKid_SCX01_17.2637.2637.2.dta
Data file K:\NewmanPaper\Piliang\3SubProteomes\Piliang3SP\mgf5ppm\SCX_3SubProteomes5ppm.mgf

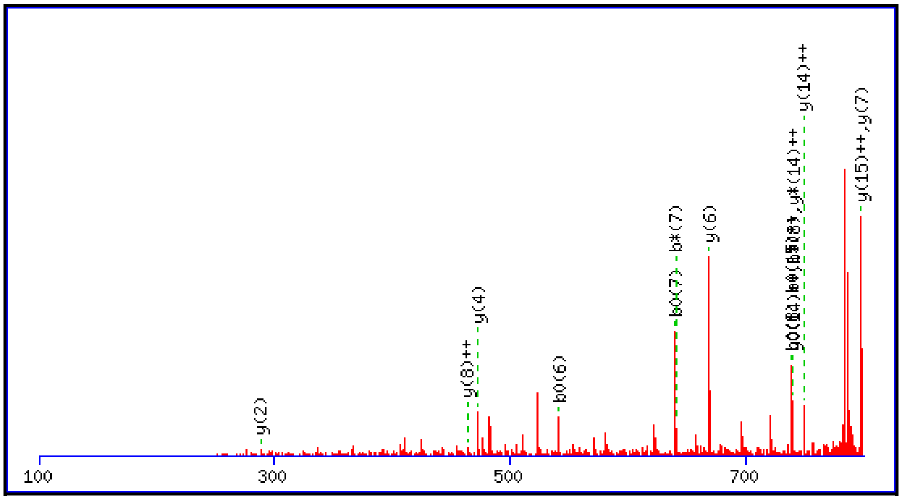

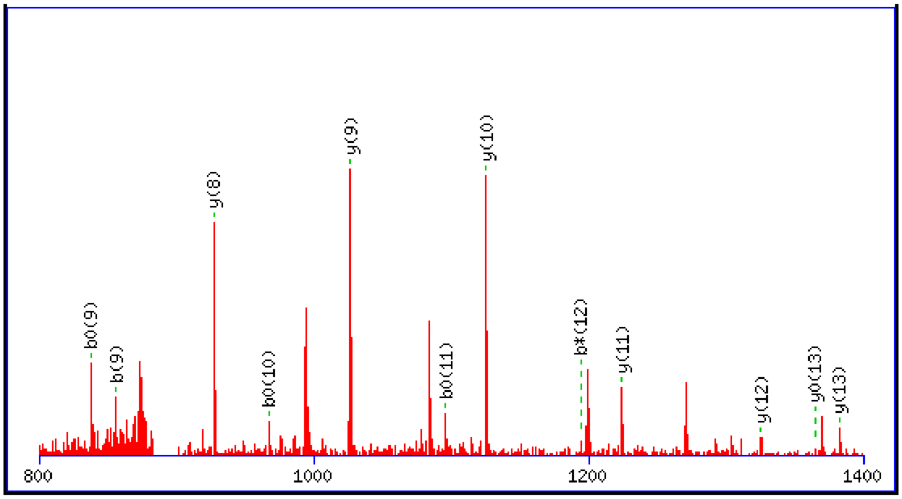

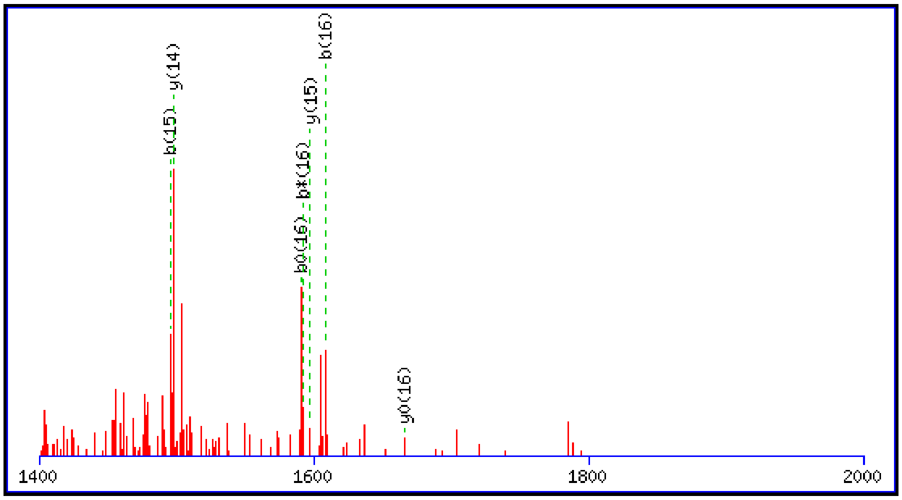

Monoisotopic mass of neutral peptide Mr(calc): 1781.9523
Fixed modifications: Carbamidomethyl (C)
Variable modifications:
N4 : Deamidated_N (N)
Ions Score: 46 **Expect:** 0.0021
Matches (Bold Red): 34/172 fragment ions using 98 most intense peaks

#	b	b^{++}	b*	b^{*++}	b^{0}	b^{0++}	Seq.	y	y^{++}	y*	y^{*++}	y^{0}	y^{0++}	#
1	100.0757	50.5415					V							17
2	187.1077	94.0575			169.0972	85.0522	S	1683.8912	842.4492	1666.8646	833.9360	**1665.8806**	833.4439	16
3	286.1761	143.5917			268.1656	134.5864	V	**1596.8592**	**798.9332**	1579.8326	790.4199	1578.8486	789.9279	15
4	401.2031	201.1052	384.1765	192.5919	383.1925	192.0999	N	**1497.7907**	**749.3990**	1480.7642	**740.8857**	1479.7802	**740.3937**	14
5	458.2245	229.6159	441.1980	221.1026	440.2140	220.6106	G	**1382.7638**	691.8855	1365.7373	683.3723	**1364.7532**	682.8803	13
6	559.2722	280.1397	542.2457	271.6265	**541.2616**	271.1345	T	**1325.7423**	663.3748	1308.7158	654.8615	1307.7318	654.3695	12
7	658.3406	329.6740	**641.3141**	321.1607	**640.3301**	320.6687	V	**1224.6947**	612.8510	1207.6681	604.3377	1206.6841	603.8457	11
8	757.4090	379.2082	**740.3825**	370.6949	**739.3985**	370.2029	V	**1125.6262**	563.3168	1108.5997	554.8035	1107.6157	554.3115	10
9	**856.4775**	428.7424	839.4509	420.2291	**838.4669**	419.7371	V	**1026.5578**	513.7826	1009.5313	505.2693	1008.5473	504.7773	9
10	985.5200	493.2637	968.4935	484.7504	**967.5095**	484.2584	E	**927.4894**	**464.2483**	910.4629	455.7351	909.4789	455.2431	8
11	1114.5626	557.7850	1097.5361	549.2717	**1096.5521**	548.7797	E	**798.4468**	399.7271	781.4203	391.2138	780.4363	390.7218	7
12	1211.6154	606.3113	**1194.5889**	597.7981	1193.6048	597.3061	P	**669.4042**	335.2058	652.3777	326.6925			6
13	1310.6838	655.8455	1293.6573	647.3323	1292.6733	646.8403	V	572.3515	286.6794	555.3249	278.1661			5
14	1367.7053	684.3563	1350.6787	675.8430	1349.6947	675.3510	G	**473.2831**	237.1452	456.2565	228.6319			4
15	**1495.7639**	748.3856	1478.7373	739.8723	1477.7533	**739.3803**	Q	416.2616	208.6344	399.2350	200.1212			3
16	**1608.8479**	804.9276	**1591.8214**	796.4143	**1590.8374**	795.9223	L	**288.2030**	144.6051	271.1765	136.0919			2
17							R	175.1190	88.0631	158.0924	79.5498			1

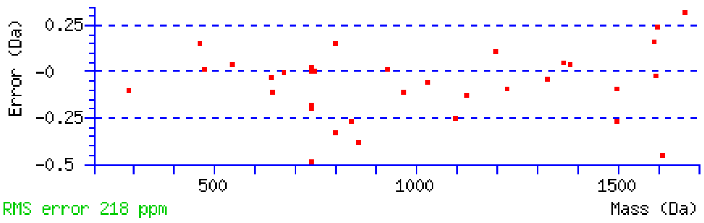

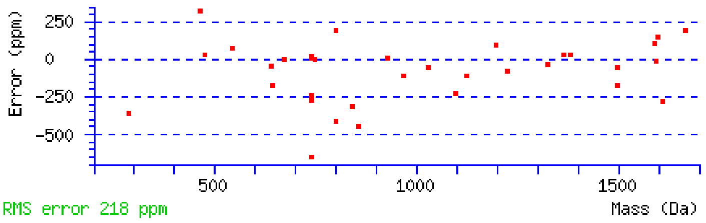

All matches to this query

Score	Mr(calc):	Delta	Sequence
46.4	1781.9523	0.0055	VSVNGTVVVEEPVGQLR
27.4	1780.9683	0.9896	VSVNGTVVVEEPVGQLR
1.3	1781.9682	-0.0104	ALTVRAEGPGGAGARLMR
0.9	1781.9570	0.0009	MEHEQKSGGLLRLLR

Spectrum No: 463; Query: 586; Rank: 1

Peptide View

MS/MS Fragmentation of **NATNVEQAFMTMAAEIK**
Found in **IPI00480673**, Tax_Id=10116 Gene_Symbol=Rab1b;MGC105830 similar to Ras-related protein Rab-1B

Match to Query 586: 1868.871428 from(935.442990,2+)
Title: 091129RatKid_SCX02_12.5293.5293.2.dta
Data file K:\NewmanPaper\Piliang\3SubProteomes\Piliang3SP\mgf5ppm\SCX_3SubProteomes5ppm.mgf

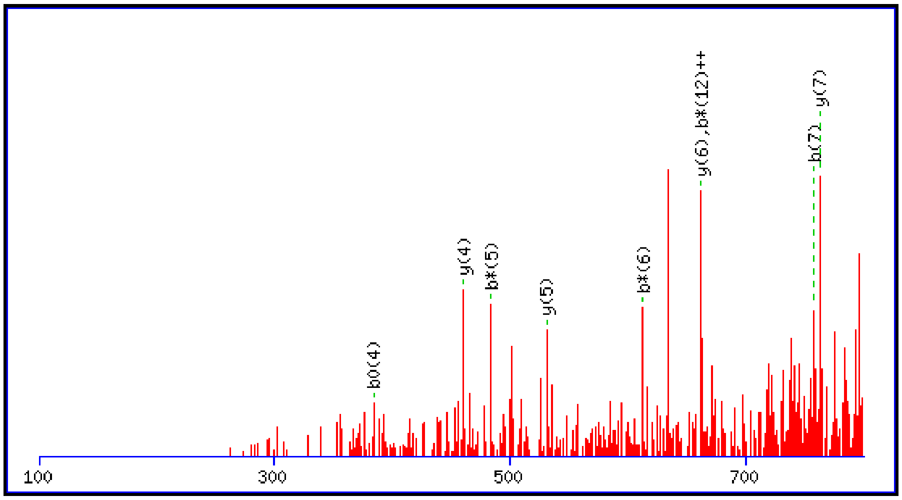

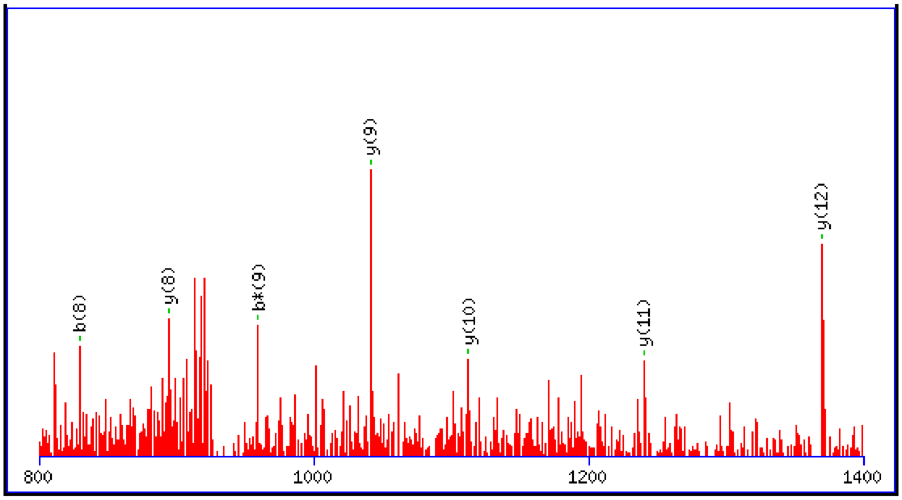

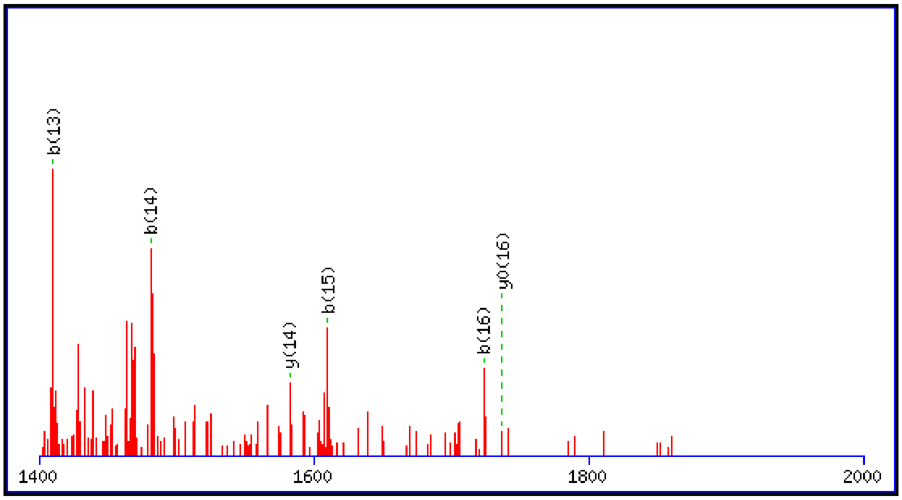

Monoisotopic mass of neutral peptide Mr(calc): 1868.8648
Fixed modifications: Carbamidomethyl (C)
Variable modifications:
N1 : Deamidated_N (N)
Ions Score: 46 **Expect:** 0.0055
Matches (Bold Red): 22/184 fragment ions using 48 most intense peaks

#	b	b^{++}	b*	b^{*++}	b^0	b^{0++}	Seq.	y	y^{++}	y*	y^{*++}	y^0	y^{0++}	#
1	116.0342	58.5207	99.0077	50.0075			N							17
2	187.0713	94.0393	170.0448	85.5260			A	1754.8452	877.9262	1737.8186	869.4129	**1736.8346**	868.9209	16
3	288.1190	144.5631	271.0925	136.0499	270.1084	135.5579	T	1683.8081	842.4077	1666.7815	833.8944	1665.7975	833.4024	15
4	402.1619	201.5846	385.1354	193.0713	**384.1514**	192.5793	N	**1582.7604**	791.8838	1565.7338	783.3706	1564.7498	782.8785	14
5	501.2303	251.1188	**484.2038**	242.6055	483.2198	242.1135	V	1468.7175	734.8624	1451.6909	726.3491	1450.7069	725.8571	13
6	630.2729	315.6401	**613.2464**	307.1268	612.2624	306.6348	E	**1369.6490**	685.3282	1352.6225	676.8149	1351.6385	676.3229	12
7	**758.3315**	379.6694	741.3050	371.1561	740.3210	370.6641	Q	**1240.6064**	620.8069	1223.5799	612.2936	1222.5959	611.8016	11
8	**829.3686**	415.1880	812.3421	406.6747	811.3581	406.1827	A	**1112.5479**	556.7776	1095.5213	548.2643	1094.5373	547.7723	10
9	976.4370	488.7222	**959.4105**	480.2089	958.4265	479.7169	F	**1041.5108**	521.2590	1024.4842	512.7457	1023.5002	512.2537	9
10	1107.4775	554.2424	1090.4510	545.7291	1089.4670	545.2371	M	**894.4423**	447.7248	877.4158	439.2115	876.4318	438.7195	8
11	1208.5252	604.7662	1191.4987	596.2530	1190.5146	595.7610	T	**763.4019**	382.2046	746.3753	373.6913	745.3913	373.1993	7
12	1339.5657	670.2865	1322.5391	**661.7732**	1321.5551	661.2812	M	**662.3542**	331.6807	645.3276	323.1675	644.3436	322.6754	6
13	**1410.6028**	705.8050	1393.5763	697.2918	1392.5922	696.7998	A	**531.3137**	266.1605	514.2871	257.6472	513.3031	257.1552	5
14	**1481.6399**	741.3236	1464.6134	732.8103	1463.6294	732.3183	A	**460.2766**	230.6419	443.2500	222.1287	442.2660	221.6366	4
15	**1610.6825**	805.8449	1593.6560	797.3316	1592.6720	796.8396	E	389.2395	195.1234	372.2129	186.6101	371.2289	186.1181	3
16	**1723.7666**	862.3869	1706.7400	853.8737	1705.7560	853.3816	I	260.1969	130.6021	243.1703	122.0888			2
17							K	147.1128	74.0600	130.0863	65.5468			1

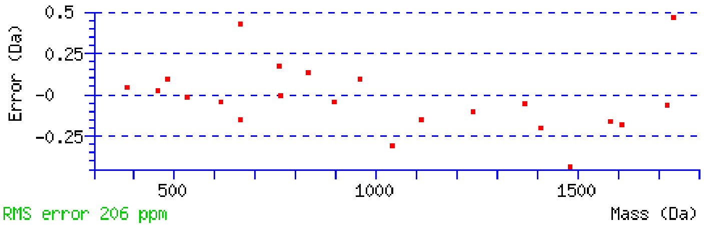

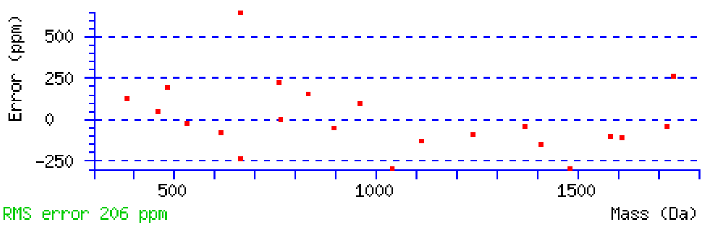

All matches to this query

Score	Mr(calc):	Delta	Sequence
46.3	1868.8648	0.0066	NATNVEQAFMTMAAEIK
39.2	1868.8648	0.0066	NATNVEQAFMTMAAEIK
30.3	1867.8808	0.9906	NATNVEQAFMTMAAEIK
7.5	1868.8768	-0.0054	NLQDTDSVSKLQAELK
5.8	1868.8768	-0.0054	NLQDTDSVSKLQAELK
5.8	1868.8768	-0.0054	NLQDTDSVSKLQAELK
5.6	1868.8597	0.0117	SIYGEKFENDSFILK
5.6	1868.8597	0.0117	SIYGEKFENDSFILK
1.5	1868.8815	-0.0101	MQSNNAKAVSARAEAIK
1.5	1868.8815	-0.0101	MQSNNAKAVSARAEAIK

Spectrum No: 464; Query: 139; Rank: 1

Peptide View

MS/MS Fragmentation of **LLNLTSPEATAR**
Found in **IPI00515854**, Tax_Id=10116 Gene_Symbol=Entpd2 Ectonucleoside triphosphate diphosphohydrolase 2

Match to Query 139: 1285.689868 from(643.852210,2+)
Title: 091127RatKid_SCX01_12.2027.2027.2.dta
Data file K:\NewmanPaper\Piliang\3SubProteomes\Piliang3SP\mgf5ppm\SCX_3SubProteomes5ppm.mgf

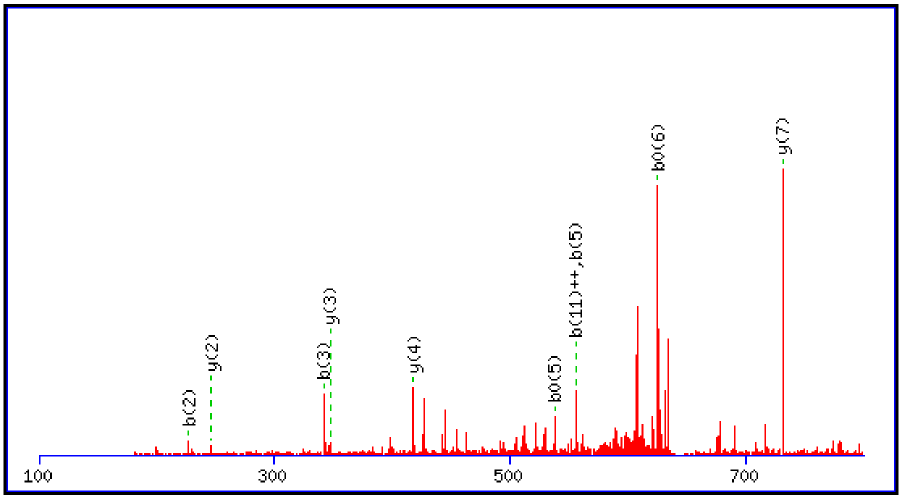

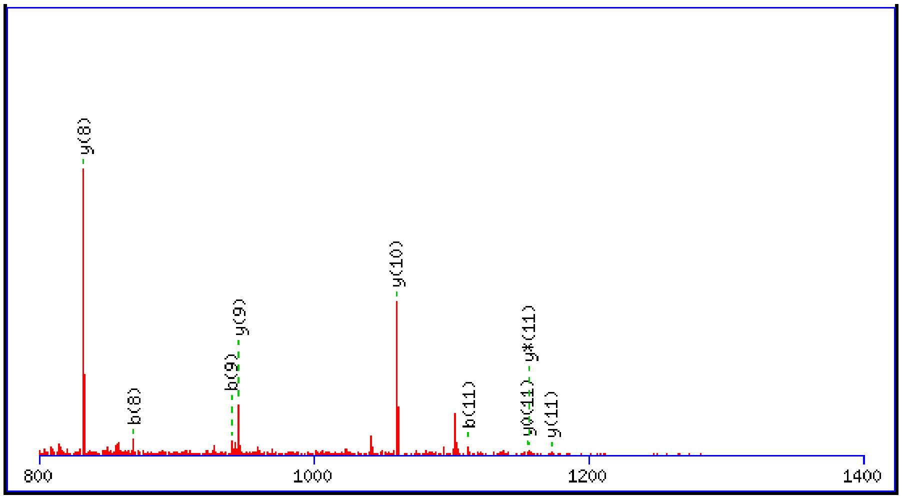

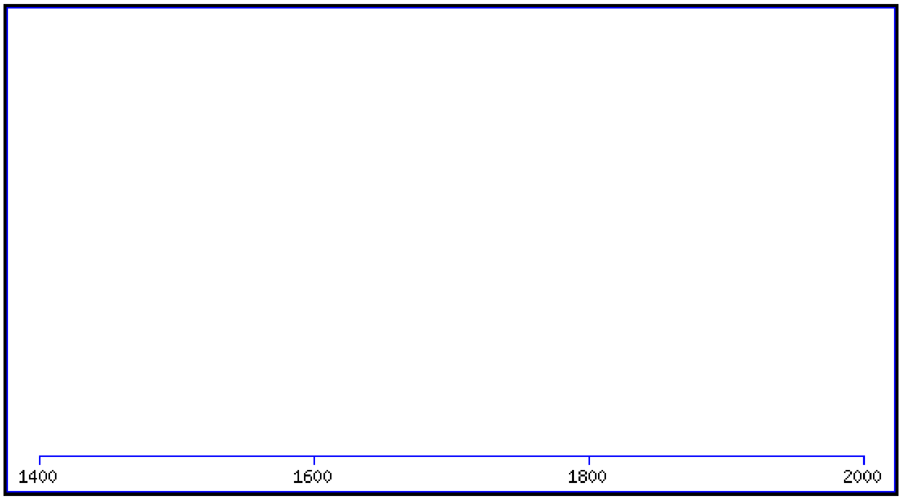

Monoisotopic mass of neutral peptide Mr(calc): 1285.6877
Fixed modifications: Carbamidomethyl (C)
Variable modifications:
N3 : Deamidated_N (N)
Ions Score: 46 **Expect:** 0.0022
Matches (Bold Red): 19/116 fragment ions using 45 most intense peaks

#	b	b^{++}	b*	b^{*++}	b^0	b^{0++}	Seq.	y	y^{++}	y*	y^{*++}	y^0	y^{0++}	#
1	114.0913	57.5493					L							12
2	**227.1754**	114.0913					L	**1173.6110**	587.3091	**1156.5844**	578.7959	**1155.6004**	578.3038	11
3	**342.2023**	171.6048	325.1758	163.0915			N	**1060.5269**	530.7671	1043.5004	522.2538	1042.5164	521.7618	10
4	455.2864	228.1468	438.2599	219.6336			L	**945.5000**	473.2536	928.4734	464.7404	927.4894	464.2483	9
5	**556.3341**	278.6707	539.3075	270.1574	**538.3235**	269.6654	T	**832.4159**	416.7116	815.3894	408.1983	814.4054	407.7063	8
6	643.3661	322.1867	626.3396	313.6734	**625.3555**	313.1814	S	**731.3682**	366.1878	714.3417	357.6745	713.3577	357.1825	7
7	740.4189	370.7131	723.3923	362.1998	722.4083	361.7078	P	644.3362	322.6717	627.3097	314.1585	626.3257	313.6665	6
8	**869.4615**	435.2344	852.4349	426.7211	851.4509	426.2291	E	547.2835	274.1454	530.2569	265.6321	529.2729	265.1401	5
9	**940.4986**	470.7529	923.4720	462.2397	922.4880	461.7476	A	**418.2409**	209.6241	401.2143	201.1108	400.2303	200.6188	4
10	1041.5463	521.2768	1024.5197	512.7635	1023.5357	512.2715	T	**347.2037**	174.1055	330.1772	165.5922	329.1932	165.1002	3
11	**1112.5834**	**556.7953**	1095.5568	548.2821	1094.5728	547.7900	A	**246.1561**	123.5817	229.1295	115.0684			2
12							R	175.1190	88.0631	158.0924	79.5498			1

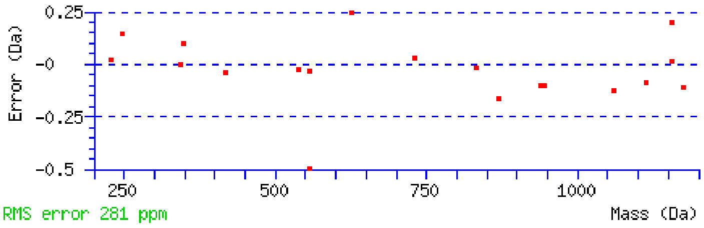

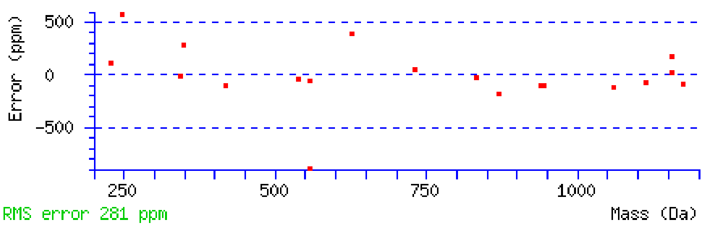

All matches to this query

Score	Mr(calc):	Delta	Sequence
45.9	1285.6877	0.0021	LLNLTSPEATAR
7.1	1285.6891	0.0008	ILIDNWNRSR
6.3	1283.6761	2.0137	EPNLVVSFPGPK
4.8	1285.6795	0.0103	AAGAAVPKPTPVK
4.6	1285.6795	0.0103	LVESFRKTVK
4.1	1285.6795	0.0103	LVESFRKTVK
3.6	1285.6853	0.0046	ILVTSFNHMPK
3.1	1285.6990	-0.0091	LAEQNILSRDK
2.4	1285.6990	-0.0091	RNADVNLKNLK
1.1	1284.6938	0.9960	NPKLSQWKQR

Spectrum No: 465; Query: 1000; Rank: 1

Peptide View

MS/MS Fragmentation of **ISDTNITAIPQGLPTSISELHLDGNK**
Found in **IPI00199861**, Tax_Id=10116 Gene_Symbol=Dcn Decorin precursor

Match to Query 1000: 2734.406862 from(912.476230,3+)
Title: 091129RatKid_SCX02_21.4941.4941.3.dta
Data file K:\NewmanPaper\Piliang\3SubProteomes\Piliang3SP\mgf5ppm\SCX_3SubProteomes5ppm.mgf

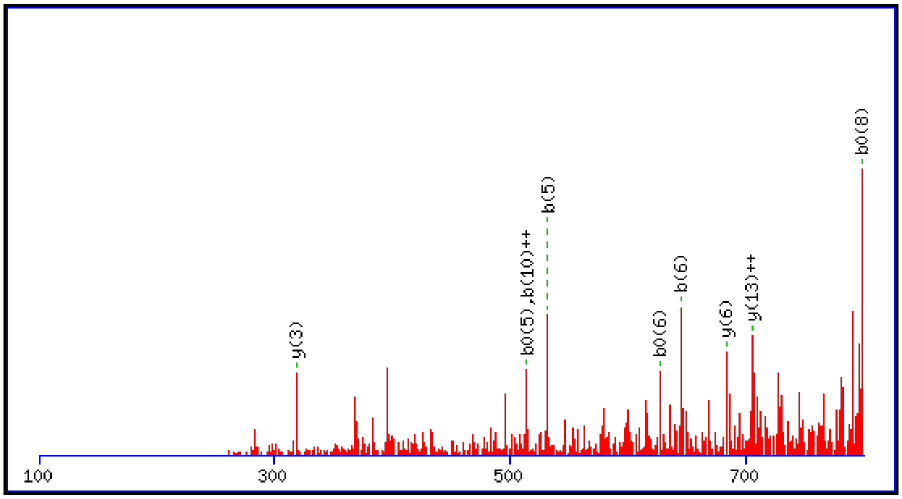

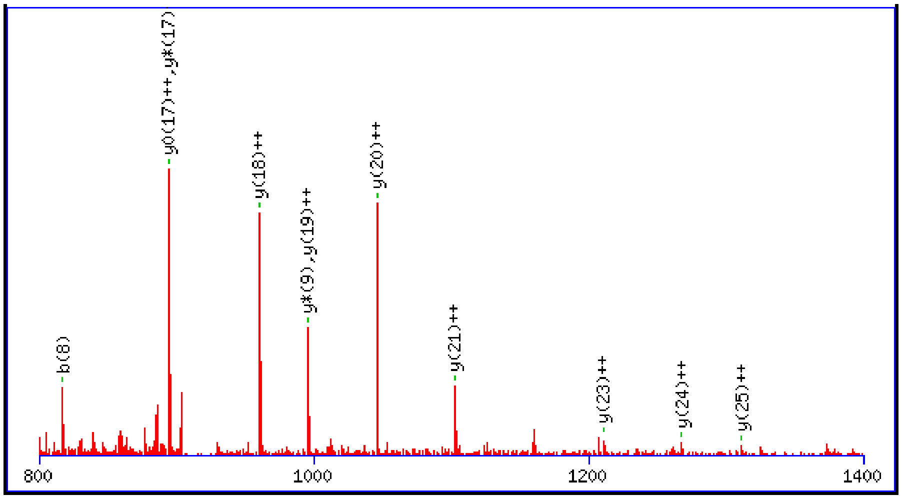

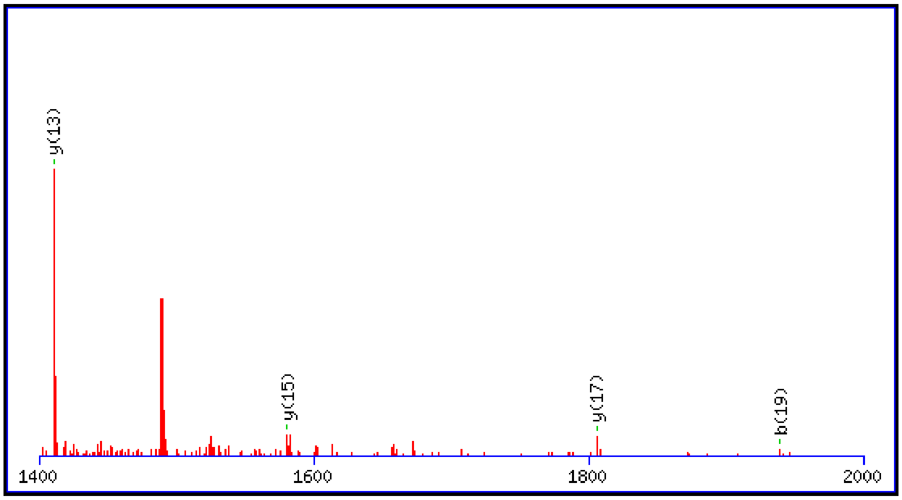

Monoisotopic mass of neutral peptide Mr(calc): 2734.4025
Fixed modifications: Carbamidomethyl (C)
Variable modifications:
N5 : Deamidated_N (N)
Ions Score: 46 **Expect:** 0.0039
Matches (Bold Red): 24/284 fragment ions using 39 most intense peaks

#	b	b^{++}	b*	b^{*++}	b^0	b^{0++}	Seq.	y	y^{++}	y*	y^{*++}	y^0	y^{0++}	#
1	114.0913	57.5493					I							26
2	201.1234	101.0653			183.1128	92.0600	S	2622.3257	**1311.6665**	2605.2992	1303.1532	2604.3151	1302.6612	25
3	316.1503	158.5788			298.1397	149.5735	D	2535.2937	**1268.1505**	2518.2671	1259.6372	2517.2831	1259.1452	24
4	417.1980	209.1026			399.1874	200.0974	T	2420.2667	**1210.6370**	2403.2402	1202.1237	2402.2562	1201.6317	23
5	**532.2249**	266.6161	515.1984	258.1028	**514.2144**	257.6108	N	2319.2191	1160.1132	2302.1925	1151.5999	2301.2085	1151.1079	22
6	**645.3090**	323.1581	628.2824	314.6449	**627.2984**	314.1529	I	2204.1921	**1102.5997**	2187.1656	1094.0864	2186.1816	1093.5944	21
7	746.3567	373.6820	729.3301	365.1687	728.3461	364.6767	T	2091.1081	**1046.0577**	2074.0815	1037.5444	2073.0975	1037.0524	20
8	**817.3938**	409.2005	800.3672	400.6873	**799.3832**	400.1952	A	1990.0604	**995.5338**	1973.0338	987.0206	1972.0498	986.5285	19
9	930.4778	465.7426	913.4513	457.2293	912.4673	456.7373	I	1919.0233	**960.0153**	1901.9967	951.5020	1901.0127	951.0100	18
10	1027.5306	**514.2689**	1010.5041	505.7557	1009.5200	505.2637	P	**1805.9392**	903.4732	1788.9127	**894.9600**	1787.9286	**894.4680**	17
11	1155.5892	578.2982	1138.5626	569.7850	1137.5786	569.2930	Q	1708.8864	854.9469	1691.8599	846.4336	1690.8759	845.9416	16
12	1212.6107	606.8090	1195.5841	598.2957	1194.6001	597.8037	G	**1580.8279**	790.9176	1563.8013	782.4043	1562.8173	781.9123	15
13	1325.6947	663.3510	1308.6682	654.8377	1307.6842	654.3457	L	1523.8064	762.4068	1506.7798	753.8936	1505.7958	753.4016	14
14	1422.7475	711.8774	1405.7209	703.3641	1404.7369	702.8721	P	**1410.7223**	**705.8648**	1393.6958	697.3515	1392.7118	696.8595	13
15	1523.7952	762.4012	1506.7686	753.8879	1505.7846	753.3959	T	1313.6696	657.3384	1296.6430	648.8251	1295.6590	648.3331	12
16	1610.8272	805.9172	1593.8006	797.4040	1592.8166	796.9119	S	1212.6219	606.8146	1195.5953	598.3013	1194.6113	597.8093	11
17	1723.9113	862.4593	1706.8847	853.9460	1705.9007	853.4540	I	1125.5899	563.2986	1108.5633	554.7853	1107.5793	554.2933	10
18	1810.9433	905.9753	1793.9167	897.4620	1792.9327	896.9700	S	1012.5058	506.7565	**995.4792**	498.2433	994.4952	497.7513	9

19	1939.9859	970.4966	1922.9593	961.9833	1921.9753	961.4913	E	925.4738	463.2405	908.4472	454.7272	907.4632	454.2352	8
20	2053.0699	1027.0386	2036.0434	1018.5253	2035.0594	1018.0333	L	796.4312	398.7192	779.4046	390.2060	778.4206	389.7139	7
21	2190.1288	1095.5681	2173.1023	1087.0548	2172.1183	1086.5628	H	683.3471	342.1772	666.3206	333.6639	665.3365	333.1719	6
22	2303.2129	1152.1101	2286.1864	1143.5968	2285.2023	1143.1048	L	546.2882	273.6477	529.2617	265.1345	528.2776	264.6425	5
23	2418.2399	1209.6236	2401.2133	1201.1103	2400.2293	1200.6183	D	433.2041	217.1057	416.1776	208.5924	415.1936	208.1004	4
24	2475.2613	1238.1343	2458.2348	1229.6210	2457.2508	1229.1290	G	318.1772	159.5922	301.1506	151.0790			3
25	2589.3042	1295.1558	2572.2777	1286.6425	2571.2937	1286.1505	N	261.1557	131.0815	244.1292	122.5682			2
26							K	147.1128	74.0600	130.0863	65.5468			1

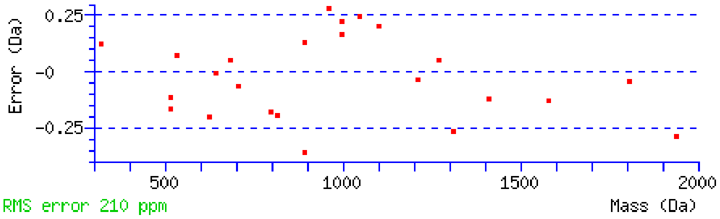

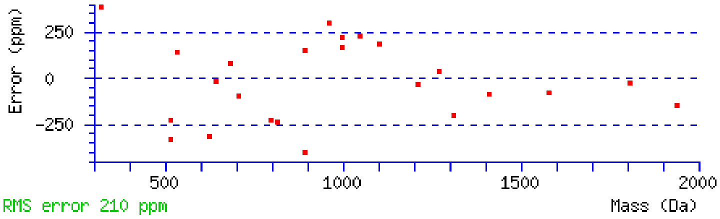

All matches to this query

Score	Mr(calc):	Delta	Sequence
45.8	2734.4025	0.0044	ISDTNITAIPQGLPTSISELHLDGNK
28.3	2733.4185	0.9884	ISDTNITAIPQGLPTSISELHLDGNK
27.5	2734.4025	0.0044	ISDTNITAIPQGLPTSISELHLDGNK
2.8	2733.4119	0.9950	TLTLLITLLLLALHTQAESPQER
2.8	2733.4119	0.9950	TLTLLITLLLLALHTQAESPQER
2.4	2732.4106	1.9962	VHYRDINKAILHGNSSANTQPLER
0.5	2733.3852	1.0216	NTLIFASVLTPLLNPMVYTFRNK
0.5	2733.3852	1.0216	NALVFATIITPLLNPMVYTFRNK
0.4	2733.4119	0.9950	TLTLLITLLLLALHTQAESPQER

Spectrum No: 466; Query: 699; Rank: 1

Peptide View

MS/MS Fragmentation of **QLLANSSALVETILGHQER**
Found in **IPI00190577**, Tax_Id=10116 Gene_Symbol=Lama5 similar to Laminin alpha-5 chain precursor

Match to Query 699: 2079.102702 from(694.041510,3+)
Title: 091127RatKid_SCX01_23.5552.5552.3.dta
Data file K:\NewmanPaper\Piliang\3SubProteomes\Piliang3SP\mgf5ppm\SCX_3SubProteomes5ppm.mgf

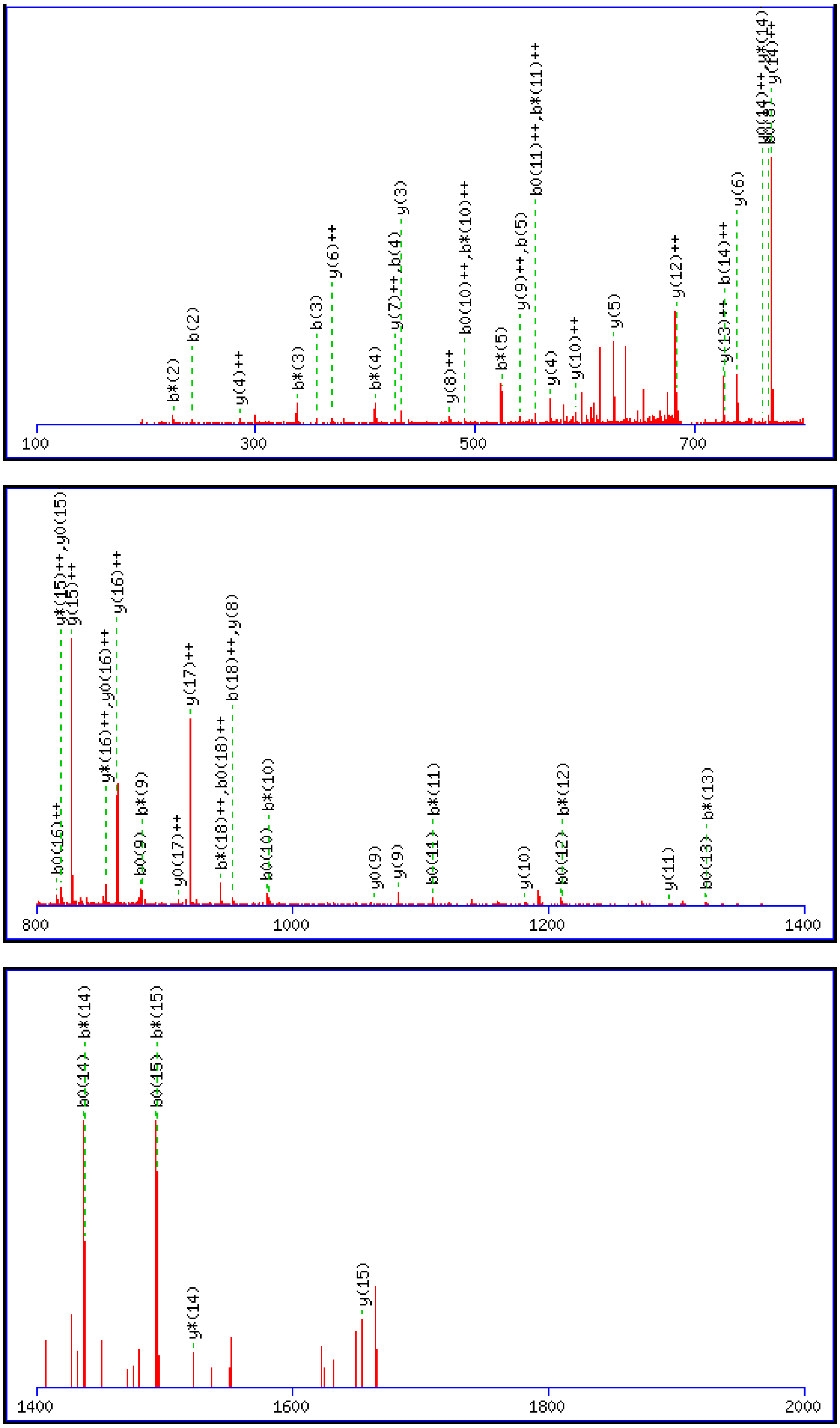

Monoisotopic mass of neutral peptide Mr(calc): 2079.0960
Fixed modifications: Carbamidomethyl (C)
Variable modifications:
N5 : Deamidated_N (N)
Ions Score: 46 **Expect:** 0.0026
Matches (Bold Red): 62/204 fragment ions using 133 most intense peaks

#	b	b^{++}	b*	b^{*++}	b^0	b^{0++}	Seq.	y	y^{++}	y*	y^{*++}	y^0	y^{0++}	#
1	129.0659	65.0366	112.0393	56.5233			Q							19
2	**242.1499**	121.5786	**225.1234**	113.0653			L	1952.0447	976.5260	1935.0182	968.0127	1934.0342	967.5207	18
3	**355.2340**	178.1206	**338.2074**	169.6074			L	1838.9607	**919.9840**	1821.9341	911.4707	1820.9501	**910.9787**	17
4	**426.2711**	213.6392	**409.2445**	205.1259			A	1725.8766	**863.4419**	1708.8500	**854.9287**	1707.8660	**854.4367**	16
5	**541.2980**	271.1527	**524.2715**	262.6394			N	**1654.8395**	**827.9234**	1637.8129	**819.4101**	1636.8289	**818.9181**	15

6	628.3301	314.6687	611.3035	306.1554	610.3195	305.6634	S	1539.8125	**770.4099**	**1522.7860**	**761.8966**	1521.8020	**761.4046**	14
7	715.3621	358.1847	698.3355	349.6714	697.3515	349.1794	S	1452.7805	**726.8939**	1435.7540	718.3806	1434.7700	717.8886	13
8	786.3992	393.7032	769.3727	385.1900	**768.3886**	384.6980	A	1365.7485	**683.3779**	1348.7219	674.8646	1347.7379	674.3726	12
9	899.4833	450.2453	**882.4567**	441.7320	**881.4727**	441.2400	L	**1294.7114**	647.8593	1277.6848	639.3461	1276.7008	638.8540	11
10	998.5517	499.7795	**981.5251**	**491.2662**	**980.5411**	**490.7742**	V	**1181.6273**	**591.3173**	1164.6008	582.8040	1163.6167	582.3120	10
11	1127.5943	564.3008	**1110.5677**	**555.7875**	**1109.5837**	**555.2955**	E	**1082.5589**	**541.7831**	1065.5324	533.2698	**1064.5483**	532.7778	9
12	1228.6420	614.8246	**1211.6154**	606.3113	**1210.6314**	605.8193	T	**953.5163**	**477.2618**	936.4898	468.7485	935.5057	468.2565	8
13	1341.7260	671.3666	**1324.6995**	662.8534	**1323.7155**	662.3614	I	852.4686	**426.7380**	835.4421	418.2247	834.4581	417.7327	7
14	1454.8101	**727.9087**	**1437.7835**	719.3954	**1436.7995**	718.9034	L	**739.3846**	**370.1959**	722.3580	361.6826	721.3740	361.1906	6
15	1511.8315	756.4194	**1494.8050**	747.9061	**1493.8210**	747.4141	G	**626.3005**	313.6539	609.2739	305.1406	608.2899	304.6486	5
16	1648.8905	824.9489	1631.8639	816.4356	1630.8799	**815.9436**	H	**569.2790**	**285.1432**	552.2525	276.6299	551.2685	276.1379	4
17	1776.9490	888.9782	1759.9225	880.4649	1758.9385	879.9729	Q	**432.2201**	216.6137	415.1936	208.1004	414.2096	207.6084	3
18	1905.9916	**953.4995**	1888.9651	**944.9862**	1887.9811	**944.4942**	E	304.1615	152.5844	287.1350	144.0711	286.1510	143.5791	2
19							R	175.1190	88.0631	158.0924	79.5498			1

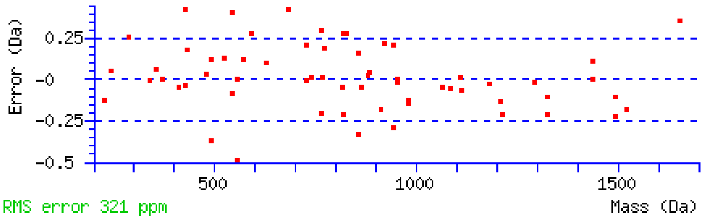

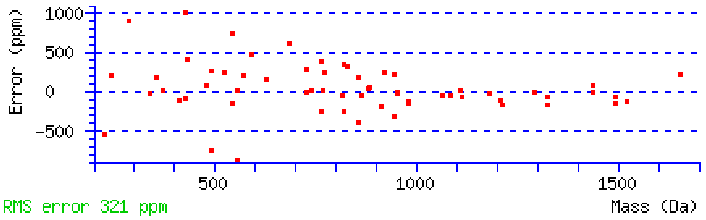

All matches to this query

Score	Mr(calc):	Delta	Sequence
45.8	2079.0960	0.0067	QLLANSSALVETILGHQER
26.5	2078.1120	0.9907	QLLANSSALVETILGHQER

Spectrum No: 467; Query: 671; Rank: 1

Peptide View

MS/MS Fragmentation of **VNLTMFFNITADGEPLGR**
Found in **IPI00364439**, Tax_Id=10116 Gene_Symbol=RGD1559682_predicted similar to Peptidyl-prolyl cis-trans isomerase A

Match to Query 671: 2010.977148 from(1006.495850,2+)
Title: 091129RatKid_SCX02_05.5885.5885.2.dta
Data file K:\NewmanPaper\Piliang\3SubProteomes\Piliang3SP\mgf5ppm\SCX_3SubProteomes5ppm.mgf

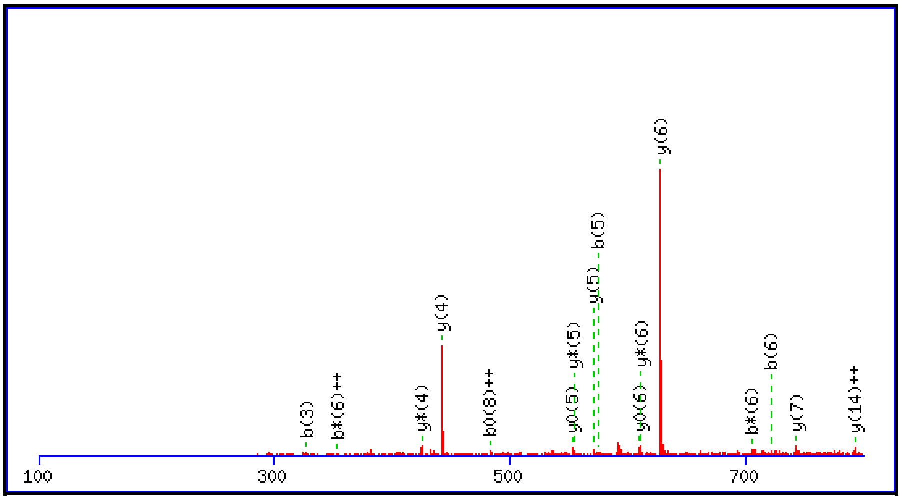

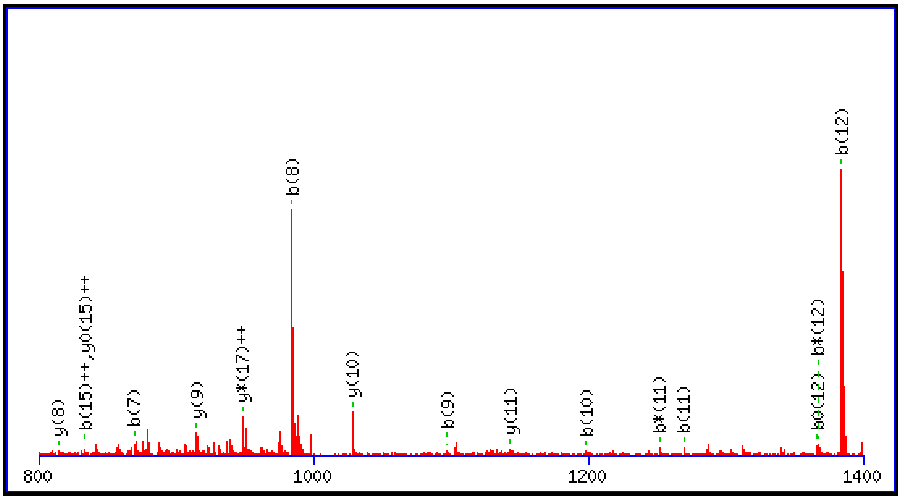

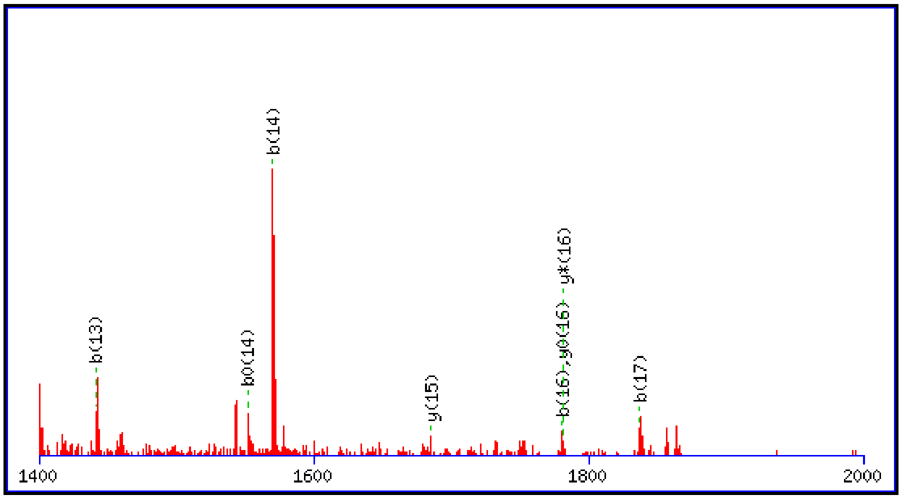

Monoisotopic mass of neutral peptide Mr(calc): 2010.9721
Fixed modifications: Carbamidomethyl (C)
Variable modifications:
M5 : Oxidation (M)
N8 : Deamidated_N (N)
Ions Score: 46 **Expect:** 0.0063
Matches (Bold Red): 40/188 fragment ions using 128 most intense peaks

#	b	b^{++}	b*	b^{*++}	b^{0}	b^{0++}	Seq.	y	y^{++}	y*	y^{*++}	y^{0}	y^{0++}	#
1	100.0757	50.5415					V							18
2	214.1186	107.5629	197.0921	99.0497			N	1912.9109	956.9591	1895.8844	**948.4458**	1894.9004	947.9538	17
3	**327.2027**	164.1050	310.1761	155.5917			L	1798.8680	899.9376	**1781.8415**	891.4244	**1780.8574**	890.9324	16
4	428.2504	214.6288	411.2238	206.1155	410.2398	205.6235	T	**1685.7839**	843.3956	1668.7574	834.8823	1667.7734	**834.3903**	15
5	**575.2858**	288.1465	558.2592	279.6332	557.2752	279.1412	M	1584.7363	**792.8718**	1567.7097	784.3585	1566.7257	783.8665	14
6	**722.3542**	361.6807	**705.3276**	**353.1675**	704.3436	352.6754	F	1437.7009	719.3541	1420.6743	710.8408	1419.6903	710.3488	13
7	**869.4226**	435.2149	852.3960	426.7017	851.4120	426.2097	F	1290.6325	645.8199	1273.6059	637.3066	1272.6219	636.8146	12
8	**984.4495**	492.7284	967.4230	484.2151	966.4390	**483.7231**	N	**1143.5640**	572.2857	1126.5375	563.7724	1125.5535	563.2804	11
9	**1097.5336**	549.2704	1080.5070	540.7572	1079.5230	540.2652	I	**1028.5371**	514.7722	1011.5106	506.2589	1010.5265	505.7669	10
10	**1198.5813**	599.7943	1181.5547	591.2810	1180.5707	590.7890	T	**915.4530**	458.2302	898.4265	449.7169	897.4425	449.2249	9
11	**1269.6184**	635.3128	**1252.5918**	626.7996	1251.6078	626.3075	A	**814.4054**	407.7063	797.3788	399.1930	796.3948	398.7010	8
12	**1384.6453**	692.8263	**1367.6188**	684.3130	**1366.6348**	683.8210	D	**743.3682**	372.1878	726.3417	363.6745	725.3577	363.1825	7
13	**1441.6668**	721.3370	1424.6402	712.8238	1423.6562	712.3318	G	**628.3413**	314.6743	**611.3148**	306.1610	**610.3307**	305.6690	6
14	**1570.7094**	785.8583	1553.6828	777.3451	**1552.6988**	776.8530	E	**571.3198**	286.1636	**554.2933**	277.6503	**553.3093**	277.1583	5
15	1667.7621	**834.3847**	1650.7356	825.8714	1649.7516	825.3794	P	**442.2772**	221.6423	**425.2507**	213.1290			4
16	**1780.8462**	890.9267	1763.8197	882.4135	1762.8356	881.9215	L	345.2245	173.1159	328.1979	164.6026			3
17	**1837.8677**	919.4375	1820.8411	910.9242	1819.8571	910.4322	G	232.1404	116.5738	215.1139	108.0606			2

18							R	175.1190	88.0631	158.0924	79.5498			1

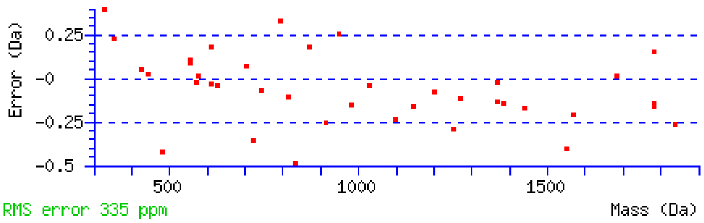

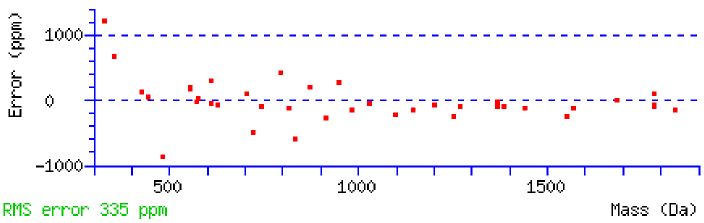

All matches to this query

Score	Mr(calc):	Delta	Sequence
45.6	2010.9721	0.0051	VNLTMFFNITADGEPLGR
21.7	2010.9721	0.0051	VNLTMFFNITADGEPLGR
9.9	2010.9637	0.0135	SSGLPDASLPGRPLGTGGHR
6.4	2009.9881	0.9891	VNLTMFFNITADGEPLGR
5.8	2010.9832	-0.0060	YTPVSIYEEKLLLKR
5.8	2010.9832	-0.0060	YTPVSIYEEKLLLKR
1.8	2010.9637	0.0135	SSGLPDASLPGRPLGTGGHR
0.6	2010.9832	-0.0060	YTPVSIYEEKLLLKR
0.5	2008.9554	2.0218	MTVVPTAHSLPSRSXQR
0.1	2010.9874	-0.0103	IVSNALGTINGSDNLSKVK

Spectrum No: 468; Query: 367; Rank: 1

Peptide View

MS/MS Fragmentation of **DFVEIWENHTSGR**
Found in **IPI00196620**, Tax_Id=10116 Gene_Symbol=Cubn Cubilin precursor

Match to Query 367: 1589.713048 from(795.863800,2+)
Title: 091129RatKid_SCX02_25.3314.3314.2.dta
Data file K:\NewmanPaper\Piliang\3SubProteomes\Piliang3SP\mgf5ppm\SCX_3SubProteomes5ppm.mgf

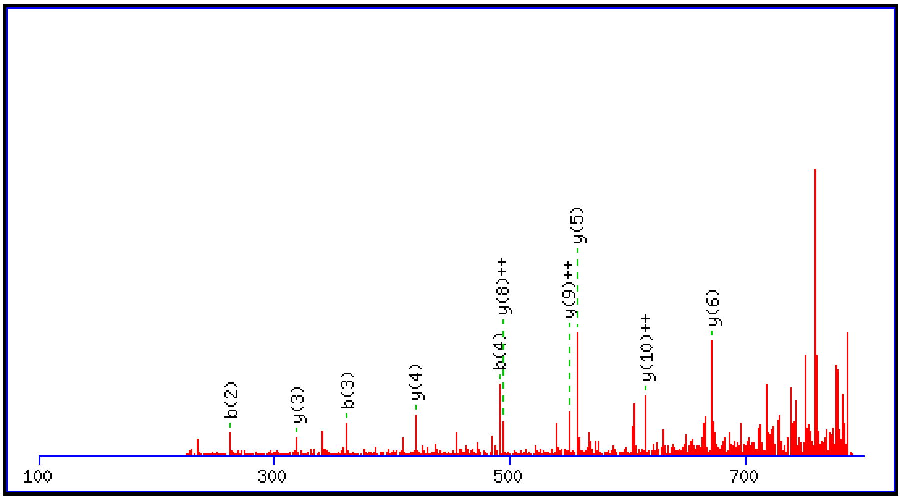

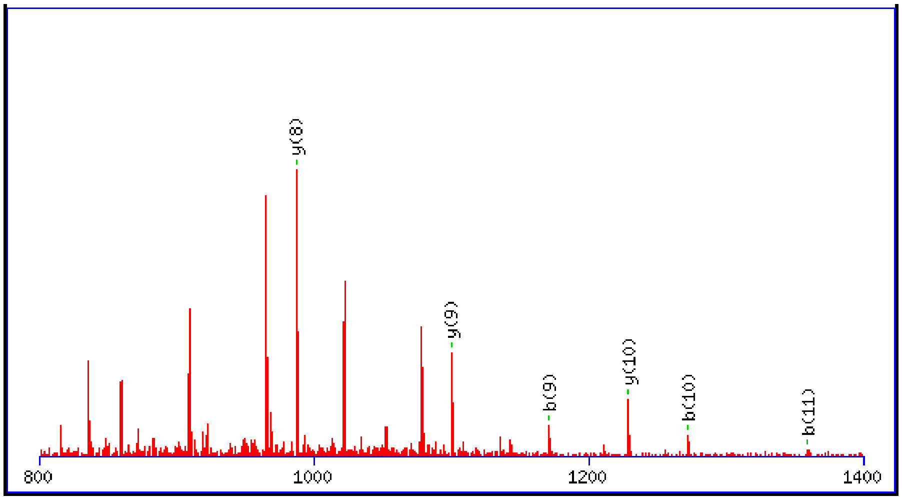

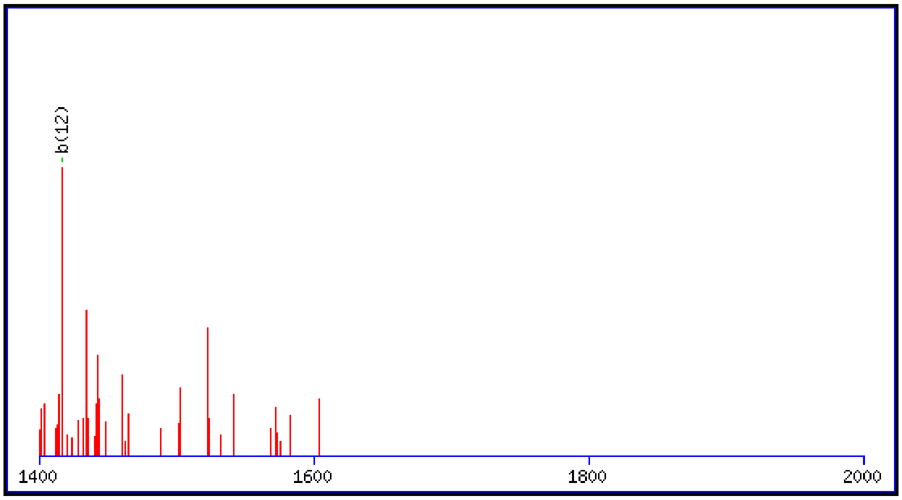

Monoisotopic mass of neutral peptide Mr(calc): 1589.7110
Fixed modifications: Carbamidomethyl (C)
Variable modifications:
N8 : Deamidated_N (N)
Ions Score: 46 **Expect:** 0.0059
Matches (Bold Red): 17/126 fragment ions using 37 most intense peaks

#	b	b^{++}	b*	b^{*++}	b^{0}	b^{0++}	Seq.	y	y^{++}	y*	y^{*++}	y^{0}	y^{0++}	#
1	116.0342	58.5207			98.0237	49.5155	D							13
2	**263.1026**	132.0550			245.0921	123.0497	F	1475.6914	738.3493	1458.6648	729.8360	1457.6808	729.3440	12
3	**362.1710**	181.5892			344.1605	172.5839	V	1328.6229	664.8151	1311.5964	656.3018	1310.6124	655.8098	11
4	**491.2136**	246.1105			473.2031	237.1052	E	**1229.5545**	**615.2809**	1212.5280	606.7676	1211.5440	606.2756	10
5	604.2977	302.6525			586.2871	293.6472	I	**1100.5119**	**550.7596**	1083.4854	542.2463	1082.5014	541.7543	9
6	790.3770	395.6921			772.3665	386.6869	W	**987.4279**	**494.2176**	970.4013	485.7043	969.4173	485.2123	8
7	919.4196	460.2134			901.4090	451.2082	E	801.3486	401.1779	784.3220	392.6646	783.3380	392.1726	7
8	1034.4465	517.7269	1017.4200	509.2136	1016.4360	508.7216	N	**672.3060**	336.6566	655.2794	328.1433	654.2954	327.6513	6
9	**1171.5055**	586.2564	1154.4789	577.7431	1153.4949	577.2511	H	**557.2790**	279.1432	540.2525	270.6299	539.2685	270.1379	5
10	**1272.5531**	636.7802	1255.5266	628.2669	1254.5426	627.7749	T	**420.2201**	210.6137	403.1936	202.1004	402.2096	201.6084	4
11	**1359.5852**	680.2962	1342.5586	671.7829	1341.5746	671.2909	S	**319.1724**	160.0899	302.1459	151.5766	301.1619	151.0846	3
12	**1416.6066**	708.8070	1399.5801	700.2937	1398.5961	699.8017	G	232.1404	116.5738	215.1139	108.0606			2
13							R	175.1190	88.0631	158.0924	79.5498			1

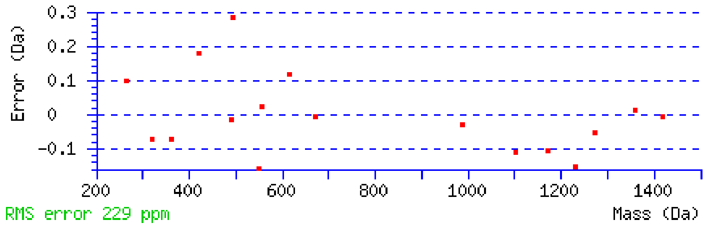

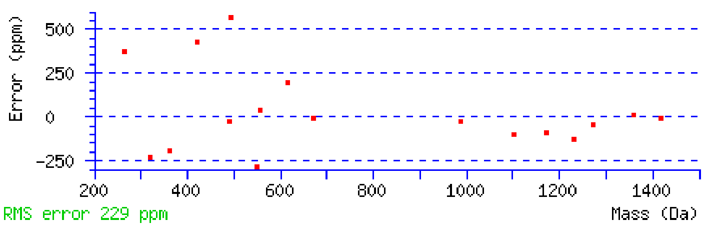

All matches to this query

Score	Mr(calc):	Delta	Sequence
45.5	1589.7110	0.0020	DFVEIWENHTSGR
6.6	1587.7020	2.0110	GNGTSMISLIIPPK
6.6	1587.7020	2.0110	GNGTSMISLIIPPK
6.6	1587.7020	2.0110	GNGTSMISLIIPPK
1.6	1589.6991	0.0140	LAGVNAVTTLVENK
1.1	1589.7143	-0.0012	NVALSGNTWILLK
0.4	1587.7222	1.9909	YTKFSDSYASVIK

Spectrum No: 469; Query: 122; Rank: 1

Peptide View

MS/MS Fragmentation of **QELSQDNATLK**
Found in **IPI00190577**, Tax_Id=10116 Gene_Symbol=Lama5 similar to Laminin alpha-5 chain precursor

Match to Query 122: 1246.604988 from(624.309770,2+)
Title: 091127RatKid_SCX01_02.757.757.2.dta
Data file K:\NewmanPaper\Piliang\3SubProteomes\Piliang3SP\mgf5ppm\SCX_3SubProteomes5ppm.mgf

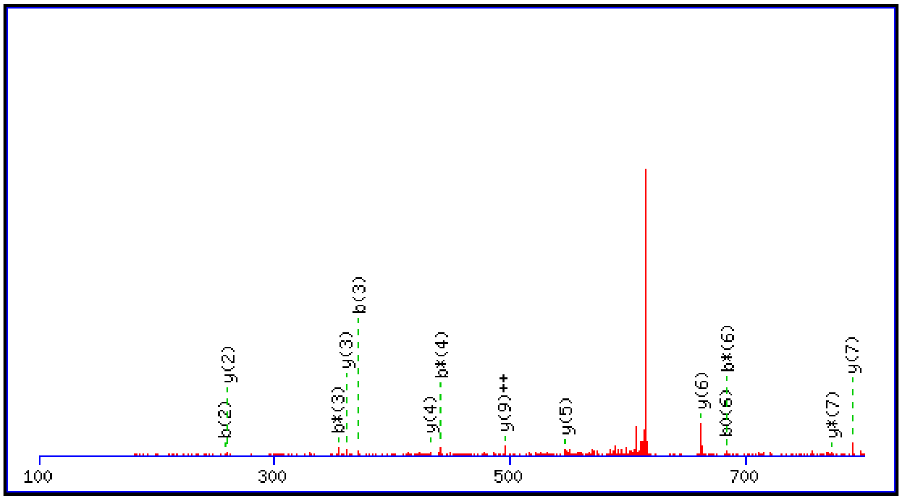

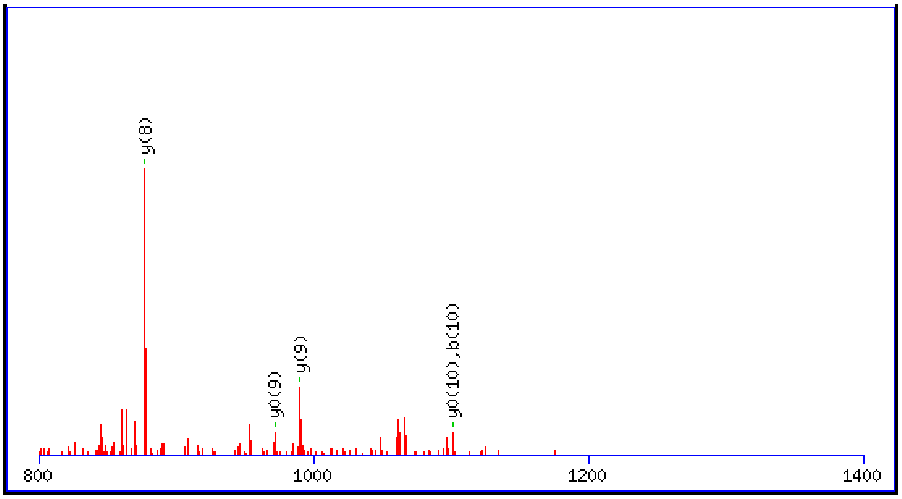

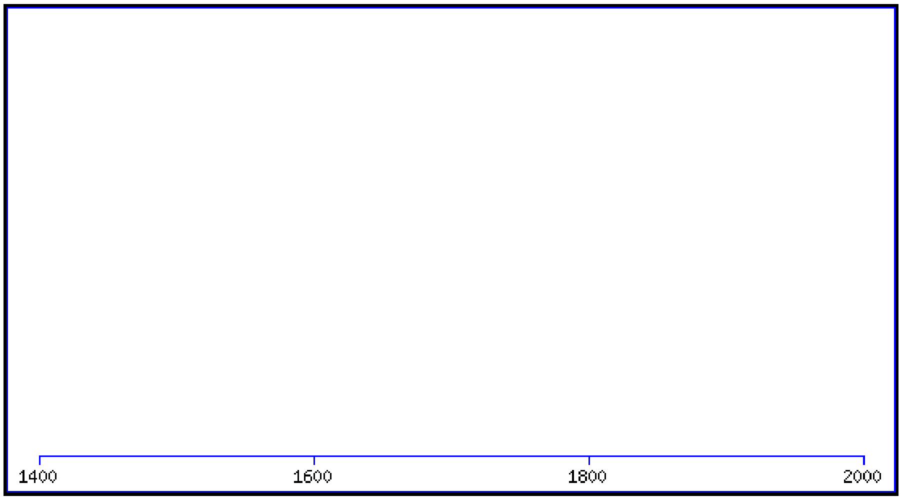

Monoisotopic mass of neutral peptide Mr(calc): 1246.6041
Fixed modifications: Carbamidomethyl (C)
Variable modifications:
N7 : Deamidated_N (N)
Ions Score: 45 **Expect:** 0.0037
Matches (Bold Red): 19/114 fragment ions using 46 most intense peaks

#	b	b^{++}	b*	b^{*++}	b^0	b^{0++}	Seq.	y	y^{++}	y*	y^{*++}	y^0	y^{0++}	#
1	129.0659	65.0366	112.0393	56.5233			Q							11
2	**258.1084**	129.5579	241.0819	121.0446	240.0979	120.5526	E	1119.5528	560.2800	1102.5263	551.7668	**1101.5422**	551.2748	10
3	**371.1925**	186.0999	**354.1660**	177.5866	353.1819	177.0946	L	**990.5102**	**495.7587**	973.4837	487.2455	**972.4996**	486.7535	9
4	458.2245	229.6159	**441.1980**	221.1026	440.2140	220.6106	S	**877.4261**	439.2167	860.3996	430.7034	859.4156	430.2114	8
5	586.2831	293.6452	569.2566	285.1319	568.2726	284.6399	Q	**790.3941**	395.7007	**773.3676**	387.1874	772.3836	386.6954	7
6	701.3101	351.1587	**684.2835**	342.6454	**683.2995**	342.1534	D	**662.3355**	331.6714	645.3090	323.1581	644.3250	322.6661	6
7	816.3370	408.6721	799.3104	400.1589	798.3264	399.6669	N	**547.3086**	274.1579	530.2820	265.6447	529.2980	265.1527	5
8	887.3741	444.1907	870.3476	435.6774	869.3635	435.1854	A	**432.2817**	216.6445	415.2551	208.1312	414.2711	207.6392	4
9	988.4218	494.7145	971.3952	486.2013	970.4112	485.7093	T	**361.2445**	181.1259	344.2180	172.6126	343.2340	172.1206	3
10	**1101.5059**	551.2566	1084.4793	542.7433	1083.4953	542.2513	L	**260.1969**	130.6021	243.1703	122.0888			2
11							K	147.1128	74.0600	130.0863	65.5468			1

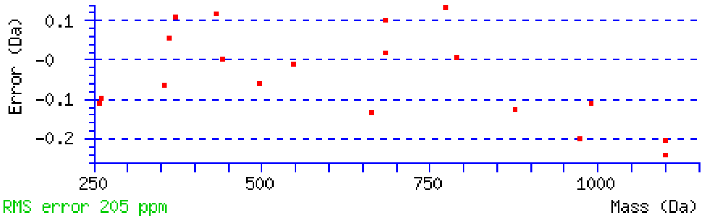

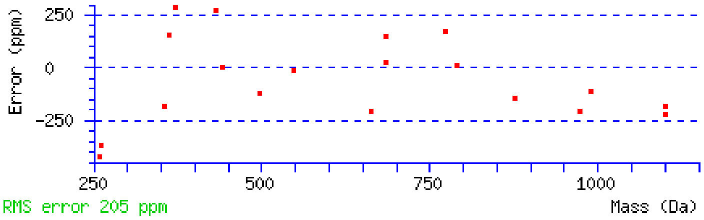

All matches to this query

Score	Mr(calc):	Delta	Sequence
45.4	1246.6041	0.0009	QELSQDNATLK
12.3	1246.5958	0.0091	KIESFGSKSGK
11.7	1246.6128	-0.0078	WALGVMAADASR
11.2	1246.6041	0.0009	AGKLSENPSESK
10.9	1246.5928	0.0122	GAELEIEETEK
6.9	1246.5958	0.0091	KIESFGSKSGK
6.6	1246.6032	0.0018	EKSMATFILK
6.5	1246.6153	-0.0103	QEKSINSEGQK
6.3	1246.6040	0.0009	EKNKEVENEK
5.3	1246.5958	0.0092	ANANQPLLTPK

Spectrum No: 470; Query: 81; Rank: 1

Peptide View

MS/MS Fragmentation of **ISSNETEIMR**
Found in **IPI00370427**, Tax_Id=10116 Gene_Symbol=Tinag Tubulointerstitial nephritis antigen

Match to Query 81: 1195.538848 from(598.776700,2+)
Title: 091127RatKid_SCX01_13.1047.1047.2.dta
Data file K:\NewmanPaper\Piliang\3SubProteomes\Piliang3SP\mgf5ppm\SCX_3SubProteomes5ppm.mgf

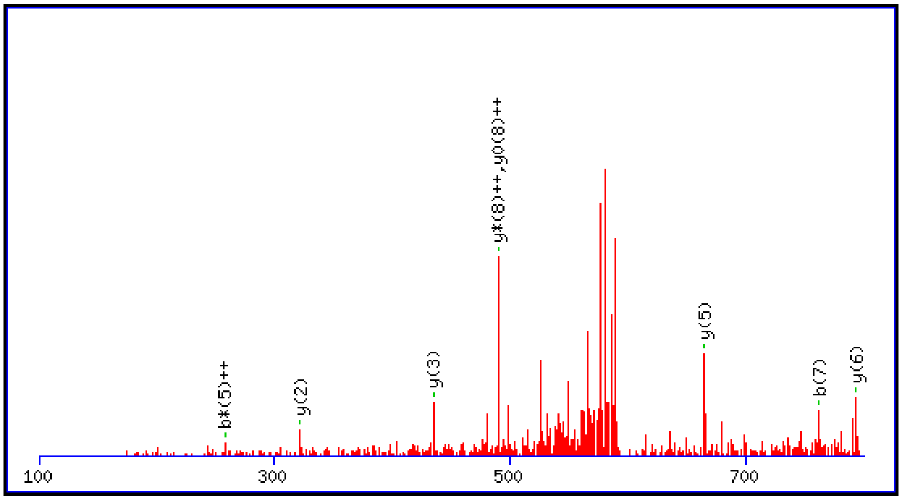

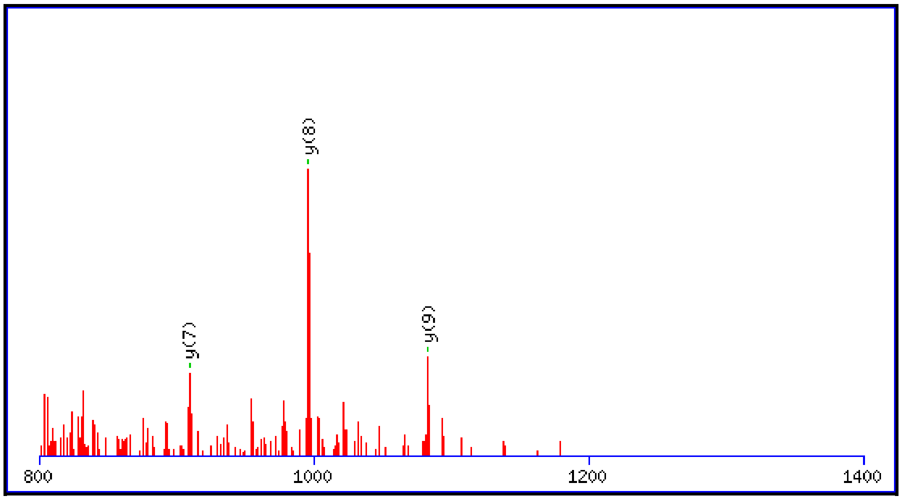

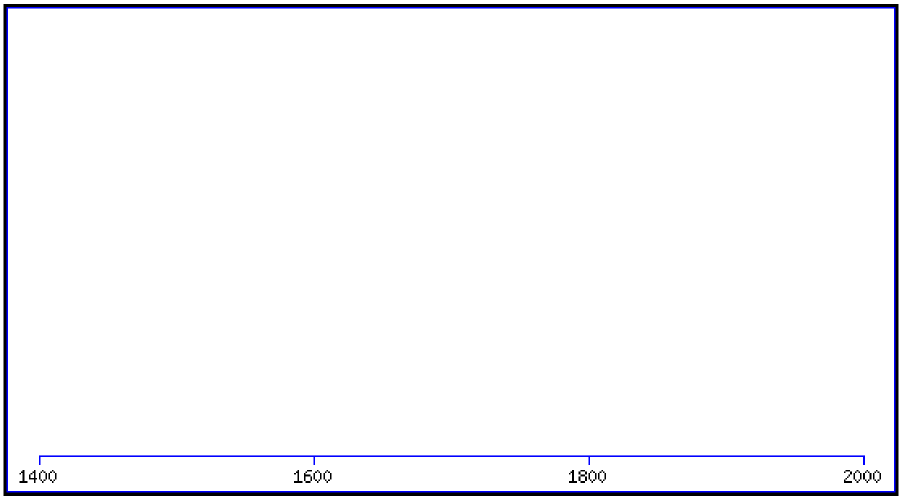

Monoisotopic mass of neutral peptide Mr(calc): 1195.5390
Fixed modifications: Carbamidomethyl (C)
Variable modifications:
N4 : Deamidated_N (N)
M9 : Oxidation (M)
Ions Score: 45 **Expect:** 0.0034
Matches (Bold Red): 11/94 fragment ions using 26 most intense peaks

#	b	b^{++}	b*	b^{*++}	b^0	b^{0++}	Seq.	y	y^{++}	y*	y^{*++}	y^0	y^{0++}	#
1	114.0913	57.5493					I							10
2	201.1234	101.0653			183.1128	92.0600	S	**1083.4623**	542.2348	1066.4357	533.7215	1065.4517	533.2295	9
3	288.1554	144.5813			270.1448	135.5761	S	**996.4302**	498.7188	979.4037	**490.2055**	978.4197	**489.7135**	8
4	403.1823	202.0948	386.1558	193.5815	385.1718	193.0895	N	**909.3982**	455.2027	892.3717	446.6895	891.3877	446.1975	7
5	532.2249	266.6161	515.1984	**258.1028**	514.2144	257.6108	E	**794.3713**	397.6893	777.3447	389.1760	776.3607	388.6840	6
6	633.2726	317.1399	616.2461	308.6267	615.2620	308.1347	T	**665.3287**	333.1680	648.3021	324.6547	647.3181	324.1627	5
7	**762.3152**	381.6612	745.2886	373.1480	744.3046	372.6560	E	564.2810	282.6441	547.2545	274.1309	546.2704	273.6389	4
8	875.3993	438.2033	858.3727	429.6900	857.3887	429.1980	I	**435.2384**	218.1228	418.2119	209.6096			3
9	1022.4347	511.7210	1005.4081	503.2077	1004.4241	502.7157	M	**322.1544**	161.5808	305.1278	153.0675			2
10							R	175.1190	88.0631	158.0924	79.5498			1

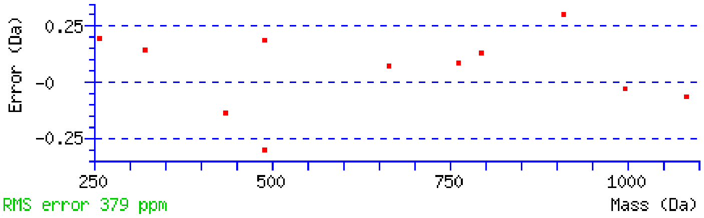

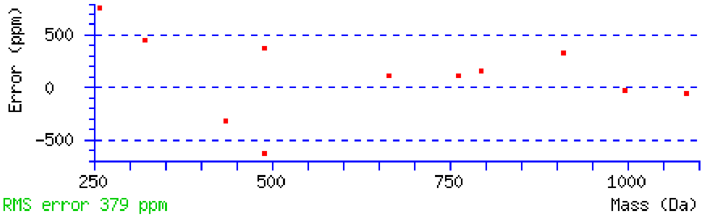

All matches to this query

Score	Mr(calc):	Delta	Sequence
45.4	1195.5390	-0.0002	ISSNETEIMR
8.0	1195.5357	0.0032	INSEFNSDLR
6.4	1195.5347	0.0042	DGSAGLGRDLR
4.5	1195.5485	-0.0097	IATEAVENIR
4.5	1194.5394	0.9994	LQNSSSAGVPR
3.6	1194.5394	0.9994	LQNSSSAGVPR
3.2	1194.5282	1.0107	LSGATPNGEAAK
2.9	1195.5459	-0.0070	RESINDRAR
2.6	1194.5282	1.0107	QASPTEVVER
0.4	1194.5394	0.9995	TAASPREVER

Spectrum No: 471; Query: 35; Rank: 1

Peptide View

MS/MS Fragmentation of **VLTLANFTTK**
Found in **IPI00188956**, Tax_Id=10116 Gene_Symbol=Thy1 Thy-1 membrane glycoprotein precursor

Match to Query 35: 1107.619968 from(554.817260,2+)
Title: 091129RatKid_SCX02_12.2553.2553.2.dta
Data file K:\NewmanPaper\Piliang\3SubProteomes\Piliang3SP\mgf5ppm\SCX_3SubProteomes5ppm.mgf

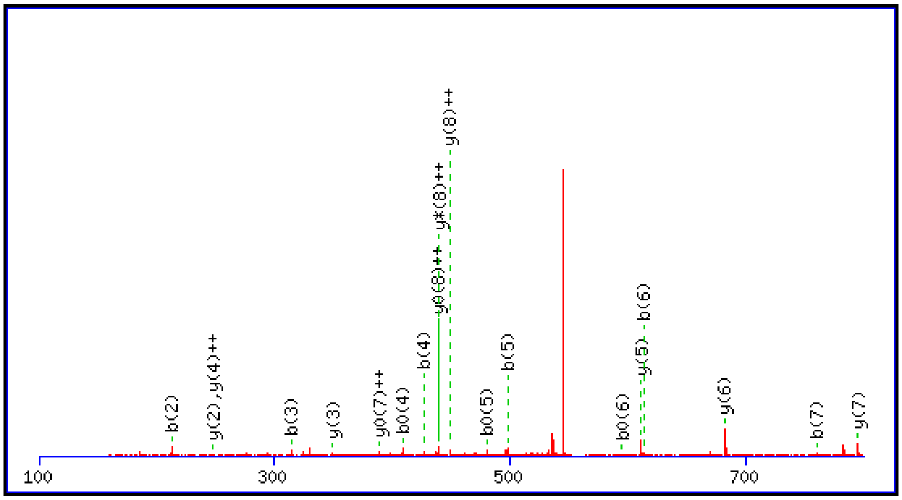

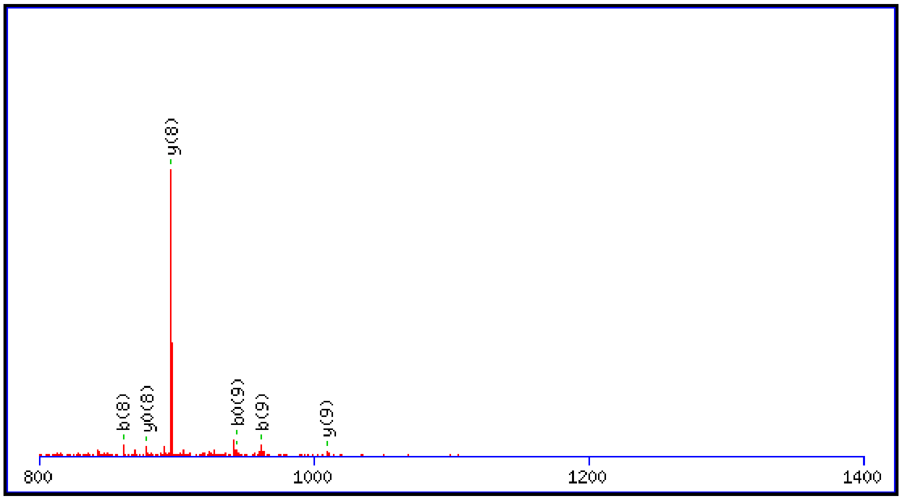

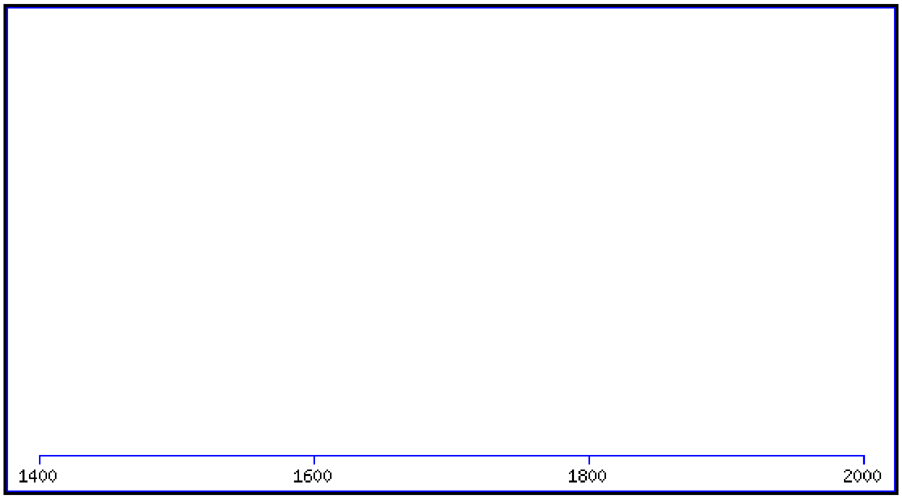

Monoisotopic mass of neutral peptide Mr(calc): 1107.6176
Fixed modifications: Carbamidomethyl (C)
Variable modifications:
N6 : Deamidated_N (N)
Ions Score: 45 **Expect:** 0.0014
Matches (Bold Red): 25/92 fragment ions using 61 most intense peaks

#	b	b^{++}	b*	b^{*++}	b^0	b^{0++}	Seq.	y	y^{++}	y*	y^{*++}	y^0	y^{0++}	#
1	100.0757	50.5415					V							10
2	**213.1598**	107.0835					L	**1009.5564**	505.2819	992.5299	496.7686	991.5459	496.2766	9
3	**314.2074**	157.6074			296.1969	148.6021	T	**896.4724**	**448.7398**	879.4458	**440.2265**	**878.4618**	**439.7345**	8
4	**427.2915**	214.1494			**409.2809**	205.1441	L	**795.4247**	398.2160	778.3981	389.7027	777.4141	**389.2107**	7
5	**498.3286**	249.6679			**480.3180**	240.6627	A	**682.3406**	341.6740	665.3141	333.1607	664.3301	332.6687	6
6	**613.3555**	307.1814	596.3290	298.6681	**595.3450**	298.1761	N	**611.3035**	306.1554	594.2770	297.6421	593.2929	297.1501	5
7	**760.4240**	380.7156	743.3974	372.2023	742.4134	371.7103	F	496.2766	**248.6419**	479.2500	240.1287	478.2660	239.6366	4
8	**861.4716**	431.2395	844.4451	422.7262	843.4611	422.2342	T	**349.2082**	175.1077	332.1816	166.5944	331.1976	166.1024	3
9	**962.5193**	481.7633	945.4928	473.2500	**944.5088**	472.7580	T	**248.1605**	124.5839	231.1339	116.0706	230.1499	115.5786	2
10							K	147.1128	74.0600	130.0863	65.5468			1

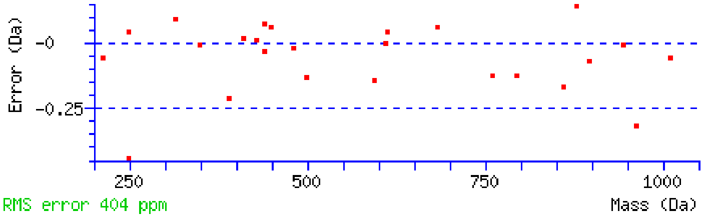

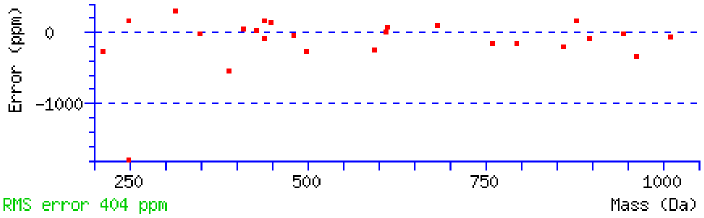

All matches to this query

Score	Mr(calc):	Delta	Sequence
45.4	1107.6176	0.0024	VLTLANFTTK
23.9	1107.6165	0.0035	NLLGKRISK
12.2	1107.6304	-0.0105	VLVDLLTKK
12.1	1107.6288	-0.0088	LVTFSASQKK
8.7	1107.6165	0.0035	AKARAILTGK
7.5	1107.6189	0.0011	VIHWNSPKK
5.4	1107.6111	0.0089	VLIFTQMTR
3.1	1106.6084	1.0116	KAILTNQYR
3.1	1107.6165	0.0035	LVGQKLSRK
2.8	1107.6165	0.0035	KAQLVRVSK

Spectrum No: 472; Query: 756; Rank: 1

Peptide View

MS/MS Fragmentation of **NGSYDIGMACGVESMSLSNR**
Found in **IPI00554106**, Tax_Id=10116 Gene_Symbol=Acaa1 Isoform 2 of 3-ketoacyl-CoA thiolase A, peroxisomal precursor

Match to Query 756: 2147.898028 from(1074.956290,2+)
Title: 091127RatKid_SCX01_13.3632.3632.2.dta
Data file K:\NewmanPaper\Piliang\3SubProteomes\Piliang3SP\mgf5ppm\SCX_3SubProteomes5ppm.mgf

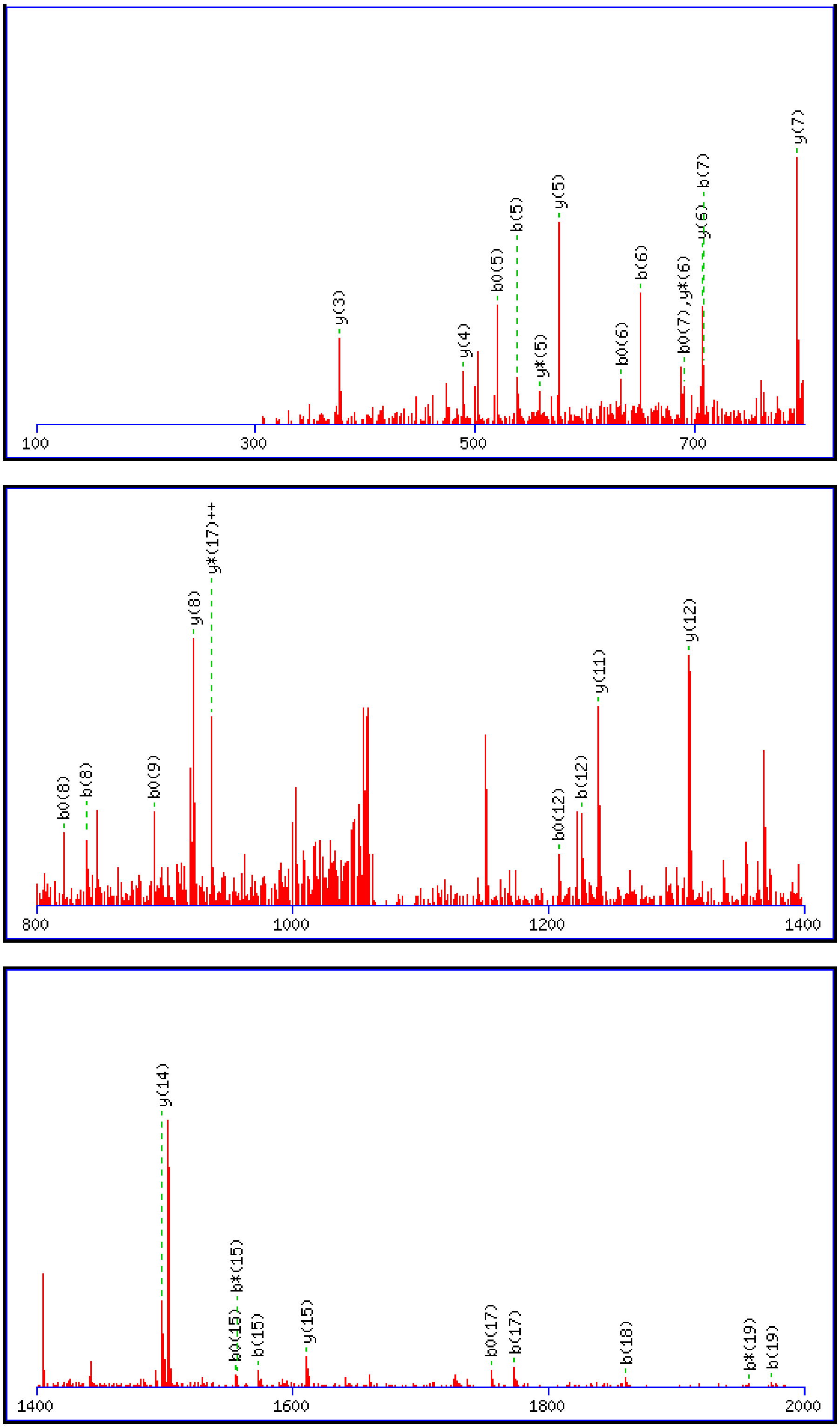

Monoisotopic mass of neutral peptide Mr(calc): 2147.8922
Fixed modifications: Carbamidomethyl (C)
Variable modifications:
N1 : Deamidated_N (N)
Ions Score: 45 **Expect:** 0.0069
Matches (Bold Red): 32/220 fragment ions using 72 most intense peaks

#	b	b++	b*	b*++	b0	b0++	Seq.	y	y++	y*	y*++	y0	y0++	#
1	116.0342	58.5207	99.0077	50.0075			N							20
2	173.0557	87.0315	156.0291	78.5182			G	2033.8725	1017.4399	2016.8460	1008.9266	2015.8620	1008.4346	19
3	260.0877	130.5475	243.0612	122.0342	242.0771	121.5422	S	1976.8511	988.9292	1959.8245	980.4159	1958.8405	979.9239	18
4	423.1510	212.0792	406.1245	203.5659	405.1405	203.0739	Y	1889.8190	945.4132	1872.7925	**936.8999**	1871.8085	936.4079	17
5	**538.1780**	269.5926	521.1514	261.0794	**520.1674**	260.5873	D	1726.7557	863.8815	1709.7292	855.3682	1708.7451	854.8762	16

**6**	**651.2620**	326.1347	634.2355	317.6214	**633.2515**	317.1294	**I**	**1611.7288**	806.3680	1594.7022	797.8547	1593.7182	797.3627	**15**
**7**	**708.2835**	354.6454	691.2570	346.1321	**690.2729**	345.6401	**G**	**1498.6447**	749.8260	1481.6181	741.3127	1480.6341	740.8207	**14**
**8**	**839.3240**	420.1656	822.2974	411.6524	**821.3134**	411.1604	**M**	1441.6232	721.3153	1424.5967	712.8020	1423.6127	712.3100	**13**
**9**	910.3611	455.6842	893.3346	447.1709	**892.3505**	446.6789	**A**	**1310.5827**	655.7950	1293.5562	647.2817	1292.5722	646.7897	**12**
**10**	1070.3918	535.6995	1053.3652	527.1862	1052.3812	526.6942	**C**	**1239.5456**	620.2765	1222.5191	611.7632	1221.5351	611.2712	**11**
**11**	1127.4132	564.2102	1110.3867	555.6970	1109.4027	555.2050	**G**	1079.5150	540.2611	1062.4884	531.7479	1061.5044	531.2558	**10**
**12**	**1226.4816**	613.7445	1209.4551	605.2312	**1208.4711**	604.7392	**V**	1022.4935	511.7504	1005.4670	503.2371	1004.4830	502.7451	**9**
**13**	1355.5242	678.2657	1338.4977	669.7525	1337.5137	669.2605	**E**	**923.4251**	462.2162	906.3986	453.7029	905.4145	453.2109	**8**
**14**	1442.5563	721.7818	1425.5297	713.2685	1424.5457	712.7765	**S**	**794.3825**	397.6949	777.3560	389.1816	776.3719	388.6896	**7**
**15**	**1573.5967**	787.3020	**1556.5702**	778.7887	**1555.5862**	778.2967	**M**	**707.3505**	354.1789	**690.3239**	345.6656	689.3399	345.1736	**6**
**16**	1660.6288	830.8180	1643.6022	822.3047	1642.6182	821.8127	**S**	**576.3100**	288.6586	**559.2835**	280.1454	558.2994	279.6534	**5**
**17**	**1773.7128**	887.3601	1756.6863	878.8468	**1755.7023**	878.3548	**L**	**489.2780**	245.1426	472.2514	236.6293	471.2674	236.1373	**4**
**18**	**1860.7449**	930.8761	1843.7183	922.3628	1842.7343	921.8708	**S**	**376.1939**	188.6006	359.1674	180.0873	358.1833	179.5953	**3**
**19**	**1974.7878**	987.8975	**1957.7612**	979.3843	1956.7772	978.8922	**N**	289.1619	145.0846	272.1353	136.5713			**2**
**20**							**R**	175.1190	88.0631	158.0924	79.5498			**1**

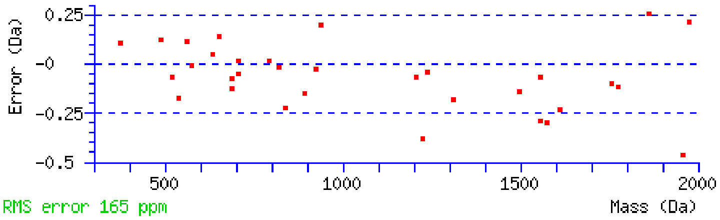

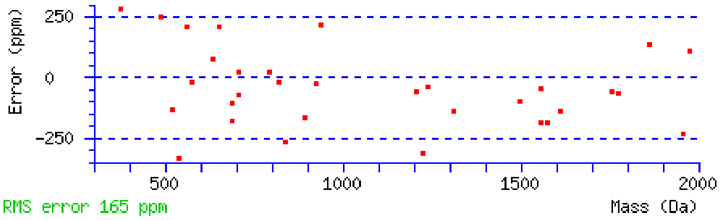

All matches to this query

Score	Mr(calc):	Delta	Sequence
45.3	2147.8922	0.0059	NGSYDIGMACGVESMSLSNR
25.7	2147.8922	0.0059	NGSYDIGMACGVESMSLSNR
22.3	2146.9082	0.9899	NGSYDIGMACGVESMSLSNR
7.2	2147.8782	0.0199	LSRPLPPATPSSMDMNSR
1.8	2147.9148	-0.0168	VFESINMDTNDMWLMMR
1.8	2147.9148	-0.0168	VFESINMDTNDMWLMMR
1.8	2147.9148	-0.0168	VFESINMDTNDMWLMMR

Spectrum No: 473; Query: 965; Rank: 1

Peptide View

MS/MS Fragmentation of **AFENVTDLQWLILDHNLLENSK**
Found in **IPI00206403**, Tax_Id=10116 Gene_Symbol=Lum Lumican precursor

Match to Query 965: 2612.321772 from(871.781200,3+)
Title: 091129RatKid_SCX02_12.4778.4778.3.dta
Data file K:\NewmanPaper\Piliang\3SubProteomes\Piliang3SP\mgf5ppm\SCX_3SubProteomes5ppm.mgf

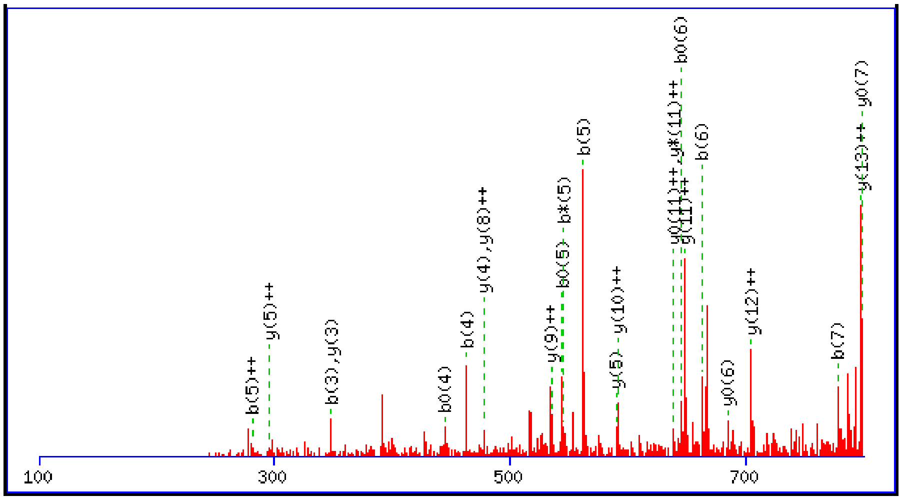

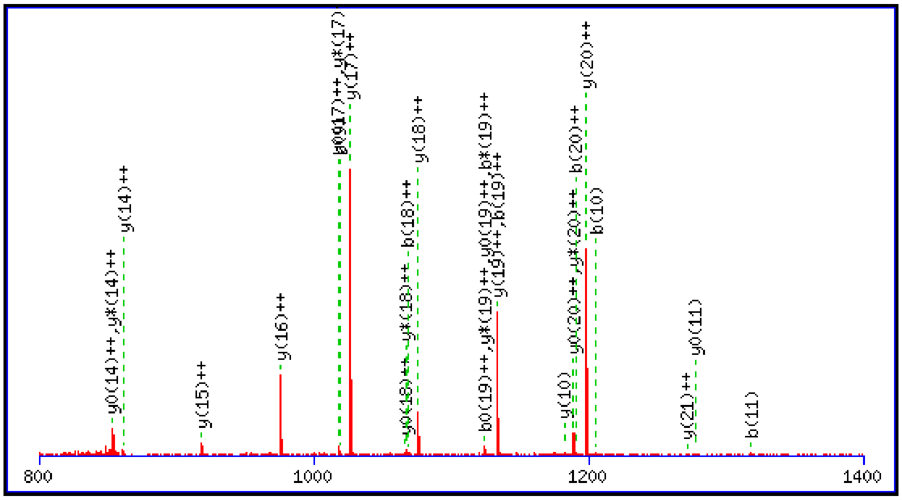

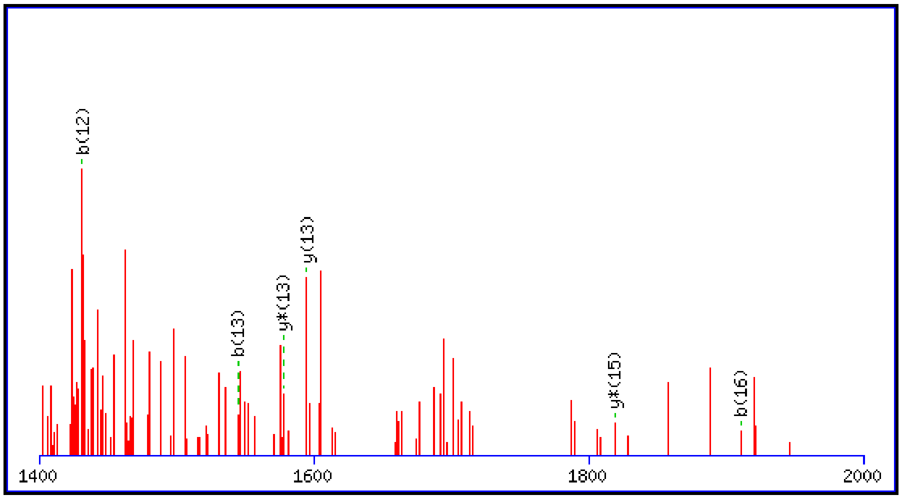

Monoisotopic mass of neutral peptide Mr(calc): 2612.3122
Fixed modifications: Carbamidomethyl (C)
Variable modifications:
N4 : Deamidated_N (N)
Ions Score: 45 **Expect:** 0.0059
Matches (Bold Red): 58/240 fragment ions using 168 most intense peaks

#	b	b++	b*	b*++	b0	b0++	Seq.	y	y++	y*	y*++	y0	y0++	#
1	72.0444	36.5258					A							22
2	219.1128	110.0600					F	2542.2824	**1271.6448**	2525.2558	1263.1316	2524.2718	1262.6395	21
3	**348.1554**	174.5813			330.1448	165.5761	E	2395.2140	**1198.1106**	2378.1874	**1189.5974**	2377.2034	**1189.1053**	20
4	**463.1823**	232.0948	446.1558	223.5815	**445.1718**	223.0895	N	2266.1714	**1133.5893**	2249.1448	**1125.0761**	2248.1608	**1124.5840**	19
5	**562.2507**	**281.6290**	**545.2242**	273.1157	**544.2402**	272.6237	V	2151.1444	**1076.0759**	2134.1179	**1067.5626**	2133.1339	**1067.0706**	18

6	663.2984	332.1529	646.2719	323.6396	645.2879	323.1476	T	2052.0760	1026.5417	2035.0495	1018.0284	2034.0655	1017.5364	17
7	778.3254	389.6663	761.2988	381.1530	760.3148	380.6610	D	1951.0284	976.0178	1934.0018	967.5045	1933.0178	967.0125	16
8	891.4094	446.2084	874.3829	437.6951	873.3989	437.2031	L	1836.0014	918.5043	1818.9749	909.9911	1817.9908	909.4991	15
9	1019.4680	510.2376	1002.4415	501.7244	1001.4574	501.2324	Q	1722.9173	861.9623	1705.8908	853.4490	1704.9068	852.9570	14
10	1205.5473	603.2773	1188.5208	594.7640	1187.5368	594.2720	W	1594.8588	797.9330	1577.8322	789.4197	1576.8482	788.9277	13
11	1318.6314	659.8193	1301.6048	651.3061	1300.6208	650.8140	L	1408.7795	704.8934	1391.7529	696.3801	1390.7689	695.8881	12
12	1431.7155	716.3614	1414.6889	707.8481	1413.7049	707.3561	I	1295.6954	648.3513	1278.6688	639.8381	1277.6848	639.3461	11
13	1544.7995	772.9034	1527.7730	764.3901	1526.7890	763.8981	L	1182.6113	591.8093	1165.5848	583.2960	1164.6008	582.8040	10
14	1659.8265	830.4169	1642.7999	821.9036	1641.8159	821.4116	D	1069.5273	535.2673	1052.5007	526.7540	1051.5167	526.2620	9
15	1796.8854	898.9463	1779.8588	890.4330	1778.8748	889.9410	H	954.5003	477.7538	937.4738	469.2405	936.4898	468.7485	8
16	1910.9283	955.9678	1893.9017	947.4545	1892.9177	946.9625	N	817.4414	409.2243	800.4149	400.7111	799.4308	400.2191	7
17	2024.0124	1012.5098	2006.9858	1003.9965	2006.0018	1003.5045	L	703.3985	352.2029	686.3719	343.6896	685.3879	343.1976	6
18	2137.0964	1069.0519	2120.0699	1060.5386	2119.0859	1060.0466	L	590.3144	295.6608	573.2879	287.1476	572.3039	286.6556	5
19	2266.1390	1133.5731	2249.1125	1125.0599	2248.1285	1124.5679	E	477.2304	239.1188	460.2038	230.6055	459.2198	230.1135	4
20	2380.1819	1190.5946	2363.1554	1182.0813	2362.1714	1181.5893	N	348.1878	174.5975	331.1612	166.0842	330.1772	165.5922	3
21	2467.2140	1234.1106	2450.1874	1225.5974	2449.2034	1225.1053	S	234.1448	117.5761	217.1183	109.0628	216.1343	108.5708	2
22							K	147.1128	74.0600	130.0863	65.5468			1

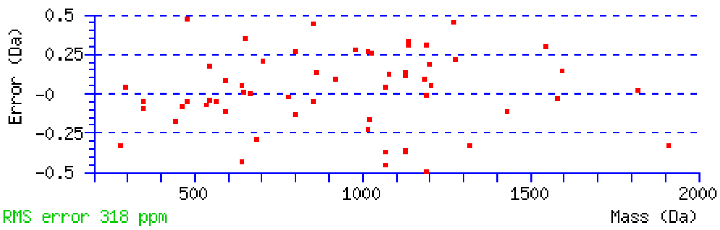

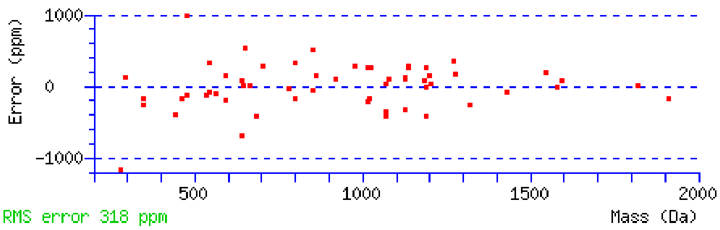

All matches to this query

Score	Mr(calc):	Delta	Sequence
45.2	2612.3122	0.0096	AFENVTDLQWLILDHNLLENSK
28.8	2612.3122	0.0096	AFENVTDLQWLILDHNLLENSK
17.4	2612.3122	0.0096	AFENVTDLQWLILDHNLLENSK
12.9	2611.3282	0.9936	AFENVTDLQWLILDHNLLENSK
5.4	2612.3082	0.0136	HPQLDADIEAVKEIYSENSVSIR
2.0	2612.3098	0.0119	LKNLPDINTLEVAGDIQLTHVQT
2.0	2612.3098	0.0119	LKNLPDINTLEVAGDIQLTHVQT
1.9	2612.3285	-0.0067	VLGPAVIFKVSANIQNMTTADVTK
1.9	2612.3285	-0.0067	VLGPAVIFKVSANIQNMTTADVTK
1.2	2610.3395	1.9823	VISLFYTLMIPMLNPIIYSFR

Spectrum No: 474; Query: 145; Rank: 1

Peptide View

MS/MS Fragmentation of **VYNGSVPFEER**
Found in **IPI00339118**, Tax_Id=10116 Gene_Symbol=Enpp1 Isoform 2 of Ectonucleotide pyrophosphatase/phosphodiesterase family member 1

Match to Query 145: 1296.597668 from(649.306110,2+)
Title: 091129RatKid_SCX02_13.1362.1362.2.dta
Data file K:\NewmanPaper\Piliang\3SubProteomes\Piliang3SP\mgf5ppm\SCX_3SubProteomes5ppm.mgf

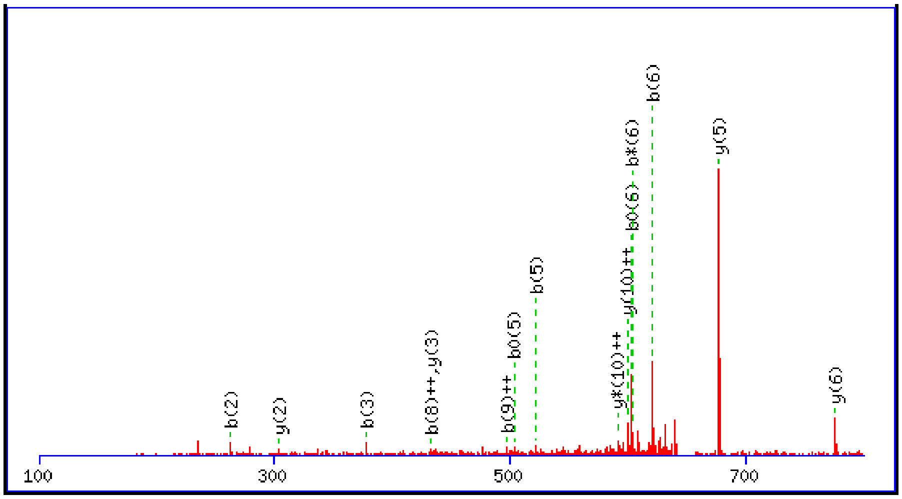

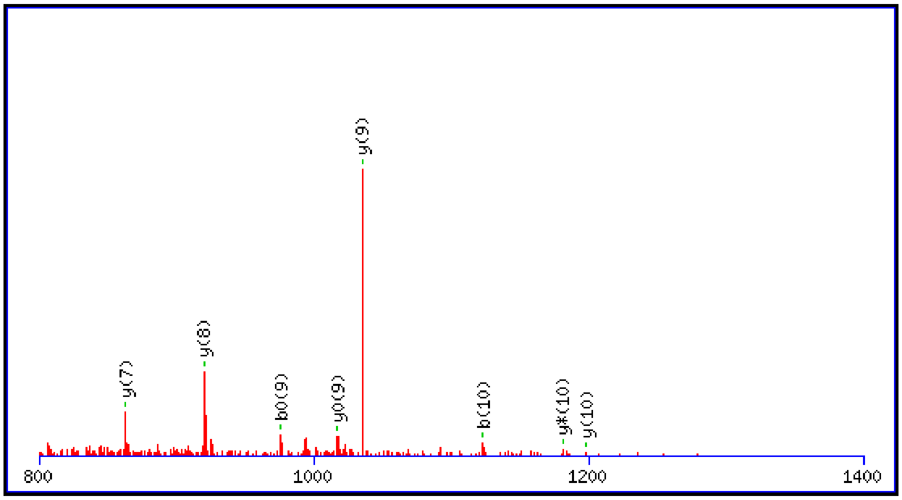

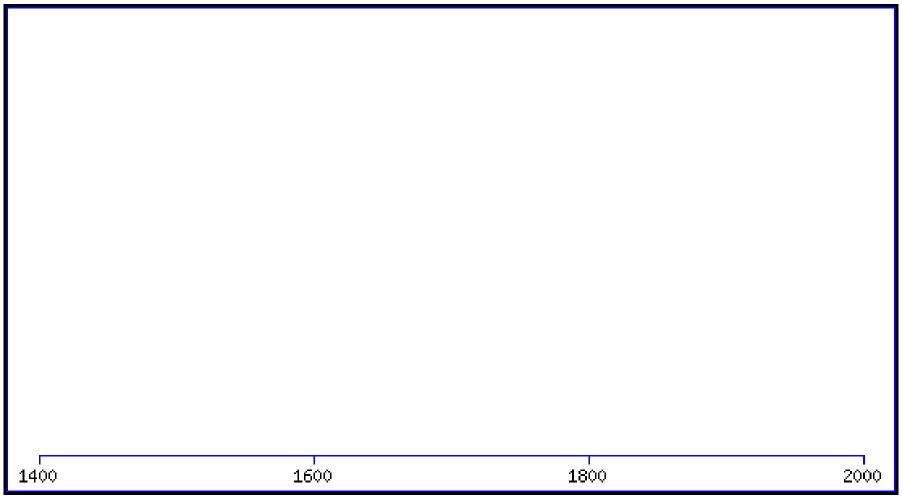

Monoisotopic mass of neutral peptide Mr(calc): 1296.5986
Fixed modifications: Carbamidomethyl (C)
Variable modifications:
N3 : Deamidated_N (N)
Ions Score: 45 **Expect:** 0.0042
Matches (Bold Red): 23/106 fragment ions using 50 most intense peaks

#	b	b^{++}	b*	b^{*++}	b^0	b^{0++}	Seq.	y	y^{++}	y*	y^{*++}	y^0	y^{0++}	#
1	100.0757	50.5415					V							11
2	**263.1390**	132.0731					Y	**1198.5375**	**599.7724**	**1181.5109**	**591.2591**	1180.5269	590.7671	10
3	**378.1660**	189.5866	361.1394	181.0733			N	**1035.4742**	518.2407	1018.4476	509.7274	**1017.4636**	509.2354	9
4	435.1874	218.0973	418.1609	209.5841			G	**920.4472**	460.7272	903.4207	452.2140	902.4367	451.7220	8
5	**522.2194**	261.6134	505.1929	253.1001	**504.2089**	252.6081	S	**863.4258**	432.2165	846.3992	423.7032	845.4152	423.2112	7

**6**	**621.2879**	311.1476	**604.2613**	302.6343	**603.2773**	302.1423	**V**	**776.3937**	388.7005	759.3672	380.1872	758.3832	379.6952	**6**
**7**	718.3406	359.6740	701.3141	351.1607	700.3301	350.6687	**P**	**677.3253**	339.1663	660.2988	330.6530	659.3148	330.1610	**5**
**8**	865.4090	**433.2082**	848.3825	424.6949	847.3985	424.2029	**F**	580.2726	290.6399	563.2460	282.1266	562.2620	281.6346	**4**
**9**	994.4516	**497.7295**	977.4251	489.2162	**976.4411**	488.7242	**E**	**433.2041**	217.1057	416.1776	208.5924	415.1936	208.1004	**3**
**10**	**1123.4942**	562.2508	1106.4677	553.7375	1105.4837	553.2455	**E**	**304.1615**	152.5844	287.1350	144.0711	286.1510	143.5791	**2**
**11**							**R**	175.1190	88.0631	158.0924	79.5498			**1**

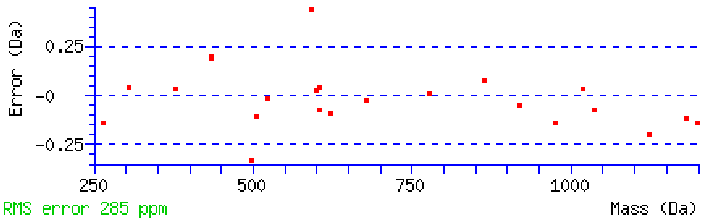

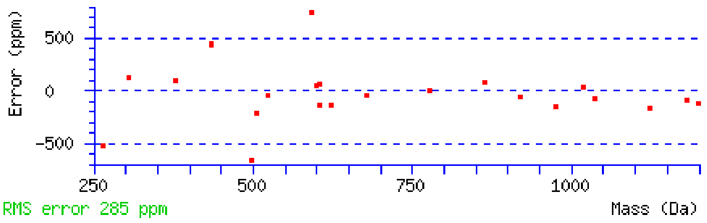

All matches to this query

Score	Mr(calc):	Delta	Sequence
45.1	1296.5986	-0.0010	VYNGSVPFEER
14.3	1296.6020	-0.0043	MDVAFLLDNSR
6.1	1296.6067	-0.0090	WRMMSLSQSR
6.0	1296.6067	-0.0090	WRMMSLSQSR
4.1	1294.5863	2.0113	WTLSNMLGDNK
3.7	1296.5992	-0.0016	VLSIQSHVVR
3.3	1294.6010	1.9967	QSTCEAVMTLR
2.5	1295.6027	0.9950	DMKSKAEDQTK
1.8	1296.6020	-0.0043	ETMVPHLPDDK
0.6	1296.6058	-0.0082	ADDRYSQSVTR

Spectrum No: 475; Query: 953; Rank: 1

Peptide View

MS/MS Fragmentation of **HHATYVNNLNVTEEKYHEALAK**
Found in **IPI00211593**, Tax_Id=10116 Gene_Symbol=Sod2 Superoxide dismutase [Mn], mitochondrial precursor

Match to Query 953: 2581.255992 from(861.425940,3+)
Title: 091127RatKid_SCX01_41.2124.2124.3.dta
Data file K:\NewmanPaper\Piliang\3SubProteomes\Piliang3SP\mgf5ppm\SCX_3SubProteomes5ppm.mgf

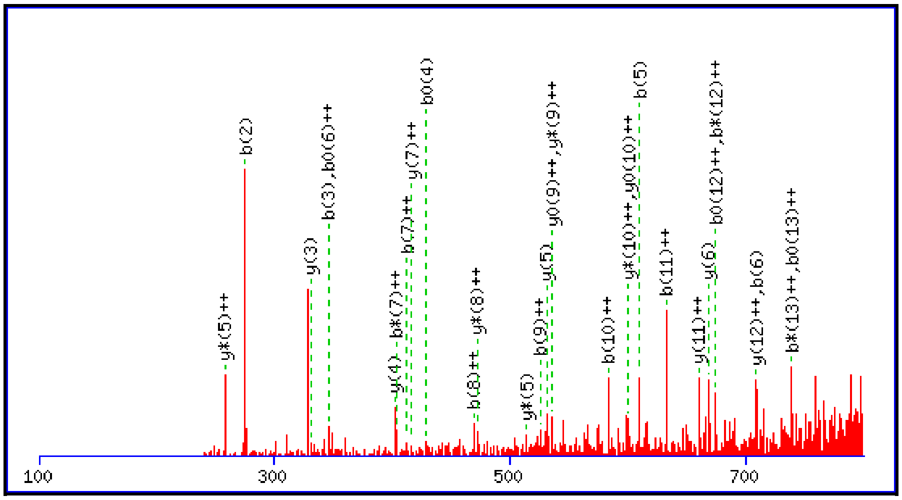

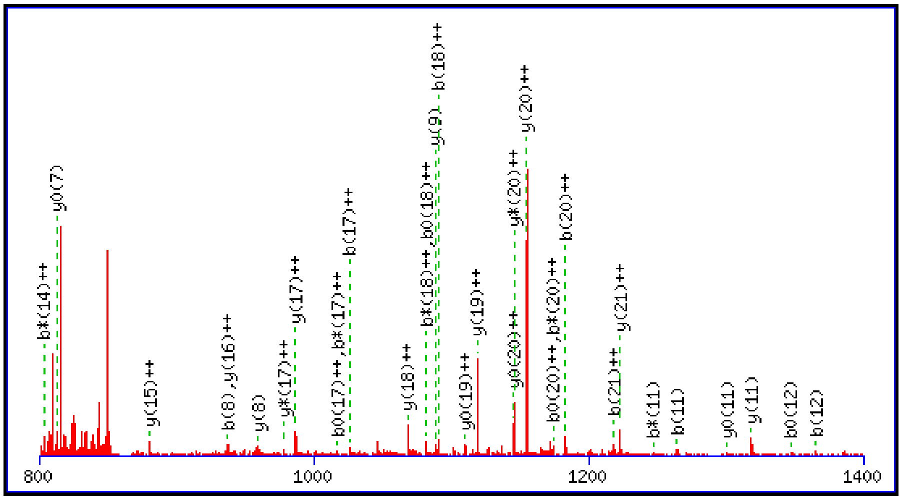

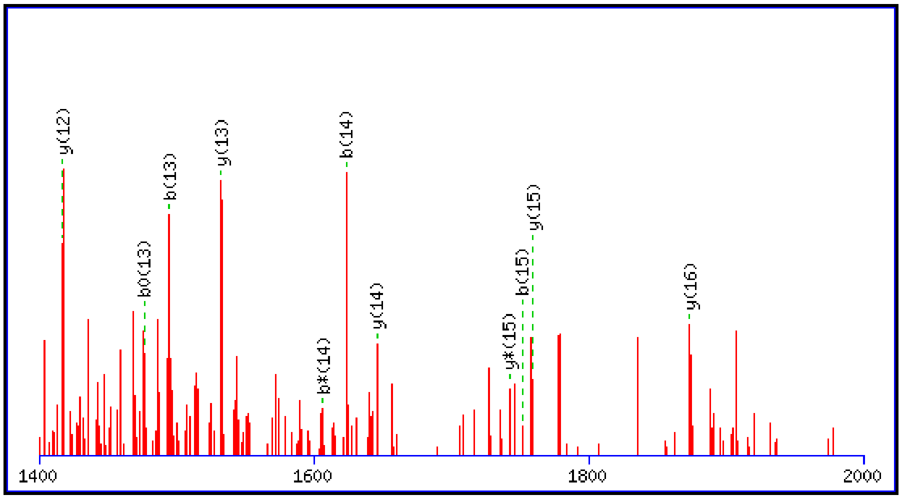

Monoisotopic mass of neutral peptide Mr(calc): 2581.2561
Fixed modifications: Carbamidomethyl (C)
Variable modifications:
N10 : Deamidated_N (N)
Ions Score: 45 **Expect:** 0.0078
Matches (Bold Red): 73/226 fragment ions using 164 most intense peaks

#	b	b^{++}	b*	b^{*++}	b^{0}	b^{0++}	Seq.	y	y^{++}	y*	y^{*++}	y^{0}	y^{0++}	#
1	138.0662	69.5367					H							22
2	**275.1251**	138.0662					H	2445.2045	**1223.1059**	2428.1779	1214.5926	2427.1939	1214.1006	21
3	**346.1622**	173.5847					A	2308.1456	**1154.5764**	2291.1190	**1146.0631**	2290.1350	**1145.5711**	20
4	447.2099	224.1086			**429.1993**	215.1033	T	2237.1084	**1119.0579**	2220.0819	1110.5446	2219.0979	**1110.0526**	19
5	**610.2732**	305.6402			592.2627	296.6350	Y	2136.0608	**1068.5340**	2119.0342	1060.0207	2118.0502	1059.5287	18
6	**709.3416**	355.1745			691.3311	**346.1692**	V	1972.9974	**987.0024**	1955.9709	**978.4891**	1954.9869	977.9971	17
7	823.3846	**412.1959**	806.3580	**403.6826**	805.3740	403.1906	N	**1873.9290**	**937.4682**	1856.9025	928.9549	1855.9185	928.4629	16
8	**937.4275**	**469.2174**	920.4009	460.7041	919.4169	460.2121	N	**1759.8861**	**880.4467**	**1742.8596**	871.9334	1741.8755	871.4414	15
9	1050.5116	**525.7594**	1033.4850	517.2461	1032.5010	516.7541	L	**1645.8432**	823.4252	1628.8166	814.9119	1627.8326	814.4199	14
10	1165.5385	**583.2729**	1148.5119	574.7596	1147.5279	574.2676	N	**1532.7591**	766.8832	1515.7326	758.3699	1514.7485	757.8779	13
11	**1264.6069**	**632.8071**	**1247.5804**	624.2938	1246.5963	623.8018	V	**1417.7322**	**709.3697**	1400.7056	700.8564	1399.7216	700.3644	12
12	**1365.6546**	683.3309	1348.6280	**674.8177**	**1347.6440**	**674.3256**	T	**1318.6638**	**659.8355**	1301.6372	651.3222	**1300.6532**	650.8302	11
13	**1494.6972**	747.8522	1477.6706	**739.3390**	**1476.6866**	**738.8469**	E	1217.6161	609.3117	1200.5895	**600.7984**	1199.6055	**600.3064**	10
14	**1623.7398**	812.3735	**1606.7132**	**803.8602**	1605.7292	803.3682	E	**1088.5735**	544.7904	1071.5469	**536.2771**	1070.5629	**535.7851**	9
15	**1751.8347**	876.4210	1734.8082	867.9077	1733.8242	867.4157	K	**959.5309**	480.2691	942.5043	**471.7558**	941.5203	471.2638	8
16	1914.8981	957.9527	1897.8715	949.4394	1896.8875	948.9474	Y	831.4359	**416.2216**	814.4094	407.7083	**813.4254**	407.2163	7
17	2051.9570	**1026.4821**	2034.9304	**1017.9689**	2033.9464	**1017.4768**	H	**668.3726**	334.6899	651.3461	326.1767	650.3620	325.6847	6
18	2180.9996	**1091.0034**	2163.9730	**1082.4901**	2162.9890	**1081.9981**	E	**531.3137**	266.1605	**514.2871**	**257.6472**	513.3031	257.1552	5

**19**	2252.0367	1126.5220	2235.0101	1118.0087	2234.0261	1117.5167	**A**	**402.2711**	201.6392	385.2445	193.1259			**4**
**20**	2365.1207	**1183.0640**	2348.0942	**1174.5507**	2347.1102	**1174.0587**	**L**	**331.2340**	166.1206	314.2074	157.6074			**3**
**21**	2436.1579	**1218.5826**	2419.1313	1210.0693	2418.1473	1209.5773	**A**	218.1499	109.5786	201.1234	101.0653			**2**
**22**							**K**	147.1128	74.0600	130.0863	65.5468			**1**

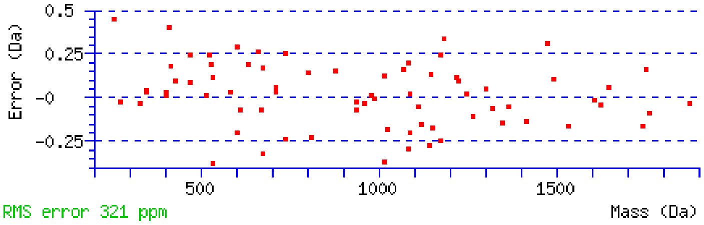

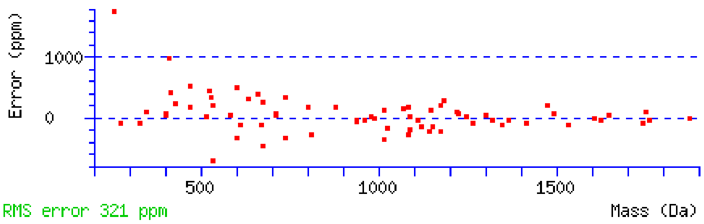

All matches to this query

Score	Mr(calc):	Delta	Sequence
45.0	2581.2561	-0.0001	HHATYVNNLNVTEEKYHEALAK
42.3	2581.2561	-0.0001	HHATYVNNLNVTEEKYHEALAK
41.9	2581.2561	-0.0001	HHATYVNNLNVTEEKYHEALAK
18.5	2580.2720	0.9839	HHATYVNNLNVTEEKYHEALAK
2.3	2581.2400	0.0160	AKRPEVPFPEITRMLGNEWSK
2.1	2580.2337	1.0223	MPPQSLLCVLVALALSGCNMAQK
1.3	2580.2632	0.9928	GLENNVNVELLNALHSHMVNKR
1.3	2581.2472	0.0088	GLENNVNVELLNALHSHMVNKR
1.3	2581.2762	-0.0202	WIASSRIGESITNSAGSGLRNGLR
1.2	2579.2493	2.0067	QLVGRTFSDGSATRTLQPPESPR

Spectrum No: 476; Query: 46; Rank: 1

Peptide View

MS/MS Fragmentation of **QANDSLTVLR**
Found in **IPI00363534**, Tax_Id=10116 Gene_Symbol=Lama1 laminin, alpha 1

Match to Query 46: 1116.578388 from(559.296470,2+)
Title: 091127RatKid_SCX01_02.1703.1703.2.dta
Data file K:\NewmanPaper\Piliang\3SubProteomes\Piliang3SP\mgf5ppm\SCX_3SubProteomes5ppm.mgf

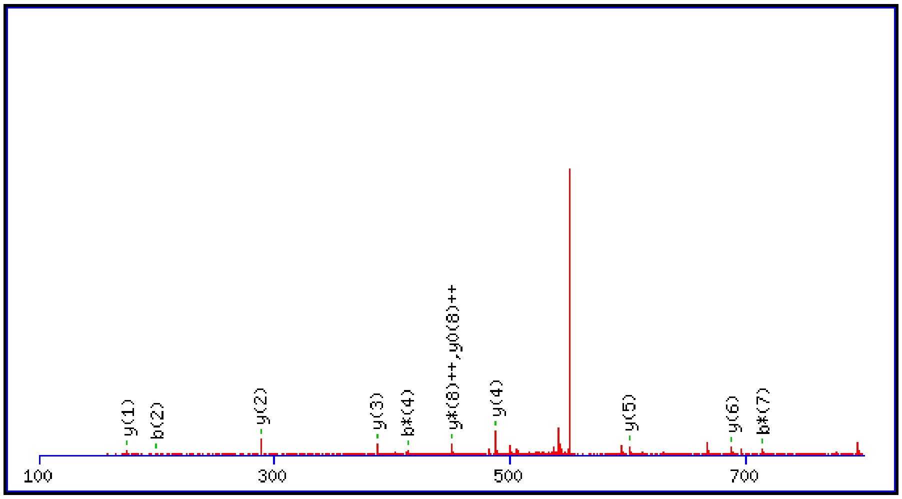

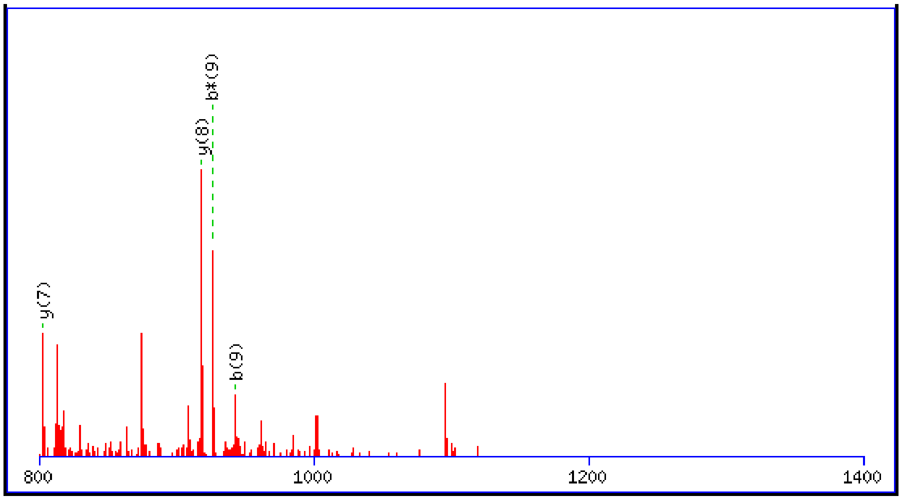

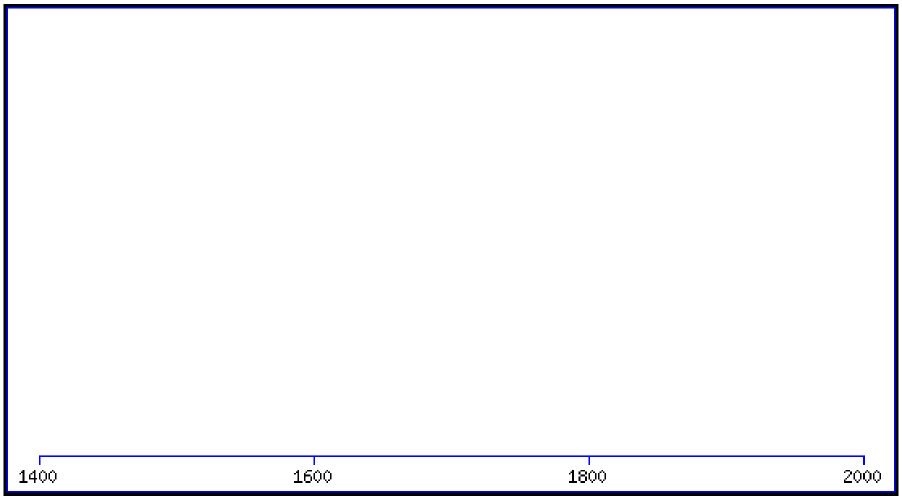

Monoisotopic mass of neutral peptide Mr(calc): 1116.5775
Fixed modifications: Carbamidomethyl (C)
Variable modifications:
N3 : Deamidated_N (N)
Ions Score: 45 **Expect:** 0.0033
Matches (Bold Red): 15/96 fragment ions using 33 most intense peaks

#	b	b^{++}	b*	b^{*++}	b^0	b^{0++}	Seq.	y	y^{++}	y*	y^{*++}	y^0	y^{0++}	#
1	129.0659	65.0366	112.0393	56.5233			Q							10
2	**200.1030**	100.5551	183.0764	92.0418			A	989.5262	495.2667	972.4996	486.7535	971.5156	486.2615	9
3	315.1299	158.0686	298.1034	149.5553			N	**918.4891**	459.7482	901.4625	**451.2349**	900.4785	**450.7429**	8
4	430.1568	215.5821	**413.1303**	207.0688	412.1463	206.5768	D	**803.4621**	402.2347	786.4356	393.7214	785.4516	393.2294	7
5	517.1889	259.0981	500.1623	250.5848	499.1783	250.0928	S	**688.4352**	344.7212	671.4087	336.2080	670.4246	335.7160	6
6	630.2729	315.6401	613.2464	307.1268	612.2624	306.6348	L	**601.4032**	301.2052	584.3766	292.6920	583.3926	292.1999	5
7	731.3206	366.1639	**714.2941**	357.6507	713.3101	357.1587	T	**488.3191**	244.6632	471.2926	236.1499	470.3085	235.6579	4
8	830.3890	415.6982	813.3625	407.1849	812.3785	406.6929	V	**387.2714**	194.1394	370.2449	185.6261			3
9	**943.4731**	472.2402	**926.4465**	463.7269	925.4625	463.2349	L	**288.2030**	144.6051	271.1765	136.0919			2
10							R	**175.1190**	88.0631	158.0924	79.5498			1

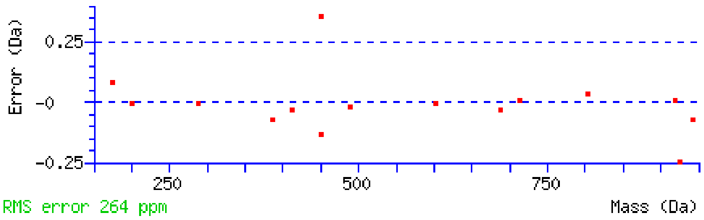

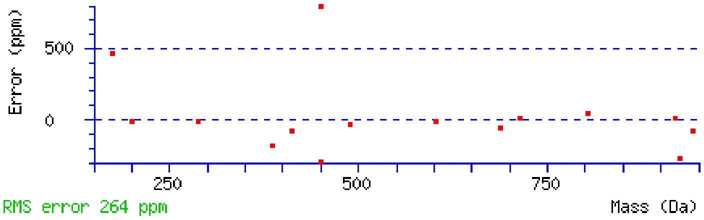

All matches to this query

Score	Mr(calc):	Delta	Sequence
45.0	1116.5775	0.0009	QANDSLTVLR
21.7	1115.5645	1.0139	LCELLTDPR
19.5	1114.5771	2.0013	LHEEGVIYR
15.1	1116.5775	0.0009	DKAKEEELR
13.6	1116.5709	0.0075	DQAAKLMANR
13.2	1115.5852	0.9932	QLISAHVLR
12.7	1116.5805	-0.0021	VLSAKHNLR
12.6	1115.5740	1.0044	NKTLKYLR
12.6	1115.5836	0.9948	SSFKGANLHR
12.4	1116.5710	0.0074	DLGCRVELR

Spectrum No: 477; Query: 1159; Rank: 1

Peptide View

MS/MS Fragmentation of **RPFGEVVYELEIDTLETTCHALDPTPLANCSVR**
Found in **IPI00327469**, Tax_Id=10116 Gene_Symbol=Ahsg Alpha-2-HS-glycoprotein precursor

Match to Query 1159: 3704.747292 from(1235.923040,3+)
Title: 091127RatKid_SCX01_22.6570.6570.3.dta
Data file K:\NewmanPaper\Piliang\3SubProteomes\Piliang3SP\mgf5ppm\SCX_3SubProteomes5ppm.mgf

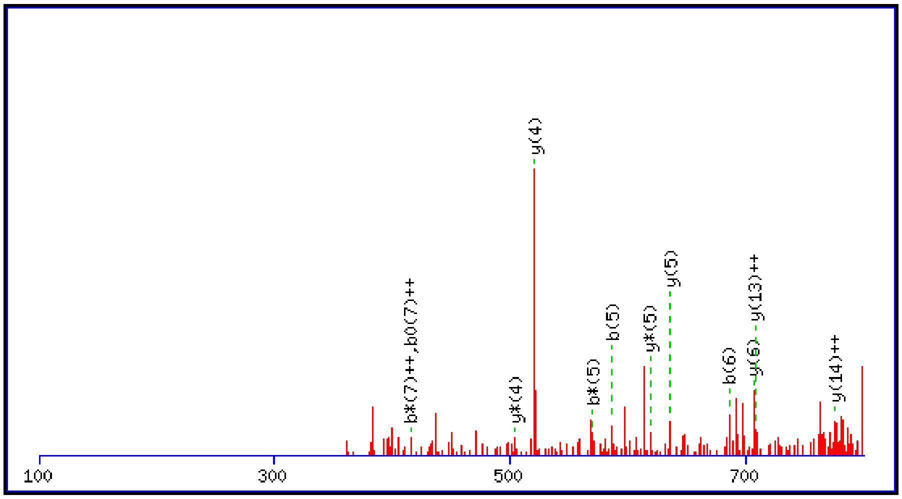

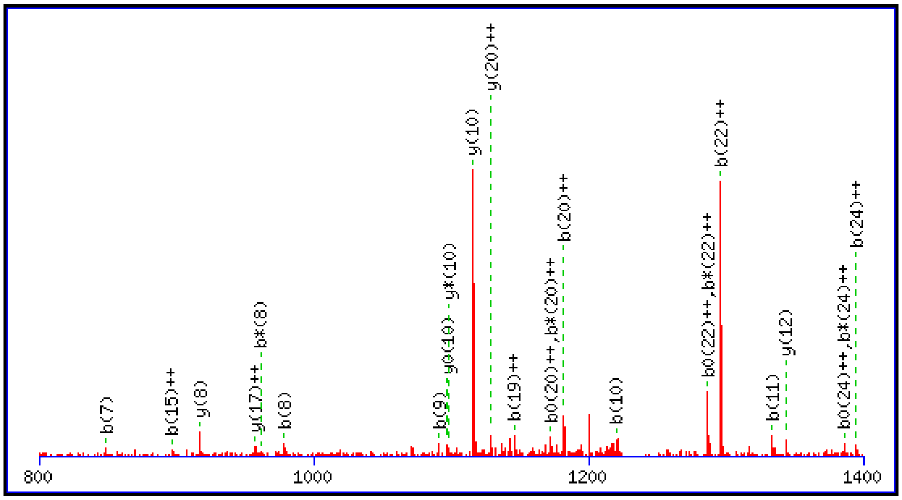

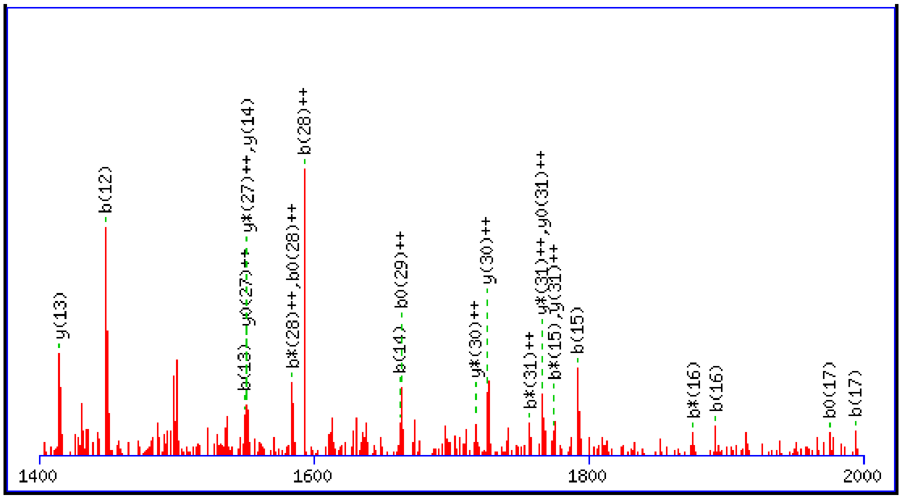

Monoisotopic mass of neutral peptide Mr(calc): 3703.7498
Fixed modifications: Carbamidomethyl (C)
Variable modifications:
N28 : Deamidated_N (N)
Ions Score: 45 **Expect:** 0.013
Matches (Bold Red): 60/360 fragment ions using 125 most intense peaks

#	b	b^{++}	b*	b^{*++}	b^{0}	b^{0++}	Seq.	y	y^{++}	y*	y^{*++}	y^{0}	y^{0++}	#
1	157.1084	79.0578	140.0818	70.5446			R							32
2	254.1612	127.5842	237.1346	119.0709			P	3548.6560	**1774.8316**	3531.6294	**1766.3184**	3530.6454	**1765.8263**	31
3	401.2296	201.1184	384.2030	192.6051			F	3451.6032	**1726.3052**	3434.5767	**1717.7920**	3433.5926	1717.3000	30
4	458.2510	229.6292	441.2245	221.1159			G	3304.5348	1652.7710	3287.5082	1644.2578	3286.5242	1643.7658	29
5	**587.2936**	294.1504	**570.2671**	285.6372	569.2831	285.1452	E	3247.5133	1624.2603	3230.4868	1615.7470	3229.5028	1615.2550	28
6	**686.3620**	343.6847	669.3355	335.1714	668.3515	334.6794	V	3118.4707	1559.7390	3101.4442	**1551.2257**	3100.4602	**1550.7337**	27
7	**849.4254**	425.2163	832.3988	**416.7030**	831.4148	**416.2110**	Y	3019.4023	1510.2048	3002.3758	1501.6915	3001.3918	1501.1995	26
8	**978.4680**	489.7376	**961.4414**	481.2243	960.4574	480.7323	E	2856.3390	1428.6731	2839.3124	1420.1599	2838.3284	1419.6679	25
9	**1091.5520**	546.2796	1074.5255	537.7664	1073.5415	537.2744	L	2727.2964	1364.1518	2710.2699	1355.6386	2709.2858	1355.1466	24
10	**1220.5946**	610.8009	1203.5681	602.2877	1202.5841	601.7957	E	2614.2123	1307.6098	2597.1858	1299.0965	2596.2018	1298.6045	23
11	**1333.6787**	667.3430	1316.6521	658.8297	1315.6681	658.3377	I	2485.1697	1243.0885	2468.1432	1234.5752	2467.1592	1234.0832	22
12	**1448.7056**	724.8564	1431.6791	716.3432	1430.6951	715.8512	D	2372.0857	1186.5465	2355.0591	1178.0332	2354.0751	1177.5412	21
13	**1549.7533**	775.3803	1532.7268	766.8670	1531.7427	766.3750	T	2257.0587	**1129.0330**	2240.0322	1120.5197	2239.0482	1120.0277	20
14	**1662.8374**	831.9223	1645.8108	823.4090	1644.8268	822.9170	L	2156.0111	1078.5092	2138.9845	1069.9959	2138.0005	1069.5039	19
15	**1791.8800**	**896.4436**	**1774.8534**	887.9303	1773.8694	887.4383	E	2042.9270	1021.9671	2025.9004	1013.4539	2024.9164	1012.9619	18
16	**1892.9276**	946.9675	**1875.9011**	938.4542	1874.9171	937.9622	T	1913.8844	**957.4458**	1896.8579	948.9326	1895.8738	948.4406	17
17	**1993.9753**	997.4913	1976.9488	988.9780	**1975.9648**	988.4860	T	1812.8367	906.9220	1795.8102	898.4087	1794.8262	897.9167	16
18	2154.0060	1077.5066	2136.9794	1068.9933	2135.9954	1068.5013	C	1711.7890	856.3982	1694.7625	847.8849	1693.7785	847.3929	15
19	2291.0649	**1146.0361**	2274.0383	1137.5228	2273.0543	1137.0308	H	**1551.7584**	**776.3828**	1534.7318	767.8696	1533.7478	767.3776	14
20	2362.1020	**1181.5546**	2345.0754	**1173.0414**	2344.0914	**1172.5494**	A	**1414.6995**	**707.8534**	1397.6729	699.3401	1396.6889	698.8481	13
21	2475.1861	1238.0967	2458.1595	1229.5834	2457.1755	1229.0914	L	**1343.6624**	672.3348	1326.6358	663.8215	1325.6518	663.3295	12
22	2590.2130	**1295.6101**	2573.1864	**1287.0969**	2572.2024	**1286.6049**	D	1230.5783	615.7928	1213.5518	607.2795	1212.5677	606.7875	11
23	2687.2658	1344.1365	2670.2392	1335.6232	2669.2552	1335.1312	P	**1115.5514**	558.2793	**1098.5248**	549.7660	**1097.5408**	549.2740	10
24	2788.3134	**1394.6604**	2771.2869	**1386.1471**	2770.3029	**1385.6551**	T	1018.4986	509.7529	1001.4721	501.2397	1000.4880	500.7477	9
25	2885.3662	1443.1867	2868.3397	1434.6735	2867.3556	1434.1815	P	**917.4509**	459.2291	900.4244	450.7158	899.4404	450.2238	8
26	2998.4503	1499.7288	2981.4237	1491.2155	2980.4397	1490.7235	L	820.3982	410.7027	803.3716	402.1894	802.3876	401.6974	7
27	3069.4874	1535.2473	3052.4608	1526.7341	3051.4768	1526.2420	A	**707.3141**	354.1607	690.2875	345.6474	689.3035	345.1554	6
28	3184.5143	**1592.7608**	3167.4878	**1584.2475**	3166.5038	**1583.7555**	N	**636.2770**	318.6421	**619.2504**	310.1289	618.2664	309.6368	5
29	3344.5450	1672.7761	3327.5184	1664.2628	3326.5344	**1663.7708**	C	**521.2500**	261.1287	**504.2235**	252.6154	503.2395	252.1234	4
30	3431.5770	1716.2921	3414.5504	1707.7789	3413.5664	1707.2869	S	361.2194	181.1133	344.1928	172.6001	343.2088	172.1081	3
31	3530.6454	**1765.8263**	3513.6189	**1757.3131**	3512.6348	1756.8211	V	274.1874	137.5973	257.1608	129.0840			2
32							R	175.1190	88.0631	158.0924	79.5498			1

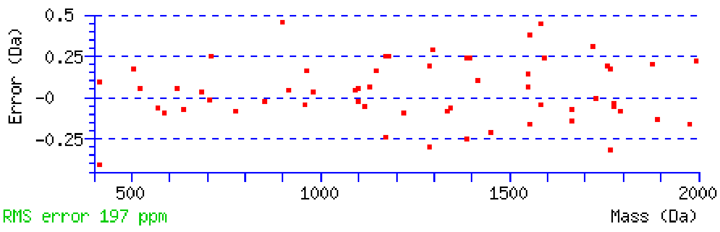

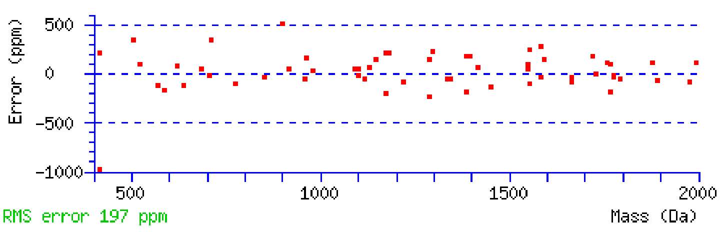

All matches to this query

Score	Mr(calc):	Delta	Sequence
45.0	3703.7498	0.9975	RPFGEVYELEIDTLETTCHALDPTPLANCSVR
18.8	3702.7658	1.9815	RPFGEVYELEIDTLETTCHALDPTPLANCSVR
7.2	3703.7090	1.0382	YLTMGGENFTRTDPPQTTVTHHPRPEGDVTLR
4.8	3703.7090	1.0382	YLTMGGENFTRTDPPQTTVTHHPRPEGDVTLR
4.7	3703.7090	1.0382	YLTMGGENFTRTDPPQTTVTHHPRPEGDVTLR
2.9	3702.7372	2.0100	GSHGERHVLIFDLGGGTFDVSILTIDDGIFEVK
2.7	3703.7075	1.0398	MAASIFMGAVRAASGILRPLNVSVSSTYRNCAR
2.7	3703.7075	1.0398	MAASIFMGAVRAASGILRPLNVSVSSTYRNCAR
2.7	3703.7484	0.9989	LAERWLLAGPATCLTPTPSPRGLHHGPQPEER
2.2	3704.7578	-0.0105	LRLYPPIKIVNVIGSVNTSSLNSSLFTMEGSK

Spectrum No: 478; Query: 304; Rank: 1

Peptide View

MS/MS Fragmentation of **NGSFQPTNEMMLR**
Found in **IPI00763831**, Tax_Id=10116 Gene_Symbol=LOC679565 similar to acyl-Coenzyme A binding domain containing 5

Match to Query 304: 1524.671828 from(763.343190,2+)
Title: 091127RatKid_SCX01_15.2511.2511.2.dta
Data file K:\NewmanPaper\Piliang\3SubProteomes\Piliang3SP\mgf5ppm\SCX_3SubProteomes5ppm.mgf

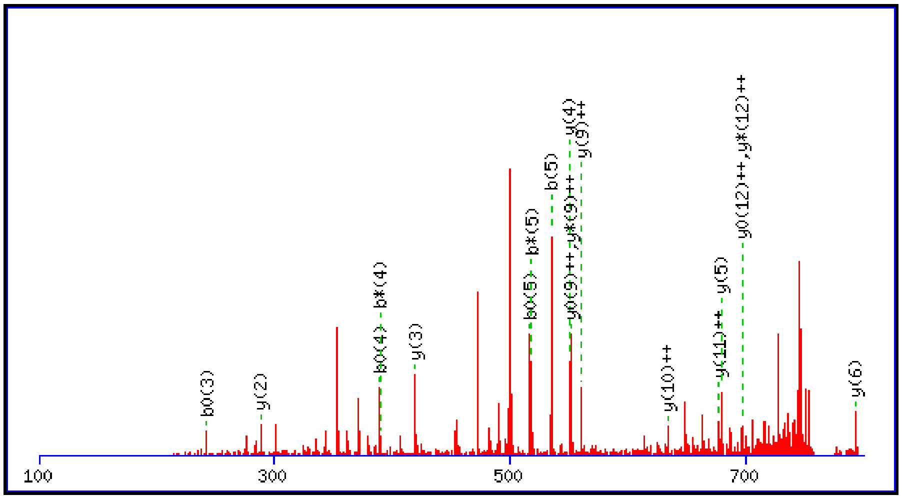

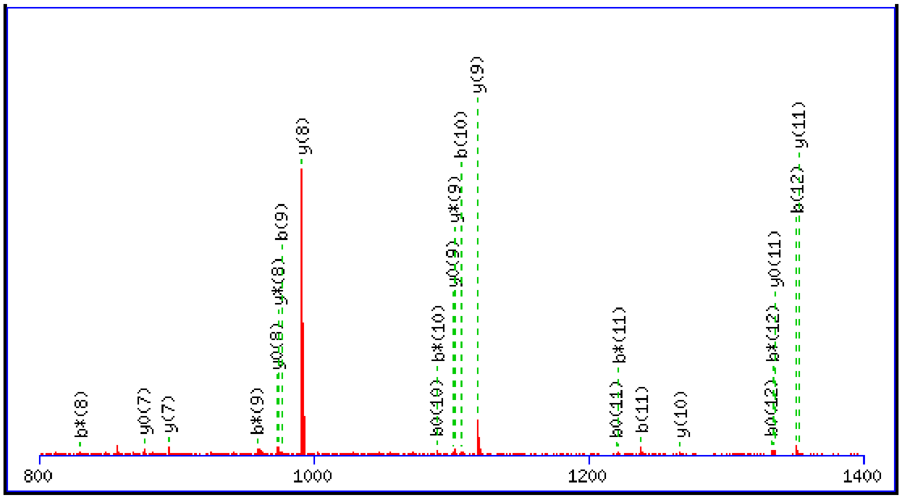

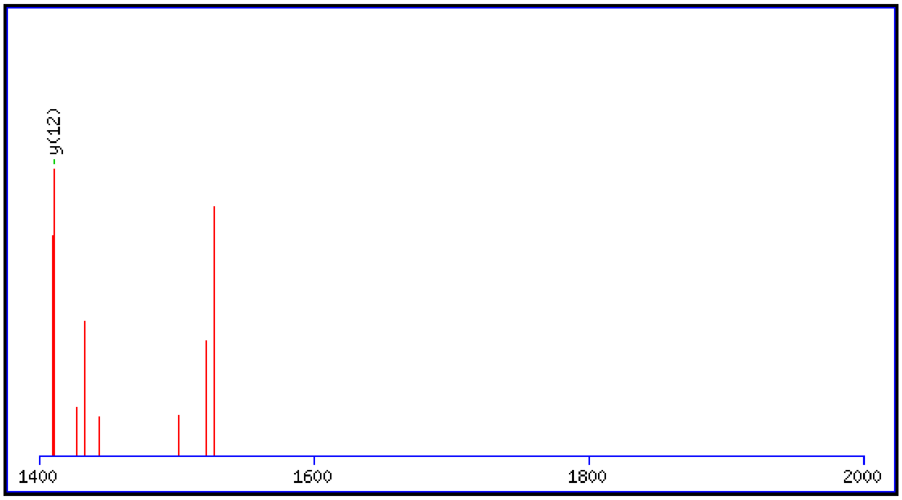

Monoisotopic mass of neutral peptide Mr(calc): 1524.6701
Fixed modifications: Carbamidomethyl (C)
Variable modifications:
N1 : Deamidated_N (N)
Ions Score: 45 **Expect:** 0.0049
Matches (Bold Red): 42/132 fragment ions using 118 most intense peaks

#	b	b^{++}	b*	b^{*++}	b^0	b^{0++}	Seq.	y	y^{++}	y*	y^{*++}	y^0	y^{0++}	#
1	116.0342	58.5207	99.0077	50.0075			N							13
2	173.0557	87.0315	156.0291	78.5182			G	**1410.6504**	705.8289	1393.6239	**697.3156**	1392.6399	**696.8236**	12
3	260.0877	130.5475	243.0612	122.0342	**242.0771**	121.5422	S	**1353.6290**	**677.3181**	1336.6024	668.8048	**1335.6184**	668.3128	11
4	407.1561	204.0817	**390.1296**	195.5684	**389.1456**	195.0764	F	**1266.5969**	**633.8021**	1249.5704	625.2888	1248.5864	624.7968	10
5	**535.2147**	268.1110	**518.1881**	259.5977	**517.2041**	259.1057	Q	**1119.5285**	**560.2679**	**1102.5020**	**551.7546**	**1101.5180**	**551.2626**	9
6	632.2675	316.6374	615.2409	308.1241	614.2569	307.6321	P	**991.4699**	496.2386	**974.4434**	487.7253	**973.4594**	487.2333	8
7	733.3151	367.1612	716.2886	358.6479	715.3046	358.1559	T	**894.4172**	447.7122	877.3906	439.1990	**876.4066**	438.7069	7
8	847.3581	424.1827	**830.3315**	415.6694	829.3475	415.1774	N	**793.3695**	397.1884	776.3430	388.6751	775.3589	388.1831	6
9	**976.4007**	488.7040	**959.3741**	480.1907	958.3901	479.6987	E	**679.3266**	340.1669	662.3000	331.6537	661.3160	331.1616	5
10	**1107.4411**	554.2242	**1090.4146**	545.7109	**1089.4306**	545.2189	M	**550.2840**	275.6456	533.2574	267.1324			4
11	**1238.4816**	619.7445	**1221.4551**	611.2312	**1220.4711**	610.7392	M	**419.2435**	210.1254	402.2170	201.6121			3
12	**1351.5657**	676.2865	**1334.5391**	667.7732	**1333.5551**	667.2812	L	**288.2030**	144.6051	271.1765	136.0919			2
13							R	175.1190	88.0631	158.0924	79.5498			1

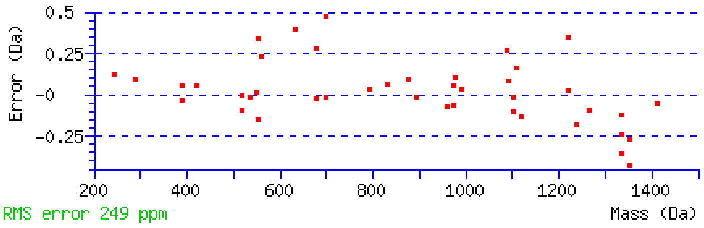

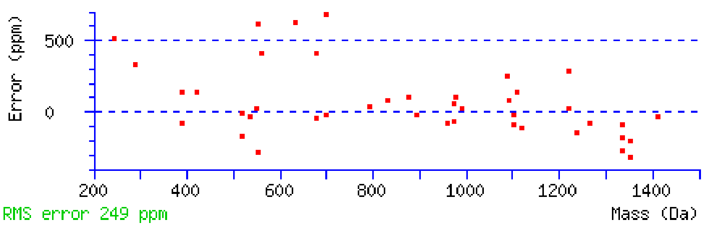

All matches to this query

Score	Mr(calc):	Delta	Sequence
44.9	1524.6701	0.0018	NGSFQPTNEMMLR
16.9	1524.6701	0.0018	NGSFQPTNEMMLR
7.4	1524.6722	-0.0004	GVLHQFSGTETNR
6.5	1524.6722	-0.0004	GVLHQFSGTETNR
6.5	1524.6722	-0.0004	GVLHQFSGTETNR
5.5	1523.6661	1.0058	KEMEQISEEEEK
2.0	1524.6804	-0.0086	SSSAATAAAWSEGSSR
1.8	1523.6602	1.0116	DVAMDFTPEEWGK
0.5	1524.6682	0.0036	EVRDTRGSSPEGR
0.5	1524.6682	0.0036	EVRDTRGSSPEGR

Spectrum No: 479; Query: 312; Rank: 1

Peptide View

MS/MS Fragmentation of **TNSSFVQGLVDHVK**
Found in **IPI00195160**, Tax_Id=10116 Gene_Symbol=Psap Sulfated glycoprotein 1 precursor

Match to Query 312: 1530.769992 from(511.263940,3+)
Title: 091129RatKid_SCX02_28.2898.2898.3.dta
Data file K:\NewmanPaper\Piliang\3SubProteomes\Piliang3SP\mgf5ppm\SCX_3SubProteomes5ppm.mgf

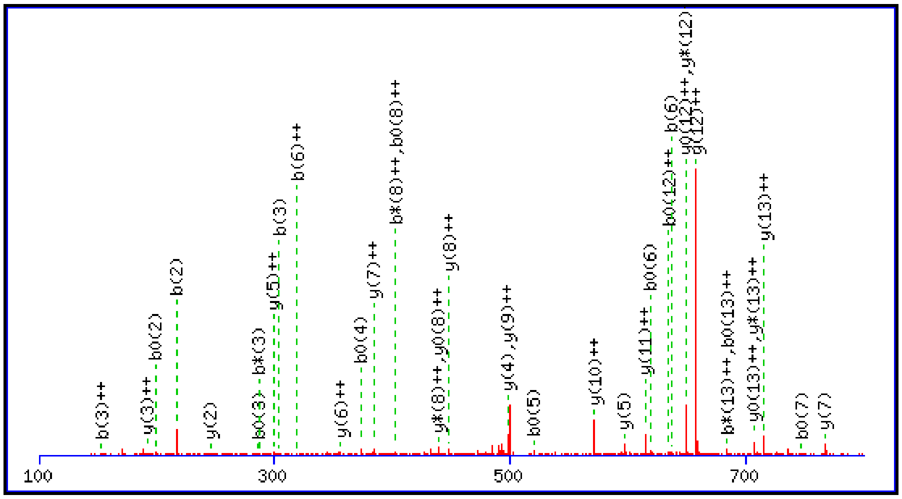

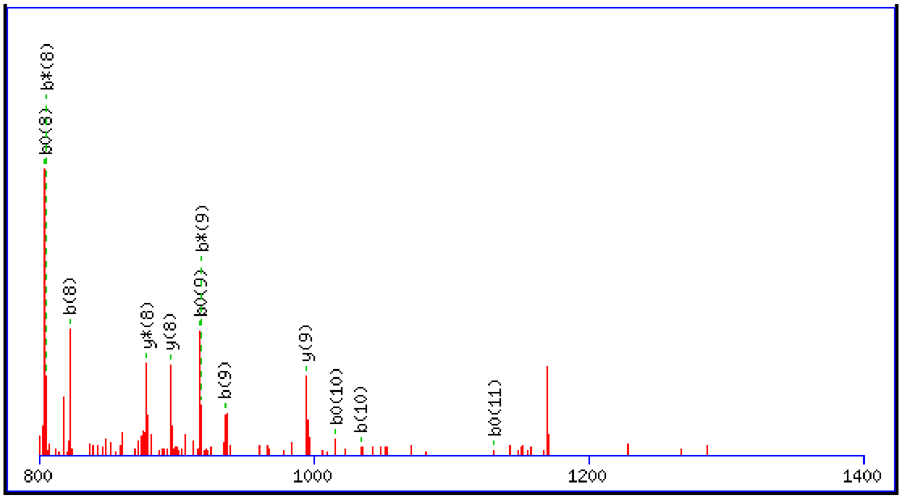

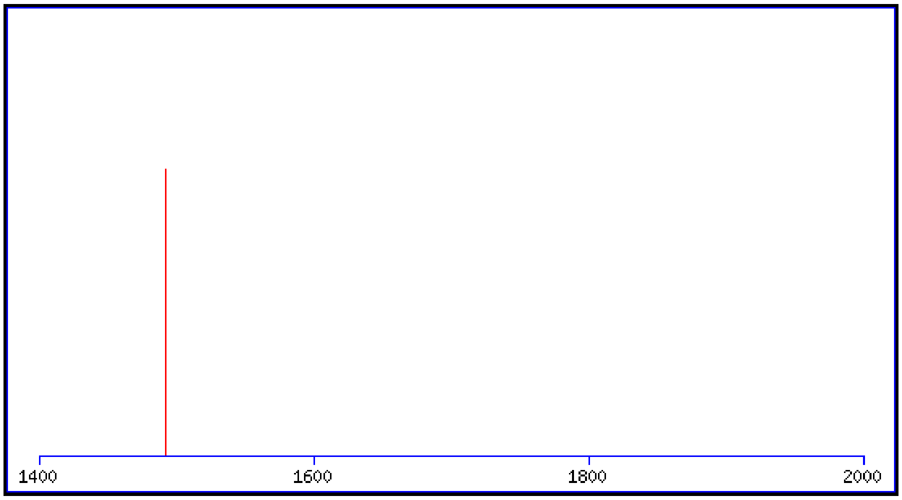

Monoisotopic mass of neutral peptide Mr(calc): 1530.7678
Fixed modifications: Carbamidomethyl (C)
Variable modifications:
N2 : Deamidated_N (N)
Ions Score: 45 **Expect:** 0.0048
Matches (Bold Red): 49/148 fragment ions using 102 most intense peaks

#	b	b^{++}	b*	b^{*++}	b^0	b^{0++}	Seq.	y	y^{++}	y*	y^{*++}	y^0	y^{0++}	#
1	102.0550	51.5311			84.0444	42.5258	T							14
2	**217.0819**	109.0446	200.0553	100.5313	**199.0713**	100.0393	N	1430.7274	**715.8673**	1413.7009	**707.3541**	1412.7168	**706.8621**	13
3	**304.1139**	**152.5606**	**287.0874**	144.0473	**286.1034**	143.5553	S	1315.7005	**658.3539**	1298.6739	**649.8406**	1297.6899	**649.3486**	12
4	391.1459	196.0766	374.1194	187.5633	**373.1354**	187.0713	S	1228.6684	**614.8379**	1211.6419	606.3246	1210.6579	605.8326	11
5	538.2144	269.6108	521.1878	261.0975	**520.2038**	260.6055	F	1141.6364	**571.3218**	1124.6099	562.8086	1123.6259	562.3166	10
6	**637.2828**	**319.1450**	620.2562	310.6318	**619.2722**	310.1397	V	**994.5680**	**497.7876**	977.5415	489.2744	976.5574	488.7824	9
7	765.3414	383.1743	748.3148	374.6610	**747.3308**	374.1690	Q	**895.4996**	**448.2534**	**878.4730**	**439.7402**	877.4890	**439.2482**	8
8	**822.3628**	411.6850	**805.3363**	**403.1718**	**804.3523**	**402.6798**	G	**767.4410**	**384.2241**	750.4145	375.7109	749.4305	375.2189	7
9	**935.4469**	468.2271	**918.4203**	459.7138	**917.4363**	459.2218	L	710.4196	**355.7134**	693.3930	347.2001	692.4090	346.7081	6
10	**1034.5153**	517.7613	1017.4887	509.2480	**1016.5047**	508.7560	V	**597.3355**	**299.1714**	580.3089	290.6581	579.3249	290.1661	5
11	1149.5422	575.2748	1132.5157	566.7615	**1131.5317**	566.2695	D	**498.2671**	249.6372	481.2405	241.1239	480.2565	240.6319	4
12	1286.6012	643.8042	1269.5746	635.2909	1268.5906	**634.7989**	H	383.2401	**192.1237**	366.2136	183.6104			3
13	1385.6696	693.3384	1368.6430	**684.8251**	1367.6590	**684.3331**	V	**246.1812**	123.5942	229.1547	115.0810			2
14							K	147.1128	74.0600	130.0863	65.5468			1

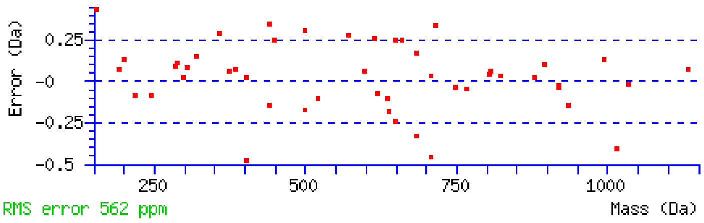

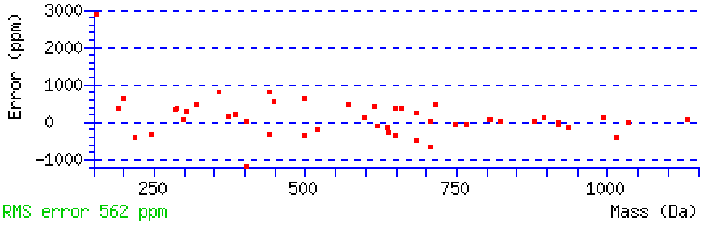

All matches to this query

Score	Mr(calc):	Delta	Sequence
44.8	1530.7678	0.0022	TNSSFVQGLVDHVK
4.7	1529.7725	0.9975	AILESGSSNAPWAVK
4.4	1528.7733	1.9967	VNSSQALPVDGSEVK
4.4	1529.7573	1.0127	VNSSQALPVDGSEVK
3.6	1528.7650	2.0050	LNKTGLPEAEIHK
2.6	1530.7555	0.0145	FGSSLVSVRSKER
2.6	1530.7555	0.0145	FGSSLVSVRSKER
2.5	1530.7695	0.0005	TDYGQTVILKVSK
2.4	1528.7763	1.9937	TSSAQFIGKLRNK
2.4	1528.7763	1.9937	TSSAQFIGKLRNK

Spectrum No: 480; Query: 157; Rank: 1

Peptide View

MS/MS Fragmentation of **WSFSNGTSWQK**
Found in **IPI00558132**, Tax_Id=10116 Gene_Symbol=Neu1 44 kDa protein

Match to Query 157: 1327.584048 from(664.799300,2+)
Title: 091129RatKid_SCX02_13.2249.2249.2.dta
Data file K:\NewmanPaper\Piliang\3SubProteomes\Piliang3SP\mgf5ppm\SCX_3SubProteomes5ppm.mgf

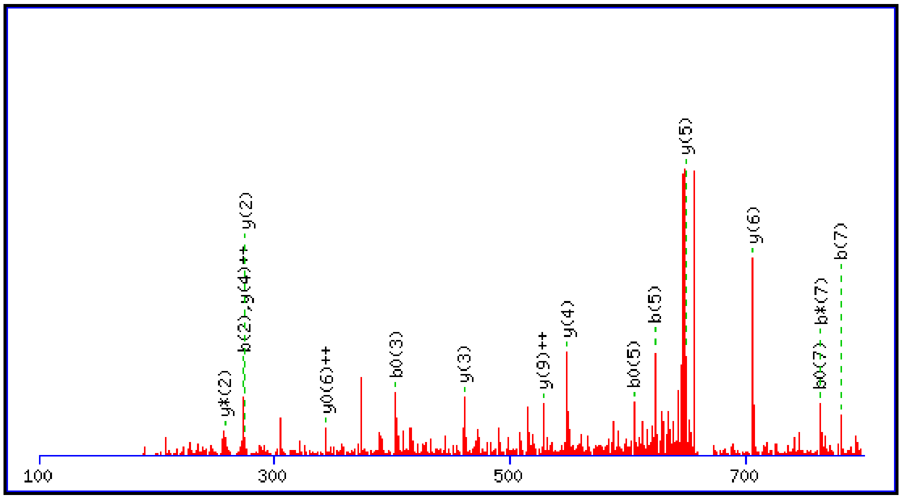

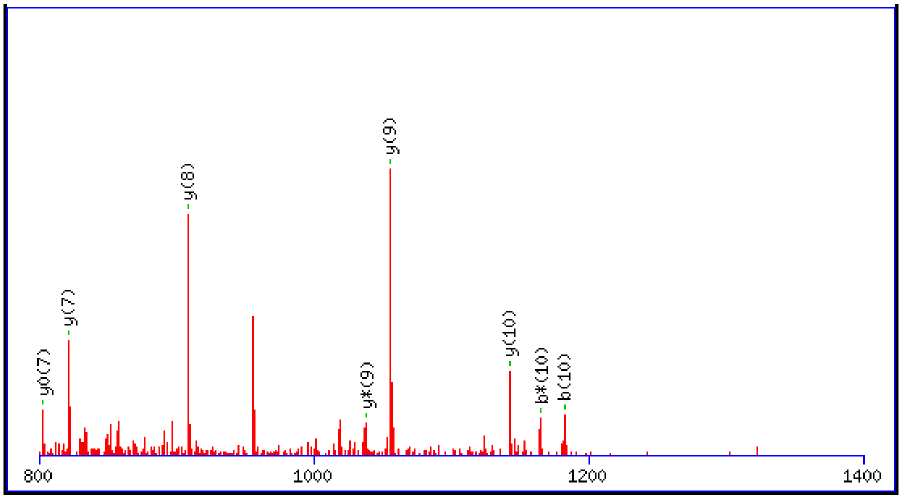

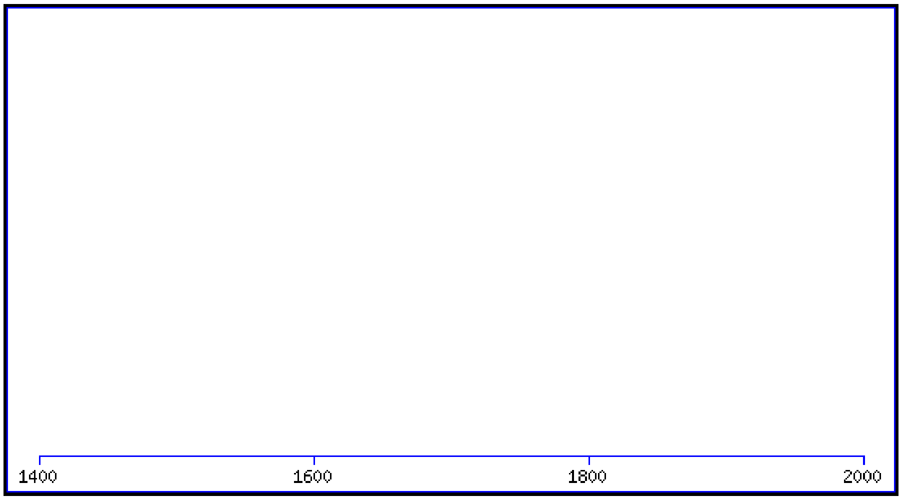

Monoisotopic mass of neutral peptide Mr(calc): 1327.5833
Fixed modifications: Carbamidomethyl (C)
Variable modifications:
N5 : Deamidated_N (N)
Ions Score: 45 **Expect:** 0.0043
Matches (Bold Red): 24/104 fragment ions using 60 most intense peaks

#	b	b^{++}	b^{*}	b^{*++}	b^{0}	b^{0++}	Seq.	y	y^{++}	y^{*}	y^{*++}	y^{0}	y^{0++}	#
1	187.0866	94.0469					W							11
2	**274.1186**	137.5629			256.1081	128.5577	S	**1142.5113**	571.7593	1125.4847	563.2460	1124.5007	562.7540	10
3	421.1870	211.0972			**403.1765**	202.0919	F	**1055.4792**	**528.2433**	**1038.4527**	519.7300	1037.4687	519.2380	9
4	508.2191	254.6132			490.2085	245.6079	S	**908.4108**	454.7091	891.3843	446.1958	890.4003	445.7038	8
5	**623.2460**	312.1266	606.2194	303.6134	**605.2354**	303.1214	N	**821.3788**	411.1930	804.3523	402.6798	**803.3682**	402.1878	7
6	680.2675	340.6374	663.2409	332.1241	662.2569	331.6321	G	**706.3519**	353.6796	689.3253	345.1663	688.3413	**344.6743**	6
7	**781.3151**	391.1612	**764.2886**	382.6479	**763.3046**	382.1559	T	**649.3304**	325.1688	632.3039	316.6556	631.3198	316.1636	5
8	868.3472	434.6772	851.3206	426.1639	850.3366	425.6719	S	**548.2827**	**274.6450**	531.2562	266.1317	530.2722	265.6397	4
9	1054.4265	527.7169	1037.3999	519.2036	1036.4159	518.7116	W	**461.2507**	231.1290	444.2241	222.6157			3
10	**1182.4851**	591.7462	**1165.4585**	583.2329	1164.4745	582.7409	Q	**275.1714**	138.0893	**258.1448**	129.5761			2
11							K	147.1128	74.0600	130.0863	65.5468			1

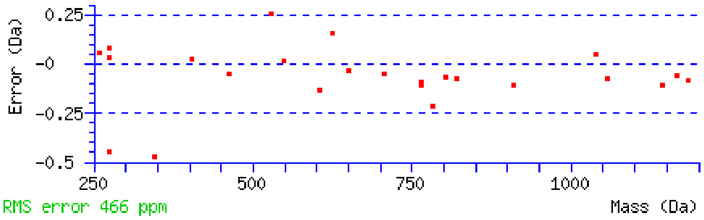

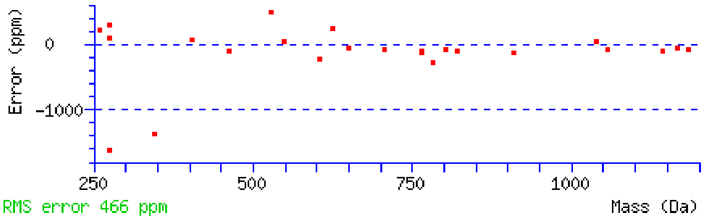

All matches to this query

Score	Mr(calc):	Delta	Sequence
44.8	1327.5833	0.0007	WSFSNGTSWQK
5.6	1326.5798	1.0042	FLFGSNPYFR
3.2	1327.5843	-0.0003	GSADNVTVMVVR
3.1	1327.5816	0.0025	SERAASGRQMR
2.6	1326.5808	1.0033	KYLSQLLMR
0.8	1326.5929	0.9912	ESRQVASSEVR
0.8	1326.5929	0.9912	ESRQVASSEVR
0.8	1327.5769	0.0071	QAATSQSATEVR
0.8	1327.5769	0.0071	QAATSQSATEVR
0.8	1327.5769	0.0071	QAATSQSATEVR

Spectrum No: 481; Query: 917; Rank: 1

Peptide View

MS/MS Fragmentation of **FTFTSHTPGDHQICLHSNSTR**
Found in **IPI00212516**, Tax_Id=10116 Gene_Symbol=Tmed4_predicted transmembrane emp24 protein transport domain containing 4

Match to Query 917: 2443.104582 from(815.375470,3+)
Title: 091127RatKid_SCX01_37.1567.1567.3.dta
Data file K:\NewmanPaper\Piliang\3SubProteomes\Piliang3SP\mgf5ppm\SCX_3SubProteomes5ppm.mgf

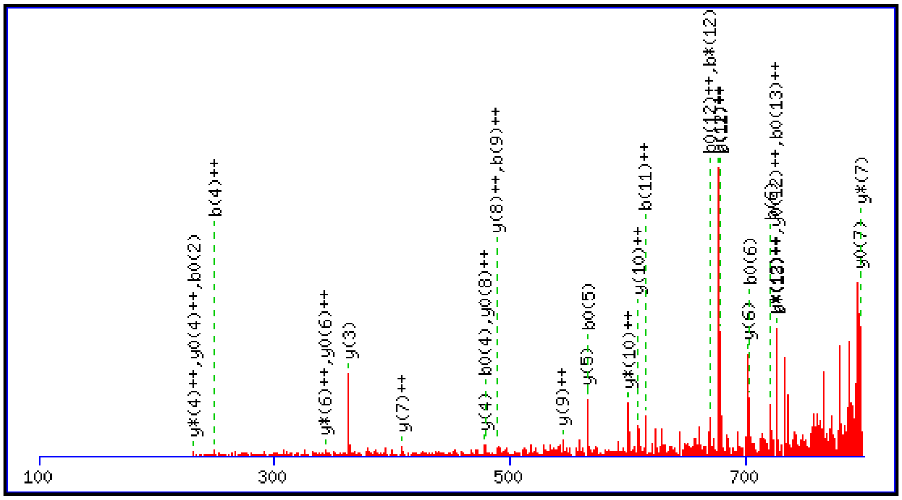

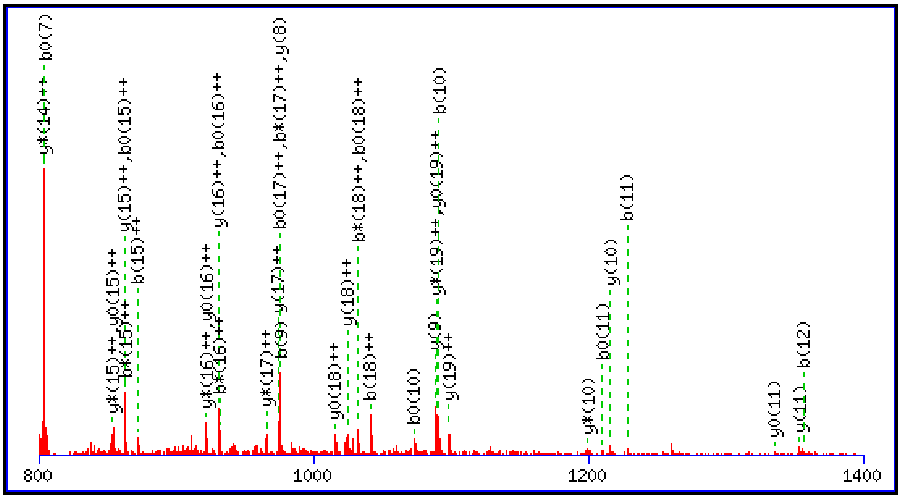

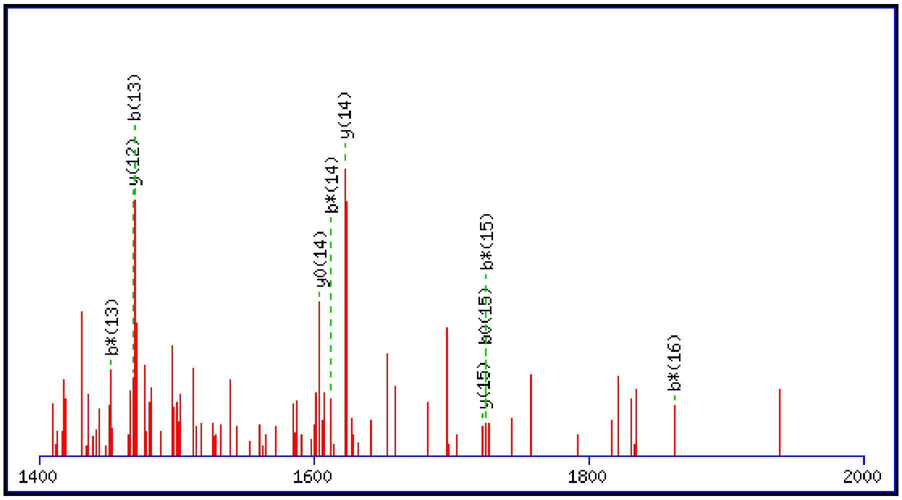

Monoisotopic mass of neutral peptide Mr(calc): 2443.0975
Fixed modifications: Carbamidomethyl (C)
Variable modifications:
N18 : Deamidated_N (N)
Ions Score: 45 **Expect:** 0.011
Matches (Bold Red): 80/214 fragment ions using 160 most intense peaks

#	b	b++	b*	b*++	b0	b0++	Seq.	y	y++	y*	y*++	y0	y0++	#
1	148.0757	74.5415					F							21
2	**249.1234**	125.0653			**231.1128**	116.0600	T	2297.0364	1149.0218	2280.0098	1140.5085	2279.0258	1140.0165	20
3	396.1918	198.5995			378.1812	189.5942	F	2195.9887	**1098.4980**	2178.9621	**1089.9847**	2177.9781	**1089.4927**	19
4	497.2395	**249.1234**			**479.2289**	240.1181	T	2048.9203	**1024.9638**	2031.8937	1016.4505	2030.9097	**1015.9585**	18
5	584.2715	292.6394			**566.2609**	283.6341	S	1947.8726	**974.4399**	1930.8460	**965.9267**	1929.8620	965.4347	17
6	**721.3304**	361.1688			**703.3198**	352.1636	H	1860.8406	**930.9239**	1843.8140	**922.4106**	1842.8300	**921.9186**	16
7	822.3781	411.6927			**804.3675**	402.6874	T	**1723.7817**	**862.3945**	1706.7551	**853.8812**	1705.7711	**853.3892**	15
8	919.4308	460.2191			901.4203	451.2138	P	**1622.7340**	811.8706	1605.7074	**803.3573**	**1604.7234**	802.8653	14
9	**976.4523**	**488.7298**			958.4417	479.7245	G	1525.6812	763.3442	1508.6547	754.8310	1507.6706	754.3390	13
10	**1091.4793**	546.2433			**1073.4687**	537.2380	D	**1468.6597**	734.8335	1451.6332	**726.3202**	1450.6492	**725.8282**	12
11	**1228.5382**	**614.7727**			**1210.5276**	605.7674	H	**1353.6328**	**677.3200**	1336.6063	668.8068	**1335.6222**	668.3148	11
12	**1356.5967**	**678.8020**	1339.5702	**670.2887**	1338.5862	**669.7967**	Q	**1216.5739**	**608.7906**	**1199.5473**	**600.2773**	1198.5633	599.7853	10
13	**1469.6808**	735.3440	**1452.6543**	**726.8308**	1451.6702	**726.3388**	I	**1088.5153**	**544.7613**	1071.4888	536.2480	1070.5047	535.7560	9
14	1629.7115	815.3594	**1612.6849**	806.8461	1611.7009	806.3541	C	**975.4312**	**488.2193**	958.4047	479.7060	957.4207	**479.2140**	8
15	1742.7955	**871.9014**	**1725.7690**	**863.3881**	**1724.7850**	**862.8961**	L	815.4006	**408.2039**	**798.3740**	399.6907	**797.3900**	399.1987	7
16	1879.8544	940.4309	**1862.8279**	**931.9176**	1861.8439	**931.4256**	H	**702.3165**	351.6619	685.2900	**343.1486**	684.3060	**342.6566**	6
17	1966.8865	983.9469	1949.8599	**975.4336**	1948.8759	**974.9416**	S	**565.2576**	283.1324	548.2311	274.6192	547.2471	274.1272	5
18	2081.9134	**1041.4603**	2064.8868	**1032.9471**	2063.9028	**1032.4551**	N	**478.2256**	239.6164	461.1990	**231.1032**	460.2150	**230.6112**	4
19	2168.9454	1084.9763	2151.9189	1076.4631	2150.9349	1075.9711	S	**363.1987**	182.1030	346.1721	173.5897	345.1881	173.0977	3
20	2269.9931	1135.5002	2252.9666	1126.9869	2251.9825	1126.4949	T	276.1666	138.5870	259.1401	130.0737	258.1561	129.5817	2
21							R	175.1190	88.0631	158.0924	79.5498			1

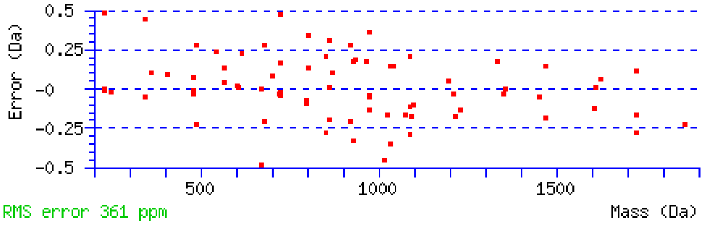

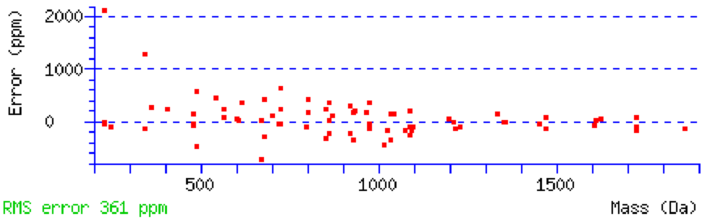

All matches to this query

Score	Mr(calc):	Delta	Sequence

44.7	2443.0975	0.0070	FTFTSHTPGDHQICLHSNSTR
5.8	2442.1135	0.9911	FTFTSHTPGDHQICLHSNSTR
2.9	2441.1025	2.0021	NKMCGHTYEEEAIVRMIESK
0.4	2442.0804	1.0242	NRKPDQYFIQIVGVMDTSR
0.4	2442.0804	1.0242	NRKPDQYFIQIVGVMDTSR
0.1	2442.0851	1.0195	AGTAGPQANKRYFLFFSFLF

Spectrum No: 482; Query: 97; Rank: 1

Peptide View

MS/MS Fragmentation of **EYNQTFGNLK**
Found in **IPI00196620**, Tax_Id=10116 Gene_Symbol=Cubn Cubilin precursor

Match to Query 97: 1213.561728 from(607.788140,2+)
Title: 091127RatKid_SCX01_14.1821.1821.2.dta
Data file K:\NewmanPaper\Piliang\3SubProteomes\Piliang3SP\mgf5ppm\SCX_3SubProteomes5ppm.mgf

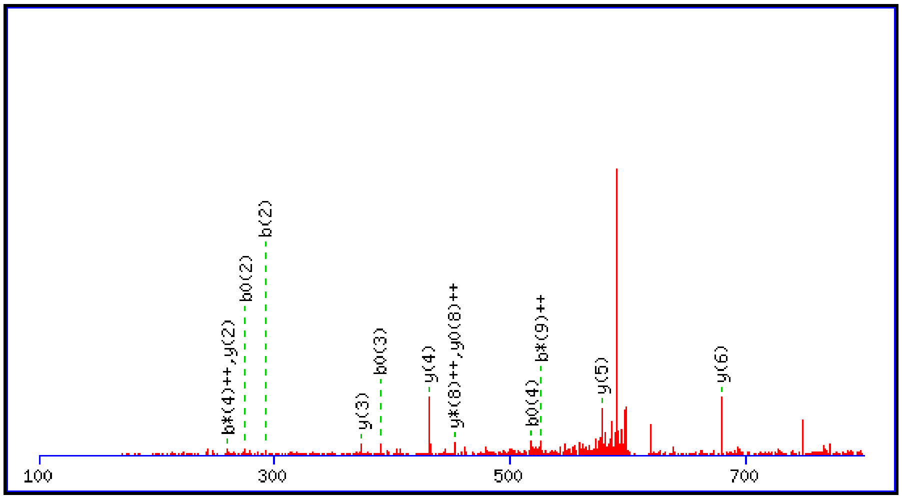

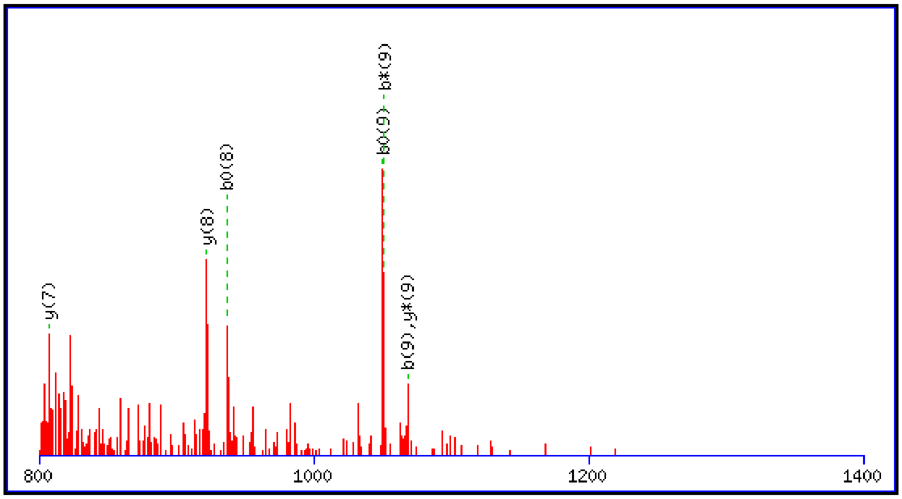

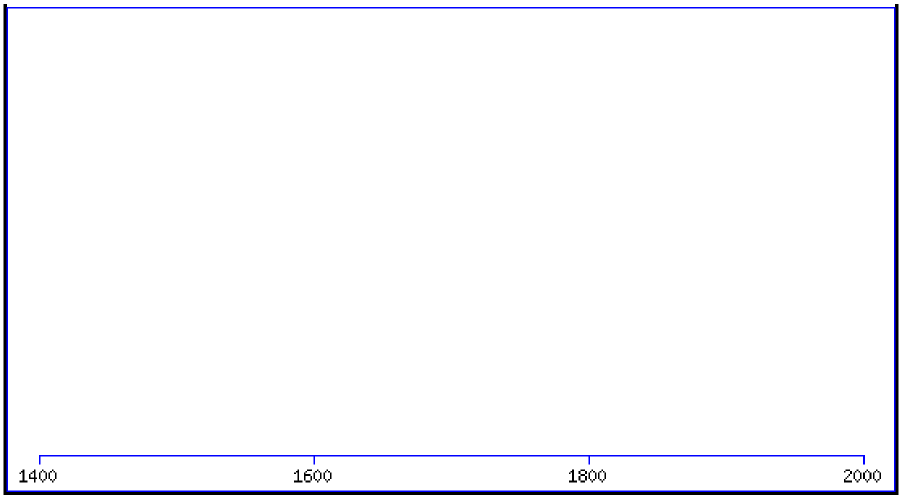

Monoisotopic mass of neutral peptide Mr(calc): 1213.5615
Fixed modifications: Carbamidomethyl (C)
Variable modifications:
N3 : Deamidated_N (N)
Ions Score: 45 **Expect:** 0.0042
Matches (Bold Red): 20/94 fragment ions using 40 most intense peaks

#	b	b^{++}	b*	b^{*++}	b^0	b^{0++}	Seq.	y	y^{++}	y*	y^{*++}	y^0	y^{0++}	#
1	130.0499	65.5286			112.0393	56.5233	E							10
2	**293.1132**	147.0602			**275.1026**	138.0550	Y	1085.5262	543.2667	**1068.4996**	534.7535	1067.5156	534.2615	9
3	408.1401	204.5737	391.1136	196.0604	**390.1296**	195.5684	N	**922.4629**	461.7351	905.4363	**453.2218**	904.4523	**452.7298**	8
4	536.1987	268.6030	519.1722	**260.0897**	**518.1881**	259.5977	Q	**807.4359**	404.2216	790.4094	395.7083	789.4254	395.2163	7
5	637.2464	319.1268	620.2198	310.6136	619.2358	310.1216	T	**679.3774**	340.1923	662.3508	331.6790	661.3668	331.1870	6
6	784.3148	392.6610	767.2883	384.1478	766.3042	383.6558	F	**578.3297**	289.6685	561.3031	281.1552			5
7	841.3363	421.1718	824.3097	412.6585	823.3257	412.1665	G	**431.2613**	216.1343	414.2347	207.6210			4
8	955.3792	478.1932	938.3526	469.6800	**937.3686**	469.1880	N	**374.2398**	187.6235	357.2132	179.1103			3
9	**1068.4633**	534.7353	**1051.4367**	**526.2220**	**1050.4527**	525.7300	L	**260.1969**	130.6021	243.1703	122.0888			2
10							K	147.1128	74.0600	130.0863	65.5468			1

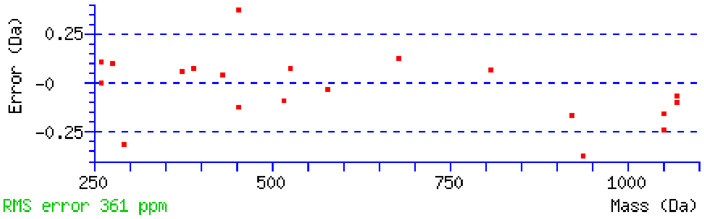

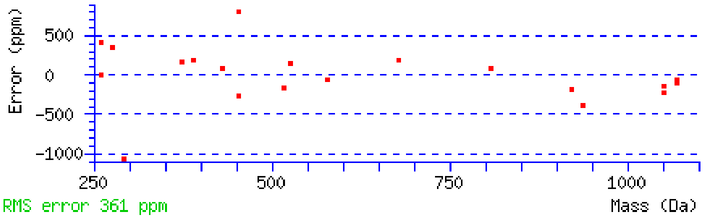

All matches to this query

Score	Mr(calc):	Delta	Sequence
44.7	1213.5615	0.0002	EYNQTFGNLK
7.8	1213.5615	0.0002	EYNQTFGNLK
6.6	1213.5591	0.0026	NTSDVISAAKK
4.9	1213.5526	0.0091	NMSVNNKSLK
3.8	1213.5649	-0.0031	EXEADLEHLK
3.2	1213.5649	-0.0032	YMSKDPTTQK
0.5	1213.5615	0.0002	EEDHAPEFIK
0.4	1213.5704	-0.0086	KTGGSSKNLGGK
0.4	1212.5639	0.9979	TLELSSQNLK
0.4	1213.5591	0.0026	ETISSILSER

Spectrum No: 483; Query: 909; Rank: 1

Peptide View

MS/MS Fragmentation of **VVLVNTTHKPFDIHVYHPYR**
Found in **IPI00205325**, Tax_Id=10116 Gene_Symbol=Lrp2 Low-density lipoprotein receptor-related protein 2 precursor

Match to Query 909: 2435.278302 from(812.766710,3+)
Title: 091127RatKid_SCX01_38.2422.2422.3.dta
Data file K:\NewmanPaper\Piliang\3SubProteomes\Piliang3SP\mgf5ppm\SCX_3SubProteomes5ppm.mgf

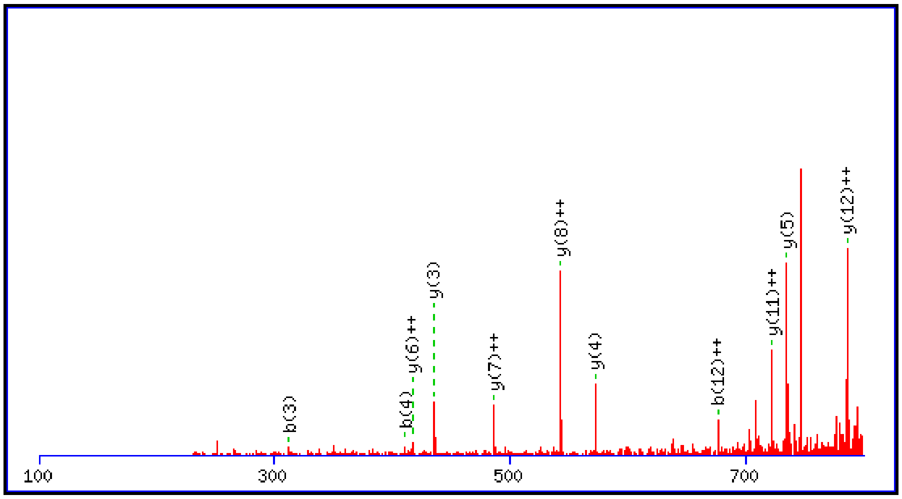

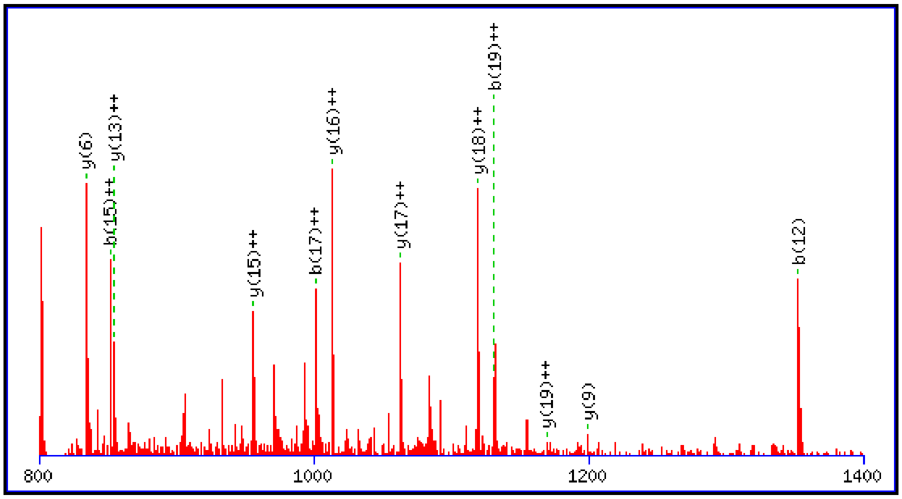

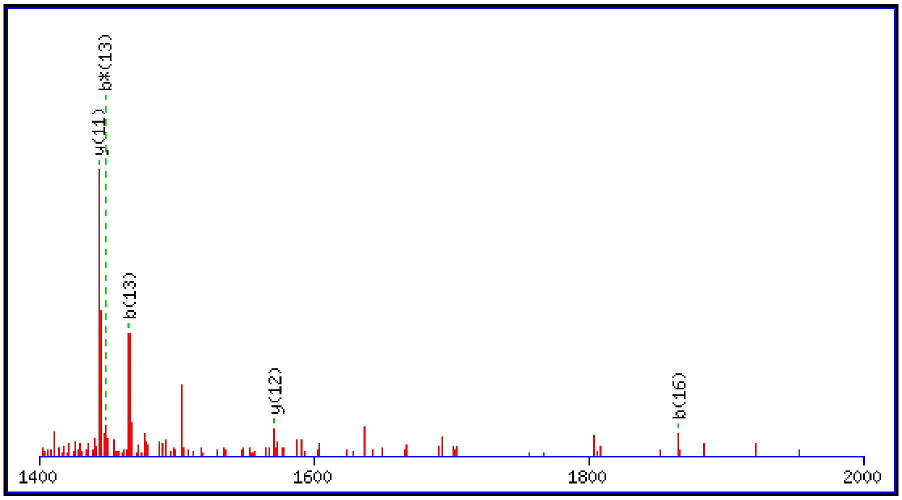

Monoisotopic mass of neutral peptide Mr(calc): 2435.2750

Fixed modifications: Carbamidomethyl (C)
Variable modifications:
N5 : Deamidated_N (N)
Ions Score: 45 **Expect:** 0.0036
Matches (Bold Red): 28/194 fragment ions using 64 most intense peaks

#	b	b^{++}	b*	b^{*++}	b^0	b^{0++}	Seq.	y	y^{++}	y*	y^{*++}	y^0	y^{0++}	#
1	100.0757	50.5415					V							20
2	199.1441	100.0757					V	2337.2139	**1169.1106**	2320.1873	1160.5973	2319.2033	1160.1053	19
3	**312.2282**	156.6177					L	2238.1454	**1119.5764**	2221.1189	1111.0631	2220.1349	1110.5711	18
4	**411.2966**	206.1519					V	2125.0614	**1063.0343**	2108.0348	1054.5211	2107.0508	1054.0290	17
5	526.3235	263.6654	509.2970	255.1521			N	2025.9930	**1013.5001**	2008.9664	1004.9868	2007.9824	1004.4948	16
6	627.3712	314.1892	610.3446	305.6760	609.3606	305.1840	T	1910.9660	**955.9867**	1893.9395	947.4734	1892.9555	946.9814	15
7	728.4189	364.7131	711.3923	356.1998	710.4083	355.7078	T	1809.9184	905.4628	1792.8918	896.9495	1791.9078	896.4575	14
8	865.4778	433.2425	848.4512	424.7293	847.4672	424.2372	H	1708.8707	**854.9390**	1691.8441	846.4257	1690.8601	845.9337	13
9	993.5728	497.2900	976.5462	488.7767	975.5622	488.2847	K	**1571.8118**	**786.4095**	1554.7852	777.8962	1553.8012	777.4042	12
10	1090.6255	545.8164	1073.5990	537.3031	1072.6149	536.8111	P	**1443.7168**	**722.3620**	1426.6902	713.8488	1425.7062	713.3568	11
11	1237.6939	619.3506	1220.6674	610.8373	1219.6834	610.3453	F	1346.6640	673.8357	1329.6375	665.3224	1328.6535	664.8304	10
12	**1352.7209**	**676.8641**	1335.6943	668.3508	1334.7103	667.8588	D	**1199.5956**	600.3014	1182.5691	591.7882	1181.5851	591.2962	9
13	**1465.8049**	733.4061	**1448.7784**	724.8928	1447.7944	724.4008	I	1084.5687	**542.7880**	1067.5421	534.2747			8
14	1602.8638	801.9356	1585.8373	793.4223	1584.8533	792.9303	H	971.4846	**486.2459**	954.4581	477.7327			7
15	1701.9323	**851.4698**	1684.9057	842.9565	1683.9217	842.4645	V	**834.4257**	**417.7165**	817.3992	409.2032			6
16	**1864.9956**	933.0014	1847.9690	924.4882	1846.9850	923.9962	Y	**735.3573**	368.1823	718.3307	359.6690			5
17	2002.0545	**1001.5309**	1985.0280	993.0176	1984.0439	992.5256	H	**572.2940**	286.6506	555.2674	278.1373			4
18	2099.1073	1050.0573	2082.0807	1041.5440	2081.0967	1041.0520	P	**435.2350**	218.1212	418.2085	209.6079			3
19	2262.1706	**1131.5889**	2245.1440	1123.0757	2244.1600	1122.5837	Y	338.1823	169.5948	321.1557	161.0815			2
20							R	175.1190	88.0631	158.0924	79.5498			1

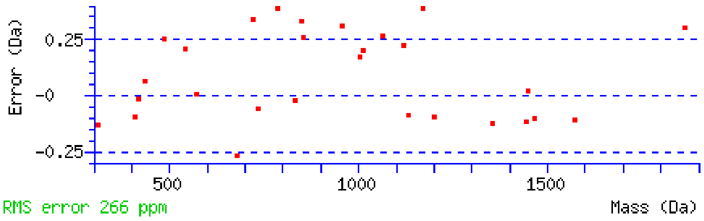

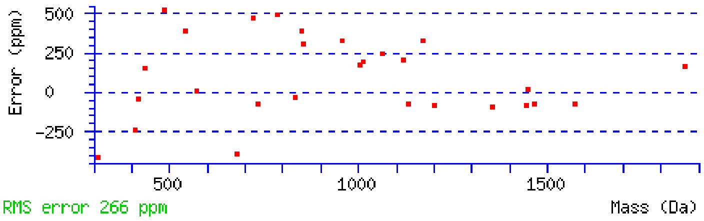

All matches to this query

Score	Mr(calc):	Delta	Sequence
44.5	2435.2750	0.0033	VVLVNTTHKPFDIHVYHPYR
16.7	2434.2910	0.9873	VVLVNTTHKPFDIHVYHPYR

Spectrum No: 484; Query: 993; Rank: 1

Peptide View

MS/MS Fragmentation of **LKEVAGIPLMDPTVNNWSQIQTFK**
Found in **IPI00204469**, Tax_Id=10116 Gene_Symbol=Sult1c2a Sulfotransferase 1C2A

Match to Query 993: 2729.410572 from(910.810800,3+)
Title: 091127RatKid_SCX01_25.5247.5247.3.dta
Data file K:\NewmanPaper\Piliang\3SubProteomes\Piliang3SP\mgf5ppm\SCX_3SubProteomes5ppm.mgf

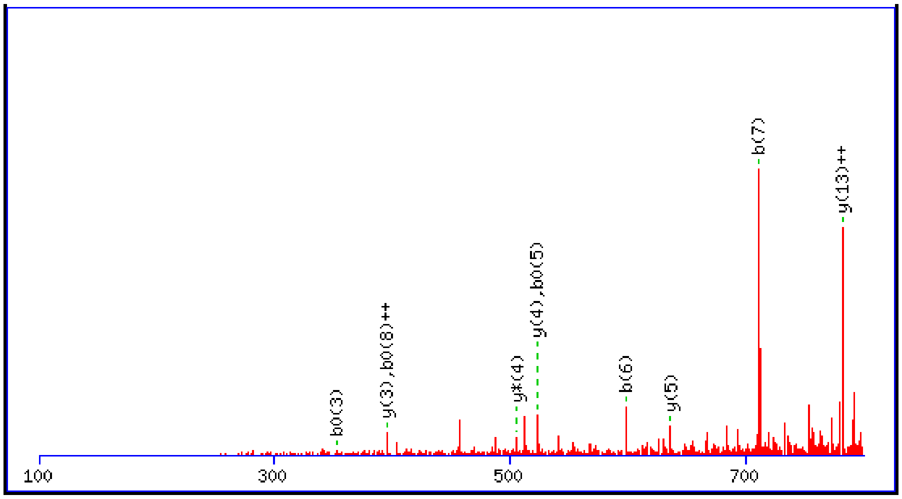

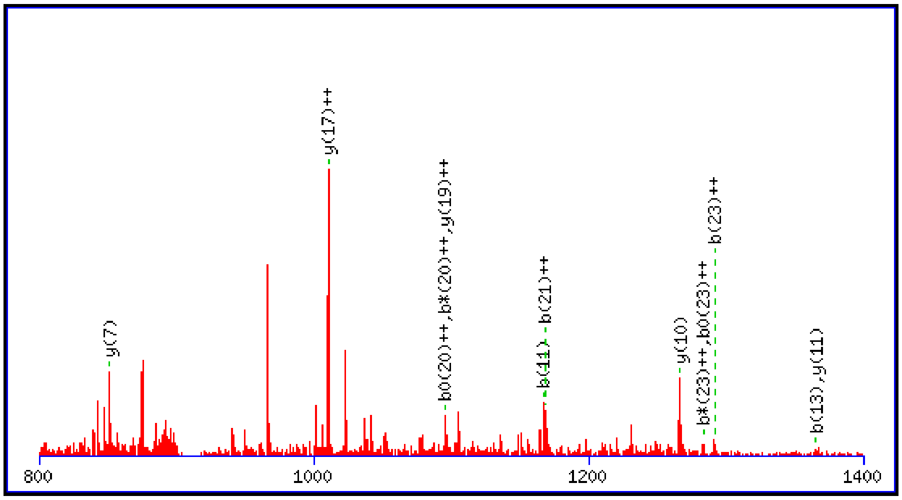

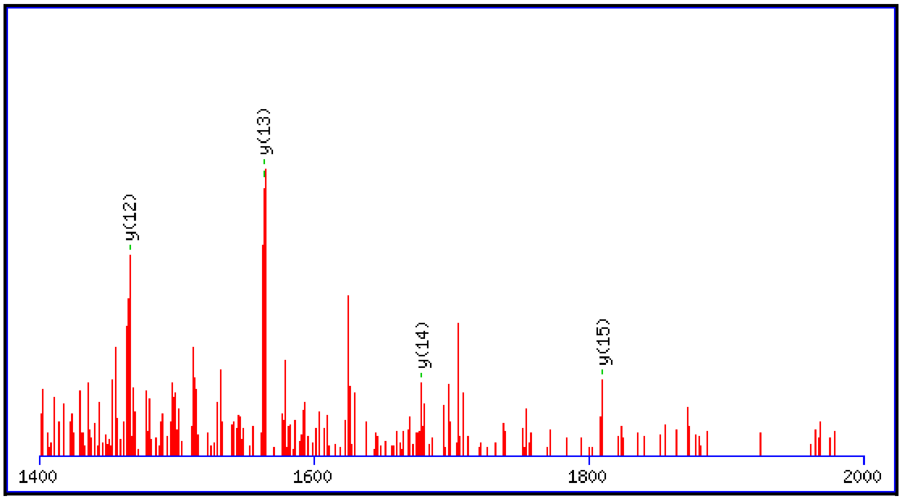

Monoisotopic mass of neutral peptide Mr(calc): 2729.4098
Fixed modifications: Carbamidomethyl (C)
Variable modifications:
N16 : Deamidated_N (N)
Ions Score: 44 **Expect:** 0.0048
Matches (Bold Red): 27/266 fragment ions using 59 most intense peaks

#	b	b^{++}	b*	b^{*++}	b^0	b^{0++}	Seq.	y	y^{++}	y*	y^{*++}	y^0	y^{0++}	#
1	114.0913	57.5493					L							24
2	242.1863	121.5968	225.1598	113.0835			K	2617.3330	1309.1702	2600.3065	1300.6569	2599.3225	1300.1649	23
3	371.2289	186.1181	354.2023	177.6048	**353.2183**	177.1128	E	2489.2381	1245.1227	2472.2115	1236.6094	2471.2275	1236.1174	22
4	470.2973	235.6523	453.2708	227.1390	452.2867	226.6470	V	2360.1955	1180.6014	2343.1689	1172.0881	2342.1849	1171.5961	21
5	541.3344	271.1709	524.3079	262.6576	**523.3239**	262.1656	A	2261.1271	1131.0672	2244.1005	1122.5539	2243.1165	1122.0619	20

6	**598.3559**	299.6816	581.3293	291.1683	580.3453	290.6763	G	2190.0900	**1095.5486**	2173.0634	1087.0353	2172.0794	1086.5433	19
7	**711.4400**	356.2236	694.4134	347.7103	693.4294	347.2183	I	2133.0685	1067.0379	2116.0419	1058.5246	2115.0579	1058.0326	18
8	808.4927	404.7500	791.4662	396.2367	790.4822	**395.7447**	P	2019.9844	**1010.4959**	2002.9579	1001.9826	2001.9739	1001.4906	17
9	921.5768	461.2920	904.5502	452.7788	903.5662	452.2867	L	1922.9317	961.9695	1905.9051	953.4562	1904.9211	952.9642	16
10	1052.6173	526.8123	1035.5907	518.2990	1034.6067	517.8070	M	**1809.8476**	905.4274	1792.8211	896.9142	1791.8370	896.4222	15
11	**1167.6442**	584.3257	1150.6177	575.8125	1149.6336	575.3205	D	**1678.8071**	839.9072	1661.7806	831.3939	1660.7966	830.9019	14
12	1264.6970	632.8521	1247.6704	624.3388	1246.6864	623.8468	P	**1563.7802**	**782.3937**	1546.7536	773.8805	1545.7696	773.3884	13
13	**1365.7447**	683.3760	1348.7181	674.8627	1347.7341	674.3707	T	**1466.7274**	733.8673	1449.7009	725.3541	1448.7168	724.8621	12
14	1464.8131	732.9102	1447.7865	724.3969	1446.8025	723.9049	V	**1365.6797**	683.3435	1348.6532	674.8302	1347.6692	674.3382	11
15	1578.8560	789.9316	1561.8294	781.4184	1560.8454	780.9264	N	**1266.6113**	633.8093	1249.5848	625.2960	1248.6008	624.8040	10
16	1693.8829	847.4451	1676.8564	838.9318	1675.8724	838.4398	N	1152.5684	576.7878	1135.5418	568.2746	1134.5578	567.7826	9
17	1879.9622	940.4848	1862.9357	931.9715	1861.9517	931.4795	W	1037.5415	519.2744	1020.5149	510.7611	1019.5309	510.2691	8
18	1966.9943	984.0008	1949.9677	975.4875	1948.9837	974.9955	S	**851.4621**	426.2347	834.4356	417.7214	833.4516	417.2294	7
19	2095.0528	1048.0301	2078.0263	1039.5168	2077.0423	1039.0248	Q	764.4301	382.7187	747.4036	374.2054	746.4196	373.7134	6
20	2208.1369	1104.5721	2191.1104	**1096.0588**	2190.1263	**1095.5668**	I	**636.3715**	318.6894	619.3450	310.1761	618.3610	309.6841	5
21	2336.1955	**1168.6014**	2319.1689	1160.0881	2318.1849	1159.5961	Q	**523.2875**	262.1474	**506.2609**	253.6341	505.2769	253.1421	4
22	2437.2432	1219.1252	2420.2166	1210.6119	2419.2326	1210.1199	T	**395.2289**	198.1181	378.2023	189.6048	377.2183	189.1128	3
23	2584.3116	**1292.6594**	2567.2850	**1284.1462**	2566.3010	**1283.6541**	F	294.1812	147.5942	277.1547	139.0810			2
24							K	147.1128	74.0600	130.0863	65.5468			1

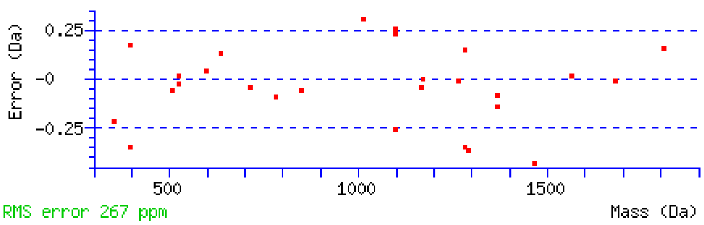

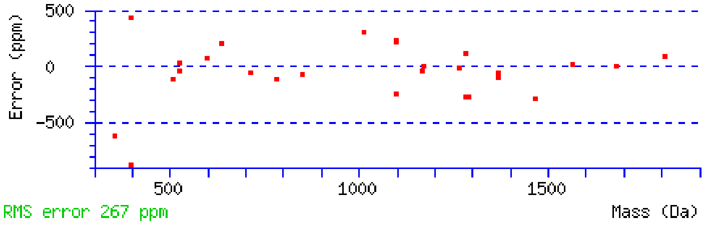

All matches to this query

Score	Mr(calc):	Delta	Sequence
44.5	2729.4098	0.0008	LKEVAGIPLMDPTVNNWSQIQTFK
44.5	2729.4098	0.0008	LKEVAGIPLMDPTVNNWSQIQTFK
24.9	2728.4258	0.9848	LKEVAGIPLMDPTVNNWSQIQTFK

Spectrum No: 485; Query: 18; Rank: 1

Peptide View

MS/MS Fragmentation of **ANITVSISQK**
Found in **IPI00388355**, Tax_Id=10116 Gene_Symbol=Manba Isoform 1 of Beta-mannosidase precursor

Match to Query 18: 1060.575868 from(531.295210,2+)
Title: 091127RatKid_SCX01_19.1215.1215.2.dta
Data file K:\NewmanPaper\Piliang\3SubProteomes\Piliang3SP\mgf5ppm\SCX_3SubProteomes5ppm.mgf

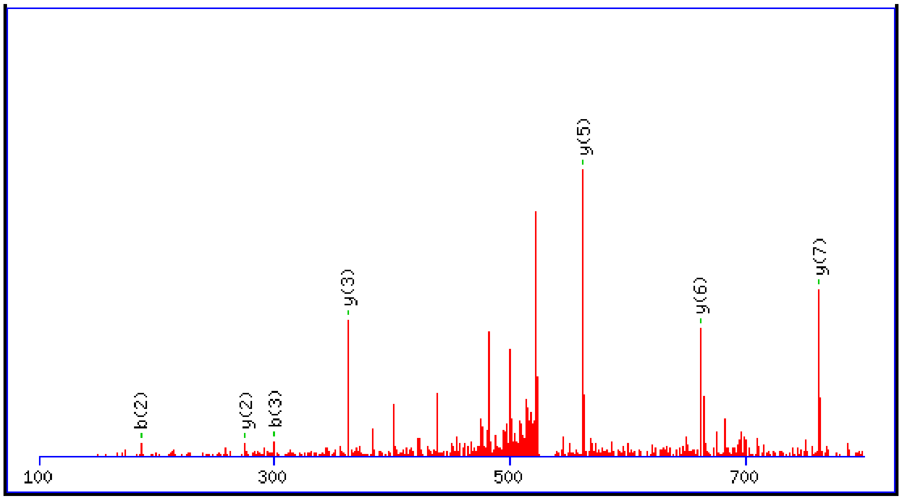

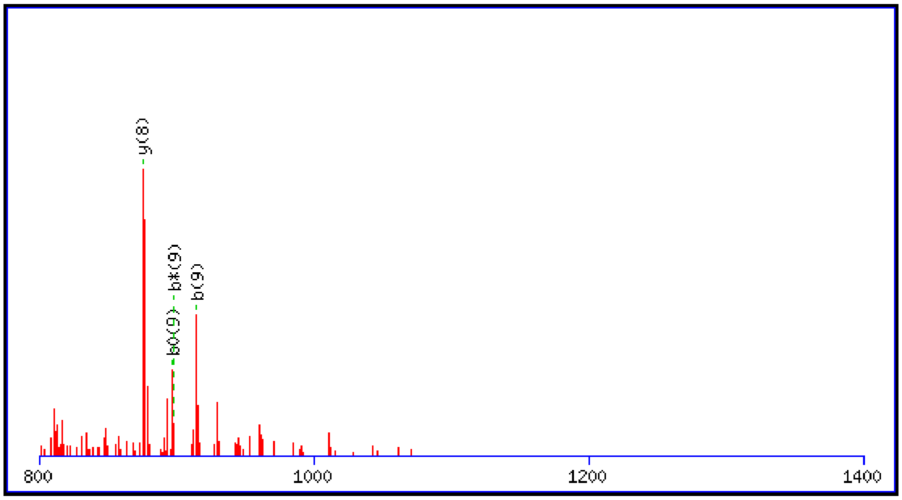

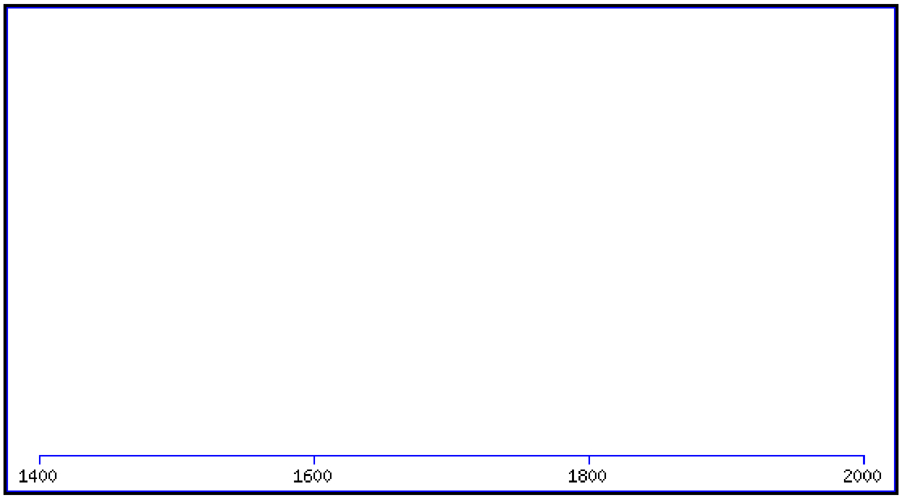

Monoisotopic mass of neutral peptide Mr(calc): 1060.5764
Fixed modifications: Carbamidomethyl (C)
Variable modifications:
N2 : Deamidated_N (N)
Ions Score: 44 **Expect:** 0.0038
Matches (Bold Red): 11/96 fragment ions using 19 most intense peaks

#	b	b^{++}	b*	b^{*++}	b^0	b^{0++}	Seq.	y	y^{++}	y*	y^{*++}	y^0	y^{0++}	#
1	72.0444	36.5258					A							10
2	**187.0713**	94.0393	170.0448	85.5260			N	990.5466	495.7769	973.5200	487.2637	972.5360	486.7717	9
3	**300.1554**	150.5813	283.1288	142.0681			I	**875.5197**	438.2635	858.4931	429.7502	857.5091	429.2582	8
4	401.2031	201.1052	384.1765	192.5919	383.1925	192.0999	T	**762.4356**	381.7214	745.4090	373.2082	744.4250	372.7162	7
5	500.2715	250.6394	483.2449	242.1261	482.2609	241.6341	V	**661.3879**	331.1976	644.3614	322.6843	643.3774	322.1923	6

**6**	587.3035	294.1554	570.2770	285.6421	569.2929	285.1501	**S**	**562.3195**	281.6634	545.2930	273.1501	544.3089	272.6581	**5**
**7**	700.3876	350.6974	683.3610	342.1842	682.3770	341.6921	**I**	475.2875	238.1474	458.2609	229.6341	457.2769	229.1421	**4**
**8**	787.4196	394.2134	770.3931	385.7002	769.4090	385.2082	**S**	**362.2034**	181.6053	345.1769	173.0921	344.1928	172.6001	**3**
**9**	**915.4782**	458.2427	**898.4516**	449.7295	**897.4676**	449.2374	**Q**	**275.1714**	138.0893	258.1448	129.5761			**2**
**10**							**K**	147.1128	74.0600	130.0863	65.5468			**1**

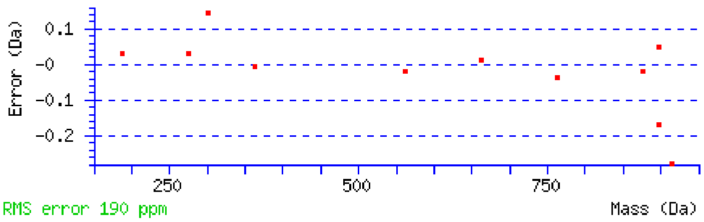

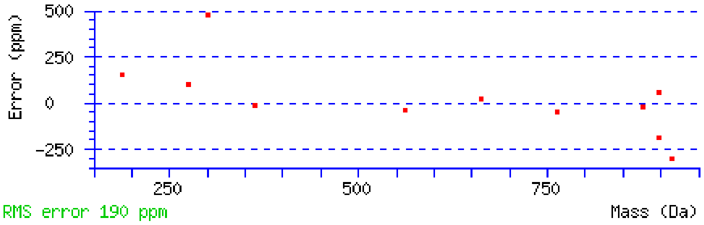

All matches to this query

Score	Mr(calc):	Delta	Sequence
44.3	1060.5764	-0.0005	ANITVSISQK
15.6	1060.5699	0.0060	GELSIQRMK
5.3	1059.5634	1.0125	ALLVSMGEPK
5.2	1060.5737	0.0021	EGLRSRSTR
5.2	1060.5764	-0.0005	EGLSNKVSVK
5.2	1060.5665	0.0094	WIRNKESK
4.3	1059.5713	1.0046	QGFKLNPQK
3.9	1058.5608	2.0151	LASDPNVSKK
3.8	1060.5699	0.0060	SVVNKMQQK
2.1	1060.5705	0.0053	IHEPFYKK

Spectrum No: 486; Query: 1120; Rank: 1

Peptide View

MS/MS Fragmentation of **SCGECIQAGPNCGWCTNTTFLQEGMPTSAR**
Found in **IPI00191681**, Tax_Id=10116 Gene_Symbol=Itgb1 Integrin beta-1 precursor

Match to Query 1120: 3391.399692 from(1131.473840,3+)
Title: 091127RatKid_SCX01_12.3528.3528.3.dta
Data file K:\NewmanPaper\Piliang\3SubProteomes\Piliang3SP\mgf5ppm\SCX_3SubProteomes5ppm.mgf

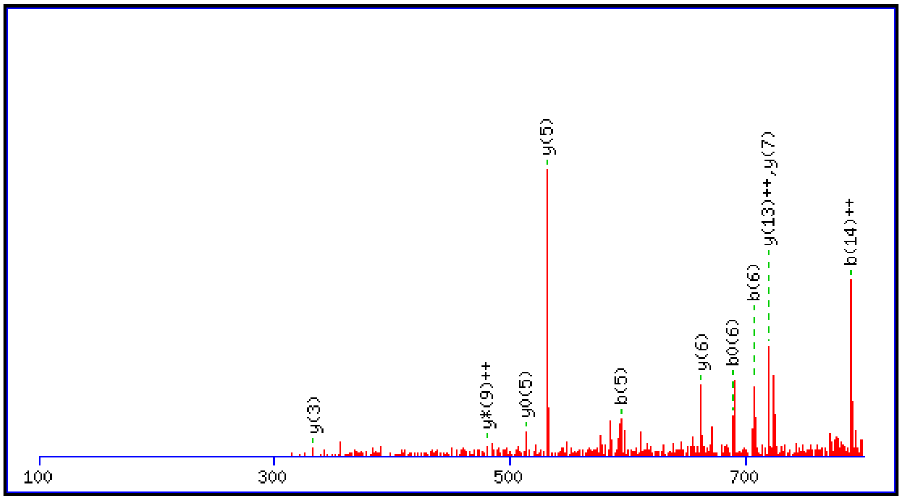

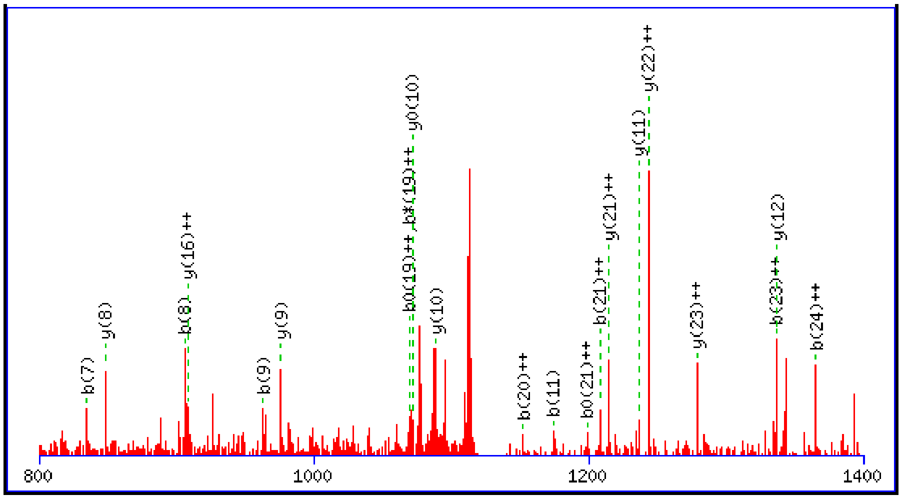

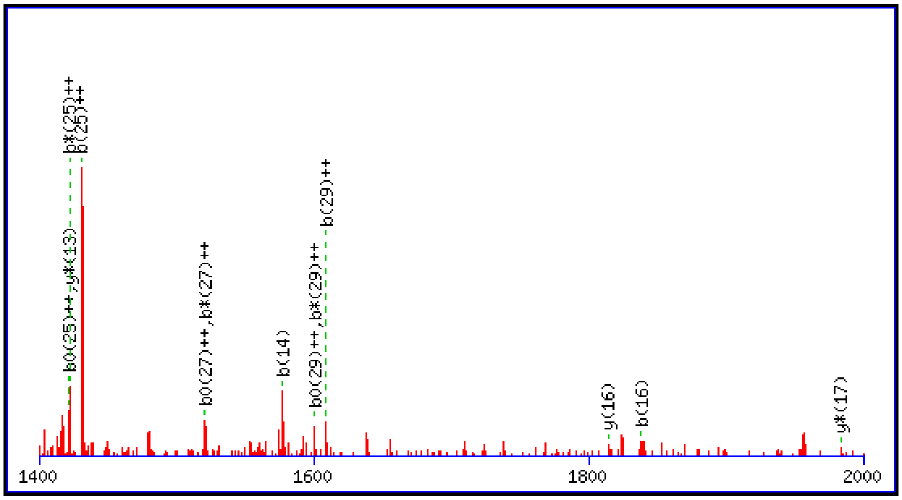

Monoisotopic mass of neutral peptide Mr(calc): 3390.3832
Fixed modifications: Carbamidomethyl (C)
Variable modifications:
N17 : Deamidated_N (N)
Ions Score: 44 **Expect:** 0.016
Matches (Bold Red): 45/332 fragment ions using 92 most intense peaks

#	b	b^{++}	b*	b^{*++}	b^0	b^{0++}	Seq.	y	y^{++}	y*	y^{*++}	y^0	y^{0++}	#
1	88.0393	44.5233			70.0287	35.5180	S							30
2	248.0700	124.5386			230.0594	115.5333	C	3304.3584	1652.6829	3287.3319	1644.1696	3286.3479	1643.6776	29
3	305.0914	153.0493			287.0809	144.0441	G	3144.3278	1572.6675	3127.3013	1564.1543	3126.3172	1563.6623	28
4	434.1340	217.5706			416.1234	208.5654	E	3087.3063	1544.1568	3070.2798	1535.6435	3069.2958	1535.1515	27
5	**594.1647**	297.5860			576.1541	288.5807	C	2958.2637	1479.6355	2941.2372	1471.1222	2940.2532	1470.6302	26
6	**707.2487**	354.1280			**689.2382**	345.1227	I	2798.2331	1399.6202	2781.2065	1391.1069	2780.2225	1390.6149	25
7	**835.3073**	418.1573	818.2808	409.6440	817.2967	409.1520	Q	2685.1490	1343.0782	2668.1225	1334.5649	2667.1385	1334.0729	24
8	**906.3444**	453.6758	889.3179	445.1626	888.3338	444.6706	A	2557.0905	**1279.0489**	2540.0639	1270.5356	2539.0799	1270.0436	23
9	**963.3659**	482.1866	946.3393	473.6733	945.3553	473.1813	G	2486.0533	**1243.5303**	2469.0268	1235.0170	2468.0428	1234.5250	22
10	1060.4186	530.7130	1043.3921	522.1997	1042.4081	521.7077	P	2429.0319	**1215.0196**	2412.0053	1206.5063	2411.0213	1206.0143	21
11	**1174.4616**	587.7344	1157.4350	579.2211	1156.4510	578.7291	N	2331.9791	1166.4932	2314.9526	1157.9799	2313.9685	1157.4879	20
12	1334.4922	667.7497	1317.4657	659.2365	1316.4817	658.7445	C	2217.9362	1109.4717	2200.9096	1100.9585	2199.9256	1100.4664	19
13	1391.5137	696.2605	1374.4871	687.7472	1373.5031	687.2552	G	2057.9055	1029.4564	2040.8790	1020.9431	2039.8950	1020.4511	18
14	**1577.5930**	**789.3001**	1560.5664	780.7869	1559.5824	780.2949	W	2000.8841	1000.9457	**1983.8575**	992.4324	1982.8735	991.9404	17
15	1737.6236	869.3155	1720.5971	860.8022	1719.6131	860.3102	C	**1814.8048**	**907.9060**	1797.7782	899.3927	1796.7942	898.9007	16
16	**1838.6713**	919.8393	1821.6448	911.3260	1820.6608	910.8340	T	1654.7741	827.8907	1637.7476	819.3774	1636.7635	818.8854	15
17	1953.6983	977.3528	1936.6717	968.8395	1935.6877	968.3475	N	1553.7264	777.3669	1536.6999	768.8536	1535.7159	768.3616	14
18	2054.7459	1027.8766	2037.7194	1019.3633	2036.7354	1018.8713	T	1438.6995	**719.8534**	**1421.6729**	711.3401	1420.6889	710.8481	13

**19**	2155.7936	1078.4004	2138.7671	**1069.8872**	2137.7831	**1069.3952**	**T**	**1337.6518**	669.3295	1320.6253	660.8163	1319.6412	660.3243	**12**
**20**	2302.8620	**1151.9347**	2285.8355	1143.4214	2284.8515	1142.9294	**F**	**1236.6041**	618.8057	1219.5776	610.2924	1218.5936	609.8004	**11**
**21**	2415.9461	**1208.4767**	2398.9195	1199.9634	2397.9355	**1199.4714**	**L**	**1089.5357**	545.2715	1072.5092	536.7582	**1071.5252**	536.2662	**10**
**22**	2544.0047	1272.5060	2526.9781	1263.9927	2525.9941	1263.5007	**Q**	**976.4517**	488.7295	959.4251	**480.2162**	958.4411	479.7242	**9**
**23**	2673.0473	**1337.0273**	2656.0207	1328.5140	2655.0367	1328.0220	**E**	**848.3931**	424.7002	831.3665	416.1869	830.3825	415.6949	**8**
**24**	2730.0687	**1365.5380**	2713.0422	1357.0247	2712.0582	1356.5327	**G**	**719.3505**	360.1789	702.3239	351.6656	701.3399	351.1736	**7**
**25**	2861.1092	**1431.0582**	2844.0827	**1422.5450**	2843.0987	**1422.0530**	**M**	**662.3290**	331.6681	645.3025	323.1549	644.3185	322.6629	**6**
**26**	2958.1620	1479.5846	2941.1354	1471.0714	2940.1514	1470.5793	**P**	**531.2885**	266.1479	514.2620	257.6346	**513.2780**	257.1426	**5**
**27**	3059.2097	1530.1085	3042.1831	**1521.5952**	3041.1991	**1521.1032**	**T**	434.2358	217.6215	417.2092	209.1083	416.2252	208.6162	**4**
**28**	3146.2417	1573.6245	3129.2151	1565.1112	3128.2311	1564.6192	**S**	**333.1881**	167.0977	316.1615	158.5844	315.1775	158.0924	**3**
**29**	3217.2788	**1609.1430**	3200.2523	**1600.6298**	3199.2682	**1600.1378**	**A**	246.1561	123.5817	229.1295	115.0684			**2**
**30**							**R**	175.1190	88.0631	158.0924	79.5498			**1**

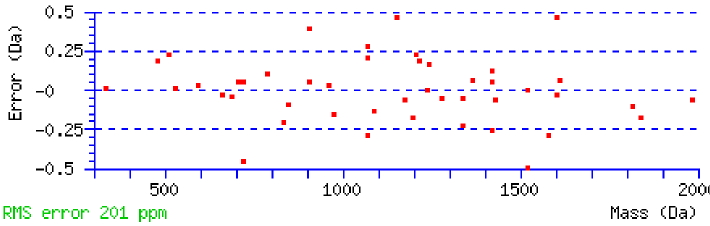

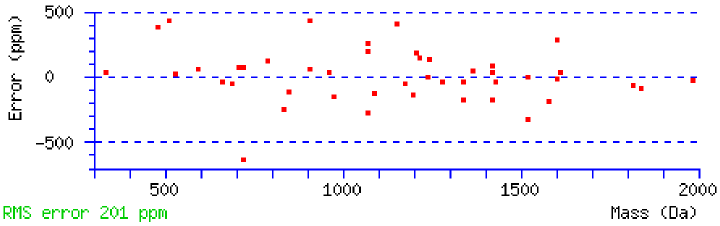

All matches to this query

Score	Mr(calc):	Delta	Sequence
44.1	3390.3832	1.0165	SCGECIQAGPNCGWCTNTTFLQEGMPTSAR
42.7	3391.3672	0.0325	SCGECIQAGPNCGWCTNTTFLQEGMPTSAR
40.4	3390.3832	1.0165	SCGECIQAGPNCGWCTNTTFLQEGMPTSAR
33.5	3389.3992	2.0005	SCGECIQAGPNCGWCTNTTFLQEGMPTSAR
3.9	3390.4238	0.9759	MHSHPSASNNSYATLGQSQTAAKRHAFASR
1.6	3389.4022	1.9975	DTHPKFPAGGKMSQYLENMNIGDTIEFR
1.6	3389.4022	1.9975	DTHPKFPAGGKMSQYLENMNIGDTIEFR
1.0	3390.4120	0.9877	ETLMDLGTKAFAMTNQILVERSVTGWK
0.9	3391.3895	0.0102	IVGYPASFMSLRCLLSDELSNVAMQVR
0.9	3391.3895	0.0102	IVGYPASFMSLRCLLSDELSNVAMQVR

Spectrum No: 487; Query: 669; Rank: 1

Peptide View

MS/MS Fragmentation of **IVAERPGTNSTGPGPMAPPR**
Found in **IPI00194567**, Tax_Id=10116 Gene_Symbol=Trim28 similar to Transcription intermediary factor 1-beta

Match to Query 669: 2005.005942 from(669.342590,3+)
Title: 091127RatKid_SCX01_28.1252.1252.3.dta
Data file K:\NewmanPaper\Piliang\3SubProteomes\Piliang3SP\mgf5ppm\SCX_3SubProteomes5ppm.mgf

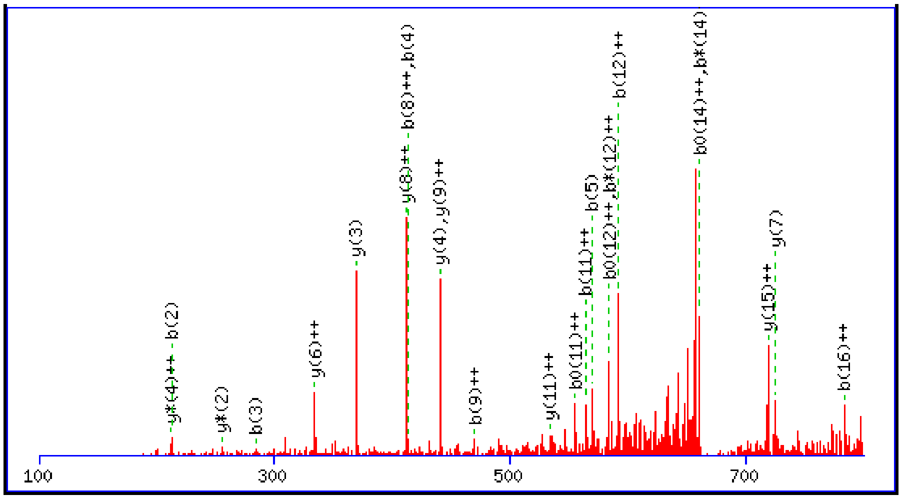

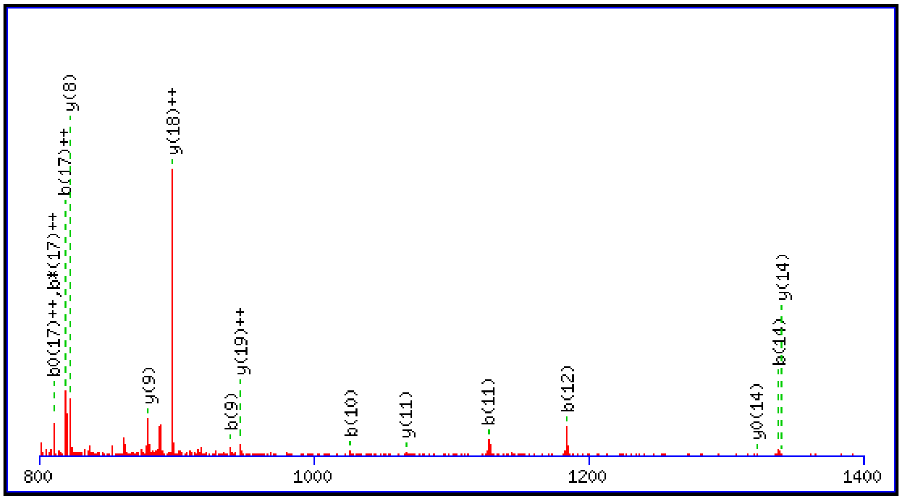

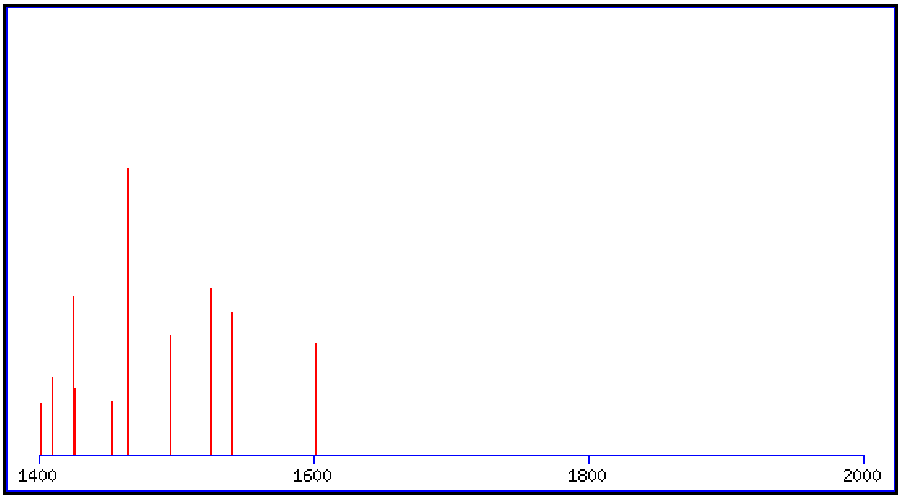

Monoisotopic mass of neutral peptide Mr(calc): 2005.0051
Fixed modifications: Carbamidomethyl (C)
Variable modifications:
N9 : Deamidated_N (N)
Ions Score: 44 **Expect:** 0.0068
Matches (Bold Red): 39/196 fragment ions using 64 most intense peaks

#	b	b++	b*	b*++	b0	b0++	Seq.	y	y++	y*	y*++	y0	y0++	#
1	114.0913	57.5493					I							20
2	**213.1598**	107.0835					V	1892.9283	**946.9678**	1875.9018	938.4545	1874.9178	937.9625	19
3	**284.1969**	142.6021					A	1793.8599	**897.4336**	1776.8334	888.9203	1775.8493	888.4283	18
4	**413.2395**	207.1234			395.2289	198.1181	E	1722.8228	861.9150	1705.7962	853.4018	1704.8122	852.9098	17
5	**569.3406**	285.1739	552.3140	276.6606	551.3300	276.1686	R	1593.7802	797.3937	1576.7536	788.8805	1575.7696	788.3885	16

**6**	666.3933	333.7003	649.3668	325.1870	648.3828	324.6950	**P**	1437.6791	**719.3432**	1420.6525	710.8299	1419.6685	710.3379	**15**
**7**	723.4148	362.2110	706.3883	353.6978	705.4042	353.2058	**G**	**1340.6263**	670.8168	1323.5998	662.3035	**1322.6158**	661.8115	**14**
**8**	824.4625	**412.7349**	807.4359	404.2216	806.4519	403.7296	**T**	1283.6049	642.3061	1266.5783	633.7928	1265.5943	633.3008	**13**
**9**	**939.4894**	**470.2483**	922.4629	461.7351	921.4789	461.2431	**N**	1182.5572	591.7822	1165.5306	583.2690	1164.5466	582.7769	**12**
**10**	**1026.5214**	513.7644	1009.4949	505.2511	1008.5109	504.7591	**S**	**1067.5302**	**534.2688**	1050.5037	525.7555	1049.5197	525.2635	**11**
**11**	**1127.5691**	**564.2882**	1110.5426	555.7749	1109.5586	**555.2829**	**T**	980.4982	490.7527	963.4717	482.2395	962.4876	481.7475	**10**
**12**	**1184.5906**	**592.7989**	1167.5640	**584.2857**	1166.5800	**583.7936**	**G**	**879.4505**	**440.2289**	862.4240	431.7156			**9**
**13**	1281.6434	641.3253	1264.6168	632.8120	1263.6328	632.3200	**P**	**822.4291**	**411.7182**	805.4025	403.2049			**8**
**14**	**1338.6648**	669.8360	1321.6383	**661.3228**	1320.6542	**660.8308**	**G**	**725.3763**	363.1918	708.3498	354.6785			**7**
**15**	1435.7176	718.3624	1418.6910	709.8492	1417.7070	709.3571	**P**	668.3548	**334.6811**	651.3283	326.1678			**6**
**16**	1566.7581	**783.8827**	1549.7315	775.3694	1548.7475	774.8774	**M**	571.3021	286.1547	554.2755	277.6414			**5**
**17**	1637.7952	**819.4012**	1620.7686	**810.8880**	1619.7846	**810.3959**	**A**	**440.2616**	220.6344	423.2350	**212.1212**			**4**
**18**	1734.8479	867.9276	1717.8214	859.4143	1716.8374	858.9223	**P**	**369.2245**	185.1159	352.1979	176.6026			**3**
**19**	1831.9007	916.4540	1814.8742	907.9407	1813.8901	907.4487	**P**	272.1717	136.5895	**255.1452**	128.0762			**2**
**20**							**R**	175.1190	88.0631	158.0924	79.5498			**1**

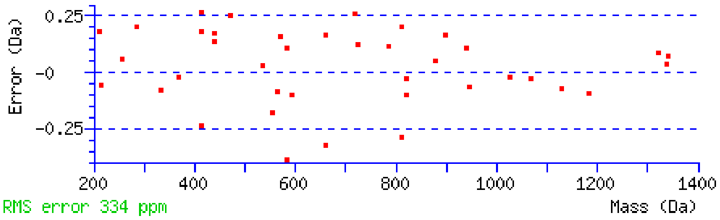

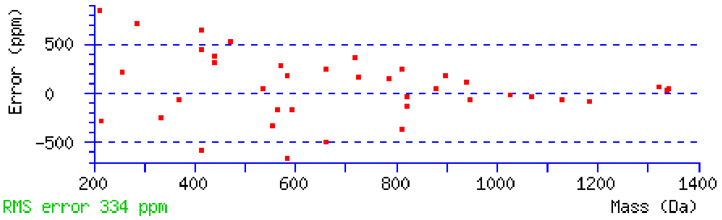
[truncated: 385,110 more chars]
